# Effects of Prenatal Exposure to Titanium Dioxide Nanoparticles on DNA Methylation and Gene Expression Profile in the Mouse Brain

**DOI:** 10.3389/ftox.2021.705910

**Published:** 2021-10-08

**Authors:** Ken Tachibana, Shotaro Kawazoe, Atsuto Onoda, Masakazu Umezawa, Ken Takeda

**Affiliations:** ^1^ Division of Toxicology and Health Science, Faculty of Pharmaceutical Sciences, Sanyo-onoda City University, Sanyo-onoda, Japan; ^2^ The Center for Environmental Health Science for the Next Generation, Research Institute for Science and Technology, Organization for Research Advancement, Tokyo University of Science, Noda, Japan; ^3^ Faculty of Pharmaceutical Sciences, Tokyo University of Science, Noda, Japan; ^4^ Department of Materials Science and Technology, Faculty of Advanced Engineering, Tokyo University of Science, Katsushika, Japan

**Keywords:** brain, DNA methylation, gene expression, titanium dioxide nanoparticle, prenatal exposure

## Abstract

**Background and Objectives:** Titanium dioxide nanoparticles (TiO_2_-NP) are important materials used in commercial practice. Reportedly, TiO_2_-NP exposure during pregnancy can affect the development of the central nervous system in mouse offspring; however, the underlying mechanism remains unknown. In the present study, we investigated the impact of prenatal TiO_2_-NP exposure on global DNA methylation and mRNA expression patterns in the brains of neonatal mice.

**Materials and Methods:** Pregnant C57BL/6J mice were intratracheally administered a TiO_2_-NP suspension (100 μg/mouse) on gestational day 10.5, and brains were collected from male and female offspring at day 1 postpartum. After extraction of methylated DNA by immunoprecipitation, the DNA methylation profile was analyzed using a mouse CpG island microarray. Total RNA was obtained, and mRNA expression profiles were comprehensively assessed using microarray analysis.

**Results:** Among genes in the CpG island microarray, DNA methylation was increased in 614 and 2,924 genes and decreased in 6,220 and 6,477 genes in male and female offspring, respectively. Combined with mRNA microarray analysis, 88 and 89 genes were upregulated (≥1.5-fold) accompanied by demethylation of CpG islands, whereas 13 and 33 genes were downregulated (≤0.67-fold) accompanied by methylation of CpG islands in male and female offspring mice, respectively. Gene Set Enrichment Analysis (GSEA) revealed that these genes were enriched in gene ontology terms related to the regulation of transcription factors, cell proliferation, and organism development. Additionally, MeSH terms related to stem cells and morphogenesis were enriched.

**Conclusion:** Prenatal TiO_2_-NP exposure induced genome-wide alterations in DNA methylation and mRNA expression in the brains of male and female offspring. Based on GSEA findings, it can be speculated that prenatal TiO_2_-NP exposure causes adverse effects on brain functions by altering the DNA methylation state of the fetal brain, especially neural stem cells, resulting in the subsequent abnormal regulation of transcription factors that modulate development and differentiation.

## Introduction

Nanomaterial research and development have gained momentum in recent years, with several new nanotechnology-based products now commercially available. Nanomaterials are characterized as objects measuring 1-100 nm in at least one plane of dimension. Notably, nanoparticulate substances impart new properties to the substance, for example, unique biokinetics and enhanced optical, catalytic, and biological activities, and are employed in several products, such as therapeutic agents, electronics, optics, and cosmetics (Oberdörster et al., 2005). Titanium dioxide nanoparticles (TiO_2_-NP) are important materials used in commercial paints for buildings (Shandilya et al., 2015), cosmetics (Kaida et al., 2004), and food additives (Weir et al., 2012). The increasing possibility of TiO_2_-NP exposure has evoked concerns regarding potential risks of TiO_2_-NP on human health (Borm et al., 2006). As possible exposure routes for TiO2-NP, inhalation, dermal and oral exposure are the most obvious (Borm et al., 2006). In particular, sunscreens and other cosmetics providing ultra violet protection may expose consumer lungs to TiO_2_-NP by inhalation because they can be available as sprayable products (Dréno et al., 2019). Pregnant women can be exposed by a similar route.

Previous studies have suggested that intranasal exposure to TiO_2_-NP (2.5 mg/kg body weight for 90 consecutive days) induces hippocampal neuroinflammation, with over-proliferation of all glial cells and impaired spatial recognition memory in adult mice (Ze et al., 2014). Induction of oxidative stress in the brain by TiO_2_-NP exposure (Shrivastava et al., 2014) would be related to these phenomena. On the other hand, following the subcutaneous administration of TiO_2_-NP to pregnant mice (500 μg/pregnant mouse), these nanoparticles were detected in the brains of offspring after birth (Takeda et al., 2009). The results have been suggested that TiO_2_-NP administered to pregnant mice can cross blood-placental barrier, and then affected the brain development. Male offspring mice exposed to TiO_2_-NP prenatally [subcutaneously injected; 100 μg/mouse on gestational day (GD) 6, 9, 12, and 15] reportedly present disruption of gene expression (Shimizu et al., 2009) and the dopaminergic system in the brain (Takahashi et al., 2010). Expression levels of genes associated with brain development were altered in 2 and 14 days after birth (Shimizu et al., 2009). Takahashi et al. (2010) showed that dopamine and its metabolites were increased in the prefrontal cortex and neostriatum in 6-week-old male pups. Furthermore, impaired learning and memory and decreased hippocampal cell proliferation were induced in offspring rats exposed to TiO_2_-NP (100 mg/kg body weight) by daily gavage administration from GD 2 to GD 21 (Mohammadipour et al., 2014). Moderate neurobehavioral alterations were observed in offspring mice following inhalation exposure of pregnant mice to a surface-coated TiO_2_-NP [1 h/day to 42 mg/m^3^ aerosolized powder (1.7 × 10^6^ particle/cm^3^)] from GD 8 to GD 18 (Hougaard et al., 2010). As the result showed in this paper, offspring mice exposed to TiO_2_-NP prenatally tended to avoid the central zone of the open filed test, and female offspring exposed to TiO_2_-NP prenatally displayed enhanced prepulse inhibition (Hougaard et al., 2010). Thus, brain toxicity appeared after birth, although the mice were exposed to TiO_2_-NP during the prenatal period. It is necessary to elucidate underlying mechanisms through which nanoparticles affect human health to assess the safety of nanoparticles in association with advances in nanotechnology.

In the present study, we focused on the impact of TiO_2_-NP exposure on DNA methylation, a crucial mechanism for stable transcriptional silencing (Bird, 2002). Abnormal regulation of DNA methylation has been observed in mental and behavioral disorders (Robertson, 2005). DNA methylation patterns are initially constructed during the fetal stage and play an important role in development and differentiation (Bird, 2002). Genome-wide, oocyte DNA tends to be hypomethylated, whereas sperm DNA tends to be hypermethylated (Monk et al., 1987). After most methylation moieties present on the original parental chromosomes are removed from DNA at the morula stage, resulting in a predominantly unmethylated genome, a wave of *de novo* methylation follows, leading to an overall increase in genome methylation levels as newly implanted embryos develop and differentiate (Kafri et al., 1992). The deletion or inhibition of DNA methyltransferases induces abnormalities in embryonic development and organogenesis (Conerly and Grady, 2010). Most DNA methylation patterns are stably maintained following DNA replication and cell division, leading to normal embryonic development (Cheedipudi et al., 2014), and regulate postnatal neurodevelopment (Szulwach et al., 2011), neuronal synaptic functions (Feng et al., 2010), and astrocyte differentiation (Takizawa et al., 2001). We hypothesized that a change in reconstitution and maintenance of DNA methylation patterns could contribute to the effects of prenatal TiO_2_-NP exposure on brain development. Herein, we assessed the effects of prenatal TiO_2_-NP exposure on global DNA methylation patterns and gene expression profiles in the mouse brain.

## Materials and Methods

### Titanium Dioxide Nanoparticles

The TiO_2_-NP suspension (rutile: anatase = 20: 80; secondary particle diameter <150 nm; primary particle diameter of starting nanopowder, 21 nm; Cat. No. 700347-25 G, Sigma-Aldrich Co. St. Louis, MO, United States) was passed through a 0.45 μm filter (Millex®-HV; Cat. No. SLHU033RS; Merck Millipore Ltd., Burlington, MA, United States) to eliminate aggregated particles and then diluted to 0.25, 1, and 4 μg/μL with distilled water. The particles in the filtered suspension were characterized by transmission electron microscopy (TEM; JEM 1200EXII, JEOL Ltd., Akishima, Tokyo, Japan) on collodion-coated 200 Cu mesh (Cat. No. 6511; Nisshin EM, Tokyo, Japan). The size distribution of secondary TiO_2_-NP in the diluted suspension was determined by dynamic light scattering (DLS) measurements, using a Zetasizer Nano ZS (Malvern Instruments Ltd., Malvern, United Kingdom). For sham-treatment, a vehicle solution without any particles was prepared by centrifugation (18000 × g for 10 min at 4°C) of the TiO_2_-NP suspension, followed by passing through an Amicon® Ultra (3 K) device (Merck Millipore). In the DLS analysis, no particulate signal was detected in the vehicle solution.

### Animals and Treatments

Pregnant C57BL/6J mice (10-week-old) at GD 6.5, 7.5, or 8.5 were purchased from Japan SLC, Inc. (Shizuoka, Japan). First, 11 pregnant mice were divided into two groups: sham exposure group: Sham (dams: *n* = 5); TiO_2_-NP (4 μg/μL) exposure group: TiO_2_-H (dams: *n* = 6) (Experiment 1). For dose-dependent analysis, a total of 19 pregnant mice were divided into TiO_2_-NP (0.25 μg/μL) exposure group: TiO_2_-L (dams: *n* = 3), TiO_2_-NP (1 μg/μL) exposure group: TiO_2_-M (Dams: *n* = 4), TiO_2_-H (Dams: *n* = 5), and Sham (Dams: *n* = 7) groups (Experiment 2). All animals were housed under controlled conditions (temperature: 22 ± 1°C, humidity: 50 ± 5%) with a 12-h light/12-h dark cycle and *ad libitum* access to food and water. On GD 10.5, 25 μL of the vehicle or TiO_2_-NP suspension was intratracheally administered to pregnant mice of Sham and TiO_2_-NP exposure groups using MicroSprayer (MicroSprayer/Syringe Assembly–MSA-250-M for Mouse, Penn-Century Inc., PA, United States). We selected GD 10.5 as the administration date because around this date is important time point for the differentiation of the mantle and marginal layers begins and eventually will form the gray matter and white matter after this date (Chen et al., 2017). Administration was performed under anesthetization with intraperitoneal injection of sodium pentobarbital according to manufacturer’s recommendation. The brains and tails of offspring mice were collected under anesthetized with isoflurane on postnatal day (PND) 1. All animal experiments were treated and handled in accordance with the Animal Research: Reporting *In Vivo* Experiments (ARRIVE) guidelines for the care and use of laboratory animals (Kilkenny et al., 2010), and with the approval of the Institutional Animal Care and Use Committee of Tokyo University of Science.

### Genomic DNA and Total RNA Extraction From the Neonatal Brain

Whole-brain tissues were homogenized in 300 μL of phosphate-buffered saline (PBS) and divided into two tubes for extracting genomic DNA (200 μL) and total RNA (100 μL). Total RNA was extracted and purified using ISOGEN-LS (Nippon Gene Co., Tokyo, Japan) according to the manufacturer’s instructions. For DNA extraction, 150 μL of PBS and 350 μL of 2× extraction buffer [20 mM Tris-HCl (pH8.0), 0.2 M EDTA, 1% SDS, and 0.6 mg/ml Proteinase K] were added and incubated overnight at 55°C. After RNase A treatment, genomic DNA was extracted by phenol/chloroform extraction and ethanol precipitation, followed by purification using NucleoSpin^®^ gDNA Clean-up (Macherey-Nagel GmbH & Co., KG, Germany), according to the manufacturer’s instructions.

### Sex Determination

For each neonatal mouse from which brain samples were collected, genomic DNA was extracted from the tails and used for sex determination. For DNA extraction, 50 μL of TE buffer [10 mM Tris-HCl (pH 8.0), 1 mM EDTA] and 1.5 μL of proteinase K (10 mg/ml) was added to small pieces of tails and incubated overnight at 55°C. DNA was purified using NucleoSpin® gDNA Clean-up (Macherey-Nagel). Extraction of the genomic DNA was confirmed by PCR amplification of the genomic sequence of the glyceraldehyde-3-phosphate dehydrogenase (*Gapdh*) with specific primers (F: 5′- CAC​CCT​GGC​ATT​TTC​TTC​CA-3′, R: 5′- GAC​CCA​GAG​ACC​TGA​ATG​CTG-3′). The sex of each offspring mouse was determined by PCR amplification of the genomic sequence of the sex-determining region Y (*Sry*) gene with specific primers (F: 5′-TGG​GAC​TGG​TGA​CAA​TTG​TC-3′, R: 5′-GAG​TAC​AGG​TGT​GCA​GCT​CT-3′). Genomic DNA obtained from male mouse was used for positive control for PCR amplification.

### Methylated DNA Immunoprecipitation

Genomic DNA obtained from the brain of offspring was pooled for each of the following four groups: Sham male, Sham female, TiO_2_-H male, TiO_2_-H female. For Sham group, 2 offspring from 5 dams were pooled all together to have one sample. For TiO_2_-H group, 2 offspring from 6 dams were pooled all together to have one sample. The pooled DNA was then sonicated with Bioruptor (Cosmo Bio. Co., Ltd., Tokyo, Japan) to produce a random fragment, mainly ranging from 200 to 800 bp. The fragmented DNA (1.5 μg/100 μL) was denatured for 10 min at 95°C and incubated in a mixture of anti-5-methyl cytosine antibody (33D3) (ab10805; Abcam Inc., Cambridge, MA, United States) and Dynabeads® M-280 sheep anti-mouse IgG (Cat. No. 11201D; Life Technologies Inc., Gaithersburg, MD, United States) in 300 μL of immunoprecipitation (IP) buffer [20 mM Tris-HCl (pH8.0), 150 mM NaCl, 2 mM EDTA, 1% Triton X-100] overnight at 4°C. The beads combined with methylated DNA fragments were collected using a magnetic separator (MagnaRack™ Magnetic Separation Rack; Cat. No. CS15000; Thermo Fisher Scientific, Inc., Waltham, MA, United States), washed with IP buffer, and then incubated with elution buffer [25 mM Tris-HCl (pH8.0), 10 mM EDTA, 0.5% SDS, 0.25 mM DTT] for 15 min at 65°C. Finally, DNA fragments were removed from beads using a magnetic separator and purified by phenol/chloroform extraction and ethanol precipitation.

### CpG Island Microarray for DNA Methylation Profiling

Input DNA and methylated DNA fragments (500 ng for each group) were labeled with Cy3 or Cy5 and purified using the SureTag DNA Labeling Kit (Agilent Technologies Inc., Santa Clara, CA, United States), respectively. The acquisition of CpG island microarray data using labeled DNA was performed with support from Agilent Technologies. Cy3-labeled input samples or Cy5-labeled methylated samples were mixed and then competitively hybridized to a Mouse CpG Island 2 × 105 K Microarray (Agilent Technologies), consisting of 88737 probes, using Oligo aCGH/ChIP-on-chip Hybridization Kit (Agilent Technologies), washed with Oligo aCGH/ChIP-on-chip Wash Buffer (Agilent Technologies), according to the manufacturer’s instructions, and scanned by SureScan G2600D (Agilent Technologies). Scanner output images were normalized and digitalized using Agilent Genomic Workbench (Agilent Technologies), and probes with a *p*-value less than 0.05 were extracted. Probes that presented higher values than the background, at least in either the Sham or TiO_2_ groups, were extracted. Relative DNA methylation was calculated by dividing the Cy5 signal by the Cy3 signal and then comparing Sham and TiO_2_-NP groups. Genes with CpG islands presenting ratios of relative DNA methylation ≥ 1.5-fold or ≤ 0.67-fold in the TiO_2_-NP group, compared with the Sham group, were determined as those demonstrating an increase or decrease in DNA methylation states, respectively. The number of flagged genes was identified using GenBank Accessions annotated on each probe in the microarray.

### DNA Methylation Analysis of Specific Loci

Enrichment of methylated DNA was performed using a Methyl Collector Ultra kit (Active Motif Inc., Carlsbad, CA, United States) according to manufacturer’s instructions. Briefly, genomic DNA was digested with restriction enzyme Mse I (Nippon genetics, Co., Ltd., Tokyo, Japan). The digested DNA (0.5 μg) was incubated with a His-tagged recombinant MBD2b/MBD3L1 protein complex to capture methylated DNA. These protein–DNA complexes were captured with nickel-coated magnetic beads. Captured methylated DNA was eluted with elution buffer supplemented with proteinase K and subsequently purified using MinElute PCR clean-up kit (QIAGEN, Hilden, Germany). Obtained methylated DNA and input DNA were applied for quantitative real-time PCR to quantitate DNA methylation level of target genes. Relative methylation levels of target genes were calculated for each sample after normalization to input signals.

### Microarray for mRNA Expression Profiling

Total RNA was equally pooled from each sample to obtain 45 μg for each of the four groups (Sham male, Sham female, TiO_2_-H male and TiO_2_-H female). For Sham group, 2 offspring from 5 dams were pooled all together to have one sample. For TiO_2_-H group, 2 offspring from 6 dams were pooled all together to have one sample. The pooled RNA was then purified using RNeasy Micro Kit (Qiagen). RNA integrity was evaluated using a Bioanalyzer 2100 (Agilent Technologies) and confirmed that the RNA integrity number (RIN) value is greater than 7. Each of the pooled RNA samples was labeled with Cy3 and hybridized to the SurePrint G3 Mouse GE 8 × 60 K Microarray (Agilent Technologies), consisting of 62976 spots, using the Gene Expression Hybridization Kit (Agilent Technologies) according to the protocol provided by Takara Bio, Inc. (Shiga, Japan). The microarray was then washed using Gene Expression Wash Pack (Agilent Technologies) and scanned using a DNA microarray scanner (G2565CA; Agilent Technologies). Scanner output images were normalized and digitalized using Feature Extraction software (Agilent Technologies) according to the Minimum Information About a Microarray Experiment (MIAME) guideline. The number of flagged genes (mRNAs) was identified using GenBank Accessions annotated on each probe in the microarray. The threshold value used to designate differentially expressed mRNAs was a fold change of ≥1.5 or ≤0.67.

### Real-Time Reverse Transcription-Polymerase Chain Reaction

In brief, total RNA (1 μg) was employed as a template to generate the first-strand cDNA using M-MLV reverse transcriptase (Thermo Fisher Scientific), according to the manufacturer’s instructions. Quantitative RT-PCR was performed using Thunderbird^TM^ SYBR^®^ qPCR Mix (Toyobo Co. Ltd., Osaka, Japan) and primers (Fasmac Co. Ltd., Kanagawa, Japan) for indicated genes (Supplementary Table S1) in Mx3000P (Stratagene, La Jolla, CA, United States). A dilution series of pooled cDNA samples was used for each primer pair to generate a relative standard curve. Relative expression levels of target genes were calculated for each sample after normalization to *Gapdh*.

### Functional Analysis of Microarray Data by Gene Ontology

To better understand the biological implications of microarray results, functional analyses were performed using gene annotation by Gene Ontology (GO). All genes printed on the microarray were annotated with GO using an annotation file (ftp://ftp.ncbi.nih.gov/gene/DATA/gene2go.gz) provided by the National Center for Biotechnology Information (NCBI; Bethesda, MD). The annotations were updated in February 2021. Differentially expressed genes were classified by GO and pathways based on their functions. Enrichment factors for each GO were defined as (nf/n)/(Nf/N), where nf is the number of flagged genes within the category, Nf is the total number of genes within the same category, n is the number of flagged genes in the entire microarray, and N is the total number of genes in the microarray. Statistical analysis was performed with Fisher’s exact test, based on a hypergeometric distribution to calculate *p* values. The categories with enrichment factors ≥ 2, nf ≥ 5, and *p* < 0.05 were extracted.

### Extraction of Cells, Biological Functions, or Brain Regions Susceptible to Prenatal TiO_2_-NP Exposure by Medical Subject Headings

All genes printed on the microarray were annotated with MeSH using the Gene-MeSH correspondence table provided by the National Center for Integrative Biotechnology Information (NCIBI). Annotations were updated in April 2018. Enrichment factors for each MeSH were defined as stated in *Functional Analysis of Microarray Data by Gene Ontology*. Statistical analysis was performed with Fisher’s exact test, based on a hypergeometric distribution to calculate *p* values. The categories with enrichment factors ≥ 2, nf ≥ 5, and *p* < 0.05 were extracted.

### Statistical Analysis

Data values are presented as mean ± standard deviation (S.D). Data on litter size were analyzed using one-way analysis of variance (ANOVA), followed by post-hoc Tukey-Kramer test. The body weight of offspring mice and quantitative RT-PCR data were analyzed using two-way ANOVA with exposure and sex as factors, followed by post-hoc Tukey-Kramer test.

## Results

### Titanium Dioxide Nanoparticle Characterization

**FIGURE 1:**
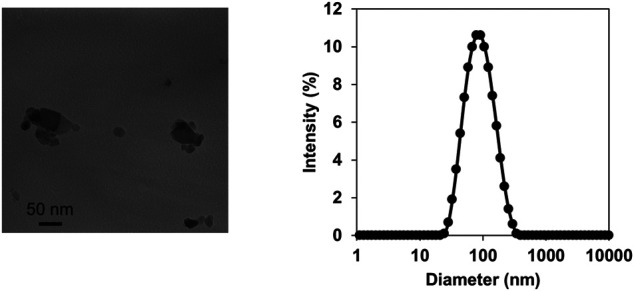
Characterization of titanium dioxide nanoparticles (TiO_2_-NPs). Transmission electron microscopy images of TiO_2_-NPs in the intratracheally administered suspension **(A)**. Size distribution of TiO_2_-NP in the suspension determined by dynamic light scattering **(B)**.

TiO_2_-NP suspension was characterized by TEM and DLS analyses. TEM analysis of the intratracheally administered suspension (4 μg/μL) revealed agglomerates ranging between 25–100 nm in diameter (Figure 1A). DLS analysis demonstrated small, agglomerated particles with a peak size of 78.6 nm (Figure 1B). The polydispersity index of 0.195 was less than 0.2, thus indicating a narrow size distribution (Tanvir et al., 2012). The observed 78.6 nm size corresponds well with the typical small agglomerate sizes of TiO_2_-NPs observed under TEM.

### Litter Size, Sex Ratio, and Body Weight of Offspring

We assessed the effects of prenatal TiO_2_-NP (TiO_2_-L, -M, and -H) exposure on the number, sex ratio, and body weight of offspring. No significant differences in the number, sex ratio, and body weight of offspring were observed between the Sham and TiO_2_-NP groups (Table 1).

**TABLE 1:** Number, sex ratio, and body weight of 1-day-old offspring.

	Group	Number of offspring per dam	Sex ratio (male %)^a^	Body weight of offspring (g)	
				Male	Female
Experiment 1	Sham (Dams: *n* = 5)	7.00 ± 0.71	42.9	1.32 ± 0.17	1.23 ± 0.04
	TiO_2_-H (Dams: *n* = 6)	7.67 ± 1.03	60.0	1.33 ± 0.08	1.32 ± 0.09
Experiment 2	Sham (Dams: *n* = 7)	7.14 ± 2.12	47.7	1.22 ± 0.10	1.22 ± 0.08
	TiO_2_-L (Dams: *n* = 3)	6.00 ± 2.00	50.0	1.40 ± 0.12	1.23 ± 0.06
	TiO_2_-M (Dams: *n* = 4)	7.50 ± 1.29	57.7	1.25 ± 0.07	1.21 ± 0.11
	TiO_2_-H (Dams: *n* = 5)	6.80 ± 1.92	46.9	1.25 ± 0.14	1.22 ± 0.09

Data are presented as mean ± SD.

a Sex ratio (%) = male/(male + female) × 100.

### DNA Methylation Profile in the Offspring Brain

**FIGURE 2:**
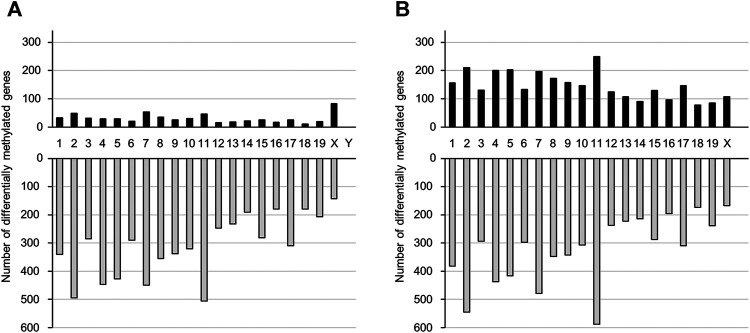
The number of genes that show altered DNA methylation states following prenatal TiO_2_-NP exposure. Number of genes presenting altered DNA methylation states between Sham and TiO_2_-H groups in the brains of 1-day-old offspring mice [**(A)**: male, **(B)**: female]. Black and gray bars indicate the number of genes with increased and decreased DNA methylation levels, respectively, in the TiO_2_-H groups when compared with the Sham group. The x-axis shows chromosome numbers.

We performed genome-wide DNA methylation analysis using a Mouse CpG island 2 × 105 K Microarray, with 88738 probes designed for 15342 CpG islands. Probes were designed for multiple regions of each gene. In the TiO_2_-H group, the number of probes with differential methylation was 11266 (≥1.5-fold: 758, ≤0.67-fold: 10508) and 15223 (≥1.5-fold: 3,779, ≤0.67-fold: 11444) in male and female offspring brains, respectively (Supplementary Tables S2, S3). Overall, DNA methylation levels were increased in 614 and 2,924 genes and were decreased in 6,220 and 6,477 genes following TiO_2_-NP exposure in the male and female offspring, respectively (Supplementary Tables S4–S7). Prenatal TiO_2_-NP exposure altered the methylation state of genes in all chromosomes. (Figure 2).

### mRNA Expression Profile in the Offspring Brain

**FIGURE 3:**
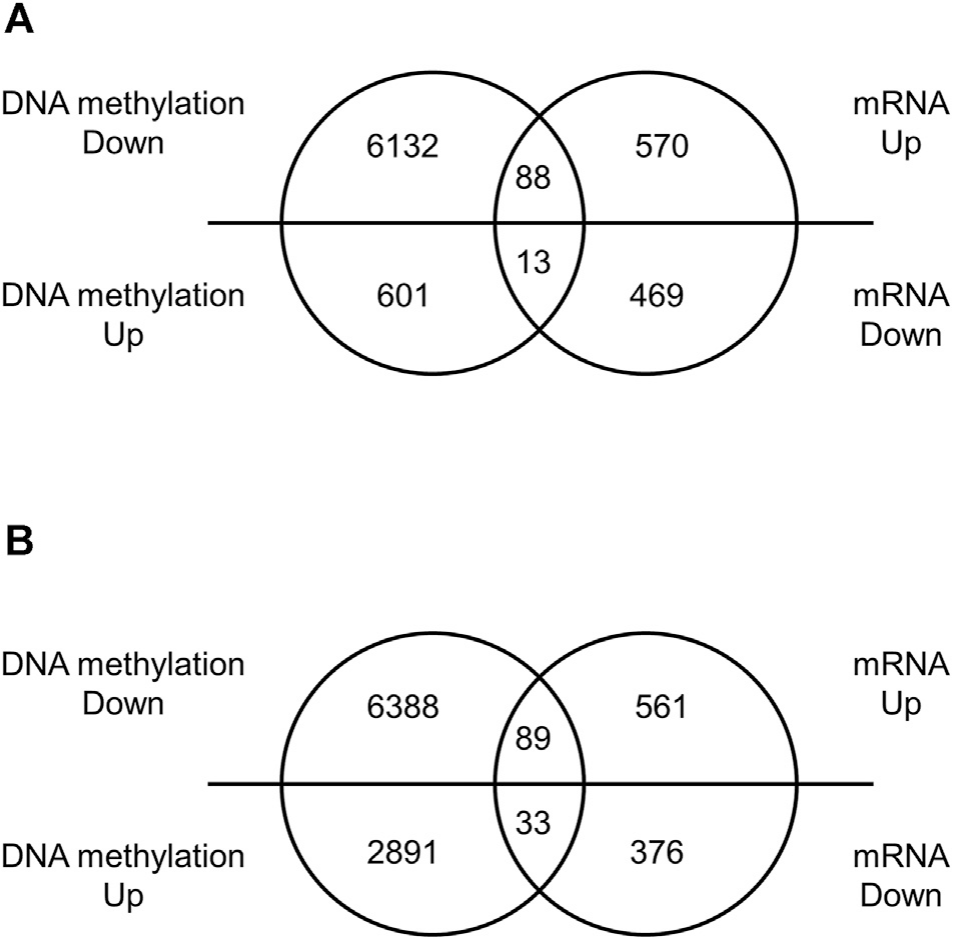
The number of differentially expressed genes with altered DNA methylation following TiO_2_-NP exposure.—Venn diagram analysis whether differentially expressed genes are accompanied by alteration of DNA methylation. Venn diagram showing the number of differentially expressed genes with altered DNA methylation following TiO_2_-NP (Sham vs. TiO_2_-H) in the brains of 1-day-old offspring mice [**(A)**: male, **(B)**: female]. The DNA methylation state and expression level of genes were identified by Mouse CpG Island Microarray and SurePrint G3 Mouse GE 8 × 60 K Microarray, respectively.

We performed a comprehensive analysis of differentially expressed mRNAs following prenatal TiO_2_-NP exposure using SurePrint G3 Mouse GE 8 × 60 K Microarray (Design ID: 028005), containing 55,681 probes. The results revealed that 658 and 650 mRNAs were upregulated (≥1.5-fold) in male and female offspring brains of the TiO_2_-H group when compared with the Sham group (Supplementary Tables S8A, S9A); in contrast, prenatal TiO_2_-NP exposure downregulated 482 and 409 mRNAs (≤0.67-fold) in male and female offspring brains (Supplementary Tables S8B, S9B). Then, differentially expressed mRNAs that correlated with changes in the DNA methylation status were extracted. In the TiO_2_-H group, 88 and 89 genes were upregulated (≥1.5-fold) accompanied by demethylation of CpG islands (Figures 3A,B; Supplementary Tables S10A,B), while 13 and 33 genes were downregulated (≤0.67-fold) accompanied by methylation of CpG islands in male and female offspring mice, respectively (Figures 3A,B; Tables 2A, B). Unexpectedly, few of these extracted genes were common in male and female offspring (Supplementary Table S11).

**TABLE 2:** The genes of which expression levels were decreased with increased methylation of CpG island in the brain of male (A) and female (B) offspring.

A				
GenBank accession	GeneSymbol	Target position of probe on CpG island microarray	mRNA	DNA methylation
			Fold change	Fold change of relative methylation
NM_001161855	4933416C03Rik	chr10:115551150–115551194	0.165	2.153
NM_008532	Epcam	chr17:88039798–88039842	0.013	1.729
NM_008192	Gucy2e	chr11:69049315–69049359	0.105	1.541
NR_027967	Hhatl	chr9:121698137–121698187	0.600	2.288
NM_213729	Inca1	chr11:70513486–70513530	0.196	1.704
NM_027397	Isl2	chr9:55394278–55394322	0.045	1.522
NM_001033250	Lemd1	chr1:134086976–134087020	0.188	1.840
NM_015743	Nr4a3	chr4:48060289–48060333	0.056	2.393
NM_008814	Pdx1	chr5:148081815–148081859	0.078	1.623
NM_009027	Rasgrf2	chr13:92901976–92902020	0.263	3.225
NM_001164704	Renbp	chrX:71167531–71167575	0.199	3.621
NM_001033415	Shisa3	chr5:67999755–67999799	0.306	1.502
NM_173429	Zfp775	chr6:48569751–48569795	0.664	1.528
NM_173429	Zfp775	chr6:48570111–48570155	0.664	1.549
NM_173429	Zfp775	chr6:48570207–48570251	0.664	2.012
NM_173429	Zfp775	chr6:48570342–48570386	0.664	2.204

B
GenBank accession	GeneSymbol	Target position of probe on CpG island microarray	mRNA	DNA methylation
			Fold change	Fold change of relative methylation
NR_024323	4930426L09Rik	chr2:18919881–18919925	0.221	1.977
NM_026185	Abhd15	chr11:77328807–77328851	0.058	1.661
NM_009681	Ap3s1	chr18:46901926–46901970	0.541	2.070
NM_001163614	Ascl4	chr10:85391455–85391499	0.513	1.767
NM_001163614	Ascl4	chr10:85391524–85391568	0.513	1.602
NM_016709	Auh	chr13:53024981–53025025	0.247	1.692
NM_009877	Cdkn2a	chr4:88940707–88940751	0.084	1.630
NM_007760	Crat	chr2:30271133–30271177	0.144	2.214
NR_028264	Dleu2	chr14:62301793–62301837	0.066	2.086
NR_028264	Dleu2	chr14:62305865–62305909	0.066	1.558
NR_028264	Dleu2	chr14:62300358–62300402	0.066	1.526
NM_133763	Dnttip1	chr2:164571271–164571315	0.282	1.520
NM_027828	Fam110c	chr12:31758655–31758699	0.105	1.608
NM_015821	Fbxl8	chr8:107788500–107788544	0.374	1.752
NM_018789	Foxo4	chrX:98450245–98450289	0.134	1.854
NM_010340	Gpr50	chrX:68917753–68917797	0.045	1.609
NM_010468	Hoxd3	chr2:74578542-74578586	0.371	1.809
NM_010468	Hoxd3	chr2:74550322–74550366	0.371	1.619
NM_028708	Jakmip3	chr7:146132486–146132530	0.036	4.012
NM_001033430	Jhdm1d	chr6:39155596–39155640	0.122	2.359
NM_001033430	Jhdm1d	chr6:39157044–39157088	0.122	1.774
NM_001037712	Kcnh6	chr11:105881863–105881907	0.153	1.967
NM_030716	Kcnip2	chr19:45890581–45890625	0.500	1.823
NM_152895	Kdm5b	chr1:136457792–136457836	0.227	1.678
NM_023061	Mcam	chr9:43943262–43943306	0.444	2.490
NM_001122667	Mkl2	chr16:13256418–13256462	0.100	1.806
NM_181860	Mkl2	chr16:13257041–13257085	0.100	1.574
NM_011037	Pax2	chr19:44830746–44830790	0.044	1.621
NM_001162929	Pom121l2	chr13:22073387–22073431	0.142	1.679
NM_001123362	Prdm12	chr2:31495757–31495801	0.420	1.676
NM_001164570	Rffl	chr11:82684465–82684509	0.474	1.706
NM_017395	Rfx5	chr3:94758251–94758295	0.221	1.892
NM_019732	Runx3	chr4:134708685–134708729	0.531	1.612
NM_011432	Snrpc	chr17:27977231–27977275	0.482	1.763
NM_054103	Stk33	chr7:116582357–116582401	0.387	1.933
NM_011517	Sycp3	chr10:87922666–87922710	0.427	1.532
NM_009331	Tcf7	chr11:52096304–52096348	0.104	1.611
NM_001085555	Tcp11	chr17:28217573–28217617	0.066	2.106
NM_013687	Tcp11	chr17:28217206–28217250	0.066	1.703
NR_001463	Xist	chrX:100677336–100677380	0.050	1.721
NR_001463	Xist	chrX:100677473–100677517	0.050	1.547

The threshold value used to designate altered mRNA expression or DNA methylation was a fold change of ≥1.5 or ≤0.67.

### DNA Methylation Analysis of the Gene That Showed Altered DNA Methylation Accompanied by Changes in mRNA Expression

**FIGURE 4:**
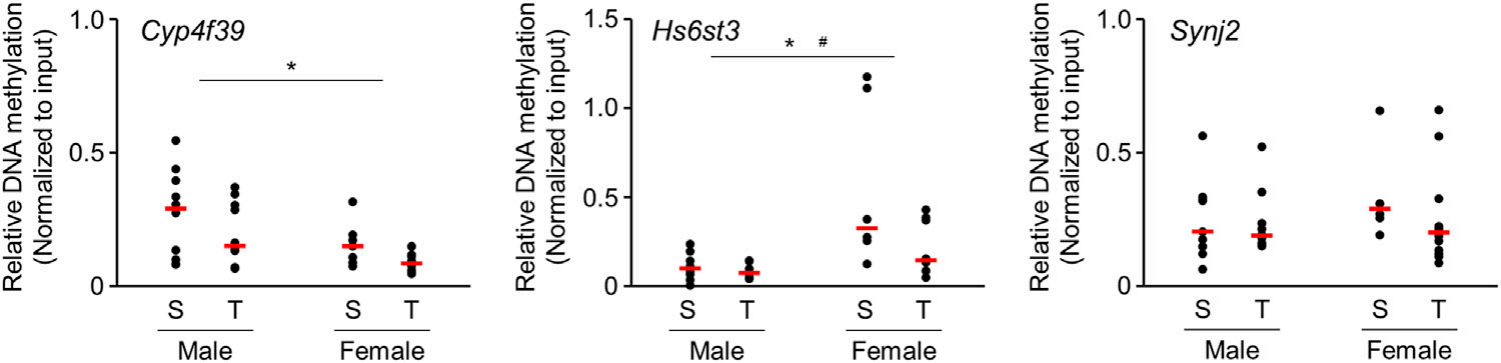
Scatter plots of brain DNA methylation levels of 1-day-old offspring mice from TiO_2_-H and Sham groups determined by Enrichment of Methylated DNA Fragments Followed by qPCR. Methylated DNA fragments were enriched from genomic DNA obtained from each sample {two offspring were randomly selected from each dam [Sham (S): *n* = 5, TiO_2_-H (T): *n* = 6]} using Methyl Collector Ultra Kit (Active Motif). Then, methylated DNA fragments were subjected to qPCR analysis. Two-way ANOVA with exposure and sex as factors, followed by post-hoc Tukey test. *p < 0.05: main effect of TiO_2_-NP exposure on relative DNA methylation; ^#^ *p* < 0.05: main effect of sex on relative DNA methylation (Sham vs. Exposure).

We further analyzed the alteration of DNA methylation using enrichment of methylated DNA fragment with methyl DNA binding proteins followed by quantitative PCR. We focused on the genes that showed altered DNA methylation accompanied by changes in mRNA expression. Experiments were performed on those genes for which PCR primers capable of specific amplification could be designed. DNA methylation state of *Cyp4f39* and *Hs6st3* genes were significantly decreased (Figure 4) in offspring brains. Significant difference of DNA methylation state was not detected in *Synj2* gene, but it seems to decrease in female offspring brains.

### Quantitative Real-Time RT-PCR Analysis of mRNA Expression

**FIGURE 5:**
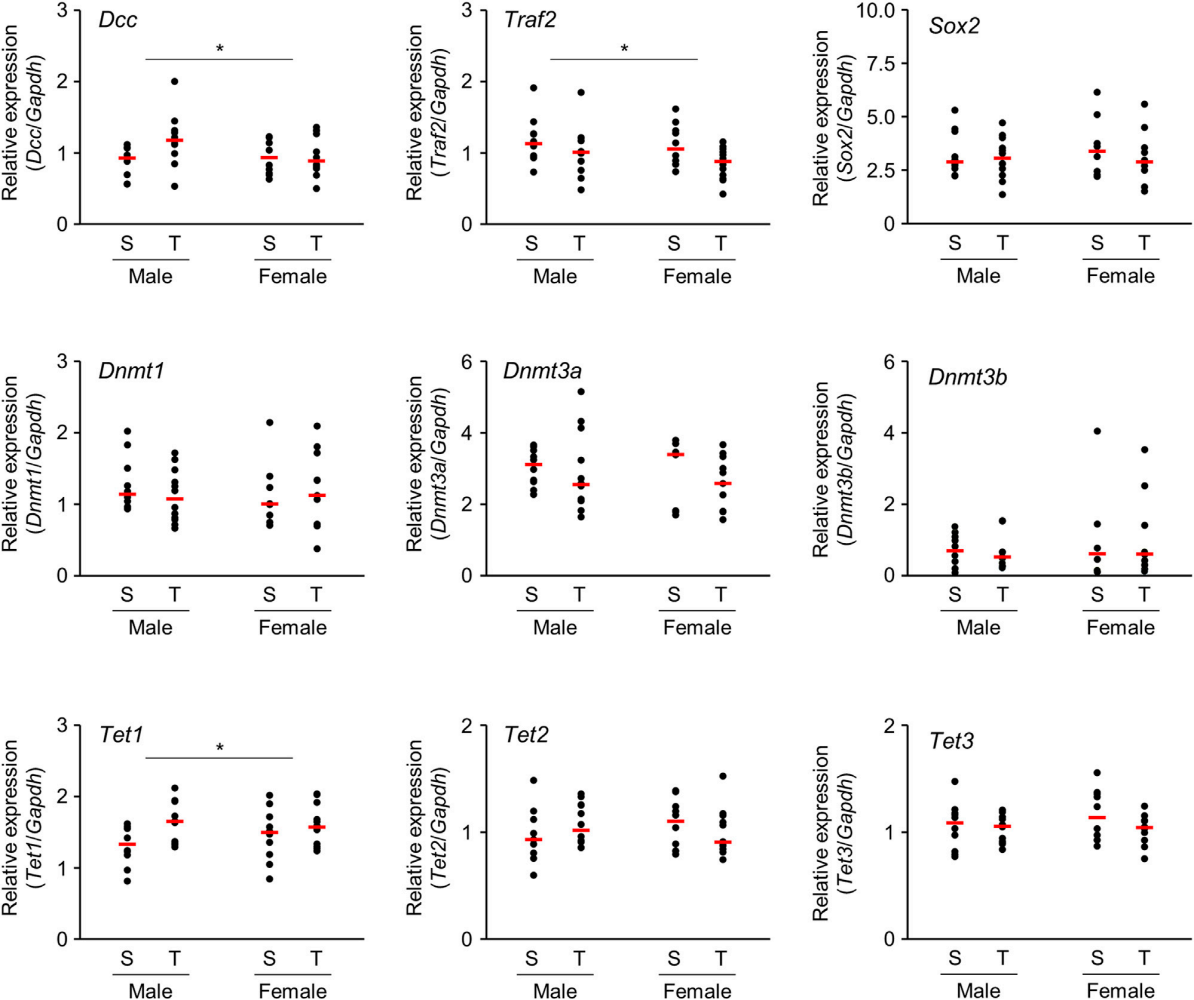
Scatter plots of brain mRNA expression levels of 1-day-old offspring mice from TiO_2_-H and Sham groups determined by qRT-PCR. Total RNA obtained from each sample {two offspring were randomly selected from each dam [Sham (S): *n* = 5, TiO_2_-H (T): *n* = 6]} was transcribed to cDNA and subjected to qRT-PCR analysis. Two-way ANOVA with exposure and sex as factors, followed by post-hoc Tukey test. *p < 0.05: main effect of TiO_2_-NP exposure on expression (Sham vs. Exposure).

Microarray analysis revealed that several mRNAs were differentially expressed in the brains of offspring prenatally exposed to TiO_2_-NP. In particular, mRNAs with altered expression levels in both males and females are speculated to be closely related to adverse health effects induced following TiO_2_-NP exposure. We performed qRT-PCR analyses of *Dcc* and *Traf2* mRNA, which demonstrate a high signal value on microarray analysis and are reportedly involved in nervous system development and inflammatory response, respectively. The tendency of expression change of these mRNAs determined by qRT-PCR was similar to those observed following the microarray analysis (Figure 5). Although we also examined gene expression levels of neural stem cell marker [SRY-box 2 (*Sox2*)] and Dnmts, no significant differences were detected (Figure 5). Further qRT-PCR analysis was performed for Ten-eleven translocation (TET) family genes associated with active DNA demethylation. The expression level of *Tet1* mRNA was significantly upregulated following prenatal TiO_2_-NP exposure (Figure 5). No statistically significant effect of sex (male *vs*. female) was observed on the expression of analyzed genes, as well as no impact of interactions between TiO_2_-NP exposure and sex on analyzed genes was detected.

### Functional Categorization of Potential Target Genes

We then aimed to obtain information regarding the biological function affected by altered DNA methylation in the brain of 1-day-old offspring mice following prenatal TiO_2_-H exposure. Accordingly, GSEA was performed for genes that showed altered DNA methylation accompanied by changes in mRNA expression. Genes upregulated accompanied by demethylation of CpG islands or those downregulated accompanied by methylation of CpG islands following TiO_2_-H exposure were functionally categorized with GO terms. The results revealed that 12 and 39 GO categories were enriched in male and female offspring, respectively (Tables 3A,B). Then, common GO terms between male and female offspring were extracted (Supplementary Table S12). The GO terms related to the regulation of transcription factors (“DNA-binding transcription factor activity,” “sequence-specific DNA binding,” and “regulation of transcription, DNA-templated”), cell proliferation (“negative regulation of cell population proliferation”), and organism development (“multicellular organism development”) were detected in genes that showed altered DNA methylation accompanied by differential mRNA expression in both male and female offspring.

**TABLE 3:** GO terms enriched in the genes that showed differential expression accompanied by altered DNA methylation in male (A) and female (B) offspring in the TiO_2_-H group.

A			
ID	GO term	Enrichment factor	*p*-value
GO:0005923	Bicellular tight junction	8.99	<0.001
GO:0015629	Actin cytoskeleton	5.07	0.003
GO:0003779	Actin binding	3.28	0.018
GO:0008285	Negative regulation of cell population proliferation	3.07	0.024
GO:0045893	Positive regulation of transcription, DNA-templated	2.71	0.014
GO:0007275	Multicellular organism development	2.63	0.001
GO:0003700	DNA-binding transcription factor activity	2.63	0.017
GO:0043565	Sequence-specific DNA binding	2.50	0.033
GO:0030054	Cell junction	2.42	0.012
GO:0006355	Regulation of transcription, DNA-templated	2.34	0.010
GO:0000981	DNA-binding transcription factor activity, RNA polymerase II-specific	2.14	0.038
GO:0045944	Positive regulation of transcription by RNA polymerase II	2.08	0.029

B
ID	GO term	Enrichment factor	*p*-value
GO:0031175	Neuron projection development	6.05	0.001
GO:0010468	Regulation of gene expression	5.20	<0.001
GO:0003700	DNA-binding transcription factor activity	5.01	<0.001
GO:0005667	Transcription regulator complex	4.94	<0.001
GO:0001228	DNA-binding transcription activator activity, RNA polymerase II-specific	4.86	<0.001
GO:1990837	Sequence-specific double-stranded DNA binding	4.71	<0.001
GO:0000977	RNA polymerase II transcription regulatory region sequence-specific DNA binding	4.67	<0.001
GO:0043565	Sequence-specific DNA binding	4.17	<0.001
GO:0031625	Ubiquitin protein ligase binding	3.97	0.004
GO:0008134	Transcription factor binding	3.91	0.002
GO:0045893	Positive regulation of transcription, DNA-templated	3.88	<0.001
GO:0007283	Spermatogenesis	3.79	0.001
GO:0045892	Negative regulation of transcription, DNA-templated	3.70	<0.001
GO:0016604	Nuclear body	3.69	0.005
GO:0045944	Positive regulation of transcription by RNA polymerase II	3.65	<0.001
GO:0008285	Negative regulation of cell population proliferation	3.58	0.003
GO:0007275	Multicellular organism development	3.48	<0.001
GO:0000978	RNA polymerase II cis-regulatory region sequence-specific DNA binding	3.47	<0.001
GO:0000981	DNA-binding transcription factor activity, RNA polymerase II-specific	3.38	<0.001
GO:0006357	Regulation of transcription by RNA polymerase II	3.36	<0.001
GO:0006355	Regulation of transcription, DNA-templated	3.31	<0.001
GO:0001701	*In utero* embryonic development	3.18	0.021
GO:0000122	Negative regulation of transcription by RNA polymerase II	2.97	<0.001
GO:0008270	Zinc ion binding	2.93	0.002
GO:0019899	Enzyme binding	2.82	0.020
GO:0043065	Positive regulation of apoptotic process	2.80	0.033
GO:0003677	DNA binding	2.80	0.000
GO:0030424	Axon	2.74	0.022
GO:0003779	Actin binding	2.73	0.036
GO:0010628	Positive regulation of gene expression	2.69	0.015
GO:0016491	Oxidoreductase activity	2.68	0.010
GO:0042803	Protein homodimerization activity	2.68	0.006
GO:0030154	Cell differentiation	2.66	0.001
GO:0019901	Protein kinase binding	2.62	0.018
GO:0042995	Cell projection	2.53	0.001
GO:0005694	Chromosome	2.43	0.037
GO:0045202	Synapse	2.39	0.013
GO:0030054	Cell junction	2.24	0.014
GO:0031410	Cytoplasmic vesicle	2.21	0.021

The enrichment factor for each category was defined as described in Materials and Methods. Statistical analysis was performed using Fisher’s exact test with hypergeometric distribution and the level of statistical significance was set at *p* < 0.05.

We also attempted to identify cells, biological functions, or brain regions susceptible to prenatal TiO_2_-NP exposure from GSEA analysis using MeSH terms (Umezawa et al., 2012) (Tables 4A,B). Supplementary Table S13 presents MeSH terms enriched in genes showing altered DNA methylation accompanied by differential mRNA expression in male and female offspring. MeSH terms possibly related to the development and differentiation of cells from stem cells (“Stem Cells” and “Morphogenesis”) and transcription factors and gene expression (“Gene Expression Regulation, Developmental,” “Transcription Factors,” and “Homeodomain Proteins”) were detected in genes showing altered DNA methylation accompanied by differential mRNA expression, common in male and female offspring. We further analyzed whether MeSH terms related to brain regions were extracted by GSEA analysis because it has not been well investigated which brain region is susceptible to the effect of prenatal nanoparticle exposure in terms of DNA methylation and gene expression. But unfortunately, MeSH terms related to brain regions were not obtained by analysis.

**TABLE 4:** MeSH terms enriched in the genes that showed differential expression accompanied by altered DNA methylation in male (A) and female (B) offspring in the TiO_2_-H group.

A			
ID	MeSH term	Enrichment factor	*p*-value
D060850	LIM-Homeodomain Proteins	8.73	<0.001
D019070	Cell Lineage	6.21	<0.001
D051792	Basic Helix-Loop-Helix Transcription Factors	4.98	0.001
D015534	Trans-Activators	4.90	0.004
D013234	Stem Cells	4.83	0.002
D012097	Repressor Proteins	4.30	0.003
D018398	Homeodomain Proteins	4.19	<0.001
D009024	Morphogenesis	3.32	0.018
D018507	Gene Expression Regulation, Developmental	3.13	<0.001
D009419	Nerve Tissue Proteins	3.09	0.013
D017403	*In Situ* Hybridization	3.07	0.005
D004622	Embryo, Mammalian	3.01	0.003
D014157	Transcription Factors	2.76	0.009

B
ID	MeSH term	Enrichment factor	*p*-value
D014566	Urogenital System	15.60	<0.001
D029961	Zebrafish Proteins	13.00	<0.001
D014212	Tretinoin	11.10	<0.001
D001755	Blastocyst	11.00	<0.001
D010641	Phenotype	9.50	<0.001
D011518	Proto-Oncogene Proteins	8.10	<0.001
D011401	Promoter Regions, Genetic	8.00	<0.001
D004475	Ectoderm	7.50	0.001
D051176	beta Catenin	7.20	0.001
D005801	Genes, Homeobox	6.80	<0.001
D038081	Organogenesis	6.70	<0.001
D009432	Neural Crest	6.40	0.001
D012249	Rhombencephalon	6.10	0.001
D051153	Wnt Proteins	5.80	<0.001
D008648	Mesoderm	5.80	<0.001
D019521	Body Patterning	5.30	<0.001
D019070	Cell Lineage	5.20	0.001
D004268	DNA-Binding Proteins	5.00	<0.001
D013234	Stem Cells	4.70	0.001
D053823	Hedgehog Proteins	4.70	0.002
D009024	Morphogenesis	4.40	<0.001
D014157	Transcription Factors	4.30	<0.001
D017403	*In Situ* Hybridization	4.20	<0.001
D009687	Nuclear Proteins	4.20	0.003
D018507	Gene Expression Regulation, Developmental	3.90	<0.001
D004622	Embryo, Mammalian	3.90	<0.001
D018398	Homeodomain Proteins	3.90	<0.001
D015398	Signal Transduction	3.60	0.002
D002454	Cell Differentiation	3.50	0.004
D002874	Chromosome Mapping	3.00	0.010
D009419	Nerve Tissue Proteins	2.60	0.030

The enrichment factor for each category was defined as described in Materials and Methods. Statistical analysis was performed using Fisher’s exact test with hypergeometric distribution and the level of statistical significance was set at *p* < 0.05.

## Discussion

DNA methylation is a pivotal mechanism in epigenetic gene regulation. The construction of DNA methylation patterns during development is crucial for morphogenesis or the acquisition of biological functions (Leeb and Wutz, 2012). Several reports have indicated that prenatal exposure to environmental agents, such as bisphenol A, vinclozolin, tobacco smoke, diesel exhaust, and traffic particles, induces altered DNA methylation and gene expression (Anway et al., 2005; Baccarelli et al., 2009; Breton et al., 2009; Ma et al., 2013; Nahar et al., 2014; Tachibana et al., 2015; Nielsen et al., 2016). Although recent studies have revealed that nanoparticle exposure alters DNA methylation (Li et al., 2016; Pogribna et al., 2020), its impact on the DNA methylation pattern during the developmental stage, especially in the brain, remains poorly understood. In the present study, we analyzed the effects of prenatal TiO_2_-NP exposure on the DNA methylation status in brain samples of mouse offspring using a CpG island microarray designed to analyze CpG islands located around the transcription start site of genes. Alteration of DNA methylation attributable to gestational exposures is thought to occur before birth. Our previous study showed that prenatal diesel exhaust exposure caused abnormal DNA methylation in the brain of mouse offspring at PND1 (Tachibana et al., 2015). Hence, to capture the impacts of early life exposure to TiO2-NP, we selected PND1 as the time point of sample collection.

GSEA analysis of genes with altered DNA methylation accompanied by changes in mRNA expression revealed that these genes were enriched in GO terms related to the regulation of transcription factors, cell proliferation, and organism development. Furthermore, the results of categorization using MeSH terms indicated that these phenomena could be associated with the proliferation and differentiation of neural stem cells. Combined with these results and findings in previous reports, alterations in DNA methylation caused by prenatal TiO_2_-NP exposure might be involved in brain dysfunction, such as impairment of learning and memory or the dopaminergic system (Takahashi et al., 2010; Mohammadipour et al., 2014), by disrupting proliferation and differentiation of neural stem cells at the developmental stage. In particular, homeodomain proteins extracted by GSEA analysis using MeSH terms are known as transcription factors that play crucial roles in several developmental processes, including in the brain, by regulating the expression of other genes during development and differentiation (Prochiantz and Di Nardo, 2015). It can be speculated that abnormal development of the nervous system could be induced by disrupted expression of genes regulated by homeodomain protein. In the present study, gene expression level of *Sox2* was not significantly affected by prenatal TiO_2_-NP exposure. Although it is just the result of the gene expression analysis, this result seems to demonstrate that the number of neural stem cells is still not significantly altered in PND1 although DNA methylation and expression of the genes related to cell proliferation and organism development were affected. Because the brain is still developing at this age, it will be necessary to investigate changes in the number of and distribution of neural stem cells in the brain occurred as the mice grow.

It is expected that genes that showed altered DNA methylation accompanied by changes in mRNA expression in male and female offspring are significantly involved in mediating the adverse effects of prenatal TiO_2_-NP exposure in the offspring. However, it remains unclear whether most of these genes, except for *Slc1a2*, are involved in brain function. *Slc1a2*, a glutamate transporter, is suggested to be involved in brain development, and its dysregulation has been associated with preterm brain injury (Pregnolato et al., 2019). Accordingly, increased *Slc1a2* expression induced by TiO_2_-NP exposure might be associated with brain injury. Interestingly, although few genes are differentially methylated in common between male and female offspring, GSEA analysis extracted common GO terms and MeSH terms for DNA methylation alterations. Previous studies have reported that sex-dependent differentially methylated regions (S-DMRs) exist in the prefrontal cortex and liver tissues, and these differences are suggested to be constructed during developmental processes (Xu et al., 2014; Ito et al., 2015). These S-DMRs might be partially associated with differences in DNA methylation between male and female offspring. Further investigations are required to address these findings.

Furthermore, we observed that GO and MeSH terms related to morphological development (GO term: “cell junction” and “actin binding,” MeSH term: “Morphology”) were enriched in the GSEA analysis. A case-control study has revealed that global DNA hypomethylation is associated with neural tube defects (Chen et al., 2010). GO and MeSH terms extracted by GSEA analysis suggested that structural changes occurred in the cranial nerve system following prenatal TiO_2_-NP exposure. We searched for brain region that is susceptible to the effect of prenatal TiO_2_-NP exposure, in terms of DNA methylation and gene expression, using GSEA analysis. Since no brain region was extracted by GSEA analysis using MeSH terms, it was not possible to identify a region that is expected to be significantly more susceptible to TiO_2_-NP exposure at this developmental stage.

Our results showed that prenatal TiO_2_-NP exposure induced genome-wide alterations in DNA methylation in the offspring brain, with a higher number of demethylated than methylated genes detected. We examined gene expression levels of *Dnmt1*, *Dnmt3a*, and *Dnmt3b*, but no significant differences were observed in these genes. A recent study has revealed that TET family proteins actively induce demethylation of 5-methylcytosine (5-mC) (Wu and Zhang, 2010). TET family proteins catalyze the hydroxylation of 5-mC into 5-hydroxymethylcytosine (5-hmC), which can further oxidize 5-hmC to 5-formylcytosine (5-fC) and 5-carboxycytosine (5-caC) (Zhu et al., 2020). Then, 5-fC and 5-caC are rapidly excised by thymine DNA glycosylase in the active demethylation state, subsequently replaced by unmodified cytosines through base excision repair mechanisms (Ji et al., 2016). The generation of 5-hmC by TET proteins has been proposed to play a pivotal role in cell differentiation by controlling the methylation status of developmentally important enhancers (Schuermann et al., 2016). It can be speculated that *Tet1* is involved in the decreased DNA methylation observed following prenatal TiO_2_-NP exposure in the present study, as qRT-PCR results revealed that, among Tet family genes, *Tet1* mRNA expression was upregulated.

Notably, DNA methylation is important for neural development during the fetal period, and it has been suggested that dysregulated DNA methylation can be associated with psychosis (Fan et al., 2001; Mill et al., 2008). Abnormal DNA methylation has been reported in the promoter regions of several genes that control neurodevelopment and mitochondrial function in the brains of patients with schizophrenia, bipolar disorder, and Alzheimer’s disease (Mill et al., 2008; Mastroeni et al., 2009). *Dcc* was differentially expressed in offspring brain following prenatal TiO_2_-NP exposure. Reportedly, *Dcc* transcription is regulated by CpG island methylation (Sato et al., 2001; Carvalho et al., 2006); however, probes for analyzing this gene were not designed in the CpG island microarray used in the present study. Interestingly, *Dcc* is reported to be involved in neuronal differentiation and neurite outgrowth (Lawlor and Narayanan, 1992; Deiner and Sretavan, 1999; Li et al., 2002). Combined with these reports, DNA demethylation caused by prenatal TiO_2_-NP exposure could be associated with central nervous system function and mental disorders.

Numerous reports have revealed that exposure to nanoparticles, including TiO_2_-NP, induces inflammation (Borm et al., 2006; Grassian et al., 2007; Cui et al., 2011). As nanoparticle exposure in the mother can be transferred to the fetus (Takeda et al., 2009; Wick et al., 2010), it is speculated that inflammation within fetal tissues is associated with adverse health effects. In the present study, *Traf2*, which is related to proinflammatory signal transduction, showed a tendency toward decreased expression. As *Traf2* reportedly affords protection against brain injury (Li et al., 2019), it can be postulated that a decrease in *Traf2* may be associated with susceptibility to brain damage. In addition, inflammation that occurs in the mother can affect fetal biological functions. Maternal inflammation has been suggested to contribute to brain outgrowth, autism-associated behaviors, and schizophrenia (Venkatasubramanian and Debnath, 2013; Le Belle et al., 2014). Furthermore, recent studies have shown that chronic inflammation causes aberrant DNA methylation (Jangiam et al., 2015; Barnicle et al., 2017). Interestingly, prenatal inflammation alters the DNA methylation status in the brain and can be associated with neurodevelopmental disorders (Basil et al., 2014). Inflammation induced by prenatal TiO_2_-NP exposure in both the fetus and mother could be involved in altered DNA methylation in the offspring brain; however, further investigations are crucial to gain a comprehensive understanding of these implications.

In the present study, we indicated that prenatal intratracheal exposure to TiO_2_-NP induces abnormal DNA methylation status and mRNA expression profile in the brain of mouse offspring at PND1. However, the brain is still developing at this stage and DNA methylation pattern is dynamically modulated during development. Actually, Cisternas et al. indicated that global DNA methylation level in the preoptic area of the hypothalamus increase during development (Cisternas et al., 2020). It would be important to investigate how the abnormal methylation pattern cause by prenatal TiO_2_-NP exposure changes with development. We attempted to find the fundamental effects of TiO_2_-NP on the brain of newborns, in terms of DNA methylation and gene expression, by extracting common changes from the analysis of each male and female samples. On the other hand, sexual dimorphism is present in the brain development (Pilgrim and Reisert, 1992). Therefore, it can be speculated that sex difference in the effect of TiO_2_-NP exposure exists between male and female. It is necessary to examine whether there is a sex difference in the effects of prenatal exposure to TiO_2_-NP on the brain development. To address these issues, it would be required to examine the effects of TiO_2_-NP at multiple developmental stages in the future study. Furthermore, to know the risks of prenatal inhalation exposure to TiO_2_-NP correctly, it is required to investigate about not only hazard but also frequency or exposure levels of it. Unfortunately, it is not well known enough about frequency or exposure levels of TiO_2_-NP inhalation exposure at the time of writing. It is required to investigate these points to assess the risk of prenatal inhalation exposure to TiO_2_-NP.

## Conclusion

Prenatal TiO_2_-NP exposure caused genome-wide alterations in DNA methylation and mRNA expression in the brains of both male and female offspring. These differential expression profiles may, in part, mediate the mechanism via which TiO_2_-NP toxicity affects brain development. GSEA analysis using GO terms suggested that these genes were associated with cell proliferation, organism development, and regulation of transcription factors. GSEA analysis using MeSH terms indicates that these phenomena are associated with the proliferation and differentiation of neural stem cells. It can be speculated that prenatal TiO_2_-NP exposure adversely impacts brain functions by altering the DNA methylation status of fetal brain tissues, especially neural stem cells, subsequently causing abnormal regulation of transcription factors that control development and differentiation, especially homeodomain protein. Future studies need to investigate molecular mechanisms underlying DNA methylation abnormalities that affect brain functions, especially from the perspective of neural stem cells.

## Data Availability

The datasets generated for this study can be found in the Gene Expression Omnibus (GSE178207) (https://www.ncbi.nlm.nih.gov/geo/query/acc.cgi?&acc=GSE178207).
