# Supplementary material for: Effects of Prenatal Exposure to Titanium Dioxide Nanoparticles on DNA Methylation and Gene Expression Profile in the Mouse Brain
Source: Front Toxicol. 2021 Oct 8;3:705910. doi: 10.3389/ftox.2021.705910 (PMC8915839; doi:10.3389/ftox.2021.705910)
Supplement: Supplementary file 1 [file Table5.pdf]

Supplementary Table 5. The genes of which DNA methylation were decreased in the brain of male offspring prenatally exposed to TiO_2-NP.

ProbeName	Target position of probe on CpG island microarray	TargetID	GeneSymbol	CpG island Description	Ratio of relative methylation (TiO_2-NP/Vehicle)	Sham group			TiO_2-H group		
						Relative methylation (Cy5/Cy3)	Cy3 signal (Input)	Cy5 signal (MeDIP)	Relative methylation (Cy5/Cy3)	Cy3 signal (Input)	Cy5 signal (MeDIP)
A_68_P28202486	chr12:4824316-4824360	NM_025323:-75	0610009D07Rik	PROMOTER	0.194	30.849	4345.01	134037.00	5.998	4690.56	28133.42
A_68_P28183963	chr11:120210170-120210214	NR_038126:201	0610009L18Rik	INSIDE	0.286	10.257	1957.16	20075.58	2.93	1664.96	4878.35
A_68_P28183962	chr11:120210037-120210081	NR_038126:67	0610009L18Rik	INSIDE	0.357	12.507	1042.64	13040.35	4.46	872.94	3893.35
A_68_P28183976	chr11:120211624-120211668	NR_038126:1655	0610009L18Rik	INSIDE	0.459	4.213	759.02	3198.10	1.933	736.97	1424.87
A_68_P31632917	chr18:38409964-38410008	NM_024179:84	0610009O20Rik	INSIDE	0.585	0.665	998.00	663.38	0.389	806.15	313.32
A_68_P27646748	chr11:23533451-23533495	NM_027860:159	0610010F05Rik	INSIDE	0.579	3.450	1143.05	3943.71	1.999	1097.41	2193.82
A_68_P24541220	chr6:72297774-72297818	NM_026696:486	0610030E20Rik	INSIDE	0.401	9.074	2910.04	26405.39	3.638	3060.46	11134.97
A_68_P25983693	chr8:80041310-80041354	NR_028125:378	0610038B21Rik	INSIDE	0.414	4.347	867.42	3770.70	1.8	792.80	1427.02
A_68_P21813623	chr2:163470481-163470525	NR_028113:-83	0610039K10Rik	PROMOTER	0.149	34.144	2768.32	94520.13	5.087	2519.52	12816.52
A_68_P23747544	chr5:64203539-64203583	NM_029554:-173	0610040J01Rik	PROMOTER	0.429	0.222	2355.00	522.49	0.095	1814.85	172.60
A_68_P28448911	chr12:55757483-55757527	NM_134054:55	1110002B05Rik	INSIDE	0.424	0.657	1011.93	664.68	0.279	814.02	226.76
A_68_P23756666	chr5:65884517-65884566	NM_133697:-467	1110003E01Rik	PROMOTER	0.471	0.327	1893.78	618.40	0.154	1187.46	182.61
A_68_P23756659	chr5:65883667-65883711	NM_133697:386	1110003E01Rik	INSIDE	0.513	3.773	7363.84	27782.82	1.936	6129.78	11867.10
A_68_P23756665	chr5:65884378-65884423	NM_133697:-326	1110003E01Rik	PROMOTER	0.626	0.398	1498.43	597.02	0.25	1102.24	275.13
A_68_P30959868	chr16:90934441-90934485	NM_026502:632	1110004E09Rik	INSIDE	0.356	0.395	1630.36	643.25	0.14	1266.69	177.69
A_68_P30959877	chr16:90935364-90935408	NM_026502:-292	1110004E09Rik	PROMOTER	0.548	0.633	908.27	574.95	0.347	758.12	262.84
A_68_P24010682	chr5:116081413-116081457	NR_027810:391	1110006O24Rik	INSIDE	0.258	0.434	1934.84	840.08	0.112	1562.92	174.87
A_68_P24010683	chr5:116081567-116081611	NR_027810:237	1110006O24Rik	INSIDE	0.651	0.328	4497.75	1474.33	0.213	3249.13	693.26
A_68_P21773774	chr2:156688896-156688940	NM_026124:61	1110008F13Rik	INSIDE	0.518	4.608	1813.99	8358.92	2.387	1553.94	3709.73
A_68_P24039805	chr5:121062634-121062678	NM_029096:-58	1110008J03Rik	DIVERGENT_PROMOTER	0.556	5.831	5847.48	34095.75	3.244	4414.24	14319.76
A_68_P21115067	chr2:32237497-32237541	NM_198001:-83	1110008P14Rik	PROMOTER	0.376	0.210	3224.53	677.23	0.079	2537.37	200.31
A_68_P21115064	chr2:32237153-32237197	NM_198001:261	1110008P14Rik	INSIDE	0.418	0.600	6050.65	3632.15	0.251	3981.36	999.84
A_68_P31306319	chr17:66798578-66798622	NM_001114098:490	1110012J17Rik	INSIDE	0.456	26.673	7609.88	202978.10	12.163	6479.32	78807.70
A_68_P31306312	chr17:66797737-66797781	NM_001114098:1332	1110012J17Rik	INSIDE	0.435	0.602	845.92	508.88	0.262	653.11	170.82
A_68_P22862528	chr4:41454176-41454220	NM_028624:599	1110017D15Rik	INSIDE	0.556	0.568	1571.60	892.16	0.316	1255.20	396.27
A_68_P28601430	chr12:86311556-86311600	NM_178065:258	1110018G07Rik	INSIDE	0.197	0.624	2887.79	1802.31	0.123	2093.69	257.76
A_68_P31129448	chr17:29854041-29854085	NM_001163741:-100	1110021J02Rik	PROMOTER	0.587	0.294	4726.84	1387.38	0.172	3320.11	571.86
A_68_P22217918	chr3:68673912-68673956	NM_001167996:427	1110032F04Rik	INSIDE	0.386	7.990	1163.42	9295.48	3.086	1154.29	3562.44
A_68_P22217916	chr3:68673757-68673801	NM_001167996:271	1110032F04Rik	INSIDE	0.41	5.336	815.44	4351.37	2.186	663.38	1450.29
A_68_P22217922	chr3:68674470-68674514	NM_001167996:985	1110032F04Rik	INSIDE	0.542	2.878	3998.19	11508.41	1.559	2930.54	4570.02
A_68_P28513315	chr12:70299066-70299110	NM_027269:328	1110034A24Rik	INSIDE	0.37	6.077	4884.17	29680.61	2.248	3762.15	8458.90
A_68_P21641570	chr2:132512792-132512837	NM_028637:-3204	1110034G24Rik	PROMOTER	0.301	15.223	4818.55	73351.83	4.577	3847.17	17606.91
A_68_P22729893	chr4:11413533-11413577	NM_001081183:450	1110037F02Rik	INSIDE	0.276	6.517	970.25	6323.49	1.797	857.74	1541.03
A_68_P31156690	chr17:35089452-35089496	NR_015536:-58	1110038B12Rik	PROMOTER	0.293	0.599	1225.91	733.76	0.175	1004.41	176.05
A_68_P27256913	chr10:74979851-74979895	NM_175133:195	1110038D17Rik	INSIDE	0.462	0.432	1079.90	466.89	0.2	862.78	172.39
A_68_P30351330	chr15:76779157-76779201	NM_054099:205	1110038F14Rik	INSIDE	0.442	0.359	1314.07	471.63	0.159	1059.92	168.24
A_68_P23331577	chr4:135543069-135543113	NM_025411:69	1110049F12Rik	INSIDE	0.489	6.608	1639.39	10833.00	3.233	1350.56	4365.97
A_68_P30608335	chr16:24393852-24393896	NR_037954:-133	1110054M08Rik	PROMOTER	0.326	8.225	991.31	8153.48	2.685	863.39	2318.07
A_68_P20091076	chr1:24012273-24012317	NM_026503:185	1110058L19Rik	INSIDE	0.651	2.787	1541.98	4297.77	1.814	1372.47	2489.53
A_68_P26892246	chr9:122859846-122859890	NM_025419:250	1110059G10Rik	INSIDE	0.222	0.420	2221.99	932.80	0.093	1853.53	172.98
A_68_P27633577	chr11:20730971-20731015	NM_173752:119	1110067D22Rik	INSIDE	0.508	12.703	1821.76	23142.07	6.458	1627.61	10510.42
A_68_P27633579	chr11:20731282-20731326	NM_173752:-193	1110067D22Rik	PROMOTER	0.436	1.438	2270.48	3265.75	0.628	1767.72	1109.74
A_68_P24864304	chr6:134878874-134878918	NR_037955:-213	1190002F15Rik	PROMOTER	0.407	7.599	1059.36	8050.56	3.091	938.46	2900.89
A_68_P29750657	chr14:79700949-79700993	NM_025427:472	1190002H23Rik	INSIDE	0.329	9.353	1471.31	13760.77	3.08	1226.26	3776.80

ProbeName	Target position of probe on CpG island microarray	TargetID	GeneSymbol	CpG island Description	Ratio of relative methylation (TiO_2-NP/Vehicle)	Sham group			TiO_2-H group		
						Relative methylation (Cy5/Cy3)	Cy3 signal (Input)	Cy5 signal (MeDIP)	Relative methylation (Cy5/Cy3)	Cy3 signal (Input)	Cy5 signal (MeDIP)
A_68_P29750659	chr14:79701095-79701139	NM_025427:326	1190002H23Rik	INSIDE	0.479	0.408	1178.97	480.49	0.195	895.35	174.64
A_68_P29750664	chr14:79701683-79701729	NM_025427:-264	1190002H23Rik	PROMOTER	0.281	0.706	1092.22	770.97	0.198	866.56	171.81
A_68_P26736551	chr9:94438921-94438965	NM_001033145:-442	1190002N15Rik	PROMOTER	0.596	0.337	2054.17	691.75	0.201	1665.12	334.40
A_68_P28744002	chr12:112664074-112664118	NM_028807:8456	1200009I06Rik	INSIDE	0.47	0.338	1923.82	651.03	0.159	1370.11	217.70
A_68_P27992496	chr11:86900314-86900358	NM_024262:-57	1200011M11Rik	PROMOTER	0.164	1.967	1545.21	3039.85	0.322	1045.01	336.57
A_68_P27992495	chr11:86900155-86900199	NM_024262:103	1200011M11Rik	INSIDE	0.501	0.271	1666.52	451.07	0.136	1331.02	180.66
A_68_P27992494	chr11:86900082-86900126	NM_024262:175	1200011M11Rik	INSIDE	0.554	0.418	3825.67	1600.32	0.232	2934.20	679.50
A_68_P27914868	chr11:72861634-72861678	NM_025818:288	1200014J11Rik	INSIDE	0.543	5.432	3787.43	20574.37	2.951	2889.51	8527.38
A_68_P27914870	chr11:72861801-72861845	NM_025818:454	1200014J11Rik	INSIDE	0.619	0.599	5663.69	3391.66	0.371	4153.41	1539.71
A_68_P20714680	chr1:153275146-153275191	NM_025819:397	1200016B10Rik	INSIDE	0.473	0.539	875.23	471.58	0.255	664.25	169.32
A_68_P27921681	chr11:74462947-74462991	NM_001081158:-28	1300001I01Rik	PROMOTER	0.154	29.396	3898.41	114598.20	4.517	3283.04	14829.53
A_68_P27921682	chr11:74463075-74463119	NM_001081158:100	1300001I01Rik	INSIDE	0.431	5.632	669.01	3767.55	2.427	641.03	1555.85
A_68_P30594370	chr16:21794748-21794792	NR_037957:351	1300002E11Rik	INSIDE	0.666	2.957	2528.25	7475.71	1.968	1968.59	3874.60
A_68_P28939817	chr13:34744234-34744278	NM_025831:294	1300014I06Rik	INSIDE	0.257	7.868	981.00	7718.28	2.021	853.07	1724.07
A_68_P28939820	chr13:34744543-34744587	NM_025831:-14	1300014I06Rik	PROMOTER	0.203	2.698	1642.41	4431.42	0.547	1270.95	695.30
A_68_P30423043	chr15:88919652-88919696	NM_027905:138	1300018J18Rik	INSIDE	0.65	3.950	455.50	1799.11	2.566	366.19	939.54
A_68_P21570237	chr2:119373578-119373622	NM_019769:158	1500003O03Rik	INSIDE	0.313	0.575	1046.40	601.31	0.18	926.15	166.53
A_68_P24006302	chr5:115263981-115264025	NR_027817:-17	1500011B03Rik	PROMOTER	0.636	2.862	1898.64	5433.68	1.821	1623.19	2956.13
A_68_P24050005	chr5:122821860-122821904	NM_026883:90	1500011H22Rik	INSIDE	0.279	9.471	784.43	7429.49	2.647	689.68	1825.57
A_68_P27978000	chr11:84339607-84339651	NR_038057:467	1500016L03Rik	INSIDE	0.601	0.232	2071.76	481.17	0.14	1449.49	202.40
A_68_P30650943	chr16:32099956-32100000	NM_025892:91	1500031L02Rik	INSIDE	0.197	18.429	5104.00	94062.43	3.639	6669.33	24270.56
A_68_P30382916	chr15:82176692-82176736	NM_026914:239	1500032L24Rik	INSIDE	0.46	0.444	1335.03	592.62	0.204	1034.05	211.11
A_68_P30382914	chr15:82176511-82176555	NM_026914:57	1500032L24Rik	INSIDE	0.566	6.779	1556.58	10552.79	3.837	1642.11	6301.31
A_68_P23227948	chr4:115816884-115816928	NM_026547:315	1520402A15Rik	INSIDE	0.203	2.357	10138.15	23900.42	0.477	6084.00	2905.06
A_68_P23227949	chr4:115817024-115817068	NM_026547:175	1520402A15Rik	INSIDE	0.581	4.077	1648.04	6719.08	2.368	1451.31	3436.82
A_68_P28854603	chr13:18085252-18085296	NM_025904:-89	1600012F09Rik	PROMOTER	0.455	0.277	1654.74	457.58	0.126	1405.43	176.67
A_68_P31062777	chr17:15080325-15080369	NM_001083880:158	1600012H06Rik	INSIDE	0.596	0.427	2709.19	1158.11	0.255	1946.47	495.60
A_68_P25065735	chr7:38968873-38968917	NM_028166:89	1600014C10Rik	INSIDE	0.227	0.703	1257.15	883.95	0.16	1096.50	175.13
A_68_P20134295	chr1:33727422-33727466	NR_033199:776	1700001G17Rik	INSIDE	0.357	16.898	1973.09	33342.06	6.028	2620.31	15795.01
A_68_P30387964	chr15:83197665-83197709	NR_027980:41	1700001L05Rik	INSIDE	0.177	20.888	6039.30	126147.60	3.691	5018.42	18523.24
A_68_P30387961	chr15:83197374-83197418	NR_027980:331	1700001L05Rik	INSIDE	0.552	0.625	1384.72	865.50	0.345	982.79	339.35
A_68_P30387965	chr15:83197791-83197835	NR_027980:-85	1700001L05Rik	PROMOTER	0.111	13.812	9916.06	136955.80	1.536	8741.50	13424.33
A_68_P21105302	chr2:30656577-30656621	NM_198000:629	1700001O22Rik	INSIDE	0.049	2.400	16309.30	39149.87	0.118	9296.62	1100.14
A_68_P21105309	chr2:30657215-30657259	NM_198000:-9	1700001O22Rik	PROMOTER	0.179	16.635	1063.74	17694.78	2.986	911.58	2721.87
A_68_P21105304	chr2:30656752-30656796	NM_198000:453	1700001O22Rik	INSIDE	0.406	1.784	3581.12	6389.04	0.724	2656.75	1924.62
A_68_P24591798	chr6:83112430-83112474	NM_027948:6055	1700003E16Rik	INSIDE	0.163	2.471	1132.77	2799.19	0.402	954.78	383.48
A_68_P30425032	chr15:89240650-89240694	NM_001162882:22	1700007E06Rik	INSIDE	0.547	4.860	782.80	3804.53	2.657	652.90	1734.57
A_68_P25505910	chr7:134655663-134655707	NR_024331:699	1700008J07Rik	INSIDE	0.616	0.565	1316.77	744.41	0.348	1010.59	351.99
A_68_P25505914	chr7:134656178-134656222	NR_024331:183	1700008J07Rik	INSIDE	0.298	5.047	2832.12	14293.18	1.505	2106.59	3169.73
A_68_P25417173	chr7:118266112-118266156	NR_015573:55	1700012D14Rik	INSIDE	0.494	0.295	1851.69	546.18	0.146	1545.50	225.13
A_68_P25417171	chr7:118265837-118265882	NR_015573:330	1700012D14Rik	INSIDE	0.602	0.597	802.97	479.32	0.359	683.60	245.57
A_68_P26541141	chr9:57109407-57109451	NM_028820:978	1700017B05Rik	INSIDE	0.187	23.006	2144.01	49326.08	4.292	2591.02	11121.93
A_68_P26541149	chr9:57110346-57110390	NM_028820:38	1700017B05Rik	INSIDE	0.387	0.732	1835.08	1342.99	0.283	1465.31	414.60
A_68_P31675575	chr18:46358675-46358719	NM_001145259:22	1700018A14Rik	INSIDE	0.371	0.317	1934.15	613.27	0.118	1562.49	183.62
A_68_P32050808	chr19:29122130-29122174	NR_028360:180	1700018L02Rik	INSIDE	0.51	5.313	3914.63	20798.10	2.709	3298.43	8936.49
A_68_P25614132	chr8:3475942-3475986	NM_029598:5103	1700019B03Rik	INSIDE	0.413	1.386	2067.59	2864.74	0.572	1516.63	867.40

ProbeName	Target position of probe on CpG island microarray	TargetID	GeneSymbol	CpG island Description	Ratio of relative methylation (TiO_2-NP/Vehicle)	Sham group			TiO_2-H group		
						Relative methylation (Cy5/Cy3)	Cy3 signal (Input)	Cy5 signal (MeDIP)	Relative methylation (Cy5/Cy3)	Cy3 signal (Input)	Cy5 signal (MeDIP)
A_68_P25614137	chr8:3476402-3476449	NM_029598:5564	1700019B03Rik	INSIDE	0.174	2.024	896.33	1814.61	0.353	566.52	200.07
A_68_P27971504	chr11:83254254-83254300	NM_025492:3081	1700020L24Rik	INSIDE	0.555	4.999	650.46	3251.60	2.776	526.58	1461.99
A_68_P27971499	chr11:83253762-83253806	NM_025492:2589	1700020L24Rik	INSIDE	0.478	6.828	290.28	1981.90	3.264	276.55	902.59
A_68_P30654978	chr16:32868216-32868260	NM_001200038:214	1700021K19Rik	INSIDE	0.207	1.680	1709.66	2871.99	0.348	1370.23	477.36
A_68_P23819410	chr5:77445261-77445305	NM_027970:235	1700023E05Rik	INSIDE	0.568	0.673	2804.38	1887.59	0.382	2086.16	797.57
A_68_P23819409	chr5:77445107-77445151	NM_027970:81	1700023E05Rik	INSIDE	0.535	3.324	1990.00	6614.67	1.78	1361.90	2423.64
A_68_P20416260	chr1:89083300-89083344	NM_027084:3255	1700027L20Rik	INSIDE	0.596	0.580	1266.99	735.23	0.346	1051.67	363.66
A_68_P27293828	chr10:82274680-82274724	NR_038042:-165	1700028I16Rik	PROMOTER	0.15	3.685	7845.96	28910.56	0.553	5741.98	3175.49
A_68_P27293831	chr10:82275065-82275109	NR_038042:219	1700028I16Rik	INSIDE	0.221	29.432	8056.09	237108.70	6.501	8354.28	54314.52
A_68_P30380760	chr15:81810911-81810955	NM_025503:-37	1700029P11Rik	PROMOTER	0.092	19.606	3615.43	70885.72	1.807	2719.96	4915.06
A_68_P26200045	chr8:119502947-119502991	NM_027103:-125	1700030J22Rik	PROMOTER	0.48	0.501	1039.64	520.39	0.24	911.95	219.23
A_68_P26200040	chr8:119502568-119502612	NM_027103:253	1700030J22Rik	INSIDE	0.654	0.407	7301.24	2970.77	0.266	5203.87	1384.21
A_68_P21633595	chr2:130985938-130985982	NM_026091:-204	1700037H04Rik	PROMOTER	0.438	0.445	1603.16	713.12	0.195	1015.66	198.04
A_68_P28450733	chr12:56225550-56225594	NM_001100116:58	1700047I17Rik2	INSIDE	0.37	11.626	1090.26	12675.10	4.301	1152.32	4956.32
A_68_P28450734	chr12:56225622-56225666	NM_001100116:130	1700047I17Rik2	INSIDE	0.466	10.524	2218.84	23350.41	4.908	1919.28	9419.67
A_68_P28450732	chr12:56225370-56225414	NM_001100116:-122	1700047I17Rik2	PROMOTER	0.465	0.707	2077.48	1468.48	0.328	1804.92	592.76
A_68_P25019420	chr7:28714464-28714508	NM_028538:-37	1700049G17Rik	PROMOTER	0.581	0.378	1213.43	459.19	0.22	1119.45	246.16
A_68_P28094671	chr11:105042686-105042730	NR_027956:39	1700052K11Rik	INSIDE	0.377	9.879	801.94	7922.32	3.721	760.76	2830.50
A_68_P20893089	chr1:185857699-185857743	NM_028516:157	1700056E22Rik	INSIDE	0.625	5.991	2528.06	15146.67	3.743	2101.24	7865.66
A_68_P31215872	chr17:48232748-48232792	NM_026625:6189	1700067P10Rik	DOWNSTREAM	0.626	0.465	1623.00	754.18	0.291	1300.31	377.96
A_68_P21101977	chr2:30093187-30093231	NR_028299:-57	1700084E18Rik	DIVERGENT_PROMOTER	0.556	3.830	3149.01	12061.76	2.129	2537.23	5401.17
A_68_P31632918	chr18:38410055-38410099	NR_027904:143	1700086O06Rik	INSIDE	0.354	0.490	1210.38	592.72	0.173	990.79	171.56
A_68_P29456087	chr14:22838983-22839027	NM_028275:71	1700112E06Rik	INSIDE	0.365	0.596	1014.37	604.89	0.218	805.48	175.55
A_68_P27317705	chr10:86521172-86521216	NM_029685:404	1700113H08Rik	INSIDE	0.254	0.325	2885.94	936.62	0.082	2059.53	169.69
A_68_P28073808	chr11:101286925-101286969	NM_026865:-299	1700113I22Rik	PROMOTER	0.657	0.284	3416.50	971.91	0.187	2515.23	470.42
A_68_P23296885	chr4:129168578-129168622	NM_001085491:167	1700125D06Rik	INSIDE	0.438	7.590	2220.92	16856.92	3.322	1952.75	6486.96
A_68_P23296944	chr4:129178242-129178286	NM_001085491:9831	1700125D06Rik	DOWNSTREAM	0.597	4.257	2313.22	9846.30	2.543	1868.00	4749.57
A_68_P23296941	chr4:129177909-129177953	NM_001085491:9497	1700125D06Rik	DOWNSTREAM	0.659	3.388	1098.70	3722.07	2.231	964.03	2150.60
A_68_P23296888	chr4:129168839-129168883	NM_001085491:427	1700125D06Rik	INSIDE	0.33	1.569	4570.27	7169.62	0.518	3594.00	1862.72
A_68_P27268621	chr10:76978260-76978304	NM_133998:276	1810008A18Rik	INSIDE	0.484	0.564	4639.09	2617.68	0.273	3093.62	844.69
A_68_P27268624	chr10:76978491-76978535	NM_133998:46	1810008A18Rik	INSIDE	0.657	0.705	1600.06	1128.55	0.463	1166.57	540.55
A_68_P28104781	chr11:106889949-106889993	NM_001163473:434	1810010H24Rik	INSIDE	0.378	8.705	968.01	8426.23	3.287	768.23	2524.97
A_68_P27945542	chr11:78639981-78640025	NM_001076681:-94	1810012P15Rik	PROMOTER	0.496	3.589	2432.27	8729.11	1.782	2212.82	3942.25
A_68_P27945543	chr11:78640074-78640118	NM_001076681:0	1810012P15Rik	INSIDE	0.66	0.388	1610.19	624.70	0.256	1487.44	380.79
A_68_P21957616	chr3:14611261-14611305	NM_001099674:-26	1810022K09Rik	PROMOTER	0.317	0.130	6802.79	884.21	0.041	4138.46	170.74
A_68_P21957615	chr3:14611165-14611209	NM_001099674:70	1810022K09Rik	INSIDE	0.57	0.273	8581.80	2342.50	0.155	5465.88	849.92
A_68_P27897438	chr11:69651419-69651463	NM_026982:-586	1810027O10Rik	DIVERGENT_PROMOTER	0.417	0.184	3374.83	622.28	0.077	2247.79	173.00
A_68_P25933301	chr8:69010356-69010400	NM_025465:28	1810029B16Rik	INSIDE	0.269	0.252	3957.52	995.44	0.068	2598.36	175.91
A_68_P20352244	chr1:75139021-75139065	NM_026977:-100	1810031K17Rik	DIVERGENT_PROMOTER	0.567	0.695	3557.16	2473.23	0.394	2452.36	966.18
A_68_P30394811	chr15:84276781-84276825	NM_001163145:725	1810041L15Rik	INSIDE	0.188	18.256	1889.93	34502.31	3.428	1664.40	5705.73
A_68_P30394506	chr15:84236917-84236961	NM_001163145:40589	1810041L15Rik	INSIDE	0.626	2.481	1544.15	3830.82	1.553	1297.94	2015.54
A_68_P28182351	chr11:119960438-119960482	NM_001110242:213	1810043H04Rik	INSIDE	0.458	0.277	1780.01	492.66	0.127	1344.35	170.45
A_68_P28182350	chr11:119960353-119960397	NM_001110242:127	1810043H04Rik	INSIDE	0.61	0.365	3175.94	1158.47	0.222	2120.74	471.57
A_68_P28532686	chr12:73637244-73637288	NM_026327:-77	1810048J11Rik	PROMOTER	0.553	0.638	1734.89	1106.10	0.352	1332.40	469.52
A_68_P31918868	chr19:3708036-3708080	NM_028077:-274	1810055G02Rik	PROMOTER	0.48	0.480	1640.65	787.44	0.23	1430.61	329.44
A_68_P31918872	chr19:3708434-3708478	NM_028077:124	1810055G02Rik	INSIDE	0.593	0.415	1365.78	566.12	0.246	1155.75	284.23

ProbeName	Target position of probe on CpG island microarray	TargetID	GeneSymbol	CpG island Description	Ratio of relative methylation (TiO_2-NP/Vehicle)	Sham group			TiO_2-H group		
						Relative methylation (Cy5/Cy3)	Cy3 signal (Input)	Cy5 signal (MeDIP)	Relative methylation (Cy5/Cy3)	Cy3 signal (Input)	Cy5 signal (MeDIP)
A_68_P22789805	chr4:25208818-25208862	NM_026194:128	1810074P20Rik	INSIDE	0.519	0.574	1506.18	864.53	0.298	1244.22	370.94
A_68_P22789806	chr4:25208898-25208942	NM_026194:48	1810074P20Rik	INSIDE	0.487	3.470	1671.47	5800.06	1.691	1477.23	2498.51
A_68_P27081458	chr10:39646733-39646777	NM_001163503:-25	2010001E11Rik	PROMOTER	0.17	2.014	4124.06	8304.38	0.341	2890.40	986.74
A_68_P31619415	chr18:35812025-35812069	NM_027222:-3025	2010001M09Rik	PROMOTER	0.644	12.405	1414.29	17544.92	7.989	1386.57	11076.80
A_68_P24127508	chr5:138220341-138220385	NM_027242:217	2010007H12Rik	INSIDE	0.397	8.140	2041.31	16615.61	3.232	1778.16	5746.73
A_68_P21863763	chr2:172171241-172171285	NM_025912:186	2010011I20Rik	INSIDE	0.551	4.238	1297.51	5498.66	2.334	1052.63	2456.94
A_68_P32145408	chr19:46764549-46764593	NM_025563:175	2010012O05Rik	INSIDE	0.181	18.753	1471.87	27601.74	3.391	1275.47	4324.57
A_68_P28747157	chr12:113205097-113205141	NM_027360:70	2010107E04Rik	INSIDE	0.628	0.483	1002.34	484.08	0.303	747.92	226.91
A_68_P29509891	chr14:31944757-31944801	NR_024069:-2662	2010107H07Rik	DIVERGENT_PROMOTER	0.038	57.614	1157.88	66710.53	2.193	993.93	2179.72
A_68_P29509872	chr14:31942367-31942412	NR_024069:-273	2010107H07Rik	DIVERGENT_PROMOTER	0.233	10.937	2520.47	27566.34	2.552	1907.23	4867.11
A_68_P29509885	chr14:31944046-31944090	NR_024069:-1952	2010107H07Rik	DIVERGENT_PROMOTER	0.332	13.826	1108.20	15321.39	4.591	945.75	4342.21
A_68_P29509887	chr14:31944357-31944401	NR_024069:-2262	2010107H07Rik	DIVERGENT_PROMOTER	0.42	0.711	1857.24	1319.65	0.299	1604.66	479.15
A_68_P29509871	chr14:31942240-31942284	NR_024069:-146	2010107H07Rik	DIVERGENT_PROMOTER	0.384	0.560	1006.06	563.43	0.215	781.15	168.03
A_68_P28336340	chr12:33063936-33063980	NM_001162903:305	2010109K11Rik	INSIDE	0.645	0.424	2377.78	1007.94	0.273	1819.75	497.23
A_68_P28336338	chr12:33063643-33063687	NM_001162903:11	2010109K11Rik	INSIDE	0.639	2.533	2129.11	5393.37	1.618	1804.96	2919.64
A_68_P23195471	chr4:108000991-108001035	NM_027250:256	2010305A19Rik	INSIDE	0.465	0.576	6022.91	3469.62	0.268	4856.87	1300.20
A_68_P26584998	chr9:64808639-64808683	NM_175153:-83	2010321M09Rik	PROMOTER	0.185	1.633	2956.14	4828.14	0.303	2255.55	683.03
A_68_P23189066	chr4:106851077-106851121	NM_025617:-127	2210012G02Rik	DIVERGENT_PROMOTER	0.562	5.301	1837.37	9739.51	2.977	1619.26	4820.62
A_68_P24006959	chr5:115392406-115392450	NM_028211:218	2210016L21Rik	INSIDE	0.658	2.708	3946.82	10687.72	1.782	2922.23	5208.83
A_68_P25357073	chr7:105804516-105804560	NM_172280:541	2210018M11Rik	INSIDE	0.095	37.445	18433.91	690255.90	3.576	24728.91	88424.40
A_68_P25357074	chr7:105804627-105804671	NM_172280:431	2210018M11Rik	INSIDE	0.376	9.022	2786.48	25138.49	3.394	2510.60	8519.92
A_68_P24172797	chr5:148086308-148086352	NR_038157:-3233	2210019I11Rik	PROMOTER	0.5	0.603	1216.85	733.98	0.302	1112.90	335.60
A_68_P30403119	chr15:85641848-85641892	NM_001164625:257	2210021J22Rik	INSIDE	0.544	0.397	1588.78	630.38	0.216	1223.56	264.27
A_68_P24950521	chr7:4740854-4740898	NM_029384:286	2210411K11Rik	INSIDE	0.309	1.572	1324.77	2082.28	0.486	998.17	484.94
A_68_P21812641	chr2:163298061-163298105	NR_028122:-198	2310001K24Rik	PROMOTER	0.4	5.320	1930.72	10271.14	2.126	1697.36	3607.90
A_68_P28157940	chr11:116060053-116060097	NM_027107:-94	2310004N24Rik	PROMOTER	0.504	0.183	2703.58	494.84	0.092	1843.39	170.13
A_68_P27282447	chr10:79783116-79783160	NM_025521:155	2310011J03Rik	INSIDE	0.585	0.435	5773.83	2513.29	0.255	3583.49	912.99
A_68_P31161375	chr17:36002953-36002997	NM_001146710:-98	2310014H01Rik	PROMOTER	0.145	22.932	4231.73	97041.41	3.324	3452.64	11475.73
A_68_P24973681	chr7:13513425-13513470	NM_029809:683	2310014L17Rik	INSIDE	0.516	0.323	1445.81	466.66	0.167	1132.74	188.62
A_68_P24973686	chr7:13514022-13514066	NM_029809:1280	2310014L17Rik	INSIDE	0.593	2.922	3446.04	10068.38	1.731	2461.50	4261.20
A_68_P24973683	chr7:13513671-13513715	NM_029809:928	2310014L17Rik	INSIDE	0.344	0.740	2424.54	1794.88	0.255	1892.17	482.51
A_68_P28573972	chr12:81233698-81233742	NR_033514:111	2310015A10Rik	INSIDE	0.578	5.984	671.43	4017.58	3.456	566.32	1957.41
A_68_P26246901	chr8:127187263-127187307	NM_175149:-15	2310022B05Rik	PROMOTER	0.33	0.287	3043.48	874.82	0.095	2246.62	212.87
A_68_P22862938	chr4:41516022-41516066	NM_001159583:516	2310028H24Rik	INSIDE	0.518	0.221	2417.11	533.28	0.114	1568.04	179.26
A_68_P23349884	chr4:138686603-138686647	NR_033745:328	2310028O11Rik	INSIDE	0.419	5.729	4633.86	26546.83	2.4	3954.54	9492.45
A_68_P23349886	chr4:138686821-138686865	NR_033745:110	2310028O11Rik	INSIDE	0.426	0.237	2208.47	523.23	0.101	1701.74	171.84
A_68_P26023270	chr8:87551092-87551137	NM_026760:383	2310036O22Rik	INSIDE	0.627	2.664	2249.34	5992.51	1.669	1834.56	3062.38
A_68_P31208848	chr17:46910008-46910052	NM_025966:447	2310039H08Rik	INSIDE	0.617	2.514	1145.74	2880.72	1.552	909.10	1411.01
A_68_P28613115	chr12:88288746-88288790	NM_173735:476	2310044G17Rik	INSIDE	0.26	0.386	2368.13	913.33	0.1	1740.73	174.32
A_68_P27892803	chr11:68873789-68873833	NM_028005:534	2310047M10Rik	INSIDE	0.615	2.597	2141.66	5561.58	1.596	1688.90	2695.47
A_68_P27288028	chr10:80657209-80657253	NR_015477:-205	2310050B05Rik	PROMOTER	0.315	6.501	2568.10	16694.83	2.051	2373.87	4868.22
A_68_P31161581	chr17:36034296-36034340	NM_001033630:5	2310061I04Rik	INSIDE	0.358	11.094	1306.38	14493.21	3.971	1067.09	4237.42
A_68_P30483167	chr15:99803669-99803713	NR_028124:28	2310068J16Rik	INSIDE	0.601	0.662	3695.49	2446.92	0.398	2651.20	1055.05
A_68_P23601494	chr5:35917751-35917795	NM_030208:-53	2310079F23Rik	DIVERGENT_PROMOTER	0.657	0.332	1347.69	447.55	0.218	1014.40	221.39
A_68_P23601493	chr5:35917636-35917680	NM_030208:61	2310079F23Rik	INSIDE	0.537	0.566	1048.80	593.36	0.304	822.91	250.22
A_68_P25376021	chr7:109054423-109054467	NM_025605:94	2400001E08Rik	INSIDE	0.534	0.363	1658.38	601.28	0.194	1305.21	252.76

ProbeName	Target position of probe on CpG island microarray	TargetID	GeneSymbol	CpG island Description	Ratio of relative methylation (TiO_2-NP/Vehicle)	Sham group			TiO_2-H group		
						Relative methylation (Cy5/Cy3)	Cy3 signal (Input)	Cy5 signal (MeDIP)	Relative methylation (Cy5/Cy3)	Cy3 signal (Input)	Cy5 signal (MeDIP)
A_68_P26158468	chr8:112217560-112217604	NM_028018:-388	2400003C14Rik	PROMOTER	0.581	0.347	1892.83	657.61	0.202	1371.90	277.04
A_68_P24430828	chr6:49023823-49023867	NM_029353:51	2410003K15Rik	INSIDE	0.49	4.147	2260.64	9374.26	2.033	1815.81	3691.98
A_68_P26631263	chr9:72951130-72951174	NM_025890:948	2410004A20Rik	INSIDE	0.476	4.960	1887.49	9362.48	2.361	1635.30	3861.06
A_68_P31609277	chr18:33954596-33954640	NR_038151:73	2410004N09Rik	INSIDE	0.628	2.170	3239.17	7027.39	1.363	2571.96	3506.32
A_68_P26760984	chr9:98765311-98765355	NM_028033:147	2410012M07Rik	INSIDE	0.558	3.215	10285.40	33067.04	1.795	7840.55	14073.05
A_68_P28595535	chr12:85292140-85292184	NM_023633:605	2410016O06Rik	INSIDE	0.58	6.576	1480.27	9733.86	3.812	1124.93	4288.29
A_68_P30345268	chr15:75812328-75812373	NM_001163518:365	2410075B13Rik	INSIDE	0.646	3.343	871.00	2911.39	2.159	684.44	1477.49
A_68_P29998441	chr15:8118989-8119033	NM_001162906:-95	2410089E03Rik	PROMOTER	0.529	3.963	834.13	3305.60	2.098	703.07	1474.83
A_68_P31992368	chr19:18745200-18745244	NM_026120:-47	2410127L17Rik	PROMOTER	0.43	15.003	750.98	11266.57	6.448	896.41	5780.04
A_68_P24025505	chr5:118695459-118695503	NM_001081236:245	2410131K14Rik	INSIDE	0.388	0.267	2172.99	579.48	0.104	1663.19	172.15
A_68_P24015125	chr5:116896779-116896823	NR_015595:8070	2410137F16Rik	INSIDE	0.507	0.693	1551.30	1075.54	0.351	1133.24	398.08
A_68_P23297489	chr4:129278119-129278163	NM_029748:190	2510006D16Rik	INSIDE	0.591	26.050	6863.68	178799.90	15.387	6430.34	98944.01
A_68_P23297490	chr4:129278201-129278245	NM_029748:272	2510006D16Rik	INSIDE	0.567	0.631	1013.22	639.02	0.358	809.47	289.40
A_68_P27288890	chr10:80788199-80788243	NM_027381:4373	2510012J08Rik	INSIDE	0.063	35.186	2489.48	87594.40	2.223	1895.64	4214.44
A_68_P27288850	chr10:80783292-80783342	NM_027381:-531	2510012J08Rik	PROMOTER	0.574	0.180	6877.60	1239.96	0.103	4380.36	452.99
A_68_P27288891	chr10:80788278-80788322	NM_027381:4453	2510012J08Rik	INSIDE	0.204	7.446	5955.39	44342.46	1.518	4664.63	7078.62
A_68_P23391467	chr4:147315560-147315604	NM_029841:579	2510039O18Rik	INSIDE	0.213	6.921	1466.78	10151.09	1.474	1232.99	1817.36
A_68_P23323199	chr4:134066870-134066914	NM_001081099:-21	2610002D18Rik	PROMOTER	0.371	6.694	1934.31	12947.65	2.482	1694.40	4205.62
A_68_P23323200	chr4:134067066-134067110	NM_001081099:175	2610002D18Rik	INSIDE	0.646	0.349	6404.91	2237.63	0.226	4041.83	912.63
A_68_P26822984	chr9:110207818-110207862	NM_001081381:145	2610002I17Rik	INSIDE	0.549	3.376	1127.33	3806.18	1.853	891.95	1652.82
A_68_P23437418	chr4:154623816-154623860	NM_001190445:-236	2610002J02Rik	PROMOTER	0.285	1.592	1666.98	2653.74	0.454	1397.84	634.23
A_68_P32373277	chrX:48194552-48194596	NM_133729:-38	2610018G03Rik	PROMOTER	0.451	6.008	935.60	5620.92	2.711	1018.23	2760.20
A_68_P25671550	chr8:13975017-13975061	NM_173744:-261	2610019F03Rik	PROMOTER	0.414	0.349	1876.08	655.17	0.145	1325.53	191.83
A_68_P30958538	chr16:90727762-90727806	NM_025642:-168	2610039C10Rik	PROMOTER	0.336	19.206	4886.50	93851.64	6.458	4350.82	28097.97
A_68_P26742258	chr9:95412445-95412489	NM_001114977:-51	2610101N10Rik	PROMOTER	0.144	75.112	11925.79	895774.60	10.851	18410.47	199780.80
A_68_P23392563	chr4:147504999-147505043	NM_027426:-213	2610109H07Rik	DIVERGENT_PROMOTER	0.624	2.784	1257.54	3500.59	1.738	1112.53	1933.54
A_68_P29699590	chr14:70553011-70553058	NM_146055:564	2610301G19Rik	INSIDE	0.27	1.571	5083.44	7986.67	0.425	3079.59	1307.66
A_68_P29699589	chr14:70552945-70552991	NM_146055:630	2610301G19Rik	INSIDE	0.292	1.363	2930.91	3995.31	0.398	1607.38	639.08
A_68_P27942255	chr11:78075391-78075435	NM_001002004:157	2610507B11Rik	INSIDE	0.353	0.449	2744.82	1231.44	0.158	1950.65	308.76
A_68_P27942253	chr11:78075063-78075107	NM_001002004:-171	2610507B11Rik	PROMOTER	0.579	8.196	2283.45	18715.86	4.743	1853.68	8791.09
A_68_P27835441	chr11:59015919-59015963	NR_037964:-7	2610507I01Rik	DIVERGENT_PROMOTER	0.607	2.394	6106.10	14619.93	1.454	4529.56	6584.36
A_68_P21503641	chr2:106814392-106814436	NM_173750:-3866	2700007P21Rik	PROMOTER	0.438	0.443	3482.80	1543.97	0.194	2480.10	481.78
A_68_P23801180	chr5:74488973-74489017	NR_015531:-113	2700023E23Rik	PROMOTER	0.611	2.972	1035.17	3076.92	1.815	907.19	1646.52
A_68_P25541691	chr7:140829138-140829185	NM_178115:-196	2700050L05Rik	PROMOTER	0.507	0.602	994.85	598.78	0.305	772.05	235.59
A_68_P31560300	chr18:24625872-24625916	NM_026529:-3477	2700062C07Rik	PROMOTER	0.379	5.141	2052.87	10554.02	1.946	1620.86	3154.59
A_68_P25592704	chr7:149246956-149247000	NM_028308:-39	2700078K21Rik	PROMOTER	0.478	8.148	1639.42	13357.89	3.898	1543.71	6017.01
A_68_P25592703	chr7:149246867-149246911	NM_028308:51	2700078K21Rik	INSIDE	0.277	6.393	1118.04	7147.61	1.769	1018.64	1802.14
A_68_P31939722	chr19:7497157-7497201	NM_175381:5064	2700081O15Rik	INSIDE	0.42	0.453	1162.03	525.99	0.19	947.62	180.02
A_68_P31939724	chr19:7497408-7497452	NM_175381:5316	2700081O15Rik	INSIDE	0.452	0.327	1640.27	535.61	0.148	1161.33	171.47
A_68_P31939711	chr19:7496090-7496134	NM_175381:3998	2700081O15Rik	INSIDE	0.463	9.177	2386.80	21902.44	4.252	2359.97	10034.06
A_68_P31939719	chr19:7496867-7496911	NM_175381:4774	2700081O15Rik	INSIDE	0.498	5.044	1930.65	9739.02	2.513	1539.12	3867.10
A_68_P31939675	chr19:7491423-7491467	NM_175381:-670	2700081O15Rik	PROMOTER	0.531	0.431	1028.97	443.92	0.229	749.33	171.75
A_68_P31939680	chr19:7491931-7491975	NM_175381:-162	2700081O15Rik	PROMOTER	0.36	4.310	1651.47	7117.52	1.552	1339.64	2078.70
A_68_P24856098	chr6:133055497-133055541	NM_001163445:262	2700089E24Rik	INSIDE	0.321	5.551	1167.06	6478.03	1.781	1163.06	2070.84
A_68_P21393924	chr2:84510645-84510689	NM_001033166:199	2700094K13Rik	INSIDE	0.324	2.033	5578.55	11342.58	0.659	3622.04	2387.31
A_68_P29309087	chr13:108834557-108834601	NM_001048250:-89	2810008M24Rik	PROMOTER	0.438	10.992	4356.19	47882.17	4.818	3682.66	17741.57

ProbeName	Target position of probe on CpG island microarray	TargetID	GeneSymbol	CpG island Description	Ratio of relative methylation (TiO_2-NP/Vehicle)	Sham group			TiO_2-H group		
						Relative methylation (Cy5/Cy3)	Cy3 signal (Input)	Cy5 signal (MeDIP)	Relative methylation (Cy5/Cy3)	Cy3 signal (Input)	Cy5 signal (MeDIP)
A_68_P27833211	chr11:58680786-58680830	NM_172403:67	2810021J22Rik	INSIDE	0.563	0.607	770.67	467.80	0.342	661.79	226.00
A_68_P28530097	chr12:73186365-73186409	NM_026038:-86	2810055F11Rik	DIVERGENT_PROMOTER	0.309	5.859	2501.74	14656.86	1.809	1978.94	3580.27
A_68_P25952315	chr8:73030423-73030467	NM_025577:194	2810428I15Rik	INSIDE	0.516	0.341	1521.58	518.96	0.176	987.45	173.74
A_68_P28788256	chr13:3477776-3477820	NR_015522:250	2810429I04Rik	INSIDE	0.481	0.565	3375.48	1907.63	0.272	2645.05	719.65
A_68_P23545746	chr5:24606967-24607011	NR_027851:196	2900005J15Rik	INSIDE	0.356	0.550	3049.21	1678.26	0.196	2140.31	419.57
A_68_P23545743	chr5:24606691-24606735	NR_027851:-80	2900005J15Rik	DIVERGENT_PROMOTER	0.093	21.508	876.73	18856.29	2.01	737.62	1482.28
A_68_P21114427	chr2:32143150-32143194	NM_175190:416	2900010J23Rik	INSIDE	0.656	0.485	921.08	447.14	0.318	794.97	253.15
A_68_P31114505	chr17:27270655-27270699	NM_026063:160	2900010M23Rik	INSIDE	0.452	23.184	4599.91	106645.20	10.468	4504.14	47151.51
A_68_P23996379	chr5:113567220-113567264	NM_172884:25091	2900026A02Rik	INSIDE	0.425	0.640	1618.09	1035.16	0.272	1155.28	314.22
A_68_P20551799	chr1:120356444-120356488	NR_027901:-624	2900060B14Rik	PROMOTER	0.379	0.317	1886.71	599.00	0.12	1419.81	170.90
A_68_P31331955	chr17:71351299-71351343	NM_026064:553	2900073G15Rik	INSIDE	0.606	3.331	3335.30	11110.10	2.017	2718.11	5483.01
A_68_P27787098	chr11:49988398-49988442	NM_026543:68	3010026O09Rik	INSIDE	0.316	0.191	3928.11	748.66	0.06	2850.92	171.57
A_68_P23023123	chr4:74923898-74923942	NM_025849:270	3110001D03Rik	INSIDE	0.506	0.635	1219.29	774.57	0.322	1024.38	329.42
A_68_P30558454	chr16:13672209-13672253	NM_025653:118	3110001I22Rik	INSIDE	0.276	7.716	1911.58	14749.22	2.127	1550.34	3296.95
A_68_P30558456	chr16:13672419-13672463	NM_025653:328	3110001I22Rik	INSIDE	0.512	5.381	701.31	3773.98	2.757	553.26	1525.16
A_68_P26973144	chr10:17742962-17743006	NM_028440:74	3110003A17Rik	INSIDE	0.605	0.214	3691.56	788.87	0.129	2509.24	324.27
A_68_P26973142	chr10:17742734-17742778	NM_028440:302	3110003A17Rik	INSIDE	0.248	0.730	3780.41	2759.07	0.181	2774.98	503.09
A_68_P24795431	chr6:119798321-119798365	NR_030776:132	3110021A11Rik	INSIDE	0.635	0.680	1054.15	717.35	0.432	859.43	371.08
A_68_P22834884	chr4:35173017-35173061	NM_001081343:91	3110043O21Rik	INSIDE	0.548	2.473	4469.14	11054.07	1.354	3681.95	4986.28
A_68_P20417972	chr1:89371861-89371906	NM_028473:5045	3110079O15Rik	INSIDE	0.108	183.914	1234.14	226976.60	19.933	583.34	11627.45
A_68_P31183537	chr17:42453381-42453428	NM_028474:509	3110082D06Rik	INSIDE	0.5	0.332	2499.17	828.80	0.166	1687.97	279.99
A_68_P24135766	chr5:139936328-139936372	NM_028469:138	3110082I17Rik	INSIDE	0.555	3.297	5227.02	17233.83	1.83	4046.47	7403.43
A_68_P28577065	chr12:81793548-81793592	NM_001177503:-17	3830431G21Rik	DIVERGENT_PROMOTER	0.359	0.573	1004.54	575.69	0.206	956.09	196.74
A_68_P20147880	chr1:36426249-36426293	NM_172652:-244	4632411B12Rik	PROMOTER	0.594	2.993	939.76	2812.42	1.777	721.68	1282.40
A_68_P21977361	chr3:19795434-19795478	NR_027985:586	4632415L05Rik	INSIDE	0.425	0.210	3514.67	736.35	0.089	2422.85	215.69
A_68_P23225501	chr4:115410210-115410254	NM_172698:-445	4732418C07Rik	PROMOTER	0.552	2.963	2606.40	7723.58	1.637	2159.42	3534.27
A_68_P29049835	chr13:54605357-54605401	NM_176987:213	4732471D19Rik	INSIDE	0.441	9.070	1716.16	15565.32	4	1515.47	6062.10
A_68_P29049838	chr13:54605642-54605686	NM_176987:499	4732471D19Rik	INSIDE	0.548	3.246	930.19	3019.14	1.78	844.63	1503.68
A_68_P28706885	chr12:106248338-106248382	NM_172500:-341	4831426I19Rik	PROMOTER	0.189	23.999	5275.40	126604.90	4.541	4767.68	21647.89
A_68_P28706884	chr12:106248180-106248224	NM_172500:-183	4831426I19Rik	PROMOTER	0.512	3.310	2040.85	6756.12	1.696	1675.58	2842.09
A_68_P31422207	chr17:87682043-87682087	NR_015506:90	4833418N02Rik	INSIDE	0.569	2.786	2416.52	6731.56	1.584	2149.17	3404.53
A_68_P29050103	chr13:54666500-54666544	NM_133797:221	4833439L19Rik	INSIDE	0.406	9.472	2442.64	23136.73	3.847	2286.26	8794.64
A_68_P28463397	chr12:58677434-58677478	NM_001037743:12355	4921506M07Rik	INSIDE	0.498	0.414	1764.18	730.56	0.206	1347.80	277.86
A_68_P28463396	chr12:58677361-58677405	NM_001037743:12281	4921506M07Rik	INSIDE	0.58	0.461	2908.17	1340.34	0.267	2198.55	587.28
A_68_P30560722	chr16:14159381-14159425	NM_001081154:-35	4921513D23Rik	DIVERGENT_PROMOTER	0.426	3.582	12446.97	44581.63	1.527	7921.49	12093.43
A_68_P22415393	chr3:108743147-108743191	NM_001078646:-306	4921515J06Rik	PROMOTER	0.327	8.131	746.17	6067.46	2.66	642.41	1708.94
A_68_P21770588	chr2:156137716-156137760	NM_027585:-470	4921517L17Rik	PROMOTER	0.583	5.020	1883.18	9453.35	2.925	1601.32	4683.35
A_68_P21900712	chr2:179711703-179711747	NR_033782:167	4921531C22Rik	INSIDE	0.334	0.549	1161.87	637.69	0.183	957.03	175.51
A_68_P27554559	chr11:3794810-3794858	NM_026150:295	4921536K21Rik	INSIDE	0.605	3.103	649.91	2016.39	1.876	513.31	962.74
A_68_P27289148	chr10:80827041-80827085	NM_001014836:1503	4930404N11Rik	INSIDE	0.552	8.669	6933.76	60107.99	4.782	5450.93	26068.35
A_68_P27289150	chr10:80827282-80827326	NM_001014836:1261	4930404N11Rik	INSIDE	0.356	1.632	5258.95	8582.41	0.581	3531.42	2051.64
A_68_P27289154	chr10:80827741-80827785	NM_001014836:803	4930404N11Rik	INSIDE	0.656	0.606	1953.13	1184.35	0.398	1435.78	570.95
A_68_P21042876	chr2:18919881-18919925	NR_024323:-43	4930426L09Rik	DIVERGENT_PROMOTER	0.604	14.903	2271.51	33853.38	8.995	2115.78	19032.05
A_68_P28755052	chr12:114394756-114394800	NM_134041:147	4930427A07Rik	INSIDE	0.515	0.243	1871.22	454.67	0.125	1391.08	174.23
A_68_P26018418	chr8:86671979-86672023	NM_001163752:64	4930432K21Rik	INSIDE	0.59	3.520	2879.48	10136.25	2.078	2185.96	4542.97
A_68_P25730019	chr8:27096869-27096915	NM_029037:7701	4930444A02Rik	INSIDE	0.499	4.482	1915.86	8587.63	2.235	1390.70	3108.12

ProbeName	Target position of probe on CpG island microarray	TargetID	GeneSymbol	CpG island Description	Ratio of relative methylation (TiO_2-NP/Vehicle)	Sham group			TiO_2-H group		
						Relative methylation (Cy5/Cy3)	Cy3 signal (Input)	Cy5 signal (MeDIP)	Relative methylation (Cy5/Cy3)	Cy3 signal (Input)	Cy5 signal (MeDIP)
A_68_P28534553	chr12:74018698-74018742	NM_029444:32	4930447C04Rik	INSIDE	0.318	8.845	6475.06	57269.33	2.816	6624.27	18656.51
A_68_P28534551	chr12:74018450-74018494	NM_029444:280	4930447C04Rik	INSIDE	0.473	11.002	2086.52	22955.46	5.206	1993.55	10379.22
A_68_P30686476	chr16:38522756-38522801	NM_024273:-31	4930455C21Rik	PROMOTER	0.335	19.519	3908.37	76286.50	6.543	3735.49	24441.40
A_68_P30686475	chr16:38522686-38522730	NM_024273:39	4930455C21Rik	INSIDE	0.632	0.446	1451.07	647.37	0.282	1157.49	326.19
A_68_P23062804	chr4:83171213-83171257	NM_001081012:-214	4930473A06Rik	PROMOTER	0.624	4.196	1106.91	4644.93	2.617	959.48	2511.44
A_68_P26073640	chr8:96337458-96337502	NR_026888:-25	4930488L21Rik	PROMOTER	0.195	10.892	843.24	9184.51	2.127	694.71	1477.47
A_68_P31122921	chr17:28660085-28660129	NM_026290:-7699	4930511I11Rik	PROMOTER	0.239	6.725	1855.53	12479.27	1.61	1387.60	2233.54
A_68_P26085413	chr8:98330883-98330927	NR_015574:175	4930513N10Rik	INSIDE	0.281	30.422	12749.69	387869.40	8.559	11443.59	97941.76
A_68_P26085411	chr8:98330678-98330722	NR_015574:-29	4930513N10Rik	PROMOTER	0.521	0.128	7002.54	896.67	0.067	4699.61	313.33
A_68_P27686040	chr11:31865634-31865678	NM_026262:24	4930524B15Rik	INSIDE	0.599	2.590	2377.96	6159.54	1.552	1860.68	2888.04
A_68_P33007146	chr9_random:49897-49941	NR_015516:-3490	4930526I15Rik	PROMOTER	0.029	92.371	2055.72	189889.90	2.712	2242.32	6080.49
A_68_P33007145	chr9_random:49764-49808	NR_015516:-3622	4930526I15Rik	PROMOTER	0.106	106.230	5527.02	587136.80	11.264	7480.18	84260.53
A_68_P33012150	chr9_random:53992-54036	NR_027950:-678	4930526I15Rik	PROMOTER	0.362	22.127	16431.24	363572.80	8	15911.47	127286.60
A_68_P33012155	chr9_random:54509-54553	NR_027950:-162	4930526I15Rik	PROMOTER	0.406	6.683	5055.25	33784.29	2.713	4165.13	11301.82
A_68_P33012178	chr9_random:57139-57183	NR_027950:2468	4930526I15Rik	DOWNSTREAM	0.413	0.242	2025.60	489.41	0.1	1697.32	169.28
A_68_P33007147	chr9_random:49997-50041	NR_015516:-3390	4930526I15Rik	PROMOTER	0.527	4.181	1515.62	6336.10	2.202	1191.06	2622.19
A_68_P33012151	chr9_random:54119-54163	NR_027950:-552	4930526I15Rik	PROMOTER	0.556	0.422	3332.11	1405.44	0.235	2516.48	590.47
A_68_P33007150	chr9_random:50340-50384	NR_015516:-3046	4930526I15Rik	PROMOTER	0.262	5.785	4307.91	24921.83	1.513	3126.19	4730.66
A_68_P22552055	chr3:135101078-135101122	NR_030689:529	4930539J05Rik	INSIDE	0.217	13.154	860.89	11324.05	2.851	897.49	2558.31
A_68_P22552056	chr3:135101186-135101230	NR_030689:421	4930539J05Rik	INSIDE	0.558	0.725	2142.64	1554.21	0.405	1608.91	651.30
A_68_P20252732	chr1:57434304-57434348	NR_037999:62	4930558J18Rik	INSIDE	0.662	0.509	1434.72	730.56	0.337	1203.44	405.82
A_68_P27910204	chr11:72028429-72028473	NM_001163728:-189	4930563E22Rik	PROMOTER	0.548	0.232	3569.23	826.98	0.127	2365.61	300.45
A_68_P30339175	chr15:74551980-74552024	NM_198607:339	4930572J05Rik	INSIDE	0.247	9.854	862.22	8496.47	2.438	882.05	2150.40
A_68_P22264735	chr3:79433039-79433083	NM_029482:60	4930579G24Rik	INSIDE	0.364	0.368	4630.62	1702.71	0.134	3633.32	485.81
A_68_P26759315	chr9:98464037-98464081	NR_029444:-28	4930579K19Rik	DIVERGENT_PROMOTER	0.399	0.358	1906.28	681.70	0.143	1379.72	196.63
A_68_P30959226	chr16:90831427-90831471	NM_001199210:345	4931408A02Rik	INSIDE	0.357	1.751	3219.74	5638.35	0.625	2673.19	1671.39
A_68_P30959217	chr16:90830454-90830498	NM_001199210:-627	4931408A02Rik	PROMOTER	0.503	3.113	2804.71	8731.09	1.567	1980.68	3103.89
A_68_P30959222	chr16:90831079-90831123	NM_001199210:-3	4931408A02Rik	PROMOTER	0.515	3.309	960.26	3177.89	1.704	855.79	1458.34
A_68_P26132762	chr8:107813304-107813348	NM_001166394:102	4931428F04Rik	INSIDE	0.392	0.469	3182.00	1493.03	0.184	2185.05	401.91
A_68_P22200393	chr3:65333036-65333080	NR_027955:278	4931440P22Rik	INSIDE	0.425	18.263	2711.64	49521.70	7.759	3283.57	25475.77
A_68_P27360775	chr10:94151238-94151282	NR_015524:98	4932415G12Rik	INSIDE	0.261	1.350	1967.11	2655.36	0.353	1394.98	492.33
A_68_P22060448	chr3:36762071-36762115	NM_172679:65	4932438A13Rik	INSIDE	0.53	4.227	1575.09	6657.86	2.238	1377.25	3082.85
A_68_P22060450	chr3:36762326-36762370	NM_172679:321	4932438A13Rik	INSIDE	0.13	0.749	7855.98	5887.33	0.098	5719.57	557.66
A_68_P28083992	chr11:103079644-103079688	NM_177801:80	4933400C05Rik	INSIDE	0.277	1.476	1964.23	2898.66	0.409	1747.47	714.10
A_68_P26202775	chr8:119960432-119960476	NM_028941:87385	4933407C03Rik	INSIDE	0.657	4.132	2398.43	9909.56	2.713	1553.32	4214.26
A_68_P23588493	chr5:33721117-33721161	NM_001081101:-206	4933407H18Rik	PROMOTER	0.372	12.593	6633.59	83536.60	4.686	6655.77	31188.57
A_68_P25834612	chr8:47248897-47248941	NM_025747:-5991	4933411K20Rik	PROMOTER	0.312	4.993	5220.99	26070.94	1.558	4141.46	6454.37
A_68_P22402613	chr3:106525217-106525262	NM_001039488:640	4933421E11Rik	INSIDE	0.638	0.392	3218.11	1261.44	0.25	2305.07	576.03
A_68_P28577649	chr12:81891588-81891632	NM_178682:65	4933426M11Rik	INSIDE	0.581	2.669	1347.98	3598.27	1.55	1105.99	1713.82
A_68_P30367720	chr15:79489229-79489273	NM_029407:136	4933432B09Rik	INSIDE	0.373	4.086	1640.21	6701.94	1.526	1400.24	2136.35
A_68_P28710687	chr12:106923624-106923668	NM_178613:85	4933433P14Rik	INSIDE	0.1	1.725	5886.75	10151.73	0.173	3579.19	618.56
A_68_P28710691	chr12:106924056-106924100	NM_178613:517	4933433P14Rik	INSIDE	0.449	0.578	1079.76	624.36	0.259	896.88	232.71
A_68_P26065121	chr8:94879862-94879906	NR_033641:135	4933436C20Rik	INSIDE	0.374	1.469	1107.29	1626.99	0.55	1055.86	580.68
A_68_P27842891	chr11:60230619-60230663	NM_025757:-6	4933439F18Rik	DIVERGENT_PROMOTER	0.192	22.260	5220.44	116207.70	4.272	4555.48	19459.76
A_68_P27842896	chr11:60231205-60231249	NM_025757:580	4933439F18Rik	INSIDE	0.247	25.566	5995.79	153288.20	6.318	5234.26	33068.95
A_68_P22130346	chr3:51364011-51364056	NR_033624:355	5031434O11Rik	INSIDE	0.613	4.357	3246.09	14142.83	2.669	2627.84	7013.68

ProbeName	Target position of probe on CpG island microarray	TargetID	GeneSymbol	CpG island Description	Ratio of relative methylation (TiO_2-NP/Vehicle)	Sham group			TiO_2-H group		
						Relative methylation (Cy5/Cy3)	Cy3 signal (Input)	Cy5 signal (MeDIP)	Relative methylation (Cy5/Cy3)	Cy3 signal (Input)	Cy5 signal (MeDIP)
A_68_P28853130	chr13:17786779-17786823	NM_138654:-201	5033411D12Rik	DIVERGENT_PROMOTER	0.223	1.754	8591.83	15066.89	0.391	6419.87	2512.42
A_68_P28853131	chr13:17786859-17786903	NM_138654:-281	5033411D12Rik	DIVERGENT_PROMOTER	0.334	0.258	3962.19	1020.85	0.086	3078.42	265.06
A_68_P28853128	chr13:17786438-17786482	NM_138654:139	5033411D12Rik	INSIDE	0.453	0.607	997.98	606.08	0.275	839.51	230.80
A_68_P28853129	chr13:17786533-17786577	NM_138654:45	5033411D12Rik	INSIDE	0.228	5.863	9270.89	54351.86	1.339	7371.60	9868.53
A_68_P30753194	chr16:50732861-50732905	NR_028300:4	5330426P16Rik	INSIDE	0.625	2.320	2027.94	4705.32	1.451	1670.92	2423.74
A_68_P21745336	chr2:151527951-151527995	NM_001160363:229	5430405G05Rik	INSIDE	0.414	20.142	865.66	17436.07	8.329	692.57	5768.25
A_68_P21745337	chr2:151528077-151528121	NM_001160363:355	5430405G05Rik	INSIDE	0.626	18.680	1901.24	35515.48	11.687	1833.46	21427.83
A_68_P21745340	chr2:151528389-151528433	NM_001160363:667	5430405G05Rik	INSIDE	0.557	2.670	1058.13	2825.19	1.486	1028.10	1527.83
A_68_P20977353	chr2:6021946-6021990	NM_001159657:29263	5430407P10Rik	INSIDE	0.387	0.511	1170.39	598.38	0.198	969.34	191.68
A_68_P20977349	chr2:6021517-6021561	NM_001159657:29693	5430407P10Rik	INSIDE	0.639	2.809	903.63	2538.23	1.795	784.06	1407.65
A_68_P23929552	chr5:100858837-100858881	NR_034038:-304	5430416N02Rik	PROMOTER	0.454	11.518	2144.98	24705.80	5.225	1707.24	8920.56
A_68_P30512103	chr16:4789758-4789802	NM_025670:155	5730403B10Rik	INSIDE	0.426	0.610	846.47	516.51	0.26	668.47	173.70
A_68_P24449884	chr6:52266647-52266691	NR_038163:-1842	5730457N03Rik	PROMOTER	0.325	32.887	5660.32	186149.80	10.674	6988.23	74592.23
A_68_P24449885	chr6:52266776-52266820	NR_038163:-1972	5730457N03Rik	PROMOTER	0.389	3.486	3115.10	10858.31	1.356	2667.11	3616.05
A_68_P29560848	chr14:41827140-41827184	NM_027464:-98	5730469M10Rik	PROMOTER	0.443	4.406	3776.91	16641.06	1.953	2973.02	5807.35
A_68_P22900661	chr4:48552753-48552797	NM_001145925:-43	5730528L13Rik	PROMOTER	0.377	5.359	894.57	4793.54	2.019	781.89	1578.86
A_68_P20644756	chr1:138131091-138131135	NM_028872:-271	5730559C18Rik	PROMOTER	0.512	12.396	1340.75	16620.30	6.341	1120.49	7105.00
A_68_P25262388	chr7:86804881-86804936	NM_029835:-173	5730590G19Rik	PROMOTER	0.633	0.364	1754.72	638.28	0.23	1248.25	287.37
A_68_P24084071	chr5:129106177-129106221	NR_015517:364	5930412G12Rik	INSIDE	0.153	12.632	1774.27	22412.96	1.93	1777.21	3429.83
A_68_P26548139	chr9:58335761-58335806	NM_176921:-626	6030419C18Rik	PROMOTER	0.642	2.354	2896.69	6817.76	1.51	2250.70	3399.63
A_68_P31498901	chr18:12279785-12279829	NM_178801:156	6030446N20Rik	INSIDE	0.448	3.896	3053.93	11896.78	1.743	2526.64	4405.01
A_68_P30024033	chr15:12754220-12754264	NM_029998:170	6030458C11Rik	INSIDE	0.595	3.345	693.19	2318.72	1.989	656.81	1306.52
A_68_P29448465	chr14:21522444-21522488	NR_029446:-145	6230400D17Rik	PROMOTER	0.468	0.323	1645.00	532.03	0.151	1261.05	190.70
A_68_P22775538	chr4:21858442-21858486	NM_175234:-8	6230409E13Rik	PROMOTER	0.569	0.676	1276.62	862.71	0.384	1092.81	420.01
A_68_P20876218	chr1:182413556-182413600	NM_001081227:57	6330403A02Rik	INSIDE	0.551	4.214	813.99	3429.87	2.323	728.06	1691.63
A_68_P27904028	chr11:70846735-70846779	NM_134022:363	6330403K07Rik	INSIDE	0.42	0.698	1171.70	818.22	0.293	1047.41	307.28
A_68_P27033509	chr10:28867346-28867390	NM_026138:3567	6330407J23Rik	INSIDE	0.427	3.281	1918.23	6294.25	1.402	1574.78	2208.06
A_68_P24975023	chr7:13863906-13863950	NM_177312:142	6330408A02Rik	INSIDE	0.233	0.339	2773.65	938.92	0.079	2174.10	171.52
A_68_P29650713	chr14:62175501-62175545	NM_025697:201	6330409N04Rik	INSIDE	0.408	6.421	927.64	5956.72	2.621	894.46	2344.18
A_68_P32151692	chr19:47806456-47806500	NM_026377:233	6330577E15Rik	INSIDE	0.107	19.388	9292.34	180158.90	2.078	7852.04	16319.65
A_68_P32151687	chr19:47805901-47805945	NM_026377:-323	6330577E15Rik	PROMOTER	0.661	0.569	2178.86	1240.65	0.376	1857.58	698.66
A_68_P32151689	chr19:47806170-47806214	NM_026377:-53	6330577E15Rik	PROMOTER	0.475	3.228	1120.34	3616.73	1.534	1005.05	1542.23
A_68_P20153982	chr1:37486790-37486834	NM_198006:136	6330578E17Rik	INSIDE	0.645	2.897	3632.44	10521.95	1.868	3140.86	5865.68
A_68_P21768923	chr2:155833524-155833568	NM_001145351:-3383	6430550D23Rik	PROMOTER	0.381	3.867	2009.01	7768.81	1.472	1535.66	2260.06
A_68_P24127315	chr5:138182692-138182736	NM_175521:-1488	6430598A04Rik	DIVERGENT_PROMOTER	0.432	6.769	1366.60	9250.50	2.926	1105.91	3236.33
A_68_P24127316	chr5:138182800-138182844	NM_175521:-1596	6430598A04Rik	DIVERGENT_PROMOTER	0.647	0.502	1555.06	781.16	0.325	1189.41	386.53
A_68_P22942710	chr4:57003066-57003110	NM_001142965:175	6430704M03Rik	INSIDE	0.655	4.430	734.50	3254.16	2.903	688.12	1997.39
A_68_P28432829	chr12:53107422-53107466	NM_029545:44	6530401N04Rik	INSIDE	0.614	2.899	2712.77	7864.76	1.78	2335.39	4157.74
A_68_P22398705	chr3:105508370-105508414	NM_001163356:17	6530418L21Rik	INSIDE	0.395	0.338	7539.28	2546.69	0.133	5237.21	698.74
A_68_P29587993	chr14:49066241-49066285	NM_172600:236	6720456H20Rik	INSIDE	0.489	4.844	2698.79	13072.53	2.37	2203.73	5222.74
A_68_P24865610	chr6:135148284-135148328	NM_028982:302	8430419L09Rik	INSIDE	0.396	0.173	8152.51	1412.26	0.069	5756.16	395.29
A_68_P24865609	chr6:135148088-135148132	NM_028982:106	8430419L09Rik	INSIDE	0.552	0.455	1043.94	475.10	0.251	901.67	226.65
A_68_P24865608	chr6:135147953-135147997	NM_028982:-30	8430419L09Rik	PROMOTER	0.49	0.624	1638.97	1022.74	0.306	1440.46	440.72
A_68_P21754954	chr2:153270097-153270141	NM_001001986:97	8430427H17Rik	INSIDE	0.34	15.947	1524.27	24307.79	5.423	1211.23	6568.42
A_68_P21755504	chr2:153354420-153354464	NM_001134300:1265	8430427H17Rik	INSIDE	0.353	15.970	2629.12	41985.91	5.633	2617.76	14746.51
A_68_P31063014	chr17:15115872-15115916	NM_001123370:67	9030025P20Rik	INSIDE	0.297	0.190	5350.74	1016.67	0.056	3868.14	218.24

ProbeName	Target position of probe on CpG island microarray	TargetID	GeneSymbol	CpG island Description	Ratio of relative methylation (TiO_2-NP/Vehicle)	Sham group			TiO_2-H group		
						Relative methylation (Cy5/Cy3)	Cy3 signal (Input)	Cy5 signal (MeDIP)	Relative methylation (Cy5/Cy3)	Cy3 signal (Input)	Cy5 signal (MeDIP)
A_68_P27085191	chr10:40402970-40403014	NM_177793:-95	9030224M15Rik	PROMOTER	0.561	3.021	1109.71	3352.53	1.695	1022.03	1732.70
A_68_P23368580	chr4:141795400-141795444	NM_001109685:-106	9030409G11Rik	PROMOTER	0.435	7.664	10908.84	83610.59	3.336	8606.03	28713.45
A_68_P23368575	chr4:141794726-141794770	NM_001109685:568	9030409G11Rik	INSIDE	0.509	3.044	4070.25	12388.06	1.548	2992.29	4632.87
A_68_P20250394	chr1:57028750-57028794	NR_024325:460	9130024F11Rik	INSIDE	0.087	3.974	4998.22	19864.48	0.347	3628.93	1258.03
A_68_P20250396	chr1:57029009-57029053	NR_024325:718	9130024F11Rik	INSIDE	0.392	0.442	6235.38	2754.46	0.173	4443.29	769.27
A_68_P20250410	chr1:57030392-57030436	NR_024325:2102	9130024F11Rik	INSIDE	0.421	0.290	3601.06	1042.85	0.122	2429.87	296.18
A_68_P20250391	chr1:57028519-57028563	NR_024325:228	9130024F11Rik	INSIDE	0.513	0.474	2584.21	1225.07	0.243	1644.22	399.83
A_68_P20250392	chr1:57028581-57028634	NR_024325:295	9130024F11Rik	INSIDE	0.068	1.376	2806.24	3861.82	0.094	1846.97	173.23
A_68_P23693365	chr5:52581836-52581880	NR_015537:-61	9230114K14Rik	DIVERGENT_PROMOTER	0.351	14.399	1320.14	19008.26	5.052	1117.46	5645.56
A_68_P28513307	chr12:70298223-70298267	NR_033222:47	9330151L19Rik	INSIDE	0.601	8.655	2343.72	20285.59	5.198	1998.27	10386.54
A_68_P21782539	chr2:158187061-158187105	NR_015463:163	9430008C03Rik	INSIDE	0.315	13.900	6040.54	83965.14	4.378	4926.39	21565.57
A_68_P21782542	chr2:158187327-158187371	NR_027887:-90	9430008C03Rik	PROMOTER	0.39	9.977	1192.13	11894.08	3.888	940.06	3654.81
A_68_P31464043	chr18:4635545-4635589	NM_001081963:640	9430020K01Rik	INSIDE	0.329	5.007	2728.23	13659.77	1.645	2297.16	3779.72
A_68_P29030706	chr13:51195741-51195786	NR_029463:333	9430083A17Rik	INSIDE	0.542	11.443	243.50	2786.48	6.202	237.92	1475.57
A_68_P23562830	chr5:28784867-28784911	NR_015562:-8635	9530036O11Rik	PROMOTER	0.435	5.693	587.72	3345.70	2.479	537.67	1332.75
A_68_P23562834	chr5:28785352-28785396	NR_015562:-8149	9530036O11Rik	PROMOTER	0.289	1.376	5354.77	7366.28	0.398	4087.02	1627.39
A_68_P26890105	chr9:122478146-122478190	NR_015610:-3450	9530059O14Rik	PROMOTER	0.305	8.373	1958.09	16394.35	2.556	1703.37	4353.59
A_68_P21774858	chr2:156866344-156866388	NM_001164663:38635	9830001H06Rik	INSIDE	0.345	8.278	2336.30	19340.03	2.854	2678.90	7644.26
A_68_P21775097	chr2:156904527-156904571	NM_001164663:453	9830001H06Rik	INSIDE	0.548	10.682	1791.92	19141.11	5.854	1483.11	8682.49
A_68_P21774859	chr2:156866427-156866471	NM_001164663:38553	9830001H06Rik	INSIDE	0.606	2.537	2468.10	6261.64	1.538	1852.39	2849.10
A_68_P21775102	chr2:156905117-156905161	NM_001164663:-137	9830001H06Rik	DIVERGENT_PROMOTER	0.521	3.100	1370.11	4247.33	1.616	1200.61	1939.79
A_68_P25286563	chr7:91234999-91235044	NM_030728:-6	9930013L23Rik	PROMOTER	0.625	2.643	895.40	2366.88	1.652	751.30	1240.87
A_68_P20935796	chr1:192849520-192849564	NR_027298:313	A230020J21Rik	INSIDE	0.46	0.403	1235.90	498.64	0.186	925.32	171.78
A_68_P23328301	chr4:134954162-134954206	NM_001168500:-42	A330049M08Rik	PROMOTER	0.455	4.333	11820.36	51214.19	1.972	8731.04	17213.40
A_68_P23328300	chr4:134954050-134954094	NM_001168500:70	A330049M08Rik	INSIDE	0.279	1.589	3477.73	5525.60	0.443	2580.05	1142.57
A_68_P31323978	chr17:69788959-69789003	NM_001145192:315	A330050F15Rik	INSIDE	0.527	0.286	3149.85	900.08	0.151	2349.25	353.74
A_68_P31323979	chr17:69789105-69789149	NM_001145192:461	A330050F15Rik	INSIDE	0.607	0.602	2965.92	1786.04	0.366	2133.31	779.92
A_68_P31145425	chr17:32533548-32533592	NR_029458:-6388	A530088E08Rik	PROMOTER	0.494	8.298	2965.78	24608.92	4.103	2866.23	11759.69
A_68_P30544728	chr16:10835113-10835157	NM_001162932:-17	A630055G03Rik	PROMOTER	0.474	9.021	1385.83	12502.07	4.277	1221.79	5225.51
A_68_P30544729	chr16:10835253-10835297	NM_001162932:123	A630055G03Rik	INSIDE	0.63	3.750	529.88	1987.11	2.363	479.89	1134.18
A_68_P32775148	chrX:155970504-155970548	NM_001033472:-73	A830080D01Rik	PROMOTER	0.449	8.810	1244.67	10965.40	3.954	1741.13	6884.87
A_68_P32775150	chrX:155970813-155970857	NM_001033472:235	A830080D01Rik	INSIDE	0.535	3.579	5281.92	18903.55	1.916	6763.38	12955.47
A_68_P32775152	chrX:155971013-155971057	NM_001033472:435	A830080D01Rik	INSIDE	0.632	5.013	1249.98	6266.55	3.167	1311.38	4153.46
A_68_P31112027	chr17:26853342-26853387	NM_029870:770	A930001N09Rik	INSIDE	0.222	9.481	2988.35	28333.07	2.108	2724.46	5743.83
A_68_P31112025	chr17:26853064-26853108	NM_029870:492	A930001N09Rik	INSIDE	0.623	0.244	2944.73	719.98	0.152	2263.71	345.07
A_68_P24067083	chr5:125956377-125956421	NM_030210:156	Aacs	INSIDE	0.456	0.609	1505.37	916.83	0.277	1014.28	281.41
A_68_P20347697	chr1:74331425-74331469	NM_001190444:-134	Aamp	DIVERGENT_PROMOTER	0.311	1.956	1382.44	2703.47	0.608	1160.16	705.17
A_68_P30214228	chr15:51872101-51872145	NM_175503:470	Aard	INSIDE	0.33	0.363	1833.53	666.26	0.12	1470.65	176.19
A_68_P31201456	chr17:45643922-45643966	NM_198608:155	Aars2	INSIDE	0.589	3.280	1352.65	4436.65	1.931	1224.60	2364.99
A_68_P27977895	chr11:84326684-84326729	NM_019816:297	Aatf	INSIDE	0.532	0.513	1343.60	689.59	0.273	993.92	271.49
A_68_P28181737	chr11:119873242-119873286	NM_001198787:29831	Aatk	INSIDE	0.223	9.494	1091.57	10363.65	2.121	837.54	1776.42
A_68_P21072052	chr2:25283953-25283999	NM_007379:-217	Abca2	PROMOTER	0.468	12.385	688.42	8525.77	5.8	657.22	3811.98
A_68_P31096787	chr17:24488644-24488688	NM_001039581:-324	Abca3	DIVERGENT_PROMOTER	0.307	0.629	1054.47	662.77	0.193	912.62	176.34
A_68_P28123546	chr11:110199057-110199101	NM_147219:-48	Abca5	PROMOTER	0.193	28.600	4304.62	123110.20	5.525	3432.27	18964.49
A_68_P23470075	chr5:8893888-8893932	NM_008830:190	Abcb4	INSIDE	0.632	2.735	1821.91	4982.46	1.729	1451.87	2510.25
A_68_P24059257	chr5:124539964-124540008	NM_019875:5821	Abcb9	INSIDE	0.648	3.275	1372.73	4495.88	2.122	1172.09	2486.79

ProbeName	Target position of probe on CpG island microarray	TargetID	GeneSymbol	CpG island Description	Ratio of relative methylation (TiO_2-NP/Vehicle)	Sham group			TiO_2-H group		
						Relative methylation (Cy5/Cy3)	Cy3 signal (Input)	Cy5 signal (MeDIP)	Relative methylation (Cy5/Cy3)	Cy3 signal (Input)	Cy5 signal (MeDIP)
A_68_P30561772	chr16:14361819-14361863	NM_008576:188	Abcc1	INSIDE	0.649	0.465	2399.02	1115.25	0.302	2018.61	608.78
A_68_P31206433	chr17:46465374-46465418	NM_170680:-424	Abcc10	PROMOTER	0.325	1.510	1023.88	1545.60	0.491	741.71	364.19
A_68_P30587614	chr16:20426497-20426541	NM_013790:-51	Abcc5	PROMOTER	0.446	0.149	3980.46	592.90	0.066	2583.49	171.52
A_68_P30587610	chr16:20425965-20426009	NM_013790:481	Abcc5	INSIDE	0.456	4.688	1781.22	8349.73	2.14	1678.92	3592.54
A_68_P30587612	chr16:20426260-20426304	NM_013790:185	Abcc5	INSIDE	0.488	0.661	6535.26	4323.00	0.323	4301.89	1389.87
A_68_P30587611	chr16:20426129-20426173	NM_013790:317	Abcc5	INSIDE	0.4	1.527	3873.60	5914.24	0.611	2762.02	1687.66
A_68_P25097409	chr7:53435456-53435500	NM_011510:-75	Abcc8	PROMOTER	0.211	10.991	868.03	9540.31	2.316	845.85	1958.92
A_68_P25097408	chr7:53435337-53435381	NM_011510:45	Abcc8	INSIDE	0.394	3.680	3191.16	11742.49	1.449	2597.83	3763.60
A_68_P30434242	chr15:91029845-91029889	NM_011994:-7628	Abcd2	PROMOTER	0.511	3.289	1802.31	5928.32	1.68	1425.20	2394.89
A_68_P30434244	chr15:91030048-91030092	NM_011994:-7832	Abcd2	PROMOTER	0.353	4.181	1559.98	6521.97	1.475	1187.10	1750.55
A_68_P23542799	chr5:24082860-24082904	NM_001190443:403	Abcf2	INSIDE	0.238	0.441	2298.50	1014.34	0.105	1800.07	189.21
A_68_P23542802	chr5:24083177-24083221	NM_001190443:87	Abcf2	INSIDE	0.523	0.456	1401.12	639.59	0.239	1048.19	250.16
A_68_P23542801	chr5:24083043-24083087	NM_001190443:221	Abcf2	INSIDE	0.598	0.617	2165.02	1336.73	0.369	1585.70	585.27
A_68_P24482547	chr6:58590413-58590457	NM_011920:43769	Abcg2	INSIDE	0.364	13.674	2132.76	29163.25	4.971	1835.40	9124.50
A_68_P26466788	chr9:44096508-44096552	NM_138955:-203	Abcg4	PROMOTER	0.419	0.651	784.60	511.03	0.273	677.70	184.97
A_68_P30728224	chr16:45742850-45742894	NM_172511:147	Abhd10	INSIDE	0.408	3.767	1804.01	6796.54	1.535	1312.07	2014.55
A_68_P21741665	chr2:150730549-150730593	NM_024465:-103	Abhd12	DIVERGENT_PROMOTER	0.161	12.406	2948.48	36577.49	1.997	2591.65	5175.81
A_68_P21741661	chr2:150730134-150730178	NM_024465:311	Abhd12	INSIDE	0.349	4.539	1488.11	6754.93	1.583	1203.26	1905.06
A_68_P31157494	chr17:35226276-35226320	NM_178592:63	Abhd16a	INSIDE	0.185	10.981	1474.84	16195.54	2.036	1176.35	2395.02
A_68_P25260061	chr7:86418376-86418420	NM_018811:247	Abhd2	INSIDE	0.428	0.621	867.94	538.88	0.265	674.24	179.01
A_68_P26888896	chr9:122260961-122261005	NM_026179:249	Abhd5	INSIDE	0.416	0.558	1766.53	985.47	0.232	1487.00	344.76
A_68_P25956922	chr8:73987506-73987550	NM_022419:28	Abhd8	INSIDE	0.417	4.380	1305.59	5718.73	1.827	1118.59	2043.21
A_68_P20268943	chr1:60466325-60466369	NM_001198570:-116	Abi2	PROMOTER	0.191	20.240	2155.76	43633.57	3.857	1818.21	7012.76
A_68_P20268947	chr1:60467002-60467046	NM_001198570:562	Abi2	INSIDE	0.269	18.882	2938.62	55485.91	5.082	2271.72	11545.38
A_68_P20268944	chr1:60466493-60466537	NM_001198570:52	Abi2	INSIDE	0.384	8.229	1999.68	16456.23	3.161	1649.21	5213.60
A_68_P20743647	chr1:158488894-158488938	NM_001136104:-1	Abl2	PROMOTER	0.278	1.413	1089.66	1539.71	0.393	1204.40	472.87
A_68_P20743645	chr1:158488645-158488689	NM_001136104:-251	Abl2	PROMOTER	0.208	8.574	846.92	7261.64	1.786	779.47	1392.18
A_68_P31760609	chr18:62071314-62071358	NM_198649:142	Ablim3	INSIDE	0.321	6.985	2009.44	14035.49	2.243	1716.82	3851.10
A_68_P27932045	chr11:76250435-76250479	NM_198018:72465	Abr	INSIDE	0.42	0.301	3809.14	1147.32	0.127	2811.21	355.79
A_68_P27932457	chr11:76308492-76308536	NM_198018:14407	Abr	INSIDE	0.654	24.286	20433.25	496239.40	15.881	18206.94	289136.50
A_68_P27932872	chr11:76384849-76384893	NM_198895:6	Abr	INSIDE	0.195	8.247	3599.00	29682.05	1.606	3012.20	4837.66
A_68_P27932455	chr11:76308282-76308326	NM_198018:14617	Abr	INSIDE	0.5	3.301	1781.90	5882.76	1.651	1563.03	2580.68
A_68_P24624506	chr6:88791877-88791921	NM_030251:31	Abtb1	INSIDE	0.124	37.885	9805.17	371466.50	4.683	10691.92	50073.97
A_68_P24624511	chr6:88792507-88792551	NM_030251:-599	Abtb1	PROMOTER	0.201	0.434	5265.31	2284.86	0.087	3137.11	273.21
A_68_P24624504	chr6:88791715-88791759	NM_030251:193	Abtb1	INSIDE	0.3	11.270	9526.24	107362.30	3.382	7628.62	25797.35
A_68_P24624505	chr6:88791782-88791826	NM_030251:125	Abtb1	INSIDE	0.513	0.161	3405.66	548.69	0.083	2112.72	174.61
A_68_P21483985	chr2:103406320-103406364	NM_178890:-124	Abtb2	PROMOTER	0.578	0.293	2483.28	727.91	0.169	1671.15	283.13
A_68_P26373433	chr9:26807126-26807170	NM_025862:-14	Acad8	DIVERGENT_PROMOTER	0.085	46.477	10292.91	478382.00	3.964	13011.26	51578.85
A_68_P22056456	chr3:35965277-35965321	NM_172678:377	Acad9	INSIDE	0.253	0.564	1862.65	1051.07	0.143	1429.68	203.97
A_68_P20305039	chr1:66909588-66909632	NM_007381:231	Acadl	INSIDE	0.575	3.758	935.73	3516.79	2.16	782.49	1690.29
A_68_P22659834	chr3:153607495-153607539	NM_007382:-120	Acadm	PROMOTER	0.271	11.696	2846.65	33295.55	3.166	2317.47	7337.93
A_68_P22659830	chr3:153607153-153607197	NM_007382:222	Acadm	INSIDE	0.588	0.467	1215.68	567.55	0.274	947.82	260.04
A_68_P22659832	chr3:153607334-153607378	NM_007382:40	Acadm	INSIDE	0.462	3.567	5083.96	18135.71	1.647	3891.89	6410.63
A_68_P24007907	chr5:115569084-115569128	NM_007383:216	Acads	INSIDE	0.349	0.341	2380.75	811.91	0.119	1669.73	198.93
A_68_P24007906	chr5:115568969-115569013	NM_007383:332	Acads	INSIDE	0.285	1.619	1200.83	1943.93	0.461	916.08	422.71
A_68_P23441289	chr4:155266283-155266327	NM_207223:321	Acap3	INSIDE	0.622	5.053	2175.04	10991.38	3.141	1723.05	5411.89

ProbeName	Target position of probe on CpG island microarray	TargetID	GeneSymbol	CpG island Description	Ratio of relative methylation (TiO_2-NP/Vehicle)	Sham group			TiO_2-H group		
						Relative methylation (Cy5/Cy3)	Cy3 signal (Input)	Cy5 signal (MeDIP)	Relative methylation (Cy5/Cy3)	Cy3 signal (Input)	Cy5 signal (MeDIP)
A_68_P23441288	chr4:155266214-155266258	NM_207223:253	Acap3	INSIDE	0.646	0.434	2115.65	917.93	0.28	1581.50	442.94
A_68_P26521623	chr9:53418301-53418345	NM_144784:133	Acat1	INSIDE	0.466	6.674	1035.36	6910.10	3.109	938.27	2917.09
A_68_P20877466	chr1:182655892-182655936	NM_133225:-259	Acbd3	PROMOTER	0.457	8.506	1640.98	13958.40	3.885	1430.10	5555.96
A_68_P21061287	chr2:22924684-22924728	NM_001102437:115	Acbd5	INSIDE	0.509	0.422	4880.74	2061.20	0.215	3409.42	733.55
A_68_P27958478	chr11:80966575-80966619	NM_007384:-191	Accn1	PROMOTER	0.327	0.219	3368.86	737.49	0.072	2443.47	174.71
A_68_P27958467	chr11:80965329-80965373	NM_007384:1055	Accn1	INSIDE	0.328	0.455	1458.90	663.31	0.149	1177.51	175.64
A_68_P27958470	chr11:80965747-80965791	NM_007384:637	Accn1	INSIDE	0.374	0.527	3011.26	1587.14	0.197	2171.28	427.88
A_68_P27963498	chr11:81782179-81782223	NM_001034013:-302	Accn1	PROMOTER	0.556	0.190	2414.38	458.68	0.106	1800.33	190.27
A_68_P27958482	chr11:80967088-80967132	NM_007384:-705	Accn1	PROMOTER	0.581	7.212	1022.38	7373.34	4.192	889.70	3729.26
A_68_P20354147	chr1:75447265-75447309	NM_183022:202	Accn4	INSIDE	0.239	1.836	1419.21	2605.30	0.439	1130.84	496.40
A_68_P28098903	chr11:105850138-105850182	NM_009598:13639	Ace	INSIDE	0.081	38.552	2160.09	83275.39	3.115	1568.09	4884.75
A_68_P28098902	chr11:105849980-105850024	NM_009598:13481	Ace	INSIDE	0.301	5.878	2272.75	13358.40	1.769	1730.76	3061.63
A_68_P28098904	chr11:105850208-105850252	NM_009598:13709	Ace	INSIDE	0.527	2.933	1280.83	3756.41	1.545	1051.59	1625.01
A_68_P23081889	chr4:86520559-86520603	NM_139306:263	Acer2	INSIDE	0.377	0.279	7623.24	2125.43	0.105	5152.00	541.04
A_68_P23081888	chr4:86520453-86520497	NM_139306:157	Acer2	INSIDE	0.492	0.469	2198.37	1031.55	0.231	1543.23	356.01
A_68_P25354926	chr7:105457776-105457820	NM_025408:239	Acer3	INSIDE	0.54	3.192	2294.20	7322.11	1.725	1970.27	3398.56
A_68_P24124994	chr5:137735588-137735632	NM_009599:6106	Ache	INSIDE	0.506	0.300	2471.09	741.11	0.152	1675.60	254.30
A_68_P28068578	chr11:100388533-100388577	NM_001199296:760	Acly	INSIDE	0.601	0.657	2071.10	1361.45	0.395	1517.37	599.42
A_68_P24219280	chr6:6906233-6906277	NM_001077713:237	Acn9	INSIDE	0.536	0.371	1940.22	720.39	0.199	1437.62	285.88
A_68_P23417004	chr4:151552321-151552365	NM_001146057:-7900	Acot7	PROMOTER	0.235	1.927	2965.49	5714.13	0.454	2022.42	917.43
A_68_P23417009	chr4:151552981-151553025	NM_001146057:-7240	Acot7	PROMOTER	0.547	3.248	1114.42	3619.68	1.775	852.83	1513.89
A_68_P28328448	chr12:31596461-31596505	NM_001110239:-5	Acp1	DIVERGENT_PROMOTER	0.423	10.008	1462.39	14635.68	4.232	1271.58	5381.71
A_68_P26749718	chr9:96789794-96789838	NM_153420:25	Acpl2	INSIDE	0.363	6.937	1502.88	10424.81	2.519	1185.95	2986.95
A_68_P26749722	chr9:96790253-96790298	NM_153420:-434	Acpl2	PROMOTER	0.648	0.366	1422.36	520.73	0.237	1075.64	255.10
A_68_P26235837	chr8:125299586-125299630	NM_144932:204	Acsf3	INSIDE	0.513	0.345	1679.65	578.94	0.177	977.96	172.98
A_68_P26235836	chr8:125299500-125299544	NM_144932:118	Acsf3	INSIDE	0.586	0.534	3722.62	1986.11	0.313	2400.04	750.90
A_68_P32707838	chrX:138825108-138825152	NM_001033600:-52	Acsl4	PROMOTER	0.623	3.089	727.07	2245.69	1.924	1147.89	2208.03
A_68_P27809324	chr11:54117377-54117421	NM_001033597:-307	Acsl6	PROMOTER	0.399	0.272	2485.18	676.57	0.109	1730.03	187.78
A_68_P27809326	chr11:54117543-54117587	NM_001033597:-141	Acsl6	PROMOTER	0.471	0.518	1063.71	550.88	0.244	915.97	223.54
A_68_P21766024	chr2:155343697-155343741	NM_019811:-60	Acss2	DIVERGENT_PROMOTER	0.455	0.296	1696.63	501.49	0.134	1276.98	171.75
A_68_P24153993	chr5:143667831-143667875	NM_007393:551	Actb	INSIDE	0.582	0.344	3766.28	1295.35	0.2	3064.36	613.14
A_68_P24153997	chr5:143668325-143668369	NM_007393:57	Actb	INSIDE	0.207	7.938	1569.73	12460.19	1.645	1250.21	2056.44
A_68_P28183961	chr11:120209795-120209839	NM_009609:-18	Actg1	DIVERGENT_PROMOTER	0.17	22.334	1802.55	40257.46	3.792	1559.25	5913.29
A_68_P22039455	chr3:32607362-32607406	NM_019673:-83	Actl6a	PROMOTER	0.654	0.450	7748.96	3484.86	0.294	5767.13	1696.51
A_68_P28574822	chr12:81361237-81361281	NM_134156:100	Actn1	INSIDE	0.282	10.091	900.07	9082.64	2.844	1029.36	2927.68
A_68_P28574815	chr12:81360407-81360451	NM_134156:930	Actn1	INSIDE	0.4	0.321	1626.88	522.96	0.129	1389.07	178.65
A_68_P28574819	chr12:81360793-81360837	NM_134156:544	Actn1	INSIDE	0.519	2.905	1065.60	3095.24	1.507	980.57	1478.15
A_68_P25024594	chr7:29747163-29747207	NM_021895:115	Actn4	INSIDE	0.496	0.577	2273.29	1312.37	0.286	1646.55	471.42
A_68_P25024595	chr7:29747291-29747335	NM_021895:-13	Actn4	PROMOTER	0.519	3.307	788.86	2608.81	1.715	661.37	1134.50
A_68_P32143657	chr19:46470296-46470340	NM_016860:-93	Actr1a	DIVERGENT_PROMOTER	0.619	0.266	1705.64	454.50	0.165	1316.50	217.12
A_68_P32143654	chr19:46469962-46470006	NM_016860:241	Actr1a	INSIDE	0.626	6.101	2936.31	17914.29	3.819	2231.25	8522.13
A_68_P27630023	chr11:20013151-20013195	NM_146243:-218	Actr2	PROMOTER	0.613	6.488	5643.42	36613.90	3.977	4722.07	18778.34
A_68_P27630019	chr11:20012621-20012665	NM_146243:312	Actr2	INSIDE	0.505	3.884	912.01	3542.27	1.961	838.10	1643.38
A_68_P20584830	chr1:127331799-127331843	NM_001205385:484	Actr3	INSIDE	0.213	9.323	7326.24	68305.88	1.983	6471.15	12830.50
A_68_P23548816	chr5:25266039-25266083	NM_001004365:217	Actr3b	INSIDE	0.575	0.510	13365.79	6809.96	0.293	8405.87	2464.47
A_68_P21784024	chr2:158450527-158450571	NM_175419:-100	Actr5	PROMOTER	0.402	0.487	987.30	481.12	0.196	890.84	174.59

ProbeName	Target position of probe on CpG island microarray	TargetID	GeneSymbol	CpG island Description	Ratio of relative methylation (TiO_2-NP/Vehicle)	Sham group			TiO_2-H group		
						Relative methylation (Cy5/Cy3)	Cy3 signal (Input)	Cy5 signal (MeDIP)	Relative methylation (Cy5/Cy3)	Cy3 signal (Input)	Cy5 signal (MeDIP)
A_68_P26872301	chr9:119336521-119336565	NM_007397:24924	Acvr2b	INSIDE	0.105	5.690	2145.41	12207.69	0.6	1569.32	941.71
A_68_P22061571	chr3:36962331-36962381	NM_009350:-221	Adad1	PROMOTER	0.372	6.005	594.43	3569.71	2.236	510.54	1141.75
A_68_P26215030	chr8:122136802-122136846	NM_029428:178	Adad2	INSIDE	0.322	9.233	1635.36	15099.33	2.97	1610.52	4783.79
A_68_P26215029	chr8:122136700-122136744	NM_029428:76	Adad2	INSIDE	0.545	3.493	2214.01	7733.27	1.904	1836.41	3496.37
A_68_P26617803	chr9:70527024-70527068	NM_007399:239	Adam10	INSIDE	0.267	10.858	3600.90	39099.53	2.897	2951.41	8550.85
A_68_P28081367	chr11:102635750-102635794	NM_001110778:13020	Adam11	INSIDE	0.428	0.630	948.00	597.41	0.27	883.23	238.23
A_68_P22318789	chr3:89153984-89154028	NM_001037722:-74	Adam15	PROMOTER	0.462	0.568	1126.74	640.46	0.262	876.59	229.96
A_68_P27765615	chr11:45869497-45869541	NM_009616:30	Adam19	INSIDE	0.476	3.604	1766.93	6367.84	1.714	1434.73	2459.75
A_68_P23467734	chr5:8367164-8367208	NM_001098225:895	Adam22	INSIDE	0.348	7.074	1703.48	12050.73	2.461	1634.80	4023.28
A_68_P23467743	chr5:8368197-8368241	NM_001098225:-137	Adam22	PROMOTER	0.466	3.883	1409.38	5472.98	1.809	1292.59	2337.86
A_68_P23467741	chr5:8368019-8368063	NM_001098225:41	Adam22	INSIDE	0.6	2.925	1279.89	3743.97	1.756	1108.29	1945.90
A_68_P23467742	chr5:8368117-8368161	NM_001098225:-57	Adam22	PROMOTER	0.439	3.957	773.37	3060.06	1.739	688.64	1197.44
A_68_P20286846	chr1:63492907-63492951	NM_001177600:451	Adam23	INSIDE	0.572	3.243	1742.18	5650.39	1.857	1543.20	2865.02
A_68_P25724843	chr8:26127513-26127557	NM_007404:-140	Adam9	DIVERGENT_PROMOTER	0.164	50.820	1149.67	58426.48	8.358	1129.13	9437.33
A_68_P25724840	chr8:26127185-26127229	NM_007404:188	Adam9	INSIDE	0.46	5.197	3178.10	16516.48	2.388	2621.45	6260.05
A_68_P25724841	chr8:26127277-26127321	NM_007404:96	Adam9	INSIDE	0.469	5.489	3121.17	17133.15	2.573	2611.31	6717.59
A_68_P30931281	chr16:85803417-85803461	NM_009621:-78	Adamts1	PROMOTER	0.612	3.544	1680.14	5954.94	2.168	1442.18	3126.07
A_68_P30931279	chr16:85803211-85803255	NM_009621:128	Adamts1	INSIDE	0.396	0.557	928.60	516.95	0.221	779.01	171.88
A_68_P26397647	chr9:30730145-30730189	NM_001024139:-129	Adamts15	PROMOTER	0.57	0.635	990.67	628.79	0.362	918.15	332.09
A_68_P26181051	chr8:116372746-116372790	NM_172466:-29	Adamts18	PROMOTER	0.468	0.429	1250.61	535.91	0.201	855.88	171.75
A_68_P31744364	chr18:58997641-58997685	NM_175506:1245	Adamts19	INSIDE	0.609	0.537	2089.54	1121.74	0.327	1682.72	550.13
A_68_P31744348	chr18:58995914-58995958	NM_175506:-481	Adamts19	PROMOTER	0.666	2.408	2790.16	6718.95	1.603	2237.18	3587.22
A_68_P27789375	chr11:50415810-50415854	NM_175643:246	Adamts2	INSIDE	0.495	4.663	3134.41	14616.63	2.309	2632.49	6078.78
A_68_P30452132	chr15:94234058-94234102	NM_177431:701	Adamts20	INSIDE	0.56	0.527	2426.14	1277.97	0.295	1737.59	512.40
A_68_P23880187	chr5:90311691-90311735	NM_001081401:647	Adamts3	INSIDE	0.256	15.559	4310.26	67062.79	3.985	3567.85	14218.31
A_68_P23880193	chr5:90312354-90312398	NM_001081401:-17	Adamts3	PROMOTER	0.522	3.428	3069.24	10522.70	1.789	2606.52	4662.09
A_68_P30931905	chr16:85901365-85901412	NM_011782:-18	Adamts5	PROMOTER	0.09	3.717	1760.58	6544.57	0.336	1227.81	412.39
A_68_P30931894	chr16:85900162-85900206	NM_011782:1186	Adamts5	INSIDE	0.618	5.067	1408.34	7136.26	3.134	1074.45	3367.18
A_68_P26716242	chr9:90057897-90057941	NM_001003911:103	Adamts7	INSIDE	0.564	0.670	1099.21	736.26	0.378	837.57	316.24
A_68_P25276872	chr7:89483764-89483808	NM_001190374:-417	Adamtsl3	PROMOTER	0.412	12.407	1775.57	22029.56	5.118	1364.99	6985.33
A_68_P27282648	chr10:79811490-79811534	NM_001113548:-321	Adamtsl5	DIVERGENT_PROMOTER	0.439	0.399	4796.66	1914.81	0.175	3258.91	571.58
A_68_P27282601	chr10:79805419-79805466	NM_001113548:5749	Adamtsl5	INSIDE	0.48	0.469	5092.12	2389.02	0.225	3241.64	729.25
A_68_P27282652	chr10:79811999-79812043	NM_001113548:-829	Adamtsl5	DIVERGENT_PROMOTER	0.565	4.071	1878.52	7647.19	2.302	1446.82	3330.61
A_68_P27282596	chr10:79804721-79804765	NM_001113548:6449	Adamtsl5	INSIDE	0.658	0.741	3042.90	2255.07	0.488	2101.57	1024.85
A_68_P27282604	chr10:79805724-79805768	NM_025629:5411	Adamtsl5	INSIDE	0.655	2.785	896.04	2495.57	1.825	800.91	1461.29
A_68_P24134879	chr5:139801058-139801102	NM_172723:338	Adap1	INSIDE	0.541	0.541	3936.51	2130.59	0.293	3112.61	911.92
A_68_P27952852	chr11:79967507-79967551	NM_172133:-135	Adap2	PROMOTER	0.418	11.002	1078.04	11860.86	4.604	963.12	4434.12
A_68_P22321213	chr3:89519038-89519082	NM_001038587:117	Adar	INSIDE	0.581	0.288	1820.27	524.38	0.167	1388.06	232.15
A_68_P22321216	chr3:89519299-89519343	NM_001038587:377	Adar	INSIDE	0.606	3.482	1073.42	3737.29	2.108	956.40	2016.31
A_68_P23294136	chr4:128639545-128639589	NM_172875:95	Adc	INSIDE	0.626	0.376	5729.19	2154.71	0.236	3816.48	898.91
A_68_P32908647	chr12:89599176-89599220	NM_028105:-46	Adck1	PROMOTER	0.575	0.437	1063.88	464.87	0.251	875.74	219.89
A_68_P24383760	chr6:39523904-39523948	NM_178873:52	Adck2	INSIDE	0.623	0.439	3958.96	1738.61	0.274	2789.82	763.42
A_68_P27571288	chr11:6963967-6964011	NM_009622:497	Adcy1	INSIDE	0.594	0.566	1156.41	654.47	0.336	905.95	304.74
A_68_P29113350	chr13:69138021-69138065	NM_153534:377	Adcy2	INSIDE	0.455	4.127	1150.25	4747.57	1.877	968.59	1818.22
A_68_P30668280	chr16:35156722-35156766	NM_001012765:1023	Adcy5	INSIDE	0.554	0.470	5162.11	2426.27	0.26	3609.15	940.04
A_68_P30668268	chr16:35154633-35154677	NM_001012765:-1067	Adcy5	PROMOTER	0.15	1.630	4004.01	6526.88	0.245	3028.83	740.67

ProbeName	Target position of probe on CpG island microarray	TargetID	GeneSymbol	CpG island Description	Ratio of relative methylation (TiO_2-NP/Vehicle)	Sham group			TiO_2-H group		
						Relative methylation (Cy5/Cy3)	Cy3 signal (Input)	Cy5 signal (MeDIP)	Relative methylation (Cy5/Cy3)	Cy3 signal (Input)	Cy5 signal (MeDIP)
A_68_P26039918	chr8:90796247-90796291	NM_007406:-33	Adcy7	PROMOTER	0.405	0.542	987.26	534.79	0.219	811.74	177.99
A_68_P30284265	chr15:64753968-64754012	NM_009623:-132	Adcy8	PROMOTER	0.211	2.071	3113.40	6448.39	0.437	2387.42	1042.73
A_68_P30284258	chr15:64753242-64753286	NM_009623:594	Adcy8	INSIDE	0.501	5.250	6000.50	31504.10	2.632	5039.46	13264.80
A_68_P24469549	chr6:55401776-55401820	NM_001025372:-175	Adcyap1r1	PROMOTER	0.662	0.325	2915.63	947.62	0.215	2084.09	448.69
A_68_P23595356	chr5:34959356-34959400	NM_001024458:43016	Add1	INSIDE	0.558	3.890	854.74	3325.03	2.171	598.94	1300.08
A_68_P32180644	chr19:53216958-53217002	NM_001164100:-265	Add3	PROMOTER	0.208	0.280	4378.53	1224.78	0.058	3433.25	199.48
A_68_P32180643	chr19:53216882-53216926	NM_001164100:-341	Add3	PROMOTER	0.381	4.104	2634.98	10814.70	1.565	2053.26	3213.74
A_68_P22568795	chr3:138105955-138105999	NM_007410:-151	Adh5	PROMOTER	0.124	18.382	6661.84	122461.20	2.278	5367.24	12224.98
A_68_P20633265	chr1:136312273-136312317	NM_028320:251	Adipor1	INSIDE	0.177	18.680	8278.87	154652.20	3.31	8788.31	29085.42
A_68_P29450644	chr14:21895153-21895197	NM_134079:23320	Adk	INSIDE	0.595	3.266	962.52	3143.30	1.945	812.35	1579.77
A_68_P21840064	chr2:168031776-168031820	NM_009628:764	Adnp	INSIDE	0.117	2.265	2778.73	6293.87	0.265	2087.10	552.26
A_68_P21840070	chr2:168032547-168032591	NM_009628:-6	Adnp	PROMOTER	0.407	0.416	2908.49	1211.20	0.17	2012.61	341.51
A_68_P21840065	chr2:168031845-168031889	NM_009628:696	Adnp	INSIDE	0.298	2.050	7331.33	15031.54	0.611	5099.65	3114.31
A_68_P21840072	chr2:168032731-168032775	NM_009628:-190	Adnp	PROMOTER	0.402	3.913	1113.40	4357.14	1.572	1073.53	1687.07
A_68_P27212538	chr10:67011354-67011398	NM_001005419:327	Ado	INSIDE	0.441	4.398	637.21	2802.12	1.939	575.89	1116.65
A_68_P27255927	chr10:74779785-74779829	NM_009630:119	Adora2a	INSIDE	0.402	0.384	1360.33	522.20	0.154	1125.47	173.69
A_68_P27255925	chr10:74779618-74779662	NM_009630:-47	Adora2a	PROMOTER	0.569	2.796	3913.36	10940.49	1.59	2968.45	4718.42
A_68_P27852919	chr11:62062182-62062226	NM_007413:-281	Adora2b	PROMOTER	0.333	0.560	3136.13	1757.05	0.186	2232.38	416.28
A_68_P26552557	chr9:59139795-59139840	NM_028121:439	Adpgk	INSIDE	0.533	0.190	2513.23	477.82	0.101	1691.91	171.47
A_68_P26552554	chr9:59139473-59139517	NM_028121:116	Adpgk	INSIDE	0.611	5.599	747.88	4187.50	3.42	740.79	2533.36
A_68_P23279474	chr4:125998794-125998839	NM_133883:131	Adprhl2	INSIDE	0.66	2.803	2017.79	5656.75	1.849	1709.35	3161.38
A_68_P21635706	chr2:131388108-131388153	NM_013460:-109	Adra1d	PROMOTER	0.577	2.799	2555.98	7153.22	1.614	1903.09	3070.85
A_68_P32186064	chr19:54121845-54121889	NM_007417:2195	Adra2a	INSIDE	0.487	0.209	4415.58	923.13	0.102	3277.97	333.67
A_68_P32186055	chr19:54120591-54120635	NM_007417:941	Adra2a	INSIDE	0.66	6.025	774.71	4667.32	3.979	627.54	2497.07
A_68_P21612946	chr2:127188781-127188825	NM_009633:-219	Adra2b	PROMOTER	0.577	0.172	5221.88	899.26	0.099	3532.07	350.70
A_68_P23599469	chr5:35623311-35623355	NM_007418:2118	Adra2c	INSIDE	0.353	13.946	1724.04	24043.59	4.921	1447.02	7121.31
A_68_P23599458	chr5:35621995-35622039	NM_007418:802	Adra2c	INSIDE	0.435	4.725	1504.33	7108.54	2.057	1071.46	2203.56
A_68_P32202573	chr19:56796579-56796623	NM_007419:-261	Adrb1	PROMOTER	0.386	7.931	1636.50	12978.71	3.059	1358.98	4157.62
A_68_P32202581	chr19:56797413-56797457	NM_007419:573	Adrb1	INSIDE	0.38	0.691	1658.52	1146.41	0.263	1354.17	356.00
A_68_P31762242	chr18:62339733-62339777	NM_007420:-141	Adrb2	PROMOTER	0.281	9.344	1573.70	14704.59	2.627	1330.02	3493.32
A_68_P31922428	chr19:4305887-4305931	NM_130863:47	Adrbk1	INSIDE	0.616	0.555	1795.46	996.54	0.342	1528.37	522.83
A_68_P21901912	chr2:179906060-179906104	NM_019822:-210	Adrm1	PROMOTER	0.345	8.076	871.81	7040.49	2.783	774.35	2155.32
A_68_P30375392	chr15:80778891-80778935	NM_009634:-36	Adsl	PROMOTER	0.329	17.074	6326.97	108028.30	5.61	5070.53	28444.53
A_68_P20861819	chr1:179726176-179726220	NM_007422:442	Adss	INSIDE	0.559	0.442	1231.19	544.04	0.247	987.61	244.16
A_68_P28751331	chr12:113858627-113858671	NM_007421:391	Adssl1	INSIDE	0.498	0.459	1642.03	754.24	0.229	1114.86	255.14
A_68_P27565299	chr11:5761759-5761803	NM_009636:-88	Aebp1	PROMOTER	0.396	0.300	2742.76	822.35	0.119	1956.30	232.46
A_68_P27565303	chr11:5762324-5762369	NM_009636:478	Aebp1	INSIDE	0.48	5.407	3426.62	18527.77	2.594	2626.17	6813.53
A_68_P23603794	chr5:36236225-36236269	NM_027373:279	Afap1	INSIDE	0.394	0.582	914.94	532.80	0.229	730.95	167.53
A_68_P31759814	chr18:61946393-61946437	NM_178928:-98	Afap1l1	PROMOTER	0.386	4.913	3825.09	18791.97	1.898	3170.46	6017.90
A_68_P31759815	chr18:61946490-61946534	NM_178928:-196	Afap1l1	PROMOTER	0.527	3.042	1624.06	4940.21	1.604	1325.09	2125.47
A_68_P32204531	chr19:57082851-57082895	NM_001177796:193	Afap1l2	INSIDE	0.172	1.731	4360.31	7547.33	0.297	3231.27	960.11
A_68_P31791928	chr18:67609055-67609099	NM_027130:-286	Afg3l2	PROMOTER	0.253	10.541	9199.88	96978.65	2.67	6801.77	18158.55
A_68_P27633200	chr11:20641586-20641630	NM_181411:-121	Aftph	PROMOTER	0.442	0.245	4346.45	1064.38	0.108	2912.09	315.53
A_68_P20429509	chr1:91351220-91351264	NM_001037136:-143	Agap1	PROMOTER	0.364	4.794	1434.04	6875.45	1.747	1259.77	2200.26
A_68_P27533997	chr10:126528077-126528121	NM_001033263:12136	Agap2	INSIDE	0.587	0.571	1456.44	832.20	0.335	1021.96	342.66
A_68_P27534006	chr10:126529092-126529136	NM_001033263:13152	Agap2	INSIDE	0.197	1.525	833.70	1271.60	0.301	561.06	168.99

ProbeName	Target position of probe on CpG island microarray	TargetID	GeneSymbol	CpG island Description	Ratio of relative methylation (TiO_2-NP/Vehicle)	Sham group			TiO_2-H group		
						Relative methylation (Cy5/Cy3)	Cy3 signal (Input)	Cy5 signal (MeDIP)	Relative methylation (Cy5/Cy3)	Cy3 signal (Input)	Cy5 signal (MeDIP)
A_68_P23542143	chr5:23988482-23988526	NM_139153:30510	Agap3	INSIDE	0.231	17.857	3854.65	68832.78	4.133	3345.90	13829.42
A_68_P23542072	chr5:23980190-23980234	NM_139153:22218	Agap3	INSIDE	0.561	3.638	1103.41	4014.60	2.043	906.41	1851.63
A_68_P24357881	chr6:34730747-34730791	NM_178630:337	Agbl3	INSIDE	0.441	9.555	347.60	3321.20	4.213	400.07	1685.67
A_68_P23205991	chr4:110070245-110070289	NM_001048189:-129	Agbl4	PROMOTER	0.581	14.500	812.56	11781.79	8.419	1177.05	9909.44
A_68_P20395062	chr1:82836520-82836565	NM_010472:485	Agfg1	INSIDE	0.381	0.319	2806.03	895.69	0.122	2271.58	276.43
A_68_P20395065	chr1:82836823-82836867	NM_010472:787	Agfg1	INSIDE	0.528	0.456	1874.53	855.58	0.241	1469.28	354.33
A_68_P24127046	chr5:138125901-138125945	NM_145566:-1	Agfg2	PROMOTER	0.409	4.407	14972.92	65986.66	1.802	11088.13	19986.01
A_68_P24127049	chr5:138126171-138126224	NM_145566:-276	Agfg2	PROMOTER	0.478	0.240	2250.02	539.54	0.115	1493.83	171.13
A_68_P24127045	chr5:138125808-138125852	NM_178162:15	Agfg2	INSIDE	0.631	0.328	2796.35	916.82	0.207	2228.88	461.18
A_68_P29240371	chr13:96145165-96145209	NM_025630:103	Aggf1	INSIDE	0.642	3.406	925.52	3152.22	2.185	817.33	1786.19
A_68_P29240369	chr13:96144912-96144956	NM_025630:355	Aggf1	INSIDE	0.625	3.705	432.35	1601.90	2.315	417.35	966.30
A_68_P22451307	chr3:116511212-116511256	NM_001081326:-150	Agl	PROMOTER	0.323	0.504	1324.35	667.65	0.163	1049.07	170.91
A_68_P21079933	chr2:26459306-26459350	NM_026212:402	Agpat2	INSIDE	0.573	3.300	837.22	2762.62	1.889	712.78	1346.69
A_68_P25715449	chr8:24318844-24318888	NM_018743:59	Agpat6	INSIDE	0.633	2.909	887.82	2582.30	1.841	707.76	1303.34
A_68_P21346286	chr2:75670175-75670220	NM_172666:-36	Agps	PROMOTER	0.245	10.174	1704.76	17343.50	2.496	1384.23	3455.41
A_68_P27794729	chr11:51398504-51398548	NM_028398:268	Agxt2l2	INSIDE	0.203	1.580	705.49	1114.52	0.32	684.25	219.00
A_68_P20872222	chr1:181733328-181733372	NM_026375:796	Ahctf1	INSIDE	0.632	5.733	555.31	3183.70	3.623	420.15	1522.21
A_68_P20872224	chr1:181733633-181733677	NM_026375:492	Ahctf1	INSIDE	0.64	3.354	1185.52	3975.65	2.147	966.20	2074.22
A_68_P23315176	chr4:132567687-132567731	NM_146155:288	Ahdc1	INSIDE	0.128	2.337	1512.84	3536.25	0.298	1143.12	340.87
A_68_P23315178	chr4:132568014-132568058	NM_146155:616	Ahdc1	INSIDE	0.319	0.428	2394.18	1024.70	0.137	1897.86	259.08
A_68_P23315382	chr4:132595283-132595327	NM_146155:27884	Ahdc1	INSIDE	0.406	7.477	1041.67	7788.72	3.032	898.00	2722.80
A_68_P23315555	chr4:132617504-132617548	NM_146155:50106	Ahdc1	INSIDE	0.548	3.660	1984.52	7262.82	2.004	1483.94	2974.26
A_68_P23315558	chr4:132617847-132617891	NM_146155:50448	Ahdc1	INSIDE	0.644	3.951	2820.22	11143.16	2.544	2120.44	5394.30
A_68_P23315180	chr4:132568310-132568354	NM_146155:912	Ahdc1	INSIDE	0.432	4.599	662.25	3045.53	1.985	619.63	1230.04
A_68_P23315717	chr4:132641057-132641101	NM_146155:73658	Ahdc1	DOWNSTREAM	0.664	0.640	1064.65	681.44	0.425	853.62	362.86
A_68_P26989977	chr10:20672484-20672528	NM_026203:154	Ahi1	INSIDE	0.318	8.161	893.93	7295.25	2.594	869.69	2256.15
A_68_P27646190	chr11:23397665-23397709	NM_172391:260	Ahsa2	INSIDE	0.528	3.201	1897.51	6074.74	1.689	1619.50	2735.29
A_68_P26530601	chr9:55174289-55174334	NM_172923:-444	AI118078	PROMOTER	0.666	7.559	2176.87	16454.92	5.038	1643.95	8281.97
A_68_P22953596	chr4:58924953-58924997	NM_172381:623	AI314180	INSIDE	0.636	5.438	341.12	1854.99	3.459	297.98	1030.58
A_68_P22953595	chr4:58924697-58924741	NM_172381:879	AI314180	INSIDE	0.373	4.220	2339.81	9873.62	1.572	1866.83	2934.83
A_68_P21492618	chr2:104966489-104966533	NR_015462:157	AI314831	INSIDE	0.439	0.450	1147.14	515.95	0.198	869.92	171.84
A_68_P21492619	chr2:104966561-104966605	NR_015462:85	AI314831	INSIDE	0.559	3.529	1031.03	3638.47	1.974	861.06	1699.77
A_68_P22862460	chr4:41445205-41445249	NM_001085515:4883	AI464131	INSIDE	0.534	0.426	1690.58	720.29	0.227	1197.03	272.14
A_68_P22862465	chr4:41445862-41445906	NM_001085515:4225	AI464131	INSIDE	0.612	0.356	1501.91	535.18	0.218	1166.99	254.31
A_68_P22862454	chr4:41444579-41444623	NM_001085515:5509	AI464131	INSIDE	0.629	0.367	2117.56	777.60	0.231	1329.78	307.29
A_68_P22862455	chr4:41444682-41444726	NM_001085515:5405	AI464131	INSIDE	0.629	0.592	2004.10	1187.10	0.373	1350.49	503.14
A_68_P27306491	chr10:84565795-84565839	NM_001013028:445	AI597468	INSIDE	0.659	0.481	3449.59	1659.67	0.317	2614.76	829.48
A_68_P31212432	chr17:47605282-47605326	NM_145489:31717	AI661453	INSIDE	0.386	6.462	613.83	3966.81	2.497	558.32	1393.98
A_68_P30473516	chr15:97998981-97999025	NM_177716:766	AI836003	INSIDE	0.509	0.226	2097.25	474.16	0.115	1490.73	171.41
A_68_P31939554	chr19:7456968-7457012	NM_001033139:526	AI846148	INSIDE	0.649	0.587	1549.94	909.27	0.381	1349.38	513.78
A_68_P31939551	chr19:7456653-7456699	NM_001033139:840	AI846148	INSIDE	0.572	3.475	412.55	1433.42	1.988	399.06	793.16
A_68_P27181919	chr10:61177928-61177972	NM_001039194:-60	Aifm2	PROMOTER	0.448	3.083	3557.58	10966.50	1.38	2623.84	3620.14
A_68_P27103326	chr10:43724148-43724192	NM_172393:482	Aim1	INSIDE	0.649	3.865	1253.73	4845.24	2.508	1106.45	2774.68
A_68_P24158560	chr5:144670456-144670500	NM_001172146:230	Aimp2	INSIDE	0.241	16.729	2475.08	41404.63	4.027	2227.97	8972.03
A_68_P24158559	chr5:144670369-144670413	NM_001172146:318	Aimp2	INSIDE	0.41	13.179	2576.58	33957.90	5.4	2109.74	11392.33
A_68_P24158558	chr5:144670295-144670339	NM_001172146:392	Aimp2	INSIDE	0.592	0.707	1108.51	783.31	0.418	876.79	366.51

ProbeName	Target position of probe on CpG island microarray	TargetID	GeneSymbol	CpG island Description	Ratio of relative methylation (TiO_2-NP/Vehicle)	Sham group			TiO_2-H group		
						Relative methylation (Cy5/Cy3)	Cy3 signal (Input)	Cy5 signal (MeDIP)	Relative methylation (Cy5/Cy3)	Cy3 signal (Input)	Cy5 signal (MeDIP)
A_68_P31053607	chr17:12934828-12934873	NR_002853:674	Airn	INSIDE	0.631	3.759	729.06	2740.79	2.371	662.15	1570.01
A_68_P23425243	chr4:152857051-152857095	NM_001099299:-133	Ajap1	PROMOTER	0.526	8.047	1672.70	13460.14	4.232	1568.13	6636.89
A_68_P23425234	chr4:152855762-152855806	NM_001099299:1155	Ajap1	INSIDE	0.6	0.523	1111.70	581.90	0.314	865.54	271.71
A_68_P28693523	chr12:103995794-103995838	NM_001142939:204	AK010878	INSIDE	0.539	0.269	1781.38	479.76	0.145	1307.32	189.77
A_68_P32050810	chr19:29122317-29122361	NM_021299:54	Ak3	INSIDE	0.636	9.563	949.84	9083.06	6.08	738.57	4490.29
A_68_P23154895	chr4:101091776-101091820	NM_001177602:-95	Ak4	PROMOTER	0.338	9.151	2534.74	23196.19	3.096	2179.45	6748.15
A_68_P23154903	chr4:101092599-101092643	NM_009647:313	Ak4	INSIDE	0.579	4.437	4087.61	18136.01	2.568	3324.19	8537.25
A_68_P23154908	chr4:101093069-101093113	NM_009647:783	Ak4	INSIDE	0.662	3.225	429.59	1385.55	2.136	363.36	776.07
A_68_P28002763	chr11:88726023-88726068	NM_001042541:-145	Akap1	PROMOTER	0.326	9.530	927.10	8834.94	3.102	822.18	2550.41
A_68_P28002760	chr11:88725701-88725745	NM_001042541:178	Akap1	INSIDE	0.476	0.562	2239.63	1257.86	0.268	1713.93	458.48
A_68_P28002759	chr11:88725539-88725583	NM_001042541:340	Akap1	INSIDE	0.605	2.694	1519.14	4092.55	1.629	1217.78	1983.87
A_68_P28002758	chr11:88725386-88725430	NM_001042541:492	Akap1	INSIDE	0.221	2.656	1798.65	4776.50	0.587	1278.28	750.17
A_68_P27851243	chr11:61743609-61743653	NM_019921:98	Akap10	INSIDE	0.53	0.219	2478.14	542.08	0.116	1962.95	227.73
A_68_P27851242	chr11:61743468-61743512	NM_019921:238	Akap10	INSIDE	0.646	0.659	3257.15	2146.56	0.426	2388.34	1017.04
A_68_P28553305	chr12:77425730-77425774	NM_001101471:-125	Akap5	PROMOTER	0.418	9.637	2243.25	21617.91	4.027	2475.28	9969.16
A_68_P31144954	chr17:32457616-32457660	NM_019774:460	Akap8	INSIDE	0.16	33.091	4510.07	149242.40	5.295	4133.55	21888.51
A_68_P31144961	chr17:32458430-32458474	NM_017476:29070	Akap8l	INSIDE	0.379	15.428	2342.26	36136.55	5.852	2149.04	12576.91
A_68_P22831249	chr4:34498566-34498610	NM_001007589:725	Akirin2	INSIDE	0.533	0.505	3053.83	1542.57	0.269	2128.59	572.65
A_68_P28751739	chr12:113912106-113912150	NM_001165894:359	Akt1	INSIDE	0.514	3.286	1136.88	3736.32	1.691	1012.74	1712.10
A_68_P25089687	chr7:52107857-52107901	NM_026270:3282	Akt1s1	INSIDE	0.325	5.753	1037.38	5967.54	1.872	680.43	1273.45
A_68_P20858583	chr1:179188004-179188048	NM_011785:-9128	Akt3	PROMOTER	0.577	0.457	1668.82	763.32	0.264	1401.87	370.09
A_68_P20858581	chr1:179187745-179187790	NM_011785:-8869	Akt3	PROMOTER	0.348	1.484	17417.69	25843.65	0.516	12721.66	6566.07
A_68_P26057102	chr8:93657583-93657627	NM_010241:1791	Aktip	INSIDE	0.3	8.983	3488.44	31335.63	2.694	3338.38	8993.09
A_68_P26057105	chr8:93657970-93658014	NM_010241:1403	Aktip	INSIDE	0.441	0.414	1291.05	534.42	0.182	956.41	174.49
A_68_P32109780	chr19:40662732-40662776	NM_019698:199	Aldh18a1	INSIDE	0.326	2.253	4810.32	10835.97	0.735	3477.83	2554.58
A_68_P24045427	chr5:122043186-122043230	NM_009656:625	Aldh2	INSIDE	0.62	0.512	1553.34	795.36	0.317	1207.96	383.37
A_68_P27847513	chr11:61080354-61080398	NM_007437:253	Aldh3a2	INSIDE	0.497	0.585	990.94	580.09	0.291	898.70	261.49
A_68_P28598366	chr12:85791678-85791722	NM_134042:200	Aldh6a1	INSIDE	0.361	0.318	7211.37	2290.21	0.115	4775.50	546.84
A_68_P31731070	chr18:56721947-56721991	NM_138600:-16	Aldh7a1	DIVERGENT_PROMOTER	0.128	50.011	16103.53	805352.20	6.406	23189.19	148549.30
A_68_P31731124	chr18:56732842-56732886	NM_001127338:-271	Aldh7a1	PROMOTER	0.129	18.996	4428.20	84119.02	2.449	3800.39	9308.30
A_68_P31731123	chr18:56732742-56732786	NM_001127338:-171	Aldh7a1	PROMOTER	0.529	0.387	1195.61	462.33	0.205	975.29	199.51
A_68_P31731071	chr18:56722024-56722068	NM_138600:-94	Aldh7a1	DIVERGENT_PROMOTER	0.338	1.599	2043.83	3268.82	0.54	1595.37	861.79
A_68_P20804630	chr1:169280208-169280252	NM_019993:109	Aldh9a1	INSIDE	0.402	4.570	2723.83	12446.88	1.836	2483.38	4559.97
A_68_P20804631	chr1:169280349-169280393	NM_019993:249	Aldh9a1	INSIDE	0.494	0.244	2178.67	531.42	0.121	1474.27	177.69
A_68_P20804634	chr1:169280619-169280663	NM_019993:519	Aldh9a1	INSIDE	0.597	0.237	4581.98	1086.65	0.142	2772.98	392.61
A_68_P25502305	chr7:133942445-133942489	NM_001177307:226	Aldoa	INSIDE	0.526	4.909	2379.74	11681.34	2.584	1772.53	4579.36
A_68_P30428912	chr15:90055009-90055053	NM_001033441:289	Alg10b	INSIDE	0.64	0.505	1377.48	695.64	0.323	1157.67	373.94
A_68_P25708990	chr8:23170949-23170993	NM_183142:-221	Alg11	DIVERGENT_PROMOTER	0.253	6.933	3353.74	23253.05	1.757	2758.90	4848.16
A_68_P32717145	chrX:140752482-140752526	NM_026247:-4	Alg13	PROMOTER	0.167	27.744	2690.32	74640.10	4.642	2963.41	13757.02
A_68_P22149461	chr3:54539488-54539532	NM_025442:50	Alg5	INSIDE	0.325	1.404	1077.11	1512.19	0.456	965.54	440.13
A_68_P26505580	chr9:50583427-50583471	NM_133981:14	Alg9	INSIDE	0.54	0.243	2162.23	525.59	0.131	1515.80	198.94
A_68_P31341464	chr17:72953935-72953979	NM_007439:-309	Alk	PROMOTER	0.297	11.661	5888.19	68661.52	3.46	5298.37	18334.04
A_68_P31341454	chr17:72953064-72953108	NM_007439:561	Alk	INSIDE	0.608	2.644	1253.30	3313.74	1.609	1077.51	1733.27
A_68_P28616635	chr12:88784339-88784383	NM_001102565:429	Alkbh1	INSIDE	0.553	2.899	3046.36	8831.83	1.604	2599.55	4169.57
A_68_P21438435	chr2:93851039-93851083	NM_026944:-173	Alkbh3	PROMOTER	0.191	14.094	744.20	10489.02	2.693	594.72	1601.76
A_68_P31259475	chr17:57137107-57137151	NM_025538:367	Alkbh7	INSIDE	0.471	0.591	4201.36	2484.09	0.279	2775.09	772.88

ProbeName	Target position of probe on CpG island microarray	TargetID	GeneSymbol	CpG island Description	Ratio of relative methylation (TiO_2-NP/Vehicle)	Sham group			TiO_2-H group		
						Relative methylation (Cy5/Cy3)	Cy3 signal (Input)	Cy5 signal (MeDIP)	Relative methylation (Cy5/Cy3)	Cy3 signal (Input)	Cy5 signal (MeDIP)
A_68_P31259472	chr17:57136735-57136779	NM_025538:-5	Alkbh7	PROMOTER	0.612	2.754	2382.33	6561.70	1.687	1934.13	3262.67
A_68_P31259474	chr17:57136971-57137015	NM_025538:231	Alkbh7	INSIDE	0.432	3.274	2824.93	9249.24	1.415	2248.60	3181.06
A_68_P27899786	chr11:70068857-70068903	NM_007440:-37	Alox12	PROMOTER	0.262	9.750	962.81	9387.80	2.557	1000.65	2559.08
A_68_P27899783	chr11:70068622-70068666	NM_007440:199	Alox12	INSIDE	0.659	2.249	2840.87	6388.97	1.482	1983.29	2939.08
A_68_P24776842	chr6:116410742-116410786	NM_009662:432	Alox5	INSIDE	0.66	0.585	1117.06	652.95	0.386	947.70	365.35
A_68_P24776845	chr6:116411172-116411216	NM_009662:2	Alox5	INSIDE	0.27	5.832	2100.63	12250.35	1.575	1794.69	2827.18
A_68_P24184312	chr5:150099157-150099201	NM_009663:22545	Alox5ap	INSIDE	0.19	1.771	2446.85	4333.55	0.337	1651.93	555.89
A_68_P24184313	chr5:150099222-150099276	NM_009663:22615	Alox5ap	INSIDE	0.649	0.716	1466.32	1050.60	0.465	1036.03	481.88
A_68_P20415724	chr1:88995295-88995339	NM_001081082:2865	Alpi	INSIDE	0.626	3.404	2123.31	7228.56	2.13	1761.92	3752.38
A_68_P20415723	chr1:88995177-88995221	NM_001081082:2983	Alpi	INSIDE	0.48	0.627	750.40	470.64	0.301	623.83	187.79
A_68_P23342100	chr4:137351824-137351868	NM_007431:446	Alpl	INSIDE	0.551	5.793	560.31	3245.87	3.195	402.12	1284.62
A_68_P26826440	chr9:110782793-110782837	NM_001146060:-11	Als2cl	PROMOTER	0.261	10.145	2031.19	20605.59	2.652	1652.21	4381.47
A_68_P22407403	chr3:107398682-107398726	NM_007441:756	Alx3	INSIDE	0.563	4.349	1582.89	6883.70	2.448	1229.92	3010.22
A_68_P21436146	chr2:93482709-93482753	NM_007442:140	Alx4	INSIDE	0.527	6.279	1727.44	10846.24	3.307	1451.58	4800.66
A_68_P21436148	chr2:93483044-93483088	NM_007442:476	Alx4	INSIDE	0.191	3.160	2773.37	8764.57	0.602	2233.81	1345.85
A_68_P21436154	chr2:93483591-93483635	NM_007442:1022	Alx4	INSIDE	0.428	5.018	509.19	2555.21	2.148	368.29	791.25
A_68_P31095748	chr17:24300500-24300544	NM_172935:178	Amdhd2	INSIDE	0.353	1.976	3444.48	6805.64	0.698	2540.90	1774.06
A_68_P26074978	chr8:96536308-96536352	NM_011787:210	Amfr	INSIDE	0.396	6.902	1486.28	10257.83	2.733	1388.98	3795.59
A_68_P32710888	chrX:139401254-139401298	NM_019496:-5	Ammecr1	PROMOTER	0.48	3.579	1260.03	4509.09	1.717	1481.47	2544.29
A_68_P32710881	chrX:139400366-139400410	NM_019496:883	Ammecr1	INSIDE	0.118	12.746	747.12	9522.63	1.505	1083.62	1630.54
A_68_P31599404	chr18:31919894-31919938	NM_153515:383	Ammecr1l	INSIDE	0.582	0.459	1164.60	534.07	0.267	927.52	247.50
A_68_P28743070	chr12:112513574-112513618	NM_033603:4275	Amn	INSIDE	0.48	5.831	791.17	4613.41	2.799	726.04	2031.93
A_68_P28743036	chr12:112509597-112509641	NM_033603:297	Amn	INSIDE	0.609	0.649	2708.39	1759.01	0.395	1997.25	789.89
A_68_P28743035	chr12:112509516-112509560	NM_033603:217	Amn	INSIDE	0.284	1.392	1777.97	2475.61	0.396	1214.53	480.84
A_68_P28743034	chr12:112509399-112509443	NM_033603:99	Amn	INSIDE	0.386	0.581	1025.24	595.74	0.224	771.04	172.79
A_68_P26318242	chr9:14419308-14419352	NM_001081395:114	Amotl1	INSIDE	0.296	0.293	3206.05	940.22	0.087	2360.33	205.16
A_68_P22410430	chr3:107889618-107889662	NM_028779:-95	Ampd2	DIVERGENT_PROMOTER	0.353	5.642	1915.39	10806.81	1.993	1653.83	3296.40
A_68_P22410425	chr3:107889046-107889090	NM_028779:477	Ampd2	INSIDE	0.623	3.158	3404.72	10751.44	1.967	2716.32	5342.97
A_68_P22410428	chr3:107889363-107889407	NM_028779:161	Ampd2	INSIDE	0.529	0.609	2365.16	1439.26	0.322	1781.17	572.96
A_68_P25415072	chr7:117911998-117912042	NM_009667:-4097	Ampd3	PROMOTER	0.653	0.226	2025.48	458.13	0.148	1631.89	241.18
A_68_P25415075	chr7:117912319-117912363	NM_009667:-3777	Ampd3	PROMOTER	0.496	4.341	595.86	2586.47	2.155	487.71	1050.89
A_68_P28859761	chr13:19040366-19040410	NM_175007:149	Amph	INSIDE	0.403	7.133	6594.53	47038.03	2.877	5148.82	14811.18
A_68_P28859763	chr13:19040669-19040713	NM_175007:451	Amph	INSIDE	0.178	9.814	792.27	7775.76	1.743	694.48	1210.74
A_68_P24143006	chr5:141211659-141211703	NM_173405:11600	Amz1	INSIDE	0.069	4.332	3795.51	16440.53	0.299	2959.07	884.58
A_68_P28118514	chr11:109287314-109287358	NM_025275:77	Amz2	INSIDE	0.33	0.439	9379.77	4118.39	0.145	6727.55	975.05
A_68_P21620795	chr2:128512977-128513021	NM_008569:133	Anapc1	INSIDE	0.561	2.736	1704.37	4663.31	1.536	1286.46	1975.38
A_68_P25996048	chr8:82235800-82235844	NM_026904:104	Anapc10	INSIDE	0.313	0.261	3883.18	1012.28	0.082	2672.35	218.21
A_68_P28185536	chr11:120459969-120460013	NM_001038230:145	Anapc11	INSIDE	0.635	0.421	1930.99	813.70	0.268	1732.35	463.83
A_68_P28185535	chr11:120459852-120459896	NM_001038230:29	Anapc11	INSIDE	0.664	3.541	833.40	2951.25	2.352	692.77	1629.24
A_68_P26781229	chr9:102528705-102528749	NM_181394:101	Anapc13	INSIDE	0.447	0.601	776.08	466.58	0.269	644.29	173.09
A_68_P21071034	chr2:25127984-25128028	NM_175300:21	Anapc2	INSIDE	0.648	0.392	1960.60	768.65	0.254	1469.93	373.31
A_68_P31150484	chr17:33918191-33918235	NM_020581:308	Angptl4	INSIDE	0.557	3.437	597.72	2054.26	1.914	467.82	895.51
A_68_P30092468	chr15:27396965-27397009	NM_020332:555	Ank	INSIDE	0.52	0.249	1853.11	461.19	0.129	1327.77	171.90
A_68_P27226083	chr10:69285075-69285119	NM_146005:288641	Ank3	INSIDE	0.617	2.509	2188.21	5490.55	1.549	1392.63	2157.05
A_68_P27912780	chr11:72503452-72503496	NM_009671:-29	Ankfy1	PROMOTER	0.451	0.281	2093.11	587.48	0.127	1414.56	179.25
A_68_P23447093	chr5:3802955-3802999	NM_001003909:133	Ankib1	INSIDE	0.243	14.546	1978.29	28777.24	3.534	1959.48	6923.90

ProbeName	Target position of probe on CpG island microarray	TargetID	GeneSymbol	CpG island Description	Ratio of relative methylation (TiO_2-NP/Vehicle)	Sham group			TiO_2-H group		
						Relative methylation (Cy5/Cy3)	Cy3 signal (Input)	Cy5 signal (MeDIP)	Relative methylation (Cy5/Cy3)	Cy3 signal (Input)	Cy5 signal (MeDIP)
A_68_P23447092	chr5:3802834-3802878	NM_001003909:253	Ankib1	INSIDE	0.595	5.980	806.78	4824.17	3.555	728.83	2591.04
A_68_P25656692	chr8:11635953-11635997	NM_133971:-220	Ankrd10	PROMOTER	0.487	0.189	2680.78	506.55	0.092	1928.56	177.56
A_68_P25656690	chr8:11635804-11635848	NM_133971:-72	Ankrd10	PROMOTER	0.499	4.199	3007.76	12628.89	2.095	2611.03	5469.54
A_68_P26237605	chr8:125566196-125566241	NM_001081379:-34	Ankrd11	PROMOTER	0.301	8.236	2761.47	22742.82	2.483	2926.30	7264.78
A_68_P26237609	chr8:125566578-125566622	NM_001081379:-416	Ankrd11	PROMOTER	0.343	0.250	2917.26	729.24	0.086	2178.85	187.04
A_68_P27937765	chr11:77285191-77285236	NM_172945:17967	Ankrd13b	INSIDE	0.54	4.452	1132.99	5044.40	2.405	918.97	2210.53
A_68_P22682770	chr3:157610734-157610778	NM_001013806:327	Ankrd13c	INSIDE	0.292	29.334	9272.17	271985.40	8.557	10819.46	92585.95
A_68_P22682769	chr3:157610620-157610664	NM_001013806:213	Ankrd13c	INSIDE	0.601	7.289	3353.47	24441.85	4.382	2864.27	12550.29
A_68_P31922243	chr19:4282842-4282886	NM_026720:273	Ankrd13d	INSIDE	0.468	0.197	3465.43	681.59	0.092	2602.73	239.50
A_68_P23882636	chr5:90794437-90794481	NM_030886:753	Ankrd17	INSIDE	0.078	1.663	8321.87	13836.28	0.13	6303.78	820.28
A_68_P23882634	chr5:90794262-90794306	NM_030886:927	Ankrd17	INSIDE	0.251	0.248	5062.16	1253.30	0.062	3650.05	227.11
A_68_P23882644	chr5:90795790-90795834	NM_030886:-601	Ankrd17	PROMOTER	0.621	2.528	4075.12	10301.11	1.571	3333.51	5236.64
A_68_P24787432	chr6:118512444-118512488	NM_001081112:-192	Ankrd26	PROMOTER	0.436	0.455	2178.03	990.26	0.198	1462.09	289.70
A_68_P29514233	chr14:32643204-32643248	NM_001024604:375	Ankrd28	INSIDE	0.341	1.559	1207.84	1882.51	0.532	1054.30	560.95
A_68_P25833746	chr8:47085665-47085709	NM_001039562:-482	Ankrd37	PROMOTER	0.523	4.131	3621.58	14961.46	2.16	2921.32	6310.88
A_68_P25833742	chr8:47085366-47085410	NM_001039562:-184	Ankrd37	PROMOTER	0.56	2.767	3010.56	8329.94	1.55	2432.96	3770.03
A_68_P20148961	chr1:36604157-36604208	NM_026241:-136	Ankrd39	PROMOTER	0.424	3.994	1522.13	6078.80	1.692	1166.14	1972.83
A_68_P28032141	chr11:94189750-94189794	NM_146024:458	Ankrd40	INSIDE	0.577	0.527	1730.07	910.89	0.304	1519.13	461.64
A_68_P27804984	chr11:53292506-53292550	NM_183173:1169	Ankrd43	INSIDE	0.56	3.698	696.66	2576.16	2.071	599.26	1240.90
A_68_P27804987	chr11:53292850-53292894	NM_183173:825	Ankrd43	INSIDE	0.247	9.435	259.59	2449.22	2.333	315.59	736.39
A_68_P20239578	chr1:54983378-54983422	NM_001081433:-169	Ankrd44	PROMOTER	0.075	26.870	7262.80	195149.40	2.014	5175.49	10421.22
A_68_P20239573	chr1:54982711-54982755	NM_001081433:499	Ankrd44	INSIDE	0.647	0.337	2696.88	908.84	0.218	2052.25	447.36
A_68_P20770260	chr1:163072965-163073010	NM_028664:145	Ankrd45	INSIDE	0.518	0.518	1176.51	608.88	0.268	954.19	255.88
A_68_P20770398	chr1:163101115-163101159	NM_028664:28294	Ankrd45	DOWNSTREAM	0.622	0.353	2426.52	855.76	0.219	1820.75	399.46
A_68_P22069519	chr3:38383514-38383558	NM_001167883:202	Ankrd50	INSIDE	0.315	9.454	3102.20	29328.63	2.979	2673.77	7964.36
A_68_P22069523	chr3:38383943-38383987	NM_001167883:-226	Ankrd50	PROMOTER	0.434	0.257	11458.35	2949.57	0.112	7403.55	826.18
A_68_P24595345	chr6:83713011-83713056	NM_029245:394	Ankrd53	INSIDE	0.379	0.578	4212.53	2434.64	0.219	2907.54	637.19
A_68_P23897556	chr5:93474347-93474391	NM_175270:-320	Ankrd56	PROMOTER	0.538	0.634	1761.77	1116.76	0.341	1435.83	489.34
A_68_P23897553	chr5:93473897-93473941	NM_175270:130	Ankrd56	INSIDE	0.229	7.020	1308.96	9189.26	1.608	1013.51	1629.40
A_68_P32321491	chrX:34588512-34588556	NM_173779:-305	Ankrd58	PROMOTER	0.357	9.297	3834.33	35649.51	3.317	3929.70	13036.38
A_68_P28741420	chr12:112215750-112215794	NM_175207:1459	Ankrd9	INSIDE	0.432	3.834	1004.36	3851.21	1.655	813.01	1345.59
A_68_P27338390	chr10:90291759-90291803	NM_001177396:252345	Anks1b	INSIDE	0.466	11.195	1225.75	13722.65	5.222	1010.62	5277.66
A_68_P27338392	chr10:90291975-90292019	NM_001177396:252561	Anks1b	INSIDE	0.564	11.878	1790.63	21269.25	6.696	1611.31	10789.95
A_68_P27338397	chr10:90292634-90292678	NM_001177396:253221	Anks1b	INSIDE	0.587	0.698	1771.95	1236.02	0.409	1443.38	590.51
A_68_P27339383	chr10:90432803-90432847	NM_001177396:393389	Anks1b	INSIDE	0.56	0.601	1114.66	669.94	0.336	969.85	326.14
A_68_P25608860	chr7:151924429-151924473	NM_178642:-67	Ano1	PROMOTER	0.61	4.093	1646.14	6736.95	2.497	1446.52	3611.71
A_68_P25608514	chr7:151864472-151864516	NM_178642:59889	Ano1	INSIDE	0.413	0.606	1028.28	623.03	0.25	796.50	199.09
A_68_P26888672	chr9:122203588-122203632	NM_133979:-118	Ano10	PROMOTER	0.477	0.484	2900.07	1404.27	0.231	2291.49	529.72
A_68_P26888673	chr9:122203754-122203798	NM_133979:-284	Ano10	PROMOTER	0.501	0.517	4856.92	2512.44	0.259	3692.67	956.14
A_68_P30459959	chr15:95620940-95620984	NM_175344:-311	Ano6	PROMOTER	0.428	7.551	3394.54	25633.06	3.232	2594.72	8387.24
A_68_P30459964	chr15:95621741-95621785	NM_175344:489	Ano6	INSIDE	0.598	0.646	1491.15	962.69	0.386	1220.25	470.75
A_68_P25957039	chr8:74005499-74005543	NM_001164679:4446	Ano8	INSIDE	0.329	12.041	669.14	8056.97	3.96	577.54	2286.76
A_68_P25957034	chr8:74004823-74004867	NM_001164679:5122	Ano8	INSIDE	0.537	9.199	3208.81	29518.88	4.939	2514.42	12419.17
A_68_P26569955	chr9:62189553-62189597	NM_009672:425	Anp32a	INSIDE	0.159	3.006	7221.72	21708.39	0.477	5020.82	2395.12
A_68_P26569948	chr9:62188707-62188751	NM_009672:-421	Anp32a	PROMOTER	0.47	0.179	3446.02	616.76	0.084	2345.07	197.15
A_68_P26569953	chr9:62189357-62189414	NM_009672:236	Anp32a	INSIDE	0.153	0.606	2485.64	1506.35	0.093	1863.51	173.19

ProbeName	Target position of probe on CpG island microarray	TargetID	GeneSymbol	CpG island Description	Ratio of relative methylation (TiO_2-NP/Vehicle)	Sham group			TiO_2-H group		
						Relative methylation (Cy5/Cy3)	Cy3 signal (Input)	Cy5 signal (MeDIP)	Relative methylation (Cy5/Cy3)	Cy3 signal (Input)	Cy5 signal (MeDIP)
A_68_P22888792	chr4:46464440-46464484	NM_130889:474	Anp32b	INSIDE	0.204	12.548	3130.00	39275.30	2.563	3142.32	8054.21
A_68_P22346557	chr3:95755188-95755232	NM_023210:22031	Anp32e	DOWNSTREAM	0.487	0.284	2592.05	736.71	0.138	1978.71	273.84
A_68_P22346556	chr3:95755077-95755121	NM_023210:21919	Anp32e	DOWNSTREAM	0.52	0.370	2712.15	1003.83	0.193	2045.71	393.81
A_68_P24615404	chr6:87285370-87285414	NM_054041:377	Antxr1	INSIDE	0.579	5.369	3989.23	21419.00	3.108	3454.43	10737.19
A_68_P23915350	chr5:98364657-98364701	NM_133738:95303	Antxr2	INSIDE	0.423	3.587	1101.44	3950.96	1.516	1160.48	1758.78
A_68_P29480016	chr14:26661518-26661562	NM_013469:-100	Anxa11	PROMOTER	0.595	0.624	982.86	613.02	0.371	866.31	321.75
A_68_P22058283	chr3:36374398-36374443	NM_009673:389	Anxa5	INSIDE	0.56	0.576	948.41	545.81	0.322	855.72	275.83
A_68_P27560709	chr11:4887252-4887296	NM_007454:367	Ap1b1	INSIDE	0.477	0.178	2572.37	457.51	0.085	2030.16	172.30
A_68_P27560706	chr11:4886974-4887018	NM_007454:89	Ap1b1	INSIDE	0.521	11.716	2548.40	29856.55	6.105	2027.28	12375.98
A_68_P27560710	chr11:4887330-4887374	NM_007454:445	Ap1b1	INSIDE	0.616	0.286	2164.43	618.98	0.176	1565.66	275.75
A_68_P26158882	chr8:112302510-112302554	NM_009677:-7	Ap1g1	PROMOTER	0.536	3.337	1295.51	4322.79	1.79	1059.87	1896.94
A_68_P25960292	chr8:74764393-74764437	NM_007456:384	Ap1m1	INSIDE	0.596	5.518	744.87	4110.48	3.288	621.84	2044.60
A_68_P25960290	chr8:74764202-74764246	NM_007456:194	Ap1m1	INSIDE	0.548	2.760	1126.60	3108.98	1.512	956.04	1445.47
A_68_P25090215	chr7:52185401-52185445	NM_001077264:-562	Ap2a1	PROMOTER	0.65	0.655	1801.44	1179.71	0.426	1462.38	622.33
A_68_P25589623	chr7:148748286-148748330	NM_007459:230	Ap2a2	INSIDE	0.331	0.369	1849.75	683.39	0.122	1462.27	178.59
A_68_P30588065	chr16:20535666-20535710	NM_009679:112	Ap2m1	INSIDE	0.497	7.429	1915.42	14230.14	3.693	1801.45	6652.06
A_68_P29234322	chr13:95129157-95129201	NM_009680:264	Ap3b1	INSIDE	0.281	0.394	2191.36	863.73	0.111	1573.17	174.34
A_68_P25712956	chr8:23916111-23916155	NM_001122820:-6	Ap3m2	PROMOTER	0.188	14.788	3058.38	45228.25	2.782	2971.41	8267.79
A_68_P31678555	chr18:46901926-46901970	NM_009681:378	Ap3s1	INSIDE	0.276	16.876	2201.88	37158.21	4.656	3362.24	15655.08
A_68_P22388171	chr3:103613470-103613514	NM_001163552:53	Ap4b1	INSIDE	0.629	5.571	844.22	4702.74	3.505	759.62	2662.26
A_68_P27339936	chr10:90545573-90545617	NM_001042558:-106	Apaf1	DIVERGENT_PROMOTER	0.568	4.326	843.78	3650.22	2.459	739.14	1817.59
A_68_P27339932	chr10:90545143-90545187	NM_001042558:324	Apaf1	INSIDE	0.634	2.751	784.93	2159.43	1.743	601.35	1048.18
A_68_P32019526	chr19:23833634-23833678	NM_177034:291	Apba1	INSIDE	0.434	0.316	3199.05	1011.68	0.137	2026.58	278.31
A_68_P32020394	chr19:23967353-23967397	NM_177034:134009	Apba1	INSIDE	0.562	0.652	3005.17	1959.66	0.367	2419.60	886.99
A_68_P32020393	chr19:23967286-23967330	NM_177034:133943	Apba1	INSIDE	0.559	0.671	1137.51	763.68	0.375	900.18	337.78
A_68_P23763025	chr5:67011439-67011483	NM_001201413:-1404	Apbb2	PROMOTER	0.25	0.396	5366.57	2127.05	0.099	3717.63	368.73
A_68_P23763011	chr5:67009509-67009553	NM_009686:380	Apbb2	INSIDE	0.536	16.129	754.98	12177.33	8.638	803.72	6942.66
A_68_P31625259	chr18:36839095-36839139	NM_146085:-96	Apbb3	PROMOTER	0.598	0.688	1834.41	1261.16	0.411	1476.34	606.92
A_68_P27282400	chr10:79777704-79777748	NM_011789:13162	Apc2	INSIDE	0.346	9.162	1131.27	10364.28	3.174	924.27	2934.09
A_68_P27282330	chr10:79767570-79767614	NM_011789:3028	Apc2	INSIDE	0.423	5.917	2652.13	15693.92	2.504	2155.16	5396.95
A_68_P27282404	chr10:79778048-79778092	NM_011789:13506	Apc2	INSIDE	0.553	6.114	1506.20	9209.21	3.384	1294.89	4381.45
A_68_P22346229	chr3:95697870-95697914	NM_146104:-26	Aph1a	DIVERGENT_PROMOTER	0.331	0.666	2030.72	1352.07	0.22	1503.98	331.32
A_68_P21481086	chr2:102914077-102914121	NM_019735:267	Apip	INSIDE	0.497	0.507	1021.97	518.37	0.252	837.99	211.11
A_68_P32362520	chrX:45387716-45387760	NM_013912:291	Apln	INSIDE	0.202	55.033	2214.34	121861.10	11.096	2614.70	29011.39
A_68_P26399340	chr9:31019173-31019217	NM_001102455:206	Aplp2	INSIDE	0.375	0.463	1209.21	559.34	0.174	995.96	172.82
A_68_P26399336	chr9:31018727-31018771	NM_001102455:652	Aplp2	INSIDE	0.631	5.929	2106.35	12488.21	3.738	1822.58	6813.65
A_68_P26399347	chr9:31020000-31020044	NM_001102455:-622	Aplp2	PROMOTER	0.653	0.668	2038.03	1360.81	0.436	1529.19	667.20
A_68_P22311543	chr3:87862306-87862350	NM_144897:89	Apoa1bp	INSIDE	0.334	0.356	2362.40	840.25	0.119	1639.93	194.58
A_68_P32538582	chrX:91612233-91612277	NM_026673:-194	Apoo	PROMOTER	0.404	6.152	340.94	2097.46	2.485	499.01	1240.16
A_68_P32538581	chrX:91612170-91612214	NM_026673:-256	Apoo	PROMOTER	0.517	3.676	1400.25	5146.88	1.9	1652.90	3140.25
A_68_P30927986	chr16:85173922-85173966	NM_001198823:8	App	INSIDE	0.476	9.726	1389.42	13512.94	4.627	1216.14	5627.16
A_68_P30927368	chr16:85080443-85080487	NM_001198823:93488	App	INSIDE	0.529	3.207	8528.77	27355.31	1.697	5386.92	9143.93
A_68_P27981860	chr11:85048235-85048279	NM_025825:366	Appbp2	INSIDE	0.56	0.326	1717.93	559.99	0.182	1340.87	244.61
A_68_P26234307	chr8:125100918-125100973	NM_009698:-138	Aprt	PROMOTER	0.419	0.428	1534.08	656.86	0.179	971.43	174.19
A_68_P26234305	chr8:125100749-125100793	NM_009698:37	Aprt	INSIDE	0.545	4.940	7805.65	38558.67	2.691	4890.72	13161.19
A_68_P26234304	chr8:125100653-125100697	NM_009698:133	Aprt	INSIDE	0.652	0.145	4514.90	656.57	0.095	2886.46	273.81

ProbeName	Target position of probe on CpG island microarray	TargetID	GeneSymbol	CpG island Description	Ratio of relative methylation (TiO_2-NP/Vehicle)	Sham group			TiO_2-H group		
						Relative methylation (Cy5/Cy3)	Cy3 signal (Input)	Cy5 signal (MeDIP)	Relative methylation (Cy5/Cy3)	Cy3 signal (Input)	Cy5 signal (MeDIP)
A_68_P25352110	chr7:104886718-104886762	NM_175105:17	Aqp11	INSIDE	0.403	0.218	4572.41	997.56	0.088	3350.12	294.83
A_68_P25352107	chr7:104886338-104886382	NM_175105:397	Aqp11	INSIDE	0.614	0.277	2201.38	610.09	0.17	1533.75	260.81
A_68_P30481041	chr15:99414682-99414726	NM_009699:5218	Aqp2	INSIDE	0.215	11.633	1533.34	17837.99	2.5	1297.93	3245.32
A_68_P22860481	chr4:41045310-41045354	NM_016689:-116	Aqp3	PROMOTER	0.517	9.679	1690.32	16360.74	5.001	1340.27	6703.27
A_68_P23742487	chr5:63157563-63157607	NM_178407:-168	Arap2	PROMOTER	0.333	10.026	1057.81	10605.39	3.337	1219.86	4070.49
A_68_P30338802	chr15:74503171-74503215	NM_018790:-192	Arc	PROMOTER	0.311	8.053	4305.23	34669.84	2.508	3354.14	8411.27
A_68_P26469462	chr9:44575608-44575652	NM_145985:261	Arcn1	INSIDE	0.575	6.676	1357.52	9062.68	3.836	1117.28	4285.91
A_68_P23887089	chr5:91568885-91568929	NM_009704:266	Areg	INSIDE	0.473	0.473	1189.06	562.53	0.224	926.93	207.44
A_68_P27835624	chr11:59040902-59040946	NM_001130408:740	Arf1	INSIDE	0.547	0.319	2680.44	856.33	0.175	2151.75	375.85
A_68_P27835630	chr11:59041516-59041560	NM_001130408:126	Arf1	INSIDE	0.644	3.317	1943.21	6445.09	2.137	1591.19	3399.69
A_68_P30476328	chr15:98593484-98593528	NM_007478:43	Arf3	INSIDE	0.469	3.895	4413.21	17187.39	1.825	3374.57	6159.61
A_68_P30476329	chr15:98593570-98593614	NM_007478:-43	Arf3	PROMOTER	0.561	0.476	3276.38	1558.73	0.267	2555.31	681.40
A_68_P30476331	chr15:98593814-98593858	NM_007478:-287	Arf3	PROMOTER	0.666	0.219	2306.22	505.29	0.146	1666.52	243.24
A_68_P30476322	chr15:98592841-98592885	NM_007478:687	Arf3	INSIDE	0.532	0.741	5681.86	4209.47	0.394	4013.30	1581.73
A_68_P21907446	chr2:180702259-180702303	NM_001177706:276	Arfgap1	INSIDE	0.583	0.467	1111.96	519.39	0.272	890.84	242.71
A_68_P30387839	chr15:83180363-83180407	NM_025445:293	Arfgap3	INSIDE	0.371	12.151	768.08	9332.71	4.513	680.10	3069.49
A_68_P20026600	chr1:10222313-10222357	NM_001102430:417	Arfgef1	INSIDE	0.578	3.869	1447.79	5601.42	2.237	1195.71	2675.03
A_68_P22291859	chr3:84386912-84386956	NM_001081093:-387	Arfip1	PROMOTER	0.47	0.545	888.59	484.14	0.256	680.89	174.47
A_68_P25637835	chr8:8689975-8690019	NM_176849:541	Arglu1	INSIDE	0.5	0.287	2350.74	675.53	0.144	1796.67	258.10
A_68_P25637837	chr8:8690176-8690220	NM_176849:339	Arglu1	INSIDE	0.639	3.949	511.74	2020.94	2.523	505.08	1274.31
A_68_P21423815	chr2:91490335-91490379	NM_146124:-3008	Arhgap1	PROMOTER	0.301	0.627	1125.46	705.27	0.188	930.72	175.27
A_68_P25983697	chr8:80041774-80041818	NM_030113:10	Arhgap10	INSIDE	0.244	6.825	10680.12	72889.35	1.665	8987.47	14968.04
A_68_P25983699	chr8:80042123-80042167	NM_030113:-338	Arhgap10	PROMOTER	0.421	0.470	1102.93	518.40	0.198	885.19	175.03
A_68_P25983698	chr8:80041949-80041993	NM_030113:-164	Arhgap10	PROMOTER	0.432	13.077	2952.21	38606.45	5.651	2401.90	13572.75
A_68_P21537143	chr2:113688822-113688866	NM_181416:-26	Arhgap11a	PROMOTER	0.536	4.018	2017.21	8104.99	2.153	1771.08	3813.04
A_68_P28050583	chr11:97301455-97301499	NM_021493:-9997	Arhgap23	PROMOTER	0.418	5.036	1199.34	6039.86	2.107	1042.70	2197.36
A_68_P28050940	chr11:97361543-97361587	NM_021493:50091	Arhgap23	INSIDE	0.56	3.426	1270.93	4354.73	1.92	1005.57	1930.28
A_68_P31315914	chr17:68353306-68353350	NM_172964:120	Arhgap28	INSIDE	0.321	10.987	2645.30	29062.82	3.524	2225.79	7844.21
A_68_P25032017	chr7:31319995-31320040	NM_178252:11	Arhgap33	INSIDE	0.263	25.281	21195.91	535863.30	6.642	22042.91	146398.80
A_68_P25032020	chr7:31320429-31320474	NM_178252:-423	Arhgap33	PROMOTER	0.393	0.246	2556.07	629.10	0.097	1794.29	173.41
A_68_P25032021	chr7:31320496-31320540	NM_178252:-490	Arhgap33	PROMOTER	0.55	11.317	3321.09	37585.47	6.223	2855.13	17766.80
A_68_P30350779	chr15:76647508-76647552	NM_001168288:1070	Arhgap39	INSIDE	0.26	8.324	1341.11	11163.26	2.168	1294.99	2807.69
A_68_P30350785	chr15:76648219-76648263	NM_001168288:360	Arhgap39	INSIDE	0.63	0.602	6059.45	3645.39	0.379	4561.18	1729.75
A_68_P30350786	chr15:76648287-76648331	NM_001168288:292	Arhgap39	INSIDE	0.528	0.609	1358.38	827.65	0.322	1217.66	391.91
A_68_P30350790	chr15:76648746-76648790	NM_001168288:-168	Arhgap39	PROMOTER	0.387	4.367	1548.20	6760.46	1.688	1303.95	2201.54
A_68_P26295538	chr9:9239294-9239338	NM_027823:-303	Arhgap42	PROMOTER	0.212	6.750	3709.82	25040.16	1.432	2910.54	4169.03
A_68_P27869724	chr11:64976171-64976215	NM_001099288:271	Arhgap44	INSIDE	0.666	3.236	1711.84	5539.12	2.156	1463.02	3154.03
A_68_P27535286	chr10:126763506-126763550	NM_146011:2746	Arhgap9	INSIDE	0.359	0.558	1171.88	653.37	0.2	882.94	176.86
A_68_P28185441	chr11:120442461-120442505	NM_133796:452	Arhgdia	INSIDE	0.666	2.890	3038.39	8781.06	1.925	2472.44	4759.83
A_68_P31108897	chr17:26337756-26337800	NM_008113:517	Arhgdig	INSIDE	0.195	16.631	3347.14	55664.70	3.251	6027.07	19591.31
A_68_P31108899	chr17:26338048-26338092	NM_008113:225	Arhgdig	INSIDE	0.388	6.810	1919.25	13069.63	2.643	1723.23	4555.21
A_68_P25677405	chr8:14911386-14911430	NM_001037736:-308	Arhgef10	PROMOTER	0.486	0.254	2039.63	517.16	0.123	1362.15	168.01
A_68_P26459688	chr9:42913910-42913954	NM_027144:-131	Arhgef12	PROMOTER	0.199	10.349	682.14	7059.17	2.058	771.40	1587.72
A_68_P23430516	chr4:153673821-153673865	NM_001112744:162	Arhgef16	INSIDE	0.368	9.165	3243.20	29723.34	3.372	2526.48	8519.77
A_68_P23430520	chr4:153674348-153674392	NM_001112744:-366	Arhgef16	PROMOTER	0.478	13.674	3399.79	46488.63	6.533	3173.06	20728.95
A_68_P25370481	chr7:108079952-108079996	NM_001081116:701	Arhgef17	INSIDE	0.464	5.471	725.01	3966.48	2.541	656.19	1667.06

ProbeName	Target position of probe on CpG island microarray	TargetID	GeneSymbol	CpG island Description	Ratio of relative methylation (TiO_2-NP/Vehicle)	Sham group			TiO_2-H group		
						Relative methylation (Cy5/Cy3)	Cy3 signal (Input)	Cy5 signal (MeDIP)	Relative methylation (Cy5/Cy3)	Cy3 signal (Input)	Cy5 signal (MeDIP)
A_68_P25370486	chr7:108080545-108080589	NM_001081116:109	Arhgef17	INSIDE	0.299	2.091	1908.97	3990.73	0.624	1614.52	1008.22
A_68_P23362920	chr4:140804003-140804047	NM_172520:5226	Arhgef19	INSIDE	0.553	9.774	3724.69	36405.27	5.401	2920.36	15771.75
A_68_P23362916	chr4:140803523-140803567	NM_172520:4746	Arhgef19	INSIDE	0.596	0.439	3251.47	1428.68	0.262	2186.12	572.16
A_68_P22314836	chr3:88425692-88425736	NM_001198912:399	Arhgef2	INSIDE	0.131	35.559	2662.05	94661.16	4.652	2101.99	9779.14
A_68_P22186543	chr3:62143850-62143894	NM_001081295:1174	Arhgef26	INSIDE	0.495	3.167	2623.35	8307.41	1.568	2025.90	3175.60
A_68_P29486550	chr14:28149011-28149055	NM_027871:97808	Arhgef3	INSIDE	0.349	1.682	1690.47	2842.75	0.587	1406.43	825.48
A_68_P29604086	chr14:52604738-52604782	NM_001145921:253	Arhgef40	INSIDE	0.457	0.190	2595.39	492.49	0.087	1979.71	171.49
A_68_P25657174	chr8:11728216-11728260	NM_001113517:134	Arhgef7	INSIDE	0.433	10.392	2045.82	21261.04	4.499	1743.99	7845.43
A_68_P25657178	chr8:11728695-11728739	NM_001113517:612	Arhgef7	INSIDE	0.506	9.351	1875.82	17541.38	4.734	1526.20	7224.88
A_68_P25657382	chr8:11758359-11758403	NM_001113518:51	Arhgef7	INSIDE	0.644	0.351	1726.43	606.56	0.226	1432.94	324.29
A_68_P25657384	chr8:11758629-11758673	NM_001113518:321	Arhgef7	INSIDE	0.645	0.727	2060.47	1497.35	0.469	1664.22	780.17
A_68_P23319341	chr4:133308432-133308476	NM_001080819:1072	Arid1a	INSIDE	0.401	4.681	1404.46	6574.22	1.876	1251.11	2347.29
A_68_P23319375	chr4:133312530-133312574	NM_001080819:-3026	Arid1a	PROMOTER	0.559	0.446	1466.38	654.50	0.249	1211.29	302.11
A_68_P31013736	chr17:4994894-4994938	NM_001085355:-157	Arid1b	PROMOTER	0.255	22.313	6116.74	136485.30	5.701	5500.12	31353.56
A_68_P31013735	chr17:4994646-4994690	NM_001085355:-405	Arid1b	PROMOTER	0.315	13.975	911.42	12736.74	4.396	798.90	3511.84
A_68_P31016358	chr17:5342260-5342304	NM_001085355:347209	Arid1b	INSIDE	0.56	5.404	1696.71	9168.42	3.023	1031.12	3117.50
A_68_P31013745	chr17:4996367-4996411	NM_001085355:1315	Arid1b	INSIDE	0.505	3.108	1066.15	3313.62	1.569	899.88	1411.78
A_68_P22864042	chr4:41678377-41678421	NM_001017362:-224	Arid3c	PROMOTER	0.105	2.389	2605.58	6224.94	0.25	1857.86	464.71
A_68_P28836964	chr13:14157071-14157115	NM_194262:1034	Arid4b	INSIDE	0.663	3.812	925.47	3527.46	2.527	791.59	2000.25
A_68_P26553629	chr9:59333798-59333842	NM_019927:361	Arih1	INSIDE	0.365	0.294	2326.04	682.99	0.107	1600.80	171.46
A_68_P29050149	chr13:54676413-54676457	NM_019968:61	Arl10	INSIDE	0.509	0.290	2180.07	632.85	0.148	1668.16	246.31
A_68_P29342439	chr13:114584551-114584595	NM_172595:-143	Arl15	PROMOTER	0.161	37.695	9408.92	354672.20	6.072	9408.06	57126.91
A_68_P29342438	chr13:114584476-114584520	NM_172595:-217	Arl15	PROMOTER	0.537	0.336	1315.30	441.29	0.18	1089.16	196.23
A_68_P29342441	chr13:114584714-114584758	NM_172595:21	Arl15	INSIDE	0.308	0.629	1776.72	1118.34	0.194	1466.85	284.68
A_68_P29342444	chr13:114585071-114585115	NM_172595:377	Arl15	INSIDE	0.531	2.619	3632.87	9514.84	1.39	2881.52	4004.09
A_68_P31932931	chr19:6140543-6140587	NM_019722:573	Arl2	INSIDE	0.561	0.561	844.81	473.74	0.314	667.24	209.79
A_68_P32144718	chr19:46647649-46647693	NM_019718:-95	Arl3	DIVERGENT_PROMOTER	0.123	34.433	2585.99	89042.10	4.241	2281.63	9675.89
A_68_P21219425	chr2:52279775-52279819	NM_182994:598	Arl5a	INSIDE	0.409	0.455	1030.59	468.59	0.186	950.63	176.65
A_68_P21219430	chr2:52280336-52280380	NM_182994:36	Arl5a	INSIDE	0.56	3.729	484.33	1805.88	2.087	422.00	880.65
A_68_P21023862	chr2:14976924-14976968	NM_029466:-42	Arl5b	DIVERGENT_PROMOTER	0.431	4.008	1674.97	6712.52	1.725	1405.08	2424.11
A_68_P30796871	chr16:59639170-59639214	NM_019665:-27	Arl6	PROMOTER	0.427	0.710	1141.19	810.49	0.303	1067.59	324.00
A_68_P24059369	chr5:124566689-124566733	NM_144509:594	Arl6ip4	INSIDE	0.631	0.331	1712.96	566.42	0.209	1201.77	250.93
A_68_P24059365	chr5:124566113-124566157	NM_144509:18	Arl6ip4	INSIDE	0.174	1.902	998.46	1899.12	0.332	858.67	284.67
A_68_P21224222	chr2:53050731-53050775	NM_022989:-365	Arl6ip6	DIVERGENT_PROMOTER	0.381	5.790	833.48	4826.10	2.207	653.54	1442.21
A_68_P20637774	chr1:137044071-137044115	NM_026823:682	Arl8a	INSIDE	0.221	23.373	9316.81	217760.70	5.172	11319.39	58544.86
A_68_P20637766	chr1:137043169-137043217	NM_026823:-218	Arl8a	PROMOTER	0.527	0.525	1077.99	565.81	0.277	839.52	232.41
A_68_P24735712	chr6:108733427-108733471	NM_026011:396	Arl8b	INSIDE	0.514	2.695	3105.15	8367.56	1.385	2465.59	3415.02
A_68_P27092475	chr10:41737966-41738010	NM_001034858:200	Armc2	INSIDE	0.489	0.586	1017.69	596.06	0.286	724.81	207.61
A_68_P28153743	chr11:115336962-115337006	NM_177778:-6	Armc7	PROMOTER	0.439	0.601	909.77	547.15	0.264	673.07	177.56
A_68_P25288546	chr7:91558357-91558401	NM_007488:91	Arnt2	INSIDE	0.631	4.215	947.34	3993.04	2.661	886.55	2359.12
A_68_P24164319	chr5:145875509-145875553	NM_023142:406	Arpc1b	INSIDE	0.452	0.353	4586.14	1619.44	0.16	3454.25	551.26
A_68_P24164317	chr5:145875247-145875291	NM_023142:144	Arpc1b	INSIDE	0.478	0.264	5144.17	1359.81	0.126	3476.33	439.15
A_68_P24164318	chr5:145875419-145875463	NM_023142:316	Arpc1b	INSIDE	0.561	0.372	10857.19	4034.47	0.208	8006.06	1669.03
A_68_P24164316	chr5:145875146-145875190	NM_023142:44	Arpc1b	INSIDE	0.518	0.635	794.01	504.28	0.329	729.61	239.83
A_68_P20347398	chr1:74283332-74283376	NM_029711:231	Arpc2	INSIDE	0.347	5.073	4474.25	22698.19	1.762	3529.81	6219.89
A_68_P20347397	chr1:74283180-74283224	NM_029711:79	Arpc2	INSIDE	0.454	0.444	5913.98	2623.32	0.201	5085.62	1024.48

ProbeName	Target position of probe on CpG island microarray	TargetID	GeneSymbol	CpG island Description	Ratio of relative methylation (TiO_2-NP/Vehicle)	Sham group			TiO_2-H group		
						Relative methylation (Cy5/Cy3)	Cy3 signal (Input)	Cy5 signal (MeDIP)	Relative methylation (Cy5/Cy3)	Cy3 signal (Input)	Cy5 signal (MeDIP)
A_68_P24050080	chr5:122842069-122842113	NM_019824:154	Arpc3	INSIDE	0.61	0.457	4659.83	2130.88	0.279	3438.11	959.23
A_68_P22029201	chr3:30498718-30498762	NM_029690:52	Arpm1	INSIDE	0.401	0.562	954.90	536.75	0.226	740.64	167.05
A_68_P27900652	chr11:70246079-70246123	NM_145429:-54	Arrb2	PROMOTER	0.573	5.407	1166.29	6306.29	3.098	910.97	2822.31
A_68_P21068908	chr2:24790503-24790547	NM_001162485:277	Arrdc1	INSIDE	0.206	12.180	3437.25	41864.51	2.507	3046.25	7637.37
A_68_P28118792	chr11:109334732-109334776	NM_028710:67	Arsg	INSIDE	0.546	4.063	2553.89	10376.97	2.219	2094.11	4646.90
A_68_P31754305	chr18:61071638-61071682	NM_001038499:-233	Arsi	PROMOTER	0.646	0.400	2206.38	881.90	0.258	1694.14	437.22
A_68_P23237353	chr4:117599338-117599382	NM_009711:3008	Artn	INSIDE	0.533	3.952	780.59	3084.84	2.105	607.68	1279.33
A_68_P30579184	chr16:18348951-18348995	NM_033474:606	Arvcf	INSIDE	0.515	3.118	3145.91	9807.42	1.605	2339.66	3755.38
A_68_P30579425	chr16:18392661-18392705	NM_033474:44316	Arvcf	INSIDE	0.626	3.474	632.67	2197.84	2.174	554.37	1205.47
A_68_P32533286	chrX:90544628-90544672	NM_007492:12666	Arx	DOWNSTREAM	0.223	17.380	1351.17	23483.81	3.884	2098.17	8150.20
A_68_P32533249	chrX:90540139-90540183	NM_007492:8176	Arx	INSIDE	0.302	6.159	1354.71	8343.20	1.862	1453.43	2706.32
A_68_P32533200	chrX:90534061-90534105	NM_007492:2098	Arx	INSIDE	0.424	44.439	3266.16	145143.40	18.854	3580.27	67501.87
A_68_P32533199	chrX:90533865-90533909	NM_007492:1902	Arx	INSIDE	0.59	3.593	1575.62	5660.86	2.12	2086.58	4422.73
A_68_P32533248	chrX:90540027-90540071	NM_007492:8064	Arx	INSIDE	0.59	2.462	2115.80	5208.13	1.452	2828.26	4107.90
A_68_P25810707	chr8:42459909-42459953	NM_019734:121	Asah1	INSIDE	0.359	1.428	1392.98	1988.59	0.512	1069.63	548.17
A_68_P30281416	chr15:64214267-64214311	NM_010026:193	Asap1	INSIDE	0.507	3.679	1222.24	4497.23	1.865	1026.85	1915.02
A_68_P30281411	chr15:64213769-64213813	NM_010026:691	Asap1	INSIDE	0.587	3.144	991.27	3116.73	1.846	822.59	1518.24
A_68_P30280959	chr15:64144695-64144739	NM_010026:69765	Asap1	INSIDE	0.636	2.791	580.35	1619.51	1.776	635.20	1128.04
A_68_P33008885	chr12:21117800-21117844	NM_001004364:206	Asap2	INSIDE	0.229	17.188	2545.90	43757.60	3.936	2257.04	8883.92
A_68_P23332631	chr4:135762030-135762074	NM_001008232:-227	Asap3	PROMOTER	0.145	1.928	4022.35	7754.17	0.279	2910.57	812.93
A_68_P23332632	chr4:135762182-135762226	NM_001008232:-75	Asap3	PROMOTER	0.151	4.326	16120.08	69727.68	0.652	11733.37	7655.58
A_68_P20443148	chr1:93437540-93437584	NM_023046:-94	Asb1	PROMOTER	0.501	0.500	940.66	469.94	0.25	823.50	206.08
A_68_P20433330	chr1:91889460-91889504	NM_139152:21670	Asb18	INSIDE	0.168	2.456	1092.40	2682.52	0.412	828.99	341.95
A_68_P27681337	chr11:30854282-30854326	NM_023906:-93	Asb3	DIVERGENT_PROMOTER	0.283	25.365	8046.42	204097.50	7.185	7609.80	54673.64
A_68_P21105464	chr2:30683897-30683941	NM_133346:-98	Asb6	PROMOTER	0.383	4.949	2323.15	11496.36	1.894	1998.46	3785.38
A_68_P21105459	chr2:30683485-30683529	NM_133346:314	Asb6	INSIDE	0.611	3.433	993.67	3411.64	2.099	780.50	1638.38
A_68_P21105458	chr2:30683380-30683424	NM_133346:418	Asb6	INSIDE	0.488	0.491	905.46	444.84	0.24	792.63	190.20
A_68_P21105462	chr2:30683743-30683787	NM_133346:56	Asb6	INSIDE	0.656	2.121	2852.54	6050.69	1.392	2224.71	3097.74
A_68_P30473398	chr15:97975961-97976005	NM_001170710:151	Asb8	INSIDE	0.224	7.646	2546.78	19471.75	1.712	2223.96	3807.40
A_68_P30473400	chr15:97976195-97976240	NM_001170710:-84	Asb8	PROMOTER	0.597	0.408	1214.04	495.43	0.244	896.31	218.31
A_68_P30473396	chr15:97975808-97975852	NM_001170710:303	Asb8	INSIDE	0.409	0.588	863.78	508.26	0.24	755.62	181.65
A_68_P27171931	chr10:59465553-59465597	NM_026937:22	Ascc1	INSIDE	0.282	7.689	2104.85	16183.86	2.169	1866.26	4047.93
A_68_P27558727	chr11:4537643-4537687	NM_029291:-131	Ascc2	DIVERGENT_PROMOTER	0.277	10.673	3195.12	34102.66	2.953	3448.02	10183.57
A_68_P27558730	chr11:4537928-4537972	NM_029291:155	Ascc2	INSIDE	0.472	17.408	1415.17	24634.89	8.209	1369.98	11246.63
A_68_P27129526	chr10:50312500-50312544	NM_001146089:48	Ascc3	INSIDE	0.206	29.406	1618.80	47602.77	6.067	1398.94	8487.91
A_68_P27320006	chr10:86956365-86956409	NM_008553:19	Ascl1	INSIDE	0.639	0.418	1195.03	498.99	0.267	976.15	260.52
A_68_P25597935	chr7:150155005-150155050	NM_008554:142	Ascl2	INSIDE	0.57	2.632	1464.25	3854.16	1.5	1175.69	1763.67
A_68_P25597928	chr7:150154114-150154158	NM_008554:1033	Ascl2	INSIDE	0.646	0.623	730.11	455.21	0.403	656.10	264.22
A_68_P27311153	chr10:85391455-85391499	NM_001163614:241	Ascl4	INSIDE	0.425	10.774	1456.97	15697.96	4.578	1348.39	6172.43
A_68_P26017300	chr8:86479980-86480024	NM_024184:410	Asf1b	INSIDE	0.481	0.244	2034.29	497.36	0.118	1499.61	176.39
A_68_P26017297	chr8:86479743-86479787	NM_024184:172	Asf1b	INSIDE	0.605	0.693	3015.12	2090.95	0.419	2342.28	982.31
A_68_P26017299	chr8:86479898-86479942	NM_024184:328	Asf1b	INSIDE	0.511	0.613	1182.23	724.28	0.313	902.86	282.38
A_68_P20231550	chr1:53409540-53409584	NM_133728:-19	Asnsd1	PROMOTER	0.418	6.719	1721.19	11564.59	2.807	1582.05	4440.25
A_68_P20231549	chr1:53409460-53409504	NM_133728:61	Asnsd1	INSIDE	0.463	10.864	1579.45	17158.58	5.031	1329.04	6686.92
A_68_P20231548	chr1:53409367-53409411	NM_133728:155	Asnsd1	INSIDE	0.648	0.322	2192.29	706.62	0.209	1763.07	368.04
A_68_P25503171	chr7:134093152-134093196	NM_001039645:-79	Asphd1	DIVERGENT_PROMOTER	0.189	8.113	2060.39	16716.67	1.532	1465.99	2245.27

ProbeName	Target position of probe on CpG island microarray	TargetID	GeneSymbol	CpG island Description	Ratio of relative methylation (TiO_2-NP/Vehicle)	Sham group			TiO_2-H group		
						Relative methylation (Cy5/Cy3)	Cy3 signal (Input)	Cy5 signal (MeDIP)	Relative methylation (Cy5/Cy3)	Cy3 signal (Input)	Cy5 signal (MeDIP)
A_68_P23991928	chr5:112823693-112823737	NM_028386:-2481	Asphd2	PROMOTER	0.141	29.357	2689.70	78960.65	4.125	2458.65	10143.01
A_68_P21109298	chr2:31325552-31325596	NM_007494:-215	Ass1	PROMOTER	0.59	0.615	1391.22	855.19	0.362	1054.89	382.30
A_68_P20753949	chr1:160293080-160293124	NM_001205204:668	Astn1	INSIDE	0.418	3.382	5789.92	19583.99	1.415	4515.28	6387.81
A_68_P21754297	chr2:153171289-153171333	NM_001039939:-564	Asxl1	PROMOTER	0.309	6.303	5042.30	31779.96	1.949	3913.71	7629.50
A_68_P31549437	chr18:22502988-22503032	NM_001167777:-579	Asxl3	PROMOTER	0.566	2.797	1304.10	3647.69	1.584	1150.97	1822.88
A_68_P30246249	chr15:57966345-57966389	NM_027435:271	Atad2	INSIDE	0.485	16.744	2155.68	36095.73	8.129	1737.41	14123.35
A_68_P28203004	chr12:4924079-4924123	NM_001099628:-58	Atad2b	DIVERGENT_PROMOTER	0.322	13.295	956.30	12714.42	4.287	914.87	3921.77
A_68_P25522112	chr7:137663073-137663117	NM_013799:-41	Ate1	PROMOTER	0.456	0.290	1514.61	439.86	0.132	1275.82	169.01
A_68_P25522114	chr7:137663333-137663377	NM_013799:-301	Ate1	PROMOTER	0.519	0.258	1849.09	476.31	0.134	1342.49	179.45
A_68_P21334154	chr2:73729891-73729935	NM_001025093:773	Atf2	INSIDE	0.202	47.307	12297.10	581737.50	9.562	16240.60	155295.50
A_68_P20936661	chr1:193006674-193006718	NM_007498:516	Atf3	INSIDE	0.414	13.600	4219.52	57387.54	5.634	3382.11	19054.22
A_68_P20936664	chr1:193006949-193006993	NM_007498:242	Atf3	INSIDE	0.602	0.611	947.23	579.00	0.368	832.23	306.47
A_68_P30371087	chr15:80085930-80085974	NM_009716:339	Atf4	INSIDE	0.356	0.223	3113.78	693.61	0.079	2168.61	171.80
A_68_P31155038	chr17:34784397-34784441	NM_017406:294	Atf6b	INSIDE	0.103	181.135	622.77	112806.10	18.667	794.84	14837.48
A_68_P31155039	chr17:34784544-34784588	NM_017406:442	Atf6b	INSIDE	0.463	3.421	2073.56	7093.21	1.583	1697.76	2687.39
A_68_P30498877	chr15:102455880-102455924	NM_146065:-50	Atf7	PROMOTER	0.508	0.657	1093.02	717.61	0.334	878.11	293.07
A_68_P24766862	chr6:114688350-114688394	NM_028835:95230	Atg7	INSIDE	0.307	9.117	2760.25	25166.17	2.803	2371.64	6647.30
A_68_P24766863	chr6:114688424-114688468	NM_028835:95304	Atg7	INSIDE	0.453	4.358	771.37	3361.63	1.975	695.44	1373.61
A_68_P31940129	chr19:7568996-7569040	NM_146091:91	Atl3	INSIDE	0.439	0.473	1013.89	479.77	0.208	862.45	179.24
A_68_P24816773	chr6:124696183-124696227	NM_007881:10301	Atn1	INSIDE	0.598	8.239	1742.74	14358.73	4.931	1473.53	7265.70
A_68_P24816767	chr6:124695514-124695558	NM_007881:10969	Atn1	INSIDE	0.211	7.605	1557.09	11841.54	1.604	1425.49	2286.38
A_68_P24816768	chr6:124695641-124695685	NM_007881:10843	Atn1	INSIDE	0.353	4.055	1784.62	7236.45	1.43	1391.80	1990.71
A_68_P24540594	chr6:72185394-72185438	NM_153778:155	Atoh8	INSIDE	0.63	4.633	1455.47	6743.86	2.919	1126.07	3287.49
A_68_P22055028	chr3:35652851-35652895	NM_029570:-187	Atp11b	PROMOTER	0.361	0.354	2854.94	1011.27	0.128	2163.39	276.48
A_68_P32414051	chrX:57657552-57657596	NM_001001798:-418	Atp11c	PROMOTER	0.318	9.381	1166.03	10938.67	2.979	1137.94	3389.47
A_68_P25947954	chr8:72315394-72315438	NM_133224:355	Atp13a1	INSIDE	0.411	8.619	950.43	8192.09	3.546	819.31	2905.60
A_68_P30641650	chr16:30388204-30388248	NM_001128094:390	Atp13a3	INSIDE	0.543	0.409	2639.83	1079.03	0.222	1938.47	430.59
A_68_P22376195	chr3:101408231-101408275	NM_144900:328	Atp1a1	INSIDE	0.261	15.877	2146.93	34087.12	4.136	2098.90	8681.54
A_68_P22376192	chr3:101407957-101408001	NM_144900:602	Atp1a1	INSIDE	0.451	5.702	657.73	3750.18	2.573	580.21	1492.99
A_68_P25007802	chr7:25790777-25790821	NM_144921:116	Atp1a3	INSIDE	0.49	0.585	1370.48	801.31	0.287	1124.10	322.34
A_68_P24050675	chr5:122951393-122951437	NM_009722:820	Atp2a2	INSIDE	0.198	1.830	1258.56	2303.71	0.362	965.00	349.71
A_68_P27914258	chr11:72775141-72775185	NM_001163336:492	Atp2a3	INSIDE	0.253	8.395	770.61	6469.44	2.127	793.41	1687.42
A_68_P27383821	chr10:98377167-98377211	NM_026482:-597	Atp2b1	PROMOTER	0.651	2.543	857.79	2181.74	1.657	647.67	1073.14
A_68_P24761302	chr6:113698581-113698626	NM_001036684:142767	Atp2b2	INSIDE	0.191	3.042	4408.14	13407.99	0.581	2901.24	1686.99
A_68_P32463053	chrX:70748921-70748965	NM_177236:518	Atp2b3	INSIDE	0.414	3.885	3303.23	12834.40	1.607	3892.93	6256.04
A_68_P26797759	chr9:105397652-105397696	NM_175025:-227	Atp2c1	PROMOTER	0.316	0.244	2994.50	730.33	0.077	2194.94	169.24
A_68_P31849681	chr18:78012952-78012997	NM_007505:468	Atp5a1	INSIDE	0.274	0.291	2558.21	743.17	0.08	2185.85	174.23
A_68_P31849682	chr18:78013054-78013098	NM_007505:570	Atp5a1	INSIDE	0.53	0.577	6724.15	3880.20	0.306	5350.17	1635.64
A_68_P20998751	chr2:10001887-10001931	NM_001112738:229	Atp5c1	INSIDE	0.204	0.562	1953.31	1097.46	0.115	1492.55	171.18
A_68_P21875562	chr2:174289603-174289647	NM_025983:-22	Atp5e	PROMOTER	0.374	7.114	1738.14	12365.53	2.661	1467.91	3905.91
A_68_P21334258	chr2:73748716-73748760	NM_175015:613	Atp5g3	INSIDE	0.567	0.477	2129.32	1015.57	0.27	1681.66	454.65
A_68_P28071366	chr11:100870700-100870746	NM_016920:-46	Atp6v0a1	PROMOTER	0.465	11.907	885.96	10549.42	5.54	836.99	4636.54
A_68_P28071367	chr11:100870778-100870822	NM_016920:32	Atp6v0a1	INSIDE	0.371	0.592	877.38	519.75	0.22	783.33	172.24
A_68_P23237067	chr4:117559785-117559829	NM_033617:128	Atp6v0b	INSIDE	0.584	0.122	4283.81	523.06	0.071	2633.95	187.83
A_68_P31095796	chr17:24306345-24306389	NR_037854:-26	Atp6v0c-ps2	PROMOTER	0.315	0.283	2529.62	715.47	0.089	1876.49	167.21
A_68_P31095792	chr17:24305949-24305993	NR_037854:370	Atp6v0c-ps2	INSIDE	0.664	0.594	10291.75	6118.01	0.395	6619.88	2612.91

ProbeName	Target position of probe on CpG island microarray	TargetID	GeneSymbol	CpG island Description	Ratio of relative methylation (TiO_2-NP/Vehicle)	Sham group			TiO_2-H group		
						Relative methylation (Cy5/Cy3)	Cy3 signal (Input)	Cy5 signal (MeDIP)	Relative methylation (Cy5/Cy3)	Cy3 signal (Input)	Cy5 signal (MeDIP)
A_68_P31095793	chr17:24306024-24306068	NR_037854:294	Atp6v0c-ps2	INSIDE	0.429	0.635	4908.54	3115.17	0.272	3174.62	863.84
A_68_P26134609	chr8:108089863-108089907	NM_013477:56	Atp6v0d1	INSIDE	0.582	2.922	3761.11	10990.97	1.699	3063.78	5206.44
A_68_P26134611	chr8:108090115-108090160	NM_013477:-197	Atp6v0d1	PROMOTER	0.621	0.264	2188.98	578.96	0.164	1461.52	240.24
A_68_P31111775	chr17:26813633-26813677	NM_025272:314	Atp6v0e	INSIDE	0.257	0.280	3044.64	852.70	0.072	2334.10	168.28
A_68_P30150544	chr15:38591583-38591627	NM_025494:-54	Atp6v1c1	PROMOTER	0.35	8.199	1552.06	12726.03	2.869	1442.73	4139.86
A_68_P30150543	chr15:38591462-38591506	NM_025494:-174	Atp6v1c1	PROMOTER	0.539	6.450	383.67	2474.74	3.476	379.46	1319.02
A_68_P22971150	chr4:63206034-63206078	NM_024173:258	Atp6v1g1	INSIDE	0.363	11.696	2488.16	29102.18	4.243	2253.30	9560.27
A_68_P31158465	chr17:35373569-35373613	NM_023179:50	Atp6v1g2	INSIDE	0.335	0.396	2034.36	804.59	0.133	1304.60	173.03
A_68_P31158463	chr17:35373372-35373416	NM_023179:-146	Atp6v1g2	DIVERGENT_PROMOTER	0.361	4.038	2907.83	11741.85	1.458	2158.07	3146.11
A_68_P23770428	chr5:68238336-68238380	NM_001038999:312	Atp8a1	INSIDE	0.44	5.129	4939.15	25334.33	2.258	4175.99	9431.23
A_68_P23770432	chr5:68238811-68238855	NM_001038999:-162	Atp8a1	PROMOTER	0.569	0.534	2501.39	1335.59	0.304	1894.21	575.47
A_68_P23770429	chr5:68238413-68238457	NM_001038999:236	Atp8a1	INSIDE	0.419	0.637	982.62	625.48	0.267	702.86	187.61
A_68_P31776707	chr18:64820614-64820658	NM_001001488:18	Atp8b1	INSIDE	0.445	5.226	854.92	4467.76	2.326	671.53	1562.29
A_68_P22322686	chr3:89766925-89766969	NM_001081182:484	Atp8b2	INSIDE	0.532	0.125	3819.65	476.67	0.066	2646.49	175.65
A_68_P21843263	chr2:168567207-168567251	NM_015731:72	Atp9a	INSIDE	0.631	3.113	524.70	1633.29	1.964	526.67	1034.62
A_68_P31867752	chr18:81130655-81130699	NM_001201569:121	Atp9b	INSIDE	0.416	6.837	3871.77	26472.11	2.842	3528.77	10029.39
A_68_P26818137	chr9:108976539-108976583	NM_172774:78	Atrip	INSIDE	0.547	0.195	4862.55	947.72	0.107	3251.02	346.52
A_68_P26818136	chr9:108976442-108976486	NM_172774:174	Atrip	INSIDE	0.553	0.376	3491.50	1312.16	0.208	2584.46	537.40
A_68_P21632194	chr2:130732229-130732273	NM_009730:19	Atrn	INSIDE	0.398	11.616	1432.49	16639.77	4.621	1291.28	5966.57
A_68_P32208220	chr19:57685997-57686041	NM_181415:495	Atrnl1	INSIDE	0.537	0.515	917.76	472.58	0.276	691.88	191.19
A_68_P32582134	chrX:103123764-103123808	NM_009530:925	Atrx	INSIDE	0.079	24.687	305.36	7538.42	1.939	409.82	794.62
A_68_P29005205	chr13:46060754-46060798	NM_009124:-431	Atxn1	PROMOTER	0.424	7.608	1221.90	9296.76	3.227	1029.85	3323.52
A_68_P29005211	chr13:46061364-46061408	NM_009124:-1041	Atxn1	PROMOTER	0.505	5.234	3191.72	16705.31	2.644	3111.43	8226.40
A_68_P29005199	chr13:46059825-46059869	NM_009124:499	Atxn1	INSIDE	0.531	5.742	1233.97	7086.02	3.052	1189.34	3629.99
A_68_P29005193	chr13:46058757-46058801	NM_009124:1567	Atxn1	INSIDE	0.541	0.185	5517.58	1020.92	0.1	3982.21	398.59
A_68_P29005200	chr13:46059987-46060031	NM_009124:337	Atxn1	INSIDE	0.584	0.436	2124.88	926.84	0.255	1625.85	414.13
A_68_P29002651	chr13:45663653-45663697	NM_009124:396671	Atxn1	INSIDE	0.27	5.658	3119.13	17647.65	1.528	2455.06	3751.67
A_68_P26158736	chr8:112261757-112261801	NM_001080930:-139	Atxn1l	PROMOTER	0.271	6.085	2011.37	12239.72	1.65	1636.60	2700.16
A_68_P24046040	chr5:122161910-122161954	NM_009125:315	Atxn2	INSIDE	0.485	4.470	1795.27	8024.61	2.169	1471.15	3191.59
A_68_P24046043	chr5:122162421-122162465	NM_009125:825	Atxn2	INSIDE	0.577	3.912	1938.90	7585.64	2.259	1569.75	3546.52
A_68_P24046045	chr5:122162695-122162739	NM_009125:1099	Atxn2	INSIDE	0.639	0.688	2600.89	1789.98	0.44	1941.80	854.28
A_68_P24046049	chr5:122163217-122163261	NM_009125:1621	Atxn2	INSIDE	0.436	1.360	2675.89	3638.37	0.593	2205.75	1307.05
A_68_P25500901	chr7:133645946-133645990	NM_183020:848	Atxn2l	INSIDE	0.209	0.516	3770.21	1946.87	0.108	2657.76	287.23
A_68_P25500905	chr7:133646409-133646453	NM_183020:386	Atxn2l	INSIDE	0.362	7.133	1783.74	12724.24	2.585	1683.88	4352.63
A_68_P25500909	chr7:133646766-133646810	NM_183020:28	Atxn2l	INSIDE	0.536	0.529	2387.50	1262.52	0.283	1843.71	522.50
A_68_P25500897	chr7:133645570-133645614	NM_183020:1224	Atxn2l	INSIDE	0.624	0.637	3532.65	2251.82	0.398	2754.98	1096.48
A_68_P25500900	chr7:133645875-133645919	NM_183020:920	Atxn2l	INSIDE	0.559	0.683	5220.41	3565.58	0.382	3929.17	1499.45
A_68_P29419197	chr14:14844919-14844966	NM_139227:-62	Atxn7	PROMOTER	0.474	5.005	1440.38	7208.47	2.37	1286.85	3049.65
A_68_P22411289	chr3:108013755-108013799	NM_175183:76	Atxn7l2	INSIDE	0.497	0.337	1426.68	480.21	0.167	1028.36	172.12
A_68_P22411290	chr3:108013838-108013882	NM_175183:-8	Atxn7l2	PROMOTER	0.609	4.341	1785.29	7750.60	2.643	1533.78	4053.90
A_68_P22411283	chr3:108013080-108013124	NM_175183:750	Atxn7l2	INSIDE	0.488	0.710	3915.59	2781.00	0.347	3033.51	1051.93
A_68_P28078744	chr11:102157408-102157452	NM_001098836:513	Atxn7l3	INSIDE	0.482	3.992	1285.82	5133.28	1.926	1098.84	2116.00
A_68_P23244381	chr4:118905195-118905239	NM_001012400:113	AU022252	INSIDE	0.341	7.196	5963.87	42916.80	2.455	4172.60	10245.31
A_68_P32233080	chrX:5659260-5659304	NM_001033211:1256	AU022751	INSIDE	0.65	2.950	367.53	1084.39	1.918	554.38	1063.30
A_68_P32233086	chrX:5659825-5659869	NM_001033211:692	AU022751	INSIDE	0.618	2.791	432.50	1207.17	1.725	710.91	1226.30
A_68_P32233088	chrX:5660010-5660054	NM_001033211:506	AU022751	INSIDE	0.413	1.720	1959.27	3369.67	0.711	2763.47	1965.04

ProbeName	Target position of probe on CpG island microarray	TargetID	GeneSymbol	CpG island Description	Ratio of relative methylation (TiO_2-NP/Vehicle)	Sham group			TiO_2-H group		
						Relative methylation (Cy5/Cy3)	Cy3 signal (Input)	Cy5 signal (MeDIP)	Relative methylation (Cy5/Cy3)	Cy3 signal (Input)	Cy5 signal (MeDIP)
A_68_P27291409	chr10:81591053-81591097	NM_001163064:317	AU041133	INSIDE	0.55	0.548	1019.00	558.28	0.301	836.15	251.90
A_68_P29040998	chr13:53024981-53025025	NM_016709:44	Auh	INSIDE	0.366	7.955	16756.53	133292.30	2.914	14427.42	42044.39
A_68_P24591144	chr6:83005100-83005144	NM_007517:476	Aup1	INSIDE	0.559	3.523	1580.49	5568.21	1.97	1138.06	2241.94
A_68_P24098215	chr5:131915714-131915758	NM_177047:1102477	Auts2	INSIDE	0.053	47.383	1798.81	85233.36	2.494	1475.66	3679.64
A_68_P24104854	chr5:133019778-133019822	NM_177047:-1587	Auts2	PROMOTER	0.286	0.233	3581.87	834.20	0.067	2717.44	181.01
A_68_P24098738	chr5:132013832-132013876	NM_177047:1004359	Auts2	INSIDE	0.328	8.122	2634.76	21398.53	2.664	2156.11	5744.24
A_68_P24098740	chr5:132014030-132014074	NM_177047:1004161	Auts2	INSIDE	0.448	7.778	5105.99	39714.78	3.486	4330.69	15097.02
A_68_P24098743	chr5:132014457-132014501	NM_177047:1003735	Auts2	INSIDE	0.49	0.548	1448.86	793.56	0.268	1067.07	286.32
A_68_P24104853	chr5:133019620-133019664	NM_177047:-1429	Auts2	PROMOTER	0.611	4.396	1129.31	4964.54	2.685	1152.24	3093.52
A_68_P24098214	chr5:131915626-131915670	NM_177047:1102565	Auts2	INSIDE	0.399	3.968	1830.72	7264.80	1.582	1339.02	2117.75
A_68_P24476446	chr6:56664815-56664859	NM_030235:-62	Avl9	PROMOTER	0.471	6.076	2611.66	15868.15	2.862	2212.44	6331.94
A_68_P27508736	chr10:121886005-121886049	NM_016847:472	Avpr1a	INSIDE	0.273	0.279	4659.60	1299.59	0.076	3733.47	284.02
A_68_P28752162	chr12:113974838-113974882	NM_001024602:14476	AW555464	INSIDE	0.129	13.505	3955.96	53424.11	1.74	3086.53	5370.93
A_68_P28752038	chr12:113960892-113960936	NM_001024602:530	AW555464	INSIDE	0.223	8.447	1419.36	11988.72	1.883	1206.51	2272.40
A_68_P28752030	chr12:113959942-113959986	NM_001024602:-420	AW555464	PROMOTER	0.304	15.851	1534.21	24318.79	4.82	1235.53	5955.18
A_68_P28752186	chr12:113977208-113977252	NM_001024602:16846	AW555464	INSIDE	0.481	9.407	3623.44	34085.04	4.525	2945.22	13326.19
A_68_P28752032	chr12:113960100-113960144	NM_001024602:-262	AW555464	PROMOTER	0.65	0.659	1456.27	959.17	0.428	1092.33	467.60
A_68_P31108560	chr17:26276183-26276227	NM_001159598:574	Axin1	INSIDE	0.475	0.692	7689.59	5319.69	0.329	5339.05	1754.11
A_68_P28115686	chr11:108782547-108782591	NM_015732:906	Axin2	INSIDE	0.19	22.708	1588.12	36062.70	4.319	1440.30	6221.14
A_68_P28115682	chr11:108781991-108782035	NM_015732:350	Axin2	INSIDE	0.572	0.329	3076.15	1011.55	0.188	2493.80	469.44
A_68_P28115679	chr11:108781544-108781588	NM_015732:-96	Axin2	PROMOTER	0.589	0.474	2420.90	1148.46	0.279	1965.85	548.93
A_68_P26528581	chr9:54798811-54798855	NM_145229:-71	AY074887	PROMOTER	0.335	7.842	478.06	3748.69	2.626	431.48	1132.88
A_68_P28182256	chr11:119948049-119948093	NM_009734:71	Azi1	INSIDE	0.659	2.833	547.64	1551.37	1.868	480.92	898.17
A_68_P26864195	chr9:117949694-117949738	NM_001048146:100	Azi2	INSIDE	0.263	46.764	2173.79	101654.80	12.314	1867.53	22996.84
A_68_P30149946	chr15:38448361-38448405	NM_001102458:639	Azin1	INSIDE	0.303	5.802	3316.45	19241.60	1.759	2747.51	4831.67
A_68_P30149944	chr15:38448175-38448219	NM_001102458:825	Azin1	INSIDE	0.469	0.421	7390.78	3112.53	0.198	5531.47	1092.93
A_68_P32573854	chrX:100810244-100810288	NR_028381:8827	B230206F22Rik	INSIDE	0.263	8.885	4836.62	42971.56	2.334	6256.06	14603.13
A_68_P32573868	chrX:100812194-100812238	NR_028381:6877	B230206F22Rik	INSIDE	0.569	3.742	633.30	2369.51	2.128	699.60	1488.72
A_68_P25588374	chr7:148547481-148547530	NR_033532:3504	B230206H07Rik	INSIDE	0.112	1.698	2490.66	4230.15	0.191	1780.08	339.71
A_68_P21073310	chr2:25463379-25463423	NM_001024616:566	B230208H17Rik	INSIDE	0.352	0.279	2441.92	682.01	0.098	1708.36	167.95
A_68_P21073314	chr2:25464001-25464045	NM_001024616:-56	B230208H17Rik	PROMOTER	0.34	2.067	2755.66	5695.23	0.702	2075.74	1457.82
A_68_P29056514	chr13:55794315-55794359	NM_181278:-148	B230219D22Rik	PROMOTER	0.257	7.240	1083.20	7842.63	1.858	910.41	1691.25
A_68_P24593643	chr6:83406064-83406108	NR_028382:14338	B230319C09Rik	DOWNSTREAM	0.582	3.578	1660.44	5941.60	2.082	1650.20	3436.51
A_68_P24593642	chr6:83405946-83405990	NR_028382:14220	B230319C09Rik	DOWNSTREAM	0.482	3.706	848.42	3144.11	1.785	739.71	1320.24
A_68_P23364702	chr4:141102564-141102608	NR_030695:510	B330016D10Rik	INSIDE	0.45	9.960	2237.93	22290.21	4.484	1798.09	8063.37
A_68_P22222029	chr3:69402614-69402658	NM_020026:147	B3galnt1	INSIDE	0.438	4.547	1804.13	8203.67	1.991	1549.99	3086.55
A_68_P28836396	chr13:14046811-14046855	NM_178640:-108	B3galnt2	PROMOTER	0.437	4.046	1866.18	7551.35	1.77	1539.98	2726.16
A_68_P28836398	chr13:14047085-14047129	NM_178640:166	B3galnt2	INSIDE	0.478	0.744	4100.82	3049.56	0.356	3099.74	1102.10
A_68_P31151211	chr17:34088231-34088275	NM_019420:181	B3galt4	INSIDE	0.406	3.665	3400.39	12461.04	1.488	2295.72	3415.39
A_68_P24186507	chr5:150480724-150480768	NM_001081204:-85	B3galtl	PROMOTER	0.549	3.463	580.45	2009.84	1.902	475.71	904.69
A_68_P27643051	chr11:22759336-22759380	NM_016888:377	B3gnt2	INSIDE	0.417	0.553	7344.89	4062.99	0.231	5140.40	1185.00
A_68_P27643047	chr11:22758992-22759036	NM_016888:721	B3gnt2	INSIDE	0.556	4.321	1530.01	6611.04	2.401	1336.75	3210.03
A_68_P25958365	chr8:74217508-74217552	NM_028189:8169	B3gnt3	INSIDE	0.251	1.983	2252.69	4468.17	0.497	1755.56	872.98
A_68_P25958358	chr8:74216704-74216748	NM_028189:8973	B3gnt3	INSIDE	0.525	0.232	2448.81	567.72	0.122	1518.48	184.70
A_68_P24056046	chr5:123958494-123958538	NM_198611:-1952	B3gnt4	PROMOTER	0.416	0.644	3478.61	2240.27	0.268	2608.74	699.50
A_68_P25354429	chr7:105342798-105342842	NM_001081167:5165	B3gnt6	INSIDE	0.636	4.631	704.81	3264.21	2.947	624.01	1839.03

ProbeName	Target position of probe on CpG island microarray	TargetID	GeneSymbol	CpG island Description	Ratio of relative methylation (TiO_2-NP/Vehicle)	Sham group			TiO_2-H group		
						Relative methylation (Cy5/Cy3)	Cy3 signal (Input)	Cy5 signal (MeDIP)	Relative methylation (Cy5/Cy3)	Cy3 signal (Input)	Cy5 signal (MeDIP)
A_68_P26132519	chr8:107778394-107778438	NM_178879:635	B3gnt9-ps	INSIDE	0.386	4.725	1789.06	8452.92	1.824	1626.54	2966.77
A_68_P26132522	chr8:107778665-107778709	NM_178879:365	B3gnt9-ps	INSIDE	0.637	0.430	1449.09	622.79	0.274	976.80	267.36
A_68_P28191921	chr11:121534336-121534380	NM_178664:107	B3gntl1	INSIDE	0.508	3.595	857.20	3081.41	1.825	780.56	1424.43
A_68_P31791364	chr18:67503647-67503691	NR_033214:89	B430212C06Rik	INSIDE	0.398	0.392	4897.33	1917.84	0.156	3679.70	573.74
A_68_P27534363	chr10:126607918-126607962	NM_027739:5054	B4galnt1	INSIDE	0.347	0.546	2803.69	1531.60	0.19	2102.15	398.72
A_68_P27534326	chr10:126603018-126603062	NM_027739:154	B4galnt1	INSIDE	0.653	3.690	1179.91	4353.36	2.409	965.39	2325.89
A_68_P28041452	chr11:95775809-95775854	NM_008081:354	B4galnt2	INSIDE	0.25	15.725	3675.93	57804.53	3.927	3139.59	12327.96
A_68_P28041223	chr11:95720113-95720157	NM_008081:56051	B4galnt2	DOWNSTREAM	0.39	0.352	3438.00	1210.91	0.137	2486.33	341.67
A_68_P24797816	chr6:120244799-120244843	NM_198884:-243	B4galnt3	PROMOTER	0.331	10.373	3441.37	35696.48	3.429	2922.85	10023.60
A_68_P24797811	chr6:120244232-120244276	NM_198884:323	B4galnt3	INSIDE	0.408	5.565	1204.19	6700.83	2.269	1158.73	2628.79
A_68_P24797812	chr6:120244301-120244345	NM_198884:255	B4galnt3	INSIDE	0.637	2.307	1710.28	3946.33	1.469	1524.83	2239.57
A_68_P25586433	chr7:148246489-148246533	NM_177897:-662	B4galnt4	PROMOTER	0.464	0.294	2169.18	637.08	0.136	1447.49	197.26
A_68_P25586434	chr7:148246645-148246689	NM_177897:-506	B4galnt4	PROMOTER	0.66	0.423	4576.17	1935.85	0.279	3017.65	842.56
A_68_P22859202	chr4:40801217-40801261	NM_022305:-207	B4galt1	PROMOTER	0.502	0.297	5386.87	1602.54	0.149	4142.84	618.66
A_68_P23237029	chr4:117555567-117555611	NM_017377:486	B4galt2	INSIDE	0.521	0.461	6366.69	2932.99	0.24	4407.64	1058.21
A_68_P20827009	chr1:173200884-173200928	NM_020579:448	B4galt3	INSIDE	0.276	18.115	1332.32	24135.45	4.999	1193.33	5965.06
A_68_P21835089	chr2:167174904-167174948	NM_019835:-248	B4galt5	PROMOTER	0.376	0.571	7525.16	4296.28	0.215	5119.97	1100.25
A_68_P21835090	chr2:167174992-167175036	NM_019835:-336	B4galt5	PROMOTER	0.383	0.675	7817.65	5277.49	0.258	5726.91	1480.14
A_68_P24248435	chr6:13627675-13627719	NM_175312:270	B630005N14Rik	INSIDE	0.295	0.279	3590.64	1002.97	0.083	2590.80	213.76
A_68_P24248434	chr6:13627597-13627641	NM_175312:348	B630005N14Rik	INSIDE	0.427	1.418	1280.71	1816.45	0.605	1148.24	695.23
A_68_P23440042	chr4:155069270-155069314	NM_178699:842	B930041F14Rik	INSIDE	0.369	4.918	2841.18	13971.86	1.815	2384.11	4328.15
A_68_P23440041	chr4:155069180-155069224	NM_178699:752	B930041F14Rik	INSIDE	0.384	0.494	2258.73	1116.25	0.19	1712.90	324.66
A_68_P24142834	chr5:141180937-141180981	NM_172724:-18	Baat1	DIVERGENT_PROMOTER	0.118	12.856	2479.60	31877.21	1.513	1798.32	2720.70
A_68_P26476278	chr9:45646743-45646787	NM_001145947:153	Bace1	INSIDE	0.57	0.567	1682.00	953.36	0.323	1427.69	461.35
A_68_P26476280	chr9:45646906-45646950	NM_001145947:317	Bace1	INSIDE	0.616	0.646	1611.21	1041.16	0.398	1266.32	504.31
A_68_P30941181	chr16:87699207-87699251	NM_007520:30	Bach1	INSIDE	0.288	1.794	5475.73	9825.50	0.517	4360.77	2253.25
A_68_P31157816	chr17:35272577-35272621	NM_057171:412	Bag6	INSIDE	0.159	0.316	4832.78	1526.57	0.05	3439.58	172.36
A_68_P28183249	chr11:120098316-120098360	NM_198423:4078	Bahcc1	INSIDE	0.376	8.051	2146.83	17283.80	3.029	1723.72	5221.30
A_68_P28183194	chr11:120092271-120092315	NM_198423:-1968	Bahcc1	PROMOTER	0.44	0.618	914.35	565.02	0.272	701.63	190.59
A_68_P21566912	chr2:118741928-118741972	NM_001045523:14600	Bahd1	INSIDE	0.369	5.952	1242.71	7396.80	2.199	1010.32	2221.34
A_68_P21566791	chr2:118727169-118727213	NM_001045523:-160	Bahd1	PROMOTER	0.526	0.652	1388.35	904.84	0.343	1196.72	410.49
A_68_P21566798	chr2:118728254-118728298	NM_001045523:926	Bahd1	INSIDE	0.38	0.687	1871.96	1286.50	0.261	1488.35	388.97
A_68_P30337605	chr15:74348245-74348289	NM_174991:1641	Bai1	INSIDE	0.096	47.138	2003.47	94440.34	4.547	2102.70	9560.64
A_68_P30337697	chr15:74359726-74359770	NM_174991:13123	Bai1	INSIDE	0.161	2.984	3326.11	9924.09	0.481	2190.32	1054.44
A_68_P30337621	chr15:74350207-74350251	NM_174991:3603	Bai1	INSIDE	0.363	5.312	2160.73	11477.17	1.931	1727.88	3336.15
A_68_P30337599	chr15:74347574-74347618	NM_174991:971	Bai1	INSIDE	0.408	14.366	1210.44	17388.55	5.868	1345.16	7893.11
A_68_P23299543	chr4:129665364-129665408	NM_001199696:3065	Bai2	INSIDE	0.201	16.546	1146.20	18964.52	3.327	912.49	3035.51
A_68_P23299524	chr4:129662900-129662944	NM_001199696:601	Bai2	INSIDE	0.229	2.112	3782.21	7986.49	0.483	2735.90	1321.87
A_68_P23299678	chr4:129683985-129684029	NM_001199696:21685	Bai2	INSIDE	0.441	4.701	883.37	4152.92	2.076	835.80	1734.75
A_68_P23299541	chr4:129665133-129665177	NM_001199696:2833	Bai2	INSIDE	0.462	0.395	1221.95	482.43	0.183	954.94	174.32
A_68_P23299525	chr4:129662999-129663043	NM_001199696:699	Bai2	INSIDE	0.173	1.368	4831.16	6609.35	0.236	3075.09	725.81
A_68_P20101394	chr1:25886869-25886913	NM_175642:-338	Bai3	PROMOTER	0.579	9.219	3037.88	28004.96	5.337	2758.94	14725.10
A_68_P28181194	chr11:119804412-119804456	NM_001037754:29	Baiap2	INSIDE	0.649	0.193	2727.17	525.72	0.125	1778.57	222.65
A_68_P24160788	chr5:145119007-145119051	NM_025833:-47	Baiap2l1	PROMOTER	0.301	6.624	3113.70	20624.33	1.995	2966.50	5918.73
A_68_P24160783	chr5:145118513-145118557	NM_025833:447	Baiap2l1	INSIDE	0.646	0.504	2487.14	1254.62	0.326	1963.71	640.34
A_68_P30365368	chr15:79091566-79091610	NM_177580:24351	Baiap2l2	INSIDE	0.498	0.421	2746.62	1157.54	0.21	2070.93	434.62

ProbeName	Target position of probe on CpG island microarray	TargetID	GeneSymbol	CpG island Description	Ratio of relative methylation (TiO_2-NP/Vehicle)	Sham group			TiO_2-H group		
						Relative methylation (Cy5/Cy3)	Cy3 signal (Input)	Cy5 signal (MeDIP)	Relative methylation (Cy5/Cy3)	Cy3 signal (Input)	Cy5 signal (MeDIP)
A_68_P30365376	chr15:79092452-79092496	NM_177580:23465	Baiap2l2	INSIDE	0.618	2.890	590.12	1705.23	1.784	524.32	935.57
A_68_P31458868	chr18:3507176-3507220	NM_026505:-756	Bambi	PROMOTER	0.131	2.625	1554.33	4080.60	0.345	1188.74	410.25
A_68_P31458876	chr18:3507944-3507988	NM_026505:12	Bambi	INSIDE	0.512	0.137	3694.06	507.34	0.07	2437.79	171.57
A_68_P21586516	chr2:122293842-122293886	NR_027919:-331	Bambi-ps1	PROMOTER	0.616	0.403	1665.19	670.79	0.248	1206.52	299.55
A_68_P21586514	chr2:122293633-122293677	NR_027919:-121	Bambi-ps1	PROMOTER	0.659	0.383	1581.38	605.90	0.253	1257.45	317.65
A_68_P31928281	chr19:5366247-5366291	NM_001038231:79	Banf1	INSIDE	0.611	0.367	1618.80	593.77	0.224	1163.24	260.83
A_68_P31928282	chr19:5366321-5366365	NM_001038231:5	Banf1	INSIDE	0.513	1.413	3688.44	5210.79	0.724	2839.98	2056.72
A_68_P26229920	chr8:124474514-124474558	NM_001110100:104	Banp	INSIDE	0.642	0.641	6585.00	4219.47	0.411	3923.30	1612.75
A_68_P26229918	chr8:124474275-124474319	NM_001110100:-136	Banp	DIVERGENT_PROMOTER	0.331	0.629	1082.70	681.23	0.209	829.53	173.01
A_68_P21094081	chr2:28764046-28764090	NM_019446:7873	Barhl1	INSIDE	0.341	5.030	1899.13	9552.90	1.718	1677.44	2881.46
A_68_P23961301	chr5:106881112-106881156	NM_001005477:6051	Barhl2	DOWNSTREAM	0.317	10.481	1141.24	11960.84	3.326	1013.40	3370.77
A_68_P23961359	chr5:106887678-106887722	NM_001005477:-515	Barhl2	PROMOTER	0.549	4.070	658.87	2681.29	2.233	566.58	1265.21
A_68_P29020699	chr13:48761332-48761376	NM_007526:2950	Barx1	INSIDE	0.492	0.585	1442.31	843.15	0.288	1109.97	319.16
A_68_P25093323	chr7:52721977-52722021	NM_007527:270	Bax	INSIDE	0.225	1.768	1770.41	3130.19	0.398	1353.83	538.60
A_68_P24115158	chr5:135663906-135663950	NM_011714:736	Baz1b	INSIDE	0.286	11.518	853.11	9826.18	3.296	792.83	2612.83
A_68_P27539535	chr10:127533910-127533954	NM_054078:4094	Baz2a	INSIDE	0.499	3.485	1854.08	6461.95	1.74	1586.16	2759.32
A_68_P27539503	chr10:127530080-127530124	NM_054078:264	Baz2a	INSIDE	0.435	1.636	3769.42	6167.96	0.711	2827.87	2011.40
A_68_P24330588	chr6:30124352-30124396	NR_038124:-2248	BB283400	DIVERGENT_PROMOTER	0.34	8.759	1147.56	10051.93	2.982	1030.24	3071.70
A_68_P24330580	chr6:30123352-30123396	NR_038124:-3248	BB283400	DIVERGENT_PROMOTER	0.623	0.457	5725.42	2616.50	0.285	3971.11	1130.45
A_68_P24980722	chr7:16885176-16885220	NM_133234:-9733	Bbc3	PROMOTER	0.585	3.186	1203.87	3835.59	1.863	1080.46	2012.68
A_68_P24980760	chr7:16895312-16895356	NM_133234:403	Bbc3	INSIDE	0.638	0.677	857.59	580.96	0.432	687.40	297.16
A_68_P27448186	chr10:110735503-110735547	NM_027914:-210	Bbs10	PROMOTER	0.224	18.234	5712.67	104165.90	4.078	4889.54	19938.80
A_68_P22062861	chr3:37211563-37211607	NM_001008502:109	Bbs12	INSIDE	0.642	0.595	1960.97	1166.48	0.382	1486.54	567.84
A_68_P26352023	chr9:22280042-22280086	NM_178415:-94	Bbs9	DIVERGENT_PROMOTER	0.624	0.681	2530.15	1722.45	0.425	1873.38	795.39
A_68_P30751459	chr16:50431707-50431751	NM_027444:774	Bbx	INSIDE	0.353	1.669	13305.32	22200.19	0.589	9735.22	5733.29
A_68_P30751458	chr16:50431622-50431666	NM_027444:858	Bbx	INSIDE	0.398	0.433	3913.34	1694.46	0.172	2500.58	431.19
A_68_P30751460	chr16:50431806-50431850	NM_027444:674	Bbx	INSIDE	0.154	1.450	1166.36	1691.38	0.223	947.70	211.15
A_68_P31126996	chr17:29406043-29406087	NM_030561:332	BC004004	INSIDE	0.299	9.927	2296.05	22793.79	2.967	2090.43	6203.03
A_68_P31126995	chr17:29405863-29405907	NM_030561:152	BC004004	INSIDE	0.509	6.144	1422.49	8739.87	3.125	1276.52	3989.19
A_68_P28889015	chr13:24892904-24892948	NM_024473:-599	BC005537	PROMOTER	0.316	9.239	600.96	5552.50	2.92	551.49	1610.62
A_68_P23952640	chr5:104937435-104937479	NM_001166581:86	BC005561	INSIDE	0.462	7.302	1100.67	8037.16	3.375	935.05	3155.77
A_68_P27279428	chr10:79337495-79337539	NM_001170935:-137	BC005764	DIVERGENT_PROMOTER	0.167	10.671	1824.69	19471.38	1.78	1361.15	2422.87
A_68_P26920734	chr10:7500514-7500558	NM_145418:179	BC013529	INSIDE	0.654	2.950	1068.10	3151.26	1.93	918.84	1773.07
A_68_P28788966	chr13:3610442-3610486	NM_134063:-110	BC016423	PROMOTER	0.387	4.172	1517.18	6329.77	1.613	1307.22	2108.51
A_68_P26349677	chr9:21748879-21748923	NM_144935:6183	BC018242	INSIDE	0.414	5.604	3548.85	19889.42	2.319	2877.10	6671.03
A_68_P26349635	chr9:21741759-21741803	NM_144935:-937	BC018242	PROMOTER	0.424	6.287	886.18	5571.76	2.669	737.22	1967.39
A_68_P26349688	chr9:21751953-21751997	NM_144935:9257	BC018242	INSIDE	0.577	0.224	2530.04	567.63	0.129	1844.13	238.55
A_68_P26254623	chr8:128434312-128434356	NM_145608:-15	BC021891	PROMOTER	0.56	0.576	5297.42	3052.78	0.323	3959.48	1277.29
A_68_P28078449	chr11:102110124-102110173	NM_153544:-47	BC030867	PROMOTER	0.276	0.550	1595.41	877.25	0.152	1163.30	176.49
A_68_P30684012	chr16:38089843-38089887	NR_033221:-518	BC031361	PROMOTER	0.481	0.454	1706.41	773.93	0.218	1467.05	319.75
A_68_P20878150	chr1:182781418-182781462	NM_145943:159	BC031781	INSIDE	0.166	2.545	2493.46	6344.99	0.421	1845.73	777.95
A_68_P29977216	chr15:3977197-3977241	NR_028266:188	BC037032	INSIDE	0.622	2.745	1179.63	3237.95	1.708	838.75	1432.27
A_68_P24129540	chr5:138705221-138705265	NM_153161:38	BC037034	INSIDE	0.194	2.184	2632.16	5748.44	0.424	2009.43	851.32
A_68_P24129534	chr5:138704628-138704672	NM_153161:630	BC037034	INSIDE	0.573	0.720	1722.67	1240.12	0.412	1403.80	579.03
A_68_P27855080	chr11:62461500-62461544	NM_198861:503	BC046404	INSIDE	0.564	2.623	2974.03	7801.82	1.479	2314.89	3424.24
A_68_P26229677	chr8:124431978-124432022	NM_001033485:268	BC048644	INSIDE	0.595	2.381	3435.13	8178.74	1.417	2614.98	3705.90

ProbeName	Target position of probe on CpG island microarray	TargetID	GeneSymbol	CpG island Description	Ratio of relative methylation (TiO_2-NP/Vehicle)	Sham group			TiO_2-H group		
						Relative methylation (Cy5/Cy3)	Cy3 signal (Input)	Cy5 signal (MeDIP)	Relative methylation (Cy5/Cy3)	Cy3 signal (Input)	Cy5 signal (MeDIP)
A_68_P20786425	chr1:165924761-165924805	NM_201364:130	BC055324	INSIDE	0.589	0.586	5433.68	3183.44	0.345	4209.85	1451.64
A_68_P24004993	chr5:115061259-115061303	NM_001004180:43021	BC057022	INSIDE	0.207	17.166	8497.55	145868.80	3.552	6800.73	24159.49
A_68_P24004683	chr5:115017800-115017844	NM_001004180:-437	BC057022	PROMOTER	0.382	45.298	2343.61	106160.80	17.302	2205.05	38152.67
A_68_P24004989	chr5:115060757-115060801	NM_001004180:42519	BC057022	INSIDE	0.387	11.454	4400.32	50402.32	4.433	3457.77	15329.18
A_68_P24004682	chr5:115017652-115017696	NM_001004180:-585	BC057022	PROMOTER	0.447	4.681	1668.42	7810.12	2.094	1552.51	3250.28
A_68_P24004680	chr5:115017431-115017475	NM_001004180:-807	BC057022	PROMOTER	0.594	27.753	917.29	25457.41	16.485	1065.24	17560.68
A_68_P24004688	chr5:115018396-115018440	NM_001004180:159	BC057022	INSIDE	0.434	0.581	1063.24	617.67	0.252	862.58	217.47
A_68_P21040965	chr2:18620391-18620435	NM_001001334:-236	BC061194	PROMOTER	0.598	0.436	1162.88	507.06	0.261	881.74	229.98
A_68_P21040894	chr2:18612064-18612108	NM_001001334:-8562	BC061194	PROMOTER	0.505	0.568	973.07	552.84	0.287	783.07	224.81
A_68_P21040929	chr2:18616201-18616245	NM_001001334:-4426	BC061194	PROMOTER	0.294	2.097	1992.00	4177.18	0.618	1879.49	1160.62
A_68_P21040925	chr2:18615733-18615777	NM_001001334:-4894	BC061194	PROMOTER	0.585	0.655	791.35	518.40	0.383	726.06	278.12
A_68_P25616602	chr8:4217183-4217227	NM_207203:108	BC068157	INSIDE	0.09	27.187	1383.92	37625.26	2.448	960.61	2351.82
A_68_P25616526	chr8:4206417-4206461	NM_207203:10874	BC068157	DOWNSTREAM	0.107	0.517	4418.94	2285.29	0.056	3158.05	175.30
A_68_P25616600	chr8:4217027-4217071	NM_207203:264	BC068157	INSIDE	0.305	1.690	4184.56	7069.86	0.516	2991.29	1542.28
A_68_P28332409	chr12:32319733-32319777	NM_001164090:-231	Bcap29	PROMOTER	0.391	26.608	1444.34	38430.43	10.392	1773.12	18426.19
A_68_P32464342	chrX:70961468-70961512	NM_012060:24	Bcap31	INSIDE	0.331	11.268	2852.47	32140.45	3.733	3439.07	12838.77
A_68_P24920738	chr6:144997199-144997243	NM_001024468:112	Bcat1	INSIDE	0.571	3.966	1581.79	6272.61	2.265	1227.79	2780.79
A_68_P25093839	chr7:52825720-52825764	NM_009737:10	Bcat2	INSIDE	0.509	4.302	2320.71	9983.62	2.189	1819.47	3982.95
A_68_P27649728	chr11:24064796-24064840	NM_001159290:84124	Bcl11a	INSIDE	0.467	5.885	1658.22	9759.19	2.746	1480.73	4065.72
A_68_P27649064	chr11:23977567-23977611	NM_001159289:-467	Bcl11a	PROMOTER	0.4	1.469	1498.92	2201.68	0.587	1228.38	720.92
A_68_P28724526	chr12:109155259-109155303	NM_001079883:86344	Bcl11b	INSIDE	0.134	13.541	2208.09	29900.34	1.815	1777.60	3225.74
A_68_P28725240	chr12:109244557-109244601	NM_001079883:-2954	Bcl11b	PROMOTER	0.304	28.894	5702.32	164761.20	8.785	6556.95	57602.73
A_68_P28724517	chr12:109154270-109154314	NM_001079883:87332	Bcl11b	INSIDE	0.314	13.740	1800.36	24737.78	4.317	2096.68	9050.86
A_68_P28724523	chr12:109154940-109154984	NM_001079883:86662	Bcl11b	INSIDE	0.525	4.499	818.88	3683.88	2.362	565.53	1335.84
A_68_P28725239	chr12:109244424-109244468	NM_001079883:-2822	Bcl11b	PROMOTER	0.545	2.697	1819.66	4906.91	1.471	1638.22	2409.29
A_68_P21617370	chr2:127952278-127952322	NM_009754:527	Bcl2l11	INSIDE	0.463	0.506	1402.23	709.89	0.235	1098.17	257.62
A_68_P24800508	chr6:120786336-120786380	NM_153516:111	Bcl2l13	INSIDE	0.341	5.241	2490.94	13054.25	1.789	2069.09	3700.66
A_68_P24995433	chr7:20408093-20408137	NM_033601:-10	Bcl3	PROMOTER	0.272	16.986	695.91	11821.05	4.626	652.18	3016.90
A_68_P30605892	chr16:23988103-23988147	NM_009744:574	Bcl6	INSIDE	0.236	21.348	3670.89	78365.26	5.04	3016.10	15201.24
A_68_P30605853	chr16:23983382-23983426	NM_009744:5294	Bcl6	INSIDE	0.422	0.311	1623.22	505.31	0.131	1346.95	176.79
A_68_P30605897	chr16:23988863-23988907	NM_009744:-186	Bcl6	PROMOTER	0.436	42.875	1831.15	78511.02	18.687	1536.40	28711.02
A_68_P30605889	chr16:23987593-23987637	NM_009744:1084	Bcl6	INSIDE	0.338	1.519	1717.62	2609.25	0.513	1537.60	788.95
A_68_P27899598	chr11:70044019-70044063	NM_007528:-740	Bcl6b	DIVERGENT_PROMOTER	0.543	4.648	787.91	3662.15	2.526	717.00	1810.86
A_68_P27899584	chr11:70042574-70042618	NM_007528:704	Bcl6b	INSIDE	0.599	4.872	729.74	3555.37	2.919	597.46	1744.27
A_68_P24055180	chr5:123794132-123794176	NM_029850:-302	Bcl7a	PROMOTER	0.444	0.220	2835.30	622.39	0.097	1776.89	173.20
A_68_P24055363	chr5:123823490-123823534	NM_029850:29056	Bcl7a	INSIDE	0.641	2.601	2208.81	5745.64	1.666	1665.99	2776.07
A_68_P24115047	chr5:135644102-135644146	NM_009745:-117	Bcl7b	PROMOTER	0.436	0.655	1317.75	862.62	0.286	1106.90	316.09
A_68_P32260338	chrX:11664552-11664596	NM_029510:-6895	Bcor	PROMOTER	0.175	13.702	507.47	6953.55	2.404	529.83	1273.56
A_68_P32260801	chrX:11735963-11736007	NM_001168321:1497	Bcor	INSIDE	0.176	28.192	1961.49	55299.04	4.949	1995.26	9873.84
A_68_P32260816	chrX:11737647-11737691	NM_001168321:-187	Bcor	PROMOTER	0.448	5.502	660.68	3635.00	2.465	830.62	2047.59
A_68_P32260573	chrX:11705501-11705545	NM_001168321:31959	Bcor	INSIDE	0.488	0.442	1320.68	583.76	0.216	1891.81	407.78
A_68_P32260334	chrX:11664151-11664195	NM_029510:-6493	Bcor	PROMOTER	0.452	3.857	1168.79	4508.01	1.745	1374.15	2397.73
A_68_P32260282	chrX:11658399-11658443	NM_029510:-741	Bcor	PROMOTER	0.159	3.929	1806.73	7097.83	0.623	1930.96	1202.52
A_68_P32260673	chrX:11717563-11717607	NM_001168321:19897	Bcor	INSIDE	0.273	6.471	668.30	4324.32	1.764	759.70	1340.06
A_68_P32260345	chrX:11665362-11665406	NM_029510:-7705	Bcor	PROMOTER	0.101	2.884	456.62	1316.67	0.292	587.60	171.59
A_68_P32260274	chrX:11657268-11657312	NM_029510:389	Bcor	INSIDE	0.387	1.843	2820.62	5198.17	0.712	3971.48	2829.17

ProbeName	Target position of probe on CpG island microarray	TargetID	GeneSymbol	CpG island Description	Ratio of relative methylation (TiO_2-NP/Vehicle)	Sham group			TiO_2-H group		
						Relative methylation (Cy5/Cy3)	Cy3 signal (Input)	Cy5 signal (MeDIP)	Relative methylation (Cy5/Cy3)	Cy3 signal (Input)	Cy5 signal (MeDIP)
A_68_P32260336	chrX:11664364-11664408	NM_029510:-6707	Bcor	PROMOTER	0.643	3.485	228.43	796.04	2.241	339.15	760.05
A_68_P32364032	chrX:45694726-45694770	NM_178782:214	Bcorl1	INSIDE	0.237	11.350	1391.46	15792.98	2.695	1560.83	4206.16
A_68_P32364027	chrX:45694166-45694210	NM_178782:-346	Bcorl1	PROMOTER	0.364	11.085	928.86	10296.44	4.032	1026.72	4140.07
A_68_P32364064	chrX:45698666-45698710	NM_178782:4154	Bcorl1	INSIDE	0.413	4.328	1122.12	4856.24	1.788	1687.75	3016.92
A_68_P32364063	chrX:45698568-45698612	NM_178782:4056	Bcorl1	INSIDE	0.482	7.597	2036.22	15468.55	3.665	2832.74	10381.65
A_68_P27254388	chr10:74523771-74523815	NM_001081412:152	Bcr	INSIDE	0.454	5.537	868.10	4807.09	2.512	796.40	2000.30
A_68_P30647296	chr16:31429742-31429786	NM_001122683:926	Bdh1	INSIDE	0.648	0.187	4034.26	755.72	0.121	2742.05	332.80
A_68_P21516613	chr2:109517788-109517832	NM_001048139:1757	Bdnf	INSIDE	0.564	0.382	1271.53	486.07	0.216	907.30	195.57
A_68_P21516744	chr2:109534011-109534055	NM_001048142:313	Bdnf	INSIDE	0.624	0.327	4866.44	1591.55	0.204	3104.13	633.11
A_68_P21516743	chr2:109533914-109533958	NM_001048142:217	Bdnf	INSIDE	0.638	5.213	1557.85	8121.61	3.327	1736.67	5777.05
A_68_P28731662	chr12:110290760-110290805	NM_001163175:15645	Begain	INSIDE	0.118	20.936	1132.63	23712.71	2.476	1118.24	2768.67
A_68_P28731660	chr12:110290621-110290665	NM_001163175:15785	Begain	INSIDE	0.26	12.956	567.92	7357.98	3.364	577.94	1943.96
A_68_P28731667	chr12:110291510-110291554	NM_001163175:14895	Begain	INSIDE	0.56	4.065	2278.81	9264.17	2.276	1868.79	4254.06
A_68_P27100527	chr10:43198488-43198532	NM_199028:-435	Bend3	PROMOTER	0.13	2.982	979.36	2920.49	0.388	797.53	309.83
A_68_P23767821	chr5:67817925-67817969	NM_001164806:1092	Bend4	INSIDE	0.16	0.327	4732.42	1545.14	0.052	3232.17	168.69
A_68_P23767820	chr5:67817841-67817885	NM_001164806:1176	Bend4	INSIDE	0.216	0.578	3608.95	2086.94	0.125	2359.90	295.43
A_68_P23767828	chr5:67819201-67819245	NM_001164806:-184	Bend4	PROMOTER	0.381	0.722	1839.29	1327.68	0.275	1590.27	437.76
A_68_P23767822	chr5:67818022-67818066	NM_001164806:994	Bend4	INSIDE	0.521	2.948	2702.94	7967.28	1.536	2483.77	3815.79
A_68_P20135584	chr1:33964894-33964938	NM_177235:-450	Bend6	PROMOTER	0.515	7.060	1116.02	7878.99	3.638	1174.60	4273.29
A_68_P25585266	chr7:148042623-148042667	NM_018742:-362	Bet1l	DIVERGENT_PROMOTER	0.629	0.642	4076.23	2615.98	0.403	2942.13	1187.03
A_68_P21969040	chr3:17955766-17955810	NM_021560:1464	Bhlhe22	INSIDE	0.206	57.716	4680.70	270149.80	11.891	5112.74	60797.59
A_68_P21969034	chr3:17954954-17954998	NM_021560:652	Bhlhe22	INSIDE	0.588	3.497	787.37	2753.30	2.057	694.22	1428.24
A_68_P21906145	chr2:180511577-180511621	NM_080641:7	Bhlhe23	INSIDE	0.592	4.099	1170.76	4798.68	2.425	1051.80	2550.12
A_68_P24735108	chr6:108610209-108610253	NM_011498:-392	Bhlhe40	PROMOTER	0.255	6.282	958.12	6018.92	1.602	931.24	1491.94
A_68_P24925489	chr6:145814071-145814115	NM_024469:-232	Bhlhe41	PROMOTER	0.536	2.977	2170.86	6463.06	1.595	1797.75	2868.20
A_68_P24800930	chr6:120866663-120866707	NM_007544:154	Bid	INSIDE	0.633	2.207	2437.44	5379.47	1.398	1954.34	2732.17
A_68_P30388927	chr15:83357675-83357720	NM_007546:406	Bik	INSIDE	0.412	0.234	2389.04	559.97	0.096	1811.76	174.78
A_68_P30388923	chr15:83357292-83357336	NM_007546:23	Bik	INSIDE	0.383	0.731	1742.68	1274.18	0.28	1483.70	415.05
A_68_P30388925	chr15:83357441-83357485	NM_007546:171	Bik	INSIDE	0.604	0.646	1155.50	746.57	0.39	909.66	355.22
A_68_P29699202	chr14:70499993-70500037	NM_021328:63	Bin3	INSIDE	0.475	4.030	2028.17	8173.03	1.913	1641.65	3141.13
A_68_P26289571	chr9:7837078-7837122	NM_007465:-1845	Birc2	PROMOTER	0.664	0.507	944.11	478.50	0.337	766.08	257.79
A_68_P28167547	chr11:117710471-117710515	NM_001012273:-58	Birc5	PROMOTER	0.548	9.427	1440.42	13578.71	5.163	1171.72	6049.61
A_68_P31351860	chr17:74928474-74928518	NM_007566:862	Birc6	INSIDE	0.265	0.401	2541.99	1019.90	0.106	1694.28	179.92
A_68_P31351861	chr17:74928573-74928617	NM_007566:960	Birc6	INSIDE	0.43	0.641	2626.09	1682.05	0.276	1976.12	544.87
A_68_P31351852	chr17:74927433-74927479	NM_007566:-178	Birc6	PROMOTER	0.614	0.614	950.76	583.37	0.377	720.46	271.33
A_68_P25267236	chr7:87679497-87679541	NM_007550:366	Blm	INSIDE	0.539	0.533	1066.63	568.71	0.288	842.05	242.20
A_68_P21040878	chr2:18610287-18610336	NM_007552:11667	Bmi1	DOWNSTREAM	0.496	8.487	398.67	3383.50	4.21	360.12	1515.91
A_68_P21040880	chr2:18610461-18610505	NM_007552:11838	Bmi1	DOWNSTREAM	0.574	0.674	1388.86	935.55	0.386	1308.97	505.81
A_68_P21040812	chr2:18602535-18602579	NM_007552:3912	Bmi1	INSIDE	0.434	4.253	996.33	4237.08	1.848	906.62	1675.23
A_68_P21646236	chr2:133380456-133380500	NM_007553:1544	Bmp2	INSIDE	0.638	5.024	2068.20	10390.65	3.205	1686.06	5404.34
A_68_P21646220	chr2:133378597-133378641	NM_007553:-316	Bmp2	PROMOTER	0.64	0.453	2075.40	939.62	0.29	1603.24	464.64
A_68_P21646214	chr2:133377926-133377970	NM_007553:-986	Bmp2	PROMOTER	0.468	5.989	375.20	2247.19	2.802	354.52	993.19
A_68_P21646233	chr2:133380149-133380193	NM_007553:1236	Bmp2	INSIDE	0.637	0.683	1350.18	921.94	0.435	1236.91	538.33
A_68_P23909957	chr5:97427058-97427102	NM_080708:373	Bmp2k	INSIDE	0.405	1.492	1539.59	2297.35	0.604	1297.78	784.12
A_68_P29575957	chr14:47007792-47007836	NM_007554:2460	Bmp4	INSIDE	0.022	8.494	5170.97	43920.79	0.19	3557.01	677.53
A_68_P29575956	chr14:47007694-47007740	NM_007554:2558	Bmp4	INSIDE	0.058	5.298	1121.59	5942.55	0.308	732.98	225.45

ProbeName	Target position of probe on CpG island microarray	TargetID	GeneSymbol	CpG island Description	Ratio of relative methylation (TiO_2-NP/Vehicle)	Sham group			TiO_2-H group		
						Relative methylation (Cy5/Cy3)	Cy3 signal (Input)	Cy5 signal (MeDIP)	Relative methylation (Cy5/Cy3)	Cy3 signal (Input)	Cy5 signal (MeDIP)
A_68_P23262157	chr4:123019781-123019825	NM_007558:244	Bmp8a	INSIDE	0.62	2.786	2262.42	6303.18	1.727	1798.22	3105.00
A_68_P23260769	chr4:122782545-122782589	NM_007559:159	Bmp8b	INSIDE	0.492	0.402	1218.82	489.48	0.198	878.60	173.66
A_68_P23260770	chr4:122782660-122782704	NM_007559:275	Bmp8b	INSIDE	0.386	3.777	2155.10	8139.71	1.457	1651.92	2406.81
A_68_P26356132	chr9:23028173-23028217	NM_028472:675	Bmper	INSIDE	0.637	0.565	1220.61	690.02	0.36	996.58	358.94
A_68_P29530305	chr14:35315556-35315600	NM_009758:154	Bmpr1a	INSIDE	0.365	9.997	2235.91	22352.38	3.652	2080.80	7598.85
A_68_P29530306	chr14:35315823-35315867	NM_009758:-112	Bmpr1a	PROMOTER	0.489	6.165	726.39	4477.99	3.011	703.14	2117.49
A_68_P20265717	chr1:59820840-59820884	NM_007561:-618	Bmpr2	PROMOTER	0.428	0.451	1406.43	633.87	0.193	1044.93	201.38
A_68_P20265713	chr1:59820408-59820452	NM_007561:-1050	Bmpr2	PROMOTER	0.443	0.328	1420.26	466.40	0.145	1181.27	171.80
A_68_P24786758	chr6:118369210-118369254	NM_194339:203	Bms1	INSIDE	0.401	4.261	3238.75	13800.05	1.709	2652.18	4532.44
A_68_P24786762	chr6:118369695-118369739	NM_194339:-281	Bms1	PROMOTER	0.645	0.627	1175.95	737.11	0.404	975.67	394.44
A_68_P24786761	chr6:118369586-118369630	NM_194339:-173	Bms1	PROMOTER	0.442	1.343	2003.93	2691.93	0.594	1661.59	986.21
A_68_P23069979	chr4:84320697-84320741	NM_172870:272	Bnc2	INSIDE	0.572	2.834	918.33	2602.37	1.62	773.63	1253.59
A_68_P31112418	chr17:26918319-26918363	NM_172149:317	Bnip1	INSIDE	0.217	12.851	4364.71	56092.84	2.794	4358.11	12178.19
A_68_P26613990	chr9:69837096-69837140	NM_016787:-189	Bnip2	PROMOTER	0.41	4.243	1642.47	6968.59	1.738	1328.42	2308.37
A_68_P25574804	chr7:146101607-146101651	NM_009760:-439	Bnip3	PROMOTER	0.171	2.925	2988.77	8743.16	0.499	2130.47	1062.66
A_68_P29682512	chr14:67627253-67627297	NM_009761:440	Bnip3l	INSIDE	0.478	6.879	2842.18	19551.47	3.288	2907.74	9560.25
A_68_P25501814	chr7:133839542-133839586	NM_175103:51	Bola2	INSIDE	0.272	0.458	1975.02	905.31	0.125	1391.06	173.59
A_68_P20241452	chr1:55419871-55419915	NM_001113367:-341	Boll	PROMOTER	0.274	1.889	3515.30	6642.09	0.517	2901.79	1500.78
A_68_P28936243	chr13:34129600-34129644	NM_026512:113	Bphl	INSIDE	0.536	3.029	1320.28	3998.73	1.624	1000.91	1625.64
A_68_P31152238	chr17:34259488-34259532	NM_010238:-818	Brd2	PROMOTER	0.051	91.383	1889.28	172647.80	4.678	1624.16	7597.16
A_68_P31152204	chr17:34255264-34255308	NR_037970:1559	Brd2	INSIDE	0.086	2.800	4230.35	11845.81	0.241	2779.17	670.53
A_68_P31152236	chr17:34259233-34259277	NM_010238:-562	Brd2	PROMOTER	0.222	38.606	2811.72	108549.20	8.554	2824.01	24157.37
A_68_P31152205	chr17:34255338-34255382	NR_037970:1485	Brd2	INSIDE	0.368	0.327	2011.88	657.03	0.12	1458.13	175.35
A_68_P31152224	chr17:34257661-34257705	NR_037970:-837	Brd2	PROMOTER	0.383	0.592	2299.28	1360.34	0.226	1346.03	304.81
A_68_P31152235	chr17:34259132-34259176	NM_010238:-462	Brd2	PROMOTER	0.551	5.812	651.04	3784.02	3.203	608.17	1947.68
A_68_P31152218	chr17:34257026-34257071	NR_037970:-203	Brd2	PROMOTER	0.334	1.743	2863.63	4991.91	0.582	2037.95	1185.17
A_68_P21084957	chr2:27331585-27331629	NM_001113573:-413	Brd3	PROMOTER	0.339	0.576	1552.46	894.13	0.195	1182.15	230.67
A_68_P21084948	chr2:27330582-27330626	NM_023336:314	Brd3	INSIDE	0.347	4.152	2118.25	8795.11	1.44	1852.18	2666.56
A_68_P24160214	chr5:145016060-145016104	NM_018772:-11	Bri3	PROMOTER	0.221	14.169	1114.39	15789.74	3.137	1066.83	3347.14
A_68_P24066928	chr5:125921960-125922004	NM_029752:45	Bri3bp	INSIDE	0.365	3.911	1870.63	7316.75	1.429	1618.13	2312.52
A_68_P28453879	chr12:56937597-56937641	NM_001037756:266	Brms1l	INSIDE	0.408	0.168	4203.46	707.48	0.069	3271.85	224.45
A_68_P28453876	chr12:56937237-56937281	NM_001037756:-94	Brms1l	PROMOTER	0.572	0.400	1962.25	784.96	0.229	1543.52	353.38
A_68_P28454005	chr12:56962212-56962256	NM_001037756:24882	Brms1l	INSIDE	0.532	2.981	3203.24	9548.66	1.585	2425.17	3843.04
A_68_P32957256	chr17:8476298-8476342	NM_018819:-357	Brp44l	PROMOTER	0.615	2.902	677.40	1965.83	1.786	633.21	1130.69
A_68_P31124371	chr17:28938550-28938594	NM_001081315:502	Brpf3	INSIDE	0.223	23.787	9729.95	231451.10	5.293	9725.33	51476.29
A_68_P24950090	chr7:4658912-4658956	NM_001003920:16405	Brsk1	INSIDE	0.37	5.176	5220.64	27024.57	1.916	4352.92	8340.51
A_68_P25591880	chr7:149136294-149136338	NM_001009929:326	Brsk2	INSIDE	0.505	15.352	609.09	9350.69	7.759	557.91	4328.78
A_68_P30990992	chr16:96303585-96303629	NM_145125:429	Brwd1	INSIDE	0.411	4.185	2679.07	11212.37	1.72	2033.25	3497.01
A_68_P30990999	chr16:96304407-96304451	NM_145125:-393	Brwd1	PROMOTER	0.589	3.302	5098.08	16833.21	1.946	4056.47	7894.86
A_68_P23296721	chr4:129138758-129138806	NM_133889:-140	Bsdc1	PROMOTER	0.193	0.411	2799.32	1149.94	0.079	2212.35	175.42
A_68_P27278360	chr10:79167439-79167483	NM_001077184:358	Bsg	INSIDE	0.644	0.526	2025.61	1066.37	0.339	1452.92	492.95
A_68_P26813123	chr9:108017642-108017686	NM_007567:75050	Bsn	INSIDE	0.232	7.651	2451.89	18760.47	1.772	1618.66	2868.00
A_68_P26813122	chr9:108017527-108017571	NM_007567:75166	Bsn	INSIDE	0.392	4.448	869.72	3868.96	1.743	612.21	1066.79
A_68_P22964669	chr4:62141433-62141477	NM_138653:354	Bspry	INSIDE	0.537	0.598	1987.89	1188.91	0.321	1472.36	473.16
A_68_P25274224	chr7:88974543-88974587	NM_146193:-247	Btbd1	PROMOTER	0.584	2.807	1421.27	3990.02	1.638	1192.91	1954.36
A_68_P27308088	chr10:84849580-84849624	NM_028709:44	Btbd11	INSIDE	0.363	5.968	505.84	3018.86	2.164	532.70	1152.66

ProbeName	Target position of probe on CpG island microarray	TargetID	GeneSymbol	CpG island Description	Ratio of relative methylation (TiO_2-NP/Vehicle)	Sham group			TiO_2-H group		
						Relative methylation (Cy5/Cy3)	Cy3 signal (Input)	Cy5 signal (MeDIP)	Relative methylation (Cy5/Cy3)	Cy3 signal (Input)	Cy5 signal (MeDIP)
A_68_P25524391	chr7:137910411-137910455	NM_001081038:-7150	Btbd16	PROMOTER	0.412	5.198	1835.29	9539.82	2.142	1445.03	3095.53
A_68_P28149790	chr11:114652964-114653008	NM_028055:4220	Btbd17	INSIDE	0.429	17.782	3081.33	54791.26	7.632	2427.64	18527.63
A_68_P21673348	chr2:138082206-138082250	NM_001025431:-91	Btbd3	PROMOTER	0.666	4.094	1744.24	7141.08	2.729	1611.60	4397.56
A_68_P31134871	chr17:30713349-30713393	NM_027060:-138	Btbd9	PROMOTER	0.334	5.754	1259.58	7247.39	1.92	1113.81	2138.83
A_68_P27371447	chr10:96079236-96079280	NM_007569:-376	Btg1	PROMOTER	0.42	5.816	627.39	3648.73	2.441	596.13	1455.21
A_68_P27371450	chr10:96079522-96079566	NM_007569:-90	Btg1	PROMOTER	0.109	0.735	5465.00	4017.31	0.08	3737.66	300.05
A_68_P30895871	chr16:78377255-78377299	NM_009770:-246	Btg3	PROMOTER	0.251	9.298	909.23	8454.07	2.331	722.63	1684.44
A_68_P28881600	chr13:23556264-23556308	NM_013483:1484	Btn1a1	INSIDE	0.378	7.816	1808.82	14136.84	2.953	1469.41	4339.58
A_68_P32139022	chr19:45616075-45616119	NM_001037758:177873	Btrc	DOWNSTREAM	0.564	4.742	606.55	2876.04	2.673	554.39	1482.12
A_68_P32139025	chr19:45616402-45616446	NM_001037758:178201	Btrc	DOWNSTREAM	0.482	0.634	1497.18	949.51	0.306	1157.24	353.63
A_68_P21564938	chr2:118423956-118424000	NM_009773:32	Bub1b	INSIDE	0.219	0.698	1614.14	1125.97	0.153	1128.93	172.46
A_68_P20258663	chr1:58450520-58450564	NM_025824:563	Bzw1	INSIDE	0.403	6.668	2543.96	16962.58	2.684	2055.39	5516.78
A_68_P32053393	chr19:29597512-29597556	NM_001081319:763	C030046E11Rik	INSIDE	0.395	0.339	1486.88	504.69	0.134	1297.41	173.90
A_68_P20411731	chr1:88256083-88256127	NR_015507:-44	C130036L24Rik	DIVERGENT_PROMOTER	0.315	15.338	3670.81	56302.52	4.83	3259.67	15743.25
A_68_P28596927	chr12:85533988-85534032	NM_001163502:955	C130039O16Rik	INSIDE	0.345	0.313	1970.82	615.89	0.108	1591.11	171.66
A_68_P28596933	chr12:85534608-85534652	NM_001163502:335	C130039O16Rik	INSIDE	0.449	8.761	1564.10	13703.84	3.936	1243.46	4894.59
A_68_P28596932	chr12:85534521-85534565	NM_001163502:423	C130039O16Rik	INSIDE	0.517	9.653	3977.73	38396.10	4.988	3056.07	15242.71
A_68_P29187496	chr13:83860499-83860543	NR_015561:-7190	C130071C03Rik	PROMOTER	0.503	8.251	1181.56	9748.55	4.148	990.02	4106.73
A_68_P24222816	chr6:7794688-7794732	NM_052993:-513	C1galt1	PROMOTER	0.582	3.926	549.15	2156.18	2.284	452.58	1033.60
A_68_P32328720	chrX:35988386-35988430	NM_021550:-88	C1galt1c1	PROMOTER	0.429	3.537	1203.21	4255.94	1.517	1642.40	2491.15
A_68_P28082431	chr11:102807578-102807622	NM_011795:175	C1ql1	INSIDE	0.585	12.097	553.53	6695.87	7.075	401.18	2838.46
A_68_P28082450	chr11:102809915-102809959	NM_011795:-2161	C1ql1	PROMOTER	0.494	3.453	2281.00	7875.28	1.706	1882.37	3211.48
A_68_P28082435	chr11:102808047-102808091	NM_011795:-293	C1ql1	PROMOTER	0.336	4.816	1263.59	6085.87	1.617	1178.83	1905.91
A_68_P20561482	chr1:122238470-122238514	NM_207233:1334	C1ql2	INSIDE	0.639	3.274	2078.14	6803.77	2.091	1698.50	3551.38
A_68_P20561486	chr1:122238913-122238957	NM_207233:1776	C1ql2	INSIDE	0.64	2.521	913.58	2302.76	1.614	780.50	1259.90
A_68_P21013699	chr2:12932692-12932736	NM_153155:-223	C1ql3	PROMOTER	0.237	34.259	1302.72	44630.10	8.115	1735.37	14081.97
A_68_P21013703	chr2:12933075-12933119	NM_153155:-605	C1ql3	PROMOTER	0.552	4.135	643.14	2659.25	2.283	509.30	1162.63
A_68_P30478152	chr15:98918819-98918863	NM_001024702:-681	C1ql4	PROMOTER	0.335	6.034	1212.17	7314.40	2.023	1025.53	2074.77
A_68_P30478144	chr15:98917975-98918019	NM_001024702:163	C1ql4	INSIDE	0.663	14.271	711.81	10157.85	9.465	672.16	6362.29
A_68_P30478150	chr15:98918593-98918637	NM_001024702:-455	C1ql4	PROMOTER	0.365	4.568	1314.17	6003.35	1.666	1424.67	2373.11
A_68_P21419450	chr2:90726161-90726205	NM_026161:240	C1qtnf4	INSIDE	0.327	11.570	2503.08	28960.75	3.786	2009.35	7608.11
A_68_P21419479	chr2:90729985-90730029	NM_026161:4064	C1qtnf4	INSIDE	0.455	28.067	622.41	17469.03	12.776	537.59	6868.19
A_68_P26536114	chr9:56265361-56265405	NM_172924:475	C230081A13Rik	INSIDE	0.444	4.912	2183.57	10726.57	2.179	1916.51	4176.11
A_68_P23351277	chr4:138908735-138908779	NM_001039200:249	C230096C10Rik	INSIDE	0.17	30.146	994.16	29970.21	5.12	891.73	4565.48
A_68_P26600872	chr9:67607645-67607689	NM_001081314:423	C2cd4b	INSIDE	0.537	5.580	1650.69	9211.53	2.995	1477.46	4424.74
A_68_P27277812	chr10:79074932-79074976	NM_198614:1809	C2cd4c	INSIDE	0.38	9.509	547.37	5204.67	3.618	659.00	2384.07
A_68_P22338541	chr3:94168285-94168329	NM_001136117:1941	C2cd4d	INSIDE	0.384	11.368	5078.60	57733.53	4.364	4296.14	18747.69
A_68_P22338534	chr3:94167626-94167670	NM_001136117:1283	C2cd4d	INSIDE	0.419	16.593	1784.18	29604.09	6.956	1838.73	12789.81
A_68_P25022916	chr7:29415707-29415751	NM_175319:306	C330005M16Rik	INSIDE	0.429	4.137	1210.85	5009.24	1.776	984.22	1748.06
A_68_P23585864	chr5:33196991-33197035	NM_194340:-133	C330019G07Rik	DIVERGENT_PROMOTER	0.43	1.397	2315.88	3235.93	0.6	1916.46	1150.33
A_68_P32385501	chrX:50410354-50410398	NR_038184:-9	C430049B03Rik	PROMOTER	0.516	3.883	2016.43	7830.03	2.004	2607.68	5224.75
A_68_P23818500	chr5:77286506-77286550	NM_001163793:16905	C530008M17Rik	INSIDE	0.155	22.025	6150.51	135467.70	3.418	4676.53	15983.63
A_68_P23818501	chr5:77286574-77286618	NM_001163793:16973	C530008M17Rik	INSIDE	0.19	9.317	2590.22	24132.85	1.77	1866.44	3303.01
A_68_P23818499	chr5:77286360-77286404	NM_001163793:16759	C530008M17Rik	INSIDE	0.577	3.543	1854.57	6571.19	2.046	1336.67	2734.18
A_68_P23818506	chr5:77287203-77287247	NM_001163793:17601	C530008M17Rik	INSIDE	0.376	5.047	1202.83	6070.12	1.898	805.69	1529.02
A_68_P24818073	chr6:124947045-124947089	NM_001145927:-1388	C530028O21Rik	PROMOTER	0.588	0.601	3758.75	2259.33	0.353	2893.67	1022.90

ProbeName	Target position of probe on CpG island microarray	TargetID	GeneSymbol	CpG island Description	Ratio of relative methylation (TiO_2-NP/Vehicle)	Sham group			TiO_2-H group		
						Relative methylation (Cy5/Cy3)	Cy3 signal (Input)	Cy5 signal (MeDIP)	Relative methylation (Cy5/Cy3)	Cy3 signal (Input)	Cy5 signal (MeDIP)
A_68_P28153096	chr11:115229081-115229125	NM_175454:-69	C630004H02Rik	PROMOTER	0.245	23.289	4537.44	105670.70	5.702	4202.80	23966.05
A_68_P28153095	chr11:115228977-115229021	NM_175454:35	C630004H02Rik	INSIDE	0.266	9.030	1026.60	9270.40	2.406	1016.81	2446.67
A_68_P28153091	chr11:115228565-115228611	NM_175454:445	C630004H02Rik	INSIDE	0.654	0.313	4449.23	1393.40	0.205	3434.93	703.82
A_68_P23295623	chr4:128900595-128900639	NM_001033189:25071	C77080	INSIDE	0.469	3.747	1107.58	4150.07	1.757	997.33	1752.02
A_68_P32576281	chrX:101395430-101395474	NM_001077354:1004	C77370	INSIDE	0.348	13.531	512.39	6933.33	4.712	590.71	2783.38
A_68_P32576286	chrX:101396172-101396216	NM_001077354:262	C77370	INSIDE	0.351	12.480	6571.38	82013.71	4.381	5761.15	25238.47
A_68_P32576282	chrX:101395577-101395621	NM_001077354:858	C77370	INSIDE	0.656	5.136	1407.38	7228.93	3.371	1682.83	5672.79
A_68_P32576283	chrX:101395692-101395736	NM_001077354:742	C77370	INSIDE	0.665	11.288	4621.00	52161.64	7.502	4579.56	34356.70
A_68_P29009048	chr13:46764994-46765038	NM_001033192:126	C78339	INSIDE	0.663	3.848	1882.86	7245.26	2.551	1612.87	4114.42
A_68_P29638535	chr14:60059777-60059821	NM_026908:-19	Cab39l	DIVERGENT_PROMOTER	0.6	0.400	1356.84	543.16	0.24	1082.58	260.08
A_68_P27257833	chr10:75130195-75130239	NM_172549:96886	Cabin1	INSIDE	0.106	41.894	1344.03	56307.31	4.457	1136.09	5063.76
A_68_P27258519	chr10:75226844-75226888	NM_172549:236	Cabin1	INSIDE	0.42	3.640	1245.52	4533.85	1.529	1169.17	1787.89
A_68_P31497275	chr18:11998719-11998763	NM_001146287:958	Cables1	INSIDE	0.448	6.001	992.84	5958.41	2.69	969.76	2608.45
A_68_P26020780	chr8:87141005-87141050	NM_007578:201765	Cacna1a	INSIDE	0.16	2.635	1961.17	5167.85	0.421	1395.90	587.37
A_68_P26019658	chr8:86939377-86939421	NM_007578:136	Cacna1a	INSIDE	0.436	8.476	1792.24	15191.33	3.699	1527.36	5649.57
A_68_P26019650	chr8:86938371-86938415	NM_007578:-870	Cacna1a	PROMOTER	0.463	4.961	798.21	3960.08	2.297	700.62	1609.64
A_68_P26020866	chr8:87163478-87163522	NM_007578:224238	Cacna1a	INSIDE	0.539	5.259	252.87	1329.87	2.837	209.27	593.67
A_68_P21067473	chr2:24534821-24534865	NM_001042528:83725	Cacna1b	INSIDE	0.427	0.699	1201.97	840.29	0.299	948.82	283.41
A_68_P24791280	chr6:119057361-119057405	NM_001159533:825	Cacna1c	INSIDE	0.409	8.605	2938.89	25289.94	3.515	2258.37	7938.79
A_68_P24791281	chr6:119057478-119057522	NM_001159533:707	Cacna1c	INSIDE	0.527	0.437	2690.74	1175.62	0.23	2248.64	517.94
A_68_P28033148	chr11:94335730-94335774	NM_001112813:-240	Cacna1g	PROMOTER	0.456	0.339	8052.87	2726.92	0.154	5260.55	812.56
A_68_P31103375	chr17:25522729-25522773	NM_001163691:47978	Cacna1h	INSIDE	0.064	33.629	2814.74	94657.34	2.16	1945.45	4202.67
A_68_P31103785	chr17:25571124-25571168	NM_001163691:-418	Cacna1h	PROMOTER	0.5	3.993	714.25	2852.00	1.995	627.78	1252.50
A_68_P31103374	chr17:25522637-25522681	NM_001163691:48070	Cacna1h	INSIDE	0.448	3.324	2200.74	7315.58	1.489	1731.93	2578.50
A_68_P26808316	chr9:107302358-107302402	NM_001174047:170	Cacna2d2	INSIDE	0.116	18.385	3243.54	59632.11	2.137	2642.89	5647.97
A_68_P26809312	chr9:107429515-107429561	NM_001174047:127328	Cacna2d2	INSIDE	0.475	0.610	1750.78	1068.25	0.29	1058.21	306.65
A_68_P28053990	chr11:97883852-97883896	NM_001159320:-4188	Cacnb1	PROMOTER	0.559	2.914	2419.96	7051.96	1.629	1991.41	3243.40
A_68_P21021376	chr2:14525863-14525907	NM_023116:-48	Cacnb2	PROMOTER	0.635	2.576	1357.19	3495.53	1.636	1199.92	1962.95
A_68_P30475601	chr15:98465084-98465128	NM_007581:-88	Cacnb3	PROMOTER	0.422	0.328	2803.49	920.39	0.138	1947.75	269.74
A_68_P21221051	chr2:52531541-52531585	NM_001037099:539	Cacnb4	INSIDE	0.453	15.981	1281.98	20486.63	7.233	1394.12	10083.60
A_68_P30357863	chr15:77950040-77950084	NM_007583:-352	Cacng2	PROMOTER	0.326	8.121	1628.11	13221.89	2.647	1424.19	3769.50
A_68_P28109101	chr11:107655547-107655591	NM_019431:210	Cacng4	INSIDE	0.516	4.770	2299.97	10971.75	2.463	1699.51	4185.43
A_68_P28109104	chr11:107655957-107656001	NM_019431:-200	Cacng4	PROMOTER	0.597	10.288	1976.14	20330.58	6.141	1707.82	10487.48
A_68_P28109099	chr11:107655337-107655381	NM_019431:420	Cacng4	INSIDE	0.456	0.542	1115.63	604.39	0.247	943.31	232.82
A_68_P33005168	chr7:3366552-3366596	NM_133189:29890	Cacng7	DOWNSTREAM	0.141	24.597	2749.66	67632.47	3.46	2176.90	7532.86
A_68_P33005171	chr7:3366221-3366265	NM_133189:29558	Cacng7	INSIDE	0.597	0.527	1003.46	528.70	0.315	805.83	253.53
A_68_P20837653	chr1:175297800-175297844	NM_053199:4	Cadm3	INSIDE	0.395	0.232	3839.40	889.00	0.092	2591.90	237.29
A_68_P25005093	chr7:25266752-25266796	NM_153112:-267	Cadm4	PROMOTER	0.572	0.523	13146.17	6881.31	0.299	8747.40	2619.20
A_68_P29413810	chr14:13654360-13654404	NM_001042617:1211	Cadps	INSIDE	0.352	10.336	2976.79	30767.83	3.633	2475.58	8994.55
A_68_P31423374	chr17:87846116-87846160	NM_007589:137	Calm2	INSIDE	0.276	0.271	3256.17	882.26	0.075	2499.18	187.02
A_68_P31423375	chr17:87846261-87846305	NM_007589:-7	Calm2	PROMOTER	0.437	0.633	3037.20	1921.80	0.276	2149.29	593.83
A_68_P31423372	chr17:87845921-87845965	NM_007589:333	Calm2	INSIDE	0.652	3.060	856.99	2622.03	1.995	573.44	1143.80
A_68_P24093838	chr5:130925235-130925279	NM_021371:595	Caln1	INSIDE	0.537	3.250	1142.90	3713.89	1.743	1039.21	1811.82
A_68_P24093843	chr5:130925785-130925831	NM_021371:1147	Caln1	INSIDE	0.518	7.018	201.60	1414.85	3.637	135.18	491.62
A_68_P20975269	chr2:5636008-5636052	NM_177343:-320	Camk1d	PROMOTER	0.356	0.417	1414.25	589.52	0.149	1151.35	171.00
A_68_P20975262	chr2:5635173-5635217	NM_177343:516	Camk1d	INSIDE	0.413	9.109	1373.86	12513.96	3.765	1076.47	4052.75

ProbeName	Target position of probe on CpG island microarray	TargetID	GeneSymbol	CpG island Description	Ratio of relative methylation (TiO_2-NP/Vehicle)	Sham group			TiO_2-H group		
						Relative methylation (Cy5/Cy3)	Cy3 signal (Input)	Cy5 signal (MeDIP)	Relative methylation (Cy5/Cy3)	Cy3 signal (Input)	Cy5 signal (MeDIP)
A_68_P20975264	chr2:5635419-5635463	NM_177343:270	Camk1d	INSIDE	0.405	0.658	715.21	470.52	0.267	627.08	167.21
A_68_P27566544	chr11:5965149-5965193	NM_001174053:581	Camk2b	INSIDE	0.103	31.487	2512.80	79121.04	3.243	2119.39	6873.85
A_68_P27566548	chr11:5966021-5966065	NM_001174053:-291	Camk2b	PROMOTER	0.595	0.651	2366.21	1539.32	0.387	1898.32	734.92
A_68_P23345840	chr4:138010011-138010055	NM_025451:-1030	Camk2n1	PROMOTER	0.3	5.595	1091.93	6109.06	1.68	914.71	1537.06
A_68_P30588535	chr16:20621603-20621647	NM_028420:-273	Camk2n2	DIVERGENT_PROMOTER	0.358	0.309	1891.84	584.61	0.11	1548.86	171.12
A_68_P30588530	chr16:20620757-20620801	NM_028420:573	Camk2n2	INSIDE	0.475	0.303	1556.21	472.16	0.144	1253.33	180.64
A_68_P30588523	chr16:20619843-20619887	NM_028420:1487	Camk2n2	INSIDE	0.333	1.385	5361.19	7425.40	0.462	3892.78	1797.28
A_68_P30588524	chr16:20619982-20620026	NM_028420:1347	Camk2n2	INSIDE	0.222	2.906	7499.33	21791.20	0.646	5057.85	3264.86
A_68_P31605184	chr18:33099024-33099068	NM_009793:352	Camk4	INSIDE	0.51	0.429	1063.84	456.63	0.219	795.73	174.31
A_68_P27914667	chr11:72832981-72833025	NM_018883:493	Camkk1	INSIDE	0.579	2.584	2572.24	6645.99	1.497	2156.47	3228.16
A_68_P24052104	chr5:123213775-123213819	NM_001199676:15623	Camkk2	INSIDE	0.076	28.420	2363.75	67177.75	2.153	1587.72	3418.43
A_68_P24052105	chr5:123213863-123213907	NM_001199676:15535	Camkk2	INSIDE	0.11	26.181	1807.01	47309.44	2.874	1173.39	3372.58
A_68_P24052194	chr5:123229578-123229622	NM_001199676:-181	Camkk2	PROMOTER	0.446	0.508	910.54	462.63	0.226	759.91	172.09
A_68_P29056138	chr13:55724621-55724665	NM_007596:277	Caml	INSIDE	0.535	3.520	1312.84	4621.82	1.884	1153.89	2173.44
A_68_P21075529	chr2:25838219-25838263	NM_001115076:562	Camsap1	INSIDE	0.651	0.389	2346.77	913.52	0.254	1741.88	441.75
A_68_P23410165	chr4:150482219-150482264	NM_001081557:753636	Camta1	INSIDE	0.57	0.751	12622.66	9484.81	0.428	8630.80	3695.08
A_68_P23415131	chr4:151235903-151235947	NM_001081557:-47	Camta1	PROMOTER	0.286	2.174	1740.70	3784.67	0.623	1388.58	864.46
A_68_P27489850	chr10:118676304-118676348	NM_027994:785	Cand1	INSIDE	0.527	5.995	1272.77	7630.83	3.161	1209.55	3823.73
A_68_P24772854	chr6:115724644-115724688	NM_025958:92	Cand2	INSIDE	0.089	1.395	2638.28	3679.97	0.125	1689.79	210.69
A_68_P28171198	chr11:118280168-118280212	NM_001025617:176	Cant1	INSIDE	0.653	0.734	2988.97	2192.62	0.479	2141.05	1025.85
A_68_P27788008	chr11:50139149-50139193	NM_001110499:5	Canx	INSIDE	0.308	7.986	5755.77	45965.27	2.462	4536.52	11168.15
A_68_P27788005	chr11:50138862-50138906	NM_001110499:291	Canx	INSIDE	0.384	6.817	5811.16	39614.81	2.615	4800.33	12553.44
A_68_P27788006	chr11:50138948-50138992	NM_001110499:205	Canx	INSIDE	0.653	6.682	5142.45	34360.58	4.364	4329.86	18896.88
A_68_P25354354	chr7:105326624-105326668	NM_007602:57	Capn5	INSIDE	0.404	11.481	1490.62	17113.11	4.64	1318.07	6115.49
A_68_P25354355	chr7:105326695-105326739	NM_007602:-13	Capn5	PROMOTER	0.422	8.630	1253.93	10820.83	3.646	1254.58	4574.08
A_68_P29511373	chr14:32150285-32150329	NM_009796:397	Capn7	INSIDE	0.449	5.981	1448.37	8662.11	2.684	1218.61	3270.98
A_68_P24942634	chr6:148844792-148844836	NM_181541:-166	Caprin2	PROMOTER	0.589	0.145	6166.70	892.58	0.085	4284.85	365.29
A_68_P24268748	chr6:17586921-17586965	NM_007604:-155	Capza2	PROMOTER	0.608	2.729	822.89	2245.71	1.66	801.79	1330.63
A_68_P23350166	chr4:138748616-138748660	NM_009798:-207	Capzb	PROMOTER	0.354	10.554	5320.54	56152.99	3.731	5013.69	18706.28
A_68_P23350170	chr4:138749124-138749168	NM_001037761:253	Capzb	INSIDE	0.478	7.171	328.36	2354.64	3.426	315.68	1081.62
A_68_P23350169	chr4:138748940-138748984	NM_001037761:69	Capzb	INSIDE	0.584	0.682	2915.76	1987.93	0.398	2385.73	949.93
A_68_P28025137	chr11:92960870-92960914	NM_028296:289	Car10	INSIDE	0.225	0.507	1882.53	955.30	0.114	1482.01	168.89
A_68_P28025142	chr11:92961341-92961387	NM_028296:761	Car10	INSIDE	0.372	0.274	2259.89	620.16	0.102	1706.36	174.34
A_68_P28025143	chr11:92961409-92961453	NM_028296:827	Car10	INSIDE	0.386	0.605	4277.66	2588.26	0.234	2988.99	698.25
A_68_P28025136	chr11:92960730-92960774	NM_028296:149	Car10	INSIDE	0.368	4.170	1723.20	7185.76	1.535	1395.84	2143.21
A_68_P25094544	chr7:52955351-52955395	NM_009800:36	Car11	INSIDE	0.627	0.501	2093.39	1049.23	0.314	1750.88	550.53
A_68_P21958957	chr3:14886237-14886281	NM_009801:-167	Car2	PROMOTER	0.554	5.636	786.61	4432.94	3.12	692.06	2159.41
A_68_P26129049	chr8:107064933-107064977	NM_053070:248	Car7	INSIDE	0.507	6.269	3302.36	20701.88	3.178	2918.38	9273.19
A_68_P30362471	chr15:78633225-78633269	NM_130859:226	Card10	INSIDE	0.431	5.927	1541.26	9135.58	2.555	1323.75	3382.45
A_68_P30362447	chr15:78629670-78629714	NM_130859:3780	Card10	INSIDE	0.457	3.370	2796.13	9424.31	1.539	2273.18	3498.22
A_68_P30533733	chr16:8672342-8672386	NM_025821:-118	Carhsp1	PROMOTER	0.379	7.778	1553.95	12087.18	2.95	1323.77	3905.44
A_68_P30533728	chr16:8671819-8671863	NM_025821:406	Carhsp1	INSIDE	0.345	1.476	1275.58	1882.89	0.509	1037.35	527.71
A_68_P25655858	chr8:11497803-11497847	NM_026995:257	Carkd	INSIDE	0.174	12.010	1776.44	21334.48	2.094	1509.47	3160.60
A_68_P31921521	chr19:4169457-4169501	NM_134148:6001	Carns1	INSIDE	0.329	4.294	5552.21	23842.23	1.414	4122.39	5827.75
A_68_P29265994	chr13:100670562-100670606	NM_001081493:54	Cartpt	INSIDE	0.554	0.316	2043.12	646.41	0.175	1598.14	280.18
A_68_P21583235	chr2:121692691-121692735	NM_001205371:-728	Casc4	PROMOTER	0.488	4.696	448.75	2107.53	2.291	450.32	1031.53

ProbeName	Target position of probe on CpG island microarray	TargetID	GeneSymbol	CpG island Description	Ratio of relative methylation (TiO_2-NP/Vehicle)	Sham group			TiO_2-H group		
						Relative methylation (Cy5/Cy3)	Cy3 signal (Input)	Cy5 signal (MeDIP)	Relative methylation (Cy5/Cy3)	Cy3 signal (Input)	Cy5 signal (MeDIP)
A_68_P21567569	chr2:118873048-118873092	NM_029617:216	Casc5	INSIDE	0.077	1.474	2170.71	3198.98	0.114	1590.03	180.56
A_68_P21567570	chr2:118873151-118873195	NM_029617:318	Casc5	INSIDE	0.46	0.682	1303.39	888.62	0.314	1055.45	331.26
A_68_P24205536	chr6:4550713-4550757	NM_145398:-331	Casd1	PROMOTER	0.238	19.764	3734.20	73802.51	4.702	3521.25	16556.29
A_68_P24205537	chr6:4550857-4550901	NM_145398:-187	Casd1	PROMOTER	0.62	0.429	1236.20	530.94	0.266	935.01	248.92
A_68_P32268351	chrX:13424038-13424082	NM_009806:-393	Cask	PROMOTER	0.227	9.986	2767.64	27636.57	2.271	3519.36	7993.94
A_68_P32268349	chrX:13423877-13423921	NM_009806:-231	Cask	PROMOTER	0.357	8.529	1284.59	10956.83	3.046	1443.95	4398.29
A_68_P32268347	chrX:13423633-13423677	NM_009806:13	Cask	INSIDE	0.373	13.211	2822.78	37292.35	4.932	3432.33	16929.01
A_68_P31097727	chr17:24642185-24642229	NM_027937:16479	Caskin1	INSIDE	0.465	3.748	3680.84	13797.26	1.744	2388.98	4166.03
A_68_P31097739	chr17:24643626-24643670	NM_027937:17921	Caskin1	INSIDE	0.516	3.373	3654.46	12328.30	1.742	2978.67	5189.44
A_68_P28155403	chr11:115664957-115665001	NM_080643:9928	Caskin2	INSIDE	0.116	51.683	3851.38	199052.70	5.971	3414.85	20390.23
A_68_P28155484	chr11:115675096-115675140	NM_080643:-212	Caskin2	DIVERGENT_PROMOTER	0.659	0.184	2911.22	535.03	0.121	2035.21	246.31
A_68_P25837064	chr8:47702719-47702763	NM_009810:-62	Casp3	DIVERGENT_PROMOTER	0.242	30.843	10478.03	323173.00	7.467	9542.52	71256.72
A_68_P25837068	chr8:47703178-47703222	NM_009810:398	Casp3	INSIDE	0.646	0.504	2655.47	1339.09	0.326	2158.43	702.81
A_68_P22522753	chr3:129604519-129604563	NM_009811:198	Casp6	INSIDE	0.483	0.148	3498.37	518.51	0.072	2607.75	186.75
A_68_P22522751	chr3:129604360-129604404	NM_009811:40	Casp6	INSIDE	0.616	0.536	5597.09	3002.22	0.331	4207.73	1391.42
A_68_P23366008	chr4:141349558-141349602	NM_015733:54	Casp9	INSIDE	0.067	4.905	6740.15	33063.28	0.328	4783.70	1571.08
A_68_P23366010	chr4:141349732-141349776	NM_015733:228	Casp9	INSIDE	0.365	0.482	1499.60	722.50	0.176	1214.61	213.54
A_68_P23366007	chr4:141349370-141349414	NM_015733:-134	Casp9	DIVERGENT_PROMOTER	0.451	0.310	3229.10	1000.06	0.14	2405.15	335.84
A_68_P29147534	chr13:74945954-74945998	NM_009817:-607	Cast	PROMOTER	0.347	0.544	1148.48	624.47	0.188	922.89	173.92
A_68_P23397281	chr4:148275240-148275284	NM_027195:96762	Casz1	INSIDE	0.113	62.221	10026.63	623866.40	7.006	13053.40	91449.69
A_68_P23396578	chr4:148179082-148179126	NM_027195:604	Casz1	INSIDE	0.282	16.237	2330.98	37847.73	4.581	2146.50	9833.67
A_68_P23397279	chr4:148275001-148275045	NM_027195:96522	Casz1	INSIDE	0.343	18.714	2830.60	52972.87	6.419	2496.11	16022.48
A_68_P23397206	chr4:148265535-148265579	NM_027195:87056	Casz1	INSIDE	0.563	10.446	2309.00	24120.42	5.883	1785.34	10503.31
A_68_P21760473	chr2:154262598-154262642	NM_009823:401	Cbfa2t2	INSIDE	0.662	0.438	1125.97	492.80	0.29	1033.92	299.75
A_68_P26235085	chr8:125202360-125202404	NM_001109873:-307	Cbfa2t3	PROMOTER	0.438	9.887	1892.08	18706.66	4.333	1653.30	7163.57
A_68_P26234726	chr8:125154578-125154622	NM_001109873:47475	Cbfa2t3	INSIDE	0.625	6.238	1790.95	11171.09	3.897	1539.90	6001.03
A_68_P26132023	chr8:107694748-107694792	NM_001161456:197	Cbfb	INSIDE	0.472	10.615	1666.07	17685.84	5.006	1702.07	8521.19
A_68_P26466435	chr9:44041858-44041902	NM_007619:249	Cbl	INSIDE	0.555	14.801	3985.06	58984.71	8.209	2970.77	24387.08
A_68_P30759540	chr16:52031753-52031797	NM_001033238:113	Cblb	INSIDE	0.421	13.307	3710.77	49377.88	5.609	3012.13	16894.19
A_68_P24995306	chr7:20382177-20382221	NM_001161844:-40	Cblc	PROMOTER	0.637	4.790	3413.87	16351.90	3.051	2815.22	8589.66
A_68_P28331595	chr12:32184383-32184427	NM_134048:1	Cbll1	INSIDE	0.308	1.891	1217.37	2301.84	0.582	976.33	568.47
A_68_P26035029	chr8:89996288-89996332	NM_019626:181	Cbln1	INSIDE	0.346	8.245	8233.06	67885.43	2.852	6667.56	19014.83
A_68_P26035009	chr8:89994219-89994263	NM_019626:2251	Cbln1	INSIDE	0.421	0.670	1138.41	762.64	0.282	884.51	249.31
A_68_P31899538	chr18:86881037-86881081	NM_172633:-1381	Cbln2	PROMOTER	0.218	0.495	14701.22	7284.10	0.108	9135.76	988.83
A_68_P30977032	chr16:93683403-93683447	NM_173047:-39	Cbr3	PROMOTER	0.399	0.307	1794.36	550.04	0.122	1425.61	174.42
A_68_P31140911	chr17:31773548-31773592	NM_178224:506	Cbs	INSIDE	0.343	9.569	7545.38	72198.44	3.284	5958.84	19568.70
A_68_P31140909	chr17:31773378-31773422	NM_178224:676	Cbs	INSIDE	0.497	0.145	3745.68	542.42	0.072	2437.82	175.54
A_68_P32026831	chr19:25036147-25036191	NM_146097:-62	Cbwd1	PROMOTER	0.551	13.971	3428.72	47903.15	7.691	2751.79	21164.97
A_68_P28175309	chr11:118884826-118884870	NM_007623:506	Cbx2	INSIDE	0.373	7.120	2113.54	15048.18	2.656	1759.07	4672.64
A_68_P28175304	chr11:118884168-118884212	NM_007623:-152	Cbx2	PROMOTER	0.372	1.860	2495.67	4642.92	0.692	1929.58	1335.67
A_68_P28175799	chr11:118951917-118951961	NM_007625:-4387	Cbx4	PROMOTER	0.273	16.718	3173.58	53055.42	4.562	2620.64	11954.55
A_68_P28175760	chr11:118947373-118947417	NM_007625:157	Cbx4	INSIDE	0.481	3.596	1634.40	5876.52	1.728	1386.19	2395.25
A_68_P30502976	chr15:103070313-103070357	NM_001076789:-87	Cbx5	DIVERGENT_PROMOTER	0.356	0.401	1628.65	653.68	0.143	1202.55	171.95
A_68_P30369297	chr15:79762789-79762833	NM_144811:266	Cbx7	INSIDE	0.211	6.583	2145.33	14122.82	1.391	1786.65	2485.08
A_68_P28175418	chr11:118898955-118898999	NM_013926:3251	Cbx8	DOWNSTREAM	0.149	26.134	2385.00	62329.48	3.888	2077.18	8075.80
A_68_P28175453	chr11:118902817-118902861	NM_013926:-611	Cbx8	PROMOTER	0.485	0.293	6059.96	1773.44	0.142	4110.36	583.40

ProbeName	Target position of probe on CpG island microarray	TargetID	GeneSymbol	CpG island Description	Ratio of relative methylation (TiO_2-NP/Vehicle)	Sham group			TiO_2-H group		
						Relative methylation (Cy5/Cy3)	Cy3 signal (Input)	Cy5 signal (MeDIP)	Relative methylation (Cy5/Cy3)	Cy3 signal (Input)	Cy5 signal (MeDIP)
A_68_P28175448	chr11:118902325-118902369	NM_013926:-119	Cbx8	PROMOTER	0.497	0.428	2146.70	918.52	0.213	1664.57	353.73
A_68_P23196907	chr4:108292428-108292472	NM_177045:-110	Cc2d1b	PROMOTER	0.482	3.031	2916.57	8840.55	1.461	2472.26	3612.47
A_68_P27275484	chr10:78215712-78215756	NM_027630:78	Ccdc105	INSIDE	0.497	0.267	2049.61	546.79	0.133	1516.22	201.07
A_68_P24951826	chr7:5008661-5008708	NM_146178:357	Ccdc106	INSIDE	0.468	4.262	1160.24	4944.85	1.993	1084.85	2162.22
A_68_P22871971	chr4:43506143-43506188	NM_001037913:-71	Ccdc107	DIVERGENT_PROMOTER	0.645	3.983	415.50	1654.94	2.569	357.08	917.49
A_68_P27169799	chr10:59079055-59079099	NM_001033259:364	Ccdc109a	INSIDE	0.398	3.955	2326.99	9202.87	1.573	1818.97	2861.44
A_68_P22523142	chr3:129672517-129672561	NM_025779:586	Ccdc109b	INSIDE	0.592	0.222	2993.04	662.98	0.131	1996.02	261.93
A_68_P27563898	chr11:5441853-5441897	NM_134033:346	Ccdc117	INSIDE	0.233	20.820	3486.46	72587.90	4.847	3399.03	16476.63
A_68_P25954611	chr8:73392845-73392889	NM_026964:4523	Ccdc124	INSIDE	0.557	0.425	1275.38	542.31	0.237	937.43	222.15
A_68_P25954643	chr8:73397351-73397395	NM_026964:17	Ccdc124	INSIDE	0.518	3.206	1845.85	5918.70	1.661	1724.20	2864.54
A_68_P29269562	chr13:101439843-101439887	NM_001168386:429	Ccdc125	INSIDE	0.496	0.496	1589.91	788.30	0.246	1131.66	278.50
A_68_P24432030	chr6:49269309-49269353	NM_175098:-21	Ccdc126	PROMOTER	0.351	0.388	1521.98	590.86	0.136	1293.29	176.44
A_68_P29145780	chr13:74487726-74487770	NM_001168659:-16	Ccdc127	DIVERGENT_PROMOTER	0.541	2.905	1985.93	5769.66	1.571	1558.11	2447.60
A_68_P26885916	chr9:121748253-121748297	NM_028384:305	Ccdc13	INSIDE	0.228	0.255	6163.35	1573.52	0.058	4100.58	239.07
A_68_P26885915	chr9:121748176-121748220	NM_028384:381	Ccdc13	INSIDE	0.363	0.283	4503.55	1275.96	0.103	3160.95	324.67
A_68_P26019095	chr8:86794255-86794299	NM_026350:-17	Ccdc130	PROMOTER	0.497	5.703	3479.83	19846.75	2.836	2715.11	7699.81
A_68_P26019094	chr8:86794146-86794190	NM_026350:91	Ccdc130	INSIDE	0.584	0.457	2592.46	1184.17	0.267	1842.21	491.12
A_68_P24326230	chr6:29353006-29353050	NM_001201378:4103	Ccdc136	INSIDE	0.557	0.307	1471.97	451.39	0.171	1150.34	196.32
A_68_P24326231	chr6:29353130-29353174	NM_001201378:4227	Ccdc136	INSIDE	0.133	2.503	644.18	1612.65	0.333	604.73	201.15
A_68_P26430110	chr9:37155994-37156038	NM_001081429:-39	Ccdc15	PROMOTER	0.206	27.621	3467.04	95762.28	5.685	2909.00	16538.62
A_68_P23895412	chr5:93104159-93104203	NM_177230:-27	Ccdc158	DIVERGENT_PROMOTER	0.482	4.788	3379.51	16182.36	2.307	3114.85	7184.88
A_68_P23895413	chr5:93104248-93104292	NM_177230:-117	Ccdc158	DIVERGENT_PROMOTER	0.6	0.688	2316.34	1594.44	0.413	1801.41	743.68
A_68_P23244137	chr4:118868027-118868071	NM_001038998:-81	Ccdc23	PROMOTER	0.485	0.377	1163.43	438.78	0.183	955.23	174.60
A_68_P20972053	chr2:5058960-5059004	NM_028804:161	Ccdc3	INSIDE	0.469	8.766	736.00	6451.89	4.108	633.18	2600.93
A_68_P23245371	chr4:119087718-119087764	NM_029286:386	Ccdc30	INSIDE	0.576	0.465	1765.93	820.50	0.268	1248.51	334.40
A_68_P23245376	chr4:119088209-119088253	NM_029286:-104	Ccdc30	PROMOTER	0.584	0.594	1005.50	597.24	0.347	729.01	252.83
A_68_P27360778	chr10:94151506-94151550	NM_029852:-6	Ccdc41	DIVERGENT_PROMOTER	0.353	0.382	1690.13	645.39	0.135	1347.93	181.74
A_68_P24039940	chr5:121081339-121081383	NM_001195094:2884	Ccdc42b	INSIDE	0.068	24.354	753.42	18348.84	1.655	659.49	1091.56
A_68_P30625227	chr16:27388952-27388996	NM_001025615:-88	Ccdc50	PROMOTER	0.515	12.389	939.46	11638.90	6.376	975.94	6223.10
A_68_P28187622	chr11:120794046-120794090	NM_027745:118	Ccdc57	INSIDE	0.63	0.525	1146.10	601.95	0.331	993.50	328.95
A_68_P27228310	chr10:69651858-69651902	NM_001111121:92012	Ccdc6	INSIDE	0.173	25.208	7326.43	184685.90	4.356	6203.63	27025.55
A_68_P27227733	chr10:69559705-69559749	NM_001111121:-142	Ccdc6	PROMOTER	0.332	9.035	2724.54	24614.93	3.001	4232.76	12702.41
A_68_P24990327	chr7:19479355-19479399	NM_001033314:16377	Ccdc61	INSIDE	0.387	7.815	3955.08	30907.87	3.027	3697.43	11193.44
A_68_P24990324	chr7:19479119-19479163	NM_001033314:16613	Ccdc61	INSIDE	0.514	0.601	3842.74	2310.42	0.309	2732.57	843.89
A_68_P24990326	chr7:19479287-19479331	NM_001033314:16445	Ccdc61	INSIDE	0.526	3.244	1504.28	4879.52	1.707	1232.07	2102.82
A_68_P24010911	chr5:116112644-116112688	NM_001080808:68902	Ccdc64	INSIDE	0.578	4.196	4301.05	18048.58	2.425	3534.14	8570.08
A_68_P24011288	chr5:116182625-116182669	NM_001080808:-1078	Ccdc64	PROMOTER	0.61	0.446	2256.61	1006.15	0.272	1874.67	509.67
A_68_P26323901	chr9:15432521-15432565	NM_181816:-165	Ccdc67	PROMOTER	0.651	0.550	2745.25	1511.18	0.358	2009.38	719.62
A_68_P26323900	chr9:15432364-15432408	NM_181816:-9	Ccdc67	PROMOTER	0.495	3.332	1354.14	4511.35	1.648	1072.75	1767.86
A_68_P26814990	chr9:108363250-108363294	NM_133744:424	Ccdc71	INSIDE	0.366	5.833	1660.21	9683.95	2.137	1517.96	3244.18
A_68_P26814987	chr9:108362953-108362997	NM_133744:126	Ccdc71	INSIDE	0.547	4.661	2033.19	9476.11	2.551	1877.30	4789.36
A_68_P26818181	chr9:108984890-108984934	NM_183250:-17	Ccdc72	DIVERGENT_PROMOTER	0.429	0.620	2809.12	1741.26	0.266	2081.09	553.09
A_68_P31373927	chr17:79234917-79234961	NM_181649:83	Ccdc75	INSIDE	0.487	4.735	2154.94	10203.52	2.308	1725.04	3981.10
A_68_P26128839	chr8:107033201-107033245	NM_180958:565	Ccdc79	INSIDE	0.599	0.464	1465.05	679.23	0.278	1255.27	348.39
A_68_P31928777	chr19:5457059-5457103	NM_198616:469	Ccdc85b	INSIDE	0.292	7.028	1482.47	10418.41	2.049	1227.97	2515.82
A_68_P28727039	chr12:109513784-109513828	NM_001159910:-179	Ccdc85c	PROMOTER	0.392	5.774	3140.62	18133.62	2.262	2653.89	6001.80

ProbeName	Target position of probe on CpG island microarray	TargetID	GeneSymbol	CpG island Description	Ratio of relative methylation (TiO_2-NP/Vehicle)	Sham group			TiO_2-H group		
						Relative methylation (Cy5/Cy3)	Cy3 signal (Input)	Cy5 signal (MeDIP)	Relative methylation (Cy5/Cy3)	Cy3 signal (Input)	Cy5 signal (MeDIP)
A_68_P27674147	chr11:29273422-29273466	NM_176841:-330	Ccdc88a	PROMOTER	0.479	4.947	1109.54	5488.48	2.367	1009.48	2389.40
A_68_P31936460	chr19:6930124-6930168	NM_001081291:2555	Ccdc88b	INSIDE	0.584	0.574	2893.46	1661.21	0.335	1958.32	656.15
A_68_P31936459	chr19:6930056-6930100	NM_001081291:2623	Ccdc88b	INSIDE	0.629	0.588	1085.10	638.52	0.37	879.97	325.73
A_68_P24980671	chr7:16871769-16871813	NM_172297:-1782	Ccdc9	PROMOTER	0.361	10.864	14817.86	160981.80	3.926	13212.72	51872.48
A_68_P24980670	chr7:16871649-16871693	NM_172297:-1662	Ccdc9	PROMOTER	0.413	7.715	11203.84	86442.65	3.187	8016.24	25548.44
A_68_P24980698	chr7:16881008-16881052	NM_001136471:-8886	Ccdc9	PROMOTER	0.646	3.116	1777.56	5539.41	2.014	1404.98	2829.57
A_68_P24980672	chr7:16871856-16871900	NM_172297:-1870	Ccdc9	PROMOTER	0.477	2.959	5333.39	15780.43	1.413	3948.89	5578.36
A_68_P28990871	chr13:43655098-43655142	NM_001081059:440	Ccdc90a	INSIDE	0.303	13.986	2235.46	31265.27	4.239	1929.19	8178.79
A_68_P25325434	chr7:99709780-99709824	NM_001162918:144	Ccdc90b	INSIDE	0.653	0.453	1004.58	455.14	0.296	836.63	247.42
A_68_P23607908	chr5:36827600-36827644	NM_025725:386	Ccdc96	INSIDE	0.309	8.693	2401.12	20872.68	2.684	1947.52	5227.38
A_68_P31159491	chr17:35654099-35654143	NM_146248:60	Cchcr1	INSIDE	0.182	2.593	4192.98	10874.03	0.473	3258.21	1540.12
A_68_P26883806	chr9:121403762-121403806	NM_031161:1023	Cck	INSIDE	0.347	0.228	2930.28	666.73	0.079	2168.82	171.22
A_68_P26883804	chr9:121403538-121403582	NM_031161:1247	Cck	INSIDE	0.664	5.614	909.60	5106.55	3.726	820.06	3055.25
A_68_P25392270	chr7:112574421-112574465	NM_007627:109	Cckbr	INSIDE	0.36	8.073	5714.55	46136.01	2.906	5389.95	15660.66
A_68_P22058858	chr3:36470838-36470882	NM_009828:58	Ccna2	INSIDE	0.573	3.758	1319.64	4958.88	2.152	1092.56	2351.43
A_68_P25610226	chr7:152128254-152128298	NM_007631:-2446	Ccnd1	PROMOTER	0.394	0.342	1709.92	584.89	0.135	1401.45	188.96
A_68_P24831080	chr6:127105235-127105279	NM_009829:-4190	Ccnd2	PROMOTER	0.641	0.525	1828.51	960.50	0.337	1447.77	487.82
A_68_P25065442	chr7:38892952-38892996	NM_007633:-465	Ccne1	PROMOTER	0.273	0.333	4859.22	1616.16	0.091	3335.73	303.30
A_68_P25065437	chr7:38892310-38892354	NM_007633:177	Ccne1	INSIDE	0.527	3.989	656.80	2619.82	2.102	583.77	1226.87
A_68_P25065443	chr7:38893032-38893076	NM_007633:-545	Ccne1	PROMOTER	0.293	5.204	2991.07	15565.60	1.522	2550.17	3881.83
A_68_P22728457	chr4:11118468-11118512	NM_001037134:-10	Ccne2	PROMOTER	0.527	4.653	878.77	4088.81	2.454	823.02	2019.50
A_68_P22728462	chr4:11118987-11119031	NM_009830:153	Ccne2	INSIDE	0.57	0.194	3866.07	749.55	0.111	2766.40	305.72
A_68_P22728452	chr4:11117767-11117811	NM_001037134:-712	Ccne2	PROMOTER	0.607	3.863	362.98	1402.22	2.343	399.51	936.23
A_68_P31096346	chr17:24388277-24388321	NM_007634:56	Ccnf	INSIDE	0.595	4.483	686.09	3075.56	2.667	593.17	1581.70
A_68_P27737179	chr11:40568476-40568520	NM_009831:290	Ccng1	INSIDE	0.533	0.613	2213.17	1357.12	0.327	1647.11	538.56
A_68_P23898892	chr5:93697057-93697101	NM_007635:480	Ccng2	INSIDE	0.417	0.485	1026.88	498.10	0.202	861.04	173.98
A_68_P32111328	chr19:40905903-40905947	NM_172839:156	Ccnj	INSIDE	0.497	3.950	878.96	3471.52	1.963	727.60	1428.04
A_68_P27750951	chr11:43342144-43342188	NM_001045530:-119	Ccnjl	PROMOTER	0.636	0.607	1087.73	659.80	0.386	789.90	304.69
A_68_P28726332	chr12:109418289-109418333	NM_009832:363	Ccnk	INSIDE	0.244	1.459	3104.04	4528.40	0.356	2274.37	810.80
A_68_P23440734	chr4:155186580-155186624	NM_207678:5	Ccnl2	INSIDE	0.375	0.710	2338.97	1660.29	0.266	1877.72	500.10
A_68_P29338034	chr13:113777821-113777865	NM_001081062:-167	Ccno	PROMOTER	0.21	12.664	650.52	8238.12	2.661	524.42	1395.62
A_68_P29338038	chr13:113778161-113778205	NM_001081062:173	Ccno	INSIDE	0.514	2.793	3745.76	10462.30	1.437	2778.15	3992.09
A_68_P31485630	chr18:9450058-9450102	NM_026484:68	Ccny	INSIDE	0.574	0.476	1295.01	616.89	0.273	1080.90	295.51
A_68_P22128529	chr3:51028612-51028656	NM_009834:266	Ccrn4l	INSIDE	0.514	0.184	4699.41	866.18	0.095	3352.62	317.37
A_68_P27479232	chr10:116500293-116500343	NM_007636:552	Cct2	INSIDE	0.572	0.360	2540.58	915.60	0.206	1176.36	242.67
A_68_P30115244	chr15:31531241-31531285	NM_007637:297	Cct5	INSIDE	0.421	0.547	879.47	480.64	0.23	737.90	169.81
A_68_P30940063	chr16:87495606-87495650	NM_009840:486	Cct8	INSIDE	0.627	0.353	3684.44	1300.97	0.221	2708.06	599.48
A_68_P25589026	chr7:148653744-148653788	NM_001111050:-921	Cd151	PROMOTER	0.059	7.390	4167.77	30798.43	0.438	3076.08	1346.02
A_68_P25589027	chr7:148653834-148653878	NM_001111050:-831	Cd151	PROMOTER	0.483	0.515	2250.51	1158.54	0.249	1771.81	440.45
A_68_P23316407	chr4:132776804-132776848	NM_027152:103	Cd164l2	INSIDE	0.37	0.669	1039.43	695.64	0.247	721.83	178.52
A_68_P20796837	chr1:167775393-167775437	NM_001113391:56603	Cd247	INSIDE	0.12	18.000	1104.23	19876.30	2.154	1003.28	2160.81
A_68_P20796835	chr1:167775162-167775206	NM_001113391:56373	Cd247	INSIDE	0.414	8.362	1485.67	12422.73	3.463	1267.50	4388.74
A_68_P27101043	chr10:43298385-43298429	NM_009846:-568	Cd24a	PROMOTER	0.395	0.489	1033.22	504.97	0.193	889.71	171.76
A_68_P27101048	chr10:43299118-43299162	NM_009846:166	Cd24a	INSIDE	0.622	0.233	3236.69	755.25	0.145	2227.95	323.47
A_68_P31186623	chr17:43013598-43013642	NM_009847:-247	Cd2ap	PROMOTER	0.563	0.345	1526.77	527.30	0.194	1197.35	232.78
A_68_P31186620	chr17:43013150-43013194	NM_009847:201	Cd2ap	INSIDE	0.527	2.947	1257.95	3706.65	1.553	1080.15	1676.95

ProbeName	Target position of probe on CpG island microarray	TargetID	GeneSymbol	CpG island Description	Ratio of relative methylation (TiO_2-NP/Vehicle)	Sham group			TiO_2-H group		
						Relative methylation (Cy5/Cy3)	Cy3 signal (Input)	Cy5 signal (MeDIP)	Relative methylation (Cy5/Cy3)	Cy3 signal (Input)	Cy5 signal (MeDIP)
A_68_P25092032	chr7:52496418-52496462	NM_007645:-2231	Cd37	PROMOTER	0.666	0.559	3359.05	1877.54	0.372	2468.76	918.51
A_68_P25091980	chr7:52489623-52489667	NM_007645:4565	Cd37	INSIDE	0.655	0.555	989.64	549.53	0.364	886.38	322.47
A_68_P24992859	chr7:19945635-19945679	NM_145822:-824	Cd3eap	DIVERGENT_PROMOTER	0.653	0.211	4122.28	871.61	0.138	2873.59	396.66
A_68_P30747800	chr16:49855504-49855548	NM_010581:-240	Cd47	PROMOTER	0.572	3.428	1292.12	4429.87	1.961	1013.50	1987.94
A_68_P27543827	chr10:128347191-128347235	NM_007653:291	Cd63	INSIDE	0.454	4.299	1509.24	6488.79	1.951	1296.20	2528.32
A_68_P27543825	chr10:128346978-128347022	NM_007653:79	Cd63	INSIDE	0.361	1.704	2333.40	3975.04	0.614	1963.80	1206.44
A_68_P30686262	chr16:38483294-38483338	NM_009855:24304	Cd80	INSIDE	0.514	7.392	3062.56	22637.71	3.8	2493.30	9474.79
A_68_P25598323	chr7:150238635-150238679	NM_133655:2	Cd81	INSIDE	0.532	6.242	386.41	2412.08	3.32	362.85	1204.51
A_68_P24820868	chr6:125444847-125444891	NM_007657:-95	Cd9	PROMOTER	0.459	26.279	5690.53	149540.00	12.074	7609.79	91882.67
A_68_P24820864	chr6:125444507-125444551	NM_007657:245	Cd9	INSIDE	0.521	6.191	907.75	5620.08	3.227	901.39	2908.97
A_68_P22449315	chr3:116126619-116126663	NM_001080818:310	Cdc14a	INSIDE	0.533	5.472	1489.96	8153.58	2.917	1226.62	3578.24
A_68_P22449313	chr3:116126350-116126394	NM_001080818:578	Cdc14a	INSIDE	0.557	0.637	1434.25	912.91	0.355	1164.73	413.22
A_68_P23240388	chr4:118109735-118109779	NM_023223:192	Cdc20	INSIDE	0.619	0.273	5585.23	1524.87	0.169	3794.64	641.15
A_68_P31613997	chr18:34810920-34810964	NM_178347:448	Cdc23	INSIDE	0.352	0.412	1752.57	722.74	0.145	1560.33	226.36
A_68_P26820784	chr9:109778541-109778586	NM_007658:481	Cdc25a	INSIDE	0.425	17.380	2365.59	41115.08	7.388	1995.59	14742.55
A_68_P27278225	chr10:79144473-79144517	NM_177613:-445	Cdc34	PROMOTER	0.476	4.379	4875.81	21348.99	2.082	3914.81	8151.45
A_68_P32050470	chr19:29065341-29065385	NM_025950:379	Cdc37l1	INSIDE	0.605	0.620	2162.05	1340.60	0.375	1677.16	629.58
A_68_P23339475	chr4:136913438-136913482	NM_009861:192	Cdc42	INSIDE	0.338	14.872	1054.36	15680.34	5.027	971.82	4885.68
A_68_P23339476	chr4:136913526-136913570	NM_009861:104	Cdc42	INSIDE	0.654	2.377	1491.88	3546.36	1.555	1398.63	2175.23
A_68_P20872965	chr1:181889796-181889840	NM_001033285:-1401	Cdc42bpa	PROMOTER	0.351	4.311	2450.33	10563.90	1.511	1967.65	2973.95
A_68_P31933852	chr19:6306118-6306162	NM_001033342:-316	Cdc42bpg	PROMOTER	0.491	0.234	2121.03	495.85	0.115	1501.11	172.31
A_68_P31933966	chr19:6320610-6320654	NM_001033342:14176	Cdc42bpg	INSIDE	0.318	5.682	1423.34	8087.09	1.804	1139.14	2055.39
A_68_P31933965	chr19:6320530-6320574	NM_001033342:14096	Cdc42bpg	INSIDE	0.56	3.195	1454.39	4646.89	1.788	1264.07	2260.50
A_68_P30362680	chr15:78673148-78673192	NM_027219:94	Cdc42ep1	INSIDE	0.185	21.208	2000.45	42424.52	3.915	1746.07	6835.79
A_68_P31376860	chr17:79754832-79754876	NM_026514:-423	Cdc42ep3	PROMOTER	0.431	0.407	1251.37	509.71	0.176	985.52	172.97
A_68_P31376853	chr17:79753911-79753955	NM_026514:499	Cdc42ep3	INSIDE	0.523	3.250	1005.60	3268.66	1.699	839.90	1426.73
A_68_P28143851	chr11:113612678-113612722	NM_020006:-226	Cdc42ep4	PROMOTER	0.514	8.991	4385.41	39430.38	4.617	3546.03	16372.77
A_68_P28143856	chr11:113613201-113613245	NM_001163346:-93	Cdc42ep4	PROMOTER	0.66	0.491	1406.11	689.81	0.324	1047.42	339.26
A_68_P22342938	chr3:95032702-95032746	NM_001038708:-149	Cdc42se1	PROMOTER	0.663	2.848	1100.87	3135.17	1.888	937.60	1770.38
A_68_P27812111	chr11:54600916-54600960	NM_178626:267	Cdc42se2	INSIDE	0.328	5.216	2804.37	14627.08	1.711	2302.70	3939.43
A_68_P27812114	chr11:54601209-54601253	NM_178626:-25	Cdc42se2	PROMOTER	0.539	4.465	997.07	4451.71	2.408	827.03	1991.58
A_68_P28059222	chr11:98768831-98768880	NM_001025779:-347	Cdc6	PROMOTER	0.212	33.015	930.06	30706.19	6.984	733.74	5124.76
A_68_P28059225	chr11:98769164-98769211	NM_001025779:-15	Cdc6	PROMOTER	0.399	0.231	2752.88	635.61	0.092	1885.97	173.69
A_68_P28059228	chr11:98769471-98769515	NM_011799:28	Cdc6	INSIDE	0.562	0.637	1752.48	1115.90	0.358	1350.98	483.61
A_68_P28059227	chr11:98769369-98769413	NM_011799:-74	Cdc6	PROMOTER	0.453	0.710	6916.39	4913.34	0.321	4454.01	1431.93
A_68_P20679676	chr1:145549881-145549925	NM_145991:-88	Cdc73	PROMOTER	0.548	2.746	5752.63	15798.02	1.505	4632.51	6971.16
A_68_P29686792	chr14:68333571-68333615	NM_001110162:75	Cdca2	INSIDE	0.25	1.937	1125.23	2180.10	0.484	821.03	397.13
A_68_P28752830	chr12:114067331-114067375	NM_028023:248	Cdca4	INSIDE	0.188	16.274	2183.85	35541.00	3.06	2091.31	6399.79
A_68_P28752833	chr12:114067608-114067652	NM_028023:-30	Cdca4	PROMOTER	0.369	10.480	2450.53	25682.27	3.862	1738.53	6714.91
A_68_P28752835	chr12:114067766-114067810	NM_028023:-188	Cdca4	PROMOTER	0.657	0.434	2299.02	997.81	0.285	1869.73	533.55
A_68_P28776182	chr12:119082285-119082329	NM_146040:-27	Cdca7l	PROMOTER	0.387	0.274	2056.70	563.73	0.106	1619.28	171.96
A_68_P28776180	chr12:119082034-119082078	NM_146040:-277	Cdca7l	PROMOTER	0.092	2.647	802.03	2122.87	0.243	720.63	174.94
A_68_P26140461	chr8:109127454-109127498	NM_009864:209	Cdh1	INSIDE	0.658	0.609	1983.81	1207.66	0.4	1453.56	582.14
A_68_P26119098	chr8:105308180-105308224	NM_009866:809	Cdh11	INSIDE	0.378	4.053	8546.02	34639.65	1.532	6024.20	9231.03
A_68_P26236514	chr8:125394402-125394446	NM_007662:22151	Cdh15	DOWNSTREAM	0.508	4.945	1768.69	8746.63	2.511	1443.29	3624.49
A_68_P26236513	chr8:125394326-125394370	NM_007662:22075	Cdh15	DOWNSTREAM	0.203	2.320	1644.43	3814.66	0.47	1153.66	542.63

ProbeName	Target position of probe on CpG island microarray	TargetID	GeneSymbol	CpG island Description	Ratio of relative methylation (TiO_2-NP/Vehicle)	Sham group			TiO_2-H group		
						Relative methylation (Cy5/Cy3)	Cy3 signal (Input)	Cy5 signal (MeDIP)	Relative methylation (Cy5/Cy3)	Cy3 signal (Input)	Cy5 signal (MeDIP)
A_68_P31524836	chr18:16965652-16965696	NM_007664:1884	Cdh2	INSIDE	0.421	8.170	1242.17	10148.37	3.44	1058.90	3642.89
A_68_P21821742	chr2:164941702-164941746	NM_174988:118513	Cdh22	INSIDE	0.55	2.909	2460.56	7156.56	1.6	1802.61	2884.77
A_68_P21822536	chr2:165059487-165059531	NM_174988:729	Cdh22	INSIDE	0.653	0.658	1326.06	872.62	0.43	1017.23	436.94
A_68_P27173579	chr10:59766022-59766066	NM_023370:393194	Cdh23	INSIDE	0.121	16.315	846.63	13812.65	1.978	747.59	1478.85
A_68_P27176099	chr10:60150136-60150180	NM_023370:9080	Cdh23	INSIDE	0.456	3.488	1117.94	3898.86	1.59	917.36	1458.73
A_68_P26140014	chr8:109035535-109035580	NM_001037809:745	Cdh3	INSIDE	0.187	11.664	9171.41	106978.50	2.182	8027.61	17513.77
A_68_P21897030	chr2:179177570-179177614	NM_009867:410	Cdh4	INSIDE	0.43	9.182	2792.08	25637.36	3.945	2153.25	8495.40
A_68_P30026213	chr15:13102919-13102963	NM_007666:454	Cdh6	INSIDE	0.396	6.502	2502.72	16273.66	2.575	2172.51	5593.89
A_68_P30026215	chr15:13103074-13103118	NM_007666:298	Cdh6	INSIDE	0.588	0.437	5506.34	2405.26	0.257	4060.96	1042.48
A_68_P26102845	chr8:101939884-101939928	NM_001039154:451	Cdh8	INSIDE	0.467	5.249	2953.65	15503.17	2.453	2063.21	5060.41
A_68_P26102842	chr8:101939617-101939661	NM_001039154:719	Cdh8	INSIDE	0.496	8.799	1533.99	13497.46	4.363	1323.93	5776.83
A_68_P26102849	chr8:101940329-101940373	NM_001039154:7	Cdh8	INSIDE	0.612	0.705	4017.61	2833.88	0.431	2796.13	1206.36
A_68_P25503356	chr7:134119822-134119866	NM_026638:-18	Cdipt	PROMOTER	0.442	3.628	966.35	3505.92	1.604	844.14	1354.39
A_68_P27351975	chr10:92623318-92623362	NM_146239:-280	Cdk17	PROMOTER	0.223	11.705	5132.61	60077.03	2.611	4930.82	12872.48
A_68_P27351976	chr10:92623453-92623497	NM_146239:-146	Cdk17	PROMOTER	0.57	10.033	754.08	7565.82	5.716	640.89	3662.99
A_68_P27083524	chr10:40069451-40069495	NM_001168304:359	Cdk19	INSIDE	0.279	0.305	6264.76	1912.16	0.085	4187.27	356.63
A_68_P27083521	chr10:40069136-40069180	NM_001168304:45	Cdk19	INSIDE	0.419	20.585	514.73	10595.62	8.626	386.71	3335.93
A_68_P27542787	chr10:128142015-128142063	NM_016756:69	Cdk2	INSIDE	0.173	1.694	1365.89	2313.57	0.293	989.41	289.76
A_68_P24060833	chr5:124803880-124803924	NM_013812:735	Cdk2ap1	INSIDE	0.412	20.972	771.72	16184.25	8.633	687.24	5932.78
A_68_P27533815	chr10:126500675-126500719	NM_009870:38	Cdk4	INSIDE	0.504	0.525	1254.97	658.46	0.265	966.39	255.76
A_68_P27954537	chr11:80290536-80290580	NM_009871:11	Cdk5r1	INSIDE	0.566	6.419	10936.56	70198.78	3.631	9125.18	33134.19
A_68_P20350886	chr1:74902924-74902968	NM_009872:1344	Cdk5r2	INSIDE	0.532	0.281	6282.17	1764.43	0.149	4219.24	630.68
A_68_P20350877	chr1:74901834-74901878	NM_009872:254	Cdk5r2	INSIDE	0.539	0.438	1040.40	455.80	0.236	905.21	213.60
A_68_P20350875	chr1:74901471-74901515	NM_009872:-110	Cdk5r2	PROMOTER	0.414	4.547	974.26	4430.07	1.881	794.09	1493.44
A_68_P23005993	chr4:70071160-70071204	NM_145990:219	Cdk5rap2	INSIDE	0.567	0.147	4949.06	726.73	0.083	3524.09	293.40
A_68_P23005994	chr4:70071277-70071321	NM_145990:103	Cdk5rap2	INSIDE	0.664	0.263	2240.90	589.43	0.175	1661.80	290.41
A_68_P23444287	chr5:3341583-3341627	NM_009873:-2707	Cdk6	PROMOTER	0.346	7.849	2581.77	20263.14	2.715	2244.07	6092.60
A_68_P24167402	chr5:147042562-147042606	NM_153599:-666	Cdk8	DIVERGENT_PROMOTER	0.597	0.497	1579.68	785.19	0.297	1236.00	366.94
A_68_P24167580	chr5:147072895-147072939	NM_153599:29666	Cdk8	INSIDE	0.619	5.660	379.19	2146.16	3.503	345.31	1209.71
A_68_P23891971	chr5:92471843-92471887	NM_177270:168	Cdkl2	INSIDE	0.564	3.861	482.99	1865.05	2.176	418.03	909.83
A_68_P31125926	chr17:29231558-29231602	NM_007669:864	Cdkn1a	INSIDE	0.3	2.268	3423.41	7764.88	0.681	2595.19	1766.35
A_68_P25601118	chr7:150651001-150651045	NM_001161624:-4067	Cdkn1c	DIVERGENT_PROMOTER	0.173	18.322	529.67	9704.43	3.176	462.05	1467.42
A_68_P25601080	chr7:150646296-150646340	NM_001161624:637	Cdkn1c	INSIDE	0.26	14.916	1195.35	17830.27	3.877	1061.87	4117.31
A_68_P25601085	chr7:150646964-150647018	NM_001161624:-35	Cdkn1c	PROMOTER	0.256	7.569	707.02	5351.67	1.939	546.95	1060.28
A_68_P23094811	chr4:88940707-88940751	NM_009877:-205	Cdkn2a	PROMOTER	0.625	3.161	604.22	1910.18	1.975	593.11	1171.53
A_68_P25843141	chr8:48799219-48799263	NM_172407:45	Cdkn2aip	INSIDE	0.477	0.483	3633.83	1756.62	0.23	2433.44	560.54
A_68_P25843136	chr8:48798707-48798751	NM_172407:557	Cdkn2aip	INSIDE	0.569	0.527	2771.69	1460.96	0.3	2211.68	662.96
A_68_P27796702	chr11:51781404-51781448	NM_029976:294	Cdkn2aipnl	INSIDE	0.63	0.356	1453.59	517.93	0.225	1158.01	260.14
A_68_P23202006	chr4:109338923-109338967	NM_007671:-967	Cdkn2c	PROMOTER	0.37	0.338	1723.95	583.48	0.125	1356.14	169.73
A_68_P23201928	chr4:109330089-109330133	NM_007671:7867	Cdkn2c	DOWNSTREAM	0.45	5.676	1055.39	5990.17	2.553	959.69	2449.94
A_68_P25471451	chr7:128125324-128125368	NM_007672:480	Cdr2	INSIDE	0.406	0.501	2837.84	1421.71	0.203	1992.31	405.40
A_68_P28153160	chr11:115243190-115243234	NM_001080929:-17	Cdr2l	PROMOTER	0.509	3.683	1435.95	5288.46	1.875	1335.48	2504.23
A_68_P28153229	chr11:115255200-115255244	NM_001080929:11993	Cdr2l	INSIDE	0.628	3.517	668.94	2352.56	2.209	489.48	1081.14
A_68_P26234239	chr8:125091584-125091628	NM_026014:-308	Cdt1	PROMOTER	0.426	3.518	2085.22	7334.98	1.5	1582.37	2373.97
A_68_P31755200	chr18:61195546-61195590	NM_009880:285	Cdx1	INSIDE	0.221	11.201	722.28	8090.13	2.475	794.46	1966.40
A_68_P31755201	chr18:61195733-61195777	NM_009880:99	Cdx1	INSIDE	0.585	0.698	3035.76	2120.11	0.409	2005.97	820.20

ProbeName	Target position of probe on CpG island microarray	TargetID	GeneSymbol	CpG island Description	Ratio of relative methylation (TiO_2-NP/Vehicle)	Sham group			TiO_2-H group		
						Relative methylation (Cy5/Cy3)	Cy3 signal (Input)	Cy5 signal (MeDIP)	Relative methylation (Cy5/Cy3)	Cy3 signal (Input)	Cy5 signal (MeDIP)
A_68_P24172963	chr5:148116041-148116085	NM_007673:2763	Cdx2	INSIDE	0.639	3.544	872.26	3091.52	2.266	721.71	1635.18
A_68_P28944866	chr13:35832734-35832778	NM_001123386:-514	Cdyl	PROMOTER	0.487	4.163	1232.12	5128.81	2.027	1092.79	2215.19
A_68_P24995570	chr7:20437597-20437641	NM_001033419:9030	Ceacam16	INSIDE	0.521	0.268	1838.15	492.22	0.14	1480.66	206.57
A_68_P25047620	chr7:35905422-35905466	NM_007678:1133	Cebpa	INSIDE	0.615	0.512	1630.16	835.33	0.315	1265.58	398.61
A_68_P21836878	chr2:167514004-167514048	NM_009883:-388	Cebpb	PROMOTER	0.537	0.471	1536.86	723.17	0.253	1231.98	311.52
A_68_P21836882	chr2:167514407-167514451	NM_009883:14	Cebpb	INSIDE	0.587	2.704	2136.50	5777.64	1.589	1693.39	2690.21
A_68_P21836888	chr2:167514985-167515029	NM_009883:592	Cebpb	INSIDE	0.371	5.788	327.85	1897.52	2.148	319.02	685.39
A_68_P30567436	chr16:15887941-15887985	NM_007679:584	Cebpd	INSIDE	0.33	13.847	733.64	10158.95	4.563	620.94	2833.16
A_68_P30567429	chr16:15887235-15887279	NM_007679:-122	Cebpd	PROMOTER	0.585	30.953	6093.78	188621.10	18.113	5712.86	103476.90
A_68_P25047313	chr7:35841354-35841398	NM_009884:209	Cebpg	INSIDE	0.526	3.028	3535.25	10703.41	1.591	2856.93	4546.54
A_68_P24799551	chr6:120615439-120615483	NM_001128151:-978	Cecr2	PROMOTER	0.474	4.269	2543.82	10860.79	2.023	2127.83	4304.58
A_68_P24798987	chr6:120481227-120481271	NM_144815:69	Cecr5	INSIDE	0.534	4.282	1509.08	6461.25	2.285	1150.60	2629.22
A_68_P24798986	chr6:120481161-120481205	NM_144815:135	Cecr5	INSIDE	0.593	2.523	1257.96	3173.68	1.495	1085.36	1623.10
A_68_P24798780	chr6:120442970-120443014	NM_033567:833	Cecr6	INSIDE	0.463	16.954	4369.27	74076.24	7.85	3106.16	24383.86
A_68_P24798783	chr6:120443507-120443551	NM_033567:297	Cecr6	INSIDE	0.666	0.436	2314.77	1008.29	0.29	1795.16	521.01
A_68_P31565718	chr18:25645741-25645785	NM_001146292:266722	Celf4	INSIDE	0.44	4.904	1561.25	7656.30	2.16	1144.42	2471.74
A_68_P31565714	chr18:25645254-25645298	NM_001146292:267208	Celf4	INSIDE	0.444	4.888	2053.16	10035.31	2.17	1638.29	3555.79
A_68_P27289987	chr10:80945075-80945119	NM_176954:358	Celf5	INSIDE	0.332	18.947	5483.64	103900.40	6.291	3725.25	23434.93
A_68_P27289858	chr10:80927485-80927529	NM_176954:17948	Celf5	INSIDE	0.367	1.340	8390.11	11240.92	0.491	5695.79	2799.19
A_68_P30404613	chr15:85862950-85862994	NM_009886:1235	Celsr1	INSIDE	0.636	4.606	1615.73	7441.87	2.931	1208.20	3541.62
A_68_P30404626	chr15:85864596-85864640	NM_009886:-411	Celsr1	PROMOTER	0.657	0.581	2083.94	1210.54	0.382	1774.91	677.21
A_68_P30404229	chr15:85809217-85809261	NM_009886:54969	Celsr1	INSIDE	0.351	5.269	1614.55	8507.35	1.848	1182.44	2185.64
A_68_P22412541	chr3:108218746-108218790	NM_001004177:-356	Celsr2	PROMOTER	0.24	15.877	2016.82	32021.64	3.814	1614.50	6157.16
A_68_P26816838	chr9:108728469-108728513	NM_080437:-160	Celsr3	PROMOTER	0.409	0.187	3640.34	681.57	0.076	2949.95	225.67
A_68_P26816837	chr9:108728364-108728408	NM_080437:-264	Celsr3	PROMOTER	0.414	0.512	8693.32	4447.53	0.212	6223.71	1319.29
A_68_P26816894	chr9:108734858-108734902	NM_080437:6230	Celsr3	INSIDE	0.491	6.222	4033.27	25093.37	3.054	3027.75	9248.06
A_68_P26816895	chr9:108734927-108734971	NM_080437:6298	Celsr3	INSIDE	0.569	8.214	2875.96	23622.34	4.673	2499.77	11681.26
A_68_P23573892	chr5:30969470-30969514	NM_007681:218	Cenpa	INSIDE	0.581	3.046	2324.66	7080.01	1.77	1988.79	3519.49
A_68_P23861886	chr5:86494686-86494730	NM_007683:-100	Cenpc1	DIVERGENT_PROMOTER	0.666	0.643	7619.39	4899.97	0.428	5698.32	2441.10
A_68_P23861882	chr5:86494317-86494361	NM_007683:270	Cenpc1	INSIDE	0.319	2.040	3291.36	6713.29	0.65	2685.93	1744.97
A_68_P29270037	chr13:101545636-101545680	NM_021886:196	Cenph	INSIDE	0.658	0.349	2288.58	798.31	0.229	1706.39	391.51
A_68_P29623591	chr14:57190486-57190530	NM_001014996:175	Cenpj	INSIDE	0.427	4.097	1211.40	4962.78	1.748	1260.50	2203.74
A_68_P29289426	chr13:105018966-105019010	NM_181061:-46	Cenpk	DIVERGENT_PROMOTER	0.595	4.743	326.02	1546.31	2.822	334.04	942.58
A_68_P21603778	chr2:125450808-125450852	NM_001081091:19	Cep152	INSIDE	0.632	0.598	6737.22	4028.77	0.378	4941.34	1866.57
A_68_P21768600	chr2:155782266-155782310	NM_001129999:-5	Cep250	PROMOTER	0.177	19.405	1864.65	36184.14	3.434	1641.80	5637.64
A_68_P32099204	chr19:38129403-38129447	NM_028293:-90	Cep55	PROMOTER	0.231	57.055	9245.04	527471.50	13.191	9890.78	130469.40
A_68_P32099202	chr19:38129262-38129306	NM_028293:-230	Cep55	PROMOTER	0.267	30.788	9046.59	278527.00	8.207	9071.33	74444.28
A_68_P26313777	chr9:13631232-13631276	NR_037622:243	Cep57	INSIDE	0.664	0.278	3866.52	1073.51	0.184	2811.76	518.03
A_68_P27630622	chr11:20149303-20149348	NM_172260:102	Cep68	INSIDE	0.282	17.107	3038.66	51981.22	4.827	2618.37	12639.74
A_68_P27630625	chr11:20149743-20149787	NM_172260:-337	Cep68	PROMOTER	0.591	0.504	1452.65	732.27	0.298	1031.53	307.34
A_68_P27630624	chr11:20149666-20149710	NM_172260:-261	Cep68	PROMOTER	0.504	0.720	1989.84	1432.55	0.363	1356.32	491.79
A_68_P26762950	chr9:99144040-99144084	NM_023873:176	Cep70	INSIDE	0.559	9.921	1401.65	13905.76	5.55	1212.50	6729.16
A_68_P29144056	chr13:74199745-74199789	NM_028959:-33	Cep72	PROMOTER	0.337	16.654	3072.90	51176.20	5.609	3388.41	19004.13
A_68_P31792809	chr18:67770642-67770686	NM_001081073:30326	Cep76	DOWNSTREAM	0.397	6.216	1089.49	6771.76	2.468	1005.06	2480.30
A_68_P31792810	chr18:67770708-67770752	NM_001081073:30260	Cep76	DOWNSTREAM	0.576	0.658	1105.71	727.22	0.379	1012.07	383.69
A_68_P31792971	chr18:67800851-67800895	NM_001081073:118	Cep76	INSIDE	0.466	3.857	720.81	2779.85	1.798	679.86	1222.12

ProbeName	Target position of probe on CpG island microarray	TargetID	GeneSymbol	CpG island Description	Ratio of relative methylation (TiO_2-NP/Vehicle)	Sham group			TiO_2-H group		
						Relative methylation (Cy5/Cy3)	Cy3 signal (Input)	Cy5 signal (MeDIP)	Relative methylation (Cy5/Cy3)	Cy3 signal (Input)	Cy5 signal (MeDIP)
A_68_P31977524	chr19:16059019-16059063	NM_198019:439	Cep78	INSIDE	0.501	0.384	2983.24	1144.87	0.192	2138.88	411.22
A_68_P31929035	chr19:5490695-5490739	NM_007687:262	Cfl1	INSIDE	0.322	11.423	3453.07	39445.90	3.674	2630.89	9665.41
A_68_P31929038	chr19:5491024-5491068	NM_007687:592	Cfl1	INSIDE	0.483	0.373	1272.67	474.73	0.18	986.48	177.69
A_68_P31929033	chr19:5490488-5490532	NM_007687:56	Cfl1	INSIDE	0.329	5.989	873.16	5229.05	1.972	753.87	1486.93
A_68_P28449510	chr12:55963628-55963672	NM_007688:214	Cfl2	INSIDE	0.572	5.500	1531.36	8422.45	3.146	1442.53	4537.63
A_68_P28449512	chr12:55963938-55963982	NM_007688:-96	Cfl2	PROMOTER	0.168	9.125	1665.60	15198.37	1.532	1307.04	2002.73
A_68_P30822062	chr16:64851258-64851302	NM_178647:-630	Cggbp1	PROMOTER	0.531	0.209	3501.21	732.46	0.111	2619.53	290.81
A_68_P30822060	chr16:64851057-64851101	NM_178647:-832	Cggbp1	PROMOTER	0.576	0.224	9221.36	2065.80	0.129	6066.40	783.08
A_68_P22340674	chr3:94590393-94590437	NM_001037711:23	Cgn	INSIDE	0.313	7.545	3002.19	22650.92	2.358	2711.27	6392.85
A_68_P22340619	chr3:94583028-94583072	NM_001037711:7387	Cgn	INSIDE	0.492	5.912	306.30	1810.85	2.908	289.58	842.15
A_68_P26624019	chr9:71619304-71619348	NM_026599:83	Cgnl1	INSIDE	0.45	4.902	1947.44	9545.76	2.206	1679.43	3704.12
A_68_P29578607	chr14:47452213-47452258	NM_026832:317	Cgrrf1	INSIDE	0.567	2.435	2532.12	6165.72	1.38	1959.46	2704.57
A_68_P30379091	chr15:81523948-81523992	NM_001164320:3747	Chadl	INSIDE	0.091	41.972	1671.69	70164.26	3.816	1462.93	5581.92
A_68_P30379103	chr15:81524969-81525013	NM_001164320:2727	Chadl	INSIDE	0.282	13.925	1681.44	23413.85	3.931	1302.78	5121.83
A_68_P30379097	chr15:81524393-81524437	NM_001164320:3303	Chadl	INSIDE	0.411	0.322	1769.37	570.33	0.133	1366.02	181.13
A_68_P30379106	chr15:81525333-81525377	NM_001164320:2363	Chadl	INSIDE	0.422	11.691	1476.74	17263.98	4.933	1194.11	5891.04
A_68_P30379101	chr15:81524789-81524833	NM_001164320:2907	Chadl	INSIDE	0.649	3.052	646.63	1973.68	1.981	583.89	1156.81
A_68_P30978142	chr16:93884445-93884489	NM_028083:321	Chaf1b	INSIDE	0.244	0.425	2301.01	978.78	0.104	1690.65	175.49
A_68_P30978138	chr16:93883923-93883967	NM_028083:-201	Chaf1b	PROMOTER	0.636	4.336	2324.51	10078.63	2.757	1919.49	5292.90
A_68_P30978140	chr16:93884209-93884253	NM_028083:85	Chaf1b	INSIDE	0.657	0.532	1619.71	861.76	0.35	1309.22	457.70
A_68_P27259492	chr10:75398810-75398854	NM_175329:515	Chchd10	INSIDE	0.409	0.186	3117.24	579.15	0.076	2302.76	174.93
A_68_P24347497	chr6:33010009-33010053	NM_025336:122	Chchd3	INSIDE	0.444	0.478	1214.83	580.57	0.212	927.20	196.82
A_68_P24639207	chr6:91423387-91423431	NM_133928:9	Chchd4	INSIDE	0.662	2.413	2010.49	4851.60	1.597	1619.53	2586.39
A_68_P22695192	chr4:3865915-3865959	NM_001190323:-98	Chchd7	DIVERGENT_PROMOTER	0.252	7.338	3153.31	23137.54	1.851	2769.80	5126.22
A_68_P22695193	chr4:3866029-3866073	NM_001190322:-10	Chchd7	PROMOTER	0.486	0.483	1131.22	546.78	0.235	920.49	216.32
A_68_P22695196	chr4:3866420-3866464	NM_001190322:382	Chchd7	INSIDE	0.659	4.869	1288.27	6272.45	3.208	989.39	3173.73
A_68_P25368311	chr7:107685728-107685772	NM_183270:137	Chchd8	INSIDE	0.277	7.571	1321.27	10003.53	2.095	1108.89	2323.37
A_68_P22355191	chr3:97414110-97414154	NM_026539:-19	Chd1l	PROMOTER	0.606	9.798	580.75	5689.89	5.94	512.93	3046.67
A_68_P22355190	chr3:97413997-97414041	NM_026539:95	Chd1l	INSIDE	0.342	6.016	765.60	4606.19	2.057	610.17	1254.83
A_68_P27894659	chr11:69182111-69182155	NM_146019:796	Chd3	INSIDE	0.577	4.082	2063.37	8422.58	2.354	1779.21	4188.85
A_68_P24818636	chr6:125046050-125046094	NM_145979:-108	Chd4	PROMOTER	0.313	5.125	6149.47	31516.36	1.602	4411.77	7068.90
A_68_P24818640	chr6:125046428-125046472	NM_145979:270	Chd4	INSIDE	0.555	2.798	862.57	2413.52	1.552	926.41	1437.45
A_68_P23418119	chr4:151713113-151713157	NM_001081376:375	Chd5	INSIDE	0.163	13.456	2790.23	37545.95	2.19	2143.06	4693.59
A_68_P21799022	chr2:160934858-160934902	NM_173368:-88	Chd6	PROMOTER	0.65	0.532	866.92	460.97	0.346	647.53	223.77
A_68_P21799020	chr2:160934676-160934731	NM_173368:89	Chd6	INSIDE	0.419	6.658	214.87	1430.64	2.792	243.39	679.64
A_68_P22715818	chr4:8619237-8619281	NM_001081417:1191	Chd7	INSIDE	0.42	0.275	1894.89	520.93	0.115	1493.17	172.27
A_68_P22715806	chr4:8617250-8617294	NM_001081417:-795	Chd7	PROMOTER	0.613	4.086	880.35	3596.76	2.504	685.49	1716.58
A_68_P22716231	chr4:8679020-8679064	NM_001081417:60975	Chd7	INSIDE	0.3	5.253	1364.86	7170.26	1.575	917.61	1445.16
A_68_P26055737	chr8:93432164-93432208	NM_177224:79453	Chd9	INSIDE	0.29	18.281	948.29	17335.94	5.302	998.94	5295.97
A_68_P26055365	chr8:93352513-93352557	NM_177224:-199	Chd9	PROMOTER	0.333	7.317	486.61	3560.55	2.439	424.34	1035.12
A_68_P32572442	chrX:100551806-100551850	NM_009767:14	Chic1	INSIDE	0.184	15.428	1926.24	29718.22	2.84	2472.37	7022.37
A_68_P23807022	chr5:75440246-75440290	NM_028850:383	Chic2	INSIDE	0.246	1.463	1049.65	1535.45	0.36	926.86	333.44
A_68_P31919589	chr19:3852192-3852236	NM_001025566:442	Chka	INSIDE	0.382	0.599	1508.85	904.16	0.229	1308.64	299.27
A_68_P26238529	chr8:125736395-125736439	NM_145606:272	Chmp1a	INSIDE	0.471	3.174	1819.47	5775.86	1.494	1353.84	2022.20
A_68_P31790565	chr18:67365128-67365172	NM_024190:138	Chmp1b	INSIDE	0.566	5.858	4765.16	27912.95	3.314	3647.28	12087.23
A_68_P31790570	chr18:67365551-67365595	NM_024190:560	Chmp1b	INSIDE	0.631	2.357	2289.45	5395.54	1.487	1860.02	2765.35

ProbeName	Target position of probe on CpG island microarray	TargetID	GeneSymbol	CpG island Description	Ratio of relative methylation (TiO_2-NP/Vehicle)	Sham group			TiO_2-H group		
						Relative methylation (Cy5/Cy3)	Cy3 signal (Input)	Cy5 signal (MeDIP)	Relative methylation (Cy5/Cy3)	Cy3 signal (Input)	Cy5 signal (MeDIP)
A_68_P24974265	chr7:13619964-13620008	NM_026885:140	Chmp2a	INSIDE	0.571	0.403	3366.75	1357.02	0.23	2286.17	526.01
A_68_P29696921	chr14:70132430-70132474	NM_134078:-75	Chmp7	PROMOTER	0.653	2.613	2372.06	6197.19	1.706	1881.65	3209.46
A_68_P24460755	chr6:53989925-53989969	NM_001163640:21	Chn2	INSIDE	0.431	0.596	858.85	511.94	0.257	669.36	171.98
A_68_P24460750	chr6:53989374-53989418	NM_001163640:-529	Chn2	PROMOTER	0.636	2.797	865.28	2420.57	1.778	748.54	1331.26
A_68_P25365140	chr7:107154438-107154482	NM_133709:-453	Chrdl2	PROMOTER	0.107	64.666	543.23	35128.59	6.893	373.70	2575.83
A_68_P28821609	chr13:10359502-10359546	NM_033269:525	Chrm3	INSIDE	0.395	0.576	1024.44	590.48	0.228	965.79	219.98
A_68_P26528975	chr9:54874310-54874354	NM_145129:34	Chrna3	INSIDE	0.618	2.598	876.18	2276.68	1.606	811.28	1302.93
A_68_P21907947	chr2:180763825-180763869	NM_015730:10036	Chrna4	INSIDE	0.331	7.261	4234.19	30742.60	2.405	2969.65	7141.43
A_68_P21907945	chr2:180763668-180763712	NM_015730:10192	Chrna4	INSIDE	0.418	7.791	1207.11	9405.10	3.261	853.24	2781.98
A_68_P21907944	chr2:180763528-180763574	NM_015730:10332	Chrna4	INSIDE	0.366	5.275	1399.03	7379.94	1.932	934.33	1805.33
A_68_P27897123	chr11:69609071-69609115	NM_009601:347	Chrnb1	INSIDE	0.559	3.350	1255.41	4205.55	1.872	1001.39	1874.88
A_68_P21429508	chr2:92440060-92440104	NM_023850:219	Chst1	INSIDE	0.566	6.433	1510.59	9717.78	3.644	1150.55	4192.37
A_68_P21429513	chr2:92440608-92440652	NM_023850:767	Chst1	INSIDE	0.598	0.308	1651.45	509.21	0.185	1118.80	206.45
A_68_P20162793	chr1:38954859-38954904	NM_145142:124	Chst10	INSIDE	0.302	19.747	1803.94	35622.22	5.973	2835.95	16938.65
A_68_P24141811	chr5:141000117-141000161	NM_021528:18576	Chst12	INSIDE	0.622	2.961	3669.43	10865.00	1.842	2656.37	4892.24
A_68_P21566993	chr2:118752216-118752260	NM_028117:6	Chst14	INSIDE	0.401	11.372	2140.97	24347.66	4.559	1697.08	7737.28
A_68_P21566994	chr2:118752369-118752413	NM_028117:158	Chst14	INSIDE	0.42	3.898	4647.71	18118.16	1.638	3371.19	5521.00
A_68_P25533785	chr7:139508177-139508221	NM_029935:640	Chst15	INSIDE	0.616	0.467	1349.27	629.68	0.287	1094.66	314.49
A_68_P26741575	chr9:95307812-95307856	NM_018763:-145	Chst2	PROMOTER	0.245	20.623	3550.66	73225.83	5.044	3073.33	15501.22
A_68_P27173119	chr10:59681994-59682038	NM_016803:-8	Chst3	PROMOTER	0.536	3.536	3467.42	12261.93	1.897	2413.42	4578.17
A_68_P27172919	chr10:59649414-59649458	NM_016803:32572	Chst3	INSIDE	0.617	3.538	1321.21	4674.07	2.184	1074.13	2345.67
A_68_P26170767	chr8:114414110-114414154	NM_019950:19967	Chst5	INSIDE	0.411	0.310	1865.94	578.25	0.127	1536.47	195.58
A_68_P26170766	chr8:114414003-114414047	NM_019950:20075	Chst5	INSIDE	0.523	0.309	2023.93	625.30	0.161	1465.82	236.69
A_68_P26170843	chr8:114434234-114434278	NM_019950:-157	Chst5	PROMOTER	0.629	3.022	1518.32	4588.59	1.902	1230.97	2341.80
A_68_P32287119	chrX:19637488-19637532	NM_021715:815	Chst7	INSIDE	0.432	10.040	1630.78	16373.84	4.337	1750.24	7590.14
A_68_P32287125	chrX:19638017-19638061	NM_021715:1343	Chst7	INSIDE	0.497	5.055	343.26	1735.23	2.515	480.13	1207.44
A_68_P25044999	chr7:35460778-35460822	NM_175140:136930	Chst8	INSIDE	0.573	0.389	4148.98	1613.42	0.223	2698.90	601.68
A_68_P25045000	chr7:35460861-35460905	NM_175140:136848	Chst8	INSIDE	0.523	0.613	1084.91	665.47	0.321	825.01	264.58
A_68_P31746201	chr18:59335430-59335474	NM_001081328:459	Chsy3	INSIDE	0.602	0.453	1585.68	718.61	0.273	1165.29	318.08
A_68_P32130475	chr19:44181890-44181934	NM_001162410:55	Chuk	INSIDE	0.558	6.616	735.01	4863.13	3.69	646.06	2383.75
A_68_P32130473	chr19:44181677-44181721	NM_001162410:269	Chuk	INSIDE	0.476	0.744	4568.08	3399.23	0.354	3182.38	1126.16
A_68_P26079748	chr8:97362202-97362246	NM_134141:16	Ciapin1	INSIDE	0.651	4.595	471.83	2167.91	2.991	360.09	1076.88
A_68_P26526448	chr9:54407958-54408002	NM_019686:-94	Cib2	PROMOTER	0.374	4.087	1069.65	4371.92	1.53	1007.22	1541.02
A_68_P25009418	chr7:26067028-26067072	NM_001110131:-147	Cic	PROMOTER	0.06	56.076	12614.10	707349.50	3.379	20306.15	68608.12
A_68_P25009422	chr7:26068018-26068068	NM_001110131:845	Cic	INSIDE	0.33	0.513	1730.62	887.16	0.169	1304.37	220.36
A_68_P25009416	chr7:26066870-26066914	NM_001110131:-305	Cic	PROMOTER	0.411	0.141	5035.75	707.79	0.058	2966.70	171.57
A_68_P25009389	chr7:26063544-26063588	NM_001110131:-3631	Cic	PROMOTER	0.578	3.300	1444.02	4764.57	1.906	1189.85	2267.65
A_68_P25009419	chr7:26067406-26067450	NM_001110131:231	Cic	INSIDE	0.587	11.482	3090.74	35486.43	6.734	2809.15	18918.15
A_68_P25948488	chr8:72407259-72407303	NM_026818:4011	Cilp2	INSIDE	0.428	4.236	1748.11	7404.67	1.814	1539.85	2793.22
A_68_P25948535	chr8:72413120-72413164	NM_026818:-1851	Cilp2	PROMOTER	0.432	0.425	1261.45	535.83	0.183	957.92	175.63
A_68_P25948536	chr8:72413250-72413294	NM_026818:-1981	Cilp2	PROMOTER	0.582	0.436	2956.64	1288.76	0.254	2137.20	542.41
A_68_P25948479	chr8:72406005-72406049	NM_026818:5265	Cilp2	INSIDE	0.643	3.411	625.37	2132.93	2.194	490.74	1076.68
A_68_P27281431	chr10:79630462-79630506	NM_007705:-101	Cirbp	PROMOTER	0.345	0.485	2374.36	1150.54	0.167	1571.35	262.72
A_68_P27281433	chr10:79630600-79630644	NM_007705:37	Cirbp	INSIDE	0.552	2.588	2085.26	5397.44	1.428	1671.11	2386.72
A_68_P26141979	chr8:109417535-109417579	NM_011574:17	Cirh1a	INSIDE	0.388	14.567	1565.24	22801.40	5.659	1324.48	7494.86
A_68_P27234932	chr10:70807397-70807441	NM_134007:179	Cisd1	INSIDE	0.55	10.321	718.93	7420.25	5.674	651.18	3694.77

ProbeName	Target position of probe on CpG island microarray	TargetID	GeneSymbol	CpG island Description	Ratio of relative methylation (TiO_2-NP/Vehicle)	Sham group			TiO_2-H group		
						Relative methylation (Cy5/Cy3)	Cy3 signal (Input)	Cy5 signal (MeDIP)	Relative methylation (Cy5/Cy3)	Cy3 signal (Input)	Cy5 signal (MeDIP)
A_68_P27234933	chr10:70807476-70807520	NM_134007:99	Cisd1	INSIDE	0.655	3.491	1438.08	5020.46	2.288	1129.33	2584.26
A_68_P28052070	chr11:97547153-97547197	NM_001085500:-91	Cisd3	PROMOTER	0.375	1.802	2334.04	4206.95	0.676	1966.90	1329.85
A_68_P26807591	chr9:107199190-107199234	NM_009895:193	Cish	INSIDE	0.309	0.598	6240.39	3729.34	0.184	4222.62	778.53
A_68_P32567806	chrX:99447101-99447145	NM_007709:-14	Cited1	PROMOTER	0.55	5.564	479.11	2665.96	3.058	586.55	1793.95
A_68_P26971606	chr10:17444088-17444133	NM_010828:1077	Cited2	INSIDE	0.458	0.391	1522.39	595.71	0.179	1010.69	181.05
A_68_P23288133	chr4:127648899-127648943	NM_001134733:-835	CK137956	PROMOTER	0.388	0.268	2588.21	692.48	0.104	1849.44	191.92
A_68_P28745339	chr12:112908725-112908769	NM_021273:1803	Ckb	INSIDE	0.464	7.542	2147.92	16198.93	3.5	1807.59	6326.02
A_68_P28745353	chr12:112910255-112910299	NM_021273:273	Ckb	INSIDE	0.497	0.262	8085.06	2116.61	0.13	5343.46	694.56
A_68_P28745355	chr12:112910496-112910540	NM_021273:31	Ckb	INSIDE	0.639	2.904	2004.53	5821.22	1.855	1646.20	3052.88
A_68_P28745354	chr12:112910412-112910456	NM_021273:115	Ckb	INSIDE	0.462	1.540	3097.84	4771.33	0.711	2580.27	1835.70
A_68_P21580440	chr2:121180697-121180741	NM_009897:-3658	Ckmt1	DIVERGENT_PROMOTER	0.537	6.913	2733.57	18898.18	3.71	1566.55	5811.17
A_68_P29033454	chr13:51740459-51740503	NM_025415:-120	Cks2	PROMOTER	0.18	0.543	2496.49	1356.80	0.098	1760.81	172.33
A_68_P26841767	chr9:113650601-113650645	NM_001114347:32	Clasp2	INSIDE	0.266	9.827	1380.42	13565.72	2.611	1168.93	3051.83
A_68_P24994312	chr7:20189858-20189902	NM_016680:-63	Clasrp	PROMOTER	0.415	0.269	2159.03	581.13	0.112	1562.07	174.37
A_68_P31921839	chr19:4214705-4214749	NM_019952:335	Clcf1	INSIDE	0.354	5.413	1299.54	7034.23	1.915	1037.92	1987.98
A_68_P30589167	chr16:20716459-20716503	NM_009900:229	Clcn2	INSIDE	0.122	2.700	3186.61	8603.47	0.33	2661.02	879.46
A_68_P30589168	chr16:20716549-20716593	NM_009900:139	Clcn2	INSIDE	0.447	0.363	1396.04	506.69	0.162	1075.87	174.38
A_68_P30589171	chr16:20717229-20717273	NM_009900:-541	Clcn2	DIVERGENT_PROMOTER	0.471	0.429	1055.51	452.69	0.202	879.81	177.83
A_68_P30589166	chr16:20716372-20716416	NM_009900:315	Clcn2	INSIDE	0.595	2.959	1623.12	4802.69	1.759	1355.77	2385.31
A_68_P22032039	chr3:31048745-31048789	NM_008770:-75	Cldn11	PROMOTER	0.606	2.931	1363.77	3997.45	1.776	1185.15	2105.37
A_68_P25782096	chr8:36889804-36889848	NM_027998:-213	Cldn23	PROMOTER	0.467	4.831	6820.01	32944.96	2.256	5433.60	12257.29
A_68_P25782094	chr8:36889597-36889641	NM_027998:-5	Cldn23	PROMOTER	0.576	0.500	1543.93	771.91	0.288	1194.16	343.63
A_68_P25782091	chr8:36889227-36889271	NM_027998:365	Cldn23	INSIDE	0.6	2.957	1803.52	5332.79	1.773	1369.75	2429.21
A_68_P30532562	chr16:8424938-8424982	NM_029070:269	Cldn26	INSIDE	0.568	0.299	2986.64	893.69	0.17	2267.53	385.16
A_68_P28167619	chr11:117721965-117722009	NM_001085535:-79	Cldn27	PROMOTER	0.646	0.597	4671.79	2786.95	0.386	3294.92	1270.53
A_68_P31092997	chr17:23816594-23816638	NM_018777:285	Cldn6	INSIDE	0.482	4.770	6391.03	30484.22	2.299	4978.32	11444.50
A_68_P31092996	chr17:23816488-23816532	NM_018777:179	Cldn6	INSIDE	0.527	0.490	1872.79	916.88	0.258	1381.95	356.24
A_68_P27898212	chr11:69779612-69779656	NM_016887:712	Cldn7	INSIDE	0.408	1.534	2007.95	3080.75	0.627	1414.57	886.36
A_68_P25086655	chr7:51560012-51560056	NM_009131:2295	Clec11a	INSIDE	0.402	0.549	1858.03	1020.11	0.221	1380.21	304.43
A_68_P30542887	chr16:10545493-10545537	NM_001204229:83	Clec16a	INSIDE	0.393	6.343	2057.42	13050.23	2.496	1789.89	4466.92
A_68_P26014851	chr8:85913804-85913848	NM_009904:37	Clgn	INSIDE	0.609	3.908	806.96	3153.22	2.381	670.02	1595.30
A_68_P23327642	chr4:134829090-134829134	NM_013885:-437	Clic4	PROMOTER	0.564	5.912	1268.14	7497.23	3.333	1249.64	4164.84
A_68_P27764454	chr11:45665659-45665703	NM_001045520:215	Clint1	INSIDE	0.475	1.393	3332.51	4640.75	0.662	2648.29	1752.98
A_68_P24057001	chr5:124133506-124133550	NM_019765:772	Clip1	INSIDE	0.572	3.698	5331.94	19716.75	2.114	4202.14	8884.08
A_68_P24057004	chr5:124133945-124133989	NM_019765:334	Clip1	INSIDE	0.35	5.488	1085.41	5956.87	1.92	864.33	1659.93
A_68_P32982272	chr5:134998625-134998669	NM_001039162:29658	Clip2	INSIDE	0.274	5.767	2497.08	14399.56	1.579	1938.66	3061.94
A_68_P31336383	chr17:72132310-72132354	NM_030179:1046	Clip4	INSIDE	0.536	0.541	1521.03	822.60	0.29	1263.69	366.62
A_68_P20258855	chr1:58480898-58480942	NM_001042634:12	Clk1	INSIDE	0.436	4.060	1682.10	6829.52	1.77	1490.74	2638.08
A_68_P20258853	chr1:58480687-58480731	NM_001042634:224	Clk1	INSIDE	0.471	4.268	1079.78	4608.42	2.012	956.84	1924.96
A_68_P20258850	chr1:58480309-58480353	NM_001042634:602	Clk1	INSIDE	0.388	4.152	1438.40	5971.80	1.61	1153.62	1857.30
A_68_P22317616	chr3:88973234-88973282	NM_001163432:4532	Clk2	INSIDE	0.102	4.108	2203.31	9050.60	0.419	1377.36	577.78
A_68_P22317617	chr3:88973343-88973387	NM_001163432:4638	Clk2	INSIDE	0.153	21.217	5983.30	126945.30	3.247	4438.93	14415.22
A_68_P26543679	chr9:57613020-57613064	NM_007713:625	Clk3	INSIDE	0.451	4.776	804.81	3843.78	2.153	721.45	1553.38
A_68_P26543678	chr9:57612898-57612942	NM_007713:747	Clk3	INSIDE	0.275	5.359	5466.59	29296.49	1.471	4415.23	6495.47
A_68_P23815279	chr5:76733734-76733778	NM_007715:-183	Clock	PROMOTER	0.66	4.034	1714.98	6917.85	2.661	1345.48	3579.90
A_68_P31259429	chr17:57129711-57129755	NM_017393:46	Clpp	INSIDE	0.269	0.583	5506.01	3210.25	0.157	3256.79	511.05

ProbeName	Target position of probe on CpG island microarray	TargetID	GeneSymbol	CpG island Description	Ratio of relative methylation (TiO_2-NP/Vehicle)	Sham group			TiO_2-H group		
						Relative methylation (Cy5/Cy3)	Cy3 signal (Input)	Cy5 signal (MeDIP)	Relative methylation (Cy5/Cy3)	Cy3 signal (Input)	Cy5 signal (MeDIP)
A_68_P31259430	chr17:57129793-57129837	NM_017393:128	Clpp	INSIDE	0.417	0.283	6239.03	1767.49	0.118	3678.62	434.71
A_68_P29140850	chr13:73741332-73741376	NM_146047:-95	Clptm1l	PROMOTER	0.615	2.555	1141.70	2917.23	1.572	969.04	1523.07
A_68_P23280756	chr4:126234616-126234660	NM_175554:415	Clspn	INSIDE	0.591	3.232	793.34	2564.21	1.911	713.57	1363.84
A_68_P23401328	chr4:148961215-148961259	NM_023051:490	Clstn1	INSIDE	0.185	0.555	9072.90	5039.10	0.103	5621.25	576.61
A_68_P23401326	chr4:148961050-148961094	NM_023051:326	Clstn1	INSIDE	0.324	0.565	4538.99	2564.66	0.183	3241.64	593.16
A_68_P23401329	chr4:148961308-148961352	NM_023051:584	Clstn1	INSIDE	0.375	0.490	8432.45	4128.96	0.184	5447.48	999.73
A_68_P23401327	chr4:148961132-148961176	NM_023051:408	Clstn1	INSIDE	0.452	0.201	3902.60	783.83	0.091	2664.89	242.11
A_68_P26756440	chr9:97933663-97933707	NM_022319:-98	Clstn2	PROMOTER	0.332	33.861	2789.71	94462.64	11.256	2462.18	27714.81
A_68_P26756438	chr9:97933240-97933284	NM_022319:324	Clstn2	INSIDE	0.339	5.591	1388.36	7762.97	1.893	1140.63	2159.72
A_68_P26756442	chr9:97933946-97933990	NM_022319:-382	Clstn2	PROMOTER	0.47	0.571	831.25	474.99	0.269	648.72	174.30
A_68_P27990839	chr11:86571533-86571577	NM_001003908:-560	Cltc	PROMOTER	0.238	8.470	1076.10	9114.69	2.014	922.78	1858.24
A_68_P23999133	chr5:114064391-114064435	NM_008153:35987	Cmklr1	INSIDE	0.122	28.317	1150.67	32583.08	3.456	798.90	2760.64
A_68_P23999132	chr5:114064289-114064333	NM_008153:36089	Cmklr1	INSIDE	0.376	4.560	3715.75	16942.92	1.715	2530.47	4339.33
A_68_P23222742	chr4:114659539-114659583	NM_025647:273	Cmpk1	INSIDE	0.321	11.746	1689.66	19846.54	3.774	1589.94	6001.18
A_68_P28302020	chr12:27154103-27154147	NM_020557:45	Cmpk2	INSIDE	0.422	0.412	2163.40	891.13	0.174	1494.19	259.60
A_68_P28302019	chr12:27154024-27154068	NM_020557:-33	Cmpk2	PROMOTER	0.653	0.432	2601.04	1122.67	0.282	1689.00	476.26
A_68_P26127987	chr8:106864391-106864435	NM_024217:-81	Cmtm3	PROMOTER	0.245	5.685	10857.31	61723.59	1.395	7671.92	10705.32
A_68_P26128337	chr8:106919324-106919368	NM_153582:250	Cmtm4	INSIDE	0.356	14.908	2970.15	44280.27	5.309	2695.43	14309.10
A_68_P26846253	chr9:114662207-114662251	NM_133978:28719	Cmtm7	DOWNSTREAM	0.653	0.440	1337.28	588.47	0.287	1096.59	315.15
A_68_P26846741	chr9:114753280-114753324	NM_027294:-32	Cmtm8	PROMOTER	0.138	25.836	9389.88	242598.20	3.554	8466.36	30093.45
A_68_P24618503	chr6:87800411-87800455	NM_001109745:668	Cnbp	INSIDE	0.382	6.101	2485.88	15165.54	2.331	2027.45	4726.63
A_68_P32769557	chrX:154481653-154481697	NM_177751:-632	Cnksr2	PROMOTER	0.491	4.427	754.91	3342.24	2.175	968.80	2107.04
A_68_P32769550	chrX:154480805-154480849	NM_177751:216	Cnksr2	INSIDE	0.544	4.754	1073.72	5104.61	2.585	1265.68	3271.24
A_68_P26898893	chr10:3134939-3134983	NM_172546:657	Cnksr3	INSIDE	0.444	5.557	3899.65	21668.81	2.469	3117.65	7695.93
A_68_P22476389	chr3:121130415-121130465	NM_028044:982	Cnn3	INSIDE	0.215	0.476	2271.50	1081.74	0.102	1697.24	173.40
A_68_P22476384	chr3:121129828-121129872	NM_028044:392	Cnn3	INSIDE	0.418	0.453	1077.59	488.04	0.189	913.46	172.73
A_68_P32126462	chr19:43515774-43515818	NM_031396:871	Cnnm1	INSIDE	0.471	5.112	1493.31	7634.49	2.408	1289.75	3105.85
A_68_P32145765	chr19:46837310-46837354	NM_001102471:1234	Cnnm2	INSIDE	0.554	0.347	1495.05	519.05	0.192	1172.44	225.51
A_68_P20148714	chr1:36568619-36568663	NM_001039551:-80	Cnnm3	PROMOTER	0.147	53.745	8553.61	459710.80	7.899	9888.23	78110.30
A_68_P20148715	chr1:36568684-36568728	NM_001039551:-14	Cnnm3	PROMOTER	0.34	6.224	3580.69	22287.92	2.117	2613.07	5532.29
A_68_P20148721	chr1:36569446-36569490	NM_001039551:748	Cnnm3	INSIDE	0.421	12.690	2248.52	28533.62	5.339	1820.57	9719.42
A_68_P20148716	chr1:36568774-36568818	NM_001039551:76	Cnnm3	INSIDE	0.559	3.388	3198.55	10837.29	1.895	2497.08	4732.09
A_68_P20148457	chr1:36528751-36528795	NM_033570:331	Cnnm4	INSIDE	0.47	0.199	2690.54	535.59	0.094	1909.55	178.61
A_68_P23609441	chr5:37139453-37139497	NM_133724:444	Cno	INSIDE	0.415	0.321	1672.99	537.77	0.133	1282.50	170.96
A_68_P26085422	chr8:98331940-98331984	NM_001205226:-600	Cnot1	PROMOTER	0.572	0.269	2060.03	555.07	0.154	1537.27	237.03
A_68_P26085416	chr8:98331248-98331292	NM_001205226:92	Cnot1	INSIDE	0.587	0.318	2656.50	844.06	0.187	1989.52	371.18
A_68_P27476522	chr10:116018238-116018282	NM_001037846:307	Cnot2	INSIDE	0.25	35.363	10319.36	364919.10	8.84	9606.38	84917.08
A_68_P27476528	chr10:116018985-116019029	NM_001037846:-439	Cnot2	PROMOTER	0.295	10.315	4469.24	46099.31	3.044	3634.81	11065.81
A_68_P27476526	chr10:116018789-116018833	NM_001037846:-243	Cnot2	PROMOTER	0.381	6.842	2068.31	14151.48	2.606	1808.40	4712.06
A_68_P27476525	chr10:116018687-116018731	NM_001037846:-141	Cnot2	PROMOTER	0.518	6.007	1004.07	6031.24	3.114	816.20	2541.68
A_68_P24946543	chr7:3597351-3597395	NM_146176:502	Cnot3	INSIDE	0.545	3.710	1058.64	3927.99	2.023	767.05	1551.76
A_68_P24359955	chr6:35083485-35083529	NM_001164412:231	Cnot4	INSIDE	0.32	9.001	4380.46	39426.65	2.885	3727.15	10751.57
A_68_P24359954	chr6:35083412-35083456	NM_001164412:303	Cnot4	INSIDE	0.091	1.468	6620.02	9718.59	0.134	5135.84	688.94
A_68_P27784449	chr11:49525483-49525527	NM_212484:708	Cnot6	INSIDE	0.311	10.907	1296.44	14139.88	3.391	1163.69	3946.21
A_68_P27784450	chr11:49525550-49525594	NM_212484:640	Cnot6	INSIDE	0.377	7.166	3749.64	26870.29	2.704	3498.92	9461.34
A_68_P25806164	chr8:41596758-41596802	NM_011135:-122	Cnot7	DIVERGENT_PROMOTER	0.374	5.277	7277.81	38406.40	1.975	5881.19	11614.18

ProbeName	Target position of probe on CpG island microarray	TargetID	GeneSymbol	CpG island Description	Ratio of relative methylation (TiO_2-NP/Vehicle)	Sham group			TiO_2-H group		
						Relative methylation (Cy5/Cy3)	Cy3 signal (Input)	Cy5 signal (MeDIP)	Relative methylation (Cy5/Cy3)	Cy3 signal (Input)	Cy5 signal (MeDIP)
A_68_P25806160	chr8:41596401-41596445	NM_011135:236	Cnot7	INSIDE	0.522	0.339	1671.34	566.07	0.177	1361.00	240.82
A_68_P27829185	chr11:57917595-57917639	NM_026949:-38	Cnot8	PROMOTER	0.381	0.725	1390.64	1008.02	0.276	1032.50	285.22
A_68_P23331196	chr4:135472765-135472809	NM_009924:21468	Cnr2	INSIDE	0.573	3.854	1697.83	6542.76	2.209	1204.66	2661.27
A_68_P20870811	chr1:181476719-181476763	NM_146105:81	Cnst	INSIDE	0.252	8.999	808.98	7279.70	2.265	745.23	1687.62
A_68_P20622147	chr1:134439405-134439449	NM_177129:91	Cntn2	INSIDE	0.276	22.365	3072.57	68716.84	6.173	2941.38	18155.92
A_68_P24420943	chr6:46994568-46994616	NM_001004357:1984533	Cntnap2	INSIDE	0.241	9.092	1945.05	17684.66	2.191	1615.76	3540.06
A_68_P21285107	chr2:65077163-65077207	NM_027225:-501	Cobll1	PROMOTER	0.213	18.146	4617.47	83790.16	3.867	4516.59	17466.60
A_68_P21070564	chr2:25066622-25066666	NM_021393:365	Cobra1	INSIDE	0.393	0.457	1458.48	665.81	0.179	1024.58	183.69
A_68_P21070567	chr2:25066910-25066954	NM_021393:77	Cobra1	INSIDE	0.597	0.575	3177.80	1827.48	0.343	2458.42	843.38
A_68_P28430598	chr12:52694641-52694685	NM_001198835:335	Coch	INSIDE	0.354	4.228	1870.62	7908.91	1.496	1593.10	2383.37
A_68_P28003125	chr11:88835210-88835254	NM_016706:-16	Coil	PROMOTER	0.616	4.857	1558.49	7569.01	2.993	1235.06	3696.92
A_68_P26665705	chr9:79566838-79566882	NM_007730:-375	Col12a1	PROMOTER	0.66	0.356	3419.79	1215.85	0.235	2331.35	546.71
A_68_P27183504	chr10:61441353-61441397	NM_007731:482	Col13a1	INSIDE	0.635	3.917	1082.03	4238.72	2.489	889.24	2213.29
A_68_P27183499	chr10:61440751-61440795	NM_007731:1084	Col13a1	INSIDE	0.567	0.745	6336.51	4717.80	0.422	4589.36	1936.48
A_68_P27183508	chr10:61441806-61441850	NM_007731:28	Col13a1	INSIDE	0.541	2.633	2851.75	7509.57	1.424	2233.02	3180.27
A_68_P23299955	chr4:129724973-129725017	NM_028266:-89	Col16a1	PROMOTER	0.299	11.884	2579.09	30649.72	3.549	2153.75	7643.56
A_68_P23299954	chr4:129724880-129724924	NM_028266:-181	Col16a1	PROMOTER	0.319	9.168	684.64	6276.43	2.923	600.23	1754.49
A_68_P27793111	chr11:51103960-51104004	NM_153393:561	Col23a1	INSIDE	0.212	0.627	1859.33	1166.27	0.133	1291.11	171.64
A_68_P22524273	chr3:129883661-129883705	NM_029838:-113	Col25a1	PROMOTER	0.376	15.912	645.29	10268.04	5.975	551.56	3295.72
A_68_P22968875	chr4:62875286-62875330	NM_025685:-1137	Col27a1	PROMOTER	0.583	3.636	824.31	2997.58	2.12	632.57	1340.96
A_68_P29246777	chr13:97312887-97312931	NM_001164222:219	Col4a3bp	INSIDE	0.485	0.545	2702.94	1473.11	0.264	2120.87	560.33
A_68_P21087493	chr2:27741935-27741979	NM_015734:12	Col5a1	INSIDE	0.575	0.536	1990.06	1066.76	0.308	1391.19	428.67
A_68_P21087491	chr2:27741628-27741672	NM_015734:-294	Col5a1	PROMOTER	0.647	0.529	1285.82	680.59	0.342	997.70	341.60
A_68_P21087494	chr2:27742066-27742110	NM_015734:144	Col5a1	INSIDE	0.428	1.639	4657.08	7633.80	0.702	3166.63	2223.19
A_68_P27263162	chr10:76086244-76086288	NM_146007:-117	Col6a2	PROMOTER	0.483	4.836	940.38	4547.50	2.334	824.88	1925.64
A_68_P27262976	chr10:76059601-76059649	NM_146007:26525	Col6a2	INSIDE	0.353	1.400	926.50	1296.82	0.494	789.43	390.20
A_68_P23279249	chr4:125963943-125963987	NM_199473:-73	Col8a2	PROMOTER	0.267	7.355	2163.60	15913.90	1.961	2144.70	4205.26
A_68_P23279343	chr4:125978444-125978488	NM_199473:14429	Col8a2	INSIDE	0.516	2.939	1725.82	5072.00	1.515	1318.90	1998.34
A_68_P21904962	chr2:180333066-180333110	NM_009936:162	Col9a3	INSIDE	0.474	0.392	3098.35	1215.61	0.186	2115.61	393.71
A_68_P27643714	chr11:22882221-22882265	NM_144514:42	Commd1	INSIDE	0.62	2.866	635.28	1820.92	1.778	571.21	1015.76
A_68_P22164786	chr3:57455644-57455688	NM_175095:-60	Commd2	PROMOTER	0.575	0.620	1422.07	881.26	0.357	1039.92	370.74
A_68_P21040743	chr2:18593952-18593996	NM_147778:-114	Commd3	PROMOTER	0.553	3.680	3026.88	11139.86	2.035	2676.80	5446.39
A_68_P29856057	chr14:102039624-102039668	NM_001033132:42	Commd6	INSIDE	0.565	0.227	4880.28	1105.73	0.128	3573.80	457.81
A_68_P25951429	chr8:72897357-72897401	NM_016685:-68	Comp	PROMOTER	0.497	7.744	1581.14	12244.55	3.85	1418.29	5460.90
A_68_P25951452	chr8:72900302-72900346	NM_016685:2878	Comp	INSIDE	0.497	0.386	1191.54	460.44	0.192	899.04	172.76
A_68_P25951438	chr8:72898648-72898692	NM_016685:1224	Comp	INSIDE	0.613	2.495	1300.02	3243.24	1.531	1136.70	1739.75
A_68_P29455231	chr14:22667975-22668019	NM_026965:136	Comtd1	INSIDE	0.324	5.101	13572.84	69239.50	1.655	10709.60	17728.26
A_68_P21605247	chr2:125684679-125684728	NM_009939:51	Cops2	INSIDE	0.551	0.512	996.58	510.17	0.282	619.08	174.72
A_68_P23930021	chr5:100947315-100947359	NM_012001:9	Cops4	INSIDE	0.54	0.497	1275.82	633.90	0.268	1046.68	280.64
A_68_P20412924	chr1:88483955-88483999	NM_172974:302	Cops7b	INSIDE	0.363	0.689	1117.63	770.04	0.25	870.11	217.38
A_68_P20437267	chr1:92500071-92500115	NM_133805:93	Cops8	INSIDE	0.467	12.001	2580.78	30972.15	5.609	2413.45	13537.29
A_68_P28047199	chr11:96723478-96723522	NM_019877:12311	Copz2	DOWNSTREAM	0.219	9.588	960.82	9212.25	2.097	761.33	1596.31
A_68_P23792212	chr5:72895290-72895334	NM_016869:135	Corin	INSIDE	0.466	0.483	1577.35	761.91	0.225	1077.74	242.51
A_68_P23792211	chr5:72895109-72895153	NM_016869:317	Corin	INSIDE	0.462	3.399	1684.48	5725.86	1.569	1283.92	2014.33
A_68_P24000798	chr5:114359230-114359275	NM_011779:-537	Coro1c	PROMOTER	0.359	14.040	9000.69	126373.00	5.041	7623.85	38435.22
A_68_P24000794	chr5:114358647-114358691	NM_011779:47	Coro1c	INSIDE	0.443	5.639	2221.54	12528.31	2.499	1889.25	4721.27

ProbeName	Target position of probe on CpG island microarray	TargetID	GeneSymbol	CpG island Description	Ratio of relative methylation (TiO_2-NP/Vehicle)	Sham group			TiO_2-H group		
						Relative methylation (Cy5/Cy3)	Cy3 signal (Input)	Cy5 signal (MeDIP)	Relative methylation (Cy5/Cy3)	Cy3 signal (Input)	Cy5 signal (MeDIP)
A_68_P24000793	chr5:114358500-114358544	NM_011779:193	Coro1c	INSIDE	0.609	4.335	673.23	2918.43	2.639	607.73	1603.97
A_68_P22889573	chr4:46614584-46614628	NM_001164804:195	Coro2a	INSIDE	0.629	0.499	1896.20	947.07	0.314	1537.99	483.45
A_68_P27937736	chr11:77281473-77281517	NM_139128:4080	Coro6	INSIDE	0.577	2.733	1407.73	3847.59	1.576	1199.36	1890.23
A_68_P30511525	chr16:4679878-4679922	NM_030205:-180	Coro7	DIVERGENT_PROMOTER	0.658	0.524	1314.65	689.12	0.345	1077.06	371.23
A_68_P24135004	chr5:139821148-139821192	NM_197980:-50	Cox19	DIVERGENT_PROMOTER	0.392	0.347	4683.33	1626.61	0.136	3200.10	436.18
A_68_P26221734	chr8:123191897-123191941	NM_010926:94	Cox4nb	INSIDE	0.205	8.958	6349.71	56880.71	1.838	5333.04	9802.11
A_68_P26542213	chr9:57368982-57369026	NM_007747:-34	Cox5a	PROMOTER	0.336	0.640	893.71	572.22	0.215	789.51	169.97
A_68_P26542218	chr9:57369471-57369515	NM_007747:454	Cox5a	INSIDE	0.438	0.605	854.24	517.19	0.265	745.01	197.54
A_68_P20149813	chr1:36748609-36748653	NM_009942:299	Cox5b	INSIDE	0.62	0.676	1744.61	1179.52	0.419	1440.90	604.23
A_68_P24009173	chr5:115798570-115798614	NM_007748:372	Cox6a1	INSIDE	0.66	0.445	2490.70	1109.55	0.294	1901.17	558.97
A_68_P30136000	chr15:35867909-35867953	NM_053071:71	Cox6c	INSIDE	0.576	0.439	1678.13	736.16	0.253	1203.89	304.24
A_68_P26665914	chr9:79607348-79607392	NM_009945:290	Cox7a2	INSIDE	0.573	0.372	2132.08	792.21	0.213	1535.77	327.09
A_68_P27934410	chr11:76660169-76660213	NM_007754:320	Cpd	INSIDE	0.641	3.807	916.74	3490.16	2.441	883.87	2157.37
A_68_P23644443	chr5:43625072-43625116	NM_001177379:393	Cpeb2	INSIDE	0.328	14.119	2221.31	31363.58	4.634	2143.06	9930.64
A_68_P32094201	chr19:37249291-37249335	NM_198300:32471	Cpeb3	INSIDE	0.094	0.448	5520.41	2472.56	0.042	4114.22	172.77
A_68_P21769580	chr2:155937622-155937666	NM_170588:57	Cpne1	INSIDE	0.643	2.976	6531.47	19437.39	1.913	4455.40	8525.27
A_68_P21769581	chr2:155937720-155937764	NM_170588:-41	Cpne1	PROMOTER	0.277	0.677	1302.27	881.81	0.188	1006.55	189.01
A_68_P31126792	chr17:29375212-29375256	NM_153166:-499	Cpne5	PROMOTER	0.42	0.290	3346.26	971.62	0.122	2414.06	294.18
A_68_P31126789	chr17:29374780-29374824	NM_153166:-67	Cpne5	PROMOTER	0.526	4.550	5748.60	26157.54	2.392	4356.20	10420.07
A_68_P31126791	chr17:29375001-29375045	NM_153166:-287	Cpne5	PROMOTER	0.576	6.169	700.91	4324.08	3.555	612.18	2176.39
A_68_P31126793	chr17:29375333-29375377	NM_153166:-619	Cpne5	PROMOTER	0.598	0.299	4973.11	1487.58	0.179	3591.59	642.21
A_68_P30431386	chr15:90509992-90510036	NM_025815:-195	Cpne8	PROMOTER	0.659	2.418	1846.49	4465.24	1.595	1338.29	2134.04
A_68_P24758532	chr6:113232291-113232335	NM_170673:12	Cpne9	INSIDE	0.607	3.004	1142.07	3430.65	1.823	1013.85	1848.15
A_68_P28688785	chr12:103214150-103214194	NM_016856:-11	Cpsf2	PROMOTER	0.559	4.124	1560.44	6434.89	2.306	1425.20	3287.19
A_68_P28688783	chr12:103213937-103213981	NM_016856:-225	Cpsf2	PROMOTER	0.568	6.788	1525.46	10354.74	3.855	1405.69	5419.09
A_68_P24164624	chr5:145928360-145928404	NM_178576:290	Cpsf4	INSIDE	0.291	1.532	1562.73	2393.37	0.446	1313.33	586.14
A_68_P27480860	chr10:116813708-116813752	NM_001013391:299	Cpsf6	INSIDE	0.651	2.814	946.02	2662.14	1.831	888.50	1626.58
A_68_P31951138	chr19:10599754-10599798	NM_001164272:43	Cpsf7	INSIDE	0.28	0.575	1973.90	1135.06	0.161	1581.81	254.33
A_68_P23601183	chr5:35868307-35868351	NM_153107:-53	Cpz	PROMOTER	0.628	0.505	2501.29	1262.37	0.317	1756.65	556.81
A_68_P26527664	chr9:54613648-54613692	NM_013496:1056	Crabp1	INSIDE	0.42	5.579	776.40	4331.19	2.342	732.00	1714.52
A_68_P26527662	chr9:54613478-54613522	NM_013496:886	Crabp1	INSIDE	0.594	0.505	5487.38	2772.90	0.3	3728.90	1119.74
A_68_P27364532	chr10:94786282-94786326	NM_009950:427	Cradd	INSIDE	0.221	0.476	2013.33	959.31	0.105	1675.08	176.45
A_68_P27364531	chr10:94786138-94786182	NM_009950:571	Cradd	INSIDE	0.349	0.293	2323.03	681.57	0.102	1688.33	172.84
A_68_P31101001	chr17:25151361-25151405	NM_020608:793	Cramp1l	INSIDE	0.434	5.458	4390.50	23962.30	2.371	3269.66	7753.24
A_68_P31101000	chr17:25151186-25151230	NM_020608:967	Cramp1l	INSIDE	0.651	0.256	9098.37	2327.64	0.166	6449.60	1073.49
A_68_P21103096	chr2:30271133-30271177	NM_007760:114	Crat	INSIDE	0.388	20.369	2961.22	60316.55	7.897	2550.62	20143.51
A_68_P21103095	chr2:30271038-30271082	NM_007760:208	Crat	INSIDE	0.572	5.031	1408.37	7084.99	2.879	1240.51	3570.93
A_68_P21144181	chr2:37649266-37649310	NM_001163566:17520	Crb2	INSIDE	0.244	10.746	2235.78	24026.48	2.627	1655.20	4348.96
A_68_P31259847	chr17:57201924-57201968	NM_177638:247	Crb3	INSIDE	0.599	0.587	2571.10	1509.47	0.352	1948.55	685.54
A_68_P24091776	chr5:130505164-130505208	NM_007761:11	Crcp	INSIDE	0.635	6.307	725.09	4573.17	4.003	637.70	2552.73
A_68_P20293016	chr1:64579676-64579720	NM_001037726:321	Creb1	INSIDE	0.082	3.676	3716.44	13662.65	0.302	3063.65	926.71
A_68_P20293011	chr1:64579120-64579164	NM_001037726:-235	Creb1	PROMOTER	0.363	0.366	3336.36	1221.74	0.133	2378.77	316.54
A_68_P21425775	chr2:91815556-91815600	NM_011957:48749	Creb3l1	DOWNSTREAM	0.251	9.822	1099.23	10796.49	2.468	870.12	2147.67
A_68_P30508953	chr16:4212906-4212950	NM_001025432:476	Crebbp	INSIDE	0.514	0.208	2247.93	468.59	0.107	1618.25	173.31
A_68_P30508958	chr16:4214152-4214196	NM_001025432:-770	Crebbp	PROMOTER	0.526	5.160	1147.44	5921.05	2.714	1096.65	2976.07
A_68_P25313627	chr7:97591983-97592027	NM_145151:714	Crebzf	INSIDE	0.663	3.686	1311.41	4833.26	2.445	1076.60	2632.20

ProbeName	Target position of probe on CpG island microarray	TargetID	GeneSymbol	CpG island Description	Ratio of relative methylation (TiO_2-NP/Vehicle)	Sham group			TiO_2-H group		
						Relative methylation (Cy5/Cy3)	Cy3 signal (Input)	Cy5 signal (MeDIP)	Relative methylation (Cy5/Cy3)	Cy3 signal (Input)	Cy5 signal (MeDIP)
A_68_P20796287	chr1:167694031-167694075	NM_011804:142	Creg1	INSIDE	0.504	4.855	2262.50	10985.43	2.445	2016.08	4929.49
A_68_P21976418	chr3:19594468-19594512	NM_205769:906	Crh	INSIDE	0.375	0.332	1763.41	585.39	0.125	1518.09	189.03
A_68_P28089255	chr11:103995098-103995142	NM_007762:925	Crhr1	INSIDE	0.657	2.809	1973.33	5543.00	1.844	1706.91	3148.03
A_68_P31369865	chr17:78599190-78599234	NM_015800:-375	Crim1	PROMOTER	0.42	8.686	2349.97	20411.08	3.645	1975.26	7200.66
A_68_P30573853	chr16:17452437-17452481	NM_007764:379	Crkl	INSIDE	0.61	0.686	1119.53	767.67	0.418	890.36	372.60
A_68_P25952284	chr8:73027155-73027199	NM_018827:10122	Crlf1	INSIDE	0.381	8.144	2579.76	21008.80	3.101	2280.48	7072.24
A_68_P23976362	chr5:109987925-109987969	NM_001164735:66	Crlf2	INSIDE	0.51	0.324	1466.27	474.48	0.165	1056.90	174.55
A_68_P27952488	chr11:79894575-79894619	NM_018776:-139	Crlf3	DIVERGENT_PROMOTER	0.399	0.329	1715.72	563.73	0.131	1305.72	171.04
A_68_P23612513	chr5:37637158-37637202	NM_007765:150	Crmp1	INSIDE	0.387	0.427	1650.56	705.41	0.166	1285.29	212.74
A_68_P23612479	chr5:37633037-37633081	NM_001136058:-260	Crmp1	PROMOTER	0.469	7.800	1639.66	12789.87	3.661	1445.56	5292.07
A_68_P23612514	chr5:37637296-37637340	NM_007765:288	Crmp1	INSIDE	0.327	0.668	1061.84	709.56	0.218	784.59	171.35
A_68_P23361673	chr4:140581289-140581333	NM_172122:28266	Crocc	INSIDE	0.334	15.554	2707.21	42106.99	5.191	2450.71	12720.41
A_68_P23361674	chr4:140581469-140581513	NM_172122:28086	Crocc	INSIDE	0.593	6.333	1495.33	9470.52	3.757	1320.63	4961.15
A_68_P23361905	chr4:140616349-140616393	NM_172122:-6794	Crocc	PROMOTER	0.557	2.937	4255.71	12500.26	1.635	3315.77	5422.82
A_68_P32121139	chr19:42505784-42505828	NM_145123:467	Crtac1	INSIDE	0.479	0.492	4151.81	2044.71	0.236	2696.15	636.18
A_68_P26844408	chr9:114299168-114299212	NM_019922:640	Crtap	INSIDE	0.251	2.634	5957.51	15694.17	0.66	4804.90	3172.55
A_68_P25268092	chr7:87833284-87833328	NM_173863:457	Crtc3	INSIDE	0.578	4.072	1553.64	6326.97	2.353	1329.15	3127.90
A_68_P30964660	chr16:91728640-91728684	NM_026994:385	Cryzl1	INSIDE	0.647	0.289	2540.49	735.24	0.187	2001.12	374.79
A_68_P27540829	chr10:127775164-127775208	NM_026444:299	Cs	INSIDE	0.528	0.663	929.56	616.68	0.35	767.80	268.93
A_68_P24850798	chr6:131338248-131338292	NM_011733:198	Csda	INSIDE	0.401	12.609	2181.97	27511.77	5.055	1956.17	9887.53
A_68_P24850799	chr6:131338430-131338474	NM_011733:16	Csda	INSIDE	0.389	4.204	2450.78	10303.38	1.634	2169.83	3544.76
A_68_P22408449	chr3:107563183-107563227	NM_001113529:183	Csf1	INSIDE	0.266	50.451	5941.35	299747.40	13.444	7769.89	104456.50
A_68_P32229409	chr19:61301021-61301065	NM_009970:3279	Csf2ra	INSIDE	0.131	2.056	2026.82	4166.78	0.269	1451.42	390.46
A_68_P31758329	chr18:61715242-61715286	NM_146087:29	Csnk1a1	INSIDE	0.568	4.287	2062.95	8843.62	2.434	1748.66	4256.80
A_68_P31758330	chr18:61715307-61715351	NM_146087:93	Csnk1a1	INSIDE	0.663	0.528	918.07	484.56	0.35	769.28	269.14
A_68_P26590184	chr9:65757067-65757111	NM_173185:272	Csnk1g1	INSIDE	0.543	15.108	2759.64	41692.76	8.197	2456.25	20134.65
A_68_P27284502	chr10:80101268-80101313	NM_001159591:8890	Csnk1g2	INSIDE	0.447	3.548	1050.23	3726.66	1.586	728.58	1155.79
A_68_P31715738	chr18:54022583-54022627	NM_152809:838	Csnk1g3	INSIDE	0.148	0.472	8836.35	4173.68	0.07	5714.25	399.61
A_68_P31715736	chr18:54022224-54022268	NM_152809:480	Csnk1g3	INSIDE	0.326	14.292	568.64	8127.05	4.665	580.68	2709.03
A_68_P31715735	chr18:54022118-54022162	NM_152809:374	Csnk1g3	INSIDE	0.381	5.539	1286.14	7124.37	2.109	1076.58	2270.90
A_68_P21748226	chr2:152053141-152053185	NM_007788:587	Csnk2a1	INSIDE	0.485	7.476	2956.63	22103.77	3.625	2567.05	9306.19
A_68_P21748223	chr2:152052880-152052924	NM_007788:327	Csnk2a1	INSIDE	0.32	1.399	2018.26	2822.73	0.448	1436.40	642.98
A_68_P31157712	chr17:35257797-35257841	NM_009975:574	Csnk2b	INSIDE	0.37	0.525	1164.49	611.44	0.194	879.76	170.73
A_68_P31157711	chr17:35257720-35257764	NM_009975:650	Csnk2b	INSIDE	0.336	4.572	2731.85	12490.93	1.537	2243.36	3449.04
A_68_P26538932	chr9:56712960-56713004	NM_139001:72	Cspg4	INSIDE	0.166	23.073	8045.17	185628.10	3.819	8694.88	33207.04
A_68_P20025490	chr1:10029865-10029909	NM_026493:1588	Cspp1	INSIDE	0.618	0.650	1250.34	812.95	0.402	939.06	377.06
A_68_P26875484	chr9:119892493-119892537	NM_153287:1262	Csrnp1	INSIDE	0.451	3.899	820.81	3199.98	1.756	686.44	1205.72
A_68_P30486141	chr15:100325298-100325342	NM_153407:350	Csrnp2	INSIDE	0.543	0.321	4029.26	1294.95	0.174	3315.81	578.58
A_68_P30486146	chr15:100325931-100325975	NM_153407:-282	Csrnp2	PROMOTER	0.54	0.630	1500.47	945.00	0.34	1135.49	386.49
A_68_P20641219	chr1:137625693-137625737	NM_007791:-59	Csrp1	PROMOTER	0.572	0.261	1878.18	489.74	0.149	1405.23	209.61
A_68_P27446111	chr10:110357053-110357097	NM_007792:-157	Csrp2	PROMOTER	0.434	0.365	1380.13	504.41	0.159	1106.64	175.40
A_68_P27446114	chr10:110357360-110357404	NM_007792:151	Csrp2	INSIDE	0.535	0.573	2855.83	1637.81	0.307	2204.80	677.10
A_68_P21706949	chr2:144219429-144219473	NM_181417:24680	Csrp2bp	INSIDE	0.408	4.915	2156.25	10597.07	2.004	1586.99	3181.10
A_68_P31928180	chr19:5349389-5349433	NM_028623:164	Cst6	INSIDE	0.105	49.406	9546.52	471656.80	5.174	9347.57	48365.33
A_68_P27274155	chr10:77888554-77888598	NM_007793:162	Cstb	INSIDE	0.641	4.113	1473.82	6062.23	2.635	1212.51	3195.37
A_68_P21863932	chr2:172196696-172196740	NM_024199:216	Cstf1	INSIDE	0.426	0.331	1642.00	543.65	0.141	1277.87	180.08

ProbeName	Target position of probe on CpG island microarray	TargetID	GeneSymbol	CpG island Description	Ratio of relative methylation (TiO_2-NP/Vehicle)	Sham group			TiO_2-H group		
						Relative methylation (Cy5/Cy3)	Cy3 signal (Input)	Cy5 signal (MeDIP)	Relative methylation (Cy5/Cy3)	Cy3 signal (Input)	Cy5 signal (MeDIP)
A_68_P32670206	chrX:130593674-130593718	NM_133196:-191	Cstf2	PROMOTER	0.549	3.467	815.06	2825.71	1.903	982.05	1868.42
A_68_P21489863	chr2:104430810-104430854	NM_001037326:192	Cstf3	INSIDE	0.548	0.658	2573.60	1693.06	0.361	1786.07	644.20
A_68_P25539010	chr7:140314266-140314310	NM_009980:878	Ctbp2	INSIDE	0.459	0.296	2119.35	626.38	0.136	1697.03	230.43
A_68_P26135037	chr8:108161197-108161241	NM_181322:781	Ctcf	INSIDE	0.464	0.584	868.86	507.56	0.271	652.33	176.94
A_68_P31864637	chr18:80666392-80666436	NM_026295:-8	Ctdp1	PROMOTER	0.4	8.549	2254.49	19274.68	3.423	1907.27	6528.03
A_68_P31864634	chr18:80666119-80666163	NM_026295:266	Ctdp1	INSIDE	0.44	9.941	936.34	9307.85	4.373	764.74	3343.90
A_68_P21583621	chr2:121782180-121782224	NM_212450:14	Ctdspl2	INSIDE	0.347	1.660	1625.03	2698.25	0.575	1271.50	731.65
A_68_P31838826	chr18:75856914-75856965	NM_201354:411	Ctif	INSIDE	0.381	0.191	3540.15	677.21	0.073	2379.53	173.62
A_68_P31838828	chr18:75857092-75857136	NM_201354:236	Ctif	INSIDE	0.385	29.051	3827.31	111186.00	11.197	3644.20	40803.31
A_68_P31838822	chr18:75856443-75856487	NM_201354:886	Ctif	INSIDE	0.641	0.313	1781.11	557.22	0.2	1411.67	283.00
A_68_P31616442	chr18:35278972-35279016	NM_009818:429	Ctnna1	INSIDE	0.559	0.219	2385.55	523.60	0.123	1678.86	205.90
A_68_P24568080	chr6:77929479-77929523	NM_001109764:161	Ctnna2	INSIDE	0.339	18.068	2123.19	38362.59	6.127	2401.98	14716.01
A_68_P24568082	chr6:77929708-77929753	NM_001109764:-69	Ctnna2	PROMOTER	0.491	4.005	814.50	3262.30	1.968	738.56	1453.46
A_68_P27190984	chr10:62892485-62892529	NM_177612:-339	Ctnna3	PROMOTER	0.376	0.265	2164.89	573.80	0.1	1739.77	173.37
A_68_P22941966	chr4:56878242-56878286	NM_018761:-181	Ctnnal1	PROMOTER	0.45	0.161	3468.15	559.76	0.073	2400.73	174.35
A_68_P22941962	chr4:56877715-56877759	NM_018761:347	Ctnnal1	INSIDE	0.547	9.294	924.09	8588.20	5.081	753.53	3828.43
A_68_P26880505	chr9:120843141-120843185	NM_001165902:467	Ctnnb1	INSIDE	0.281	0.201	4393.19	883.31	0.056	3231.79	182.45
A_68_P26880500	chr9:120842272-120842316	NM_007614:-223	Ctnnb1	PROMOTER	0.598	3.519	2695.00	9482.95	2.105	2084.96	4389.00
A_68_P26880506	chr9:120843223-120843267	NM_001165902:549	Ctnnb1	INSIDE	0.32	0.661	1691.69	1118.35	0.211	1353.62	286.23
A_68_P26880510	chr9:120843613-120843657	NM_001165902:939	Ctnnb1	INSIDE	0.442	3.723	1304.58	4857.16	1.647	1064.60	1753.08
A_68_P23400905	chr4:148892711-148892755	NM_023465:383	Ctnnbip1	INSIDE	0.513	0.223	2204.99	491.56	0.114	1765.40	201.91
A_68_P21778674	chr2:157563392-157563436	NM_025680:278	Ctnnbl1	INSIDE	0.115	1.370	1484.06	2033.63	0.157	1089.68	171.50
A_68_P30109622	chr15:30576916-30576960	NM_008729:474591	Ctnnd2	INSIDE	0.343	0.665	7274.99	4838.91	0.228	5336.05	1217.09
A_68_P30109621	chr15:30576825-30576869	NM_008729:474499	Ctnnd2	INSIDE	0.498	1.412	3205.43	4527.23	0.703	2395.59	1683.65
A_68_P23252015	chr4:120242852-120242896	NM_016748:7	Ctps	INSIDE	0.506	0.340	1424.43	484.21	0.172	1261.21	216.73
A_68_P23252011	chr4:120242395-120242439	NM_016748:465	Ctps	INSIDE	0.539	0.519	1328.18	689.34	0.28	948.94	265.27
A_68_P25416676	chr7:118172200-118172244	NM_009431:-242	Ctr9	PROMOTER	0.314	5.977	2155.75	12885.95	1.878	2030.94	3813.94
A_68_P25416681	chr7:118172726-118172772	NM_009431:284	Ctr9	INSIDE	0.607	0.722	2768.56	2000.00	0.438	2051.76	899.41
A_68_P29659269	chr14:63741273-63741317	NM_007798:-7	Ctsb	PROMOTER	0.351	0.498	4573.60	2275.53	0.175	3441.04	600.79
A_68_P29659271	chr14:63741571-63741615	NM_007798:291	Ctsb	INSIDE	0.447	0.482	907.80	437.39	0.215	775.90	167.13
A_68_P29098528	chr13:64471285-64471329	NM_009984:308	Ctsl	INSIDE	0.491	0.664	1385.91	919.73	0.326	1294.72	422.25
A_68_P22394779	chr3:104855704-104855748	NM_001163332:145	Cttnbp2nl	INSIDE	0.635	0.310	1782.56	552.57	0.197	1417.95	278.91
A_68_P25616898	chr8:4259319-4259363	NM_183315:-66	Ctxn1	PROMOTER	0.188	19.462	4310.53	83889.43	3.658	3651.96	13358.12
A_68_P27998413	chr11:88007252-88007296	NM_001172099:24208	Cuedc1	INSIDE	0.114	2.423	2882.88	6985.05	0.277	2076.83	575.99
A_68_P27997846	chr11:87913149-87913193	NM_198013:523	Cuedc1	INSIDE	0.371	0.509	1000.62	509.27	0.189	904.21	170.82
A_68_P27997843	chr11:87912671-87912715	NM_198013:45	Cuedc1	INSIDE	0.405	9.525	3333.44	31750.35	3.853	2797.55	10780.06
A_68_P27998412	chr11:88007125-88007169	NM_001172099:24080	Cuedc1	INSIDE	0.199	1.846	1272.13	2348.18	0.367	906.05	332.19
A_68_P24423143	chr6:47403815-47403859	NM_012042:-486	Cul1	PROMOTER	0.402	11.188	1137.22	12723.62	4.497	945.31	4251.02
A_68_P31458282	chr18:3383392-3383436	NM_029402:192	Cul2	INSIDE	0.418	6.798	2281.72	15511.64	2.844	1880.61	5348.06
A_68_P31208134	chr17:46790061-46790105	NM_025611:2796	Cul7	INSIDE	0.317	6.538	3192.33	20871.53	2.072	2148.82	4452.97
A_68_P31208112	chr17:46787505-46787549	NM_025611:240	Cul7	INSIDE	0.414	9.393	1487.55	13971.99	3.884	1263.76	4909.06
A_68_P31208133	chr17:46789942-46789986	NM_025611:2678	Cul7	INSIDE	0.482	2.824	3715.78	10493.33	1.36	2579.36	3508.38
A_68_P31207500	chr17:46683388-46683432	NM_001081335:-73	Cul9	PROMOTER	0.364	0.390	3471.13	1353.26	0.142	2061.11	292.17
A_68_P31207496	chr17:46682947-46682991	NM_001081335:369	Cul9	INSIDE	0.518	0.237	2703.44	641.15	0.123	1865.70	229.18
A_68_P32128434	chr19:43827858-43827902	NM_001113562:368	Cutc	INSIDE	0.324	7.670	2635.80	20215.51	2.486	2250.70	5595.39
A_68_P32128432	chr19:43827652-43827696	NM_001113562:162	Cutc	INSIDE	0.547	0.266	5834.22	1553.87	0.146	4124.20	600.48

ProbeName	Target position of probe on CpG island microarray	TargetID	GeneSymbol	CpG island Description	Ratio of relative methylation (TiO_2-NP/Vehicle)	Sham group			TiO_2-H group		
						Relative methylation (Cy5/Cy3)	Cy3 signal (Input)	Cy5 signal (MeDIP)	Relative methylation (Cy5/Cy3)	Cy3 signal (Input)	Cy5 signal (MeDIP)
A_68_P26317495	chr9:14305304-14305348	NM_023153:264	Cwc15	INSIDE	0.597	2.435	1609.59	3919.90	1.454	1259.40	1831.62
A_68_P23797494	chr5:73797444-73797488	NM_181323:150	Cwh43	INSIDE	0.453	3.550	2319.29	8233.48	1.607	1893.84	3043.21
A_68_P24780081	chr6:117118598-117118642	NM_021704:68	Cxcl12	INSIDE	0.579	0.146	3358.43	489.86	0.084	2507.04	211.74
A_68_P29060390	chr13:56397734-56397778	NM_019568:156	Cxcl14	INSIDE	0.411	6.804	3822.82	26010.69	2.798	3018.25	8444.56
A_68_P31620539	chr18:36020271-36020315	NM_133687:30821	Cxxc5	INSIDE	0.252	17.460	1481.47	25866.44	4.401	1400.03	6161.88
A_68_P31620307	chr18:35990504-35990548	NM_133687:1055	Cxxc5	INSIDE	0.652	4.829	1250.28	6037.72	3.149	1022.64	3220.21
A_68_P22411201	chr3:108003559-108003603	NM_001081320:172	Cyb561d1	INSIDE	0.351	0.386	1898.24	733.06	0.136	1323.73	179.69
A_68_P22411200	chr3:108003435-108003479	NM_001081320:296	Cyb561d1	INSIDE	0.541	0.741	2842.43	2107.49	0.401	2080.31	833.74
A_68_P26233345	chr8:124956729-124956773	NM_007806:90	Cyba	INSIDE	0.577	0.587	3880.94	2277.85	0.339	2752.43	932.11
A_68_P30349758	chr15:76490310-76490354	NM_019396:159	Cyhr1	INSIDE	0.335	9.416	4580.06	43127.39	3.151	3456.10	10889.31
A_68_P30349756	chr15:76490140-76490184	NM_019396:329	Cyhr1	INSIDE	0.363	1.837	11144.20	20472.54	0.668	7634.34	5096.87
A_68_P31378939	chr17:80115332-80115376	NM_009994:-973	Cyp1b1	PROMOTER	0.597	4.292	502.02	2154.57	2.564	475.75	1219.75
A_68_P21853424	chr2:170322747-170322791	NM_009996:-130	Cyp24a1	PROMOTER	0.5	3.199	4753.69	15208.84	1.598	3215.45	5139.58
A_68_P32097107	chr19:37773571-37773615	NM_007811:1295	Cyp26a1	INSIDE	0.61	0.362	1320.72	477.64	0.221	994.28	219.31
A_68_P28727385	chr12:109572280-109572324	NM_010010:-288	Cyp46a1	PROMOTER	0.492	4.631	4210.48	19497.48	2.279	3257.31	7422.64
A_68_P31145617	chr17:32589667-32589711	NM_177307:21	Cyp4f39	INSIDE	0.12	2.371	1504.08	3566.06	0.285	1127.42	321.30
A_68_P31145618	chr17:32589827-32589872	NM_177307:182	Cyp4f39	INSIDE	0.193	0.449	2912.05	1306.87	0.087	2088.99	181.11
A_68_P31145776	chr17:32629460-32629505	NM_177307:39815	Cyp4f39	INSIDE	0.623	2.462	1107.20	2726.28	1.535	870.36	1336.12
A_68_P28291022	chr12:25365866-25365910	NM_001004455:772	Cys1	INSIDE	0.337	13.271	14254.71	189169.80	4.468	10704.52	47827.01
A_68_P28291023	chr12:25365989-25366033	NM_001004455:650	Cys1	INSIDE	0.452	27.862	5422.52	151081.60	12.597	4119.47	51894.63
A_68_P28291019	chr12:25365387-25365431	NM_001004455:1252	Cys1	INSIDE	0.601	0.572	6071.39	3474.86	0.344	4342.00	1492.50
A_68_P28291020	chr12:25365549-25365593	NM_001004455:1090	Cys1	INSIDE	0.385	0.698	1912.47	1334.76	0.269	1599.88	429.81
A_68_P24157329	chr5:144383488-144383532	NM_011182:195	Cyth3	INSIDE	0.451	13.191	2328.35	30713.57	5.948	2158.73	12841.01
A_68_P24157332	chr5:144383775-144383819	NM_011182:481	Cyth3	INSIDE	0.63	0.507	2427.97	1231.82	0.32	2013.63	643.50
A_68_P24157328	chr5:144383397-144383441	NM_011182:103	Cyth3	INSIDE	0.633	2.907	848.95	2467.84	1.841	712.48	1311.55
A_68_P27272484	chr10:77632233-77632277	NM_138601:259	D10Jhu81e	INSIDE	0.639	0.435	1509.80	656.28	0.278	1271.90	353.39
A_68_P28143433	chr11:113546017-113546061	NM_177777:313	D11Wsu47e	INSIDE	0.35	0.432	1942.39	839.98	0.152	1450.70	219.88
A_68_P28143432	chr11:113545919-113545963	NM_177777:215	D11Wsu47e	INSIDE	0.603	2.662	3133.96	8343.07	1.606	2360.29	3790.01
A_68_P28143424	chr11:113544952-113544996	NM_138598:491	D11Wsu99e	INSIDE	0.57	0.214	2879.24	615.66	0.122	2251.73	274.48
A_68_P28889295	chr13:24936978-24937022	NM_001081051:1	D130043K22Rik	INSIDE	0.484	0.679	1023.62	695.20	0.329	867.26	284.95
A_68_P29487113	chr14:28242620-28242665	NM_001114879:610	D14Abb1e	INSIDE	0.499	4.238	868.16	3679.47	2.113	754.70	1594.80
A_68_P31794495	chr18:68092683-68092727	NM_172631:-206	D18Ertd653e	PROMOTER	0.607	7.279	1055.77	7684.66	4.418	816.45	3606.82
A_68_P32040826	chr19:27504142-27504186	NM_177474:146	D19Bwg1357e	INSIDE	0.526	0.497	4637.45	2303.19	0.261	3351.48	876.29
A_68_P32040827	chr19:27504259-27504303	NM_177474:30	D19Bwg1357e	INSIDE	0.542	0.389	9667.95	3759.92	0.211	6413.12	1352.58
A_68_P32121691	chr19:42593347-42593391	NM_177464:74	D19Ertd386e	INSIDE	0.413	0.290	2184.32	632.98	0.12	1389.43	166.36
A_68_P32121690	chr19:42593224-42593268	NM_177464:-48	D19Ertd386e	PROMOTER	0.512	3.051	1442.79	4401.50	1.563	1135.42	1774.19
A_68_P20166302	chr1:39593044-39593088	NM_028043:420	D1Bwg0212e	INSIDE	0.453	5.492	835.53	4588.94	2.489	795.46	1979.74
A_68_P20166303	chr1:39593180-39593224	NM_028043:556	D1Bwg0212e	INSIDE	0.662	3.322	693.48	2304.07	2.2	635.04	1397.08
A_68_P20166299	chr1:39592701-39592745	NM_028043:76	D1Bwg0212e	INSIDE	0.593	0.611	756.55	462.18	0.362	664.89	240.71
A_68_P20472155	chr1:99558144-99558188	NM_133825:429	D1Ertd622e	INSIDE	0.329	7.701	2441.43	18801.09	2.537	2150.06	5454.26
A_68_P20472150	chr1:99557453-99557497	NM_133825:1121	D1Ertd622e	INSIDE	0.398	6.349	2041.21	12960.51	2.527	1696.26	4286.05
A_68_P20910364	chr1:188791866-188791912	NM_033077:594	D1Pas1	INSIDE	0.656	9.330	1303.29	12159.80	6.124	1252.64	7671.44
A_68_P21566329	chr2:118640037-118640081	NM_026412:320	D2Ertd750e	INSIDE	0.077	2.300	16381.02	37678.23	0.178	11054.12	1965.48
A_68_P22454038	chr3:117062948-117062993	NM_177664:824	D3Bwg0562e	INSIDE	0.632	4.016	958.52	3849.34	2.537	802.13	2035.17
A_68_P28767699	chr12:117643707-117643751	NR_028421:-94	D430020J02Rik	DIVERGENT_PROMOTER	0.617	0.429	2037.08	874.35	0.265	1444.33	382.36
A_68_P25496230	chr7:132851736-132851780	NM_001081022:369	D430042O09Rik	INSIDE	0.539	0.362	2454.82	888.17	0.195	1786.11	348.16

ProbeName	Target position of probe on CpG island microarray	TargetID	GeneSymbol	CpG island Description	Ratio of relative methylation (TiO_2-NP/Vehicle)	Sham group			TiO_2-H group		
						Relative methylation (Cy5/Cy3)	Cy3 signal (Input)	Cy5 signal (MeDIP)	Relative methylation (Cy5/Cy3)	Cy3 signal (Input)	Cy5 signal (MeDIP)
A_68_P25496227	chr7:132851431-132851475	NM_001081022:63	D430042O09Rik	INSIDE	0.624	4.440	489.46	2173.21	2.769	466.29	1290.99
A_68_P25496229	chr7:132851609-132851653	NM_001081022:241	D430042O09Rik	INSIDE	0.153	1.405	1060.76	1489.95	0.215	813.51	174.77
A_68_P29134803	chr13:72766020-72766064	NR_030701:-30	D430050G20	DIVERGENT_PROMOTER	0.617	0.506	3248.06	1644.28	0.312	2563.93	800.55
A_68_P23362370	chr4:140695629-140695673	NM_001025608:61	D4Ertd22e	INSIDE	0.538	0.459	2363.59	1084.52	0.247	1796.75	443.95
A_68_P23362371	chr4:140695718-140695762	NM_001025608:-29	D4Ertd22e	DIVERGENT_PROMOTER	0.659	3.059	1078.44	3299.01	2.016	949.33	1914.00
A_68_P23241428	chr4:118293645-118293692	NR_029469:-5340	D4Ertd617e	PROMOTER	0.417	0.511	993.19	507.83	0.213	798.63	170.18
A_68_P23325561	chr4:134479965-134480009	NM_023665:447	D4Wsu53e	INSIDE	0.53	6.088	2326.27	14163.40	3.227	1976.11	6377.12
A_68_P23609090	chr5:37086922-37086966	NM_001081232:264	D5Ertd579e	INSIDE	0.238	23.644	4520.27	106878.30	5.617	4569.91	25668.50
A_68_P23609089	chr5:37086829-37086873	NM_001081232:358	D5Ertd579e	INSIDE	0.564	3.300	2083.76	6876.78	1.861	1662.14	3092.48
A_68_P23609093	chr5:37087355-37087399	NM_001081232:-168	D5Ertd579e	PROMOTER	0.623	5.078	3228.04	16392.35	3.162	2377.72	7517.28
A_68_P23609088	chr5:37086702-37086746	NM_001081232:484	D5Ertd579e	INSIDE	0.353	4.341	1154.20	5010.80	1.532	1062.38	1627.28
A_68_P22942460	chr4:56960340-56960384	NM_175518:-61	D730040F13Rik	PROMOTER	0.323	8.400	1757.11	14758.92	2.714	1590.68	4317.24
A_68_P22942456	chr4:56959870-56959914	NM_175518:409	D730040F13Rik	INSIDE	0.565	11.302	868.64	9817.03	6.385	717.33	4580.31
A_68_P23262658	chr4:123089142-123089186	NM_001167918:-10	D830031N03Rik	PROMOTER	0.266	31.158	1746.71	54423.81	8.297	1576.78	13082.16
A_68_P23262662	chr4:123089650-123089695	NM_001167918:-518	D830031N03Rik	PROMOTER	0.298	11.766	2861.54	33669.52	3.51	2405.78	8444.63
A_68_P23262657	chr4:123089026-123089070	NM_001167918:106	D830031N03Rik	INSIDE	0.499	0.528	844.12	445.36	0.263	663.76	174.78
A_68_P25784087	chr8:37210103-37210147	NM_172911:52243	D8Ertd82e	INSIDE	0.187	15.940	5919.52	94359.74	2.984	4299.44	12831.17
A_68_P25784086	chr8:37209993-37210038	NM_172911:52134	D8Ertd82e	INSIDE	0.124	13.200	1587.80	20959.58	1.631	999.05	1629.66
A_68_P25784088	chr8:37210172-37210216	NM_172911:52313	D8Ertd82e	INSIDE	0.641	3.319	420.63	1395.89	2.127	339.78	722.82
A_68_P25409557	chr7:116847217-116847263	NM_020616:-10	D930014E17Rik	PROMOTER	0.637	0.574	1221.63	700.80	0.366	1016.56	371.65
A_68_P22288618	chr3:83843229-83843273	NM_172681:833	D930015E06Rik	INSIDE	0.347	0.464	1948.25	904.25	0.161	1460.55	235.01
A_68_P22288626	chr3:83844024-83844068	NM_172681:37	D930015E06Rik	INSIDE	0.583	5.213	540.29	2816.34	3.037	485.13	1473.14
A_68_P22288630	chr3:83844564-83844615	NM_172681:-506	D930015E06Rik	PROMOTER	0.608	0.510	1361.75	694.44	0.31	1029.51	319.13
A_68_P27795131	chr11:51464219-51464263	NR_027958:-215	D930048N14Rik	PROMOTER	0.508	0.477	1163.28	554.68	0.242	805.01	195.15
A_68_P28528483	chr12:72932910-72932958	NM_026102:870	Daam1	INSIDE	0.471	16.035	681.28	10924.35	7.554	471.49	3561.64
A_68_P21134948	chr2:35516613-35516657	NM_001114124:39134	Dab2ip	INSIDE	0.216	8.180	2037.57	16666.78	1.77	1618.00	2863.21
A_68_P21134365	chr2:35438082-35438126	NM_001114125:24127	Dab2ip	INSIDE	0.462	0.476	939.12	446.95	0.22	850.35	186.88
A_68_P21134366	chr2:35438211-35438255	NM_001114125:24255	Dab2ip	INSIDE	0.549	16.600	1336.70	22188.83	9.117	1111.06	10129.56
A_68_P21134954	chr2:35517488-35517532	NM_001114124:40010	Dab2ip	INSIDE	0.403	0.569	955.61	543.28	0.229	786.16	180.21
A_68_P21134361	chr2:35437675-35437719	NM_001114125:23719	Dab2ip	INSIDE	0.575	0.465	954.38	443.79	0.267	690.85	184.61
A_68_P28525473	chr12:72410602-72410646	NM_001190466:-246	Dact1	PROMOTER	0.291	15.837	4553.68	72114.96	4.602	4120.54	18961.94
A_68_P28525474	chr12:72410830-72410874	NM_001190466:-18	Dact1	PROMOTER	0.542	3.448	6753.69	23287.90	1.87	5030.80	9408.97
A_68_P31058444	chr17:14340634-14340678	NM_172826:182	Dact2	INSIDE	0.518	4.039	1991.98	8044.72	2.092	1713.22	3583.31
A_68_P24983771	chr7:17470567-17470612	NM_001081655:9924	Dact3	INSIDE	0.639	3.453	835.06	2883.06	2.205	701.54	1546.76
A_68_P24156632	chr5:144225585-144225629	NM_144915:246	Daglb	INSIDE	0.416	0.611	1005.49	614.79	0.254	726.11	184.74
A_68_P26815510	chr9:108472099-108472144	NM_026378:-101	Dalrd3	PROMOTER	0.597	0.294	2571.09	755.20	0.175	2167.32	379.96
A_68_P26022160	chr8:87356049-87356093	NM_201227:-9348	Dand5	PROMOTER	0.482	0.645	1958.52	1263.48	0.311	1443.93	448.92
A_68_P30113003	chr15:31154105-31154149	NM_146057:-13	Dap	PROMOTER	0.502	5.187	3792.24	19670.51	2.601	3267.94	8501.27
A_68_P22316449	chr3:88754686-88754730	NM_001164533:-504	Dap3	PROMOTER	0.513	0.527	2815.02	1482.56	0.27	2095.62	566.46
A_68_P29086263	chr13:60704015-60704059	NM_134062:465	Dapk1	INSIDE	0.431	0.385	1532.58	590.67	0.166	1042.19	173.27
A_68_P26591484	chr9:66005801-66005855	NM_010019:-204	Dapk2	PROMOTER	0.198	0.334	3967.89	1323.61	0.066	2553.50	168.46
A_68_P26591858	chr9:66068690-66068734	NM_010019:62680	Dapk2	INSIDE	0.523	3.487	4650.35	16217.89	1.825	3486.01	6361.46
A_68_P20600403	chr1:130313627-130313671	NM_177445:345	Dars	INSIDE	0.577	0.270	1809.49	488.79	0.156	1491.99	232.36
A_68_P20600401	chr1:130313411-130313455	NM_177445:561	Dars	INSIDE	0.589	0.274	3318.08	909.62	0.162	2455.72	396.62
A_68_P20600406	chr1:130314008-130314052	NM_177445:-37	Dars	PROMOTER	0.554	3.099	1251.91	3880.16	1.717	1083.37	1860.29
A_68_P31150931	chr17:34048707-34048751	NM_001199733:2183	Daxx	INSIDE	0.231	12.294	647.12	7955.69	2.836	508.64	1442.69

ProbeName	Target position of probe on CpG island microarray	TargetID	GeneSymbol	CpG island Description	Ratio of relative methylation (TiO_2-NP/Vehicle)	Sham group			TiO_2-H group		
						Relative methylation (Cy5/Cy3)	Cy3 signal (Input)	Cy5 signal (MeDIP)	Relative methylation (Cy5/Cy3)	Cy3 signal (Input)	Cy5 signal (MeDIP)
A_68_P31150915	chr17:34046487-34046531	NM_001199733:-37	Daxx	PROMOTER	0.383	0.465	3538.10	1646.34	0.178	2378.10	423.75
A_68_P30486712	chr15:100446399-100446443	NM_011873:328	Dazap2	INSIDE	0.653	0.324	2710.06	877.67	0.212	1943.11	411.22
A_68_P31227866	chr17:50432495-50432539	NM_010021:408	Dazl	INSIDE	0.456	4.404	888.80	3914.26	2.008	715.02	1435.56
A_68_P29055349	chr13:55589582-55589626	NM_001177371:-167	Dbn1	PROMOTER	0.244	30.542	3496.49	106791.00	7.455	3213.59	23956.83
A_68_P29055348	chr13:55589381-55589425	NM_001177371:35	Dbn1	INSIDE	0.358	7.134	18369.07	131039.00	2.557	15419.62	39432.89
A_68_P29055347	chr13:55589235-55589279	NM_001177371:181	Dbn1	INSIDE	0.394	5.961	1065.06	6348.38	2.35	953.01	2239.64
A_68_P26240290	chr8:126039221-126039265	NM_001170976:113	Dbndd1	INSIDE	0.605	0.341	2120.77	722.98	0.206	1721.06	355.20
A_68_P26240288	chr8:126039003-126039047	NM_001170976:331	Dbndd1	INSIDE	0.615	2.477	2080.25	5152.24	1.522	1626.01	2475.06
A_68_P21818151	chr2:164311938-164311982	NM_001048228:-416	Dbndd2	PROMOTER	0.277	2.435	3784.77	9217.63	0.675	2741.63	1851.91
A_68_P27564951	chr11:5688365-5688409	NM_001146308:-99	Dbnl	PROMOTER	0.455	15.923	2401.53	38238.50	7.244	2110.05	15284.98
A_68_P25113826	chr7:56892177-56892221	NM_001005232:7	Dbx1	INSIDE	0.457	0.298	2470.93	736.55	0.136	1723.68	234.81
A_68_P30459322	chr15:95484732-95484776	NM_207533:448	Dbx2	INSIDE	0.564	3.075	1095.13	3367.27	1.735	908.05	1575.78
A_68_P22882382	chr4:45355016-45355060	NM_153167:66	Dcaf10	INSIDE	0.248	7.033	1859.15	13075.52	1.743	1448.89	2525.34
A_68_P22861615	chr4:41261631-41261675	NM_026893:282	Dcaf12	INSIDE	0.433	23.648	4193.24	99163.75	10.24	3770.80	38611.90
A_68_P32351254	chrX:42143356-42143400	NM_178739:-4	Dcaf12l1	PROMOTER	0.165	13.903	2915.72	40537.66	2.289	3557.54	8143.55
A_68_P32351252	chrX:42143188-42143232	NM_001190718:128	Dcaf12l1	INSIDE	0.318	21.577	2278.05	49153.15	6.868	2874.17	19739.36
A_68_P32351250	chrX:42142934-42142979	NM_001190718:382	Dcaf12l1	INSIDE	0.647	2.623	1042.05	2733.41	1.698	1366.22	2320.25
A_68_P21317258	chr2:70894105-70894149	NM_001165982:326	Dcaf17	INSIDE	0.229	1.878	685.65	1287.51	0.43	660.09	283.74
A_68_P20794600	chr1:167390414-167390458	NM_028759:158	Dcaf6	INSIDE	0.53	3.043	3012.19	9165.54	1.612	2299.87	3706.25
A_68_P20831241	chr1:174078163-174078207	NM_153555:39	Dcaf8	INSIDE	0.438	0.431	1147.09	494.95	0.189	905.44	171.25
A_68_P28082865	chr11:102878532-102878576	NM_026551:-93	Dcakd	PROMOTER	0.46	9.560	4783.64	45731.95	4.395	4096.92	18007.55
A_68_P30791337	chr16:58408386-58408430	NM_028523:-239	Dcbld2	PROMOTER	0.467	6.272	3568.85	22382.07	2.927	2932.92	8585.37
A_68_P28313702	chr12:29236975-29237019	NM_001177964:-3794	Dcdc2c	PROMOTER	0.415	7.770	1633.09	12689.17	3.222	1363.32	4393.03
A_68_P28313705	chr12:29237270-29237314	NM_001177964:-4090	Dcdc2c	PROMOTER	0.519	4.698	1190.65	5594.05	2.437	967.34	2357.64
A_68_P25394242	chr7:112911135-112911179	NM_001162943:24908	Dchs1	INSIDE	0.615	2.795	2894.30	8088.42	1.719	2059.40	3539.33
A_68_P25394241	chr7:112911036-112911080	NM_001162943:25006	Dchs1	INSIDE	0.206	3.197	3585.70	11462.16	0.658	2418.98	1592.50
A_68_P22152359	chr3:55046645-55046689	NM_001111053:219	Dclk1	INSIDE	0.303	8.749	6859.61	60014.81	2.649	6472.14	17142.87
A_68_P22152358	chr3:55046521-55046565	NM_001111053:95	Dclk1	INSIDE	0.644	0.606	1276.15	772.82	0.39	1022.32	398.62
A_68_P22305772	chr3:86723682-86723726	NM_001195496:1102	Dclk2	INSIDE	0.4	0.625	927.68	579.68	0.25	691.59	172.95
A_68_P22388169	chr3:103613288-103613332	NM_001025312:0	Dclre1b	INSIDE	0.517	15.311	1664.62	25487.03	7.91	1394.24	11028.96
A_68_P24791602	chr6:119125266-119125312	NM_001033379:18	Dcp1b	INSIDE	0.525	10.998	3550.37	39048.49	5.773	3190.00	18416.10
A_68_P31666414	chr18:44540418-44540462	NM_027490:287	Dcp2	INSIDE	0.512	0.348	1799.47	625.56	0.178	1427.51	253.90
A_68_P22318793	chr3:89154296-89154340	NM_029974:14843	Dcst1	INSIDE	0.599	0.432	1640.11	709.10	0.259	1174.76	304.42
A_68_P25845477	chr8:49195740-49195784	NM_001161515:396	Dctd	INSIDE	0.032	323.808	1628.66	527373.50	10.284	1739.52	17888.47
A_68_P27534905	chr10:126703422-126703466	NM_001190453:127	Dctn2	INSIDE	0.204	14.133	3482.31	49215.90	2.885	2487.36	7175.26
A_68_P27534904	chr10:126703309-126703353	NM_001190453:13	Dctn2	INSIDE	0.401	0.554	3230.13	1790.77	0.222	2102.14	467.24
A_68_P25772218	chr8:35171274-35171318	NM_011722:269	Dctn6	INSIDE	0.26	5.825	2103.22	12251.53	1.516	1540.45	2334.72
A_68_P25504836	chr7:134404187-134404231	NM_023203:-27	Dctpp1	PROMOTER	0.484	0.255	2013.31	513.23	0.123	1393.97	171.84
A_68_P25504835	chr7:134404064-134404108	NM_023203:95	Dctpp1	INSIDE	0.65	3.964	4662.59	18481.05	2.577	3374.00	8696.03
A_68_P23797911	chr5:73882756-73882800	NM_001190733:514	Dcun1d4	INSIDE	0.062	6.439	11008.92	70884.21	0.402	8520.39	3421.00
A_68_P23797912	chr5:73883055-73883099	NM_001190733:812	Dcun1d4	INSIDE	0.075	28.203	15266.92	430570.40	2.119	14251.19	30193.34
A_68_P23797913	chr5:73883146-73883190	NM_001190733:904	Dcun1d4	INSIDE	0.174	21.160	13763.10	291227.70	3.671	11169.76	41008.77
A_68_P23797905	chr5:73882068-73882112	NM_001190733:-174	Dcun1d4	PROMOTER	0.469	0.641	804.77	515.58	0.301	680.94	204.73
A_68_P26286958	chr9:7184623-7184667	NM_029775:79	Dcun1d5	INSIDE	0.36	8.667	600.98	5208.75	3.118	604.61	1885.14
A_68_P28186436	chr11:120588411-120588455	NM_026428:163	Dcxr	INSIDE	0.399	0.200	2955.28	591.86	0.08	2128.55	170.09
A_68_P22610589	chr3:145421770-145421814	NM_026993:137	Ddah1	INSIDE	0.45	5.864	2797.07	16403.12	2.637	2330.25	6145.65

ProbeName	Target position of probe on CpG island microarray	TargetID	GeneSymbol	CpG island Description	Ratio of relative methylation (TiO_2-NP/Vehicle)	Sham group			TiO_2-H group		
						Relative methylation (Cy5/Cy3)	Cy3 signal (Input)	Cy5 signal (MeDIP)	Relative methylation (Cy5/Cy3)	Cy3 signal (Input)	Cy5 signal (MeDIP)
A_68_P31157314	chr17:35196243-35196287	NM_001190449:285	Ddah2	INSIDE	0.399	1.627	3268.87	5317.91	0.648	2467.34	1599.73
A_68_P29571979	chr14:46277367-46277411	NM_001039106:430	Ddhd1	INSIDE	0.411	10.022	7838.84	78558.79	4.115	6513.02	26804.03
A_68_P25728861	chr8:26864655-26864699	NM_028102:76	Ddhd2	INSIDE	0.264	11.336	2293.48	25997.79	2.988	1950.92	5828.56
A_68_P23365645	chr4:141279495-141279539	NM_001017966:-182	Ddi2	PROMOTER	0.132	17.908	1213.28	21727.21	2.361	1002.17	2365.81
A_68_P27535079	chr10:126727356-126727401	NM_007837:-470	Ddit3	DIVERGENT_PROMOTER	0.462	4.564	1745.63	7967.43	2.108	1257.85	2651.32
A_68_P27171643	chr10:59415089-59415133	NM_029083:-592	Ddit4	PROMOTER	0.372	0.309	1903.83	588.52	0.115	1471.91	169.09
A_68_P22564417	chr3:137286650-137286694	NM_030143:37	Ddit4l	INSIDE	0.638	2.460	1423.87	3502.19	1.57	1243.61	1952.75
A_68_P30476596	chr15:98637471-98637515	NM_001013741:864	Ddn	INSIDE	0.431	7.112	1852.08	13172.41	3.067	1502.45	4608.33
A_68_P31160531	chr17:35837972-35838016	NM_001198833:-459	Ddr1	PROMOTER	0.493	0.410	1927.63	789.47	0.202	1593.50	321.55
A_68_P26519504	chr9:53055815-53055859	NM_029936:381	Ddx10	INSIDE	0.091	7.885	11952.62	94246.19	0.717	8271.68	5930.36
A_68_P30367146	chr15:79376680-79376724	NM_199079:287	Ddx17	INSIDE	0.405	3.900	1702.08	6637.67	1.579	1236.09	1952.26
A_68_P20567558	chr1:123464212-123464256	NM_025860:323	Ddx18	INSIDE	0.234	1.570	1789.67	2809.15	0.367	1426.47	523.84
A_68_P26166134	chr8:113551628-113551672	NM_001190800:20	Ddx19b	INSIDE	0.63	4.436	2211.50	9809.13	2.796	1779.54	4976.10
A_68_P22398585	chr3:105490135-105490179	NM_017397:333	Ddx20	INSIDE	0.484	6.710	1321.30	8865.42	3.247	1141.50	3706.15
A_68_P26016158	chr8:86239347-86239391	NM_197982:293	Ddx39	INSIDE	0.226	0.310	5014.24	1554.21	0.07	3640.02	255.44
A_68_P32265732	chrX:12859122-12859166	NM_010028:997	Ddx3x	INSIDE	0.573	3.598	724.48	2606.66	2.063	915.37	1888.62
A_68_P32265727	chrX:12858577-12858621	NM_010028:451	Ddx3x	INSIDE	0.562	0.655	821.03	537.77	0.368	1204.65	443.29
A_68_P32265729	chrX:12858825-12858869	NM_010028:699	Ddx3x	INSIDE	0.467	3.610	661.98	2389.75	1.685	986.81	1662.34
A_68_P32265730	chrX:12858920-12858964	NM_010028:795	Ddx3x	INSIDE	0.462	4.906	287.65	1411.29	2.268	356.23	807.90
A_68_P29335931	chr13:113442236-113442280	NM_001145885:260	Ddx4	INSIDE	0.579	4.909	1076.86	5286.76	2.841	989.81	2811.90
A_68_P29335930	chr13:113442131-113442175	NM_001145885:366	Ddx4	INSIDE	0.416	3.482	2258.55	7863.71	1.449	1756.08	2544.04
A_68_P29055657	chr13:55638051-55638100	NM_134059:-56	Ddx41	PROMOTER	0.399	0.628	964.35	605.26	0.251	690.81	173.17
A_68_P26659378	chr9:78243891-78243935	NM_001191044:329	Ddx43	INSIDE	0.585	7.303	1536.34	11220.55	4.274	1291.75	5520.44
A_68_P28103521	chr11:106649919-106649963	NM_007840:-132	Ddx5	DIVERGENT_PROMOTER	0.139	45.067	9198.80	414562.10	6.26	13912.83	87092.01
A_68_P27187154	chr10:62113629-62113673	NM_053183:296	Ddx50	INSIDE	0.124	0.450	4852.59	2185.45	0.056	3482.73	193.74
A_68_P27187162	chr10:62114413-62114457	NM_053183:-488	Ddx50	PROMOTER	0.226	0.572	1753.08	1002.26	0.129	1352.62	175.10
A_68_P27187161	chr10:62114275-62114319	NM_053183:-350	Ddx50	PROMOTER	0.496	0.398	1607.48	639.72	0.197	1284.21	253.45
A_68_P27187153	chr10:62113528-62113572	NM_053183:396	Ddx50	INSIDE	0.551	3.245	1107.59	3594.01	1.788	1002.60	1792.24
A_68_P23981086	chr5:111082431-111082475	NM_027156:-17	Ddx51	DIVERGENT_PROMOTER	0.361	6.970	789.45	5502.19	2.517	696.94	1753.86
A_68_P23981092	chr5:111083016-111083060	NM_027156:569	Ddx51	INSIDE	0.437	0.300	1630.05	488.41	0.131	1346.74	176.16
A_68_P23981091	chr5:111082933-111082977	NM_027156:485	Ddx51	INSIDE	0.537	0.501	2127.71	1066.46	0.269	1491.08	401.41
A_68_P20645802	chr1:138311830-138311874	NM_026500:5	Ddx59	INSIDE	0.521	5.839	650.08	3795.51	3.041	549.49	1671.18
A_68_P26468513	chr9:44413201-44413245	NM_001110826:248	Ddx6	INSIDE	0.424	10.879	9224.69	100351.10	4.613	7085.37	32685.89
A_68_P26468515	chr9:44413405-44413449	NM_001110826:452	Ddx6	INSIDE	0.459	0.452	2280.14	1031.53	0.208	1626.84	337.74
A_68_P26885056	chr9:121619308-121619352	NM_026794:-176	Deb1	DIVERGENT_PROMOTER	0.521	3.723	775.58	2887.44	1.939	710.60	1377.53
A_68_P22752472	chr4:15872547-15872591	NM_026172:86	Decr1	INSIDE	0.119	39.957	2408.67	96242.29	4.75	2160.21	10262.00
A_68_P22752471	chr4:15872402-15872446	NM_026172:230	Decr1	INSIDE	0.532	5.502	3132.49	17235.99	2.93	2522.66	7390.14
A_68_P25009065	chr7:26004933-26004977	NM_207677:-76	Dedd2	DIVERGENT_PROMOTER	0.323	7.153	6885.05	49248.39	2.308	5664.48	13073.28
A_68_P25009060	chr7:26004436-26004480	NM_207677:420	Dedd2	INSIDE	0.631	0.700	2854.88	1997.00	0.441	1958.50	864.23
A_68_P26239891	chr8:125967011-125967055	NM_054046:147	Def8	INSIDE	0.541	0.334	3707.19	1238.43	0.181	2691.08	486.29
A_68_P20885892	chr1:184212460-184212504	NM_007853:408	Degs1	INSIDE	0.114	17.948	543.77	9759.45	2.045	463.51	947.70
A_68_P29011724	chr13:47201824-47201868	NM_025900:-257	Dek	PROMOTER	0.42	0.279	2109.84	589.48	0.117	1590.23	186.46
A_68_P21147278	chr2:38142391-38142435	NM_146122:492	Dennd1a	INSIDE	0.449	0.456	4337.31	1976.77	0.205	3339.93	683.31
A_68_P20660113	chr1:140860423-140860467	NM_181347:-3853	Dennd1b	PROMOTER	0.382	8.761	841.45	7371.74	3.345	740.23	2476.04
A_68_P24383687	chr6:39507859-39507903	NM_172477:-47	Dennd2a	PROMOTER	0.457	3.647	2528.24	9221.71	1.669	1927.46	3216.24
A_68_P22401916	chr3:106285683-106285727	NM_001093754:356	Dennd2d	INSIDE	0.395	0.659	6745.20	4445.56	0.261	4900.81	1277.30

ProbeName	Target position of probe on CpG island microarray	TargetID	GeneSymbol	CpG island Description	Ratio of relative methylation (TiO_2-NP/Vehicle)	Sham group			TiO_2-H group		
						Relative methylation (Cy5/Cy3)	Cy3 signal (Input)	Cy5 signal (MeDIP)	Relative methylation (Cy5/Cy3)	Cy3 signal (Input)	Cy5 signal (MeDIP)
A_68_P30330811	chr15:73342672-73342717	NM_001081066:-295	Dennd3	PROMOTER	0.643	0.729	1609.56	1172.67	0.469	1273.10	596.73
A_68_P26584123	chr9:64659420-64659464	NM_001162917:625	Dennd4a	INSIDE	0.359	0.270	4137.13	1118.65	0.097	3013.55	292.63
A_68_P26584118	chr9:64659001-64659045	NM_001162917:205	Dennd4a	INSIDE	0.284	1.568	8897.12	13948.25	0.446	7041.90	3140.06
A_68_P22324271	chr3:90069155-90069199	NM_201407:-1279	Dennd4b	PROMOTER	0.445	0.569	2686.69	1528.34	0.253	1954.74	495.02
A_68_P23081278	chr4:86395175-86395219	NM_001081014:738	Dennd4c	INSIDE	0.229	0.526	3200.32	1681.99	0.12	2439.53	293.69
A_68_P23081271	chr4:86394332-86394376	NM_001081014:-104	Dennd4c	PROMOTER	0.627	2.373	1574.51	3736.34	1.488	1363.29	2028.42
A_68_P25410984	chr7:117103504-117103548	NM_021494:410	Dennd5a	INSIDE	0.53	3.767	1225.14	4615.07	1.995	999.80	1995.09
A_68_P24058184	chr5:124357527-124357571	NM_026603:265	Denr	INSIDE	0.555	0.463	1514.28	701.37	0.257	1305.54	335.70
A_68_P24058180	chr5:124357134-124357178	NM_026603:-127	Denr	PROMOTER	0.596	0.454	3377.27	1532.03	0.27	2848.07	769.71
A_68_P29310806	chr13:109106501-109106545	NM_178683:-8	Depdc1b	PROMOTER	0.435	0.418	3675.73	1535.07	0.182	2718.30	493.38
A_68_P23585886	chr5:33206293-33206337	NM_177786:-55	Depdc5	DIVERGENT_PROMOTER	0.647	2.331	1799.56	4194.97	1.509	1584.44	2390.16
A_68_P30230393	chr15:54944017-54944061	NM_145470:1	Deptor	INSIDE	0.619	0.248	2399.95	595.03	0.153	1836.11	281.63
A_68_P30244836	chr15:57723668-57723712	NM_024207:283	Derl1	INSIDE	0.567	3.214	1404.45	4514.49	1.823	1222.06	2227.63
A_68_P27259217	chr10:75355978-75356022	NM_024440:-142	Derl3	PROMOTER	0.21	0.624	6040.04	3768.93	0.131	4003.56	525.26
A_68_P23398684	chr4:148478173-148478217	NM_010044:-67	Dffa	DIVERGENT_PROMOTER	0.642	0.628	3648.23	2291.78	0.403	2619.50	1056.67
A_68_P30348811	chr15:76341995-76342039	NM_010046:232	Dgat1	INSIDE	0.6	2.780	3924.72	10911.99	1.669	3012.44	5027.84
A_68_P27543002	chr10:128181219-128181263	NM_016811:-128	Dgka	DIVERGENT_PROMOTER	0.481	0.238	2633.70	626.76	0.114	1962.97	224.49
A_68_P30599074	chr16:22656977-22657021	NM_138650:306	Dgkg	INSIDE	0.607	0.588	1230.63	723.43	0.357	959.91	342.33
A_68_P30599077	chr16:22657267-22657311	NM_138650:16	Dgkg	INSIDE	0.542	3.115	1765.89	5500.20	1.69	1521.65	2570.96
A_68_P29747537	chr14:79124881-79124925	NM_001081336:-6	Dgkh	PROMOTER	0.572	3.626	1006.54	3649.66	2.072	745.91	1545.86
A_68_P24371747	chr6:37249971-37250015	NM_001081206:-16	Dgki	PROMOTER	0.335	6.393	2254.33	14412.02	2.14	2041.71	4369.81
A_68_P21425706	chr2:91805641-91805685	NM_138306:-1942	Dgkz	PROMOTER	0.651	2.639	777.03	2050.67	1.717	762.90	1310.17
A_68_P25603236	chr7:151015869-151015913	NM_007856:6819	Dhcr7	INSIDE	0.623	0.607	1330.03	807.58	0.378	1006.46	380.57
A_68_P25603237	chr7:151015959-151016003	NM_007856:6909	Dhcr7	INSIDE	0.598	2.534	1306.38	3310.32	1.516	1126.15	1706.89
A_68_P23320471	chr4:133556862-133556906	NM_026144:-105	Dhdds	PROMOTER	0.54	11.936	813.30	9707.32	6.445	665.36	4288.38
A_68_P27979838	chr11:84642248-84642292	NM_177564:235	Dhrs11	INSIDE	0.656	0.553	3173.74	1753.63	0.363	2352.49	852.96
A_68_P23379974	chr4:144482718-144482768	NM_011303:-297	Dhrs3	PROMOTER	0.376	0.368	1790.23	658.30	0.138	1246.50	172.26
A_68_P33007785	chr4_random:127689-127733	NM_001033326:17909	Dhrsx	INSIDE	0.102	64.425	8286.35	533849.80	6.567	11135.68	73124.40
A_68_P33007787	chr4_random:127945-127989	NM_001033326:18165	Dhrsx	INSIDE	0.134	16.068	7130.96	114583.00	2.146	6525.85	14005.12
A_68_P33007786	chr4_random:127770-127814	NM_001033326:17991	Dhrsx	INSIDE	0.141	31.561	10966.91	346127.50	4.455	11105.85	49482.05
A_68_P33007798	chr4_random:129389-129433	NM_001033326:19609	Dhrsx	INSIDE	0.184	18.031	1802.85	32507.13	3.326	1528.65	5083.70
A_68_P33008826	chr4_random:110027-110071	NM_001033326:247	Dhrsx	INSIDE	0.382	13.727	1803.35	24754.46	5.245	1560.27	8184.30
A_68_P33007792	chr4_random:128621-128665	NM_001033326:18841	Dhrsx	INSIDE	0.405	6.909	4644.84	32090.81	2.801	3903.58	10935.14
A_68_P33007796	chr4_random:129068-129112	NM_001033326:19289	Dhrsx	INSIDE	0.417	17.938	8042.91	144271.80	7.478	6325.12	47299.27
A_68_P33007795	chr4_random:128995-129039	NM_001033326:19215	Dhrsx	INSIDE	0.425	15.826	3871.96	61278.76	6.727	2721.39	18307.87
A_68_P33007797	chr4_random:129202-129246	NM_001033326:19423	Dhrsx	INSIDE	0.432	7.521	1095.16	8236.50	3.251	1101.20	3580.38
A_68_P33008823	chr4_random:109636-109680	NM_001033326:-143	Dhrsx	PROMOTER	0.443	12.008	5296.78	63601.85	5.316	5681.98	30208.22
A_68_P33007789	chr4_random:128217-128261	NM_001033326:18437	Dhrsx	INSIDE	0.395	4.172	2211.97	9227.52	1.647	1665.24	2742.65
A_68_P33007788	chr4_random:128082-128126	NM_001033326:18303	Dhrsx	INSIDE	0.128	11.744	7505.04	88140.66	1.506	6219.22	9363.54
A_68_P23693364	chr5:52581717-52581761	NM_007839:20	Dhx15	INSIDE	0.216	2.334	1018.27	2376.18	0.505	945.10	476.85
A_68_P31161462	chr17:36016904-36016948	NM_026987:204	Dhx16	INSIDE	0.48	6.575	741.26	4873.42	3.153	620.96	1958.11
A_68_P31161463	chr17:36016977-36017021	NM_026987:276	Dhx16	INSIDE	0.573	4.052	978.26	3964.01	2.323	909.08	2112.12
A_68_P26822031	chr9:110019702-110019746	NM_133347:-1638	Dhx30	PROMOTER	0.63	2.773	1298.46	3600.71	1.748	1129.09	1973.38
A_68_P26822035	chr9:110020257-110020301	NM_133347:-2192	Dhx30	PROMOTER	0.441	3.165	2643.15	8364.72	1.396	2282.66	3186.87
A_68_P25542609	chr7:140968445-140968489	NM_133941:20	Dhx32	INSIDE	0.453	4.230	1253.34	5302.09	1.915	1147.00	2196.60
A_68_P27903827	chr11:70817840-70817884	NM_178367:72	Dhx33	INSIDE	0.38	6.036	770.98	4653.98	2.293	663.32	1520.80

ProbeName	Target position of probe on CpG island microarray	TargetID	GeneSymbol	CpG island Description	Ratio of relative methylation (TiO_2-NP/Vehicle)	Sham group			TiO_2-H group		
						Relative methylation (Cy5/Cy3)	Cy3 signal (Input)	Cy5 signal (MeDIP)	Relative methylation (Cy5/Cy3)	Cy3 signal (Input)	Cy5 signal (MeDIP)
A_68_P21785050	chr2:158620772-158620816	NM_145742:239	Dhx35	INSIDE	0.655	0.481	999.00	480.27	0.315	828.79	260.84
A_68_P26157751	chr8:112089473-112089517	NM_178380:7	Dhx38	INSIDE	0.239	0.456	2369.59	1080.27	0.109	1708.93	185.95
A_68_P31630972	chr18:38094826-38094871	NM_007858:217	Diap1	INSIDE	0.503	7.146	532.32	3803.99	3.593	417.01	1498.11
A_68_P28705225	chr12:105989571-105989615	NM_148948:570	Dicer1	INSIDE	0.561	0.381	1625.20	618.45	0.214	1240.61	264.93
A_68_P21905697	chr2:180444518-180444564	NM_175551:-7735	Dido1	PROMOTER	0.017	21.434	3271.19	70114.96	0.371	2462.67	912.87
A_68_P30483526	chr15:99869552-99869596	NM_001159361:480	Dip2b	INSIDE	0.52	5.082	914.30	4646.46	2.641	792.66	2093.57
A_68_P30483524	chr15:99869292-99869336	NM_001159361:220	Dip2b	INSIDE	0.542	4.811	1564.39	7525.50	2.608	1533.11	3997.99
A_68_P27286934	chr10:80484978-80485022	NM_145217:3122	Diras1	INSIDE	0.477	0.444	1163.92	516.58	0.212	899.20	190.20
A_68_P30672037	chr16:35769289-35769333	NM_153550:132	Dirc2	INSIDE	0.454	7.041	2040.44	14367.07	3.196	1643.83	5253.46
A_68_P29841645	chr14:99499049-99499093	NM_028315:-81	Dis3	PROMOTER	0.535	3.502	659.14	2308.31	1.872	651.39	1219.36
A_68_P26581355	chr9:64188489-64188533	NM_172519:-246	Dis3l	PROMOTER	0.303	0.469	1682.53	788.63	0.142	1302.98	184.92
A_68_P26249219	chr8:127578258-127578302	NM_174853:186	Disc1	INSIDE	0.327	10.409	15893.16	165434.10	3.404	13119.40	44663.73
A_68_P33015623	chr1_random:50870-50914	NM_026866:134446	Disp1	INSIDE	0.243	6.700	1619.39	10849.99	1.631	1109.79	1809.71
A_68_P26505085	chr9:50507371-50507415	NM_178118:28697	Dixdc1	INSIDE	0.422	0.447	1070.80	478.83	0.189	901.26	169.96
A_68_P25422972	chr7:119301935-119301979	NM_015814:615	Dkk3	INSIDE	0.051	1.981	7428.89	14720.23	0.101	4719.05	477.76
A_68_P25422973	chr7:119302042-119302086	NM_015814:507	Dkk3	INSIDE	0.35	0.591	1347.09	795.53	0.207	1017.41	210.48
A_68_P26504861	chr9:50467491-50467535	NM_145614:373	Dlat	INSIDE	0.487	6.733	2608.96	17565.28	3.277	2287.11	7494.61
A_68_P28330749	chr12:32035950-32035994	NM_007861:330	Dld	INSIDE	0.242	16.471	1472.89	24260.65	3.986	1285.79	5124.58
A_68_P29651397	chr14:62301793-62301837	NR_028264:-604	Dleu2	PROMOTER	0.133	33.820	1801.01	60910.22	4.512	1575.92	7110.65
A_68_P29651387	chr14:62300610-62300654	NR_028264:578	Dleu2	INSIDE	0.418	0.303	3539.51	1074.17	0.127	2486.23	315.26
A_68_P29651430	chr14:62305865-62305909	NR_028264:-4676	Dleu2	PROMOTER	0.483	8.241	1149.58	9473.90	3.981	1073.08	4271.50
A_68_P29651385	chr14:62300358-62300402	NR_028264:830	Dleu2	INSIDE	0.568	4.961	3998.31	19835.99	2.816	3117.20	8778.21
A_68_P29651395	chr14:62301504-62301548	NR_028264:-316	Dleu2	PROMOTER	0.49	3.345	1117.35	3737.30	1.639	1016.54	1665.98
A_68_P30648664	chr16:31663664-31663708	NM_007862:-438	Dlg1	PROMOTER	0.39	0.222	3867.55	859.99	0.087	3038.80	263.39
A_68_P29470473	chr14:25064406-25064450	NM_001163513:714	Dlg5	INSIDE	0.498	46.795	415.21	19429.75	23.291	552.06	12857.76
A_68_P29470478	chr14:25065176-25065220	NM_001163513:-56	Dlg5	PROMOTER	0.591	3.651	888.55	3244.33	2.159	734.67	1585.92
A_68_P31330059	chr17:71007053-71007097	NM_001128181:95988	Dlgap1	INSIDE	0.036	90.238	2860.03	258083.40	3.226	2685.13	8661.43
A_68_P31329272	chr17:70865492-70865536	NM_001128180:-5935	Dlgap1	PROMOTER	0.422	4.556	1875.93	8546.88	1.921	1246.99	2395.79
A_68_P31330058	chr17:71006929-71006973	NM_001128181:95864	Dlgap1	INSIDE	0.509	3.470	801.76	2782.45	1.767	595.12	1051.65
A_68_P25672261	chr8:14096967-14097011	NM_001145965:1114	Dlgap2	INSIDE	0.343	7.244	3945.87	28584.58	2.483	3166.93	7864.34
A_68_P25672257	chr8:14096396-14096440	NM_001145965:544	Dlgap2	INSIDE	0.459	7.008	889.96	6236.66	3.217	801.54	2578.26
A_68_P23284203	chr4:126911090-126911134	NM_198618:64598	Dlgap3	INSIDE	0.513	3.832	1142.23	4377.61	1.967	936.95	1843.29
A_68_P23284202	chr4:126910975-126911019	NM_198618:64482	Dlgap3	INSIDE	0.654	2.468	2079.05	5131.43	1.615	1657.09	2675.78
A_68_P23284102	chr4:126891893-126891937	NM_198618:45400	Dlgap3	INSIDE	0.148	4.805	4749.31	22818.46	0.712	3542.45	2523.44
A_68_P21772346	chr2:156440160-156440204	NM_146128:742	Dlgap4	INSIDE	0.645	0.373	1732.86	646.62	0.241	1273.89	306.50
A_68_P29582179	chr14:48037872-48037916	NM_144553:188	Dlgap5	INSIDE	0.627	0.501	3253.48	1629.81	0.314	2361.43	741.19
A_68_P28732711	chr12:110692025-110692069	NM_001190703:382	Dlk1	INSIDE	0.246	9.275	10239.29	94972.59	2.285	8356.84	19098.28
A_68_P28732710	chr12:110691897-110691941	NM_001190703:254	Dlk1	INSIDE	0.298	0.395	2246.16	888.20	0.118	1618.51	190.79
A_68_P28732704	chr12:110691154-110691198	NM_001190703:-488	Dlk1	PROMOTER	0.613	0.315	1592.39	502.08	0.193	1130.44	218.33
A_68_P31065266	chr17:15513473-15513517	NM_007865:-707	Dll1	PROMOTER	0.295	13.539	1323.19	17914.92	3.998	1101.70	4404.50
A_68_P31065261	chr17:15512852-15512896	NM_007865:-87	Dll1	PROMOTER	0.612	0.530	1563.73	828.15	0.324	1193.55	387.08
A_68_P31065253	chr17:15511986-15512030	NM_007865:779	Dll1	INSIDE	0.636	2.992	808.76	2420.02	1.903	632.74	1204.01
A_68_P31065249	chr17:15511628-15511672	NM_007865:1137	Dll1	INSIDE	0.552	0.551	856.24	472.03	0.304	682.74	207.77
A_68_P25021043	chr7:29086277-29086321	NM_007866:506	Dll3	INSIDE	0.267	0.351	3738.75	1312.09	0.094	2985.06	279.36
A_68_P25021007	chr7:29079751-29079795	NM_007866:7032	Dll3	INSIDE	0.599	7.724	1433.21	11069.61	4.628	1262.28	5841.41
A_68_P21569032	chr2:119148703-119148747	NM_019454:-2795	Dll4	PROMOTER	0.456	4.856	1530.44	7431.54	2.215	1247.18	2762.04

ProbeName	Target position of probe on CpG island microarray	TargetID	GeneSymbol	CpG island Description	Ratio of relative methylation (TiO_2-NP/Vehicle)	Sham group			TiO_2-H group		
						Relative methylation (Cy5/Cy3)	Cy3 signal (Input)	Cy5 signal (MeDIP)	Relative methylation (Cy5/Cy3)	Cy3 signal (Input)	Cy5 signal (MeDIP)
A_68_P21569026	chr2:119148000-119148050	NM_019454:-3495	Dll4	PROMOTER	0.376	0.627	908.40	569.61	0.236	790.48	186.60
A_68_P21569151	chr2:119163754-119163798	NM_019454:12257	Dll4	DOWNSTREAM	0.51	3.415	1257.37	4293.57	1.743	1189.51	2072.88
A_68_P21319994	chr2:71367531-71367575	NM_010053:51	Dlx1	INSIDE	0.577	2.507	8298.51	20800.57	1.445	6612.22	9556.67
A_68_P21320094	chr2:71379207-71379251	NR_002854:-3280	Dlx1as	PROMOTER	0.285	1.596	3665.41	5851.22	0.456	2656.84	1210.58
A_68_P28036958	chr11:95006743-95006787	NM_007867:351	Dlx4	INSIDE	0.638	0.300	3997.38	1201.17	0.192	2943.26	564.37
A_68_P24218861	chr6:6829299-6829343	NM_010056:2748	Dlx5	INSIDE	0.285	7.114	2021.83	14382.96	2.028	1612.54	3270.84
A_68_P24218878	chr6:6831381-6831425	NM_010056:666	Dlx5	INSIDE	0.36	4.228	1687.87	7136.31	1.522	1443.23	2197.15
A_68_P24218728	chr6:6813006-6813052	NM_010057:-305	Dlx6	PROMOTER	0.059	51.134	427.69	21869.28	3.023	360.22	1089.01
A_68_P24218732	chr6:6813819-6813863	NM_010057:507	Dlx6	INSIDE	0.18	0.280	12673.06	3549.11	0.05	8542.92	429.61
A_68_P23236051	chr4:117354667-117354711	NM_023178:142	Dmap1	INSIDE	0.507	0.190	2404.48	455.99	0.096	1817.66	174.72
A_68_P23236052	chr4:117354732-117354776	NM_023178:76	Dmap1	INSIDE	0.585	2.668	2920.06	7791.93	1.56	2392.93	3733.24
A_68_P23226835	chr4:115616658-115616702	NM_001025567:-4149	Dmbx1	PROMOTER	0.487	5.990	3268.99	19580.02	2.916	2581.11	7526.92
A_68_P23226836	chr4:115616847-115616895	NM_001025567:-4339	Dmbx1	PROMOTER	0.089	2.491	1113.50	2773.65	0.221	788.76	174.38
A_68_P32030090	chr19:25580813-25580857	NM_015826:639	Dmrt1	INSIDE	0.629	0.414	4812.90	1992.83	0.26	3409.31	887.88
A_68_P32030095	chr19:25581366-25581410	NM_015826:1193	Dmrt1	INSIDE	0.345	5.960	529.90	3158.39	2.055	517.51	1063.69
A_68_P32031149	chr19:25746643-25746687	NM_145831:-236	Dmrt2	PROMOTER	0.581	7.249	1336.82	9690.69	4.21	1075.64	4528.09
A_68_P23203782	chr4:109656380-109656424	NM_172296:5773	Dmrta2	DOWNSTREAM	0.208	10.106	1407.13	14220.11	2.099	1464.29	3073.70
A_68_P23203724	chr4:109649844-109649888	NM_172296:-763	Dmrta2	PROMOTER	0.291	121.929	8082.71	985513.10	35.514	15549.77	552236.40
A_68_P23203774	chr4:109655335-109655379	NM_172296:4727	Dmrta2	INSIDE	0.507	7.550	3077.05	23232.37	3.831	2522.57	9664.16
A_68_P23203768	chr4:109654665-109654709	NM_172296:4057	Dmrta2	INSIDE	0.657	2.954	954.12	2818.81	1.94	756.62	1468.12
A_68_P23203773	chr4:109655219-109655263	NM_172296:4611	Dmrta2	INSIDE	0.51	0.665	1139.66	758.39	0.34	951.45	323.09
A_68_P23203770	chr4:109654911-109654955	NM_172296:4303	Dmrta2	INSIDE	0.538	2.867	1248.79	3580.84	1.542	1086.84	1676.19
A_68_P23471513	chr5:9161567-9161611	NM_001110327:188	Dmtf1	INSIDE	0.617	5.639	1528.65	8619.96	3.479	1372.97	4776.48
A_68_P26526109	chr9:54349056-54349100	NM_172771:355	Dmxl2	INSIDE	0.288	9.155	2698.03	24701.73	2.639	2665.42	7033.24
A_68_P26526112	chr9:54349310-54349354	NM_172771:101	Dmxl2	INSIDE	0.623	5.160	1932.73	9972.70	3.214	1531.74	4922.86
A_68_P26527193	chr9:54547061-54547105	NM_021422:-283	Dnaja4	PROMOTER	0.623	0.446	6973.54	3108.14	0.278	4447.90	1234.44
A_68_P26015683	chr8:86132210-86132254	NM_018808:159	Dnajb1	INSIDE	0.47	7.191	2673.40	19225.00	3.382	2084.68	7050.44
A_68_P26015679	chr8:86131823-86131867	NM_018808:-229	Dnajb1	PROMOTER	0.564	0.303	4023.76	1217.48	0.171	2917.97	498.07
A_68_P26015682	chr8:86132133-86132177	NM_018808:81	Dnajb1	INSIDE	0.515	0.579	1364.60	789.48	0.298	1070.77	318.80
A_68_P20352841	chr1:75233173-75233217	NM_001159885:-82	Dnajb2	PROMOTER	0.288	0.257	3327.35	853.97	0.074	2427.45	179.60
A_68_P20422167	chr1:90102351-90102395	NM_008299:-49	Dnajb3	DIVERGENT_PROMOTER	0.311	17.249	1709.35	29485.20	5.359	1612.74	8642.06
A_68_P20422165	chr1:90102210-90102254	NM_008299:91	Dnajb3	INSIDE	0.65	4.023	787.18	3166.91	2.615	642.81	1681.22
A_68_P22869097	chr4:42963079-42963123	NM_019874:-2865	Dnajb5	PROMOTER	0.273	1.643	9149.84	15036.31	0.449	7435.32	3337.94
A_68_P28396964	chr12:45311296-45311340	NM_013760:-263	Dnajb9	PROMOTER	0.47	4.711	1125.02	5300.14	2.216	963.24	2135.01
A_68_P28396959	chr12:45310845-45310889	NM_013760:189	Dnajb9	INSIDE	0.62	0.375	1932.72	724.84	0.233	1354.14	315.03
A_68_P21039675	chr2:18314331-18314375	NM_001190817:105	Dnajc1	INSIDE	0.351	7.825	2043.12	15986.49	2.747	1867.52	5130.76
A_68_P21374004	chr2:80155781-80155825	NM_024181:180	Dnajc10	INSIDE	0.488	3.006	2570.47	7726.38	1.466	2308.90	3385.12
A_68_P23415470	chr4:151308129-151308173	NM_172704:322	Dnajc11	INSIDE	0.648	3.443	526.10	1811.38	2.232	469.64	1048.00
A_68_P26790788	chr9:104165092-104165136	NM_001163026:146	Dnajc13	INSIDE	0.206	16.631	1978.08	32897.61	3.427	1919.77	6579.55
A_68_P26790789	chr9:104165231-104165275	NM_001163026:8	Dnajc13	INSIDE	0.373	0.240	2466.83	593.23	0.09	1912.01	171.58
A_68_P26790786	chr9:104164871-104164915	NM_001163026:368	Dnajc13	INSIDE	0.472	0.433	1920.59	830.74	0.204	1602.66	327.24
A_68_P26790784	chr9:104164628-104164672	NM_001163026:610	Dnajc13	INSIDE	0.497	0.515	1212.70	624.92	0.256	998.56	255.73
A_68_P23365986	chr4:141346174-141346218	NM_172338:363	Dnajc16	INSIDE	0.409	0.400	1789.07	715.09	0.164	1294.99	211.78
A_68_P23365990	chr4:141346639-141346683	NM_172338:-101	Dnajc16	DIVERGENT_PROMOTER	0.523	3.452	2002.55	6913.20	1.805	1717.60	3099.53
A_68_P30010737	chr15:10399801-10399845	NM_030046:449	Dnajc21	INSIDE	0.608	7.805	1522.65	11883.78	4.745	1284.58	6095.83
A_68_P30478229	chr15:98928779-98928823	NM_176835:-1114	Dnajc22	PROMOTER	0.113	66.058	5624.69	371554.10	7.457	5794.86	43214.53

ProbeName	Target position of probe on CpG island microarray	TargetID	GeneSymbol	CpG island Description	Ratio of relative methylation (TiO_2-NP/Vehicle)	Sham group			TiO_2-H group		
						Relative methylation (Cy5/Cy3)	Cy3 signal (Input)	Cy5 signal (MeDIP)	Relative methylation (Cy5/Cy3)	Cy3 signal (Input)	Cy5 signal (MeDIP)
A_68_P30478193	chr15:98924227-98924271	NM_176835:-5666	Dnajc22	DIVERGENT_PROMOTER	0.26	14.367	5607.89	80570.22	3.743	5092.45	19058.76
A_68_P22954051	chr4:59016462-59016506	NM_001033165:420	Dnajc25	INSIDE	0.575	0.417	1082.29	451.58	0.24	870.88	208.79
A_68_P28198624	chr12:4082843-4082887	NM_153082:291	Dnajc27	INSIDE	0.462	0.139	6389.61	890.76	0.064	4254.90	273.87
A_68_P28198625	chr12:4082923-4082967	NM_153082:371	Dnajc27	INSIDE	0.602	0.246	2468.77	608.25	0.148	1742.88	258.39
A_68_P24114557	chr5:135540470-135540514	NM_025362:417	Dnajc30	INSIDE	0.535	3.528	2056.67	7255.39	1.889	1733.73	3274.88
A_68_P21911108	chr2:181255242-181255287	NM_016775:55	Dnajc5	INSIDE	0.581	3.155	1675.78	5287.68	1.832	1401.14	2566.73
A_68_P23155326	chr4:101169630-101169674	NM_198412:400	Dnajc6	INSIDE	0.641	0.565	2181.97	1232.96	0.362	1629.31	590.61
A_68_P29446595	chr14:21207859-21207903	NM_134081:252	Dnajc9	INSIDE	0.571	0.275	2077.12	572.02	0.157	1491.82	234.62
A_68_P28596399	chr12:85455453-85455497	NM_028821:197	Dnalc1	INSIDE	0.033	13.866	1911.42	26504.15	0.451	1640.56	739.58
A_68_P20405120	chr1:84692097-84692141	NM_152915:678	Dner	INSIDE	0.641	2.937	1204.93	3538.73	1.884	976.32	1839.23
A_68_P20778066	chr1:164408081-164408125	NM_001038619:59	Dnm3	INSIDE	0.637	3.284	1051.87	3454.03	2.092	903.45	1890.08
A_68_P26344762	chr9:20757074-20757118	NM_001199433:-29	Dnmt1	PROMOTER	0.429	0.702	1993.79	1400.52	0.301	1427.76	430.21
A_68_P21819501	chr2:164571271-164571315	NM_133763:-222	Dnttip1	DIVERGENT_PROMOTER	0.497	3.502	6852.01	23993.92	1.742	5440.97	9476.78
A_68_P21819502	chr2:164571363-164571407	NM_133763:-130	Dnttip1	DIVERGENT_PROMOTER	0.627	0.522	1533.02	799.94	0.327	1194.18	390.83
A_68_P27928203	chr11:75608556-75608600	NM_007873:981	Doc2b	INSIDE	0.237	32.112	383.77	12323.56	7.613	369.88	2815.93
A_68_P27928208	chr11:75609122-75609166	NM_007873:415	Doc2b	INSIDE	0.33	1.724	1452.03	2503.85	0.57	1206.84	687.54
A_68_P25547702	chr7:141862476-141862520	NM_001033420:129	Dock1	INSIDE	0.532	3.303	524.98	1734.18	1.756	714.12	1254.34
A_68_P20383167	chr1:80754578-80754622	NM_175291:528	Dock10	INSIDE	0.479	0.366	3570.56	1305.26	0.175	2764.56	484.17
A_68_P20383169	chr1:80754785-80754829	NM_175291:322	Dock10	INSIDE	0.635	0.353	1510.44	533.37	0.224	1205.31	270.37
A_68_P32315765	chrX:33428989-33429033	NM_001009947:184	Dock11	INSIDE	0.236	18.324	588.72	10787.83	4.316	729.77	3149.80
A_68_P32316566	chrX:33565537-33565581	NM_001009947:136732	Dock11	INSIDE	0.459	8.094	850.60	6884.36	3.716	826.54	3071.20
A_68_P32316569	chrX:33565828-33565872	NM_001009947:137024	Dock11	INSIDE	0.526	3.351	516.25	1729.77	1.762	707.45	1246.26
A_68_P26807068	chr9:107095792-107095844	NM_153413:38422	Dock3	INSIDE	0.352	5.434	507.40	2757.23	1.915	445.80	853.56
A_68_P28378468	chr12:41173035-41173079	NM_172803:417	Dock4	INSIDE	0.357	0.530	1076.55	570.66	0.189	929.65	176.01
A_68_P23142414	chr4:98787172-98787216	NM_026082:412	Dock7	INSIDE	0.59	4.153	834.47	3465.39	2.451	771.28	1890.78
A_68_P32027007	chr19:25073867-25073911	NM_028785:-130	Dock8	PROMOTER	0.542	6.927	332.35	2302.08	3.753	301.16	1130.38
A_68_P24590984	chr6:82983529-82983573	NM_010070:-85	Dok1	DIVERGENT_PROMOTER	0.593	7.372	1183.26	8723.34	4.371	925.74	4046.44
A_68_P24590979	chr6:82982955-82982999	NM_010070:489	Dok1	INSIDE	0.555	0.717	1800.35	1291.45	0.398	1300.49	517.94
A_68_P26080011	chr8:97400151-97400195	NM_053246:40	Dok4	INSIDE	0.545	4.275	975.30	4169.73	2.331	787.85	1836.79
A_68_P21102300	chr2:30141879-30141923	NM_177648:-26	Dolk	DIVERGENT_PROMOTER	0.409	4.771	2649.77	12641.10	1.95	2061.55	4020.85
A_68_P21102918	chr2:30248319-30248363	NM_020329:405	Dolpp1	INSIDE	0.408	10.216	1927.09	19687.94	4.167	1604.25	6684.18
A_68_P21102914	chr2:30247811-30247855	NM_020329:-103	Dolpp1	PROMOTER	0.621	0.366	4699.42	1717.82	0.227	3180.84	722.04
A_68_P31156013	chr17:34974519-34974563	NM_001163770:577	Dom3z	INSIDE	0.334	4.400	3237.77	14247.30	1.472	2585.03	3804.56
A_68_P26699344	chr9:86361211-86361255	NM_177208:472	Dopey1	INSIDE	0.642	3.307	976.28	3228.41	2.124	821.56	1745.33
A_68_P27281161	chr10:79594591-79594635	NM_001195268:5520	Dos	INSIDE	0.395	5.536	2139.90	11847.02	2.187	1703.36	3725.22
A_68_P27281217	chr10:79602092-79602136	NR_036582:-2	Dos	DIVERGENT_PROMOTER	0.562	2.860	1947.65	5569.42	1.608	1668.53	2682.56
A_68_P27285229	chr10:80217921-80217965	NM_199322:-8	Dot1l	PROMOTER	0.445	6.892	2508.33	17287.75	3.066	2007.67	6155.52
A_68_P27285230	chr10:80218002-80218046	NM_199322:74	Dot1l	INSIDE	0.427	3.953	1226.89	4850.29	1.687	1015.29	1712.33
A_68_P26467020	chr9:44135161-44135205	NM_007875:255	Dpagt1	INSIDE	0.206	0.610	2871.95	1752.56	0.126	1700.55	213.70
A_68_P26467018	chr9:44134953-44134997	NM_007875:47	Dpagt1	INSIDE	0.343	1.916	1697.71	3253.43	0.657	1401.80	921.22
A_68_P26137127	chr8:108502919-108502963	NM_027960:379	Dpep3	INSIDE	0.255	1.816	2161.81	3926.54	0.464	1619.13	750.58
A_68_P26137129	chr8:108503182-108503226	NM_027960:115	Dpep3	INSIDE	0.278	11.125	6493.86	72245.71	3.093	4188.51	12955.84
A_68_P26137128	chr8:108503069-108503114	NM_027960:228	Dpep3	INSIDE	0.237	8.878	869.18	7716.98	2.105	642.37	1352.27
A_68_P25026497	chr7:30093255-30093301	NM_013874:4255	Dpf1	INSIDE	0.614	0.644	1195.12	769.71	0.395	931.55	368.34
A_68_P28593012	chr12:84827504-84827548	NM_058212:1132	Dpf3	INSIDE	0.647	0.179	5407.48	967.52	0.116	3580.43	414.76
A_68_P23237102	chr4:117564195-117564239	NM_026344:392	Dph2	INSIDE	0.229	0.408	5819.32	2371.87	0.093	4218.79	393.88

ProbeName	Target position of probe on CpG island microarray	TargetID	GeneSymbol	CpG island Description	Ratio of relative methylation (TiO_2-NP/Vehicle)	Sham group			TiO_2-H group		
						Relative methylation (Cy5/Cy3)	Cy3 signal (Input)	Cy5 signal (MeDIP)	Relative methylation (Cy5/Cy3)	Cy3 signal (Input)	Cy5 signal (MeDIP)
A_68_P23237103	chr4:117564318-117564368	NM_026344:266	Dph2	INSIDE	0.367	0.733	2939.28	2154.03	0.269	1881.26	505.59
A_68_P31925904	chr19:4928163-4928207	NM_133803:103	Dpp3	INSIDE	0.591	0.634	2854.44	1809.43	0.375	1926.28	721.40
A_68_P26585437	chr9:64880753-64880797	NM_028906:510	Dpp8	INSIDE	0.403	0.215	2856.42	613.70	0.086	1985.83	171.74
A_68_P26362426	chr9:24307746-24307790	NM_172920:-184	Dpy19l1	PROMOTER	0.638	4.675	232.69	1087.90	2.982	196.65	586.43
A_68_P25051002	chr7:36539157-36539201	NM_178704:295	Dpy19l3	INSIDE	0.447	0.509	5151.65	2621.48	0.228	4057.59	923.15
A_68_P25051005	chr7:36539482-36539526	NM_178704:-31	Dpy19l3	PROMOTER	0.537	0.345	2771.84	956.61	0.185	2169.64	402.29
A_68_P25051003	chr7:36539278-36539322	NM_178704:173	Dpy19l3	INSIDE	0.536	3.416	1149.53	3926.61	1.831	894.95	1638.83
A_68_P22729159	chr4:11248834-11248879	NM_001081201:422	Dpy19l4	INSIDE	0.642	0.489	2895.40	1415.73	0.314	2216.54	695.77
A_68_P31350938	chr17:74715809-74715853	NM_001146223:-57	Dpy30	PROMOTER	0.397	9.108	742.87	6766.43	3.616	668.58	2417.42
A_68_P31350937	chr17:74715727-74715771	NM_001146222:-14	Dpy30	PROMOTER	0.183	1.596	688.31	1098.72	0.292	666.70	194.73
A_68_P29681787	chr14:67486496-67486540	NM_009955:919	Dpysl2	INSIDE	0.505	0.428	1738.13	743.30	0.216	1290.82	278.95
A_68_P31661239	chr18:43552742-43552786	NM_009468:221	Dpysl3	INSIDE	0.291	1.841	1045.63	1925.16	0.536	899.31	482.42
A_68_P23574101	chr5:31013677-31013721	NM_023047:-569	Dpysl5	PROMOTER	0.664	4.037	572.68	2311.69	2.68	463.56	1242.56
A_68_P31928602	chr19:5424535-5424579	NM_024176:360	Drap1	INSIDE	0.454	7.032	6106.47	42940.80	3.19	5159.74	16457.16
A_68_P31928601	chr19:5424408-5424452	NM_024176:486	Drap1	INSIDE	0.479	0.527	1770.09	933.39	0.252	1236.88	312.17
A_68_P25587953	chr7:148479688-148479732	NM_007878:1806	Drd4	INSIDE	0.415	0.273	2739.67	748.29	0.113	1787.88	202.52
A_68_P25587940	chr7:148478056-148478100	NM_007878:174	Drd4	INSIDE	0.61	0.224	4292.84	962.27	0.137	2837.40	388.21
A_68_P27551031	chr11:3166132-3166176	NM_007879:235	Drg1	INSIDE	0.229	2.516	8542.66	21496.64	0.577	5917.33	3415.16
A_68_P26473565	chr9:45238529-45238573	NM_001081270:175	Dscaml1	INSIDE	0.437	0.689	1524.89	1050.94	0.301	901.62	271.83
A_68_P30981932	chr16:94747888-94747932	NM_007834:326	Dscr3	INSIDE	0.665	0.506	3973.27	2008.56	0.336	2831.37	952.28
A_68_P21774638	chr2:156832712-156832756	NM_025853:77	Dsn1	INSIDE	0.519	0.572	2261.46	1292.49	0.296	1720.00	509.82
A_68_P28958812	chr13:38243505-38243549	NM_023842:364	Dsp	INSIDE	0.556	3.539	650.74	2302.74	1.969	605.05	1191.37
A_68_P21704233	chr2:143740849-143740894	NM_019771:-195	Dstn	PROMOTER	0.514	6.233	3563.67	22213.22	3.207	3002.60	9629.19
A_68_P21704238	chr2:143741481-143741525	NM_019771:436	Dstn	INSIDE	0.531	3.865	1081.15	4178.71	2.051	1004.34	2059.98
A_68_P20621314	chr1:134314077-134314121	NM_172516:69	Dstyk	INSIDE	0.37	19.833	700.81	13899.32	7.342	691.20	5074.80
A_68_P20621319	chr1:134314633-134314681	NM_172516:627	Dstyk	INSIDE	0.51	0.179	2570.23	459.08	0.091	1898.50	173.01
A_68_P21708094	chr2:144425869-144425913	NM_025314:202	Dtd1	INSIDE	0.243	8.863	2210.45	19590.95	2.153	1986.50	4277.02
A_68_P28195719	chr12:3572230-3572274	NM_001162465:-138	Dtnb	PROMOTER	0.661	3.739	1110.87	4153.84	2.473	948.99	2346.87
A_68_P28999556	chr13:45097278-45097322	NM_025772:165	Dtnbp1	INSIDE	0.178	1.731	990.83	1715.12	0.308	848.28	261.67
A_68_P31694513	chr18:49915007-49915051	NM_001170960:227	Dtwd2	INSIDE	0.221	16.309	2202.36	35919.34	3.608	1820.78	6569.51
A_68_P24040306	chr5:121144444-121144488	NM_008052:17212	Dtx1	INSIDE	0.284	0.579	1387.80	802.87	0.164	1044.47	171.49
A_68_P24040430	chr5:121161722-121161766	NM_008052:-66	Dtx1	PROMOTER	0.593	2.814	2844.45	8005.30	1.669	2237.80	3736.01
A_68_P31961135	chr19:12575854-12575898	NM_172442:610	Dtx4	INSIDE	0.665	2.427	1302.43	3161.39	1.613	1105.36	1783.00
A_68_P20457519	chr1:95698419-95698463	NM_001105667:71	Dtymk	INSIDE	0.528	0.255	1970.84	503.39	0.135	1568.39	211.40
A_68_P20457518	chr1:95698322-95698366	NM_001105667:167	Dtymk	INSIDE	0.616	0.673	1805.00	1214.25	0.415	1504.20	623.67
A_68_P21585601	chr2:122127327-122127371	NM_025777:2713	Duoxa2	INSIDE	0.623	0.416	4013.85	1671.38	0.259	2744.06	711.44
A_68_P29454311	chr14:22506206-22506253	NM_001013826:27569	Dupd1	INSIDE	0.447	4.822	437.00	2107.24	2.157	467.42	1008.26
A_68_P31258228	chr17:56904354-56904398	NM_144858:203	Dus3l	INSIDE	0.294	0.415	1966.70	815.74	0.122	1422.43	173.46
A_68_P31110713	chr17:26645297-26645341	NM_013642:99	Dusp1	INSIDE	0.517	0.124	4256.78	529.14	0.064	2625.45	168.81
A_68_P24607367	chr6:85911667-85911711	NM_028099:-27	Dusp11	PROMOTER	0.364	7.044	1399.11	9855.28	2.562	1234.87	3163.48
A_68_P24607366	chr6:85911499-85911543	NM_028099:141	Dusp11	INSIDE	0.452	0.507	910.37	461.44	0.229	754.60	173.01
A_68_P20825163	chr1:172815285-172815329	NM_023173:365	Dusp12	INSIDE	0.663	0.560	1448.89	811.35	0.371	1216.87	452.00
A_68_P21752056	chr2:152768692-152768736	NM_001159376:8604	Dusp15	INSIDE	0.517	0.457	979.91	448.21	0.237	721.50	170.68
A_68_P21612764	chr2:127162058-127162102	NM_010090:186	Dusp2	INSIDE	0.533	4.894	1626.05	7958.25	2.611	1485.39	3878.29
A_68_P20834309	chr1:174562662-174562706	NM_026725:421	Dusp23	INSIDE	0.384	10.573	15277.02	161528.10	4.058	13869.07	56280.77
A_68_P25776099	chr8:35872603-35872647	NM_176933:1961	Dusp4	INSIDE	0.26	7.488	746.27	5587.92	1.945	570.98	1110.78

ProbeName	Target position of probe on CpG island microarray	TargetID	GeneSymbol	CpG island Description	Ratio of relative methylation (TiO_2-NP/Vehicle)	Sham group			TiO_2-H group		
						Relative methylation (Cy5/Cy3)	Cy3 signal (Input)	Cy5 signal (MeDIP)	Relative methylation (Cy5/Cy3)	Cy3 signal (Input)	Cy5 signal (MeDIP)
A_68_P27386026	chr10:98725041-98725087	NM_026268:-800	Dusp6	PROMOTER	0.369	0.665	818.77	544.63	0.246	692.63	170.05
A_68_P26802905	chr9:106270040-106270084	NM_153459:-900	Dusp7	PROMOTER	0.457	13.059	6602.50	86221.55	5.971	6705.15	40037.82
A_68_P25592946	chr7:149281230-149281274	NM_008748:-63	Dusp8	PROMOTER	0.478	0.710	2261.27	1604.42	0.339	1587.62	538.86
A_68_P25592938	chr7:149280400-149280444	NM_008748:767	Dusp8	INSIDE	0.305	6.726	792.34	5329.17	2.05	622.43	1275.70
A_68_P25592835	chr7:149268541-149268585	NM_008748:12627	Dusp8	INSIDE	0.645	2.990	759.02	2269.26	1.927	544.80	1049.88
A_68_P23440963	chr4:155222080-155222124	NM_010091:582	Dvl1	INSIDE	0.665	2.711	758.14	2055.41	1.803	678.26	1223.21
A_68_P30588038	chr16:20531403-20531447	NM_007889:14288	Dvl3	INSIDE	0.098	20.831	5802.22	120867.10	2.04	5084.57	10372.67
A_68_P31834477	chr18:75178883-75178927	NM_027727:479	Dym	INSIDE	0.573	6.432	1505.18	9681.12	3.684	1262.35	4650.03
A_68_P28739206	chr12:111839872-111839916	NM_030238:290	Dync1h1	INSIDE	0.57	5.851	1179.41	6901.11	3.338	1049.09	3501.45
A_68_P24213704	chr6:5955887-5955931	NM_001191023:280270	Dync1i1	INSIDE	0.523	3.995	1327.07	5301.65	2.088	1330.86	2778.28
A_68_P26128587	chr8:106966661-106966705	NM_001013380:265	Dync1li2	INSIDE	0.581	4.815	3468.32	16700.52	2.799	2869.92	8033.58
A_68_P24008929	chr5:115751222-115751266	NM_019682:-245	Dynll1	PROMOTER	0.299	12.262	2422.69	29707.45	3.664	2139.11	7837.32
A_68_P27997247	chr11:87800951-87800995	NM_001168472:-885	Dynll2	PROMOTER	0.445	4.861	1949.26	9475.56	2.163	1602.52	3466.15
A_68_P27997248	chr11:87801126-87801170	NM_026556:-113	Dynll2	PROMOTER	0.094	5.585	2031.77	11347.32	0.527	1437.17	756.86
A_68_P25020569	chr7:28964516-28964561	NM_010092:26	Dyrk1b	INSIDE	0.065	3.147	1025.61	3227.71	0.204	823.60	168.06
A_68_P25020574	chr7:28965243-28965287	NM_010092:752	Dyrk1b	INSIDE	0.434	7.385	2802.97	20700.36	3.206	2345.49	7519.89
A_68_P20613763	chr1:133026416-133026460	NM_145508:8373	Dyrk3	INSIDE	0.39	7.982	1320.71	10541.57	3.116	923.70	2878.32
A_68_P26765226	chr9:99530146-99530190	NM_028258:155	Dzip1l	INSIDE	0.252	1.647	945.08	1556.12	0.415	765.78	317.56
A_68_P30743456	chr16:48994162-48994206	NM_001110017:41	Dzip3	INSIDE	0.325	7.350	2933.66	21562.66	2.387	2660.28	6349.02
A_68_P30743455	chr16:48994040-48994084	NM_001110017:163	Dzip3	INSIDE	0.499	0.739	2363.02	1745.77	0.369	1904.91	702.33
A_68_P28051732	chr11:97489553-97489597	NM_175332:1456	E130012A19Rik	INSIDE	0.274	40.898	3942.88	161257.60	11.186	5389.68	60290.04
A_68_P26135515	chr8:108231965-108232009	NM_198299:82	E130303B06Rik	INSIDE	0.426	4.649	5816.72	27042.27	1.981	4452.55	8820.22
A_68_P27080339	chr10:39451377-39451421	NR_038037:415	E130307A14Rik	INSIDE	0.405	4.840	3442.55	16661.05	1.962	3121.68	6123.49
A_68_P22957376	chr4:59638970-59639014	NM_153158:-100	E130308A19Rik	PROMOTER	0.572	3.355	1274.23	4275.33	1.92	1214.20	2331.63
A_68_P27921611	chr11:74451961-74452005	NM_001013784:18876	E130309D14Rik	INSIDE	0.163	28.020	3433.46	96207.16	4.576	2764.15	12647.78
A_68_P27921508	chr11:74433189-74433233	NM_001013784:104	E130309D14Rik	INSIDE	0.229	10.430	3784.22	39471.16	2.385	3419.13	8154.43
A_68_P27921507	chr11:74433104-74433148	NM_001013784:20	E130309D14Rik	INSIDE	0.579	0.359	2374.87	853.55	0.208	1834.45	382.00
A_68_P27921510	chr11:74433466-74433510	NM_001013784:382	E130309D14Rik	INSIDE	0.644	14.844	2517.81	37374.31	9.561	2197.74	21012.85
A_68_P27921506	chr11:74432896-74432940	NM_001013784:-188	E130309D14Rik	PROMOTER	0.56	2.550	1701.80	4339.18	1.428	1520.17	2170.44
A_68_P22903295	chr4:49072454-49072498	NM_178756:143	E130309F12Rik	INSIDE	0.482	0.424	1082.84	459.25	0.205	855.05	174.96
A_68_P22903296	chr4:49072520-49072564	NM_178756:209	E130309F12Rik	INSIDE	0.535	3.163	2425.52	7672.68	1.694	1901.37	3220.44
A_68_P22194169	chr3:63733044-63733088	NM_177856:241	E130311K13Rik	INSIDE	0.368	0.667	887.46	591.86	0.245	753.91	185.02
A_68_P22194043	chr3:63709285-63709329	NM_177856:24001	E130311K13Rik	DOWNSTREAM	0.51	3.458	805.63	2786.11	1.763	793.54	1398.63
A_68_P27279294	chr10:79318023-79318067	NR_029447:-328	E130317F20Rik	PROMOTER	0.163	34.900	1553.94	54232.57	5.704	1341.34	7651.26
A_68_P28914784	chr13:30077124-30077168	NM_010093:786	E2f3	INSIDE	0.263	9.354	1229.21	11497.89	2.46	965.82	2375.69
A_68_P28914786	chr13:30077338-30077382	NM_010093:572	E2f3	INSIDE	0.43	0.225	3140.30	708.04	0.097	2361.54	228.88
A_68_P28914790	chr13:30077733-30077777	NM_010093:178	E2f3	INSIDE	0.664	0.726	2990.03	2170.27	0.482	2327.55	1122.10
A_68_P28914780	chr13:30076726-30076770	NM_010093:1184	E2f3	INSIDE	0.622	0.564	855.68	482.30	0.35	674.32	236.33
A_68_P28266976	chr12:16818150-16818194	NM_033270:402	E2f6	INSIDE	0.453	0.244	2539.10	620.28	0.111	1991.19	220.50
A_68_P28266974	chr12:16817834-16817878	NM_033270:86	E2f6	INSIDE	0.48	6.153	1480.99	9112.01	2.953	1270.58	3752.49
A_68_P27445213	chr10:110182319-110182363	NM_178609:-180	E2f7	PROMOTER	0.13	22.417	979.95	21967.84	2.905	796.01	2312.34
A_68_P27445215	chr10:110182553-110182597	NM_178609:54	E2f7	INSIDE	0.519	0.288	1571.99	452.57	0.149	1123.51	167.91
A_68_P25108786	chr7:56137106-56137150	NM_001013368:-717	E2f8	PROMOTER	0.353	9.692	2961.86	28705.55	3.422	2564.28	8776.02
A_68_P24388784	chr6:40385730-40385774	NM_175528:380	E330009J07Rik	INSIDE	0.444	15.276	5431.10	82967.24	6.785	4073.31	27636.83
A_68_P30253796	chr15:59205558-59205602	NM_153548:127	E430025E21Rik	INSIDE	0.337	18.021	2963.70	53409.95	6.071	3611.32	21925.66
A_68_P31097445	chr17:24592379-24592423	NM_007893:-144	E4f1	PROMOTER	0.484	0.160	3007.77	481.06	0.077	2171.91	168.28

ProbeName	Target position of probe on CpG island microarray	TargetID	GeneSymbol	CpG island Description	Ratio of relative methylation (TiO_2-NP/Vehicle)	Sham group			TiO_2-H group		
						Relative methylation (Cy5/Cy3)	Cy3 signal (Input)	Cy5 signal (MeDIP)	Relative methylation (Cy5/Cy3)	Cy3 signal (Input)	Cy5 signal (MeDIP)
A_68_P31097441	chr17:24591842-24591886	NM_007893:392	E4f1	INSIDE	0.666	0.506	871.83	441.17	0.337	699.72	235.70
A_68_P30677693	chr16:36875711-36875755	NM_134111:-820	Eaf2	DIVERGENT_PROMOTER	0.491	0.578	842.77	486.75	0.283	768.84	217.84
A_68_P28449065	chr12:55796514-55796558	NM_025456:316	Eapp	INSIDE	0.351	1.489	1388.06	2066.84	0.522	1114.33	582.18
A_68_P27756960	chr11:44434061-44434105	NM_007897:2447	Ebf1	INSIDE	0.355	0.441	1492.34	657.59	0.156	1146.11	179.17
A_68_P29683667	chr14:67850834-67850879	NM_010095:-1272	Ebf2	PROMOTER	0.351	21.247	266.20	5655.93	7.468	253.76	1895.06
A_68_P25564249	chr7:144390277-144390323	NM_001113414:115828	Ebf3	INSIDE	0.174	21.337	2387.99	50952.21	3.717	1555.72	5782.73
A_68_P25565202	chr7:144505325-144505370	NM_001113414:781	Ebf3	INSIDE	0.295	20.549	4019.96	82604.76	6.066	3820.65	23176.83
A_68_P25564250	chr7:144390350-144390394	NM_001113414:115756	Ebf3	INSIDE	0.421	5.434	2363.38	12843.10	2.289	1717.02	3929.41
A_68_P25565208	chr7:144505952-144505996	NM_001113414:154	Ebf3	INSIDE	0.495	4.850	774.79	3757.56	2.402	702.73	1688.28
A_68_P25565281	chr7:144514141-144514185	NM_001113414:-8034	Ebf3	PROMOTER	0.634	0.564	1993.40	1124.82	0.358	1677.16	600.23
A_68_P25564251	chr7:144390467-144390511	NM_001113414:115640	Ebf3	INSIDE	0.088	19.292	2478.23	47810.94	1.698	1741.96	2958.28
A_68_P21628998	chr2:130120631-130120675	NM_001110513:-1022	Ebf4	PROMOTER	0.601	0.737	2576.62	1898.00	0.443	1949.46	863.34
A_68_P21629321	chr2:130188203-130188247	NM_001110513:66550	Ebf4	INSIDE	0.428	0.740	2270.76	1679.36	0.316	1663.46	526.39
A_68_P23342966	chr4:137514008-137514052	NM_199307:95879	Ece1	INSIDE	0.653	2.677	2940.99	7874.44	1.748	2022.63	3535.19
A_68_P30588586	chr16:20630082-20630126	NM_139293:181	Ece2	INSIDE	0.574	4.454	2037.90	9076.91	2.555	1813.63	4634.20
A_68_P25582340	chr7:147302368-147302412	NM_053119:-68	Echs1	DIVERGENT_PROMOTER	0.522	0.223	4549.95	1014.31	0.116	3149.68	366.59
A_68_P22009690	chr3:27052485-27052529	NM_001177625:270	Ect2	INSIDE	0.395	3.586	5317.18	19067.98	1.418	4064.00	5762.16
A_68_P26543338	chr9:57556763-57556807	NM_153799:409	Edc3	INSIDE	0.449	15.539	3960.11	61536.92	6.974	2926.51	20409.19
A_68_P26543337	chr9:57556573-57556620	NM_153799:221	Edc3	INSIDE	0.661	0.643	2021.04	1299.56	0.425	1478.24	628.77
A_68_P26543336	chr9:57556435-57556479	NM_153799:81	Edc3	INSIDE	0.571	0.685	2606.91	1785.76	0.391	2052.09	803.20
A_68_P26136484	chr8:108404850-108404894	NM_181594:22	Edc4	INSIDE	0.519	0.342	3293.70	1125.21	0.177	2225.20	394.87
A_68_P24735989	chr6:108778919-108778963	NM_138677:306	Edem1	INSIDE	0.621	7.337	533.75	3915.99	4.555	451.21	2055.29
A_68_P20716449	chr1:153602522-153602566	NM_001039644:41	Edem3	INSIDE	0.567	2.954	1812.78	5355.08	1.676	1486.38	2490.47
A_68_P21877294	chr2:174586233-174586277	NM_007903:-19	Edn3	PROMOTER	0.138	25.665	1138.40	29216.91	3.542	951.93	3371.74
A_68_P25310578	chr7:97128916-97128960	NM_021876:548	Eed	INSIDE	0.368	0.307	5086.20	1562.78	0.113	3195.11	361.75
A_68_P28962023	chr13:38751002-38751046	NM_025380:-127	Eef1e1	PROMOTER	0.462	5.075	4001.95	20310.04	2.347	3156.14	7406.67
A_68_P25470625	chr7:127986822-127986866	NM_007908:448	Eef2k	INSIDE	0.36	6.377	1151.41	7342.53	2.298	976.40	2243.90
A_68_P20863709	chr1:180336540-180336584	NM_026626:551	Efcab2	INSIDE	0.486	0.135	4102.98	552.80	0.065	2969.71	194.43
A_68_P20863707	chr1:180336323-180336367	NM_026626:333	Efcab2	INSIDE	0.575	0.694	1167.18	810.30	0.399	972.58	388.06
A_68_P25588978	chr7:148647831-148647875	NM_001025103:860	Efcab4a	INSIDE	0.351	0.658	1350.40	888.46	0.231	1103.03	254.42
A_68_P21756890	chr2:153604913-153604957	NR_036629:-1680	Efcab8	PROMOTER	0.53	0.522	1498.42	782.75	0.277	1195.55	330.97
A_68_P29631109	chr14:58617905-58617949	NM_028643:173	Efha1	INSIDE	0.385	6.051	797.18	4823.98	2.331	765.20	1784.04
A_68_P25804901	chr8:41393403-41393447	NM_030110:20	Efha2	INSIDE	0.602	5.979	546.66	3268.69	3.601	547.98	1973.17
A_68_P20075374	chr1:20941893-20941937	NM_027974:208	Efhc1	INSIDE	0.628	0.376	3072.26	1154.33	0.236	2394.83	565.07
A_68_P20416757	chr1:89160862-89160906	NM_028889:-54	Efhd1	PROMOTER	0.07	70.804	33225.71	2352525.00	4.954	88834.39	440091.60
A_68_P23366442	chr4:141430855-141430899	NM_025994:-41	Efhd2	PROMOTER	0.27	14.192	1252.71	17778.64	3.828	1094.27	4188.37
A_68_P23366440	chr4:141430540-141430584	NM_025994:273	Efhd2	INSIDE	0.582	4.341	1102.38	4784.97	2.524	1020.16	2575.17
A_68_P22318338	chr3:89084974-89085018	NM_001162425:-123	Efna1	PROMOTER	0.335	0.630	938.16	590.58	0.211	814.52	171.57
A_68_P27281569	chr10:79649352-79649396	NM_007909:7148	Efna2	INSIDE	0.348	1.537	1339.60	2059.33	0.535	1087.45	582.05
A_68_P27281570	chr10:79649434-79649479	NM_007909:7230	Efna2	INSIDE	0.063	11.606	5431.24	63037.40	0.737	3814.41	2810.60
A_68_P22318564	chr3:89125165-89125211	NM_010108:1613	Efna3	INSIDE	0.63	3.830	364.94	1397.75	2.412	291.88	703.94
A_68_P22318693	chr3:89141626-89141670	NM_007910:302	Efna4	INSIDE	0.576	3.254	628.01	2043.52	1.874	562.93	1054.93
A_68_P31286852	chr17:63231463-63231507	NM_010109:-818	Efna5	PROMOTER	0.565	5.046	2882.54	14546.12	2.851	2041.94	5820.86
A_68_P31286846	chr17:63230685-63230729	NM_010109:-40	Efna5	PROMOTER	0.45	0.751	6935.04	5209.99	0.338	4758.40	1609.23
A_68_P25637623	chr8:8661349-8661393	NM_010111:-597	Efnb2	PROMOTER	0.32	6.742	979.41	6603.47	2.156	876.20	1889.19
A_68_P25637627	chr8:8661837-8661882	NM_010111:-1086	Efnb2	PROMOTER	0.53	0.342	1371.96	469.86	0.182	947.69	172.13

ProbeName	Target position of probe on CpG island microarray	TargetID	GeneSymbol	CpG island Description	Ratio of relative methylation (TiO_2-NP/Vehicle)	Sham group			TiO_2-H group		
						Relative methylation (Cy5/Cy3)	Cy3 signal (Input)	Cy5 signal (MeDIP)	Relative methylation (Cy5/Cy3)	Cy3 signal (Input)	Cy5 signal (MeDIP)
A_68_P27895787	chr11:69373754-69373798	NM_007911:-37	Efnb3	PROMOTER	0.562	0.172	11727.39	2017.88	0.097	7543.67	728.92
A_68_P28198379	chr12:4038471-4038515	NM_001082483:423	Efr3b	INSIDE	0.255	12.393	3498.45	43355.13	3.156	3243.67	10237.78
A_68_P28198046	chr12:3982083-3982127	NM_001082483:56811	Efr3b	INSIDE	0.494	4.403	3336.94	14692.98	2.176	2659.29	5787.26
A_68_P29615096	chr14:55545666-55545710	NM_010112:-63	Efs	DIVERGENT_PROMOTER	0.422	49.495	2662.56	131783.70	20.882	2165.63	45222.08
A_68_P29615089	chr14:55545056-55545100	NM_010112:547	Efs	INSIDE	0.62	3.963	4140.98	16412.57	2.456	3196.81	7850.12
A_68_P29615085	chr14:55544633-55544677	NM_010112:971	Efs	INSIDE	0.503	0.504	854.19	430.19	0.253	663.11	168.03
A_68_P22019968	chr3:28981553-28981597	NM_001167748:76	Egfem1	INSIDE	0.547	4.852	732.43	3554.01	2.657	643.51	1709.52
A_68_P22019967	chr3:28981485-28981529	NM_001167748:8	Egfem1	INSIDE	0.402	5.238	353.72	1852.80	2.106	397.52	836.99
A_68_P26248668	chr8:127473510-127473554	NM_053207:-378	Egln1	PROMOTER	0.664	4.371	661.86	2893.01	2.9	537.13	1557.90
A_68_P31615126	chr18:35020689-35020733	NM_007913:-150	Egr1	PROMOTER	0.378	0.568	990.51	562.40	0.214	816.92	175.14
A_68_P27212423	chr10:66998199-66998243	NM_010118:-2396	Egr2	PROMOTER	0.413	4.107	3019.86	12403.14	1.696	2438.29	4135.25
A_68_P29699021	chr14:70477881-70477925	NM_018781:651	Egr3	INSIDE	0.217	0.543	3160.00	1717.26	0.118	2181.42	257.22
A_68_P29699040	chr14:70480133-70480177	NM_018781:2903	Egr3	DOWNSTREAM	0.218	28.718	3036.85	87212.91	6.262	2696.36	16884.80
A_68_P29699033	chr14:70479382-70479426	NM_018781:2153	Egr3	INSIDE	0.441	0.228	2303.41	524.62	0.101	1703.99	171.32
A_68_P29699027	chr14:70478576-70478620	NM_018781:1347	Egr3	INSIDE	0.611	4.016	2707.17	10872.53	2.452	2145.40	5260.73
A_68_P29698999	chr14:70474927-70474971	NM_018781:-2303	Egr3	PROMOTER	0.285	6.138	794.51	4876.83	1.749	727.45	1271.98
A_68_P27640630	chr11:22186736-22186780	NM_153078:-917	Ehbp1	PROMOTER	0.625	3.768	1578.63	5947.50	2.354	1396.45	3287.43
A_68_P31933638	chr19:6277193-6277237	NM_010119:319	Ehd1	INSIDE	0.296	0.331	4989.95	1653.94	0.098	3522.46	345.49
A_68_P31933641	chr19:6277445-6277489	NM_010119:571	Ehd1	INSIDE	0.351	6.389	2532.70	16181.42	2.245	2206.54	4953.99
A_68_P31933640	chr19:6277337-6277381	NM_010119:463	Ehd1	INSIDE	0.627	0.523	2016.75	1054.88	0.328	1523.82	499.82
A_68_P21573668	chr2:119980528-119980572	NM_133838:-239	Ehd4	PROMOTER	0.518	0.212	2233.09	472.40	0.11	1571.57	172.19
A_68_P21573666	chr2:119980254-119980299	NM_133838:35	Ehd4	INSIDE	0.627	0.395	10069.02	3976.76	0.248	6504.30	1610.84
A_68_P31156414	chr17:35042317-35042361	NM_145830:6420	Ehmt2	INSIDE	0.421	7.146	1391.15	9941.16	3.008	935.62	2814.57
A_68_P28067423	chr11:100181873-100181917	NM_011508:585	Eif1	INSIDE	0.587	4.952	682.43	3379.33	2.907	595.24	1730.14
A_68_P28067422	chr11:100181743-100181787	NM_011508:455	Eif1	INSIDE	0.639	0.311	1870.18	581.86	0.199	1481.16	294.53
A_68_P31677781	chr18:46757255-46757299	NM_010120:-81	Eif1a	DIVERGENT_PROMOTER	0.549	0.397	4289.70	1703.57	0.218	3382.56	737.72
A_68_P26878190	chr9:120401831-120401875	NM_026892:129	Eif1b	INSIDE	0.483	0.428	2341.74	1001.92	0.207	1658.98	342.89
A_68_P26878194	chr9:120402272-120402316	NM_026892:571	Eif1b	INSIDE	0.579	0.580	944.12	547.40	0.336	807.30	271.01
A_68_P23232037	chr4:116692073-116692117	NM_175135:82	Eif2b3	INSIDE	0.611	3.455	1324.62	4577.04	2.11	1178.25	2486.07
A_68_P23280649	chr4:126210358-126210402	NM_153177:322	Eif2c4	INSIDE	0.309	7.068	1603.91	11336.43	2.186	1607.89	3514.33
A_68_P21762932	chr2:154718551-154718596	NM_026030:69	Eif2s2	INSIDE	0.286	10.338	2683.09	27738.43	2.956	2161.98	6390.25
A_68_P21762929	chr2:154718235-154718279	NM_026030:386	Eif2s2	INSIDE	0.325	0.677	1090.01	738.04	0.22	843.90	185.47
A_68_P25501141	chr7:133709739-133709783	NM_146200:120	Eif3c	INSIDE	0.63	0.491	3445.34	1689.94	0.309	2674.03	826.07
A_68_P25405197	chr7:116094538-116094582	NM_025344:16632	Eif3f	DOWNSTREAM	0.269	6.889	1731.30	11926.63	1.855	1574.20	2919.92
A_68_P26344425	chr9:20702979-20703023	NM_016876:34	Eif3g	INSIDE	0.308	0.271	2893.80	783.64	0.084	2097.59	175.17
A_68_P30213289	chr15:51697145-51697189	NM_080635:-159	Eif3h	PROMOTER	0.374	7.055	640.97	4521.97	2.64	580.33	1531.88
A_68_P27896437	chr11:69484692-69484736	NM_001159375:1211	Eif4a1	INSIDE	0.514	0.617	1103.79	680.76	0.317	851.53	270.20
A_68_P22569291	chr3:138190828-138190872	NM_007917:1696	Eif4e	INSIDE	0.374	0.369	1359.77	501.66	0.138	1242.93	171.67
A_68_P22569284	chr3:138189919-138189963	NM_007917:786	Eif4e	INSIDE	0.461	7.730	1272.85	9839.55	3.563	1045.88	3726.64
A_68_P24687558	chr6:99616754-99616798	NM_025829:-11	Eif4e3	PROMOTER	0.253	1.740	1243.58	2164.40	0.44	1051.58	462.30
A_68_P27180609	chr10:60915255-60915299	NM_010124:141	Eif4ebp2	INSIDE	0.647	2.238	1651.43	3696.51	1.448	1424.98	2063.05
A_68_P27550687	chr11:3102424-3102468	NM_023743:-139	Eif4enif1	PROMOTER	0.48	3.154	1370.58	4322.26	1.513	1163.20	1760.38
A_68_P30588855	chr16:20673605-20673650	NM_001005331:806	Eif4g1	INSIDE	0.41	0.266	2025.71	539.60	0.109	1532.74	167.45
A_68_P25416987	chr7:118226039-118226083	NM_001040131:484	Eif4g2	INSIDE	0.459	4.981	1129.00	5623.55	2.284	999.89	2284.17
A_68_P23343151	chr4:137549520-137549564	NM_172703:158	Eif4g3	INSIDE	0.536	0.650	2604.57	1693.99	0.349	1930.62	673.56
A_68_P23343150	chr4:137549405-137549449	NM_172703:42	Eif4g3	INSIDE	0.534	0.576	946.81	545.25	0.308	693.19	213.20

ProbeName	Target position of probe on CpG island microarray	TargetID	GeneSymbol	CpG island Description	Ratio of relative methylation (TiO_2-NP/Vehicle)	Sham group			TiO_2-H group		
						Relative methylation (Cy5/Cy3)	Cy3 signal (Input)	Cy5 signal (MeDIP)	Relative methylation (Cy5/Cy3)	Cy3 signal (Input)	Cy5 signal (MeDIP)
A_68_P32982443	chr5:135115150-135115194	NM_033561:26	Eif4h	INSIDE	0.591	0.556	1596.44	888.28	0.329	1252.95	411.72
A_68_P32982444	chr5:135115258-135115302	NM_033561:-82	Eif4h	PROMOTER	0.652	3.251	2202.82	7162.43	2.119	1880.78	3985.80
A_68_P28744646	chr12:112776044-112776088	NM_173363:-245	Eif5	PROMOTER	0.472	0.292	2963.42	865.80	0.138	2159.00	297.81
A_68_P27897963	chr11:69734825-69734869	NM_001166595:-69	Eif5a	PROMOTER	0.047	4.336	6910.82	29962.82	0.205	4611.64	946.52
A_68_P27897958	chr11:69734258-69734302	NM_001166590:-148	Eif5a	PROMOTER	0.172	14.029	6402.64	89824.87	2.417	5746.65	13890.47
A_68_P27897961	chr11:69734599-69734643	NM_001166596:-33	Eif5a	PROMOTER	0.359	0.344	2065.80	711.09	0.123	1447.09	178.64
A_68_P27897964	chr11:69734972-69735016	NM_001166589:-106	Eif5a	PROMOTER	0.501	0.614	3202.60	1967.17	0.308	2345.28	721.62
A_68_P22019078	chr3:28680105-28680149	NM_177586:-106	Eif5a2	PROMOTER	0.352	6.185	3411.61	21100.71	2.177	2906.89	6327.09
A_68_P22019079	chr3:28680302-28680346	NM_177586:92	Eif5a2	INSIDE	0.391	15.799	1661.00	26241.40	6.178	1514.36	9355.38
A_68_P20157022	chr1:38055055-38055099	NM_198303:222	Eif5b	INSIDE	0.662	0.404	1567.51	632.84	0.267	1211.38	323.85
A_68_P21767848	chr2:155651937-155651981	NM_010579:703	Eif6	INSIDE	0.588	0.313	2692.86	842.90	0.184	1625.16	299.01
A_68_P31827572	chr18:73914245-73914289	NM_053255:-133	Elac1	PROMOTER	0.42	0.580	1720.24	997.68	0.243	1415.22	344.38
A_68_P23103759	chr4:91041388-91041432	NM_010486:-2664	Elavl2	PROMOTER	0.495	0.586	2540.60	1489.80	0.29	1928.59	559.55
A_68_P23103735	chr4:91038685-91038729	NM_010486:40	Elavl2	INSIDE	0.575	7.470	2803.23	20940.94	4.293	2468.40	10595.95
A_68_P26350213	chr9:21855003-21855047	NM_010487:1443	Elavl3	INSIDE	0.41	7.923	2629.52	20834.43	3.249	2114.97	6871.46
A_68_P26350214	chr9:21855080-21855124	NM_010487:1365	Elavl3	INSIDE	0.585	2.981	1316.48	3925.00	1.745	1124.80	1962.35
A_68_P26350216	chr9:21855292-21855336	NM_010487:1153	Elavl3	INSIDE	0.505	4.291	753.28	3232.32	2.166	517.23	1120.19
A_68_P23205807	chr4:110024246-110024290	NM_001163397:248	Elavl4	INSIDE	0.356	0.520	1206.22	627.17	0.185	1001.49	185.13
A_68_P23205808	chr4:110024387-110024431	NM_001163397:108	Elavl4	INSIDE	0.63	0.458	1440.13	659.36	0.288	1136.72	327.63
A_68_P22128837	chr3:51081149-51081193	NM_023502:48739	Elf2	INSIDE	0.242	0.357	2832.38	1012.22	0.087	2020.41	174.93
A_68_P22128839	chr3:51081429-51081473	NM_023502:48459	Elf2	INSIDE	0.27	19.593	1389.14	27217.97	5.291	1433.08	7582.12
A_68_P22128832	chr3:51080406-51080450	NM_023502:49481	Elf2	INSIDE	0.39	4.559	3023.51	13783.43	1.778	2390.05	4250.32
A_68_P22128834	chr3:51080739-51080783	NM_023502:49149	Elf2	INSIDE	0.398	11.927	3179.26	37917.70	4.741	2733.37	12959.40
A_68_P24138655	chr5:140448297-140448346	NM_175522:64425	Elfn1	INSIDE	0.521	10.016	1341.34	13434.61	5.216	977.49	5098.59
A_68_P24138666	chr5:140449442-140449486	NM_175522:65568	Elfn1	INSIDE	0.599	3.494	2262.08	7903.52	2.093	1640.20	3432.18
A_68_P24138654	chr5:140448211-140448259	NM_175522:64338	Elfn1	INSIDE	0.325	5.593	1260.89	7052.73	1.815	933.20	1694.06
A_68_P24138255	chr5:140384458-140384502	NM_175522:584	Elfn1	INSIDE	0.436	5.201	378.20	1966.97	2.269	374.69	850.00
A_68_P24138292	chr5:140388982-140389026	NM_175522:5108	Elfn1	INSIDE	0.552	1.333	5128.84	6837.46	0.735	3662.78	2693.37
A_68_P30361565	chr15:78503546-78503590	NM_183141:44975	Elfn2	INSIDE	0.159	16.703	784.62	13105.85	2.655	523.15	1389.09
A_68_P30361566	chr15:78503626-78503670	NM_183141:44895	Elfn2	INSIDE	0.162	28.618	1775.59	50813.45	4.627	1443.11	6676.69
A_68_P30361918	chr15:78549192-78549236	NM_183141:-671	Elfn2	PROMOTER	0.655	3.000	2785.14	8355.97	1.966	2073.22	4075.04
A_68_P27352992	chr10:92773316-92773360	NM_013508:566	Elk3	INSIDE	0.427	3.990	3898.63	15556.10	1.702	3263.63	5555.05
A_68_P25952517	chr8:73062831-73062889	NM_007924:-713	Ell	PROMOTER	0.454	0.241	1964.80	472.53	0.109	1572.63	171.80
A_68_P29150855	chr13:75845818-75845862	NM_138953:909	Ell2	INSIDE	0.395	0.155	3951.55	614.05	0.061	2811.48	172.60
A_68_P29150847	chr13:75844848-75844893	NM_138953:-61	Ell2	PROMOTER	0.282	0.682	1166.97	795.48	0.192	883.32	169.72
A_68_P28865541	chr13:20183122-20183166	NM_080288:769	Elmo1	INSIDE	0.433	0.207	2258.52	467.88	0.09	1910.49	171.29
A_68_P28865538	chr13:20182700-20182744	NM_080288:347	Elmo1	INSIDE	0.482	6.300	1929.69	12156.71	3.037	1914.47	5814.15
A_68_P28865540	chr13:20183015-20183059	NM_080288:661	Elmo1	INSIDE	0.559	0.332	1556.77	516.59	0.185	1297.00	240.59
A_68_P28865539	chr13:20182875-20182919	NM_080288:521	Elmo1	INSIDE	0.55	0.615	5407.23	3326.13	0.338	4207.06	1423.58
A_68_P26014555	chr8:85856129-85856173	NM_001170691:235	Elmod2	INSIDE	0.341	13.263	3003.85	39839.69	4.522	2637.03	11924.19
A_68_P26685702	chr9:83699787-83699831	NM_001145974:41	Elovl4	INSIDE	0.588	19.406	8364.67	162324.50	11.418	7310.91	83473.79
A_68_P26657027	chr9:77765735-77765781	NM_134255:587	Elovl5	INSIDE	0.45	4.528	1699.60	7696.50	2.038	1355.55	2763.24
A_68_P26656976	chr9:77758360-77758404	NM_134255:-6789	Elovl5	PROMOTER	0.429	3.795	923.05	3503.07	1.627	711.41	1157.53
A_68_P22520451	chr3:129236724-129236768	NM_130450:1443	Elovl6	INSIDE	0.54	5.192	513.19	2664.64	2.806	485.70	1362.78
A_68_P21497226	chr2:105744559-105744603	NM_023876:77	Elp4	INSIDE	0.404	9.492	3531.32	33520.20	3.83	3064.70	11739.00
A_68_P29361885	chr13:118009364-118009408	NM_010330:7	Emb	INSIDE	0.5	5.189	1126.27	5843.97	2.596	938.97	2437.30

ProbeName	Target position of probe on CpG island microarray	TargetID	GeneSymbol	CpG island Description	Ratio of relative methylation (TiO_2-NP/Vehicle)	Sham group			TiO_2-H group		
						Relative methylation (Cy5/Cy3)	Cy3 signal (Input)	Cy5 signal (MeDIP)	Relative methylation (Cy5/Cy3)	Cy3 signal (Input)	Cy5 signal (MeDIP)
A_68_P29361890	chr13:118009924-118009968	NM_010330:567	Emb	INSIDE	0.503	2.924	1651.22	4828.68	1.47	1363.71	2004.64
A_68_P24123515	chr5:137358798-137358842	NM_024474:157	Emid2	INSIDE	0.433	5.247	2053.22	10773.15	2.271	1648.20	3742.74
A_68_P24123512	chr5:137358486-137358530	NM_024474:469	Emid2	INSIDE	0.446	0.469	1151.88	540.22	0.209	843.40	176.34
A_68_P24123511	chr5:137358417-137358461	NM_024474:539	Emid2	INSIDE	0.466	0.456	6255.40	2854.37	0.212	4107.58	872.52
A_68_P24123513	chr5:137358561-137358605	NM_024474:395	Emid2	INSIDE	0.476	0.498	2429.93	1209.22	0.237	1841.67	436.09
A_68_P24123508	chr5:137357952-137357996	NM_024474:1003	Emid2	INSIDE	0.595	4.171	1521.09	6344.50	2.481	1267.70	3145.80
A_68_P31333820	chr17:71660018-71660062	NM_145158:265	Emilin2	INSIDE	0.317	10.032	1101.64	11051.42	3.182	1135.43	3612.94
A_68_P31398414	chr17:83750191-83750235	NM_001114361:-58	Eml4	PROMOTER	0.623	3.139	735.81	2309.96	1.957	615.22	1204.15
A_68_P28672821	chr12:100139294-100139338	NM_001081191:378	Eml5	INSIDE	0.631	2.403	1453.07	3491.18	1.515	1262.87	1913.14
A_68_P27677360	chr11:29924702-29924746	NM_146016:1309	Eml6	INSIDE	0.611	2.473	6704.14	16579.08	1.512	4802.32	7259.60
A_68_P30541580	chr16:10313819-10313863	NM_007929:221	Emp2	INSIDE	0.21	0.465	7071.89	3289.10	0.098	4370.11	427.19
A_68_P24603559	chr6:85137815-85137859	NM_010131:-88	Emx1	PROMOTER	0.524	13.012	2471.87	32164.52	6.817	1983.08	13518.39
A_68_P24603556	chr6:85137482-85137526	NM_010131:-420	Emx1	PROMOTER	0.257	6.750	1720.97	11615.93	1.734	1516.02	2629.00
A_68_P32219569	chr19:59533670-59533714	NM_010132:513	Emx2	INSIDE	0.639	4.411	751.09	3312.96	2.82	593.46	1673.49
A_68_P32219573	chr19:59534086-59534130	NM_010132:929	Emx2	INSIDE	0.357	0.624	1603.08	1000.96	0.223	1223.71	272.44
A_68_P32219575	chr19:59534436-59534480	NM_010132:1279	Emx2	INSIDE	0.578	0.617	1162.37	717.29	0.357	943.22	336.53
A_68_P32219657	chr19:59543612-59543656	NM_010132:10455	Emx2	DOWNSTREAM	0.305	6.617	657.74	4352.48	2.02	591.45	1194.75
A_68_P20884849	chr1:183950484-183950528	NM_001083120:-395	Enah	PROMOTER	0.335	4.212	3460.14	14575.45	1.412	3051.03	4309.19
A_68_P29250236	chr13:98010968-98011012	NM_007930:-69	Enc1	PROMOTER	0.387	17.089	1550.45	26494.92	6.619	1118.27	7402.22
A_68_P29250235	chr13:98010886-98010930	NM_007930:-151	Enc1	PROMOTER	0.527	4.667	1649.16	7697.38	2.462	1465.44	3607.38
A_68_P29250239	chr13:98011276-98011320	NM_007930:239	Enc1	INSIDE	0.299	0.702	1251.11	878.43	0.21	954.41	200.36
A_68_P29250243	chr13:98011697-98011741	NM_007930:659	Enc1	INSIDE	0.566	0.586	1323.43	775.89	0.332	941.48	312.46
A_68_P26317084	chr9:14185441-14185485	NM_028013:224	Endod1	INSIDE	0.593	2.891	956.09	2764.34	1.714	872.74	1495.66
A_68_P28171535	chr11:118338434-118338478	NM_172573:183	Engase	INSIDE	0.505	0.714	3113.19	2222.52	0.36	2533.24	912.59
A_68_P22863377	chr4:41586983-41587027	NM_027147:331	Enho	INSIDE	0.562	0.427	2488.66	1063.82	0.24	1929.43	463.77
A_68_P23404992	chr4:149611119-149611164	NM_023119:-164	Eno1	PROMOTER	0.339	0.369	1929.69	711.55	0.125	1478.54	184.71
A_68_P23404990	chr4:149610826-149610870	NM_023119:-457	Eno1	PROMOTER	0.648	0.415	1665.56	690.86	0.269	1403.49	377.46
A_68_P24816933	chr6:124719608-124719652	NM_013509:-103	Eno2	PROMOTER	0.05	1.581	6519.70	10309.01	0.079	4434.36	352.50
A_68_P32573188	chrX:100689079-100689123	NR_015508:186	Enox	INSIDE	0.221	18.153	2531.94	45962.15	4.012	2671.89	10718.50
A_68_P32573186	chrX:100688845-100688889	NR_015508:-48	Enox	PROMOTER	0.255	22.720	1393.18	31653.55	5.799	1582.72	9177.79
A_68_P27010234	chr10:24431792-24431836	NM_008813:94	Enpp1	INSIDE	0.371	13.199	2581.35	34070.65	4.9	2096.70	10274.84
A_68_P27010233	chr10:24431686-24431730	NM_008813:200	Enpp1	INSIDE	0.591	0.687	1001.55	688.00	0.406	832.98	338.36
A_68_P28175186	chr11:118861003-118861047	NM_001030291:11523	Enpp7	DOWNSTREAM	0.275	1.391	1867.91	2597.56	0.382	1369.19	523.52
A_68_P21071854	chr2:25251918-25251962	NM_009849:547	Entpd2	INSIDE	0.256	12.090	2575.41	31135.52	3.092	2150.57	6649.71
A_68_P29695818	chr14:69955482-69955526	NM_026174:297	Entpd4	INSIDE	0.381	4.259	1479.14	6299.78	1.624	1130.94	1836.46
A_68_P28598113	chr12:85749685-85749729	NM_001026214:273	Entpd5	INSIDE	0.341	11.123	2704.93	30087.60	3.79	2475.18	9381.37
A_68_P28598112	chr12:85749605-85749649	NM_001026214:353	Entpd5	INSIDE	0.448	9.518	2268.13	21587.34	4.262	1858.42	7920.01
A_68_P32128058	chr19:43764232-43764276	NM_053103:76	Entpd7	INSIDE	0.524	0.496	1447.72	718.17	0.26	1176.65	306.15
A_68_P30378498	chr15:81415923-81415967	NM_177821:-699	Ep300	PROMOTER	0.406	0.219	2512.77	550.30	0.089	1908.37	169.55
A_68_P30378505	chr15:81416613-81416657	NM_177821:-9	Ep300	PROMOTER	0.58	3.751	800.48	3002.47	2.174	719.50	1563.92
A_68_P30378500	chr15:81416070-81416114	NM_177821:-551	Ep300	PROMOTER	0.5	0.561	1474.79	827.92	0.281	1083.99	304.46
A_68_P30378507	chr15:81416859-81416903	NM_177821:237	Ep300	INSIDE	0.509	2.956	3158.82	9338.85	1.506	2500.65	3766.12
A_68_P23309924	chr4:131604876-131604920	NM_001128606:95	Epb4.1	INSIDE	0.284	11.018	1979.51	21810.15	3.133	2156.98	6758.17
A_68_P23309921	chr4:131604501-131604545	NM_001128606:471	Epb4.1	INSIDE	0.623	7.245	2333.47	16906.60	4.517	2015.94	9105.56
A_68_P31610600	chr18:34165600-34165644	NM_013512:1238	Epb4.1l4a	INSIDE	0.255	17.685	5320.33	94091.75	4.51	6017.10	27137.76
A_68_P31610604	chr18:34166101-34166145	NM_013512:738	Epb4.1l4a	INSIDE	0.589	0.359	3036.85	1089.35	0.211	2194.39	463.45

ProbeName	Target position of probe on CpG island microarray	TargetID	GeneSymbol	CpG island Description	Ratio of relative methylation (TiO_2-NP/Vehicle)	Sham group			TiO_2-H group		
						Relative methylation (Cy5/Cy3)	Cy3 signal (Input)	Cy5 signal (MeDIP)	Relative methylation (Cy5/Cy3)	Cy3 signal (Input)	Cy5 signal (MeDIP)
A_68_P20558189	chr1:121545562-121545606	NM_145506:-7	Epb4.1l5	PROMOTER	0.608	0.513	1099.49	563.82	0.312	1000.69	312.03
A_68_P31473580	chr18:6490337-6490381	NM_027497:496	Epc1	INSIDE	0.568	2.586	1730.38	4474.29	1.469	1441.81	2118.12
A_68_P21202304	chr2:49307386-49307430	NM_172663:403	Epc2	INSIDE	0.436	0.313	2584.01	809.75	0.136	1819.89	248.40
A_68_P21202301	chr2:49307033-49307077	NM_172663:49	Epc2	INSIDE	0.444	4.974	1836.07	9132.72	2.21	1649.07	3644.79
A_68_P21202298	chr2:49306701-49306745	NM_172663:-283	Epc2	PROMOTER	0.458	4.303	806.59	3470.77	1.971	725.25	1429.59
A_68_P28863236	chr13:19711521-19711565	NM_134065:157	Epdr1	INSIDE	0.548	0.599	987.20	591.78	0.329	788.55	259.14
A_68_P23270846	chr4:124559573-124559617	NM_177671:566	Epha10	INSIDE	0.178	1.915	12164.25	23297.14	0.342	8002.88	2735.82
A_68_P23270845	chr4:124559481-124559525	NM_177671:474	Epha10	INSIDE	0.468	0.521	9488.65	4942.85	0.244	6764.70	1649.03
A_68_P23270847	chr4:124559714-124559758	NM_177671:708	Epha10	INSIDE	0.526	0.522	2414.62	1259.56	0.274	1818.71	498.69
A_68_P23270910	chr4:124571819-124571863	NM_177671:12812	Epha10	DOWNSTREAM	0.462	1.517	7443.31	11294.07	0.701	5094.91	3570.32
A_68_P23363269	chr4:140864959-140865003	NM_010139:7826	Epha2	INSIDE	0.211	10.036	3252.79	32644.13	2.114	2360.02	4989.13
A_68_P23363270	chr4:140865058-140865102	NM_010139:7926	Epha2	INSIDE	0.426	6.445	1169.23	7535.23	2.744	663.55	1820.48
A_68_P23363268	chr4:140864869-140864913	NM_010139:7736	Epha2	INSIDE	0.476	4.808	1408.11	6770.53	2.289	1137.61	2603.55
A_68_P30817172	chr16:63857062-63857106	NM_010140:6899	Epha3	INSIDE	0.231	8.919	2440.75	21769.50	2.06	1983.97	4086.01
A_68_P20366327	chr1:77511720-77511764	NM_007936:-79	Epha4	PROMOTER	0.465	0.635	5367.05	3407.24	0.295	3817.69	1127.11
A_68_P23854797	chr5:84846399-84846443	NM_007937:-13	Epha5	PROMOTER	0.434	4.007	1176.56	4714.05	1.738	1210.33	2103.73
A_68_P26779727	chr9:102256967-102257011	NM_001168296:35	Ephb1	INSIDE	0.059	2.231	4566.96	10189.52	0.133	3276.99	434.71
A_68_P23336241	chr4:136391071-136391115	NM_010142:758	Ephb2	INSIDE	0.205	12.078	3553.86	42924.61	2.477	2836.70	7026.98
A_68_P23335794	chr4:136326887-136326931	NM_010142:64942	Ephb2	INSIDE	0.344	6.665	3283.90	21887.63	2.294	2436.67	5589.13
A_68_P23335297	chr4:136250002-136250046	NM_010142:141826	Ephb2	INSIDE	0.354	4.198	2213.18	9291.28	1.485	1669.64	2478.92
A_68_P30590967	chr16:21205377-21205421	NM_010143:531	Ephb3	INSIDE	0.358	34.900	1027.96	35875.76	12.51	965.36	12076.20
A_68_P30590962	chr16:21204783-21204827	NM_010143:-63	Ephb3	PROMOTER	0.386	5.390	817.09	4404.46	2.082	718.80	1496.36
A_68_P24125311	chr5:137791525-137791569	NM_001159571:210	Ephb4	INSIDE	0.595	0.409	4971.76	2034.98	0.244	3491.94	850.67
A_68_P24125309	chr5:137791367-137791411	NM_001159571:52	Ephb4	INSIDE	0.206	1.898	1266.87	2404.99	0.392	1041.07	407.73
A_68_P27849286	chr11:61393305-61393349	NM_010148:-173	Epn2	PROMOTER	0.27	12.658	3215.04	40697.20	3.417	2890.16	9875.89
A_68_P27849285	chr11:61393132-61393176	NM_010148:-1	Epn2	PROMOTER	0.652	3.918	2672.87	10471.73	2.555	2060.15	5263.19
A_68_P27849284	chr11:61392956-61393000	NM_010148:175	Epn2	INSIDE	0.402	0.653	811.95	529.83	0.262	667.58	174.93
A_68_P23200236	chr4:108953010-108953054	NM_007943:153	Eps15	INSIDE	0.48	0.464	2234.17	1036.89	0.223	1849.75	411.73
A_68_P23200238	chr4:108953316-108953360	NM_007943:459	Eps15	INSIDE	0.552	3.128	735.72	2301.60	1.727	759.08	1311.10
A_68_P23200234	chr4:108952731-108952775	NM_007943:-127	Eps15	PROMOTER	0.462	2.988	2393.83	7153.75	1.379	2075.68	2863.28
A_68_P23571444	chr5:30559486-30559531	NM_027652:351	Ept1	INSIDE	0.314	5.256	1327.07	6975.27	1.65	1190.33	1964.40
A_68_P28056291	chr11:98274041-98274085	NM_001003817:265	Erbb2	INSIDE	0.241	12.656	820.36	10382.44	3.054	629.14	1921.48
A_68_P28056288	chr11:98273791-98273835	NM_001003817:15	Erbb2	INSIDE	0.302	0.514	4851.63	2493.44	0.155	3713.58	576.32
A_68_P29287630	chr13:104711739-104711783	NM_001005868:-1166	Erbb2ip	PROMOTER	0.438	13.548	2962.07	40129.85	5.928	2591.35	15362.63
A_68_P29287623	chr13:104710322-104710366	NM_001005868:250	Erbb2ip	INSIDE	0.631	0.431	1483.04	638.91	0.272	1316.64	358.09
A_68_P20317726	chr1:69154255-69154299	NM_010154:357	Erbb4	INSIDE	0.6	0.514	5204.13	2676.20	0.309	4079.23	1259.03
A_68_P24795429	chr6:119797862-119797906	NM_053204:284	Erc1	INSIDE	0.323	5.903	1312.07	7745.55	1.908	1114.75	2126.52
A_68_P24795427	chr6:119797721-119797765	NM_053204:426	Erc1	INSIDE	0.479	3.019	2956.66	8925.84	1.447	2463.29	3563.37
A_68_P29488026	chr14:28435956-28436000	NM_177814:351	Erc2	INSIDE	0.111	1.853	1225.17	2270.13	0.205	856.04	175.52
A_68_P29488024	chr14:28435732-28435776	NM_177814:127	Erc2	INSIDE	0.365	11.324	1557.29	17634.33	4.133	1235.41	5106.51
A_68_P29488027	chr14:28436033-28436077	NM_177814:427	Erc2	INSIDE	0.362	1.734	3404.93	5904.79	0.627	2270.83	1424.01
A_68_P24992765	chr7:19927675-19927719	NM_001127324:-2723	Ercc1	PROMOTER	0.5	15.299	1261.08	19293.71	7.656	963.79	7378.38
A_68_P30555522	chr16:13110157-13110201	NM_015769:350	Ercc4	INSIDE	0.581	5.396	2542.99	13721.66	3.137	2061.91	6467.44
A_68_P30555521	chr16:13110069-13110113	NM_015769:262	Ercc4	INSIDE	0.456	0.691	1057.95	730.63	0.315	896.16	282.30
A_68_P20192816	chr1:44204826-44204870	NM_011729:260	Ercc5	INSIDE	0.505	12.080	1909.39	23066.39	6.096	1463.77	8923.56
A_68_P20192817	chr1:44204965-44205009	NM_011729:398	Ercc5	INSIDE	0.391	4.386	2045.98	8972.74	1.714	1934.97	3316.70

ProbeName	Target position of probe on CpG island microarray	TargetID	GeneSymbol	CpG island Description	Ratio of relative methylation (TiO_2-NP/Vehicle)	Sham group			TiO_2-H group		
						Relative methylation (Cy5/Cy3)	Cy3 signal (Input)	Cy5 signal (MeDIP)	Relative methylation (Cy5/Cy3)	Cy3 signal (Input)	Cy5 signal (MeDIP)
A_68_P32567492	chrX:99352458-99352502	NM_146235:-50	Ercc6l	PROMOTER	0.362	16.180	567.04	9174.78	5.854	614.16	3595.25
A_68_P21768928	chr2:155834064-155834108	NM_025516:226	Ergic3	INSIDE	0.527	0.396	2346.42	929.46	0.209	1688.81	352.79
A_68_P28576808	chr12:81744517-81744561	NM_007951:310	Erh	INSIDE	0.315	11.954	1260.18	15063.69	3.764	1721.71	6480.22
A_68_P25780173	chr8:36558550-36558594	NM_026067:15	Eri1	INSIDE	0.514	4.763	754.71	3594.44	2.447	717.97	1756.73
A_68_P25780168	chr8:36557978-36558022	NM_026067:587	Eri1	INSIDE	0.647	0.429	1898.39	814.57	0.278	1444.71	401.08
A_68_P23235083	chr4:117223023-117223067	NM_080469:-54	Eri3	PROMOTER	0.388	10.474	761.48	7975.61	4.065	640.08	2601.64
A_68_P23235082	chr4:117222946-117222990	NM_080469:-130	Eri3	PROMOTER	0.489	3.272	1244.62	4072.15	1.601	1053.42	1686.27
A_68_P27681336	chr11:30854073-30854117	NM_025745:37	Erlec1	INSIDE	0.431	15.294	1201.40	18374.23	6.588	1212.06	7984.66
A_68_P28832915	chr13:12658494-12658538	NM_026184:367	Ero1lb	INSIDE	0.512	2.906	2804.12	8147.78	1.488	2181.41	3246.35
A_68_P22899537	chr4:48292195-48292239	NM_029572:245	Erp44	INSIDE	0.328	20.199	1918.62	38753.41	6.627	1700.90	11272.16
A_68_P22899538	chr4:48292337-48292381	NM_029572:103	Erp44	INSIDE	0.437	4.752	5227.92	24843.35	2.074	3973.13	8241.76
A_68_P23408691	chr4:150229048-150229092	NM_133753:-129	Errfi1	PROMOTER	0.258	13.398	1914.87	25654.75	3.461	1888.53	6536.88
A_68_P31490817	chr18:10609887-10609931	NM_001081222:442	Esco1	INSIDE	0.574	2.875	1192.44	3428.37	1.65	968.91	1598.64
A_68_P29723690	chr14:75131937-75131981	NM_016903:-193	Esd	PROMOTER	0.208	14.700	4498.44	66126.47	3.058	3785.84	11575.92
A_68_P23416733	chr4:151506350-151506394	NM_019585:-3338	Espn	PROMOTER	0.355	7.677	1821.50	13984.02	2.727	1384.91	3776.43
A_68_P23416723	chr4:151505009-151505053	NM_019585:-1996	Espn	PROMOTER	0.424	0.557	1965.02	1094.64	0.236	1593.26	375.96
A_68_P23416878	chr4:151527866-151527910	NM_207687:-1572	Espn	DIVERGENT_PROMOTER	0.498	0.232	2318.31	537.01	0.115	1545.10	178.17
A_68_P23416730	chr4:151506015-151506059	NM_019585:-3002	Espn	PROMOTER	0.643	0.545	3268.94	1782.97	0.351	2509.66	880.03
A_68_P23416732	chr4:151506202-151506248	NM_019585:-3190	Espn	PROMOTER	0.434	0.497	1206.92	600.03	0.216	797.00	172.07
A_68_P23416728	chr4:151505797-151505841	NM_019585:-2784	Espn	PROMOTER	0.411	3.916	1781.12	6975.66	1.61	1524.64	2454.57
A_68_P20441857	chr1:93241429-93241473	NM_001033292:22799	Espnl	INSIDE	0.389	1.662	1817.71	3021.66	0.647	1322.49	855.26
A_68_P22729559	chr4:11315357-11315401	NM_194055:-1448	Esrp1	PROMOTER	0.511	3.636	1054.86	3835.03	1.857	930.36	1727.49
A_68_P26137945	chr8:108660566-108660610	NM_176838:286	Esrp2	INSIDE	0.65	0.324	2548.68	824.70	0.21	1810.20	381.01
A_68_P26137942	chr8:108660258-108660302	NM_176838:594	Esrp2	INSIDE	0.495	1.404	2065.36	2900.47	0.695	1833.10	1273.28
A_68_P31936680	chr19:6996479-6996523	NM_007953:-202	Esrra	PROMOTER	0.472	0.374	1883.14	705.08	0.177	1455.58	257.50
A_68_P31936679	chr19:6996326-6996370	NM_007953:-50	Esrra	PROMOTER	0.356	5.529	1094.00	6048.69	1.971	880.80	1735.82
A_68_P28610003	chr12:87763757-87763801	NM_011934:1185	Esrrb	INSIDE	0.416	0.533	2274.77	1211.82	0.222	1852.24	410.35
A_68_P32684276	chrX:133653076-133653120	NM_007957:1767	Esx1	INSIDE	0.347	17.983	1100.34	19787.72	6.234	1174.61	7321.97
A_68_P32684282	chrX:133653782-133653826	NM_007957:1061	Esx1	INSIDE	0.537	6.061	3063.70	18568.97	3.252	3479.29	11316.30
A_68_P27541865	chr10:127962373-127962417	NM_011843:521	Esyt1	INSIDE	0.431	0.548	2754.52	1510.81	0.236	1989.60	469.96
A_68_P28767111	chr12:117519591-117519635	NM_028731:-82	Esyt2	PROMOTER	0.4	5.554	1374.77	7636.04	2.22	1195.26	2653.87
A_68_P22264731	chr3:79432542-79432586	NM_025794:125	Etfdh	INSIDE	0.435	0.628	1450.12	911.34	0.274	1156.32	316.42
A_68_P25005645	chr7:25370764-25370808	NM_023154:-1775	Ethe1	PROMOTER	0.079	39.582	7587.64	300332.50	3.107	6440.93	20013.85
A_68_P25005644	chr7:25370658-25370702	NM_023154:-1881	Ethe1	PROMOTER	0.134	15.520	6529.11	101333.40	2.078	4937.82	10260.18
A_68_P24908780	chr6:143116314-143116358	NM_029250:587	Etnk1	INSIDE	0.226	13.949	8225.02	114733.00	3.146	6017.84	18931.90
A_68_P30988901	chr16:95923763-95923807	NM_011809:-229	Ets2	PROMOTER	0.589	0.387	1816.56	702.70	0.228	1492.15	340.03
A_68_P28075715	chr11:101645928-101645972	NM_008815:674	Etv4	INSIDE	0.377	4.974	1094.81	5445.73	1.874	1081.42	2026.10
A_68_P24859179	chr6:133986298-133986342	NM_007961:596	Etv6	INSIDE	0.21	17.584	2416.10	42484.00	3.696	2498.86	9236.19
A_68_P24859175	chr6:133985925-133985969	NM_007961:222	Etv6	INSIDE	0.219	15.081	3526.56	53184.74	3.303	3088.19	10200.62
A_68_P23969232	chr5:108297963-108298007	NM_007964:6142	Evi5	INSIDE	0.365	7.762	3189.43	24757.36	2.837	2817.18	7992.27
A_68_P28728718	chr12:109792810-109792854	NM_001163394:-97	Evl	PROMOTER	0.259	11.010	8267.83	91030.21	2.857	7380.15	21085.03
A_68_P28158070	chr11:116083313-116083358	NM_025276:16070	Evpl	INSIDE	0.228	20.723	1782.84	36945.26	4.722	2000.67	9447.97
A_68_P28158149	chr11:116093971-116094015	NM_025276:5413	Evpl	INSIDE	0.218	1.612	1818.40	2930.65	0.351	1366.37	479.53
A_68_P28158150	chr11:116094047-116094091	NM_025276:5337	Evpl	INSIDE	0.267	1.690	1758.24	2971.87	0.452	1098.68	496.31
A_68_P24449862	chr6:52264017-52264061	NM_007966:547	Evx1	INSIDE	0.331	8.966	2241.50	20097.16	2.964	1807.29	5356.27
A_68_P24449831	chr6:52260423-52260467	NM_007966:-3047	Evx1	PROMOTER	0.468	6.727	3488.81	23470.92	3.148	3200.57	10076.31

ProbeName	Target position of probe on CpG island microarray	TargetID	GeneSymbol	CpG island Description	Ratio of relative methylation (TiO_2-NP/Vehicle)	Sham group			TiO_2-H group		
						Relative methylation (Cy5/Cy3)	Cy3 signal (Input)	Cy5 signal (MeDIP)	Relative methylation (Cy5/Cy3)	Cy3 signal (Input)	Cy5 signal (MeDIP)
A_68_P21338573	chr2:74497229-74497276	NM_007967:224	Evx2	INSIDE	0.464	0.453	1299.52	588.98	0.21	899.21	189.10
A_68_P21338574	chr2:74497327-74497371	NM_007967:128	Evx2	INSIDE	0.61	0.403	3941.56	1588.83	0.246	2804.61	689.41
A_68_P27561404	chr11:4998804-4998848	NM_007968:254	Ewsr1	INSIDE	0.355	5.405	1328.62	7181.41	1.919	1108.56	2127.78
A_68_P27561405	chr11:4998913-4998957	NM_007968:146	Ewsr1	INSIDE	0.457	3.024	3596.47	10875.99	1.381	2790.68	3853.92
A_68_P21570230	chr2:119372732-119372776	NM_172857:609	Exd1	INSIDE	0.59	0.405	2373.45	961.84	0.239	1774.28	423.93
A_68_P28575794	chr12:81564131-81564176	NM_133798:72	Exd2	INSIDE	0.321	0.471	1319.78	622.20	0.151	1135.68	171.87
A_68_P28575798	chr12:81564728-81564777	NM_133798:671	Exd2	INSIDE	0.574	14.237	1169.38	16648.60	8.169	1072.06	8757.40
A_68_P28920307	chr13:31065636-31065680	NM_025588:258	Exoc2	INSIDE	0.648	3.109	3193.62	9929.31	2.016	2610.24	5262.42
A_68_P28920310	chr13:31065956-31066000	NM_025588:-62	Exoc2	PROMOTER	0.606	2.761	742.02	2048.96	1.674	665.08	1113.60
A_68_P32096195	chr19:37625247-37625291	NM_175353:361	Exoc6	INSIDE	0.62	4.482	768.98	3446.73	2.779	836.16	2323.83
A_68_P32096197	chr19:37625488-37625532	NM_175353:603	Exoc6	INSIDE	0.508	0.675	823.05	555.60	0.343	676.46	232.19
A_68_P24602879	chr6:85019299-85019343	NM_177077:187	Exoc6b	INSIDE	0.645	0.276	2806.97	775.79	0.178	2468.89	440.04
A_68_P26248326	chr8:127421066-127421110	NM_198103:517	Exoc8	INSIDE	0.483	5.642	1642.28	9265.19	2.725	1236.75	3369.64
A_68_P26248330	chr8:127421598-127421642	NM_198103:-15	Exoc8	DIVERGENT_PROMOTER	0.54	9.312	1767.78	16460.99	5.028	1354.38	6810.04
A_68_P26248322	chr8:127420595-127420639	NM_198103:989	Exoc8	INSIDE	0.571	0.663	1022.83	677.79	0.379	788.59	298.57
A_68_P26248320	chr8:127420438-127420486	NM_198103:1143	Exoc8	INSIDE	0.454	0.634	771.13	488.65	0.288	624.80	179.66
A_68_P26248321	chr8:127420511-127420555	NM_198103:1073	Exoc8	INSIDE	0.645	0.589	961.84	566.73	0.38	646.50	245.74
A_68_P26166328	chr8:113580417-113580461	NM_028274:200	Exosc6	INSIDE	0.522	14.354	535.31	7684.14	7.498	443.88	3328.15
A_68_P26166332	chr8:113580916-113580960	NM_028274:700	Exosc6	INSIDE	0.375	0.661	1867.68	1233.62	0.248	1515.81	375.88
A_68_P26893205	chr9:123022399-123022443	NM_001081188:72	Exosc7	INSIDE	0.382	0.362	1632.29	591.07	0.138	1258.68	174.19
A_68_P30221123	chr15:53177744-53177788	NM_010162:-28	Ext1	PROMOTER	0.52	5.931	1011.38	5998.95	3.084	818.78	2524.93
A_68_P30221122	chr15:53177633-53177677	NM_010162:84	Ext1	INSIDE	0.659	7.075	2034.17	14390.97	4.665	1885.25	8794.00
A_68_P29671484	chr14:65695263-65695307	NM_018788:21659	Extl3	INSIDE	0.199	10.797	2118.30	22870.95	2.145	1521.00	3262.68
A_68_P29671618	chr14:65716583-65716627	NM_018788:339	Extl3	INSIDE	0.603	0.622	1136.78	707.47	0.375	1040.53	390.25
A_68_P20045407	chr1:14300000-14300044	NM_010164:258	Eya1	INSIDE	0.383	0.584	1873.15	1093.49	0.223	1445.08	322.70
A_68_P28072728	chr11:101087284-101087328	NM_007970:458	Ezh1	INSIDE	0.377	0.195	4464.89	872.57	0.074	2904.42	213.77
A_68_P28072730	chr11:101087587-101087631	NM_007970:156	Ezh1	INSIDE	0.581	0.635	2717.95	1724.72	0.369	2156.99	795.67
A_68_P20827965	chr1:173367690-173367734	NM_172647:21	F11r	INSIDE	0.505	0.394	1646.68	648.47	0.199	1325.54	263.38
A_68_P29241611	chr13:96388009-96388053	NM_010169:358	F2r	INSIDE	0.446	0.403	1512.63	609.78	0.18	1179.95	212.15
A_68_P29241618	chr13:96388753-96388804	NM_010169:-390	F2r	PROMOTER	0.562	0.605	1665.46	1007.42	0.34	1211.77	411.78
A_68_P22478234	chr3:121426543-121426587	NM_010171:110	F3	INSIDE	0.589	0.527	1882.21	992.19	0.31	1569.77	487.40
A_68_P22478235	chr3:121426699-121426743	NM_010171:266	F3	INSIDE	0.618	4.292	962.14	4129.90	2.655	857.04	2275.28
A_68_P29630110	chr14:58444745-58444790	NM_198605:233	F630043A04Rik	INSIDE	0.356	0.419	1521.46	637.88	0.149	1174.29	175.21
A_68_P27289092	chr10:80820623-80820667	NM_028657:330	F630110N24Rik	INSIDE	0.584	0.700	1989.85	1392.01	0.408	1507.20	615.46
A_68_P28340238	chr12:33832153-33832197	NR_015602:277	F730043M19Rik	INSIDE	0.65	0.604	1690.78	1020.69	0.392	1206.41	473.15
A_68_P27865444	chr11:64249527-64249571	NR_028445:628	F930015N05Rik	INSIDE	0.58	4.074	1161.35	4730.79	2.362	1035.29	2444.96
A_68_P26168094	chr8:113917651-113917695	NM_178086:49	Fa2h	INSIDE	0.59	0.622	1446.84	899.72	0.367	1231.28	452.01
A_68_P23227298	chr4:115690557-115690601	NM_010173:-71	Faah	PROMOTER	0.64	3.537	687.61	2432.34	2.264	611.15	1383.39
A_68_P23301443	chr4:129985869-129985913	NM_010174:-131	Fabp3	PROMOTER	0.371	0.431	2388.78	1029.20	0.16	1856.20	297.07
A_68_P23301437	chr4:129985225-129985269	NM_010174:-775	Fabp3	PROMOTER	0.429	0.173	3211.87	554.63	0.074	2397.13	177.50
A_68_P23301445	chr4:129986078-129986122	NM_010174:79	Fabp3	INSIDE	0.613	0.213	2396.24	509.94	0.13	1544.72	201.42
A_68_P21940379	chr3:10012439-10012483	NM_010634:-145	Fabp5	PROMOTER	0.17	12.762	3940.87	50292.20	2.164	3406.52	7370.36
A_68_P21940380	chr3:10012548-10012592	NM_010634:-35	Fabp5	PROMOTER	0.613	0.167	3118.08	522.11	0.103	2016.44	207.13
A_68_P31948840	chr19:10257815-10257859	NM_146094:459	Fads1	INSIDE	0.641	0.223	3251.53	725.75	0.143	2314.52	330.98
A_68_P31948062	chr19:10115784-10115828	NM_021890:-231	Fads3	PROMOTER	0.136	53.512	675.28	36135.81	7.254	538.97	3909.75
A_68_P28152627	chr11:115158906-115158950	NM_178035:-68	Fads6	DIVERGENT_PROMOTER	0.362	0.345	1814.15	625.62	0.125	1389.79	173.65

ProbeName	Target position of probe on CpG island microarray	TargetID	GeneSymbol	CpG island Description	Ratio of relative methylation (TiO_2-NP/Vehicle)	Sham group			TiO_2-H group		
						Relative methylation (Cy5/Cy3)	Cy3 signal (Input)	Cy5 signal (MeDIP)	Relative methylation (Cy5/Cy3)	Cy3 signal (Input)	Cy5 signal (MeDIP)
A_68_P23202060	chr4:109349261-109349305	NM_007983:51	Faf1	INSIDE	0.324	7.287	1033.60	7532.28	2.363	916.52	2165.59
A_68_P29050433	chr13:54723497-54723542	NM_178397:375	Faf2	INSIDE	0.426	0.293	1840.87	539.38	0.125	1359.40	169.71
A_68_P29050429	chr13:54723129-54723173	NM_178397:6	Faf2	INSIDE	0.54	4.661	776.85	3620.96	2.518	730.93	1840.77
A_68_P25289619	chr7:91754548-91754592	NM_010176:-118	Fah	PROMOTER	0.22	1.926	10481.68	20189.63	0.424	7413.86	3139.90
A_68_P24064187	chr5:125491813-125491857	NM_028443:7990	Fam101a	INSIDE	0.391	12.231	2576.68	31514.55	4.778	2006.76	9587.94
A_68_P27929565	chr11:75840897-75840941	NM_029658:366	Fam101b	INSIDE	0.651	0.600	903.27	542.41	0.391	739.44	289.27
A_68_P21115867	chr2:32391280-32391324	NM_153560:424	Fam102a	INSIDE	0.25	11.855	1161.69	13772.42	2.963	957.39	2836.57
A_68_P21115862	chr2:32390731-32390776	NM_153560:-125	Fam102a	PROMOTER	0.34	0.479	1392.49	666.87	0.163	1056.01	171.76
A_68_P21115865	chr2:32391002-32391046	NM_153560:146	Fam102a	INSIDE	0.654	3.058	1322.22	4043.78	2	1218.78	2437.91
A_68_P25273848	chr7:88908491-88908535	NM_025997:674	Fam103a1	INSIDE	0.453	9.960	745.57	7426.03	4.51	643.87	2903.97
A_68_P25273840	chr7:88907783-88907827	NM_025997:-34	Fam103a1	PROMOTER	0.542	0.432	1152.24	497.87	0.234	774.79	181.59
A_68_P30093918	chr15:27611330-27611374	NM_198301:-99	Fam105a	PROMOTER	0.59	3.943	616.17	2429.72	2.328	622.18	1448.34
A_68_P30093919	chr15:27611395-27611439	NM_198301:-163	Fam105a	PROMOTER	0.634	13.679	2164.67	29610.62	8.669	1666.78	14449.73
A_68_P30093655	chr15:27560477-27560521	NM_001013792:-50	Fam105b	PROMOTER	0.217	12.921	4138.03	53468.42	2.802	3611.60	10121.13
A_68_P20963945	chr2:3630790-3630834	NM_025626:83	Fam107b	INSIDE	0.38	5.514	743.35	4098.46	2.095	703.73	1474.48
A_68_P32007552	chr19:21727793-21727837	NM_146096:16	Fam108b	INSIDE	0.371	12.493	500.87	6257.19	4.629	385.83	1786.09
A_68_P32007551	chr19:21727608-21727652	NM_146096:-168	Fam108b	DIVERGENT_PROMOTER	0.543	2.990	1810.11	5411.62	1.624	1492.96	2424.59
A_68_P25286992	chr7:91299923-91299967	NM_133722:459	Fam108c	INSIDE	0.48	0.578	833.87	481.69	0.277	628.97	174.41
A_68_P24046892	chr5:122303021-122303065	NM_175474:4006	Fam109a	INSIDE	0.629	3.215	1662.21	5344.82	2.022	1128.77	2282.66
A_68_P24046891	chr5:122302921-122302965	NM_175474:3906	Fam109a	INSIDE	0.656	3.181	1148.22	3651.93	2.087	863.35	1802.11
A_68_P24046831	chr5:122293021-122293065	NM_175474:-5994	Fam109a	DIVERGENT_PROMOTER	0.64	5.738	264.38	1517.13	3.67	240.81	883.83
A_68_P30382876	chr15:82171483-82171527	NM_177391:-104	Fam109b	DIVERGENT_PROMOTER	0.653	0.570	939.25	535.26	0.372	731.21	271.91
A_68_P21746889	chr2:151796098-151796142	NM_028666:3590	Fam110a	INSIDE	0.397	6.316	3931.16	24827.43	2.51	2729.19	6849.39
A_68_P22702205	chr4:5571555-5571599	NM_173426:251	Fam110b	INSIDE	0.288	20.981	1867.39	39178.74	6.034	1477.46	8915.62
A_68_P22702206	chr4:5571678-5571722	NM_173426:375	Fam110b	INSIDE	0.474	0.544	2365.07	1287.68	0.258	1875.84	484.11
A_68_P28329059	chr12:31758655-31758699	NM_027828:-156	Fam110c	PROMOTER	0.285	9.323	1356.78	12649.50	2.653	1128.26	2993.70
A_68_P23753715	chr5:65361156-65361200	NM_026667:-135	Fam114a1	DIVERGENT_PROMOTER	0.305	12.213	2772.68	33862.73	3.725	2407.61	8967.16
A_68_P27826023	chr11:57331958-57332002	NM_001168667:166	Fam114a2	INSIDE	0.574	0.249	3911.32	972.79	0.143	2759.51	394.29
A_68_P28038133	chr11:95198233-95198277	NM_172543:-77	Fam117a	PROMOTER	0.133	1.817	3393.51	6167.50	0.242	2636.14	636.86
A_68_P28038137	chr11:95198780-95198824	NM_172543:471	Fam117a	INSIDE	0.212	1.782	6413.08	11428.75	0.377	4390.86	1657.53
A_68_P28038138	chr11:95198892-95198936	NM_172543:583	Fam117a	INSIDE	0.493	0.600	7771.69	4665.31	0.296	5185.97	1534.88
A_68_P20266458	chr1:59969786-59969830	NM_001037725:-41	Fam117b	PROMOTER	0.253	27.529	2144.41	59033.32	6.953	2043.61	14208.35
A_68_P30398340	chr15:84867516-84867560	NM_177067:47	Fam118a	INSIDE	0.561	3.108	1914.79	5951.72	1.745	1510.18	2634.55
A_68_P26421597	chr9:35075221-35075265	NM_194257:110	Fam118b	INSIDE	0.509	0.553	1287.25	711.76	0.282	961.60	270.85
A_68_P26421598	chr9:35075374-35075418	NM_175411:-31	Fam118b	DIVERGENT_PROMOTER	0.353	4.898	1139.72	5582.50	1.729	1021.75	1766.46
A_68_P32745112	chrX:147779006-147779050	NM_198105:263	Fam120c	INSIDE	0.286	6.947	1515.24	10526.29	1.988	1842.26	3662.60
A_68_P32023822	chr19:24552159-24552210	NM_026520:-220	Fam122a	PROMOTER	0.147	2.023	821.81	1662.71	0.298	595.27	177.23
A_68_P32386813	chrX:50622802-50622846	NM_001166365:158	Fam122b	INSIDE	0.372	19.216	3324.66	63888.20	7.147	4022.16	28746.31
A_68_P29644196	chr14:60998046-60998090	NM_001164705:946	Fam123a	INSIDE	0.465	0.400	1155.68	461.78	0.186	933.77	173.43
A_68_P29644193	chr14:60997735-60997779	NM_001164705:634	Fam123a	INSIDE	0.62	5.234	1279.32	6696.27	3.244	1051.92	3412.79
A_68_P32541781	chrX:92639710-92639754	NM_175179:447	Fam123b	INSIDE	0.463	3.418	1157.33	3955.47	1.581	1487.14	2351.07
A_68_P21124370	chr2:33742800-33742844	NM_175184:644	Fam125b	INSIDE	0.099	20.037	3217.55	64468.49	1.984	3456.08	6857.89
A_68_P21124367	chr2:33742451-33742495	NM_175184:994	Fam125b	INSIDE	0.57	0.506	994.72	503.51	0.289	795.25	229.57
A_68_P23539566	chr5:23536151-23536195	NM_053090:329	Fam126a	INSIDE	0.516	7.790	2376.19	18510.80	4.018	1989.03	7992.75
A_68_P23539565	chr5:23536003-23536047	NM_053090:477	Fam126a	INSIDE	0.607	0.496	2627.65	1302.76	0.301	2239.75	674.47
A_68_P20259677	chr1:58643036-58643080	NM_172513:119	Fam126b	INSIDE	0.371	14.373	4736.05	68072.24	5.339	4052.83	21639.03

ProbeName	Target position of probe on CpG island microarray	TargetID	GeneSymbol	CpG island Description	Ratio of relative methylation (TiO_2-NP/Vehicle)	Sham group			TiO_2-H group		
						Relative methylation (Cy5/Cy3)	Cy3 signal (Input)	Cy5 signal (MeDIP)	Relative methylation (Cy5/Cy3)	Cy3 signal (Input)	Cy5 signal (MeDIP)
A_68_P25957748	chr8:74126235-74126279	NM_001166213:4712	Fam129c	INSIDE	0.448	0.255	1969.03	502.88	0.114	1544.60	176.77
A_68_P25957751	chr8:74126551-74126595	NM_001166213:5028	Fam129c	INSIDE	0.509	4.911	2077.60	10202.27	2.498	1557.89	3890.87
A_68_P25957754	chr8:74126853-74126897	NM_001166213:5330	Fam129c	INSIDE	0.354	4.288	10947.26	46944.34	1.516	8094.19	12273.11
A_68_P25957750	chr8:74126449-74126493	NM_001166213:4926	Fam129c	INSIDE	0.659	0.635	1208.97	767.13	0.418	971.80	406.32
A_68_P30589003	chr16:20693683-20693727	NM_133778:-1425	Fam131a	PROMOTER	0.466	0.545	3913.83	2132.10	0.254	2777.55	705.57
A_68_P24397652	chr6:42273788-42273832	NM_001113327:829	Fam131b	INSIDE	0.483	3.786	4334.81	16413.53	1.828	3019.67	5518.59
A_68_P24397660	chr6:42274774-42274818	NM_001113327:-157	Fam131b	PROMOTER	0.596	3.977	3185.98	12671.07	2.372	2627.56	6232.55
A_68_P23363642	chr4:140924465-140924509	NM_001085513:352	Fam131c	INSIDE	0.552	0.372	2413.93	897.70	0.205	1997.32	409.73
A_68_P23363727	chr4:140938341-140938385	NM_001085513:14228	Fam131c	INSIDE	0.608	0.424	1651.05	699.35	0.258	1396.34	359.70
A_68_P20442011	chr1:93263227-93263272	NM_173395:243	Fam132b	INSIDE	0.25	11.867	2853.82	33865.38	2.968	2339.24	6943.54
A_68_P20442008	chr1:93262992-93263036	NM_173395:8	Fam132b	INSIDE	0.642	0.443	3617.60	1601.55	0.284	2724.40	774.66
A_68_P24609728	chr6:86315617-86315661	NM_025591:-38	Fam136a	PROMOTER	0.603	0.505	4146.69	2093.70	0.305	3243.57	988.18
A_68_P24609727	chr6:86315530-86315574	NM_025591:-124	Fam136a	PROMOTER	0.665	0.473	6645.97	3145.06	0.315	5018.17	1578.79
A_68_P25644699	chr8:9770615-9770659	NM_173446:387	Fam155a	INSIDE	0.393	14.544	3210.88	46700.39	5.709	2772.40	15827.16
A_68_P25644704	chr8:9771350-9771394	NM_173446:-349	Fam155a	PROMOTER	0.51	23.890	15314.31	365866.00	12.186	15118.96	184234.70
A_68_P23195768	chr4:108055890-108055934	NM_001099303:42	Fam159a	INSIDE	0.546	6.363	1832.76	11661.22	3.475	1518.30	5276.65
A_68_P23195767	chr4:108055814-108055858	NM_001099303:118	Fam159a	INSIDE	0.614	0.717	1519.60	1089.25	0.44	1152.63	506.88
A_68_P25392116	chr7:112548270-112548314	NM_199009:233	Fam160a2	INSIDE	0.309	7.158	3210.79	22983.31	2.214	2843.74	6296.31
A_68_P25392118	chr7:112548478-112548522	NM_199009:25	Fam160a2	INSIDE	0.575	4.697	1624.09	7628.66	2.703	1375.05	3716.50
A_68_P21082466	chr2:26968285-26968329	NM_175427:29691	Fam163b	INSIDE	0.238	6.910	5277.62	36466.67	1.642	3582.35	5882.96
A_68_P21929042	chr3:7503409-7503453	NM_173181:5	Fam164a	INSIDE	0.063	6.880	10628.77	73129.27	0.431	7633.56	3292.49
A_68_P29661298	chr14:64055156-64055200	NM_177628:-52	Fam167a	PROMOTER	0.377	0.322	1634.56	527.04	0.121	1412.44	171.52
A_68_P25369174	chr7:107855061-107855105	NM_178764:-133	Fam168a	PROMOTER	0.061	8.379	4418.98	37025.17	0.509	3518.56	1791.08
A_68_P25369178	chr7:107855525-107855569	NM_178764:331	Fam168a	INSIDE	0.184	8.790	9958.72	87540.65	1.616	7595.81	12278.41
A_68_P20140952	chr1:34899643-34899687	NM_001160235:231	Fam168b	INSIDE	0.373	8.145	1218.00	9921.16	3.039	1194.06	3628.58
A_68_P29249234	chr13:97841159-97841203	NM_001100458:-417	Fam169a	PROMOTER	0.451	0.384	1952.57	750.58	0.174	1527.91	265.13
A_68_P28079628	chr11:102308536-102308580	NM_199200:419	Fam171a2	INSIDE	0.542	0.247	4370.86	1081.57	0.134	2985.07	400.62
A_68_P28079629	chr11:102308671-102308715	NM_199200:285	Fam171a2	INSIDE	0.553	3.750	1804.78	6768.47	2.075	1578.42	3274.43
A_68_P28079567	chr11:102300117-102300165	NM_199200:8837	Fam171a2	INSIDE	0.564	0.501	1165.21	583.70	0.282	893.59	252.26
A_68_P29160014	chr13:77847868-77847912	NM_001163419:-60	Fam172a	PROMOTER	0.524	0.172	3061.04	527.12	0.09	2252.69	203.20
A_68_P31106282	chr17:25929216-25929260	NM_145410:-9	Fam173a	PROMOTER	0.253	16.391	4190.51	68686.74	4.143	3416.11	14153.56
A_68_P25230685	chr7:80885691-80885735	NM_001162532:520	Fam174b	INSIDE	0.649	0.686	1204.03	825.76	0.445	963.39	428.76
A_68_P23931642	chr5:101249853-101249897	NM_172405:80	Fam175a	INSIDE	0.615	0.715	1417.59	1013.71	0.44	1061.25	467.05
A_68_P25537310	chr7:140050852-140050896	NM_198017:-33	Fam175b	DIVERGENT_PROMOTER	0.505	5.833	1063.62	6203.99	2.945	885.03	2606.06
A_68_P24586428	chr6:81991724-81991768	NM_145570:125	Fam176a	INSIDE	0.653	0.443	1613.20	714.78	0.289	1361.48	393.64
A_68_P23278482	chr4:125826960-125827005	NM_172145:1736	Fam176b	INSIDE	0.621	22.693	1358.59	30830.98	14.099	1663.80	23457.48
A_68_P32135373	chr19:45005375-45005419	NM_001081225:-212	Fam178a	PROMOTER	0.543	4.524	3330.33	15065.27	2.455	2792.92	6856.45
A_68_P32135377	chr19:45005799-45005843	NM_001081225:212	Fam178a	INSIDE	0.572	0.435	2835.54	1232.54	0.248	2261.84	562.06
A_68_P32135379	chr19:45006013-45006057	NM_001081225:426	Fam178a	INSIDE	0.569	0.608	1873.04	1139.46	0.346	1506.04	521.50
A_68_P32135378	chr19:45005914-45005958	NM_001081225:328	Fam178a	INSIDE	0.663	0.576	1457.25	838.65	0.382	1105.90	422.21
A_68_P25328173	chr7:100228372-100228416	NM_021427:6	Fam181b	INSIDE	0.196	11.151	1094.17	12200.94	2.183	942.20	2057.21
A_68_P25328176	chr7:100228697-100228741	NM_021427:330	Fam181b	INSIDE	0.433	5.090	2554.05	12998.83	2.201	2207.70	4859.78
A_68_P25328180	chr7:100229175-100229219	NM_021427:808	Fam181b	INSIDE	0.421	3.729	3081.17	11488.85	1.569	2465.07	3868.07
A_68_P23657737	chr5:46030747-46030791	NM_021416:-28	Fam184b	PROMOTER	0.625	3.001	1599.40	4799.13	1.875	1384.95	2596.92
A_68_P21010977	chr2:12340687-12340731	NM_024185:379	Fam188a	INSIDE	0.571	0.636	5553.86	3530.67	0.363	4077.86	1480.15
A_68_P25181446	chr7:72301608-72301652	NM_183087:-216	Fam189a1	PROMOTER	0.561	0.280	1872.20	523.98	0.157	1482.98	233.02

ProbeName	Target position of probe on CpG island microarray	TargetID	GeneSymbol	CpG island Description	Ratio of relative methylation (TiO_2-NP/Vehicle)	Sham group			TiO_2-H group		
						Relative methylation (Cy5/Cy3)	Cy3 signal (Input)	Cy5 signal (MeDIP)	Relative methylation (Cy5/Cy3)	Cy3 signal (Input)	Cy5 signal (MeDIP)
A_68_P22317741	chr3:88990976-88991020	NM_001014995:3852	Fam189b	INSIDE	0.596	0.436	1085.81	473.64	0.26	661.71	172.02
A_68_P27856224	chr11:62692825-62692869	NM_026210:-145	Fam18b	PROMOTER	0.35	25.405	28151.52	715189.10	8.885	30155.74	267944.50
A_68_P27856225	chr11:62692980-62693024	NM_026210:11	Fam18b	INSIDE	0.504	0.285	3339.69	950.67	0.144	2494.90	358.18
A_68_P27856223	chr11:62692744-62692788	NM_026210:-225	Fam18b	PROMOTER	0.425	1.365	2786.51	3804.57	0.58	2155.59	1249.48
A_68_P27856226	chr11:62693049-62693093	NM_026210:79	Fam18b	INSIDE	0.573	2.689	2213.25	5950.44	1.541	1760.06	2712.63
A_68_P26078518	chr8:97125424-97125468	NM_028221:180	Fam192a	INSIDE	0.602	4.124	909.70	3751.62	2.482	785.98	1950.67
A_68_P29055901	chr13:55672343-55672387	NM_001162881:-1028	Fam193b	PROMOTER	0.427	7.458	2344.06	17481.71	3.183	2037.96	6486.86
A_68_P28185237	chr11:120411070-120411114	NM_001033231:-51	Fam195b	PROMOTER	0.454	1.329	5275.53	7011.11	0.604	3292.58	1988.11
A_68_P25549531	chr7:142129256-142129301	NM_001143802:835	Fam196a	INSIDE	0.25	13.841	1371.29	18979.54	3.462	1024.49	3547.17
A_68_P32683852	chrX:133584183-133584227	NM_146261:72	Fam199x	INSIDE	0.266	21.281	25444.91	541499.60	5.664	28224.05	159849.10
A_68_P30414445	chr15:87454455-87454499	NM_134096:-1183	Fam19a5	PROMOTER	0.509	0.600	1080.33	647.93	0.305	781.02	238.49
A_68_P30347857	chr15:76199220-76199264	NM_021555:-85	Fam203a	PROMOTER	0.361	0.318	2154.47	684.48	0.115	1724.20	197.66
A_68_P28120304	chr11:109582790-109582834	NM_153782:758	Fam20a	INSIDE	0.469	0.348	1313.73	456.84	0.163	1048.04	170.88
A_68_P24131504	chr5:139231132-139231176	NM_030565:120	Fam20c	INSIDE	0.472	3.072	2019.17	6202.19	1.449	1575.46	2283.21
A_68_P25960243	chr8:74743668-74743712	NM_026455:62	Fam32a	INSIDE	0.394	0.612	1753.49	1073.79	0.241	1543.75	372.53
A_68_P26234161	chr8:125074980-125075024	NM_001037298:227	Fam38a	INSIDE	0.533	5.610	705.29	3956.91	2.988	629.16	1880.10
A_68_P26234162	chr8:125075132-125075176	NM_001037298:75	Fam38a	INSIDE	0.542	4.820	1042.60	5025.81	2.614	886.44	2317.13
A_68_P26234163	chr8:125075221-125075265	NM_001037298:-13	Fam38a	PROMOTER	0.533	2.617	2088.51	5465.30	1.396	1774.13	2475.82
A_68_P31769415	chr18:63546129-63546173	NM_001039485:687	Fam38b	INSIDE	0.377	5.784	2318.40	13409.99	2.18	1857.21	4048.60
A_68_P31769416	chr18:63546211-63546255	NM_001039485:605	Fam38b	INSIDE	0.51	0.554	1248.31	691.41	0.282	988.60	279.15
A_68_P24290981	chr6:22306235-22306279	NM_138587:-175	Fam3c	PROMOTER	0.473	3.360	982.00	3299.06	1.589	848.23	1347.41
A_68_P24329145	chr6:29866863-29866907	NM_001037740:-128	Fam40b	PROMOTER	0.233	20.339	311.84	6342.68	4.73	288.25	1363.28
A_68_P30642683	chr16:30601337-30601381	NM_177632:1550	Fam43a	INSIDE	0.216	1.538	746.16	1147.44	0.332	623.80	207.21
A_68_P32227328	chr19:60887531-60887575	NM_001167829:80	Fam45a	INSIDE	0.627	0.248	2126.86	527.73	0.156	1621.74	252.22
A_68_P32227329	chr19:60887627-60887671	NM_001167829:176	Fam45a	INSIDE	0.45	0.528	879.31	463.88	0.238	721.80	171.45
A_68_P26693191	chr9:85220481-85220525	NM_001160378:229	Fam46a	INSIDE	0.505	0.491	4516.13	2216.78	0.248	3357.76	832.08
A_68_P26693200	chr9:85221504-85221548	NM_001160379:-769	Fam46a	PROMOTER	0.348	4.078	6944.85	28321.09	1.421	6358.16	9036.06
A_68_P23317811	chr4:133036049-133036093	NM_175307:23	Fam46b	INSIDE	0.594	0.337	2131.02	717.39	0.2	1801.93	360.16
A_68_P22149162	chr3:54496870-54496914	NM_019995:-134	Fam48a	PROMOTER	0.226	0.179	6128.20	1099.31	0.04	4294.48	173.78
A_68_P28242246	chr12:12269143-12269187	NM_001146119:220	Fam49a	INSIDE	0.499	1.343	4330.97	5817.73	0.67	3342.73	2239.94
A_68_P30278749	chr15:63829759-63829803	NM_144846:62230	Fam49b	INSIDE	0.365	9.357	2671.05	24993.40	3.415	2414.01	8244.50
A_68_P30278744	chr15:63829220-63829264	NM_144846:62768	Fam49b	INSIDE	0.461	4.890	4166.04	20370.35	2.254	3444.13	7762.09
A_68_P30278743	chr15:63829112-63829156	NM_144846:62876	Fam49b	INSIDE	0.603	0.581	2484.16	1443.02	0.35	1866.87	653.71
A_68_P23590170	chr5:33972447-33972492	NM_178390:-185	Fam53a	PROMOTER	0.14	25.321	7451.91	188691.00	3.547	7135.63	25313.44
A_68_P23590169	chr5:33972359-33972403	NM_178390:-96	Fam53a	PROMOTER	0.33	8.423	5598.11	47150.39	2.784	4725.71	13154.16
A_68_P23590167	chr5:33972133-33972177	NM_178390:130	Fam53a	INSIDE	0.509	0.549	899.80	494.37	0.279	617.63	172.60
A_68_P25537056	chr7:140005623-140005667	NM_212473:-765	Fam53b	PROMOTER	0.524	5.720	1814.14	10377.75	2.995	1497.14	4484.01
A_68_P23323339	chr4:134091201-134091245	NM_029759:-2555	Fam54b	PROMOTER	0.5	5.261	1489.12	7834.89	2.632	1374.86	3618.83
A_68_P23323334	chr4:134090623-134090667	NM_029759:-1977	Fam54b	PROMOTER	0.518	4.628	337.86	1563.74	2.397	336.94	807.49
A_68_P27930554	chr11:76015406-76015450	NM_027773:-129	Fam57a	PROMOTER	0.382	4.960	3039.67	15078.27	1.895	2594.73	4916.46
A_68_P25502374	chr7:133952923-133952967	NM_026884:-7454	Fam57b	DIVERGENT_PROMOTER	0.634	0.402	1144.30	460.11	0.255	853.39	217.53
A_68_P27945140	chr11:78565152-78565196	NM_197989:57	Fam58b	INSIDE	0.594	4.234	1038.07	4395.58	2.514	853.57	2145.97
A_68_P31544271	chr18:21458929-21458973	NM_001033445:-310	Fam59a	PROMOTER	0.218	39.778	11525.00	458439.70	8.682	10709.19	92982.43
A_68_P31544268	chr18:21458551-21458595	NM_001033445:68	Fam59a	INSIDE	0.43	3.842	989.68	3802.72	1.653	937.54	1550.13
A_68_P24942904	chr6:148895207-148895251	NM_019643:-274	Fam60a	PROMOTER	0.388	12.510	2506.49	31356.58	4.857	2021.03	9817.04
A_68_P24942894	chr6:148893997-148894041	NM_019643:936	Fam60a	INSIDE	0.543	7.344	3137.61	23042.87	3.989	2769.87	11048.82

ProbeName	Target position of probe on CpG island microarray	TargetID	GeneSymbol	CpG island Description	Ratio of relative methylation (TiO_2-NP/Vehicle)	Sham group			TiO_2-H group		
						Relative methylation (Cy5/Cy3)	Cy3 signal (Input)	Cy5 signal (MeDIP)	Relative methylation (Cy5/Cy3)	Cy3 signal (Input)	Cy5 signal (MeDIP)
A_68_P24942897	chr6:148894399-148894443	NM_019643:534	Fam60a	INSIDE	0.663	5.557	1884.51	10472.32	3.685	2098.64	7734.40
A_68_P24942887	chr6:148893096-148893140	NM_019643:1836	Fam60a	INSIDE	0.346	1.905	3403.85	6484.20	0.658	2673.38	1759.94
A_68_P26617701	chr9:70504626-70504670	NM_172772:333	Fam63b	INSIDE	0.359	11.632	2112.42	24571.98	4.173	1729.45	7216.24
A_68_P26617706	chr9:70505115-70505159	NM_172772:-155	Fam63b	PROMOTER	0.654	4.778	1834.16	8764.04	3.124	1653.59	5165.14
A_68_P27909076	chr11:71855740-71855784	NM_144526:-241	Fam64a	DIVERGENT_PROMOTER	0.403	9.758	2472.06	24122.00	3.929	2129.64	8367.52
A_68_P23969768	chr5:108415971-108416015	NM_026062:104	Fam69a	INSIDE	0.433	4.199	4138.24	17375.57	1.819	3254.02	5917.85
A_68_P21080069	chr2:26483908-26483952	NM_019833:-46	Fam69b	PROMOTER	0.46	4.007	1924.36	7711.46	1.842	1538.61	2833.59
A_68_P25087741	chr7:51751968-51752015	NM_028169:34	Fam71e1	INSIDE	0.541	0.683	1086.63	742.31	0.37	832.77	307.71
A_68_P21102744	chr2:30220027-30220071	NM_175392:194	Fam73b	INSIDE	0.363	0.465	1380.64	642.62	0.169	1086.56	183.64
A_68_P23314728	chr4:132478506-132478550	NM_001163792:-62	Fam76a	PROMOTER	0.401	0.632	1418.51	897.09	0.254	1193.84	303.06
A_68_P26313788	chr9:13632555-13632599	NM_176836:406	Fam76b	INSIDE	0.476	3.500	1125.46	3939.40	1.666	989.72	1648.90
A_68_P21113215	chr2:31939833-31939877	NM_175511:-629	Fam78a	PROMOTER	0.356	14.821	1281.97	19000.45	5.271	1200.35	6327.57
A_68_P20802634	chr1:168931803-168931847	NM_001160261:277	Fam78b	INSIDE	0.375	0.736	2188.79	1612.00	0.277	1693.90	468.44
A_68_P30373737	chr15:80502053-80502097	NM_145986:-202	Fam83f	PROMOTER	0.386	3.749	1979.10	7419.20	1.448	1944.36	2815.05
A_68_P30345428	chr15:75834117-75834161	NM_001168253:5790	Fam83h	INSIDE	0.463	14.784	2386.94	35288.85	6.838	2134.93	14598.51
A_68_P30261330	chr15:60655945-60655989	NM_001162926:669	Fam84b	INSIDE	0.494	6.289	838.99	5276.14	3.105	695.96	2161.27
A_68_P30514665	chr16:5255833-5255877	NM_027446:195	Fam86	INSIDE	0.6	2.888	1452.84	4195.36	1.734	1226.79	2126.84
A_68_P31930484	chr19:5729356-5729400	NM_023166:288	Fam89b	INSIDE	0.511	0.656	977.68	641.18	0.335	855.78	286.72
A_68_P26129632	chr8:107165445-107165490	NM_026753:161	Fam96b	INSIDE	0.412	0.284	5994.17	1700.66	0.117	4153.80	485.32
A_68_P26129634	chr8:107165624-107165668	NM_026753:-18	Fam96b	PROMOTER	0.388	0.559	1031.31	576.54	0.217	801.23	173.79
A_68_P21556816	chr2:117075560-117075604	NM_026620:108	Fam98b	INSIDE	0.251	8.944	1861.55	16649.58	2.247	1374.48	3087.83
A_68_P25025638	chr7:29940590-29940634	NM_001146023:617	Fam98c	INSIDE	0.468	0.301	3070.87	924.42	0.141	2357.05	332.26
A_68_P31121750	chr17:28450482-28450526	NM_001163819:30	Fance	INSIDE	0.582	0.426	1874.88	798.46	0.248	1349.69	334.71
A_68_P22869480	chr4:43023362-43023406	NM_001163233:-211	Fancg	PROMOTER	0.661	0.253	2407.11	609.76	0.167	1746.34	292.23
A_68_P25260794	chr7:86537246-86537290	NM_145946:45	Fanci	INSIDE	0.541	0.209	2239.41	469.15	0.113	1527.29	173.24
A_68_P27662606	chr11:26287134-26287178	NM_025923:73	Fancl	INSIDE	0.341	1.445	1848.32	2670.27	0.492	1490.34	733.37
A_68_P28493839	chr12:66176820-66176864	NM_178912:250	Fancm	INSIDE	0.658	2.915	4426.51	12903.88	1.918	3598.23	6902.67
A_68_P24937826	chr6:147995940-147995984	NM_178797:25	Far2	INSIDE	0.611	0.491	2931.14	1439.22	0.3	1926.11	577.44
A_68_P29953204	chr14:121435005-121435049	NM_134082:231	Farp1	INSIDE	0.518	4.890	1259.57	6159.59	2.534	1088.02	2756.73
A_68_P28947125	chr13:36209443-36209487	NM_001039189:185	Fars2	INSIDE	0.291	9.172	2895.01	26552.57	2.673	2505.89	6697.17
A_68_P29110678	chr13:68721295-68721339	NM_027123:192	Fastkd3	INSIDE	0.408	5.486	2114.90	11603.06	2.236	1817.95	4065.73
A_68_P25828113	chr8:46129756-46129800	NM_001081286:94217	Fat1	INSIDE	0.472	4.957	2254.47	11175.19	2.338	1549.89	3622.88
A_68_P25828173	chr8:46136798-46136846	NM_001081286:101261	Fat1	INSIDE	0.41	3.857	1883.33	7263.89	1.583	1276.02	2019.71
A_68_P25828057	chr8:46122480-46122524	NM_001081286:86941	Fat1	INSIDE	0.648	2.457	1388.90	3413.09	1.593	1059.16	1686.75
A_68_P22071594	chr3:38784504-38784548	NM_183221:-1335	Fat4	PROMOTER	0.408	0.524	965.34	506.22	0.214	839.27	179.60
A_68_P28157767	chr11:116029485-116029529	NM_172571:-14	Fbf1	PROMOTER	0.032	134.819	287.55	38767.85	4.339	237.78	1031.63
A_68_P30399218	chr15:85036672-85036716	NM_010180:257	Fbln1	INSIDE	0.137	11.438	2321.81	26557.43	1.562	2495.85	3897.39
A_68_P24637495	chr6:91162688-91162734	NM_001081437:-47	Fbln2	PROMOTER	0.583	0.601	792.13	476.38	0.351	692.57	242.83
A_68_P21621960	chr2:128718146-128718190	NM_024237:28501	Fbln7	INSIDE	0.589	2.643	1351.49	3571.34	1.558	1147.92	1788.00
A_68_P21621794	chr2:128690006-128690050	NM_024237:361	Fbln7	INSIDE	0.307	5.675	732.00	4154.46	1.744	647.16	1128.70
A_68_P21603039	chr2:125331555-125331599	NM_007993:598	Fbn1	INSIDE	0.338	0.654	960.39	628.30	0.221	785.27	173.85
A_68_P31741000	chr18:58369346-58369390	NM_010181:212	Fbn2	INSIDE	0.316	10.588	2946.85	31201.84	3.344	2818.31	9424.53
A_68_P23979337	chr5:110807040-110807084	NM_028596:9084	Fbrsl1	INSIDE	0.169	11.403	3623.47	41317.83	1.929	2678.47	5166.05
A_68_P23979846	chr5:110877794-110877838	NM_001142642:-294	Fbrsl1	PROMOTER	0.602	0.525	2997.12	1573.22	0.316	2436.30	770.39
A_68_P23979845	chr5:110877631-110877675	NM_001142642:-130	Fbrsl1	PROMOTER	0.418	0.606	2755.20	1671.00	0.253	2316.87	587.05
A_68_P24793340	chr6:119429030-119429074	NM_133940:-633	Fbxl14	PROMOTER	0.327	0.364	5600.41	2036.19	0.119	3829.51	455.14

ProbeName	Target position of probe on CpG island microarray	TargetID	GeneSymbol	CpG island Description	Ratio of relative methylation (TiO_2-NP/Vehicle)	Sham group			TiO_2-H group		
						Relative methylation (Cy5/Cy3)	Cy3 signal (Input)	Cy5 signal (MeDIP)	Relative methylation (Cy5/Cy3)	Cy3 signal (Input)	Cy5 signal (MeDIP)
A_68_P32143277	chr19:46403804-46403848	NM_133694:1153	Fbxl15	INSIDE	0.642	2.993	1531.12	4582.41	1.921	1269.11	2437.81
A_68_P31106388	chr17:25946298-25946342	NM_001164225:291	Fbxl16	INSIDE	0.312	0.417	1708.39	711.60	0.13	1415.10	184.10
A_68_P31106474	chr17:25956330-25956374	NM_001164225:10323	Fbxl16	INSIDE	0.363	0.464	1434.61	665.43	0.168	1020.47	171.71
A_68_P31106476	chr17:25956604-25956648	NM_001164225:10597	Fbxl16	INSIDE	0.353	0.637	969.93	618.17	0.225	759.15	170.72
A_68_P25507036	chr7:134890617-134890661	NM_172748:350	Fbxl19	INSIDE	0.591	0.572	3218.00	1842.02	0.338	2254.09	762.32
A_68_P25507079	chr7:134895761-134895805	NM_172748:5494	Fbxl19	INSIDE	0.643	2.990	2144.60	6411.55	1.922	1698.71	3265.60
A_68_P25507025	chr7:134889380-134889424	NM_172748:-886	Fbxl19	PROMOTER	0.165	0.733	2722.86	1996.17	0.121	1892.05	228.45
A_68_P28054695	chr11:98011746-98011790	NM_028149:-838	Fbxl20	PROMOTER	0.312	7.010	1175.04	8237.59	2.188	1144.24	2503.81
A_68_P26593507	chr9:66359284-66359328	NM_175206:3094	Fbxl22	INSIDE	0.515	0.652	740.79	482.77	0.335	622.45	208.78
A_68_P29863770	chr14:103498866-103498910	NM_015822:-162	Fbxl3	PROMOTER	0.425	4.122	1046.46	4313.45	1.753	937.25	1642.62
A_68_P22777889	chr4:22284762-22284806	NM_172988:73	Fbxl4	INSIDE	0.533	5.296	1018.61	5394.52	2.824	974.16	2750.59
A_68_P23647569	chr5:44173172-44173216	NM_001159963:194	Fbxl5	INSIDE	0.185	26.774	3222.13	86269.49	4.952	2926.02	14490.04
A_68_P23647570	chr5:44173303-44173347	NM_001159963:64	Fbxl5	INSIDE	0.519	8.375	1991.84	16682.25	4.35	1643.85	7151.11
A_68_P30089765	chr15:26824750-26824795	NM_176959:547	Fbxl7	INSIDE	0.28	1.374	5591.35	7682.76	0.384	3698.34	1420.71
A_68_P26132607	chr8:107792187-107792231	NM_015821:3661	Fbxl8	INSIDE	0.585	0.561	1743.11	978.06	0.328	1309.65	429.71
A_68_P26132587	chr8:107788500-107788544	NM_015821:-25	Fbxl8	DIVERGENT_PROMOTER	0.62	6.688	818.66	5475.04	4.149	720.00	2986.96
A_68_P25023290	chr7:29517813-29517857	NM_015796:16026	Fbxo17	INSIDE	0.418	0.402	1521.33	611.36	0.168	1250.00	209.78
A_68_P25023288	chr7:29517538-29517582	NM_015796:15752	Fbxo17	INSIDE	0.596	0.570	1029.43	587.08	0.34	832.20	283.05
A_68_P21007905	chr2:11699155-11699199	NM_015792:-22	Fbxo18	DIVERGENT_PROMOTER	0.633	0.132	7006.88	922.42	0.083	4577.66	381.74
A_68_P25671122	chr8:13907695-13907739	NM_025785:-89	Fbxo25	PROMOTER	0.404	0.445	1277.51	568.09	0.18	937.32	168.56
A_68_P25023126	chr7:29478641-29478685	NM_001163702:500	Fbxo27	INSIDE	0.486	0.563	1387.12	780.63	0.274	1145.25	313.41
A_68_P25023124	chr7:29478378-29478422	NM_001163702:238	Fbxo27	INSIDE	0.513	2.876	2026.53	5827.78	1.474	1730.28	2550.33
A_68_P21486778	chr2:103867915-103867959	NM_020593:-19	Fbxo3	PROMOTER	0.402	0.473	1103.43	521.42	0.19	925.00	175.79
A_68_P30246699	chr15:58046420-58046464	NM_026346:-9	Fbxo32	PROMOTER	0.4	3.789	1331.63	5046.20	1.517	1085.96	1647.00
A_68_P28470857	chr12:60320041-60320085	NM_001033156:408	Fbxo33	INSIDE	0.291	7.841	1083.99	8499.82	2.284	889.53	2032.13
A_68_P31764311	chr18:62708175-62708219	NM_134136:201	Fbxo38	INSIDE	0.417	0.158	4975.61	787.31	0.066	3171.92	209.47
A_68_P31764310	chr18:62708047-62708091	NM_134136:329	Fbxo38	INSIDE	0.546	0.193	2853.89	551.85	0.106	1924.01	203.07
A_68_P31764312	chr18:62708242-62708286	NM_134136:133	Fbxo38	INSIDE	0.612	5.148	9265.65	47703.57	3.151	7273.27	22920.84
A_68_P27910787	chr11:72128038-72128082	NM_001099688:115	Fbxo39	INSIDE	0.623	5.361	1370.43	7347.23	3.338	1159.75	3870.82
A_68_P23362414	chr4:140703695-140703739	NM_172518:-120	Fbxo42	DIVERGENT_PROMOTER	0.598	3.749	1176.32	4410.39	2.243	970.75	2177.03
A_68_P23362420	chr4:140704425-140704469	NM_172518:610	Fbxo42	INSIDE	0.407	0.529	870.36	460.47	0.215	780.37	168.06
A_68_P30137020	chr15:36094444-36094488	NM_001081253:173	Fbxo43	INSIDE	0.341	16.880	2899.25	48937.93	5.76	2384.82	13736.90
A_68_P23392752	chr4:147534277-147534321	NM_001161852:-125	Fbxo44	DIVERGENT_PROMOTER	0.125	0.378	6289.52	2374.87	0.047	3934.69	186.45
A_68_P24991559	chr7:19703647-19703691	NM_175530:-1539	Fbxo46	PROMOTER	0.385	6.037	1110.06	6701.04	2.325	964.72	2243.29
A_68_P28053229	chr11:97745535-97745579	NM_001081435:-88	Fbxo47	PROMOTER	0.361	19.323	5566.02	107552.90	6.969	5310.18	37007.41
A_68_P28053223	chr11:97744951-97744995	NM_001081435:496	Fbxo47	INSIDE	0.442	33.030	5115.90	168978.20	14.597	6474.28	94505.26
A_68_P23392700	chr4:147525826-147525870	NM_001163704:186	Fbxo6	INSIDE	0.296	13.030	4591.60	59830.66	3.861	3966.95	15318.05
A_68_P27311667	chr10:85484851-85484895	NM_153195:199	Fbxo7	INSIDE	0.352	1.491	989.79	1476.06	0.524	973.95	510.68
A_68_P26658155	chr9:77956059-77956103	NM_023605:383	Fbxo9	INSIDE	0.27	27.969	3822.62	106914.40	7.546	3316.71	25029.24
A_68_P26658154	chr9:77955972-77956016	NM_023605:469	Fbxo9	INSIDE	0.462	0.556	1642.13	913.02	0.257	1249.46	321.24
A_68_P26658159	chr9:77956440-77956484	NM_023605:1	Fbxo9	INSIDE	0.643	5.768	1187.16	6847.55	3.707	997.51	3698.27
A_68_P22293161	chr3:84619085-84619129	NM_001177773:-392	Fbxw7	PROMOTER	0.128	43.147	2671.49	115267.70	5.537	2901.05	16064.09
A_68_P26023482	chr8:87584261-87584305	NM_026791:265	Fbxw9	INSIDE	0.528	0.671	2283.54	1532.81	0.354	1708.04	604.89
A_68_P20826002	chr1:172995322-172995366	NM_010188:-5810	Fcgr3	PROMOTER	0.254	0.483	36209.92	17487.70	0.123	24470.33	3005.28
A_68_P20825999	chr1:172994938-172994982	NM_010188:-5426	Fcgr3	PROMOTER	0.332	0.110	58296.28	6397.06	0.036	37505.03	1368.36
A_68_P20825996	chr1:172994474-172994518	NM_010188:-4962	Fcgr3	PROMOTER	0.44	0.391	12476.45	4878.90	0.172	9152.24	1575.82

ProbeName	Target position of probe on CpG island microarray	TargetID	GeneSymbol	CpG island Description	Ratio of relative methylation (TiO_2-NP/Vehicle)	Sham group			TiO_2-H group		
						Relative methylation (Cy5/Cy3)	Cy3 signal (Input)	Cy5 signal (MeDIP)	Relative methylation (Cy5/Cy3)	Cy3 signal (Input)	Cy5 signal (MeDIP)
A_68_P20825998	chr1:172994760-172994804	NM_010188:-5248	Fcgr3	PROMOTER	0.636	0.206	7929.36	1637.27	0.131	6673.30	876.07
A_68_P20826006	chr1:172995780-172995824	NM_010188:-6268	Fcgr3	PROMOTER	0.661	0.465	14721.57	6849.05	0.308	11937.52	3671.06
A_68_P20825994	chr1:172994223-172994267	NM_010188:-4710	Fcgr3	PROMOTER	0.548	1.327	20433.59	27106.95	0.727	14810.87	10769.90
A_68_P29259683	chr13:99585179-99585223	NM_172591:204	Fcho2	INSIDE	0.487	9.015	1145.03	10322.85	4.389	1056.32	4636.48
A_68_P25371475	chr7:108256912-108256956	NM_001146010:-354	Fchsd2	PROMOTER	0.437	5.134	1834.54	9419.39	2.245	1509.14	3388.41
A_68_P25371480	chr7:108257652-108257696	NM_001146010:386	Fchsd2	INSIDE	0.501	0.090	6497.46	582.30	0.045	3805.06	170.94
A_68_P22317217	chr3:88905983-88906028	NM_134469:-138	Fdps	PROMOTER	0.541	0.251	1784.33	448.65	0.136	1285.31	174.78
A_68_P26512426	chr9:51771455-51771499	NM_007996:162	Fdx1	INSIDE	0.578	5.063	1862.15	9427.74	2.925	1630.65	4769.98
A_68_P26572466	chr9:62659190-62659234	NM_010193:243	Fem1b	INSIDE	0.614	2.819	1555.36	4384.68	1.73	1209.32	2092.41
A_68_P26572467	chr9:62659271-62659315	NM_010193:163	Fem1b	INSIDE	0.334	4.991	1054.19	5261.81	1.668	939.05	1566.66
A_68_P31677413	chr18:46685542-46685586	NM_173423:61	Fem1c	INSIDE	0.446	5.211	1702.60	8872.81	2.324	1472.98	3422.83
A_68_P31677409	chr18:46684937-46684981	NM_173423:667	Fem1c	INSIDE	0.621	0.254	1911.12	485.40	0.158	1233.54	194.61
A_68_P20351049	chr1:74932092-74932136	NM_153111:-132	Fev	PROMOTER	0.521	0.595	3267.02	1943.39	0.31	2377.94	736.61
A_68_P20351021	chr1:74929129-74929173	NM_153111:2832	Fev	INSIDE	0.619	5.870	1780.93	10454.00	3.635	1496.83	5440.77
A_68_P20351017	chr1:74928629-74928673	NM_153111:3332	Fev	INSIDE	0.359	1.369	3706.49	5073.77	0.492	2667.29	1311.92
A_68_P20351050	chr1:74932235-74932279	NM_153111:-274	Fev	PROMOTER	0.341	0.751	4934.27	3705.15	0.256	3228.15	825.92
A_68_P31371532	chr17:78817452-78817496	NM_199448:-31	Fez2	PROMOTER	0.486	9.016	7718.43	69588.63	4.384	6124.71	26847.78
A_68_P31371531	chr17:78817355-78817399	NM_199448:67	Fez2	INSIDE	0.55	0.538	987.46	531.69	0.296	837.68	247.98
A_68_P24295389	chr6:23198493-23198537	NM_028462:-250	Fezf1	PROMOTER	0.412	4.627	2155.94	9974.80	1.905	1753.19	3339.79
A_68_P24295432	chr6:23203617-23203661	NM_028462:-5374	Fezf1	PROMOTER	0.476	10.452	2245.73	23473.24	4.97	2002.41	9952.80
A_68_P29410675	chr14:13178814-13178858	NM_080433:-457	Fezf2	PROMOTER	0.217	14.766	1197.16	17677.36	3.211	1038.89	3335.93
A_68_P32743933	chrX:147481461-147481505	NM_008001:-293	Fgd1	PROMOTER	0.467	6.866	2226.50	15287.65	3.204	2542.07	8145.78
A_68_P29369781	chr13:119503243-119503287	NM_008002:-241	Fgf10	PROMOTER	0.258	14.292	7312.58	104508.50	3.69	6227.51	22977.91
A_68_P30630415	chr16:28446508-28446552	NM_183064:-1217	Fgf12	PROMOTER	0.66	2.535	2411.53	6113.21	1.674	2134.53	3572.38
A_68_P30630419	chr16:28446947-28446991	NM_183064:-1655	Fgf12	PROMOTER	0.444	4.544	531.93	2417.14	2.017	496.50	1001.39
A_68_P32408601	chrX:56388072-56388116	NM_010200:-481	Fgf13	PROMOTER	0.618	3.064	965.40	2958.24	1.895	1082.71	2051.70
A_68_P25609917	chr7:152082446-152082490	NM_008003:32	Fgf15	INSIDE	0.542	4.215	3006.87	12673.36	2.283	2528.97	5772.87
A_68_P27278709	chr10:79217725-79217769	NM_023304:-117	Fgf22	DIVERGENT_PROMOTER	0.197	2.856	5628.12	16071.45	0.562	3723.45	2090.72
A_68_P24830596	chr6:127030399-127030443	NM_022657:7501	Fgf23	INSIDE	0.239	2.276	2785.47	6339.70	0.543	2304.56	1251.49
A_68_P25609544	chr7:152024886-152024930	NM_008007:392	Fgf3	INSIDE	0.315	6.684	1469.15	9819.30	2.107	1237.72	2607.39
A_68_P25609547	chr7:152025243-152025287	NM_008007:748	Fgf3	INSIDE	0.534	0.377	1970.21	742.29	0.201	1402.79	282.08
A_68_P25609683	chr7:152047109-152047153	NM_010202:-160	Fgf4	PROMOTER	0.364	0.234	2931.27	686.16	0.085	2058.54	175.57
A_68_P25609686	chr7:152047482-152047526	NM_010202:214	Fgf4	INSIDE	0.478	11.583	1243.44	14402.95	5.536	1073.19	5941.27
A_68_P25609690	chr7:152047867-152047911	NM_010202:598	Fgf4	INSIDE	0.56	0.396	1532.94	607.38	0.222	1150.17	255.40
A_68_P23917031	chr5:98683201-98683245	NM_010203:20	Fgf5	INSIDE	0.281	0.476	1898.36	902.96	0.133	1257.93	167.89
A_68_P32140182	chr19:45811402-45811446	NM_001166361:5950	Fgf8	INSIDE	0.195	18.935	2505.67	47445.07	3.686	2352.52	8671.54
A_68_P29631462	chr14:58689892-58689936	NM_013518:-1608	Fgf9	PROMOTER	0.159	16.795	1021.85	17161.86	2.663	893.96	2380.35
A_68_P29631499	chr14:58694324-58694368	NM_013518:2824	Fgf9	INSIDE	0.278	7.191	3328.63	23936.56	2	2711.75	5423.60
A_68_P29631513	chr14:58695681-58695725	NM_013518:4180	Fgf9	INSIDE	0.478	0.387	1645.55	636.95	0.185	1351.33	250.08
A_68_P29631470	chr14:58691022-58691066	NM_013518:-478	Fgf9	PROMOTER	0.486	14.271	1657.06	23648.57	6.937	1402.36	9728.70
A_68_P29631503	chr14:58694761-58694806	NM_013518:3261	Fgf9	INSIDE	0.543	3.851	4352.72	16762.62	2.092	2972.15	6216.44
A_68_P29631498	chr14:58694189-58694233	NM_013518:2688	Fgf9	INSIDE	0.556	0.092	5259.79	484.26	0.051	3418.86	174.99
A_68_P29631505	chr14:58694946-58694991	NM_013518:3446	Fgf9	INSIDE	0.525	3.258	1755.12	5718.72	1.71	1436.89	2456.79
A_68_P32092666	chr19:36993233-36993277	NM_028263:835	Fgfbp3	INSIDE	0.248	16.944	1816.27	30774.48	4.208	1718.49	7231.51
A_68_P32092668	chr19:36993413-36993457	NM_028263:655	Fgfbp3	INSIDE	0.409	7.580	1133.08	8588.41	3.103	1013.98	3145.97
A_68_P32092667	chr19:36993325-36993369	NM_028263:743	Fgfbp3	INSIDE	0.617	4.072	762.42	3104.58	2.511	632.75	1588.68

ProbeName	Target position of probe on CpG island microarray	TargetID	GeneSymbol	CpG island Description	Ratio of relative methylation (TiO_2-NP/Vehicle)	Sham group			TiO_2-H group		
						Relative methylation (Cy5/Cy3)	Cy3 signal (Input)	Cy5 signal (MeDIP)	Relative methylation (Cy5/Cy3)	Cy3 signal (Input)	Cy5 signal (MeDIP)
A_68_P25727484	chr8:26629202-26629246	NM_001079908:-19	Fgfr1	PROMOTER	0.302	7.807	1501.83	11724.49	2.358	1291.85	3045.73
A_68_P25520723	chr7:137409825-137409869	NM_010207:476	Fgfr2	INSIDE	0.633	3.088	996.99	3078.95	1.955	844.51	1651.22
A_68_P23590785	chr5:34065022-34065066	NM_001163216:90	Fgfr3	INSIDE	0.571	2.962	1572.73	4657.66	1.691	1320.28	2232.52
A_68_P29053522	chr13:55254151-55254195	NM_008011:-6	Fgfr4	PROMOTER	0.211	21.830	1839.54	40156.61	4.596	1586.41	7290.99
A_68_P29053525	chr13:55254569-55254613	NM_008011:412	Fgfr4	INSIDE	0.635	0.537	3946.66	2120.52	0.341	2875.49	980.71
A_68_P23973608	chr5:109123109-109123155	NM_001164259:-115	Fgfrl1	PROMOTER	0.45	0.348	6038.26	2101.15	0.157	4201.76	658.13
A_68_P23973612	chr5:109123736-109123780	NM_001164259:511	Fgfrl1	INSIDE	0.208	0.641	1764.96	1130.69	0.133	1300.31	173.33
A_68_P23973609	chr5:109123219-109123263	NM_001164259:-7	Fgfrl1	PROMOTER	0.425	0.705	2032.62	1432.90	0.299	1675.83	501.58
A_68_P20849149	chr1:177555403-177555447	NM_010209:342	Fh1	INSIDE	0.28	9.617	1870.38	17987.20	2.697	1757.70	4741.27
A_68_P20849151	chr1:177555721-177555765	NM_010209:24	Fh1	INSIDE	0.346	6.839	6751.95	46174.11	2.369	4962.41	11758.03
A_68_P20849150	chr1:177555582-177555626	NM_010209:162	Fh1	INSIDE	0.567	0.579	1485.54	860.23	0.329	1149.69	377.70
A_68_P29403726	chr14:11994369-11994413	NM_010210:156	Fhit	INSIDE	0.288	9.744	6040.88	58859.64	2.806	4740.65	13302.99
A_68_P20187367	chr1:43220695-43220739	NM_010212:90	Fhl2	INSIDE	0.28	20.256	1252.61	25373.45	5.669	1120.49	6351.75
A_68_P20187366	chr1:43220621-43220665	NM_010212:164	Fhl2	INSIDE	0.479	3.951	782.01	3090.09	1.892	639.71	1210.40
A_68_P23269776	chr4:124378137-124378181	NM_010213:216	Fhl3	INSIDE	0.363	6.795	689.38	4684.20	2.467	686.39	1693.16
A_68_P23269774	chr4:124377782-124377826	NM_010213:-138	Fhl3	DIVERGENT_PROMOTER	0.457	0.220	3540.88	777.65	0.1	2581.36	258.87
A_68_P23269775	chr4:124377958-124378002	NM_010213:38	Fhl3	INSIDE	0.649	0.560	3094.54	1733.12	0.364	2503.46	910.38
A_68_P31561526	chr18:24868000-24868044	NM_175276:77	Fhod3	INSIDE	0.504	9.659	1495.34	14443.42	4.873	1332.27	6491.52
A_68_P21111665	chr2:31702261-31702305	NM_178887:-757	Fibcd1	PROMOTER	0.559	0.460	2461.32	1131.23	0.257	1778.32	457.06
A_68_P21111657	chr2:31701223-31701267	NM_178887:281	Fibcd1	INSIDE	0.356	0.656	878.42	576.53	0.234	735.34	171.83
A_68_P24607724	chr6:85978997-85979041	NM_012013:11834	Figla	DOWNSTREAM	0.508	0.313	1453.61	455.21	0.159	1048.16	166.87
A_68_P30786987	chr16:57549528-57549572	NM_001177871:196	Filip1l	INSIDE	0.192	14.652	588.94	8629.19	2.813	533.66	1500.92
A_68_P23803966	chr5:74931407-74931451	NM_001159573:-78	Fip1l1	DIVERGENT_PROMOTER	0.541	0.307	2430.82	746.56	0.166	2079.66	345.32
A_68_P21812643	chr2:163298309-163298353	NM_173397:35	Fitm2	INSIDE	0.585	0.612	2173.06	1330.33	0.358	1844.42	660.13
A_68_P24951534	chr7:4960187-4960231	NM_001110330:4636	Fiz1	INSIDE	0.632	3.740	833.43	3117.02	2.364	842.17	1991.29
A_68_P21477090	chr2:102291323-102291367	NM_010218:605	Fjx1	INSIDE	0.337	1.619	1396.55	2260.70	0.545	1192.27	649.58
A_68_P21744416	chr2:151368526-151368570	NM_008019:314	Fkbp1a	INSIDE	0.46	0.398	1157.71	460.21	0.183	948.27	173.29
A_68_P31937108	chr19:7054737-7054781	NM_008020:172	Fkbp2	INSIDE	0.464	5.469	4058.24	22194.95	2.537	3421.09	8678.41
A_68_P31937106	chr19:7054484-7054528	NM_008020:424	Fkbp2	INSIDE	0.643	0.341	4590.59	1564.47	0.219	3043.90	666.89
A_68_P31937105	chr19:7054354-7054400	NM_008020:554	Fkbp2	INSIDE	0.27	1.616	1745.97	2821.14	0.436	1576.23	686.47
A_68_P31937107	chr19:7054605-7054649	NM_008020:304	Fkbp2	INSIDE	0.102	1.354	12555.98	17002.83	0.138	8584.28	1184.61
A_68_P31937111	chr19:7054987-7055031	NM_001166368:-57	Fkbp2	PROMOTER	0.52	2.949	1068.59	3151.34	1.535	930.90	1428.74
A_68_P24477004	chr6:56782166-56782210	NM_012056:136	Fkbp9	INSIDE	0.338	8.196	2179.75	17866.11	2.772	1869.07	5181.68
A_68_P26406146	chr9:32348818-32348862	NM_008026:113	Fli1	INSIDE	0.477	0.471	3707.44	1746.18	0.225	2626.42	590.06
A_68_P26406147	chr9:32348923-32348967	NM_008026:9	Fli1	INSIDE	0.53	16.047	1716.14	27538.81	8.502	1468.95	12488.89
A_68_P26406144	chr9:32348552-32348596	NM_008026:379	Fli1	INSIDE	0.506	0.553	1063.49	587.86	0.28	777.05	217.54
A_68_P29386047	chr14:8650522-8650566	NM_134080:74	Flnb	INSIDE	0.35	0.405	1618.21	655.09	0.142	1204.56	170.50
A_68_P31161104	chr17:35960060-35960104	NM_008027:-219	Flot1	PROMOTER	0.542	10.115	1427.69	14440.63	5.482	1280.98	7022.68
A_68_P28656384	chr12:96934105-96934150	NM_201518:3692	Flrt2	INSIDE	0.244	10.932	1259.32	13767.25	2.672	1153.19	3081.73
A_68_P24175500	chr5:148537455-148537499	NM_010228:88	Flt1	INSIDE	0.408	4.458	2363.82	10536.99	1.819	2002.69	3643.33
A_68_P24175497	chr5:148537110-148537154	NM_010228:432	Flt1	INSIDE	0.652	0.410	2412.06	989.49	0.267	1817.92	485.97
A_68_P24173496	chr5:148211773-148211817	NM_010229:271	Flt3	INSIDE	0.561	3.894	4934.58	19216.54	2.184	3700.56	8083.11
A_68_P27783947	chr11:49447973-49448018	NM_008029:24815	Flt4	INSIDE	0.199	1.884	2592.80	4885.70	0.375	1879.71	705.30
A_68_P20842596	chr1:176433328-176433372	NM_019445:1395	Fmn2	INSIDE	0.514	0.625	3560.51	2226.01	0.321	2499.17	803.37
A_68_P32441102	chrX:65932146-65932191	NM_008031:439	Fmr1	INSIDE	0.061	79.612	863.93	68779.64	4.838	1100.11	5322.37
A_68_P20332286	chr1:71699573-71699617	NM_010233:151	Fn1	INSIDE	0.476	0.454	1214.83	551.95	0.216	881.96	190.84

ProbeName	Target position of probe on CpG island microarray	TargetID	GeneSymbol	CpG island Description	Ratio of relative methylation (TiO_2-NP/Vehicle)	Sham group			TiO_2-H group		
						Relative methylation (Cy5/Cy3)	Cy3 signal (Input)	Cy5 signal (MeDIP)	Relative methylation (Cy5/Cy3)	Cy3 signal (Input)	Cy5 signal (MeDIP)
A_68_P21107218	chr2:30997212-30997256	NM_001038700:294	Fnbp1	INSIDE	0.663	3.485	1159.37	4040.31	2.312	996.38	2303.18
A_68_P21107216	chr2:30997021-30997065	NM_001038700:486	Fnbp1	INSIDE	0.663	2.122	2031.14	4310.82	1.407	1620.01	2278.54
A_68_P22483663	chr3:122321938-122321982	NM_001114665:625	Fnbp1l	INSIDE	0.389	15.921	1039.67	16552.94	6.196	820.63	5084.25
A_68_P22483665	chr3:122322229-122322273	NM_001114665:335	Fnbp1l	INSIDE	0.501	5.207	1088.81	5669.31	2.609	988.41	2579.20
A_68_P29713891	chr14:73109647-73109691	NM_207636:142	Fndc3a	INSIDE	0.237	9.821	1348.47	13243.63	2.324	1132.61	2632.08
A_68_P29713892	chr14:73109778-73109822	NM_207636:10	Fndc3a	INSIDE	0.344	10.265	13839.32	142063.40	3.526	12155.77	42867.09
A_68_P23577610	chr5:31597885-31597929	NM_022424:344	Fndc4	INSIDE	0.481	3.876	2752.14	10667.55	1.864	2262.72	4217.11
A_68_P23577613	chr5:31598167-31598211	NM_022424:62	Fndc4	INSIDE	0.574	3.079	1441.87	4439.37	1.767	1181.80	2088.55
A_68_P27810090	chr11:54251929-54251973	NM_173753:270	Fnip1	INSIDE	0.261	7.954	2068.67	16454.92	2.073	1619.88	3357.35
A_68_P27810087	chr11:54251592-54251636	NM_173753:-66	Fnip1	PROMOTER	0.4	0.694	2971.60	2061.06	0.277	2261.99	627.61
A_68_P28604394	chr12:86827035-86827079	NM_010234:12206	Fos	DOWNSTREAM	0.555	6.086	1484.00	9032.17	3.376	1256.43	4242.17
A_68_P24992615	chr7:19895551-19895595	NM_008036:-178	Fosb	PROMOTER	0.278	1.384	1330.77	1841.72	0.384	1033.93	397.39
A_68_P24992603	chr7:19894118-19894162	NM_008036:1254	Fosb	INSIDE	0.652	0.647	937.07	605.99	0.422	765.94	323.17
A_68_P23582164	chr5:32439192-32439236	NM_008037:370	Fosl2	INSIDE	0.438	5.630	2352.80	13247.34	2.464	2001.74	4931.33
A_68_P23582152	chr5:32437568-32437612	NM_008037:-1254	Fosl2	PROMOTER	0.594	7.937	791.55	6282.38	4.717	735.72	3470.66
A_68_P23582162	chr5:32438904-32438948	NM_008037:82	Fosl2	INSIDE	0.436	0.710	1651.22	1172.32	0.309	1329.52	411.27
A_68_P23582160	chr5:32438700-32438744	NM_008037:-122	Fosl2	PROMOTER	0.602	0.596	822.70	490.01	0.359	719.14	258.00
A_68_P28463190	chr12:58643591-58643635	NM_008259:3496	Foxa1	INSIDE	0.158	15.644	1325.88	20742.12	2.476	1278.01	3164.06
A_68_P28463194	chr12:58643991-58644035	NM_008259:3096	Foxa1	INSIDE	0.592	2.906	1441.61	4188.94	1.721	1200.29	2066.01
A_68_P21727904	chr2:147870499-147870543	NM_010446:2185	Foxa2	INSIDE	0.413	0.298	2007.38	598.65	0.123	1411.39	173.94
A_68_P21727901	chr2:147870136-147870180	NM_010446:2547	Foxa2	INSIDE	0.605	0.164	4403.84	723.54	0.099	3016.30	299.71
A_68_P31982709	chr19:16947995-16948040	NM_008023:303	Foxb2	INSIDE	0.443	0.474	976.06	462.86	0.21	817.77	171.89
A_68_P31982710	chr19:16948092-16948136	NM_008023:206	Foxb2	INSIDE	0.488	0.612	1899.51	1161.97	0.298	1639.82	489.07
A_68_P28925097	chr13:31898615-31898660	NM_008592:123	Foxc1	INSIDE	0.582	2.603	1630.93	4244.97	1.515	1490.87	2259.28
A_68_P28925107	chr13:31900007-31900051	NM_008592:1514	Foxc1	INSIDE	0.269	5.495	1158.52	6365.60	1.475	1102.05	1625.91
A_68_P26224615	chr8:123641830-123641874	NM_013519:1782	Foxc2	INSIDE	0.505	0.312	1449.04	452.43	0.158	1065.72	168.08
A_68_P29257223	chr13:99136439-99136483	NM_008242:12261	Foxd1	DOWNSTREAM	0.222	32.878	2081.36	68430.25	7.311	2015.04	14731.69
A_68_P23145500	chr4:99317555-99317599	NM_010425:-5413	Foxd3	PROMOTER	0.421	0.288	2616.70	754.15	0.121	1965.49	238.55
A_68_P23145552	chr4:99323494-99323538	NM_010425:527	Foxd3	INSIDE	0.528	0.564	1457.91	822.32	0.298	1233.70	367.20
A_68_P23145560	chr4:99324186-99324230	NM_010425:1219	Foxd3	INSIDE	0.588	4.867	1999.33	9729.87	2.86	1725.55	4934.40
A_68_P23145563	chr4:99324552-99324596	NM_010425:1585	Foxd3	INSIDE	0.324	4.813	2447.77	11781.11	1.557	2105.03	3278.05
A_68_P32026412	chr19:24971921-24971965	NM_008022:3857	Foxd4	DOWNSTREAM	0.436	0.271	1853.06	501.62	0.118	1496.21	176.51
A_68_P32026433	chr19:24974465-24974509	NM_008022:1313	Foxd4	INSIDE	0.4	0.737	2695.99	1986.81	0.295	2207.77	651.03
A_68_P22888266	chr4:46358077-46358121	NM_183298:1033	Foxe1	INSIDE	0.633	4.144	2190.68	9077.89	2.624	1744.79	4577.54
A_68_P23222364	chr4:114598169-114598213	NM_015758:428	Foxe3	INSIDE	0.434	0.237	2299.20	543.97	0.103	1832.81	188.23
A_68_P28924022	chr13:31712233-31712277	NM_010225:-5430	Foxf2	PROMOTER	0.648	0.672	949.79	638.38	0.435	833.11	362.61
A_68_P28419968	chr12:50486468-50486512	NM_008241:2497	Foxg1	INSIDE	0.29	0.367	3366.09	1236.07	0.106	2328.34	247.72
A_68_P28419964	chr12:50485980-50486024	NM_008241:2009	Foxg1	INSIDE	0.413	0.655	2280.13	1493.63	0.271	1787.04	483.68
A_68_P24533915	chr6:70907176-70907220	NM_001101464:599	Foxi3	INSIDE	0.274	1.841	3441.50	6335.32	0.504	2499.18	1259.50
A_68_P24533907	chr6:70906387-70906431	NM_001101464:-191	Foxi3	PROMOTER	0.461	0.327	4996.50	1634.16	0.151	3620.74	545.71
A_68_P24533908	chr6:70906504-70906548	NM_001101464:-73	Foxi3	PROMOTER	0.558	0.385	1769.83	680.83	0.215	1429.16	306.93
A_68_P24810537	chr6:122761310-122761354	NM_021899:-8869	Foxj2	PROMOTER	0.242	1.710	783.75	1340.49	0.414	690.96	285.92
A_68_P28189606	chr11:121121098-121121142	NM_001080932:-180	Foxk2	PROMOTER	0.348	5.718	2350.34	13439.24	1.991	2044.75	4070.94
A_68_P26224704	chr8:123652972-123653016	NM_008024:1410	Foxl1	INSIDE	0.66	0.401	1419.70	569.26	0.265	1115.88	295.21
A_68_P26761428	chr9:98856199-98856243	NM_012020:195	Foxl2	INSIDE	0.448	6.841	1510.84	10336.09	3.068	1238.63	3800.07
A_68_P26761439	chr9:98857310-98857354	NM_012020:1307	Foxl2	INSIDE	0.614	0.233	3054.19	712.86	0.143	2387.31	342.29

ProbeName	Target position of probe on CpG island microarray	TargetID	GeneSymbol	CpG island Description	Ratio of relative methylation (TiO_2-NP/Vehicle)	Sham group			TiO_2-H group		
						Relative methylation (Cy5/Cy3)	Cy3 signal (Input)	Cy5 signal (MeDIP)	Relative methylation (Cy5/Cy3)	Cy3 signal (Input)	Cy5 signal (MeDIP)
A_68_P26761440	chr9:98857399-98857443	NM_012020:1395	Foxl2	INSIDE	0.448	3.714	1698.08	6305.88	1.664	1317.25	2192.27
A_68_P26761418	chr9:98855113-98855157	NR_003248:595	Foxl2os	INSIDE	0.517	0.681	982.79	669.53	0.352	881.52	310.55
A_68_P24838573	chr6:128313280-128313324	NM_008021:291	Foxm1	INSIDE	0.532	0.308	2159.55	665.68	0.164	1579.24	258.89
A_68_P28675864	chr12:100630527-100630571	NM_183186:57736	Foxn3	INSIDE	0.647	0.263	1709.47	449.21	0.17	1345.47	228.62
A_68_P22134615	chr3:52073411-52073455	NM_019739:1174	Foxo1	INSIDE	0.159	1.775	7725.16	13710.01	0.282	5184.75	1462.71
A_68_P22134618	chr3:52073824-52073868	NM_019739:1588	Foxo1	INSIDE	0.599	4.027	570.55	2297.75	2.414	519.99	1255.19
A_68_P27094083	chr10:41996329-41996375	NM_019740:196	Foxo3	INSIDE	0.549	6.616	906.72	5998.57	3.634	744.89	2706.70
A_68_P27094078	chr10:41995833-41995877	NM_019740:694	Foxo3	INSIDE	0.488	3.158	1287.91	4066.75	1.542	1065.76	1643.62
A_68_P32563078	chrX:98450245-98450289	NM_018789:400	Foxo4	INSIDE	0.419	4.799	2172.30	10423.83	2.011	2654.85	5339.44
A_68_P23250309	chr4:119940503-119940547	NM_194060:19342	Foxo6	INSIDE	0.312	7.644	1442.21	11023.78	2.386	1441.46	3439.49
A_68_P23250457	chr4:119959679-119959723	NM_194060:166	Foxo6	INSIDE	0.451	4.815	766.35	3690.27	2.172	768.35	1669.15
A_68_P23250423	chr4:119955345-119955389	NM_194060:4500	Foxo6	INSIDE	0.468	2.934	2848.89	8358.62	1.373	2199.56	3020.49
A_68_P24683080	chr6:98978547-98978591	NM_001197322:-276	Foxp1	PROMOTER	0.343	4.148	1681.19	6974.41	1.422	1446.78	2057.49
A_68_P24253394	chr6:14851545-14851589	NM_053242:218	Foxp2	INSIDE	0.542	0.632	2056.09	1300.30	0.343	1694.70	580.56
A_68_P31214913	chr17:48046778-48046822	NM_001110824:14781	Foxp4	INSIDE	0.393	12.576	5432.99	68325.44	4.936	4172.51	20597.22
A_68_P31214657	chr17:48009489-48009535	NM_001110824:52069	Foxp4	INSIDE	0.445	0.598	2724.91	1630.35	0.267	1899.07	506.12
A_68_P28923686	chr13:31651815-31651859	NM_008239:1798	Foxq1	INSIDE	0.259	8.790	1076.75	9464.54	2.275	1085.90	2470.17
A_68_P28923679	chr13:31651001-31651045	NM_008239:984	Foxq1	INSIDE	0.59	0.500	2086.85	1043.04	0.295	1548.12	456.29
A_68_P28923670	chr13:31649941-31649985	NM_008239:-76	Foxq1	PROMOTER	0.638	0.572	1508.25	863.27	0.365	1236.15	451.09
A_68_P28923672	chr13:31650159-31650203	NM_008239:142	Foxq1	INSIDE	0.603	0.644	820.41	528.25	0.388	656.90	254.91
A_68_P26467611	chr9:44248858-44248902	NM_001033469:71	Foxr1	INSIDE	0.526	3.419	1165.43	3984.24	1.8	902.53	1624.16
A_68_P30356791	chr15:77786971-77787015	NM_001017983:160	Foxred2	INSIDE	0.501	3.832	3127.71	11986.52	1.92	2519.78	4838.52
A_68_P30356784	chr15:77786172-77786216	NM_001017983:958	Foxred2	INSIDE	0.613	0.414	1629.63	675.06	0.254	1148.36	291.39
A_68_P32117383	chr19:41905096-41905140	NM_008043:659	Frat1	INSIDE	0.41	6.826	1965.36	13415.00	2.798	1620.10	4532.39
A_68_P32117376	chr19:41904363-41904407	NM_008043:-75	Frat1	PROMOTER	0.525	0.523	2134.34	1115.30	0.274	1695.74	465.29
A_68_P32117486	chr19:41921651-41921695	NM_177603:950	Frat2	INSIDE	0.418	0.326	2336.02	760.74	0.136	1590.21	216.58
A_68_P23018909	chr4:73659645-73659689	NM_172869:160	Frmd3	INSIDE	0.468	4.905	923.62	4530.27	2.295	800.53	1837.35
A_68_P20967784	chr2:4322707-4322751	NM_001177843:707	Frmd4a	INSIDE	0.189	16.821	7527.32	126614.00	3.176	10461.68	33222.13
A_68_P24674231	chr6:97567120-97567164	NM_145148:509	Frmd4b	INSIDE	0.53	0.372	5806.70	2162.43	0.197	4399.25	868.33
A_68_P21582988	chr2:121632726-121632770	NM_172673:45	Frmd5	INSIDE	0.453	20.750	4897.55	101624.80	9.406	4143.55	38975.12
A_68_P21582987	chr2:121632660-121632704	NM_172673:111	Frmd5	INSIDE	0.563	2.746	4388.39	12050.75	1.547	3599.42	5566.79
A_68_P28522738	chr12:71926826-71926870	NM_028127:348	Frmd6	INSIDE	0.422	4.704	724.75	3409.49	1.987	592.70	1177.70
A_68_P32815030	chrX:164561176-164561220	NM_001033330:453967	Frmpd4	INSIDE	0.378	4.388	2690.99	11808.96	1.658	3484.59	5776.30
A_68_P27479719	chr10:116584876-116584920	NM_177798:632	Frs2	INSIDE	0.666	0.711	1986.56	1411.91	0.473	1559.59	737.76
A_68_P27479722	chr10:116585133-116585177	NM_177798:376	Frs2	INSIDE	0.561	2.606	4551.75	11861.79	1.461	3467.57	5066.63
A_68_P31213683	chr17:47831917-47831961	NM_144939:-217	Frs3	DIVERGENT_PROMOTER	0.305	9.159	3544.93	32466.79	2.798	2953.21	8261.78
A_68_P23796637	chr5:73647888-73647932	NM_028194:-53	Fryl	PROMOTER	0.425	0.520	1724.51	896.51	0.221	1292.85	285.95
A_68_P23796632	chr5:73647376-73647420	NM_028194:459	Fryl	INSIDE	0.493	4.801	1052.79	5054.89	2.368	984.02	2330.00
A_68_P24154219	chr5:143723667-143723712	NM_007984:1656	Fscn1	INSIDE	0.478	7.196	1552.66	11173.16	3.438	1226.30	4216.55
A_68_P24154206	chr5:143722119-143722163	NM_007984:107	Fscn1	INSIDE	0.665	3.359	1763.26	5923.65	2.233	1345.15	3004.16
A_68_P27278867	chr10:79240093-79240137	NM_031380:96	Fstl3	INSIDE	0.077	1.565	8031.39	12570.87	0.12	5164.75	618.52
A_68_P27800933	chr11:52578613-52578657	NM_177059:427	Fstl4	INSIDE	0.526	0.411	2551.81	1049.67	0.216	1880.60	407.03
A_68_P31129059	chr17:29797809-29797853	NM_028791:285	Ftsjd2	INSIDE	0.635	9.117	1696.61	15468.22	5.789	1709.09	9893.73
A_68_P21109951	chr2:31428059-31428103	NM_001033389:-164	Fubp3	PROMOTER	0.426	27.351	1075.75	29422.88	11.651	1202.14	14006.08
A_68_P21109952	chr2:31428160-31428204	NM_001033389:-62	Fubp3	PROMOTER	0.385	1.729	1792.26	3098.41	0.665	1460.45	970.88
A_68_P23331228	chr4:135476820-135476864	NM_024243:202	Fuca1	INSIDE	0.6	0.260	1758.33	457.32	0.156	1391.38	217.06

ProbeName	Target position of probe on CpG island microarray	TargetID	GeneSymbol	CpG island Description	Ratio of relative methylation (TiO_2-NP/Vehicle)	Sham group			TiO_2-H group		
						Relative methylation (Cy5/Cy3)	Cy3 signal (Input)	Cy5 signal (MeDIP)	Relative methylation (Cy5/Cy3)	Cy3 signal (Input)	Cy5 signal (MeDIP)
A_68_P25266551	chr7:87547096-87547140	NM_011046:530	Furin	INSIDE	0.272	50.346	868.92	43746.91	13.704	1807.75	24774.12
A_68_P25266552	chr7:87547168-87547212	NM_011046:458	Furin	INSIDE	0.349	14.752	1249.08	18425.87	5.144	1184.69	6093.72
A_68_P29448409	chr14:21514700-21514744	NM_028428:533	Fut11	INSIDE	0.566	3.543	986.93	3496.59	2.007	904.75	1815.67
A_68_P28558333	chr12:78339907-78339951	NM_016893:-79	Fut8	PROMOTER	0.328	9.596	1608.63	15436.55	3.147	1495.21	4705.57
A_68_P28558338	chr12:78340507-78340551	NM_016893:521	Fut8	INSIDE	0.605	0.632	2602.05	1645.70	0.383	2079.16	795.58
A_68_P28558336	chr12:78340320-78340364	NM_016893:335	Fut8	INSIDE	0.534	3.597	856.63	3081.42	1.921	687.91	1321.56
A_68_P28558329	chr12:78339501-78339545	NM_016893:-485	Fut8	PROMOTER	0.18	9.757	780.65	7617.04	1.757	613.25	1077.65
A_68_P22791943	chr4:25726823-25726867	NM_010243:306	Fut9	INSIDE	0.365	0.283	3529.38	998.20	0.103	2714.49	280.35
A_68_P22791942	chr4:25726695-25726739	NM_010243:434	Fut9	INSIDE	0.31	0.684	1364.26	933.55	0.212	1188.21	252.41
A_68_P25089992	chr7:52151653-52151697	NM_027376:226	Fuz	INSIDE	0.485	0.436	1983.11	863.90	0.211	1386.80	293.08
A_68_P32022553	chr19:24355018-24355062	NM_008044:36	Fxn	INSIDE	0.525	6.187	1213.16	7505.30	3.247	954.44	3099.50
A_68_P22045621	chr3:33919264-33919308	NM_001113188:286	Fxr1	INSIDE	0.216	22.222	1583.87	35196.04	4.792	1287.89	6171.01
A_68_P22045622	chr3:33919516-33919560	NM_001113188:538	Fxr1	INSIDE	0.637	2.252	2817.10	6343.72	1.434	2576.68	3695.97
A_68_P27896201	chr11:69446248-69446292	NM_011814:-202	Fxr2	PROMOTER	0.347	0.310	2306.57	716.14	0.108	1629.22	175.41
A_68_P25034759	chr7:31837288-31837332	NM_052991:2562	Fxyd1	INSIDE	0.497	0.300	1558.47	466.84	0.149	1189.68	177.09
A_68_P27077942	chr10:39090283-39090342	NM_001122892:708	Fyn	INSIDE	0.124	1.530	3941.07	6030.98	0.189	2769.65	524.37
A_68_P27077941	chr10:39090193-39090237	NM_001122892:610	Fyn	INSIDE	0.573	0.639	2419.94	1547.16	0.367	1994.98	731.19
A_68_P30655031	chr16:32877641-32877685	NM_001159349:-207	Fyttd1	DIVERGENT_PROMOTER	0.388	0.485	1114.23	539.88	0.188	920.93	173.35
A_68_P30655034	chr16:32877951-32877995	NM_001159349:103	Fyttd1	INSIDE	0.573	3.797	1931.35	7332.85	2.174	1515.61	3294.44
A_68_P28080438	chr11:102467204-102467248	NM_020510:1482	Fzd2	INSIDE	0.547	0.486	1616.82	786.38	0.266	1283.77	341.67
A_68_P25307423	chr7:96553263-96553307	NM_008055:409	Fzd4	INSIDE	0.53	3.048	1175.70	3583.86	1.614	1045.70	1687.87
A_68_P20294249	chr1:64782318-64782364	NM_001042659:1984	Fzd5	INSIDE	0.262	9.512	1401.83	13334.23	2.488	920.93	2290.97
A_68_P20294244	chr1:64781733-64781777	NM_001042659:2570	Fzd5	INSIDE	0.602	5.101	575.98	2937.95	3.068	569.96	1748.74
A_68_P20294248	chr1:64782204-64782248	NM_001042659:2098	Fzd5	INSIDE	0.586	2.630	1533.36	4033.12	1.542	1163.27	1793.85
A_68_P20264199	chr1:59540719-59540763	NM_008057:1750	Fzd7	INSIDE	0.581	2.819	876.08	2469.66	1.638	806.10	1320.64
A_68_P31484544	chr18:9212850-9212894	NM_008058:19	Fzd8	INSIDE	0.389	7.252	966.81	7011.68	2.823	868.88	2452.65
A_68_P31484557	chr18:9214262-9214306	NM_008058:1431	Fzd8	INSIDE	0.428	0.394	1629.94	641.53	0.169	1292.72	218.00
A_68_P24115508	chr5:135726136-135726180	NM_010246:759	Fzd9	INSIDE	0.221	0.363	3077.48	1117.24	0.08	2147.33	172.50
A_68_P24115511	chr5:135726586-135726630	NM_010246:309	Fzd9	INSIDE	0.488	0.267	2434.43	649.52	0.13	1814.97	236.48
A_68_P24115510	chr5:135726402-135726446	NM_010246:493	Fzd9	INSIDE	0.563	0.416	1648.41	684.97	0.234	1285.17	300.70
A_68_P24115514	chr5:135726995-135727039	NM_010246:-99	Fzd9	PROMOTER	0.484	0.641	850.92	545.48	0.31	736.85	228.41
A_68_P27289250	chr10:80840838-80840882	NM_019757:255	Fzr1	INSIDE	0.503	0.377	1169.09	440.53	0.19	920.10	174.45
A_68_P27289251	chr10:80840907-80840951	NM_019757:187	Fzr1	INSIDE	0.561	0.607	8113.33	4922.97	0.341	6043.09	2057.76
A_68_P27289254	chr10:80841374-80841418	NM_019757:-281	Fzr1	DIVERGENT_PROMOTER	0.36	1.943	9174.02	17828.13	0.7	5589.37	3914.09
A_68_P23892217	chr5:92512328-92512372	NM_001080797:-407	G3bp2	PROMOTER	0.245	11.491	2576.55	29608.31	2.814	2192.39	6169.38
A_68_P23892219	chr5:92512481-92512525	NM_001080797:-559	G3bp2	PROMOTER	0.312	1.783	7799.34	13904.26	0.557	5517.59	3072.77
A_68_P23892215	chr5:92512150-92512194	NM_001080797:-229	G3bp2	PROMOTER	0.338	5.944	1919.01	11406.71	2.009	1944.11	3906.22
A_68_P23892218	chr5:92512396-92512440	NM_001080797:-475	G3bp2	PROMOTER	0.457	0.296	1711.96	507.01	0.135	1244.05	168.37
A_68_P27897193	chr11:69617732-69617776	NR_027913:584	G630025P09Rik	INSIDE	0.353	0.191	3619.85	690.41	0.067	2557.37	172.07
A_68_P28176853	chr11:119129394-119129438	NM_008064:95	Gaa	INSIDE	0.517	2.988	1053.18	3146.47	1.544	963.66	1487.46
A_68_P27898357	chr11:69805074-69805118	NM_019749:225	Gabarap	INSIDE	0.504	0.125	6403.09	798.62	0.063	3963.44	248.96
A_68_P26171007	chr8:114465131-114465175	NM_026693:535	Gabarapl2	INSIDE	0.135	187.032	1837.26	343627.60	25.185	2661.22	67023.62
A_68_P31166955	chr17:37183328-37183372	NM_019439:440	Gabbr1	INSIDE	0.546	0.182	6567.77	1197.77	0.1	4593.09	457.16
A_68_P31166953	chr17:37183108-37183152	NM_019439:220	Gabbr1	INSIDE	0.483	5.207	494.20	2573.43	2.517	385.40	969.87
A_68_P31166952	chr17:37183039-37183083	NM_019439:150	Gabbr1	INSIDE	0.531	3.256	1518.21	4942.58	1.727	1237.75	2137.94
A_68_P30925866	chr16:84835027-84835071	NM_008065:-320	Gabpa	PROMOTER	0.619	0.299	3781.56	1129.27	0.185	2904.43	537.16

ProbeName	Target position of probe on CpG island microarray	TargetID	GeneSymbol	CpG island Description	Ratio of relative methylation (TiO_2-NP/Vehicle)	Sham group			TiO_2-H group		
						Relative methylation (Cy5/Cy3)	Cy3 signal (Input)	Cy5 signal (MeDIP)	Relative methylation (Cy5/Cy3)	Cy3 signal (Input)	Cy5 signal (MeDIP)
A_68_P21609427	chr2:126500825-126500869	NM_010249:376	Gabpb1	INSIDE	0.412	8.743	1050.96	9188.85	3.599	998.18	3592.58
A_68_P23787605	chr5:72049192-72049236	NM_010251:333	Gabra4	INSIDE	0.551	7.889	993.89	7840.85	4.343	849.95	3691.34
A_68_P25151144	chr7:64764458-64764502	NM_176942:899	Gabra5	INSIDE	0.293	9.248	1897.13	17544.88	2.711	2047.40	5551.45
A_68_P25151575	chr7:64847050-64847094	NM_008071:530	Gabrb3	INSIDE	0.404	0.424	3468.55	1469.31	0.171	2636.97	451.77
A_68_P32457442	chrX:69520267-69520311	NM_017369:-228	Gabre	PROMOTER	0.311	11.686	2613.67	30543.72	3.633	2822.07	10252.28
A_68_P25150559	chr7:64642303-64642347	NM_008074:-83	Gabrg3	DIVERGENT_PROMOTER	0.27	0.668	997.77	666.74	0.18	956.73	172.55
A_68_P21059051	chr2:22478980-22479024	NM_008078:1156	Gad2	INSIDE	0.561	0.530	2670.91	1416.84	0.298	2028.38	603.54
A_68_P21059041	chr2:22477785-22477829	NM_008078:-40	Gad2	PROMOTER	0.438	0.720	9901.76	7125.49	0.315	6746.09	2125.46
A_68_P27286395	chr10:80391635-80391679	NM_008655:-1179	Gadd45b	PROMOTER	0.452	0.601	1445.67	868.49	0.272	1106.37	300.52
A_68_P27286403	chr10:80392697-80392741	NM_008655:-117	Gadd45b	PROMOTER	0.518	0.562	826.90	464.86	0.291	588.86	171.56
A_68_P29034741	chr13:51941420-51941464	NM_011817:-601	Gadd45g	PROMOTER	0.471	6.776	2471.76	16749.05	3.189	2032.65	6483.08
A_68_P29034752	chr13:51942644-51942688	NM_011817:623	Gadd45g	INSIDE	0.544	0.610	5685.95	3470.58	0.332	4173.85	1386.26
A_68_P29034750	chr13:51942448-51942492	NM_011817:427	Gadd45g	INSIDE	0.563	0.402	6802.48	2736.25	0.227	4371.48	990.46
A_68_P29034746	chr13:51942012-51942056	NM_011817:-9	Gadd45g	PROMOTER	0.49	3.335	1694.58	5651.45	1.633	1453.81	2373.79
A_68_P28669125	chr12:99497683-99497727	NM_008079:-157	Galc	DIVERGENT_PROMOTER	0.322	8.292	1409.66	11689.27	2.673	1229.54	3286.98
A_68_P28669124	chr12:99497596-99497640	NM_008079:-71	Galc	DIVERGENT_PROMOTER	0.47	4.249	1459.93	6202.74	1.997	1204.70	2405.30
A_68_P28669118	chr12:99496934-99496978	NM_008079:591	Galc	INSIDE	0.348	0.670	836.84	561.07	0.233	792.73	184.80
A_68_P22892659	chr4:47105046-47105090	NM_172693:244	Galnt12	INSIDE	0.355	10.172	1003.17	10204.31	3.606	785.60	2833.17
A_68_P22892661	chr4:47105201-47105245	NM_172693:398	Galnt12	INSIDE	0.446	5.717	4336.11	24787.40	2.549	3894.42	9927.72
A_68_P31347831	chr17:74059828-74059872	NM_027864:-59	Galnt14	PROMOTER	0.352	7.268	2660.90	19338.79	2.557	2039.03	5214.45
A_68_P31347829	chr17:74059599-74059643	NM_027864:171	Galnt14	INSIDE	0.486	10.315	2015.33	20788.29	5.013	1913.21	9591.43
A_68_P31347825	chr17:74059155-74059199	NM_027864:615	Galnt14	INSIDE	0.666	0.720	7551.82	5433.89	0.479	4699.23	2251.34
A_68_P27385039	chr10:98569849-98569893	NM_015737:-898	Galnt4	PROMOTER	0.233	11.635	804.42	9359.60	2.715	643.78	1747.77
A_68_P30487413	chr15:100559897-100559941	NM_001161767:-111	Galnt6	PROMOTER	0.527	0.357	2648.00	945.00	0.188	2017.48	379.44
A_68_P25892525	chr8:60132078-60132122	NM_001167981:-248	Galnt7	PROMOTER	0.281	1.671	2534.53	4234.44	0.47	2112.33	991.99
A_68_P25892520	chr8:60131247-60131291	NM_001167981:584	Galnt7	INSIDE	0.498	5.662	904.42	5120.98	2.82	780.72	2201.65
A_68_P25892513	chr8:60130467-60130511	NM_001167981:1364	Galnt7	INSIDE	0.511	3.224	1102.10	3553.19	1.647	868.20	1429.71
A_68_P23980414	chr5:110973587-110973631	NM_198306:245	Galnt9	INSIDE	0.442	7.774	2008.29	15612.26	3.436	1700.57	5842.70
A_68_P23980413	chr5:110973506-110973550	NM_198306:165	Galnt9	INSIDE	0.555	3.903	1118.28	4364.94	2.168	979.25	2123.23
A_68_P23980412	chr5:110973428-110973472	NM_198306:87	Galnt9	INSIDE	0.407	3.511	1807.41	6345.05	1.428	1480.28	2114.22
A_68_P28576082	chr12:81619771-81619815	NM_001081421:-184	Galntl1	PROMOTER	0.406	5.436	1013.19	5508.09	2.207	950.71	2098.66
A_68_P28576088	chr12:81620372-81620416	NM_001081421:418	Galntl1	INSIDE	0.46	4.556	1818.93	8287.05	2.094	1568.25	3284.45
A_68_P28576092	chr12:81620747-81620791	NM_001081421:792	Galntl1	INSIDE	0.654	0.414	1744.47	722.82	0.271	1435.89	389.35
A_68_P25420899	chr7:118923736-118923780	NM_173739:-267	Galntl4	PROMOTER	0.332	8.964	2631.83	23591.71	2.976	2526.26	7519.38
A_68_P25420896	chr7:118923422-118923466	NM_173739:47	Galntl4	INSIDE	0.631	0.611	1330.92	813.32	0.385	1083.76	417.61
A_68_P30364007	chr15:78873556-78873600	NM_015738:1264	Galr3	INSIDE	0.609	3.135	1875.79	5879.70	1.91	1612.64	3079.91
A_68_P22864172	chr4:41702487-41702531	NM_016658:383	Galt	INSIDE	0.558	0.395	2337.88	922.60	0.22	1771.17	390.16
A_68_P27282039	chr10:79723618-79723662	NM_010255:73	Gamt	INSIDE	0.49	0.398	1230.19	489.11	0.195	1006.46	196.21
A_68_P27282040	chr10:79723724-79723768	NM_010255:-33	Gamt	DIVERGENT_PROMOTER	0.621	0.297	3735.52	1109.67	0.184	2460.85	453.80
A_68_P27282041	chr10:79723845-79723889	NM_010255:-153	Gamt	DIVERGENT_PROMOTER	0.234	0.663	1398.62	927.46	0.155	1088.73	168.63
A_68_P24819104	chr6:125115336-125115380	NM_008084:243	Gapdh	INSIDE	0.205	0.227	6173.14	1404.04	0.047	3749.46	174.54
A_68_P24819105	chr6:125115461-125115505	NM_008084:119	Gapdh	INSIDE	0.335	0.558	6802.88	3795.55	0.187	4388.22	819.41
A_68_P24819103	chr6:125115217-125115261	NM_008084:363	Gapdh	INSIDE	0.663	0.253	2902.06	733.42	0.168	2086.89	349.89
A_68_P21129593	chr2:34609363-34609407	NM_025709:1368	Gapvd1	INSIDE	0.652	0.272	4319.16	1175.04	0.177	3142.20	557.06
A_68_P22522346	chr3:129533887-129533931	NM_026578:406	Gar1	INSIDE	0.433	0.204	2666.52	543.73	0.088	1986.14	175.51
A_68_P24466829	chr6:54988330-54988374	NM_180678:358	Gars	INSIDE	0.622	0.265	2550.94	677.06	0.165	1923.10	317.45

ProbeName	Target position of probe on CpG island microarray	TargetID	GeneSymbol	CpG island Description	Ratio of relative methylation (TiO_2-NP/Vehicle)	Sham group			TiO_2-H group		
						Relative methylation (Cy5/Cy3)	Cy3 signal (Input)	Cy5 signal (MeDIP)	Relative methylation (Cy5/Cy3)	Cy3 signal (Input)	Cy5 signal (MeDIP)
A_68_P27561184	chr11:4965305-4965349	NM_001190406:4	Gas2l1	INSIDE	0.228	30.997	5880.61	182278.40	7.082	6315.26	44723.20
A_68_P27561149	chr11:4961389-4961433	NM_001190406:3920	Gas2l1	INSIDE	0.358	0.467	3323.39	1551.09	0.167	2316.28	386.88
A_68_P27561153	chr11:4961885-4961929	NM_001190406:3424	Gas2l1	INSIDE	0.619	0.677	1434.71	971.99	0.419	1094.47	459.05
A_68_P27561152	chr11:4961738-4961782	NM_001190406:3570	Gas2l1	INSIDE	0.403	1.435	1879.76	2698.34	0.578	1397.91	807.91
A_68_P20769690	chr1:162964920-162964964	NR_002840:-354	Gas5	DIVERGENT_PROMOTER	0.27	0.420	4540.97	1909.28	0.114	3460.19	393.32
A_68_P20769692	chr1:162965131-162965175	NR_002840:-144	Gas5	DIVERGENT_PROMOTER	0.272	7.720	2261.90	17462.71	2.1	1713.50	3598.47
A_68_P25668725	chr8:13494426-13494470	NM_019521:87	Gas6	INSIDE	0.292	0.330	3199.94	1055.89	0.096	2366.19	227.66
A_68_P24620292	chr6:88140898-88140951	NM_008090:-7733	Gata2	PROMOTER	0.026	8.546	1561.74	13346.42	0.224	1150.55	257.71
A_68_P24620367	chr6:88149612-88149656	NM_008090:977	Gata2	INSIDE	0.495	3.533	2392.40	8452.21	1.748	2057.41	3595.44
A_68_P24620288	chr6:88140437-88140481	NM_008090:-8199	Gata2	PROMOTER	0.508	4.881	3206.69	15650.88	2.479	2575.13	6383.56
A_68_P24620316	chr6:88143988-88144032	NM_008090:-4647	Gata2	PROMOTER	0.541	5.843	2143.77	12525.75	3.163	1795.02	5678.02
A_68_P24620291	chr6:88140777-88140827	NM_008090:-7855	Gata2	PROMOTER	0.117	1.640	1269.70	2082.58	0.192	915.53	175.58
A_68_P24620282	chr6:88139868-88139912	NM_008090:-8767	Gata2	PROMOTER	0.555	0.636	1562.10	994.00	0.353	1359.45	480.16
A_68_P24620345	chr6:88147159-88147203	NM_008090:-1477	Gata2	PROMOTER	0.518	3.659	891.72	3262.82	1.896	739.99	1402.79
A_68_P20997431	chr2:9796515-9796559	NM_008091:3691	Gata3	INSIDE	0.661	4.341	5467.48	23734.93	2.869	4250.59	12193.23
A_68_P29660043	chr14:63859471-63859515	NM_008092:4605	Gata4	INSIDE	0.282	10.779	1424.85	15358.87	3.036	1502.62	4562.46
A_68_P29660136	chr14:63870354-63870398	NM_008092:-6279	Gata4	PROMOTER	0.365	0.373	2569.19	958.26	0.136	1877.55	255.90
A_68_P29660046	chr14:63859832-63859876	NM_008092:4243	Gata4	INSIDE	0.619	3.742	900.18	3368.69	2.315	899.96	2083.61
A_68_P29660080	chr14:63863910-63863954	NM_008092:165	Gata4	INSIDE	0.547	3.219	1127.01	3627.81	1.761	917.65	1615.93
A_68_P21903062	chr2:180068497-180068541	NM_008093:866	Gata5	INSIDE	0.529	4.680	784.04	3669.63	2.474	726.50	1797.46
A_68_P31493063	chr18:11054641-11054685	NM_010258:2155	Gata6	INSIDE	0.554	4.031	912.64	3678.79	2.233	817.62	1825.38
A_68_P31493066	chr18:11054906-11054950	NM_010258:2421	Gata6	INSIDE	0.559	7.659	2562.46	19626.71	4.279	2096.68	8970.77
A_68_P31493065	chr18:11054816-11054860	NM_010258:2331	Gata6	INSIDE	0.55	3.366	1109.21	3733.28	1.85	995.55	1841.84
A_68_P31493046	chr18:11052798-11052842	NM_010258:313	Gata6	INSIDE	0.123	2.841	956.35	2717.13	0.351	809.26	283.89
A_68_P25949121	chr8:72498323-72498367	NM_001113346:-62	Gatad2a	PROMOTER	0.458	11.577	4190.62	48514.25	5.297	3625.57	19204.65
A_68_P24111498	chr5:134575433-134575477	NM_030719:-163	Gatsl2	PROMOTER	0.249	11.990	1179.42	14140.74	2.982	1212.40	3615.48
A_68_P27556422	chr11:4118442-4118486	NM_028022:211	Gatsl3	INSIDE	0.398	19.020	1337.68	25442.36	7.567	1130.11	8551.50
A_68_P27556420	chr11:4118164-4118208	NM_028022:-67	Gatsl3	PROMOTER	0.482	5.606	4072.71	22833.39	2.704	3340.03	9032.55
A_68_P27556423	chr11:4118542-4118586	NM_028022:311	Gatsl3	INSIDE	0.658	0.677	1738.17	1177.53	0.446	1537.93	685.86
A_68_P30849196	chr16:70314241-70314285	NM_028803:-69	Gbe1	PROMOTER	0.362	6.078	2664.57	16195.37	2.201	2393.22	5267.64
A_68_P30849195	chr16:70314120-70314164	NM_028803:-189	Gbe1	PROMOTER	0.598	3.136	2105.27	6601.98	1.875	1731.07	3246.56
A_68_P20432927	chr1:91819381-91819425	NM_010262:8349	Gbx2	DOWNSTREAM	0.238	35.625	1750.91	62376.73	8.481	3320.42	28160.92
A_68_P20432996	chr1:91827206-91827250	NM_010262:523	Gbx2	INSIDE	0.373	4.144	4490.63	18610.07	1.546	3521.21	5443.08
A_68_P20432981	chr1:91825501-91825545	NM_010262:2229	Gbx2	INSIDE	0.551	2.905	1336.63	3882.95	1.6	1172.01	1875.61
A_68_P21270820	chr2:62502454-62502498	NM_145523:93	Gca	INSIDE	0.633	0.491	1226.25	602.09	0.311	896.10	278.42
A_68_P21270819	chr2:62502267-62502311	NM_145523:-95	Gca	PROMOTER	0.561	2.927	2441.42	7145.55	1.641	1956.78	3210.56
A_68_P30960667	chr16:91044987-91045031	NM_026110:-384	Gcfc1	PROMOTER	0.194	13.540	1639.79	22202.91	2.627	1341.35	3523.62
A_68_P21568184	chr2:118993532-118993576	NM_177157:31	Gchfr	INSIDE	0.414	6.675	2422.91	16172.69	2.765	2299.42	6356.93
A_68_P22481380	chr3:121949179-121949223	NM_008129:691	Gclm	INSIDE	0.636	0.628	854.72	536.92	0.399	693.97	277.08
A_68_P24010273	chr5:116015592-116015636	NM_172719:343	Gcn1l1	INSIDE	0.375	0.376	1579.65	593.63	0.141	1253.55	176.89
A_68_P31985486	chr19:17431193-17431237	NM_173442:-57	Gcnt1	PROMOTER	0.267	7.135	4674.89	33356.77	1.908	4288.84	8183.35
A_68_P22367899	chr3:99966585-99966629	NM_010269:166	Gdap2	INSIDE	0.406	1.630	2820.59	4598.57	0.661	2278.58	1506.63
A_68_P25459669	chr7:125849289-125849333	NM_019580:-58	Gde1	DIVERGENT_PROMOTER	0.466	5.810	2536.44	14736.27	2.708	1958.48	5303.74
A_68_P25951136	chr8:72854054-72854098	NM_008107:413	Gdf1	INSIDE	0.617	2.689	870.64	2340.93	1.659	696.33	1155.04
A_68_P29526837	chr14:34736673-34736717	NM_145741:-78	Gdf10	PROMOTER	0.552	4.409	3318.05	14630.38	2.434	2730.66	6646.48
A_68_P27543728	chr10:128328158-128328202	NM_010272:594	Gdf11	INSIDE	0.545	0.588	2530.20	1488.68	0.32	1928.98	618.00

ProbeName	Target position of probe on CpG island microarray	TargetID	GeneSymbol	CpG island Description	Ratio of relative methylation (TiO_2-NP/Vehicle)	Sham group			TiO_2-H group		
						Relative methylation (Cy5/Cy3)	Cy3 signal (Input)	Cy5 signal (MeDIP)	Relative methylation (Cy5/Cy3)	Cy3 signal (Input)	Cy5 signal (MeDIP)
A_68_P22722447	chr4:9771366-9771410	NM_013526:-130	Gdf6	PROMOTER	0.324	5.839	23323.31	136191.10	1.891	17517.31	33128.66
A_68_P22722446	chr4:9771240-9771284	NM_013526:-256	Gdf6	PROMOTER	0.494	0.607	1330.65	807.90	0.3	1119.98	335.61
A_68_P22722448	chr4:9771474-9771518	NM_013526:-22	Gdf6	PROMOTER	0.49	3.221	2599.45	8373.76	1.58	2223.16	3511.75
A_68_P28221329	chr12:8309070-8309114	NM_013527:-332	Gdf7	PROMOTER	0.09	20.305	6061.23	123070.60	1.818	5200.90	9453.14
A_68_P29996479	chr15:7760686-7760730	NM_010275:-302	Gdnf	PROMOTER	0.563	22.032	1220.67	26893.79	12.408	1141.06	14157.80
A_68_P29996481	chr15:7760813-7760857	NM_010275:-176	Gdnf	PROMOTER	0.607	8.241	1273.89	10497.94	4.999	1036.16	5180.26
A_68_P29996501	chr15:7762967-7763011	NM_010275:1978	Gdnf	INSIDE	0.626	0.281	2639.98	743.13	0.176	1961.07	345.74
A_68_P29996473	chr15:7760094-7760138	NM_010275:-894	Gdnf	PROMOTER	0.431	0.510	922.07	470.15	0.22	789.18	173.57
A_68_P29996524	chr15:7765607-7765652	NM_010275:4619	Gdnf	INSIDE	0.428	0.541	1038.98	561.84	0.231	765.72	177.05
A_68_P27992419	chr11:86887561-86887605	NM_025638:57	Gdpd1	INSIDE	0.627	2.928	1012.82	2965.10	1.835	740.62	1358.83
A_68_P25361481	chr7:106530607-106530651	NM_201352:570	Gdpd5	INSIDE	0.528	0.561	1639.40	919.75	0.296	1222.98	361.97
A_68_P27930669	chr11:76030890-76030934	NM_177367:162	Gemin4	INSIDE	0.631	0.598	1083.90	648.45	0.378	962.65	363.40
A_68_P24994094	chr7:20158457-20158510	NM_027189:209	Gemin7	INSIDE	0.499	0.581	755.56	439.27	0.29	606.39	176.08
A_68_P28237416	chr12:11272565-11272609	NM_177331:7	Gen1	INSIDE	0.466	0.465	2466.98	1147.70	0.217	1801.37	390.55
A_68_P28082095	chr11:102754803-102754847	NM_010277:3690	Gfap	INSIDE	0.464	0.648	1072.49	695.48	0.301	743.69	223.94
A_68_P23968440	chr5:108154062-108154106	NM_010278:-721	Gfi1	PROMOTER	0.471	0.339	1534.89	521.01	0.16	1094.55	174.91
A_68_P23968450	chr5:108155208-108155253	NM_010278:-1867	Gfi1	PROMOTER	0.477	6.014	692.06	4161.92	2.867	516.34	1480.28
A_68_P23968426	chr5:108152356-108152400	NM_010278:985	Gfi1	INSIDE	0.639	0.178	3371.59	601.63	0.114	2289.08	261.20
A_68_P26135759	chr8:108282466-108282510	NM_027469:19	Gfod2	INSIDE	0.652	2.465	1592.62	3925.56	1.607	1141.70	1834.72
A_68_P24613524	chr6:86992684-86992728	NM_013528:-133	Gfpt1	PROMOTER	0.662	4.107	1151.38	4728.38	2.717	1014.21	2755.70
A_68_P27784883	chr11:49607746-49607790	NM_013529:112	Gfpt2	INSIDE	0.582	0.417	1646.30	686.87	0.243	1194.95	290.24
A_68_P27784886	chr11:49608055-49608100	NM_013529:421	Gfpt2	INSIDE	0.627	0.222	2188.53	486.49	0.139	1581.26	220.55
A_68_P32213514	chr19:58529502-58529546	NM_010279:-568	Gfra1	PROMOTER	0.496	0.190	2363.86	448.47	0.094	1780.92	167.50
A_68_P32213516	chr19:58529694-58529738	NM_010279:-760	Gfra1	PROMOTER	0.578	0.236	3058.91	720.64	0.136	2108.65	287.35
A_68_P29703989	chr14:71289781-71289825	NM_008115:-134	Gfra2	PROMOTER	0.318	19.731	2080.46	41049.71	6.275	1854.56	11638.09
A_68_P28154359	chr11:115465196-115465240	NM_173048:2	Gga3	INSIDE	0.521	7.300	844.03	6161.44	3.801	721.44	2741.93
A_68_P22767955	chr4:19969272-19969316	NM_010281:96	Ggh	INSIDE	0.309	7.466	667.75	4985.63	2.306	650.39	1499.54
A_68_P22767953	chr4:19968999-19969043	NM_010281:-178	Ggh	PROMOTER	0.477	0.470	1889.06	887.65	0.224	1463.79	327.91
A_68_P25025741	chr7:29956446-29956490	NM_182696:1060	Ggn	INSIDE	0.228	1.939	2967.74	5753.56	0.442	2459.62	1087.28
A_68_P25025751	chr7:29957972-29958016	NM_182696:2586	Ggn	INSIDE	0.227	1.631	2582.70	4212.72	0.37	1775.81	656.58
A_68_P25025743	chr7:29956717-29956761	NM_182696:1330	Ggn	INSIDE	0.218	3.302	4130.19	13639.94	0.72	2597.12	1870.93
A_68_P28836952	chr13:14155652-14155696	NM_010282:-6	Ggps1	DIVERGENT_PROMOTER	0.429	0.233	2713.51	631.73	0.1	1778.55	177.77
A_68_P21765995	chr2:155340656-155340700	NM_144786:-96	Ggt7	DIVERGENT_PROMOTER	0.327	11.316	768.11	8691.89	3.697	645.31	2385.79
A_68_P21765994	chr2:155340501-155340545	NM_144786:60	Ggt7	INSIDE	0.498	3.950	1611.98	6366.81	1.968	1388.55	2733.22
A_68_P29543501	chr14:37948355-37948400	NM_001199122:-52	Ghitm	PROMOTER	0.604	0.538	1256.15	675.88	0.325	1012.40	329.06
A_68_P29975444	chr15:3533365-3533409	NM_010284:-156	Ghr	PROMOTER	0.399	17.326	1091.49	18911.05	6.907	979.34	6764.21
A_68_P22010771	chr3:27271350-27271394	NM_177330:1100	Ghsr	INSIDE	0.409	0.282	3197.08	901.15	0.115	2083.48	240.35
A_68_P22010766	chr3:27270872-27270916	NM_177330:622	Ghsr	INSIDE	0.541	0.463	2069.29	957.84	0.25	1554.93	389.18
A_68_P22010767	chr3:27270950-27270994	NM_177330:700	Ghsr	INSIDE	0.577	0.299	1597.62	477.37	0.172	1050.59	181.02
A_68_P24126098	chr5:137952294-137952338	NM_031408:-7791	Gigyf1	DIVERGENT_PROMOTER	0.568	3.085	3504.39	10812.43	1.751	2773.02	4855.83
A_68_P20417068	chr1:89223852-89223896	NM_001110212:271	Gigyf2	INSIDE	0.611	7.190	1615.62	11617.02	4.396	1409.91	6197.48
A_68_P20417070	chr1:89224090-89224134	NM_001110212:509	Gigyf2	INSIDE	0.625	4.583	920.16	4217.36	2.864	794.55	2275.68
A_68_P26221254	chr8:123113358-123113402	NM_178856:-405	Gins2	PROMOTER	0.382	21.160	4111.26	86994.01	8.073	3539.38	28575.03
A_68_P26221250	chr8:123112916-123112960	NM_178856:37	Gins2	INSIDE	0.474	3.286	2756.27	9056.83	1.558	2285.32	3560.88
A_68_P26084323	chr8:98157470-98157514	NM_030198:34	Gins3	INSIDE	0.499	0.294	1992.07	586.42	0.147	1539.97	226.43
A_68_P27288983	chr10:80800571-80800615	NM_148951:5419	Gipc3	INSIDE	0.347	9.819	2168.99	21297.23	3.408	1888.36	6434.75

ProbeName	Target position of probe on CpG island microarray	TargetID	GeneSymbol	CpG island Description	Ratio of relative methylation (TiO_2-NP/Vehicle)	Sham group			TiO_2-H group		
						Relative methylation (Cy5/Cy3)	Cy3 signal (Input)	Cy5 signal (MeDIP)	Relative methylation (Cy5/Cy3)	Cy3 signal (Input)	Cy5 signal (MeDIP)
A_68_P27289008	chr10:80806150-80806194	NM_148951:-161	Gipc3	PROMOTER	0.252	2.574	5183.04	13339.99	0.648	3959.80	2567.02
A_68_P27937920	chr11:77307416-77307460	NM_001004144:525	Git1	INSIDE	0.548	3.232	3497.56	11302.98	1.772	2764.12	4898.37
A_68_P29625743	chr14:57655656-57655700	NM_016975:21104	Gja3	INSIDE	0.599	3.763	1509.15	5678.80	2.256	1052.85	2375.03
A_68_P32563826	chrX:98580104-98580148	NM_008124:7451	Gjb1	INSIDE	0.38	9.532	430.33	4101.74	3.623	607.47	2201.16
A_68_P29626122	chr14:57723066-57723110	NM_008125:451	Gjb2	INSIDE	0.526	0.593	2017.74	1197.51	0.312	1571.07	490.77
A_68_P28059762	chr11:98843848-98843892	NM_178596:460	Gjd3	INSIDE	0.658	5.536	596.58	3302.56	3.642	553.31	2015.35
A_68_P29072412	chr13:58376159-58376204	NM_019832:-632	Gkap1	PROMOTER	0.127	2.655	1362.62	3617.64	0.337	970.98	326.88
A_68_P26844443	chr9:114310274-114310318	NM_009752:60	Glb1	INSIDE	0.492	0.266	4618.31	1227.16	0.131	3057.03	399.89
A_68_P24226577	chr6:8460033-8460077	NM_133236:459	Glcci1	INSIDE	0.583	4.831	1617.13	7812.71	2.817	1349.88	3802.65
A_68_P24226570	chr6:8459130-8459177	NM_133236:-442	Glcci1	PROMOTER	0.488	0.560	881.44	493.78	0.273	798.76	218.36
A_68_P26167381	chr8:113782707-113782751	NM_009149:374	Glg1	INSIDE	0.599	0.406	1237.06	502.04	0.243	1150.52	279.70
A_68_P26167383	chr8:113783057-113783101	NM_009149:24	Glg1	INSIDE	0.661	13.766	2316.15	31884.71	9.096	2359.23	21458.40
A_68_P23190563	chr4:107107680-107107724	NM_147221:379	Glis1	INSIDE	0.477	3.690	1564.37	5772.69	1.76	1365.98	2404.64
A_68_P23190564	chr4:107107777-107107821	NM_147221:475	Glis1	INSIDE	0.617	0.601	771.05	463.15	0.371	690.12	255.97
A_68_P32048649	chr19:28752873-28752917	NM_175459:1673	Glis3	INSIDE	0.461	0.396	2310.71	915.11	0.183	1807.64	330.17
A_68_P31136610	chr17:31038660-31038704	NM_021332:-129	Glp1r	PROMOTER	0.512	7.204	1567.97	11295.01	3.688	1380.01	5089.48
A_68_P22271218	chr3:80717646-80717690	NM_010298:-122	Glrb	PROMOTER	0.411	8.709	3164.68	27560.49	3.582	2354.69	8433.32
A_68_P20226307	chr1:52289184-52289228	NM_001081081:870	Gls	INSIDE	0.638	3.774	1498.09	5653.57	2.408	1254.88	3021.53
A_68_P20226306	chr1:52289040-52289084	NM_001081081:1014	Gls	INSIDE	0.503	0.552	1003.87	554.19	0.278	800.54	222.38
A_68_P27540020	chr10:127631671-127631715	NM_001033264:2	Gls2	INSIDE	0.494	4.376	1413.43	6184.64	2.162	1217.09	2630.92
A_68_P27540023	chr10:127631990-127632034	NM_001033264:322	Gls2	INSIDE	0.512	0.690	2514.21	1735.23	0.353	1986.39	701.87
A_68_P25957809	chr8:74135189-74135233	NM_146211:288	Glt25d1	INSIDE	0.561	4.594	1144.94	5259.29	2.578	916.92	2364.07
A_68_P25957808	chr8:74135004-74135048	NM_146211:104	Glt25d1	INSIDE	0.617	4.619	1507.38	6961.87	2.85	1270.62	3621.13
A_68_P20720545	chr1:154247264-154247308	NM_177756:290	Glt25d2	INSIDE	0.245	1.681	945.35	1589.56	0.411	823.88	338.73
A_68_P22299774	chr3:85691661-85691705	NM_177130:-242	Glt28d2	PROMOTER	0.142	36.209	1929.94	69880.48	5.145	1886.60	9706.08
A_68_P29529251	chr14:35124024-35124068	NM_008133:134	Glud1	INSIDE	0.227	10.189	2528.48	25763.31	2.309	2187.17	5049.12
A_68_P29529256	chr14:35124536-35124580	NM_008133:646	Glud1	INSIDE	0.38	4.774	2822.10	13473.25	1.816	2448.32	4446.40
A_68_P30347362	chr15:76125061-76125105	NM_001195537:219	Gm10345	INSIDE	0.51	0.715	1438.60	1028.17	0.365	1251.41	456.52
A_68_P30347360	chr15:76124857-76124901	NM_001195537:15	Gm10345	INSIDE	0.643	0.626	1141.46	715.00	0.403	933.41	375.72
A_68_P20941836	chr1:193960762-193960806	NR_033536:5	Gm10516	INSIDE	0.165	10.464	1965.20	20564.72	1.727	1614.02	2787.08
A_68_P25533783	chr7:139508008-139508052	NR_028578:684	Gm10584	INSIDE	0.178	13.059	1087.11	14196.28	2.331	787.91	1836.38
A_68_P27267011	chr10:76721843-76721887	NR_026944:104	Gm10941	INSIDE	0.564	0.618	2224.01	1375.12	0.349	1649.58	575.10
A_68_P22737302	chr4:12833974-12834018	NM_001171801:13	Gm11818	INSIDE	0.382	12.115	1598.48	19364.76	4.628	1648.33	7629.20
A_68_P22737304	chr4:12834151-12834195	NM_001171801:189	Gm11818	INSIDE	0.45	4.623	645.52	2984.57	2.083	624.18	1300.08
A_68_P28058704	chr11:98671394-98671438	NR_033551:-13	Gm12359	PROMOTER	0.468	8.199	8459.75	69358.97	3.836	6892.18	26436.53
A_68_P28058705	chr11:98671479-98671523	NR_033551:-97	Gm12359	PROMOTER	0.609	0.501	911.00	456.68	0.305	651.64	199.05
A_68_P23219033	chr4:114079567-114079611	NM_001085549:260	Gm12824	INSIDE	0.517	0.641	3355.45	2151.91	0.332	2660.09	881.91
A_68_P20122141	chr1:31006006-31006050	NR_002688:527	Gm13363	INSIDE	0.499	6.899	1128.03	7781.86	3.445	928.23	3197.50
A_68_P21565618	chr2:118529436-118529480	NM_001081971:241	Gm1337	INSIDE	0.27	0.493	1749.84	862.56	0.133	1312.43	174.78
A_68_P21565620	chr2:118529638-118529682	NM_001081971:39	Gm1337	INSIDE	0.433	7.013	903.27	6334.40	3.04	811.92	2468.17
A_68_P21565608	chr2:118528286-118528330	NM_001081971:1391	Gm1337	INSIDE	0.579	0.358	1867.84	669.00	0.207	1376.07	285.47
A_68_P21052792	chr2:20891136-20891180	NR_033225:658	Gm13375	INSIDE	0.537	6.748	1780.96	12018.05	3.627	1518.60	5507.25
A_68_P21052788	chr2:20890736-20890780	NR_033225:258	Gm13375	INSIDE	0.487	3.404	4511.88	15359.15	1.658	3374.33	5596.32
A_68_P21099289	chr2:29607138-29607182	NM_001177392:-9887	Gm13547	PROMOTER	0.437	0.404	1351.52	545.48	0.176	986.55	174.08
A_68_P21438071	chr2:93797315-93797359	NM_001145034:-79	Gm13889	PROMOTER	0.226	9.529	3199.60	30488.02	2.155	2520.05	5431.02
A_68_P21438069	chr2:93797146-93797190	NM_001145034:89	Gm13889	INSIDE	0.372	6.775	2127.62	14414.08	2.522	1881.80	4745.47

ProbeName	Target position of probe on CpG island microarray	TargetID	GeneSymbol	CpG island Description	Ratio of relative methylation (TiO_2-NP/Vehicle)	Sham group			TiO_2-H group		
						Relative methylation (Cy5/Cy3)	Cy3 signal (Input)	Cy5 signal (MeDIP)	Relative methylation (Cy5/Cy3)	Cy3 signal (Input)	Cy5 signal (MeDIP)
A_68_P25616822	chr8:4249449-4249493	NM_001195258:1257	Gm14378	INSIDE	0.434	0.337	3447.36	1162.98	0.147	2555.09	374.40
A_68_P29575892	chr14:47000105-47000149	NR_037981:928	Gm15217	INSIDE	0.489	5.975	2935.49	17539.11	2.921	2243.04	6551.66
A_68_P28080758	chr11:102526866-102526910	NM_001127576:-13	Gm1564	PROMOTER	0.325	7.711	2154.14	16611.32	2.507	1806.19	4528.10
A_68_P28080759	chr11:102527010-102527054	NM_001127576:131	Gm1564	INSIDE	0.421	4.304	1459.72	6283.06	1.814	1326.03	2405.59
A_68_P28080761	chr11:102527193-102527237	NM_001127576:313	Gm1564	INSIDE	0.308	5.381	788.36	4241.96	1.657	675.67	1119.50
A_68_P28577474	chr12:81859527-81859571	NM_001008423:2154	Gm1568	INSIDE	0.55	5.464	1065.24	5820.79	3.004	907.72	2726.34
A_68_P28577469	chr12:81858735-81858779	NM_001008423:2946	Gm1568	INSIDE	0.663	5.062	1589.35	8045.37	3.355	1239.57	4159.16
A_68_P24225087	chr6:8209485-8209529	NR_033518:219	Gm16039	INSIDE	0.544	0.251	2446.14	613.16	0.136	1860.92	253.74
A_68_P27845567	chr11:60745252-60745296	NR_027800:95	Gm16516	INSIDE	0.281	12.309	950.20	11696.09	3.461	742.58	2569.84
A_68_P23591967	chr5:34326256-34326300	NM_001033458:156	Gm1673	INSIDE	0.647	0.222	4414.50	981.23	0.144	3189.89	458.66
A_68_P23591963	chr5:34325816-34325860	NM_001033458:-284	Gm1673	PROMOTER	0.616	0.721	1572.43	1133.96	0.444	1049.30	466.00
A_68_P26156365	chr8:111864724-111864768	NR_002928:62	Gm1943	INSIDE	0.321	6.015	1845.53	11099.98	1.933	1391.34	2689.21
A_68_P26156364	chr8:111864649-111864693	NR_002928:138	Gm1943	INSIDE	0.328	6.192	1198.30	7419.66	2.03	826.37	1677.93
A_68_P28744405	chr12:112724265-112724309	NM_001033248:-252	Gm266	PROMOTER	0.47	0.554	1026.79	568.84	0.26	819.16	213.36
A_68_P27813685	chr11:54911498-54911542	NM_010299:34	Gm2a	INSIDE	0.483	7.685	2286.38	17570.58	3.709	2024.86	7510.07
A_68_P27813684	chr11:54911411-54911457	NM_010299:-52	Gm2a	PROMOTER	0.653	2.363	1578.93	3730.86	1.544	1369.70	2114.98
A_68_P21046072	chr2:19579131-19579175	NR_033642:372	Gm3230	INSIDE	0.606	0.645	2066.54	1333.14	0.391	1660.51	649.10
A_68_P25766300	chr8:33994475-33994519	NM_001177589:66002	Gm3985	DOWNSTREAM	0.219	12.170	767.79	9343.79	2.669	665.15	1775.25
A_68_P25766302	chr8:33994797-33994841	NM_001177589:65680	Gm3985	DOWNSTREAM	0.36	26.085	581.32	15163.82	9.392	652.44	6127.58
A_68_P25766304	chr8:33995018-33995062	NM_001177589:65458	Gm3985	DOWNSTREAM	0.609	0.476	2627.00	1250.26	0.29	1877.13	544.18
A_68_P25362934	chr7:106774303-106774347	NM_001195529:1289	Gm4980	INSIDE	0.508	0.513	1501.71	770.63	0.261	1134.78	296.10
A_68_P26248333	chr8:127422026-127422070	NM_001111141:263	Gm505	INSIDE	0.567	3.208	2296.87	7368.00	1.82	1889.63	3438.54
A_68_P20875118	chr1:182262286-182262330	NR_003623:-1628	Gm5069	PROMOTER	0.577	4.951	2315.80	11465.41	2.858	1725.01	4930.61
A_68_P32571132	chrX:100129205-100129249	NR_026596:336	Gm5126	INSIDE	0.604	5.673	1209.63	6862.38	3.425	1425.38	4882.61
A_68_P26587299	chr9:65219825-65219869	NM_001111145:10980	Gm514	INSIDE	0.303	12.901	3786.15	48846.87	3.913	3639.38	14242.37
A_68_P28492940	chr12:66018887-66018931	NM_001025605:11	Gm527	INSIDE	0.282	5.863	1355.77	7948.81	1.651	1174.89	1940.03
A_68_P24131920	chr5:139296987-139297031	NM_001195128:975	Gm5294	INSIDE	0.613	0.494	2073.89	1024.82	0.303	1495.15	453.08
A_68_P29751456	chr14:79851782-79851826	NM_001034882:163	Gm5465	INSIDE	0.28	6.546	3778.59	24735.20	1.831	2911.44	5330.77
A_68_P26912794	chr10:5914339-5914383	NR_002891:-3014	Gm5512	PROMOTER	0.503	0.608	1349.11	819.68	0.306	1077.70	329.38
A_68_P25661493	chr8:12431257-12431301	NR_027975:45508	Gm5607	INSIDE	0.265	15.231	12003.20	182815.50	4.036	12247.10	49429.15
A_68_P25661164	chr8:12385360-12385404	NR_027975:-388	Gm5607	PROMOTER	0.386	4.110	1230.54	5057.56	1.586	893.83	1418.03
A_68_P23792661	chr5:72978442-72978486	NM_001024147:325	Gm5868	INSIDE	0.229	1.847	944.96	1745.46	0.423	811.47	343.09
A_68_P30720009	chr16:44173533-44173577	NM_001029889:45	Gm608	INSIDE	0.542	5.801	1169.51	6784.49	3.142	1048.72	3295.30
A_68_P21932364	chr3:8463041-8463085	NR_033512:36	Gm6194	INSIDE	0.195	25.196	5365.55	135188.40	4.913	6241.04	30661.47
A_68_P21932363	chr3:8462950-8462994	NR_033512:126	Gm6194	INSIDE	0.338	14.466	3042.24	44010.47	4.889	2523.39	12336.00
A_68_P31458147	chr18:3336797-3336841	NR_033457:405	Gm6225	INSIDE	0.509	0.560	780.75	437.06	0.285	606.60	172.88
A_68_P29338084	chr13:113784193-113784237	NM_001037914:139	Gm6320	INSIDE	0.554	0.401	1728.83	693.17	0.222	1433.39	318.36
A_68_P29338086	chr13:113784385-113784429	NM_001037914:331	Gm6320	INSIDE	0.247	1.460	2515.61	3672.83	0.36	2066.65	745.01
A_68_P21345225	chr2:75497497-75497541	NR_033513:172	Gm6793	INSIDE	0.377	5.666	2543.00	14409.88	2.138	1994.29	4264.24
A_68_P28159316	chr11:116295859-116295905	NR_003376:356	Gm7367	INSIDE	0.257	7.650	651.77	4986.28	1.967	484.36	952.87
A_68_P31162860	chr17:36305369-36305413	NM_001081032:92	Gm8909	INSIDE	0.183	13.072	4693.47	61351.91	2.39	4598.19	10990.42
A_68_P26886028	chr9:121766761-121766805	NM_001112668:-133	Gm9790	PROMOTER	0.343	17.016	4265.60	72584.03	5.842	3511.22	20512.38
A_68_P26886029	chr9:121766854-121766898	NM_001112668:-227	Gm9790	PROMOTER	0.556	3.778	1727.10	6524.21	2.1	1453.08	3051.48
A_68_P31949295	chr19:10315203-10315247	NM_001033481:14	Gm98	INSIDE	0.469	4.122	3109.08	12814.66	1.934	2721.18	5263.67
A_68_P22323963	chr3:90017329-90017373	NM_001190258:219	Gm9846	INSIDE	0.532	0.603	1624.59	979.47	0.321	1221.38	392.11
A_68_P27292918	chr10:82092906-82092950	NR_037190:342	Gm9855	INSIDE	0.447	3.555	1836.65	6530.12	1.59	1532.72	2436.96

ProbeName	Target position of probe on CpG island microarray	TargetID	GeneSymbol	CpG island Description	Ratio of relative methylation (TiO_2-NP/Vehicle)	Sham group			TiO_2-H group		
						Relative methylation (Cy5/Cy3)	Cy3 signal (Input)	Cy5 signal (MeDIP)	Relative methylation (Cy5/Cy3)	Cy3 signal (Input)	Cy5 signal (MeDIP)
A_68_P21073104	chr2:25434737-25434781	NM_001005424:861	Gm996	INSIDE	0.663	0.568	3422.45	1944.41	0.377	2558.14	964.01
A_68_P25150562	chr7:64642665-64642709	NR_033504:45	Gm9962	INSIDE	0.619	0.147	3091.74	453.42	0.091	2218.58	201.28
A_68_P24611767	chr6:86683275-86683319	NM_011818:76	Gmcl1	INSIDE	0.276	18.118	5463.07	98982.12	4.994	4789.71	23917.66
A_68_P28928667	chr13:32430111-32430155	NM_146041:281	Gmds	INSIDE	0.076	40.359	1329.57	53659.65	3.056	1445.44	4417.57
A_68_P21909702	chr2:181022305-181022349	NM_198169:345	Gmeb2	INSIDE	0.531	2.823	1962.74	5540.02	1.5	1588.92	2383.40
A_68_P29578547	chr14:47441814-47441858	NM_022023:81	Gmfb	INSIDE	0.58	0.626	787.09	492.84	0.363	662.26	240.56
A_68_P28888886	chr13:24853737-24853781	NM_020567:48	Gmnn	INSIDE	0.53	0.736	4031.54	2968.66	0.39	3079.81	1202.00
A_68_P22194401	chr3:63780425-63780469	NM_001033300:382	Gmps	INSIDE	0.394	0.577	1013.24	585.03	0.228	847.48	192.88
A_68_P24143542	chr5:141305948-141305992	NM_010302:415	Gna12	INSIDE	0.437	0.409	1381.25	565.33	0.179	1133.90	202.97
A_68_P27290145	chr10:80965609-80965653	NM_010304:21340	Gna15	INSIDE	0.367	0.593	948.70	562.68	0.217	785.43	170.77
A_68_P23510646	chr5:17866146-17866190	NM_010305:63	Gnai1	INSIDE	0.542	5.226	1238.02	6470.42	2.834	1063.10	3013.19
A_68_P26810120	chr9:107537483-107537527	NM_008138:169	Gnai2	INSIDE	0.59	0.639	1915.96	1224.34	0.377	1384.35	522.29
A_68_P31790105	chr18:67293085-67293129	NM_010307:-373	Gnal	PROMOTER	0.654	2.449	897.11	2197.25	1.602	834.89	1337.47
A_68_P26073617	chr8:96334994-96335038	NM_001113384:279	Gnao1	INSIDE	0.285	18.185	8727.79	158714.60	5.184	7633.60	39571.44
A_68_P26073619	chr8:96335224-96335268	NM_001113384:509	Gnao1	INSIDE	0.347	0.493	4341.46	2138.19	0.171	2969.92	507.27
A_68_P26073620	chr8:96335296-96335343	NM_001113384:582	Gnao1	INSIDE	0.564	0.483	4629.65	2235.91	0.273	3034.71	827.27
A_68_P21874744	chr2:174153010-174153054	NR_003258:-382	Gnas	PROMOTER	0.26	2.241	5066.86	11357.07	0.582	3426.22	1993.33
A_68_P21874393	chr2:174110277-174110321	NM_019690:478	Gnas	INSIDE	0.618	3.956	2342.57	9267.78	2.444	1863.83	4555.98
A_68_P21874764	chr2:174156135-174156179	NM_001077510:567	Gnas	INSIDE	0.623	0.289	3251.12	940.13	0.18	2377.04	428.49
A_68_P21874768	chr2:174156475-174156519	NM_001077510:907	Gnas	INSIDE	0.194	1.462	5532.60	8089.57	0.283	4201.30	1190.70
A_68_P21874504	chr2:174124338-174124382	NM_201617:1001	Gnas	INSIDE	0.65	2.705	1746.33	4724.44	1.759	1477.74	2598.71
A_68_P26810382	chr9:107578840-107578884	NM_008140:3061	Gnat1	INSIDE	0.451	0.224	2309.00	516.24	0.101	1730.08	174.28
A_68_P26810381	chr9:107578748-107578792	NM_008140:3153	Gnat1	INSIDE	0.509	0.338	3276.25	1108.89	0.172	2379.31	409.51
A_68_P26810380	chr9:107578632-107578676	NM_008140:3269	Gnat1	INSIDE	0.51	0.146	3614.16	528.66	0.075	2343.75	174.69
A_68_P23438941	chr4:154865679-154865723	NM_001160016:231	Gnb1	INSIDE	0.471	6.123	2474.40	15150.75	2.882	2376.96	6850.77
A_68_P23438946	chr4:154866208-154866252	NM_001160016:761	Gnb1	INSIDE	0.586	0.344	2041.75	702.08	0.201	1627.62	327.90
A_68_P30580075	chr16:18498740-18498784	NM_001081682:-98	Gnb1l	PROMOTER	0.233	11.286	3899.87	44012.36	2.628	3212.14	8442.37
A_68_P30580079	chr16:18499124-18499168	NM_023120:183	Gnb1l	INSIDE	0.514	4.274	1920.77	8208.65	2.198	1614.58	3548.22
A_68_P24126253	chr5:137973169-137973213	NM_010312:1267	Gnb2	INSIDE	0.396	0.197	3284.21	647.78	0.078	2461.82	192.41
A_68_P24126241	chr5:137971926-137971970	NM_010312:2509	Gnb2	INSIDE	0.611	0.548	2088.66	1144.83	0.335	1629.94	545.61
A_68_P22039054	chr3:32515641-32515685	NM_013531:-205	Gnb4	PROMOTER	0.184	1.430	6565.63	9387.31	0.263	4896.37	1286.64
A_68_P22875167	chr4:44085230-44085274	NM_001190414:293	Gne	INSIDE	0.507	5.053	1838.45	9289.36	2.56	1573.62	4028.53
A_68_P22954219	chr4:59048073-59048117	NM_025277:67	Gng10	INSIDE	0.308	8.890	1152.93	10249.48	2.741	1356.65	3718.53
A_68_P22954220	chr4:59048156-59048200	NM_025277:151	Gng10	INSIDE	0.492	8.377	994.41	8329.70	4.12	954.08	3930.83
A_68_P24518615	chr6:66846390-66846434	NM_001177558:-472	Gng12	PROMOTER	0.57	4.627	2125.04	9832.26	2.636	1848.22	4871.51
A_68_P28835611	chr13:13876643-13876687	NM_010317:-141	Gng4	PROMOTER	0.307	0.401	1526.46	611.85	0.123	1405.03	172.94
A_68_P27286894	chr10:80477702-80477746	NM_010319:-54	Gng7	PROMOTER	0.507	0.388	1199.14	465.42	0.197	887.34	174.78
A_68_P27286725	chr10:80448582-80448626	NM_001038655:15506	Gng7	INSIDE	0.612	0.537	2061.52	1107.00	0.329	1708.56	561.29
A_68_P27286724	chr10:80448504-80448548	NM_001038655:15584	Gng7	INSIDE	0.178	9.096	5126.54	46628.50	1.619	4826.00	7812.57
A_68_P24983830	chr7:17480526-17480570	NM_010320:3414	Gng8	INSIDE	0.552	0.219	2558.44	560.18	0.121	1994.66	240.89
A_68_P31162043	chr17:36117444-36117488	NM_008136:567	Gnl1	INSIDE	0.287	0.357	2156.93	770.19	0.102	1689.83	172.90
A_68_P32743829	chrX:147451959-147452003	NM_198110:-115	Gnl3l	PROMOTER	0.544	3.121	1267.59	3956.52	1.699	1413.60	2401.16
A_68_P23778718	chr5:69983636-69983680	NM_001038015:-134	Gnpda2	PROMOTER	0.405	0.560	1096.88	614.12	0.227	972.73	220.33
A_68_P27503065	chr10:120802501-120802545	NM_029364:377	Gns	INSIDE	0.63	3.461	1122.84	3885.78	2.179	838.06	1826.14
A_68_P23978151	chr5:110605818-110605862	NM_008146:121	Golga3	INSIDE	0.509	0.426	1742.61	742.32	0.217	1273.89	275.98
A_68_P30021002	chr15:12251634-12251678	NM_025673:406	Golph3	INSIDE	0.664	0.549	1634.20	896.50	0.364	1226.02	446.60

ProbeName	Target position of probe on CpG island microarray	TargetID	GeneSymbol	CpG island Description	Ratio of relative methylation (TiO_2-NP/Vehicle)	Sham group			TiO_2-H group		
						Relative methylation (Cy5/Cy3)	Cy3 signal (Input)	Cy5 signal (MeDIP)	Relative methylation (Cy5/Cy3)	Cy3 signal (Input)	Cy5 signal (MeDIP)
A_68_P24904258	chr6:142336086-142336130	NM_025872:346	Golt1b	INSIDE	0.455	3.912	939.07	3673.34	1.782	833.84	1485.59
A_68_P26875190	chr9:119846515-119846559	NM_028976:140	Gorasp1	INSIDE	0.605	0.206	4076.33	841.76	0.125	2840.59	354.61
A_68_P21315049	chr2:70499932-70499976	NM_027352:389	Gorasp2	INSIDE	0.39	7.711	560.01	4318.25	3.007	551.75	1659.05
A_68_P30641169	chr16:30309242-30309286	NM_008148:1601	Gp5	INSIDE	0.627	0.718	1508.68	1083.39	0.45	1373.54	617.99
A_68_P30347594	chr15:76161371-76161427	NM_010331:-325	Gpaa1	PROMOTER	0.496	0.413	2862.57	1182.26	0.205	1896.42	388.53
A_68_P32191983	chr19:55174072-55174116	NM_008149:-157	Gpam	PROMOTER	0.512	0.317	1548.67	490.50	0.162	1244.54	201.69
A_68_P20911845	chr1:189039493-189039537	NM_026367:125	Gpatch2	INSIDE	0.435	0.524	903.89	473.25	0.228	761.05	173.31
A_68_P23318268	chr4:133130953-133130997	NM_172876:315	Gpatch3	INSIDE	0.375	6.933	1372.63	9516.87	2.598	1068.95	2777.19
A_68_P28080239	chr11:102417477-102417521	NM_001159492:-26	Gpatch8	PROMOTER	0.247	22.248	6350.92	141292.60	5.489	5300.60	29093.34
A_68_P29329250	chr13:112279384-112279428	NM_001122963:843	Gpbp1	INSIDE	0.487	37.059	9632.40	356965.70	18.055	9553.14	172478.40
A_68_P29329254	chr13:112280052-112280096	NM_001122963:175	Gpbp1	INSIDE	0.63	0.613	6139.64	3761.00	0.386	3820.80	1473.56
A_68_P20451406	chr1:94729272-94729316	NM_016696:1032	Gpc1	INSIDE	0.606	9.419	2171.26	20451.62	5.713	1686.34	9633.36
A_68_P20451396	chr1:94727977-94728021	NM_016696:-264	Gpc1	PROMOTER	0.62	0.438	3142.99	1376.76	0.272	2569.68	698.21
A_68_P24129654	chr5:138721309-138721353	NM_172412:-165	Gpc2	DIVERGENT_PROMOTER	0.418	1.435	3665.23	5258.97	0.599	2747.01	1646.63
A_68_P32383369	chrX:49967083-49967127	NM_016697:47	Gpc3	INSIDE	0.412	7.590	3210.43	24367.94	3.124	3727.30	11642.36
A_68_P32383368	chrX:49966913-49966957	NM_016697:217	Gpc3	INSIDE	0.571	4.514	1366.81	6169.90	2.579	1517.99	3915.36
A_68_P32383370	chrX:49967260-49967304	NM_016697:-131	Gpc3	PROMOTER	0.654	3.655	6131.96	22412.89	2.391	7696.73	18406.15
A_68_P32380519	chrX:49518713-49518757	NM_008150:-634	Gpc4	PROMOTER	0.334	10.272	1650.01	16949.59	3.431	1706.87	5855.84
A_68_P32380511	chrX:49517673-49517717	NM_008150:406	Gpc4	INSIDE	0.467	4.847	1072.93	5200.91	2.266	1413.93	3204.08
A_68_P29928902	chr14:117325279-117325323	NM_001079844:764	Gpc6	INSIDE	0.645	0.631	792.47	500.33	0.407	613.24	249.71
A_68_P26847149	chr9:114843024-114843068	NM_175380:59	Gpd1l	INSIDE	0.353	11.103	1866.68	20725.45	3.916	1836.68	7192.17
A_68_P26847147	chr9:114842779-114842823	NM_175380:305	Gpd1l	INSIDE	0.237	2.811	3387.17	9522.58	0.667	2505.57	1672.17
A_68_P32805174	chrX:162677268-162677312	NM_001177961:416	Gpm6b	INSIDE	0.388	5.114	895.95	4582.29	1.985	1307.68	2595.77
A_68_P23578383	chr5:31797238-31797282	NM_133756:127	Gpn1	INSIDE	0.592	0.341	1345.08	458.58	0.202	985.84	198.83
A_68_P23318324	chr4:133140609-133140653	NM_133884:343	Gpn2	INSIDE	0.313	0.642	1632.91	1049.04	0.201	1231.47	247.76
A_68_P32401470	chrX:54756780-54756824	NM_001033360:132	Gpr101	INSIDE	0.492	5.001	581.05	2905.56	2.462	792.41	1950.54
A_68_P32401468	chrX:54756573-54756617	NM_001033360:340	Gpr101	INSIDE	0.626	2.434	745.16	1813.82	1.525	990.99	1510.80
A_68_P21107276	chr2:31008080-31008124	NM_178760:267	Gpr107	INSIDE	0.319	0.529	1269.28	671.92	0.169	1043.37	176.24
A_68_P25580590	chr7:147020054-147020098	NM_177469:4	Gpr123	INSIDE	0.627	0.705	1574.67	1110.08	0.442	1319.83	583.50
A_68_P25736450	chr8:28196606-28196651	NM_054044:316	Gpr124	INSIDE	0.344	29.149	2976.49	86760.58	10.021	2502.82	25081.23
A_68_P25736445	chr8:28195931-28195975	NM_054044:-360	Gpr124	PROMOTER	0.25	0.609	1432.91	872.01	0.152	1166.24	177.36
A_68_P23681262	chr5:50450445-50450489	NM_133911:-231	Gpr125	PROMOTER	0.348	21.765	1503.93	32733.35	7.58	1351.39	10243.36
A_68_P23681257	chr5:50449672-50449716	NM_133911:541	Gpr125	INSIDE	0.505	5.712	6712.57	38339.98	2.883	4960.44	14298.62
A_68_P23681258	chr5:50449746-50449790	NM_133911:467	Gpr125	INSIDE	0.572	5.187	4401.94	22832.67	2.969	3267.83	9702.39
A_68_P28530000	chr12:73171697-73171741	NM_181752:260	Gpr135	INSIDE	0.401	0.361	2637.92	953.39	0.145	1783.79	258.27
A_68_P28833516	chr13:13485759-13485803	NM_031999:111	Gpr137b	INSIDE	0.365	0.466	2075.81	967.98	0.17	1803.12	306.95
A_68_P29569514	chr14:45839639-45839683	NM_027518:269	Gpr137c	INSIDE	0.184	17.341	1178.45	20435.32	3.192	1129.27	3604.69
A_68_P23417714	chr4:151657357-151657401	NM_178406:8908	Gpr153	INSIDE	0.496	8.413	952.51	8013.73	4.17	782.50	3263.26
A_68_P23417713	chr4:151657232-151657276	NM_178406:8784	Gpr153	INSIDE	0.57	2.979	1127.10	3357.65	1.698	933.35	1584.38
A_68_P21331224	chr2:73224675-73224719	NM_001190297:-241	Gpr155	PROMOTER	0.285	1.874	7415.80	13896.36	0.534	5216.30	2784.54
A_68_P21331223	chr2:73224563-73224607	NM_001190297:-129	Gpr155	PROMOTER	0.523	0.247	1855.13	458.40	0.129	1329.39	171.70
A_68_P23404232	chr4:149461503-149461547	NM_177366:-87	Gpr157	PROMOTER	0.449	0.405	10540.94	4271.23	0.182	7641.42	1390.47
A_68_P23404231	chr4:149461398-149461442	NM_177366:-191	Gpr157	PROMOTER	0.606	0.370	13075.60	4833.85	0.224	8936.39	2002.49
A_68_P23404235	chr4:149461799-149461843	NM_177366:209	Gpr157	INSIDE	0.546	0.705	3750.36	2642.87	0.384	3163.27	1216.07
A_68_P23404325	chr4:149475753-149475801	NM_177366:14165	Gpr157	INSIDE	0.543	3.998	617.34	2467.94	2.172	450.47	978.55
A_68_P24817421	chr6:124811227-124811271	NM_013533:2687	Gpr162	INSIDE	0.6	5.518	1212.96	6693.58	3.312	920.01	3047.40

ProbeName	Target position of probe on CpG island microarray	TargetID	GeneSymbol	CpG island Description	Ratio of relative methylation (TiO_2-NP/Vehicle)	Sham group			TiO_2-H group		
						Relative methylation (Cy5/Cy3)	Cy3 signal (Input)	Cy5 signal (MeDIP)	Relative methylation (Cy5/Cy3)	Cy3 signal (Input)	Cy5 signal (MeDIP)
A_68_P30349002	chr15:76369497-76369541	NM_029643:146	Gpr172b	INSIDE	0.361	0.557	1571.86	875.06	0.201	1146.53	230.36
A_68_P30349001	chr15:76369402-76369446	NM_029643:52	Gpr172b	INSIDE	0.232	2.587	6777.90	17533.63	0.601	4155.94	2498.94
A_68_P29936846	chr14:118536719-118536763	NM_021434:392	Gpr180	INSIDE	0.658	0.310	5106.93	1584.75	0.204	3041.77	620.89
A_68_P29936842	chr14:118536259-118536304	NM_021434:-67	Gpr180	DIVERGENT_PROMOTER	0.638	0.689	2485.09	1713.16	0.44	2027.47	891.64
A_68_P20644872	chr1:138156299-138156343	NM_001101516:1130	Gpr25	INSIDE	0.38	0.469	1320.82	618.87	0.178	979.16	174.42
A_68_P23316365	chr4:132768551-132768595	NM_008154:-121	Gpr3	DIVERGENT_PROMOTER	0.278	13.679	3123.72	42727.95	3.804	2779.06	10570.30
A_68_P31953772	chr19:11015670-11015714	NM_009962:4043	Gpr44	INSIDE	0.582	0.732	2229.65	1632.57	0.426	1798.69	766.89
A_68_P20186163	chr1:43010333-43010377	NM_053107:638	Gpr45	INSIDE	0.218	0.218	9723.69	2115.25	0.048	5955.94	283.05
A_68_P32454734	chrX:68917679-68917723	NM_010340:759	Gpr50	INSIDE	0.155	20.170	1269.34	25602.54	3.134	1531.22	4799.51
A_68_P27087305	chr10:40792189-40792233	NM_199058:-820	Gpr6	PROMOTER	0.557	6.011	1710.35	10281.43	3.35	1391.10	4660.35
A_68_P26803570	chr9:106367374-106367418	NM_001159652:875	Gpr62	INSIDE	0.497	10.172	903.30	9188.31	5.053	722.25	3649.19
A_68_P28684687	chr12:102146364-102146408	NM_001177674:-8652	Gpr68	PROMOTER	0.486	7.314	1923.33	14066.79	3.555	1773.10	6302.57
A_68_P24249109	chr6:13788674-13788718	NM_145066:1152	Gpr85	INSIDE	0.632	3.770	946.95	3569.63	2.384	763.62	1820.23
A_68_P22448215	chr3:115955466-115955511	NM_022427:914	Gpr88	INSIDE	0.395	6.076	5680.41	34514.62	2.401	4720.45	11332.92
A_68_P22351745	chr3:96708815-96708859	NM_026229:385	Gpr89	INSIDE	0.346	1.502	2209.37	3319.36	0.52	1866.08	970.31
A_68_P32678434	chrX:132373604-132373648	NM_001163015:54	Gprasp2	INSIDE	0.543	3.276	755.78	2475.96	1.779	913.54	1624.99
A_68_P24864944	chr6:135015833-135015878	NM_181444:176	Gprc5a	INSIDE	0.438	18.625	1860.40	34649.78	8.156	1460.73	11913.48
A_68_P24864942	chr6:135015624-135015668	NM_181444:-33	Gprc5a	PROMOTER	0.431	3.372	1395.04	4704.66	1.455	1159.85	1687.73
A_68_P28150134	chr11:114713892-114713936	NM_147217:571	Gprc5c	INSIDE	0.282	6.611	1233.63	8155.35	1.867	1163.31	2172.43
A_68_P24486168	chr6:59376435-59376479	NM_183183:-172	Gprin3	PROMOTER	0.274	5.714	1878.34	10732.82	1.564	1603.98	2507.91
A_68_P21077785	chr2:26171010-26171055	NM_001199146:-20	Gpsm1	PROMOTER	0.259	31.857	2382.51	75900.46	8.266	2467.51	20395.37
A_68_P30350021	chr15:76528223-76528267	NM_182805:1051	Gpt	INSIDE	0.507	0.400	2561.27	1023.42	0.203	1951.07	395.28
A_68_P26025056	chr8:88017117-88017161	NM_173866:623	Gpt2	INSIDE	0.464	0.187	2690.46	502.06	0.087	1974.24	170.79
A_68_P27280645	chr10:79516852-79516896	NM_001037741:93	Gpx4	INSIDE	0.487	10.295	546.45	5625.78	5.016	453.88	2276.89
A_68_P25035344	chr7:31935750-31935794	NM_027898:297	Gramd1a	INSIDE	0.314	0.290	2562.13	743.04	0.091	1941.66	176.96
A_68_P25035342	chr7:31935428-31935472	NM_027898:619	Gramd1a	INSIDE	0.64	0.534	4880.07	2604.44	0.341	3218.51	1098.69
A_68_P26443399	chr9:40263199-40263243	NM_172768:129	Gramd1b	INSIDE	0.53	5.189	563.12	2922.00	2.751	603.66	1660.72
A_68_P26443402	chr9:40263578-40263622	NM_172768:-251	Gramd1b	PROMOTER	0.461	3.718	1141.14	4242.40	1.716	890.73	1528.20
A_68_P31730289	chr18:56591860-56591904	NM_026240:97	Gramd3	INSIDE	0.633	2.439	1509.60	3682.31	1.544	1323.43	2043.89
A_68_P30404769	chr15:85889475-85889519	NM_001205353:340	Gramd4	INSIDE	0.438	4.310	2378.69	10253.35	1.888	1960.07	3699.81
A_68_P30490489	chr15:101062456-101062514	NM_019518:7847	Grasp	INSIDE	0.256	43.024	774.23	33310.39	11.015	1157.16	12745.62
A_68_P30490486	chr15:101062223-101062267	NM_019518:7607	Grasp	INSIDE	0.598	6.171	1681.68	10377.19	3.693	1394.24	5148.89
A_68_P30490485	chr15:101062094-101062138	NM_019518:7479	Grasp	INSIDE	0.611	3.356	1778.96	5970.07	2.052	1177.17	2415.50
A_68_P27594327	chr11:11937393-11937437	NM_001177629:-9440	Grb10	PROMOTER	0.139	50.173	10824.78	543112.40	6.956	11582.27	80567.90
A_68_P21283787	chr2:64861006-64861050	NM_016719:-205	Grb14	PROMOTER	0.588	0.395	1902.11	750.52	0.232	1589.10	368.76
A_68_P28056595	chr11:98319477-98319521	NM_010346:11351	Grb7	DOWNSTREAM	0.408	0.322	2922.90	941.86	0.131	2188.86	287.66
A_68_P28056588	chr11:98318831-98318875	NM_010346:10705	Grb7	DOWNSTREAM	0.413	0.550	966.58	531.66	0.227	766.59	174.05
A_68_P28266913	chr12:16807387-16807431	NM_015764:284	Greb1	INSIDE	0.542	0.732	8523.03	6242.06	0.397	5842.99	2319.88
A_68_P31489152	chr18:10325147-10325191	NM_001083628:-8	Greb1l	PROMOTER	0.62	7.009	1588.16	11130.92	4.347	1481.65	6440.09
A_68_P31489154	chr18:10325470-10325514	NM_001083628:316	Greb1l	INSIDE	0.223	1.527	2908.88	4441.05	0.341	2098.56	715.06
A_68_P21536555	chr2:113598914-113598958	NM_011824:-131	Grem1	PROMOTER	0.276	20.773	8418.03	174867.10	5.724	8046.88	46059.13
A_68_P28290299	chr12:25257426-25257470	NM_145890:-8135	Grhl1	PROMOTER	0.306	6.131	1783.35	10934.05	1.874	1344.66	2519.73
A_68_P30142979	chr15:37162921-37162965	NM_026496:152	Grhl2	INSIDE	0.322	4.458	1807.73	8058.34	1.437	1641.73	2358.60
A_68_P23329301	chr4:135126386-135126430	NM_001013756:3127	Grhl3	INSIDE	0.407	0.308	1607.54	494.35	0.125	1371.29	171.72
A_68_P29531956	chr14:35633743-35633787	NM_008166:443	Grid1	INSIDE	0.514	0.495	1473.76	728.99	0.254	1168.82	296.95
A_68_P29531957	chr14:35633864-35633908	NM_008166:565	Grid1	INSIDE	0.638	0.315	3523.50	1108.34	0.201	2246.02	451.00

ProbeName	Target position of probe on CpG island microarray	TargetID	GeneSymbol	CpG island Description	Ratio of relative methylation (TiO_2-NP/Vehicle)	Sham group			TiO_2-H group		
						Relative methylation (Cy5/Cy3)	Cy3 signal (Input)	Cy5 signal (MeDIP)	Relative methylation (Cy5/Cy3)	Cy3 signal (Input)	Cy5 signal (MeDIP)
A_68_P29531946	chr14:35632404-35632448	NM_008166:-895	Grid1	PROMOTER	0.644	2.821	750.83	2118.01	1.817	548.21	996.24
A_68_P24501242	chr6:63207663-63207707	NM_008167:834	Grid2	INSIDE	0.131	30.133	1851.65	55794.82	3.943	1759.87	6938.32
A_68_P24501230	chr6:63206331-63206375	NM_008167:-498	Grid2	PROMOTER	0.241	0.606	1508.49	914.89	0.146	1180.95	172.97
A_68_P24501227	chr6:63205942-63205990	NM_008167:-884	Grid2	PROMOTER	0.285	14.387	203.67	2930.16	4.098	163.85	671.38
A_68_P24156175	chr5:144119353-144119397	NM_133355:-6212	Grid2ip	PROMOTER	0.228	13.069	4620.79	60387.04	2.978	5637.83	16789.43
A_68_P24156215	chr5:144126161-144126205	NM_133355:596	Grid2ip	INSIDE	0.586	0.210	2959.50	622.80	0.123	1981.07	244.10
A_68_P24156243	chr5:144131687-144131731	NM_133355:6122	Grid2ip	INSIDE	0.612	3.395	5570.72	18910.80	2.078	4294.89	8926.70
A_68_P27127237	chr10:49508470-49508514	NM_001111268:68	Grik2	INSIDE	0.489	5.204	1094.33	5695.15	2.544	960.14	2442.78
A_68_P27127238	chr10:49508642-49508686	NM_001111268:-104	Grik2	PROMOTER	0.641	0.605	1115.84	675.54	0.388	881.53	342.05
A_68_P23274589	chr4:125167651-125167695	NM_001081097:-402	Grik3	PROMOTER	0.508	0.408	1298.11	530.22	0.208	1124.28	233.45
A_68_P23274592	chr4:125168108-125168152	NM_001081097:56	Grik3	INSIDE	0.589	0.388	1483.86	575.77	0.228	1108.83	253.21
A_68_P26458694	chr9:42752015-42752059	NM_175481:418	Grik4	INSIDE	0.218	1.884	17401.53	32792.59	0.41	11246.89	4614.66
A_68_P26458693	chr9:42751946-42751990	NM_175481:486	Grik4	INSIDE	0.485	0.628	9874.86	6196.69	0.304	6634.49	2017.41
A_68_P25008201	chr7:25857783-25857827	NM_008168:-416	Grik5	DIVERGENT_PROMOTER	0.132	11.424	4908.53	56077.17	1.504	3581.93	5386.44
A_68_P25008202	chr7:25857869-25857913	NM_008168:-502	Grik5	DIVERGENT_PROMOTER	0.636	0.636	1055.57	670.82	0.404	834.79	337.27
A_68_P21071262	chr2:25160552-25160596	NM_001177656:14109	Grin1	INSIDE	0.369	0.462	1051.63	486.21	0.171	1021.12	174.21
A_68_P21071361	chr2:25174135-25174179	NM_001177656:527	Grin1	INSIDE	0.476	0.362	1298.74	470.03	0.172	1001.42	172.39
A_68_P30540368	chr16:9995820-9995865	NM_008170:-3216	Grin2a	PROMOTER	0.289	48.957	5587.85	273567.00	14.158	4843.32	68571.05
A_68_P24868564	chr6:135683145-135683189	NM_008171:440363	Grin2b	INSIDE	0.384	4.222	905.19	3821.63	1.623	759.81	1233.34
A_68_P28152428	chr11:115128243-115128287	NM_010350:293	Grin2c	INSIDE	0.615	3.228	1735.54	5603.06	1.985	1395.58	2769.90
A_68_P28152412	chr11:115126609-115126660	NM_010350:1923	Grin2c	INSIDE	0.376	0.558	1204.42	672.04	0.21	826.67	173.34
A_68_P25095390	chr7:53088658-53088702	NM_008172:33371	Grin2d	INSIDE	0.274	11.489	1196.48	13746.95	3.144	1052.85	3310.47
A_68_P30347073	chr15:76077636-76077680	NM_023168:422	Grina	INSIDE	0.529	0.184	4811.17	882.98	0.097	3289.54	319.45
A_68_P26622299	chr9:71333296-71333340	NM_001164793:-553	Grinl1a	PROMOTER	0.562	3.662	1188.42	4352.38	2.058	1057.13	2175.26
A_68_P26622301	chr9:71333539-71333583	NM_001164793:-795	Grinl1a	PROMOTER	0.584	4.369	1312.31	5733.46	2.551	1152.64	2940.10
A_68_P23595699	chr5:35003466-35003510	NM_001080743:461	Grk4	INSIDE	0.574	0.497	1231.55	611.63	0.285	962.03	274.46
A_68_P32227733	chr19:60965534-60965578	NM_018869:-95	Grk5	PROMOTER	0.097	2.449	1100.36	2694.77	0.239	983.32	234.71
A_68_P32227741	chr19:60966626-60966673	NM_018869:998	Grk5	INSIDE	0.368	0.207	3096.97	642.47	0.076	2245.14	171.58
A_68_P26804350	chr9:106549989-106550033	NM_001160353:8430	Grm2	INSIDE	0.279	8.076	1674.87	13526.21	2.252	1141.71	2571.62
A_68_P26804349	chr9:106549876-106549920	NM_001160353:8542	Grm2	INSIDE	0.444	6.096	2193.42	13370.08	2.707	1478.58	4002.47
A_68_P26804348	chr9:106549759-106549803	NM_001160353:8660	Grm2	INSIDE	0.472	3.771	1295.65	4886.22	1.78	984.82	1753.42
A_68_P31116317	chr17:27571773-27571817	NM_001013385:68455	Grm4	INSIDE	0.524	3.457	1117.79	3863.66	1.811	894.63	1620.10
A_68_P31116576	chr17:27610068-27610113	NM_001013385:30159	Grm4	INSIDE	0.242	5.812	3722.98	21637.15	1.407	2716.10	3820.61
A_68_P24318684	chr6:28083743-28083787	NM_008174:605	Grm8	INSIDE	0.523	0.708	1487.28	1052.63	0.37	1204.11	445.73
A_68_P28703478	chr12:105710469-105710513	NM_010351:956	Gsc	INSIDE	0.535	0.397	2463.23	978.90	0.213	1930.01	410.49
A_68_P28703487	chr12:105711415-105711459	NM_010351:10	Gsc	INSIDE	0.47	0.573	1147.12	657.47	0.269	935.38	252.07
A_68_P30576539	chr16:17915177-17915221	NM_029469:-46	Gsc2	PROMOTER	0.647	0.469	1225.31	574.14	0.303	989.77	300.03
A_68_P30576536	chr16:17914943-17914987	NM_029469:188	Gsc2	INSIDE	0.104	4.270	874.20	3732.83	0.442	791.06	349.74
A_68_P26220483	chr8:123012034-123012078	NM_198671:-709	Gse1	PROMOTER	0.239	14.603	1216.14	17759.59	3.483	1024.06	3567.12
A_68_P26220485	chr8:123012259-123012305	NM_198671:-483	Gse1	PROMOTER	0.355	0.477	1583.91	756.29	0.17	1025.10	173.82
A_68_P26220843	chr8:123060419-123060463	NM_001145896:-868	Gse1	PROMOTER	0.489	4.382	2738.69	11999.78	2.144	2157.47	4626.13
A_68_P26220844	chr8:123060496-123060540	NM_001145896:-790	Gse1	PROMOTER	0.544	0.302	2711.67	819.84	0.164	1881.50	309.45
A_68_P26220850	chr8:123061186-123061230	NM_001145896:-100	Gse1	PROMOTER	0.578	5.213	981.48	5116.57	3.016	777.72	2345.42
A_68_P26220858	chr8:123061951-123061995	NM_001145896:664	Gse1	INSIDE	0.579	6.979	1181.93	8248.55	4.039	1157.61	4675.15
A_68_P26220864	chr8:123062616-123062660	NM_001145896:1330	Gse1	INSIDE	0.606	5.862	840.45	4926.71	3.553	706.75	2511.27
A_68_P26220480	chr8:123011650-123011694	NM_198671:-1093	Gse1	PROMOTER	0.607	4.867	936.02	4556.05	2.953	830.37	2452.12

ProbeName	Target position of probe on CpG island microarray	TargetID	GeneSymbol	CpG island Description	Ratio of relative methylation (TiO_2-NP/Vehicle)	Sham group			TiO_2-H group		
						Relative methylation (Cy5/Cy3)	Cy3 signal (Input)	Cy5 signal (MeDIP)	Relative methylation (Cy5/Cy3)	Cy3 signal (Input)	Cy5 signal (MeDIP)
A_68_P25498532	chr7:133225682-133225726	NM_001101488:221	Gsg1l	INSIDE	0.415	15.989	11785.03	188433.00	6.63	11223.69	74409.58
A_68_P21132488	chr2:35112210-35112254	NM_001206367:354	Gsn	INSIDE	0.466	0.341	1457.10	496.82	0.159	1080.47	171.85
A_68_P21132680	chr2:35138083-35138127	NM_146120:205	Gsn	INSIDE	0.654	3.020	862.33	2604.64	1.977	698.14	1379.91
A_68_P30547081	chr16:11253703-11253747	NM_001130008:694	Gspt1	INSIDE	0.651	0.216	2698.43	581.74	0.14	1876.57	263.32
A_68_P25770033	chr8:34764110-34764154	NM_010344:423	Gsr	INSIDE	0.19	25.257	3735.47	94346.14	4.809	3566.68	17152.30
A_68_P25770032	chr8:34763961-34764005	NM_010344:273	Gsr	INSIDE	0.298	10.628	2685.30	28539.80	3.166	2448.10	7751.02
A_68_P21766453	chr2:155418540-155418584	NM_008180:-120	Gss	PROMOTER	0.337	7.990	617.29	4931.97	2.692	493.71	1329.24
A_68_P22540995	chr3:132754697-132754741	NM_026231:-14	Gstcd	DIVERGENT_PROMOTER	0.649	4.037	595.63	2404.51	2.618	473.32	1239.38
A_68_P32152318	chr19:47929317-47929361	NM_010362:-140	Gsto1	PROMOTER	0.639	0.463	1455.41	673.73	0.296	928.36	274.80
A_68_P28614428	chr12:88487974-88488018	NM_010363:-671	Gstz1	PROMOTER	0.587	0.314	1978.57	620.32	0.184	1418.73	261.21
A_68_P23807227	chr5:75471945-75471989	NM_133256:341	Gsx2	INSIDE	0.459	0.319	1455.62	464.33	0.146	1200.85	175.92
A_68_P23807226	chr5:75471874-75471918	NM_133256:271	Gsx2	INSIDE	0.496	0.325	2388.43	776.69	0.161	1816.43	292.80
A_68_P24757354	chr6:113026958-113027002	NR_027010:258	Gt(ROSA)26Sor	INSIDE	0.484	4.496	1802.80	8105.08	2.176	1451.70	3159.46
A_68_P24757355	chr6:113027040-113027084	NR_027010:176	Gt(ROSA)26Sor	INSIDE	0.536	0.480	2044.80	981.45	0.257	1453.86	374.00
A_68_P24757356	chr6:113027164-113027208	NR_027010:52	Gt(ROSA)26Sor	INSIDE	0.598	0.731	1810.41	1323.73	0.437	1529.90	669.26
A_68_P30681056	chr16:37539468-37539512	NM_028812:365	Gtf2e1	INSIDE	0.418	4.552	2060.25	9377.99	1.903	1606.61	3057.15
A_68_P31259561	chr17:57151014-57151058	NM_133801:-325	Gtf2f1	PROMOTER	0.439	0.611	944.17	576.76	0.268	847.31	227.10
A_68_P29731240	chr14:76410493-76410537	NM_026816:158	Gtf2f2	INSIDE	0.388	1.525	1912.28	2915.77	0.592	1440.03	851.87
A_68_P29268695	chr13:101262403-101262447	NM_022011:140	Gtf2h2	INSIDE	0.64	0.575	3647.85	2097.80	0.368	2948.48	1086.01
A_68_P29268696	chr13:101262505-101262549	NM_022011:38	Gtf2h2	INSIDE	0.632	0.648	1354.04	876.97	0.409	1037.52	424.46
A_68_P24112572	chr5:134790667-134790711	NM_001080746:-72	Gtf2i	PROMOTER	0.129	12.729	1467.43	18678.71	1.641	1243.95	2041.38
A_68_P24111875	chr5:134660308-134660352	NM_053266:423	Gtf2ird2	INSIDE	0.34	0.562	2938.40	1651.33	0.191	2311.99	441.48
A_68_P25496222	chr7:132850904-132850948	NM_207239:276	Gtf3c1	INSIDE	0.391	3.869	4293.94	16613.61	1.512	3357.21	5075.98
A_68_P21093574	chr2:28695671-28695715	NM_001166033:188	Gtf3c4	INSIDE	0.538	12.856	1087.65	13982.45	6.915	941.61	6510.80
A_68_P21092000	chr2:28438292-28438336	NM_148928:485	Gtf3c5	INSIDE	0.46	0.332	2587.58	859.12	0.153	1882.72	287.44
A_68_P21092002	chr2:28438501-28438545	NM_148928:277	Gtf3c5	INSIDE	0.528	5.551	1292.81	7176.76	2.932	1096.95	3215.73
A_68_P27083091	chr10:39977457-39977501	NM_026113:-7	Gtf3c6	PROMOTER	0.454	6.615	3183.08	21055.75	3.003	2515.06	7552.74
A_68_P30367927	chr15:79521233-79521289	NM_013818:-65	Gtpbp1	DIVERGENT_PROMOTER	0.28	11.681	2288.35	26730.24	3.272	2033.95	6655.64
A_68_P30367929	chr15:79521459-79521503	NM_013818:155	Gtpbp1	INSIDE	0.581	3.547	1222.28	4335.99	2.061	1307.26	2693.62
A_68_P30367930	chr15:79521605-79521649	NM_013818:301	Gtpbp1	INSIDE	0.623	0.345	2604.32	898.00	0.215	2091.72	449.21
A_68_P25957096	chr8:74013396-74013440	NM_032544:1417	Gtpbp3	INSIDE	0.13	2.218	4534.58	10055.97	0.288	2727.25	784.23
A_68_P28813934	chr13:8995156-8995200	NM_027000:80	Gtpbp4	INSIDE	0.666	0.304	1830.01	555.65	0.202	1409.53	285.13
A_68_P23977825	chr5:110537297-110537341	NM_145147:-102	Gtpbp6	DIVERGENT_PROMOTER	0.402	0.228	2728.39	620.74	0.091	2078.78	190.21
A_68_P26274325	chr9:3532341-3532385	NM_001033322:9	Gucy1a2	INSIDE	0.579	0.384	3908.35	1501.05	0.222	2804.85	623.69
A_68_P27835392	chr11:59005058-59005102	NM_001159410:-3837	Guk1	PROMOTER	0.476	0.507	1601.92	812.80	0.242	1375.53	332.32
A_68_P20193921	chr1:44608865-44608909	NM_028450:371	Gulp1	INSIDE	0.49	0.221	2300.21	508.64	0.108	1656.59	179.52
A_68_P30446024	chr15:93105588-93105632	NM_001033275:-95	Gxylt1	PROMOTER	0.326	1.911	4323.84	8263.36	0.623	3746.84	2334.33
A_68_P21978659	chr3:20054868-20054912	NM_013755:105	Gyg	INSIDE	0.445	7.488	1259.03	9427.42	3.332	1028.86	3428.21
A_68_P32507212	chrX:83022021-83022065	NM_008194:116	Gyk	INSIDE	0.146	26.601	17194.68	457396.20	3.871	21902.74	84778.35
A_68_P32507214	chrX:83022249-83022295	NM_008194:-114	Gyk	PROMOTER	0.344	4.295	4360.39	18727.04	1.476	5156.15	7612.52
A_68_P21428052	chr2:92211160-92211204	NM_001166633:11	Gyltl1b	INSIDE	0.403	4.639	1282.40	5949.35	1.87	1124.23	2102.80
A_68_P31603477	chr18:32719222-32719266	NM_001048207:444	Gypc	INSIDE	0.28	0.429	2063.54	884.32	0.12	1429.60	171.79
A_68_P24904453	chr6:142363298-142363342	NM_145572:58309	Gys2	DOWNSTREAM	0.456	0.715	1454.52	1040.15	0.326	1207.20	393.40
A_68_P21750488	chr2:152495422-152495466	NM_001159553:248	H13	INSIDE	0.251	1.719	898.66	1544.80	0.431	763.18	329.28
A_68_P24619080	chr6:87930668-87930712	NM_198622:786	H1fx	INSIDE	0.652	0.340	2472.15	840.80	0.222	1936.27	429.60
A_68_P27568543	chr11:6344478-6344522	NM_029938:-54	H2afv	PROMOTER	0.377	0.340	1983.44	674.83	0.128	1408.62	180.62

ProbeName	Target position of probe on CpG island microarray	TargetID	GeneSymbol	CpG island Description	Ratio of relative methylation (TiO_2-NP/Vehicle)	Sham group			TiO_2-H group		
						Relative methylation (Cy5/Cy3)	Cy3 signal (Input)	Cy5 signal (MeDIP)	Relative methylation (Cy5/Cy3)	Cy3 signal (Input)	Cy5 signal (MeDIP)
A_68_P27568544	chr11:6344567-6344611	NM_029938:-142	H2afv	PROMOTER	0.636	0.465	1341.96	624.37	0.296	1012.70	299.51
A_68_P26467074	chr9:44143508-44143552	NM_010436:733	H2afx	INSIDE	0.408	0.444	1186.35	527.18	0.181	972.09	176.06
A_68_P26467075	chr9:44143629-44143673	NM_010436:853	H2afx	INSIDE	0.614	4.096	1431.25	5862.71	2.517	1093.42	2751.62
A_68_P27182284	chr10:61246043-61246087	NM_207000:548	H2afy2	INSIDE	0.314	9.348	4056.25	37916.29	2.936	3492.48	10253.82
A_68_P27182287	chr10:61246439-61246483	NM_207000:152	H2afy2	INSIDE	0.378	6.467	1958.38	12665.06	2.443	1658.48	4051.32
A_68_P27182283	chr10:61245921-61245965	NM_207000:670	H2afy2	INSIDE	0.609	0.413	1967.07	811.85	0.251	1647.49	413.87
A_68_P22565464	chr3:137527246-137527290	NM_016750:-294	H2afz	PROMOTER	0.483	0.498	2535.72	1262.74	0.24	2245.52	539.68
A_68_P22565463	chr3:137527117-137527161	NM_016750:-424	H2afz	PROMOTER	0.656	0.248	3125.98	774.95	0.163	2669.40	434.07
A_68_P31162404	chr17:36221085-36221129	NM_008199:88	H2-Bl	INSIDE	0.514	4.394	3179.98	13972.88	2.257	2373.15	5356.52
A_68_P31159077	chr17:35516814-35516858	NM_001143689:275	H2-Gs10	INSIDE	0.539	3.417	3679.33	12573.35	1.842	3005.95	5536.06
A_68_P31158869	chr17:35457566-35457610	NM_010390:86	H2-Q1	INSIDE	0.475	5.762	2539.44	14632.92	2.735	2319.46	6343.19
A_68_P31158874	chr17:35458237-35458281	NM_010390:756	H2-Q1	INSIDE	0.499	0.539	1144.81	617.09	0.269	836.48	225.08
A_68_P31159262	chr17:35607004-35607048	NM_010391:-7	H2-Q10	PROMOTER	0.2	8.084	1210.58	9786.28	1.616	993.48	1604.98
A_68_P20877951	chr1:182742690-182742734	NM_008210:1022	H3f3a	INSIDE	0.151	18.742	2513.01	47097.58	2.833	2015.95	5712.06
A_68_P20877853	chr1:182724319-182724363	NM_008210:19394	H3f3a	DOWNSTREAM	0.486	0.447	1126.64	504.08	0.217	771.05	167.60
A_68_P28156895	chr11:115885168-115885212	NM_008211:628	H3f3b	INSIDE	0.611	3.015	971.02	2927.41	1.841	937.34	1725.58
A_68_P29097470	chr13:64263694-64263738	NM_019986:543	Habp4	INSIDE	0.626	0.527	2107.43	1111.32	0.33	1687.00	556.80
A_68_P27111732	chr10:45297581-45297625	NM_172473:-32	Hace1	PROMOTER	0.566	3.283	1107.82	3636.49	1.858	970.39	1802.56
A_68_P27111735	chr10:45297945-45297989	NM_172473:332	Hace1	INSIDE	0.313	1.809	1829.34	3308.69	0.566	1825.88	1034.30
A_68_P22531299	chr3:130975017-130975061	NM_008212:-83	Hadh	PROMOTER	0.301	41.650	8778.36	365618.90	12.523	9092.29	113865.60
A_68_P22531298	chr3:130974889-130974933	NM_008212:45	Hadh	INSIDE	0.633	0.235	3552.14	833.06	0.149	2431.21	361.16
A_68_P22531296	chr3:130974638-130974682	NM_008212:295	Hadh	INSIDE	0.337	5.011	1863.39	9338.08	1.69	1553.33	2625.87
A_68_P31099857	chr17:24987665-24987709	NM_001159626:75	Hagh	INSIDE	0.493	0.290	1556.30	450.76	0.143	1190.42	170.07
A_68_P27827732	chr11:57641072-57641118	NM_008213:4555	Hand1	DOWNSTREAM	0.33	0.189	4384.34	829.12	0.062	2794.41	174.37
A_68_P27827767	chr11:57645283-57645327	NM_008213:345	Hand1	INSIDE	0.45	0.309	3744.02	1157.56	0.139	2660.39	370.35
A_68_P27827764	chr11:57644954-57644998	NM_008213:673	Hand1	INSIDE	0.418	0.579	796.38	461.18	0.242	721.17	174.76
A_68_P25890515	chr8:59799750-59799801	NM_010402:-4	Hand2	PROMOTER	0.491	0.509	1349.59	686.47	0.25	1073.24	268.30
A_68_P25890518	chr8:59800166-59800210	NM_010402:409	Hand2	INSIDE	0.504	0.701	1244.56	872.55	0.353	1052.77	371.70
A_68_P25949728	chr8:72607980-72608024	NM_177900:575	Hapln4	INSIDE	0.616	0.341	2269.69	772.97	0.21	1812.79	380.31
A_68_P31075179	chr17:17987266-17987310	NM_008215:4864	Has1	INSIDE	0.581	2.922	1525.40	4457.41	1.697	1352.07	2294.49
A_68_P31075177	chr17:17987055-17987099	NM_008215:5076	Has1	INSIDE	0.664	2.098	2850.27	5978.69	1.394	2369.22	3301.97
A_68_P26141795	chr8:109394997-109395041	NM_008217:877	Has3	INSIDE	0.585	3.327	877.17	2918.42	1.946	642.65	1250.37
A_68_P21319207	chr2:71227345-71227389	NM_026115:50	Hat1	INSIDE	0.507	0.360	1242.72	447.20	0.182	965.14	175.99
A_68_P31849646	chr18:78006372-78006416	NM_146089:125	Haus1	INSIDE	0.612	0.455	2189.81	997.24	0.279	1727.22	481.65
A_68_P21576348	chr2:120454904-120454948	NM_025475:19755	Haus2	DOWNSTREAM	0.443	0.550	3497.08	1922.07	0.244	2629.21	640.83
A_68_P25955959	chr8:73796363-73796407	NM_001163042:105	Haus8	INSIDE	0.38	1.673	2303.92	3853.60	0.636	1752.89	1115.41
A_68_P31624332	chr18:36675376-36675420	NM_010415:61	Hbegf	INSIDE	0.443	5.245	1800.79	9444.64	2.325	1499.99	3487.56
A_68_P28334080	chr12:32634604-32634648	NM_153198:774	Hbp1	INSIDE	0.324	13.325	568.38	7573.39	4.321	470.49	2032.94
A_68_P28334082	chr12:32634899-32634943	NM_153198:480	Hbp1	INSIDE	0.617	3.210	1745.62	5603.41	1.981	1435.26	2842.98
A_68_P28334079	chr12:32634445-32634489	NM_153198:934	Hbp1	INSIDE	0.433	0.556	964.02	536.42	0.241	794.91	191.61
A_68_P32820569	chrX:165758461-165758505	NM_008222:-207	Hccs	PROMOTER	0.564	4.283	519.63	2225.42	2.416	745.30	1800.48
A_68_P21753005	chr2:152934198-152934242	NM_001172117:17	Hck	INSIDE	0.469	0.681	1181.61	804.69	0.32	1003.63	320.66
A_68_P27278461	chr10:79180701-79180746	NM_008226:1345	Hcn2	INSIDE	0.484	4.164	2340.20	9743.98	2.016	1781.80	3591.85
A_68_P27278520	chr10:79187527-79187571	NM_008226:8170	Hcn2	INSIDE	0.623	22.870	1189.04	27193.51	14.243	1050.53	14963.06
A_68_P27278521	chr10:79187599-79187649	NM_008226:8246	Hcn2	INSIDE	0.273	6.861	980.97	6730.24	1.875	602.40	1129.66
A_68_P27278518	chr10:79187320-79187368	NM_008226:7966	Hcn2	INSIDE	0.645	4.968	177.15	880.01	3.205	190.86	611.75

ProbeName	Target position of probe on CpG island microarray	TargetID	GeneSymbol	CpG island Description	Ratio of relative methylation (TiO_2-NP/Vehicle)	Sham group			TiO_2-H group		
						Relative methylation (Cy5/Cy3)	Cy3 signal (Input)	Cy5 signal (MeDIP)	Relative methylation (Cy5/Cy3)	Cy3 signal (Input)	Cy5 signal (MeDIP)
A_68_P26550035	chr9:58671367-58671411	NM_001081192:70	Hcn4	INSIDE	0.495	3.593	2133.88	7667.67	1.779	1610.75	2865.05
A_68_P26550037	chr9:58671573-58671617	NM_001081192:276	Hcn4	INSIDE	0.321	0.547	1131.29	618.76	0.175	976.33	171.31
A_68_P26550025	chr9:58670099-58670144	NM_001081192:-1197	Hcn4	PROMOTER	0.147	1.622	1161.93	1884.44	0.238	881.33	210.06
A_68_P26550039	chr9:58671843-58671887	NM_001081192:546	Hcn4	INSIDE	0.523	3.016	983.49	2966.56	1.577	829.24	1307.57
A_68_P23300608	chr4:129815629-129815674	NM_001163027:144	Hcrtr1	INSIDE	0.393	6.966	614.43	4280.19	2.741	713.03	1954.45
A_68_P23300616	chr4:129816532-129816576	NM_198959:-148	Hcrtr1	PROMOTER	0.556	0.198	7853.64	1558.28	0.11	5354.20	591.01
A_68_P23300612	chr4:129816147-129816191	NM_001163027:-373	Hcrtr1	PROMOTER	0.397	3.523	3720.86	13108.15	1.398	3072.11	4296.04
A_68_P26648623	chr9:76171335-76171379	NM_198962:29	Hcrtr2	INSIDE	0.464	4.003	1175.58	4706.16	1.857	1097.69	2038.65
A_68_P23297200	chr4:129219696-129219740	NM_008228:172	Hdac1	INSIDE	0.166	0.385	6590.63	2537.30	0.064	4746.96	303.75
A_68_P24637122	chr6:91107049-91107096	NM_144919:264	Hdac11	INSIDE	0.034	3.174	2422.63	7689.29	0.108	1633.35	176.01
A_68_P24637121	chr6:91106926-91106970	NM_144919:140	Hdac11	INSIDE	0.317	0.596	1206.74	718.70	0.189	923.26	174.50
A_68_P27067359	chr10:36693905-36693949	NM_008229:-423	Hdac2	PROMOTER	0.518	4.334	2835.53	12288.01	2.244	2263.39	5079.76
A_68_P31253728	chr17:56219108-56219152	NM_008233:51	Hdgfrp2	INSIDE	0.545	2.589	2558.01	6621.65	1.411	2033.94	2870.84
A_68_P25274810	chr7:89078740-89078784	NM_013886:583	Hdgfrp3	INSIDE	0.353	6.275	1311.28	8228.27	2.212	1080.55	2390.19
A_68_P25274814	chr7:89079181-89079225	NM_013886:143	Hdgfrp3	INSIDE	0.45	6.436	3005.13	19341.77	2.899	2568.61	7445.94
A_68_P25274817	chr7:89079514-89079558	NM_013886:-191	Hdgfrp3	PROMOTER	0.574	14.333	2728.46	39107.26	8.225	2238.90	18414.98
A_68_P31845028	chr18:77182898-77182942	NM_001039201:-236	Hdhd2	PROMOTER	0.205	11.715	3053.65	35772.05	2.398	2668.84	6399.28
A_68_P31845029	chr18:77182994-77183040	NM_001039201:-140	Hdhd2	PROMOTER	0.48	3.581	1373.49	4917.88	1.72	1094.64	1883.08
A_68_P22964787	chr4:62163343-62163387	NM_024257:-130	Hdhd3	PROMOTER	0.5	0.206	2693.60	554.91	0.103	2026.34	208.81
A_68_P22964786	chr4:62163240-62163284	NM_024257:-28	Hdhd3	PROMOTER	0.613	0.123	4544.34	557.85	0.075	3227.90	242.78
A_68_P20455835	chr1:95374821-95374865	NM_133808:543	Hdlbp	INSIDE	0.252	21.863	1641.39	35885.35	5.513	1553.66	8565.40
A_68_P20455837	chr1:95375021-95375065	NM_133808:343	Hdlbp	INSIDE	0.481	0.338	2888.38	977.65	0.163	2397.49	390.68
A_68_P20455651	chr1:95342033-95342077	NM_133808:33331	Hdlbp	INSIDE	0.644	2.679	1747.31	4681.35	1.724	1468.56	2532.06
A_68_P24133859	chr5:139625867-139625911	NM_001081265:-288	Heatr2	PROMOTER	0.206	2.529	10625.35	26867.62	0.521	7252.67	3781.35
A_68_P24133861	chr5:139626021-139626065	NM_001081265:-134	Heatr2	PROMOTER	0.327	14.750	14452.78	213173.20	4.822	11343.23	54701.27
A_68_P24133858	chr5:139625772-139625819	NM_001081265:-381	Heatr2	PROMOTER	0.526	7.488	1040.73	7792.57	3.939	505.93	1992.65
A_68_P24133860	chr5:139625951-139625996	NM_001081265:-203	Heatr2	PROMOTER	0.292	4.837	5194.57	25127.28	1.413	2931.70	4141.60
A_68_P26039225	chr8:90661714-90661758	NM_172757:-47	Heatr3	PROMOTER	0.408	4.620	3666.96	16940.39	1.887	2985.46	5632.95
A_68_P28432638	chr12:53072168-53072212	NM_177171:118	Heatr5a	INSIDE	0.55	0.427	2112.74	901.09	0.234	1807.79	423.72
A_68_P28432636	chr12:53071968-53072012	NM_177171:318	Heatr5a	INSIDE	0.589	4.370	3285.24	14357.79	2.573	2602.22	6694.56
A_68_P28431905	chr12:52930950-52930994	NM_144788:-449	Hectd1	PROMOTER	0.431	5.932	742.76	4406.16	2.557	650.61	1663.77
A_68_P28431901	chr12:52930414-52930458	NM_144788:87	Hectd1	INSIDE	0.62	3.725	941.58	3507.00	2.308	811.98	1874.11
A_68_P28431895	chr12:52929741-52929785	NM_144788:761	Hectd1	INSIDE	0.523	3.929	471.13	1850.83	2.055	417.24	857.61
A_68_P20235844	chr1:54251715-54251759	NM_001001883:142	Hecw2	INSIDE	0.494	3.996	1172.16	4683.99	1.975	999.05	1972.82
A_68_P30659187	chr16:33684576-33684620	NM_175256:47	Heg1	INSIDE	0.467	11.606	2904.55	33710.47	5.419	2428.71	13160.77
A_68_P27495356	chr10:119549659-119549703	NM_080446:341	Helb	INSIDE	0.519	3.696	1984.87	7336.08	1.918	1776.38	3406.32
A_68_P23931501	chr5:101227554-101227598	NM_001081107:43	Helq	INSIDE	0.493	0.687	885.81	608.77	0.339	718.75	243.49
A_68_P25835333	chr8:47377718-47377762	NM_173789:2285	Helt	INSIDE	0.647	0.388	1629.76	632.28	0.251	1314.19	329.79
A_68_P28107584	chr11:107408339-107408383	NM_198298:-913	Helz	PROMOTER	0.537	0.277	1664.59	461.48	0.149	1172.69	174.50
A_68_P28107590	chr11:107409081-107409125	NM_198298:-171	Helz	PROMOTER	0.38	4.164	2818.39	11737.05	1.583	2334.80	3694.91
A_68_P26807873	chr9:107240712-107240756	NM_133984:-53	Hemk1	DIVERGENT_PROMOTER	0.492	0.552	2410.42	1329.71	0.272	1869.43	507.86
A_68_P26807872	chr9:107240552-107240596	NM_133984:107	Hemk1	INSIDE	0.593	0.268	1763.67	471.96	0.159	1443.30	228.89
A_68_P26807870	chr9:107240416-107240460	NM_133984:243	Hemk1	INSIDE	0.503	0.594	943.97	560.49	0.299	823.36	246.04
A_68_P26430295	chr9:37192346-37192390	NM_175189:17178	Hepacam	INSIDE	0.617	2.931	1242.31	3640.65	1.809	1003.58	1815.85
A_68_P24483218	chr6:58781700-58781744	NM_028705:-1971	Herc3	PROMOTER	0.526	3.273	954.60	3124.68	1.722	818.52	1409.32
A_68_P26077217	chr8:96910495-96910539	NM_022331:117	Herpud1	INSIDE	0.342	13.190	2565.41	33837.97	4.507	2149.97	9690.51

ProbeName	Target position of probe on CpG island microarray	TargetID	GeneSymbol	CpG island Description	Ratio of relative methylation (TiO_2-NP/Vehicle)	Sham group			TiO_2-H group		
						Relative methylation (Cy5/Cy3)	Cy3 signal (Input)	Cy5 signal (MeDIP)	Relative methylation (Cy5/Cy3)	Cy3 signal (Input)	Cy5 signal (MeDIP)
A_68_P30639626	chr16:30064558-30064602	NM_008235:-862	Hes1	PROMOTER	0.38	8.693	2306.54	20051.64	3.305	1972.99	6520.56
A_68_P30639627	chr16:30064633-30064677	NM_008235:-788	Hes1	PROMOTER	0.367	0.611	1361.17	832.21	0.225	1126.74	253.01
A_68_P23417739	chr4:151660365-151660409	NM_008237:5385	Hes3	INSIDE	0.645	2.597	4746.14	12325.37	1.676	3047.20	5105.66
A_68_P23417770	chr4:151664569-151664614	NM_008237:1180	Hes3	INSIDE	0.629	0.664	852.40	566.25	0.418	670.87	280.39
A_68_P20442328	chr1:93309360-93309404	NM_019479:417	Hes6	INSIDE	0.502	0.697	1778.32	1240.26	0.35	1506.30	526.86
A_68_P27893215	chr11:68935929-68935973	NM_033041:1996	Hes7	INSIDE	0.495	0.497	5916.12	2940.40	0.246	3985.07	980.90
A_68_P27893214	chr11:68935855-68935899	NM_033041:1922	Hes7	INSIDE	0.038	1.343	7920.47	10640.04	0.051	4879.46	249.53
A_68_P28083427	chr11:102977675-102977719	NM_138753:58	Hexim1	INSIDE	0.411	0.304	1839.15	558.66	0.125	1383.55	172.59
A_68_P28083517	chr11:102994122-102994166	NM_001130516:-508	Hexim2	PROMOTER	0.498	5.407	2192.67	11856.64	2.692	1761.39	4741.82
A_68_P21933421	chr3:8667100-8667144	NM_010423:-84	Hey1	PROMOTER	0.388	5.658	614.06	3474.22	2.195	652.36	1432.15
A_68_P27041764	chr10:30562697-30562741	NM_013904:-129	Hey2	PROMOTER	0.319	9.269	346.46	3211.26	2.958	370.32	1095.37
A_68_P23964040	chr5:107354969-107355013	NM_177873:-81	Hfm1	PROMOTER	0.593	0.487	2482.71	1208.85	0.289	2162.09	624.19
A_68_P28184739	chr11:120329033-120329077	NM_001159328:106	Hgs	INSIDE	0.436	3.905	2232.94	8719.56	1.702	2063.40	3512.27
A_68_P25729953	chr8:27086780-27086824	NM_029884:414	Hgsnat	INSIDE	0.286	8.180	1564.06	12793.88	2.338	1299.35	3037.95
A_68_P20945385	chr1:194596745-194596789	NM_144881:647	Hhat	INSIDE	0.631	2.753	3309.90	9113.81	1.737	2528.30	4391.43
A_68_P32095553	chr19:37509433-37509477	NM_008245:124	Hhex	INSIDE	0.335	8.485	3993.14	33881.85	2.841	3198.22	9086.96
A_68_P25997733	chr8:82581462-82581506	NM_020259:423	Hhip	INSIDE	0.586	0.405	2250.09	911.88	0.238	1891.96	449.61
A_68_P28727298	chr12:109557168-109557212	NM_001044380:12711	Hhipl1	INSIDE	0.314	5.578	977.29	5451.23	1.751	750.86	1314.66
A_68_P22450560	chr3:116365543-116365587	NM_008246:31	Hiat1	INSIDE	0.198	17.471	1432.24	25023.23	3.453	1228.92	4242.89
A_68_P22450556	chr3:116365109-116365153	NM_008246:465	Hiat1	INSIDE	0.429	7.209	733.42	5287.09	3.093	672.50	2080.28
A_68_P22450555	chr3:116364918-116364962	NM_008246:655	Hiat1	INSIDE	0.605	4.102	1338.61	5491.50	2.48	1118.94	2774.89
A_68_P24451816	chr6:52590190-52590234	NM_145567:82	Hibadh	INSIDE	0.655	0.721	1735.81	1252.15	0.473	1489.72	704.01
A_68_P27924602	chr11:74984051-74984095	NM_010430:-315	Hic1	DIVERGENT_PROMOTER	0.183	19.888	1652.73	32868.68	3.631	1678.06	6092.22
A_68_P27924582	chr11:74981827-74981871	NM_001098203:-194	Hic1	PROMOTER	0.433	0.477	4246.15	2027.22	0.207	2986.27	616.86
A_68_P27924600	chr11:74983847-74983891	NM_010430:-111	Hic1	DIVERGENT_PROMOTER	0.497	4.318	971.09	4193.66	2.148	919.26	1974.56
A_68_P27924590	chr11:74982634-74982678	NM_001098203:-1002	Hic1	PROMOTER	0.641	3.948	1060.59	4187.26	2.529	916.36	2317.76
A_68_P27924566	chr11:74979860-74979904	NM_001098203:1772	Hic1	INSIDE	0.513	0.621	1543.63	958.02	0.319	1165.94	371.37
A_68_P27924569	chr11:74980234-74980278	NM_001098203:1398	Hic1	INSIDE	0.6	0.671	1636.02	1097.06	0.403	1372.84	552.72
A_68_P27924599	chr11:74983746-74983790	NM_010430:-11	Hic1	DIVERGENT_PROMOTER	0.308	1.820	2305.96	4197.79	0.56	1588.99	890.17
A_68_P27924570	chr11:74980316-74980360	NM_001098203:1316	Hic1	INSIDE	0.496	0.505	945.92	477.99	0.25	689.26	172.62
A_68_P30572653	chr16:17233627-17233671	NM_178922:-31	Hic2	PROMOTER	0.302	12.445	2297.73	28595.64	3.761	1898.42	7139.92
A_68_P28540462	chr12:75009562-75009606	NM_010431:731	Hif1a	INSIDE	0.332	9.804	1876.72	18399.44	3.254	1756.38	5714.59
A_68_P27040510	chr10:30337995-30338039	NM_025798:156	Hint3	INSIDE	0.425	7.280	1206.29	8781.89	3.09	938.87	2901.56
A_68_P24116580	chr5:135964648-135964692	NM_146001:56322	Hip1	INSIDE	0.383	15.193	2283.18	34687.93	5.811	1991.82	11575.14
A_68_P24116811	chr5:136020789-136020833	NM_146001:182	Hip1	INSIDE	0.459	0.306	1711.51	523.75	0.14	1248.87	175.35
A_68_P24058535	chr5:124423625-124423669	NM_145070:10	Hip1r	INSIDE	0.313	7.355	2396.38	17625.96	2.302	2000.57	4604.78
A_68_P22388055	chr3:103594904-103594948	NM_010432:272	Hipk1	INSIDE	0.337	0.507	1403.59	710.94	0.171	1077.12	184.08
A_68_P22388058	chr3:103595426-103595470	NM_010432:-250	Hipk1	PROMOTER	0.436	0.650	1861.81	1210.07	0.283	1625.81	460.62
A_68_P24380025	chr6:38826655-38826699	NM_010433:-487	Hipk2	PROMOTER	0.499	4.138	1462.86	6052.62	2.064	1272.01	2625.20
A_68_P21489528	chr2:104334070-104334114	NM_001145824:554	Hipk3	INSIDE	0.516	3.319	1257.86	4174.89	1.714	1185.29	2031.60
A_68_P26954155	chr10:13685730-13685774	NM_010437:-432	Hivep2	PROMOTER	0.163	18.094	3983.63	72080.82	2.947	3668.21	10811.27
A_68_P26954148	chr10:13684931-13684975	NM_010437:-1232	Hivep2	PROMOTER	0.372	11.818	1911.70	22591.53	4.397	1901.72	8361.35
A_68_P26954164	chr10:13687012-13687056	NM_010437:850	Hivep2	INSIDE	0.585	5.661	888.05	5027.48	3.311	799.37	2647.04
A_68_P26954156	chr10:13685876-13685920	NM_010437:-286	Hivep2	PROMOTER	0.592	2.770	2655.91	7356.14	1.639	1959.55	3211.82
A_68_P26954153	chr10:13685444-13685488	NM_010437:-718	Hivep2	PROMOTER	0.415	3.528	1426.40	5032.53	1.462	1267.20	1853.18
A_68_P27185079	chr10:61732442-61732486	NM_001146100:70705	Hk1	INSIDE	0.358	5.186	3663.08	18996.14	1.856	2365.73	4391.54

ProbeName	Target position of probe on CpG island microarray	TargetID	GeneSymbol	CpG island Description	Ratio of relative methylation (TiO_2-NP/Vehicle)	Sham group			TiO_2-H group		
						Relative methylation (Cy5/Cy3)	Cy3 signal (Input)	Cy5 signal (MeDIP)	Relative methylation (Cy5/Cy3)	Cy3 signal (Input)	Cy5 signal (MeDIP)
A_68_P27185080	chr10:61732562-61732606	NM_001146100:70585	Hk1	INSIDE	0.574	21.237	2914.56	61897.34	12.193	2377.54	28989.19
A_68_P27185476	chr10:61802626-61802671	NM_001146100:521	Hk1	INSIDE	0.454	0.677	2861.20	1937.66	0.308	2117.44	651.59
A_68_P24589794	chr6:82723294-82723338	NM_013820:1132	Hk2	INSIDE	0.356	0.256	2175.77	557.57	0.091	1885.48	172.02
A_68_P24589795	chr6:82723374-82723418	NM_013820:1052	Hk2	INSIDE	0.446	0.687	2554.65	1755.07	0.306	2229.60	682.80
A_68_P21978078	chr3:19957968-19958012	NM_144959:180	Hltf	INSIDE	0.131	33.226	5464.91	181574.70	4.368	5318.58	23230.84
A_68_P20897050	chr1:186542380-186542424	NM_008250:13970	Hlx	DOWNSTREAM	0.449	5.144	1415.69	7282.56	2.308	1250.52	2886.21
A_68_P20897049	chr1:186542249-186542293	NM_008250:14102	Hlx	DOWNSTREAM	0.594	0.488	1327.74	647.62	0.29	1139.57	330.15
A_68_P20897052	chr1:186542532-186542576	NM_008250:13818	Hlx	DOWNSTREAM	0.432	0.509	941.78	479.40	0.22	816.29	179.55
A_68_P27289029	chr10:80809838-80809882	NM_001163165:3311	Hmg20b	INSIDE	0.266	16.925	1854.76	31391.82	4.505	1274.50	5741.75
A_68_P27289055	chr10:80813338-80813382	NM_001163166:-158	Hmg20b	DIVERGENT_PROMOTER	0.43	6.851	5741.55	39337.41	2.946	4085.62	12034.82
A_68_P27289051	chr10:80812913-80812957	NM_001163165:237	Hmg20b	INSIDE	0.568	0.369	1489.33	549.71	0.21	1183.52	248.28
A_68_P27289026	chr10:80809521-80809565	NM_001163165:3629	Hmg20b	INSIDE	0.599	5.368	1920.92	10312.00	3.217	1791.67	5764.31
A_68_P27289052	chr10:80813045-80813089	NM_001163165:105	Hmg20b	INSIDE	0.618	0.549	1560.79	856.29	0.339	1217.17	412.93
A_68_P31117105	chr17:27693117-27693161	NM_001025427:-380	Hmga1	PROMOTER	0.595	10.623	898.14	9541.14	6.322	762.59	4820.81
A_68_P31117097	chr17:27692164-27692208	NM_001025427:-1332	Hmga1	PROMOTER	0.6	6.284	1209.90	7602.54	3.767	1051.27	3960.53
A_68_P31117117	chr17:27694485-27694529	NM_001166476:909	Hmga1-rs1	INSIDE	0.476	7.577	4539.78	34397.05	3.605	3276.07	11808.89
A_68_P27497678	chr10:119913602-119913646	NM_010441:367	Hmga2	INSIDE	0.234	36.455	884.15	32231.96	8.518	1384.54	11793.17
A_68_P27497681	chr10:119914095-119914139	NM_010441:-125	Hmga2	PROMOTER	0.267	7.840	1987.12	15578.65	2.095	1722.23	3608.59
A_68_P27497666	chr10:119912069-119912113	NM_010441:1901	Hmga2	INSIDE	0.618	0.280	2463.22	689.41	0.173	1661.37	287.53
A_68_P27497667	chr10:119912187-119912231	NM_010441:1783	Hmga2	INSIDE	0.523	0.477	939.19	447.68	0.249	689.79	171.96
A_68_P32454254	chrX:68810106-68810150	NM_008253:961	Hmgb3	INSIDE	0.162	18.039	436.64	7876.60	2.917	591.35	1725.19
A_68_P23331345	chr4:135502447-135502491	NM_008254:101	Hmgcl	INSIDE	0.654	0.608	1367.65	831.00	0.398	1049.18	417.24
A_68_P30991207	chr16:96348653-96348697	NM_008251:658	Hmgn1	INSIDE	0.289	7.886	5024.79	39623.60	2.279	3933.67	8964.38
A_68_P30991213	chr16:96349447-96349491	NM_008251:-136	Hmgn1	PROMOTER	0.597	0.436	4737.17	2063.08	0.26	3741.26	973.10
A_68_P30991212	chr16:96349324-96349368	NM_008251:-14	Hmgn1	PROMOTER	0.447	1.663	6448.44	10724.32	0.744	5013.27	3730.00
A_68_P23320290	chr4:133524024-133524068	NM_016957:-140	Hmgn2	PROMOTER	0.496	4.606	2875.39	13243.59	2.283	2416.42	5515.89
A_68_P26682076	chr9:83039410-83039454	NM_026122:782	Hmgn3	INSIDE	0.625	0.463	1642.78	760.06	0.289	1237.20	357.97
A_68_P25971363	chr8:77517838-77517882	NM_178017:259	Hmgxb4	INSIDE	0.622	5.667	1304.00	7390.26	3.525	1090.59	3844.49
A_68_P23600267	chr5:35740761-35740805	NM_010445:9017	Hmx1	DOWNSTREAM	0.543	0.358	1608.62	575.65	0.194	1263.66	245.40
A_68_P23600191	chr5:35731800-35731844	NM_010445:57	Hmx1	INSIDE	0.447	3.596	902.38	3244.72	1.606	791.01	1270.17
A_68_P25528895	chr7:138685558-138685602	NM_008257:-896	Hmx3	PROMOTER	0.204	15.034	811.86	12205.31	3.068	712.80	2186.93
A_68_P25528898	chr7:138685827-138685871	NM_008257:-628	Hmx3	PROMOTER	0.395	8.652	323.81	2801.55	3.419	308.96	1056.24
A_68_P25528897	chr7:138685710-138685754	NM_008257:-744	Hmx3	PROMOTER	0.55	6.961	2094.81	14582.64	3.828	1707.81	6537.35
A_68_P21345221	chr2:75497073-75497117	NM_053263:-221	Hnrnpa3	PROMOTER	0.609	0.665	808.30	537.82	0.405	716.29	290.38
A_68_P29604760	chr14:52723420-52723464	NM_001170981:261	Hnrnpc	INSIDE	0.449	5.877	2695.94	15843.90	2.642	2393.20	6322.00
A_68_P23927133	chr5:100407179-100407223	NM_001077265:757	Hnrnpd	INSIDE	0.192	0.661	8208.25	5425.04	0.127	5011.45	636.97
A_68_P23927140	chr5:100408133-100408177	NM_001077265:-197	Hnrnpd	PROMOTER	0.235	6.833	1933.32	13209.58	1.604	1529.46	2453.81
A_68_P24783935	chr6:117857266-117857310	NM_001166428:-533	Hnrnpf	PROMOTER	0.313	13.451	3197.36	43008.23	4.213	2590.15	10912.60
A_68_P24784010	chr6:117867385-117867429	NM_001166427:95	Hnrnpf	INSIDE	0.423	12.559	2033.11	25534.01	5.309	1853.52	9839.65
A_68_P24784008	chr6:117867198-117867242	NM_001166427:-91	Hnrnpf	PROMOTER	0.481	4.328	3074.48	13306.78	2.082	2613.45	5439.97
A_68_P24784006	chr6:117866996-117867040	NM_001166427:-293	Hnrnpf	PROMOTER	0.509	6.218	2874.88	17875.18	3.167	2570.96	8141.43
A_68_P24783941	chr6:117857819-117857863	NM_001166427:-9471	Hnrnpf	PROMOTER	0.564	12.162	744.48	9054.49	6.859	907.09	6221.42
A_68_P27788199	chr11:50190689-50190733	NM_021510:-510	Hnrnph1	PROMOTER	0.173	28.444	4066.40	115663.80	4.911	3731.46	18325.90
A_68_P27788210	chr11:50191797-50191841	NM_021510:598	Hnrnph1	INSIDE	0.592	0.476	2356.37	1120.75	0.281	1778.98	500.75
A_68_P27189024	chr10:62485962-62486006	NM_001079824:613	Hnrnph3	INSIDE	0.653	0.502	3207.96	1610.80	0.328	2394.68	785.23
A_68_P25023673	chr7:29596377-29596421	NM_177301:490	Hnrnpl	INSIDE	0.311	7.520	2200.99	16550.83	2.336	1825.02	4263.84

ProbeName	Target position of probe on CpG island microarray	TargetID	GeneSymbol	CpG island Description	Ratio of relative methylation (TiO_2-NP/Vehicle)	Sham group			TiO_2-H group		
						Relative methylation (Cy5/Cy3)	Cy3 signal (Input)	Cy5 signal (MeDIP)	Relative methylation (Cy5/Cy3)	Cy3 signal (Input)	Cy5 signal (MeDIP)
A_68_P25023656	chr7:29594118-29594162	NM_177301:-1768	Hnrnpl	PROMOTER	0.472	4.505	1315.37	5925.28	2.128	1051.94	2238.01
A_68_P25023658	chr7:29594290-29594344	NM_177301:-1592	Hnrnpl	PROMOTER	0.496	8.739	1109.92	9700.05	4.335	1287.04	5579.80
A_68_P23333240	chr4:135866700-135866744	NM_028871:-168	Hnrnpr	PROMOTER	0.513	3.851	1413.45	5442.72	1.977	1214.01	2400.42
A_68_P23927384	chr5:100467950-100467994	NM_016690:269	Hnrpdl	INSIDE	0.134	43.715	3714.95	162398.80	5.868	4379.64	25700.04
A_68_P23927390	chr5:100468503-100468547	NM_016690:-283	Hnrpdl	DIVERGENT_PROMOTER	0.659	4.712	879.10	4142.33	3.106	757.38	2352.36
A_68_P23927389	chr5:100468389-100468433	NM_016690:-169	Hnrpdl	DIVERGENT_PROMOTER	0.337	6.405	693.79	4443.46	2.159	558.94	1206.75
A_68_P31381047	chr17:80461465-80461509	NM_144802:188	Hnrpll	INSIDE	0.406	15.690	1257.37	19727.94	6.375	1046.22	6670.04
A_68_P31381046	chr17:80461343-80461387	NM_144802:310	Hnrpll	INSIDE	0.414	7.379	1379.59	10180.16	3.057	1129.01	3451.28
A_68_P31381042	chr17:80460659-80460703	NM_144802:994	Hnrpll	INSIDE	0.474	0.410	1544.79	632.75	0.194	1168.53	226.70
A_68_P31381048	chr17:80461571-80461615	NM_144802:82	Hnrpll	INSIDE	0.478	4.538	2034.89	9234.84	2.169	1586.21	3440.13
A_68_P29614701	chr14:55482992-55483036	NM_001177705:-19	Homez	PROMOTER	0.459	5.566	2920.09	16251.83	2.557	2550.01	6520.29
A_68_P23126053	chr4:95633810-95633854	NM_030014:-237	Hook1	PROMOTER	0.425	0.250	2611.23	651.82	0.106	1969.93	209.10
A_68_P26023052	chr8:87514587-87514631	NM_001167991:115	Hook2	INSIDE	0.46	0.377	1391.61	525.31	0.174	1095.09	190.06
A_68_P26023020	chr8:87509915-87509959	NM_001167991:-4557	Hook2	DIVERGENT_PROMOTER	0.608	0.364	1244.68	453.02	0.221	1004.59	222.48
A_68_P25730819	chr8:27229628-27229672	NM_207659:46	Hook3	INSIDE	0.41	5.149	1514.23	7796.30	2.113	1283.95	2712.44
A_68_P25730815	chr8:27229276-27229320	NM_207659:398	Hook3	INSIDE	0.428	4.570	1147.82	5245.98	1.956	1203.31	2354.26
A_68_P25730814	chr8:27229164-27229208	NM_207659:510	Hook3	INSIDE	0.605	0.386	1911.28	738.63	0.234	1542.31	360.44
A_68_P25730817	chr8:27229449-27229493	NM_207659:226	Hook3	INSIDE	0.626	3.415	2837.07	9689.55	2.137	2199.55	4701.18
A_68_P24448669	chr6:52108855-52108899	NM_010449:-560	Hoxa1	PROMOTER	0.598	0.338	2086.70	706.14	0.202	1716.31	347.33
A_68_P24449298	chr6:52184077-52184121	NM_008263:840	Hoxa10	INSIDE	0.367	12.663	1195.87	15143.50	4.649	931.42	4330.16
A_68_P24449291	chr6:52183467-52183515	NM_008263:1448	Hoxa10	INSIDE	0.573	0.257	2769.56	711.49	0.147	1872.60	275.52
A_68_P24449398	chr6:52195787-52195837	NM_010450:-46	Hoxa11	PROMOTER	0.393	0.340	1621.71	551.18	0.134	1309.12	174.90
A_68_P24449403	chr6:52196333-52196377	NM_010450:-588	Hoxa11	PROMOTER	0.41	4.278	1791.22	7663.59	1.754	1486.73	2607.06
A_68_P24449396	chr6:52195498-52195542	NM_010450:246	Hoxa11	INSIDE	0.218	2.593	2456.92	6370.88	0.565	1856.40	1049.58
A_68_P24449533	chr6:52211079-52211123	NM_008264:-226	Hoxa13	PROMOTER	0.184	27.325	2779.56	75951.19	5.025	2595.49	13043.32
A_68_P24449527	chr6:52210204-52210248	NM_008264:648	Hoxa13	INSIDE	0.306	8.045	1325.53	10664.32	2.463	1358.88	3347.26
A_68_P24449526	chr6:52210086-52210130	NM_008264:766	Hoxa13	INSIDE	0.583	0.612	1543.12	944.21	0.357	1192.89	425.77
A_68_P24448783	chr6:52122417-52122461	NM_010452:40628	Hoxa3	INSIDE	0.373	0.361	2282.94	824.36	0.135	1770.34	238.52
A_68_P24448786	chr6:52122729-52122773	NM_010452:40316	Hoxa3	INSIDE	0.647	5.949	2940.50	17492.75	3.85	2448.92	9427.52
A_68_P24449232	chr6:52176211-52176255	NM_010456:1137	Hoxa9	INSIDE	0.418	4.667	4308.80	20109.61	1.951	3552.25	6930.77
A_68_P24449229	chr6:52175914-52175958	NM_010456:1433	Hoxa9	INSIDE	0.645	2.952	1625.79	4799.94	1.906	1360.77	2593.18
A_68_P28043961	chr11:96207239-96207283	NM_010458:2178	Hoxb3	INSIDE	0.116	2.713	1957.52	5310.17	0.314	1554.32	488.80
A_68_P28043962	chr11:96207315-96207359	NM_010458:2254	Hoxb3	INSIDE	0.182	2.019	3111.63	6281.84	0.368	2332.86	859.27
A_68_P28043848	chr11:96194036-96194080	NM_001079869:9619	Hoxb3	INSIDE	0.66	0.335	2594.32	870.23	0.221	1726.37	382.37
A_68_P28043737	chr11:96180501-96180545	NM_010459:942	Hoxb4	INSIDE	0.035	1.745	4244.68	7406.77	0.061	2806.90	171.78
A_68_P28043617	chr11:96166483-96166527	NM_008268:1679	Hoxb5	INSIDE	0.426	5.525	7634.92	42183.91	2.351	5844.08	13741.38
A_68_P28043432	chr11:96144310-96144354	NM_010461:1114	Hoxb8	INSIDE	0.326	8.789	684.92	6019.69	2.863	581.39	1664.65
A_68_P28043442	chr11:96145448-96145492	NM_010461:2252	Hoxb8	INSIDE	0.639	4.287	2430.10	10416.96	2.74	2032.91	5569.52
A_68_P28043342	chr11:96133436-96133480	NM_008270:815	Hoxb9	INSIDE	0.382	10.017	2588.77	25931.84	3.827	2366.46	9057.44
A_68_P30501057	chr15:102796954-102796998	NM_010462:-250	Hoxc10	PROMOTER	0.622	0.627	1951.61	1223.01	0.39	1587.51	618.69
A_68_P30500976	chr15:102786820-102786864	NM_001024842:1886	Hoxc11	INSIDE	0.51	0.527	3255.94	1716.96	0.269	2488.92	669.16
A_68_P30500682	chr15:102752309-102752353	NM_010464:769	Hoxc13	INSIDE	0.576	3.756	504.80	1896.02	2.163	464.17	1004.06
A_68_P30501631	chr15:102865426-102865470	NM_013553:623	Hoxc4	INSIDE	0.54	0.372	2997.88	1114.74	0.201	2366.66	475.13
A_68_P21339341	chr2:74591156-74591200	NM_010467:-9858	Hoxd1	PROMOTER	0.269	11.931	3358.33	40069.74	3.213	2895.54	9304.74
A_68_P21339410	chr2:74601035-74601079	NM_010467:20	Hoxd1	INSIDE	0.636	0.650	1129.14	733.98	0.414	902.76	373.35
A_68_P21338754	chr2:74520144-74520188	NM_008273:-283	Hoxd11	PROMOTER	0.29	10.090	1152.53	11628.88	2.927	1130.00	3307.47

ProbeName	Target position of probe on CpG island microarray	TargetID	GeneSymbol	CpG island Description	Ratio of relative methylation (TiO_2-NP/Vehicle)	Sham group			TiO_2-H group		
						Relative methylation (Cy5/Cy3)	Cy3 signal (Input)	Cy5 signal (MeDIP)	Relative methylation (Cy5/Cy3)	Cy3 signal (Input)	Cy5 signal (MeDIP)
A_68_P21338756	chr2:74520344-74520390	NM_008273:-83	Hoxd11	PROMOTER	0.644	0.497	922.46	458.51	0.32	703.39	225.13
A_68_P21338643	chr2:74506367-74506411	NM_008275:22	Hoxd13	INSIDE	0.451	4.076	3412.48	13910.78	1.84	2661.02	4895.26
A_68_P21338645	chr2:74506594-74506638	NM_008275:250	Hoxd13	INSIDE	0.626	0.326	3711.37	1210.24	0.204	2726.26	556.72
A_68_P21339293	chr2:74585053-74585097	NM_010468:35025	Hoxd3	INSIDE	0.24	0.294	3664.04	1077.55	0.07	2458.65	173.22
A_68_P21339240	chr2:74578542-74578586	NM_010468:28515	Hoxd3	INSIDE	0.484	4.150	1611.23	6686.36	2.008	1490.45	2992.35
A_68_P21339003	chr2:74549994-74550038	NM_010468:-33	Hoxd3	PROMOTER	0.535	5.669	390.69	2214.89	3.03	356.78	1081.16
A_68_P21339006	chr2:74550322-74550366	NM_010468:295	Hoxd3	INSIDE	0.657	3.767	1900.04	7157.20	2.473	1647.51	4074.33
A_68_P21338948	chr2:74542744-74542788	NM_008276:-779	Hoxd8	PROMOTER	0.132	30.145	1083.35	32657.17	3.984	1246.28	4965.44
A_68_P21338950	chr2:74542960-74543004	NM_008276:-563	Hoxd8	PROMOTER	0.506	5.803	959.04	5565.40	2.935	795.92	2335.65
A_68_P23344531	chr4:137772915-137772959	NM_001122897:-109	Hp1bp3	PROMOTER	0.453	46.508	5205.70	242105.10	21.045	4402.29	92647.28
A_68_P23344527	chr4:137772352-137772396	NM_001122896:-167	Hp1bp3	PROMOTER	0.55	3.310	1887.70	6249.09	1.82	1593.82	2900.41
A_68_P23295046	chr4:128798355-128798400	NM_010471:158	Hpca	INSIDE	0.499	8.433	1029.71	8683.63	4.206	997.52	4195.85
A_68_P23295047	chr4:128798459-128798503	NM_010471:55	Hpca	INSIDE	0.529	3.339	1064.16	3552.78	1.767	891.83	1576.08
A_68_P28271881	chr12:17697954-17697998	NM_016677:357	Hpcal1	INSIDE	0.606	0.179	5858.35	1048.37	0.108	3889.39	421.75
A_68_P23261234	chr4:122866488-122866532	NM_174998:5764	Hpcal4	INSIDE	0.248	22.031	7416.31	163385.30	5.473	7757.84	42458.31
A_68_P23261232	chr4:122866227-122866271	NM_174998:5502	Hpcal4	INSIDE	0.434	7.854	3244.64	25482.17	3.409	2517.96	8583.65
A_68_P25885333	chr8:58773316-58773360	NM_008278:-10	Hpgd	PROMOTER	0.489	3.538	1767.13	6252.74	1.729	1377.65	2381.37
A_68_P32385147	chrX:50341550-50341594	NM_013556:318	Hprt	INSIDE	0.283	11.322	1408.15	15942.46	3.2	1739.25	5565.11
A_68_P32385145	chrX:50341393-50341437	NM_013556:160	Hprt	INSIDE	0.664	7.736	907.96	7023.88	5.14	1142.58	5873.30
A_68_P21978014	chr3:19935050-19935094	NM_001146323:238	Hps3	INSIDE	0.363	0.678	995.89	675.54	0.246	770.72	189.69
A_68_P25101365	chr7:54051013-54051057	NM_001005247:217	Hps5	INSIDE	0.637	0.386	3602.83	1391.76	0.246	2634.05	648.51
A_68_P32141596	chr19:46078435-46078479	NM_176785:489	Hps6	INSIDE	0.504	0.097	5378.81	520.77	0.049	3786.92	184.82
A_68_P29702038	chr14:70954648-70954692	NM_021877:808	Hr	INSIDE	0.506	0.731	2602.65	1901.52	0.369	2160.25	798.11
A_68_P29702031	chr14:70953904-70953948	NM_021877:64	Hr	INSIDE	0.504	2.850	6755.28	19254.55	1.437	5072.39	7291.46
A_68_P30634660	chr16:29210031-29210075	NM_013751:272	Hrasls	INSIDE	0.038	6.808	4090.06	27844.91	0.256	3070.62	785.46
A_68_P30634661	chr16:29210118-29210162	NM_013751:360	Hrasls	INSIDE	0.207	1.598	1610.09	2573.39	0.331	1239.09	410.17
A_68_P29048046	chr13:54287380-54287425	NM_008286:-95	Hrh2	PROMOTER	0.459	6.019	1033.33	6219.25	2.761	884.34	2441.84
A_68_P21901458	chr2:179839037-179839081	NM_133849:-131	Hrh3	PROMOTER	0.664	0.593	7196.23	4270.60	0.394	5222.48	2059.23
A_68_P24025038	chr5:118620106-118620150	NM_007545:356	Hrk	INSIDE	0.405	5.568	1838.36	10236.71	2.253	1390.75	3133.72
A_68_P24025031	chr5:118619337-118619381	NM_007545:-414	Hrk	PROMOTER	0.6	0.302	2608.62	789.09	0.181	1852.22	335.97
A_68_P22603827	chr3:144232599-144232643	NM_011828:560	Hs2st1	INSIDE	0.491	0.525	1798.73	943.85	0.257	1361.95	350.57
A_68_P22603829	chr3:144232802-144232849	NM_011828:355	Hs2st1	INSIDE	0.409	4.233	1464.18	6197.45	1.731	1097.38	1899.86
A_68_P23627551	chr5:40035622-40035667	NM_010474:226	Hs3st1	INSIDE	0.402	3.845	4406.23	16940.34	1.547	3554.46	5497.59
A_68_P25474216	chr7:128643864-128643910	NM_001081327:108077	Hs3st2	INSIDE	0.271	23.314	987.10	23013.58	6.307	1326.15	8364.54
A_68_P25473609	chr7:128536673-128536717	NM_001081327:885	Hs3st2	INSIDE	0.446	0.319	1616.53	515.90	0.142	1331.88	189.60
A_68_P27862290	chr11:63734698-63734742	NM_018805:1066	Hs3st3b1	INSIDE	0.259	29.304	2551.94	74782.77	7.579	2667.89	20219.22
A_68_P27862275	chr11:63732951-63732995	NM_018805:2814	Hs3st3b1	INSIDE	0.568	8.484	722.88	6132.87	4.819	596.33	2873.75
A_68_P27862296	chr11:63735472-63735516	NM_018805:292	Hs3st3b1	INSIDE	0.665	0.241	2218.35	534.22	0.16	1689.01	270.32
A_68_P31099381	chr17:24889759-24889803	NM_001012402:-167	Hs3st6	PROMOTER	0.433	0.377	1437.71	542.48	0.163	1078.25	175.97
A_68_P20146151	chr1:36125666-36125710	NM_015818:444	Hs6st1	INSIDE	0.522	4.377	955.86	4183.66	2.283	747.57	1707.05
A_68_P32378028	chrX:49034553-49034597	NM_015819:-920	Hs6st2	PROMOTER	0.049	33.281	991.44	32995.99	1.629	1273.83	2075.54
A_68_P29942854	chr14:119537426-119537470	NM_015820:-38	Hs6st3	PROMOTER	0.635	4.136	1214.23	5021.90	2.627	1037.33	2724.78
A_68_P29942852	chr14:119537211-119537255	NM_015820:-254	Hs6st3	PROMOTER	0.592	2.925	777.84	2275.28	1.731	742.20	1284.88
A_68_P31863131	chr18:80433374-80433418	NM_001136181:10445	Hsbp1l1	INSIDE	0.48	0.365	1365.02	497.74	0.175	1002.66	175.52
A_68_P26134297	chr8:108042949-108042993	NM_008289:325	Hsd11b2	INSIDE	0.417	5.234	654.69	3426.55	2.181	607.34	1324.76
A_68_P28071796	chr11:100941137-100941181	NM_010475:1434	Hsd17b1	INSIDE	0.376	0.429	1533.39	658.38	0.162	1070.95	173.02

ProbeName	Target position of probe on CpG island microarray	TargetID	GeneSymbol	CpG island Description	Ratio of relative methylation (TiO_2-NP/Vehicle)	Sham group			TiO_2-H group		
						Relative methylation (Cy5/Cy3)	Cy3 signal (Input)	Cy5 signal (MeDIP)	Relative methylation (Cy5/Cy3)	Cy3 signal (Input)	Cy5 signal (MeDIP)
A_68_P20819669	chr1:171899278-171899322	NM_010476:36	Hsd17b7	INSIDE	0.365	0.453	2407.09	1089.48	0.165	1486.78	245.35
A_68_P20819668	chr1:171899210-171899254	NM_010476:104	Hsd17b7	INSIDE	0.608	0.661	3478.82	2299.86	0.402	2308.79	927.99
A_68_P26214830	chr8:122098835-122098879	NM_175185:244	Hsdl1	INSIDE	0.36	0.686	976.71	670.43	0.247	850.02	210.30
A_68_P22957168	chr4:59594155-59594199	NM_024255:-258	Hsdl2	PROMOTER	0.606	2.798	2946.60	8245.50	1.697	2125.33	3605.96
A_68_P30348596	chr15:76307956-76308000	NM_008296:104	Hsf1	INSIDE	0.456	0.329	2695.12	887.04	0.15	1974.23	296.35
A_68_P27160596	chr10:57206710-57206754	NM_008297:542	Hsf2	INSIDE	0.656	2.874	912.61	2622.59	1.885	799.26	1506.65
A_68_P26132621	chr8:107793840-107793884	NM_011939:89	Hsf4	INSIDE	0.625	3.372	1203.70	4058.35	2.107	941.61	1984.28
A_68_P27995284	chr11:87452873-87452917	NM_001045527:22229	Hsf5	INSIDE	0.392	12.151	1975.52	24003.92	4.759	1993.58	9487.26
A_68_P27995151	chr11:87430824-87430868	NM_001045527:181	Hsf5	INSIDE	0.539	4.320	1230.71	5316.17	2.328	1094.48	2548.05
A_68_P27995154	chr11:87431401-87431445	NM_001045527:757	Hsf5	INSIDE	0.374	4.696	1225.21	5753.72	1.758	943.36	1658.90
A_68_P27995153	chr11:87431204-87431248	NM_001045527:561	Hsf5	INSIDE	0.447	3.834	1357.53	5204.98	1.713	1038.76	1778.89
A_68_P28739824	chr12:111934703-111934747	NM_010480:-119	Hsp90aa1	PROMOTER	0.348	29.054	1407.62	40896.73	10.118	1432.84	14496.78
A_68_P31201910	chr17:45710240-45710284	NM_008302:-52	Hsp90ab1	PROMOTER	0.272	6.393	2650.05	16943.05	1.738	2212.71	3844.69
A_68_P28553815	chr12:77504735-77504779	NM_001002012:-406	Hspa2	PROMOTER	0.334	6.979	2825.42	19718.34	2.33	2677.11	6236.68
A_68_P28553818	chr12:77505144-77505188	NM_008301:-190	Hspa2	PROMOTER	0.474	7.039	2117.11	14902.49	3.335	1803.55	6014.91
A_68_P27804074	chr11:53113101-53113145	NM_008300:859	Hspa4	INSIDE	0.319	10.167	6557.04	66663.24	3.244	5943.61	19280.61
A_68_P27804084	chr11:53114187-53114231	NM_008300:-227	Hspa4	PROMOTER	0.663	0.365	1310.43	478.09	0.242	1130.30	273.34
A_68_P22081210	chr3:40548895-40548939	NM_011020:-618	Hspa4l	PROMOTER	0.175	12.126	1087.83	13191.32	2.116	1274.93	2698.02
A_68_P22081217	chr3:40549685-40549729	NM_011020:172	Hspa4l	INSIDE	0.501	4.333	1714.35	7427.61	2.17	1487.79	3229.13
A_68_P22081213	chr3:40549234-40549278	NM_011020:-278	Hspa4l	PROMOTER	0.537	4.132	1009.91	4173.25	2.219	909.99	2018.91
A_68_P22081212	chr3:40549078-40549122	NM_011020:-434	Hspa4l	PROMOTER	0.137	3.206	890.25	2853.76	0.44	776.70	341.71
A_68_P21129651	chr2:34627506-34627550	NM_001163434:-81	Hspa5	PROMOTER	0.378	0.649	1035.15	671.54	0.245	854.67	209.66
A_68_P26445380	chr9:40609023-40609067	NM_031165:-311	Hspa8	PROMOTER	0.213	11.513	2138.33	24617.57	2.452	1811.11	4440.18
A_68_P23364007	chr4:140981800-140981844	NM_013868:5129	Hspb7	DOWNSTREAM	0.323	0.335	2220.04	742.98	0.108	1612.02	174.39
A_68_P20240337	chr1:55144375-55144419	NM_010477:380	Hspd1	INSIDE	0.49	0.389	2843.83	1107.19	0.191	2252.86	430.11
A_68_P20240346	chr1:55145446-55145490	NM_008303:477	Hspe1	INSIDE	0.404	10.485	1395.32	14629.35	4.234	1212.37	5133.08
A_68_P23340287	chr4:137074439-137074483	NM_008305:49743	Hspg2	INSIDE	0.24	12.071	2269.66	27398.09	2.896	1810.99	5244.94
A_68_P23339949	chr4:137024813-137024857	NM_008305:117	Hspg2	INSIDE	0.406	0.575	2239.64	1288.49	0.233	1758.02	410.41
A_68_P23339947	chr4:137024556-137024600	NM_008305:-139	Hspg2	PROMOTER	0.56	3.391	2396.70	8128.03	1.898	1868.78	3546.50
A_68_P23340288	chr4:137074524-137074568	NM_008305:49829	Hspg2	INSIDE	0.125	11.251	3055.83	34381.26	1.412	2251.40	3178.98
A_68_P24186280	chr5:150438607-150438651	NM_013559:262	Hsph1	INSIDE	0.605	0.445	2805.76	1248.55	0.269	1999.08	538.14
A_68_P26674977	chr9:81526156-81526200	NM_010482:-19	Htr1b	PROMOTER	0.561	0.466	1418.38	660.48	0.261	1057.15	276.25
A_68_P23333958	chr4:135999081-135999125	NM_008309:19664	Htr1d	INSIDE	0.324	7.772	2928.54	22759.19	2.521	2250.36	5672.78
A_68_P23333957	chr4:135998981-135999032	NM_008309:19568	Htr1d	INSIDE	0.639	5.482	240.30	1317.21	3.504	187.29	656.28
A_68_P30823193	chr16:65105676-65105720	NM_008310:-88	Htr1f	PROMOTER	0.263	13.181	453.51	5977.76	3.46	371.85	1286.73
A_68_P23349533	chr4:138631065-138631109	NM_021358:-382	Htr6	PROMOTER	0.443	4.680	3115.79	14581.77	2.075	2430.71	5044.30
A_68_P23349526	chr4:138630332-138630376	NM_021358:350	Htr6	INSIDE	0.618	0.387	3999.32	1547.42	0.239	2661.55	636.77
A_68_P23349532	chr4:138630943-138630988	NM_021358:-261	Htr6	PROMOTER	0.418	0.612	3230.53	1975.73	0.255	2281.27	582.52
A_68_P32087899	chr19:36132232-36132276	NM_008315:-404	Htr7	PROMOTER	0.422	6.368	2128.49	13554.08	2.684	1913.48	5136.19
A_68_P32087889	chr19:36131064-36131112	NM_008315:762	Htr7	INSIDE	0.532	0.527	858.42	452.33	0.28	624.24	175.06
A_68_P30956559	chr16:90387283-90387327	NM_015755:663	Hunk	INSIDE	0.46	0.233	3352.55	779.56	0.107	2523.63	269.68
A_68_P26809623	chr9:107471781-107471825	NM_010489:309	Hyal2	INSIDE	0.506	5.159	4467.22	23048.09	2.613	3746.07	9788.24
A_68_P26162181	chr8:112889462-112889508	NM_172916:98608	Hydin	INSIDE	0.31	5.710	701.32	4004.42	1.767	522.91	924.17
A_68_P28280007	chr12:21322457-21322501	NM_026347:226	Iah1	INSIDE	0.479	3.366	2887.91	9719.69	1.611	2406.55	3877.25
A_68_P26695696	chr9:85642551-85642595	NM_001081282:369	Ibtk	INSIDE	0.427	0.281	1787.12	503.05	0.12	1456.91	175.12
A_68_P26695698	chr9:85642712-85642756	NM_001081282:207	Ibtk	INSIDE	0.587	5.949	621.89	3699.55	3.492	618.78	2160.68

ProbeName	Target position of probe on CpG island microarray	TargetID	GeneSymbol	CpG island Description	Ratio of relative methylation (TiO_2-NP/Vehicle)	Sham group			TiO_2-H group		
						Relative methylation (Cy5/Cy3)	Cy3 signal (Input)	Cy5 signal (MeDIP)	Relative methylation (Cy5/Cy3)	Cy3 signal (Input)	Cy5 signal (MeDIP)
A_68_P26345150	chr9:20836502-20836546	NM_008319:43	Icam5	INSIDE	0.298	13.156	4238.44	55762.66	3.925	3696.19	14506.28
A_68_P26658192	chr9:77961262-77961306	NM_019987:240	Ick	INSIDE	0.17	40.681	9071.49	369035.60	6.93	16858.96	116833.10
A_68_P26658189	chr9:77960962-77961006	NM_019987:-60	Ick	PROMOTER	0.557	4.250	1134.97	4823.69	2.369	965.33	2286.53
A_68_P26658190	chr9:77961055-77961099	NM_019987:32	Ick	INSIDE	0.426	0.682	1105.94	754.07	0.29	887.22	257.60
A_68_P27271929	chr10:77531813-77531858	NM_015790:-277	Icosl	PROMOTER	0.659	2.891	1246.98	3605.45	1.905	1083.22	2063.09
A_68_P27271934	chr10:77532267-77532311	NM_015790:176	Icosl	INSIDE	0.284	0.598	2334.17	1394.82	0.169	1631.55	276.47
A_68_P27271933	chr10:77532197-77532241	NM_015790:106	Icosl	INSIDE	0.392	1.820	6797.94	12371.89	0.713	5027.73	3582.32
A_68_P21750890	chr2:152560836-152560880	NM_010495:-1151	Id1	PROMOTER	0.509	0.532	2192.45	1167.06	0.271	1621.39	438.98
A_68_P21750891	chr2:152560945-152560989	NM_010495:-1043	Id1	PROMOTER	0.524	0.511	921.79	470.72	0.268	666.54	178.42
A_68_P21750931	chr2:152565335-152565379	NM_010495:3347	Id1	DOWNSTREAM	0.383	1.631	1472.39	2401.90	0.624	1166.84	728.48
A_68_P28293506	chr12:25781121-25781165	NM_010496:-185	Id2	PROMOTER	0.564	3.433	4629.95	15895.97	1.936	3315.96	6420.21
A_68_P28293548	chr12:25785727-25785771	NM_010496:-4791	Id2	PROMOTER	0.656	2.798	2853.81	7985.62	1.835	2310.39	4239.36
A_68_P23332340	chr4:135704489-135704533	NM_008321:4774	Id3	DOWNSTREAM	0.599	0.320	2201.19	703.53	0.192	1634.58	313.05
A_68_P23332312	chr4:135700753-135700797	NM_008321:1038	Id3	INSIDE	0.446	3.609	2257.86	8148.05	1.609	2065.82	3324.69
A_68_P23332310	chr4:135700553-135700600	NM_008321:840	Id3	INSIDE	0.313	13.243	200.24	2651.70	4.14	188.46	780.16
A_68_P23332339	chr4:135704351-135704395	NM_008321:4636	Id3	DOWNSTREAM	0.545	2.721	3422.92	9314.21	1.482	2513.25	3724.44
A_68_P29018477	chr13:48357119-48357169	NM_031166:349	Id4	INSIDE	0.414	0.263	2417.74	637.03	0.109	1701.23	185.59
A_68_P25264893	chr7:87260180-87260224	NM_173011:34	Idh2	INSIDE	0.406	11.747	891.99	10478.05	4.774	846.08	4039.24
A_68_P25264894	chr7:87260253-87260297	NM_173011:-38	Idh2	PROMOTER	0.425	6.352	700.60	4450.26	2.697	594.99	1604.80
A_68_P25264896	chr7:87260469-87260513	NM_173011:-254	Idh2	PROMOTER	0.583	0.665	1503.69	1000.58	0.388	1157.43	449.10
A_68_P25264890	chr7:87259796-87259840	NM_173011:418	Idh2	INSIDE	0.361	4.278	6810.67	29138.43	1.546	5157.58	7974.00
A_68_P26526551	chr9:54434255-54434299	NM_029573:-41	Idh3a	PROMOTER	0.379	0.439	3703.96	1627.04	0.166	2832.09	471.14
A_68_P26526552	chr9:54434359-54434404	NM_029573:64	Idh3a	INSIDE	0.649	0.509	6675.40	3397.48	0.33	4601.58	1519.80
A_68_P26526554	chr9:54434551-54434595	NM_029573:255	Idh3a	INSIDE	0.649	0.277	2048.76	567.74	0.18	1613.34	290.18
A_68_P26020946	chr8:87180770-87180814	NM_010499:5959	Ier2	DOWNSTREAM	0.526	0.185	2366.67	438.80	0.097	1758.83	171.40
A_68_P20735838	chr1:156946384-156946428	NM_010500:360	Ier5	INSIDE	0.594	2.825	1332.41	3764.42	1.678	1438.38	2413.00
A_68_P21103442	chr2:30328841-30328885	NM_030244:857	Ier5l	INSIDE	0.607	23.916	3361.79	80399.59	14.51	2933.67	42566.58
A_68_P21103418	chr2:30325823-30325867	NM_030244:3875	Ier5l	DOWNSTREAM	0.535	0.617	852.42	526.04	0.33	591.16	195.32
A_68_P21103437	chr2:30328220-30328264	NM_030244:1477	Ier5l	INSIDE	0.498	3.034	1014.83	3079.15	1.511	1137.59	1718.90
A_68_P23352769	chr4:139130258-139130302	NM_001205173:-354	Iffo2	PROMOTER	0.586	0.358	1781.71	638.35	0.21	1334.49	280.28
A_68_P26809753	chr9:107489861-107489907	NM_025903:-164	Ifrd2	PROMOTER	0.621	0.156	3321.26	518.12	0.097	2147.41	208.01
A_68_P21809984	chr2:162843268-162843312	NM_172150:83	Ift52	INSIDE	0.66	3.471	954.84	3314.14	2.291	845.26	1936.40
A_68_P24051357	chr5:123064217-123064261	NM_009879:289	Ift81	INSIDE	0.614	3.796	1327.98	5041.41	2.329	993.80	2314.67
A_68_P26585801	chr9:64949612-64949656	NM_020043:333	Igdcc4	INSIDE	0.467	0.365	1740.16	635.33	0.17	1366.39	232.84
A_68_P26585802	chr9:64949678-64949722	NM_020043:399	Igdcc4	INSIDE	0.623	2.710	2645.64	7169.06	1.689	2158.49	3646.37
A_68_P28041915	chr11:95866931-95866976	NM_009951:305	Igf2bp1	INSIDE	0.238	36.239	5007.01	181448.10	8.64	4839.69	41813.43
A_68_P28041918	chr11:95867212-95867256	NM_009951:24	Igf2bp1	INSIDE	0.469	0.406	1664.35	676.14	0.19	1233.14	234.71
A_68_P28041914	chr11:95866847-95866891	NM_009951:390	Igf2bp1	INSIDE	0.508	0.469	1627.41	762.56	0.238	1227.03	291.97
A_68_P28041927	chr11:95868155-95868199	NM_009951:-918	Igf2bp1	PROMOTER	0.555	0.542	1344.96	728.31	0.301	1077.68	323.96
A_68_P28041932	chr11:95868837-95868881	NM_009951:-1600	Igf2bp1	PROMOTER	0.56	0.391	3060.47	1195.25	0.219	2028.11	443.83
A_68_P28041910	chr11:95866408-95866452	NM_009951:828	Igf2bp1	INSIDE	0.237	0.729	1735.24	1265.16	0.173	1259.26	217.90
A_68_P28041933	chr11:95868905-95868949	NM_009951:-1668	Igf2bp1	PROMOTER	0.372	3.881	2748.76	10669.31	1.446	2109.42	3049.75
A_68_P30596432	chr16:22162719-22162763	NM_183029:632	Igf2bp2	INSIDE	0.238	16.068	1143.71	18377.39	3.817	948.27	3619.91
A_68_P30596436	chr16:22163233-22163277	NM_183029:118	Igf2bp2	INSIDE	0.543	10.093	941.58	9503.77	5.483	768.01	4211.01
A_68_P24431596	chr6:49163809-49163853	NM_023670:1123	Igf2bp3	INSIDE	0.495	0.342	2254.13	771.90	0.169	1602.58	271.56
A_68_P24431605	chr6:49164853-49164897	NM_023670:79	Igf2bp3	INSIDE	0.64	2.446	1200.20	2935.23	1.564	904.04	1413.99

ProbeName	Target position of probe on CpG island microarray	TargetID	GeneSymbol	CpG island Description	Ratio of relative methylation (TiO_2-NP/Vehicle)	Sham group			TiO_2-H group		
						Relative methylation (Cy5/Cy3)	Cy3 signal (Input)	Cy5 signal (MeDIP)	Relative methylation (Cy5/Cy3)	Cy3 signal (Input)	Cy5 signal (MeDIP)
A_68_P31053794	chr17:12962250-12962294	NM_010515:300	Igf2r	INSIDE	0.571	2.806	909.27	2551.70	1.603	737.76	1182.40
A_68_P20339424	chr1:72920224-72920268	NM_010518:1193	Igfbp5	INSIDE	0.494	0.539	837.59	451.40	0.266	663.31	176.54
A_68_P25082512	chr7:50721931-50721975	NM_001164518:23441	Iglon5	DOWNSTREAM	0.315	13.920	1621.44	22570.20	4.381	1661.34	7277.54
A_68_P25082509	chr7:50721683-50721727	NM_001164518:23689	Iglon5	DOWNSTREAM	0.422	0.347	1566.64	543.24	0.146	1157.36	169.51
A_68_P23356005	chr4:139665180-139665224	NM_198610:137524	Igsf21	INSIDE	0.512	0.487	1416.44	689.49	0.249	1136.35	283.18
A_68_P22375206	chr3:101254965-101255009	NM_207205:73939	Igsf3	INSIDE	0.246	7.953	1208.33	9609.83	1.957	922.74	1805.43
A_68_P22375207	chr3:101255068-101255112	NM_207205:74043	Igsf3	INSIDE	0.65	2.901	1115.46	3236.07	1.886	926.56	1747.79
A_68_P20832289	chr1:174242640-174242684	NM_080419:125	Igsf8	INSIDE	0.631	0.392	3689.98	1447.67	0.247	2802.52	693.29
A_68_P20351507	chr1:74997610-74997654	NM_010544:593	Ihh	INSIDE	0.478	0.690	1656.90	1143.72	0.33	1282.44	423.08
A_68_P20351513	chr1:74998432-74998476	NM_010544:-229	Ihh	PROMOTER	0.351	4.578	2899.96	13275.51	1.607	2471.08	3970.40
A_68_P27339938	chr10:90545771-90545815	NM_026166:9	Ikbip	INSIDE	0.529	3.291	1593.85	5245.38	1.742	1258.26	2191.73
A_68_P27591949	chr11:11585605-11585649	NM_001025597:-589	Ikzf1	PROMOTER	0.596	12.762	351.87	4490.46	7.602	344.11	2615.95
A_68_P27542462	chr10:128082827-128082871	NM_011772:201	Ikzf4	INSIDE	0.47	13.309	3088.95	41110.10	6.26	2729.69	17086.53
A_68_P25528245	chr7:138553702-138553746	NM_175115:268	Ikzf5	INSIDE	0.658	0.713	1720.92	1227.00	0.469	1320.08	619.57
A_68_P25954269	chr8:73340672-73340716	NM_008353:8347	Il12rb1	INSIDE	0.035	5.004	3144.10	15733.39	0.173	2490.45	430.72
A_68_P25954190	chr8:73328850-73328894	NM_008353:-3475	Il12rb1	PROMOTER	0.652	0.603	3195.01	1927.03	0.393	2412.25	948.60
A_68_P32317010	chrX:33652351-33652395	NM_133990:239	Il13ra1	INSIDE	0.447	3.246	3200.83	10389.80	1.451	4193.97	6084.60
A_68_P29628416	chr14:58143558-58143602	NM_145837:-85	Il17d	PROMOTER	0.253	7.796	1589.63	12392.26	1.971	1460.23	2878.24
A_68_P24798577	chr6:120413598-120413642	NM_008359:406	Il17ra	INSIDE	0.482	0.314	1932.85	606.46	0.151	1661.27	251.21
A_68_P24759767	chr6:113432661-113432705	NM_134159:11234	Il17rc	INSIDE	0.244	1.703	1847.65	3146.59	0.415	1456.32	604.52
A_68_P24759766	chr6:113432543-113432587	NM_134159:11116	Il17rc	INSIDE	0.429	7.578	1851.07	14027.48	3.253	1872.12	6090.91
A_68_P24759668	chr6:113419933-113419977	NM_001034029:11066	Il17re	INSIDE	0.589	3.547	1494.55	5301.10	2.088	1306.49	2727.72
A_68_P20170470	chr1:40323556-40323600	NM_001123382:148	Il1r1	INSIDE	0.207	41.897	1157.58	48499.53	8.68	1507.43	13083.92
A_68_P30621546	chr16:26581967-26582011	NM_134103:198	Il1rap	INSIDE	0.636	5.367	2008.65	10780.79	3.411	1704.95	5816.21
A_68_P30621544	chr16:26581734-26581778	NM_134103:-34	Il1rap	PROMOTER	0.535	0.563	889.14	501.00	0.301	744.09	224.18
A_68_P26017803	chr8:86566236-86566280	NM_016671:216	Il27ra	INSIDE	0.646	0.470	3994.59	1878.06	0.304	2955.02	897.99
A_68_P25089584	chr7:52095285-52095329	NM_010215:3649	Il4i1	INSIDE	0.614	4.420	1944.00	8593.42	2.713	1535.92	4167.13
A_68_P25495415	chr7:132725071-132725115	NM_001008700:29297	Il4ra	DOWNSTREAM	0.428	6.849	3961.94	27135.26	2.931	3334.80	9775.35
A_68_P25495193	chr7:132695650-132695696	NM_001008700:-123	Il4ra	PROMOTER	0.639	0.132	4626.35	610.06	0.084	3153.96	265.87
A_68_P22322383	chr3:89716803-89716848	NM_010559:259	Il6ra	INSIDE	0.241	1.884	1240.42	2337.20	0.454	979.48	444.97
A_68_P29334871	chr13:113254322-113254366	NM_010560:67	Il6st	INSIDE	0.235	17.455	3215.98	56135.71	4.094	2685.55	10994.51
A_68_P29334874	chr13:113254711-113254755	NM_010560:455	Il6st	INSIDE	0.642	0.329	1513.02	497.06	0.211	1155.11	243.72
A_68_P30676660	chr16:36694130-36694174	NM_134109:29	Ildr1	INSIDE	0.391	0.296	1709.33	506.13	0.116	1497.35	173.31
A_68_P26346744	chr9:21172242-21172286	NM_001042708:-182	Ilf3	PROMOTER	0.608	0.520	1557.47	809.84	0.316	1271.68	401.86
A_68_P27274853	chr10:78037676-78037720	NM_173751:454	Ilvbl	INSIDE	0.417	0.622	3936.86	2447.17	0.259	2783.18	721.47
A_68_P27274847	chr10:78037023-78037067	NM_173751:-200	Ilvbl	PROMOTER	0.316	1.564	1428.58	2234.13	0.494	1141.12	563.58
A_68_P28382007	chr12:41750810-41750854	NM_053122:156	Immp2l	INSIDE	0.662	0.446	2327.54	1038.51	0.296	1836.58	542.83
A_68_P31791019	chr18:67449170-67449214	NM_053261:316	Impa2	INSIDE	0.326	0.243	7053.67	1714.70	0.079	4672.54	370.16
A_68_P31791020	chr18:67449254-67449298	NM_053261:400	Impa2	INSIDE	0.349	0.240	4473.25	1075.64	0.084	2739.69	229.72
A_68_P31791016	chr18:67448771-67448815	NM_053261:-84	Impa2	PROMOTER	0.309	5.012	20624.26	103364.90	1.551	13635.60	21147.84
A_68_P22698893	chr4:4719656-4719700	NM_177730:775	Impad1	INSIDE	0.572	0.540	3177.34	1715.82	0.309	2435.65	751.73
A_68_P24325256	chr6:29166419-29166463	NM_011829:-4169	Impdh1	PROMOTER	0.429	0.212	2614.27	554.58	0.091	1928.67	175.62
A_68_P24325225	chr6:29162476-29162520	NM_011829:-227	Impdh1	PROMOTER	0.556	0.372	2683.66	999.52	0.207	1745.27	361.59
A_68_P28751168	chr12:113838436-113838480	NM_198411:11464	Inf2	INSIDE	0.359	7.364	1328.70	9784.61	2.64	1204.17	3179.19
A_68_P25656232	chr8:11558421-11558465	NM_011919:2377	Ing1	INSIDE	0.467	0.425	1461.32	620.90	0.199	1260.25	250.19
A_68_P25656224	chr8:11557705-11557749	NM_011919:1661	Ing1	INSIDE	0.473	9.078	2896.25	26292.38	4.297	2428.61	10435.22

ProbeName	Target position of probe on CpG island microarray	TargetID	GeneSymbol	CpG island Description	Ratio of relative methylation (TiO_2-NP/Vehicle)	Sham group			TiO_2-H group		
						Relative methylation (Cy5/Cy3)	Cy3 signal (Input)	Cy5 signal (MeDIP)	Relative methylation (Cy5/Cy3)	Cy3 signal (Input)	Cy5 signal (MeDIP)
A_68_P25656230	chr8:11558245-11558289	NM_011919:2201	Ing1	INSIDE	0.478	0.204	3020.02	616.19	0.098	2361.76	230.49
A_68_P25656226	chr8:11557934-11557978	NM_011919:1891	Ing1	INSIDE	0.648	0.546	1033.64	564.85	0.354	897.42	317.67
A_68_P25656210	chr8:11555759-11555803	NM_011919:-285	Ing1	DIVERGENT_PROMOTER	0.477	1.373	9017.62	12381.28	0.655	5590.31	3659.65
A_68_P25842937	chr8:48759109-48759153	NM_023503:1383	Ing2	INSIDE	0.278	11.244	11000.72	123695.80	3.125	10972.11	34283.09
A_68_P25842954	chr8:48761152-48761196	NM_023503:-661	Ing2	PROMOTER	0.354	8.314	724.93	6026.78	2.945	668.79	1969.30
A_68_P25842947	chr8:48760467-48760511	NM_023503:25	Ing2	INSIDE	0.41	0.513	956.23	490.19	0.21	833.48	175.16
A_68_P25842949	chr8:48760597-48760641	NM_023503:-105	Ing2	PROMOTER	0.566	4.366	2121.54	9262.43	2.471	1828.02	4517.60
A_68_P24288408	chr6:21900000-21900044	NM_023626:408	Ing3	INSIDE	0.42	7.093	5371.62	38101.50	2.976	4782.62	14232.34
A_68_P20457532	chr1:95700895-95700939	NM_025454:375	Ing5	INSIDE	0.571	14.238	2220.37	31613.45	8.127	2131.13	17319.50
A_68_P20457529	chr1:95700443-95700487	NM_025454:-77	Ing5	DIVERGENT_PROMOTER	0.61	19.804	2725.91	53983.97	12.09	3606.40	43599.99
A_68_P20457526	chr1:95700053-95700097	NM_025454:-467	Ing5	DIVERGENT_PROMOTER	0.663	4.810	1220.12	5868.39	3.189	1009.03	3217.41
A_68_P20556969	chr1:121317983-121318027	NM_008381:821	Inhbb	INSIDE	0.107	41.670	1872.06	78007.86	4.476	1721.12	7703.15
A_68_P20556967	chr1:121317480-121317524	NM_008381:1323	Inhbb	INSIDE	0.275	27.802	1256.28	34926.84	7.659	1256.92	9627.07
A_68_P20556963	chr1:121316999-121317043	NM_008381:1805	Inhbb	INSIDE	0.549	0.566	1350.37	764.25	0.311	1091.12	338.81
A_68_P21569910	chr2:119303067-119303111	NM_026574:277	Ino80	INSIDE	0.279	21.684	4438.93	96253.65	6.044	3950.27	23876.86
A_68_P21569912	chr2:119303420-119303464	NM_026574:-77	Ino80	PROMOTER	0.624	0.142	4321.30	611.75	0.088	2933.78	259.28
A_68_P31558687	chr18:24280026-24280070	NM_172625:272	Ino80c	INSIDE	0.162	9.425	3130.67	29506.96	1.527	2895.10	4421.16
A_68_P20285212	chr1:63160117-63160161	NM_001081436:703	Ino80d	INSIDE	0.443	0.471	983.83	463.57	0.209	824.08	172.20
A_68_P20285216	chr1:63160901-63160945	NM_001081436:-81	Ino80d	PROMOTER	0.462	4.591	1508.33	6925.12	2.12	1331.07	2821.57
A_68_P20229131	chr1:52874158-52874202	NM_008384:352	Inpp1	INSIDE	0.443	0.570	2852.41	1624.61	0.253	2027.86	512.04
A_68_P20229132	chr1:52874292-52874338	NM_008384:218	Inpp1	INSIDE	0.614	2.479	1883.64	4669.43	1.522	1555.88	2367.69
A_68_P20153136	chr1:37357143-37357187	NM_030266:427	Inpp4a	INSIDE	0.639	0.639	1224.87	783.17	0.409	958.56	391.58
A_68_P25577541	chr7:146574687-146574731	NM_001127363:-299	Inpp5a	PROMOTER	0.351	0.260	5312.41	1379.76	0.091	3808.95	347.66
A_68_P23270092	chr4:124428943-124428987	NM_008385:9843	Inpp5b	INSIDE	0.323	0.616	6449.98	3970.22	0.199	4622.63	918.80
A_68_P21078497	chr2:26264502-26264546	NM_033134:184	Inpp5e	INSIDE	0.457	4.112	1057.70	4349.29	1.881	902.36	1697.09
A_68_P25512009	chr7:135831848-135831892	NM_178641:76992	Inpp5f	INSIDE	0.554	2.775	1826.23	5068.24	1.537	1497.98	2301.92
A_68_P25375722	chr7:108985190-108985234	NM_001122739:1126	Inppl1	INSIDE	0.565	3.131	1994.81	6246.43	1.769	1677.64	2967.99
A_68_P25375721	chr7:108985015-108985059	NM_001122739:1302	Inppl1	INSIDE	0.6	0.554	4797.81	2659.12	0.333	2846.53	946.77
A_68_P25375729	chr7:108986068-108986112	NM_001122739:248	Inppl1	INSIDE	0.604	0.338	5847.50	1975.62	0.204	3893.45	795.06
A_68_P23560503	chr5:28397698-28397742	NM_153526:-231	Insig1	PROMOTER	0.663	3.833	888.29	3404.66	2.542	720.32	1831.25
A_68_P20566310	chr1:123224253-123224297	NM_133748:65	Insig2	INSIDE	0.38	1.368	3542.34	4844.79	0.52	2743.57	1426.11
A_68_P25958332	chr8:74213234-74213278	NM_013564:106	Insl3	INSIDE	0.654	3.629	521.54	1892.79	2.373	451.43	1071.42
A_68_P21717098	chr2:146047771-146047815	NM_016889:60	Insm1	INSIDE	0.165	13.395	1053.78	14115.07	2.213	976.69	2161.45
A_68_P21717097	chr2:146047662-146047707	NM_016889:-48	Insm1	PROMOTER	0.509	3.387	1926.61	6524.97	1.723	1753.67	3021.36
A_68_P28452657	chr12:56700590-56700634	NM_020287:709	Insm2	INSIDE	0.521	14.282	1195.61	17075.86	7.441	1080.33	8038.87
A_68_P28452665	chr12:56701584-56701628	NM_020287:1703	Insm2	INSIDE	0.559	0.476	1111.46	528.88	0.266	981.43	261.14
A_68_P28452664	chr12:56701514-56701558	NM_020287:1633	Insm2	INSIDE	0.59	0.332	1411.45	468.18	0.196	997.24	195.03
A_68_P28452663	chr12:56701421-56701465	NM_020287:1539	Insm2	INSIDE	0.391	3.882	2430.24	9433.13	1.517	2039.70	3093.58
A_68_P22310075	chr3:87600813-87600858	NM_011832:-37	Insrr	DIVERGENT_PROMOTER	0.207	23.052	3449.55	79519.62	4.773	3917.19	18697.95
A_68_P22310076	chr3:87600886-87600930	NM_011832:36	Insrr	INSIDE	0.353	0.637	2015.51	1283.07	0.225	1616.44	363.56
A_68_P20938882	chr1:193399613-193399657	NM_178632:22	Ints7	INSIDE	0.577	0.267	1736.12	462.97	0.154	1134.88	174.70
A_68_P26812405	chr9:107905152-107905196	NM_013785:196	Ip6k1	INSIDE	0.38	7.425	1003.94	7453.83	2.823	916.59	2587.36
A_68_P27234953	chr10:70810954-70810998	NM_027184:436	Ipmk	INSIDE	0.342	0.443	3195.92	1414.56	0.151	2274.36	343.92
A_68_P29952571	chr14:121309972-121310016	NM_023579:-421	Ipo5	PROMOTER	0.649	3.538	1798.95	6365.45	2.296	1661.44	3814.40
A_68_P20639342	chr1:137326653-137326697	NM_153774:394	Ipo9	INSIDE	0.208	1.731	8171.43	14147.53	0.36	5225.48	1883.26
A_68_P20639343	chr1:137326748-137326792	NM_153774:298	Ipo9	INSIDE	0.62	0.209	5870.25	1225.02	0.129	4153.60	537.32

ProbeName	Target position of probe on CpG island microarray	TargetID	GeneSymbol	CpG island Description	Ratio of relative methylation (TiO_2-NP/Vehicle)	Sham group			TiO_2-H group		
						Relative methylation (Cy5/Cy3)	Cy3 signal (Input)	Cy5 signal (MeDIP)	Relative methylation (Cy5/Cy3)	Cy3 signal (Input)	Cy5 signal (MeDIP)
A_68_P23297597	chr4:129296176-129296220	NM_198026:139	Iqcc	INSIDE	0.418	0.219	2721.88	596.95	0.092	1849.92	169.76
A_68_P24142813	chr5:141177968-141178012	NM_028833:342	Iqce	INSIDE	0.406	0.372	1740.77	647.72	0.151	1408.17	212.94
A_68_P24142816	chr5:141178207-141178251	NM_028833:104	Iqce	INSIDE	0.591	3.718	924.54	3437.70	2.197	727.76	1598.83
A_68_P24142814	chr5:141178050-141178094	NM_028833:260	Iqce	INSIDE	0.633	0.178	3165.61	563.89	0.113	2205.24	248.58
A_68_P25460625	chr7:125999193-125999237	NM_001081446:-74	Iqck	DIVERGENT_PROMOTER	0.341	5.817	2678.66	15581.81	1.983	2329.61	4620.53
A_68_P25268780	chr7:87947830-87947874	NM_016721:365	Iqgap1	INSIDE	0.483	3.201	2043.16	6540.89	1.547	1650.86	2553.95
A_68_P29243355	chr13:96661950-96661994	NM_027711:-95	Iqgap2	PROMOTER	0.264	27.287	3759.45	102583.80	7.214	3596.67	25947.03
A_68_P24634427	chr6:90686065-90686109	NM_001134384:74031	Iqsec1	INSIDE	0.466	0.364	2031.02	739.08	0.17	1518.11	257.68
A_68_P24804132	chr6:121363263-121363307	NM_001033354:60412	Iqsec3	INSIDE	0.377	4.656	1327.55	6181.45	1.757	1115.25	1959.63
A_68_P24804559	chr6:121423187-121423231	NM_001033354:488	Iqsec3	INSIDE	0.666	2.929	686.18	2009.53	1.952	628.57	1226.79
A_68_P26680223	chr9:82723595-82723639	NM_001168240:204	Irak1bp1	INSIDE	0.421	0.528	886.85	467.89	0.222	779.23	172.88
A_68_P27495833	chr10:119638332-119638376	NM_028679:239	Irak3	INSIDE	0.596	0.389	1228.30	477.30	0.232	971.88	225.24
A_68_P30452934	chr15:94374073-94374117	NM_029926:4	Irak4	INSIDE	0.417	3.858	1290.72	4979.19	1.609	1150.77	1851.71
A_68_P25837693	chr8:47826718-47826762	NM_008391:1642	Irf2	INSIDE	0.604	5.897	405.22	2389.66	3.561	351.73	1252.47
A_68_P24990858	chr7:19589997-19590041	NM_178757:605	Irf2bp1	INSIDE	0.544	0.554	4609.76	2553.78	0.301	3409.84	1026.83
A_68_P24990862	chr7:19590451-19590495	NM_178757:1059	Irf2bp1	INSIDE	0.609	0.501	2079.31	1040.97	0.305	1420.36	433.39
A_68_P24990865	chr7:19590861-19590905	NM_178757:1469	Irf2bp1	INSIDE	0.643	3.396	1532.94	5205.87	2.183	1355.98	2960.64
A_68_P26258433	chr8:129118351-129118402	NM_001164598:-1040	Irf2bp2	PROMOTER	0.339	11.397	1627.24	18545.09	3.865	1622.28	6270.18
A_68_P26258437	chr8:129118704-129118748	NM_001164598:-1390	Irf2bp2	PROMOTER	0.382	0.564	922.41	519.87	0.215	808.29	173.93
A_68_P26258415	chr8:129116306-129116350	NM_001164598:1008	Irf2bp2	INSIDE	0.244	5.997	6570.39	39405.48	1.464	5191.38	7598.11
A_68_P25090529	chr7:52253176-52253220	NM_016849:169	Irf3	INSIDE	0.614	4.074	1319.91	5377.81	2.5	1173.69	2934.68
A_68_P28918888	chr13:30842515-30842559	NM_013674:1410	Irf4	INSIDE	0.408	0.323	1970.94	636.43	0.132	1425.95	188.06
A_68_P24327113	chr6:29477125-29477169	NM_012057:414	Irf5	INSIDE	0.58	10.413	1452.59	15125.86	6.043	2305.01	13928.90
A_68_P25004862	chr7:25217754-25217798	NM_199013:12925	Irgc1	INSIDE	0.523	3.254	2833.34	9219.24	1.701	1930.10	3283.73
A_68_P25004866	chr7:25218198-25218242	NM_199013:12481	Irgc1	INSIDE	0.412	3.368	3328.63	11210.64	1.387	2485.15	3447.44
A_68_P25005401	chr7:25319057-25319101	NM_153134:3412	Irgq	INSIDE	0.351	20.526	1074.41	22052.98	7.199	856.23	6164.20
A_68_P25005396	chr7:25318615-25318659	NM_153134:2970	Irgq	INSIDE	0.607	3.770	3005.50	11330.11	2.288	2155.49	4931.92
A_68_P25005400	chr7:25318962-25319006	NM_153134:3318	Irgq	INSIDE	0.628	4.003	2042.00	8173.99	2.515	1680.68	4226.88
A_68_P25005399	chr7:25318886-25318930	NM_153134:3242	Irgq	INSIDE	0.479	1.356	2843.81	3857.23	0.649	2134.11	1385.39
A_68_P20391718	chr1:82287606-82287650	NM_010570:386	Irs1	INSIDE	0.371	11.600	2884.61	33460.27	4.309	2642.31	11385.69
A_68_P20391723	chr1:82288408-82288452	NM_010570:-416	Irs1	PROMOTER	0.582	0.359	1614.42	579.61	0.209	1163.49	242.97
A_68_P25652455	chr8:11009184-11009228	NM_001081212:-776	Irs2	PROMOTER	0.475	4.612	2100.96	9689.31	2.192	1716.80	3762.79
A_68_P25652449	chr8:11008424-11008468	NM_001081212:-16	Irs2	PROMOTER	0.553	7.070	1197.25	8464.67	3.91	1076.38	4208.25
A_68_P32704868	chrX:138159961-138160005	NM_010572:-222	Irs4	PROMOTER	0.289	5.875	951.64	5591.31	1.697	1179.34	2001.84
A_68_P29130726	chr13:72101391-72101435	NM_010573:-241	Irx1	PROMOTER	0.374	6.857	1193.72	8185.37	2.567	978.90	2512.86
A_68_P29134822	chr13:72768633-72768679	NM_010574:2231	Irx2	INSIDE	0.232	7.335	5009.05	36743.20	1.703	3838.90	6536.18
A_68_P29134821	chr13:72768527-72768571	NM_010574:2123	Irx2	INSIDE	0.425	0.581	1135.07	659.37	0.247	855.80	211.16
A_68_P26061528	chr8:94324564-94324608	NM_008393:687	Irx3	INSIDE	0.552	0.465	1669.40	776.24	0.257	1170.76	300.65
A_68_P26061524	chr8:94323959-94324003	NM_008393:1293	Irx3	INSIDE	0.57	0.574	6760.10	3882.27	0.327	5042.84	1651.06
A_68_P26061503	chr8:94321755-94321804	NM_008393:3494	Irx3	DOWNSTREAM	0.391	0.632	925.26	584.72	0.247	712.56	176.28
A_68_P29138629	chr13:73397900-73397944	NM_018885:-22	Irx4	PROMOTER	0.583	3.681	1710.04	6294.18	2.145	1482.96	3181.08
A_68_P26065137	chr8:94881817-94881861	NM_018826:144	Irx5	INSIDE	0.202	13.254	1519.77	20143.64	2.683	1299.29	3486.08
A_68_P26065139	chr8:94882073-94882117	NM_018826:400	Irx5	INSIDE	0.329	0.387	1801.93	697.17	0.127	1373.92	174.78
A_68_P26065141	chr8:94882239-94882283	NM_018826:566	Irx5	INSIDE	0.488	2.870	2523.37	7241.93	1.4	2041.46	2858.11
A_68_P23999970	chr5:114222262-114222309	NM_025526:-535	Iscu	DIVERGENT_PROMOTER	0.211	12.075	212.47	2565.44	2.543	242.02	615.41
A_68_P22310778	chr3:87734179-87734223	NM_177663:-35	Isg20l2	DIVERGENT_PROMOTER	0.181	1.781	2925.96	5210.95	0.322	2314.48	744.59

ProbeName	Target position of probe on CpG island microarray	TargetID	GeneSymbol	CpG island Description	Ratio of relative methylation (TiO_2-NP/Vehicle)	Sham group			TiO_2-H group		
						Relative methylation (Cy5/Cy3)	Cy3 signal (Input)	Cy5 signal (MeDIP)	Relative methylation (Cy5/Cy3)	Cy3 signal (Input)	Cy5 signal (MeDIP)
A_68_P26531872	chr9:55390256-55390300	NM_027397:1323	Isl2	INSIDE	0.414	10.579	1217.45	12878.94	4.378	938.91	4111.01
A_68_P26531904	chr9:55394018-55394062	NM_027397:5085	Isl2	DOWNSTREAM	0.462	0.326	1560.43	508.95	0.151	1311.11	197.69
A_68_P26546334	chr9:58046265-58046309	NM_001161535:3236	Islr2	INSIDE	0.392	0.539	2556.06	1378.13	0.211	1918.58	405.69
A_68_P21680294	chr2:139504121-139504165	NM_001126490:229	Ism1	INSIDE	0.55	3.557	796.59	2833.82	1.957	784.35	1534.93
A_68_P31743422	chr18:58818798-58818854	NM_025478:-309	Isoc1	PROMOTER	0.489	5.284	329.92	1743.31	2.582	277.99	717.66
A_68_P24618420	chr6:87788600-87788644	NM_133934:131	Isy1	INSIDE	0.251	7.685	1328.59	10210.09	1.926	1095.98	2111.30
A_68_P25952868	chr8:73118866-73118910	NM_023627:509	Isyna1	INSIDE	0.245	1.790	1883.37	3371.17	0.438	1427.14	624.72
A_68_P25952861	chr8:73118130-73118174	NM_023627:-227	Isyna1	PROMOTER	0.455	4.194	3472.61	14565.09	1.91	3053.17	5832.41
A_68_P25952869	chr8:73118953-73118997	NM_023627:595	Isyna1	INSIDE	0.561	3.350	2214.30	7417.17	1.88	1815.32	3413.70
A_68_P25952871	chr8:73119135-73119179	NM_023627:777	Isyna1	INSIDE	0.201	1.608	2058.67	3310.71	0.324	1429.51	463.17
A_68_P28079748	chr11:102328467-102328511	NM_010575:2709	Itga2b	INSIDE	0.647	0.627	2262.61	1419.22	0.406	1638.68	664.68
A_68_P28036505	chr11:94937282-94937326	NM_013565:724	Itga3	INSIDE	0.596	0.558	1412.04	787.76	0.333	1060.31	352.82
A_68_P21367543	chr2:79095483-79095527	NM_010576:-78	Itga4	PROMOTER	0.117	21.370	2289.27	48921.29	2.49	1909.88	4755.31
A_68_P30503777	chr15:103196605-103196649	NM_010577:553	Itga5	INSIDE	0.655	0.605	1535.75	928.68	0.396	1311.19	519.71
A_68_P27543981	chr10:128370721-128370765	NM_008398:-126	Itga7	PROMOTER	0.621	0.455	1960.48	892.28	0.283	1642.02	464.24
A_68_P21010259	chr2:12223211-12223256	NM_001001309:314	Itga8	INSIDE	0.617	0.568	1489.17	845.50	0.35	1228.66	430.17
A_68_P21010261	chr2:12223501-12223545	NM_001001309:25	Itga8	INSIDE	0.613	0.530	1051.83	556.96	0.324	963.76	312.73
A_68_P26867372	chr9:118515925-118515969	NM_133721:120	Itga9	INSIDE	0.523	6.368	405.58	2582.90	3.328	373.62	1243.27
A_68_P21389130	chr2:83564617-83564661	NM_008402:85	Itgav	INSIDE	0.503	4.261	1384.64	5900.57	2.142	1083.81	2322.01
A_68_P26270752	chr8:131210063-131210107	NM_010578:531	Itgb1	INSIDE	0.582	0.498	2472.70	1231.91	0.29	1927.34	558.79
A_68_P28156775	chr11:115866293-115866337	NM_001005608:30276	Itgb4	INSIDE	0.665	5.231	1294.88	6773.39	3.479	1080.66	3759.61
A_68_P28156587	chr11:115836535-115836579	NM_001005608:518	Itgb4	INSIDE	0.525	3.412	963.13	3286.51	1.791	807.49	1445.91
A_68_P21631064	chr2:130493650-130493694	NM_025922:96	Itpa	INSIDE	0.298	2.064	4791.59	9890.47	0.615	3285.60	2021.26
A_68_P28693227	chr12:103943323-103943367	NM_172584:-265	Itpk1	PROMOTER	0.492	10.242	904.96	9268.19	5.036	796.13	4009.56
A_68_P21571253	chr2:119569096-119569140	NM_146125:1046	Itpka	INSIDE	0.186	22.215	2709.16	60183.55	4.137	2120.51	8772.03
A_68_P25016218	chr7:28013554-28013598	NM_181593:40	Itpkc	INSIDE	0.424	16.169	833.68	13480.08	6.849	671.27	4597.69
A_68_P32152778	chr19:47993679-47993723	NM_001001738:89	Itprip	INSIDE	0.329	5.622	650.31	3655.92	1.848	674.75	1247.26
A_68_P25458530	chr7:125633291-125633335	NM_001033380:2177	Itpripl2	INSIDE	0.492	0.503	2854.22	1434.94	0.247	1990.52	492.02
A_68_P25458544	chr7:125634804-125634848	NM_001033380:663	Itpripl2	INSIDE	0.512	0.401	1280.54	513.28	0.205	1037.94	213.16
A_68_P30964671	chr16:91730311-91730355	NM_001110276:717	Itsn1	INSIDE	0.601	0.443	3873.75	1716.56	0.266	2635.96	701.69
A_68_P31601053	chr18:32227655-32227699	NM_173441:289	Iws1	INSIDE	0.601	0.627	1223.68	767.83	0.377	920.90	347.10
A_68_P27284931	chr10:80165309-80165353	NM_027829:-107	Izumo4	DIVERGENT_PROMOTER	0.619	3.797	3907.29	14834.83	2.349	3037.74	7136.60
A_68_P21666835	chr2:136940626-136940670	NM_013822:1608	Jag1	INSIDE	0.623	0.563	1111.48	625.25	0.35	832.34	291.65
A_68_P25958256	chr8:74203690-74203734	NM_001190830:3431	Jak3	INSIDE	0.137	27.220	842.86	22942.59	3.719	707.30	2630.20
A_68_P23611439	chr5:37482314-37482358	NM_178394:40241	Jakmip1	INSIDE	0.33	0.692	2000.66	1383.82	0.228	1495.74	341.67
A_68_P30925479	chr16:84774664-84774708	NM_023844:319	Jam2	INSIDE	0.58	6.481	959.24	6216.45	3.761	880.55	3312.08
A_68_P30925477	chr16:84774396-84774440	NM_023844:51	Jam2	INSIDE	0.406	0.657	3115.12	2047.97	0.267	2295.00	612.21
A_68_P26374351	chr9:26962634-26962678	NM_023277:350	Jam3	INSIDE	0.662	4.887	725.17	3544.05	3.238	601.20	1946.46
A_68_P28997663	chr13:44826717-44826761	NM_001205043:99	Jarid2	INSIDE	0.226	20.566	4994.44	102713.30	4.655	4289.58	19966.52
A_68_P28997682	chr13:44829049-44829093	NM_001205043:2431	Jarid2	INSIDE	0.348	30.171	10760.54	324652.40	10.487	10207.91	107055.20
A_68_P28998903	chr13:44998308-44998352	NM_001205043:171691	Jarid2	INSIDE	0.435	6.846	3166.83	21678.85	2.977	2520.40	7502.30
A_68_P28605007	chr12:86940319-86940363	NM_030887:-25	Jdp2	PROMOTER	0.179	45.373	5116.31	232143.00	8.133	5396.53	43889.39
A_68_P28605005	chr12:86939999-86940043	NM_001205052:-34	Jdp2	PROMOTER	0.661	2.920	1330.16	3883.50	1.929	1103.85	2129.85
A_68_P24381989	chr6:39155596-39155640	NM_001033430:1154	Jhdm1d	INSIDE	0.222	23.176	3823.62	88617.98	5.138	4429.59	22761.16
A_68_P24381994	chr6:39156178-39156223	NM_001033430:572	Jhdm1d	INSIDE	0.586	0.647	1059.83	685.78	0.379	839.27	317.97
A_68_P28530101	chr12:73186989-73187033	NM_001205067:185	Jkamp	INSIDE	0.252	22.635	1946.75	44064.46	5.7	2060.00	11742.81

ProbeName	Target position of probe on CpG island microarray	TargetID	GeneSymbol	CpG island Description	Ratio of relative methylation (TiO_2-NP/Vehicle)	Sham group			TiO_2-H group		
						Relative methylation (Cy5/Cy3)	Cy3 signal (Input)	Cy5 signal (MeDIP)	Relative methylation (Cy5/Cy3)	Cy3 signal (Input)	Cy5 signal (MeDIP)
A_68_P28530098	chr12:73186548-73186592	NM_001205067:-255	Jkamp	DIVERGENT_PROMOTER	0.303	5.710	1286.00	7343.38	1.727	1000.61	1728.43
A_68_P27836979	chr11:59263493-59263537	NM_001205068:-32	Jmjd4	DIVERGENT_PROMOTER	0.617	0.404	1241.18	500.96	0.249	1015.58	252.77
A_68_P28161573	chr11:116704755-116704799	NM_033398:-13	Jmjd6	DIVERGENT_PROMOTER	0.268	0.379	2236.17	847.18	0.101	1699.33	172.25
A_68_P28161572	chr11:116704633-116704677	NM_033398:109	Jmjd6	INSIDE	0.565	4.099	2190.15	8978.22	2.318	1796.52	4164.00
A_68_P21572764	chr2:119853205-119853250	NM_001114637:9	Jmjd7	INSIDE	0.408	0.603	2059.89	1242.09	0.246	1678.45	412.65
A_68_P30367903	chr15:79518094-79518138	NM_028792:186	Josd1	INSIDE	0.584	5.818	1310.26	7623.08	3.397	1055.39	3584.75
A_68_P26228657	chr8:124277495-124277539	NM_020605:23058	Jph3	INSIDE	0.354	5.501	3487.99	19186.51	1.948	2204.93	4294.49
A_68_P26228499	chr8:124254393-124254437	NM_020605:-44	Jph3	PROMOTER	0.299	5.446	8497.77	46276.12	1.626	6690.67	10877.92
A_68_P26228652	chr8:124276907-124276951	NM_020605:22470	Jph3	INSIDE	0.216	7.245	1923.37	13935.40	1.564	1431.14	2238.10
A_68_P22324052	chr3:90035906-90035950	NM_206924:410	Jtb	INSIDE	0.66	2.304	1445.62	3330.58	1.522	1113.35	1693.99
A_68_P29613087	chr14:55194747-55194791	NM_010590:1730	Jub	INSIDE	0.285	7.831	2751.01	21541.86	2.229	2476.38	5519.09
A_68_P29613086	chr14:55194659-55194703	NM_010590:1818	Jub	INSIDE	0.358	7.023	2275.71	15981.85	2.516	1954.89	4919.23
A_68_P23120719	chr4:94719459-94719503	NM_010591:-567	Jun	PROMOTER	0.513	3.781	7295.38	27583.54	1.94	5427.99	10532.67
A_68_P23120696	chr4:94716722-94716767	NM_010591:2169	Jun	INSIDE	0.643	0.547	1318.68	720.76	0.351	1165.55	409.66
A_68_P23120705	chr4:94717778-94717822	NM_010591:1113	Jun	INSIDE	0.664	3.621	1111.05	4023.42	2.404	999.06	2401.58
A_68_P23120704	chr4:94717633-94717677	NM_010591:1259	Jun	INSIDE	0.459	0.601	1396.71	839.61	0.276	946.56	261.46
A_68_P23120712	chr4:94718521-94718565	NM_010591:371	Jun	INSIDE	0.431	0.556	985.27	548.08	0.24	727.06	174.50
A_68_P25953471	chr8:73223632-73223676	NM_010592:2017	Jund	INSIDE	0.612	7.930	1425.47	11304.46	4.851	1222.03	5927.98
A_68_P28067852	chr11:100258833-100258877	NM_010593:250	Jup	INSIDE	0.264	19.103	2901.44	55426.13	5.051	2654.17	13407.18
A_68_P32028426	chr19:25311621-25311665	NM_181404:-49	Kank1	PROMOTER	0.492	0.370	1495.52	553.18	0.182	1221.74	222.56
A_68_P26348961	chr9:21602785-21602833	NM_145611:182	Kank2	INSIDE	0.171	0.362	4326.83	1564.86	0.062	3172.52	196.39
A_68_P26348962	chr9:21602957-21603001	NM_145611:12	Kank2	INSIDE	0.411	5.962	2033.57	12123.94	2.45	1632.51	3999.50
A_68_P26348964	chr9:21603129-21603173	NM_145611:-160	Kank2	PROMOTER	0.631	2.863	1128.16	3229.66	1.808	1057.41	1911.46
A_68_P31150606	chr17:33954625-33954670	NM_030697:7180	Kank3	INSIDE	0.289	6.053	689.19	4171.81	1.749	621.90	1087.45
A_68_P28069613	chr11:100569782-100569828	NM_001038010:3977	Kat2a	INSIDE	0.293	13.585	501.08	6807.32	3.986	337.99	1347.24
A_68_P28069617	chr11:100570105-100570149	NM_001038010:3655	Kat2a	INSIDE	0.43	4.825	1099.78	5306.49	2.074	927.52	1923.39
A_68_P31244142	chr17:53707297-53707341	NM_020005:1023	Kat2b	INSIDE	0.527	3.991	868.65	3466.82	2.105	755.15	1589.70
A_68_P31244136	chr17:53706802-53706846	NM_020005:529	Kat2b	INSIDE	0.619	2.746	768.35	2110.09	1.7	600.24	1020.37
A_68_P24182264	chr5:149740161-149740215	NM_153572:35	Katnal1	INSIDE	0.471	0.380	1311.63	499.03	0.179	940.46	168.38
A_68_P24182263	chr5:149739992-149740036	NM_153572:209	Katnal1	INSIDE	0.504	5.361	2609.49	13989.98	2.703	2053.42	5549.88
A_68_P26081249	chr8:97605091-97605135	NM_028805:12	Katnb1	INSIDE	0.245	0.412	2396.49	987.25	0.101	1694.72	171.15
A_68_P26081251	chr8:97605349-97605393	NM_028805:270	Katnb1	INSIDE	0.665	0.564	4282.02	2413.21	0.375	3026.25	1133.68
A_68_P32136345	chr19:45154436-45154480	NM_178929:3830	Kazald1	DOWNSTREAM	0.521	2.901	3774.36	10949.40	1.512	2928.13	4427.24
A_68_P25678290	chr8:15028480-15028524	NM_029116:17478	Kbtbd11	INSIDE	0.286	28.325	1548.81	43870.60	8.089	1357.34	10979.26
A_68_P25678159	chr8:15011427-15011471	NM_029116:424	Kbtbd11	INSIDE	0.388	5.831	3028.76	17659.41	2.265	2360.44	5346.24
A_68_P25678160	chr8:15011568-15011612	NM_029116:566	Kbtbd11	INSIDE	0.489	0.212	2977.21	632.21	0.104	2183.12	226.58
A_68_P25678286	chr8:15027955-15027999	NM_029116:16952	Kbtbd11	INSIDE	0.576	3.456	737.63	2549.35	1.991	745.14	1483.78
A_68_P26587421	chr9:65238821-65238865	NM_028974:617	Kbtbd13	INSIDE	0.239	13.355	803.00	10723.86	3.19	729.63	2327.81
A_68_P24476912	chr6:56747694-56747738	NM_145958:91	Kbtbd2	INSIDE	0.469	4.412	1110.01	4897.46	2.071	953.86	1975.12
A_68_P26885522	chr9:121686826-121686870	NM_028202:124	Kbtbd5	INSIDE	0.6	4.204	523.35	2200.08	2.524	461.95	1165.74
A_68_P24660677	chr6:95068035-95068079	NM_001008785:157	Kbtbd8	INSIDE	0.549	0.529	4497.14	2380.29	0.291	3435.60	998.43
A_68_P24660675	chr6:95067758-95067802	NM_001008785:-119	Kbtbd8	PROMOTER	0.576	0.433	1506.39	651.60	0.249	1254.16	312.24
A_68_P24828018	chr6:126594962-126595006	NM_010595:835	Kcna1	INSIDE	0.664	0.253	2561.43	647.69	0.168	1806.70	303.50
A_68_P22404196	chr3:106904415-106904459	NM_008417:-48	Kcna2	PROMOTER	0.458	5.734	2433.95	13957.27	2.629	2125.46	5587.19
A_68_P25092915	chr7:52661625-52661669	NM_010596:317	Kcna7	INSIDE	0.423	0.309	1776.92	549.92	0.131	1315.91	172.22
A_68_P25092916	chr7:52661724-52661768	NM_010596:417	Kcna7	INSIDE	0.582	7.510	1765.36	13257.75	4.372	1372.68	6001.24

ProbeName	Target position of probe on CpG island microarray	TargetID	GeneSymbol	CpG island Description	Ratio of relative methylation (TiO_2-NP/Vehicle)	Sham group			TiO_2-H group		
						Relative methylation (Cy5/Cy3)	Cy3 signal (Input)	Cy5 signal (MeDIP)	Relative methylation (Cy5/Cy3)	Cy3 signal (Input)	Cy5 signal (MeDIP)
A_68_P25092919	chr7:52661992-52662036	NM_010596:685	Kcna7	INSIDE	0.612	4.137	948.95	3925.84	2.532	763.47	1932.83
A_68_P27894336	chr11:69139970-69140014	NM_010599:233	Kcnab3	INSIDE	0.225	3.058	7713.08	23585.02	0.689	5318.43	3666.06
A_68_P21834182	chr2:167015184-167015228	NM_008420:-907	Kcnb1	PROMOTER	0.248	18.516	3238.05	59954.17	4.583	2705.09	12398.09
A_68_P21833675	chr2:166930471-166930515	NM_008420:83807	Kcnb1	INSIDE	0.382	4.859	2978.21	14471.27	1.857	2217.62	4118.91
A_68_P21833676	chr2:166930579-166930623	NM_008420:83699	Kcnb1	INSIDE	0.376	5.091	1233.40	6278.83	1.913	856.46	1638.79
A_68_P21833672	chr2:166930250-166930294	NM_008420:84027	Kcnb1	INSIDE	0.642	2.750	1746.90	4803.47	1.764	1257.15	2218.09
A_68_P21834179	chr2:167014887-167014931	NM_008420:-609	Kcnb1	PROMOTER	0.408	0.589	936.89	552.21	0.24	725.54	174.39
A_68_P21834170	chr2:167013596-167013640	NM_008420:681	Kcnb1	INSIDE	0.376	4.129	1655.95	6836.63	1.553	1137.84	1767.44
A_68_P25099054	chr7:53683157-53683201	NM_001112739:31312	Kcnc1	INSIDE	0.156	20.408	2045.73	41750.30	3.193	1296.96	4140.88
A_68_P25098809	chr7:53651661-53651705	NM_008421:-155	Kcnc1	PROMOTER	0.251	12.831	613.13	7866.82	3.218	528.92	1702.26
A_68_P25098815	chr7:53652229-53652273	NM_001112739:384	Kcnc1	INSIDE	0.389	6.118	2466.46	15088.97	2.377	2121.18	5041.57
A_68_P25098823	chr7:53653169-53653213	NM_001112739:1324	Kcnc1	INSIDE	0.427	5.938	1314.17	7803.02	2.534	1162.48	2945.43
A_68_P25098813	chr7:53652081-53652125	NM_001112739:236	Kcnc1	INSIDE	0.506	7.381	1069.23	7891.83	3.731	990.87	3697.05
A_68_P25088287	chr7:51847145-51847189	NM_008422:911	Kcnc3	INSIDE	0.435	0.301	1686.00	508.02	0.131	1307.74	171.35
A_68_P25088286	chr7:51846961-51847005	NM_008422:727	Kcnc3	INSIDE	0.451	13.256	3014.90	39964.72	5.984	2426.53	14519.46
A_68_P25088268	chr7:51844686-51844730	NM_008422:-1547	Kcnc3	PROMOTER	0.4	3.413	3444.51	11756.75	1.366	2959.22	4041.90
A_68_P22406469	chr3:107262382-107262426	NM_145922:-588	Kcnc4	PROMOTER	0.27	2.279	8052.46	18352.02	0.615	5880.26	3618.37
A_68_P22406475	chr3:107263123-107263167	NM_145922:-1328	Kcnc4	PROMOTER	0.55	0.709	1174.05	832.14	0.39	955.17	372.54
A_68_P24284108	chr6:21165672-21165716	NM_019697:-414	Kcnd2	PROMOTER	0.445	0.286	1718.01	491.13	0.127	1371.31	174.60
A_68_P24284102	chr6:21164876-21164920	NM_019697:-1210	Kcnd2	PROMOTER	0.588	0.292	1604.96	469.17	0.172	1216.51	209.08
A_68_P22397067	chr3:105255865-105255909	NM_019931:-5847	Kcnd3	PROMOTER	0.319	6.074	1535.86	9329.24	1.937	1366.98	2648.32
A_68_P22397063	chr3:105255456-105255500	NM_019931:-6255	Kcnd3	PROMOTER	0.337	0.555	1948.77	1081.71	0.187	1561.59	292.06
A_68_P28269092	chr12:17182452-17182496	NM_201531:1220	Kcnf1	INSIDE	0.316	0.554	1301.55	720.46	0.175	960.04	167.83
A_68_P21840390	chr2:168088577-168088621	NM_001081134:6233	Kcng1	INSIDE	0.57	4.297	4361.24	18739.16	2.45	2906.36	7120.49
A_68_P21840418	chr2:168094705-168094749	NM_001081134:105	Kcng1	INSIDE	0.588	3.640	983.52	3580.12	2.139	739.33	1581.57
A_68_P21840415	chr2:168094408-168094454	NM_001081134:401	Kcng1	INSIDE	0.409	4.282	2421.00	10365.90	1.749	1511.91	2645.07
A_68_P31863637	chr18:80519734-80519778	NM_001190373:41237	Kcng2	INSIDE	0.203	14.789	4055.94	59984.47	3	3920.61	11763.00
A_68_P31863848	chr18:80559585-80559629	NM_001190373:1387	Kcng2	INSIDE	0.417	0.394	1542.32	607.28	0.164	1124.70	184.51
A_68_P31863635	chr18:80519494-80519538	NM_001190373:41477	Kcng2	INSIDE	0.64	3.831	441.64	1691.72	2.452	409.67	1004.58
A_68_P23540978	chr5:23830927-23830971	NM_013569:26474	Kcnh2	INSIDE	0.096	18.023	2213.80	39899.76	1.728	1657.58	2864.20
A_68_P30478814	chr15:99054189-99054233	NM_010601:-1196	Kcnh3	PROMOTER	0.61	8.345	2950.99	24627.05	5.093	2417.99	12314.27
A_68_P30478815	chr15:99054306-99054350	NM_010601:-1078	Kcnh3	PROMOTER	0.496	3.058	1369.67	4187.86	1.515	1145.96	1736.21
A_68_P27697249	chr11:33743483-33743527	NM_001190885:81	Kcnip1	INSIDE	0.27	13.568	5255.63	71308.33	3.669	4708.97	17276.87
A_68_P32140683	chr19:45890581-45890625	NM_030716:-309	Kcnip2	PROMOTER	0.268	5.417	2890.15	15656.76	1.453	2399.06	3485.83
A_68_P21613747	chr2:127308014-127308058	NM_001111331:199	Kcnip3	INSIDE	0.32	0.420	3906.05	1640.46	0.134	2692.95	361.81
A_68_P21613749	chr2:127308338-127308382	NM_001111331:-125	Kcnip3	PROMOTER	0.549	19.367	1113.17	21559.32	10.641	1073.31	11420.83
A_68_P20832484	chr1:174270940-174270984	NM_001039484:-378	Kcnj10	PROMOTER	0.045	7.362	3003.23	22108.38	0.333	2186.16	728.77
A_68_P20832486	chr1:174271151-174271195	NM_001039484:-168	Kcnj10	PROMOTER	0.386	0.345	1768.62	610.59	0.133	1283.95	171.26
A_68_P20832488	chr1:174271323-174271367	NM_001039484:4	Kcnj10	INSIDE	0.464	0.571	2294.72	1311.16	0.265	1607.46	426.14
A_68_P20832487	chr1:174271228-174271272	NM_001039484:-90	Kcnj10	PROMOTER	0.624	0.626	4056.32	2539.02	0.391	2832.93	1106.93
A_68_P25096865	chr7:53356200-53356244	NM_001204411:-88	Kcnj11	PROMOTER	0.631	12.183	7227.34	88051.30	7.683	6783.89	52118.43
A_68_P25095297	chr7:53075356-53075400	NM_145963:4739	Kcnj14	INSIDE	0.318	7.997	3274.23	26185.67	2.541	2912.29	7399.42
A_68_P31425152	chr17:88197017-88197061	NM_199251:296	Kcnk12	INSIDE	0.344	8.799	1680.83	14790.38	3.027	1520.29	4601.60
A_68_P31425147	chr17:88196405-88196449	NM_199251:908	Kcnk12	INSIDE	0.618	0.647	873.49	565.25	0.4	724.27	289.76
A_68_P28679268	chr12:101203451-101203495	NM_001164426:764	Kcnk13	INSIDE	0.615	0.270	2349.47	634.17	0.166	1725.51	286.27
A_68_P21814762	chr2:163679681-163679725	NM_001030292:217	Kcnk15	INSIDE	0.545	4.843	783.66	3795.20	2.639	755.23	1993.03

ProbeName	Target position of probe on CpG island microarray	TargetID	GeneSymbol	CpG island Description	Ratio of relative methylation (TiO_2-NP/Vehicle)	Sham group			TiO_2-H group		
						Relative methylation (Cy5/Cy3)	Cy3 signal (Input)	Cy5 signal (MeDIP)	Relative methylation (Cy5/Cy3)	Cy3 signal (Input)	Cy5 signal (MeDIP)
A_68_P21814796	chr2:163684382-163684426	NM_001030292:4919	Kcnk15	INSIDE	0.583	5.186	2225.56	11541.72	3.024	1687.71	5102.91
A_68_P20925497	chr1:191167558-191167602	NM_001159850:-348	Kcnk2	PROMOTER	0.487	3.001	1686.08	5059.42	1.461	1493.73	2181.65
A_68_P23573357	chr5:30891361-30891405	NM_010608:840	Kcnk3	INSIDE	0.518	0.498	1527.80	761.11	0.258	1279.20	330.27
A_68_P23573588	chr5:30924821-30924865	NM_010608:34300	Kcnk3	INSIDE	0.656	2.609	747.65	1950.84	1.713	754.51	1292.25
A_68_P31936764	chr19:7008234-7008278	NM_008431:749	Kcnk4	INSIDE	0.599	3.393	2278.42	7730.47	2.033	1811.88	3684.46
A_68_P31936715	chr19:7000722-7000766	NM_008431:8261	Kcnk4	INSIDE	0.652	3.800	1603.23	6091.50	2.477	1392.89	3450.48
A_68_P29445598	chr14:21001028-21001072	NM_021542:-46	Kcnk5	PROMOTER	0.396	9.403	4267.38	40125.03	3.719	3768.27	14015.40
A_68_P29469015	chr14:24824174-24824218	NM_010610:-769	Kcnma1	PROMOTER	0.344	13.965	975.81	13626.95	4.801	800.56	3843.67
A_68_P29469016	chr14:24824250-24824294	NM_010610:-845	Kcnma1	PROMOTER	0.533	3.383	2132.94	7215.90	1.803	1735.56	3129.79
A_68_P27475788	chr10:115911221-115911265	NM_021452:-663	Kcnmb4	PROMOTER	0.649	4.957	1928.13	9557.83	3.218	1642.97	5287.17
A_68_P25954525	chr8:73380746-73380790	NM_032397:139	Kcnn1	INSIDE	0.122	0.384	5751.18	2206.98	0.047	3705.75	173.22
A_68_P25954496	chr8:73376577-73376621	NM_032397:4309	Kcnn1	INSIDE	0.194	10.913	1768.20	19295.67	2.122	1247.22	2646.32
A_68_P25954526	chr8:73380907-73380951	NM_032397:-21	Kcnn1	PROMOTER	0.376	1.452	2643.16	3837.42	0.545	2222.04	1212.12
A_68_P25954583	chr8:73389397-73389441	NM_032397:-8511	Kcnn1	PROMOTER	0.389	1.411	2073.78	2925.19	0.549	1561.47	856.59
A_68_P25954428	chr8:73366677-73366723	NM_032397:14207	Kcnn1	INSIDE	0.223	14.866	222.35	3305.43	3.31	187.16	619.41
A_68_P31672396	chr18:45718914-45718958	NM_080465:-871	Kcnn2	PROMOTER	0.38	0.357	1552.40	553.72	0.136	1267.20	171.95
A_68_P31672403	chr18:45719784-45719828	NM_080465:-1	Kcnn2	PROMOTER	0.457	0.284	3369.40	955.33	0.13	2465.33	319.79
A_68_P31672402	chr18:45719605-45719649	NM_080465:-181	Kcnn2	PROMOTER	0.519	7.294	3937.16	28718.46	3.782	3434.67	12991.30
A_68_P31672401	chr18:45719495-45719539	NM_080465:-291	Kcnn2	PROMOTER	0.561	0.630	2229.96	1405.55	0.354	1769.64	625.77
A_68_P25598742	chr7:150293216-150293260	NM_008434:80	Kcnq1	INSIDE	0.267	29.120	591.18	17215.29	7.79	724.91	5646.69
A_68_P21908760	chr2:180869636-180869680	NM_001003824:272	Kcnq2	INSIDE	0.653	3.255	653.35	2126.76	2.124	565.28	1200.85
A_68_P30290903	chr15:66118372-66118416	NM_152923:-608	Kcnq3	PROMOTER	0.462	4.613	996.07	4594.83	2.132	875.95	1867.84
A_68_P23253010	chr4:120419437-120419481	NM_001081142:323	Kcnq4	INSIDE	0.168	14.857	1280.43	19023.78	2.501	1107.55	2769.56
A_68_P23252661	chr4:120370848-120370892	NM_001081142:48911	Kcnq4	INSIDE	0.216	8.670	2546.81	22080.43	1.877	2150.48	4035.80
A_68_P23252895	chr4:120403760-120403804	NM_001081142:15999	Kcnq4	INSIDE	0.344	0.393	2305.34	904.90	0.135	1703.61	230.22
A_68_P20080972	chr1:21951304-21951348	NM_001160139:697	Kcnq5	INSIDE	0.421	0.455	2016.48	917.26	0.191	1561.90	298.98
A_68_P20080973	chr1:21951507-21951551	NM_001160139:495	Kcnq5	INSIDE	0.642	3.011	1926.30	5800.88	1.932	1755.16	3390.94
A_68_P21816594	chr2:163993596-163993640	NM_008435:3231	Kcns1	INSIDE	0.613	0.372	3288.05	1224.32	0.228	2325.65	530.97
A_68_P21816616	chr2:163996879-163996923	NM_008435:-51	Kcns1	PROMOTER	0.631	2.709	1230.53	3334.09	1.71	1009.44	1726.58
A_68_P21816593	chr2:163993465-163993515	NM_008435:3359	Kcns1	INSIDE	0.214	2.250	1057.67	2380.19	0.482	824.56	397.51
A_68_P30130472	chr15:34767602-34767646	NM_181317:489	Kcns2	INSIDE	0.586	0.643	1481.50	953.16	0.377	986.92	372.27
A_68_P28236792	chr12:11157416-11157460	NM_173417:-259	Kcns3	PROMOTER	0.582	18.817	2607.36	49062.42	10.951	2413.67	26433.12
A_68_P21074758	chr2:25733280-25733324	NM_175462:-78	Kcnt1	PROMOTER	0.443	1.445	1959.51	2831.41	0.641	1431.07	916.86
A_68_P30183902	chr15:44945966-44946010	NM_026200:492	Kcnv1	INSIDE	0.293	22.059	1443.67	31846.39	6.474	1904.83	12332.45
A_68_P31515588	chr18:15222716-15222760	NM_001142731:-647	Kctd1	PROMOTER	0.209	14.582	1698.98	24774.45	3.052	1474.68	4500.34
A_68_P31515584	chr18:15221979-15222023	NM_001142731:91	Kctd1	INSIDE	0.431	5.735	1240.96	7116.87	2.472	1143.39	2826.01
A_68_P31515586	chr18:15222359-15222403	NM_001142731:-289	Kctd1	PROMOTER	0.565	3.981	1349.06	5371.15	2.251	1063.40	2394.02
A_68_P29863083	chr14:103380887-103380931	NM_177715:946	Kctd12	INSIDE	0.55	0.532	1629.94	867.49	0.293	1236.06	362.15
A_68_P29863087	chr14:103381523-103381567	NM_177715:310	Kctd12	INSIDE	0.621	11.845	1906.41	22582.14	7.352	2046.13	15042.13
A_68_P25503042	chr7:134072295-134072339	NM_172747:-76	Kctd13	PROMOTER	0.595	3.359	3482.36	11697.06	1.999	2772.64	5542.07
A_68_P25503044	chr7:134072503-134072547	NM_172747:132	Kctd13	INSIDE	0.614	10.313	1902.82	19624.25	6.335	1570.54	9948.65
A_68_P25044855	chr7:35438249-35438293	NM_146188:-410	Kctd15	PROMOTER	0.386	0.521	1542.65	804.30	0.201	1238.78	249.18
A_68_P30359963	chr15:78267288-78267332	NM_001081367:8253	Kctd17	INSIDE	0.438	5.161	2401.27	12392.81	2.261	1710.70	3867.61
A_68_P30359964	chr15:78267418-78267462	NM_001081367:8383	Kctd17	INSIDE	0.227	3.083	7871.01	24269.27	0.701	5502.28	3857.48
A_68_P31125154	chr17:29089463-29089507	NM_025888:-676	Kctd20	PROMOTER	0.319	5.834	1733.35	10112.97	1.861	1558.60	2900.49
A_68_P25349933	chr7:104480292-104480336	NM_001039039:-518	Kctd21	DIVERGENT_PROMOTER	0.388	0.641	773.80	496.07	0.249	696.26	173.21

ProbeName	Target position of probe on CpG island microarray	TargetID	GeneSymbol	CpG island Description	Ratio of relative methylation (TiO_2-NP/Vehicle)	Sham group			TiO_2-H group		
						Relative methylation (Cy5/Cy3)	Cy3 signal (Input)	Cy5 signal (MeDIP)	Relative methylation (Cy5/Cy3)	Cy3 signal (Input)	Cy5 signal (MeDIP)
A_68_P29388379	chr14:9046505-9046549	NM_027782:-135	Kctd6	PROMOTER	0.532	11.768	4201.61	49445.11	6.265	3553.49	22262.29
A_68_P23777458	chr5:69732840-69732884	NM_175519:86	Kctd8	INSIDE	0.269	70.740	5009.52	354371.80	19.023	4684.44	89112.15
A_68_P30366908	chr15:79341974-79342018	NM_134090:-4841	Kdelr3	DIVERGENT_PROMOTER	0.633	0.642	1622.80	1041.80	0.406	1090.42	443.23
A_68_P30366912	chr15:79342432-79342476	NM_134090:-4383	Kdelr3	DIVERGENT_PROMOTER	0.22	2.296	1656.91	3804.60	0.505	1233.72	622.62
A_68_P30366909	chr15:79342087-79342131	NM_134090:-4729	Kdelr3	DIVERGENT_PROMOTER	0.151	2.534	669.24	1695.62	0.383	583.03	223.43
A_68_P23334806	chr4:136158309-136158353	NM_133872:308	Kdm1a	INSIDE	0.238	35.282	3402.69	120054.60	8.395	3248.48	27271.42
A_68_P29011271	chr13:47138796-47138840	NM_172262:-89	Kdm1b	DIVERGENT_PROMOTER	0.247	2.550	1605.38	4093.90	0.629	1396.03	878.70
A_68_P31922932	chr19:4396607-4396651	NM_001001984:449	Kdm2a	INSIDE	0.27	8.036	2934.24	23579.01	2.172	2800.53	6081.63
A_68_P31922938	chr19:4397627-4397671	NM_001001984:-571	Kdm2a	PROMOTER	0.415	7.168	2019.31	14474.82	2.978	1739.43	5180.65
A_68_P24052812	chr5:123350466-123350510	NM_013910:-129	Kdm2b	PROMOTER	0.268	10.856	954.16	10358.64	2.905	844.16	2452.17
A_68_P24053343	chr5:123439281-123439325	NM_001003953:-201	Kdm2b	PROMOTER	0.352	0.357	1785.27	636.75	0.126	1371.05	172.16
A_68_P24053342	chr5:123439126-123439170	NM_001003953:-47	Kdm2b	PROMOTER	0.402	0.300	2701.33	810.93	0.121	1901.61	229.44
A_68_P24052813	chr5:123350619-123350663	NM_013910:-281	Kdm2b	PROMOTER	0.538	5.215	614.74	3206.04	2.803	441.49	1237.62
A_68_P24053344	chr5:123439364-123439408	NM_001003953:-285	Kdm2b	PROMOTER	0.611	2.960	8209.73	24299.30	1.808	5894.51	10656.80
A_68_P24053328	chr5:123437535-123437579	NM_001005866:986	Kdm2b	INSIDE	0.486	0.696	1047.11	728.51	0.338	829.56	280.28
A_68_P24052806	chr5:123349603-123349647	NM_013910:735	Kdm2b	INSIDE	0.64	2.523	771.54	1946.25	1.615	743.18	1200.35
A_68_P24537468	chr6:71583029-71583078	NM_001038695:-154	Kdm3a	PROMOTER	0.525	4.327	1426.92	6174.18	2.271	1014.14	2303.49
A_68_P24537472	chr6:71583440-71583484	NM_001038695:-563	Kdm3a	PROMOTER	0.633	0.482	2058.11	992.98	0.306	1631.59	498.67
A_68_P24537464	chr6:71582547-71582591	NM_173001:112	Kdm3a	INSIDE	0.104	2.436	1017.81	2478.93	0.253	745.19	188.81
A_68_P31614616	chr18:34937211-34937255	NM_001081256:571	Kdm3b	INSIDE	0.518	5.783	1612.31	9323.51	2.995	1680.36	5032.97
A_68_P31255286	chr17:56465347-56465391	NM_172132:-104	Kdm4b	PROMOTER	0.26	9.069	2095.20	19002.17	2.355	1747.58	4114.94
A_68_P24798118	chr6:120314755-120314799	NM_145997:660	Kdm5a	INSIDE	0.565	0.546	2553.61	1393.58	0.308	1785.39	550.25
A_68_P24798119	chr6:120314850-120314894	NM_145997:756	Kdm5a	INSIDE	0.356	1.495	2480.72	3708.93	0.533	1993.27	1061.64
A_68_P20634033	chr1:136457792-136457836	NM_152895:1060	Kdm5b	INSIDE	0.6	4.849	4196.57	20349.16	2.908	3252.45	9457.53
A_68_P32281781	chrX:17739765-17739810	NM_009483:-5	Kdm6a	PROMOTER	0.255	1.999	1682.78	3364.70	0.51	2186.26	1115.14
A_68_P32281785	chrX:17740258-17740302	NM_009483:488	Kdm6a	INSIDE	0.431	4.335	1102.87	4780.53	1.869	1400.87	2617.95
A_68_P32281788	chrX:17740669-17740713	NM_009483:898	Kdm6a	INSIDE	0.657	0.513	999.58	512.80	0.337	1490.43	502.24
A_68_P32281783	chrX:17740018-17740062	NM_009483:248	Kdm6a	INSIDE	0.365	0.536	1084.69	581.61	0.196	1370.44	268.06
A_68_P32281782	chrX:17739949-17739993	NM_009483:178	Kdm6a	INSIDE	0.613	2.405	1665.71	4006.39	1.475	1959.61	2889.88
A_68_P27894946	chr11:69224331-69224375	NM_001017426:2825	Kdm6b	INSIDE	0.469	0.708	4665.06	3300.55	0.332	2811.40	932.84
A_68_P23812530	chr5:76374553-76374597	NM_010612:-121	Kdr	PROMOTER	0.342	9.974	5202.07	51884.59	3.413	4223.18	14411.64
A_68_P20510207	chr1:108656347-108656391	NM_027534:-49	Kdsr	PROMOTER	0.249	6.201	2322.88	14403.23	1.546	1935.91	2992.03
A_68_P26346113	chr9:21043510-21043554	NM_001110305:244	Keap1	INSIDE	0.442	5.048	1263.25	6376.65	2.232	1110.11	2477.33
A_68_P26346080	chr9:21037928-21037972	NM_001110305:5826	Keap1	INSIDE	0.451	3.647	1857.59	6775.04	1.646	1307.28	2151.82
A_68_P20127130	chr1:32229462-32229506	NM_133235:-166	Khdrbs2	PROMOTER	0.385	4.886	2172.36	10614.90	1.882	1956.23	3682.04
A_68_P20127131	chr1:32229533-32229577	NM_133235:-96	Khdrbs2	PROMOTER	0.475	5.389	635.18	3422.71	2.557	521.28	1333.03
A_68_P29620447	chr14:56504073-56504117	NM_027143:289	Khnyn	INSIDE	0.452	0.569	4692.73	2670.57	0.257	3139.14	807.26
A_68_P31259695	chr17:57170806-57170850	NM_010613:102	Khsrp	INSIDE	0.258	16.053	2332.85	37448.62	4.135	2131.99	8815.79
A_68_P31259625	chr17:57161925-57161969	NM_010613:8984	Khsrp	INSIDE	0.375	5.849	4876.38	28523.05	2.191	3711.55	8132.93
A_68_P31259627	chr17:57162083-57162127	NM_010613:8826	Khsrp	INSIDE	0.349	4.962	1360.31	6749.79	1.734	1033.29	1791.65
A_68_P32095251	chr19:37450692-37450736	NM_010615:-178	Kif11	PROMOTER	0.446	0.169	3201.80	539.81	0.075	2282.31	171.56
A_68_P29010691	chr13:47024841-47024885	NM_010617:225	Kif13a	INSIDE	0.313	9.844	2514.98	24757.73	3.082	2075.48	6396.77
A_68_P29010697	chr13:47025669-47025713	NM_010617:-603	Kif13a	PROMOTER	0.186	0.663	2095.59	1389.32	0.123	1403.51	172.90
A_68_P20646088	chr1:138363089-138363133	NM_001081258:-1424	Kif14	PROMOTER	0.428	8.733	2093.00	18279.05	3.736	1830.56	6838.21
A_68_P20453282	chr1:94998673-94998717	NM_001110315:-252	Kif1a	PROMOTER	0.25	7.282	2757.13	20078.38	1.817	2848.62	5177.21
A_68_P20453280	chr1:94998488-94998532	NM_001110315:-68	Kif1a	PROMOTER	0.314	6.846	1402.53	9601.14	2.153	1206.85	2598.27

ProbeName	Target position of probe on CpG island microarray	TargetID	GeneSymbol	CpG island Description	Ratio of relative methylation (TiO_2-NP/Vehicle)	Sham group			TiO_2-H group		
						Relative methylation (Cy5/Cy3)	Cy3 signal (Input)	Cy5 signal (MeDIP)	Relative methylation (Cy5/Cy3)	Cy3 signal (Input)	Cy5 signal (MeDIP)
A_68_P20453264	chr1:94996840-94996884	NM_001110315:1580	Kif1a	INSIDE	0.463	0.417	1100.70	458.77	0.193	908.75	175.35
A_68_P20453278	chr1:94998327-94998371	NM_001110315:94	Kif1a	INSIDE	0.533	0.171	2679.34	457.95	0.091	1929.05	175.81
A_68_P20453265	chr1:94996931-94996975	NM_001110315:1490	Kif1a	INSIDE	0.625	4.489	2277.51	10224.81	2.807	1894.47	5318.38
A_68_P23399812	chr4:148681667-148681711	NM_207682:119	Kif1b	INSIDE	0.314	14.365	1498.57	21526.57	4.504	1286.63	5795.43
A_68_P23399815	chr4:148682002-148682046	NM_207682:-217	Kif1b	PROMOTER	0.53	0.297	5374.37	1596.06	0.157	3732.21	587.00
A_68_P31613843	chr18:34784053-34784097	NM_001166407:-203	Kif20a	PROMOTER	0.499	0.352	1271.53	447.80	0.176	977.46	171.85
A_68_P32082884	chr19:34996996-34997040	NM_183046:171	Kif20b	INSIDE	0.437	0.322	1661.98	535.27	0.141	1479.69	208.06
A_68_P32082882	chr19:34996734-34996778	NM_183046:-91	Kif20b	PROMOTER	0.324	5.358	736.49	3945.86	1.736	703.96	1222.07
A_68_P30433473	chr15:90880476-90880520	NM_001109040:-119	Kif21a	PROMOTER	0.304	10.777	4706.66	50724.13	3.276	4716.99	15455.03
A_68_P30433470	chr15:90880052-90880096	NM_001109040:305	Kif21a	INSIDE	0.652	2.926	1474.02	4313.34	1.909	1154.64	2204.22
A_68_P20644107	chr1:138036961-138037006	NM_001039472:9006	Kif21b	INSIDE	0.253	48.932	2946.34	144171.30	12.39	3635.69	45046.02
A_68_P20644037	chr1:138028308-138028352	NM_001039472:353	Kif21b	INSIDE	0.663	0.350	2218.73	775.75	0.232	1671.00	387.36
A_68_P22862283	chr4:41411609-41411653	NM_024241:251	Kif24	INSIDE	0.401	0.555	2046.47	1134.82	0.223	1628.68	362.60
A_68_P28748276	chr12:113400089-113400133	NM_001097621:15692	Kif26a	INSIDE	0.326	6.178	3747.61	23154.53	2.016	2633.00	5307.30
A_68_P28748141	chr12:113384025-113384069	NM_001097621:-372	Kif26a	PROMOTER	0.63	0.598	2069.50	1237.52	0.377	1770.69	666.92
A_68_P28748144	chr12:113384449-113384493	NM_001097621:52	Kif26a	INSIDE	0.54	3.617	851.97	3081.55	1.952	774.72	1512.43
A_68_P28748275	chr12:113399969-113400013	NM_001097621:15572	Kif26a	INSIDE	0.209	7.682	2756.31	21175.17	1.608	1860.32	2991.25
A_68_P20864287	chr1:180457911-180457955	NM_001161665:-1323	Kif26b	PROMOTER	0.507	0.176	2559.91	450.86	0.089	1910.65	170.75
A_68_P20864304	chr1:180459747-180459791	NM_001161665:513	Kif26b	INSIDE	0.535	0.260	2545.58	660.76	0.139	1868.64	259.42
A_68_P20864306	chr1:180459903-180459947	NM_001161665:669	Kif26b	INSIDE	0.268	0.592	1492.57	883.27	0.158	1098.57	174.02
A_68_P29072860	chr13:58460543-58460587	NM_175214:-4341	Kif27	PROMOTER	0.648	0.171	4611.61	789.67	0.111	3200.26	354.97
A_68_P27805436	chr11:53381255-53381299	NM_008443:396	Kif3a	INSIDE	0.474	0.351	1485.88	522.11	0.167	1127.81	187.99
A_68_P27534887	chr10:126700073-126700117	NM_001039000:325	Kif5a	INSIDE	0.459	0.247	3645.87	902.07	0.113	2605.77	295.74
A_68_P31472616	chr18:6241499-6241543	NM_008448:2	Kif5b	INSIDE	0.367	52.116	1248.26	65054.65	19.129	1274.37	24376.90
A_68_P21203206	chr2:49475493-49475538	NM_008449:682	Kif5c	INSIDE	0.27	6.064	1425.87	8646.00	1.638	1304.51	2136.40
A_68_P21203199	chr2:49474675-49474719	NM_008449:-137	Kif5c	PROMOTER	0.39	4.270	752.93	3215.26	1.664	680.83	1132.70
A_68_P31223848	chr17:49754556-49754600	NM_177052:82	Kif6	INSIDE	0.393	0.387	1849.38	715.58	0.152	1229.78	187.06
A_68_P25262701	chr7:86855086-86855130	NM_010626:3964	Kif7	INSIDE	0.574	5.930	446.10	2645.27	3.404	353.53	1203.48
A_68_P30349763	chr15:76491001-76491045	NM_010630:-48	Kifc2	DIVERGENT_PROMOTER	0.398	13.616	2723.23	37078.76	5.418	2476.48	13416.31
A_68_P25031610	chr7:31242110-31242154	NM_172898:402	Kirrel2	INSIDE	0.421	39.449	1316.05	51916.35	16.6	1699.24	28206.49
A_68_P25031592	chr7:31239092-31239136	NM_172898:3420	Kirrel2	INSIDE	0.644	0.534	1216.70	649.59	0.344	1045.48	359.56
A_68_P26416478	chr9:34296474-34296518	NM_001190913:181	Kirrel3	INSIDE	0.513	0.583	1182.38	689.03	0.299	960.61	286.91
A_68_P32383986	chrX:50097843-50097887	NR_003188:-94	Kis2	PROMOTER	0.349	5.786	8280.59	47908.36	2.021	9638.08	19481.23
A_68_P27279739	chr10:79384461-79384505	NM_053244:4767	Kiss1r	INSIDE	0.483	0.359	1394.62	500.59	0.173	1182.77	204.97
A_68_P27279734	chr10:79383882-79383926	NM_053244:4189	Kiss1r	INSIDE	0.624	4.801	2768.17	13290.42	2.995	2320.87	6950.62
A_68_P23809987	chr5:75970827-75970871	NM_001122733:-163	Kit	PROMOTER	0.241	2.635	2626.85	6922.63	0.635	1989.73	1262.89
A_68_P23756018	chr5:65771113-65771157	NM_031180:31485	Klb	INSIDE	0.496	7.838	1216.91	9537.76	3.886	1164.00	4523.08
A_68_P28745894	chr12:112997376-112997420	NM_008450:339	Klc1	INSIDE	0.475	3.260	6367.80	20756.28	1.548	4881.01	7555.90
A_68_P31927058	chr19:5118339-5118383	NM_008451:48	Klc2	INSIDE	0.494	3.818	1109.19	4234.86	1.885	950.57	1791.62
A_68_P31927053	chr19:5117800-5117844	NM_008451:586	Klc2	INSIDE	0.608	0.524	960.53	503.18	0.318	764.66	243.51
A_68_P24993125	chr7:19989502-19989546	NM_146182:-4319	Klc3	DIVERGENT_PROMOTER	0.354	14.418	648.38	9348.06	5.102	493.59	2518.52
A_68_P24993122	chr7:19989220-19989264	NM_146182:-4037	Klc3	DIVERGENT_PROMOTER	0.401	13.907	886.35	12326.03	5.583	749.76	4185.77
A_68_P26022557	chr8:87427077-87427121	NM_010635:1272	Klf1	INSIDE	0.413	5.170	1798.28	9297.68	2.137	1485.91	3176.05
A_68_P30148791	chr15:38229661-38229705	NM_013692:780	Klf10	INSIDE	0.381	10.905	1451.12	15824.66	4.157	1529.86	6359.60
A_68_P30148784	chr15:38228806-38228850	NM_013692:1634	Klf10	INSIDE	0.504	4.856	2459.22	11941.20	2.447	2126.00	5201.77
A_68_P28290823	chr12:25335979-25336023	NM_178357:-235	Klf11	PROMOTER	0.258	17.722	4395.79	77901.66	4.577	3973.86	18187.96

ProbeName	Target position of probe on CpG island microarray	TargetID	GeneSymbol	CpG island Description	Ratio of relative methylation (TiO_2-NP/Vehicle)	Sham group			TiO_2-H group		
						Relative methylation (Cy5/Cy3)	Cy3 signal (Input)	Cy5 signal (MeDIP)	Relative methylation (Cy5/Cy3)	Cy3 signal (Input)	Cy5 signal (MeDIP)
A_68_P28290825	chr12:25336262-25336306	NM_178357:49	Klf11	INSIDE	0.606	3.402	1048.34	3566.20	2.061	863.97	1780.32
A_68_P25174396	chr7:71082541-71082585	NM_021366:1239	Klf13	INSIDE	0.25	18.327	11937.12	218772.50	4.581	11367.83	52076.42
A_68_P25174399	chr7:71082777-71082821	NM_021366:1003	Klf13	INSIDE	0.339	9.387	2650.33	24878.85	3.184	2197.99	6998.53
A_68_P25174077	chr7:71036413-71036457	NM_021366:47367	Klf13	INSIDE	0.474	4.516	2962.07	13376.75	2.14	2307.35	4937.92
A_68_P25174406	chr7:71083847-71083891	NM_021366:-67	Klf13	PROMOTER	0.487	9.514	1398.56	13306.22	4.63	1409.54	6526.61
A_68_P24334780	chr6:30908766-30908810	NM_001135093:202	Klf14	INSIDE	0.598	7.070	409.29	2893.56	4.227	365.84	1546.22
A_68_P24632565	chr6:90412944-90412988	NM_023184:347	Klf15	INSIDE	0.231	7.821	1533.19	11990.76	1.81	1377.05	2491.99
A_68_P24632571	chr6:90413720-90413764	NM_023184:1123	Klf15	INSIDE	0.365	8.519	1995.49	16999.43	3.106	1772.00	5503.47
A_68_P24632567	chr6:90413191-90413235	NM_023184:593	Klf15	INSIDE	0.594	0.299	2689.64	804.41	0.178	1962.08	348.86
A_68_P27284036	chr10:80031664-80031708	NM_078477:8355	Klf16	INSIDE	0.483	6.322	2428.79	15355.87	3.056	2045.92	6253.13
A_68_P27284038	chr10:80031829-80031873	NM_078477:8191	Klf16	INSIDE	0.556	4.481	1642.29	7358.92	2.492	1160.64	2892.49
A_68_P27284101	chr10:80040251-80040295	NM_078477:-231	Klf16	PROMOTER	0.372	0.651	3165.58	2061.57	0.242	2376.86	576.29
A_68_P27284104	chr10:80040680-80040724	NM_078477:-661	Klf16	PROMOTER	0.648	0.649	2286.92	1484.04	0.42	1754.91	737.89
A_68_P27284037	chr10:80031748-80031792	NM_078477:8271	Klf16	INSIDE	0.566	2.685	1725.80	4633.84	1.519	1385.63	2104.25
A_68_P25960687	chr8:74843237-74843281	NM_008452:298	Klf2	INSIDE	0.506	0.610	2973.05	1814.99	0.309	2073.77	640.51
A_68_P25960693	chr8:74844062-74844106	NM_008452:1124	Klf2	INSIDE	0.595	0.523	872.78	456.71	0.311	776.03	241.50
A_68_P23753093	chr5:65213285-65213329	NM_008453:18545	Klf3	INSIDE	0.445	4.946	1027.51	5082.37	2.203	792.73	1746.53
A_68_P23753094	chr5:65213362-65213406	NM_008453:18623	Klf3	INSIDE	0.533	4.177	2271.44	9486.65	2.228	1774.70	3953.37
A_68_P22934435	chr4:55545469-55545513	NM_010637:-143	Klf4	PROMOTER	0.243	17.596	1835.93	32305.50	4.284	1474.32	6316.10
A_68_P22934428	chr4:55544566-55544610	NM_010637:759	Klf4	INSIDE	0.423	0.439	1069.20	469.77	0.186	902.40	167.52
A_68_P22934417	chr4:55543327-55543371	NM_010637:1999	Klf4	INSIDE	0.423	4.565	2174.30	9925.34	1.933	1653.39	3195.18
A_68_P22934423	chr4:55544008-55544052	NM_010637:1317	Klf4	INSIDE	0.66	0.612	3148.06	1927.76	0.404	2298.95	929.46
A_68_P29842698	chr14:99698055-99698099	NM_009769:167	Klf5	INSIDE	0.37	1.531	3680.85	5635.64	0.567	3351.95	1899.28
A_68_P29842694	chr14:99697586-99697630	NM_009769:-301	Klf5	PROMOTER	0.497	0.662	2080.32	1376.82	0.329	1553.43	511.43
A_68_P29842699	chr14:99698157-99698201	NM_009769:269	Klf5	INSIDE	0.438	0.677	1579.18	1068.39	0.296	1420.87	420.76
A_68_P32016047	chr19:23210802-23210846	NM_010638:-4891	Klf9	PROMOTER	0.234	11.470	3471.51	39819.07	2.684	2796.32	7504.81
A_68_P32016035	chr19:23209504-23209553	NM_010638:-6187	Klf9	PROMOTER	0.4	0.524	1387.27	726.80	0.209	914.50	191.44
A_68_P32016043	chr19:23210311-23210355	NM_010638:-5383	Klf9	PROMOTER	0.543	7.699	713.90	5496.23	4.182	726.19	3037.23
A_68_P32016039	chr19:23209957-23210001	NM_010638:-5737	Klf9	PROMOTER	0.591	6.789	3967.47	26936.11	4.01	3747.86	15029.50
A_68_P28513502	chr12:70342816-70342860	NM_178253:20	Klhdc1	INSIDE	0.556	3.937	916.23	3607.32	2.191	810.62	1775.78
A_68_P24332082	chr6:30409887-30409931	NM_029742:58000	Klhdc10	DOWNSTREAM	0.623	0.634	1167.19	740.30	0.395	1022.73	404.42
A_68_P24331758	chr6:30352294-30352338	NM_029742:408	Klhdc10	INSIDE	0.478	0.651	727.48	473.26	0.311	619.50	192.79
A_68_P28513756	chr12:70398098-70398142	NM_027117:453	Klhdc2	INSIDE	0.423	0.430	1136.84	489.37	0.182	984.69	179.41
A_68_P24932468	chr6:147040234-147040278	NM_001081237:660	Klhdc5	INSIDE	0.403	8.342	492.84	4111.15	3.363	482.77	1623.39
A_68_P24932466	chr6:147039958-147040002	NM_001081237:384	Klhdc5	INSIDE	0.404	4.494	1427.84	6416.18	1.816	1204.42	2187.07
A_68_P20620518	chr1:134195183-134195227	NM_144810:2	Klhdc8a	INSIDE	0.485	4.697	1444.73	6785.27	2.278	1322.58	3013.07
A_68_P20633482	chr1:136352339-136352383	NM_153128:229	Klhl12	INSIDE	0.6	2.825	657.39	1857.41	1.695	692.63	1173.86
A_68_P32299549	chrX:22942071-22942115	NM_026167:87	Klhl13	INSIDE	0.609	3.914	343.03	1342.60	2.384	490.28	1168.88
A_68_P26823921	chr9:110379166-110379210	NM_177771:10	Klhl18	INSIDE	0.578	0.382	2012.34	768.57	0.221	1555.14	343.36
A_68_P25926103	chr8:67373325-67373369	NM_178633:370	Klhl2	INSIDE	0.492	0.663	2096.45	1389.30	0.326	1658.60	541.28
A_68_P23415961	chr4:151383718-151383762	NM_001033352:741	Klhl21	INSIDE	0.546	3.900	915.52	3570.34	2.128	713.47	1518.53
A_68_P30575507	chr16:17759672-17759716	NM_145479:-19	Klhl22	PROMOTER	0.475	0.740	3612.47	2673.55	0.352	3467.97	1220.08
A_68_P30585937	chr16:20098035-20098079	NM_029436:430	Klhl24	INSIDE	0.264	9.914	1789.92	17745.53	2.613	1554.39	4061.57
A_68_P30585939	chr16:20098297-20098341	NM_029436:692	Klhl24	INSIDE	0.491	3.846	1282.59	4932.63	1.889	1065.10	2011.79
A_68_P28206071	chr12:5382006-5382050	NM_001164493:460	Klhl29	INSIDE	0.581	0.277	3449.82	955.37	0.161	2574.20	414.24
A_68_P28206077	chr12:5382789-5382833	NM_001164493:-322	Klhl29	DIVERGENT_PROMOTER	0.534	3.564	1037.55	3698.33	1.903	791.99	1507.17

ProbeName	Target position of probe on CpG island microarray	TargetID	GeneSymbol	CpG island Description	Ratio of relative methylation (TiO_2-NP/Vehicle)	Sham group			TiO_2-H group		
						Relative methylation (Cy5/Cy3)	Cy3 signal (Input)	Cy5 signal (MeDIP)	Relative methylation (Cy5/Cy3)	Cy3 signal (Input)	Cy5 signal (MeDIP)
A_68_P22787931	chr4:24778185-24778229	NM_001033531:27	Klhl32	INSIDE	0.434	4.507	5368.11	24194.28	1.957	4691.34	9181.28
A_68_P25362045	chr7:106617271-106617315	NM_028145:2779	Klhl35	INSIDE	0.639	4.654	648.90	3019.75	2.973	490.49	1458.41
A_68_P23949339	chr5:104339900-104339944	NM_178741:326	Klhl8	INSIDE	0.285	5.873	1184.58	6957.11	1.676	988.08	1656.07
A_68_P25084097	chr7:51052779-51052823	NM_008940:-146	Klk8	PROMOTER	0.387	0.635	1096.82	696.23	0.246	885.63	217.45
A_68_P31428327	chr17:88929344-88929388	NM_028658:-97	Klraq1	PROMOTER	0.304	28.816	2037.74	58719.99	8.76	2009.20	17600.09
A_68_P31428328	chr17:88929429-88929473	NM_028658:-13	Klraq1	PROMOTER	0.497	13.045	715.66	9335.68	6.488	565.24	3667.33
A_68_P24378362	chr6:38586500-38586544	NM_001033171:717	Klrg2	INSIDE	0.338	0.455	1365.55	621.75	0.154	1136.26	174.65
A_68_P24378364	chr6:38586770-38586814	NM_001033171:447	Klrg2	INSIDE	0.584	3.319	1524.13	5057.87	1.938	1299.48	2518.92
A_68_P23226465	chr4:115563121-115563165	NM_001039124:6138	Kncn	DOWNSTREAM	0.471	0.462	1055.68	487.80	0.218	799.48	174.02
A_68_P22219388	chr3:68931188-68931232	NM_008467:-196	Kpna4	PROMOTER	0.073	2.057	13854.49	28505.50	0.15	9238.92	1388.77
A_68_P28049252	chr11:97049849-97049893	NM_008379:-664	Kpnb1	PROMOTER	0.261	9.297	1000.62	9302.49	2.422	1047.32	2536.53
A_68_P28049241	chr11:97048390-97048434	NM_008379:794	Kpnb1	INSIDE	0.58	0.321	8256.35	2649.53	0.186	5813.37	1082.12
A_68_P24535484	chr6:71221790-71221834	NM_145568:-200	Krcc1	DIVERGENT_PROMOTER	0.259	23.736	3112.47	73877.84	6.148	2722.08	16734.84
A_68_P24535483	chr6:71221618-71221662	NM_145568:-372	Krcc1	DIVERGENT_PROMOTER	0.458	14.427	685.51	9889.51	6.602	699.88	4620.51
A_68_P31093373	chr17:23879194-23879238	NM_028416:3580	Kremen2	INSIDE	0.262	2.554	5329.80	13613.01	0.67	3680.43	2465.98
A_68_P26346371	chr9:21092727-21092771	NM_145416:-335	Kri1	PROMOTER	0.628	0.602	3347.25	2015.55	0.378	2359.00	892.67
A_68_P26346367	chr9:21092297-21092341	NM_145416:95	Kri1	INSIDE	0.657	0.514	911.29	468.79	0.338	805.11	271.98
A_68_P23447098	chr5:3803414-3803458	NR_033173:256	Krit1	INSIDE	0.514	10.899	1880.62	20497.50	5.603	1759.17	9856.43
A_68_P30495553	chr15:101858609-101858653	NM_010664:-16	Krt18	PROMOTER	0.172	8.667	1530.43	13264.64	1.491	1318.32	1964.97
A_68_P28066375	chr11:100006570-100006614	NM_008471:641	Krt19	INSIDE	0.499	4.175	5592.43	23346.31	2.082	4511.45	9391.14
A_68_P23577316	chr5:31554254-31554298	NM_027221:198	Krtcap3	INSIDE	0.32	13.995	2525.27	35341.94	4.484	2385.63	10697.93
A_68_P27946741	chr11:78858490-78858534	NM_013571:101344	Ksr1	INSIDE	0.223	19.093	3693.03	70512.30	4.257	2824.95	12025.58
A_68_P27946742	chr11:78858560-78858604	NM_013571:101274	Ksr1	INSIDE	0.339	7.933	2639.61	20941.02	2.692	1975.45	5318.55
A_68_P24020426	chr5:117863550-117863594	NM_001034873:-436	Ksr2	PROMOTER	0.294	1.822	3302.93	6018.79	0.535	2569.88	1376.05
A_68_P21809534	chr2:162769378-162769422	NM_001081338:200	L3mbtl1	INSIDE	0.154	14.252	1075.20	15324.21	2.196	996.64	2188.66
A_68_P30378912	chr15:81494223-81494269	NM_145993:-119	L3mbtl2	PROMOTER	0.3	5.223	1163.86	6078.34	1.565	1155.88	1809.30
A_68_P27019249	chr10:26095302-26095346	NM_172787:-333	L3mbtl3	PROMOTER	0.2	9.784	3662.02	35830.58	1.961	2832.92	5555.66
A_68_P31317441	chr17:68623070-68623114	NM_177278:-44	L3mbtl4	PROMOTER	0.546	0.286	5092.04	1456.66	0.156	3286.63	513.66
A_68_P20042287	chr1:13650338-13650382	NM_145381:230	Lactb2	INSIDE	0.527	7.904	2299.26	18172.28	4.168	2141.63	8925.88
A_68_P31313935	chr17:68046587-68046631	NM_008480:4	Lama1	INSIDE	0.41	0.543	954.05	518.43	0.223	779.53	173.81
A_68_P21902329	chr2:179960496-179960540	NM_001081171:46	Lama5	INSIDE	0.336	6.364	1142.50	7270.33	2.135	1033.74	2207.13
A_68_P21902328	chr2:179960330-179960374	NM_001081171:212	Lama5	INSIDE	0.391	6.734	784.97	5286.25	2.63	637.65	1677.24
A_68_P21902323	chr2:179959846-179959890	NM_001081171:696	Lama5	INSIDE	0.497	0.199	2394.50	477.18	0.099	1765.94	175.05
A_68_P21902326	chr2:179960119-179960164	NM_001081171:423	Lama5	INSIDE	0.524	0.405	1412.76	571.52	0.212	1084.88	229.90
A_68_P21902331	chr2:179960697-179960741	NM_001081171:-154	Lama5	PROMOTER	0.385	4.164	1602.68	6673.21	1.602	1409.02	2257.87
A_68_P28330136	chr12:31950421-31950465	NM_008482:284	Lamb1	INSIDE	0.53	0.650	1422.66	925.42	0.345	1219.99	420.48
A_68_P20726280	chr1:155180208-155180252	NM_010683:-314	Lamc1	PROMOTER	0.163	25.860	16203.73	419027.90	4.213	15757.72	66385.65
A_68_P20726276	chr1:155179693-155179737	NM_010683:202	Lamc1	INSIDE	0.203	7.878	1241.17	9778.35	1.598	1318.89	2107.60
A_68_P20725330	chr1:155033404-155033448	NM_008485:151	Lamc2	INSIDE	0.468	0.341	1319.35	449.29	0.16	1068.22	170.41
A_68_P20306027	chr1:67085323-67085367	NM_001190984:64	Lancl1	INSIDE	0.131	14.879	2655.91	39517.13	1.943	2376.55	4617.08
A_68_P24479790	chr6:57652980-57653024	NM_133737:554	Lancl2	INSIDE	0.456	4.502	1005.99	4529.21	2.053	906.63	1861.30
A_68_P32245740	chrX:8777550-8777594	NM_173414:474	Lancl3	INSIDE	0.349	9.592	1484.17	14236.01	3.347	1969.47	6591.74
A_68_P28225274	chr12:8928368-8928412	NM_008640:278	Laptm4a	INSIDE	0.569	3.627	2798.59	10150.02	2.063	2199.44	4537.06
A_68_P28225280	chr12:8929060-8929104	NM_008640:970	Laptm4a	INSIDE	0.485	0.579	944.95	546.86	0.28	848.32	237.93
A_68_P30127461	chr15:34168406-34168450	NM_033521:648	Laptm4b	INSIDE	0.622	3.197	1526.56	4880.56	1.989	1230.31	2447.01
A_68_P25965605	chr8:75876686-75876730	NM_010687:-253	Large	PROMOTER	0.251	9.088	840.32	7637.16	2.278	683.67	1557.57

ProbeName	Target position of probe on CpG island microarray	TargetID	GeneSymbol	CpG island Description	Ratio of relative methylation (TiO_2-NP/Vehicle)	Sham group			TiO_2-H group		
						Relative methylation (Cy5/Cy3)	Cy3 signal (Input)	Cy5 signal (MeDIP)	Relative methylation (Cy5/Cy3)	Cy3 signal (Input)	Cy5 signal (MeDIP)
A_68_P25965606	chr8:75876811-75876855	NM_010687:-377	Large	PROMOTER	0.539	4.281	2551.74	10925.25	2.309	2121.95	4898.95
A_68_P25965603	chr8:75876434-75876478	NM_010687:-1	Large	PROMOTER	0.336	0.606	977.07	591.99	0.204	877.59	178.72
A_68_P27828613	chr11:57822962-57823006	NM_028451:419	Larp1	INSIDE	0.191	22.907	528.51	12106.68	4.372	443.33	1938.12
A_68_P27828609	chr11:57822259-57822303	NM_028451:-285	Larp1	PROMOTER	0.405	4.652	2208.52	10274.83	1.885	1845.00	3477.85
A_68_P27828619	chr11:57823508-57823552	NM_028451:965	Larp1	INSIDE	0.587	2.725	2359.55	6429.00	1.598	1986.28	3174.53
A_68_P22082254	chr3:40753323-40753367	NM_001040399:-1208	Larp1b	PROMOTER	0.266	0.307	2638.23	810.51	0.082	2116.40	172.97
A_68_P22082256	chr3:40753573-40753617	NM_001040399:-958	Larp1b	PROMOTER	0.319	5.647	22877.88	129182.70	1.802	17749.01	31990.78
A_68_P22082255	chr3:40753446-40753490	NM_001040399:-1084	Larp1b	PROMOTER	0.341	0.411	3817.27	1570.69	0.14	2531.94	355.16
A_68_P22082262	chr3:40754266-40754310	NM_001040399:-264	Larp1b	PROMOTER	0.657	3.591	391.95	1407.61	2.358	337.21	795.13
A_68_P28814402	chr13:9093309-9093353	NM_172585:180	Larp4b	INSIDE	0.376	9.157	942.75	8633.03	3.441	780.80	2686.51
A_68_P28814398	chr13:9092754-9092798	NM_172585:-374	Larp4b	PROMOTER	0.58	0.635	2612.19	1658.14	0.368	2073.66	763.46
A_68_P28052725	chr11:97660923-97660967	NM_010688:-41	Lasp1	PROMOTER	0.258	16.486	2771.70	45693.91	4.261	2551.51	10872.38
A_68_P30482201	chr15:99602827-99602871	NM_028015:98	Lass5	INSIDE	0.263	11.774	3094.04	36429.22	3.097	2922.19	9049.87
A_68_P30482203	chr15:99603119-99603163	NM_028015:-194	Lass5	PROMOTER	0.564	0.331	2892.18	958.14	0.187	2085.71	389.64
A_68_P21305453	chr2:68700233-68700278	NM_172856:642	Lass6	INSIDE	0.355	9.019	2732.94	24647.57	3.199	2154.80	6892.76
A_68_P21305451	chr2:68700007-68700051	NM_172856:415	Lass6	INSIDE	0.506	0.451	1020.96	460.81	0.228	755.77	172.56
A_68_P26920199	chr10:7401013-7401057	NM_010690:28	Lats1	INSIDE	0.596	4.139	1850.72	7660.54	2.467	1579.74	3896.59
A_68_P29629404	chr14:58310425-58310469	NM_015771:54514	Lats2	INSIDE	0.537	6.484	1765.64	11448.43	3.483	1261.68	4394.29
A_68_P29629463	chr14:58318497-58318541	NM_015771:46442	Lats2	INSIDE	0.602	5.385	1068.87	5755.93	3.242	999.54	3240.01
A_68_P31343015	chr17:73268700-73268744	NM_029999:1078	Lbh	INSIDE	0.518	3.357	1573.77	5283.40	1.74	1264.07	2199.45
A_68_P32137222	chr19:45309238-45309283	NM_010691:466	Lbx1	INSIDE	0.365	10.586	1659.62	17567.90	3.865	1443.45	5579.08
A_68_P32137213	chr19:45308119-45308163	NM_010691:1586	Lbx1	DOWNSTREAM	0.636	0.368	5457.04	2009.37	0.234	4276.70	1001.24
A_68_P32137226	chr19:45309781-45309825	NM_010691:-76	Lbx1	PROMOTER	0.639	21.413	1573.67	33696.85	13.678	1477.24	20206.26
A_68_P26683681	chr9:83341600-83341644	NM_029434:-6888	Lca5	PROMOTER	0.202	2.800	2335.68	6539.34	0.565	1860.48	1051.89
A_68_P21080307	chr2:26517162-26517206	NM_010695:9618	Lcn4	DOWNSTREAM	0.586	3.588	1457.17	5228.30	2.103	1265.11	2660.56
A_68_P23658784	chr5:46247513-46247557	NM_172153:257	Lcorl	INSIDE	0.569	0.668	13526.02	9037.86	0.38	9307.36	3535.86
A_68_P23658795	chr5:46248998-46249042	NM_178142:-241	Lcorl	PROMOTER	0.651	2.722	1260.23	3429.91	1.771	1078.57	1910.09
A_68_P29530592	chr14:35362336-35362380	NM_001039074:39435	Ldb3	INSIDE	0.495	3.705	1133.75	4201.09	1.835	951.86	1746.76
A_68_P26348588	chr9:21528254-21528298	NM_010700:239	Ldlr	INSIDE	0.624	4.640	420.71	1952.05	2.895	350.96	1016.16
A_68_P21475553	chr2:102026602-102026646	NM_178886:-90	Ldlrad3	PROMOTER	0.469	0.279	2002.60	559.61	0.131	1299.81	170.45
A_68_P21475551	chr2:102026439-102026483	NM_178886:74	Ldlrad3	INSIDE	0.31	5.301	3475.26	18420.90	1.644	3401.38	5592.18
A_68_P23324724	chr4:134323586-134323630	NM_145554:311	Ldlrap1	INSIDE	0.4	1.466	3483.36	5107.14	0.587	2645.97	1552.38
A_68_P23324728	chr4:134324024-134324068	NM_145554:-127	Ldlrap1	PROMOTER	0.211	1.987	1157.63	2300.52	0.42	934.26	392.16
A_68_P30395524	chr15:84388079-84388123	NM_177630:153	Ldoc1l	INSIDE	0.296	27.570	2339.53	64500.72	8.159	2155.00	17583.38
A_68_P30395523	chr15:84387987-84388031	NM_177630:245	Ldoc1l	INSIDE	0.508	4.837	1529.89	7399.47	2.456	1276.49	3135.57
A_68_P22530123	chr3:130809073-130809117	NM_010703:-4294	Lef1	PROMOTER	0.464	20.818	1229.38	25593.15	9.664	1033.56	9987.96
A_68_P22530177	chr3:130815462-130815506	NM_010703:2096	Lef1	INSIDE	0.524	5.006	2693.03	13480.68	2.625	2115.21	5551.97
A_68_P20878385	chr1:182824965-182825009	NM_177099:1737	Lefty2	INSIDE	0.409	1.577	2504.29	3948.52	0.645	1888.14	1217.32
A_68_P22201288	chr3:65469873-65469924	NM_001166659:-251	Lekr1	PROMOTER	0.331	0.389	1730.71	673.35	0.129	1331.58	171.44
A_68_P22201243	chr3:65463433-65463477	NM_001166659:-6695	Lekr1	PROMOTER	0.574	5.218	812.52	4239.66	2.995	820.63	2457.54
A_68_P22201292	chr3:65470413-65470457	NM_001037923:278	Lekr1	INSIDE	0.491	3.144	2437.75	7664.93	1.545	2105.82	3252.58
A_68_P31114987	chr17:27341486-27341531	NM_146075:-125	Lemd2	PROMOTER	0.555	2.963	1111.65	3294.20	1.646	894.15	1471.45
A_68_P27500779	chr10:120416497-120416541	NM_001081193:-132	Lemd3	PROMOTER	0.442	10.152	1061.90	10779.95	4.486	818.06	3669.66
A_68_P27500776	chr10:120416131-120416176	NM_001081193:233	Lemd3	INSIDE	0.665	6.361	279.40	1777.24	4.229	266.84	1128.36
A_68_P27500772	chr10:120415468-120415512	NM_001081193:896	Lemd3	INSIDE	0.56	0.623	972.25	605.52	0.349	795.51	277.58
A_68_P24946692	chr7:3616894-3616938	NM_027203:526	Leng1	INSIDE	0.409	0.328	1934.21	634.64	0.134	1507.99	202.39

ProbeName	Target position of probe on CpG island microarray	TargetID	GeneSymbol	CpG island Description	Ratio of relative methylation (TiO_2-NP/Vehicle)	Sham group			TiO_2-H group		
						Relative methylation (Cy5/Cy3)	Cy3 signal (Input)	Cy5 signal (MeDIP)	Relative methylation (Cy5/Cy3)	Cy3 signal (Input)	Cy5 signal (MeDIP)
A_68_P24947962	chr7:4098539-4098584	NM_172736:9904	Leng8	INSIDE	0.437	8.662	1500.55	12998.22	3.782	1434.60	5426.27
A_68_P23244387	chr4:118905895-118905939	NM_001042411:-517	Lepre1	PROMOTER	0.54	5.820	1630.56	9490.42	3.145	1577.43	4961.31
A_68_P30619231	chr16:26105726-26105770	NM_173379:122	Leprel1	INSIDE	0.232	7.300	2904.48	21203.66	1.693	2238.65	3789.06
A_68_P24817390	chr6:124807543-124807587	NM_013534:141	Leprel2	INSIDE	0.158	19.837	760.14	15079.30	3.141	623.90	1959.81
A_68_P28067972	chr11:100275705-100275749	NM_176830:407	Leprel4	INSIDE	0.469	10.251	1184.43	12141.63	4.805	1064.28	5113.63
A_68_P28067976	chr11:100276206-100276250	NM_176830:-95	Leprel4	DIVERGENT_PROMOTER	0.504	4.752	1108.90	5269.69	2.397	1022.83	2452.00
A_68_P28067969	chr11:100275377-100275421	NM_176830:735	Leprel4	INSIDE	0.532	4.572	1167.79	5339.03	2.432	944.53	2296.64
A_68_P23156220	chr4:101320226-101320270	NM_175036:-139	Leprot	PROMOTER	0.358	3.895	6581.36	25635.68	1.396	4816.22	6723.82
A_68_P25772430	chr8:35210035-35210079	NM_026609:-263	Leprotl1	DIVERGENT_PROMOTER	0.362	4.404	1295.42	5704.72	1.594	1092.59	1741.30
A_68_P23591177	chr5:34124884-34124928	NM_019694:447	Letm1	INSIDE	0.527	6.020	1675.69	10088.25	3.175	1278.02	4057.78
A_68_P25727984	chr8:26707935-26707980	NM_173012:2	Letm2	INSIDE	0.458	0.253	5426.56	1375.09	0.116	3967.34	460.52
A_68_P25727982	chr8:26707741-26707785	NM_173012:197	Letm2	INSIDE	0.516	10.892	1180.03	12852.69	5.621	1015.89	5710.19
A_68_P30485949	chr15:100299584-100299628	NM_134093:142	Letmd1	INSIDE	0.446	0.426	1453.67	618.72	0.19	1270.51	241.17
A_68_P30485951	chr15:100299759-100299803	NM_134093:316	Letmd1	INSIDE	0.597	0.306	3279.87	1004.91	0.183	2418.29	442.44
A_68_P30485948	chr15:100299486-100299530	NM_134093:44	Letmd1	INSIDE	0.476	3.525	2085.36	7351.45	1.677	1566.54	2626.56
A_68_P24142219	chr5:141083562-141083606	NM_008494:290	Lfng	INSIDE	0.563	0.663	870.16	576.50	0.373	738.22	275.42
A_68_P29581905	chr14:47993552-47993596	NM_001145953:40	Lgals3	INSIDE	0.647	4.124	2821.85	11637.06	2.667	2372.65	6326.72
A_68_P23695553	chr5:52957300-52957344	NM_144945:197	Lgi2	INSIDE	0.254	23.712	6419.20	152212.80	6.024	5748.51	34631.75
A_68_P25034849	chr7:31852601-31852645	NM_144556:7669	Lgi4	INSIDE	0.622	2.883	1235.29	3561.33	1.794	1048.81	1881.10
A_68_P20637314	chr1:136982170-136982214	NM_001033409:19661	Lgr6	INSIDE	0.498	0.664	2984.86	1980.98	0.33	2376.55	785.05
A_68_P20637315	chr1:136982242-136982286	NM_001033409:19589	Lgr6	INSIDE	0.636	3.779	485.63	1835.20	2.404	426.83	1025.91
A_68_P22139473	chr3:52845232-52845276	NM_175386:-214	Lhfp	PROMOTER	0.314	7.474	1788.38	13365.59	2.349	1407.91	3306.98
A_68_P22139474	chr3:52845318-52845362	NM_175386:-128	Lhfp	PROMOTER	0.488	0.333	2414.37	804.70	0.163	1756.47	285.46
A_68_P29232482	chr13:94827759-94827803	NM_172589:30	Lhfpl2	INSIDE	0.6	3.174	800.74	2541.60	1.903	847.01	1611.89
A_68_P23532885	chr5:22251850-22251894	NM_001081231:-138	Lhfpl3	PROMOTER	0.424	0.625	875.47	546.80	0.265	744.84	197.25
A_68_P24758100	chr6:113144398-113144442	NM_177763:958	Lhfpl4	INSIDE	0.223	2.280	5603.62	12778.98	0.508	4208.04	2136.49
A_68_P25535615	chr7:139802512-139802556	NM_029609:209	Lhpp	INSIDE	0.415	0.414	1645.08	681.37	0.172	1293.10	222.05
A_68_P21147688	chr2:38212213-38212257	NM_010710:5407	Lhx2	INSIDE	0.38	0.246	2624.45	644.92	0.093	1858.02	173.49
A_68_P21147689	chr2:38212332-38212376	NM_010710:5527	Lhx2	INSIDE	0.6	0.253	2188.33	554.17	0.152	1694.41	257.40
A_68_P21147643	chr2:38206924-38206968	NM_010710:119	Lhx2	INSIDE	0.47	0.604	1053.39	636.05	0.284	909.49	258.08
A_68_P21077007	chr2:26058001-26058045	NM_001039653:4054	Lhx3	INSIDE	0.565	0.610	1097.85	669.61	0.345	733.89	253.06
A_68_P20739614	chr1:157590081-157590125	NM_010712:-945	Lhx4	PROMOTER	0.583	0.510	6681.45	3405.52	0.297	4433.06	1316.30
A_68_P20739570	chr1:157584953-157584997	NM_010712:4183	Lhx4	INSIDE	0.615	0.197	9479.99	1870.50	0.121	6309.21	765.50
A_68_P24038706	chr5:120886174-120886218	NM_008499:4302	Lhx5	INSIDE	0.339	7.008	1478.06	10358.38	2.379	1307.77	3110.79
A_68_P24038737	chr5:120890387-120890431	NM_008499:8514	Lhx5	INSIDE	0.446	0.261	2010.22	524.76	0.116	1471.69	171.19
A_68_P24038700	chr5:120885515-120885559	NM_008499:3642	Lhx5	INSIDE	0.635	0.314	4706.35	1475.46	0.199	3504.64	697.15
A_68_P21137836	chr2:35960496-35960540	NM_001083126:-938	Lhx6	PROMOTER	0.099	4.104	8539.48	35044.27	0.406	6106.23	2477.57
A_68_P21137838	chr2:35960787-35960831	NM_001083126:-1228	Lhx6	PROMOTER	0.473	6.347	1668.93	10592.28	3.004	1599.57	4804.34
A_68_P21137822	chr2:35958973-35959017	NM_001083126:586	Lhx6	INSIDE	0.481	0.224	2085.71	466.64	0.108	1626.21	175.07
A_68_P21137747	chr2:35949642-35949686	NM_001083126:9916	Lhx6	INSIDE	0.571	0.185	2641.60	487.73	0.105	1628.97	171.85
A_68_P21137837	chr2:35960605-35960649	NM_001083126:-1046	Lhx6	PROMOTER	0.475	2.859	2960.05	8462.42	1.359	2480.01	3370.41
A_68_P22661977	chr3:153991290-153991334	NM_010713:2212	Lhx8	INSIDE	0.505	4.801	2818.87	13533.70	2.426	2300.00	5580.56
A_68_P22661990	chr3:153992513-153992557	NM_010713:990	Lhx8	INSIDE	0.414	0.680	1588.88	1080.80	0.282	1025.30	288.68
A_68_P29992340	chr15:7079603-7079647	NM_001113386:53	Lifr	INSIDE	0.261	9.644	893.28	8615.15	2.515	798.79	2008.66
A_68_P29992343	chr15:7080001-7080045	NM_001113386:451	Lifr	INSIDE	0.109	6.046	2283.25	13805.45	0.658	1626.51	1070.81
A_68_P25645821	chr8:9976314-9976358	NM_176953:-13	Lig4	DIVERGENT_PROMOTER	0.362	22.621	7317.00	165515.10	8.199	8460.67	69365.40

ProbeName	Target position of probe on CpG island microarray	TargetID	GeneSymbol	CpG island Description	Ratio of relative methylation (TiO_2-NP/Vehicle)	Sham group			TiO_2-H group		
						Relative methylation (Cy5/Cy3)	Cy3 signal (Input)	Cy5 signal (MeDIP)	Relative methylation (Cy5/Cy3)	Cy3 signal (Input)	Cy5 signal (MeDIP)
A_68_P25645820	chr8:9976227-9976271	NM_176953:75	Lig4	INSIDE	0.518	0.420	3197.44	1341.72	0.217	2477.51	538.84
A_68_P30482737	chr15:99705642-99705686	NM_001113545:223	Lima1	INSIDE	0.608	5.061	1494.32	7562.47	3.075	1401.44	4310.01
A_68_P23763603	chr5:67136839-67136883	NM_001001980:-218	Limch1	PROMOTER	0.251	21.113	1520.74	32107.16	5.302	1307.08	6929.68
A_68_P23763606	chr5:67137204-67137248	NM_001001980:148	Limch1	INSIDE	0.66	0.608	758.18	461.00	0.402	717.05	287.90
A_68_P24112797	chr5:135164410-135164454	NM_010717:28	Limk1	INSIDE	0.405	3.706	1361.24	5044.90	1.501	1100.44	1652.15
A_68_P27163123	chr10:57786395-57786439	NM_026148:203	Lims1	INSIDE	0.202	15.351	2405.51	36927.26	3.1	2455.49	7611.64
A_68_P27163122	chr10:57786302-57786346	NM_026148:111	Lims1	INSIDE	0.611	5.131	802.49	4117.90	3.136	665.99	2088.38
A_68_P23929939	chr5:100929613-100929657	NM_001115010:-2042	Lin54	PROMOTER	0.204	14.516	2417.70	35094.18	2.955	2088.35	6171.33
A_68_P25092719	chr7:52625927-52625971	NM_011698:-14	Lin7b	PROMOTER	0.478	4.640	3331.18	15456.06	2.22	2764.31	6136.33
A_68_P25092718	chr7:52625828-52625872	NM_011698:84	Lin7b	INSIDE	0.612	0.585	9089.15	5319.05	0.358	6555.24	2349.11
A_68_P20877047	chr1:182571834-182571878	NM_175186:392	Lin9	INSIDE	0.472	8.571	583.51	5001.08	4.043	447.99	1811.24
A_68_P20877048	chr1:182571948-182571992	NM_175186:506	Lin9	INSIDE	0.511	0.416	1065.92	443.04	0.212	876.73	186.20
A_68_P26537382	chr9:56483766-56483810	NM_181074:49272	Lingo1	INSIDE	0.283	7.696	3256.56	25061.48	2.181	3258.69	7107.66
A_68_P26537258	chr9:56467405-56467449	NM_181074:65634	Lingo1	INSIDE	0.284	11.987	2295.73	27518.58	3.401	1478.74	5028.59
A_68_P26537259	chr9:56467537-56467581	NM_181074:65502	Lingo1	INSIDE	0.347	6.905	2802.13	19350.00	2.393	1961.27	4693.39
A_68_P22843572	chr4:36897429-36897473	NM_001165999:1327	Lingo2	INSIDE	0.611	0.678	1692.51	1146.94	0.414	1366.16	565.62
A_68_P25010077	chr7:26164977-26165021	NM_001039507:10127	Lipe	INSIDE	0.163	18.894	2490.30	47052.76	3.084	2028.76	6256.06
A_68_P25010203	chr7:26181112-26181156	NM_010719:-128	Lipe	PROMOTER	0.297	5.703	3113.46	17756.42	1.693	2663.24	4509.22
A_68_P25010202	chr7:26180987-26181031	NM_010719:-2	Lipe	PROMOTER	0.414	8.888	4038.52	35893.13	3.679	3660.94	13467.10
A_68_P20156379	chr1:37932274-37932318	NM_001037918:3246	Lipt1	INSIDE	0.207	8.305	1628.04	13521.32	1.716	1121.49	1924.64
A_68_P22350154	chr3:96405147-96405191	NM_001163170:113	Lix1l	INSIDE	0.446	1.459	2985.64	4355.04	0.65	2395.75	1558.21
A_68_P28155817	chr11:115719757-115719801	NM_145438:34407	Llgl2	DOWNSTREAM	0.523	0.451	1056.04	476.63	0.236	857.06	202.43
A_68_P28155824	chr11:115720427-115720471	NM_145438:35077	Llgl2	DOWNSTREAM	0.526	0.505	2227.88	1125.69	0.266	1524.34	404.75
A_68_P28155559	chr11:115685801-115685845	NM_145438:451	Llgl2	INSIDE	0.533	0.420	2850.18	1198.05	0.224	2092.28	468.57
A_68_P27495998	chr10:119664180-119664224	NM_025431:87	Llph	INSIDE	0.543	0.463	1448.90	671.41	0.252	1034.77	260.46
A_68_P31784507	chr18:66162242-66162286	NM_027400:25	Lman1	INSIDE	0.374	5.215	1332.00	6946.77	1.953	1259.87	2460.42
A_68_P20148354	chr1:36501974-36502018	NM_001013374:88	Lman2l	INSIDE	0.226	0.499	7468.27	3727.18	0.113	5000.20	564.40
A_68_P20148355	chr1:36502065-36502109	NM_001013374:-2	Lman2l	PROMOTER	0.429	6.857	7851.19	53835.34	2.941	5664.76	16662.73
A_68_P20148353	chr1:36501896-36501940	NM_001013374:166	Lman2l	INSIDE	0.464	0.340	2293.34	778.85	0.158	1754.38	276.60
A_68_P23567296	chr5:29704564-29704608	NM_020295:344	Lmbr1	INSIDE	0.435	0.501	3897.45	1951.08	0.218	2799.72	609.67
A_68_P23567299	chr5:29704950-29704994	NM_020295:-42	Lmbr1	PROMOTER	0.627	0.432	1311.24	566.30	0.271	1119.30	303.32
A_68_P30477336	chr15:98748107-98748151	NM_029098:401	Lmbr1l	INSIDE	0.574	3.246	1588.21	5155.56	1.864	1339.28	2496.12
A_68_P20094994	chr1:24685459-24685503	NM_026719:98	Lmbrd1	INSIDE	0.218	91.869	3001.82	275773.00	20.013	3259.09	65225.73
A_68_P30656039	chr16:33062880-33062924	NM_172823:296	Lmln	INSIDE	0.191	0.609	2846.06	1733.93	0.116	2108.32	244.99
A_68_P22314130	chr3:88306601-88306645	NM_019390:-9401	Lmna	PROMOTER	0.573	0.483	2310.36	1115.81	0.277	1689.08	467.45
A_68_P31731708	chr18:56868611-56868655	NM_010721:1166	Lmnb1	INSIDE	0.415	0.238	2453.40	583.19	0.099	1768.13	174.31
A_68_P31731697	chr18:56867382-56867426	NM_010721:-62	Lmnb1	PROMOTER	0.645	3.522	634.40	2234.67	2.272	700.03	1590.76
A_68_P27286341	chr10:80380635-80380679	NM_010722:334	Lmnb2	INSIDE	0.656	0.425	1675.54	712.62	0.279	1245.15	347.59
A_68_P22601420	chr3:143865390-143865434	NM_001161769:-52	Lmo4	PROMOTER	0.305	13.562	6965.17	94461.32	4.134	5745.36	23751.89
A_68_P22601440	chr3:143867896-143867940	NM_001161769:-2558	Lmo4	PROMOTER	0.374	7.263	4736.97	34403.52	2.716	3483.88	9462.06
A_68_P29856570	chr14:102129092-102129136	NM_201529:-30	Lmo7	PROMOTER	0.334	0.559	1464.54	818.17	0.187	1215.21	227.08
A_68_P24159372	chr5:144861058-144861106	NM_001081109:-222	Lmtk2	PROMOTER	0.51	4.691	1566.54	7348.48	2.394	1369.91	3279.40
A_68_P24159378	chr5:144861753-144861797	NM_001081109:470	Lmtk2	INSIDE	0.349	1.371	2363.34	3240.54	0.478	1891.31	904.22
A_68_P20806523	chr1:169619018-169619062	NM_033652:-648	Lmx1a	PROMOTER	0.359	6.936	1652.18	11459.28	2.487	1309.48	3257.03
A_68_P21122756	chr2:33495852-33495899	NM_010725:156	Lmx1b	INSIDE	0.539	4.191	724.55	3036.50	2.258	678.28	1531.66
A_68_P21122760	chr2:33496334-33496378	NM_010725:-325	Lmx1b	PROMOTER	0.604	4.163	1475.40	6141.44	2.516	1316.81	3312.90

ProbeName	Target position of probe on CpG island microarray	TargetID	GeneSymbol	CpG island Description	Ratio of relative methylation (TiO_2-NP/Vehicle)	Sham group			TiO_2-H group		
						Relative methylation (Cy5/Cy3)	Cy3 signal (Input)	Cy5 signal (MeDIP)	Relative methylation (Cy5/Cy3)	Cy3 signal (Input)	Cy5 signal (MeDIP)
A_68_P21338178	chr2:74416574-74416618	NM_001110209:409	Lnp	INSIDE	0.453	0.351	1342.69	470.68	0.159	1064.19	169.06
A_68_P31074422	chr17:17761131-17761175	NM_172827:301	Lnpep	INSIDE	0.334	0.246	3460.67	852.77	0.082	2372.90	195.44
A_68_P24171753	chr5:147888406-147888450	NM_080795:-280	Lnx2	DIVERGENT_PROMOTER	0.261	10.128	8346.79	84535.60	2.639	6768.30	17862.45
A_68_P24171749	chr5:147887960-147888004	NM_080795:166	Lnx2	INSIDE	0.505	5.432	1903.82	10341.25	2.745	1674.73	4597.20
A_68_P25021326	chr7:29135937-29135981	NR_028129:-600	LOC100302567	PROMOTER	0.656	0.628	2196.37	1378.97	0.412	1729.26	711.70
A_68_P30347565	chr15:76157913-76157957	NR_028283:29	LOC100302626	INSIDE	0.484	0.598	1248.85	747.00	0.29	1103.04	319.52
A_68_P30512787	chr16:4939335-4939379	NR_037666:229	LOC100503704	INSIDE	0.637	0.343	6087.73	2085.69	0.218	3904.97	851.66
A_68_P27533642	chr10:126478457-126478501	NM_001205036:-28	LOC100504608	DIVERGENT_PROMOTER	0.548	6.109	2582.96	15780.57	3.346	1930.12	6458.34
A_68_P26445384	chr9:40609412-40609456	NR_015570:-3251	LOC624853	PROMOTER	0.418	0.312	3914.52	1221.81	0.13	2473.95	322.53
A_68_P24862701	chr6:134591261-134591305	NM_026371:325	Loh12cr1	INSIDE	0.414	5.341	1209.36	6458.72	2.209	1138.20	2514.37
A_68_P31708185	chr18:52688188-52688232	NM_010728:1152	Lox	INSIDE	0.242	1.429	3874.34	5537.06	0.345	2975.55	1027.80
A_68_P26547031	chr9:58159806-58159850	NM_010729:1191	Loxl1	INSIDE	0.193	2.275	2297.87	5227.04	0.439	1775.89	779.51
A_68_P26547037	chr9:58160685-58160729	NM_010729:313	Loxl1	INSIDE	0.509	8.306	860.28	7145.57	4.232	775.53	3281.83
A_68_P29696151	chr14:70009216-70009260	NM_033325:-44	Loxl2	PROMOTER	0.61	0.450	2552.86	1148.39	0.274	1881.60	516.49
A_68_P29696148	chr14:70008926-70008970	NM_033325:-334	Loxl2	PROMOTER	0.085	1.372	3270.31	4487.18	0.117	2383.69	278.41
A_68_P32122315	chr19:42687286-42687330	NM_001164311:-12	Loxl4	PROMOTER	0.143	29.923	2275.04	68076.62	4.281	1945.35	8327.81
A_68_P22951804	chr4:58566187-58566231	NM_172989:-43	Lpar1	PROMOTER	0.465	28.499	1857.66	52941.65	13.259	1608.59	21328.25
A_68_P22951806	chr4:58566399-58566443	NM_010336:-57	Lpar1	PROMOTER	0.566	0.436	3628.95	1581.88	0.247	2707.67	667.52
A_68_P22951801	chr4:58565861-58565905	NM_172989:283	Lpar1	INSIDE	0.617	0.698	1198.78	837.11	0.431	906.91	391.00
A_68_P22613734	chr3:145947914-145947958	NM_022983:64012	Lpar3	INSIDE	0.173	9.703	1884.45	18283.99	1.678	1549.21	2599.18
A_68_P24818549	chr6:125031882-125031926	NM_001163268:10610	Lpar5	INSIDE	0.398	0.628	907.02	569.49	0.25	678.96	169.71
A_68_P29140001	chr13:73605046-73605090	NM_145376:238	Lpcat1	INSIDE	0.135	24.310	4515.67	109775.90	3.275	4125.66	13512.63
A_68_P26068285	chr8:95379185-95379229	NM_173014:-42	Lpcat2	PROMOTER	0.64	10.461	2816.51	29462.88	6.694	2570.22	17204.16
A_68_P24816267	chr6:124612992-124613036	NM_145130:-107	Lpcat3	PROMOTER	0.291	6.859	5590.52	38343.38	1.999	5177.33	10348.96
A_68_P24816271	chr6:124613291-124613335	NM_145130:191	Lpcat3	INSIDE	0.371	0.150	4489.72	674.18	0.056	3161.30	175.96
A_68_P20939666	chr1:193542578-193542622	NM_001134829:327	Lpgat1	INSIDE	0.309	6.568	1479.44	9716.82	2.031	1499.71	3046.33
A_68_P20939662	chr1:193541860-193541904	NM_172266:-20	Lpgat1	PROMOTER	0.538	4.272	997.11	4259.60	2.298	957.52	2200.43
A_68_P26016967	chr8:86424037-86424081	NM_181039:62	Lphn1	INSIDE	0.239	13.664	2200.08	30060.83	3.262	1840.35	6002.37
A_68_P26016964	chr8:86423687-86423732	NM_181039:-287	Lphn1	PROMOTER	0.623	0.181	3917.41	708.75	0.113	2824.93	318.62
A_68_P23837529	chr5:81450446-81450490	NM_198702:-149	Lphn3	PROMOTER	0.241	1.934	4911.69	9501.65	0.466	3588.94	1673.47
A_68_P23837533	chr5:81450893-81450938	NM_198702:298	Lphn3	INSIDE	0.371	0.258	2807.16	723.66	0.096	1819.39	173.90
A_68_P23837522	chr5:81449591-81449635	NM_198702:-1005	Lphn3	PROMOTER	0.455	9.254	1760.99	16296.12	4.21	1538.61	6477.68
A_68_P23837517	chr5:81448669-81448713	NM_198702:-1927	Lphn3	PROMOTER	0.57	3.298	829.46	2735.66	1.879	763.93	1435.57
A_68_P23839618	chr5:81758787-81758831	NM_198702:308191	Lphn3	INSIDE	0.65	2.366	1467.51	3472.65	1.538	1171.30	1801.91
A_68_P25945210	chr8:71405256-71405300	NM_008509:825	Lpl	INSIDE	0.539	0.475	2293.81	1090.18	0.256	1900.05	486.62
A_68_P27996402	chr11:87630757-87630801	NM_080420:8838	Lpo	INSIDE	0.402	6.448	2215.84	14287.79	2.594	1939.32	5029.74
A_68_P22301528	chr3:86028273-86028317	NM_001077687:-317	Lrba	PROMOTER	0.564	6.070	796.06	4832.22	3.426	692.63	2372.97
A_68_P22301529	chr3:86028379-86028423	NM_001077687:-211	Lrba	PROMOTER	0.64	0.236	2156.75	509.86	0.151	1729.16	261.64
A_68_P32730437	chrX:143988143-143988187	NM_001081173:460	Lrch2	INSIDE	0.31	11.471	7108.66	81543.79	3.555	7613.44	27062.05
A_68_P30655247	chr16:32914173-32914217	NM_001081255:9	Lrch3	INSIDE	0.098	2.805	1686.32	4729.57	0.274	1457.09	399.30
A_68_P24126717	chr5:138070748-138070792	NM_001168652:420	Lrch4	INSIDE	0.483	0.320	3778.42	1210.53	0.155	2657.87	411.25
A_68_P25021976	chr7:29237431-29237475	NM_001141921:196	Lrfn1	INSIDE	0.102	6.594	7481.67	49337.40	0.673	5569.13	3750.77
A_68_P31220008	chr17:49071618-49071662	NM_027452:-266	Lrfn2	PROMOTER	0.169	31.137	5792.05	180346.20	5.273	7865.26	41473.44
A_68_P31220014	chr17:49072235-49072279	NM_027452:350	Lrfn2	INSIDE	0.405	0.384	1474.77	566.54	0.156	1102.24	171.59
A_68_P31220004	chr17:49070978-49071022	NM_027452:-906	Lrfn2	PROMOTER	0.445	5.276	3529.11	18618.44	2.35	2680.59	6298.91
A_68_P25031213	chr7:31145836-31145880	NM_175478:1933	Lrfn3	INSIDE	0.306	21.868	2305.52	50417.28	6.695	1828.93	12244.41

ProbeName	Target position of probe on CpG island microarray	TargetID	GeneSymbol	CpG island Description	Ratio of relative methylation (TiO_2-NP/Vehicle)	Sham group			TiO_2-H group		
						Relative methylation (Cy5/Cy3)	Cy3 signal (Input)	Cy5 signal (MeDIP)	Relative methylation (Cy5/Cy3)	Cy3 signal (Input)	Cy5 signal (MeDIP)
A_68_P25031211	chr7:31145626-31145670	NM_175478:2143	Lrfn3	INSIDE	0.472	5.103	1554.60	7933.87	2.411	1049.26	2529.57
A_68_P28480019	chr12:62623999-62624043	NM_178714:-1598	Lrfn5	PROMOTER	0.189	0.635	2124.17	1349.23	0.12	1427.21	171.46
A_68_P28480018	chr12:62623914-62623958	NM_178714:-1682	Lrfn5	PROMOTER	0.621	0.662	1690.07	1119.24	0.411	1292.42	531.60
A_68_P22391858	chr3:104315425-104315469	NM_001025067:333	Lrig2	INSIDE	0.3	14.553	663.70	9658.50	4.371	537.12	2347.84
A_68_P27528046	chr10:125403373-125403417	NM_177152:120	Lrig3	INSIDE	0.539	5.542	1610.37	8924.70	2.988	1399.07	4180.43
A_68_P27528047	chr10:125403454-125403498	NM_177152:202	Lrig3	INSIDE	0.178	8.287	1384.08	11470.44	1.475	1232.79	1818.71
A_68_P27537125	chr10:127058908-127058952	NM_008512:-726	Lrp1	PROMOTER	0.323	13.451	2712.02	36479.67	4.34	2732.90	11861.63
A_68_P27537124	chr10:127058798-127058842	NM_008512:-616	Lrp1	PROMOTER	0.187	7.885	5013.78	39532.69	1.477	4128.01	6095.19
A_68_P29612551	chr14:55083412-55083456	NM_022993:451	Lrp10	INSIDE	0.132	1.881	2456.32	4621.55	0.248	1764.35	438.01
A_68_P29612547	chr14:55083035-55083079	NM_022993:73	Lrp10	INSIDE	0.505	4.300	673.63	2896.69	2.173	636.70	1383.58
A_68_P30156691	chr15:39775111-39775155	NM_172814:171	Lrp12	INSIDE	0.21	53.975	1015.59	54816.81	11.32	975.29	11040.61
A_68_P31918778	chr19:3685913-3685957	NM_008513:630	Lrp5	INSIDE	0.486	0.634	718.49	455.63	0.308	564.86	174.17
A_68_P23192848	chr4:107475278-107475322	NM_001080926:437	Lrp8	INSIDE	0.628	2.490	1142.64	2844.65	1.564	863.30	1350.61
A_68_P23598368	chr5:35448526-35448576	NM_013587:-204	Lrpap1	PROMOTER	0.544	0.383	1276.91	488.84	0.208	831.61	173.29
A_68_P23598365	chr5:35448131-35448175	NM_013587:194	Lrpap1	INSIDE	0.602	0.487	1297.03	631.92	0.293	943.89	276.86
A_68_P31950785	chr19:10531094-10531138	NM_001111140:821	Lrrc10b	INSIDE	0.643	3.153	1144.95	3610.39	2.028	956.13	1938.94
A_68_P28886522	chr13:24373081-24373125	NM_026825:-443	Lrrc16a	PROMOTER	0.273	11.659	1692.30	19731.23	3.177	1473.71	4682.57
A_68_P28886519	chr13:24372543-24372587	NM_026825:95	Lrrc16a	INSIDE	0.472	0.312	1704.62	531.38	0.147	1198.61	176.50
A_68_P28886518	chr13:24372431-24372475	NM_026825:207	Lrrc16a	INSIDE	0.524	0.136	8062.33	1095.20	0.071	4901.77	348.67
A_68_P28886516	chr13:24372229-24372273	NM_026825:409	Lrrc16a	INSIDE	0.383	0.588	1796.59	1056.54	0.225	1450.37	326.60
A_68_P29618044	chr14:56110165-56110209	NM_001024645:257	Lrrc16b	INSIDE	0.438	5.185	4024.82	20867.27	2.27	3288.96	7465.00
A_68_P27180700	chr10:60938374-60938418	NM_153542:-184	Lrrc20	PROMOTER	0.516	0.417	1469.37	612.24	0.215	1036.86	222.92
A_68_P27180702	chr10:60938602-60938646	NM_153542:44	Lrrc20	INSIDE	0.2	9.011	1478.95	13326.77	1.803	1194.90	2153.95
A_68_P30350212	chr15:76553038-76553082	NM_198119:-457	Lrrc24	PROMOTER	0.349	0.470	1354.51	637.02	0.164	1066.14	174.76
A_68_P30350167	chr15:76546156-76546200	NM_198119:6425	Lrrc24	INSIDE	0.437	0.528	938.97	496.14	0.231	720.09	166.16
A_68_P25194961	chr7:74790016-74790060	NM_175124:84	Lrrc28	INSIDE	0.639	0.478	1833.84	877.09	0.306	1377.77	421.07
A_68_P26133049	chr8:107850154-107850198	NM_177449:0	Lrrc29	INSIDE	0.158	17.564	1173.39	20608.99	2.779	1057.87	2940.29
A_68_P27270908	chr10:77365226-77365270	NM_145152:33	Lrrc3	INSIDE	0.149	4.160	4975.53	20698.02	0.621	3717.96	2309.54
A_68_P30651244	chr16:32165418-32165462	NM_146069:122	Lrrc33	INSIDE	0.235	1.511	3321.92	5017.82	0.355	2390.74	848.31
A_68_P23374714	chr4:142940380-142940424	NM_001162983:750	Lrrc38	INSIDE	0.636	0.625	947.67	592.09	0.398	758.50	301.62
A_68_P24322963	chr6:28783645-28783689	NM_138682:-1919	Lrrc4	PROMOTER	0.199	15.176	1598.75	24263.15	3.014	1587.56	4784.47
A_68_P24322961	chr6:28783190-28783234	NM_138682:-1465	Lrrc4	PROMOTER	0.577	5.045	513.03	2587.96	2.909	462.52	1345.49
A_68_P23227558	chr4:115748182-115748226	NM_153521:135	Lrrc41	INSIDE	0.253	10.390	1573.01	16344.11	2.628	1402.84	3686.16
A_68_P23189554	chr4:106926184-106926228	NM_029985:-68	Lrrc42	DIVERGENT_PROMOTER	0.406	0.483	6893.85	3332.12	0.196	4926.58	965.73
A_68_P23189553	chr4:106926047-106926091	NM_029985:70	Lrrc42	INSIDE	0.361	4.000	1986.40	7946.22	1.442	1682.91	2427.23
A_68_P25087548	chr7:51717376-51717420	NM_198250:19542	Lrrc4b	INSIDE	0.322	7.388	1204.34	8897.81	2.379	824.06	1960.15
A_68_P25587300	chr7:148380302-148380347	NM_153777:-717	Lrrc56	PROMOTER	0.549	0.174	3251.25	566.68	0.096	2122.23	203.08
A_68_P30682957	chr16:37868699-37868743	NM_177093:235	Lrrc58	INSIDE	0.563	4.357	1393.57	6072.36	2.451	1145.49	2807.84
A_68_P28033987	chr11:94490892-94490936	NM_133807:-223	Lrrc59	PROMOTER	0.409	0.284	1932.60	548.17	0.116	1498.38	173.85
A_68_P28033988	chr11:94491055-94491099	NM_133807:-61	Lrrc59	PROMOTER	0.431	0.482	1058.25	509.84	0.208	826.90	171.72
A_68_P29726036	chr14:75530896-75530940	NM_027581:-228	Lrrc63	DIVERGENT_PROMOTER	0.516	0.531	1105.93	587.80	0.274	1023.69	280.96
A_68_P24994044	chr7:20147793-20147837	NM_199149:-67	Lrrc68	PROMOTER	0.562	0.280	1825.32	511.77	0.158	1532.01	241.53
A_68_P24993843	chr7:20118011-20118055	NM_199149:29715	Lrrc68	INSIDE	0.344	5.232	1150.64	6020.66	1.797	849.76	1527.31
A_68_P22686351	chr3:158224713-158224757	NM_001081358:451	Lrrc7	INSIDE	0.395	0.625	1538.12	961.57	0.247	1311.36	324.16
A_68_P21102112	chr2:30112106-30112150	NM_177725:18840	Lrrc8a	INSIDE	0.646	3.503	723.21	2533.17	2.261	556.17	1257.66
A_68_P23955154	chr5:105844706-105844750	NM_001033550:-65	Lrrc8b	PROMOTER	0.293	10.790	244.10	2633.90	3.166	270.86	857.46

ProbeName	Target position of probe on CpG island microarray	TargetID	GeneSymbol	CpG island Description	Ratio of relative methylation (TiO_2-NP/Vehicle)	Sham group			TiO_2-H group		
						Relative methylation (Cy5/Cy3)	Cy3 signal (Input)	Cy5 signal (MeDIP)	Relative methylation (Cy5/Cy3)	Cy3 signal (Input)	Cy5 signal (MeDIP)
A_68_P23956789	chr5:106129618-106129662	NM_178701:-188	Lrrc8d	PROMOTER	0.517	0.345	4166.85	1436.84	0.178	2464.70	439.30
A_68_P23956961	chr5:106161428-106161472	NM_178701:31622	Lrrc8d	INSIDE	0.17	1.415	5582.70	7897.24	0.24	3824.84	919.71
A_68_P20440041	chr1:92949532-92949576	NM_001111311:-466	Lrrfip1	PROMOTER	0.404	0.404	1372.48	555.05	0.164	1069.44	174.86
A_68_P20440043	chr1:92949729-92949773	NM_001111311:-270	Lrrfip1	PROMOTER	0.564	0.455	2119.83	965.49	0.257	1753.18	450.11
A_68_P26827666	chr9:111020550-111020594	NM_001164838:-42	Lrrfip2	PROMOTER	0.304	16.611	3524.23	58539.49	5.047	3153.52	15916.71
A_68_P25187997	chr7:73532946-73532990	NM_146191:259	Lrrk1	INSIDE	0.493	3.768	3158.28	11900.54	1.856	2715.44	5040.95
A_68_P25187993	chr7:73532543-73532587	NM_146191:663	Lrrk1	INSIDE	0.532	3.248	3581.61	11632.99	1.727	3114.01	5377.04
A_68_P24728933	chr6:107479987-107480031	NM_008516:289	Lrrn1	INSIDE	0.507	0.528	2795.65	1475.24	0.268	1837.82	491.70
A_68_P24728936	chr6:107480232-107480276	NM_008516:535	Lrrn1	INSIDE	0.564	0.505	2045.11	1033.62	0.285	1496.84	426.81
A_68_P24563510	chr6:77193700-77193744	NM_028880:1012	Lrrtm1	INSIDE	0.575	0.419	2931.13	1229.01	0.241	2281.35	550.31
A_68_P24792500	chr6:119280593-119280637	NM_001172207:-1392	Lrtm2	PROMOTER	0.5	3.958	2243.32	8879.50	1.98	1753.31	3472.20
A_68_P24792501	chr6:119280733-119280777	NM_001172207:-1532	Lrtm2	PROMOTER	0.585	0.608	2659.11	1615.80	0.355	2011.27	714.90
A_68_P27765093	chr11:45758539-45758583	NM_028185:-123	Lsm11	PROMOTER	0.353	9.754	3430.10	33455.97	3.446	2987.92	10297.46
A_68_P21900913	chr2:179759394-179759438	NM_177727:-275	Lsm14b	PROMOTER	0.173	23.955	4888.74	117108.40	4.14	4326.28	17911.92
A_68_P21900919	chr2:179760085-179760129	NM_177727:415	Lsm14b	INSIDE	0.568	6.876	1939.13	13333.97	3.907	1598.52	6245.70
A_68_P31156879	chr17:35119358-35119402	NM_001204273:306	Lsm2	INSIDE	0.523	0.524	1512.08	791.60	0.274	1297.13	355.28
A_68_P25953306	chr8:73197314-73197358	NM_015816:207	Lsm4	INSIDE	0.321	0.309	2817.85	871.78	0.099	1728.61	171.92
A_68_P24476402	chr6:56654577-56654621	NM_025520:95	Lsm5	INSIDE	0.493	4.295	1419.02	6094.99	2.117	1168.93	2474.17
A_68_P25991120	chr8:81344808-81344852	NM_001191004:221	Lsm6	INSIDE	0.359	0.696	1019.26	709.18	0.25	845.49	211.32
A_68_P25034375	chr7:31758234-31758278	NM_001164184:232	Lsr	INSIDE	0.402	9.123	1443.07	13165.64	3.669	1167.10	4281.62
A_68_P27262526	chr10:75994451-75994495	NM_146006:101	Lss	INSIDE	0.378	14.080	6425.93	90477.43	5.322	5329.23	28359.91
A_68_P27353694	chr10:92916374-92916418	NM_008517:256	Lta4h	INSIDE	0.445	0.410	1170.50	480.40	0.183	937.50	171.38
A_68_P31158194	chr17:35333102-35333146	NM_008518:1673	Ltb	INSIDE	0.515	0.545	1432.03	780.89	0.281	955.12	268.15
A_68_P29619799	chr14:56386649-56386693	NM_008519:1872	Ltb4r1	INSIDE	0.612	7.915	735.21	5819.29	4.846	741.68	3593.91
A_68_P29619756	chr14:56381232-56381276	NM_020490:990	Ltb4r2	INSIDE	0.345	5.562	897.44	4991.78	1.92	806.52	1548.47
A_68_P31354400	chr17:75404960-75405004	NM_019919:114	Ltbp1	INSIDE	0.498	0.445	3775.29	1678.25	0.221	3029.98	670.94
A_68_P28600849	chr12:86217266-86217310	NM_013589:157	Ltbp2	INSIDE	0.546	0.584	823.70	481.06	0.319	678.19	216.13
A_68_P28600848	chr12:86217110-86217154	NM_013589:313	Ltbp2	INSIDE	0.52	3.120	998.46	3115.14	1.623	796.19	1292.34
A_68_P31930563	chr19:5742280-5742324	NM_008520:1399	Ltbp3	INSIDE	0.646	4.488	979.33	4395.29	2.9	820.07	2378.51
A_68_P25016756	chr7:28119811-28119855	NM_001113549:-1165	Ltbp4	PROMOTER	0.141	42.098	2999.77	126285.20	5.954	2854.40	16996.54
A_68_P25016747	chr7:28118579-28118623	NM_001113549:67	Ltbp4	INSIDE	0.637	5.449	3176.01	17304.89	3.469	2482.67	8611.15
A_68_P25016714	chr7:28114472-28114516	NM_001113549:4173	Ltbp4	INSIDE	0.658	0.602	2497.06	1503.05	0.396	1878.71	743.91
A_68_P27787506	chr11:50050605-50050649	NM_008521:1347	Ltc4s	INSIDE	0.571	0.303	2006.05	607.92	0.173	1560.60	270.26
A_68_P21571366	chr2:119584202-119584246	NM_008523:37	Ltk	INSIDE	0.538	0.466	1507.11	702.03	0.251	1170.98	293.68
A_68_P23334085	chr4:136025641-136025685	NM_024452:-13	Luzp1	PROMOTER	0.22	21.123	7545.46	159381.30	4.655	7336.11	34149.96
A_68_P22210127	chr3:67267701-67267745	NM_016753:107	Lxn	INSIDE	0.501	0.257	7056.69	1810.48	0.128	4741.17	609.03
A_68_P30340463	chr15:74785980-74786024	NM_001164040:467	Ly6e	INSIDE	0.394	4.165	20258.82	84383.59	1.641	13715.32	22512.76
A_68_P30342689	chr15:75396844-75396888	NM_001135689:212	Ly6h	INSIDE	0.4	4.154	1598.42	6639.32	1.663	1344.88	2236.13
A_68_P30339717	chr15:74630092-74630141	NM_029627:282	Ly6k	INSIDE	0.322	10.065	480.52	4836.29	3.24	354.65	1149.21
A_68_P21258086	chr2:60221286-60221330	NM_013825:-20	Ly75	PROMOTER	0.45	5.866	3497.23	20513.67	2.641	3109.62	8211.24
A_68_P26021276	chr8:87229573-87229617	NM_008535:4239	Lyl1	DOWNSTREAM	0.221	0.745	3072.24	2288.91	0.165	2307.01	380.46
A_68_P22693715	chr4:3605587-3605631	NM_001111096:341	Lyn	INSIDE	0.45	0.443	1055.27	467.42	0.199	881.01	175.69
A_68_P20587179	chr1:127808497-127808541	NM_145100:273	Lypd1	INSIDE	0.621	2.893	876.14	2535.08	1.798	766.61	1378.38
A_68_P21206004	chr2:49921862-49921906	NM_177139:-97	Lypd6	PROMOTER	0.521	3.227	2394.08	7724.53	1.68	2045.96	3436.90
A_68_P21204319	chr2:49643540-49643584	NM_027990:357	Lypd6b	INSIDE	0.142	21.321	1601.58	34147.29	3.021	1610.27	4865.23
A_68_P20007252	chr1:4797954-4797998	NM_008866:3	Lypla1	INSIDE	0.478	4.775	1069.45	5106.36	2.281	859.84	1961.23

ProbeName	Target position of probe on CpG island microarray	TargetID	GeneSymbol	CpG island Description	Ratio of relative methylation (TiO_2-NP/Vehicle)	Sham group			TiO_2-H group		
						Relative methylation (Cy5/Cy3)	Cy3 signal (Input)	Cy5 signal (MeDIP)	Relative methylation (Cy5/Cy3)	Cy3 signal (Input)	Cy5 signal (MeDIP)
A_68_P20007250	chr1:4797717-4797761	NM_008866:-235	Lypla1	PROMOTER	0.618	4.059	603.83	2451.13	2.509	499.11	1252.10
A_68_P23331510	chr4:135528254-135528298	NM_011942:233	Lypla2	INSIDE	0.647	2.254	1559.64	3514.85	1.458	1320.09	1924.78
A_68_P20905056	chr1:187941218-187941262	NM_146106:-51	Lyplal1	PROMOTER	0.308	9.616	1200.81	11546.52	2.957	1191.57	3523.73
A_68_P28946344	chr13:36083419-36083472	NM_201358:125781	Lyrm4	INSIDE	0.414	7.422	844.99	6271.42	3.07	570.97	1752.81
A_68_P27812425	chr11:54674397-54674456	NM_029327:-333	Lyrm7	DIVERGENT_PROMOTER	0.521	14.532	344.36	5004.24	7.577	388.89	2946.68
A_68_P32135961	chr19:45100549-45100593	NM_001130526:7946	Lzts2	INSIDE	0.543	2.936	1470.58	4317.62	1.596	1049.71	1674.94
A_68_P23264207	chr4:123341175-123341219	NM_001199136:20407	Macf1	INSIDE	0.371	5.532	1458.50	8067.78	2.054	1178.19	2419.63
A_68_P23264212	chr4:123341892-123341936	NM_001199136:19689	Macf1	INSIDE	0.611	10.964	2472.16	27105.89	6.698	2250.52	15072.89
A_68_P23264337	chr4:123364300-123364344	NM_001199136:-2719	Macf1	PROMOTER	0.537	5.806	356.63	2070.72	3.121	306.51	956.52
A_68_P31938325	chr19:7240713-7240757	NM_134147:109477	Macrod1	INSIDE	0.252	0.465	1909.55	887.99	0.117	1458.61	171.11
A_68_P31938321	chr19:7239801-7239845	NM_134147:108565	Macrod1	INSIDE	0.38	16.280	779.70	12693.18	6.192	726.34	4497.65
A_68_P31938323	chr19:7240213-7240257	NM_134147:108977	Macrod1	INSIDE	0.498	7.999	2981.27	23847.61	3.987	2750.16	10964.07
A_68_P21683849	chr2:140221825-140221869	NM_028387:676	Macrod2	INSIDE	0.449	14.317	1232.61	17646.76	6.429	1273.21	8185.06
A_68_P23392617	chr4:147514830-147514874	NM_027985:254	Mad2l2	INSIDE	0.633	0.419	1910.07	800.61	0.265	1476.80	391.99
A_68_P21421182	chr2:91022680-91022724	NM_001177720:-3224	Madd	PROMOTER	0.264	8.445	2899.87	24488.93	2.226	2882.43	6417.65
A_68_P21421185	chr2:91022943-91022987	NM_001177720:-3486	Madd	PROMOTER	0.283	8.740	1467.67	12826.73	2.477	1261.85	3125.56
A_68_P21421187	chr2:91023173-91023217	NM_001177720:-3716	Madd	PROMOTER	0.491	5.237	1290.11	6756.13	2.569	1032.59	2653.24
A_68_P23588236	chr5:33678555-33678599	NM_021500:356	Maea	INSIDE	0.191	17.675	2530.06	44717.93	3.369	2601.50	8763.33
A_68_P23588238	chr5:33678734-33678778	NM_021500:536	Maea	INSIDE	0.307	11.425	2167.96	24769.17	3.511	2535.27	8901.25
A_68_P26192688	chr8:118232099-118232143	NM_001025577:-1326	Maf	PROMOTER	0.657	0.190	2838.91	538.50	0.125	2085.80	260.09
A_68_P26192683	chr8:118231489-118231533	NM_001025577:-716	Maf	PROMOTER	0.423	5.309	592.93	3148.12	2.248	526.12	1182.62
A_68_P30347740	chr15:76181682-76181726	NM_001164607:-19	Maf1	DIVERGENT_PROMOTER	0.41	0.221	2769.03	610.79	0.09	1897.87	171.50
A_68_P30343816	chr15:75578159-75578203	NM_194350:172	Mafa	INSIDE	0.245	9.964	2709.67	26999.66	2.442	2089.56	5103.67
A_68_P30343819	chr15:75578604-75578648	NM_194350:-274	Mafa	PROMOTER	0.462	6.083	1264.26	7690.13	2.813	1040.80	2927.84
A_68_P30365871	chr15:79178186-79178230	NM_010755:101	Maff	INSIDE	0.242	1.531	2130.10	3261.92	0.371	1570.55	582.99
A_68_P30365939	chr15:79188220-79188264	NM_010755:10135	Maff	INSIDE	0.476	2.948	2025.66	5971.59	1.404	1815.50	2549.60
A_68_P24137624	chr5:140267260-140267304	NM_010757:-207	Mafk	PROMOTER	0.397	0.402	1582.04	636.76	0.16	1138.64	181.86
A_68_P21583618	chr2:121781814-121781858	NM_008545:-8	Mageb3	DIVERGENT_PROMOTER	0.542	3.301	3811.57	12580.17	1.79	3111.88	5571.81
A_68_P32539367	chrX:91787344-91787392	NM_019791:45	Maged1	INSIDE	0.573	2.976	2920.98	8693.26	1.705	3854.87	6570.69
A_68_P32742983	chrX:147247480-147247524	NM_001199246:744	Maged2	INSIDE	0.29	14.530	1894.38	27526.01	4.21	2257.26	9503.99
A_68_P32751751	chrX:149472100-149472144	NM_023788:-16	Mageh1	PROMOTER	0.224	32.130	7696.09	247278.30	7.19	9640.83	69321.28
A_68_P24656008	chr6:94234014-94234058	NM_001029850:-138	Magi1	PROMOTER	0.532	4.748	2368.94	11247.21	2.526	2175.93	5496.34
A_68_P24652024	chr6:93628341-93628385	NM_001083320:235322	Magi1	INSIDE	0.653	3.376	894.64	3020.03	2.204	767.35	1691.62
A_68_P24656000	chr6:94233185-94233229	NM_001029850:692	Magi1	INSIDE	0.326	4.885	5237.43	25583.96	1.592	4135.59	6583.54
A_68_P23513990	chr5:18732536-18732580	NM_001170746:-305	Magi2	PROMOTER	0.075	65.454	6912.95	452476.70	4.912	8241.07	40481.29
A_68_P23513989	chr5:18732427-18732471	NM_001170746:-415	Magi2	PROMOTER	0.296	12.409	1297.07	16095.34	3.678	1207.93	4442.88
A_68_P23513988	chr5:18732289-18732333	NM_001170746:-553	Magi2	PROMOTER	0.34	0.332	1843.36	611.41	0.113	1547.69	174.49
A_68_P23513996	chr5:18733426-18733470	NM_001170746:585	Magi2	INSIDE	0.641	0.715	5245.02	3749.67	0.459	3734.41	1712.63
A_68_P32582402	chrX:103206930-103206974	NM_001190409:286	Magt1	INSIDE	0.285	6.819	430.29	2934.30	1.947	469.63	914.20
A_68_P21614869	chr2:127481863-127481907	NM_001171187:547	Mal	INSIDE	0.543	0.598	1374.86	822.43	0.325	1083.43	352.17
A_68_P31930914	chr19:5803350-5803394	NR_002847:-701	Malat1	PROMOTER	0.452	5.734	2684.41	15392.82	2.591	2135.47	5532.58
A_68_P31781218	chr18:65590541-65590585	NM_172833:-88	Malt1	PROMOTER	0.504	8.260	11701.60	96651.59	4.16	9139.36	38022.61
A_68_P27787814	chr11:50105808-50105852	NM_175334:8	Maml1	INSIDE	0.433	5.731	1755.59	10061.15	2.482	1407.63	3494.36
A_68_P27787812	chr11:50105519-50105563	NM_175334:298	Maml1	INSIDE	0.608	5.774	503.47	2907.09	3.509	460.95	1617.56
A_68_P27787811	chr11:50105373-50105417	NM_175334:444	Maml1	INSIDE	0.393	4.991	550.88	2749.71	1.962	496.94	975.22
A_68_P22133096	chr3:51811618-51811662	NM_001004176:97288	Maml3	INSIDE	0.342	12.144	568.56	6904.67	4.154	490.96	2039.64

ProbeName	Target position of probe on CpG island microarray	TargetID	GeneSymbol	CpG island Description	Ratio of relative methylation (TiO_2-NP/Vehicle)	Sham group			TiO_2-H group		
						Relative methylation (Cy5/Cy3)	Cy3 signal (Input)	Cy5 signal (MeDIP)	Relative methylation (Cy5/Cy3)	Cy3 signal (Input)	Cy5 signal (MeDIP)
A_68_P22133744	chr3:51906476-51906520	NM_001004176:2430	Maml3	INSIDE	0.538	0.410	1078.83	441.92	0.22	768.88	169.46
A_68_P32451589	chrX:68303308-68303352	NM_001081354:-100	Mamld1	PROMOTER	0.572	4.438	1505.62	6681.86	2.54	1931.20	4906.19
A_68_P27145715	chr10:53795287-53795331	NM_008548:294	Man1a	INSIDE	0.38	4.958	2671.49	13246.60	1.886	2168.98	4090.89
A_68_P23324350	chr4:134260533-134260577	NM_207237:-349	Man1c1	PROMOTER	0.321	19.942	5003.90	99787.64	6.408	4159.51	26652.78
A_68_P31296462	chr17:64950677-64950721	NM_008549:-290	Man2a1	PROMOTER	0.429	5.549	804.63	4465.22	2.379	696.99	1657.98
A_68_P25266322	chr7:87515822-87515866	NM_172903:417	Man2a2	INSIDE	0.56	0.274	4802.20	1315.21	0.153	3544.35	543.76
A_68_P25266277	chr7:87508028-87508072	NM_172903:8211	Man2a2	INSIDE	0.554	2.619	1734.29	4541.42	1.45	1255.27	1820.32
A_68_P23609898	chr5:37221813-37221857	NM_008550:54	Man2b2	INSIDE	0.654	2.997	412.63	1236.83	1.962	433.02	849.45
A_68_P26540423	chr9:56978779-56978823	NM_028636:217	Man2c1	INSIDE	0.658	3.960	801.25	3172.80	2.606	753.09	1962.19
A_68_P22793481	chr4:26274038-26274083	NM_172865:-261	Manea	PROMOTER	0.311	6.065	2711.56	16445.35	1.884	2310.71	4354.36
A_68_P23270750	chr4:124538805-124538849	NM_001007573:589	Maneal	INSIDE	0.061	8.010	3440.02	27553.32	0.491	2535.58	1246.18
A_68_P24862663	chr6:134582475-134582519	NM_026345:10	Mansc1	INSIDE	0.433	5.125	1936.85	9927.15	2.218	1798.79	3990.47
A_68_P21764750	chr2:155102799-155102843	NM_025735:641	Map1lc3a	INSIDE	0.49	4.385	4307.23	18887.10	2.148	3514.34	7547.10
A_68_P26227606	chr8:124109508-124109552	NM_026160:-4837	Map1lc3b	DIVERGENT_PROMOTER	0.4	0.438	1199.20	525.81	0.175	980.12	171.77
A_68_P26227634	chr8:124114601-124114645	NM_026160:255	Map1lc3b	INSIDE	0.603	0.522	2123.02	1109.21	0.315	1631.37	513.67
A_68_P26580975	chr9:64101634-64101678	NM_008927:-244	Map2k1	PROMOTER	0.464	0.297	2077.34	616.27	0.138	1605.92	221.00
A_68_P26580973	chr9:64101378-64101422	NM_008927:12	Map2k1	INSIDE	0.562	4.372	882.16	3856.47	2.458	797.56	1960.31
A_68_P29330800	chr13:112598036-112598080	NM_011945:1133	Map3k1	INSIDE	0.105	26.349	1186.05	31251.77	2.771	1174.60	3254.93
A_68_P29330809	chr13:112599173-112599217	NM_011945:-3	Map3k1	PROMOTER	0.465	0.585	1309.83	765.93	0.272	1198.71	325.72
A_68_P29330398	chr13:112545643-112545687	NM_011945:53527	Map3k1	INSIDE	0.627	2.701	1153.51	3115.24	1.694	822.52	1392.99
A_68_P25018407	chr7:28442995-28443039	NM_001081292:16601	Map3k10	INSIDE	0.027	86.462	1113.78	96299.86	2.296	971.87	2231.25
A_68_P25018412	chr7:28443528-28443572	NM_001081292:16067	Map3k10	INSIDE	0.651	0.459	1339.91	614.72	0.299	1088.23	325.03
A_68_P30498333	chr15:102347359-102347404	NM_001163643:54	Map3k12	INSIDE	0.449	0.688	1942.88	1337.60	0.309	1470.80	454.28
A_68_P28084317	chr11:103128474-103128518	NM_016896:219	Map3k14	INSIDE	0.596	0.454	3227.12	1463.87	0.27	2341.20	633.10
A_68_P32777676	chrX:156426760-156426804	NM_001163085:418	Map3k15	INSIDE	0.514	5.651	2715.87	15346.63	2.907	3046.51	8854.83
A_68_P32777673	chrX:156426105-156426149	NM_001163085:-238	Map3k15	PROMOTER	0.18	11.486	279.96	3215.67	2.07	425.72	881.28
A_68_P28099478	chr11:105946211-105946255	NM_011947:17	Map3k3	INSIDE	0.239	28.094	15969.16	448632.40	6.724	16021.48	107730.80
A_68_P31051121	chr17:12511550-12511594	NM_011948:-46	Map3k4	PROMOTER	0.656	2.636	1507.08	3972.04	1.73	1185.29	2050.04
A_68_P26983894	chr10:19654073-19654117	NM_008580:-237	Map3k5	PROMOTER	0.417	10.144	1115.49	11315.26	4.235	988.92	4188.13
A_68_P26983899	chr10:19654713-19654757	NM_008580:403	Map3k5	INSIDE	0.467	4.167	3540.13	14751.75	1.946	2946.61	5734.17
A_68_P26983898	chr10:19654588-19654632	NM_008580:279	Map3k5	INSIDE	0.465	3.408	2042.61	6961.11	1.584	1622.72	2570.75
A_68_P26983891	chr10:19653749-19653793	NM_008580:-561	Map3k5	PROMOTER	0.53	2.794	1894.80	5294.45	1.481	1702.51	2520.82
A_68_P23316586	chr4:132807259-132807303	NM_016693:10548	Map3k6	INSIDE	0.274	8.256	1556.79	12852.33	2.259	1341.91	3031.42
A_68_P23316515	chr4:132797012-132797056	NM_016693:302	Map3k6	INSIDE	0.51	7.870	1013.37	7974.99	4.012	997.14	4000.40
A_68_P23316517	chr4:132797307-132797351	NM_016693:596	Map3k6	INSIDE	0.596	0.473	3478.96	1644.15	0.282	2357.19	664.00
A_68_P22818618	chr4:32050821-32050865	NM_009316:-239	Map3k7	PROMOTER	0.468	0.217	2523.37	548.78	0.102	1779.29	180.95
A_68_P28582806	chr12:82881553-82881597	NM_001174107:583	Map3k9	INSIDE	0.325	1.361	3489.75	4747.82	0.442	2529.30	1117.34
A_68_P31934083	chr19:6341265-6341309	NM_009006:37	Map4k2	INSIDE	0.498	0.616	1076.33	663.29	0.307	772.14	236.83
A_68_P31383814	chr17:80976741-80976785	NM_001081357:150671	Map4k3	DOWNSTREAM	0.439	0.486	1519.42	739.03	0.213	1256.91	268.23
A_68_P20168277	chr1:39957569-39957613	NM_008696:-167	Map4k4	PROMOTER	0.321	12.803	1517.83	19432.07	4.11	1793.64	7371.56
A_68_P30571313	chr16:16983889-16983933	NM_001038663:436	Mapk1	INSIDE	0.428	5.887	4073.90	23984.47	2.522	3237.17	8165.22
A_68_P30423478	chr15:88979773-88979817	NM_011161:242	Mapk11	INSIDE	0.48	4.376	2613.63	11436.73	2.102	1964.48	4128.71
A_68_P30423405	chr15:88970834-88970878	NM_013871:277	Mapk12	INSIDE	0.503	0.508	1499.04	760.81	0.255	1103.96	282.04
A_68_P30423403	chr15:88970637-88970681	NM_013871:475	Mapk12	INSIDE	0.633	0.295	1947.81	574.59	0.187	1572.17	293.36
A_68_P31123750	chr17:28826626-28826670	NM_001168508:-1638	Mapk14	PROMOTER	0.43	5.066	1189.47	6026.43	2.177	1054.75	2296.20
A_68_P31123769	chr17:28828900-28828944	NM_001168513:-590	Mapk14	PROMOTER	0.557	0.607	1290.68	784.06	0.338	1118.14	378.49

ProbeName	Target position of probe on CpG island microarray	TargetID	GeneSymbol	CpG island Description	Ratio of relative methylation (TiO_2-NP/Vehicle)	Sham group			TiO_2-H group		
						Relative methylation (Cy5/Cy3)	Cy3 signal (Input)	Cy5 signal (MeDIP)	Relative methylation (Cy5/Cy3)	Cy3 signal (Input)	Cy5 signal (MeDIP)
A_68_P31123770	chr17:28829032-28829076	NM_001168513:-458	Mapk14	PROMOTER	0.458	0.726	2017.18	1463.77	0.332	1613.15	535.63
A_68_P31123766	chr17:28828497-28828541	NM_001168513:-994	Mapk14	PROMOTER	0.529	3.894	434.60	1692.20	2.061	379.53	782.07
A_68_P30345385	chr15:75829377-75829421	NM_177922:5200	Mapk15	INSIDE	0.621	2.862	4479.57	12821.58	1.778	3593.24	6389.98
A_68_P30345384	chr15:75829239-75829283	NM_177922:5062	Mapk15	INSIDE	0.64	3.480	1737.40	6047.00	2.226	1423.99	3169.53
A_68_P31829410	chr18:74224381-74224425	NM_172632:201	Mapk4	INSIDE	0.457	0.566	1435.77	812.37	0.259	1115.19	288.34
A_68_P31829408	chr18:74224160-74224204	NM_172632:421	Mapk4	INSIDE	0.657	0.725	3255.75	2360.53	0.477	2406.03	1146.95
A_68_P31829405	chr18:74223815-74223859	NM_172632:767	Mapk4	INSIDE	0.43	3.437	1723.84	5925.21	1.48	1489.27	2203.42
A_68_P21428205	chr2:92229093-92229137	NM_001202445:3726	Mapk8ip1	INSIDE	0.466	3.368	2283.65	7692.17	1.568	1806.50	2832.77
A_68_P30425259	chr15:89284711-89284755	NM_021921:391	Mapk8ip2	INSIDE	0.458	0.495	3233.23	1599.42	0.227	2209.40	500.47
A_68_P30425255	chr15:89284136-89284180	NM_021921:-183	Mapk8ip2	PROMOTER	0.542	0.556	3937.48	2190.35	0.302	3176.78	957.88
A_68_P31100481	chr17:25073380-25073424	NM_001163447:520	Mapk8ip3	INSIDE	0.539	0.221	2053.29	454.44	0.119	1450.03	172.93
A_68_P24045146	chr5:121995041-121995098	NM_010765:832	Mapkapk5	INSIDE	0.573	5.427	279.38	1516.11	3.109	246.94	767.79
A_68_P31556965	chr18:23962258-23962302	NM_153058:-190	Mapre2	PROMOTER	0.406	5.892	1668.99	9833.96	2.393	1444.19	3456.40
A_68_P31556969	chr18:23962757-23962801	NM_153058:308	Mapre2	INSIDE	0.454	0.145	7107.70	1029.10	0.066	4380.54	288.07
A_68_P28089851	chr11:104093898-104093942	NM_001038609:1171	Mapt	INSIDE	0.451	6.172	2740.74	16916.38	2.781	2141.65	5956.53
A_68_P30086024	chr15:26238596-26238640	NM_177597:-208	Marchf11	PROMOTER	0.395	0.496	4344.32	2156.18	0.196	2980.77	584.44
A_68_P30086025	chr15:26238685-26238729	NM_177597:-120	Marchf11	PROMOTER	0.388	0.639	8018.27	5123.72	0.248	6078.48	1507.12
A_68_P30086030	chr15:26239396-26239440	NM_177597:592	Marchf11	INSIDE	0.529	2.677	1822.00	4876.61	1.416	1517.22	2147.74
A_68_P31150250	chr17:33855758-33855802	NM_145486:-165	Marchf2	PROMOTER	0.528	4.266	2967.14	12659.28	2.253	2447.61	5514.90
A_68_P20337461	chr1:72582629-72582673	NM_001045533:488	Marchf4	INSIDE	0.254	27.854	2544.21	70866.77	7.082	3225.79	22845.53
A_68_P20337470	chr1:72583581-72583625	NM_001045533:-464	Marchf4	PROMOTER	0.26	12.123	2539.98	30792.07	3.154	2025.32	6388.41
A_68_P32094489	chr19:37308834-37308878	NM_027314:26822	Marchf5	DOWNSTREAM	0.394	0.521	994.79	518.73	0.205	846.17	173.64
A_68_P30114903	chr15:31460728-31460772	NM_172606:42	Marchf6	INSIDE	0.54	3.033	2497.09	7574.21	1.637	2042.04	3343.72
A_68_P23297033	chr4:129190774-129190818	NM_010807:-28	Marcksl1	PROMOTER	0.176	26.757	8573.76	229406.30	4.716	7217.99	34040.56
A_68_P23297034	chr4:129190844-129190888	NM_010807:42	Marcksl1	INSIDE	0.304	22.473	20159.53	453042.60	6.825	26780.04	182765.90
A_68_P31939393	chr19:7416055-7416099	NM_001080388:258	Mark2	INSIDE	0.617	4.153	5950.38	24711.91	2.563	4671.16	11973.90
A_68_P28744817	chr12:112812887-112812931	NM_021516:188	Mark3	INSIDE	0.528	3.505	1062.69	3724.50	1.85	1009.97	1868.02
A_68_P24993426	chr7:20043771-20043815	NM_172279:51	Mark4	INSIDE	0.486	0.433	4507.64	1951.17	0.21	3344.54	703.22
A_68_P24993423	chr7:20043402-20043446	NM_172279:419	Mark4	INSIDE	0.494	0.291	3492.57	1015.28	0.144	2783.00	399.99
A_68_P27535217	chr10:126749029-126749081	NM_001003913:-212	Mars	PROMOTER	0.477	0.482	996.78	480.45	0.23	739.51	169.85
A_68_P20241002	chr1:55294197-55294241	NM_175439:198	Mars2	INSIDE	0.427	5.374	2283.85	12272.68	2.294	1728.60	3966.00
A_68_P32119293	chr19:42222595-42222639	NM_183195:738	Marveld1	INSIDE	0.48	0.646	1029.89	664.90	0.31	803.01	248.62
A_68_P32119294	chr19:42222686-42222730	NM_183195:830	Marveld1	INSIDE	0.329	4.533	2919.40	13233.54	1.49	2335.28	3479.00
A_68_P29269301	chr13:101386489-101386533	NM_178410:389	Marveld2	INSIDE	0.26	1.765	3100.44	5472.10	0.459	2256.34	1036.25
A_68_P29269300	chr13:101386400-101386447	NM_178410:476	Marveld2	INSIDE	0.169	1.783	651.62	1161.68	0.301	586.59	176.61
A_68_P26159961	chr8:112485994-112486038	NM_212447:89	Marveld3	INSIDE	0.346	5.213	1351.68	7046.13	1.802	1133.55	2042.34
A_68_P26022615	chr8:87436867-87436911	NM_019945:24364	Mast1	INSIDE	0.41	0.367	1556.05	570.92	0.15	1137.58	171.01
A_68_P26022617	chr8:87437152-87437196	NM_019945:24078	Mast1	INSIDE	0.486	0.555	1475.15	818.54	0.269	1094.54	294.91
A_68_P26022687	chr8:87447829-87447873	NM_019945:13402	Mast1	INSIDE	0.554	4.282	3009.37	12887.33	2.371	2220.73	5266.00
A_68_P23229430	chr4:116136663-116136707	NM_001042743:104	Mast2	INSIDE	0.573	3.875	347.13	1345.30	2.22	368.43	818.01
A_68_P25954148	chr8:73321826-73321870	NM_199308:-5516	Mast3	PROMOTER	0.27	12.095	5056.21	61156.39	3.264	3980.91	12995.17
A_68_P25954142	chr8:73321226-73321270	NM_199308:-4916	Mast3	PROMOTER	0.55	3.859	2678.64	10336.04	2.121	2107.02	4468.76
A_68_P25954101	chr8:73316001-73316050	NM_199308:307	Mast3	INSIDE	0.599	0.291	1787.48	519.49	0.174	1017.68	177.13
A_68_P25954141	chr8:73321121-73321165	NM_199308:-4810	Mast3	PROMOTER	0.615	0.607	2211.77	1341.47	0.373	1584.22	591.21
A_68_P25954143	chr8:73321298-73321342	NM_199308:-4988	Mast3	PROMOTER	0.323	1.501	2649.06	3975.94	0.485	1755.26	851.34
A_68_P25954066	chr8:73311865-73311909	NM_199308:4446	Mast3	INSIDE	0.637	2.387	3158.61	7539.91	1.521	2443.16	3715.87

ProbeName	Target position of probe on CpG island microarray	TargetID	GeneSymbol	CpG island Description	Ratio of relative methylation (TiO_2-NP/Vehicle)	Sham group			TiO_2-H group		
						Relative methylation (Cy5/Cy3)	Cy3 signal (Input)	Cy5 signal (MeDIP)	Relative methylation (Cy5/Cy3)	Cy3 signal (Input)	Cy5 signal (MeDIP)
A_68_P29284632	chr13:104123896-104123940	NM_175171:654	Mast4	INSIDE	0.494	0.506	1214.73	614.74	0.25	966.06	241.67
A_68_P29284641	chr13:104125004-104125048	NM_175171:-454	Mast4	PROMOTER	0.663	0.481	1658.63	797.94	0.319	1270.46	405.19
A_68_P21061711	chr2:23011327-23011371	NM_025979:196	Mastl	INSIDE	0.372	0.665	2600.21	1730.05	0.247	2031.23	502.52
A_68_P24541702	chr6:72388860-72388904	NM_145569:670	Mat2a	INSIDE	0.275	8.109	863.19	6999.29	2.229	1039.02	2316.02
A_68_P24541704	chr6:72389176-72389220	NM_145569:354	Mat2a	INSIDE	0.301	11.436	2172.30	24842.27	3.441	1849.32	6362.74
A_68_P27736855	chr11:40508399-40508443	NM_134017:-2236	Mat2b	PROMOTER	0.602	3.252	1593.37	5182.06	1.956	1228.22	2402.71
A_68_P27288382	chr10:80720130-80720174	NM_010768:-137	Matk	PROMOTER	0.424	20.262	2854.65	57841.95	8.593	2300.38	19766.92
A_68_P23303541	chr4:130506143-130506190	NM_010769:5867	Matn1	INSIDE	0.447	6.948	442.25	3072.63	3.103	387.60	1202.61
A_68_P30127809	chr15:34236397-34236441	NM_016762:-17	Matn2	PROMOTER	0.439	5.815	3737.21	21730.89	2.55	3068.17	7823.31
A_68_P30127808	chr15:34236313-34236357	NM_016762:-101	Matn2	PROMOTER	0.364	1.809	2725.20	4931.12	0.658	2124.67	1398.31
A_68_P21817647	chr2:164229981-164230025	NM_013592:112	Matn4	INSIDE	0.634	0.358	1481.17	530.91	0.227	1272.08	289.03
A_68_P31618899	chr18:35721824-35721868	NM_010771:35	Matr3	INSIDE	0.394	4.151	2558.74	10621.30	1.635	2207.80	3608.95
A_68_P28556950	chr12:78062975-78063019	NM_001146176:239	Max	INSIDE	0.219	0.293	4325.01	1265.43	0.064	2678.14	171.51
A_68_P25503658	chr7:134170789-134170833	NM_010772:-817	Maz	PROMOTER	0.276	0.602	19783.56	11918.67	0.166	11811.76	1966.26
A_68_P27282936	chr10:79858095-79858140	NM_013595:4107	Mbd3	INSIDE	0.28	2.610	4813.12	12564.17	0.73	3177.82	2318.87
A_68_P27535068	chr10:126725665-126725709	NM_033072:141	Mbd6	INSIDE	0.274	4.949	14531.18	71921.44	1.358	9192.33	12478.68
A_68_P24129310	chr5:138635652-138635696	NM_177878:133	Mblac1	INSIDE	0.495	0.323	1797.41	580.54	0.16	1435.83	229.74
A_68_P28915541	chr13:30228560-30228604	NM_153546:224	Mboat1	INSIDE	0.371	10.736	4790.51	51429.42	3.988	4566.35	18211.17
A_68_P28291801	chr12:25516771-25516815	NM_001083341:329	Mboat2	INSIDE	0.492	3.671	791.02	2903.89	1.806	693.02	1251.52
A_68_P24946816	chr7:3637239-3637283	NM_029934:7867	Mboat7	INSIDE	0.258	19.125	2504.59	47900.21	4.928	2018.48	9947.75
A_68_P24946819	chr7:3637588-3637632	NM_029934:7517	Mboat7	INSIDE	0.401	8.766	2627.66	23035.25	3.512	2092.90	7349.97
A_68_P31876422	chr18:82644757-82644801	NM_001025245:264	Mbp	INSIDE	0.486	5.328	1431.99	7629.59	2.59	1045.76	2708.19
A_68_P31876428	chr18:82645431-82645475	NM_001025245:938	Mbp	INSIDE	0.485	0.673	1081.99	727.73	0.327	878.17	286.73
A_68_P28029575	chr11:93747831-93747875	NM_134012:320	Mbtd1	INSIDE	0.36	10.162	2123.53	21578.33	3.659	1981.51	7250.03
A_68_P26214707	chr8:122082424-122082468	NM_001167910:215	Mbtps1	INSIDE	0.351	7.802	9604.49	74933.46	2.735	8609.03	23543.82
A_68_P32767195	chrX:154036840-154036884	NM_172307:-215	Mbtps2	PROMOTER	0.652	4.827	417.07	2013.38	3.147	622.68	1959.52
A_68_P26465911	chr9:43943262-43943306	NM_023061:544	Mcam	INSIDE	0.333	12.210	1718.71	20985.06	4.062	1384.23	5622.67
A_68_P22882678	chr4:45412906-45412950	NM_001009949:8710	Mcart1	INSIDE	0.448	7.117	1373.56	9776.28	3.186	1107.08	3526.66
A_68_P22882724	chr4:45421232-45421276	NM_001009949:384	Mcart1	INSIDE	0.501	3.673	1929.53	7086.32	1.84	1570.20	2889.70
A_68_P22882726	chr4:45421447-45421491	NM_001009949:170	Mcart1	INSIDE	0.631	3.630	1981.67	7192.47	2.289	1532.93	3509.30
A_68_P22056223	chr3:35899395-35899439	NM_023644:184	Mccc1	INSIDE	0.616	0.400	5125.83	2048.61	0.246	3737.33	920.19
A_68_P25664800	chr8:12915822-12915866	NM_178076:-48	Mcf2l	PROMOTER	0.428	0.273	1966.51	535.93	0.117	1481.99	172.78
A_68_P22344954	chr3:95463365-95463409	NM_008562:744	Mcl1	INSIDE	0.535	5.217	801.31	4180.19	2.789	758.09	2114.67
A_68_P24624872	chr6:88848652-88848696	NM_008564:100	Mcm2	INSIDE	0.438	5.674	2297.68	13036.55	2.486	1807.44	4492.51
A_68_P27143272	chr10:53349951-53349995	NM_027830:273	Mcm9	INSIDE	0.191	1.638	5152.57	8441.77	0.314	3394.92	1064.89
A_68_P27143271	chr10:53349847-53349891	NM_027830:377	Mcm9	INSIDE	0.411	1.546	16171.53	24998.16	0.636	11051.09	7029.36
A_68_P31161218	chr17:35978447-35978494	NM_001010833:28	Mdc1	INSIDE	0.281	18.353	6201.78	113823.70	5.154	6426.22	33119.70
A_68_P31130151	chr17:29979095-29979139	NM_001081160:45711	Mdga1	INSIDE	0.297	0.521	4055.41	2112.77	0.155	2881.24	446.42
A_68_P31130464	chr17:30025085-30025129	NM_001081160:-279	Mdga1	PROMOTER	0.653	9.733	2706.21	26338.31	6.352	2349.99	14926.13
A_68_P31130455	chr17:30024049-30024093	NM_001081160:757	Mdga1	INSIDE	0.665	3.834	1726.56	6620.00	2.549	1531.21	3903.07
A_68_P22822698	chr4:32744460-32744504	NM_001081392:389	Mdn1	INSIDE	0.559	0.462	2792.48	1291.31	0.259	2048.41	529.83
A_68_P31827985	chr18:73975089-73975133	NM_145494:-64	Me2	PROMOTER	0.065	57.954	999.74	57938.87	3.771	1008.46	3803.24
A_68_P25308743	chr7:96781285-96781329	NM_181407:-21	Me3	PROMOTER	0.553	6.256	868.71	5434.74	3.46	832.44	2880.64
A_68_P23272064	chr4:124762396-124762440	NM_027310:28	Meaf6	INSIDE	0.438	11.852	2018.53	23922.96	5.189	1725.41	8952.98
A_68_P22028773	chr3:30407847-30407891	NM_021442:541	Mecom	INSIDE	0.283	44.123	2522.00	111278.00	12.481	2417.37	30171.52
A_68_P22028772	chr3:30407701-30407745	NM_021442:687	Mecom	INSIDE	0.571	4.660	1111.47	5179.14	2.662	1054.62	2807.00

ProbeName	Target position of probe on CpG island microarray	TargetID	GeneSymbol	CpG island Description	Ratio of relative methylation (TiO_2-NP/Vehicle)	Sham group			TiO_2-H group		
						Relative methylation (Cy5/Cy3)	Cy3 signal (Input)	Cy5 signal (MeDIP)	Relative methylation (Cy5/Cy3)	Cy3 signal (Input)	Cy5 signal (MeDIP)
A_68_P32466325	chrX:71330416-71330460	NM_010788:494	Mecp2	INSIDE	0.333	6.806	2005.59	13650.66	2.263	2683.14	6072.30
A_68_P23308624	chr4:131399356-131399400	NM_025297:-7	Mecr	DIVERGENT_PROMOTER	0.353	0.426	2371.23	1009.60	0.15	1615.93	242.92
A_68_P22171318	chr3:58810124-58810168	NM_177855:-753	Med12l	PROMOTER	0.519	6.773	1045.86	7083.22	3.512	862.56	3029.62
A_68_P22171315	chr3:58809811-58809855	NM_177855:-1067	Med12l	PROMOTER	0.532	5.021	727.95	3654.82	2.67	646.12	1725.28
A_68_P27988570	chr11:86171604-86171648	NM_001080931:-599	Med13	PROMOTER	0.396	0.385	3099.33	1193.03	0.152	2303.48	351.03
A_68_P27988567	chr11:86170969-86171013	NM_001080931:37	Med13	INSIDE	0.398	0.587	2631.85	1543.99	0.233	2007.40	468.66
A_68_P24027251	chr5:119010546-119010590	NM_172424:-159	Med13l	PROMOTER	0.567	10.954	1227.99	13451.13	6.208	1211.00	7518.11
A_68_P24027248	chr5:119010105-119010149	NM_172424:-601	Med13l	PROMOTER	0.574	4.604	1788.46	8233.53	2.642	1614.13	4264.57
A_68_P32263511	chrX:12338951-12338995	NM_001048208:127	Med14	INSIDE	0.362	7.268	1126.92	8189.90	2.633	1147.77	3022.10
A_68_P32263512	chrX:12339036-12339080	NM_001048208:41	Med14	INSIDE	0.647	12.249	703.71	8619.50	7.926	926.72	7345.63
A_68_P30575359	chr16:17722895-17722939	NM_001040683:107	Med15	INSIDE	0.335	4.942	2261.57	11176.66	1.654	1945.39	3218.06
A_68_P27279588	chr10:79361859-79361903	NM_001163276:9803	Med16	INSIDE	0.368	6.447	3496.68	22542.65	2.37	2609.92	6186.09
A_68_P27279643	chr10:79371605-79371649	NM_001163276:57	Med16	INSIDE	0.628	4.140	5613.44	23242.10	2.599	4199.47	10914.67
A_68_P27279589	chr10:79361980-79362024	NM_001163276:9681	Med16	INSIDE	0.345	5.165	1415.33	7310.07	1.782	987.21	1758.92
A_68_P24929768	chr6:146591129-146591173	NM_025315:50	Med21	INSIDE	0.448	0.322	1672.11	538.97	0.144	1195.65	172.62
A_68_P24929770	chr6:146591301-146591345	NM_025315:222	Med21	INSIDE	0.518	7.192	900.29	6474.61	3.727	781.40	2912.19
A_68_P25089971	chr7:52147810-52147854	NM_029365:-96	Med25	DIVERGENT_PROMOTER	0.219	14.412	767.19	11057.09	3.163	702.40	2221.40
A_68_P25089969	chr7:52147509-52147554	NM_029365:205	Med25	INSIDE	0.644	3.690	1616.10	5963.83	2.376	1302.29	3094.39
A_68_P25962045	chr8:75071657-75071701	NM_027485:531	Med26	INSIDE	0.629	4.316	3433.96	14822.61	2.717	2819.82	7661.01
A_68_P25962046	chr8:75071784-75071828	NM_027485:403	Med26	INSIDE	0.386	0.637	1555.40	991.26	0.246	1227.26	302.01
A_68_P23657073	chr5:45911523-45911567	NM_025895:77	Med28	INSIDE	0.661	4.434	887.68	3935.81	2.931	798.95	2341.80
A_68_P27767873	chr11:46250861-46250905	NM_001104556:420	Med7	INSIDE	0.449	0.320	1535.64	490.78	0.143	1208.18	173.21
A_68_P23240178	chr4:118082173-118082217	NM_020000:253	Med8	INSIDE	0.364	0.255	7300.01	1858.24	0.093	4583.73	424.43
A_68_P29186150	chr13:83662891-83662935	NM_001170537:19880	Mef2c	INSIDE	0.24	10.851	447.23	4852.85	2.6	397.83	1034.16
A_68_P31734231	chr18:57292496-57292540	NM_001001979:-225	Megf10	PROMOTER	0.296	0.421	1719.34	724.23	0.125	1402.66	174.97
A_68_P31734234	chr18:57292707-57292751	NM_001001979:-15	Megf10	PROMOTER	0.445	0.311	1555.24	483.97	0.138	1231.98	170.42
A_68_P26581612	chr9:64233974-64234018	NM_172522:564	Megf11	INSIDE	0.446	0.491	1112.93	546.65	0.219	961.58	210.45
A_68_P26581610	chr9:64233768-64233812	NM_172522:358	Megf11	INSIDE	0.653	0.398	2461.38	978.91	0.26	1940.60	503.88
A_68_P23006650	chr4:70195415-70195459	NM_172694:526	Megf9	INSIDE	0.345	10.688	9060.64	96839.65	3.688	6797.84	25067.31
A_68_P23006649	chr4:70195250-70195294	NM_172694:690	Megf9	INSIDE	0.375	0.502	1621.97	814.94	0.188	1403.30	264.15
A_68_P23006657	chr4:70196174-70196218	NM_172694:-234	Megf9	PROMOTER	0.508	0.525	2419.26	1270.32	0.267	1789.16	477.34
A_68_P30381264	chr15:81900574-81900618	NM_028897:50	Mei1	INSIDE	0.456	0.639	2524.80	1612.69	0.291	1856.49	540.84
A_68_P27622849	chr11:18771565-18771609	NM_001193271:147386	Meis1	DOWNSTREAM	0.068	3.225	1238.52	3993.78	0.219	978.72	214.57
A_68_P24980083	chr7:16760918-16760963	NM_008627:212	Meis3	INSIDE	0.601	2.696	2076.52	5598.79	1.621	1699.10	2753.68
A_68_P31350850	chr17:74695143-74695187	NM_133771:-961	Memo1	PROMOTER	0.011	207.364	483.88	100338.60	2.384	378.21	901.76
A_68_P31934033	chr19:6335488-6335532	NM_001168490:472	Men1	INSIDE	0.449	0.572	2322.96	1328.83	0.257	1852.07	475.28
A_68_P31934035	chr19:6335704-6335748	NM_001168490:688	Men1	INSIDE	0.545	0.373	3440.46	1284.85	0.203	2377.57	483.65
A_68_P24127560	chr5:138227659-138227706	NM_144913:247	Mepce	INSIDE	0.271	1.531	1876.04	2872.39	0.414	1473.01	610.09
A_68_P25285350	chr7:91040523-91040567	NM_023403:35	Mesdc2	INSIDE	0.363	0.368	4649.86	1710.77	0.134	3282.50	438.40
A_68_P22569096	chr3:138152576-138152620	NM_175224:-252	Metap1	PROMOTER	0.58	2.519	2898.86	7303.55	1.461	2628.67	3840.67
A_68_P27356283	chr10:93351892-93351936	NM_019648:2033	Metap2	INSIDE	0.073	5.984	1524.46	9121.76	0.435	1393.16	605.72
A_68_P31106324	chr17:25934049-25934093	NM_133719:-80	Metrn	PROMOTER	0.331	6.469	1534.53	9926.76	2.144	1235.68	2648.81
A_68_P31106322	chr17:25933806-25933850	NM_133719:162	Metrn	INSIDE	0.523	3.659	784.81	2872.02	1.914	649.88	1243.57
A_68_P28192109	chr11:121564851-121564895	NM_144797:1132	Metrnl	INSIDE	0.415	4.695	2930.29	13758.20	1.948	2296.79	4473.20
A_68_P28192096	chr11:121563476-121563520	NM_144797:-242	Metrnl	PROMOTER	0.527	0.626	1307.78	818.99	0.33	1028.24	339.53
A_68_P27533648	chr10:126479103-126479147	NM_010792:137	Mettl1	INSIDE	0.633	0.627	2430.18	1524.16	0.397	1794.65	712.88

ProbeName	Target position of probe on CpG island microarray	TargetID	GeneSymbol	CpG island Description	Ratio of relative methylation (TiO_2-NP/Vehicle)	Sham group			TiO_2-H group		
						Relative methylation (Cy5/Cy3)	Cy3 signal (Input)	Cy5 signal (MeDIP)	Relative methylation (Cy5/Cy3)	Cy3 signal (Input)	Cy5 signal (MeDIP)
A_68_P21105336	chr2:30663318-30663362	NM_170592:-156	Mettl11a	DIVERGENT_PROMOTER	0.455	0.492	14256.57	7010.46	0.224	9464.63	2118.77
A_68_P21105338	chr2:30663536-30663580	NM_170592:62	Mettl11a	INSIDE	0.586	0.308	2271.22	700.12	0.181	1511.78	273.12
A_68_P21105337	chr2:30663394-30663438	NM_170592:-80	Mettl11a	DIVERGENT_PROMOTER	0.665	0.601	7065.78	4245.08	0.4	4800.18	1919.04
A_68_P21105335	chr2:30663189-30663233	NM_170592:-286	Mettl11a	DIVERGENT_PROMOTER	0.201	1.357	10787.37	14634.56	0.273	6626.55	1811.21
A_68_P20778372	chr1:164478561-164478605	NM_144877:-106	Mettl13	PROMOTER	0.391	7.228	7653.33	55318.14	2.827	6438.02	18203.18
A_68_P20778374	chr1:164478713-164478757	NM_144877:-258	Mettl13	PROMOTER	0.556	0.266	2041.11	543.14	0.148	1589.47	234.96
A_68_P22487800	chr3:123088652-123088696	NM_201638:234	Mettl14	INSIDE	0.299	22.576	7098.51	160259.20	6.744	6139.90	41409.68
A_68_P28515346	chr12:70683995-70684039	NM_001033236:-1	Mettl21d	PROMOTER	0.495	6.730	2074.78	13964.01	3.334	1792.66	5976.00
A_68_P28515345	chr12:70683875-70683919	NM_001033236:119	Mettl21d	INSIDE	0.607	0.739	3309.30	2446.31	0.449	2583.72	1159.55
A_68_P30532721	chr16:8470714-8470758	NM_146247:-169	Mettl22	PROMOTER	0.404	0.587	902.31	529.68	0.237	721.61	171.24
A_68_P29605899	chr14:52924704-52924752	NM_019721:71	Mettl3	INSIDE	0.38	8.202	464.47	3809.81	3.115	445.29	1387.14
A_68_P25471703	chr7:128178649-128178693	NM_021554:712	Mettl9	INSIDE	0.325	29.657	1473.10	43687.54	9.653	1359.53	13123.49
A_68_P22314306	chr3:88335633-88335677	NM_001029890:-662	Mex3a	PROMOTER	0.324	0.324	2523.85	817.24	0.105	1608.53	168.89
A_68_P25279941	chr7:90014673-90014717	NM_175366:-1148	Mex3b	PROMOTER	0.359	11.848	4563.36	54066.36	4.249	3655.66	15533.36
A_68_P31826505	chr18:73732011-73732055	NM_001039214:-326	Mex3c	PROMOTER	0.63	2.335	3205.79	7486.07	1.471	2393.53	3519.73
A_68_P27282830	chr10:79844326-79844370	NM_198615:6048	Mex3d	INSIDE	0.415	4.248	2981.56	12666.68	1.763	2229.36	3930.02
A_68_P27282834	chr10:79844741-79844785	NM_198615:5634	Mex3d	INSIDE	0.481	4.090	1338.10	5472.92	1.967	1183.66	2328.33
A_68_P27282831	chr10:79844492-79844536	NM_198615:5882	Mex3d	INSIDE	0.619	9.614	1963.34	18875.82	5.948	1617.24	9619.99
A_68_P27282840	chr10:79845290-79845334	NM_198615:5084	Mex3d	INSIDE	0.586	3.033	1828.09	5545.13	1.779	1390.87	2473.99
A_68_P27282875	chr10:79849919-79849963	NM_198615:456	Mex3d	INSIDE	0.405	4.259	1618.70	6894.82	1.726	1281.50	2212.50
A_68_P27282838	chr10:79845116-79845160	NM_198615:5258	Mex3d	INSIDE	0.665	2.585	1483.80	3836.13	1.72	1265.13	2175.79
A_68_P27826025	chr11:57332162-57332206	NM_145426:18	Mfap3	INSIDE	0.542	0.549	1153.51	633.80	0.298	894.00	266.29
A_68_P25259449	chr7:86294014-86294058	NM_001045489:-90	Mfge8	PROMOTER	0.583	2.674	1049.42	2805.85	1.558	996.58	1552.77
A_68_P25780633	chr8:36651446-36651490	NM_001081279:617	Mfhas1	INSIDE	0.49	4.894	1282.15	6274.51	2.397	1018.45	2441.56
A_68_P22038555	chr3:32428477-32428521	NM_024200:95	Mfn1	INSIDE	0.532	6.202	1423.26	8826.69	3.302	1215.48	4013.24
A_68_P22038556	chr3:32428603-32428647	NM_024200:221	Mfn1	INSIDE	0.523	0.719	1559.33	1120.53	0.376	1280.93	481.22
A_68_P23972004	chr5:108877831-108877875	NM_172883:58	Mfsd7a	INSIDE	0.316	0.481	1601.26	770.51	0.152	1137.43	173.18
A_68_P20173639	chr1:40847463-40847507	NM_172499:18	Mfsd9	INSIDE	0.26	21.933	1135.89	24914.04	5.703	1020.09	5817.75
A_68_P21571997	chr2:119722828-119722872	NM_001164274:-113	Mga	PROMOTER	0.257	1.553	748.40	1162.07	0.399	716.30	285.85
A_68_P27782124	chr11:49064057-49064101	NM_001110150:25	Mgat1	INSIDE	0.353	0.641	1759.78	1127.20	0.226	1426.98	322.38
A_68_P27782066	chr11:49057568-49057612	NM_001110148:-102	Mgat1	PROMOTER	0.642	0.719	3484.12	2505.13	0.462	2583.02	1192.59
A_68_P27782067	chr11:49057649-49057693	NM_001110148:-22	Mgat1	PROMOTER	0.392	1.618	2096.80	3391.97	0.634	1877.30	1190.87
A_68_P27787453	chr11:50044668-50044712	NM_145926:5854	Mgat4b	INSIDE	0.336	6.424	1667.52	10712.42	2.158	1380.60	2978.72
A_68_P24623660	chr6:88674554-88674598	NM_001166250:-121	Mgll	PROMOTER	0.236	23.515	2426.83	57065.65	5.551	2399.63	13320.86
A_68_P25562263	chr7:144086164-144086208	NM_008598:-107	Mgmt	PROMOTER	0.357	0.319	4903.22	1561.85	0.114	3218.98	365.64
A_68_P30512463	chr16:4886291-4886335	NM_029657:61	Mgrn1	INSIDE	0.379	0.675	1999.47	1348.68	0.256	1458.57	372.85
A_68_P23990789	chr5:112656382-112656426	NR_003718:1564	Miat	INSIDE	0.273	11.438	2965.05	33913.02	3.123	2685.93	8388.19
A_68_P23439858	chr4:155028864-155028908	NM_145124:14450	Mib2	INSIDE	0.592	3.094	4077.96	12616.73	1.83	3209.32	5874.58
A_68_P32252887	chrX:10294418-10294462	NM_001166635:-50	Mid1ip1	PROMOTER	0.393	5.153	992.00	5111.38	2.025	1198.99	2427.40
A_68_P27281298	chr10:79613010-79613054	NM_021565:1998	Midn	INSIDE	0.46	0.442	1167.17	515.82	0.203	838.52	170.42
A_68_P27281304	chr10:79613780-79613824	NM_021565:2768	Midn	INSIDE	0.635	0.378	2704.66	1022.79	0.24	1938.29	465.59
A_68_P27281299	chr10:79613231-79613275	NM_021565:2218	Midn	INSIDE	0.405	5.276	771.29	4069.55	2.134	644.93	1376.46
A_68_P23164325	chr4:102787321-102787365	NM_001039081:-4652	Mier1	PROMOTER	0.068	78.630	8987.55	706694.20	5.339	14019.98	74855.87
A_68_P23164324	chr4:102787243-102787287	NM_001039081:-4730	Mier1	PROMOTER	0.169	11.293	1428.35	16129.76	1.914	1298.23	2484.75
A_68_P23164323	chr4:102787124-102787168	NM_001039081:-4848	Mier1	PROMOTER	0.616	0.396	2024.81	802.00	0.244	1560.07	380.56
A_68_P27277569	chr10:79017872-79017916	NM_027422:-58	Mier2	PROMOTER	0.281	9.772	1987.07	19417.24	2.747	1702.44	4676.65

ProbeName	Target position of probe on CpG island microarray	TargetID	GeneSymbol	CpG island Description	Ratio of relative methylation (TiO_2-NP/Vehicle)	Sham group			TiO_2-H group		
						Relative methylation (Cy5/Cy3)	Cy3 signal (Input)	Cy5 signal (MeDIP)	Relative methylation (Cy5/Cy3)	Cy3 signal (Input)	Cy5 signal (MeDIP)
A_68_P29330297	chr13:112515612-112515656	NM_172593:39249	Mier3	DOWNSTREAM	0.389	7.064	3966.04	28016.35	2.747	3560.39	9779.19
A_68_P27259065	chr10:75322775-75322819	NM_010798:199	Mif	INSIDE	0.542	0.475	1509.09	717.50	0.258	1209.83	311.87
A_68_P28154418	chr11:115474202-115474246	NM_027162:-407	Mif4gd	PROMOTER	0.261	0.463	2170.12	1004.82	0.121	1415.03	171.14
A_68_P28154417	chr11:115474024-115474068	NM_027162:-229	Mif4gd	PROMOTER	0.529	3.364	5090.39	17126.30	1.78	3803.94	6772.08
A_68_P28154416	chr11:115473786-115473830	NM_027162:9	Mif4gd	INSIDE	0.563	5.152	1432.34	7378.98	2.9	1192.50	3458.37
A_68_P32070425	chr19:32560187-32560231	NM_010799:-50	Minpp1	PROMOTER	0.502	4.073	1788.69	7284.71	2.045	1481.25	3029.60
A_68_P32383984	chrX:50097577-50097621	NR_029657:-1854	Mir106a	PROMOTER	0.522	3.244	599.85	1945.72	1.692	706.81	1196.03
A_68_P32016082	chr19:23215759-23215803	NR_035423:-8140	Mir1192	PROMOTER	0.135	2.117	2850.82	6035.95	0.285	2397.44	683.85
A_68_P32016092	chr19:23216941-23216985	NR_035423:-6958	Mir1192	PROMOTER	0.44	0.689	1602.14	1103.53	0.303	1236.50	374.51
A_68_P26017626	chr8:86535448-86535492	NR_035431:62	Mir1199	INSIDE	0.602	6.003	1483.23	8903.27	3.613	1231.41	4449.27
A_68_P29668497	chr14:65206255-65206299	NR_029813:-3217	Mir124a-1	PROMOTER	0.624	4.732	6463.42	30587.16	2.952	4681.80	13821.97
A_68_P21906910	chr2:180629073-180629117	NR_029538:350	Mir124a-3	DOWNSTREAM	0.082	2.607	2188.73	5704.96	0.215	1497.18	321.85
A_68_P21394386	chr2:84583579-84583623	NR_029544:-2265	Mir130a	PROMOTER	0.53	0.243	3999.71	972.34	0.129	2696.91	347.57
A_68_P24443350	chr6:51220702-51220746	NR_029719:-815	Mir148a	PROMOTER	0.654	2.823	1500.36	4235.41	1.846	1341.47	2476.02
A_68_P20451522	chr1:94745253-94745297	NR_029559:-1680	Mir149	PROMOTER	0.212	1.869	1413.57	2641.83	0.397	1053.88	418.04
A_68_P28047118	chr11:96711362-96711406	NR_029562:-322	Mir152	PROMOTER	0.15	1.480	1673.31	2476.48	0.222	1299.06	287.93
A_68_P22218631	chr3:68809558-68809602	NR_029529:-4113	Mir15b	PROMOTER	0.491	3.919	2074.32	8129.82	1.926	1601.53	3084.00
A_68_P22218636	chr3:68810008-68810052	NR_029529:-3663	Mir15b	PROMOTER	0.453	3.436	1325.60	4555.37	1.555	1013.98	1576.99
A_68_P29919366	chr14:115439544-115439588	NR_029785:-3326	Mir17	PROMOTER	0.522	7.460	1236.85	9226.71	3.893	1052.90	4098.61
A_68_P26018642	chr8:86707908-86707952	NR_029821:-5070	Mir181c	PROMOTER	0.154	35.978	2565.51	92301.31	5.531	2143.91	11858.40
A_68_P26345157	chr9:20837581-20837625	NR_035438:-830	Mir1900	PROMOTER	0.353	6.031	1219.11	7352.60	2.128	1048.80	2231.68
A_68_P27950594	chr11:79524651-79524695	NR_029579:-798	Mir193	PROMOTER	0.343	0.574	2333.21	1338.17	0.197	1682.25	330.58
A_68_P27950601	chr11:79525364-79525408	NR_029579:-84	Mir193	PROMOTER	0.408	8.319	1919.16	15965.01	3.39	1763.23	5977.24
A_68_P27950596	chr11:79524897-79524941	NR_029579:-552	Mir193	PROMOTER	0.503	3.155	2151.72	6787.75	1.588	1604.22	2547.16
A_68_P27351984	chr10:92624465-92624509	NR_035452:-1043	Mir1931	PROMOTER	0.221	0.520	1980.92	1030.74	0.115	1526.86	175.96
A_68_P30557335	chr16:13448659-13448703	NR_030549:-935	Mir193b	PROMOTER	0.609	0.375	3612.55	1354.05	0.228	2414.28	550.95
A_68_P29240037	chr13:96095372-96095416	NR_035461:-5140	Mir1940	PROMOTER	0.584	0.391	2515.05	983.47	0.228	1988.06	453.93
A_68_P26134879	chr8:108137855-108137899	NR_035492:-1489	Mir1966	PROMOTER	0.474	0.424	1902.82	807.42	0.201	1425.43	286.68
A_68_P26134826	chr8:108129362-108129406	NR_035492:-9981	Mir1966	PROMOTER	0.557	5.655	2094.24	11843.50	3.152	1923.98	6065.27
A_68_P26134881	chr8:108138096-108138140	NR_035492:-1247	Mir1966	PROMOTER	0.3	4.987	1410.42	7033.62	1.497	1456.10	2179.66
A_68_P27285698	chr10:80289762-80289807	NR_035503:-1757	Mir1982	PROMOTER	0.225	2.117	1332.34	2821.00	0.475	1191.13	566.24
A_68_P21114613	chr2:32168350-32168394	NR_029811:-5607	Mir199b	PROMOTER	0.256	0.499	1975.35	985.17	0.128	1366.12	174.74
A_68_P21114609	chr2:32167955-32167999	NR_029811:-6003	Mir199b	PROMOTER	0.505	9.639	12622.64	121668.10	4.871	11254.23	54814.25
A_68_P21114610	chr2:32168059-32168103	NR_029811:-5899	Mir199b	PROMOTER	0.627	7.410	8050.30	59652.90	4.646	7124.71	33104.31
A_68_P22858433	chr4:40669503-40669547	NR_029594:-425	Mir207	PROMOTER	0.534	0.416	2240.52	932.07	0.222	1810.04	402.07
A_68_P27924625	chr11:74986839-74986883	NR_029794:-29	Mir212	DIVERGENT_PROMOTER	0.664	0.458	2659.01	1216.68	0.304	2133.01	648.50
A_68_P31151557	chr17:34162040-34162084	NR_029800:-25	Mir219-1	PROMOTER	0.527	0.510	3497.10	1782.51	0.269	2712.00	729.12
A_68_P21099810	chr2:29702001-29702045	NR_029838:-775	Mir219-2	PROMOTER	0.307	0.472	1777.47	839.54	0.145	1395.19	202.49
A_68_P21099803	chr2:29701365-29701409	NR_029838:-139	Mir219-2	PROMOTER	0.437	0.521	2170.48	1130.39	0.227	1655.25	376.32
A_68_P21099801	chr2:29701142-29701186	NR_029838:83	Mir219-2	INSIDE	0.587	0.593	1855.13	1099.99	0.348	1225.98	426.75
A_68_P21099804	chr2:29701460-29701504	NR_029838:-235	Mir219-2	PROMOTER	0.577	0.692	1390.36	962.22	0.399	1038.93	414.75
A_68_P26018712	chr8:86722479-86722523	NR_029740:-9916	Mir23a	PROMOTER	0.448	0.489	950.20	464.79	0.219	791.79	173.66
A_68_P20348302	chr1:74438798-74438842	NR_029743:-2063	Mir26b	PROMOTER	0.51	4.748	921.21	4374.18	2.422	1016.16	2461.35
A_68_P27365819	chr10:95026630-95026674	NR_037212:-4697	Mir3058	PROMOTER	0.382	11.846	6512.50	77149.59	4.524	5507.76	24914.77
A_68_P28103517	chr11:106649497-106649541	NR_037224:-5445	Mir3064	PROMOTER	0.658	0.618	1386.18	856.41	0.407	1113.61	452.82
A_68_P28269969	chr12:17355438-17355482	NR_037226:-6737	Mir3066	PROMOTER	0.609	3.440	695.59	2392.72	2.094	625.64	1310.29

ProbeName	Target position of probe on CpG island microarray	TargetID	GeneSymbol	CpG island Description	Ratio of relative methylation (TiO_2-NP/Vehicle)	Sham group			TiO_2-H group		
						Relative methylation (Cy5/Cy3)	Cy3 signal (Input)	Cy5 signal (MeDIP)	Relative methylation (Cy5/Cy3)	Cy3 signal (Input)	Cy5 signal (MeDIP)
A_68_P21104807	chr2:30567268-30567312	NR_037272:-9439	Mir3089	PROMOTER	0.532	2.774	1801.34	4996.17	1.475	1524.28	2248.08
A_68_P22312502	chr3:88018703-88018747	NR_037276:-368	Mir3093	PROMOTER	0.446	5.834	1698.19	9907.63	2.605	1520.88	3961.46
A_68_P25015934	chr7:27963629-27963675	NR_037283:-2540	Mir3101	PROMOTER	0.539	4.982	10559.19	52603.08	2.687	8140.43	21874.99
A_68_P24333876	chr6:30688404-30688448	NR_029900:-2872	Mir335	PROMOTER	0.454	4.972	1778.62	8842.73	2.255	1396.31	3149.37
A_68_P24135185	chr5:139847981-139848025	NR_029768:-2303	Mir339	DIVERGENT_PROMOTER	0.432	6.893	2605.73	17960.72	2.975	2316.27	6890.47
A_68_P24992976	chr7:19967388-19967432	NR_030759:-4581	Mir343	PROMOTER	0.579	0.580	1264.71	733.48	0.336	856.33	287.71
A_68_P30501452	chr15:102844615-102844659	NR_030526:-704	Mir615	PROMOTER	0.233	0.451	2250.72	1016.11	0.105	1616.05	170.20
A_68_P30501459	chr15:102845371-102845415	NR_030526:52	Mir615	INSIDE	0.107	1.368	1575.52	2155.29	0.146	1185.42	172.81
A_68_P27539432	chr10:127520351-127520395	NR_030442:-1969	Mir677	PROMOTER	0.665	0.680	1073.80	729.65	0.452	953.85	430.86
A_68_P32240004	chrX:7327982-7328026	NR_030454:-2210	Mir684-1	DIVERGENT_PROMOTER	0.325	9.805	1351.43	13251.11	3.191	1453.31	4637.30
A_68_P29613301	chr14:55236421-55236465	NR_030457:-820	Mir686	PROMOTER	0.523	0.371	1791.10	664.04	0.194	1399.46	271.10
A_68_P30499098	chr15:102502893-102502944	NR_030460:-621	Mir688	PROMOTER	0.36	0.356	1943.08	691.15	0.128	1370.69	175.41
A_68_P24796449	chr6:119987179-119987223	NR_030487:-2871	Mir706	PROMOTER	0.648	3.767	1164.46	4386.17	2.442	1011.95	2470.71
A_68_P22481656	chr3:121996594-121996638	NR_030439:5	Mir760	INSIDE	0.24	14.696	1904.45	27988.15	3.524	1758.36	6195.61
A_68_P22481653	chr3:121996304-121996348	NR_030439:295	Mir760	DOWNSTREAM	0.63	6.174	369.96	2284.23	3.889	333.02	1295.04
A_68_P25506858	chr7:134851780-134851824	NR_030428:-198	Mir762	PROMOTER	0.655	0.576	3874.50	2231.45	0.377	2650.16	999.48
A_68_P27788127	chr11:50172534-50172578	NR_030529:-1175	Mir804	PROMOTER	0.354	0.458	1285.94	589.24	0.162	1061.14	172.00
A_68_P27788131	chr11:50172882-50172926	NR_030529:-1523	Mir804	PROMOTER	0.557	3.018	2423.16	7313.91	1.68	2167.61	3642.37
A_68_P29187684	chr13:83884882-83884926	NR_029545:6486	Mir9-2	DOWNSTREAM	0.571	0.320	1365.76	436.55	0.183	1116.82	203.96
A_68_P25261415	chr7:86644791-86644835	NR_029818:-5337	Mir9-3	PROMOTER	0.465	4.863	1401.78	6817.46	2.263	1269.49	2872.94
A_68_P25261466	chr7:86650289-86650333	NR_029818:161	Mir9-3	DOWNSTREAM	0.556	4.021	2681.78	10783.01	2.237	2071.92	4634.22
A_68_P25261496	chr7:86653957-86654001	NR_029818:3829	Mir9-3	DOWNSTREAM	0.576	0.646	957.89	618.69	0.372	714.33	265.87
A_68_P29020011	chr13:48641397-48641441	NR_029725:-7777	Mirlet7a-1	PROMOTER	0.186	24.817	3161.68	78462.14	4.619	3116.20	14394.91
A_68_P29020016	chr13:48641931-48641975	NR_029725:-8311	Mirlet7a-1	PROMOTER	0.548	0.705	1778.10	1253.01	0.386	1349.95	521.60
A_68_P27903938	chr11:70833216-70833260	NM_025993:126	Mis12	INSIDE	0.506	0.395	2180.90	861.40	0.2	1619.85	323.94
A_68_P28494358	chr12:66273553-66273597	NM_172578:-7	Mis18bp1	PROMOTER	0.151	1.714	1115.66	1912.74	0.259	860.45	222.84
A_68_P30556199	chr16:13256418-13256462	NM_001122667:-133	Mkl2	PROMOTER	0.35	6.790	1629.00	11061.32	2.373	1423.55	3378.69
A_68_P30556205	chr16:13257041-13257085	NM_181860:-8109	Mkl2	PROMOTER	0.419	6.948	4866.64	33813.50	2.909	4367.76	12706.97
A_68_P24337493	chr6:31348815-31348859	NM_013791:9	Mkln1	INSIDE	0.5	11.424	1393.73	15922.24	5.718	1263.86	7226.32
A_68_P24337499	chr6:31349464-31349508	NM_013791:659	Mkln1	INSIDE	0.531	7.922	3078.64	24390.51	4.206	2695.41	11336.19
A_68_P23226097	chr4:115511687-115511731	NM_021461:-142	Mknk1	PROMOTER	0.654	0.252	2571.17	647.09	0.165	1830.83	301.43
A_68_P27284768	chr10:80139566-80139610	NM_021462:-4867	Mknk2	PROMOTER	0.663	2.198	1743.27	3831.17	1.457	1275.95	1858.43
A_68_P24383013	chr6:39369919-39369963	NM_018810:428	Mkrn1	INSIDE	0.492	5.929	1726.04	10234.14	2.919	1386.34	4046.18
A_68_P24383009	chr6:39369612-39369656	NM_018810:734	Mkrn1	INSIDE	0.545	8.420	888.00	7477.02	4.587	747.60	3429.44
A_68_P24383012	chr6:39369835-39369879	NM_018810:512	Mkrn1	INSIDE	0.576	3.823	1502.98	5745.59	2.202	1241.75	2733.98
A_68_P25169129	chr7:69564808-69564852	NM_011746:195	Mkrn3	INSIDE	0.604	3.751	1084.06	4065.90	2.265	935.21	2118.52
A_68_P22209745	chr3:67177879-67177923	NM_001039543:-118	Mlf1	PROMOTER	0.433	0.610	847.54	517.09	0.264	675.31	178.20
A_68_P24817835	chr6:124881155-124881199	NM_001170341:-229	Mlf2	PROMOTER	0.516	3.473	3810.56	13234.11	1.79	3364.47	6024.08
A_68_P24817834	chr6:124881062-124881106	NM_001170341:-321	Mlf2	PROMOTER	0.098	4.947	1295.76	6410.00	0.484	1205.14	583.07
A_68_P26167758	chr8:113861821-113861865	NM_029005:-39	Mlkl	PROMOTER	0.279	0.402	2063.93	830.33	0.112	1514.28	170.23
A_68_P23536416	chr5:22940274-22940318	NM_026984:50	Mll5	INSIDE	0.498	3.551	1255.26	4457.28	1.769	990.94	1753.10
A_68_P31259248	chr17:57074657-57074701	NM_022328:133	Mllt1	INSIDE	0.492	0.278	2419.65	672.53	0.137	1789.32	244.77
A_68_P21038062	chr2:17979342-17979387	NM_010804:2467	Mllt10	INSIDE	0.241	0.375	2426.96	910.56	0.09	1917.93	173.29
A_68_P21038125	chr2:17987169-17987213	NM_010804:10293	Mllt10	INSIDE	0.477	4.477	1630.97	7301.34	2.133	1256.39	2680.45
A_68_P21038121	chr2:17986435-17986479	NM_010804:9559	Mllt10	INSIDE	0.227	0.642	2206.60	1417.54	0.146	1675.53	244.03
A_68_P23088444	chr4:87677993-87678037	NM_027326:1297	Mllt3	INSIDE	0.17	2.479	3514.11	8711.65	0.421	2617.59	1100.97

ProbeName	Target position of probe on CpG island microarray	TargetID	GeneSymbol	CpG island Description	Ratio of relative methylation (TiO_2-NP/Vehicle)	Sham group			TiO_2-H group		
						Relative methylation (Cy5/Cy3)	Cy3 signal (Input)	Cy5 signal (MeDIP)	Relative methylation (Cy5/Cy3)	Cy3 signal (Input)	Cy5 signal (MeDIP)
A_68_P28051873	chr11:97521768-97521812	NM_139311:-2935	Mllt6	PROMOTER	0.516	8.306	616.49	5120.89	4.284	604.56	2590.04
A_68_P24114755	chr5:135583039-135583083	NM_021455:278	Mlxipl	INSIDE	0.582	0.521	1318.08	686.65	0.303	1039.41	314.98
A_68_P24152507	chr5:143084713-143084757	NM_175217:-28	Mmd2	PROMOTER	0.192	0.581	2230.04	1296.42	0.112	1563.20	174.47
A_68_P22190750	chr3:63100022-63100066	NM_008604:251	Mme	INSIDE	0.276	10.390	1542.40	16026.10	2.867	1298.10	3721.88
A_68_P22190746	chr3:63099501-63099545	NM_008604:-271	Mme	PROMOTER	0.288	0.295	4355.10	1283.87	0.085	3612.81	306.22
A_68_P22190748	chr3:63099748-63099793	NM_008604:-23	Mme	PROMOTER	0.354	0.594	968.10	575.05	0.21	817.49	171.73
A_68_P27855086	chr11:62462305-62462349	NM_175002:161	Mmgt2	INSIDE	0.374	0.444	1499.83	665.42	0.166	1010.60	167.69
A_68_P26082829	chr8:97876209-97876253	NM_008609:-6	Mmp15	PROMOTER	0.529	4.510	1400.02	6313.53	2.384	1155.24	2754.50
A_68_P24089922	chr5:130089988-130090034	NM_011846:-78	Mmp17	PROMOTER	0.412	30.984	3412.34	105727.10	12.775	3403.04	43474.18
A_68_P21767513	chr2:155601572-155601616	NM_010808:515	Mmp24	INSIDE	0.119	2.271	8112.30	18424.15	0.271	5367.53	1452.54
A_68_P31092804	chr17:23781905-23781949	NM_001033339:310	Mmp25	INSIDE	0.189	1.741	1629.63	2836.38	0.329	1227.60	404.03
A_68_P27971658	chr11:83276246-83276290	NM_080453:195	Mmp28	INSIDE	0.642	3.104	703.24	2183.16	1.994	618.52	1233.18
A_68_P27971522	chr11:83256374-83256418	NM_080453:20067	Mmp28	INSIDE	0.573	2.523	1772.80	4472.32	1.445	1577.41	2278.91
A_68_P21820679	chr2:164775318-164775362	NM_013599:1590	Mmp9	INSIDE	0.372	0.463	1315.76	609.04	0.172	1018.09	175.15
A_68_P21820707	chr2:164778880-164778924	NM_013599:5152	Mmp9	INSIDE	0.535	0.681	902.60	615.08	0.365	714.38	260.48
A_68_P32118288	chr19:42055682-42055726	NM_028152:-78	Mms19	DIVERGENT_PROMOTER	0.514	3.902	1346.81	5255.75	2.005	1236.96	2480.30
A_68_P22785943	chr4:24423379-24423423	NM_199467:-208	Mms22l	PROMOTER	0.532	3.774	1526.65	5761.71	2.008	1328.16	2667.08
A_68_P23985683	chr5:111849467-111849511	NM_001081235:2303	Mn1	INSIDE	0.259	10.459	688.33	7199.54	2.709	572.82	1551.84
A_68_P23985655	chr5:111846403-111846447	NM_001081235:-761	Mn1	PROMOTER	0.61	4.213	945.06	3981.47	2.571	836.77	2151.52
A_68_P23985651	chr5:111845888-111845932	NM_001081235:-1275	Mn1	PROMOTER	0.235	1.634	1325.93	2166.79	0.384	1022.69	392.23
A_68_P22289190	chr3:83959655-83959699	NM_029797:32	Mnd1	INSIDE	0.548	0.725	2990.75	2167.02	0.397	2419.22	960.46
A_68_P26627767	chr9:72286337-72286381	NM_008613:23	Mns1	INSIDE	0.368	0.625	1009.94	631.07	0.23	764.76	175.81
A_68_P27922696	chr11:74645200-74645244	NM_010813:797	Mnt	INSIDE	0.252	10.725	694.65	7449.89	2.697	580.05	1564.55
A_68_P27922751	chr11:74651412-74651456	NM_010813:7009	Mnt	INSIDE	0.342	11.049	2332.06	25767.92	3.774	1987.40	7501.22
A_68_P27922756	chr11:74651953-74651997	NM_010813:7549	Mnt	INSIDE	0.407	5.905	2776.38	16394.53	2.406	2278.48	5481.98
A_68_P27922692	chr11:74644684-74644728	NM_010813:281	Mnt	INSIDE	0.418	6.940	2179.64	15125.77	2.902	1858.67	5394.01
A_68_P27922700	chr11:74645583-74645627	NM_010813:1179	Mnt	INSIDE	0.432	0.699	1375.58	961.56	0.302	988.67	298.35
A_68_P23567791	chr5:29805138-29805182	NM_019944:-150	Mnx1	PROMOTER	0.178	17.738	2291.31	40643.41	3.16	1840.17	5814.48
A_68_P23567792	chr5:29805215-29805259	NM_019944:-226	Mnx1	PROMOTER	0.454	0.261	2479.91	648.22	0.119	1911.14	226.83
A_68_P23567786	chr5:29804560-29804604	NM_019944:428	Mnx1	INSIDE	0.48	5.235	1586.83	8307.66	2.513	1363.35	3426.35
A_68_P23873662	chr5:89150059-89150103	NM_026735:185	Mobkl1a	INSIDE	0.342	20.193	3041.63	61421.08	6.915	2517.00	17404.73
A_68_P23873665	chr5:89150336-89150380	NM_026735:463	Mobkl1a	INSIDE	0.427	1.396	2896.28	4043.45	0.596	2209.38	1317.84
A_68_P24592782	chr6:83275935-83275979	NM_145571:-76	Mobkl1b	DIVERGENT_PROMOTER	0.611	3.478	1014.27	3527.39	2.126	852.49	1812.28
A_68_P23226013	chr4:115500963-115501007	NM_175308:288	Mobkl2c	INSIDE	0.579	0.737	2374.32	1748.69	0.426	1856.47	791.20
A_68_P23226010	chr4:115500724-115500768	NM_175308:50	Mobkl2c	INSIDE	0.664	0.551	1112.32	613.23	0.366	917.38	335.84
A_68_P20240461	chr1:55187949-55187993	NM_025283:-118	Mobkl3	PROMOTER	0.4	4.746	1084.20	5145.66	1.899	974.97	1851.88
A_68_P20240462	chr1:55188131-55188175	NM_025283:64	Mobkl3	INSIDE	0.275	5.569	1368.88	7623.68	1.534	1064.37	1632.68
A_68_P21840226	chr2:168056561-168056605	NM_001160330:461	Mocs3	INSIDE	0.424	8.767	459.21	4025.98	3.717	445.51	1655.75
A_68_P21840223	chr2:168056306-168056350	NM_001160330:207	Mocs3	INSIDE	0.529	5.556	502.57	2792.32	2.94	408.14	1199.95
A_68_P21840225	chr2:168056474-168056518	NM_001160330:375	Mocs3	INSIDE	0.541	3.585	739.16	2649.94	1.938	618.43	1198.80
A_68_P24591516	chr6:83065809-83065853	NM_020619:331	Mogs	INSIDE	0.417	3.558	1357.52	4829.48	1.484	1350.00	2003.40
A_68_P26811667	chr9:107803499-107803544	NM_028369:13062	Mon1a	INSIDE	0.147	35.845	1299.78	46590.01	5.271	1185.93	6250.71
A_68_P27512350	chr10:122513515-122513559	NM_001163024:25	Mon2	INSIDE	0.224	21.028	2602.11	54717.66	4.708	2375.13	11181.54
A_68_P27512349	chr10:122513403-122513447	NM_001163024:137	Mon2	INSIDE	0.475	7.389	2421.52	17893.38	3.51	2036.13	7147.15
A_68_P27553117	chr11:3549252-3549296	NM_001159288:-222	Morc2a	DIVERGENT_PROMOTER	0.61	0.263	1689.83	445.08	0.161	1329.54	213.46
A_68_P26716012	chr9:90009520-90009564	NM_001039147:116	Morf4l1	INSIDE	0.495	0.700	4601.79	3223.35	0.347	3379.36	1171.99

ProbeName	Target position of probe on CpG island microarray	TargetID	GeneSymbol	CpG island Description	Ratio of relative methylation (TiO_2-NP/Vehicle)	Sham group			TiO_2-H group		
						Relative methylation (Cy5/Cy3)	Cy3 signal (Input)	Cy5 signal (MeDIP)	Relative methylation (Cy5/Cy3)	Cy3 signal (Input)	Cy5 signal (MeDIP)
A_68_P31382195	chr17:80689441-80689485	NM_194269:-89	Morn2	PROMOTER	0.268	13.105	1218.17	15963.82	3.512	1152.31	4046.59
A_68_P20897789	chr1:186669350-186669394	NM_133684:354	Mosc2	INSIDE	0.158	15.632	5620.81	87861.90	2.469	4684.37	11564.24
A_68_P24126542	chr5:138041887-138041931	NM_030037:360	Mospd3	INSIDE	0.318	0.542	3610.33	1957.90	0.173	3050.06	526.71
A_68_P24126543	chr5:138042018-138042062	NM_030037:228	Mospd3	INSIDE	0.538	0.520	1645.41	854.80	0.28	1269.11	355.01
A_68_P24126541	chr5:138041805-138041849	NM_030037:442	Mospd3	INSIDE	0.612	0.083	7389.10	616.22	0.051	5168.69	263.95
A_68_P31677060	chr18:46625364-46625408	NM_028086:84	Mospd4	INSIDE	0.18	2.770	2856.79	7912.30	0.498	2310.71	1149.82
A_68_P22393343	chr3:104620941-104620985	NM_001163441:261	Mov10	INSIDE	0.319	0.506	1270.69	643.24	0.162	1047.75	169.32
A_68_P23051091	chr4:81088361-81088405	NM_010820:327	Mpdz	INSIDE	0.205	11.655	10529.43	122723.40	2.385	9551.56	22781.92
A_68_P25176744	chr7:71536910-71536954	NM_026483:190	Mphosph10	INSIDE	0.618	2.574	2038.22	5246.48	1.591	1557.27	2477.57
A_68_P28077433	chr11:101948728-101948772	NM_016695:1079	Mpp2	INSIDE	0.314	0.297	4112.29	1221.84	0.093	2491.50	232.15
A_68_P28565714	chr12:79850102-79850146	NM_019579:191	Mpp5	INSIDE	0.283	5.338	1378.34	7357.55	1.511	1225.74	1851.99
A_68_P31790832	chr18:67404825-67404869	NM_172630:638	Mppe1	INSIDE	0.237	6.475	2007.76	13001.15	1.533	1650.16	2529.52
A_68_P30359772	chr15:78241077-78241121	NM_138670:3565	Mpst	INSIDE	0.62	0.635	1534.70	975.17	0.394	1070.64	421.61
A_68_P26764036	chr9:99336932-99336976	NM_008624:177	Mras	INSIDE	0.406	7.841	3431.79	26908.26	3.187	2799.93	8924.09
A_68_P26319360	chr9:14589324-14589368	NM_018736:196	Mre11a	INSIDE	0.246	13.984	5131.82	71762.65	3.434	4331.26	14874.07
A_68_P25394539	chr7:112959728-112959772	NM_025301:-149	Mrpl17	PROMOTER	0.585	0.175	10857.93	1902.30	0.103	7131.32	731.36
A_68_P23579049	chr5:31916560-31916604	NM_025796:259	Mrpl33	INSIDE	0.601	0.591	854.82	504.93	0.355	752.00	266.75
A_68_P23188353	chr4:106739305-106739349	NM_025500:145	Mrpl37	INSIDE	0.592	0.723	1482.67	1072.16	0.428	1289.58	551.93
A_68_P28157575	chr11:115999733-115999777	NM_024177:428	Mrpl38	INSIDE	0.277	1.360	2372.16	3226.34	0.377	1807.04	680.56
A_68_P26344986	chr9:20807641-20807685	NM_023167:482	Mrpl4	INSIDE	0.457	0.419	2562.53	1072.48	0.191	1838.80	351.39
A_68_P25368691	chr7:107757091-107757135	NR_003559:-602	Mrpl48	PROMOTER	0.33	0.484	1601.95	775.17	0.16	1226.33	196.07
A_68_P24591425	chr6:83051529-83051573	NM_026744:-7551	Mrpl53	PROMOTER	0.389	0.448	1527.82	684.99	0.174	1100.92	192.09
A_68_P20765302	chr1:162125324-162125368	NM_025474:-44	Mrps14	PROMOTER	0.629	21.409	4047.51	86652.27	13.461	4112.07	55352.12
A_68_P23931503	chr5:101227718-101227762	NM_026826:-37	Mrps18c	DIVERGENT_PROMOTER	0.395	0.328	2436.45	798.72	0.13	1640.28	212.58
A_68_P26759567	chr9:98501957-98502001	NM_025485:120	Mrps22	INSIDE	0.348	6.257	1469.70	9195.37	2.176	1239.45	2697.07
A_68_P27998475	chr11:88018009-88018053	NM_024174:111	Mrps23	INSIDE	0.48	0.282	2405.16	677.29	0.135	1698.42	229.61
A_68_P21630447	chr2:130390003-130390047	NM_207207:532	Mrps26	INSIDE	0.072	32.650	1939.58	63328.33	2.359	1571.25	3707.26
A_68_P21934677	chr3:8923780-8923824	NM_025434:55	Mrps28	INSIDE	0.588	3.719	1338.59	4977.89	2.187	1187.09	2596.25
A_68_P29368026	chr13:119175523-119175567	NM_021556:515	Mrps30	INSIDE	0.273	9.208	1088.47	10023.10	2.514	856.55	2153.60
A_68_P24385095	chr6:39760829-39760873	NM_001010930:85	Mrps33	INSIDE	0.47	0.712	3420.69	2434.71	0.334	2710.92	906.07
A_68_P28154361	chr11:115465384-115465428	NM_025305:-58	Mrps7	DIVERGENT_PROMOTER	0.424	0.668	815.19	544.45	0.283	639.34	180.93
A_68_P20185544	chr1:42908420-42908464	NM_023514:365	Mrps9	INSIDE	0.393	0.526	1053.88	554.65	0.207	828.80	171.58
A_68_P20185543	chr1:42908342-42908386	NM_023514:287	Mrps9	INSIDE	0.572	0.289	1784.00	516.04	0.166	1331.06	220.35
A_68_P31157234	chr17:35183191-35183235	NM_013600:339	Msh5	INSIDE	0.37	4.956	1062.51	5265.27	1.833	949.28	1740.47
A_68_P24009622	chr5:115896965-115897009	NM_008629:17293	Msi1	INSIDE	0.507	8.516	639.15	5442.84	4.319	467.97	2021.01
A_68_P24009562	chr5:115886952-115886996	NM_008629:7281	Msi1	INSIDE	0.598	3.455	455.65	1574.34	2.065	391.78	808.96
A_68_P28058586	chr11:98656221-98656265	NM_028722:-840	Msl1	PROMOTER	0.382	0.286	5337.08	1527.85	0.109	3414.89	373.88
A_68_P26772685	chr9:100978043-100978087	NM_001100451:-386	Msl2	PROMOTER	0.535	0.200	2351.25	469.41	0.107	1649.39	176.18
A_68_P32817209	chrX:165111905-165111949	NM_010832:-92	Msl3	PROMOTER	0.306	7.186	757.40	5442.37	2.199	931.82	2049.40
A_68_P27154484	chr10:55835338-55835382	NM_001163833:8638	Msl3l2	INSIDE	0.146	4.084	6078.34	24822.20	0.595	4652.99	2768.39
A_68_P21044522	chr2:19293408-19293452	NM_029619:168	Msrb2	INSIDE	0.441	0.198	2889.29	572.07	0.087	1998.02	174.48
A_68_P27500269	chr10:120335461-120335505	NM_177092:545	Msrb3	INSIDE	0.434	3.899	2111.37	8232.64	1.693	1719.39	2910.31
A_68_P29044041	chr13:53558129-53558173	NM_013601:9999	Msx2	DOWNSTREAM	0.59	8.528	1118.99	9542.65	5.028	771.19	3877.56
A_68_P25092690	chr7:52622810-52622854	NR_027802:230	Mtag2	INSIDE	0.309	8.735	2435.59	21275.01	2.699	2060.03	5559.04
A_68_P21579980	chr2:121115168-121115212	NM_032393:-147	Mtap1a	PROMOTER	0.571	0.671	1569.84	1053.97	0.384	1188.73	456.07
A_68_P29263993	chr13:100286596-100286640	NM_008634:-61	Mtap1b	PROMOTER	0.649	2.943	1197.47	3524.38	1.91	969.09	1850.65

ProbeName	Target position of probe on CpG island microarray	TargetID	GeneSymbol	CpG island Description	Ratio of relative methylation (TiO_2-NP/Vehicle)	Sham group			TiO_2-H group		
						Relative methylation (Cy5/Cy3)	Cy3 signal (Input)	Cy5 signal (MeDIP)	Relative methylation (Cy5/Cy3)	Cy3 signal (Input)	Cy5 signal (MeDIP)
A_68_P29263992	chr13:100286417-100286461	NM_008634:119	Mtap1b	INSIDE	0.482	3.556	1346.76	4789.54	1.714	1241.29	2127.29
A_68_P25954856	chr8:73430275-73430319	NM_173013:424	Mtap1s	INSIDE	0.019	1.903	7734.20	14719.17	0.036	4897.58	176.30
A_68_P25954897	chr8:73438085-73438129	NM_173013:8234	Mtap1s	INSIDE	0.57	8.199	4707.81	38600.73	4.672	3053.63	14265.22
A_68_P26821066	chr9:109834856-109834900	NM_001205331:601	Mtap4	INSIDE	0.266	0.149	6203.87	922.14	0.04	4381.75	173.28
A_68_P26821065	chr9:109834739-109834783	NM_001205331:483	Mtap4	INSIDE	0.449	0.547	2693.17	1473.96	0.246	2155.28	530.18
A_68_P26821058	chr9:109833931-109833975	NM_001205331:-325	Mtap4	PROMOTER	0.611	2.959	1437.11	4251.69	1.807	1262.52	2281.62
A_68_P25360757	chr7:106417697-106417741	NM_001043355:863	Mtap6	INSIDE	0.521	8.783	2466.05	21659.86	4.579	2227.02	10197.98
A_68_P25360742	chr7:106416261-106416305	NM_001043355:-573	Mtap6	PROMOTER	0.301	0.707	1197.08	846.93	0.213	949.60	202.53
A_68_P25360741	chr7:106416185-106416229	NM_001043355:-649	Mtap6	PROMOTER	0.565	0.611	848.42	518.03	0.345	699.10	241.33
A_68_P26985109	chr10:19868572-19868616	NM_001198635:-131	Mtap7	PROMOTER	0.351	13.871	1039.74	14422.10	4.871	913.19	4447.81
A_68_P23278957	chr4:125914290-125914334	NM_001145970:19251	Mtap7d1	INSIDE	0.344	5.891	1505.06	8866.39	2.026	1519.59	3078.45
A_68_P23278955	chr4:125914068-125914112	NM_001145970:19473	Mtap7d1	INSIDE	0.41	4.490	1638.00	7354.78	1.842	1646.56	3032.50
A_68_P23279068	chr4:125932630-125932674	NM_001145970:911	Mtap7d1	INSIDE	0.42	0.372	6210.38	2307.54	0.156	4088.77	638.75
A_68_P32774619	chrX:155852596-155852640	NM_001081124:109	Mtap7d2	INSIDE	0.521	4.757	937.89	4461.50	2.478	1107.55	2744.71
A_68_P22278905	chr3:82162143-82162187	NM_001081230:171	Mtap9	INSIDE	0.444	0.402	1391.87	559.51	0.179	1036.93	185.22
A_68_P22278904	chr3:82162070-82162114	NM_001081230:99	Mtap9	INSIDE	0.512	38.631	804.21	31067.63	19.785	1118.44	22128.60
A_68_P30232957	chr15:55389253-55389297	NM_001168250:312	Mtbp	INSIDE	0.665	0.548	2715.69	1487.23	0.364	1897.42	690.55
A_68_P31127484	chr17:29484749-29484793	NM_019880:79	Mtch1	INSIDE	0.515	6.177	1359.77	8399.43	3.184	1143.48	3640.84
A_68_P29106491	chr13:67034356-67034400	NM_025547:-370	Mterfd1	PROMOTER	0.56	4.029	534.17	2152.17	2.256	534.77	1206.35
A_68_P20454693	chr1:95202297-95202341	NM_178051:129	Mterfd2	INSIDE	0.658	0.447	3350.82	1496.37	0.294	2379.30	699.51
A_68_P21973912	chr3:19088304-19088348	NM_026182:83	Mtfr1	INSIDE	0.187	2.610	3631.34	9476.04	0.489	2644.68	1293.72
A_68_P27674920	chr11:29426294-29426338	NM_133767:-140	Mtif2	PROMOTER	0.553	0.482	1472.68	709.92	0.266	1174.59	312.91
A_68_P24171031	chr5:147775224-147775268	NM_029581:104	Mtif3	INSIDE	0.443	0.506	3644.80	1846.03	0.224	2536.71	568.80
A_68_P32453217	chrX:68617827-68617871	NM_016985:-86	Mtmr1	PROMOTER	0.242	13.437	1953.32	26247.50	3.247	2256.72	7326.90
A_68_P25176079	chr7:71432668-71432712	NM_172742:135	Mtmr10	INSIDE	0.485	4.020	1592.95	6403.62	1.948	1247.80	2430.94
A_68_P30020394	chr15:12134922-12134966	NM_172958:96	Mtmr12	INSIDE	0.465	4.082	942.82	3848.56	1.899	867.82	1648.38
A_68_P24758234	chr6:113187868-113187912	NM_026849:54	Mtmr14	INSIDE	0.39	0.215	3153.46	677.65	0.084	2065.13	173.07
A_68_P31462885	chr18:4375737-4375781	NM_026157:169	Mtpap	INSIDE	0.605	0.453	1370.29	621.18	0.274	1001.43	274.53
A_68_P24362057	chr6:35489865-35489909	NM_008098:2	Mtpn	INSIDE	0.23	11.848	2004.06	23743.21	2.726	2014.68	5491.32
A_68_P26905236	chr10:4522611-4522655	NM_175374:35	Mtrf1l	INSIDE	0.464	7.259	1636.62	11880.41	3.37	1379.61	4649.49
A_68_P29110675	chr13:68720936-68720980	NM_172480:40	Mtrr	INSIDE	0.451	0.350	2022.52	707.42	0.158	1425.71	224.75
A_68_P30252145	chr15:58913581-58913625	NM_001146180:-21	Mtss1	PROMOTER	0.25	13.211	3144.28	41539.66	3.298	2840.09	9367.04
A_68_P30252139	chr15:58912975-58913019	NM_001146180:585	Mtss1	INSIDE	0.552	8.307	2403.37	19964.47	4.586	2081.23	9544.50
A_68_P30252138	chr15:58912850-58912894	NM_001146180:709	Mtss1	INSIDE	0.514	2.983	1156.44	3449.76	1.535	949.89	1457.62
A_68_P26164572	chr8:113262808-113262852	NM_198625:17447	Mtss1l	INSIDE	0.017	193.881	1840.55	356846.70	3.239	2084.88	6753.17
A_68_P26164573	chr8:113262875-113262919	NM_198625:17513	Mtss1l	INSIDE	0.021	105.656	3149.55	332767.40	2.261	2838.32	6418.79
A_68_P26164568	chr8:113262460-113262504	NM_198625:17099	Mtss1l	INSIDE	0.411	26.427	5706.20	150795.70	10.854	4326.60	46958.93
A_68_P26164571	chr8:113262720-113262764	NM_198625:17359	Mtss1l	INSIDE	0.532	3.377	904.35	3054.45	1.798	678.95	1220.54
A_68_P25809389	chr8:42218752-42218796	NM_001005863:306	Mtus1	INSIDE	0.25	2.070	2426.59	5023.81	0.517	1885.11	974.86
A_68_P25809394	chr8:42219374-42219418	NM_001005863:-316	Mtus1	PROMOTER	0.65	0.680	1593.53	1083.59	0.442	1258.88	556.11
A_68_P24176699	chr5:148769334-148769378	NM_029920:461	Mtus2	INSIDE	0.307	20.897	4254.09	88899.27	6.406	3833.29	24556.72
A_68_P24176695	chr5:148768968-148769012	NM_029920:95	Mtus2	INSIDE	0.665	0.566	1745.54	988.58	0.376	1468.09	552.62
A_68_P24176700	chr5:148769468-148769512	NM_029920:595	Mtus2	INSIDE	0.5	0.646	1699.14	1097.18	0.323	1281.07	413.73
A_68_P24176698	chr5:148769249-148769293	NM_029920:375	Mtus2	INSIDE	0.559	0.467	1047.25	489.00	0.261	815.38	212.72
A_68_P29226007	chr13:93614754-93614798	NM_001162945:35	Mtx3	INSIDE	0.146	22.334	1251.02	27940.75	3.262	1134.97	3702.12
A_68_P23345719	chr4:137990637-137990681	NM_026689:72	Mul1	INSIDE	0.537	3.336	3149.25	10506.10	1.79	2473.46	4428.47

ProbeName	Target position of probe on CpG island microarray	TargetID	GeneSymbol	CpG island Description	Ratio of relative methylation (TiO_2-NP/Vehicle)	Sham group			TiO_2-H group		
						Relative methylation (Cy5/Cy3)	Cy3 signal (Input)	Cy5 signal (MeDIP)	Relative methylation (Cy5/Cy3)	Cy3 signal (Input)	Cy5 signal (MeDIP)
A_68_P27281785	chr10:79689246-79689290	NM_023431:-74	Mum1	PROMOTER	0.509	6.414	6822.97	43760.36	3.266	5081.53	16598.51
A_68_P28961841	chr13:38726802-38726846	NM_139063:154	Muted	INSIDE	0.442	0.642	2275.12	1461.65	0.284	1757.32	498.83
A_68_P23231034	chr4:116480288-116480332	NM_001159581:-28	Mutyh	DIVERGENT_PROMOTER	0.472	3.420	3295.33	11270.52	1.613	2643.21	4262.61
A_68_P24611353	chr6:86619098-86619142	NM_010751:33	Mxd1	INSIDE	0.353	13.366	10836.90	144842.90	4.72	9986.21	47139.07
A_68_P29054388	chr13:55431309-55431353	NM_016662:-239	Mxd3	PROMOTER	0.585	6.457	1210.70	7817.59	3.778	1170.70	4422.70
A_68_P23592883	chr5:34529870-34529914	NM_010753:467	Mxd4	INSIDE	0.117	2.098	1356.88	2846.45	0.245	1181.48	289.47
A_68_P32181743	chr19:53388066-53388111	NM_001008542:3093	Mxi1	INSIDE	0.271	0.397	1925.78	763.86	0.107	1585.55	170.17
A_68_P32181854	chr19:53403918-53403962	NM_001008543:-995	Mxi1	PROMOTER	0.294	5.539	1075.23	5955.43	1.626	975.99	1586.84
A_68_P26991379	chr10:20879243-20879287	NM_001198914:1526	Myb	INSIDE	0.575	7.373	1565.43	11542.36	4.237	1389.64	5887.99
A_68_P30267513	chr15:61816737-61816781	NM_001177352:-137	Myc	PROMOTER	0.147	14.380	3677.40	52880.90	2.116	3248.65	6874.03
A_68_P30267538	chr15:61819548-61819592	NM_001177352:2675	Myc	INSIDE	0.389	3.907	3138.99	12264.02	1.521	2562.81	3898.58
A_68_P30267527	chr15:61818322-61818366	NM_001177352:1449	Myc	INSIDE	0.382	1.390	1245.38	1731.34	0.531	1068.24	567.42
A_68_P29865209	chr14:103745828-103745872	NM_207215:167	Mycbp2	INSIDE	0.572	0.717	2081.61	1493.20	0.41	1532.16	628.17
A_68_P29865204	chr14:103745283-103745327	NM_207215:713	Mycbp2	INSIDE	0.476	1.410	1757.03	2477.14	0.671	1480.02	992.67
A_68_P23260191	chr4:122672828-122672872	NM_008506:-491	Mycl1	PROMOTER	0.555	21.944	3224.91	70768.91	12.183	3065.06	37340.68
A_68_P23260196	chr4:122673426-122673470	NM_008506:107	Mycl1	INSIDE	0.125	12.941	3046.31	39423.20	1.615	3553.71	5740.89
A_68_P23260198	chr4:122673656-122673700	NM_008506:337	Mycl1	INSIDE	0.158	9.716	1076.52	10459.42	1.535	914.51	1403.55
A_68_P28246307	chr12:12948424-12948468	NM_008709:196	Mycn	INSIDE	0.37	0.516	1057.00	544.99	0.191	916.74	174.89
A_68_P28246269	chr12:12944121-12944165	NM_008709:4500	Mycn	INSIDE	0.474	6.381	779.67	4975.32	3.022	669.38	2022.59
A_68_P28246291	chr12:12946601-12946645	NM_008709:2020	Mycn	INSIDE	0.624	0.624	2212.10	1380.89	0.389	1506.87	586.72
A_68_P28246293	chr12:12946958-12947002	NM_008709:1662	Mycn	INSIDE	0.452	0.636	982.02	624.86	0.288	836.84	240.93
A_68_P28246270	chr12:12944193-12944237	NM_008709:4428	Mycn	INSIDE	0.558	2.705	1461.52	3952.69	1.51	1162.05	1754.25
A_68_P32231475	chrX:5045956-5046000	NM_010850:405	Mycs	INSIDE	0.579	3.136	452.37	1418.66	1.814	515.62	935.50
A_68_P20450137	chr1:94538493-94538537	NR_028108:11	Myeov2	INSIDE	0.589	0.180	3499.92	631.69	0.106	2770.20	294.64
A_68_P30497295	chr15:102162385-102162429	NM_021713:267	Mygl	INSIDE	0.536	0.453	3249.04	1471.44	0.243	2375.77	577.07
A_68_P27890688	chr11:68505670-68505714	NM_175260:276	Myh10	INSIDE	0.329	0.517	4356.62	2254.50	0.17	3124.73	532.02
A_68_P27890689	chr11:68505755-68505799	NM_175260:360	Myh10	INSIDE	0.421	1.525	3915.85	5972.21	0.642	2834.94	1821.08
A_68_P25088768	chr7:51924794-51924838	NM_028021:-3943	Myh14	PROMOTER	0.211	15.416	1825.84	28146.26	3.258	1429.82	4658.40
A_68_P25088776	chr7:51926206-51926250	NM_028021:-5355	Myh14	PROMOTER	0.663	12.010	1394.28	16744.59	7.957	1172.68	9331.54
A_68_P21766702	chr2:155455115-155455159	NM_001085378:18189	Myh7b	INSIDE	0.464	6.282	3010.77	18914.98	2.918	2848.95	8313.32
A_68_P30356228	chr15:77672759-77672803	NM_022410:-235	Myh9	PROMOTER	0.241	19.914	7216.80	143716.80	4.804	6059.97	29113.77
A_68_P30356227	chr15:77672647-77672691	NM_022410:-123	Myh9	PROMOTER	0.271	11.950	5006.12	59822.93	3.238	4383.52	14193.62
A_68_P29001573	chr13:45484920-45484964	NM_153789:-168	Mylip	PROMOTER	0.519	3.960	1841.04	7289.92	2.056	1532.36	3150.05
A_68_P22029229	chr3:30501750-30501794	NM_030557:764	Mynn	INSIDE	0.64	0.613	1888.13	1156.60	0.392	1555.51	609.73
A_68_P30082169	chr15:25552086-25552130	NM_019472:-196	Myo10	PROMOTER	0.569	3.366	14492.29	48780.82	1.916	10889.22	20861.56
A_68_P30082783	chr15:25643838-25643882	NM_019472:91556	Myo10	INSIDE	0.314	1.836	2350.96	4317.50	0.577	1648.10	951.27
A_68_P27927426	chr11:75469605-75469649	NM_001080775:110	Myo1c	INSIDE	0.255	8.039	797.52	6411.55	2.047	645.36	1320.90
A_68_P27927427	chr11:75469683-75469727	NM_001080775:188	Myo1c	INSIDE	0.292	4.752	4891.53	23245.95	1.39	3727.55	5181.06
A_68_P27568981	chr11:6428736-6428780	NM_178440:-7797	Myo1g	PROMOTER	0.432	0.413	1277.90	527.96	0.178	991.75	176.86
A_68_P26641829	chr9:74918724-74918768	NM_010864:-266	Myo5a	PROMOTER	0.563	0.625	1686.43	1053.79	0.352	1376.76	484.42
A_68_P26641831	chr9:74919027-74919071	NM_010864:36	Myo5a	INSIDE	0.508	0.497	878.96	437.07	0.253	740.52	187.03
A_68_P31831023	chr18:74602569-74602613	NM_201600:318	Myo5b	INSIDE	0.191	14.686	469.93	6901.27	2.8	441.80	1236.91
A_68_P26555224	chr9:59598868-59598912	NM_173018:-90	Myo9a	DIVERGENT_PROMOTER	0.191	0.256	4671.39	1195.67	0.049	3527.30	172.45
A_68_P29448043	chr14:21459839-21459883	NM_021508:15902	Myoz1	DOWNSTREAM	0.202	1.820	6470.88	11777.94	0.367	4777.25	1753.79
A_68_P24990788	chr7:19577261-19577305	NM_145579:689	Mypop	INSIDE	0.469	0.270	1986.25	535.83	0.126	1417.64	179.24
A_68_P25713191	chr8:23969515-23969559	NM_001081149:-474	Myst3	PROMOTER	0.516	3.950	553.11	2184.96	2.037	591.03	1204.08

ProbeName	Target position of probe on CpG island microarray	TargetID	GeneSymbol	CpG island Description	Ratio of relative methylation (TiO_2-NP/Vehicle)	Sham group			TiO_2-H group		
						Relative methylation (Cy5/Cy3)	Cy3 signal (Input)	Cy5 signal (MeDIP)	Relative methylation (Cy5/Cy3)	Cy3 signal (Input)	Cy5 signal (MeDIP)
A_68_P29453025	chr14:22319120-22319164	NM_017479:136	Myst4	INSIDE	0.322	0.360	2412.67	869.60	0.116	1703.23	197.85
A_68_P29453036	chr14:22320298-22320342	NM_017479:1314	Myst4	INSIDE	0.613	5.171	1494.11	7725.64	3.17	1339.32	4246.18
A_68_P29453027	chr14:22319300-22319344	NM_017479:316	Myst4	INSIDE	0.615	0.468	2190.32	1025.05	0.288	1686.73	485.09
A_68_P29453037	chr14:22320470-22320514	NM_017479:1486	Myst4	INSIDE	0.616	16.195	5806.59	94039.73	9.977	5067.49	50556.74
A_68_P29453022	chr14:22318726-22318770	NM_001205241:-243	Myst4	PROMOTER	0.617	0.612	2935.38	1795.20	0.377	2104.24	794.22
A_68_P22868863	chr4:42929796-42929840	NM_172690:-304	N28178	PROMOTER	0.593	3.316	570.40	1891.47	1.967	528.59	1039.52
A_68_P23757989	chr5:66155461-66155505	NM_001024917:723	N4bp2	INSIDE	0.368	7.259	1692.23	12284.20	2.671	1437.58	3839.27
A_68_P23912411	chr5:97821152-97821196	NM_001033191:175	Naa11	INSIDE	0.606	3.772	1414.03	5333.83	2.285	1092.98	2497.99
A_68_P22129522	chr3:51220411-51220455	NM_053089:495	Naa15	INSIDE	0.333	7.913	2752.13	21778.76	2.636	2590.45	6828.50
A_68_P29080348	chr13:59686650-59686694	NM_030153:-21	Naa35	PROMOTER	0.259	22.863	1997.25	45663.33	5.927	1619.67	9600.59
A_68_P20227608	chr1:52557632-52557676	NM_008667:-362	Nab1	PROMOTER	0.516	3.175	1515.43	4811.50	1.638	1379.50	2260.18
A_68_P27537384	chr10:127104122-127104166	NM_001122895:-385	Nab2	DIVERGENT_PROMOTER	0.631	0.603	1096.99	661.29	0.38	956.88	363.85
A_68_P27539155	chr10:127472881-127472925	NM_001113199:501	Naca	INSIDE	0.61	0.423	1367.98	578.81	0.258	1110.02	286.28
A_68_P27539153	chr10:127472665-127472709	NM_001113199:285	Naca	INSIDE	0.621	0.723	4190.81	3028.70	0.449	2684.43	1204.30
A_68_P27569379	chr11:6505657-6505701	NM_001081652:379	Nacad	INSIDE	0.232	35.660	3979.22	141899.00	8.259	3910.03	32291.72
A_68_P23439342	chr4:154938332-154938376	NM_001159637:431	Nadk	INSIDE	0.639	0.469	1458.15	683.82	0.3	1189.20	356.32
A_68_P25934950	chr8:69384793-69384837	NM_001163564:699	Naf1	INSIDE	0.549	2.752	2119.25	5832.72	1.51	1997.96	3017.46
A_68_P30382866	chr15:82169438-82169482	NM_008669:-204	Naga	DIVERGENT_PROMOTER	0.429	3.981	1002.77	3991.89	1.71	853.42	1459.09
A_68_P28071734	chr11:100931359-100931403	NM_013792:-27	Naglu	PROMOTER	0.336	5.038	1188.94	5989.80	1.694	1061.19	1797.71
A_68_P30514378	chr16:5203992-5204036	NM_013796:91	Nagpa	INSIDE	0.545	6.661	3376.19	22488.63	3.629	2947.54	10696.80
A_68_P30514376	chr16:5203769-5203813	NM_013796:315	Nagpa	INSIDE	0.465	3.257	1369.30	4460.46	1.514	1033.53	1564.34
A_68_P28077768	chr11:102007243-102007287	NM_178053:-415	Nags	PROMOTER	0.607	0.233	1914.30	445.58	0.141	1210.67	171.19
A_68_P32226993	chr19:60831736-60831780	NM_178421:-131	Nanos1	PROMOTER	0.198	25.627	9787.45	250827.80	5.084	8785.24	44665.91
A_68_P32227001	chr19:60832863-60832907	NM_178421:995	Nanos1	INSIDE	0.394	0.274	2197.98	601.42	0.108	1633.49	176.11
A_68_P32226999	chr19:60832637-60832681	NM_178421:769	Nanos1	INSIDE	0.588	0.579	1817.98	1051.75	0.34	1410.41	479.50
A_68_P21742434	chr2:150864733-150864777	NM_026086:361	Nanp	INSIDE	0.247	10.563	852.90	9009.42	2.611	683.71	1785.26
A_68_P22889024	chr4:46502472-46502516	NM_053179:294	Nans	INSIDE	0.187	0.379	11568.22	4387.92	0.071	8142.36	577.58
A_68_P22889023	chr4:46502385-46502429	NM_053179:206	Nans	INSIDE	0.347	0.299	7126.86	2130.11	0.104	4817.26	499.71
A_68_P22889022	chr4:46502288-46502332	NM_053179:110	Nans	INSIDE	0.611	0.408	1653.33	674.08	0.249	1239.56	308.85
A_68_P25601671	chr7:150734944-150734988	NM_008672:29	Nap1l4	INSIDE	0.505	0.619	2951.63	1827.48	0.313	2493.99	779.51
A_68_P24979588	chr7:16683856-16683900	NM_025898:-113	Napa	PROMOTER	0.552	0.396	1921.39	761.43	0.219	1502.47	328.54
A_68_P24979593	chr7:16684397-16684441	NM_025898:427	Napa	INSIDE	0.648	0.484	1728.52	836.25	0.313	1423.04	446.05
A_68_P23527254	chr5:21181777-21181821	NM_178728:25365	Napepld	INSIDE	0.311	4.439	3823.06	16969.91	1.38	2411.76	3328.97
A_68_P23527255	chr5:21181848-21181895	NM_178728:25292	Napepld	INSIDE	0.541	3.669	584.15	2143.50	1.984	400.49	794.75
A_68_P30344754	chr15:75724482-75724526	NM_172607:407	Naprt1	INSIDE	0.525	0.324	1977.77	640.17	0.17	1579.36	268.41
A_68_P30344755	chr15:75724584-75724628	NM_172607:305	Naprt1	INSIDE	0.541	0.317	5450.65	1729.04	0.172	3968.47	680.70
A_68_P30344756	chr15:75724658-75724702	NM_172607:231	Naprt1	INSIDE	0.654	0.264	4065.70	1074.06	0.173	2855.64	493.66
A_68_P30344676	chr15:75714693-75714737	NM_172607:10197	Naprt1	DOWNSTREAM	0.347	1.415	1575.12	2228.67	0.491	1370.77	672.60
A_68_P30506992	chr16:3884518-3884562	NM_029090:-78	Nat15	PROMOTER	0.607	2.477	1320.75	3271.31	1.503	1052.27	1581.39
A_68_P23592057	chr5:34343782-34343826	NM_001001985:5172	Nat8l	INSIDE	0.258	27.305	11547.77	315315.90	7.042	13848.50	97519.56
A_68_P23592019	chr5:34339056-34339100	NM_001001985:446	Nat8l	INSIDE	0.454	0.532	3556.52	1891.58	0.241	2527.11	609.87
A_68_P23592017	chr5:34338746-34338790	NM_001001985:136	Nat8l	INSIDE	0.562	0.605	1210.36	732.41	0.34	843.35	287.05
A_68_P23592026	chr5:34340043-34340087	NM_001001985:1432	Nat8l	INSIDE	0.655	3.630	413.70	1501.55	2.376	390.95	928.97
A_68_P20640026	chr1:137429314-137429358	NM_173437:52596	Nav1	INSIDE	0.483	6.512	2271.82	14793.51	3.145	1994.12	6270.63
A_68_P20640400	chr1:137481754-137481798	NM_173437:156	Nav1	INSIDE	0.644	2.835	1100.91	3121.40	1.825	1033.12	1885.40
A_68_P20640025	chr1:137429239-137429283	NM_173437:52672	Nav1	INSIDE	0.401	3.588	2759.00	9898.07	1.44	1981.09	2852.38

ProbeName	Target position of probe on CpG island microarray	TargetID	GeneSymbol	CpG island Description	Ratio of relative methylation (TiO_2-NP/Vehicle)	Sham group			TiO_2-H group		
						Relative methylation (Cy5/Cy3)	Cy3 signal (Input)	Cy5 signal (MeDIP)	Relative methylation (Cy5/Cy3)	Cy3 signal (Input)	Cy5 signal (MeDIP)
A_68_P25111224	chr7:56501224-56501268	NM_175272:-312	Nav2	PROMOTER	0.108	3.907	2064.35	8065.19	0.42	1535.32	645.25
A_68_P22157978	chr3:55987140-55987184	NM_030595:461	Nbea	INSIDE	0.561	8.086	1297.98	10495.14	4.534	1054.17	4780.10
A_68_P22157981	chr3:55987567-55987611	NM_030595:35	Nbea	INSIDE	0.655	4.632	1531.89	7095.84	3.034	1285.81	3900.77
A_68_P22157982	chr3:55987695-55987739	NM_030595:-93	Nbea	PROMOTER	0.364	3.992	2641.91	10546.02	1.455	2028.54	2950.88
A_68_P26824824	chr9:110532605-110532649	NM_183276:24039	Nbeal2	INSIDE	0.382	4.705	3282.21	15444.19	1.796	2716.56	4877.96
A_68_P26825022	chr9:110556586-110556630	NM_183276:57	Nbeal2	INSIDE	0.369	1.452	1661.02	2411.96	0.536	1260.76	675.89
A_68_P22752524	chr4:15885248-15885292	NM_013752:157	Nbn	INSIDE	0.274	12.495	1486.88	18578.71	3.429	1308.66	4487.30
A_68_P22752527	chr4:15885475-15885519	NM_013752:383	Nbn	INSIDE	0.547	0.507	1173.18	595.04	0.277	980.94	272.14
A_68_P26500013	chr9:49606044-49606088	NM_001081445:1108	Ncam1	INSIDE	0.301	20.268	5898.36	119548.20	6.097	4574.99	27895.24
A_68_P26500007	chr9:49605368-49605412	NM_001081445:1784	Ncam1	INSIDE	0.533	3.901	1140.33	4448.77	2.078	1037.64	2155.76
A_68_P26500009	chr9:49605633-49605677	NM_001081445:1520	Ncam1	INSIDE	0.548	0.402	1145.20	460.38	0.22	785.22	172.84
A_68_P25949903	chr8:72635987-72636031	NM_007789:8735	Ncan	INSIDE	0.471	5.904	2933.55	17318.80	2.78	2492.03	6927.56
A_68_P25949910	chr8:72636709-72636753	NM_007789:8013	Ncan	INSIDE	0.573	0.415	1865.75	773.94	0.238	1415.27	336.17
A_68_P30424721	chr15:89186360-89186404	NM_001115132:230	Ncaph2	INSIDE	0.136	28.479	3458.44	98491.17	3.877	2896.90	11232.48
A_68_P23281848	chr4:126430490-126430534	NM_011986:161	Ncdn	INSIDE	0.386	0.242	2370.16	573.17	0.093	1843.55	172.25
A_68_P26770183	chr9:100446090-100446134	NM_010878:360	Nck1	INSIDE	0.401	4.466	1380.73	6166.28	1.791	1094.73	1960.92
A_68_P20188961	chr1:43503040-43503084	NM_010879:467	Nck2	INSIDE	0.445	4.367	2316.61	10115.83	1.941	1947.43	3780.04
A_68_P21375628	chr2:80421369-80421413	NM_016965:-268	Nckap1	PROMOTER	0.265	6.820	76905.88	524509.10	1.808	75077.69	135759.90
A_68_P21375629	chr2:80421545-80421589	NM_016965:-444	Nckap1	PROMOTER	0.666	3.131	1623.05	5082.09	2.084	1354.39	2822.61
A_68_P30480085	chr15:99256217-99256261	NM_001001884:31941	Nckap5l	INSIDE	0.233	15.731	1705.75	26832.42	3.663	1580.55	5789.30
A_68_P30480282	chr15:99287858-99287902	NM_001001884:299	Nckap5l	INSIDE	0.597	0.185	6301.60	1164.77	0.11	4218.41	465.80
A_68_P30480281	chr15:99287653-99287697	NM_001001884:505	Nckap5l	INSIDE	0.631	2.400	2254.69	5410.69	1.514	1503.20	2276.11
A_68_P27290094	chr10:80959100-80959144	NM_134009:-14	Ncln	PROMOTER	0.421	8.579	2459.66	21101.18	3.61	2053.09	7410.88
A_68_P20040932	chr1:13363158-13363202	NM_001077695:984	Ncoa2	INSIDE	0.423	4.337	1127.25	4889.44	1.836	1059.23	1945.12
A_68_P21826986	chr2:165818945-165818989	NM_008679:830	Ncoa3	INSIDE	0.08	2.698	6374.16	17197.15	0.215	4410.67	949.18
A_68_P21826984	chr2:165818646-165818690	NM_008679:532	Ncoa3	INSIDE	0.124	13.620	12843.30	174919.50	1.684	8024.59	13515.65
A_68_P21826985	chr2:165818808-165818852	NM_008679:694	Ncoa3	INSIDE	0.154	15.115	14430.03	218112.70	2.325	10179.76	23671.07
A_68_P21826983	chr2:165818573-165818617	NM_008679:458	Ncoa3	INSIDE	0.269	18.216	5947.57	108339.50	4.902	4972.87	24377.71
A_68_P21826979	chr2:165818066-165818110	NM_008679:-48	Ncoa3	PROMOTER	0.435	6.723	1522.74	10237.25	2.928	1451.10	4248.49
A_68_P21826982	chr2:165818442-165818486	NM_008679:328	Ncoa3	INSIDE	0.372	5.386	717.80	3866.25	2.005	746.50	1497.07
A_68_P21821252	chr2:164859772-164859816	NM_144892:485	Ncoa5	INSIDE	0.656	0.506	1147.16	580.26	0.332	859.81	285.34
A_68_P24064307	chr5:125507269-125507313	NM_011424:152294	Ncor2	INSIDE	0.063	58.036	7291.77	423182.10	3.661	8079.84	29580.49
A_68_P24064309	chr5:125507455-125507499	NM_011424:152108	Ncor2	INSIDE	0.512	3.995	433.59	1731.99	2.044	414.05	846.26
A_68_P31075109	chr17:17975189-17975234	NM_001162909:7273	Ncrna00085	INSIDE	0.444	5.830	477.00	2781.12	2.589	382.96	991.51
A_68_P20830847	chr1:174013137-174013181	NM_021607:-278	Ncstn	PROMOTER	0.478	0.329	3203.62	1052.55	0.157	2321.67	364.74
A_68_P31334901	chr17:71876213-71876257	NM_023294:-37	Ndc80	PROMOTER	0.486	5.497	1961.49	10782.79	2.67	1597.00	4264.22
A_68_P30560748	chr16:14163592-14163636	NM_001114085:247	Nde1	INSIDE	0.35	0.297	3580.72	1063.66	0.104	2526.63	262.93
A_68_P30560747	chr16:14163515-14163559	NM_001114085:169	Nde1	INSIDE	0.42	11.550	15138.60	174851.70	4.847	11819.43	57291.89
A_68_P30560749	chr16:14163667-14163711	NM_001114085:321	Nde1	INSIDE	0.503	0.604	5743.11	3467.18	0.304	4051.26	1231.38
A_68_P30560744	chr16:14163261-14163305	NM_001114085:-85	Nde1	PROMOTER	0.601	2.588	1076.80	2787.25	1.554	884.31	1374.62
A_68_P31633859	chr18:38578724-38578769	NM_022996:118	Ndfip1	INSIDE	0.519	4.365	1315.07	5740.56	2.267	1304.26	2956.92
A_68_P25168663	chr7:69493375-69493419	NM_010882:234	Ndn	INSIDE	0.128	31.326	1608.43	50385.34	4.001	1663.76	6656.33
A_68_P25179904	chr7:72017616-72017660	NM_023239:288	Ndnl2	INSIDE	0.316	6.239	995.54	6210.73	1.969	879.48	1731.82
A_68_P21070911	chr2:25110830-25110874	NM_001082476:82	Ndor1	INSIDE	0.478	0.316	1531.68	484.65	0.151	1160.37	175.65
A_68_P30295277	chr15:66801273-66801317	NM_008681:-91	Ndrg1	PROMOTER	0.373	0.521	2003.32	1043.17	0.194	1590.22	308.65
A_68_P30295269	chr15:66800435-66800479	NM_008681:747	Ndrg1	INSIDE	0.485	11.363	2614.78	29711.21	5.507	2356.24	12974.70

ProbeName	Target position of probe on CpG island microarray	TargetID	GeneSymbol	CpG island Description	Ratio of relative methylation (TiO_2-NP/Vehicle)	Sham group			TiO_2-H group		
						Relative methylation (Cy5/Cy3)	Cy3 signal (Input)	Cy5 signal (MeDIP)	Relative methylation (Cy5/Cy3)	Cy3 signal (Input)	Cy5 signal (MeDIP)
A_68_P30295278	chr15:66801372-66801416	NM_008681:-191	Ndrg1	PROMOTER	0.549	0.574	967.64	555.23	0.315	807.17	254.09
A_68_P29603743	chr14:52532832-52532876	NM_001145959:309	Ndrg2	INSIDE	0.66	0.278	3459.08	960.99	0.183	2385.53	437.18
A_68_P32322121	chrX:34731184-34731228	NM_019443:27	Ndufa1	INSIDE	0.303	5.054	966.91	4886.47	1.534	1546.38	2371.58
A_68_P25948620	chr8:72427025-72427069	NM_023312:-589	Ndufa13	PROMOTER	0.305	10.679	862.27	9207.91	3.253	775.12	2521.75
A_68_P27536264	chr10:126948850-126948894	NM_001098789:-3122	Ndufa4l2	PROMOTER	0.116	28.470	1072.48	30533.48	3.292	1105.74	3639.85
A_68_P27536275	chr10:126950036-126950080	NM_001098789:-1936	Ndufa4l2	PROMOTER	0.55	0.629	1221.07	768.11	0.346	973.94	336.92
A_68_P24302044	chr6:24477440-24477484	NM_026614:225	Ndufa5	INSIDE	0.546	0.236	3311.46	782.61	0.129	2359.40	304.52
A_68_P30382971	chr15:82184657-82184701	NM_025987:43	Ndufa6	INSIDE	0.349	19.256	3679.93	70861.36	6.717	3520.96	23651.41
A_68_P30382969	chr15:82184423-82184467	NM_025987:277	Ndufa6	INSIDE	0.465	0.467	1002.34	468.56	0.217	795.30	172.72
A_68_P29309820	chr13:108948751-108948795	NM_001127346:47	Ndufaf2	INSIDE	0.449	0.169	11178.31	1893.75	0.076	7221.17	549.56
A_68_P24383895	chr6:39542839-39542883	NM_026612:279	Ndufb2	INSIDE	0.663	0.403	1163.17	468.46	0.267	789.95	210.89
A_68_P26015449	chr8:86090778-86090822	NM_025843:144	Ndufb7	INSIDE	0.553	2.952	1093.68	3228.74	1.632	949.37	1549.77
A_68_P22129479	chr3:51212850-51212894	NM_025523:5	Ndufc1	INSIDE	0.624	0.465	1382.49	643.32	0.291	1181.27	343.23
A_68_P22129477	chr3:51212635-51212679	NM_025523:221	Ndufc1	INSIDE	0.533	0.621	1738.60	1079.87	0.331	1455.66	482.14
A_68_P20285511	chr1:63223372-63223416	NM_001160038:2	Ndufs1	INSIDE	0.557	0.457	968.14	442.14	0.254	799.15	203.17
A_68_P21419564	chr2:90744694-90744738	NM_026688:162	Ndufs3	INSIDE	0.462	0.682	3159.48	2155.03	0.315	2360.62	744.29
A_68_P29139134	chr13:73465946-73465990	NM_010888:-38	Ndufs6	DIVERGENT_PROMOTER	0.118	1.497	1952.79	2923.75	0.177	1279.44	226.27
A_68_P31304302	chr17:66450798-66450842	NM_028388:11	Ndufv2	INSIDE	0.452	5.245	850.84	4462.85	2.371	783.17	1857.26
A_68_P31304303	chr17:66450941-66450985	NM_028388:-131	Ndufv2	DIVERGENT_PROMOTER	0.543	4.438	341.78	1516.97	2.408	296.11	712.99
A_68_P31931196	chr19:5845867-5845911	NR_003513:-410	Neat1	PROMOTER	0.644	0.271	4089.18	1107.93	0.174	2804.98	489.26
A_68_P26628950	chr9:72510550-72510594	NM_010890:419	Nedd4	INSIDE	0.595	0.561	1333.10	747.60	0.334	1157.43	386.11
A_68_P31777748	chr18:65047791-65047835	NM_001114386:403	Nedd4l	INSIDE	0.455	4.344	1521.13	6607.43	1.975	1346.81	2659.66
A_68_P28977626	chr13:41454570-41454617	NM_017464:-223	Nedd9	PROMOTER	0.312	23.320	596.64	13913.27	7.267	753.83	5477.97
A_68_P29689081	chr14:68702832-68702876	NM_010910:914	Nefl	INSIDE	0.499	0.263	2060.88	542.36	0.131	1532.25	201.05
A_68_P29689074	chr14:68701856-68701900	NM_010910:-62	Nefl	PROMOTER	0.605	0.269	1678.36	452.05	0.163	1277.45	208.14
A_68_P20658346	chr1:140515636-140515682	NM_021605:615	Nek7	INSIDE	0.451	0.292	1628.88	476.07	0.132	1313.01	172.92
A_68_P20658349	chr1:140515986-140516030	NM_021605:265	Nek7	INSIDE	0.617	3.842	1650.05	6338.98	2.372	1373.02	3256.17
A_68_P21069553	chr2:24909996-24910040	NM_001039386:120	Nelf	INSIDE	0.645	12.491	1079.55	13485.03	8.051	1149.52	9254.74
A_68_P30458578	chr15:95358757-95358801	NM_016743:359	Nell2	INSIDE	0.499	4.404	5109.58	22505.05	2.196	4233.25	9296.71
A_68_P20937430	chr1:193141794-193141838	NM_025424:181	Nenf	INSIDE	0.503	5.260	903.83	4753.94	2.645	802.00	2121.30
A_68_P26551398	chr9:58883897-58883941	NM_001042752:330	Neo1	INSIDE	0.637	4.150	777.53	3226.66	2.642	703.61	1858.82
A_68_P22310998	chr3:87774969-87775013	NM_016701:-24	Nes	PROMOTER	0.655	0.246	2865.31	705.86	0.161	2156.26	347.75
A_68_P28790554	chr13:3917102-3917146	NM_019671:342	Net1	INSIDE	0.278	1.951	4052.83	7905.88	0.543	2838.92	1540.91
A_68_P31156585	chr17:35068472-35068516	NM_010893:297	Neu1	INSIDE	0.37	0.495	1014.82	501.93	0.183	940.47	171.98
A_68_P32148153	chr19:47254029-47254073	NM_021360:741	Neurl1a	INSIDE	0.348	0.415	2026.15	840.96	0.145	1608.45	232.46
A_68_P32148598	chr19:47327938-47327982	NM_001163480:24628	Neurl1a	INSIDE	0.46	6.345	386.21	2450.60	2.92	352.48	1029.32
A_68_P32148144	chr19:47253081-47253125	NM_021360:-207	Neurl1a	PROMOTER	0.466	4.204	1639.84	6893.77	1.96	1535.68	3010.41
A_68_P32148149	chr19:47253623-47253667	NM_021360:335	Neurl1a	INSIDE	0.502	4.853	1262.96	6128.81	2.434	1041.90	2535.75
A_68_P32148151	chr19:47253878-47253922	NM_021360:591	Neurl1a	INSIDE	0.516	4.278	2634.78	11270.61	2.209	2360.82	5215.09
A_68_P31110244	chr17:26575843-26575887	NM_001081656:23955	Neurl1b	INSIDE	0.574	5.335	348.51	1859.16	3.064	441.45	1352.82
A_68_P21368904	chr2:79294386-79294430	NM_010894:2385	Neurod1	INSIDE	0.301	2.179	2093.25	4562.04	0.657	1584.45	1040.41
A_68_P28055686	chr11:98188438-98188482	NM_010895:2499	Neurod2	INSIDE	0.288	10.632	1942.97	20657.70	3.059	3056.82	9351.32
A_68_P28055696	chr11:98189596-98189640	NM_010895:1341	Neurod2	INSIDE	0.637	2.765	784.84	2170.15	1.762	617.98	1088.84
A_68_P28055690	chr11:98188811-98188855	NM_010895:2127	Neurod2	INSIDE	0.658	3.312	400.92	1327.88	2.178	339.98	740.57
A_68_P29060115	chr13:56353410-56353454	NM_010896:92	Neurog1	INSIDE	0.498	0.383	3239.29	1239.24	0.19	2356.87	448.76
A_68_P29060176	chr13:56360497-56360541	NM_010896:-6994	Neurog1	PROMOTER	0.659	0.224	2315.77	518.76	0.148	1728.74	255.30

ProbeName	Target position of probe on CpG island microarray	TargetID	GeneSymbol	CpG island Description	Ratio of relative methylation (TiO_2-NP/Vehicle)	Sham group			TiO_2-H group		
						Relative methylation (Cy5/Cy3)	Cy3 signal (Input)	Cy5 signal (MeDIP)	Relative methylation (Cy5/Cy3)	Cy3 signal (Input)	Cy5 signal (MeDIP)
A_68_P29060118	chr13:56353718-56353762	NM_010896:-216	Neurog1	PROMOTER	0.32	0.614	1542.28	946.22	0.196	1264.18	248.07
A_68_P29060175	chr13:56360319-56360363	NM_010896:-6816	Neurog1	PROMOTER	0.275	0.639	1271.81	812.57	0.175	971.58	170.49
A_68_P22509735	chr3:127337358-127337402	NM_009718:1318	Neurog2	INSIDE	0.337	1.743	5601.73	9762.48	0.588	3839.53	2257.47
A_68_P22509720	chr3:127335433-127335477	NM_009718:-608	Neurog2	PROMOTER	0.561	3.202	903.88	2894.18	1.797	797.87	1434.11
A_68_P27560010	chr11:4749422-4749466	NM_010898:86	Nf2	INSIDE	0.559	3.269	3759.12	12286.85	1.826	3179.59	5806.33
A_68_P27560006	chr11:4749005-4749049	NM_010898:504	Nf2	INSIDE	0.492	3.373	1363.62	4599.38	1.658	1073.49	1780.16
A_68_P26143993	chr8:109817338-109817382	NM_018823:-9	Nfat5	PROMOTER	0.343	5.039	1829.94	9221.48	1.73	1635.44	2829.08
A_68_P31866219	chr18:80894683-80894727	NM_001164111:10208	Nfatc1	INSIDE	0.216	12.780	2035.02	26007.20	2.761	1634.42	4513.11
A_68_P31866345	chr18:80909646-80909690	NM_001164111:-4756	Nfatc1	PROMOTER	0.425	8.808	4112.32	36223.16	3.747	3107.60	11643.24
A_68_P31866342	chr18:80909356-80909400	NM_001164111:-4466	Nfatc1	PROMOTER	0.431	5.681	1199.37	6813.49	2.45	1088.29	2666.60
A_68_P31866300	chr18:80904546-80904590	NM_001164111:344	Nfatc1	INSIDE	0.578	4.621	725.60	3352.71	2.67	730.02	1949.24
A_68_P31866306	chr18:80905336-80905380	NM_001164111:-446	Nfatc1	PROMOTER	0.631	3.247	1144.24	3715.40	2.048	973.67	1994.51
A_68_P21842271	chr2:168396389-168396433	NM_001136073:19281	Nfatc2	INSIDE	0.282	6.908	3048.11	21056.47	1.948	2529.71	4927.51
A_68_P21842272	chr2:168396475-168396519	NM_001136073:19195	Nfatc2	INSIDE	0.339	5.172	3385.24	17509.07	1.753	2818.87	4940.67
A_68_P21842270	chr2:168396257-168396301	NM_001136073:19413	Nfatc2	INSIDE	0.574	4.099	1596.65	6544.04	2.351	1163.11	2735.04
A_68_P21842488	chr2:168427130-168427174	NM_001037178:3	Nfatc2	INSIDE	0.642	4.490	4171.85	18730.44	2.881	3164.34	9115.15
A_68_P21842487	chr2:168427037-168427082	NM_001037178:96	Nfatc2	INSIDE	0.372	0.724	2443.08	1769.22	0.269	1936.66	521.42
A_68_P25500257	chr7:133539825-133539869	NM_010900:405	Nfatc2ip	INSIDE	0.49	0.275	4412.28	1213.80	0.135	3227.23	434.80
A_68_P26137548	chr8:108583970-108584014	NM_010901:490	Nfatc3	INSIDE	0.56	11.038	2434.95	26875.81	6.18	1748.50	10805.21
A_68_P29620076	chr14:56445768-56445812	NM_001168346:2159	Nfatc4	INSIDE	0.53	11.745	3614.20	42448.87	6.219	2890.61	17977.55
A_68_P29620045	chr14:56442427-56442471	NM_001168346:-1183	Nfatc4	PROMOTER	0.511	5.786	407.11	2355.72	2.956	317.22	937.78
A_68_P29620075	chr14:56445685-56445729	NM_001168346:2075	Nfatc4	INSIDE	0.287	1.749	1506.44	2634.98	0.502	1123.27	563.76
A_68_P21345534	chr2:75542163-75542207	NM_010902:514	Nfe2l2	INSIDE	0.497	0.525	3075.90	1615.98	0.261	2249.17	587.23
A_68_P24444193	chr6:51382790-51382834	NM_010903:144	Nfe2l3	INSIDE	0.528	0.729	1893.21	1379.86	0.385	1293.98	498.02
A_68_P23134369	chr4:97445584-97445628	NM_001122953:1290	Nfia	INSIDE	0.43	0.463	1070.36	495.89	0.199	855.00	170.25
A_68_P23134316	chr4:97438997-97439041	NM_001122953:-5298	Nfia	PROMOTER	0.463	4.056	2680.83	10873.67	1.877	1982.51	3720.53
A_68_P23134368	chr4:97445476-97445520	NM_001122953:1182	Nfia	INSIDE	0.559	3.582	2784.93	9974.79	2.002	2389.50	4783.02
A_68_P23057176	chr4:82151139-82151183	NM_001113209:52	Nfib	INSIDE	0.391	0.330	1869.09	616.40	0.129	1337.80	172.40
A_68_P23057168	chr4:82150339-82150383	NM_001113209:852	Nfib	INSIDE	0.517	3.404	1649.54	5615.10	1.759	1356.58	2386.09
A_68_P27289568	chr10:80882945-80882989	NM_008688:6952	Nfic	INSIDE	0.664	3.658	1823.73	6671.19	2.43	1568.48	3810.97
A_68_P29041280	chr13:53076043-53076087	NM_017373:344	Nfil3	INSIDE	0.24	1.799	896.73	1613.52	0.431	714.08	307.99
A_68_P26021284	chr8:87230641-87230685	NM_001081981:67606	Nfix	DOWNSTREAM	0.333	11.660	4981.59	58087.59	3.889	4195.03	16312.64
A_68_P26021287	chr8:87230962-87231012	NM_001081981:67282	Nfix	DOWNSTREAM	0.622	10.542	893.18	9415.44	6.559	726.21	4763.02
A_68_P31201770	chr17:45692568-45692612	NM_008690:-74	Nfkbie	DIVERGENT_PROMOTER	0.227	37.318	8887.28	331655.00	8.478	10161.13	86145.20
A_68_P31158451	chr17:35371961-35372005	NM_010909:778	Nfkbil1	INSIDE	0.58	8.555	4924.99	42132.90	4.959	4541.31	22520.14
A_68_P31158453	chr17:35372182-35372226	NM_010909:556	Nfkbil1	INSIDE	0.599	0.443	3503.53	1553.16	0.266	2514.34	667.78
A_68_P30777937	chr16:55822273-55822317	NM_001159394:-43	Nfkbiz	PROMOTER	0.456	3.572	2191.77	7828.68	1.629	1734.26	2825.88
A_68_P26400214	chr9:31193743-31193787	NM_172766:-12	Nfrkb	PROMOTER	0.628	3.245	1217.65	3950.90	2.038	1064.90	2169.98
A_68_P24613352	chr6:86959758-86959802	NM_001170591:-49	Nfu1	PROMOTER	0.177	21.636	2939.88	63607.49	3.82	2422.74	9254.97
A_68_P31217293	chr17:48549071-48549115	NM_001110832:53	Nfya	INSIDE	0.626	0.632	5257.62	3322.28	0.395	3696.04	1461.68
A_68_P27293632	chr10:82226781-82226825	NM_010914:84	Nfyb	INSIDE	0.294	0.387	2243.73	869.13	0.114	1479.04	168.38
A_68_P27293629	chr10:82226162-82226206	NM_010914:702	Nfyb	INSIDE	0.555	0.475	2155.53	1023.52	0.264	1611.26	424.79
A_68_P28614148	chr12:88443473-88443517	NM_022414:-5	Ngb	PROMOTER	0.255	0.585	2310.43	1352.54	0.149	1809.69	270.13
A_68_P28614145	chr12:88443030-88443074	NM_022414:437	Ngb	INSIDE	0.332	0.377	4379.42	1653.02	0.125	2907.70	364.08
A_68_P22381100	chr3:102273874-102273918	NM_001112698:46	Ngf	INSIDE	0.511	0.658	1415.40	931.85	0.337	1289.62	434.10
A_68_P29428095	chr14:17075686-17075730	NM_021504:-6119	Ngly1	PROMOTER	0.374	0.678	846.93	574.10	0.253	692.36	175.41

ProbeName	Target position of probe on CpG island microarray	TargetID	GeneSymbol	CpG island Description	Ratio of relative methylation (TiO_2-NP/Vehicle)	Sham group			TiO_2-H group		
						Relative methylation (Cy5/Cy3)	Cy3 signal (Input)	Cy5 signal (MeDIP)	Relative methylation (Cy5/Cy3)	Cy3 signal (Input)	Cy5 signal (MeDIP)
A_68_P25265632	chr7:87406421-87406465	NM_031375:342	Ngrn	INSIDE	0.261	7.316	679.21	4969.41	1.911	606.99	1160.01
A_68_P20352133	chr1:75121786-75121830	NM_029342:-8	Nhej1	PROMOTER	0.599	5.239	3063.74	16050.80	3.136	2370.17	7432.34
A_68_P20830643	chr1:173984062-173984107	NM_010916:3643	Nhlh1	INSIDE	0.316	0.410	3467.82	1421.76	0.129	2660.89	344.49
A_68_P22378552	chr3:101816741-101816785	NM_178777:2695	Nhlh2	INSIDE	0.512	9.241	3911.69	36146.64	4.734	4109.68	19454.39
A_68_P22378540	chr3:101815556-101815600	NM_178777:1511	Nhlh2	INSIDE	0.47	3.073	1741.84	5352.33	1.445	1397.40	2018.78
A_68_P29011114	chr13:47109579-47109623	NM_175340:619	Nhlrc1	INSIDE	0.45	0.483	1269.77	613.21	0.217	964.99	209.81
A_68_P32201471	chr19:56623197-56623241	NM_025811:468	Nhlrc2	INSIDE	0.557	3.969	717.90	2849.64	2.211	559.90	1238.01
A_68_P27794924	chr11:51433455-51433500	NM_026631:203	Nhp2	INSIDE	0.458	9.857	4475.98	44120.24	4.512	3361.27	15164.94
A_68_P27794923	chr11:51433377-51433421	NM_026631:124	Nhp2	INSIDE	0.587	0.203	5265.55	1066.93	0.119	3376.50	401.88
A_68_P32786683	chrX:158346162-158346206	NM_001081052:251538	Nhs	INSIDE	0.45	7.088	1681.66	11919.76	3.189	1982.16	6320.56
A_68_P32565869	chrX:99044829-99044873	NM_001163610:127	Nhsl2	INSIDE	0.465	7.985	799.76	6386.04	3.716	988.31	3672.92
A_68_P32565872	chrX:99045232-99045276	NM_001163610:531	Nhsl2	INSIDE	0.303	5.776	1308.33	7557.35	1.753	1458.87	2557.02
A_68_P32565875	chrX:99045611-99045655	NM_001163610:909	Nhsl2	INSIDE	0.26	6.056	1100.16	6662.80	1.577	1575.40	2483.94
A_68_P32565873	chrX:99045311-99045356	NM_001163610:610	Nhsl2	INSIDE	0.541	2.891	722.32	2088.17	1.565	1169.86	1830.77
A_68_P29443159	chr14:20570751-20570795	NM_008695:294	Nid2	INSIDE	0.457	0.398	1200.76	478.07	0.182	941.00	171.17
A_68_P28518531	chr12:71213190-71213234	NM_008697:-300	Nin	PROMOTER	0.478	5.415	2061.35	11161.33	2.591	1967.58	5097.48
A_68_P29023779	chr13:49283004-49283048	NM_013610:111	Ninj1	INSIDE	0.29	13.879	3481.65	48321.28	4.03	2771.27	11166.86
A_68_P25144564	chr7:63274831-63274875	NM_153578:91	Nipa1	INSIDE	0.539	9.088	4070.28	36990.41	4.895	3323.25	16265.73
A_68_P25144266	chr7:63217700-63217744	NM_023647:98	Nipa2	INSIDE	0.6	2.662	1358.98	3617.97	1.597	1139.05	1819.52
A_68_P23328804	chr4:135050696-135050741	NM_028995:-299	Nipal3	DIVERGENT_PROMOTER	0.532	3.806	2610.23	9935.07	2.023	2233.58	4518.58
A_68_P30000046	chr15:8394229-8394273	NM_027707:213	Nipbl	INSIDE	0.499	0.248	2697.71	667.81	0.124	2196.74	271.52
A_68_P30000045	chr15:8394103-8394149	NM_027707:337	Nipbl	INSIDE	0.506	0.518	1117.19	578.37	0.262	798.15	209.14
A_68_P22920590	chr4:53025131-53025175	NM_025623:357	Nipsnap3b	INSIDE	0.206	0.560	10475.14	5867.79	0.115	6766.69	780.64
A_68_P22920589	chr4:53024957-53025001	NM_025623:183	Nipsnap3b	INSIDE	0.392	0.482	3402.69	1639.34	0.189	2167.24	409.72
A_68_P29510442	chr14:32019467-32019511	NM_022656:524	Nisch	INSIDE	0.096	3.541	5906.63	20912.64	0.341	3987.25	1361.17
A_68_P29510250	chr14:31989817-31989861	NM_022656:30174	Nisch	INSIDE	0.486	3.651	2426.73	8860.46	1.774	1879.25	3334.18
A_68_P29510249	chr14:31989749-31989793	NM_022656:30242	Nisch	INSIDE	0.612	3.771	3111.65	11734.73	2.307	2104.36	4854.98
A_68_P29510443	chr14:32019604-32019648	NM_022656:386	Nisch	INSIDE	0.641	0.518	1688.69	874.43	0.332	1213.28	402.47
A_68_P27050627	chr10:32609677-32609721	NM_001013411:23	Nkain2	INSIDE	0.368	24.737	4079.18	100907.80	9.097	3408.48	31006.78
A_68_P27050630	chr10:32610114-32610158	NM_001013411:-415	Nkain2	PROMOTER	0.504	0.280	3607.54	1008.97	0.141	2584.79	364.18
A_68_P22771644	chr4:20705685-20705729	NM_172987:109	Nkain3	INSIDE	0.645	0.430	2626.42	1128.41	0.277	2023.30	561.00
A_68_P21907361	chr2:180689354-180689398	NM_001141933:28	Nkain4	INSIDE	0.656	0.156	4928.05	766.40	0.102	3255.60	331.96
A_68_P21907359	chr2:180689094-180689138	NM_001141933:288	Nkain4	INSIDE	0.331	5.707	1384.59	7902.08	1.889	1114.76	2105.55
A_68_P32321829	chrX:34667041-34667085	NM_025937:305	Nkap	INSIDE	0.507	3.127	630.52	1971.66	1.584	900.97	1427.23
A_68_P28873012	chr13:21560534-21560582	NM_025719:-188	Nkapl	PROMOTER	0.63	9.861	784.72	7738.18	6.215	673.85	4188.18
A_68_P28873011	chr13:21560407-21560451	NM_025719:-58	Nkapl	PROMOTER	0.649	7.033	2394.40	16840.05	4.566	2088.69	9536.99
A_68_P26041379	chr8:91045377-91045421	NM_001163660:-6133	Nkd1	PROMOTER	0.42	11.401	5071.43	57820.92	4.784	4077.82	19508.96
A_68_P26041378	chr8:91045300-91045344	NM_001163660:-6209	Nkd1	PROMOTER	0.578	4.337	538.07	2333.87	2.505	574.02	1437.96
A_68_P26041381	chr8:91045707-91045751	NM_001163660:-5803	Nkd1	PROMOTER	0.495	1.493	7560.62	11290.25	0.74	5562.55	4115.86
A_68_P29142604	chr13:73984411-73984456	NM_028186:646	Nkd2	INSIDE	0.438	4.886	1546.45	7556.67	2.138	1315.74	2813.48
A_68_P29437788	chr14:19103568-19103612	NM_023526:-65	Nkiras1	DIVERGENT_PROMOTER	0.642	0.549	804.74	441.76	0.352	639.87	225.36
A_68_P24993775	chr7:20108712-20108756	NM_027116:4655	Nkpd1	INSIDE	0.46	5.174	1826.86	9452.17	2.38	1648.04	3921.96
A_68_P28457455	chr12:57637056-57637100	NM_001146198:-985	Nkx2-1	PROMOTER	0.256	0.340	2657.43	903.15	0.087	2045.88	178.03
A_68_P28457457	chr12:57637250-57637294	NM_001146198:-1179	Nkx2-1	PROMOTER	0.433	0.172	3414.35	586.84	0.074	2308.87	171.73
A_68_P28457433	chr12:57634159-57634203	NM_001146198:1913	Nkx2-1	INSIDE	0.437	9.371	2054.35	19250.91	4.095	1835.58	7516.56
A_68_P21722817	chr2:147019650-147019694	NM_001077632:-7534	Nkx2-2	PROMOTER	0.261	8.137	1733.54	14105.00	2.127	1602.07	3408.38

ProbeName	Target position of probe on CpG island microarray	TargetID	GeneSymbol	CpG island Description	Ratio of relative methylation (TiO_2-NP/Vehicle)	Sham group			TiO_2-H group		
						Relative methylation (Cy5/Cy3)	Cy3 signal (Input)	Cy5 signal (MeDIP)	Relative methylation (Cy5/Cy3)	Cy3 signal (Input)	Cy5 signal (MeDIP)
A_68_P21722826	chr2:147020543-147020587	NM_001077632:-8426	Nkx2-2	PROMOTER	0.628	7.919	1016.60	8050.39	4.975	1029.27	5120.29
A_68_P21722727	chr2:147009567-147009611	NR_030769:-230	Nkx2-2as	PROMOTER	0.628	5.155	367.62	1895.17	3.24	341.48	1106.34
A_68_P21722730	chr2:147009969-147010013	NR_030769:172	Nkx2-2as	INSIDE	0.463	0.652	1075.49	701.07	0.302	881.04	265.70
A_68_P21722728	chr2:147009701-147009745	NR_030769:-96	Nkx2-2as	PROMOTER	0.532	3.029	976.25	2957.54	1.612	835.70	1346.76
A_68_P32127607	chr19:43691200-43691244	NM_008699:4408	Nkx2-3	DOWNSTREAM	0.617	0.680	1225.57	833.76	0.42	1122.07	471.31
A_68_P21722283	chr2:146909704-146909748	NM_023504:1355	Nkx2-4	INSIDE	0.357	24.441	1977.10	48322.77	8.734	2308.60	20163.81
A_68_P21722284	chr2:146909810-146909854	NM_023504:1249	Nkx2-4	INSIDE	0.561	4.038	665.38	2686.79	2.263	619.35	1401.86
A_68_P31112722	chr17:26978019-26978063	NM_008700:470	Nkx2-5	INSIDE	0.315	7.467	1792.28	13382.51	2.353	1521.12	3579.00
A_68_P31112710	chr17:26976655-26976699	NM_008700:1834	Nkx2-5	INSIDE	0.523	2.877	1768.16	5087.29	1.505	1500.18	2257.29
A_68_P28457808	chr12:57713134-57713178	NM_008701:1115	Nkx2-9	INSIDE	0.267	14.732	1200.77	17689.43	3.932	987.09	3880.83
A_68_P23637417	chr5:42154620-42154664	NM_007524:817	Nkx3-2	INSIDE	0.359	4.189	3864.90	16189.79	1.504	3176.98	4778.06
A_68_P23936334	chr5:102093547-102093591	NM_144955:162	Nkx6-1	INSIDE	0.58	4.212	2521.18	10619.86	2.443	1974.42	4824.33
A_68_P23936342	chr5:102094436-102094480	NM_144955:-728	Nkx6-1	PROMOTER	0.396	0.553	1115.14	616.49	0.219	942.62	206.38
A_68_P23936340	chr5:102094274-102094318	NM_144955:-566	Nkx6-1	PROMOTER	0.156	11.627	1446.15	16814.93	1.818	1209.71	2199.09
A_68_P23936338	chr5:102094059-102094103	NM_144955:-350	Nkx6-1	PROMOTER	0.588	0.659	835.84	551.23	0.388	697.70	270.55
A_68_P25578920	chr7:146770604-146770648	NR_027857:-1930	Nkx6-2	PROMOTER	0.311	49.669	1337.28	66421.38	15.457	1221.97	18888.29
A_68_P25578890	chr7:146767389-146767434	NR_027857:1285	Nkx6-2	INSIDE	0.411	0.193	3293.94	634.45	0.079	2164.76	171.50
A_68_P25578901	chr7:146768634-146768678	NR_027857:40	Nkx6-2	INSIDE	0.54	9.953	4461.07	44398.86	5.37	3552.49	19076.38
A_68_P25578895	chr7:146768050-146768094	NR_027857:624	Nkx6-2	INSIDE	0.56	5.980	1981.70	11850.74	3.35	1544.38	5173.00
A_68_P25715145	chr8:24268305-24268349	NM_029002:4584	Nkx6-3	INSIDE	0.389	11.930	1692.05	20185.67	4.637	1416.13	6566.50
A_68_P27969032	chr11:82721609-82721653	NM_145431:267	Nle1	INSIDE	0.64	0.637	1634.68	1041.90	0.408	1275.09	519.74
A_68_P27969033	chr11:82721735-82721779	NM_145431:141	Nle1	INSIDE	0.43	1.530	2330.49	3566.62	0.658	1825.16	1200.84
A_68_P22006134	chr3:26230520-26230564	NM_138666:289	Nlgn1	INSIDE	0.391	0.574	1757.74	1009.26	0.225	1366.86	306.96
A_68_P22222507	chr3:69525834-69525878	NM_133787:-120	Nmd3	PROMOTER	0.325	10.646	9105.78	96938.22	3.464	7798.13	27009.25
A_68_P22222508	chr3:69525922-69525966	NM_133787:-32	Nmd3	PROMOTER	0.617	0.364	1555.03	565.39	0.224	1097.87	246.18
A_68_P28029989	chr11:93817362-93817406	NM_008705:-63	Nme2	PROMOTER	0.123	26.780	2655.28	71107.23	3.291	2215.75	7293.03
A_68_P31108289	chr17:26232390-26232434	NM_019731:3	Nme4	INSIDE	0.635	4.440	796.63	3536.95	2.818	760.29	2142.47
A_68_P20788410	chr1:166237694-166237738	NM_178071:-89	Nme7	DIVERGENT_PROMOTER	0.309	12.094	631.18	7633.34	3.735	546.38	2040.66
A_68_P20961515	chr2:3201949-3201993	NM_008708:411	Nmt2	INSIDE	0.64	0.568	1655.99	940.02	0.363	593.47	215.52
A_68_P21777851	chr2:157386103-157386147	NM_010923:279	Nnat	INSIDE	0.151	17.161	4277.47	73403.65	2.592	3325.14	8617.58
A_68_P21777850	chr2:157385964-157386008	NM_010923:141	Nnat	INSIDE	0.157	3.827	1567.94	5999.97	0.602	1236.91	744.48
A_68_P29372955	chr13:120197789-120197833	NM_008710:8	Nnt	INSIDE	0.583	0.408	2565.80	1045.69	0.238	1858.87	441.94
A_68_P26144747	chr8:109949061-109949105	NM_026277:-144	Nob1	PROMOTER	0.562	3.890	1531.84	5959.37	2.185	1194.34	2609.62
A_68_P24466341	chr6:54894000-54894044	NM_001171007:27633	Nod1	INSIDE	0.413	3.424	1682.55	5760.32	1.414	1511.80	2137.28
A_68_P28004910	chr11:89163525-89163569	NM_008711:327	Nog	INSIDE	0.09	21.710	2367.84	51404.87	1.96	1992.65	3905.11
A_68_P28004885	chr11:89160628-89160672	NM_008711:3223	Nog	DOWNSTREAM	0.612	0.624	1589.54	991.91	0.382	1233.74	470.86
A_68_P28269967	chr12:17355126-17355170	NM_001008421:-150	Nol10	PROMOTER	0.593	2.395	2944.51	7052.57	1.421	2295.60	3262.86
A_68_P28105679	chr11:107050342-107050386	NM_001161329:331	Nol11	INSIDE	0.237	8.081	3296.09	26634.14	1.916	2616.05	5012.78
A_68_P30363285	chr15:78765693-78765737	NM_133800:352	Nol12	INSIDE	0.599	4.528	942.00	4265.29	2.714	857.93	2328.24
A_68_P30363284	chr15:78765610-78765654	NM_133800:270	Nol12	INSIDE	0.653	0.503	950.05	478.26	0.329	874.71	287.47
A_68_P26132669	chr8:107800240-107800284	NM_030152:-84	Nol3	PROMOTER	0.633	0.691	9516.51	6578.33	0.438	6741.36	2949.47
A_68_P26132670	chr8:107800360-107800404	NM_030152:36	Nol3	INSIDE	0.555	0.720	1356.17	975.88	0.399	1098.86	438.73
A_68_P31552995	chr18:23197987-23198031	NM_001161483:-844	Nol4	PROMOTER	0.463	4.856	4197.94	20385.93	2.249	3539.10	7959.97
A_68_P31553001	chr18:23198592-23198636	NM_001161483:-1450	Nol4	PROMOTER	0.629	3.115	949.51	2957.68	1.958	753.86	1476.42
A_68_P28989932	chr13:43493670-43493714	NM_023554:-52	Nol7	PROMOTER	0.235	0.695	1242.94	864.13	0.163	1059.95	172.92
A_68_P23416174	chr4:151413616-151413660	NM_001159599:203	Nol9	INSIDE	0.359	0.662	1332.79	881.76	0.237	1033.59	245.20

ProbeName	Target position of probe on CpG island microarray	TargetID	GeneSymbol	CpG island Description	Ratio of relative methylation (TiO_2-NP/Vehicle)	Sham group			TiO_2-H group		
						Relative methylation (Cy5/Cy3)	Cy3 signal (Input)	Cy5 signal (MeDIP)	Relative methylation (Cy5/Cy3)	Cy3 signal (Input)	Cy5 signal (MeDIP)
A_68_P32142001	chr19:46150433-46150477	NM_001039351:102	Nolc1	INSIDE	0.229	9.099	2359.33	21467.81	2.086	1959.06	4086.94
A_68_P23567513	chr5:29761841-29761885	NM_001033457:656	Nom1	INSIDE	0.617	0.445	1702.54	756.86	0.274	1418.49	388.89
A_68_P23567510	chr5:29761543-29761587	NM_001033457:358	Nom1	INSIDE	0.535	0.633	3091.56	1957.96	0.339	2338.98	793.05
A_68_P21628851	chr2:130099810-130099854	NM_024193:-315	Nop56	PROMOTER	0.208	1.354	1658.74	2246.48	0.281	1363.95	383.63
A_68_P21079204	chr2:26358051-26358095	NM_008714:1270	Notch1	INSIDE	0.591	0.578	1752.28	1013.57	0.342	1392.33	475.75
A_68_P22357701	chr3:97817894-97817938	NM_010928:456	Notch2	INSIDE	0.512	0.147	3414.87	500.48	0.075	2299.13	172.60
A_68_P24990655	chr7:19548437-19548481	NM_001029877:37222	Nova2	INSIDE	0.438	0.524	953.74	499.86	0.229	786.70	180.45
A_68_P25297066	chr7:94395079-94395123	NM_015760:-204	Nox4	PROMOTER	0.27	12.651	1904.13	24088.97	3.411	1525.24	5201.92
A_68_P25297065	chr7:94394966-94395010	NM_015760:-316	Nox4	PROMOTER	0.271	0.556	1506.57	838.00	0.15	1146.30	172.50
A_68_P25297067	chr7:94395194-94395238	NM_015760:-88	Nox4	PROMOTER	0.436	0.681	4495.76	3063.01	0.297	3325.58	987.11
A_68_P25297068	chr7:94395278-94395322	NM_015760:-4	Nox4	PROMOTER	0.265	5.878	1999.15	11751.30	1.556	1782.30	2773.71
A_68_P31099061	chr17:24833259-24833303	NM_027988:102	Noxo1	INSIDE	0.504	2.761	3800.91	10492.99	1.39	3165.23	4401.17
A_68_P24981712	chr7:17061914-17061958	NM_008718:193	Npas1	INSIDE	0.58	0.475	2904.27	1379.89	0.275	2024.98	557.78
A_68_P28445674	chr12:55169509-55169553	NM_013780:819867	Npas3	INSIDE	0.597	5.206	1462.92	7615.34	3.108	1339.74	4164.27
A_68_P31926197	chr19:4990111-4990155	NM_153553:-161	Npas4	PROMOTER	0.353	6.474	1222.90	7916.65	2.287	977.54	2235.59
A_68_P28185591	chr11:120470102-120470146	NM_153288:334	Npb	INSIDE	0.355	5.410	3205.24	17338.84	1.92	2612.05	5015.17
A_68_P28185588	chr11:120469756-120469808	NM_153288:-8	Npb	PROMOTER	0.623	0.177	2687.01	474.46	0.11	1881.60	207.09
A_68_P20010817	chr1:5907429-5907473	NM_010342:29	Npbwr1	INSIDE	0.492	0.258	1725.05	444.78	0.127	1354.24	171.74
A_68_P31499629	chr18:12395086-12395130	NM_008720:-213	Npc1	PROMOTER	0.238	21.550	3402.20	73318.83	5.13	3023.15	15508.87
A_68_P28600138	chr12:86114028-86114072	NM_023409:12	Npc2	INSIDE	0.46	0.237	2161.95	513.04	0.109	1571.66	171.67
A_68_P30368805	chr15:79659228-79659272	NM_001013360:5513	Npcd	INSIDE	0.593	5.782	1236.96	7151.81	3.431	1111.20	3812.75
A_68_P30368843	chr15:79664263-79664307	NM_001013360:479	Npcd	INSIDE	0.544	1.363	6481.54	8833.00	0.741	4357.32	3229.42
A_68_P21071913	chr2:25258906-25258950	NM_008721:326	Npdc1	INSIDE	0.139	17.554	2181.10	38286.51	2.437	1847.93	4503.43
A_68_P21071911	chr2:25258679-25258723	NM_008721:98	Npdc1	INSIDE	0.359	0.514	1545.55	794.64	0.185	1205.80	222.51
A_68_P21071909	chr2:25258527-25258571	NM_008721:-54	Npdc1	PROMOTER	0.291	2.333	4875.49	11374.09	0.678	3855.83	2613.25
A_68_P21873559	chr2:173946554-173946598	NM_213733:10725	Npepl1	INSIDE	0.35	5.104	1348.35	6881.92	1.788	1033.29	1847.79
A_68_P27181450	chr10:61088648-61088692	NM_001177511:30439	Npffr1	INSIDE	0.404	0.518	1924.51	996.82	0.209	1457.03	305.01
A_68_P26789122	chr9:103904643-103904687	NM_172460:-209	Nphp3	PROMOTER	0.55	0.288	4715.33	1358.72	0.158	3399.71	538.68
A_68_P26789125	chr9:103905125-103905169	NM_172460:273	Nphp3	INSIDE	0.613	0.595	2100.16	1250.59	0.365	1586.19	579.41
A_68_P26789124	chr9:103904969-103905013	NM_172460:117	Nphp3	INSIDE	0.33	1.976	5669.05	11204.36	0.652	3896.97	2539.81
A_68_P26789123	chr9:103904786-103904830	NM_172460:-65	Nphp3	PROMOTER	0.472	1.416	9209.55	13036.32	0.668	5971.48	3990.32
A_68_P25031675	chr7:31250465-31250509	NR_004443:124	Nphs1as	INSIDE	0.533	0.366	2168.92	793.02	0.195	1630.11	317.58
A_68_P28184543	chr11:120298753-120298797	NM_001195023:240	Nploc4	INSIDE	0.431	4.035	12173.16	49118.74	1.738	9211.39	16011.99
A_68_P22325410	chr3:90270013-90270057	NM_008727:-246	Npr1	PROMOTER	0.439	4.875	1593.01	7765.51	2.142	1249.12	2676.11
A_68_P22872800	chr4:43644731-43644775	NM_173788:-54	Npr2	PROMOTER	0.354	0.608	4537.89	2759.18	0.215	2628.48	565.31
A_68_P22872799	chr4:43644616-43644660	NM_173788:-168	Npr2	PROMOTER	0.306	1.510	3989.89	6023.00	0.461	2694.36	1243.33
A_68_P30018903	chr15:11836061-11836105	NM_001039181:-653	Npr3	PROMOTER	0.661	0.473	1950.79	922.98	0.313	1573.85	491.98
A_68_P30018904	chr15:11836154-11836198	NM_001039181:-747	Npr3	PROMOTER	0.304	5.758	1783.79	10271.45	1.748	1611.43	2817.16
A_68_P26548747	chr9:58430661-58430705	NM_009145:636	Nptn	INSIDE	0.499	0.367	1284.33	470.83	0.183	1002.96	183.65
A_68_P28178599	chr11:119410964-119411008	NM_008730:-1852	Nptx1	PROMOTER	0.326	5.934	1720.80	10211.95	1.934	1380.81	2670.43
A_68_P28178578	chr11:119408323-119408368	NM_008730:789	Nptx1	INSIDE	0.596	0.322	3494.07	1126.50	0.192	2456.94	472.42
A_68_P28178598	chr11:119410881-119410925	NM_008730:-1768	Nptx1	PROMOTER	0.609	0.606	1432.17	867.71	0.369	1383.18	510.55
A_68_P24434512	chr6:49772812-49772856	NM_023456:107	Npy	INSIDE	0.426	4.931	3294.21	16244.79	2.099	2427.74	5096.37
A_68_P24434511	chr6:49772705-49772749	NM_023456:-1	Npy	PROMOTER	0.533	0.736	2189.44	1610.63	0.392	1559.49	611.85
A_68_P24434508	chr6:49772350-49772395	NM_023456:-355	Npy	PROMOTER	0.576	4.067	462.84	1882.26	2.342	405.06	948.81
A_68_P24434509	chr6:49772474-49772518	NM_023456:-231	Npy	PROMOTER	0.236	1.526	804.45	1227.73	0.36	628.74	226.11

ProbeName	Target position of probe on CpG island microarray	TargetID	GeneSymbol	CpG island Description	Ratio of relative methylation (TiO_2-NP/Vehicle)	Sham group			TiO_2-H group		
						Relative methylation (Cy5/Cy3)	Cy3 signal (Input)	Cy5 signal (MeDIP)	Relative methylation (Cy5/Cy3)	Cy3 signal (Input)	Cy5 signal (MeDIP)
A_68_P29437678	chr14:19071659-19071703	NM_011584:-60	Nr1d2	PROMOTER	0.309	1.708	5735.36	9794.93	0.528	4165.97	2197.81
A_68_P29437679	chr14:19071747-19071791	NM_011584:-148	Nr1d2	PROMOTER	0.338	0.595	1326.13	788.41	0.201	885.80	177.75
A_68_P25088125	chr7:51807692-51807736	NM_009473:1579	Nr1h2	INSIDE	0.06	45.256	6124.76	277181.50	2.728	5354.14	14605.14
A_68_P25088126	chr7:51807778-51807822	NM_009473:1493	Nr1h2	INSIDE	0.082	48.836	5011.53	244744.90	4.008	4299.09	17230.36
A_68_P25088136	chr7:51808933-51808977	NM_009473:339	Nr1h2	INSIDE	0.229	22.535	10921.18	246112.50	5.153	10635.21	54802.82
A_68_P27357767	chr10:93610648-93610692	NM_011629:-5	Nr2c1	PROMOTER	0.618	4.891	3680.06	17999.77	3.021	3083.36	9314.46
A_68_P24643143	chr6:92042164-92042208	NM_011630:775	Nr2c2	INSIDE	0.499	4.082	1422.51	5807.19	2.038	1104.82	2251.52
A_68_P27095712	chr10:42303728-42303772	NM_152229:-356	Nr2e1	PROMOTER	0.255	62.638	8325.78	521511.30	15.997	8379.40	134048.60
A_68_P27095676	chr10:42299357-42299401	NM_152229:4016	Nr2e1	INSIDE	0.292	13.823	3183.87	44011.44	4.035	2999.90	12105.90
A_68_P27095770	chr10:42311388-42311432	NM_152229:-8016	Nr2e1	PROMOTER	0.428	0.414	1661.77	687.90	0.177	1265.41	224.02
A_68_P27095710	chr10:42303461-42303505	NM_152229:-88	Nr2e1	PROMOTER	0.546	0.548	1911.84	1048.26	0.299	1435.69	429.90
A_68_P27095769	chr10:42311289-42311333	NM_152229:-7916	Nr2e1	PROMOTER	0.569	3.072	753.72	2315.49	1.749	593.55	1038.14
A_68_P29162267	chr13:78334860-78334904	NM_010151:3361	Nr2f1	INSIDE	0.221	0.389	6558.08	2552.63	0.086	4038.21	347.14
A_68_P25210716	chr7:77496064-77496108	NM_009697:9393	Nr2f2	DOWNSTREAM	0.363	3.716	3606.36	13400.93	1.35	3075.23	4152.40
A_68_P25956629	chr8:73904896-73904940	NM_010150:933	Nr2f6	INSIDE	0.478	0.200	2556.06	510.33	0.095	1801.77	171.79
A_68_P25956630	chr8:73905073-73905117	NM_010150:757	Nr2f6	INSIDE	0.3	0.633	1129.83	714.65	0.189	913.03	172.97
A_68_P31640479	chr18:39649428-39649472	NM_008173:-2551	Nr3c1	PROMOTER	0.515	0.621	705.28	437.83	0.32	532.99	170.55
A_68_P25979817	chr8:79425933-79425977	NM_001083906:-452	Nr3c2	DIVERGENT_PROMOTER	0.592	0.604	932.10	562.94	0.357	798.88	285.56
A_68_P25981540	chr8:79709769-79709813	NM_001083906:283384	Nr3c2	INSIDE	0.109	20.110	639.26	12855.60	2.185	427.93	934.94
A_68_P21240085	chr2:56976720-56976764	NM_001139509:-328	Nr4a2	PROMOTER	0.599	3.716	1222.46	4543.02	2.226	1147.64	2554.09
A_68_P21240022	chr2:56969332-56969376	NM_013613:-1905	Nr4a2	PROMOTER	0.646	3.098	1519.54	4708.16	2.002	1291.94	2586.20
A_68_P22898222	chr4:48064747-48064791	NM_015743:649	Nr4a3	INSIDE	0.338	5.198	2920.07	15179.05	1.757	2302.71	4046.67
A_68_P22898162	chr4:48057954-48057998	NM_015743:-6143	Nr4a3	PROMOTER	0.648	0.237	2002.91	473.77	0.153	1487.38	227.84
A_68_P21149827	chr2:38544490-38544534	NM_139051:25550	Nr5a1	DOWNSTREAM	0.548	7.045	1813.04	12772.41	3.859	1685.38	6504.55
A_68_P21150017	chr2:38567253-38567297	NM_139051:2788	Nr5a1	INSIDE	0.56	4.863	2076.42	10097.60	2.721	1697.95	4620.07
A_68_P21151183	chr2:38781338-38781382	NM_010264:621	Nr6a1	INSIDE	0.663	0.528	2382.09	1256.91	0.35	1833.88	641.37
A_68_P21151181	chr2:38781046-38781090	NM_010264:913	Nr6a1	INSIDE	0.432	1.521	2427.00	3692.42	0.657	1989.57	1307.05
A_68_P21070358	chr2:25036517-25036561	NM_025980:261	Nrarp	INSIDE	0.506	4.466	897.58	4008.82	2.261	796.83	1801.97
A_68_P27211071	chr10:66748168-66748213	NM_001036293:-161	Nrbf2	PROMOTER	0.475	0.529	1658.00	877.22	0.251	1343.10	337.73
A_68_P23577231	chr5:31543485-31543529	NM_147201:216	Nrbp1	INSIDE	0.498	5.367	1621.10	8700.73	2.674	1273.16	3404.95
A_68_P30346015	chr15:75921006-75921050	NM_144847:-585	Nrbp2	PROMOTER	0.241	8.705	3058.82	26625.67	2.097	2702.78	5667.30
A_68_P30346013	chr15:75920805-75920849	NM_144847:-383	Nrbp2	PROMOTER	0.277	7.205	2431.10	17515.78	1.998	2105.37	4207.54
A_68_P28397452	chr12:45429719-45429763	NM_001146031:-131	Nrcam	PROMOTER	0.425	5.271	1107.69	5838.85	2.241	972.97	2180.73
A_68_P24329792	chr6:29998675-29998719	NM_001164230:709	Nrf1	INSIDE	0.444	3.712	1469.61	5455.56	1.649	1245.59	2054.38
A_68_P31621449	chr18:36178281-36178325	NM_001167891:178512	Nrg2	INSIDE	0.422	4.343	3842.15	16686.84	1.832	2967.01	5435.91
A_68_P31621446	chr18:36177796-36177840	NM_001167891:178996	Nrg2	INSIDE	0.441	18.345	1274.49	23380.37	8.095	1182.84	9574.69
A_68_P31622624	chr18:36356271-36356315	NM_001167891:522	Nrg2	INSIDE	0.49	5.751	2485.76	14294.83	2.818	2050.19	5776.54
A_68_P31621448	chr18:36178193-36178239	NM_001167891:178598	Nrg2	INSIDE	0.556	12.103	4804.38	58147.81	6.733	4345.50	29256.46
A_68_P31621829	chr18:36227658-36227702	NM_001167891:129134	Nrg2	INSIDE	0.599	2.847	961.69	2737.82	1.705	755.32	1288.06
A_68_P28950705	chr13:36816445-36816489	NM_153529:9857	Nrn1	DOWNSTREAM	0.148	40.311	3981.54	160501.10	5.974	3512.43	20982.29
A_68_P28950790	chr13:36827018-36827062	NM_153529:-717	Nrn1	PROMOTER	0.568	2.527	4822.60	12187.79	1.436	3690.97	5300.89
A_68_P20282811	chr1:62749708-62749752	NM_001077406:-160	Nrp2	PROMOTER	0.303	22.058	3116.08	68733.06	6.673	2738.95	18275.69
A_68_P31438308	chr17:90854876-90854920	NM_020252:637244	Nrxn1	INSIDE	0.522	0.673	1789.80	1205.27	0.351	1402.90	492.81
A_68_P31935125	chr19:6497430-6497474	NM_001205234:78715	Nrxn2	INSIDE	0.52	4.166	1142.61	4759.61	2.167	949.70	2057.76
A_68_P31935415	chr19:6532017-6532061	NM_001205234:113301	Nrxn2	INSIDE	0.553	0.258	1928.09	498.12	0.143	1354.18	193.37
A_68_P31935410	chr19:6531540-6531584	NM_001205234:112825	Nrxn2	INSIDE	0.659	0.577	1907.46	1101.02	0.381	1340.08	510.08

ProbeName	Target position of probe on CpG island microarray	TargetID	GeneSymbol	CpG island Description	Ratio of relative methylation (TiO_2-NP/Vehicle)	Sham group			TiO_2-H group		
						Relative methylation (Cy5/Cy3)	Cy3 signal (Input)	Cy5 signal (MeDIP)	Relative methylation (Cy5/Cy3)	Cy3 signal (Input)	Cy5 signal (MeDIP)
A_68_P31934666	chr19:6428995-6429039	NM_001205234:10279	Nrxn2	INSIDE	0.33	1.937	4836.47	9367.25	0.64	3239.76	2073.03
A_68_P31935127	chr19:6497654-6497698	NM_001205234:78939	Nrxn2	INSIDE	0.263	2.585	2736.40	7072.29	0.681	2172.33	1479.01
A_68_P23617871	chr5:38550739-38550783	NM_010942:-54	Nsg1	PROMOTER	0.329	10.944	4175.85	45700.36	3.598	3548.73	12767.99
A_68_P23617869	chr5:38550496-38550540	NM_010942:188	Nsg1	INSIDE	0.637	0.710	1987.09	1410.59	0.452	1487.18	672.75
A_68_P22705748	chr4:6380821-6380865	NM_010945:576	Nsmaf	INSIDE	0.42	3.696	1170.75	4327.39	1.554	989.08	1536.67
A_68_P25522280	chr7:137690501-137690545	NM_001162855:373	Nsmce4a	INSIDE	0.372	0.380	2317.15	880.71	0.141	1698.29	239.90
A_68_P25522281	chr7:137690595-137690639	NM_001162855:279	Nsmce4a	INSIDE	0.545	0.389	1169.31	454.95	0.212	871.10	184.87
A_68_P23227486	chr4:115726398-115726442	NM_028142:61	Nsun4	INSIDE	0.36	0.195	4148.25	808.00	0.07	2701.00	189.29
A_68_P23760679	chr5:66651534-66651578	NM_027602:193	Nsun7	INSIDE	0.131	2.525	2681.53	6770.13	0.331	1761.27	583.73
A_68_P23760677	chr5:66651381-66651425	NM_027602:39	Nsun7	INSIDE	0.496	3.219	1611.69	5188.34	1.596	1310.00	2090.26
A_68_P23760676	chr5:66651307-66651351	NM_027602:-35	Nsun7	PROMOTER	0.648	0.510	905.12	461.50	0.33	731.09	241.61
A_68_P23760674	chr5:66651117-66651161	NM_027602:-225	Nsun7	PROMOTER	0.622	0.663	874.08	579.66	0.413	769.24	317.43
A_68_P32147200	chr19:47089737-47089781	NM_001164363:-79	Nt5c2	PROMOTER	0.567	6.906	767.42	5300.14	3.918	634.82	2487.15
A_68_P27056941	chr10:34138297-34138341	NM_176968:16	Nt5dc1	INSIDE	0.215	0.566	5092.58	2880.23	0.122	3390.74	413.21
A_68_P29509959	chr14:31952078-31952122	NM_027289:4062	Nt5dc2	INSIDE	0.321	10.066	4899.76	49319.37	3.235	3283.42	10622.75
A_68_P27315977	chr10:86241971-86242015	NM_175331:243	Nt5dc3	INSIDE	0.354	24.494	2104.38	51544.07	8.677	1868.15	16209.37
A_68_P30559120	chr16:13819791-13819845	NM_010946:449	Ntan1	INSIDE	0.298	11.498	720.52	8284.46	3.424	568.52	1946.44
A_68_P25092988	chr7:52671206-52671250	NM_198190:2164	Ntf5	INSIDE	0.404	0.685	2026.27	1388.51	0.277	1357.91	376.12
A_68_P25092986	chr7:52670982-52671026	NM_198190:1940	Ntf5	INSIDE	0.517	4.733	506.81	2398.72	2.446	418.74	1024.37
A_68_P25092990	chr7:52671388-52671432	NM_198190:2346	Ntf5	INSIDE	0.662	2.842	940.30	2672.18	1.882	753.32	1417.53
A_68_P25092987	chr7:52671096-52671140	NM_198190:2054	Ntf5	INSIDE	0.493	1.388	2877.89	3994.94	0.684	2100.49	1436.64
A_68_P31098618	chr17:24769679-24769723	NM_008743:74	Nthl1	INSIDE	0.167	3.861	1894.53	7315.67	0.645	1554.24	1001.76
A_68_P27887968	chr11:68026330-68026374	NM_008744:173976	Ntn1	INSIDE	0.224	29.224	4129.55	120683.70	6.555	4151.68	27215.64
A_68_P27889095	chr11:68200015-68200060	NM_008744:291	Ntn1	INSIDE	0.386	0.482	1272.93	614.17	0.186	917.14	170.95
A_68_P27889094	chr11:68199927-68199971	NM_008744:380	Ntn1	INSIDE	0.442	0.650	3640.90	2366.21	0.287	2795.25	802.49
A_68_P25094516	chr7:52949587-52949631	NM_001033356:9825	Ntn5	INSIDE	0.291	22.910	3440.59	78824.36	6.658	2200.81	14653.29
A_68_P22422573	chr3:109946012-109946056	NM_001163348:356	Ntng1	INSIDE	0.6	0.548	1361.28	746.44	0.329	1095.19	360.57
A_68_P22422570	chr3:109945667-109945711	NM_001163348:702	Ntng1	INSIDE	0.092	16.948	2123.04	35980.28	1.563	1821.54	2846.42
A_68_P21095673	chr2:29050527-29050571	NM_133500:53012	Ntng2	INSIDE	0.251	0.393	2535.37	997.28	0.099	1817.28	179.27
A_68_P26253528	chr8:128258194-128258238	NM_025636:114	Ntpcr	INSIDE	0.374	8.234	4091.79	33690.40	3.082	3218.72	9920.73
A_68_P26253526	chr8:128258037-128258081	NM_025636:-44	Ntpcr	PROMOTER	0.232	6.836	1908.56	13047.68	1.586	1625.68	2578.00
A_68_P22310062	chr3:87599138-87599182	NM_001033124:-76	Ntrk1	DIVERGENT_PROMOTER	0.421	11.026	1007.03	11103.28	4.642	762.42	3539.47
A_68_P22310063	chr3:87599240-87599284	NM_001033124:-178	Ntrk1	DIVERGENT_PROMOTER	0.287	0.600	2295.65	1376.87	0.172	1624.96	279.34
A_68_P29075362	chr13:58909694-58909740	NM_001025074:523	Ntrk2	INSIDE	0.352	7.359	647.17	4762.47	2.593	506.67	1313.64
A_68_P29075346	chr13:58907905-58907950	NM_008745:-29	Ntrk2	PROMOTER	0.467	0.243	2056.18	500.24	0.114	1516.35	172.35
A_68_P29075348	chr13:58908057-58908101	NM_001025074:-1115	Ntrk2	PROMOTER	0.498	0.141	4100.58	577.28	0.07	2446.10	171.33
A_68_P29075350	chr13:58908196-58908240	NM_001025074:-975	Ntrk2	PROMOTER	0.635	26.662	8517.08	227082.40	16.937	7482.81	126733.00
A_68_P23543337	chr5:24191628-24191672	NM_016736:18	Nub1	INSIDE	0.463	0.287	4834.89	1388.88	0.133	2959.02	393.70
A_68_P30542115	chr16:10411996-10412040	NM_011955:-12	Nubp1	PROMOTER	0.335	0.564	1090.10	615.31	0.189	908.91	171.68
A_68_P28433223	chr12:53198848-53198892	NM_029760:138	Nubpl	INSIDE	0.52	3.447	2020.88	6966.46	1.794	1650.30	2960.76
A_68_P25093531	chr7:52765530-52765574	NM_001163662:226	Nucb1	INSIDE	0.467	0.519	2890.71	1498.92	0.242	2137.16	517.47
A_68_P23318161	chr4:133101757-133101801	NM_010948:164	Nudc	INSIDE	0.571	10.450	3080.14	32186.97	5.965	2673.64	15947.66
A_68_P27567294	chr11:6099870-6099914	NM_173748:562	Nudcd3	INSIDE	0.262	2.000	2225.27	4451.02	0.523	1785.58	934.43
A_68_P26795688	chr9:105033875-105033919	NM_029385:239	Nudt16	INSIDE	0.012	203.975	576.83	117659.00	2.399	426.44	1022.96
A_68_P25049946	chr7:36340772-36340816	NM_033080:153	Nudt19	INSIDE	0.595	5.475	2268.58	12419.67	3.257	1917.20	6244.42
A_68_P31117527	chr17:27760515-27760559	NM_019837:-139	Nudt3	PROMOTER	0.642	2.678	704.83	1887.89	1.719	661.19	1136.69

ProbeName	Target position of probe on CpG island microarray	TargetID	GeneSymbol	CpG island Description	Ratio of relative methylation (TiO_2-NP/Vehicle)	Sham group			TiO_2-H group		
						Relative methylation (Cy5/Cy3)	Cy3 signal (Input)	Cy5 signal (MeDIP)	Relative methylation (Cy5/Cy3)	Cy3 signal (Input)	Cy5 signal (MeDIP)
A_68_P22063510	chr3:37318580-37318625	NM_153561:-90	Nudt6	DIVERGENT_PROMOTER	0.635	2.286	6968.69	15929.59	1.453	4674.85	6790.54
A_68_P23950024	chr5:104475955-104475999	NM_028794:-53	Nudt9	PROMOTER	0.608	3.501	890.14	3116.24	2.128	753.08	1602.68
A_68_P27938848	chr11:77500486-77500530	NM_001024205:868	Nufip2	INSIDE	0.344	4.464	2147.89	9588.86	1.535	1796.34	2757.21
A_68_P27483044	chr10:117229909-117229953	NM_134010:-169	Nup107	PROMOTER	0.295	7.828	5192.53	40648.95	2.307	3845.52	8869.74
A_68_P29009458	chr13:46822289-46822335	NM_175749:906	Nup153	INSIDE	0.226	7.408	2218.41	16432.93	1.675	1729.67	2896.42
A_68_P29009460	chr13:46822473-46822517	NM_175749:724	Nup153	INSIDE	0.4	3.832	3956.24	15161.45	1.533	3021.28	4630.77
A_68_P29009466	chr13:46823130-46823174	NM_175749:66	Nup153	INSIDE	0.594	2.711	1851.45	5019.40	1.611	1473.74	2374.03
A_68_P29998115	chr15:8059531-8059575	NM_133227:240	Nup155	INSIDE	0.598	0.285	2330.93	665.33	0.171	1626.47	277.41
A_68_P21418380	chr2:90519239-90519283	NM_021512:1889	Nup160	INSIDE	0.332	18.086	6782.75	122670.20	6.006	5940.62	35682.06
A_68_P21418382	chr2:90519554-90519598	NM_021512:2205	Nup160	INSIDE	0.361	16.604	6101.25	101305.10	5.991	6189.93	37086.19
A_68_P24360134	chr6:35127844-35127888	NM_027513:251	Nup205	INSIDE	0.61	4.052	1340.92	5433.28	2.473	1078.18	2665.95
A_68_P24360135	chr6:35127937-35127981	NM_027513:343	Nup205	INSIDE	0.644	0.395	1167.59	460.62	0.254	918.89	233.45
A_68_P24636902	chr6:91066754-91066798	NM_018815:44	Nup210	INSIDE	0.438	9.384	1083.64	10169.05	4.111	885.74	3641.48
A_68_P23894271	chr5:92863563-92863607	NM_183392:641	Nup54	INSIDE	0.478	5.751	2516.69	14472.77	2.752	2170.98	5973.89
A_68_P32697224	chrX:136596652-136596696	NM_001081668:433	Nup62cl	INSIDE	0.555	4.242	2006.00	8510.10	2.352	2644.37	6220.86
A_68_P26076074	chr8:96738369-96738413	NM_172410:-110	Nup93	PROMOTER	0.339	0.587	1135.20	666.86	0.199	911.68	181.42
A_68_P27931783	chr11:76212254-76212298	NM_008750:367	Nxn	INSIDE	0.475	4.066	961.47	3909.63	1.93	809.69	1562.75
A_68_P29031120	chr13:51266739-51266783	NM_029173:367	Nxnl2	INSIDE	0.385	8.118	2552.68	20723.69	3.124	2146.50	6705.41
A_68_P28039255	chr11:95375266-95375310	NM_130858:591	Nxph3	INSIDE	0.216	25.981	6314.19	164045.80	5.62	5878.50	33037.56
A_68_P28039256	chr11:95375387-95375431	NM_130858:471	Nxph3	INSIDE	0.452	0.587	8493.79	4984.50	0.265	5854.98	1551.89
A_68_P28039252	chr11:95374960-95375004	NM_130858:897	Nxph3	INSIDE	0.632	5.451	3174.08	17302.23	3.443	2361.14	8129.37
A_68_P27536454	chr10:126971360-126971404	NM_183297:233	Nxph4	INSIDE	0.171	2.398	2023.28	4852.81	0.411	1414.25	581.01
A_68_P26460524	chr9:43048046-43048090	NM_178644:-169	Oaf	PROMOTER	0.504	0.197	2592.80	510.80	0.099	1977.12	196.26
A_68_P26588950	chr9:65524268-65524312	NM_010952:-64	Oaz2-ps	PROMOTER	0.513	0.644	1034.04	666.15	0.33	828.51	273.74
A_68_P27835027	chr11:58949650-58949694	NM_001171512:205	Obscn	INSIDE	0.272	0.520	1460.24	759.87	0.141	1196.70	169.32
A_68_P27835010	chr11:58947926-58947970	NM_001171512:1929	Obscn	INSIDE	0.38	10.105	1866.07	18855.90	3.836	1561.03	5987.57
A_68_P20354543	chr1:75501853-75501897	NM_178884:1153	Obsl1	INSIDE	0.464	0.501	1489.61	746.05	0.232	1075.69	249.78
A_68_P20354552	chr1:75502961-75503005	NM_178884:45	Obsl1	INSIDE	0.578	0.466	1605.97	747.87	0.269	1165.75	313.76
A_68_P20354547	chr1:75502266-75502310	NM_178884:739	Obsl1	INSIDE	0.537	0.583	1351.84	788.21	0.313	1038.20	325.15
A_68_P29269015	chr13:101322806-101322850	NM_008756:-375	Ocln	PROMOTER	0.407	0.577	2297.39	1325.03	0.235	1472.76	345.37
A_68_P29269014	chr13:101322735-101322779	NM_008756:-303	Ocln	PROMOTER	0.562	0.399	5134.24	2048.17	0.224	3359.35	752.93
A_68_P29269012	chr13:101322550-101322594	NM_008756:-119	Ocln	PROMOTER	0.324	4.535	896.75	4067.00	1.471	1110.84	1633.54
A_68_P28271091	chr12:17551967-17552011	NM_013614:310	Odc1	INSIDE	0.348	6.048	2852.57	17251.29	2.106	2346.28	4941.16
A_68_P28271087	chr12:17551396-17551440	NM_013614:-260	Odc1	PROMOTER	0.646	0.497	1751.27	870.06	0.321	1203.10	386.08
A_68_P22606649	chr3:144781741-144781785	NM_025714:199	Odf2l	INSIDE	0.285	14.518	4727.28	68630.81	4.142	3794.73	15719.34
A_68_P27710860	chr11:35952997-35953056	NM_011856:804719	Odz2	INSIDE	0.346	0.484	2429.40	1175.52	0.167	1562.01	261.42
A_68_P27710218	chr11:35860780-35860826	NM_011856:896943	Odz2	INSIDE	0.315	4.937	1923.99	9497.90	1.553	1299.69	2018.82
A_68_P25848475	chr8:49640835-49640879	NM_001145937:119188	Odz3	INSIDE	0.369	0.581	1916.22	1112.69	0.214	1487.56	318.56
A_68_P25848476	chr8:49641034-49641078	NM_001145937:118988	Odz3	INSIDE	0.457	0.549	4165.93	2285.66	0.251	2781.98	697.24
A_68_P25343512	chr7:103359060-103359104	NM_011858:-64	Odz4	PROMOTER	0.397	38.101	2285.45	87077.32	15.127	2165.61	32758.66
A_68_P27567701	chr11:6191334-6191378	NM_010956:-243	Ogdh	PROMOTER	0.478	0.735	2173.78	1597.99	0.352	1542.47	542.49
A_68_P29516971	chr14:33135063-33135107	NM_001081130:-120	Ogdhl	PROMOTER	0.659	0.624	1007.18	628.21	0.411	913.51	375.43
A_68_P21904884	chr2:180324017-180324061	NM_031373:-73	Ogfr	PROMOTER	0.592	2.863	1045.20	2992.04	1.695	992.83	1682.89
A_68_P24758832	chr6:113276968-113277012	NM_010957:21	Ogg1	INSIDE	0.372	10.029	10699.42	107305.60	3.734	8012.92	29921.16
A_68_P21330227	chr2:73052203-73052247	NM_025942:280	Ola1	INSIDE	0.545	0.582	2866.94	1668.53	0.317	2220.20	704.53
A_68_P21089690	chr2:28060930-28060989	NM_001038612:-249	Olfm1	PROMOTER	0.359	0.564	1500.00	846.40	0.203	1119.31	227.04

ProbeName	Target position of probe on CpG island microarray	TargetID	GeneSymbol	CpG island Description	Ratio of relative methylation (TiO_2-NP/Vehicle)	Sham group			TiO_2-H group		
						Relative methylation (Cy5/Cy3)	Cy3 signal (Input)	Cy5 signal (MeDIP)	Relative methylation (Cy5/Cy3)	Cy3 signal (Input)	Cy5 signal (MeDIP)
A_68_P21089590	chr2:28048879-28048923	NM_001038614:288	Olfm1	INSIDE	0.235	7.088	1513.87	10730.84	1.663	1290.91	2146.84
A_68_P21089695	chr2:28061711-28061755	NM_001038612:524	Olfm1	INSIDE	0.622	2.619	1781.67	4665.33	1.63	1436.98	2341.80
A_68_P26343627	chr9:20532217-20532261	NM_173777:420	Olfm2	INSIDE	0.339	8.213	1862.67	15297.24	2.786	1835.77	5114.73
A_68_P26343622	chr9:20531703-20531748	NM_173777:933	Olfm2	INSIDE	0.621	0.319	3075.19	980.37	0.198	2381.44	471.48
A_68_P26343617	chr9:20530951-20530995	NM_173777:1686	Olfm2	INSIDE	0.433	0.518	1058.10	548.06	0.224	783.61	175.55
A_68_P27833087	chr11:58660940-58660984	NM_146537:6345	Olfr311	DOWNSTREAM	0.421	0.424	1129.41	479.43	0.179	965.28	172.54
A_68_P30961698	chr16:91227011-91227055	NM_016967:1238	Olig2	INSIDE	0.375	0.464	1249.35	579.60	0.174	1062.44	185.01
A_68_P30961697	chr16:91226847-91226891	NM_016967:1074	Olig2	INSIDE	0.394	6.542	5567.01	36422.09	2.578	4249.40	10954.07
A_68_P30961642	chr16:91220360-91220404	NM_016967:-5412	Olig2	PROMOTER	0.496	0.283	1587.40	449.33	0.14	1246.74	175.07
A_68_P30961686	chr16:91225569-91225613	NM_016967:-204	Olig2	PROMOTER	0.64	0.304	2629.07	800.21	0.195	1875.02	364.98
A_68_P26980660	chr10:19077044-19077088	NM_053008:722	Olig3	INSIDE	0.222	0.599	7506.11	4498.61	0.133	4946.21	658.21
A_68_P23165465	chr4:102986727-102986771	NM_025909:298	Oma1	INSIDE	0.5	6.059	1403.97	8506.77	3.029	1382.51	4186.96
A_68_P23165462	chr4:102986330-102986374	NM_025909:-98	Oma1	PROMOTER	0.514	0.461	1519.70	701.05	0.237	1143.07	271.17
A_68_P23165464	chr4:102986645-102986689	NM_025909:216	Oma1	INSIDE	0.549	0.467	1189.22	555.07	0.256	974.75	249.59
A_68_P26640627	chr9:74710721-74710765	NM_008262:1015	Onecut1	INSIDE	0.24	0.480	2957.01	1420.21	0.115	2078.59	239.48
A_68_P26640632	chr9:74711201-74711245	NM_008262:1495	Onecut1	INSIDE	0.358	8.849	3472.56	30729.65	3.167	2743.16	8687.56
A_68_P26640623	chr9:74710266-74710310	NM_008262:561	Onecut1	INSIDE	0.496	7.179	2147.83	15419.09	3.564	1773.00	6318.86
A_68_P31774686	chr18:64500029-64500073	NM_194268:33	Onecut2	INSIDE	0.219	0.628	5931.93	3723.46	0.138	3936.79	541.98
A_68_P20849642	chr1:177622972-177623016	NM_010098:-273	Opn3	DIVERGENT_PROMOTER	0.636	5.353	3471.86	18585.79	3.406	3086.55	10512.64
A_68_P20849643	chr1:177623101-177623145	NM_010098:-401	Opn3	DIVERGENT_PROMOTER	0.268	2.264	4445.38	10062.20	0.607	3322.23	2017.76
A_68_P24053482	chr5:123464905-123464949	NM_175423:-156	Orai1	PROMOTER	0.136	14.484	1060.55	15360.69	1.975	960.72	1897.46
A_68_P24119778	chr5:136646540-136646584	NM_178751:-36	Orai2	PROMOTER	0.45	5.842	836.92	4889.29	2.627	733.34	1926.26
A_68_P25507195	chr7:134913265-134913309	NM_198424:-42	Orai3	PROMOTER	0.613	0.222	2175.93	483.65	0.136	1638.56	223.10
A_68_P20259227	chr1:58561519-58561563	NM_001025378:-3280	Orc2	PROMOTER	0.589	4.259	686.39	2923.54	2.511	568.86	1428.17
A_68_P21199929	chr2:48804619-48804663	NR_033442:147	Orc4	INSIDE	0.477	0.491	1925.98	946.40	0.234	1453.81	340.76
A_68_P27280137	chr10:79441295-79441339	NM_001003949:5759	ORF61	INSIDE	0.208	11.511	1260.63	14511.06	2.399	787.90	1889.86
A_68_P27280132	chr10:79440564-79440611	NM_001003949:6488	ORF61	INSIDE	0.539	5.788	293.67	1699.88	3.123	256.51	801.00
A_68_P27534166	chr10:126558114-126558159	NM_001171026:80	Os9	INSIDE	0.224	0.515	2423.47	1249.02	0.115	1797.69	207.29
A_68_P27553494	chr11:3605439-3605483	NM_152818:158446	Osbp2	INSIDE	0.598	2.585	1511.05	3905.42	1.546	1122.76	1736.14
A_68_P31503253	chr18:13020852-13020896	NM_207530:79355	Osbpl1a	INSIDE	0.584	3.815	1063.76	4058.62	2.227	992.87	2211.00
A_68_P21901578	chr2:179854064-179854108	NM_144500:16	Osbpl2	INSIDE	0.09	3.704	6455.20	23912.12	0.333	4839.36	1611.83
A_68_P21350016	chr2:76244453-76244497	NM_145525:-120	Osbpl6	PROMOTER	0.655	0.424	2305.20	976.93	0.278	1736.07	482.02
A_68_P27447375	chr10:110602439-110602483	NM_001003717:603	Osbpl8	INSIDE	0.651	0.467	1566.98	731.98	0.304	1219.16	370.75
A_68_P22752864	chr4:15941113-15941157	NM_145950:-110	Osgin2	PROMOTER	0.235	16.835	1721.03	28973.48	3.949	1747.87	6901.82
A_68_P30132612	chr15:35225823-35225867	NM_054049:-22	Osr2	PROMOTER	0.416	4.479	1599.47	7164.60	1.863	1329.92	2477.58
A_68_P30132613	chr15:35225986-35226030	NM_054049:142	Osr2	INSIDE	0.608	0.360	1403.60	504.98	0.219	1072.54	234.70
A_68_P30132591	chr15:35223546-35223590	NM_054049:-2298	Osr2	PROMOTER	0.562	0.596	807.32	481.14	0.335	693.25	232.09
A_68_P23575371	chr5:31210102-31210146	NM_001134692:-29	Ost4	PROMOTER	0.605	0.470	1426.47	670.70	0.284	963.93	274.06
A_68_P23575369	chr5:31209729-31209773	NM_001134692:345	Ost4	INSIDE	0.587	2.833	1452.17	4113.31	1.662	1177.96	1957.94
A_68_P31992143	chr19:18706329-18706373	NM_017375:-47	Ostf1	DIVERGENT_PROMOTER	0.066	45.820	705.63	32332.11	3.031	606.12	1837.27
A_68_P27096130	chr10:42398668-42398712	NM_172416:-31	Ostm1	PROMOTER	0.236	34.151	4846.33	165506.70	8.06	5276.02	42523.68
A_68_P23618578	chr5:38668746-38668790	NM_172709:126	Otop1	INSIDE	0.377	5.384	2020.57	10877.80	2.031	1656.47	3364.31
A_68_P23618575	chr5:38668403-38668447	NM_172709:-218	Otop1	PROMOTER	0.64	2.865	3587.41	10278.44	1.834	2748.74	5040.53
A_68_P29237596	chr13:95653350-95653394	NM_011021:7791	Otp	INSIDE	0.361	6.564	1630.03	10699.32	2.369	1420.95	3366.45
A_68_P29237544	chr13:95647510-95647554	NM_011021:1951	Otp	INSIDE	0.418	8.459	803.47	6796.36	3.535	662.45	2342.04
A_68_P29237593	chr13:95652973-95653017	NM_011021:7413	Otp	INSIDE	0.434	0.354	1409.35	499.16	0.154	1121.49	172.26

ProbeName	Target position of probe on CpG island microarray	TargetID	GeneSymbol	CpG island Description	Ratio of relative methylation (TiO_2-NP/Vehicle)	Sham group			TiO_2-H group		
						Relative methylation (Cy5/Cy3)	Cy3 signal (Input)	Cy5 signal (MeDIP)	Relative methylation (Cy5/Cy3)	Cy3 signal (Input)	Cy5 signal (MeDIP)
A_68_P28697278	chr12:104627062-104627106	NM_001177841:193	Otub2	INSIDE	0.472	4.197	1689.66	7091.67	1.981	1421.72	2816.69
A_68_P23348674	chr4:138469719-138469763	NM_028453:122	Otud3	INSIDE	0.592	7.623	345.24	2631.58	4.513	260.28	1174.73
A_68_P25995588	chr8:82163767-82163811	NM_001081164:214	Otud4	INSIDE	0.374	10.673	527.04	5624.89	3.991	449.80	1795.00
A_68_P25995580	chr8:82162925-82162969	NM_001081164:-628	Otud4	PROMOTER	0.644	2.173	2281.28	4957.91	1.4	1862.88	2608.89
A_68_P22747167	chr4:14753409-14753453	NM_152812:304	Otud6b	INSIDE	0.41	0.624	3781.30	2359.60	0.256	3006.44	770.04
A_68_P22347475	chr3:95908828-95908872	NM_001025613:401	Otud7b	INSIDE	0.483	0.144	3567.04	513.91	0.07	2509.03	174.46
A_68_P22347472	chr3:95908537-95908581	NM_001025613:109	Otud7b	INSIDE	0.428	0.702	1435.41	1008.30	0.3	1199.47	360.22
A_68_P22347474	chr3:95908723-95908767	NM_001025613:295	Otud7b	INSIDE	0.584	0.736	5681.96	4184.31	0.43	4335.36	1863.95
A_68_P22347470	chr3:95908249-95908293	NM_001025613:-179	Otud7b	PROMOTER	0.547	3.399	597.16	2029.46	1.861	492.41	916.13
A_68_P27638910	chr11:21901664-21901708	NM_011023:-32	Otx1	PROMOTER	0.58	0.286	2168.11	620.05	0.166	1582.44	262.52
A_68_P27638837	chr11:21893202-21893246	NM_011023:8430	Otx1	DOWNSTREAM	0.656	2.965	862.32	2556.39	1.945	728.67	1417.23
A_68_P27638841	chr11:21893689-21893733	NM_011023:7944	Otx1	DOWNSTREAM	0.591	3.335	834.04	2781.36	1.969	636.89	1254.36
A_68_P27924667	chr11:74992384-74992428	NM_027136:-96	Ovca2	PROMOTER	0.399	14.983	2776.92	41605.28	5.972	2340.53	13978.79
A_68_P21706641	chr2:144156468-144156512	NM_152947:608	Ovol2	INSIDE	0.375	11.109	3392.51	37685.84	4.167	3852.27	16051.29
A_68_P21706649	chr2:144157501-144157545	NM_152947:-424	Ovol2	PROMOTER	0.392	0.355	1504.90	534.44	0.139	1212.79	168.91
A_68_P30167378	chr15:41620567-41620611	NM_001130163:-472	Oxr1	PROMOTER	0.359	13.937	1467.08	20446.09	5	1165.76	5828.55
A_68_P21630523	chr2:130402275-130402319	NM_011025:388	Oxt	INSIDE	0.606	3.229	3293.35	10632.68	1.955	2662.77	5207.04
A_68_P24051802	chr5:123157935-123157979	NM_011026:391	P2rx4	INSIDE	0.602	3.859	1353.86	5223.91	2.325	1147.60	2667.73
A_68_P22180925	chr3:60806609-60806653	NM_008772:-86	P2ry1	PROMOTER	0.563	3.096	1840.23	5697.39	1.744	1497.25	2611.95
A_68_P25370889	chr7:108147504-108147552	NM_008773:12977	P2ry2	INSIDE	0.194	2.385	3640.34	8683.04	0.462	2464.78	1139.41
A_68_P25370888	chr7:108147418-108147462	NM_008773:13065	P2ry2	INSIDE	0.325	9.426	1621.03	15280.04	3.061	1119.29	3426.53
A_68_P25370976	chr7:108160468-108160512	NM_008773:15	P2ry2	INSIDE	0.573	6.174	2020.15	12472.53	3.535	1785.01	6310.80
A_68_P25370975	chr7:108160402-108160446	NM_008773:81	P2ry2	INSIDE	0.293	0.708	2778.38	1968.12	0.207	2200.73	456.30
A_68_P27168078	chr10:58786437-58786481	NM_011030:415	P4ha1	INSIDE	0.128	28.226	3736.78	105473.00	3.622	3684.07	13342.36
A_68_P27542078	chr10:128003336-128003380	NM_011119:-368	Pa2g4	PROMOTER	0.278	5.312	4430.26	23532.50	1.477	3126.35	4616.50
A_68_P30139524	chr15:36538383-36538427	NM_008774:324	Pabpc1	INSIDE	0.15	0.479	8879.46	4255.60	0.072	5877.48	423.28
A_68_P30139531	chr15:36539130-36539174	NM_008774:-424	Pabpc1	PROMOTER	0.552	3.642	1319.91	4807.70	2.011	1111.23	2234.29
A_68_P30139527	chr15:36538715-36538759	NM_008774:-8	Pabpc1	PROMOTER	0.505	2.852	3355.60	9568.90	1.441	2718.06	3916.37
A_68_P32625282	chrX:117040767-117040811	NM_053114:-16	Pabpc5	PROMOTER	0.385	9.730	1798.22	17496.54	3.745	2107.84	7893.89
A_68_P31117689	chr17:27792526-27792570	NM_011861:-78	Pacsin1	PROMOTER	0.361	7.965	1179.05	9390.92	2.879	910.40	2620.94
A_68_P31117838	chr17:27822326-27822370	NM_178365:150	Pacsin1	INSIDE	0.476	6.484	4742.78	30750.53	3.087	3710.82	11454.31
A_68_P31117690	chr17:27792675-27792719	NM_011861:70	Pacsin1	INSIDE	0.567	0.641	1887.62	1210.79	0.364	1351.59	491.59
A_68_P21421545	chr2:91095901-91095945	NM_030880:-1047	Pacsin3	PROMOTER	0.381	11.652	1128.44	13148.06	4.437	1013.17	4495.89
A_68_P27922136	chr11:74536967-74537011	NR_037610:372	Pafah1b1	INSIDE	0.539	0.710	1265.32	898.65	0.383	1009.15	386.22
A_68_P26477175	chr9:45792523-45792567	NM_008775:410	Pafah1b2	INSIDE	0.645	2.810	2243.23	6303.73	1.813	1741.72	3157.73
A_68_P25009543	chr7:26082789-26082833	NM_008776:164	Pafah1b3	INSIDE	0.45	7.151	2824.78	20201.14	3.221	2067.08	6658.69
A_68_P21939654	chr3:9833650-9833694	NM_001195031:7	Pag1	INSIDE	0.541	0.381	1729.65	658.87	0.206	1385.24	285.29
A_68_P23819021	chr5:77380379-77380423	NM_025939:-35	Paics	PROMOTER	0.451	0.304	8363.93	2541.60	0.137	5303.09	726.55
A_68_P23819018	chr5:77380092-77380136	NM_025939:-321	Paics	PROMOTER	0.619	0.307	2695.99	826.54	0.19	1819.59	345.12
A_68_P24595787	chr6:83781567-83781611	NM_146169:147	Paip2b	INSIDE	0.335	1.710	2043.91	3494.24	0.573	1733.27	993.73
A_68_P28975465	chr13:41095865-41095909	NM_026550:-492	Pak1ip1	PROMOTER	0.53	0.518	1243.95	644.54	0.274	883.89	242.58
A_68_P30650846	chr16:32079318-32079362	NM_177326:19	Pak2	INSIDE	0.313	26.788	3755.46	100599.90	8.386	3458.00	29000.34
A_68_P30650841	chr16:32078788-32078844	NM_177326:543	Pak2	INSIDE	0.589	9.849	225.80	2223.77	5.805	176.28	1023.40
A_68_P25022782	chr7:29383016-29383060	NM_027470:165	Pak4	INSIDE	0.244	0.401	5364.48	2152.33	0.098	3842.95	376.42
A_68_P25022784	chr7:29383198-29383242	NM_027470:-17	Pak4	PROMOTER	0.501	5.118	1812.93	9277.71	2.566	1531.53	3930.21
A_68_P25911981	chr8:64238686-64238730	NM_001081390:142779	Palld	INSIDE	0.638	4.294	916.27	3934.11	2.74	861.27	2360.27

ProbeName	Target position of probe on CpG island microarray	TargetID	GeneSymbol	CpG island Description	Ratio of relative methylation (TiO_2-NP/Vehicle)	Sham group			TiO_2-H group		
						Relative methylation (Cy5/Cy3)	Cy3 signal (Input)	Cy5 signal (MeDIP)	Relative methylation (Cy5/Cy3)	Cy3 signal (Input)	Cy5 signal (MeDIP)
A_68_P25910880	chr8:64070053-64070097	NM_001081390:311413	Palld	INSIDE	0.646	9.035	3129.17	28272.33	5.839	2664.18	15556.16
A_68_P25911983	chr8:64238848-64238892	NM_001081390:142617	Palld	INSIDE	0.594	2.541	2100.35	5336.70	1.51	1569.52	2369.59
A_68_P26017663	chr8:86545319-86545363	NM_028877:-32	Palm3	DIVERGENT_PROMOTER	0.206	18.549	2207.36	40944.76	3.822	1887.34	7213.31
A_68_P26017662	chr8:86545209-86545255	NM_028877:-140	Palm3	DIVERGENT_PROMOTER	0.183	2.268	1245.14	2824.33	0.414	780.82	323.35
A_68_P20474869	chr1:99992118-99992162	NM_013626:69	Pam	INSIDE	0.379	5.052	1169.79	5910.16	1.913	1078.00	2061.93
A_68_P24173631	chr5:148241874-148241918	NM_028291:-259	Pan3	PROMOTER	0.599	5.143	3363.07	17296.50	3.081	2658.09	8188.84
A_68_P27708451	chr11:35582769-35582813	NM_145962:-206	Pank3	PROMOTER	0.318	0.523	1397.59	731.27	0.167	1056.64	176.04
A_68_P27708454	chr11:35583064-35583108	NM_145962:90	Pank3	INSIDE	0.338	5.069	6332.95	32101.38	1.712	5201.17	8906.74
A_68_P27708453	chr11:35582983-35583027	NM_145962:8	Pank3	INSIDE	0.539	0.239	1971.65	471.67	0.129	1361.41	175.45
A_68_P27708457	chr11:35583330-35583374	NM_145962:356	Pank3	INSIDE	0.635	3.373	600.18	2024.63	2.144	558.04	1196.29
A_68_P23435277	chr4:154338248-154338292	NM_172990:29	Pank4	INSIDE	0.535	12.199	5930.10	72342.88	6.523	4994.15	32579.02
A_68_P23435279	chr4:154338485-154338529	NM_172990:265	Pank4	INSIDE	0.599	0.554	7413.89	4104.02	0.331	5560.93	1842.69
A_68_P30422819	chr15:88890786-88890830	NM_001002005:653	Panx2	INSIDE	0.538	6.417	1565.46	10045.75	3.454	1402.24	4843.98
A_68_P30422817	chr15:88890519-88890563	NM_001002005:385	Panx2	INSIDE	0.6	3.165	766.69	2426.33	1.899	687.84	1306.22
A_68_P26039507	chr8:90723531-90723575	NM_001164497:441	Papd5	INSIDE	0.35	16.172	2495.74	40361.61	5.663	2223.68	12592.88
A_68_P29116251	chr13:69672583-69672627	NM_001169131:138	Papd7	INSIDE	0.173	12.721	449.70	5720.77	2.197	343.31	754.19
A_68_P27648124	chr11:23795033-23795077	NM_172555:216	Papolg	INSIDE	0.18	0.369	3919.35	1448.16	0.067	2619.80	174.63
A_68_P27648126	chr11:23795318-23795362	NM_172555:-70	Papolg	PROMOTER	0.347	7.697	4639.05	35706.53	2.673	4139.32	11063.87
A_68_P22532369	chr3:131227486-131227530	NM_011863:-223	Papss1	PROMOTER	0.447	0.287	1722.67	493.94	0.128	1338.38	171.68
A_68_P31093353	chr17:23876731-23876775	NM_023824:545	Paqr4	INSIDE	0.401	1.474	1677.42	2472.02	0.591	1241.24	733.31
A_68_P22313388	chr3:88170026-88170070	NM_198410:1538	Paqr6	INSIDE	0.038	16.223	4686.10	76023.95	0.616	3353.18	2065.33
A_68_P20074998	chr1:20880874-20880920	NM_028829:194	Paqr8	INSIDE	0.428	0.450	2148.42	966.56	0.193	1616.23	311.47
A_68_P26742500	chr9:95460884-95460928	NM_198414:671	Paqr9	INSIDE	0.489	3.956	1484.25	5871.13	1.934	1243.67	2404.78
A_68_P26742492	chr9:95459743-95459787	NM_198414:-471	Paqr9	PROMOTER	0.234	0.703	2517.68	1768.84	0.164	1907.99	313.17
A_68_P26742494	chr9:95459899-95459943	NM_198414:-315	Paqr9	PROMOTER	0.492	3.265	1239.71	4047.93	1.606	937.98	1506.42
A_68_P26261064	chr8:129588848-129588893	NM_001122850:915	Pard3	INSIDE	0.497	0.523	4128.19	2160.81	0.26	2853.95	743.11
A_68_P26261059	chr8:129588231-129588275	NM_001122850:297	Pard3	INSIDE	0.55	5.323	1834.66	9765.90	2.929	1410.85	4132.46
A_68_P26261061	chr8:129588412-129588456	NM_001122850:479	Pard3	INSIDE	0.55	0.619	2397.64	1483.39	0.34	1976.40	672.18
A_68_P21839353	chr2:167906491-167906535	NM_021409:9	Pard6b	INSIDE	0.193	21.921	2334.74	51178.77	4.238	2119.24	8981.94
A_68_P21839351	chr2:167906222-167906266	NM_021409:-259	Pard6b	PROMOTER	0.362	13.741	1204.19	16547.25	4.973	1182.24	5879.23
A_68_P30587017	chr16:20302514-20302558	NM_001005767:-101	Parl	PROMOTER	0.534	0.385	2393.82	922.71	0.206	1882.98	387.91
A_68_P20876659	chr1:182498959-182499003	NM_007415:-125	Parp1	PROMOTER	0.561	7.485	2330.79	17446.08	4.202	2065.89	8681.63
A_68_P24832848	chr6:127403924-127403968	NM_181402:206	Parp11	INSIDE	0.665	2.149	1688.07	3627.60	1.43	1441.79	2061.21
A_68_P24381543	chr6:39067895-39067939	NM_172893:432	Parp12	INSIDE	0.391	5.853	2016.33	11801.11	2.291	1647.17	3774.16
A_68_P26554375	chr9:59465216-59465260	NM_001205239:148	Parp6	INSIDE	0.455	9.165	2997.05	27467.10	4.173	2346.25	9791.85
A_68_P29360867	chr13:117813151-117813195	NM_001081009:1151	Parp8	INSIDE	0.542	4.343	2108.72	9159.16	2.354	1783.52	4198.80
A_68_P27551117	chr11:3189431-3189475	NM_019574:-1007	Patz1	PROMOTER	0.065	2.211	3385.85	7487.73	0.144	2638.23	379.08
A_68_P27551126	chr11:3190443-3190488	NM_019574:6	Patz1	INSIDE	0.547	2.862	1777.35	5086.40	1.565	1447.92	2266.69
A_68_P27551118	chr11:3189560-3189605	NM_019574:-877	Patz1	PROMOTER	0.198	6.930	4036.75	27975.50	1.371	3438.14	4714.94
A_68_P21723786	chr2:147191194-147191239	NM_008780:487	Pax1	INSIDE	0.665	20.511	670.62	13755.08	13.634	655.27	8933.76
A_68_P32134165	chr19:44825072-44825116	NM_011037:-6789	Pax2	PROMOTER	0.378	0.503	2070.40	1042.38	0.19	1376.71	261.96
A_68_P32134233	chr19:44833666-44833710	NM_011037:1805	Pax2	INSIDE	0.591	0.442	4629.59	2045.77	0.261	3281.49	856.37
A_68_P32134818	chr19:44911149-44911193	NM_011037:79287	Pax2	INSIDE	0.65	0.194	6713.00	1300.47	0.126	4614.66	581.06
A_68_P32134209	chr19:44830746-44830790	NM_011037:-1115	Pax2	PROMOTER	0.663	2.719	5207.22	14158.57	1.802	4079.18	7350.84
A_68_P32134813	chr19:44910513-44910557	NM_011037:78651	Pax2	INSIDE	0.281	0.723	2104.44	1521.21	0.203	1548.23	314.14
A_68_P22878697	chr4:44723513-44723557	NM_008782:-222	Pax5	PROMOTER	0.471	7.043	1725.62	12152.83	3.319	1327.63	4406.15

ProbeName	Target position of probe on CpG island microarray	TargetID	GeneSymbol	CpG island Description	Ratio of relative methylation (TiO_2-NP/Vehicle)	Sham group			TiO_2-H group		
						Relative methylation (Cy5/Cy3)	Cy3 signal (Input)	Cy5 signal (MeDIP)	Relative methylation (Cy5/Cy3)	Cy3 signal (Input)	Cy5 signal (MeDIP)
A_68_P22878646	chr4:44717054-44717098	NM_008782:6236	Pax5	INSIDE	0.533	6.133	1632.46	10011.37	3.268	1241.78	4058.51
A_68_P21495913	chr2:105509959-105510003	NR_002867:507	Pax6os1	INSIDE	0.542	0.478	1459.96	698.18	0.259	1162.57	301.44
A_68_P25948238	chr8:72356673-72356717	NM_001024954:92	Pbx4	INSIDE	0.581	0.291	1622.17	472.37	0.169	1136.78	192.41
A_68_P24610520	chr6:86475945-86475989	NM_011865:193	Pcbp1	INSIDE	0.406	6.884	2278.48	15684.45	2.796	1795.30	5019.68
A_68_P24610513	chr6:86475289-86475333	NM_011865:849	Pcbp1	INSIDE	0.591	5.513	1592.12	8777.15	3.261	1305.26	4255.89
A_68_P30498007	chr15:102301316-102301361	NM_001103165:276	Pcbp2	INSIDE	0.6	0.535	1892.09	1012.28	0.321	1369.34	439.88
A_68_P27265105	chr10:76424885-76424929	NM_021568:-214	Pcbp3	PROMOTER	0.301	60.852	7248.18	441069.30	18.327	7274.33	133313.50
A_68_P27265104	chr10:76424807-76424851	NM_021568:-136	Pcbp3	PROMOTER	0.449	0.214	2525.51	539.36	0.096	1781.10	170.89
A_68_P27265101	chr10:76424431-76424475	NM_021568:240	Pcbp3	INSIDE	0.658	2.946	4474.32	13182.71	1.938	3546.01	6870.54
A_68_P26803472	chr9:106355981-106356025	NM_021567:-185	Pcbp4	PROMOTER	0.423	0.487	4136.53	2014.62	0.206	3021.83	622.23
A_68_P26803474	chr9:106356284-106356328	NM_021567:119	Pcbp4	INSIDE	0.461	3.852	12225.09	47096.56	1.777	9829.10	17462.05
A_68_P26772513	chr9:100935215-100935259	NM_025835:58	Pccb	INSIDE	0.308	13.213	6626.56	87559.59	4.076	6324.75	25778.49
A_68_P31632704	chr18:38371400-38371444	NM_029357:-2006	Pcdh1	PROMOTER	0.41	7.700	1854.85	14282.33	3.155	1638.91	5171.49
A_68_P31632591	chr18:38357539-38357583	NM_029357:11856	Pcdh1	INSIDE	0.361	5.411	1318.83	7136.02	1.952	925.57	1806.74
A_68_P31632606	chr18:38358924-38358968	NM_029357:10470	Pcdh1	INSIDE	0.529	3.137	1577.52	4948.22	1.661	1118.83	1857.86
A_68_P22101775	chr3:45185176-45185220	NM_011043:2879	Pcdh10	INSIDE	0.563	4.813	1031.57	4964.96	2.709	886.35	2401.00
A_68_P22101761	chr3:45183553-45183597	NM_011043:1255	Pcdh10	INSIDE	0.487	3.461	2322.96	8039.93	1.687	1843.89	3110.86
A_68_P22101773	chr3:45184916-45184960	NM_011043:2619	Pcdh10	INSIDE	0.434	3.413	1906.87	6508.29	1.48	1674.26	2477.41
A_68_P29773654	chr14:84846918-84846962	NM_001013753:3571	Pcdh17	INSIDE	0.54	0.159	3338.06	529.70	0.086	1991.07	170.49
A_68_P29773624	chr14:84843322-84843371	NM_001013753:-23	Pcdh17	PROMOTER	0.559	3.936	754.25	2968.52	2.199	559.14	1229.38
A_68_P29753340	chr14:80169561-80169605	NM_001042726:1537	Pcdh8	INSIDE	0.421	0.413	1704.59	703.93	0.174	1327.17	230.53
A_68_P29753373	chr14:80173634-80173678	NM_001042726:-2537	Pcdh8	PROMOTER	0.427	0.262	1902.41	499.27	0.112	1523.53	170.73
A_68_P29753346	chr14:80170179-80170223	NM_001042726:919	Pcdh8	INSIDE	0.564	2.791	871.53	2432.14	1.575	768.35	1210.25
A_68_P31626159	chr18:37091821-37091865	NM_198117:-7016	Pcdha2	PROMOTER	0.494	5.357	634.05	3396.85	2.647	538.83	1426.16
A_68_P31626317	chr18:37114129-37114173	NM_009959:-5943	Pcdha5	PROMOTER	0.463	4.326	2315.20	10015.39	2.004	1589.34	3185.05
A_68_P31626864	chr18:37249859-37249905	NM_001003671:93	Pcdhac1	INSIDE	0.416	0.500	1041.32	520.72	0.208	823.88	171.49
A_68_P31630106	chr18:37965667-37965711	NM_033581:-375	Pcdhgc3	PROMOTER	0.643	0.466	1514.64	706.44	0.3	1307.46	392.29
A_68_P31630110	chr18:37966049-37966093	NM_033582:-8662	Pcdhgc4	PROMOTER	0.661	0.488	2038.82	994.99	0.323	1607.29	518.55
A_68_P31630116	chr18:37966805-37966849	NM_033582:-7906	Pcdhgc4	PROMOTER	0.613	0.624	937.34	585.31	0.383	813.20	311.43
A_68_P25326014	chr7:99817883-99817941	NM_029078:510	Pcf11	INSIDE	0.379	8.419	805.85	6784.74	3.195	700.22	2237.02
A_68_P25326017	chr7:99818284-99818328	NM_029078:116	Pcf11	INSIDE	0.321	2.236	5195.96	11618.03	0.718	3522.71	2529.81
A_68_P24591291	chr6:83027998-83028042	NM_197992:-363	Pcgf1	DIVERGENT_PROMOTER	0.647	0.572	1747.29	999.98	0.37	1462.91	541.51
A_68_P23972071	chr5:108890061-108890105	NM_172716:-268	Pcgf3	PROMOTER	0.451	3.560	2483.95	8842.31	1.607	1902.13	3055.81
A_68_P32089884	chr19:36484605-36484649	NM_029508:31070	Pcgf5	INSIDE	0.449	6.768	307.52	2081.21	3.04	267.90	814.47
A_68_P32089882	chr19:36484156-36484200	NM_029508:30622	Pcgf5	INSIDE	0.507	2.870	3713.21	10655.91	1.454	3064.11	4455.91
A_68_P25666154	chr8:13104856-13104900	NM_178708:465	Pcid2	INSIDE	0.313	1.454	1030.93	1499.28	0.456	838.07	382.05
A_68_P21820253	chr2:164705104-164705148	NM_146129:259	Pcif1	INSIDE	0.61	4.877	1774.67	8654.58	2.974	1443.95	4294.59
A_68_P21820252	chr2:164704950-164704994	NM_146129:105	Pcif1	INSIDE	0.623	0.512	3429.51	1756.11	0.319	2458.11	784.06
A_68_P29618378	chr14:56159260-56159304	NM_028994:180	Pck2	INSIDE	0.579	0.715	4052.08	2898.03	0.414	2880.19	1193.46
A_68_P25809871	chr8:42325399-42325443	NM_023662:308	Pcm1	INSIDE	0.388	0.356	1996.55	710.70	0.138	1690.27	233.57
A_68_P25809870	chr8:42325280-42325324	NM_023662:190	Pcm1	INSIDE	0.598	20.372	600.65	12236.76	12.174	474.91	5781.50
A_68_P20015305	chr1:7078905-7078949	NM_183028:-74	Pcmtd1	PROMOTER	0.433	4.291	1433.68	6152.17	1.859	1324.33	2462.57
A_68_P26254550	chr8:128421964-128422008	NM_175561:207	Pcnxl2	INSIDE	0.656	3.055	1821.17	5564.46	2.005	1508.63	3024.68
A_68_P31930177	chr19:5688405-5688449	NM_144868:482	Pcnxl3	INSIDE	0.483	0.243	2046.69	496.61	0.117	1472.61	172.73
A_68_P32240903	chrX:7500233-7500277	NM_013892:3307	Pcsk1n	INSIDE	0.473	6.294	1017.24	6402.24	2.98	1184.74	3529.96
A_68_P31988616	chr19:17911313-17911358	NM_001190483:787	Pcsk5	INSIDE	0.45	0.337	1443.70	486.23	0.152	1119.48	169.81

ProbeName	Target position of probe on CpG island microarray	TargetID	GeneSymbol	CpG island Description	Ratio of relative methylation (TiO_2-NP/Vehicle)	Sham group			TiO_2-H group		
						Relative methylation (Cy5/Cy3)	Cy3 signal (Input)	Cy5 signal (MeDIP)	Relative methylation (Cy5/Cy3)	Cy3 signal (Input)	Cy5 signal (MeDIP)
A_68_P31923511	chr19:4510848-4510892	NM_001162946:399	Pcx	INSIDE	0.488	0.532	1414.40	753.06	0.26	1096.72	284.98
A_68_P31923505	chr19:4510153-4510197	NM_001162946:-297	Pcx	PROMOTER	0.625	0.718	2297.18	1650.27	0.449	1500.34	673.19
A_68_P31759277	chr18:61867112-61867156	NM_172832:155	Pcyox1l	INSIDE	0.567	3.870	3761.24	14556.32	2.195	2992.97	6570.01
A_68_P31759276	chr18:61867012-61867056	NM_172832:255	Pcyox1l	INSIDE	0.66	0.708	3106.76	2199.06	0.467	2249.29	1051.40
A_68_P30652427	chr16:32431113-32431157	NM_001163160:-181	Pcyt1a	PROMOTER	0.252	0.372	2644.62	983.12	0.094	1838.39	171.98
A_68_P30652428	chr16:32431215-32431259	NM_001163160:-79	Pcyt1a	PROMOTER	0.297	0.482	2116.12	1020.56	0.143	1564.64	223.97
A_68_P30652424	chr16:32430803-32430847	NM_009981:-182	Pcyt1a	PROMOTER	0.639	0.580	2424.65	1405.91	0.371	1787.92	662.81
A_68_P30652425	chr16:32430923-32430967	NM_009981:-62	Pcyt1a	PROMOTER	0.426	0.682	2737.46	1867.17	0.291	1691.80	491.66
A_68_P24164494	chr5:145900797-145900843	NM_001033313:138	Pdap1	INSIDE	0.316	7.415	5487.08	40685.70	2.341	4441.77	10397.62
A_68_P22246600	chr3:75360381-75360425	NM_019745:319	Pdcd10	INSIDE	0.606	3.055	7609.69	23251.20	1.853	5322.36	9862.28
A_68_P31066146	chr17:15663980-15664024	NM_008799:263	Pdcd2	INSIDE	0.39	5.868	969.56	5688.96	2.287	799.67	1829.10
A_68_P25050417	chr7:36432045-36432089	NM_019746:435	Pdcd5	INSIDE	0.664	0.425	1179.48	501.46	0.282	981.05	276.98
A_68_P29145568	chr13:74454823-74454867	NM_011051:-70	Pdcd6	PROMOTER	0.64	0.705	3326.22	2345.03	0.451	2534.64	1143.93
A_68_P29145569	chr13:74454902-74454946	NM_011051:-150	Pdcd6	PROMOTER	0.355	1.387	1068.42	1481.85	0.492	887.53	436.83
A_68_P26587151	chr9:65194360-65194404	NM_016688:508	Pdcd7	INSIDE	0.572	6.096	504.72	3076.89	3.485	494.57	1723.39
A_68_P20163207	chr1:39044543-39044587	NM_026850:-94	Pdcl3	PROMOTER	0.343	0.505	1441.50	728.03	0.173	1149.55	199.12
A_68_P31029172	chr17:8994701-8994745	NM_011866:113	Pde10a	INSIDE	0.379	4.533	4186.23	18975.07	1.719	3483.54	5988.31
A_68_P25373531	chr7:108599782-108599826	NR_026574:-17	Pde2a	PROMOTER	0.519	0.370	5109.07	1888.07	0.192	3728.98	714.54
A_68_P25373532	chr7:108599866-108599910	NR_026574:67	Pde2a	INSIDE	0.57	0.255	2628.54	671.15	0.145	1842.47	268.08
A_68_P25373530	chr7:108599609-108599653	NM_001143849:-135	Pde2a	PROMOTER	0.652	0.689	2865.24	1973.60	0.449	2143.41	962.23
A_68_P24898883	chr6:141198105-141198149	NM_018779:337	Pde3a	INSIDE	0.409	4.231	1405.88	5947.70	1.73	1153.49	1995.38
A_68_P24898887	chr6:141198621-141198665	NM_018779:853	Pde3a	INSIDE	0.348	4.322	1811.05	7826.88	1.505	1348.40	2029.04
A_68_P25435757	chr7:121559995-121560039	NM_011055:1249	Pde3b	INSIDE	0.429	7.146	1742.10	12448.95	3.065	1398.90	4287.13
A_68_P25435753	chr7:121559659-121559703	NM_011055:913	Pde3b	INSIDE	0.487	3.110	1263.06	3928.14	1.514	1114.35	1686.83
A_68_P25435752	chr7:121559588-121559632	NM_011055:843	Pde3b	INSIDE	0.488	3.049	1388.17	4232.17	1.486	1204.26	1790.11
A_68_P26345770	chr9:20970026-20970070	NM_183408:-109	Pde4a	PROMOTER	0.569	7.554	1112.68	8405.08	4.298	889.97	3824.89
A_68_P26345851	chr9:20988585-20988629	NM_183408:18449	Pde4a	INSIDE	0.584	2.524	2409.20	6081.60	1.475	1981.96	2923.83
A_68_P25953728	chr8:73267438-73267482	NM_201607:19498	Pde4c	INSIDE	0.307	5.992	1118.37	6700.93	1.841	970.15	1785.97
A_68_P29314989	chr13:109906595-109906639	NM_011056:462246	Pde4d	INSIDE	0.637	0.605	1717.73	1038.81	0.385	1351.37	520.42
A_68_P29314992	chr13:109906946-109906990	NM_011056:462598	Pde4d	INSIDE	0.346	2.018	10150.78	20483.50	0.698	7906.44	5521.04
A_68_P22356816	chr3:97671824-97671868	NM_177145:389	Pde4dip	INSIDE	0.108	0.583	3541.46	2063.68	0.063	2669.53	168.57
A_68_P22484168	chr3:122432681-122432725	NM_153422:627	Pde5a	INSIDE	0.471	8.095	1777.89	14392.46	3.814	1529.93	5834.51
A_68_P29239503	chr13:95992534-95992578	NM_001170669:449	Pde8b	INSIDE	0.622	8.204	1422.05	11666.24	5.106	1214.95	6203.03
A_68_P31139421	chr17:31523560-31523605	NM_001163748:404	Pde9a	INSIDE	0.462	0.480	1719.00	825.19	0.222	1303.97	289.18
A_68_P31139420	chr17:31523449-31523493	NM_001163748:292	Pde9a	INSIDE	0.659	0.613	1884.57	1155.80	0.404	1565.75	632.34
A_68_P24132957	chr5:139469288-139469332	NM_008808:1597	Pdgfa	INSIDE	0.559	0.604	2147.47	1296.16	0.337	1577.16	532.02
A_68_P24132973	chr5:139471053-139471097	NM_008808:-167	Pdgfa	PROMOTER	0.323	0.635	1899.29	1206.37	0.205	1326.51	272.22
A_68_P24132963	chr5:139469932-139469976	NM_008808:953	Pdgfa	INSIDE	0.296	5.390	4372.21	23564.20	1.594	3536.25	5638.33
A_68_P30369715	chr15:79843444-79843488	NM_011057:1772	Pdgfb	INSIDE	0.457	0.627	731.90	458.83	0.286	597.95	171.26
A_68_P22271772	chr3:80841440-80841484	NM_019971:1125	Pdgfc	INSIDE	0.529	0.278	1777.49	494.85	0.147	1183.10	174.27
A_68_P23807625	chr5:75548984-75549028	NM_001083316:-3184	Pdgfra	PROMOTER	0.529	6.698	2568.52	17204.50	3.543	2020.43	7157.85
A_68_P23807627	chr5:75549167-75549211	NM_001083316:-3002	Pdgfra	PROMOTER	0.649	0.335	1714.13	574.87	0.218	1296.09	282.12
A_68_P32778409	chrX:156576281-156576325	NM_008810:-34	Pdha1	PROMOTER	0.48	16.506	557.58	9203.49	7.923	646.04	5118.87
A_68_P29388212	chr14:9005175-9005219	NM_024221:310	Pdhb	INSIDE	0.59	0.650	3662.54	2381.19	0.383	2672.32	1024.60
A_68_P30670431	chr16:35490952-35490996	NM_028295:-15	Pdia5	PROMOTER	0.551	0.388	1699.98	659.14	0.214	1408.36	301.15
A_68_P30670423	chr16:35490181-35490225	NM_028295:757	Pdia5	INSIDE	0.662	0.162	4058.80	656.69	0.107	2935.56	314.63

ProbeName	Target position of probe on CpG island microarray	TargetID	GeneSymbol	CpG island Description	Ratio of relative methylation (TiO_2-NP/Vehicle)	Sham group			TiO_2-H group		
						Relative methylation (Cy5/Cy3)	Cy3 signal (Input)	Cy5 signal (MeDIP)	Relative methylation (Cy5/Cy3)	Cy3 signal (Input)	Cy5 signal (MeDIP)
A_68_P30670429	chr16:35490684-35490728	NM_028295:253	Pdia5	INSIDE	0.356	0.649	7367.00	4777.89	0.231	5180.93	1195.34
A_68_P23322029	chr4:133843071-133843115	NM_001163794:76	Pdik1l	INSIDE	0.381	5.853	10137.97	59336.73	2.23	8273.17	18448.61
A_68_P23322026	chr4:133842693-133842737	NM_001163794:454	Pdik1l	INSIDE	0.662	3.106	1060.70	3294.43	2.056	913.87	1878.85
A_68_P23322032	chr4:133843514-133843558	NM_001163794:-368	Pdik1l	PROMOTER	0.097	17.777	748.47	13305.30	1.723	1255.60	2163.19
A_68_P21322072	chr2:71711713-71711757	NM_172665:406	Pdk1	INSIDE	0.491	7.754	570.64	4424.47	3.81	492.57	1876.89
A_68_P28036286	chr11:94902671-94902715	NM_133667:-7	Pdk2	PROMOTER	0.533	3.694	1625.21	6004.20	1.968	1351.91	2660.51
A_68_P32536210	chrX:91077357-91077401	NM_145630:56	Pdk3	INSIDE	0.537	6.230	1146.82	7144.60	3.347	1333.01	4461.83
A_68_P24210894	chr6:5446053-5446097	NM_013743:204	Pdk4	INSIDE	0.129	20.420	707.54	14448.01	2.631	585.76	1541.22
A_68_P32107651	chr19:40346260-40346304	NM_016861:-176	Pdlim1	PROMOTER	0.629	0.552	882.82	487.14	0.347	730.85	253.51
A_68_P31095624	chr17:24278101-24278145	NM_001080773:434	Pdpk1	INSIDE	0.355	5.833	3189.21	18601.60	2.068	2769.00	5727.13
A_68_P21752417	chr2:152841063-152841107	NM_178939:35	Pdrg1	INSIDE	0.443	0.338	1554.73	524.86	0.15	1148.52	171.73
A_68_P24172759	chr5:148081959-148082003	NM_008814:274	Pdx1	INSIDE	0.67	0.173	3429.37	594.67	0.116	2570.26	298.82
A_68_P30363152	chr15:78744712-78744756	NM_020271:386	Pdxp	INSIDE	0.607	6.807	1048.38	7136.82	4.134	840.81	3475.76
A_68_P21626037	chr2:129514047-129514091	NM_018863:1536	Pdyn	INSIDE	0.444	7.988	2883.12	23030.88	3.546	1823.95	6467.65
A_68_P21626036	chr2:129513877-129513921	NM_018863:1706	Pdyn	INSIDE	0.654	3.055	2358.70	7206.16	1.999	1920.54	3838.54
A_68_P32464984	chrX:71070091-71070135	NM_001029868:196	Pdzd4	INSIDE	0.287	13.677	720.77	9857.79	3.923	813.65	3192.02
A_68_P32218816	chr19:59419677-59419721	NM_001033222:572	Pdzd8	INSIDE	0.658	0.479	1183.09	566.66	0.315	975.31	307.55
A_68_P24697529	chr6:101327967-101328012	NM_018884:-98	Pdzrn3	PROMOTER	0.563	8.650	1183.07	10233.63	4.874	902.46	4398.20
A_68_P22309854	chr3:87572957-87573001	NM_001032413:-103	Pear1	PROMOTER	0.231	7.991	1373.70	10976.56	1.844	1231.18	2270.21
A_68_P20335741	chr1:72301294-72301338	NM_023523:29572	Pecr	DOWNSTREAM	0.522	4.101	2305.36	9455.12	2.143	2048.03	4388.60
A_68_P25169318	chr7:69609337-69609381	NM_013788:38	Peg12	INSIDE	0.269	7.906	921.86	7288.09	2.129	878.06	1869.31
A_68_P25169316	chr7:69609111-69609155	NM_013788:264	Peg12	INSIDE	0.648	3.145	973.29	3060.76	2.039	897.40	1830.07
A_68_P24956666	chr7:6682979-6683023	NM_008817:130	Peg3	INSIDE	0.45	5.688	2026.99	11529.03	2.56	1672.44	4282.05
A_68_P27635062	chr11:20991838-20991882	NM_023324:534	Peli1	INSIDE	0.382	5.561	1848.19	10277.19	2.126	1499.58	3187.37
A_68_P29585968	chr14:48741266-48741310	NM_033602:745	Peli2	INSIDE	0.57	0.654	1236.78	809.08	0.373	1046.96	390.37
A_68_P29585964	chr14:48740853-48740897	NM_033602:331	Peli2	INSIDE	0.319	2.085	4166.77	8686.10	0.665	2809.61	1868.44
A_68_P31926007	chr19:4942924-4942968	NM_172835:146	Peli3	INSIDE	0.385	0.577	7161.92	4135.20	0.222	5433.61	1207.22
A_68_P31926004	chr19:4942668-4942712	NM_172835:402	Peli3	INSIDE	0.505	0.456	2962.07	1351.60	0.231	2142.21	494.05
A_68_P29350211	chr13:115880342-115880386	NM_134058:2	Pelo	INSIDE	0.492	3.792	1722.47	6532.41	1.865	1344.02	2505.99
A_68_P27900566	chr11:70223756-70223800	NM_029231:-245	Pelp1	PROMOTER	0.619	0.307	2775.70	851.51	0.19	1948.56	370.09
A_68_P27900563	chr11:70223515-70223559	NM_029231:-3	Pelp1	PROMOTER	0.402	0.687	1378.84	947.46	0.276	1043.92	288.21
A_68_P27840490	chr11:59859741-59859785	NM_008819:-9421	Pemt	PROMOTER	0.545	0.476	2154.54	1025.41	0.259	1549.95	401.69
A_68_P22696144	chr4:4066014-4066058	NM_001002927:-444	Penk	PROMOTER	0.497	11.460	1986.64	22766.13	5.693	1751.35	9969.61
A_68_P25046335	chr7:35697613-35697657	NM_008820:209	Pepd	INSIDE	0.622	2.561	1639.47	4197.96	1.592	1272.31	2025.65
A_68_P20442663	chr1:93355837-93355883	NM_011066:45	Per2	INSIDE	0.503	7.227	5953.81	43025.53	3.638	5029.32	18295.82
A_68_P26977750	chr10:18565037-18565081	NM_022032:182	Perp	INSIDE	0.415	11.293	2649.31	29918.96	4.684	2256.84	10570.54
A_68_P26977748	chr10:18564800-18564844	NM_022032:-54	Perp	PROMOTER	0.562	9.320	1025.94	9561.95	5.241	876.84	4595.29
A_68_P21706811	chr2:144194639-144194683	NM_001164813:-58	Pet117	PROMOTER	0.474	0.596	820.72	489.33	0.283	609.27	172.35
A_68_P24815139	chr6:124364839-124364883	NM_008995:225	Pex5	INSIDE	0.578	0.228	5007.41	1139.56	0.132	3661.63	481.76
A_68_P31208519	chr17:46848833-46848877	NM_145488:443	Pex6	INSIDE	0.413	6.793	1065.32	7236.73	2.805	917.93	2575.19
A_68_P21853525	chr2:170336931-170336975	NM_001110152:223	Pfdn4	INSIDE	0.464	0.257	3568.54	916.20	0.119	2660.15	317.22
A_68_P21853526	chr2:170337089-170337133	NM_001110152:381	Pfdn4	INSIDE	0.593	0.474	5414.35	2564.64	0.281	4067.13	1143.32
A_68_P21006641	chr2:11457093-11457137	NM_001177758:18442	Pfkfb3	INSIDE	0.538	3.518	1839.73	6471.73	1.892	1470.87	2782.16
A_68_P21006733	chr2:11475422-11475466	NM_001177758:112	Pfkfb3	INSIDE	0.601	0.549	1055.19	579.03	0.33	801.94	264.39
A_68_P27271614	chr10:77472403-77472447	NM_008826:117	Pfkl	INSIDE	0.574	0.419	3071.53	1286.17	0.24	2271.02	546.10
A_68_P28803336	chr13:6648044-6648088	NM_019703:-96	Pfkp	PROMOTER	0.44	4.368	1268.68	5541.50	1.922	1057.06	2031.83

ProbeName	Target position of probe on CpG island microarray	TargetID	GeneSymbol	CpG island Description	Ratio of relative methylation (TiO_2-NP/Vehicle)	Sham group			TiO_2-H group		
						Relative methylation (Cy5/Cy3)	Cy3 signal (Input)	Cy5 signal (MeDIP)	Relative methylation (Cy5/Cy3)	Cy3 signal (Input)	Cy5 signal (MeDIP)
A_68_P27901897	chr11:70468130-70468174	NM_011072:0	Pfn1	INSIDE	0.275	43.491	1949.71	84794.26	11.972	2059.17	24652.77
A_68_P27901890	chr11:70467341-70467385	NM_011072:790	Pfn1	INSIDE	0.351	7.939	1342.53	10657.78	2.788	1279.59	3567.95
A_68_P27901889	chr11:70467270-70467315	NM_011072:860	Pfn1	INSIDE	0.398	0.387	2469.92	956.93	0.154	1833.26	282.98
A_68_P27901895	chr11:70467926-70467970	NM_011072:204	Pfn1	INSIDE	0.585	0.254	3873.96	982.31	0.148	2528.53	375.03
A_68_P27901903	chr11:70468945-70468989	NM_011072:-814	Pfn1	DIVERGENT_PROMOTER	0.39	5.415	651.92	3530.14	2.113	565.44	1194.81
A_68_P22165658	chr3:57651977-57652021	NM_019410:-319	Pfn2	PROMOTER	0.579	0.688	2556.09	1758.37	0.398	2078.55	827.45
A_68_P32117861	chr19:41986212-41986256	NM_023418:-126	Pgam1	PROMOTER	0.506	4.623	2453.59	11343.61	2.338	2074.59	4851.02
A_68_P32117863	chr19:41986389-41986433	NM_023418:50	Pgam1	INSIDE	0.648	0.197	2990.13	588.66	0.128	2024.69	258.21
A_68_P28056218	chr11:98261638-98261682	NM_001033537:144	Pgap3	INSIDE	0.387	4.266	1502.31	6409.04	1.65	1208.58	1994.29
A_68_P28872847	chr13:21532533-21532577	NM_001162919:25	Pgbd1	INSIDE	0.432	0.552	909.20	502.10	0.239	747.14	178.41
A_68_P26245549	chr8:126958481-126958525	NM_171824:-666	Pgbd5	PROMOTER	0.655	7.721	1071.47	8272.96	5.057	932.52	4715.60
A_68_P31676054	chr18:46440414-46440458	NM_172627:68	Pggt1b	INSIDE	0.305	7.501	3190.02	23928.25	2.288	2819.49	6449.70
A_68_P25957670	chr8:74115606-74115650	NM_025396:-454	Pgls	PROMOTER	0.147	1.598	5209.98	8324.02	0.235	3612.54	850.33
A_68_P25957707	chr8:74119888-74119932	NM_025396:3828	Pgls	INSIDE	0.352	0.579	1088.27	630.26	0.204	883.31	180.27
A_68_P23749120	chr5:64484497-64484543	NM_025700:332	Pgm1	INSIDE	0.311	12.546	2558.83	32102.38	3.896	2308.76	8994.89
A_68_P23146552	chr4:99602526-99602570	NM_028132:493	Pgm2	INSIDE	0.189	26.464	10392.97	275037.20	5.009	8892.29	44538.52
A_68_P23146551	chr4:99602438-99602482	NM_028132:405	Pgm2	INSIDE	0.491	0.571	5152.67	2940.60	0.28	3804.95	1066.30
A_68_P31097493	chr17:24606963-24607007	NM_025954:-433	Pgp	PROMOTER	0.623	15.399	2600.81	40050.20	9.594	2580.53	24757.08
A_68_P25953245	chr8:73183706-73183750	NM_023217:-148	Pgpep1	PROMOTER	0.517	2.631	8023.77	21113.74	1.361	5522.16	7517.41
A_68_P22083003	chr3:40886734-40886778	NM_027558:212	Pgrmc2	INSIDE	0.461	7.849	24393.22	191460.10	3.621	18660.36	67572.34
A_68_P28987052	chr13:43011760-43011804	NM_001005740:206833	Phactr1	INSIDE	0.431	0.584	2821.85	1647.06	0.252	2106.93	529.95
A_68_P28987053	chr13:43011826-43011870	NM_001005740:206899	Phactr1	INSIDE	0.452	0.410	1120.22	459.20	0.185	912.89	168.98
A_68_P26951409	chr10:13193710-13193754	NM_001195065:470	Phactr2	INSIDE	0.478	0.149	3501.68	520.93	0.071	2434.43	173.28
A_68_P26951412	chr10:13194053-13194097	NM_001195065:128	Phactr2	INSIDE	0.501	3.394	901.85	3060.54	1.699	823.23	1398.79
A_68_P26951410	chr10:13193842-13193886	NM_001195065:338	Phactr2	INSIDE	0.627	0.578	827.23	477.98	0.362	628.28	227.51
A_68_P21888793	chr2:177877242-177877286	NM_028806:627	Phactr3	INSIDE	0.252	39.592	1666.92	65997.16	9.961	3068.04	30562.13
A_68_P21888635	chr2:177853739-177853783	NM_001177789:81	Phactr3	INSIDE	0.394	76.279	1809.71	138043.50	30.024	1774.78	53286.71
A_68_P21888792	chr2:177877156-177877200	NM_028806:541	Phactr3	INSIDE	0.479	4.708	936.36	4408.66	2.256	796.52	1797.12
A_68_P24816531	chr6:124662333-124662377	NM_007531:48	Phb2	INSIDE	0.383	0.440	1287.71	567.04	0.169	1026.66	173.00
A_68_P23292757	chr4:128404849-128404893	NM_001195083:56	Phc2	INSIDE	0.486	4.454	590.17	2628.52	2.162	575.56	1244.56
A_68_P23292758	chr4:128405111-128405155	NM_001195083:318	Phc2	INSIDE	0.58	3.647	536.67	1957.41	2.116	495.84	1048.97
A_68_P22031102	chr3:30868233-30868277	NM_001165954:83	Phc3	INSIDE	0.349	4.074	2822.68	11499.68	1.421	2373.31	3372.60
A_68_P31113238	chr17:27070074-27070118	NM_009343:25	Phf1	INSIDE	0.653	0.570	1319.93	752.10	0.372	1108.21	412.32
A_68_P31062920	chr17:15098814-15098858	NM_024250:-599	Phf10	PROMOTER	0.26	0.598	11698.05	6992.56	0.155	8093.89	1255.59
A_68_P23415887	chr4:151369933-151369977	NM_172705:334	Phf13	INSIDE	0.54	3.908	8796.02	34378.46	2.111	7288.53	15384.85
A_68_P27796195	chr11:51671777-51671821	NM_199299:-815	Phf15	PROMOTER	0.223	13.406	10080.01	135127.60	2.993	8945.15	26775.78
A_68_P27796185	chr11:51670574-51670621	NM_199299:386	Phf15	INSIDE	0.461	0.565	1675.00	946.75	0.261	1170.10	304.86
A_68_P27796190	chr11:51671242-51671287	NM_199299:-281	Phf15	PROMOTER	0.427	5.765	435.01	2507.71	2.463	371.11	914.16
A_68_P22085575	chr3:41369640-41369684	NM_001130186:861	Phf17	INSIDE	0.162	32.989	1447.85	47762.86	5.335	1444.20	7704.69
A_68_P22085568	chr3:41368782-41368826	NM_001130186:3	Phf17	INSIDE	0.498	53.076	2858.64	151724.80	26.427	2735.21	72284.81
A_68_P21130446	chr2:34769487-34769531	NM_028716:-12	Phf19	PROMOTER	0.529	10.232	3741.33	38281.43	5.408	3524.29	19059.40
A_68_P30292508	chr15:66409642-66409686	NM_001081409:531	Phf20l1	INSIDE	0.447	6.051	1826.08	11049.52	2.706	1440.84	3898.94
A_68_P30292506	chr15:66409382-66409426	NM_001081409:271	Phf20l1	INSIDE	0.603	3.870	809.08	3131.53	2.333	797.57	1860.91
A_68_P30397291	chr15:84684245-84684289	NM_001081166:2293	Phf21b	INSIDE	0.393	10.083	2702.97	27253.92	3.965	2534.32	10048.42
A_68_P30397299	chr15:84685568-84685612	NM_001081166:969	Phf21b	INSIDE	0.574	0.190	2682.21	510.36	0.109	1847.85	201.88
A_68_P27898392	chr11:69810209-69810253	NM_030064:958	Phf23	INSIDE	0.166	3.646	10595.95	38634.62	0.607	7472.55	4534.43

ProbeName	Target position of probe on CpG island microarray	TargetID	GeneSymbol	CpG island Description	Ratio of relative methylation (TiO_2-NP/Vehicle)	Sham group			TiO_2-H group		
						Relative methylation (Cy5/Cy3)	Cy3 signal (Input)	Cy5 signal (MeDIP)	Relative methylation (Cy5/Cy3)	Cy3 signal (Input)	Cy5 signal (MeDIP)
A_68_P27898385	chr11:69809540-69809584	NM_030064:290	Phf23	INSIDE	0.197	0.589	3058.27	1800.92	0.116	2147.57	249.46
A_68_P27898388	chr11:69809807-69809851	NM_030064:556	Phf23	INSIDE	0.293	0.297	2805.74	834.42	0.087	1998.34	174.11
A_68_P27898391	chr11:69810124-69810168	NM_030064:874	Phf23	INSIDE	0.321	18.097	15139.65	273985.70	5.81	11712.78	68056.12
A_68_P27898395	chr11:69810513-69810557	NM_030064:1262	Phf23	INSIDE	0.357	0.389	3534.42	1375.80	0.139	2540.93	352.63
A_68_P27898394	chr11:69810400-69810444	NM_030064:1150	Phf23	INSIDE	0.382	5.908	5938.97	35087.35	2.255	4440.29	10010.75
A_68_P27898389	chr11:69809872-69809916	NM_030064:622	Phf23	INSIDE	0.443	0.259	2722.41	704.74	0.115	2097.39	240.66
A_68_P27898381	chr11:69809079-69809123	NM_030064:-172	Phf23	PROMOTER	0.506	3.304	3768.24	12450.48	1.672	3213.60	5373.05
A_68_P27898384	chr11:69809416-69809460	NM_030064:166	Phf23	INSIDE	0.649	0.584	3824.57	2234.63	0.379	2597.87	985.52
A_68_P27898393	chr11:69810295-69810339	NM_030064:1044	Phf23	INSIDE	0.132	1.594	2742.91	4372.06	0.21	1909.58	401.01
A_68_P27898390	chr11:69809983-69810027	NM_030064:732	Phf23	INSIDE	0.495	1.393	2557.98	3563.32	0.69	1847.74	1274.88
A_68_P32384750	chrX:50265645-50265689	NM_027642:224	Phf6	INSIDE	0.543	7.354	346.85	2550.60	3.996	488.01	1950.17
A_68_P32745693	chrX:147954890-147954934	NM_177201:-302	Phf8	PROMOTER	0.453	3.238	2293.83	7428.00	1.467	2864.33	4203.35
A_68_P27449326	chr10:110943860-110943904	NM_009344:541	Phlda1	INSIDE	0.429	7.412	603.25	4471.39	3.183	574.10	1827.31
A_68_P27449323	chr10:110943535-110943582	NM_009344:217	Phlda1	INSIDE	0.53	3.948	1452.27	5734.25	2.092	1281.38	2681.02
A_68_P25601380	chr7:150688561-150688605	NM_009434:-153	Phlda2	PROMOTER	0.491	0.545	4073.59	2221.28	0.268	2655.22	711.25
A_68_P20641449	chr1:137662705-137662749	NM_013750:65	Phlda3	INSIDE	0.51	7.135	5182.48	36978.83	3.637	4035.33	14675.46
A_68_P20641447	chr1:137662511-137662555	NM_013750:-129	Phlda3	PROMOTER	0.054	10.328	2142.23	22124.64	0.556	1254.64	697.80
A_68_P26469279	chr9:44541771-44541815	NM_153537:1485	Phldb1	INSIDE	0.184	0.525	3623.11	1903.46	0.097	2489.64	240.50
A_68_P26469100	chr9:44516622-44516666	NM_153537:26633	Phldb1	INSIDE	0.397	73.524	3381.87	248650.50	29.18	3442.32	100446.30
A_68_P26469197	chr9:44529171-44529218	NM_153537:14083	Phldb1	INSIDE	0.621	0.529	973.07	515.01	0.329	808.66	265.78
A_68_P25005873	chr7:25413942-25413986	NM_001102613:17618	Phldb3	INSIDE	0.479	0.374	3133.63	1171.30	0.179	2198.51	394.03
A_68_P20506808	chr1:108069211-108069255	NM_133821:787	Phlpp1	INSIDE	0.28	25.154	2021.30	50844.64	7.038	1557.05	10958.65
A_68_P20506810	chr1:108069429-108069473	NM_133821:1005	Phlpp1	INSIDE	0.388	9.761	2928.95	28589.68	3.783	2302.71	8711.33
A_68_P26159430	chr8:112393252-112393296	NM_001122594:772	Phlpp2	INSIDE	0.56	0.580	4581.39	2655.34	0.325	3166.80	1028.60
A_68_P26159425	chr8:112392612-112392656	NM_001122594:132	Phlpp2	INSIDE	0.65	4.506	1708.50	7697.70	2.931	1297.91	3803.64
A_68_P25375581	chr7:108966881-108966925	NM_008887:76	Phox2a	INSIDE	0.394	0.374	2951.61	1104.96	0.148	2052.41	302.95
A_68_P25375583	chr7:108967177-108967221	NM_008887:372	Phox2a	INSIDE	0.404	11.603	3414.21	39615.71	4.683	3054.75	14304.46
A_68_P25375611	chr7:108970306-108970350	NM_008887:3502	Phox2a	INSIDE	0.449	4.530	746.55	3381.92	2.034	658.53	1339.49
A_68_P23765940	chr5:67488811-67488855	NM_008888:1533	Phox2b	INSIDE	0.581	0.609	1772.38	1079.21	0.354	1406.82	498.05
A_68_P21073067	chr2:25430182-25430226	NM_029293:187	Phpt1	INSIDE	0.24	15.582	7003.56	109129.80	3.733	6290.67	23480.70
A_68_P25587734	chr7:148445714-148445758	NM_001081118:31050	Phrf1	INSIDE	0.391	19.006	1514.49	28783.80	7.433	1034.04	7686.38
A_68_P23523151	chr5:20387300-20387344	NM_172992:620	Phtf2	INSIDE	0.496	4.429	1376.17	6095.61	2.197	1104.05	2425.63
A_68_P23523156	chr5:20387967-20388011	NM_172992:-46	Phtf2	DIVERGENT_PROMOTER	0.405	5.191	588.95	3057.11	2.1	507.67	1066.03
A_68_P27230702	chr10:70061703-70061747	NM_001162846:-6164	Phyhipl	PROMOTER	0.305	11.273	2285.06	25759.85	3.444	1801.89	6205.21
A_68_P27230709	chr10:70062556-70062600	NM_178621:-539	Phyhipl	PROMOTER	0.523	4.632	472.95	2190.91	2.422	457.03	1106.79
A_68_P27230712	chr10:70062823-70062867	NM_178621:-805	Phyhipl	PROMOTER	0.574	2.851	1186.63	3383.60	1.637	973.79	1593.85
A_68_P32118991	chr19:42165475-42165519	NM_145501:572	Pi4k2a	INSIDE	0.64	0.379	3153.55	1195.75	0.243	2187.46	531.14
A_68_P32118987	chr19:42164891-42164935	NM_145501:-12	Pi4k2a	DIVERGENT_PROMOTER	0.538	2.963	1531.45	4538.41	1.596	1148.55	1832.87
A_68_P23696524	chr5:53132760-53132804	NM_025951:-30	Pi4k2b	PROMOTER	0.282	1.585	1937.20	3070.64	0.446	1775.82	792.78
A_68_P26573394	chr9:62827923-62827967	NM_019663:742	Pias1	INSIDE	0.586	0.302	6982.96	2107.82	0.177	4611.90	816.42
A_68_P26573399	chr9:62828500-62828544	NM_019663:164	Pias1	INSIDE	0.573	2.403	2503.33	6015.92	1.376	2286.68	3147.15
A_68_P27287761	chr10:80616838-80616882	NM_021501:13605	Pias4	INSIDE	0.155	15.406	2458.38	37874.14	2.392	1900.87	4547.52
A_68_P27287760	chr10:80616735-80616779	NM_021501:13709	Pias4	INSIDE	0.386	8.332	1631.33	13592.50	3.22	1375.50	4428.64
A_68_P27287763	chr10:80617060-80617104	NM_021501:13383	Pias4	INSIDE	0.468	8.197	1124.44	9217.45	3.837	968.35	3715.98
A_68_P27287757	chr10:80616441-80616485	NM_021501:14003	Pias4	DOWNSTREAM	0.116	13.429	6725.42	90318.46	1.562	4762.57	7436.99
A_68_P27287756	chr10:80616365-80616414	NM_021501:14076	Pias4	DOWNSTREAM	0.507	3.757	948.09	3562.29	1.903	566.60	1078.44

ProbeName	Target position of probe on CpG island microarray	TargetID	GeneSymbol	CpG island Description	Ratio of relative methylation (TiO_2-NP/Vehicle)	Sham group			TiO_2-H group		
						Relative methylation (Cy5/Cy3)	Cy3 signal (Input)	Cy5 signal (MeDIP)	Relative methylation (Cy5/Cy3)	Cy3 signal (Input)	Cy5 signal (MeDIP)
A_68_P32798239	chrX:160857687-160857731	NM_011081:-10	Piga	PROMOTER	0.431	5.858	1300.25	7616.29	2.524	1556.34	3928.43
A_68_P20774854	chr1:163899508-163899552	NM_001039045:212	Pigc	INSIDE	0.588	3.344	1227.56	4105.14	1.965	1053.54	2070.55
A_68_P31420721	chr17:87424605-87424649	NM_008838:115	Pigf	INSIDE	0.66	3.124	1113.55	3478.42	2.06	893.77	1841.43
A_68_P28567643	chr12:80190635-80190679	NM_029988:1	Pigh	INSIDE	0.641	0.646	1502.42	969.96	0.414	1253.38	519.04
A_68_P20832749	chr1:174307088-174307133	NM_026234:449	Pigm	INSIDE	0.317	0.265	3326.17	882.85	0.084	2135.28	179.66
A_68_P20832748	chr1:174307013-174307057	NM_026234:373	Pigm	INSIDE	0.608	0.488	1963.27	958.54	0.297	1414.72	420.11
A_68_P30980968	chr16:94592282-94592326	NM_001159618:-147	Pigp	PROMOTER	0.609	4.669	2584.85	12067.39	2.843	2308.31	6562.89
A_68_P30980976	chr16:94593244-94593288	NM_001159617:-644	Pigp	PROMOTER	0.37	0.730	1827.64	1334.79	0.27	1500.91	405.57
A_68_P27942686	chr11:78142057-78142101	NM_201406:155	Pigs	INSIDE	0.46	4.073	3766.39	15341.33	1.872	2984.95	5587.69
A_68_P21818281	chr2:164332907-164332953	NM_133779:9906	Pigt	INSIDE	0.291	7.655	1300.80	9957.93	2.23	852.47	1900.93
A_68_P21818280	chr2:164332796-164332840	NM_133779:9794	Pigt	INSIDE	0.486	4.371	1306.84	5712.28	2.124	860.11	1827.18
A_68_P21818223	chr2:164322720-164322769	NM_133779:-280	Pigt	PROMOTER	0.51	0.523	913.94	477.82	0.266	684.91	182.51
A_68_P23318794	chr4:133228762-133228806	NM_001145955:-222	Pigv	PROMOTER	0.409	4.087	1347.24	5506.32	1.671	1219.68	2038.54
A_68_P27980117	chr11:84693941-84693985	NM_001077636:-175	Pigw	PROMOTER	0.182	52.694	3882.16	204565.50	9.569	5945.42	56891.86
A_68_P24479719	chr6:57641697-57641742	NM_025574:353	Pigy	INSIDE	0.335	5.793	2023.36	11722.28	1.941	1631.97	3167.70
A_68_P26350663	chr9:21961255-21961299	NM_001082532:-13	Pigyl	PROMOTER	0.227	8.448	1232.58	10412.78	1.921	1041.97	2001.87
A_68_P30650136	chr16:31933025-31933069	NM_172822:-890	Pigz	PROMOTER	0.504	0.265	1767.81	469.31	0.134	1277.62	171.04
A_68_P25091451	chr7:52409634-52409678	NM_029406:-111	Pih1d1	PROMOTER	0.564	4.063	1762.43	7160.94	2.29	1312.38	3005.86
A_68_P32114803	chr19:41459691-41459735	NM_031376:-152	Pik3ap1	PROMOTER	0.482	4.671	2613.57	12207.69	2.251	2091.72	4708.47
A_68_P25448125	chr7:123586995-123587039	NM_011083:-44	Pik3c2a	PROMOTER	0.235	0.240	4514.05	1082.78	0.056	3029.76	171.09
A_68_P20625254	chr1:134942880-134942924	NM_001099276:314	Pik3c2b	INSIDE	0.505	3.674	1150.08	4225.12	1.855	918.94	1704.23
A_68_P20625253	chr1:134942691-134942735	NM_001099276:124	Pik3c2b	INSIDE	0.499	3.555	1048.91	3728.65	1.774	990.83	1757.69
A_68_P31592065	chr18:30432515-30432561	NM_181414:-11	Pik3c3	PROMOTER	0.13	2.891	3678.52	10635.71	0.375	2394.20	898.98
A_68_P26762516	chr9:99040770-99040814	NM_029094:-162	Pik3cb	PROMOTER	0.327	6.431	1655.42	10645.47	2.105	1483.05	3122.47
A_68_P23401742	chr4:149029621-149029665	NM_001164052:7783	Pik3cd	INSIDE	0.434	4.739	3227.30	15295.16	2.056	2465.50	5070.21
A_68_P27551374	chr11:3232269-3232313	NM_178149:1557	Pik3ip1	INSIDE	0.494	4.064	979.65	3981.59	2.006	788.31	1581.22
A_68_P29275481	chr13:102538276-102538320	NM_001077495:-126	Pik3r1	PROMOTER	0.486	0.162	2915.65	473.13	0.079	2192.26	172.99
A_68_P25953980	chr8:73300616-73300660	NM_008841:-27	Pik3r2	DIVERGENT_PROMOTER	0.278	12.436	809.26	10064.22	3.457	697.19	2409.90
A_68_P26798484	chr9:105547053-105547097	NM_001081309:1750	Pik3r4	INSIDE	0.441	3.849	1959.10	7541.47	1.699	1184.01	2011.58
A_68_P26798485	chr9:105547166-105547210	NM_001081309:1864	Pik3r4	INSIDE	0.542	2.535	3190.76	8088.27	1.373	2134.77	2931.10
A_68_P31128230	chr17:29627256-29627303	NM_008842:-710	Pim1	PROMOTER	0.017	5.145	2572.23	13234.98	0.088	2009.63	177.35
A_68_P31128229	chr17:29627153-29627197	NM_008842:-815	Pim1	PROMOTER	0.277	0.316	7599.60	2398.95	0.087	4856.63	424.95
A_68_P31128228	chr17:29627044-29627088	NM_008842:-923	Pim1	PROMOTER	0.412	0.454	4485.02	2035.29	0.187	3117.04	582.07
A_68_P31128240	chr17:29628771-29628815	NM_008842:803	Pim1	INSIDE	0.466	0.584	821.26	479.34	0.272	637.33	173.27
A_68_P30421719	chr15:88693018-88693062	NM_145478:417	Pim3	INSIDE	0.501	8.591	786.51	6756.88	4.305	654.69	2818.36
A_68_P23345204	chr4:137881562-137881606	NM_026880:627	Pink1	INSIDE	0.574	0.694	5665.38	3931.78	0.398	3962.70	1577.34
A_68_P29664074	chr14:64479020-64479064	NM_028228:-106	Pinx1	PROMOTER	0.5	6.036	3060.25	18470.54	3.017	2680.70	8087.83
A_68_P23524646	chr5:20692316-20692360	NM_175437:254	Pion	INSIDE	0.377	0.400	1359.36	543.83	0.151	1145.88	173.00
A_68_P27534649	chr10:126648747-126648791	NM_054097:-90	Pip4k2c	PROMOTER	0.576	3.113	2201.33	6853.29	1.792	1793.56	3213.90
A_68_P32024327	chr19:24630294-24630338	NM_008846:1	Pip5k1b	INSIDE	0.436	0.554	3788.76	2100.33	0.242	2822.06	682.18
A_68_P27288654	chr10:80756115-80756159	NM_001146687:420	Pip5k1c	INSIDE	0.631	0.389	1273.69	495.97	0.246	1052.36	258.67
A_68_P31003040	chr17:3084893-3084937	NR_003519:-731	Pisd-ps2	PROMOTER	0.137	57.380	9688.39	555915.20	7.842	14183.87	111235.30
A_68_P27926998	chr11:75401820-75401864	NM_008850:233	Pitpna	INSIDE	0.214	0.720	1377.59	991.45	0.154	1138.10	175.25
A_68_P23985067	chr5:111759832-111759876	NM_019640:72	Pitpnb	INSIDE	0.39	0.507	2976.19	1509.07	0.198	2108.94	416.52
A_68_P23985069	chr5:111760075-111760119	NM_019640:314	Pitpnb	INSIDE	0.642	4.396	1669.73	7339.69	2.82	1430.10	4033.06
A_68_P28107262	chr11:107332069-107332113	NM_145823:-56	Pitpnc1	DIVERGENT_PROMOTER	0.175	52.747	1881.36	99235.72	9.221	1811.77	16705.94

ProbeName	Target position of probe on CpG island microarray	TargetID	GeneSymbol	CpG island Description	Ratio of relative methylation (TiO_2-NP/Vehicle)	Sham group			TiO_2-H group		
						Relative methylation (Cy5/Cy3)	Cy3 signal (Input)	Cy5 signal (MeDIP)	Relative methylation (Cy5/Cy3)	Cy3 signal (Input)	Cy5 signal (MeDIP)
A_68_P28107247	chr11:107330576-107330620	NM_145823:1436	Pitpnc1	INSIDE	0.255	8.952	1767.68	15823.68	2.287	1446.65	3307.90
A_68_P31921046	chr19:4100132-4100176	NM_008851:-467	Pitpnm1	PROMOTER	0.476	0.502	1677.53	841.80	0.239	1305.95	311.77
A_68_P31921054	chr19:4101079-4101123	NM_008851:479	Pitpnm1	INSIDE	0.638	5.453	924.78	5042.96	3.482	713.35	2483.59
A_68_P31921047	chr19:4100251-4100295	NM_008851:-349	Pitpnm1	PROMOTER	0.603	0.721	2728.58	1967.89	0.435	1824.77	793.86
A_68_P29057249	chr13:55926961-55927005	NM_011097:5804	Pitx1	INSIDE	0.351	0.348	1992.95	693.84	0.122	1424.16	173.83
A_68_P29057257	chr13:55927883-55927927	NM_011097:4882	Pitx1	INSIDE	0.639	0.329	1995.88	655.69	0.21	1477.08	310.31
A_68_P29057339	chr13:55937443-55937487	NM_011097:-4678	Pitx1	PROMOTER	0.66	0.388	1354.13	525.04	0.256	1033.48	264.53
A_68_P29057258	chr13:55927956-55928000	NM_011097:4808	Pitx1	INSIDE	0.59	0.529	843.86	446.64	0.312	604.44	188.71
A_68_P22518659	chr3:128907292-128907336	NM_001042502:-9540	Pitx2	PROMOTER	0.505	6.243	1767.20	11032.23	3.151	1538.06	4846.81
A_68_P22518617	chr3:128902587-128902631	NM_011098:-227	Pitx2	PROMOTER	0.514	0.483	2337.83	1129.65	0.248	1736.43	431.21
A_68_P32555655	chrX:96666023-96666067	NM_001083110:30	Pja1	INSIDE	0.273	7.102	1277.35	9071.57	1.937	1523.97	2952.02
A_68_P31295068	chr17:64681131-64681175	NM_001025309:71	Pja2	INSIDE	0.375	0.350	3014.25	1054.52	0.131	2325.24	304.75
A_68_P23952342	chr5:104888645-104888689	NM_008861:191	Pkd2	INSIDE	0.549	4.715	1537.23	7247.83	2.588	1339.06	3465.07
A_68_P31612701	chr18:34569089-34569133	NM_001163004:34	Pkd2l2	INSIDE	0.636	0.328	1527.22	500.57	0.208	1191.08	248.16
A_68_P31612702	chr18:34569207-34569251	NM_001163004:152	Pkd2l2	INSIDE	0.424	0.604	837.07	505.44	0.256	701.65	179.51
A_68_P31397730	chr17:83614079-83614123	NM_134117:-522	Pkdcc	PROMOTER	0.49	0.728	3730.04	2714.20	0.357	2980.81	1063.09
A_68_P31397731	chr17:83614185-83614229	NM_134117:-416	Pkdcc	PROMOTER	0.499	1.341	6543.80	8772.55	0.668	5063.68	3384.43
A_68_P31093271	chr17:23863763-23863807	NM_023058:482	Pkmyt1	INSIDE	0.574	0.229	3527.72	806.86	0.131	2574.35	338.09
A_68_P22593186	chr3:142544706-142544750	NM_178654:240	Pkn2	INSIDE	0.521	0.520	2269.16	1179.81	0.271	1875.39	507.86
A_68_P22593188	chr3:142544925-142544969	NM_178654:22	Pkn2	INSIDE	0.616	4.019	508.48	2043.65	2.477	451.95	1119.41
A_68_P31140479	chr17:31701231-31701275	NM_016670:-465	Pknox1	PROMOTER	0.498	0.370	1268.12	468.71	0.184	937.33	172.50
A_68_P31140482	chr17:31701718-31701762	NM_016670:23	Pknox1	INSIDE	0.55	4.512	1511.54	6820.34	2.481	1242.28	3081.84
A_68_P31140484	chr17:31702098-31702142	NM_016670:403	Pknox1	INSIDE	0.631	3.692	1076.92	3975.86	2.33	878.13	2046.47
A_68_P26429021	chr9:36954305-36954349	NM_001029838:525	Pknox2	INSIDE	0.503	7.486	1016.38	7608.18	3.763	1083.61	4077.45
A_68_P26429019	chr9:36953992-36954036	NM_001029838:837	Pknox2	INSIDE	0.418	1.501	2262.45	3396.24	0.627	1680.96	1054.33
A_68_P20642551	chr1:137815771-137815815	NM_019645:-191	Pkp1	PROMOTER	0.441	5.454	2399.13	13085.60	2.406	1921.63	4624.38
A_68_P20642552	chr1:137815854-137815898	NM_019645:-275	Pkp1	PROMOTER	0.558	0.457	2336.80	1067.53	0.255	1820.21	463.99
A_68_P30568393	chr16:16213292-16213336	NM_026163:-123	Pkp2	PROMOTER	0.189	9.630	1159.20	11162.65	1.817	1196.20	2172.95
A_68_P21250388	chr2:58999667-58999711	NM_026361:782	Pkp4	INSIDE	0.214	35.035	2268.46	79475.51	7.494	2024.24	15168.80
A_68_P21250385	chr2:58999390-58999434	NM_026361:506	Pkp4	INSIDE	0.499	10.276	3508.04	36048.67	5.125	3278.06	16798.53
A_68_P21250386	chr2:58999473-58999517	NM_026361:588	Pkp4	INSIDE	0.59	3.497	1547.70	5413.03	2.064	1509.30	3114.86
A_68_P22522614	chr3:129581824-129581868	NM_183423:-2255	Pla2g12a	PROMOTER	0.239	1.752	4134.24	7242.39	0.418	3080.98	1289.25
A_68_P22522616	chr3:129582033-129582077	NM_183423:-2047	Pla2g12a	PROMOTER	0.471	6.890	10442.24	71949.27	3.243	8042.95	26085.59
A_68_P26138002	chr8:108674112-108674156	NM_133792:-164	Pla2g15	PROMOTER	0.31	0.569	4762.86	2709.43	0.176	3213.56	566.51
A_68_P30365773	chr15:79158644-79158688	NM_001199023:135	Pla2g6	INSIDE	0.452	0.221	4905.22	1081.93	0.1	3732.77	371.90
A_68_P29480407	chr14:26722558-26722602	NM_207229:-201	Plac9	PROMOTER	0.639	0.250	8892.07	2225.30	0.16	6775.33	1084.23
A_68_P29480406	chr14:26722448-26722492	NM_207229:-91	Plac9	PROMOTER	0.374	0.640	3597.92	2301.93	0.239	2828.77	676.60
A_68_P22695183	chr4:3864597-3864641	NM_019969:934	Plag1	INSIDE	0.356	1.953	2583.12	5044.34	0.695	2078.55	1445.00
A_68_P26949005	chr10:12810608-12810652	NM_009538:37	Plagl1	INSIDE	0.265	18.094	959.03	17352.27	4.791	1216.96	5830.91
A_68_P26949004	chr10:12810446-12810490	NM_009538:-125	Plagl1	PROMOTER	0.416	5.980	694.78	4154.71	2.488	617.15	1535.17
A_68_P21753788	chr2:153067075-153067119	NM_018807:-2	Plagl2	DIVERGENT_PROMOTER	0.524	10.564	1539.62	16264.83	5.538	1350.43	7478.49
A_68_P24039106	chr5:120953388-120953432	NM_023625:222	Plbd2	INSIDE	0.511	8.777	920.64	8080.82	4.485	807.62	3622.16
A_68_P21652760	chr2:134611895-134611939	NM_001145830:17	Plcb1	INSIDE	0.563	0.423	1380.01	583.66	0.238	1137.35	270.68
A_68_P21565686	chr2:118537255-118537299	NM_177568:16898	Plcb2	INSIDE	0.54	0.635	1015.65	644.52	0.343	807.57	276.76
A_68_P28083319	chr11:102962554-102962598	NM_152813:396	Plcd3	INSIDE	0.642	0.394	2909.55	1147.04	0.253	2109.40	533.53
A_68_P28083321	chr11:102962810-102962854	NM_152813:140	Plcd3	INSIDE	0.647	2.388	1137.71	2717.33	1.545	1007.27	1556.26

ProbeName	Target position of probe on CpG island microarray	TargetID	GeneSymbol	CpG island Description	Ratio of relative methylation (TiO_2-NP/Vehicle)	Sham group			TiO_2-H group		
						Relative methylation (Cy5/Cy3)	Cy3 signal (Input)	Cy5 signal (MeDIP)	Relative methylation (Cy5/Cy3)	Cy3 signal (Input)	Cy5 signal (MeDIP)
A_68_P21796699	chr2:160557598-160557642	NM_021280:575	Plcg1	INSIDE	0.427	5.062	608.93	3082.17	2.161	568.33	1227.93
A_68_P26203094	chr8:120022462-120022506	NM_172285:294	Plcg2	INSIDE	0.485	5.523	847.34	4679.43	2.677	701.15	1877.05
A_68_P30729955	chr16:46010463-46010507	NM_001134480:42	Plcxd2	INSIDE	0.469	7.767	669.95	5203.46	3.641	669.50	2437.72
A_68_P20852908	chr1:178205507-178205551	NM_176916:-85	Pld5	PROMOTER	0.664	0.437	1870.98	817.69	0.29	1543.03	447.49
A_68_P21587817	chr2:122564114-122564158	NM_019788:-104	Pldn	PROMOTER	0.551	8.335	1421.46	11848.38	4.592	1198.23	5502.68
A_68_P30346633	chr15:76014154-76014198	NM_201394:11964	Plec	INSIDE	0.226	11.748	2053.12	24120.89	2.659	1509.41	4013.61
A_68_P30346604	chr15:76010482-76010526	NM_201394:15636	Plec	INSIDE	0.339	10.277	1640.52	16860.15	3.484	1272.05	4431.79
A_68_P30346980	chr15:76063274-76063318	NM_001163542:-1488	Plec	PROMOTER	0.414	0.219	3853.54	844.42	0.091	2792.91	253.13
A_68_P30346610	chr15:76011282-76011326	NM_201394:14836	Plec	INSIDE	0.468	9.111	2790.03	25420.21	4.266	2443.19	10422.82
A_68_P30346599	chr15:76009927-76009971	NM_201394:16192	Plec	INSIDE	0.497	8.843	1956.77	17303.72	4.392	1633.83	7175.34
A_68_P30346762	chr15:76028710-76028754	NM_201393:-93	Plec	PROMOTER	0.503	5.923	2450.91	14516.94	2.978	1891.93	5634.95
A_68_P30346619	chr15:76012289-76012333	NM_201394:13830	Plec	INSIDE	0.513	10.738	1434.37	15402.07	5.506	1159.68	6385.55
A_68_P30346603	chr15:76010310-76010354	NM_201394:15808	Plec	INSIDE	0.543	4.905	961.49	4716.08	2.662	807.41	2149.71
A_68_P30346634	chr15:76014247-76014291	NM_201394:11872	Plec	INSIDE	0.604	4.067	926.44	3768.10	2.455	746.46	1832.64
A_68_P30346596	chr15:76009591-76009635	NM_201394:16528	Plec	INSIDE	0.63	4.703	609.73	2867.45	2.964	445.53	1320.38
A_68_P30346760	chr15:76028479-76028523	NM_201394:-2360	Plec	PROMOTER	0.65	0.613	1237.98	759.18	0.399	1012.30	403.55
A_68_P28566637	chr12:80012969-80013013	NM_013738:-5065	Plek2	PROMOTER	0.447	4.278	3387.80	14494.01	1.913	2498.63	4779.52
A_68_P28566598	chr12:80007781-80007825	NM_013738:123	Plek2	INSIDE	0.626	0.459	1555.93	713.42	0.287	1208.20	346.60
A_68_P28566640	chr12:80013209-80013253	NM_013738:-5305	Plek2	PROMOTER	0.629	2.541	2776.88	7056.32	1.599	2231.34	3566.86
A_68_P24894485	chr6:140373203-140373247	NM_144920:605	Plekha5	INSIDE	0.497	3.844	4497.33	17289.78	1.909	3516.52	6712.97
A_68_P22727584	chr4:10935394-10935438	NM_175175:-650	Plekhf2	PROMOTER	0.574	5.750	2712.79	15598.02	3.298	2277.95	7513.81
A_68_P22727582	chr4:10935187-10935231	NM_175175:-442	Plekhf2	PROMOTER	0.603	0.228	2113.71	481.38	0.137	1683.46	231.29
A_68_P26133402	chr8:107898609-107898653	NM_001081333:-650	Plekhg4	PROMOTER	0.612	2.851	3039.37	8665.39	1.746	2366.70	4132.54
A_68_P28072309	chr11:101025372-101025416	NM_146030:7222	Plekhh3	INSIDE	0.564	0.645	951.73	613.73	0.364	781.18	284.37
A_68_P27285555	chr10:80260794-80260838	NM_023900:555	Plekhj1	INSIDE	0.158	23.409	11019.46	257951.60	3.709	12675.68	47008.31
A_68_P27285560	chr10:80261376-80261420	NM_023900:-27	Plekhj1	DIVERGENT_PROMOTER	0.442	4.846	1202.93	5829.57	2.144	922.45	1977.41
A_68_P28085163	chr11:103273626-103273670	NM_183034:330	Plekhm1	INSIDE	0.134	2.744	2841.21	7797.57	0.366	1922.36	704.52
A_68_P28085165	chr11:103273873-103273917	NM_183034:84	Plekhm1	INSIDE	0.61	0.515	1722.07	886.76	0.314	1303.77	409.40
A_68_P22346838	chr3:95799917-95799961	NM_023320:-176	Plekho1	PROMOTER	0.504	5.714	1141.45	6521.89	2.879	1178.79	3393.30
A_68_P26588443	chr9:65427955-65427999	NM_153119:-135	Plekho2	DIVERGENT_PROMOTER	0.28	9.385	4801.39	45060.09	2.624	4058.99	10650.31
A_68_P25477639	chr7:129303337-129303384	NM_011121:410	Plk1	INSIDE	0.621	0.630	1176.14	741.12	0.391	935.48	366.10
A_68_P29322927	chr13:111184916-111184960	NM_152804:-313	Plk2	PROMOTER	0.494	3.432	2287.71	7850.49	1.696	1950.48	3307.78
A_68_P27282704	chr10:79819796-79819840	NM_183152:615	Plk5	INSIDE	0.31	0.453	1761.03	798.45	0.141	1228.22	172.81
A_68_P22282897	chr3:82859249-82859293	NM_016784:-189	Plrg1	DIVERGENT_PROMOTER	0.25	6.089	3863.13	23520.79	1.523	3271.48	4982.21
A_68_P26744225	chr9:95745638-95745682	NM_001033210:38	Pls1	INSIDE	0.568	4.480	3735.02	16731.53	2.543	2920.87	7428.76
A_68_P26744223	chr9:95745481-95745525	NM_001033210:196	Pls1	INSIDE	0.626	3.667	2793.23	10243.12	2.295	2477.91	5686.74
A_68_P21820162	chr2:164683144-164683188	NM_011125:42	Pltp	INSIDE	0.65	0.441	1712.17	755.16	0.287	1434.13	410.95
A_68_P25957202	chr8:74031641-74031685	NM_032398:4006	Plvap	INSIDE	0.531	5.078	435.87	2213.15	2.698	397.99	1073.82
A_68_P28053756	chr11:97847867-97847911	NM_001163608:-128	Plxdc1	PROMOTER	0.393	11.939	830.50	9915.23	4.692	684.88	3213.30
A_68_P24344494	chr6:32537701-32537745	NM_175750:470	Plxna4	INSIDE	0.592	7.862	1068.36	8399.66	4.657	923.09	4298.69
A_68_P26818266	chr9:108998036-108998080	NM_172775:109	Plxnb1	INSIDE	0.421	5.352	701.71	3755.20	2.253	648.34	1460.50
A_68_P26818267	chr9:108998137-108998181	NM_172775:209	Plxnb1	INSIDE	0.503	7.555	1089.73	8232.98	3.798	873.87	3319.31
A_68_P30423567	chr15:88991606-88991650	NM_001159521:12652	Plxnb2	INSIDE	0.195	16.901	8433.43	142530.80	3.291	7079.71	23302.35
A_68_P30423673	chr15:89004061-89004105	NM_001159521:198	Plxnb2	INSIDE	0.481	4.895	1883.89	9222.11	2.354	1570.29	3696.63
A_68_P30423564	chr15:88991163-88991207	NM_001159521:13096	Plxnb2	INSIDE	0.623	4.460	833.84	3719.29	2.78	702.71	1953.79
A_68_P30423730	chr15:89010897-89010941	NM_001159521:-6638	Plxnb2	PROMOTER	0.577	2.732	1262.57	3449.71	1.577	879.48	1386.68

ProbeName	Target position of probe on CpG island microarray	TargetID	GeneSymbol	CpG island Description	Ratio of relative methylation (TiO_2-NP/Vehicle)	Sham group			TiO_2-H group		
						Relative methylation (Cy5/Cy3)	Cy3 signal (Input)	Cy5 signal (MeDIP)	Relative methylation (Cy5/Cy3)	Cy3 signal (Input)	Cy5 signal (MeDIP)
A_68_P27362366	chr10:94407709-94407753	NM_018797:-518	Plxnc1	PROMOTER	0.217	42.207	234.31	9889.50	9.139	198.95	1818.17
A_68_P24774311	chr6:115944633-115944678	NM_026376:368	Plxnd1	INSIDE	0.39	16.337	3214.22	52511.56	6.372	2471.36	15746.92
A_68_P24774306	chr6:115943883-115943927	NM_026376:1119	Plxnd1	INSIDE	0.485	11.333	1106.07	12534.67	5.497	1383.00	7602.58
A_68_P22825645	chr4:33276560-33276604	NM_001034867:130	Pm20d2	INSIDE	0.661	2.684	600.64	1612.20	1.773	590.12	1046.37
A_68_P27857775	chr11:62945991-62946035	NM_008885:1001	Pmp22	INSIDE	0.192	10.393	1769.02	18385.28	1.998	1494.83	2987.05
A_68_P27857781	chr11:62946689-62946733	NM_008885:1699	Pmp22	INSIDE	0.573	0.233	2504.88	584.73	0.134	1666.52	222.98
A_68_P20231212	chr1:53353853-53353897	NM_153556:-34	Pms1	DIVERGENT_PROMOTER	0.421	9.592	1489.65	14288.44	4.043	1317.53	5326.38
A_68_P32464005	chrX:70905148-70905192	NM_001199351:23	Pnck	INSIDE	0.616	2.852	851.64	2428.53	1.757	1087.71	1910.68
A_68_P20347960	chr1:74379230-74379274	NM_019999:69	Pnkd	INSIDE	0.382	1.466	1572.00	2303.77	0.56	1170.01	654.62
A_68_P28596606	chr12:85489129-85489173	NM_027438:289	Pnma1	INSIDE	0.346	0.536	3092.20	1656.06	0.185	2107.56	390.50
A_68_P29681988	chr14:67530140-67530184	NM_175498:118	Pnma2	INSIDE	0.442	0.292	1655.47	482.92	0.129	1332.49	171.74
A_68_P29681987	chr14:67530058-67530102	NM_175498:36	Pnma2	INSIDE	0.645	0.735	2691.80	1978.50	0.474	2134.64	1011.81
A_68_P27614819	chr11:17111398-17111442	NM_025443:172	Pno1	INSIDE	0.339	5.406	1583.56	8560.84	1.83	1306.05	2390.71
A_68_P25588916	chr7:148641001-148641045	NM_001163689:-64	Pnpla2	PROMOTER	0.577	2.955	1129.93	3339.02	1.705	878.83	1498.41
A_68_P25614471	chr8:3537772-3537816	NM_001122818:22411	Pnpla6	INSIDE	0.168	12.242	1592.29	19492.17	2.054	1359.54	2792.23
A_68_P25614473	chr8:3537974-3538018	NM_001122818:22613	Pnpla6	INSIDE	0.636	2.270	1775.72	4031.16	1.443	1429.79	2063.06
A_68_P22825899	chr4:33334854-33334898	NM_001033225:886	Pnrc1	INSIDE	0.256	0.408	2397.57	977.95	0.104	1645.37	171.83
A_68_P22825901	chr4:33335015-33335059	NM_001033225:726	Pnrc1	INSIDE	0.178	1.469	792.72	1164.64	0.261	653.65	170.80
A_68_P23330986	chr4:135430015-135430060	NM_026383:-276	Pnrc2	PROMOTER	0.474	1.402	1820.51	2552.59	0.664	1514.89	1006.63
A_68_P27385038	chr10:98569722-98569766	NM_027740:-60	Poc1b	PROMOTER	0.602	3.098	683.65	2118.01	1.864	569.75	1061.75
A_68_P24338521	chr6:31513401-31513445	NM_013723:515	Podxl	INSIDE	0.46	11.340	1099.14	12464.31	5.213	940.67	4904.07
A_68_P24624718	chr6:88824525-88824569	NM_176973:-508	Podxl2	PROMOTER	0.324	0.293	3118.72	914.73	0.095	2345.27	222.72
A_68_P20800091	chr1:168339580-168339624	NM_001142948:357	Pogk	INSIDE	0.378	11.590	8607.21	99759.48	4.381	6925.17	30336.49
A_68_P22340919	chr3:94642155-94642199	NM_001165948:688	Pogz	INSIDE	0.373	17.304	1660.07	28725.60	6.451	1218.90	7863.01
A_68_P25088095	chr7:51804301-51804346	NM_011131:-138	Pold1	PROMOTER	0.384	5.520	3324.86	18352.93	2.119	2614.04	5539.68
A_68_P27565430	chr11:5778421-5778465	NM_008894:-183	Pold2	PROMOTER	0.473	0.678	2167.20	1468.51	0.32	1711.56	548.48
A_68_P22964956	chr4:62185874-62185918	NM_021498:152	Pole3	INSIDE	0.636	0.539	2503.40	1349.55	0.343	1904.11	652.39
A_68_P25261270	chr7:86610733-86610777	NM_017462:405	Polg	INSIDE	0.645	3.370	2568.92	8658.13	2.174	2046.11	4448.13
A_68_P28103448	chr11:106640841-106640885	NM_015810:-11	Polg2	PROMOTER	0.322	5.021	1270.28	6378.51	1.616	1028.30	1661.85
A_68_P31205623	chr17:46339517-46339561	NM_030715:36	Polh	INSIDE	0.59	0.497	2549.61	1268.43	0.294	1963.68	576.59
A_68_P30678526	chr16:37011868-37011912	NM_001159369:19	Polq	INSIDE	0.618	4.006	1317.57	5278.82	2.475	1050.69	2600.68
A_68_P21623003	chr2:128926712-128926756	NM_009086:3	Polr1b	INSIDE	0.454	4.534	2538.32	11509.42	2.057	2008.22	4131.54
A_68_P21623005	chr2:128926868-128926912	NM_009086:159	Polr1b	INSIDE	0.591	0.626	891.38	558.17	0.37	723.34	267.55
A_68_P27896914	chr11:69572502-69572546	NM_009089:-799	Polr2a	PROMOTER	0.419	0.441	1570.95	693.56	0.185	1265.65	234.30
A_68_P27896907	chr11:69571625-69571671	NM_009089:77	Polr2a	INSIDE	0.16	2.453	1058.38	2595.96	0.394	908.40	357.54
A_68_P23820901	chr5:77739843-77739887	NM_153798:356	Polr2b	INSIDE	0.536	0.718	1680.74	1206.98	0.385	1337.27	514.50
A_68_P26079858	chr8:97381751-97381795	NM_009090:423	Polr2c	INSIDE	0.612	2.834	1641.04	4651.18	1.734	1473.97	2555.96
A_68_P31599575	chr18:31948663-31948707	NM_027002:-128	Polr2d	PROMOTER	0.416	3.934	4404.54	17325.76	1.638	3654.87	5986.75
A_68_P30589177	chr16:20718065-20718109	NM_145632:188	Polr2h	INSIDE	0.37	6.874	1813.67	12467.72	2.54	1483.78	3769.07
A_68_P30589173	chr16:20717602-20717646	NM_145632:-274	Polr2h	DIVERGENT_PROMOTER	0.356	0.624	877.04	547.03	0.222	763.70	169.46
A_68_P25030468	chr7:31017900-31017944	NM_027259:830	Polr2i	INSIDE	0.482	0.399	1420.32	566.24	0.192	934.33	179.40
A_68_P24119515	chr5:136592514-136592559	NM_011293:-24	Polr2j	PROMOTER	0.63	0.483	4323.18	2087.26	0.304	2616.34	795.89
A_68_P29701295	chr14:70843090-70843134	NM_001164082:-78	Polr3d	PROMOTER	0.247	0.636	1674.47	1065.15	0.157	1265.38	198.53
A_68_P25471037	chr7:128061761-128061805	NM_001164096:525	Polr3e	INSIDE	0.64	0.712	1639.80	1167.97	0.456	1203.18	548.86
A_68_P25471034	chr7:128061307-128061351	NM_001164096:71	Polr3e	INSIDE	0.477	0.515	935.41	481.68	0.245	725.06	177.99
A_68_P30380497	chr15:81756399-81756443	NM_030229:223	Polr3h	INSIDE	0.428	1.429	2293.36	3278.18	0.612	1756.62	1075.11

ProbeName	Target position of probe on CpG island microarray	TargetID	GeneSymbol	CpG island Description	Ratio of relative methylation (TiO_2-NP/Vehicle)	Sham group			TiO_2-H group		
						Relative methylation (Cy5/Cy3)	Cy3 signal (Input)	Cy5 signal (MeDIP)	Relative methylation (Cy5/Cy3)	Cy3 signal (Input)	Cy5 signal (MeDIP)
A_68_P27278598	chr10:79198902-79198960	NM_172551:10396	Polrmt	INSIDE	0.552	4.807	740.39	3559.27	2.656	640.79	1701.96
A_68_P27278628	chr10:79202047-79202091	NM_172551:7258	Polrmt	INSIDE	0.526	2.911	1358.85	3955.25	1.532	1182.89	1811.96
A_68_P24116186	chr5:135870022-135870066	NM_148932:372	Pom121	INSIDE	0.115	13.618	1646.66	22423.52	1.569	1600.49	2511.52
A_68_P28875600	chr13:22073387-22073431	NM_001162929:346	Pom121l2	INSIDE	0.521	4.596	2683.11	12332.36	2.396	2012.63	4821.61
A_68_P28197877	chr12:3960273-3960317	NM_008895:5344	Pomc	INSIDE	0.498	0.206	2197.34	453.30	0.103	1717.83	176.59
A_68_P21114136	chr2:32092306-32092350	NM_145145:126	Pomt1	INSIDE	0.565	2.951	1891.23	5581.27	1.667	1610.80	2685.22
A_68_P24117608	chr5:136209662-136209706	NM_008898:44601	Por	INSIDE	0.181	10.332	3038.55	31393.97	1.865	2076.18	3872.82
A_68_P24117359	chr5:136164594-136164638	NM_008898:-467	Por	PROMOTER	0.461	0.298	1782.05	531.31	0.137	1258.03	172.74
A_68_P24117361	chr5:136164752-136164796	NM_008898:-309	Por	PROMOTER	0.546	9.589	2177.46	20878.79	5.236	1914.85	10026.02
A_68_P24117609	chr5:136209766-136209810	NM_008898:44705	Por	INSIDE	0.535	4.017	767.73	3084.09	2.151	566.80	1219.06
A_68_P24117607	chr5:136209485-136209529	NM_008898:44423	Por	INSIDE	0.439	3.226	2989.33	9645.02	1.416	2259.15	3199.48
A_68_P20797864	chr1:167933679-167933723	NM_011137:-935	Pou2f1	PROMOTER	0.474	11.169	1554.10	17357.40	5.294	1399.86	7411.20
A_68_P26460308	chr9:43013714-43013758	NM_011139:102	Pou2f3	INSIDE	0.204	1.435	1816.19	2607.09	0.293	1437.86	420.73
A_68_P26460307	chr9:43013586-43013630	NM_011139:230	Pou2f3	INSIDE	0.241	3.062	5560.16	17023.88	0.739	4550.98	3363.56
A_68_P23269516	chr4:124335391-124335435	NM_011141:524	Pou3f1	INSIDE	0.336	9.005	1443.44	12997.70	3.022	1355.45	4096.45
A_68_P23269526	chr4:124336792-124336836	NM_011141:1926	Pou3f1	INSIDE	0.496	0.344	6618.55	2274.65	0.171	4445.77	758.31
A_68_P23269522	chr4:124336272-124336316	NM_011141:1406	Pou3f1	INSIDE	0.343	1.740	2076.23	3613.18	0.598	1760.83	1052.14
A_68_P22778510	chr4:22415241-22415285	NM_008899:16	Pou3f2	INSIDE	0.544	0.391	2107.39	824.79	0.213	1663.65	353.90
A_68_P22778521	chr4:22416923-22416967	NM_008899:-1666	Pou3f2	PROMOTER	0.539	0.622	822.76	511.47	0.335	634.98	212.88
A_68_P20184680	chr1:42755426-42755470	NM_008900:1458	Pou3f3	INSIDE	0.429	17.782	4072.45	72416.80	7.622	4099.35	31245.41
A_68_P20184674	chr1:42754743-42754787	NM_008900:774	Pou3f3	INSIDE	0.609	0.593	2034.26	1205.46	0.361	1555.61	561.49
A_68_P29871680	chr14:104872642-104872686	NM_011143:-5448	Pou4f1	PROMOTER	0.209	52.150	4740.71	247225.60	10.897	4514.38	49195.20
A_68_P25988740	chr8:80959381-80959425	NM_138944:1149	Pou4f2	INSIDE	0.151	1.540	13955.02	21493.13	0.232	8368.32	1939.93
A_68_P31655269	chr18:42554834-42554878	NM_138945:606	Pou4f3	INSIDE	0.625	0.441	2025.91	892.64	0.275	1499.75	413.05
A_68_P29336883	chr13:113591639-113591683	NM_008903:530	Ppap2a	INSIDE	0.544	0.593	2283.63	1353.95	0.322	1912.62	616.80
A_68_P27277398	chr10:78996156-78996200	NM_015817:354	Ppap2c	INSIDE	0.144	4.409	1718.05	7574.22	0.637	1333.98	849.22
A_68_P25728673	chr8:26830531-26830575	NM_028000:33	Ppapdc1b	INSIDE	0.292	8.777	2196.63	19278.84	2.563	1849.34	4739.13
A_68_P25728674	chr8:26830692-26830736	NM_028000:195	Ppapdc1b	INSIDE	0.656	0.670	1428.07	957.13	0.439	1080.84	474.91
A_68_P32050411	chr19:29049575-29049620	NM_028922:11188	Ppapdc2	DOWNSTREAM	0.49	0.639	754.78	482.52	0.313	633.51	198.51
A_68_P30402716	chr15:85566360-85566404	NM_011144:177	Ppara	INSIDE	0.385	0.329	1738.36	572.53	0.127	1333.40	169.13
A_68_P30402711	chr15:85565654-85565698	NM_001113418:-317	Ppara	PROMOTER	0.458	0.511	919.18	469.78	0.234	732.13	171.25
A_68_P31121256	chr17:28369519-28369563	NM_011145:-158	Ppard	PROMOTER	0.535	2.862	2483.10	7107.74	1.531	1918.90	2937.62
A_68_P24770499	chr6:115312049-115312093	NM_001127330:832	Pparg	INSIDE	0.353	4.126	1543.15	6366.58	1.457	1149.46	1674.46
A_68_P31757526	chr18:61558105-61558149	NM_133249:1959	Ppargc1b	INSIDE	0.379	5.728	4256.60	24379.78	2.171	3385.49	7350.66
A_68_P31757530	chr18:61558577-61558621	NM_133249:1487	Ppargc1b	INSIDE	0.509	0.227	3369.00	764.95	0.116	2313.21	267.30
A_68_P31757535	chr18:61559223-61559268	NM_133249:840	Ppargc1b	INSIDE	0.647	2.812	5430.66	15270.23	1.819	4172.25	7587.30
A_68_P23819023	chr5:77380662-77380706	NM_172146:-81	Ppat	PROMOTER	0.276	8.298	3676.70	30508.73	2.291	3140.00	7193.87
A_68_P25092685	chr7:52622314-52622358	NM_029741:53	Ppfia3	INSIDE	0.164	17.299	1478.94	25584.70	2.83	1291.89	3655.76
A_68_P25092648	chr7:52616935-52616979	NM_029741:5433	Ppfia3	INSIDE	0.43	0.360	2045.80	736.44	0.155	1522.41	235.79
A_68_P25092682	chr7:52621989-52622033	NM_029741:379	Ppfia3	INSIDE	0.558	4.595	1392.60	6398.41	2.562	1231.04	3154.06
A_68_P25092684	chr7:52622218-52622262	NM_029741:149	Ppfia3	INSIDE	0.612	3.364	1074.10	3613.66	2.059	934.85	1925.29
A_68_P27568397	chr11:6315971-6316015	NM_008907:120	Ppia	INSIDE	0.584	5.032	2503.18	12596.68	2.937	2126.45	6245.58
A_68_P27568398	chr11:6316069-6316113	NM_008907:218	Ppia	INSIDE	0.589	7.384	4050.31	29909.23	4.352	3279.79	14272.93
A_68_P26590952	chr9:65908094-65908138	NM_011149:141	Ppib	INSIDE	0.288	16.430	1869.26	30711.29	4.737	1550.75	7345.68
A_68_P31713137	chr18:53577351-53577395	NM_008908:289	Ppic	INSIDE	0.573	0.214	8818.02	1890.04	0.123	5882.07	722.96
A_68_P29479229	chr14:26513511-26513556	NM_134084:-122	Ppif	PROMOTER	0.582	0.277	1826.32	506.33	0.161	1504.75	242.94

ProbeName	Target position of probe on CpG island microarray	TargetID	GeneSymbol	CpG island Description	Ratio of relative methylation (TiO_2-NP/Vehicle)	Sham group			TiO_2-H group		
						Relative methylation (Cy5/Cy3)	Cy3 signal (Input)	Cy5 signal (MeDIP)	Relative methylation (Cy5/Cy3)	Cy3 signal (Input)	Cy5 signal (MeDIP)
A_68_P23244929	chr4:118993213-118993257	NM_028677:-106	Ppih	PROMOTER	0.649	0.521	2026.80	1055.06	0.338	1542.53	521.22
A_68_P31126967	chr17:29400949-29400993	NM_026845:-54	Ppil1	DIVERGENT_PROMOTER	0.627	3.702	1073.72	3975.37	2.322	925.55	2148.69
A_68_P20258947	chr1:58502118-58502162	NM_027351:190	Ppil3	INSIDE	0.361	0.279	2470.29	689.35	0.101	1768.09	178.24
A_68_P27089555	chr10:41210220-41210264	NM_028430:-2	Ppil6	DIVERGENT_PROMOTER	0.561	0.713	1762.19	1256.05	0.4	1340.79	535.67
A_68_P30513991	chr16:5132312-5132356	NM_008909:240	Ppl	INSIDE	0.53	0.562	5415.45	3044.06	0.298	4165.18	1241.18
A_68_P30513988	chr16:5132000-5132044	NM_008909:552	Ppl	INSIDE	0.642	0.305	2063.75	629.58	0.196	1408.36	275.65
A_68_P28533645	chr12:73862367-73862411	NM_008910:191	Ppm1a	INSIDE	0.4	7.813	3411.99	26657.79	3.122	2809.36	8771.36
A_68_P31407761	chr17:85357878-85357922	NM_001159496:560	Ppm1b	INSIDE	0.25	13.811	901.58	12451.38	3.455	797.45	2755.07
A_68_P27993897	chr11:87172172-87172216	NM_177167:302	Ppm1e	INSIDE	0.367	7.524	1691.21	12724.86	2.761	1419.79	3920.31
A_68_P23577162	chr5:31523190-31523234	NM_008014:-294	Ppm1g	PROMOTER	0.5	0.328	1437.81	471.55	0.164	1047.96	171.87
A_68_P23577160	chr5:31522948-31522992	NM_008014:-52	Ppm1g	PROMOTER	0.583	8.862	3065.63	27168.74	5.171	2698.63	13954.10
A_68_P27509905	chr10:122115624-122115668	NM_176919:-171	Ppm1h	PROMOTER	0.413	0.346	1706.44	590.47	0.143	1214.48	173.64
A_68_P22393107	chr3:104584043-104584087	NM_027982:91	Ppm1j	INSIDE	0.315	0.298	2426.55	724.12	0.094	1859.20	174.92
A_68_P22393109	chr3:104584285-104584329	NM_027982:333	Ppm1j	INSIDE	0.537	15.569	2500.03	38921.97	8.362	2174.36	18181.62
A_68_P22220267	chr3:69120847-69120891	NM_178726:29	Ppm1l	INSIDE	0.66	0.358	2916.72	1043.10	0.236	2394.70	565.15
A_68_P22220265	chr3:69120566-69120610	NM_178726:-251	Ppm1l	PROMOTER	0.659	0.639	2269.43	1449.66	0.421	1675.42	704.89
A_68_P24992443	chr7:19865221-19865265	NM_177691:156	Ppm1n	INSIDE	0.377	4.065	1250.25	5082.41	1.531	1154.90	1767.86
A_68_P31921666	chr19:4192011-4192055	NM_031868:-141	Ppp1ca	DIVERGENT_PROMOTER	0.539	0.572	1919.29	1097.91	0.308	1614.27	497.29
A_68_P31921667	chr19:4192216-4192260	NM_031868:65	Ppp1ca	INSIDE	0.552	0.542	2474.68	1340.84	0.299	1970.46	589.10
A_68_P23584119	chr5:32761375-32761419	NM_172707:54	Ppp1cb	INSIDE	0.522	6.523	5572.54	36350.01	3.407	4639.94	15808.09
A_68_P24048831	chr5:122608072-122608116	NM_013636:-193	Ppp1cc	PROMOTER	0.644	2.715	2745.16	7453.73	1.748	2260.82	3950.81
A_68_P27430008	chr10:107599648-107599692	NM_027892:215	Ppp1r12a	INSIDE	0.647	4.061	1526.10	6197.06	2.626	1270.34	3336.52
A_68_P20636473	chr1:136852289-136852333	NM_001081307:207	Ppp1r12b	INSIDE	0.326	15.818	1902.64	30096.17	5.159	1589.06	8197.65
A_68_P24992918	chr7:19955528-19955572	NM_001010836:8986	Ppp1r13l	INSIDE	0.603	2.499	1169.52	2922.70	1.506	1014.37	1527.81
A_68_P25026383	chr7:30074297-30074341	NM_026731:-20	Ppp1r14a	DIVERGENT_PROMOTER	0.612	4.710	1431.36	6742.01	2.885	1184.09	3416.07
A_68_P25026386	chr7:30074743-30074787	NM_026731:426	Ppp1r14a	INSIDE	0.484	0.671	847.44	568.42	0.324	530.14	171.96
A_68_P31937057	chr19:7049099-7049143	NM_008889:-417	Ppp1r14b	DIVERGENT_PROMOTER	0.54	0.209	2396.80	500.89	0.113	1723.92	194.53
A_68_P20625773	chr1:135028384-135028428	NM_133819:664	Ppp1r15b	INSIDE	0.592	0.401	1435.44	575.17	0.237	1172.41	277.93
A_68_P20625767	chr1:135027845-135027889	NM_133819:124	Ppp1r15b	INSIDE	0.522	0.697	2508.78	1747.81	0.363	1895.67	688.95
A_68_P30349855	chr15:76502255-76502299	NM_033371:167	Ppp1r16a	INSIDE	0.546	10.440	4083.59	42633.47	5.701	3855.50	21979.22
A_68_P21784241	chr2:158493133-158493177	NM_001159662:284	Ppp1r16b	INSIDE	0.283	0.236	4732.26	1116.97	0.067	3217.17	214.96
A_68_P21784240	chr2:158492976-158493020	NM_001159662:128	Ppp1r16b	INSIDE	0.58	0.360	2120.45	764.03	0.209	1797.42	375.58
A_68_P28055832	chr11:98209796-98209843	NM_144828:-232	Ppp1r1b	PROMOTER	0.227	1.803	1408.55	2540.03	0.409	928.28	379.58
A_68_P30646578	chr16:31275334-31275378	NM_025800:7	Ppp1r2	INSIDE	0.2	11.668	2788.76	32538.38	2.338	2356.89	5511.18
A_68_P21890601	chr2:178148869-178148913	NM_001085501:287	Ppp1r3d	INSIDE	0.558	22.506	2154.23	48483.33	12.559	2294.34	28814.06
A_68_P28946211	chr13:36060705-36060749	NM_029628:952	Ppp1r3g	INSIDE	0.266	7.148	917.76	6560.38	1.898	845.58	1604.89
A_68_P23314346	chr4:132399235-132399279	NM_146154:-172	Ppp1r8	PROMOTER	0.642	0.722	1960.31	1415.94	0.464	1512.67	701.92
A_68_P28035919	chr11:94852484-94852528	NM_172261:-19	Ppp1r9b	PROMOTER	0.621	5.740	701.44	4026.28	3.563	655.97	2337.10
A_68_P28035927	chr11:94853399-94853443	NM_172261:895	Ppp1r9b	INSIDE	0.636	6.288	606.41	3813.08	4.002	495.49	1983.02
A_68_P28035916	chr11:94852207-94852251	NM_172261:-297	Ppp1r9b	PROMOTER	0.664	0.697	2842.56	1980.93	0.463	2087.21	966.41
A_68_P28035922	chr11:94852837-94852881	NM_172261:333	Ppp1r9b	INSIDE	0.632	2.510	1136.77	2853.42	1.588	944.22	1499.01
A_68_P29682921	chr14:67691110-67691154	NM_001205188:176	Ppp2r2a	INSIDE	0.262	0.170	6397.46	1084.75	0.044	4803.79	213.56
A_68_P29682917	chr14:67690619-67690663	NM_001205188:668	Ppp2r2a	INSIDE	0.594	14.113	2435.77	34375.09	8.383	2204.27	18479.41
A_68_P23610086	chr5:37259995-37260039	NM_172994:208	Ppp2r2c	INSIDE	0.438	11.014	2582.42	28443.98	4.823	1995.29	9623.79
A_68_P23610095	chr5:37260897-37260941	NM_172994:1110	Ppp2r2c	INSIDE	0.516	5.453	695.53	3792.80	2.815	653.94	1840.67
A_68_P25574426	chr7:146038756-146038800	NM_026391:710	Ppp2r2d	INSIDE	0.657	3.635	773.34	2810.95	2.388	696.51	1663.32

ProbeName	Target position of probe on CpG island microarray	TargetID	GeneSymbol	CpG island Description	Ratio of relative methylation (TiO_2-NP/Vehicle)	Sham group			TiO_2-H group		
						Relative methylation (Cy5/Cy3)	Cy3 signal (Input)	Cy5 signal (MeDIP)	Relative methylation (Cy5/Cy3)	Cy3 signal (Input)	Cy5 signal (MeDIP)
A_68_P26773477	chr9:101153798-101153842	NM_001161362:342	Ppp2r3a	INSIDE	0.425	3.958	819.45	3243.52	1.682	774.08	1302.28
A_68_P33012167	chr9_random:55784-55828	NM_011154:-774	Ppp2r3d	PROMOTER	0.448	0.336	4242.99	1424.99	0.15	3167.36	476.68
A_68_P33012160	chr9_random:55072-55116	NM_011154:-62	Ppp2r3d	PROMOTER	0.472	5.249	3128.53	16420.36	2.478	2413.88	5982.19
A_68_P20937889	chr1:193221406-193221450	NM_144880:-508	Ppp2r5a	PROMOTER	0.315	5.800	1288.39	7472.35	1.829	989.90	1810.50
A_68_P31933357	chr19:6235648-6235692	NM_198168:170	Ppp2r5b	INSIDE	0.374	4.957	802.99	3980.75	1.855	635.64	1179.19
A_68_P28738222	chr12:111685697-111685741	NM_001135001:330	Ppp2r5c	INSIDE	0.363	1.387	2953.34	4095.97	0.503	2514.36	1265.01
A_68_P28738216	chr12:111684724-111684768	NM_001135001:-642	Ppp2r5c	PROMOTER	0.49	3.390	3912.71	13263.31	1.66	3370.77	5594.48
A_68_P28738217	chr12:111684829-111684873	NM_001135001:-538	Ppp2r5c	PROMOTER	0.203	7.908	1681.31	13296.40	1.604	2104.87	3375.66
A_68_P28549425	chr12:76697618-76697662	NM_012024:-453	Ppp2r5e	PROMOTER	0.523	3.840	3336.00	12808.60	2.008	2748.52	5519.67
A_68_P28549422	chr12:76697372-76697416	NM_012024:-207	Ppp2r5e	PROMOTER	0.629	0.644	4695.46	3024.70	0.405	3699.68	1499.29
A_68_P22558745	chr3:136333964-136334008	NM_008913:253	Ppp3ca	INSIDE	0.65	0.745	2676.85	1993.21	0.484	1857.76	899.35
A_68_P22558741	chr3:136333499-136333554	NM_008913:-207	Ppp3ca	PROMOTER	0.651	3.192	459.06	1465.28	2.078	380.92	791.54
A_68_P29700473	chr14:70689417-70689461	NM_008915:-182	Ppp3cc	PROMOTER	0.542	2.975	771.38	2294.97	1.614	713.08	1150.58
A_68_P27614517	chr11:17059599-17059643	NM_024459:320	Ppp3r1	INSIDE	0.283	6.398	2908.18	18606.77	1.811	2466.91	4467.17
A_68_P31302643	chr17:66132754-66132798	NM_001114131:82	Ppp4r1	INSIDE	0.346	6.280	3857.08	24222.78	2.172	3292.08	7150.77
A_68_P21871010	chr2:173484993-173485037	NR_027957:26	Ppp4r1l-ps	INSIDE	0.411	9.287	2064.72	19175.46	3.816	1801.11	6872.69
A_68_P21871011	chr2:173485139-173485183	NR_027957:-120	Ppp4r1l-ps	DIVERGENT_PROMOTER	0.6	0.377	1285.09	484.87	0.227	960.35	217.53
A_68_P24984543	chr7:17612915-17612959	NM_011155:327	Ppp5c	INSIDE	0.664	3.691	1055.36	3895.75	2.452	934.96	2292.96
A_68_P24949868	chr7:4610619-4610663	NM_172894:-88	Ppp6r1	PROMOTER	0.663	0.643	1670.29	1074.66	0.427	1322.38	564.43
A_68_P31918147	chr19:3575682-3575726	NM_001164159:45	Ppp6r3	INSIDE	0.538	0.164	4289.07	703.75	0.088	2937.32	259.44
A_68_P31918148	chr19:3575782-3575826	NM_001164159:-55	Ppp6r3	PROMOTER	0.508	2.942	7004.83	20611.47	1.496	5031.46	7526.72
A_68_P30380957	chr15:81846273-81846317	NM_134095:276	Pppde2	INSIDE	0.288	6.828	2369.30	16177.16	1.965	2069.58	4066.70
A_68_P30380961	chr15:81846636-81846680	NM_134095:-88	Pppde2	DIVERGENT_PROMOTER	0.494	0.082	7904.43	648.06	0.041	5489.92	222.38
A_68_P32141867	chr19:46131714-46131758	NM_001081214:708	Pprc1	INSIDE	0.257	0.539	3616.58	1951.08	0.138	2799.71	387.55
A_68_P23351039	chr4:138866532-138866576	NM_145384:61	Pqlc2	INSIDE	0.473	0.445	1055.46	469.76	0.211	810.85	170.77
A_68_P32239859	chrX:7305710-7305754	NM_138602:36	Praf2	INSIDE	0.374	4.428	706.00	3126.29	1.657	884.39	1465.77
A_68_P23374912	chr4:142984306-142984350	NM_031377:-15	Pramel1	PROMOTER	0.185	17.768	4460.54	79254.39	3.291	3162.67	10408.06
A_68_P25265834	chr7:87439359-87439403	NM_145150:30	Prc1	INSIDE	0.369	0.247	4920.70	1216.96	0.091	3560.89	324.82
A_68_P25265832	chr7:87439097-87439141	NM_145150:-232	Prc1	PROMOTER	0.607	8.026	2723.51	21857.93	4.869	2372.66	11552.24
A_68_P25265836	chr7:87439680-87439724	NM_145150:352	Prc1	INSIDE	0.661	0.644	1363.99	878.62	0.426	1112.17	473.71
A_68_P21432198	chr2:92885847-92885891	NM_001177536:433	Prdm11	INSIDE	0.427	5.289	3023.10	15989.88	2.261	2238.96	5061.45
A_68_P21431995	chr2:92855411-92855455	NM_001177536:30869	Prdm11	INSIDE	0.59	2.717	1286.95	3497.04	1.603	1005.85	1612.73
A_68_P21110348	chr2:31491307-31491351	NM_001123362:-4228	Prdm12	PROMOTER	0.383	0.201	10562.22	2118.78	0.077	6120.66	470.58
A_68_P21110386	chr2:31495757-31495801	NM_001123362:222	Prdm12	INSIDE	0.576	6.497	3228.70	20975.42	3.739	2586.37	9671.65
A_68_P21110351	chr2:31491732-31491776	NM_001123362:-3802	Prdm12	PROMOTER	0.346	0.665	1764.65	1172.67	0.23	1419.36	326.21
A_68_P21110366	chr2:31493479-31493523	NM_001123362:-2056	Prdm12	PROMOTER	0.463	3.794	1553.31	5893.20	1.755	1458.55	2559.78
A_68_P22774300	chr4:21606032-21606076	NM_001080771:7056	Prdm13	INSIDE	0.559	5.640	604.33	3408.35	3.151	543.35	1712.21
A_68_P22774296	chr4:21605518-21605562	NM_001080771:7570	Prdm13	INSIDE	0.448	4.182	559.41	2339.56	1.875	546.00	1023.88
A_68_P23430799	chr4:153715942-153715986	NM_001177995:295018	Prdm16	INSIDE	0.219	12.606	3980.75	50180.00	2.758	2948.33	8132.86
A_68_P23430797	chr4:153715735-153715779	NM_001177995:295226	Prdm16	INSIDE	0.367	8.994	3790.60	34091.07	3.297	3094.69	10202.19
A_68_P23430798	chr4:153715842-153715886	NM_001177995:295118	Prdm16	INSIDE	0.438	15.483	4044.38	62621.07	6.781	2778.82	18843.17
A_68_P23373725	chr4:142752302-142752346	NM_001081355:50288	Prdm2	INSIDE	0.587	0.294	1755.01	515.31	0.172	1512.32	260.77
A_68_P27311096	chr10:85379833-85379877	NM_181650:-164	Prdm4	PROMOTER	0.383	7.743	1347.35	10432.13	2.963	1152.25	3413.71
A_68_P24513815	chr6:65728982-65729026	NM_027547:49	Prdm5	INSIDE	0.014	316.300	900.26	284753.20	4.533	664.10	3010.54
A_68_P31713378	chr18:53623569-53623613	NM_001033281:-609	Prdm6	PROMOTER	0.328	0.369	1791.95	660.48	0.121	1426.89	172.47
A_68_P31713385	chr18:53624187-53624231	NM_001033281:9	Prdm6	INSIDE	0.471	4.147	1866.19	7739.13	1.952	1508.85	2945.29

ProbeName	Target position of probe on CpG island microarray	TargetID	GeneSymbol	CpG island Description	Ratio of relative methylation (TiO_2-NP/Vehicle)	Sham group			TiO_2-H group		
						Relative methylation (Cy5/Cy3)	Cy3 signal (Input)	Cy5 signal (MeDIP)	Relative methylation (Cy5/Cy3)	Cy3 signal (Input)	Cy5 signal (MeDIP)
A_68_P23916648	chr5:98615578-98615622	NM_029947:5713	Prdm8	INSIDE	0.276	0.395	2460.18	971.48	0.109	1558.51	169.79
A_68_P23916646	chr5:98615304-98615348	NM_029947:5439	Prdm8	INSIDE	0.457	4.574	933.25	4268.89	2.089	837.89	1750.10
A_68_P23916639	chr5:98614275-98614319	NM_029947:4409	Prdm8	INSIDE	0.413	1.445	3015.19	4358.31	0.598	2294.59	1371.19
A_68_P32227660	chr19:60950291-60950335	NM_007452:129	Prdx3	INSIDE	0.563	2.641	3903.54	10308.49	1.487	3244.29	4825.19
A_68_P20770872	chr1:163180941-163180985	NM_007453:335	Prdx6	INSIDE	0.599	3.326	897.81	2986.07	1.994	842.95	1680.63
A_68_P23575728	chr5:31262393-31262437	NM_016703:320	Preb	INSIDE	0.471	0.378	2531.40	956.47	0.178	1714.35	304.81
A_68_P31652785	chr18:42110533-42110577	NM_029942:294	Prelid2	INSIDE	0.469	0.416	1733.74	721.18	0.195	1333.89	260.32
A_68_P27109370	chr10:44787342-44787386	NM_011156:345	Prep	INSIDE	0.53	4.646	964.77	4482.79	2.463	890.10	2191.88
A_68_P21831536	chr2:166539405-166539449	NM_177782:-94	Prex1	PROMOTER	0.434	0.550	13307.77	7325.71	0.239	8195.33	1958.08
A_68_P21831533	chr2:166539060-166539104	NM_177782:250	Prex1	INSIDE	0.628	0.685	969.04	663.77	0.43	808.08	347.51
A_68_P20029518	chr1:10983714-10983758	NM_029525:191	Prex2	INSIDE	0.304	17.273	2587.03	44685.94	5.248	2184.92	11466.39
A_68_P20029522	chr1:10984124-10984168	NM_029525:601	Prex2	INSIDE	0.467	0.158	3225.98	510.48	0.074	2288.69	169.14
A_68_P30447922	chr15:93426512-93426556	NM_001033217:-212	Prickle1	PROMOTER	0.067	62.567	26467.23	1655987.00	4.22	57745.57	243694.80
A_68_P30447388	chr15:93349565-93349609	NM_001033217:76736	Prickle1	INSIDE	0.481	3.884	2269.48	8815.13	1.868	1800.37	3363.79
A_68_P30447265	chr15:93331280-93331324	NM_001033217:95020	Prickle1	INSIDE	0.178	3.237	3107.79	10059.46	0.576	2348.61	1352.52
A_68_P30447392	chr15:93349977-93350021	NM_001033217:76324	Prickle1	INSIDE	0.294	6.314	1269.25	8013.82	1.856	1192.10	2212.87
A_68_P28696317	chr12:104480189-104480236	NM_133364:144	Prima1	INSIDE	0.235	2.372	5523.18	13103.63	0.556	3984.60	2217.08
A_68_P23176712	chr4:104782284-104782328	NM_178143:197	Prkaa2	INSIDE	0.223	24.256	12747.99	309214.30	5.399	15756.39	85071.52
A_68_P24012823	chr5:116474520-116474564	NM_031869:-105	Prkab1	PROMOTER	0.393	0.356	3223.01	1146.40	0.14	2287.23	319.64
A_68_P22355467	chr3:97462102-97462146	NM_182997:-10	Prkab2	PROMOTER	0.279	14.890	7856.55	116987.80	4.159	6926.46	28804.75
A_68_P22355468	chr3:97462189-97462238	NM_182997:79	Prkab2	INSIDE	0.571	2.763	1498.03	4138.89	1.579	1098.49	1734.50
A_68_P22616951	chr3:146475944-146475988	NM_011100:-56	Prkacb	PROMOTER	0.579	4.241	2636.73	11181.43	2.454	2190.83	5376.51
A_68_P23544553	chr5:24414393-24414437	NM_001170555:-87	Prkag2	PROMOTER	0.445	6.232	1345.78	8387.03	2.774	1158.73	3214.87
A_68_P23544552	chr5:24414202-24414246	NM_001170555:103	Prkag2	INSIDE	0.448	22.387	7731.40	173082.90	10.028	7038.83	70583.25
A_68_P23544551	chr5:24414128-24414172	NM_001170555:177	Prkag2	INSIDE	0.569	5.815	830.01	4826.18	3.306	788.55	2606.86
A_68_P24133762	chr5:139605565-139605609	NM_008923:120	Prkar1b	INSIDE	0.256	10.552	4217.40	44503.13	2.702	3340.18	9024.74
A_68_P24133761	chr5:139605471-139605515	NM_008923:214	Prkar1b	INSIDE	0.246	2.824	4331.48	12233.15	0.694	3074.04	2133.35
A_68_P26816174	chr9:108594858-108594902	NM_008924:407	Prkar2a	INSIDE	0.389	6.777	1372.64	9302.42	2.638	1183.89	3122.57
A_68_P28334784	chr12:32745555-32745599	NM_011158:568	Prkar2b	INSIDE	0.577	0.530	7450.17	3948.74	0.306	5938.26	1814.64
A_68_P28334787	chr12:32745879-32745923	NM_011158:244	Prkar2b	INSIDE	0.641	3.108	1261.42	3920.80	1.991	1166.18	2322.27
A_68_P25478239	chr7:129432560-129432604	NM_008855:-56	Prkcb	PROMOTER	0.57	4.957	632.50	3135.11	2.825	509.43	1439.12
A_68_P29507005	chr14:31438638-31438682	NM_011103:734	Prkcd	INSIDE	0.651	2.549	5843.10	14893.64	1.66	4852.86	8054.41
A_68_P29507007	chr14:31438823-31438867	NM_011103:550	Prkcd	INSIDE	0.115	1.496	4483.95	6708.90	0.172	2856.46	490.83
A_68_P25392587	chr7:112630270-112630314	NM_028444:419	Prkcdbp	INSIDE	0.382	0.463	1357.90	628.88	0.177	1100.85	194.53
A_68_P25392588	chr7:112630344-112630388	NM_028444:345	Prkcdbp	INSIDE	0.629	6.082	1457.53	8864.05	3.826	1286.77	4923.07
A_68_P31415143	chr17:86567807-86567851	NM_011104:704	Prkce	INSIDE	0.399	5.725	1678.71	9610.37	2.287	1401.91	3205.98
A_68_P31415139	chr17:86567438-86567482	NM_011104:336	Prkce	INSIDE	0.644	0.473	1427.23	674.44	0.304	1127.50	343.29
A_68_P28538319	chr12:74685927-74685971	NM_008856:-79	Prkch	PROMOTER	0.656	6.638	437.32	2903.11	4.355	340.57	1483.18
A_68_P28538317	chr12:74685599-74685643	NM_008856:-407	Prkch	PROMOTER	0.283	7.514	414.56	3115.17	2.126	445.28	946.50
A_68_P28426162	chr12:51750316-51750360	NM_008858:-128	Prkd1	PROMOTER	0.432	6.120	4139.04	25331.10	2.643	3647.40	9639.96
A_68_P28426163	chr12:51750413-51750457	NM_008858:-224	Prkd1	PROMOTER	0.636	0.524	1861.64	974.69	0.333	1598.89	532.73
A_68_P24983574	chr7:17428226-17428270	NM_178900:-165	Prkd2	PROMOTER	0.285	0.554	3067.71	1700.05	0.158	2102.08	332.31
A_68_P24983575	chr7:17428409-17428455	NM_178900:19	Prkd2	INSIDE	0.425	0.388	1361.56	528.41	0.165	1044.25	172.04
A_68_P24983629	chr7:17441116-17441160	NM_178900:12725	Prkd2	INSIDE	0.496	5.812	872.82	5072.69	2.882	712.50	2053.09
A_68_P24983630	chr7:17441193-17441237	NM_178900:12801	Prkd2	INSIDE	0.562	3.508	2124.41	7453.45	1.973	1552.90	3063.81
A_68_P31375073	chr17:79420257-79420301	NM_001171004:-122	Prkd3	PROMOTER	0.182	10.151	1153.77	11711.84	1.85	1224.29	2265.17

ProbeName	Target position of probe on CpG island microarray	TargetID	GeneSymbol	CpG island Description	Ratio of relative methylation (TiO_2-NP/Vehicle)	Sham group			TiO_2-H group		
						Relative methylation (Cy5/Cy3)	Cy3 signal (Input)	Cy5 signal (MeDIP)	Relative methylation (Cy5/Cy3)	Cy3 signal (Input)	Cy5 signal (MeDIP)
A_68_P30566363	chr16:15638071-15638115	NM_011159:134	Prkdc	INSIDE	0.412	3.977	1163.16	4626.20	1.638	995.00	1629.82
A_68_P21351539	chr2:76486029-76486073	NM_011871:1	Prkra	INSIDE	0.472	5.368	1307.62	7019.35	2.535	1038.94	2634.09
A_68_P21351540	chr2:76486169-76486213	NM_011871:-139	Prkra	DIVERGENT_PROMOTER	0.492	0.524	6794.51	3560.68	0.258	4531.34	1169.24
A_68_P21351543	chr2:76486449-76486493	NM_011871:-419	Prkra	DIVERGENT_PROMOTER	0.131	10.790	4736.26	51106.26	1.409	3883.48	5473.54
A_68_P21351534	chr2:76485547-76485591	NM_011871:483	Prkra	INSIDE	0.487	4.089	491.61	2010.32	1.993	424.36	845.73
A_68_P21351542	chr2:76486311-76486355	NM_011871:-281	Prkra	DIVERGENT_PROMOTER	0.481	2.818	3783.40	10662.80	1.355	3236.07	4383.43
A_68_P24119913	chr5:136674484-136674528	NM_025774:318	Prkrip1	INSIDE	0.525	0.151	2995.52	453.30	0.079	2220.05	176.22
A_68_P25114625	chr7:57033846-57033890	NM_133740:141	Prmt3	INSIDE	0.467	3.632	1067.56	3877.69	1.696	802.62	1360.85
A_68_P30577450	chr16:18088960-18089004	NM_011172:301	Prodh	INSIDE	0.385	6.252	3003.99	18781.34	2.406	2308.27	5552.68
A_68_P30577454	chr16:18089367-18089416	NM_011172:-108	Prodh	PROMOTER	0.494	0.183	2977.65	545.31	0.09	1920.85	173.63
A_68_P30577452	chr16:18089136-18089180	NM_011172:125	Prodh	INSIDE	0.605	0.449	1627.51	730.10	0.271	1209.79	328.28
A_68_P23649335	chr5:44492359-44492403	NM_001163584:-6373	Prom1	PROMOTER	0.534	2.831	3731.91	10565.79	1.512	3138.34	4745.01
A_68_P25736226	chr8:28152855-28152901	NM_001039077:-148	Prosc	PROMOTER	0.299	13.323	2552.44	34006.57	3.987	2589.09	10322.20
A_68_P25736232	chr8:28153444-28153488	NM_001039077:440	Prosc	INSIDE	0.403	0.337	1884.48	635.32	0.136	1345.51	182.96
A_68_P21224216	chr2:53050084-53050128	NM_018785:115	Prpf40a	INSIDE	0.597	0.231	1937.96	448.17	0.138	1257.64	173.68
A_68_P30479249	chr15:99125815-99125859	NM_018786:-3	Prpf40b	DIVERGENT_PROMOTER	0.339	4.173	5198.95	21694.89	1.414	3515.55	4969.87
A_68_P28941009	chr13:34967839-34967883	NM_013830:498	Prpf4b	INSIDE	0.604	2.386	2106.02	5025.49	1.442	1716.71	2475.53
A_68_P27926352	chr11:75300162-75300206	NM_138659:-94	Prpf8	PROMOTER	0.369	5.512	3080.77	16980.74	2.032	2544.97	5172.46
A_68_P30477986	chr15:98885749-98885793	NM_001163588:166	Prph	INSIDE	0.569	4.784	981.67	4696.14	2.722	918.40	2500.04
A_68_P30477985	chr15:98885628-98885672	NM_001163588:46	Prph	INSIDE	0.6	0.530	3488.99	1848.15	0.318	2482.47	788.73
A_68_P28159701	chr11:116359924-116359968	NM_026364:-8286	Prpsap1	PROMOTER	0.475	4.878	3169.25	15460.20	2.316	2474.88	5732.92
A_68_P28159663	chr11:116351729-116351773	NM_026364:-90	Prpsap1	PROMOTER	0.55	22.545	3700.01	83415.17	12.389	3011.08	37305.19
A_68_P25505713	chr7:134615484-134615528	NM_145589:379	Prr14	INSIDE	0.25	14.476	1950.70	28237.49	3.617	1999.69	7233.34
A_68_P25505707	chr7:134614706-134614750	NM_145589:-399	Prr14	PROMOTER	0.561	0.521	5800.50	3024.69	0.292	4384.59	1282.06
A_68_P25505711	chr7:134615245-134615289	NM_145589:139	Prr14	INSIDE	0.653	3.043	1226.71	3732.73	1.988	956.66	1901.68
A_68_P32957129	chr17:8534343-8534387	NM_178774:761	Prr18	INSIDE	0.457	0.371	1374.72	510.06	0.17	1023.69	173.62
A_68_P26760867	chr9:98743140-98743184	NM_001134660:157	Prr23a	INSIDE	0.592	6.581	2914.61	19182.09	3.897	2403.08	9365.98
A_68_P24980575	chr7:16858057-16858101	NM_001136270:963	Prr24	INSIDE	0.407	0.539	1474.82	795.55	0.219	994.31	218.04
A_68_P24980579	chr7:16858527-16858571	NM_001136270:493	Prr24	INSIDE	0.49	0.275	11937.56	3284.75	0.135	8647.03	1164.95
A_68_P31162033	chr17:36116300-36116344	NR_028516:-354	Prr3	PROMOTER	0.371	0.303	1913.26	580.52	0.112	1542.74	173.46
A_68_P31162035	chr17:36116527-36116571	NM_145487:-136	Prr3	PROMOTER	0.549	0.307	2006.16	616.67	0.169	1613.23	272.32
A_68_P31162036	chr17:36116621-36116665	NM_145487:-230	Prr3	PROMOTER	0.658	0.506	995.46	504.02	0.333	765.90	255.13
A_68_P30396220	chr15:84511228-84511272	NM_146061:-177	Prr5	PROMOTER	0.406	0.317	1988.73	629.91	0.128	1496.23	192.18
A_68_P29055232	chr13:55574226-55574270	NM_001030296:8621	Prr7	INSIDE	0.192	14.894	1414.12	21061.62	2.861	1088.22	3113.78
A_68_P29055171	chr13:55565839-55565883	NM_001030296:233	Prr7	INSIDE	0.557	0.406	1369.66	556.30	0.226	1080.16	244.58
A_68_P29055227	chr13:55573644-55573688	NM_001030296:8039	Prr7	INSIDE	0.615	2.588	923.28	2389.15	1.592	817.01	1300.31
A_68_P31735662	chr18:57522846-57522891	NM_028447:8482	Prrc1	INSIDE	0.171	12.342	2797.48	34527.06	2.106	2164.47	4558.56
A_68_P31157969	chr17:35293395-35293439	NM_001199044:8406	Prrc2a	INSIDE	0.218	13.444	5312.02	71416.80	2.929	4431.94	12980.25
A_68_P21491162	chr2:104689920-104689964	NM_178695:65	Prrg4	INSIDE	0.624	3.505	1523.59	5340.27	2.188	1418.23	3103.54
A_68_P21491163	chr2:104690040-104690084	NM_178695:-55	Prrg4	PROMOTER	0.461	0.577	813.25	468.97	0.266	634.74	168.62
A_68_P31154948	chr17:34769360-34769404	NM_030890:2752	Prrt1	DOWNSTREAM	0.207	0.566	1996.44	1129.68	0.117	1471.41	172.00
A_68_P31154933	chr17:34767732-34767776	NM_030890:1124	Prrt1	INSIDE	0.336	0.532	2637.75	1402.42	0.179	2147.67	383.99
A_68_P31154947	chr17:34769261-34769305	NM_030890:2652	Prrt1	DOWNSTREAM	0.342	5.254	2457.70	12913.71	1.797	2109.48	3789.89
A_68_P25503619	chr7:134164872-134164916	NM_001102563:-169	Prrt2	PROMOTER	0.644	0.471	2117.54	996.99	0.303	1571.45	476.48
A_68_P24325042	chr6:29129526-29129570	NM_001101443:36	Prrt4	INSIDE	0.635	0.479	967.87	463.51	0.304	820.40	249.62
A_68_P29518733	chr14:33411046-33411090	NM_001001796:-2044	Prrxl1	PROMOTER	0.398	4.082	2020.46	8246.63	1.625	1713.66	2784.89

ProbeName	Target position of probe on CpG island microarray	TargetID	GeneSymbol	CpG island Description	Ratio of relative methylation (TiO_2-NP/Vehicle)	Sham group			TiO_2-H group		
						Relative methylation (Cy5/Cy3)	Cy3 signal (Input)	Cy5 signal (MeDIP)	Relative methylation (Cy5/Cy3)	Cy3 signal (Input)	Cy5 signal (MeDIP)
A_68_P28875767	chr13:22097925-22097969	NM_019429:3664	Prss16	INSIDE	0.26	9.874	1177.53	11627.21	2.565	1158.86	2972.50
A_68_P25508222	chr7:135079466-135079510	NM_001081374:10751	Prss36	INSIDE	0.518	8.155	1726.95	14083.44	4.222	1652.41	6977.25
A_68_P26826274	chr9:110760774-110760818	NM_146227:326	Prss50	INSIDE	0.437	0.683	889.64	607.70	0.298	810.72	241.99
A_68_P25017609	chr7:28293338-28293382	NM_019412:9018	Prx	INSIDE	0.63	0.134	5077.72	678.81	0.084	3445.19	290.23
A_68_P25017608	chr7:28293225-28293269	NM_019412:8904	Prx	INSIDE	0.282	0.638	15571.94	9930.58	0.18	9846.51	1771.96
A_68_P32143181	chr19:46392296-46392341	NM_028627:9328	Psd	INSIDE	0.241	9.651	1811.96	17488.09	2.329	1495.30	3482.84
A_68_P31621137	chr18:36124949-36124993	NM_028707:482	Psd2	INSIDE	0.557	0.452	1129.83	510.59	0.252	780.76	196.47
A_68_P20874688	chr1:182186063-182186107	NM_001128605:347	Psen2	INSIDE	0.625	0.586	4354.86	2550.75	0.366	3079.78	1127.23
A_68_P23062645	chr4:83131826-83131870	NM_133948:446	Psip1	INSIDE	0.647	3.379	1664.25	5622.74	2.187	1451.95	3175.41
A_68_P28523608	chr12:72076024-72076068	NM_011184:437	Psma3	INSIDE	0.576	3.539	714.66	2529.47	2.037	695.08	1416.17
A_68_P28523609	chr12:72076112-72076156	NM_011184:525	Psma3	INSIDE	0.58	2.688	1281.28	3444.17	1.56	1022.93	1595.42
A_68_P28451632	chr12:56500204-56500248	NM_011968:415	Psma6	INSIDE	0.388	0.533	1213.14	646.26	0.207	1074.15	222.00
A_68_P31065956	chr17:15635037-15635081	NM_011185:182	Psmb1	INSIDE	0.331	7.976	3653.45	29141.72	2.643	2916.48	7708.56
A_68_P31065957	chr17:15635193-15635237	NM_011185:26	Psmb1	INSIDE	0.557	0.406	1102.11	447.29	0.226	790.56	178.63
A_68_P23281396	chr4:126355179-126355223	NM_011970:314	Psmb2	INSIDE	0.56	0.645	1304.87	841.78	0.362	1047.34	378.65
A_68_P22341205	chr3:94690730-94690774	NM_008945:128	Psmb4	INSIDE	0.561	3.582	1841.01	6594.36	2.009	1459.49	2931.97
A_68_P21149539	chr2:38499296-38499340	NM_011187:108	Psmb7	INSIDE	0.235	0.470	1748.77	822.77	0.111	1588.08	175.55
A_68_P21149540	chr2:38499397-38499441	NM_011187:8	Psmb7	INSIDE	0.306	6.449	2094.85	13509.21	1.974	1752.46	3458.58
A_68_P28679987	chr12:101348579-101348623	NM_008947:-1940	Psmc1	PROMOTER	0.546	0.571	938.05	535.98	0.312	889.56	277.61
A_68_P21420369	chr2:90894125-90894169	NM_008948:-26	Psmc3	PROMOTER	0.096	39.443	16104.40	635204.20	3.804	23468.94	89268.29
A_68_P29419901	chr14:14953551-14953595	NM_025550:-154	Psmd6	PROMOTER	0.287	13.566	693.19	9403.92	3.892	891.07	3467.88
A_68_P30990393	chr16:96212588-96212632	NM_019537:-100	Psmg1	PROMOTER	0.245	12.247	2005.04	24555.50	3.005	1726.16	5186.85
A_68_P28936994	chr13:34255135-34255179	NM_001101430:311	Psmg4	INSIDE	0.373	1.794	3594.63	6448.61	0.669	2840.65	1900.19
A_68_P29624689	chr14:57396463-57396507	NM_025682:669	Pspc1	INSIDE	0.185	22.333	4565.37	101958.30	4.133	4138.28	17104.60
A_68_P24090826	chr5:130293163-130293207	NM_133900:-55	Psph	DIVERGENT_PROMOTER	0.391	7.070	1853.85	13107.54	2.765	1590.58	4398.32
A_68_P31849787	chr18:78033303-78033347	NM_013831:36	Pstpip2	INSIDE	0.66	0.170	3874.13	658.57	0.112	2827.84	317.10
A_68_P31849791	chr18:78033691-78033735	NM_013831:424	Pstpip2	INSIDE	0.359	1.876	5810.60	10900.82	0.673	3986.54	2683.68
A_68_P32019128	chr19:23762244-23762288	NM_028208:377	Ptar1	INSIDE	0.447	0.619	1848.52	1144.46	0.277	1450.49	401.64
A_68_P22467635	chr3:119486116-119486160	NM_019550:168	Ptbp2	INSIDE	0.551	0.695	1495.59	1039.80	0.383	1184.60	453.97
A_68_P29094155	chr13:63664480-63664524	NM_008957:2326	Ptch1	INSIDE	0.274	12.316	1113.69	13716.51	3.37	1015.86	3423.88
A_68_P29094163	chr13:63665381-63665425	NM_008957:1426	Ptch1	INSIDE	0.586	4.077	2328.57	9493.97	2.391	1676.42	4008.77
A_68_P29094157	chr13:63664712-63664756	NM_008957:2094	Ptch1	INSIDE	0.631	0.534	1691.12	903.48	0.337	1329.87	448.24
A_68_P29094159	chr13:63664958-63665003	NM_008957:1848	Ptch1	INSIDE	0.608	0.602	1122.76	675.98	0.366	865.33	316.92
A_68_P32760538	chrX:152057229-152057273	NM_001093750:620	Ptchd1	INSIDE	0.328	24.528	2249.38	55173.80	8.057	2267.62	18270.83
A_68_P32760547	chrX:152058267-152058311	NM_001093750:-418	Ptchd1	PROMOTER	0.436	3.512	765.63	2688.62	1.531	949.26	1453.77
A_68_P23393512	chr4:147662081-147662125	NM_001083342:-28	Ptchd2	PROMOTER	0.245	7.494	1581.12	11848.32	1.833	1406.69	2578.68
A_68_P25586914	chr7:148317021-148317066	NM_013782:-141	Ptdss2	PROMOTER	0.52	3.345	1810.92	6056.80	1.739	1545.29	2686.94
A_68_P25586915	chr7:148317107-148317151	NM_013782:-56	Ptdss2	PROMOTER	0.657	2.616	1046.76	2738.14	1.718	952.74	1637.02
A_68_P32071916	chr19:32830947-32830991	NM_008960:-1098	Pten	PROMOTER	0.467	0.107	5322.53	569.82	0.05	3777.15	189.03
A_68_P32071925	chr19:32832040-32832084	NM_008960:-4	Pten	PROMOTER	0.661	0.521	2336.66	1217.86	0.344	1674.36	576.78
A_68_P32071915	chr19:32830782-32830826	NM_008960:-1262	Pten	PROMOTER	0.588	0.607	1109.11	672.77	0.357	873.77	311.80
A_68_P21044973	chr2:19367231-19367275	NM_018809:-37	Ptf1a	PROMOTER	0.591	2.858	786.86	2248.95	1.688	684.27	1155.26
A_68_P26015929	chr8:86192596-86192640	NM_013641:2080	Ptger1	INSIDE	0.459	11.587	5305.56	61475.54	5.315	4602.21	24461.88
A_68_P22680840	chr3:157229758-157229802	NM_011196:-75	Ptger3	PROMOTER	0.278	9.755	1604.12	15648.43	2.714	1358.23	3686.42
A_68_P21115159	chr2:32252005-32252049	NM_133783:617	Ptges2	INSIDE	0.592	3.019	903.74	2728.84	1.787	776.13	1387.31
A_68_P22373271	chr3:100914318-100914362	NM_011197:-251	Ptgfrn	PROMOTER	0.48	3.067	1807.95	5544.68	1.471	1414.66	2081.29

ProbeName	Target position of probe on CpG island microarray	TargetID	GeneSymbol	CpG island Description	Ratio of relative methylation (TiO_2-NP/Vehicle)	Sham group			TiO_2-H group		
						Relative methylation (Cy5/Cy3)	Cy3 signal (Input)	Cy5 signal (MeDIP)	Relative methylation (Cy5/Cy3)	Cy3 signal (Input)	Cy5 signal (MeDIP)
A_68_P21834483	chr2:167066012-167066056	NM_008968:3	Ptgis	INSIDE	0.541	0.216	2353.51	507.95	0.117	1497.47	174.96
A_68_P21834481	chr2:167065786-167065830	NM_008968:229	Ptgis	INSIDE	0.573	0.349	1877.66	655.51	0.2	1519.30	304.12
A_68_P26825469	chr9:110629781-110629825	NM_001083936:15312	Pth1r	INSIDE	0.152	2.996	6180.74	18515.58	0.454	4290.88	1949.83
A_68_P26825587	chr9:110645381-110645425	NM_001083936:-288	Pth1r	PROMOTER	0.536	0.273	2266.20	618.67	0.146	1496.12	219.12
A_68_P26825589	chr9:110645592-110645636	NM_001083936:-500	Pth1r	PROMOTER	0.613	0.258	2146.88	554.64	0.158	1721.26	272.41
A_68_P26825596	chr9:110646504-110646548	NM_001083935:-336	Pth1r	PROMOTER	0.624	0.398	1593.26	633.92	0.248	1178.52	292.71
A_68_P26825588	chr9:110645482-110645526	NM_001083936:-390	Pth1r	PROMOTER	0.524	0.683	3623.83	2475.84	0.358	2691.32	964.34
A_68_P26825597	chr9:110646599-110646643	NM_001083935:-430	Pth1r	PROMOTER	0.624	0.730	2240.81	1636.05	0.456	1744.84	795.52
A_68_P26825466	chr9:110629375-110629419	NM_001083936:15718	Pth1r	INSIDE	0.323	5.052	2738.63	13836.13	1.634	1975.78	3228.94
A_68_P25091647	chr7:52436916-52436960	NM_053256:574	Pth2	INSIDE	0.27	0.524	5418.87	2837.03	0.141	3563.08	502.95
A_68_P25091653	chr7:52437711-52437755	NM_053256:1368	Pth2	DOWNSTREAM	0.509	4.641	650.08	3017.13	2.363	500.39	1182.28
A_68_P30330337	chr15:73253106-73253150	NM_007982:493	Ptk2	INSIDE	0.468	0.176	5904.49	1039.96	0.082	4155.07	342.66
A_68_P30330343	chr15:73253736-73253780	NM_007982:-137	Ptk2	PROMOTER	0.488	7.856	792.60	6226.57	3.836	657.85	2523.38
A_68_P31207975	chr17:46766402-46766446	NM_175168:29	Ptk7	INSIDE	0.525	5.271	13135.56	69237.90	2.766	10620.74	29374.66
A_68_P20412593	chr1:88422665-88422709	NM_008972:-624	Ptma	PROMOTER	0.27	20.385	3696.88	75360.08	5.511	4036.99	22249.27
A_68_P20412615	chr1:88425095-88425139	NM_008972:1806	Ptma	INSIDE	0.645	0.372	1629.33	605.92	0.24	1127.57	270.47
A_68_P20412584	chr1:88421724-88421768	NM_008972:-1564	Ptma	PROMOTER	0.508	0.615	1238.92	762.29	0.313	1014.25	317.15
A_68_P20412608	chr1:88424274-88424318	NM_008972:986	Ptma	INSIDE	0.601	2.884	904.77	2609.45	1.735	819.47	1421.37
A_68_P25089823	chr7:52124557-52124601	NM_133949:580	Ptov1	INSIDE	0.095	5.857	2930.64	17164.45	0.554	2284.77	1264.67
A_68_P23298639	chr4:129497779-129497823	NM_001164745:-151	Ptp4a2	PROMOTER	0.3	6.461	1710.26	11049.71	1.938	1463.73	2836.24
A_68_P23298637	chr4:129497229-129497273	NM_008974:-472	Ptp4a2	PROMOTER	0.578	2.917	1696.06	4948.18	1.687	1367.31	2307.32
A_68_P21019042	chr2:13977187-13977231	NM_001012396:454	Ptpla	INSIDE	0.287	0.570	9012.69	5135.50	0.164	6283.10	1028.73
A_68_P26585377	chr9:64869052-64869096	NM_021345:450	Ptplad1	INSIDE	0.422	0.480	1106.41	531.29	0.203	921.68	186.97
A_68_P30667506	chr16:35022484-35022528	NM_023587:0	Ptplb	INSIDE	0.272	7.707	1215.68	9369.57	2.093	1096.32	2294.46
A_68_P21838403	chr2:167758113-167758157	NM_011201:308	Ptpn1	INSIDE	0.208	0.532	2119.88	1126.99	0.11	1551.62	171.31
A_68_P23524104	chr5:20561279-20561323	NM_011203:315	Ptpn12	INSIDE	0.314	7.917	1336.21	10578.73	2.487	1184.48	2945.80
A_68_P23524108	chr5:20561799-20561843	NM_011203:-205	Ptpn12	PROMOTER	0.441	4.907	4378.04	21484.22	2.165	3302.39	7149.40
A_68_P20927655	chr1:191551708-191551752	NM_008976:-416	Ptpn14	PROMOTER	0.376	0.224	2807.97	627.62	0.084	2038.12	171.50
A_68_P20927662	chr1:191552465-191552509	NM_008976:340	Ptpn14	INSIDE	0.436	0.546	1055.36	576.04	0.238	870.99	207.07
A_68_P28671530	chr12:99927621-99927665	NM_011877:45213	Ptpn21	INSIDE	0.366	5.755	1592.79	9167.12	2.107	1186.26	2499.97
A_68_P28671841	chr12:99975831-99975875	NM_001146199:-237	Ptpn21	DIVERGENT_PROMOTER	0.399	9.478	3886.78	36838.76	3.784	3072.85	11627.72
A_68_P22944675	chr4:57313204-57313248	NM_011207:1483	Ptpn3	INSIDE	0.459	7.311	701.42	5128.13	3.353	652.13	2186.31
A_68_P25103254	chr7:54387461-54387505	NM_001163565:486	Ptpn5	INSIDE	0.071	31.574	1838.90	58061.76	2.228	1616.12	3601.49
A_68_P26539664	chr9:56843551-56843595	NM_019651:798	Ptpn9	INSIDE	0.552	3.374	621.79	2098.15	1.863	513.05	955.63
A_68_P21629885	chr2:130276440-130276484	NM_001163688:449	Ptpra	INSIDE	0.435	0.334	1630.16	543.85	0.145	1326.95	192.54
A_68_P27474704	chr10:115738319-115738363	NM_029928:-89	Ptprb	PROMOTER	0.127	1.579	1921.57	3033.58	0.2	1870.32	373.78
A_68_P23029295	chr4:76095885-76095929	NM_011211:144297	Ptprd	INSIDE	0.295	16.882	2196.77	37086.54	4.979	1636.13	8145.78
A_68_P25553144	chr7:142729766-142729810	NM_011212:282	Ptpre	INSIDE	0.652	0.595	2166.38	1289.60	0.388	1802.32	699.57
A_68_P23238885	chr4:117884835-117884879	NM_011213:79146	Ptprf	INSIDE	0.173	22.297	2032.58	45320.75	3.854	1528.96	5893.29
A_68_P23239476	chr4:117963343-117963387	NM_011213:638	Ptprf	INSIDE	0.443	6.586	1180.45	7774.63	2.92	1100.41	3213.53
A_68_P29405439	chr14:12386172-12386216	NM_008981:128	Ptprg	INSIDE	0.448	0.466	2390.49	1114.81	0.209	1790.31	374.14
A_68_P29405441	chr14:12386359-12386403	NM_008981:314	Ptprg	INSIDE	0.544	0.718	1461.88	1050.01	0.391	1182.67	462.35
A_68_P21417962	chr2:90420971-90421015	NM_008982:-188	Ptprj	PROMOTER	0.178	15.593	2553.41	39814.77	2.769	2156.20	5971.22
A_68_P21417961	chr2:90420785-90420829	NM_008982:-2	Ptprj	PROMOTER	0.476	13.553	652.91	8849.08	6.455	523.13	3376.75
A_68_P21417957	chr2:90420274-90420318	NM_008982:508	Ptprj	INSIDE	0.532	4.627	1854.70	8580.82	2.463	1521.14	3746.38
A_68_P21417960	chr2:90420716-90420760	NM_008982:66	Ptprj	INSIDE	0.635	7.816	1205.37	9421.14	4.962	1031.43	5118.41

ProbeName	Target position of probe on CpG island microarray	TargetID	GeneSymbol	CpG island Description	Ratio of relative methylation (TiO_2-NP/Vehicle)	Sham group			TiO_2-H group		
						Relative methylation (Cy5/Cy3)	Cy3 signal (Input)	Cy5 signal (MeDIP)	Relative methylation (Cy5/Cy3)	Cy3 signal (Input)	Cy5 signal (MeDIP)
A_68_P31311926	chr17:67704196-67704240	NM_008984:-419	Ptprm	PROMOTER	0.326	21.269	2942.60	62587.27	6.94	2719.79	18874.76
A_68_P31311922	chr17:67703725-67703769	NM_008984:53	Ptprm	INSIDE	0.47	15.356	1640.96	25198.52	7.214	1433.44	10340.11
A_68_P31311916	chr17:67703103-67703147	NM_008984:675	Ptprm	INSIDE	0.487	0.526	1615.27	848.97	0.256	1246.50	318.89
A_68_P31311920	chr17:67703577-67703621	NM_008984:201	Ptprm	INSIDE	0.648	0.532	1152.25	612.48	0.344	886.52	305.37
A_68_P31311912	chr17:67702632-67702676	NM_008984:1145	Ptprm	INSIDE	0.608	0.676	976.11	660.18	0.411	815.63	335.26
A_68_P20352935	chr1:75248212-75248256	NM_008985:12549	Ptprn	INSIDE	0.538	4.043	2186.64	8840.29	2.174	1735.38	3772.70
A_68_P28768143	chr12:117724447-117724491	NM_011215:276	Ptprn2	INSIDE	0.157	39.030	3862.17	150741.30	6.123	3124.72	19133.84
A_68_P24877027	chr6:137200894-137200938	NM_001164401:97	Ptpro	INSIDE	0.251	21.881	4116.11	90062.86	5.485	3529.44	19359.62
A_68_P31256054	chr17:56568541-56568585	NM_011218:47341	Ptprs	INSIDE	0.118	18.760	1854.56	34791.94	2.216	1462.99	3242.15
A_68_P31256434	chr17:56615114-56615158	NM_011218:767	Ptprs	INSIDE	0.171	0.455	10481.57	4768.19	0.078	6163.81	480.21
A_68_P31255958	chr17:56556525-56556570	NM_011218:59356	Ptprs	INSIDE	0.306	7.555	3642.57	27518.50	2.31	2378.09	5493.44
A_68_P31256336	chr17:56601995-56602039	NM_011218:13887	Ptprs	INSIDE	0.448	0.411	7479.27	3072.71	0.184	5055.17	930.14
A_68_P31256333	chr17:56601586-56601630	NM_011218:14295	Ptprs	INSIDE	0.584	3.575	883.06	3156.54	2.086	746.97	1558.44
A_68_P31256024	chr17:56564825-56564869	NM_011218:51057	Ptprs	INSIDE	0.566	2.728	1088.07	2968.67	1.545	994.21	1536.05
A_68_P31256047	chr17:56567658-56567702	NM_011218:48223	Ptprs	INSIDE	0.494	3.219	942.82	3035.25	1.591	732.83	1166.01
A_68_P23308580	chr4:131392321-131392365	NM_001083119:1851	Ptpru	INSIDE	0.527	4.544	3074.25	13969.52	2.394	2555.44	6118.88
A_68_P23308588	chr4:131393415-131393459	NM_001083119:757	Ptpru	INSIDE	0.624	2.811	16370.49	46015.01	1.754	9681.65	16981.78
A_68_P23308584	chr4:131392713-131392757	NM_001083119:1459	Ptpru	INSIDE	0.648	0.631	11428.85	7210.40	0.409	5778.40	2361.17
A_68_P28071212	chr11:100830764-100830808	NM_008986:1145	Ptrf	INSIDE	0.155	1.737	8427.13	14637.97	0.269	5612.01	1508.29
A_68_P28071210	chr11:100830506-100830550	NM_008986:1403	Ptrf	INSIDE	0.224	1.764	4296.44	7580.02	0.395	3356.42	1325.66
A_68_P28071133	chr11:100820100-100820144	NM_008986:11809	Ptrf	INSIDE	0.643	5.573	2471.99	13777.32	3.586	1786.65	6406.05
A_68_P28071211	chr11:100830670-100830714	NM_008986:1239	Ptrf	INSIDE	0.321	0.733	6925.73	5076.71	0.235	4409.15	1037.16
A_68_P31622955	chr18:36441203-36441247	NM_008989:409	Pura	INSIDE	0.636	0.743	3289.40	2444.45	0.473	2347.88	1109.82
A_68_P25768631	chr8:34496720-34496764	NM_152821:-54	Purg	DIVERGENT_PROMOTER	0.633	0.213	7604.72	1620.37	0.135	5221.15	704.12
A_68_P23441284	chr4:155265872-155265916	NM_001033490:-23	Pusl1	DIVERGENT_PROMOTER	0.392	4.679	1864.53	8724.33	1.832	1739.03	3185.79
A_68_P23441280	chr4:155265412-155265456	NM_001033490:437	Pusl1	INSIDE	0.647	0.664	2371.53	1574.93	0.43	1733.88	745.04
A_68_P26463567	chr9:43552486-43552530	NM_021424:-150	Pvrl1	PROMOTER	0.436	0.500	2207.24	1104.63	0.218	1747.47	381.46
A_68_P26463576	chr9:43553455-43553499	NM_021424:818	Pvrl1	INSIDE	0.255	7.258	1255.96	9115.18	1.85	1066.43	1973.30
A_68_P26464017	chr9:43611785-43611829	NM_021424:59148	Pvrl1	INSIDE	0.13	15.039	1229.29	18486.93	1.962	634.95	1245.57
A_68_P26464016	chr9:43611710-43611754	NM_021424:59074	Pvrl1	INSIDE	0.148	10.161	1775.58	18041.40	1.506	1216.62	1831.86
A_68_P24995078	chr7:20334418-20334462	NM_008990:482	Pvrl2	INSIDE	0.013	8.208	3838.47	31507.40	0.107	2499.01	267.74
A_68_P24995082	chr7:20334905-20334949	NM_008990:-4	Pvrl2	PROMOTER	0.394	0.608	5012.37	3048.37	0.24	3979.00	953.42
A_68_P24995083	chr7:20334983-20335030	NM_008990:-84	Pvrl2	PROMOTER	0.557	0.537	3948.80	2118.90	0.299	2623.64	783.68
A_68_P24995084	chr7:20335086-20335130	NM_008990:-186	Pvrl2	PROMOTER	0.662	0.598	2208.44	1320.34	0.396	1521.52	602.11
A_68_P24995077	chr7:20334338-20334382	NM_008990:562	Pvrl2	INSIDE	0.146	1.554	1154.21	1793.13	0.227	774.53	175.66
A_68_P27310783	chr10:85334508-85334552	NM_133993:-45	Pwp1	PROMOTER	0.358	11.249	2174.07	24455.18	4.029	2079.98	8380.80
A_68_P23978770	chr5:110715093-110715137	NM_008993:73	Pxmp2	INSIDE	0.056	32.431	1785.94	57920.70	1.811	1789.91	3240.72
A_68_P23978771	chr5:110715204-110715248	NM_008993:-39	Pxmp2	DIVERGENT_PROMOTER	0.393	4.675	1469.93	6871.59	1.837	1256.84	2308.33
A_68_P20878434	chr1:182834646-182834690	NM_133705:264	Pycr2	INSIDE	0.544	4.099	736.36	3018.69	2.23	577.16	1286.79
A_68_P21740964	chr2:150612823-150612867	NM_153781:313	Pygb	INSIDE	0.445	10.068	1373.12	13823.87	4.483	1330.42	5964.12
A_68_P28519206	chr12:71328237-71328281	NM_133198:412	Pygl	INSIDE	0.664	0.383	1611.91	617.39	0.254	1245.88	316.95
A_68_P26630339	chr9:72773100-72773144	NM_028116:-334	Pygo1	PROMOTER	0.351	3.918	2863.66	11220.02	1.376	2332.82	3209.57
A_68_P22319359	chr3:89234506-89234550	NM_026869:-230	Pygo2	PROMOTER	0.266	13.992	3052.05	42702.78	3.726	2686.95	10011.19
A_68_P28077570	chr11:101968399-101968443	NM_145435:670	Pyy	INSIDE	0.409	0.554	826.81	458.46	0.227	776.13	175.82
A_68_P31375199	chr17:79451854-79451898	NM_027455:631	Qpct	INSIDE	0.464	0.341	1413.98	482.52	0.158	1063.43	168.26
A_68_P31375202	chr17:79452069-79452113	NM_027455:845	Qpct	INSIDE	0.486	3.967	1952.77	7747.16	1.926	1842.85	3550.21

ProbeName	Target position of probe on CpG island microarray	TargetID	GeneSymbol	CpG island Description	Ratio of relative methylation (TiO_2-NP/Vehicle)	Sham group			TiO_2-H group		
						Relative methylation (Cy5/Cy3)	Cy3 signal (Input)	Cy5 signal (MeDIP)	Relative methylation (Cy5/Cy3)	Cy3 signal (Input)	Cy5 signal (MeDIP)
A_68_P26815242	chr9:108420124-108420168	NM_001114119:729	Qrich1	INSIDE	0.546	0.234	3745.43	875.06	0.128	2787.63	355.73
A_68_P26815232	chr9:108418960-108419004	NM_001114119:-435	Qrich1	PROMOTER	0.556	2.966	2243.87	6654.63	1.648	1630.32	2686.18
A_68_P21490977	chr2:104656727-104656771	NM_001123327:105	Qser1	INSIDE	0.181	45.786	3159.56	144665.00	8.309	2661.15	22112.38
A_68_P21077278	chr2:26093092-26093136	NM_153559:-174	Qsox2	PROMOTER	0.443	1.670	5176.58	8643.49	0.74	3858.13	2854.55
A_68_P29696766	chr14:70107048-70107092	NM_001146012:317	R3hcc1	INSIDE	0.504	0.387	1434.31	554.51	0.195	1219.93	237.47
A_68_P20598625	chr1:129999853-129999897	NM_181750:-8	R3hdm1	DIVERGENT_PROMOTER	0.32	8.714	3510.52	30589.00	2.788	2870.45	8001.90
A_68_P20598628	chr1:130000152-130000196	NM_181750:292	R3hdm1	INSIDE	0.381	5.412	1253.63	6784.43	2.061	1082.73	2231.16
A_68_P28194540	chr12:3309680-3309724	NM_016676:267	Rab10	INSIDE	0.184	15.532	1571.80	24412.68	2.859	1436.06	4105.69
A_68_P25737020	chr8:28284902-28284946	NM_001080813:194	Rab11fip1	INSIDE	0.589	0.689	2103.30	1448.70	0.405	1769.79	717.52
A_68_P31107657	chr17:26122228-26122272	NM_153140:36555	Rab11fip3	DOWNSTREAM	0.596	0.700	1470.29	1028.85	0.417	1300.03	541.87
A_68_P27949793	chr11:79404552-79404596	NM_175543:-139	Rab11fip4	PROMOTER	0.531	4.497	1258.89	5661.57	2.39	1083.23	2589.20
A_68_P24604715	chr6:85324050-85324094	NM_001003955:556	Rab11fip5	INSIDE	0.575	19.701	3005.45	59209.63	11.326	3246.19	36767.77
A_68_P31306704	chr17:66868707-66868751	NM_024448:282	Rab12	INSIDE	0.107	1.895	2003.75	3796.23	0.204	1459.60	297.06
A_68_P31306703	chr17:66868585-66868629	NM_024448:404	Rab12	INSIDE	0.496	0.378	1786.40	675.84	0.188	1223.00	229.37
A_68_P31306702	chr17:66868481-66868525	NM_024448:508	Rab12	INSIDE	0.556	0.376	3041.87	1142.50	0.209	2194.83	458.04
A_68_P22323989	chr3:90024711-90024755	NM_026677:-6	Rab13	DIVERGENT_PROMOTER	0.526	0.694	3254.29	2257.40	0.365	2310.95	843.67
A_68_P21132198	chr2:35056008-35056052	NM_026697:610	Rab14	INSIDE	0.409	5.832	1437.36	8382.91	2.386	1042.47	2487.45
A_68_P21132202	chr2:35056385-35056429	NM_026697:234	Rab14	INSIDE	0.648	3.448	3263.21	11251.83	2.236	2668.65	5966.79
A_68_P27468929	chr10:114752147-114752191	NM_024454:479	Rab21	INSIDE	0.659	2.641	819.51	2164.61	1.739	645.14	1122.22
A_68_P21871012	chr2:173485328-173485372	NM_024436:5	Rab22a	INSIDE	0.08	25.437	1141.67	29040.85	2.035	1861.28	3787.47
A_68_P29054323	chr13:55423060-55423104	NM_009000:259	Rab24	INSIDE	0.45	4.973	2008.43	9987.92	2.236	1618.64	3619.89
A_68_P29054322	chr13:55422929-55422973	NM_009000:391	Rab24	INSIDE	0.542	0.321	4235.05	1359.66	0.174	3169.38	551.05
A_68_P31807637	chr18:70212806-70212850	NM_030554:195	Rab27b	INSIDE	0.621	0.573	1332.73	763.07	0.356	1142.22	406.28
A_68_P22715106	chr4:8463176-8463220	NM_021518:408	Rab2a	INSIDE	0.356	6.577	1279.04	8412.38	2.34	1226.77	2870.52
A_68_P29605724	chr14:52898950-52898996	NM_172601:98	Rab2b	INSIDE	0.485	0.648	1498.74	970.80	0.314	1058.95	332.69
A_68_P29605620	chr14:52877501-52877545	NM_172601:21548	Rab2b	DOWNSTREAM	0.509	3.582	601.77	2155.26	1.822	511.31	931.66
A_68_P31302582	chr17:66121758-66121802	NM_133685:312	Rab31	INSIDE	0.331	6.405	5430.81	34783.59	2.121	4432.75	9401.57
A_68_P31302584	chr17:66121929-66121973	NM_133685:142	Rab31	INSIDE	0.477	9.646	2594.50	25026.92	4.601	2155.74	9917.68
A_68_P26936200	chr10:10277608-10277652	NM_026405:375	Rab32	INSIDE	0.612	0.400	5005.76	2002.88	0.245	3827.58	937.46
A_68_P22129906	chr3:51287666-51287710	NM_016858:-199	Rab33b	PROMOTER	0.364	8.169	3235.80	26433.17	2.978	2825.05	8411.60
A_68_P22129907	chr3:51287845-51287889	NM_016858:-21	Rab33b	PROMOTER	0.446	3.977	1831.64	7285.32	1.773	1491.40	2644.31
A_68_P27941849	chr11:78002317-78002361	NM_033475:89	Rab34	INSIDE	0.357	1.691	1645.39	2782.65	0.603	1233.96	744.57
A_68_P26522114	chr9:53513817-53513861	NM_175562:499	Rab39	INSIDE	0.442	0.330	1581.68	522.71	0.146	1199.61	175.24
A_68_P26522120	chr9:53514529-53514573	NM_175562:-213	Rab39	PROMOTER	0.545	0.515	3118.70	1605.21	0.281	2233.35	626.66
A_68_P25953806	chr8:73278838-73278882	NM_001166399:283	Rab3a	INSIDE	0.605	0.201	5085.85	1021.64	0.121	3013.76	365.99
A_68_P23198163	chr4:108551696-108551740	NM_023537:44	Rab3b	INSIDE	0.596	2.524	1214.08	3064.80	1.504	1173.22	1764.30
A_68_P26349531	chr9:21722321-21722365	NM_031874:223	Rab3d	INSIDE	0.479	5.379	1023.10	5503.36	2.576	798.60	2057.10
A_68_P26349532	chr9:21722448-21722492	NM_031874:95	Rab3d	INSIDE	0.429	0.750	3737.57	2804.05	0.322	2775.73	892.49
A_68_P26349533	chr9:21722608-21722652	NM_031874:-65	Rab3d	PROMOTER	0.464	3.060	4098.24	12542.05	1.421	2943.62	4182.59
A_68_P20597394	chr1:129765721-129765775	NM_178690:399	Rab3gap1	INSIDE	0.537	0.664	793.43	526.68	0.357	646.33	230.60
A_68_P31947893	chr19:10092733-10092777	NM_144538:37	Rab3il1	INSIDE	0.598	3.651	2743.61	10016.55	2.184	2107.93	4604.76
A_68_P27478502	chr10:116386947-116386991	NM_001003950:468	Rab3ip	INSIDE	0.435	0.419	1339.14	561.26	0.182	1107.99	201.79
A_68_P24618289	chr6:87762138-87762182	NM_001039394:-387	Rab43	PROMOTER	0.471	0.452	4179.28	1889.82	0.213	2791.44	594.32
A_68_P24618284	chr6:87761631-87761675	NM_133717:-1904	Rab43	PROMOTER	0.55	7.961	4332.47	34492.59	4.378	3934.41	17225.35
A_68_P27542730	chr10:128133098-128133142	NM_177411:204	Rab5b	INSIDE	0.343	0.502	1345.14	675.09	0.172	1169.94	201.22
A_68_P27542729	chr10:128133023-128133067	NM_177411:280	Rab5b	INSIDE	0.415	4.627	2910.18	13464.68	1.921	2311.04	4439.93

ProbeName	Target position of probe on CpG island microarray	TargetID	GeneSymbol	CpG island Description	Ratio of relative methylation (TiO_2-NP/Vehicle)	Sham group			TiO_2-H group		
						Relative methylation (Cy5/Cy3)	Cy3 signal (Input)	Cy5 signal (MeDIP)	Relative methylation (Cy5/Cy3)	Cy3 signal (Input)	Cy5 signal (MeDIP)
A_68_P26783902	chr9:103014760-103014804	NM_173781:379	Rab6b	INSIDE	0.273	1.941	4529.23	8792.63	0.53	3167.67	1679.24
A_68_P26783904	chr9:103014963-103015007	NM_173781:581	Rab6b	INSIDE	0.384	13.872	2587.22	35888.99	5.33	2774.85	14791.03
A_68_P25959941	chr8:74685000-74685047	NM_023126:-75	Rab8a	PROMOTER	0.494	0.315	1637.31	515.70	0.156	1121.68	174.52
A_68_P25959940	chr8:74684842-74684886	NM_023126:-234	Rab8a	PROMOTER	0.496	0.284	3172.28	900.18	0.141	2139.17	301.03
A_68_P27902925	chr11:70658259-70658303	NM_019400:16	Rabep1	INSIDE	0.649	2.421	2013.55	4873.95	1.57	1686.88	2648.14
A_68_P25500413	chr7:133572890-133572934	NM_030566:632	Rabep2	INSIDE	0.392	0.342	4851.39	1659.45	0.134	3439.45	460.69
A_68_P21142183	chr2:37307736-37307780	NM_001033960:-15	Rabgap1	PROMOTER	0.446	9.916	3651.58	36208.05	4.42	3192.94	14113.65
A_68_P21142185	chr2:37307958-37308002	NM_001033960:207	Rabgap1	INSIDE	0.517	0.739	1990.50	1470.13	0.382	1526.11	583.26
A_68_P24092504	chr5:130647587-130647631	NM_001199059:-80	Rabgef1	PROMOTER	0.446	5.519	1321.64	7294.70	2.46	1152.48	2835.23
A_68_P29619514	chr14:56341028-56341072	NM_019519:-37	Rabggta	PROMOTER	0.082	5.023	1571.51	7893.49	0.411	1273.96	524.04
A_68_P29619515	chr14:56341110-56341154	NM_019519:-119	Rabggta	PROMOTER	0.662	2.278	1268.43	2889.57	1.509	1039.85	1568.81
A_68_P30426169	chr15:89422304-89422348	NM_026817:28	Rabl2	INSIDE	0.652	4.513	1153.57	5206.58	2.941	867.27	2550.53
A_68_P24156969	chr5:144288624-144288668	NM_009007:215	Rac1	INSIDE	0.537	4.608	1857.37	8558.74	2.473	1369.78	3387.22
A_68_P28186391	chr11:120582930-120582974	NM_133223:171	Rac3	INSIDE	0.554	3.314	2214.97	7340.34	1.836	1588.72	2916.56
A_68_P30010836	chr15:10415930-10415974	NM_011232:164	Rad1	INSIDE	0.389	0.584	1494.98	873.51	0.228	1244.27	283.15
A_68_P30213989	chr15:51823378-51823422	NM_009009:-94	Rad21	PROMOTER	0.524	3.868	720.05	2785.36	2.029	634.52	1287.29
A_68_P22933509	chr4:55363107-55363151	NM_009011:215	Rad23b	INSIDE	0.237	1.662	2784.46	4626.53	0.394	2165.64	853.14
A_68_P22933503	chr4:55362594-55362638	NM_009011:-297	Rad23b	PROMOTER	0.643	3.209	1822.31	5848.06	2.064	1614.64	3332.82
A_68_P22933507	chr4:55362962-55363006	NM_009011:71	Rad23b	INSIDE	0.589	0.506	1152.14	583.35	0.298	908.97	271.28
A_68_P27806202	chr11:53520643-53520687	NM_009012:157	Rad50	INSIDE	0.541	0.658	1028.88	676.59	0.356	919.09	327.11
A_68_P28238663	chr12:11462730-11462774	NM_001111118:-132	Rad51ap2	PROMOTER	0.617	3.567	3579.91	12769.26	2.2	3008.78	6618.02
A_68_P24152257	chr5:143026729-143026773	NM_178702:281	Radil	INSIDE	0.532	4.652	2326.96	10824.39	2.476	1967.65	4871.76
A_68_P21867064	chr2:172826009-172826053	NM_175112:413	Rae1	INSIDE	0.432	0.477	1207.13	576.20	0.206	956.24	197.21
A_68_P26997097	chr10:21871019-21871063	NM_009016:-7374	Raet1a	PROMOTER	0.209	2.399	3269.75	7844.94	0.501	2489.50	1248.33
A_68_P27840810	chr11:59919914-59919958	NM_009021:1422	Rai1	INSIDE	0.371	19.625	3112.25	61079.23	7.289	2474.58	18038.22
A_68_P27840803	chr11:59919168-59919212	NM_009021:676	Rai1	INSIDE	0.374	0.259	5814.91	1506.59	0.097	4461.44	432.26
A_68_P27841039	chr11:59953284-59953328	NM_001037764:-277	Rai1	PROMOTER	0.456	0.338	1719.66	581.33	0.154	1427.86	220.01
A_68_P27840799	chr11:59918534-59918578	NM_009021:42	Rai1	INSIDE	0.507	0.474	2690.56	1276.21	0.241	1759.41	423.15
A_68_P27840800	chr11:59918733-59918777	NM_009021:240	Rai1	INSIDE	0.539	5.901	691.86	4082.82	3.179	631.31	2007.20
A_68_P27840876	chr11:59928639-59928683	NM_009021:10146	Rai1	INSIDE	0.612	0.280	3296.34	922.72	0.171	2453.15	420.12
A_68_P27841325	chr11:59989438-59989482	NM_001037764:35877	Rai1	INSIDE	0.459	0.715	1814.75	1298.26	0.328	1397.10	458.66
A_68_P30012295	chr15:10643929-10643973	NM_030690:-655	Rai14	PROMOTER	0.567	0.359	3987.37	1433.16	0.204	2959.65	602.96
A_68_P30012299	chr15:10644337-10644381	NM_030690:-1063	Rai14	PROMOTER	0.543	3.680	634.83	2336.20	2	465.60	931.11
A_68_P20557414	chr1:121401510-121401554	NM_022327:-173	Ralb	PROMOTER	0.556	0.351	2120.98	745.37	0.195	1577.09	308.12
A_68_P21782731	chr2:158236154-158236198	NM_177658:588	Ralgapb	INSIDE	0.124	15.145	2329.18	35274.44	1.884	1970.53	3711.95
A_68_P21782725	chr2:158235581-158235625	NM_177658:14	Ralgapb	INSIDE	0.398	6.776	1218.48	8256.97	2.7	1210.16	3267.65
A_68_P21119569	chr2:32985913-32985957	NM_175211:241064	Ralgps1	DOWNSTREAM	0.534	0.664	4351.26	2890.99	0.355	3222.92	1143.76
A_68_P21762357	chr2:154616385-154616429	NM_001139511:-439	Raly	PROMOTER	0.371	0.363	5694.62	2065.65	0.134	4512.46	606.63
A_68_P21762364	chr2:154617306-154617350	NM_001139511:483	Raly	INSIDE	0.481	22.124	3048.25	67440.53	10.651	2679.44	28537.50
A_68_P21762360	chr2:154616838-154616882	NM_001139511:15	Raly	INSIDE	0.493	4.964	2336.39	11598.55	2.447	1863.53	4560.55
A_68_P21762359	chr2:154616708-154616752	NM_001139511:-115	Raly	PROMOTER	0.489	0.621	3190.50	1980.02	0.303	2437.02	739.23
A_68_P28072819	chr11:101107662-101107706	NM_019444:37	Ramp2	INSIDE	0.358	6.202	402.02	2493.37	2.218	436.22	967.62
A_68_P24086565	chr5:129526623-129526667	NM_009391:614	Ran	INSIDE	0.484	7.038	1680.94	11830.84	3.405	1272.35	4331.77
A_68_P24086564	chr5:129526514-129526558	NM_009391:506	Ran	INSIDE	0.518	8.228	1861.13	15313.54	4.258	1650.36	7027.68
A_68_P30578589	chr16:18248400-18248444	NM_011239:365	Ranbp1	INSIDE	0.655	2.574	2608.12	6714.49	1.686	2123.74	3580.20
A_68_P27163773	chr10:57909758-57909803	NM_011240:181	Ranbp2	INSIDE	0.332	5.402	1144.20	6180.90	1.795	995.21	1786.33

ProbeName	Target position of probe on CpG island microarray	TargetID	GeneSymbol	CpG island Description	Ratio of relative methylation (TiO_2-NP/Vehicle)	Sham group			TiO_2-H group		
						Relative methylation (Cy5/Cy3)	Cy3 signal (Input)	Cy5 signal (MeDIP)	Relative methylation (Cy5/Cy3)	Cy3 signal (Input)	Cy5 signal (MeDIP)
A_68_P27163774	chr10:57909910-57909954	NM_011240:333	Ranbp2	INSIDE	0.412	1.450	1306.65	1894.56	0.597	1120.44	669.26
A_68_P32054982	chr19:29887617-29887661	NM_177721:-174	Ranbp6	PROMOTER	0.604	0.365	1461.18	533.17	0.22	788.52	173.70
A_68_P27921006	chr11:74335943-74335987	NM_001015046:67696	Rap1gap2	INSIDE	0.43	0.517	2375.82	1227.66	0.222	1862.75	413.67
A_68_P22572454	chr3:138738598-138738642	NM_001040690:-457	Rap1gds1	PROMOTER	0.298	20.616	1476.65	30442.31	6.135	1401.36	8597.68
A_68_P22572455	chr3:138738714-138738758	NM_001040690:-573	Rap1gds1	PROMOTER	0.448	0.392	1708.32	669.76	0.175	1382.05	242.54
A_68_P22572447	chr3:138737478-138737522	NM_001040690:663	Rap1gds1	INSIDE	0.567	4.597	2377.80	10930.75	2.606	2042.14	5321.26
A_68_P22182581	chr3:61168376-61168420	NM_028712:-30	Rap2b	PROMOTER	0.38	8.436	4310.45	36363.72	3.209	3282.49	10532.29
A_68_P32374346	chrX:48371757-48371801	NM_172413:-583	Rap2c	PROMOTER	0.583	4.521	833.89	3770.41	2.634	959.94	2528.28
A_68_P21099264	chr2:29603006-29603050	NM_001039086:127789	Rapgef1	DOWNSTREAM	0.553	3.111	1565.20	4870.10	1.721	1286.63	2214.83
A_68_P21099263	chr2:29602898-29602942	NM_001039086:127681	Rapgef1	DOWNSTREAM	0.101	4.569	1108.67	5065.63	0.46	989.32	454.99
A_68_P28774232	chr12:118772473-118772517	NM_175930:17543	Rapgef5	INSIDE	0.527	4.272	746.29	3188.01	2.252	618.89	1393.44
A_68_P27810583	chr11:54336349-54336393	NM_175258:22	Rapgef6	INSIDE	0.649	3.179	1124.78	3575.78	2.064	877.71	1811.57
A_68_P28058844	chr11:98698263-98698307	NM_001080925:186	Rapgefl1	INSIDE	0.457	4.779	2950.56	14099.85	2.185	2197.43	4800.73
A_68_P28058842	chr11:98698054-98698098	NM_001080925:-22	Rapgefl1	PROMOTER	0.079	8.711	5231.71	45574.25	0.691	3934.93	2719.37
A_68_P20269871	chr1:60624074-60624118	NM_001045513:-487	Raph1	PROMOTER	0.61	0.304	1545.57	469.47	0.185	1094.11	202.73
A_68_P28059422	chr11:98797644-98797688	NM_009024:-1343	Rara	PROMOTER	0.045	8.229	2727.20	22442.87	0.374	2038.18	762.41
A_68_P28059604	chr11:98821830-98821874	NM_001176528:68	Rara	INSIDE	0.22	43.731	2510.01	109764.20	9.62	2442.25	23493.43
A_68_P28059432	chr11:98798988-98799032	NM_001177302:-21	Rara	PROMOTER	0.442	0.367	1564.13	573.69	0.162	1247.35	202.23
A_68_P28059420	chr11:98797438-98797483	NM_009024:-1549	Rara	PROMOTER	0.213	3.016	1978.17	5966.04	0.643	1557.56	1002.11
A_68_P29430092	chr14:17408235-17408279	NM_011243:-532	Rarb	PROMOTER	0.613	0.204	3111.01	636.14	0.125	2515.90	315.30
A_68_P29430091	chr14:17408166-17408210	NM_011243:-464	Rarb	PROMOTER	0.557	0.702	1593.38	1119.32	0.391	1302.44	509.67
A_68_P29430090	chr14:17408053-17408097	NM_011243:-350	Rarb	PROMOTER	0.269	0.694	1190.95	826.73	0.186	915.27	170.66
A_68_P30496837	chr15:102077508-102077552	NM_001042727:-747	Rarg	PROMOTER	0.421	0.455	2924.92	1329.53	0.191	2294.99	439.03
A_68_P30496838	chr15:102077585-102077629	NM_001042727:-823	Rarg	PROMOTER	0.605	0.451	1825.25	822.34	0.272	1483.54	404.16
A_68_P30496912	chr15:102087767-102087811	NM_011244:126	Rarg	INSIDE	0.424	3.472	1312.65	4557.58	1.471	1214.45	1786.48
A_68_P27708863	chr11:35648179-35648223	NM_025936:-170	Rars	PROMOTER	0.426	5.002	1445.96	7233.07	2.132	1142.09	2435.26
A_68_P26747582	chr9:96437440-96437484	NM_053268:94460	Rasa2	DOWNSTREAM	0.324	5.321	1565.13	8328.03	1.725	1389.25	2396.74
A_68_P25669970	chr8:13676888-13676932	NM_009025:677	Rasa3	INSIDE	0.328	6.096	2021.53	12322.74	1.998	1412.47	2822.00
A_68_P25669976	chr8:13677580-13677624	NM_009025:-15	Rasa3	PROMOTER	0.634	4.600	1489.45	6850.75	2.915	1326.36	3866.78
A_68_P24040030	chr5:121099321-121099365	NM_013832:512	Rasal1	INSIDE	0.346	5.941	6050.80	35950.15	2.058	4897.56	10077.57
A_68_P20748460	chr1:159342861-159342905	NM_177644:-156	Rasal2	PROMOTER	0.52	0.633	3665.67	2318.90	0.329	2803.36	922.10
A_68_P25972618	chr8:77737845-77737889	NM_029182:24	Rasd2	INSIDE	0.106	38.638	1291.79	49912.11	4.104	1050.47	4311.43
A_68_P25972617	chr8:77737759-77737803	NM_029182:-62	Rasd2	PROMOTER	0.522	3.635	1357.60	4934.54	1.896	1194.94	2265.59
A_68_P25972612	chr8:77737217-77737261	NM_029182:-604	Rasd2	PROMOTER	0.365	0.579	1023.01	592.77	0.212	821.80	173.95
A_68_P23018376	chr4:73436995-73437039	NM_001017427:-510	Rasef	PROMOTER	0.189	38.609	1435.83	55435.42	7.296	1153.76	8418.16
A_68_P23922674	chr5:99681727-99681771	NM_145839:198	Rasgef1b	INSIDE	0.538	2.721	2087.72	5680.22	1.463	1807.65	2644.07
A_68_P26714906	chr9:89805323-89805367	NM_001039655:732	Rasgrf1	INSIDE	0.585	0.318	2720.21	864.88	0.186	2131.34	396.44
A_68_P29222291	chr13:92901092-92901136	NM_009027:335	Rasgrf2	INSIDE	0.395	0.582	1094.37	636.64	0.23	846.82	194.52
A_68_P21557415	chr2:117168712-117168756	NM_011246:-121	Rasgrp1	PROMOTER	0.623	0.383	1740.34	665.95	0.239	1362.08	324.93
A_68_P21557414	chr2:117168572-117168616	NM_011246:19	Rasgrp1	INSIDE	0.432	4.147	715.72	2967.99	1.791	616.99	1105.31
A_68_P31934467	chr19:6399578-6399622	NM_011242:-982	Rasgrp2	PROMOTER	0.302	13.734	2666.65	36622.50	4.15	2457.35	10198.32
A_68_P31934550	chr19:6413237-6413281	NM_011242:12676	Rasgrp2	INSIDE	0.33	5.098	4443.36	22652.63	1.685	3337.01	5621.76
A_68_P31934470	chr19:6400140-6400185	NM_011242:-420	Rasgrp2	PROMOTER	0.545	0.202	2184.44	440.73	0.11	1554.57	170.97
A_68_P31934551	chr19:6413350-6413399	NM_011242:12792	Rasgrp2	INSIDE	0.537	4.700	632.96	2974.75	2.523	461.82	1165.35
A_68_P25094128	chr7:52883995-52884039	NM_028544:1110	Rasip1	INSIDE	0.08	4.932	2009.90	9912.39	0.397	1614.54	640.34
A_68_P27561123	chr11:4958083-4958127	NM_145216:-26	Rasl10a	PROMOTER	0.436	4.940	1637.72	8090.22	2.155	1442.08	3107.13

ProbeName	Target position of probe on CpG island microarray	TargetID	GeneSymbol	CpG island Description	Ratio of relative methylation (TiO_2-NP/Vehicle)	Sham group			TiO_2-H group		
						Relative methylation (Cy5/Cy3)	Cy3 signal (Input)	Cy5 signal (MeDIP)	Relative methylation (Cy5/Cy3)	Cy3 signal (Input)	Cy5 signal (MeDIP)
A_68_P27561122	chr11:4958001-4958045	NM_145216:-108	Rasl10a	PROMOTER	0.626	3.419	1202.96	4113.24	2.141	1130.47	2420.44
A_68_P23801768	chr5:74591245-74591289	NM_026878:-84	Rasl11b	PROMOTER	0.323	13.986	3561.47	49812.52	4.523	3145.02	14223.97
A_68_P23801769	chr5:74591333-74591377	NM_026878:4	Rasl11b	INSIDE	0.457	0.262	1823.17	478.53	0.12	1426.79	171.25
A_68_P26809495	chr9:107453757-107453801	NM_019713:-3208	Rassf1	PROMOTER	0.634	0.387	7365.54	2852.24	0.245	5014.26	1230.30
A_68_P25427821	chr7:120097865-120097909	NM_175279:411	Rassf10	INSIDE	0.641	2.533	1643.42	4162.07	1.624	1471.94	2389.76
A_68_P25427818	chr7:120097419-120097463	NM_175279:-35	Rassf10	PROMOTER	0.517	2.752	1926.09	5301.57	1.423	1513.77	2153.60
A_68_P27503761	chr10:120913745-120913792	NM_138956:-462	Rassf3	PROMOTER	0.252	15.160	349.18	5293.47	3.824	285.74	1092.76
A_68_P20614486	chr1:133141955-133141999	NM_018750:-221	Rassf5	PROMOTER	0.413	5.669	1391.30	7887.67	2.34	1184.46	2771.07
A_68_P20614485	chr1:133141876-133141920	NM_018750:-143	Rassf5	PROMOTER	0.414	0.706	1286.75	908.54	0.292	1068.44	312.12
A_68_P23152967	chr4:100741468-100741512	NM_183024:-152	Raver2	PROMOTER	0.284	0.566	1615.03	914.69	0.161	1380.87	221.74
A_68_P23152974	chr4:100742276-100742320	NM_183024:656	Raver2	INSIDE	0.512	0.431	2459.71	1059.50	0.221	2052.86	453.06
A_68_P23152972	chr4:100742061-100742105	NM_183024:440	Raver2	INSIDE	0.605	0.294	3743.03	1100.46	0.178	2510.29	446.19
A_68_P23152973	chr4:100742175-100742219	NM_183024:554	Raver2	INSIDE	0.307	0.680	3209.04	2182.71	0.208	2040.94	425.50
A_68_P23152968	chr4:100741662-100741706	NM_183024:42	Raver2	INSIDE	0.546	3.284	543.85	1785.95	1.792	529.47	948.96
A_68_P31784091	chr18:66094837-66094881	NM_013833:3885	Rax	INSIDE	0.379	18.432	3460.71	63787.54	6.986	3359.06	23466.09
A_68_P31784121	chr18:66098398-66098442	NM_013833:323	Rax	INSIDE	0.492	3.312	826.47	2737.52	1.629	765.02	1246.28
A_68_P29716897	chr14:73725909-73725953	NM_009029:-332	Rb1	PROMOTER	0.473	3.135	1686.90	5289.23	1.484	1535.71	2278.61
A_68_P24155330	chr5:143942270-143942314	NM_001045482:130	Rbak	INSIDE	0.568	4.719	829.17	3913.10	2.681	684.50	1835.31
A_68_P23296219	chr4:129012748-129012792	NM_009030:-156	Rbbp4	DIVERGENT_PROMOTER	0.143	22.530	3808.66	85810.59	3.219	3408.34	10970.36
A_68_P23296217	chr4:129012516-129012560	NM_009030:76	Rbbp4	INSIDE	0.271	8.387	1470.12	12330.21	2.272	1283.67	2916.85
A_68_P23296214	chr4:129012152-129012196	NM_009030:440	Rbbp4	INSIDE	0.269	0.603	1270.88	765.76	0.162	1129.83	183.29
A_68_P23296216	chr4:129012377-129012421	NM_009030:216	Rbbp4	INSIDE	0.62	2.391	1301.35	3111.39	1.482	1132.09	1678.06
A_68_P25481826	chr7:130114107-130114151	NM_175023:51	Rbbp6	INSIDE	0.438	0.427	1658.25	708.24	0.187	1293.41	241.79
A_68_P25481817	chr7:130113128-130113172	NM_175023:-927	Rbbp6	PROMOTER	0.658	0.491	2354.63	1157.06	0.323	1895.45	613.11
A_68_P31496424	chr18:11815840-11815884	NM_001081223:-488	Rbbp8	PROMOTER	0.221	18.025	955.70	17226.32	3.984	1063.59	4237.07
A_68_P31862861	chr18:80389593-80389637	NM_199197:7744	Rbfa	INSIDE	0.639	4.141	703.34	2912.61	2.644	543.45	1437.11
A_68_P30520835	chr16:6349110-6349154	NM_021477:464247	Rbfox1	INSIDE	0.347	0.386	2027.80	782.39	0.134	1541.04	206.52
A_68_P30520836	chr16:6349205-6349249	NM_021477:464341	Rbfox1	INSIDE	0.617	0.314	2729.62	856.06	0.194	2051.37	397.23
A_68_P30353506	chr15:77137384-77137428	NM_001110827:77	Rbfox2	INSIDE	0.386	0.687	1113.01	765.05	0.265	949.51	251.81
A_68_P28172265	chr11:118430608-118430652	NM_001024931:340256	Rbfox3	INSIDE	0.284	0.544	1618.19	880.81	0.155	1094.22	169.39
A_68_P28174620	chr11:118768747-118768791	NM_001024931:2118	Rbfox3	INSIDE	0.395	11.268	5303.70	59759.60	4.45	5242.25	23326.89
A_68_P23579557	chr5:32000096-32000140	NM_153196:-135	Rbks	DIVERGENT_PROMOTER	0.476	0.438	1131.18	495.20	0.209	845.18	176.26
A_68_P21775763	chr2:157030686-157030730	NM_011249:-438	Rbl1	PROMOTER	0.162	30.092	942.89	28373.19	4.885	686.38	3352.91
A_68_P31925249	chr19:4811087-4811131	NM_019869:526	Rbm14	INSIDE	0.435	4.568	2690.55	12289.88	1.986	2219.67	4407.99
A_68_P22405709	chr3:107135696-107135740	NM_001045807:489	Rbm15	INSIDE	0.355	9.538	4039.79	38532.99	3.385	3465.41	11731.39
A_68_P22405721	chr3:107137047-107137091	NM_001045807:-861	Rbm15	PROMOTER	0.663	3.033	838.24	2542.70	2.012	760.82	1530.88
A_68_P26805694	chr9:106789235-106789279	NM_175402:75	Rbm15b	INSIDE	0.51	5.776	406.01	2345.06	2.946	350.91	1033.65
A_68_P26805680	chr9:106787523-106787567	NM_175402:1787	Rbm15b	INSIDE	0.369	1.408	1778.68	2504.86	0.52	1245.10	647.39
A_68_P21138067	chr2:35992191-35992235	NM_001159635:12	Rbm18	INSIDE	0.62	0.585	1624.14	950.81	0.363	1272.33	462.00
A_68_P32183823	chr19:53751563-53751607	NM_001170847:-211	Rbm20	PROMOTER	0.429	0.331	1569.35	520.05	0.142	1265.54	179.91
A_68_P32183825	chr19:53751761-53751805	NM_001170847:-13	Rbm20	PROMOTER	0.485	4.317	1595.18	6886.09	2.093	1217.52	2548.10
A_68_P29875607	chr14:105575770-105575816	NM_134077:752	Rbm26	INSIDE	0.45	0.553	1975.20	1091.96	0.249	1420.45	353.40
A_68_P31654583	chr18:42435328-42435372	NM_172626:344	Rbm27	INSIDE	0.455	6.151	3320.18	20423.61	2.798	2766.67	7742.30
A_68_P23562013	chr5:28643983-28644027	NM_028234:276	Rbm33	INSIDE	0.462	5.386	870.84	4690.49	2.489	982.58	2445.53
A_68_P21769931	chr2:156005366-156005410	NM_133242:588	Rbm39	INSIDE	0.232	13.974	1406.13	19649.39	3.243	1342.49	4353.31
A_68_P31925086	chr19:4787882-4787926	NM_009032:5973	Rbm4	INSIDE	0.483	4.644	2152.43	9995.03	2.243	1546.42	3468.47

ProbeName	Target position of probe on CpG island microarray	TargetID	GeneSymbol	CpG island Description	Ratio of relative methylation (TiO_2-NP/Vehicle)	Sham group			TiO_2-H group		
						Relative methylation (Cy5/Cy3)	Cy3 signal (Input)	Cy5 signal (MeDIP)	Relative methylation (Cy5/Cy3)	Cy3 signal (Input)	Cy5 signal (MeDIP)
A_68_P21349791	chr2:76208360-76208404	NM_153405:342	Rbm45	INSIDE	0.619	0.589	1176.42	692.52	0.365	858.91	313.18
A_68_P31924879	chr19:4756746-4756790	NM_025717:244	Rbm4b	INSIDE	0.127	1.624	1127.37	1830.72	0.206	887.37	182.99
A_68_P26811550	chr9:107775126-107775170	NM_011251:2	Rbm6	INSIDE	0.609	5.723	787.38	4506.14	3.488	698.71	2437.00
A_68_P26492138	chr9:48303203-48303247	NM_144948:211	Rbm7	INSIDE	0.227	8.038	1539.52	12373.93	1.827	1521.44	2780.24
A_68_P21261139	chr2:60719156-60719200	NM_001141931:317	Rbms1	INSIDE	0.313	8.137	616.13	5013.28	2.545	531.68	1353.06
A_68_P21261715	chr2:60800332-60800376	NM_020296:907	Rbms1	INSIDE	0.386	0.483	1444.11	697.22	0.187	1141.07	212.85
A_68_P21261718	chr2:60800604-60800648	NM_020296:635	Rbms1	INSIDE	0.562	2.729	1025.82	2799.94	1.533	974.51	1493.57
A_68_P32365934	chrX:46048429-46048473	NM_173376:270	Rbmx2	INSIDE	0.272	58.723	2282.45	134032.80	15.969	2472.44	39481.60
A_68_P32365932	chrX:46048174-46048218	NM_173376:16	Rbmx2	INSIDE	0.563	2.922	520.22	1520.30	1.646	824.75	1357.53
A_68_P32099689	chr19:38199320-38199364	NM_011255:-41	Rbp4	PROMOTER	0.37	6.530	790.03	5158.60	2.419	642.59	1554.39
A_68_P23701554	chr5:53982361-53982405	NM_001080927:570	Rbpj	INSIDE	0.525	3.358	2406.61	8081.91	1.764	2260.44	3987.11
A_68_P23701542	chr5:53980937-53980981	NM_009035:-495	Rbpj	PROMOTER	0.451	3.791	779.18	2953.58	1.71	634.53	1084.92
A_68_P21817620	chr2:164226294-164226338	NM_009036:-2377	Rbpjl	PROMOTER	0.639	2.983	437.30	1304.63	1.907	433.73	827.02
A_68_P26588687	chr9:65478956-65479000	NM_028030:590	Rbpms2	INSIDE	0.394	14.086	1241.41	17486.32	5.549	1104.64	6129.26
A_68_P30378087	chr15:81296943-81296987	NM_019712:219	Rbx1	INSIDE	0.38	1.537	3248.11	4991.92	0.584	2458.50	1436.29
A_68_P20768946	chr1:162837198-162837242	NM_001024952:679	Rc3h1	INSIDE	0.57	5.293	807.61	4274.62	3.019	628.08	1895.92
A_68_P29637346	chr14:59820048-59820093	NM_027764:6	Rcbtb1	INSIDE	0.371	5.294	450.43	2384.55	1.963	404.25	793.56
A_68_P29715887	chr14:73543290-73543334	NR_033185:721	Rcbtb2	INSIDE	0.403	4.467	797.72	3563.17	1.802	641.35	1155.75
A_68_P23311885	chr4:131901472-131901516	NM_001197082:171	Rcc1	INSIDE	0.538	3.120	1477.74	4610.08	1.679	1280.28	2150.02
A_68_P31924141	chr19:4625447-4625491	NM_023131:149	Rce1	INSIDE	0.31	0.443	1835.20	812.13	0.137	1447.38	198.86
A_68_P21494381	chr2:105239586-105239630	NM_009037:-132	Rcn1	PROMOTER	0.377	6.643	1141.03	7579.42	2.506	1034.65	2592.47
A_68_P28741731	chr12:112278009-112278053	NM_198023:22	Rcor1	INSIDE	0.442	7.156	8362.73	59843.73	3.164	7907.72	25016.53
A_68_P28741728	chr12:112276886-112276930	NM_198023:-1100	Rcor1	PROMOTER	0.602	0.611	1583.69	966.89	0.367	1221.13	448.76
A_68_P20941842	chr1:193961625-193961669	NM_144814:23	Rcor3	INSIDE	0.186	1.562	1010.97	1579.40	0.29	954.51	277.11
A_68_P20053922	chr1:16095556-16095600	NM_133832:-384	Rdh10	DIVERGENT_PROMOTER	0.576	0.430	1945.20	835.94	0.248	2108.90	522.25
A_68_P20053921	chr1:16095417-16095461	NM_133832:-524	Rdh10	DIVERGENT_PROMOTER	0.6	0.453	3873.83	1753.78	0.272	3167.22	860.05
A_68_P22874082	chr4:43888324-43888368	NM_016678:-55	Reck	PROMOTER	0.276	16.830	1356.28	22825.94	4.638	1107.84	5137.88
A_68_P30350128	chr15:76540931-76540975	NM_058214:37	Recql4	INSIDE	0.407	13.384	3297.82	44138.29	5.452	2532.14	13805.33
A_68_P24537771	chr6:71657856-71657900	NM_178608:204	Reep1	INSIDE	0.392	8.539	1855.64	15844.95	3.344	1419.87	4747.51
A_68_P24537769	chr6:71657624-71657668	NM_178608:-28	Reep1	PROMOTER	0.608	5.499	698.19	3839.67	3.344	604.85	2022.87
A_68_P29701956	chr14:70944977-70945021	NM_180588:-59	Reep4	PROMOTER	0.281	5.653	985.96	5573.77	1.587	1005.53	1595.95
A_68_P31612548	chr18:34533141-34533185	NM_007874:-93	Reep5	PROMOTER	0.522	7.573	2010.58	15226.28	3.957	1697.32	6715.74
A_68_P27647504	chr11:23671033-23671077	NM_009044:-84	Rel	PROMOTER	0.187	15.560	2250.42	35016.13	2.906	1816.34	5277.87
A_68_P27647502	chr11:23670649-23670693	NM_009044:300	Rel	INSIDE	0.494	0.559	2490.00	1392.09	0.276	1976.79	545.75
A_68_P24994377	chr7:20204860-20204904	NM_009046:9905	Relb	INSIDE	0.513	4.493	2576.35	11575.14	2.306	1972.30	4547.36
A_68_P31631093	chr18:38115542-38115586	NM_153793:352	Rell2	INSIDE	0.416	0.659	960.81	633.22	0.274	713.91	195.84
A_68_P23531274	chr5:21850512-21850556	NM_011261:-11	Reln	PROMOTER	0.178	22.193	3405.06	75569.33	3.943	3361.31	13252.76
A_68_P29612657	chr14:55098262-55098306	NM_080726:3348	Rem2	INSIDE	0.665	0.659	784.60	517.41	0.439	717.69	314.74
A_68_P24428193	chr6:48544163-48544207	NM_001079901:303	Repin1	INSIDE	0.34	7.737	1168.19	9038.70	2.627	998.88	2624.41
A_68_P32790431	chrX:159081429-159081473	NM_178256:83	Reps2	INSIDE	0.611	3.250	761.28	2474.53	1.986	1211.18	2404.97
A_68_P23405735	chr4:149745973-149746017	NM_001085492:89970	Rere	INSIDE	0.447	0.461	1060.76	488.76	0.206	823.68	169.75
A_68_P23405733	chr4:149745839-149745883	NM_001085492:89836	Rere	INSIDE	0.479	4.040	4955.23	20019.55	1.934	3612.39	6988.12
A_68_P23405236	chr4:149655404-149655448	NM_001085492:-598	Rere	PROMOTER	0.538	3.865	616.95	2384.53	2.078	530.46	1102.46
A_68_P23820630	chr5:77694944-77694988	NM_011263:448	Rest	INSIDE	0.388	4.363	5805.59	25327.01	1.692	5013.89	8483.06
A_68_P23820628	chr5:77694649-77694693	NM_011263:152	Rest	INSIDE	0.416	0.414	2214.91	917.38	0.172	1783.11	306.87
A_68_P20157818	chr1:38186099-38186143	NM_019570:387	Rev1	INSIDE	0.363	9.920	438.13	4346.33	3.601	367.82	1324.45

ProbeName	Target position of probe on CpG island microarray	TargetID	GeneSymbol	CpG island Description	Ratio of relative methylation (TiO_2-NP/Vehicle)	Sham group			TiO_2-H group		
						Relative methylation (Cy5/Cy3)	Cy3 signal (Input)	Cy5 signal (MeDIP)	Relative methylation (Cy5/Cy3)	Cy3 signal (Input)	Cy5 signal (MeDIP)
A_68_P20157823	chr1:38186758-38186802	NM_019570:-273	Rev1	PROMOTER	0.412	4.911	1462.82	7184.29	2.025	1187.76	2404.86
A_68_P27080342	chr10:39452039-39452083	NM_011264:95	Rev3l	INSIDE	0.595	3.261	1153.02	3760.53	1.942	938.62	1822.54
A_68_P27283985	chr10:80024218-80024262	NM_025852:65	Rexo1	INSIDE	0.26	6.078	1681.51	10220.73	1.58	1423.57	2248.61
A_68_P24020335	chr5:117838986-117839030	NM_028128:24	Rfc5	INSIDE	0.398	8.682	1274.30	11064.05	3.457	1077.10	3723.51
A_68_P27968802	chr11:82684465-82684509	NM_001164570:-240	Rffl	PROMOTER	0.306	12.092	75451.67	912370.80	3.705	75627.29	280208.80
A_68_P31227302	chr17:50329542-50329586	NM_181397:258	Rftn1	INSIDE	0.557	6.496	1837.38	11936.49	3.619	1503.42	5440.21
A_68_P31227303	chr17:50329691-50329735	NM_181397:110	Rftn1	INSIDE	0.542	2.899	1315.82	3814.09	1.57	1172.43	1840.84
A_68_P20759145	chr1:161162430-161162474	NM_011931:-4	Rfwd2	PROMOTER	0.454	6.997	1750.85	12250.56	3.177	1472.92	4679.26
A_68_P20759140	chr1:161161812-161161856	NM_011931:-622	Rfwd2	PROMOTER	0.247	7.504	948.32	7116.27	1.856	901.20	1672.79
A_68_P26017904	chr8:86591013-86591057	NM_009055:300	Rfx1	INSIDE	0.244	20.887	1693.86	35379.67	5.089	1497.29	7618.96
A_68_P26017907	chr8:86591308-86591352	NM_009055:596	Rfx1	INSIDE	0.369	0.542	1500.36	812.79	0.2	1146.94	228.98
A_68_P31258630	chr17:56969214-56969258	NM_009056:1195	Rfx2	INSIDE	0.088	48.008	3818.31	183311.00	4.245	4371.30	18556.10
A_68_P22341452	chr3:94758251-94758295	NM_017395:-664	Rfx5	PROMOTER	0.506	4.120	1429.59	5889.30	2.086	1155.06	2409.21
A_68_P26628308	chr9:72380438-72380482	NM_001033536:414	Rfx7	INSIDE	0.666	0.662	880.09	582.69	0.441	706.63	311.64
A_68_P22149943	chr3:54611747-54611791	NM_133231:-55	Rfxap	PROMOTER	0.326	13.547	1254.37	16992.80	4.416	1042.53	4603.62
A_68_P30778982	chr16:56037716-56037760	NM_029092:149	Rg9mtd1	INSIDE	0.46	4.831	1668.53	8060.93	2.223	1516.55	3370.90
A_68_P22567229	chr3:137806950-137806994	NM_175389:471	Rg9mtd2	INSIDE	0.539	3.552	514.26	1826.44	1.915	425.21	814.23
A_68_P31151052	chr17:34067414-34067462	NM_009059:600	Rgl2	INSIDE	0.03	3.337	2866.57	9566.15	0.099	1768.58	174.51
A_68_P31151050	chr17:34067192-34067236	NM_009059:376	Rgl2	INSIDE	0.214	7.444	4761.17	35442.67	1.595	3832.98	6112.83
A_68_P25228678	chr7:80521046-80521090	NM_177740:663	Rgma	INSIDE	0.62	3.324	920.53	3060.04	2.061	860.57	1773.29
A_68_P25228675	chr7:80520597-80520641	NM_177740:213	Rgma	INSIDE	0.156	14.423	580.97	8379.37	2.255	559.63	1261.73
A_68_P25228921	chr7:80554435-80554479	NM_177740:34051	Rgma	INSIDE	0.214	6.992	3369.48	23560.11	1.496	2541.73	3802.47
A_68_P31067776	chr17:15968184-15968228	NM_178615:-4656	Rgmb	PROMOTER	0.366	5.977	1656.09	9898.39	2.185	1394.25	3046.20
A_68_P31067738	chr17:15963543-15963587	NM_178615:-14	Rgmb	PROMOTER	0.455	3.833	8115.38	31108.04	1.746	6611.76	11542.98
A_68_P29256277	chr13:98976271-98976315	NM_012026:-172	Rgnef	PROMOTER	0.529	5.664	1608.02	9107.79	2.994	1459.00	4368.49
A_68_P31108919	chr17:26340559-26340603	NM_001081069:674	Rgs11	INSIDE	0.579	0.682	1229.14	838.60	0.395	1009.76	398.59
A_68_P23597150	chr5:35292277-35292321	NM_173402:202	Rgs12	INSIDE	0.561	17.300	339.73	5877.26	9.702	301.42	2924.35
A_68_P26904675	chr10:4424482-4424526	NM_001161822:364	Rgs17	INSIDE	0.347	5.226	1843.19	9632.28	1.811	1705.28	3088.76
A_68_P28587838	chr12:83718593-83718637	NM_015812:590	Rgs6	INSIDE	0.385	0.471	1653.72	778.87	0.181	1373.35	249.00
A_68_P20848445	chr1:177422484-177422528	NM_001199003:170	Rgs7	INSIDE	0.359	9.124	2262.55	20642.37	3.272	2456.38	8037.73
A_68_P29294054	chr13:105844417-105844461	NM_029879:572	Rgs7bp	INSIDE	0.608	8.637	1089.17	9407.65	5.253	921.49	4840.20
A_68_P25050058	chr7:36370084-36370128	NM_145840:495	Rgs9bp	INSIDE	0.355	6.935	789.46	5474.66	2.462	639.71	1574.67
A_68_P25050056	chr7:36369864-36369908	NM_145840:715	Rgs9bp	INSIDE	0.364	9.461	1220.26	11545.36	3.442	1057.30	3638.79
A_68_P25050063	chr7:36370599-36370643	NM_145840:-19	Rgs9bp	DIVERGENT_PROMOTER	0.511	4.326	2625.31	11355.89	2.211	2421.43	5354.76
A_68_P27561411	chr11:4999553-4999597	NM_177370:134	Rhbdd3	INSIDE	0.421	5.840	862.66	5038.17	2.458	789.13	1939.80
A_68_P27687498	chr11:32121990-32122034	NM_010117:281	Rhbdf1	INSIDE	0.572	3.911	1338.09	5233.86	2.236	1135.25	2538.57
A_68_P27687433	chr11:32113469-32113513	NM_010117:8803	Rhbdf1	INSIDE	0.634	2.817	709.74	1999.27	1.786	762.11	1361.14
A_68_P28160475	chr11:116485728-116485781	NM_001167680:-188	Rhbdf2	PROMOTER	0.349	0.438	1602.52	702.62	0.153	1144.91	175.02
A_68_P28160474	chr11:116485631-116485675	NM_001167680:-86	Rhbdf2	PROMOTER	0.619	4.052	5239.83	21233.13	2.508	4416.02	11076.95
A_68_P27953576	chr11:80114526-80114570	NM_139228:135	Rhbdl3	INSIDE	0.233	19.290	2033.34	39222.77	4.491	2272.18	10205.44
A_68_P27953582	chr11:80115193-80115237	NM_139228:801	Rhbdl3	INSIDE	0.593	0.607	995.19	604.06	0.36	801.18	288.40
A_68_P23544150	chr5:24348013-24348057	NM_053075:145	Rheb	INSIDE	0.415	0.201	2859.82	575.06	0.083	2079.43	173.32
A_68_P23544155	chr5:24348677-24348721	NM_053075:-519	Rheb	PROMOTER	0.435	4.526	3081.97	13950.00	1.969	2398.77	4722.44
A_68_P23544153	chr5:24348440-24348484	NM_053075:-283	Rheb	PROMOTER	0.506	0.444	3223.86	1430.26	0.224	2355.18	528.44
A_68_P23544149	chr5:24347845-24347889	NM_053075:313	Rheb	INSIDE	0.67	3.850	1334.59	5138.40	2.58	1128.52	2911.69
A_68_P30477110	chr15:98711473-98711517	NM_026967:351	Rhebl1	INSIDE	0.482	0.254	3894.13	990.18	0.123	2605.10	319.53

ProbeName	Target position of probe on CpG island microarray	TargetID	GeneSymbol	CpG island Description	Ratio of relative methylation (TiO_2-NP/Vehicle)	Sham group			TiO_2-H group		
						Relative methylation (Cy5/Cy3)	Cy3 signal (Input)	Cy5 signal (MeDIP)	Relative methylation (Cy5/Cy3)	Cy3 signal (Input)	Cy5 signal (MeDIP)
A_68_P26814202	chr9:108209138-108209182	NM_016802:625	Rhoa	INSIDE	0.307	39.272	4239.46	166490.60	12.056	5206.00	62761.70
A_68_P28222996	chr12:8506600-8506644	NM_007483:169	Rhob	INSIDE	0.184	161.995	2950.86	478024.90	29.755	7203.39	214334.50
A_68_P27222447	chr10:68675289-68675333	NM_001081347:-94	Rhobtb1	PROMOTER	0.618	3.573	1151.97	4116.12	2.207	1052.26	2322.50
A_68_P27222783	chr10:68732909-68732953	NM_001081347:57526	Rhobtb1	INSIDE	0.614	2.730	1628.29	4445.35	1.676	1245.43	2086.93
A_68_P29697194	chr14:70196895-70196939	NM_153514:8436	Rhobtb2	INSIDE	0.242	8.035	2572.59	20670.41	1.945	1852.08	3601.81
A_68_P29152220	chr13:76081481-76081525	NM_028493:-230	Rhobtb3	PROMOTER	0.418	6.213	987.90	6137.65	2.597	957.65	2486.71
A_68_P24054155	chr5:123591978-123592024	NM_175092:-9362	Rhof	PROMOTER	0.223	16.947	489.04	8287.69	3.778	419.41	1584.51
A_68_P24054149	chr5:123591328-123591372	NM_175092:-8712	Rhof	PROMOTER	0.497	0.299	1459.96	436.57	0.149	1159.69	172.26
A_68_P24054157	chr5:123592214-123592258	NM_175092:-9598	Rhof	PROMOTER	0.564	0.481	2289.59	1101.85	0.271	1817.54	493.30
A_68_P24054154	chr5:123591908-123591952	NM_175092:-9292	Rhof	PROMOTER	0.604	0.729	3569.95	2602.05	0.44	2612.47	1149.36
A_68_P25377912	chr7:109398432-109398476	NM_019566:178	Rhog	INSIDE	0.187	10.105	3032.42	30642.26	1.887	2710.32	5113.83
A_68_P25377910	chr7:109398192-109398236	NM_019566:418	Rhog	INSIDE	0.438	0.469	1876.50	880.94	0.206	1553.19	319.22
A_68_P31420286	chr17:87362969-87363013	NM_145491:540	Rhoq	INSIDE	0.347	12.694	3374.14	42831.20	4.401	2746.89	12090.36
A_68_P26240713	chr8:126177658-126177702	NM_133955:-148	Rhou	PROMOTER	0.547	3.956	2751.98	10885.63	2.165	2261.03	4894.38
A_68_P21568741	chr2:119096977-119097021	NM_145530:-36	Rhov	PROMOTER	0.384	6.159	1883.64	11601.46	2.367	1855.11	4391.51
A_68_P30343472	chr15:75534891-75534935	NM_001163465:195	Rhpn1	INSIDE	0.26	6.014	1306.09	7854.76	1.565	1097.30	1717.45
A_68_P25048762	chr7:36119539-36119583	NM_027897:305	Rhpn2	INSIDE	0.485	0.620	5555.01	3444.86	0.3	4258.67	1279.73
A_68_P25585270	chr7:148043114-148043158	NM_053194:-159	Ric8	DIVERGENT_PROMOTER	0.242	0.298	3875.04	1155.70	0.072	2426.49	175.07
A_68_P25585272	chr7:148043313-148043357	NM_053194:39	Ric8	INSIDE	0.382	3.809	1269.69	4835.97	1.456	1118.98	1628.75
A_68_P27305481	chr10:84380679-84380723	NM_001013441:300	Ric8b	INSIDE	0.406	0.568	878.35	498.48	0.23	748.39	172.29
A_68_P29989754	chr15:6659137-6659181	NM_030168:778	Rictor	INSIDE	0.596	0.680	917.48	623.92	0.405	665.95	269.70
A_68_P21217118	chr2:51928841-51928885	NM_175238:506	Rif1	INSIDE	0.569	0.595	3917.29	2331.58	0.338	3156.03	1068.21
A_68_P27926518	chr11:75324220-75324264	NM_001029938:647	Rilp	INSIDE	0.108	33.801	3288.10	111141.00	3.637	2893.24	10521.49
A_68_P27926516	chr11:75323969-75324013	NM_001029938:395	Rilp	INSIDE	0.411	10.816	899.94	9733.36	4.444	831.33	3694.52
A_68_P27926514	chr11:75323805-75323849	NM_001029938:231	Rilp	INSIDE	0.572	4.062	748.97	3041.95	2.323	671.63	1560.00
A_68_P24061765	chr5:124981116-124981160	NM_021430:262	Rilpl1	INSIDE	0.47	0.497	987.05	490.74	0.233	785.34	183.35
A_68_P24085128	chr5:129287634-129287678	NM_001081388:171581	Rimbp2	INSIDE	0.487	4.459	506.69	2259.21	2.173	425.31	924.02
A_68_P30572558	chr16:17210568-17210612	NM_001033338:2363	Rimbp3	INSIDE	0.654	0.282	2029.93	572.50	0.185	1561.58	288.11
A_68_P30572557	chr16:17210481-17210525	NM_001033338:2275	Rimbp3	INSIDE	0.373	0.629	816.11	513.02	0.234	724.66	169.77
A_68_P30572552	chr16:17209857-17209901	NM_001033338:1651	Rimbp3	INSIDE	0.361	0.656	897.37	589.10	0.237	730.16	172.97
A_68_P23245805	chr4:119164931-119164975	NM_177572:251	Rimkla	INSIDE	0.449	4.635	1019.97	4727.32	2.082	770.77	1604.61
A_68_P20085263	chr1:22812453-22812497	NM_001012623:89	Rims1	INSIDE	0.179	1.870	2696.03	5041.18	0.335	1864.70	623.90
A_68_P20085264	chr1:22812557-22812601	NM_001012623:-15	Rims1	PROMOTER	0.259	10.289	4947.17	50900.68	2.667	4380.63	11683.94
A_68_P20083595	chr1:22540893-22540937	NM_001012623:271649	Rims1	INSIDE	0.304	0.547	1221.71	668.62	0.166	1036.90	172.52
A_68_P30152564	chr15:39028639-39028683	NM_053271:-1217	Rims2	PROMOTER	0.481	0.481	1366.81	657.22	0.231	951.29	220.15
A_68_P30152571	chr15:39029657-39029701	NM_053271:-199	Rims2	PROMOTER	0.559	0.623	2972.98	1851.96	0.348	2218.02	771.94
A_68_P28690583	chr12:103521466-103521510	NM_001161365:-362	Rin3	PROMOTER	0.433	4.644	2162.32	10042.56	2.012	1981.58	3986.38
A_68_P31151553	chr17:34161608-34161652	NM_009066:-5	Ring1	PROMOTER	0.647	2.212	3117.56	6896.96	1.431	2534.09	3626.12
A_68_P28958211	chr13:38129415-38129459	NM_024242:579	Riok1	INSIDE	0.615	0.705	1971.54	1390.59	0.434	1582.57	686.78
A_68_P31072977	chr17:17511065-17511109	NM_025934:-209	Riok2	PROMOTER	0.579	0.506	1064.57	538.26	0.293	925.84	271.12
A_68_P28936016	chr13:34094703-34094748	NM_009068:-17	Ripk1	DIVERGENT_PROMOTER	0.375	15.960	3543.80	56557.75	5.987	2640.53	15809.99
A_68_P22753435	chr4:16090304-16090348	NM_138952:319	Ripk2	INSIDE	0.544	3.654	1210.40	4422.58	1.988	1107.11	2200.82
A_68_P31000878	chr16:97985324-97985368	NM_023663:16	Ripk4	INSIDE	0.45	0.404	2684.93	1084.93	0.182	2099.62	382.19
A_68_P31000877	chr16:97985236-97985280	NM_023663:104	Ripk4	INSIDE	0.461	0.243	1900.11	461.68	0.112	1559.73	174.56
A_68_P22315261	chr3:88520728-88520772	NM_001163310:-25	Rit1	PROMOTER	0.306	11.631	2239.03	26042.87	3.556	2136.92	7598.66
A_68_P32575424	chrX:101176475-101176519	NM_011276:127	Rlim	INSIDE	0.557	4.956	7279.61	36077.17	2.761	6551.88	18092.29

ProbeName	Target position of probe on CpG island microarray	TargetID	GeneSymbol	CpG island Description	Ratio of relative methylation (TiO_2-NP/Vehicle)	Sham group			TiO_2-H group		
						Relative methylation (Cy5/Cy3)	Cy3 signal (Input)	Cy5 signal (MeDIP)	Relative methylation (Cy5/Cy3)	Cy3 signal (Input)	Cy5 signal (MeDIP)
A_68_P26135404	chr8:108218075-108218119	NM_001033320:3291	Rltpr	INSIDE	0.35	4.335	11616.29	50354.34	1.519	8602.76	13063.87
A_68_P29073064	chr13:58503963-58504007	NM_001168248:27	Rmi1	INSIDE	0.636	0.399	2549.66	1018.02	0.254	1919.51	487.12
A_68_P29073057	chr13:58503189-58503233	NR_031761:-398	Rmi1	DIVERGENT_PROMOTER	0.654	0.377	3808.08	1437.35	0.247	2955.21	729.22
A_68_P29073065	chr13:58504155-58504199	NM_001168248:219	Rmi1	INSIDE	0.396	0.647	2004.25	1296.31	0.256	1642.08	420.89
A_68_P24424174	chr6:47601153-47601197	NR_002841:3919	Rn4.5s	DOWNSTREAM	0.617	0.681	10862.09	7397.97	0.42	7550.37	3173.78
A_68_P26022869	chr8:87489816-87489860	NM_027187:72	Rnaseh2a	INSIDE	0.57	0.567	1145.61	649.83	0.323	895.36	289.29
A_68_P29654922	chr14:62951071-62951115	NM_026001:151	Rnaseh2b	INSIDE	0.61	3.021	1081.64	3268.12	1.845	857.52	1581.74
A_68_P31929627	chr19:5602038-5602082	NM_026616:188	Rnaseh2c	INSIDE	0.6	11.041	2200.29	24292.80	6.619	1810.45	11983.22
A_68_P31023590	chr17:7182740-7182784	NM_026611:-446	Rnaset2b	PROMOTER	0.156	17.257	15522.29	267874.10	2.7	12991.05	35071.29
A_68_P31023591	chr17:7182888-7182932	NM_026611:-298	Rnaset2b	PROMOTER	0.204	9.607	4526.31	43486.38	1.959	3437.95	6733.37
A_68_P31023593	chr17:7183066-7183110	NM_026611:-120	Rnaset2b	PROMOTER	0.402	0.678	7988.11	5417.89	0.273	6776.83	1847.49
A_68_P31023594	chr17:7183213-7183257	NM_026611:26	Rnaset2b	INSIDE	0.392	1.443	4827.05	6966.93	0.565	3799.87	2148.30
A_68_P28074048	chr11:101329727-101329771	NM_009708:97	Rnd2	INSIDE	0.191	0.622	2024.55	1258.59	0.119	1467.10	173.92
A_68_P21212081	chr2:51003539-51003583	NM_028810:1071	Rnd3	INSIDE	0.631	0.245	5281.23	1292.73	0.154	3451.55	532.72
A_68_P21212091	chr2:51004685-51004729	NM_028810:-75	Rnd3	PROMOTER	0.638	5.557	283.68	1576.37	3.543	201.31	713.21
A_68_P23201329	chr4:109148711-109148755	NM_013876:378	Rnf11	INSIDE	0.448	5.446	13831.98	75331.74	2.439	12448.27	30364.51
A_68_P26616836	chr9:70350943-70350987	NM_033604:568	Rnf111	INSIDE	0.586	2.393	2245.79	5374.95	1.403	1913.55	2683.86
A_68_P31542302	chr18:21120354-21120398	NM_026301:17251	Rnf125	INSIDE	0.496	4.340	1907.77	8280.46	2.153	1459.57	3142.49
A_68_P27278802	chr10:79229397-79229442	NM_144528:247	Rnf126	INSIDE	0.312	13.917	5535.31	77032.25	4.346	4338.90	18855.56
A_68_P27278806	chr10:79229855-79229899	NR_027505:-190	Rnf126	PROMOTER	0.427	0.205	3237.47	662.78	0.087	2326.84	203.44
A_68_P27278801	chr10:79229322-79229366	NM_144528:322	Rnf126	INSIDE	0.616	2.837	1463.18	4151.63	1.748	1235.59	2160.32
A_68_P27278810	chr10:79230292-79230336	NR_027505:-628	Rnf126	DIVERGENT_PROMOTER	0.495	0.680	969.60	659.50	0.337	741.97	249.99
A_68_P32694935	chrX:136145294-136145338	NM_023270:158	Rnf128	INSIDE	0.507	4.755	989.47	4704.93	2.412	1139.39	2748.48
A_68_P22165084	chr3:57539624-57539668	NM_011883:-341	Rnf13	PROMOTER	0.604	0.348	1894.88	658.73	0.21	1531.76	321.89
A_68_P31542490	chr18:21160400-21160444	NM_019706:581	Rnf138	INSIDE	0.368	0.451	3046.78	1375.54	0.166	2206.69	366.92
A_68_P31542491	chr18:21160534-21160578	NM_019706:715	Rnf138	INSIDE	0.388	16.024	6335.04	101514.80	6.225	5214.47	32460.84
A_68_P30250719	chr15:58720548-58720592	NM_175226:-213	Rnf139	PROMOTER	0.533	8.455	992.10	8388.26	4.503	814.77	3669.29
A_68_P30250724	chr15:58721209-58721253	NM_175226:447	Rnf139	INSIDE	0.664	3.265	3107.94	10148.70	2.167	2402.36	5205.76
A_68_P28301681	chr12:27100085-27100129	NM_001081977:15	Rnf144a	INSIDE	0.319	11.711	2328.57	27269.81	3.73	2055.05	7665.90
A_68_P28301678	chr12:27099581-27099625	NM_001081977:519	Rnf144a	INSIDE	0.537	0.212	4006.26	851.18	0.114	2914.36	332.56
A_68_P28301682	chr12:27100225-27100269	NM_001081977:-125	Rnf144a	PROMOTER	0.609	3.080	2302.21	7090.53	1.875	1913.97	3589.07
A_68_P29011800	chr13:47218349-47218393	NM_146042:282	Rnf144b	INSIDE	0.654	0.579	1024.65	593.04	0.378	899.46	340.26
A_68_P20502873	chr1:107252976-107253020	NM_001160368:289	Rnf152	INSIDE	0.487	5.736	2080.90	11936.69	2.792	1850.31	5166.27
A_68_P25365011	chr7:107128763-107128807	NM_175388:184	Rnf169	INSIDE	0.549	3.766	931.22	3506.74	2.067	955.13	1974.54
A_68_P29622736	chr14:57021701-57021745	NM_001033043:189	Rnf17	INSIDE	0.412	4.847	1414.69	6857.48	1.999	1167.89	2334.29
A_68_P29622735	chr14:57021569-57021613	NM_001033043:57	Rnf17	INSIDE	0.48	4.939	1975.11	9755.91	2.37	1600.64	3794.28
A_68_P28991174	chr13:43711396-43711440	NM_183204:253	Rnf182	INSIDE	0.17	21.222	2087.41	44298.75	3.615	1941.40	7018.23
A_68_P28991175	chr13:43711506-43711550	NM_183204:363	Rnf182	INSIDE	0.512	3.743	862.13	3226.68	1.915	820.52	1570.97
A_68_P27833619	chr11:58752222-58752266	NM_022423:164	Rnf187	INSIDE	0.515	3.754	558.96	2098.26	1.934	480.28	928.62
A_68_P30137691	chr15:36213148-36213192	NM_013923:-268	Rnf19a	PROMOTER	0.274	32.305	445.42	14389.43	8.837	1292.86	11424.69
A_68_P23294679	chr4:128735519-128735563	NM_029219:26	Rnf19b	INSIDE	0.512	4.535	1529.12	6934.44	2.321	1385.42	3215.13
A_68_P22906127	chr4:49645190-49645234	NM_001163263:281	Rnf20	INSIDE	0.566	0.511	1327.38	677.92	0.289	1036.09	299.31
A_68_P23417945	chr4:151687256-151687300	NM_001033489:5456	Rnf207	INSIDE	0.354	0.600	1760.43	1056.56	0.213	1356.25	288.48
A_68_P21070804	chr2:25097923-25097967	NM_176834:-504	Rnf208	DIVERGENT_PROMOTER	0.523	0.708	1178.71	835.00	0.371	813.61	301.60
A_68_P27045332	chr10:31328778-31328822	NM_001146349:731	Rnf217	INSIDE	0.419	3.704	883.54	3272.82	1.554	841.42	1307.20
A_68_P23234760	chr4:117167587-117167631	NM_025739:1912	Rnf220	INSIDE	0.52	6.839	2937.95	20092.45	3.554	2220.64	7892.76

ProbeName	Target position of probe on CpG island microarray	TargetID	GeneSymbol	CpG island Description	Ratio of relative methylation (TiO_2-NP/Vehicle)	Sham group			TiO_2-H group		
						Relative methylation (Cy5/Cy3)	Cy3 signal (Input)	Cy5 signal (MeDIP)	Relative methylation (Cy5/Cy3)	Cy3 signal (Input)	Cy5 signal (MeDIP)
A_68_P23234771	chr4:117169165-117169209	NM_025739:334	Rnf220	INSIDE	0.643	0.247	2128.46	526.55	0.159	1549.72	246.35
A_68_P23233435	chr4:116962137-116962181	NM_025739:207362	Rnf220	INSIDE	0.652	0.584	2392.16	1395.88	0.381	1861.34	708.58
A_68_P29618705	chr14:56210981-56211025	NM_194346:376	Rnf31	INSIDE	0.454	0.380	1353.20	513.71	0.172	1041.78	179.47
A_68_P23566422	chr5:29522137-29522181	NM_021470:-2387	Rnf32	PROMOTER	0.309	0.492	2601.62	1279.35	0.152	1750.48	265.88
A_68_P22875663	chr4:44181273-44181317	NM_001038993:-139	Rnf38	PROMOTER	0.602	3.584	562.19	2015.00	2.159	535.36	1155.78
A_68_P31166410	chr17:37084116-37084160	NM_001099632:4143	Rnf39	INSIDE	0.249	19.915	866.35	17253.39	4.955	706.65	3501.29
A_68_P23593765	chr5:34669759-34669803	NM_011278:-9258	Rnf4	PROMOTER	0.332	0.514	1541.23	792.14	0.171	1004.29	171.31
A_68_P23593767	chr5:34669934-34669978	NM_011278:-9082	Rnf4	PROMOTER	0.474	1.481	3944.20	5842.07	0.703	2879.97	2024.01
A_68_P29050814	chr13:54788639-54788683	NM_001146025:509	Rnf44	INSIDE	0.062	27.456	1216.48	33399.78	1.695	926.66	1570.25
A_68_P31154750	chr17:34741259-34741304	NM_019403:-775	Rnf5	DIVERGENT_PROMOTER	0.254	0.443	2061.33	912.63	0.112	1577.67	177.43
A_68_P26747279	chr9:96379047-96379091	NM_011279:-54	Rnf7	PROMOTER	0.604	2.534	2025.91	5132.96	1.531	1488.76	2279.74
A_68_P27989203	chr11:86298068-86298112	NM_029788:-68	Rnft1	PROMOTER	0.158	14.908	2966.41	44223.39	2.36	2976.60	7024.49
A_68_P22826133	chr4:33397493-33397537	NM_011884:229	Rngtt	INSIDE	0.159	15.167	1904.43	28884.30	2.409	1677.23	4041.17
A_68_P25587156	chr7:148354570-148354614	NM_001172101:-7	Rnh1	PROMOTER	0.649	0.266	2206.60	585.97	0.172	1609.40	277.48
A_68_P27930798	chr11:76057462-76057506	NM_183263:247	Rnmtl1	INSIDE	0.443	0.552	991.05	547.21	0.245	778.94	190.52
A_68_P20638513	chr1:137180458-137180502	NM_001159624:181	Rnpep	INSIDE	0.322	4.694	1377.17	6464.01	1.51	1492.42	2253.06
A_68_P20451965	chr1:94807505-94807549	NM_181405:125	Rnpepl1	INSIDE	0.441	5.870	1548.03	9087.58	2.589	1266.31	3278.29
A_68_P20451969	chr1:94807947-94807991	NM_181405:567	Rnpepl1	INSIDE	0.511	0.535	1688.55	902.54	0.273	1332.44	363.70
A_68_P20451967	chr1:94807773-94807817	NM_181405:393	Rnpepl1	INSIDE	0.385	4.234	1443.51	6111.13	1.632	1160.36	1893.60
A_68_P22301214	chr3:85946197-85946241	NR_004418:-1609	Rnu73b	PROMOTER	0.647	0.433	1183.61	512.76	0.28	957.74	268.48
A_68_P26430635	chr9:37240928-37240972	NM_001164767:-190	Robo3	PROMOTER	0.28	18.396	2537.72	46683.47	5.156	2744.80	14151.15
A_68_P31488614	chr18:10181658-10181702	NM_009071:110	Rock1	INSIDE	0.537	12.341	2717.38	33536.55	6.625	2340.76	15507.87
A_68_P31488617	chr18:10181982-10182026	NM_009071:-214	Rock1	PROMOTER	0.65	0.580	1458.84	846.38	0.377	1232.44	464.50
A_68_P22957033	chr4:59562622-59562666	NM_144904:-408	Rod1	PROMOTER	0.425	0.512	1062.72	544.14	0.218	811.20	176.48
A_68_P30513218	chr16:5013781-5013827	NM_133185:-158	Rogdi	PROMOTER	0.502	7.236	2307.05	16693.76	3.633	1868.98	6789.54
A_68_P23147506	chr4:99768281-99768325	NM_013845:-93	Ror1	PROMOTER	0.394	0.652	809.54	528.06	0.257	787.03	202.35
A_68_P23147511	chr4:99768962-99769006	NM_013845:589	Ror1	INSIDE	0.524	4.067	514.42	2092.27	2.13	493.78	1051.97
A_68_P29043022	chr13:53381392-53381436	NM_013846:64	Ror2	INSIDE	0.419	6.860	660.35	4530.21	2.876	619.15	1780.47
A_68_P29041999	chr13:53214193-53214237	NM_013846:167264	Ror2	INSIDE	0.499	3.598	2532.47	9112.96	1.794	1890.85	3392.72
A_68_P26609884	chr9:69138237-69138281	NM_013646:636650	Rora	INSIDE	0.108	27.228	1239.31	33744.02	2.935	1064.86	3125.57
A_68_P26609891	chr9:69138894-69138938	NM_013646:637308	Rora	INSIDE	0.28	0.482	1962.33	944.90	0.135	1427.14	192.09
A_68_P26605679	chr9:68502496-68502540	NM_013646:910	Rora	INSIDE	0.558	0.272	1996.70	543.07	0.152	1380.80	209.44
A_68_P26605669	chr9:68501280-68501324	NM_013646:-306	Rora	PROMOTER	0.147	0.734	3092.11	2271.12	0.108	2324.91	250.68
A_68_P26609887	chr9:69138471-69138515	NM_013646:636884	Rora	INSIDE	0.555	3.724	828.81	3086.28	2.066	723.37	1494.26
A_68_P26352003	chr9:22272492-22272536	NM_018739:286	Rp9	INSIDE	0.339	18.727	2293.11	42943.43	6.35	2097.46	13319.22
A_68_P23313959	chr4:132324309-132324353	NM_011284:56	Rpa2	INSIDE	0.519	0.310	2193.65	680.46	0.161	1770.70	285.23
A_68_P23313962	chr4:132324543-132324587	NM_011284:290	Rpa2	INSIDE	0.586	0.256	4197.08	1073.47	0.15	3046.55	456.63
A_68_P20304095	chr1:66747574-66747618	NM_025683:130	Rpe	INSIDE	0.586	0.207	2601.77	537.27	0.121	2152.99	260.73
A_68_P22615131	chr3:146184129-146184173	NM_027371:237	Rpf1	INSIDE	0.49	0.518	1928.27	998.59	0.254	1454.09	369.11
A_68_P29604814	chr14:52738859-52738903	NM_001168515:8303	Rpgrip1	INSIDE	0.505	0.455	1582.58	720.39	0.23	1134.72	260.71
A_68_P31121837	chr17:28465985-28466029	NM_011287:591	Rpl10a	INSIDE	0.445	4.799	2961.29	14211.33	2.133	2473.56	5276.74
A_68_P31121839	chr17:28466226-28466270	NM_011287:833	Rpl10a	INSIDE	0.501	0.524	1371.14	717.90	0.262	1168.63	306.29
A_68_P31121836	chr17:28465889-28465933	NM_011287:495	Rpl10a	INSIDE	0.613	0.687	1005.85	691.03	0.421	833.96	351.14
A_68_P31121838	chr17:28466106-28466150	NM_011287:713	Rpl10a	INSIDE	0.639	2.559	1046.11	2676.97	1.634	836.82	1367.68
A_68_P23331869	chr4:135609312-135609356	NM_025919:-48	Rpl11	PROMOTER	0.566	5.952	1166.35	6941.95	3.37	938.02	3161.15
A_68_P23331867	chr4:135609136-135609180	NM_025919:128	Rpl11	INSIDE	0.537	3.081	1172.32	3612.22	1.656	1008.57	1670.22

ProbeName	Target position of probe on CpG island microarray	TargetID	GeneSymbol	CpG island Description	Ratio of relative methylation (TiO_2-NP/Vehicle)	Sham group			TiO_2-H group		
						Relative methylation (Cy5/Cy3)	Cy3 signal (Input)	Cy5 signal (MeDIP)	Relative methylation (Cy5/Cy3)	Cy3 signal (Input)	Cy5 signal (MeDIP)
A_68_P25091299	chr7:52384642-52384686	NM_009438:-559	Rpl13a	PROMOTER	0.309	1.964	2380.17	4674.16	0.608	1891.93	1149.53
A_68_P26878665	chr9:120486576-120486620	NM_025974:5965	Rpl14	DOWNSTREAM	0.434	4.003	1518.47	6078.95	1.737	1211.74	2104.56
A_68_P23418024	chr4:151700327-151700371	NM_009079:362	Rpl22	INSIDE	0.188	22.183	1550.58	34396.87	4.167	1346.53	5610.95
A_68_P22019227	chr3:28704574-28704618	NM_026517:164	Rpl22l1	INSIDE	0.575	0.505	1306.69	659.33	0.29	1055.22	306.13
A_68_P25408483	chr7:116662453-116662497	NM_011975:-234	Rpl27a	PROMOTER	0.45	0.348	2305.35	802.07	0.157	1706.44	267.13
A_68_P26803290	chr9:106331991-106332035	NM_009082:143	Rpl29	INSIDE	0.553	2.631	7925.55	20850.19	1.454	6389.13	9289.27
A_68_P30128581	chr15:34373372-34373416	NM_001163485:-363	Rpl30	DIVERGENT_PROMOTER	0.404	5.892	2755.46	16234.42	2.381	2301.77	5481.64
A_68_P30128580	chr15:34373273-34373317	NM_001163485:-263	Rpl30	DIVERGENT_PROMOTER	0.423	0.397	1824.14	724.93	0.168	1344.95	225.99
A_68_P20165362	chr1:39424508-39424553	NM_053257:-165	Rpl31	PROMOTER	0.553	6.584	615.35	4051.50	3.638	562.31	2045.68
A_68_P20165367	chr1:39425174-39425218	NM_053257:501	Rpl31	INSIDE	0.619	0.346	1655.86	572.29	0.214	1238.15	265.09
A_68_P21151631	chr2:38860577-38860621	NM_025592:53	Rpl35	INSIDE	0.582	4.609	2911.63	13419.21	2.684	2300.59	6175.07
A_68_P30655993	chr16:33056579-33056624	NM_021338:2	Rpl35a	INSIDE	0.473	3.134	1847.44	5789.03	1.483	1534.97	2276.64
A_68_P28149045	chr11:114530045-114530089	NM_001048058:-28	Rpl38	PROMOTER	0.14	1.481	2640.77	3910.89	0.207	1810.70	374.31
A_68_P28149043	chr11:114529888-114529932	NM_001048058:-184	Rpl38	PROMOTER	0.436	0.514	997.65	512.69	0.224	764.52	171.46
A_68_P27541976	chr10:127986184-127986228	NM_018860:18	Rpl41	INSIDE	0.356	0.353	3206.78	1133.47	0.126	2214.88	278.39
A_68_P30351089	chr15:76734484-76734528	NM_012053:6	Rpl8	INSIDE	0.173	0.581	3096.84	1798.05	0.101	2143.99	215.53
A_68_P30351091	chr15:76734692-76734736	NM_012053:214	Rpl8	INSIDE	0.486	0.308	1728.44	532.36	0.15	1188.90	177.79
A_68_P24010226	chr5:116009731-116009775	NM_007475:277	Rplp0	INSIDE	0.62	3.015	1157.91	3490.80	1.869	927.84	1734.05
A_68_P24619664	chr6:88035091-88035135	NM_133933:646	Rpn1	INSIDE	0.541	5.774	782.78	4519.63	3.124	608.45	1900.87
A_68_P31163327	chr17:36394876-36394920	NM_026308:-107	Rpp21	PROMOTER	0.342	15.373	1216.24	18697.89	5.253	1066.74	5603.70
A_68_P26542152	chr9:57352020-57352064	NM_133982:134	Rpp25	INSIDE	0.623	0.410	1348.97	553.46	0.256	1025.33	262.28
A_68_P21780565	chr2:157854409-157854453	NM_027434:-99	Rprd1b	PROMOTER	0.11	16.854	3826.39	64488.23	1.854	2922.26	5418.75
A_68_P21780563	chr2:157854132-157854176	NM_027434:-375	Rprd1b	PROMOTER	0.57	6.088	994.44	6054.36	3.47	818.96	2841.53
A_68_P22345830	chr3:95622664-95622708	NM_001081293:190	Rprd2	INSIDE	0.219	1.512	1089.67	1648.07	0.331	944.70	312.56
A_68_P28086208	chr11:103510960-103511004	NM_001033212:160	Rprml	INSIDE	0.631	0.721	1681.18	1211.44	0.455	1425.68	648.19
A_68_P25091259	chr7:52379751-52379795	NM_013725:-13	Rps11	PROMOTER	0.499	0.464	1373.95	637.04	0.231	1095.56	253.49
A_68_P25456593	chr7:125259495-125259539	NM_170669:145	Rps15a	INSIDE	0.354	14.448	1414.01	20430.20	5.122	1806.14	9250.36
A_68_P25007073	chr7:25669913-25669957	NM_023133:202	Rps19	INSIDE	0.152	14.538	1608.63	23386.54	2.207	1366.81	3016.65
A_68_P25007070	chr7:25669613-25669657	NM_023133:-98	Rps19	PROMOTER	0.61	0.740	3185.18	2355.69	0.451	2321.96	1047.36
A_68_P22694582	chr4:3762328-3762372	NM_026147:397	Rps20	INSIDE	0.51	0.538	1858.89	1000.46	0.275	1428.48	392.41
A_68_P21902494	chr2:179991894-179991938	NM_025587:-167	Rps21	PROMOTER	0.638	18.338	2705.41	49611.95	11.705	2314.11	27085.52
A_68_P26596129	chr9:66793897-66793941	NM_026467:-6	Rps27l	PROMOTER	0.629	0.649	789.64	512.35	0.408	651.37	266.01
A_68_P28513108	chr12:70260224-70260268	NM_009093:-73	Rps29	DIVERGENT_PROMOTER	0.326	5.931	1679.86	9962.69	1.935	1391.30	2691.93
A_68_P24973639	chr7:13507675-13507719	NM_009095:37	Rps5	INSIDE	0.464	6.075	388.59	2360.77	2.821	336.68	949.72
A_68_P31024851	chr17:7374165-7374209	NM_011299:-277	Rps6ka2	PROMOTER	0.448	0.302	2317.05	700.25	0.135	1794.94	243.17
A_68_P31936363	chr19:6914626-6914670	NM_019924:443	Rps6ka4	INSIDE	0.227	0.500	2039.11	1019.05	0.113	1574.09	178.37
A_68_P31936365	chr19:6914837-6914881	NM_019924:233	Rps6ka4	INSIDE	0.577	0.266	1956.69	520.17	0.153	1575.51	241.71
A_68_P31936369	chr19:6915237-6915281	NM_019924:-167	Rps6ka4	PROMOTER	0.638	0.407	1185.19	482.34	0.26	947.33	246.05
A_68_P28683722	chr12:101962780-101962824	NM_153587:436	Rps6ka5	INSIDE	0.486	0.528	2157.54	1138.45	0.256	1682.36	431.45
A_68_P32599598	chrX:108576717-108576761	NM_025949:74816	Rps6ka6	INSIDE	0.51	3.085	731.69	2256.93	1.573	890.99	1401.57
A_68_P28314231	chr12:29320191-29320235	NM_011300:606	Rps7	INSIDE	0.217	6.336	2673.21	16937.75	1.373	2906.76	3990.97
A_68_P24946923	chr7:3656384-3656428	NM_029767:764	Rps9	INSIDE	0.477	0.282	2863.93	806.48	0.134	2193.11	294.80
A_68_P22824535	chr4:33070523-33070567	NM_027491:572	Rragd	INSIDE	0.313	0.567	1445.46	819.16	0.178	1195.33	212.32
A_68_P25434388	chr7:121260597-121260641	NM_025846:677	Rras2	INSIDE	0.313	6.577	2077.90	13665.91	2.059	1835.47	3779.26
A_68_P21704892	chr2:143836307-143836351	NM_024281:671	Rrbp1	INSIDE	0.178	2.350	2030.18	4771.76	0.419	1560.92	653.87
A_68_P21704898	chr2:143837238-143837282	NM_024281:-261	Rrbp1	PROMOTER	0.406	5.698	1486.26	8468.90	2.315	1516.64	3511.11

ProbeName	Target position of probe on CpG island microarray	TargetID	GeneSymbol	CpG island Description	Ratio of relative methylation (TiO_2-NP/Vehicle)	Sham group			TiO_2-H group		
						Relative methylation (Cy5/Cy3)	Cy3 signal (Input)	Cy5 signal (MeDIP)	Relative methylation (Cy5/Cy3)	Cy3 signal (Input)	Cy5 signal (MeDIP)
A_68_P28956729	chr13:37918014-37918058	NM_001177868:-744	Rreb1	PROMOTER	0.348	5.020	2308.13	11585.84	1.748	1847.22	3229.62
A_68_P28957528	chr13:38022622-38022667	NM_001177869:41905	Rreb1	INSIDE	0.62	2.904	814.66	2365.55	1.799	596.37	1073.06
A_68_P28291147	chr12:25393381-25393425	NM_009104:284	Rrm2	INSIDE	0.6	0.343	2790.42	957.38	0.206	2209.20	454.99
A_68_P28291143	chr12:25392901-25392945	NM_009104:-196	Rrm2	PROMOTER	0.423	0.647	4815.13	3115.75	0.273	3761.10	1028.33
A_68_P31143302	chr17:32173587-32173631	NM_028244:502	Rrp1b	INSIDE	0.314	18.119	2787.67	50510.74	5.696	2814.73	16033.01
A_68_P31208271	chr17:46811147-46811191	NM_144857:36	Rrp36	INSIDE	0.439	4.216	5040.76	21250.47	1.849	4306.92	7965.26
A_68_P30386713	chr15:82953055-82953099	NM_029101:155	Rrp7a	INSIDE	0.574	0.649	3025.07	1962.44	0.373	2264.48	843.87
A_68_P25351151	chr7:104728309-104728353	NM_001081267:-75	Rsf1	DIVERGENT_PROMOTER	0.608	0.334	4624.77	1542.75	0.203	3248.42	659.37
A_68_P25351157	chr7:104729030-104729074	NM_001081267:647	Rsf1	INSIDE	0.527	3.732	903.58	3372.26	1.966	794.66	1561.99
A_68_P30546791	chr16:11203150-11203194	NM_025546:213	Rsl1d1	INSIDE	0.482	5.165	5654.18	29205.26	2.492	4654.34	11598.90
A_68_P24991219	chr7:19650979-19651023	NM_001159671:10965	Rsph6a	INSIDE	0.525	9.095	4047.08	36808.89	4.773	3107.96	14832.81
A_68_P23271474	chr4:124663490-124663534	NM_138683:-161	Rspo1	PROMOTER	0.459	10.100	1037.73	10480.83	4.635	986.54	4572.54
A_68_P23271504	chr4:124667418-124667462	NM_138683:3767	Rspo1	INSIDE	0.536	0.431	5276.89	2274.32	0.231	3784.04	874.32
A_68_P26078523	chr8:97125990-97126034	NM_026274:172	Rspry1	INSIDE	0.585	4.051	1785.52	7232.72	2.369	1389.96	3292.47
A_68_P26022781	chr8:87469555-87469599	NM_144929:-1313	Rtbdn	DIVERGENT_PROMOTER	0.419	0.514	983.75	505.69	0.215	811.88	174.66
A_68_P24591678	chr6:83092414-83092458	NM_009106:5359	Rtkn	INSIDE	0.462	0.402	1708.60	686.72	0.186	1175.53	218.37
A_68_P27215086	chr10:67442552-67442596	NM_001081346:229	Rtkn2	INSIDE	0.508	3.967	1490.27	5912.28	2.017	1317.87	2658.03
A_68_P27215083	chr10:67442230-67442274	NM_001081346:-93	Rtkn2	PROMOTER	0.559	4.236	8244.42	34922.94	2.368	5613.62	13295.19
A_68_P24992460	chr7:19868360-19868404	NM_001025364:-8065	Rtn2	PROMOTER	0.501	0.413	2113.73	873.17	0.207	1634.28	338.45
A_68_P31940080	chr19:7557684-7557728	NM_053076:65	Rtn3	INSIDE	0.513	0.579	784.59	454.49	0.297	591.30	175.79
A_68_P27675742	chr11:29593934-29593982	NM_194051:185	Rtn4	INSIDE	0.376	0.381	1837.39	699.14	0.143	1210.19	173.32
A_68_P30577646	chr16:18127970-18128014	NM_022982:194	Rtn4r	INSIDE	0.604	0.376	1638.74	616.49	0.227	1184.28	269.11
A_68_P27924777	chr11:75008575-75008619	NM_177708:1102	Rtn4rl1	INSIDE	0.198	0.339	4912.53	1665.71	0.067	3102.47	208.35
A_68_P27924762	chr11:75006792-75006836	NM_177708:-680	Rtn4rl1	DIVERGENT_PROMOTER	0.599	0.252	4979.75	1255.09	0.151	3603.38	543.89
A_68_P27924905	chr11:75027578-75027622	NM_177708:20106	Rtn4rl1	INSIDE	0.6	0.636	1520.47	967.61	0.382	1191.96	455.41
A_68_P21395235	chr2:84729286-84729330	NM_199223:-2459	Rtn4rl2	PROMOTER	0.056	2.076	2809.16	5831.72	0.116	1973.51	228.67
A_68_P21395236	chr2:84729363-84729407	NM_199223:-2535	Rtn4rl2	PROMOTER	0.076	1.707	2317.36	3956.25	0.129	1508.46	194.70
A_68_P21395110	chr2:84712227-84712271	NM_199223:14601	Rtn4rl2	INSIDE	0.345	12.961	2834.90	36743.06	4.466	2534.35	11317.36
A_68_P21395215	chr2:84726754-84726798	NM_199223:73	Rtn4rl2	INSIDE	0.368	0.404	1526.41	616.37	0.149	1142.52	169.83
A_68_P21395229	chr2:84728472-84728517	NM_199223:-1645	Rtn4rl2	PROMOTER	0.233	1.928	1315.40	2535.51	0.448	1090.47	488.98
A_68_P21395214	chr2:84726634-84726678	NM_199223:193	Rtn4rl2	INSIDE	0.433	1.370	2335.26	3198.31	0.593	1839.48	1091.08
A_68_P31909673	chr18:89141458-89141502	NM_175542:299	Rttn	INSIDE	0.416	0.167	3789.33	631.49	0.069	2510.12	173.97
A_68_P31909672	chr18:89141358-89141413	NM_175542:204	Rttn	INSIDE	0.458	0.483	1081.71	522.97	0.221	775.31	171.62
A_68_P28073802	chr11:101286306-101286350	NM_172566:-70	Rundc1	PROMOTER	0.333	10.625	4366.13	46391.26	3.541	3995.13	14145.36
A_68_P28079339	chr11:102260809-102260853	NM_016759:6082	Rundc3a	INSIDE	0.024	11.191	3349.14	37480.81	0.269	2382.09	640.17
A_68_P28079292	chr11:102254981-102255025	NM_016759:254	Rundc3a	INSIDE	0.137	1.750	1018.94	1782.68	0.239	871.52	208.48
A_68_P23468796	chr5:8622618-8622662	NM_198620:312	Rundc3b	INSIDE	0.236	9.506	2070.16	19679.79	2.246	1740.61	3910.05
A_68_P30970267	chr16:92606200-92606244	NM_001111023:91351	Runx1	INSIDE	0.134	2.535	3470.72	8799.83	0.34	2598.87	883.04
A_68_P30970265	chr16:92605955-92605999	NM_001111023:91597	Runx1	INSIDE	0.133	4.865	1646.43	8010.43	0.649	1409.60	914.16
A_68_P30970952	chr16:92697859-92697903	NM_001111023:-307	Runx1	PROMOTER	0.319	1.900	1252.70	2379.54	0.606	1166.09	706.42
A_68_P31196997	chr17:44873430-44873474	NM_001145920:145	Runx2	INSIDE	0.541	4.250	1208.78	5137.89	2.301	1097.15	2524.36
A_68_P31196991	chr17:44872774-44872818	NM_001145920:801	Runx2	INSIDE	0.659	0.727	2709.00	1968.73	0.479	2095.26	1003.58
A_68_P31196993	chr17:44872985-44873029	NM_001145920:591	Runx2	INSIDE	0.599	0.663	987.64	654.32	0.397	876.10	347.81
A_68_P23326917	chr4:134708685-134708729	NM_019732:32147	Runx3	INSIDE	0.512	6.904	3885.39	26825.84	3.532	3039.98	10736.17
A_68_P23326916	chr4:134708430-134708475	NM_019732:31893	Runx3	INSIDE	0.626	4.864	260.86	1268.90	3.043	269.36	819.60
A_68_P22317129	chr3:88894411-88894455	NM_001083808:-479	Rusc1	PROMOTER	0.377	7.916	2872.15	22737.08	2.988	2249.68	6722.41

ProbeName	Target position of probe on CpG island microarray	TargetID	GeneSymbol	CpG island Description	Ratio of relative methylation (TiO_2-NP/Vehicle)	Sham group			TiO_2-H group		
						Relative methylation (Cy5/Cy3)	Cy3 signal (Input)	Cy5 signal (MeDIP)	Relative methylation (Cy5/Cy3)	Cy3 signal (Input)	Cy5 signal (MeDIP)
A_68_P22871354	chr4:43394705-43394749	NM_001037709:-127	Rusc2	PROMOTER	0.308	7.560	1047.07	7915.96	2.33	1027.65	2394.13
A_68_P26700013	chr9:86465522-86465566	NM_001145969:-50	Rwdd2a	DIVERGENT_PROMOTER	0.296	1.776	2769.39	4919.01	0.525	2306.18	1210.58
A_68_P25842222	chr8:48619150-48619194	NM_203507:174	Rwdd4a	INSIDE	0.599	0.439	1287.28	564.78	0.263	1069.96	281.33
A_68_P30013962	chr15:10966866-10966910	NM_178717:835	Rxfp3	INSIDE	0.384	12.376	3024.76	37434.95	4.748	2652.57	12593.33
A_68_P21085998	chr2:27532103-27532149	NM_011305:-594	Rxra	PROMOTER	0.427	0.545	1804.65	983.61	0.233	1375.65	320.42
A_68_P21086006	chr2:27533318-27533362	NM_011305:620	Rxra	INSIDE	0.623	5.439	1315.61	7155.05	3.388	1084.55	3674.72
A_68_P24691293	chr6:100237792-100237836	NM_019743:-462	Rybp	PROMOTER	0.261	14.916	2055.04	30652.63	3.895	1817.03	7077.70
A_68_P24691294	chr6:100237868-100237912	NM_019743:-538	Rybp	PROMOTER	0.373	8.881	669.90	5949.24	3.315	603.05	1998.82
A_68_P24691288	chr6:100237003-100237047	NM_019743:328	Rybp	INSIDE	0.64	4.719	1032.72	4872.93	3.02	924.48	2792.05
A_68_P28830400	chr13:12198333-12198377	NM_023868:858	Ryr2	INSIDE	0.537	0.372	1812.19	674.09	0.2	1552.39	310.36
A_68_P28830403	chr13:12198675-12198719	NM_023868:516	Ryr2	INSIDE	0.581	3.735	1063.44	3972.17	2.171	885.97	1923.72
A_68_P22336986	chr3:93359142-93359186	NM_009112:126	S100a10	INSIDE	0.612	2.839	1737.25	4932.13	1.736	1572.34	2729.77
A_68_P22336828	chr3:93324960-93325004	NM_016740:565	S100a11	INSIDE	0.552	0.683	901.76	615.53	0.377	834.50	314.26
A_68_P26344891	chr9:20780545-20780589	NM_010333:671	S1pr2	INSIDE	0.261	5.632	2033.75	11454.52	1.467	1714.83	2516.32
A_68_P29032311	chr13:51504011-51504055	NM_010101:46	S1pr3	INSIDE	0.635	0.417	3748.54	1563.60	0.265	2785.80	737.42
A_68_P31932823	chr19:6118207-6118251	NM_133678:358	Sac3d1	INSIDE	0.47	3.525	1080.85	3809.71	1.655	900.03	1489.57
A_68_P29648702	chr14:61792214-61792258	NM_172809:34943	Sacs	INSIDE	0.405	0.628	897.83	564.00	0.254	778.33	197.78
A_68_P24981209	chr7:16973109-16973153	NM_019748:4	Sae1	INSIDE	0.57	4.718	1704.34	8041.02	2.688	1342.46	3608.64
A_68_P31257082	chr17:56724139-56724183	NM_001029979:-154	Safb2	DIVERGENT_PROMOTER	0.443	5.400	1573.14	8495.49	2.391	1356.65	3243.72
A_68_P31257075	chr17:56723467-56723511	NM_001029979:518	Safb2	INSIDE	0.522	0.477	4679.57	2232.96	0.249	3157.61	786.13
A_68_P26044785	chr8:91566664-91566708	NM_021390:1375	Sall1	INSIDE	0.554	6.166	1436.93	8860.52	3.414	1236.93	4222.95
A_68_P26044645	chr8:91549654-91549698	NM_021390:18385	Sall1	DOWNSTREAM	0.493	1.432	2948.71	4223.94	0.706	2292.52	1618.33
A_68_P31868115	chr18:81184119-81184163	NM_178280:-823	Sall3	PROMOTER	0.517	0.512	2867.29	1468.64	0.265	2153.85	570.39
A_68_P31868107	chr18:81182856-81182900	NM_178280:439	Sall3	INSIDE	0.566	5.157	3708.02	19120.81	2.916	3019.58	8806.17
A_68_P31868010	chr18:81171392-81171436	NM_178280:11903	Sall3	INSIDE	0.631	4.796	1236.88	5932.56	3.025	1065.01	3222.15
A_68_P31868114	chr18:81184015-81184059	NM_178280:-719	Sall3	PROMOTER	0.219	2.079	991.64	2061.64	0.455	858.83	390.69
A_68_P31867990	chr18:81169207-81169251	NM_178280:14089	Sall3	INSIDE	0.581	2.418	1949.25	4714.21	1.404	1663.98	2336.29
A_68_P21843418	chr2:168591344-168591388	NM_175303:1335	Sall4	INSIDE	0.26	5.668	1391.82	7889.47	1.473	1195.18	1761.08
A_68_P21911581	chr2:181334008-181334052	NM_172676:-178	Samd10	DIVERGENT_PROMOTER	0.417	0.521	966.50	503.60	0.217	783.67	170.12
A_68_P30224202	chr15:53734012-53734057	NM_177225:-43	Samd12	PROMOTER	0.663	0.413	1206.15	498.54	0.274	976.51	267.51
A_68_P28036074	chr11:94871790-94871834	NM_146025:620	Samd14	INSIDE	0.418	5.804	2468.23	14326.48	2.427	1805.32	4382.41
A_68_P29578866	chr14:47501811-47501855	NM_001037221:-807	Samd4	PROMOTER	0.415	5.047	1814.27	9156.95	2.096	1631.39	3419.32
A_68_P29578876	chr14:47503079-47503123	NM_001037221:461	Samd4	INSIDE	0.461	7.248	1458.44	10571.13	3.343	1202.81	4021.40
A_68_P29578888	chr14:47504466-47504510	NM_001037221:1849	Samd4	INSIDE	0.492	0.368	3060.90	1126.13	0.181	2296.67	415.48
A_68_P25021894	chr7:29221444-29221488	NM_175021:-256	Samd4b	DIVERGENT_PROMOTER	0.576	5.393	2380.23	12836.48	3.105	1883.61	5848.84
A_68_P29454669	chr14:22569927-22569971	NM_026283:196	Samd8	INSIDE	0.628	3.707	1125.54	4172.01	2.329	1055.85	2459.17
A_68_P21775423	chr2:156960457-156960501	NM_001139520:480	Samhd1	INSIDE	0.337	1.534	1491.70	2288.64	0.518	1099.04	568.83
A_68_P30393147	chr15:84022777-84022821	NM_178614:136	Samm50	INSIDE	0.542	0.276	4482.86	1238.45	0.15	3083.17	461.38
A_68_P25891489	chr8:59966195-59966239	NM_021788:441	Sap30	INSIDE	0.625	2.791	1688.06	4710.90	1.743	1439.23	2508.71
A_68_P22412729	chr3:108247976-108248020	NM_001204979:179	Sars	INSIDE	0.635	0.265	1778.91	471.83	0.169	1301.67	219.36
A_68_P23999965	chr5:114221529-114221573	NM_016926:108	Sart3	INSIDE	0.407	6.367	1214.70	7733.49	2.593	1048.84	2719.86
A_68_P26926726	chr10:8605914-8605958	NM_175155:-68	Sash1	PROMOTER	0.06	30.907	2025.15	62591.28	1.85	1771.27	3276.68
A_68_P31236178	chr17:51952223-51952267	NM_001163630:-865	Satb1	PROMOTER	0.121	37.030	2464.00	91242.21	4.497	2287.29	10285.18
A_68_P31236181	chr17:51952553-51952597	NM_001163630:-1195	Satb1	PROMOTER	0.604	3.846	466.83	1795.45	2.325	411.03	955.56
A_68_P20250386	chr1:57027876-57027920	NM_139146:280	Satb2	INSIDE	0.529	0.295	4681.28	1381.97	0.156	3361.58	524.98
A_68_P20250374	chr1:57026684-57026728	NM_139146:1472	Satb2	INSIDE	0.476	0.540	1087.63	586.84	0.257	871.60	224.08

ProbeName	Target position of probe on CpG island microarray	TargetID	GeneSymbol	CpG island Description	Ratio of relative methylation (TiO_2-NP/Vehicle)	Sham group			TiO_2-H group		
						Relative methylation (Cy5/Cy3)	Cy3 signal (Input)	Cy5 signal (MeDIP)	Relative methylation (Cy5/Cy3)	Cy3 signal (Input)	Cy5 signal (MeDIP)
A_68_P28517710	chr12:71088293-71088337	NM_022028:-325	Sav1	PROMOTER	0.432	5.385	1490.63	8027.44	2.326	1417.04	3296.14
A_68_P28517706	chr12:71087715-71087759	NM_022028:253	Sav1	INSIDE	0.662	0.358	1483.04	530.78	0.237	1068.38	253.21
A_68_P24092946	chr5:130731035-130731079	NM_023248:276	Sbds	INSIDE	0.348	0.160	5681.93	906.48	0.055	4031.69	223.53
A_68_P25499633	chr7:133416815-133416859	NM_145587:704	Sbk1	INSIDE	0.65	0.490	1172.88	575.25	0.319	1006.54	320.79
A_68_P24061239	chr5:124875960-124876004	NM_001081203:-59	Sbno1	PROMOTER	0.266	0.545	3322.89	1812.14	0.145	2544.78	368.76
A_68_P24061235	chr5:124875362-124875406	NM_001081203:539	Sbno1	INSIDE	0.455	6.532	1811.23	11831.63	2.97	1560.38	4634.48
A_68_P24061238	chr5:124875857-124875901	NM_001081203:45	Sbno1	INSIDE	0.16	2.549	1258.20	3206.66	0.407	985.26	400.90
A_68_P25090599	chr7:52262741-52262785	NM_001008422:8857	Scaf1	INSIDE	0.369	5.262	2199.20	11573.13	1.941	1407.76	2732.51
A_68_P25090672	chr7:52271527-52271571	NM_001008422:71	Scaf1	INSIDE	0.471	0.406	1217.20	494.01	0.191	906.17	173.40
A_68_P25090668	chr7:52271191-52271235	NM_001008422:407	Scaf1	INSIDE	0.441	0.637	1923.56	1224.64	0.281	1560.92	438.02
A_68_P25090600	chr7:52262845-52262889	NM_001008422:8753	Scaf1	INSIDE	0.482	3.526	1568.51	5531.24	1.699	1118.58	1900.42
A_68_P30463770	chr15:96290889-96290933	NM_028148:364	Scaf11	INSIDE	0.56	3.613	1615.67	5837.36	2.024	1457.51	2949.74
A_68_P30463766	chr15:96290439-96290483	NM_028148:814	Scaf11	INSIDE	0.415	4.095	1628.56	6668.76	1.698	1279.05	2171.51
A_68_P30463773	chr15:96291502-96291546	NM_028148:-250	Scaf11	PROMOTER	0.633	2.728	1397.12	3810.80	1.727	1138.18	1965.83
A_68_P21152636	chr2:39045852-39045896	NM_178778:376	Scai	INSIDE	0.174	28.316	14689.69	415958.70	4.917	15309.73	75281.39
A_68_P21152639	chr2:39046148-39046192	NM_178778:80	Scai	INSIDE	0.361	6.847	1035.03	7087.34	2.474	947.97	2345.23
A_68_P21152635	chr2:39045739-39045783	NM_178778:490	Scai	INSIDE	0.511	0.454	3393.06	1540.16	0.232	2628.93	609.71
A_68_P29233990	chr13:95055582-95055626	NM_029153:-368	Scamp1	PROMOTER	0.346	5.618	3309.45	18592.68	1.946	2466.64	4799.34
A_68_P26542457	chr9:57408653-57408697	NM_022813:-76	Scamp2	DIVERGENT_PROMOTER	0.423	20.366	2500.86	50933.05	8.606	2434.73	20952.46
A_68_P27284238	chr10:80065633-80065677	NM_019575:28	Scamp4	INSIDE	0.324	6.938	2006.11	13919.30	2.245	1691.72	3798.05
A_68_P27284240	chr10:80065867-80065911	NM_019575:262	Scamp4	INSIDE	0.604	0.413	1214.28	502.05	0.25	911.55	227.71
A_68_P26823386	chr9:110283633-110283677	NM_001001144:47858	Scap	INSIDE	0.442	5.002	3934.82	19680.33	2.212	2835.87	6272.53
A_68_P26533458	chr9:55785595-55785639	NM_001081341:306	Scaper	INSIDE	0.286	4.704	6442.04	30301.48	1.347	5520.86	7439.09
A_68_P24065931	chr5:125748080-125748124	NM_001205082:73362	Scarb1	DOWNSTREAM	0.593	0.693	2022.80	1401.07	0.411	1444.99	593.50
A_68_P30575813	chr16:17807076-17807120	NM_153790:9724	Scarf2	INSIDE	0.592	3.898	4992.91	19462.69	2.307	3820.96	8813.61
A_68_P30575781	chr16:17803149-17803193	NM_153790:5796	Scarf2	INSIDE	0.361	4.851	1503.45	7292.82	1.751	1322.66	2316.00
A_68_P30575879	chr16:17816053-17816097	NM_153790:18700	Scarf2	DOWNSTREAM	0.634	0.587	891.91	523.85	0.373	796.48	296.69
A_68_P23803939	chr5:74927453-74927497	NM_001114660:300	Scfd2	INSIDE	0.552	0.546	1058.08	578.12	0.302	844.52	254.83
A_68_P22215798	chr3:68299063-68299107	NR_036665:970	Schip1	INSIDE	0.191	19.854	1849.51	36720.29	3.794	1466.42	5563.98
A_68_P28376737	chr12:40860793-40860837	NM_001146196:1	Scin	INSIDE	0.311	31.667	1583.71	50152.05	9.835	1313.46	12917.85
A_68_P28376736	chr12:40860722-40860766	NM_001146196:71	Scin	INSIDE	0.553	0.514	2437.62	1251.97	0.284	1859.84	528.38
A_68_P22086571	chr3:41546266-41546310	NM_001081411:148	Sclt1	INSIDE	0.651	3.960	470.62	1863.60	2.576	412.05	1061.46
A_68_P20441527	chr1:93194765-93194809	NM_016717:-128	Scly	PROMOTER	0.611	6.828	3561.39	24318.47	4.169	2840.17	11840.30
A_68_P25035179	chr7:31911519-31911563	NM_011322:424	Scn1b	INSIDE	0.322	0.610	3044.38	1857.24	0.196	2291.90	450.00
A_68_P26471636	chr9:44946722-44946766	NM_001013390:-380	Scn4b	DIVERGENT_PROMOTER	0.573	0.674	1524.09	1026.85	0.386	1206.43	465.82
A_68_P26873354	chr9:119487614-119487658	NM_021544:498	Scn5a	INSIDE	0.438	0.447	1057.80	473.28	0.196	883.75	173.10
A_68_P26873361	chr9:119488531-119488575	NM_021544:-418	Scn5a	PROMOTER	0.466	0.501	1047.98	524.93	0.234	854.30	199.54
A_68_P26872698	chr9:119394966-119395010	NM_021544:93146	Scn5a	INSIDE	0.574	3.233	900.07	2909.77	1.854	785.18	1455.93
A_68_P26872699	chr9:119395083-119395128	NM_021544:93029	Scn5a	INSIDE	0.051	31.146	1336.36	41621.74	1.601	939.86	1504.95
A_68_P26873357	chr9:119487930-119487974	NM_021544:182	Scn5a	INSIDE	0.525	1.373	5392.46	7402.09	0.721	3971.19	2862.30
A_68_P30489328	chr15:100884313-100884357	NM_011323:117633	Scn8a	DOWNSTREAM	0.514	4.470	1247.33	5575.41	2.296	1132.87	2601.52
A_68_P30489331	chr15:100884729-100884773	NM_011323:118049	Scn8a	DOWNSTREAM	0.231	1.592	2429.59	3868.69	0.367	1721.51	631.95
A_68_P30489220	chr15:100870918-100870964	NM_011323:104239	Scn8a	INSIDE	0.133	13.682	1065.07	14572.30	1.826	681.72	1244.61
A_68_P26015153	chr8:85966434-85966478	NM_019708:91	Scoc	INSIDE	0.434	0.561	1124.21	630.30	0.243	862.25	209.73
A_68_P28003086	chr11:88816762-88816806	NM_029023:-28	Scpep1	PROMOTER	0.226	1.353	1975.12	2672.77	0.306	1469.37	450.10
A_68_P30345853	chr15:75900100-75900144	NM_134089:38	Scrib	INSIDE	0.361	13.356	4297.18	57393.92	4.819	3727.65	17964.15

ProbeName	Target position of probe on CpG island microarray	TargetID	GeneSymbol	CpG island Description	Ratio of relative methylation (TiO_2-NP/Vehicle)	Sham group			TiO_2-H group		
						Relative methylation (Cy5/Cy3)	Cy3 signal (Input)	Cy5 signal (MeDIP)	Relative methylation (Cy5/Cy3)	Cy3 signal (Input)	Cy5 signal (MeDIP)
A_68_P24464123	chr6:54516358-54516402	NM_027268:-4	Scrn1	PROMOTER	0.563	2.581	1684.14	4347.55	1.452	1424.54	2068.80
A_68_P30348909	chr15:76354254-76354298	NM_130893:-1717	Scrt1	PROMOTER	0.254	8.486	910.26	7724.37	2.152	716.37	1541.71
A_68_P30348857	chr15:76348429-76348487	NM_130893:4101	Scrt1	INSIDE	0.293	0.449	2097.85	941.41	0.131	1329.20	174.48
A_68_P30348853	chr15:76348038-76348082	NM_130893:4499	Scrt1	INSIDE	0.593	0.449	1563.83	702.50	0.266	1221.92	325.52
A_68_P21747518	chr2:151908021-151908065	NM_001160410:778	Scrt2	INSIDE	0.208	21.962	5536.29	121590.10	4.568	5287.55	24154.67
A_68_P21747609	chr2:151919690-151919734	NM_001160410:12448	Scrt2	INSIDE	0.448	12.565	1162.98	14613.23	5.631	787.97	4436.92
A_68_P21747605	chr2:151919256-151919300	NM_001160410:12014	Scrt2	INSIDE	0.569	3.117	1608.91	5014.20	1.772	1389.89	2463.10
A_68_P21747603	chr2:151918965-151919009	NM_001160410:11722	Scrt2	INSIDE	0.578	3.163	920.63	2912.03	1.828	692.48	1265.56
A_68_P21747621	chr2:151920967-151921011	NM_001160410:13724	Scrt2	INSIDE	0.364	1.686	1745.11	2942.59	0.614	1362.72	836.82
A_68_P30390278	chr15:83555204-83555248	NM_022723:225	Scube1	INSIDE	0.285	13.489	2075.41	27994.71	3.844	1790.71	6883.39
A_68_P30390280	chr15:83555410-83555454	NM_022723:19	Scube1	INSIDE	0.526	4.330	822.95	3563.69	2.276	735.27	1673.79
A_68_P30390276	chr15:83554904-83554948	NM_022723:525	Scube1	INSIDE	0.611	0.524	1253.23	656.66	0.32	986.81	315.82
A_68_P30390277	chr15:83555041-83555085	NM_022723:389	Scube1	INSIDE	0.326	5.324	1704.57	9075.48	1.736	1345.64	2336.56
A_68_P31120665	chr17:28278842-28278886	NM_001004366:-606	Scube3	PROMOTER	0.397	7.659	1487.84	11394.73	3.043	1113.05	3386.68
A_68_P28224331	chr12:8779590-8779634	NM_011519:1411	Sdc1	INSIDE	0.643	0.338	3446.53	1166.25	0.218	2546.27	553.94
A_68_P28224314	chr12:8777965-8778009	NM_011519:-215	Sdc1	PROMOTER	0.648	0.321	2549.24	817.65	0.208	1886.28	392.34
A_68_P28224332	chr12:8779734-8779778	NM_011519:1555	Sdc1	INSIDE	0.523	0.678	1060.90	719.47	0.355	798.88	283.39
A_68_P22705223	chr4:6292852-6292896	NM_001098227:48	Sdcbp	INSIDE	0.622	4.126	4357.31	17976.09	2.564	3521.59	9029.89
A_68_P30572147	chr16:17132218-17132262	NM_022324:236	Sdf2l1	INSIDE	0.236	0.255	4322.78	1101.57	0.06	2781.54	166.93
A_68_P30572142	chr16:17131635-17131679	NM_022324:820	Sdf2l1	INSIDE	0.431	4.518	2159.02	9754.10	1.948	1747.09	3403.15
A_68_P30572146	chr16:17132077-17132121	NM_022324:378	Sdf2l1	INSIDE	0.515	0.551	1613.72	889.50	0.284	1245.03	353.48
A_68_P30572145	chr16:17131918-17131970	NM_022324:532	Sdf2l1	INSIDE	0.501	0.686	1190.30	816.77	0.344	865.91	297.79
A_68_P30572148	chr16:17132335-17132379	NM_022324:120	Sdf2l1	INSIDE	0.296	4.912	10225.57	50227.23	1.452	8694.48	12625.17
A_68_P23441760	chr4:155367175-155367219	NM_011341:174	Sdf4	INSIDE	0.661	0.155	3357.46	519.15	0.102	2443.54	249.87
A_68_P29145778	chr13:74487536-74487580	NM_023281:130	Sdha	INSIDE	0.487	0.319	1500.22	479.11	0.155	1130.10	175.67
A_68_P28163122	chr11:116976258-116976302	NR_029459:-205	Sec14l1	PROMOTER	0.439	0.178	2815.12	501.45	0.078	2186.67	171.08
A_68_P21078768	chr2:26301082-26301126	NM_153125:-368	Sec16a	PROMOTER	0.225	20.041	8245.31	165243.40	4.505	7995.13	36017.64
A_68_P21078766	chr2:26300840-26300884	NM_153125:-126	Sec16a	PROMOTER	0.394	0.673	2954.36	1987.24	0.265	2275.49	603.46
A_68_P21078762	chr2:26300450-26300494	NM_153125:264	Sec16a	INSIDE	0.488	0.482	1032.41	497.33	0.235	739.01	173.67
A_68_P21078770	chr2:26301305-26301349	NM_153125:-590	Sec16a	PROMOTER	0.238	8.103	742.65	6017.72	1.93	635.99	1227.49
A_68_P30669634	chr16:35363730-35363774	NM_133704:252	Sec22a	INSIDE	0.385	6.448	1680.87	10837.47	2.482	1453.31	3606.69
A_68_P30669636	chr16:35364019-35364063	NM_133704:-36	Sec22a	PROMOTER	0.433	0.389	1197.07	466.22	0.169	1004.19	169.40
A_68_P26885027	chr9:121614483-121614527	NM_001164562:-357	Sec22c	DIVERGENT_PROMOTER	0.467	0.529	935.79	494.67	0.247	710.71	175.59
A_68_P28469855	chr12:60112957-60113001	NM_009147:26	Sec23a	INSIDE	0.532	4.176	785.48	3280.01	2.223	882.81	1962.18
A_68_P29448259	chr14:21493732-21493776	NM_001168273:212	Sec24c	INSIDE	0.597	0.315	2794.17	881.20	0.188	2128.45	400.71
A_68_P27611243	chr11:16407800-16407844	NM_001109971:665	Sec61g	INSIDE	0.51	0.501	1530.09	766.02	0.256	1291.52	330.04
A_68_P22030179	chr3:30692442-30692486	NM_027016:667	Sec62	INSIDE	0.469	0.233	2861.68	668.12	0.11	2311.37	253.13
A_68_P27096532	chr10:42482055-42482099	NM_153055:775	Sec63	INSIDE	0.054	2.663	9901.14	26371.04	0.145	6332.66	916.70
A_68_P27096531	chr10:42481959-42482003	NM_153055:679	Sec63	INSIDE	0.275	2.170	2606.39	5656.42	0.597	1535.20	916.93
A_68_P29033511	chr13:51746883-51746927	NM_029279:-178	Secisbp2	PROMOTER	0.432	4.729	3348.80	15837.10	2.043	2633.05	5380.04
A_68_P23699207	chr5:53604122-53604166	NM_172710:547	Sel1l3	INSIDE	0.48	0.283	1754.23	496.43	0.136	1298.38	176.36
A_68_P27552432	chr11:3417267-3417311	NM_053267:2584	Selm	INSIDE	0.396	6.685	679.35	4541.59	2.649	590.05	1562.97
A_68_P26809841	chr9:107501260-107501304	NM_009153:6451	Sema3b	INSIDE	0.335	4.633	1352.97	6268.20	1.554	879.63	1367.02
A_68_P23491409	chr5:14025282-14025326	NM_011348:29	Sema3e	INSIDE	0.146	10.068	2540.45	25576.63	1.466	2012.11	2950.24
A_68_P25265269	chr7:87343641-87343685	NM_013659:11936	Sema4b	INSIDE	0.564	2.895	2542.83	7360.25	1.633	2022.68	3302.93
A_68_P20149051	chr1:36614518-36614562	NM_001126047:686	Sema4c	INSIDE	0.366	0.479	1438.95	689.70	0.175	1110.06	194.80

ProbeName	Target position of probe on CpG island microarray	TargetID	GeneSymbol	CpG island Description	Ratio of relative methylation (TiO_2-NP/Vehicle)	Sham group			TiO_2-H group		
						Relative methylation (Cy5/Cy3)	Cy3 signal (Input)	Cy5 signal (MeDIP)	Relative methylation (Cy5/Cy3)	Cy3 signal (Input)	Cy5 signal (MeDIP)
A_68_P20149055	chr1:36615091-36615135	NM_001126047:114	Sema4c	INSIDE	0.414	6.213	1341.44	8335.01	2.572	1098.81	2825.84
A_68_P20149047	chr1:36614040-36614084	NM_001126047:1164	Sema4c	INSIDE	0.467	0.698	1022.22	713.22	0.326	784.75	255.83
A_68_P24590571	chr6:82889627-82889671	NM_011350:96	Sema4f	INSIDE	0.365	10.139	1543.55	15650.78	3.698	1375.80	5088.03
A_68_P32135694	chr19:45063945-45063989	NM_011976:133	Sema4g	INSIDE	0.138	18.588	1178.76	21910.65	2.573	834.88	2148.04
A_68_P32135692	chr19:45063608-45063652	NM_011976:-203	Sema4g	PROMOTER	0.581	0.365	3922.89	1432.24	0.212	2719.81	577.18
A_68_P30670707	chr16:35542109-35542153	NM_013661:683	Sema5b	INSIDE	0.136	0.515	6535.40	3367.83	0.07	4524.58	317.55
A_68_P30670710	chr16:35542481-35542525	NM_013661:1055	Sema5b	INSIDE	0.484	0.496	1190.61	590.54	0.24	977.90	234.98
A_68_P31682229	chr18:47526349-47526393	NM_018744:2152	Sema6a	INSIDE	0.532	0.655	1774.42	1163.13	0.349	1435.14	500.24
A_68_P31254209	chr17:56288172-56288216	NM_001130456:-8428	Sema6b	PROMOTER	0.264	0.219	4161.65	913.20	0.058	2970.56	171.88
A_68_P31254208	chr17:56288089-56288133	NM_001130456:-8344	Sema6b	PROMOTER	0.269	1.753	6190.51	10852.68	0.472	3905.85	1843.60
A_68_P31254018	chr17:56263505-56263549	NM_013662:9749	Sema6b	INSIDE	0.457	6.582	2669.68	17570.65	3.011	2173.74	6544.45
A_68_P31254024	chr17:56264131-56264175	NM_013662:9123	Sema6b	INSIDE	0.564	3.770	2677.39	10092.73	2.126	2135.22	4539.08
A_68_P31254020	chr17:56263726-56263770	NM_013662:9527	Sema6b	INSIDE	0.595	7.975	3837.91	30607.46	4.743	3089.12	14652.10
A_68_P31254210	chr17:56288310-56288354	NM_001130456:-8566	Sema6b	PROMOTER	0.197	2.125	1680.54	3570.47	0.418	1244.87	520.18
A_68_P22342541	chr3:94965423-94965467	NM_011351:-2851	Sema6c	PROMOTER	0.185	11.963	1753.45	20975.77	2.21	1499.13	3313.31
A_68_P22342633	chr3:94977194-94977238	NM_011351:8921	Sema6c	INSIDE	0.466	4.925	1927.06	9491.62	2.294	1705.08	3912.06
A_68_P22342539	chr3:94965148-94965192	NM_011351:-3125	Sema6c	PROMOTER	0.626	0.440	2816.48	1239.50	0.275	2059.45	567.24
A_68_P30595531	chr16:22009697-22009741	NM_029457:162	Senp2	INSIDE	0.149	0.532	4186.72	2228.25	0.079	2397.66	190.21
A_68_P30779064	chr16:56075886-56075930	NM_001003972:387	Senp7	INSIDE	0.373	13.475	2124.98	28634.42	5.029	2047.57	10297.22
A_68_P26555222	chr9:59598670-59598714	NM_001172068:-236	Senp8	DIVERGENT_PROMOTER	0.428	6.875	1681.01	11556.61	2.942	1406.47	4138.16
A_68_P25504895	chr7:134417127-134417171	NM_009266:425	Sephs2	INSIDE	0.315	6.346	1478.26	9381.36	2.002	1457.50	2918.21
A_68_P27167594	chr10:58684574-58684618	NM_001024910:-1	Septin10	DIVERGENT_PROMOTER	0.435	13.067	4317.45	56416.18	5.685	3863.85	21966.69
A_68_P27167591	chr10:58684147-58684191	NM_001024910:427	Septin10	INSIDE	0.504	0.249	2008.68	499.40	0.125	1369.75	171.55
A_68_P27167593	chr10:58684415-58684459	NM_001024910:159	Septin10	INSIDE	0.645	3.677	587.10	2158.75	2.371	411.95	976.58
A_68_P23897742	chr5:93522042-93522086	NM_001009818:-418	Septin11	PROMOTER	0.495	0.671	1656.00	1110.92	0.332	1335.10	442.91
A_68_P23897752	chr5:93523254-93523298	NM_001009818:794	Septin11	INSIDE	0.469	0.603	767.13	462.61	0.283	655.77	185.57
A_68_P27994864	chr11:87392137-87392181	NM_011129:-2474	Septin4	PROMOTER	0.643	0.723	1534.82	1109.96	0.465	1265.72	588.28
A_68_P26365997	chr9:25060616-25060660	NM_001205367:599	Septin7	INSIDE	0.329	6.278	2054.76	12898.97	2.068	1703.00	3521.15
A_68_P26365989	chr9:25059719-25059763	NM_001205367:-299	Septin7	PROMOTER	0.592	6.017	4214.92	25359.73	3.56	3400.39	12106.25
A_68_P28164254	chr11:117151837-117151881	NM_001113487:24299	Septin9	INSIDE	0.263	15.463	3624.64	56046.70	4.066	3858.10	15686.75
A_68_P28163660	chr11:117060956-117061000	NM_001113486:4	Septin9	INSIDE	0.409	7.035	1843.89	12971.58	2.875	1521.64	4375.22
A_68_P31020947	chr17:6079672-6079716	NM_001111017:45	Serac1	INSIDE	0.479	5.172	2613.79	13518.49	2.476	2219.69	5495.05
A_68_P24520694	chr6:67217359-67217403	NM_001113564:408	Serbp1	INSIDE	0.645	3.310	612.14	2026.20	2.135	561.04	1197.95
A_68_P21580978	chr2:121275537-121275581	NM_011354:595	Serf2	INSIDE	0.275	7.980	3373.92	26923.05	2.192	2886.88	6328.70
A_68_P21580976	chr2:121275325-121275369	NM_011354:383	Serf2	INSIDE	0.643	4.699	1353.46	6359.38	3.022	1157.58	3498.15
A_68_P28936013	chr13:34094245-34094289	NR_028309:352	Serpinb6a	INSIDE	0.601	3.232	1357.63	4387.50	1.943	1111.67	2159.99
A_68_P25017511	chr7:28271855-28271899	NM_018820:-95	Sertad1	PROMOTER	0.475	6.413	3626.00	23254.60	3.049	2819.18	8594.37
A_68_P27632486	chr11:20531998-20532042	NM_001038625:41	Sertad2	INSIDE	0.311	0.520	1333.81	693.09	0.162	1070.91	173.23
A_68_P27631990	chr11:20443463-20443507	NM_021372:229	Sertad2	INSIDE	0.528	0.592	2126.85	1258.60	0.312	1637.20	511.22
A_68_P27631993	chr11:20443834-20443878	NM_021372:601	Sertad2	INSIDE	0.553	16.862	4797.16	80888.09	9.332	10810.09	100882.60
A_68_P27632484	chr11:20531833-20531877	NM_001038625:-125	Sertad2	PROMOTER	0.605	5.213	5956.20	31047.82	3.154	4585.13	14460.13
A_68_P27631991	chr11:20443663-20443707	NM_021372:429	Sertad2	INSIDE	0.62	3.967	1030.68	4089.01	2.458	875.72	2152.93
A_68_P27632488	chr11:20532242-20532286	NM_001038625:285	Sertad2	INSIDE	0.545	0.562	1036.84	582.35	0.306	880.08	269.26
A_68_P27631992	chr11:20443745-20443789	NM_021372:511	Sertad2	INSIDE	0.648	0.676	995.72	672.69	0.438	757.99	331.66
A_68_P25017446	chr7:28258643-28258687	NM_133210:-194	Sertad3	PROMOTER	0.5	0.273	2182.54	594.87	0.136	1616.26	220.30
A_68_P20945779	chr1:194661281-194661325	NM_001177794:16639	Sertad4	DOWNSTREAM	0.449	0.525	1801.81	946.80	0.236	1036.70	244.65

ProbeName	Target position of probe on CpG island microarray	TargetID	GeneSymbol	CpG island Description	Ratio of relative methylation (TiO_2-NP/Vehicle)	Sham group			TiO_2-H group		
						Relative methylation (Cy5/Cy3)	Cy3 signal (Input)	Cy5 signal (MeDIP)	Relative methylation (Cy5/Cy3)	Cy3 signal (Input)	Cy5 signal (MeDIP)
A_68_P20945912	chr1:194681174-194681218	NM_001177794:-3255	Sertad4	PROMOTER	0.564	5.529	555.36	3070.73	3.117	587.87	1832.48
A_68_P27091644	chr10:41607176-41607220	NM_001013370:-46	Sesn1	PROMOTER	0.2	30.415	2642.04	80357.11	6.098	2450.35	14941.85
A_68_P27091229	chr10:41530226-41530270	NM_001162908:-131	Sesn1	DIVERGENT_PROMOTER	0.377	13.232	3212.91	42512.76	4.983	2825.88	14080.45
A_68_P27091227	chr10:41530014-41530058	NM_001162908:-343	Sesn1	DIVERGENT_PROMOTER	0.651	0.503	1239.08	623.18	0.327	925.01	302.89
A_68_P21101134	chr2:29922658-29922702	NM_001204875:687	Set	INSIDE	0.333	0.709	1763.03	1250.49	0.236	1454.45	343.75
A_68_P21101133	chr2:29922340-29922384	NM_001204875:369	Set	INSIDE	0.567	0.683	966.14	659.94	0.387	776.54	300.71
A_68_P31857881	chr18:79305640-79305684	NM_053099:468	Setbp1	INSIDE	0.65	0.451	5517.63	2487.88	0.293	3734.81	1093.80
A_68_P25507242	chr7:134920047-134920091	NM_178029:-834	Setd1a	PROMOTER	0.226	19.436	493.61	9593.67	4.385	414.23	1816.36
A_68_P25507247	chr7:134920536-134920580	NM_178029:-344	Setd1a	PROMOTER	0.599	0.148	3606.11	532.60	0.089	2611.17	231.20
A_68_P26824202	chr9:110434375-110434420	NM_001081340:-703	Setd2	PROMOTER	0.516	4.760	294.29	1400.70	2.458	305.21	750.17
A_68_P28726321	chr12:109416856-109416900	NM_028262:616	Setd3	INSIDE	0.252	10.574	1245.92	13174.61	2.659	1002.01	2664.78
A_68_P28726325	chr12:109417342-109417386	NM_028262:130	Setd3	INSIDE	0.406	8.599	1175.09	10105.07	3.492	1189.12	4152.12
A_68_P28726327	chr12:109417619-109417664	NM_028262:-147	Setd3	DIVERGENT_PROMOTER	0.52	8.841	1960.12	17329.44	4.601	1548.66	7126.06
A_68_P28726319	chr12:109416700-109416744	NM_028262:772	Setd3	INSIDE	0.609	8.701	892.91	7768.85	5.299	712.29	3774.26
A_68_P30976720	chr16:93603886-93603930	NM_145482:152	Setd4	INSIDE	0.21	16.338	4238.43	69249.46	3.431	3898.83	13375.52
A_68_P24757359	chr6:113027455-113027499	NM_028385:-156	Setd5	DIVERGENT_PROMOTER	0.564	5.716	2786.01	15924.61	3.225	2410.92	7776.20
A_68_P26084861	chr8:98239992-98240036	NM_001035123:202	Setd6	INSIDE	0.253	7.049	3379.22	23821.73	1.786	2810.53	5018.48
A_68_P22130348	chr3:51364282-51364326	NM_080793:441	Setd7	INSIDE	0.285	15.363	2357.60	36219.01	4.374	2346.63	10263.83
A_68_P22130354	chr3:51364924-51364968	NM_080793:-201	Setd7	PROMOTER	0.49	0.578	792.60	458.17	0.283	726.00	205.60
A_68_P24061285	chr5:124889815-124889859	NM_030241:-102	Setd8	PROMOTER	0.111	59.861	859.03	51422.41	6.661	600.93	4003.06
A_68_P22343684	chr3:95161081-95161125	NM_001163641:22	Setdb1	INSIDE	0.615	2.674	1231.55	3293.21	1.644	946.69	1556.43
A_68_P21095252	chr2:28980951-28980995	NM_198033:461	Setx	INSIDE	0.345	5.843	2493.43	14569.91	2.015	2406.84	4849.73
A_68_P21095250	chr2:28980805-28980849	NM_198033:315	Setx	INSIDE	0.128	12.604	1437.09	18112.98	1.61	1340.66	2158.30
A_68_P23993057	chr5:113006567-113006611	NM_019982:-383	Sez6l	PROMOTER	0.531	0.497	1047.96	520.79	0.264	693.28	182.92
A_68_P31934224	chr19:6364358-6364402	NM_001110791:691	Sf1	INSIDE	0.357	44.030	1416.05	62348.05	15.736	1278.26	20115.07
A_68_P27556014	chr11:4060531-4060575	NM_026175:196	Sf3a1	INSIDE	0.427	3.449	2797.13	9646.12	1.474	2096.81	3090.03
A_68_P23269838	chr4:124386319-124386363	NM_029157:-5764	Sf3a3	PROMOTER	0.596	2.889	1068.46	3086.42	1.722	862.82	1485.84
A_68_P31927885	chr19:5295364-5295408	NM_030109:69	Sf3b2	INSIDE	0.646	2.931	901.21	2641.50	1.893	655.96	1241.73
A_68_P22347940	chr3:95976314-95976359	NM_153053:-136	Sf3b4	PROMOTER	0.358	0.580	1181.55	685.50	0.207	950.02	197.08
A_68_P22347939	chr3:95976249-95976293	NM_153053:-202	Sf3b4	PROMOTER	0.445	0.633	1249.94	790.95	0.281	956.12	268.96
A_68_P26948616	chr10:12728440-12728484	NM_175102:207	Sf3b5	INSIDE	0.289	0.333	2534.71	844.38	0.096	1788.72	172.04
A_68_P29507580	chr14:31529106-31529150	NM_001166532:328	Sfmbt1	INSIDE	0.413	9.873	1094.16	10802.99	4.077	925.62	3773.93
A_68_P29507582	chr14:31529438-31529482	NM_001166532:660	Sfmbt1	INSIDE	0.552	2.969	2544.96	7556.09	1.639	2204.48	3614.20
A_68_P21000515	chr2:10296432-10296476	NM_001198809:2392	Sfmbt2	INSIDE	0.514	0.413	1281.14	529.68	0.213	1127.09	239.74
A_68_P21000493	chr2:10294275-10294319	NM_001198809:234	Sfmbt2	INSIDE	0.362	3.939	1570.79	6186.73	1.427	1379.69	1969.01
A_68_P23318415	chr4:133155946-133155990	NM_018754:2115	Sfn	DOWNSTREAM	0.418	5.386	2660.32	14328.67	2.249	2093.60	4708.21
A_68_P23318413	chr4:133155649-133155693	NM_018754:2413	Sfn	DOWNSTREAM	0.414	3.647	1311.73	4784.25	1.51	1007.83	1521.82
A_68_P23283136	chr4:126699461-126699505	NM_023603:938	Sfpq	INSIDE	0.055	2.786	1345.22	3747.53	0.153	1100.24	168.04
A_68_P23283133	chr4:126698748-126698792	NM_023603:226	Sfpq	INSIDE	0.511	5.996	1835.88	11008.32	3.066	1541.13	4724.61
A_68_P23283135	chr4:126699373-126699417	NM_023603:850	Sfpq	INSIDE	0.629	10.723	6779.66	72700.71	6.747	4945.00	33364.39
A_68_P25716360	chr8:24521956-24522000	NM_013834:5	Sfrp1	INSIDE	0.424	22.978	1992.42	45780.78	9.737	2013.55	19606.23
A_68_P22286847	chr3:83570542-83570586	NM_009144:322	Sfrp2	INSIDE	0.53	0.389	1508.89	587.27	0.206	1042.36	215.10
A_68_P22286851	chr3:83570878-83570922	NM_009144:658	Sfrp2	INSIDE	0.617	0.366	1328.72	485.86	0.225	1055.29	237.95
A_68_P32119644	chr19:42276497-42276541	NM_018780:224	Sfrp5	INSIDE	0.65	2.689	1919.50	5162.35	1.748	1653.86	2891.29
A_68_P32119640	chr19:42276046-42276090	NM_018780:674	Sfrp5	INSIDE	0.248	0.710	1095.15	777.46	0.176	992.07	174.76
A_68_P20793343	chr1:167124694-167124739	NM_145512:-152	Sft2d2	PROMOTER	0.547	3.824	975.07	3729.10	2.094	811.01	1698.00

ProbeName	Target position of probe on CpG island microarray	TargetID	GeneSymbol	CpG island Description	Ratio of relative methylation (TiO_2-NP/Vehicle)	Sham group			TiO_2-H group		
						Relative methylation (Cy5/Cy3)	Cy3 signal (Input)	Cy5 signal (MeDIP)	Relative methylation (Cy5/Cy3)	Cy3 signal (Input)	Cy5 signal (MeDIP)
A_68_P31600224	chr18:32070540-32070584	NM_026006:995	Sft2d3	INSIDE	0.495	11.043	2686.51	29666.39	5.463	2166.95	11837.24
A_68_P29047306	chr13:54167631-54167675	NM_027324:439	Sfxn1	INSIDE	0.613	0.321	1832.84	589.06	0.197	1499.66	295.42
A_68_P32136240	chr19:45139857-45139901	NM_001178012:17813	Sfxn3	DOWNSTREAM	0.326	5.682	1740.28	9888.72	1.855	1607.12	2980.42
A_68_P24604451	chr6:85283005-85283049	NM_178639:390	Sfxn5	INSIDE	0.363	1.686	1658.97	2797.55	0.611	1185.99	725.21
A_68_P25797392	chr8:39723329-39723373	NM_145841:1212	Sgcz	INSIDE	0.441	73.024	1406.20	102686.50	32.184	2001.79	64424.65
A_68_P26996379	chr10:21714219-21714263	NM_011361:-231	Sgk1	PROMOTER	0.336	1.499	2001.99	3001.10	0.503	1708.17	860.05
A_68_P20024136	chr1:9788012-9788056	NM_133220:-176	Sgk3	PROMOTER	0.5	6.334	1031.83	6535.47	3.164	1133.83	3587.67
A_68_P32070015	chr19:32462327-32462371	NM_144792:596	Sgms1	INSIDE	0.409	1.506	3440.03	5181.96	0.616	2622.05	1616.43
A_68_P31244849	chr17:53828675-53828719	NM_028232:-56	Sgol1	PROMOTER	0.486	4.297	2110.37	9069.26	2.09	1787.18	3735.90
A_68_P31244848	chr17:53828528-53828572	NM_028232:90	Sgol1	INSIDE	0.335	0.592	1348.66	798.22	0.198	1103.45	218.88
A_68_P28550068	chr12:76836769-76836813	NM_030750:-74	Sgpp1	PROMOTER	0.357	0.645	1614.73	1042.21	0.23	1034.79	238.46
A_68_P20370950	chr1:78306691-78306735	NM_001004173:-208	Sgpp2	PROMOTER	0.399	0.225	2698.76	606.08	0.09	2029.14	181.69
A_68_P23997432	chr5:113739494-113739538	NM_172718:290	Sgsm1	INSIDE	0.389	7.640	3609.71	27577.42	2.976	3233.51	9621.84
A_68_P23997435	chr5:113739797-113739841	NM_172718:-12	Sgsm1	PROMOTER	0.404	0.590	1257.06	741.36	0.238	1025.37	244.17
A_68_P27923093	chr11:74710538-74710582	NM_197943:22	Sgsm2	INSIDE	0.523	3.838	2014.72	7732.11	2.008	1808.41	3631.27
A_68_P29288687	chr13:104899925-104899970	NM_144838:78	Sgtb	INSIDE	0.623	0.459	3803.84	1747.20	0.286	2921.93	836.11
A_68_P24120143	chr5:136720679-136720723	NM_018825:73	Sh2b2	INSIDE	0.629	2.912	1531.74	4460.46	1.831	1303.73	2387.58
A_68_P24046791	chr5:122286329-122286373	NM_008507:460	Sh2b3	INSIDE	0.264	0.466	1726.12	804.70	0.123	1351.57	166.39
A_68_P24046730	chr5:122278610-122278654	NM_008507:8178	Sh2b3	INSIDE	0.444	6.699	937.07	6277.09	2.975	731.32	2175.72
A_68_P21117161	chr2:32597158-32597202	NM_013781:20589	Sh2d3c	INSIDE	0.422	6.622	1984.58	13142.66	2.795	1611.98	4506.04
A_68_P23344730	chr4:137806358-137806402	NM_001099631:55	Sh2d5	INSIDE	0.597	0.259	2531.11	655.76	0.155	1815.77	280.87
A_68_P26684174	chr9:83442213-83442257	NM_172507:290	Sh3bgrl2	INSIDE	0.187	9.065	1760.84	15961.54	1.697	1491.47	2531.67
A_68_P23594807	chr5:34868707-34868751	NM_011893:296	Sh3bp2	INSIDE	0.15	1.851	3976.36	7359.05	0.277	2749.22	761.55
A_68_P23594805	chr5:34868409-34868453	NM_011893:-2	Sh3bp2	PROMOTER	0.512	52.477	3688.71	193572.60	26.884	4262.09	114580.20
A_68_P27830412	chr11:58144420-58144464	NM_001161338:223	Sh3bp5l	INSIDE	0.635	2.439	1305.05	3183.30	1.548	1062.66	1645.17
A_68_P31253474	chr17:56175980-56176024	NM_013664:-3	Sh3gl1	DIVERGENT_PROMOTER	0.131	31.459	7444.54	234194.90	4.124	7326.39	30212.16
A_68_P32150072	chr19:47538682-47538726	NM_001164717:197	Sh3pxd2a	INSIDE	0.612	0.645	1760.40	1135.58	0.395	1340.64	529.18
A_68_P32149065	chr19:47388402-47388446	NM_001164717:150477	Sh3pxd2a	INSIDE	0.623	3.537	400.11	1415.08	2.204	324.69	715.73
A_68_P27688273	chr11:32248073-32248117	NM_177364:284	Sh3pxd2b	INSIDE	0.539	0.671	5260.44	3527.37	0.362	4176.44	1510.54
A_68_P25908468	chr8:63703117-63703161	NM_021506:171	Sh3rf1	INSIDE	0.568	3.392	2336.59	7926.42	1.927	2188.74	4216.89
A_68_P27165498	chr10:58275060-58275104	NM_172788:-1024	Sh3rf3	PROMOTER	0.457	4.578	8141.16	37273.25	2.094	5777.23	12096.18
A_68_P27165512	chr10:58276626-58276670	NM_172788:542	Sh3rf3	INSIDE	0.618	4.377	979.13	4285.71	2.704	964.58	2608.64
A_68_P27165497	chr10:58274956-58275000	NM_172788:-1128	Sh3rf3	PROMOTER	0.537	0.457	1074.77	491.12	0.245	814.59	199.89
A_68_P28328449	chr12:31596572-31596616	NM_013709:61	Sh3yl1	INSIDE	0.616	0.466	1228.16	572.73	0.287	940.82	270.05
A_68_P25086960	chr7:51607818-51607862	NM_001034115:42207	Shank1	INSIDE	0.279	10.961	6501.73	71268.62	3.055	6680.07	20408.74
A_68_P25086855	chr7:51591550-51591594	NM_001034115:25939	Shank1	INSIDE	0.397	8.565	2601.89	22284.29	3.403	2049.50	6973.43
A_68_P25086970	chr7:51608696-51608740	NM_001034115:43085	Shank1	INSIDE	0.488	0.447	1316.34	588.72	0.218	935.29	204.30
A_68_P25086973	chr7:51609094-51609138	NM_001034115:43483	Shank1	INSIDE	0.307	1.627	3158.44	5139.86	0.499	2098.70	1047.18
A_68_P25086993	chr7:51612401-51612445	NM_001034115:46789	Shank1	INSIDE	0.589	0.602	2803.45	1687.35	0.355	1991.09	705.97
A_68_P30425837	chr15:89378103-89378147	NM_021423:47837	Shank3	INSIDE	0.248	1.759	3752.30	6599.53	0.437	2812.98	1229.05
A_68_P30425531	chr15:89329734-89329778	NM_021423:-531	Shank3	PROMOTER	0.341	0.433	2392.38	1036.55	0.148	1708.70	252.46
A_68_P30425530	chr15:89329634-89329678	NM_021423:-631	Shank3	PROMOTER	0.354	0.389	1916.37	745.78	0.138	1272.46	175.29
A_68_P30425529	chr15:89329556-89329600	NM_021423:-709	Shank3	PROMOTER	0.39	5.027	1658.38	8336.83	1.961	1526.83	2994.82
A_68_P30425839	chr15:89378376-89378420	NM_021423:48111	Shank3	INSIDE	0.264	1.360	2837.83	3860.63	0.359	1739.34	625.18
A_68_P30425916	chr15:89388272-89388316	NM_021423:58007	Shank3	INSIDE	0.302	5.886	1077.18	6339.77	1.78	894.50	1591.79
A_68_P22883653	chr4:45543529-45543573	NM_001033306:150	Shb	INSIDE	0.292	9.867	1094.08	10795.81	2.882	781.32	2251.62

ProbeName	Target position of probe on CpG island microarray	TargetID	GeneSymbol	CpG island Description	Ratio of relative methylation (TiO_2-NP/Vehicle)	Sham group			TiO_2-H group		
						Relative methylation (Cy5/Cy3)	Cy3 signal (Input)	Cy5 signal (MeDIP)	Relative methylation (Cy5/Cy3)	Cy3 signal (Input)	Cy5 signal (MeDIP)
A_68_P22883647	chr4:45542639-45542683	NM_001033306:1040	Shb	INSIDE	0.493	7.258	835.70	6065.18	3.578	826.92	2958.73
A_68_P22883652	chr4:45543353-45543397	NM_001033306:326	Shb	INSIDE	0.216	7.018	1413.36	9919.59	1.519	1206.72	1833.07
A_68_P27277981	chr10:79100167-79100211	NM_001024539:475	Shc2	INSIDE	0.364	0.433	1534.33	664.89	0.158	1070.97	168.86
A_68_P27277984	chr10:79100620-79100664	NM_001024539:21	Shc2	INSIDE	0.555	3.622	2102.56	7615.93	2.009	1718.62	3452.40
A_68_P29033142	chr13:51662332-51662377	NM_009167:99	Shc3	INSIDE	0.388	5.536	2770.47	15336.94	2.147	2172.90	4665.06
A_68_P31253054	chr17:56109780-56109824	NM_001159523:-102	Shd	PROMOTER	0.437	0.412	2328.20	958.09	0.18	1808.33	324.95
A_68_P31253055	chr17:56109896-56109940	NM_001159523:14	Shd	INSIDE	0.612	0.376	5273.30	1981.68	0.23	3978.26	915.32
A_68_P22321898	chr3:89634943-89634987	NM_172530:-327	She	PROMOTER	0.206	11.500	1292.82	14867.42	2.373	1188.13	2820.02
A_68_P22321899	chr3:89635034-89635078	NM_172530:-235	She	PROMOTER	0.658	8.314	1545.46	12848.45	5.469	1447.86	7918.31
A_68_P21585947	chr2:122174316-122174360	NM_001013829:20316	Shf	DOWNSTREAM	0.296	5.405	1209.03	6535.32	1.6	996.65	1594.88
A_68_P23768850	chr5:67999647-67999692	NM_001033415:548	Shisa3	INSIDE	0.408	4.683	1759.23	8238.34	1.91	1406.89	2686.51
A_68_P23768847	chr5:67999277-67999321	NM_001033415:177	Shisa3	INSIDE	0.563	0.633	772.62	489.39	0.357	674.82	240.66
A_68_P20638991	chr1:137271378-137271422	NM_175259:240	Shisa4	INSIDE	0.224	0.174	6423.69	1117.98	0.039	4263.38	166.10
A_68_P24950762	chr7:4788423-4788469	NM_172737:7852	Shisa7	INSIDE	0.529	6.789	1929.91	13102.79	3.594	1356.23	4874.35
A_68_P24950760	chr7:4788206-4788250	NM_172737:8070	Shisa7	INSIDE	0.588	7.049	732.08	5160.73	4.146	736.41	3052.89
A_68_P24950759	chr7:4788111-4788155	NM_172737:8166	Shisa7	INSIDE	0.621	4.371	1191.87	5209.93	2.714	1008.51	2736.63
A_68_P24950724	chr7:4781725-4781769	NM_172737:14552	Shisa7	INSIDE	0.487	3.387	3272.08	11083.24	1.651	2714.68	4480.70
A_68_P30550104	chr16:11984103-11984147	NM_001174086:-81	Shisa9	PROMOTER	0.135	12.731	9176.63	116832.20	1.715	7469.81	12810.96
A_68_P32185492	chr19:54018463-54018507	NM_001168505:-311	Shoc2	PROMOTER	0.137	21.032	396.12	8330.95	2.878	434.92	1251.77
A_68_P32185499	chr19:54019439-54019483	NM_019658:95	Shoc2	INSIDE	0.428	1.399	1267.94	1773.90	0.599	1202.18	719.71
A_68_P22207889	chr3:66784973-66785017	NM_013665:699	Shox2	INSIDE	0.382	0.522	2397.58	1251.08	0.199	1882.51	375.08
A_68_P22207824	chr3:66777458-66777502	NM_013665:8213	Shox2	INSIDE	0.63	2.518	3490.16	8787.59	1.587	2944.89	4673.70
A_68_P27915805	chr11:73013090-73013134	NM_029031:129	Shpk	INSIDE	0.412	8.157	953.47	7777.44	3.357	849.11	2850.46
A_68_P27804897	chr11:53277337-53277381	NM_027917:6652	Shroom1	INSIDE	0.633	4.544	600.90	2730.59	2.878	591.07	1701.38
A_68_P32234207	chrX:5977686-5977730	NM_001040459:446	Shroom4	INSIDE	0.129	16.582	744.12	12339.33	2.138	1001.84	2141.56
A_68_P32234202	chrX:5977172-5977216	NM_001040459:-68	Shroom4	PROMOTER	0.373	6.338	1532.78	9715.36	2.363	1758.02	4154.78
A_68_P26031095	chr8:89269939-89269983	NM_009172:-55	Siah1a	PROMOTER	0.655	4.399	426.05	1874.08	2.883	455.75	1313.75
A_68_P32796350	chrX:160514136-160514180	NM_009173:-177	Siah1b	PROMOTER	0.229	12.860	1208.95	15547.29	2.951	1769.35	5221.04
A_68_P22169673	chr3:58495733-58495777	NM_009174:556	Siah2	INSIDE	0.567	3.590	1245.46	4471.05	2.037	1075.95	2191.71
A_68_P22169679	chr3:58496414-58496458	NM_009174:-126	Siah2	PROMOTER	0.4	0.553	1062.20	587.76	0.221	888.28	196.59
A_68_P26477010	chr9:45763375-45763419	NM_172257:-64	Sidt2	PROMOTER	0.661	0.621	1049.63	651.58	0.411	795.66	326.64
A_68_P31850942	chr18:78244032-78244076	NM_001101038:9953	Siglec15	INSIDE	0.571	4.696	465.22	2184.50	2.679	511.26	1369.72
A_68_P31850954	chr18:78245651-78245695	NM_001101038:8335	Siglec15	INSIDE	0.444	3.743	1736.13	6497.61	1.663	1522.18	2531.48
A_68_P22864089	chr4:41687680-41687724	NM_011014:484	Sigmar1	INSIDE	0.474	0.248	2503.00	621.17	0.118	1890.70	222.53
A_68_P31142312	chr17:31992898-31992942	NM_010831:-183	Sik1	PROMOTER	0.601	0.482	1818.98	876.21	0.29	1487.58	430.88
A_68_P31142309	chr17:31992389-31992433	NM_010831:327	Sik1	INSIDE	0.356	0.716	4413.93	3158.28	0.255	3279.48	836.39
A_68_P31142266	chr17:31986924-31986968	NM_010831:5791	Sik1	INSIDE	0.238	2.007	1090.53	2188.18	0.478	833.65	398.13
A_68_P26506990	chr9:50816646-50816690	NM_178710:510	Sik2	INSIDE	0.503	0.672	1628.63	1094.26	0.338	1294.56	437.73
A_68_P26506991	chr9:50816720-50816764	NM_178710:436	Sik2	INSIDE	0.513	2.689	3771.68	10142.20	1.379	3030.87	4178.37
A_68_P26477292	chr9:45821526-45821572	NM_027498:646	Sik3	INSIDE	0.625	8.570	926.50	7940.16	5.357	793.63	4251.53
A_68_P22383782	chr3:102799932-102799976	NM_025679:292	Sike1	INSIDE	0.247	8.584	3585.39	30777.25	2.122	2547.28	5405.25
A_68_P26540098	chr9:56924586-56924630	NM_001110350:426	Sin3a	INSIDE	0.544	0.613	3977.29	2437.32	0.334	3107.69	1036.89
A_68_P26540100	chr9:56924835-56924879	NM_001110350:674	Sin3a	INSIDE	0.657	0.593	937.70	556.48	0.39	857.98	334.42
A_68_P25962756	chr8:75273840-75273884	NM_009188:26676	Sin3b	INSIDE	0.347	5.760	1028.65	5925.31	1.996	795.41	1587.88
A_68_P31929912	chr19:5654628-5654672	NM_001164480:9057	Sipa1	INSIDE	0.659	0.593	1901.34	1128.13	0.391	1234.93	483.00
A_68_P31929910	chr19:5654473-5654517	NM_001164480:9213	Sipa1	INSIDE	0.25	5.596	4344.72	24310.99	1.4	3787.09	5301.82

ProbeName	Target position of probe on CpG island microarray	TargetID	GeneSymbol	CpG island Description	Ratio of relative methylation (TiO_2-NP/Vehicle)	Sham group			TiO_2-H group		
						Relative methylation (Cy5/Cy3)	Cy3 signal (Input)	Cy5 signal (MeDIP)	Relative methylation (Cy5/Cy3)	Cy3 signal (Input)	Cy5 signal (MeDIP)
A_68_P28585119	chr12:83270170-83270214	NM_001167983:-810	Sipa1l1	PROMOTER	0.513	19.116	1183.65	22626.43	9.805	1007.89	9882.41
A_68_P28585121	chr12:83270447-83270491	NM_001167983:-534	Sipa1l1	PROMOTER	0.073	1.567	2823.57	4425.88	0.115	2025.68	232.16
A_68_P28585130	chr12:83271372-83271416	NM_001167983:392	Sipa1l1	INSIDE	0.631	0.662	1432.44	947.99	0.417	1162.02	485.02
A_68_P25026631	chr7:30117136-30117180	NM_001081028:173321	Sipa1l3	INSIDE	0.181	11.388	2921.14	33264.66	2.056	1955.80	4020.36
A_68_P21625558	chr2:129418959-129419003	NM_001177647:50	Sirpa	INSIDE	0.324	8.829	1245.58	10996.65	2.863	1057.85	3028.71
A_68_P25585439	chr7:148067707-148067751	NM_001127351:40	Sirt3	INSIDE	0.511	0.182	4799.28	874.23	0.093	3218.55	299.73
A_68_P25585440	chr7:148067788-148067832	NM_001127351:-42	Sirt3	PROMOTER	0.254	2.420	5916.50	14316.09	0.616	4741.91	2919.75
A_68_P24009835	chr5:115942032-115942076	NM_133760:-7561	Sirt4	PROMOTER	0.416	0.649	5467.43	3550.52	0.27	3681.75	995.48
A_68_P28751514	chr12:113883371-113883415	NM_001161737:354	Siva1	INSIDE	0.358	0.558	1172.51	654.06	0.2	874.57	174.52
A_68_P28535268	chr12:74147049-74147093	NM_009189:629	Six1	INSIDE	0.576	0.495	1419.93	703.57	0.285	1042.55	297.58
A_68_P28535257	chr12:74145983-74146027	NM_009189:1695	Six1	INSIDE	0.655	4.019	982.34	3947.92	2.631	851.00	2238.63
A_68_P31412114	chr17:86086928-86086972	NM_011380:644	Six2	INSIDE	0.304	13.015	1597.76	20795.58	3.954	1430.45	5656.20
A_68_P31412155	chr17:86092046-86092090	NM_011380:-4474	Six2	PROMOTER	0.409	0.296	1756.29	520.02	0.121	1420.77	171.86
A_68_P31412116	chr17:86087149-86087193	NM_011380:424	Six2	INSIDE	0.558	4.908	741.39	3638.39	2.738	707.33	1936.83
A_68_P31411779	chr17:86022957-86023001	NM_011381:2805	Six3	INSIDE	0.595	4.461	1342.19	5987.56	2.653	1429.32	3792.07
A_68_P31411712	chr17:86014601-86014645	NR_038085:3114	Six3os1	INSIDE	0.318	9.717	10273.14	99827.86	3.091	7725.37	23881.48
A_68_P28535622	chr12:74213314-74213358	NM_011382:896	Six4	INSIDE	0.104	0.392	5498.39	2152.84	0.041	4292.50	174.41
A_68_P28535628	chr12:74213900-74213944	NM_011382:310	Six4	INSIDE	0.292	0.423	2875.82	1217.02	0.123	1971.95	243.45
A_68_P28535623	chr12:74213467-74213511	NM_011382:744	Six4	INSIDE	0.62	0.277	1809.29	501.42	0.172	1015.45	174.58
A_68_P24991405	chr7:19680434-19680478	NM_011383:564	Six5	INSIDE	0.434	0.626	751.45	470.51	0.272	634.33	172.26
A_68_P28534667	chr12:74041473-74041517	NM_011384:563	Six6	INSIDE	0.4	0.585	949.68	555.89	0.234	744.83	174.18
A_68_P31830207	chr18:74367434-74367478	NM_001164355:16	Ska1	INSIDE	0.349	0.459	1376.40	631.32	0.16	1067.62	170.74
A_68_P24447786	chr6:51962237-51962281	NM_018773:290	Skap2	INSIDE	0.407	0.424	2170.54	920.35	0.173	1652.40	285.26
A_68_P23437240	chr4:154596449-154596493	NM_011385:174	Ski	INSIDE	0.257	9.407	1575.26	14818.71	2.414	1453.57	3509.40
A_68_P23437242	chr4:154597147-154597191	NM_011385:-524	Ski	PROMOTER	0.616	3.277	413.53	1355.19	2.02	409.16	826.46
A_68_P22031739	chr3:30993591-30993635	NM_001039090:-370	Skil	PROMOTER	0.496	4.434	1055.85	4681.81	2.197	925.85	2034.53
A_68_P22031742	chr3:30994277-30994321	NM_001039090:316	Skil	INSIDE	0.529	0.441	5998.94	2645.47	0.233	4304.00	1003.50
A_68_P22031740	chr3:30993764-30993808	NM_001039090:-196	Skil	PROMOTER	0.599	3.150	539.48	1699.23	1.888	470.05	887.32
A_68_P31156111	chr17:34987166-34987210	NM_021337:-39	Skiv2l	DIVERGENT_PROMOTER	0.48	8.801	896.18	7887.38	4.227	875.59	3701.06
A_68_P26574053	chr9:62986483-62986527	NM_172446:8283	Skor1	INSIDE	0.388	21.249	353.95	7521.13	8.239	276.94	2281.83
A_68_P26574137	chr9:62997201-62997245	NM_001163758:-454	Skor1	PROMOTER	0.546	6.685	881.08	5890.19	3.648	708.70	2585.49
A_68_P26574111	chr9:62993729-62993773	NM_172446:1037	Skor1	INSIDE	0.366	6.693	496.67	3324.03	2.452	438.49	1074.96
A_68_P31844540	chr18:77098052-77098096	NM_001109743:2931	Skor2	INSIDE	0.478	7.802	2093.95	16336.46	3.73	1816.15	6774.82
A_68_P31844550	chr18:77099205-77099249	NM_001109743:4083	Skor2	INSIDE	0.625	0.323	4990.29	1610.35	0.202	3042.20	613.93
A_68_P31844544	chr18:77098440-77098484	NM_001109743:3319	Skor2	INSIDE	0.324	1.371	2975.82	4079.68	0.445	2425.40	1078.73
A_68_P23794550	chr5:73305332-73305376	NM_001113423:-201	Slain2	PROMOTER	0.646	2.350	1936.76	4551.74	1.518	1457.36	2212.50
A_68_P23590298	chr5:33995400-33995444	NM_009193:-467	Slbp	PROMOTER	0.312	6.572	4585.69	30138.39	2.053	3829.61	7863.65
A_68_P25989168	chr8:81033011-81033055	NM_029736:-201	Slc10a7	DIVERGENT_PROMOTER	0.277	1.395	1461.72	2039.57	0.387	1097.15	424.60
A_68_P30485732	chr15:100253831-100253875	NM_008732:-366	Slc11a2	PROMOTER	0.563	0.565	3034.38	1714.61	0.318	2275.44	723.35
A_68_P31738737	chr18:58039024-58039069	NM_009194:715	Slc12a2	INSIDE	0.526	3.909	820.82	3208.83	2.058	748.27	1540.08
A_68_P31738728	chr18:58037843-58037887	NM_009194:-467	Slc12a2	PROMOTER	0.494	3.332	1467.47	4890.07	1.648	1281.34	2111.34
A_68_P26137064	chr8:108489850-108489894	NM_009195:67	Slc12a4	INSIDE	0.629	0.631	2083.23	1313.72	0.397	1570.95	623.13
A_68_P21820794	chr2:164793741-164793785	NM_020333:275	Slc12a5	INSIDE	0.337	26.873	4930.95	132507.70	9.043	6853.92	61980.75
A_68_P21820992	chr2:164823190-164823234	NM_020333:29725	Slc12a5	INSIDE	0.497	9.013	1694.84	15275.83	4.478	1249.45	5595.64
A_68_P21820797	chr2:164794239-164794283	NM_020333:773	Slc12a5	INSIDE	0.613	5.299	639.76	3390.24	3.247	496.03	1610.42
A_68_P21820817	chr2:164796549-164796593	NM_020333:3083	Slc12a5	INSIDE	0.463	0.698	1730.06	1207.33	0.323	1414.90	457.24

ProbeName	Target position of probe on CpG island microarray	TargetID	GeneSymbol	CpG island Description	Ratio of relative methylation (TiO_2-NP/Vehicle)	Sham group			TiO_2-H group		
						Relative methylation (Cy5/Cy3)	Cy3 signal (Input)	Cy5 signal (MeDIP)	Relative methylation (Cy5/Cy3)	Cy3 signal (Input)	Cy5 signal (MeDIP)
A_68_P21820739	chr2:164786459-164786503	NM_020333:-7007	Slc12a5	PROMOTER	0.431	4.919	844.63	4154.70	2.121	662.02	1403.82
A_68_P21820991	chr2:164823054-164823098	NM_020333:29589	Slc12a5	INSIDE	0.412	3.738	1805.92	6750.66	1.542	1791.03	2761.46
A_68_P21820793	chr2:164793533-164793577	NM_020333:67	Slc12a5	INSIDE	0.544	2.634	3391.28	8932.75	1.434	2458.40	3525.23
A_68_P29141936	chr13:73901069-73901113	NM_011390:-54	Slc12a7	PROMOTER	0.118	32.934	1613.23	53129.70	3.895	1325.01	5160.40
A_68_P29141935	chr13:73901004-73901048	NM_011390:-118	Slc12a7	PROMOTER	0.557	4.262	3282.09	13989.70	2.374	2548.77	6050.33
A_68_P24078663	chr5:128097296-128097340	NM_133895:444	Slc15a4	INSIDE	0.619	3.087	614.40	1896.37	1.91	537.33	1026.19
A_68_P22392370	chr3:104442843-104442887	NM_009196:274	Slc16a1	INSIDE	0.483	6.456	1407.99	9090.06	3.116	1227.02	3823.22
A_68_P22392371	chr3:104442921-104442965	NM_009196:352	Slc16a1	INSIDE	0.626	0.386	1465.46	566.38	0.242	1274.09	308.04
A_68_P32081999	chr19:34821540-34821584	NM_172838:39	Slc16a12	INSIDE	0.583	0.529	1315.86	695.76	0.308	1004.37	309.60
A_68_P27899519	chr11:70034182-70034226	NM_172371:292	Slc16a13	INSIDE	0.641	0.324	1565.83	507.01	0.208	1103.68	229.02
A_68_P32574197	chrX:100890665-100890709	NM_009197:126641	Slc16a2	DOWNSTREAM	0.315	1.904	1132.49	2156.26	0.599	1711.20	1024.90
A_68_P28187724	chr11:120812178-120812222	NM_001038653:1820	Slc16a3	INSIDE	0.522	0.299	2214.02	661.22	0.156	1563.77	243.56
A_68_P28187710	chr11:120810691-120810735	NM_001038653:332	Slc16a3	INSIDE	0.505	3.000	1170.72	3512.26	1.514	955.55	1446.52
A_68_P28118794	chr11:109334955-109334999	NM_001029842:-66	Slc16a6	PROMOTER	0.387	4.439	2630.53	11676.20	1.719	2101.79	3613.44
A_68_P28118793	chr11:109334829-109334873	NM_001029842:60	Slc16a6	INSIDE	0.378	1.436	1139.50	1635.92	0.543	1037.64	563.52
A_68_P27228617	chr10:69707740-69707784	NM_025807:-261	Slc16a9	PROMOTER	0.179	9.797	3144.99	30810.94	1.751	2501.21	4379.18
A_68_P27228619	chr10:69707994-69708038	NM_025807:-7	Slc16a9	PROMOTER	0.55	15.837	1618.75	25635.75	8.704	1504.39	13093.78
A_68_P25091604	chr7:52431430-52431474	NM_182993:12162	Slc17a7	INSIDE	0.38	0.442	7594.95	3360.72	0.168	4071.35	684.56
A_68_P25091507	chr7:52417775-52417819	NM_182993:-1494	Slc17a7	PROMOTER	0.409	8.732	2697.36	23552.26	3.574	2574.00	9199.22
A_68_P25091605	chr7:52431556-52431600	NM_182993:12288	Slc17a7	DOWNSTREAM	0.357	1.356	9000.55	12204.20	0.484	6068.21	2937.91
A_68_P25946260	chr8:71612907-71612951	NM_153054:193	Slc18a1	INSIDE	0.649	0.558	832.01	464.20	0.362	678.38	245.68
A_68_P27265504	chr10:76495615-76495659	NM_031196:-320	Slc19a1	PROMOTER	0.292	2.074	4712.37	9772.15	0.605	3574.02	2163.40
A_68_P20788009	chr1:166179184-166179228	NM_054087:21	Slc19a2	INSIDE	0.351	0.405	3794.18	1538.11	0.142	3055.85	435.03
A_68_P20788007	chr1:166178862-166178906	NM_054087:-301	Slc19a2	PROMOTER	0.577	0.226	2066.81	467.83	0.13	1509.83	197.02
A_68_P20788011	chr1:166179434-166179478	NM_054087:271	Slc19a2	INSIDE	0.461	1.381	1471.47	2031.48	0.637	1271.99	810.24
A_68_P21478259	chr2:102498900-102498944	NM_001077514:83	Slc1a2	INSIDE	0.58	0.484	1239.71	599.68	0.28	1037.42	290.83
A_68_P30001153	chr15:8586301-8586345	NM_148938:74485	Slc1a3	INSIDE	0.515	5.240	824.55	4320.80	2.698	671.80	1812.50
A_68_P27630973	chr11:20232357-20232401	NM_018861:338	Slc1a4	INSIDE	0.528	0.706	1377.04	972.57	0.373	1063.63	396.61
A_68_P24983214	chr7:17367558-17367602	NM_009201:886	Slc1a5	INSIDE	0.613	0.430	2356.48	1012.36	0.263	1749.54	460.61
A_68_P21623516	chr2:129024287-129024331	NM_001159593:-200	Slc20a1	PROMOTER	0.359	6.009	1879.39	11293.74	2.159	1657.36	3577.44
A_68_P21623523	chr2:129025044-129025088	NM_001159593:558	Slc20a1	INSIDE	0.19	1.541	1009.83	1556.30	0.293	858.59	251.74
A_68_P28938191	chr13:34436110-34436154	NM_001033167:919	Slc22a23	INSIDE	0.295	8.969	4281.69	38402.14	2.648	3062.90	8111.00
A_68_P27807844	chr11:53841558-53841603	NM_019687:12	Slc22a4	INSIDE	0.575	0.572	1750.54	1000.60	0.329	1337.83	439.62
A_68_P27807186	chr11:53705037-53705081	NM_011396:147	Slc22a5	INSIDE	0.553	2.872	3290.34	9451.29	1.589	2519.74	4004.61
A_68_P24039145	chr5:120961107-120961151	NM_001177594:-72	Slc24a6	DIVERGENT_PROMOTER	0.613	2.581	5449.23	14066.54	1.582	3993.33	6317.74
A_68_P28184908	chr11:120353660-120353704	NM_013770:532	Slc25a10	INSIDE	0.437	0.640	1704.38	1090.48	0.28	1265.83	354.02
A_68_P27901831	chr11:70460715-70460759	NM_024211:-241	Slc25a11	DIVERGENT_PROMOTER	0.154	14.381	1754.75	25234.36	2.219	1429.90	3172.75
A_68_P30377507	chr15:81190981-81191029	NM_011399:191	Slc25a17	INSIDE	0.145	1.809	800.98	1448.95	0.261	660.67	172.72
A_68_P28154496	chr11:115489226-115489270	NM_026071:205	Slc25a19	INSIDE	0.322	0.348	2792.67	971.50	0.112	2015.96	225.91
A_68_P26816015	chr9:108564655-108564699	NM_020520:248	Slc25a20	INSIDE	0.44	4.988	1631.14	8136.49	2.195	1381.79	3032.84
A_68_P25588799	chr7:148623331-148623375	NM_001177576:-2859	Slc25a22	PROMOTER	0.569	0.358	2994.05	1072.98	0.204	2033.30	414.36
A_68_P25588800	chr7:148623401-148623445	NM_001177576:-2929	Slc25a22	PROMOTER	0.574	0.722	2008.57	1449.57	0.414	1539.81	638.23
A_68_P25588801	chr7:148623566-148623613	NM_001177576:-3096	Slc25a22	PROMOTER	0.499	3.239	1050.14	3401.27	1.616	929.14	1501.40
A_68_P31259834	chr17:57199854-57199898	NM_025877:-590	Slc25a23	DIVERGENT_PROMOTER	0.249	8.628	1851.23	15972.05	2.145	1452.38	3114.88
A_68_P32127978	chr19:43749225-43749269	NM_145156:125	Slc25a28	INSIDE	0.337	9.672	4767.17	46105.84	3.256	4149.56	13512.10
A_68_P28730238	chr12:110073635-110073679	NM_181328:430	Slc25a29	INSIDE	0.54	0.630	1453.93	915.32	0.34	1147.03	389.73

ProbeName	Target position of probe on CpG island microarray	TargetID	GeneSymbol	CpG island Description	Ratio of relative methylation (TiO_2-NP/Vehicle)	Sham group			TiO_2-H group		
						Relative methylation (Cy5/Cy3)	Cy3 signal (Input)	Cy5 signal (MeDIP)	Relative methylation (Cy5/Cy3)	Cy3 signal (Input)	Cy5 signal (MeDIP)
A_68_P23402414	chr4:149148446-149148491	NM_027460:-92	Slc25a33	PROMOTER	0.334	16.405	4873.22	79942.86	5.486	4274.75	23450.02
A_68_P29695467	chr14:69902363-69902407	NM_026331:776	Slc25a37	INSIDE	0.327	1.896	2334.71	4425.71	0.62	1745.34	1082.93
A_68_P29695466	chr14:69902277-69902321	NM_026331:862	Slc25a37	INSIDE	0.421	1.599	2278.00	3642.92	0.672	1789.57	1203.45
A_68_P26876104	chr9:120020031-120020078	NM_144793:538	Slc25a38	INSIDE	0.449	3.725	848.74	3161.44	1.672	702.11	1174.24
A_68_P25834907	chr8:47296398-47296442	NM_007450:-57	Slc25a4	PROMOTER	0.647	0.501	1598.02	799.97	0.324	939.14	303.99
A_68_P32319888	chrX:34283671-34283715	NM_001085497:39	Slc25a43	INSIDE	0.349	6.883	2519.25	17339.68	2.4	2023.86	4857.96
A_68_P31598790	chr18:31769072-31769116	NM_026165:462	Slc25a46	INSIDE	0.433	10.601	1006.97	10675.03	4.59	1010.75	4639.80
A_68_P31598791	chr18:31769247-31769291	NM_026165:288	Slc25a46	INSIDE	0.478	6.929	1151.43	7978.72	3.309	972.22	3217.24
A_68_P23973506	chr5:109106670-109106714	NM_174870:-2024	Slc26a1	PROMOTER	0.464	0.315	4302.76	1354.94	0.146	2778.35	405.53
A_68_P28177384	chr11:119217045-119217089	NM_178743:196	Slc26a11	INSIDE	0.51	0.497	1671.09	830.85	0.254	1336.47	339.20
A_68_P28177386	chr11:119217235-119217283	NM_178743:388	Slc26a11	INSIDE	0.524	5.591	1935.77	10822.04	2.931	1525.85	4471.54
A_68_P28332010	chr12:32244021-32244065	NM_011867:792	Slc26a4	INSIDE	0.466	5.125	807.09	4136.68	2.388	676.91	1616.68
A_68_P25957546	chr8:74094381-74094431	NM_011977:1581	Slc27a1	INSIDE	0.168	0.738	2797.77	2065.35	0.124	2048.84	253.96
A_68_P21608666	chr2:126378873-126378917	NM_011978:135	Slc27a2	INSIDE	0.259	6.556	808.33	5299.02	1.697	834.43	1416.22
A_68_P22324942	chr3:90193856-90193900	NM_011988:-29	Slc27a3	PROMOTER	0.479	6.118	960.33	5874.99	2.931	769.28	2254.56
A_68_P22324939	chr3:90193494-90193538	NM_011988:333	Slc27a3	INSIDE	0.593	3.792	1298.80	4925.30	2.25	1065.92	2398.60
A_68_P21099538	chr2:29658204-29658248	NM_011989:27	Slc27a4	INSIDE	0.225	0.446	2253.00	1004.55	0.1	1708.28	171.17
A_68_P31202059	chr17:45732650-45732694	NM_001199116:-151	Slc29a1	PROMOTER	0.517	0.460	2913.28	1341.48	0.238	2115.33	503.37
A_68_P31926384	chr19:5024260-5024304	NM_007854:277	Slc29a2	INSIDE	0.484	0.475	1966.71	935.16	0.23	1569.34	361.44
A_68_P24153011	chr5:143194631-143194675	NM_146257:16598	Slc29a4	INSIDE	0.224	6.011	6344.96	38139.95	1.349	4446.52	5998.52
A_68_P23243653	chr4:118781349-118781393	NM_011400:21	Slc2a1	INSIDE	0.391	0.539	2541.84	1368.84	0.21	1617.51	340.35
A_68_P30436287	chr15:91403612-91403656	NM_001033633:58	Slc2a13	INSIDE	0.421	19.472	4168.66	81173.45	8.197	3773.79	30933.86
A_68_P27898121	chr11:69761400-69761444	NM_009204:270	Slc2a4	INSIDE	0.073	4.222	1460.49	6166.78	0.309	1026.80	316.95
A_68_P27898124	chr11:69761728-69761772	NM_009204:-58	Slc2a4	PROMOTER	0.277	0.417	11628.89	4853.51	0.116	7608.48	879.93
A_68_P20940733	chr1:193730478-193730522	NM_009579:-159	Slc30a1	PROMOTER	0.634	2.342	2407.96	5640.00	1.484	1856.91	2756.09
A_68_P23322292	chr4:133899412-133899456	NM_001039677:450	Slc30a2	INSIDE	0.508	0.408	2752.68	1123.50	0.207	1827.11	378.83
A_68_P23576450	chr5:31396001-31396045	NM_011773:-122	Slc30a3	PROMOTER	0.214	1.947	3951.78	7693.18	0.416	2743.87	1142.19
A_68_P23576447	chr5:31395717-31395761	NM_011773:162	Slc30a3	INSIDE	0.434	7.776	1766.69	13737.23	3.373	1457.31	4915.89
A_68_P23576449	chr5:31395933-31395977	NM_011773:-54	Slc30a3	PROMOTER	0.406	1.693	5007.10	8477.13	0.687	3946.31	2709.73
A_68_P21587592	chr2:122528587-122528631	NM_011774:-209	Slc30a4	PROMOTER	0.159	25.581	1702.69	43556.69	4.059	1548.85	6287.05
A_68_P21587589	chr2:122528219-122528263	NM_011774:159	Slc30a4	INSIDE	0.532	3.093	1108.79	3429.47	1.645	913.54	1502.55
A_68_P29270336	chr13:101602642-101602692	NM_022885:716	Slc30a5	INSIDE	0.322	20.384	1100.44	22430.95	6.564	847.81	5565.00
A_68_P31351306	chr17:74795028-74795072	NM_144798:79	Slc30a6	INSIDE	0.45	0.405	1154.03	467.67	0.182	948.97	172.95
A_68_P22963537	chr4:61947638-61947682	NM_025286:182	Slc31a2	INSIDE	0.52	3.324	4896.97	16275.66	1.73	3921.15	6782.82
A_68_P22963535	chr4:61947389-61947433	NM_025286:-68	Slc31a2	PROMOTER	0.557	3.484	1111.75	3872.77	1.94	944.05	1831.32
A_68_P21783928	chr2:158436941-158436985	NM_009508:469	Slc32a1	INSIDE	0.181	29.114	14996.71	436611.40	5.271	16820.22	88661.24
A_68_P21783953	chr2:158439712-158439756	NM_009508:3241	Slc32a1	INSIDE	0.405	0.161	3981.05	639.05	0.065	2654.73	172.76
A_68_P21783917	chr2:158435595-158435639	NM_009508:-877	Slc32a1	PROMOTER	0.452	0.093	6111.51	570.88	0.042	4033.42	170.38
A_68_P21783954	chr2:158439815-158439861	NM_009508:3345	Slc32a1	INSIDE	0.491	0.228	2520.65	574.17	0.112	1663.17	185.86
A_68_P22194351	chr3:63768401-63768445	NM_015728:233	Slc33a1	INSIDE	0.481	0.411	1246.80	512.84	0.198	989.48	195.71
A_68_P22450819	chr3:116414740-116414784	NM_144902:436	Slc35a3	INSIDE	0.366	1.367	1864.89	2549.32	0.501	1739.25	871.08
A_68_P31201839	chr17:45701335-45701379	NM_028662:256	Slc35b2	INSIDE	0.554	2.784	1794.01	4995.31	1.542	1531.12	2360.79
A_68_P28963697	chr13:39052368-39052412	NM_001170430:16	Slc35b3	INSIDE	0.474	3.559	2302.28	8192.98	1.687	1958.86	3304.00
A_68_P24354189	chr6:34126897-34126941	NM_021435:136	Slc35b4	INSIDE	0.642	0.618	1721.62	1064.21	0.397	1306.18	517.99
A_68_P23165023	chr4:102888044-102888088	NM_177732:-577	Slc35d1	PROMOTER	0.328	1.611	1410.75	2272.84	0.529	1101.54	582.85
A_68_P26983368	chr10:19569800-19569844	NM_029529:1443	Slc35d3	INSIDE	0.625	0.452	1701.95	769.14	0.282	1358.54	383.51

ProbeName	Target position of probe on CpG island microarray	TargetID	GeneSymbol	CpG island Description	Ratio of relative methylation (TiO_2-NP/Vehicle)	Sham group			TiO_2-H group		
						Relative methylation (Cy5/Cy3)	Cy3 signal (Input)	Cy5 signal (MeDIP)	Relative methylation (Cy5/Cy3)	Cy3 signal (Input)	Cy5 signal (MeDIP)
A_68_P26983377	chr10:19570989-19571033	NM_029529:255	Slc35d3	INSIDE	0.464	3.432	2047.16	7026.53	1.592	1596.30	2541.29
A_68_P27554683	chr11:3813912-3813956	NM_153142:733	Slc35e4	INSIDE	0.302	6.056	5203.76	31515.42	1.827	4266.49	7795.66
A_68_P27554682	chr11:3813819-3813863	NM_153142:827	Slc35e4	INSIDE	0.488	3.984	3481.57	13871.58	1.946	2569.06	4998.35
A_68_P27138493	chr10:52410426-52410470	NM_178675:142	Slc35f1	INSIDE	0.602	4.569	2398.89	10960.25	2.749	2340.94	6435.97
A_68_P27138496	chr10:52410751-52410795	NM_178675:466	Slc35f1	INSIDE	0.384	4.090	1888.03	7721.63	1.57	1555.69	2443.17
A_68_P26256736	chr8:128822425-128822469	NM_175434:-32	Slc35f3	PROMOTER	0.442	0.265	4066.54	1075.99	0.117	2853.48	333.41
A_68_P20585375	chr1:127456906-127456950	NM_028787:-664	Slc35f5	PROMOTER	0.449	11.503	1748.27	20110.30	5.159	1613.02	8321.73
A_68_P20585376	chr1:127456998-127457042	NM_028787:-572	Slc35f5	PROMOTER	0.581	0.360	1398.66	503.43	0.209	1022.14	213.84
A_68_P20585382	chr1:127457522-127457566	NM_028787:-48	Slc35f5	PROMOTER	0.593	6.394	1131.57	7235.56	3.793	1030.12	3907.32
A_68_P31138852	chr17:31433001-31433045	NM_153062:595	Slc37a1	INSIDE	0.349	0.564	1730.34	976.44	0.197	1323.48	260.43
A_68_P24382792	chr6:39327695-39327739	NM_028123:-10	Slc37a3	DIVERGENT_PROMOTER	0.445	0.512	1063.19	544.83	0.228	938.11	213.90
A_68_P30464706	chr15:96472084-96472128	NM_134086:287	Slc38a1	INSIDE	0.43	0.497	1137.01	565.10	0.214	878.03	187.74
A_68_P30464716	chr15:96473367-96473411	NM_001166458:-44	Slc38a1	PROMOTER	0.578	0.614	2168.50	1331.30	0.355	1539.61	546.55
A_68_P30465077	chr15:96529839-96529883	NM_175121:269	Slc38a2	INSIDE	0.534	2.618	2067.46	5412.61	1.399	1793.58	2509.59
A_68_P20203509	chr1:46909562-46909606	NM_172653:770	Slc39a10	INSIDE	0.596	0.509	1373.09	698.70	0.303	1150.61	348.80
A_68_P21420493	chr2:90910689-90910733	NM_026721:-332	Slc39a13	PROMOTER	0.261	12.838	3650.51	46863.91	3.344	2985.87	9985.84
A_68_P29700848	chr14:70750798-70750842	NM_001135152:-2580	Slc39a14	PROMOTER	0.303	0.627	1650.41	1035.16	0.19	1193.48	226.98
A_68_P31560903	chr18:24761642-24761686	NM_139143:654	Slc39a6	INSIDE	0.339	7.099	2729.12	19374.04	2.408	2467.88	5943.63
A_68_P31560905	chr18:24761962-24762006	NM_139143:334	Slc39a6	INSIDE	0.509	4.928	1341.48	6611.05	2.506	1075.63	2695.86
A_68_P31943154	chr19:8787857-8787901	NM_008577:509	Slc3a2	INSIDE	0.518	0.551	1326.04	730.75	0.285	1166.47	333.00
A_68_P31943158	chr19:8788397-8788441	NM_008577:-31	Slc3a2	PROMOTER	0.612	4.707	1804.66	8493.88	2.881	1498.30	4316.92
A_68_P20617850	chr1:133724396-133724440	NM_173865:-170	Slc41a1	PROMOTER	0.348	0.274	3584.13	982.94	0.095	2461.52	234.84
A_68_P22923084	chr4:53453754-53453798	NM_001159633:492	Slc44a1	INSIDE	0.197	18.588	3346.21	62198.84	3.656	2657.29	9715.04
A_68_P23407530	chr4:150026271-150026315	NM_173774:-9	Slc45a1	PROMOTER	0.431	12.611	848.79	10704.51	5.429	803.59	4362.92
A_68_P27943494	chr11:78279579-78279623	NM_026740:398	Slc46a1	INSIDE	0.425	12.199	6528.71	79640.88	5.184	5706.41	29579.39
A_68_P27943490	chr11:78279217-78279261	NM_026740:36	Slc46a1	INSIDE	0.406	1.574	1621.38	2551.60	0.639	1428.04	912.23
A_68_P21631241	chr2:130523070-130523114	NM_001081162:163	Slc4a11	INSIDE	0.278	10.025	868.22	8703.72	2.788	801.89	2235.54
A_68_P23541735	chr5:23931232-23931276	NM_009207:205	Slc4a2	INSIDE	0.478	0.436	1088.83	474.19	0.208	823.76	171.46
A_68_P20354814	chr1:75542871-75542915	NM_009208:52	Slc4a3	INSIDE	0.121	23.504	2431.65	57153.29	2.851	2080.10	5930.51
A_68_P20354818	chr1:75543316-75543360	NM_009208:498	Slc4a3	INSIDE	0.404	9.897	4981.69	49304.31	3.998	4554.15	18207.95
A_68_P29421307	chr14:15536004-15536048	NM_001033270:488	Slc4a7	INSIDE	0.64	5.139	809.97	4162.26	3.29	747.28	2458.52
A_68_P27327917	chr10:88348711-88348755	NM_145423:-4	Slc5a8	PROMOTER	0.202	20.755	1522.29	31595.58	4.184	1398.24	5849.87
A_68_P24764863	chr6:114232692-114232736	NM_178703:86	Slc6a1	INSIDE	0.271	1.590	3849.50	6121.86	0.431	2880.77	1241.81
A_68_P24764867	chr6:114233070-114233114	NM_178703:464	Slc6a1	INSIDE	0.474	3.014	2246.96	6772.79	1.429	1766.54	2523.81
A_68_P27406628	chr10:102830256-102830300	NM_175328:-198	Slc6a15	PROMOTER	0.455	0.608	2389.03	1452.70	0.277	1894.58	523.86
A_68_P27406629	chr10:102830356-102830400	NM_175328:-98	Slc6a15	PROMOTER	0.254	2.394	2950.70	7063.49	0.608	2134.34	1297.66
A_68_P26068970	chr8:95484812-95484856	NM_009209:-111	Slc6a2	PROMOTER	0.598	6.545	317.46	2077.85	3.916	237.22	929.01
A_68_P27935204	chr11:76811878-76811922	NM_010484:-198	Slc6a4	PROMOTER	0.365	5.713	1387.74	7927.47	2.085	1286.59	2683.04
A_68_P25115500	chr7:57167143-57167187	NM_148931:95	Slc6a5	INSIDE	0.412	0.486	1098.37	533.61	0.2	856.33	171.53
A_68_P25115504	chr7:57167691-57167735	NM_148931:643	Slc6a5	INSIDE	0.429	0.471	2537.49	1194.41	0.202	1718.90	347.46
A_68_P32464061	chrX:70918592-70918636	NM_001142809:143	Slc6a8	INSIDE	0.606	8.556	1555.52	13309.22	5.183	1950.56	10108.87
A_68_P23236661	chr4:117508209-117508255	NM_008135:370	Slc6a9	INSIDE	0.24	0.303	4098.47	1243.51	0.073	2444.37	177.80
A_68_P23236660	chr4:117508118-117508162	NM_008135:278	Slc6a9	INSIDE	0.31	0.403	3167.64	1277.95	0.125	2129.47	265.96
A_68_P23236670	chr4:117509289-117509333	NM_008135:1448	Slc6a9	INSIDE	0.349	9.632	2421.70	23325.77	3.358	1994.34	6697.05
A_68_P23236917	chr4:117540757-117540801	NM_008135:32916	Slc6a9	INSIDE	0.429	4.154	2324.66	9657.02	1.783	1937.72	3455.48
A_68_P23236665	chr4:117508655-117508699	NM_008135:814	Slc6a9	INSIDE	0.547	3.122	2474.39	7726.08	1.706	1632.51	2785.86

ProbeName	Target position of probe on CpG island microarray	TargetID	GeneSymbol	CpG island Description	Ratio of relative methylation (TiO_2-NP/Vehicle)	Sham group			TiO_2-H group		
						Relative methylation (Cy5/Cy3)	Cy3 signal (Input)	Cy5 signal (MeDIP)	Relative methylation (Cy5/Cy3)	Cy3 signal (Input)	Cy5 signal (MeDIP)
A_68_P23236664	chr4:117508530-117508574	NM_008135:690	Slc6a9	INSIDE	0.343	4.702	1879.56	8838.23	1.611	1694.97	2731.00
A_68_P25048054	chr7:35970299-35970343	NM_017394:-1083	Slc7a10	PROMOTER	0.452	0.588	2533.37	1489.96	0.266	1646.65	438.00
A_68_P25807719	chr8:41947388-41947432	NM_007514:-310	Slc7a2	PROMOTER	0.201	24.936	4237.04	105656.80	5.023	5343.37	26840.43
A_68_P26229674	chr8:124431610-124431654	NM_011404:-46	Slc7a5	DIVERGENT_PROMOTER	0.433	4.786	3723.50	17819.10	2.07	2905.12	6015.02
A_68_P26138122	chr8:108692733-108692777	NM_178798:-20	Slc7a6	PROMOTER	0.387	28.841	2528.24	72917.60	11.151	2539.59	28319.56
A_68_P26138124	chr8:108693007-108693051	NM_178798:254	Slc7a6	INSIDE	0.523	0.577	2790.28	1609.39	0.302	2096.69	633.09
A_68_P26138372	chr8:108734689-108734733	NM_001007567:123	Slc7a6os	INSIDE	0.648	4.770	1409.93	6725.36	3.093	1244.13	3847.48
A_68_P29614298	chr14:55400102-55400146	NM_016972:599	Slc7a8	INSIDE	0.378	0.288	2374.56	683.49	0.109	1643.20	178.92
A_68_P24979913	chr7:16730734-16730778	NM_148946:15108	Slc8a2	INSIDE	0.445	6.366	4370.43	27824.25	2.834	3721.43	10547.02
A_68_P24980002	chr7:16744202-16744246	NM_148946:28576	Slc8a2	INSIDE	0.365	0.697	1718.95	1198.69	0.255	1399.58	356.59
A_68_P23317226	chr4:132925776-132925820	NM_016981:112	Slc9a1	INSIDE	0.318	0.312	2793.50	870.82	0.099	2172.60	215.43
A_68_P20172913	chr1:40738293-40738337	NM_001033289:-242	Slc9a2	PROMOTER	0.495	3.568	620.92	2215.36	1.768	587.94	1039.32
A_68_P26133225	chr8:107873334-107873378	NM_001081332:1199	Slc9a5	INSIDE	0.576	0.630	1972.18	1242.39	0.363	1528.75	554.93
A_68_P26133220	chr8:107872806-107872850	NM_001081332:671	Slc9a5	INSIDE	0.658	2.242	1560.60	3499.31	1.475	1254.24	1849.89
A_68_P32396713	chrX:53862851-53862895	NM_172780:-139	Slc9a6	PROMOTER	0.523	9.509	1106.81	10524.94	4.976	1279.05	6364.30
A_68_P32287972	chrX:19869101-19869145	NM_177353:-232	Slc9a7	PROMOTER	0.554	4.493	1197.28	5379.45	2.491	1426.88	3554.22
A_68_P26783273	chr9:102910754-102910801	NM_033314:-41	Slco2a1	PROMOTER	0.478	0.133	4354.79	579.95	0.064	2740.78	174.64
A_68_P25235399	chr7:81699616-81699660	NM_001038643:28	Slco3a1	INSIDE	0.623	7.709	1065.28	8212.05	4.801	923.64	4434.64
A_68_P20038598	chr1:12982722-12982766	NM_172841:-1528	Slco5a1	PROMOTER	0.226	0.472	2190.54	1033.05	0.106	1598.88	170.27
A_68_P20038584	chr1:12980774-12980818	NM_172841:420	Slco5a1	INSIDE	0.629	0.470	1005.03	472.27	0.295	901.35	266.25
A_68_P20038625	chr1:12986560-12986610	NM_172841:-5368	Slco5a1	PROMOTER	0.396	1.375	2545.68	3500.83	0.545	1750.50	953.83
A_68_P23668870	chr5:48377571-48377615	NM_178804:3199	Slit2	INSIDE	0.379	5.782	2273.35	13145.51	2.19	2010.74	4403.22
A_68_P22236489	chr3:72861536-72861580	NM_198864:-693	Slitrk3	PROMOTER	0.351	6.020	8763.30	52758.28	2.111	6452.19	13617.50
A_68_P22236487	chr3:72861315-72861359	NM_198864:-471	Slitrk3	PROMOTER	0.403	3.480	2431.63	8460.95	1.403	1984.19	2784.57
A_68_P32427541	chrX:61528987-61529031	NM_178740:1163	Slitrk4	INSIDE	0.491	3.020	1465.31	4425.97	1.483	2035.70	3018.86
A_68_P29482222	chr14:27353534-27353578	NM_032008:-330	Slmap	PROMOTER	0.373	6.911	1775.01	12266.26	2.575	1413.60	3640.72
A_68_P29482229	chr14:27354402-27354446	NM_032008:-1198	Slmap	PROMOTER	0.267	2.296	2302.60	5286.47	0.613	1853.86	1136.99
A_68_P31791990	chr18:67624448-67624492	NM_144867:-32	Slmo1	PROMOTER	0.527	4.872	799.23	3894.15	2.569	703.45	1807.15
A_68_P31841968	chr18:76400994-76401038	NM_010754:-562	Smad2	PROMOTER	0.662	3.759	790.84	2972.69	2.488	782.28	1946.28
A_68_P29062977	chr13:56805003-56805048	NM_001164042:-3	Smad5	PROMOTER	0.212	17.542	2796.88	49063.25	3.724	2594.73	9662.27
A_68_P29062978	chr13:56805103-56805147	NM_001164042:96	Smad5	INSIDE	0.546	0.343	1657.23	568.61	0.187	1263.29	236.65
A_68_P22149790	chr3:54590088-54590132	NM_019483:30607	Smad9	INSIDE	0.401	8.574	3894.07	33385.96	3.438	2801.68	9632.19
A_68_P30486832	chr15:100467219-100467263	NM_174992:46	Smagp	INSIDE	0.339	6.654	943.29	6277.07	2.256	829.56	1871.82
A_68_P30486834	chr15:100467426-100467470	NM_001033872:-152	Smagp	PROMOTER	0.389	4.251	1846.52	7848.79	1.654	1448.11	2395.13
A_68_P23254536	chr4:120689836-120689880	NM_133716:-6	Smap2	PROMOTER	0.316	4.620	2617.04	12091.59	1.459	2389.05	3485.88
A_68_P32036088	chr19:26681356-26681400	NM_011416:1729	Smarca2	INSIDE	0.182	13.709	697.21	9557.84	2.501	610.17	1526.28
A_68_P32036078	chr19:26680160-26680204	NM_011416:533	Smarca2	INSIDE	0.324	8.775	608.78	5342.11	2.845	560.40	1594.10
A_68_P32036068	chr19:26679131-26679177	NM_011416:-495	Smarca2	PROMOTER	0.552	0.297	2545.94	757.28	0.164	1737.56	285.40
A_68_P32036092	chr19:26681843-26681887	NM_011416:2215	Smarca2	INSIDE	0.642	0.350	2413.54	845.56	0.225	1580.72	355.37
A_68_P32036067	chr19:26678985-26679030	NM_011416:-642	Smarca2	PROMOTER	0.651	0.253	3581.52	906.17	0.165	2378.11	391.54
A_68_P32036081	chr19:26680484-26680528	NM_011416:857	Smarca2	INSIDE	0.434	0.592	1557.75	922.16	0.257	1328.34	341.65
A_68_P32036079	chr19:26680265-26680310	NM_011416:638	Smarca2	INSIDE	0.596	2.477	2011.24	4981.15	1.475	1656.17	2443.24
A_68_P26348417	chr9:21490581-21490625	NM_001174078:69990	Smarca4	INSIDE	0.356	5.168	2468.09	12756.14	1.841	1940.26	3572.85
A_68_P27259388	chr10:75383953-75383997	NM_001161853:385	Smarcb1	INSIDE	0.486	3.775	838.93	3167.39	1.834	723.20	1326.31
A_68_P27541473	chr10:127896021-127896065	NM_001114096:-249	Smarcc2	PROMOTER	0.649	2.443	1830.66	4472.31	1.586	1519.34	2410.26
A_68_P23542997	chr5:24110453-24110497	NM_025891:-2654	Smarcd3	PROMOTER	0.384	0.463	1175.02	544.18	0.178	955.91	170.11

ProbeName	Target position of probe on CpG island microarray	TargetID	GeneSymbol	CpG island Description	Ratio of relative methylation (TiO_2-NP/Vehicle)	Sham group			TiO_2-H group		
						Relative methylation (Cy5/Cy3)	Cy3 signal (Input)	Cy5 signal (MeDIP)	Relative methylation (Cy5/Cy3)	Cy3 signal (Input)	Cy5 signal (MeDIP)
A_68_P23542998	chr5:24110558-24110602	NM_025891:-2760	Smarcd3	PROMOTER	0.433	16.460	4308.31	70915.24	7.132	4900.85	34954.33
A_68_P23542977	chr5:24108129-24108173	NM_025891:-330	Smarcd3	PROMOTER	0.61	3.314	4069.30	13486.67	2.022	2945.29	5956.21
A_68_P28237421	chr12:11273151-11273195	NM_025695:481	Smc6	INSIDE	0.187	13.777	939.08	12937.77	2.581	943.87	2435.93
A_68_P28237417	chr12:11272676-11272720	NM_025695:7	Smc6	INSIDE	0.59	0.434	1822.24	790.67	0.256	1312.19	335.82
A_68_P30370941	chr15:80064556-80064600	NM_178719:69	Smcr7l	INSIDE	0.645	0.343	5045.16	1728.90	0.221	3632.03	802.34
A_68_P28685879	chr12:102321594-102321638	NM_001160214:296	Smek1	INSIDE	0.385	4.288	1968.83	8442.53	1.651	1570.20	2592.04
A_68_P28685877	chr12:102321356-102321400	NM_001160214:534	Smek1	INSIDE	0.396	1.767	3537.25	6249.12	0.7	2796.17	1957.22
A_68_P25457391	chr7:125387085-125387129	NM_001031814:45	Smg1	INSIDE	0.331	13.966	12638.80	176516.30	4.628	12475.46	57736.43
A_68_P27923261	chr11:74739468-74739512	NM_001002764:117	Smg6	INSIDE	0.456	0.358	1305.44	467.21	0.163	1051.23	171.40
A_68_P20723679	chr1:154749232-154749276	NM_001005507:522	Smg7	INSIDE	0.202	2.179	4550.42	9913.49	0.441	3436.92	1513.98
A_68_P20723682	chr1:154749623-154749667	NM_001005507:132	Smg7	INSIDE	0.346	0.501	1162.33	582.07	0.173	1004.07	173.85
A_68_P20723684	chr1:154750088-154750132	NM_001005507:-334	Smg7	PROMOTER	0.64	4.347	1157.82	5033.32	2.781	1103.91	3070.25
A_68_P32182255	chr19:53465351-53465395	NM_172429:-309	Smndc1	PROMOTER	0.603	0.729	1885.30	1374.76	0.439	1204.91	529.54
A_68_P32182251	chr19:53464993-53465037	NM_172429:49	Smndc1	INSIDE	0.602	0.664	1164.91	773.16	0.4	843.53	337.14
A_68_P24328142	chr6:29685305-29685349	NM_176996:-170	Smo	PROMOTER	0.207	22.318	2834.26	63256.23	4.628	3526.71	16322.13
A_68_P24328151	chr6:29686313-29686357	NM_176996:838	Smo	INSIDE	0.33	0.667	1588.49	1059.44	0.22	1205.12	265.19
A_68_P31058946	chr17:14416584-14416628	NM_022315:94	Smoc2	INSIDE	0.516	0.379	1740.50	660.48	0.196	1364.69	267.17
A_68_P31058947	chr17:14416697-14416741	NM_022315:206	Smoc2	INSIDE	0.598	2.779	1752.62	4871.34	1.663	1452.33	2415.80
A_68_P31058955	chr17:14417592-14417636	NM_022315:1102	Smoc2	INSIDE	0.642	2.425	1610.56	3905.96	1.556	1433.62	2230.55
A_68_P21635209	chr2:131317684-131317728	NM_001177833:109	Smox	INSIDE	0.306	20.198	1795.54	36266.77	6.179	1686.77	10422.75
A_68_P21635215	chr2:131318208-131318252	NM_001177833:633	Smox	INSIDE	0.592	2.859	1518.29	4340.50	1.692	1257.46	2127.77
A_68_P30574719	chr16:17619415-17619459	NM_001164609:-10	Smpd4	PROMOTER	0.452	6.782	1075.21	7292.30	3.066	1004.04	3078.56
A_68_P32766765	chrX:153929388-153929432	NM_009214:568	Sms	INSIDE	0.072	79.600	1452.53	115620.40	5.755	1320.61	7599.69
A_68_P32766772	chrX:153930279-153930323	NM_009214:-322	Sms	PROMOTER	0.232	13.436	666.61	8956.81	3.12	845.01	2636.47
A_68_P32766764	chrX:153929309-153929353	NM_009214:648	Sms	INSIDE	0.297	12.292	1197.81	14723.36	3.645	1439.66	5247.86
A_68_P27552578	chr11:3435772-3435816	NM_001159284:3501	Smtn	INSIDE	0.642	2.838	1078.60	3060.70	1.822	945.63	1722.56
A_68_P27911313	chr11:72224759-72224803	NM_177776:435	Smtnl2	INSIDE	0.361	0.375	1718.11	643.49	0.135	1289.82	174.22
A_68_P27911318	chr11:72225271-72225315	NM_177776:-77	Smtnl2	PROMOTER	0.614	0.588	2818.73	1656.50	0.361	2277.54	821.31
A_68_P27911312	chr11:72224674-72224718	NM_177776:519	Smtnl2	INSIDE	0.516	1.385	5723.61	7927.76	0.714	4039.92	2885.46
A_68_P22858652	chr4:40704660-40704704	NM_021535:236	Smu1	INSIDE	0.612	0.424	2922.73	1238.01	0.259	2249.20	583.30
A_68_P30502555	chr15:102999581-102999625	NM_027885:-5887	Smug1	PROMOTER	0.607	0.312	3774.05	1176.01	0.189	2638.36	498.73
A_68_P20929009	chr1:191745833-191745877	NM_026796:313	Smyd2	INSIDE	0.588	0.498	966.61	481.33	0.293	740.30	216.73
A_68_P20870658	chr1:181448224-181448272	NM_027188:-114	Smyd3	PROMOTER	0.386	7.518	403.41	3032.77	2.899	401.11	1162.83
A_68_P24604962	chr6:85381989-85382033	NM_144918:41	Smyd5	INSIDE	0.242	0.435	7225.66	3144.00	0.105	4966.28	522.38
A_68_P21836028	chr2:167364137-167364181	NM_011427:432	Snai1	INSIDE	0.081	5.602	5151.27	28856.89	0.451	3904.21	1761.31
A_68_P21836027	chr2:167363971-167364015	NM_011427:266	Snai1	INSIDE	0.179	0.581	2127.20	1235.05	0.104	1734.31	179.91
A_68_P21836025	chr2:167363730-167363774	NM_011427:26	Snai1	INSIDE	0.539	3.881	1853.76	7195.30	2.094	1431.63	2997.72
A_68_P30573672	chr16:17405621-17405665	NM_023348:-450	Snap29	PROMOTER	0.213	0.384	3505.04	1346.68	0.082	2288.37	186.98
A_68_P30573671	chr16:17405548-17405592	NM_023348:-522	Snap29	PROMOTER	0.521	0.362	12618.44	4569.64	0.189	8101.82	1529.92
A_68_P26701864	chr9:86774762-86774806	NM_013669:423	Snap91	INSIDE	0.53	4.604	2650.91	12204.73	2.442	2135.65	5215.77
A_68_P26701866	chr9:86774905-86774949	NM_013669:281	Snap91	INSIDE	0.535	0.506	2058.87	1041.19	0.271	1509.81	408.48
A_68_P25616848	chr8:4253392-4253436	NM_133968:313	Snapc2	INSIDE	0.658	0.552	6601.73	3644.66	0.363	4322.41	1571.18
A_68_P21078285	chr2:26236006-26236050	NM_172339:145	Snapc4	INSIDE	0.555	5.818	4068.14	23670.15	3.23	3519.84	11368.40
A_68_P26580558	chr9:64027298-64027342	NM_183316:217	Snapc5	INSIDE	0.134	3.044	3137.48	9549.74	0.407	2343.71	954.67
A_68_P31709379	chr18:52927884-52927928	NM_001199154:109	Sncaip	INSIDE	0.528	0.386	3409.45	1316.33	0.204	2717.88	554.13
A_68_P29051301	chr13:54867120-54867164	NM_033610:659	Sncb	INSIDE	0.223	11.149	3002.74	33476.62	2.485	2459.80	6113.32

ProbeName	Target position of probe on CpG island microarray	TargetID	GeneSymbol	CpG island Description	Ratio of relative methylation (TiO_2-NP/Vehicle)	Sham group			TiO_2-H group		
						Relative methylation (Cy5/Cy3)	Cy3 signal (Input)	Cy5 signal (MeDIP)	Relative methylation (Cy5/Cy3)	Cy3 signal (Input)	Cy5 signal (MeDIP)
A_68_P24320419	chr6:28430125-28430169	NM_019776:-201	Snd1	PROMOTER	0.158	15.933	1099.32	17515.59	2.52	913.55	2302.59
A_68_P28707014	chr12:106270570-106270614	NR_003145:-103	Snhg10	DIVERGENT_PROMOTER	0.556	5.347	929.62	4970.50	2.971	793.66	2358.17
A_68_P23271975	chr4:124744170-124744214	NM_175246:256	Snip1	INSIDE	0.491	0.354	1525.28	540.26	0.174	1257.49	218.92
A_68_P30546007	chr16:11066704-11066748	NM_009223:336	Snn	INSIDE	0.524	0.559	2007.11	1122.01	0.293	1578.33	462.07
A_68_P30546005	chr16:11066391-11066435	NM_009223:22	Snn	INSIDE	0.453	3.180	1801.03	5727.97	1.441	1430.35	2060.77
A_68_P28052666	chr11:97643069-97643113	NR_028078:-10	Snora21	PROMOTER	0.285	14.773	616.51	9108.00	4.207	531.98	2238.10
A_68_P22488493	chr3:123211082-123211126	NR_028573:-120	Snora24	PROMOTER	0.666	0.582	1115.16	649.16	0.388	905.49	350.90
A_68_P23801182	chr5:74489320-74489364	NR_031758:-212	Snora26	PROMOTER	0.523	2.692	2497.29	6723.24	1.407	1993.42	2805.65
A_68_P28744649	chr12:112776399-112776444	NR_033168:-2735	Snora28	PROMOTER	0.53	5.232	8730.39	45678.01	2.774	7630.71	21167.08
A_68_P28744650	chr12:112776572-112776616	NR_033168:-2562	Snora28	PROMOTER	0.656	4.307	1024.48	4412.95	2.828	837.98	2369.67
A_68_P25408486	chr7:116662855-116662899	NR_028079:-769	Snora3	PROMOTER	0.314	4.615	2348.62	10839.78	1.448	1996.36	2891.62
A_68_P25588870	chr7:148633634-148633678	NR_034049:-1075	Snora52	PROMOTER	0.527	4.458	6465.15	28821.91	2.349	4454.11	10464.27
A_68_P25362157	chr7:106632148-106632192	NR_002172:-728	Snord15a	PROMOTER	0.471	11.794	561.47	6621.76	5.552	490.09	2720.92
A_68_P25091293	chr7:52383927-52383971	NR_000002:-1115	Snord32a	PROMOTER	0.281	0.322	2829.31	909.74	0.09	1895.64	171.39
A_68_P23232782	chr4:116828713-116828757	NR_030704:-281	Snord55	PROMOTER	0.34	9.572	511.10	4892.19	3.25	398.17	1294.26
A_68_P20411724	chr1:88255322-88255366	NR_002851:-2442	Snord82	PROMOTER	0.474	9.423	1536.45	14477.65	4.465	1451.30	6480.35
A_68_P20024912	chr1:9934064-9934108	NR_004410:-1462	Snord87	PROMOTER	0.428	0.135	3966.62	534.62	0.058	3038.84	175.19
A_68_P20024913	chr1:9934160-9934204	NR_004410:-1558	Snord87	PROMOTER	0.651	0.556	1117.95	621.59	0.362	975.05	352.84
A_68_P21745020	chr2:151457975-151458019	NM_198214:273	Snph	INSIDE	0.658	6.586	472.55	3112.25	4.334	416.14	1803.38
A_68_P26887498	chr9:122026335-122026379	NM_001164572:-27	Snrk	PROMOTER	0.217	11.597	3141.76	36435.59	2.512	2439.50	6128.63
A_68_P26887499	chr9:122026546-122026590	NM_001164572:185	Snrk	INSIDE	0.537	3.528	972.93	3432.51	1.894	919.55	1741.79
A_68_P21612035	chr2:127034187-127034231	NM_177214:69	Snrnp200	INSIDE	0.662	0.718	1313.49	943.06	0.475	1072.77	509.89
A_68_P25092755	chr7:52631986-52632030	NM_009224:19009	Snrnp70	INSIDE	0.387	10.824	1130.96	12241.60	4.184	773.33	3235.63
A_68_P25092756	chr7:52632188-52632232	NM_009224:18807	Snrnp70	INSIDE	0.66	4.607	1895.08	8731.55	3.043	1598.42	4864.31
A_68_P26142251	chr8:109460182-109460226	NM_009229:555	Sntb2	INSIDE	0.636	2.321	4107.32	9532.27	1.475	3322.65	4900.66
A_68_P20022051	chr1:9290036-9290080	NM_027671:-247	Sntg1	PROMOTER	0.309	5.622	1717.99	9657.83	1.736	1560.12	2707.92
A_68_P28325482	chr12:31058380-31058424	NM_172951:-162	Sntg2	PROMOTER	0.406	0.499	1556.86	776.31	0.202	1105.56	223.58
A_68_P26591323	chr9:65972506-65972550	NM_019727:165	Snx1	INSIDE	0.515	5.208	1288.16	6709.08	2.68	1037.84	2781.87
A_68_P28351016	chr12:35731926-35731970	NM_001014973:88	Snx13	INSIDE	0.425	14.375	1732.80	24908.32	6.108	1509.54	9219.88
A_68_P28351013	chr12:35731615-35731659	NM_001014973:-224	Snx13	PROMOTER	0.473	0.497	1190.52	591.77	0.235	988.09	232.41
A_68_P26710064	chr9:88333383-88333427	NM_172926:385	Snx14	INSIDE	0.528	3.570	1707.29	6095.83	1.886	1643.71	3099.45
A_68_P23576986	chr5:31495832-31495876	NM_153680:178	Snx17	INSIDE	0.563	3.205	2302.97	7382.16	1.804	1908.52	3443.58
A_68_P26042151	chr8:91151730-91151774	NM_027840:8275	Snx20	INSIDE	0.441	0.614	850.77	522.22	0.271	720.04	195.10
A_68_P26591015	chr9:65917041-65917085	NM_001025612:476	Snx22	INSIDE	0.429	9.475	1279.96	12127.39	4.069	1089.34	4432.06
A_68_P22339767	chr3:94386067-94386111	NM_029721:536	Snx27	INSIDE	0.573	0.425	6518.14	2769.21	0.243	5148.40	1253.51
A_68_P22339768	chr3:94386146-94386190	NM_029721:456	Snx27	INSIDE	0.621	0.202	11436.09	2306.99	0.125	7841.33	982.67
A_68_P27095211	chr10:42222280-42222324	NM_017472:443	Snx3	INSIDE	0.299	6.207	1549.09	9615.81	1.856	1410.44	2617.34
A_68_P27095209	chr10:42222002-42222046	NM_017472:165	Snx3	INSIDE	0.566	7.629	1114.58	8502.80	4.318	1004.25	4335.96
A_68_P26539387	chr9:56775942-56775986	NM_175483:214	Snx33	INSIDE	0.453	0.389	1257.61	489.57	0.176	958.00	168.78
A_68_P30656969	chr16:33251415-33251459	NM_080557:-105	Snx4	PROMOTER	0.408	27.187	2831.71	76986.63	11.095	2881.85	31973.48
A_68_P30656972	chr16:33251779-33251823	NM_080557:259	Snx4	INSIDE	0.552	0.270	1803.21	486.24	0.149	1300.60	193.66
A_68_P28449264	chr12:55896516-55896560	NM_026998:111	Snx6	INSIDE	0.616	9.543	1283.44	12247.70	5.882	1078.64	6344.10
A_68_P24141205	chr5:140865282-140865326	NM_172277:-103	Snx8	PROMOTER	0.216	23.637	9783.02	231241.60	5.098	9897.74	50457.56
A_68_P31019379	chr17:5841808-5841852	NM_025664:451	Snx9	INSIDE	0.461	4.417	1491.23	6586.62	2.038	1390.78	2834.36
A_68_P27098030	chr10:42741219-42741263	NM_175407:153096	Sobp	INSIDE	0.48	8.160	373.08	3044.23	3.915	410.64	1607.62
A_68_P27098028	chr10:42740979-42741023	NM_175407:153336	Sobp	INSIDE	0.533	0.401	3043.00	1219.42	0.214	2287.09	488.48

ProbeName	Target position of probe on CpG island microarray	TargetID	GeneSymbol	CpG island Description	Ratio of relative methylation (TiO_2-NP/Vehicle)	Sham group			TiO_2-H group		
						Relative methylation (Cy5/Cy3)	Cy3 signal (Input)	Cy5 signal (MeDIP)	Relative methylation (Cy5/Cy3)	Cy3 signal (Input)	Cy5 signal (MeDIP)
A_68_P27099065	chr10:42894301-42894345	NM_175407:14	Sobp	INSIDE	0.633	6.909	3896.52	26921.78	4.374	2963.48	12961.24
A_68_P27099056	chr10:42893322-42893366	NM_175407:992	Sobp	INSIDE	0.549	0.606	1859.35	1127.59	0.333	1411.93	469.73
A_68_P30544459	chr16:10785615-10785659	NM_009896:-7	Socs1	PROMOTER	0.203	19.143	3039.73	58189.53	3.89	3625.07	14101.38
A_68_P30544453	chr16:10785046-10785090	NM_009896:561	Socs1	INSIDE	0.589	0.449	2679.68	1204.06	0.265	1935.07	511.90
A_68_P30544457	chr16:10785415-10785459	NM_009896:193	Socs1	INSIDE	0.61	0.332	2828.04	938.03	0.202	2170.50	439.04
A_68_P27365029	chr10:94878409-94878453	NM_001168656:-312	Socs2	PROMOTER	0.223	13.181	1245.41	16415.86	2.94	1039.55	3056.69
A_68_P27365030	chr10:94878524-94878568	NM_001168656:-428	Socs2	PROMOTER	0.373	6.560	1093.33	7172.50	2.445	934.54	2285.36
A_68_P27365031	chr10:94878605-94878649	NM_001168656:-508	Socs2	PROMOTER	0.382	4.964	1939.45	9626.72	1.895	1727.76	3274.51
A_68_P27365028	chr10:94878277-94878321	NM_001168656:-180	Socs2	PROMOTER	0.579	0.582	848.29	493.84	0.337	687.89	231.95
A_68_P31421198	chr17:87506616-87506660	NM_019654:-380	Socs5	PROMOTER	0.521	0.733	2296.38	1683.06	0.382	1712.39	653.32
A_68_P28050117	chr11:97224316-97224360	NM_138657:474	Socs7	INSIDE	0.375	0.426	1441.96	614.45	0.16	1079.91	172.56
A_68_P28050112	chr11:97223519-97223563	NM_138657:-324	Socs7	PROMOTER	0.663	3.264	1124.23	3669.05	2.163	967.33	2091.97
A_68_P28050120	chr11:97224658-97224702	NM_138657:816	Socs7	INSIDE	0.619	0.580	1565.12	908.08	0.359	1240.45	445.47
A_68_P31054932	chr17:13200778-13200822	NM_013671:96	Sod2	INSIDE	0.431	7.803	1330.80	10383.88	3.362	1191.45	4006.21
A_68_P29699987	chr14:70606016-70606060	NM_011366:1406	Sorbs3	INSIDE	0.175	21.525	2530.59	54470.58	3.761	2066.87	7774.50
A_68_P29699885	chr14:70591939-70591983	NM_011366:15484	Sorbs3	INSIDE	0.609	0.527	1143.35	602.40	0.321	769.27	246.75
A_68_P32168785	chr19:50752526-50752570	NM_021377:554	Sorcs1	INSIDE	0.443	14.019	4391.82	61569.29	6.216	3649.68	22687.58
A_68_P32168786	chr19:50752601-50752645	NM_021377:480	Sorcs1	INSIDE	0.542	0.425	1670.57	710.31	0.23	1201.36	276.82
A_68_P23607369	chr5:36740782-36740826	NM_030889:-16	Sorcs2	PROMOTER	0.381	15.471	5117.20	79169.25	5.891	4123.64	24290.65
A_68_P22411695	chr3:108086638-108086682	NM_019972:-389	Sort1	PROMOTER	0.557	19.733	3612.14	71278.76	11.001	2622.22	28845.93
A_68_P31383417	chr17:80882569-80882613	NM_009231:-2797	Sos1	PROMOTER	0.322	0.626	1303.49	815.46	0.202	952.58	191.98
A_68_P28515845	chr12:70782400-70782444	NM_001135559:417	Sos2	INSIDE	0.34	6.612	1440.51	9525.15	2.248	1675.07	3766.11
A_68_P28357647	chr12:37043910-37043954	NM_025312:3177	Sostdc1	INSIDE	0.547	0.697	1698.83	1183.92	0.381	1316.72	502.17
A_68_P25661260	chr8:12397084-12397128	NM_009233:1588	Sox1	INSIDE	0.27	1.810	2602.44	4710.81	0.489	2121.75	1038.07
A_68_P25661253	chr8:12396004-12396049	NM_009233:508	Sox1	INSIDE	0.41	0.342	2027.52	693.03	0.14	1660.66	232.48
A_68_P30364761	chr15:78993943-78993987	NM_011437:956	Sox10	INSIDE	0.55	2.593	1913.59	4961.26	1.426	1604.39	2287.73
A_68_P28307263	chr12:28027320-28027364	NM_009234:241	Sox11	INSIDE	0.156	103.203	3259.87	336428.00	16.093	3150.51	50700.29
A_68_P28307270	chr12:28028339-28028383	NM_009234:-777	Sox11	PROMOTER	0.342	5.657	4899.40	27717.95	1.934	3744.01	7242.26
A_68_P28307255	chr12:28026369-28026413	NM_009234:1193	Sox11	INSIDE	0.661	3.332	1641.80	5470.89	2.204	1388.04	3059.62
A_68_P28307233	chr12:28023729-28023773	NM_009234:3833	Sox11	INSIDE	0.214	6.879	2093.01	14397.38	1.474	1637.57	2414.18
A_68_P21749123	chr2:152223588-152223632	NM_011438:172	Sox12	INSIDE	0.393	5.835	1739.99	10152.56	2.291	1548.22	3547.68
A_68_P26766649	chr9:99775472-99775516	NM_011440:1095	Sox14	INSIDE	0.206	2.567	3598.45	9235.58	0.529	2666.34	1411.62
A_68_P26766650	chr9:99775559-99775603	NM_011440:1009	Sox14	INSIDE	0.61	0.464	2068.85	959.84	0.283	1665.96	471.59
A_68_P26766660	chr9:99776718-99776762	NM_011440:-151	Sox14	PROMOTER	0.644	0.416	1413.09	587.69	0.268	1250.45	334.78
A_68_P20005810	chr1:4483711-4483755	NM_011441:2762	Sox17	INSIDE	0.32	13.492	885.17	11943.04	4.32	857.33	3703.58
A_68_P22049386	chr3:34548677-34548721	NM_011443:-228	Sox2	PROMOTER	0.352	5.555	2691.89	14952.66	1.958	2182.76	4273.41
A_68_P22049390	chr3:34549137-34549181	NM_011443:232	Sox2	INSIDE	0.593	0.378	2625.88	992.93	0.224	1939.84	434.88
A_68_P22049387	chr3:34548813-34548857	NM_011443:-92	Sox2	PROMOTER	0.652	3.744	1580.98	5919.50	2.442	1304.64	3185.36
A_68_P22049376	chr3:34547722-34547766	NM_011443:-1182	Sox2	PROMOTER	0.501	0.686	1624.65	1114.27	0.344	1262.60	434.06
A_68_P22049395	chr3:34549827-34549871	NM_011443:922	Sox2	INSIDE	0.419	0.612	1064.82	651.41	0.256	839.42	214.94
A_68_P29937469	chr14:118633887-118633936	NM_177753:2341	Sox21	INSIDE	0.587	5.995	503.43	3018.09	3.52	437.99	1541.65
A_68_P29937483	chr14:118635646-118635690	NM_177753:584	Sox21	INSIDE	0.611	0.327	2353.72	770.23	0.2	1733.91	346.63
A_68_P32415817	chrX:58147657-58147701	NM_009237:-1073	Sox3	PROMOTER	0.396	10.797	998.49	10780.88	4.275	942.82	4030.79
A_68_P32415805	chrX:58146252-58146296	NM_009237:331	Sox3	INSIDE	0.569	3.555	752.86	2676.58	2.023	957.38	1937.19
A_68_P28909030	chr13:29051600-29051644	NM_009238:-6071	Sox4	PROMOTER	0.26	12.007	3531.25	42401.00	3.125	2709.29	8466.92
A_68_P28908966	chr13:29044288-29044332	NM_009238:1241	Sox4	INSIDE	0.4	8.519	2096.89	17864.26	3.411	1944.60	6633.73

ProbeName	Target position of probe on CpG island microarray	TargetID	GeneSymbol	CpG island Description	Ratio of relative methylation (TiO_2-NP/Vehicle)	Sham group			TiO_2-H group		
						Relative methylation (Cy5/Cy3)	Cy3 signal (Input)	Cy5 signal (MeDIP)	Relative methylation (Cy5/Cy3)	Cy3 signal (Input)	Cy5 signal (MeDIP)
A_68_P28908970	chr13:29044714-29044758	NM_009238:815	Sox4	INSIDE	0.343	0.634	1008.27	639.16	0.217	796.77	173.19
A_68_P24912678	chr6:143782013-143782057	NM_001113559:376044	Sox5	INSIDE	0.382	5.597	1547.89	8663.64	2.138	1251.56	2676.23
A_68_P25445817	chr7:123177303-123177347	NM_001025560:-2763	Sox6	PROMOTER	0.248	12.802	2651.77	33948.16	3.171	2843.58	9017.24
A_68_P25445803	chr7:123175462-123175506	NM_001025560:-923	Sox6	PROMOTER	0.61	0.687	1410.28	969.42	0.419	1211.72	508.01
A_68_P29664626	chr14:64562881-64562925	NM_011446:360	Sox7	INSIDE	0.403	7.142	1803.76	12882.79	2.881	1423.64	4101.32
A_68_P31104792	chr17:25707704-25707748	NM_011447:-95	Sox8	DIVERGENT_PROMOTER	0.412	14.886	1392.61	20730.61	6.131	1551.40	9512.24
A_68_P28137828	chr11:112643707-112643751	NM_011448:205	Sox9	INSIDE	0.386	7.846	3446.36	27040.08	3.029	2836.24	8590.64
A_68_P28137844	chr11:112645388-112645432	NM_011448:1887	Sox9	INSIDE	0.554	0.264	1723.84	455.79	0.146	1270.92	186.03
A_68_P28137853	chr11:112646459-112646503	NM_011448:2957	Sox9	INSIDE	0.637	0.396	6314.11	2500.50	0.252	4360.43	1100.54
A_68_P28137856	chr11:112646719-112646763	NM_011448:3217	Sox9	INSIDE	0.351	0.630	5261.07	3314.66	0.221	3892.09	861.02
A_68_P21328883	chr2:72818359-72818403	NM_001098425:-1042	Sp3	PROMOTER	0.308	8.933	32205.15	287680.70	2.754	32382.12	89188.84
A_68_P21328888	chr2:72818835-72818879	NM_001018042:-353	Sp3	PROMOTER	0.614	2.612	854.11	2230.59	1.605	781.34	1253.86
A_68_P28778756	chr12:119539812-119539856	NM_001166385:79	Sp4	INSIDE	0.251	0.535	1618.47	865.55	0.134	1259.85	169.29
A_68_P28778754	chr12:119539626-119539670	NM_001166385:265	Sp4	INSIDE	0.391	5.206	5877.59	30597.97	2.034	3981.67	8098.09
A_68_P28048173	chr11:96879906-96879950	NM_031183:5046	Sp6	INSIDE	0.212	38.177	4626.93	176640.20	8.078	4093.57	33069.18
A_68_P30497455	chr15:102189021-102189065	NM_130458:7660	Sp7	INSIDE	0.479	0.625	1051.32	656.60	0.299	838.98	250.76
A_68_P28781893	chr12:120087514-120087558	NM_177082:2735	Sp8	INSIDE	0.312	18.501	2059.05	38094.48	5.772	1767.06	10199.17
A_68_P28781792	chr12:120075230-120075274	NM_177082:-9549	Sp8	PROMOTER	0.385	6.190	3647.18	22574.69	2.384	3375.21	8047.28
A_68_P28781896	chr12:120087840-120087884	NM_177082:3061	Sp8	INSIDE	0.574	5.137	1586.31	8148.51	2.946	1331.88	3924.24
A_68_P28781791	chr12:120075046-120075090	NM_177082:-9733	Sp8	PROMOTER	0.575	2.873	2502.06	7189.62	1.653	2156.18	3565.15
A_68_P21330556	chr2:73115402-73115446	NM_001005343:5442	Sp9	DOWNSTREAM	0.542	7.338	4678.20	34327.20	3.98	3886.55	15466.91
A_68_P21330519	chr2:73111290-73111334	NM_001005343:1330	Sp9	INSIDE	0.563	0.348	2147.04	748.19	0.196	1601.39	314.39
A_68_P21330530	chr2:73112578-73112622	NM_001005343:2618	Sp9	INSIDE	0.332	0.549	1097.63	602.89	0.183	941.29	171.83
A_68_P30137263	chr15:36140403-36140447	NM_012031:31140	Spag1	INSIDE	0.482	8.408	1790.19	15052.41	4.053	1408.04	5707.29
A_68_P21769255	chr2:155891652-155891696	NM_139151:726	Spag4	INSIDE	0.615	0.535	1774.69	949.27	0.329	1421.86	467.60
A_68_P27942521	chr11:78115275-78115319	NM_017407:204	Spag5	INSIDE	0.335	16.766	1771.39	29698.17	5.615	1687.68	9476.14
A_68_P27942517	chr11:78114890-78114934	NM_017407:-180	Spag5	PROMOTER	0.533	0.162	3084.85	499.25	0.086	2123.21	183.18
A_68_P27942519	chr11:78115075-78115119	NM_017407:4	Spag5	INSIDE	0.228	8.469	1162.44	9844.94	1.93	860.31	1660.03
A_68_P31351023	chr17:74738587-74738631	NM_001162870:282	Spast	INSIDE	0.517	7.640	623.43	4763.15	3.952	658.38	2601.62
A_68_P22614971	chr3:146162605-146162649	NM_027617:91	Spata1	INSIDE	0.371	6.845	855.20	5853.87	2.542	829.01	2107.13
A_68_P21835810	chr2:167318513-167318557	NM_170756:-160	Spata2	PROMOTER	0.599	0.212	2531.34	536.10	0.127	1772.05	224.75
A_68_P23212277	chr4:111392547-111392591	NM_026470:-46	Spata6	PROMOTER	0.606	0.574	2706.61	1552.60	0.348	2306.75	802.12
A_68_P30478338	chr15:98957605-98957662	NM_139140:358	Spats2	INSIDE	0.286	1.449	1939.60	2811.39	0.414	1398.25	579.14
A_68_P30478336	chr15:98957457-98957502	NM_139140:204	Spats2	INSIDE	0.184	3.312	6359.01	21058.04	0.609	4680.12	2850.94
A_68_P21307670	chr2:69044152-69044196	NM_001199123:73	Spc25	INSIDE	0.465	8.314	1840.59	15302.46	3.869	1557.52	6025.92
A_68_P27851932	chr11:61890556-61890600	NM_001029936:-20	Specc1	PROMOTER	0.432	0.369	1476.33	545.37	0.159	1083.54	172.78
A_68_P27851930	chr11:61890326-61890370	NM_001029936:-250	Specc1	PROMOTER	0.618	2.395	7250.65	17368.42	1.479	5633.34	8334.09
A_68_P27851931	chr11:61890435-61890479	NM_001029936:-142	Specc1	PROMOTER	0.158	4.381	3469.95	15203.14	0.694	2141.83	1485.77
A_68_P21633642	chr2:130997305-130997349	NM_027641:3220	Spef1	INSIDE	0.444	3.522	1540.97	5427.74	1.562	1232.85	1926.10
A_68_P20353634	chr1:75378425-75378469	NM_001085371:-242	Speg	PROMOTER	0.29	0.453	5876.43	2661.35	0.131	3950.90	518.23
A_68_P20353933	chr1:75419052-75419096	NM_001085370:36890	Speg	INSIDE	0.61	0.489	3269.59	1599.86	0.299	2249.05	671.35
A_68_P20353935	chr1:75419232-75419276	NM_001085370:37070	Speg	INSIDE	0.619	0.457	7924.28	3617.88	0.283	5372.85	1518.28
A_68_P20353688	chr1:75385168-75385212	NM_001085370:3006	Speg	INSIDE	0.347	1.421	1447.58	2056.59	0.494	1195.13	589.92
A_68_P23364649	chr4:141094339-141094383	NM_019763:152	Spen	INSIDE	0.647	3.140	2734.34	8586.71	2.031	2255.10	4580.05
A_68_P23364273	chr4:141028839-141028883	NM_019763:65652	Spen	INSIDE	0.493	3.431	2378.24	8158.90	1.693	1733.72	2934.54
A_68_P23364274	chr4:141028926-141028970	NM_019763:65564	Spen	INSIDE	0.51	3.648	887.46	3237.06	1.86	682.78	1269.74

ProbeName	Target position of probe on CpG island microarray	TargetID	GeneSymbol	CpG island Description	Ratio of relative methylation (TiO_2-NP/Vehicle)	Sham group			TiO_2-H group		
						Relative methylation (Cy5/Cy3)	Cy3 signal (Input)	Cy5 signal (MeDIP)	Relative methylation (Cy5/Cy3)	Cy3 signal (Input)	Cy5 signal (MeDIP)
A_68_P21584534	chr2:121944006-121944050	NM_145531:94	Spgl1	INSIDE	0.54	3.536	671.89	2376.09	1.908	588.62	1123.13
A_68_P21584536	chr2:121944215-121944259	NM_145531:-114	Spgl1	PROMOTER	0.657	2.137	2817.11	6019.45	1.404	2073.16	2910.78
A_68_P21584533	chr2:121943910-121943954	NM_145531:190	Spgl1	INSIDE	0.592	2.835	697.44	1977.49	1.679	645.75	1084.01
A_68_P26587800	chr9:65308975-65309019	NM_138584:253	Spg21	INSIDE	0.666	6.458	6488.78	41903.93	4.301	4694.43	20192.73
A_68_P28159847	chr11:116392890-116392934	NM_001172475:-312	Sphk1	PROMOTER	0.543	0.599	1841.73	1103.25	0.325	1391.64	452.77
A_68_P21568536	chr2:119063328-119063372	NM_016907:255	Spint1	INSIDE	0.56	3.173	3403.62	10801.20	1.776	2471.55	4390.22
A_68_P21568538	chr2:119063550-119063594	NM_016907:477	Spint1	INSIDE	0.439	0.550	855.56	470.86	0.242	709.67	171.53
A_68_P25026344	chr7:30066373-30066417	NM_001082548:602	Spint2	INSIDE	0.43	1.476	5378.55	7937.48	0.634	4274.11	2709.97
A_68_P26239286	chr8:125857009-125857053	NM_172287:418	Spire2	INSIDE	0.342	0.254	2831.39	720.31	0.087	2101.99	183.10
A_68_P21100500	chr2:29821292-29821336	NM_001076554:235	Spna2	INSIDE	0.327	17.872	3160.10	56478.54	5.84	2532.28	14788.68
A_68_P21100496	chr2:29820843-29820887	NM_001076554:-215	Spna2	PROMOTER	0.185	8.876	3243.39	28788.00	1.638	2832.75	4639.45
A_68_P28555624	chr12:77810657-77810701	NM_013675:856	Spnb1	INSIDE	0.345	0.215	3091.93	664.61	0.074	2382.64	176.62
A_68_P28554952	chr12:77707425-77707469	NM_013675:104088	Spnb1	INSIDE	0.633	4.505	1219.24	5492.35	2.851	1089.76	3106.85
A_68_P25017009	chr7:28160497-28160541	NM_001199235:19007	Spnb4	INSIDE	0.338	5.690	4048.84	23038.68	1.922	3451.72	6635.78
A_68_P25017170	chr7:28193438-28193482	NM_032610:38148	Spnb4	INSIDE	0.655	5.502	1770.19	9739.16	3.603	1522.09	5484.83
A_68_P25017007	chr7:28160302-28160347	NM_001199235:19201	Spnb4	INSIDE	0.327	0.668	1094.70	731.29	0.218	792.26	172.80
A_68_P25016939	chr7:28149566-28149610	NM_001199235:29937	Spnb4	INSIDE	0.563	3.476	873.82	3037.44	1.956	778.34	1522.68
A_68_P25500162	chr7:133520602-133520646	NM_023712:824	Spns1	INSIDE	0.501	4.614	650.93	3003.24	2.31	552.69	1276.81
A_68_P27911818	chr11:72302266-72302310	NM_153060:1118	Spns2	INSIDE	0.475	0.443	1103.11	488.43	0.21	813.54	171.23
A_68_P27911828	chr11:72303091-72303135	NM_153060:294	Spns2	INSIDE	0.479	3.577	2827.95	10116.19	1.714	2149.10	3684.30
A_68_P24007303	chr5:115460994-115461038	NM_029012:-516	Sppl3	PROMOTER	0.373	4.632	1783.22	8259.04	1.728	1456.82	2517.27
A_68_P24603257	chr6:85087734-85087778	NM_011467:2	Spr	INSIDE	0.226	0.526	2042.05	1074.10	0.119	1436.46	170.42
A_68_P24603258	chr6:85087813-85087857	NM_011467:-76	Spr	PROMOTER	0.411	0.531	1979.14	1050.64	0.218	1366.14	298.30
A_68_P21556049	chr2:116946411-116946455	NM_033524:-753	Spred1	PROMOTER	0.131	39.299	5910.89	232290.90	5.147	5771.43	29706.72
A_68_P21556050	chr2:116946481-116946525	NM_033524:-683	Spred1	PROMOTER	0.347	7.810	5392.71	42115.70	2.711	4421.52	11985.30
A_68_P27628926	chr11:19825568-19825612	NM_033523:1146	Spred2	INSIDE	0.47	13.531	1779.04	24072.84	6.356	1431.38	9097.91
A_68_P25025683	chr7:29946879-29946923	NM_182927:6766	Spred3	INSIDE	0.371	4.861	1180.76	5739.64	1.803	997.07	1797.96
A_68_P30496205	chr15:101966489-101966533	NM_001033277:136	Spryd3	INSIDE	0.462	5.874	3541.42	20800.60	2.711	2869.16	7777.40
A_68_P23403482	chr4:149328861-149328905	NM_029035:233	Spsb1	INSIDE	0.415	1.394	6305.63	8791.00	0.579	4240.23	2454.87
A_68_P23403484	chr4:149329133-149329178	NM_029035:-40	Spsb1	PROMOTER	0.656	2.259	1446.81	3267.93	1.482	1119.36	1658.90
A_68_P28615786	chr12:88729140-88729184	NM_011479:18	Sptlc2	INSIDE	0.375	17.400	774.55	13476.88	6.526	757.97	4946.72
A_68_P28615783	chr12:88728757-88728801	NM_011479:402	Sptlc2	INSIDE	0.406	4.967	1158.90	5755.72	2.017	1017.67	2052.99
A_68_P28615267	chr12:88640639-88640683	NM_011479:88520	Sptlc2	DOWNSTREAM	0.605	4.716	699.39	3298.52	2.855	682.87	1949.59
A_68_P28615271	chr12:88641179-88641223	NM_011479:87980	Sptlc2	DOWNSTREAM	0.664	0.168	3968.25	665.40	0.111	2707.98	301.72
A_68_P25102503	chr7:54263926-54263970	NM_175318:-164	Spty2d1	PROMOTER	0.404	0.342	2201.62	753.89	0.138	1781.20	246.15
A_68_P25102500	chr7:54263669-54263713	NM_175318:94	Spty2d1	INSIDE	0.454	6.467	3039.27	19656.11	2.938	2376.88	6982.53
A_68_P21587981	chr2:122591010-122591054	NM_001162503:-62	Sqrdl	PROMOTER	0.252	30.517	2179.99	66525.79	7.693	2040.56	15698.75
A_68_P21587980	chr2:122590894-122590938	NM_001162503:-178	Sqrdl	PROMOTER	0.471	0.235	2211.21	520.59	0.111	1558.62	172.66
A_68_P27787321	chr11:50023806-50023850	NM_011018:464	Sqstm1	INSIDE	0.627	6.929	5593.53	38759.49	4.343	4686.81	20353.84
A_68_P31625185	chr18:36829798-36829843	NM_001164406:145	Sra1	INSIDE	0.417	0.428	1743.38	746.09	0.179	1217.44	217.43
A_68_P21777116	chr2:157282885-157282929	NM_001025395:32878	Src	INSIDE	0.649	3.222	1800.13	5800.61	2.092	1413.63	2957.28
A_68_P28051255	chr11:97407967-97408011	NM_018873:28452	Srcin1	INSIDE	0.317	8.625	1287.69	11106.43	2.735	1015.31	2776.77
A_68_P28051298	chr11:97413135-97413179	NM_018873:23284	Srcin1	INSIDE	0.482	4.338	1189.66	5160.49	2.093	980.54	2052.00
A_68_P28051188	chr11:97398038-97398082	NM_018873:38380	Srcin1	INSIDE	0.504	4.913	1153.69	5668.10	2.478	943.27	2337.66
A_68_P28051170	chr11:97396005-97396049	NM_018873:40414	Srcin1	INSIDE	0.549	4.521	1309.04	5918.14	2.482	1111.12	2758.33
A_68_P24118744	chr5:136438405-136438449	NM_001160366:11920	Srcrb4d	INSIDE	0.296	0.215	3782.94	813.03	0.064	2700.39	171.50

ProbeName	Target position of probe on CpG island microarray	TargetID	GeneSymbol	CpG island Description	Ratio of relative methylation (TiO_2-NP/Vehicle)	Sham group			TiO_2-H group		
						Relative methylation (Cy5/Cy3)	Cy3 signal (Input)	Cy5 signal (MeDIP)	Relative methylation (Cy5/Cy3)	Cy3 signal (Input)	Cy5 signal (MeDIP)
A_68_P24118747	chr5:136438697-136438741	NM_001160366:11628	Srcrb4d	INSIDE	0.476	7.883	1549.56	12215.52	3.753	1231.39	4621.59
A_68_P24118745	chr5:136438525-136438569	NM_001160366:11800	Srcrb4d	INSIDE	0.423	0.731	4377.74	3200.32	0.309	3132.85	969.07
A_68_P29116722	chr13:69750305-69750349	NM_175283:15	Srd5a1	INSIDE	0.481	3.428	1868.32	6404.75	1.65	1356.24	2238.09
A_68_P31349753	chr17:74397223-74397267	NM_053188:12	Srd5a2	INSIDE	0.291	6.484	3013.79	19540.63	1.89	2485.46	4697.10
A_68_P23814358	chr5:76569347-76569391	NM_020611:71	Srd5a3	INSIDE	0.342	7.018	1335.00	9369.30	2.404	1129.73	2715.53
A_68_P27841686	chr11:60033953-60033997	NM_011480:132	Srebf1	INSIDE	0.552	0.264	1741.07	460.04	0.146	1188.00	173.23
A_68_P27841610	chr11:60023907-60023951	NM_011480:10178	Srebf1	INSIDE	0.612	0.728	2074.77	1510.39	0.445	1696.82	755.50
A_68_P27841688	chr11:60034191-60034235	NM_011480:-106	Srebf1	PROMOTER	0.606	2.765	1403.36	3880.53	1.676	1102.97	1848.15
A_68_P30381701	chr15:81977690-81977734	NM_033218:14	Srebf2	INSIDE	0.371	6.379	8215.56	52408.15	2.369	6171.03	14618.79
A_68_P30381702	chr15:81977800-81977844	NM_033218:124	Srebf2	INSIDE	0.574	0.458	1364.87	625.72	0.263	1024.85	269.87
A_68_P30381703	chr15:81977883-81977927	NM_033218:206	Srebf2	INSIDE	0.583	0.216	7521.51	1625.00	0.126	5172.89	651.05
A_68_P29286848	chr13:104564132-104564176	NM_172592:-9492	Srek1	PROMOTER	0.405	0.472	2136.96	1008.92	0.191	1802.32	345.01
A_68_P29292645	chr13:105607251-105607295	NM_026075:-46	Srek1ip1	DIVERGENT_PROMOTER	0.64	0.386	1304.00	503.88	0.247	1120.17	277.20
A_68_P31207575	chr17:46691962-46692006	NM_020493:1127	Srf	INSIDE	0.385	0.268	8520.05	2279.70	0.103	5509.04	568.20
A_68_P31207578	chr17:46692314-46692358	NM_020493:775	Srf	INSIDE	0.434	0.355	1581.74	560.85	0.154	1126.85	173.48
A_68_P31207574	chr17:46691890-46691934	NM_020493:1199	Srf	INSIDE	0.586	0.555	4633.97	2571.44	0.325	3359.52	1092.89
A_68_P31207579	chr17:46692454-46692498	NM_020493:635	Srf	INSIDE	0.594	4.499	2250.07	10123.89	2.671	1949.78	5207.91
A_68_P31207577	chr17:46692178-46692222	NM_020493:911	Srf	INSIDE	0.593	0.733	4237.18	3105.48	0.435	3055.51	1329.00
A_68_P31707756	chr18:52625256-52625301	NM_026040:-68	Srfbp1	PROMOTER	0.418	0.634	748.62	474.97	0.265	669.37	177.64
A_68_P24755319	chr6:112677559-112677603	NM_080448:219680	Srgap3	INSIDE	0.355	2.003	3894.57	7802.11	0.711	2713.45	1929.44
A_68_P31612299	chr18:34490607-34490651	NM_025527:-170	Srp19	PROMOTER	0.384	6.366	1794.84	11425.33	2.448	1422.63	3482.01
A_68_P31612301	chr18:34490799-34490843	NM_025527:22	Srp19	INSIDE	0.569	3.093	1264.91	3912.00	1.758	984.56	1731.08
A_68_P28450919	chr12:56331687-56331731	NM_001100110:244	Srp54c	INSIDE	0.431	0.494	1129.23	557.63	0.213	853.72	181.71
A_68_P28158413	chr11:116135476-116135520	NM_146032:33	Srp68	INSIDE	0.296	0.444	7277.56	3229.84	0.131	4487.06	589.75
A_68_P23819150	chr5:77403737-77403781	NM_025691:33	Srp72	INSIDE	0.598	9.753	3631.82	35420.19	5.835	2981.44	17398.05
A_68_P20885446	chr1:184055324-184055368	NM_012058:479	Srp9	INSIDE	0.495	0.183	2689.96	493.40	0.091	2007.08	182.07
A_68_P20885441	chr1:184054789-184054833	NM_012058:-57	Srp9	PROMOTER	0.345	5.066	1395.93	7071.10	1.749	1364.20	2386.36
A_68_P27923259	chr11:74739230-74739274	NM_001163311:48	Srr	INSIDE	0.565	0.379	1387.74	525.97	0.214	964.63	206.54
A_68_P27923257	chr11:74738967-74739011	NM_001163311:312	Srr	INSIDE	0.245	1.637	706.67	1156.85	0.402	693.66	278.66
A_68_P23991577	chr5:112772027-112772071	NM_027323:12	Srrd	INSIDE	0.356	9.834	15906.55	156425.20	3.499	13275.67	46454.18
A_68_P23328080	chr4:134908744-134908788	NM_001130477:363	Srrm1	INSIDE	0.298	6.905	1479.01	10212.29	2.056	1416.95	2913.82
A_68_P31093720	chr17:23940883-23940927	NM_175229:751	Srrm2	INSIDE	0.277	10.108	1958.51	19795.94	2.804	1623.79	4553.91
A_68_P23330841	chr4:135411994-135412038	NM_001080387:10	Srsf10	INSIDE	0.369	0.261	2410.39	629.17	0.096	1808.11	174.16
A_68_P22683257	chr3:157694741-157694785	NM_001093753:-44	Srsf11	PROMOTER	0.425	0.311	2276.09	708.64	0.132	1835.40	242.58
A_68_P22825734	chr4:33296280-33296324	NM_177774:337	Srsf12	INSIDE	0.243	10.272	1327.10	13631.74	2.501	1158.65	2897.71
A_68_P22825735	chr4:33296468-33296513	NM_177774:525	Srsf12	INSIDE	0.39	0.424	3081.14	1306.95	0.165	2467.00	407.73
A_68_P22825736	chr4:33296596-33296640	NM_177774:653	Srsf12	INSIDE	0.405	5.363	5715.09	30649.37	2.169	4247.43	9214.69
A_68_P28161643	chr11:116713965-116714009	NM_011358:422	Srsf2	INSIDE	0.571	4.954	653.05	3235.36	2.827	587.39	1660.35
A_68_P28161641	chr11:116713805-116713849	NM_011358:582	Srsf2	INSIDE	0.139	12.148	1791.94	21767.64	1.69	1609.92	2720.95
A_68_P28161647	chr11:116714367-116714411	NM_011358:20	Srsf2	INSIDE	0.379	1.815	3435.71	6236.16	0.688	2475.39	1703.22
A_68_P23308817	chr4:131429521-131429565	NM_020587:-11	Srsf4	PROMOTER	0.448	7.422	682.52	5065.55	3.323	611.89	2033.13
A_68_P23308822	chr4:131430090-131430134	NM_020587:559	Srsf4	INSIDE	0.466	0.389	1501.90	584.45	0.181	1123.42	203.62
A_68_P23308821	chr4:131429974-131430018	NM_020587:443	Srsf4	INSIDE	0.589	2.694	1073.69	2891.99	1.586	1010.39	1602.42
A_68_P28578485	chr12:82046655-82046699	NM_001079694:186	Srsf5	INSIDE	0.55	0.284	2669.10	759.28	0.156	2118.75	331.45
A_68_P28578486	chr12:82046738-82046782	NM_001079694:270	Srsf5	INSIDE	0.114	0.697	4739.14	3303.91	0.079	3149.86	249.86
A_68_P24009031	chr5:115777029-115777073	NM_025573:-135	Srsf9	PROMOTER	0.489	6.783	2016.18	13676.53	3.315	1821.64	6038.89

ProbeName	Target position of probe on CpG island microarray	TargetID	GeneSymbol	CpG island Description	Ratio of relative methylation (TiO_2-NP/Vehicle)	Sham group			TiO_2-H group		
						Relative methylation (Cy5/Cy3)	Cy3 signal (Input)	Cy5 signal (MeDIP)	Relative methylation (Cy5/Cy3)	Cy3 signal (Input)	Cy5 signal (MeDIP)
A_68_P21747681	chr2:151931454-151931498	NM_029688:11	Srxn1	INSIDE	0.496	0.440	2389.36	1052.18	0.218	1890.55	412.54
A_68_P21747680	chr2:151931345-151931389	NM_029688:-99	Srxn1	PROMOTER	0.658	0.402	3257.66	1309.65	0.265	2470.05	653.77
A_68_P31513737	chr18:14841164-14841208	NM_001161369:237	Ss18	INSIDE	0.573	10.602	1362.45	14444.14	6.08	1228.99	7472.10
A_68_P24389050	chr6:40421239-40421283	NM_028358:-153	Ssbp1	PROMOTER	0.539	0.582	1868.79	1086.97	0.314	1644.64	516.06
A_68_P24389053	chr6:40421556-40421600	NM_028358:165	Ssbp1	INSIDE	0.494	3.584	896.15	3212.16	1.772	740.07	1311.17
A_68_P29218716	chr13:91599885-91599929	NM_024186:-795	Ssbp2	PROMOTER	0.102	174.625	672.69	117469.20	17.854	442.39	7898.70
A_68_P29218720	chr13:91600319-91600363	NM_024186:-361	Ssbp2	PROMOTER	0.191	18.334	2704.99	49594.22	3.511	2556.31	8974.46
A_68_P29218721	chr13:91600629-91600673	NM_024186:-51	Ssbp2	PROMOTER	0.284	7.627	1494.61	11399.36	2.167	1221.49	2646.45
A_68_P29218725	chr13:91601454-91601498	NM_024186:775	Ssbp2	INSIDE	0.56	0.731	3313.02	2422.51	0.409	2465.42	1009.39
A_68_P23187158	chr4:106584284-106584328	NM_023672:232	Ssbp3	INSIDE	0.348	1.573	1848.55	2907.47	0.547	1467.94	802.48
A_68_P25952971	chr8:73131884-73131928	NM_133772:307	Ssbp4	INSIDE	0.612	0.537	2730.85	1465.88	0.328	1965.90	645.76
A_68_P24001336	chr5:114443642-114443686	NM_198109:102	Ssh1	INSIDE	0.09	33.204	956.51	31759.58	2.987	895.25	2674.46
A_68_P27937051	chr11:77161599-77161643	NM_177710:131694	Ssh2	INSIDE	0.183	17.644	1507.63	26600.26	3.226	1329.41	4288.45
A_68_P27936388	chr11:77029801-77029845	NM_177710:-104	Ssh2	PROMOTER	0.559	0.649	4703.86	3050.84	0.362	3184.38	1154.16
A_68_P31922172	chr19:4269137-4269181	NM_198113:14	Ssh3	INSIDE	0.5	0.490	1022.56	501.35	0.245	697.88	171.17
A_68_P21071033	chr2:25127873-25127917	NM_023464:44	Ssna1	INSIDE	0.436	5.907	2569.10	15176.02	2.573	2065.33	5314.07
A_68_P28957970	chr13:38085587-38085631	NM_025965:451	Ssr1	INSIDE	0.445	0.239	4223.06	1009.36	0.106	3072.11	326.86
A_68_P28957974	chr13:38085985-38086029	NM_025965:53	Ssr1	INSIDE	0.536	0.315	1627.74	512.03	0.169	1324.03	223.20
A_68_P32464770	chrX:71032514-71032558	NM_009279:55	Ssr4	INSIDE	0.628	0.601	775.11	465.90	0.378	1005.70	379.91
A_68_P21395954	chr2:84877657-84877701	NM_001136081:71	Ssrp1	INSIDE	0.455	0.426	1121.19	477.29	0.194	860.96	166.70
A_68_P21395950	chr2:84877269-84877313	NM_182990:-67	Ssrp1	DIVERGENT_PROMOTER	0.642	0.127	4495.70	571.40	0.082	2994.17	244.23
A_68_P28466190	chr12:59313609-59313653	NM_009216:840	Sstr1	INSIDE	0.508	6.620	1165.22	7713.75	3.36	1156.44	3886.09
A_68_P30360706	chr15:78370035-78370079	NM_009218:4719	Sstr3	INSIDE	0.286	9.952	1579.74	15721.33	2.845	1304.84	3712.66
A_68_P23440100	chr4:155079091-155079135	NM_026899:189	Ssu72	INSIDE	0.154	21.946	7516.46	164959.10	3.381	6348.83	21462.90
A_68_P22614372	chr3:146067861-146067905	NM_138744:232	Ssx2ip	INSIDE	0.592	8.704	926.58	8064.60	5.153	788.61	4063.97
A_68_P22614368	chr3:146067514-146067558	NM_138744:-114	Ssx2ip	PROMOTER	0.596	0.485	1134.89	550.24	0.289	983.15	284.10
A_68_P26398844	chr9:30939486-30939530	NM_011176:-124	St14	PROMOTER	0.216	14.902	5684.63	84710.67	3.219	4778.31	15380.51
A_68_P30296517	chr15:67008766-67008810	NM_009177:-344	St3gal1	PROMOTER	0.521	0.625	3318.88	2075.33	0.326	2483.44	808.67
A_68_P30296514	chr15:67008443-67008487	NM_009177:-20	St3gal1	PROMOTER	0.61	2.819	1097.04	3092.86	1.72	924.90	1591.29
A_68_P26165552	chr8:113443992-113444036	NM_009179:250	St3gal2	INSIDE	0.228	15.119	2206.22	33355.75	3.455	2054.16	7096.33
A_68_P26165834	chr8:113494088-113494132	NM_009179:50346	St3gal2	INSIDE	0.662	0.395	1300.08	514.05	0.262	1034.52	270.70
A_68_P23238480	chr4:117807649-117807693	NM_001161774:-151	St3gal3	PROMOTER	0.577	3.996	2930.07	11707.11	2.304	2454.68	5655.30
A_68_P24539865	chr6:72047349-72047393	NM_001035228:-236	St3gal5	PROMOTER	0.521	0.422	1304.79	550.03	0.219	1068.88	234.61
A_68_P24539866	chr6:72047427-72047471	NM_001035228:-158	St3gal5	PROMOTER	0.629	2.538	1532.42	3889.86	1.597	1295.00	2068.25
A_68_P30792109	chr16:58523405-58523449	NM_018784:-1	St3gal6	PROMOTER	0.511	0.725	2050.70	1487.64	0.37	1644.36	609.20
A_68_P30601918	chr16:23225193-23225237	NM_145933:380	St6gal1	INSIDE	0.415	5.971	2056.46	12280.02	2.481	1713.72	4250.91
A_68_P30601919	chr16:23225265-23225309	NM_145933:452	St6gal1	INSIDE	0.202	8.080	1586.76	12821.04	1.635	1403.68	2294.32
A_68_P22658557	chr3:153388050-153388094	NM_011372:25	St6galnac3	INSIDE	0.386	6.342	3858.07	24468.67	2.448	3094.73	7574.95
A_68_P21116241	chr2:32442826-32442870	NM_011373:-161	St6galnac4	PROMOTER	0.593	0.280	3698.80	1036.90	0.166	2304.16	383.17
A_68_P21116407	chr2:32470667-32470711	NM_001025310:8208	St6galnac6	INSIDE	0.471	5.167	1411.27	7291.65	2.435	984.56	2397.46
A_68_P21116392	chr2:32467870-32467914	NM_001025310:5412	St6galnac6	INSIDE	0.523	3.246	955.30	3101.19	1.696	755.54	1281.66
A_68_P24907676	chr6:142912659-142912703	NM_011374:292	St8sia1	INSIDE	0.365	0.252	4450.76	1119.46	0.092	2824.49	259.56
A_68_P24907678	chr6:142912941-142912985	NM_011374:10	St8sia1	INSIDE	0.045	35.016	2360.18	82645.11	1.575	1877.24	2957.50
A_68_P31774240	chr18:64425610-64425654	NM_009182:11620	St8sia3	INSIDE	0.58	0.255	2977.95	760.20	0.148	2477.38	367.06
A_68_P31846334	chr18:77424014-77424058	NM_013666:-549	St8sia5	PROMOTER	0.482	0.289	1625.22	469.07	0.139	1237.29	171.98
A_68_P31846729	chr18:77493099-77493143	NM_013666:68535	St8sia5	INSIDE	0.555	4.099	1499.76	6148.10	2.274	1143.08	2599.72

ProbeName	Target position of probe on CpG island microarray	TargetID	GeneSymbol	CpG island Description	Ratio of relative methylation (TiO_2-NP/Vehicle)	Sham group			TiO_2-H group		
						Relative methylation (Cy5/Cy3)	Cy3 signal (Input)	Cy5 signal (MeDIP)	Relative methylation (Cy5/Cy3)	Cy3 signal (Input)	Cy5 signal (MeDIP)
A_68_P31846338	chr18:77424406-77424450	NM_013666:-157	St8sia5	PROMOTER	0.606	3.004	970.36	2915.18	1.821	1005.23	1830.79
A_68_P28054168	chr11:97913868-97913912	NM_146028:886	Stac2	INSIDE	0.568	4.071	9519.30	38748.93	2.311	6996.43	16167.87
A_68_P28054165	chr11:97913498-97913542	NM_146028:1256	Stac2	INSIDE	0.489	3.530	1343.95	4743.72	1.727	1157.77	1999.16
A_68_P26770654	chr9:100544043-100544087	NM_009282:23	Stag1	INSIDE	0.281	0.634	1164.96	738.69	0.178	975.90	173.81
A_68_P32341125	chrX:39502207-39502251	NM_001077712:-360	Stag2	PROMOTER	0.113	23.329	573.78	13385.56	2.638	782.73	2065.03
A_68_P21221436	chr2:52601404-52601448	NM_019667:-243	Stam2	PROMOTER	0.436	4.089	1929.37	7888.42	1.783	1685.76	3005.35
A_68_P25372610	chr7:108469852-108469896	NM_019990:42	Stard10	INSIDE	0.23	24.513	14694.86	360212.20	5.633	12104.94	68183.90
A_68_P25372613	chr7:108470115-108470159	NM_019990:304	Stard10	INSIDE	0.522	0.425	2143.49	911.03	0.222	1609.67	357.39
A_68_P28055912	chr11:98219735-98219779	NM_021547:59	Stard3	INSIDE	0.56	0.482	2503.02	1206.41	0.27	2025.66	546.42
A_68_P28055911	chr11:98219606-98219650	NM_021547:-69	Stard3	PROMOTER	0.644	0.419	2385.51	998.46	0.27	1924.84	518.80
A_68_P28055914	chr11:98219976-98220020	NM_021547:301	Stard3	INSIDE	0.387	5.054	909.09	4594.51	1.955	829.48	1621.67
A_68_P28862223	chr13:19487305-19487349	NM_024270:295	Stard3nl	INSIDE	0.597	3.841	1162.52	4465.63	2.295	997.80	2290.03
A_68_P21612440	chr2:127096105-127096149	NM_139308:162	Stard7	INSIDE	0.582	5.553	978.66	5434.17	3.229	935.23	3020.10
A_68_P32553141	chrX:96238064-96238108	NM_199018:167	Stard8	INSIDE	0.303	11.547	2154.10	24873.26	3.498	2286.14	7998.04
A_68_P32553143	chrX:96238297-96238345	NM_199018:401	Stard8	INSIDE	0.576	16.383	181.69	2976.60	9.44	259.72	2451.64
A_68_P23457345	chr5:5749151-5749195	NM_027399:145	Steap1	INSIDE	0.182	23.290	5675.45	132181.20	4.247	4774.64	20277.72
A_68_P23457021	chr5:5694320-5694364	NM_001103156:-265	Steap2	PROMOTER	0.487	6.675	2220.02	14817.92	3.247	1899.53	6168.35
A_68_P23457020	chr5:5694251-5694295	NM_001103156:-195	Steap2	PROMOTER	0.616	0.747	3671.27	2741.46	0.46	2980.38	1371.39
A_68_P23222803	chr4:114672646-114672690	NM_009185:-54	Stil	PROMOTER	0.413	5.635	514.54	2899.54	2.328	514.38	1197.62
A_68_P23222804	chr4:114672804-114672848	NM_009185:104	Stil	INSIDE	0.553	0.555	944.55	524.32	0.307	706.34	216.97
A_68_P25377989	chr7:109416038-109416082	NM_009287:-277	Stim1	PROMOTER	0.389	11.561	1217.60	14076.90	4.497	1245.84	5602.65
A_68_P25377988	chr7:109415960-109416004	NM_009287:-355	Stim1	PROMOTER	0.486	7.247	1613.31	11692.16	3.52	1412.95	4973.92
A_68_P25377993	chr7:109416492-109416536	NM_009287:177	Stim1	INSIDE	0.378	0.627	841.39	527.61	0.237	720.94	171.11
A_68_P23703749	chr5:54390129-54390173	NM_001081103:389	Stim2	INSIDE	0.611	7.994	1441.68	11524.60	4.881	1177.34	5746.12
A_68_P31937446	chr19:7114469-7114513	NM_016737:26	Stip1	INSIDE	0.389	1.728	3196.57	5523.47	0.671	2545.37	1708.99
A_68_P27689365	chr11:32432848-32432892	NM_009288:-395	Stk10	DIVERGENT_PROMOTER	0.462	0.562	1029.60	578.75	0.26	828.76	215.10
A_68_P27281038	chr10:79578186-79578230	NM_011492:-1072	Stk11	PROMOTER	0.199	1.775	953.82	1693.02	0.353	753.36	266.15
A_68_P27281041	chr10:79578414-79578458	NM_011492:-844	Stk11	PROMOTER	0.553	0.621	756.13	469.28	0.343	648.76	222.49
A_68_P20352666	chr1:75207636-75207680	NM_011494:255	Stk16	INSIDE	0.613	0.395	1563.73	618.28	0.242	1227.98	297.45
A_68_P31156004	chr17:34973557-34973601	NM_019442:270	Stk19	INSIDE	0.552	3.869	3317.82	12836.81	2.137	2714.35	5799.51
A_68_P29955473	chr14:121778112-121778156	NM_145465:318	Stk24	INSIDE	0.497	3.836	1232.28	4727.12	1.908	1138.38	2171.56
A_68_P29955475	chr14:121778359-121778403	NM_145465:72	Stk24	INSIDE	0.544	0.606	882.88	534.91	0.33	825.12	272.11
A_68_P20456742	chr1:95532281-95532325	NM_021537:2	Stk25	INSIDE	0.151	31.244	4798.25	149918.80	4.706	4280.30	20141.23
A_68_P20456741	chr1:95532187-95532231	NM_021537:96	Stk25	INSIDE	0.318	7.816	1565.60	12237.24	2.489	1386.94	3452.66
A_68_P20456738	chr1:95531890-95531934	NM_021537:392	Stk25	INSIDE	0.523	0.461	2836.07	1307.03	0.241	2385.55	574.90
A_68_P24432387	chr6:49345594-49345638	NM_029916:14	Stk31	INSIDE	0.334	15.659	5686.33	89040.63	5.232	6212.99	32506.31
A_68_P24432388	chr6:49345736-49345780	NM_029916:156	Stk31	INSIDE	0.625	2.312	1667.02	3853.76	1.446	1308.97	1892.68
A_68_P31659996	chr18:43367102-43367146	NM_178749:-226	Stk32a	PROMOTER	0.395	14.983	2828.40	42378.51	5.917	2304.88	13637.45
A_68_P25576307	chr7:146374240-146374284	NM_021302:131	Stk32c	INSIDE	0.269	15.387	1056.02	16249.25	4.144	942.87	3907.07
A_68_P25576402	chr7:146399285-146399329	NM_001162540:-100	Stk32c	PROMOTER	0.488	0.330	2089.52	689.84	0.161	1745.04	281.27
A_68_P25408016	chr7:116582357-116582401	NM_054103:189	Stk33	INSIDE	0.33	8.335	1844.82	15376.30	2.753	1624.21	4471.70
A_68_P21303499	chr2:68309684-68309728	NM_016866:332	Stk39	INSIDE	0.37	0.326	1897.48	618.01	0.121	1449.36	174.79
A_68_P23322940	chr4:134024241-134024287	NM_019641:30	Stmn1	INSIDE	0.422	0.432	1153.28	498.12	0.182	961.04	175.02
A_68_P23322942	chr4:134024472-134024516	NM_019641:260	Stmn1	INSIDE	0.461	0.226	2522.41	570.75	0.104	1664.75	173.52
A_68_P23322941	chr4:134024349-134024393	NM_019641:136	Stmn1	INSIDE	0.493	5.564	2832.99	15763.14	2.741	2197.19	6022.58
A_68_P28635988	chr12:92885896-92885940	NM_175367:138958	Ston2	INSIDE	0.378	4.627	925.93	4284.14	1.75	719.88	1259.90

ProbeName	Target position of probe on CpG island microarray	TargetID	GeneSymbol	CpG island Description	Ratio of relative methylation (TiO_2-NP/Vehicle)	Sham group			TiO_2-H group		
						Relative methylation (Cy5/Cy3)	Cy3 signal (Input)	Cy5 signal (MeDIP)	Relative methylation (Cy5/Cy3)	Cy3 signal (Input)	Cy5 signal (MeDIP)
A_68_P27187531	chr10:62188832-62188876	NM_001033260:-7	Stox1	PROMOTER	0.438	6.096	1939.86	11824.88	2.67	1656.47	4421.98
A_68_P27187528	chr10:62188471-62188515	NM_001033260:355	Stox1	INSIDE	0.51	5.133	1621.93	8325.76	2.619	1641.72	4300.12
A_68_P25840857	chr8:48373280-48373324	NM_001114311:1454	Stox2	INSIDE	0.645	0.339	1993.07	676.63	0.219	1485.65	325.14
A_68_P25840861	chr8:48373757-48373801	NM_001114311:978	Stox2	INSIDE	0.333	0.622	4454.41	2768.75	0.207	3012.25	623.10
A_68_P28100097	chr11:106055038-106055082	NM_028126:-198	Strada	PROMOTER	0.343	0.213	3165.58	675.03	0.073	2400.09	175.45
A_68_P31373355	chr17:79135650-79135694	NM_011500:228	Strn	INSIDE	0.256	0.500	2372.08	1186.95	0.128	1702.51	218.00
A_68_P31373358	chr17:79136029-79136073	NM_011500:-150	Strn	PROMOTER	0.425	6.001	1127.60	6766.50	2.548	1071.44	2729.78
A_68_P31373354	chr17:79135563-79135607	NM_011500:316	Strn	INSIDE	0.273	1.330	5810.44	7725.01	0.362	4252.47	1541.50
A_68_P31106582	chr17:25969835-25969879	NM_019719:450	Stub1	INSIDE	0.599	3.208	1371.43	4399.72	1.923	1153.84	2218.49
A_68_P31106584	chr17:25970012-25970056	NM_019719:272	Stub1	INSIDE	0.556	0.608	3984.84	2424.17	0.338	2602.93	880.08
A_68_P24114435	chr5:135518130-135518174	NM_016801:18711	Stx1a	INSIDE	0.132	35.841	8353.11	299381.30	4.728	7492.82	35427.42
A_68_P24114431	chr5:135517742-135517786	NM_016801:18323	Stx1a	INSIDE	0.362	1.747	2305.55	4027.04	0.633	1669.20	1056.22
A_68_P25507562	chr7:134967999-134968043	NM_024414:25	Stx1b	INSIDE	0.376	5.694	6040.06	34392.47	2.141	5067.13	10848.90
A_68_P25507560	chr7:134967764-134967808	NM_024414:259	Stx1b	INSIDE	0.381	0.646	8045.51	5198.03	0.246	5440.19	1338.54
A_68_P25507649	chr7:134985559-134985603	NM_009294:259	Stx4a	INSIDE	0.443	0.304	4126.89	1255.92	0.135	2715.30	365.96
A_68_P31943308	chr19:8815842-8815886	NM_001167799:-49	Stx5a	PROMOTER	0.617	0.322	3042.10	980.33	0.199	2102.17	417.85
A_68_P20736177	chr1:157006243-157006287	NM_021433:432	Stx6	INSIDE	0.161	1.491	945.99	1410.78	0.24	743.64	178.73
A_68_P26932995	chr10:9620910-9620954	NM_001081344:-94	Stxbp5	PROMOTER	0.456	5.875	697.93	4100.38	2.677	668.92	1790.54
A_68_P30680167	chr16:37384851-37384895	NM_001114611:172	Stxbp5l	INSIDE	0.429	0.482	2109.56	1016.26	0.207	1613.95	333.70
A_68_P28402112	chr12:46175110-46175154	NM_144552:338	Stxbp6	INSIDE	0.233	6.723	3350.74	22527.40	1.565	2656.67	4157.77
A_68_P28402111	chr12:46175015-46175059	NM_144552:434	Stxbp6	INSIDE	0.529	2.763	8643.98	23881.18	1.462	6562.63	9591.75
A_68_P29718152	chr14:73952682-73952726	NM_011506:112	Sucla2	INSIDE	0.647	9.240	1843.41	17033.81	5.981	1522.85	9108.78
A_68_P29752204	chr14:79987400-79987444	NM_026474:-75	Sugt1	PROMOTER	0.194	21.182	3054.32	64697.14	4.105	2767.81	11362.74
A_68_P24731710	chr6:108090490-108090549	NM_145937:45058	Sumf1	INSIDE	0.509	13.474	241.71	3256.82	6.857	197.85	1356.71
A_68_P28154074	chr11:115397049-115397093	NM_133354:474	Sumo2	INSIDE	0.259	29.466	10963.04	323041.50	7.622	10328.40	78720.07
A_68_P28154071	chr11:115396739-115396783	NM_133354:784	Sumo2	INSIDE	0.663	0.454	2167.75	984.35	0.301	1771.19	532.94
A_68_P24134133	chr5:139676870-139676914	NM_024451:269	Sun1	INSIDE	0.333	7.186	1395.73	10029.60	2.39	1226.25	2931.31
A_68_P24134129	chr5:139676494-139676538	NM_024451:-107	Sun1	PROMOTER	0.547	0.278	2355.64	653.84	0.152	1695.71	257.66
A_68_P24134128	chr5:139676346-139676390	NM_024451:-255	Sun1	PROMOTER	0.552	0.334	2216.33	740.22	0.184	1493.43	275.16
A_68_P30368252	chr15:79573047-79573091	NM_001205345:-102	Sun2	PROMOTER	0.38	5.130	2073.14	10635.10	1.948	1762.98	3434.60
A_68_P27542559	chr10:128105989-128106033	NM_173733:4964	Suox	DOWNSTREAM	0.338	13.369	8718.14	116551.10	4.521	7972.00	36039.49
A_68_P27542562	chr10:128106343-128106387	NM_173733:4610	Suox	DOWNSTREAM	0.461	0.346	1797.31	621.27	0.159	1418.49	225.90
A_68_P29605230	chr14:52816435-52816479	NM_033618:458	Supt16h	INSIDE	0.359	14.921	2830.37	42232.40	5.36	2329.61	12487.65
A_68_P29605237	chr14:52817103-52817147	NM_033618:-210	Supt16h	PROMOTER	0.491	3.078	2303.84	7090.94	1.51	2022.27	3053.78
A_68_P31197210	chr17:44914016-44914060	NM_178652:-81	Supt3h	PROMOTER	0.2	20.449	2123.79	43428.43	4.089	1867.40	7636.72
A_68_P27995861	chr11:87550950-87550994	NM_009296:-94	Supt4h1	PROMOTER	0.23	26.080	2079.81	54241.14	6.002	1854.30	11130.31
A_68_P27995858	chr11:87550476-87550520	NM_009296:-568	Supt4h1	PROMOTER	0.648	0.648	852.48	552.41	0.42	691.43	290.42
A_68_P21081132	chr2:26771837-26771881	NM_013678:-82	Surf2	PROMOTER	0.419	0.365	1669.71	608.66	0.153	1123.65	171.78
A_68_P21081272	chr2:26789177-26789221	NM_011512:-167	Surf4	DIVERGENT_PROMOTER	0.393	0.425	10136.32	4303.22	0.167	6995.54	1166.56
A_68_P21081268	chr2:26788702-26788746	NM_011512:307	Surf4	INSIDE	0.638	2.782	1016.38	2827.59	1.776	899.72	1597.46
A_68_P29024153	chr13:49343267-49343311	NM_025491:244	Susd3	INSIDE	0.524	0.629	828.18	520.52	0.329	670.85	221.02
A_68_P26843529	chr9:113966624-113966668	NM_001101510:175	Susd5	INSIDE	0.495	3.427	915.33	3136.92	1.695	879.89	1491.49
A_68_P32241633	chrX:7651543-7651587	NM_011514:322	Suv39h1	INSIDE	0.65	4.203	772.23	3245.64	2.734	1065.32	2912.35
A_68_P27952010	chr11:79806374-79806418	NM_001163018:-211	Suz12	PROMOTER	0.573	0.520	1357.98	706.09	0.298	1004.96	299.66
A_68_P27952012	chr11:79806597-79806641	NM_001163018:11	Suz12	INSIDE	0.384	1.585	1560.51	2473.39	0.609	1229.58	748.96
A_68_P25239159	chr7:82453955-82453999	NM_001109753:-704	Sv2b	PROMOTER	0.253	13.494	1864.28	25157.06	3.415	1776.50	6065.86

ProbeName	Target position of probe on CpG island microarray	TargetID	GeneSymbol	CpG island Description	Ratio of relative methylation (TiO_2-NP/Vehicle)	Sham group			TiO_2-H group		
						Relative methylation (Cy5/Cy3)	Cy3 signal (Input)	Cy5 signal (MeDIP)	Relative methylation (Cy5/Cy3)	Cy3 signal (Input)	Cy5 signal (MeDIP)
A_68_P29244724	chr13:96902709-96902753	NM_029210:-198	Sv2c	PROMOTER	0.62	0.296	7090.20	2097.89	0.183	5648.62	1035.85
A_68_P29244710	chr13:96901056-96901100	NM_029210:1454	Sv2c	INSIDE	0.583	0.704	4741.83	3339.71	0.411	3585.88	1472.69
A_68_P29244723	chr13:96902535-96902579	NM_029210:-24	Sv2c	PROMOTER	0.343	5.005	1292.96	6471.90	1.719	1057.08	1816.67
A_68_P22949805	chr4:58219063-58219107	NM_022814:384	Svep1	INSIDE	0.358	7.466	1289.98	9631.17	2.675	1016.98	2720.62
A_68_P25126430	chr7:59261254-59261298	NR_027704:-192	Svip	PROMOTER	0.257	9.933	6758.50	67131.00	2.552	5763.62	14707.79
A_68_P24001851	chr5:114541342-114541386	NM_026805:25	Svop	INSIDE	0.465	12.756	2929.99	37375.02	5.933	2572.75	15264.66
A_68_P25412183	chr7:117365487-117365531	NM_009302:292	Swap70	INSIDE	0.219	23.750	5107.12	121294.60	5.202	4472.30	23264.24
A_68_P25412179	chr7:117365148-117365192	NM_009302:-46	Swap70	PROMOTER	0.381	3.789	2060.35	7806.15	1.445	1757.78	2540.39
A_68_P32791361	chrX:159326363-159326407	NM_025932:10	Syap1	INSIDE	0.289	12.715	788.75	10029.03	3.675	1309.32	4811.62
A_68_P26022398	chr8:87396278-87396322	NM_001168244:144	Syce2	INSIDE	0.423	0.367	3258.84	1194.90	0.155	2207.30	342.71
A_68_P26022397	chr8:87396203-87396247	NM_001168244:68	Syce2	INSIDE	0.204	1.501	7622.26	11440.23	0.307	4769.98	1462.61
A_68_P21890546	chr2:178142021-178142065	NM_177191:321	Sycp2	INSIDE	0.277	11.108	5967.97	66290.52	3.079	4676.45	14396.86
A_68_P21890548	chr2:178142227-178142271	NM_177191:115	Sycp2	INSIDE	0.356	11.296	7147.79	80743.11	4.021	5733.90	23053.65
A_68_P21890547	chr2:178142154-178142198	NM_177191:187	Sycp2	INSIDE	0.384	5.678	2012.08	11423.98	2.178	1581.23	3443.22
A_68_P27325555	chr10:87922666-87922710	NM_011517:357	Sycp3	INSIDE	0.289	20.936	4872.95	102020.80	6.056	4351.11	26348.51
A_68_P27325556	chr10:87922749-87922793	NM_011517:439	Sycp3	INSIDE	0.347	21.086	6259.31	131981.00	7.308	4940.52	36106.55
A_68_P27325554	chr10:87922543-87922587	NM_011517:233	Sycp3	INSIDE	0.434	6.826	1971.69	13458.42	2.962	1655.42	4903.21
A_68_P22611968	chr3:145651227-145651271	NM_001166064:415	Syde2	INSIDE	0.666	4.458	853.73	3806.05	2.971	782.59	2324.98
A_68_P24769159	chr6:115085098-115085142	NM_013681:201	Syn2	INSIDE	0.316	0.487	1505.60	732.78	0.154	1241.74	190.72
A_68_P24769158	chr6:115084990-115085034	NM_013681:93	Syn2	INSIDE	0.46	0.550	1339.45	736.48	0.253	1046.67	264.52
A_68_P26710370	chr9:88377975-88378019	NM_019796:-761	Syncrip	PROMOTER	0.124	38.068	1508.99	57443.43	4.703	1311.06	6165.33
A_68_P26710354	chr9:88376160-88376210	NM_019796:1051	Syncrip	INSIDE	0.521	0.210	3214.22	675.19	0.109	2237.69	244.70
A_68_P28550368	chr12:76919708-76919752	NM_001005510:426	Syne2	INSIDE	0.359	0.557	1030.90	574.66	0.2	873.08	174.60
A_68_P28167342	chr11:117671257-117671301	NM_009304:298	Syngr2	INSIDE	0.549	4.078	964.68	3933.59	2.24	888.40	1989.60
A_68_P31099001	chr17:24824425-24824469	NM_011522:2448	Syngr3	INSIDE	0.287	28.056	8971.75	251707.60	8.065	11775.36	94964.08
A_68_P31020009	chr17:5941768-5941812	NM_001113352:511	Synj2	INSIDE	0.232	0.412	11320.76	4668.84	0.096	7065.51	677.11
A_68_P31020008	chr17:5941641-5941685	NM_001113352:383	Synj2	INSIDE	0.521	0.289	1802.63	521.06	0.151	1374.25	206.83
A_68_P25195705	chr7:74903906-74903950	NM_183312:700	Synm	INSIDE	0.562	4.050	1848.05	7485.45	2.274	1473.41	3351.05
A_68_P25195709	chr7:74904507-74904551	NM_183312:100	Synm	INSIDE	0.659	0.223	3588.25	798.57	0.147	2521.00	369.91
A_68_P31752323	chr18:60755666-60755710	NM_177340:28271	Synpo	INSIDE	0.377	0.308	2648.93	816.74	0.116	1944.63	226.20
A_68_P31752537	chr18:60784125-60784169	NM_177340:-187	Synpo	PROMOTER	0.508	0.550	3208.71	1764.92	0.28	2489.57	696.05
A_68_P22411406	chr3:108029457-108029501	NM_008596:39	Sypl2	INSIDE	0.374	5.566	2981.04	16592.25	2.083	2211.77	4606.55
A_68_P22411405	chr3:108029325-108029369	NM_008596:171	Sypl2	INSIDE	0.641	0.503	4473.85	2250.19	0.322	3493.37	1126.39
A_68_P30427167	chr15:89672007-89672051	NM_018803:263	Syt10	INSIDE	0.339	0.090	10755.66	972.72	0.031	7899.47	241.94
A_68_P31923331	chr19:4476940-4476984	NM_134164:181	Syt12	INSIDE	0.18	2.477	2280.26	5647.72	0.447	1761.04	787.26
A_68_P31923328	chr19:4476573-4476617	NM_134164:549	Syt12	INSIDE	0.529	0.361	2442.89	882.16	0.191	1747.21	333.84
A_68_P31923329	chr19:4476649-4476693	NM_134164:473	Syt12	INSIDE	0.598	0.235	2195.87	517.01	0.141	1524.74	214.63
A_68_P21431327	chr2:92755255-92755299	NM_030725:19	Syt13	INSIDE	0.48	5.720	882.39	5047.34	2.743	761.48	2088.81
A_68_P20946922	chr1:194862087-194862136	NM_181546:-219	Syt14	PROMOTER	0.115	1.910	6975.73	13320.24	0.22	4013.16	881.17
A_68_P20946920	chr1:194861857-194861901	NM_181546:14	Syt14	INSIDE	0.533	5.146	829.13	4266.34	2.745	785.70	2156.72
A_68_P20946924	chr1:194862258-194862302	NM_181546:-388	Syt14	PROMOTER	0.661	0.504	5259.24	2650.57	0.333	3658.86	1219.75
A_68_P25458299	chr7:125591289-125591333	NM_138649:-4244	Syt17	PROMOTER	0.386	10.839	2547.52	27613.11	4.188	2302.25	9640.87
A_68_P20634933	chr1:136605811-136605855	NM_009307:62575	Syt2	INSIDE	0.421	5.078	1897.53	9635.76	2.138	1323.73	2830.76
A_68_P20634931	chr1:136605584-136605628	NM_009307:62349	Syt2	INSIDE	0.594	0.237	1885.67	446.76	0.141	1236.24	174.07
A_68_P22386748	chr3:103380204-103380248	NM_018800:1023	Syt6	INSIDE	0.435	4.069	1559.63	6345.54	1.77	1370.81	2426.49
A_68_P31950560	chr19:10500789-10500833	NM_018801:37231	Syt7	INSIDE	0.614	2.397	1612.41	3865.74	1.473	1281.14	1887.21

ProbeName	Target position of probe on CpG island microarray	TargetID	GeneSymbol	CpG island Description	Ratio of relative methylation (TiO_2-NP/Vehicle)	Sham group			TiO_2-H group		
						Relative methylation (Cy5/Cy3)	Cy3 signal (Input)	Cy5 signal (MeDIP)	Relative methylation (Cy5/Cy3)	Cy3 signal (Input)	Cy5 signal (MeDIP)
A_68_P23240175	chr4:118081682-118081726	NM_198170:164	Szt2	INSIDE	0.604	0.229	2402.50	551.35	0.139	1915.43	265.72
A_68_P32958522	chr17:8627015-8627059	NM_009309:-251	T	PROMOTER	0.425	4.880	1464.90	7149.03	2.074	1082.31	2245.19
A_68_P32958527	chr17:8627631-8627675	NM_009309:365	T	INSIDE	0.574	0.194	2641.09	513.10	0.111	2000.46	222.90
A_68_P32506209	chrX:82819101-82819145	NM_025729:-238	Tab3	PROMOTER	0.469	11.876	1216.28	14444.67	5.565	1540.83	8574.91
A_68_P24221481	chr6:7505151-7505195	NM_009311:102	Tac1	INSIDE	0.405	0.554	1056.85	585.96	0.225	897.41	201.57
A_68_P24521963	chr6:67485504-67485548	NM_020047:290	Tacstd2	INSIDE	0.473	0.375	1348.90	506.48	0.177	1002.42	177.89
A_68_P20799887	chr1:168309926-168309970	NM_030245:651	Tada1	INSIDE	0.374	0.353	1882.85	665.09	0.132	1366.92	180.43
A_68_P23607900	chr5:36826413-36826457	NM_001170454:500	Tada2b	INSIDE	0.38	4.776	4833.82	23087.36	1.815	3577.27	6493.85
A_68_P24759109	chr6:113327446-113327490	NM_133932:46	Tada3	INSIDE	0.527	6.313	1269.55	8014.61	3.325	1133.13	3767.78
A_68_P23311001	chr4:131830273-131830317	NM_025579:5	Taf12	INSIDE	0.38	28.479	2299.70	65492.25	10.832	2990.47	32391.58
A_68_P27971718	chr11:83286618-83286662	NM_027427:31	Taf15	INSIDE	0.618	11.464	1383.49	15859.68	7.083	1151.42	8155.26
A_68_P26322118	chr9:15110644-15110688	NM_029248:9	Taf1d	INSIDE	0.577	0.200	2373.51	474.24	0.115	1651.29	190.53
A_68_P30230212	chr15:54903756-54903800	NM_001081288:-71	Taf2	PROMOTER	0.3	11.448	1788.59	20475.17	3.433	1766.33	6064.12
A_68_P20998559	chr2:9970073-9970117	NM_027748:142	Taf3	INSIDE	0.346	10.045	2947.51	29609.12	3.476	2396.69	8329.85
A_68_P21900382	chr2:179666703-179666747	NM_001081092:44627	Taf4a	INSIDE	0.609	5.157	691.46	3565.60	3.14	639.49	2007.82
A_68_P26242940	chr8:126544834-126544878	NM_133966:353	Taf5l	INSIDE	0.393	10.906	1070.38	11674.08	4.29	1010.88	4336.37
A_68_P26242939	chr8:126544720-126544764	NM_133966:467	Taf5l	INSIDE	0.591	10.690	493.72	5277.67	6.321	603.44	3814.54
A_68_P32672214	chrX:131010586-131010630	NM_028958:421	Taf7l	INSIDE	0.045	65.374	1115.96	72954.09	2.942	1597.35	4698.67
A_68_P32672211	chrX:131010176-131010220	NM_028958:831	Taf7l	INSIDE	0.465	3.786	2288.45	8663.75	1.761	2850.66	5018.73
A_68_P32672217	chrX:131010986-131011030	NM_028958:21	Taf7l	INSIDE	0.455	3.587	757.16	2716.04	1.632	1042.98	1701.90
A_68_P32672209	chrX:131010003-131010047	NM_028958:1005	Taf7l	INSIDE	0.609	2.368	1744.11	4129.79	1.441	2200.65	3172.10
A_68_P29269476	chr13:101421663-101421707	NM_001015889:123	Taf9	INSIDE	0.582	0.343	2070.05	709.46	0.2	1375.51	274.55
A_68_P32583453	chrX:103415136-103415180	NM_001001176:23	Taf9b	INSIDE	0.427	15.269	675.90	10320.31	6.516	796.47	5189.83
A_68_P31023474	chr17:7165620-7165665	NM_147155:-137	Tagap1	PROMOTER	0.465	0.684	5919.13	4048.81	0.318	4413.67	1404.51
A_68_P20833435	chr1:174430368-174430412	NM_178598:14	Tagln2	INSIDE	0.475	0.433	1115.02	483.17	0.206	842.98	173.38
A_68_P23223128	chr4:114729001-114729045	NM_011527:-3109	Tal1	PROMOTER	0.508	0.412	2616.66	1077.58	0.209	2009.19	420.00
A_68_P23223131	chr4:114729380-114729424	NM_011527:-2729	Tal1	PROMOTER	0.546	2.588	4268.67	11048.55	1.413	3401.31	4806.60
A_68_P28096908	chr11:105451551-105451595	NM_181071:273	Tanc2	INSIDE	0.432	3.804	3370.44	12821.32	1.642	2675.98	4395.20
A_68_P21265457	chr2:61494997-61495041	NM_001164071:63865	Tank	DOWNSTREAM	0.358	0.368	1667.20	612.71	0.132	1301.76	171.33
A_68_P21265454	chr2:61494500-61494549	NM_001164071:63371	Tank	DOWNSTREAM	0.296	0.727	1516.13	1101.96	0.215	1234.38	265.80
A_68_P27938548	chr11:77420609-77420653	NM_144825:687	Taok1	INSIDE	0.415	0.283	2526.22	715.70	0.118	2014.47	236.88
A_68_P27938551	chr11:77420898-77420942	NM_144825:397	Taok1	INSIDE	0.477	0.605	1353.50	819.02	0.289	1199.59	346.38
A_68_P31152726	chr17:34341832-34341876	NM_011530:431	Tap2	INSIDE	0.486	0.480	1209.72	580.66	0.233	904.93	210.99
A_68_P31150991	chr17:34056894-34056938	NM_001025313:494	Tapbp	INSIDE	0.549	0.677	2254.49	1527.17	0.372	1627.88	605.47
A_68_P31151041	chr17:34066176-34066220	NM_001025313:9776	Tapbp	INSIDE	0.632	0.663	1719.57	1140.76	0.419	1139.16	477.62
A_68_P23395558	chr4:148000660-148000704	NM_001003898:423	Tardbp	INSIDE	0.628	2.963	1168.97	3463.12	1.859	954.82	1775.33
A_68_P23416140	chr4:151406689-151406733	NM_031867:5889	Tas1r1	INSIDE	0.617	3.249	1622.87	5273.41	2.005	1058.51	2122.32
A_68_P23416139	chr4:151406623-151406667	NM_031867:5955	Tas1r1	INSIDE	0.42	3.638	2515.95	9151.97	1.527	1718.84	2623.83
A_68_P24760941	chr6:113647011-113647055	NM_001033463:-460	Tatdn2	PROMOTER	0.278	6.479	1189.58	7707.06	1.799	969.23	1743.35
A_68_P20936016	chr1:192886793-192886837	NM_001163421:-3	Tatdn3	DIVERGENT_PROMOTER	0.409	5.793	12163.44	70459.89	2.367	11334.29	26832.08
A_68_P24452215	chr6:52664021-52664065	NM_025816:320	Tax1bp1	INSIDE	0.418	14.018	3019.86	42331.38	5.853	2942.36	17222.28
A_68_P24452213	chr6:52663843-52663887	NM_025816:142	Tax1bp1	INSIDE	0.572	0.656	1253.16	822.40	0.375	1078.99	405.12
A_68_P23749456	chr5:64551627-64551671	NM_019636:199	Tbc1d1	INSIDE	0.195	35.635	8568.08	305321.70	6.941	9715.48	67431.21
A_68_P23749879	chr5:64621748-64621793	NM_019636:70321	Tbc1d1	INSIDE	0.336	10.562	6386.03	67452.32	3.55	6537.95	23211.67
A_68_P23749454	chr5:64551417-64551461	NM_019636:-11	Tbc1d1	PROMOTER	0.642	2.866	1827.38	5236.48	1.841	1511.04	2781.79
A_68_P23749880	chr5:64621862-64621906	NM_019636:70435	Tbc1d1	INSIDE	0.162	1.514	1606.75	2433.17	0.246	1340.78	329.25

ProbeName	Target position of probe on CpG island microarray	TargetID	GeneSymbol	CpG island Description	Ratio of relative methylation (TiO_2-NP/Vehicle)	Sham group			TiO_2-H group		
						Relative methylation (Cy5/Cy3)	Cy3 signal (Input)	Cy5 signal (MeDIP)	Relative methylation (Cy5/Cy3)	Cy3 signal (Input)	Cy5 signal (MeDIP)
A_68_P23608585	chr5:36924742-36924786	NM_133910:282	Tbc1d14	INSIDE	0.344	6.075	12143.67	73773.52	2.093	9588.68	20065.82
A_68_P23608244	chr5:36873039-36873083	NM_001113364:212	Tbc1d14	INSIDE	0.612	0.469	2138.78	1002.86	0.287	1537.10	441.23
A_68_P23608581	chr5:36924292-36924336	NM_133910:732	Tbc1d14	INSIDE	0.446	0.637	4895.51	3116.78	0.284	3849.15	1092.07
A_68_P27468585	chr10:114688513-114688557	NM_025706:15	Tbc1d15	INSIDE	0.497	0.354	1244.04	440.56	0.176	956.59	168.42
A_68_P27468588	chr10:114688762-114688806	NM_025706:-235	Tbc1d15	PROMOTER	0.66	0.463	1296.89	600.39	0.305	937.61	286.37
A_68_P28176626	chr11:119089661-119089705	NM_172443:131	Tbc1d16	INSIDE	0.364	6.236	2157.65	13454.20	2.268	1676.01	3800.57
A_68_P23702720	chr5:54200876-54200920	NM_144517:33	Tbc1d19	INSIDE	0.54	0.342	1561.96	533.96	0.185	1188.69	219.42
A_68_P21748557	chr2:152119766-152119810	NM_024196:181	Tbc1d20	INSIDE	0.458	12.090	3509.40	42427.00	5.534	3204.15	17731.53
A_68_P21748558	chr2:152119855-152119899	NM_024196:269	Tbc1d20	INSIDE	0.471	7.793	3346.34	26078.70	3.672	2780.79	10210.81
A_68_P21748556	chr2:152119601-152119645	NM_024196:15	Tbc1d20	INSIDE	0.256	9.321	521.77	4863.34	2.388	428.37	1023.14
A_68_P30405824	chr15:86045163-86045207	NM_145476:296	Tbc1d22a	INSIDE	0.083	3.410	4843.13	16515.90	0.283	3426.01	970.03
A_68_P30405823	chr15:86045014-86045058	NM_145476:148	Tbc1d22a	INSIDE	0.188	2.156	5983.50	12900.16	0.406	3919.00	1591.17
A_68_P31096039	chr17:24342230-24342274	NM_001163847:255	Tbc1d24	INSIDE	0.575	0.394	13422.85	5292.28	0.227	8641.67	1959.23
A_68_P31096042	chr17:24342530-24342574	NM_001163847:-45	Tbc1d24	PROMOTER	0.35	5.291	1246.79	6596.58	1.85	1010.18	1868.70
A_68_P26717019	chr9:90165367-90165411	NM_194334:219	Tbc1d2b	INSIDE	0.587	0.502	1679.92	843.73	0.295	1363.08	402.06
A_68_P27502792	chr10:120747561-120747605	NM_029057:663	Tbc1d30	INSIDE	0.646	2.864	2890.32	8277.53	1.849	2310.94	4273.08
A_68_P31232583	chr17:51318678-51318722	NM_028162:-26	Tbc1d5	PROMOTER	0.349	8.673	1547.86	13425.11	3.027	1375.87	4164.15
A_68_P29236942	chr13:95558754-95558798	NM_009321:-121	Tbca	PROMOTER	0.262	18.244	2742.42	50032.37	4.784	2383.12	11400.63
A_68_P25030460	chr7:31017038-31017082	NM_025548:-12	Tbcb	DIVERGENT_PROMOTER	0.371	0.370	2003.76	741.66	0.137	1431.88	196.66
A_68_P25030456	chr7:31016600-31016644	NM_025548:426	Tbcb	INSIDE	0.51	0.301	1744.04	525.35	0.154	1191.04	182.97
A_68_P31209391	chr17:47027623-47027667	NM_178385:75	Tbcc	INSIDE	0.518	0.276	1913.68	527.85	0.143	1376.31	196.79
A_68_P30600190	chr16:22857403-22857447	NM_001081368:218	Tbccd1	INSIDE	0.343	0.311	2055.14	638.28	0.107	1630.40	173.79
A_68_P28836851	chr13:14131718-14131762	NM_178337:127	Tbce	INSIDE	0.48	1.452	3653.01	5304.49	0.698	3015.52	2103.49
A_68_P22538542	chr3:132347071-132347115	NM_173032:-15	Tbck	DIVERGENT_PROMOTER	0.592	0.266	2651.91	706.10	0.158	2003.10	315.72
A_68_P27504343	chr10:121023890-121023934	NM_019786:-62	Tbk1	PROMOTER	0.528	11.528	1246.05	14364.42	6.082	1160.29	7056.93
A_68_P28048904	chr11:97000172-97000216	NM_198100:10832	Tbkbp1	INSIDE	0.25	14.358	971.03	13942.24	3.596	860.94	3096.24
A_68_P28048905	chr11:97000299-97000343	NM_198100:10706	Tbkbp1	INSIDE	0.259	8.875	1054.08	9355.28	2.296	922.77	2118.37
A_68_P21986766	chr3:21976041-21976085	NM_030732:489	Tbl1xr1	INSIDE	0.569	4.628	1710.11	7914.53	2.631	1456.55	3832.84
A_68_P24114953	chr5:135624970-135625015	NM_013763:-588	Tbl2	PROMOTER	0.242	1.909	3553.24	6783.82	0.462	2916.28	1346.09
A_68_P21266439	chr2:61649898-61649942	NM_009322:7411	Tbr1	INSIDE	0.63	4.145	488.29	2023.74	2.612	449.65	1174.31
A_68_P21266476	chr2:61654143-61654187	NM_009322:11655	Tbr1	DOWNSTREAM	0.5	3.396	795.05	2699.89	1.699	758.19	1288.19
A_68_P21266440	chr2:61650101-61650145	NM_009322:7613	Tbr1	INSIDE	0.524	3.542	585.62	2074.53	1.858	525.83	976.85
A_68_P30580602	chr16:18586865-18586909	NM_011532:176	Tbx1	INSIDE	0.503	7.133	2128.82	15184.47	3.588	1960.73	7035.55
A_68_P30580630	chr16:18590465-18590509	NM_011532:-3424	Tbx1	PROMOTER	0.514	0.697	1426.75	994.92	0.358	1070.75	383.82
A_68_P22363128	chr3:99057574-99057618	NM_009323:-86	Tbx15	PROMOTER	0.295	8.407	5320.47	44730.30	2.477	4922.61	12195.54
A_68_P27985642	chr11:85658143-85658187	NM_009324:12048	Tbx2	DOWNSTREAM	0.447	0.263	2040.30	537.52	0.118	1483.65	174.86
A_68_P27985546	chr11:85647033-85647077	NM_009324:938	Tbx2	INSIDE	0.458	8.801	4343.31	38223.78	4.028	3406.52	13722.09
A_68_P27985620	chr11:85655489-85655533	NM_009324:9394	Tbx2	DOWNSTREAM	0.609	5.313	1817.14	9653.90	3.236	1580.19	5113.47
A_68_P27985536	chr11:85645744-85645788	NM_009324:-350	Tbx2	PROMOTER	0.443	3.398	2950.93	10026.32	1.505	2242.07	3373.90
A_68_P28048726	chr11:96976651-96976695	NM_019507:-27	Tbx21	PROMOTER	0.59	0.294	1767.70	519.82	0.174	1464.88	254.26
A_68_P24033996	chr5:120121006-120121050	NM_011535:351	Tbx3	INSIDE	0.477	3.672	3781.07	13883.47	1.751	2869.98	5025.96
A_68_P24034076	chr5:120130713-120130757	NM_011535:10057	Tbx3	INSIDE	0.535	0.378	1455.07	550.12	0.202	1177.54	238.07
A_68_P24033995	chr5:120120847-120120895	NM_011535:193	Tbx3	INSIDE	0.57	0.202	2220.12	447.71	0.115	1498.71	172.39
A_68_P27288939	chr10:80795142-80795186	NM_009325:3689	Tbxa2r	INSIDE	0.641	3.761	891.23	3351.91	2.412	730.81	1763.09
A_68_P27288944	chr10:80795734-80795778	NM_009325:4281	Tbxa2r	INSIDE	0.379	3.637	3047.87	11085.19	1.378	2069.68	2852.34
A_68_P21912035	chr2:181415320-181415364	NM_009326:328	Tcea2	INSIDE	0.375	0.438	1755.11	768.50	0.164	1293.50	212.48

ProbeName	Target position of probe on CpG island microarray	TargetID	GeneSymbol	CpG island Description	Ratio of relative methylation (TiO_2-NP/Vehicle)	Sham group			TiO_2-H group		
						Relative methylation (Cy5/Cy3)	Cy3 signal (Input)	Cy5 signal (MeDIP)	Relative methylation (Cy5/Cy3)	Cy3 signal (Input)	Cy5 signal (MeDIP)
A_68_P23332903	chr4:135804530-135804574	NM_011542:681	Tcea3	INSIDE	0.048	1.826	3260.77	5953.46	0.088	2214.32	195.60
A_68_P23332904	chr4:135804629-135804673	NM_011542:779	Tcea3	INSIDE	0.104	2.007	9144.81	18354.43	0.209	6751.74	1409.41
A_68_P32806692	chrX:162947996-162948040	NM_001007577:392	Tceanc	INSIDE	0.396	5.439	1079.01	5868.45	2.153	1260.43	2713.48
A_68_P23331724	chr4:135577368-135577412	NM_013736:174	Tceb3	INSIDE	0.182	20.897	4745.41	99163.77	3.811	4095.71	15608.79
A_68_P23331727	chr4:135577835-135577879	NM_013736:-292	Tceb3	PROMOTER	0.461	0.599	2895.09	1735.37	0.276	1945.71	537.09
A_68_P31655789	chr18:42671379-42671423	NM_001039474:260	Tcergl	INSIDE	0.245	18.512	9172.13	169792.70	4.538	13650.79	61940.73
A_68_P31655791	chr18:42671723-42671767	NM_001039474:604	Tcergl	INSIDE	0.667	2.980	2106.68	6277.19	1.986	1697.33	3371.33
A_68_P25571662	chr7:145589207-145589251	NM_183289:185	Tcergl1	INSIDE	0.219	53.499	2890.62	154645.50	11.702	3051.36	35707.91
A_68_P25571660	chr7:145588945-145588989	NM_183289:447	Tcergl1	INSIDE	0.409	0.226	2633.77	594.36	0.092	1941.16	178.96
A_68_P26626138	chr9:71959964-71960008	NM_011544:-360	Tcf12	PROMOTER	0.338	7.084	1883.56	13343.23	2.393	1771.55	4239.38
A_68_P26626136	chr9:71959653-71959697	NM_011544:-48	Tcf12	PROMOTER	0.527	3.979	528.68	2103.83	2.098	448.27	940.63
A_68_P31159487	chr17:35653700-35653744	NM_001163763:47	Tcf19	INSIDE	0.313	6.385	2048.94	13081.55	2	1570.11	3140.89
A_68_P30385400	chr15:82742401-82742445	NM_001114140:142	Tcf20	INSIDE	0.409	0.500	5541.12	2771.74	0.205	3630.93	743.61
A_68_P26239530	chr8:125898229-125898273	NM_001037877:516	Tcf25	INSIDE	0.332	0.391	2794.84	1093.94	0.13	1968.05	255.60
A_68_P26239531	chr8:125898300-125898344	NM_001037877:588	Tcf25	INSIDE	0.432	8.630	11570.42	99857.40	3.729	8980.69	33485.36
A_68_P26239529	chr8:125898146-125898191	NM_001037877:434	Tcf25	INSIDE	0.557	0.535	3226.03	1726.65	0.298	2447.20	729.45
A_68_P26239532	chr8:125898381-125898425	NM_001037877:668	Tcf25	INSIDE	0.125	3.254	1421.78	4625.90	0.406	968.17	392.99
A_68_P24543633	chr6:72738096-72738140	NM_001079822:832	Tcf7l1	INSIDE	0.116	19.252	3388.56	65237.42	2.242	2764.83	6200.11
A_68_P24543644	chr6:72739438-72739482	NM_001079822:-510	Tcf7l1	PROMOTER	0.269	0.462	2844.61	1314.03	0.124	1927.43	239.32
A_68_P32196178	chr19:55817586-55817630	NM_001142918:1309	Tcf7l2	INSIDE	0.105	3.733	4010.11	14968.30	0.392	2598.80	1019.38
A_68_P32196169	chr19:55816232-55816276	NM_001142918:-45	Tcf7l2	PROMOTER	0.192	10.882	4572.26	49756.32	2.094	4259.01	8917.84
A_68_P32196175	chr19:55817146-55817190	NM_001142918:869	Tcf7l2	INSIDE	0.473	0.249	2300.40	571.72	0.118	1487.47	175.00
A_68_P28974164	chr13:40824040-40824084	NM_001122948:1750	Tcfap2a	INSIDE	0.27	13.043	3946.53	51473.22	3.519	3196.59	11248.19
A_68_P28974119	chr13:40818320-40818364	NM_001122948:7470	Tcfap2a	INSIDE	0.38	0.509	4239.38	2156.20	0.193	2816.68	544.65
A_68_P28974187	chr13:40826726-40826770	NM_001122948:-936	Tcfap2a	PROMOTER	0.394	6.289	3512.43	22088.26	2.481	3030.25	7516.84
A_68_P28974246	chr13:40833476-40833520	NM_011547:-4306	Tcfap2a	PROMOTER	0.102	1.481	1420.29	2103.33	0.15	1260.51	189.65
A_68_P28974208	chr13:40829120-40829164	NM_001122948:-3330	Tcfap2a	PROMOTER	0.377	1.579	1145.92	1809.85	0.595	1030.74	613.26
A_68_P21864915	chr2:172375828-172375872	NM_001159696:-640	Tcfap2c	PROMOTER	0.377	5.141	2122.54	10912.07	1.937	1786.69	3461.55
A_68_P21864909	chr2:172375260-172375304	NM_001159696:-1208	Tcfap2c	PROMOTER	0.508	4.774	857.80	4095.00	2.425	673.51	1633.23
A_68_P21864924	chr2:172377062-172377106	NM_001159696:594	Tcfap2c	INSIDE	0.657	0.364	4544.19	1656.14	0.24	3087.93	739.58
A_68_P21864923	chr2:172376959-172377003	NM_001159696:490	Tcfap2c	INSIDE	0.29	2.164	7458.25	16141.87	0.628	5449.51	3419.99
A_68_P30510788	chr16:4558960-4559004	NM_031182:738	Tcfap4	INSIDE	0.492	0.203	4811.34	977.90	0.1	3391.16	338.79
A_68_P30510674	chr16:4542026-4542070	NM_031182:17672	Tcfap4	DOWNSTREAM	0.066	5.396	691.83	3733.47	0.356	575.94	204.89
A_68_P30486392	chr15:100382126-100382170	NM_033476:230	Tcfcp2	INSIDE	0.604	3.330	646.72	2153.59	2.01	565.17	1136.03
A_68_P31213969	chr17:47875277-47875321	NM_001161723:419	Tcfeb	INSIDE	0.64	6.032	367.39	2216.08	3.859	322.20	1243.24
A_68_P22336409	chr3:93249587-93249631	NM_001163098:3357	Tchh	INSIDE	0.395	0.086	8077.76	692.50	0.034	5725.83	193.82
A_68_P22336429	chr3:93251869-93251913	NM_001163098:5639	Tchh	INSIDE	0.471	0.212	3971.15	840.83	0.1	3045.14	303.73
A_68_P31120338	chr17:28217573-28217617	NM_001085555:-10	Tcp11	PROMOTER	0.346	13.584	4030.36	54748.28	4.702	3008.57	14146.85
A_68_P31120334	chr17:28216998-28217042	NM_013687:509	Tcp11	INSIDE	0.404	0.615	802.42	493.40	0.249	695.30	172.80
A_68_P31201639	chr17:45672060-45672104	NM_013688:11700	Tcte1	INSIDE	0.517	3.720	1149.43	4276.04	1.924	954.42	1836.61
A_68_P31056399	chr17:13898251-13898295	NM_022311:129	Tcte2	INSIDE	0.433	4.769	2961.93	14124.00	2.064	2334.14	4816.75
A_68_P31056398	chr17:13898109-13898153	NM_022311:271	Tcte2	INSIDE	0.602	2.569	1940.06	4983.53	1.547	1809.67	2799.29
A_68_P32109943	chr19:40686882-40686926	NM_026260:-199	Tctn3	PROMOTER	0.613	0.575	970.42	557.89	0.352	798.91	281.58
A_68_P28678695	chr12:101122821-101122865	NM_028354:118	Tdp1	INSIDE	0.415	7.347	533.18	3917.09	3.047	461.07	1405.04
A_68_P32203265	chr19:56896867-56896911	NM_001002238:-3810	Tdrd1	DIVERGENT_PROMOTER	0.66	0.222	2265.84	503.88	0.147	1771.15	260.15
A_68_P32203282	chr19:56901047-56901091	NM_001002241:-4133	Tdrd1	PROMOTER	0.501	3.517	1489.84	5240.28	1.763	1170.22	2063.39

ProbeName	Target position of probe on CpG island microarray	TargetID	GeneSymbol	CpG island Description	Ratio of relative methylation (TiO_2-NP/Vehicle)	Sham group			TiO_2-H group		
						Relative methylation (Cy5/Cy3)	Cy3 signal (Input)	Cy5 signal (MeDIP)	Relative methylation (Cy5/Cy3)	Cy3 signal (Input)	Cy5 signal (MeDIP)
A_68_P25049869	chr7:36322564-36322608	NM_028034:177	Tdrd12	INSIDE	0.619	3.598	1204.97	4335.94	2.229	882.05	1965.80
A_68_P29789069	chr14:87817144-87817188	NM_172605:777	Tdrd3	INSIDE	0.209	2.165	848.45	1836.84	0.452	772.94	349.11
A_68_P20742449	chr1:158233625-158233669	NM_001134741:-167	Tdrd5	DIVERGENT_PROMOTER	0.289	29.999	2710.44	81311.18	8.673	2484.37	21545.74
A_68_P31190361	chr17:43766788-43766832	NM_001161366:438	Tdrd6	INSIDE	0.434	4.654	1113.03	5179.56	2.022	904.62	1828.91
A_68_P22338876	chr3:94217322-94217366	NM_028307:105	Tdrkh	INSIDE	0.513	0.409	9800.19	4008.53	0.21	6340.61	1330.42
A_68_P25091865	chr7:52471363-52471407	NM_011565:262	Tead2	INSIDE	0.479	0.334	5420.27	1810.59	0.16	3889.48	621.76
A_68_P31121999	chr17:28487626-28487670	NM_001098226:-103	Tead3	PROMOTER	0.414	5.045	4520.81	22806.81	2.09	3213.02	6714.37
A_68_P31121931	chr17:28478403-28478447	NM_001204156:8867	Tead3	INSIDE	0.58	0.415	1782.98	740.05	0.241	1244.01	299.29
A_68_P31122000	chr17:28487865-28487909	NM_011566:-136	Tead3	PROMOTER	0.585	0.264	4560.45	1203.48	0.154	3076.88	475.11
A_68_P24160101	chr5:144984767-144984811	NM_027410:-341	Tecpr1	PROMOTER	0.651	2.954	1297.78	3833.00	1.923	1042.01	2003.51
A_68_P28740917	chr12:112127699-112127743	NM_001081057:247	Tecpr2	INSIDE	0.603	0.694	1552.07	1077.29	0.419	1254.33	525.14
A_68_P26015636	chr8:86118488-86118532	NM_027179:-120	Tecr	PROMOTER	0.458	9.145	1098.59	10046.78	4.191	873.61	3661.07
A_68_P30379756	chr15:81641379-81641423	NM_017376:-443	Tef	PROMOTER	0.168	17.378	10654.80	185160.20	2.923	10571.02	30903.49
A_68_P30379760	chr15:81641863-81641907	NM_017376:41	Tef	INSIDE	0.412	5.676	1001.47	5684.48	2.337	917.13	2143.06
A_68_P26082617	chr8:97844517-97844561	NM_028532:9026	Tepp	INSIDE	0.434	0.324	3486.29	1128.07	0.14	2599.44	365.02
A_68_P22349233	chr3:96218809-96218853	NR_001579:-74	Terc	PROMOTER	0.662	0.192	2942.58	564.18	0.127	2313.97	293.88
A_68_P26143096	chr8:109620241-109620285	NM_009353:183	Terf2	INSIDE	0.376	17.207	4723.60	81278.80	6.467	3894.76	25186.26
A_68_P24024080	chr5:118478322-118478366	NM_021344:512	Tesc	INSIDE	0.454	10.452	1030.87	10774.63	4.748	938.17	4454.18
A_68_P24024083	chr5:118478594-118478638	NM_021344:784	Tesc	INSIDE	0.549	7.559	4420.04	33410.55	4.15	3856.94	16007.83
A_68_P22543376	chr3:133207147-133207191	NM_001040400:186	Tet2	INSIDE	0.44	0.189	4010.45	759.75	0.083	3200.21	266.50
A_68_P22543381	chr3:133207644-133207688	NM_001040400:-312	Tet2	PROMOTER	0.527	2.680	1937.46	5191.86	1.412	1619.33	2287.00
A_68_P22900481	chr4:48486343-48486387	NM_172304:-70	Tex10	PROMOTER	0.614	4.002	582.42	2330.93	2.459	569.63	1400.81
A_68_P26504312	chr9:50369341-50369385	NM_025687:11	Tex12	INSIDE	0.601	5.524	1744.63	9636.69	3.322	1491.29	4953.83
A_68_P28102658	chr11:106473614-106473658	NM_198292:608	Tex2	INSIDE	0.175	0.629	2316.62	1457.52	0.11	1521.27	167.36
A_68_P26804666	chr9:106588275-106588319	NM_001081654:-17	Tex264	PROMOTER	0.547	4.292	1944.94	8346.91	2.346	1489.94	3495.53
A_68_P26804663	chr9:106587992-106588036	NM_011573:-16	Tex264	PROMOTER	0.644	2.753	1150.34	3166.63	1.772	959.85	1701.16
A_68_P26804662	chr9:106587823-106587867	NM_011573:154	Tex264	INSIDE	0.302	7.160	494.40	3539.94	2.164	461.63	999.07
A_68_P26628115	chr9:72339784-72339828	NM_009359:-43	Tex9	PROMOTER	0.611	0.479	923.58	442.10	0.292	796.64	232.86
A_68_P27234347	chr10:70700994-70701038	NM_009360:-224	Tfam	PROMOTER	0.633	0.502	4368.79	2192.04	0.318	3184.49	1011.73
A_68_P26745887	chr9:96096585-96096629	NM_001184706:-87	Tfdp2	PROMOTER	0.631	3.177	1415.17	4495.65	2.004	1162.14	2328.60
A_68_P30782600	chr16:56717318-56717362	NM_019678:127	Tfg	INSIDE	0.623	2.713	1052.83	2856.10	1.69	839.37	1418.80
A_68_P30653349	chr16:32609415-32609459	NM_011638:455	Tfrc	INSIDE	0.461	0.256	2046.51	523.35	0.118	1646.44	194.15
A_68_P30653348	chr16:32609325-32609369	NM_011638:365	Tfrc	INSIDE	0.476	0.634	1428.00	905.47	0.302	1188.86	359.06
A_68_P30653344	chr16:32608841-32608885	NM_011638:-119	Tfrc	PROMOTER	0.504	1.359	5826.51	7918.40	0.685	4304.81	2949.15
A_68_P29936814	chr14:118531918-118531962	NM_029578:47	Tgds	INSIDE	0.476	3.377	909.96	3073.38	1.606	799.06	1283.42
A_68_P24608765	chr6:86145281-86145325	NM_031199:58	Tgfa	INSIDE	0.216	17.717	231.97	4109.69	3.821	237.92	909.05
A_68_P25011349	chr7:26474463-26474507	NM_011577:2464	Tgfb1	INSIDE	0.207	17.971	1056.05	18977.85	3.715	933.96	3470.06
A_68_P25011329	chr7:26472470-26472514	NM_011577:472	Tgfb1	INSIDE	0.274	8.253	5584.03	46084.32	2.26	4182.51	9452.83
A_68_P25011324	chr7:26471878-26471922	NM_011577:-120	Tgfb1	PROMOTER	0.516	4.550	2698.07	12277.28	2.349	2201.23	5169.88
A_68_P29062337	chr13:56710941-56710985	NM_009369:-1	Tgfbi	PROMOTER	0.355	9.599	5803.96	55714.75	3.412	4662.61	15906.64
A_68_P29062336	chr13:56710843-56710887	NM_009369:-99	Tgfbi	PROMOTER	0.587	0.718	5070.50	3642.52	0.422	3639.79	1535.14
A_68_P22894261	chr4:47365847-47365891	NM_009370:-308	Tgfbr1	PROMOTER	0.567	10.769	1144.36	12323.61	6.102	945.49	5769.23
A_68_P22894263	chr4:47366193-47366237	NM_009370:38	Tgfbr1	INSIDE	0.555	0.710	2143.75	1521.46	0.394	1938.66	763.60
A_68_P22894259	chr4:47365606-47365650	NM_009370:-548	Tgfbr1	PROMOTER	0.136	1.376	3727.85	5128.32	0.187	2428.47	453.85
A_68_P20186937	chr1:43149031-43149075	NM_001013025:6415	Tgfbrap1	INSIDE	0.574	0.714	9908.23	7071.57	0.409	6781.44	2775.75
A_68_P20186939	chr1:43149198-43149242	NM_001013025:6247	Tgfbrap1	INSIDE	0.48	2.966	2358.80	6995.25	1.423	1997.88	2842.03

ProbeName	Target position of probe on CpG island microarray	TargetID	GeneSymbol	CpG island Description	Ratio of relative methylation (TiO_2-NP/Vehicle)	Sham group			TiO_2-H group		
						Relative methylation (Cy5/Cy3)	Cy3 signal (Input)	Cy5 signal (MeDIP)	Relative methylation (Cy5/Cy3)	Cy3 signal (Input)	Cy5 signal (MeDIP)
A_68_P21773626	chr2:156665615-156665659	NM_173396:-176	Tgif2	PROMOTER	0.458	8.673	1767.34	15328.38	3.974	1485.20	5902.60
A_68_P21773631	chr2:156666079-156666123	NM_173396:288	Tgif2	INSIDE	0.323	1.980	6374.22	12623.59	0.639	4634.40	2961.43
A_68_P25731030	chr8:27269296-27269340	NM_199042:678	Thap1	INSIDE	0.588	2.921	924.59	2701.00	1.719	815.77	1402.34
A_68_P25731031	chr8:27269366-27269410	NM_199042:748	Thap1	INSIDE	0.654	2.719	887.41	2412.46	1.777	672.72	1195.74
A_68_P26136330	chr8:108379864-108379908	NM_021513:884	Thap11	INSIDE	0.483	3.641	506.86	1845.25	1.758	580.50	1020.79
A_68_P27469298	chr10:114821572-114821616	NM_025780:-103	Thap2	DIVERGENT_PROMOTER	0.539	3.000	5320.67	15963.03	1.616	4478.92	7237.94
A_68_P20457301	chr1:95651221-95651265	NM_025920:173	Thap4	INSIDE	0.542	21.250	1246.81	26494.83	11.519	1057.88	12185.53
A_68_P30574323	chr16:17530866-17530910	NM_026909:257	Thap7	INSIDE	0.615	0.397	1489.41	591.49	0.244	1150.18	280.77
A_68_P29225755	chr13:93564632-93564676	NM_011582:119	Thbs4	INSIDE	0.624	0.709	2007.16	1422.38	0.442	1683.21	744.79
A_68_P29225757	chr13:93564876-93564920	NM_011582:-125	Thbs4	PROMOTER	0.309	4.635	2863.76	13273.80	1.432	2452.25	3512.07
A_68_P21054135	chr2:21127530-21127574	NM_001001297:202	Thnsl1	INSIDE	0.48	0.300	6492.61	1947.72	0.144	4404.38	633.71
A_68_P24534825	chr6:71094013-71094057	NM_001033929:336	Thnsl2	INSIDE	0.578	0.225	2124.48	477.30	0.13	1557.07	202.28
A_68_P31487558	chr18:9957999-9958043	NM_153552:-157	Thoc1	PROMOTER	0.526	0.426	1843.66	784.65	0.224	1254.37	280.62
A_68_P29418922	chr14:14794169-14794213	NM_025435:-451	Thoc7	PROMOTER	0.454	4.942	2190.83	10827.34	2.243	1661.00	3725.13
A_68_P29418923	chr14:14794239-14794283	NM_025435:-521	Thoc7	PROMOTER	0.482	0.207	2108.36	437.07	0.1	1667.73	166.61
A_68_P29418915	chr14:14793361-14793405	NM_025435:357	Thoc7	INSIDE	0.531	0.545	1099.33	598.71	0.289	904.73	261.56
A_68_P27287202	chr10:80532912-80532956	NM_022653:107	Thop1	INSIDE	0.415	0.453	1082.07	490.69	0.188	906.68	170.83
A_68_P27287274	chr10:80542595-80542639	NM_022653:9789	Thop1	INSIDE	0.435	8.528	1511.84	12892.21	3.707	1131.97	4196.19
A_68_P27287200	chr10:80532719-80532763	NM_022653:-87	Thop1	DIVERGENT_PROMOTER	0.526	0.478	1115.50	533.50	0.251	822.43	206.78
A_68_P25510954	chr7:135604766-135604810	NM_009383:239	Tial1	INSIDE	0.366	0.606	941.02	570.63	0.222	769.46	170.76
A_68_P24485184	chr6:59158961-59159005	NM_001081145:119	Tigd2	INSIDE	0.365	0.549	998.69	548.76	0.201	870.37	174.58
A_68_P30344892	chr15:75741160-75741204	NM_178646:1018	Tigd5	INSIDE	0.441	0.402	3776.82	1517.89	0.177	2619.10	464.45
A_68_P30344883	chr15:75740087-75740131	NM_178646:-56	Tigd5	DIVERGENT_PROMOTER	0.443	8.989	2220.24	19956.75	3.983	1822.60	7260.19
A_68_P27540232	chr10:127669379-127669423	NM_011589:-5721	Timeless	PROMOTER	0.574	4.758	451.46	2147.94	2.732	453.97	1240.19
A_68_P27286217	chr10:80363400-80363444	NM_013895:292	Timm13	INSIDE	0.601	0.577	2581.43	1489.59	0.347	1978.14	686.41
A_68_P20638619	chr1:137210199-137210243	NM_011590:94	Timm17a	INSIDE	0.368	0.719	1778.51	1279.48	0.265	1296.06	343.09
A_68_P32672454	chrX:131076324-131076368	NM_013898:-178	Timm8a1	PROMOTER	0.652	5.603	653.84	3663.71	3.656	777.20	2841.19
A_68_P27313251	chr10:85763083-85763128	NM_011595:-51	Timp3	PROMOTER	0.448	0.498	1575.43	784.17	0.223	1143.75	255.07
A_68_P26421202	chr9:35007602-35007646	NM_001177846:-74	Tirap	PROMOTER	0.544	0.527	1317.83	694.87	0.287	1076.40	308.80
A_68_P25182469	chr7:72516013-72516058	NM_001163574:95	Tjp1	INSIDE	0.469	3.299	1368.64	4514.79	1.548	1268.22	1962.92
A_68_P32021816	chr19:24205473-24205517	NM_001198985:43136	Tjp2	INSIDE	0.524	4.826	1351.96	6524.45	2.53	894.01	2262.13
A_68_P32021818	chr19:24205720-24205764	NM_001198985:42888	Tjp2	INSIDE	0.623	5.235	2439.68	12771.47	3.263	2049.05	6686.72
A_68_P32021817	chr19:24205542-24205588	NM_001198985:43066	Tjp2	INSIDE	0.661	3.726	1186.01	4419.04	2.462	677.04	1667.03
A_68_P32022083	chr19:24248437-24248481	NM_001198985:172	Tjp2	INSIDE	0.6	2.756	2320.67	6396.59	1.655	1896.03	3137.02
A_68_P27288599	chr10:80748500-80748544	NM_013769:5490	Tjp3	INSIDE	0.519	3.807	966.46	3679.46	1.976	850.41	1680.67
A_68_P23012913	chr4:71860924-71860968	NM_011599:1007	Tle1	INSIDE	0.304	6.951	2590.65	18007.08	2.113	1987.73	4199.84
A_68_P27290609	chr10:81038162-81038206	NM_019725:-92	Tle2	PROMOTER	0.434	0.644	1396.48	899.67	0.28	1016.97	284.33
A_68_P27290695	chr10:81050342-81050386	NM_019725:12088	Tle2	INSIDE	0.362	1.716	1903.27	3266.39	0.622	1348.19	838.39
A_68_P27290790	chr10:81063642-81063689	NM_053254:-20	Tle6	DIVERGENT_PROMOTER	0.234	18.140	7066.68	128192.70	4.24	6006.87	25471.42
A_68_P32136800	chr19:45228638-45228682	NM_021901:3456	Tlx1	INSIDE	0.472	6.584	918.77	6049.38	3.108	802.80	2494.97
A_68_P32136769	chr19:45224972-45225016	NM_021901:-210	Tlx1	PROMOTER	0.576	0.667	1789.84	1194.09	0.384	1140.04	438.16
A_68_P24591244	chr6:83019585-83019631	NM_009392:611	Tlx2	INSIDE	0.387	0.329	2238.17	737.06	0.127	1368.53	174.27
A_68_P24591254	chr6:83020596-83020640	NM_009392:-399	Tlx2	DIVERGENT_PROMOTER	0.606	0.429	3675.15	1578.13	0.26	2826.61	735.39
A_68_P24591247	chr6:83019894-83019938	NM_009392:303	Tlx2	INSIDE	0.6	0.697	1081.67	753.75	0.418	874.68	365.92
A_68_P27693341	chr11:33101001-33101045	NM_019916:2566	Tlx3	INSIDE	0.107	96.416	8051.74	776319.60	10.278	7890.96	81101.33
A_68_P27693355	chr11:33102836-33102880	NM_019916:730	Tlx3	INSIDE	0.142	18.170	1307.87	23764.26	2.589	1176.07	3044.85

ProbeName	Target position of probe on CpG island microarray	TargetID	GeneSymbol	CpG island Description	Ratio of relative methylation (TiO_2-NP/Vehicle)	Sham group			TiO_2-H group		
						Relative methylation (Cy5/Cy3)	Cy3 signal (Input)	Cy5 signal (MeDIP)	Relative methylation (Cy5/Cy3)	Cy3 signal (Input)	Cy5 signal (MeDIP)
A_68_P27693346	chr11:33101716-33101763	NM_019916:1849	Tlx3	INSIDE	0.476	12.349	560.41	6920.58	5.875	413.97	2431.97
A_68_P27693349	chr11:33102051-33102095	NM_019916:1516	Tlx3	INSIDE	0.476	0.517	1789.35	924.59	0.246	1444.79	355.69
A_68_P27693347	chr11:33101808-33101852	NM_019916:1758	Tlx3	INSIDE	0.6	6.719	678.13	4556.16	4.033	555.04	2238.52
A_68_P27693350	chr11:33102180-33102224	NM_019916:1386	Tlx3	INSIDE	0.359	1.649	5402.44	8908.16	0.593	3434.10	2035.45
A_68_P25724849	chr8:26128143-26128187	NM_027194:482	Tm2d2	INSIDE	0.245	0.481	1840.08	884.48	0.118	1453.21	171.01
A_68_P25724844	chr8:26127584-26127628	NM_027194:-76	Tm2d2	DIVERGENT_PROMOTER	0.383	6.709	1458.42	9784.54	2.573	1086.41	2795.16
A_68_P25184109	chr7:72838329-72838373	NM_026795:48	Tm2d3	INSIDE	0.656	0.393	2252.22	884.61	0.258	1644.02	423.77
A_68_P24929724	chr6:146582793-146582837	NM_026281:300	Tm7sf3	INSIDE	0.555	4.395	1122.76	4934.32	2.439	908.24	2215.27
A_68_P29619051	chr14:56262543-56262587	NM_028780:79	Tm9sf1	INSIDE	0.543	4.031	405.44	1634.35	2.188	313.76	686.42
A_68_P29959675	chr14:122506713-122506757	NM_080556:431	Tm9sf2	INSIDE	0.497	2.701	5108.74	13801.07	1.342	4342.02	5828.33
A_68_P32114042	chr19:41337768-41337812	NM_133352:704	Tm9sf3	INSIDE	0.479	0.275	1746.78	479.90	0.132	1293.87	170.34
A_68_P27495880	chr10:119645922-119645966	NM_026617:63	Tmbim4	INSIDE	0.589	0.262	3551.55	930.18	0.154	2661.92	410.32
A_68_P30479870	chr15:99223391-99223435	NM_001171035:-237	Tmbim6	PROMOTER	0.459	3.027	2638.99	7988.00	1.389	1822.35	2530.96
A_68_P28167167	chr11:117641287-117641331	NM_181321:627	Tmc6	INSIDE	0.39	1.638	1868.72	3060.13	0.639	1440.74	920.85
A_68_P25459064	chr7:125728226-125728270	NM_172476:-48	Tmc7	PROMOTER	0.315	9.534	1980.82	18885.00	3.001	1667.38	5003.63
A_68_P28167195	chr11:117644840-117644884	NM_001195088:891	Tmc8	INSIDE	0.548	0.568	1808.48	1027.56	0.312	1281.42	399.22
A_68_P28167194	chr11:117644703-117644747	NM_001195088:753	Tmc8	INSIDE	0.395	0.599	986.25	590.64	0.237	735.83	174.18
A_68_P20620973	chr1:134257439-134257483	NM_178874:30398	Tmcc2	INSIDE	0.177	9.517	2388.69	22733.30	1.684	1665.87	2804.72
A_68_P20620970	chr1:134257180-134257224	NM_178874:30656	Tmcc2	INSIDE	0.266	6.028	2560.53	15436.10	1.606	1950.91	3132.71
A_68_P20620974	chr1:134257507-134257551	NM_178874:30330	Tmcc2	INSIDE	0.165	10.506	1549.51	16279.63	1.735	1020.05	1769.83
A_68_P27359835	chr10:93978114-93978158	NM_172051:535	Tmcc3	INSIDE	0.311	5.488	788.88	4329.78	1.704	752.18	1281.98
A_68_P27359833	chr10:93977922-93977966	NM_172051:343	Tmcc3	INSIDE	0.584	2.981	849.55	2532.13	1.741	677.41	1179.37
A_68_P20804423	chr1:169238910-169238954	NM_001039483:132	Tmco1	INSIDE	0.282	1.871	1099.58	2057.40	0.528	994.03	524.59
A_68_P23348948	chr4:138528822-138528867	NM_029857:25	Tmco4	INSIDE	0.473	0.391	1135.29	443.45	0.185	945.15	174.78
A_68_P26140913	chr8:109207201-109207245	NM_173037:255	Tmco7	INSIDE	0.334	0.559	1524.39	852.03	0.186	1261.35	235.15
A_68_P23974100	chr5:109203943-109203987	NM_026109:20418	Tmed11	DOWNSTREAM	0.405	5.774	1712.08	9886.38	2.338	1252.47	2927.85
A_68_P23974103	chr5:109204294-109204338	NM_026109:20066	Tmed11	DOWNSTREAM	0.664	5.458	353.45	1929.18	3.627	308.01	1117.04
A_68_P26713768	chr9:89599588-89599632	NM_025360:271	Tmed3	INSIDE	0.583	3.562	591.75	2107.87	2.077	546.24	1134.78
A_68_P28614745	chr12:88540669-88540713	NM_001033475:489	Tmed8	INSIDE	0.544	8.065	2948.87	23783.33	4.387	2858.07	12538.43
A_68_P28614749	chr12:88541116-88541160	NM_001033475:41	Tmed8	INSIDE	0.551	3.888	664.02	2581.48	2.142	571.75	1224.88
A_68_P22900943	chr4:48598769-48598813	NM_021436:726	Tmeff1	INSIDE	0.267	0.483	3027.22	1461.41	0.129	1987.89	256.00
A_68_P22900940	chr4:48598477-48598521	NM_021436:434	Tmeff1	INSIDE	0.548	2.957	1439.88	4257.16	1.621	1266.55	2053.43
A_68_P20218128	chr1:50984412-50984456	NM_019790:68	Tmeff2	INSIDE	0.543	0.425	4058.92	1725.90	0.231	2998.17	692.76
A_68_P20218125	chr1:50984012-50984056	NM_019790:-332	Tmeff2	PROMOTER	0.598	0.472	2014.25	951.46	0.283	1550.99	438.43
A_68_P28151868	chr11:115048748-115048792	NM_001033393:-30	Tmem104	DIVERGENT_PROMOTER	0.125	18.772	3719.65	69827.15	2.34	3137.54	7340.92
A_68_P26787849	chr9:103664052-103664096	NM_178638:93	Tmem108	INSIDE	0.518	1.341	4084.50	5477.06	0.695	3466.31	2409.31
A_68_P27845266	chr11:60692244-60692288	NM_001168507:274	Tmem11	INSIDE	0.203	7.656	1920.40	14702.31	1.551	1425.01	2209.97
A_68_P29508103	chr14:31638633-31638679	NM_028839:-123	Tmem110	PROMOTER	0.358	0.578	959.54	554.71	0.207	822.54	170.30
A_68_P30453398	chr15:94459261-94459305	NM_178789:-333	Tmem117	PROMOTER	0.388	9.803	7988.03	78309.62	3.799	6806.18	25859.53
A_68_P24117669	chr5:136219992-136220036	NM_172541:28	Tmem120a	INSIDE	0.658	3.980	879.92	3501.71	2.619	812.80	2129.06
A_68_P28755242	chr12:114426423-114426467	NM_153776:2331	Tmem121	INSIDE	0.145	3.118	1371.17	4275.94	0.453	880.28	398.47
A_68_P26289277	chr9:7763854-7763898	NM_133739:-200	Tmem123	PROMOTER	0.505	4.325	10329.97	44680.41	2.183	7527.24	16430.26
A_68_P26289278	chr9:7763954-7763998	NM_133739:-100	Tmem123	PROMOTER	0.588	0.744	3257.35	2423.47	0.437	2489.70	1088.37
A_68_P21612305	chr2:127073731-127073775	NM_175145:42	Tmem127	INSIDE	0.52	0.560	1874.65	1049.86	0.291	1521.94	443.42
A_68_P21612309	chr2:127074111-127074155	NM_175145:422	Tmem127	INSIDE	0.635	0.209	2784.04	581.25	0.133	2224.28	294.90
A_68_P24162428	chr5:145522451-145522495	NM_177735:-25	Tmem130	DIVERGENT_PROMOTER	0.6	0.346	2753.08	951.46	0.207	2133.65	442.15

ProbeName	Target position of probe on CpG island microarray	TargetID	GeneSymbol	CpG island Description	Ratio of relative methylation (TiO_2-NP/Vehicle)	Sham group			TiO_2-H group		
						Relative methylation (Cy5/Cy3)	Cy3 signal (Input)	Cy5 signal (MeDIP)	Relative methylation (Cy5/Cy3)	Cy3 signal (Input)	Cy5 signal (MeDIP)
A_68_P20151305	chr1:36996651-36996695	NM_018872:-300	Tmem131	PROMOTER	0.378	6.749	1322.50	8925.82	2.549	1149.07	2929.05
A_68_P31953349	chr19:10944024-10944068	NM_133804:223	Tmem132a	INSIDE	0.556	0.579	1305.52	755.96	0.322	959.59	308.70
A_68_P24083090	chr5:128938897-128938941	NM_172885:19	Tmem132d	INSIDE	0.29	7.447	1193.01	8884.56	2.162	1005.68	2174.25
A_68_P27966237	chr11:82248208-82248252	NM_023438:45829	Tmem132e	INSIDE	0.305	7.573	1570.32	11891.34	2.313	1312.11	3035.12
A_68_P27965917	chr11:82200919-82200963	NM_023438:-1461	Tmem132e	PROMOTER	0.331	27.849	2136.92	59510.48	9.207	1818.74	16744.97
A_68_P27965931	chr11:82202507-82202551	NM_023438:127	Tmem132e	INSIDE	0.374	13.834	5415.05	74912.48	5.172	4748.40	24560.59
A_68_P25009653	chr7:26100013-26100057	NM_183311:8908	Tmem145	INSIDE	0.141	2.404	14992.69	36037.19	0.34	11158.22	3793.58
A_68_P25009654	chr7:26100103-26100147	NM_183311:8998	Tmem145	INSIDE	0.268	1.674	7216.44	12081.47	0.449	5423.28	2433.05
A_68_P25009660	chr7:26100723-26100767	NM_183311:9618	Tmem145	INSIDE	0.606	0.388	2820.08	1095.09	0.235	2181.15	513.45
A_68_P25009594	chr7:26091283-26091327	NM_183311:178	Tmem145	INSIDE	0.592	0.616	3009.63	1853.99	0.365	2225.78	811.57
A_68_P25009661	chr7:26100795-26100839	NM_183311:9690	Tmem145	INSIDE	0.266	1.476	4834.69	7136.14	0.392	3539.88	1388.40
A_68_P28975559	chr13:41111578-41111622	NM_025387:-18	Tmem14c	PROMOTER	0.241	0.705	6037.14	4256.58	0.17	4574.58	778.62
A_68_P31201735	chr17:45686486-45686530	NM_001013749:118	Tmem151b	INSIDE	0.425	10.595	4074.84	43171.88	4.499	3265.38	14692.44
A_68_P31201732	chr17:45686195-45686239	NM_001013749:410	Tmem151b	INSIDE	0.332	1.766	1870.40	3302.28	0.586	1471.70	862.99
A_68_P31201733	chr17:45686337-45686381	NM_001013749:268	Tmem151b	INSIDE	0.624	2.241	2075.39	4650.67	1.399	1663.84	2327.00
A_68_P26894026	chr9:123168701-123168745	NM_001002267:1185	Tmem158	INSIDE	0.457	7.087	2042.78	14477.81	3.241	1655.11	5364.69
A_68_P26894035	chr9:123169998-123170042	NM_001002267:-113	Tmem158	PROMOTER	0.507	10.440	1637.03	17089.91	5.292	1360.18	7197.90
A_68_P26894025	chr9:123168633-123168677	NM_001002267:1253	Tmem158	INSIDE	0.478	0.698	1210.71	844.88	0.333	931.20	310.35
A_68_P25467117	chr7:127246125-127246169	NM_145586:207	Tmem159	INSIDE	0.4	0.168	3469.94	584.10	0.067	2505.18	168.88
A_68_P25467116	chr7:127246039-127246083	NM_145586:121	Tmem159	INSIDE	0.651	0.704	2527.48	1780.36	0.458	1796.40	823.40
A_68_P29189772	chr13:84361804-84361848	NM_175187:-74	Tmem161b	PROMOTER	0.604	0.338	2524.23	854.19	0.204	1890.80	386.39
A_68_P32709177	chrX:139116370-139116414	NM_001199360:124	Tmem164	INSIDE	0.128	13.855	2170.98	30078.15	1.779	3007.14	5348.28
A_68_P32709174	chrX:139116023-139116067	NM_001199360:-224	Tmem164	PROMOTER	0.254	15.826	1855.57	29367.03	4.022	2254.65	9068.42
A_68_P23814584	chr5:76613501-76613545	NM_011626:618	Tmem165	INSIDE	0.269	9.295	879.14	8171.63	2.498	725.42	1812.27
A_68_P29211817	chr13:90229380-90229424	NM_025335:131	Tmem167	INSIDE	0.521	0.551	1916.41	1055.95	0.287	1377.86	395.41
A_68_P22413373	chr3:108365105-108365149	NM_026198:258	Tmem167b	INSIDE	0.295	18.995	8898.02	169017.80	5.612	8057.95	45224.74
A_68_P22413375	chr3:108365261-108365305	NM_026198:102	Tmem167b	INSIDE	0.574	0.708	3611.83	2555.42	0.406	2566.84	1043.33
A_68_P24248064	chr6:13557588-13557632	NM_028990:453	Tmem168	INSIDE	0.46	0.516	2080.19	1074.30	0.237	1668.85	396.20
A_68_P27641464	chr11:22412537-22412581	NM_153596:273	Tmem17	INSIDE	0.634	3.622	780.00	2824.76	2.298	643.08	1477.64
A_68_P27641465	chr11:22412657-22412701	NM_153596:393	Tmem17	INSIDE	0.47	0.648	1021.32	662.15	0.304	840.04	255.77
A_68_P23973160	chr5:109058811-109058855	NM_001163531:4	Tmem175	INSIDE	0.627	4.226	513.33	2169.35	2.648	405.30	1073.24
A_68_P31385964	chr17:81344186-81344230	NM_026516:237	Tmem178	INSIDE	0.563	2.640	2512.48	6633.17	1.486	2258.91	3355.95
A_68_P32143397	chr19:46423267-46423311	NM_029186:-8081	Tmem180	PROMOTER	0.362	0.391	1512.94	591.97	0.142	1232.75	174.71
A_68_P32143438	chr19:46431466-46431510	NM_029186:119	Tmem180	INSIDE	0.661	2.639	973.95	2570.38	1.745	734.62	1282.27
A_68_P20632910	chr1:136241239-136241283	NM_001042485:17316	Tmem183a	DOWNSTREAM	0.658	2.305	2382.19	5492.01	1.517	1919.40	2912.31
A_68_P30366260	chr15:79233510-79233554	NM_172608:201	Tmem184b	INSIDE	0.545	3.928	1419.11	5573.95	2.14	1259.10	2694.42
A_68_P30366258	chr15:79233359-79233403	NM_172608:353	Tmem184b	INSIDE	0.397	3.706	2220.01	8227.52	1.472	1732.71	2550.37
A_68_P20557524	chr1:121422451-121422495	NM_146103:-271	Tmem185b	PROMOTER	0.494	0.741	2448.54	1813.17	0.366	1880.43	687.69
A_68_P30572936	chr16:17276365-17276409	NM_177473:-6	Tmem191c	PROMOTER	0.227	44.372	1020.19	45267.85	10.068	958.07	9645.53
A_68_P30572953	chr16:17278195-17278239	NM_177473:1824	Tmem191c	INSIDE	0.428	0.412	1164.71	479.94	0.176	972.05	171.41
A_68_P30572951	chr16:17278027-17278071	NM_177473:1656	Tmem191c	INSIDE	0.623	3.377	990.52	3345.19	2.102	806.69	1696.04
A_68_P30572940	chr16:17276845-17276889	NM_177473:474	Tmem191c	INSIDE	0.626	2.707	2331.16	6309.91	1.694	1990.76	3371.40
A_68_P25926491	chr8:67471091-67471135	NM_001163747:29	Tmem192	INSIDE	0.302	8.354	3239.39	27061.78	2.519	2758.96	6950.86
A_68_P27537442	chr10:127114141-127114185	NM_173732:42	Tmem194	INSIDE	0.664	0.212	5612.37	1189.25	0.141	3773.02	530.48
A_68_P20354344	chr1:75476279-75476323	NM_177056:194	Tmem198	INSIDE	0.407	0.239	2579.82	616.40	0.097	1830.31	178.15
A_68_P32008156	chr19:21853273-21853317	NM_001033759:463	Tmem2	INSIDE	0.253	25.214	3784.82	95432.40	6.368	3811.50	24272.01

ProbeName	Target position of probe on CpG island microarray	TargetID	GeneSymbol	CpG island Description	Ratio of relative methylation (TiO_2-NP/Vehicle)	Sham group			TiO_2-H group		
						Relative methylation (Cy5/Cy3)	Cy3 signal (Input)	Cy5 signal (MeDIP)	Relative methylation (Cy5/Cy3)	Cy3 signal (Input)	Cy5 signal (MeDIP)
A_68_P32008153	chr19:21852916-21852960	NM_001033759:107	Tmem2	INSIDE	0.374	7.182	1322.07	9495.04	2.689	1278.46	3438.38
A_68_P27017611	chr10:25798724-25798768	NM_029881:112	Tmem200a	INSIDE	0.478	5.988	270.98	1622.63	2.863	246.37	705.46
A_68_P23309116	chr4:131477634-131477678	NM_001201367:-29	Tmem200b	PROMOTER	0.294	8.700	690.80	6009.97	2.561	649.09	1662.45
A_68_P31320370	chr17:69186260-69186304	NM_001206661:-193	Tmem200c	PROMOTER	0.238	0.556	1719.09	956.11	0.133	1301.66	172.64
A_68_P31320413	chr17:69190632-69190676	NM_001206661:4179	Tmem200c	INSIDE	0.534	5.009	1378.07	6903.44	2.675	1278.70	3420.88
A_68_P31320372	chr17:69186496-69186540	NM_001206661:43	Tmem200c	INSIDE	0.626	4.099	919.72	3769.58	2.567	865.10	2221.13
A_68_P31320371	chr17:69186413-69186457	NM_001206661:-41	Tmem200c	PROMOTER	0.397	0.641	1918.27	1229.03	0.255	1484.38	377.88
A_68_P31320416	chr17:69191045-69191089	NM_001206661:4591	Tmem200c	INSIDE	0.511	3.651	673.59	2458.96	1.865	688.80	1284.76
A_68_P23402215	chr4:149111795-149111839	NM_001025106:327	Tmem201	INSIDE	0.445	16.572	1441.67	23890.87	7.38	1270.59	9377.05
A_68_P20937464	chr1:193150232-193150276	NM_025864:411	Tmem206	INSIDE	0.288	1.468	1792.34	2630.91	0.423	1316.40	556.96
A_68_P26133052	chr8:107850532-107850576	NM_025486:291	Tmem208	INSIDE	0.267	8.549	7159.82	61209.05	2.284	6331.58	14458.71
A_68_P23575088	chr5:31172158-31172203	NM_144525:161	Tmem214	INSIDE	0.627	0.259	1756.72	454.42	0.162	1055.03	171.19
A_68_P26770338	chr9:100471400-100471444	NM_001101483:82	Tmem22	INSIDE	0.646	2.889	867.28	2505.38	1.866	777.91	1451.59
A_68_P25957474	chr8:74082197-74082241	NM_001100462:552	Tmem221	INSIDE	0.489	0.522	1504.94	786.26	0.255	1126.48	287.69
A_68_P25957473	chr8:74082049-74082093	NM_001100462:700	Tmem221	INSIDE	0.584	0.615	821.63	505.67	0.359	600.29	215.65
A_68_P23316767	chr4:132833610-132833654	NM_025667:73	Tmem222	INSIDE	0.496	6.799	1012.23	6881.83	3.372	883.33	2978.74
A_68_P24304143	chr6:24905716-24905760	NM_177013:387	Tmem229a	INSIDE	0.294	7.698	1461.17	11247.84	2.261	1491.93	3373.25
A_68_P28567140	chr12:80108151-80108195	NM_178745:92	Tmem229b	INSIDE	0.263	25.377	8916.01	226262.20	6.669	7548.11	50341.03
A_68_P23766883	chr5:67652121-67652165	NM_028975:252	Tmem33	INSIDE	0.618	0.519	1049.40	544.75	0.321	897.43	287.77
A_68_P25962147	chr8:75095836-75095880	NM_144534:-143	Tmem38a	DIVERGENT_PROMOTER	0.3	7.842	1345.95	10555.38	2.355	1261.07	2969.42
A_68_P25962148	chr8:75095909-75095953	NM_144534:-71	Tmem38a	DIVERGENT_PROMOTER	0.38	10.363	4465.03	46269.56	3.939	3690.29	14535.95
A_68_P23298058	chr4:129373895-129373939	NM_199305:166	Tmem39b	INSIDE	0.349	6.958	3057.48	21274.87	2.43	2861.17	6953.66
A_68_P23298057	chr4:129373770-129373814	NM_199305:290	Tmem39b	INSIDE	0.403	3.624	1807.99	6552.53	1.46	1437.68	2098.96
A_68_P30595261	chr16:21947632-21947676	NM_025693:-29	Tmem41a	PROMOTER	0.617	2.893	1256.19	3633.66	1.786	1074.19	1918.16
A_68_P25411125	chr7:117129431-117129475	NM_153525:292	Tmem41b	INSIDE	0.123	11.575	4584.48	53066.92	1.42	3413.63	4846.92
A_68_P26892620	chr9:122930391-122930435	NM_001164823:-31	Tmem42	PROMOTER	0.634	2.564	1851.59	4748.15	1.626	1515.72	2464.51
A_68_P30642390	chr16:30550650-30550694	NM_172614:-8	Tmem44	PROMOTER	0.238	10.087	574.43	5794.35	2.404	460.81	1107.58
A_68_P30642387	chr16:30550318-30550362	NM_172614:324	Tmem44	INSIDE	0.377	4.340	2343.51	10171.71	1.634	1863.95	3045.99
A_68_P32488667	chrX:78316560-78316604	NM_138751:600	Tmem47	INSIDE	0.364	9.770	3736.22	36502.81	3.557	4584.15	16303.61
A_68_P32488661	chrX:78315918-78315962	NM_138751:-42	Tmem47	PROMOTER	0.515	6.359	1495.91	9512.26	3.276	1540.05	5045.95
A_68_P23190148	chr4:107040619-107040663	NM_028355:252	Tmem48	INSIDE	0.559	16.085	1282.45	20627.92	8.992	1067.63	9600.44
A_68_P23190150	chr4:107040819-107040863	NM_028355:452	Tmem48	INSIDE	0.579	0.654	1441.41	942.88	0.379	1029.63	390.26
A_68_P27507134	chr10:121533967-121534011	NM_153059:170	Tmem5	INSIDE	0.383	7.575	20524.39	155477.00	2.899	17853.07	51758.16
A_68_P27507137	chr10:121534278-121534322	NM_153059:-142	Tmem5	PROMOTER	0.585	2.895	2466.96	7141.65	1.694	1911.49	3238.85
A_68_P23233186	chr4:116924691-116924735	NM_026837:119	Tmem53	INSIDE	0.555	0.473	1223.37	579.07	0.263	980.03	257.60
A_68_P23294939	chr4:128782828-128782872	NM_025452:77	Tmem54	INSIDE	0.228	7.414	3310.08	24542.52	1.693	2551.47	4320.85
A_68_P23294938	chr4:128782726-128782770	NM_025452:-25	Tmem54	PROMOTER	0.457	8.139	2930.77	23852.26	3.721	2553.34	9499.98
A_68_P29599189	chr14:51550570-51550614	NM_001033271:-68	Tmem55b	PROMOTER	0.217	25.358	4891.25	124034.70	5.507	4781.19	26328.79
A_68_P29599187	chr14:51550282-51550326	NM_001033271:220	Tmem55b	INSIDE	0.421	20.208	786.60	15896.01	8.517	995.08	8474.90
A_68_P22475538	chr3:120965447-120965491	NM_178936:766	Tmem56	INSIDE	0.54	5.628	1426.56	8028.14	3.037	1280.79	3889.84
A_68_P23325212	chr4:134408532-134408576	NM_025382:706	Tmem57	INSIDE	0.64	2.773	2056.23	5701.01	1.774	1814.54	3218.63
A_68_P25952182	chr8:73013396-73013440	NM_182991:-2161	Tmem59l	DIVERGENT_PROMOTER	0.178	11.840	1750.32	20723.04	2.104	1499.60	3155.63
A_68_P31202525	chr17:45822791-45822835	NM_198167:355	Tmem63b	INSIDE	0.241	8.639	1267.86	10953.44	2.086	1034.41	2157.66
A_68_P22749333	chr4:15193561-15193605	NM_181401:616	Tmem64	INSIDE	0.529	0.395	2602.34	1027.26	0.209	1907.84	398.71
A_68_P30250398	chr15:58654714-58654758	NM_175212:246	Tmem65	INSIDE	0.664	2.611	674.70	1761.92	1.733	634.00	1098.67
A_68_P25772480	chr8:35217817-35217861	NM_026432:222	Tmem66	INSIDE	0.459	3.774	1308.99	4940.73	1.733	1196.71	2073.93

ProbeName	Target position of probe on CpG island microarray	TargetID	GeneSymbol	CpG island Description	Ratio of relative methylation (TiO_2-NP/Vehicle)	Sham group			TiO_2-H group		
						Relative methylation (Cy5/Cy3)	Cy3 signal (Input)	Cy5 signal (MeDIP)	Relative methylation (Cy5/Cy3)	Cy3 signal (Input)	Cy5 signal (MeDIP)
A_68_P22313145	chr3:88136905-88136949	NM_024246:1429	Tmem79	INSIDE	0.582	4.405	1759.64	7751.48	2.565	1243.81	3190.56
A_68_P31108362	chr17:26250244-26250288	NM_021793:6	Tmem8	INSIDE	0.509	4.113	1706.38	7017.93	2.092	1422.94	2976.35
A_68_P25588178	chr7:148514084-148514128	NR_024509:78	Tmem80	INSIDE	0.084	24.609	2565.37	63131.64	2.055	2214.69	4551.27
A_68_P21621499	chr2:128643821-128643865	NM_028248:-196	Tmem87b	PROMOTER	0.068	2.992	1713.07	5124.76	0.204	1273.81	259.35
A_68_P23440605	chr4:155158694-155158738	NM_001033394:1267	Tmem88b	INSIDE	0.517	0.407	1277.10	519.44	0.21	1035.94	217.90
A_68_P22873079	chr4:43682099-43682143	NM_001085508:278	Tmem8b	INSIDE	0.256	10.648	2460.90	26204.62	2.729	2170.74	5923.37
A_68_P28599774	chr12:86039811-86039855	NM_001033334:-75	Tmem90a	PROMOTER	0.378	4.512	2658.19	11995.07	1.704	2128.36	3627.33
A_68_P28599772	chr12:86039566-86039610	NM_001033334:169	Tmem90a	INSIDE	0.38	5.985	2837.29	16982.16	2.277	2615.82	5956.27
A_68_P28599771	chr12:86039461-86039505	NM_001033334:275	Tmem90a	INSIDE	0.564	2.966	1309.61	3883.86	1.672	1099.44	1837.86
A_68_P27915637	chr11:72990334-72990378	NM_001168470:154	Tmem93	INSIDE	0.665	2.880	1176.03	3386.91	1.915	983.77	1884.05
A_68_P27956496	chr11:80623682-80623726	NM_029537:-212	Tmem98	PROMOTER	0.398	0.548	963.57	528.18	0.218	817.98	178.63
A_68_P27956499	chr11:80624028-80624072	NM_029537:134	Tmem98	INSIDE	0.38	1.414	1155.29	1633.98	0.537	1082.04	581.44
A_68_P22886521	chr4:46051942-46051986	NM_021883:-129	Tmod1	PROMOTER	0.337	5.604	2526.95	14160.11	1.888	2346.85	4430.35
A_68_P27340458	chr10:90644261-90644305	NM_001080129:-9955	Tmpo	PROMOTER	0.409	0.486	1063.40	517.27	0.199	849.06	168.99
A_68_P30999920	chr16:97832492-97832536	NM_015775:288	Tmprss2	INSIDE	0.348	0.582	1791.24	1042.51	0.203	1338.42	271.34
A_68_P24544425	chr6:72908782-72908826	NM_001190327:-62	Tmsb10	PROMOTER	0.52	5.198	1709.89	8887.22	2.701	1391.61	3758.81
A_68_P27416798	chr10:105011030-105011074	NM_177368:483	Tmtc2	INSIDE	0.634	2.428	1654.31	4017.21	1.54	1382.22	2129.21
A_68_P27392328	chr10:99950341-99950385	NM_001110013:-381	Tmtc3	DIVERGENT_PROMOTER	0.548	0.422	4037.31	1703.24	0.231	2906.99	672.67
A_68_P29964713	chr14:123381923-123381967	NM_028651:539	Tmtc4	INSIDE	0.594	9.272	1127.80	10457.54	5.51	983.30	5418.19
A_68_P29964714	chr14:123382020-123382064	NM_028651:441	Tmtc4	INSIDE	0.664	5.492	363.50	1996.21	3.647	278.18	1014.45
A_68_P23541916	chr5:23953751-23953795	NM_022418:-108	Tmub1	DIVERGENT_PROMOTER	0.328	0.492	1965.10	966.42	0.162	1426.03	230.37
A_68_P23541913	chr5:23953475-23953519	NM_022418:168	Tmub1	INSIDE	0.612	4.386	1203.36	5277.55	2.686	1014.42	2724.67
A_68_P28520555	chr12:71554358-71554402	NM_028339:240	Tmx1	INSIDE	0.045	8.464	1518.30	12850.62	0.385	1285.03	494.57
A_68_P31915394	chr18:90679335-90679379	NM_198295:-189	Tmx3	PROMOTER	0.625	0.682	891.08	607.43	0.426	719.80	306.74
A_68_P28744115	chr12:112680803-112680847	NM_009396:-47	Tnfaip2	PROMOTER	0.638	0.710	1804.36	1281.50	0.453	1392.86	630.78
A_68_P26523853	chr9:53916041-53916085	NM_001033535:156	Tnfaip8l3	INSIDE	0.617	0.709	1251.44	887.81	0.438	1063.38	465.32
A_68_P31092981	chr17:23813793-23813837	NM_001161746:602	Tnfrsf12a	INSIDE	0.514	4.322	3393.40	14667.34	2.223	2817.50	6263.63
A_68_P31092979	chr17:23813505-23813549	NM_001161746:890	Tnfrsf12a	INSIDE	0.536	0.266	2348.19	624.69	0.143	1622.35	231.23
A_68_P31092980	chr17:23813651-23813695	NM_001161746:744	Tnfrsf12a	INSIDE	0.564	0.107	4339.41	465.40	0.06	2838.89	171.66
A_68_P27846772	chr11:60960787-60960831	NM_021349:6472	Tnfrsf13b	INSIDE	0.124	3.188	2291.03	7303.32	0.395	1736.25	686.66
A_68_P29647966	chr14:61656769-61656813	NM_001164155:34	Tnfrsf19	INSIDE	0.416	3.329	6945.07	23118.85	1.384	5492.60	7600.46
A_68_P24820215	chr6:125311848-125311892	NM_011609:12130	Tnfrsf1a	INSIDE	0.607	5.962	885.09	5277.24	3.619	690.14	2497.94
A_68_P24820216	chr6:125311932-125311976	NM_011609:12214	Tnfrsf1a	INSIDE	0.618	0.527	1365.02	719.29	0.325	1061.45	345.42
A_68_P31187135	chr17:43153738-43153782	NM_178589:257	Tnfrsf21	INSIDE	0.516	7.506	2106.67	15812.32	3.877	1661.17	6439.85
A_68_P31187140	chr17:43154341-43154385	NM_178589:859	Tnfrsf21	INSIDE	0.644	0.391	4120.10	1610.04	0.252	2750.37	691.98
A_68_P31187133	chr17:43153476-43153520	NM_178589:-5	Tnfrsf21	PROMOTER	0.446	3.625	981.34	3557.05	1.617	805.54	1302.89
A_68_P29745018	chr14:78707731-78707775	NM_011613:98	Tnfsf11	INSIDE	0.567	6.794	2233.52	15174.63	3.853	1801.02	6939.85
A_68_P31260061	chr17:57244849-57244893	NM_009404:63	Tnfsf9	INSIDE	0.425	0.278	2052.86	570.07	0.118	1459.95	172.12
A_68_P27812935	chr11:54769524-54769568	NM_001199275:78	Tnip1	INSIDE	0.453	3.224	1604.15	5172.50	1.462	1358.52	1986.03
A_68_P23594734	chr5:34856584-34856628	NM_139064:22	Tnip2	INSIDE	0.574	0.493	2572.46	1267.61	0.283	1987.28	561.91
A_68_P30653563	chr16:32644599-32644643	NM_001110147:-108	Tnk2	PROMOTER	0.483	0.456	1095.38	499.73	0.22	909.46	200.39
A_68_P25777209	chr8:36028037-36028081	NM_175091:686	Tnks	INSIDE	0.422	0.574	2763.96	1586.46	0.242	1924.84	466.31
A_68_P21396039	chr2:84888658-84888702	NM_001081260:-1936	Tnks1bp1	PROMOTER	0.198	0.316	3722.50	1177.13	0.063	2978.83	186.42
A_68_P21396040	chr2:84888732-84888776	NM_001081260:-1862	Tnks1bp1	PROMOTER	0.323	0.403	1626.88	656.20	0.13	1310.37	170.89
A_68_P21396036	chr2:84888342-84888387	NM_001081260:-2252	Tnks1bp1	PROMOTER	0.402	0.171	8581.65	1464.96	0.069	6077.22	416.81
A_68_P32092159	chr19:36908711-36908755	NM_001163635:11	Tnks2	INSIDE	0.606	3.473	859.55	2985.20	2.103	660.38	1388.73

ProbeName	Target position of probe on CpG island microarray	TargetID	GeneSymbol	CpG island Description	Ratio of relative methylation (TiO_2-NP/Vehicle)	Sham group			TiO_2-H group		
						Relative methylation (Cy5/Cy3)	Cy3 signal (Input)	Cy5 signal (MeDIP)	Relative methylation (Cy5/Cy3)	Cy3 signal (Input)	Cy5 signal (MeDIP)
A_68_P29260367	chr13:99696244-99696288	NM_178716:73	Tnpo1	INSIDE	0.634	2.741	1308.08	3586.09	1.739	1086.83	1889.51
A_68_P26023340	chr8:87562055-87562099	NM_001122843:1010	Tnpo2	INSIDE	0.564	3.659	3374.39	12347.17	2.065	2565.94	5298.06
A_68_P26023335	chr8:87561478-87561522	NM_001122843:434	Tnpo2	INSIDE	0.16	1.404	4726.94	6636.08	0.224	3358.97	752.52
A_68_P26023334	chr8:87561401-87561445	NM_001122843:356	Tnpo2	INSIDE	0.319	0.733	15628.91	11460.78	0.234	10690.96	2502.35
A_68_P24327618	chr6:29559429-29559473	NM_177296:157	Tnpo3	INSIDE	0.488	0.375	1305.50	489.76	0.183	941.43	172.44
A_68_P24153558	chr5:143580250-143580294	NM_001122730:-1206	Tnrc18	PROMOTER	0.332	9.682	897.85	8692.93	3.214	772.06	2481.23
A_68_P24153530	chr5:143576863-143576907	NM_001122730:2182	Tnrc18	INSIDE	0.479	0.684	2136.63	1460.96	0.328	1759.22	576.49
A_68_P24153574	chr5:143581988-143582032	NM_001122730:-2944	Tnrc18	PROMOTER	0.207	2.695	2735.12	7369.82	0.558	2005.54	1119.83
A_68_P24153528	chr5:143576639-143576683	NM_001122730:2406	Tnrc18	INSIDE	0.612	2.758	700.40	1931.98	1.687	629.58	1062.09
A_68_P25482572	chr7:130267793-130267837	NM_144925:416	Tnrc6a	INSIDE	0.468	3.642	716.98	2611.43	1.706	652.87	1113.63
A_68_P30374043	chr15:80542551-80542595	NM_144812:830	Tnrc6b	INSIDE	0.133	112.875	2840.44	320614.30	14.991	2573.79	38584.84
A_68_P30374042	chr15:80542447-80542491	NM_144812:726	Tnrc6b	INSIDE	0.275	0.322	2707.92	871.68	0.088	1936.59	171.14
A_68_P30374041	chr15:80542355-80542399	NM_144812:634	Tnrc6b	INSIDE	0.554	0.179	2586.17	461.86	0.099	1960.30	194.05
A_68_P28166382	chr11:117515746-117515790	NM_198022:166	Tnrc6c	INSIDE	0.404	0.318	1728.61	550.43	0.129	1370.32	176.43
A_68_P28166391	chr11:117517129-117517173	NM_198022:1548	Tnrc6c	INSIDE	0.62	4.025	3118.10	12549.69	2.495	2399.34	5985.44
A_68_P27579284	chr11:8564125-8564169	NM_001083587:392	Tns3	INSIDE	0.156	36.115	1589.21	57394.03	5.643	1508.83	8514.28
A_68_P27579286	chr11:8564350-8564394	NM_001083587:166	Tns3	INSIDE	0.353	0.499	1334.80	666.62	0.176	975.09	171.74
A_68_P27579285	chr11:8564248-8564292	NM_001083587:268	Tns3	INSIDE	0.629	2.330	2351.62	5478.42	1.465	1712.12	2507.59
A_68_P31155254	chr17:34817160-34817204	NM_031176:9703	Tnxb	INSIDE	0.553	3.067	1904.72	5841.78	1.695	1471.73	2494.94
A_68_P28031425	chr11:94073133-94073177	NM_009427:387	Tob1	INSIDE	0.374	0.597	867.94	518.38	0.224	757.15	169.32
A_68_P28031422	chr11:94072689-94072733	NM_009427:-57	Tob1	PROMOTER	0.234	6.539	3149.27	20591.70	1.528	2636.85	4030.19
A_68_P28031427	chr11:94073436-94073480	NM_009427:691	Tob1	INSIDE	0.64	2.800	629.15	1761.61	1.791	671.49	1202.80
A_68_P30380067	chr15:81688792-81688836	NM_020507:-58	Tob2	PROMOTER	0.179	21.293	2374.53	50560.36	3.807	2014.73	7670.88
A_68_P30380065	chr15:81688607-81688651	NM_020507:128	Tob2	INSIDE	0.61	0.112	3973.88	444.83	0.068	2740.27	187.00
A_68_P25591624	chr7:149088169-149088213	NM_023764:121	Tollip	INSIDE	0.562	2.617	1276.58	3341.35	1.472	1125.43	1656.49
A_68_P25971564	chr8:77557785-77557829	NM_001136259:222	Tom1	INSIDE	0.553	0.520	1080.80	562.52	0.288	781.92	225.08
A_68_P28013349	chr11:90548688-90548732	NM_028011:205	Tom1l1	INSIDE	0.233	1.840	1262.31	2322.54	0.429	992.28	425.51
A_68_P28013351	chr11:90548942-90548986	NM_028011:-49	Tom1l1	PROMOTER	0.517	2.867	1444.15	4140.43	1.482	1369.25	2029.56
A_68_P27842503	chr11:60166349-60166393	NM_001039092:37	Tom1l2	INSIDE	0.339	5.341	4009.41	21414.98	1.812	3569.69	6468.11
A_68_P21815977	chr2:163896377-163896421	NM_025996:440	Tomm34	INSIDE	0.333	0.602	2950.43	1774.69	0.2	2067.23	414.37
A_68_P24994887	chr7:20300662-20300706	NM_001109748:94	Tomm40	INSIDE	0.632	0.717	10300.91	7384.59	0.453	7771.63	3522.75
A_68_P31213639	chr17:47826066-47826110	NM_001164729:-753	Tomm6	DIVERGENT_PROMOTER	0.435	1.628	6013.85	9789.13	0.707	4310.25	3049.35
A_68_P30784286	chr16:57122130-57122174	NM_138599:326	Tomm70a	INSIDE	0.253	13.268	1752.38	23250.74	3.361	1570.51	5279.04
A_68_P30784285	chr16:57122018-57122062	NM_138599:214	Tomm70a	INSIDE	0.27	40.722	3732.15	151981.90	10.996	3611.09	39706.31
A_68_P30349539	chr15:76462358-76462402	NM_183091:7979	Tonsl	INSIDE	0.212	30.616	6521.23	199654.90	6.504	6693.08	43532.96
A_68_P21796146	chr2:160471717-160471761	NM_009408:106	Top1	INSIDE	0.47	0.316	1848.24	584.65	0.149	1349.19	200.59
A_68_P21796147	chr2:160471841-160471885	NM_009408:230	Top1	INSIDE	0.458	0.665	986.43	656.45	0.305	856.90	261.12
A_68_P29428732	chr14:17198090-17198134	NM_009409:393	Top2b	INSIDE	0.476	7.214	854.61	6165.09	3.433	692.42	2376.91
A_68_P29428731	chr14:17197979-17198023	NM_009409:281	Top2b	INSIDE	0.529	3.610	1639.46	5918.14	1.91	1397.51	2668.76
A_68_P30570745	chr16:16871029-16871073	NM_011624:67	Top3b	INSIDE	0.604	3.451	1907.71	6584.09	2.083	1638.04	3412.33
A_68_P22856580	chr4:40216716-40216760	NM_134097:136	Topors	INSIDE	0.515	0.509	1360.31	692.41	0.262	1102.10	289.03
A_68_P22856570	chr4:40215530-40215574	NM_134097:1322	Topors	INSIDE	0.408	0.588	901.22	529.90	0.24	711.45	170.83
A_68_P21106251	chr2:30822965-30823009	NM_144884:452	Tor1a	INSIDE	0.406	0.254	3522.14	893.42	0.103	2502.37	257.40
A_68_P20741243	chr1:157883361-157883407	NM_001160182:219	Tor1aip2	INSIDE	0.423	0.383	1508.62	577.07	0.162	1045.63	169.36
A_68_P21106144	chr2:30808670-30808714	NM_133673:172	Tor1b	INSIDE	0.611	0.640	989.26	633.41	0.392	752.85	294.76
A_68_P21117289	chr2:32612990-32613034	NM_152800:222	Tor2a	INSIDE	0.378	15.608	1375.65	21471.14	5.893	1434.81	8454.75

ProbeName	Target position of probe on CpG island microarray	TargetID	GeneSymbol	CpG island Description	Ratio of relative methylation (TiO_2-NP/Vehicle)	Sham group			TiO_2-H group		
						Relative methylation (Cy5/Cy3)	Cy3 signal (Input)	Cy5 signal (MeDIP)	Relative methylation (Cy5/Cy3)	Cy3 signal (Input)	Cy5 signal (MeDIP)
A_68_P21117286	chr2:32612762-32612806	NM_152800:-6	Tor2a	PROMOTER	0.65	6.598	1893.50	12493.94	4.289	1584.66	6795.89
A_68_P20744276	chr1:158604245-158604289	NM_023141:204	Tor3a	INSIDE	0.405	0.371	1435.21	532.09	0.15	1106.57	166.13
A_68_P22708703	chr4:6917953-6917997	NM_145711:-104	Tox	PROMOTER	0.512	6.673	5323.35	35524.89	3.419	4566.98	15612.38
A_68_P26696146	chr9:85737161-85737205	NM_001164792:724	Tpbg	INSIDE	0.361	0.238	8628.63	2057.76	0.086	5667.50	487.59
A_68_P26696145	chr9:85737079-85737123	NM_001164792:642	Tpbg	INSIDE	0.578	0.567	3144.98	1781.96	0.328	2440.16	799.75
A_68_P26696139	chr9:85736432-85736476	NM_001164792:-4	Tpbg	PROMOTER	0.418	4.537	844.81	3833.31	1.899	753.71	1430.94
A_68_P26696141	chr9:85736583-85736627	NM_001164792:146	Tpbg	INSIDE	0.214	2.716	1224.87	3326.23	0.58	987.46	572.98
A_68_P21910960	chr2:181231996-181232040	NM_025482:61	Tpd52l2	INSIDE	0.341	5.440	815.45	4436.17	1.855	786.81	1459.34
A_68_P26596714	chr9:66891475-66891519	NM_001164252:182	Tpm1	INSIDE	0.392	7.049	866.05	6104.98	2.764	768.62	2124.32
A_68_P26596750	chr9:66896100-66896144	NM_001164252:-4444	Tpm1	PROMOTER	0.491	0.310	1620.63	502.39	0.152	1147.51	174.68
A_68_P26596760	chr9:66897350-66897394	NM_001164249:-352	Tpm1	PROMOTER	0.378	0.597	900.28	537.83	0.226	741.73	167.37
A_68_P26596757	chr9:66897011-66897055	NM_001164249:-12	Tpm1	PROMOTER	0.51	1.397	3767.27	5261.26	0.712	2996.00	2133.43
A_68_P22872185	chr4:43536399-43536443	NM_009416:-160	Tpm2	PROMOTER	0.464	3.209	1930.10	6193.54	1.489	1582.63	2356.58
A_68_P22323362	chr3:89884192-89884236	NM_022314:7599	Tpm3	INSIDE	0.433	3.913	5344.46	20915.18	1.694	3927.64	6654.70
A_68_P25959798	chr8:74659599-74659643	NM_001001491:430	Tpm4	INSIDE	0.448	0.152	3740.26	570.33	0.068	2571.11	175.59
A_68_P25959792	chr8:74658995-74659039	NM_001001491:-174	Tpm4	PROMOTER	0.619	0.191	8003.40	1525.75	0.118	5199.51	613.25
A_68_P29011270	chr13:47138723-47138767	NM_016785:-158	Tpmt	DIVERGENT_PROMOTER	0.211	9.508	4376.17	41608.75	2.006	4031.00	8085.64
A_68_P20191548	chr1:43991025-43991069	NM_009418:195	Tpp2	INSIDE	0.352	13.406	942.15	12630.36	4.725	769.08	3633.53
A_68_P29143670	chr13:74147870-74147914	NM_182839:1026	Tppp	INSIDE	0.231	14.355	976.04	14010.85	3.319	933.12	3096.58
A_68_P29143669	chr13:74147784-74147828	NM_182839:940	Tppp	INSIDE	0.561	0.437	1526.28	666.69	0.245	1164.50	285.30
A_68_P26134000	chr8:107995392-107995436	NM_026481:-92	Tppp3	PROMOTER	0.199	15.484	2639.26	40866.47	3.086	3013.48	9299.17
A_68_P23429475	chr4:153535140-153535184	NM_026388:-369	Tprgl	DIVERGENT_PROMOTER	0.341	0.217	2974.64	646.19	0.074	2313.46	171.34
A_68_P24092031	chr5:130555095-130555139	NM_001130476:-239	Tpst1	PROMOTER	0.458	5.569	796.40	4434.88	2.551	689.68	1759.44
A_68_P24092036	chr5:130555652-130555696	NM_001130476:319	Tpst1	INSIDE	0.597	0.425	1851.35	786.24	0.254	1394.10	353.43
A_68_P23991345	chr5:112736931-112736975	NM_009419:31226	Tpst2	INSIDE	0.538	4.764	1112.34	5299.03	2.565	880.04	2257.47
A_68_P23991123	chr5:112705779-112705823	NM_009419:74	Tpst2	INSIDE	0.662	4.030	936.59	3774.58	2.666	801.63	2137.48
A_68_P24431872	chr6:49213460-49213504	NM_198102:569	Tra2a	INSIDE	0.318	0.521	1990.66	1036.78	0.166	1478.70	245.04
A_68_P30596855	chr16:22266102-22266148	NM_009186:-122	Tra2b	PROMOTER	0.651	0.377	1975.21	745.12	0.246	1456.10	357.54
A_68_P30596854	chr16:22265994-22266038	NM_009186:-14	Tra2b	PROMOTER	0.334	0.632	3059.54	1934.50	0.211	2265.06	478.23
A_68_P21072876	chr2:25402210-25402254	NM_009422:182	Traf2	INSIDE	0.449	0.409	3098.55	1266.69	0.184	2253.42	413.94
A_68_P20442836	chr1:93391186-93391230	NM_028718:-36	Traf3ip1	PROMOTER	0.636	2.559	1105.60	2828.68	1.626	1050.47	1708.11
A_68_P22492339	chr3:124023918-124023962	NM_146140:-14	Tram1l1	PROMOTER	0.302	6.544	1821.77	11922.35	1.977	1633.03	3229.14
A_68_P26828805	chr9:111214091-111214135	NM_001164659:-130	Trank1	PROMOTER	0.508	3.754	1684.45	6323.56	1.907	1446.14	2757.24
A_68_P27273026	chr10:77707095-77707139	NM_001081055:271	Trappc10	INSIDE	0.35	0.282	2199.14	619.51	0.099	1755.30	173.11
A_68_P25615130	chr8:3679220-3679265	NM_025701:2766	Trappc5	INSIDE	0.176	14.925	2141.88	31968.12	2.623	1818.66	4771.21
A_68_P30328130	chr15:72888613-72888657	NM_029640:-2392	Trappc9	PROMOTER	0.308	5.144	985.80	5070.76	1.586	809.44	1284.17
A_68_P27466129	chr10:114238135-114238179	NM_146241:270	Trhde	INSIDE	0.37	4.510	882.51	3980.43	1.669	792.33	1322.38
A_68_P30255510	chr15:59479785-59479829	NM_144549:-402	Trib1	PROMOTER	0.42	4.719	1562.35	7372.48	1.984	1432.83	2842.82
A_68_P30255506	chr15:59479250-59479294	NM_144549:-936	Trib1	PROMOTER	0.477	14.703	7431.83	109272.00	7.019	7428.50	52140.49
A_68_P30255512	chr15:59479961-59480005	NM_144549:-226	Trib1	PROMOTER	0.531	3.128	3052.36	9546.55	1.66	2531.62	4202.71
A_68_P28261138	chr12:15822842-15822886	NM_144551:727	Trib2	INSIDE	0.186	12.707	839.03	10661.90	2.359	763.57	1801.40
A_68_P24459338	chr6:53768811-53768855	NM_025817:1987	Tril	INSIDE	0.236	19.721	3198.56	63077.89	4.653	3682.30	17135.05
A_68_P24459356	chr6:53770927-53770974	NM_025817:-131	Tril	PROMOTER	0.606	0.706	1367.82	965.32	0.428	962.63	411.91
A_68_P27833865	chr11:58791958-58792002	NM_053168:363	Trim11	INSIDE	0.479	6.198	2819.36	17474.02	2.966	2198.80	6522.71
A_68_P27833862	chr11:58791684-58791728	NM_053168:89	Trim11	INSIDE	0.666	0.667	1598.81	1066.08	0.444	1188.21	527.71
A_68_P29650879	chr14:62218392-62218436	NM_023233:66	Trim13	INSIDE	0.4	1.367	3835.86	5244.48	0.546	3126.68	1708.71

ProbeName	Target position of probe on CpG island microarray	TargetID	GeneSymbol	CpG island Description	Ratio of relative methylation (TiO_2-NP/Vehicle)	Sham group			TiO_2-H group		
						Relative methylation (Cy5/Cy3)	Cy3 signal (Input)	Cy5 signal (MeDIP)	Relative methylation (Cy5/Cy3)	Cy3 signal (Input)	Cy5 signal (MeDIP)
A_68_P22889131	chr4:46520297-46520341	NM_029077:28698	Trim14	INSIDE	0.294	7.422	1371.02	10176.38	2.179	1068.84	2329.35
A_68_P22889129	chr4:46520101-46520145	NM_029077:28894	Trim14	INSIDE	0.553	4.782	1212.32	5796.77	2.646	993.21	2628.48
A_68_P22289430	chr3:83995003-83995047	NM_030706:29775	Trim2	INSIDE	0.486	0.711	1690.55	1202.64	0.346	1290.21	446.50
A_68_P31165740	chr17:36973820-36973864	NM_001025599:-243	Trim26	PROMOTER	0.568	0.164	2937.31	480.36	0.093	1853.77	172.06
A_68_P28871505	chr13:21272743-21272787	NM_009054:937	Trim27	INSIDE	0.451	8.154	1589.09	12957.55	3.678	1485.18	5462.04
A_68_P28871497	chr13:21271956-21272000	NM_009054:151	Trim27	INSIDE	0.53	3.399	847.80	2881.60	1.801	760.63	1370.13
A_68_P22982499	chr4:65266160-65266204	NM_001161782:163	Trim32	INSIDE	0.602	0.610	10896.95	6648.01	0.367	6969.68	2560.81
A_68_P22982500	chr4:65266278-65266324	NM_001161782:281	Trim32	INSIDE	0.281	5.837	5587.75	32618.48	1.642	3908.81	6417.89
A_68_P22385231	chr3:103083438-103083482	NM_001079830:245	Trim33	INSIDE	0.637	0.599	1304.98	782.08	0.382	982.78	375.33
A_68_P31675573	chr18:46358367-46358411	NM_001170855:84	Trim36	INSIDE	0.309	7.480	1377.02	10299.86	2.314	1133.78	2623.97
A_68_P27992700	chr11:86940726-86940770	NM_197987:170	Trim37	INSIDE	0.446	7.723	311.70	2407.35	3.444	280.60	966.38
A_68_P27992698	chr11:86940439-86940483	NM_197987:-118	Trim37	PROMOTER	0.45	0.610	1929.14	1177.30	0.274	1678.48	460.65
A_68_P26752043	chr9:97266499-97266543	NM_030219:3857	Trim42	INSIDE	0.423	5.152	1754.21	9038.35	2.179	1123.69	2448.20
A_68_P21476827	chr2:102241389-102241433	NM_020267:-353	Trim44	PROMOTER	0.623	0.418	1848.56	772.14	0.26	1437.23	374.26
A_68_P22218905	chr3:68848719-68848763	NM_025863:-76	Trim59	PROMOTER	0.399	8.875	976.85	8669.19	3.544	903.02	3199.91
A_68_P23293634	chr4:128561007-128561051	NM_178110:-355	Trim62	PROMOTER	0.388	1.822	2801.07	5102.29	0.707	2122.11	1500.28
A_68_P26247734	chr8:127317918-127317962	NM_198632:1022	Trim67	INSIDE	0.178	27.423	4225.45	115875.00	4.875	3664.56	17864.27
A_68_P26247724	chr8:127316847-127316891	NM_198632:-50	Trim67	PROMOTER	0.662	0.274	3558.78	973.90	0.181	2494.90	451.88
A_68_P27780217	chr11:48651539-48651583	NM_053166:11921	Trim7	INSIDE	0.347	1.469	2034.11	2987.75	0.509	1821.65	927.17
A_68_P26845370	chr9:114471876-114471920	NM_001042503:1589	Trim71	INSIDE	0.396	5.137	1100.73	5654.24	2.034	881.67	1793.47
A_68_P25508612	chr7:135153559-135153603	NM_001079932:5689	Trim72	INSIDE	0.552	5.536	6317.78	34972.87	3.053	4731.10	14445.62
A_68_P25508611	chr7:135153487-135153531	NM_001079932:5617	Trim72	INSIDE	0.604	3.628	1654.02	6000.29	2.19	1148.93	2516.34
A_68_P25508613	chr7:135153669-135153713	NM_001079932:5799	Trim72	INSIDE	0.485	3.258	4638.12	15109.16	1.578	3555.66	5612.53
A_68_P32144304	chr19:46576472-46576516	NM_053100:357	Trim8	INSIDE	0.66	3.632	886.66	3220.62	2.398	733.32	1758.37
A_68_P32144298	chr19:46575400-46575444	NM_053100:-715	Trim8	PROMOTER	0.664	5.483	1545.33	8472.53	3.638	1234.78	4491.97
A_68_P32144302	chr19:46576160-46576204	NM_053100:45	Trim8	INSIDE	0.323	16.520	163.10	2694.42	5.334	141.01	752.19
A_68_P28520026	chr12:71448401-71448445	NM_001110202:179	Trim9	INSIDE	0.304	0.644	925.72	595.74	0.196	898.54	175.82
A_68_P30094308	chr15:27677507-27677551	NM_001081302:278075	Trio	INSIDE	0.514	4.006	2156.18	8636.87	2.06	1455.25	2998.28
A_68_P30363491	chr15:78798091-78798135	NM_001039155:19959	Triobp	INSIDE	0.201	8.567	3060.74	26220.30	1.72	2289.66	3938.74
A_68_P30363492	chr15:78798174-78798218	NM_001039155:20043	Triobp	INSIDE	0.413	9.290	2430.05	22575.18	3.836	1791.15	6871.01
A_68_P30363494	chr15:78798409-78798453	NM_001039155:20277	Triobp	INSIDE	0.464	4.656	4441.63	20678.56	2.159	3364.77	7262.97
A_68_P30363493	chr15:78798329-78798373	NM_001039155:20197	Triobp	INSIDE	0.494	31.544	1054.42	33260.79	15.571	488.06	7599.52
A_68_P30363604	chr15:78813243-78813287	NM_001024716:-221	Triobp	PROMOTER	0.62	0.617	3301.33	2038.24	0.383	2551.90	977.32
A_68_P30363681	chr15:78823328-78823373	NM_001024716:9865	Triobp	INSIDE	0.608	2.765	1262.82	3491.42	1.681	879.07	1477.70
A_68_P28688419	chr12:103150855-103150899	NM_028446:505	Trip11	INSIDE	0.188	18.335	5879.80	107806.90	3.441	6105.52	21011.29
A_68_P20405967	chr1:84835781-84835825	NM_133975:77	Trip12	INSIDE	0.426	0.464	2212.25	1027.11	0.198	1691.06	334.56
A_68_P20405963	chr1:84835382-84835426	NM_133975:475	Trip12	INSIDE	0.578	0.685	1522.52	1043.35	0.396	1213.41	480.33
A_68_P20405961	chr1:84835228-84835272	NM_133975:629	Trip12	INSIDE	0.193	1.336	5341.29	7135.66	0.258	3648.02	940.37
A_68_P20405960	chr1:84835061-84835105	NM_133975:797	Trip12	INSIDE	0.41	3.666	1235.96	4531.30	1.502	1053.12	1582.02
A_68_P29143148	chr13:74074758-74074802	NM_027182:435	Trip13	INSIDE	0.582	0.524	6983.73	3661.09	0.305	4529.14	1381.18
A_68_P29143149	chr13:74074834-74074878	NM_027182:359	Trip13	INSIDE	0.659	0.273	4240.45	1158.11	0.18	2977.08	535.97
A_68_P30578596	chr16:18249157-18249201	NM_001080999:203	Trmt2a	INSIDE	0.489	0.685	1915.85	1311.78	0.335	1317.31	441.46
A_68_P23317940	chr4:133054507-133054551	NM_001081156:-63	Trnp1	PROMOTER	0.502	0.157	3016.53	473.14	0.079	2228.61	175.58
A_68_P24725198	chr6:106719039-106719083	NM_027296:-105	Trnt1	PROMOTER	0.445	10.964	1343.93	14734.38	4.879	1229.38	5998.37
A_68_P21433080	chr2:93028458-93028502	NM_001025246:740	Trp53i11	INSIDE	0.485	0.300	1498.58	450.25	0.146	1206.42	175.85
A_68_P22728294	chr4:11083669-11083713	NM_001199105:103	Trp53inp1	INSIDE	0.578	0.402	2031.76	817.47	0.233	1773.46	412.41

ProbeName	Target position of probe on CpG island microarray	TargetID	GeneSymbol	CpG island Description	Ratio of relative methylation (TiO_2-NP/Vehicle)	Sham group			TiO_2-H group		
						Relative methylation (Cy5/Cy3)	Cy3 signal (Input)	Cy5 signal (MeDIP)	Relative methylation (Cy5/Cy3)	Cy3 signal (Input)	Cy5 signal (MeDIP)
A_68_P21765248	chr2:155207645-155207689	NM_178111:75	Trp53inp2	INSIDE	0.359	6.062	513.07	3110.36	2.177	495.56	1078.96
A_68_P21765289	chr2:155212485-155212529	NM_178111:4915	Trp53inp2	INSIDE	0.339	1.910	3004.53	5737.90	0.647	2156.84	1394.83
A_68_P23428914	chr4:153455643-153455687	NM_001126330:15618	Trp73	INSIDE	0.499	0.216	2436.95	527.53	0.108	1620.54	174.91
A_68_P23428913	chr4:153455504-153455548	NM_001126330:15756	Trp73	INSIDE	0.581	0.267	2598.01	694.51	0.155	1756.36	272.84
A_68_P21767078	chr2:155518080-155518124	NM_001163452:18	Trpc4ap	INSIDE	0.462	8.259	894.44	7387.28	3.82	713.20	2724.25
A_68_P21767075	chr2:155517577-155517621	NM_001163452:522	Trpc4ap	INSIDE	0.622	0.097	4922.71	475.10	0.06	3575.68	214.62
A_68_P26292118	chr9:8544562-8544606	NM_013838:443	Trpc6	INSIDE	0.397	12.204	846.87	10334.97	4.849	723.75	3509.22
A_68_P32010020	chr19:22213374-22213418	NM_001035239:-210	Trpm3	PROMOTER	0.524	4.474	1125.60	5035.93	2.343	1040.25	2436.87
A_68_P21610504	chr2:126701750-126701794	NM_001164325:225	Trpm7	INSIDE	0.495	3.701	1860.61	6885.61	1.832	1548.09	2836.60
A_68_P24162469	chr5:145529670-145529714	NM_001081362:32	Trrap	INSIDE	0.417	5.753	5281.38	30385.58	2.401	4356.13	10461.21
A_68_P24162463	chr5:145529030-145529074	NM_001081362:-608	Trrap	DIVERGENT_PROMOTER	0.622	4.582	3665.54	16795.20	2.849	3019.60	8603.87
A_68_P21099454	chr2:29642948-29642997	NM_145520:219	Trub2	INSIDE	0.37	0.549	924.17	507.03	0.203	862.71	175.13
A_68_P21092309	chr2:28496355-28496399	NM_022887:-386	Tsc1	PROMOTER	0.315	0.551	1853.30	1021.05	0.173	1451.70	251.68
A_68_P29733544	chr14:76814636-76814680	NM_207652:-969	Tsc22d1	PROMOTER	0.169	1.930	3386.77	6535.55	0.326	2314.11	754.95
A_68_P29733553	chr14:76816095-76816139	NM_207652:489	Tsc22d1	INSIDE	0.289	9.553	3456.50	33019.08	2.764	2825.40	7809.91
A_68_P29733542	chr14:76814361-76814405	NM_207652:-1245	Tsc22d1	PROMOTER	0.482	5.868	586.23	3440.03	2.826	479.84	1356.04
A_68_P29733545	chr14:76814748-76814792	NM_207652:-857	Tsc22d1	PROMOTER	0.587	0.621	1598.43	992.46	0.365	1252.12	456.57
A_68_P32700068	chrX:137134513-137134557	NM_001077364:527	Tsc22d3	INSIDE	0.449	5.130	5944.70	30498.89	2.303	7180.11	16536.71
A_68_P32700067	chrX:137134400-137134444	NM_001077364:639	Tsc22d3	INSIDE	0.451	6.133	3425.77	21009.56	2.767	3788.04	10481.17
A_68_P32700064	chrX:137133979-137134023	NM_001077364:1061	Tsc22d3	INSIDE	0.63	3.174	744.94	2364.09	2	856.32	1712.92
A_68_P32700063	chrX:137133904-137133948	NM_001077364:1135	Tsc22d3	INSIDE	0.598	3.879	316.60	1228.20	2.322	509.56	1183.02
A_68_P24771681	chr6:115494875-115494919	NM_199033:175	Tsen2	INSIDE	0.294	14.118	1512.89	21358.93	4.151	2416.80	10032.40
A_68_P24946870	chr7:3646016-3646060	NM_024168:-71	Tsen34	PROMOTER	0.341	5.080	3491.47	17736.04	1.731	2895.41	5010.50
A_68_P24946869	chr7:3645931-3645975	NM_024168:-157	Tsen34	PROMOTER	0.517	0.337	1839.91	620.44	0.174	1508.13	262.88
A_68_P25102014	chr7:54175443-54175487	NM_021884:-164	Tsg101	PROMOTER	0.552	3.507	1424.48	4994.98	1.937	1247.19	2415.83
A_68_P25102013	chr7:54175369-54175413	NM_021884:-90	Tsg101	PROMOTER	0.64	2.745	991.35	2721.16	1.756	866.74	1522.17
A_68_P20156310	chr1:37921470-37921514	NM_207228:432	Tsga10	INSIDE	0.312	1.860	5367.40	9985.04	0.581	4334.17	2518.67
A_68_P20156312	chr1:37921635-37921679	NM_207228:268	Tsga10	INSIDE	0.558	0.449	2636.43	1182.54	0.25	2207.76	553.01
A_68_P25056449	chr7:37481732-37481776	NM_172298:-1382	Tshz3	PROMOTER	0.258	16.491	6436.68	106147.30	4.258	5467.08	23278.94
A_68_P25355275	chr7:105509998-105510042	NM_001168539:-182	Tsku	PROMOTER	0.046	2.072	3011.61	6241.40	0.094	1938.74	183.00
A_68_P25355276	chr7:105510079-105510123	NM_001168539:-262	Tsku	PROMOTER	0.047	5.549	1709.69	9486.98	0.261	1318.34	343.74
A_68_P25355271	chr7:105509578-105509622	NM_001024619:198	Tsku	INSIDE	0.599	0.594	1207.63	717.15	0.356	1019.20	362.34
A_68_P20551183	chr1:120207585-120207629	NM_011650:103	Tsn	INSIDE	0.645	0.612	2139.88	1308.59	0.394	1646.87	649.15
A_68_P26248943	chr8:127536584-127536628	NM_016909:-290	Tsnax	PROMOTER	0.309	0.530	1477.65	783.17	0.164	1057.41	173.46
A_68_P29560590	chr14:41779806-41779850	NM_145928:268	Tspan14	INSIDE	0.356	10.058	6546.96	65849.12	3.582	5189.04	18586.35
A_68_P29560588	chr14:41779590-41779634	NM_145928:484	Tspan14	INSIDE	0.529	0.536	2081.48	1115.92	0.284	1483.94	420.74
A_68_P27184871	chr10:61693322-61693366	NM_197996:622	Tspan15	INSIDE	0.428	0.549	866.09	475.83	0.235	750.03	176.25
A_68_P27184877	chr10:61693989-61694033	NM_197996:-44	Tspan15	PROMOTER	0.388	1.470	2221.02	3264.22	0.57	1675.81	954.55
A_68_P29051405	chr13:54890823-54890867	NM_028841:79	Tspan17	INSIDE	0.446	9.364	480.73	4501.43	4.173	390.54	1629.55
A_68_P22382656	chr3:102538778-102538822	NM_027533:108	Tspan2	INSIDE	0.325	10.790	9467.85	102159.20	3.509	7488.68	26280.67
A_68_P22382660	chr3:102539302-102539346	NM_027533:632	Tspan2	INSIDE	0.628	0.559	2367.81	1322.52	0.351	1838.22	644.70
A_68_P22570418	chr3:138405498-138405542	NM_019571:362	Tspan5	INSIDE	0.337	11.691	1502.74	17568.13	3.939	1409.44	5551.75
A_68_P24836602	chr6:127984334-127984378	NM_175414:109240	Tspan9	INSIDE	0.587	3.260	1211.24	3949.13	1.913	1006.07	1924.58
A_68_P32749012	chrX:148777026-148777070	NM_029836:-21	Tspyl2	PROMOTER	0.193	30.468	16973.12	517138.50	5.878	22304.88	131113.10
A_68_P21753655	chr2:153050987-153051031	NM_198617:169	Tspyl3	INSIDE	0.457	0.609	1495.88	910.46	0.278	1055.58	293.52
A_68_P30125732	chr15:33617536-33617580	NM_001085421:80	Tspyl5	INSIDE	0.589	0.574	942.93	541.55	0.338	741.94	250.99

ProbeName	Target position of probe on CpG island microarray	TargetID	GeneSymbol	CpG island Description	Ratio of relative methylation (TiO_2-NP/Vehicle)	Sham group			TiO_2-H group		
						Relative methylation (Cy5/Cy3)	Cy3 signal (Input)	Cy5 signal (MeDIP)	Relative methylation (Cy5/Cy3)	Cy3 signal (Input)	Cy5 signal (MeDIP)
A_68_P25598465	chr7:150255389-150255433	NM_001115085:138	Tssc4	INSIDE	0.471	0.208	3250.53	675.38	0.098	2256.73	220.74
A_68_P25598469	chr7:150255890-150255934	NM_001115085:640	Tssc4	INSIDE	0.588	0.599	5213.06	3124.57	0.352	3258.66	1147.64
A_68_P25948613	chr8:72426109-72426153	NM_032004:17	Tssk6	INSIDE	0.524	0.459	3428.13	1572.94	0.24	2391.65	574.97
A_68_P30345040	chr15:75760067-75760111	NM_031201:72	Tsta3	INSIDE	0.496	0.591	5896.80	3482.19	0.293	4244.46	1242.73
A_68_P31206907	chr17:46581950-46581994	NM_001162864:42652	Ttbk1	INSIDE	0.234	1.725	4372.47	7540.54	0.404	3040.88	1228.71
A_68_P31206981	chr17:46592555-46592609	NM_001162864:32042	Ttbk1	INSIDE	0.144	1.606	2997.41	4815.00	0.231	1882.94	434.33
A_68_P31206908	chr17:46582089-46582133	NM_001162864:42514	Ttbk1	INSIDE	0.173	4.256	9357.05	39827.77	0.736	6778.45	4992.25
A_68_P27752129	chr11:43561327-43561371	NM_133795:127	Ttc1	INSIDE	0.326	0.432	1486.55	642.85	0.141	1233.18	173.70
A_68_P28314928	chr12:29435138-29435182	NM_001161410:99	Ttc15	INSIDE	0.663	0.626	1705.92	1068.68	0.415	1404.47	582.95
A_68_P21291249	chr2:66094636-66094680	NM_001047604:16	Ttc21b	INSIDE	0.539	0.647	1123.30	726.52	0.349	857.28	299.11
A_68_P23982396	chr5:111309011-111309055	NM_024477:211	Ttc28	INSIDE	0.112	38.572	4600.44	177447.30	4.338	4497.50	19509.57
A_68_P29982442	chr15:5135931-5135975	NM_026213:393	Ttc33	INSIDE	0.424	0.251	2225.11	557.63	0.106	1668.54	177.34
A_68_P30176278	chr15:43309169-43309213	NM_025736:416	Ttc35	INSIDE	0.421	25.836	2135.91	55182.54	10.872	2587.27	28129.36
A_68_P30176273	chr15:43308698-43308742	NM_025736:-54	Ttc35	PROMOTER	0.534	4.062	1739.51	7065.81	2.167	1442.43	3126.40
A_68_P31501880	chr18:12802925-12802969	NM_028341:905	Ttc39c	INSIDE	0.612	3.461	2371.03	8206.25	2.118	1765.30	3739.09
A_68_P31422209	chr17:87682307-87682351	NM_028639:103	Ttc7	INSIDE	0.33	9.975	661.31	6596.70	3.295	640.24	2109.31
A_68_P26685864	chr9:83728415-83728459	NM_001110265:141	Ttk	INSIDE	0.536	2.741	4201.15	11513.40	1.47	3321.28	4882.37
A_68_P21622816	chr2:128891587-128891631	NM_027192:-74	Ttl	PROMOTER	0.417	9.612	2427.05	23329.29	4.006	1926.42	7717.62
A_68_P30388745	chr15:83319976-83320026	NM_178869:21337	Ttll1	INSIDE	0.644	2.992	969.10	2899.88	1.928	713.86	1376.17
A_68_P23441977	chr4:155409010-155409054	NM_029264:15894	Ttll10	INSIDE	0.666	0.408	1128.86	460.92	0.272	932.70	253.65
A_68_P20349748	chr1:74708251-74708295	NM_001014974:-55	Ttll4	PROMOTER	0.215	9.283	1550.04	14389.51	1.999	1370.92	2739.81
A_68_P20349750	chr1:74708445-74708489	NM_001014974:139	Ttll4	INSIDE	0.573	0.292	2247.13	655.21	0.167	1702.79	284.36
A_68_P30422255	chr15:88785176-88785220	NM_172818:-350	Ttll8	PROMOTER	0.488	6.204	1398.23	8674.98	3.03	1365.34	4136.81
A_68_P30422253	chr15:88784916-88784960	NM_172818:-90	Ttll8	PROMOTER	0.303	1.940	4277.78	8300.67	0.588	3257.18	1916.56
A_68_P21813371	chr2:163428304-163428348	NM_029512:277	Ttpal	INSIDE	0.272	0.342	2445.99	836.27	0.093	1860.25	173.25
A_68_P20352745	chr1:75216487-75216531	NM_009447:-680	Tuba4a	PROMOTER	0.488	0.190	2677.13	507.46	0.093	1906.83	176.49
A_68_P20352744	chr1:75216331-75216375	NM_009447:-524	Tuba4a	PROMOTER	0.513	0.470	1002.88	471.65	0.241	768.24	185.35
A_68_P21875504	chr2:174282882-174282926	NM_001080971:6709	Tubb1	INSIDE	0.478	5.031	1558.76	7841.86	2.406	1026.48	2469.78
A_68_P28936501	chr13:34169775-34169819	NM_009450:81	Tubb2a	INSIDE	0.475	0.339	1833.43	621.40	0.161	1293.09	208.13
A_68_P21070656	chr2:25080752-25080796	NM_146116:-552	Tubb2c	PROMOTER	0.34	13.933	2674.67	37265.21	4.738	2101.61	9958.46
A_68_P21070644	chr2:25079420-25079464	NM_146116:780	Tubb2c	INSIDE	0.406	8.083	2216.47	17915.21	3.284	1693.63	5562.06
A_68_P21070645	chr2:25079554-25079599	NM_146116:646	Tubb2c	INSIDE	0.479	0.395	1231.34	486.71	0.189	948.99	179.72
A_68_P21070648	chr2:25079927-25079971	NM_146116:274	Tubb2c	INSIDE	0.527	0.240	2957.25	709.72	0.126	2137.98	270.26
A_68_P31161191	chr17:35974038-35974082	NM_011655:1186	Tubb5	INSIDE	0.664	3.365	892.95	3004.75	2.235	786.75	1758.55
A_68_P31791603	chr18:67550248-67550292	NM_026473:-114	Tubb6	PROMOTER	0.321	7.013	1680.77	11786.62	2.252	1244.25	2802.68
A_68_P31791666	chr18:67561362-67561406	NM_026473:11000	Tubb6	INSIDE	0.529	2.644	3309.81	8752.45	1.398	2174.39	3040.34
A_68_P25663081	chr8:12672535-12672579	NM_198031:-456	Tubgcp3	PROMOTER	0.505	6.466	2552.56	16503.67	3.262	2284.20	7451.73
A_68_P30423271	chr15:88953473-88953517	NM_001163319:86	Tubgcp6	INSIDE	0.043	4.196	3973.70	16672.95	0.182	2516.49	458.60
A_68_P31122079	chr17:28501420-28501464	NM_021478:646	Tulp1	INSIDE	0.43	0.548	858.81	470.82	0.236	730.02	171.94
A_68_P24838525	chr6:128306097-128306141	NM_011657:-249	Tulp3	PROMOTER	0.556	3.361	2550.00	8571.74	1.868	2181.20	4073.43
A_68_P24838521	chr6:128305667-128305711	NM_011657:181	Tulp3	INSIDE	0.652	0.168	7292.48	1226.23	0.11	4936.87	541.12
A_68_P23111973	chr4:93001689-93001733	NM_026954:492	Tusc1	INSIDE	0.61	3.404	601.08	2045.96	2.077	510.03	1059.51
A_68_P25798746	chr8:40068990-40069034	NM_030254:92	Tusc3	INSIDE	0.472	6.510	1202.48	7828.14	3.07	951.06	2919.66
A_68_P30453204	chr15:94420006-94420050	NM_008971:227	Twf1	INSIDE	0.636	3.677	2299.31	8454.98	2.338	2057.03	4809.61
A_68_P28344357	chr12:34643155-34643199	NM_011658:641	Twist1	INSIDE	0.296	0.299	2558.09	765.96	0.089	1984.73	175.87
A_68_P28344355	chr12:34642855-34642899	NM_011658:341	Twist1	INSIDE	0.657	3.850	702.80	2705.68	2.531	605.39	1532.02

ProbeName	Target position of probe on CpG island microarray	TargetID	GeneSymbol	CpG island Description	Ratio of relative methylation (TiO_2-NP/Vehicle)	Sham group			TiO_2-H group		
						Relative methylation (Cy5/Cy3)	Cy3 signal (Input)	Cy5 signal (MeDIP)	Relative methylation (Cy5/Cy3)	Cy3 signal (Input)	Cy5 signal (MeDIP)
A_68_P20444766	chr1:93698474-93698518	NM_007855:443	Twist2	INSIDE	0.452	4.733	2410.29	11408.30	2.141	1842.76	3944.96
A_68_P23297754	chr4:129318346-129318390	NM_001199695:-59	Txlna	PROMOTER	0.626	3.668	1785.51	6549.05	2.297	1285.76	2953.12
A_68_P23297751	chr4:129318057-129318101	NM_001005506:-29	Txlna	PROMOTER	0.317	5.088	5807.29	29549.93	1.615	4077.45	6585.41
A_68_P32791134	chrX:159266854-159266898	NM_178935:455	Txlng	INSIDE	0.543	2.910	4348.46	12652.54	1.579	5890.97	9304.41
A_68_P22948244	chr4:57969284-57969328	NM_011660:-23	Txn1	PROMOTER	0.492	6.029	679.88	4099.18	2.963	608.21	1802.36
A_68_P27910150	chr11:72020411-72020455	NM_026559:-623	Txndc17	DIVERGENT_PROMOTER	0.555	0.457	1213.97	555.17	0.254	969.72	246.20
A_68_P28961200	chr13:38619778-38619822	NM_145367:529	Txndc5	INSIDE	0.453	6.011	5121.71	30786.14	2.724	4146.44	11295.16
A_68_P31771010	chr18:63852103-63852147	NM_016792:-111	Txnl1	PROMOTER	0.603	0.643	1345.04	864.35	0.387	1238.07	479.62
A_68_P31862938	chr18:80403841-80403885	NM_001042408:257	Txnl4a	INSIDE	0.482	0.249	4855.19	1208.20	0.12	3726.93	446.95
A_68_P24629448	chr6:89594761-89594805	NM_001178058:801	Txnrd3	INSIDE	0.28	2.010	6299.79	12663.20	0.562	4324.98	2430.42
A_68_P24629447	chr6:89594662-89594706	NM_001178058:703	Txnrd3	INSIDE	0.242	1.598	12733.49	20342.89	0.386	8128.02	3141.29
A_68_P26345607	chr9:20936048-20936095	NM_001205312:-352	Tyk2	PROMOTER	0.597	2.947	630.66	1858.86	1.76	554.07	975.26
A_68_P23570613	chr5:30400253-30400312	NM_021288:-117	Tyms	PROMOTER	0.354	8.293	163.62	1356.92	2.937	196.53	577.16
A_68_P21571594	chr2:119625143-119625187	NM_019392:-85	Tyro3	PROMOTER	0.297	28.500	3915.12	111582.60	8.451	3211.22	27138.52
A_68_P27181843	chr10:61158699-61158743	NM_027912:459	Tysnd1	INSIDE	0.521	6.670	764.65	5100.16	3.478	858.82	2987.15
A_68_P27181846	chr10:61159115-61159159	NM_027912:875	Tysnd1	INSIDE	0.584	3.473	1123.98	3903.99	2.029	969.38	1967.14
A_68_P31962487	chr19:12869271-12869315	NR_024093:1321	U05342	INSIDE	0.422	6.383	2425.49	15482.06	2.696	2044.23	5511.60
A_68_P31962490	chr19:12869772-12869816	NR_024093:819	U05342	INSIDE	0.524	4.672	1009.15	4715.07	2.45	914.61	2240.79
A_68_P31962486	chr19:12869204-12869248	NR_024093:1387	U05342	INSIDE	0.625	0.377	2199.47	829.46	0.236	1807.16	425.79
A_68_P24951871	chr7:5013926-5013970	NM_001205231:204	U2af2	INSIDE	0.205	2.615	5596.70	14634.09	0.536	3711.59	1988.35
A_68_P24951860	chr7:5012759-5012803	NM_001205231:-964	U2af2	PROMOTER	0.358	0.318	2136.64	678.94	0.114	1510.70	171.87
A_68_P24951873	chr7:5014151-5014195	NM_001205231:428	U2af2	INSIDE	0.429	8.495	2056.68	17471.74	3.645	1830.75	6673.51
A_68_P26560916	chr9:60642500-60642544	NM_028283:168	Uaca	INSIDE	0.638	0.621	1034.48	641.98	0.396	808.81	320.42
A_68_P20820922	chr1:172105070-172105114	NM_133806:-15	Uap1	PROMOTER	0.557	3.982	1542.79	6144.12	2.218	1283.64	2846.65
A_68_P20820920	chr1:172104780-172104824	NM_133806:275	Uap1	INSIDE	0.58	0.668	1465.71	978.62	0.387	1278.34	495.04
A_68_P24671729	chr6:97155626-97155670	NM_001111106:-12	Uba3	DIVERGENT_PROMOTER	0.439	9.842	7120.08	70073.38	4.32	5403.44	23342.03
A_68_P21075771	chr2:25877080-25877124	NM_133835:178	Ubac1	INSIDE	0.375	8.585	800.14	6869.01	3.222	718.20	2314.12
A_68_P29958338	chr14:122278102-122278146	NM_026861:297	Ubac2	INSIDE	0.307	14.251	2618.41	37315.27	4.371	2126.74	9295.05
A_68_P29958337	chr14:122277966-122278010	NM_026861:161	Ubac2	INSIDE	0.475	4.528	1122.24	5081.21	2.149	1097.75	2358.77
A_68_P29958335	chr14:122277673-122277717	NM_026861:-133	Ubac2	PROMOTER	0.487	3.474	1599.41	5556.43	1.692	1326.49	2244.90
A_68_P22861754	chr4:41296077-41296121	NM_023305:70	Ubap1	INSIDE	0.181	1.822	8995.94	16387.40	0.329	6236.55	2054.03
A_68_P22861756	chr4:41296379-41296423	NM_023305:372	Ubap1	INSIDE	0.59	3.244	2009.90	6519.69	1.913	1648.19	3152.80
A_68_P22861397	chr4:41222317-41222368	NM_026872:-174	Ubap2	PROMOTER	0.486	0.619	873.21	540.94	0.301	582.68	175.33
A_68_P22323188	chr3:89856392-89856436	NM_028475:-17	Ubap2l	PROMOTER	0.579	3.150	9771.78	30779.79	1.823	7195.58	13118.35
A_68_P22323185	chr3:89855913-89855957	NM_028475:463	Ubap2l	INSIDE	0.641	0.473	1217.32	576.05	0.303	885.71	268.60
A_68_P26447525	chr9:40965613-40965657	NM_176860:-57	Ubash3b	PROMOTER	0.576	0.356	1445.72	514.41	0.205	1250.10	256.31
A_68_P26447523	chr9:40965396-40965440	NM_176860:159	Ubash3b	INSIDE	0.586	2.628	2147.70	5644.37	1.541	1733.67	2671.07
A_68_P27854524	chr11:62364466-62364510	NM_011664:-517	Ubb	PROMOTER	0.484	0.466	4400.41	2052.18	0.226	3101.27	699.88
A_68_P24066623	chr5:125869716-125869760	NM_019639:649	Ubc	INSIDE	0.463	0.125	6429.18	803.62	0.058	4337.05	251.25
A_68_P24066624	chr5:125869903-125869947	NM_019639:463	Ubc	INSIDE	0.648	2.561	2217.68	5679.07	1.659	1820.53	3021.04
A_68_P32320545	chrX:34415031-34415075	NM_019668:692	Ube2a	INSIDE	0.434	7.141	1108.21	7914.06	3.097	1290.92	3997.85
A_68_P32320541	chrX:34414442-34414486	NM_019668:104	Ube2a	INSIDE	0.536	3.919	815.12	3194.34	2.101	918.09	1928.68
A_68_P21819631	chr2:164595113-164595157	NM_026785:-294	Ube2c	PROMOTER	0.239	6.461	2547.06	16457.69	1.542	2041.51	3147.20
A_68_P27234633	chr10:70747499-70747543	NM_145420:490	Ube2d1	INSIDE	0.531	0.392	4694.90	1841.84	0.208	3179.23	662.56
A_68_P31619972	chr18:35930966-35931010	NM_019912:-224	Ube2d2	PROMOTER	0.544	0.549	2007.64	1102.70	0.299	1593.86	476.44
A_68_P22552063	chr3:135102173-135102217	NM_025356:472	Ube2d3	INSIDE	0.331	0.325	2047.70	664.75	0.108	1598.90	171.89

ProbeName	Target position of probe on CpG island microarray	TargetID	GeneSymbol	CpG island Description	Ratio of relative methylation (TiO_2-NP/Vehicle)	Sham group			TiO_2-H group		
						Relative methylation (Cy5/Cy3)	Cy3 signal (Input)	Cy5 signal (MeDIP)	Relative methylation (Cy5/Cy3)	Cy3 signal (Input)	Cy5 signal (MeDIP)
A_68_P29440563	chr14:19726488-19726532	NM_144839:-369	Ube2e2	PROMOTER	0.294	0.638	15052.49	9608.33	0.187	9262.80	1735.84
A_68_P21365118	chr2:78709482-78709526	NM_009454:301	Ube2e3	INSIDE	0.329	8.998	1573.03	14153.71	2.963	1544.67	4576.64
A_68_P27912376	chr11:72420588-72420632	NM_025985:-152	Ube2g1	PROMOTER	0.475	6.359	1096.16	6971.01	3.021	988.34	2986.17
A_68_P23756857	chr5:65928719-65928763	NM_016786:241	Ube2k	INSIDE	0.158	0.522	9691.95	5056.31	0.082	6728.44	554.46
A_68_P30572505	chr16:17201070-17201114	NM_009456:493	Ube2l3	INSIDE	0.493	3.191	2306.86	7362.04	1.572	1851.34	2909.86
A_68_P24974292	chr7:13623438-13623482	NM_145578:-133	Ube2m	PROMOTER	0.502	4.314	1077.12	4646.18	2.163	1007.76	2180.10
A_68_P24974288	chr7:13622741-13622791	NM_145578:561	Ube2m	INSIDE	0.188	3.149	2050.30	6456.84	0.593	1439.19	854.10
A_68_P27365515	chr10:94978265-94978309	NM_080560:491	Ube2n	INSIDE	0.299	10.331	953.93	9854.63	3.089	871.40	2691.60
A_68_P27365516	chr10:94978402-94978446	NM_080560:629	Ube2n	INSIDE	0.426	4.884	2045.02	9987.90	2.083	1734.03	3611.76
A_68_P28160203	chr11:116442887-116442931	NM_173755:-147	Ube2o	PROMOTER	0.486	5.666	3606.33	20433.94	2.753	3018.10	8309.29
A_68_P28160202	chr11:116442703-116442747	NM_173755:37	Ube2o	INSIDE	0.633	2.456	2322.71	5705.40	1.556	1934.32	3009.20
A_68_P22321608	chr3:89577074-89577118	NM_027315:-434	Ube2q1	DIVERGENT_PROMOTER	0.43	4.100	2235.48	9164.41	1.762	1903.71	3353.64
A_68_P26529572	chr9:54997117-54997161	NM_180600:-37	Ube2q2	PROMOTER	0.201	61.848	2238.27	138431.50	12.426	1991.93	24752.62
A_68_P26529571	chr9:54996925-54996969	NM_180600:-229	Ube2q2	PROMOTER	0.308	18.463	996.35	18395.37	5.686	946.54	5382.45
A_68_P29117519	chr13:69878102-69878146	NM_001145162:651	Ube2ql1	INSIDE	0.549	7.357	4737.78	34856.33	4.039	3859.43	15588.87
A_68_P29117525	chr13:69878696-69878740	NM_001145162:57	Ube2ql1	INSIDE	0.587	5.203	1462.87	7611.13	3.056	1231.82	3764.24
A_68_P29117514	chr13:69877429-69877473	NM_001145162:1325	Ube2ql1	INSIDE	0.45	0.613	976.92	598.71	0.276	793.90	219.09
A_68_P24950639	chr7:4764364-4764408	NM_133777:-444	Ube2s	PROMOTER	0.242	0.393	10422.05	4095.97	0.095	6400.51	609.46
A_68_P24950633	chr7:4763653-4763698	NM_133777:267	Ube2s	INSIDE	0.569	0.445	2336.46	1039.89	0.253	1636.76	414.18
A_68_P21836569	chr2:167457610-167457654	NM_023230:-127	Ube2v1	PROMOTER	0.493	5.897	791.94	4670.09	2.906	726.28	2110.85
A_68_P21836563	chr2:167456985-167457029	NM_023230:499	Ube2v1	INSIDE	0.576	3.524	1940.80	6838.82	2.03	1674.02	3398.98
A_68_P30566186	chr16:15594125-15594170	NM_001159351:464	Ube2v2	INSIDE	0.52	0.391	2049.05	801.92	0.203	1540.80	313.41
A_68_P30566189	chr16:15594510-15594554	NM_001159351:79	Ube2v2	INSIDE	0.398	3.746	2432.86	9114.13	1.491	1874.12	2794.59
A_68_P28042227	chr11:95926026-95926070	NM_172300:630	Ube2z	INSIDE	0.493	0.487	7188.82	3502.89	0.24	5618.64	1349.39
A_68_P28042228	chr11:95926131-95926175	NM_172300:526	Ube2z	INSIDE	0.609	0.196	5721.94	1121.19	0.119	3943.31	470.33
A_68_P25158144	chr7:66483973-66484017	NM_173010:-125	Ube3a	PROMOTER	0.588	0.375	2242.56	841.24	0.221	1783.96	393.80
A_68_P24003597	chr5:114830959-114831004	NM_054093:366	Ube3b	INSIDE	0.241	0.253	4341.07	1097.59	0.061	2995.62	182.44
A_68_P24003598	chr5:114831037-114831081	NM_054093:443	Ube3b	INSIDE	0.464	0.416	6627.17	2757.61	0.193	4444.96	858.62
A_68_P23568204	chr5:29895570-29895614	NM_133907:-189	Ube3c	PROMOTER	0.367	0.475	1295.66	615.72	0.175	997.14	174.02
A_68_P23400514	chr4:148800490-148800534	NM_022022:228	Ube4b	INSIDE	0.617	2.289	5177.47	11852.33	1.412	4073.89	5750.92
A_68_P25477108	chr7:129210764-129210808	NM_138589:75	Ubfd1	INSIDE	0.154	23.947	1694.42	40575.72	3.678	1503.20	5528.84
A_68_P25477112	chr7:129211243-129211287	NM_138589:553	Ubfd1	INSIDE	0.575	9.190	811.48	7457.89	5.287	746.43	3946.61
A_68_P23394412	chr4:147818678-147818722	NM_027873:160	Ubiad1	INSIDE	0.579	2.784	1527.74	4253.54	1.611	1183.16	1906.59
A_68_P24180071	chr5:149364369-149364413	NM_011908:-26	Ubl3	PROMOTER	0.506	0.610	3898.41	2379.51	0.309	2736.57	844.74
A_68_P32467744	chrX:71613927-71613971	NM_145405:-82	Ubl4	PROMOTER	0.268	11.787	1474.52	17380.07	3.16	1800.58	5689.28
A_68_P24377219	chr6:38384972-38385016	NM_177185:1070	Ubn2	INSIDE	0.418	0.653	695.58	454.20	0.273	618.05	168.50
A_68_P32753662	chrX:149932604-149932648	NM_018798:-148	Ubqln2	PROMOTER	0.472	4.829	353.53	1707.34	2.28	504.62	1150.58
A_68_P31210123	chr17:47147224-47147268	NM_001177374:235	Ubr2	INSIDE	0.494	5.859	1680.79	9847.55	2.893	1361.37	3938.02
A_68_P21311590	chr2:69735990-69736034	NM_001081548:710	Ubr3	INSIDE	0.208	1.482	7251.83	10748.61	0.308	5292.04	1631.70
A_68_P23351448	chr4:138936736-138936780	NM_001160319:185	Ubr4	INSIDE	0.367	0.607	1068.30	648.89	0.223	997.37	222.25
A_68_P28693527	chr12:103996226-103996270	NM_025666:64	Ubr7	INSIDE	0.397	4.332	2078.40	9003.99	1.718	1781.22	3059.83
A_68_P32118295	chr19:42056730-42056774	NM_145500:500	Ubtd1	INSIDE	0.338	20.304	988.56	20072.19	6.862	804.12	5517.79
A_68_P32118291	chr19:42056116-42056160	NM_145500:-114	Ubtd1	DIVERGENT_PROMOTER	0.615	0.329	3097.75	1018.03	0.202	2361.85	477.62
A_68_P27688946	chr11:32355594-32355638	NM_173784:245	Ubtd2	INSIDE	0.19	28.601	5209.89	149010.30	5.421	4748.29	25740.71
A_68_P27688945	chr11:32355498-32355542	NM_173784:149	Ubtd2	INSIDE	0.484	0.546	1734.28	947.24	0.265	1425.13	377.10
A_68_P28078893	chr11:102178182-102178226	NM_001044383:397	Ubtf	INSIDE	0.178	3.235	2504.79	8102.22	0.575	2092.62	1203.83

ProbeName	Target position of probe on CpG island microarray	TargetID	GeneSymbol	CpG island Description	Ratio of relative methylation (TiO_2-NP/Vehicle)	Sham group			TiO_2-H group		
						Relative methylation (Cy5/Cy3)	Cy3 signal (Input)	Cy5 signal (MeDIP)	Relative methylation (Cy5/Cy3)	Cy3 signal (Input)	Cy5 signal (MeDIP)
A_68_P28078907	chr11:102180135-102180179	NM_001044383:-1555	Ubtf	PROMOTER	0.621	0.578	1183.42	683.47	0.358	873.60	313.14
A_68_P22704455	chr4:6118180-6118224	NM_026534:-49	Ubxn2b	PROMOTER	0.616	0.422	2361.50	996.79	0.26	1702.39	442.49
A_68_P22704457	chr4:6118424-6118468	NM_026534:195	Ubxn2b	INSIDE	0.353	0.596	926.50	551.78	0.21	820.51	172.58
A_68_P20599492	chr1:130141015-130141059	NM_026390:279	Ubxn4	INSIDE	0.566	0.371	5094.67	1890.51	0.21	3951.43	829.47
A_68_P20599495	chr1:130141420-130141464	NM_026390:685	Ubxn4	INSIDE	0.431	0.524	976.86	512.12	0.226	759.43	171.57
A_68_P31253704	chr17:56213984-56214028	NM_024432:406	Ubxn6	INSIDE	0.469	4.893	13838.65	67713.64	2.297	9829.10	22579.97
A_68_P30651945	chr16:32332864-32332908	NM_177633:549	Ubxn7	INSIDE	0.224	2.611	2953.26	7710.31	0.586	2231.18	1306.51
A_68_P23763269	chr5:67068133-67068180	NM_011670:797	Uchl1	INSIDE	0.617	0.527	1662.19	875.34	0.325	1401.92	455.48
A_68_P29856116	chr14:102053116-102053160	NM_016723:-45	Uchl3	PROMOTER	0.462	0.353	1362.99	481.77	0.163	1025.46	167.35
A_68_P20803942	chr1:169156556-169156600	NM_030724:58680	Uck2	INSIDE	0.062	31.042	3768.01	116966.70	1.925	2710.74	5219.33
A_68_P20803943	chr1:169156624-169156668	NM_030724:58612	Uck2	INSIDE	0.124	26.520	2920.43	77450.41	3.295	2152.54	7092.25
A_68_P21911426	chr2:181316540-181316584	NM_026765:116	Uckl1	INSIDE	0.567	4.596	1683.94	7739.48	2.604	1292.03	3364.26
A_68_P21911428	chr2:181316733-181316777	NM_026765:-76	Uckl1	PROMOTER	0.356	0.631	2793.98	1763.07	0.224	2180.26	489.32
A_68_P23576671	chr5:31441022-31441066	NM_021290:224	Ucn	INSIDE	0.58	4.313	2403.30	10365.56	2.5	1961.62	4903.76
A_68_P22955050	chr4:59202567-59202613	NM_011673:169	Ugcg	INSIDE	0.432	0.465	1148.08	533.49	0.201	854.13	171.54
A_68_P22955051	chr4:59202692-59202736	NM_011673:293	Ugcg	INSIDE	0.433	0.476	1516.57	722.07	0.206	1248.52	257.69
A_68_P22955049	chr4:59202246-59202290	NM_011673:-153	Ugcg	PROMOTER	0.566	3.929	615.15	2417.21	2.224	531.33	1181.92
A_68_P23756355	chr5:65826987-65827031	NM_009466:73	Ugdh	INSIDE	0.643	3.100	475.49	1473.90	1.992	405.65	808.03
A_68_P27636577	chr11:21270655-21270699	NM_139297:206	Ugp2	INSIDE	0.213	79.936	2208.96	176574.20	16.996	4744.49	80635.91
A_68_P27636579	chr11:21270946-21270990	NM_139297:-86	Ugp2	PROMOTER	0.563	0.458	3791.47	1737.75	0.258	3064.67	790.64
A_68_P27636581	chr11:21271108-21271152	NM_139297:-248	Ugp2	PROMOTER	0.301	1.533	1643.56	2519.76	0.461	1408.82	649.53
A_68_P20821183	chr1:172145120-172145164	NM_010633:382	Uhmk1	INSIDE	0.36	5.209	1932.99	10069.26	1.876	1660.33	3115.44
A_68_P31255143	chr17:56443802-56443846	NM_001111078:89	Uhrf1	INSIDE	0.247	0.478	2123.74	1014.44	0.118	1618.34	190.75
A_68_P31255141	chr17:56443624-56443668	NM_001111078:-89	Uhrf1	PROMOTER	0.25	0.198	9914.40	1960.09	0.049	6936.55	343.35
A_68_P31255142	chr17:56443737-56443781	NM_001111078:23	Uhrf1	INSIDE	0.417	0.330	2373.17	783.57	0.138	1778.96	244.83
A_68_P31118855	chr17:27993453-27993497	NM_001080769:23	Uhrf1bp1	INSIDE	0.429	5.652	964.71	5452.25	2.422	773.09	1872.73
A_68_P31118859	chr17:27993828-27993872	NM_001080769:399	Uhrf1bp1	INSIDE	0.432	0.604	3644.68	2202.79	0.261	2810.33	733.36
A_68_P27332821	chr10:89207726-89207770	NM_029166:13	Uhrf1bp1l	INSIDE	0.196	12.406	2626.81	32588.73	2.435	2413.37	5876.43
A_68_P32056099	chr19:30105106-30105150	NM_144873:126	Uhrf2	INSIDE	0.435	15.491	1731.27	26819.19	6.737	1783.33	12014.07
A_68_P32056100	chr19:30105268-30105312	NM_144873:288	Uhrf2	INSIDE	0.66	2.476	1791.43	4435.76	1.634	1433.42	2342.74
A_68_P27850822	chr11:61668093-61668137	NM_013881:480	Ulk2	INSIDE	0.246	40.230	3359.80	135163.60	9.896	4434.13	43882.38
A_68_P27850828	chr11:61668703-61668747	NM_013881:-130	Ulk2	PROMOTER	0.491	17.197	2241.70	38550.71	8.449	2267.35	19157.74
A_68_P26542618	chr9:57437330-57437374	NM_027895:94	Ulk3	INSIDE	0.572	5.579	1153.09	6433.44	3.189	952.13	3036.45
A_68_P27942762	chr11:78157060-78157104	NM_011676:59	Unc119	INSIDE	0.443	10.608	4713.98	50007.22	4.702	4436.26	20857.16
A_68_P24007966	chr5:115577255-115577310	NM_175352:7702	Unc119b	INSIDE	0.315	6.127	1117.73	6848.68	1.931	803.34	1551.35
A_68_P25958205	chr8:74195695-74195739	NM_001029873:-60	Unc13a	DIVERGENT_PROMOTER	0.147	15.613	7880.89	123046.50	2.302	6870.17	15815.29
A_68_P25958090	chr8:74178710-74178754	NM_001029873:16924	Unc13a	INSIDE	0.355	0.394	1942.44	764.56	0.14	1294.46	181.01
A_68_P25958206	chr8:74195761-74195810	NM_001029873:-129	Unc13a	DIVERGENT_PROMOTER	0.514	0.192	2433.00	467.79	0.099	1750.14	172.98
A_68_P22869767	chr4:43072121-43072165	NM_021468:198	Unc13b	INSIDE	0.577	2.696	1250.52	3370.99	1.555	940.37	1462.10
A_68_P25266128	chr7:87485010-87485054	NM_133952:73	Unc45a	INSIDE	0.329	0.598	1675.81	1001.95	0.196	1233.73	242.35
A_68_P27969172	chr11:82744813-82744857	NM_178680:20080	Unc45b	INSIDE	0.501	3.803	3969.26	15095.12	1.905	3547.15	6757.86
A_68_P29052709	chr13:55098533-55098577	NM_153131:47762	Unc5a	INSIDE	0.167	16.583	788.24	13071.43	2.773	785.78	2179.26
A_68_P29052710	chr13:55098681-55098725	NM_153131:47910	Unc5a	INSIDE	0.516	3.318	1526.43	5065.02	1.713	1297.66	2223.03
A_68_P29052335	chr13:55051056-55051100	NM_153131:286	Unc5a	INSIDE	0.432	0.609	834.47	508.28	0.263	648.87	170.90
A_68_P27177056	chr10:60293675-60293719	NM_029770:633	Unc5b	INSIDE	0.355	7.952	1707.08	13574.75	2.819	1395.46	3933.81
A_68_P27177053	chr10:60293340-60293384	NM_029770:967	Unc5b	INSIDE	0.223	7.373	1547.85	11411.61	1.645	1260.73	2074.18

ProbeName	Target position of probe on CpG island microarray	TargetID	GeneSymbol	CpG island Description	Ratio of relative methylation (TiO_2-NP/Vehicle)	Sham group			TiO_2-H group		
						Relative methylation (Cy5/Cy3)	Cy3 signal (Input)	Cy5 signal (MeDIP)	Relative methylation (Cy5/Cy3)	Cy3 signal (Input)	Cy5 signal (MeDIP)
A_68_P25747455	chr8:30329725-30329769	NM_153135:362	Unc5d	INSIDE	0.223	29.747	7015.13	208678.60	6.632	6327.55	41964.02
A_68_P28694455	chr12:104187509-104187553	NM_001081017:462	Unc79	INSIDE	0.596	0.461	1643.72	757.08	0.275	1366.91	375.43
A_68_P28694458	chr12:104187832-104187876	NM_001081017:786	Unc79	INSIDE	0.277	1.471	1517.82	2233.33	0.407	1256.75	511.43
A_68_P20302824	chr1:66515076-66515120	NM_175510:78	Unc80	INSIDE	0.187	0.445	2926.51	1302.92	0.083	2155.88	179.75
A_68_P20303908	chr1:66724124-66724168	NM_175510:209126	Unc80	INSIDE	0.297	45.747	10144.14	464059.40	13.59	9245.74	125648.70
A_68_P20302822	chr1:66514910-66514954	NM_175510:-88	Unc80	PROMOTER	0.487	3.646	1296.54	4726.53	1.775	1018.82	1808.67
A_68_P24136157	chr5:140020297-140020341	NM_013702:467	Uncx	INSIDE	0.378	7.690	2735.53	21035.63	2.909	2431.98	7073.73
A_68_P24136156	chr5:140020220-140020264	NM_013702:391	Uncx	INSIDE	0.578	4.804	1623.73	7800.91	2.778	1267.08	3520.29
A_68_P24136155	chr5:140020104-140020148	NM_013702:275	Uncx	INSIDE	0.452	4.290	415.56	1782.79	1.937	435.66	843.99
A_68_P24002094	chr5:114581485-114581529	NM_011677:342	Ung	INSIDE	0.248	0.540	2473.90	1336.67	0.134	1856.91	248.73
A_68_P31102167	chr17:25325180-25325224	NM_001197024:-142	Unkl	PROMOTER	0.355	7.681	1143.92	8786.32	2.73	1041.02	2842.13
A_68_P31102174	chr17:25326044-25326091	NM_001197024:723	Unkl	INSIDE	0.366	0.365	1802.61	658.65	0.134	1293.94	173.18
A_68_P31102168	chr17:25325357-25325401	NM_001197024:34	Unkl	INSIDE	0.37	4.285	17844.48	76455.40	1.587	16840.51	26720.24
A_68_P20976549	chr2:5872087-5872131	NM_001081132:-406	Upf2	DIVERGENT_PROMOTER	0.604	0.217	2460.79	533.96	0.131	1782.96	233.49
A_68_P25670554	chr8:13785750-13785794	NM_025924:158	Upf3a	INSIDE	0.611	7.105	2314.73	16446.13	4.34	1848.79	8023.05
A_68_P25670551	chr8:13785477-13785521	NM_025924:-116	Upf3a	PROMOTER	0.633	3.275	1463.75	4793.70	2.072	1082.54	2242.75
A_68_P21768463	chr2:155755683-155755727	NM_018888:342	Uqcc	INSIDE	0.507	4.577	3153.32	14433.86	2.32	2430.23	5639.13
A_68_P27283011	chr10:79869405-79869449	NM_025650:140	Uqcr11	INSIDE	0.483	0.494	1213.96	599.47	0.239	844.24	201.47
A_68_P26817224	chr9:108838919-108838963	NM_025407:280	Uqcrc1	INSIDE	0.266	9.574	4933.57	47232.61	2.547	4242.02	10804.94
A_68_P25469455	chr7:127778730-127778774	NM_025899:50	Uqcrc2	INSIDE	0.282	0.417	1801.67	751.29	0.118	1414.72	166.50
A_68_P27804722	chr11:53244281-53244325	NM_025352:31	Uqcrq	INSIDE	0.642	3.053	715.21	2183.24	1.959	669.01	1310.57
A_68_P30959122	chr16:90810767-90810811	NM_029497:-130	Urb1	PROMOTER	0.326	1.720	7203.96	12392.99	0.56	5600.60	3136.50
A_68_P20827835	chr1:173341606-173341650	NM_009480:-183	Usf1	PROMOTER	0.515	3.650	1106.49	4038.76	1.878	996.76	1872.35
A_68_P25034259	chr7:31742709-31742753	NM_011680:-908	Usf2	PROMOTER	0.56	0.302	1632.50	493.00	0.169	1225.09	207.25
A_68_P25034246	chr7:31740698-31740742	NM_011680:1102	Usf2	INSIDE	0.504	3.320	1110.87	3687.77	1.672	1040.73	1740.36
A_68_P25034247	chr7:31740799-31740843	NM_011680:1002	Usf2	INSIDE	0.451	3.619	990.99	3586.64	1.633	872.97	1425.56
A_68_P28152781	chr11:115183238-115183282	NM_176847:-28	Ush1g	PROMOTER	0.153	38.563	5518.96	212828.80	5.89	9897.43	58292.24
A_68_P28152750	chr11:115179759-115179803	NM_176847:3452	Ush1g	INSIDE	0.364	0.682	1807.60	1233.48	0.248	1497.44	371.84
A_68_P32147605	chr19:47164876-47164920	NM_023211:217	Usmg5	INSIDE	0.618	0.700	1728.07	1209.69	0.432	1282.08	554.37
A_68_P23892504	chr5:92567234-92567278	NM_019490:293	Uso1	INSIDE	0.456	1.373	1978.54	2716.25	0.626	1719.24	1075.47
A_68_P26217005	chr8:122480667-122480711	NM_009462:45937	Usp10	INSIDE	0.113	24.920	6984.46	174055.60	2.819	5820.23	16408.36
A_68_P31487968	chr18:10030044-10030088	NM_001038589:81	Usp14	INSIDE	0.48	0.717	3348.88	2401.55	0.344	2436.85	839.25
A_68_P24803059	chr6:121195955-121195999	NM_011909:53	Usp18	INSIDE	0.253	8.105	1162.70	9423.85	2.055	1081.67	2222.32
A_68_P32975180	chr9:108393143-108393187	NM_027804:158	Usp19	INSIDE	0.57	3.862	1446.94	5587.74	2.202	1165.90	2567.49
A_68_P26465575	chr9:43892820-43892864	NM_198091:-160	Usp2	PROMOTER	0.435	0.485	981.69	476.04	0.211	809.13	170.69
A_68_P20827151	chr1:173217930-173217974	NM_013919:140	Usp21	INSIDE	0.359	1.958	2982.46	5840.73	0.703	2649.78	1861.85
A_68_P27846975	chr11:60987984-60988028	NM_001004143:555	Usp22	INSIDE	0.476	0.431	5170.89	2230.40	0.205	3616.36	742.69
A_68_P23183546	chr4:105988404-105988448	NM_183225:-391	Usp24	PROMOTER	0.585	0.495	1562.33	772.90	0.289	1186.52	343.13
A_68_P26494984	chr9:48793240-48793284	NM_175482:-227	Usp28	PROMOTER	0.244	1.402	7010.19	9827.68	0.342	4904.73	1676.99
A_68_P26494985	chr9:48793375-48793419	NM_175482:-93	Usp28	PROMOTER	0.388	1.392	7579.24	10546.68	0.539	5805.48	3130.47
A_68_P26494989	chr9:48793954-48794001	NM_175482:488	Usp28	INSIDE	0.563	0.501	897.93	449.83	0.282	713.23	201.23
A_68_P26593991	chr9:66440276-66440320	NM_144937:489	Usp3	INSIDE	0.576	5.936	716.89	4255.84	3.417	667.22	2279.95
A_68_P27981393	chr11:84953057-84953101	NM_001029934:379	Usp32	INSIDE	0.306	1.562	16362.56	25565.40	0.478	12773.99	6102.77
A_68_P22650044	chr3:152009738-152009782	NM_001076676:316	Usp33	INSIDE	0.653	5.228	786.11	4110.09	3.415	662.34	2261.62
A_68_P25349897	chr7:104474502-104474546	NM_001177412:-50	Usp35	DIVERGENT_PROMOTER	0.51	3.201	2097.29	6712.78	1.633	1627.33	2658.00
A_68_P28170438	chr11:118151273-118151317	NM_001033528:264	Usp36	INSIDE	0.483	0.206	3290.31	678.34	0.1	2105.36	209.48

ProbeName	Target position of probe on CpG island microarray	TargetID	GeneSymbol	CpG island Description	Ratio of relative methylation (TiO_2-NP/Vehicle)	Sham group			TiO_2-H group		
						Relative methylation (Cy5/Cy3)	Cy3 signal (Input)	Cy5 signal (MeDIP)	Relative methylation (Cy5/Cy3)	Cy3 signal (Input)	Cy5 signal (MeDIP)
A_68_P28170437	chr11:118151196-118151240	NM_001033528:340	Usp36	INSIDE	0.381	0.729	2839.97	2070.31	0.278	1876.40	521.53
A_68_P26814408	chr9:108250538-108250582	NM_011678:399	Usp4	INSIDE	0.477	0.450	1307.37	588.64	0.215	1070.12	229.65
A_68_P26814406	chr9:108250276-108250320	NM_011678:137	Usp4	INSIDE	0.503	0.245	1869.63	457.55	0.123	1526.10	187.83
A_68_P26814405	chr9:108250089-108250133	NM_011678:-51	Usp4	PROMOTER	0.525	6.024	1538.15	9266.21	3.161	1242.00	3926.20
A_68_P20421182	chr1:89904977-89905021	NM_001033291:128	Usp40	INSIDE	0.267	9.377	4266.98	40009.58	2.506	3702.54	9279.79
A_68_P24158012	chr5:144493603-144493647	NM_029749:-475	Usp42	PROMOTER	0.368	0.545	1475.76	804.92	0.201	1308.55	263.00
A_68_P27886200	chr11:67735519-67735563	NM_173754:115	Usp43	INSIDE	0.557	3.474	1391.90	4835.16	1.935	1213.41	2347.55
A_68_P27886202	chr11:67735776-67735820	NM_173754:-143	Usp43	PROMOTER	0.313	1.468	1317.77	1934.66	0.459	1125.23	517.01
A_68_P27886201	chr11:67735651-67735695	NM_173754:-17	Usp43	PROMOTER	0.435	4.729	446.81	2113.06	2.059	427.61	880.29
A_68_P27355931	chr10:93296360-93296404	NM_183199:2083	Usp44	INSIDE	0.599	0.475	1411.40	671.09	0.285	1062.83	302.93
A_68_P27355937	chr10:93297003-93297047	NM_183199:2725	Usp44	INSIDE	0.621	2.803	1930.38	5410.32	1.74	1608.41	2797.89
A_68_P23801063	chr5:74464594-74464638	NM_177561:-180	Usp46	PROMOTER	0.553	0.452	2382.84	1077.88	0.25	1784.36	446.76
A_68_P23340749	chr4:137150280-137150324	NM_130879:199	Usp48	INSIDE	0.431	0.458	1331.25	610.35	0.198	1180.72	233.30
A_68_P31213521	chr17:47809690-47809734	NM_198421:42074	Usp49	INSIDE	0.522	4.586	2288.15	10492.34	2.393	2035.79	4872.52
A_68_P31213274	chr17:47766353-47766397	NM_198421:-1264	Usp49	PROMOTER	0.454	0.668	1360.10	909.15	0.303	1028.76	312.12
A_68_P31213275	chr17:47766487-47766531	NM_198421:-1130	Usp49	PROMOTER	0.425	1.345	8293.25	11154.79	0.571	5439.96	3107.51
A_68_P24817210	chr6:124779618-124779662	NM_013700:-175	Usp5	DIVERGENT_PROMOTER	0.564	0.244	2817.97	686.36	0.137	1909.71	262.38
A_68_P32751543	chrX:149442021-149442065	NM_001137547:1031	Usp51	INSIDE	0.382	9.961	995.35	9914.25	3.809	1377.41	5246.05
A_68_P21609529	chr2:126533259-126533303	NM_019729:182	Usp8	INSIDE	0.482	0.640	887.08	568.06	0.308	758.30	233.87
A_68_P24183692	chr5:149996457-149996501	NM_001013378:343	Uspl1	INSIDE	0.442	1.502	4845.24	7276.85	0.664	3331.99	2212.19
A_68_P26924749	chr10:8238335-8238379	NM_177387:267	Ust	INSIDE	0.609	0.398	3258.08	1298.29	0.243	2681.70	650.88
A_68_P25581335	chr7:147129738-147129782	NM_009482:6	Utf1	INSIDE	0.586	0.281	2515.92	707.88	0.165	1935.41	319.21
A_68_P32363781	chrX:45638888-45638932	NM_028276:28800	Utp14a	DOWNSTREAM	0.645	5.807	3357.09	19493.72	3.748	3802.11	14248.69
A_68_P20372363	chr1:78654178-78654222	NM_001001981:-199	Utp14b	PROMOTER	0.093	26.790	1278.64	34254.76	2.486	1142.92	2841.70
A_68_P20372367	chr1:78654608-78654652	NM_001136226:-384	Utp14b	PROMOTER	0.501	6.005	1602.32	9621.33	3.011	1344.53	4048.06
A_68_P28029564	chr11:93746411-93746455	NM_001013375:648	Utp18	INSIDE	0.256	9.139	6959.49	63605.10	2.344	6746.99	15813.23
A_68_P26947987	chr10:12588699-12588743	NM_011682:-7187	Utrn	PROMOTER	0.38	0.650	1122.31	729.59	0.247	1001.24	247.31
A_68_P20191095	chr1:43884838-43884882	NM_026430:-307	Uxs1	PROMOTER	0.283	0.521	1434.72	747.53	0.147	1173.02	172.94
A_68_P26163689	chr8:113142496-113142540	NM_146216:-19	Vac14	PROMOTER	0.481	0.362	1270.64	460.11	0.174	993.47	173.05
A_68_P24819389	chr6:125165905-125165949	NM_001080557:328	Vamp1	INSIDE	0.556	3.845	1140.19	4383.50	2.138	983.93	2103.59
A_68_P20778496	chr1:164501404-164501449	NM_016796:468	Vamp4	INSIDE	0.392	6.988	878.02	6135.81	2.738	680.98	1864.57
A_68_P24541411	chr6:72330618-72330663	NM_001080742:-178	Vamp5	PROMOTER	0.562	0.510	1278.88	652.66	0.287	959.20	275.29
A_68_P31156940	chr17:35137702-35137746	NM_011690:-127	Vars	PROMOTER	0.552	0.340	1410.73	479.16	0.188	1001.84	187.96
A_68_P31156941	chr17:35137890-35137934	NM_011690:61	Vars	INSIDE	0.426	3.987	975.92	3891.01	1.698	807.25	1370.69
A_68_P24992394	chr7:19856763-19856807	NM_009499:419	Vasp	INSIDE	0.294	5.209	1530.72	7973.63	1.531	1246.47	1907.98
A_68_P21084626	chr2:27282675-27282719	NM_009500:-351	Vav2	PROMOTER	0.512	4.115	1005.73	4139.00	2.106	886.53	1866.84
A_68_P21084621	chr2:27281926-27281970	NM_009500:397	Vav2	INSIDE	0.611	4.640	1258.84	5841.58	2.835	1060.10	3005.64
A_68_P21084617	chr2:27281328-27281372	NM_009500:995	Vav2	INSIDE	0.661	0.630	3003.28	1893.12	0.417	2295.80	957.05
A_68_P32217690	chr19:59244706-59244750	NM_009501:-209	Vax1	PROMOTER	0.374	5.288	3436.05	18169.27	1.979	3155.62	6244.40
A_68_P32217657	chr19:59240762-59240806	NM_009501:3735	Vax1	INSIDE	0.507	0.308	1698.24	522.72	0.156	1106.35	172.75
A_68_P32217704	chr19:59246215-59246259	NM_009501:-1717	Vax1	PROMOTER	0.581	4.554	3344.34	15228.67	2.647	2570.32	6803.99
A_68_P32217658	chr19:59240861-59240905	NM_009501:3637	Vax1	INSIDE	0.618	0.554	1146.76	635.07	0.342	1030.08	352.77
A_68_P32217702	chr19:59246036-59246083	NM_009501:-1540	Vax1	PROMOTER	0.183	2.413	1341.76	3238.23	0.441	943.20	415.91
A_68_P32217685	chr19:59244013-59244057	NM_009501:485	Vax1	INSIDE	0.447	0.597	806.34	481.48	0.267	642.80	171.68
A_68_P24594973	chr6:83652821-83652865	NR_002873:8084	Vax2os1	INSIDE	0.593	3.747	1169.85	4383.01	2.223	965.36	2145.99
A_68_P29449770	chr14:21748700-21748744	NM_009502:68	Vcl	INSIDE	0.429	8.204	4020.15	32980.67	3.521	3297.88	11612.28

ProbeName	Target position of probe on CpG island microarray	TargetID	GeneSymbol	CpG island Description	Ratio of relative methylation (TiO_2-NP/Vehicle)	Sham group			TiO_2-H group		
						Relative methylation (Cy5/Cy3)	Cy3 signal (Input)	Cy5 signal (MeDIP)	Relative methylation (Cy5/Cy3)	Cy3 signal (Input)	Cy5 signal (MeDIP)
A_68_P29449769	chr14:21748618-21748662	NM_009502:-14	Vcl	PROMOTER	0.636	0.338	4290.40	1452.10	0.215	3005.68	647.13
A_68_P27798802	chr11:52174768-52174812	NM_011694:174	Vdac1	INSIDE	0.163	2.682	1028.93	2759.30	0.438	906.19	397.01
A_68_P29455108	chr14:22650786-22650830	NM_011695:26	Vdac2	INSIDE	0.514	13.124	822.06	10788.91	6.748	690.90	4661.87
A_68_P29455105	chr14:22650386-22650430	NM_011695:-374	Vdac2	PROMOTER	0.351	4.280	2435.52	10425.08	1.501	2235.30	3355.37
A_68_P30471846	chr15:97738704-97738748	NM_009504:1	Vdr	INSIDE	0.21	0.462	2858.32	1320.86	0.097	2076.91	201.51
A_68_P30471844	chr15:97738350-97738394	NM_009504:355	Vdr	INSIDE	0.357	0.292	2329.31	679.96	0.104	1729.66	180.14
A_68_P30471845	chr15:97738527-97738571	NM_009504:179	Vdr	INSIDE	0.382	0.276	2381.94	657.98	0.105	1640.41	172.99
A_68_P31204668	chr17:46168904-46168948	NM_001110266:-6814	Vegfa	PROMOTER	0.406	0.479	2341.79	1121.77	0.195	1760.34	342.67
A_68_P31204664	chr17:46168459-46168503	NM_001110266:-6368	Vegfa	PROMOTER	0.615	6.709	2358.58	15823.53	4.124	2130.40	8786.20
A_68_P31204659	chr17:46167970-46168014	NM_001110266:-5880	Vegfa	PROMOTER	0.635	0.397	2738.50	1085.98	0.252	1898.00	478.22
A_68_P31937159	chr19:7062178-7062222	NM_001185164:-59	Vegfb	PROMOTER	0.323	10.485	3940.23	41311.66	3.383	3110.36	10520.87
A_68_P27357055	chr10:93498582-93498626	NM_172538:-111	Vezt	DIVERGENT_PROMOTER	0.443	0.166	3716.71	616.98	0.074	2769.04	203.63
A_68_P24124227	chr5:137507750-137507794	NM_001039385:1608	Vgf	INSIDE	0.507	9.433	499.83	4714.95	4.783	521.99	2496.69
A_68_P24124211	chr5:137505950-137505994	NM_001039385:-192	Vgf	PROMOTER	0.405	4.267	1070.76	4568.45	1.726	830.78	1433.97
A_68_P27135755	chr10:51747513-51747557	NM_153786:5043	Vgll2	INSIDE	0.489	1.384	3160.43	4373.09	0.677	2699.44	1827.98
A_68_P27135713	chr10:51742583-51742627	NM_153786:113	Vgll2	INSIDE	0.446	1.439	1699.31	2445.21	0.642	1452.97	933.45
A_68_P30826541	chr16:65815730-65815774	NM_028572:-125	Vgll3	PROMOTER	0.558	6.091	847.50	5162.17	3.397	760.84	2584.54
A_68_P24760552	chr6:113574106-113574150	NM_009507:114	Vhl	INSIDE	0.361	0.482	1238.43	596.52	0.174	985.11	171.45
A_68_P21017264	chr2:13495981-13496025	NM_011701:65	Vim	INSIDE	0.436	7.670	3027.34	23220.35	3.346	2527.76	8458.13
A_68_P21017265	chr2:13496059-13496103	NM_011701:143	Vim	INSIDE	0.471	0.561	1071.73	601.09	0.264	879.94	232.67
A_68_P28765897	chr12:117316101-117316145	NM_009511:-73	Vipr2	PROMOTER	0.538	0.314	2312.97	725.55	0.169	1857.53	313.53
A_68_P24091293	chr5:130418207-130418251	NM_001001327:246	Vkorc1l1	INSIDE	0.337	14.562	1401.25	20405.02	4.91	1286.23	6314.99
A_68_P24091286	chr5:130417459-130417503	NM_001001327:-502	Vkorc1l1	PROMOTER	0.28	1.625	9001.68	14623.46	0.454	5478.56	2487.71
A_68_P31257914	chr17:56856378-56856423	NM_178926:-56	Vmac	PROMOTER	0.358	10.857	1274.93	13841.94	3.883	1457.09	5657.88
A_68_P31257916	chr17:56856619-56856667	NM_178926:-298	Vmac	PROMOTER	0.049	1.466	4395.06	6441.24	0.072	2470.41	177.21
A_68_P24630038	chr6:89785494-89785538	NM_053221:10015	Vmn1r42	DOWNSTREAM	0.205	13.011	1310.26	17047.42	2.667	1012.65	2700.36
A_68_P24630036	chr6:89785323-89785367	NM_053221:10187	Vmn1r42	DOWNSTREAM	0.226	8.776	1196.35	10498.54	1.984	957.41	1899.19
A_68_P24630040	chr6:89785772-89785816	NM_053221:9737	Vmn1r42	DOWNSTREAM	0.324	6.657	1548.13	10306.38	2.16	1516.70	3276.24
A_68_P24480348	chr6:57774597-57774643	NM_146168:499	Vopp1	INSIDE	0.607	0.655	1035.62	678.34	0.397	827.49	328.79
A_68_P26805304	chr9:106724237-106724281	NM_001015507:-48	Vprbp	PROMOTER	0.654	0.559	1581.47	883.32	0.365	1169.58	427.22
A_68_P26805306	chr9:106724414-106724458	NM_001015507:130	Vprbp	INSIDE	0.3	1.454	3471.08	5046.43	0.436	3013.62	1314.19
A_68_P31982219	chr19:16855558-16855602	NM_173028:-163	Vps13a	PROMOTER	0.527	7.738	1726.93	13362.57	4.074	1473.37	6002.89
A_68_P30133053	chr15:35301385-35301429	NM_177151:106	Vps13b	INSIDE	0.359	0.675	903.65	609.62	0.242	715.60	173.07
A_68_P30133052	chr15:35301302-35301346	NM_177151:24	Vps13b	INSIDE	0.644	2.684	715.80	1921.11	1.728	608.95	1052.33
A_68_P26601300	chr9:67688155-67688199	NM_177184:-26	Vps13c	DIVERGENT_PROMOTER	0.334	9.346	3134.86	29297.93	3.122	2896.51	9043.62
A_68_P23381901	chr4:144785001-144785047	NM_001128198:-4529	Vps13d	PROMOTER	0.086	29.295	972.55	28490.65	2.518	847.36	2133.83
A_68_P23381898	chr4:144784634-144784678	NM_001128198:-4161	Vps13d	PROMOTER	0.445	5.282	1153.26	6091.41	2.352	986.39	2319.78
A_68_P24536895	chr6:71493871-71493915	NM_025783:45	Vps24	INSIDE	0.284	18.313	2835.51	51925.97	5.2	2771.85	14412.25
A_68_P24536901	chr6:71494350-71494394	NM_025783:525	Vps24	INSIDE	0.407	0.646	773.03	499.11	0.262	658.17	172.74
A_68_P27186307	chr10:61949576-61949620	NM_133672:-45	Vps26a	PROMOTER	0.309	8.332	1278.18	10650.14	2.573	1163.90	2994.17
A_68_P24049903	chr5:122804592-122804636	NM_019780:193	Vps29	INSIDE	0.628	2.615	1950.40	5100.00	1.642	1601.18	2629.69
A_68_P25806165	chr8:41596899-41596943	NM_033560:-216	Vps37a	DIVERGENT_PROMOTER	0.444	3.941	3225.29	12710.11	1.75	2437.83	4266.35
A_68_P26142730	chr8:109555781-109555825	NM_126165:577	Vps4a	INSIDE	0.635	3.645	801.51	2921.40	2.316	627.07	1452.45
A_68_P27635752	chr11:21139299-21139343	NM_139061:429	Vps54	INSIDE	0.504	8.505	1716.75	14600.85	4.283	1454.87	6231.57
A_68_P27635747	chr11:21138559-21138603	NM_139061:-311	Vps54	PROMOTER	0.358	0.650	3920.25	2547.82	0.233	2793.68	650.24
A_68_P30592417	chr16:21423184-21423228	NM_001081366:16	Vps8	INSIDE	0.626	3.405	710.09	2418.06	2.133	733.28	1564.24

ProbeName	Target position of probe on CpG island microarray	TargetID	GeneSymbol	CpG island Description	Ratio of relative methylation (TiO_2-NP/Vehicle)	Sham group			TiO_2-H group		
						Relative methylation (Cy5/Cy3)	Cy3 signal (Input)	Cy5 signal (MeDIP)	Relative methylation (Cy5/Cy3)	Cy3 signal (Input)	Cy5 signal (MeDIP)
A_68_P28712491	chr12:107248439-107248483	NM_001029843:-12	Vrk1	PROMOTER	0.619	0.317	1521.11	482.70	0.197	1173.76	230.73
A_68_P27663517	chr11:26493581-26493625	NM_027260:318	Vrk2	INSIDE	0.531	2.914	1597.12	4654.19	1.547	1507.61	2332.12
A_68_P20833903	chr1:174493113-174493157	NR_027644:3098	Vsig8	INSIDE	0.55	3.062	2433.69	7451.77	1.684	2084.88	3511.43
A_68_P25075288	chr7:48157851-48157898	NM_021387:3227	Vstm2b	INSIDE	0.576	0.422	1087.53	458.80	0.243	806.10	195.71
A_68_P21780087	chr2:157769902-157769946	NM_198627:29536	Vstm2l	INSIDE	0.055	9.334	2107.58	19672.56	0.518	1581.10	819.02
A_68_P28599061	chr12:85911065-85911109	NM_007701:285	Vsx2	INSIDE	0.639	0.339	1748.42	593.53	0.217	1463.05	317.31
A_68_P26958515	chr10:14425185-14425230	NM_025418:88	Vta1	INSIDE	0.359	0.523	1471.28	770.08	0.188	1107.04	207.74
A_68_P28567992	chr12:80273136-80273180	NM_016800:287	Vti1b	INSIDE	0.576	0.480	2432.19	1167.40	0.276	1959.67	541.32
A_68_P32203587	chr19:56948670-56948714	NM_172840:-213	Vwa2	PROMOTER	0.545	5.186	2940.63	15249.80	2.824	2146.29	6060.69
A_68_P32203589	chr19:56948890-56948934	NM_172840:7	Vwa2	INSIDE	0.558	0.521	1747.23	911.04	0.291	1478.97	430.00
A_68_P25470426	chr7:127953525-127953569	NM_177697:70476	Vwa3a	DOWNSTREAM	0.509	0.610	4828.66	2945.58	0.31	3300.86	1024.12
A_68_P30588341	chr16:20591588-20591632	NM_182636:1956	Vwa5b2	INSIDE	0.215	11.729	2114.48	24801.46	2.521	1810.32	4563.05
A_68_P30588338	chr16:20591336-20591380	NM_182636:1704	Vwa5b2	INSIDE	0.369	6.945	2226.40	15461.78	2.563	1806.56	4629.48
A_68_P27589636	chr11:11014080-11014124	NM_177033:84	Vwc2	INSIDE	0.629	0.640	1533.69	980.99	0.402	1370.23	550.99
A_68_P27589635	chr11:11013930-11013974	NM_177033:-66	Vwc2	PROMOTER	0.58	4.048	575.79	2330.61	2.346	535.50	1256.10
A_68_P31479221	chr18:7868637-7868681	NR_027465:-197	Wac	PROMOTER	0.418	5.342	747.68	3993.78	2.233	666.84	1489.14
A_68_P31479225	chr18:7869058-7869102	NM_001146298:-114	Wac	PROMOTER	0.534	7.118	3456.63	24603.15	3.799	2823.26	10726.12
A_68_P31479228	chr18:7869362-7869406	NM_001146298:190	Wac	INSIDE	0.446	3.636	2819.43	10252.17	1.622	2314.17	3753.24
A_68_P31479223	chr18:7868850-7868894	NM_001146298:-322	Wac	PROMOTER	0.631	2.581	1324.18	3417.19	1.63	1161.22	1892.38
A_68_P29531321	chr14:35487082-35487126	NM_001004436:-9	Wapal	PROMOTER	0.595	0.308	4406.71	1357.11	0.183	3008.35	551.13
A_68_P29531324	chr14:35487544-35487588	NM_001004436:453	Wapal	INSIDE	0.232	1.528	2522.03	3854.20	0.355	2180.15	774.43
A_68_P29531317	chr14:35486617-35486661	NM_001004436:-475	Wapal	PROMOTER	0.506	0.635	866.37	549.96	0.321	780.40	250.77
A_68_P22362402	chr3:98945208-98945252	NM_027462:218	Wars2	INSIDE	0.433	0.449	1100.29	493.62	0.194	882.20	171.23
A_68_P23315906	chr4:132686467-132686511	NM_153423:-59	Wasf2	PROMOTER	0.45	0.732	3986.67	2919.21	0.33	3125.51	1030.76
A_68_P24168282	chr5:147197029-147197073	NM_145155:469	Wasf3	INSIDE	0.581	0.610	2292.66	1399.19	0.355	1841.46	653.01
A_68_P24302940	chr6:24615184-24615228	NM_028459:-211	Wasl	PROMOTER	0.339	6.211	4818.32	29924.94	2.107	4029.34	8489.21
A_68_P24874653	chr6:136776590-136776634	NM_021714:125	Wbp11	INSIDE	0.454	0.397	2483.75	985.22	0.18	1814.91	326.54
A_68_P29751622	chr14:79881067-79881111	NM_018765:-13	Wbp4	PROMOTER	0.64	0.505	1964.99	992.14	0.323	1592.47	514.21
A_68_P29751627	chr14:79881898-79881942	NM_018765:-845	Wbp4	PROMOTER	0.65	3.347	880.72	2948.07	2.175	926.86	2016.24
A_68_P24111847	chr5:134652378-134652422	NM_033572:237	Wbscr16	INSIDE	0.198	1.854	2299.18	4262.18	0.367	1790.35	657.14
A_68_P24097673	chr5:131783669-131783713	NM_145218:-298	Wbscr17	PROMOTER	0.499	0.665	1597.90	1062.92	0.332	1253.76	416.00
A_68_P20377583	chr1:79758129-79758173	NM_001111279:194	Wdfy1	INSIDE	0.604	0.601	1463.61	879.92	0.363	1147.36	416.76
A_68_P23938671	chr5:102499208-102499252	NM_172882:-290	Wdfy3	PROMOTER	0.173	14.534	917.57	13335.85	2.518	734.25	1848.83
A_68_P23938669	chr5:102498892-102498936	NM_172882:26	Wdfy3	INSIDE	0.397	0.728	5028.58	3661.48	0.289	3833.51	1106.86
A_68_P25516577	chr7:136735544-136735588	NM_172255:190	Wdr11	INSIDE	0.35	6.359	1587.50	10094.35	2.227	1367.20	3044.71
A_68_P32241861	chrX:7706354-7706398	NR_029428:3597	Wdr13	INSIDE	0.634	3.461	555.13	1921.05	2.193	762.62	1672.51
A_68_P28739967	chr12:111976055-111976099	NM_027149:-82	Wdr20a	PROMOTER	0.354	7.535	1485.41	11192.80	2.665	1288.43	3433.82
A_68_P28739965	chr12:111975801-111975845	NM_027149:-336	Wdr20a	PROMOTER	0.437	0.571	1522.70	869.82	0.25	1160.39	289.62
A_68_P20880198	chr1:183142413-183142457	NM_145514:-325	Wdr26	PROMOTER	0.327	6.178	926.12	5721.75	2.02	840.62	1698.13
A_68_P22964621	chr4:62131699-62131744	NM_023597:185	Wdr31	INSIDE	0.244	1.513	1254.33	1897.58	0.369	770.68	284.28
A_68_P31599635	chr18:31963656-31963700	NM_001170966:-32	Wdr33	PROMOTER	0.513	0.303	1475.30	447.60	0.156	1118.46	174.08
A_68_P31599639	chr18:31964071-31964115	NM_001170966:382	Wdr33	INSIDE	0.326	0.576	1194.31	688.37	0.188	989.24	185.91
A_68_P21101048	chr2:29904402-29904446	NM_001008498:-25	Wdr34	PROMOTER	0.184	11.266	1300.02	14645.92	2.078	1068.39	2220.20
A_68_P28225578	chr12:8980743-8980787	NM_001159527:-42	Wdr35	PROMOTER	0.403	5.805	7569.68	43945.40	2.338	6186.31	14460.89
A_68_P31140217	chr17:31649105-31649149	NM_021322:306	Wdr4	INSIDE	0.405	1.441	1602.70	2309.36	0.583	1264.82	737.56
A_68_P31335312	chr17:71965459-71965503	NM_175639:-74	Wdr43	PROMOTER	0.419	0.355	1462.92	518.90	0.149	1233.46	183.24

ProbeName	Target position of probe on CpG island microarray	TargetID	GeneSymbol	CpG island Description	Ratio of relative methylation (TiO_2-NP/Vehicle)	Sham group			TiO_2-H group		
						Relative methylation (Cy5/Cy3)	Cy3 signal (Input)	Cy5 signal (MeDIP)	Relative methylation (Cy5/Cy3)	Cy3 signal (Input)	Cy5 signal (MeDIP)
A_68_P28190095	chr11:121215875-121215919	NM_025793:-135	Wdr45l	PROMOTER	0.531	19.509	2340.27	45656.25	10.359	2116.89	21928.48
A_68_P28190096	chr11:121215946-121215990	NM_025793:-207	Wdr45l	PROMOTER	0.586	0.635	1829.26	1161.11	0.372	1330.60	494.59
A_68_P31151139	chr17:34078067-34078111	NM_020603:421	Wdr46	INSIDE	0.207	0.341	3799.71	1296.95	0.071	2367.94	166.98
A_68_P31151137	chr17:34077801-34077845	NM_020603:155	Wdr46	INSIDE	0.626	0.644	1487.98	958.75	0.403	1153.56	465.27
A_68_P22413525	chr3:108394707-108394751	NM_181400:533	Wdr47	INSIDE	0.424	0.249	2304.89	573.11	0.106	1660.59	175.19
A_68_P22413522	chr3:108394356-108394400	NM_181400:183	Wdr47	INSIDE	0.531	0.647	1898.17	1228.80	0.344	1584.66	544.49
A_68_P22413523	chr3:108394473-108394517	NM_181400:299	Wdr47	INSIDE	0.479	3.528	1048.81	3700.31	1.691	902.24	1525.69
A_68_P26815583	chr9:108480765-108480809	NM_031392:215	Wdr6	INSIDE	0.345	0.462	5089.97	2351.11	0.159	3376.18	537.94
A_68_P26815586	chr9:108481063-108481107	NM_031392:-83	Wdr6	PROMOTER	0.397	5.140	2071.78	10648.06	2.041	1604.07	3273.50
A_68_P26815582	chr9:108480653-108480697	NM_031392:327	Wdr6	INSIDE	0.401	1.409	13201.65	18597.52	0.565	8763.28	4952.59
A_68_P29998080	chr15:8049104-8049148	NM_001081402:83	Wdr70	INSIDE	0.603	0.711	3529.05	2508.22	0.429	2725.13	1168.87
A_68_P23164318	chr4:102786543-102786587	NM_146254:340	Wdr78	INSIDE	0.284	1.585	921.57	1460.80	0.45	729.85	328.31
A_68_P23429335	chr4:153515771-153515815	NM_021499:-688	Wdr8	DIVERGENT_PROMOTER	0.549	0.260	1994.20	519.38	0.143	1248.83	178.57
A_68_P26023613	chr8:87604137-87604181	NM_026399:487	Wdr83	INSIDE	0.554	0.497	2185.31	1085.52	0.275	1616.52	444.82
A_68_P26023614	chr8:87604286-87604330	NM_026399:337	Wdr83	INSIDE	0.436	0.543	853.48	463.61	0.237	723.00	171.24
A_68_P28549762	chr12:76770236-76770280	NM_028203:266	Wdr89	INSIDE	0.629	0.229	2304.01	526.63	0.144	1733.11	249.37
A_68_P32152243	chr19:47911627-47911671	NM_027559:203	Wdr96	INSIDE	0.373	4.673	2170.92	10143.70	1.741	1864.07	3245.19
A_68_P23317106	chr4:132895104-132895148	NM_199306:104	Wdtc1	INSIDE	0.66	6.305	11701.67	73775.12	4.158	8398.29	34920.00
A_68_P23317102	chr4:132894698-132894742	NM_199306:510	Wdtc1	INSIDE	0.259	6.185	2325.72	14383.71	1.599	1875.13	2997.84
A_68_P25411826	chr7:117266404-117266448	NM_009516:854	Wee1	INSIDE	0.575	3.702	1116.99	4135.61	2.128	944.19	2009.28
A_68_P25411827	chr7:117266477-117266521	NM_009516:926	Wee1	INSIDE	0.605	4.146	910.18	3773.83	2.51	733.64	1841.24
A_68_P23610941	chr5:37379997-37380041	NM_011716:203	Wfs1	INSIDE	0.501	4.787	1509.10	7224.37	2.397	1209.06	2898.00
A_68_P25272788	chr7:88716073-88716117	NM_001004185:-83	Whamm	DIVERGENT_PROMOTER	0.11	24.435	786.25	19212.31	2.678	599.16	1604.68
A_68_P22970869	chr4:63155665-63155709	NM_001008791:1299	Whrn	INSIDE	0.244	0.464	3019.31	1401.60	0.113	1984.72	224.59
A_68_P22970352	chr4:63077484-63077528	NM_001008797:16850	Whrn	INSIDE	0.493	4.527	1434.69	6494.22	2.231	1044.73	2330.30
A_68_P22970873	chr4:63156014-63156058	NM_001008791:949	Whrn	INSIDE	0.371	0.567	957.02	542.21	0.21	816.53	171.74
A_68_P22970876	chr4:63156377-63156421	NM_001008791:587	Whrn	INSIDE	0.549	0.658	1037.46	682.36	0.361	892.75	322.65
A_68_P22970353	chr4:63077625-63077669	NM_001008797:16710	Whrn	INSIDE	0.42	3.395	2697.89	9158.23	1.426	1801.74	2569.37
A_68_P27543031	chr10:128185237-128185281	NM_001170869:-319	Wibg	PROMOTER	0.583	4.865	306.48	1491.03	2.835	260.53	738.52
A_68_P27501021	chr10:120471044-120471088	NM_011915:7	Wif1	INSIDE	0.597	0.570	1336.61	762.49	0.341	1140.69	388.58
A_68_P28058995	chr11:98724946-98724990	NM_197940:57	Wipf2	INSIDE	0.562	7.420	785.56	5828.54	4.173	737.94	3079.77
A_68_P28119631	chr11:109473059-109473103	NM_145940:-377	Wipi1	PROMOTER	0.216	13.648	11035.45	150616.70	2.945	10320.41	30397.09
A_68_P28119630	chr11:109472954-109472998	NM_145940:-273	Wipi1	PROMOTER	0.401	0.329	10745.04	3534.95	0.132	8000.49	1055.48
A_68_P28119629	chr11:109472837-109472881	NM_145940:-155	Wipi1	PROMOTER	0.192	7.370	5577.41	41103.93	1.418	4433.13	6287.17
A_68_P24152597	chr5:143106025-143106069	NM_178398:509	Wipi2	INSIDE	0.222	11.227	1652.99	18558.22	2.487	1403.67	3490.67
A_68_P24152591	chr5:143105523-143105567	NM_178398:7	Wipi2	INSIDE	0.489	8.034	1261.67	10136.29	3.931	1076.55	4231.53
A_68_P29023536	chr13:49241942-49241986	NM_029361:1419	Wnk2	INSIDE	0.223	1.591	1548.58	2463.92	0.354	1308.62	463.78
A_68_P28072948	chr11:101126562-101126606	NM_175638:4704	Wnk4	INSIDE	0.401	0.679	1070.03	726.13	0.272	865.95	235.47
A_68_P30476474	chr15:98622103-98622147	NM_021279:1837	Wnt1	INSIDE	0.55	0.347	2166.81	751.02	0.191	1577.94	300.94
A_68_P20350529	chr1:74838384-74838428	NM_009518:-186	Wnt10a	PROMOTER	0.375	8.378	4881.71	40900.31	3.145	4179.01	13144.18
A_68_P20350627	chr1:74849814-74849858	NM_009518:11244	Wnt10a	INSIDE	0.588	6.354	3596.33	22850.01	3.739	2878.67	10762.00
A_68_P20350626	chr1:74849701-74849745	NM_009518:11130	Wnt10a	INSIDE	0.498	0.588	1136.07	667.63	0.293	870.37	254.67
A_68_P22394252	chr3:104770874-104770918	NM_009520:-6269	Wnt2b	PROMOTER	0.584	4.240	1528.40	6480.49	2.477	1285.17	3183.53
A_68_P28087051	chr11:103634875-103634919	NM_009521:-592	Wnt3	PROMOTER	0.372	5.191	2340.70	12151.38	1.93	1972.33	3806.04
A_68_P28087058	chr11:103635743-103635787	NM_009521:276	Wnt3	INSIDE	0.525	0.573	2978.55	1706.93	0.301	1963.40	591.06
A_68_P28087325	chr11:103671166-103671210	NM_009521:35700	Wnt3	INSIDE	0.644	4.443	1423.09	6323.34	2.861	1176.17	3365.44

ProbeName	Target position of probe on CpG island microarray	TargetID	GeneSymbol	CpG island Description	Ratio of relative methylation (TiO_2-NP/Vehicle)	Sham group			TiO_2-H group		
						Relative methylation (Cy5/Cy3)	Cy3 signal (Input)	Cy5 signal (MeDIP)	Relative methylation (Cy5/Cy3)	Cy3 signal (Input)	Cy5 signal (MeDIP)
A_68_P28087340	chr11:103672887-103672931	NM_009521:37420	Wnt3	INSIDE	0.37	1.535	5185.16	7958.48	0.568	3846.16	2184.98
A_68_P27836016	chr11:59102534-59102578	NM_009522:1697	Wnt3a	INSIDE	0.427	5.467	676.52	3698.70	2.336	600.87	1403.78
A_68_P27836028	chr11:59103858-59103902	NM_009522:373	Wnt3a	INSIDE	0.45	5.035	1402.31	7060.71	2.265	1114.32	2524.12
A_68_P27835858	chr11:59077515-59077559	NM_009522:26717	Wnt3a	INSIDE	0.551	3.726	2440.13	9090.96	2.054	2067.24	4245.55
A_68_P27836018	chr11:59102804-59102848	NM_009522:1427	Wnt3a	INSIDE	0.531	3.068	3276.09	10050.35	1.629	2678.94	4363.13
A_68_P23339116	chr4:136852284-136852328	NM_009523:18757	Wnt4	INSIDE	0.242	8.064	2043.81	16480.33	1.95	1532.91	2989.55
A_68_P29493847	chr14:29318173-29318217	NM_009524:-464	Wnt5a	PROMOTER	0.547	5.930	378.48	2244.33	3.241	317.23	1028.24
A_68_P29493853	chr14:29318903-29318947	NM_009524:266	Wnt5a	INSIDE	0.564	0.470	1668.73	783.61	0.265	1221.18	323.20
A_68_P24793727	chr6:119494261-119494305	NM_009525:83	Wnt5b	INSIDE	0.601	3.335	699.84	2333.71	2.005	624.46	1252.13
A_68_P20350376	chr1:74818349-74818393	NM_009526:-95	Wnt6	PROMOTER	0.363	4.412	3721.32	16418.52	1.6	3141.85	5027.64
A_68_P24638868	chr6:91361534-91361578	NM_009527:-193	Wnt7a	PROMOTER	0.022	127.907	1809.64	231465.70	2.863	1680.91	4812.19
A_68_P24638863	chr6:91360911-91360955	NM_009527:431	Wnt7a	INSIDE	0.571	3.842	1568.17	6025.34	2.192	1334.11	2924.93
A_68_P30401591	chr15:85408071-85408115	NM_001163634:408	Wnt7b	INSIDE	0.653	2.469	814.19	2010.24	1.613	719.26	1159.83
A_68_P28086889	chr11:103611261-103611305	NM_011719:-147	Wnt9b	PROMOTER	0.243	10.527	936.16	9854.61	2.562	947.30	2427.14
A_68_P28086886	chr11:103610912-103610956	NM_011719:201	Wnt9b	INSIDE	0.653	0.488	1610.49	785.77	0.319	1379.32	439.56
A_68_P28931383	chr13:32894479-32894523	NM_030215:602	Wrnip1	INSIDE	0.252	11.611	1751.88	20341.37	2.931	1455.46	4266.13
A_68_P28931384	chr13:32894661-32894705	NM_030215:784	Wrnip1	INSIDE	0.605	7.417	1628.39	12078.28	4.485	1329.27	5961.32
A_68_P28931385	chr13:32894778-32894822	NM_030215:902	Wrnip1	INSIDE	0.65	0.541	1334.45	721.90	0.351	990.75	348.12
A_68_P27907296	chr11:71564250-71564294	NM_177618:68	Wscd1	INSIDE	0.443	9.144	4220.52	38591.95	4.047	3527.84	14278.89
A_68_P27907301	chr11:71564787-71564831	NM_177618:604	Wscd1	INSIDE	0.262	6.593	1212.15	7991.98	1.729	1053.46	1821.54
A_68_P21492621	chr2:104966817-104966861	NM_144783:153	Wt1	INSIDE	0.348	11.179	8166.27	91293.24	3.889	7275.83	28292.98
A_68_P31054977	chr17:13185351-13185395	NM_001113533:-247	Wtap	PROMOTER	0.398	6.084	1000.88	6089.31	2.419	947.67	2292.62
A_68_P27709779	chr11:35793475-35793519	NM_170779:95	Wwc1	INSIDE	0.464	0.734	1753.17	1286.47	0.341	1274.85	434.15
A_68_P25844867	chr8:49075341-49075385	NM_133791:543	Wwc2	INSIDE	0.295	14.564	3575.04	52065.85	4.292	3948.66	16948.62
A_68_P25844872	chr8:49076065-49076109	NM_133791:-181	Wwc2	PROMOTER	0.431	6.787	3714.95	25214.01	2.924	3213.68	9395.34
A_68_P22766407	chr4:19635843-19635887	NM_177327:276	Wwp1	INSIDE	0.446	0.553	1627.85	900.16	0.247	1447.23	356.79
A_68_P22766408	chr4:19636033-19636077	NM_177327:86	Wwp1	INSIDE	0.448	6.671	1591.26	10615.58	2.987	1458.50	4356.67
A_68_P22164435	chr3:57379593-57379637	NM_133784:184	Wwtr1	INSIDE	0.401	0.397	1308.74	519.81	0.159	1061.90	169.05
A_68_P22164439	chr3:57380171-57380215	NM_001168281:-360	Wwtr1	PROMOTER	0.509	0.476	1156.64	550.58	0.242	944.24	228.96
A_68_P32340723	chrX:39421307-39421351	NM_009688:316	Xiap	INSIDE	0.231	12.851	1713.78	22024.00	2.971	2236.71	6645.29
A_68_P26875613	chr9:119920587-119920631	NM_011724:12108	Xirp1	DOWNSTREAM	0.349	5.578	2730.75	15230.90	1.949	2439.09	4753.46
A_68_P32573118	chrX:100677473-100677517	NR_001463:1078	Xist	INSIDE	0.483	4.529	1685.95	7635.19	2.189	2219.48	4857.73
A_68_P32573117	chrX:100677336-100677380	NR_001463:1214	Xist	INSIDE	0.394	4.157	2538.89	10555.06	1.638	3070.32	5028.57
A_68_P20002764	chr1:3660776-3660820	NM_001011874:781	Xkr4	INSIDE	0.408	0.511	1071.55	547.94	0.208	886.61	184.80
A_68_P29662310	chr14:64225813-64225857	NM_173393:468	Xkr6	INSIDE	0.615	0.312	3067.00	955.64	0.192	2308.69	442.40
A_68_P21752521	chr2:152857720-152857764	NM_001011732:155	Xkr7	INSIDE	0.304	13.817	3341.98	46175.58	4.194	2910.81	12208.10
A_68_P21752666	chr2:152880620-152880664	NM_001011732:23055	Xkr7	INSIDE	0.472	6.356	1801.47	11449.75	3.003	1605.59	4820.97
A_68_P27644767	chr11:23156427-23156471	NM_001035226:408	Xpo1	INSIDE	0.633	4.712	433.65	2043.36	2.983	346.05	1032.20
A_68_P27644759	chr11:23155418-23155462	NM_001035226:-600	Xpo1	PROMOTER	0.405	4.238	1251.56	5303.65	1.718	1027.19	1764.36
A_68_P25499304	chr7:133344026-133344070	NM_028816:-126	Xpo6	PROMOTER	0.628	37.151	6173.64	229358.10	23.32	6848.80	159715.30
A_68_P25499303	chr7:133343931-133343975	NM_028816:-30	Xpo6	PROMOTER	0.648	3.149	1643.56	5175.00	2.042	1432.71	2925.04
A_68_P29703309	chr14:71165897-71165941	NM_023045:517	Xpo7	INSIDE	0.569	0.396	5015.39	1987.01	0.225	3665.86	826.55
A_68_P27504467	chr10:121043954-121043999	NM_001081056:19396	Xpot	INSIDE	0.313	6.640	1770.72	11756.74	2.078	1292.70	2686.19
A_68_P20737711	chr1:157264084-157264128	NM_011273:468	Xpr1	INSIDE	0.327	6.735	1520.11	10237.20	2.202	1239.20	2729.21
A_68_P20737716	chr1:157264675-157264719	NM_011273:-122	Xpr1	PROMOTER	0.36	5.717	1556.49	8897.86	2.059	1326.53	2731.43
A_68_P20737714	chr1:157264468-157264512	NM_011273:84	Xpr1	INSIDE	0.456	9.356	10743.13	100514.30	4.265	8556.76	36492.69

ProbeName	Target position of probe on CpG island microarray	TargetID	GeneSymbol	CpG island Description	Ratio of relative methylation (TiO_2-NP/Vehicle)	Sham group			TiO_2-H group		
						Relative methylation (Cy5/Cy3)	Cy3 signal (Input)	Cy5 signal (MeDIP)	Relative methylation (Cy5/Cy3)	Cy3 signal (Input)	Cy5 signal (MeDIP)
A_68_P20737715	chr1:157264567-157264611	NM_011273:-14	Xpr1	PROMOTER	0.62	2.675	1802.36	4820.78	1.659	1416.57	2349.84
A_68_P25005464	chr7:25332066-25332110	NM_009532:-80	Xrcc1	DIVERGENT_PROMOTER	0.252	13.265	2086.45	27677.00	3.345	1555.16	5201.66
A_68_P28746254	chr12:113052004-113052048	NM_028875:26	Xrcc3	INSIDE	0.593	0.223	4240.97	945.46	0.132	3037.69	401.37
A_68_P27532842	chr10:126338342-126338386	NM_026858:63	Xrcc6bp1	INSIDE	0.624	6.772	3111.90	21073.50	4.227	2593.86	10962.95
A_68_P21721815	chr2:146838797-146838841	NM_011917:23	Xrn2	INSIDE	0.66	2.462	2032.46	5003.02	1.626	1723.13	2801.38
A_68_P26871926	chr9:119266492-119266536	NM_001033209:16	Xylb	INSIDE	0.617	0.605	1842.76	1115.11	0.374	1325.42	495.09
A_68_P28034316	chr11:94538398-94538442	NM_145828:387	Xylt2	INSIDE	0.373	4.621	1984.57	9170.79	1.723	1653.50	2849.64
A_68_P30446389	chr15:93167185-93167229	NM_024189:160	Yaf2	INSIDE	0.614	4.981	787.78	3923.84	3.058	698.95	2137.42
A_68_P30446395	chr15:93167839-93167883	NM_024189:-494	Yaf2	PROMOTER	0.512	0.630	1392.46	877.35	0.322	1049.12	338.34
A_68_P26290455	chr9:8003868-8003912	NM_001171147:706	Yap1	INSIDE	0.119	0.580	4279.99	2480.28	0.069	3014.92	208.43
A_68_P26290461	chr9:8004643-8004687	NM_001171147:-68	Yap1	PROMOTER	0.456	13.274	3997.64	53063.55	6.05	3697.92	22371.76
A_68_P23295442	chr4:128867260-128867304	NM_134151:244	Yars	INSIDE	0.476	0.304	3726.64	1132.74	0.145	2577.90	372.99
A_68_P23295439	chr4:128866891-128866935	NM_134151:-126	Yars	DIVERGENT_PROMOTER	0.482	0.564	1918.86	1082.58	0.272	1499.91	407.94
A_68_P23244790	chr4:118967092-118967136	NM_011732:4	Ybx1	INSIDE	0.351	4.761	3996.26	19028.16	1.672	3153.34	5273.34
A_68_P23244789	chr4:118966777-118966821	NM_011732:320	Ybx1	INSIDE	0.591	8.279	1654.08	13693.95	4.894	1526.68	7471.33
A_68_P23244791	chr4:118967175-118967219	NM_011732:-78	Ybx1	PROMOTER	0.651	0.199	7723.78	1538.23	0.13	4873.79	631.57
A_68_P23244786	chr4:118966226-118966270	NM_011732:870	Ybx1	INSIDE	0.423	0.515	986.12	507.54	0.218	818.69	178.27
A_68_P27898025	chr11:69749877-69749926	NM_016875:501	Ybx2	INSIDE	0.554	0.421	2058.08	867.16	0.234	1532.56	357.94
A_68_P23189866	chr4:106987036-106987080	NM_001205156:91	Yipf1	INSIDE	0.636	0.562	979.75	550.87	0.358	806.61	288.62
A_68_P31644533	chr18:40378844-40378888	NM_023311:187	Yipf5	INSIDE	0.349	0.395	12578.36	4970.76	0.138	7341.75	1012.71
A_68_P31644534	chr18:40378957-40379001	NM_023311:75	Yipf5	INSIDE	0.548	0.439	3724.92	1634.89	0.241	2603.82	626.34
A_68_P31644532	chr18:40378757-40378801	NM_023311:275	Yipf5	INSIDE	0.586	0.525	6449.68	3383.27	0.308	4086.89	1257.06
A_68_P31644535	chr18:40379053-40379097	NM_023311:-21	Yipf5	PROMOTER	0.339	1.485	1966.22	2920.69	0.503	1606.03	807.55
A_68_P27565810	chr11:5855603-5855647	NM_019661:-136	Ykt6	DIVERGENT_PROMOTER	0.327	0.708	1197.77	847.82	0.231	1079.51	249.77
A_68_P20611349	chr1:132613579-132613625	NM_178691:-301	Yod1	DIVERGENT_PROMOTER	0.656	4.171	475.98	1985.07	2.735	389.80	1066.15
A_68_P30571778	chr16:17070255-17070299	NM_023249:-127	Ypel1	PROMOTER	0.371	4.086	3880.56	15855.81	1.518	4572.45	6940.11
A_68_P27992049	chr11:86807064-86807108	NM_001005341:178	Ypel2	INSIDE	0.434	0.500	1002.37	501.62	0.217	888.74	193.19
A_68_P23865420	chr5:87233089-87233133	NM_177680:-404	Ythdc1	PROMOTER	0.435	3.537	1794.78	6348.61	1.538	1448.30	2227.88
A_68_P31669249	chr18:44988742-44988786	NM_001163013:446	Ythdc2	INSIDE	0.546	0.287	1637.61	469.22	0.156	1106.09	172.92
A_68_P23310689	chr4:131767916-131767960	NM_145393:233	Ythdf2	INSIDE	0.269	18.893	7431.48	140401.90	5.079	6305.15	32026.80
A_68_P21962515	chr3:16083047-16083091	NM_001145919:-114	Ythdf3	PROMOTER	0.466	0.468	1251.31	585.31	0.218	967.96	211.04
A_68_P21962516	chr3:16083176-16083220	NM_001145919:16	Ythdf3	INSIDE	0.169	3.169	1760.89	5579.49	0.535	1390.62	744.13
A_68_P21962520	chr3:16083485-16083529	NM_001145919:324	Ythdf3	INSIDE	0.483	0.693	1104.33	765.71	0.335	754.23	252.37
A_68_P21815513	chr2:163821038-163821082	NM_018753:128	Ywhab	INSIDE	0.651	0.367	1585.32	582.32	0.239	1377.55	329.17
A_68_P27927819	chr11:75547027-75547071	NM_009536:660	Ywhae	INSIDE	0.612	0.186	5096.88	948.39	0.114	3829.26	436.15
A_68_P24118624	chr5:136409884-136409928	NM_018871:605	Ywhag	INSIDE	0.551	0.674	3636.60	2450.64	0.371	2721.07	1010.88
A_68_P23586715	chr5:33361518-33361562	NM_011738:76	Ywhah	INSIDE	0.173	10.863	609.77	6624.19	1.878	519.15	974.71
A_68_P30140505	chr15:36723538-36723582	NM_011740:733	Ywhaz	INSIDE	0.328	6.634	2028.35	13456.22	2.173	1919.78	4171.96
A_68_P28729922	chr12:110031214-110031258	NM_009537:-284	Yy1	PROMOTER	0.541	0.499	3334.54	1665.13	0.27	2401.53	648.81
A_68_P28729930	chr12:110032477-110032521	NM_009537:978	Yy1	INSIDE	0.644	2.855	1236.34	3529.54	1.84	1091.73	2008.52
A_68_P31886142	chr18:84261746-84261790	NM_146090:4219	Zadh2	INSIDE	0.573	0.464	979.87	454.54	0.266	758.60	201.67
A_68_P23792610	chr5:72971627-72971672	NM_174877:674	Zar1	INSIDE	0.629	2.495	2106.55	5256.23	1.569	1798.33	2821.68
A_68_P30421089	chr15:88581968-88582012	NM_181412:-150	Zbed4	PROMOTER	0.551	5.606	1024.65	5744.38	3.092	866.09	2677.64
A_68_P28553596	chr12:77471058-77471102	NM_178744:-172	Zbtb1	DIVERGENT_PROMOTER	0.542	0.447	2470.84	1105.66	0.243	1833.29	444.74
A_68_P21936419	chr3:9251754-9251798	NM_177660:1210	Zbtb10	INSIDE	0.412	5.161	5514.32	28459.85	2.127	5033.87	10707.83
A_68_P21936415	chr3:9251283-9251327	NM_177660:738	Zbtb10	INSIDE	0.452	7.899	1047.66	8275.47	3.567	858.80	3063.58

ProbeName	Target position of probe on CpG island microarray	TargetID	GeneSymbol	CpG island Description	Ratio of relative methylation (TiO_2-NP/Vehicle)	Sham group			TiO_2-H group		
						Relative methylation (Cy5/Cy3)	Cy3 signal (Input)	Cy5 signal (MeDIP)	Relative methylation (Cy5/Cy3)	Cy3 signal (Input)	Cy5 signal (MeDIP)
A_68_P21936418	chr3:9251625-9251669	NM_177660:1080	Zbtb10	INSIDE	0.51	0.626	4811.89	3013.40	0.319	3633.06	1159.38
A_68_P26494151	chr9:48644017-48644061	NM_001033324:12	Zbtb16	INSIDE	0.178	10.225	2135.94	21839.73	1.822	1895.43	3454.02
A_68_P26494153	chr9:48644221-48644265	NM_001033324:-192	Zbtb16	PROMOTER	0.33	0.567	4912.39	2785.64	0.187	3560.34	666.55
A_68_P26494143	chr9:48643240-48643284	NM_001033324:788	Zbtb16	INSIDE	0.467	13.161	2092.41	27538.51	6.143	1791.48	11004.45
A_68_P23364088	chr4:141001073-141001117	NM_009541:507	Zbtb17	INSIDE	0.63	0.727	4891.49	3554.15	0.458	3585.11	1640.41
A_68_P31150957	chr17:34053104-34053148	NM_020625:6	Zbtb22	INSIDE	0.639	7.579	10250.51	77688.67	4.842	9088.49	44010.86
A_68_P27089285	chr10:41170167-41170211	NM_153398:-13	Zbtb24	PROMOTER	0.49	0.587	1730.74	1015.21	0.287	1318.49	378.97
A_68_P28553591	chr12:77470382-77470426	NM_001172104:47	Zbtb25	INSIDE	0.642	3.424	1190.91	4077.48	2.199	1068.76	2349.71
A_68_P28553588	chr12:77470008-77470052	NM_001172104:421	Zbtb25	INSIDE	0.534	2.848	1239.06	3528.73	1.521	1159.28	1762.99
A_68_P32326603	chrX:35543022-35543066	NM_001079513:75	Zbtb33	INSIDE	0.251	12.365	664.66	8218.42	3.103	847.57	2630.18
A_68_P32326600	chrX:35542695-35542742	NM_001079513:-251	Zbtb33	PROMOTER	0.466	4.951	338.18	1674.24	2.309	490.07	1131.43
A_68_P20769689	chr1:162964733-162964777	NM_173424:-364	Zbtb37	DIVERGENT_PROMOTER	0.42	0.498	3120.28	1555.22	0.209	2443.89	511.00
A_68_P27896951	chr11:69579684-69579728	NM_029348:293	Zbtb4	INSIDE	0.183	33.874	764.61	25900.01	6.209	804.48	4994.92
A_68_P27896952	chr11:69579759-69579803	NM_029348:367	Zbtb4	INSIDE	0.487	0.348	1387.90	482.58	0.169	1011.70	171.47
A_68_P27896956	chr11:69580125-69580169	NM_029348:733	Zbtb4	INSIDE	0.566	0.530	1328.27	703.39	0.3	1081.72	323.99
A_68_P23337642	chr4:136604574-136604621	NM_198248:13	Zbtb40	INSIDE	0.515	0.530	1200.55	635.99	0.273	860.94	234.82
A_68_P28751782	chr12:113917645-113917689	NM_001100460:616	Zbtb42	INSIDE	0.601	0.562	2356.14	1325.08	0.338	1712.57	578.84
A_68_P28751775	chr12:113916856-113916901	NM_001100460:-172	Zbtb42	DIVERGENT_PROMOTER	0.659	2.423	1124.74	2724.88	1.596	911.01	1453.67
A_68_P28751778	chr12:113917322-113917366	NM_001100460:294	Zbtb42	INSIDE	0.526	3.320	567.05	1882.44	1.745	583.20	1017.64
A_68_P21121774	chr2:33323833-33323877	NM_001025594:198	Zbtb43	INSIDE	0.488	4.099	2033.48	8335.69	2	1737.98	3476.41
A_68_P26398232	chr9:30838076-30838120	NM_001115130:-130	Zbtb44	PROMOTER	0.284	14.281	4552.42	65015.08	4.055	4696.97	19047.91
A_68_P24974109	chr7:13593102-13593146	NM_001024699:2025	Zbtb45	INSIDE	0.13	19.198	1225.05	23519.13	2.496	1242.95	3102.73
A_68_P24974110	chr7:13593210-13593254	NM_001024699:1917	Zbtb45	INSIDE	0.555	2.756	2989.28	8239.68	1.53	2417.82	3699.58
A_68_P21910759	chr2:181195175-181195219	NM_028125:-1065	Zbtb46	PROMOTER	0.258	0.509	1963.59	999.44	0.131	1393.02	182.83
A_68_P22880598	chr4:45025167-45025211	NM_001163283:96	Zbtb5	INSIDE	0.106	22.987	843.74	19394.94	2.426	668.90	1623.00
A_68_P22880600	chr4:45025359-45025403	NM_001163283:-96	Zbtb5	DIVERGENT_PROMOTER	0.437	0.618	755.55	466.83	0.27	650.65	175.57
A_68_P27287713	chr10:80611280-80611324	NM_010731:12287	Zbtb7a	INSIDE	0.158	10.609	4036.57	42825.63	1.674	3077.26	5150.63
A_68_P27287617	chr10:80598897-80598941	NM_010731:-97	Zbtb7a	PROMOTER	0.201	13.495	1633.06	22037.63	2.718	1392.77	3784.91
A_68_P27287631	chr10:80600637-80600681	NM_010731:1643	Zbtb7a	INSIDE	0.268	8.189	2301.25	18845.33	2.191	2011.20	4405.99
A_68_P27287609	chr10:80598116-80598160	NM_010731:-877	Zbtb7a	PROMOTER	0.455	0.334	1684.92	563.50	0.152	1147.05	174.56
A_68_P27287712	chr10:80611089-80611133	NM_010731:12095	Zbtb7a	INSIDE	0.553	6.117	1575.42	9636.18	3.382	1525.95	5160.37
A_68_P27287686	chr10:80607070-80607114	NM_010731:8077	Zbtb7a	INSIDE	0.577	3.747	1828.70	6851.41	2.161	1355.36	2928.99
A_68_P27287625	chr10:80599890-80599934	NM_010731:897	Zbtb7a	INSIDE	0.644	0.482	2797.79	1349.20	0.311	2035.18	631.94
A_68_P27287610	chr10:80598190-80598234	NM_010731:-803	Zbtb7a	PROMOTER	0.605	0.708	2307.02	1632.27	0.428	1556.31	666.41
A_68_P27287714	chr10:80611346-80611390	NM_010731:12353	Zbtb7a	INSIDE	0.189	7.971	1575.86	12561.40	1.509	1135.97	1714.50
A_68_P22319131	chr3:89200123-89200167	NM_009565:-3019	Zbtb7b	PROMOTER	0.115	15.403	3466.80	53397.43	1.764	2884.93	5089.04
A_68_P22319099	chr3:89196394-89196438	NM_009565:709	Zbtb7b	INSIDE	0.53	0.386	1477.56	570.24	0.205	1038.90	212.56
A_68_P22319094	chr3:89195751-89195795	NM_009565:1353	Zbtb7b	INSIDE	0.574	0.541	1519.82	821.53	0.31	1218.24	378.03
A_68_P31839435	chr18:75979484-75979528	NM_145356:-325	Zbtb7c	PROMOTER	0.253	11.475	2547.03	29225.94	2.905	2074.82	6026.34
A_68_P31839436	chr18:75979559-75979603	NM_145356:-251	Zbtb7c	PROMOTER	0.525	6.033	1442.38	8701.42	3.166	1254.22	3970.50
A_68_P31839441	chr18:75980377-75980421	NM_145356:567	Zbtb7c	INSIDE	0.65	3.739	973.02	3637.92	2.43	890.25	2163.59
A_68_P31113562	chr17:27109927-27109971	NM_001005916:-175	Zbtb9	PROMOTER	0.567	0.473	1367.80	646.85	0.268	1192.89	319.67
A_68_P31113563	chr17:27110040-27110084	NM_001005916:-61	Zbtb9	PROMOTER	0.5	0.584	874.77	511.28	0.292	747.05	218.30
A_68_P31113564	chr17:27110129-27110173	NM_001005916:27	Zbtb9	INSIDE	0.193	1.671	560.70	936.97	0.322	518.55	167.00
A_68_P27541965	chr10:127984764-127984808	NM_134003:14	Zc3h10	INSIDE	0.616	2.392	2345.64	5610.15	1.473	1932.29	2846.34
A_68_P23272336	chr4:124804630-124804674	NM_153159:473	Zc3h12a	INSIDE	0.232	34.611	6707.85	232165.00	8.02	7862.82	63061.51

ProbeName	Target position of probe on CpG island microarray	TargetID	GeneSymbol	CpG island Description	Ratio of relative methylation (TiO_2-NP/Vehicle)	Sham group			TiO_2-H group		
						Relative methylation (Cy5/Cy3)	Cy3 signal (Input)	Cy5 signal (MeDIP)	Relative methylation (Cy5/Cy3)	Cy3 signal (Input)	Cy5 signal (MeDIP)
A_68_P23272342	chr4:124805193-124805237	NM_153159:-89	Zc3h12a	PROMOTER	0.289	8.678	1384.34	12013.33	2.508	1239.79	3109.66
A_68_P23272339	chr4:124804897-124804941	NM_153159:207	Zc3h12a	INSIDE	0.423	0.539	872.52	470.23	0.228	775.04	176.57
A_68_P29726978	chr14:75684076-75684120	NM_026083:-81	Zc3h13	PROMOTER	0.602	15.853	2013.70	31922.26	9.544	1669.86	15937.61
A_68_P24981260	chr7:16985638-16985682	NM_198631:-884	Zc3h4	PROMOTER	0.353	0.631	1990.76	1257.12	0.223	1441.66	321.72
A_68_P21622283	chr2:128792891-128792935	NM_178404:-225	Zc3h6	PROMOTER	0.488	14.353	14051.55	201684.20	7.01	12751.01	89382.71
A_68_P30379485	chr15:81602412-81602456	NM_001081016:27157	Zc3h7b	INSIDE	0.319	5.933	1015.88	6027.55	1.893	891.79	1688.26
A_68_P21622216	chr2:128769433-128769477	NM_020594:302	Zc3h8	INSIDE	0.656	0.655	3993.28	2617.40	0.43	3006.44	1292.54
A_68_P27804178	chr11:53138130-53138174	NM_026479:-38	Zcchc10	PROMOTER	0.319	8.298	2590.69	21498.50	2.645	2359.62	6240.66
A_68_P23196094	chr4:108132526-108132570	NM_175472:518	Zcchc11	INSIDE	0.284	29.990	3955.07	118611.60	8.523	3948.37	33650.84
A_68_P23196091	chr4:108132080-108132124	NM_175472:72	Zcchc11	INSIDE	0.558	4.541	2052.51	9319.48	2.535	1672.66	4239.36
A_68_P23196100	chr4:108133295-108133339	NM_175472:1286	Zcchc11	INSIDE	0.277	0.703	1601.03	1125.21	0.195	1096.65	213.54
A_68_P26228075	chr8:124175832-124175876	NM_080855:-21	Zcchc14	PROMOTER	0.57	0.350	2196.33	767.63	0.199	1564.54	311.75
A_68_P20505897	chr1:107890521-107890565	NM_001122675:3560	Zcchc2	INSIDE	0.185	13.593	1999.28	27176.55	2.52	1914.60	4824.80
A_68_P20505871	chr1:107887105-107887149	NM_001122675:144	Zcchc2	INSIDE	0.54	3.842	1354.17	5202.58	2.073	1176.78	2439.83
A_68_P21749221	chr2:152240295-152240339	NM_175126:464	Zcchc3	INSIDE	0.411	6.635	2925.17	19409.69	2.726	2490.88	6789.81
A_68_P23696773	chr5:53174399-53174443	NM_030185:115	Zcchc4	INSIDE	0.405	6.044	2787.50	16848.57	2.445	2432.11	5947.18
A_68_P24127578	chr5:138229494-138229544	NM_001005426:489	Zcwpw1	INSIDE	0.387	8.595	583.15	5012.21	3.324	424.96	1412.51
A_68_P24127577	chr5:138229403-138229447	NM_001005426:395	Zcwpw1	INSIDE	0.216	6.717	5078.36	34112.24	1.452	3349.00	4863.84
A_68_P21101318	chr2:29949237-29949281	NM_001037762:-103	Zdhhc12	PROMOTER	0.553	0.654	1891.41	1236.08	0.361	1566.60	566.11
A_68_P25108145	chr7:56044314-56044358	NM_028031:-36	Zdhhc13	PROMOTER	0.299	0.267	3148.45	842.16	0.08	2462.41	197.00
A_68_P32578104	chrX:101866109-101866153	NM_175358:273	Zdhhc15	INSIDE	0.254	10.527	3324.52	34996.18	2.673	3587.79	9590.16
A_68_P32578106	chrX:101866324-101866368	NM_175358:57	Zdhhc15	INSIDE	0.279	6.169	594.51	3667.22	1.723	890.33	1533.99
A_68_P27446660	chr10:110446716-110446760	NM_172554:384	Zdhhc17	INSIDE	0.53	2.671	3922.03	10474.56	1.417	3309.91	4688.79
A_68_P25805590	chr8:41509132-41509176	NM_178395:-14	Zdhhc2	PROMOTER	0.4	9.273	741.01	6871.19	3.713	714.20	2652.00
A_68_P25805592	chr8:41509363-41509407	NM_178395:216	Zdhhc2	INSIDE	0.517	5.422	2176.18	11799.49	2.802	1992.11	5581.72
A_68_P23059231	chr4:82505707-82505751	NM_026647:-163	Zdhhc21	PROMOTER	0.409	4.798	3418.07	16399.04	1.96	2776.86	5443.05
A_68_P28613411	chr12:88331320-88331364	NM_001080943:-1716	Zdhhc22	PROMOTER	0.387	0.521	1196.06	622.75	0.201	865.74	174.23
A_68_P28613409	chr12:88331117-88331161	NM_001080943:-1512	Zdhhc22	PROMOTER	0.507	5.875	1167.70	6860.22	2.98	1093.20	3257.86
A_68_P28613410	chr12:88331231-88331275	NM_001080943:-1626	Zdhhc22	PROMOTER	0.586	2.529	2638.70	6673.82	1.482	1974.45	2925.24
A_68_P31925604	chr19:4878831-4878875	NM_001168517:163	Zdhhc24	INSIDE	0.662	2.627	2902.92	7625.30	1.738	2404.89	4179.64
A_68_P26893203	chr9:123022219-123022263	NM_026917:83	Zdhhc3	INSIDE	0.649	2.624	635.40	1667.25	1.704	724.27	1233.87
A_68_P21394209	chr2:84555435-84555479	NM_144887:-135	Zdhhc5	PROMOTER	0.198	36.607	6143.31	224885.20	7.234	5942.24	42988.50
A_68_P21394210	chr2:84555540-84555584	NM_144887:-241	Zdhhc5	PROMOTER	0.535	3.046	1478.37	4502.79	1.629	1220.80	1989.16
A_68_P32193377	chr19:55390489-55390533	NM_001033573:12	Zdhhc6	INSIDE	0.495	5.463	1186.76	6482.89	2.701	1135.90	3068.54
A_68_P26217897	chr8:122625576-122625620	NM_133967:-226	Zdhhc7	PROMOTER	0.375	6.017	1493.99	8989.72	2.254	1154.27	2601.25
A_68_P26217895	chr8:122625358-122625402	NM_133967:-8	Zdhhc7	PROMOTER	0.417	9.669	1131.89	10944.45	4.035	1115.42	4500.70
A_68_P26217898	chr8:122625658-122625702	NM_133967:-308	Zdhhc7	PROMOTER	0.643	0.451	1487.19	671.46	0.29	1257.00	364.67
A_68_P30578495	chr16:18235059-18235103	NM_172151:149	Zdhhc8	INSIDE	0.287	10.125	1979.11	20038.45	2.906	1497.71	4351.94
A_68_P30578494	chr16:18234940-18234984	NM_172151:267	Zdhhc8	INSIDE	0.335	7.121	5706.27	40635.87	2.385	4566.68	10891.98
A_68_P30578497	chr16:18235357-18235401	NM_172151:-149	Zdhhc8	PROMOTER	0.495	5.079	2752.05	13977.44	2.514	2130.17	5354.30
A_68_P31469349	chr18:5591373-5591417	NM_011546:-494	Zeb1	PROMOTER	0.372	5.586	1452.00	8110.23	2.075	1176.56	2441.46
A_68_P31469353	chr18:5591872-5591916	NM_011546:6	Zeb1	INSIDE	0.479	0.246	2214.41	544.36	0.118	1636.77	192.66
A_68_P31469354	chr18:5592137-5592181	NM_011546:270	Zeb1	INSIDE	0.656	4.018	1995.10	8015.57	2.638	1563.96	4125.00
A_68_P20352373	chr1:75165429-75165473	NM_026846:142	Zfand2b	INSIDE	0.191	0.496	2379.96	1179.30	0.094	1766.67	166.78
A_68_P30302244	chr15:68090487-68090531	NM_001145888:-90	Zfat	PROMOTER	0.449	0.589	1376.47	810.77	0.265	1159.55	307.01
A_68_P27469308	chr10:114822753-114822797	NM_001033261:760	Zfc3h1	INSIDE	0.569	0.629	1729.95	1088.92	0.358	1430.19	512.00

ProbeName	Target position of probe on CpG island microarray	TargetID	GeneSymbol	CpG island Description	Ratio of relative methylation (TiO_2-NP/Vehicle)	Sham group			TiO_2-H group		
						Relative methylation (Cy5/Cy3)	Cy3 signal (Input)	Cy5 signal (MeDIP)	Relative methylation (Cy5/Cy3)	Cy3 signal (Input)	Cy5 signal (MeDIP)
A_68_P26152542	chr8:111238708-111238758	NM_007496:189	Zfhx3	INSIDE	0.043	2.820	2476.04	6982.12	0.121	1424.62	172.03
A_68_P26152544	chr8:111239109-111239153	NM_007496:587	Zfhx3	INSIDE	0.42	0.204	6229.38	1270.80	0.086	4264.77	365.10
A_68_P26154344	chr8:111480628-111480672	NM_007496:242107	Zfhx3	INSIDE	0.527	3.299	8812.01	29071.96	1.739	6218.15	10815.05
A_68_P26154270	chr8:111471142-111471186	NM_007496:232621	Zfhx3	INSIDE	0.652	4.715	2049.93	9666.05	3.072	1646.02	5056.85
A_68_P26152541	chr8:111238563-111238607	NM_007496:41	Zfhx3	INSIDE	0.491	3.243	1176.68	3816.01	1.592	1005.73	1601.52
A_68_P21920096	chr3:5219661-5219705	NM_030708:1129	Zfhx4	INSIDE	0.259	10.866	1606.65	17457.84	2.815	1497.25	4214.16
A_68_P24596103	chr6:83864434-83864478	NM_001166371:110	Zfml	INSIDE	0.409	0.248	4689.15	1164.94	0.102	3016.55	306.87
A_68_P24596102	chr6:83864366-83864410	NM_001166371:42	Zfml	INSIDE	0.5	0.186	2774.78	516.86	0.093	1922.33	179.10
A_68_P26169432	chr8:114167808-114167852	NM_001037665:488	Zfp1	INSIDE	0.232	17.625	1990.90	35089.03	4.095	2504.02	10253.26
A_68_P26892104	chr9:122832365-122832409	NM_009544:191	Zfp105	INSIDE	0.534	5.938	2277.12	13522.67	3.173	1819.73	5773.70
A_68_P21575995	chr2:120389596-120389640	NM_011743:-51	Zfp106	DIVERGENT_PROMOTER	0.34	4.186	5139.42	21512.98	1.423	4204.87	5983.79
A_68_P24973210	chr7:13420359-13420403	NM_022981:222	Zfp110	INSIDE	0.469	0.590	830.71	490.08	0.277	653.34	180.68
A_68_P24129053	chr5:138596838-138596882	NM_019747:112	Zfp113	INSIDE	0.363	0.606	1372.63	831.23	0.22	1096.32	240.83
A_68_P31092441	chr17:23713283-23713327	NM_011747:23150	Zfp13	INSIDE	0.658	3.039	1709.23	5194.00	2.001	1216.93	2434.64
A_68_P31092442	chr17:23713394-23713438	NM_011747:23038	Zfp13	INSIDE	0.617	2.353	1954.88	4599.91	1.452	1465.36	2127.48
A_68_P25029683	chr7:30836003-30836047	NM_011748:391	Zfp14	INSIDE	0.602	0.694	939.24	651.99	0.418	727.87	304.31
A_68_P20349427	chr1:74634546-74634590	NM_029888:34	Zfp142	INSIDE	0.661	2.463	2313.89	5699.77	1.628	1818.34	2959.53
A_68_P25411545	chr7:117205191-117205235	NM_009281:-3	Zfp143	PROMOTER	0.278	7.577	10205.16	77320.15	2.105	8148.83	17150.06
A_68_P31084703	chr17:21146020-21146064	NM_145483:138	Zfp160	INSIDE	0.474	0.487	910.39	443.60	0.231	744.18	171.89
A_68_P31323668	chr17:69733260-69733304	NM_009547:-35	Zfp161	PROMOTER	0.458	10.618	2408.01	25567.43	4.858	2356.18	11446.16
A_68_P26891892	chr9:122795954-122795998	NM_001177505:-1612	Zfp167	PROMOTER	0.637	3.025	1965.24	5944.57	1.927	1655.55	3190.15
A_68_P31558278	chr18:24178812-24178856	NM_021559:438	Zfp191	INSIDE	0.427	1.454	1657.93	2410.20	0.62	1458.87	905.15
A_68_P31092272	chr17:23683887-23683931	NM_001033496:17285	Zfp213	DOWNSTREAM	0.252	0.332	3226.36	1070.58	0.083	2251.20	187.97
A_68_P21851419	chr2:169956951-169956995	NM_001033299:-252	Zfp217	PROMOTER	0.554	4.193	1168.35	4898.36	2.322	959.49	2228.30
A_68_P21851410	chr2:169955855-169955909	NM_001033299:838	Zfp217	INSIDE	0.605	3.585	907.44	3252.83	2.17	820.90	1781.26
A_68_P21851412	chr2:169956064-169956108	NM_001033299:634	Zfp217	INSIDE	0.59	3.613	461.24	1666.47	2.133	403.67	861.01
A_68_P20859688	chr1:179372159-179372203	NM_001012330:-2611	Zfp238	PROMOTER	0.412	0.724	1653.30	1196.43	0.298	1368.48	408.08
A_68_P24786877	chr6:118405159-118405203	NM_028335:344	Zfp248	INSIDE	0.399	4.345	3063.08	13309.66	1.734	2549.33	4421.26
A_68_P24786878	chr6:118405236-118405280	NM_028335:266	Zfp248	INSIDE	0.517	0.454	1123.84	510.77	0.235	855.95	201.03
A_68_P30350974	chr15:76701808-76701852	NM_001007568:35	Zfp251	INSIDE	0.628	3.521	1479.04	5208.16	2.212	1315.66	2910.36
A_68_P26342514	chr9:20264309-20264353	NM_011753:274	Zfp26	INSIDE	0.225	23.286	10102.27	235239.80	5.24	10171.73	53297.95
A_68_P26342513	chr9:20264224-20264268	NM_011753:358	Zfp26	INSIDE	0.41	0.293	1721.25	503.79	0.12	1484.07	178.25
A_68_P26342786	chr9:20326146-20326190	NM_001082485:-305	Zfp266	PROMOTER	0.406	0.130	5943.44	774.21	0.053	4233.63	224.02
A_68_P26238816	chr8:125778247-125778291	NM_020497:174	Zfp276	INSIDE	0.347	24.510	3325.16	81498.42	8.513	3380.16	28776.64
A_68_P24955199	chr7:6335923-6335967	NM_175247:-83	Zfp28	PROMOTER	0.298	0.374	1853.70	693.84	0.112	1538.27	171.77
A_68_P24955200	chr7:6336041-6336085	NM_175247:35	Zfp28	INSIDE	0.403	0.489	3061.49	1497.87	0.197	2158.04	425.05
A_68_P32365466	chrX:45947510-45947554	NM_153532:-2222	Zfp280c	PROMOTER	0.214	22.188	746.05	16553.50	4.748	1313.40	6235.82
A_68_P24424708	chr6:47827424-47827468	NM_146175:-107	Zfp282	PROMOTER	0.624	3.092	728.07	2251.11	1.928	566.69	1092.83
A_68_P22833088	chr4:34830058-34830102	NM_013889:117	Zfp292	INSIDE	0.453	4.929	2006.08	9888.46	2.233	1729.84	3862.09
A_68_P22833091	chr4:34830379-34830423	NM_013889:-203	Zfp292	PROMOTER	0.626	3.311	2242.04	7423.12	2.071	1823.68	3777.74
A_68_P31002108	chr16:98183316-98183360	NM_001081684:448	Zfp295	INSIDE	0.528	5.885	2276.96	13399.36	3.106	1843.59	5726.69
A_68_P31002112	chr16:98183669-98183713	NM_001081684:96	Zfp295	INSIDE	0.555	0.668	1184.31	790.72	0.37	1008.64	373.66
A_68_P27902559	chr11:70577608-70577652	NM_177565:-318	Zfp3	PROMOTER	0.405	0.574	912.87	524.39	0.233	739.99	172.09
A_68_P24155791	chr5:144031328-144031372	NM_017467:351	Zfp316	INSIDE	0.401	0.160	5020.24	804.44	0.064	3347.88	215.14
A_68_P24155694	chr5:144016617-144016661	NM_017467:15063	Zfp316	INSIDE	0.419	10.127	3357.67	34003.19	4.246	2909.93	12355.27
A_68_P24155790	chr5:144031255-144031299	NM_017467:425	Zfp316	INSIDE	0.468	0.473	1283.13	607.32	0.222	903.32	200.17

ProbeName	Target position of probe on CpG island microarray	TargetID	GeneSymbol	CpG island Description	Ratio of relative methylation (TiO_2-NP/Vehicle)	Sham group			TiO_2-H group		
						Relative methylation (Cy5/Cy3)	Cy3 signal (Input)	Cy5 signal (MeDIP)	Relative methylation (Cy5/Cy3)	Cy3 signal (Input)	Cy5 signal (MeDIP)
A_68_P24155687	chr5:144015862-144015906	NM_017467:15817	Zfp316	INSIDE	0.557	5.059	1230.10	6223.05	2.819	1094.11	3084.66
A_68_P24155695	chr5:144016767-144016811	NM_017467:14913	Zfp316	INSIDE	0.593	2.872	2786.85	8004.61	1.703	2116.04	3603.23
A_68_P31206539	chr17:46520782-46520826	NM_207671:90	Zfp318	INSIDE	0.563	2.981	824.26	2457.04	1.68	757.13	1271.60
A_68_P28881068	chr13:23461198-23461242	NM_001111107:-149	Zfp322a	PROMOTER	0.606	0.342	1472.86	504.41	0.208	1200.74	249.33
A_68_P24973879	chr7:13551564-13551609	NM_178732:374	Zfp324	INSIDE	0.233	51.187	6451.35	330223.90	11.917	8469.74	100930.50
A_68_P23957854	chr5:106305322-106305366	NM_018759:-242	Zfp326	PROMOTER	0.508	0.329	1355.51	446.33	0.167	1002.95	167.77
A_68_P23957855	chr5:106305400-106305444	NM_018759:-164	Zfp326	PROMOTER	0.241	2.194	1976.07	4336.38	0.528	1546.24	816.09
A_68_P21823566	chr2:165213753-165213797	NM_178411:-15	Zfp334	PROMOTER	0.369	0.554	1037.17	574.86	0.204	847.21	173.11
A_68_P21823503	chr2:165205763-165205807	NM_178411:7975	Zfp334	INSIDE	0.592	2.744	1443.76	3961.27	1.624	1110.56	1803.03
A_68_P29053295	chr13:55206803-55206847	NM_012017:155	Zfp346	INSIDE	0.359	0.341	4353.31	1482.67	0.122	3196.00	391.16
A_68_P27792049	chr11:50872760-50872804	NM_009329:24	Zfp354a	INSIDE	0.603	0.505	1455.68	734.53	0.304	1235.42	375.86
A_68_P27792052	chr11:50873003-50873047	NM_009329:266	Zfp354a	INSIDE	0.654	6.552	3393.45	22233.00	4.287	3095.95	13273.79
A_68_P25614225	chr8:3495864-3495908	NM_080461:2749	Zfp358	INSIDE	0.394	7.981	807.54	6444.89	3.141	623.66	1959.12
A_68_P25021510	chr7:29163296-29163340	NM_011756:929	Zfp36	INSIDE	0.258	9.160	2654.22	24313.81	2.366	1973.49	4669.69
A_68_P29097416	chr13:64254085-64254129	NM_175494:401	Zfp367	INSIDE	0.147	14.226	1047.87	14906.98	2.085	926.66	1932.38
A_68_P29097413	chr13:64253752-64253796	NM_175494:733	Zfp367	INSIDE	0.504	0.332	1927.61	640.04	0.167	1473.87	246.79
A_68_P31403204	chr17:84587274-84587318	NM_001001806:-9	Zfp36l2	PROMOTER	0.368	0.177	3754.94	663.21	0.065	2792.85	181.64
A_68_P31403206	chr17:84587417-84587461	NM_001001806:-151	Zfp36l2	PROMOTER	0.598	0.437	1279.86	559.92	0.262	1052.89	275.42
A_68_P25030071	chr7:30928622-30928666	NM_001081007:21678	Zfp382	DOWNSTREAM	0.1	2.642	913.05	2412.12	0.263	712.88	187.61
A_68_P24818153	chr6:124958879-124958923	NM_175557:-922	Zfp384	PROMOTER	0.507	0.622	1307.59	813.15	0.315	1131.03	356.58
A_68_P30503506	chr15:103153692-103153736	NM_013866:16803	Zfp385a	INSIDE	0.285	1.757	9211.34	16180.72	0.5	6774.33	3386.98
A_68_P30503505	chr15:103153548-103153598	NM_013866:16945	Zfp385a	INSIDE	0.542	0.494	2473.16	1222.65	0.268	1797.61	481.91
A_68_P30503432	chr15:103144711-103144755	NM_013866:25785	Zfp385a	INSIDE	0.63	2.485	1189.61	2956.33	1.565	794.17	1242.82
A_68_P21359595	chr2:77654503-77654547	NM_178723:349	Zfp385b	INSIDE	0.256	16.111	894.90	14417.27	4.121	843.44	3475.70
A_68_P28069222	chr11:100501295-100501339	NM_177790:10691	Zfp385c	INSIDE	0.499	0.613	902.69	553.45	0.306	721.11	220.49
A_68_P27833411	chr11:58717772-58717816	NM_011758:-67	Zfp39	PROMOTER	0.147	26.738	5800.81	155102.90	3.932	6440.76	25327.23
A_68_P27833410	chr11:58717675-58717719	NM_011758:31	Zfp39	INSIDE	0.63	0.228	4167.97	950.05	0.144	2823.16	405.16
A_68_P29672987	chr14:65977066-65977114	NM_199029:-422	Zfp395	PROMOTER	0.237	19.521	425.31	8302.50	4.621	447.34	2067.38
A_68_P29672990	chr14:65977418-65977462	NM_199029:-72	Zfp395	PROMOTER	0.629	0.393	1440.14	566.19	0.247	1207.82	298.85
A_68_P29672993	chr14:65977836-65977880	NM_199029:346	Zfp395	INSIDE	0.604	0.620	1479.81	917.71	0.375	1212.45	454.15
A_68_P31889610	chr18:84759292-84759336	NM_001033341:-418	Zfp407	PROMOTER	0.326	5.399	1198.36	6470.22	1.762	1006.08	1772.24
A_68_P30342960	chr15:75447074-75447118	NM_001044718:-18	Zfp41	PROMOTER	0.623	0.576	6231.42	3590.13	0.359	4329.92	1554.37
A_68_P28597562	chr12:85658591-85658635	NM_144833:533	Zfp410	INSIDE	0.392	0.541	1061.60	574.43	0.212	852.74	180.74
A_68_P26036153	chr8:90184081-90184125	NM_033327:299392	Zfp423	DOWNSTREAM	0.189	10.543	2363.56	24919.65	1.991	2027.33	4036.72
A_68_P26038373	chr8:90485269-90485313	NM_033327:-1796	Zfp423	PROMOTER	0.192	26.790	680.95	18242.61	5.148	845.76	4353.84
A_68_P26036154	chr8:90184192-90184236	NM_033327:299280	Zfp423	DOWNSTREAM	0.399	0.314	7403.14	2327.67	0.125	5032.66	631.18
A_68_P26038364	chr8:90484174-90484218	NM_033327:-702	Zfp423	PROMOTER	0.415	6.225	1394.24	8679.11	2.586	1231.56	3185.05
A_68_P26038355	chr8:90482696-90482740	NM_033327:776	Zfp423	INSIDE	0.492	3.899	1369.58	5340.31	1.919	1248.10	2395.09
A_68_P26038239	chr8:90467780-90467824	NM_033327:15692	Zfp423	INSIDE	0.613	0.564	1560.94	880.73	0.346	1289.81	446.06
A_68_P26038357	chr8:90483082-90483126	NM_033327:390	Zfp423	INSIDE	0.629	3.851	780.29	3005.11	2.423	678.18	1643.01
A_68_P26038371	chr8:90485107-90485151	NM_033327:-1634	Zfp423	PROMOTER	0.663	0.387	1552.82	600.70	0.256	1189.31	304.89
A_68_P26038384	chr8:90486654-90486698	NM_033327:-3182	Zfp423	PROMOTER	0.489	0.501	929.85	466.02	0.245	690.86	169.19
A_68_P31468071	chr18:5334781-5334825	NM_178722:-365	Zfp438	PROMOTER	0.252	0.483	6785.18	3277.13	0.122	4478.59	545.33
A_68_P31468065	chr18:5334203-5334247	NM_178722:213	Zfp438	INSIDE	0.552	5.535	1423.08	7876.34	3.056	1181.30	3609.60
A_68_P31468070	chr18:5334710-5334760	NM_178722:-297	Zfp438	PROMOTER	0.467	0.567	907.27	514.26	0.265	661.86	175.13
A_68_P22931104	chr4:54958261-54958305	NM_172867:-2534	Zfp462	PROMOTER	0.41	5.504	2244.29	12353.32	2.259	1821.80	4114.54

ProbeName	Target position of probe on CpG island microarray	TargetID	GeneSymbol	CpG island Description	Ratio of relative methylation (TiO_2-NP/Vehicle)	Sham group			TiO_2-H group		
						Relative methylation (Cy5/Cy3)	Cy3 signal (Input)	Cy5 signal (MeDIP)	Relative methylation (Cy5/Cy3)	Cy3 signal (Input)	Cy5 signal (MeDIP)
A_68_P24427169	chr6:48388853-48388897	NM_001085415:6215	Zfp467	INSIDE	0.375	0.416	2030.80	844.69	0.156	1345.85	209.91
A_68_P24427166	chr6:48388473-48388517	NM_001085415:6595	Zfp467	INSIDE	0.441	3.344	3082.85	10308.21	1.476	2411.10	3559.22
A_68_P31147863	chr17:33103125-33103169	NM_153063:371	Zfp472	INSIDE	0.662	0.324	1577.42	511.24	0.215	1272.08	273.09
A_68_P29455874	chr14:22804989-22805033	NM_145459:3813	Zfp503	INSIDE	0.007	756.184	700.71	529864.90	5.651	579.29	3273.36
A_68_P29455867	chr14:22804205-22804249	NM_145459:4597	Zfp503	INSIDE	0.345	5.991	7375.08	44187.60	2.066	5464.57	11290.66
A_68_P29455876	chr14:22805255-22805299	NM_145459:3547	Zfp503	INSIDE	0.38	14.565	5489.67	79957.10	5.528	4677.07	25856.19
A_68_P29455869	chr14:22804369-22804413	NM_145459:4433	Zfp503	INSIDE	0.621	0.570	1621.00	923.93	0.354	1140.35	403.81
A_68_P29455871	chr14:22804612-22804656	NM_145459:4189	Zfp503	INSIDE	0.661	5.874	3121.55	18335.59	3.884	2448.48	9510.18
A_68_P25051309	chr7:36588072-36588116	NM_177739:-86	Zfp507	PROMOTER	0.664	4.466	538.18	2403.49	2.963	438.71	1300.11
A_68_P23577056	chr5:31504361-31504406	NM_001177901:32	Zfp513	INSIDE	0.37	0.545	6730.20	3670.60	0.202	4327.21	872.94
A_68_P23577042	chr5:31502796-31502840	NM_001177901:1597	Zfp513	INSIDE	0.493	5.420	4681.35	25373.72	2.672	3795.62	10141.27
A_68_P31879479	chr18:83126499-83126543	NM_001177464:42497	Zfp516	INSIDE	0.507	17.603	2919.73	51394.69	8.931	2477.46	22125.38
A_68_P31879115	chr18:83079129-83079173	NM_183033:-1120	Zfp516	PROMOTER	0.616	0.309	1518.87	468.91	0.19	1099.75	209.03
A_68_P31879119	chr18:83079657-83079701	NM_183033:-592	Zfp516	PROMOTER	0.476	0.615	1550.50	953.35	0.293	1137.94	333.22
A_68_P32111692	chr19:40969658-40969702	NM_028319:486	Zfp518a	INSIDE	0.53	3.484	6126.17	21345.01	1.846	4860.83	8971.56
A_68_P23622265	chr5:39075785-39075829	NM_001081144:259	Zfp518b	INSIDE	0.494	3.856	1102.86	4252.39	1.904	978.40	1863.31
A_68_P23622266	chr5:39075940-39075984	NM_001081144:103	Zfp518b	INSIDE	0.548	0.548	1168.43	640.76	0.3	779.05	234.10
A_68_P31086417	chr17:21669593-21669637	NM_144515:-2888	Zfp52	PROMOTER	0.27	9.839	2084.85	20513.02	2.654	1832.60	4864.57
A_68_P31120939	chr17:28314640-28314684	NM_172617:300	Zfp523	INSIDE	0.439	4.229	5514.91	23321.52	1.858	4160.94	7732.45
A_68_P31120935	chr17:28314314-28314358	NM_172617:-26	Zfp523	PROMOTER	0.521	0.547	1353.59	740.26	0.285	1047.92	298.69
A_68_P31120933	chr17:28314032-28314076	NM_172617:-308	Zfp523	PROMOTER	0.613	3.594	684.82	2461.06	2.204	637.36	1405.01
A_68_P24951576	chr7:4967342-4967386	NM_025324:255	Zfp524	INSIDE	0.202	14.183	2053.75	29128.82	2.862	1800.97	5153.48
A_68_P25009105	chr7:26010680-26010724	NM_175436:4233	Zfp526	INSIDE	0.27	6.914	2580.38	17841.68	1.867	2047.57	3822.63
A_68_P25009109	chr7:26011119-26011163	NM_175436:4671	Zfp526	INSIDE	0.16	10.814	1521.76	16456.45	1.735	1161.35	2014.58
A_68_P31086236	chr17:21626256-21626300	NM_013843:327	Zfp53	INSIDE	0.531	2.898	1895.58	5493.17	1.538	1545.00	2375.49
A_68_P31782005	chr18:65740650-65740694	NM_207255:789	Zfp532	INSIDE	0.387	12.322	3872.62	47719.12	4.773	3404.33	16247.81
A_68_P25063502	chr7:38555339-38555383	NM_172385:-589	Zfp536	PROMOTER	0.391	0.456	9916.38	4523.94	0.178	7162.99	1277.13
A_68_P24979385	chr7:16647414-16647458	NM_001099277:-9854	Zfp541	PROMOTER	0.371	9.507	2746.06	26108.04	3.531	2272.32	8023.73
A_68_P25504683	chr7:134376847-134376891	NM_146201:-88	Zfp553	DIVERGENT_PROMOTER	0.331	15.655	568.24	8895.95	5.177	480.02	2485.11
A_68_P25008269	chr7:25866719-25866763	NM_175477:4476	Zfp574	INSIDE	0.151	23.491	3696.85	86841.91	3.549	3400.58	12068.27
A_68_P25008265	chr7:25866336-25866380	NM_175477:4094	Zfp574	INSIDE	0.472	5.670	2917.37	16542.04	2.674	2116.14	5658.82
A_68_P25008235	chr7:25862727-25862771	NM_175477:484	Zfp574	INSIDE	0.307	1.453	2476.28	3598.12	0.445	1547.92	689.55
A_68_P25008229	chr7:25861867-25861911	NM_001168506:-335	Zfp574	DIVERGENT_PROMOTER	0.383	4.326	1113.67	4817.49	1.656	1001.54	1658.21
A_68_P25008231	chr7:25862140-25862187	NM_001168506:-60	Zfp574	DIVERGENT_PROMOTER	0.475	0.583	820.50	478.30	0.277	645.82	178.79
A_68_P25005689	chr7:25378891-25378935	NM_001033205:-6252	Zfp575	PROMOTER	0.165	33.757	2252.05	76022.70	5.586	2117.67	11829.71
A_68_P24951459	chr7:4945480-4945524	NM_026741:2201	Zfp579	INSIDE	0.494	4.487	1280.83	5747.66	2.216	1002.19	2221.17
A_68_P24951458	chr7:4945401-4945445	NM_026741:2281	Zfp579	INSIDE	0.194	8.422	3274.55	27578.06	1.638	2537.02	4155.10
A_68_P24951457	chr7:4945305-4945349	NM_026741:2377	Zfp579	INSIDE	0.549	2.953	1990.81	5878.89	1.62	1477.55	2393.36
A_68_P24951471	chr7:4947350-4947394	NM_026741:331	Zfp579	INSIDE	0.454	1.444	4635.29	6695.36	0.656	3415.49	2240.23
A_68_P31098880	chr17:24806951-24806995	NM_183149:276	Zfp598	INSIDE	0.338	0.640	871.57	557.66	0.216	800.54	173.03
A_68_P26351028	chr9:22064295-22064339	NM_181419:23	Zfp599	INSIDE	0.424	0.319	1657.29	529.49	0.135	1289.40	174.46
A_68_P31722245	chr18:55147700-55147744	NM_175751:2112	Zfp608	INSIDE	0.395	0.450	1514.12	680.91	0.177	1091.69	193.72
A_68_P26160581	chr8:112603963-112604007	NM_175480:351	Zfp612	INSIDE	0.613	0.549	2764.34	1517.47	0.337	1938.77	652.79
A_68_P25959077	chr8:74446861-74446905	NM_133358:159	Zfp617	INSIDE	0.312	16.672	2407.48	40137.12	5.207	1971.16	10262.98
A_68_P27781838	chr11:49016880-49016924	NM_001024846:-99	Zfp62	PROMOTER	0.547	0.238	5367.39	1275.26	0.13	4132.54	537.45
A_68_P27781839	chr11:49017033-49017077	NM_001024846:53	Zfp62	INSIDE	0.461	3.659	2540.33	9295.37	1.688	2373.33	4005.83

ProbeName	Target position of probe on CpG island microarray	TargetID	GeneSymbol	CpG island Description	Ratio of relative methylation (TiO_2-NP/Vehicle)	Sham group			TiO_2-H group		
						Relative methylation (Cy5/Cy3)	Cy3 signal (Input)	Cy5 signal (MeDIP)	Relative methylation (Cy5/Cy3)	Cy3 signal (Input)	Cy5 signal (MeDIP)
A_68_P30084544	chr15:25914715-25914759	NM_144523:616	Zfp622	INSIDE	0.378	4.754	2650.50	12600.73	1.795	2255.05	4047.97
A_68_P24951043	chr7:4866972-4867016	NM_170759:176	Zfp628	INSIDE	0.271	5.831	864.02	5038.02	1.579	884.70	1397.27
A_68_P25506497	chr7:134754654-134754698	NM_177226:3271	Zfp629	INSIDE	0.446	3.959	1145.38	4534.04	1.766	849.77	1500.93
A_68_P22038453	chr3:32409465-32409509	NM_144519:-27	Zfp639	PROMOTER	0.493	0.521	2415.56	1258.84	0.257	2009.45	516.73
A_68_P21844345	chr2:168780474-168780518	NM_009564:591	Zfp64	INSIDE	0.6	3.543	1202.65	4261.04	2.125	932.86	1982.27
A_68_P21844347	chr2:168780711-168780761	NM_009564:351	Zfp64	INSIDE	0.467	4.783	666.43	3187.51	2.234	533.72	1192.21
A_68_P30474027	chr15:98126645-98126689	NM_173769:-152	Zfp641	PROMOTER	0.425	3.319	2048.04	6798.11	1.41	1810.43	2552.14
A_68_P23962878	chr5:107125443-107125487	NM_026856:385	Zfp644	INSIDE	0.268	16.071	4782.83	76863.17	4.314	4987.03	21513.49
A_68_P20730829	chr1:156051855-156051899	NM_001204908:3560	Zfp648	INSIDE	0.131	4.095	5231.91	21426.70	0.538	3768.72	2029.03
A_68_P20730836	chr1:156052430-156052474	NM_001204908:4136	Zfp648	INSIDE	0.375	0.428	1494.58	639.68	0.16	1078.37	172.94
A_68_P20730831	chr1:156052030-156052074	NM_001204908:3736	Zfp648	INSIDE	0.498	0.584	1900.23	1110.07	0.291	1416.98	411.97
A_68_P20730830	chr1:156051942-156051986	NM_001204908:3648	Zfp648	INSIDE	0.339	0.745	4743.29	3536.02	0.253	3081.93	779.94
A_68_P26350251	chr9:21860554-21860598	NM_177318:15213	Zfp653	INSIDE	0.584	3.853	1380.52	5319.27	2.251	1101.07	2478.54
A_68_P26350325	chr9:21875273-21875317	NM_177318:495	Zfp653	INSIDE	0.401	1.427	4977.41	7101.47	0.571	3658.47	2090.79
A_68_P24063275	chr5:125343770-125343814	NM_001081750:718	Zfp664	INSIDE	0.639	4.844	2298.29	11133.67	3.096	1910.48	5914.66
A_68_P24954797	chr7:6237978-6238022	NM_001024928:-181	Zfp667	PROMOTER	0.52	0.268	3000.13	803.94	0.139	2178.68	303.45
A_68_P25507825	chr7:135020108-135020152	NM_146259:207	Zfp668	INSIDE	0.592	6.285	1404.48	8826.69	3.721	1267.05	4714.84
A_68_P27830380	chr11:58136423-58136467	NM_178761:404	Zfp672	INSIDE	0.41	9.885	4353.21	43032.60	4.05	3488.11	14125.80
A_68_P24130913	chr5:139060841-139060885	NM_001044747:109	Zfp68	INSIDE	0.468	0.484	2209.14	1069.77	0.226	1753.11	396.97
A_68_P22341853	chr3:94819143-94819187	NM_030074:-4	Zfp687	PROMOTER	0.299	16.095	846.55	13624.91	4.816	806.50	3884.07
A_68_P25505557	chr7:134565221-134565265	NM_026999:306	Zfp688	INSIDE	0.308	0.488	2151.03	1049.15	0.15	1438.30	216.07
A_68_P23254147	chr4:120624114-120624158	NM_001005788:170	Zfp69	INSIDE	0.607	2.552	2306.99	5887.24	1.549	1743.43	2700.56
A_68_P23244004	chr4:118846518-118846562	NM_001145935:-79	Zfp691	PROMOTER	0.451	0.635	1607.08	1020.69	0.286	1227.37	351.53
A_68_P22360096	chr3:98232313-98232357	NM_172863:45931	Zfp697	INSIDE	0.423	0.571	940.70	536.71	0.241	707.16	170.51
A_68_P25735809	chr8:28089115-28089159	NM_001101502:1329	Zfp703	INSIDE	0.427	4.645	14604.29	67830.69	1.986	14441.44	28673.71
A_68_P25735800	chr8:28087824-28087868	NM_001101502:39	Zfp703	INSIDE	0.47	0.417	2304.99	960.84	0.196	1541.77	302.16
A_68_P25735818	chr8:28090001-28090046	NM_001101502:2216	Zfp703	INSIDE	0.285	0.712	1575.55	1121.15	0.203	1321.40	267.63
A_68_P25735811	chr8:28089322-28089366	NM_001101502:1537	Zfp703	INSIDE	0.635	3.286	723.68	2378.21	2.087	729.40	1522.42
A_68_P25735806	chr8:28088778-28088822	NM_001101502:993	Zfp703	INSIDE	0.361	1.520	1842.05	2799.86	0.549	1493.82	820.12
A_68_P30141812	chr15:36936605-36936649	NM_026521:531	Zfp706	INSIDE	0.479	5.799	250.12	1450.57	2.78	206.54	574.12
A_68_P25264700	chr7:87226640-87226684	NM_175433:55549	Zfp710	INSIDE	0.187	12.741	1708.87	21772.02	2.383	1022.46	2436.62
A_68_P25264340	chr7:87172009-87172053	NM_175433:917	Zfp710	INSIDE	0.575	3.941	1746.40	6883.01	2.267	1315.18	2981.11
A_68_P25264699	chr7:87226574-87226619	NM_175433:55483	Zfp710	INSIDE	0.233	8.937	895.67	8004.38	2.079	610.44	1269.19
A_68_P32603687	chrX:109715096-109715140	NM_177747:984	Zfp711	INSIDE	0.245	15.671	1477.62	23155.34	3.837	1608.66	6173.19
A_68_P29107216	chr13:67161978-67162022	NM_001166218:106	Zfp712	INSIDE	0.459	0.318	1639.99	521.51	0.146	1317.04	192.18
A_68_P25505251	chr7:134487065-134487109	NM_146202:1742	Zfp768	INSIDE	0.31	5.999	1993.65	11960.13	1.86	1651.80	3071.80
A_68_P25505266	chr7:134488622-134488666	NM_146202:184	Zfp768	INSIDE	0.649	0.217	3739.21	810.92	0.141	2431.81	342.08
A_68_P25505268	chr7:134488780-134488824	NM_146202:26	Zfp768	INSIDE	0.28	0.734	5120.10	3759.66	0.206	3552.74	730.37
A_68_P21538596	chr2:114027103-114027147	NM_175466:44	Zfp770	INSIDE	0.572	0.363	1424.85	516.94	0.208	1260.36	261.62
A_68_P25504787	chr7:134397287-134397332	NM_177362:9270	Zfp771	INSIDE	0.589	0.391	1123.45	438.99	0.23	899.72	207.16
A_68_P27291280	chr10:81233564-81233608	NM_199062:301	Zfp781	INSIDE	0.583	0.227	3839.17	869.87	0.132	3090.06	407.98
A_68_P24424497	chr6:47780471-47780515	NM_177882:12	Zfp786	INSIDE	0.459	3.629	1942.11	7047.29	1.666	1618.94	2696.50
A_68_P24954289	chr7:6106868-6106913	NM_001013012:683	Zfp787	INSIDE	0.215	16.756	1597.64	26770.05	3.611	1417.97	5120.07
A_68_P24954137	chr7:6083887-6083931	NM_001013012:23665	Zfp787	INSIDE	0.229	12.361	4331.65	53543.80	2.827	3297.93	9323.84
A_68_P24954294	chr7:6107601-6107645	NM_001013012:-49	Zfp787	PROMOTER	0.338	12.439	5459.95	67917.29	4.21	4556.41	19183.30
A_68_P24954292	chr7:6107153-6107197	NM_001013012:399	Zfp787	INSIDE	0.418	4.422	1488.33	6582.05	1.848	1265.44	2338.38

ProbeName	Target position of probe on CpG island microarray	TargetID	GeneSymbol	CpG island Description	Ratio of relative methylation (TiO_2-NP/Vehicle)	Sham group			TiO_2-H group		
						Relative methylation (Cy5/Cy3)	Cy3 signal (Input)	Cy5 signal (MeDIP)	Relative methylation (Cy5/Cy3)	Cy3 signal (Input)	Cy5 signal (MeDIP)
A_68_P24954135	chr7:6083578-6083622	NM_001013012:23973	Zfp787	INSIDE	0.465	3.287	1690.65	5556.83	1.527	1115.37	1703.42
A_68_P31147212	chr17:32966976-32967020	NM_177359:195	Zfp799	INSIDE	0.562	10.188	2381.93	24266.48	5.729	2106.33	12066.59
A_68_P31147211	chr17:32966831-32966875	NM_177359:341	Zfp799	INSIDE	0.592	3.135	1182.37	3706.89	1.856	1138.48	2113.11
A_68_P24319362	chr6:28210996-28211040	NM_001081678:583	Zfp800	INSIDE	0.535	2.687	1900.06	5105.06	1.438	1540.35	2214.29
A_68_P21382117	chr2:81893227-81893271	NM_175513:-566	Zfp804a	PROMOTER	0.296	0.159	5001.19	793.66	0.047	3575.95	167.76
A_68_P31148785	chr17:33495528-33495572	NM_207541:273	Zfp81	INSIDE	0.374	5.558	3644.49	20255.24	2.078	2861.71	5946.11
A_68_P25029775	chr7:30857776-30857820	NM_177889:44	Zfp82	INSIDE	0.355	5.909	1356.87	8018.10	2.099	1143.65	2400.16
A_68_P25992691	chr8:81636666-81636710	NM_178267:84353	Zfp827	INSIDE	0.266	20.442	4682.79	95726.33	5.437	3326.37	18085.89
A_68_P25992690	chr8:81636588-81636632	NM_178267:84275	Zfp827	INSIDE	0.296	57.403	15357.31	881555.80	16.995	11258.76	191345.50
A_68_P25992689	chr8:81636479-81636523	NM_178267:84165	Zfp827	INSIDE	0.327	28.202	8524.10	240396.70	9.227	7163.49	66099.21
A_68_P25992132	chr8:81553538-81553582	NM_178267:1225	Zfp827	INSIDE	0.304	2.217	9188.56	20368.76	0.673	6680.46	4497.67
A_68_P25670894	chr8:13870055-13870099	NM_181854:436	Zfp828	INSIDE	0.609	2.481	1254.15	3111.15	1.511	1020.05	1541.57
A_68_P28740684	chr12:112088879-112088923	NM_001199785:412	Zfp839	INSIDE	0.481	3.293	2951.81	9719.22	1.585	2449.04	3880.62
A_68_P24951681	chr7:4982839-4982883	NM_001033383:10883	Zfp865	INSIDE	0.415	6.419	1765.97	11335.35	2.666	1359.54	3625.01
A_68_P24951673	chr7:4981991-4982035	NM_001033383:10035	Zfp865	INSIDE	0.598	7.280	2511.67	18285.45	4.356	2053.22	8943.64
A_68_P24951675	chr7:4982142-4982186	NM_001033383:10187	Zfp865	INSIDE	0.477	3.119	2198.84	6857.53	1.489	1634.18	2432.58
A_68_P25947574	chr8:72149082-72149126	NM_001045553:-30	Zfp868	PROMOTER	0.542	0.538	1822.77	980.12	0.292	1675.63	488.70
A_68_P25947784	chr8:72240592-72240636	NM_181274:-229	Zfp869	PROMOTER	0.51	3.244	2504.49	8124.55	1.656	2136.81	3538.04
A_68_P31147009	chr17:32924787-32924831	NM_172458:424	Zfp871	INSIDE	0.338	12.789	5589.51	71483.83	4.319	5520.83	23842.80
A_68_P26350760	chr9:21992704-21992748	NM_001033813:117	Zfp872	INSIDE	0.538	2.863	5663.59	16212.23	1.541	5243.17	8077.34
A_68_P25959007	chr8:74432656-74432700	NM_001166645:174	Zfp882	INSIDE	0.416	0.527	926.01	488.35	0.22	831.67	182.67
A_68_P31087861	chr17:22058135-22058179	NM_001199048:41275	Zfp942	DOWNSTREAM	0.102	32.051	8198.71	262777.90	3.256	6842.64	22280.27
A_68_P31090923	chr17:23013560-23013604	NM_001110254:-9481	Zfp945	PROMOTER	0.067	44.514	1304.97	58089.41	2.971	1206.20	3583.43
A_68_P31090924	chr17:23013653-23013697	NM_001110254:-9573	Zfp945	PROMOTER	0.478	6.586	2152.40	14175.36	3.149	1957.09	6161.95
A_68_P25947839	chr8:72273658-72273702	NM_001200023:181	Zfp963	INSIDE	0.576	0.308	2533.06	780.27	0.178	2056.74	365.12
A_68_P25947613	chr8:72178336-72178380	NM_001177527:-96	Zfp964	PROMOTER	0.264	18.228	2085.40	38013.32	4.821	2199.77	10604.31
A_68_P31932619	chr19:6084791-6084835	NM_024231:79	Zfpl1	INSIDE	0.346	0.591	3651.86	2156.43	0.204	2635.83	538.86
A_68_P30160398	chr15:40486792-40486836	NM_011766:227	Zfpm2	INSIDE	0.506	7.198	7105.08	51140.23	3.645	5615.96	20472.51
A_68_P30019884	chr15:12048005-12048049	NM_011767:421	Zfr	INSIDE	0.619	0.514	1950.82	1003.57	0.318	1385.51	441.13
A_68_P30019883	chr15:12047938-12047982	NM_011767:355	Zfr	INSIDE	0.46	1.544	5344.90	8252.53	0.711	4369.14	3104.86
A_68_P27288263	chr10:80702990-80703034	NM_001034895:7105	Zfr2	INSIDE	0.538	5.713	600.43	3430.12	3.074	495.93	1524.53
A_68_P27288225	chr10:80695805-80695849	NM_001034895:-81	Zfr2	DIVERGENT_PROMOTER	0.644	0.425	5747.10	2441.83	0.274	4280.50	1171.40
A_68_P32537532	chrX:91368506-91368550	NM_001044386:218	Zfx	INSIDE	0.175	21.432	2404.92	51542.39	3.757	2478.79	9313.05
A_68_P32537531	chrX:91368428-91368472	NM_001044386:296	Zfx	INSIDE	0.41	10.475	759.67	7957.33	4.29	1001.92	4298.47
A_68_P32537534	chrX:91368825-91368869	NM_001044386:-100	Zfx	PROMOTER	0.583	4.503	1053.41	4743.59	2.625	1277.08	3352.04
A_68_P28593634	chr12:84937743-84937787	NM_183154:333	Zfyve1	INSIDE	0.431	1.374	3829.52	5260.96	0.592	2794.19	1655.50
A_68_P29224475	chr13:93300495-93300539	NM_173392:249	Zfyve16	INSIDE	0.614	0.657	2361.86	1551.97	0.403	2126.17	857.67
A_68_P24643833	chr6:92164652-92164696	NM_030081:131	Zfyve20	INSIDE	0.313	0.668	4844.02	3235.50	0.209	3519.97	735.16
A_68_P28746261	chr12:113052815-113052859	NM_026752:456	Zfyve21	INSIDE	0.273	9.437	1503.11	14185.03	2.577	1294.91	3337.63
A_68_P26345262	chr9:20871580-20871624	NM_001103168:-65	Zglp1	PROMOTER	0.345	5.722	1207.85	6911.08	1.971	902.97	1779.98
A_68_P21910181	chr2:181099844-181099888	NM_144894:231	Zgpat	INSIDE	0.524	0.659	1336.39	880.37	0.345	1143.15	394.38
A_68_P30245925	chr15:57908333-57908377	NM_001042438:-310	Zhx1	PROMOTER	0.401	0.430	4868.91	2091.80	0.172	3668.62	631.32
A_68_P30245918	chr15:57907631-57907675	NM_001042438:392	Zhx1	INSIDE	0.401	5.692	1199.71	6828.30	2.282	990.02	2259.41
A_68_P30245926	chr15:57908463-57908507	NM_001042438:-440	Zhx1	PROMOTER	0.363	0.677	1352.46	915.48	0.246	996.72	245.05
A_68_P30245927	chr15:57908540-57908584	NM_001042438:-518	Zhx1	PROMOTER	0.394	0.562	953.43	535.40	0.221	791.69	175.00
A_68_P30245923	chr15:57908171-57908215	NM_001042438:-148	Zhx1	PROMOTER	0.565	0.546	886.61	484.14	0.309	802.31	247.56

ProbeName	Target position of probe on CpG island microarray	TargetID	GeneSymbol	CpG island Description	Ratio of relative methylation (TiO_2-NP/Vehicle)	Sham group			TiO_2-H group		
						Relative methylation (Cy5/Cy3)	Cy3 signal (Input)	Cy5 signal (MeDIP)	Relative methylation (Cy5/Cy3)	Cy3 signal (Input)	Cy5 signal (MeDIP)
A_68_P30243549	chr15:57526400-57526444	NM_199449:201	Zhx2	INSIDE	0.562	7.089	1327.69	9412.04	3.983	1293.75	5153.26
A_68_P29961928	chr14:122873448-122873492	NM_009574:-1135	Zic2	DIVERGENT_PROMOTER	0.604	0.228	2511.84	571.86	0.138	2225.37	306.16
A_68_P32403976	chrX:55291949-55291993	NM_009575:8166	Zic3	DOWNSTREAM	0.563	3.678	1336.91	4917.67	2.071	1651.21	3420.18
A_68_P26721663	chr9:91278946-91278990	NM_009576:15159	Zic4	INSIDE	0.296	10.992	800.65	8800.42	3.25	660.80	2147.93
A_68_P24966280	chr7:11080607-11080656	NM_009577:99	Zik1	INSIDE	0.321	0.295	2470.30	728.06	0.095	1811.96	171.52
A_68_P24956426	chr7:6649059-6649103	NM_011769:63	Zim1	INSIDE	0.59	0.627	1814.77	1137.60	0.37	1559.31	576.39
A_68_P22953710	chr4:58956479-58956523	NM_001099323:1	Zkscan16	INSIDE	0.409	0.465	1117.49	520.17	0.19	934.25	177.80
A_68_P22953712	chr4:58956665-58956709	NM_001099323:187	Zkscan16	INSIDE	0.555	4.085	1525.46	6230.94	2.268	1295.60	2938.35
A_68_P27837321	chr11:59319755-59319799	NM_001130529:366	Zkscan17	INSIDE	0.541	0.425	1461.94	621.91	0.23	1028.74	236.60
A_68_P28872660	chr13:21494324-21494368	NM_001145778:278	Zkscan3	INSIDE	0.352	0.590	1925.17	1136.24	0.208	1451.63	301.52
A_68_P32674751	chrX:131543547-131543591	NM_175446:180	Zmat1	INSIDE	0.524	40.111	3521.20	141237.30	21.019	3919.63	82387.01
A_68_P32674749	chrX:131543372-131543416	NM_175446:354	Zmat1	INSIDE	0.667	4.035	435.75	1758.39	2.69	632.93	1702.58
A_68_P29478601	chr14:26427680-26427724	NM_183208:149032	Zmiz1	INSIDE	0.322	9.180	2487.28	22832.59	2.957	2013.49	5953.76
A_68_P32564024	chrX:98615209-98615253	NM_001177985:23	Zmym3	INSIDE	0.476	5.288	491.12	2597.15	2.518	658.60	1658.25
A_68_P32564025	chrX:98615354-98615398	NM_001177985:-123	Zmym3	PROMOTER	0.421	3.518	958.20	3371.01	1.483	1176.17	1743.94
A_68_P23282960	chr4:126645057-126645101	NM_001114399:89	Zmym4	INSIDE	0.467	3.769	2182.81	8227.52	1.76	2099.61	3695.66
A_68_P23282958	chr4:126644783-126644827	NM_001114399:363	Zmym4	INSIDE	0.572	8.598	4294.76	36927.70	4.917	3776.01	18565.30
A_68_P28818477	chr13:9763394-9763438	NM_001199141:1144	Zmynd11	INSIDE	0.304	6.989	2045.03	14293.39	2.123	1775.82	3769.94
A_68_P21826274	chr2:165671183-165671231	NM_027230:38982	Zmynd8	INSIDE	0.562	2.986	1104.63	3298.67	1.679	685.34	1150.61
A_68_P21911521	chr2:181327466-181327510	NM_001164597:-322	Znf512b	PROMOTER	0.34	10.186	1400.03	14261.33	3.46	1311.09	4535.87
A_68_P21911518	chr2:181326975-181327019	NM_001164597:170	Znf512b	INSIDE	0.631	10.607	1022.62	10846.77	6.689	992.12	6635.95
A_68_P31932458	chr19:6061153-6061197	NM_013859:-32	Znhit2-ps	PROMOTER	0.353	0.341	1875.32	640.29	0.121	1461.78	176.36
A_68_P27980352	chr11:84729732-84729776	NM_001005223:104	Znhit3	INSIDE	0.588	0.659	3790.63	2497.74	0.387	2877.36	1113.95
A_68_P27563479	chr11:5344842-5344886	NM_001080924:-14	Znrf3	PROMOTER	0.146	11.768	3101.91	36504.15	1.713	2643.88	4530.14
A_68_P27563481	chr11:5345058-5345102	NM_001080924:-230	Znrf3	PROMOTER	0.506	0.238	4336.98	1033.23	0.12	3024.56	364.35
A_68_P31256671	chr17:56651263-56651307	NM_011483:622	Znrf4	INSIDE	0.516	5.782	1417.70	8196.64	2.985	1129.25	3370.97
A_68_P28057142	chr11:98412381-98412425	NM_001166494:-8	Zpbp2	DIVERGENT_PROMOTER	0.378	18.905	3602.38	68103.84	7.148	3296.85	23566.15
A_68_P28057144	chr11:98412585-98412629	NM_001166494:196	Zpbp2	INSIDE	0.644	0.183	2926.84	535.78	0.118	2291.00	270.08
A_68_P28872465	chr13:21454901-21454949	NM_016684:236	Zscan12	INSIDE	0.539	7.835	235.43	1844.67	4.22	240.82	1016.21
A_68_P25269100	chr7:88020745-88020789	NM_009553:13773	Zscan2	INSIDE	0.461	5.377	1034.82	5563.89	2.479	794.04	1968.41
A_68_P23231372	chr4:116550094-116550138	NM_001029912:110	Zswim5	INSIDE	0.607	6.159	2895.89	17836.71	3.736	2342.00	8750.24
A_68_P29308210	chr13:108681440-108681484	NM_145456:-1204	Zswim6	PROMOTER	0.169	17.018	1274.94	21697.33	2.882	1146.83	3304.62
A_68_P29308200	chr13:108680217-108680261	NM_145456:20	Zswim6	INSIDE	0.589	6.191	1222.47	7568.90	3.647	1154.99	4212.69
A_68_P29308203	chr13:108680619-108680663	NM_145456:-382	Zswim6	PROMOTER	0.624	0.576	11774.47	6782.52	0.359	7516.87	2701.47
A_68_P27054330	chr10:33670738-33670782	NM_028287:258	Zufsp	INSIDE	0.3	0.540	1362.95	735.67	0.162	1060.42	171.70
A_68_P26495379	chr9:48863756-48863800	NM_012039:93	Zw10	INSIDE	0.637	0.485	1510.54	732.02	0.309	1208.78	373.30
A_68_P24631969	chr6:90320325-90320370	NM_030260:860	Zxdc	INSIDE	0.517	0.430	1236.83	531.50	0.222	969.17	215.44
A_68_P24631961	chr6:90319416-90319460	NM_030260:-49	Zxdc	PROMOTER	0.282	5.527	1505.56	8321.12	1.56	1240.83	1935.08
A_68_P23195047	chr4:107889859-107889903	NM_001167936:647	Zyg11a	INSIDE	0.176	23.274	13940.83	324454.60	4.088	13191.37	53932.38
A_68_P23195054	chr4:107890703-107890747	NM_001167936:-197	Zyg11a	PROMOTER	0.359	1.705	8237.25	14046.49	0.612	5763.79	3529.03
A_68_P24397781	chr6:42299527-42299571	NM_011777:-278	Zyx	PROMOTER	0.497	0.550	1090.25	599.96	0.274	836.90	228.96
