# Supplementary material for: Effects of Prenatal Exposure to Titanium Dioxide Nanoparticles on DNA Methylation and Gene Expression Profile in the Mouse Brain
Source: Front Toxicol. 2021 Oct 8;3:705910. doi: 10.3389/ftox.2021.705910 (PMC8915839; doi:10.3389/ftox.2021.705910)
Supplement: Supplementary file 2 [file Table4.pdf]

Supplementary Table 4. The genes of which DNA methylation were increased in the brain of male offspring prenatally exposed to TiO_2-NP.

ProbeName	Target position of probe on CpG island microarray	TargetID	GeneSymbol	CpG island Description	Ratio of relative methylation (TiO_2-NP/Vehicle)	Sham group			TiO_2-H group		
						Relative methylation (Cy5/Cy3)	Cy3 signal (Input)	Cy5 signal (MeDIP)	Relative methylation (Cy5/Cy3)	Cy3 signal (Input)	Cy5 signal (MeDIP)
A_68_P28202484	chr12:4824078-4824122	NM_025323:-313	0610009D07Rik	PROMOTER	1.732	0.265	2234.03	592.81	0.459	1788.98	821.98
A_68_P21641569	chr2:132512720-132512764	NM_028637:-3276	1110034G24Rik	PROMOTER	1.548	0.350	1952.87	683.47	0.542	1693.38	917.50
A_68_P30382917	chr15:82176766-82176812	NM_026914:313	1500032L24Rik	INSIDE	2.098	2.644	631.38	1669.31	5.547	551.68	3060.30
A_68_P32683271	chrX:133462182-133462226	NM_027067:341	1700014N06Rik	INSIDE	3.021	2.158	821.50	1772.67	6.518	1230.09	8018.31
A_68_P28607942	chr12:87423668-87423712	NM_001199843:180	1700019E19Rik	INSIDE	1.577	0.227	2092.79	474.48	0.358	1592.66	569.46
A_68_P25933298	chr8:69010075-69010123	NM_025465:308	1810029B16Rik	INSIDE	4.022	1.912	403.87	772.20	7.690	290.64	2235.19
A_68_P30568280	chr16:16146642-16146686	NM_146068:262	2310008H04Rik	INSIDE	1.544	2.496	1629.22	4067.01	3.854	1384.77	5337.16
A_68_P30778641	chr16:55974831-55974875	NR_027965:-122	2310061J03Rik	PROMOTER	1.820	2.660	3051.78	8118.32	4.842	2453.14	11878.15
A_68_P27288895	chr10:80788735-80788779	NM_027381:4909	2510012J08Rik	INSIDE	1.682	1.954	425.68	831.64	3.286	321.71	1057.14
A_68_P32070028	chr19:32463753-32463797	NR_033198:69	2700046G09Rik	INSIDE	2.032	0.300	1668.43	500.16	0.609	1234.31	751.89
A_68_P32226223	chr19:60656220-60656264	NM_001172096:684	2700078E11Rik	INSIDE	1.533	0.220	2860.62	630.53	0.338	2091.20	706.46
A_68_P28182974	chr11:120050877-120050921	NR_030682:272	2810410L24Rik	INSIDE	1.885	5.036	1021.71	5145.36	9.492	783.76	7439.62
A_68_P23996382	chr5:113567569-113567615	NM_172884:24741	2900026A02Rik	INSIDE	1.838	0.310	2228.96	691.26	0.570	1857.69	1058.77
A_68_P26215962	chr8:122292157-122292201	NM_028883:10138	4632415K11Rik	INSIDE	1.518	1.472	696.02	1024.85	2.236	559.07	1249.89
A_68_P21823609	chr2:165221660-165221704	NM_029021:4212	4833422F24Rik	INSIDE	1.748	2.230	594.07	1324.93	3.899	539.25	2102.65
A_68_P30560720	chr16:14159038-14159083	NM_001081154:307	4921513D23Rik	INSIDE	1.582	0.244	2275.29	554.47	0.386	1666.31	642.52
A_68_P25730016	chr8:27096591-27096635	NM_029037:7981	4930444A02Rik	INSIDE	1.685	1.666	858.07	1429.54	2.808	702.41	1972.07
A_68_P22129414	chr3:51200267-51200311	NM_026358:181	4930583H14Rik	INSIDE	2.500	5.279	1233.49	6511.29	13.198	872.47	11514.77
A_68_P27473560	chr10:115551150-115551194	NM_001161855:-199	4933416C03Rik	PROMOTER	2.153	1.966	708.88	1393.92	4.233	540.85	2289.42
A_68_P26065128	chr8:94880726-94880770	NR_033641:-729	4933436C20Rik	DIVERGENT_PROMOTER	1.553	0.351	2445.88	857.95	0.545	1753.69	955.28
A_68_P21754953	chr2:153269988-153270032	NM_001001986:205	8430427H17Rik	INSIDE	1.627	4.589	2156.90	9898.03	7.466	1736.38	12964.18
A_68_P21782541	chr2:158187217-158187261	NR_015463:7	9430008C03Rik	INSIDE	1.706	0.236	2003.52	473.16	0.403	1240.63	499.96
A_68_P21774735	chr2:156846821-156846865	NM_001164663:58159	9830001H06Rik	INSIDE	1.642	1.603	495.12	793.91	2.633	417.25	1098.49
A_68_P21774854	chr2:156865842-156865886	NM_001164663:39137	9830001H06Rik	INSIDE	1.533	1.558	521.91	812.91	2.388	432.79	1033.39
A_68_P26344263	chr9:20672999-20673043	NM_175687:-65	A230050P20Rik	PROMOTER	1.636	7.477	836.66	6255.73	12.233	690.61	8448.31
A_68_P32775151	chrX:155970887-155970931	NM_001033472:309	A830080D01Rik	INSIDE	1.536	0.258	1739.12	449.18	0.397	2453.34	973.31
A_68_P24513205	chr6:65622381-65622425	NM_172399:798	A930038C07Rik	INSIDE	1.550	0.118	4061.98	481.18	0.184	2778.12	510.25
A_68_P28123544	chr11:110198866-110198910	NM_147219:142	Abca5	INSIDE	2.491	3.885	461.84	1794.05	9.675	441.97	4276.21
A_68_P32577124	chrX:101609142-101609186	NM_009592:21	Abcb7	INSIDE	2.883	0.193	1097.20	212.25	0.558	1604.97	895.04
A_68_P21741660	chr2:150730004-150730048	NM_024465:441	Abhd12	INSIDE	1.535	0.142	3194.54	454.68	0.218	2540.11	554.83
A_68_P31491179	chr18:10706877-10706921	NM_134130:-204	Abhd3	PROMOTER	1.623	0.207	2949.55	611.31	0.336	2040.67	686.49
A_68_P26870880	chr9:119071809-119071853	NM_146230:-5619	Acaa1b	PROMOTER	1.752	2.371	214.86	509.47	4.155	234.49	974.38
A_68_P27958481	chr11:80966909-80966953	NM_007384:-525	Accn1	PROMOTER	1.504	0.414	1496.46	619.82	0.623	1191.67	742.41
A_68_P23417006	chr4:151552589-151552633	NM_001146057:-7632	Acot7	PROMOTER	1.656	0.299	1702.59	509.26	0.495	1356.58	671.96
A_68_P27765617	chr11:45869706-45869750	NM_009616:240	Adam19	INSIDE	1.677	0.172	2823.73	486.49	0.289	1961.13	566.57
A_68_P26397640	chr9:30729401-30729445	NM_001024139:615	Adamts15	INSIDE	2.160	1.424	1059.29	1508.30	3.075	891.62	2741.86
A_68_P25276874	chr7:89484048-89484092	NM_001190374:-133	Adamtsl3	PROMOTER	1.574	18.961	862.19	16348.22	29.848	879.16	26241.57
A_68_P30284264	chr15:64753856-64753901	NM_009623:-20	Adcy8	PROMOTER	1.610	0.349	3013.77	1051.94	0.562	2141.26	1203.36
A_68_P31922425	chr19:4305540-4305585	NM_130863:393	Adrbk1	INSIDE	1.562	0.418	2712.76	1132.82	0.652	2083.86	1359.66
A_68_P23424871	chr4:152806869-152806913	NM_001099299:50049	Ajap1	INSIDE	1.931	1.860	657.91	1224.04	3.592	471.45	1693.38
A_68_P32717148	chrX:140752921-140752965	NM_026247:434	Alg13	INSIDE	5.797	0.491	4385.26	2155.07	2.849	5164.89	14713.45
A_68_P32710891	chrX:139401598-139401642	NM_019496:-349	Ammecr1	PROMOTER	3.177	3.070	901.91	2768.52	9.753	1089.13	10622.67
A_68_P32710890	chrX:139401516-139401560	NM_019496:-267	Ammecr1	PROMOTER	2.217	2.823	286.77	809.65	6.259	396.02	2478.75

ProbeName	Target position of probe on CpG island microarray	TargetID	GeneSymbol	CpG island Description	Ratio of relative methylation (TiO_2-NP/Vehicle)	Sham group			TiO_2-H group		
						Relative methylation (Cy5/Cy3)	Cy3 signal (Input)	Cy5 signal (MeDIP)	Relative methylation (Cy5/Cy3)	Cy3 signal (Input)	Cy5 signal (MeDIP)
A_68_P31599408	chr18:31920327-31920372	NM_153515:816	Ammecr1l	INSIDE	3.933	0.506	964.60	487.95	1.990	689.52	1371.92
A_68_P32321494	chrX:34588841-34588885	NM_173779:23	Ankrd58	INSIDE	3.596	0.508	536.14	272.46	1.827	800.77	1463.16
A_68_P28741418	chr12:112215432-112215476	NM_175207:1777	Ankrd9	INSIDE	1.565	2.047	1357.37	2778.67	3.204	1039.98	3331.74
A_68_P28741417	chr12:112215277-112215321	NM_175207:1933	Ankrd9	INSIDE	1.813	2.012	421.16	847.49	3.649	328.77	1199.66
A_68_P30512950	chr16:4964504-4964548	NM_028301:-196	Anks3	PROMOTER	1.541	0.136	3798.00	515.96	0.209	2488.68	521.14
A_68_P30459963	chr15:95621546-95621590	NM_175344:295	Ano6	INSIDE	1.584	0.231	2539.28	587.51	0.367	1826.40	669.48
A_68_P32538585	chrX:91612495-91612539	NM_001199337:24	Apoo	INSIDE	3.027	0.188	1992.20	374.32	0.569	2559.37	1455.83
A_68_P27835628	chr11:59041341-59041385	NM_001130408:302	Arfl	INSIDE	2.261	2.342	666.77	1561.72	5.295	505.35	2675.83
A_68_P32369309	chrX:46847140-46847184	NM_001081123:23536	Arhgap36	INSIDE	5.435	0.128	2155.34	275.35	0.694	2870.86	1993.46
A_68_P25677778	chr8:14962588-14962634	NM_001037736:50894	Arhgef10	INSIDE	1.558	1.769	895.76	1584.48	2.755	660.31	1819.28
A_68_P23358589	chr4:140071100-140071144	NM_001112723:133549	Arhgef10l	INSIDE	1.776	1.709	474.60	811.00	3.035	406.30	1233.20
A_68_P25614032	chr8:3451969-3452013	NM_133962:58983	Arhgef18	INSIDE	1.553	2.272	821.70	1866.53	3.528	687.55	2425.37
A_68_P22314993	chr3:88447939-88447984	NM_001198912:22646	Arhgef2	INSIDE	1.785	1.673	740.23	1238.66	2.987	650.76	1944.13
A_68_P30462697	chr15:96118805-96118849	NM_175251:874	Arid2	INSIDE	2.730	0.661	898.10	593.90	1.805	600.20	1083.54
A_68_P32678078	chrX:132277552-132277596	NM_001009575:344	Armcx5	INSIDE	3.479	0.513	1635.75	839.37	1.785	2346.21	4187.96
A_68_P20789366	chr1:166388627-166388671	NM_009721:-162	Atp1b1	PROMOTER	1.503	0.401	2682.17	1076.58	0.603	2176.29	1312.79
A_68_P24763306	chr6:113991628-113991672	NM_009723:424	Atp2b2	INSIDE	2.132	0.074	8276.90	609.06	0.157	4814.09	755.17
A_68_P32262594	chrX:12164926-12164970	NM_027439:64	Atp6ap2	INSIDE	4.317	0.101	1878.74	190.33	0.437	2616.86	1144.46
A_68_P32233091	chrX:5660358-5660402	NM_001033211:158	AU022751	INSIDE	1.908	2.380	272.91	649.47	4.540	403.61	1832.32
A_68_P24098211	chr5:131915292-131915336	NM_177047:1102899	Auts2	INSIDE	1.764	1.814	777.47	1410.48	3.201	711.14	2276.02
A_68_P25958415	chr8:74225497-74225541	NM_028189:181	B3gnt3	INSIDE	1.614	0.242	2052.69	497.30	0.391	1379.50	539.29
A_68_P26920733	chr10:7500333-7500377	NM_145418:361	BC013529	INSIDE	1.764	0.232	2535.06	586.93	0.408	1864.52	761.54
A_68_P25020989	chr7:29076252-29076296	NM_175033:-121	BC089491	PROMOTER	1.647	0.216	2033.38	439.83	0.356	1466.96	522.67
A_68_P22384844	chr3:102975721-102975767	NM_026602:111	Bcas2	INSIDE	1.891	1.428	987.01	1409.12	2.700	677.36	1829.15
A_68_P30480363	chr15:99301136-99301180	NM_029236:4003	Bcdin3d	INSIDE	1.787	1.554	599.21	930.96	2.777	461.69	1282.07
A_68_P25508016	chr7:135047821-135047865	NM_009739:256	Bckdk	INSIDE	1.542	0.364	1335.83	485.95	0.561	1061.10	595.23
A_68_P27649722	chr11:24064060-24064104	NM_001159290:83388	Bcl11a	INSIDE	2.538	2.525	192.38	485.74	6.409	134.34	861.06
A_68_P28724521	chr12:109154706-109154750	NM_001079883:86896	Bcl11b	INSIDE	2.493	2.102	445.62	936.66	5.240	395.98	2074.80
A_68_P28724520	chr12:109154600-109154644	NM_001079883:87002	Bcl11b	INSIDE	1.617	2.480	1098.32	2724.25	4.012	947.29	3800.08
A_68_P28724516	chr12:109154117-109154161	NM_001079883:87486	Bcl11b	INSIDE	1.875	1.461	719.33	1050.81	2.740	531.10	1455.00
A_68_P32260575	chrX:11705762-11705806	NM_001168321:31697	Bcor	INSIDE	1.659	0.268	2609.74	699.09	0.445	2866.64	1274.23
A_68_P32260250	chrX:11654399-11654443	NM_029510:3259	Bcor	INSIDE	8.005	0.077	3083.96	237.31	0.616	3350.61	2063.97
A_68_P32364066	chrX:45698978-45699030	NM_178782:4470	Bcorl1	INSIDE	17.870	0.299	906.62	271.43	5.350	1281.69	6857.13
A_68_P32364050	chrX:45697107-45697151	NM_178782:2594	Bcorl1	INSIDE	2.310	1.888	8344.09	15754.93	4.361	8516.29	37139.67
A_68_P32364037	chrX:45695522-45695566	NM_178782:1010	Bcorl1	INSIDE	1.612	0.210	2061.59	433.79	0.339	2647.51	897.87
A_68_P32364035	chrX:45695205-45695249	NM_178782:692	Bcorl1	INSIDE	1.548	3.670	5012.89	18396.86	5.679	6406.74	36386.35
A_68_P32364052	chrX:45697351-45697395	NM_178782:2838	Bcorl1	INSIDE	9.266	0.194	506.06	98.31	1.800	745.77	1342.50
A_68_P28731504	chr12:110272212-110272256	NM_001163175:34193	Begain	INSIDE	1.571	2.918	478.65	1396.53	4.583	373.70	1712.51
A_68_P28731503	chr12:110272067-110272111	NM_001163175:34339	Begain	INSIDE	1.519	2.353	722.34	1699.64	3.574	578.02	2065.63
A_68_P27100735	chr10:43231159-43231203	NM_199028:32235	Bend3	INSIDE	1.882	2.038	861.53	1755.85	3.836	612.04	2347.96
A_68_P27100736	chr10:43231246-43231290	NM_199028:32323	Bend3	INSIDE	1.705	1.921	799.67	1535.84	3.275	751.16	2460.31
A_68_P21906146	chr2:180511719-180511763	NM_080641:-135	Bhlhe23	PROMOTER	1.618	0.381	1734.71	661.43	0.617	1407.63	868.39
A_68_P27233861	chr10:70622146-70622190	NM_031397:214	Bicc1	INSIDE	2.843	0.620	1556.32	965.12	1.763	1183.74	2087.16
A_68_P29024947	chr13:49478476-49478520	NM_001039179:41581	Bicd2	INSIDE	1.671	2.133	233.03	496.98	3.564	197.56	704.06
A_68_P28959944	chr13:38437327-38437371	NM_007556:-236	Bmp6	PROMOTER	1.959	0.216	2341.21	506.63	0.424	1669.47	707.81

ProbeName	Target position of probe on CpG island microarray	TargetID	GeneSymbol	CpG island Description	Ratio of relative methylation (TiO_2-NP/Vehicle)	Sham group			TiO_2-H group		
						Relative methylation (Cy5/Cy3)	Cy3 signal (Input)	Cy5 signal (MeDIP)	Relative methylation (Cy5/Cy3)	Cy3 signal (Input)	Cy5 signal (MeDIP)
A_68_P31152226	chr17:34257950-34257994	NR_037970:-1127	Brd2	PROMOTER	1.652	0.313	3155.24	987.92	0.517	2093.02	1082.94
A_68_P23296719	chr4:129138535-129138579	NM_133889:-366	Bsdc1	PROMOTER	1.777	2.524	257.67	650.34	4.485	232.11	1041.10
A_68_P26813047	chr9:108008622-108008666	NM_007567:84070	Bsn	INSIDE	1.882	2.202	813.15	1790.15	4.142	688.49	2851.86
A_68_P21673353	chr2:138082625-138082669	NM_001025431:327	Btbd3	INSIDE	1.644	0.264	2671.76	705.92	0.434	2204.93	957.51
A_68_P32137924	chr19:45438347-45438391	NM_001037758:145	Btrc	INSIDE	1.821	0.240	3569.15	855.22	0.436	2453.40	1070.74
A_68_P31001860	chr16:98144127-98144171	NM_174847:95	C2cd2	INSIDE	1.669	0.354	1662.37	589.29	0.592	1271.53	752.40
A_68_P23818508	chr5:77287446-77287490	NM_001163793:17845	C530008M17Rik	INSIDE	1.671	1.383	979.12	1353.77	2.311	680.53	1572.60
A_68_P24818072	chr6:124946958-124947002	NM_001145927:-1474	C530028O21Rik	PROMOTER	1.509	0.142	6089.60	866.83	0.215	4238.32	910.63
A_68_P32576278	chrX:101395079-101395123	NM_001077354:1356	C77370	INSIDE	5.811	0.451	392.85	177.07	2.619	482.00	1262.41
A_68_P30371309	chr15:80118848-80118907	NM_001044308:1210	Cacna1i	INSIDE	2.024	2.193	492.37	1079.74	4.440	239.54	1063.46
A_68_P25961388	chr8:74967563-74967607	NM_029782:-8529	Calr3	PROMOTER	1.519	0.381	1833.69	698.67	0.579	1318.55	762.96
A_68_P20975268	chr2:5635854-5635898	NM_177343:-166	Camk1d	PROMOTER	1.910	1.570	441.71	693.26	2.998	365.20	1094.94
A_68_P25354352	chr7:105326428-105326472	NM_007602:253	Capn5	INSIDE	1.958	0.284	2352.49	668.53	0.556	1727.60	961.11
A_68_P21485631	chr2:103637120-103637164	NM_001111289:93	Caprin1	INSIDE	1.502	0.376	1745.83	655.96	0.565	1212.22	684.33
A_68_P30533731	chr16:8672077-8672121	NM_025821:148	Carhsp1	INSIDE	1.672	0.146	3146.51	460.63	0.245	2201.25	538.75
A_68_P23396583	chr4:148179764-148179808	NM_027195:1286	Casz1	INSIDE	1.713	0.271	2501.03	678.08	0.464	1694.65	786.98
A_68_P23396589	chr4:148180346-148180401	NM_027195:1873	Casz1	INSIDE	2.563	2.263	157.20	355.69	5.798	158.00	916.16
A_68_P23321453	chr4:133742651-133742697	NM_144527:326	Ccdc21	INSIDE	1.880	0.057	10640.00	604.37	0.107	7099.09	758.19
A_68_P23245374	chr4:119088045-119088089	NM_029286:60	Ccdc30	INSIDE	1.971	0.321	1956.19	627.93	0.633	1382.91	875.07
A_68_P26467525	chr9:44225880-44225924	NM_201372:188	Ccdc84	INSIDE	1.525	0.332	3304.29	1097.27	0.506	2174.23	1101.07
A_68_P26467526	chr9:44226004-44226048	NM_201372:64	Ccdc84	INSIDE	1.649	0.388	1972.34	765.18	0.640	1325.77	848.27
A_68_P21576978	chr2:120557105-120557149	NM_026891:127	Cdan1	INSIDE	1.570	0.285	3303.82	940.94	0.447	2692.91	1204.22
A_68_P22449311	chr3:116126122-116126166	NM_001080818:806	Cdc14a	INSIDE	1.691	0.245	1918.75	469.38	0.414	1518.17	628.04
A_68_P21325833	chr2:72314668-72314712	NM_025866:415	Cdca7	INSIDE	1.551	0.290	1575.87	457.67	0.451	1228.83	553.63
A_68_P26236516	chr8:125394674-125394718	NM_007662:22423	Cdh15	DOWNSTREAM	1.590	2.976	1299.16	3866.90	4.732	1076.60	5094.76
A_68_P29613441	chr14:55260479-55260523	NM_199470:-299	Cdh24	PROMOTER	1.539	0.216	3125.93	676.17	0.333	2189.69	729.00
A_68_P30025417	chr15:12980979-12981031	NM_007666:122390	Cdh6	INSIDE	1.763	1.647	512.34	843.97	2.904	383.52	1113.55
A_68_P31125955	chr17:29235539-29235583	NM_007669:4844	Cdkn1a	INSIDE	1.531	1.882	670.31	1261.45	2.880	550.86	1586.62
A_68_P30778319	chr16:55905763-55905807	NM_028815:29177	Cep97	INSIDE	1.598	1.563	990.59	1548.31	2.498	782.50	1954.51
A_68_P22402364	chr3:106350168-106350212	NM_133869:489	Cept1	INSIDE	1.572	0.222	2445.83	542.46	0.349	1738.70	606.10
A_68_P24631555	chr6:90259775-90259819	NM_027928:15383	Chst13	INSIDE	1.596	1.739	623.35	1084.11	2.775	545.54	1513.97
A_68_P24631552	chr6:90259348-90259392	NM_027928:15809	Chst13	INSIDE	2.462	1.710	452.86	774.52	4.211	374.31	1576.36
A_68_P32130472	chr19:44181487-44181534	NM_001162410:457	Chuk	INSIDE	1.825	0.327	5135.69	1677.71	0.596	3118.05	1858.58
A_68_P32567810	chrX:99447558-99447602	NM_007709:-472	Cited1	PROMOTER	3.080	0.207	1239.88	256.43	0.637	1780.68	1134.14
A_68_P22413865	chr3:108456942-108456986	NM_001177770:57	Clcc1	INSIDE	1.864	0.241	2252.56	543.78	0.450	1605.12	722.39
A_68_P23609440	chr5:37139337-37139381	NM_133724:560	Cno	INSIDE	1.529	0.242	2046.23	495.77	0.371	1537.15	569.55
A_68_P24946537	chr7:3596654-3596698	NM_146176:-194	Cnot3	PROMOTER	1.534	0.208	3351.90	698.25	0.319	2377.64	759.59
A_68_P24359960	chr6:35084098-35084143	NM_001164412:-383	Cnot4	PROMOTER	1.520	2.253	427.91	963.94	3.423	256.87	879.31
A_68_P22893528	chr4:47258456-47258500	NM_009928:37595	Col15a1	INSIDE	1.522	1.663	468.23	778.66	2.531	379.79	961.29
A_68_P21629541	chr2:130223202-130223246	NM_019696:141	Cpxm1	INSIDE	1.601	0.280	2046.86	573.02	0.448	1504.46	674.27
A_68_P31157716	chr17:35258201-35258245	NM_009975:170	Csnk2b	INSIDE	1.985	0.153	3762.87	576.81	0.304	2574.40	783.50
A_68_P26875426	chr9:119883221-119883265	NM_153287:10534	Csrnp1	INSIDE	1.720	2.451	1093.10	2679.13	4.217	802.54	3384.01
A_68_P25539016	chr7:140315036-140315089	NM_009980:104	Ctbp2	INSIDE	2.003	2.608	6932.15	18081.65	5.225	4442.76	23212.76
A_68_P26880499	chr9:120842203-120842247	NM_007614:-293	Ctnnb1	PROMOTER	1.542	3.009	2567.49	7725.82	4.640	1727.24	8014.87
A_68_P25416834	chr7:118199064-118199108	NM_009431:26622	Ctr9	INSIDE	1.755	1.489	700.82	1043.42	2.613	551.07	1439.80

ProbeName	Target position of probe on CpG island microarray	TargetID	GeneSymbol	CpG island Description	Ratio of relative methylation (TiO_2-NP/Vehicle)	Sham group			TiO_2-H group		
						Relative methylation (Cy5/Cy3)	Cy3 signal (Input)	Cy5 signal (MeDIP)	Relative methylation (Cy5/Cy3)	Cy3 signal (Input)	Cy5 signal (MeDIP)
A_68_P25083573	chr7:50930678-50930722	NM_145582:3300	Ctu1	INSIDE	1.878	1.646	1003.66	1652.20	3.092	792.73	2451.30
A_68_P32328377	chrX:35918417-35918470	NM_001110142:10930	Cul4b	INSIDE	17.143	0.162	532.97	86.41	2.779	814.00	2262.38
A_68_P24120341	chr5:136751221-136751265	NM_198602:292033	Cux1	INSIDE	1.594	2.390	540.71	1292.55	3.811	381.48	1453.75
A_68_P24120337	chr5:136750787-136750831	NM_198602:292467	Cux1	INSIDE	1.580	2.240	334.43	749.01	3.538	319.82	1131.52
A_68_P27913317	chr11:72609127-72609171	NM_001024926:193	Cyb5d2	INSIDE	1.695	0.215	2830.15	607.14	0.364	2067.92	751.72
A_68_P29874397	chr14:105339298-105339349	NR_015593:300960	D130009I18Rik	INSIDE	1.924	1.521	567.06	862.71	2.927	459.99	1346.31
A_68_P32145200	chr19:46728723-46728767	NM_001177813:30854	D19Wsu162e	INSIDE	1.514	1.640	976.15	1600.61	2.483	751.59	1866.24
A_68_P20910361	chr1:188791550-188791594	NM_033077:278	D1Pas1	INSIDE	1.682	2.248	960.00	2158.23	3.782	797.59	3016.45
A_68_P20910363	chr1:188791777-188791821	NM_033077:504	D1Pas1	INSIDE	1.814	1.467	667.22	978.63	2.661	604.52	1608.39
A_68_P23325564	chr4:134480330-134480374	NM_023665:813	D4Wsu53e	INSIDE	1.584	0.244	3057.39	744.91	0.386	1836.84	708.77
A_68_P28525539	chr12:72419195-72419245	NM_001190466:8350	Dact1	INSIDE	1.767	2.627	467.90	1229.28	4.641	368.31	1709.50
A_68_P31150930	chr17:34048600-34048644	NM_001199733:2077	Daxx	INSIDE	1.763	1.564	487.56	762.60	2.758	369.37	1018.60
A_68_P31227869	chr17:50432800-50432844	NM_010021:102	Dazl	INSIDE	2.150	1.933	738.63	1427.62	4.155	602.36	2502.83
A_68_P20560374	chr1:122017670-122017714	NM_007830:-19	Dbi	DIVERGENT_PROMOTER	1.526	0.212	2689.91	570.67	0.324	2156.88	698.14
A_68_P25094601	chr7:52962152-52962196	NM_016974:1558	Dbp	INSIDE	2.345	0.208	2271.06	472.85	0.488	1663.32	812.26
A_68_P25394427	chr7:112936384-112936433	NM_001162943:-344	Dchs1	PROMOTER	3.028	1.604	528.58	847.78	4.857	345.57	1678.51
A_68_P32395899	chrX:53708448-53708501	NM_172779:459	Ddx26b	INSIDE	11.436	0.200	465.56	93.05	2.286	596.72	1363.81
A_68_P23981088	chr5:111082625-111082669	NM_027156:177	Ddx51	INSIDE	1.574	0.330	2106.83	696.10	0.520	1562.17	812.51
A_68_P25093408	chr7:52744206-52744250	NM_027903:-62	Dhdh	PROMOTER	1.554	0.316	2097.87	662.37	0.491	1541.75	756.48
A_68_P30672035	chr16:35769023-35769067	NM_153550:398	Dirc2	INSIDE	1.823	0.244	2104.43	513.87	0.445	1598.90	711.60
A_68_P33015620	chr1_random:50449-50497	NM_026866:134866	Disp1	INSIDE	2.133	1.701	352.30	599.12	3.628	263.73	956.84
A_68_P33015621	chr1_random:50610-50662	NM_026866:134702	Disp1	INSIDE	2.087	1.420	808.65	1148.55	2.964	567.90	1683.36
A_68_P25422971	chr7:119301817-119301861	NM_015814:733	Dkk3	INSIDE	2.190	3.136	3496.34	10964.25	6.869	2815.34	19338.78
A_68_P26504859	chr9:50467279-50467323	NM_145614:585	Dlat	INSIDE	2.072	0.210	2240.02	470.46	0.435	1713.12	745.43
A_68_P29651209	chr14:62267312-62267356	NR_028264:33876	Dleu2	INSIDE	1.668	0.215	2631.81	566.47	0.359	1978.19	710.00
A_68_P25021026	chr7:29083763-29083810	NM_007866:3018	Dll3	INSIDE	1.812	3.280	272.38	893.50	5.944	265.45	1577.90
A_68_P21569149	chr2:119163546-119163590	NM_019454:12049	Dll4	DOWNSTREAM	2.340	2.253	1574.09	3547.00	5.274	1325.48	6990.45
A_68_P23192163	chr4:107356485-107356529	NM_019872:261	Dmrtb1	INSIDE	1.685	2.808	415.51	1166.68	4.732	366.10	1732.21
A_68_P27928205	chr11:75608766-75608811	NM_007873:771	Doc2b	INSIDE	1.696	0.319	1994.14	635.34	0.540	1600.45	865.04
A_68_P25026465	chr7:30089302-30089346	NM_013874:301	Dpf1	INSIDE	1.713	0.358	1868.51	669.59	0.614	1280.73	786.10
A_68_P25587962	chr7:148480819-148480877	NM_007878:2944	Drd4	INSIDE	1.726	1.860	348.96	648.99	3.211	289.29	928.85
A_68_P30981935	chr16:94748214-94748258	NM_007834:0	Dscr3	INSIDE	2.783	0.671	1402.73	940.97	1.867	1033.99	1930.43
A_68_P21585602	chr2:122127398-122127442	NM_025777:2785	Duoxa2	INSIDE	1.563	0.114	4204.83	479.12	0.178	2958.28	526.81
A_68_P21585603	chr2:122127484-122127528	NM_025777:2871	Duoxa2	INSIDE	1.603	0.421	2088.59	879.01	0.675	1524.50	1028.42
A_68_P24863577	chr6:134742556-134742600	NM_001048054:68	Dusp16	INSIDE	1.567	0.223	2441.07	544.37	0.350	1732.81	605.64
A_68_P24596644	chr6:83958657-83958701	NM_001077694:95	Dysf	INSIDE	2.356	2.367	269.48	637.86	5.577	182.83	1019.63
A_68_P31252967	chr17:56095790-56095834	NM_015766:3767	Ebi3	INSIDE	1.598	1.688	665.34	1122.94	2.697	484.33	1306.29
A_68_P23342965	chr4:137513918-137513962	NM_199307:95789	Ece1	INSIDE	2.003	2.220	214.85	477.07	4.447	141.05	627.21
A_68_P20416033	chr1:89051506-89051553	NM_021306:73	Ecel1	INSIDE	4.247	0.146	1120.48	163.91	0.621	1467.78	911.93
A_68_P21908932	chr2:180891704-180891748	NM_007906:-6	Eef1a2	PROMOTER	1.836	0.204	2968.38	604.30	0.374	2100.95	785.23
A_68_P23366443	chr4:141430949-141430993	NM_025994:-135	Efhd2	PROMOTER	1.559	2.287	630.10	1441.25	3.566	498.78	1778.50
A_68_P32553704	chrX:96332183-96332227	NM_010110:736	Efnb1	INSIDE	10.685	0.064	1361.04	87.02	0.683	2035.53	1390.49
A_68_P27212434	chr10:66999516-66999563	NM_010118:-1077	Egr2	PROMOTER	2.674	0.241	3139.30	755.22	0.643	2120.74	1364.36
A_68_P29699020	chr14:70477756-70477800	NM_018781:527	Egr3	INSIDE	1.651	0.223	2097.74	468.42	0.369	1575.88	581.14
A_68_P23280027	chr4:126106647-126106691	NM_153402:118	Eif2c3	INSIDE	1.637	0.374	1530.92	573.05	0.613	1160.71	711.12

ProbeName	Target position of probe on CpG island microarray	TargetID	GeneSymbol	CpG island Description	Ratio of relative methylation (TiO_2-NP/Vehicle)	Sham group			TiO_2-H group		
						Relative methylation (Cy5/Cy3)	Cy3 signal (Input)	Cy5 signal (MeDIP)	Relative methylation (Cy5/Cy3)	Cy3 signal (Input)	Cy5 signal (MeDIP)
A_68_P32364768	chrX:45807315-45807359	NM_019680:8973	Elf4	INSIDE	8.200	0.167	1849.80	308.14	1.366	2377.43	3247.39
A_68_P32364760	chrX:45806092-45806136	NM_019680:10195	Elf4	INSIDE	3.044	0.558	559.37	312.10	1.698	753.01	1278.76
A_68_P25952523	chr8:73063414-73063459	NM_007924:-137	Ell	PROMOTER	1.683	0.270	1813.60	490.19	0.455	1275.50	580.29
A_68_P20884270	chr1:183853884-183853931	NM_001083120:96204	Enah	INSIDE	2.593	3.063	166.71	510.59	7.942	178.16	1414.96
A_68_P31418978	chr17:87152455-87152501	NM_010137:-725	Epas1	PROMOTER	1.663	0.353	2221.64	784.05	0.587	1898.75	1114.31
A_68_P31424359	chr17:88039798-88039842	NM_008532:4502	Epcam	INSIDE	1.729	1.980	800.19	1584.07	3.422	598.90	2049.64
A_68_P23363267	chr4:140864773-140864817	NM_010139:7640	Epha2	INSIDE	2.088	1.537	1059.68	1629.05	3.210	785.18	2520.79
A_68_P28575795	chr12:81564283-81564327	NM_133798:223	Exd2	INSIDE	1.675	2.365	4896.40	11578.73	3.960	3661.54	14500.29
A_68_P29241525	chr13:96374455-96374499	NM_010169:13912	F2r	INSIDE	1.519	2.412	310.99	750.01	3.664	265.74	973.81
A_68_P29241524	chr13:96374376-96374423	NM_010169:13989	F2r	INSIDE	1.575	1.802	361.54	651.63	2.839	278.87	791.75
A_68_P23227295	chr4:115690207-115690251	NM_010173:279	Faah	INSIDE	1.722	0.350	2630.89	920.09	0.602	2040.46	1228.88
A_68_P22415887	chr3:108830521-108830565	NM_001163567:-17	Fam102b	PROMOTER	1.852	20.640	400.36	8263.26	38.234	341.40	13053.19
A_68_P27284174	chr10:80049350-80049394	NM_145421:3714	Fam108a	INSIDE	2.079	1.607	515.22	827.96	3.340	442.41	1477.84
A_68_P24046894	chr5:122303239-122303283	NM_175474:4224	Fam109a	INSIDE	1.789	1.939	269.97	523.60	3.470	184.59	640.48
A_68_P29644189	chr14:60997251-60997301	NM_001164705:154	Fam123a	INSIDE	1.541	1.684	536.98	904.21	2.595	444.10	1152.31
A_68_P25957394	chr8:74067191-74067235	NM_028617:384	Fam125a	INSIDE	1.758	0.273	3039.25	828.37	0.479	1670.19	800.38
A_68_P25641011	chr8:9207733-9207777	NM_173446:563269	Fam155a	INSIDE	1.545	2.130	368.77	785.31	3.289	363.86	1196.84
A_68_P23195766	chr4:108055710-108055754	NM_001099303:222	Fam159a	INSIDE	1.714	3.555	592.30	2105.86	6.094	439.28	2676.84
A_68_P20741943	chr1:158134761-158134805	NM_177838:374	Fam163a	INSIDE	1.693	0.263	2869.64	754.65	0.445	1812.02	806.75
A_68_P23317853	chr4:133042110-133042154	NM_175307:6085	Fam46b	INSIDE	1.562	1.674	434.02	726.59	2.615	367.20	960.25
A_68_P23317854	chr4:133042232-133042276	NM_175307:6207	Fam46b	INSIDE	1.698	1.413	1009.88	1427.21	2.400	720.35	1728.53
A_68_P28940414	chr13:34838852-34838896	NM_138746:7364	Fam50b	INSIDE	1.742	1.574	689.16	1084.99	2.743	604.58	1658.24
A_68_P25827954	chr8:46109471-46109515	NM_001081286:73931	Fat1	INSIDE	1.601	2.260	335.56	758.44	3.619	296.17	1071.77
A_68_P25827948	chr8:46108836-46108887	NM_001081286:73300	Fat1	INSIDE	1.725	1.680	466.67	784.00	2.899	425.62	1233.75
A_68_P25827947	chr8:46108650-46108696	NM_001081286:73111	Fat1	INSIDE	1.510	1.742	344.66	600.52	2.630	309.34	813.66
A_68_P22071595	chr3:38784639-38784683	NM_183221:-1201	Fat4	PROMOTER	1.722	0.178	2961.97	526.82	0.306	2064.19	632.37
A_68_P26227387	chr8:124078337-124078381	NM_133765:24348	Fbxo31	INSIDE	1.904	1.515	1027.42	1556.91	2.886	530.05	1529.47
A_68_P27311664	chr10:85484474-85484519	NM_153195:-177	Fbxo7	DIVERGENT_PROMOTER	4.235	0.674	2000.06	1348.16	2.854	1567.98	4475.60
A_68_P20351044	chr1:74931604-74931648	NM_153111:356	Fev	INSIDE	1.728	0.267	2685.54	716.56	0.461	1784.15	822.48
A_68_P24642400	chr6:91929179-91929223	NM_172731:-7903	Fgd5	PROMOTER	1.629	0.395	2597.95	1026.01	0.643	1718.17	1105.19
A_68_P32408595	chrX:56387162-56387206	NM_010200:429	Fgf13	INSIDE	1.979	0.311	1261.91	392.17	0.615	1698.91	1044.83
A_68_P21278824	chr2:63936695-63936739	NM_021716:-647	Fign	PROMOTER	1.661	2.118	538.99	1141.77	3.518	411.11	1446.40
A_68_P24115931	chr5:135825936-135825980	NM_033571:-97	Fkbp6	DIVERGENT_PROMOTER	1.824	1.574	704.41	1109.04	2.872	547.06	1570.95
A_68_P32441101	chrX:65932010-65932054	NM_008031:303	Fmr1	INSIDE	1.992	0.297	1599.72	474.71	0.591	2304.18	1362.31
A_68_P29712960	chr14:72932250-72932294	NM_207636:177538	Fndc3a	DOWNSTREAM	1.664	0.301	1859.19	559.88	0.501	1521.85	762.55
A_68_P22134616	chr3:52073560-52073612	NM_019739:1328	Foxo1	INSIDE	1.940	0.183	2769.06	506.36	0.355	2060.18	730.76
A_68_P27094080	chr10:41996065-41996109	NM_019740:462	Foxo3	INSIDE	3.189	0.488	1655.90	808.58	1.557	1214.85	1891.52
A_68_P32563074	chrX:98449869-98449914	NM_018789:25	Foxo4	INSIDE	1.514	2.626	281.88	740.27	3.976	328.81	1307.51
A_68_P23250319	chr4:119941839-119941885	NM_194060:18004	Foxo6	INSIDE	1.757	2.373	474.54	1126.11	4.169	328.99	1371.62
A_68_P31927969	chr19:5307468-5307512	NM_001024717:9160	Gal3st3	INSIDE	1.899	2.270	533.63	1211.34	4.310	441.31	1901.92
A_68_P24620361	chr6:88149067-88149115	NM_008090:433	Gata2	INSIDE	1.736	2.264	284.27	643.48	3.930	220.78	867.73
A_68_P23968414	chr5:108150872-108150916	NM_010278:2469	Gfi1	INSIDE	1.570	1.690	810.46	1369.39	2.653	672.04	1783.15
A_68_P28989506	chr13:43399105-43399149	NM_001033399:415	Gfod1	INSIDE	1.571	2.029	435.09	882.67	3.186	346.76	1104.88
A_68_P20553865	chr1:120734246-120734290	NM_001081125:215928	Gli2	INSIDE	2.177	1.864	939.88	1751.56	4.057	806.68	3272.86
A_68_P21565612	chr2:118528731-118528775	NM_001081971:947	Gm1337	INSIDE	1.584	0.199	4636.43	923.64	0.316	2941.44	928.25

ProbeName	Target position of probe on CpG island microarray	TargetID	GeneSymbol	CpG island Description	Ratio of relative methylation (TiO_2-NP/Vehicle)	Sham group			TiO_2-H group		
						Relative methylation (Cy5/Cy3)	Cy3 signal (Input)	Cy5 signal (MeDIP)	Relative methylation (Cy5/Cy3)	Cy3 signal (Input)	Cy5 signal (MeDIP)
A_68_P21565607	chr2:118528199-118528243	NM_001081971:1479	Gm1337	INSIDE	2.449	0.255	2260.52	577.06	0.625	1654.55	1034.37
A_68_P21393860	chr2:84499188-84499232	NM_001145100:-25	Gm13718	DIVERGENT_PROMOTER	1.565	1.819	519.35	944.47	2.846	426.74	1214.56
A_68_P28577475	chr12:81859600-81859644	NM_001008423:2080	Gm1568	INSIDE	1.609	1.836	768.46	1410.83	2.953	652.26	1926.39
A_68_P28577470	chr12:81858836-81858880	NM_001008423:2844	Gm1568	INSIDE	1.507	2.963	556.94	1650.35	4.466	420.40	1877.31
A_68_P24225086	chr6:8209405-8209449	NR_033518:139	Gm16039	INSIDE	1.837	0.140	5642.36	790.47	0.257	3767.88	969.76
A_68_P26156363	chr8:111864572-111864616	NR_002928:214	Gm1943	INSIDE	1.599	1.488	582.11	866.47	2.380	532.26	1266.95
A_68_P26886025	chr9:121766341-121766385	NM_001112668:287	Gm9790	INSIDE	1.516	0.157	3381.31	530.35	0.238	2281.90	542.74
A_68_P29002205	chr13:45602874-45602918	NM_025508:59	Gmpr	INSIDE	1.531	0.356	1557.92	554.04	0.545	1301.95	708.93
A_68_P21874746	chr2:174153286-174153330	NR_003258:-106	Gnas	PROMOTER	1.724	1.934	675.21	1305.65	3.334	531.82	1773.19
A_68_P21874752	chr2:174154225-174154269	NM_001077510:-1343	Gnas	PROMOTER	1.602	2.507	808.27	2026.45	4.017	672.22	2700.54
A_68_P21874518	chr2:174125914-174125958	NM_201617:2577	Gnas	INSIDE	1.728	1.699	452.44	768.55	2.936	400.19	1174.77
A_68_P21874507	chr2:174124666-174124710	NM_201617:1329	Gnas	INSIDE	3.046	0.619	738.53	457.38	1.886	622.75	1174.72
A_68_P30641174	chr16:30309847-30309891	NM_008148:997	Gp5	INSIDE	1.766	0.320	1564.95	500.53	0.565	1214.54	685.85
A_68_P28080237	chr11:102417265-102417309	NM_001159492:186	Gpatch8	INSIDE	1.677	0.216	3121.03	675.32	0.363	2300.98	834.83
A_68_P32380514	chrX:49518048-49518092	NM_008150:30	Gpc4	INSIDE	61.524	0.065	1399.00	90.81	3.994	1906.71	7614.88
A_68_P32380515	chrX:49518249-49518293	NM_008150:-170	Gpc4	PROMOTER	10.005	0.221	1132.83	250.79	2.215	1539.10	3409.03
A_68_P32239706	chrX:7274518-7274562	NM_173747:281	Gpkow	INSIDE	6.175	0.327	901.99	295.18	2.021	1162.22	2348.67
A_68_P24135223	chr5:139856324-139856369	NM_030258:-274	Gpr146	PROMOTER	1.662	1.968	274.18	539.44	3.269	202.68	662.60
A_68_P24863969	chr6:134820135-134820179	NM_001167697:17721	Gpr19	INSIDE	1.843	4.031	579.69	2336.83	7.431	428.98	3187.78
A_68_P22351747	chr3:96709002-96709046	NM_026229:197	Gpr89	INSIDE	1.871	1.576	533.53	841.00	2.950	437.29	1290.01
A_68_P27280641	chr10:79516441-79516485	NM_001037741:-319	Gpx4	PROMOTER	1.528	0.176	2834.81	497.87	0.268	1951.86	523.91
A_68_P28056597	chr11:98319612-98319657	NM_010346:11487	Grb7	DOWNSTREAM	1.578	0.138	3883.85	535.77	0.218	2521.03	548.76
A_68_P24156244	chr5:144131752-144131796	NM_133355:6188	Grid2ip	INSIDE	1.694	0.303	1815.43	549.57	0.513	1340.64	687.37
A_68_P28152301	chr11:115111293-115111337	NM_010350:17243	Grin2c	INSIDE	3.224	1.760	793.83	1397.33	5.676	698.84	3966.33
A_68_P26804377	chr9:106553386-106553432	NM_001160353:5032	Grm2	INSIDE	1.656	1.807	377.80	682.59	2.992	294.56	881.45
A_68_P26804376	chr9:106553310-106553354	NM_001160353:5108	Grm2	INSIDE	1.848	1.497	648.69	970.98	2.766	402.07	1112.10
A_68_P25301151	chr7:95278746-95278790	NM_001081414:546091	Grm5	INSIDE	2.120	1.363	1351.23	1841.59	2.890	1033.99	2987.83
A_68_P29731241	chr14:76410609-76410653	NM_026816:42	Gtf2f2	INSIDE	1.795	0.312	1724.64	538.92	0.561	1194.18	669.73
A_68_P23977822	chr5:110536969-110537013	NM_145147:226	Gtpbp6	INSIDE	1.699	0.315	2420.61	761.52	0.534	1757.54	939.16
A_68_P27893905	chr11:69049315-69049359	NM_008192:1188	Gucy2e	INSIDE	1.541	0.419	2068.04	866.60	0.646	1463.12	944.98
A_68_P32507213	chrX:83022104-83022148	NM_008194:32	Gyk	INSIDE	5.864	0.336	2422.30	813.29	1.969	2706.02	5327.99
A_68_P24904451	chr6:142363030-142363089	NM_145572:58570	Gys2	DOWNSTREAM	5.355	0.662	863.47	571.55	3.545	537.27	1904.41
A_68_P30473862	chr15:98087209-98087253	NM_027304:508	H1fnt	INSIDE	1.606	1.620	954.26	1546.11	2.602	873.30	2272.54
A_68_P32465786	chrX:71211813-71211857	NM_008224:-180	Hcfc1	PROMOTER	2.939	0.230	1366.39	314.92	0.677	1731.22	1172.57
A_68_P32241074	chrX:7524906-7524950	NM_001130416:87	Hdac6	INSIDE	4.492	0.607	3825.27	2320.66	2.725	4633.10	12626.24
A_68_P32241075	chrX:7525044-7525088	NM_001130416:-51	Hdac6	PROMOTER	3.194	0.509	1639.57	833.72	1.624	2095.91	3403.82
A_68_P31253724	chr17:56218702-56218746	NM_008233:-355	Hdgfrp2	DIVERGENT_PROMOTER	2.469	0.141	7388.62	1040.37	0.348	4630.24	1609.56
A_68_P26885589	chr9:121698137-121698187	NR_027967:2076	Hhatl	INSIDE	2.288	1.626	500.78	814.04	3.719	414.01	1539.80
A_68_P27812443	chr11:54679796-54679846	NM_008248:-119	Hint1	DIVERGENT_PROMOTER	1.895	2.380	248.26	590.93	4.510	173.83	783.91
A_68_P32672782	chrX:131135885-131135929	NM_019868:82	Hnrnph2	INSIDE	2.464	0.562	2657.04	1493.76	1.385	3488.49	4832.40
A_68_P31149893	chr17:33787074-33787118	NM_001109913:35307	Hnrnpm	INSIDE	1.808	1.668	589.13	982.79	3.016	574.68	1733.42
A_68_P31149892	chr17:33786930-33786974	NM_001109913:35451	Hnrnpm	INSIDE	2.297	1.398	963.35	1346.75	3.211	802.72	2577.27
A_68_P24449293	chr6:52183630-52183674	NM_008263:1286	Hoxa10	INSIDE	1.588	0.293	2830.25	830.02	0.466	1972.81	918.58
A_68_P21338760	chr2:74520854-74520898	NM_008273:427	Hoxd11	INSIDE	1.503	1.606	794.93	1277.00	2.414	550.14	1328.16
A_68_P23295049	chr4:128798702-128798746	NM_010471:-189	Hpca	PROMOTER	1.586	0.278	2390.01	665.10	0.441	1676.39	739.71

ProbeName	Target position of probe on CpG island microarray	TargetID	GeneSymbol	CpG island Description	Ratio of relative methylation (TiO_2-NP/Vehicle)	Sham group			TiO_2-H group		
						Relative methylation (Cy5/Cy3)	Cy3 signal (Input)	Cy5 signal (MeDIP)	Relative methylation (Cy5/Cy3)	Cy3 signal (Input)	Cy5 signal (MeDIP)
A_68_P20146386	chr1:36160613-36160657	NM_015818:35390	Hs6st1	INSIDE	1.584	1.853	925.69	1715.49	2.936	730.38	2144.55
A_68_P32378025	chrX:49034174-49034218	NM_015819:-542	Hs6st2	PROMOTER	5.909	0.369	513.47	189.23	2.178	863.98	1881.51
A_68_P32378022	chrX:49033742-49033786	NM_015819:-110	Hs6st2	PROMOTER	2.175	0.640	2173.56	1390.68	1.392	2795.09	3889.74
A_68_P24118365	chr5:136364296-136364340	NM_013560:530	Hspb1	INSIDE	1.523	0.187	2755.66	514.34	0.284	1786.65	507.87
A_68_P32399260	chrX:54306606-54306650	NM_028242:-118	Htatsf1	PROMOTER	4.864	0.135	1244.97	168.41	0.658	1743.33	1147.10
A_68_P32746464	chrX:148238102-148238146	NM_021523:300	Huwe1	INSIDE	2.779	0.180	2429.92	438.56	0.502	3154.13	1582.19
A_68_P32746468	chrX:148238435-148238479	NM_021523:632	Huwe1	INSIDE	1.845	0.347	1722.71	597.79	0.640	1999.73	1280.02
A_68_P26658578	chr9:78023836-78023880	NM_019987:62814	Ick	DOWNSTREAM	1.638	0.336	1825.54	612.89	0.550	1395.07	766.97
A_68_P20296825	chr1:65225492-65225536	NM_010497:202	Idh1	INSIDE	1.515	0.410	1695.33	695.45	0.622	1416.10	880.13
A_68_P21103419	chr2:30325956-30326000	NM_030244:3741	Ier5l	DOWNSTREAM	1.605	0.343	2161.39	740.55	0.550	1642.82	903.44
A_68_P20833279	chr1:174412349-174412393	NM_033608:-77	Igsf9	PROMOTER	1.830	0.193	2408.90	465.85	0.354	1925.94	681.49
A_68_P27340040	chr10:90564963-90565014	NM_027078:19205	Ikbip	INSIDE	2.268	2.131	412.10	878.02	4.833	314.48	1519.95
A_68_P27902198	chr11:70513486-70513530	NM_213729:149	Inca1	INSIDE	1.704	0.373	1682.66	626.86	0.635	1299.08	824.54
A_68_P25656209	chr8:11555685-11555729	NM_011919:-359	Ing1	DIVERGENT_PROMOTER	1.740	0.270	5636.96	1523.50	0.470	3196.92	1503.60
A_68_P25656260	chr8:11561678-11561724	NM_011919:5635	Ing1	INSIDE	1.948	1.552	1273.73	1976.26	3.023	996.74	3013.09
A_68_P25613343	chr8:3279377-3279421	NM_010568:219	Insr	INSIDE	1.543	0.255	1912.01	487.08	0.393	1470.64	578.08
A_68_P22080609	chr3:40439835-40439879	NM_175515:168	Intu	INSIDE	1.596	1.973	428.44	845.19	3.149	386.19	1216.25
A_68_P24634428	chr6:90686241-90686285	NM_001134384:73855	Iqsec1	INSIDE	1.650	0.353	3009.87	1062.80	0.582	2298.95	1339.06
A_68_P32748065	chrX:148579255-148579299	NM_001114664:466	Iqsec2	INSIDE	2.361	0.659	796.04	524.81	1.557	945.39	1471.80
A_68_P32466000	chrX:71269156-71269200	NM_001177975:79	Irak1	INSIDE	1.524	1.825	346.10	631.57	2.781	389.37	1082.67
A_68_P26258349	chr8:129107965-129108009	NM_001164598:9350	Irf2bp2	DOWNSTREAM	1.591	2.285	885.07	2022.28	3.636	712.81	2591.75
A_68_P26258430	chr8:129118076-129118120	NM_001164598:-762	Irf2bp2	PROMOTER	1.563	0.396	2117.82	838.03	0.619	1574.73	974.21
A_68_P25004860	chr7:25217492-25217536	NM_199013:13187	Irgc1	INSIDE	2.142	1.731	440.08	761.58	3.707	320.09	1186.70
A_68_P26531906	chr9:55394278-55394322	NM_027397:5345	Isl2	DOWNSTREAM	1.522	0.234	1850.22	433.38	0.356	1441.72	513.96
A_68_P28036506	chr11:94937357-94937401	NM_013565:650	Itga3	INSIDE	1.670	0.113	4784.33	540.29	0.189	3225.48	608.12
A_68_P29717288	chr14:73784719-73784763	NM_008410:338	Itm2b	INSIDE	1.584	3.100	6962.05	21584.94	4.910	4986.58	24483.19
A_68_P20058120	chr1:17082054-17082098	NM_020604:5894	Jph1	INSIDE	1.650	1.440	1229.62	1770.80	2.376	920.75	2187.68
A_68_P29616405	chr14:55733423-55733480	NM_177049:2321	Jph4	INSIDE	2.280	2.002	1122.21	2246.68	4.564	773.50	3529.98
A_68_P29613096	chr14:55195734-55195778	NM_010590:742	Jub	INSIDE	1.924	5.119	2649.16	13561.20	9.847	2311.01	22755.52
A_68_P32029537	chr19:25484593-25484638	NM_181404:172924	Kank1	INSIDE	1.507	2.285	2394.53	5471.72	3.443	1589.63	5473.83
A_68_P26348936	chr9:21599583-21599627	NM_145611:3386	Kank2	INSIDE	1.759	1.910	501.90	958.76	3.361	448.01	1505.68
A_68_P22403732	chr3:106838896-106838940	NM_008418:-161	Kcna3	PROMOTER	1.789	0.142	3571.14	507.56	0.254	2358.78	599.69
A_68_P25099052	chr7:53682950-53683004	NM_001112739:31110	Kcnc1	INSIDE	1.564	1.650	407.92	672.90	2.581	344.82	889.84
A_68_P22397119	chr3:105261830-105261874	NM_019931:119	Kcnd3	INSIDE	1.731	1.781	303.17	539.90	3.083	250.77	773.21
A_68_P32707348	chrX:138739964-138740008	NM_021487:755	Kcne1l	INSIDE	3.176	0.708	1822.01	1290.10	2.249	2308.97	5191.85
A_68_P30366726	chr15:79315953-79315997	NM_008427:19697	Kcnj4	INSIDE	2.331	2.447	581.72	1423.23	5.702	398.66	2273.14
A_68_P20832394	chr1:174256462-174256506	NM_008429:2910	Kcnj9	INSIDE	1.738	0.292	1705.00	498.67	0.508	1191.95	605.77
A_68_P29469017	chr14:24824321-24824365	NM_010610:-915	Kcnma1	PROMOTER	2.399	2.574	193.74	498.61	6.174	149.01	919.99
A_68_P27475787	chr10:115911149-115911205	NM_021452:-597	Kcnmb4	PROMOTER	1.517	2.621	290.22	760.53	3.975	215.55	856.77
A_68_P25954564	chr8:73386897-73386941	NM_032397:-6011	Kcnn1	PROMOTER	1.500	6.261	840.75	5263.93	9.393	619.46	5818.55
A_68_P25599895	chr7:150481208-150481262	NR_001461:1218	Kcnq1ot1	INSIDE	1.654	1.374	2449.59	3366.09	2.272	1823.34	4142.94
A_68_P25599898	chr7:150481545-150481589	NR_001461:886	Kcnq1ot1	INSIDE	2.601	0.682	986.10	672.52	1.774	700.25	1242.20
A_68_P26346083	chr9:21038393-21038437	NM_001110305:5362	Keap1	INSIDE	1.594	1.654	1336.36	2210.62	2.636	945.49	2492.56
A_68_P28748396	chr12:113414015-113414059	NM_001097621:29618	Kif26a	INSIDE	1.730	1.975	900.70	1779.15	3.418	683.57	2336.22
A_68_P32560535	chrX:97821487-97821531	NM_008446:105	Kif4	INSIDE	2.286	0.260	1904.88	495.30	0.594	2503.48	1487.94

ProbeName	Target position of probe on CpG island microarray	TargetID	GeneSymbol	CpG island Description	Ratio of relative methylation (TiO_2-NP/Vehicle)	Sham group			TiO_2-H group		
						Relative methylation (Cy5/Cy3)	Cy3 signal (Input)	Cy5 signal (MeDIP)	Relative methylation (Cy5/Cy3)	Cy3 signal (Input)	Cy5 signal (MeDIP)
A_68_P28745984	chr12:113012732-113012779	NM_008450:15696	Klc1	INSIDE	1.516	1.643	686.42	1127.46	2.490	533.02	1326.97
A_68_P25952090	chr8:73000940-73000985	NM_001122830:-120	Klhl26	PROMOTER	1.669	0.330	1622.05	534.58	0.550	1096.92	603.54
A_68_P25951933	chr8:72975932-72975976	NM_001122830:24888	Klhl26	INSIDE	1.959	1.948	433.02	843.53	3.816	354.97	1354.58
A_68_P25951936	chr8:72976257-72976301	NM_001122830:24564	Klhl26	INSIDE	1.835	1.784	393.61	702.20	3.273	368.26	1205.30
A_68_P26216539	chr8:122394248-122394292	NM_146219:8066	Klhl36	INSIDE	1.596	1.599	505.80	808.68	2.552	435.56	1111.54
A_68_P21111931	chr2:31742789-31742833	NM_011836:10	Lamc3	INSIDE	1.682	2.699	2216.13	5982.30	4.540	1867.86	8480.68
A_68_P29629465	chr14:58318680-58318724	NM_015771:46258	Lats2	INSIDE	2.607	1.658	689.44	1142.92	4.322	532.08	2299.53
A_68_P25101651	chr7:54101747-54101791	NM_001136069:-683	Ldha	PROMOTER	1.729	2.175	339.47	738.22	3.760	284.90	1071.22
A_68_P20619890	chr1:134086976-134087020	NM_001033250:-1014	Lemd1	PROMOTER	1.840	1.810	1646.03	2980.13	3.332	1149.32	3829.46
A_68_P27470613	chr10:115024518-115024562	NM_010195:296	Lgr5	INSIDE	1.839	0.245	2171.00	532.17	0.451	1610.90	726.18
A_68_P20637452	chr1:137001893-137001941	NM_001033409:-63	Lgr6	PROMOTER	2.422	4.446	4998.07	22220.63	10.766	3343.21	35991.99
A_68_P21137825	chr2:35959267-35959311	NM_001083126:292	Lhx6	INSIDE	1.802	0.194	2317.86	450.57	0.350	1731.51	606.54
A_68_P26537272	chr9:56468941-56468985	NM_181074:64098	Lingo1	INSIDE	1.653	3.085	633.99	1956.15	5.099	577.55	2944.92
A_68_P29054563	chr13:55463630-55463674	NM_025828:492	Lman2	INSIDE	1.805	0.217	2875.17	625.02	0.392	1990.88	781.29
A_68_P27286342	chr10:80380790-80380834	NM_010722:178	Lmnb2	INSIDE	1.586	0.325	1581.34	513.81	0.515	1088.62	561.15
A_68_P25406573	chr7:116318129-116318173	NM_057173:-4328	Lmo1	PROMOTER	2.469	0.252	1759.47	444.02	0.623	1325.32	825.90
A_68_P24159376	chr5:144861468-144861512	NM_001081109:186	Lmtk2	INSIDE	1.574	0.403	1643.20	661.93	0.634	1281.72	812.68
A_68_P25031176	chr7:31140849-31140896	NM_175478:6919	Lrfn3	INSIDE	1.619	1.958	404.54	791.98	3.170	318.58	1009.97
A_68_P25031175	chr7:31140774-31140818	NM_175478:6995	Lrfn3	INSIDE	1.994	1.603	781.27	1252.73	3.197	605.49	1935.65
A_68_P24658378	chr6:94649175-94649219	NM_008377:943	Lrig1	INSIDE	2.581	0.229	2084.00	477.63	0.592	1595.82	943.95
A_68_P25048198	chr7:35988774-35988818	NM_001024707:11568	Lrp3	INSIDE	1.769	1.947	586.59	1141.93	3.444	500.55	1723.88
A_68_P25048197	chr7:35988690-35988734	NM_001024707:11652	Lrp3	INSIDE	1.551	1.372	1452.98	1992.91	2.127	1076.89	2290.20
A_68_P27180703	chr10:60938705-60938749	NM_153542:146	Lrrc20	INSIDE	2.288	9.024	1247.99	11261.26	20.643	1159.24	23929.67
A_68_P27270906	chr10:77365060-77365104	NM_145152:199	Lrrc3	INSIDE	1.548	3.432	3586.26	12307.29	5.313	2736.76	14541.29
A_68_P21101982	chr2:30093777-30093821	NM_177725:510	Lrrc8a	INSIDE	4.251	1.957	955.12	1869.22	8.320	574.18	4777.13
A_68_P21102113	chr2:30112262-30112306	NM_177725:18996	Lrrc8a	INSIDE	2.022	1.769	407.46	720.73	3.576	320.25	1145.16
A_68_P20624277	chr1:134777288-134777332	NM_010732:379	Lrrn2	INSIDE	1.718	0.294	1764.76	519.54	0.506	1301.43	658.12
A_68_P27765091	chr11:45758223-45758267	NM_028185:193	Lsm11	INSIDE	1.534	0.288	2141.80	616.33	0.441	1630.18	719.40
A_68_P32539364	chrX:91786968-91787022	NM_019791:419	Maged1	INSIDE	11.572	0.339	339.65	114.98	3.917	412.04	1614.14
A_68_P32742985	chrX:147247678-147247726	NM_001199246:544	Maged2	INSIDE	5.935	0.285	892.44	254.21	1.691	1026.15	1734.92
A_68_P31050851	chr17:12464717-12464771	NM_011948:46782	Map3k4	INSIDE	1.845	1.626	385.90	627.60	3.001	330.30	991.17
A_68_P32375460	chrX:48558753-48558797	NM_134163:235	Mbnl3	INSIDE	2.964	0.214	1536.57	329.20	0.635	2032.11	1290.50
A_68_P24946817	chr7:3637393-3637437	NM_029934:7713	Mboat7	INSIDE	1.777	3.262	2159.27	7043.79	5.798	1525.34	8843.73
A_68_P32767191	chrX:154036331-154036375	NM_172307:295	Mbtps2	INSIDE	3.803	0.459	567.72	260.66	1.746	880.69	1537.88
A_68_P25614334	chr8:3515198-3515255	NM_053177:14708	Mcoln1	INSIDE	2.210	0.207	2436.92	505.26	0.458	1788.27	819.41
A_68_P26676070	chr9:81757205-81757249	NM_175213:-124	Mei4	PROMOTER	1.621	3.082	1470.15	4531.51	4.995	1183.85	5913.82
A_68_P27922355	chr11:74584384-74584428	NM_026197:42	Mettl16	INSIDE	1.530	0.178	2736.63	487.93	0.273	2018.83	550.68
A_68_P20293513	chr1:64663621-64663665	NM_025964:100	Mettl21a	INSIDE	1.599	4.913	1637.15	8044.00	7.858	1502.99	11811.15
A_68_P27787411	chr11:50039460-50039504	NM_145926:646	Mgat4b	INSIDE	1.980	4.502	382.43	1721.71	8.913	278.84	2485.20
A_68_P32252886	chrX:10294260-10294304	NM_001166635:-208	Mid1ip1	PROMOTER	2.203	0.318	1574.06	501.13	0.701	2058.97	1444.01
A_68_P30547090	chr16:11254627-11254671	NR_035466:-110	Mir1945	PROMOTER	1.567	0.316	2248.78	711.56	0.496	1709.84	847.57
A_68_P25025268	chr7:29871232-29871276	NR_035489:-2541	Mir1963	PROMOTER	1.638	1.764	415.65	733.30	2.890	357.18	1032.21
A_68_P20348301	chr1:74438711-74438755	NR_029743:-2151	Mir26b	PROMOTER	2.978	2.357	827.27	1950.27	7.021	706.00	4957.01
A_68_P32385499	chrX:50410041-50410085	NR_029756:-2536	Mir322	PROMOTER	49.785	0.204	5800.27	1184.88	10.170	6244.37	63505.16
A_68_P22209749	chr3:67178298-67178342	NM_001039543:302	Mlf1	INSIDE	2.876	1.779	1046.06	1860.89	5.117	666.83	3412.09

ProbeName	Target position of probe on CpG island microarray	TargetID	GeneSymbol	CpG island Description	Ratio of relative methylation (TiO_2-NP/Vehicle)	Sham group			TiO_2-H group		
						Relative methylation (Cy5/Cy3)	Cy3 signal (Input)	Cy5 signal (MeDIP)	Relative methylation (Cy5/Cy3)	Cy3 signal (Input)	Cy5 signal (MeDIP)
A_68_P21840230	chr2:168056944-168056988	NM_001160330:845	Mocs3	INSIDE	1.535	1.895	487.01	922.94	2.910	414.82	1207.07
A_68_P32696393	chrX:136405600-136405644	NM_001193309:571	Morc4	INSIDE	1.677	0.399	1891.42	754.02	0.668	2472.22	1652.64
A_68_P30422455	chr15:88813114-88813158	NM_031260:-287	Mov10l1	DIVERGENT_PROMOTER	1.602	1.486	2470.29	3671.14	2.380	1879.55	4473.90
A_68_P32470782	chrX:72376286-72376330	NM_008621:-20	Mpp1	PROMOTER	2.259	0.195	2064.33	401.99	0.440	2710.33	1192.38
A_68_P28077443	chr11:101949878-101949922	NM_016695:-71	Mpp2	PROMOTER	2.098	0.193	3210.39	619.78	0.405	2128.88	862.08
A_68_P25394537	chr7:112959576-112959620	NM_025301:3	Mrpl17	INSIDE	1.529	2.362	2548.18	6019.48	3.613	2008.51	7256.28
A_68_P21069122	chr2:24830308-24830352	NM_001031808:288	Mrpl41	INSIDE	2.280	0.723	2687.27	1941.63	1.648	2071.68	3413.36
A_68_P32135814	chr19:45080366-45080410	NM_053164:544	Mrpl43	INSIDE	2.299	0.250	1799.59	449.76	0.575	1291.80	742.32
A_68_P29223483	chr13:93125415-93125459	NM_010829:-478	Msh3	PROMOTER	1.569	0.239	1971.36	470.53	0.375	1596.63	597.95
A_68_P25581954	chr7:147234659-147234703	NM_010836:307	Msx3	INSIDE	2.295	0.216	2207.42	477.63	0.497	1627.52	808.22
A_68_P25954894	chr8:73437692-73437736	NM_173013:7842	Mtap1s	INSIDE	1.991	2.071	445.74	923.08	4.122	366.91	1512.58
A_68_P25954889	chr8:73437195-73437239	NM_173013:7344	Mtap1s	INSIDE	1.503	2.443	1002.78	2450.27	3.673	734.12	2696.25
A_68_P23279071	chr4:125932970-125933014	NM_001145970:571	Mtap7d1	INSIDE	1.552	0.154	3150.35	486.29	0.240	2341.62	560.84
A_68_P23278936	chr4:125911491-125911535	NM_001145970:22051	Mtap7d1	INSIDE	1.543	1.770	605.01	1070.80	2.731	472.27	1289.92
A_68_P23278937	chr4:125911565-125911609	NM_001145970:21977	Mtap7d1	INSIDE	1.787	1.469	1469.61	2158.84	2.626	1098.38	2883.83
A_68_P26224550	chr8:123632450-123632508	NM_001166482:-199	Mthfsd	DIVERGENT_PROMOTER	2.575	8.435	7313.59	61693.66	21.723	5216.59	113321.80
A_68_P22317890	chr3:89017814-89017858	NM_001161824:421	Mtx1	INSIDE	1.817	0.158	4322.03	684.84	0.288	2702.35	778.07
A_68_P29591680	chr14:49685967-49686011	NM_144535:-181	Mudeng	PROMOTER	1.729	2.017	1046.28	2109.90	3.486	737.81	2572.08
A_68_P29591677	chr14:49685584-49685628	NM_144535:-563	Mudeng	PROMOTER	1.509	0.260	2330.59	605.85	0.392	1785.02	700.21
A_68_P28961844	chr13:38727138-38727182	NM_139063:-182	Muted	PROMOTER	1.531	1.475	950.24	1401.85	2.259	748.62	1690.97
A_68_P32181719	chr19:53384898-53384942	NM_001008542:-75	Mxi1	PROMOTER	1.508	0.240	2140.35	514.43	0.362	1497.42	542.63
A_68_P28246310	chr12:12948750-12948795	NM_008709:-130	Mycn	PROMOTER	2.008	3.679	260.59	958.63	7.387	267.60	1976.74
A_68_P27880945	chr11:66908117-66908162	NM_001099635:16338	Myh3	INSIDE	1.521	2.111	436.07	920.67	3.212	351.00	1127.30
A_68_P30083232	chr15:25710801-25710845	NM_019472:158518	Myo10	INSIDE	2.003	2.319	305.15	707.70	4.645	231.43	1075.06
A_68_P30083236	chr15:25711164-25711208	NM_019472:158882	Myo10	INSIDE	1.869	1.508	789.52	1190.24	2.818	644.68	1816.39
A_68_P30083235	chr15:25711092-25711136	NM_019472:158810	Myo10	INSIDE	2.020	1.501	740.92	1112.48	3.033	587.23	1780.88
A_68_P27290092	chr10:80958931-80958975	NM_134009:156	Ncln	INSIDE	1.516	0.354	4239.80	1501.56	0.537	2783.29	1494.70
A_68_P24810881	chr6:122824922-122824967	NM_026267:370	Necap1	INSIDE	1.664	0.234	2510.08	587.64	0.390	2010.55	783.17
A_68_P27349651	chr10:92185236-92185280	NM_008682:-95	Nedd1	PROMOTER	1.594	3.868	1026.85	3972.04	6.166	844.64	5207.63
A_68_P31779044	chr18:65243606-65243650	NM_031881:60448	Nedd4l	INSIDE	1.789	1.879	684.61	1286.05	3.361	570.07	1915.77
A_68_P31779045	chr18:65243748-65243792	NM_031881:60590	Nedd4l	INSIDE	1.620	1.872	371.61	695.75	3.033	328.71	996.84
A_68_P32148154	chr19:47254136-47254180	NM_021360:849	Neurl1a	INSIDE	2.454	3.531	10959.86	38703.66	8.665	7399.64	64118.75
A_68_P27184377	chr10:61596546-61596590	NM_009719:731	Neurog3	INSIDE	1.705	0.112	4629.82	517.03	0.190	2985.56	568.41
A_68_P29428097	chr14:17075904-17075948	NM_021504:-5901	Ngly1	PROMOTER	1.502	0.405	1376.50	557.39	0.608	1114.37	677.95
A_68_P32786690	chrX:158346840-158346884	NM_001081052:250860	Nhs	INSIDE	3.772	0.436	477.00	207.86	1.644	686.46	1128.29
A_68_P32788273	chrX:158597915-158597959	NM_001081052:-214	Nhs	PROMOTER	4.380	0.464	411.34	190.82	2.032	592.38	1203.58
A_68_P27944883	chr11:78510477-78510521	NM_008702:429	Nlk	INSIDE	2.545	0.696	2646.93	1841.40	1.770	1946.36	3445.82
A_68_P32564062	chrX:98625083-98625127	NM_023144:115	Nono	INSIDE	2.829	0.587	1493.95	876.95	1.661	1905.39	3164.27
A_68_P28442990	chr12:54808173-54808232	NM_013780:458539	Npas3	INSIDE	2.604	2.556	1021.39	2610.68	6.655	682.44	4541.62
A_68_P31926196	chr19:4989991-4990035	NM_153553:-41	Npas4	PROMOTER	1.980	2.362	406.11	959.10	4.676	263.63	1232.82
A_68_P22540268	chr3:132612964-132613008	NM_001029836:269	Npnt	INSIDE	1.747	1.714	1731.25	2966.97	2.994	1385.21	4146.86
A_68_P22325407	chr3:90269682-90269726	NM_008727:84	Npr1	INSIDE	1.772	0.206	2332.55	480.37	0.365	1707.74	623.32
A_68_P28058390	chr11:98629362-98629406	NM_145434:7172	Nr1d1	INSIDE	1.616	1.621	778.67	1262.07	2.619	614.34	1609.16
A_68_P25088121	chr7:51807262-51807306	NM_009473:2009	Nr1h2	INSIDE	1.682	1.730	1979.26	3424.77	2.910	1609.46	4683.19
A_68_P25981542	chr8:79709972-79710016	NM_001083906:283588	Nr3c2	INSIDE	1.531	1.782	349.27	622.44	2.729	324.79	886.38

ProbeName	Target position of probe on CpG island microarray	TargetID	GeneSymbol	CpG island Description	Ratio of relative methylation (TiO_2-NP/Vehicle)	Sham group			TiO_2-H group		
						Relative methylation (Cy5/Cy3)	Cy3 signal (Input)	Cy5 signal (MeDIP)	Relative methylation (Cy5/Cy3)	Cy3 signal (Input)	Cy5 signal (MeDIP)
A_68_P22898182	chr4:48060289-48060333	NM_015743:-3809	Nr4a3	PROMOTER	2.393	1.659	756.90	1255.55	3.969	625.18	2481.39
A_68_P21070362	chr2:25036916-25036960	NM_025980:661	Nrarp	INSIDE	1.635	0.183	2638.15	482.61	0.299	1731.34	517.99
A_68_P32691811	chrX:135448869-135448913	NM_013724:-78	Nrk	PROMOTER	2.396	0.569	7328.70	4171.94	1.364	9260.75	12630.82
A_68_P32691812	chrX:135449017-135449063	NM_013724:72	Nrk	INSIDE	8.865	0.368	493.89	181.82	3.264	842.52	2749.69
A_68_P31442544	chr17:91487486-91487530	NM_020252:4634	Nrxn1	INSIDE	2.018	2.302	485.64	1117.90	4.646	424.97	1974.58
A_68_P31442543	chr17:91487324-91487368	NM_020252:4796	Nrxn1	INSIDE	1.817	1.943	1145.42	2225.01	3.529	881.58	3111.41
A_68_P31442547	chr17:91487821-91487865	NM_020252:4300	Nrxn1	INSIDE	1.594	2.062	468.22	965.36	3.286	452.02	1485.32
A_68_P31442548	chr17:91487890-91487934	NM_020252:4230	Nrxn1	INSIDE	1.850	1.683	641.82	1080.50	3.114	507.56	1580.79
A_68_P22422568	chr3:109945463-109945507	NM_001163348:906	Ntng1	INSIDE	1.972	0.319	1982.73	633.03	0.629	1424.00	896.34
A_68_P22309972	chr3:87587929-87587973	NM_001033124:11134	Ntrk1	INSIDE	2.199	1.569	2044.81	3208.63	3.451	1530.24	5280.39
A_68_P30542118	chr16:10412308-10412352	NM_011955:300	Nubp1	INSIDE	2.576	0.734	3573.45	2623.49	1.891	2645.25	5003.00
A_68_P22063505	chr3:37318090-37318134	NM_153561:400	Nudt6	INSIDE	4.156	0.120	2067.96	247.88	0.498	1795.61	894.47
A_68_P21102303	chr2:30142233-30142277	NM_198304:302	Nup188	INSIDE	1.638	0.152	3059.33	466.03	0.249	2138.98	533.67
A_68_P28039261	chr11:95376208-95376252	NM_130858:-351	Nxph3	PROMOTER	2.040	0.324	2834.73	917.67	0.660	1960.35	1294.53
A_68_P32266426	chrX:13063861-13063905	NM_173415:19085	Nyx	INSIDE	1.681	1.889	334.08	631.22	3.176	589.98	1873.51
A_68_P21100044	chr2:29744774-29744818	NM_001113214:-443	Odf2	PROMOTER	1.790	0.191	4382.86	836.92	0.342	2890.64	988.22
A_68_P25846165	chr8:49314031-49314075	NM_001145937:445992	Odz3	INSIDE	3.322	1.784	431.03	769.15	5.928	367.59	2179.09
A_68_P25343508	chr7:103358546-103358591	NM_011858:-578	Odz4	PROMOTER	2.010	1.498	4344.14	6507.77	3.011	3344.48	10070.19
A_68_P27280128	chr10:79440201-79440245	NM_001003949:6853	ORF61	INSIDE	1.659	2.195	695.17	1526.00	3.641	559.28	2036.57
A_68_P23199842	chr4:108874395-108874439	NM_133885:461	Osbpl9	INSIDE	1.776	0.200	3571.63	712.93	0.355	2515.70	892.03
A_68_P32240442	chrX:7418743-7418787	NM_138604:-192	Otud5	PROMOTER	4.783	0.117	2334.38	273.05	0.559	3436.31	1922.30
A_68_P25173479	chr7:70902673-70902717	NM_130880:313037	Otud7a	INSIDE	1.567	0.327	1352.09	442.30	0.513	1046.27	536.41
A_68_P23819019	chr5:77380180-77380224	NM_025939:-233	Paics	PROMOTER	1.758	0.241	2192.33	527.64	0.423	1553.11	657.01
A_68_P32071013	chr19:32670325-32670369	NM_001201470:142	Papss2	INSIDE	1.822	0.205	2227.45	457.03	0.374	1521.46	568.70
A_68_P31041951	chr17:11033242-11033286	NM_016694:15	Park2	INSIDE	1.772	0.287	1910.66	548.03	0.508	1440.01	731.73
A_68_P26554373	chr9:59464987-59465031	NM_001205239:-82	Parp6	PROMOTER	1.526	3.340	845.14	2822.99	5.099	648.15	3304.86
A_68_P21127262	chr2:34226792-34226836	NM_016768:742	Pbx3	INSIDE	1.616	0.303	1723.18	522.71	0.490	1308.56	641.41
A_68_P32668459	chrX:130222079-130222123	NM_001105245:1432	Pcdh19	INSIDE	1.730	2.137	285.14	609.41	3.698	250.74	927.10
A_68_P23720362	chr5:58110309-58110354	NM_018764:1072	Pcdh7	INSIDE	3.482	0.639	2287.11	1460.71	2.224	1835.76	4082.13
A_68_P20015303	chr1:7078680-7078724	NM_183028:-298	Pcmtd1	PROMOTER	1.689	2.187	2152.62	4707.41	3.694	1689.57	6241.42
A_68_P30778928	chr16:56029876-56029920	NM_001024622:-68	Pcnp	PROMOTER	1.527	0.256	1773.65	454.80	0.392	1345.79	527.11
A_68_P27261658	chr10:75837803-75837847	NM_008787:67833	Pcnt	INSIDE	1.614	2.954	164.05	484.64	4.767	218.99	1044.01
A_68_P32240898	chrX:7499680-7499725	NM_013892:2755	Pcsk1n	INSIDE	6.679	0.245	824.75	202.36	1.639	1055.20	1729.15
A_68_P32240899	chrX:7499781-7499825	NM_013892:2855	Pcsk1n	INSIDE	1.976	0.332	2036.08	676.01	0.656	2725.85	1788.51
A_68_P32778406	chrX:156575977-156576021	NM_008810:270	Pdha1	INSIDE	2.072	0.276	3065.39	846.09	0.572	4295.24	2456.84
A_68_P24172758	chr5:148081815-148081859	NM_008814:130	Pdx1	INSIDE	1.623	0.218	2266.47	494.36	0.354	1608.93	569.41
A_68_P25587733	chr7:148445537-148445581	NM_001081118:30872	Phrf1	INSIDE	1.986	2.754	845.36	2328.09	5.468	611.19	3342.18
A_68_P32798240	chrX:160857773-160857818	NM_011081:77	Piga	INSIDE	8.287	0.056	1524.74	85.80	0.466	2229.20	1039.60
A_68_P26798472	chr9:105545510-105545554	NM_001081309:208	Pik3r4	INSIDE	1.512	0.310	1822.33	565.49	0.469	1292.67	606.40
A_68_P26949007	chr10:12810795-12810839	NM_009538:223	Plagl1	INSIDE	1.571	1.604	502.13	805.41	2.520	473.66	1193.50
A_68_P30346606	chr15:76010797-76010841	NM_201394:15322	Plec	INSIDE	1.584	2.227	522.79	1164.31	3.529	471.45	1663.62
A_68_P30346558	chr15:76004732-76004776	NM_201394:21386	Plec	INSIDE	1.757	2.136	282.12	602.52	3.752	235.14	882.35
A_68_P30346601	chr15:76010142-76010186	NM_201394:15976	Plec	INSIDE	2.270	1.702	549.07	934.42	3.864	398.89	1541.12
A_68_P30346580	chr15:76007443-76007487	NM_201394:18676	Plec	INSIDE	1.750	1.639	487.93	799.57	2.867	377.14	1081.42
A_68_P30346556	chr15:76004532-76004576	NM_201394:21586	Plec	INSIDE	1.862	1.743	380.99	664.23	3.246	275.55	894.45

ProbeName	Target position of probe on CpG island microarray	TargetID	GeneSymbol	CpG island Description	Ratio of relative methylation (TiO_2-NP/Vehicle)	Sham group			TiO_2-H group		
						Relative methylation (Cy5/Cy3)	Cy3 signal (Input)	Cy5 signal (MeDIP)	Relative methylation (Cy5/Cy3)	Cy3 signal (Input)	Cy5 signal (MeDIP)
A_68_P30346557	chr15:76004653-76004697	NM_201394:21466	Plec	INSIDE	2.121	1.500	534.42	801.39	3.180	465.60	1480.79
A_68_P25957206	chr8:74031971-74032015	NM_032398:3676	Plvap	INSIDE	1.819	0.236	1907.23	450.22	0.429	1399.19	600.73
A_68_P30423622	chr15:88998029-88998073	NM_001159521:6230	Plxnb2	INSIDE	2.411	1.607	643.21	1033.76	3.875	470.78	1824.37
A_68_P20347698	chr1:74331582-74331626	NM_025580:-3	Pnkd	DIVERGENT_PROMOTER	1.765	0.305	1703.19	519.82	0.539	1221.58	658.22
A_68_P20347699	chr1:74331738-74331785	NM_025580:154	Pnkd	INSIDE	1.694	1.725	655.05	1130.24	2.923	482.29	1409.71
A_68_P24117611	chr5:136209975-136210019	NM_008898:44913	Por	INSIDE	2.184	1.547	890.21	1376.88	3.378	673.17	2273.97
A_68_P20797862	chr1:167933482-167933526	NM_011137:-739	Pou2f1	PROMOTER	2.040	0.179	2526.00	452.13	0.365	1730.81	631.83
A_68_P23269531	chr4:124337466-124337523	NM_011141:2606	Pou3f1	INSIDE	1.952	4.260	241.30	1027.91	8.317	93.99	781.72
A_68_P32598389	chrX:108010047-108010091	NM_008901:265	Pou3f4	INSIDE	5.265	0.298	3935.65	1171.48	1.567	4717.83	7394.34
A_68_P25092565	chr7:52602361-52602407	NM_029741:20005	Ppfia3	INSIDE	2.535	11.831	2987.65	35347.23	29.987	1937.28	58093.45
A_68_P22393108	chr3:104584185-104584229	NM_027982:233	Ppm1j	INSIDE	1.972	0.246	2316.75	569.48	0.485	1672.75	810.97
A_68_P27430001	chr10:107598764-107598808	NM_027892:-669	Ppp1r12a	PROMOTER	1.832	0.175	3327.76	582.55	0.321	2329.62	747.04
A_68_P32238978	chrX:7151494-7151538	NM_138605:-270	Ppp1r3f	DIVERGENT_PROMOTER	5.965	0.429	1256.22	539.47	2.562	1721.03	4408.97
A_68_P32238976	chrX:7151221-7151265	NM_138605:4	Ppp1r3f	INSIDE	1.617	1.495	674.16	1007.90	2.417	789.03	1906.97
A_68_P21432203	chr2:92886316-92886360	NM_001177536:-37	Prdm11	PROMOTER	1.510	1.879	971.84	1826.31	2.838	846.08	2401.58
A_68_P23916649	chr5:98615733-98615777	NM_029947:5867	Prdm8	INSIDE	1.516	0.147	3951.21	579.69	0.222	2634.54	585.82
A_68_P27110004	chr10:44878135-44878179	NM_011156:91137	Prep	INSIDE	1.759	1.456	657.43	957.17	2.561	534.61	1369.23
A_68_P24983572	chr7:17428015-17428059	NM_178900:-377	Prkd2	PROMOTER	1.730	0.202	2521.34	508.68	0.349	1818.88	634.89
A_68_P20930616	chr1:191994691-191994750	NM_008937:-161	Prox1	PROMOTER	1.668	2.216	242.53	537.35	3.696	169.99	628.26
A_68_P32699217	chrX:136991521-136991565	NM_021463:401	Prps1	INSIDE	2.973	0.211	1266.95	267.82	0.629	1920.29	1206.99
A_68_P31162032	chr17:36116207-36116251	NR_028516:-260	Prr3	PROMOTER	1.603	4.207	1691.40	7116.57	6.744	1419.06	9570.31
A_68_P30396225	chr15:84511839-84511883	NM_146061:433	Prr5	INSIDE	1.620	0.312	2530.29	789.78	0.506	1745.30	882.49
A_68_P29055231	chr13:55574123-55574167	NM_001030296:8517	Prr7	INSIDE	1.608	0.364	1763.37	641.56	0.585	1317.49	770.58
A_68_P21420371	chr2:90894443-90894487	NM_008948:292	Psmc3	INSIDE	2.173	1.785	2443.15	4360.47	3.878	1878.91	7286.54
A_68_P28107298	chr11:107341035-107341079	NM_025894:215	Psmd12	INSIDE	2.060	0.238	1905.14	454.14	0.491	1652.60	811.49
A_68_P32071922	chr19:32831644-32831688	NM_008960:-400	Pten	PROMOTER	1.586	2.778	1292.51	3590.86	4.406	1141.86	5031.26
A_68_P22373052	chr3:100881123-100881167	NM_011197:32945	Ptgfrn	INSIDE	1.502	1.645	671.72	1105.19	2.472	527.59	1304.09
A_68_P24983897	chr7:17492695-17492739	NM_008967:878	Ptgir	INSIDE	1.528	1.588	600.61	953.87	2.426	465.57	1129.69
A_68_P24983894	chr7:17492409-17492453	NM_008967:592	Ptgir	INSIDE	2.814	0.651	708.64	461.56	1.833	558.46	1023.67
A_68_P31256055	chr17:56568653-56568712	NM_011218:47221	Ptprs	INSIDE	5.356	3.108	245.36	762.48	16.645	231.78	3857.90
A_68_P23308583	chr4:131392567-131392611	NM_001083119:1605	Ptpru	INSIDE	2.257	0.269	2368.25	636.01	0.606	1582.03	958.96
A_68_P23308456	chr4:131375306-131375350	NM_001083119:18865	Ptpru	INSIDE	1.729	1.597	1042.14	1664.19	2.761	864.47	2386.37
A_68_P28071137	chr11:100820628-100820677	NM_008986:11279	Ptrf	INSIDE	2.707	2.201	210.73	463.80	5.958	211.95	1262.72
A_68_P31107655	chr17:26122056-26122100	NM_153140:36727	Rab11fip3	DOWNSTREAM	1.685	6.674	899.31	6001.87	11.249	635.66	7150.21
A_68_P25015938	chr7:27964028-27964072	NM_029391:-148	Rab4b	PROMOTER	4.680	0.126	4937.38	624.26	0.592	2873.46	1700.28
A_68_P32806533	chrX:162917564-162917608	NM_019773:213	Rab9	INSIDE	4.426	0.339	1345.44	456.69	1.502	1695.97	2548.07
A_68_P30012296	chr15:10644051-10644095	NM_030690:-777	Rai14	PROMOTER	2.486	0.080	6721.26	535.19	0.198	4187.07	828.76
A_68_P32374343	chrX:48371227-48371271	NM_172413:-53	Rap2c	PROMOTER	1.855	2.627	2565.97	6739.85	4.874	3133.60	15272.22
A_68_P22210462	chr3:67319224-67319268	NM_001164763:199	Rarres1	INSIDE	1.553	1.481	1178.17	1744.78	2.300	947.12	2178.81
A_68_P29194040	chr13:85429148-85429192	NM_145452:-79	Rasa1	PROMOTER	1.518	0.247	2692.68	663.92	0.374	1962.77	734.81
A_68_P29222297	chr13:92901976-92902020	NM_009027:-549	Rasgrf2	PROMOTER	3.225	6.389	1014.97	6484.30	20.601	890.04	18335.31
A_68_P32790793	chrX:159198362-159198406	NM_009031:81	Rbbp7	INSIDE	1.645	2.460	233.74	575.00	4.047	231.53	937.09
A_68_P32790792	chrX:159198218-159198262	NM_009031:-63	Rbbp7	PROMOTER	2.446	0.590	2086.11	1231.58	1.444	2947.80	4256.55
A_68_P31862858	chr18:80389262-80389306	NM_199197:8074	Rbfa	INSIDE	2.232	1.732	287.56	498.14	3.866	239.31	925.26
A_68_P22405767	chr3:107145905-107145949	NM_001045807:-9719	Rbm15	PROMOTER	2.015	1.595	464.07	740.00	3.213	316.43	1016.53

ProbeName	Target position of probe on CpG island microarray	TargetID	GeneSymbol	CpG island Description	Ratio of relative methylation (TiO_2-NP/Vehicle)	Sham group			TiO_2-H group		
						Relative methylation (Cy5/Cy3)	Cy3 signal (Input)	Cy5 signal (MeDIP)	Relative methylation (Cy5/Cy3)	Cy3 signal (Input)	Cy5 signal (MeDIP)
A_68_P31654584	chr18:42435490-42435534	NM_172626:506	Rbm27	INSIDE	2.095	0.187	3474.90	651.48	0.393	2384.05	936.27
A_68_P25032744	chr7:31435111-31435155	NM_133693:115	Rbm42	INSIDE	2.009	3.085	839.39	2589.48	6.197	704.41	4365.24
A_68_P21750311	chr2:152460303-152460347	NM_009047:7581	Rem1	INSIDE	1.536	1.814	1543.47	2799.99	2.787	1315.22	3665.33
A_68_P32465511	chrX:71167531-71167575	NM_001164704:8637	Renbp	INSIDE	3.621	0.192	1381.64	264.79	0.694	1856.56	1288.37
A_68_P24428221	chr6:48547445-48547489	NM_001079901:3585	Repin1	INSIDE	2.148	1.780	691.69	1231.51	3.824	554.78	2121.41
A_68_P24428220	chr6:48547266-48547310	NM_001079901:3407	Repin1	INSIDE	1.645	1.685	663.81	1118.38	2.771	534.14	1480.00
A_68_P26018119	chr8:86618743-86618787	NM_009055:28030	Rfx1	INSIDE	1.624	1.389	979.05	1359.71	2.255	697.14	1572.22
A_68_P20008184	chr1:5009350-5009394	NM_021374:135	Rgs20	INSIDE	1.824	1.630	429.72	700.65	2.974	327.89	975.19
A_68_P22393168	chr3:104591684-104591728	NM_007484:-245	Rhoc	PROMOTER	2.088	1.741	3023.33	5263.26	3.635	2294.56	8340.07
A_68_P24061483	chr5:124928214-124928258	NM_030259:8	Rilpl2	INSIDE	1.526	0.363	1350.56	490.35	0.554	1065.78	590.43
A_68_P32322124	chrX:34731543-34731587	NM_153503:227	Rnf113a1	INSIDE	2.962	0.512	1233.97	632.16	1.518	1646.58	2498.92
A_68_P27278803	chr10:79229502-79229546	NM_144528:142	Rnf126	INSIDE	1.847	0.279	2767.60	771.27	0.515	1817.83	935.64
A_68_P20715106	chr1:153347232-153347276	NM_011277:699	Rnf2	INSIDE	1.624	0.175	4372.86	764.02	0.284	3016.28	855.76
A_68_P30513214	chr16:5013320-5013364	NM_133185:304	Rogdi	INSIDE	1.728	0.251	2249.34	563.58	0.433	1525.30	660.29
A_68_P29043020	chr13:53381133-53381177	NM_013846:324	Ror2	INSIDE	2.073	0.233	1983.66	461.48	0.482	1614.55	778.74
A_68_P31121831	chr17:28465275-28465320	NM_011287:-118	Rpl10a	PROMOTER	2.165	7.985	692.24	5527.91	17.290	589.46	10192.03
A_68_P32321655	chrX:34625187-34625231	NM_026055:-29	Rpl39	PROMOTER	21.396	0.085	1013.99	86.41	1.823	1556.92	2838.82
A_68_P32773806	chrX:155694082-155694126	NM_148945:53	Rps6ka3	INSIDE	1.926	0.244	2420.69	589.55	0.469	3876.93	1818.22
A_68_P32773804	chrX:155693758-155693802	NM_148945:-271	Rps6ka3	PROMOTER	1.882	2.042	3195.43	6523.51	3.843	4290.60	16489.08
A_68_P31936308	chr19:6906036-6906086	NM_019924:9031	Rps6ka4	INSIDE	2.654	1.459	1216.81	1775.48	3.873	779.02	3017.13
A_68_P20348983	chr1:74552547-74552591	NM_021383:-65	Rqcd1	PROMOTER	1.514	0.174	2740.05	477.34	0.264	1867.95	492.63
A_68_P28956732	chr13:37918318-37918362	NM_001177868:-440	Rreb1	PROMOTER	1.968	0.192	2689.39	517.44	0.379	1794.61	679.43
A_68_P24992504	chr7:19878717-19878763	NM_001025364:2293	Rtn2	INSIDE	1.710	2.102	784.71	1649.76	3.595	597.71	2148.90
A_68_P27924771	chr11:75007879-75007923	NM_177708:406	Rtn4rl1	INSIDE	1.602	0.271	2789.24	755.49	0.434	2280.03	989.42
A_68_P29648696	chr14:61791453-61791497	NM_172809:34181	Sacs	INSIDE	1.557	1.992	836.79	1666.59	3.101	725.41	2249.71
A_68_P31257221	chr17:56743113-56743157	NM_001163300:18730	Safb	INSIDE	1.905	1.514	1938.43	2935.29	2.884	1412.45	4074.11
A_68_P31256956	chr17:56705945-56705989	NM_001029979:18040	Safb2	INSIDE	1.645	1.951	909.41	1774.22	3.210	754.39	2421.77
A_68_P21843350	chr2:168581447-168581491	NM_175303:11233	Sall4	INSIDE	1.543	1.449	1210.08	1753.35	2.235	914.42	2043.79
A_68_P21843327	chr2:168578173-168578217	NM_175303:14507	Sall4	INSIDE	1.599	1.529	583.90	892.75	2.445	471.65	1153.34
A_68_P21843351	chr2:168581527-168581571	NM_175303:11153	Sall4	INSIDE	1.674	1.382	1585.45	2190.77	2.313	1173.46	2714.67
A_68_P20250385	chr1:57027773-57027817	NM_139146:384	Satb2	INSIDE	2.570	0.246	2235.81	549.83	0.632	1644.99	1039.49
A_68_P25499766	chr7:133435827-133435871	NM_145587:19716	Sbk1	INSIDE	1.999	2.022	559.32	1130.85	4.042	450.99	1822.70
A_68_P25499767	chr7:133435936-133435980	NM_145587:19826	Sbk1	INSIDE	1.642	2.270	580.62	1318.11	3.729	536.44	2000.20
A_68_P25090603	chr7:52263124-52263168	NM_001008422:8473	Scaf1	INSIDE	1.773	1.772	837.70	1484.02	3.142	678.79	2132.59
A_68_P30348874	chr15:76350196-76350240	NM_130893:2341	Scrt1	INSIDE	2.076	0.131	10867.94	1424.35	0.272	6340.97	1725.64
A_68_P31120666	chr17:28279127-28279172	NM_001004366:-321	Scube3	PROMOTER	2.745	0.571	4769.03	2723.59	1.568	2991.69	4690.23
A_68_P23441761	chr4:155367293-155367337	NM_011341:292	Sdf4	INSIDE	1.571	0.321	1513.03	485.85	0.504	1152.48	581.34
A_68_P28164255	chr11:117151945-117151989	NM_001113487:24407	Septin9	INSIDE	1.782	1.947	363.47	707.70	3.471	295.72	1026.33
A_68_P32926577	chr13:93380980-93381024	NM_172588:-90	Serinc5	PROMOTER	1.611	0.158	7100.57	1119.19	0.254	5051.02	1282.79
A_68_P27550645	chr11:3093025-3093069	NM_030207:420	Sfi1	INSIDE	1.895	1.406	3558.17	5003.50	2.665	3254.57	8672.89
A_68_P27550647	chr11:3093206-3093251	NM_030207:238	Sfi1	INSIDE	1.872	1.403	1094.27	1535.67	2.627	929.28	2441.55
A_68_P23283131	chr4:126698502-126698546	NM_023603:-20	Sfpq	PROMOTER	1.599	7.855	272.36	2139.24	12.563	196.30	2466.18
A_68_P29047303	chr13:54167121-54167165	NM_027324:-71	Sfxn1	PROMOTER	1.561	2.052	1709.35	3506.97	3.202	1465.24	4691.95
A_68_P32070012	chr19:32462008-32462052	NM_144792:914	Sgms1	INSIDE	1.688	0.367	2034.86	745.92	0.619	1583.37	979.49
A_68_P23594804	chr5:34868325-34868369	NM_011893:-86	Sh3bp2	PROMOTER	2.525	2.153	1339.96	2885.60	5.438	1184.98	6443.67

ProbeName	Target position of probe on CpG island microarray	TargetID	GeneSymbol	CpG island Description	Ratio of relative methylation (TiO_2-NP/Vehicle)	Sham group			TiO_2-H group		
						Relative methylation (Cy5/Cy3)	Cy3 signal (Input)	Cy5 signal (MeDIP)	Relative methylation (Cy5/Cy3)	Cy3 signal (Input)	Cy5 signal (MeDIP)
A_68_P32149066	chr19:47388501-47388545	NM_001164717:150379	Sh3pxd2a	INSIDE	1.583	0.333	2750.14	915.69	0.527	1718.01	905.35
A_68_P23768851	chr5:67999755-67999799	NM_001033415:655	Shisa3	INSIDE	1.502	0.275	1881.37	517.27	0.413	1478.53	610.77
A_68_P27877796	chr11:66340159-66340203	NM_001034874:-552	Shisa6	PROMOTER	1.831	1.519	1562.74	2373.39	2.780	1295.79	3602.52
A_68_P23896973	chr5:93371964-93372008	NM_001077596:44963	Shroom3	INSIDE	2.365	2.100	408.11	856.83	4.966	332.56	1651.58
A_68_P23896968	chr5:93371161-93371205	NM_001077596:44159	Shroom3	INSIDE	1.608	1.917	1053.60	2019.89	3.082	826.42	2546.97
A_68_P23896967	chr5:93371085-93371129	NM_001077596:44083	Shroom3	INSIDE	1.590	1.563	696.42	1088.74	2.485	602.34	1496.86
A_68_P32234209	chrX:5977910-5977954	NM_001040459:670	Shroom4	INSIDE	2.004	0.343	2044.80	701.11	0.687	2663.69	1830.68
A_68_P26540099	chr9:56924722-56924766	NM_001110350:562	Sin3a	INSIDE	1.584	0.287	1708.00	489.86	0.454	1323.87	601.34
A_68_P31844515	chr18:77095020-77095064	NM_001109743:-101	Skor2	PROMOTER	1.508	0.323	1356.52	438.61	0.488	1062.22	518.02
A_68_P28187725	chr11:120812339-120812383	NM_001038653:1980	Slc16a3	INSIDE	1.647	0.229	2113.89	483.75	0.377	1532.58	577.78
A_68_P27630976	chr11:20232710-20232754	NM_018861:-16	Slc1a4	PROMOTER	1.646	0.327	1958.54	640.30	0.538	1457.79	784.40
A_68_P21115505	chr2:32306645-32306689	NM_001164357:324	Slc25a25	INSIDE	1.502	1.850	878.89	1626.27	2.779	652.53	1813.39
A_68_P25957633	chr8:74108592-74108640	NM_011977:15791	Slc27a1	INSIDE	1.628	1.543	768.24	1185.18	2.512	539.64	1355.69
A_68_P32240694	chrX:7461707-7461754	NM_001083937:361	Slc35a2	INSIDE	12.416	0.120	941.20	113.40	1.496	1263.13	1889.59
A_68_P30467109	chr15:96884897-96884941	NM_027052:1469	Slc38a4	INSIDE	1.592	1.712	895.77	1533.73	2.725	748.90	2040.91
A_68_P27287001	chr10:80494270-80494314	NM_134135:2365	Slc39a3	INSIDE	1.630	1.767	862.94	1525.04	2.881	622.02	1792.32
A_68_P27286999	chr10:80494095-80494139	NM_134135:2541	Slc39a3	INSIDE	1.555	2.055	930.54	1912.41	3.196	754.80	2412.12
A_68_P27926647	chr11:75344750-75344794	NM_001199284:-423	Slc43a2	PROMOTER	1.762	0.159	3975.62	633.93	0.281	2785.27	782.43
A_68_P28151695	chr11:115024532-115024576	NM_012030:-100	Slc9a3r1	PROMOTER	1.671	0.282	1630.69	459.78	0.471	1205.14	567.95
A_68_P32427553	chrX:61530261-61530305	NM_178740:-111	Slitrk4	PROMOTER	2.267	0.619	3195.79	1978.14	1.403	4275.36	5998.39
A_68_P31841966	chr18:76400802-76400846	NM_010754:-754	Smad2	PROMOTER	1.641	0.240	2882.94	691.34	0.393	2107.21	829.06
A_68_P26578207	chr9:63606312-63606356	NM_016769:-533	Smad3	PROMOTER	1.501	0.280	2870.02	804.38	0.421	2141.43	900.73
A_68_P31836532	chr18:75527265-75527309	NM_001042660:268	Smad7	INSIDE	1.839	0.168	3019.38	506.90	0.309	2303.56	711.01
A_68_P32182254	chr19:53465262-53465306	NM_172429:-221	Smndc1	PROMOTER	1.590	0.206	4922.77	1013.14	0.327	3156.19	1032.49
A_68_P21635426	chr2:131346286-131346330	NM_001177833:28711	Smox	INSIDE	1.550	1.698	659.66	1120.13	2.632	448.43	1180.16
A_68_P20022045	chr1:9289343-9289387	NM_027671:447	Sntg1	INSIDE	1.765	3.599	3399.54	12235.12	6.352	2815.61	17886.00
A_68_P26042148	chr8:91151286-91151330	NM_027840:8719	Snx20	INSIDE	1.599	2.728	217.07	592.28	4.363	191.56	835.79
A_68_P28050118	chr11:97224447-97224491	NM_138657:604	Socs7	INSIDE	1.511	0.313	1559.60	488.66	0.473	1136.17	537.84
A_68_P30964219	chr16:91660394-91660440	NM_019973:12348	Son	INSIDE	2.750	1.459	914.91	1334.67	4.011	646.45	2593.12
A_68_P21585133	chr2:122060313-122060357	NM_146126:-240	Sord	PROMOTER	1.521	0.373	1837.63	684.78	0.567	1449.10	821.49
A_68_P32415800	chrX:58145373-58145417	NM_009237:1211	Sox3	INSIDE	1.865	2.191	259.66	568.79	4.086	310.19	1267.33
A_68_P28137854	chr11:112646538-112646582	NM_011448:3037	Sox9	INSIDE	1.615	0.200	15378.05	3070.87	0.323	9983.62	3220.41
A_68_P29509082	chr14:31814863-31814907	NM_026911:-32	Spcs1	PROMOTER	1.770	4.059	489.82	1988.26	7.185	437.64	3144.61
A_68_P31335035	chr17:71901365-71901409	NM_001142631:-14	Spdya	PROMOTER	1.598	1.774	425.98	755.53	2.835	324.11	918.71
A_68_P23364326	chr4:141034893-141034937	NM_019763:59598	Spen	INSIDE	1.537	1.574	1020.25	1605.88	2.419	800.35	1935.71
A_68_P23364325	chr4:141034764-141034809	NM_019763:59726	Spen	INSIDE	1.641	2.009	298.14	598.94	3.297	270.41	891.44
A_68_P28159867	chr11:116395295-116395339	NM_011451:-737	Sphk1	PROMOTER	1.598	0.314	2168.91	680.12	0.501	1488.89	745.85
A_68_P25094658	chr7:52968623-52968667	NM_020011:1933	Sphk2	INSIDE	1.554	1.691	643.79	1088.66	2.627	518.03	1361.13
A_68_P21866928	chr2:172805309-172805353	NM_001083959:-12	Spo11	PROMOTER	2.015	1.547	582.53	901.17	3.117	494.94	1542.80
A_68_P26750366	chr9:96896367-96896411	NM_145134:22386	Spsb4	INSIDE	1.725	1.856	566.28	1051.28	3.202	489.07	1566.15
A_68_P28450915	chr12:56256240-56256284	NM_011899:-64	Srp54a	PROMOTER	2.060	0.293	1566.37	459.68	0.604	1133.80	685.33
A_68_P31093844	chr17:23956989-23957033	NM_175229:16857	Srrm2	INSIDE	2.292	1.378	1318.88	1817.02	3.157	811.12	2560.97
A_68_P27997513	chr11:87861967-87862012	NM_001078167:817	Srsf1	INSIDE	17.221	0.140	648.36	90.54	2.405	447.32	1075.72
A_68_P30360709	chr15:78370273-78370318	NM_009218:4480	Sstr3	INSIDE	1.783	1.666	582.46	970.35	2.970	447.44	1328.90
A_68_P21116406	chr2:32470512-32470556	NM_001025310:8054	St6galnac6	INSIDE	1.731	2.288	358.67	820.74	3.961	284.96	1128.59

ProbeName	Target position of probe on CpG island microarray	TargetID	GeneSymbol	CpG island Description	Ratio of relative methylation (TiO_2-NP/Vehicle)	Sham group			TiO_2-H group		
						Relative methylation (Cy5/Cy3)	Cy3 signal (Input)	Cy5 signal (MeDIP)	Relative methylation (Cy5/Cy3)	Cy3 signal (Input)	Cy5 signal (MeDIP)
A_68_P27316752	chr10:86371693-86371737	NM_138673:98973	Stab2	INSIDE	2.122	2.927	9338.35	27331.90	6.211	6520.23	40497.84
A_68_P32341131	chrX:39503134-39503178	NM_021465:-721	Stag2	PROMOTER	13.004	0.178	2207.41	393.97	2.321	2820.54	6545.97
A_68_P25950576	chr8:72757876-72757920	NM_001168290:-226	Sugp2	PROMOTER	1.610	0.309	2012.33	621.99	0.498	1348.88	671.26
A_68_P32241634	chrX:7651662-7651714	NM_011514:198	Suv39h1	INSIDE	10.451	0.239	364.48	87.10	2.498	517.22	1291.81
A_68_P21890549	chr2:178142357-178142402	NM_177191:-16	Sycp2	PROMOTER	1.520	2.826	231.63	654.59	4.296	230.14	988.75
A_68_P28550370	chr12:76919981-76920025	NM_001005510:698	Syne2	INSIDE	1.740	0.195	2841.57	554.76	0.340	2189.70	743.85
A_68_P32239400	chrX:7215668-7215712	NM_009305:-15	Syp	PROMOTER	2.608	0.180	5125.89	922.11	0.469	5731.52	2688.86
A_68_P28096909	chr11:105451661-105451705	NM_181071:383	Tanc2	INSIDE	1.546	0.378	1891.76	715.96	0.585	1507.47	882.01
A_68_P21682258	chr2:139892550-139892594	NM_001159640:-51	Tasp1	PROMOTER	1.691	0.358	1664.46	596.21	0.606	1402.58	849.45
A_68_P28176530	chr11:119072055-119072099	NM_172443:17737	Tbc1d16	INSIDE	1.812	12.586	1786.95	22490.69	22.804	1619.18	36924.48
A_68_P21266388	chr2:61643903-61643948	NM_009322:1416	Tbr1	INSIDE	1.784	0.355	1982.06	703.06	0.633	1308.33	827.97
A_68_P27798408	chr11:52096304-52096348	NM_009331:-574	Tcf7	PROMOTER	1.514	1.363	2891.10	3941.51	2.065	2257.87	4661.79
A_68_P20066646	chr1:19204206-19204250	NM_001025305:2094	Tcfap2b	INSIDE	1.766	0.311	4912.11	1525.68	0.548	3553.92	1949.08
A_68_P20552784	chr1:120524752-120524796	NM_023755:253	Tcfcp2l1	INSIDE	1.656	0.287	2154.08	618.23	0.475	1761.18	836.84
A_68_P22336407	chr3:93249382-93249426	NM_001163098:3153	Tchh	INSIDE	2.254	0.162	2806.80	453.59	0.364	2119.88	772.12
A_68_P22336399	chr3:93248419-93248463	NM_001163098:2189	Tchh	INSIDE	1.547	49.363	1152.98	56914.80	76.343	1409.58	107611.20
A_68_P30379759	chr15:81641797-81641841	NM_017376:-25	Tef	PROMOTER	1.521	1.570	413.49	649.22	2.388	313.65	748.92
A_68_P26504313	chr9:50369450-50369494	NM_025687:-99	Tex12	DIVERGENT_PROMOTER	2.564	1.670	595.94	995.40	4.283	497.92	2132.59
A_68_P23991446	chr5:112755370-112755414	NM_018783:4	Tfip11	INSIDE	1.503	0.238	1951.80	463.90	0.357	1333.45	476.26
A_68_P25011335	chr7:26473118-26473162	NM_011577:1120	Tgfb1	INSIDE	1.719	3.422	493.96	1690.29	5.884	412.39	2426.41
A_68_P32340124	chrX:39264668-39264712	NM_001033422:388	Thoc2	INSIDE	5.531	0.379	789.49	299.23	2.096	1208.76	2534.12
A_68_P29049666	chr13:54569805-54569849	NM_028597:375	Thoc3	INSIDE	1.617	2.235	11703.62	26157.32	3.614	6830.35	24686.83
A_68_P30953523	chr16:89885140-89885184	NM_009384:75910	Tiam1	INSIDE	1.909	1.563	459.65	718.24	2.983	319.79	953.89
A_68_P27286214	chr10:80363023-80363067	NM_013895:670	Timm13	INSIDE	1.777	0.239	2267.77	540.95	0.424	1599.29	677.80
A_68_P32240782	chrX:7476676-7476720	NM_011591:175	Timm17b	INSIDE	2.030	0.309	1357.54	419.03	0.626	1773.99	1111.37
A_68_P27693351	chr11:33102254-33102303	NM_019916:1310	Tlx3	INSIDE	1.997	1.851	1352.30	2502.54	3.696	734.82	2716.23
A_68_P27567653	chr11:6174835-6174879	NM_134020:-16	Tmed4	PROMOTER	1.665	0.110	5812.34	641.98	0.184	4030.60	741.25
A_68_P20218129	chr1:50984521-50984565	NM_019790:176	Tmeff2	INSIDE	1.788	0.328	2119.57	694.49	0.586	1600.50	937.73
A_68_P23590340	chr5:34000396-34000440	NM_026698:63	Tmem129	INSIDE	1.506	0.268	1998.42	535.52	0.404	1330.61	536.91
A_68_P31926782	chr19:5082847-5082891	NM_001001885:2609	Tmem151a	INSIDE	1.970	2.443	698.39	1706.27	4.812	594.37	2860.14
A_68_P32709179	chrX:139116571-139116615	NM_001199360:324	Tmem164	INSIDE	5.083	0.468	2513.19	1176.81	2.380	3268.80	7779.73
A_68_P21070914	chr2:25111268-25111312	NM_177344:332	Tmem203	INSIDE	1.743	0.151	3164.81	478.67	0.264	2143.55	565.17
A_68_P32488665	chrX:78316348-78316392	NM_138751:388	Tmem47	INSIDE	1.604	0.254	2353.73	597.05	0.407	3251.49	1322.72
A_68_P23523160	chr5:20388333-20388392	NM_177601:92	Tmem60	INSIDE	2.231	1.509	541.80	817.53	3.366	338.81	1140.40
A_68_P25102684	chr7:54305881-54305925	NM_026436:-107	Tmem86a	PROMOTER	1.869	1.527	543.73	830.13	2.853	554.78	1582.91
A_68_P27340413	chr10:90634459-90634503	NM_001080129:-153	Tmpo	PROMOTER	1.883	0.314	1872.50	588.32	0.592	1528.15	904.17
A_68_P30484944	chr15:100111219-100111263	NM_183109:-27	Tmprss12	PROMOTER	1.650	2.002	494.51	989.99	3.304	430.45	1422.16
A_68_P30484946	chr15:100111437-100111481	NM_183109:191	Tmprss12	INSIDE	1.619	1.739	1151.96	2003.20	2.815	938.91	2642.65
A_68_P21652098	chr2:134469794-134469838	NM_029148:41	Tmx4	INSIDE	1.554	0.379	1662.61	629.68	0.588	1179.99	694.37
A_68_P22015879	chr3:28162505-28162549	NM_001163009:391	Tnik	INSIDE	1.526	0.327	1429.22	467.12	0.499	1024.73	511.09
A_68_P30380080	chr15:81690283-81690327	NM_020507:-1548	Tob2	PROMOTER	1.543	0.302	2348.56	709.08	0.466	1709.12	796.35
A_68_P29428730	chr14:17197854-17197898	NM_009409:157	Top2b	INSIDE	2.689	0.183	2298.70	420.51	0.492	1847.83	908.83
A_68_P28470051	chr12:60162088-60162132	NM_030057:338	Trappc6b	INSIDE	1.608	0.221	3539.41	780.78	0.355	2721.52	965.22
A_68_P24974188	chr7:13609447-13609491	NM_011588:-32	Trim28	PROMOTER	1.799	7.313	835.47	6109.48	13.158	753.92	9919.86
A_68_P22372025	chr3:100726540-100726584	NM_001165953:147	Trim45	INSIDE	3.751	0.092	5529.70	508.00	0.345	3495.54	1204.59

ProbeName	Target position of probe on CpG island microarray	TargetID	GeneSymbol	CpG island Description	Ratio of relative methylation (TiO_2-NP/Vehicle)	Sham group			TiO_2-H group		
						Relative methylation (Cy5/Cy3)	Cy3 signal (Input)	Cy5 signal (MeDIP)	Relative methylation (Cy5/Cy3)	Cy3 signal (Input)	Cy5 signal (MeDIP)
A_68_P28157433	chr11:115971055-115971099	NM_001205081:473	Trim47	INSIDE	1.725	5.091	5170.14	26322.32	8.782	3817.22	33524.51
A_68_P29880580	chr14:106505493-106505537	NM_198601:100	Trim52	INSIDE	1.755	2.108	518.00	1092.14	3.700	395.37	1462.87
A_68_P29880583	chr14:106505860-106505904	NM_198601:468	Trim52	INSIDE	1.709	2.239	287.69	644.21	3.828	221.37	847.33
A_68_P26247733	chr8:127317798-127317842	NM_198632:902	Trim67	INSIDE	1.504	0.233	3066.14	714.57	0.351	2096.77	735.01
A_68_P30094306	chr15:27677312-27677364	NM_001081302:278265	Trio	INSIDE	1.502	2.243	997.20	2236.39	3.369	801.14	2698.99
A_68_P31260730	chr17:57388800-57388844	NM_134125:-51	Trip10	DIVERGENT_PROMOTER	1.631	0.256	3650.49	934.72	0.418	2504.21	1046.13
A_68_P20405966	chr1:84835679-84835723	NM_133975:179	Trip12	INSIDE	1.580	0.338	2282.17	772.45	0.535	1822.84	974.98
A_68_P22059401	chr3:36570355-36570399	NM_019510:18713	Trpc3	INSIDE	1.700	1.747	840.14	1467.61	2.970	787.56	2338.68
A_68_P25092433	chr7:52582778-52582822	NM_175130:6350	Trpm4	INSIDE	1.554	2.236	1288.18	2880.47	3.474	996.52	3462.35
A_68_P32700072	chrX:137134843-137134887	NM_001077364:197	Tsc22d3	INSIDE	3.226	3.164	1997.35	6319.86	10.209	2177.27	22226.92
A_68_P25355269	chr7:105509376-105509420	NM_001024619:400	Tsku	INSIDE	1.548	0.145	4000.65	581.14	0.225	2564.41	576.78
A_68_P23982393	chr5:111308521-111308565	NM_024477:-279	Ttc28	PROMOTER	1.902	3.194	198.86	635.17	6.075	172.72	1049.32
A_68_P23982395	chr5:111308888-111308932	NM_024477:89	Ttc28	INSIDE	1.694	2.668	340.56	908.75	4.521	237.19	1072.43
A_68_P23984844	chr5:111706164-111706208	NM_024477:397365	Ttc28	INSIDE	1.770	1.542	1479.85	2281.56	2.729	1118.60	3052.93
A_68_P23984176	chr5:111612120-111612164	NM_024477:303321	Ttc28	INSIDE	1.695	1.505	1079.85	1624.98	2.551	796.72	2032.28
A_68_P30388853	chr15:83341230-83341274	NM_178869:85	Ttll1	INSIDE	1.671	0.332	1781.62	590.68	0.554	1213.30	672.35
A_68_P32791140	chrX:159267555-159267599	NM_178935:-245	Txlng	PROMOTER	2.343	0.241	1253.80	302.40	0.565	1704.19	963.03
A_68_P32791135	chrX:159266933-159266977	NM_178935:377	Txlng	INSIDE	2.261	0.628	2796.29	1755.34	1.420	3526.59	5006.40
A_68_P30356632	chr15:77759535-77759579	NM_019913:-132	Txn2	PROMOTER	2.219	0.114	4801.38	548.13	0.253	3063.19	776.05
A_68_P28159934	chr11:116403055-116403099	NM_173755:39685	Ube2o	INSIDE	1.561	3.072	1274.06	3913.69	4.794	978.30	4690.02
A_68_P22314483	chr3:88357962-88358006	NM_033526:347	Ubqln4	INSIDE	1.671	1.563	4922.57	7695.81	2.612	3964.44	10354.34
A_68_P30651947	chr16:32333125-32333169	NM_177633:809	Ubxn7	INSIDE	1.633	2.079	455.69	947.25	3.395	349.78	1187.46
A_68_P25958124	chr8:74184428-74184472	NM_001029873:11206	Unc13a	INSIDE	1.621	0.164	3754.13	614.72	0.265	2588.49	687.20
A_68_P22584284	chr3:141128445-141128489	NM_009472:-61	Unc5c	PROMOTER	1.551	0.255	2975.85	758.83	0.395	1961.62	775.69
A_68_P27564657	chr11:5617071-5617115	NM_178623:24087	Urgcp	INSIDE	2.355	2.436	705.57	1718.85	5.737	499.08	2863.32
A_68_P32289745	chrX:20281291-20281340	NM_145628:281	Usp11	INSIDE	27.746	0.147	585.44	85.89	4.071	955.71	3890.55
A_68_P32289747	chrX:20281555-20281599	NM_145628:542	Usp11	INSIDE	3.597	0.193	1625.82	314.34	0.696	2294.48	1595.85
A_68_P32289746	chrX:20281381-20281426	NM_145628:369	Usp11	INSIDE	11.256	0.505	675.37	341.38	5.689	884.13	5030.22
A_68_P27356011	chr10:93308901-93308948	NM_183199:14625	Usp44	INSIDE	1.536	2.412	499.45	1204.85	3.704	426.66	1580.55
A_68_P27356009	chr10:93308576-93308623	NM_183199:14300	Usp44	INSIDE	1.827	1.592	529.10	842.17	2.909	453.10	1317.95
A_68_P32363782	chrX:45639057-45639101	NM_028276:28968	Utp14a	DOWNSTREAM	3.521	0.176	1319.61	232.35	0.620	1763.97	1093.58
A_68_P21017270	chr2:13496599-13496643	NM_011701:683	Vim	INSIDE	1.621	0.082	6678.46	550.72	0.134	4542.28	607.33
A_68_P21568844	chr2:119119741-119119785	NM_172269:5285	Vps18	INSIDE	1.805	2.271	503.93	1144.60	4.099	423.20	1734.62
A_68_P25075289	chr7:48157966-48158010	NM_021387:3341	Vstm2b	INSIDE	1.899	0.287	1707.59	489.65	0.545	1239.87	675.27
A_68_P31140216	chr17:31649029-31649073	NM_021322:382	Wdr4	INSIDE	1.916	0.277	1615.37	446.67	0.530	1152.78	610.77
A_68_P27947945	chr11:79068066-79068110	NM_019653:109	Wsb1	INSIDE	1.574	0.314	1771.06	556.97	0.495	1263.90	625.78
A_68_P32340722	chrX:39421221-39421265	NM_009688:230	Xiap	INSIDE	3.424	0.695	1431.58	994.36	2.378	1849.87	4399.72
A_68_P20002774	chr1:3662119-3662163	NM_001011874:-561	Xkr4	PROMOTER	1.549	0.370	2511.88	930.49	0.574	1903.13	1091.73
A_68_P29703312	chr14:71166447-71166491	NM_023045:-33	Xpo7	DIVERGENT_PROMOTER	2.005	2.141	476.78	1020.60	4.291	423.92	1819.16
A_68_P24118492	chr5:136387366-136387410	NM_018871:23123	Ywhag	INSIDE	1.517	1.841	684.51	1259.91	2.792	570.54	1593.10
A_68_P24118629	chr5:136410508-136410552	NM_018871:-19	Ywhag	PROMOTER	1.555	1.573	1243.73	1956.40	2.446	1088.59	2662.76
A_68_P23364203	chr4:141020484-141020528	NM_009541:19919	Zbtb17	INSIDE	2.058	1.590	789.70	1255.95	3.272	619.89	2028.53
A_68_P28553587	chr12:77469901-77469960	NM_001172104:521	Zbtb25	INSIDE	1.894	1.822	365.41	665.81	3.450	245.61	847.38
A_68_P31841472	chr18:76305759-76305803	NM_145356:325949	Zbtb7c	INSIDE	1.650	1.498	831.82	1246.11	2.472	691.68	1709.80
A_68_P32542578	chrX:92907030-92907074	NM_001034907:36	Zc3h12b	INSIDE	1.685	1.411	1150.99	1624.49	2.379	1422.01	3382.68

ProbeName	Target position of probe on CpG island microarray	TargetID	GeneSymbol	CpG island Description	Ratio of relative methylation (TiO_2-NP/Vehicle)	Sham group			TiO_2-H group		
						Relative methylation (Cy5/Cy3)	Cy3 signal (Input)	Cy5 signal (MeDIP)	Relative methylation (Cy5/Cy3)	Cy3 signal (Input)	Cy5 signal (MeDIP)
A_68_P21394205	chr2:84554801-84554845	NM_144887:499	Zdhhc5	INSIDE	1.812	0.267	2885.30	770.98	0.484	2065.95	1000.18
A_68_P21394211	chr2:84555629-84555673	NM_144887:-329	Zdhhc5	PROMOTER	1.618	0.133	4957.00	658.84	0.215	3396.61	730.59
A_68_P29616218	chr14:55710458-55710502	NM_001039198:405	Zfhx2	INSIDE	1.533	0.389	2644.87	1028.06	0.596	2164.12	1289.75
A_68_P26153173	chr8:111324170-111324214	NM_007496:85649	Zfhx3	INSIDE	1.551	2.009	632.58	1270.69	3.116	445.30	1387.73
A_68_P25029686	chr7:30836414-30836458	NM_011748:-21	Zfp14	PROMOTER	1.721	0.324	2527.42	818.75	0.558	1723.15	960.79
A_68_P31092362	chr17:23694847-23694891	NM_001033496:6325	Zfp213	INSIDE	1.729	1.803	713.38	1286.19	3.118	601.25	1874.66
A_68_P32462127	chrX:70588115-70588159	NM_001160229:177	Zfp275	INSIDE	8.456	0.519	2966.77	1538.49	4.385	3385.03	14843.76
A_68_P24424840	chr6:47855086-47855130	NM_146175:27555	Zfp282	INSIDE	1.625	1.496	923.18	1381.37	2.432	720.93	1753.37
A_68_P24155795	chr5:144031809-144031857	NM_017467:-131	Zfp316	PROMOTER	1.660	0.329	1913.01	630.08	0.547	1443.98	789.70
A_68_P24973912	chr7:13556599-13556643	NM_178732:5408	Zfp324	INSIDE	2.378	1.721	736.15	1267.14	4.093	587.18	2403.51
A_68_P24973913	chr7:13556719-13556763	NM_178732:5528	Zfp324	INSIDE	1.565	1.604	533.79	856.25	2.511	400.72	1006.01
A_68_P25614226	chr8:3495954-3495998	NM_080461:2839	Zfp358	INSIDE	1.971	2.538	517.85	1314.41	5.003	404.01	2021.19
A_68_P25614227	chr8:3496122-3496166	NM_080461:3007	Zfp358	INSIDE	1.721	2.260	486.82	1100.25	3.890	447.45	1740.49
A_68_P25614229	chr8:3496319-3496363	NM_080461:3203	Zfp358	INSIDE	1.516	1.676	1376.54	2306.53	2.540	1074.47	2729.19
A_68_P29455881	chr14:22805841-22805885	NM_145459:2961	Zfp503	INSIDE	1.513	0.347	1498.64	519.66	0.525	1110.85	582.74
A_68_P31879113	chr18:83078968-83079012	NM_183033:-1280	Zfp516	PROMOTER	1.503	0.335	7196.37	2414.20	0.504	4540.73	2289.47
A_68_P31879481	chr18:83126670-83126714	NM_001177464:42669	Zfp516	INSIDE	1.781	1.623	1174.94	1906.40	2.889	905.86	2617.09
A_68_P25506496	chr7:134754584-134754628	NM_177226:3341	Zfp629	INSIDE	1.839	2.172	607.26	1318.89	3.994	438.16	1749.85
A_68_P30351141	chr15:76741990-76742034	NM_001168276:13866	Zfp647	INSIDE	1.607	2.385	350.58	836.06	3.831	309.61	1186.20
A_68_P30351140	chr15:76741911-76741955	NM_001168276:13946	Zfp647	INSIDE	1.561	2.963	993.98	2945.01	4.626	782.11	3618.19
A_68_P30351139	chr15:76741794-76741838	NM_001168276:14062	Zfp647	INSIDE	1.822	1.629	833.74	1358.50	2.968	654.49	1942.66
A_68_P30351142	chr15:76742082-76742126	NM_001168276:13774	Zfp647	INSIDE	1.603	1.493	752.04	1122.60	2.393	600.17	1436.20
A_68_P26885415	chr9:121672022-121672066	NM_001166644:2894	Zfp651	INSIDE	1.872	2.020	462.48	934.42	3.782	345.42	1306.30
A_68_P26350330	chr9:21875518-21875562	NM_177318:249	Zfp653	INSIDE	1.538	0.384	1654.20	635.45	0.591	1180.45	697.20
A_68_P24954799	chr7:6238258-6238302	NM_001024928:99	Zfp667	INSIDE	2.041	0.248	3830.73	948.82	0.506	2759.75	1395.27
A_68_P25505622	chr7:134587895-134587939	NM_175163:4756	Zfp689	INSIDE	1.674	2.210	702.77	1552.86	3.698	587.12	2171.29
A_68_P25505623	chr7:134587980-134588024	NM_175163:4670	Zfp689	INSIDE	1.538	5.898	1196.95	7060.06	9.071	1019.62	9248.95
A_68_P32603686	chrX:109714974-109715018	NM_177747:862	Zfp711	INSIDE	1.564	1.966	220.76	433.99	3.074	313.10	962.39
A_68_P29109187	chr13:67653829-67653873	NM_001035231:238	Zfp748	INSIDE	1.538	0.304	1694.03	514.77	0.467	1325.27	619.47
A_68_P25505248	chr7:134486671-134486730	NM_146202:2128	Zfp768	INSIDE	2.019	2.895	246.64	713.95	5.843	173.77	1015.44
A_68_P24428351	chr6:48570207-48570251	NM_173429:7050	Zfp775	INSIDE	2.012	1.780	864.07	1538.00	3.581	728.59	2609.17
A_68_P24428350	chr6:48570111-48570155	NM_173429:6954	Zfp775	INSIDE	1.549	1.999	1323.59	2646.24	3.098	983.15	3045.57
A_68_P24428347	chr6:48569751-48569795	NM_173429:6594	Zfp775	INSIDE	1.528	1.728	709.09	1225.03	2.640	595.89	1573.25
A_68_P24428352	chr6:48570342-48570386	NM_173429:7186	Zfp775	INSIDE	2.204	1.796	319.13	573.30	3.959	296.38	1173.33
A_68_P24954141	chr7:6084325-6084369	NM_001013012:23227	Zfp787	INSIDE	1.652	1.816	707.21	1284.10	2.999	635.80	1906.85
A_68_P24951677	chr7:4982359-4982403	NM_001033383:10403	Zfp865	INSIDE	1.711	1.571	765.01	1202.04	2.688	647.70	1740.82
A_68_P32462526	chrX:70667546-70667590	NM_009566:11127	Zfp92	INSIDE	1.543	1.420	939.56	1333.78	2.190	1156.79	2533.90
A_68_P32403906	chrX:55284045-55284104	NM_009575:270	Zic3	INSIDE	9.281	0.334	328.10	109.52	3.098	667.60	2068.30
A_68_P23195055	chr4:107890802-107890861	NM_001167936:-304	Zyg11a	PROMOTER	1.750	1.967	828.18	1628.84	3.443	646.09	2224.34
A_68_P24397783	chr6:42299752-42299796	NM_011777:-52	Zyx	PROMOTER	2.065	0.117	4896.28	575.25	0.243	3371.39	817.83
