# Supplementary material for: Effects of Prenatal Exposure to Titanium Dioxide Nanoparticles on DNA Methylation and Gene Expression Profile in the Mouse Brain
Source: Front Toxicol. 2021 Oct 8;3:705910. doi: 10.3389/ftox.2021.705910 (PMC8915839; doi:10.3389/ftox.2021.705910)
Supplement: Supplementary file 3 [file Table11.PDF]

Supplementary Table 11.
The genes that showed differential expression accompanied by altered DNA methylation commonly in male (M) and female (F) offspring

GenBank Accession	GeneSymbol	Sex	Target position of probe on CpG island microarray	mRNA	DNA methylation
				Fold change	Fold change of relative methylation
NM_001163145	1810041L15Rik	M	chr15:84276781-84276825	1.861	0.188
			chr15:84236917-84236961	1.861	0.626
		F	chr15:84237151-84237195	1.886	0.495
			chr15:84237076-84237131	1.886	0.649
NM_009767	Chic1	M	chrX:100551806-100551850	1.781	0.184
		F	chrX:100551713-100551757	2.065	0.123
NM_177307	Cyp4f39	M	chr17:32589667-32589711	2.094	0.120
			chr17:32589827-32589872	2.094	0.193
			chr17:32629460-32629505	2.094	0.623
		F	chr17:32589667-32589711	2.594	0.369
			chr17:32589827-32589872	2.594	0.131
NM_015820	Hs6st3	M	chr14:119537211-119537255	2.048	0.592
			chr14:119537426-119537470	2.048	0.635
		F	chr14:119537625-119537669	1.672	0.226
NM_001077514	Slc1a2	M	chr2:102498900-102498944	1.912	0.580
		F	chr2:102499439-102499483	1.670	0.349
NM_139308	Stard7	M	chr2:127096105-127096149	1.788	0.582
		F	chr2:127096615-127096659	3.769	0.234
NM_001113352	Synj2	M	chr17:5941768-5941812	2.072	0.232
			chr17:5941641-5941685	2.072	0.521
		F	chr17:5941641-5941685	3.461	0.428
