# Supplementary material for: Effects of Prenatal Exposure to Titanium Dioxide Nanoparticles on DNA Methylation and Gene Expression Profile in the Mouse Brain
Source: Front Toxicol. 2021 Oct 8;3:705910. doi: 10.3389/ftox.2021.705910 (PMC8915839; doi:10.3389/ftox.2021.705910)
Supplement: Supplementary file 4 [file Table1.PDF]

Supplementary Table 1

(A) Sequences of primers for DNA methylation analysis

Gene	Accession No.	Sequence (5' to 3')
Cyp4f39	NC_000083	F: GTCCAGCGACTGTGGCCTTA<br>R: ACACCAGAGCGCGCTCAGGA
Hs6st3	NC_000080	F: GAGCCTCATGCAGCTCCTGT<br>R: GCTCCTCTTGTACCTGGACG
Synj2	NC_000083	F: CTTGTCCGCGGGAGTACTGGGT<br>R: CAGTTGGCTCTCCAAGAACAGA

(B) Sequences of primers for qRT-PCR

Gene	Accession No.	Sequence (5' to 3')
Dcc	NM_007831	F: TACCAATGCGAGGCATCCTT<br>R: GTATCTCCCATGAAGGCTGT
Traf2	NM_009422	F: ACGGAGTGTCCTGCATGTAA<br>R: GCATGCTCTAACATGGTCCTGA
Sox2	NM_011443	F: GGAAAGGGTTCTTGCTGGGT<br>R: ACGAAAACGGTCTTGCCAGT
Dnmt1	NM_010066	F: TCCAGAGCCCTATCGCATCG<br>R: CGTTGTAGGACCTGTGGGTAT
Dnmt3a	NM_007872	F: CTGCAAGAGTGTCTGGAGCA<br>R: TCAGTGCACCACAGGATGTC
Dnmt3b	NM_010068	F: AGTCGAAGACGCACAACCAATG<br>R: GTGACTTCAGAAGCCATCCGT
Tet1	NM_001253857	F: TGCTGGAGACTGTCGACTTG<br>R: CGAATCAACGTACACACCACG
Tet2	NM_001040400	F: GGCTGCCCTGTAGGATTTGT<br>R: AATGAATCCAGCAGCACCGT
Tet3	NM_183138	F: AATGATGACCGGACCTGTGC<br>R: GGAACTTTCGTGGCGTCTTG
Gapdh	NM_008084	F: TGTGCAGTGCCAGCCTCGTC<br>R: GGATGCATTGCTGACAATCT
