# Supplementary material for: Effects of Prenatal Exposure to Titanium Dioxide Nanoparticles on DNA Methylation and Gene Expression Profile in the Mouse Brain
Source: Front Toxicol. 2021 Oct 8;3:705910. doi: 10.3389/ftox.2021.705910 (PMC8915839; doi:10.3389/ftox.2021.705910)
Supplement: Supplementary file 5 [file Table2.pdf]

Supplementary Table 2. Alteration of DNA methylation state of the brain of male offspring prenatally exposed to TiO_2-NP.

The relative DNA methylation was calculated by dividing Cy5 signal by Cy3 signal obtained from CpG island microarray and then compared between Sham and TiO_2-NP groups.

ProbeName	Target position of probe on CpG island microarray	TargetID	GeneSymbol	CpG island Description	Ratio of relative methylation (TiO_2-NP/Vehicle)	Sham group			TiO_2-H group		
						Relative methylation (Cy5/Cy3)	Cy3 signal (Input)	Cy5 signal (MeDIP)	Relative methylation (Cy5/Cy3)	Cy3 signal (Input)	Cy5 signal (MeDIP)
A_68_P32380514	chrX:49518048-49518092	NM_008150:30	Gpc4	INSIDE	61.524	0.065	1399.00	90.81	3.994	1906.71	7614.88
A_68_P32385499	chrX:50410041-50410085	NR_029756:-2536	Mir322	PROMOTER	49.785	0.204	5800.27	1184.88	10.170	6244.37	63505.16
A_68_P32289745	chrX:20281291-20281340	NM_145628:281	Usp11	INSIDE	27.746	0.147	585.44	85.89	4.071	955.71	3890.55
A_68_P32321655	chrX:34625187-34625231	NM_026055:-29	Rpl39	PROMOTER	21.396	0.085	1013.99	86.41	1.823	1556.92	2838.82
A_68_P32364066	chrX:45698978-45699030	NM_178782:4470	Bcorl1	INSIDE	17.870	0.299	906.62	271.43	5.350	1281.69	6857.13
A_68_P27997513	chr11:87861967-87862012	NM_001078167:817	Srsf1	INSIDE	17.221	0.140	648.36	90.54	2.405	447.32	1075.72
A_68_P32328377	chrX:35918417-35918470	NM_001110142:10930	Cul4b	INSIDE	17.143	0.162	532.97	86.41	2.779	814.00	2262.38
A_68_P32341131	chrX:39503134-39503178	NM_021465:-721	Stag2	PROMOTER	13.004	0.178	2207.41	393.97	2.321	2820.54	6545.97
A_68_P32240694	chrX:7461707-7461754	NM_001083937:361	Slc35a2	INSIDE	12.416	0.120	941.20	113.40	1.496	1263.13	1889.59
A_68_P32539364	chrX:91786968-91787022	NM_019791:419	Maged1	INSIDE	11.572	0.339	339.65	114.98	3.917	412.04	1614.14
A_68_P32395899	chrX:53708448-53708501	NM_172779:459	Ddx26b	INSIDE	11.436	0.200	465.56	93.05	2.286	596.72	1363.81
A_68_P32289746	chrX:20281381-20281426	NM_145628:369	Usp11	INSIDE	11.256	0.505	675.37	341.38	5.689	884.13	5030.22
A_68_P32553704	chrX:96332183-96332227	NM_010110:736	Efnb1	INSIDE	10.685	0.064	1361.04	87.02	0.683	2035.53	1390.49
A_68_P32241634	chrX:7651662-7651714	NM_011514:198	Suv39h1	INSIDE	10.451	0.239	364.48	87.10	2.498	517.22	1291.81
A_68_P32380515	chrX:49518249-49518293	NM_008150:-170	Gpc4	PROMOTER	10.005	0.221	1132.83	250.79	2.215	1539.10	3409.03
A_68_P32403906	chrX:55284045-55284104	NM_009575:270	Zic3	INSIDE	9.281	0.334	328.10	109.52	3.098	667.60	2068.30
A_68_P32364052	chrX:45697351-45697395	NM_178782:2838	Bcorl1	INSIDE	9.266	0.194	506.06	98.31	1.800	745.77	1342.50
A_68_P32691812	chrX:135449017-135449063	NM_013724:72	Nrk	INSIDE	8.865	0.368	493.89	181.82	3.264	842.52	2749.69
A_68_P32462127	chrX:70588115-70588159	NM_001160229:177	Zfp275	INSIDE	8.456	0.519	2966.77	1538.49	4.385	3385.03	14843.76
A_68_P32798240	chrX:160857773-160857818	NM_011081:77	Piga	INSIDE	8.287	0.056	1524.74	85.80	0.466	2229.20	1039.60
A_68_P32364768	chrX:45807315-45807359	NM_019680:8973	Elf4	INSIDE	8.200	0.167	1849.80	308.14	1.366	2377.43	3247.39
A_68_P32260250	chrX:11654399-11654443	NM_029510:3259	Bcor	INSIDE	8.005	0.077	3083.96	237.31	0.616	3350.61	2063.97
A_68_P32240898	chrX:7499680-7499725	NM_013892:2755	Pcsk1n	INSIDE	6.679	0.245	824.75	202.36	1.639	1055.20	1729.15
A_68_P32239706	chrX:7274518-7274562	NM_173747:281	Gpkow	INSIDE	6.175	0.327	901.99	295.18	2.021	1162.22	2348.67
A_68_P32543330	chrX:93163948-93164001			Unknown	6.123	1.578	867.83	1369.34	9.662	918.74	8876.91
A_68_P32238978	chrX:7151494-7151538	NM_138605:-270	Ppp1r3f	DIVERGENT_PROMOTER	5.965	0.429	1256.22	539.47	2.562	1721.03	4408.97
A_68_P32742985	chrX:147247678-147247726	NM_001199246:544	Maged2	INSIDE	5.935	0.285	892.44	254.21	1.691	1026.15	1734.92
A_68_P32378025	chrX:49034174-49034218	NM_015819:-542	Hs6st2	PROMOTER	5.909	0.369	513.47	189.23	2.178	863.98	1881.51
A_68_P32507213	chrX:83022104-83022148	NM_008194:32	Gyk	INSIDE	5.864	0.336	2422.30	813.29	1.969	2706.02	5327.99
A_68_P32576278	chrX:101395079-101395123	NM_001077354:1356	C77370	INSIDE	5.811	0.451	392.85	177.07	2.619	482.00	1262.41
A_68_P32717148	chrX:140752921-140752965	NM_026247:434	Alg13	INSIDE	5.797	0.491	4385.26	2155.07	2.849	5164.89	14713.45
A_68_P32340124	chrX:39264668-39264712	NM_001033422:388	Thoc2	INSIDE	5.531	0.379	789.49	299.23	2.096	1208.76	2534.12
A_68_P32369309	chrX:46847140-46847184	NM_001081123:23536	Arhgap36	INSIDE	5.435	0.128	2155.34	275.35	0.694	2870.86	1993.46
A_68_P31256055	chr17:56568653-56568712	NM_011218:47221	Ptprs	INSIDE	5.356	3.108	245.36	762.48	16.645	231.78	3857.90
A_68_P24904451	chr6:142363030-142363089	NM_145572:58570	Gys2	DOWNSTREAM	5.355	0.662	863.47	571.55	3.545	537.27	1904.41
A_68_P32598389	chrX:108010047-108010091	NM_008901:265	Pou3f4	INSIDE	5.265	0.298	3935.65	1171.48	1.567	4717.83	7394.34
A_68_P32709179	chrX:139116571-139116615	NM_001199360:324	Tmem164	INSIDE	5.083	0.468	2513.19	1176.81	2.380	3268.80	7779.73
A_68_P32399260	chrX:54306606-54306650	NM_028242:-118	Htatsf1	PROMOTER	4.864	0.135	1244.97	168.41	0.658	1743.33	1147.10
A_68_P32240442	chrX:7418743-7418787	NM_138604:-192	Otud5	PROMOTER	4.783	0.117	2334.38	273.05	0.559	3436.31	1922.30
A_68_P25015938	chr7:27964028-27964072	NM_029391:-148	Rab4b	PROMOTER	4.680	0.126	4937.38	624.26	0.592	2873.46	1700.28
A_68_P32241074	chrX:7524906-7524950	NM_001130416:87	Hdac6	INSIDE	4.492	0.607	3825.27	2320.66	2.725	4633.10	12626.24
A_68_P32601449	chrX:109009111-109009155			Unknown	4.434	0.330	1963.34	647.03	1.461	2907.10	4247.92
A_68_P32806533	chrX:162917564-162917608	NM_019773:213	Rab9	INSIDE	4.426	0.339	1345.44	456.69	1.502	1695.97	2548.07
A_68_P32788273	chrX:158597915-158597959	NM_001081052:-214	Nhs	PROMOTER	4.380	0.464	411.34	190.82	2.032	592.38	1203.58
A_68_P32262594	chrX:12164926-12164970	NM_027439:64	Atp6ap2	INSIDE	4.317	0.101	1878.74	190.33	0.437	2616.86	1144.46
A_68_P21101982	chr2:30093777-30093821	NM_177725:510	Lrrc8a	INSIDE	4.251	1.957	955.12	1869.22	8.320	574.18	4777.13
A_68_P20416033	chr1:89051506-89051553	NM_021306:73	Ecel1	INSIDE	4.247	0.146	1120.48	163.91	0.621	1467.78	911.93
A_68_P27311664	chr10:85484474-85484519	NM_153195:-177	Fbxo7	DIVERGENT_PROMOTER	4.235	0.674	2000.06	1348.16	2.854	1567.98	4475.60
A_68_P22063505	chr3:37318090-37318134	NM_153561:400	Nudt6	INSIDE	4.156	0.120	2067.96	247.88	0.498	1795.61	894.47
A_68_P25933298	chr8:69010075-69010123	NM_025465:308	1810029B16Rik	INSIDE	4.022	1.912	403.87	772.20	7.690	290.64	2235.19
A_68_P31599408	chr18:31920327-31920372	NM_153515:816	Ammecr1l	INSIDE	3.933	0.506	964.60	487.95	1.990	689.52	1371.92
A_68_P32767191	chrX:154036331-154036375	NM_172307:295	Mbtps2	INSIDE	3.803	0.459	567.72	260.66	1.746	880.69	1537.88
A_68_P32786690	chrX:158346840-158346884	NM_001081052:250860	Nhs	INSIDE	3.772	0.436	477.00	207.86	1.644	686.46	1128.29
A_68_P22372025	chr3:100726540-100726584	NM_001165953:147	Trim45	INSIDE	3.751	0.092	5529.70	508.00	0.345	3495.54	1204.59
A_68_P32465511	chrX:71167531-71167575	NM_001164704:8637	Renbp	INSIDE	3.621	0.192	1381.64	264.79	0.694	1856.56	1288.37
A_68_P32289747	chrX:20281555-20281599	NM_145628:542	Usp11	INSIDE	3.597	0.193	1625.82	314.34	0.696	2294.48	1595.85
A_68_P32321494	chrX:34588841-34588885	NM_173779:23	Ankrd58	INSIDE	3.596	0.508	536.14	272.46	1.827	800.77	1463.16
A_68_P32363782	chrX:45639057-45639101	NM_028276:28968	Utp14a	DOWNSTREAM	3.521	0.176	1319.61	232.35	0.620	1763.97	1093.58
A_68_P23720362	chr5:58110309-58110354	NM_018764:1072	Pcdh7	INSIDE	3.482	0.639	2287.11	1460.71	2.224	1835.76	4082.13
A_68_P32678078	chrX:132277552-132277596	NM_001009575:344	Armcx5	INSIDE	3.479	0.513	1635.75	839.37	1.785	2346.21	4187.96
A_68_P32340722	chrX:39421221-39421265	NM_009688:230	Xiap	INSIDE	3.424	0.695	1431.58	994.36	2.378	1849.87	4399.72

ProbeName	Target position of probe on CpG island microarray	TargetID	GeneSymbol	CpG island Description	Ratio of relative methylation (TiO_2-NP/Vehicle)	Sham group			TiO_2-H group		
						Relative methylation (Cy5/Cy3)	Cy3 signal (Input)	Cy5 signal (MeDIP)	Relative methylation (Cy5/Cy3)	Cy3 signal (Input)	Cy5 signal (MeDIP)
A_68_P25846165	chr8:49314031-49314075	NM_001145937:445992	Odz3	INSIDE	3.322	1.784	431.03	769.15	5.928	367.59	2179.09
A_68_P32700072	chrX:137134843-137134887	NM_001077364:197	Tsc22d3	INSIDE	3.226	3.164	1997.35	6319.86	10.209	2177.27	22226.92
A_68_P29222297	chr13:92901976-92902020	NM_009027:-549	Rasgrf2	PROMOTER	3.225	6.389	1014.97	6484.30	20.601	890.04	18335.31
A_68_P28152301	chr11:115111293-115111337	NM_010350:17243	Grin2c	INSIDE	3.224	1.760	793.83	1397.33	5.676	698.84	3966.33
A_68_P23588918	chr5:33785513-33785557			Unknown	3.206	1.547	1862.72	2882.22	4.960	1071.25	5313.56
A_68_P32241075	chrX:7525044-7525088	NM_001130416:-51	Hdac6	PROMOTER	3.194	0.509	1639.57	833.72	1.624	2095.91	3403.82
A_68_P27094080	chr10:41996065-41996109	NM_019740:462	Foxo3	INSIDE	3.189	0.488	1655.90	808.58	1.557	1214.85	1891.52
A_68_P32710891	chrX:139401598-139401642	NM_019496:-349	Ammecr1	PROMOTER	3.177	3.070	901.91	2768.52	9.753	1089.13	10622.67
A_68_P32707348	chrX:138739964-138740008	NM_021487:755	Kcne1l	INSIDE	3.176	0.708	1822.01	1290.10	2.249	2308.97	5191.85
A_68_P32567810	chrX:99447558-99447602	NM_007709:-472	Cited1	PROMOTER	3.080	0.207	1239.88	256.43	0.637	1780.68	1134.14
A_68_P21874507	chr2:174124666-174124710	NM_201617:1329	Gnas	INSIDE	3.046	0.619	738.53	457.38	1.886	622.75	1174.72
A_68_P32364760	chrX:45806092-45806136	NM_019680:10195	Elf4	INSIDE	3.044	0.558	559.37	312.10	1.698	753.01	1278.76
A_68_P25394427	chr7:112936384-112936433	NM_001162943:-344	Dchs1	PROMOTER	3.028	1.604	528.58	847.78	4.857	345.57	1678.51
A_68_P32538585	chrX:91612495-91612539	NM_001199337:24	Apoo	INSIDE	3.027	0.188	1992.20	374.32	0.569	2559.37	1455.83
A_68_P32683271	chrX:133462182-133462226	NM_027067:341	1700014N06Rik	INSIDE	3.021	2.158	821.50	1772.67	6.518	1230.09	8018.31
A_68_P20348301	chr1:74438711-74438755	NR_029743:-2151	Mir26b	PROMOTER	2.978	2.357	827.27	1950.27	7.021	706.00	4957.01
A_68_P32699217	chrX:136991521-136991565	NM_021463:401	Prps1	INSIDE	2.973	0.211	1266.95	267.82	0.629	1920.29	1206.99
A_68_P32698228	chrX:136812874-136812918	ENSMUST00000130153:-495		PROMOTER	2.967	0.213	2882.36	614.51	0.633	3594.99	2274.27
A_68_P32375460	chrX:48558753-48558797	NM_134163:235	Mbnl3	INSIDE	2.964	0.214	1536.57	329.20	0.635	2032.11	1290.50
A_68_P32322124	chrX:34731543-34731587	NM_153503:227	Rnf113a1	INSIDE	2.962	0.512	1233.97	632.16	1.518	1646.58	2498.92
A_68_P32465786	chrX:71211813-71211857	NM_008224:-180	Hcfc1	PROMOTER	2.939	0.230	1366.39	314.92	0.677	1731.22	1172.57
A_68_P23588919	chr5:33785607-33785651			Unknown	2.925	0.191	2204.79	420.13	0.557	1614.85	899.93
A_68_P32577124	chrX:101609142-101609186	NM_009592:21	Abcb7	INSIDE	2.883	0.193	1097.20	212.25	0.558	1604.97	895.04
A_68_P22209749	chr3:67178298-67178342	NM_001039543:302	Mlf1	INSIDE	2.876	1.779	1046.06	1860.89	5.117	666.83	3412.09
A_68_P27233861	chr10:70622146-70622190	NM_031397:214	Bicc1	INSIDE	2.843	0.620	1556.32	965.12	1.763	1183.74	2087.16
A_68_P32564062	chrX:98625083-98625127	NM_023144:115	Nono	INSIDE	2.829	0.587	1493.95	876.95	1.661	1905.39	3164.27
A_68_P24983894	chr7:17492409-17492453	NM_008967:592	Ptgir	INSIDE	2.814	0.651	708.64	461.56	1.833	558.46	1023.67
A_68_P30981935	chr16:94748214-94748258	NM_007834:0	Dscr3	INSIDE	2.783	0.671	1402.73	940.97	1.867	1033.99	1930.43
A_68_P32746464	chrX:148238102-148238146	NM_021523:300	Huwe1	INSIDE	2.779	0.180	2429.92	438.56	0.502	3154.13	1582.19
A_68_P30964219	chr16:91660394-91660440	NM_019973:12348	Son	INSIDE	2.750	1.459	914.91	1334.67	4.011	646.45	2593.12
A_68_P31120666	chr17:28279127-28279172	NM_001004366:-321	Scube3	PROMOTER	2.745	0.571	4769.03	2723.59	1.568	2991.69	4690.23
A_68_P30462697	chr15:96118805-96118849	NM_175251:874	Arid2	INSIDE	2.730	0.661	898.10	593.90	1.805	600.20	1083.54
A_68_P28071137	chr11:100820628-100820677	NM_008986:11279	Ptrf	INSIDE	2.707	2.201	210.73	463.80	5.958	211.95	1262.72
A_68_P29428730	chr14:17197854-17197898	NM_009409:157	Top2b	INSIDE	2.689	0.183	2298.70	420.51	0.492	1847.83	908.83
A_68_P27212434	chr10:66999516-66999563	NM_010118:-1077	Egr2	PROMOTER	2.674	0.241	3139.30	755.22	0.643	2120.74	1364.36
A_68_P31936308	chr19:6906036-6906086	NM_019924:9031	Rps6ka4	INSIDE	2.654	1.459	1216.81	1775.48	3.873	779.02	3017.13
A_68_P32239400	chrX:7215668-7215712	NM_009305:-15	Syp	PROMOTER	2.608	0.180	5125.89	922.11	0.469	5731.52	2688.86
A_68_P29629465	chr14:58318680-58318724	NM_015771:46258	Lats2	INSIDE	2.607	1.658	689.44	1142.92	4.322	532.08	2299.53
A_68_P28442990	chr12:54808173-54808232	NM_013780:458539	Npas3	INSIDE	2.604	2.556	1021.39	2610.68	6.655	682.44	4541.62
A_68_P25599898	chr7:150481545-150481589	NR_001461:886	Kcnq1ot1	INSIDE	2.601	0.682	986.10	672.52	1.774	700.25	1242.20
A_68_P20884270	chr1:183853884-183853931	NM_001083120:96204	Enah	INSIDE	2.593	3.063	166.71	510.59	7.942	178.16	1414.96
A_68_P24658378	chr6:94649175-94649219	NM_008377:943	Lrig1	INSIDE	2.581	0.229	2084.00	477.63	0.592	1595.82	943.95
A_68_P30542118	chr16:10412308-10412352	NM_011955:300	Nubp1	INSIDE	2.576	0.734	3573.45	2623.49	1.891	2645.25	5003.00
A_68_P26224550	chr8:123632450-123632508	NM_001166482:-199	Mthfsd	DIVERGENT_PROMOTER	2.575	8.435	7313.59	61693.66	21.723	5216.59	113321.80
A_68_P20250385	chr1:57027773-57027817	NM_139146:384	Satb2	INSIDE	2.570	0.246	2235.81	549.83	0.632	1644.99	1039.49
A_68_P26504313	chr9:50369450-50369494	NM_025687:-99	Tex12	DIVERGENT_PROMOTER	2.564	1.670	595.94	995.40	4.283	497.92	2132.59
A_68_P23396589	chr4:148180346-148180401	NM_027195:1873	Casz1	INSIDE	2.563	2.263	157.20	355.69	5.798	158.00	916.16
A_68_P27944883	chr11:78510477-78510521	NM_008702:429	Nlk	INSIDE	2.545	0.696	2646.93	1841.40	1.770	1946.36	3445.82
A_68_P27649722	chr11:24064060-24064104	NM_001159290:83388	Bcl11a	INSIDE	2.538	2.525	192.38	485.74	6.409	134.34	861.06
A_68_P25092565	chr7:52602361-52602407	NM_029741:20005	Ppfia3	INSIDE	2.535	11.831	2987.65	35347.23	29.987	1937.28	58093.45
A_68_P23594804	chr5:34868325-34868369	NM_011893:-86	Sh3bp2	PROMOTER	2.525	2.153	1339.96	2885.60	5.438	1184.98	6443.67
A_68_P22129414	chr3:51200267-51200311	NM_026358:181	4930583H14Rik	INSIDE	2.500	5.279	1233.49	6511.29	13.198	872.47	11514.77
A_68_P28724521	chr12:109154706-109154750	NM_001079883:86896	Bcl11b	INSIDE	2.493	2.102	445.62	936.66	5.240	395.98	2074.80
A_68_P28123544	chr11:110198866-110198910	NM_147219:142	Abca5	INSIDE	2.491	3.885	461.84	1794.05	9.675	441.97	4276.21
A_68_P30012296	chr15:10644051-10644095	NM_030690:-777	Rai14	PROMOTER	2.486	0.080	6721.26	535.19	0.198	4187.07	828.76
A_68_P31253724	chr17:56218702-56218746	NM_008233:-355	Hdgfrp2	DIVERGENT_PROMOTER	2.469	0.141	7388.62	1040.37	0.348	4630.24	1609.56
A_68_P25406573	chr7:116318129-116318173	NM_057173:-4328	Lmo1	PROMOTER	2.469	0.252	1759.47	444.02	0.623	1325.32	825.90
A_68_P32672782	chrX:131135885-131135929	NM_019868:82	Hnrnph2	INSIDE	2.464	0.562	2657.04	1493.76	1.385	3488.49	4832.40
A_68_P24631552	chr6:90259348-90259392	NM_027928:15809	Chst13	INSIDE	2.462	1.710	452.86	774.52	4.211	374.31	1576.36
A_68_P32148154	chr19:47254136-47254180	NM_021360:849	Neurl1a	INSIDE	2.454	3.531	10959.86	38703.66	8.665	7399.64	64118.75
A_68_P21565607	chr2:118528199-118528243	NM_001081971:1479	Gm1337	INSIDE	2.449	0.255	2260.52	577.06	0.625	1654.55	1034.37
A_68_P32790792	chrX:159198218-159198262	NM_009031:-63	Rbbp7	PROMOTER	2.446	0.590	2086.11	1231.58	1.444	2947.80	4256.55
A_68_P20637452	chr1:137001893-137001941	NM_001033409:-63	Lgr6	PROMOTER	2.422	4.446	4998.07	22220.63	10.766	3343.21	35991.99
A_68_P30423622	chr15:88998029-88998073	NM_001159521:6230	Plxnb2	INSIDE	2.411	1.607	643.21	1033.76	3.875	470.78	1824.37
A_68_P29469017	chr14:24824321-24824365	NM_010610:-915	Kcnma1	PROMOTER	2.399	2.574	193.74	498.61	6.174	149.01	919.99

ProbeName	Target position of probe on CpG island microarray	TargetID	GeneSymbol	CpG island Description	Ratio of relative methylation (TiO_2-NP/Vehicle)	Sham group			TiO_2-H group		
						Relative methylation (Cy5/Cy3)	Cy3 signal (Input)	Cy5 signal (MeDIP)	Relative methylation (Cy5/Cy3)	Cy3 signal (Input)	Cy5 signal (MeDIP)
A_68_P32691811	chrX:135448869-135448913	NM_013724:-78	Nrk	PROMOTER	2.396	0.569	7328.70	4171.94	1.364	9260.75	12630.82
A_68_P22898182	chr4:48060289-48060333	NM_015743:-3809	Nr4a3	PROMOTER	2.393	1.659	756.90	1255.55	3.969	625.18	2481.39
A_68_P24973912	chr7:13556599-13556643	NM_178732:5408	Zfp324	INSIDE	2.378	1.721	736.15	1267.14	4.093	587.18	2403.51
A_68_P23896973	chr5:93371964-93372008	NM_001077596:44963	Shroom3	INSIDE	2.365	2.100	408.11	856.83	4.966	332.56	1651.58
A_68_P32748065	chrX:148579255-148579299	NM_001114664:466	Iqsec2	INSIDE	2.361	0.659	796.04	524.81	1.557	945.39	1471.80
A_68_P24596644	chr6:83958657-83958701	NM_001077694:95	Dysf	INSIDE	2.356	2.367	269.48	637.86	5.577	182.83	1019.63
A_68_P27564657	chr11:5617071-5617115	NM_178623:24087	Urgcp	INSIDE	2.355	2.436	705.57	1718.85	5.737	499.08	2863.32
A_68_P25094601	chr7:52962152-52962196	NM_016974:1558	Dbp	INSIDE	2.345	0.208	2271.06	472.85	0.488	1663.32	812.26
A_68_P32791140	chrX:159267555-159267599	NM_178935:-245	Txlng	PROMOTER	2.343	0.241	1253.80	302.40	0.565	1704.19	963.03
A_68_P21569149	chr2:119163546-119163590	NM_019454:12049	Dll4	DOWNSTREAM	2.340	2.253	1574.09	3547.00	5.274	1325.48	6990.45
A_68_P30366726	chr15:79315953-79315997	NM_008427:19697	Kcnj4	INSIDE	2.331	2.447	581.72	1423.23	5.702	398.66	2273.14
A_68_P31175232	chr17:39983513-39983557	ENSMUST00000157647:-536		PROMOTER	2.327	0.749	4175.81	3126.30	1.742	3082.65	5369.94
A_68_P32364050	chrX:45697107-45697151	NM_178782:2594	Bcorl1	INSIDE	2.310	1.888	8344.09	15754.93	4.361	8516.29	37139.67
A_68_P32135814	chr19:45080366-45080410	NM_053164:544	Mrpl43	INSIDE	2.299	0.250	1799.59	449.76	0.575	1291.80	742.32
A_68_P31149892	chr17:33786930-33786974	NM_001109913:35451	Hnrnpm	INSIDE	2.297	1.398	963.35	1346.75	3.211	802.72	2577.27
A_68_P25581954	chr7:147234659-147234703	NM_010836:307	Msx3	INSIDE	2.295	0.216	2207.42	477.63	0.497	1627.52	808.22
A_68_P24026284	chr5:118830994-118831046			Unknown	2.295	3.521	3038.53	10697.23	8.080	1888.70	15261.42
A_68_P31093844	chr17:23956989-23957033	NM_175229:16857	Srrm2	INSIDE	2.292	1.378	1318.88	1817.02	3.157	811.12	2560.97
A_68_P27180703	chr10:60938705-60938749	NM_153542:146	Lrrc20	INSIDE	2.288	9.024	1247.99	11261.26	20.643	1159.24	23929.67
A_68_P26885589	chr9:121698137-121698187	NR_027967:2076	Hhatl	INSIDE	2.288	1.626	500.78	814.04	3.719	414.01	1539.80
A_68_P32560535	chrX:97821487-97821531	NM_008446:105	Kif4	INSIDE	2.286	0.260	1904.88	495.30	0.594	2503.48	1487.94
A_68_P29616405	chr14:55733423-55733480	NM_177049:2321	Jph4	INSIDE	2.280	2.002	1122.21	2246.68	4.564	773.50	3529.98
A_68_P21069122	chr2:24830308-24830352	NM_001031808:288	Mrpl41	INSIDE	2.280	0.723	2687.27	1941.63	1.648	2071.68	3413.36
A_68_P30346601	chr15:76010142-76010186	NM_201394:15976	Plec	INSIDE	2.270	1.702	549.07	934.42	3.864	398.89	1541.12
A_68_P27340040	chr10:90564963-90565014	NM_027078:19205	Ikbip	INSIDE	2.268	2.131	412.10	878.02	4.833	314.48	1519.95
A_68_P32427553	chrX:61530261-61530305	NM_178740:-111	Slitrk4	PROMOTER	2.267	0.619	3195.79	1978.14	1.403	4275.36	5998.39
A_68_P32791135	chrX:159266933-159266977	NM_178935:377	Txlng	INSIDE	2.261	0.628	2796.29	1755.34	1.420	3526.59	5006.40
A_68_P27835628	chr11:59041341-59041385	NM_001130408:302	Arfl	INSIDE	2.261	2.342	666.77	1561.72	5.295	505.35	2675.83
A_68_P32470782	chrX:72376286-72376330	NM_008621:-20	Mpp1	PROMOTER	2.259	0.195	2064.33	401.99	0.440	2710.33	1192.38
A_68_P23308583	chr4:131392567-131392611	NM_001083119:1605	Ptpru	INSIDE	2.257	0.269	2368.25	636.01	0.606	1582.03	958.96
A_68_P22336407	chr3:93249382-93249426	NM_001163098:3153	Tchh	INSIDE	2.254	0.162	2806.80	453.59	0.364	2119.88	772.12
A_68_P31862858	chr18:80389262-80389306	NM_199197:8074	Rbfa	INSIDE	2.232	1.732	287.56	498.14	3.866	239.31	925.26
A_68_P23523160	chr5:20388333-20388392	NM_177601:92	Tmem60	INSIDE	2.231	1.509	541.80	817.53	3.366	338.81	1140.40
A_68_P30356632	chr15:77759535-77759579	NM_019913:-132	Txn2	PROMOTER	2.219	0.114	4801.38	548.13	0.253	3063.19	776.05
A_68_P32710890	chrX:139401516-139401560	NM_019496:-267	Ammecr1	PROMOTER	2.217	2.823	286.77	809.65	6.259	396.02	2478.75
A_68_P25614334	chr8:3515198-3515255	NM_053177:14708	Mcoln1	INSIDE	2.210	0.207	2436.92	505.26	0.458	1788.27	819.41
A_68_P24428352	chr6:48570342-48570386	NM_173429:7186	Zfp775	INSIDE	2.204	1.796	319.13	573.30	3.959	296.38	1173.33
A_68_P32252886	chrX:10294260-10294304	NM_001166635:-208	Mid1ip1	PROMOTER	2.203	0.318	1574.06	501.13	0.701	2058.97	1444.01
A_68_P22309972	chr3:87587929-87587973	NM_001033124:11134	Ntrk1	INSIDE	2.199	1.569	2044.81	3208.63	3.451	1530.24	5280.39
A_68_P25422971	chr7:119301817-119301861	NM_015814:733	Dkk3	INSIDE	2.190	3.136	3496.34	10964.25	6.869	2815.34	19338.78
A_68_P24117611	chr5:136209975-136210019	NM_008898:44913	Por	INSIDE	2.184	1.547	890.21	1376.88	3.378	673.17	2273.97
A_68_P20553865	chr1:120734246-120734290	NM_001081125:215928	Gli2	INSIDE	2.177	1.864	939.88	1751.56	4.057	806.68	3272.86
A_68_P32378022	chrX:49033742-49033786	NM_015819:-110	Hs6st2	PROMOTER	2.175	0.640	2173.56	1390.68	1.392	2795.09	3889.74
A_68_P21420371	chr2:90894443-90894487	NM_008948:292	Psmc3	INSIDE	2.173	1.785	2443.15	4360.47	3.878	1878.91	7286.54
A_68_P31121831	chr17:28465275-28465320	NM_011287:-118	Rpl10a	PROMOTER	2.165	7.985	692.24	5527.91	17.290	589.46	10192.03
A_68_P26397640	chr9:30729401-30729445	NM_001024139:615	Adamts15	INSIDE	2.160	1.424	1059.29	1508.30	3.075	891.62	2741.86
A_68_P27473560	chr10:115551150-115551194	NM_001161855:-199	4933416C03Rik	PROMOTER	2.153	1.966	708.88	1393.92	4.233	540.85	2289.42
A_68_P31227869	chr17:50432800-50432844	NM_010021:102	Dazl	INSIDE	2.150	1.933	738.63	1427.62	4.155	602.36	2502.83
A_68_P24428221	chr6:48547445-48547489	NM_001079901:3585	Repin1	INSIDE	2.148	1.780	691.69	1231.51	3.824	554.78	2121.41
A_68_P25004860	chr7:25217492-25217536	NM_199013:13187	Irgc1	INSIDE	2.142	1.731	440.08	761.58	3.707	320.09	1186.70
A_68_P33015620	chr1_random:50449-50497	NM_026866:134866	Disp1	INSIDE	2.133	1.701	352.30	599.12	3.628	263.73	956.84
A_68_P24763306	chr6:113991628-113991672	NM_009723:424	Atp2b2	INSIDE	2.132	0.074	8276.90	609.06	0.157	4814.09	755.17
A_68_P27316752	chr10:86371693-86371737	NM_138673:98973	Stab2	INSIDE	2.122	2.927	9338.35	27331.90	6.211	6520.23	40497.84
A_68_P30346557	chr15:76004653-76004697	NM_201394:21466	Plec	INSIDE	2.121	1.500	534.42	801.39	3.180	465.60	1480.79
A_68_P25301151	chr7:95278746-95278790	NM_001081414:546091	Grm5	INSIDE	2.120	1.363	1351.23	1841.59	2.890	1033.99	2987.83
A_68_P22630575	chr3:148652818-148652862	ENSMUST00000098518:-560		PROMOTER	2.101	6.740	913.99	6160.19	14.161	763.84	10816.45
A_68_P30382917	chr15:82176766-82176812	NM_026914:313	1500032L24Rik	INSIDE	2.098	2.644	631.38	1669.31	5.547	551.68	3060.30
A_68_P28077443	chr11:101949878-101949922	NM_016695:-71	Mpp2	PROMOTER	2.098	0.193	3210.39	619.78	0.405	2128.88	862.08
A_68_P31654584	chr18:42435490-42435534	NM_172626:506	Rbm27	INSIDE	2.095	0.187	3474.90	651.48	0.393	2384.05	936.27
A_68_P23363267	chr4:140864773-140864817	NM_010139:7640	Epha2	INSIDE	2.088	1.537	1059.68	1629.05	3.210	785.18	2520.79
A_68_P22393168	chr3:104591684-104591728	NM_007484:-245	Rhoc	PROMOTER	2.088	1.741	3023.33	5263.26	3.635	2294.56	8340.07
A_68_P33015621	chr1_random:50610-50662	NM_026866:134702	Disp1	INSIDE	2.087	1.420	808.65	1148.55	2.964	567.90	1683.36
A_68_P27284174	chr10:80049350-80049394	NM_145421:3714	Fam108a	INSIDE	2.079	1.607	515.22	827.96	3.340	442.41	1477.84
A_68_P30348874	chr15:76350196-76350240	NM_130893:2341	Scrt1	INSIDE	2.076	0.131	10867.94	1424.35	0.272	6340.97	1725.64
A_68_P21594127	chr2:123916191-123916235	ENSMUST00000137172:508		INSIDE	2.076	0.205	6166.41	1264.61	0.426	4195.88	1786.08

ProbeName	Target position of probe on CpG island microarray	TargetID	GeneSymbol	CpG island Description	Ratio of relative methylation (TiO_2-NP/Vehicle)	Sham group			TiO_2-H group		
						Relative methylation (Cy5/Cy3)	Cy3 signal (Input)	Cy5 signal (MeDIP)	Relative methylation (Cy5/Cy3)	Cy3 signal (Input)	Cy5 signal (MeDIP)
A_68_P29043020	chr13:53381133-53381177	NM_013846:324	Ror2	INSIDE	2.073	0.233	1983.66	461.48	0.482	1614.55	778.74
A_68_P32778406	chrX:156575977-156576021	NM_008810:270	Pdha1	INSIDE	2.072	0.276	3065.39	846.09	0.572	4295.24	2456.84
A_68_P26504859	chr9:50467279-50467323	NM_145614:585	Dlat	INSIDE	2.072	0.210	2240.02	470.46	0.435	1713.12	745.43
A_68_P24397783	chr6:42299752-42299796	NM_011777:-52	Zyx	PROMOTER	2.065	0.117	4896.28	575.25	0.243	3371.39	817.83
A_68_P28450915	chr12:56256240-56256284	NM_011899:-64	Srp54a	PROMOTER	2.060	0.293	1566.37	459.68	0.604	1133.80	685.33
A_68_P28107298	chr11:107341035-107341079	NM_025894:215	Psmd12	INSIDE	2.060	0.238	1905.14	454.14	0.491	1652.60	811.49
A_68_P23364203	chr4:141020484-141020528	NM_009541:19919	Zbtb17	INSIDE	2.058	1.590	789.70	1255.95	3.272	619.89	2028.53
A_68_P24954799	chr7:6238258-6238302	NM_001024928:99	Zfp667	INSIDE	2.041	0.248	3830.73	948.82	0.506	2759.75	1395.27
A_68_P28039261	chr11:95376208-95376252	NM_130858:-351	Nxph3	PROMOTER	2.040	0.324	2834.73	917.67	0.660	1960.35	1294.53
A_68_P20797862	chr1:167933482-167933526	NM_011137:-739	Pou2f1	PROMOTER	2.040	0.179	2526.00	452.13	0.365	1730.81	631.83
A_68_P28713546	chr12:107412574-107412618	BC156654:374736		DOWNSTREAM	2.038	0.259	2052.74	532.01	0.528	1538.56	812.54
A_68_P32070028	chr19:32463753-32463797	NR_033198:69	2700046G09Rik	INSIDE	2.032	0.300	1668.43	500.16	0.609	1234.31	751.89
A_68_P32240782	chrX:7476676-7476720	NM_011591:175	Timm17b	INSIDE	2.030	0.309	1357.54	419.03	0.626	1773.99	1111.37
A_68_P30371309	chr15:80118848-80118907	NM_001044308:1210	Cacna1i	INSIDE	2.024	2.193	492.37	1079.74	4.440	239.54	1063.46
A_68_P21102113	chr2:30112262-30112306	NM_177725:18996	Lrrc8a	INSIDE	2.022	1.769	407.46	720.73	3.576	320.25	1145.16
A_68_P30083235	chr15:25711092-25711136	NM_019472:158810	Myo10	INSIDE	2.020	1.501	740.92	1112.48	3.033	587.23	1780.88
A_68_P25505248	chr7:134486671-134486730	NM_146202:2128	Zfp768	INSIDE	2.019	2.895	246.64	713.95	5.843	173.77	1015.44
A_68_P31442544	chr17:91487486-91487530	NM_020252:4634	Nrxn1	INSIDE	2.018	2.302	485.64	1117.90	4.646	424.97	1974.58
A_68_P22405767	chr3:107145905-107145949	NM_001045807:-9719	Rbm15	PROMOTER	2.015	1.595	464.07	740.00	3.213	316.43	1016.53
A_68_P21866928	chr2:172805309-172805353	NM_001083959:-12	Spo11	PROMOTER	2.015	1.547	582.53	901.17	3.117	494.94	1542.80
A_68_P24428351	chr6:48570207-48570251	NM_173429:7050	Zfp775	INSIDE	2.012	1.780	864.07	1538.00	3.581	728.59	2609.17
A_68_P25343508	chr7:103358546-103358591	NM_011858:-578	Odz4	PROMOTER	2.010	1.498	4344.14	6507.77	3.011	3344.48	10070.19
A_68_P25032744	chr7:31435111-31435155	NM_133693:115	Rbm42	INSIDE	2.009	3.085	839.39	2589.48	6.197	704.41	4365.24
A_68_P28246310	chr12:12948750-12948795	NM_008709:-130	Mycn	PROMOTER	2.008	3.679	260.59	958.63	7.387	267.60	1976.74
A_68_P29703312	chr14:71166447-71166491	NM_023045:-33	Xpo7	DIVERGENT_PROMOTER	2.005	2.141	476.78	1020.60	4.291	423.92	1819.16
A_68_P32234209	chrX:5977910-5977954	NM_001040459:670	Shroom4	INSIDE	2.004	0.343	2044.80	701.11	0.687	2663.69	1830.68
A_68_P30083232	chr15:25710801-25710845	NM_019472:158518	Myo10	INSIDE	2.003	2.319	305.15	707.70	4.645	231.43	1075.06
A_68_P25539016	chr7:140315036-140315089	NM_009980:104	Ctbp2	INSIDE	2.003	2.608	6932.15	18081.65	5.225	4442.76	23212.76
A_68_P23342965	chr4:137513918-137513962	NM_199307:95789	Ece1	INSIDE	2.003	2.220	214.85	477.07	4.447	141.05	627.21
A_68_P25499766	chr7:133435827-133435871	NM_145587:19716	Sbk1	INSIDE	1.999	2.022	559.32	1130.85	4.042	450.99	1822.70
A_68_P27693351	chr11:33102254-33102303	NM_019916:1310	Tlx3	INSIDE	1.997	1.851	1352.30	2502.54	3.696	734.82	2716.23
A_68_P25031175	chr7:31140774-31140818	NM_175478:6995	Lrfn3	INSIDE	1.994	1.603	781.27	1252.73	3.197	605.49	1935.65
A_68_P32441101	chrX:65932010-65932054	NM_008031:303	Fmr1	INSIDE	1.992	0.297	1599.72	474.71	0.591	2304.18	1362.31
A_68_P25954894	chr8:73437692-73437736	NM_173013:7842	Mtap1s	INSIDE	1.991	2.071	445.74	923.08	4.122	366.91	1512.58
A_68_P25587733	chr7:148445537-148445581	NM_001081118:30872	Phrf1	INSIDE	1.986	2.754	845.36	2328.09	5.468	611.19	3342.18
A_68_P31157716	chr17:35258201-35258245	NM_009975:170	Csnk2b	INSIDE	1.985	0.153	3762.87	576.81	0.304	2574.40	783.50
A_68_P31926196	chr19:4989991-4990035	NM_153553:-41	Npas4	PROMOTER	1.980	2.362	406.11	959.10	4.676	263.63	1232.82
A_68_P27787411	chr11:50039460-50039504	NM_145926:646	Mgat4b	INSIDE	1.980	4.502	382.43	1721.71	8.913	278.84	2485.20
A_68_P32408595	chrX:56387162-56387206	NM_010200:429	Fgf13	INSIDE	1.979	0.311	1261.91	392.17	0.615	1698.91	1044.83
A_68_P32240899	chrX:7499781-7499825	NM_013892:2855	Pcsk1n	INSIDE	1.976	0.332	2036.08	676.01	0.656	2725.85	1788.51
A_68_P29090576	chr13:63066071-63066115	ENSMUST00000170896:-160		PROMOTER	1.973	3.275	947.47	3103.12	6.462	692.35	4473.91
A_68_P22422568	chr3:109945463-109945507	NM_001163348:906	Ntng1	INSIDE	1.972	0.319	1982.73	633.03	0.629	1424.00	896.34
A_68_P22393108	chr3:104584185-104584229	NM_027982:233	Ppm1j	INSIDE	1.972	0.246	2316.75	569.48	0.485	1672.75	810.97
A_68_P25614226	chr8:3495954-3495998	NM_080461:2839	Zfp358	INSIDE	1.971	2.538	517.85	1314.41	5.003	404.01	2021.19
A_68_P23245374	chr4:119088045-119088089	NM_029286:60	Ccdc30	INSIDE	1.971	0.321	1956.19	627.93	0.633	1382.91	875.07
A_68_P31926782	chr19:5082847-5082891	NM_001001885:2609	Tmem151a	INSIDE	1.970	2.443	698.39	1706.27	4.812	594.37	2860.14
A_68_P21047273	chr2:19831293-19831337	ENSMUST00000114610:-92		PROMOTER	1.969	2.237	5125.79	11465.38	4.405	3385.86	14914.46
A_68_P28956732	chr13:37918318-37918362	NM_001177868:-440	Rreb1	PROMOTER	1.968	0.192	2689.39	517.44	0.379	1794.61	679.43
A_68_P21485723	chr2:103659521-103659569	ENSMUST00000167989:89		INSIDE	1.961	2.496	182.53	455.65	4.895	181.30	887.47
A_68_P28959944	chr13:38437327-38437371	NM_007556:-236	Bmp6	PROMOTER	1.959	0.216	2341.21	506.63	0.424	1669.47	707.81
A_68_P25951933	chr8:72975932-72975976	NM_001122830:24888	Klhl26	INSIDE	1.959	1.948	433.02	843.53	3.816	354.97	1354.58
A_68_P25354352	chr7:105326428-105326472	NM_007602:253	Capn5	INSIDE	1.958	0.284	2352.49	668.53	0.556	1727.60	961.11
A_68_P23269531	chr4:124337466-124337523	NM_011141:2606	Pou3f1	INSIDE	1.952	4.260	241.30	1027.91	8.317	93.99	781.72
A_68_P25656260	chr8:11561678-11561724	NM_011919:5635	Ing1	INSIDE	1.948	1.552	1273.73	1976.26	3.023	996.74	3013.09
A_68_P22134616	chr3:52073560-52073612	NM_019739:1328	Foxo1	INSIDE	1.940	0.183	2769.06	506.36	0.355	2060.18	730.76
A_68_P23424871	chr4:152806869-152806913	NM_001099299:50049	Ajap1	INSIDE	1.931	1.860	657.91	1224.04	3.592	471.45	1693.38
A_68_P30988237	chr16:95807509-95807553	ENSMUST00000121809:670		INSIDE	1.927	0.115	3966.20	455.79	0.221	2643.76	585.32
A_68_P32773806	chrX:155694082-155694126	NM_148945:53	Rps6ka3	INSIDE	1.926	0.244	2420.69	589.55	0.469	3876.93	1818.22
A_68_P29874397	chr14:105339298-105339349	NR_015593:300960	D130009I18Rik	INSIDE	1.924	1.521	567.06	862.71	2.927	459.99	1346.31
A_68_P29613096	chr14:55195734-55195778	NM_010590:742	Jub	INSIDE	1.924	5.119	2649.16	13561.20	9.847	2311.01	22755.52
A_68_P31140216	chr17:31649029-31649073	NM_021322:382	Wdr4	INSIDE	1.916	0.277	1615.37	446.67	0.530	1152.78	610.77
A_68_P26663736	chr9:79190551-79190595			Unknown	1.914	1.699	749.13	1272.91	3.252	610.66	1985.84
A_68_P20975268	chr2:5635854-5635898	NM_177343:-166	Camk1d	PROMOTER	1.910	1.570	441.71	693.26	2.998	365.20	1094.94
A_68_P30953523	chr16:89885140-89885184	NM_009384:75910	Tiam1	INSIDE	1.909	1.563	459.65	718.24	2.983	319.79	953.89
A_68_P32233091	chrX:5660358-5660402	NM_001033211:158	AU022751	INSIDE	1.908	2.380	272.91	649.47	4.540	403.61	1832.32

ProbeName	Target position of probe on CpG island microarray	TargetID	GeneSymbol	CpG island Description	Ratio of relative methylation (TiO_2-NP/Vehicle)	Sham group			TiO_2-H group		
						Relative methylation (Cy5/Cy3)	Cy3 signal (Input)	Cy5 signal (MeDIP)	Relative methylation (Cy5/Cy3)	Cy3 signal (Input)	Cy5 signal (MeDIP)
A_68_P31257221	chr17:56743113-56743157	NM_001163300:18730	Safb	INSIDE	1.905	1.514	1938.43	2935.29	2.884	1412.45	4074.11
A_68_P26227387	chr8:124078337-124078381	NM_133765:24348	Fbxo31	INSIDE	1.904	1.515	1027.42	1556.91	2.886	530.05	1529.47
A_68_P23982393	chr5:111308521-111308565	NM_024477:-279	Ttc28	PROMOTER	1.902	3.194	198.86	635.17	6.075	172.72	1049.32
A_68_P31927969	chr19:5307468-5307512	NM_001024717:9160	Gal3st3	INSIDE	1.899	2.270	533.63	1211.34	4.310	441.31	1901.92
A_68_P25075289	chr7:48157966-48158010	NM_021387:3341	Vstm2b	INSIDE	1.899	0.287	1707.59	489.65	0.545	1239.87	675.27
A_68_P27812443	chr11:54679796-54679846	NM_008248:-119	Hint1	DIVERGENT_PROMOTER	1.895	2.380	248.26	590.93	4.510	173.83	783.91
A_68_P27550645	chr11:3093025-3093069	NM_030207:420	Sfi1	INSIDE	1.895	1.406	3558.17	5003.50	2.665	3254.57	8672.89
A_68_P28553587	chr12:77469901-77469960	NM_001172104:521	Zbtb25	INSIDE	1.894	1.822	365.41	665.81	3.450	245.61	847.38
A_68_P22384844	chr3:102975721-102975767	NM_026602:111	Bcas2	INSIDE	1.891	1.428	987.01	1409.12	2.700	677.36	1829.15
A_68_P28182974	chr11:120050877-120050921	NR_030682:272	2810410L24Rik	INSIDE	1.885	5.036	1021.71	5145.36	9.492	783.76	7439.62
A_68_P27340413	chr10:90634459-90634503	NM_001080129:-153	Tmpo	PROMOTER	1.883	0.314	1872.50	588.32	0.592	1528.15	904.17
A_68_P32773804	chrX:155693758-155693802	NM_148945:-271	Rps6ka3	PROMOTER	1.882	2.042	3195.43	6523.51	3.843	4290.60	16489.08
A_68_P27100735	chr10:43231159-43231203	NM_199028:32235	Bend3	INSIDE	1.882	2.038	861.53	1755.85	3.836	612.04	2347.96
A_68_P26813047	chr9:108008622-108008666	NM_007567:84070	Bsn	INSIDE	1.882	2.202	813.15	1790.15	4.142	688.49	2851.86
A_68_P23321453	chr4:133742651-133742697	NM_144527:326	Ccdc21	INSIDE	1.880	0.057	10640.00	604.37	0.107	7099.09	758.19
A_68_P25083573	chr7:50930678-50930722	NM_145582:3300	Ctu1	INSIDE	1.878	1.646	1003.66	1652.20	3.092	792.73	2451.30
A_68_P28724516	chr12:109154117-109154161	NM_001079883:87486	Bcl11b	INSIDE	1.875	1.461	719.33	1050.81	2.740	531.10	1455.00
A_68_P27550647	chr11:3093206-3093251	NM_030207:238	Sfi1	INSIDE	1.872	1.403	1094.27	1535.67	2.627	929.28	2441.55
A_68_P26885415	chr9:121672022-121672066	NM_001166644:2894	Zfp651	INSIDE	1.872	2.020	462.48	934.42	3.782	345.42	1306.30
A_68_P22351747	chr3:96709002-96709046	NM_026229:197	Gpr89	INSIDE	1.871	1.576	533.53	841.00	2.950	437.29	1290.01
A_68_P30083236	chr15:25711164-25711208	NM_019472:158882	Myo10	INSIDE	1.869	1.508	789.52	1190.24	2.818	644.68	1816.39
A_68_P25102684	chr7:54305881-54305925	NM_026436:-107	Tmem86a	PROMOTER	1.869	1.527	543.73	830.13	2.853	554.78	1582.91
A_68_P32415800	chrX:58145373-58145417	NM_009237:1211	Sox3	INSIDE	1.865	2.191	259.66	568.79	4.086	310.19	1267.33
A_68_P22413865	chr3:108456942-108456986	NM_001177770:57	Clcc1	INSIDE	1.864	0.241	2252.56	543.78	0.450	1605.12	722.39
A_68_P30346556	chr15:76004532-76004576	NM_201394:21586	Plec	INSIDE	1.862	1.743	380.99	664.23	3.246	275.55	894.45
A_68_P32374343	chrX:48371227-48371271	NM_172413:-53	Rap2c	PROMOTER	1.855	2.627	2565.97	6739.85	4.874	3133.60	15272.22
A_68_P22415887	chr3:108830521-108830565	NM_001163567:-17	Fam102b	PROMOTER	1.852	20.640	400.36	8263.26	38.234	341.40	13053.19
A_68_P31442548	chr17:91487890-91487934	NM_020252:4230	Nrxn1	INSIDE	1.850	1.683	641.82	1080.50	3.114	507.56	1580.79
A_68_P26804376	chr9:106553310-106553354	NM_001160353:5108	Grm2	INSIDE	1.848	1.497	648.69	970.98	2.766	402.07	1112.10
A_68_P27278803	chr10:79229502-79229546	NM_144528:142	Rnf126	INSIDE	1.847	0.279	2767.60	771.27	0.515	1817.83	935.64
A_68_P32746468	chrX:148238435-148238479	NM_021523:632	Huwe1	INSIDE	1.845	0.347	1722.71	597.79	0.640	1999.73	1280.02
A_68_P31050851	chr17:12464717-12464771	NM_011948:46782	Map3k4	INSIDE	1.845	1.626	385.90	627.60	3.001	330.30	991.17
A_68_P24863969	chr6:134820135-134820179	NM_001167697:17721	Gpr19	INSIDE	1.843	4.031	579.69	2336.83	7.431	428.98	3187.78
A_68_P32565628	chrX:98989774-98989818	ENSMUST00000151231:-198		DIVERGENT_PROMOTER	1.840	2.425	150.71	365.40	4.462	276.66	1234.39
A_68_P20619890	chr1:134086976-134087020	NM_001033250:-1014	Lemd1	PROMOTER	1.840	1.810	1646.03	2980.13	3.332	1149.32	3829.46
A_68_P31836532	chr18:75527265-75527309	NM_001042660:268	Smad7	INSIDE	1.839	0.168	3019.38	506.90	0.309	2303.56	711.01
A_68_P27470613	chr10:115024518-115024562	NM_010195:296	Lgr5	INSIDE	1.839	0.245	2171.00	532.17	0.451	1610.90	726.18
A_68_P25506496	chr7:134754584-134754628	NM_177226:3341	Zfp629	INSIDE	1.839	2.172	607.26	1318.89	3.994	438.16	1749.85
A_68_P23996382	chr5:113567569-113567615	NM_172884:24741	2900026A02Rik	INSIDE	1.838	0.310	2228.96	691.26	0.570	1857.69	1058.77
A_68_P24225086	chr6:8209405-8209449	NR_033518:139	Gm16039	INSIDE	1.837	0.140	5642.36	790.47	0.257	3767.88	969.76
A_68_P21908932	chr2:180891704-180891748	NM_007906:-6	Eef1a2	PROMOTER	1.836	0.204	2968.38	604.30	0.374	2100.95	785.23
A_68_P25951936	chr8:72976257-72976301	NM_001122830:24564	Klhl26	INSIDE	1.835	1.784	393.61	702.20	3.273	368.26	1205.30
A_68_P27430001	chr10:107598764-107598808	NM_027892:-669	Ppp1r12a	PROMOTER	1.832	0.175	3327.76	582.55	0.321	2329.62	747.04
A_68_P27877796	chr11:66340159-66340203	NM_001034874:-552	Shisa6	PROMOTER	1.831	1.519	1562.74	2373.39	2.780	1295.79	3602.52
A_68_P23694987	chr5:52867888-52867932			Unknown	1.830	0.337	2158.42	726.74	0.616	1507.62	929.16
A_68_P20833279	chr1:174412349-174412393	NM_033608:-77	Igsf9	PROMOTER	1.830	0.193	2408.90	465.85	0.354	1925.94	681.49
A_68_P25827306	chr8:46023782-46023826	AK079043:-3291		PROMOTER	1.828	0.225	3579.14	806.03	0.412	2270.26	934.44
A_68_P27356009	chr10:93308576-93308623	NM_183199:14300	Usp44	INSIDE	1.827	1.592	529.10	842.17	2.909	453.10	1317.95
A_68_P32130472	chr19:44181487-44181534	NM_001162410:457	Chuk	INSIDE	1.825	0.327	5135.69	1677.71	0.596	3118.05	1858.58
A_68_P24115931	chr5:135825936-135825980	NM_033571:-97	Fkbp6	DIVERGENT_PROMOTER	1.824	1.574	704.41	1109.04	2.872	547.06	1570.95
A_68_P20008184	chr1:5009350-5009394	NM_021374:135	Rgs20	INSIDE	1.824	1.630	429.72	700.65	2.974	327.89	975.19
A_68_P30672035	chr16:35769023-35769067	NM_153550:398	Dirc2	INSIDE	1.823	0.244	2104.43	513.87	0.445	1598.90	711.60
A_68_P32071013	chr19:32670325-32670369	NM_001201470:142	Papss2	INSIDE	1.822	0.205	2227.45	457.03	0.374	1521.46	568.70
A_68_P30351139	chr15:76741794-76741838	NM_001168276:14062	Zfp647	INSIDE	1.822	1.629	833.74	1358.50	2.968	654.49	1942.66
A_68_P32137924	chr19:45438347-45438391	NM_001037758:145	Btrc	INSIDE	1.821	0.240	3569.15	855.22	0.436	2453.40	1070.74
A_68_P30778641	chr16:55974831-55974875	NR_027965:-122	2310061J03Rik	PROMOTER	1.820	2.660	3051.78	8118.32	4.842	2453.14	11878.15
A_68_P25957206	chr8:74031971-74032015	NM_032398:3676	Plvap	INSIDE	1.819	0.236	1907.23	450.22	0.429	1399.19	600.73
A_68_P31442543	chr17:91487324-91487368	NM_020252:4796	Nrxn1	INSIDE	1.817	1.943	1145.42	2225.01	3.529	881.58	3111.41
A_68_P22317890	chr3:89017814-89017858	NM_001161824:421	Mtx1	INSIDE	1.817	0.158	4322.03	684.84	0.288	2702.35	778.07
A_68_P20910363	chr1:188791777-188791821	NM_033077:504	D1Pas1	INSIDE	1.814	1.467	667.22	978.63	2.661	604.52	1608.39
A_68_P28741417	chr12:112215277-112215321	NM_175207:1933	Ankrd9	INSIDE	1.813	2.012	421.16	847.49	3.649	328.77	1199.66
A_68_P28176530	chr11:119072055-119072099	NM_172443:17737	Tbc1d16	INSIDE	1.812	12.586	1786.95	22490.69	22.804	1619.18	36924.48
A_68_P25021026	chr7:29083763-29083810	NM_007866:3018	Dll3	INSIDE	1.812	3.280	272.38	893.50	5.944	265.45	1577.90
A_68_P21394205	chr2:84554801-84554845	NM_144887:499	Zdhhc5	INSIDE	1.812	0.267	2885.30	770.98	0.484	2065.95	1000.18
A_68_P31149893	chr17:33787074-33787118	NM_001109913:35307	Hnrnpm	INSIDE	1.808	1.668	589.13	982.79	3.016	574.68	1733.42

ProbeName	Target position of probe on CpG island microarray	TargetID	GeneSymbol	CpG island Description	Ratio of relative methylation (TiO_2-NP/Vehicle)	Sham group			TiO_2-H group		
						Relative methylation (Cy5/Cy3)	Cy3 signal (Input)	Cy5 signal (MeDIP)	Relative methylation (Cy5/Cy3)	Cy3 signal (Input)	Cy5 signal (MeDIP)
A_68_P29054563	chr13:55463630-55463674	NM_025828:492	Lman2	INSIDE	1.805	0.217	2875.17	625.02	0.392	1990.88	781.29
A_68_P24944609	chr6:149310012-149310056	ENSMUST00000120260:-173		DIVERGENT_PROMOTER	1.805	1.637	759.41	1243.52	2.956	581.59	1719.18
A_68_P21568844	chr2:119119741-119119785	NM_172269:5285	Vps18	INSIDE	1.805	2.271	503.93	1144.60	4.099	423.20	1734.62
A_68_P21137825	chr2:35959267-35959311	NM_001083126:292	Lhx6	INSIDE	1.802	0.194	2317.86	450.57	0.350	1731.51	606.54
A_68_P24974188	chr7:13609447-13609491	NM_011588:-32	Trim28	PROMOTER	1.799	7.313	835.47	6109.48	13.158	753.92	9919.86
A_68_P29731241	chr14:76410609-76410653	NM_026816:42	Gtf2f2	INSIDE	1.795	0.312	1724.64	538.92	0.561	1194.18	669.73
A_68_P21100044	chr2:29744774-29744818	NM_001113214:-443	Odf2	PROMOTER	1.790	0.191	4382.86	836.92	0.342	2890.64	988.22
A_68_P31779044	chr18:65243606-65243650	NM_031881:60448	Nedd4l	INSIDE	1.789	1.879	684.61	1286.05	3.361	570.07	1915.77
A_68_P24046894	chr5:122303239-122303283	NM_175474:4224	Fam109a	INSIDE	1.789	1.939	269.97	523.60	3.470	184.59	640.48
A_68_P22403732	chr3:106838896-106838940	NM_008418:-161	Kcna3	PROMOTER	1.789	0.142	3571.14	507.56	0.254	2358.78	599.69
A_68_P20218129	chr1:50984521-50984565	NM_019790:176	Tmeff2	INSIDE	1.788	0.328	2119.57	694.49	0.586	1600.50	937.73
A_68_P30480363	chr15:99301136-99301180	NM_029236:4003	Bcdin3d	INSIDE	1.787	1.554	599.21	930.96	2.777	461.69	1282.07
A_68_P23278937	chr4:125911565-125911609	NM_001145970:21977	Mtap7d1	INSIDE	1.787	1.469	1469.61	2158.84	2.626	1098.38	2883.83
A_68_P22314993	chr3:88447939-88447984	NM_001198912:22646	Arhgef2	INSIDE	1.785	1.673	740.23	1238.66	2.987	650.76	1944.13
A_68_P21266388	chr2:61643903-61643948	NM_009322:1416	Tbr1	INSIDE	1.784	0.355	1982.06	703.06	0.633	1308.33	827.97
A_68_P30360709	chr15:78370273-78370318	NM_009218:4480	Sstr3	INSIDE	1.783	1.666	582.46	970.35	2.970	447.44	1328.90
A_68_P28164255	chr11:117151945-117151989	NM_001113487:24407	Septin9	INSIDE	1.782	1.947	363.47	707.70	3.471	295.72	1026.33
A_68_P31879481	chr18:83126670-83126714	NM_001177464:42669	Zfp516	INSIDE	1.781	1.623	1174.94	1906.40	2.889	905.86	2617.09
A_68_P27286214	chr10:80363023-80363067	NM_013895:670	Timm13	INSIDE	1.777	0.239	2267.77	540.95	0.424	1599.29	677.80
A_68_P24946817	chr7:3637393-3637437	NM_029934:7713	Mboat7	INSIDE	1.777	3.262	2159.27	7043.79	5.798	1525.34	8843.73
A_68_P23296719	chr4:129138535-129138579	NM_133889:-366	Bsdc1	PROMOTER	1.777	2.524	257.67	650.34	4.485	232.11	1041.10
A_68_P23358589	chr4:140071100-140071144	NM_001112723:133549	Arhgef10l	INSIDE	1.776	1.709	474.60	811.00	3.035	406.30	1233.20
A_68_P23199842	chr4:108874395-108874439	NM_133885:461	Osbpl9	INSIDE	1.776	0.200	3571.63	712.93	0.355	2515.70	892.03
A_68_P25090603	chr7:52263124-52263168	NM_001008422:8473	Scaf1	INSIDE	1.773	1.772	837.70	1484.02	3.142	678.79	2132.59
A_68_P31041951	chr17:11033242-11033286	NM_016694:15	Park2	INSIDE	1.772	0.287	1910.66	548.03	0.508	1440.01	731.73
A_68_P22325407	chr3:90269682-90269726	NM_008727:84	Npr1	INSIDE	1.772	0.206	2332.55	480.37	0.365	1707.74	623.32
A_68_P29509082	chr14:31814863-31814907	NM_026911:-32	Spcs1	PROMOTER	1.770	4.059	489.82	1988.26	7.185	437.64	3144.61
A_68_P23984844	chr5:111706164-111706208	NM_024477:397365	Ttc28	INSIDE	1.770	1.542	1479.85	2281.56	2.729	1118.60	3052.93
A_68_P25048198	chr7:35988774-35988818	NM_001024707:11568	Lrp3	INSIDE	1.769	1.947	586.59	1141.93	3.444	500.55	1723.88
A_68_P28525539	chr12:72419195-72419245	NM_001190466:8350	Dact1	INSIDE	1.767	2.627	467.90	1229.28	4.641	368.31	1709.50
A_68_P30641174	chr16:30309847-30309891	NM_008148:997	Gp5	INSIDE	1.766	0.320	1564.95	500.53	0.565	1214.54	685.85
A_68_P20066646	chr1:19204206-19204250	NM_001025305:2094	Tcfap2b	INSIDE	1.766	0.311	4912.11	1525.68	0.548	3553.92	1949.08
A_68_P20347698	chr1:74331582-74331626	NM_025580:-3	Pnkd	DIVERGENT_PROMOTER	1.765	0.305	1703.19	519.82	0.539	1221.58	658.22
A_68_P20022045	chr1:9289343-9289387	NM_027671:447	Sntg1	INSIDE	1.765	3.599	3399.54	12235.12	6.352	2815.61	17886.00
A_68_P26920733	chr10:7500333-7500377	NM_145418:361	BC013529	INSIDE	1.764	0.232	2535.06	586.93	0.408	1864.52	761.54
A_68_P24098211	chr5:131915292-131915336	NM_177047:1102899	Auts2	INSIDE	1.764	1.814	777.47	1410.48	3.201	711.14	2276.02
A_68_P31150930	chr17:34048600-34048644	NM_001199733:2077	Daxx	INSIDE	1.763	1.564	487.56	762.60	2.758	369.37	1018.60
A_68_P30025417	chr15:12980979-12981031	NM_007666:122390	Cdh6	INSIDE	1.763	1.647	512.34	843.97	2.904	383.52	1113.55
A_68_P27926647	chr11:75344750-75344794	NM_001199284:-423	Slc43a2	PROMOTER	1.762	0.159	3975.62	633.93	0.281	2785.27	782.43
A_68_P27110004	chr10:44878135-44878179	NM_011156:91137	Prep	INSIDE	1.759	1.456	657.43	957.17	2.561	534.61	1369.23
A_68_P26348936	chr9:21599583-21599627	NM_145611:3386	Kank2	INSIDE	1.759	1.910	501.90	958.76	3.361	448.01	1505.68
A_68_P25957394	chr8:74067191-74067235	NM_028617:384	Fam125a	INSIDE	1.758	0.273	3039.25	828.37	0.479	1670.19	800.38
A_68_P25761256	chr8:33120256-33120300	ENSMUST00000080782:-697		PROMOTER	1.758	5.073	3771.00	19130.24	8.916	2946.08	26268.20
A_68_P23819019	chr5:77380180-77380224	NM_025939:-233	Paics	PROMOTER	1.758	0.241	2192.33	527.64	0.423	1553.11	657.01
A_68_P30346558	chr15:76004732-76004776	NM_201394:21386	Plec	INSIDE	1.757	2.136	282.12	602.52	3.752	235.14	882.35
A_68_P23250319	chr4:119941839-119941885	NM_194060:18004	Foxo6	INSIDE	1.757	2.373	474.54	1126.11	4.169	328.99	1371.62
A_68_P29880580	chr14:106505493-106505537	NM_198601:100	Trim52	INSIDE	1.755	2.108	518.00	1092.14	3.700	395.37	1462.87
A_68_P25416834	chr7:118199064-118199108	NM_009431:26622	Ctr9	INSIDE	1.755	1.489	700.82	1043.42	2.613	551.07	1439.80
A_68_P26870880	chr9:119071809-119071853	NM_146230:-5619	Acaa1b	PROMOTER	1.752	2.371	214.86	509.47	4.155	234.49	974.38
A_68_P30346580	chr15:76007443-76007487	NM_201394:18676	Plec	INSIDE	1.750	1.639	487.93	799.57	2.867	377.14	1081.42
A_68_P23195055	chr4:107890802-107890861	NM_001167936:-304	Zyg11a	PROMOTER	1.750	1.967	828.18	1628.84	3.443	646.09	2224.34
A_68_P25729013	A_68_P25729013			Unknown	1.749	2.201	219.76	483.64	3.848	128.99	496.36
A_68_P21823609	chr2:165221660-165221704	NM_029021:4212	4833422F24Rik	INSIDE	1.748	2.230	594.07	1324.93	3.899	539.25	2102.65
A_68_P22540268	chr3:132612964-132613008	NM_001029836:269	Npnt	INSIDE	1.747	1.714	1731.25	2966.97	2.994	1385.21	4146.86
A_68_P21070914	chr2:25111268-25111312	NM_177344:332	Tmem203	INSIDE	1.743	0.151	3164.81	478.67	0.264	2143.55	565.17
A_68_P28940414	chr13:34838852-34838896	NM_138746:7364	Fam50b	INSIDE	1.742	1.574	689.16	1084.99	2.743	604.58	1658.24
A_68_P28550370	chr12:76919981-76920025	NM_001005510:698	Syne2	INSIDE	1.740	0.195	2841.57	554.76	0.340	2189.70	743.85
A_68_P25656209	chr8:11555685-11555729	NM_011919:-359	Ing1	DIVERGENT_PROMOTER	1.740	0.270	5636.96	1523.50	0.470	3196.92	1503.60
A_68_P20832394	chr1:174256462-174256506	NM_008429:2910	Kcnj9	INSIDE	1.738	0.292	1705.00	498.67	0.508	1191.95	605.77
A_68_P24620361	chr6:88149067-88149115	NM_008090:433	Gata2	INSIDE	1.736	2.264	284.27	643.48	3.930	220.78	867.73
A_68_P22148834	chr3:54437446-54437490	ENSMUST00000172420:2108		INSIDE	1.733	3.146	1027.70	3233.13	5.453	782.58	4267.43
A_68_P28202484	chr12:4824078-4824122	NM_025323:-313	0610009D07Rik	PROMOTER	1.732	0.265	2234.03	592.81	0.459	1788.98	821.98
A_68_P22397119	chr3:105261830-105261874	NM_019931:119	Kcnd3	INSIDE	1.731	1.781	303.17	539.90	3.083	250.77	773.21
A_68_P21116406	chr2:32470512-32470556	NM_001025310:8054	St6galnac6	INSIDE	1.731	2.288	358.67	820.74	3.961	284.96	1128.59
A_68_P32668459	chrX:130222079-130222123	NM_001105245:1432	Pcdh19	INSIDE	1.730	2.137	285.14	609.41	3.698	250.74	927.10

ProbeName	Target position of probe on CpG island microarray	TargetID	GeneSymbol	CpG island Description	Ratio of relative methylation (TiO_2-NP/Vehicle)	Sham group			TiO_2-H group		
						Relative methylation (Cy5/Cy3)	Cy3 signal (Input)	Cy5 signal (MeDIP)	Relative methylation (Cy5/Cy3)	Cy3 signal (Input)	Cy5 signal (MeDIP)
A_68_P28748396	chr12:113414015-113414059	NM_001097621:29618	Kif26a	INSIDE	1.730	1.975	900.70	1779.15	3.418	683.57	2336.22
A_68_P24983572	chr7:17428015-17428059	NM_178900:-377	Prkd2	PROMOTER	1.730	0.202	2521.34	508.68	0.349	1818.88	634.89
A_68_P31424359	chr17:88039798-88039842	NM_008532:4502	Epcam	INSIDE	1.729	1.980	800.19	1584.07	3.422	598.90	2049.64
A_68_P31092362	chr17:23694847-23694891	NM_001033496:6325	Zfp213	INSIDE	1.729	1.803	713.38	1286.19	3.118	601.25	1874.66
A_68_P29591680	chr14:49685967-49686011	NM_144535:-181	Mudeng	PROMOTER	1.729	2.017	1046.28	2109.90	3.486	737.81	2572.08
A_68_P25101651	chr7:54101747-54101791	NM_001136069:-683	Ldha	PROMOTER	1.729	2.175	339.47	738.22	3.760	284.90	1071.22
A_68_P23308456	chr4:131375306-131375350	NM_001083119:18865	Ptpru	INSIDE	1.729	1.597	1042.14	1664.19	2.761	864.47	2386.37
A_68_P30513214	chr16:5013320-5013364	NM_133185:304	Rogdi	INSIDE	1.728	0.251	2249.34	563.58	0.433	1525.30	660.29
A_68_P26226906	chr8:123992825-123992870	ENSMUST00000127664:1558107		INSIDE	1.728	1.688	615.85	1039.39	2.917	472.80	1379.09
A_68_P21874518	chr2:174125914-174125958	NM_201617:2577	Gnas	INSIDE	1.728	1.699	452.44	768.55	2.936	400.19	1174.77
A_68_P20351044	chr1:74931604-74931648	NM_153111:356	Fev	INSIDE	1.728	0.267	2685.54	716.56	0.461	1784.15	822.48
A_68_P25587962	chr7:148480819-148480877	NM_007878:2944	Drd4	INSIDE	1.726	1.860	348.96	648.99	3.211	289.29	928.85
A_68_P28157433	chr11:115971055-115971099	NM_001205081:473	Trim47	INSIDE	1.725	5.091	5170.14	26322.32	8.782	3817.22	33524.51
A_68_P26750366	chr9:96896367-96896411	NM_145134:22386	Spsb4	INSIDE	1.725	1.856	566.28	1051.28	3.202	489.07	1566.15
A_68_P25827948	chr8:46108836-46108887	NM_001081286:73300	Fat1	INSIDE	1.725	1.680	466.67	784.00	2.899	425.62	1233.75
A_68_P21874746	chr2:174153286-174153330	NR_003258:-106	Gnas	PROMOTER	1.724	1.934	675.21	1305.65	3.334	531.82	1773.19
A_68_P23227295	chr4:115690207-115690251	NM_010173:279	Faah	INSIDE	1.722	0.350	2630.89	920.09	0.602	2040.46	1228.88
A_68_P22071595	chr3:38784639-38784683	NM_183221:-1201	Fat4	PROMOTER	1.722	0.178	2961.97	526.82	0.306	2064.19	632.37
A_68_P25614227	chr8:3496122-3496166	NM_080461:3007	Zfp358	INSIDE	1.721	2.260	486.82	1100.25	3.890	447.45	1740.49
A_68_P25029686	chr7:30836414-30836458	NM_011748:-21	Zfp14	PROMOTER	1.721	0.324	2527.42	818.75	0.558	1723.15	960.79
A_68_P26875426	chr9:119883221-119883265	NM_153287:10534	Csrnp1	INSIDE	1.720	2.451	1093.10	2679.13	4.217	802.54	3384.01
A_68_P25011335	chr7:26473118-26473162	NM_011577:1120	Tgfb1	INSIDE	1.719	3.422	493.96	1690.29	5.884	412.39	2426.41
A_68_P20624277	chr1:134777288-134777332	NM_010732:379	Lrrn2	INSIDE	1.718	0.294	1764.76	519.54	0.506	1301.43	658.12
A_68_P23195766	chr4:108055710-108055754	NM_001099303:222	Fam159a	INSIDE	1.714	3.555	592.30	2105.86	6.094	439.28	2676.84
A_68_P25026465	chr7:30089302-30089346	NM_013874:301	Dpf1	INSIDE	1.713	0.358	1868.51	669.59	0.614	1280.73	786.10
A_68_P23396583	chr4:148179764-148179808	NM_027195:1286	Casz1	INSIDE	1.713	0.271	2501.03	678.08	0.464	1694.65	786.98
A_68_P24951677	chr7:4982359-4982403	NM_001033383:10403	Zfp865	INSIDE	1.711	1.571	765.01	1202.04	2.688	647.70	1740.82
A_68_P24992504	chr7:19878717-19878763	NM_001025364:2293	Rtn2	INSIDE	1.710	2.102	784.71	1649.76	3.595	597.71	2148.90
A_68_P24686655	chr6:99472528-99472572	ENSMUST00000155466:144241		INSIDE	1.710	0.110	4849.00	532.00	0.188	3077.81	577.52
A_68_P29880583	chr14:106505860-106505904	NM_198601:468	Trim52	INSIDE	1.709	2.239	287.69	644.21	3.828	221.37	847.33
A_68_P21782541	chr2:158187217-158187261	NR_015463:7	9430008C03Rik	INSIDE	1.706	0.236	2003.52	473.16	0.403	1240.63	499.96
A_68_P27184377	chr10:61596546-61596590	NM_009719:731	Neurog3	INSIDE	1.705	0.112	4629.82	517.03	0.190	2985.56	568.41
A_68_P27100736	chr10:43231246-43231290	NM_199028:32323	Bend3	INSIDE	1.705	1.921	799.67	1535.84	3.275	751.16	2460.31
A_68_P27902198	chr11:70513486-70513530	NM_213729:149	Inca1	INSIDE	1.704	0.373	1682.66	626.86	0.635	1299.08	824.54
A_68_P28272752	chr12:17888520-17888564	ENSMUST00000169996:-460		PROMOTER	1.700	0.287	2387.44	686.31	0.489	1848.97	903.42
A_68_P22059401	chr3:36570355-36570399	NM_019510:18713	Trpc3	INSIDE	1.700	1.747	840.14	1467.61	2.970	787.56	2338.68
A_68_P23977822	chr5:110536969-110537013	NM_145147:226	Gtpbp6	INSIDE	1.699	0.315	2420.61	761.52	0.534	1757.54	939.16
A_68_P23317854	chr4:133042232-133042276	NM_175307:6207	Fam46b	INSIDE	1.698	1.413	1009.88	1427.21	2.400	720.35	1728.53
A_68_P27928205	chr11:75608766-75608811	NM_007873:771	Doc2b	INSIDE	1.696	0.319	1994.14	635.34	0.540	1600.45	865.04
A_68_P27913317	chr11:72609127-72609171	NM_001024926:193	Cyb5d2	INSIDE	1.695	0.215	2830.15	607.14	0.364	2067.92	751.72
A_68_P24837693	chr6:128150794-128150838	ENSMUST00000071101:-175		PROMOTER	1.695	0.312	4102.06	1279.11	0.529	3031.90	1602.73
A_68_P23984176	chr5:111612120-111612164	NM_024477:303321	Ttc28	INSIDE	1.695	1.505	1079.85	1624.98	2.551	796.72	2032.28
A_68_P24156244	chr5:144131752-144131796	NM_133355:6188	Grid2ip	INSIDE	1.694	0.303	1815.43	549.57	0.513	1340.64	687.37
A_68_P23982395	chr5:111308888-111308932	NM_024477:89	Ttc28	INSIDE	1.694	2.668	340.56	908.75	4.521	237.19	1072.43
A_68_P20347699	chr1:74331738-74331785	NM_025580:154	Pnkd	INSIDE	1.694	1.725	655.05	1130.24	2.923	482.29	1409.71
A_68_P20741943	chr1:158134761-158134805	NM_177838:374	Fam163a	INSIDE	1.693	0.263	2869.64	754.65	0.445	1812.02	806.75
A_68_P22449311	chr3:116126122-116126166	NM_001080818:806	Cdc14a	INSIDE	1.691	0.245	1918.75	469.38	0.414	1518.17	628.04
A_68_P21682258	chr2:139892550-139892594	NM_001159640:-51	Tasp1	PROMOTER	1.691	0.358	1664.46	596.21	0.606	1402.58	849.45
A_68_P20015303	chr1:7078680-7078724	NM_183028:-298	Pcmtd1	PROMOTER	1.689	2.187	2152.62	4707.41	3.694	1689.57	6241.42
A_68_P32070012	chr19:32462008-32462052	NM_144792:914	Sgms1	INSIDE	1.688	0.367	2034.86	745.92	0.619	1583.37	979.49
A_68_P32542578	chrX:92907030-92907074	NM_001034907:36	Zc3h12b	INSIDE	1.685	1.411	1150.99	1624.49	2.379	1422.01	3382.68
A_68_P31107655	chr17:26122056-26122100	NM_153140:36727	Rab11fip3	DOWNSTREAM	1.685	6.674	899.31	6001.87	11.249	635.66	7150.21
A_68_P25730016	chr8:27096591-27096635	NM_029037:7981	4930444A02Rik	INSIDE	1.685	1.666	858.07	1429.54	2.808	702.41	1972.07
A_68_P23192163	chr4:107356485-107356529	NM_019872:261	Dmrtb1	INSIDE	1.685	2.808	415.51	1166.68	4.732	366.10	1732.21
A_68_P25952523	chr8:73063414-73063459	NM_007924:-137	Ell	PROMOTER	1.683	0.270	1813.60	490.19	0.455	1275.50	580.29
A_68_P20141345	chr1:34986894-34986938			Unknown	1.683	1.997	974.32	1946.03	3.362	887.68	2984.15
A_68_P27288895	chr10:80788735-80788779	NM_027381:4909	2510012J08Rik	INSIDE	1.682	1.954	425.68	831.64	3.286	321.71	1057.14
A_68_P25088121	chr7:51807262-51807306	NM_009473:2009	Nr1h2	INSIDE	1.682	1.730	1979.26	3424.77	2.910	1609.46	4683.19
A_68_P21111931	chr2:31742789-31742833	NM_011836:10	Lamc3	INSIDE	1.682	2.699	2216.13	5982.30	4.540	1867.86	8480.68
A_68_P20910361	chr1:188791550-188791594	NM_033077:278	D1Pas1	INSIDE	1.682	2.248	960.00	2158.23	3.782	797.59	3016.45
A_68_P32266426	chrX:13063861-13063905	NM_173415:19085	Nyx	INSIDE	1.681	1.889	334.08	631.22	3.176	589.98	1873.51
A_68_P32543327	chrX:93163621-93163665			Unknown	1.678	2.439	1011.71	2467.70	4.093	1285.90	5262.57
A_68_P32696393	chrX:136405600-136405644	NM_001193309:571	Morc4	INSIDE	1.677	0.399	1891.42	754.02	0.668	2472.22	1652.64
A_68_P28080237	chr11:102417265-102417309	NM_001159492:186	Gpatch8	INSIDE	1.677	0.216	3121.03	675.32	0.363	2300.98	834.83
A_68_P27765617	chr11:45869706-45869750	NM_009616:240	Adam19	INSIDE	1.677	0.172	2823.73	486.49	0.289	1961.13	566.57

ProbeName	Target position of probe on CpG island microarray	TargetID	GeneSymbol	CpG island Description	Ratio of relative methylation (TiO_2-NP/Vehicle)	Sham group			TiO_2-H group		
						Relative methylation (Cy5/Cy3)	Cy3 signal (Input)	Cy5 signal (MeDIP)	Relative methylation (Cy5/Cy3)	Cy3 signal (Input)	Cy5 signal (MeDIP)
A_68_P28575795	chr12:81564283-81564327	NM_133798:223	Exd2	INSIDE	1.675	2.365	4896.40	11578.73	3.960	3661.54	14500.29
A_68_P25505622	chr7:134587895-134587939	NM_175163:4756	Zfp689	INSIDE	1.674	2.210	702.77	1552.86	3.698	587.12	2171.29
A_68_P21843351	chr2:168581527-168581571	NM_175303:11153	Sall4	INSIDE	1.674	1.382	1585.45	2190.77	2.313	1173.46	2714.67
A_68_P30533731	chr16:8672077-8672121	NM_025821:148	Carhsp1	INSIDE	1.672	0.146	3146.51	460.63	0.245	2201.25	538.75
A_68_P30388853	chr15:83341230-83341274	NM_178869:85	Ttll1	INSIDE	1.671	0.332	1781.62	590.68	0.554	1213.30	672.35
A_68_P29024947	chr13:49478476-49478520	NM_001039179:41581	Bicd2	INSIDE	1.671	2.133	233.03	496.98	3.564	197.56	704.06
A_68_P28151695	chr11:115024532-115024576	NM_012030:-100	Slc9a3r1	PROMOTER	1.671	0.282	1630.69	459.78	0.471	1205.14	567.95
A_68_P23818508	chr5:77287446-77287490	NM_001163793:17845	C530008M17Rik	INSIDE	1.671	1.383	979.12	1353.77	2.311	680.53	1572.60
A_68_P22314483	chr3:88357962-88358006	NM_033526:347	Ubqln4	INSIDE	1.671	1.563	4922.57	7695.81	2.612	3964.44	10354.34
A_68_P28036506	chr11:94937357-94937401	NM_013565:650	Itga3	INSIDE	1.670	0.113	4784.33	540.29	0.189	3225.48	608.12
A_68_P31001860	chr16:98144127-98144171	NM_174847:95	C2cd2	INSIDE	1.669	0.354	1662.37	589.29	0.592	1271.53	752.40
A_68_P25952090	chr8:73000940-73000985	NM_001122830:-120	Klhl26	PROMOTER	1.669	0.330	1622.05	534.58	0.550	1096.92	603.54
A_68_P29651209	chr14:62267312-62267356	NR_028264:33876	Dleu2	INSIDE	1.668	0.215	2631.81	566.47	0.359	1978.19	710.00
A_68_P20930616	chr1:191994691-191994750	NM_008937:-161	Prox1	PROMOTER	1.668	2.216	242.53	537.35	3.696	169.99	628.26
A_68_P23588862	chr5:33778061-33778105	ENSMUST00000030994:-4238		PROMOTER	1.666	0.413	4343.86	1792.73	0.688	2983.03	2051.08
A_68_P27567653	chr11:6174835-6174879	NM_134020:-16	Tmed4	PROMOTER	1.665	0.110	5812.34	641.98	0.184	4030.60	741.25
A_68_P29712960	chr14:72932250-72932294	NM_207636:177538	Fndc3a	DOWNSTREAM	1.664	0.301	1859.19	559.88	0.501	1521.85	762.55
A_68_P24810881	chr6:122824922-122824967	NM_026267:370	Necap1	INSIDE	1.664	0.234	2510.08	587.64	0.390	2010.55	783.17
A_68_P31418978	chr17:87152455-87152501	NM_010137:-725	Epas1	PROMOTER	1.663	0.353	2221.64	784.05	0.587	1898.75	1114.31
A_68_P24135223	chr5:139856324-139856369	NM_030258:-274	Gpr146	PROMOTER	1.662	1.968	274.18	539.44	3.269	202.68	662.60
A_68_P21278824	chr2:63936695-63936739	NM_021716:-647	Fign	PROMOTER	1.661	2.118	538.99	1141.77	3.518	411.11	1446.40
A_68_P24155795	chr5:144031809-144031857	NM_017467:-131	Zfp316	PROMOTER	1.660	0.329	1913.01	630.08	0.547	1443.98	789.70
A_68_P32260575	chrX:11705762-11705806	NM_001168321:31697	Bcor	INSIDE	1.659	0.268	2609.74	699.09	0.445	2866.64	1274.23
A_68_P27280128	chr10:79440201-79440245	NM_001003949:6853	ORF61	INSIDE	1.659	2.195	695.17	1526.00	3.641	559.28	2036.57
A_68_P26804377	chr9:106553386-106553432	NM_001160353:5032	Grm2	INSIDE	1.656	1.807	377.80	682.59	2.992	294.56	881.45
A_68_P25183344	chr7:72672119-72672163			Unknown	1.656	0.306	2307.21	706.04	0.507	1891.50	958.67
A_68_P23417006	chr4:151552589-151552633	NM_001146057:-7632	Acot7	PROMOTER	1.656	0.299	1702.59	509.26	0.495	1356.58	671.96
A_68_P20552784	chr1:120524752-120524796	NM_023755:253	Tcfcp2l1	INSIDE	1.656	0.287	2154.08	618.23	0.475	1761.18	836.84
A_68_P25599895	chr7:150481208-150481262	NR_001461:1218	Kcnq1ot1	INSIDE	1.654	1.374	2449.59	3366.09	2.272	1823.34	4142.94
A_68_P26537272	chr9:56468941-56468985	NM_181074:64098	Lingo1	INSIDE	1.653	3.085	633.99	1956.15	5.099	577.55	2944.92
A_68_P31152226	chr17:34257950-34257994	NR_037970:-1127	Brd2	PROMOTER	1.652	0.313	3155.24	987.92	0.517	2093.02	1082.94
A_68_P24954141	chr7:6084325-6084369	NM_001013012:23227	Zfp787	INSIDE	1.652	1.816	707.21	1284.10	2.999	635.80	1906.85
A_68_P29699020	chr14:70477756-70477800	NM_018781:527	Egr3	INSIDE	1.651	0.223	2097.74	468.42	0.369	1575.88	581.14
A_68_P31841472	chr18:76305759-76305803	NM_145356:325949	Zbtb7c	INSIDE	1.650	1.498	831.82	1246.11	2.472	691.68	1709.80
A_68_P30484944	chr15:100111219-100111263	NM_183109:-27	Tmprss12	PROMOTER	1.650	2.002	494.51	989.99	3.304	430.45	1422.16
A_68_P24634428	chr6:90686241-90686285	NM_001134384:73855	Iqsec1	INSIDE	1.650	0.353	3009.87	1062.80	0.582	2298.95	1339.06
A_68_P20058120	chr1:17082054-17082098	NM_020604:5894	Jph1	INSIDE	1.650	1.440	1229.62	1770.80	2.376	920.75	2187.68
A_68_P26467526	chr9:44226004-44226048	NM_201372:64	Ccdc84	INSIDE	1.649	0.388	1972.34	765.18	0.640	1325.77	848.27
A_68_P28187725	chr11:120812339-120812383	NM_001038653:1980	Slc16a3	INSIDE	1.647	0.229	2113.89	483.75	0.377	1532.58	577.78
A_68_P25020989	chr7:29076252-29076296	NM_175033:-121	BC089491	PROMOTER	1.647	0.216	2033.38	439.83	0.356	1466.96	522.67
A_68_P27630976	chr11:20232710-20232754	NM_018861:-16	Slc1a4	PROMOTER	1.646	0.327	1958.54	640.30	0.538	1457.79	784.40
A_68_P23339579	chr4:136944180-136944224	ENSMUST00000078305:-7005		PROMOTER	1.646	0.193	2341.37	452.28	0.318	1685.96	536.12
A_68_P32790793	chrX:159198362-159198406	NM_009031:81	Rbbp7	INSIDE	1.645	2.460	233.74	575.00	4.047	231.53	937.09
A_68_P31256956	chr17:56705945-56705989	NM_001029979:18040	Safb2	INSIDE	1.645	1.951	909.41	1774.22	3.210	754.39	2421.77
A_68_P24428220	chr6:48547266-48547310	NM_001079901:3407	Repin1	INSIDE	1.645	1.685	663.81	1118.38	2.771	534.14	1480.00
A_68_P21673353	chr2:138082625-138082669	NM_001025431:327	Btbd3	INSIDE	1.644	0.264	2671.76	705.92	0.434	2204.93	957.51
A_68_P25499767	chr7:133435936-133435980	NM_145587:19826	Sbk1	INSIDE	1.642	2.270	580.62	1318.11	3.729	536.44	2000.20
A_68_P21774735	chr2:156846821-156846865	NM_001164663:58159	9830001H06Rik	INSIDE	1.642	1.603	495.12	793.91	2.633	417.25	1098.49
A_68_P31841966	chr18:76400802-76400846	NM_010754:-754	Smad2	PROMOTER	1.641	0.240	2882.94	691.34	0.393	2107.21	829.06
A_68_P23364325	chr4:141034764-141034809	NM_019763:59726	Spen	INSIDE	1.641	2.009	298.14	598.94	3.297	270.41	891.44
A_68_P26658578	chr9:78023836-78023880	NM_019987:62814	Ick	DOWNSTREAM	1.638	0.336	1825.54	612.89	0.550	1395.07	766.97
A_68_P25025268	chr7:29871232-29871276	NR_035489:-2541	Mir1963	PROMOTER	1.638	1.764	415.65	733.30	2.890	357.18	1032.21
A_68_P21102303	chr2:30142233-30142277	NM_198304:302	Nup188	INSIDE	1.638	0.152	3059.33	466.03	0.249	2138.98	533.67
A_68_P23280027	chr4:126106647-126106691	NM_153402:118	Eif2c3	INSIDE	1.637	0.374	1530.92	573.05	0.613	1160.71	711.12
A_68_P26344263	chr9:20672999-20673043	NM_175687:-65	A230050P20Rik	PROMOTER	1.636	7.477	836.66	6255.73	12.233	690.61	8448.31
A_68_P21070362	chr2:25036916-25036960	NM_025980:661	Nrarp	INSIDE	1.635	0.183	2638.15	482.61	0.299	1731.34	517.99
A_68_P30651947	chr16:32333125-32333169	NM_177633:809	Ubxn7	INSIDE	1.633	2.079	455.69	947.25	3.395	349.78	1187.46
A_68_P31260730	chr17:57388800-57388844	NM_134125:-51	Trip10	DIVERGENT_PROMOTER	1.631	0.256	3650.49	934.72	0.418	2504.21	1046.13
A_68_P27287001	chr10:80494270-80494314	NM_134135:2365	Slc39a3	INSIDE	1.630	1.767	862.94	1525.04	2.881	622.02	1792.32
A_68_P24642400	chr6:91929179-91929223	NM_172731:-7903	Fgd5	PROMOTER	1.629	0.395	2597.95	1026.01	0.643	1718.17	1105.19
A_68_P25957633	chr8:74108592-74108640	NM_011977:15791	Slc27a1	INSIDE	1.628	1.543	768.24	1185.18	2.512	539.64	1355.69
A_68_P21754953	chr2:153269988-153270032	NM_001001986:205	8430427H17Rik	INSIDE	1.627	4.589	2156.90	9898.03	7.466	1736.38	12964.18
A_68_P24424840	chr6:47855086-47855130	NM_146175:27555	Zfp282	INSIDE	1.625	1.496	923.18	1381.37	2.432	720.93	1753.37
A_68_P26018119	chr8:86618743-86618787	NM_009055:28030	Rfx1	INSIDE	1.624	1.389	979.05	1359.71	2.255	697.14	1572.22
A_68_P20715106	chr1:153347232-153347276	NM_011277:699	Rnf2	INSIDE	1.624	0.175	4372.86	764.02	0.284	3016.28	855.76

ProbeName	Target position of probe on CpG island microarray	TargetID	GeneSymbol	CpG island Description	Ratio of relative methylation (TiO_2-NP/Vehicle)	Sham group			TiO_2-H group		
						Relative methylation (Cy5/Cy3)	Cy3 signal (Input)	Cy5 signal (MeDIP)	Relative methylation (Cy5/Cy3)	Cy3 signal (Input)	Cy5 signal (MeDIP)
A_68_P31491179	chr18:10706877-10706921	NM_134130:-204	Abhd3	PROMOTER	1.623	0.207	2949.55	611.31	0.336	2040.67	686.49
A_68_P24172758	chr5:148081815-148081859	NM_008814:130	Pdx1	INSIDE	1.623	0.218	2266.47	494.36	0.354	1608.93	569.41
A_68_P23611143	chr5:37420486-37420530	ENSMUST00000165557:12377		DOWNSTREAM	1.622	0.277	1634.49	452.43	0.449	1241.90	557.60
A_68_P26676070	chr9:81757205-81757249	NM_175213:-124	Mei4	PROMOTER	1.621	3.082	1470.15	4531.51	4.995	1183.85	5913.82
A_68_P25958124	chr8:74184428-74184472	NM_001029873:11206	Unc13a	INSIDE	1.621	0.164	3754.13	614.72	0.265	2588.49	687.20
A_68_P21017270	chr2:13496599-13496643	NM_011701:683	Vim	INSIDE	1.621	0.082	6678.46	550.72	0.134	4542.28	607.33
A_68_P31779045	chr18:65243748-65243792	NM_031881:60590	Nedd4l	INSIDE	1.620	1.872	371.61	695.75	3.033	328.71	996.84
A_68_P30396225	chr15:84511839-84511883	NM_146061:433	Prr5	INSIDE	1.620	0.312	2530.29	789.78	0.506	1745.30	882.49
A_68_P30484946	chr15:100111437-100111481	NM_183109:191	Tmprss12	INSIDE	1.619	1.739	1151.96	2003.20	2.815	938.91	2642.65
A_68_P25031176	chr7:31140849-31140896	NM_175478:6919	Lrfn3	INSIDE	1.619	1.958	404.54	791.98	3.170	318.58	1009.97
A_68_P21326874	chr2:72472122-72472166	ENSMUST00000120282:152		INSIDE	1.619	1.997	635.97	1270.33	3.233	447.42	1446.62
A_68_P21906146	chr2:180511719-180511763	NM_080641:-135	Bhlhe23	PROMOTER	1.618	0.381	1734.71	661.43	0.617	1407.63	868.39
A_68_P21394211	chr2:84555629-84555673	NM_144887:-329	Zdhhc5	PROMOTER	1.618	0.133	4957.00	658.84	0.215	3396.61	730.59
A_68_P32238976	chrX:7151221-7151265	NM_138605:4	Ppp1r3f	INSIDE	1.617	1.495	674.16	1007.90	2.417	789.03	1906.97
A_68_P29049666	chr13:54569805-54569849	NM_028597:375	Thoc3	INSIDE	1.617	2.235	11703.62	26157.32	3.614	6830.35	24686.83
A_68_P28724520	chr12:109154600-109154644	NM_001079883:87002	Bcl11b	INSIDE	1.617	2.480	1098.32	2724.25	4.012	947.29	3800.08
A_68_P31554070	chr18:23469238-23469282	X95227:535		INSIDE	1.616	0.380	1553.32	589.99	0.614	1166.18	715.95
A_68_P28058390	chr11:98629362-98629406	NM_145434:7172	Nr1d1	INSIDE	1.616	1.621	778.67	1262.07	2.619	614.34	1609.16
A_68_P21127262	chr2:34226792-34226836	NM_016768:742	Pbx3	INSIDE	1.616	0.303	1723.18	522.71	0.490	1308.56	641.41
A_68_P28137854	chr11:112646538-112646582	NM_011448:3037	Sox9	INSIDE	1.615	0.200	15378.05	3070.87	0.323	9983.62	3220.41
A_68_P27261658	chr10:75837803-75837847	NM_008787:67833	Pcnt	INSIDE	1.614	2.954	164.05	484.64	4.767	218.99	1044.01
A_68_P25958415	chr8:74225497-74225541	NM_028189:181	B3gnt3	INSIDE	1.614	0.242	2052.69	497.30	0.391	1379.50	539.29
A_68_P32364037	chrX:45695522-45695566	NM_178782:1010	Bcorl1	INSIDE	1.612	0.210	2061.59	433.79	0.339	2647.51	897.87
A_68_P32926577	chr13:93380980-93381024	NM_172588:-90	Serinc5	PROMOTER	1.611	0.158	7100.57	1119.19	0.254	5051.02	1282.79
A_68_P30284264	chr15:64753856-64753901	NM_009623:-20	Adcy8	PROMOTER	1.610	0.349	3013.77	1051.94	0.562	2141.26	1203.36
A_68_P25950576	chr8:72757876-72757920	NM_001168290:-226	Sugp2	PROMOTER	1.610	0.309	2012.33	621.99	0.498	1348.88	671.26
A_68_P23971467	chr5:108795517-108795561	ENSMUST00000100944:-3338		PROMOTER	1.610	0.264	2710.11	716.24	0.426	1827.65	777.70
A_68_P28577475	chr12:81859600-81859644	NM_001008423:2080	Gm1568	INSIDE	1.609	1.836	768.46	1410.83	2.953	652.26	1926.39
A_68_P29055231	chr13:55574123-55574167	NM_001030296:8517	Prr7	INSIDE	1.608	0.364	1763.37	641.56	0.585	1317.49	770.58
A_68_P28470051	chr12:60162088-60162132	NM_030057:338	Trappc6b	INSIDE	1.608	0.221	3539.41	780.78	0.355	2721.52	965.22
A_68_P23896968	chr5:93371161-93371205	NM_001077596:44159	Shroom3	INSIDE	1.608	1.917	1053.60	2019.89	3.082	826.42	2546.97
A_68_P30351141	chr15:76741990-76742034	NM_001168276:13866	Zfp647	INSIDE	1.607	2.385	350.58	836.06	3.831	309.61	1186.20
A_68_P30473862	chr15:98087209-98087253	NM_027304:508	H1fnt	INSIDE	1.606	1.620	954.26	1546.11	2.602	873.30	2272.54
A_68_P21103419	chr2:30325956-30326000	NM_030244:3741	Ier5l	DOWNSTREAM	1.605	0.343	2161.39	740.55	0.550	1642.82	903.44
A_68_P32488665	chrX:78316348-78316392	NM_138751:388	Tmem47	INSIDE	1.604	0.254	2353.73	597.05	0.407	3251.49	1322.72
A_68_P31162032	chr17:36116207-36116251	NR_028516:-260	Prr3	PROMOTER	1.603	4.207	1691.40	7116.57	6.744	1419.06	9570.31
A_68_P30351142	chr15:76742082-76742126	NM_001168276:13774	Zfp647	INSIDE	1.603	1.493	752.04	1122.60	2.393	600.17	1436.20
A_68_P21585603	chr2:122127484-122127528	NM_025777:2871	Duoxa2	INSIDE	1.603	0.421	2088.59	879.01	0.675	1524.50	1028.42
A_68_P30422455	chr15:88813114-88813158	NM_031260:-287	Mov10l1	DIVERGENT_PROMOTER	1.602	1.486	2470.29	3671.14	2.380	1879.55	4473.90
A_68_P27924771	chr11:75007879-75007923	NM_177708:406	Rtn4rl1	INSIDE	1.602	0.271	2789.24	755.49	0.434	2280.03	989.42
A_68_P21874752	chr2:174154225-174154269	NM_001077510:-1343	Gnas	PROMOTER	1.602	2.507	808.27	2026.45	4.017	672.22	2700.54
A_68_P25827954	chr8:46109471-46109515	NM_001081286:73931	Fat1	INSIDE	1.601	2.260	335.56	758.44	3.619	296.17	1071.77
A_68_P21629541	chr2:130223202-130223246	NM_019696:141	Cpxm1	INSIDE	1.601	0.280	2046.86	573.02	0.448	1504.46	674.27
A_68_P26156363	chr8:111864572-111864616	NR_002928:214	Gm1943	INSIDE	1.599	1.488	582.11	866.47	2.380	532.26	1266.95
A_68_P26042148	chr8:91151286-91151330	NM_027840:8719	Snx20	INSIDE	1.599	2.728	217.07	592.28	4.363	191.56	835.79
A_68_P23283131	chr4:126698502-126698546	NM_023603:-20	Sfpq	PROMOTER	1.599	7.855	272.36	2139.24	12.563	196.30	2466.18
A_68_P21843327	chr2:168578173-168578217	NM_175303:14507	Sall4	INSIDE	1.599	1.529	583.90	892.75	2.445	471.65	1153.34
A_68_P20293513	chr1:64663621-64663665	NM_025964:100	Mettl21a	INSIDE	1.599	4.913	1637.15	8044.00	7.858	1502.99	11811.15
A_68_P31335035	chr17:71901365-71901409	NM_001142631:-14	Spdya	PROMOTER	1.598	1.774	425.98	755.53	2.835	324.11	918.71
A_68_P31252967	chr17:56095790-56095834	NM_015766:3767	Ebi3	INSIDE	1.598	1.688	665.34	1122.94	2.697	484.33	1306.29
A_68_P30778319	chr16:55905763-55905807	NM_028815:29177	Cep97	INSIDE	1.598	1.563	990.59	1548.31	2.498	782.50	1954.51
A_68_P28159867	chr11:116395295-116395339	NM_011451:-737	Sphk1	PROMOTER	1.598	0.314	2168.91	680.12	0.501	1488.89	745.85
A_68_P26216539	chr8:122394248-122394292	NM_146219:8066	Klhl36	INSIDE	1.596	1.599	505.80	808.68	2.552	435.56	1111.54
A_68_P24631555	chr6:90259775-90259819	NM_027928:15383	Chst13	INSIDE	1.596	1.739	623.35	1084.11	2.775	545.54	1513.97
A_68_P22080609	chr3:40439835-40439879	NM_175515:168	Intu	INSIDE	1.596	1.973	428.44	845.19	3.149	386.19	1216.25
A_68_P31442547	chr17:91487821-91487865	NM_020252:4300	Nrxn1	INSIDE	1.594	2.062	468.22	965.36	3.286	452.02	1485.32
A_68_P27349651	chr10:92185236-92185280	NM_008682:-95	Nedd1	PROMOTER	1.594	3.868	1026.85	3972.04	6.166	844.64	5207.63
A_68_P26346083	chr9:21038393-21038437	NM_001110305:5362	Keap1	INSIDE	1.594	1.654	1336.36	2210.62	2.636	945.49	2492.56
A_68_P24120341	chr5:136751221-136751265	NM_198602:292033	Cux1	INSIDE	1.594	2.390	540.71	1292.55	3.811	381.48	1453.75
A_68_P30467109	chr15:96884897-96884941	NM_027052:1469	Slc38a4	INSIDE	1.592	1.712	895.77	1533.73	2.725	748.90	2040.91
A_68_P26258349	chr8:129107965-129108009	NM_001164598:9350	Irf2bp2	DOWNSTREAM	1.591	2.285	885.07	2022.28	3.636	712.81	2591.75
A_68_P32182254	chr19:53465262-53465306	NM_172429:-221	Smndc1	PROMOTER	1.590	0.206	4922.77	1013.14	0.327	3156.19	1032.49
A_68_P26236516	chr8:125394674-125394718	NM_007662:22423	Cdh15	DOWNSTREAM	1.590	2.976	1299.16	3866.90	4.732	1076.60	5094.76
A_68_P23896967	chr5:93371085-93371129	NM_001077596:44083	Shroom3	INSIDE	1.590	1.563	696.42	1088.74	2.485	602.34	1496.86
A_68_P24449293	chr6:52183630-52183674	NM_008263:1286	Hoxa10	INSIDE	1.588	0.293	2830.25	830.02	0.466	1972.81	918.58

ProbeName	Target position of probe on CpG island microarray	TargetID	GeneSymbol	CpG island Description	Ratio of relative methylation (TiO_2-NP/Vehicle)	Sham group			TiO_2-H group		
						Relative methylation (Cy5/Cy3)	Cy3 signal (Input)	Cy5 signal (MeDIP)	Relative methylation (Cy5/Cy3)	Cy3 signal (Input)	Cy5 signal (MeDIP)
A_68_P32071922	chr19:32831644-32831688	NM_008960:-400	Pten	PROMOTER	1.586	2.778	1292.51	3590.86	4.406	1141.86	5031.26
A_68_P27286342	chr10:80380790-80380834	NM_010722:178	Lmnb2	INSIDE	1.586	0.325	1581.34	513.81	0.515	1088.62	561.15
A_68_P23295049	chr4:128798702-128798746	NM_010471:-189	Hpca	PROMOTER	1.586	0.278	2390.01	665.10	0.441	1676.39	739.71
A_68_P30459963	chr15:95621546-95621590	NM_175344:295	Ano6	INSIDE	1.584	0.231	2539.28	587.51	0.367	1826.40	669.48
A_68_P30346606	chr15:76010797-76010841	NM_201394:15322	Plec	INSIDE	1.584	2.227	522.79	1164.31	3.529	471.45	1663.62
A_68_P29717288	chr14:73784719-73784763	NM_008410:338	Itm2b	INSIDE	1.584	3.100	6962.05	21584.94	4.910	4986.58	24483.19
A_68_P26540099	chr9:56924722-56924766	NM_001110350:562	Sin3a	INSIDE	1.584	0.287	1708.00	489.86	0.454	1323.87	601.34
A_68_P23325564	chr4:134480330-134480374	NM_023665:813	D4Wsu53e	INSIDE	1.584	0.244	3057.39	744.91	0.386	1836.84	708.77
A_68_P21565612	chr2:118528731-118528775	NM_001081971:947	Gm1337	INSIDE	1.584	0.199	4636.43	923.64	0.316	2941.44	928.25
A_68_P20146386	chr1:36160613-36160657	NM_015818:35390	Hs6st1	INSIDE	1.584	1.853	925.69	1715.49	2.936	730.38	2144.55
A_68_P32149066	chr19:47388501-47388545	NM_001164717:150379	Sh3pxd2a	INSIDE	1.583	0.333	2750.14	915.69	0.527	1718.01	905.35
A_68_P20319169	chr1:69423807-69423851			Unknown	1.583	2.431	609.74	1482.57	3.850	518.55	1996.22
A_68_P30560720	chr16:14159038-14159083	NM_001081154:307	4921513D23Rik	INSIDE	1.582	0.244	2275.29	554.47	0.386	1666.31	642.52
A_68_P24120337	chr5:136750787-136750831	NM_198602:292467	Cux1	INSIDE	1.580	2.240	334.43	749.01	3.538	319.82	1131.52
A_68_P20405966	chr1:84835679-84835723	NM_133975:179	Trip12	INSIDE	1.580	0.338	2282.17	772.45	0.535	1822.84	974.98
A_68_P28056597	chr11:98319612-98319657	NM_010346:11487	Grb7	DOWNSTREAM	1.578	0.138	3883.85	535.77	0.218	2521.03	548.76
A_68_P28607942	chr12:87423668-87423712	NM_001199843:180	1700019E19Rik	INSIDE	1.577	0.227	2092.79	474.48	0.358	1592.66	569.46
A_68_P29241524	chr13:96374376-96374423	NM_010169:13989	F2r	INSIDE	1.575	1.802	361.54	651.63	2.839	278.87	791.75
A_68_P27947945	chr11:79068066-79068110	NM_019653:109	Wsb1	INSIDE	1.574	0.314	1771.06	556.97	0.495	1263.90	625.78
A_68_P25276874	chr7:89484048-89484092	NM_001190374:-133	Adamtsl3	PROMOTER	1.574	18.961	862.19	16348.22	29.848	879.16	26241.57
A_68_P24159376	chr5:144861468-144861512	NM_001081109:186	Lmtk2	INSIDE	1.574	0.403	1643.20	661.93	0.634	1281.72	812.68
A_68_P23981088	chr5:111082625-111082669	NM_027156:177	Ddx51	INSIDE	1.574	0.330	2106.83	696.10	0.520	1562.17	812.51
A_68_P22402364	chr3:106350168-106350212	NM_133869:489	Cept1	INSIDE	1.572	0.222	2445.83	542.46	0.349	1738.70	606.10
A_68_P28989506	chr13:43399105-43399149	NM_001033399:415	Gfod1	INSIDE	1.571	2.029	435.09	882.67	3.186	346.76	1104.88
A_68_P28731504	chr12:110272212-110272256	NM_001163175:34193	Begain	INSIDE	1.571	2.918	478.65	1396.53	4.583	373.70	1712.51
A_68_P26949007	chr10:12810795-12810839	NM_009538:223	Plagl1	INSIDE	1.571	1.604	502.13	805.41	2.520	473.66	1193.50
A_68_P23441761	chr4:155367293-155367337	NM_011341:292	Sdf4	INSIDE	1.571	0.321	1513.03	485.85	0.504	1152.48	581.34
A_68_P23968414	chr5:108150872-108150916	NM_010278:2469	Gfi1	INSIDE	1.570	1.690	810.46	1369.39	2.653	672.04	1783.15
A_68_P21576978	chr2:120557105-120557149	NM_026891:127	Cdan1	INSIDE	1.570	0.285	3303.82	940.94	0.447	2692.91	1204.22
A_68_P29223483	chr13:93125415-93125459	NM_010829:-478	Msh3	PROMOTER	1.569	0.239	1971.36	470.53	0.375	1596.63	597.95
A_68_P30547090	chr16:11254627-11254671	NR_035466:-110	Mir1945	PROMOTER	1.567	0.316	2248.78	711.56	0.496	1709.84	847.57
A_68_P25173479	chr7:70902673-70902717	NM_130880:313037	Otud7a	INSIDE	1.567	0.327	1352.09	442.30	0.513	1046.27	536.41
A_68_P24863577	chr6:134742556-134742600	NM_001048054:68	Dusp16	INSIDE	1.567	0.223	2441.07	544.37	0.350	1732.81	605.64
A_68_P28741418	chr12:112215432-112215476	NM_175207:1777	Ankrd9	INSIDE	1.565	2.047	1357.37	2778.67	3.204	1039.98	3331.74
A_68_P24973913	chr7:13556719-13556763	NM_178732:5528	Zfp324	INSIDE	1.565	1.604	533.79	856.25	2.511	400.72	1006.01
A_68_P21393860	chr2:84499188-84499232	NM_001145100:-25	Gm13718	DIVERGENT_PROMOTER	1.565	1.819	519.35	944.47	2.846	426.74	1214.56
A_68_P32603686	chrX:109714974-109715018	NM_177747:862	Zfp711	INSIDE	1.564	1.966	220.76	433.99	3.074	313.10	962.39
A_68_P25099052	chr7:53682950-53683004	NM_001112739:31110	Kcnc1	INSIDE	1.564	1.650	407.92	672.90	2.581	344.82	889.84
A_68_P26258430	chr8:129118076-129118120	NM_001164598:-762	Irf2bp2	PROMOTER	1.563	0.396	2117.82	838.03	0.619	1574.73	974.21
A_68_P21585602	chr2:122127398-122127442	NM_025777:2785	Duoxa2	INSIDE	1.563	0.114	4204.83	479.12	0.178	2958.28	526.81
A_68_P31922425	chr19:4305540-4305585	NM_130863:393	Adrbk1	INSIDE	1.562	0.418	2712.76	1132.82	0.652	2083.86	1359.66
A_68_P26568894	chr9:61969694-61969738	mmu-mir-5133:685		DOWNSTREAM	1.562	0.211	4224.32	889.81	0.329	3162.54	1040.44
A_68_P23317853	chr4:133042110-133042154	NM_175307:6085	Fam46b	INSIDE	1.562	1.674	434.02	726.59	2.615	367.20	960.25
A_68_P30351140	chr15:76741911-76741955	NM_001168276:13946	Zfp647	INSIDE	1.561	2.963	993.98	2945.01	4.626	782.11	3618.19
A_68_P29047303	chr13:54167121-54167165	NM_027324:-71	Sfxn1	PROMOTER	1.561	2.052	1709.35	3506.97	3.202	1465.24	4691.95
A_68_P28159934	chr11:116403055-116403099	NM_173755:39685	Ube2o	INSIDE	1.561	3.072	1274.06	3913.69	4.794	978.30	4690.02
A_68_P23366443	chr4:141430949-141430993	NM_025994:-135	Efhd2	PROMOTER	1.559	2.287	630.10	1441.25	3.566	498.78	1778.50
A_68_P25677778	chr8:14962588-14962634	NM_001037736:50894	Arhgef10	INSIDE	1.558	1.769	895.76	1584.48	2.755	660.31	1819.28
A_68_P29648696	chr14:61791453-61791497	NM_172809:34181	Sacs	INSIDE	1.557	1.992	836.79	1666.59	3.101	725.41	2249.71
A_68_P27286999	chr10:80494095-80494139	NM_134135:2541	Slc39a3	INSIDE	1.555	2.055	930.54	1912.41	3.196	754.80	2412.12
A_68_P24118629	chr5:136410508-136410552	NM_018871:-19	Ywhag	PROMOTER	1.555	1.573	1243.73	1956.40	2.446	1088.59	2662.76
A_68_P25094658	chr7:52968623-52968667	NM_020011:1933	Sphk2	INSIDE	1.554	1.691	643.79	1088.66	2.627	518.03	1361.13
A_68_P25093408	chr7:52744206-52744250	NM_027903:-62	Dhdh	PROMOTER	1.554	0.316	2097.87	662.37	0.491	1541.75	756.48
A_68_P25092433	chr7:52582778-52582822	NM_175130:6350	Trpm4	INSIDE	1.554	2.236	1288.18	2880.47	3.474	996.52	3462.35
A_68_P21652098	chr2:134469794-134469838	NM_029148:41	Tmx4	INSIDE	1.554	0.379	1662.61	629.68	0.588	1179.99	694.37
A_68_P21149699	chr2:38527835-38527879	ENSMUST00000152645:-200		PROMOTER	1.554	1.507	924.70	1393.76	2.342	754.59	1767.01
A_68_P26065128	chr8:94880726-94880770	NR_033641:-729	4933436C20Rik	DIVERGENT_PROMOTER	1.553	0.351	2445.88	857.95	0.545	1753.69	955.28
A_68_P25614032	chr8:3451969-3452013	NM_133962:58983	Arhgef18	INSIDE	1.553	2.272	821.70	1866.53	3.528	687.55	2425.37
A_68_P22210462	chr3:67319224-67319268	NM_001164763:199	Rarres1	INSIDE	1.553	1.481	1178.17	1744.78	2.300	947.12	2178.81
A_68_P23279071	chr4:125932970-125933014	NM_001145970:571	Mtap7d1	INSIDE	1.552	0.154	3150.35	486.29	0.240	2341.62	560.84
A_68_P22349550	chr3:96279054-96279098	AK136676:11082		DOWNSTREAM	1.552	1.458	2619.71	3819.02	2.263	2034.29	4603.85
A_68_P28736910	chr12:111426306-111426350	AK044800:543		INSIDE	1.551	11.585	2520.93	29205.16	17.965	2360.68	42408.73
A_68_P26153173	chr8:111324170-111324214	NM_007496:85649	Zfhx3	INSIDE	1.551	2.009	632.58	1270.69	3.116	445.30	1387.73
A_68_P25048197	chr7:35988690-35988734	NM_001024707:11652	Lrp3	INSIDE	1.551	1.372	1452.98	1992.91	2.127	1076.89	2290.20
A_68_P22584284	chr3:141128445-141128489	NM_009472:-61	Unc5c	PROMOTER	1.551	0.255	2975.85	758.83	0.395	1961.62	775.69

ProbeName	Target position of probe on CpG island microarray	TargetID	GeneSymbol	CpG island Description	Ratio of relative methylation (TiO_2-NP/Vehicle)	Sham group			TiO_2-H group		
						Relative methylation (Cy5/Cy3)	Cy3 signal (Input)	Cy5 signal (MeDIP)	Relative methylation (Cy5/Cy3)	Cy3 signal (Input)	Cy5 signal (MeDIP)
A_68_P21325833	chr2:72314668-72314712	NM_025866:415	Cdca7	INSIDE	1.551	0.290	1575.87	457.67	0.451	1228.83	553.63
A_68_P24513205	chr6:65622381-65622425	NM_172399:798	A930038C07Rik	INSIDE	1.550	0.118	4061.98	481.18	0.184	2778.12	510.25
A_68_P21635426	chr2:131346286-131346330	NM_001177833:28711	Smox	INSIDE	1.550	1.698	659.66	1120.13	2.632	448.43	1180.16
A_68_P24428350	chr6:48570111-48570155	NM_173429:6954	Zfp775	INSIDE	1.549	1.999	1323.59	2646.24	3.098	983.15	3045.57
A_68_P20002774	chr1:3662119-3662163	NM_001011874:-561	Xkr4	PROMOTER	1.549	0.370	2511.88	930.49	0.574	1903.13	1091.73
A_68_P32741378	chrX:146801546-146801590	ENSMUST00000117777:891		DOWNSTREAM	1.548	2.247	478.43	1075.27	3.478	681.33	2369.81
A_68_P32364035	chrX:45695205-45695249	NM_178782:692	Bcorl1	INSIDE	1.548	3.670	5012.89	18396.86	5.679	6406.74	36386.35
A_68_P27270906	chr10:77365060-77365104	NM_145152:199	Lrrc3	INSIDE	1.548	3.432	3586.26	12307.29	5.313	2736.76	14541.29
A_68_P25355269	chr7:105509376-105509420	NM_001024619:400	Tsku	INSIDE	1.548	0.145	4000.65	581.14	0.225	2564.41	576.78
A_68_P21641569	chr2:132512720-132512764	NM_028637:-3276	1110034G24Rik	PROMOTER	1.548	0.350	1952.87	683.47	0.542	1693.38	917.50
A_68_P22336399	chr3:93248419-93248463	NM_001163098:2189	Tchh	INSIDE	1.547	49.363	1152.98	56914.80	76.343	1409.58	107611.20
A_68_P28096909	chr11:105451661-105451705	NM_181071:383	Tanc2	INSIDE	1.546	0.378	1891.76	715.96	0.585	1507.47	882.01
A_68_P25641011	chr8:9207733-9207777	NM_173446:563269	Fam155a	INSIDE	1.545	2.130	368.77	785.31	3.289	363.86	1196.84
A_68_P30568280	chr16:16146642-16146686	NM_146068:262	2310008H04Rik	INSIDE	1.544	2.496	1629.22	4067.01	3.854	1384.77	5337.16
A_68_P32462526	chrX:70667546-70667590	NM_009566:11127	Zfp92	INSIDE	1.543	1.420	939.56	1333.78	2.190	1156.79	2533.90
A_68_P30380080	chr15:81690283-81690327	NM_020507:-1548	Tob2	PROMOTER	1.543	0.302	2348.56	709.08	0.466	1709.12	796.35
A_68_P28435270	chr12:53604591-53604635	AK131622:-346		PROMOTER	1.543	0.344	4896.20	1682.09	0.530	3462.18	1835.74
A_68_P25613343	chr8:3279377-3279421	NM_010568:219	Insr	INSIDE	1.543	0.255	1912.01	487.08	0.393	1470.64	578.08
A_68_P23278936	chr4:125911491-125911535	NM_001145970:22051	Mtap7d1	INSIDE	1.543	1.770	605.01	1070.80	2.731	472.27	1289.92
A_68_P21843350	chr2:168581447-168581491	NM_175303:11233	Sall4	INSIDE	1.543	1.449	1210.08	1753.35	2.235	914.42	2043.79
A_68_P26880499	chr9:120842203-120842247	NM_007614:-293	Ctnnb1	PROMOTER	1.542	3.009	2567.49	7725.82	4.640	1727.24	8014.87
A_68_P25508016	chr7:135047821-135047865	NM_009739:256	Bckdk	INSIDE	1.542	0.364	1335.83	485.95	0.561	1061.10	595.23
A_68_P30512950	chr16:4964504-4964548	NM_028301:-196	Anks3	PROMOTER	1.541	0.136	3798.00	515.96	0.209	2488.68	521.14
A_68_P29644189	chr14:60997251-60997301	NM_001164705:154	Fam123a	INSIDE	1.541	1.684	536.98	904.21	2.595	444.10	1152.31
A_68_P27893905	chr11:69049315-69049359	NM_008192:1188	Gucy2e	INSIDE	1.541	0.419	2068.04	866.60	0.646	1463.12	944.98
A_68_P29613441	chr14:55260479-55260523	NM_199470:-299	Cdh24	PROMOTER	1.539	0.216	3125.93	676.17	0.333	2189.69	729.00
A_68_P29109187	chr13:67653829-67653873	NM_001035231:238	Zfp748	INSIDE	1.538	0.304	1694.03	514.77	0.467	1325.27	619.47
A_68_P26766898	chr9:99847864-99847908	ENSMUST00000104307:3759		DOWNSTREAM	1.538	1.878	457.53	859.36	2.888	421.24	1216.62
A_68_P26350330	chr9:21875518-21875562	NM_177318:249	Zfp653	INSIDE	1.538	0.384	1654.20	635.45	0.591	1180.45	697.20
A_68_P25505623	chr7:134587980-134588024	NM_175163:4670	Zfp689	INSIDE	1.538	5.898	1196.95	7060.06	9.071	1019.62	9248.95
A_68_P32126393	chr19:43502105-43502149			Unknown	1.537	2.290	651.63	1492.49	3.520	554.64	1952.26
A_68_P23364326	chr4:141034893-141034937	NM_019763:59598	Spen	INSIDE	1.537	1.574	1020.25	1605.88	2.419	800.35	1935.71
A_68_P32775151	chrX:155970887-155970931	NM_001033472:309	A830080D01Rik	INSIDE	1.536	0.258	1739.12	449.18	0.397	2453.34	973.31
A_68_P27356011	chr10:93308901-93308948	NM_183199:14625	Usp44	INSIDE	1.536	2.412	499.45	1204.85	3.704	426.66	1580.55
A_68_P21750311	chr2:152460303-152460347	NM_009047:7581	Rem1	INSIDE	1.536	1.814	1543.47	2799.99	2.787	1315.22	3665.33
A_68_P21840230	chr2:168056944-168056988	NM_001160330:845	Mocs3	INSIDE	1.535	1.895	487.01	922.94	2.910	414.82	1207.07
A_68_P21741660	chr2:150730004-150730048	NM_024465:441	Abhd12	INSIDE	1.535	0.142	3194.54	454.68	0.218	2540.11	554.83
A_68_P27765091	chr11:45758223-45758267	NM_028185:193	Lsm11	INSIDE	1.534	0.288	2141.80	616.33	0.441	1630.18	719.40
A_68_P24946537	chr7:3596654-3596698	NM_146176:-194	Cnot3	PROMOTER	1.534	0.208	3351.90	698.25	0.319	2377.64	759.59
A_68_P32226223	chr19:60656220-60656264	NM_001172096:233	2700078E11Rik	INSIDE	1.533	0.220	2860.62	630.53	0.338	2091.20	706.46
A_68_P29616218	chr14:55710458-55710502	NM_001039198:405	Zfhx2	INSIDE	1.533	0.389	2644.87	1028.06	0.596	2164.12	1289.75
A_68_P21774854	chr2:156865842-156865886	NM_001164663:39137	9830001H06Rik	INSIDE	1.533	1.558	521.91	812.91	2.388	432.79	1033.39
A_68_P31125955	chr17:29235539-29235583	NM_007669:4844	Cdkn1a	INSIDE	1.531	1.882	670.31	1261.45	2.880	550.86	1586.62
A_68_P29002205	chr13:45602874-45602918	NM_025508:59	Gmpr	INSIDE	1.531	0.356	1557.92	554.04	0.545	1301.95	708.93
A_68_P28961844	chr13:38727138-38727182	NM_139063:-182	Muted	PROMOTER	1.531	1.475	950.24	1401.85	2.259	748.62	1690.97
A_68_P25981542	chr8:79709972-79710016	NM_001083906:283588	Nr3c2	INSIDE	1.531	1.782	349.27	622.44	2.729	324.79	886.38
A_68_P27922355	chr11:74584384-74584428	NM_026197:42	Mettl16	INSIDE	1.530	0.178	2736.63	487.93	0.273	2018.83	550.68
A_68_P25394537	chr7:112959576-112959620	NM_025301:3	Mrpl17	INSIDE	1.529	2.362	2548.18	6019.48	3.613	2008.51	7256.28
A_68_P23609440	chr5:37139337-37139381	NM_133724:560	Cno	INSIDE	1.529	0.242	2046.23	495.77	0.371	1537.15	569.55
A_68_P27280641	chr10:79516441-79516485	NM_001037741:-319	Gpx4	PROMOTER	1.528	0.176	2834.81	497.87	0.268	1951.86	523.91
A_68_P24983897	chr7:17492695-17492739	NM_008967:878	Ptgir	INSIDE	1.528	1.588	600.61	953.87	2.426	465.57	1129.69
A_68_P24428347	chr6:48569751-48569795	NM_173429:6594	Zfp775	INSIDE	1.528	1.728	709.09	1225.03	2.640	595.89	1573.25
A_68_P30778928	chr16:56029876-56029920	NM_001024622:-68	Pcnp	PROMOTER	1.527	0.256	1773.65	454.80	0.392	1345.79	527.11
A_68_P26554373	chr9:59464987-59465031	NM_001205239:-82	Parp6	PROMOTER	1.526	3.340	845.14	2822.99	5.099	648.15	3304.86
A_68_P24061483	chr5:124928214-124928258	NM_030259:8	Rilpl2	INSIDE	1.526	0.363	1350.56	490.35	0.554	1065.78	590.43
A_68_P22015879	chr3:28162505-28162549	NM_001163009:391	Tnik	INSIDE	1.526	0.327	1429.22	467.12	0.499	1024.73	511.09
A_68_P20560374	chr1:122017670-122017714	NM_007830:-19	Dbi	DIVERGENT_PROMOTER	1.526	0.212	2689.91	570.67	0.324	2156.88	698.14
A_68_P26467525	chr9:44225880-44225924	NM_201372:188	Ccdc84	INSIDE	1.525	0.332	3304.29	1097.27	0.506	2174.23	1101.07
A_68_P32466000	chrX:71269156-71269200	NM_001177975:79	Irak1	INSIDE	1.524	1.825	346.10	631.57	2.781	389.37	1082.67
A_68_P24118365	chr5:136364296-136364340	NM_013560:530	Hspb1	INSIDE	1.523	0.187	2755.66	514.34	0.284	1786.65	507.87
A_68_P26531906	chr9:55394278-55394322	NM_027397:5345	Isl2	DOWNSTREAM	1.522	0.234	1850.22	433.38	0.356	1441.72	513.96
A_68_P22893528	chr4:47258456-47258500	NM_009928:37595	Col15a1	INSIDE	1.522	1.663	468.23	778.66	2.531	379.79	961.29
A_68_P30379759	chr15:81641797-81641841	NM_017376:-25	Tef	PROMOTER	1.521	1.570	413.49	649.22	2.388	313.65	748.92
A_68_P27880945	chr11:66908117-66908162	NM_001099635:16338	Myh3	INSIDE	1.521	2.111	436.07	920.67	3.212	351.00	1127.30
A_68_P21585133	chr2:122060313-122060357	NM_146126:-240	Sord	PROMOTER	1.521	0.373	1837.63	684.78	0.567	1449.10	821.49

ProbeName	Target position of probe on CpG island microarray	TargetID	GeneSymbol	CpG island Description	Ratio of relative methylation (TiO_2-NP/Vehicle)	Sham group			TiO_2-H group		
						Relative methylation (Cy5/Cy3)	Cy3 signal (Input)	Cy5 signal (MeDIP)	Relative methylation (Cy5/Cy3)	Cy3 signal (Input)	Cy5 signal (MeDIP)
A_68_P20319174	chr1:69424426-69424470			Unknown	1.521	1.608	573.14	921.67	2.445	462.84	1131.79
A_68_P24359960	chr6:35084098-35084143	NM_001164412:-383	Cnot4	PROMOTER	1.520	2.253	427.91	963.94	3.423	256.87	879.31
A_68_P21890549	chr2:178142357-178142402	NM_177191:-16	Sycp2	PROMOTER	1.520	2.826	231.63	654.59	4.296	230.14	988.75
A_68_P29241525	chr13:96374455-96374499	NM_010169:13912	F2r	INSIDE	1.519	2.412	310.99	750.01	3.664	265.74	973.81
A_68_P28731503	chr12:110272067-110272111	NM_001163175:34339	Begain	INSIDE	1.519	2.353	722.34	1699.64	3.574	578.02	2065.63
A_68_P25961388	chr8:74967563-74967607	NM_029782:-8529	Calr3	PROMOTER	1.519	0.381	1833.69	698.67	0.579	1318.55	762.96
A_68_P29194040	chr13:85429148-85429192	NM_145452:-79	Rasa1	PROMOTER	1.518	0.247	2692.68	663.92	0.374	1962.77	734.81
A_68_P26215962	chr8:122292157-122292201	NM_028883:10138	4632415K11Rik	INSIDE	1.518	1.472	696.02	1024.85	2.236	559.07	1249.89
A_68_P27475787	chr10:115911149-115911205	NM_021452:-597	Kcnmb4	PROMOTER	1.517	2.621	290.22	760.53	3.975	215.55	856.77
A_68_P24118492	chr5:136387366-136387410	NM_018871:23123	Ywhag	INSIDE	1.517	1.841	684.51	1259.91	2.792	570.54	1593.10
A_68_P28745984	chr12:113012732-113012779	NM_008450:15696	Klc1	INSIDE	1.516	1.643	686.42	1127.46	2.490	533.02	1326.97
A_68_P27290092	chr10:80958931-80958975	NM_134009:156	Ncln	INSIDE	1.516	0.354	4239.80	1501.56	0.537	2783.29	1494.70
A_68_P26886025	chr9:121766341-121766385	NM_001112668:287	Gm9790	INSIDE	1.516	0.157	3381.31	530.35	0.238	2281.90	542.74
A_68_P25614229	chr8:3496319-3496363	NM_080461:3203	Zfp358	INSIDE	1.516	1.676	1376.54	2306.53	2.540	1074.47	2729.19
A_68_P23916649	chr5:98615733-98615777	NM_029947:5867	Prdm8	INSIDE	1.516	0.147	3951.21	579.69	0.222	2634.54	585.82
A_68_P20296825	chr1:65225492-65225536	NM_010497:202	Idh1	INSIDE	1.515	0.410	1695.33	695.45	0.622	1416.10	880.13
A_68_P32563074	chrX:98449869-98449914	NM_018789:25	Foxo4	INSIDE	1.514	2.626	281.88	740.27	3.976	328.81	1307.51
A_68_P32145200	chr19:46728723-46728767	NM_001177813:30854	D19Wsu162e	INSIDE	1.514	1.640	976.15	1600.61	2.483	751.59	1866.24
A_68_P27798408	chr11:52096304-52096348	NM_009331:-574	Tcf7	PROMOTER	1.514	1.363	2891.10	3941.51	2.065	2257.87	4661.79
A_68_P20348983	chr1:74552547-74552591	NM_021383:-65	Rqcd1	PROMOTER	1.514	0.174	2740.05	477.34	0.264	1867.95	492.63
A_68_P29455881	chr14:22805841-22805885	NM_145459:2961	Zfp503	INSIDE	1.513	0.347	1498.64	519.66	0.525	1110.85	582.74
A_68_P26798472	chr9:105545510-105545554	NM_001081309:208	Pik3r4	INSIDE	1.512	0.310	1822.33	565.49	0.469	1292.67	606.40
A_68_P28050118	chr11:97224447-97224491	NM_138657:604	Socs7	INSIDE	1.511	0.313	1559.60	488.66	0.473	1136.17	537.84
A_68_P25827947	chr8:46108650-46108696	NM_001081286:73111	Fat1	INSIDE	1.510	1.742	344.66	600.52	2.630	309.34	813.66
A_68_P21432203	chr2:92886316-92886360	NM_001177536:-37	Prdm11	PROMOTER	1.510	1.879	971.84	1826.31	2.838	846.08	2401.58
A_68_P29591677	chr14:49685584-49685628	NM_144535:-563	Mudeng	PROMOTER	1.509	0.260	2330.59	605.85	0.392	1785.02	700.21
A_68_P24818072	chr6:124946958-124947002	NM_001145927:-1474	C530028O21Rik	PROMOTER	1.509	0.142	6089.60	866.83	0.215	4238.32	910.63
A_68_P32181719	chr19:53384898-53384942	NM_001008542:-75	Mxi1	PROMOTER	1.508	0.240	2140.35	514.43	0.362	1497.42	542.63
A_68_P31844515	chr18:77095020-77095064	NM_001109743:-101	Skor2	PROMOTER	1.508	0.323	1356.52	438.61	0.488	1062.22	518.02
A_68_P32029537	chr19:25484593-25484638	NM_181404:172924	Kank1	INSIDE	1.507	2.285	2394.53	5471.72	3.443	1589.63	5473.83
A_68_P28577470	chr12:81858836-81858880	NM_001008423:2844	Gm1568	INSIDE	1.507	2.963	556.94	1650.35	4.466	420.40	1877.31
A_68_P23590340	chr5:34000396-34000440	NM_026698:63	Tmem129	INSIDE	1.506	0.268	1998.42	535.52	0.404	1330.61	536.91
A_68_P27958481	chr11:80966909-80966953	NM_007384:-525	Accn1	PROMOTER	1.504	0.414	1496.46	619.82	0.623	1191.67	742.41
A_68_P26247733	chr8:127317798-127317842	NM_198632:902	Trim67	INSIDE	1.504	0.233	3066.14	714.57	0.351	2096.77	735.01
A_68_P31879113	chr18:83078968-83079012	NM_183033:-1280	Zfp516	PROMOTER	1.503	0.335	7196.37	2414.20	0.504	4540.73	2289.47
A_68_P25954889	chr8:73437195-73437239	NM_173013:7344	Mtap1s	INSIDE	1.503	2.443	1002.78	2450.27	3.673	734.12	2696.25
A_68_P23991446	chr5:112755370-112755414	NM_018783:4	Tfip11	INSIDE	1.503	0.238	1951.80	463.90	0.357	1333.45	476.26
A_68_P21338760	chr2:74520854-74520898	NM_008273:427	Hoxd11	INSIDE	1.503	1.606	794.93	1277.00	2.414	550.14	1328.16
A_68_P20789366	chr1:166388627-166388671	NM_009721:-162	Atp1b1	PROMOTER	1.503	0.401	2682.17	1076.58	0.603	2176.29	1312.79
A_68_P30094306	chr15:27677312-27677364	NM_001081302:278265	Trio	INSIDE	1.502	2.243	997.20	2236.39	3.369	801.14	2698.99
A_68_P29428097	chr14:17075904-17075948	NM_021504:-5901	Ngly1	PROMOTER	1.502	0.405	1376.50	557.39	0.608	1114.37	677.95
A_68_P23768851	chr5:67999755-67999799	NM_001033415:655	Shisa3	INSIDE	1.502	0.275	1881.37	517.27	0.413	1478.53	610.77
A_68_P22373052	chr3:100881123-100881167	NM_011197:32945	Ptgfrn	INSIDE	1.502	1.645	671.72	1105.19	2.472	527.59	1304.09
A_68_P21485631	chr2:103637120-103637164	NM_001111289:93	Caprin1	INSIDE	1.502	0.376	1745.83	655.96	0.565	1212.22	684.33
A_68_P21115505	chr2:32306645-32306689	NM_001164357:324	Slc25a25	INSIDE	1.502	1.850	878.89	1626.27	2.779	652.53	1813.39
A_68_P26578207	chr9:63606312-63606356	NM_016769:-533	Smad3	PROMOTER	1.501	0.280	2870.02	804.38	0.421	2141.43	900.73
A_68_P25954564	chr8:73386897-73386941	NM_032397:-6011	Kcnn1	PROMOTER	1.500	6.261	840.75	5263.93	9.393	619.46	5818.55
A_68_P29560589	chr14:41779724-41779768	NM_145928:350	Tspan14	INSIDE	1.498	0.311	2156.30	671.09	0.466	1756.14	818.69
A_68_P31762245	chr18:62340024-62340068	NM_007420:-433	Adrb2	PROMOTER	1.497	0.286	5597.12	1599.09	0.428	3678.35	1573.60
A_68_P28157577	chr11:115999946-115999990	NM_024177:214	Mrpl38	INSIDE	1.496	0.146	4213.24	615.21	0.218	2899.57	633.40
A_68_P25961590	chr8:74998787-74998831	NM_138585:324	Cherp	INSIDE	1.496	0.362	1851.66	670.40	0.542	1371.16	742.64
A_68_P30423838	chr15:89026454-89026498	NM_027081:429	Fam116b	INSIDE	1.495	0.287	2312.65	662.95	0.429	1612.81	691.41
A_68_P28744028	chr12:112667836-112667880	NM_028807:12218	1200009I06Rik	INSIDE	1.495	0.218	2027.14	442.13	0.326	1484.02	483.74
A_68_P28323159	chr12:30687550-30687594	NM_181395:64672	Pxdn	INSIDE	1.495	1.522	1278.87	1946.33	2.275	930.64	2116.85
A_68_P28053987	chr11:97883567-97883611	NM_001159320:-3902	Cacnb1	PROMOTER	1.495	0.412	1795.08	739.36	0.616	1382.62	851.43
A_68_P27290579	chr10:81033785-81033829	NM_019725:-4470	Tle2	PROMOTER	1.495	0.223	3792.39	844.11	0.333	2763.30	919.66
A_68_P24798787	chr6:120444078-120444122	NM_033567:-275	Cecr6	PROMOTER	1.495	1.560	620.17	967.69	2.332	424.24	989.39
A_68_P21776096	chr2:157104650-157104694	NM_019642:-161	Rpn2	PROMOTER	1.495	0.383	1150.39	440.03	0.572	940.28	537.65
A_68_P31932217	chr19:6015773-6015817	NM_007600:-547	Capn1	PROMOTER	1.494	0.222	2524.93	560.52	0.332	1773.39	588.10
A_68_P26714899	chr9:89804436-89804480	NM_001039655:-154	Rasgrf1	PROMOTER	1.494	0.407	1766.08	718.53	0.608	1267.68	770.30
A_68_P20632245	chr1:136131391-136131435	NM_001008533:592	Adora1	INSIDE	1.494	0.228	3375.96	770.44	0.341	2233.62	761.32
A_68_P31928782	chr19:5457485-5457529	NM_198616:43	Ccdc85b	INSIDE	1.493	1.660	560.01	929.53	2.479	445.61	1104.47
A_68_P30511520	chr16:4679389-4679433	NM_030205:310	Coro7	INSIDE	1.493	2.515	179.13	450.45	3.753	130.50	489.80
A_68_P28737553	chr12:111517859-111517903	NM_172119:441	Dio3	INSIDE	1.493	0.394	1607.95	633.51	0.588	1262.64	742.74
A_68_P24050685	chr5:122952811-122952855	NM_009722:-598	Atp2a2	PROMOTER	1.493	0.267	2360.21	629.74	0.398	1663.02	662.67

ProbeName	Target position of probe on CpG island microarray	TargetID	GeneSymbol	CpG island Description	Ratio of relative methylation (TiO_2-NP/Vehicle)	Sham group			TiO_2-H group		
						Relative methylation (Cy5/Cy3)	Cy3 signal (Input)	Cy5 signal (MeDIP)	Relative methylation (Cy5/Cy3)	Cy3 signal (Input)	Cy5 signal (MeDIP)
A_68_P23955548	chr5:105910054-105910098	NM_001033550:65283	Lrrc8b	INSIDE	1.493	1.755	928.19	1629.41	2.622	664.51	1742.14
A_68_P20038599	chr1:12982807-12982851	NM_172841:-1612	Slco5a1	PROMOTER	1.493	2.100	6705.62	14083.00	3.136	4703.65	14752.82
A_68_P28752829	chr12:114067223-114067267	NM_028023:356	Cdca4	INSIDE	1.492	0.204	3445.97	701.86	0.304	2245.65	682.24
A_68_P24768720	chr6:114987629-114987673	NM_026894:242	1500001M20Rik	INSIDE	1.492	0.310	1626.85	503.87	0.462	1311.80	606.24
A_68_P23188358	chr4:106739774-106739823	NM_025500:-327	Mrpl37	DIVERGENT_PROMOTER	1.492	0.222	2543.79	564.54	0.331	1881.77	623.06
A_68_P21867219	chr2:172847604-172847648	NM_019547:224	Rbm38	INSIDE	1.492	0.299	1981.76	592.34	0.446	1198.76	534.71
A_68_P25499768	chr7:133436020-133436064	NM_145587:19910	Sbk1	INSIDE	1.491	2.371	750.14	1778.46	3.535	682.32	2411.94
A_68_P23295722	chr4:128916103-128916147	NM_001033189:9563	C77080	INSIDE	1.491	0.242	2777.08	670.86	0.360	1912.04	688.74
A_68_P32053974	chr19:29700925-29700969	NM_001081213:21964	Ermp1	INSIDE	1.490	1.437	14870.57	21368.74	2.141	10056.66	21529.80
A_68_P31412092	chr17:86084426-86084470	NM_011380:3146	Six2	INSIDE	1.490	0.304	2298.26	697.67	0.452	1579.89	714.42
A_68_P23344532	chr4:137773013-137773058	NM_001122897:-10	Hp1bp3	PROMOTER	1.490	0.332	1469.03	487.61	0.494	1216.64	601.62
A_68_P20058119	chr1:17081907-17081951	NM_020604:6042	Jph1	INSIDE	1.489	2.146	530.95	1139.20	3.196	474.77	1517.24
A_68_P31206536	chr17:46520399-46520443	NM_207671:-294	Zfp318	PROMOTER	1.488	0.309	1845.88	569.79	0.459	1433.35	658.47
A_68_P24793359	chr6:119431189-119431233	NM_133940:1525	Fbxl14	INSIDE	1.487	0.145	3293.49	477.92	0.216	2414.79	521.20
A_68_P22510090	chr3:127398655-127398699	NM_027808:84769	Alpk1	INSIDE	1.487	0.263	1713.52	449.90	0.390	1307.97	510.71
A_68_P32456123	chrX:69207672-69207716	NM_001081135:-665	Prrg3	PROMOTER	1.486	1.715	521.27	893.92	2.549	611.88	1559.40
A_68_P29269472	chr13:101421231-101421275	NM_027139:-45	Taf9	DIVERGENT_PROMOTER	1.486	0.201	2977.38	598.20	0.298	2320.27	692.55
A_68_P21840386	chr2:168088202-168088246	NM_001081134:6607	Kcng1	INSIDE	1.486	1.802	375.56	676.85	2.679	316.31	847.29
A_68_P21005390	chr2:11212009-11212053	NM_008859:118022	Prkcq	INSIDE	1.486	1.996	465.40	928.98	2.967	427.72	1268.93
A_68_P20593980	chr1:129102101-129102145	NM_145128:560	Mgat5	INSIDE	1.486	0.384	2405.19	924.39	0.571	1914.13	1092.94
A_68_P23424870	chr4:152806765-152806809	NM_001099299:50153	Ajap1	INSIDE	1.485	1.667	1071.95	1787.43	2.475	801.26	1983.50
A_68_P20349425	chr1:74634360-74634404	NM_029888:220	Zfp142	INSIDE	1.485	0.211	2847.42	602.04	0.314	2206.03	692.71
A_68_P31119041	chr17:28024335-28024379	NM_001080769:30905	Uhrf1bp1	INSIDE	1.484	1.932	1024.68	1979.50	2.866	802.44	2300.13
A_68_P20561481	chr1:122238357-122238401	NM_207233:1220	C1ql2	INSIDE	1.484	0.122	5126.44	623.20	0.180	3362.12	606.34
A_68_P29448170	chr14:21480684-21480728	NM_175132:6822	Synpo2l	INSIDE	1.483	1.696	430.91	730.67	2.514	349.81	879.38
A_68_P27924588	chr11:74982421-74982465	NM_001098203:-788	Hic1	PROMOTER	1.483	0.223	2789.76	622.65	0.331	1786.26	591.23
A_68_P26527191	chr9:54546796-54546840	NM_021422:-547	Dnaja4	PROMOTER	1.483	0.457	3364.50	1538.70	0.678	2288.87	1551.86
A_68_P24117822	chr5:136253735-136253779	NM_029659:499	Styxl1	INSIDE	1.483	0.327	1575.25	515.05	0.485	1214.89	589.07
A_68_P30480361	chr15:99300972-99301016	NM_029236:4167	Bcdin3d	INSIDE	1.482	1.698	755.14	1282.56	2.517	565.91	1424.31
A_68_P30349378	chr15:76437639-76437683	NM_001164173:361	Cpsf1	INSIDE	1.482	0.293	1802.58	528.55	0.434	1328.22	577.00
A_68_P26750484	chr9:96919484-96919528	NM_145134:-732	Spsb4	PROMOTER	1.482	1.581	1387.59	2193.63	2.343	1037.77	2431.98
A_68_P25954892	chr8:73437474-73437518	NM_173013:7624	Mtap1s	INSIDE	1.482	2.138	659.65	1410.59	3.169	504.98	1600.14
A_68_P20186159	chr1:43009715-43009759	NM_053107:20	Gpr45	INSIDE	1.482	0.442	3575.55	1582.14	0.656	2387.26	1565.97
A_68_P28600846	chr12:86216896-86216940	NR_028577:167	D030025P21Rik	INSIDE	1.481	1.761	352.76	621.04	2.607	249.52	650.40
A_68_P24798990	chr6:120481585-120481629	NM_144815:-289	Cecr5	PROMOTER	1.481	0.223	2334.95	521.75	0.331	1678.15	555.28
A_68_P21753656	chr2:153051085-153051129	NM_198617:71	Tspyl3	INSIDE	1.481	0.312	1707.80	532.78	0.462	1310.27	605.32
A_68_P29102523	chr13:65361108-65361152	ENSMUST00000099425:284		INSIDE	1.480	1.831	537.69	984.76	2.711	457.80	1241.13
A_68_P28525536	chr12:72418903-72418947	NM_001190466:8054	Dact1	INSIDE	1.480	1.625	692.80	1125.76	2.405	605.88	1457.35
A_68_P24287932	chr6:21801985-21802029	NM_173007:509	Tspan12	INSIDE	1.479	0.177	2617.51	462.38	0.261	1969.84	514.64
A_68_P29484384	chr14:27783854-27783898	NM_145221:-139	Appl1	PROMOTER	1.478	0.416	2215.21	921.88	0.615	1650.48	1015.43
A_68_P24138647	chr5:140447479-140447523	NM_175522:63604	Elfn1	INSIDE	1.478	2.014	975.97	1965.46	2.977	871.55	2594.99
A_68_P32103772	chr19:38893658-38893702	NM_021315:47	Noc3l	INSIDE	1.477	0.129	3613.42	466.31	0.191	2516.37	479.59
A_68_P26429693	chr9:37062879-37062923	NM_001145960:423	Slc37a2	INSIDE	1.477	0.310	2929.52	908.09	0.458	1999.90	915.53
A_68_P32132942	chr19:44629636-44629680	NM_026061:247	Ndufb8	INSIDE	1.476	0.254	2687.58	682.13	0.375	1827.03	684.54
A_68_P31058944	chr17:14416346-14416390	NM_022315:-144	Smoc2	PROMOTER	1.476	0.355	1502.36	533.10	0.524	1017.77	533.14
A_68_P28694110	chr12:104116592-104116636	NM_172806:2	Btbd7	INSIDE	1.476	2.194	428.52	940.25	3.238	409.84	1326.94
A_68_P25950467	chr8:72736364-72736408	NM_001007570:-206	Slc25a42	PROMOTER	1.476	0.280	2400.13	670.88	0.413	1749.72	721.99
A_68_P27826628	chr11:57458677-57458721	NM_134189:-245	Galnt10	PROMOTER	1.475	0.368	2311.20	850.87	0.543	1679.48	912.06
A_68_P23837519	chr5:81449181-81449225	NM_198702:-1415	Lphn3	PROMOTER	1.475	1.885	891.41	1679.91	2.780	740.39	2058.40
A_68_P21840388	chr2:168088389-168088433	NM_001081134:6421	Kcng1	INSIDE	1.475	1.551	1101.21	1708.13	2.288	811.30	1856.15
A_68_P30425291	chr15:89288179-89288223	NM_021921:3859	Mapk8ip2	INSIDE	1.474	1.697	1294.85	2197.20	2.501	1029.80	2575.61
A_68_P27160892	chr10:57251955-57251999	NM_019760:359	Serinc1	INSIDE	1.474	0.401	1962.12	787.60	0.592	1514.33	895.89
A_68_P26997093	chr10:21870689-21870733	NM_009016:-7704	Raet1a	PROMOTER	1.474	0.304	1554.03	473.06	0.449	1067.37	478.88
A_68_P25010075	chr7:26164748-26164792	NM_001039507:10355	Lipe	INSIDE	1.474	0.269	2153.44	578.72	0.396	1674.69	663.53
A_68_P22311356	chr3:87827542-87827586	NM_022031:3869	Hapln2	INSIDE	1.474	0.319	2906.19	925.63	0.470	2056.44	965.53
A_68_P31256955	chr17:56705842-56705886	NM_001029979:18142	Safb2	INSIDE	1.473	1.365	2049.07	2796.10	2.010	1660.34	3337.70
A_68_P23185612	chr4:106311841-106311885	NM_177667:16809	Ttc22	INSIDE	1.473	2.053	1374.58	2822.08	3.025	1133.36	3427.99
A_68_P29613438	chr14:55260166-55260210	NM_199470:13	Cdh24	INSIDE	1.472	1.490	944.28	1407.35	2.194	693.17	1520.53
A_68_P20613815	chr1:133034491-133034535	NM_145508:299	Dyrk3	INSIDE	1.472	0.319	2079.97	662.91	0.469	1667.59	782.47
A_68_P20510206	chr1:108656242-108656286	NM_027534:55	Kdsr	INSIDE	1.472	0.324	3033.64	982.43	0.477	2091.65	997.31
A_68_P32126392	chr19:43502033-43502077			Unknown	1.471	2.225	881.98	1962.28	3.274	661.94	2167.11
A_68_P31373925	chr17:79234724-79234768	NM_001081179:-25	Heatr5b	DIVERGENT_PROMOTER	1.471	0.271	1815.91	491.99	0.399	1360.14	542.04
A_68_P23807228	chr5:75472072-75472116	NM_133256:469	Gsx2	INSIDE	1.470	0.389	2239.96	872.28	0.572	1597.95	914.67
A_68_P21766452	chr2:155418422-155418466	NM_008180:-2	Gss	PROMOTER	1.470	0.289	1828.49	527.90	0.425	1375.27	583.84
A_68_P32029536	chr19:25484468-25484512	NM_181404:172799	Kank1	INSIDE	1.468	1.942	1131.13	2196.25	2.850	901.39	2568.88

ProbeName	Target position of probe on CpG island microarray	TargetID	GeneSymbol	CpG island Description	Ratio of relative methylation (TiO_2-NP/Vehicle)	Sham group			TiO_2-H group		
						Relative methylation (Cy5/Cy3)	Cy3 signal (Input)	Cy5 signal (MeDIP)	Relative methylation (Cy5/Cy3)	Cy3 signal (Input)	Cy5 signal (MeDIP)
A_68_P24034078	chr5:120130938-120130982	NM_011535:10283	Tbx3	INSIDE	1.468	0.317	2251.38	714.73	0.466	1499.98	699.25
A_68_P26891189	chr9:122656317-122656361	NM_001199736:-125	Gm9524	PROMOTER	1.467	1.891	267.91	506.63	2.774	252.70	700.86
A_68_P26891187	chr9:122656143-122656187	NM_001199736:-299	Gm9524	PROMOTER	1.467	1.793	950.60	1704.26	2.630	742.34	1952.09
A_68_P25957393	chr8:74067094-74067138	NM_028617:288	Fam125a	INSIDE	1.467	0.290	2292.09	665.51	0.426	1776.41	756.89
A_68_P24863967	chr6:134819942-134819986	NM_001167697:17913	Gpr19	INSIDE	1.467	1.994	598.92	1194.23	2.926	467.39	1367.39
A_68_P33012181	chr9_random:57403-57447	NR_027950:2732	4930526I15Rik	DOWNSTREAM	1.466	0.369	1779.20	656.88	0.541	1298.22	702.63
A_68_P30559115	chr16:13819208-13819252	NM_010946:-139	Ntan1	PROMOTER	1.466	0.278	1848.89	513.98	0.407	1317.47	536.84
A_68_P21081131	chr2:26771732-26771776	NM_013678:-186	Surf2	PROMOTER	1.466	0.388	1285.05	498.01	0.568	1038.40	589.78
A_68_P27568396	chr11:6315839-6315883	NM_008907:-12	Ppia	PROMOTER	1.464	0.244	2060.69	502.42	0.357	1452.93	518.76
A_68_P26667556	chr9:79915402-79915446	NM_146003:715	Senp6	INSIDE	1.464	0.392	1468.25	575.70	0.574	1044.63	599.56
A_68_P24838282	chr6:128250695-128250739	NM_001080979:115	Tead4	INSIDE	1.464	0.076	7095.42	538.99	0.111	5395.04	599.85
A_68_P20224252	chr1:51972666-51972710	NM_001161817:130	Myo1b	INSIDE	1.464	0.115	4570.39	524.83	0.168	3119.15	524.53
A_68_P32233092	chrX:5660427-5660471	NM_001033211:90	AU022751	INSIDE	1.463	1.728	399.75	690.65	2.528	531.11	1342.85
A_68_P23295627	chr4:128901057-128901101	NM_001033189:24609	C77080	INSIDE	1.462	1.615	967.28	1562.22	2.362	714.02	1686.30
A_68_P31084705	chr17:21146214-21146258	NM_145483:332	Zfp160	INSIDE	1.460	0.340	3278.27	1115.15	0.497	2376.09	1180.36
A_68_P21820025	chr2:164659401-164659445	NM_001082974:-326	Neurl2	PROMOTER	1.460	0.262	2567.96	671.78	0.382	1999.39	763.39
A_68_P20405971	chr1:84836410-84836454	NM_025386:17	Fbxo36	INSIDE	1.460	1.784	1517.11	2705.86	2.604	1125.36	2930.68
A_68_P20149939	chr1:36766403-36766447	NM_146107:346	Actr1b	INSIDE	1.460	0.174	3005.28	521.50	0.253	2295.79	581.80
A_68_P30500861	chr15:102773323-102773367	NM_010463:6061	Hoxc12	DOWNSTREAM	1.459	0.350	1879.01	658.47	0.511	1333.67	681.77
A_68_P28037998	chr11:95171463-95171507	NM_001195003:76	Myst2	INSIDE	1.459	0.335	2082.59	698.04	0.489	1691.45	826.93
A_68_P31668416	chr18:44821733-44821777	NM_001085374:166	Mcc	INSIDE	1.458	3.135	1511.76	4739.71	4.571	1495.40	6834.87
A_68_P31634885	chr18:38761130-38761174	NM_011898:-230	Spry4	PROMOTER	1.458	2.425	310.67	753.24	3.535	261.06	922.83
A_68_P30895372	chr16:78302001-78302045	NM_001025192:87	Cxadr	INSIDE	1.458	0.272	1726.82	470.51	0.397	1177.72	468.02
A_68_P30255522	chr15:59481111-59481155	NM_144549:924	Trib1	INSIDE	1.458	0.262	3244.64	851.06	0.382	2232.25	853.81
A_68_P27704166	chr11:34935291-34935335	NM_011412:355	Slit3	INSIDE	1.458	0.287	2142.64	613.88	0.418	1614.54	674.64
A_68_P25730825	chr8:27230377-27230421	NM_029965:547	Rnf170	INSIDE	1.458	1.895	299.19	566.91	2.762	296.58	819.22
A_68_P24269923	chr6:17790613-17790657	NM_001083315:91419	St7	INSIDE	1.458	1.970	635.04	1251.07	2.873	548.18	1574.71
A_68_P31425149	chr17:88196609-88196653	NM_199251:704	Kcnk12	INSIDE	1.457	1.778	445.17	791.55	2.591	353.72	916.39
A_68_P30575742	chr16:17798080-17798124	NM_153790:728	Scarf2	INSIDE	1.457	0.348	1807.80	629.33	0.507	1378.11	699.04
A_68_P27897910	chr11:69727956-69728000	NM_019726:285	Gps2	INSIDE	1.457	0.189	2677.58	506.30	0.275	2078.72	572.52
A_68_P25637619	chr8:8660957-8661001	NM_010111:-205	Efnb2	PROMOTER	1.457	0.337	1939.01	654.25	0.492	1459.46	717.66
A_68_P25090607	chr7:52263642-52263686	NM_001008422:7955	Scaf1	INSIDE	1.457	1.447	770.73	1115.21	2.108	646.02	1361.52
A_68_P24863968	chr6:134820038-134820082	NM_001167697:17817	Gpr19	INSIDE	1.457	1.436	998.29	1433.34	2.092	807.29	1688.85
A_68_P24760939	chr6:113646833-113646877	NM_001033463:-638	Tatdn2	PROMOTER	1.457	0.214	2098.05	448.47	0.311	1556.25	484.64
A_68_P22483671	chr3:122323019-122323074	NM_001114665:-461	Fnbp1l	PROMOTER	1.457	2.883	722.61	2083.63	4.202	656.22	2757.69
A_68_P21783958	chr2:158440228-158440272	NM_009508:3757	Slc32a1	INSIDE	1.457	0.383	1564.31	599.23	0.558	1190.87	664.87
A_68_P29098140	chr13:64375537-64375581	NM_172587:738	Cdc14b	INSIDE	1.456	0.413	1301.25	537.40	0.601	1157.75	695.93
A_68_P27356012	chr10:93309036-93309080	NM_183199:14759	Usp44	INSIDE	1.456	1.609	1114.25	1792.38	2.343	963.41	2257.08
A_68_P30244924	chr15:57743688-57743732	NM_001167679:-43	Wdr67	PROMOTER	1.455	0.415	1676.36	695.60	0.604	1266.67	764.71
A_68_P24771973	chr6:115552217-115552261	NM_023290:283	Mkrn2	INSIDE	1.455	0.244	4188.74	1023.94	0.356	3027.77	1076.57
A_68_P29055509	chr13:55614601-55614645	NM_001114088:178	Pdlim7	INSIDE	1.454	0.138	3572.18	492.36	0.200	2517.06	504.44
A_68_P27913322	chr11:72609852-72609896	NM_001045536:147	Zzef1	INSIDE	1.454	0.242	2172.23	524.63	0.351	1651.45	580.12
A_68_P26867068	chr9:118468753-118468797	NM_018748:53339	Golga4	INSIDE	1.454	2.311	1290.27	2981.63	3.359	1100.50	3696.71
A_68_P24025035	chr5:118619751-118619795	NM_007545:0	Hrk	INSIDE	1.454	1.427	2488.24	3549.70	2.074	1846.17	3828.29
A_68_P21102108	chr2:30111813-30111859	NM_177725:18548	Lrrc8a	INSIDE	1.454	1.701	354.95	603.85	2.474	274.85	679.86
A_68_P32054107	chr19:29722618-29722662	NM_001081213:270	Ermp1	INSIDE	1.453	0.411	1390.11	571.51	0.597	1153.53	689.23
A_68_P30510672	chr16:4541712-4541756	NM_031182:17986	Tcfap4	DOWNSTREAM	1.453	0.294	2107.96	620.66	0.428	1487.34	636.36
A_68_P28752184	chr12:113977018-113977062	NM_001024602:16656	AW555464	INSIDE	1.453	1.591	1371.26	2181.14	2.312	1034.77	2392.12
A_68_P28727032	chr12:109512870-109512914	NM_001159910:735	Ccdc85c	INSIDE	1.453	2.704	1089.52	2946.28	3.928	783.45	3077.46
A_68_P26037062	chr8:90305072-90305116	NM_033327:178400	Zfp423	INSIDE	1.453	1.866	763.14	1423.87	2.712	583.10	1581.15
A_68_P24540589	chr6:72184688-72184732	NM_153778:861	Atoh8	INSIDE	1.453	0.181	3571.94	647.15	0.263	2632.80	692.91
A_68_P24052414	chr5:123270992-123271036	NM_001042491:334	Anapc5	INSIDE	1.453	27.981	2422.47	67783.89	40.662	2751.94	111899.60
A_68_P24039105	chr5:120953280-120953324	NM_023625:330	Plbd2	INSIDE	1.453	1.750	351.45	615.07	2.543	260.57	662.60
A_68_P21054134	chr2:21127399-21127443	NM_001001297:70	Thnsl1	INSIDE	1.453	0.314	2218.94	696.83	0.456	1706.96	778.72
A_68_P30397298	chr15:84685375-84685419	NM_001081166:1163	Phf21b	INSIDE	1.452	0.329	1796.30	590.50	0.477	1342.54	640.98
A_68_P29309818	chr13:108948386-108948430	NM_001127346:411	Ndufaf2	INSIDE	1.452	0.322	2264.22	728.08	0.467	1490.35	695.71
A_68_P27564655	chr11:5616910-5616954	NM_178623:24247	Urgcp	INSIDE	1.452	1.888	482.96	912.02	2.742	471.85	1293.64
A_68_P22941606	chr4:56815104-56815148	NM_001081420:-74	BC026590	PROMOTER	1.452	0.217	2039.97	443.65	0.316	1317.03	415.85
A_68_P22629195	chr3:148480715-148480769	NM_001081298:136857	Lphn2	INSIDE	1.452	1.522	532.09	809.97	2.211	364.52	805.84
A_68_P30232954	chr15:55388910-55388954	NM_001168250:-30	Mtbp	DIVERGENT_PROMOTER	1.451	0.204	3223.79	656.96	0.296	2013.26	595.38
A_68_P24631554	chr6:90259619-90259663	NM_027928:15539	Chst13	INSIDE	1.450	1.624	697.70	1133.18	2.356	576.80	1358.75
A_68_P31093842	chr17:23956720-23956764	NM_175229:16589	Srrm2	INSIDE	1.449	2.102	794.32	1669.99	3.045	632.05	1924.81
A_68_P25833744	chr8:47085514-47085558	NM_001039562:-332	Ankrd37	PROMOTER	1.449	0.125	5969.60	744.53	0.181	3564.14	644.01
A_68_P25614335	chr8:3515288-3515332	NM_001122818:-73	Pnpla6	PROMOTER	1.449	0.224	2507.71	562.64	0.325	2030.39	660.31
A_68_P26373638	chr9:26837452-26837496	NM_178027:205	Vps26b	INSIDE	1.448	0.278	2454.97	681.59	0.402	1687.85	678.67

ProbeName	Target position of probe on CpG island microarray	TargetID	GeneSymbol	CpG island Description	Ratio of relative methylation (TiO_2-NP/Vehicle)	Sham group			TiO_2-H group		
						Relative methylation (Cy5/Cy3)	Cy3 signal (Input)	Cy5 signal (MeDIP)	Relative methylation (Cy5/Cy3)	Cy3 signal (Input)	Cy5 signal (MeDIP)
A_68_P21910753	chr2:181194478-181194522	NM_028125:-369	Zbtb46	PROMOTER	1.448	2.531	332.72	842.15	3.666	285.08	1044.98
A_68_P26844379	chr9:114294023-114294067	NM_019922:5786	Crtap	INSIDE	1.447	1.494	940.08	1404.03	2.161	739.98	1599.43
A_68_P31105766	chr17:25854648-25854692	NM_022422:554	Gng13	INSIDE	1.446	0.296	1599.55	473.29	0.428	1332.45	570.00
A_68_P29751623	chr14:79881191-79881235	NM_018765:-137	Wbp4	PROMOTER	1.446	2.158	464.48	1002.13	3.120	363.83	1134.98
A_68_P24542637	chr6:72566597-72566641	NM_009444:124	Tgoln2	INSIDE	1.446	0.366	1297.01	475.06	0.530	962.50	509.71
A_68_P31977525	chr19:16059111-16059155	NM_198019:347	Cep78	INSIDE	1.445	0.377	2040.10	770.05	0.545	1584.63	864.11
A_68_P30346541	chr15:76002795-76002839	NM_201394:23324	Plec	INSIDE	1.445	1.498	906.89	1358.49	2.165	726.68	1572.94
A_68_P26154294	chr8:111474065-111474109	NM_007496:235543	Zfhx3	INSIDE	1.445	1.620	714.32	1157.35	2.341	540.02	1264.18
A_68_P25565217	chr7:144507254-144507298	NM_001113414:-1148	Ebf3	PROMOTER	1.445	0.385	1281.87	493.78	0.557	955.01	531.71
A_68_P25025186	chr7:29856672-29856716	NM_009109:53476	Ryr1	INSIDE	1.445	1.872	603.41	1129.75	2.705	393.75	1064.92
A_68_P29177385	chr13:81850332-81850377	NM_028372:-61	Mblac2	DIVERGENT_PROMOTER	1.444	0.274	2862.63	784.32	0.396	2291.99	906.84
A_68_P24688741	chr6:99828483-99828532	ENSMUST00000074759:-80		PROMOTER	1.444	1.669	382.03	637.50	2.410	325.71	785.01
A_68_P32200577	chr19:56471803-56471847	NM_007611:206	Casp7	INSIDE	1.443	0.247	2201.57	544.84	0.357	1772.55	632.99
A_68_P26153110	chr8:111316387-111316431	NM_007496:77865	Zfhx3	INSIDE	1.443	1.643	1314.23	2159.27	2.370	1051.31	2491.81
A_68_P24372701	chr6:37391699-37391743	NM_178661:428	Creb3l2	INSIDE	1.443	0.281	1656.88	465.23	0.405	1233.94	500.14
A_68_P25736705	chr8:28232196-28232240	NM_054044:35906	Gpr124	INSIDE	1.442	0.388	1321.67	512.23	0.559	1027.91	574.44
A_68_P21334155	chr2:73729976-73730020	NM_001025093:687	Atf2	INSIDE	1.442	0.393	2833.56	1114.90	0.567	2154.41	1222.10
A_68_P26015685	chr8:86132504-86132548	NM_018808:453	Dnajb1	INSIDE	1.440	0.358	1340.95	479.43	0.515	1125.30	579.35
A_68_P23192160	chr4:107356153-107356197	NM_019872:593	Dmrtb1	INSIDE	1.440	3.072	768.36	2360.64	4.424	691.31	3058.31
A_68_P32320216	chrX:34347853-34347897	NM_007451:12228	Slc25a5	DOWNSTREAM	1.439	1.973	251.15	495.48	2.839	277.34	787.28
A_68_P23221708	chr4:114494316-114494360	NR_033617:14	Gm12830	INSIDE	1.439	0.288	1547.23	445.04	0.414	1177.50	487.31
A_68_P22006135	chr3:26230635-26230679	NM_138666:175	Nlgn1	INSIDE	1.439	0.360	2308.93	830.94	0.518	1588.70	822.58
A_68_P21075525	chr2:25837701-25837745	NM_001115076:1080	Camsap1	INSIDE	1.439	0.137	5181.77	711.83	0.198	3575.31	706.81
A_68_P20947554	chr1:194979527-194979571	NM_016851:243	Irf6	INSIDE	1.439	0.376	3591.68	1352.10	0.542	2977.07	1612.41
A_68_P25615129	chr8:3679115-3679159	NM_025701:2660	Trappc5	INSIDE	1.438	2.787	716.41	1996.56	4.007	664.95	2664.63
A_68_P22318571	chr3:89125906-89125950	NM_010108:873	Efna3	INSIDE	1.438	0.306	10722.21	3281.89	0.440	6905.81	3038.75
A_68_P32565868	chrX:99044658-99044702	NM_001163610:-43	Nhsl2	PROMOTER	1.437	2.733	1170.32	3198.52	3.929	1391.97	5468.53
A_68_P31213957	chr17:47873861-47873905	NM_001161722:-103	Tcfeb	PROMOTER	1.437	0.316	1801.82	569.81	0.454	1342.94	610.19
A_68_P20829087	chr1:173609897-173609941	NM_007649:-2267	Cd48	PROMOTER	1.436	1.632	885.82	1445.40	2.342	731.68	1713.94
A_68_P20151303	chr1:36996367-36996411	NM_018872:-16	Tmem131	PROMOTER	1.436	1.598	2800.54	4474.22	2.294	1930.35	4428.46
A_68_P30350127	chr15:76540848-76540892	NM_058214:119	Recql4	INSIDE	1.435	0.380	1944.42	738.13	0.545	1442.06	785.38
A_68_P28033281	chr11:94352708-94352752	NM_027984:8558	Epn3	INSIDE	1.435	0.368	2071.26	762.89	0.528	1527.51	807.25
A_68_P23226776	chr4:115608983-115609027	NM_001025567:3527	Dmbx1	INSIDE	1.435	0.414	2108.29	873.75	0.595	1546.94	920.24
A_68_P28621836	chr12:90498827-90498871	NM_172544:307015	Nrxn3	INSIDE	1.434	1.397	1192.91	1666.21	2.002	880.82	1763.71
A_68_P30970947	chr16:92697153-92697197	NM_001111023:399	Runx1	INSIDE	1.433	0.228	2946.20	671.98	0.327	2008.55	656.30
A_68_P30483165	chr15:99803409-99803453	NM_001024526:220	Larp4	INSIDE	1.433	0.292	3114.27	909.15	0.418	2161.31	904.19
A_68_P24819102	chr6:125115139-125115183	NM_008084:441	Gapdh	INSIDE	1.433	0.440	2706.78	1189.88	0.630	2077.64	1308.50
A_68_P28052175	chr11:97560549-97560593	NM_001163307:128	Pcgf2	INSIDE	1.432	2.185	297.64	650.34	3.129	250.93	785.27
A_68_P26017400	chr8:86496806-86496851	NM_008854:-48	Prkaca	PROMOTER	1.432	0.240	2685.65	644.26	0.343	1924.06	660.89
A_68_P24596101	chr6:83864271-83864315	NM_001166371:-54	Zfml	PROMOTER	1.432	2.751	650.79	1790.06	3.938	540.18	2127.33
A_68_P22973922	chr4:63678515-63678559	NM_011607:29513	Tnc	INSIDE	1.432	1.878	2125.58	3991.52	2.690	1607.60	4324.41
A_68_P22832217	chr4:34663673-34663717	NM_027041:13761	1700003M02Rik	INSIDE	1.432	1.944	296.21	575.71	2.783	254.95	709.55
A_68_P28457446	chr12:57635707-57635751	NM_001146198:365	Nkx2-1	INSIDE	1.431	0.410	1902.21	780.82	0.588	1469.91	863.64
A_68_P24058179	chr5:124357031-124357075	NM_026603:-231	Denr	PROMOTER	1.431	0.287	2802.58	802.95	0.410	2378.02	975.22
A_68_P21840389	chr2:168088474-168088518	NM_001081134:6335	Kcng1	INSIDE	1.431	1.943	897.82	1744.77	2.780	612.13	1701.83
A_68_P31305846	chr17:66728820-66728864	NM_001114098:70248	1110012J17Rik	INSIDE	1.430	1.397	2057.48	2874.55	1.998	1414.62	2826.30
A_68_P26345767	chr9:20969656-20969700	NM_183408:-479	Pde4a	PROMOTER	1.430	0.415	2231.83	926.82	0.594	1651.27	980.46
A_68_P23927132	chr5:100407097-100407141	NM_001077265:839	Hnrnpd	INSIDE	1.430	0.196	3256.22	639.09	0.281	2214.94	621.65
A_68_P23358590	chr4:140071282-140071326	NM_001112723:133367	Arhgef10l	INSIDE	1.430	1.726	596.26	1029.16	2.468	523.31	1291.72
A_68_P20596874	chr1:129670673-129670717	NM_028399:-46	Ccnt2	PROMOTER	1.430	0.319	3055.82	973.75	0.456	2267.80	1033.20
A_68_P31152228	chr17:34258124-34258168	NR_037970:-1301	Brd2	PROMOTER	1.429	0.207	2882.30	596.79	0.296	1892.76	559.99
A_68_P23296400	chr4:129054411-129054455	NM_028603:840	Zbtb8a	INSIDE	1.429	0.194	4022.63	780.14	0.277	2649.70	734.25
A_68_P20796834	chr1:167775041-167775085	NM_001113391:56251	Cd247	INSIDE	1.429	1.677	418.03	701.11	2.397	365.20	875.56
A_68_P20352246	chr1:75139198-75139242	NM_170755:-139	Fam134a	DIVERGENT_PROMOTER	1.429	0.174	3131.97	544.76	0.249	2388.80	593.87
A_68_P32375458	chrX:48558403-48558447	NM_134163:585	Mbnl3	INSIDE	1.428	2.177	406.92	885.95	3.110	495.98	1542.51
A_68_P31863849	chr18:80559674-80559719	NM_001190373:1297	Kcng2	INSIDE	1.428	0.406	2142.53	870.50	0.580	1467.81	851.45
A_68_P26022006	chr8:87324594-87324638	NM_001081982:-377	Nfix	PROMOTER	1.428	0.096	5465.98	525.08	0.137	3632.77	498.45
A_68_P31632590	chr18:38357395-38357439	NM_029357:12000	Pcdh1	INSIDE	1.427	1.638	510.42	835.96	2.337	466.74	1090.60
A_68_P28040434	chr11:95573914-95573958	ENSMUST00000107717:-50		PROMOTER	1.427	0.246	1855.63	455.90	0.351	1503.76	527.09
A_68_P25278635	chr7:89796803-89796847	NM_177894:214	Fam154b	INSIDE	1.427	0.339	1362.44	461.37	0.483	1140.29	551.00
A_68_P20553863	chr1:120734057-120734104	NM_001081125:216116	Gli2	INSIDE	1.427	1.506	943.42	1420.38	2.148	707.74	1520.02
A_68_P31629193	chr18:37829497-37829541	NM_033586:-4470	Pcdhga3	PROMOTER	1.426	2.186	477.10	1043.06	3.117	470.25	1465.82
A_68_P30346577	chr15:76007094-76007138	NM_201394:19024	Plec	INSIDE	1.426	1.568	659.32	1033.87	2.237	503.45	1126.05
A_68_P27949796	chr11:79404983-79405027	NM_175543:291	Rab11fip4	INSIDE	1.426	0.321	1696.61	545.01	0.458	1297.82	594.59
A_68_P22383504	chr3:102739949-102739993	NM_011516:53	Sycp1	INSIDE	1.426	1.816	418.65	760.33	2.590	292.83	758.29

ProbeName	Target position of probe on CpG island microarray	TargetID	GeneSymbol	CpG island Description	Ratio of relative methylation (TiO_2-NP/Vehicle)	Sham group			TiO_2-H group		
						Relative methylation (Cy5/Cy3)	Cy3 signal (Input)	Cy5 signal (MeDIP)	Relative methylation (Cy5/Cy3)	Cy3 signal (Input)	Cy5 signal (MeDIP)
A_68_P31114337	chr17:27250538-27250582	NM_080553:56312	Itpr3	INSIDE	1.425	1.404	1180.78	1657.81	2.001	1011.85	2024.24
A_68_P28525542	chr12:72419588-72419632	NM_001190466:8740	Dact1	INSIDE	1.425	2.764	864.22	2388.29	3.938	675.06	2658.06
A_68_P28378464	chr12:41172641-41172685	NM_172803:23	Dock4	INSIDE	1.425	2.044	389.11	795.22	2.912	318.82	928.45
A_68_P27975821	chr11:83993178-83993222	ENSMUST00000136463:-112		PROMOTER	1.425	0.420	1548.49	650.88	0.599	1238.66	741.67
A_68_P24990312	chr7:19477759-19477803	NM_001033314:17973	Ccdc61	INSIDE	1.425	0.289	1787.16	516.40	0.412	1504.23	619.46
A_68_P22570423	chr3:138406072-138406116	NM_019571:936	Tspan5	INSIDE	1.425	0.172	5442.88	933.54	0.244	3784.70	924.85
A_68_P30778453	chr16:55934660-55934704	NM_028815:279	Cep97	INSIDE	1.424	0.322	1386.99	446.42	0.458	996.15	456.44
A_68_P30476587	chr15:98636550-98636594	NM_001013741:1784	Ddn	INSIDE	1.424	0.395	1375.76	543.45	0.563	1063.82	598.48
A_68_P25416680	chr7:118172636-118172680	NM_009431:194	Ctr9	INSIDE	1.424	0.331	2590.73	856.30	0.471	1887.50	888.68
A_68_P28566344	chr12:79963184-79963228	NM_026114:148	Eif2s1	INSIDE	1.423	0.277	1721.65	477.63	0.395	1420.68	560.96
A_68_P31256957	chr17:56706054-56706098	NM_001029979:17930	Safb2	INSIDE	1.422	1.731	501.32	867.57	2.461	429.55	1057.04
A_68_P23226466	chr4:115563188-115563232	NM_001039124:6206	Kncn	DOWNSTREAM	1.422	0.364	2109.62	766.94	0.517	1694.70	875.94
A_68_P28377242	chr12:40949783-40949827	NM_013562:-28	Ifrd1	PROMOTER	1.421	0.188	2981.74	559.39	0.267	2066.43	551.00
A_68_P24160217	chr5:145016703-145016747	NM_018772:631	Bri3	INSIDE	1.421	0.188	3508.79	660.47	0.267	2340.32	625.99
A_68_P22150381	chr3:54703304-54703351	NM_177854:16482	6030405A18Rik	INSIDE	1.421	1.991	276.63	550.85	2.830	276.43	782.43
A_68_P21775756	chr2:157029918-157029962	NM_011249:330	Rbl1	INSIDE	1.421	1.363	1181.98	1611.55	1.937	1042.53	2019.44
A_68_P32710882	chrX:139400446-139400490	NM_019496:803	Ammecr1	INSIDE	1.420	3.560	464.43	1653.22	5.056	669.31	3384.12
A_68_P22383501	chr3:102739596-102739640	NM_011516:405	Sycp1	INSIDE	1.420	2.247	1377.48	3095.09	3.191	1048.47	3345.46
A_68_P22356139	chr3:97555701-97555745	NM_178080:16215	Pde4dip	INSIDE	1.420	2.218	847.15	1878.74	3.148	694.53	2186.51
A_68_P32092597	chr19:36979014-36979058			Unknown	1.419	0.344	2294.95	789.67	0.488	1782.72	870.17
A_68_P26710352	chr9:88376001-88376047	NM_019796:1211	Syncrip	INSIDE	1.419	0.189	2385.88	450.80	0.268	1758.74	471.49
A_68_P24597191	chr6:84055754-84055806	NM_021469:86398	Dysf	INSIDE	1.419	2.436	208.04	506.86	3.457	149.71	517.61
A_68_P31175186	A_68_P31175186			Unknown	1.418	0.405	37690.54	15251.15	0.574	30699.18	17618.32
A_68_P27473559	chr10:115551010-115551054	NM_001161855:-59	4933416C03Rik	PROMOTER	1.418	2.421	1826.63	4422.78	3.432	1416.61	4862.28
A_68_P25616894	chr8:4258857-4258901	NM_183315:396	Ctxn1	INSIDE	1.418	0.403	1895.86	763.26	0.571	1464.72	836.43
A_68_P22488132	chr3:123150107-123150151	NM_008939:298	Prss12	INSIDE	1.418	0.348	2053.44	714.21	0.493	1668.09	822.80
A_68_P28813939	chr13:8995639-8995683	NM_027000:-402	Gtpbp4	PROMOTER	1.417	0.299	2825.72	844.32	0.423	2180.33	923.20
A_68_P23576980	chr5:31495225-31495269	NM_001127355:266	Eif2b4	INSIDE	1.417	0.225	3582.64	807.29	0.319	2532.82	808.99
A_68_P21084623	chr2:27282256-27282300	NM_009500:67	Vav2	INSIDE	1.417	0.305	3430.20	1046.07	0.432	2326.50	1005.64
A_68_P30477337	chr15:98748179-98748223	NM_029098:329	Lmbr1l	INSIDE	1.415	0.347	1619.20	562.00	0.491	1280.20	628.72
A_68_P28598116	chr12:85750091-85750135	NM_025525:-8037	Rnf113a2	PROMOTER	1.415	4.869	1025.26	4992.01	6.890	722.02	4974.40
A_68_P28577467	chr12:81858507-81858551	NM_001008423:3174	Gm1568	INSIDE	1.415	1.793	919.69	1649.42	2.538	749.79	1902.67
A_68_P27893182	chr11:68931694-68931738	NM_033041:-2238	Hes7	PROMOTER	1.415	0.366	4376.57	1603.33	0.518	3102.25	1608.30
A_68_P27176090	chr10:60149152-60149197	NM_023370:10064	Cdh23	INSIDE	1.415	6.913	243.14	1680.82	9.782	242.58	2372.93
A_68_P26221248	chr8:123112748-123112792	NM_178856:205	Gins2	INSIDE	1.415	0.368	2214.22	814.50	0.520	1578.57	821.50
A_68_P25042342	chr7:34918494-34918538	NM_207212:-229	Wtip	PROMOTER	1.415	0.213	3291.79	702.64	0.302	2478.07	748.59
A_68_P24464804	chr6:54631784-54631828	NM_026629:41	2410066E13Rik	INSIDE	1.415	0.321	1521.98	488.84	0.454	1260.22	572.58
A_68_P20241449	chr1:55419481-55419525	NM_001113367:49	Boll	INSIDE	1.415	1.458	720.84	1050.76	2.063	610.33	1258.95
A_68_P25798748	chr8:40069239-40069283	NM_030254:340	Tusc3	INSIDE	1.414	0.343	1872.53	641.62	0.484	1483.70	718.63
A_68_P24620342	chr6:88146929-88146973	NM_008090:-1707	Gata2	PROMOTER	1.414	0.251	2957.95	742.11	0.355	2142.40	760.29
A_68_P22303235	chr3:86350759-86350806	NM_011839:1423	Mab21l2	INSIDE	1.414	0.298	1694.67	505.55	0.422	1166.10	491.76
A_68_P21084947	chr2:27330469-27330516	NM_023336:426	Brd3	INSIDE	1.414	0.178	3844.88	685.71	0.252	2448.35	617.34
A_68_P32116953	chr19:41818287-41818331	NM_015748:38	Slit1	INSIDE	1.413	0.217	4695.96	1019.05	0.307	3074.27	942.37
A_68_P25588642	chr7:148600091-148600135	NM_172116:-88	Pddc1	PROMOTER	1.413	0.236	6753.25	1595.21	0.334	4541.06	1515.22
A_68_P25393400	chr7:112781499-112781543	NM_018880:493	Trim3	INSIDE	1.413	0.209	5693.83	1191.30	0.296	3483.47	1029.88
A_68_P24118896	chr5:136470915-136470959	NM_023742:190	Dtx2	INSIDE	1.413	0.352	2939.46	1033.54	0.497	2270.19	1127.99
A_68_P21073109	chr2:25435334-25435378	NM_001005424:263	Gm996	INSIDE	1.413	0.284	2218.96	629.77	0.401	1557.98	624.82
A_68_P31116776	chr17:27640013-27640058	NM_001013385:214	Grm4	INSIDE	1.412	1.871	298.01	557.68	2.642	276.98	731.85
A_68_P30514884	chr16:5307097-5307141			Unknown	1.412	0.350	2271.66	793.99	0.494	1693.98	836.19
A_68_P25951937	chr8:72976378-72976422	NM_001122830:24442	Klhl26	INSIDE	1.412	1.775	443.80	787.84	2.507	402.96	1010.39
A_68_P27877787	chr11:66338783-66338827	NM_001034874:824	Shisa6	INSIDE	1.411	0.395	2282.10	900.72	0.557	1682.38	937.15
A_68_P21731904	chr2:148557983-148558027	NM_019632:152	Napb	INSIDE	1.411	0.229	3548.62	813.64	0.323	2559.38	827.85
A_68_P26400215	chr9:31193897-31193941	NM_172766:142	Nfrkb	INSIDE	1.410	2.701	6751.91	18237.79	3.808	4733.50	18025.78
A_68_P24397786	chr6:42300193-42300237	NM_011777:388	Zyx	INSIDE	1.410	0.189	3068.13	579.50	0.266	2299.43	612.19
A_68_P22893527	chr4:47258362-47258406	NM_009928:37501	Col15a1	INSIDE	1.410	1.783	725.16	1292.65	2.513	473.16	1189.22
A_68_P25279963	chr7:90017868-90017912	NM_175366:2048	Mex3b	INSIDE	1.409	0.422	1539.82	650.13	0.595	1212.98	721.80
A_68_P22609989	chr3:145316674-145316720	NM_010516:-3747	Cyr61	PROMOTER	1.409	1.612	600.54	968.14	2.271	492.94	1119.42
A_68_P21102110	chr2:30111958-30112002	NM_177725:18692	Lrrc8a	INSIDE	1.409	1.448	1685.82	2441.76	2.041	964.84	1969.17
A_68_P20939668	chr1:193542953-193542997	NM_001134829:701	Lpgat1	INSIDE	1.409	0.320	1526.07	488.57	0.451	1165.14	525.71
A_68_P28748395	chr12:113413871-113413915	NM_001097621:29474	Kif26a	INSIDE	1.408	1.492	675.08	1007.22	2.101	507.92	1067.18
A_68_P28730242	chr12:110074008-110074052	NM_181328:56	Slc25a29	INSIDE	1.408	0.375	1549.96	581.95	0.528	1143.89	604.50
A_68_P23185610	chr4:106311623-106311667	NM_177667:16591	Ttc22	INSIDE	1.408	1.781	1536.53	2736.86	2.508	1177.51	2953.43
A_68_P26821061	chr9:109834270-109834314	NM_001205331:15	Mtap4	INSIDE	1.407	0.391	1220.29	476.85	0.550	1016.15	558.84
A_68_P31305847	chr17:66728898-66728942	NM_001114098:70170	1110012J17Rik	INSIDE	1.406	1.528	1625.87	2484.28	2.148	1174.71	2522.77
A_68_P30086027	chr15:26239002-26239046	NM_177597:198	Marchf11	INSIDE	1.406	0.331	2691.83	890.37	0.465	1853.82	862.16

ProbeName	Target position of probe on CpG island microarray	TargetID	GeneSymbol	CpG island Description	Ratio of relative methylation (TiO_2-NP/Vehicle)	Sham group			TiO_2-H group		
						Relative methylation (Cy5/Cy3)	Cy3 signal (Input)	Cy5 signal (MeDIP)	Relative methylation (Cy5/Cy3)	Cy3 signal (Input)	Cy5 signal (MeDIP)
A_68_P24120336	chr5:136750661-136750705	NM_198602:292593	Cux1	INSIDE	1.406	2.200	501.35	1103.08	3.093	525.68	1626.06
A_68_P21835385	chr2:167247037-167247081	NM_148929:-162	Slc9a8	PROMOTER	1.406	0.279	2101.19	585.79	0.392	1484.86	582.20
A_68_P31620879	chr18:36078772-36078816	AK021191:6009		DOWNSTREAM	1.405	0.327	1362.31	445.37	0.459	1060.52	487.11
A_68_P24377210	chr6:38383835-38383879	NM_177185:-68	Ubn2	PROMOTER	1.405	2.165	297.56	644.12	3.042	223.10	678.60
A_68_P23997436	chr5:113739937-113739981	NM_172718:-152	Sgsm1	PROMOTER	1.405	0.355	1261.83	447.93	0.499	984.60	490.94
A_68_P23438939	chr4:154865343-154865387	NM_001160016:-105	Gnb1	PROMOTER	1.405	0.262	1883.24	492.98	0.368	1523.79	560.43
A_68_P23145554	chr4:99323682-99323726	NM_010425:715	Foxd3	INSIDE	1.405	0.212	3328.80	706.05	0.298	2333.35	695.32
A_68_P28313703	chr12:29237082-29237126	NM_001177964:-3902	Dcdc2c	PROMOTER	1.404	1.474	676.69	997.33	2.069	542.34	1121.94
A_68_P25957926	chr8:74152893-74152939	NM_001029873:42740	Unc13a	INSIDE	1.404	2.051	264.99	543.58	2.879	198.00	570.06
A_68_P32536864	chrX:91226158-91226202	NR_028320:12186	AU015836	DOWNSTREAM	1.403	2.416	332.30	802.69	3.388	498.64	1689.30
A_68_P22059402	chr3:36570442-36570486	NM_019510:18625	Trpc3	INSIDE	1.403	1.730	1228.45	2125.02	2.427	1001.33	2430.30
A_68_P20058116	chr1:17081618-17081662	NM_020604:6330	Jph1	INSIDE	1.403	1.905	902.12	1718.20	2.673	758.57	2027.57
A_68_P25499765	chr7:133435758-133435803	NM_145587:19648	Sbk1	INSIDE	1.402	1.828	542.37	991.71	2.564	494.97	1269.02
A_68_P23615699	chr5:38212879-38212923	NR_027920:1098	Msx1as	INSIDE	1.402	0.328	2189.25	718.43	0.460	1614.75	743.07
A_68_P32140286	chr19:45824101-45824145	NM_008723:-69	Npm3	PROMOTER	1.401	1.342	2157.87	2895.47	1.880	1853.71	3485.59
A_68_P30423733	chr15:89011473-89011517	NM_138749:-276	Plxnb2	PROMOTER	1.401	0.330	1703.53	562.35	0.463	1284.82	594.25
A_68_P20087503	chr1:23263704-23263748	ENSMUST00000097807:-481		PROMOTER	1.401	0.365	2293.07	835.97	0.511	1973.38	1007.83
A_68_P29675223	chr14:66349464-66349508	NM_001168318:64247	Scara5	INSIDE	1.400	1.688	500.10	844.34	2.364	348.46	823.67
A_68_P25361263	chr7:106497545-106497589	NM_001111043:4183	Serpinh1	INSIDE	1.400	1.630	620.22	1011.20	2.283	533.03	1216.93
A_68_P24904259	chr6:142336167-142336211	NM_025872:426	Golt1b	INSIDE	1.400	0.293	1653.62	484.15	0.410	1256.65	515.24
A_68_P31886106	chr18:84257584-84257628	NM_146090:57	Zadh2	INSIDE	1.399	0.158	3050.58	482.20	0.221	2257.76	499.25
A_68_P30479873	chr15:99223693-99223737	NM_001171035:65	Tmbim6	INSIDE	1.399	0.221	2733.32	603.87	0.309	2225.08	687.81
A_68_P30447287	chr15:93333954-93333998	NM_001033217:92346	Prickle1	INSIDE	1.399	3.033	772.52	2343.24	4.243	650.04	2757.93
A_68_P27392942	chr10:100051680-100051724	NM_175128:191	4930430F08Rik	INSIDE	1.399	0.385	1516.06	583.26	0.538	1224.22	659.06
A_68_P23266750	chr4:123856175-123856229			Unknown	1.399	0.275	2033.73	559.45	0.385	1423.97	548.10
A_68_P23154898	chr4:101092046-101092090	NM_009647:-239	Ak4	PROMOTER	1.399	0.447	1917.86	857.90	0.626	1472.35	921.47
A_68_P20560373	chr1:122017586-122017630	NM_001037999:-112	Dbi	PROMOTER	1.399	0.177	3532.97	625.55	0.248	2346.33	581.08
A_68_P28575258	chr12:81440823-81440867	NM_177267:96744	Dcaf5	INSIDE	1.398	1.494	576.80	861.94	2.089	518.63	1083.67
A_68_P25435185	chr7:121419707-121419751	NM_011965:-98	Psma1	PROMOTER	1.398	0.314	3069.76	962.79	0.438	2304.30	1010.20
A_68_P21901050	chr2:179777293-179777337	NM_178750:127	Ss18l1	INSIDE	1.398	0.187	2324.83	434.76	0.262	1638.91	428.60
A_68_P33015615	chr1_random:49917-49962	NM_026866:135399	Disp1	INSIDE	1.397	1.959	225.29	441.30	2.737	220.67	603.88
A_68_P25008200	chr7:25857700-25857744	NM_008168:-334	Grik5	DIVERGENT_PROMOTER	1.397	0.388	2298.77	892.26	0.542	1685.73	914.05
A_68_P20066615	chr1:19200195-19200239	NM_001025305:-1918	Tcfap2b	PROMOTER	1.397	0.395	1537.08	606.60	0.551	1258.67	693.87
A_68_P31351304	chr17:74794846-74794890	NM_144798:-103	Slc30a6	PROMOTER	1.396	0.396	1187.56	470.14	0.553	937.41	518.17
A_68_P31125954	chr17:29235470-29235514	NM_007669:4776	Cdkn1a	INSIDE	1.396	1.605	433.05	694.83	2.241	398.24	892.34
A_68_P27709960	chr11:35821756-35821800	NM_011856:935967	Odz2	INSIDE	1.396	2.172	821.55	1784.24	3.031	655.42	1986.51
A_68_P20149760	chr1:36739833-36739877	NM_001126046:174	Fam178b	INSIDE	1.396	1.964	1379.93	2710.09	2.742	1221.80	3349.91
A_68_P28032110	chr11:94183065-94183109	NM_026313:139	Luc7l3	INSIDE	1.395	0.362	1634.60	591.22	0.505	1080.90	545.37
A_68_P26400213	chr9:31193594-31193638	NM_172766:-160	Nfrkb	PROMOTER	1.395	0.387	1812.91	701.65	0.540	1460.95	788.52
A_68_P26345697	chr9:20954349-20954393	NM_016742:-20	Cdc37	PROMOTER	1.395	0.351	1276.81	448.68	0.490	1063.56	521.53
A_68_P25708739	chr8:23123882-23123926	NM_007511:46642	Atp7b	INSIDE	1.395	1.544	1027.15	1585.93	2.153	804.97	1733.22
A_68_P24397656	chr6:42274272-42274316	NM_001113327:345	Fam131b	INSIDE	1.395	0.283	1654.51	468.37	0.395	1127.97	445.46
A_68_P27287199	chr10:80532582-80532627	NM_022653:-223	Thop1	DIVERGENT_PROMOTER	1.394	3.812	175.73	669.89	5.316	153.88	817.98
A_68_P24001850	chr5:114541225-114541269	NM_026805:143	Svop	INSIDE	1.393	2.244	556.87	1249.81	3.126	381.86	1193.78
A_68_P23417961	chr4:151689896-151689940	NM_001033489:2816	Rnf207	INSIDE	1.393	0.291	1520.86	443.27	0.406	963.97	391.40
A_68_P23226957	chr4:115635357-115635401	ENSMUST00000125761:54902		DOWNSTREAM	1.393	0.206	2444.91	502.89	0.287	1714.56	491.40
A_68_P23212280	chr4:111392824-111392869	NM_026470:232	Spata6	INSIDE	1.393	0.231	2077.25	480.09	0.322	1753.16	564.36
A_68_P31251408	chr17:55621977-55622021	NM_172829:36984	St6gal2	INSIDE	1.392	1.978	860.05	1700.90	2.753	769.03	2117.40
A_68_P30633252	chr16:28929399-28929443	NM_177718:364	1600021P15Rik	INSIDE	1.392	0.193	3429.05	663.19	0.269	2441.79	657.49
A_68_P27222449	chr10:68675460-68675504	NM_001081347:78	Rhobtb1	INSIDE	1.392	0.304	1523.06	462.71	0.423	1225.33	518.16
A_68_P24511775	chr6:65331286-65331330	NM_175524:21	C130060K24Rik	INSIDE	1.392	0.260	2263.10	588.36	0.362	1772.42	641.27
A_68_P25957040	chr8:74005621-74005665	NM_001164679:4324	Ano8	INSIDE	1.391	2.842	1616.20	4593.17	3.954	1209.94	4784.17
A_68_P23416685	chr4:151500131-151500175	NM_019585:2882	Espn	INSIDE	1.391	1.392	1695.58	2360.44	1.936	1180.44	2285.52
A_68_P28748413	chr12:113416336-113416380	NM_001097621:31940	Kif26a	INSIDE	1.390	1.804	1786.95	3224.36	2.508	1385.30	3474.33
A_68_P26853659	chr9:116084433-116084477	NM_009371:27	Tgfbr2	INSIDE	1.390	0.212	3617.62	767.01	0.295	2386.30	703.09
A_68_P24129051	chr5:138596643-138596687	NM_019747:308	Zfp113	INSIDE	1.390	0.330	1920.15	634.36	0.459	1334.93	612.91
A_68_P30083238	chr15:25711367-25711411	NM_019472:159084	Myo10	INSIDE	1.389	1.508	1078.74	1626.25	2.094	982.36	2057.15
A_68_P29288689	chr13:104900121-104900165	NM_144838:273	Sgtb	INSIDE	1.389	0.229	3480.75	798.17	0.318	2396.32	762.99
A_68_P27901894	chr11:70467828-70467872	NM_011072:302	Pfn1	INSIDE	1.389	0.404	1663.38	672.11	0.561	1237.11	694.49
A_68_P26320797	chr9:14849991-14850035	NM_019482:-90	Panx1	PROMOTER	1.389	0.410	1370.09	561.59	0.569	1130.76	643.61
A_68_P25023458	chr7:29551865-29551909	NM_001122765:116	Sirt2	INSIDE	1.389	0.385	1690.71	651.51	0.535	1402.52	750.61
A_68_P24763847	chr6:114081056-114081100	NM_172890:-156	Slc6a11	PROMOTER	1.389	0.454	1603.73	728.88	0.631	1291.71	815.31
A_68_P28457439	chr12:57634754-57634798	NM_001146198:1317	Nkx2-1	INSIDE	1.388	0.232	1882.13	436.56	0.322	1251.29	402.81
A_68_P22336393	chr3:93247754-93247798	NM_001163098:1525	Tchh	INSIDE	1.388	1.862	630.24	1173.79	2.585	498.01	1287.51
A_68_P20935551	chr1:192803342-192803386	NR_027352:-189	Vash2	PROMOTER	1.388	2.770	1230.90	3410.13	3.844	945.61	3635.38

ProbeName	Target position of probe on CpG island microarray	TargetID	GeneSymbol	CpG island Description	Ratio of relative methylation (TiO_2-NP/Vehicle)	Sham group			TiO_2-H group		
						Relative methylation (Cy5/Cy3)	Cy3 signal (Input)	Cy5 signal (MeDIP)	Relative methylation (Cy5/Cy3)	Cy3 signal (Input)	Cy5 signal (MeDIP)
A_68_P31932936	chr19:6141162-6141206	NM_019722:-47	Arl2	PROMOTER	1.387	1.398	936.91	1310.17	1.940	678.40	1316.16
A_68_P30141816	chr15:36937216-36937260	NM_026521:-81	Zfp706	PROMOTER	1.387	0.295	2733.18	807.16	0.410	1821.13	745.80
A_68_P29477473	chr14:26277283-26277327	NM_183208:-1366	Zmiz1	PROMOTER	1.387	0.405	1232.68	499.37	0.562	969.37	544.67
A_68_P27094094	chr10:41997864-41997908	NM_019740:-1338	Foxo3	PROMOTER	1.387	0.361	1399.66	505.63	0.501	1032.09	517.32
A_68_P26142834	chr8:109572130-109572174	NM_026513:362	Pdf	INSIDE	1.387	0.238	2802.17	665.89	0.330	1916.22	631.76
A_68_P22522345	chr3:129533741-129533785	NM_026578:552	Gar1	INSIDE	1.387	0.198	2336.91	462.94	0.275	1812.06	498.04
A_68_P29773647	chr14:84846157-84846201	NM_001013753:2809	Pcdh17	INSIDE	1.386	0.360	3217.20	1159.35	0.499	2469.15	1233.03
A_68_P25031174	chr7:31140698-31140742	NM_175478:7071	Lrfn3	INSIDE	1.386	1.559	573.40	893.92	2.160	487.10	1052.33
A_68_P24819777	chr6:125236044-125236088	NM_009446:-6	Tuba3a	PROMOTER	1.386	1.674	789.67	1321.93	2.320	718.75	1667.29
A_68_P22871974	chr4:43506583-43506627	NM_001037913:368	Ccdc107	INSIDE	1.386	0.268	1658.08	444.53	0.371	1198.89	445.37
A_68_P21084946	chr2:27330392-27330443	NM_023336:501	Brd3	INSIDE	1.386	0.348	1478.52	514.68	0.482	1058.25	510.42
A_68_P30588528	chr16:20620501-20620545	NM_028420:829	Camk2n2	INSIDE	1.385	0.259	3531.41	916.20	0.359	2305.63	828.49
A_68_P27897804	chr11:69714837-69714881	NM_027419:-370	2810408A11Rik	DIVERGENT_PROMOTER	1.385	0.185	2872.10	532.22	0.257	2036.60	522.69
A_68_P27287002	chr10:80494365-80494409	NM_134135:2271	Slc39a3	INSIDE	1.385	1.578	1506.25	2377.32	2.187	1054.94	2306.88
A_68_P25025265	chr7:29870930-29870974	NR_035489:-2239	Mir1963	PROMOTER	1.385	1.986	679.00	1348.77	2.752	551.46	1517.72
A_68_P22060449	chr3:36762158-36762202	NM_172679:153	4932438A13Rik	INSIDE	1.385	0.157	3898.93	611.85	0.217	2825.36	614.28
A_68_P21906911	chr2:180629180-180629224	NR_029538:458	Mir124a-3	DOWNSTREAM	1.385	0.228	2929.01	669.21	0.316	2277.48	720.44
A_68_P20455838	chr1:95375093-95375137	NM_133808:271	Hdlbp	INSIDE	1.385	0.369	1597.96	588.88	0.510	1326.68	677.12
A_68_P27172912	chr10:59648749-59648793	NM_016803:33238	Chst3	INSIDE	1.384	1.754	2305.55	4042.85	2.428	1927.57	4679.31
A_68_P25090466	chr7:52241494-52241538	NM_019830:186	Prmt1	INSIDE	1.384	0.299	2489.30	744.64	0.414	1648.05	682.15
A_68_P24151266	chr5:142843739-142843783			Unknown	1.384	1.694	613.20	1038.48	2.344	505.37	1184.54
A_68_P24055190	chr5:123795453-123795497	NM_029850:1018	Bcl7a	INSIDE	1.384	0.349	2532.98	885.05	0.483	1848.18	893.53
A_68_P27489859	chr10:118677390-118677434	NM_027994:-301	Cand1	PROMOTER	1.383	1.567	577.95	905.51	2.166	418.53	906.61
A_68_P24793131	chr6:119390505-119390549	NM_009525:103839	Wnt5b	INSIDE	1.382	1.592	1111.57	1769.34	2.199	893.46	1965.11
A_68_P21428050	chr2:92210966-92211010	NM_001166633:205	Gyltl1b	INSIDE	1.382	0.318	1659.65	527.06	0.439	1266.00	555.60
A_68_P31412119	chr17:86087840-86087884	NM_011380:-268	Six2	PROMOTER	1.381	1.751	705.61	1235.27	2.417	561.32	1356.87
A_68_P24170389	chr5:147656737-147656781	NM_026864:112	Rasl11a	INSIDE	1.381	0.397	1403.34	556.43	0.547	1121.23	613.86
A_68_P27940380	chr11:77744288-77744332	NM_021286:-134	Sez6	DIVERGENT_PROMOTER	1.380	0.222	2858.55	633.45	0.306	1963.67	600.40
A_68_P30403120	chr15:85641984-85642028	NM_001164625:121	2210021J22Rik	INSIDE	1.379	0.223	2909.95	648.25	0.307	2071.91	636.60
A_68_P29554799	chr14:40287043-40287087	NM_001190187:-688	Nrg3	PROMOTER	1.379	0.398	1625.88	647.49	0.549	1126.15	618.32
A_68_P28043947	chr11:96205604-96205648	NM_010458:544	Hoxb3	INSIDE	1.379	0.190	2592.18	493.52	0.262	1894.32	497.24
A_68_P22314994	chr3:88448009-88448053	NM_001198912:22715	Arhgef2	INSIDE	1.379	1.931	1148.33	2217.22	2.662	862.53	2296.39
A_68_P32678565	chrX:132420534-132420578	NM_001098222:167	Bhlhb9	INSIDE	1.378	2.106	993.88	2093.14	2.902	1337.70	3881.82
A_68_P30579428	chr16:18393075-18393119	NM_033474:44730	Arvcf	INSIDE	1.378	0.397	1739.04	691.19	0.548	1250.46	685.05
A_68_P30480288	chr15:99288482-99288527	NM_001001884:-325	Nckap5l	PROMOTER	1.378	2.655	278.04	738.11	3.659	244.86	895.96
A_68_P27649724	chr11:24064291-24064335	NM_001159290:83618	Bcl11a	INSIDE	1.378	1.917	504.26	966.87	2.641	427.79	1129.91
A_68_P28452658	chr12:56700808-56700852	NM_020287:927	Insm2	INSIDE	1.377	1.670	411.50	687.05	2.299	348.83	802.04
A_68_P22683294	chr3:157699823-157699867	NM_024194:178	Lrrc40	INSIDE	1.377	0.210	2752.62	576.94	0.289	2179.50	629.10
A_68_P30346617	chr15:76012075-76012119	NM_201394:14044	Plec	INSIDE	1.376	1.766	1115.55	1969.79	2.430	756.49	1837.97
A_68_P28731505	chr12:110272292-110272336	NM_001163175:34113	Begain	INSIDE	1.376	1.807	806.58	1457.81	2.487	717.92	1785.63
A_68_P28356086	chr12:36768656-36768700	NM_025359:387	Tspan13	INSIDE	1.376	0.164	4490.16	737.74	0.226	3548.70	802.53
A_68_P28047897	chr11:96838592-96838636	NM_001080964:388	Sp2	INSIDE	1.376	3.819	1142.33	4363.08	5.254	884.56	4647.34
A_68_P23985682	chr5:111849354-111849398	NM_001081235:2191	Mn1	INSIDE	1.376	1.842	860.04	1584.08	2.534	679.27	1721.45
A_68_P32099207	chr19:38129688-38129732	NM_001164362:179	Cep55	INSIDE	1.375	0.421	1845.82	776.97	0.579	1446.49	837.46
A_68_P26805312	chr9:106725024-106725068	NM_001015507:740	Vprbp	INSIDE	1.375	0.280	1972.49	553.14	0.386	1587.71	612.32
A_68_P25958570	chr8:74249375-74249419	NM_028715:184	Fcho1	INSIDE	1.375	0.420	3429.75	1440.80	0.578	2593.09	1498.26
A_68_P28956456	chr13:37870834-37870878	ENSMUST00000122842:588		INSIDE	1.374	0.254	1910.09	485.21	0.349	1393.14	486.20
A_68_P27291023	chr10:81105955-81106002	NM_027480:14694	Ankrd24	INSIDE	1.374	0.321	1411.89	452.65	0.441	1109.82	488.95
A_68_P26631268	chr9:72951661-72951705	NM_025890:1478	2410004A20Rik	INSIDE	1.374	1.935	454.13	878.54	2.658	410.23	1090.45
A_68_P22061573	chr3:36962572-36962616	NM_009350:17	Adad1	INSIDE	1.374	1.898	669.61	1270.72	2.607	553.69	1443.31
A_68_P32111689	chr19:40969301-40969345	NM_028319:128	Zfp518a	INSIDE	1.373	0.293	2355.05	689.55	0.402	1709.13	686.87
A_68_P31161346	chr17:35998590-35998640	NM_134122:352	Nrm	INSIDE	1.373	0.236	2993.81	706.39	0.324	1917.04	621.10
A_68_P30673613	chr16:36071461-36071505	NM_027342:119	Fam162a	INSIDE	1.373	0.177	3588.75	635.58	0.243	2652.58	645.01
A_68_P25171969	chr7:70590173-70590217	NM_130880:537	Otud7a	INSIDE	1.373	0.253	2097.41	529.64	0.347	1706.34	591.63
A_68_P24155561	chr5:143997211-143997255	NM_177681:346	Zfp12	INSIDE	1.373	0.303	2939.86	892.02	0.416	2119.23	882.65
A_68_P22314351	chr3:88340661-88340705	NR_035434:-378	Mir1905	PROMOTER	1.373	0.405	2190.98	887.32	0.556	1585.61	881.79
A_68_P20842584	chr1:176431990-176432034	NM_019445:57	Fmn2	INSIDE	1.373	0.320	2238.51	715.48	0.439	1820.32	799.09
A_68_P32132940	chr19:44629409-44629454	NM_026061:474	Ndufb8	INSIDE	1.372	0.392	4500.37	1765.05	0.538	3036.93	1634.35
A_68_P26787844	chr9:103663476-103663520	NM_178638:669	Tmem108	INSIDE	1.372	4.657	2174.50	10126.10	6.389	1535.28	9809.06
A_68_P24639210	chr6:91423696-91423740	NM_028766:-26	Tmem43	DIVERGENT_PROMOTER	1.372	2.190	1086.00	2378.87	3.005	839.32	2522.53
A_68_P24466344	chr6:54894258-54894302	NM_001171007:27375	Nod1	INSIDE	1.372	1.576	928.53	1463.03	2.161	727.74	1572.76
A_68_P27804076	chr11:53113307-53113351	NM_008300:653	Hspa4	INSIDE	1.370	0.317	1593.77	504.99	0.434	1353.88	587.65
A_68_P25761249	chr8:33119399-33119443	ENSMUST00000080782:161		INSIDE	1.370	0.493	3400.45	1676.37	0.675	2487.43	1679.72
A_68_P25457393	chr7:125387260-125387304	NM_001031814:-131	Smg1	DIVERGENT_PROMOTER	1.370	1.355	2326.44	3151.82	1.856	1389.27	2578.93
A_68_P23298636	chr4:129497126-129497170	NM_008974:-574	Ptp4a2	PROMOTER	1.370	1.419	2042.79	2899.52	1.944	1543.98	3001.92

ProbeName	Target position of probe on CpG island microarray	TargetID	GeneSymbol	CpG island Description	Ratio of relative methylation (TiO_2-NP/Vehicle)	Sham group			TiO_2-H group		
						Relative methylation (Cy5/Cy3)	Cy3 signal (Input)	Cy5 signal (MeDIP)	Relative methylation (Cy5/Cy3)	Cy3 signal (Input)	Cy5 signal (MeDIP)
A_68_P31755198	chr18:61195223-61195267	NM_009880:609	Cdx1	INSIDE	1.369	1.883	457.32	860.98	2.577	414.65	1068.64
A_68_P29249639	chr13:97908050-97908094	NM_177266:181	Gfm2	INSIDE	1.369	0.261	1774.08	462.51	0.357	1242.89	443.64
A_68_P27968102	chr11:82578176-82578220	NM_025884:352	Zfp830	INSIDE	1.369	0.220	3085.32	678.84	0.301	2148.93	647.44
A_68_P23627553	chr5:40035844-40035888	NM_010474:4	Hs3st1	INSIDE	1.369	0.398	1842.07	733.31	0.545	1285.84	700.60
A_68_P22778505	chr4:22414595-22414639	NM_008899:662	Pou3f2	INSIDE	1.369	0.270	5014.99	1356.55	0.370	3398.24	1258.09
A_68_P29671483	chr14:65695129-65695173	NM_018788:21793	Extl3	INSIDE	1.368	1.500	964.60	1446.62	2.052	787.30	1615.67
A_68_P28728198	chr12:109701817-109701861	NM_001043335:40813	Eml1	INSIDE	1.368	1.937	608.07	1178.07	2.650	551.37	1461.07
A_68_P27502790	chr10:120747317-120747361	NM_029057:907	Tbc1d30	INSIDE	1.368	0.257	5948.50	1527.90	0.351	3760.39	1321.46
A_68_P23837532	chr5:81450776-81450820	NM_198702:181	Lphn3	INSIDE	1.368	0.283	1702.24	482.25	0.387	1169.93	453.27
A_68_P21309980	chr2:69424250-69424294	NM_001081088:-148	Lrp2	PROMOTER	1.368	0.209	3217.77	673.09	0.286	2332.30	667.32
A_68_P27480106	chr10:116661162-116661206	NM_026570:379	Yeats4	INSIDE	1.367	0.235	1993.45	468.93	0.322	1463.33	470.60
A_68_P26022272	chr8:87370728-87370772	NM_007591:80	Calr	INSIDE	1.367	0.149	5417.33	806.28	0.203	3548.25	722.03
A_68_P24761383	chr6:113708921-113708965	NM_001036684:132428	Atp2b2	INSIDE	1.367	1.443	696.52	1005.08	1.973	567.45	1119.74
A_68_P32111334	chr19:40906541-40906585	NM_172839:794	Ccnj	INSIDE	1.366	0.285	1924.96	548.73	0.389	1393.97	542.89
A_68_P29645530	chr14:61253653-61253697	NM_001033272:109	Spata13	INSIDE	1.366	0.437	2159.18	943.06	0.596	1678.97	1001.44
A_68_P28084316	chr11:103128366-103128410	NM_016896:327	Map3k14	INSIDE	1.366	0.244	2551.88	621.46	0.333	1858.34	618.05
A_68_P27581769	chr11:9050712-9050764	ENSMUST00000121861:-138		PROMOTER	1.366	2.374	282.29	670.26	3.243	247.72	803.40
A_68_P22449312	chr3:116126248-116126292	NM_001080818:680	Cdc14a	INSIDE	1.366	0.272	2269.81	617.91	0.372	1692.99	629.52
A_68_P22336424	chr3:93251272-93251316	NM_001163098:5043	Tchh	INSIDE	1.366	0.303	2062.99	624.51	0.413	1687.53	697.70
A_68_P30752013	chr16:50529490-50529534	ENSMUST00000150937:898		INSIDE	1.365	0.400	1139.83	455.37	0.545	926.36	505.32
A_68_P30472484	chr15:97835084-97835128	NM_001113515:49	Col2a1	INSIDE	1.365	0.358	1476.14	528.50	0.489	1112.69	543.60
A_68_P30279146	chr15:63891463-63891507	NM_144846:526	Fam49b	INSIDE	1.365	0.299	1479.26	442.41	0.408	1195.67	488.16
A_68_P25950069	chr8:72663704-72663748	NM_001145552:115	2310045N01Rik	INSIDE	1.365	0.261	2619.04	683.94	0.356	1744.60	621.94
A_68_P29626124	chr14:57723397-57723441	NM_008125:121	Gjb2	INSIDE	1.364	0.454	1560.28	708.94	0.620	1199.08	743.39
A_68_P31152955	chr17:34402022-34402066	NM_207105:1873	H2-Ab1	INSIDE	1.363	0.298	4790.83	1427.92	0.406	3642.90	1480.01
A_68_P28736924	chr12:111427791-111427835	AK044800:2027		INSIDE	1.363	0.422	1113.82	470.36	0.576	981.50	564.99
A_68_P25499763	chr7:133435519-133435563	NM_145587:19408	Sbk1	INSIDE	1.363	1.937	1354.46	2622.97	2.640	947.53	2501.15
A_68_P22157975	chr3:55986717-55986761	NM_030595:885	Nbea	INSIDE	1.363	0.281	1819.35	512.12	0.384	1358.62	521.40
A_68_P23327636	chr4:134828309-134828353	NM_013885:345	Clic4	INSIDE	1.362	0.431	1625.17	699.73	0.586	1384.59	811.88
A_68_P22310776	chr3:87733936-87733980	NM_153562:159	Rrnad1	INSIDE	1.362	0.125	3662.01	458.81	0.171	2538.02	433.25
A_68_P31983174	chr19:17030658-17030702	NM_181348:73	Prune2	INSIDE	1.361	0.517	4041.20	2088.08	0.703	2910.48	2046.39
A_68_P29051304	chr13:54867489-54867533	NM_033610:291	Sncb	INSIDE	1.361	0.428	1884.67	805.85	0.582	1415.37	823.90
A_68_P28091681	chr11:104432890-104432934	NM_010858:20936	Myl4	INSIDE	1.361	0.199	3193.22	637.01	0.272	2460.07	668.13
A_68_P27954278	chr11:80242652-80242696	NM_178616:558	Psmd11	INSIDE	1.361	0.229	2162.85	495.37	0.312	1676.19	522.32
A_68_P22509730	chr3:127336734-127336778	NM_009718:694	Neurog2	INSIDE	1.361	5.205	1071.80	5578.23	7.083	993.35	7036.16
A_68_P31487556	chr18:9957739-9957784	NM_153552:-416	Thoc1	PROMOTER	1.360	1.667	514.93	858.36	2.266	449.85	1019.49
A_68_P30602964	A_68_P30602964			Unknown	1.360	2.585	585.29	1512.88	3.516	475.97	1673.49
A_68_P29054896	chr13:55516436-55516480	NM_029303:135	Pfn3	INSIDE	1.360	0.274	2253.89	617.13	0.372	1614.02	601.14
A_68_P28104644	chr11:106860905-106860949	NM_010655:-87	Kpna2	PROMOTER	1.360	1.775	347.18	616.38	2.415	308.63	745.32
A_68_P21835856	chr2:167329663-167329708	NM_030743:11541	Rnf114	INSIDE	1.360	0.275	1783.23	490.21	0.374	1150.76	430.14
A_68_P31117189	chr17:27702653-27702697	NM_001002895:-2	AI413582	PROMOTER	1.359	0.377	1491.64	562.08	0.512	1202.06	615.72
A_68_P26171282	chr8:114516157-114516201	NM_013925:24	Adat1	INSIDE	1.359	0.409	1322.61	541.39	0.556	994.08	553.12
A_68_P33015613	chr1_random:49705-49759	NM_026866:135606	Disp1	INSIDE	1.358	1.737	568.35	987.13	2.358	499.11	1176.91
A_68_P30879835	chr16:75593202-75593246	NM_198302:89	Rbm11	INSIDE	1.358	0.382	1173.58	448.35	0.519	928.04	481.53
A_68_P26802099	chr9:106150157-106150201	NM_020559:107	Alas1	INSIDE	1.358	0.409	1778.84	726.90	0.555	1285.60	713.36
A_68_P25357075	chr7:105804717-105804761	NM_172280:341	2210018M11Rik	INSIDE	1.358	1.632	1109.67	1811.05	2.217	855.68	1896.70
A_68_P23656661	chr5:45841349-45841393	NM_024236:98	Qdpr	INSIDE	1.358	0.329	1491.20	490.78	0.447	1121.66	501.35
A_68_P21835087	chr2:167174410-167174454	NM_019835:246	B4galt5	INSIDE	1.358	0.433	1767.67	765.32	0.588	1235.50	726.18
A_68_P30751463	chr16:50432288-50432332	NM_027444:192	Bbx	INSIDE	1.357	2.643	409.47	1082.28	3.587	306.97	1101.19
A_68_P27141570	chr10:53018974-53019019	NM_001204983:80661	Gm9766	INSIDE	1.357	1.617	472.54	764.32	2.195	409.98	900.08
A_68_P26772687	chr9:100978284-100978328	NM_001100451:-144	Msl2	PROMOTER	1.357	2.990	1149.27	3436.21	4.059	696.46	2826.67
A_68_P25048201	chr7:35989266-35989310	NM_001024707:11076	Lrp3	INSIDE	1.357	2.280	930.93	2122.37	3.093	694.60	2148.61
A_68_P24428223	chr6:48547614-48547658	NM_001079901:3755	Repin1	INSIDE	1.357	3.042	1077.72	3278.41	4.128	957.10	3950.99
A_68_P21121521	chr2:33286475-33286519	NM_001085507:348	Zbtb34	INSIDE	1.357	0.365	1222.38	446.24	0.495	998.50	494.63
A_68_P29161996	chr13:78302959-78303003	NM_001163419:455030	Fam172a	INSIDE	1.356	0.310	1424.72	441.96	0.421	1046.69	440.33
A_68_P23244925	chr4:118992833-118992877	NM_028677:274	Ppih	INSIDE	1.356	0.307	1795.96	552.14	0.417	1322.75	551.45
A_68_P23196910	chr4:108292768-108292812	NM_177045:230	Cc2d1b	INSIDE	1.356	0.338	4497.10	1518.03	0.458	3305.37	1512.71
A_68_P31661524	chr18:43597803-43597847	ENSMUST00000121805:116		INSIDE	1.355	0.423	1276.93	540.08	0.573	1070.12	613.31
A_68_P28149599	chr11:114626559-114626603	NM_001102615:-122	Kif19a	PROMOTER	1.355	0.470	2333.32	1096.51	0.637	1828.20	1164.15
A_68_P24383682	chr6:39507280-39507324	NM_172477:531	Dennd2a	INSIDE	1.355	0.438	3038.79	1330.64	0.593	2239.09	1328.67
A_68_P23417688	chr4:151654062-151654106	NM_178406:5614	Gpr153	INSIDE	1.355	1.545	1198.09	1850.71	2.094	869.88	1821.25
A_68_P23364639	chr4:141092925-141092969	NM_019763:1566	Spen	INSIDE	1.355	0.386	1174.04	453.74	0.524	907.65	475.19
A_68_P22356140	chr3:97555778-97555823	NM_178080:16137	Pde4dip	INSIDE	1.355	2.401	620.70	1490.59	3.254	554.26	1803.67
A_68_P23617865	chr5:38550138-38550182	NM_010942:546	Nsg1	INSIDE	1.354	0.129	4685.43	605.45	0.175	3091.84	541.01
A_68_P29241522	chr13:96374151-96374199	NM_010169:14214	F2r	INSIDE	1.353	2.386	775.93	1851.20	3.228	656.51	2119.16

ProbeName	Target position of probe on CpG island microarray	TargetID	GeneSymbol	CpG island Description	Ratio of relative methylation (TiO_2-NP/Vehicle)	Sham group			TiO_2-H group		
						Relative methylation (Cy5/Cy3)	Cy3 signal (Input)	Cy5 signal (MeDIP)	Relative methylation (Cy5/Cy3)	Cy3 signal (Input)	Cy5 signal (MeDIP)
A_68_P28940417	chr13:34839281-34839325	NM_138746:7792	Fam50b	INSIDE	1.353	2.415	1218.09	2941.09	3.268	936.08	3059.08
A_68_P28035934	chr11:94854133-94854177	NM_172261:1629	Ppp1r9b	INSIDE	1.353	0.471	3820.58	1798.95	0.637	2865.07	1825.38
A_68_P24591679	chr6:83092501-83092545	NM_009106:5445	Rtkn	INSIDE	1.353	0.429	2749.62	1178.83	0.580	1872.00	1086.14
A_68_P32318066	chrX:33868905-33868949	NM_028894:523	Lonrf3	INSIDE	1.352	2.763	252.26	697.10	3.736	334.76	1250.78
A_68_P27096133	chr10:42399029-42399073	NM_172416:329	Ostm1	INSIDE	1.352	1.536	1038.91	1596.16	2.078	836.30	1737.63
A_68_P26519508	chr9:53056259-53056305	NM_029936:-65	Ddx10	PROMOTER	1.352	0.184	2581.63	474.49	0.248	1671.26	415.23
A_68_P24994776	chr7:20282146-20282190	NM_009696:2347	Apoe	INSIDE	1.352	2.736	745.00	2038.43	3.700	641.29	2372.65
A_68_P24135225	chr5:139856460-139856504	NM_030258:-138	Gpr146	PROMOTER	1.352	0.356	1613.36	573.70	0.481	1163.64	559.63
A_68_P23694282	chr5:52759804-52759848	NM_011435:4784	Sod3	INSIDE	1.352	1.588	568.62	902.82	2.147	478.72	1027.65
A_68_P28593011	chr12:84827402-84827446	NM_058212:1234	Dpf3	INSIDE	1.350	0.072	8389.48	600.92	0.097	5223.75	505.09
A_68_P26232281	chr8:124805601-124805645	NM_009569:-418	Zfpm1	PROMOTER	1.350	1.502	5122.77	7695.11	2.028	3049.58	6184.94
A_68_P31930503	chr19:5731362-5731406	NM_020491:337	Sssca1	INSIDE	1.349	0.287	2087.81	599.56	0.387	1488.41	576.49
A_68_P29113347	chr13:69137713-69137757	NM_153534:685	Adcy2	INSIDE	1.349	0.285	3396.58	969.00	0.385	2665.48	1026.00
A_68_P24467400	chr6:55067624-55067668	NM_009953:15320	Crhr2	INSIDE	1.349	0.235	2646.36	620.64	0.316	2012.68	636.63
A_68_P24206536	chr6:4697665-4697715	NM_001040611:385	Peg10	INSIDE	1.349	1.684	432.60	728.64	2.272	398.12	904.53
A_68_P28513104	chr12:70259759-70259803	NM_009093:393	Rps29	INSIDE	1.348	0.391	1483.00	580.19	0.527	1090.29	574.85
A_68_P28307257	chr12:28026632-28026676	NM_009234:929	Sox11	INSIDE	1.348	2.042	1733.87	3540.26	2.752	1449.66	3990.13
A_68_P24595551	chr6:83745218-83745262	NM_001164187:89	Nagk	INSIDE	1.348	0.162	6956.80	1126.72	0.218	4385.47	957.53
A_68_P22892656	chr4:47104671-47104715	NM_172693:-132	Galnt12	PROMOTER	1.348	0.237	3387.92	801.36	0.319	2444.43	779.26
A_68_P28221292	chr12:8305005-8305049	NM_013527:3734	Gdf7	INSIDE	1.347	0.164	3003.05	492.93	0.221	2007.26	443.77
A_68_P20941835	chr1:193960556-193960600	NR_033536:-201	Gm10516	PROMOTER	1.347	0.395	1763.48	697.25	0.532	1305.32	695.05
A_68_P31927966	chr19:5307193-5307237	NM_001024717:8884	Gal3st3	INSIDE	1.346	1.642	1188.23	1951.06	2.210	866.90	1915.82
A_68_P31402644	chr17:84496087-84496131	AK043195:-103		PROMOTER	1.346	0.279	2357.91	658.03	0.376	1841.96	691.80
A_68_P31304340	chr17:66460894-66460938	NM_001037757:-68	ORF19	PROMOTER	1.346	0.458	1546.65	707.72	0.616	1215.25	748.36
A_68_P30015972	chr15:11313703-11313747	NM_033074:15689	Tars	INSIDE	1.346	1.638	1225.31	2007.66	2.205	1038.16	2289.30
A_68_P25585260	chr7:148041975-148042019	NM_018742:286	Bet1l	INSIDE	1.346	0.331	1687.30	558.80	0.446	1253.90	558.85
A_68_P24059409	chr5:124571234-124571278	NM_011256:95171	Pitpnm2	INSIDE	1.346	2.190	688.90	1508.92	2.948	514.37	1516.57
A_68_P27826626	chr11:57458476-57458520	NM_134189:-445	Galnt10	PROMOTER	1.345	0.347	1424.90	494.34	0.467	1062.72	495.82
A_68_P20553864	chr1:120734165-120734209	NM_001081125:216010	Gli2	INSIDE	1.345	1.961	930.33	1824.23	2.638	791.43	2087.67
A_68_P32240058	chrX:7339999-7340043	NM_001105196:234	Tcfe3	INSIDE	1.344	1.585	492.98	781.48	2.131	540.24	1151.06
A_68_P31207758	chr17:46728606-46728650	NM_175168:37825	Ptk7	INSIDE	1.344	1.942	1043.91	2027.37	2.610	833.43	2175.10
A_68_P30665796	chr16:34745100-34745144	ENSMUST00000155268:-173		PROMOTER	1.344	3.162	300.17	949.05	4.251	292.22	1242.09
A_68_P27261659	chr10:75837873-75837917	NM_008787:67763	Pcnt	INSIDE	1.344	2.530	215.69	545.71	3.399	205.76	699.48
A_68_P26818264	chr9:108997741-108997785	NM_172775:-187	Plxnb1	PROMOTER	1.344	1.885	556.69	1049.62	2.535	433.03	1097.60
A_68_P25083571	chr7:50930445-50930489	NM_145582:3066	Ctu1	INSIDE	1.344	2.462	800.78	1971.64	3.310	493.13	1632.23
A_68_P25022019	chr7:29244304-29244350	NM_001141921:7070	Lrfn1	INSIDE	1.344	1.488	810.56	1205.95	1.999	683.67	1366.93
A_68_P32749011	chrX:148776892-148776936	NM_029836:113	Tspyl2	INSIDE	1.343	0.303	2056.57	623.31	0.407	2711.86	1103.60
A_68_P31254139	chr17:56278984-56279028	NM_013662:-5731	Sema6b	PROMOTER	1.343	0.271	2667.27	723.68	0.364	1917.33	698.39
A_68_P30961946	chr16:91270379-91270423	NM_016968:387	Olig1	INSIDE	1.343	1.429	3045.18	4351.91	1.919	2309.55	4432.08
A_68_P29060392	chr13:56397929-56397973	NM_019568:-38	Cxcl14	PROMOTER	1.343	2.123	14903.11	31645.12	2.851	9661.94	27547.52
A_68_P26595475	chr9:66683088-66683132	NM_026674:-597	Aph1c	PROMOTER	1.343	0.196	3328.17	651.23	0.263	2468.06	648.79
A_68_P25362046	chr7:106617483-106617527	NM_028145:2991	Klhl35	INSIDE	1.343	6.042	483.64	2922.08	8.114	430.35	3491.94
A_68_P22729550	chr4:11314133-11314177	NM_194055:-224	Esrp1	PROMOTER	1.343	0.200	3701.99	740.02	0.268	2997.41	804.64
A_68_P21221950	chr2:52717844-52717888	NM_172409:965	Fmnl2	INSIDE	1.343	0.422	1709.26	721.25	0.567	1368.24	775.52
A_68_P26466977	chr9:44127873-44127917	NM_027909:471	C2cd2l	INSIDE	1.342	0.430	4350.60	1871.12	0.577	3002.60	1732.57
A_68_P24136132	chr5:140016559-140016603	NM_013702:-3271	Uncx	PROMOTER	1.342	0.328	2745.59	899.60	0.440	1988.93	874.83
A_68_P30332045	chr15:73526210-73526254	NM_173365:11703	Gpr20	INSIDE	1.341	1.534	2360.67	3621.55	2.057	1712.03	3521.81
A_68_P24828616	chr6:126689917-126689961	NM_013568:754	Kcna6	INSIDE	1.341	0.357	1553.53	554.26	0.478	1131.44	541.38
A_68_P24828013	chr6:126594315-126594359	NM_010595:1483	Kcna1	INSIDE	1.341	0.342	2061.46	704.72	0.458	1544.52	708.07
A_68_P21817648	chr2:164230100-164230144	NM_013592:-8	Matn4	PROMOTER	1.341	0.297	2244.98	666.48	0.398	1709.65	680.45
A_68_P20880195	chr1:183141802-183141846	NM_145514:285	Wdr26	INSIDE	1.341	3.743	2174.89	8140.83	5.019	1616.27	8111.24
A_68_P32217858	chr19:59268636-59268680			Unknown	1.340	0.343	2421.10	830.68	0.460	1757.01	807.54
A_68_P29484662	chr14:27852087-27852131	NM_134437:-78	Il17rd	PROMOTER	1.340	0.244	2002.55	488.15	0.327	1452.38	474.45
A_68_P27285769	chr10:80298722-80298766	NM_001013758:8040	Lingo3	INSIDE	1.340	1.983	1219.52	2418.83	2.658	901.37	2395.61
A_68_P25031430	chr7:31206709-31206753	NM_172142:-1592	Nfkbid	DIVERGENT_PROMOTER	1.340	1.931	312.53	603.45	2.588	253.29	655.58
A_68_P32698229	chrX:136813030-136813074	ENSMUST00000130153:-651		PROMOTER	1.339	0.302	2143.64	646.91	0.404	2796.66	1129.90
A_68_P32397421	chrX:53985333-53985377	NM_001077361:221	Fhl1	INSIDE	1.339	1.697	586.25	994.61	2.271	725.04	1646.46
A_68_P30346591	chr15:76008957-76009001	NM_201394:17162	Plec	INSIDE	1.339	1.849	867.28	1603.84	2.477	664.33	1645.32
A_68_P25586497	chr7:148254408-148254452	NM_177897:7258	B4galnt4	INSIDE	1.339	1.951	403.93	788.00	2.612	325.49	850.13
A_68_P23074276	chr4:85160312-85160356	ENSMUST00000147066:104		INSIDE	1.339	0.280	1627.98	456.41	0.375	1338.27	502.45
A_68_P32147505	chr19:47149484-47149530	NM_177342:7269	Taf5	INSIDE	1.338	1.946	266.87	519.42	2.604	245.24	638.65
A_68_P26164569	chr8:113262564-113262608	NM_198625:17203	Mtss1l	INSIDE	1.338	2.191	337.98	740.35	2.932	335.20	982.67
A_68_P26144744	chr8:109948750-109948794	NM_026277:166	Nob1	INSIDE	1.338	0.363	1537.35	558.82	0.486	1284.35	624.49
A_68_P24618848	chr6:87863953-87863997	NM_173737:5	8430410A17Rik	INSIDE	1.338	0.192	2328.73	447.35	0.257	1956.76	503.06
A_68_P22862463	chr4:41445595-41445641	NM_001085515:4491	AI464131	INSIDE	1.337	0.377	2265.64	853.92	0.504	1509.91	761.11

ProbeName	Target position of probe on CpG island microarray	TargetID	GeneSymbol	CpG island Description	Ratio of relative methylation (TiO_2-NP/Vehicle)	Sham group			TiO_2-H group		
						Relative methylation (Cy5/Cy3)	Cy3 signal (Input)	Cy5 signal (MeDIP)	Relative methylation (Cy5/Cy3)	Cy3 signal (Input)	Cy5 signal (MeDIP)
A_68_P22412513	chr3:108215712-108215756	NM_001004177:2678	Celsr2	INSIDE	1.337	2.188	920.60	2014.50	2.926	772.27	2259.77
A_68_P22151536	chr3:54916119-54916163	NM_001144987:53	Spg20	INSIDE	1.337	0.405	1483.94	601.12	0.541	1217.30	659.14
A_68_P21221053	chr2:52531826-52531870	NM_001037099:253	Cacnb4	INSIDE	1.337	0.390	1585.58	617.60	0.521	1156.23	602.11
A_68_P20793342	chr1:167124624-167124668	NM_145512:-82	Sft2d2	PROMOTER	1.337	0.368	1550.94	571.12	0.492	1233.21	607.23
A_68_P31126969	chr17:29401134-29401178	NM_026845:-240	Ppil1	DIVERGENT_PROMOTER	1.336	13.017	805.72	10487.86	17.387	859.18	14938.59
A_68_P30979318	chr16:94085768-94085812	NM_011377:286	Sim2	INSIDE	1.336	0.319	1531.90	489.36	0.427	1031.81	440.48
A_68_P27473557	chr10:115550809-115550853	NM_001161855:143	4933416C03Rik	INSIDE	1.336	1.800	927.69	1670.12	2.405	836.44	2011.26
A_68_P25016961	chr7:28152919-28152968	NM_001199235:26582	Spnb4	INSIDE	1.336	3.431	790.96	2713.69	4.584	603.28	2765.21
A_68_P23931758	chr5:101275769-101275813	NM_172715:543	Agpat9	INSIDE	1.336	0.348	1425.83	496.33	0.465	1240.95	577.07
A_68_P32233090	chrX:5660210-5660254	NM_001033211:306	AU022751	INSIDE	1.335	2.003	525.42	1052.68	2.675	683.86	1829.42
A_68_P31162031	chr17:36116088-36116132	NR_028516:-142	Prr3	PROMOTER	1.335	0.448	1301.30	583.35	0.599	1071.92	641.72
A_68_P24116035	chr5:135845821-135845865	NM_145414:20	Nsun5	INSIDE	1.335	15.258	2483.83	37899.03	20.366	1966.73	40055.44
A_68_P23308458	chr4:131375508-131375552	NM_001083119:18663	Ptpru	INSIDE	1.335	1.461	956.12	1396.76	1.950	781.48	1523.87
A_68_P25953597	chr8:73240870-73240914	NM_001195253:-1549	Gm3336	PROMOTER	1.334	1.558	793.84	1236.54	2.078	647.94	1346.27
A_68_P24777346	chr6:116578724-116578768	NM_026057:249	Zfp422	INSIDE	1.334	0.326	3421.32	1114.42	0.435	2220.50	964.90
A_68_P24735710	chr6:108733170-108733214	NM_026011:140	Arl8b	INSIDE	1.334	0.318	1696.19	539.68	0.425	1297.49	550.90
A_68_P23404994	chr4:149611310-149611354	NM_001025388:-100	Gm5506	PROMOTER	1.334	0.345	4227.31	1459.33	0.461	2795.38	1287.35
A_68_P29701298	chr14:70843425-70843469	NR_029802:130	Mir320	DOWNSTREAM	1.333	0.236	3625.09	854.64	0.314	2717.84	853.95
A_68_P27056145	chr10:34002444-34002488	NM_009433:471	Tspyl1	INSIDE	1.333	0.287	2342.06	672.25	0.383	1757.82	672.73
A_68_P22941963	chr4:56877865-56877909	NM_018761:197	Ctnnal1	INSIDE	1.333	0.302	3548.51	1070.53	0.402	2895.70	1164.28
A_68_P28741416	chr12:112215114-112215158	NM_175207:2095	Ankrd9	INSIDE	1.332	2.813	668.21	1879.91	3.748	597.33	2238.78
A_68_P28577472	chr12:81859091-81859135	NM_001008423:2590	Gm1568	INSIDE	1.332	2.785	490.38	1365.61	3.708	385.83	1430.79
A_68_P26986177	chr10:20032526-20032570	NM_001025392:274	Bclaf1	INSIDE	1.332	0.239	1949.06	466.44	0.319	1491.84	475.64
A_68_P25953725	chr8:73267162-73267206	NM_201607:19222	Pde4c	INSIDE	1.332	0.527	4561.73	2403.15	0.702	2906.03	2039.24
A_68_P24428222	chr6:48547522-48547566	NM_001079901:3663	Repin1	INSIDE	1.332	2.462	660.24	1625.33	3.278	568.56	1863.62
A_68_P21395955	chr2:84877762-84877806	NM_001136081:177	Ssrp1	INSIDE	1.332	0.284	1565.21	444.37	0.378	1115.44	421.76
A_68_P31621445	chr18:36177706-36177750	NM_001167891:179086	Nrg2	INSIDE	1.331	2.858	280.82	802.66	3.803	240.68	915.34
A_68_P27913320	chr11:72609515-72609559	NM_001045536:-191	Zzef1	DIVERGENT_PROMOTER	1.331	3.863	2792.85	10787.80	5.140	2299.14	11817.40
A_68_P26600191	chr9:67481194-67481238			Unknown	1.331	0.293	2009.53	589.70	0.390	1622.33	633.46
A_68_P23440735	chr4:155186739-155186783	NM_207678:163	Ccnl2	INSIDE	1.331	0.146	4911.38	719.31	0.195	3012.84	587.40
A_68_P22970883	chr4:63157206-63157250	NM_001008791:-243	Whrn	PROMOTER	1.331	1.784	542.45	967.72	2.375	382.79	909.14
A_68_P22148836	chr3:54437650-54437694	ENSMUST00000172420:2312		INSIDE	1.331	2.395	192.02	459.91	3.187	209.40	667.38
A_68_P31113145	chr17:27054067-27054111	NM_053173:53	Kifc5b	INSIDE	1.330	0.391	1584.65	620.28	0.520	1290.84	671.83
A_68_P30346546	chr15:76003525-76003570	NM_201394:22593	Plec	INSIDE	1.330	1.706	779.93	1330.52	2.268	622.25	1411.44
A_68_P25031177	chr7:31140943-31140987	NM_175478:6827	Lrfn3	INSIDE	1.330	1.893	844.89	1599.40	2.518	626.76	1578.04
A_68_P24990654	chr7:19548318-19548362	NM_001029877:37104	Nova2	INSIDE	1.330	0.242	2534.43	612.30	0.321	1859.43	597.48
A_68_P22128831	chr3:51080337-51080381	NM_023502:49551	Elf2	INSIDE	1.330	1.635	676.36	1105.78	2.175	424.48	923.07
A_68_P25827950	chr8:46108981-46109030	NM_001081286:73444	Fat1	INSIDE	1.329	2.462	292.26	719.54	3.273	268.65	879.29
A_68_P25370884	chr7:108146949-108146996	NM_008773:13533	P2ry2	INSIDE	1.329	1.481	994.20	1472.50	1.969	765.59	1507.16
A_68_P31677414	chr18:46685686-46685730	NM_173423:-83	Fem1c	PROMOTER	1.328	1.371	4409.41	6045.94	1.821	3157.20	5748.04
A_68_P30377509	chr15:81191134-81191178	NM_011399:39	Slc25a17	INSIDE	1.328	0.272	2055.42	560.01	0.362	1543.73	558.50
A_68_P26017291	chr8:86479163-86479209	NM_024184:-406	Asf1b	PROMOTER	1.328	0.348	1545.38	538.53	0.463	1170.57	541.51
A_68_P22373053	chr3:100881249-100881294	NM_011197:32818	Ptgfrn	INSIDE	1.328	2.081	646.83	1345.89	2.764	544.35	1504.62
A_68_P28614436	chr12:88488867-88488911	NM_153415:-36	Pomt2	PROMOTER	1.327	0.293	2237.64	656.16	0.389	1733.37	674.31
A_68_P23748515	chr5:64359726-64359770	NM_145923:388	Rell1	INSIDE	1.327	0.358	4289.80	1535.64	0.475	3026.35	1437.07
A_68_P23345554	chr4:137951181-137951225	NM_001081672:1171	Fam43b	INSIDE	1.327	2.178	471.86	1027.61	2.889	361.25	1043.79
A_68_P20412616	chr1:88425208-88425252	NM_008972:1920	Ptma	INSIDE	1.327	0.180	2736.62	492.31	0.239	1845.24	440.53
A_68_P30346612	chr15:76011489-76011533	NM_201394:14630	Plec	INSIDE	1.326	2.367	1003.57	2375.78	3.138	885.83	2780.04
A_68_P27534775	chr10:126676335-126676379	NM_001039000:24063	Kif5a	INSIDE	1.326	1.405	1048.96	1474.14	1.864	890.21	1659.46
A_68_P26682081	chr9:83040074-83040118	NM_026122:118	Hmgn3	INSIDE	1.326	0.347	1459.06	505.70	0.459	1092.01	501.72
A_68_P25601079	chr7:150645980-150646024	NM_001161624:953	Cdkn1c	INSIDE	1.326	2.270	1289.41	2927.00	3.010	981.14	2952.91
A_68_P21839205	chr2:167875907-167875952			Unknown	1.326	0.332	1425.80	473.15	0.440	1128.99	496.96
A_68_P25494916	chr7:132635012-132635056	NM_026330:22	Nsmce1	INSIDE	1.325	0.202	2384.34	482.12	0.268	1775.33	475.57
A_68_P24946818	chr7:3637479-3637523	NM_029934:7627	Mboat7	INSIDE	1.325	2.143	1838.63	3939.96	2.839	1372.20	3895.37
A_68_P24629445	chr6:89594448-89594492	NM_001178058:489	Txnrd3	INSIDE	1.325	0.468	2780.20	1300.59	0.620	2046.31	1268.65
A_68_P24049493	chr5:122735201-122735245	NM_177242:816	Pptc7	INSIDE	1.325	0.188	3191.84	599.94	0.249	2469.62	615.05
A_68_P24008924	chr5:115750457-115750502	NM_019682:520	Dynll1	INSIDE	1.325	0.237	2994.88	709.41	0.314	2155.30	676.67
A_68_P31940079	chr19:7557572-7557616	NM_053076:177	Rtn3	INSIDE	1.324	1.918	2396.30	4596.96	2.541	1800.96	4575.59
A_68_P31295067	A_68_P31295067			Unknown	1.324	2.011	1241.61	2497.03	2.663	920.89	2452.63
A_68_P29064232	chr13:56989171-56989218	NM_012035:7755	Trpc7	INSIDE	1.324	1.684	1176.16	1980.06	2.229	899.33	2004.92
A_68_P28684632	chr12:102137444-102137488	NM_001177674:268	Gpr68	INSIDE	1.324	0.219	2941.79	644.71	0.290	2024.31	587.44
A_68_P27258520	chr10:75226966-75227010	NM_172549:114	Cabin1	INSIDE	1.324	0.439	1476.85	649.02	0.582	1153.68	671.33
A_68_P22410841	chr3:107948966-107949010	NM_010306:44	Gnai3	INSIDE	1.324	2.564	1011.46	2593.09	3.394	855.37	2902.74
A_68_P22286829	chr3:83568459-83568506	NM_009144:-1760	Sfrp2	PROMOTER	1.324	0.379	1412.76	535.56	0.502	967.45	485.72
A_68_P21018171	chr2:13715028-13715072	NM_145838:97	St8sia6	INSIDE	1.324	0.372	3139.76	1167.35	0.492	2343.32	1153.78

ProbeName	Target position of probe on CpG island microarray	TargetID	GeneSymbol	CpG island Description	Ratio of relative methylation (TiO_2-NP/Vehicle)	Sham group			TiO_2-H group		
						Relative methylation (Cy5/Cy3)	Cy3 signal (Input)	Cy5 signal (MeDIP)	Relative methylation (Cy5/Cy3)	Cy3 signal (Input)	Cy5 signal (MeDIP)
A_68_P32572889	chrX:100643328-100643372	NR_002844:16495	Tsix	INSIDE	1.323	1.375	3968.83	5459.02	1.819	4954.81	9013.97
A_68_P27278592	chr10:79198242-79198286	NM_008226:18886	Hcn2	INSIDE	1.323	1.835	1282.77	2353.34	2.427	1018.67	2471.96
A_68_P25004348	chr7:25118652-25118696	NM_001163810:310	1700008P20Rik	INSIDE	1.323	1.467	868.40	1273.97	1.941	749.18	1453.82
A_68_P24995081	chr7:20334787-20334831	NM_008990:114	Pvrl2	INSIDE	1.323	0.423	2386.47	1009.48	0.560	2033.07	1137.80
A_68_P23316765	chr4:132833435-132833480	NM_025667:248	Tmem222	INSIDE	1.323	0.246	2946.45	725.95	0.326	2128.82	693.70
A_68_P23118557	chr4:94269595-94269639	NM_172695:322	Plaa	INSIDE	1.323	0.403	1650.84	665.73	0.533	1240.94	662.00
A_68_P20834311	chr1:174562855-174562899	NM_026725:229	Dusp23	INSIDE	1.323	0.477	1649.80	787.47	0.632	1302.32	822.57
A_68_P20005842	chr1:4487520-4487564	NM_011441:-1048	Sox17	PROMOTER	1.323	0.230	2030.68	467.46	0.305	1584.80	482.83
A_68_P32127028	chr19:43598925-43598969	NM_010324:149	Got1	INSIDE	1.322	0.395	1639.16	647.15	0.522	1345.55	702.23
A_68_P26814409	chr9:108250644-108250688	NM_011678:505	Usp4	INSIDE	1.322	0.222	3255.73	722.81	0.293	2312.82	678.61
A_68_P31438310	chr17:90855140-90855185	NM_020252:636980	Nrxn1	INSIDE	1.321	2.801	518.65	1452.54	3.699	394.51	1459.12
A_68_P27468583	chr10:114688211-114688255	NM_025706:317	Tbc1d15	INSIDE	1.321	1.842	1404.07	2586.99	2.435	1237.42	3012.92
A_68_P26363431	chr9:24578905-24578949	NM_194263:-179	Tbx20	PROMOTER	1.321	1.479	777.73	1150.18	1.954	629.45	1229.92
A_68_P23396094	chr4:148098432-148098477	ENSMUST00000139895:10643		DOWNSTREAM	1.321	0.432	2146.58	928.34	0.571	1816.39	1037.77
A_68_P21309975	chr2:69423669-69423713	NM_001081088:434	Lrp2	INSIDE	1.321	0.434	1111.06	482.20	0.573	964.66	552.96
A_68_P20945776	chr1:194660855-194660899	NM_001177794:17065	Sertad4	DOWNSTREAM	1.321	1.974	2562.42	5058.19	2.608	1975.88	5152.90
A_68_P31383394	chr17:80879061-80879105	NM_009231:711	Sos1	INSIDE	1.320	0.253	2477.76	627.85	0.335	1830.12	612.36
A_68_P30496839	chr15:102077744-102077788	NM_001042727:-983	Rarg	PROMOTER	1.320	0.402	1196.50	480.58	0.530	1028.25	545.33
A_68_P28863266	chr13:19714869-19714913	NM_016687:-153	Sfrp4	DIVERGENT_PROMOTER	1.320	0.273	2014.46	550.36	0.361	1509.88	544.40
A_68_P21005392	chr2:11212245-11212289	NM_008859:118258	Prkcq	INSIDE	1.320	1.611	827.56	1333.33	2.127	768.65	1635.20
A_68_P31929450	chr19:5560018-5560062	NM_019935:535	Ovol1	INSIDE	1.319	0.235	2281.88	536.09	0.310	1721.44	533.57
A_68_P26808318	chr9:107302722-107302766	NM_001174047:534	Cacna2d2	INSIDE	1.319	0.393	1614.10	635.05	0.519	1145.92	594.49
A_68_P22128838	chr3:51081294-51081338	NM_023502:48593	Elf2	INSIDE	1.319	2.796	8211.97	22959.71	3.686	5624.35	20733.52
A_68_P26750365	chr9:96896276-96896320	NM_145134:22476	Spsb4	INSIDE	1.318	2.172	364.38	791.50	2.863	367.54	1052.12
A_68_P25266278	chr7:87508116-87508160	NM_172903:8123	Man2a2	INSIDE	1.318	1.838	884.46	1625.25	2.423	737.55	1786.93
A_68_P24325222	chr6:29162054-29162098	NM_011829:195	Impdh1	INSIDE	1.318	0.219	2643.04	578.00	0.288	1725.02	497.21
A_68_P23904968	chr5:96590192-96590236	NM_178854:560	Cnot6l	INSIDE	1.318	1.698	651.10	1105.26	2.237	536.99	1201.17
A_68_P23277864	chr4:125724511-125724555	NM_025544:361	Mrps15	INSIDE	1.318	0.437	1692.65	738.90	0.575	1074.63	618.19
A_68_P22411476	chr3:108042892-108042936	ENSMUST00000119163:-85		PROMOTER	1.318	1.812	871.23	1578.94	2.389	727.32	1737.78
A_68_P21101779	chr2:30033906-30033950	NM_172660:51	D2Wsu81e	INSIDE	1.318	0.425	3975.88	1688.85	0.560	3095.18	1732.95
A_68_P32672207	chrX:131009751-131009795	NM_028958:1257	Taf7l	INSIDE	1.317	2.021	601.67	1215.86	2.662	771.08	2052.52
A_68_P32180646	chr19:53217182-53217226	NM_001164100:-41	Add3	PROMOTER	1.317	0.417	2307.13	961.88	0.549	1881.12	1033.21
A_68_P29604237	chr14:52626582-52626626	NM_001033805:-9935	G630016D24Rik	PROMOTER	1.317	1.595	649.79	1036.41	2.100	509.33	1069.78
A_68_P26567742	chr9:61762149-61762193	NM_018853:147	Rplp1	INSIDE	1.317	0.362	1490.42	539.29	0.477	1112.39	530.23
A_68_P26345766	chr9:20969493-20969537	NM_183408:-643	Pde4a	PROMOTER	1.317	0.281	1875.01	527.14	0.370	1544.80	571.86
A_68_P26216376	chr8:122364529-122364573	NM_028071:-71	Cotl1	PROMOTER	1.317	0.363	1731.01	628.86	0.479	1445.85	691.99
A_68_P25016757	chr7:28119959-28120009	NM_001113549:-1317	Ltbp4	PROMOTER	1.317	2.483	303.80	754.33	3.271	344.86	1128.01
A_68_P24089919	chr5:130089703-130089747	NM_011846:-364	Mmp17	PROMOTER	1.317	0.387	2930.55	1134.73	0.510	2134.34	1088.71
A_68_P23621603	chr5:38952428-38952472	NM_011715:384	Wdr1	INSIDE	1.317	0.393	1410.85	554.31	0.517	1025.95	530.72
A_68_P20644879	chr1:138157097-138157141	NM_001101516:332	Gpr25	INSIDE	1.317	1.924	326.83	628.93	2.534	299.25	758.39
A_68_P27280126	chr10:79440000-79440044	NM_001003949:7053	ORF61	INSIDE	1.316	5.447	2080.13	11330.92	7.168	1648.85	11818.67
A_68_P21842275	chr2:168396762-168396807	NM_001136073:18907	Nfatc2	INSIDE	1.316	2.364	888.73	2101.28	3.112	827.72	2575.78
A_68_P28744398	chr12:112723379-112723423	NM_001033248:634	Gm266	INSIDE	1.315	0.303	2180.78	660.56	0.398	1861.44	741.25
A_68_P26809314	chr9:107429770-107429814	NM_001174047:127582	Cacna2d2	INSIDE	1.315	1.727	5487.02	9473.39	2.270	3671.48	8332.51
A_68_P25951932	chr8:72975857-72975901	NM_001122830:24964	Klhl26	INSIDE	1.315	2.234	837.40	1870.73	2.937	655.00	1923.50
A_68_P20346376	chr1:74083629-74083673	NM_027884:87371	Tns1	INSIDE	1.315	0.475	1756.53	834.89	0.625	1589.87	993.40
A_68_P30570064	chr16:16599861-16599905	ENSMUST00000162671:189		INSIDE	1.314	0.462	1622.83	749.16	0.606	1421.85	862.26
A_68_P27286998	chr10:80494022-80494066	NM_134135:2613	Slc39a3	INSIDE	1.314	2.236	1451.44	3245.12	2.938	1239.83	3643.13
A_68_P24118628	chr5:136410394-136410438	NM_018871:95	Ywhag	INSIDE	1.314	1.917	1360.13	2607.75	2.520	1155.29	2911.35
A_68_P23393509	chr4:147661752-147661796	NM_001083342:300	Ptchd2	INSIDE	1.314	0.203	3276.87	664.41	0.266	2378.68	633.80
A_68_P22998987	chr4:68614912-68614956	NM_019967:497	Dbc1	INSIDE	1.314	0.348	4055.65	1410.99	0.457	3247.95	1484.77
A_68_P25656686	chr8:11635354-11635398	NR_030781:-340	Ankrd10	PROMOTER	1.313	1.566	1125.95	1763.77	2.056	799.96	1644.84
A_68_P24870736	chr6:135994225-135994269	NM_008171:129283	Grin2b	INSIDE	1.313	1.632	1082.07	1766.38	2.144	990.22	2122.99
A_68_P23451681	chr5:4756586-4756630	NM_021457:1608	Fzd1	INSIDE	1.313	0.524	4179.30	2191.23	0.689	3094.53	2130.87
A_68_P23310402	chr4:131700231-131700275	NM_013622:149	Oprd1	INSIDE	1.313	0.226	3644.99	824.98	0.297	2347.37	697.36
A_68_P21345229	chr2:75497988-75498032	NR_033513:664	Gm6793	INSIDE	1.313	0.215	2944.20	634.25	0.283	2232.59	631.64
A_68_P29681788	chr14:67486601-67486645	NM_009955:815	Dpysl2	INSIDE	1.312	0.127	5089.13	646.56	0.167	3366.07	561.14
A_68_P27629545	chr11:19920766-19920810	NM_033523:96344	Spred2	INSIDE	1.312	1.678	648.45	1088.06	2.201	597.89	1315.80
A_68_P27290415	chr10:81007993-81008037	NM_010301:-223	Gna11	PROMOTER	1.312	0.384	2232.54	857.04	0.503	1758.21	885.24
A_68_P23069061	chr4:84191429-84191473	NM_172870:129540	Bnc2	INSIDE	1.312	2.024	1082.47	2190.48	2.656	796.86	2116.11
A_68_P20877855	chr1:182724593-182724637	NM_008210:19120	H3f3a	DOWNSTREAM	1.312	0.424	1770.96	751.47	0.557	1384.31	770.93
A_68_P27311665	chr10:85484572-85484616	NM_153195:-79	Fbxo7	DIVERGENT_PROMOTER	1.311	2.852	177.92	507.41	3.740	154.22	576.79
A_68_P25272114	chr7:88600003-88600048	NM_007755:-463	Cpeb1	PROMOTER	1.311	1.691	715.94	1210.90	2.218	530.14	1175.95
A_68_P24025033	chr5:118619514-118619558	NM_007545:-236	Hrk	PROMOTER	1.311	0.324	2818.19	912.69	0.425	2040.04	866.03
A_68_P23320879	chr4:133628861-133628905	NM_001162970:4516	Aim1l	INSIDE	1.311	1.613	972.62	1568.77	2.114	804.80	1701.17

ProbeName	Target position of probe on CpG island microarray	TargetID	GeneSymbol	CpG island Description	Ratio of relative methylation (TiO_2-NP/Vehicle)	Sham group			TiO_2-H group		
						Relative methylation (Cy5/Cy3)	Cy3 signal (Input)	Cy5 signal (MeDIP)	Relative methylation (Cy5/Cy3)	Cy3 signal (Input)	Cy5 signal (MeDIP)
A_68_P22406466	chr3:107262060-107262104	NM_145922:-266	Kcnc4	PROMOTER	1.311	0.370	1744.14	645.42	0.485	1247.11	604.84
A_68_P30346608	chr15:76011006-76011051	NM_201394:15112	Plec	INSIDE	1.310	2.462	287.45	707.83	3.226	213.67	689.25
A_68_P28944868	chr13:35832908-35832952	NM_001123386:-340	Cdyl	PROMOTER	1.310	0.313	1498.67	469.18	0.410	1194.12	489.59
A_68_P25091867	chr7:52471545-52471589	NM_011565:444	Tead2	INSIDE	1.310	17.182	3543.32	60881.34	22.503	3355.12	75499.59
A_68_P22889660	chr4:46626863-46626907	NM_198664:36187	Tbc1d2	INSIDE	1.310	3.039	978.82	2974.40	3.980	734.67	2924.01
A_68_P31620319	chr18:35991681-35991725	NM_133687:2231	Cxxc5	INSIDE	1.309	2.293	373.89	857.49	3.002	302.32	907.50
A_68_P28573970	chr12:81233412-81233456	NR_033514:397	2310015A10Rik	INSIDE	1.309	0.387	1427.97	551.95	0.506	1018.35	515.23
A_68_P26202777	chr8:119960582-119960626	NM_028941:87535	4933407C03Rik	INSIDE	1.309	1.829	660.47	1207.96	2.394	513.95	1230.35
A_68_P24519506	chr6:66986584-66986628	NM_007836:795	Gadd45a	INSIDE	1.309	0.233	3658.79	852.37	0.305	2660.62	811.40
A_68_P31096791	chr17:24489119-24489163	NM_001039581:150	Abca3	INSIDE	1.308	0.456	1424.81	649.66	0.596	1105.14	658.96
A_68_P30113930	chr15:31296769-31296813	NM_001164441:724	Ankrd33b	INSIDE	1.308	0.166	2956.50	491.73	0.218	2128.16	463.09
A_68_P28066376	chr11:100006643-100006687	NM_008471:569	Krt19	INSIDE	1.308	0.334	1649.84	551.55	0.437	1328.21	580.71
A_68_P31257262	chr17:56749026-56749070	NM_153152:146	2410015M20Rik	INSIDE	1.307	0.213	4498.76	958.41	0.278	3077.95	856.75
A_68_P27535211	chr10:126748493-126748537	NM_001003913:328	Mars	INSIDE	1.306	0.153	4612.44	705.86	0.200	2996.95	598.76
A_68_P23080770	chr4:86257737-86257781	NM_173400:168	Haus6	INSIDE	1.306	0.297	1761.64	523.29	0.388	1403.00	544.12
A_68_P22309969	chr3:87587605-87587650	NM_001033124:11457	Ntrk1	INSIDE	1.306	2.331	303.51	707.43	3.044	259.67	790.31
A_68_P20849636	chr1:177622240-177622284	NM_021350:-3778	Chml	PROMOTER	1.306	0.215	2710.45	583.66	0.281	1960.54	551.39
A_68_P31206429	chr17:46464876-46464920	NM_145140:-2183	Abcc10	PROMOTER	1.305	0.399	1239.53	495.19	0.521	1091.95	569.18
A_68_P25091031	chr7:52332036-52332080	NM_001163684:14260	Nosip	INSIDE	1.305	1.993	1190.75	2372.84	2.601	801.35	2084.49
A_68_P21628854	chr2:130100093-130100137	NM_024193:-33	Nop56	PROMOTER	1.305	0.251	1917.24	480.28	0.327	1416.48	462.94
A_68_P31933117	chr19:6184699-6184743	NM_001165919:311	1700123I01Rik	INSIDE	1.304	2.190	262.79	575.42	2.855	210.77	601.85
A_68_P30480285	chr15:99288147-99288191	NM_001001884:11	Nckap5l	INSIDE	1.304	0.414	1572.95	651.31	0.540	1151.06	621.60
A_68_P29753353	chr14:80170990-80171034	NM_001042726:107	Pcdh8	INSIDE	1.304	0.350	1638.51	574.27	0.457	1308.35	598.05
A_68_P29083949	chr13:60278471-60278515	NM_008086:404	Gas1	INSIDE	1.304	0.293	2567.99	753.59	0.383	2111.34	807.83
A_68_P23328509	chr4:134989508-134989552	NM_022980:190	Rcan3	INSIDE	1.304	0.316	1630.66	515.86	0.412	1232.17	508.13
A_68_P21812328	chr2:163244957-163245001	NM_025699:228	3230401D17Rik	INSIDE	1.304	6.779	1919.21	13010.32	8.838	1791.47	15832.50
A_68_P21617369	chr2:127952159-127952203	NM_009754:407	Bcl2l11	INSIDE	1.304	0.324	2287.50	740.65	0.422	1719.17	725.92
A_68_P31836527	chr18:75526699-75526743	NM_001042660:-298	Smad7	PROMOTER	1.303	1.615	417.34	674.14	2.105	342.31	720.67
A_68_P30346592	chr15:76009110-76009154	NM_201394:17008	Plec	INSIDE	1.303	3.265	461.92	1508.37	4.253	430.44	1830.80
A_68_P27798797	chr11:52174094-52174138	NM_011694:-500	Vdac1	PROMOTER	1.303	0.312	1548.22	482.60	0.406	1141.90	463.94
A_68_P24995608	chr7:20443293-20443337	NM_001033419:3334	Ceacam16	INSIDE	1.303	1.472	1276.46	1879.08	1.919	1008.81	1935.46
A_68_P24816526	chr6:124661846-124661896	NM_013536:326	Emg1	INSIDE	1.303	0.386	2119.75	817.73	0.503	1490.93	749.20
A_68_P23309105	chr4:131476142-131476186	NM_001201367:-1521	Tmem200b	PROMOTER	1.303	0.401	1453.97	582.58	0.522	1045.35	545.77
A_68_P22900935	chr4:48597734-48597778	NM_021436:-308	Tmeff1	PROMOTER	1.303	0.460	1372.62	631.21	0.599	1091.80	654.22
A_68_P21839461	chr2:167924453-167924497	NM_021409:17971	Pard6b	INSIDE	1.303	2.360	1665.83	3931.70	3.076	1248.74	3840.90
A_68_P21492627	chr2:104967572-104967616	NM_144783:909	Wt1	INSIDE	1.303	0.352	1474.42	518.53	0.458	1098.29	503.24
A_68_P30003954	chr15:9000908-9000952	NM_001040395:-78	1110020G09Rik	PROMOTER	1.302	0.277	2116.37	587.00	0.361	1418.65	512.49
A_68_P29568494	chr14:45608721-45608765	NM_008964:957	Ptger2	INSIDE	1.302	0.217	2225.43	482.42	0.282	1591.99	449.46
A_68_P27567649	chr11:6174389-6174433	NM_134020:430	Tmed4	INSIDE	1.302	0.367	2396.20	880.18	0.478	1827.38	873.79
A_68_P24759222	chr6:113342962-113343006	NM_133923:494	Ttll3	INSIDE	1.302	0.203	2526.49	512.23	0.264	1697.60	448.10
A_68_P23202061	chr4:109349433-109349477	NM_007983:223	Faf1	INSIDE	1.302	0.260	1791.29	466.61	0.339	1379.64	467.79
A_68_P32691810	chrX:135448778-135448822	NM_013724:-168	Nrk	PROMOTER	1.301	0.385	3970.43	1529.09	0.501	5464.84	2738.88
A_68_P32328376	chrX:35918353-35918397	NM_001110142:10999	Cul4b	INSIDE	1.301	2.803	3487.45	9776.16	3.648	4131.05	15071.25
A_68_P31545966	chr18:21811604-21811648	NM_001081403:-757	Klhl14	PROMOTER	1.301	0.193	3321.83	642.14	0.252	2383.05	599.46
A_68_P31206979	chr17:46592282-46592326	NM_001162864:32320	Ttbk1	INSIDE	1.301	0.245	2886.81	706.84	0.319	2133.13	679.67
A_68_P31160312	chr17:35804249-35804293	NM_175137:267	Vars2	INSIDE	1.301	0.362	2203.36	797.33	0.471	1599.39	753.10
A_68_P27844725	chr11:60591042-60591086	NM_175491:38	Smcr8	INSIDE	1.301	0.332	1436.11	476.89	0.432	1131.64	488.87
A_68_P27500272	chr10:120335821-120335865	NM_177092:185	Msrb3	INSIDE	1.301	0.334	1478.22	494.38	0.435	1247.18	542.58
A_68_P25262217	chr7:86762595-86762639	NM_019799:-73	Rhcg	PROMOTER	1.301	20.862	1474.45	30759.52	27.135	1460.37	39626.33
A_68_P25004262	chr7:25101544-25101588	NM_001199321:119	Zfp94	INSIDE	1.301	0.408	1660.69	677.11	0.531	1257.67	667.33
A_68_P21769695	chr2:155970095-155970139	NM_025946:37	Romo1	INSIDE	1.301	0.433	2141.07	927.68	0.564	1660.50	936.20
A_68_P20411445	chr1:88200193-88200237	NM_145222:419	B3gnt7	INSIDE	1.301	0.431	1836.70	790.70	0.560	1517.28	849.60
A_68_P31676224	chr18:46471665-46471709	NM_001160399:-104	Ccdc112	PROMOTER	1.300	4.166	5865.44	24434.63	5.415	4336.97	23484.22
A_68_P29513185	chr14:32454394-32454438	NM_025295:174	Btd	INSIDE	1.300	0.425	1264.83	537.92	0.553	1015.41	561.19
A_68_P28872662	chr13:21494612-21494656	NM_001145778:-10	Zkscan3	PROMOTER	1.300	0.305	2628.86	800.86	0.396	2106.94	834.35
A_68_P23981860	chr5:111209639-111209683	NM_001025562:-26	Pus1	PROMOTER	1.300	0.416	1512.58	629.70	0.541	1170.10	633.24
A_68_P32150744	chr19:47653811-47653855	NM_001164639:-676	Slk	PROMOTER	1.299	1.507	1276.59	1923.71	1.957	891.75	1745.01
A_68_P29659270	chr14:63741403-63741447	NM_007798:123	Ctsb	INSIDE	1.299	0.215	2384.47	511.87	0.279	1873.90	522.54
A_68_P28608874	chr12:87585109-87585153	NM_027405:2303	1700020O03Rik	INSIDE	1.299	1.853	1418.39	2627.86	2.408	1076.17	2590.94
A_68_P27278193	chr10:79138046-79138092	NM_148934:5914	Gm16517	INSIDE	1.299	2.145	276.22	592.36	2.785	214.61	597.71
A_68_P27184368	chr10:61595655-61595699	NM_009719:-161	Neurog3	PROMOTER	1.299	0.286	2299.34	658.07	0.372	1727.61	642.15
A_68_P25948512	chr8:72410079-72410123	NM_026818:1191	Cilp2	INSIDE	1.299	0.180	2762.66	498.59	0.234	1986.05	465.45
A_68_P25447598	chr7:123478008-123478053	NM_026533:-326	Rps13	PROMOTER	1.299	0.286	2323.63	663.98	0.371	1618.98	600.78
A_68_P32119204	chr19:42203483-42203527	NM_027106:-21	Avpi1	PROMOTER	1.298	0.292	2105.64	615.59	0.379	1533.59	581.90
A_68_P30346597	chr15:76009753-76009797	NM_201394:16366	Plec	INSIDE	1.298	1.552	681.66	1057.90	2.015	538.31	1084.79

ProbeName	Target position of probe on CpG island microarray	TargetID	GeneSymbol	CpG island Description	Ratio of relative methylation (TiO_2-NP/Vehicle)	Sham group			TiO_2-H group		
						Relative methylation (Cy5/Cy3)	Cy3 signal (Input)	Cy5 signal (MeDIP)	Relative methylation (Cy5/Cy3)	Cy3 signal (Input)	Cy5 signal (MeDIP)
A_68_P28149754	chr11:114647920-114647964	NM_001102615:21240	Kif19a	INSIDE	1.298	1.886	536.88	1012.50	2.448	441.27	1080.37
A_68_P24125310	chr5:137791448-137791492	NM_001159571:134	Ephb4	INSIDE	1.298	0.206	4503.91	926.26	0.267	3246.01	866.65
A_68_P23410163	chr4:150482039-150482083	NM_001081557:753817	Camta1	INSIDE	1.298	0.280	5984.16	1675.87	0.364	3987.72	1449.67
A_68_P21797520	chr2:160698110-160698154	NM_177263:594	Zhx3	INSIDE	1.298	0.115	4670.71	537.22	0.149	3264.78	487.43
A_68_P28540453	chr12:75008595-75008639	NM_010431:-237	Hif1a	PROMOTER	1.297	0.252	1858.57	469.16	0.327	1482.34	485.40
A_68_P26891188	chr9:122656242-122656286	NM_001199736:-199	Gm9524	PROMOTER	1.297	3.515	643.80	2262.74	4.558	549.93	2506.31
A_68_P25989170	chr8:81033243-81033287	NM_029736:31	Slc10a7	INSIDE	1.297	0.403	1833.99	739.27	0.523	1317.44	688.69
A_68_P20474867	chr1:99991841-99991885	NM_013626:347	Pam	INSIDE	1.297	0.399	1708.89	681.03	0.517	1349.11	697.31
A_68_P30290896	chr15:66117380-66117424	NM_152923:384	Kcnq3	INSIDE	1.296	0.185	2678.24	496.25	0.240	2094.95	503.12
A_68_P28787943	chr13:3358419-3358463			Unknown	1.296	0.217	2208.39	479.62	0.281	1732.17	487.49
A_68_P25354129	chr7:105293827-105293871	NM_011010:109	Omp	INSIDE	1.296	1.752	463.76	812.44	2.271	464.29	1054.49
A_68_P25091594	chr7:52430274-52430318	NM_182993:11006	Slc17a7	INSIDE	1.296	0.444	1265.00	562.19	0.576	999.07	575.29
A_68_P25089829	chr7:52125162-52125206	NM_133949:-26	Ptov1	PROMOTER	1.296	0.444	1731.79	768.80	0.575	1240.77	713.84
A_68_P25083584	chr7:50932175-50932219	NM_145582:4796	Ctu1	INSIDE	1.296	2.713	616.90	1673.93	3.515	514.08	1807.25
A_68_P23600261	chr5:35740010-35740054	NM_010445:8267	Hmx1	DOWNSTREAM	1.296	0.106	8048.35	853.31	0.137	5498.87	755.37
A_68_P22341989	chr3:94846418-94846462	NM_008951:27	Psmd4	INSIDE	1.296	0.297	1720.14	510.94	0.385	1334.25	513.60
A_68_P21906905	chr2:180628389-180628433	NR_029538:-334	Mir124a-3	PROMOTER	1.296	0.424	1328.05	562.43	0.549	1090.91	598.78
A_68_P20454938	chr1:95239228-95239272	NM_080850:115	Pask	INSIDE	1.296	0.224	2775.84	622.58	0.291	2143.12	622.95
A_68_P30602965	chr16:23431568-23431612	NM_001004151:2385	Rtp1	INSIDE	1.295	1.754	1303.27	2285.33	2.271	1098.51	2494.21
A_68_P25949497	chr8:72567016-72567060	NM_027481:327	Sugp1	INSIDE	1.295	0.147	3436.06	505.09	0.190	2178.13	414.63
A_68_P21874751	chr2:174154053-174154097	NM_001077510:-1515	Gnas	PROMOTER	1.295	1.894	786.73	1490.41	2.453	633.22	1553.30
A_68_P32126461	chr19:43515670-43515714	NM_031396:767	Cnnm1	INSIDE	1.294	3.081	159.00	489.96	3.987	151.54	604.27
A_68_P29075347	chr13:58907978-58908022	NM_001025074:-1193	Ntrk2	PROMOTER	1.294	0.142	6505.53	924.79	0.184	3925.32	722.21
A_68_P27883391	chr11:67269235-67269279	ENSMUST00000136796:31		INSIDE	1.294	0.282	1652.68	466.58	0.365	1237.65	452.04
A_68_P26567746	chr9:61762612-61762656	NM_018853:-317	Rplp1	PROMOTER	1.294	0.187	2345.30	437.76	0.242	1718.32	415.04
A_68_P26346146	chr9:21049162-21049206	NM_053190:3703	S1pr5	INSIDE	1.294	0.228	2162.55	492.89	0.295	1718.82	506.84
A_68_P23896971	chr5:93371578-93371622	NM_001077596:44577	Shroom3	INSIDE	1.294	3.305	646.80	2137.83	4.276	551.87	2359.53
A_68_P21419732	chr2:90780813-90780857	NM_017368:220	Celf1	INSIDE	1.294	0.551	3437.79	1895.04	0.713	2484.04	1771.98
A_68_P30671369	chr16:35651290-35651334	NM_013661:109865	Sema5b	INSIDE	1.293	2.247	454.10	1020.41	2.905	421.02	1223.21
A_68_P28724511	chr12:109153539-109153583	NM_001079883:88064	Bcl11b	INSIDE	1.293	1.544	1788.38	2761.93	1.997	1461.38	2918.96
A_68_P26932993	chr10:9620586-9620630	NM_001081344:230	Stxbp5	INSIDE	1.293	14.404	1367.86	19703.19	18.628	1239.31	23086.17
A_68_P26022317	chr8:87381112-87381157	NM_025648:250	Farsa	INSIDE	1.293	3.088	313.32	967.43	3.991	278.72	1112.50
A_68_P20454941	chr1:95239500-95239544	NM_080850:-157	Pask	DIVERGENT_PROMOTER	1.293	0.174	3182.46	553.28	0.225	2288.19	514.24
A_68_P29292644	chr13:105607137-105607182	NM_026072:-126	Cwc27	DIVERGENT_PROMOTER	1.292	0.405	1573.21	637.03	0.523	1288.44	674.22
A_68_P23270865	chr4:124562969-124563014	NM_177671:3963	Epha10	INSIDE	1.292	2.230	455.89	1016.47	2.881	384.22	1106.80
A_68_P21483987	chr2:103406555-103406599	NM_178890:110	Abtb2	INSIDE	1.292	1.993	319.01	635.67	2.575	240.66	619.63
A_68_P29715884	chr14:73542895-73542939	NR_033185:325	Rcbtb2	INSIDE	1.291	1.728	593.02	1024.51	2.230	591.52	1319.26
A_68_P28778765	chr12:119540713-119540757	NM_001166385:-821	Sp4	PROMOTER	1.291	0.131	4105.49	538.03	0.169	3065.02	518.76
A_68_P26543677	chr9:57612772-57612816	NM_007713:873	Clk3	INSIDE	1.291	0.364	2904.78	1057.75	0.470	2183.38	1026.68
A_68_P23609675	chr5:37187966-37188010	NM_026242:5	Mrfap1	INSIDE	1.291	0.255	2037.43	519.66	0.329	1425.34	469.37
A_68_P22412463	chr3:108209838-108209882	NM_001004177:8552	Celsr2	INSIDE	1.291	2.894	2559.35	7405.74	3.736	1771.85	6619.86
A_68_P31777744	chr18:65047479-65047523	NM_001114386:91	Nedd4l	INSIDE	1.290	5.680	941.20	5346.41	7.328	858.36	6289.79
A_68_P31201713	chr17:45683888-45683932	NM_001013749:2716	Tmem151b	INSIDE	1.290	1.692	599.04	1013.44	2.183	501.46	1094.45
A_68_P30575874	chr16:17815530-17815575	NM_153790:18178	Scarf2	DOWNSTREAM	1.290	2.682	1484.94	3981.96	3.458	1083.32	3746.05
A_68_P27940743	chr11:77796384-77796428	NM_174852:89	Phf12	INSIDE	1.290	0.208	4551.59	945.02	0.268	2944.50	788.72
A_68_P26744826	chr9:95855075-95855119	NM_011916:-82	Xrn1	PROMOTER	1.290	0.524	2662.76	1394.68	0.676	1943.91	1313.87
A_68_P26018643	chr8:86708025-86708069	NR_029821:-5186	Mir181c	PROMOTER	1.290	0.513	3349.34	1718.75	0.662	2464.63	1631.42
A_68_P21106329	chr2:30838095-30838139	NM_028846:318	Usp20	INSIDE	1.290	0.474	1907.64	904.50	0.612	1474.45	901.77
A_68_P20354314	chr1:75472567-75472611	NM_001001565:3458	Chpf	INSIDE	1.290	1.961	847.44	1661.95	2.529	666.32	1685.46
A_68_P26949658	chr10:12912982-12913026	NM_181470:-61	Ltv1	PROMOTER	1.289	0.415	1251.61	519.47	0.535	983.51	526.17
A_68_P24643638	chr6:92133742-92133786	NM_025578:253	Mrps25	INSIDE	1.289	0.280	2002.52	560.41	0.361	1564.11	564.12
A_68_P24589802	chr6:82723996-82724040	NM_013820:430	Hk2	INSIDE	1.289	0.304	1784.03	542.69	0.392	1450.22	568.76
A_68_P24596104	chr6:83864539-83864583	NM_001166371:214	Zfml	INSIDE	1.288	0.432	2319.11	1002.13	0.557	1802.13	1002.90
A_68_P24172965	chr5:148116273-148116317	NM_007673:2531	Cdx2	INSIDE	1.288	0.239	2213.21	528.76	0.308	1629.21	501.25
A_68_P24089368	chr5:130007907-130007951	NM_172276:823	Sfswap	INSIDE	1.288	0.435	1786.15	776.10	0.560	1305.79	731.00
A_68_P26896524	chr9:123626729-123626773	NM_033322:-75	Lztfl1	PROMOTER	1.287	0.125	4366.97	547.89	0.162	3007.71	485.76
A_68_P25232272	chr7:81159033-81159078	NM_009181:-487	St8sia2	PROMOTER	1.287	0.291	3581.87	1044.01	0.375	2712.34	1017.60
A_68_P24444428	chr6:51419499-51419543	NM_016806:373	Hnrnpa2b1	INSIDE	1.287	0.475	1424.05	677.05	0.612	1110.65	679.55
A_68_P24428219	chr6:48547191-48547235	NM_001079901:3331	Repin1	INSIDE	1.287	1.870	1352.94	2529.51	2.405	983.32	2365.22
A_68_P23430519	chr4:153674176-153674220	NM_001112744:-194	Arhgef16	PROMOTER	1.287	0.188	3389.84	636.21	0.242	2489.16	601.41
A_68_P20786426	chr1:165924837-165924881	NM_201364:54	BC055324	INSIDE	1.287	0.469	1721.65	807.48	0.604	1362.76	822.82
A_68_P20029520	chr1:10983953-10983997	NM_029525:429	Prex2	INSIDE	1.287	0.314	1592.01	499.55	0.404	1229.65	496.47
A_68_P31093845	chr17:23957117-23957161	NM_175229:16985	Srrm2	INSIDE	1.286	1.860	451.32	839.32	2.392	346.38	828.63
A_68_P30671880	chr16:35735579-35735623	NM_153550:33842	Dirc2	INSIDE	1.286	1.726	1610.02	2778.64	2.219	1261.43	2798.70
A_68_P30339720	chr15:74630372-74630416	NM_029627:4	Ly6k	INSIDE	1.286	2.020	1108.73	2239.87	2.598	742.65	1929.31

ProbeName	Target position of probe on CpG island microarray	TargetID	GeneSymbol	CpG island Description	Ratio of relative methylation (TiO_2-NP/Vehicle)	Sham group			TiO_2-H group		
						Relative methylation (Cy5/Cy3)	Cy3 signal (Input)	Cy5 signal (MeDIP)	Relative methylation (Cy5/Cy3)	Cy3 signal (Input)	Cy5 signal (MeDIP)
A_68_P30015973	chr15:11313872-11313916	NM_033074:15519	Tars	INSIDE	1.286	1.941	351.06	681.40	2.496	286.84	715.96
A_68_P28071861	chr11:100948728-100948772	NM_001159384:147	Mlx	INSIDE	1.286	0.393	2630.50	1033.17	0.505	2104.17	1063.07
A_68_P22753437	chr4:16090490-16090534	NM_138952:133	Ripk2	INSIDE	1.286	0.322	3967.53	1279.45	0.415	2996.42	1242.91
A_68_P20409777	chr1:87914871-87914915	NM_027029:-3624	Spata3	PROMOTER	1.286	0.357	2414.22	860.68	0.459	1752.72	803.78
A_68_P31110189	chr17:26568886-26568930	NM_001081656:16999	Neurl1b	INSIDE	1.285	1.833	2342.37	4294.55	2.356	1846.26	4350.00
A_68_P28003593	chr11:88922175-88922219	NM_019505:-134	Dgke	PROMOTER	1.285	0.150	4029.39	606.34	0.193	2730.09	527.72
A_68_P27086292	chr10:40604319-40604365	NM_031877:1003	Wasf1	INSIDE	1.285	3.335	253.64	845.83	4.286	180.46	773.54
A_68_P26554291	chr9:59451119-59451163	NM_175235:24997	Celf6	INSIDE	1.285	1.841	737.69	1357.90	2.366	634.13	1500.21
A_68_P25597927	chr7:150153952-150153996	NM_008554:1195	Ascl2	INSIDE	1.285	0.411	3483.77	1431.55	0.528	2633.06	1390.80
A_68_P25581342	chr7:147130697-147130741	NM_009482:964	Utf1	INSIDE	1.285	0.171	2838.49	483.98	0.219	1797.28	393.92
A_68_P24385305	chr6:39822036-39822080			Unknown	1.285	0.357	2133.78	761.15	0.458	1493.78	684.69
A_68_P23363215	chr4:140857342-140857386	NM_010139:210	Epha2	INSIDE	1.285	0.438	2947.70	1289.85	0.562	2188.14	1229.93
A_68_P32131187	chr19:44322861-44322905	NM_024450:45105	Scd3	DOWNSTREAM	1.284	0.326	1587.01	517.06	0.418	1310.55	548.28
A_68_P30588527	chr16:20620400-20620445	NM_028420:929	Camk2n2	INSIDE	1.284	0.125	4137.73	519.10	0.161	2515.72	405.37
A_68_P27643050	chr11:22759249-22759293	NM_016888:465	B3gnt2	INSIDE	1.284	0.290	4004.32	1159.74	0.372	2650.74	985.84
A_68_P26020945	chr8:87180658-87180702	NM_010499:6071	Ier2	DOWNSTREAM	1.284	0.481	1382.24	664.51	0.617	1112.70	686.72
A_68_P23637424	chr5:42155415-42155459	NM_007524:23	Nkx3-2	INSIDE	1.284	0.308	5211.60	1607.03	0.396	3773.54	1494.42
A_68_P27954543	chr11:80291234-80291279	NM_009871:709	Cdk5r1	INSIDE	1.283	0.061	7386.67	454.19	0.079	4446.67	350.74
A_68_P25458767	chr7:125676796-125676840	NM_009940:9	Coq7	INSIDE	1.283	0.319	1982.61	632.28	0.409	1452.91	594.26
A_68_P25394534	chr7:112959304-112959348	NM_025301:275	Mrpl17	INSIDE	1.283	0.406	1297.87	527.10	0.521	990.48	515.91
A_68_P25034847	chr7:31852339-31852383	NM_144556:7407	Lgi4	INSIDE	1.283	1.565	808.51	1265.61	2.009	681.35	1368.60
A_68_P23981083	chr5:111082064-111082108	NM_153570:315	Noc4l	INSIDE	1.283	0.420	1503.50	630.88	0.538	1167.25	628.36
A_68_P21870545	chr2:173412385-173412429	NR_027957:72634	Ppp4r1l-ps	INSIDE	1.283	2.392	671.43	1605.84	3.068	540.42	1657.78
A_68_P32957266	chr17:8477443-8477487	NM_018819:787	Brp44l	INSIDE	1.282	0.261	2203.74	576.14	0.335	1705.46	571.56
A_68_P31258174	chr17:56896255-56896299	NM_008738:677	Nrtn	INSIDE	1.282	7.823	1462.06	11438.42	10.029	1451.35	14555.22
A_68_P30896823	chr16:78577179-78577223	NM_025967:-287	D16Ertd472e	PROMOTER	1.282	0.425	1328.10	564.02	0.544	1128.44	614.17
A_68_P28696867	chr12:104563817-104563863	NM_023049:30371	Asb2	INSIDE	1.282	6.426	493.34	3170.14	8.241	471.98	3889.61
A_68_P26463562	chr9:43551825-43551869	NM_021424:-812	Pvrl1	PROMOTER	1.282	1.704	581.32	990.63	2.185	423.46	925.26
A_68_P25957635	chr8:74108819-74108863	NM_011977:16015	Slc27a1	INSIDE	1.282	2.414	808.79	1952.03	3.094	690.38	2135.92
A_68_P25351148	chr7:104727864-104727908	NM_001177945:121	1810020D17Rik	INSIDE	1.282	0.240	1993.20	478.36	0.308	1385.99	426.46
A_68_P24156971	chr5:144288853-144288897	NM_009007:-13	Rac1	PROMOTER	1.282	0.455	2436.32	1109.03	0.584	1783.19	1040.78
A_68_P24141307	chr5:140894611-140894655	NM_133916:-626	Eif3b	PROMOTER	1.282	0.264	1870.73	493.54	0.338	1405.37	475.17
A_68_P31790836	chr18:67405160-67405204	NM_172630:302	Mppe1	INSIDE	1.281	0.431	1295.46	557.75	0.551	1082.04	596.58
A_68_P27787485	chr11:50048388-50048432	NM_145926:9574	Mgat4b	INSIDE	1.281	1.604	749.61	1202.25	2.055	632.99	1300.93
A_68_P26844412	chr9:114299556-114299600	NM_019922:252	Crtap	INSIDE	1.281	3.082	371.68	1145.63	3.950	305.72	1207.52
A_68_P21866930	chr2:172805522-172805566	NM_001083959:202	Spo11	INSIDE	1.281	2.460	632.08	1554.93	3.151	594.37	1872.98
A_68_P21776626	chr2:157193205-157193256	NM_026968:-99	Manbal	PROMOTER	1.281	2.217	237.41	526.31	2.840	206.99	587.83
A_68_P28607920	chr12:87420588-87420632	NM_009368:-619	Tgfb3	DIVERGENT_PROMOTER	1.280	0.450	2154.58	968.91	0.575	1626.10	935.74
A_68_P22871359	chr4:43395245-43395289	NM_001037709:413	Rusc2	INSIDE	1.280	0.284	1891.64	538.15	0.364	1339.95	488.11
A_68_P20137271	chr1:34234808-34234852	NM_133833:166161	Dst	INSIDE	1.280	3.833	549.21	2105.12	4.907	417.96	2050.91
A_68_P31933382	chr19:6241388-6241441	NM_194348:-253	Atg2a	DIVERGENT_PROMOTER	1.279	0.301	1821.44	549.14	0.386	1340.19	516.87
A_68_P31849792	chr18:78033867-78033918	NM_013831:604	Pstpip2	INSIDE	1.279	0.197	2305.12	455.22	0.253	1672.55	422.35
A_68_P27673279	chr11:29072650-29072694	NM_134034:-234	Smek2	PROMOTER	1.279	0.341	1800.92	613.39	0.436	1230.73	536.29
A_68_P27172913	chr10:59648822-59648866	NM_016803:33164	Chst3	INSIDE	1.279	1.934	340.50	658.62	2.474	308.66	763.60
A_68_P31791018	chr18:67449050-67449094	NM_053261:196	Impa2	INSIDE	1.278	0.219	4323.62	948.96	0.280	3072.70	861.75
A_68_P31092364	chr17:23695056-23695100	NM_001033496:6115	Zfp213	INSIDE	1.278	1.510	792.94	1197.09	1.929	669.21	1291.17
A_68_P30350125	chr15:76540600-76540644	NM_058214:367	Recql4	INSIDE	1.278	0.457	1728.37	790.09	0.584	1718.40	1004.22
A_68_P28988894	chr13:43295868-43295912	NM_001033399:103651	Gfod1	INSIDE	1.278	1.764	658.97	1162.68	2.256	553.59	1248.74
A_68_P26543014	chr9:57493074-57493118	NM_007783:-109	Csk	PROMOTER	1.278	0.376	1434.76	539.82	0.481	1187.15	570.99
A_68_P23695550	chr5:52957024-52957068	NM_144945:473	Lgi2	INSIDE	1.278	1.596	495.76	791.18	2.040	414.40	845.19
A_68_P22485525	chr3:122687267-122687311	NM_133857:77	Usp53	INSIDE	1.278	0.453	2830.01	1283.15	0.580	1917.23	1111.19
A_68_P33015611	chr1_random:49523-49567	NM_026866:135794	Disp1	INSIDE	1.277	1.732	514.63	891.22	2.211	397.01	877.97
A_68_P31541309	chr18:20904102-20904146	NM_019737:781	B4galt6	INSIDE	1.277	0.164	2801.93	459.79	0.210	2045.35	428.73
A_68_P31254017	chr17:56263391-56263435	NM_013662:9863	Sema6b	INSIDE	1.277	1.542	698.14	1076.52	1.970	579.78	1141.95
A_68_P28291796	chr12:25516059-25516103	NM_001083341:-383	Mboat2	PROMOTER	1.277	2.399	726.32	1742.45	3.063	610.12	1868.69
A_68_P27910560	chr11:72080885-72080933	NM_001004148:-802	Slc13a5	PROMOTER	1.277	0.299	1628.71	486.66	0.382	1109.78	423.42
A_68_P26519506	chr9:53055980-53056024	NM_029936:215	Ddx10	INSIDE	1.277	0.452	1361.38	615.41	0.577	1064.27	614.19
A_68_P25033085	chr7:31514372-31514419	NM_027215:158	Tmem147	INSIDE	1.277	2.214	633.90	1403.21	2.827	590.43	1669.40
A_68_P24863574	chr6:134742286-134742330	NM_001048054:338	Dusp16	INSIDE	1.277	1.879	749.47	1408.59	2.401	618.43	1484.59
A_68_P23297927	chr4:129349792-129349836	NM_008468:197	Kpna6	INSIDE	1.277	0.318	2263.69	719.89	0.406	1664.09	675.86
A_68_P26761639	chr9:98886751-98886795	NM_001122851:-19	Faim	PROMOTER	1.276	0.231	3458.83	797.74	0.294	2734.99	805.03
A_68_P24084082	chr5:129107740-129107784	NM_175284:782	Fzd10	INSIDE	1.276	0.188	2724.39	512.14	0.240	2004.73	480.80
A_68_P21070952	chr2:25118487-25118531	NM_175286:391	Tprn	INSIDE	1.276	0.449	2631.15	1182.13	0.573	1863.52	1068.34
A_68_P20826000	chr1:172995096-172995140	NM_010188:-5584	Fcgr3	PROMOTER	1.276	1.546	17985.76	27810.48	1.972	13452.32	26533.76
A_68_P31929125	chr19:5503714-5503758	NM_023781:51	1700020D05Rik	INSIDE	1.275	1.634	795.13	1299.02	2.083	617.86	1287.26

ProbeName	Target position of probe on CpG island microarray	TargetID	GeneSymbol	CpG island Description	Ratio of relative methylation (TiO_2-NP/Vehicle)	Sham group			TiO_2-H group		
						Relative methylation (Cy5/Cy3)	Cy3 signal (Input)	Cy5 signal (MeDIP)	Relative methylation (Cy5/Cy3)	Cy3 signal (Input)	Cy5 signal (MeDIP)
A_68_P31075037	chr17:17964256-17964300	NR_029536:-2873	Mir99b	PROMOTER	1.275	0.308	2779.43	857.07	0.393	2166.23	851.61
A_68_P30657580	chr16:33381562-33381606	NM_011749:724	Zfp148	INSIDE	1.275	0.384	4590.60	1762.98	0.490	3259.30	1596.15
A_68_P26347005	chr9:21216579-21216623	NM_021888:320	Qtrt1	INSIDE	1.275	0.465	1751.96	814.52	0.593	1327.31	786.69
A_68_P25022015	chr7:29243791-29243835	NM_001141921:6556	Lrfn1	INSIDE	1.275	1.441	954.11	1374.77	1.838	788.00	1448.07
A_68_P25018411	chr7:28443445-28443489	NM_001081292:16151	Map3k10	INSIDE	1.275	2.548	3688.85	9399.74	3.248	2862.10	9296.56
A_68_P23240688	chr4:118159059-118159103	NM_011587:3374	Tie1	INSIDE	1.275	1.656	1322.60	2189.96	2.112	1023.93	2162.20
A_68_P21842274	chr2:168396695-168396739	NM_001136073:18975	Nfatc2	INSIDE	1.275	2.976	520.82	1549.77	3.793	507.12	1923.28
A_68_P30370807	chr15:80041905-80041949	NM_010795:37776	Mgat3	INSIDE	1.274	1.606	1226.96	1971.10	2.046	930.84	1904.76
A_68_P29054504	chr13:55452758-55452802	NM_025828:11364	Lman2	INSIDE	1.274	1.606	754.51	1211.43	2.046	626.17	1281.32
A_68_P23968432	chr5:108153037-108153081	NM_010278:305	Gfi1	INSIDE	1.274	0.258	2832.74	731.96	0.329	2163.89	712.33
A_68_P23185132	chr4:106233810-106233854	NM_053272:190	Dhcr24	INSIDE	1.274	0.293	2432.47	712.60	0.373	1584.16	591.14
A_68_P31620976	chr18:36093025-36093069			Unknown	1.273	0.167	5962.18	996.05	0.213	4266.32	907.07
A_68_P30377738	chr15:81230455-81230499	NM_177310:-141	Xpnpep3	DIVERGENT_PROMOTER	1.273	0.281	3522.27	990.07	0.358	2530.65	905.55
A_68_P27267071	chr10:76728631-76728675	NM_030262:6608	Pofut2	INSIDE	1.273	1.685	853.54	1438.16	2.145	751.24	1611.12
A_68_P24176178	chr5:148674084-148674130	NM_025624:1903	Pomp	INSIDE	1.273	1.647	656.54	1081.44	2.096	444.26	931.25
A_68_P21070912	chr2:25110973-25111017	NM_177344:36	Tmem203	INSIDE	1.273	0.230	2138.54	492.65	0.293	1657.71	486.30
A_68_P20411732	chr1:88256159-88256203	NR_015507:32	C130036L24Rik	INSIDE	1.273	0.223	3192.90	711.14	0.283	2197.17	622.76
A_68_P29614297	chr14:55400013-55400059	NM_016972:687	Slc7a8	INSIDE	1.272	0.498	1677.79	835.64	0.633	1200.52	760.33
A_68_P26834222	chr9:112137879-112137923	NM_001177619:-555	Arpp21	PROMOTER	1.272	0.299	1718.53	513.99	0.380	1399.79	532.48
A_68_P23308590	chr4:131393615-131393659	NM_001083119:557	Ptpru	INSIDE	1.272	0.229	1977.61	452.84	0.291	1475.58	429.66
A_68_P22394202	chr3:104764921-104764965	NM_009520:-315	Wnt2b	PROMOTER	1.272	0.299	1664.44	498.31	0.381	1264.57	481.60
A_68_P22291219	chr3:84284726-84284770	NM_001033301:-387	Fhdc1	PROMOTER	1.272	0.333	2056.45	685.42	0.424	1599.67	678.00
A_68_P28745895	chr12:112997453-112997497	NM_008450:415	Klc1	INSIDE	1.271	0.251	5195.71	1304.49	0.319	3738.17	1193.14
A_68_P25736230	chr8:28153302-28153346	NM_001039077:298	Prosc	INSIDE	1.271	0.164	9119.77	1494.09	0.208	6244.35	1300.47
A_68_P25087543	chr7:51716772-51716816	NM_198250:18938	Lrrc4b	INSIDE	1.271	1.907	1340.41	2556.81	2.424	1156.96	2804.00
A_68_P23952344	chr5:104888893-104888937	NM_008861:439	Pkd2	INSIDE	1.271	1.604	607.89	974.87	2.038	498.79	1016.41
A_68_P31254442	chr17:56323432-56323476	NM_080837:-111	D17Wsu104e	PROMOTER	1.270	0.167	2904.84	484.32	0.212	2151.05	455.50
A_68_P26372146	chr9:26541704-26541748	ENSMUST00000067362:291		INSIDE	1.270	0.415	3385.70	1405.32	0.527	2388.41	1258.99
A_68_P25094514	chr7:52949352-52949396	NM_001033356:9591	Ntn5	INSIDE	1.270	2.907	819.72	2382.83	3.691	589.76	2176.90
A_68_P23197627	chr4:108453252-108453296	ENSMUST00000106658:-166		PROMOTER	1.270	1.911	885.84	1693.23	2.427	736.90	1788.82
A_68_P31256421	chr17:56613568-56613612	NM_011218:2313	Ptprs	INSIDE	1.269	0.304	2060.58	626.04	0.386	1476.99	569.50
A_68_P29480509	chr14:26746634-26746678	NM_025311:80	D14Ertd449e	INSIDE	1.269	0.149	4005.12	595.77	0.189	2871.46	541.93
A_68_P26240286	chr8:126038854-126038898	NM_001170976:479	Dbndd1	INSIDE	1.269	0.448	1620.05	726.32	0.569	1214.65	690.94
A_68_P26065130	chr8:94880881-94880925	NM_018826:-792	Irx5	DIVERGENT_PROMOTER	1.269	0.139	4720.07	654.40	0.176	3087.10	543.25
A_68_P23340286	chr4:137074294-137074338	NM_008305:49599	Hspg2	INSIDE	1.269	1.833	779.09	1427.74	2.325	639.69	1487.53
A_68_P32140500	chr19:45857855-45857899	NM_023799:-95	Mgea5	PROMOTER	1.268	9.578	863.69	8272.36	12.146	648.07	7871.67
A_68_P26559434	chr9:60369978-60370022	ENSMUST00000140824:-147		PROMOTER	1.268	2.501	373.98	935.18	3.171	331.95	1052.62
A_68_P26169910	chr8:114255749-114255793	NM_001198839:266	Bcar1	INSIDE	1.268	1.805	987.26	1781.87	2.288	830.61	1900.60
A_68_P32543773	chrX:93291458-93291502	NM_010833:97	Msn	INSIDE	1.267	0.292	1655.31	484.14	0.371	2463.02	913.07
A_68_P29683699	chr14:67854572-67854616	NM_010095:2466	Ebf2	INSIDE	1.267	0.408	2269.28	926.45	0.517	1642.47	849.66
A_68_P28301679	chr12:27099772-27099816	NM_001081977:327	Rnf144a	INSIDE	1.267	0.350	3556.01	1245.88	0.444	2575.57	1143.47
A_68_P27243858	chr10:72562474-72562518	ENSMUST00000053544:295		INSIDE	1.267	1.471	1057.77	1556.20	1.864	808.17	1506.55
A_68_P27181839	chr10:61158255-61158299	NM_027912:15	Tysnd1	INSIDE	1.267	0.294	1721.17	505.32	0.372	1461.03	543.66
A_68_P24248438	chr6:13628084-13628128	NM_175312:-140	B630005N14Rik	PROMOTER	1.267	0.341	3320.98	1132.48	0.432	2545.64	1099.81
A_68_P23335296	chr4:136249920-136249964	NM_010142:141908	Ephb2	INSIDE	1.267	1.676	821.11	1376.27	2.123	654.20	1388.74
A_68_P23185611	chr4:106311721-106311765	NM_177667:16689	Ttc22	INSIDE	1.267	2.008	820.24	1647.08	2.543	625.22	1590.12
A_68_P21752415	chr2:152840795-152840839	NM_178939:303	Pdrg1	INSIDE	1.267	0.217	2239.70	486.90	0.275	1553.48	427.89
A_68_P32104298	chr19:39005890-39005934	NM_008234:433	Hells	INSIDE	1.266	0.349	1258.99	439.77	0.442	944.15	417.59
A_68_P30345339	chr15:75824110-75824154	NM_177922:-66	Mapk15	PROMOTER	1.266	1.703	1001.21	1705.03	2.156	730.35	1574.79
A_68_P28091895	chr11:104469498-104469542	NM_016780:207	Itgb3	INSIDE	1.266	0.119	4531.19	537.04	0.150	3158.81	473.97
A_68_P26203092	chr8:120022171-120022215	NM_172285:2	Plcg2	INSIDE	1.266	0.402	1291.65	519.73	0.509	957.00	487.42
A_68_P23339578	chr4:136944088-136944132	ENSMUST00000078305:-7097		PROMOTER	1.266	0.326	1635.56	532.88	0.413	1271.58	524.53
A_68_P31092624	chr17:23747448-23747492	NM_001033425:9648	Zscan10	INSIDE	1.265	1.409	2169.51	3057.70	1.783	1615.22	2879.77
A_68_P26215510	chr8:122223902-122223946	NM_026922:16	Atp2c2	INSIDE	1.265	2.462	673.10	1657.07	3.113	458.97	1428.96
A_68_P25144267	chr7:63217811-63217855	NM_023647:-12	Nipa2	DIVERGENT_PROMOTER	1.265	0.315	2262.49	713.05	0.399	1595.64	636.27
A_68_P20937463	chr1:193150081-193150125	NM_025864:259	Tmem206	INSIDE	1.265	0.263	4424.89	1163.49	0.333	3106.46	1033.42
A_68_P20228140	chr1:52687403-52687448	NM_001142647:-123	Tmem194b	PROMOTER	1.265	1.701	1341.73	2281.84	2.151	1109.52	2386.35
A_68_P27674921	chr11:29426434-29426478	NM_133767:0	Mtif2	INSIDE	1.264	0.247	2329.84	575.37	0.312	1717.06	536.06
A_68_P27083323	chr10:40020837-40020881	NM_007444:1134	Amd2	INSIDE	1.264	0.333	1838.49	612.25	0.421	1345.20	566.27
A_68_P25285284	chr7:91030916-91030960	NM_030705:1913	Mesdc1	INSIDE	1.264	1.537	1167.91	1794.53	1.942	912.99	1773.30
A_68_P24008164	chr5:115608110-115608154	NM_175403:53	Mlec	INSIDE	1.264	0.286	1983.70	567.77	0.362	1546.42	559.61
A_68_P23311150	chr4:131864462-131864506	NR_029468:-108	Snhg12	DIVERGENT_PROMOTER	1.264	0.428	1302.02	557.06	0.541	1015.30	548.91
A_68_P23240958	chr4:118214708-118214752	NM_172383:1601	Tmem125	INSIDE	1.264	2.005	1377.65	2762.51	2.534	1014.47	2571.00
A_68_P21261721	chr2:60800858-60800902	NM_020296:381	Rbms1	INSIDE	1.264	2.191	697.80	1528.96	2.769	601.69	1666.26
A_68_P31791665	chr18:67561243-67561287	NM_026473:10880	Tubb6	INSIDE	1.263	1.567	1129.96	1770.95	1.979	833.98	1650.65

ProbeName	Target position of probe on CpG island microarray	TargetID	GeneSymbol	CpG island Description	Ratio of relative methylation (TiO_2-NP/Vehicle)	Sham group			TiO_2-H group		
						Relative methylation (Cy5/Cy3)	Cy3 signal (Input)	Cy5 signal (MeDIP)	Relative methylation (Cy5/Cy3)	Cy3 signal (Input)	Cy5 signal (MeDIP)
A_68_P28863269	chr13:19715200-19715245	NM_016687:179	Sfrp4	INSIDE	1.263	2.339	303.53	709.88	2.953	226.36	668.44
A_68_P27255928	chr10:74779853-74779897	NM_009630:187	Adora2a	INSIDE	1.263	0.497	3628.86	1804.15	0.628	2809.92	1764.39
A_68_P25265199	chr7:87331654-87331698	NM_013659:-50	Sema4b	PROMOTER	1.263	0.350	1342.94	470.59	0.442	1034.99	457.95
A_68_P22281942	chr3:82707839-82707883	NM_023624:36	Lrat	INSIDE	1.263	0.172	6596.99	1136.54	0.218	4538.71	987.30
A_68_P21818285	chr2:164333404-164333448	NM_133779:10402	Pigt	INSIDE	1.263	1.879	1019.02	1914.90	2.373	854.88	2029.01
A_68_P32326386	chrX:35474728-35474772	NM_001185002:734	Rhox13	INSIDE	1.262	2.173	338.99	736.47	2.741	458.94	1257.81
A_68_P29094230	chr13:63673702-63673746	NM_008957:-6896	Ptch1	PROMOTER	1.262	0.417	1214.88	506.68	0.527	968.06	509.72
A_68_P29040996	chr13:53024696-53024740	NM_016709:328	Auh	INSIDE	1.262	0.260	2543.90	661.78	0.328	1831.51	601.19
A_68_P26844288	chr9:114277438-114277482	NM_019922:22370	Crtap	DOWNSTREAM	1.262	0.339	1596.67	540.48	0.427	1248.47	533.29
A_68_P21612304	chr2:127073616-127073660	NM_175145:-72	Tmem127	DIVERGENT_PROMOTER	1.262	22.276	4925.41	109718.40	28.102	4844.06	136126.50
A_68_P28054684	chr11:98010545-98010589	NM_028149:364	Fbxl20	INSIDE	1.261	0.132	3676.24	484.96	0.166	2954.94	491.56
A_68_P24658387	chr6:94650132-94650176	NM_008377:-15	Lrig1	PROMOTER	1.261	0.400	1487.67	595.03	0.504	1174.16	592.05
A_68_P22417419	chr3:109143833-109143877	NM_020505:254	Vav3	INSIDE	1.261	1.792	823.83	1476.30	2.259	689.86	1558.42
A_68_P20156459	chr1:37947346-37947390	NM_027098:-29	Mrpl30	DIVERGENT_PROMOTER	1.261	0.536	2466.79	1321.06	0.675	1947.93	1315.04
A_68_P32140229	chr19:45817346-45817390	NM_001166361:6	Fgf8	INSIDE	1.260	2.516	1166.78	2936.02	3.170	969.53	3073.40
A_68_P29032310	chr13:51503858-51503902	NM_010101:-106	S1pr3	PROMOTER	1.260	6.755	929.95	6282.22	8.512	687.76	5854.52
A_68_P28936822	chr13:34222312-34222356	NM_023716:-111	Tubb2b	PROMOTER	1.260	0.147	5168.74	761.52	0.186	2944.71	546.61
A_68_P25957752	chr8:74126620-74126664	NM_001166213:5098	Fam129c	INSIDE	1.260	0.303	1801.91	545.62	0.381	1362.17	519.55
A_68_P25089828	chr7:52125095-52125139	NM_133949:42	Ptov1	INSIDE	1.260	1.361	1690.20	2299.80	1.714	1334.15	2287.01
A_68_P24620314	chr6:88143770-88143814	NM_008090:-4865	Gata2	PROMOTER	1.260	0.233	2086.67	486.58	0.294	1619.18	475.81
A_68_P24519509	chr6:66986858-66986902	NM_007836:521	Gadd45a	INSIDE	1.260	0.464	2689.34	1248.13	0.585	1857.87	1086.08
A_68_P23441291	chr4:155266628-155266672	NM_207223:667	Acap3	INSIDE	1.260	0.390	1366.95	532.62	0.491	1001.80	492.01
A_68_P30721027	chr16:44347501-44347545	NM_144550:9	Spice1	INSIDE	1.259	0.361	1438.86	520.15	0.455	1143.31	520.37
A_68_P30402710	chr15:85565486-85565530	NM_001113418:-485	Ppara	PROMOTER	1.259	0.417	2390.10	996.93	0.525	1784.97	937.67
A_68_P29134804	chr13:72766185-72766229	NR_030701:-194	D430050G20	DIVERGENT_PROMOTER	1.259	0.355	2032.48	721.40	0.447	1492.76	667.19
A_68_P28743717	chr12:112615357-112615401	NM_183016:551	Cdc42bpb	INSIDE	1.259	7.915	1249.34	9888.00	9.966	1085.67	10819.89
A_68_P27956332	chr11:80593019-80593063	NM_177390:487	Myo1d	INSIDE	1.259	0.151	3686.11	555.27	0.190	2557.01	485.07
A_68_P26873360	chr9:119488455-119488499	NM_021544:-342	Scn5a	PROMOTER	1.259	0.397	4633.05	1839.55	0.500	3115.63	1557.49
A_68_P26551402	chr9:58884501-58884545	NM_001042752:-274	Neo1	PROMOTER	1.259	1.696	1990.17	3375.82	2.136	1599.97	3418.12
A_68_P24995079	chr7:20334543-20334587	NM_008990:358	Pvrl2	INSIDE	1.259	0.424	1201.90	509.40	0.534	999.24	533.12
A_68_P23416138	chr4:151406540-151406584	NM_031867:6037	Tas1r1	INSIDE	1.259	1.405	1302.93	1830.74	1.769	1022.31	1808.96
A_68_P22339769	chr3:94386307-94386351	NM_029721:296	Snx27	INSIDE	1.259	0.108	4724.52	511.45	0.136	3256.64	443.94
A_68_P20433333	chr1:91889782-91889826	NM_139152:21348	Asb18	INSIDE	1.259	1.528	756.04	1155.13	1.924	594.54	1144.01
A_68_P32977629	A_68_P32977629			Unknown	1.258	2.209	3058.50	6755.03	2.778	1640.70	4557.60
A_68_P32533203	chrX:90534565-90534609	NM_007492:2602	Arx	INSIDE	1.258	1.845	486.23	897.00	2.320	710.57	1648.57
A_68_P30370811	chr15:80042269-80042313	NM_010795:38140	Mgat3	INSIDE	1.258	2.410	1012.24	2439.21	3.030	816.61	2474.61
A_68_P30083239	chr15:25711450-25711494	NM_019472:159168	Myo10	INSIDE	1.258	2.328	263.90	614.30	2.928	231.30	677.14
A_68_P27594246	chr11:11926108-11926153	NM_001177629:1844	Grb10	INSIDE	1.258	2.066	2282.26	4716.21	2.600	1727.04	4490.68
A_68_P26467961	chr9:44306644-44306688	NM_030256:-552	Bcl9l	PROMOTER	1.258	0.329	1891.32	621.89	0.414	1135.06	469.69
A_68_P26254624	chr8:128434410-128434454	NM_145608:83	BC021891	INSIDE	1.258	0.197	4504.68	889.36	0.248	3138.93	779.85
A_68_P25181448	chr7:72301812-72301856	NM_183087:-420	Fam189a1	PROMOTER	1.258	0.166	3362.16	557.37	0.209	2381.53	496.77
A_68_P23544554	chr5:24414480-24414524	NM_001170555:-175	Prkag2	PROMOTER	1.258	1.802	372.05	670.61	2.268	316.36	717.42
A_68_P23227951	chr4:115817250-115817294	NM_026547:-51	1520402A15Rik	DIVERGENT_PROMOTER	1.258	0.462	1519.10	701.25	0.581	1226.04	711.94
A_68_P20644035	chr1:138028029-138028073	NM_001039472:73	Kif21b	INSIDE	1.258	1.440	882.36	1270.76	1.811	688.69	1247.37
A_68_P28099128	chr11:105880140-105880184	NM_001037712:10646	Kcnh6	INSIDE	1.257	1.468	679.35	996.99	1.845	556.19	1026.37
A_68_P25833939	chr8:47120895-47120939	NM_207213:88584	Snx25	INSIDE	1.257	1.486	629.31	934.92	1.867	604.73	1128.89
A_68_P25097405	chr7:53435026-53435070	NM_011510:355	Abcc8	INSIDE	1.257	0.499	1889.96	943.33	0.627	1467.15	920.14
A_68_P23398659	chr4:148473653-148473697	NM_019781:247	Pex14	INSIDE	1.257	2.427	1003.36	2434.82	3.050	984.08	3001.20
A_68_P22855812	chr4:40090603-40090647	NM_007386:327	Aco1	INSIDE	1.257	0.435	1213.29	527.46	0.546	956.17	522.44
A_68_P22185878	chr3:61962959-61963003			Unknown	1.257	2.196	506.59	1112.43	2.760	433.68	1196.76
A_68_P20319167	chr1:69423580-69423624			Unknown	1.257	1.702	750.79	1277.49	2.139	708.41	1515.62
A_68_P31655787	chr18:42671213-42671257	NM_001039474:94	Tcergl	INSIDE	1.256	0.201	2692.25	540.73	0.252	2063.25	520.34
A_68_P30364132	chr15:78893377-78893421	NM_144849:-109	Ankrd54	PROMOTER	1.256	0.453	1273.00	576.60	0.569	995.12	566.07
A_68_P30093796	chr15:27595252-27595296	NM_198301:15979	Fam105a	INSIDE	1.256	0.458	1644.42	753.14	0.575	1234.71	710.48
A_68_P26465906	chr9:43942731-43942775	NM_023061:12	Mcam	INSIDE	1.256	0.413	1280.28	529.07	0.519	1030.78	535.01
A_68_P24059412	chr5:124571591-124571635	NM_011256:94815	Pitpnm2	INSIDE	1.256	2.461	943.34	2321.27	3.089	747.58	2309.57
A_68_P23576669	chr5:31440849-31440893	NM_021290:398	Ucn	INSIDE	1.256	1.686	647.64	1091.72	2.117	452.53	957.99
A_68_P23364638	chr4:141092840-141092884	NM_019763:1650	Spen	INSIDE	1.256	0.282	1658.06	467.41	0.354	1166.32	412.89
A_68_P23336494	chr4:136436600-136436644	NM_009777:5470	C1qb	INSIDE	1.256	2.062	660.32	1361.29	2.589	561.21	1453.18
A_68_P23255468	chr4:120887443-120887487	ENSMUST00000056635:225		INSIDE	1.256	0.246	3324.90	816.36	0.308	2472.47	762.23
A_68_P20997455	chr2:9799133-9799177	NM_008091:1073	Gata3	INSIDE	1.256	1.619	1747.55	2828.95	2.033	1216.60	2473.06
A_68_P32454246	chrX:68808773-68808817	NM_008253:-373	Hmgb3	PROMOTER	1.255	2.507	752.58	1886.53	3.147	1038.59	3268.66
A_68_P31140915	chr17:31773909-31773953	NM_178224:146	Cbs	INSIDE	1.255	0.468	2094.34	980.32	0.587	1720.97	1011.05
A_68_P22030513	chr3:30755322-30755366	NM_001134385:473	Gpr160	INSIDE	1.255	0.333	2314.28	770.84	0.418	1651.80	690.56
A_68_P28924077	chr13:31718533-31718577	NM_010225:870	Foxf2	INSIDE	1.254	0.331	1624.25	538.43	0.416	1143.30	475.39

ProbeName	Target position of probe on CpG island microarray	TargetID	GeneSymbol	CpG island Description	Ratio of relative methylation (TiO_2-NP/Vehicle)	Sham group			TiO_2-H group		
						Relative methylation (Cy5/Cy3)	Cy3 signal (Input)	Cy5 signal (MeDIP)	Relative methylation (Cy5/Cy3)	Cy3 signal (Input)	Cy5 signal (MeDIP)
A_68_P28178206	chr11:119352324-119352368	NM_001164636:-314	A730011L01Rik	PROMOTER	1.254	0.450	1641.13	739.09	0.565	1074.32	606.50
A_68_P26003886	chr8:83866274-83866319	ENSMUST00000172167:-158		PROMOTER	1.254	0.311	1555.23	483.97	0.390	1232.39	481.02
A_68_P25952415	chr8:73046804-73046848	NM_029366:253	2810422J05Rik	INSIDE	1.254	0.231	3166.10	730.40	0.289	2279.51	659.51
A_68_P24785198	chr6:118088913-118088957	NM_030165:224	Csgalnact2	INSIDE	1.254	1.568	548.30	859.72	1.967	505.03	993.22
A_68_P24636903	chr6:91066915-91066959	NM_018815:-116	Nup210	PROMOTER	1.254	0.441	1637.37	722.25	0.553	1199.83	663.43
A_68_P24327617	chr6:29559260-29559304	NM_177296:325	Tnpo3	INSIDE	1.254	0.104	7030.42	730.37	0.130	4645.09	605.13
A_68_P24117613	chr5:136210132-136210176	NM_008898:45071	Por	INSIDE	1.254	1.757	716.32	1258.62	2.203	565.62	1245.93
A_68_P21615857	chr2:127657373-127657417	NM_001113179:201	Bub1	INSIDE	1.254	0.359	1951.60	701.24	0.451	1647.10	742.31
A_68_P21350022	chr2:76245198-76245242	NM_145525:626	Osbpl6	INSIDE	1.254	0.237	3617.14	858.13	0.298	2452.75	729.95
A_68_P30575778	chr16:17802918-17802962	NM_153790:5566	Scarf2	INSIDE	1.253	1.777	488.58	868.31	2.227	434.78	968.39
A_68_P27287691	chr10:80607646-80607690	NM_010731:8653	Zbtb7a	INSIDE	1.253	2.358	971.39	2290.94	2.956	778.69	2301.55
A_68_P27282403	chr10:79777962-79778006	NM_011789:13420	Apc2	INSIDE	1.253	1.531	615.27	942.13	1.918	426.02	817.11
A_68_P26237922	chr8:125626652-125626696	NR_028128:-283	Snord68	PROMOTER	1.253	0.445	1633.12	727.13	0.558	1299.15	724.95
A_68_P26044774	chr8:91565521-91565568	NM_021390:2517	Sall1	INSIDE	1.253	3.868	268.56	1038.90	4.847	225.63	1093.71
A_68_P25089776	chr7:52119038-52119082	NM_133949:6098	Ptov1	INSIDE	1.253	1.923	3295.47	6336.54	2.410	2159.92	5205.11
A_68_P24333875	chr6:30688293-30688337	NR_029900:-2984	Mir335	PROMOTER	1.253	1.553	738.35	1146.91	1.947	695.03	1353.05
A_68_P23404989	chr4:149610705-149610749	NM_023119:-579	Eno1	PROMOTER	1.253	0.200	4465.83	894.24	0.251	3272.18	821.26
A_68_P21819741	chr2:164611806-164611850	NM_133924:308	Snx21	INSIDE	1.253	1.600	1447.60	2316.22	2.004	1097.96	2200.61
A_68_P31118767	chr17:27977094-27977138	NM_011432:85	Snrpc	INSIDE	1.252	0.459	2181.90	1001.03	0.574	1686.04	968.25
A_68_P26600198	chr9:67481973-67482017			Unknown	1.252	1.442	1007.25	1452.24	1.805	775.80	1400.18
A_68_P32562267	chrX:98254914-98254958	NM_031384:42	Tex11	INSIDE	1.251	1.743	1107.91	1931.57	2.181	1396.66	3046.47
A_68_P29696922	chr14:70132513-70132557	NM_134078:-157	Chmp7	PROMOTER	1.251	0.478	1930.12	922.95	0.598	1447.76	866.20
A_68_P24059258	chr5:124540048-124540092	NM_019875:5737	Abcb9	INSIDE	1.251	1.602	919.97	1474.25	2.005	743.84	1491.70
A_68_P21831539	chr2:166539726-166539770	NM_177782:-416	Prex1	PROMOTER	1.251	0.300	1471.13	441.36	0.375	1151.85	432.44
A_68_P21576347	chr2:120454717-120454761	NM_025475:19567	Haus2	DOWNSTREAM	1.251	1.616	2554.85	4127.53	2.021	1851.53	3741.96
A_68_P20370402	chr1:78193529-78193573	NM_001159520:161	Pax3	INSIDE	1.251	0.363	1600.37	581.70	0.455	1480.80	673.47
A_68_P32142233	chr19:46210854-46210898	NM_008852:11939	Pitx3	INSIDE	1.250	0.181	3163.74	571.19	0.226	2288.60	516.65
A_68_P28582490	chr12:82833202-82833246	NM_001174107:48933	Map3k9	INSIDE	1.250	2.215	298.11	660.40	2.769	278.73	771.75
A_68_P28431152	chr12:52792067-52792111	NM_021710:136	Ap4s1	INSIDE	1.250	0.311	2106.65	655.04	0.389	1589.70	618.05
A_68_P28059766	chr11:98844320-98844364	NM_178596:-12	Gjd3	PROMOTER	1.250	0.183	2670.84	489.27	0.229	2060.64	471.80
A_68_P27431035	chr10:107769451-107769495	NM_054056:228	Pawr	INSIDE	1.250	0.280	2166.28	605.51	0.349	1773.04	619.51
A_68_P27167602	chr10:58685294-58685338	NM_172939:647	Ankrd57	INSIDE	1.250	1.764	450.25	794.12	2.204	378.28	833.64
A_68_P23896972	chr5:93371791-93371835	NM_001077596:44789	Shroom3	INSIDE	1.250	3.628	728.13	2641.99	4.534	664.37	3012.26
A_68_P20464815	chr1:97564271-97564315	NM_009183:-121	St8sia4	PROMOTER	1.250	0.166	3237.05	536.55	0.207	2258.99	468.05
A_68_P30959228	chr16:90831647-90831691	NM_001199210:565	4931408A02Rik	INSIDE	1.249	0.264	1768.42	466.44	0.329	1298.29	427.71
A_68_P29592278	chr14:49792394-49792438	NM_001081430:515	Naa30	INSIDE	1.249	6.982	436.33	3046.52	8.719	404.54	3527.40
A_68_P28737547	chr12:111517314-111517358	NM_172119:-103	Dio3	DIVERGENT_PROMOTER	1.249	0.256	2029.57	519.33	0.320	1627.81	520.36
A_68_P28598156	chr12:85758502-85758546	NM_025525:375	Rnf113a2	INSIDE	1.249	0.441	1662.43	732.66	0.551	1267.41	697.76
A_68_P24137625	chr5:140267376-140267420	NM_010757:-91	Mafk	PROMOTER	1.249	0.123	7943.36	978.53	0.154	5023.40	772.68
A_68_P24066628	chr5:125870372-125870416	NM_019639:-7	Ubc	PROMOTER	1.249	0.229	2188.43	500.80	0.286	1652.29	472.20
A_68_P22350868	chr3:96531418-96531462	NM_028925:-78	Polr3c	DIVERGENT_PROMOTER	1.249	0.366	1729.27	633.44	0.458	1389.67	636.04
A_68_P20616245	chr1:133423785-133423829	NM_001081011:132	Srgap2	INSIDE	1.249	0.133	10318.50	1368.45	0.166	6913.37	1145.51
A_68_P31632592	chr18:38357632-38357676	NM_029357:11762	Pcdh1	INSIDE	1.248	2.175	872.27	1896.91	2.714	704.22	1910.91
A_68_P30602963	chr16:23431338-23431382	NM_001004151:2155	Rtp1	INSIDE	1.248	2.074	295.91	613.69	2.588	245.68	635.76
A_68_P28190286	chr11:121249176-121249220	NM_139147:367	Rab40b	INSIDE	1.248	0.337	1571.31	530.02	0.421	1260.17	530.50
A_68_P27286201	chr10:80360945-80360989	NM_001081688:18406	Tmprss9	INSIDE	1.248	0.493	2079.48	1025.27	0.615	1294.38	796.46
A_68_P26216538	chr8:122394181-122394225	NM_146219:7998	Klhl36	INSIDE	1.248	1.981	400.75	793.99	2.473	372.61	921.44
A_68_P23590344	chr5:34000777-34000821	NM_001040435:3	Tacc3	INSIDE	1.248	0.420	1693.98	712.05	0.525	1261.76	661.84
A_68_P21568845	chr2:119119812-119119856	NM_172269:5357	Vps18	INSIDE	1.248	2.098	598.17	1254.66	2.618	565.33	1479.76
A_68_P21566327	chr2:118639858-118639902	NM_026412:142	D2Ertd750e	INSIDE	1.248	0.455	2899.42	1318.79	0.568	2005.13	1138.16
A_68_P30379107	chr15:81525500-81525544	NM_001164320:2195	Chadl	INSIDE	1.247	1.420	2652.98	3766.22	1.770	1981.59	3508.32
A_68_P28175493	chr11:118908126-118908170	NM_013926:-5921	Cbx8	PROMOTER	1.247	2.495	1353.24	3375.83	3.110	1113.49	3463.25
A_68_P27901908	chr11:70469503-70469547	NM_001136062:-1190	Eno3	DIVERGENT_PROMOTER	1.247	1.710	731.54	1250.91	2.132	519.27	1107.12
A_68_P23195770	chr4:108056055-108056099	NM_001099303:-122	Fam159a	PROMOTER	1.247	0.247	3844.04	947.97	0.307	2877.66	884.85
A_68_P30977036	chr16:93683818-93683862	NM_173047:377	Cbr3	INSIDE	1.246	0.214	2892.88	619.81	0.267	2036.32	543.60
A_68_P29030710	chr13:51196400-51196444	NM_146043:156	Spin1	INSIDE	1.246	0.432	1629.64	703.36	0.538	1356.22	729.40
A_68_P27571287	chr11:6963887-6963931	NM_009622:417	Adcy1	INSIDE	1.246	0.421	1158.80	488.01	0.525	927.36	486.47
A_68_P26940377	chr10:11063537-11063581	NM_010146:316	Epm2a	INSIDE	1.246	0.407	1738.65	708.34	0.507	1435.06	728.25
A_68_P26018419	chr8:86672061-86672105	NM_001163752:146	4930432K21Rik	INSIDE	1.246	0.414	2261.54	936.50	0.516	1554.40	801.82
A_68_P25100396	chr7:53895073-53895117	NM_013789:83	Sergef	INSIDE	1.246	1.889	971.47	1834.96	2.354	666.59	1569.39
A_68_P23896966	chr5:93370979-93371023	NM_001077596:43977	Shroom3	INSIDE	1.246	3.019	733.80	2215.28	3.760	726.43	2731.57
A_68_P30363896	chr15:78858665-78858709	NM_008197:45	H1f0	INSIDE	1.245	1.381	1302.59	1799.34	1.720	1011.11	1738.76
A_68_P28079343	chr11:102261298-102261342	NM_016759:6572	Rundc3a	INSIDE	1.245	0.459	1760.60	808.78	0.572	1421.40	813.24
A_68_P26998899	chr10:22365156-22365200	NM_178934:362	Slc2a12	INSIDE	1.245	0.336	3295.59	1106.36	0.418	2390.66	999.49
A_68_P24974127	chr7:13595301-13595345	NM_001024699:-173	Zbtb45	PROMOTER	1.245	0.464	1835.96	851.16	0.577	1301.72	751.44

ProbeName	Target position of probe on CpG island microarray	TargetID	GeneSymbol	CpG island Description	Ratio of relative methylation (TiO_2-NP/Vehicle)	Sham group			TiO_2-H group		
						Relative methylation (Cy5/Cy3)	Cy3 signal (Input)	Cy5 signal (MeDIP)	Relative methylation (Cy5/Cy3)	Cy3 signal (Input)	Cy5 signal (MeDIP)
A_68_P23538623	chr5:23350008-23350052	NM_025394:-67	Tomm7	DIVERGENT_PROMOTER	1.245	0.202	4457.80	900.45	0.251	2944.93	740.41
A_68_P23260768	chr4:122782372-122782416	NM_007559:-13	Bmp8b	PROMOTER	1.245	0.458	1519.47	695.20	0.570	1139.26	648.97
A_68_P21626548	chr2:129627604-129627648	NR_029452:-431	4932416H05Rik	PROMOTER	1.245	0.353	2415.76	852.16	0.439	1786.23	784.47
A_68_P21013690	chr2:12931726-12931771	NM_153155:743	C1ql3	INSIDE	1.245	0.368	2652.22	976.06	0.458	2070.22	948.61
A_68_P31554066	chr18:23468709-23468753	X95227:5		INSIDE	1.244	0.321	1440.18	461.93	0.399	1092.01	435.80
A_68_P31493068	chr18:11055096-11055140	NM_010258:2611	Gata6	INSIDE	1.244	0.383	1931.55	740.44	0.477	1472.98	702.48
A_68_P31255961	chr17:56556971-56557015	NM_011218:58911	Ptprs	INSIDE	1.244	2.151	2599.19	5590.02	2.675	1767.23	4727.77
A_68_P30514377	chr16:5203908-5203952	NM_013796:175	Nagpa	INSIDE	1.244	0.249	3313.51	823.89	0.309	2460.62	761.38
A_68_P30346600	chr15:76010013-76010057	NM_201394:16106	Plec	INSIDE	1.244	3.979	754.51	3002.39	4.949	572.19	2831.49
A_68_P29056640	chr13:55819313-55819357	NM_175150:3324	Txndc15	INSIDE	1.244	1.766	669.53	1182.19	2.196	653.81	1435.91
A_68_P26521207	chr9:53345172-53345216	NM_001081152:43	Npat	INSIDE	1.244	1.773	1575.74	2793.36	2.206	1208.92	2666.71
A_68_P25654579	chr8:11312543-11312587	NM_009931:262	Col4a1	INSIDE	1.244	1.868	1513.74	2827.95	2.324	1251.60	2908.82
A_68_P25025682	chr7:29946707-29946751	NM_182927:6938	Spred3	INSIDE	1.244	1.563	2033.33	3177.47	1.943	1567.08	3045.39
A_68_P24980758	chr7:16895019-16895063	NM_133234:109	Bbc3	INSIDE	1.244	0.353	5935.79	2097.38	0.440	4029.03	1770.89
A_68_P29629466	chr14:58318760-58318804	NM_015771:46178	Lats2	INSIDE	1.243	2.532	568.58	1439.49	3.147	519.91	1635.90
A_68_P28050942	chr11:97361786-97361830	NM_021493:50335	Arhgap23	INSIDE	1.243	0.446	2288.07	1020.17	0.554	1922.63	1065.50
A_68_P27537443	chr10:127114329-127114373	NM_173732:230	Tmem194	INSIDE	1.243	1.907	528.87	1008.76	2.371	475.85	1128.16
A_68_P26626260	chr9:71981061-71981105			Unknown	1.243	2.478	736.83	1825.85	3.080	660.41	2034.03
A_68_P26466561	chr9:44061469-44061513	NM_001163743:14780	Nlrx1	INSIDE	1.243	1.562	728.65	1138.36	1.942	614.70	1193.91
A_68_P25804898	chr8:41393101-41393145	NM_030110:-282	Efha2	PROMOTER	1.243	2.027	459.92	932.25	2.519	410.18	1033.08
A_68_P22194397	chr3:63780079-63780123	NM_001033300:36	Gmps	INSIDE	1.243	0.291	2478.12	721.52	0.362	1950.35	705.90
A_68_P29616407	chr14:55733626-55733670	NM_177049:2124	Jph4	INSIDE	1.242	2.392	6646.44	15901.51	2.971	4814.51	14303.12
A_68_P26696144	chr9:85736977-85737021	NM_001164792:540	Tpbg	INSIDE	1.242	0.197	3615.95	712.15	0.245	2488.52	608.92
A_68_P24155686	chr5:144015791-144015835	NM_017467:15889	Zfp316	INSIDE	1.242	1.516	685.73	1039.46	1.882	679.29	1278.56
A_68_P23897548	chr5:93473182-93473226	NM_175270:844	Ankrd56	INSIDE	1.242	0.378	2534.98	959.40	0.470	1951.95	917.30
A_68_P23569024	chr5:30063021-30063065	NR_028426:-566	Gm5129	PROMOTER	1.242	0.557	4336.91	2416.40	0.692	2993.55	2071.65
A_68_P21569059	chr2:119151913-119151957	NR_030683:-1	Gm14207	PROMOTER	1.242	0.213	2762.49	587.18	0.264	1994.86	526.84
A_68_P33007149	chr9_random:50218-50262	NR_015516:-3168	4930526I15Rik	PROMOTER	1.241	1.724	1246.66	2149.57	2.140	1063.93	2276.92
A_68_P32982273	chr5:134998822-134998866	NM_001039162:29460	Clip2	INSIDE	1.241	1.880	2332.51	4385.25	2.333	1804.03	4209.28
A_68_P32319275	chrX:34138849-34138893	NM_016783:651	Pgrmc1	INSIDE	1.241	2.095	505.50	1059.20	2.601	769.33	2001.13
A_68_P31786172	chr18:66451549-66451593	NM_178793:-78	Ccbe1	PROMOTER	1.241	1.446	735.82	1063.73	1.794	577.35	1035.63
A_68_P31156853	chr17:35115138-35115182	NM_013558:5513	Hspa1l	INSIDE	1.241	1.992	541.80	1079.23	2.473	420.94	1040.90
A_68_P29615091	chr14:55545244-55545288	NM_010112:359	Efs	INSIDE	1.241	0.156	2962.99	463.33	0.194	2217.30	430.41
A_68_P27094073	chr10:41995265-41995309	NM_019740:1262	Foxo3	INSIDE	1.241	1.775	482.69	856.93	2.204	444.48	979.45
A_68_P23996932	chr5:113649483-113649527	NM_001033428:-6424	Tmem211	PROMOTER	1.241	0.290	1624.45	470.61	0.360	1217.52	437.89
A_68_P31207759	chr17:46728766-46728810	NM_175168:37665	Ptk7	INSIDE	1.240	4.137	826.86	3421.06	5.129	723.64	3711.35
A_68_P31166377	chr17:37079998-37080042	NM_001099632:25	Rnf39	INSIDE	1.240	0.197	2318.65	456.00	0.244	1975.57	481.83
A_68_P26242535	chr8:126473140-126473184	NM_172288:3	Nup133	INSIDE	1.240	3.415	1466.25	5007.94	4.236	1200.12	5083.63
A_68_P25362041	chr7:106616835-106616879	NM_028145:2343	Klhl35	INSIDE	1.240	1.957	730.28	1428.94	2.426	592.41	1437.21
A_68_P23763027	chr5:67011694-67011738	NM_001201413:-1660	Apbb2	PROMOTER	1.240	0.397	1535.66	609.99	0.492	990.08	487.62
A_68_P22248557	chr3:75750620-75750664	NM_175193:10111	Golim4	INSIDE	1.240	1.883	492.11	926.71	2.334	532.68	1243.39
A_68_P21839458	chr2:167924133-167924177	NM_021409:17651	Pard6b	INSIDE	1.240	1.772	1452.91	2575.09	2.198	1071.13	2354.82
A_68_P21740322	chr2:150493841-150493885	NM_080575:114	Acss1	INSIDE	1.240	0.409	1314.57	537.92	0.507	1153.82	585.55
A_68_P21350018	chr2:76244804-76244848	NM_145525:232	Osbpl6	INSIDE	1.240	0.309	1521.12	469.30	0.383	1174.05	449.29
A_68_P32817206	chrX:165111558-165111602	NM_010832:254	Msl3	INSIDE	1.239	1.630	1243.05	2026.16	2.019	1572.35	3174.91
A_68_P31493012	chr18:11048533-11048577	NM_010258:-3953	Gata6	PROMOTER	1.239	0.344	1514.18	520.19	0.426	1280.04	544.83
A_68_P30511777	chr16:4726653-4726697	NM_001136066:314	Hmox2	INSIDE	1.239	0.362	3257.86	1180.91	0.449	2688.73	1207.29
A_68_P23696525	chr5:53132855-53132899	NM_025951:64	Pi4k2b	INSIDE	1.239	5.001	1736.26	8682.58	6.196	1387.02	8593.79
A_68_P21794497	chr2:160192823-160192867	NM_010658:-43	Mafb	PROMOTER	1.239	1.914	548.00	1048.61	2.372	496.01	1176.32
A_68_P31161702	chr17:36053831-36053875	NM_175934:-3	Ppp1r10	DIVERGENT_PROMOTER	1.238	0.320	3411.25	1090.14	0.396	2663.04	1053.59
A_68_P30631085	chr16:28564344-28564388	NM_010199:188922	Fgf12	INSIDE	1.238	0.335	1333.55	447.24	0.415	1155.28	479.51
A_68_P25947476	chr8:71923403-71923447	NR_038043:-3981	Gm10033	PROMOTER	1.238	0.279	3552.44	989.69	0.345	2650.08	913.67
A_68_P24432386	chr6:49345471-49345515	NM_029916:-110	Stk31	PROMOTER	1.238	2.964	1143.99	3390.62	3.670	920.09	3376.90
A_68_P23894275	chr5:92864051-92864096	NM_183392:152	Nup54	INSIDE	1.238	0.121	6262.74	756.13	0.150	3884.67	580.76
A_68_P21345223	chr2:75497311-75497355	NR_033513:-14	Gm6793	PROMOTER	1.238	2.896	675.59	1956.44	3.584	505.61	1811.93
A_68_P21322066	chr2:71711031-71711075	NM_172665:-276	Pdk1	PROMOTER	1.238	0.230	2384.96	547.98	0.284	1719.16	489.01
A_68_P32237968	chrX:6896043-6896087	ENSMUST00000132126:232		INSIDE	1.237	1.867	554.61	1035.19	2.308	619.28	1429.39
A_68_P29130689	chr13:72096834-72096878	NM_010573:4315	Irx1	INSIDE	1.237	0.285	1608.48	457.79	0.352	1160.60	408.48
A_68_P28112961	chr11:108286908-108286952	NM_029606:351	Ccdc46	INSIDE	1.237	0.384	2064.70	793.77	0.475	1436.79	683.09
A_68_P26596761	chr9:66897452-66897496	NM_001164249:-454	Tpm1	PROMOTER	1.237	0.485	1989.91	964.75	0.600	1493.45	896.02
A_68_P32260265	chrX:11656150-11656206	NM_029510:1501	Bcor	INSIDE	1.236	1.834	410.52	752.96	2.268	354.63	804.18
A_68_P32206528	chr19:57389489-57389533	ENSMUST00000156316:-101		PROMOTER	1.236	0.432	1731.95	747.37	0.533	1299.72	693.19
A_68_P29454308	chr14:22505922-22505966	NM_001013826:27854	Dupd1	INSIDE	1.236	0.391	1419.53	555.42	0.484	1177.56	569.41
A_68_P28159656	chr11:116351010-116351061	NM_026364:625	Prpsap1	INSIDE	1.236	22.728	3075.42	69897.39	28.093	2269.63	63761.20
A_68_P27504341	chr10:121023507-121023551	NM_019786:322	Tbk1	INSIDE	1.236	1.593	991.72	1579.79	1.969	832.82	1639.73

ProbeName	Target position of probe on CpG island microarray	TargetID	GeneSymbol	CpG island Description	Ratio of relative methylation (TiO_2-NP/Vehicle)	Sham group			TiO_2-H group		
						Relative methylation (Cy5/Cy3)	Cy3 signal (Input)	Cy5 signal (MeDIP)	Relative methylation (Cy5/Cy3)	Cy3 signal (Input)	Cy5 signal (MeDIP)
A_68_P24759667	chr6:113419865-113419909	NM_001034029:10998	Il17re	INSIDE	1.236	3.952	1345.74	5318.57	4.884	1235.18	6032.45
A_68_P22449707	chr3:116211186-116211230	NM_025517:-115	Rtcd1	DIVERGENT_PROMOTER	1.236	0.296	1893.59	560.07	0.365	1525.59	557.55
A_68_P21489860	chr2:104430467-104430511	NM_001037326:-152	Cstf3	PROMOTER	1.236	0.233	2792.79	650.42	0.288	1953.47	562.11
A_68_P32775564	chrX:156064880-156064924	NM_001135727:-437	Sh3kbp1	PROMOTER	1.235	1.799	1837.87	3306.11	2.223	2427.98	5396.20
A_68_P27965916	chr11:82200818-82200862	NM_023438:-1561	Tmem132e	PROMOTER	1.235	0.350	1404.84	491.60	0.432	1125.75	486.32
A_68_P26897424	A_68_P26897424			Unknown	1.235	1.754	987.87	1732.45	2.165	764.53	1655.50
A_68_P25805594	chr8:41509577-41509621	NM_178395:430	Zdhhc2	INSIDE	1.235	0.252	2192.48	552.67	0.311	1738.74	541.11
A_68_P25553143	chr7:142729512-142729556	NM_011212:28	Ptpre	INSIDE	1.235	3.064	1174.93	3599.72	3.782	911.49	3447.47
A_68_P23470208	chr5:8912735-8912783	NM_008830:19038	Abcb4	INSIDE	1.235	2.932	759.35	2226.36	3.620	656.30	2375.48
A_68_P31746195	chr18:59334616-59334660	NM_001081328:-355	Chsy3	PROMOTER	1.234	4.054	831.12	3369.67	5.004	722.92	3617.34
A_68_P29071677	chr13:58230562-58230606	NM_029872:-667	Hnrnpa0	PROMOTER	1.234	0.211	3457.23	730.15	0.261	2459.47	640.90
A_68_P27354561	chr10:93052055-93052099	NM_027246:327	Snrpf	INSIDE	1.234	0.367	1508.73	553.85	0.453	1106.79	501.27
A_68_P24158010	chr5:144493382-144493426	NM_029749:-255	Usp42	PROMOTER	1.234	0.420	1530.57	642.39	0.518	1226.33	635.25
A_68_P23331511	chr4:135528428-135528472	NM_011942:59	Lypla2	INSIDE	1.234	0.287	2166.31	620.97	0.354	1781.83	630.28
A_68_P32022084	chr19:24248544-24248588	NM_001198985:64	Tjp2	INSIDE	1.233	1.712	770.31	1318.72	2.111	594.61	1255.15
A_68_P31286847	chr17:63230901-63230945	NM_010109:-256	Efna5	PROMOTER	1.233	0.499	2342.27	1167.78	0.615	1753.58	1077.63
A_68_P29613716	chr14:55305799-55305843	NM_001085473:-52	Acin1	PROMOTER	1.233	0.239	2349.03	560.27	0.294	1789.81	526.42
A_68_P23560507	chr5:28398167-28398211	NM_153526:237	Insig1	INSIDE	1.233	0.157	2971.39	467.22	0.194	2133.04	413.54
A_68_P21431331	chr2:92755584-92755628	NM_030725:349	Syt13	INSIDE	1.233	0.332	2927.36	971.98	0.410	1985.95	813.33
A_68_P20613814	chr1:133034401-133034445	NM_145508:389	Dyrk3	INSIDE	1.233	0.253	2071.56	524.93	0.312	1601.32	500.29
A_68_P31514145	chr18:14942138-14942182	NM_001100449:407	Taf4b	INSIDE	1.232	2.687	423.18	1137.13	3.310	311.84	1032.27
A_68_P30572509	chr16:17201580-17201624	NM_009456:-17	Ube2l3	DIVERGENT_PROMOTER	1.232	1.553	1033.40	1605.37	1.914	843.56	1614.40
A_68_P30481999	chr15:99556228-99556272	NM_183256:202	2310016M24Rik	INSIDE	1.232	0.285	2001.52	570.26	0.351	1469.79	516.09
A_68_P29211816	chr13:90229293-90229337	NM_025335:43	Tmem167	INSIDE	1.232	0.183	2702.92	494.11	0.225	2093.81	471.39
A_68_P29095533	chr13:63916826-63916870	NM_023507:207	0610007P08Rik	INSIDE	1.232	1.624	1285.44	2088.02	2.001	1087.53	2176.00
A_68_P28101900	chr11:106349136-106349180	NM_023913:-48	Ern1	PROMOTER	1.232	0.304	2331.82	708.17	0.374	1869.85	699.78
A_68_P25083585	chr7:50932262-50932306	NM_145582:4884	Ctu1	INSIDE	1.232	2.153	1020.97	2197.68	2.653	767.21	2035.30
A_68_P23541889	chr5:23950730-23950774	NM_023229:301	Fastk	INSIDE	1.232	0.225	2361.18	530.33	0.277	1766.48	488.72
A_68_P22872184	chr4:43536297-43536341	NM_009416:-58	Tpm2	PROMOTER	1.232	0.368	1585.64	583.56	0.453	1329.58	602.84
A_68_P22445154	chr3:115417852-115417896	NM_007901:99	S1pr1	INSIDE	1.232	3.284	180.54	592.83	4.044	149.01	602.60
A_68_P22415391	chr3:108742937-108742981	NM_025723:-43	4921515J06Rik	PROMOTER	1.232	1.486	832.62	1237.36	1.831	683.79	1251.83
A_68_P20061791	chr1:18048589-18048633			Unknown	1.232	0.322	1491.39	480.28	0.397	1220.35	484.03
A_68_P32203591	chr19:56949104-56949148	NM_172840:221	Vwa2	INSIDE	1.231	0.433	1378.34	596.33	0.532	1218.32	648.72
A_68_P31256043	chr17:56567276-56567320	NM_011218:48605	Ptprs	INSIDE	1.231	3.273	3861.77	12638.48	4.029	2909.55	11722.15
A_68_P31003198	chr17:3115122-3115166	NM_134123:173	Scaf8	INSIDE	1.231	0.313	2153.85	674.99	0.386	1751.24	675.63
A_68_P30961711	chr16:91228596-91228641	NM_016967:2824	Olig2	INSIDE	1.231	0.374	2239.50	837.06	0.460	1578.53	726.41
A_68_P30931274	chr16:85802529-85802573	NM_009621:810	Adamts1	INSIDE	1.231	0.350	1431.11	500.77	0.431	1104.54	475.89
A_68_P29651206	chr14:62266896-62266940	NR_028264:34292	Dleu2	INSIDE	1.231	0.457	1724.00	787.11	0.562	1410.33	792.67
A_68_P29482227	chr14:27354230-27354274	NM_032008:-1026	Slmap	PROMOTER	1.231	0.131	3310.42	435.01	0.162	2421.68	391.80
A_68_P28575260	chr12:81440954-81440998	NM_177267:96612	Dcaf5	INSIDE	1.231	1.496	1250.58	1870.93	1.841	903.98	1664.32
A_68_P23295630	chr4:128901440-128901484	NM_001033189:24225	C77080	INSIDE	1.231	1.437	1342.77	1929.47	1.769	1192.70	2109.35
A_68_P32218217	chr19:59335443-59335487	NM_172523:97	Slc18a2	INSIDE	1.230	0.338	2175.18	735.44	0.416	1556.44	647.17
A_68_P31251409	chr17:55622134-55622178	NM_172829:37142	St6gal2	INSIDE	1.230	1.453	829.93	1206.24	1.787	716.97	1281.50
A_68_P31092444	chr17:23713564-23713608	NM_011747:22868	Zfp13	INSIDE	1.230	2.272	1009.72	2294.24	2.794	763.06	2132.10
A_68_P29696916	chr14:70131894-70131938	NM_134078:461	Chmp7	INSIDE	1.230	0.520	1743.11	906.95	0.640	1340.05	857.88
A_68_P29470471	chr14:25064223-25064267	NM_001163513:898	Dlg5	INSIDE	1.230	1.787	841.71	1504.44	2.198	627.20	1378.55
A_68_P26430752	chr9:37267961-37268005			Unknown	1.230	0.303	2592.09	784.38	0.372	2116.89	787.68
A_68_P26233343	chr8:124956522-124956566	NM_007806:296	Cyba	INSIDE	1.230	1.938	1044.86	2025.28	2.384	742.14	1769.22
A_68_P26030504	chr8:89148137-89148181	NM_025827:217	Lonp2	INSIDE	1.230	0.359	1248.41	448.63	0.442	912.30	403.27
A_68_P24138646	chr5:140447394-140447438	NM_175522:63520	Elfn1	INSIDE	1.230	1.709	2624.93	4487.06	2.103	1850.57	3892.50
A_68_P20596471	chr1:129574483-129574527	NM_028135:94	Tmem163	INSIDE	1.230	0.380	2026.97	769.76	0.467	1709.00	798.29
A_68_P30477112	chr15:98711721-98711765	NM_026967:103	Rhebl1	INSIDE	1.229	0.390	1456.86	567.67	0.479	1106.76	530.06
A_68_P30425030	chr15:89240388-89240432	NM_001162882:284	1700007E06Rik	INSIDE	1.229	2.862	1193.04	3414.03	3.517	907.32	3191.07
A_68_P29248565	chr13:97695108-97695152	NM_001166065:487	Gcnt4	INSIDE	1.229	0.214	2226.23	475.60	0.263	1896.57	498.04
A_68_P25614230	chr8:3496425-3496469	NM_080461:3309	Zfp358	INSIDE	1.229	1.881	2609.91	4910.51	2.313	2003.09	4633.64
A_68_P20141344	chr1:34986763-34986807			Unknown	1.229	1.684	705.09	1187.12	2.069	588.79	1218.31
A_68_P31465563	chr18:4920525-4920569	ENSMUST00000131210:-18		PROMOTER	1.228	1.578	578.78	913.19	1.937	481.31	932.25
A_68_P30370815	chr15:80042691-80042735	NM_010795:38562	Mgat3	INSIDE	1.228	2.272	437.21	993.29	2.789	334.32	932.32
A_68_P27933360	chr11:76479988-76480032	NM_177182:-5961	Bhlha9	PROMOTER	1.228	3.293	552.02	1817.58	4.043	561.86	2271.49
A_68_P24946862	chr7:3645247-3645291	NM_024168:-841	Tsen34	PROMOTER	1.228	0.369	4149.34	1533.12	0.454	2679.76	1215.50
A_68_P23917033	chr5:98683430-98683474	NM_010203:250	Fgf5	INSIDE	1.228	0.353	1647.77	582.29	0.434	1322.58	574.16
A_68_P20935795	chr1:192849358-192849402	NR_027298:151	A230020J21Rik	INSIDE	1.228	0.323	1731.26	558.98	0.396	1431.26	567.38
A_68_P20734232	chr1:156646813-156646857	AK046991:-6961		PROMOTER	1.228	3.469	587.31	2037.19	4.258	429.14	1827.18
A_68_P20241448	chr1:55419388-55419432	NM_001113367:141	Boll	INSIDE	1.228	1.444	730.01	1054.03	1.774	627.26	1112.49
A_68_P32241968	chrX:7722430-7722474	NR_033561:-100	2900002K06Rik	PROMOTER	1.227	0.376	1221.03	458.81	0.461	1768.06	815.40

ProbeName	Target position of probe on CpG island microarray	TargetID	GeneSymbol	CpG island Description	Ratio of relative methylation (TiO_2-NP/Vehicle)	Sham group			TiO_2-H group		
						Relative methylation (Cy5/Cy3)	Cy3 signal (Input)	Cy5 signal (MeDIP)	Relative methylation (Cy5/Cy3)	Cy3 signal (Input)	Cy5 signal (MeDIP)
A_68_P31117096	chr17:27692021-27692065	NM_001025427:-1476	Hmga1	PROMOTER	1.227	0.452	1287.58	582.47	0.555	1032.69	573.11
A_68_P30594367	chr16:21794433-21794477	NR_037957:35	1300002E11Rik	INSIDE	1.227	0.510	2204.70	1124.79	0.626	1756.80	1099.43
A_68_P29660083	chr14:63864272-63864316	NM_008092:-197	Gata4	PROMOTER	1.227	0.194	3999.87	775.34	0.238	2698.72	641.81
A_68_P28157765	chr11:116029229-116029273	NM_172571:242	Fbf1	INSIDE	1.227	0.282	2790.15	785.78	0.346	2021.09	698.61
A_68_P26812954	chr9:107996445-107996489	NM_146226:345	Apeh	INSIDE	1.227	0.264	3402.94	899.10	0.324	2327.18	754.28
A_68_P25958569	chr8:74249306-74249350	NM_028715:252	Fcho1	INSIDE	1.227	0.281	2065.09	581.14	0.345	1646.34	568.28
A_68_P24735708	chr6:108732909-108732953	NM_026011:-122	Arl8b	PROMOTER	1.227	0.482	2919.63	1407.48	0.592	2224.36	1315.81
A_68_P23335797	chr4:136327232-136327276	NM_010142:64596	Ephb2	INSIDE	1.227	1.729	1005.33	1738.47	2.123	778.69	1652.82
A_68_P22314992	chr3:88447835-88447879	NM_001198912:22541	Arhgef2	INSIDE	1.227	1.747	575.74	1005.68	2.143	502.19	1076.23
A_68_P31202057	chr17:45732420-45732464	NM_001199113:-1853	Slc29a1	PROMOTER	1.226	0.546	2637.90	1441.45	0.670	1789.07	1198.50
A_68_P30508954	chr16:4213021-4213065	NM_001025432:362	Crebbp	INSIDE	1.226	0.282	2936.26	827.16	0.345	2092.22	722.39
A_68_P30382912	chr15:82176300-82176344	NM_026914:-153	1500032L24Rik	PROMOTER	1.226	1.914	522.40	1000.07	2.347	457.94	1074.93
A_68_P28600137	chr12:86113873-86113917	NM_023409:168	Npc2	INSIDE	1.226	0.140	4197.99	587.68	0.172	3161.57	542.63
A_68_P27222454	chr10:68676238-68676282	NM_001081347:856	Rhobtb1	INSIDE	1.226	1.445	1133.60	1638.28	1.772	891.81	1580.72
A_68_P25094126	chr7:52883769-52883813	NM_028544:884	Rasip1	INSIDE	1.226	1.675	598.51	1002.55	2.053	507.39	1041.83
A_68_P20864291	chr1:180458344-180458388	NM_001161665:-889	Kif26b	PROMOTER	1.226	0.195	3883.83	758.30	0.239	2956.41	707.82
A_68_P31304162	chr17:66425686-66425730	NM_001025572:678	Ankrd12	INSIDE	1.225	0.260	2814.48	732.18	0.319	2090.64	666.37
A_68_P27226082	chr10:69284986-69285030	NM_146005:288553	Ank3	INSIDE	1.225	1.724	693.24	1195.38	2.112	573.15	1210.39
A_68_P27172914	chr10:59648890-59648934	NM_016803:33096	Chst3	INSIDE	1.225	2.696	324.98	876.11	3.303	308.20	1018.03
A_68_P26132671	chr8:107800458-107800502	NM_030152:134	Nol3	INSIDE	1.225	1.462	4668.59	6826.82	1.791	3389.41	6069.38
A_68_P24142833	chr5:141180856-141180900	NM_172724:-98	Baat1	DIVERGENT_PROMOTER	1.225	0.443	1911.22	845.95	0.542	1462.64	793.28
A_68_P21570454	chr2:119420252-119420296	NR_015473:243	1700020I14Rik	INSIDE	1.225	0.385	2359.35	908.59	0.472	1948.31	918.88
A_68_P31201771	chr17:45692645-45692689	NM_008690:2	Nfkbie	INSIDE	1.224	0.259	2349.08	607.60	0.317	1651.29	522.73
A_68_P30542067	chr16:10395304-10395348	NM_001099275:215	Tekt5	INSIDE	1.224	0.472	2067.83	976.87	0.578	1595.28	922.57
A_68_P24874649	chr6:136776165-136776209	NM_021714:551	Wbp11	INSIDE	1.224	1.637	1360.15	2226.67	2.004	1054.90	2113.88
A_68_P23978152	chr5:110605959-110606003	NM_008146:261	Golga3	INSIDE	1.224	0.190	3991.32	759.83	0.233	2912.96	678.71
A_68_P22210465	chr3:67319575-67319619	NM_001164763:-151	Rarres1	PROMOTER	1.224	0.336	1405.35	471.94	0.411	1173.71	482.40
A_68_P28675866	chr12:100630793-100630837	NM_183186:57470	Foxn3	INSIDE	1.223	0.413	1577.77	651.89	0.505	1279.43	646.38
A_68_P28456271	chr12:57446391-57446435	NM_145442:469	Mbip	INSIDE	1.223	0.492	1634.61	804.85	0.602	1198.77	721.95
A_68_P25956521	chr8:73891201-73891245	NM_025917:107	Use1	INSIDE	1.223	0.308	1637.65	504.83	0.377	1259.92	475.00
A_68_P24465594	chr6:54766604-54766648	NM_199143:-283	Znrf2	PROMOTER	1.223	0.305	2000.18	610.82	0.374	1588.66	593.46
A_68_P24052416	chr5:123271272-123271316	NM_001042491:54	Anapc5	INSIDE	1.223	0.339	1324.47	448.39	0.414	1164.88	482.12
A_68_P23365307	chr4:141220027-141220071	NM_001033150:-18	Plekhm2	PROMOTER	1.223	1.782	541.05	964.05	2.180	433.37	944.62
A_68_P23303540	chr4:130506009-130506056	NM_010769:5733	Matn1	INSIDE	1.223	1.880	581.15	1092.80	2.301	618.76	1423.54
A_68_P31104079	chr17:25611509-25611553	NM_027951:2996	Tekt4	INSIDE	1.222	0.204	2493.07	508.94	0.249	1648.56	411.28
A_68_P29734164	chr14:76904319-76904363	NM_009366:24	Tsc22d1	INSIDE	1.222	1.887	696.18	1313.53	2.306	499.26	1151.17
A_68_P28818473	chr13:9762982-9763026	NM_001199141:1556	Zmynd11	INSIDE	1.222	0.124	5025.46	624.53	0.152	3432.42	521.24
A_68_P26845368	chr9:114471438-114471482	NM_001042503:2027	Trim71	INSIDE	1.222	1.730	691.69	1196.34	2.114	492.23	1040.46
A_68_P26824826	chr9:110532797-110532841	NM_183276:23847	Nbeal2	INSIDE	1.222	1.689	2354.63	3977.11	2.065	1639.02	3384.18
A_68_P26822034	chr9:110020069-110020113	NM_133347:-2004	Dhx30	PROMOTER	1.222	2.276	710.13	1616.59	2.781	560.85	1559.67
A_68_P26402553	chr9:31721424-31721468	NM_013800:-576	Barx2	PROMOTER	1.222	0.285	2676.04	763.89	0.349	2142.30	747.50
A_68_P25956547	chr8:73895356-73895400	NM_029865:182	Ocel1	INSIDE	1.222	0.423	1353.06	572.97	0.517	1080.09	558.81
A_68_P25952417	chr8:73047003-73047047	NM_029366:55	2810422J05Rik	INSIDE	1.222	2.155	1198.81	2582.97	2.632	935.61	2462.81
A_68_P25708988	chr8:23170683-23170727	NM_007511:-158	Atp7b	DIVERGENT_PROMOTER	1.222	0.461	1210.19	558.24	0.564	1046.96	590.24
A_68_P24325043	chr6:29129618-29129662	NM_001101443:-56	Prrt4	PROMOTER	1.222	6.341	7959.01	50471.28	7.748	4929.96	38198.39
A_68_P22291215	chr3:84284164-84284208	NM_001205355:-773	Fhdc1	PROMOTER	1.222	0.204	3644.28	743.10	0.249	2527.87	629.91
A_68_P20141348	chr1:34987329-34987373			Unknown	1.222	1.482	1462.77	2167.50	1.811	1302.42	2358.27
A_68_P32410977	chrX:56820526-56820570	ENSMUST00000124402:101		INSIDE	1.221	4.446	640.91	2849.80	5.429	594.78	3229.29
A_68_P32289636	chrX:20265804-20265848	NM_011049:208	Cdk16	INSIDE	1.221	3.018	347.64	1049.17	3.686	410.26	1512.27
A_68_P32119983	chr19:42330356-42330400	NM_027694:150	Golga7b	INSIDE	1.221	0.312	2003.28	624.36	0.380	1516.08	576.76
A_68_P29616399	chr14:55732726-55732770	NM_177049:3024	Jph4	INSIDE	1.221	2.167	1011.37	2191.68	2.645	777.31	2056.14
A_68_P29152753	chr13:76194078-76194122	NM_175495:344	Gpr150	INSIDE	1.221	1.733	576.50	999.09	2.115	465.63	984.92
A_68_P28164528	chr11:117193656-117193700	NM_001113488:52	Septin9	INSIDE	1.221	0.260	1870.58	487.11	0.318	1293.19	411.33
A_68_P27143268	chr10:53349438-53349484	NM_027830:785	Mcm9	INSIDE	1.221	2.063	328.30	677.42	2.520	284.18	716.10
A_68_P25953468	chr8:73223214-73223258	NM_010592:1599	Jund	INSIDE	1.221	0.432	1171.90	506.84	0.528	925.21	488.67
A_68_P25574803	chr7:146101482-146101526	NM_009760:-315	Bnip3	PROMOTER	1.221	0.175	7737.19	1357.01	0.214	5359.51	1147.85
A_68_P25394039	chr7:112885744-112885788	NM_133951:133	Rrp8	INSIDE	1.221	0.336	1786.55	599.51	0.410	1376.64	563.86
A_68_P25094417	chr7:52934632-52934676	NM_019934:274	Sec1	INSIDE	1.221	2.041	1128.99	2303.94	2.491	810.20	2018.09
A_68_P31258633	chr17:56969523-56969567	NM_009056:887	Rfx2	INSIDE	1.220	0.522	2502.82	1306.73	0.637	1636.33	1042.31
A_68_P30094472	chr15:27703695-27703739	NM_001081302:251887	Trio	INSIDE	1.220	2.379	1438.49	3422.40	2.904	1191.15	3458.71
A_68_P27995152	chr11:87431026-87431070	NM_001045527:383	Hsf5	INSIDE	1.220	2.987	1008.07	3011.45	3.646	802.93	2927.31
A_68_P22359703	chr3:98143412-98143460	NM_016966:456	Phgdh	INSIDE	1.220	0.267	1767.58	472.53	0.326	1285.11	419.12
A_68_P21116281	chr2:32449587-32449631	NM_001025311:-5620	St6galnac6	PROMOTER	1.220	1.774	981.64	1741.01	2.164	754.19	1632.41
A_68_P20149049	chr1:36614326-36614370	NM_001126047:878	Sema4c	INSIDE	1.220	9.408	5550.05	52216.09	11.478	4207.05	48287.19
A_68_P30595258	chr16:21947351-21947395	NM_025693:253	Tmem41a	INSIDE	1.219	0.212	5133.85	1087.85	0.258	3778.43	976.19

ProbeName	Target position of probe on CpG island microarray	TargetID	GeneSymbol	CpG island Description	Ratio of relative methylation (TiO_2-NP/Vehicle)	Sham group			TiO_2-H group		
						Relative methylation (Cy5/Cy3)	Cy3 signal (Input)	Cy5 signal (MeDIP)	Relative methylation (Cy5/Cy3)	Cy3 signal (Input)	Cy5 signal (MeDIP)
A_68_P29249638	chr13:97907886-97907931	NM_177266:17	Gfm2	INSIDE	1.219	7.255	428.52	3108.84	8.843	362.41	3204.74
A_68_P28071220	chr11:100831835-100831879	NM_008986:75	Ptrf	INSIDE	1.219	0.498	2611.15	1300.73	0.607	1990.09	1208.48
A_68_P27880662	chr11:66866069-66866113	NM_025510:30	2310004I24Rik	INSIDE	1.219	0.221	2345.30	517.47	0.269	1580.43	425.21
A_68_P26643862	chr9:75257222-75257266	NM_027418:-78	Mapk6	PROMOTER	1.219	1.940	1056.41	2049.55	2.365	873.47	2065.70
A_68_P26014849	chr8:85913580-85913624	NM_009904:-187	Clgn	PROMOTER	1.219	0.395	1376.75	543.28	0.481	1173.40	564.67
A_68_P22311003	chr3:87775500-87775544	NM_016701:508	Nes	INSIDE	1.219	0.531	1653.72	878.44	0.648	1387.87	898.87
A_68_P20641451	chr1:137662928-137662972	NM_013750:289	Phlda3	INSIDE	1.219	1.816	840.57	1526.18	2.212	751.79	1663.31
A_68_P30575506	chr16:17759553-17759597	NM_145479:-139	Klhl22	PROMOTER	1.218	0.296	1608.55	476.78	0.361	1423.97	514.08
A_68_P28529995	chr12:73171043-73171090	NM_181752:912	Gpr135	INSIDE	1.218	3.065	738.98	2264.87	3.734	594.81	2220.74
A_68_P25962580	chr8:75247857-75247901	NM_001113248:692	Sin3b	INSIDE	1.218	0.512	1865.97	954.52	0.623	1487.24	926.69
A_68_P21814245	chr2:163575768-163575812	NM_007398:123	Ada	INSIDE	1.218	0.207	2150.90	446.10	0.253	1540.54	389.02
A_68_P21269076	chr2:62249843-62249887	NM_010074:424	Dpp4	INSIDE	1.218	2.014	521.37	1049.95	2.452	439.76	1078.46
A_68_P31863473	chr18:80492422-80492466	NM_001190373:68549	Kcng2	INSIDE	1.217	2.319	676.58	1568.75	2.823	586.71	1656.03
A_68_P30018898	chr15:11835366-11835410	NM_001039181:41	Npr3	INSIDE	1.217	0.434	3161.08	1370.76	0.528	2311.15	1219.44
A_68_P25608862	chr7:151924611-151924655	NM_178642:-249	Ano1	PROMOTER	1.217	0.518	1540.13	797.98	0.631	1273.33	803.16
A_68_P25082496	chr7:50720344-50720388	NM_026695:20854	Etfb	DOWNSTREAM	1.217	0.252	3641.91	918.21	0.307	2723.97	835.63
A_68_P24093837	chr5:130925156-130925200	NM_021371:517	Caln1	INSIDE	1.217	1.517	1392.32	2111.54	1.846	1204.23	2223.39
A_68_P28731497	chr12:110271448-110271492	NM_001163175:34957	Begain	INSIDE	1.216	1.443	919.34	1326.66	1.755	803.95	1411.12
A_68_P26081252	chr8:97605501-97605545	NM_028805:422	Katnb1	INSIDE	1.216	1.476	996.14	1470.66	1.796	729.42	1309.84
A_68_P25263202	chr7:86937639-86937683	NM_008588:816	Mesp1	INSIDE	1.216	1.757	970.33	1704.78	2.137	802.37	1714.67
A_68_P23281732	chr4:126413411-126413455	NM_198960:81	Tcfap2e	INSIDE	1.216	0.302	2761.25	833.26	0.367	1819.61	667.74
A_68_P22037671	chr3:32264236-32264280	NR_027966:-152	4930429B21Rik	PROMOTER	1.216	0.407	1637.90	666.24	0.495	1506.25	744.96
A_68_P32408597	chrX:56387379-56387423	NM_010200:213	Fgf13	INSIDE	1.215	2.150	218.90	470.66	2.613	304.91	796.78
A_68_P30939274	chr16:87354456-87354500	NM_001159331:49	N6amt1	INSIDE	1.215	2.217	643.66	1426.86	2.693	551.34	1484.57
A_68_P29612545	chr14:55082897-55082941	NM_022993:-65	Lrp10	PROMOTER	1.215	0.222	2277.03	505.36	0.270	1701.85	458.84
A_68_P28748382	chr12:113412288-113412332	NM_001097621:27892	Kif26a	INSIDE	1.215	2.017	688.83	1389.19	2.449	585.53	1434.25
A_68_P28181190	chr11:119803788-119803832	NM_001037754:-595	Baiap2	PROMOTER	1.215	0.226	3474.27	784.86	0.275	2646.36	726.44
A_68_P28149048	chr11:114530351-114530395	NM_001048058:278	Rpl38	INSIDE	1.215	0.316	1567.45	495.97	0.384	1247.40	479.60
A_68_P27897481	chr11:69659900-69659944	NM_023564:-196	Plscr3	PROMOTER	1.215	0.305	2931.77	894.45	0.371	2177.49	807.11
A_68_P25486801	chr7:131127425-131127469	ENSMUST00000106437:1064		INSIDE	1.215	0.318	1581.46	502.70	0.386	1230.64	475.21
A_68_P23600259	chr5:35739868-35739912	NM_010445:8125	Hmx1	DOWNSTREAM	1.215	0.423	2176.94	920.59	0.514	1680.77	863.52
A_68_P23320286	chr4:133523495-133523539	NM_016957:390	Hmgn2	INSIDE	1.215	0.191	3344.10	637.34	0.232	2363.78	547.39
A_68_P29531975	chr14:35636100-35636144	NM_008166:2801	Grid1	INSIDE	1.214	0.364	1707.15	621.27	0.442	1199.16	529.74
A_68_P26713770	chr9:89599836-89599880	NM_025360:23	Tmed3	INSIDE	1.214	0.386	2039.77	788.19	0.469	1648.45	773.22
A_68_P23804519	chr5:75016038-75016082	NM_001159578:57837	Lnx1	INSIDE	1.214	1.554	690.64	1073.03	1.887	497.35	938.26
A_68_P23752944	chr5:65194780-65194824	NM_008453:41	Klf3	INSIDE	1.214	0.533	2168.14	1155.01	0.647	1661.66	1074.97
A_68_P22069323	chr3:38354784-38354828	NM_001167883:28932	Ankrd50	INSIDE	1.214	1.415	836.46	1183.62	1.718	686.67	1180.04
A_68_P28033246	chr11:94347284-94347328	NM_144827:-682	Spata20	PROMOTER	1.213	0.429	1094.03	469.58	0.520	884.81	460.52
A_68_P26811066	chr9:107673364-107673408	NM_148930:-53	Rbm5	PROMOTER	1.213	0.375	2534.94	951.68	0.455	1806.99	823.04
A_68_P31162851	chr17:36304426-36304470	NM_001081032:1034	Gm8909	INSIDE	1.212	0.263	2732.01	717.32	0.318	1974.55	628.55
A_68_P29620446	chr14:56503972-56504016	NM_027143:189	Khnyn	INSIDE	1.212	0.201	3182.28	639.04	0.243	2561.03	623.11
A_68_P28054171	chr11:97914188-97914232	NM_146028:566	Stac2	INSIDE	1.212	0.412	1827.52	753.76	0.500	1366.91	683.41
A_68_P25089581	chr7:52094932-52094976	NM_010215:3297	Il4i1	INSIDE	1.212	3.248	2344.90	7616.81	3.936	1923.09	7568.65
A_68_P24602909	chr6:85026381-85026425	NR_002702:268	Npm3-ps1	INSIDE	1.212	1.416	1307.30	1850.96	1.716	1050.03	1801.77
A_68_P22481535	chr3:121977658-121977702	NM_153806:349	Dnttip2	INSIDE	1.212	0.434	2595.32	1126.84	0.526	1874.35	986.69
A_68_P21933400	chr3:8664630-8664674	NM_010423:2386	Hey1	INSIDE	1.212	0.371	2105.80	781.37	0.450	1675.46	753.70
A_68_P21646218	chr2:133378320-133378364	NM_007553:-592	Bmp2	PROMOTER	1.212	0.422	2139.81	901.98	0.511	1659.65	848.21
A_68_P20157010	chr1:38053691-38053737	NM_172054:339	Txndc9	INSIDE	1.212	2.310	324.13	748.58	2.799	283.38	793.33
A_68_P31933116	chr19:6184605-6184649	NM_001165919:217	1700123I01Rik	INSIDE	1.211	2.315	397.59	920.22	2.803	364.23	1020.76
A_68_P31757541	chr18:61560019-61560063	NM_133249:45	Ppargc1b	INSIDE	1.211	2.510	297.96	747.90	3.039	298.43	906.89
A_68_P31631086	chr18:38114645-38114689	NM_010411:-24	Hdac3	DIVERGENT_PROMOTER	1.211	0.206	2248.85	464.31	0.250	1718.36	429.55
A_68_P31112725	chr17:26978307-26978351	NM_008700:182	Nkx2-5	INSIDE	1.211	0.257	1819.82	468.26	0.312	1352.32	421.39
A_68_P30397307	chr15:84686477-84686521	NM_001081166:61	Phf21b	INSIDE	1.211	3.050	475.45	1449.91	3.694	422.82	1561.76
A_68_P28058440	chr11:98635015-98635059	NM_145434:1520	Nr1d1	INSIDE	1.211	1.841	386.64	711.78	2.230	327.60	730.41
A_68_P26887820	chr9:122075655-122075699	NM_001164572:49293	Snrk	INSIDE	1.211	1.692	2234.85	3781.60	2.049	1613.67	3306.42
A_68_P25574930	chr7:146132695-146132739	NM_028708:284	Jakmip3	INSIDE	1.211	0.187	6719.69	1254.53	0.226	4236.82	957.54
A_68_P23439845	chr4:155027518-155027562	NM_011985:-47	Mmp23	PROMOTER	1.211	0.466	1399.98	652.78	0.565	1001.00	565.18
A_68_P28727350	chr12:109566414-109566458	NM_001044380:21957	Hhipl1	INSIDE	1.210	0.458	1188.84	544.19	0.554	953.79	528.19
A_68_P28187705	chr11:120810221-120810265	NM_001038653:-138	Slc16a3	PROMOTER	1.210	0.449	1245.90	559.87	0.544	1022.40	555.90
A_68_P26158627	chr8:112248255-112248299	NM_029468:18289	Zfp821	INSIDE	1.210	2.173	2460.53	5345.89	2.629	1668.51	4386.37
A_68_P26019015	chr8:86780899-86780943	NM_026423:303	Mri1	INSIDE	1.210	1.637	1240.16	2029.86	1.981	1024.56	2029.87
A_68_P25586657	chr7:148278318-148278362	NM_023059:8105	Sigirr	INSIDE	1.210	0.351	1413.74	496.88	0.425	1015.59	431.87
A_68_P25024699	chr7:29766684-29766728	NM_028659:127	Eif3k	INSIDE	1.210	0.410	1805.29	739.70	0.496	1276.41	632.73
A_68_P24757357	chr6:113027235-113027279	NR_027010:-18	Gt(ROSA)26Sor	DIVERGENT_PROMOTER	1.210	1.694	815.10	1380.87	2.049	636.56	1304.34
A_68_P23577043	chr5:31502922-31502966	NM_001177901:1471	Zfp513	INSIDE	1.210	1.429	2812.49	4020.40	1.729	2212.28	3825.83

ProbeName	Target position of probe on CpG island microarray	TargetID	GeneSymbol	CpG island Description	Ratio of relative methylation (TiO_2-NP/Vehicle)	Sham group			TiO_2-H group		
						Relative methylation (Cy5/Cy3)	Cy3 signal (Input)	Cy5 signal (MeDIP)	Relative methylation (Cy5/Cy3)	Cy3 signal (Input)	Cy5 signal (MeDIP)
A_68_P22970870	chr4:63155745-63155789	NM_001008791:1219	Whrn	INSIDE	1.210	0.429	2769.24	1187.59	0.519	2112.61	1096.19
A_68_P22344500	chr3:95363378-95363422	NM_026489:-198	Hormad1	PROMOTER	1.210	1.711	713.83	1221.57	2.070	582.22	1205.47
A_68_P32135815	chr19:45080453-45080497	NM_053164:458	Mrpl43	INSIDE	1.209	0.122	5439.85	665.58	0.148	3434.22	508.00
A_68_P29643099	chr14:60795734-60795778	ENSMUST00000140924:620		INSIDE	1.209	0.489	1735.64	848.43	0.591	1364.34	806.03
A_68_P26217007	chr8:122480845-122480889	NM_009462:46115	Usp10	INSIDE	1.209	2.663	1472.27	3920.41	3.220	1195.76	3850.75
A_68_P24322954	chr6:28782175-28782219	NM_138682:-449	Lrrc4	PROMOTER	1.209	0.319	1526.63	487.61	0.386	1341.45	517.95
A_68_P24186284	chr5:150438995-150439039	NM_013559:-126	Hsph1	PROMOTER	1.209	0.356	1401.32	499.15	0.431	1127.22	485.44
A_68_P22207890	chr3:66785065-66785109	NM_013665:607	Shox2	INSIDE	1.209	5.664	653.19	3699.57	6.849	588.23	4028.74
A_68_P21752058	chr2:152768919-152768963	NM_001159376:8378	Dusp15	INSIDE	1.209	1.952	470.86	919.15	2.360	398.50	940.46
A_68_P31940699	chr19:7686892-7686936	NM_025731:-144	Hrasls5	DIVERGENT_PROMOTER	1.208	0.332	2765.98	919.28	0.401	2109.68	846.96
A_68_P30762452	chr16:52452809-52452853	NM_009655:280	Alcam	INSIDE	1.208	0.395	2252.61	888.65	0.477	1654.41	788.58
A_68_P28744821	chr12:112813339-112813383	NM_021516:640	Mark3	INSIDE	1.208	0.369	1416.32	522.06	0.445	1150.65	512.54
A_68_P28549421	chr12:76697259-76697303	NM_012024:-93	Ppp2r5e	PROMOTER	1.208	0.286	3449.50	988.07	0.346	2404.89	832.00
A_68_P28267484	chr12:16901480-16901524	NM_009072:-281	Rock2	PROMOTER	1.208	1.695	1580.31	2678.09	2.047	1188.06	2432.56
A_68_P27288893	chr10:80788499-80788543	NM_027381:4673	2510012J08Rik	INSIDE	1.208	1.675	2203.42	3691.43	2.024	1499.36	3034.94
A_68_P24469553	chr6:55402157-55402201	NM_001025372:205	Adcyap1r1	INSIDE	1.208	1.814	1467.32	2662.13	2.191	1130.30	2476.27
A_68_P21078907	chr2:26321004-26321048	NM_008714:38316	Notch1	INSIDE	1.208	1.836	1554.48	2853.81	2.217	1161.53	2575.60
A_68_P20558185	chr1:121545078-121545122	NM_145506:477	Epb4.1l5	INSIDE	1.208	0.311	1429.32	445.04	0.376	1107.74	416.55
A_68_P29855890	chr14:102007762-102007806	NM_001081278:624	Tbc1d4	INSIDE	1.207	0.235	2583.18	606.07	0.283	2019.12	571.66
A_68_P24792873	chr6:119339010-119339056	NM_197985:28469	Adipor2	INSIDE	1.207	0.271	2638.21	715.31	0.327	2101.54	687.50
A_68_P24536898	chr6:71494106-71494150	NM_025783:281	Vps24	INSIDE	1.207	2.303	788.48	1816.12	2.781	639.74	1778.80
A_68_P21177699	chr2:44412715-44412759	NM_172662:304406	Gtdc1	DOWNSTREAM	1.207	0.504	1664.86	839.17	0.608	1248.72	759.55
A_68_P20740950	chr1:157820522-157820566	NM_001039184:-159	Cep350	PROMOTER	1.207	1.857	2041.57	3790.34	2.242	1556.98	3490.14
A_68_P31227870	chr17:50432887-50432931	NM_010021:16	Dazl	INSIDE	1.206	2.117	1399.11	2961.75	2.553	1261.61	3220.33
A_68_P29116719	chr13:69750004-69750048	NM_175283:315	Srd5a1	INSIDE	1.206	0.298	2638.87	786.99	0.360	2086.18	750.48
A_68_P28170165	chr11:118110109-118110153	NM_001112699:-224	Cyth1	PROMOTER	1.206	2.196	1181.59	2594.29	2.649	1252.73	3318.30
A_68_P26890110	chr9:122478797-122478841	NR_015610:-2800	9530059O14Rik	PROMOTER	1.206	0.379	2191.09	829.73	0.457	1396.37	637.47
A_68_P25579476	chr7:146846040-146846084	ENSMUST00000118750:-8		PROMOTER	1.206	2.233	2714.71	6063.11	2.694	2058.94	5546.25
A_68_P25505762	chr7:134622580-134622624	NM_010183:-6132	Fbrs	PROMOTER	1.206	2.489	393.60	979.55	3.000	320.21	960.72
A_68_P24769157	chr6:115084895-115084939	NM_013681:-3	Syn2	PROMOTER	1.206	2.718	1914.24	5202.17	3.277	1552.24	5086.53
A_68_P23222369	chr4:114598689-114598733	NM_015758:-92	Foxe3	PROMOTER	1.206	1.723	1932.01	3329.67	2.078	1658.22	3446.52
A_68_P22733170	chr4:12015068-12015112	NM_177861:14	Tmem67	INSIDE	1.206	0.260	1889.03	491.51	0.314	1425.83	447.58
A_68_P22570417	chr3:138405329-138405373	NM_019571:192	Tspan5	INSIDE	1.206	2.422	279.64	677.21	2.920	223.35	652.12
A_68_P21079937	chr2:26459672-26459716	NM_026212:36	Agpat2	INSIDE	1.206	1.433	9973.52	14290.83	1.728	7332.92	12667.68
A_68_P32536863	chrX:91225992-91226036	NR_028320:12020	AU015836	DOWNSTREAM	1.205	2.189	319.89	700.34	2.637	490.12	1292.63
A_68_P30000044	chr15:8393984-8394028	NM_027707:457	Nipbl	INSIDE	1.205	1.848	489.20	904.14	2.228	443.53	988.07
A_68_P28881067	chr13:23461111-23461155	NM_001111107:-61	Zfp322a	PROMOTER	1.205	0.213	2438.98	520.61	0.257	1804.57	464.03
A_68_P28184541	chr11:120298583-120298627	NM_001195023:410	Nploc4	INSIDE	1.205	0.452	2081.25	941.46	0.545	1730.26	943.28
A_68_P26822985	chr9:110207937-110207981	NM_001081381:263	2610002I17Rik	INSIDE	1.205	0.201	2201.44	442.62	0.242	1720.73	416.88
A_68_P26748151	chr9:96531280-96531324	NM_053268:620	Rasa2	INSIDE	1.205	0.459	2770.06	1272.72	0.554	2343.52	1297.69
A_68_P26345602	chr9:20935593-20935637	NM_001205312:105	Tyk2	INSIDE	1.205	0.549	1811.60	994.36	0.662	1361.16	900.55
A_68_P25410469	chr7:117008961-117009005	NM_020052:211	Scube2	INSIDE	1.205	3.563	1046.59	3729.15	4.294	877.42	3767.53
A_68_P21569908	chr2:119302816-119302860	NM_026574:527	Ino80	INSIDE	1.205	0.358	1783.85	638.49	0.431	1408.56	607.67
A_68_P32026208	chr19:24936124-24936168	NM_175013:186	Pgm5	INSIDE	1.204	0.420	1048.58	440.90	0.506	820.62	415.38
A_68_P31257917	chr17:56856713-56856757	NM_178926:-390	Vmac	PROMOTER	1.204	0.216	4758.42	1026.60	0.260	3054.12	793.29
A_68_P31206428	chr17:46464777-46464821	NM_145140:-2083	Abcc10	PROMOTER	1.204	0.374	1541.53	576.39	0.450	1265.31	569.76
A_68_P27552973	chr11:3529636-3529680	ENSMUST00000121615:832		INSIDE	1.204	0.303	1552.06	471.01	0.365	1150.43	420.26
A_68_P26991381	chr10:20879458-20879502	NM_001198914:1310	Myb	INSIDE	1.204	1.699	2649.30	4502.22	2.047	1211.58	2479.94
A_68_P26803572	chr9:106367744-106367788	NM_001159652:505	Gpr62	INSIDE	1.204	1.475	1065.49	1571.41	1.776	813.87	1445.14
A_68_P26783276	chr9:102911099-102911143	NM_033314:302	Slco2a1	INSIDE	1.204	0.435	1241.42	540.14	0.524	951.93	498.76
A_68_P26585941	chr9:64971903-64971947	NM_020043:22623	Igdcc4	INSIDE	1.204	1.749	949.08	1659.80	2.106	634.24	1335.92
A_68_P25609691	chr7:152047946-152047990	NM_010202:678	Fgf4	INSIDE	1.204	0.284	1641.62	465.71	0.341	1177.33	402.05
A_68_P24908781	chr6:143116381-143116425	NM_029250:653	Etnk1	INSIDE	1.204	0.176	2774.05	487.16	0.211	1869.09	395.27
A_68_P23991341	chr5:112736511-112736555	NM_009419:30806	Tpst2	INSIDE	1.204	1.510	821.95	1240.82	1.817	641.41	1165.58
A_68_P22889661	chr4:46627029-46627073	NM_198664:36021	Tbc1d2	INSIDE	1.204	2.432	1152.89	2804.25	2.930	979.64	2870.07
A_68_P21571901	chr2:119698597-119698641	ENSMUST00000120952:5521		DOWNSTREAM	1.204	0.542	2312.12	1252.99	0.652	1847.25	1204.98
A_68_P30497078	chr15:102126593-102126637	NM_001014976:-109	Espl1	PROMOTER	1.203	0.477	1360.38	649.44	0.574	999.09	573.97
A_68_P30337992	chr15:74394645-74394689	NM_174991:48041	Bai1	INSIDE	1.203	0.372	1877.10	698.43	0.448	1372.60	614.36
A_68_P29668832	chr14:65271520-65271564	NM_001081177:175	Kif13b	INSIDE	1.203	0.371	3085.61	1144.65	0.446	2470.66	1102.34
A_68_P26816898	chr9:108735159-108735203	NM_080437:6530	Celsr3	INSIDE	1.203	0.556	2450.47	1361.43	0.668	1927.36	1288.15
A_68_P21640034	chr2:132210975-132211019	NM_144944:187	Prokr2	INSIDE	1.203	0.323	2172.71	700.84	0.388	1625.00	630.38
A_68_P31868104	chr18:81182542-81182586	NM_178280:753	Sall3	INSIDE	1.202	0.354	1255.58	444.73	0.426	950.61	404.79
A_68_P28221298	chr12:8305600-8305644	NM_013527:3138	Gdf7	INSIDE	1.202	2.082	1726.95	3596.34	2.502	1337.95	3347.93
A_68_P27756066	chr11:44283825-44283869	NM_024475:204	Ublcp1	INSIDE	1.202	0.392	1753.48	687.03	0.471	1306.87	615.46
A_68_P27077934	chr10:39089328-39089372	NM_001122892:-254	Fyn	PROMOTER	1.202	0.296	9694.68	2871.25	0.356	6086.34	2166.36

ProbeName	Target position of probe on CpG island microarray	TargetID	GeneSymbol	CpG island Description	Ratio of relative methylation (TiO_2-NP/Vehicle)	Sham group			TiO_2-H group		
						Relative methylation (Cy5/Cy3)	Cy3 signal (Input)	Cy5 signal (MeDIP)	Relative methylation (Cy5/Cy3)	Cy3 signal (Input)	Cy5 signal (MeDIP)
A_68_P25532700	chr7:139346324-139346368	NM_018867:78	Cpxm2	INSIDE	1.202	3.841	2104.28	8082.00	4.618	1958.18	9043.76
A_68_P25398385	chr7:114353404-114353448	NM_029660:468	Rbmxl2	INSIDE	1.202	1.589	1054.21	1675.11	1.910	1050.13	2006.16
A_68_P25015864	chr7:27951357-27951401	NM_053208:443	Egln2	INSIDE	1.202	0.170	3522.58	599.91	0.205	2527.01	517.37
A_68_P24377801	chr6:38484955-38484999	NM_025363:116	1110001J03Rik	INSIDE	1.202	0.431	1602.63	690.35	0.518	1192.09	617.01
A_68_P23966390	chr5:107760143-107760187	NM_001079873:-48	Brdt	PROMOTER	1.202	1.959	1306.55	2559.29	2.355	1199.83	2825.20
A_68_P21774855	chr2:156866013-156866057	NM_001164663:38967	9830001H06Rik	INSIDE	1.202	2.541	554.80	1409.93	3.055	492.55	1504.74
A_68_P20638618	chr1:137210111-137210155	NM_011590:182	Timm17a	INSIDE	1.202	0.273	2589.31	706.61	0.328	1649.59	541.32
A_68_P20296826	chr1:65225580-65225625	NM_010497:114	Idh1	INSIDE	1.202	0.374	1397.53	521.98	0.449	1058.00	474.88
A_68_P30580603	chr16:18586991-18587035	NM_011532:50	Tbx1	INSIDE	1.201	0.360	2071.59	746.02	0.433	1487.07	643.38
A_68_P29055508	chr13:55614427-55614471	NM_001114088:352	Pdlim7	INSIDE	1.201	0.567	3067.32	1737.68	0.681	2242.24	1525.91
A_68_P26543018	chr9:57493508-57493552	NM_007783:-543	Csk	PROMOTER	1.201	1.496	721.58	1079.84	1.798	606.18	1089.81
A_68_P24981705	chr7:17060954-17060998	NM_008718:1153	Npas1	INSIDE	1.201	0.224	3744.80	837.27	0.269	2942.71	790.44
A_68_P24864234	chr6:134870563-134870607	NM_009875:166	Cdkn1b	INSIDE	1.201	0.476	3963.73	1886.23	0.572	2830.26	1617.77
A_68_P31217294	chr17:48549181-48549225	NM_001110832:-57	Nfya	DIVERGENT_PROMOTER	1.200	0.318	10935.71	3477.58	0.382	7384.42	2818.81
A_68_P31006136	chr17:3557684-3557728	NM_146074:7	Tfb1m	INSIDE	1.200	0.354	3178.70	1125.87	0.425	2137.29	908.35
A_68_P28164253	chr11:117151685-117151737	NM_001113487:24151	Septin9	INSIDE	1.200	2.475	212.44	525.73	2.970	217.13	644.98
A_68_P26276226	chr9:4260571-4260615			Unknown	1.200	1.674	1397.36	2339.40	2.009	1118.25	2246.17
A_68_P25394536	chr7:112959464-112959508	NM_025301:115	Mrpl17	INSIDE	1.200	0.394	2723.32	1074.28	0.473	1940.93	918.55
A_68_P25261545	chr7:86660396-86660440	ENSMUST00000150985:301		INSIDE	1.200	1.521	1512.18	2299.49	1.825	1131.75	2065.97
A_68_P24994417	chr7:20214353-20214397	NM_009046:413	Relb	INSIDE	1.200	0.398	3398.68	1353.16	0.478	2331.50	1113.84
A_68_P22049383	chr3:34548418-34548462	NM_011443:-486	Sox2	PROMOTER	1.200	0.150	3667.76	549.91	0.180	2699.83	485.93
A_68_P20348980	chr1:74552200-74552244	NM_021383:-411	Rqcd1	PROMOTER	1.200	0.163	5136.77	838.28	0.196	3847.01	753.11
A_68_P32536213	chrX:91077623-91077667	NM_145630:-210	Pdk3	PROMOTER	1.199	2.264	757.29	1714.32	2.715	776.53	2108.38
A_68_P32364065	chrX:45698842-45698886	NM_178782:4330	Bcorl1	INSIDE	1.199	2.293	633.42	1452.22	2.749	766.99	2108.65
A_68_P31112416	chr17:26918138-26918182	NM_172149:137	Bnip1	INSIDE	1.199	0.223	2345.85	523.44	0.267	1733.93	463.74
A_68_P28291793	chr12:25515677-25515721	NM_001083341:-765	Mboat2	PROMOTER	1.199	16.018	1261.34	20204.30	19.207	1498.06	28773.75
A_68_P26971602	chr10:17443634-17443678	NM_010828:623	Cited2	INSIDE	1.199	0.233	4223.75	984.84	0.280	3168.59	886.11
A_68_P25022018	chr7:29244235-29244279	NM_001141921:7000	Lrfn1	INSIDE	1.199	2.281	786.29	1793.56	2.734	613.44	1677.14
A_68_P23210414	chr4:111087603-111087647	NM_026279:14	Bend5	INSIDE	1.199	1.887	2131.70	4022.14	2.261	1651.39	3734.60
A_68_P31834370	chr18:75160579-75160623	NR_028552:-121	Snord58b	PROMOTER	1.198	0.252	2136.70	538.59	0.302	1743.51	526.70
A_68_P29613713	chr14:55305483-55305527	NM_001085473:264	Acin1	INSIDE	1.198	1.520	1637.40	2489.30	1.822	1194.13	2175.36
A_68_P28056108	chr11:98248019-98248063	NM_008890:95	Pnmt	INSIDE	1.198	0.402	2122.65	852.60	0.481	1679.16	807.77
A_68_P25092513	chr7:52594761-52594805	NM_029741:27607	Ppfia3	INSIDE	1.198	1.587	640.62	1016.48	1.901	467.52	888.96
A_68_P31601517	chr18:32322542-32322586	NM_011946:-178	Map3k2	PROMOTER	1.197	2.261	465.55	1052.63	2.707	416.86	1128.47
A_68_P28155402	chr11:115664810-115664854	NM_080643:10074	Caskin2	INSIDE	1.197	1.818	994.11	1807.49	2.176	690.47	1502.52
A_68_P28003251	chr11:88860482-88860526	NM_009546:-212	Trim25	PROMOTER	1.197	0.488	1602.92	781.62	0.584	1195.66	698.08
A_68_P25724985	chr8:26149132-26149176	NM_001081187:280	Htra4	INSIDE	1.197	2.500	950.00	2375.43	2.993	862.89	2582.85
A_68_P25094661	chr7:52968968-52969012	NM_020011:1587	Sphk2	INSIDE	1.197	1.414	1175.57	1661.68	1.691	965.85	1633.60
A_68_P24617030	chr6:87540705-87540749	NM_021381:-31	Prokr1	PROMOTER	1.197	0.308	3575.60	1102.94	0.369	2514.43	928.39
A_68_P31958296	chr19:11952929-11952973	NM_001011775:-7246	Olfr1419	PROMOTER	1.196	1.574	1350.64	2125.56	1.883	1136.18	2139.27
A_68_P31142305	chr17:31991956-31992000	NM_010831:759	Sik1	INSIDE	1.196	0.425	1809.98	768.91	0.508	1392.42	707.26
A_68_P31029165	chr17:8993570-8993614	NM_011866:-1017	Pde10a	PROMOTER	1.196	1.723	2033.33	3503.98	2.062	1493.65	3079.64
A_68_P30360708	chr15:78370197-78370241	NM_009218:4557	Sstr3	INSIDE	1.196	1.502	1015.87	1525.96	1.797	875.19	1572.94
A_68_P28924080	chr13:31718919-31718963	NM_010225:1256	Foxf2	INSIDE	1.196	0.268	3113.15	833.39	0.320	2195.73	702.85
A_68_P28530004	chr12:73172194-73172239	NM_181752:-238	Gpr135	PROMOTER	1.196	0.443	1336.89	592.86	0.530	1049.76	556.56
A_68_P27540751	chr10:127759837-127759881	NM_019953:344	Cnpy2	INSIDE	1.196	0.439	3478.05	1528.30	0.525	2512.70	1320.10
A_68_P25890689	chr8:59819744-59819788	ENSMUST00000132988:8852		DOWNSTREAM	1.196	2.799	1054.82	2952.26	3.347	727.08	2433.72
A_68_P25780170	chr8:36558186-36558230	NM_026067:379	Eri1	INSIDE	1.196	0.494	1689.38	833.92	0.591	1308.63	772.78
A_68_P23574737	chr5:31117105-31117149	NM_133350:-2	Mapre3	PROMOTER	1.196	1.541	613.68	945.92	1.844	512.57	945.05
A_68_P23341303	chr4:137237621-137237665	NM_001081155:25	Rap1gap	INSIDE	1.196	0.159	3133.15	496.87	0.190	2335.90	442.96
A_68_P22199717	chr3:65196220-65196264	NM_026155:233	Ssr3	INSIDE	1.196	0.174	7469.40	1297.17	0.208	5185.42	1077.14
A_68_P21680286	chr2:139503286-139503330	NM_001126490:-605	Ism1	PROMOTER	1.196	0.258	3001.58	774.40	0.309	2202.59	679.61
A_68_P23574106	chr5:31014351-31014395	NM_023047:105	Dpysl5	INSIDE	1.195	0.273	5138.30	1404.40	0.326	3413.44	1114.48
A_68_P23270673	chr4:124528328-124528372	NM_153566:348	Yrdc	INSIDE	1.195	4.452	179.05	797.22	5.322	132.78	706.60
A_68_P21099539	chr2:29658324-29658368	NM_011989:147	Slc27a4	INSIDE	1.195	0.552	3098.36	1711.19	0.660	2201.71	1453.58
A_68_P20616242	chr1:133423393-133423437	NM_001081011:524	Srgap2	INSIDE	1.195	0.275	3029.11	832.82	0.329	2025.26	665.36
A_68_P28563523	chr12:79327207-79327251	NM_145965:-413	Gphn	PROMOTER	1.194	0.445	1394.89	620.15	0.531	1284.30	681.66
A_68_P27995148	chr11:87430476-87430520	NM_001045527:-167	Hsf5	PROMOTER	1.194	2.724	710.37	1935.09	3.254	632.21	2057.12
A_68_P27937764	chr11:77285096-77285140	NM_172945:18062	Ankrd13b	INSIDE	1.194	1.397	1523.46	2127.97	1.667	1142.59	1905.22
A_68_P26018414	chr8:86671587-86671631	NM_145970:44	Cc2d1a	INSIDE	1.194	0.332	1988.97	660.05	0.396	1501.56	595.05
A_68_P25843139	chr8:48798999-48799043	NM_172407:265	Cdkn2aip	INSIDE	1.194	0.474	1328.32	629.40	0.566	988.30	559.22
A_68_P25512274	A_68_P25512274			Unknown	1.194	1.584	931.85	1476.37	1.892	763.24	1443.93
A_68_P24141721	chr5:140981647-140981691	NM_021528:106	Chst12	INSIDE	1.194	0.383	4743.33	1815.18	0.457	3521.73	1609.30
A_68_P22286850	chr3:83570771-83570815	NM_009144:550	Sfrp2	INSIDE	1.194	0.460	1302.53	599.45	0.549	943.08	518.13
A_68_P32565191	chrX:98891432-98891476	ENSMUST00000073812:-4265		PROMOTER	1.193	2.391	474.10	1133.65	2.852	750.55	2140.88

ProbeName	Target position of probe on CpG island microarray	TargetID	GeneSymbol	CpG island Description	Ratio of relative methylation (TiO_2-NP/Vehicle)	Sham group			TiO_2-H group		
						Relative methylation (Cy5/Cy3)	Cy3 signal (Input)	Cy5 signal (MeDIP)	Relative methylation (Cy5/Cy3)	Cy3 signal (Input)	Cy5 signal (MeDIP)
A_68_P30718912	chr16:43979481-43979525	NM_001007460:-339	Zdhhc23	PROMOTER	1.193	0.338	1409.19	476.02	0.403	1175.13	473.60
A_68_P29671485	chr14:65695365-65695412	NM_018788:21555	Extl3	INSIDE	1.193	1.596	635.02	1013.19	1.903	436.42	830.45
A_68_P29130723	chr13:72100965-72101009	NM_010573:185	Irx1	INSIDE	1.193	0.560	2578.54	1442.88	0.668	2005.41	1338.87
A_68_P26023615	chr8:87604389-87604433	NM_026399:235	Wdr83	INSIDE	1.193	0.304	4658.02	1414.46	0.362	3187.00	1154.29
A_68_P25496221	chr7:132850823-132850867	NM_207239:358	Gtf3c1	INSIDE	1.193	0.102	4310.39	440.04	0.122	3187.31	388.13
A_68_P24151769	chr5:142939785-142939829	NM_172725:-78	C330006K01Rik	PROMOTER	1.193	0.490	1963.57	962.48	0.585	1385.86	810.61
A_68_P22888257	chr4:46356845-46356889	NM_183298:-199	Foxe1	PROMOTER	1.193	0.349	1485.26	518.90	0.417	1193.11	497.10
A_68_P20646991	chr1:138521544-138521595	NM_001160251:92	Zfp281	INSIDE	1.193	1.711	487.78	834.66	2.041	400.73	817.73
A_68_P31867986	chr18:81168668-81168712	NM_178280:14627	Sall3	INSIDE	1.192	1.797	824.78	1481.80	2.142	659.84	1413.45
A_68_P30966735	chr16:92059004-92059048	NM_080456:446	Mrps6	INSIDE	1.192	0.353	2214.24	782.70	0.421	1602.29	675.31
A_68_P29222294	chr13:92901468-92901512	NM_009027:-41	Rasgrf2	PROMOTER	1.192	0.432	1301.99	563.07	0.515	1066.45	549.72
A_68_P24743884	chr6:110595643-110595687	NM_177328:73	Grm7	INSIDE	1.192	0.169	3812.96	644.57	0.202	2827.30	569.91
A_68_P22071617	chr3:38787100-38787144	NM_183221:1261	Fat4	INSIDE	1.192	0.276	1781.59	491.08	0.329	1276.02	419.32
A_68_P31151048	chr17:34066906-34066950	NM_009059:90	Rgl2	INSIDE	1.191	0.376	3099.73	1166.90	0.448	2089.05	936.67
A_68_P29941247	chr14:119254329-119254375	NM_001160099:181	Cldn10	INSIDE	1.191	2.131	395.91	843.87	2.538	362.86	920.96
A_68_P29675225	chr14:66349795-66349839	NM_001168318:64577	Scara5	INSIDE	1.191	1.816	990.35	1798.09	2.163	811.45	1754.77
A_68_P27259491	chr10:75398745-75398789	NM_175329:449	Chchd10	INSIDE	1.191	0.220	2501.66	549.30	0.261	1816.74	474.98
A_68_P27256916	chr10:74980191-74980235	NM_175133:-145	1110038D17Rik	DIVERGENT_PROMOTER	1.191	0.355	1487.55	527.53	0.423	1267.35	535.48
A_68_P27094093	chr10:41997793-41997837	NM_019740:-1266	Foxo3	PROMOTER	1.191	0.452	1901.95	858.90	0.538	1510.34	812.41
A_68_P25780627	chr8:36650640-36650684	NM_001081279:-189	Mfhas1	PROMOTER	1.191	0.306	1869.13	572.00	0.365	1433.66	522.69
A_68_P25505917	chr7:134656518-134656562	NR_024331:-157	1700008J07Rik	PROMOTER	1.191	0.420	1449.26	609.25	0.501	1109.24	555.55
A_68_P24593027	chr6:83318037-83318081	NM_183138:73614	Tet3	INSIDE	1.191	1.501	726.11	1090.14	1.788	703.04	1256.71
A_68_P32567037	chrX:99266374-99266418	NM_183318:250	Rgag4	INSIDE	1.190	1.936	1798.24	3482.15	2.304	2307.36	5316.97
A_68_P26273049	chr9:3199691-3199735	NR_027988:101	4930433N12Rik	INSIDE	1.190	7.651	3269.02	25010.24	9.101	2174.29	19787.41
A_68_P25837692	chr8:47826584-47826628	NM_008391:1508	Irf2	INSIDE	1.190	1.546	547.86	846.78	1.840	511.85	941.66
A_68_P23311790	chr4:131885707-131885751	NM_027925:-275	Trnau1ap	PROMOTER	1.190	0.413	1937.79	801.10	0.492	1532.28	753.52
A_68_P23091881	chr4:88367948-88367992	NM_172871:442	Klhl9	INSIDE	1.190	1.372	1702.46	2335.31	1.632	1349.34	2201.96
A_68_P22408057	chr3:107499480-107499524	NM_145542:-36	Ahcyl1	PROMOTER	1.190	0.219	2415.64	529.02	0.261	1659.14	432.42
A_68_P20268941	chr1:60466118-60466162	NM_001198570:-322	Abi2	PROMOTER	1.190	0.346	1424.77	492.29	0.411	1195.03	491.50
A_68_P31097619	chr17:24625915-24625959	NM_027937:209	Caskin1	INSIDE	1.189	4.370	2619.40	11446.77	5.194	2335.25	12130.40
A_68_P27997244	chr11:87800515-87800559	NM_001168472:-449	Dynll2	PROMOTER	1.189	1.517	541.40	821.04	1.804	539.94	973.96
A_68_P27880946	chr11:66908183-66908227	NM_001099635:16403	Myh3	INSIDE	1.189	2.432	311.34	757.26	2.893	392.78	1136.30
A_68_P27784888	chr11:49608283-49608327	NM_013529:648	Gfpt2	INSIDE	1.189	0.352	1425.01	501.96	0.419	1077.50	451.11
A_68_P27289087	chr10:80820153-80820197	NM_028657:-140	F630110N24Rik	DIVERGENT_PROMOTER	1.189	1.817	1252.98	2276.46	2.160	1068.78	2308.52
A_68_P20161229	chr1:38683722-38683766	NM_010678:38056	Aff3	INSIDE	1.189	0.424	1609.29	682.81	0.504	1172.37	591.29
A_68_P28452660	chr12:56701063-56701107	NM_020287:1181	Insm2	INSIDE	1.188	0.340	2062.19	700.67	0.404	1614.75	651.86
A_68_P27674922	chr11:29426519-29426563	NM_133767:84	Mtif2	INSIDE	1.188	0.457	1146.49	524.35	0.543	1029.62	559.26
A_68_P27446113	chr10:110357232-110357276	NM_007792:23	Csrp2	INSIDE	1.188	0.487	2721.42	1325.33	0.579	2129.44	1232.26
A_68_P26659639	chr9:78296196-78296240	NM_026658:202	Mto1	INSIDE	1.188	2.245	925.24	2076.86	2.666	536.64	1430.48
A_68_P26550030	chr9:58670732-58670776	NM_001081192:-564	Hcn4	PROMOTER	1.188	2.571	546.67	1405.49	3.055	455.60	1391.89
A_68_P24059429	chr5:124573689-124573733	NM_011256:92717	Pitpnm2	INSIDE	1.188	2.884	830.26	2394.56	3.425	647.48	2217.61
A_68_P21489524	chr2:104333565-104333609	NM_001145824:1060	Hipk3	INSIDE	1.188	1.897	5429.51	10298.87	2.253	3848.77	8669.53
A_68_P30364603	chr15:78971259-78971303	NM_138581:401	1700088E04Rik	INSIDE	1.187	0.416	1076.77	447.82	0.494	807.07	398.40
A_68_P29737459	chr14:77436746-77436790	NM_175369:190	Ccdc122	INSIDE	1.187	0.378	1274.15	481.56	0.449	1055.83	473.62
A_68_P27846981	chr11:60988586-60988630	NM_001004143:-47	Usp22	PROMOTER	1.187	0.212	3657.66	774.00	0.251	2694.37	676.62
A_68_P25021461	chr7:29156631-29156675	NM_001083912:1029	Plekhg2	INSIDE	1.187	0.386	1815.27	700.94	0.458	1430.38	655.73
A_68_P24298747	chr6:23789611-23789655	NM_153163:-332	Cadps2	PROMOTER	1.187	1.450	2518.00	3650.78	1.721	1871.47	3220.56
A_68_P24163027	chr5:145612055-145612099	NM_001081362:82416	Trrap	INSIDE	1.187	1.716	773.17	1326.76	2.036	590.17	1201.71
A_68_P21754299	chr2:153171488-153171547	NM_001039939:-357	Asxl1	PROMOTER	1.187	3.699	149.65	553.61	4.390	150.68	661.46
A_68_P32260813	chrX:11737388-11737432	NM_001168321:71	Bcor	INSIDE	1.186	2.041	380.77	777.48	2.421	380.77	921.99
A_68_P31102615	chr17:25392896-25392940	NM_001163270:391	Baiap3	INSIDE	1.186	0.295	1778.57	524.11	0.350	1255.63	438.84
A_68_P29638536	chr14:60059932-60059976	NM_026908:137	Cab39l	INSIDE	1.186	0.347	2733.07	949.11	0.412	1940.70	799.04
A_68_P28931376	chr13:32893858-32893902	NM_030215:-18	Wrnip1	PROMOTER	1.186	0.241	1994.53	480.89	0.286	1422.58	406.72
A_68_P27894433	chr11:69154419-69154463	NR_027827:170	A030009H04Rik	INSIDE	1.186	0.597	4647.66	2776.79	0.708	3289.95	2330.53
A_68_P27287684	chr10:80606810-80606854	NM_010731:7817	Zbtb7a	INSIDE	1.186	2.237	398.15	890.56	2.654	333.72	885.56
A_68_P23979843	chr5:110877476-110877520	NM_001142642:24	Fbrsl1	INSIDE	1.186	0.289	1864.55	539.50	0.343	1341.62	460.47
A_68_P21311407	chr2:69699641-69699685	NM_001110145:44	Ssb	INSIDE	1.186	0.290	1710.32	496.14	0.344	1451.38	499.31
A_68_P32099206	chr19:38129609-38129653	NM_001164362:99	Cep55	INSIDE	1.185	0.440	1018.37	448.12	0.521	819.62	427.38
A_68_P31143943	chr17:32280673-32280717	NM_008716:23103	Notch3	INSIDE	1.185	0.353	2380.94	840.83	0.419	1708.01	714.89
A_68_P30346023	chr15:75922119-75922163	NM_144847:-1697	Nrbp2	PROMOTER	1.185	0.445	2190.81	974.97	0.528	1612.26	850.49
A_68_P29699019	chr14:70477604-70477648	NM_018781:375	Egr3	INSIDE	1.185	0.198	2217.60	439.04	0.235	1608.31	377.24
A_68_P29075345	chr13:58907821-58907865	NM_008745:-114	Ntrk2	PROMOTER	1.185	0.239	1987.29	474.19	0.283	1461.66	413.46
A_68_P28470861	chr12:60320508-60320552	NM_001033156:-60	Fbxo33	PROMOTER	1.185	0.457	1336.51	610.65	0.542	1134.72	614.46
A_68_P27892697	chr11:68853623-68853667	NM_011496:-5500	Aurkb	PROMOTER	1.185	0.478	1243.98	594.60	0.567	955.98	541.59
A_68_P27033723	chr10:28916701-28916745	NM_026138:52921	6330407J23Rik	INSIDE	1.185	2.017	1853.51	3738.08	2.389	1528.16	3650.73

ProbeName	Target position of probe on CpG island microarray	TargetID	GeneSymbol	CpG island Description	Ratio of relative methylation (TiO_2-NP/Vehicle)	Sham group			TiO_2-H group		
						Relative methylation (Cy5/Cy3)	Cy3 signal (Input)	Cy5 signal (MeDIP)	Relative methylation (Cy5/Cy3)	Cy3 signal (Input)	Cy5 signal (MeDIP)
A_68_P26816846	chr9:108729372-108729416	NM_080437:744	Celsr3	INSIDE	1.185	0.139	3678.16	509.61	0.164	2615.21	429.48
A_68_P26663737	chr9:79190663-79190707			Unknown	1.185	1.485	2733.28	4058.19	1.759	2078.71	3656.55
A_68_P23891543	chr5:92392231-92392285	NM_026557:-164	Rchy1	PROMOTER	1.185	1.508	698.96	1054.26	1.787	546.86	977.10
A_68_P21755508	chr2:153355057-153355101	NM_001134300:629	8430427H17Rik	INSIDE	1.185	0.194	4974.64	966.20	0.230	3249.40	748.04
A_68_P31897794	chr18:86565038-86565082	NM_144946:717	Neto1	INSIDE	1.184	0.585	3384.86	1978.50	0.692	2409.97	1668.38
A_68_P31135104	chr17:30749389-30749433	NM_001113560:194	Glo1	INSIDE	1.184	3.481	689.10	2398.47	4.120	522.05	2150.64
A_68_P30280956	chr15:64144021-64144065	NM_010026:70439	Asap1	INSIDE	1.184	0.406	1759.31	714.23	0.481	1313.29	631.06
A_68_P29220644	chr13:91947270-91947314	NM_145453:9	Zcchc9	INSIDE	1.184	0.381	1921.00	731.52	0.451	1639.46	739.39
A_68_P28950041	chr13:36670784-36670828	AK135154:191265		INSIDE	1.184	0.326	1675.62	546.33	0.386	1342.97	518.60
A_68_P28376185	chr12:40764703-40764747	NM_001039515:-150	Arl4a	PROMOTER	1.184	1.771	435.81	771.62	2.096	467.21	979.36
A_68_P28084545	chr11:103173341-103173385			Unknown	1.184	0.335	1579.78	529.37	0.397	1248.63	495.59
A_68_P27629548	chr11:19921114-19921161	NM_033523:96693	Spred2	INSIDE	1.184	3.147	272.22	856.61	3.727	274.54	1023.30
A_68_P26630945	chr9:72887670-72887714	NM_018889:-186	Pigb	DIVERGENT_PROMOTER	1.184	1.767	848.08	1498.97	2.092	808.35	1691.20
A_68_P26374354	chr9:26962948-26962992	NM_023277:36	Jam3	INSIDE	1.184	0.564	2647.75	1492.68	0.667	2067.00	1379.38
A_68_P25948521	chr8:72411450-72411494	NM_026818:-181	Cilp2	PROMOTER	1.184	0.308	2563.57	788.33	0.364	1875.65	682.98
A_68_P25585441	chr7:148067881-148067925	NM_001127351:-134	Sirt3	PROMOTER	1.184	0.296	2231.87	659.73	0.350	1745.19	610.64
A_68_P25409558	chr7:116847343-116847387	NM_020616:114	D930014E17Rik	INSIDE	1.184	0.265	1743.29	462.58	0.314	1400.54	440.15
A_68_P21703962	chr2:143688269-143688313	NM_009751:619	Bfsp1	INSIDE	1.184	0.184	3297.93	608.04	0.218	2456.15	536.03
A_68_P32290860	chrX:20539394-20539453	NR_027446:184	A230072C01Rik	INSIDE	1.183	2.278	386.92	881.45	2.695	356.80	961.61
A_68_P28248077	chr12:13255581-13255625	NM_134040:378	Ddx1	INSIDE	1.183	0.470	1819.93	856.14	0.556	1337.62	744.26
A_68_P26013517	chr8:85689748-85689792	NM_001111304:520	Tbc1d9	INSIDE	1.183	0.284	2129.00	604.84	0.336	1555.92	522.80
A_68_P25090602	chr7:52263044-52263088	NM_001008422:8553	Scaf1	INSIDE	1.183	2.075	1270.29	2635.58	2.454	858.07	2106.09
A_68_P23397710	chr4:148325835-148325879	NM_001159344:147356	Caszl	INSIDE	1.183	0.522	2520.96	1315.01	0.617	1797.54	1108.84
A_68_P23364322	chr4:141034341-141034385	NM_019763:60150	Spen	INSIDE	1.183	1.715	882.16	1512.81	2.029	771.26	1565.08
A_68_P23335793	chr4:136326759-136326803	NM_010142:65070	Ephb2	INSIDE	1.183	1.990	1284.61	2556.52	2.354	987.74	2325.57
A_68_P20625766	chr1:135027721-135027765	NM_133819:0	Ppp1r15b	INSIDE	1.183	0.553	2527.27	1396.34	0.654	1865.30	1219.11
A_68_P31151558	chr17:34162154-34162198	NR_029800:-139	Mir219-1	PROMOTER	1.182	0.129	3886.31	502.51	0.153	2552.29	389.96
A_68_P28072079	chr11:100980744-100980788	NM_026501:372	Fam134c	INSIDE	1.182	1.814	5546.84	10064.69	2.144	3954.99	8480.60
A_68_P27287762	chr10:80616963-80617007	NM_021501:13481	Pias4	INSIDE	1.182	2.127	879.17	1870.05	2.514	824.56	2072.57
A_68_P26846410	chr9:114690555-114690599	NM_133978:371	Cmtm7	INSIDE	1.182	0.273	2003.56	546.81	0.323	1528.30	493.03
A_68_P25951146	chr8:72855029-72855073	NM_008107:1387	Gdf1	INSIDE	1.182	0.408	1136.54	463.71	0.482	902.25	435.00
A_68_P25715446	chr8:24318402-24318446	NM_018743:501	Agpat6	INSIDE	1.182	0.366	2397.04	876.38	0.432	1766.68	763.60
A_68_P25357342	chr7:105851579-105851623	NM_028410:-272	Prkrir	PROMOTER	1.182	0.261	2454.40	640.93	0.309	1850.60	570.97
A_68_P24773305	chr6:115803441-115803485	NM_001167763:-83	Ift122	DIVERGENT_PROMOTER	1.182	0.139	3916.26	542.41	0.164	2491.83	407.96
A_68_P24455806	chr6:53237701-53237745	NR_015553:434	9430076C15Rik	INSIDE	1.182	0.276	2340.23	646.81	0.327	1748.07	570.98
A_68_P21775758	chr2:157030220-157030264	NM_011249:28	Rbl1	INSIDE	1.182	0.361	1705.55	615.45	0.427	1282.20	546.90
A_68_P20935541	chr1:192802311-192802355	NM_144879:545	Vash2	INSIDE	1.182	0.523	3295.08	1722.84	0.618	2306.33	1424.86
A_68_P32114751	chr19:41450414-41450458	NM_031376:9124	Pik3ap1	INSIDE	1.181	2.139	637.12	1362.57	2.525	608.22	1535.99
A_68_P31097729	chr17:24642452-24642496	NM_027937:16747	Caskin1	INSIDE	1.181	1.642	943.39	1549.19	1.940	726.47	1409.40
A_68_P30595154	chr16:21926561-21926606	NM_172821:34542	Map3k13	INSIDE	1.181	2.902	202.96	588.94	3.428	154.65	530.12
A_68_P28914773	chr13:30075688-30075732	NM_010093:2222	E2f3	INSIDE	1.181	0.201	2545.78	512.77	0.238	2063.17	490.93
A_68_P28554953	chr12:77707492-77707536	NM_013675:104020	Spnb1	INSIDE	1.181	2.161	249.69	539.53	2.552	250.48	639.31
A_68_P26824823	chr9:110532457-110532506	NM_183276:24184	Nbeal2	INSIDE	1.181	1.881	375.94	707.05	2.222	307.79	683.88
A_68_P22889891	chr4:46663047-46663091	NM_198664:3	Tbc1d2	INSIDE	1.181	0.377	2237.25	844.28	0.446	1678.11	748.16
A_68_P22310619	chr3:87710732-87710776	NM_008231:512	Hdgf	INSIDE	1.181	1.631	1567.60	2556.00	1.926	1257.82	2422.36
A_68_P21736750	chr2:149656913-149656958	NM_001085521:417	Tmem90b	INSIDE	1.181	0.267	2198.39	585.93	0.315	1868.05	587.98
A_68_P31092363	chr17:23694927-23694971	NM_001033496:6245	Zfp213	INSIDE	1.180	1.819	1228.78	2234.71	2.146	909.88	1952.25
A_68_P28185375	chr11:120434093-120434137	NM_011032:136	P4hb	INSIDE	1.180	0.350	1498.47	523.87	0.413	1027.12	423.77
A_68_P28102659	chr11:106473733-106473777	NM_198292:490	Tex2	INSIDE	1.180	0.186	4562.11	850.70	0.220	2792.82	614.49
A_68_P27865441	chr11:64249280-64249324	NM_178870:469	Hs3st3a1	INSIDE	1.180	0.516	1281.93	661.39	0.609	1083.91	659.67
A_68_P26238528	chr8:125736269-125736313	NM_145606:398	Chmp1a	INSIDE	1.180	0.370	2564.67	947.81	0.436	1700.72	741.47
A_68_P23998981	chr5:114037991-114038035	NM_177292:118546	Wscd2	INSIDE	1.180	2.143	1598.94	3427.07	2.530	1289.76	3262.99
A_68_P23223139	chr4:114730373-114730417	NM_011527:-1737	Tal1	PROMOTER	1.180	0.414	1361.93	564.14	0.489	1103.14	539.17
A_68_P20429508	chr1:91351074-91351118	NM_001037136:-289	Agap1	PROMOTER	1.180	0.465	1159.28	539.24	0.549	922.00	506.01
A_68_P33015612	chr1_random:49603-49647	NM_026866:135714	Disp1	INSIDE	1.179	1.968	1345.66	2648.54	2.321	1046.09	2428.27
A_68_P32465794	chrX:71212588-71212632	NM_008224:-956	Hcfc1	PROMOTER	1.179	1.473	762.22	1122.71	1.736	1032.39	1792.71
A_68_P31675568	chr18:46357840-46357884	NM_001170855:610	Trim36	INSIDE	1.179	3.438	627.58	2157.72	4.055	527.62	2139.40
A_68_P31629950	chr18:37927496-37927540	NM_033595:2285	Pcdhga12	INSIDE	1.179	2.288	528.09	1208.40	2.697	440.85	1189.10
A_68_P29338085	chr13:113784265-113784309	NM_001037914:211	Gm6320	INSIDE	1.179	0.396	1288.22	510.14	0.467	1078.82	503.49
A_68_P24112568	chr5:134790208-134790252	NM_001080746:386	Gtf2i	INSIDE	1.179	0.381	2706.59	1032.45	0.450	2015.01	906.05
A_68_P23336931	chr4:136501821-136501865	NM_007939:10889	Epha8	INSIDE	1.179	1.861	1219.06	2268.71	2.194	968.48	2125.29
A_68_P31095629	chr17:24278798-24278842	NM_011062:-259	Pdpk1	PROMOTER	1.178	1.715	1449.97	2486.99	2.020	1135.52	2293.57
A_68_P28724519	chr12:109154477-109154521	NM_001079883:87126	Bcl11b	INSIDE	1.178	2.828	3017.68	8534.54	3.330	2220.17	7394.25
A_68_P27900565	chr11:70223680-70223724	NM_029231:-169	Pelp1	PROMOTER	1.178	0.322	3385.85	1091.77	0.380	2736.24	1038.93
A_68_P25954495	chr8:73376456-73376500	NM_032397:4429	Kcnn1	INSIDE	1.178	2.658	511.63	1359.84	3.131	449.64	1407.79

ProbeName	Target position of probe on CpG island microarray	TargetID	GeneSymbol	CpG island Description	Ratio of relative methylation (TiO_2-NP/Vehicle)	Sham group			TiO_2-H group		
						Relative methylation (Cy5/Cy3)	Cy3 signal (Input)	Cy5 signal (MeDIP)	Relative methylation (Cy5/Cy3)	Cy3 signal (Input)	Cy5 signal (MeDIP)
A_68_P29863558	chr14:103469714-103469758	NM_001033242:304	Cln5	INSIDE	1.177	0.321	1775.36	570.25	0.378	1325.51	501.18
A_68_P27633575	chr11:20730716-20730761	NM_173752:373	1110067D22Rik	INSIDE	1.177	1.791	1004.39	1799.21	2.109	789.28	1664.30
A_68_P25959799	chr8:74659667-74659711	NM_001001491:498	Tpm4	INSIDE	1.177	0.512	1654.34	846.28	0.602	1172.25	705.80
A_68_P25095112	chr7:53050502-53050546	NM_001005511:11208	Lmtk3	INSIDE	1.177	2.665	1098.83	2928.83	3.136	962.31	3018.24
A_68_P24994777	chr7:20282272-20282316	NM_009696:2221	Apoe	INSIDE	1.177	1.671	772.80	1291.16	1.967	617.23	1214.12
A_68_P24643141	chr6:92041701-92041746	NM_011630:312	Nr2c2	INSIDE	1.177	2.293	494.81	1134.54	2.699	425.57	1148.65
A_68_P22859195	chr4:40800489-40800533	NM_022305:521	B4galt1	INSIDE	1.177	1.591	683.01	1086.38	1.873	659.43	1234.89
A_68_P21763873	chr2:154899789-154899833	NM_016661:423	Ahcy	INSIDE	1.177	0.421	1122.53	472.64	0.496	920.22	456.21
A_68_P20056645	chr1:16655542-16655586	NM_026392:293	Tmem70	INSIDE	1.177	0.435	1285.38	558.56	0.511	1202.71	614.96
A_68_P31162042	chr17:36117354-36117398	NM_008136:477	Gnl1	INSIDE	1.176	0.564	4675.50	2635.21	0.663	3306.06	2191.82
A_68_P31058439	chr17:14340006-14340050	NM_172826:810	Dact2	INSIDE	1.176	0.493	1371.99	677.03	0.581	1281.60	744.03
A_68_P28875776	chr13:22098904-22098948	NM_019429:2684	Prss16	INSIDE	1.176	2.814	2548.17	7169.65	3.308	1915.86	6337.90
A_68_P27845442	chr11:60727111-60727155	NM_025294:162	Gm16515	INSIDE	1.176	0.447	1827.23	817.65	0.526	1402.50	738.35
A_68_P27541867	chr10:127962642-127962686	NM_011843:251	Esyt1	INSIDE	1.176	0.308	1949.43	600.46	0.362	1388.71	503.04
A_68_P26546363	chr9:58049670-58049721	NM_001161540:-89	Islr2	PROMOTER	1.176	0.331	3107.29	1028.86	0.389	2118.30	824.77
A_68_P25365013	chr7:107128942-107128986	NM_175388:4	Rnf169	INSIDE	1.176	0.460	1458.57	670.25	0.541	1136.87	614.49
A_68_P24658381	chr6:94649584-94649628	NM_008377:533	Lrig1	INSIDE	1.176	0.507	2534.01	1285.16	0.596	2050.01	1222.35
A_68_P23720370	chr5:58111147-58111191	NM_018764:1909	Pcdh7	INSIDE	1.176	0.171	4374.70	746.19	0.201	3275.31	656.79
A_68_P23513994	chr5:18733193-18733237	NM_001170746:351	Magi2	INSIDE	1.176	1.587	878.05	1393.26	1.866	719.58	1342.45
A_68_P23272068	chr4:124762857-124762901	NM_027310:488	Meaf6	INSIDE	1.176	0.169	2940.21	497.08	0.199	2039.83	405.54
A_68_P21438060	chr2:93796133-93796185	NM_001145034:1099	Gm13889	INSIDE	1.176	0.331	1500.77	496.74	0.389	1030.10	401.01
A_68_P20441530	chr1:93195184-93195228	NM_016717:292	Scly	INSIDE	1.176	0.217	3070.54	665.94	0.255	2379.22	606.73
A_68_P30359768	chr15:78240674-78240718	NM_138670:3163	Mpst	INSIDE	1.175	0.441	2424.32	1069.69	0.518	1628.45	843.98
A_68_P21566331	chr2:118640254-118640305	NM_026412:541	D2Ertd750e	INSIDE	1.175	2.571	276.35	710.53	3.021	245.92	742.82
A_68_P21092313	chr2:28496774-28496818	NM_022887:34	Tsc1	INSIDE	1.175	0.403	1852.04	747.25	0.474	1437.41	681.25
A_68_P32488666	chrX:78316434-78316478	NM_138751:474	Tmem47	INSIDE	1.174	0.480	1419.11	681.42	0.564	1852.35	1044.34
A_68_P30364009	chr15:78873840-78873884	NM_015738:1548	Galr3	INSIDE	1.174	0.240	2535.61	609.08	0.282	1777.92	501.26
A_68_P28950723	chr13:36818496-36818540	NM_153529:7805	Nrn1	INSIDE	1.174	0.297	1614.11	479.50	0.349	1100.22	383.86
A_68_P28039686	chr11:95446719-95446763	NM_033217:2272	Ngfr	INSIDE	1.174	0.369	1745.23	643.59	0.433	1343.04	581.38
A_68_P25693344	chr8:17535329-17535373	NM_053171:35	Csmd1	INSIDE	1.174	0.446	3792.04	1692.68	0.524	2909.46	1524.71
A_68_P25098820	chr7:53652793-53652837	NM_001112739:948	Kcnc1	INSIDE	1.174	0.390	2470.19	964.58	0.458	1671.21	765.95
A_68_P24377897	chr6:38501394-38501438	NM_001170849:-27	Luc7l2	PROMOTER	1.174	0.266	2860.98	761.77	0.313	2343.13	732.64
A_68_P21765246	chr2:155207391-155207435	NM_178111:-179	Trp53inp2	PROMOTER	1.174	0.162	3642.40	591.34	0.191	2857.35	544.52
A_68_P21151182	chr2:38781242-38781286	NM_010264:717	Nr6a1	INSIDE	1.174	0.331	2401.57	796.10	0.389	1741.43	677.48
A_68_P20774313	chr1:163806678-163806722	NM_172645:92	AI848100	INSIDE	1.174	0.232	4092.85	947.70	0.272	3038.27	825.71
A_68_P30575358	chr16:17722742-17722786	NM_001040683:259	Med15	INSIDE	1.173	0.191	2350.93	448.70	0.224	1815.81	406.61
A_68_P30544452	chr16:10784908-10784952	NM_009896:699	Socs1	INSIDE	1.173	0.140	5609.63	783.46	0.164	3988.39	653.64
A_68_P26373432	chr9:26807056-26807100	NM_025862:56	Acad8	INSIDE	1.173	0.348	2805.54	977.12	0.409	1841.97	752.46
A_68_P26319145	chr9:14556214-14556258	NM_010242:330	Fut4	INSIDE	1.173	0.378	1235.79	467.67	0.444	1029.49	457.18
A_68_P25843138	chr8:48798881-48798925	NM_172407:383	Cdkn2aip	INSIDE	1.173	0.444	1219.79	540.98	0.520	979.56	509.46
A_68_P31369877	chr17:78600790-78600834	NM_015800:1225	Crim1	INSIDE	1.172	0.270	1791.05	483.34	0.316	1266.09	400.41
A_68_P26458702	chr9:42753013-42753057	NM_175481:-580	Grik4	PROMOTER	1.172	0.427	1199.32	511.91	0.500	929.93	465.22
A_68_P24950807	chr7:4796189-4796233	NM_172737:88	Shisa7	INSIDE	1.172	0.175	3580.32	626.50	0.205	2493.63	511.34
A_68_P22829976	chr4:34136877-34136921	NM_026293:144	Spaca1	INSIDE	1.172	2.150	355.16	763.62	2.520	305.89	770.97
A_68_P22503483	chr3:126300156-126300200	NM_001025438:288	Camk2d	INSIDE	1.172	1.654	1107.25	1831.65	1.940	952.94	1848.30
A_68_P31708189	chr18:52688590-52688634	NM_010728:750	Lox	INSIDE	1.171	1.974	1149.46	2268.69	2.311	984.80	2275.50
A_68_P30600196	chr16:22858132-22858176	NM_001190804:237	Dnajb11	INSIDE	1.171	0.375	1693.38	635.09	0.439	1244.18	546.57
A_68_P28670941	chr12:99812671-99812719	NM_029911:3456	Kcnk10	INSIDE	1.171	2.551	803.82	2050.63	2.988	690.18	2062.35
A_68_P23991346	chr5:112737009-112737053	NM_009419:31304	Tpst2	INSIDE	1.171	1.795	2388.15	4287.34	2.103	1855.04	3900.57
A_68_P23962030	chr5:106990411-106990455	ENSMUST00000131029:13302		INSIDE	1.171	0.266	2433.69	647.97	0.312	1910.01	595.68
A_68_P23134366	chr4:97445300-97445344	NM_001122953:1006	Nfia	INSIDE	1.171	3.275	699.35	2290.60	3.835	521.40	1999.69
A_68_P31936611	chr19:6984134-6984178	NM_012021:-21	Prdx5	DIVERGENT_PROMOTER	1.170	0.430	1720.57	739.11	0.503	1476.95	742.42
A_68_P27865440	chr11:64249134-64249178	NM_178870:323	Hs3st3a1	INSIDE	1.170	0.372	2386.05	887.45	0.435	1883.30	819.60
A_68_P27845571	chr11:60745673-60745717	NM_008928:136	Map2k3	INSIDE	1.170	0.431	2184.95	941.37	0.504	1695.82	854.53
A_68_P26845894	chr9:114598017-114598061	NM_146229:90	Dync1li1	INSIDE	1.170	0.443	1103.18	488.43	0.518	988.49	512.12
A_68_P25352336	chr7:104937141-104937185	ENSMUST00000172423:112		INSIDE	1.170	0.273	3048.70	832.76	0.319	2394.93	765.14
A_68_P23698174	chr5:53440423-53440467	NM_011402:-147	Slc34a2	PROMOTER	1.170	0.252	2145.45	540.49	0.295	1615.04	475.88
A_68_P23252506	chr4:120339576-120339620	NM_019563:431	Cited4	INSIDE	1.170	0.477	1253.44	597.86	0.558	1017.65	567.76
A_68_P31927963	chr19:5306888-5306932	NM_001024717:8580	Gal3st3	INSIDE	1.169	1.743	1710.94	2981.81	2.037	1292.63	2632.72
A_68_P28036776	chr11:94982434-94982478	NM_010055:1026	Dlx3	INSIDE	1.169	2.488	2918.87	7262.24	2.910	2040.20	5936.45
A_68_P27995155	chr11:87431487-87431532	NM_001045527:844	Hsf5	INSIDE	1.169	2.349	381.64	896.29	2.746	253.68	696.68
A_68_P27765618	chr11:45869772-45869816	NM_009616:306	Adam19	INSIDE	1.169	0.310	2272.22	704.70	0.362	1806.30	654.75
A_68_P27551032	chr11:3166258-3166302	NM_007879:109	Drg1	INSIDE	1.169	0.144	3582.63	514.95	0.168	2753.98	462.62
A_68_P26885085	chr9:121628083-121628127	NM_010918:-194	Nktr	PROMOTER	1.169	0.447	1608.10	718.94	0.523	1089.46	569.44
A_68_P26346746	chr9:21172514-21172558	NM_001042708:90	Ilf3	INSIDE	1.169	0.265	2090.22	554.34	0.310	1424.78	441.62

ProbeName	Target position of probe on CpG island microarray	TargetID	GeneSymbol	CpG island Description	Ratio of relative methylation (TiO_2-NP/Vehicle)	Sham group			TiO_2-H group		
						Relative methylation (Cy5/Cy3)	Cy3 signal (Input)	Cy5 signal (MeDIP)	Relative methylation (Cy5/Cy3)	Cy3 signal (Input)	Cy5 signal (MeDIP)
A_68_P20729268	chr1:155747036-155747080	NM_008131:-16	Glul	PROMOTER	1.169	1.473	1771.46	2609.16	1.721	1277.58	2199.34
A_68_P29613141	chr14:55203509-55203559	NM_028890:21211	4931414P19Rik	INSIDE	1.168	1.986	635.81	1262.81	2.320	543.37	1260.45
A_68_P28076812	chr11:101846260-101846304	NM_028207:-177	Dusp3	DIVERGENT_PROMOTER	1.168	0.411	1305.52	536.49	0.480	1080.01	518.38
A_68_P27806465	chr11:53583822-53583866	NM_001159396:-130	Irf1	PROMOTER	1.168	1.410	1186.66	1673.78	1.648	927.87	1529.21
A_68_P27288652	chr10:80755895-80755939	NM_001146687:200	Pip5k1c	INSIDE	1.168	0.235	3144.58	739.80	0.275	2340.09	642.95
A_68_P27267070	chr10:76728491-76728535	NM_030262:6468	Pofut2	INSIDE	1.168	1.931	1300.39	2510.79	2.255	992.32	2237.95
A_68_P26630975	chr9:72892792-72892836	NM_023635:143	Rab27a	INSIDE	1.168	0.440	2420.41	1064.42	0.514	1735.76	891.75
A_68_P26038379	chr8:90486004-90486048	NM_033327:-2532	Zfp423	PROMOTER	1.168	0.182	2519.84	457.57	0.212	1726.89	366.34
A_68_P24880090	chr6:137684038-137684082	NM_011499:458	Strap	INSIDE	1.168	0.231	2185.12	503.87	0.269	1629.31	438.91
A_68_P23577159	chr5:31522755-31522799	NM_008014:142	Ppm1g	INSIDE	1.168	0.507	1636.63	830.54	0.593	1293.12	766.49
A_68_P21018168	chr2:13714756-13714800	NM_145838:369	St8sia6	INSIDE	1.168	0.270	2808.60	759.17	0.316	2014.32	636.07
A_68_P32008255	chr19:21867281-21867325	NM_001033759:14471	Tmem2	INSIDE	1.167	2.606	1502.12	3914.51	3.042	1134.07	3450.19
A_68_P31156943	chr17:35138141-35138185	NM_011690:311	Vars	INSIDE	1.167	2.313	409.66	947.74	2.699	341.88	922.72
A_68_P30586702	chr16:20241285-20241329	NM_198599:125	Map6d1	INSIDE	1.167	1.647	733.83	1208.91	1.922	617.80	1187.32
A_68_P28158071	chr11:116083426-116083470	NM_025276:15957	Evpl	INSIDE	1.167	1.812	726.83	1317.15	2.114	646.83	1367.45
A_68_P27690051	chr11:32543056-32543100	NM_134015:204	Fbxw11	INSIDE	1.167	0.475	1336.53	634.38	0.554	1101.65	610.03
A_68_P26804351	chr9:106550055-106550099	NM_001160353:8364	Grm2	INSIDE	1.167	1.772	811.87	1438.53	2.068	676.12	1398.03
A_68_P25581957	chr7:147235055-147235099	NM_010836:-89	Msx3	PROMOTER	1.167	0.214	2621.77	560.82	0.250	1876.61	468.28
A_68_P24046036	chr5:122161287-122161331	NM_009125:-309	Atxn2	PROMOTER	1.167	0.305	2562.67	781.29	0.356	2038.64	725.30
A_68_P20354530	chr1:75500243-75500287	NM_178884:2763	Obsl1	INSIDE	1.167	2.027	1592.96	3229.46	2.365	1352.15	3198.39
A_68_P20348297	chr1:74438060-74438104	NM_153088:-100	Ctdsp1	PROMOTER	1.167	0.570	2242.53	1279.04	0.666	1882.33	1252.94
A_68_P31678447	chr18:46887518-46887562	NM_033037:456	Cdo1	INSIDE	1.166	0.094	5396.74	505.43	0.109	3705.51	404.76
A_68_P30422457	chr15:88813275-88813319	NM_031260:-127	Mov10l1	DIVERGENT_PROMOTER	1.166	2.150	582.23	1252.02	2.507	445.59	1117.12
A_68_P28752832	chr12:114067540-114067584	NM_028023:38	Cdca4	INSIDE	1.166	0.236	3500.60	825.63	0.275	2620.41	720.42
A_68_P28685876	chr12:102321244-102321288	NR_038047:606	D130020L05Rik	INSIDE	1.166	1.406	1010.75	1420.87	1.639	884.53	1449.63
A_68_P28183243	chr11:120097692-120097736	NM_198423:3454	Bahcc1	INSIDE	1.166	0.418	2180.80	911.18	0.487	1659.71	808.82
A_68_P27927425	chr11:75469530-75469574	NM_001080775:36	Myo1c	INSIDE	1.166	2.012	483.41	972.40	2.345	410.78	963.28
A_68_P27894313	chr11:69137244-69137288	NM_172560:109	Cntrob	INSIDE	1.166	0.125	4432.25	554.54	0.146	3100.72	452.34
A_68_P27839286	chr11:59653097-59653142	NM_011991:150	Cops3	INSIDE	1.166	0.478	1737.74	831.48	0.558	1410.51	787.13
A_68_P25528910	chr7:138687411-138687455	NM_008257:956	Hmx3	INSIDE	1.166	0.263	2612.44	686.66	0.306	1882.30	576.73
A_68_P24326758	chr6:29423877-29423921	NM_001145360:2462	Gm9047	DOWNSTREAM	1.166	0.354	1476.53	523.42	0.413	1059.62	437.81
A_68_P24117614	chr5:136210246-136210290	NM_008898:45185	Por	INSIDE	1.166	2.066	1336.15	2761.08	2.409	1110.32	2674.33
A_68_P23440310	chr4:155113949-155113993	NM_001101506:5058	Gm5151	INSIDE	1.166	1.525	746.06	1137.56	1.778	653.20	1161.53
A_68_P21070791	chr2:25096537-25096581	NM_026415:-1141	2310002J15Rik	DIVERGENT_PROMOTER	1.166	0.321	1903.91	611.89	0.375	1469.45	550.63
A_68_P30456841	chr15:95059370-95059414	NM_016743:299745	Nell2	INSIDE	1.165	1.995	422.91	843.53	2.324	315.28	732.69
A_68_P28998905	chr13:44998485-44998530	NM_001205043:171868	Jarid2	INSIDE	1.165	1.632	660.14	1077.64	1.901	485.27	922.67
A_68_P26585803	chr9:64949765-64949813	NM_020043:487	Igdcc4	INSIDE	1.165	0.492	2762.05	1359.87	0.574	1974.35	1132.86
A_68_P26546339	chr9:58046840-58046884	NM_001161535:2660	Islr2	INSIDE	1.165	0.377	2541.89	958.29	0.439	1656.28	727.22
A_68_P25958199	chr8:74194916-74194960	NM_001029873:718	Unc13a	INSIDE	1.165	0.218	2944.37	642.98	0.254	1885.63	479.84
A_68_P25505712	chr7:134615346-134615390	NM_145589:241	Prr14	INSIDE	1.165	0.324	1883.25	610.90	0.378	1517.05	573.53
A_68_P21745018	chr2:151457749-151457793	NM_198214:499	Snph	INSIDE	1.165	0.299	1740.58	520.89	0.349	1362.15	475.02
A_68_P21132485	chr2:35111777-35111821	NM_001206367:-80	Gsn	PROMOTER	1.165	0.238	1838.97	438.17	0.278	1288.69	357.70
A_68_P20023664	chr1:9690196-9690240	NM_008651:72	Mybl1	INSIDE	1.165	8.541	1038.71	8871.47	9.949	808.97	8048.80
A_68_P32795515	chrX:160347015-160347059	NM_026887:-55	Ap1s2	PROMOTER	1.164	1.676	830.92	1392.22	1.951	1117.86	2180.85
A_68_P31156856	chr17:35115532-35115587	NM_013558:5912	Hspa1l	INSIDE	1.164	1.757	2299.51	4040.45	2.045	1716.35	3509.95
A_68_P30931896	chr16:85900309-85900353	NM_011782:1040	Adamts5	INSIDE	1.164	0.430	1594.61	685.33	0.500	1296.59	648.72
A_68_P29152749	chr13:76193665-76193709	NM_175495:758	Gpr150	INSIDE	1.164	1.457	1386.67	2020.45	1.696	1011.54	1715.14
A_68_P28003255	chr11:88860965-88861009	NM_009546:270	Trim25	INSIDE	1.164	0.383	1799.46	689.08	0.446	1289.83	574.90
A_68_P26017561	chr8:86525454-86525498	NM_183097:-128	1700067K01Rik	PROMOTER	1.164	1.543	833.04	1285.48	1.796	674.63	1211.94
A_68_P23445682	chr5:3543936-3543980	NM_001042501:126	Fam133b	INSIDE	1.164	0.321	1818.44	584.49	0.374	1251.82	468.32
A_68_P23363726	chr4:140938216-140938260	NM_001085513:14104	Fam131c	INSIDE	1.164	1.555	648.18	1007.87	1.810	542.93	982.76
A_68_P23335295	chr4:136249748-136249792	NM_010142:142080	Ephb2	INSIDE	1.164	1.683	1638.15	2756.33	1.959	1128.47	2210.88
A_68_P32543771	chrX:93291182-93291226	NM_010833:-179	Msn	PROMOTER	1.163	1.365	1453.78	1983.78	1.587	2171.29	3445.62
A_68_P31502464	chr18:12899809-12899853	NM_001042418:-33	Cabyr	PROMOTER	1.163	0.318	2017.19	642.29	0.370	1341.16	496.43
A_68_P31097726	chr17:24642065-24642109	NM_027937:16359	Caskin1	INSIDE	1.163	1.931	746.44	1441.08	2.246	548.78	1232.68
A_68_P29269917	chr13:101514369-101514413	NM_001190264:224	Mrps36	INSIDE	1.163	0.385	1700.02	653.83	0.447	1258.59	563.07
A_68_P28731502	chr12:110271962-110272006	NM_001163175:34443	Begain	INSIDE	1.163	2.318	1259.78	2919.60	2.696	1081.61	2916.30
A_68_P27718622	chr11:37050106-37050153	ENSMUST00000157030:-663		PROMOTER	1.163	0.155	4391.13	682.06	0.181	3046.02	550.40
A_68_P25050057	chr7:36369976-36370020	NM_145840:603	Rgs9bp	INSIDE	1.163	2.037	829.25	1689.00	2.370	664.77	1575.27
A_68_P21820014	chr2:164658302-164658346	NM_001038492:-48	Ctsa	PROMOTER	1.163	0.476	1342.64	638.44	0.553	1042.53	576.72
A_68_P31627037	chr18:37303374-37303418	NM_001003672:-226	Pcdhac2	PROMOTER	1.162	0.413	1294.55	534.54	0.480	1009.22	484.13
A_68_P31215625	chr17:48179041-48179085	AK007241:-3819		PROMOTER	1.162	0.409	2066.06	844.93	0.475	1592.34	756.50
A_68_P29047193	chr13:54150633-54150677	NM_010076:373	Drd1a	INSIDE	1.162	0.296	2597.85	768.22	0.344	1986.54	682.72
A_68_P28693524	chr12:103995935-103995979	NM_001142939:64	AK010878	INSIDE	1.162	0.400	1172.38	468.67	0.465	893.91	415.31
A_68_P26643352	chr9:75161879-75161923	NM_138719:-148	Gnb5	PROMOTER	1.162	2.050	2175.74	4459.93	2.381	1760.22	4191.18

ProbeName	Target position of probe on CpG island microarray	TargetID	GeneSymbol	CpG island Description	Ratio of relative methylation (TiO_2-NP/Vehicle)	Sham group			TiO_2-H group		
						Relative methylation (Cy5/Cy3)	Cy3 signal (Input)	Cy5 signal (MeDIP)	Relative methylation (Cy5/Cy3)	Cy3 signal (Input)	Cy5 signal (MeDIP)
A_68_P25368682	chr7:107756133-107756177	NM_001163663:55	Rab6	INSIDE	1.162	1.380	1083.40	1495.18	1.603	873.60	1400.64
A_68_P25264703	chr7:87226955-87226999	NM_175433:55863	Zfp710	INSIDE	1.162	2.583	1875.43	4844.58	3.002	1383.97	4154.49
A_68_P24819298	chr6:125141713-125141757	NM_146171:-130	Ncapd2	DIVERGENT_PROMOTER	1.162	0.306	1780.91	545.22	0.356	1377.72	490.17
A_68_P23394410	chr4:147818444-147818488	NM_027873:394	Ubiad1	INSIDE	1.162	0.545	2219.41	1208.58	0.633	1802.49	1140.33
A_68_P23223134	chr4:114729741-114729785	NM_011527:-2369	Tal1	PROMOTER	1.162	0.444	6051.50	2688.19	0.516	3719.35	1920.39
A_68_P22872581	chr4:43591992-43592036	NM_172692:-278	Gba2	PROMOTER	1.162	0.231	1946.45	450.36	0.269	1641.39	441.45
A_68_P32224194	chr19:60302569-60302613	NM_029648:10	D19Ertd737e	INSIDE	1.161	1.820	769.30	1400.24	2.113	617.53	1304.84
A_68_P30645474	chr16:31081440-31081484	NM_198626:56	AI480653	INSIDE	1.161	3.490	992.79	3464.80	4.053	789.95	3201.47
A_68_P30605415	chr16:23890655-23890699	NM_009215:254	Sst	INSIDE	1.161	1.974	505.45	997.62	2.292	361.97	829.72
A_68_P25470558	chr7:127974492-127974536			Unknown	1.161	0.359	1785.76	641.77	0.417	1406.43	586.90
A_68_P24034079	chr5:120131042-120131086	NM_011535:10387	Tbx3	INSIDE	1.161	0.284	2067.27	587.47	0.330	1488.08	491.08
A_68_P32717153	chrX:140753652-140753696	NM_026247:1166	Alg13	INSIDE	1.160	1.355	2512.50	3404.61	1.573	3941.41	6198.04
A_68_P31980264	chr19:16511462-16511506	NM_008137:1328	Gna14	INSIDE	1.160	0.425	1114.44	473.46	0.493	892.24	439.82
A_68_P31978254	chr19:16207846-16207890	NM_008139:548	Gnaq	INSIDE	1.160	0.337	1739.28	586.09	0.391	1249.19	488.47
A_68_P30963823	chr16:91597831-91597875	NM_030018:73	Tmem50b	INSIDE	1.160	2.078	1442.41	2997.19	2.410	1137.86	2741.80
A_68_P28030167	chr11:93857753-93857797	NM_001199203:370	Spag9	INSIDE	1.160	1.627	624.83	1016.64	1.888	539.05	1017.71
A_68_P27841324	chr11:59989338-59989382	NM_001037764:35777	Rai1	INSIDE	1.160	0.414	1229.90	509.34	0.481	935.26	449.44
A_68_P27340038	chr10:90564802-90564859	NM_027078:19047	Ikbip	INSIDE	1.160	3.020	443.60	1339.58	3.502	354.33	1240.86
A_68_P26345408	chr9:20896439-20896483	NR_038081:-8	Raver1-fdx1l	PROMOTER	1.160	0.390	1454.10	566.52	0.452	1186.90	536.34
A_68_P26238593	chr8:125749038-125749082	NM_194444:320	Cdk10	INSIDE	1.160	0.277	2211.52	611.59	0.321	1709.17	548.31
A_68_P25020923	chr7:29062778-29062822	NM_001177427:76	Eid2b	INSIDE	1.160	0.395	1227.41	484.49	0.458	862.31	394.84
A_68_P32185226	chr19:53978356-53978400	NM_001168491:538	Pdcd4	INSIDE	1.159	0.436	1533.71	667.97	0.505	1237.83	624.93
A_68_P31982213	chr19:16854797-16854841	NM_173028:599	Vps13a	INSIDE	1.159	0.491	2215.43	1088.66	0.569	1800.91	1025.30
A_68_P30392998	chr15:83998328-83998372	NM_054088:105	Pnpla3	INSIDE	1.159	0.489	1152.57	564.07	0.567	1029.65	584.25
A_68_P27533591	chr10:126467810-126467854	NM_025537:38	Tsfm	INSIDE	1.159	0.516	1589.37	820.23	0.598	1296.21	775.39
A_68_P27253670	chr10:74430232-74430276	NM_010311:279	Gnaz	INSIDE	1.159	1.568	1195.39	1874.23	1.816	936.72	1701.54
A_68_P27215509	chr10:67504533-67504577	NM_001081346:62209	Rtkn2	INSIDE	1.159	1.745	575.04	1003.32	2.023	531.14	1074.28
A_68_P26221731	chr8:123191547-123191591	NM_010926:444	Cox4nb	INSIDE	1.159	1.627	778.03	1266.17	1.886	672.28	1267.96
A_68_P25022915	chr7:29415604-29415648	NM_175319:408	C330005M16Rik	INSIDE	1.159	0.552	1534.93	846.87	0.639	1415.81	905.35
A_68_P23328078	chr4:134908468-134908512	NM_001130477:639	Srrm1	INSIDE	1.159	0.389	1445.43	562.23	0.451	1151.40	519.26
A_68_P32669432	chrX:130433013-130433072	NM_019656:-74	Tspan6	DIVERGENT_PROMOTER	1.158	3.293	149.86	493.50	3.814	183.41	699.48
A_68_P32259782	chrX:11583929-11583973			Unknown	1.158	0.380	1370.73	520.44	0.440	1892.15	831.68
A_68_P32203280	chr19:56900735-56900779	NM_001002240:-8	Tdrd1	PROMOTER	1.158	2.115	1052.37	2225.57	2.448	803.98	1968.37
A_68_P30476460	chr15:98620564-98620608	NM_021279:299	Wnt1	INSIDE	1.158	1.550	1242.85	1926.75	1.795	909.11	1632.26
A_68_P24192244	chr5:151476355-151476399	NM_175310:-25	Pds5b	DIVERGENT_PROMOTER	1.158	1.899	2085.53	3960.73	2.199	1713.12	3767.06
A_68_P24137873	chr5:140302698-140302742	NM_025604:77	Psmg3	INSIDE	1.158	0.353	1474.45	520.61	0.409	1165.84	476.73
A_68_P24044263	chr5:121835304-121835348	NM_001163470:298	Trafd1	INSIDE	1.158	0.380	1881.73	714.15	0.440	1359.98	597.86
A_68_P23588840	chr5:33775538-33775582	ENSMUST00000030994:-1716		PROMOTER	1.158	3.339	956.20	3192.98	3.867	837.07	3236.89
A_68_P21875215	chr2:174241468-174241512	NM_020580:186	Th1l	INSIDE	1.158	0.287	1571.58	451.11	0.332	1224.23	406.90
A_68_P21628929	chr2:130109758-130109802	NM_130884:407	Idh3b	INSIDE	1.158	0.485	2909.30	1409.60	0.561	1944.76	1090.70
A_68_P21129653	chr2:34627681-34627725	NM_001163434:93	Hspa5	INSIDE	1.158	0.215	2472.31	532.55	0.249	1728.91	431.21
A_68_P21116405	chr2:32470425-32470469	NM_001025310:7966	St6galnac6	INSIDE	1.158	2.210	1442.03	3186.25	2.559	1029.46	2634.80
A_68_P31123428	chr17:28758772-28758816	NM_016795:605	Srpk1	INSIDE	1.157	0.458	2088.59	956.97	0.530	1569.62	831.94
A_68_P27809328	chr11:54117787-54117831	NM_001033597:103	Acsl6	INSIDE	1.157	0.344	2212.45	761.16	0.398	1731.62	689.16
A_68_P24994775	chr7:20282050-20282094	NM_009696:2443	Apoe	INSIDE	1.157	2.291	1157.48	2651.44	2.651	959.80	2544.35
A_68_P23547921	chr5:25035545-25035589	NR_027388:-269	1700096K18Rik	PROMOTER	1.157	0.218	3042.97	662.30	0.252	2397.17	603.66
A_68_P21240002	chr2:56966945-56966989	NM_013613:483	Nr4a2	INSIDE	1.157	0.187	3697.74	692.27	0.217	2757.04	597.32
A_68_P29725072	chr14:75346702-75346746	NM_001033439:960	Lrch1	INSIDE	1.156	1.446	1014.34	1466.65	1.671	880.62	1471.36
A_68_P29613383	chr14:55251294-55251338	NM_199470:8885	Cdh24	INSIDE	1.156	1.544	1571.50	2426.64	1.785	1162.37	2074.35
A_68_P28935716	chr13:34027599-34027643	NM_001164117:332	Serpinb6a	INSIDE	1.156	0.480	1151.12	552.48	0.555	974.81	540.89
A_68_P26025443	chr8:88079254-88079298	NM_019794:-106	Dnaja2	PROMOTER	1.156	0.488	2248.22	1098.18	0.565	1765.13	996.96
A_68_P25090605	chr7:52263349-52263393	NM_001008422:8249	Scaf1	INSIDE	1.156	1.576	661.91	1043.16	1.821	593.57	1081.15
A_68_P25087550	chr7:51717698-51717742	NM_198250:19864	Lrrc4b	INSIDE	1.156	1.990	1087.39	2163.71	2.300	943.69	2170.27
A_68_P23541414	chr5:23889472-23889516	NM_008713:18858	Nos3	INSIDE	1.156	1.796	1125.34	2021.22	2.077	851.50	1768.17
A_68_P23268973	chr4:124252578-124252622	ENSMUST00000164308:6168		INSIDE	1.156	0.243	2886.00	701.17	0.281	2121.13	595.70
A_68_P22649277	chr3:151872936-151872980	NM_027287:305	Dnajb4	INSIDE	1.156	2.333	1408.35	3286.34	2.698	1172.59	3163.39
A_68_P22165085	chr3:57539702-57539746	NM_011883:-263	Rnf13	PROMOTER	1.156	0.392	2165.21	849.67	0.453	1876.80	851.02
A_68_P30330813	chr15:73342857-73342901	NM_001081066:-111	Dennd3	PROMOTER	1.155	1.427	3185.18	4543.84	1.648	2476.27	4080.01
A_68_P30128575	chr15:34372745-34372789	NM_009083:214	Rpl30	INSIDE	1.155	0.313	1638.04	512.14	0.361	1432.37	517.22
A_68_P29007695	chr13:46514396-46514440	NM_001081425:750	Rbm24	INSIDE	1.155	1.948	638.40	1243.69	2.249	482.45	1085.16
A_68_P28938194	chr13:34436403-34436447	NM_001033167:627	Slc22a23	INSIDE	1.155	1.427	1287.33	1836.50	1.648	1081.40	1782.10
A_68_P28862224	chr13:19487507-19487551	NM_024270:93	Stard3nl	INSIDE	1.155	1.845	680.83	1256.04	2.131	566.44	1206.90
A_68_P26710068	A_68_P26710068			Unknown	1.155	0.606	3125.41	1894.18	0.700	2328.50	1629.99
A_68_P26248944	chr8:127536746-127536790	NM_016909:-128	Tsnax	PROMOTER	1.155	0.521	2198.72	1145.04	0.601	1506.01	905.50
A_68_P26232278	chr8:124805221-124805265	NM_009569:-798	Zfpm1	PROMOTER	1.155	0.289	2987.84	863.19	0.334	2046.94	683.00

ProbeName	Target position of probe on CpG island microarray	TargetID	GeneSymbol	CpG island Description	Ratio of relative methylation (TiO_2-NP/Vehicle)	Sham group			TiO_2-H group		
						Relative methylation (Cy5/Cy3)	Cy3 signal (Input)	Cy5 signal (MeDIP)	Relative methylation (Cy5/Cy3)	Cy3 signal (Input)	Cy5 signal (MeDIP)
A_68_P26142833	chr8:109572050-109572094	NM_026513:442	Pdf	INSIDE	1.155	0.377	1271.14	479.66	0.436	924.51	403.07
A_68_P25092883	chr7:52650953-52650997	NM_009224:43	Snrnp70	INSIDE	1.155	0.437	2027.22	886.31	0.505	1520.43	767.95
A_68_P23456155	chr5:5514599-5514644	NM_001193660:168	Cldn12	INSIDE	1.155	0.408	1295.64	528.92	0.472	1052.81	496.50
A_68_P21862264	chr2:171868280-171868324	NM_175631:664	Cbln4	INSIDE	1.155	0.215	2797.42	600.66	0.248	1885.76	467.57
A_68_P21425372	chr2:91762936-91762980	NM_007699:613	Chrm4	INSIDE	1.155	0.450	2052.64	923.70	0.520	1534.82	797.83
A_68_P32462207	chrX:70599165-70599209	NM_001160229:11227	Zfp275	INSIDE	1.154	2.422	376.68	912.33	2.794	480.37	1342.32
A_68_P32050807	chr19:29122028-29122072	NR_028360:78	1700018L02Rik	INSIDE	1.154	0.306	2629.23	805.65	0.354	2031.11	718.10
A_68_P30884150	chr16:76373795-76373839	NM_173440:-522	Nrip1	PROMOTER	1.154	0.357	1354.84	483.15	0.412	995.26	409.73
A_68_P27708164	chr11:35521558-35521602	NM_011412:586623	Slit3	INSIDE	1.154	1.590	981.84	1561.11	1.835	698.59	1281.89
A_68_P27349650	chr10:92185045-92185089	NM_008682:97	Nedd1	INSIDE	1.154	0.353	1323.08	467.52	0.408	1066.30	434.93
A_68_P25392336	chr7:112583704-112583750	NM_007627:9393	Cckbr	INSIDE	1.154	2.084	725.24	1511.66	2.405	566.24	1361.87
A_68_P24592784	chr6:83276117-83276161	NM_145571:106	Mobkl1b	INSIDE	1.154	0.389	1962.63	763.79	0.449	1476.02	662.96
A_68_P22674484	chr3:156225009-156225062	NM_177274:113	Negr1	INSIDE	1.154	8.014	1065.30	8536.85	9.246	961.54	8890.71
A_68_P31053606	chr17:12934729-12934773	NR_002853:574	Airn	INSIDE	1.153	2.532	827.95	2096.57	2.921	790.39	2308.42
A_68_P30346611	chr15:76011370-76011414	NM_201394:14748	Plec	INSIDE	1.153	3.260	577.71	1883.28	3.760	548.92	2063.80
A_68_P29937482	chr14:118635523-118635567	NM_177753:708	Sox21	INSIDE	1.153	0.201	3873.48	776.71	0.231	2577.92	595.89
A_68_P28203006	chr12:4924358-4924402	NM_001099628:222	Atad2b	INSIDE	1.153	2.282	258.49	589.86	2.631	236.74	622.83
A_68_P26612672	chr9:69607372-69607416	NM_022378:1353	Foxb1	INSIDE	1.153	0.315	1701.96	535.94	0.363	1309.96	475.54
A_68_P26229921	chr8:124474582-124474626	NM_001110100:172	Banp	INSIDE	1.153	6.238	4284.41	26726.32	7.193	3300.59	23740.87
A_68_P24956667	chr7:6683071-6683115	NM_008817:38	Peg3	INSIDE	1.153	2.078	1909.50	3968.59	2.395	1646.24	3943.31
A_68_P22314347	chr3:88340273-88340317	NR_035434:10	Mir1905	INSIDE	1.153	0.531	2278.35	1208.81	0.612	1430.11	874.64
A_68_P21900704	chr2:179710019-179710063	NM_001081092:1311	Taf4a	INSIDE	1.153	0.493	1162.13	573.00	0.569	1004.74	571.28
A_68_P20852904	chr1:178205035-178205079	NM_176916:387	Pld5	INSIDE	1.153	0.474	3129.70	1482.32	0.546	2234.83	1220.24
A_68_P20138941	chr1:34516537-34516581	NM_011206:-32	Ptpn18	PROMOTER	1.153	0.465	1225.85	570.03	0.536	937.91	502.93
A_68_P31927968	chr19:5307358-5307402	NM_001024717:9050	Gal3st3	INSIDE	1.152	1.766	1418.19	2504.79	2.034	1174.75	2389.54
A_68_P31920645	chr19:4012393-4012437	NM_133666:311	Ndufv1	INSIDE	1.152	0.174	2667.99	465.41	0.201	2002.56	402.26
A_68_P31399352	chr17:83913503-83913547	NM_001159529:149	Cox7a2l	INSIDE	1.152	2.014	913.45	1839.67	2.321	778.36	1806.42
A_68_P30578587	chr16:18248121-18248165	NM_011239:645	Ranbp1	INSIDE	1.152	0.422	1309.96	553.19	0.487	1060.61	516.20
A_68_P30332048	chr15:73526555-73526599	NM_173365:11359	Gpr20	INSIDE	1.152	2.735	810.83	2217.73	3.150	642.81	2025.12
A_68_P28940415	chr13:34838970-34839014	NM_138746:7482	Fam50b	INSIDE	1.152	5.359	1047.06	5611.04	6.173	826.79	5103.88
A_68_P22180927	chr3:60806813-60806857	NM_008772:118	P2ry1	INSIDE	1.152	0.406	1672.30	678.36	0.468	1253.91	586.21
A_68_P21783960	chr2:158440477-158440521	NM_009508:4005	Slc32a1	INSIDE	1.152	0.510	1551.29	790.87	0.587	1267.44	744.54
A_68_P21489014	chr2:104250060-104250104	NM_001033347:409	D430041D05Rik	INSIDE	1.152	0.476	1534.53	729.93	0.548	1257.43	689.10
A_68_P21072311	chr2:25316393-25316437	NM_153557:200	BC029214	INSIDE	1.152	0.188	2491.22	469.52	0.217	1657.97	359.98
A_68_P32464980	chrX:71069698-71069742	NM_001029868:588	Pdzd4	INSIDE	1.151	4.180	297.43	1243.36	4.810	289.87	1394.39
A_68_P31940127	chr19:7568796-7568840	NM_146091:-109	Atl3	PROMOTER	1.151	0.270	2042.23	551.90	0.311	1381.60	429.88
A_68_P31183535	chr17:42453211-42453255	NM_028474:337	3110082D06Rik	INSIDE	1.151	0.241	3833.29	925.06	0.278	2563.50	711.80
A_68_P31162046	chr17:36117706-36117750	NM_008136:829	Gnl1	INSIDE	1.151	0.500	1767.18	883.88	0.576	1214.40	699.07
A_68_P30639681	chr16:30070804-30070848	NM_008235:5384	Hes1	DOWNSTREAM	1.151	1.413	5120.71	7233.25	1.626	3907.73	6354.85
A_68_P27594240	chr11:11925551-11925597	NM_001177629:2400	Grb10	INSIDE	1.151	1.736	939.53	1631.11	1.999	899.55	1798.22
A_68_P26350211	chr9:21854807-21854851	NM_010487:1639	Elavl3	INSIDE	1.151	0.383	1424.06	545.59	0.441	1102.14	485.81
A_68_P25951143	chr8:72854752-72854796	NM_008107:1111	Gdf1	INSIDE	1.151	0.421	1666.46	701.20	0.484	1252.96	606.89
A_68_P24604720	chr6:85324839-85324883	NM_001003955:-232	Rab11fip5	PROMOTER	1.151	0.432	1674.01	723.64	0.498	1283.95	639.01
A_68_P22649285	chr3:151874067-151874111	NM_057172:667	Fubp1	INSIDE	1.151	0.547	2035.33	1113.22	0.629	1722.96	1084.57
A_68_P22336391	chr3:93247585-93247629	NM_001163098:1355	Tchh	INSIDE	1.151	1.550	1690.69	2620.96	1.784	1448.66	2584.61
A_68_P21433076	chr2:93028001-93028045	NM_001025246:282	Trp53i11	INSIDE	1.151	0.441	1417.66	625.38	0.508	1185.99	602.09
A_68_P32554229	chrX:96412142-96412186			Unknown	1.150	1.624	502.42	815.79	1.867	738.16	1378.41
A_68_P31207972	chr17:46766049-46766093	NM_175168:383	Ptk7	INSIDE	1.150	0.222	3386.16	751.94	0.255	2488.23	635.49
A_68_P30421723	chr15:88693456-88693500	NM_145478:855	Pim3	INSIDE	1.150	0.516	1552.37	801.73	0.594	1202.90	714.21
A_68_P30018890	chr15:11834572-11834616	NM_001039181:835	Npr3	INSIDE	1.150	0.185	2976.96	550.11	0.212	2026.04	430.47
A_68_P29879699	chr14:106294307-106294351	NM_011897:1708	Spry2	INSIDE	1.150	0.162	3956.75	640.18	0.186	2595.09	483.04
A_68_P28100096	chr11:106054873-106054917	NM_028126:-32	Strada	PROMOTER	1.150	0.265	2310.69	612.24	0.305	1641.69	500.26
A_68_P27968098	chr11:82577707-82577751	NM_009839:62	Cct6b	INSIDE	1.150	0.513	1435.91	736.38	0.590	1088.75	642.06
A_68_P27926233	chr11:75275698-75275742	NR_029739:-1497	Mir22	PROMOTER	1.150	2.728	277.05	755.93	3.137	193.98	608.47
A_68_P27288892	chr10:80788388-80788440	NM_027381:4567	2510012J08Rik	INSIDE	1.150	2.657	898.50	2387.25	3.057	737.34	2253.75
A_68_P27284271	chr10:80069708-80069752	NM_019575:4104	Scamp4	INSIDE	1.150	2.487	1329.52	3306.83	2.859	1014.69	2901.32
A_68_P25502555	chr7:133991327-133991371	NM_010069:282	Doc2a	INSIDE	1.150	0.365	1374.52	501.54	0.420	1099.33	461.43
A_68_P21905657	chr2:180436282-180436326	NM_175551:501	Dido1	INSIDE	1.150	0.255	1998.35	510.26	0.294	1372.14	402.84
A_68_P21017267	chr2:13496235-13496279	NM_011701:319	Vim	INSIDE	1.150	0.328	2129.59	698.68	0.377	1499.09	565.73
A_68_P32696392	chrX:136405520-136405564	NM_001193309:651	Morc4	INSIDE	1.149	1.609	4720.96	7597.08	1.849	6272.37	11595.94
A_68_P30473042	chr15:97923469-97923513	NM_144851:346	Senp1	INSIDE	1.149	0.338	1458.34	492.26	0.388	1104.48	428.55
A_68_P24076587	chr5:127722499-127722543	NM_175432:325	Tmem132c	INSIDE	1.149	0.357	2341.18	835.00	0.410	1684.14	689.97
A_68_P24004707	chr5:115021632-115021676	NM_001004180:3395	BC057022	INSIDE	1.149	0.171	3244.04	556.30	0.197	2405.66	473.96
A_68_P21115868	chr2:32391385-32391429	NM_153560:528	Fam102a	INSIDE	1.149	0.441	2121.98	935.07	0.506	1602.19	810.92
A_68_P20221656	chr1:51535340-51535384	NM_028696:-119	Obfc2a	PROMOTER	1.149	0.259	5570.24	1443.76	0.298	4377.69	1303.35

ProbeName	Target position of probe on CpG island microarray	TargetID	GeneSymbol	CpG island Description	Ratio of relative methylation (TiO_2-NP/Vehicle)	Sham group			TiO_2-H group		
						Relative methylation (Cy5/Cy3)	Cy3 signal (Input)	Cy5 signal (MeDIP)	Relative methylation (Cy5/Cy3)	Cy3 signal (Input)	Cy5 signal (MeDIP)
A_68_P31424878	chr17:88145892-88145936	NM_199251:51420	Kcnk12	INSIDE	1.148	1.532	1476.95	2263.30	1.760	1162.87	2046.52
A_68_P31201706	chr17:45682619-45682663	NM_001013749:3986	Tmem151b	INSIDE	1.148	0.406	1823.87	739.90	0.466	1154.65	537.87
A_68_P31157970	chr17:35293556-35293603	NM_001199044:8243	Prrc2a	INSIDE	1.148	5.570	267.40	1489.46	6.395	123.09	787.17
A_68_P26807153	chr9:107133795-107133839	NM_153413:424	Dock3	INSIDE	1.148	0.509	1290.63	656.96	0.584	1099.25	642.40
A_68_P24994423	chr7:20214968-20215012	NM_009046:-203	Relb	PROMOTER	1.148	0.421	1603.84	675.46	0.483	1203.92	581.87
A_68_P24928967	chr6:146450896-146450940	NM_010586:-484	Itpr2	PROMOTER	1.148	0.339	2169.07	735.31	0.389	1769.95	688.59
A_68_P23339119	chr4:136852669-136852713	NM_009523:19141	Wnt4	INSIDE	1.148	2.733	697.88	1907.36	3.137	616.52	1933.89
A_68_P21774856	chr2:156866097-156866141	NM_001164663:38883	9830001H06Rik	INSIDE	1.148	3.051	605.06	1846.00	3.501	511.55	1790.97
A_68_P20768477	chr1:162723220-162723264	NM_013862:-173	Rabgap1l	PROMOTER	1.148	19.370	8266.08	160112.30	22.230	8115.50	180408.80
A_68_P32461572	chrX:70474075-70474119	NM_007978:452	F8a	INSIDE	1.147	1.659	436.78	724.76	1.904	521.16	992.26
A_68_P31830206	chr18:74367357-74367401	NM_001164355:94	Ska1	INSIDE	1.147	0.420	2584.85	1085.36	0.482	1765.85	850.45
A_68_P30588849	chr16:20672661-20672705	NM_001005331:-139	Eif4g1	PROMOTER	1.147	0.331	3340.70	1107.23	0.380	2323.65	883.29
A_68_P29696150	chr14:70009101-70009145	NM_033325:-160	Loxl2	PROMOTER	1.147	0.239	4706.81	1126.77	0.275	3529.23	969.20
A_68_P29134817	chr13:72767989-72768033	NM_010574:1585	Irx2	INSIDE	1.147	0.436	1564.72	682.02	0.500	1203.02	601.44
A_68_P27290090	chr10:80958685-80958729	NM_134009:402	Ncln	INSIDE	1.147	0.303	2194.61	665.24	0.348	1525.34	530.21
A_68_P26640504	chr9:74696320-74696364	ENSMUST00000160842:-459		PROMOTER	1.147	0.186	3306.19	613.80	0.213	2119.64	451.54
A_68_P25954893	chr8:73437576-73437620	NM_173013:7726	Mtap1s	INSIDE	1.147	2.732	619.98	1693.92	3.133	459.36	1439.03
A_68_P23968409	chr5:108150354-108150398	NM_010278:2987	Gfi1	INSIDE	1.147	0.147	3043.22	448.51	0.169	2501.33	422.86
A_68_P23893303	chr5:92706870-92706914	NM_001163687:315	Naaa	INSIDE	1.147	0.282	2056.38	578.98	0.323	1401.05	452.57
A_68_P23436702	chr4:154524641-154524685	NM_011385:71982	Ski	DOWNSTREAM	1.147	0.249	2465.51	614.13	0.286	1642.70	469.29
A_68_P21837475	chr2:167603130-167603174	ENSMUST00000124850:-4400		PROMOTER	1.147	1.764	648.02	1143.42	2.023	595.24	1204.23
A_68_P31117836	chr17:27822046-27822090	NM_178365:-130	Pacsin1	PROMOTER	1.146	0.275	1758.90	483.67	0.315	1439.46	453.64
A_68_P30670705	chr16:35541905-35541949	NM_013661:479	Sema5b	INSIDE	1.146	0.504	2062.10	1039.00	0.577	1642.58	948.23
A_68_P29335935	chr13:113442657-113442701	NM_001145885:-160	Ddx4	DIVERGENT_PROMOTER	1.146	2.258	585.70	1322.37	2.588	500.49	1295.29
A_68_P26601253	chr9:67679488-67679532	NM_001163143:627	C2cd4a	INSIDE	1.146	0.440	2460.40	1081.52	0.504	1851.56	932.98
A_68_P26238821	chr8:125778775-125778819	NM_020497:702	Zfp276	INSIDE	1.146	0.548	1654.30	905.75	0.628	1335.08	837.90
A_68_P26022868	chr8:87489665-87489709	NM_027187:224	Rnaseh2a	INSIDE	1.146	0.244	2326.90	567.54	0.280	1643.97	459.67
A_68_P25776088	chr8:35871210-35871254	NM_176933:569	Dusp4	INSIDE	1.146	0.362	2563.63	927.66	0.415	1911.00	792.49
A_68_P25545210	chr7:141416870-141416914	NM_007400:-112	Adam12	PROMOTER	1.146	0.426	3451.12	1468.87	0.488	2224.28	1084.56
A_68_P24424710	chr6:47827670-47827714	NM_146175:139	Zfp282	INSIDE	1.146	1.467	845.45	1240.45	1.681	715.37	1202.47
A_68_P23794549	chr5:73305228-73305272	NM_001113423:-305	Slain2	PROMOTER	1.146	1.632	1475.69	2408.87	1.871	1180.83	2208.94
A_68_P21421301	chr2:91042892-91042936	NM_007387:-154	Acp2	DIVERGENT_PROMOTER	1.146	0.124	3665.06	453.48	0.142	2634.53	373.56
A_68_P21147617	chr2:38203630-38203674	NM_010710:-3175	Lhx2	PROMOTER	1.146	0.523	1997.40	1044.31	0.599	1401.09	839.72
A_68_P31711733	chr18:53336104-53336148	NM_026386:108	Snx2	INSIDE	1.145	2.197	720.10	1581.78	2.516	532.38	1339.25
A_68_P26145654	chr8:110112041-110112085	NM_010817:320	Psmd7	INSIDE	1.145	0.576	2511.87	1446.99	0.660	1988.46	1311.78
A_68_P26134606	chr8:108089561-108089605	NM_013477:358	Atp6v0d1	INSIDE	1.145	0.453	2217.96	1004.25	0.519	1551.63	804.69
A_68_P21495905	chr2:105509058-105509107	NR_002867:1405	Pax6os1	INSIDE	1.145	0.518	1495.50	774.52	0.593	1110.08	658.36
A_68_P20636645	chr1:136884255-136884299	NM_001033409:117577	Lgr6	INSIDE	1.145	13.440	2188.74	29416.00	15.391	1822.59	28050.68
A_68_P32341136	chrX:39503692-39503736	NM_021465:-163	Stag2	PROMOTER	1.144	2.167	485.90	1052.86	2.479	567.28	1406.39
A_68_P29616395	chr14:55732380-55732424	NM_177049:3370	Jph4	INSIDE	1.144	2.150	914.68	1966.94	2.461	777.90	1914.32
A_68_P28751328	chr12:113858149-113858193	NM_007421:-87	Adssl1	PROMOTER	1.144	1.767	1502.31	2653.87	2.021	1098.69	2220.67
A_68_P27889187	chr11:68213377-68213421	ENSMUST00000108674:927		INSIDE	1.144	0.480	2995.16	1437.15	0.549	2140.83	1174.82
A_68_P27794870	chr11:51420420-51420464	NM_001048061:-59	Hnrnpab	PROMOTER	1.144	0.570	3299.83	1880.33	0.652	2199.78	1433.84
A_68_P27287711	chr10:80610869-80610913	NM_010731:11875	Zbtb7a	INSIDE	1.144	2.040	1690.04	3447.99	2.335	1178.46	2751.26
A_68_P26136633	chr8:108424693-108424737	NM_173432:341	Pskh1	INSIDE	1.144	0.263	2073.32	544.95	0.301	1534.28	461.27
A_68_P23513995	chr5:18733341-18733385	NM_001170746:499	Magi2	INSIDE	1.144	0.319	1905.36	608.16	0.365	1276.19	465.80
A_68_P23365310	chr4:141220298-141220342	NM_001033150:-290	Plekhm2	PROMOTER	1.144	0.311	3240.98	1006.88	0.355	2315.26	822.68
A_68_P31219171	chr17:48925588-48925633			Unknown	1.143	1.897	447.77	849.36	2.167	403.86	875.26
A_68_P31110246	chr17:26576080-26576124	NM_001081656:24193	Neurl1b	INSIDE	1.143	1.990	887.94	1766.93	2.274	796.62	1811.18
A_68_P30500796	chr15:102765447-102765491	NM_010463:-1815	Hoxc12	PROMOTER	1.143	0.340	2107.50	716.27	0.388	1727.48	671.07
A_68_P23998980	chr5:114037914-114037958	NM_177292:118470	Wscd2	INSIDE	1.143	2.134	1572.41	3355.75	2.439	1201.55	2930.21
A_68_P22401913	chr3:106285353-106285397	NM_001093754:26	Dennd2d	INSIDE	1.143	2.571	1697.74	4365.47	2.939	1449.48	4260.05
A_68_P21817622	chr2:164226486-164226530	NM_009036:-2185	Rbpjl	PROMOTER	1.143	0.165	2863.43	471.20	0.188	1917.49	360.68
A_68_P21775424	chr2:156960529-156960573	NM_001139520:408	Samhd1	INSIDE	1.143	0.371	1287.36	477.34	0.424	1070.35	453.66
A_68_P21150010	chr2:38566321-38566365	NM_139051:3720	Nr5a1	INSIDE	1.143	0.381	1607.40	612.28	0.435	1233.58	537.15
A_68_P20377582	chr1:79758038-79758082	NM_001111279:284	Wdfy1	INSIDE	1.143	0.341	4164.25	1421.55	0.390	2986.27	1164.90
A_68_P31836526	chr18:75526561-75526605	NM_001042660:-436	Smad7	PROMOTER	1.142	0.207	3103.18	643.82	0.237	2281.77	540.83
A_68_P31121929	chr17:28478200-28478244	NM_001204156:9069	Tead3	INSIDE	1.142	0.487	1530.12	744.55	0.556	1225.76	681.15
A_68_P31092440	chr17:23713162-23713206	NM_011747:23270	Zfp13	INSIDE	1.142	2.363	1378.21	3256.14	2.697	1092.52	2946.77
A_68_P29701214	chr14:70828882-70828926	NM_021308:-3	Piwil2	PROMOTER	1.142	3.012	1291.02	3888.68	3.438	964.61	3316.73
A_68_P28083441	chr11:102979217-102979261	NM_138753:1600	Hexim1	INSIDE	1.142	0.489	1583.73	773.85	0.558	1159.26	646.74
A_68_P26682080	chr9:83039935-83039979	NM_026122:258	Hmgn3	INSIDE	1.142	0.409	2020.80	826.09	0.467	1620.78	756.41
A_68_P23993056	chr5:113006473-113006517	NM_019982:-289	Sez6l	PROMOTER	1.142	0.295	1848.41	545.21	0.337	1404.04	472.98
A_68_P23593807	chr5:34678844-34678888	NM_011278:-172	Rnf4	PROMOTER	1.142	0.421	1474.56	620.92	0.481	1285.95	618.36
A_68_P22968877	chr4:62875556-62875600	NM_025685:-867	Col27a1	PROMOTER	1.142	0.253	5541.78	1399.61	0.288	3879.82	1119.01

ProbeName	Target position of probe on CpG island microarray	TargetID	GeneSymbol	CpG island Description	Ratio of relative methylation (TiO_2-NP/Vehicle)	Sham group			TiO_2-H group		
						Relative methylation (Cy5/Cy3)	Cy3 signal (Input)	Cy5 signal (MeDIP)	Relative methylation (Cy5/Cy3)	Cy3 signal (Input)	Cy5 signal (MeDIP)
A_68_P22958317	chr4:59796668-59796712	NM_001013577:37	1110054O05Rik	INSIDE	1.142	0.136	5896.95	801.32	0.155	4481.30	695.30
A_68_P21839459	chr2:167924216-167924266	NM_021409:17737	Pard6b	INSIDE	1.142	2.003	469.45	940.42	2.287	339.46	776.41
A_68_P20264196	chr1:59540370-59540414	NM_008057:1402	Fzd7	INSIDE	1.142	0.119	5232.61	622.55	0.136	3752.67	510.09
A_68_P32288521	chrX:20002973-20003017	ENSMUST00000136093:81		INSIDE	1.141	2.275	1191.68	2711.06	2.595	1560.05	4047.93
A_68_P31157498	chr17:35226674-35226718	NM_178592:461	Abhd16a	INSIDE	1.141	0.449	2010.49	901.79	0.512	1465.00	749.47
A_68_P31100079	chr17:25023157-25023201	NM_011956:117	Nubp2	INSIDE	1.141	0.174	5378.38	934.62	0.198	3679.70	729.42
A_68_P30693870	chr16:39985032-39985076	AK082117:347		INSIDE	1.141	0.501	4129.42	2066.86	0.571	2900.30	1656.91
A_68_P29455232	chr14:22668131-22668175	NM_026965:-20	Comtd1	PROMOTER	1.141	0.161	3098.66	499.49	0.184	2141.19	393.66
A_68_P28744814	chr12:112812598-112812642	NM_021516:-100	Mark3	PROMOTER	1.141	0.361	2417.00	872.15	0.412	1830.30	753.53
A_68_P27539536	chr10:127534079-127534123	NM_054078:4262	Baz2a	INSIDE	1.141	1.484	754.18	1118.92	1.693	619.51	1048.69
A_68_P24771682	chr6:115494959-115495004	NM_199033:260	Tsen2	INSIDE	1.141	2.403	311.78	749.19	2.741	248.27	680.55
A_68_P24605096	chr6:85401513-85401557	NM_007638:36	Cct7	INSIDE	1.141	0.458	1148.71	525.82	0.522	913.78	477.16
A_68_P23284714	chr4:127003444-127003488	NM_008126:2903	Gjb3	INSIDE	1.141	1.522	1073.58	1633.91	1.737	822.31	1428.11
A_68_P23253637	chr4:120527431-120527475	ENSMUST00000106283:-6929		PROMOTER	1.141	1.636	1516.03	2480.37	1.867	1125.74	2101.82
A_68_P23156637	chr4:101390213-101390257	NM_010704:223	Lepr	INSIDE	1.141	0.195	2429.19	472.76	0.222	1780.02	395.33
A_68_P22376196	chr3:101408366-101408410	NM_144900:192	Atp1a1	INSIDE	1.141	2.363	726.63	1716.75	2.696	564.75	1522.72
A_68_P21571541	chr2:119613237-119613281	NM_001163701:15	Rpap1	INSIDE	1.141	0.393	1506.71	591.46	0.448	1285.60	575.79
A_68_P20377581	chr1:79757938-79757982	NM_001111279:384	Wdfy1	INSIDE	1.141	0.394	3087.38	1216.64	0.450	2260.96	1017.00
A_68_P31161378	chr17:36003437-36003481	NM_175242:-101	2310014H01Rik	PROMOTER	1.140	0.373	1846.34	689.09	0.426	1521.55	647.51
A_68_P30716740	chr16:43618606-43618656	NM_019778:371234	Zbtb20	INSIDE	1.140	2.507	244.43	612.76	2.857	223.79	639.30
A_68_P28186833	chr11:120657765-120657809	NM_026824:-77	Dus1l	PROMOTER	1.140	0.530	1869.00	990.98	0.604	1356.90	819.98
A_68_P27979771	chr11:84632822-84632866	NM_145433:173	Mrm1	INSIDE	1.140	0.272	6246.23	1700.79	0.310	3937.09	1222.03
A_68_P27751867	chr11:43519036-43519080	NM_027557:23559	Pwwp2a	INSIDE	1.140	1.543	707.59	1091.59	1.758	662.83	1165.35
A_68_P27284175	chr10:80049466-80049510	NM_145421:3598	Fam108a	INSIDE	1.140	4.671	1080.57	5047.50	5.323	1054.43	5612.80
A_68_P27278630	chr10:79202255-79202299	NM_172551:7050	Polrmt	INSIDE	1.140	1.875	1690.84	3170.42	2.138	1392.30	2977.15
A_68_P27266462	chr10:76628409-76628453	NM_009929:845	Col18a1	INSIDE	1.140	0.501	1630.24	816.97	0.572	1272.14	727.05
A_68_P25435069	chr7:121398243-121398287	NM_033370:-70	Copb1	PROMOTER	1.140	0.358	1238.45	442.86	0.408	1018.03	415.12
A_68_P24328146	chr6:29685667-29685711	NM_176996:192	Smo	INSIDE	1.140	3.943	1188.88	4688.28	4.497	954.73	4293.21
A_68_P23586711	chr5:33361070-33361114	NM_011738:-372	Ywhah	PROMOTER	1.140	0.416	1257.28	522.43	0.474	984.51	466.49
A_68_P31415133	chr17:86566520-86566564	NM_011104:-582	Prkce	PROMOTER	1.139	0.423	1871.02	791.26	0.482	1450.89	698.94
A_68_P31187141	chr17:43154407-43154452	NM_178589:926	Tnfrsf21	INSIDE	1.139	0.388	5598.12	2169.99	0.442	3695.46	1632.11
A_68_P28450510	chr12:56181387-56181431	NM_011899:-74	Srp54a	DIVERGENT_PROMOTER	1.139	0.475	1773.00	842.82	0.541	1266.42	685.53
A_68_P27633195	chr11:20640941-20640985	NM_181411:525	Aftph	INSIDE	1.139	6.466	1258.02	8134.69	7.366	1024.84	7548.83
A_68_P26918730	chr10:6980045-6980089	NM_133485:325	Ppp1r14c	INSIDE	1.139	0.397	2983.27	1183.78	0.452	2033.25	918.94
A_68_P26810384	chr9:107579026-107579070	NM_008140:2875	Gnat1	INSIDE	1.139	0.318	1888.92	600.30	0.362	1566.72	567.33
A_68_P26749716	chr9:96789602-96789646	NM_153420:217	Acpl2	INSIDE	1.139	0.227	8600.24	1953.02	0.259	5764.23	1490.65
A_68_P26721431	chr9:91250815-91250859	NM_009573:9801	Zic1	DOWNSTREAM	1.139	2.374	586.78	1393.30	2.704	551.35	1490.93
A_68_P24828016	chr6:126594679-126594723	NM_010595:1119	Kcna1	INSIDE	1.139	0.257	1795.81	461.70	0.293	1435.52	420.20
A_68_P24059430	chr5:124573768-124573812	NM_011256:92637	Pitpnm2	INSIDE	1.139	2.028	853.64	1731.08	2.309	641.70	1481.64
A_68_P21769577	chr2:155937298-155937342	NM_170588:381	Cpne1	INSIDE	1.139	2.335	3039.96	7097.65	2.659	2614.85	6952.96
A_68_P21395113	chr2:84712639-84712683	NM_199223:14189	Rtn4rl2	INSIDE	1.139	1.790	597.37	1069.51	2.039	498.96	1017.54
A_68_P32217856	chr19:59268460-59268504			Unknown	1.138	0.418	3132.72	1310.62	0.476	2522.83	1201.15
A_68_P31067774	chr17:15967950-15967994	NM_178615:-4422	Rgmb	PROMOTER	1.138	0.302	3329.67	1004.99	0.344	2462.59	846.14
A_68_P28115684	chr11:108782304-108782348	NM_015732:664	Axin2	INSIDE	1.138	0.190	3686.44	700.78	0.216	2784.13	602.41
A_68_P27445216	chr10:110182710-110182754	NM_178609:212	E2f7	INSIDE	1.138	0.303	2031.04	615.54	0.345	1719.87	593.40
A_68_P26847148	chr9:114842896-114842940	NM_175380:187	Gpd1l	INSIDE	1.138	0.244	2149.29	523.72	0.277	1620.97	449.62
A_68_P26710359	chr9:88376679-88376723	NM_019796:535	Syncrip	INSIDE	1.138	1.937	678.74	1314.93	2.205	560.22	1235.52
A_68_P26567943	chr9:61794500-61794544	NM_024245:84	Kif23	INSIDE	1.138	1.480	1549.00	2292.83	1.684	1146.12	1929.77
A_68_P25981541	chr8:79709878-79709922	NM_001083906:283494	Nr3c2	INSIDE	1.138	2.378	1010.56	2403.21	2.707	856.67	2319.17
A_68_P22601418	chr3:143865112-143865156	NM_001161770:162	Lmo4	INSIDE	1.138	1.862	3427.90	6383.27	2.119	2461.32	5215.26
A_68_P22321903	chr3:89635681-89635725	NM_172530:411	She	INSIDE	1.138	2.202	554.65	1221.41	2.505	482.48	1208.84
A_68_P32260375	chrX:11668929-11668973	NM_001168321:68531	Bcor	INSIDE	1.137	1.847	469.66	867.63	2.100	494.20	1037.71
A_68_P30346631	chr15:76013891-76013935	NM_201394:12228	Plec	INSIDE	1.137	1.666	1412.39	2353.48	1.894	1031.30	1953.33
A_68_P28914111	chr13:29947064-29947108	NM_144536:371	Cdkal1	INSIDE	1.137	3.418	1153.16	3941.10	3.887	982.18	3817.37
A_68_P23962876	chr5:107125079-107125123	NM_026856:749	Zfp644	INSIDE	1.137	0.253	2692.00	681.12	0.288	1905.38	548.33
A_68_P32697221	chrX:136596305-136596349	NM_001081668:781	Nup62cl	INSIDE	1.136	1.885	567.33	1069.32	2.142	657.84	1409.07
A_68_P32129171	chr19:43954139-43954183	NM_028029:32722	Dnmbp	INSIDE	1.136	0.189	3118.74	588.54	0.214	2388.89	512.00
A_68_P30540361	chr16:9994922-9994966	NM_008170:-2318	Grin2a	PROMOTER	1.136	0.340	1560.49	530.32	0.386	1220.84	471.14
A_68_P30330816	chr15:73343260-73343304	NM_001081066:293	Dennd3	INSIDE	1.136	0.422	1689.47	712.62	0.479	1324.94	634.69
A_68_P27615063	chr11:17157655-17157699	NM_020558:56	C1d	INSIDE	1.136	0.136	4186.19	569.60	0.155	2746.59	424.50
A_68_P27098027	chr10:42740871-42740915	NM_175407:153444	Sobp	INSIDE	1.136	0.383	1397.27	534.83	0.435	1050.66	456.82
A_68_P25050066	chr7:36371059-36371104	NM_178263:-184	Ankrd27	DIVERGENT_PROMOTER	1.136	0.309	1822.88	562.75	0.351	1247.91	437.81
A_68_P25017512	chr7:28271931-28271975	NM_018820:-19	Sertad1	PROMOTER	1.136	0.241	2565.06	617.63	0.274	1860.51	509.08
A_68_P23600187	chr5:35731282-35731326	NM_010445:-461	Hmx1	PROMOTER	1.136	0.393	2114.82	830.42	0.446	1591.32	710.03
A_68_P21909035	chr2:180922349-180922393	NM_025598:323	Ppdpf	INSIDE	1.136	0.534	2021.92	1079.08	0.606	1620.58	982.44

ProbeName	Target position of probe on CpG island microarray	TargetID	GeneSymbol	CpG island Description	Ratio of relative methylation (TiO_2-NP/Vehicle)	Sham group			TiO_2-H group		
						Relative methylation (Cy5/Cy3)	Cy3 signal (Input)	Cy5 signal (MeDIP)	Relative methylation (Cy5/Cy3)	Cy3 signal (Input)	Cy5 signal (MeDIP)
A_68_P20832387	chr1:174255748-174255792	NM_008429:3624	Kcnj9	INSIDE	1.136	2.910	3488.32	10149.39	3.304	2641.16	8726.65
A_68_P20584833	chr1:127332293-127332337	NM_001205385:-10	Actr3	PROMOTER	1.136	3.489	248.94	868.52	3.963	209.44	829.91
A_68_P20085267	chr1:22813048-22813092	NM_001012623:-507	Rims1	PROMOTER	1.136	0.218	2100.76	457.02	0.247	1570.51	388.20
A_68_P31123145	chr17:28712577-28712621	NM_026571:-65	Lhfpl5	PROMOTER	1.135	1.833	898.42	1646.54	2.080	756.54	1573.66
A_68_P30127810	chr15:34236528-34236572	NM_016762:115	Matn2	INSIDE	1.135	0.299	2540.56	759.60	0.339	1739.91	590.52
A_68_P29664621	chr14:64562219-64562263	NM_011446:-302	Sox7	PROMOTER	1.135	0.319	2187.32	696.97	0.362	1585.99	573.76
A_68_P28177063	chr11:119161761-119161805	NM_138669:-425	Eif4a3	PROMOTER	1.135	6.891	3354.03	23112.34	7.818	2858.45	22348.69
A_68_P28078648	chr11:102145969-102146013	NM_028076:-284	Tmub2	PROMOTER	1.135	0.100	5208.54	518.92	0.113	3858.28	436.19
A_68_P28033149	chr11:94335822-94335866	NM_001112813:-332	Cacna1g	PROMOTER	1.135	0.141	4723.66	667.83	0.160	3157.31	506.58
A_68_P27543986	chr10:128371189-128371233	NM_008398:342	Itga7	INSIDE	1.135	0.201	2663.36	535.09	0.228	1968.78	448.87
A_68_P26467844	chr9:44291603-44291647	ENSMUST00000136322:4764		DOWNSTREAM	1.135	0.239	2604.80	623.45	0.272	1852.48	503.37
A_68_P24991222	chr7:19651391-19651435	NM_001159671:11377	Rsph6a	INSIDE	1.135	0.497	1720.52	855.83	0.565	1279.38	722.42
A_68_P23271159	chr4:124613810-124613854	NM_026560:329	Cdca8	INSIDE	1.135	0.233	2366.27	550.37	0.264	1768.10	466.56
A_68_P23196909	chr4:108292640-108292684	NM_177045:102	Cc2d1b	INSIDE	1.135	0.419	1109.55	465.37	0.476	947.91	451.13
A_68_P22340673	chr3:94590302-94590346	NM_001037711:113	Cgn	INSIDE	1.135	0.253	4514.55	1144.41	0.288	3470.70	998.75
A_68_P22185877	chr3:61962834-61962878			Unknown	1.135	1.918	386.73	741.56	2.176	391.81	852.51
A_68_P21833060	chr2:166821691-166821735	NM_001109905:66	Stau1	INSIDE	1.135	0.446	6844.94	3052.76	0.506	4963.40	2512.36
A_68_P21612948	chr2:127188974-127189018	NM_009633:-25	Adra2b	PROMOTER	1.135	0.378	1323.23	500.19	0.429	1031.68	442.74
A_68_P30588542	chr16:20622241-20622285	NM_028420:-911	Camk2n2	DIVERGENT_PROMOTER	1.134	0.358	1428.00	511.31	0.406	1004.76	408.07
A_68_P28835606	chr13:13876193-13876237	NM_010317:-591	Gng4	PROMOTER	1.134	1.474	735.15	1083.28	1.671	635.72	1062.17
A_68_P27172917	chr10:59649185-59649231	NM_016803:32800	Chst3	INSIDE	1.134	1.598	991.22	1584.31	1.812	705.29	1278.12
A_68_P26164570	chr8:113262638-113262682	NM_198625:17277	Mtss1l	INSIDE	1.134	2.065	396.98	819.96	2.343	420.34	984.68
A_68_P25957634	chr8:74108737-74108781	NM_011977:15933	Slc27a1	INSIDE	1.134	2.558	766.62	1960.69	2.901	576.09	1671.52
A_68_P24248437	chr6:13627972-13628016	NM_175312:-28	B630005N14Rik	PROMOTER	1.134	0.428	2870.51	1229.05	0.486	1980.71	961.76
A_68_P32567036	chrX:99266249-99266293	NM_183318:376	Rgag4	INSIDE	1.133	8.152	2685.80	21895.36	9.237	3174.52	29324.03
A_68_P32229422	chr19:61304541-61304585	NM_009970:-241	Csf2ra	PROMOTER	1.133	2.075	1002.48	2080.34	2.350	930.10	2186.19
A_68_P31383664	chr17:80942827-80942871	NM_001033443:20326	Cdkl4	INSIDE	1.133	1.664	1311.24	2182.34	1.886	1026.28	1935.53
A_68_P31097947	chr17:24670396-24670440	NM_177375:274	Rab26	INSIDE	1.133	0.390	1380.25	538.60	0.442	1185.45	523.89
A_68_P30361554	chr15:78502409-78502453	NM_183141:46113	Elfn2	INSIDE	1.133	1.892	841.45	1592.21	2.143	659.46	1413.51
A_68_P29193442	chr13:85329134-85329178	NM_023243:75	Ccnh	INSIDE	1.133	2.611	804.22	2099.59	2.959	602.35	1782.44
A_68_P28181984	chr11:119908317-119908361	NM_001198787:-5243	Aatk	PROMOTER	1.133	1.679	779.83	1309.13	1.902	645.21	1227.02
A_68_P28069996	chr11:100621272-100621316	NM_001081194:-202	Kcnh4	PROMOTER	1.133	5.363	749.37	4019.15	6.077	591.14	3592.26
A_68_P27942518	chr11:78114962-78115006	NM_017407:-108	Spag5	PROMOTER	1.133	0.311	1820.25	566.92	0.353	1394.72	492.16
A_68_P27643872	chr11:22908277-22908321	NM_028672:-5088	Fam161a	PROMOTER	1.133	0.559	1516.49	848.16	0.634	1226.73	777.23
A_68_P27287710	chr10:80610694-80610738	NM_010731:11701	Zbtb7a	INSIDE	1.133	4.081	875.33	3572.04	4.624	721.52	3336.04
A_68_P27254397	chr10:74524869-74524913	NM_001081412:1250	Bcr	INSIDE	1.133	0.398	2802.76	1114.96	0.451	2053.77	925.66
A_68_P26170600	chr8:114378284-114378328	NM_011801:-96	Cfdp1	PROMOTER	1.133	0.346	1415.98	489.62	0.392	1147.97	449.59
A_68_P25007068	chr7:25669351-25669396	NM_023133:-359	Rps19	PROMOTER	1.133	0.437	2257.91	985.65	0.494	1726.76	853.73
A_68_P24990419	chr7:19495688-19495732	NM_001033314:43	Ccdc61	INSIDE	1.133	0.397	1406.84	558.82	0.450	1130.94	509.04
A_68_P23590586	chr5:34037967-34038013	AK172441:87396		INSIDE	1.133	3.447	238.36	821.53	3.903	177.52	692.94
A_68_P23401332	chr4:148961707-148961751	NM_023051:982	Clstn1	INSIDE	1.133	0.233	2179.29	508.50	0.264	1717.91	454.03
A_68_P32775568	chrX:156065624-156065668	NM_001135727:307	Sh3kbp1	INSIDE	1.132	1.436	961.54	1381.12	1.625	1169.90	1901.43
A_68_P29578364	chr14:47407974-47408018	NM_009919:36	Cnih	INSIDE	1.132	0.566	3391.44	1919.19	0.641	2521.86	1616.05
A_68_P29446598	chr14:21208189-21208233	NM_134081:-78	Dnajc9	PROMOTER	1.132	2.163	1179.76	2551.47	2.447	990.82	2424.75
A_68_P24449792	chr6:52256062-52256106	NR_038163:8742	5730457N03Rik	DOWNSTREAM	1.132	0.422	1148.01	484.97	0.478	926.36	442.81
A_68_P22408056	chr3:107499381-107499425	NM_145542:64	Ahcyl1	INSIDE	1.132	2.920	818.42	2390.11	3.307	623.56	2061.82
A_68_P20275804	chr1:61685813-61685857	NM_001081050:437	Pard3b	INSIDE	1.132	1.465	966.65	1416.20	1.658	804.02	1332.96
A_68_P31208325	chr17:46817475-46817519	NM_001163729:383	Klhdc3	INSIDE	1.131	1.710	1903.33	3254.53	1.934	1099.27	2126.44
A_68_P30345479	chr15:75839800-75839844	NM_001168253:106	Fam83h	INSIDE	1.131	4.591	1955.06	8975.36	5.194	1581.01	8211.31
A_68_P29645533	chr14:61254039-61254083	NM_001033272:495	Spata13	INSIDE	1.131	0.540	1756.49	949.37	0.611	1299.49	794.36
A_68_P29628537	chr14:58161247-58161291	NM_145837:17603	Il17d	INSIDE	1.131	2.083	967.13	2014.77	2.357	870.32	2051.47
A_68_P28457454	chr12:57636900-57636944	NM_001146198:-829	Nkx2-1	PROMOTER	1.131	0.188	2685.57	506.02	0.213	1944.00	414.16
A_68_P22317892	chr3:89018127-89018171	NM_001161824:109	Mtx1	INSIDE	1.131	0.377	2144.98	808.73	0.426	1678.55	715.50
A_68_P29614877	chr14:55513260-55513304	NM_019402:303	Pabpn1	INSIDE	1.130	12.911	2184.23	28200.89	14.588	1941.38	28320.05
A_68_P28833519	chr13:12742906-12742950	NR_003568:-266	Gpr137b-ps	PROMOTER	1.130	0.124	4730.26	584.94	0.140	3522.12	492.26
A_68_P28693443	chr12:103981804-103981848	NM_001142937:44	Moap1	INSIDE	1.130	1.451	961.69	1395.06	1.639	705.36	1155.77
A_68_P28202485	chr12:4824209-4824253	NM_025323:-183	0610009D07Rik	PROMOTER	1.130	0.269	1926.48	517.93	0.304	1523.54	462.81
A_68_P28056292	chr11:98274199-98274243	NM_001003817:423	Erbb2	INSIDE	1.130	0.331	1485.02	491.88	0.374	1097.00	410.47
A_68_P28013004	chr11:90499848-90499892	NM_199008:373	Cox11	INSIDE	1.130	1.583	965.69	1529.10	1.789	797.38	1426.64
A_68_P26239401	chr8:125877966-125878010	NM_172287:21376	Spire2	INSIDE	1.130	2.572	1174.72	3021.15	2.905	871.73	2532.33
A_68_P26164574	chr8:113263014-113263058	NM_198625:17653	Mtss1l	INSIDE	1.130	2.610	820.00	2140.46	2.950	678.90	2002.68
A_68_P26157753	chr8:112089724-112089768	NM_175646:-139	Txnl4b	DIVERGENT_PROMOTER	1.130	0.343	1269.52	434.82	0.387	987.35	382.13
A_68_P25481819	chr7:130113409-130113453	NM_175023:-647	Rbbp6	PROMOTER	1.130	1.703	773.93	1317.87	1.924	643.74	1238.32
A_68_P24771970	chr6:115551815-115551859	NM_023290:-119	Mkrn2	DIVERGENT_PROMOTER	1.130	1.722	688.75	1185.89	1.946	593.38	1154.62
A_68_P24639212	chr6:91423890-91423934	NM_028766:168	Tmem43	INSIDE	1.130	0.486	1146.39	557.43	0.549	959.64	527.28

ProbeName	Target position of probe on CpG island microarray	TargetID	GeneSymbol	CpG island Description	Ratio of relative methylation (TiO_2-NP/Vehicle)	Sham group			TiO_2-H group		
						Relative methylation (Cy5/Cy3)	Cy3 signal (Input)	Cy5 signal (MeDIP)	Relative methylation (Cy5/Cy3)	Cy3 signal (Input)	Cy5 signal (MeDIP)
A_68_P31131060	chr17:30141259-30141303	NM_148926:-751	Zfand3	PROMOTER	1.129	0.367	1343.43	492.85	0.414	1112.20	460.53
A_68_P30547082	chr16:11253796-11253840	NM_001130008:600	Gspt1	INSIDE	1.129	1.902	1416.32	2694.16	2.147	1146.29	2461.60
A_68_P27990833	chr11:86570909-86570954	NM_001003908:63	Cltc	INSIDE	1.129	0.239	5458.58	1304.26	0.270	3622.73	977.06
A_68_P26766596	chr9:99769415-99769459	NM_011440:7153	Sox14	DOWNSTREAM	1.129	0.407	1264.73	514.37	0.459	1002.28	460.38
A_68_P26372145	chr9:26541609-26541653	ENSMUST00000067362:195		INSIDE	1.129	0.310	2424.49	751.27	0.350	1824.32	637.94
A_68_P25599899	chr7:150481613-150481657	NR_001461:818	Kcnq1ot1	INSIDE	1.129	1.513	1226.62	1855.91	1.708	1080.75	1846.26
A_68_P24973911	chr7:13556484-13556528	NM_178732:5294	Zfp324	INSIDE	1.129	3.742	2699.19	10101.38	4.225	2600.21	10986.28
A_68_P24954288	chr7:6106767-6106811	NM_001013012:785	Zfp787	INSIDE	1.129	1.458	1876.27	2734.94	1.645	1536.82	2528.77
A_68_P24444427	chr6:51419364-51419408	NM_016806:507	Hnrnpa2b1	INSIDE	1.129	0.083	8097.05	668.55	0.093	5757.75	536.72
A_68_P23362917	chr4:140803601-140803645	NM_172520:4824	Arhgef19	INSIDE	1.129	0.422	3145.49	1327.86	0.476	2379.16	1133.51
A_68_P22311001	chr3:87775341-87775385	NM_016701:348	Nes	INSIDE	1.129	0.424	1061.29	450.16	0.479	810.47	388.17
A_68_P20793719	chr1:167212029-167212073			Unknown	1.129	0.392	1357.57	532.28	0.443	1202.87	532.51
A_68_P31828825	chr18:74129885-74129929	NM_172632:94697	Mapk4	INSIDE	1.128	1.547	1007.15	1557.59	1.745	809.73	1412.62
A_68_P30349814	chr15:76497752-76497796	NM_010630:6704	Kifc2	INSIDE	1.128	2.156	837.87	1806.08	2.431	667.90	1623.92
A_68_P30328265	chr15:72921187-72921231	NM_053068:367	Chrac1	INSIDE	1.128	0.545	1515.33	826.00	0.615	1174.51	722.00
A_68_P30012297	chr15:10644132-10644176	NM_030690:-859	Rai14	PROMOTER	1.128	0.155	3393.72	527.49	0.175	2427.50	425.47
A_68_P28161639	chr11:116713577-116713621	NM_011358:810	Srsf2	INSIDE	1.128	0.291	2680.67	781.01	0.329	2025.35	665.36
A_68_P28158435	chr11:116142974-116143018	NM_010254:744	Galr2	INSIDE	1.128	0.317	3004.84	951.05	0.357	2071.76	739.68
A_68_P28152793	chr11:115184885-115184929	NM_176847:-1674	Ush1g	PROMOTER	1.128	0.426	1140.94	486.31	0.481	821.92	395.03
A_68_P27921684	chr11:74463331-74463375	NM_001081158:356	1300001I01Rik	INSIDE	1.128	2.536	732.59	1857.59	2.859	584.42	1671.03
A_68_P27289149	chr10:80827197-80827241	NM_001014836:1347	4930404N11Rik	INSIDE	1.128	0.311	3181.51	989.96	0.351	2362.47	829.42
A_68_P27045328	chr10:31328333-31328377	NM_001146349:1177	Rnf217	INSIDE	1.128	1.650	480.86	793.21	1.861	516.90	961.95
A_68_P25955964	chr8:73796836-73796880	NM_001142322:246	Myo9b	INSIDE	1.128	0.256	2740.32	701.96	0.289	1978.92	571.76
A_68_P25661492	chr8:12431084-12431128	NR_027975:45336	Gm5607	INSIDE	1.128	0.307	3487.80	1069.39	0.346	2727.24	943.54
A_68_P25586658	chr7:148278423-148278467	NM_023059:8001	Sigirr	INSIDE	1.128	0.498	3584.49	1785.61	0.562	2632.31	1478.72
A_68_P23575370	chr5:31209922-31209966	NM_001134692:151	Ost4	INSIDE	1.128	0.111	4332.62	479.70	0.125	2641.69	330.03
A_68_P21761443	chr2:154438790-154438834	NM_199304:-291	Zfp341	DIVERGENT_PROMOTER	1.128	0.307	1454.20	446.37	0.346	1147.69	397.53
A_68_P21727917	chr2:147872064-147872108	NM_010446:619	Foxa2	INSIDE	1.128	0.191	3370.65	642.75	0.215	2438.91	524.76
A_68_P31227872	chr17:50433053-50433097	NM_010021:-150	Dazl	PROMOTER	1.127	1.454	1204.17	1750.27	1.638	909.61	1490.36
A_68_P30988902	chr16:95923853-95923897	NM_011809:-139	Ets2	PROMOTER	1.127	0.464	2650.34	1229.22	0.523	2100.92	1097.86
A_68_P27889186	chr11:68213303-68213358	ENSMUST00000108674:995		INSIDE	1.127	0.474	1843.92	874.76	0.535	1142.96	611.33
A_68_P27257832	chr10:75129986-75130030	NM_172549:97094	Cabin1	INSIDE	1.127	0.320	2032.34	649.86	0.360	1327.70	478.50
A_68_P27176097	chr10:60149838-60149882	NM_023370:9378	Cdh23	INSIDE	1.127	0.489	1524.79	746.03	0.551	1045.11	576.22
A_68_P25957676	chr8:74116134-74116178	NM_025396:74	Pgls	INSIDE	1.127	2.167	374.49	811.44	2.442	307.64	751.35
A_68_P21909038	chr2:180922669-180922713	NM_025598:643	Ppdpf	INSIDE	1.127	0.305	1736.62	529.89	0.344	1263.98	434.55
A_68_P21906900	chr2:180627890-180627934	NR_029538:-832	Mir124a-3	PROMOTER	1.127	0.204	3246.69	662.22	0.230	2284.00	525.15
A_68_P21421467	chr2:91077045-91077089	NM_028119:157	Ddb2	INSIDE	1.127	0.330	2738.04	904.40	0.372	1962.56	730.73
A_68_P21078344	chr2:26245012-26245056	NM_173180:167	Pmpca	INSIDE	1.127	0.166	3408.65	566.10	0.187	2299.93	430.48
A_68_P32785479	chrX:158155132-158155176	NM_198409:-330	Rai2	PROMOTER	1.126	1.564	1129.16	1766.33	1.761	1400.44	2466.66
A_68_P32774623	chrX:155853132-155853176	NM_001081124:645	Mtap7d2	INSIDE	1.126	0.612	2442.59	1495.35	0.690	3255.88	2245.16
A_68_P31464038	chr18:4635009-4635053	NM_001081963:104	9430020K01Rik	INSIDE	1.126	0.220	3367.86	739.27	0.247	2415.32	597.14
A_68_P31167786	chr17:37334030-37334074	NM_001011518:859	Olfr94	INSIDE	1.126	0.429	1511.48	648.89	0.484	1120.23	541.74
A_68_P30481816	chr15:99532450-99532494	NM_031842:-245	Smarcd1	PROMOTER	1.126	0.358	1223.57	437.62	0.403	1059.11	426.67
A_68_P30421913	chr15:88721305-88721349	ENSMUST00000172019:63522		INSIDE	1.126	0.279	4362.64	1216.78	0.314	3054.68	959.00
A_68_P28194541	chr12:3309848-3309892	NM_016676:99	Rab10	INSIDE	1.126	0.243	2243.94	545.73	0.274	1685.62	461.46
A_68_P28159935	chr11:116403194-116403238	NM_173755:39545	Ube2o	INSIDE	1.126	1.884	1223.43	2305.15	2.121	941.47	1996.88
A_68_P27913316	chr11:72608989-72609033	NM_001024926:331	Cyb5d2	INSIDE	1.126	0.392	1839.39	721.40	0.442	1391.52	614.68
A_68_P27282554	chr10:79798280-79798324	NM_001204931:5413	Reep6	INSIDE	1.126	2.120	868.15	1840.71	2.387	730.16	1742.71
A_68_P26920086	chr10:7382947-7382991	NR_030698:-200	A630066F11Rik	PROMOTER	1.126	0.481	5185.52	2495.51	0.542	3553.23	1924.70
A_68_P25957071	chr8:74009830-74009874	NM_001164679:114	Ano8	INSIDE	1.126	0.464	2077.95	963.76	0.522	1636.75	854.55
A_68_P25023220	chr7:29501806-29501850	NM_015796:20	Fbxo17	INSIDE	1.126	0.555	1854.77	1030.09	0.625	1505.72	941.32
A_68_P21420490	chr2:90910289-90910333	NM_026721:68	Slc39a13	INSIDE	1.126	1.399	2630.86	3680.82	1.575	2056.30	3239.43
A_68_P21077040	chr2:26061806-26061850	NM_001039653:248	Lhx3	INSIDE	1.126	0.440	1366.93	601.64	0.496	994.94	493.07
A_68_P20599491	chr1:130140934-130140978	NM_026390:199	Ubxn4	INSIDE	1.126	0.368	2450.27	901.14	0.414	1838.74	761.58
A_68_P32111332	chr19:40906336-40906380	NM_172839:590	Ccnj	INSIDE	1.125	0.610	3868.34	2358.27	0.686	2932.59	2010.74
A_68_P31112704	chr17:26975881-26975926	NM_008700:2607	Nkx2-5	INSIDE	1.125	0.222	3083.57	686.05	0.250	2359.01	590.63
A_68_P30621545	chr16:26581869-26581913	NM_134103:100	Il1rap	INSIDE	1.125	0.336	1775.86	596.17	0.378	1435.28	542.18
A_68_P30339172	chr15:74551721-74551765	NM_198607:79	4930572J05Rik	INSIDE	1.125	0.514	1700.41	873.40	0.578	1480.77	855.41
A_68_P29694908	chr14:69809045-69809089	NM_010921:318	Nkx3-1	INSIDE	1.125	0.481	1363.19	655.42	0.541	949.42	513.47
A_68_P28198045	chr12:3981964-3982008	NM_001082483:56929	Efr3b	INSIDE	1.125	0.248	4043.17	1001.92	0.279	2760.04	769.47
A_68_P28056086	chr11:98245656-98245700	NM_011540:554	Tcap	INSIDE	1.125	1.838	1059.97	1948.35	2.068	896.40	1853.43
A_68_P27894948	chr11:69224589-69224633	NM_001017426:2567	Kdm6b	INSIDE	1.125	0.506	2175.74	1101.52	0.569	1399.96	797.23
A_68_P27541017	chr10:127814266-127814310	NM_172790:109	Ankrd52	INSIDE	1.125	0.312	1450.45	452.13	0.351	1055.35	370.15
A_68_P26612674	chr9:69607679-69607723	NM_022378:1047	Foxb1	INSIDE	1.125	1.989	390.65	776.99	2.237	314.04	702.46
A_68_P25020985	chr7:29075914-29075958	NM_175033:217	BC089491	INSIDE	1.125	0.354	4981.21	1765.49	0.399	3443.66	1372.73

ProbeName	Target position of probe on CpG island microarray	TargetID	GeneSymbol	CpG island Description	Ratio of relative methylation (TiO_2-NP/Vehicle)	Sham group			TiO_2-H group		
						Relative methylation (Cy5/Cy3)	Cy3 signal (Input)	Cy5 signal (MeDIP)	Relative methylation (Cy5/Cy3)	Cy3 signal (Input)	Cy5 signal (MeDIP)
A_68_P24633501	chr6:90555093-90555137	NM_027868:241	Slc41a3	INSIDE	1.125	0.341	4539.89	1548.52	0.384	3360.64	1289.13
A_68_P24449405	chr6:52196592-52196638	NM_010450:-848	Hoxa11	PROMOTER	1.125	2.649	252.47	668.78	2.979	192.18	572.48
A_68_P24159893	chr5:144952289-144952338	NM_010800:1159	Bhlha15	INSIDE	1.125	2.882	608.89	1754.55	3.243	487.84	1581.96
A_68_P23230003	chr4:116269931-116269975	NR_029450:283	C530005A16Rik	INSIDE	1.125	0.412	1712.52	705.70	0.463	1220.31	565.51
A_68_P21907443	chr2:180701912-180701956	NM_001177706:-70	Arfgap1	PROMOTER	1.125	0.416	1181.49	492.06	0.469	1011.45	473.88
A_68_P32217859	chr19:59268751-59268795			Unknown	1.124	0.318	1653.58	526.05	0.358	1236.71	442.24
A_68_P31482454	chr18:8694075-8694120	ENSMUST00000171469:23		INSIDE	1.124	0.389	1875.26	729.07	0.437	1272.44	555.83
A_68_P30463768	chr15:96290631-96290675	NM_028148:622	Scaf11	INSIDE	1.124	0.329	1407.62	463.55	0.370	986.00	365.13
A_68_P29699037	chr14:70479901-70479945	NM_018781:2671	Egr3	INSIDE	1.124	3.920	1088.92	4268.18	4.407	930.42	4100.04
A_68_P27926564	chr11:75329061-75329105	NM_001004157:2040	Scarf1	INSIDE	1.124	1.900	1090.85	2072.42	2.136	809.80	1729.87
A_68_P26742255	chr9:95412044-95412088	NM_001114977:349	2610101N10Rik	INSIDE	1.124	0.463	1582.99	732.31	0.520	1382.94	718.85
A_68_P26587470	chr9:65246468-65246513	NM_001033158:196	Rasl12	INSIDE	1.124	1.369	1457.12	1994.93	1.539	1137.98	1751.38
A_68_P24995609	chr7:20443379-20443423	NM_001033419:3248	Ceacam16	INSIDE	1.124	1.608	866.27	1392.65	1.807	720.28	1301.88
A_68_P23929476	chr5:100844949-100844993	NM_026969:283	Sec31a	INSIDE	1.124	1.836	921.25	1691.42	2.064	771.13	1591.61
A_68_P22009787	chr3:27081688-27081732	NM_178772:-215	Nceh1	PROMOTER	1.124	0.389	2098.37	816.81	0.437	1541.93	674.51
A_68_P31939559	chr19:7457501-7457545	NM_001033139:-6	AI846148	PROMOTER	1.123	0.427	2111.89	901.99	0.480	1496.41	717.98
A_68_P31921019	chr19:4097126-4097170	NM_026373:-202	Cdk2ap2	PROMOTER	1.123	0.321	2481.81	797.33	0.361	1916.80	691.51
A_68_P31677778	chr18:46756824-46756868	NM_025698:343	Tmed7	INSIDE	1.123	2.791	730.15	2037.69	3.134	580.92	1820.56
A_68_P31301690	chr17:65963450-65963494	NM_013933:-577	Vapa	PROMOTER	1.123	8.841	1068.28	9445.11	9.926	938.61	9316.25
A_68_P31058441	chr17:14340236-14340280	NM_172826:580	Dact2	INSIDE	1.123	0.370	1501.35	555.62	0.416	1262.27	524.56
A_68_P26086063	chr8:98475827-98475871			Unknown	1.123	1.354	1668.51	2259.36	1.521	1463.00	2225.48
A_68_P25844871	chr8:49075873-49075917	NM_133791:11	Wwc2	INSIDE	1.123	1.833	968.02	1774.16	2.058	847.06	1743.40
A_68_P25598468	chr7:150255797-150255841	NM_001115085:546	Tssc4	INSIDE	1.123	0.198	5312.63	1050.27	0.222	3363.42	746.39
A_68_P24126243	chr5:137972158-137972202	NM_010312:2277	Gnb2	INSIDE	1.123	0.414	2555.20	1058.12	0.465	1788.47	831.56
A_68_P23254532	chr4:120689345-120689392	NM_133716:484	Smap2	INSIDE	1.123	0.216	2105.68	455.51	0.243	1423.06	345.60
A_68_P22071592	chr3:38784302-38784349	NM_183221:-1536	Fat4	PROMOTER	1.123	0.519	2246.13	1165.50	0.583	1631.84	950.83
A_68_P29330802	chr13:112598180-112598224	NM_011945:989	Map3k1	INSIDE	1.122	2.293	1470.35	3372.16	2.573	1189.17	3059.27
A_68_P29288685	chr13:104899639-104899683	NM_029447:34	Nln	INSIDE	1.122	0.159	4003.46	636.94	0.179	3055.13	545.38
A_68_P26948442	chr10:12684445-12684489	NM_001163590:-401	Stx11	PROMOTER	1.122	0.279	1868.80	522.05	0.313	1504.52	471.51
A_68_P23341302	chr4:137237494-137237538	NM_001081155:-101	Rap1gap	PROMOTER	1.122	0.458	2073.72	950.44	0.514	1698.15	873.65
A_68_P21833422	chr2:166888209-166888253	NM_001081005:-203	1500012F01Rik	PROMOTER	1.122	2.687	630.48	1694.39	3.016	591.17	1782.93
A_68_P20799612	chr1:168239545-168239589	NM_001164528:55297	Ildr2	INSIDE	1.122	0.539	1375.81	741.48	0.605	1147.87	694.14
A_68_P20770874	chr1:163181125-163181169	NM_007453:151	Prdx6	INSIDE	1.122	0.290	1516.43	440.23	0.326	1184.97	386.05
A_68_P32700071	chrX:137134752-137134796	NM_001077364:287	Tsc22d3	INSIDE	1.121	2.608	3579.24	9335.49	2.924	4290.32	12542.95
A_68_P30346595	chr15:76009519-76009563	NM_201394:16600	Plec	INSIDE	1.121	3.342	1106.65	3698.51	3.746	908.93	3404.66
A_68_P27325553	chr10:87922455-87922499	NM_011517:145	Sycp3	INSIDE	1.121	2.650	916.13	2427.51	2.970	714.83	2123.24
A_68_P25827401	chr8:46036029-46036073	NM_001081286:489	Fat1	INSIDE	1.121	1.525	1445.33	2203.55	1.709	1105.28	1889.01
A_68_P25737279	chr8:28338376-28338420	NM_013462:1662	Adrb3	INSIDE	1.121	0.480	4070.88	1953.86	0.538	2898.19	1559.17
A_68_P25661263	chr8:12397648-12397692	NM_009233:2152	Sox1	INSIDE	1.121	0.538	1331.16	716.76	0.604	1080.53	652.39
A_68_P25276880	chr7:89484688-89484732	NM_001190374:507	Adamtsl3	INSIDE	1.121	0.351	3063.74	1075.47	0.393	2204.86	867.57
A_68_P21901918	chr2:179906691-179906735	NM_019822:420	Adrm1	INSIDE	1.121	0.445	1738.90	773.23	0.499	1460.38	728.27
A_68_P30094307	chr15:27677421-27677465	NM_001081302:278161	Trio	INSIDE	1.120	2.937	3436.95	10093.05	3.289	2318.21	7623.58
A_68_P29055222	chr13:55573097-55573141	NM_001030296:7491	Prr7	INSIDE	1.120	1.482	725.21	1074.47	1.660	709.63	1177.87
A_68_P28958208	chr13:38129074-38129118	NM_024242:239	Riok1	INSIDE	1.120	0.150	3616.82	541.41	0.168	2551.20	427.64
A_68_P27551372	chr11:3231986-3232030	NM_178149:1275	Pik3ip1	INSIDE	1.120	1.414	1000.32	1414.73	1.584	770.16	1219.62
A_68_P26891191	chr9:122656494-122656538	NM_001199736:53	Gm9524	INSIDE	1.120	2.157	1214.10	2618.44	2.415	943.30	2278.32
A_68_P26458363	chr9:42705778-42705822	NM_175481:46654	Grik4	INSIDE	1.120	0.138	6076.86	837.51	0.154	3910.35	603.67
A_68_P25478245	chr7:129433344-129433388	NM_008855:728	Prkcb	INSIDE	1.120	0.269	1959.32	527.76	0.302	1483.20	447.36
A_68_P25032390	chr7:31373515-31373559	NM_029274:209	Wbp7	INSIDE	1.120	1.399	1458.40	2040.30	1.567	1246.80	1953.12
A_68_P24804564	chr6:121423714-121423758	NM_001033354:-40	Iqsec3	PROMOTER	1.120	3.897	2016.96	7859.52	4.362	1444.40	6301.03
A_68_P24333874	chr6:30688204-30688248	NR_029900:-3072	Mir335	PROMOTER	1.120	2.843	510.11	1450.42	3.185	426.95	1359.84
A_68_P24040307	chr5:121144585-121144629	NM_008052:17072	Dtx1	INSIDE	1.120	2.089	648.67	1354.98	2.340	525.84	1230.47
A_68_P22049393	chr3:34549575-34549619	NM_011443:670	Sox2	INSIDE	1.120	0.299	1851.13	553.34	0.335	1447.35	484.49
A_68_P21635428	chr2:131346491-131346535	NM_001177833:28915	Smox	INSIDE	1.120	1.425	1910.33	2722.53	1.597	1577.16	2518.46
A_68_P33003690	chr1_random:342987-343031	NM_177389:-95	Mia3	PROMOTER	1.119	0.292	3553.61	1037.14	0.327	2721.40	888.59
A_68_P32461571	chrX:70473910-70473954	NM_007978:288	F8a	INSIDE	1.119	2.440	511.51	1248.03	2.730	673.63	1839.14
A_68_P32053973	chr19:29700840-29700884	NM_001081213:22048	Ermp1	INSIDE	1.119	1.711	5846.27	10001.11	1.913	3894.49	7451.76
A_68_P30512788	chr16:4939417-4939461	NR_037666:147	LOC100503704	INSIDE	1.119	0.246	3730.97	916.14	0.275	2535.45	696.48
A_68_P29716895	chr14:73725625-73725669	NM_009029:-48	Rb1	PROMOTER	1.119	3.136	279.15	875.31	3.508	202.65	710.92
A_68_P27280127	chr10:79440093-79440137	NM_001003949:6961	ORF61	INSIDE	1.119	2.312	871.16	2014.30	2.587	758.16	1961.47
A_68_P27176094	chr10:60149519-60149563	NM_023370:9698	Cdh23	INSIDE	1.119	0.380	2536.31	962.91	0.425	1801.08	765.07
A_68_P26467960	chr9:44306562-44306606	NM_030256:-634	Bcl9l	PROMOTER	1.119	0.387	2909.15	1124.52	0.433	1989.93	860.96
A_68_P25782095	chr8:36889705-36889749	NM_027998:-113	Cldn23	PROMOTER	1.119	0.194	5341.53	1034.02	0.217	4010.58	868.74
A_68_P23702721	chr5:54200945-54200989	NM_144517:101	Tbc1d19	INSIDE	1.119	0.621	2420.68	1503.35	0.695	1800.50	1250.92
A_68_P21144177	chr2:37648771-37648815	NM_001163566:17024	Crb2	INSIDE	1.119	1.866	1140.08	2127.17	2.089	981.62	2050.14

ProbeName	Target position of probe on CpG island microarray	TargetID	GeneSymbol	CpG island Description	Ratio of relative methylation (TiO_2-NP/Vehicle)	Sham group			TiO_2-H group		
						Relative methylation (Cy5/Cy3)	Cy3 signal (Input)	Cy5 signal (MeDIP)	Relative methylation (Cy5/Cy3)	Cy3 signal (Input)	Cy5 signal (MeDIP)
A_68_P32241972	chrX:7722879-7722923	NM_016809:28	Rbm3	INSIDE	1.118	1.411	1302.58	1838.39	1.577	1667.17	2629.45
A_68_P31928721	chr19:5447928-5447972	NM_010235:253	Fosl1	INSIDE	1.118	0.257	2359.61	606.72	0.287	1704.65	490.03
A_68_P29747596	chr14:79132794-79132838	NM_001081336:-7920	Dgkh	PROMOTER	1.118	0.232	3359.88	778.28	0.259	2257.77	584.68
A_68_P28675862	chr12:100630292-100630340	NM_183186:57968	Foxn3	INSIDE	1.118	2.573	306.88	789.70	2.877	261.15	751.20
A_68_P28183958	chr11:120209420-120209464	NM_009609:356	Actg1	INSIDE	1.118	1.426	1276.10	1819.17	1.593	1009.20	1607.74
A_68_P26494988	chr9:48793866-48793910	NM_175482:399	Usp28	INSIDE	1.118	0.289	2683.53	774.29	0.322	2027.14	653.67
A_68_P26468514	chr9:44413289-44413333	NM_001110826:336	Ddx6	INSIDE	1.118	0.415	2971.88	1233.98	0.464	2436.58	1130.94
A_68_P26153174	chr8:111324296-111324340	NM_007496:85775	Zfhx3	INSIDE	1.118	1.455	1375.33	2000.89	1.627	1105.88	1799.18
A_68_P24605101	chr6:85402012-85402056	NR_028081:-70	1700040I03Rik	PROMOTER	1.118	0.394	1213.29	477.70	0.440	1018.03	448.26
A_68_P23410166	chr4:150482290-150482334	NM_001081557:753565	Camta1	INSIDE	1.118	0.140	6499.62	912.61	0.157	4637.77	727.76
A_68_P23349525	chr4:138630261-138630305	NM_021358:422	Htr6	INSIDE	1.118	0.424	1774.39	752.37	0.474	1335.43	633.12
A_68_P21071713	chr2:25228424-25228468	NM_001081085:606	2010317E24Rik	INSIDE	1.118	2.792	606.66	1693.61	3.121	536.67	1675.11
A_68_P20842597	chr1:176433418-176433462	NM_019445:1485	Fmn2	INSIDE	1.118	0.327	2411.95	789.30	0.366	1603.87	587.04
A_68_P32603677	chrX:109714158-109714202	NM_177747:46	Zfp711	INSIDE	1.117	2.329	316.32	736.87	2.602	438.23	1140.35
A_68_P32465797	chrX:71212951-71212995	NM_008224:-1318	Hcfc1	PROMOTER	1.117	0.516	1491.43	770.29	0.577	2001.07	1154.75
A_68_P32134819	chr19:44911268-44911312	NM_011037:79407	Pax2	INSIDE	1.117	0.466	1415.44	659.86	0.521	1201.67	625.63
A_68_P29244711	chr13:96901122-96901166	NM_029210:1388	Sv2c	INSIDE	1.117	1.507	2586.25	3897.85	1.684	2113.39	3558.19
A_68_P27992593	chr11:86922410-86922454	NM_175563:-216	Prr11	DIVERGENT_PROMOTER	1.117	0.158	3908.32	616.99	0.176	2649.50	467.19
A_68_P26574085	chr9:62990414-62990458	NM_172446:4351	Skor1	INSIDE	1.117	0.159	4163.22	660.00	0.177	2802.95	496.21
A_68_P25065936	chr7:39006581-39006625	NM_024413:6411	Plekhf1	INSIDE	1.117	2.018	2672.72	5393.44	2.255	1940.76	4375.92
A_68_P24113930	chr5:135408345-135408389	NM_024479:124	Wbscr27	INSIDE	1.117	0.281	2441.35	685.05	0.313	1794.06	562.31
A_68_P23231032	chr4:116480068-116480112	NM_026654:74	Toe1	INSIDE	1.117	0.354	1813.52	642.01	0.395	1214.92	480.45
A_68_P22907354	chr4:49858702-49858746	NM_001033351:-771	Grin3a	PROMOTER	1.117	0.375	1699.78	637.65	0.419	1278.64	536.01
A_68_P20354207	chr1:75455378-75455422	NM_183022:8316	Accn4	INSIDE	1.117	1.582	611.50	967.25	1.767	569.08	1005.45
A_68_P20319175	chr1:69424592-69424636			Unknown	1.117	1.669	1331.20	2221.54	1.865	1132.14	2111.24
A_68_P32561247	chrX:97972457-97972501	NM_001177780:-131	Dlg3	PROMOTER	1.116	2.442	416.06	1016.18	2.726	502.75	1370.72
A_68_P31918873	chr19:3708527-3708572	NM_028077:217	1810055G02Rik	INSIDE	1.116	0.158	9798.04	1552.42	0.177	6208.52	1098.21
A_68_P23407012	chr4:149943746-149943790	NM_001085492:287744	Rere	INSIDE	1.116	1.943	585.32	1137.12	2.168	480.25	1041.34
A_68_P23325610	chr4:134487232-134487276	NM_026780:360	Syf2	INSIDE	1.116	30.434	7436.08	226310.00	33.962	7074.44	240260.20
A_68_P22856642	chr4:40226159-40226203	NM_001033305:221	Ndufb6	INSIDE	1.116	0.296	1976.44	584.46	0.330	1490.86	492.00
A_68_P22200389	chr3:65332655-65332699	NM_178892:308	Tiparp	INSIDE	1.116	0.363	2144.42	778.70	0.405	1698.37	688.48
A_68_P22030511	chr3:30755072-30755116	NM_001134385:223	Gpr160	INSIDE	1.116	4.162	959.83	3995.28	4.644	841.86	3909.34
A_68_P21820675	chr2:164774897-164774941	NM_013599:1168	Mmp9	INSIDE	1.116	0.213	2121.46	451.37	0.237	1500.04	356.16
A_68_P21114429	chr2:32143411-32143455	NM_175190:156	2900010J23Rik	INSIDE	1.116	0.498	2156.76	1074.94	0.556	1717.54	955.66
A_68_P20644038	chr1:138028419-138028463	NM_001039472:463	Kif21b	INSIDE	1.116	0.482	1401.85	675.99	0.538	1238.13	666.31
A_68_P32820570	chrX:165758553-165758597	NM_008222:-299	Hccs	PROMOTER	1.115	2.660	185.27	492.74	2.965	215.14	637.78
A_68_P32410978	chrX:56820599-56820643	ENSMUST00000124402:29		INSIDE	1.115	2.919	458.97	1339.88	3.254	451.66	1469.69
A_68_P31256669	chr17:56651021-56651065	NM_011483:864	Znrf4	INSIDE	1.115	0.208	3205.42	665.88	0.232	2218.40	513.72
A_68_P28175804	chr11:118952556-118952600	NM_007625:-5027	Cbx4	PROMOTER	1.115	0.347	1481.02	513.51	0.386	1119.30	432.58
A_68_P28051746	chr11:97491089-97491133	NM_175332:-80	E130012A19Rik	PROMOTER	1.115	0.438	1904.29	834.69	0.489	1507.51	736.87
A_68_P28037459	chr11:95084648-95084692	ENSMUST00000150818:2276		INSIDE	1.115	4.147	1593.18	6606.31	4.623	1695.12	7836.45
A_68_P26804375	chr9:106553157-106553201	NM_001160353:5262	Grm2	INSIDE	1.115	2.355	937.52	2207.83	2.625	767.78	2015.60
A_68_P26766610	chr9:99771206-99771250	NM_011440:5361	Sox14	DOWNSTREAM	1.115	0.362	1933.73	699.36	0.403	1366.35	550.75
A_68_P26141794	chr8:109394848-109394892	NM_008217:729	Has3	INSIDE	1.115	0.447	1888.67	844.80	0.499	1381.87	689.21
A_68_P25506338	chr7:134732367-134732411	NM_172281:177	Rnf40	INSIDE	1.115	0.182	4126.44	749.63	0.202	2972.70	601.88
A_68_P25114497	chr7:57014275-57014319	NM_001146049:-179	Htatip2	PROMOTER	1.115	0.303	1557.04	471.14	0.337	1120.05	377.85
A_68_P25016729	chr7:28116317-28116361	NM_001113549:2329	Ltbp4	INSIDE	1.115	1.584	916.00	1450.57	1.766	753.90	1331.09
A_68_P25004864	chr7:25218003-25218050	NM_199013:12675	Irgc1	INSIDE	1.115	1.995	489.22	975.83	2.224	453.24	1007.79
A_68_P22317894	chr3:89018391-89018435	NM_001161824:-155	Mtx1	DIVERGENT_PROMOTER	1.115	0.617	3486.57	2150.07	0.688	2353.16	1618.14
A_68_P31619631	chr18:35862603-35862647	NM_029669:174	Dnajc18	INSIDE	1.114	0.398	1143.08	454.77	0.443	939.78	416.68
A_68_P29478589	chr14:26426247-26426291	NM_183208:147598	Zmiz1	INSIDE	1.114	0.464	1522.33	707.01	0.517	1087.64	562.53
A_68_P28814400	chr13:9093008-9093052	NM_172585:-120	Larp4b	PROMOTER	1.114	0.407	1806.37	734.83	0.453	1426.49	646.16
A_68_P26350702	chr9:21969587-21969631	NM_001082532:8319	Pigyl	DOWNSTREAM	1.114	1.481	1635.55	2422.96	1.651	1401.31	2313.39
A_68_P23152971	chr4:100741981-100742025	NM_183024:360	Raver2	INSIDE	1.114	0.459	1101.82	506.15	0.512	919.33	470.43
A_68_P20411738	chr1:88256632-88256676	NR_015507:506	C130036L24Rik	INSIDE	1.114	1.509	940.40	1418.76	1.681	696.45	1170.66
A_68_P20121784	chr1:30930609-30930653	ENSMUST00000046443:-91		PROMOTER	1.114	2.480	810.75	2010.57	2.763	766.23	2116.79
A_68_P31349750	chr17:74396973-74397017	NM_053188:262	Srd5a2	INSIDE	1.113	0.314	1886.88	592.90	0.350	1429.22	499.68
A_68_P30501454	chr15:102844844-102844888	NR_030526:-474	Mir615	PROMOTER	1.113	1.373	1889.81	2594.82	1.528	1383.09	2112.90
A_68_P30423620	chr15:88997813-88997857	NM_001159521:6446	Plxnb2	INSIDE	1.113	1.384	1472.60	2038.48	1.540	1091.55	1681.34
A_68_P28670942	chr12:99812802-99812846	NM_029911:3326	Kcnk10	INSIDE	1.113	0.152	5346.54	812.45	0.169	3698.57	625.40
A_68_P28206069	chr12:5381867-5381911	NM_001164493:600	Klhl29	INSIDE	1.113	0.415	1618.36	672.28	0.462	1203.04	556.04
A_68_P26815690	chr9:108499968-108500012	NM_028944:-59	P4htm	PROMOTER	1.113	1.983	751.58	1490.67	2.208	634.42	1401.07
A_68_P25587562	chr7:148414623-148414668	NM_001081118:-41	Phrf1	DIVERGENT_PROMOTER	1.113	2.248	670.73	1507.93	2.502	755.59	1890.58
A_68_P25447475	chr7:123451082-123451126	ENSMUST00000106605:786		INSIDE	1.113	0.502	2677.52	1343.17	0.558	2143.52	1197.07
A_68_P25259447	chr7:86293791-86293835	NM_001045489:134	Mfge8	INSIDE	1.113	2.104	1416.34	2980.44	2.343	1341.03	3141.95

ProbeName	Target position of probe on CpG island microarray	TargetID	GeneSymbol	CpG island Description	Ratio of relative methylation (TiO_2-NP/Vehicle)	Sham group			TiO_2-H group		
						Relative methylation (Cy5/Cy3)	Cy3 signal (Input)	Cy5 signal (MeDIP)	Relative methylation (Cy5/Cy3)	Cy3 signal (Input)	Cy5 signal (MeDIP)
A_68_P23985677	chr5:111848812-111848856	NM_001081235:1649	Mn1	INSIDE	1.113	1.417	1412.83	2002.14	1.577	1030.36	1625.08
A_68_P23314731	chr4:132478927-132478977	NM_001163792:-486	Fam76a	PROMOTER	1.113	3.754	738.20	2771.49	4.180	495.74	2072.42
A_68_P23255466	chr4:120887199-120887243	ENSMUST00000056635:469		INSIDE	1.113	0.225	1964.08	441.90	0.250	1583.77	396.49
A_68_P28190094	chr11:121215805-121215849	NM_025793:-65	Wdr45l	PROMOTER	1.112	0.529	2387.55	1263.95	0.589	1905.59	1122.12
A_68_P27987615	chr11:86014727-86014771	NM_178309:-53	Brip1	PROMOTER	1.112	0.304	2087.80	633.72	0.337	1438.01	485.29
A_68_P24127563	chr5:138227997-138228041	NM_144913:-89	Mepce	DIVERGENT_PROMOTER	1.112	0.513	1274.82	653.97	0.571	1011.25	577.04
A_68_P23361671	chr4:140581070-140581114	NM_172122:28484	Crocc	INSIDE	1.112	2.326	1017.04	2366.01	2.586	825.62	2134.98
A_68_P32745115	chrX:147779348-147779392	NM_198105:605	Fam120c	INSIDE	1.111	0.458	1880.41	861.73	0.509	2660.68	1354.51
A_68_P27926999	chr11:75401904-75401948	NM_008850:317	Pitpna	INSIDE	1.111	0.430	4852.70	2087.36	0.478	3561.26	1702.53
A_68_P27842501	chr11:60166165-60166209	NM_001039092:221	Tom1l2	INSIDE	1.111	0.428	4446.54	1903.18	0.475	3126.60	1486.32
A_68_P26846117	chr9:114640435-114640479	NM_026036:136	Cmtm6	INSIDE	1.111	0.514	2635.07	1355.05	0.571	1984.40	1133.82
A_68_P23396577	chr4:148178997-148179041	NM_027195:518	Casz1	INSIDE	1.111	0.465	4247.04	1972.76	0.516	3146.90	1623.88
A_68_P23094809	chr4:88940440-88940484	NM_009877:61	Cdkn2a	INSIDE	1.111	0.536	1654.60	886.47	0.595	1305.81	777.02
A_68_P21486779	chr2:103868017-103868061	NM_020593:83	Fbxo3	INSIDE	1.111	0.266	3671.46	974.86	0.295	2944.25	868.35
A_68_P20612315	chr1:132774907-132774951	NM_026976:12575	Faim3	INSIDE	1.111	0.262	1735.46	453.86	0.291	1337.21	388.59
A_68_P32134212	chr19:44831155-44831199	NM_011037:-707	Pax2	PROMOTER	1.110	0.538	2253.99	1212.03	0.597	1559.84	930.80
A_68_P31302581	chr17:66121665-66121709	NM_133685:406	Rab31	INSIDE	1.110	0.441	2716.96	1198.64	0.490	1927.01	943.73
A_68_P30243544	chr15:57525714-57525758	NM_199449:-485	Zhx2	PROMOTER	1.110	3.503	1871.99	6556.72	3.887	1484.97	5771.70
A_68_P29493964	chr14:29331522-29331566	NM_009524:12886	Wnt5a	INSIDE	1.110	1.655	1351.37	2235.87	1.836	1124.74	2065.52
A_68_P28818475	chr13:9763163-9763207	NM_001199141:1376	Zmynd11	INSIDE	1.110	0.355	1397.82	495.98	0.394	1148.73	452.37
A_68_P27922752	chr11:74651542-74651586	NM_010813:7139	Mnt	INSIDE	1.110	2.458	517.44	1271.93	2.727	326.15	889.53
A_68_P27628922	chr11:19825101-19825145	NM_033523:678	Spred2	INSIDE	1.110	0.337	3659.67	1233.68	0.374	2597.89	972.12
A_68_P27209838	chr10:66558947-66558991	NM_001204915:768	Reep3	INSIDE	1.110	0.490	1209.72	593.06	0.544	978.98	532.75
A_68_P26023616	chr8:87604506-87604561	NM_026399:112	Wdr83	INSIDE	1.110	0.481	1228.12	590.99	0.534	955.47	510.52
A_68_P25910162	chr8:63966799-63966843	NM_145595:290	Cbr4	INSIDE	1.110	0.464	1393.00	646.55	0.515	1173.15	604.56
A_68_P25094160	chr7:52888072-52888116	NM_028544:5188	Rasip1	INSIDE	1.110	1.638	909.62	1489.61	1.817	777.10	1412.06
A_68_P24966279	chr7:11080522-11080566	NM_009577:186	Zik1	INSIDE	1.110	0.260	3250.83	843.76	0.288	2207.70	636.12
A_68_P24818548	chr6:125031803-125031847	NM_001163268:10530	Lpar5	INSIDE	1.110	1.581	1868.08	2953.70	1.755	1410.66	2476.21
A_68_P22829266	chr4:34011372-34011416	NM_007726:-212	Cnr1	PROMOTER	1.110	0.448	1868.44	836.94	0.497	1415.07	703.68
A_68_P32414052	chrX:57657724-57657768	NM_001001798:-590	Atp11c	PROMOTER	1.109	1.679	478.68	803.87	1.863	606.67	1130.01
A_68_P30961940	chr16:91269862-91269906	NM_016968:-129	Olig1	PROMOTER	1.109	0.328	1470.37	482.10	0.364	1255.49	456.52
A_68_P30358260	chr15:78004836-78004880	NM_025931:-320	Ift27	PROMOTER	1.109	0.239	2333.00	558.64	0.265	1880.15	499.08
A_68_P28175491	chr11:118907932-118907976	NM_013926:-5727	Cbx8	PROMOTER	1.109	1.841	630.97	1161.78	2.041	570.62	1164.86
A_68_P27563766	chr11:5420720-5420764	NM_013842:-227	Xbp1	PROMOTER	1.109	0.489	1366.29	667.68	0.542	1044.16	565.99
A_68_P27542229	chr10:128026612-128026657	NM_010153:-77	Erbb3	PROMOTER	1.109	1.750	898.36	1571.98	1.941	769.38	1493.27
A_68_P25604012	chr7:151164433-151164477			Unknown	1.109	0.224	2660.44	594.73	0.248	1919.36	475.66
A_68_P25578898	chr7:146768350-146768394	NR_027857:324	Nkx6-2	INSIDE	1.109	0.341	1595.34	543.90	0.378	1120.82	423.67
A_68_P25261264	chr7:86610001-86610045	NM_017462:1137	Polg	INSIDE	1.109	0.459	1216.41	558.65	0.509	990.24	504.17
A_68_P23591876	chr5:34278835-34278879	NM_011914:51	Whsc2	INSIDE	1.109	0.380	1360.82	516.89	0.421	1125.87	474.35
A_68_P23440547	chr4:155148630-155148674	NM_147776:18	Vwa1	INSIDE	1.109	0.293	2628.37	770.43	0.325	1934.37	628.77
A_68_P23361907	chr4:140616661-140616705	NM_001145958:-222	Crocc	PROMOTER	1.109	0.393	1826.04	717.62	0.436	1337.03	582.93
A_68_P21423681	chr2:91470003-91470047	NM_010168:6547	F2	INSIDE	1.109	1.707	539.04	920.10	1.894	502.20	950.98
A_68_P20441861	chr1:93241894-93241938	NM_001033292:23265	Espnl	INSIDE	1.109	1.498	810.16	1213.39	1.662	667.32	1108.80
A_68_P20349521	chr1:74647661-74647705	NM_021313:289	Rnf25	INSIDE	1.109	0.136	6113.89	829.41	0.150	4479.19	673.61
A_68_P30346951	chr15:76060156-76060200	NM_201385:-251	Plec	PROMOTER	1.108	0.336	1789.68	601.41	0.372	1485.92	553.33
A_68_P27979770	chr11:84632693-84632737	NM_145433:303	Mrm1	INSIDE	1.108	0.492	1343.97	660.63	0.545	973.51	530.14
A_68_P27937691	chr11:77275964-77276008	NM_139128:-1428	Coro6	PROMOTER	1.108	0.209	4451.93	928.40	0.231	2809.57	649.43
A_68_P26039231	chr8:90662304-90662348	NM_172757:543	Heatr3	INSIDE	1.108	1.757	1433.83	2519.86	1.947	1148.41	2235.62
A_68_P23192161	chr4:107356258-107356302	NM_019872:487	Dmrtb1	INSIDE	1.108	1.977	982.66	1942.46	2.190	808.33	1770.19
A_68_P29613385	chr14:55251451-55251495	NM_199470:8729	Cdh24	INSIDE	1.107	1.824	1694.53	3091.64	2.020	1282.18	2590.64
A_68_P28159196	chr11:116273617-116273661	NM_027258:708	Rnf157	INSIDE	1.107	0.376	1451.09	546.07	0.417	1043.15	434.53
A_68_P27041530	chr10:30522669-30522713	NM_172495:223	Ncoa7	INSIDE	1.107	0.433	1834.54	793.92	0.479	1452.38	695.51
A_68_P26537376	chr9:56482845-56482889	NM_181074:50194	Lingo1	INSIDE	1.107	0.368	2087.25	767.14	0.407	1601.65	651.59
A_68_P25026345	chr7:30066535-30066579	NM_001082548:440	Spint2	INSIDE	1.107	0.545	2082.96	1134.67	0.603	1619.17	976.05
A_68_P25005382	chr7:25316864-25316908	NM_153134:1220	Irgq	INSIDE	1.107	0.317	1627.83	516.23	0.351	1206.83	423.61
A_68_P24025453	chr5:118687359-118687403	NM_172998:7490	Rnft2	INSIDE	1.107	3.142	890.78	2798.54	3.477	680.09	2364.39
A_68_P22793479	chr4:26273870-26273915	NM_172865:-93	Manea	PROMOTER	1.107	0.180	2616.42	472.13	0.200	2024.39	404.28
A_68_P22393610	chr3:104667906-104667950	NM_153091:-70	St7l	DIVERGENT_PROMOTER	1.107	0.285	1712.93	489.02	0.316	1296.25	409.62
A_68_P21929617	chr3:7612894-7612938	NM_008371:511	Il7	INSIDE	1.107	0.401	1255.65	504.12	0.444	1025.88	455.89
A_68_P21490976	chr2:104656612-104656656	NM_001123327:219	Qser1	INSIDE	1.107	0.322	2158.06	695.21	0.357	1649.73	588.14
A_68_P29747539	chr14:79125062-79125106	NM_001081336:-188	Dgkh	PROMOTER	1.106	2.805	1179.16	3308.12	3.102	968.33	3003.91
A_68_P28575259	chr12:81440889-81440933	NM_177267:96678	Dcaf5	INSIDE	1.106	2.568	1857.66	4771.33	2.840	1549.29	4399.42
A_68_P28195051	chr12:3427564-3427608	NM_172421:703	Asxl2	INSIDE	1.106	0.243	1819.53	442.08	0.269	1364.88	366.70
A_68_P28187623	chr11:120794145-120794189	NM_027745:20	Ccdc57	INSIDE	1.106	0.543	1317.16	714.59	0.600	1044.35	626.89
A_68_P27996046	chr11:87578150-87578194	NM_172449:4130	Bzrap1	INSIDE	1.106	2.091	1103.79	2307.97	2.312	868.36	2007.97

ProbeName	Target position of probe on CpG island microarray	TargetID	GeneSymbol	CpG island Description	Ratio of relative methylation (TiO_2-NP/Vehicle)	Sham group			TiO_2-H group		
						Relative methylation (Cy5/Cy3)	Cy3 signal (Input)	Cy5 signal (MeDIP)	Relative methylation (Cy5/Cy3)	Cy3 signal (Input)	Cy5 signal (MeDIP)
A_68_P27351983	chr10:92624393-92624437	NR_035452:-1115	Mir1931	PROMOTER	1.106	0.541	1967.72	1064.91	0.599	1395.43	835.42
A_68_P25009650	chr7:26099741-26099785	NM_183311:8636	Tmem145	INSIDE	1.106	0.432	1584.23	684.18	0.478	1045.82	499.67
A_68_P24912679	chr6:143782088-143782132	NM_001113559:375968	Sox5	INSIDE	1.106	1.770	1524.08	2697.88	1.958	1199.21	2348.55
A_68_P20353687	chr1:75384995-75385039	NM_001085370:2832	Speg	INSIDE	1.106	0.398	1784.85	709.58	0.440	1483.72	652.17
A_68_P31054930	chr17:13201009-13201053	NM_013671:326	Sod2	INSIDE	1.105	1.392	1312.49	1826.65	1.538	976.97	1503.04
A_68_P30546424	chr16:11134701-11134745	NM_029582:-97	Txndc11	PROMOTER	1.105	0.506	2753.46	1392.09	0.559	1788.73	999.58
A_68_P30347742	chr15:76181910-76181954	NM_001164607:209	Mafl	INSIDE	1.105	0.389	1514.71	588.73	0.429	1170.54	502.54
A_68_P29334866	chr13:113253950-113253994	NM_010560:-305	Il6st	PROMOTER	1.105	1.808	1029.38	1861.12	1.999	806.91	1612.68
A_68_P28397455	chr12:45430084-45430128	NM_001146031:235	Nrcam	INSIDE	1.105	0.267	3998.96	1066.46	0.295	2784.03	820.51
A_68_P28078916	chr11:102181390-102181434	NM_011551:-1002	Ubtf	PROMOTER	1.105	0.314	3436.86	1077.63	0.346	2262.82	783.88
A_68_P28033280	chr11:94352619-94352663	NM_027984:8648	Epn3	INSIDE	1.105	13.067	1271.49	16614.50	14.435	1188.27	17152.23
A_68_P27562402	chr11:5161353-5161397	NM_032396:239	Kremen1	INSIDE	1.105	0.442	1343.69	593.69	0.488	1099.26	536.49
A_68_P27282339	chr10:79769158-79769202	NM_011789:4616	Apc2	INSIDE	1.105	2.014	808.64	1628.97	2.227	615.96	1371.71
A_68_P24928970	chr6:146451188-146451232	NM_010586:-776	Itpr2	PROMOTER	1.105	0.299	3872.86	1156.64	0.330	2939.48	969.67
A_68_P24295434	chr6:23203943-23203987	NM_028462:-5700	Fezf1	PROMOTER	1.105	0.157	3078.47	484.21	0.174	2409.94	418.92
A_68_P24114330	chr5:135499738-135499782	NM_016801:319	Stx1a	INSIDE	1.105	0.524	3483.36	1824.90	0.579	2358.36	1364.70
A_68_P23467993	chr5:8422278-8422322	NM_001190717:416	Dbf4	INSIDE	1.105	0.592	1801.82	1066.38	0.654	1517.02	992.48
A_68_P23245424	chr4:119094947-119094991	NM_026494:57	Ppcs	INSIDE	1.105	0.501	1317.62	660.52	0.554	1063.73	589.39
A_68_P22220270	chr3:69121308-69121352	NM_178726:491	Ppm1l	INSIDE	1.105	0.322	1520.90	489.59	0.356	1169.69	415.95
A_68_P21419567	chr2:90745097-90745145	NM_025991:178	Kbtbd4	INSIDE	1.105	0.561	1506.02	845.17	0.620	1223.04	758.71
A_68_P32668461	chrX:130222350-130222394	NM_001105245:1160	Pcdh19	INSIDE	1.104	2.748	228.56	628.15	3.034	249.23	756.11
A_68_P32340726	chrX:39421609-39421653	NM_009688:618	Xiap	INSIDE	1.104	1.882	439.85	828.00	2.078	588.47	1222.72
A_68_P31097896	chr17:24664666-24664710	NM_001172113:195	Traf7	INSIDE	1.104	0.177	5646.95	1000.47	0.196	4132.17	808.29
A_68_P30479790	chr15:99200609-99200653	NM_011711:267	Fmnl3	INSIDE	1.104	0.227	4671.40	1062.34	0.251	3321.16	834.15
A_68_P28534709	chr12:74046481-74046525	NM_011384:5571	Six6	DOWNSTREAM	1.104	0.579	1999.71	1158.77	0.640	1504.79	962.91
A_68_P28222997	chr12:8506759-8506803	NM_007483:11	Rhob	INSIDE	1.104	1.604	948.80	1522.29	1.771	725.07	1284.13
A_68_P25025266	chr7:29871013-29871057	NR_035489:-2321	Mir1963	PROMOTER	1.104	2.419	877.75	2123.31	2.671	742.66	1983.82
A_68_P22888515	chr4:46402231-46402275	NM_029086:43	5830415F09Rik	INSIDE	1.104	0.498	4041.86	2013.06	0.550	2916.30	1603.69
A_68_P21938382	chr3:9608958-9609002	NM_133218:1105	Zfp704	INSIDE	1.104	0.108	6032.07	650.45	0.119	4160.01	495.35
A_68_P20430407	chr1:91477732-91477776	NM_001037136:126369	Agap1	INSIDE	1.104	2.375	260.01	617.47	2.621	250.82	657.38
A_68_P29697251	chr14:70205056-70205100	NM_153514:274	Rhobtb2	INSIDE	1.103	0.601	2671.90	1605.59	0.663	2013.38	1334.39
A_68_P29654694	chr14:62911407-62911451	NM_173419:388	Dleu7	INSIDE	1.103	0.407	1299.76	529.01	0.449	1072.60	481.40
A_68_P29613381	chr14:55251101-55251145	NM_199470:9079	Cdh24	INSIDE	1.103	2.210	1356.14	2997.33	2.437	961.56	2343.74
A_68_P29062972	chr13:56804405-56804449	NM_001164042:-602	Smad5	PROMOTER	1.103	1.884	494.88	932.55	2.079	482.09	1002.08
A_68_P27897675	chr11:69694588-69694632	NM_153143:-123	Kctd11	PROMOTER	1.103	1.507	1666.35	2510.99	1.662	1350.69	2245.13
A_68_P26544128	chr9:57684324-57684368	NM_019689:-2305	Arid3b	PROMOTER	1.103	0.362	1933.11	699.06	0.399	1545.87	616.69
A_68_P26495378	chr9:48863573-48863617	NM_012039:-91	Zw10	PROMOTER	1.103	0.516	1384.30	714.11	0.569	1086.86	618.44
A_68_P26137946	chr8:108660718-108660762	NM_176838:134	Esrp2	INSIDE	1.103	0.314	6601.99	2074.40	0.347	4047.10	1402.69
A_68_P25357077	chr7:105804935-105804979	NM_172280:123	2210018M11Rik	INSIDE	1.103	0.588	1736.48	1021.56	0.649	1484.11	962.66
A_68_P25353896	chr7:105260083-105260127	NM_008663:7899	Myo7a	INSIDE	1.103	0.424	1460.71	619.62	0.468	1190.08	556.76
A_68_P25031178	chr7:31141047-31141091	NM_175478:6723	Lrfn3	INSIDE	1.103	1.967	688.55	1354.37	2.169	539.87	1170.82
A_68_P24427164	chr6:48388295-48388339	NM_001085415:6773	Zfp467	INSIDE	1.103	1.698	1366.27	2319.91	1.873	1096.40	2054.06
A_68_P23590585	chr5:34037821-34037865	AK172441:87544		INSIDE	1.103	0.354	1604.79	568.88	0.391	1242.92	486.01
A_68_P23310049	chr4:131630823-131630867	NM_183428:-2832	Epb4.1	PROMOTER	1.103	1.510	1013.40	1530.50	1.666	850.15	1416.44
A_68_P21418960	chr2:90623108-90623152	NM_178755:230	Agbl2	INSIDE	1.103	0.403	1605.48	646.67	0.444	1331.61	591.35
A_68_P21144178	chr2:37648888-37648932	NM_001163566:17142	Crb2	INSIDE	1.103	2.564	1413.81	3624.76	2.829	1122.49	3175.43
A_68_P20735842	chr1:156946788-156946833	NM_010500:-44	Ier5	PROMOTER	1.103	0.268	3672.38	982.74	0.295	2235.82	659.87
A_68_P20038597	chr1:12982588-12982632	NM_172841:-1394	Slco5a1	PROMOTER	1.103	0.356	1221.05	434.46	0.392	977.42	383.61
A_68_P28577654	chr12:81892119-81892163	NM_178682:595	4933426M11Rik	INSIDE	1.102	2.224	522.90	1162.88	2.450	478.41	1171.96
A_68_P28469868	chr12:60114568-60114612	NM_025656:211	Sip1	INSIDE	1.102	3.509	621.94	2182.17	3.865	526.32	2034.40
A_68_P28445676	chr12:55169720-55169764	NM_013780:820079	Npas3	INSIDE	1.102	1.909	2087.72	3985.25	2.103	1694.89	3564.69
A_68_P28314292	chr12:29334596-29334640	NM_011275:152	Rnaseh1	INSIDE	1.102	0.457	1415.33	647.21	0.504	1185.96	597.72
A_68_P28054685	chr11:98010627-98010671	NM_028149:282	Fbxl20	INSIDE	1.102	0.294	2575.38	757.13	0.324	1901.39	615.97
A_68_P26128335	chr8:106919047-106919091	NM_153582:528	Cmtm4	INSIDE	1.102	2.157	1074.39	2317.48	2.378	853.77	2029.99
A_68_P25447478	chr7:123451427-123451471	ENSMUST00000106605:442		INSIDE	1.102	0.416	1531.59	637.06	0.459	1171.05	536.96
A_68_P24982503	chr7:17200478-17200522	NM_172739:-158	Grlf1	PROMOTER	1.102	0.482	1290.16	621.23	0.531	1018.58	540.68
A_68_P24537469	chr6:71583121-71583165	NM_001038695:-243	Kdm3a	PROMOTER	1.102	0.141	4508.21	634.16	0.155	2782.05	431.17
A_68_P24152140	chr5:143005995-143006039	NM_019943:14	Papolb	INSIDE	1.102	2.032	1224.47	2487.96	2.238	1036.92	2321.01
A_68_P23442480	chr4:155483981-155484025	NM_145557:-104	9430015G10Rik	PROMOTER	1.102	1.723	648.95	1118.35	1.900	535.02	1016.33
A_68_P23416137	chr4:151406428-151406472	NM_031867:6149	Tas1r1	INSIDE	1.102	2.461	740.95	1823.80	2.713	586.67	1591.56
A_68_P20900753	chr1:187187399-187187443	NM_029735:447	Eprs	INSIDE	1.102	0.351	2061.23	723.82	0.387	1490.09	576.67
A_68_P30486145	chr15:100325764-100325808	NM_153407:-116	Csrnp2	PROMOTER	1.101	0.564	1583.58	892.87	0.621	1207.26	749.18
A_68_P29335933	chr13:113442449-113442493	NM_001145885:48	Ddx4	INSIDE	1.101	4.721	1008.49	4761.07	5.197	889.08	4620.32
A_68_P27285629	chr10:80269928-80269972	NM_007445:1958	Amh	INSIDE	1.101	2.500	835.86	2089.26	2.752	697.80	1920.65
A_68_P25392272	chr7:112574662-112574706	NM_007627:351	Cckbr	INSIDE	1.101	0.469	2069.32	971.21	0.517	1792.00	925.91

ProbeName	Target position of probe on CpG island microarray	TargetID	GeneSymbol	CpG island Description	Ratio of relative methylation (TiO_2-NP/Vehicle)	Sham group			TiO_2-H group		
						Relative methylation (Cy5/Cy3)	Cy3 signal (Input)	Cy5 signal (MeDIP)	Relative methylation (Cy5/Cy3)	Cy3 signal (Input)	Cy5 signal (MeDIP)
A_68_P24125354	chr5:137799067-137799111	NM_001159571:7752	Ephb4	INSIDE	1.101	2.129	981.13	2089.03	2.343	749.86	1757.13
A_68_P22820141	chr4:32326208-32326252	ENSMUST00000165661:-4477		PROMOTER	1.101	1.677	1093.54	1833.77	1.846	883.86	1631.51
A_68_P22393113	chr3:104584603-104584647	NM_027982:651	Ppm1j	INSIDE	1.101	0.467	1567.49	732.67	0.515	1209.44	622.54
A_68_P20877310	chr1:182626954-182626998	NM_013729:189	Mixl1	INSIDE	1.101	0.225	2590.98	583.86	0.248	2035.96	505.24
A_68_P31934468	chr19:6399999-6400043	NM_011242:-562	Rasgrp2	PROMOTER	1.100	0.164	4787.65	784.94	0.180	3568.60	643.30
A_68_P30151559	chr15:38838244-38838288	NM_008056:388	Fzd6	INSIDE	1.100	0.348	1344.80	468.56	0.383	1102.33	422.36
A_68_P27954544	chr11:80291421-80291465	NM_009871:895	Cdk5r1	INSIDE	1.100	0.390	1737.44	678.43	0.430	1254.26	538.75
A_68_P27163120	chr10:57786106-57786150	NM_026148:-85	Lims1	PROMOTER	1.100	0.509	2853.44	1452.18	0.560	2125.27	1189.57
A_68_P24770497	chr6:115311740-115311784	NM_001127330:524	Pparg	INSIDE	1.100	0.352	2522.93	888.69	0.388	1978.34	766.69
A_68_P24622499	chr6:88450317-88450361	NM_019685:34922	Ruvbl1	DOWNSTREAM	1.100	0.567	4432.87	2514.48	0.624	3391.75	2115.67
A_68_P20409047	chr1:87791215-87791259	NM_022417:152	Itm2c	INSIDE	1.100	0.501	2446.38	1226.62	0.552	1688.92	931.53
A_68_P30490428	chr15:101054889-101054933	NM_019518:273	Grasp	INSIDE	1.099	0.507	1684.39	853.27	0.557	1185.96	660.50
A_68_P29224480	chr13:93301031-93301075	NM_173392:-287	Zfyve16	PROMOTER	1.099	0.400	1600.73	639.95	0.439	1199.35	527.05
A_68_P28451629	chr12:56499790-56499834	NM_011968:1	Psma6	INSIDE	1.099	0.471	1254.07	591.17	0.518	872.84	452.33
A_68_P27973305	chr11:83567284-83567328	NM_145432:168	Heatr6	INSIDE	1.099	0.543	1583.02	859.36	0.597	1275.65	761.02
A_68_P27844241	chr11:60513360-60513404	NM_001159404:191	Llgl1	INSIDE	1.099	1.629	1028.71	1676.13	1.791	818.90	1466.73
A_68_P25711726	chr8:23704183-23704227	NM_001198998:81	Vdac3	INSIDE	1.099	0.372	1530.42	568.79	0.409	1089.06	444.92
A_68_P24008812	chr5:115729714-115729758	NM_026504:26	Coq5	INSIDE	1.099	1.621	777.59	1260.46	1.782	760.79	1355.89
A_68_P23894643	chr5:92934119-92934163	NM_007644:494	Scarb2	INSIDE	1.099	0.494	2167.01	1070.99	0.543	1548.96	841.48
A_68_P23091884	chr4:88368371-88368415	NM_172871:20	Klhl9	INSIDE	1.099	0.597	2079.37	1240.83	0.656	1622.21	1063.72
A_68_P23080771	chr4:86257888-86257932	NM_173400:16	Haus6	INSIDE	1.099	0.385	2681.51	1031.22	0.423	2114.50	893.52
A_68_P21875631	chr2:174298018-174298062	NM_025531:402	Slmo2	INSIDE	1.099	0.338	1508.72	509.79	0.371	1013.00	376.02
A_68_P30630410	chr16:28445868-28445912	NM_183064:-577	Fgf12	PROMOTER	1.098	0.415	2690.57	1117.09	0.456	1866.18	850.76
A_68_P29625865	chr14:57676961-57677005	NM_016975:-200	Gja3	PROMOTER	1.098	0.314	1694.20	532.14	0.345	1243.96	429.18
A_68_P27473558	chr10:115550897-115550941	NM_001161855:55	4933416C03Rik	INSIDE	1.098	1.773	1294.54	2295.81	1.946	1194.81	2325.58
A_68_P26239285	chr8:125856857-125856901	NM_172287:266	Spire2	INSIDE	1.098	0.297	2313.91	686.98	0.326	1636.59	533.63
A_68_P24129312	chr5:138635953-138635997	NM_177878:433	Mblac1	INSIDE	1.098	0.359	1533.42	550.79	0.395	1226.96	484.10
A_68_P23547919	chr5:25035293-25035337	NR_027388:-521	1700096K18Rik	PROMOTER	1.098	0.235	4048.40	952.43	0.258	2923.60	755.33
A_68_P23356008	chr4:139665546-139665590	NM_198610:137158	Igsf21	INSIDE	1.098	0.485	1011.63	490.22	0.532	876.55	466.55
A_68_P23221710	chr4:114494540-114494590	NR_033617:240	Gm12830	INSIDE	1.098	0.286	2037.43	582.42	0.314	1457.85	457.72
A_68_P31930919	chr19:5803763-5803807	NR_002847:-1113	Malat1	PROMOTER	1.097	3.896	3965.50	15448.17	4.273	3016.15	12888.07
A_68_P31836528	chr18:75526822-75526866	NM_001042660:-174	Smad7	PROMOTER	1.097	2.762	659.77	1822.55	3.031	556.81	1687.88
A_68_P28055831	chr11:98209650-98209695	NM_144828:-379	Ppp1r1b	PROMOTER	1.097	0.209	4699.15	984.09	0.230	2974.88	683.55
A_68_P27926228	chr11:75275176-75275220	NR_029739:-2019	Mir22	PROMOTER	1.097	0.568	1983.62	1126.03	0.622	1511.41	940.82
A_68_P27154487	chr10:55835680-55835724	NM_001163833:8980	Msl3l2	INSIDE	1.097	0.414	1125.30	465.78	0.454	966.03	438.60
A_68_P22970878	chr4:63156551-63156595	NM_001008791:413	Whrn	INSIDE	1.097	0.320	4175.17	1334.00	0.350	2978.94	1043.96
A_68_P22948240	chr4:57968866-57968910	NM_011660:395	Txn1	INSIDE	1.097	0.481	1318.20	634.60	0.528	1162.25	613.90
A_68_P20969135	chr2:4525120-4525164	NM_001177844:44343	Frmd4a	INSIDE	1.097	1.850	1330.09	2460.61	2.029	1111.00	2254.39
A_68_P32786684	chrX:158346258-158346302	NM_001081052:251442	Nhs	INSIDE	1.096	1.731	472.18	817.43	1.898	544.40	1033.34
A_68_P32234206	chrX:5977596-5977640	NM_001040459:356	Shroom4	INSIDE	1.096	1.700	380.75	647.37	1.864	529.35	986.89
A_68_P29644190	chr14:60997369-60997413	NM_001164705:268	Fam123a	INSIDE	1.096	0.275	2152.09	590.82	0.301	1589.37	478.14
A_68_P28055691	chr11:98188926-98188970	NM_010895:2011	Neurod2	INSIDE	1.096	1.674	850.01	1423.01	1.835	728.37	1336.48
A_68_P26445382	chr9:40609227-40609271	NM_031165:-107	Hspa8	PROMOTER	1.096	0.251	2990.86	750.10	0.275	2044.34	561.81
A_68_P25957632	chr8:74108446-74108490	NM_011977:15643	Slc27a1	INSIDE	1.096	1.429	1251.87	1789.33	1.567	1039.97	1629.36
A_68_P25728676	chr8:26830919-26830963	NM_028000:421	Ppapdc1b	INSIDE	1.096	0.284	2361.12	671.33	0.312	1706.32	531.75
A_68_P25661487	chr8:12430676-12430720	NR_027975:44928	Gm5607	INSIDE	1.096	0.306	1857.02	567.49	0.335	1416.91	474.43
A_68_P25505621	chr7:134587797-134587841	NM_175163:4854	Zfp689	INSIDE	1.096	1.910	1537.70	2936.88	2.093	1110.02	2322.87
A_68_P25471457	chr7:128125949-128125995	NM_028955:-50	4933427G17Rik	DIVERGENT_PROMOTER	1.096	3.883	213.15	827.69	4.255	189.86	807.93
A_68_P23571063	chr5:30481709-30481753	NM_145558:-131	Hadhb	DIVERGENT_PROMOTER	1.096	0.365	1495.34	545.52	0.400	1258.72	503.24
A_68_P23245373	chr4:119087968-119088012	NM_029286:136	Ccdc30	INSIDE	1.096	0.200	2468.94	494.39	0.219	1750.79	384.08
A_68_P23005030	chr4:69910440-69910484	NM_145990:160939	Cdk5rap2	INSIDE	1.096	2.849	733.87	2090.82	3.122	637.01	1988.64
A_68_P22363132	chr3:99058041-99058085	NM_009323:380	Tbx15	INSIDE	1.096	0.357	2834.62	1011.03	0.391	2038.96	797.38
A_68_P20530739	chr1:114313105-114313149			Unknown	1.096	0.520	3001.77	1560.28	0.570	2352.05	1340.04
A_68_P20186988	chr1:43155340-43155384	NM_001013025:105	Tgfbrap1	INSIDE	1.096	0.458	1741.02	796.82	0.502	1475.88	740.35
A_68_P29961930	chr14:122873605-122873656	NM_009574:-975	Zic2	DIVERGENT_PROMOTER	1.095	3.226	476.11	1535.78	3.533	444.03	1568.84
A_68_P29258645	chr13:99383273-99383317	AK135687:193159		INSIDE	1.095	2.316	432.27	1001.15	2.536	396.26	1005.03
A_68_P28185532	chr11:120459254-120459298	NM_011568:403	Thoc4	INSIDE	1.095	1.582	890.37	1408.65	1.733	742.27	1286.33
A_68_P27282702	chr10:79819562-79819606	NM_183152:381	Plk5	INSIDE	1.095	3.493	1901.32	6641.44	3.825	1566.62	5992.95
A_68_P25947576	chr8:72149308-72149352	NM_001045553:-256	Zfp868	PROMOTER	1.095	0.461	4550.59	2099.27	0.505	3622.75	1829.22
A_68_P25581338	chr7:147130178-147130222	NM_009482:446	Utf1	INSIDE	1.095	0.426	1325.00	565.02	0.467	1007.99	470.81
A_68_P24451818	chr6:52590402-52590446	NM_145567:-130	Hibadh	PROMOTER	1.095	2.511	934.11	2345.81	2.749	766.92	2108.09
A_68_P24054545	chr5:123678394-123678438	NM_026000:218	Psmd9	INSIDE	1.095	0.291	2198.69	639.59	0.318	1590.21	506.38
A_68_P23820887	chr5:77738314-77738358	NM_019836:775	2610024G14Rik	INSIDE	1.095	2.815	855.65	2408.87	3.084	662.39	2042.67
A_68_P23351224	chr4:138902115-138902159	NM_023536:6077	Mrto4	DOWNSTREAM	1.095	1.502	1678.30	2520.00	1.644	1346.70	2214.30
A_68_P20633261	chr1:136311870-136311914	NM_028320:-151	Adipor1	PROMOTER	1.095	0.354	1479.06	523.61	0.388	1187.97	460.35

ProbeName	Target position of probe on CpG island microarray	TargetID	GeneSymbol	CpG island Description	Ratio of relative methylation (TiO_2-NP/Vehicle)	Sham group			TiO_2-H group		
						Relative methylation (Cy5/Cy3)	Cy3 signal (Input)	Cy5 signal (MeDIP)	Relative methylation (Cy5/Cy3)	Cy3 signal (Input)	Cy5 signal (MeDIP)
A_68_P20165368	chr1:39425278-39425322	NM_053257:605	Rpl31	INSIDE	1.095	0.178	5773.97	1028.11	0.195	3996.55	779.14
A_68_P31891632	chr18:85121550-85121594	NM_025969:-656	1700034H14Rik	PROMOTER	1.094	0.238	3185.89	758.94	0.261	2489.95	649.08
A_68_P27535082	chr10:126727745-126727789	NM_007837:-82	Ddit3	DIVERGENT_PROMOTER	1.094	0.353	2060.73	728.15	0.387	1694.25	654.91
A_68_P27449324	chr10:110943649-110943693	NM_009344:329	Phlda1	INSIDE	1.094	0.387	1331.75	514.89	0.423	1046.44	442.72
A_68_P27282650	chr10:79811706-79811750	NM_001113548:-537	Adamtsl5	DIVERGENT_PROMOTER	1.094	0.462	2525.29	1166.90	0.505	1777.01	898.07
A_68_P24385308	chr6:39822333-39822377			Unknown	1.094	0.346	1362.29	471.56	0.379	1095.44	414.66
A_68_P24138652	chr5:140448047-140448091	NM_175522:64172	Elfn1	INSIDE	1.094	1.510	1810.71	2733.60	1.652	1293.19	2135.95
A_68_P22737305	chr4:12834278-12834322	NM_001171801:317	Gm11818	INSIDE	1.094	2.105	344.03	724.34	2.304	335.02	771.93
A_68_P21492620	chr2:104966668-104966712	NM_144783:5	Wt1	INSIDE	1.094	0.465	1475.58	685.65	0.509	1220.95	620.90
A_68_P31412124	chr17:86088425-86088469	NM_011380:-852	Six2	PROMOTER	1.093	0.331	4944.01	1638.69	0.362	3497.57	1266.57
A_68_P31256044	chr17:56567422-56567466	NM_011218:48459	Ptprs	INSIDE	1.093	2.444	907.26	2216.99	2.670	695.18	1856.10
A_68_P30424871	chr15:89208941-89208985	NM_001013022:723	Odf3b	INSIDE	1.093	0.412	1848.79	762.19	0.451	1341.74	604.83
A_68_P30370942	chr15:80064643-80064687	NM_178719:155	Smcr7l	INSIDE	1.093	0.324	1843.06	596.98	0.354	1430.71	506.28
A_68_P28747156	chr12:113205010-113205054	NM_027360:156	2010107E04Rik	INSIDE	1.093	0.437	1559.85	681.69	0.478	1307.09	624.62
A_68_P28206058	chr12:5380617-5380662	NM_001164493:1849	Klhl29	INSIDE	1.093	0.344	1663.16	571.61	0.376	1272.16	477.90
A_68_P27776268	chr11:47802068-47802112	ENSMUST00000128558:381		INSIDE	1.093	0.392	1908.83	748.49	0.429	1436.68	615.73
A_68_P25102011	chr7:54175094-54175138	NM_021884:184	Tsg101	INSIDE	1.093	3.292	1048.97	3453.48	3.599	1018.88	3666.50
A_68_P23536407	chr5:22939195-22939239	NM_026984:-1030	Mll5	PROMOTER	1.093	0.377	5695.43	2146.94	0.412	3839.16	1582.14
A_68_P30348994	chr15:76368510-76368555	NM_013909:644	Fbxl6	INSIDE	1.092	0.251	1851.36	464.93	0.274	1420.16	389.37
A_68_P29050104	chr13:54666624-54666668	NM_133797:97	4833439L19Rik	INSIDE	1.092	0.414	1788.38	739.50	0.451	1295.42	584.85
A_68_P28158411	chr11:116135230-116135274	NM_146032:279	Srp68	INSIDE	1.092	0.362	1277.88	462.62	0.395	969.36	383.06
A_68_P26119099	chr8:105308249-105308294	NM_009866:740	Cdh11	INSIDE	1.092	0.379	3787.05	1437.03	0.414	2835.43	1174.48
A_68_P26065140	chr8:94882146-94882190	NM_018826:474	Irx5	INSIDE	1.092	0.424	1721.65	730.27	0.463	1483.26	687.11
A_68_P25393298	chr7:112766252-112766301	NM_018880:15737	Trim3	INSIDE	1.092	1.709	740.47	1265.11	1.866	509.53	950.97
A_68_P25005239	chr7:25292778-25292822	NM_146183:695	Zfp428	INSIDE	1.092	0.232	2253.62	523.27	0.253	1783.03	451.99
A_68_P23424872	chr4:152806992-152807036	NM_001099299:49925	Ajap1	INSIDE	1.092	1.746	1941.96	3389.70	1.906	1476.20	2813.27
A_68_P21622284	chr2:128792983-128793027	NM_178404:-133	Zc3h6	PROMOTER	1.092	0.642	2733.46	1755.20	0.701	2179.40	1527.52
A_68_P21579147	chr2:120966036-120966080	NM_177846:376	Lcmt2	INSIDE	1.092	2.586	299.52	774.64	2.825	265.53	750.00
A_68_P21084949	chr2:27330700-27330744	NM_023336:196	Brd3	INSIDE	1.092	36.589	294.55	10777.04	39.942	329.34	13154.64
A_68_P20937886	chr1:193221052-193221096	NM_144880:-154	Ppp2r5a	PROMOTER	1.092	0.351	1501.90	526.59	0.383	1210.98	463.83
A_68_P20852906	chr1:178205311-178205355	NM_176916:111	Pld5	INSIDE	1.092	0.187	3626.98	678.22	0.204	2888.63	590.07
A_68_P31827988	chr18:73975355-73975399	NM_145494:-330	Me2	PROMOTER	1.091	0.168	3065.20	514.98	0.183	2241.30	410.86
A_68_P28751166	chr12:113838256-113838300	NM_198411:11284	Inf2	INSIDE	1.091	1.478	1973.43	2915.94	1.612	1551.98	2501.84
A_68_P28678694	chr12:101122653-101122697	NM_028354:-50	Tdp1	DIVERGENT_PROMOTER	1.091	3.497	583.81	2041.65	3.814	487.52	1859.60
A_68_P27279732	chr10:79383697-79383741	NM_053244:4003	Kiss1r	INSIDE	1.091	0.382	2203.02	841.97	0.417	1766.69	736.92
A_68_P26467271	chr9:44187775-44187819	NM_021395:224	Hyou1	INSIDE	1.091	0.320	1999.40	640.58	0.350	1429.29	499.62
A_68_P26055739	chr8:93432325-93432369	NM_177224:79613	Chd9	INSIDE	1.091	0.398	1367.22	543.56	0.434	874.24	379.17
A_68_P24620370	chr6:88149892-88149936	NM_008090:1257	Gata2	INSIDE	1.091	0.484	1163.32	562.49	0.528	980.07	517.12
A_68_P24428218	chr6:48547068-48547112	NM_001079901:3209	Repin1	INSIDE	1.091	1.977	1732.24	3424.73	2.157	1357.20	2927.85
A_68_P24084069	chr5:129106010-129106054	NR_015517:530	5930412G12Rik	INSIDE	1.091	0.392	1563.52	612.64	0.427	1243.90	531.71
A_68_P23578164	chr5:31754796-31754840	NM_172993:10	Zfp512	INSIDE	1.091	0.522	1657.99	865.78	0.569	1125.51	640.93
A_68_P31442550	chr17:91488113-91488157	NM_020252:4008	Nrxn1	INSIDE	1.090	2.813	1523.85	4285.90	3.066	1204.75	3694.12
A_68_P30345226	chr15:75805450-75805494	NM_001081065:5858	Zfp707	INSIDE	1.090	1.765	2568.19	4533.19	1.924	1813.40	3488.97
A_68_P29699005	chr14:70475525-70475569	NM_018781:-1705	Egr3	PROMOTER	1.090	1.968	1533.01	3016.89	2.144	1271.93	2727.20
A_68_P28033655	chr11:94410319-94410363	NM_001013381:181	Rsad1	INSIDE	1.090	0.310	1593.23	494.57	0.338	1282.65	434.02
A_68_P27896513	chr11:69495025-69495071	NM_030702:424	Senp3	INSIDE	1.090	0.489	1219.10	596.18	0.533	939.96	501.23
A_68_P26429009	chr9:36953011-36953055	NM_001029838:1819	Pknox2	INSIDE	1.090	0.532	1721.00	915.53	0.580	1359.86	788.39
A_68_P24537461	chr6:71582317-71582361	NM_173001:342	Kdm3a	INSIDE	1.090	0.502	2426.82	1218.93	0.547	1984.96	1086.46
A_68_P23873438	chr5:89104430-89104474	NM_001098476:153	Grsf1	INSIDE	1.090	0.464	1243.29	576.29	0.505	1107.67	559.57
A_68_P23118613	chr4:94281110-94281154	NM_026319:-78	Ift74	PROMOTER	1.090	0.346	2392.75	827.68	0.377	1931.12	728.34
A_68_P21752520	chr2:152857602-152857646	NM_001011732:37	Xkr7	INSIDE	1.090	10.939	1230.96	13465.12	11.922	860.75	10262.16
A_68_P32364026	chrX:45694076-45694120	NM_178782:-436	Bcorl1	PROMOTER	1.089	1.526	638.22	974.10	1.662	949.04	1577.70
A_68_P32321656	chrX:34625307-34625351	NM_026055:-149	Rpl39	PROMOTER	1.089	1.538	480.75	739.47	1.676	693.25	1161.72
A_68_P31793419	chr18:67883751-67883795	NM_001127177:503	Ptpn2	INSIDE	1.089	0.532	1547.49	824.02	0.580	1223.40	709.64
A_68_P31746197	chr18:59334824-59334868	NM_001081328:-147	Chsy3	PROMOTER	1.089	0.383	2714.26	1039.18	0.417	2024.90	843.87
A_68_P31422206	chr17:87681968-87682012	NR_015506:164	4833418N02Rik	INSIDE	1.089	2.145	1228.54	2635.08	2.337	817.90	1911.05
A_68_P31075038	chr17:17964345-17964389	NR_029536:-2785	Mir99b	PROMOTER	1.089	0.554	3197.27	1771.20	0.603	2365.68	1427.49
A_68_P28577466	chr12:81858386-81858430	NM_001008423:3294	Gm1568	INSIDE	1.089	1.743	1217.63	2122.79	1.898	891.81	1692.37
A_68_P27892737	chr11:68859470-68859514	NM_011496:348	Aurkb	INSIDE	1.089	0.492	1317.45	648.28	0.536	984.71	527.69
A_68_P23931499	chr5:101227260-101227304	NM_001081107:337	Helq	INSIDE	1.089	3.062	2336.96	7155.12	3.335	1882.15	6277.21
A_68_P23349527	chr4:138630455-138630499	NM_021358:228	Htr6	INSIDE	1.089	0.450	1119.28	503.90	0.490	748.15	366.95
A_68_P23189069	chr4:106851457-106851501	NM_029565:244	Tmem59	INSIDE	1.089	0.427	2407.53	1027.63	0.465	1812.78	842.81
A_68_P22522615	chr3:129581952-129581996	NM_183423:-2127	Pla2g12a	PROMOTER	1.089	4.116	5189.68	21360.57	4.484	3186.84	14288.86
A_68_P21019039	chr2:13976814-13976858	NM_001012396:826	Ptpla	INSIDE	1.089	0.318	2558.54	813.58	0.346	2048.90	709.48
A_68_P32744218	chrX:147530920-147530964	NM_001164578:144	Tsr2	INSIDE	1.088	2.843	1160.26	3299.17	3.094	1368.05	4233.34

ProbeName	Target position of probe on CpG island microarray	TargetID	GeneSymbol	CpG island Description	Ratio of relative methylation (TiO_2-NP/Vehicle)	Sham group			TiO_2-H group		
						Relative methylation (Cy5/Cy3)	Cy3 signal (Input)	Cy5 signal (MeDIP)	Relative methylation (Cy5/Cy3)	Cy3 signal (Input)	Cy5 signal (MeDIP)
A_68_P31626662	chr18:37166261-37166305	NM_009960:-4229	Pcdha11	PROMOTER	1.088	2.151	647.88	1393.90	2.340	514.41	1203.88
A_68_P31381750	chr17:80606382-80606426	NM_001195485:241	Srsf7	INSIDE	1.088	0.455	1484.67	676.00	0.495	1155.84	572.55
A_68_P27594243	chr11:11925830-11925874	NM_001177629:2122	Grb10	INSIDE	1.088	1.361	1513.04	2058.64	1.480	1249.37	1848.81
A_68_P26801799	chr9:106101397-106101441	NM_198931:-148	Ppm1m	PROMOTER	1.088	2.407	514.15	1237.39	2.619	437.08	1144.85
A_68_P26343621	chr9:20531598-20531642	NM_173777:1038	Olfm2	INSIDE	1.088	0.493	2880.07	1418.64	0.536	2217.13	1188.74
A_68_P26021881	chr8:87308180-87308224	NM_001081981:-9934	Nfix	PROMOTER	1.088	0.294	1842.88	542.36	0.320	1454.01	465.63
A_68_P23451693	chr5:4758175-4758219	NM_021457:20	Fzd1	INSIDE	1.088	1.737	1410.81	2450.48	1.889	1144.28	2161.74
A_68_P23254629	chr4:120712162-120712206	NM_007741:14	Col9a2	INSIDE	1.088	0.320	1921.30	614.53	0.348	1574.98	547.99
A_68_P23239263	chr4:117933446-117933490	NM_011213:30534	Ptprf	INSIDE	1.088	5.274	3731.73	19679.44	5.738	2788.33	15999.25
A_68_P21908931	chr2:180891580-180891624	NM_007906:118	Eef1a2	INSIDE	1.088	1.883	1528.84	2879.09	2.049	1212.50	2484.95
A_68_P20350605	chr1:74847285-74847329	NM_009518:8714	Wnt10a	INSIDE	1.088	1.751	793.91	1389.91	1.905	646.81	1231.94
A_68_P32569614	chrX:99839705-99839761	NM_008832:-147	Phka1	PROMOTER	1.087	1.882	395.28	744.02	2.046	439.81	899.70
A_68_P31746196	chr18:59334709-59334753	NM_001081328:-263	Chsy3	PROMOTER	1.087	0.366	2425.46	887.87	0.398	1980.93	788.53
A_68_P31243777	chr17:53618701-53618745	NM_025887:164	Rab5a	INSIDE	1.087	0.484	2132.66	1032.75	0.527	1716.05	903.53
A_68_P31099849	chr17:24986693-24986737	NM_023480:533	Fahd1	INSIDE	1.087	1.560	727.95	1135.31	1.696	869.60	1474.55
A_68_P30963194	chr16:91485638-91485682	NM_010508:201	Ifnar1	INSIDE	1.087	1.871	887.37	1660.34	2.034	808.87	1645.06
A_68_P30686668	chr16:38562990-38563034	NM_001205287:86	Tmem39a	INSIDE	1.087	0.523	1316.39	688.37	0.568	1062.48	603.73
A_68_P30284260	chr15:64753424-64753468	NM_009623:412	Adcy8	INSIDE	1.087	0.458	1459.69	668.99	0.498	1180.26	587.85
A_68_P29130721	chr13:72100670-72100714	NM_010573:479	Irx1	INSIDE	1.087	0.612	2230.77	1364.55	0.665	1552.28	1031.98
A_68_P25956632	chr8:73905289-73905333	NM_010150:541	Nr2f6	INSIDE	1.087	2.205	490.00	1080.41	2.398	382.53	917.20
A_68_P25510686	chr7:135561251-135561295	NM_026418:414	Rgs10	INSIDE	1.087	0.441	1571.72	693.52	0.480	1245.21	597.48
A_68_P25007549	chr7:25739920-25739970			Unknown	1.087	0.426	2230.47	949.36	0.463	1651.03	763.67
A_68_P21533268	chr2:113057184-113057228	ENSMUST00000099579:-3		PROMOTER	1.087	3.481	218.08	759.21	3.783	186.79	706.64
A_68_P31116775	chr17:27639850-27639894	NM_001013385:377	Grm4	INSIDE	1.086	1.656	1596.06	2643.64	1.799	1082.44	1947.28
A_68_P28080762	chr11:102527342-102527386	NM_001127576:463	Gm1564	INSIDE	1.086	2.452	999.72	2451.73	2.664	922.71	2457.77
A_68_P27902560	chr11:70577738-70577782	NM_177565:-188	Zfp3	PROMOTER	1.086	0.252	2540.55	640.48	0.274	1908.71	522.65
A_68_P27551122	chr11:3190063-3190107	NM_019574:-375	Patz1	PROMOTER	1.086	4.738	2135.80	10118.88	5.146	1786.05	9191.66
A_68_P23373719	chr4:142751586-142751630	NM_001081355:51004	Prdm2	INSIDE	1.086	0.234	2409.96	564.70	0.255	1905.33	484.91
A_68_P22411088	chr3:107989646-107989690	NM_001004293:416	Amigo1	INSIDE	1.086	0.429	1042.33	446.93	0.466	829.53	386.23
A_68_P21077276	chr2:26092871-26092915	NM_153559:48	Qsox2	INSIDE	1.086	0.521	1337.89	696.57	0.566	1142.26	646.15
A_68_P32820568	chrX:165758296-165758340	NM_008222:-43	Hccs	PROMOTER	1.085	2.584	365.43	944.24	2.804	395.08	1107.74
A_68_P31097159	chr17:24551794-24551838	NM_001080127:197	Rnps1	INSIDE	1.085	0.417	1904.48	794.59	0.453	1501.84	680.13
A_68_P29750655	chr14:79700758-79700803	NM_025427:662	1190002H23Rik	INSIDE	1.085	0.370	2297.81	849.56	0.401	1660.79	666.05
A_68_P28197875	chr12:3960097-3960141	NM_008895:5168	Pomc	INSIDE	1.085	0.260	2222.37	577.75	0.282	1673.23	471.93
A_68_P28177062	chr11:119161599-119161643	NM_138669:-263	Eif4a3	PROMOTER	1.085	0.542	1282.74	694.87	0.588	1063.76	625.44
A_68_P28068582	chr11:100389027-100389071	NM_001199296:266	Acly	INSIDE	1.085	0.524	2797.07	1465.22	0.568	2081.94	1182.97
A_68_P27843265	chr11:60290482-60290526	NM_001103171:7664	Myo15	INSIDE	1.085	1.910	493.24	941.89	2.071	432.82	896.37
A_68_P27835480	chr11:59021879-59021923	NM_024210:38	2310033P09Rik	INSIDE	1.085	0.242	2830.58	685.64	0.263	2084.23	547.69
A_68_P27635749	chr11:21138708-21138752	NM_139061:-161	Vps54	PROMOTER	1.085	0.511	1122.21	573.48	0.554	1013.56	562.00
A_68_P26346148	chr9:21049372-21049416	NM_053190:3493	S1pr5	INSIDE	1.085	0.477	6400.66	3050.99	0.517	4360.12	2254.41
A_68_P25954900	chr8:73438460-73438504	NM_173013:8610	Mtap1s	INSIDE	1.085	2.408	1357.25	3268.62	2.612	1008.45	2634.10
A_68_P24053562	chr5:123479427-123479471	NM_175423:14366	Orai1	INSIDE	1.085	2.965	1690.16	5010.64	3.216	1232.43	3963.79
A_68_P21707654	chr2:144353449-144353493	NM_029763:-10	Polr3f	DIVERGENT_PROMOTER	1.085	0.262	4161.37	1089.10	0.284	3317.22	942.00
A_68_P29057275	chr13:55929910-55929954	NM_011097:2854	Pitx1	INSIDE	1.084	0.571	2063.66	1178.87	0.619	1535.26	950.94
A_68_P27941031	chr11:77845557-77845601	NM_183286:-236	Dhrs13	PROMOTER	1.084	0.444	3150.32	1399.23	0.481	2397.66	1153.91
A_68_P26681180	chr9:82869404-82869448	NM_001081216:-330	Phip	PROMOTER	1.084	0.474	1667.26	789.60	0.514	1103.21	566.52
A_68_P26554121	chr9:59425750-59425794	NM_175235:-371	Celf6	PROMOTER	1.084	0.161	8287.80	1330.33	0.174	5697.30	991.58
A_68_P25958356	chr8:74216542-74216586	NM_028189:9135	B3gnt3	INSIDE	1.084	0.546	2269.32	1239.98	0.593	1463.93	867.46
A_68_P24834772	chr6:127719462-127719506	NM_201371:-737	Prmt8	PROMOTER	1.084	0.382	2746.34	1050.43	0.415	1736.38	720.17
A_68_P23916645	chr5:98615219-98615263	NM_029947:5353	Prdm8	INSIDE	1.084	1.759	841.79	1480.68	1.907	655.80	1250.90
A_68_P23342103	chr4:137352189-137352233	NM_007431:82	Alpl	INSIDE	1.084	2.093	1062.44	2223.17	2.269	990.14	2246.21
A_68_P20830644	chr1:173984270-173984314	NM_010916:3435	Nhlh1	INSIDE	1.084	1.684	684.35	1152.71	1.826	565.00	1031.91
A_68_P20827191	chr1:173224816-173224860	NM_025388:275	Ufc1	INSIDE	1.084	0.365	2505.59	915.61	0.396	1764.06	698.64
A_68_P20229134	chr1:52874442-52874486	NM_008384:68	Inpp1	INSIDE	1.084	0.467	2183.23	1019.53	0.506	1823.31	923.34
A_68_P32364765	chrX:45806673-45806717	NM_019680:9615	Elf4	INSIDE	1.083	2.888	838.74	2422.12	3.129	957.26	2995.10
A_68_P30347361	chr15:76124944-76124988	NM_001195537:103	Gm10345	INSIDE	1.083	0.329	1923.99	633.26	0.356	1509.49	538.07
A_68_P30156695	chr15:39775632-39775677	NM_172814:-351	Lrp12	PROMOTER	1.083	1.921	762.17	1464.23	2.080	671.18	1395.99
A_68_P29613710	chr14:55305167-55305211	NM_021437:181	1700123O20Rik	INSIDE	1.083	0.384	1366.67	524.42	0.415	1157.80	481.04
A_68_P29154208	chr13:76522545-76522589	NM_030174:158	Mctp1	INSIDE	1.083	0.474	943.33	447.29	0.514	814.28	418.25
A_68_P28889017	chr13:24893261-24893305	NM_024473:-243	BC005537	PROMOTER	1.083	0.283	3720.51	1054.58	0.307	2497.52	767.02
A_68_P26329352	chr9:16306770-16306815			Unknown	1.083	0.588	1532.89	900.90	0.637	1249.12	795.24
A_68_P26037061	chr8:90304956-90305000	NM_033327:178516	Zfp423	INSIDE	1.083	2.057	857.52	1763.85	2.228	792.07	1764.39
A_68_P25047615	chr7:35904906-35904950	NM_007678:617	Cebpa	INSIDE	1.083	0.475	2392.34	1137.34	0.515	1928.02	992.31
A_68_P25021325	chr7:29135852-29135896	NR_028129:-684	LOC100302567	PROMOTER	1.083	1.491	1820.87	2714.46	1.614	1405.40	2268.44
A_68_P22286828	chr3:83568363-83568407	NM_009144:-1858	Sfrp2	PROMOTER	1.083	0.472	1165.22	550.08	0.511	995.49	508.92

ProbeName	Target position of probe on CpG island microarray	TargetID	GeneSymbol	CpG island Description	Ratio of relative methylation (TiO_2-NP/Vehicle)	Sham group			TiO_2-H group		
						Relative methylation (Cy5/Cy3)	Cy3 signal (Input)	Cy5 signal (MeDIP)	Relative methylation (Cy5/Cy3)	Cy3 signal (Input)	Cy5 signal (MeDIP)
A_68_P20189690	chr1:43610952-43610996	NM_010879:108379	Nck2	INSIDE	1.083	1.611	845.62	1362.71	1.746	623.47	1088.41
A_68_P32326382	chrX:35474320-35474364	NM_001185002:326	Rhox13	INSIDE	1.082	1.973	432.09	852.51	2.134	762.89	1628.18
A_68_P31144956	chr17:32457857-32457902	NM_019774:219	Akap8	INSIDE	1.082	0.323	1591.42	514.37	0.350	1213.17	424.40
A_68_P31022091	chr17:6256702-6256746	ENSMUST00000121576:2822		DOWNSTREAM	1.082	0.210	2571.74	540.08	0.227	2024.50	460.08
A_68_P30811312	chr16:62846773-62846817	NM_026577:72	Arl13b	INSIDE	1.082	0.331	2951.83	976.86	0.358	2175.36	778.79
A_68_P28601431	chr12:86311677-86311721	NM_178065:138	1110018G07Rik	INSIDE	1.082	0.564	2717.44	1532.18	0.610	2038.78	1243.25
A_68_P27093482	chr10:41917067-41917111	NM_019740:79460	Foxo3	INSIDE	1.082	2.039	1209.71	2467.19	2.207	929.74	2052.11
A_68_P26885917	chr9:121748367-121748412	NM_028384:190	Ccdc13	INSIDE	1.082	0.449	1305.50	586.37	0.486	980.83	476.83
A_68_P26749720	chr9:96790099-96790143	NM_153420:-279	Acpl2	PROMOTER	1.082	3.270	717.07	2344.86	3.537	628.12	2221.39
A_68_P26228654	chr8:124277231-124277275	NM_020605:22794	Jph3	INSIDE	1.082	2.388	1181.74	2821.82	2.585	965.62	2495.70
A_68_P25616319	chr8:4166672-4166716	NM_001039578:128	Evi5l	INSIDE	1.082	0.414	3135.72	1298.39	0.448	2403.84	1076.64
A_68_P25615118	chr8:3676568-3676612	NM_025701:114	Trappc5	INSIDE	1.082	0.479	964.33	462.11	0.518	863.47	447.64
A_68_P25393299	chr7:112766364-112766408	NM_018880:15627	Trim3	INSIDE	1.082	2.166	1023.22	2216.48	2.344	760.54	1782.85
A_68_P24126247	chr5:137972597-137972641	NM_010312:1839	Gnb2	INSIDE	1.082	2.834	513.34	1455.01	3.067	419.07	1285.31
A_68_P22405708	chr3:107135601-107135645	NM_001045807:585	Rbm15	INSIDE	1.082	0.333	1512.52	503.85	0.361	1262.53	455.16
A_68_P22336402	chr3:93248746-93248790	NM_001163098:2517	Tchh	INSIDE	1.082	0.399	1783.91	711.51	0.432	1369.57	591.30
A_68_P22009691	chr3:27052647-27052692	NM_001177625:107	Ect2	INSIDE	1.082	0.515	1996.67	1027.36	0.557	1410.66	785.19
A_68_P21843325	chr2:168577925-168577969	NM_175303:14755	Sall4	INSIDE	1.082	2.358	1449.80	3418.38	2.551	1110.51	2833.01
A_68_P20779533	chr1:164670297-164670341	NM_001081290:369	Prrc2c	INSIDE	1.082	1.606	898.75	1443.53	1.738	749.51	1302.87
A_68_P32319552	chrX:34185014-34185058	NM_001081956:373	Akap17b	INSIDE	1.081	1.895	435.82	825.90	2.049	564.81	1157.11
A_68_P31201698	chr17:45681700-45681744	NM_001013749:4904	Tmem151b	INSIDE	1.081	1.690	720.68	1218.15	1.827	565.03	1032.47
A_68_P30960423	chr16:91011703-91011747	NM_001164483:-171	Synj1	PROMOTER	1.081	0.528	2229.39	1177.59	0.571	1725.34	985.31
A_68_P28514078	chr12:70457923-70457967	NM_025441:209	Nemf	INSIDE	1.081	2.715	595.14	1615.50	2.934	464.41	1362.56
A_68_P26781225	chr9:102528131-102528175	NM_001081122:302	Cep63	INSIDE	1.081	2.222	2116.69	4703.14	2.403	1709.51	4107.14
A_68_P24617811	chr6:87680675-87680719	NM_001159697:-166	Ccdc48	PROMOTER	1.081	0.156	4868.17	761.81	0.169	3237.31	547.61
A_68_P24218731	chr6:6813573-6813617	NM_010057:261	Dlx6	INSIDE	1.081	0.298	4312.27	1286.66	0.322	3187.23	1027.77
A_68_P23396587	chr4:148180164-148180208	NM_027195:1686	Caszl	INSIDE	1.081	0.437	3747.67	1637.11	0.472	2862.22	1351.55
A_68_P22383500	chr3:102739435-102739479	NM_011516:567	Sycp1	INSIDE	1.081	4.506	948.02	4272.17	4.871	750.73	3656.63
A_68_P21839349	chr2:167906063-167906107	NM_021409:-419	Pard6b	PROMOTER	1.081	0.331	1880.23	621.57	0.357	1358.09	485.26
A_68_P21611137	chr2:126834338-126834382	NM_175550:-86	Ap4e1	PROMOTER	1.081	0.345	3064.52	1057.77	0.373	2207.13	823.21
A_68_P32561248	chrX:97972645-97972689	NM_001177780:57	Dlg3	INSIDE	1.080	2.114	490.02	1036.06	2.283	663.38	1514.31
A_68_P31873837	chr18:82113134-82113178	AK046168:311		INSIDE	1.080	1.830	1409.22	2578.36	1.975	1099.17	2171.39
A_68_P31351685	chr17:74888837-74888881	NM_026417:26	Yipf4	INSIDE	1.080	0.519	2166.44	1124.19	0.560	1756.19	984.15
A_68_P31096038	chr17:24342160-24342204	NM_001163847:325	Tbc1d24	INSIDE	1.080	0.255	4961.69	1267.46	0.276	3116.90	859.74
A_68_P30574557	chr16:17576828-17576872	NM_144852:-86	Slc7a4	PROMOTER	1.080	0.383	1665.39	637.05	0.413	1167.23	482.21
A_68_P30357051	chr15:77825627-77825674	NM_007583:124060	Cacng2	INSIDE	1.080	2.408	521.86	1256.73	2.602	407.89	1061.31
A_68_P30135995	chr15:35867423-35867467	NM_053071:557	Cox6c	INSIDE	1.080	0.353	2594.78	916.61	0.381	2155.84	822.29
A_68_P29348447	chr13:115608397-115608441	NM_001113374:-26	Mocs2	PROMOTER	1.080	0.251	2621.79	658.31	0.271	1892.39	513.22
A_68_P28613112	chr12:88288445-88288489	NM_173735:174	2310044G17Rik	INSIDE	1.080	0.619	2602.17	1609.59	0.668	1991.17	1330.20
A_68_P28149795	chr11:114653533-114653577	NM_028055:3652	Btbd17	INSIDE	1.080	0.484	1016.99	492.43	0.523	866.33	452.96
A_68_P26742254	chr9:95411930-95411974	NM_001114977:463	2610101N10Rik	INSIDE	1.080	0.540	4068.97	2197.61	0.583	2775.46	1618.40
A_68_P26154343	chr8:111480556-111480607	NM_007496:242038	Zfhx3	INSIDE	1.080	1.973	7636.56	15065.37	2.131	4838.45	10309.00
A_68_P25948616	chr8:72426603-72426647	NM_023312:-167	Ndufa13	PROMOTER	1.080	0.393	1460.22	573.21	0.424	983.77	417.22
A_68_P24874567	chr6:136765429-136765473	NM_021714:11287	Wbp11	INSIDE	1.080	1.711	1736.79	2972.44	1.848	1527.97	2824.33
A_68_P24707915	chr6:103462130-103462174	NM_007697:1283	Chl1	INSIDE	1.080	0.377	2515.16	948.72	0.407	1787.39	728.26
A_68_P24624900	chr6:88852416-88852460	NM_011906:194	Tpra1	INSIDE	1.080	0.279	1893.30	528.33	0.301	1422.46	428.69
A_68_P23120295	chr4:94645754-94645798	NM_177239:15	Mysm1	INSIDE	1.080	0.411	1371.96	563.73	0.444	1003.74	445.52
A_68_P22964668	chr4:62141329-62141373	NM_138653:250	Bspry	INSIDE	1.080	0.534	1280.63	684.01	0.577	1019.43	587.92
A_68_P22592456	chr3:142428233-142428277	NM_145546:44	Gtf2b	INSIDE	1.080	0.472	1130.99	533.27	0.509	945.80	481.48
A_68_P21751448	chr2:152657132-152657176	NM_009743:264	Bcl2l1	INSIDE	1.080	1.960	573.83	1124.97	2.117	549.94	1164.17
A_68_P30630406	chr16:28445546-28445590	NM_183064:-255	Fgf12	PROMOTER	1.079	3.544	1409.96	4997.31	3.826	1071.05	4097.38
A_68_P27894658	chr11:69182004-69182048	NM_146019:902	Chd3	INSIDE	1.079	0.391	2427.30	948.07	0.422	1715.05	722.98
A_68_P25591879	chr7:149136030-149136074	NM_001009929:62	Brsk2	INSIDE	1.079	0.318	2015.11	640.32	0.343	1645.01	563.98
A_68_P25369176	chr7:107855309-107855353	NM_178764:115	Fam168a	INSIDE	1.079	0.407	1349.59	548.88	0.439	1165.19	511.40
A_68_P25353646	chr7:105227755-105227799	NM_008663:40227	Myo7a	INSIDE	1.079	1.694	1062.03	1798.58	1.827	850.38	1553.93
A_68_P24602877	chr6:85019040-85019084	NM_177077:445	Exoc6b	INSIDE	1.079	0.327	1390.53	454.42	0.352	1113.01	392.30
A_68_P24158346	chr5:144578888-144578932	NM_013557:250	Eif2ak1	INSIDE	1.079	0.211	3260.08	689.36	0.228	2225.89	507.66
A_68_P24085130	chr5:129287884-129287928	NM_001081388:171331	Rimbp2	INSIDE	1.079	1.512	1227.92	1857.20	1.631	985.93	1608.48
A_68_P22963157	chr4:61869358-61869402	NM_009554:200	Zfp37	INSIDE	1.079	0.255	3607.12	919.17	0.275	2976.63	818.32
A_68_P21874766	chr2:174156274-174156318	NM_001077510:707	Gnas	INSIDE	1.079	0.329	2042.93	671.28	0.355	1537.23	545.27
A_68_P21489530	chr2:104334257-104334301	NM_001145824:368	Hipk3	INSIDE	1.079	0.510	3337.49	1702.31	0.550	2145.51	1180.32
A_68_P20510205	chr1:108656151-108656195	NM_027534:147	Kdsr	INSIDE	1.079	0.464	1680.30	780.28	0.501	1194.12	598.49
A_68_P32395552	chrX:53627636-53627680	NR_028086:-104	Ncrna00086	PROMOTER	1.078	1.413	2375.02	3355.74	1.523	3128.62	4764.24
A_68_P32136711	chr19:45217923-45217967	NM_021901:-7260	Tlx1	PROMOTER	1.078	0.480	1647.73	790.90	0.517	1244.43	643.77
A_68_P31933968	chr19:6320770-6320814	NM_001033342:14336	Cdc42bpg	INSIDE	1.078	1.574	861.45	1355.80	1.697	715.08	1213.41

ProbeName	Target position of probe on CpG island microarray	TargetID	GeneSymbol	CpG island Description	Ratio of relative methylation (TiO_2-NP/Vehicle)	Sham group			TiO_2-H group		
						Relative methylation (Cy5/Cy3)	Cy3 signal (Input)	Cy5 signal (MeDIP)	Relative methylation (Cy5/Cy3)	Cy3 signal (Input)	Cy5 signal (MeDIP)
A_68_P31866349	chr18:80910082-80910126	NM_001164110:-294	Nfatc1	PROMOTER	1.078	0.190	2445.14	465.55	0.205	1879.35	385.73
A_68_P30476457	chr15:98620052-98620096	NM_021279:-213	Wnt1	PROMOTER	1.078	0.214	3307.21	708.66	0.231	2433.98	562.18
A_68_P29453038	chr14:22320578-22320622	NM_017479:1594	Myst4	INSIDE	1.078	9.192	6178.05	56787.98	9.905	4311.08	42700.71
A_68_P28189463	chr11:121098621-121098665	NM_026272:93	Narf	INSIDE	1.078	0.267	1700.56	454.30	0.288	1120.76	322.70
A_68_P27985585	chr11:85651342-85651386	NM_009324:5248	Tbx2	INSIDE	1.078	3.250	697.89	2268.40	3.505	559.88	1962.46
A_68_P27291025	chr10:81106120-81106164	NM_027480:14858	Ankrd24	INSIDE	1.078	0.569	2514.66	1430.12	0.613	1977.73	1212.45
A_68_P26346144	chr9:21048991-21049035	NM_053190:3875	S1pr5	INSIDE	1.078	0.373	3283.12	1225.46	0.403	2539.18	1022.02
A_68_P25574802	chr7:146101366-146101410	NM_009760:-199	Bnip3	PROMOTER	1.078	0.274	8279.31	2269.63	0.295	5830.81	1722.99
A_68_P25511160	chr7:135666886-135666930	NM_013863:-188	Bag3	PROMOTER	1.078	0.501	1736.97	870.65	0.540	1244.47	672.33
A_68_P24908452	chr6:143048181-143048225	NM_001109688:425	5730419I09Rik	INSIDE	1.078	0.271	2181.47	592.15	0.293	1787.60	523.05
A_68_P23409061	chr4:150288454-150288498	NM_020569:-4446	Park7	DIVERGENT_PROMOTER	1.078	0.581	1481.44	860.03	0.626	1241.92	776.89
A_68_P22650017	chr3:152003054-152003098	NM_001162375:295	Fam73a	INSIDE	1.078	0.485	1123.32	545.16	0.523	881.66	461.13
A_68_P32143075	chr19:46380056-46380100	NM_019408:-106	Nfkb2	PROMOTER	1.077	0.445	1319.88	587.27	0.479	1196.35	573.56
A_68_P27643055	chr11:22759832-22759876	NM_016888:-119	B3gnt2	PROMOTER	1.077	0.467	2682.06	1251.36	0.502	2057.55	1033.79
A_68_P27259219	chr10:75356127-75356171	NM_024440:6	Derl3	INSIDE	1.077	0.274	1895.51	519.40	0.295	1209.34	357.00
A_68_P27143973	chr10:53471309-53471353	NM_001081428:-615	Fam184a	PROMOTER	1.077	0.239	6424.40	1537.55	0.258	4570.42	1177.58
A_68_P26075129	chr8:96561318-96561362	NM_001093757:243	Ogfod1	INSIDE	1.077	0.326	2197.33	717.07	0.352	1794.21	630.81
A_68_P23991126	chr5:112706061-112706105	NM_009419:356	Tpst2	INSIDE	1.077	0.350	2245.69	786.85	0.377	1565.61	590.90
A_68_P23155322	chr4:101169271-101169315	NM_198412:40	Dnajc6	INSIDE	1.077	0.250	1784.58	445.81	0.269	1397.80	376.06
A_68_P22503482	chr3:126300061-126300105	NM_001025438:192	Camk2d	INSIDE	1.077	1.518	1060.31	1610.04	1.636	913.63	1494.61
A_68_P21579145	chr2:120965882-120965926	NM_177846:530	Lcmt2	INSIDE	1.077	2.313	631.34	1460.50	2.492	532.97	1328.15
A_68_P21258085	chr2:60221190-60221234	NM_013825:76	Ly75	INSIDE	1.077	0.264	3884.30	1023.85	0.284	2806.16	796.66
A_68_P31934227	chr19:6364618-6364662	NM_001110791:951	Sf1	INSIDE	1.076	1.811	832.39	1507.14	1.949	684.50	1333.94
A_68_P31544269	chr18:21458728-21458772	NM_001033445:-110	Fam59a	PROMOTER	1.076	0.421	1299.33	547.37	0.453	1163.44	527.32
A_68_P31515574	chr18:15220674-15220718	NM_001142731:1395	Kctd1	INSIDE	1.076	0.409	2802.51	1146.47	0.440	2137.33	940.48
A_68_P31092956	chr17:23810793-23810838	NM_001008425:-78	Thoc6	PROMOTER	1.076	0.333	1725.93	574.68	0.358	1342.02	480.78
A_68_P29228406	chr13:94073700-94073744	NM_011982:-727	Homer1	PROMOTER	1.076	1.989	597.90	1189.27	2.140	417.28	892.81
A_68_P25168660	chr7:69493069-69493113	NM_010882:-72	Ndn	PROMOTER	1.076	0.473	1659.56	785.53	0.510	1281.97	653.19
A_68_P23468798	chr5:8623006-8623050	NM_198620:-76	Rundc3b	PROMOTER	1.076	0.234	2551.77	595.85	0.251	2005.27	503.97
A_68_P22305771	chr3:86723508-86723553	NM_001195496:1276	Dclk2	INSIDE	1.076	0.450	1242.93	559.86	0.485	940.00	455.61
A_68_P20141347	chr1:34987195-34987239			Unknown	1.076	1.742	1267.15	2207.24	1.875	1031.52	1933.96
A_68_P31781216	chr18:65590296-65590340	NM_172833:-332	Malt1	PROMOTER	1.075	4.459	3003.21	13391.98	4.794	2138.67	10253.36
A_68_P31601519	chr18:32322742-32322786	NM_011946:22	Map3k2	INSIDE	1.075	1.674	980.22	1640.54	1.799	876.16	1576.39
A_68_P31556683	chr18:23910867-23910911	NM_001162941:-1337	Mapre2	PROMOTER	1.075	0.294	1554.52	456.41	0.316	1148.25	362.31
A_68_P29855885	chr14:102007169-102007213	NM_001081278:1218	Tbc1d4	INSIDE	1.075	0.303	2758.90	835.00	0.325	1605.49	522.16
A_68_P28549414	chr12:76696397-76696441	NM_012024:769	Ppp2r5e	INSIDE	1.075	0.545	1370.97	747.39	0.586	1042.60	610.83
A_68_P28047528	chr11:96777981-96778025	NM_030248:-207	Cdk5rap3	PROMOTER	1.075	0.341	1665.59	568.09	0.367	1284.68	470.92
A_68_P28032140	chr11:94189670-94189714	NM_146024:378	Ankrd40	INSIDE	1.075	0.231	4084.29	944.56	0.249	2805.86	697.73
A_68_P27673731	chr11:29147082-29147126	NM_025740:168	Ccdc104	INSIDE	1.075	0.408	2810.35	1147.13	0.439	2290.80	1004.88
A_68_P26579683	chr9:63870481-63870525	NM_008542:-636	Smad6	PROMOTER	1.075	2.648	3147.92	8337.02	2.848	2373.94	6761.53
A_68_P24859169	chr6:133985084-133985128	NM_007961:-618	Etv6	PROMOTER	1.075	1.848	1760.97	3253.99	1.986	1491.83	2962.50
A_68_P23985662	chr5:111847249-111847293	NM_001081235:85	Mn1	INSIDE	1.075	0.457	1647.69	752.25	0.491	1182.17	580.08
A_68_P23333245	chr4:135867254-135867298	NM_028871:386	Hnrnpr	INSIDE	1.075	1.518	1297.93	1970.65	1.633	927.50	1514.48
A_68_P22503487	chr3:126300546-126300590	NM_001025438:678	Camk2d	INSIDE	1.075	0.256	2135.74	547.14	0.275	1741.73	479.62
A_68_P21085075	chr2:27370596-27370640	NM_080848:-48	Wdr5	PROMOTER	1.075	0.275	5374.91	1476.11	0.295	3630.20	1071.81
A_68_P20744278	chr1:158604428-158604472	NM_023141:20	Tor3a	INSIDE	1.075	0.420	1469.93	616.90	0.451	1202.73	542.72
A_68_P20293018	chr1:64580121-64580165	NM_001037726:765	Creb1	INSIDE	1.075	2.176	1123.34	2444.72	2.339	905.77	2118.17
A_68_P20091642	chr1:24106929-24106973	NM_026604:230	Fam135a	INSIDE	1.075	0.385	1744.14	671.69	0.414	1476.55	611.24
A_68_P30964221	chr16:91660638-91660682	NM_019973:12592	Son	INSIDE	1.074	1.397	5134.84	7170.97	1.500	4066.06	6098.78
A_68_P28944867	chr13:35832810-35832854	NM_001123386:-438	Cdyl	PROMOTER	1.074	0.316	1521.82	480.88	0.340	1243.04	422.02
A_68_P28513155	chr12:70269942-70269986	NM_001081406:164	Lrr1	INSIDE	1.074	0.393	1683.75	662.36	0.422	1372.18	579.61
A_68_P28206064	chr12:5381209-5381253	NM_001164493:1258	Klhl29	INSIDE	1.074	0.357	2234.46	796.59	0.383	1577.86	603.93
A_68_P27288863	chr10:80785224-80785268	NM_027381:1399	2510012J08Rik	INSIDE	1.074	1.590	1333.79	2120.61	1.708	1006.32	1718.65
A_68_P27285904	chr10:80317637-80317681	NM_025349:296	Lsm7	INSIDE	1.074	0.270	2925.45	791.05	0.290	1902.09	552.24
A_68_P25452562	chr7:124525096-124525140	NM_175645:626	Xylt1	INSIDE	1.074	0.478	982.50	469.21	0.513	784.13	402.14
A_68_P21842273	chr2:168396596-168396647	NM_001136073:19070	Nfatc2	INSIDE	1.074	5.445	465.08	2532.48	5.849	283.33	1657.11
A_68_P21603045	chr2:125332058-125332102	NM_007993:94	Fbn1	INSIDE	1.074	0.600	1922.41	1154.37	0.645	1612.80	1040.39
A_68_P20937880	chr1:193219969-193220013	NM_144880:930	Ppp2r5a	INSIDE	1.074	0.432	3125.08	1349.93	0.464	2190.86	1016.38
A_68_P31150390	chr17:33896983-33897027	NM_008997:427	Rab11b	INSIDE	1.073	0.216	3806.56	820.57	0.231	2906.55	672.03
A_68_P30337591	chr15:74346432-74346476	NM_174991:-171	Bai1	PROMOTER	1.073	0.326	1668.45	544.01	0.350	1212.73	424.47
A_68_P28732761	chr12:110697987-110698031	NM_001190703:6344	Dlk1	INSIDE	1.073	1.673	1351.04	2259.69	1.795	1083.40	1944.97
A_68_P27612233	chr11:16652100-16652144	NM_007912:-83	Egfr	PROMOTER	1.073	0.151	3925.46	591.11	0.162	2975.92	480.97
A_68_P26765225	chr9:99530050-99530094	NM_028258:59	Dzip1l	INSIDE	1.073	0.416	1764.06	734.12	0.447	1316.43	587.84
A_68_P26224353	chr8:123607977-123608021	NM_010426:-375	Foxf1a	PROMOTER	1.073	0.216	2104.23	455.00	0.232	1718.92	398.99
A_68_P25953241	chr8:73183221-73183265	NM_023217:338	Pgpep1	INSIDE	1.073	0.194	2571.47	499.76	0.209	1670.56	348.42

ProbeName	Target position of probe on CpG island microarray	TargetID	GeneSymbol	CpG island Description	Ratio of relative methylation (TiO_2-NP/Vehicle)	Sham group			TiO_2-H group		
						Relative methylation (Cy5/Cy3)	Cy3 signal (Input)	Cy5 signal (MeDIP)	Relative methylation (Cy5/Cy3)	Cy3 signal (Input)	Cy5 signal (MeDIP)
A_68_P24951777	chr7:5003100-5003144	NM_026900:-11	Zfp580	PROMOTER	1.073	0.340	1815.57	616.55	0.364	1204.09	438.67
A_68_P24758107	chr6:113145188-113145232	NM_177763:168	Lhfpl4	INSIDE	1.073	0.565	1630.49	920.58	0.606	1342.77	813.26
A_68_P24605097	chr6:85401585-85401629	NM_007638:108	Cct7	INSIDE	1.073	0.236	2013.98	474.62	0.253	1587.56	401.55
A_68_P21152001	chr2:38921133-38921177	NM_029793:-228	Golga1	PROMOTER	1.073	1.835	568.71	1043.35	1.969	428.57	843.71
A_68_P21114928	chr2:32218406-32218450	NM_028412:-325	Ciz1	DIVERGENT_PROMOTER	1.073	1.734	621.98	1078.33	1.861	497.46	925.67
A_68_P21041406	chr2:18689012-18689056	ENSMUST00000119424:8424		DOWNSTREAM	1.073	0.338	2352.35	794.49	0.362	1784.64	646.56
A_68_P20897156	chr1:186555676-186555720	NM_008250:674	Hlx	INSIDE	1.073	0.389	5417.63	2107.51	0.417	3816.56	1592.69
A_68_P31864632	chr18:80665927-80665971	NM_026295:458	Ctdp1	INSIDE	1.072	0.310	2489.58	771.83	0.332	1874.41	622.71
A_68_P29480508	chr14:26746521-26746565	NM_025311:194	D14Ertd449e	INSIDE	1.072	0.081	5802.37	470.56	0.087	4404.28	382.80
A_68_P28940416	chr13:34839156-34839200	NM_138746:7668	Fam50b	INSIDE	1.072	2.132	897.90	1913.92	2.285	641.07	1465.06
A_68_P28748149	chr12:113384890-113384934	NM_001097621:494	Kif26a	INSIDE	1.072	0.501	1675.87	840.04	0.537	1252.05	672.82
A_68_P28458301	chr12:57798039-57798083	NM_011041:1435	Pax9	INSIDE	1.072	0.665	3044.63	2025.03	0.713	2345.24	1672.11
A_68_P28078911	chr11:102180801-102180845	NM_011551:-412	Ubtf	PROMOTER	1.072	0.395	1662.53	656.08	0.423	1105.74	467.92
A_68_P23637425	chr5:42155503-42155547	NM_007524:-65	Nkx3-2	PROMOTER	1.072	0.221	2391.15	528.68	0.237	1802.02	427.26
A_68_P22372021	chr3:100726069-100726113	NM_001165953:-325	Trim45	PROMOTER	1.072	0.213	3059.91	652.15	0.229	2205.36	504.05
A_68_P21842276	chr2:168396951-168396995	NM_001136073:18719	Nfatc2	INSIDE	1.072	1.943	531.14	1031.78	2.082	462.34	962.57
A_68_P21749219	chr2:152240057-152240101	NM_175126:702	Zcchc3	INSIDE	1.072	0.377	1828.88	690.07	0.405	1566.79	633.82
A_68_P32453218	chrX:68617930-68617974	NM_016985:18	Mtmr1	INSIDE	1.071	2.569	413.63	1062.59	2.752	475.47	1308.70
A_68_P31928725	chr19:5448265-5448309	NM_010235:589	Fosl1	INSIDE	1.071	0.335	2767.43	925.82	0.358	1906.92	683.17
A_68_P31130477	chr17:30026875-30026919	NM_001081160:-2069	Mdga1	PROMOTER	1.071	2.036	734.45	1495.17	2.181	591.88	1290.85
A_68_P31099059	chr17:24832929-24832973	NM_023040:151	Gfer	INSIDE	1.071	2.149	492.67	1058.72	2.302	421.49	970.42
A_68_P30596433	chr16:22162850-22162894	NM_183029:500	Igf2bp2	INSIDE	1.071	1.915	768.77	1472.12	2.051	680.49	1395.85
A_68_P30500795	chr15:102765355-102765399	NM_010463:-1907	Hoxc12	PROMOTER	1.071	0.521	1021.20	531.82	0.558	949.35	529.56
A_68_P30344887	chr15:75740593-75740637	NM_178646:450	Tigd5	INSIDE	1.071	0.416	3271.53	1361.67	0.446	2288.42	1019.88
A_68_P28397454	chr12:45430004-45430048	NM_001146031:155	Nrcam	INSIDE	1.071	0.545	2968.99	1617.72	0.583	2234.11	1303.43
A_68_P27927393	chr11:75465155-75465200	NM_001080774:-474	Myo1c	PROMOTER	1.071	0.429	1774.31	761.74	0.460	1291.36	593.64
A_68_P26323063	chr9:15297918-15297962	AK146267:65		INSIDE	1.071	2.773	395.23	1096.12	2.970	350.16	1040.02
A_68_P25581871	chr7:147222691-147222735	NM_027201:423	Zfp511	INSIDE	1.071	0.535	1687.50	902.14	0.572	1244.43	712.28
A_68_P24117827	chr5:136254305-136254349	NM_029659:-71	Styxl1	DIVERGENT_PROMOTER	1.071	0.325	1899.44	616.39	0.348	1424.78	495.24
A_68_P23972005	chr5:108877904-108877948	NM_172883:-16	Mfsd7a	PROMOTER	1.071	0.462	1285.88	593.76	0.495	932.23	461.10
A_68_P21838231	chr2:167720809-167720853			Unknown	1.071	0.449	1294.42	580.62	0.481	1026.85	493.42
A_68_P20351170	chr1:74947336-74947380	NR_029876:-63	Mir375	PROMOTER	1.071	0.299	2808.98	839.55	0.320	2090.58	669.43
A_68_P30350209	chr15:76552567-76552611	NM_198119:15	Lrrc24	INSIDE	1.070	0.467	1227.73	573.48	0.500	1008.82	504.42
A_68_P28752688	chr12:114047423-114047467	NM_145450:259	BC022687	INSIDE	1.070	1.698	1136.31	1929.39	1.817	885.04	1607.75
A_68_P26531723	chr9:55359728-55359772	NM_145615:300	Etfa	INSIDE	1.070	0.581	1894.70	1101.07	0.622	1488.10	925.22
A_68_P25591882	chr7:149136496-149136540	NM_001009929:528	Brsk2	INSIDE	1.070	1.503	1027.52	1544.08	1.607	877.12	1409.82
A_68_P24287933	chr6:21802094-21802138	NM_173007:399	Tspan12	INSIDE	1.070	0.420	2125.80	892.24	0.449	1666.00	748.24
A_68_P23308596	chr4:131394388-131394432	NM_001083119:-217	Ptpru	DIVERGENT_PROMOTER	1.070	0.451	1258.87	568.13	0.483	919.44	443.79
A_68_P22309971	chr3:87587854-87587898	NM_001033124:11208	Ntrk1	INSIDE	1.070	1.712	1023.57	1752.08	1.831	831.83	1523.43
A_68_P21825959	chr2:165622131-165622175	NM_027230:88036	Zmynd8	INSIDE	1.070	1.734	12399.91	21498.46	1.854	9200.08	17059.98
A_68_P21739505	chr2:150326509-150326553	ENSMUST00000121441:51		INSIDE	1.070	1.923	957.77	1841.53	2.057	818.70	1684.24
A_68_P21600379	chr2:124949302-124949346	NM_001162417:72	Myef2	INSIDE	1.070	4.435	719.36	3190.18	4.746	617.51	2930.60
A_68_P31257382	chr17:56766395-56766439	NM_028782:-90	Lonp1	DIVERGENT_PROMOTER	1.069	2.314	5126.00	11862.87	2.475	3231.24	7996.14
A_68_P30506931	chr16:3872466-3872510	NM_001033159:-114	Zfp597	PROMOTER	1.069	0.180	3042.93	549.08	0.193	1961.33	378.16
A_68_P30475231	chr15:98397707-98397751	NR_028442:-26	9330020H09Rik	PROMOTER	1.069	0.200	2365.41	472.71	0.214	1844.10	393.91
A_68_P29023542	chr13:49242589-49242633	NM_029361:773	Wnk2	INSIDE	1.069	2.196	1197.26	2628.77	2.348	958.48	2250.54
A_68_P28160473	chr11:116485490-116485534	NM_001167680:54	Rhbdf2	INSIDE	1.069	0.440	1560.35	687.00	0.471	1192.93	561.51
A_68_P28098603	chr11:105805256-105805300	NM_007805:183	Cyb561	INSIDE	1.069	3.212	1622.65	5212.33	3.433	1191.05	4088.79
A_68_P26079046	chr8:97219612-97219656	NM_026385:508	Pllp	INSIDE	1.069	3.789	748.88	2837.53	4.052	654.20	2650.54
A_68_P25656683	chr8:11634954-11634998	NR_030781:60	Ankrd10	INSIDE	1.069	0.598	3035.54	1816.51	0.640	2257.50	1443.79
A_68_P25614134	chr8:3476114-3476158	NM_029598:5275	1700019B03Rik	INSIDE	1.069	0.402	1403.58	564.04	0.430	935.08	401.68
A_68_P24933418	chr6:147212829-147212873	NM_008970:-243	Pthlh	PROMOTER	1.069	0.475	1121.42	532.97	0.508	937.16	476.24
A_68_P24773308	chr6:115803699-115803744	NM_001167763:176	Ift122	INSIDE	1.069	0.672	4267.34	2867.05	0.718	3092.07	2219.80
A_68_P24098213	chr5:131915558-131915602	NM_177047:1102633	Auts2	INSIDE	1.069	1.567	2275.33	3566.11	1.675	1616.87	2708.49
A_68_P22422565	chr3:109945185-109945229	NM_001163348:1184	Ntng1	INSIDE	1.069	2.497	2153.83	5377.78	2.668	1718.16	4584.72
A_68_P21812072	chr2:163201808-163201852	NM_021566:21879	Jph2	INSIDE	1.069	2.141	927.66	1985.99	2.290	787.69	1803.52
A_68_P31106680	chr17:25981271-25981315	NM_145999:504	Rhot2	INSIDE	1.068	0.547	3939.58	2155.56	0.584	2670.68	1560.82
A_68_P30591825	chr16:21333336-21333380	AK018285:71		INSIDE	1.068	2.526	2125.19	5369.29	2.698	1641.95	4429.77
A_68_P30387340	chr15:83081767-83081811	NM_001170954:373	A4galt	INSIDE	1.068	0.486	1825.79	886.74	0.519	1440.89	747.34
A_68_P28194146	chr12:3235815-3235859	NR_015551:46	1700012B15Rik	INSIDE	1.068	0.521	1462.27	761.95	0.556	1151.43	640.50
A_68_P28182345	chr11:119959914-119959958	NM_183137:109	2410002I01Rik	INSIDE	1.068	2.161	1017.18	2198.02	2.307	775.64	1789.48
A_68_P28103510	chr11:106648827-106648871	NR_037224:-4775	Mir3064	PROMOTER	1.068	0.266	3869.77	1030.95	0.285	2701.50	768.89
A_68_P25995584	chr8:82163380-82163424	NM_001081164:-172	Otud4	PROMOTER	1.068	0.478	1855.16	886.92	0.511	1425.74	728.09
A_68_P25499769	chr7:133436111-133436155	NM_145587:20000	Sbk1	INSIDE	1.068	2.915	804.46	2345.25	3.113	637.94	1985.61
A_68_P24130914	chr5:139060935-139060979	NM_001044747:15	Zfp68	INSIDE	1.068	0.364	2172.73	791.53	0.389	1663.22	647.13

ProbeName	Target position of probe on CpG island microarray	TargetID	GeneSymbol	CpG island Description	Ratio of relative methylation (TiO_2-NP/Vehicle)	Sham group			TiO_2-H group		
						Relative methylation (Cy5/Cy3)	Cy3 signal (Input)	Cy5 signal (MeDIP)	Relative methylation (Cy5/Cy3)	Cy3 signal (Input)	Cy5 signal (MeDIP)
A_68_P23592016	chr5:34338666-34338710	NM_001001985:56	Nat8l	INSIDE	1.068	0.426	2902.18	1235.28	0.454	2101.05	954.75
A_68_P23451690	chr5:4757789-4757833	NM_021457:406	Fzd1	INSIDE	1.068	0.559	1534.67	858.37	0.597	1332.37	795.71
A_68_P23342099	chr4:137351703-137351747	NM_007431:568	Alpl	INSIDE	1.068	0.358	2837.14	1014.86	0.382	2090.37	798.26
A_68_P22728295	chr4:11083786-11083830	NM_001199105:221	Trp53inp1	INSIDE	1.068	0.370	2052.68	758.67	0.395	1570.76	620.20
A_68_P21840413	chr2:168094194-168094238	NM_001081134:615	Kcng1	INSIDE	1.068	1.753	1343.77	2355.15	1.872	984.12	1842.06
A_68_P20937462	chr1:193150005-193150049	NM_025864:183	Tmem206	INSIDE	1.068	0.278	3000.63	835.21	0.297	2205.31	655.86
A_68_P20433237	chr1:91865288-91865332	NM_139152:45842	Asb18	INSIDE	1.068	0.427	1149.68	490.47	0.455	973.36	443.31
A_68_P32694936	chrX:136145461-136145505	NM_023270:324	Rnf128	INSIDE	1.067	2.112	284.98	602.01	2.255	416.55	939.32
A_68_P31490815	chr18:10609631-10609677	NM_001081222:696	Esco1	INSIDE	1.067	1.953	1047.68	2045.85	2.084	785.37	1636.75
A_68_P30631086	chr16:28564499-28564543	NM_010199:188768	Fgf12	INSIDE	1.067	2.797	978.17	2735.90	2.984	748.26	2232.65
A_68_P29285955	chr13:104383068-104383112	BC099526:114623		INSIDE	1.067	1.906	686.18	1308.16	2.035	555.81	1130.98
A_68_P28068129	chr11:100302265-100302309	NM_026561:117	Nt5c3l	INSIDE	1.067	0.450	1729.01	778.42	0.480	1274.73	612.46
A_68_P28054175	chr11:97914595-97914639	NM_146028:160	Stac2	INSIDE	1.067	0.286	3816.45	1093.04	0.306	2746.75	839.77
A_68_P28046787	chr11:96650859-96650903	NM_007622:431	Cbx1	INSIDE	1.067	0.210	2512.74	526.81	0.224	1862.92	416.61
A_68_P27303375	chr10:84039721-84039765	NM_146008:51	Tcp11l2	INSIDE	1.067	1.814	732.89	1329.10	1.936	723.04	1399.67
A_68_P24994095	chr7:20158628-20158672	NM_027189:42	Gemin7	INSIDE	1.067	0.411	3139.57	1290.52	0.439	2235.76	980.67
A_68_P24951591	chr7:4969560-4969604	NM_025324:2473	Zfp524	INSIDE	1.067	2.043	1612.76	3294.57	2.180	1293.37	2819.09
A_68_P24819885	chr6:125264177-125264221	NM_010736:-310	Ltbr	DIVERGENT_PROMOTER	1.067	0.457	1261.86	576.20	0.487	908.08	442.41
A_68_P24448666	chr6:52108435-52108479	NM_010449:-140	Hoxa1	PROMOTER	1.067	0.477	1637.69	781.71	0.509	1346.21	685.46
A_68_P24130230	chr5:138882841-138882885	NM_028130:159	Zfp157	INSIDE	1.067	0.341	2039.04	695.01	0.364	1534.02	558.08
A_68_P23229785	chr4:116230013-116230057	NM_029868:-297	Gpbp1l1	DIVERGENT_PROMOTER	1.067	0.500	1550.31	775.15	0.534	1139.49	608.09
A_68_P31149281	chr17:33661172-33661216	NM_172619:54	Adamts10	INSIDE	1.066	0.603	1544.75	931.46	0.643	1259.66	809.61
A_68_P29448404	chr14:21514121-21514165	NM_028428:-47	Fut11	PROMOTER	1.066	0.394	1796.01	707.15	0.420	1257.57	527.76
A_68_P28083251	chr11:102941489-102941533	NM_152813:21462	Plcd3	INSIDE	1.066	0.551	2319.36	1278.57	0.588	1765.03	1037.68
A_68_P27483042	chr10:117229727-117229771	NM_134010:13	Nup107	INSIDE	1.066	1.601	917.58	1469.06	1.707	696.19	1188.24
A_68_P26016161	chr8:86239606-86239651	NM_197982:553	Ddx39	INSIDE	1.066	0.513	1392.80	714.27	0.547	1161.27	634.85
A_68_P24799554	chr6:120615829-120615873	NM_001128151:-588	Cecr2	PROMOTER	1.066	0.140	3754.85	526.78	0.150	2756.21	412.30
A_68_P21707841	chr2:144382255-144382299	NM_019787:264	Sec23b	INSIDE	1.066	2.829	1172.27	3316.41	3.016	930.44	2806.03
A_68_P20877854	chr1:182724487-182724531	NM_008210:19226	H3f3a	DOWNSTREAM	1.066	0.156	8514.08	1324.18	0.166	5787.34	959.60
A_68_P31712049	chr18:53405446-53405490	NM_029394:153	Snx24	INSIDE	1.065	0.475	1168.60	554.96	0.506	889.56	449.85
A_68_P31175203	chr17:39980581-39980625	ENSMUST00000102050:-478		PROMOTER	1.065	0.261	4433.17	1158.75	0.278	3720.09	1035.28
A_68_P31106586	chr17:25970234-25970278	NM_019719:50	Stub1	INSIDE	1.065	0.344	1697.84	584.28	0.367	1228.68	450.34
A_68_P30991209	chr16:96348941-96348985	NM_008251:370	Hmgn1	INSIDE	1.065	0.487	2665.21	1297.21	0.518	1914.62	992.71
A_68_P29941732	chr14:119324506-119324550	NM_025943:-2139	Dzip1	PROMOTER	1.065	0.265	1760.32	467.30	0.283	1434.00	405.27
A_68_P29725076	chr14:75347080-75347124	NM_001033439:582	Lrch1	INSIDE	1.065	4.701	973.85	4578.53	5.009	825.10	4133.23
A_68_P28066379	chr11:100006994-100007038	NM_008471:217	Krt19	INSIDE	1.065	0.582	1994.44	1161.22	0.620	1491.95	925.39
A_68_P27542071	chr10:128002533-128002577	NM_011119:436	Pa2g4	INSIDE	1.065	0.139	5604.04	781.56	0.149	4028.08	598.29
A_68_P27287759	chr10:80616606-80616650	NM_021501:13837	Pias4	DOWNSTREAM	1.065	3.536	851.31	3010.09	3.766	730.72	2751.76
A_68_P26643861	chr9:75257109-75257153	NM_027418:36	Mapk6	INSIDE	1.065	6.778	871.04	5903.73	7.217	779.25	5623.58
A_68_P24125091	chr5:137748534-137748578	NM_001109909:346	Srrt	INSIDE	1.065	0.144	3357.87	485.01	0.154	2380.23	366.19
A_68_P24118278	chr5:136344278-136344322	NM_021403:50325	Srrm3	INSIDE	1.065	2.384	642.15	1531.17	2.538	584.58	1483.88
A_68_P24066887	chr5:125914212-125914256	NM_203319:184	Dhx37	INSIDE	1.065	0.386	2315.32	892.99	0.411	1890.76	776.35
A_68_P31793418	chr18:67883645-67883689	NM_001127177:609	Ptpn2	INSIDE	1.064	0.405	3110.58	1259.24	0.431	2494.25	1074.55
A_68_P31152223	chr17:34257573-34257617	NR_037970:-749	Brd2	PROMOTER	1.064	0.416	1984.19	824.44	0.442	1480.77	654.76
A_68_P31118766	chr17:27976996-27977040	NM_011432:-13	Snrpc	PROMOTER	1.064	0.199	2675.68	532.40	0.212	2005.67	424.52
A_68_P30000055	chr15:8395316-8395364	NM_027707:-877	Nipbl	PROMOTER	1.064	12.591	6665.76	83929.01	13.399	6273.59	84062.63
A_68_P27630331	chr11:20101120-20101164	NM_008996:-462	Rab1	PROMOTER	1.064	0.452	1283.69	580.12	0.481	1048.30	503.91
A_68_P25268784	chr7:87948360-87948404	NM_016721:-165	Iqgap1	PROMOTER	1.064	0.430	1608.55	691.25	0.457	1331.22	608.78
A_68_P24318694	chr6:28085042-28085086	NM_008174:-695	Grm8	PROMOTER	1.064	1.448	1929.74	2794.67	1.541	1398.37	2155.40
A_68_P24135896	chr5:139960379-139960423	NM_001159908:45	Zfand2a	INSIDE	1.064	0.430	2431.94	1044.55	0.457	1787.05	816.42
A_68_P21629723	chr2:130249901-130249945	NM_178762:102	Fam113a	INSIDE	1.064	0.438	1877.07	822.51	0.466	1465.58	683.50
A_68_P21571598	chr2:119625749-119625793	NM_019392:521	Tyro3	INSIDE	1.064	0.329	4737.05	1559.92	0.350	2971.58	1041.19
A_68_P30413820	chr15:87375020-87375064	ENSMUST00000169110:139		INSIDE	1.063	2.085	869.86	1813.24	2.215	706.51	1564.81
A_68_P29410629	chr14:13173646-13173690	NM_080433:4711	Fezf2	DOWNSTREAM	1.063	0.398	1250.13	497.89	0.423	1174.67	497.28
A_68_P29034740	chr13:51941294-51941338	NM_011817:-727	Gadd45g	PROMOTER	1.063	0.396	1625.64	644.53	0.421	1102.68	464.62
A_68_P28175793	chr11:118951344-118951390	NM_007625:-3815	Cbx4	PROMOTER	1.063	0.572	4043.12	2310.91	0.608	2558.23	1554.29
A_68_P27558729	chr11:4537823-4537867	NM_029291:49	Ascc2	INSIDE	1.063	0.501	1327.47	665.71	0.533	1154.04	615.04
A_68_P25271554	chr7:88489875-88489919	NM_009092:224	Rps17	INSIDE	1.063	1.517	994.81	1508.81	1.612	732.89	1181.07
A_68_P24656006	chr6:94233732-94233776	NM_001029850:144	Magi1	INSIDE	1.063	1.654	1095.27	1811.45	1.758	1005.68	1767.91
A_68_P24053678	chr5:123508053-123508097	ENSMUST00000119611:-8176		PROMOTER	1.063	0.167	3271.06	546.48	0.178	2120.38	376.41
A_68_P23417689	chr4:151654150-151654194	NM_178406:5702	Gpr153	INSIDE	1.063	2.013	571.40	1150.38	2.140	401.64	859.65
A_68_P31626772	chr18:37181762-37181806	NM_138663:1901	Pcdha12	INSIDE	1.062	2.195	2202.86	4834.38	2.330	1647.15	3837.23
A_68_P31472610	chr18:6240800-6240844	NM_008448:700	Kif5b	INSIDE	1.062	0.362	2039.64	738.43	0.384	1566.28	601.97
A_68_P30988899	chr16:95923500-95923544	NM_011809:-491	Ets2	PROMOTER	1.062	1.785	883.88	1577.42	1.894	732.35	1387.44
A_68_P30380066	chr15:81688707-81688754	NM_020507:26	Tob2	INSIDE	1.062	0.246	2154.97	529.81	0.261	1383.55	361.39

ProbeName	Target position of probe on CpG island microarray	TargetID	GeneSymbol	CpG island Description	Ratio of relative methylation (TiO_2-NP/Vehicle)	Sham group			TiO_2-H group		
						Relative methylation (Cy5/Cy3)	Cy3 signal (Input)	Cy5 signal (MeDIP)	Relative methylation (Cy5/Cy3)	Cy3 signal (Input)	Cy5 signal (MeDIP)
A_68_P28044023	chr11:96214672-96214716	NM_134032:1749	Hoxb2	INSIDE	1.062	2.100	1159.37	2435.22	2.231	905.56	2020.41
A_68_P26809635	chr9:107473258-107473302	NM_010489:1787	Hyal2	INSIDE	1.062	2.610	923.19	2409.92	2.772	717.80	1990.04
A_68_P26667549	chr9:79914528-79914572	NM_146003:-159	Senp6	PROMOTER	1.062	2.147	402.18	863.39	2.280	341.22	778.05
A_68_P26138085	chr8:108687070-108687114	NM_133792:12794	Pla2g15	INSIDE	1.062	1.713	1731.77	2967.17	1.819	1289.44	2345.39
A_68_P23912413	chr5:97821466-97821510	NM_001033191:-139	Naa11	PROMOTER	1.062	2.851	581.06	1656.82	3.027	427.64	1294.35
A_68_P21749118	chr2:152222671-152222715	NM_011438:1090	Sox12	INSIDE	1.062	0.539	2677.10	1442.53	0.572	2010.05	1149.91
A_68_P21485378	chr2:103601161-103601205	NM_153126:225	Nat10	INSIDE	1.062	0.263	3207.72	842.93	0.279	2171.14	605.86
A_68_P21076474	chr2:25977754-25977798	NM_026495:555	Nacc2	INSIDE	1.062	0.344	1332.28	458.82	0.366	1097.09	401.36
A_68_P20240341	chr1:55144942-55144986	NM_008303:-27	Hspe1	DIVERGENT_PROMOTER	1.062	0.492	1921.35	945.66	0.523	1551.98	810.94
A_68_P31038473	chr17:10512600-10512644	NR_037588:-161	B930003M22Rik	DIVERGENT_PROMOTER	1.061	2.022	602.85	1219.06	2.146	438.02	939.89
A_68_P30421087	chr15:88581789-88581833	NM_181412:-330	Zbed4	PROMOTER	1.061	1.707	661.39	1128.85	1.811	554.28	1004.07
A_68_P28170155	chr11:118108984-118109028	NM_001112699:900	Cyth1	INSIDE	1.061	0.452	1620.94	733.32	0.480	1071.26	514.40
A_68_P27286997	chr10:80493927-80493971	NM_134135:2709	Slc39a3	INSIDE	1.061	2.021	1871.16	3782.14	2.145	1562.61	3351.37
A_68_P25949911	chr8:72636783-72636827	NM_007789:7939	Ncan	INSIDE	1.061	0.300	2211.93	664.25	0.318	1758.44	560.04
A_68_P25011515	chr7:26503925-26503969	NM_028771:126	Ccdc97	INSIDE	1.061	0.232	2103.27	488.56	0.246	1644.41	405.09
A_68_P22518620	chr3:128902934-128902978	NM_001042504:115	Pitx2	INSIDE	1.061	0.434	2583.17	1120.84	0.461	2096.50	965.48
A_68_P32668456	chrX:130221722-130221766	NM_001105245:1788	Pcdh19	INSIDE	1.060	1.861	739.29	1376.04	1.973	864.36	1705.03
A_68_P32321831	chrX:34667243-34667287	NM_025937:507	Nkap	INSIDE	1.060	1.802	393.15	708.61	1.910	600.13	1146.20
A_68_P31932622	chr19:6085142-6085186	NM_026410:68	Cdca5	INSIDE	1.060	0.321	2536.18	813.51	0.340	1982.31	674.10
A_68_P31630976	chr18:38095246-38095290	NM_007858:-203	Diap1	PROMOTER	1.060	0.436	1045.36	455.57	0.462	881.79	407.35
A_68_P30544730	chr16:10835415-10835459	NM_001162932:285	A630055G03Rik	INSIDE	1.060	1.905	424.68	808.90	2.019	458.54	925.71
A_68_P27838142	chr11:59475681-59475726	NM_012027:-293	Mprip	PROMOTER	1.060	0.307	2862.37	879.14	0.325	2291.92	745.85
A_68_P27564592	chr11:5607482-5607526	NM_026080:198	Mrps24	INSIDE	1.060	4.475	794.23	3554.35	4.745	632.25	3000.08
A_68_P27289009	chr10:80806292-80806336	NM_148951:-303	Gipc3	PROMOTER	1.060	0.542	1960.43	1061.72	0.574	1542.95	886.10
A_68_P27184376	chr10:61596437-61596481	NM_009719:621	Neurog3	INSIDE	1.060	0.132	3948.45	519.62	0.140	2583.09	360.38
A_68_P25954898	chr8:73438225-73438269	NM_173013:8374	Mtap1s	INSIDE	1.060	1.743	786.50	1370.98	1.848	654.65	1209.78
A_68_P25087377	chr7:51684556-51684600	ENSMUST00000127790:191		INSIDE	1.060	0.446	2399.69	1071.41	0.473	1695.67	802.84
A_68_P24159039	chr5:144775595-144775639	NM_177682:106	Ccz1	INSIDE	1.060	0.297	2268.79	673.16	0.315	1516.50	477.01
A_68_P23961351	chr5:106886587-106886631	NM_001005477:577	Barhl2	INSIDE	1.060	4.270	5755.15	24573.08	4.527	4066.46	18409.85
A_68_P23777454	chr5:69732466-69732510	NM_175519:460	Kctd8	INSIDE	1.060	0.377	1307.82	492.56	0.399	1094.66	436.86
A_68_P23392782	chr4:147538404-147538448	NM_176848:3650	Fbxo2	INSIDE	1.060	1.726	1048.70	1809.63	1.829	883.37	1615.39
A_68_P21393859	chr2:84499121-84499165	NM_001145100:-93	Gm13718	DIVERGENT_PROMOTER	1.060	2.134	1525.97	3256.91	2.262	1303.41	2948.43
A_68_P20739566	chr1:157584400-157584444	NM_010712:4735	Lhx4	INSIDE	1.060	0.298	2382.02	710.69	0.316	1633.94	516.93
A_68_P20456743	chr1:95532366-95532410	NM_021537:-84	Stk25	PROMOTER	1.060	0.385	1552.76	597.09	0.407	1213.78	494.54
A_68_P20262086	chr1:59177080-59177125	NM_001033449:-162	Als2cr4	PROMOTER	1.060	0.409	2781.43	1137.68	0.433	2172.02	941.55
A_68_P32455383	chrX:69062125-69062169	NM_001081356:29	Vma21	INSIDE	1.059	1.569	679.19	1065.49	1.662	925.66	1538.35
A_68_P32290254	chrX:20425761-20425805	NM_001159645:114	Araf	INSIDE	1.059	1.727	1516.09	2618.63	1.829	2075.38	3796.62
A_68_P31442546	chr17:91487752-91487796	NM_020252:4368	Nrxn1	INSIDE	1.059	4.552	853.30	3884.42	4.821	711.30	3429.18
A_68_P31120337	chr17:28217277-28217321	NM_013687:231	Tcp11	INSIDE	1.059	0.370	4305.03	1593.64	0.392	3097.79	1214.07
A_68_P29338045	chr13:113778996-113779044	NM_001081062:1011	Ccno	INSIDE	1.059	3.420	811.14	2774.32	3.624	661.99	2398.79
A_68_P28156898	chr11:115885554-115885598	NM_008211:242	H3f3b	INSIDE	1.059	0.568	1945.35	1104.82	0.602	1440.44	866.68
A_68_P27897169	chr11:69615077-69615121	NM_010198:29	Fgf11	INSIDE	1.059	0.484	2157.60	1043.77	0.512	1586.74	812.97
A_68_P25948475	chr8:72405501-72405545	NM_026818:5769	Cilp2	INSIDE	1.059	2.662	528.47	1406.92	2.820	396.79	1118.85
A_68_P24648071	chr6:92893655-92893699	NR_015530:3101	9530026P05Rik	INSIDE	1.059	2.055	553.73	1137.81	2.176	551.84	1200.54
A_68_P24155679	chr5:144014938-144014982	NM_017467:16741	Zfp316	INSIDE	1.059	1.649	752.48	1241.20	1.747	670.08	1170.92
A_68_P24132971	chr5:139470887-139470931	NM_008808:-1	Pdgfa	PROMOTER	1.059	10.792	11443.00	123490.80	11.432	10853.40	124075.50
A_68_P23984845	chr5:111706231-111706275	NM_024477:397431	Ttc28	INSIDE	1.059	2.718	892.14	2424.95	2.878	695.79	2002.54
A_68_P23865427	chr5:87233816-87233860	NM_177680:324	Ythdc1	INSIDE	1.059	1.732	523.27	906.34	1.834	482.21	884.43
A_68_P23527252	chr5:21181527-21181571	NM_178728:25615	Napepld	INSIDE	1.059	1.949	1063.57	2073.24	2.064	729.43	1505.67
A_68_P22336395	chr3:93247993-93248037	NM_001163098:1763	Tchh	INSIDE	1.059	0.450	4476.29	2013.62	0.476	3550.11	1690.43
A_68_P21796148	chr2:160471963-160472007	NM_009408:352	Top1	INSIDE	1.059	0.281	2217.23	623.57	0.298	1651.08	491.53
A_68_P33003688	chr1_random:342766-342810	NM_177389:125	Mia3	INSIDE	1.058	0.404	3440.91	1390.83	0.428	2426.55	1037.76
A_68_P31939721	chr19:7497057-7497101	NM_175381:4964	2700081O15Rik	INSIDE	1.058	0.451	3538.58	1595.62	0.477	2459.77	1173.84
A_68_P31925432	chr19:4855210-4855254	NM_019861:104	Ctsf	INSIDE	1.058	0.410	1569.12	644.06	0.434	1161.53	504.26
A_68_P31207757	chr17:46728534-46728578	NM_175168:37897	Ptk7	INSIDE	1.058	2.033	1324.26	2691.64	2.150	1011.12	2173.68
A_68_P30633251	chr16:28929310-28929354	NM_177718:452	1600021P15Rik	INSIDE	1.058	1.741	1360.69	2369.11	1.843	1057.82	1949.22
A_68_P30588850	chr16:20672735-20672779	NM_001005331:-65	Eif4g1	PROMOTER	1.058	0.380	3470.55	1317.66	0.402	2485.24	998.20
A_68_P30347860	chr15:76199569-76199613	NM_021555:263	Fam203a	INSIDE	1.058	0.391	1356.78	530.37	0.414	1066.68	441.27
A_68_P28356088	chr12:36768830-36768874	NM_025359:213	Tspan13	INSIDE	1.058	0.154	3175.15	487.90	0.163	2256.95	366.97
A_68_P28155138	chr11:115626737-115626781	NM_028014:12	2310067B10Rik	INSIDE	1.058	0.362	1310.12	473.93	0.383	936.93	358.63
A_68_P27896369	chr11:69476075-69476120	NM_011900:47	Mpdu1	INSIDE	1.058	0.459	1531.00	702.79	0.486	1285.75	624.25
A_68_P27750956	chr11:43342693-43342737	NM_001045530:429	Ccnjl	INSIDE	1.058	0.334	2252.04	752.46	0.354	1772.04	626.48
A_68_P27261657	chr10:75837670-75837714	NM_008787:67965	Pcnt	INSIDE	1.058	2.921	859.15	2509.51	3.092	820.71	2537.43
A_68_P27171640	chr10:59414686-59414730	NM_029083:-190	Ddit4	PROMOTER	1.058	0.426	1909.88	813.61	0.451	1534.23	691.56
A_68_P26423570	chr9:35382219-35382263	NM_001167603:8	Gm6762	INSIDE	1.058	3.650	821.64	2998.70	3.863	634.80	2451.93

ProbeName	Target position of probe on CpG island microarray	TargetID	GeneSymbol	CpG island Description	Ratio of relative methylation (TiO_2-NP/Vehicle)	Sham group			TiO_2-H group		
						Relative methylation (Cy5/Cy3)	Cy3 signal (Input)	Cy5 signal (MeDIP)	Relative methylation (Cy5/Cy3)	Cy3 signal (Input)	Cy5 signal (MeDIP)
A_68_P23593766	chr5:34669863-34669907	NM_011278:-9154	Rnf4	PROMOTER	1.058	0.335	2857.24	958.01	0.355	2087.20	740.36
A_68_P23345552	chr4:137950860-137950904	NM_001081672:1491	Fam43b	INSIDE	1.058	0.472	1154.21	545.14	0.500	909.50	454.52
A_68_P21059044	chr2:22478107-22478151	NM_008078:282	Gad2	INSIDE	1.058	0.280	3162.53	884.56	0.296	2375.54	703.20
A_68_P20960553	chr2:3036013-3036057	NM_001081161:375	Fam171a1	INSIDE	1.058	2.170	688.42	1494.19	2.296	599.65	1376.65
A_68_P32403977	chrX:55292060-55292104	NM_009575:8278	Zic3	DOWNSTREAM	1.057	2.328	1433.02	3335.79	2.461	1968.56	4844.77
A_68_P31926781	chr19:5082721-5082765	NM_001001885:2735	Tmem151a	INSIDE	1.057	1.813	746.05	1352.68	1.917	563.47	1080.34
A_68_P31779043	chr18:65243488-65243532	NM_031881:60330	Nedd4l	INSIDE	1.057	2.331	1516.40	3533.99	2.463	1232.81	3036.01
A_68_P31400337	chr17:84105870-84105914	NM_001171053:390	Mta3	INSIDE	1.057	0.444	1094.69	486.31	0.469	790.83	371.17
A_68_P30244925	chr15:57743868-57743912	NM_001167679:137	Wdr67	INSIDE	1.057	0.329	3166.79	1041.47	0.347	2248.17	781.16
A_68_P28248078	chr12:13255679-13255723	NM_134040:280	Ddx1	INSIDE	1.057	0.434	1310.54	569.23	0.459	939.51	431.33
A_68_P28183252	chr11:120098764-120098808	NM_198423:4526	Bahcc1	INSIDE	1.057	1.627	873.99	1422.37	1.719	696.88	1198.22
A_68_P25007799	chr7:25790496-25790540	NM_144921:396	Atp1a3	INSIDE	1.057	0.242	4314.24	1045.27	0.256	3173.59	812.67
A_68_P20876660	chr1:182499047-182499091	NM_007415:-37	Parp1	PROMOTER	1.057	2.181	465.38	1015.02	2.305	380.82	877.82
A_68_P31655270	chr18:42554978-42555022	NM_138945:750	Pou4f3	INSIDE	1.056	0.464	1692.92	785.49	0.490	1297.67	635.52
A_68_P31412120	chr17:86087935-86087979	NM_011380:-362	Six2	PROMOTER	1.056	10.037	4122.90	41382.66	10.597	3521.03	37310.67
A_68_P31323856	chr17:69766821-69766865	NR_026848:1056	C030034I22Rik	INSIDE	1.056	0.452	2871.19	1297.47	0.477	2095.99	999.99
A_68_P30296518	chr15:67008847-67008891	NM_009177:-424	St3gal1	PROMOTER	1.056	0.401	3786.05	1518.45	0.424	2720.53	1152.68
A_68_P30127811	chr15:34236594-34236638	NM_016762:181	Matn2	INSIDE	1.056	0.489	3056.15	1494.07	0.516	2137.50	1103.96
A_68_P28524527	chr12:72237618-72237662	NM_001024853:22	Timm9	INSIDE	1.056	0.451	2342.07	1055.28	0.476	1756.84	836.02
A_68_P28048724	chr11:96976323-96976367	NM_019507:301	Tbx21	INSIDE	1.056	0.538	2065.34	1110.56	0.568	1377.82	782.47
A_68_P27804274	chr11:53163809-53163853	NM_033565:-438	Aff4	PROMOTER	1.056	0.466	1367.96	637.09	0.492	1098.32	540.08
A_68_P27649723	chr11:24064200-24064244	NM_001159290:83528	Bcl11a	INSIDE	1.056	2.837	1322.34	3751.89	2.996	1147.20	3436.79
A_68_P27416806	chr10:105012019-105012063	NR_038032:434	9330159K06	INSIDE	1.056	0.480	1270.98	610.60	0.507	970.82	492.52
A_68_P27277400	chr10:78996361-78996405	NM_015817:150	Ppap2c	INSIDE	1.056	0.592	10992.16	6502.02	0.625	6895.41	4308.36
A_68_P26844378	chr9:114293915-114293959	NM_019922:5894	Crtap	INSIDE	1.056	2.175	557.04	1211.49	2.296	519.73	1193.38
A_68_P25971361	chr8:77517617-77517661	NM_178017:37	Hmgxb4	INSIDE	1.056	0.431	1116.33	481.32	0.455	922.27	420.00
A_68_P25452560	chr7:124524755-124524799	NM_175645:284	Xylt1	INSIDE	1.056	3.283	979.99	3217.76	3.469	792.10	2747.65
A_68_P25196754	chr7:75097245-75097289	NM_010513:124	Igf1r	INSIDE	1.056	0.278	3364.79	933.82	0.293	2384.60	698.85
A_68_P23444292	chr5:3342226-3342270	NM_009873:-2063	Cdk6	PROMOTER	1.056	0.488	1524.85	744.65	0.516	1319.56	680.42
A_68_P23437243	chr4:154597315-154597359	NM_011385:-692	Ski	PROMOTER	1.056	0.393	1937.00	762.16	0.415	1427.04	592.69
A_68_P23364089	chr4:141001208-141001252	NM_009541:643	Zbtb17	INSIDE	1.056	1.668	1739.23	2901.36	1.762	1272.49	2242.26
A_68_P23223132	chr4:114729449-114729493	NM_011527:-2661	Tal1	PROMOTER	1.056	0.169	4549.89	769.24	0.178	3450.48	615.79
A_68_P21710711	chr2:144993855-144993899	ENSMUST00000153249:222		INSIDE	1.056	1.964	721.84	1417.51	2.074	495.36	1027.20
A_68_P32707836	chrX:138824951-138824995	NM_001033600:106	Acsl4	INSIDE	1.055	2.751	559.99	1540.81	2.903	701.51	2036.36
A_68_P30954380	chr16:90011810-90011858	ENSMUST00000089084:132180		INSIDE	1.055	2.062	398.81	822.38	2.175	361.37	785.92
A_68_P29746359	chr14:78936378-78936422	NM_001164503:267	Akap11	INSIDE	1.055	0.361	1797.76	648.36	0.380	1508.37	573.65
A_68_P28696318	chr12:104480308-104480352	NM_133364:26	Prima1	INSIDE	1.055	0.610	2158.92	1316.40	0.643	1657.14	1065.79
A_68_P28067417	chr11:100180968-100181012	NM_011508:-319	Eif1	PROMOTER	1.055	0.367	2430.29	892.04	0.387	1899.91	735.45
A_68_P28049002	chr11:97012100-97012144	NM_198100:-1096	Tbkbp1	PROMOTER	1.055	0.327	7129.49	2333.93	0.345	4725.36	1632.54
A_68_P24008169	chr5:115608781-115608825	NM_175403:-617	Mlec	PROMOTER	1.055	0.442	1170.64	517.82	0.467	984.15	459.26
A_68_P22129913	chr3:51288449-51288493	NM_016858:583	Rab33b	INSIDE	1.055	0.335	1717.44	575.82	0.354	1466.51	518.83
A_68_P21617373	chr2:127952615-127952659	NM_009754:863	Bcl2l11	INSIDE	1.055	0.408	1535.54	626.23	0.430	1161.22	499.56
A_68_P21094157	chr2:28773265-28773309	NM_001164186:-1326	Barhl1	PROMOTER	1.055	0.294	2597.09	762.54	0.310	1878.42	582.04
A_68_P32464972	chrX:71068859-71068903	NM_001029868:1428	Pdzd4	INSIDE	1.054	0.524	1340.87	703.00	0.553	1602.83	885.67
A_68_P32181858	chr19:53404585-53404629	NM_001008543:-329	Mxi1	PROMOTER	1.054	1.851	4359.74	8070.26	1.952	3297.05	6434.58
A_68_P31924143	chr19:4625643-4625687	NM_023131:-47	Rce1	PROMOTER	1.054	1.486	1990.78	2959.11	1.567	1643.64	2576.16
A_68_P31599397	chr18:31919152-31919196	NM_153515:-359	Ammecr1l	PROMOTER	1.054	0.468	2732.69	1278.39	0.493	2046.79	1009.62
A_68_P30574322	chr16:17530755-17530799	NM_026909:369	Thap7	INSIDE	1.054	0.521	2662.02	1385.94	0.549	1827.13	1002.79
A_68_P30351094	chr15:76734935-76734983	NM_012053:458	Rpl8	INSIDE	1.054	2.582	531.28	1371.88	2.722	444.26	1209.30
A_68_P29240038	chr13:96095444-96095488	NR_035461:-5068	Mir1940	PROMOTER	1.054	0.265	2182.34	579.03	0.280	1886.01	527.36
A_68_P28615064	chr12:88607770-88607814	NM_146036:115	Ahsa1	INSIDE	1.054	0.623	1669.81	1039.97	0.657	1362.78	894.92
A_68_P27898021	chr11:69749163-69749207	NM_016875:-216	Ybx2	PROMOTER	1.054	0.536	3492.13	1870.05	0.565	2558.80	1444.58
A_68_P27873632	chr11:65621027-65621071	NM_026107:303	Zkscan6	INSIDE	1.054	3.878	803.87	3117.46	4.086	683.48	2792.41
A_68_P26954150	chr10:13685112-13685158	NM_010437:-1050	Hivep2	PROMOTER	1.054	2.906	1389.29	4036.74	3.063	1166.58	3572.78
A_68_P26897437	A_68_P26897437			Unknown	1.054	0.483	1446.17	698.30	0.509	1125.45	572.73
A_68_P26814948	chr9:108354920-108354964	NM_133744:-7906	Ccdc71	PROMOTER	1.054	0.469	1517.93	711.22	0.494	1162.04	574.14
A_68_P26021285	chr8:87230722-87230773	NM_001081981:67521	Nfix	DOWNSTREAM	1.054	0.515	1690.10	870.41	0.543	1162.63	630.92
A_68_P25285286	chr7:91031153-91031197	NM_030705:1677	Mesdc1	INSIDE	1.054	2.180	866.76	1889.60	2.298	676.25	1554.37
A_68_P25032101	chr7:31337274-31337318	NM_183321:-5	BC053749	DIVERGENT_PROMOTER	1.054	0.547	2264.42	1239.13	0.577	1585.67	914.27
A_68_P24449045	chr6:52154649-52154693	NM_010453:-84	Hoxa5	PROMOTER	1.054	1.679	1034.30	1736.48	1.769	882.85	1562.05
A_68_P23278477	chr4:125826476-125826520	NM_172145:1252	Fam176b	INSIDE	1.054	0.543	1227.54	666.84	0.573	1124.44	643.84
A_68_P23265704	chr4:123668100-123668144			Unknown	1.054	0.315	1402.07	442.29	0.332	1091.09	362.78
A_68_P21776097	chr2:157104828-157104872	NM_019642:17	Rpn2	INSIDE	1.054	0.456	3463.45	1579.28	0.481	2697.78	1296.30
A_68_P21736749	chr2:149656814-149656858	NM_001085521:318	Tmem90b	INSIDE	1.054	0.481	1346.95	647.50	0.507	1077.83	546.20
A_68_P21203200	chr2:49474749-49474793	NM_008449:-63	Kif5c	PROMOTER	1.054	0.388	1252.49	485.99	0.409	1008.17	412.31

ProbeName	Target position of probe on CpG island microarray	TargetID	GeneSymbol	CpG island Description	Ratio of relative methylation (TiO_2-NP/Vehicle)	Sham group Relative methylation (Cy5/Cy3)	Sham group Cy3 signal (Input)	Sham group Cy5 signal (MeDIP)	TiO_2-H group Relative methylation (Cy5/Cy3)	TiO_2-H group Cy3 signal (Input)	TiO_2-H group Cy5 signal (MeDIP)
A_68_P31932456	chr19:6060898-6060957	NM_013859:-279	Znhit2-ps	PROMOTER	1.053	11.335	12903.78	146258.90	11.930	12436.61	148365.70
A_68_P31110054	chr17:26552202-26552246	NM_001081656:315	Neurl1b	INSIDE	1.053	6.910	1430.88	9887.10	7.279	1022.67	7443.67
A_68_P30348598	chr15:76308251-76308295	NM_008296:398	Hsf1	INSIDE	1.053	0.572	1847.48	1056.02	0.602	1453.74	875.07
A_68_P30348454	chr15:76288272-76288316	NM_198885:427	Scx	INSIDE	1.053	0.474	2259.15	1070.84	0.499	1668.90	832.74
A_68_P29385917	chr14:8624251-8624295	ENSMUST00000081331:-107		DIVERGENT_PROMOTER	1.053	2.190	1323.08	2898.02	2.306	1117.40	2577.06
A_68_P29050859	chr13:54795572-54795616	NR_027395:-273	Rnf44	DIVERGENT_PROMOTER	1.053	0.277	3303.62	915.62	0.292	2376.88	693.87
A_68_P27542784	chr10:128141723-128141767	NM_016756:363	Cdk2	INSIDE	1.053	0.367	1829.82	670.76	0.386	1576.60	608.47
A_68_P27278459	chr10:79180444-79180488	NM_008226:1088	Hcn2	INSIDE	1.053	0.357	1345.24	480.44	0.376	1140.91	428.90
A_68_P22322663	chr3:89764566-89764610	NM_001081182:2842	Atp8b2	INSIDE	1.053	2.248	617.83	1389.00	2.368	505.72	1197.53
A_68_P21822532	chr2:165058963-165059007	NM_174988:1253	Cdh22	INSIDE	1.053	0.506	2849.12	1442.04	0.533	2233.77	1190.60
A_68_P29001576	chr13:45485361-45485405	NM_181043:57	Mylip	INSIDE	1.052	1.937	1772.12	3431.99	2.037	1316.39	2681.69
A_68_P28917611	chr13:30637211-30637255	NM_025710:-47	Uqcrfs1	PROMOTER	1.052	0.332	1401.32	465.46	0.349	1120.83	391.65
A_68_P28598365	chr12:85791528-85791574	NM_134042:350	Aldh6a1	INSIDE	1.052	0.360	1392.25	500.73	0.378	946.81	358.26
A_68_P27882410	chr11:67112201-67112246	NM_177369:21598	Myh8	INSIDE	1.052	2.396	301.16	721.59	2.522	306.09	771.86
A_68_P27288888	chr10:80788031-80788075	NM_027381:4205	2510012J08Rik	INSIDE	1.052	2.365	1033.60	2444.79	2.487	866.02	2153.92
A_68_P26973141	chr10:17742602-17742646	NM_028440:434	3110003A17Rik	INSIDE	1.052	0.383	3356.74	1285.56	0.403	2301.82	927.18
A_68_P25655776	chr8:11478793-11478843	NM_011227:-319	Rab20	PROMOTER	1.052	5.066	3959.58	20060.63	5.328	2328.20	12404.53
A_68_P25095551	chr7:53121751-53121795	NM_008172:279	Grin2d	INSIDE	1.052	1.996	674.47	1346.54	2.101	571.04	1199.74
A_68_P24127041	chr5:138125324-138125368	NM_178162:499	Agfg2	INSIDE	1.052	0.527	2428.91	1279.11	0.554	1854.67	1027.71
A_68_P22373077	chr3:100884149-100884197	NM_011197:29917	Ptgfrn	INSIDE	1.052	0.495	1549.90	767.53	0.521	1092.56	569.10
A_68_P20616246	chr1:133423879-133423923	NM_001081011:38	Srgap2	INSIDE	1.052	0.105	6359.93	667.77	0.110	4263.79	470.97
A_68_P29237614	chr13:95655500-95655544	NM_011021:9941	Otp	DOWNSTREAM	1.051	3.429	287.95	987.44	3.603	224.97	810.50
A_68_P28740918	chr12:112127848-112127892	NM_001081057:397	Tecpr2	INSIDE	1.051	0.363	2180.09	790.41	0.381	1458.69	555.96
A_68_P27559369	chr11:4646659-4646703	NM_138948:101	Cabp7	INSIDE	1.051	0.523	1343.44	702.95	0.550	1026.94	564.96
A_68_P26818138	chr9:108976646-108976690	NM_172774:-30	Atrip	PROMOTER	1.051	0.279	2285.60	637.01	0.293	1535.26	449.67
A_68_P25996047	chr8:82235719-82235763	NM_026904:22	Anapc10	INSIDE	1.051	0.151	6019.94	911.02	0.159	3743.45	595.29
A_68_P24850991	chr6:131383858-131383902			Unknown	1.051	0.458	2592.61	1186.17	0.481	2082.47	1001.50
A_68_P24759852	chr6:113445089-113445133	NM_172487:6702	Prrt3	INSIDE	1.051	1.911	1326.85	2535.73	2.009	1063.98	2137.05
A_68_P21820159	chr2:164682781-164682825	NM_011125:406	Pltp	INSIDE	1.051	3.466	524.17	1816.53	3.643	404.10	1472.00
A_68_P31561529	chr18:24868269-24868313	NM_175276:345	Fhod3	INSIDE	1.050	1.527	934.10	1426.13	1.603	792.91	1270.65
A_68_P31540308	chr18:20716974-20717018	NM_007883:380	Dsg2	INSIDE	1.050	0.332	1651.66	547.84	0.348	1162.14	404.80
A_68_P31052339	chr17:12700068-12700112	NM_011395:480	Slc22a3	INSIDE	1.050	0.433	2018.42	873.58	0.454	1382.22	627.87
A_68_P30481497	chr15:99481812-99481856	NM_012025:218	Racgap1	INSIDE	1.050	0.424	7793.93	3303.31	0.445	5823.43	2592.14
A_68_P29733552	chr14:76816020-76816064	NM_207652:415	Tsc22d1	INSIDE	1.050	0.455	1961.47	893.01	0.478	1415.26	676.60
A_68_P25596369	chr7:149846534-149846578	NM_001122736:384	Igf2	INSIDE	1.050	0.384	1867.57	717.46	0.403	1474.67	594.97
A_68_P25578902	chr7:146768759-146768803	NR_027857:-84	Nkx6-2	PROMOTER	1.050	0.397	1284.05	509.95	0.417	1021.49	425.83
A_68_P24477003	chr6:56781975-56782019	NM_012056:-56	Fkbp9	PROMOTER	1.050	3.267	861.11	2813.19	3.430	698.74	2396.35
A_68_P24338523	chr6:31513635-31513679	NM_013723:281	Podxl	INSIDE	1.050	0.529	1454.84	769.90	0.555	1220.74	678.10
A_68_P22303234	chr3:86350689-86350733	NM_011839:1495	Mab21l2	INSIDE	1.050	0.242	3107.88	751.28	0.254	2288.37	580.72
A_68_P21334161	chr2:73730794-73730838	NM_001025093:-131	Atf2	PROMOTER	1.050	0.290	1559.25	452.04	0.305	1091.75	332.48
A_68_P21110488	chr2:31509792-31509836	NM_001123362:14258	Prdm12	INSIDE	1.050	0.329	3165.39	1040.87	0.345	2716.35	937.84
A_68_P21107212	chr2:30996536-30996580	NM_001038700:970	Fnbp1	INSIDE	1.050	0.321	1791.14	575.66	0.338	1301.59	439.36
A_68_P21004675	chr2:11093849-11093893	NM_008859:-138	Prkcq	PROMOTER	1.050	1.758	907.02	1594.38	1.845	675.25	1246.00
A_68_P32040193	chr19:27398062-27398106	NM_183179:976	Kcnv2	INSIDE	1.049	1.609	1947.47	3133.66	1.688	1600.87	2702.61
A_68_P31334607	chr17:71824822-71824866	NM_028887:-161	Smchd1	PROMOTER	1.049	1.638	1188.26	1945.85	1.718	969.58	1665.65
A_68_P29052703	chr13:55097861-55097905	NM_153131:47090	Unc5a	INSIDE	1.049	1.733	952.52	1651.03	1.819	807.31	1468.19
A_68_P28587835	chr12:83718196-83718240	NM_015812:194	Rgs6	INSIDE	1.049	0.438	1773.81	776.77	0.459	1511.61	694.49
A_68_P27473556	chr10:115550730-115550774	NM_001161855:221	4933416C03Rik	INSIDE	1.049	2.139	577.24	1234.85	2.244	502.42	1127.29
A_68_P26845381	chr9:114473232-114473276	NM_001042503:233	Trim71	INSIDE	1.049	9.185	657.33	6037.71	9.634	612.52	5901.16
A_68_P26421596	chr9:35075089-35075133	NM_194257:242	Fam118b	INSIDE	1.049	0.490	1056.19	517.59	0.514	827.51	425.32
A_68_P25954407	chr8:73363491-73363535	NM_027560:107	Arrdc2	INSIDE	1.049	0.188	4530.78	853.53	0.198	2614.60	516.52
A_68_P25409556	chr7:116847071-116847115	NM_020616:-158	D930014E17Rik	PROMOTER	1.049	0.308	2814.73	866.47	0.323	2032.70	656.29
A_68_P25046332	chr7:35697322-35697366	NM_008820:-81	Pepd	PROMOTER	1.049	4.446	3266.03	14520.50	4.663	2500.78	11661.35
A_68_P21113256	chr2:31944358-31944408	NM_175511:-5157	Fam78a	DIVERGENT_PROMOTER	1.049	0.271	1721.61	467.14	0.285	1237.71	352.20
A_68_P31933635	chr19:6276972-6277016	NM_010119:99	Ehd1	INSIDE	1.048	1.676	1345.94	2256.06	1.757	1027.64	1805.41
A_68_P29982087	chr15:5066180-5066224	NM_026069:-410	Rpl37	DIVERGENT_PROMOTER	1.048	0.433	1790.28	775.42	0.454	1173.36	532.59
A_68_P27278227	chr10:79144709-79144753	NM_177613:-209	Cdc34	PROMOTER	1.048	0.488	2278.94	1111.44	0.511	1770.89	905.01
A_68_P25262740	chr7:86860262-86860307	NM_010626:-1212	Kif7	PROMOTER	1.048	0.554	1968.17	1090.08	0.581	1534.69	891.10
A_68_P23588237	chr5:33678644-33678688	NM_021500:446	Maea	INSIDE	1.048	3.096	1524.85	4720.51	3.245	1244.89	4039.92
A_68_P22550732	chr3:134875583-134875627	NM_173762:78	Cenpe	INSIDE	1.048	0.471	1188.96	560.26	0.494	955.24	471.74
A_68_P21747903	chr2:151969066-151969110	NM_009328:-256	Tcf15	PROMOTER	1.048	0.452	1183.00	535.29	0.474	906.03	429.77
A_68_P21666834	chr2:136940556-136940600	NM_013822:1678	Jag1	INSIDE	1.048	0.571	2187.26	1248.24	0.598	1651.17	987.26
A_68_P21061191	chr2:22895186-22895230	NM_001077190:552	Abi1	INSIDE	1.048	0.335	1498.10	502.49	0.351	1302.17	457.69
A_68_P33007144	chr9_random:49674-49720	NR_015516:-3712	4930526I15Rik	PROMOTER	1.047	26.196	545.09	14278.92	27.434	313.56	8602.31
A_68_P30425290	chr15:89288093-89288138	NM_021921:3774	Mapk8ip2	INSIDE	1.047	1.620	1150.92	1864.06	1.695	950.05	1610.33

ProbeName	Target position of probe on CpG island microarray	TargetID	GeneSymbol	CpG island Description	Ratio of relative methylation (TiO_2-NP/Vehicle)	Sham group			TiO_2-H group		
						Relative methylation (Cy5/Cy3)	Cy3 signal (Input)	Cy5 signal (MeDIP)	Relative methylation (Cy5/Cy3)	Cy3 signal (Input)	Cy5 signal (MeDIP)
A_68_P29075361	chr13:58909630-58909674	NM_001025074:459	Ntrk2	INSIDE	1.047	0.282	1774.52	500.73	0.296	1398.04	413.14
A_68_P28925106	chr13:31899841-31899885	NM_008592:1348	Foxc1	INSIDE	1.047	1.630	7676.40	12512.61	1.707	6285.91	10729.88
A_68_P27568394	chr11:6315574-6315618	NM_008907:-276	Ppia	PROMOTER	1.047	0.153	3425.99	524.85	0.160	2460.98	394.92
A_68_P26876196	chr9:120036691-120036735	NM_011029:-171	Rpsa	PROMOTER	1.047	1.821	461.71	840.95	1.907	506.44	965.83
A_68_P26084863	chr8:98240210-98240254	NM_001035123:420	Setd6	INSIDE	1.047	0.331	2006.21	663.65	0.346	1425.74	493.86
A_68_P25411817	chr7:117265589-117265633	NM_009516:38	Wee1	INSIDE	1.047	0.295	2179.41	643.56	0.309	1556.60	481.08
A_68_P24942895	chr6:148894085-148894129	NM_019643:848	Fam60a	INSIDE	1.047	0.526	1004.15	527.68	0.550	909.37	500.48
A_68_P24543641	chr6:72739120-72739164	NM_001079822:-192	Tcf7l1	PROMOTER	1.047	0.400	3632.65	1451.47	0.418	2528.89	1057.66
A_68_P24222133	chr6:7643105-7643149	NM_012055:56	Asns	INSIDE	1.047	0.618	1628.51	1007.08	0.648	1314.69	851.45
A_68_P24055345	chr5:123821191-123821247	NM_029850:26762	Bcl7a	INSIDE	1.047	1.814	591.32	1072.89	1.899	511.42	971.42
A_68_P23561108	chr5:28492702-28492746	NM_010134:489	En2	INSIDE	1.047	0.499	1298.59	648.53	0.523	1068.12	558.51
A_68_P31317443	chr17:68623323-68623367	NM_177278:208	L3mbtl4	INSIDE	1.046	0.487	2197.81	1070.46	0.510	1604.63	817.80
A_68_P31100173	chr17:25033768-25033812	NM_019730:346	Nme3	INSIDE	1.046	0.398	1681.94	669.54	0.417	1217.75	507.25
A_68_P30961703	chr16:91227699-91227743	NM_016967:1926	Olig2	INSIDE	1.046	0.542	3383.85	1834.96	0.567	2477.53	1405.40
A_68_P30956056	chr16:90283863-90283907	NM_178923:786	Scaf4	INSIDE	1.046	1.890	1116.02	2108.88	1.977	949.03	1876.01
A_68_P29689304	chr14:68742856-68742900	NM_008691:183	Nefm	INSIDE	1.046	0.639	1907.91	1219.60	0.669	1668.32	1115.36
A_68_P27894483	chr11:69160115-69160159	NM_146019:22792	Chd3	INSIDE	1.046	1.731	525.65	910.08	1.811	500.42	906.42
A_68_P25956626	chr8:73904570-73904614	NM_010150:1259	Nr2f6	INSIDE	1.046	2.431	815.18	1981.98	2.543	649.32	1651.22
A_68_P25574422	chr7:146038351-146038395	NM_026391:304	Ppp2r2d	INSIDE	1.046	0.674	3842.92	2591.89	0.706	2962.56	2090.64
A_68_P24459343	chr6:53769427-53769471	NM_025817:1371	Tril	INSIDE	1.046	0.307	1827.17	561.67	0.321	1545.89	496.89
A_68_P23270359	chr4:124479600-124479644	NM_008636:-170	Mtf1	PROMOTER	1.046	1.450	1238.41	1795.20	1.516	1116.10	1692.25
A_68_P22880596	chr4:45024872-45024916	NM_001163283:390	Zbtb5	INSIDE	1.046	0.650	2974.93	1932.87	0.679	2174.76	1477.52
A_68_P22321219	chr3:89519590-89519634	NM_001038587:669	Adar	INSIDE	1.046	0.503	5965.23	3002.12	0.526	4257.31	2240.89
A_68_P21436189	chr2:93487479-93487523	NM_007442:4910	Alx4	INSIDE	1.046	1.455	2888.44	4202.76	1.522	2303.53	3504.97
A_68_P31106382	chr17:25945536-25945580	NM_001164225:-471	Fbxl16	PROMOTER	1.045	0.208	2687.99	558.74	0.217	1871.05	406.59
A_68_P30970946	chr16:92697084-92697128	NM_001111023:467	Runx1	INSIDE	1.045	0.548	1403.81	769.21	0.572	1073.70	614.58
A_68_P29668499	chr14:65206535-65206579	NR_029813:-2937	Mir124a-1	PROMOTER	1.045	0.109	5036.82	547.12	0.114	3794.88	430.79
A_68_P29102444	chr13:65342538-65342582			Unknown	1.045	0.426	1556.79	662.93	0.445	1201.03	534.34
A_68_P27033724	chr10:28916766-28916810	NM_026138:52987	6330407J23Rik	INSIDE	1.045	1.937	1990.19	3854.35	2.024	1485.80	3006.65
A_68_P27003030	chr10:23069823-23069869	NM_010167:-137	Eya4	PROMOTER	1.045	4.050	512.07	2073.66	4.231	481.51	2037.32
A_68_P26388504	chr9:29219516-29219560	NM_172290:551176	Ntm	INSIDE	1.045	0.504	1936.52	976.10	0.527	1450.96	764.40
A_68_P26203093	chr8:120022311-120022355	NM_172285:142	Plcg2	INSIDE	1.045	0.325	1517.28	493.25	0.340	1190.61	404.65
A_68_P25422111	chr7:119166689-119166733	NM_133758:-309	Usp47	PROMOTER	1.045	0.549	1495.47	820.76	0.574	1283.57	736.15
A_68_P24979911	chr7:16730509-16730553	NM_148946:14882	Slc8a2	INSIDE	1.045	1.385	1481.72	2051.87	1.447	1234.62	1786.14
A_68_P24049902	chr5:122804456-122804500	NM_019780:57	Vps29	INSIDE	1.045	0.626	2934.58	1837.27	0.655	2108.90	1380.32
A_68_P23542950	chr5:24104639-24104683	NM_025891:3160	Smarcd3	INSIDE	1.045	0.297	3777.66	1122.40	0.311	2484.45	771.46
A_68_P22481651	chr3:121996059-121996103	NR_030439:541	Mir760	DOWNSTREAM	1.045	3.008	680.46	2046.57	3.142	493.29	1549.94
A_68_P21867217	chr2:172847468-172847512	NM_019547:88	Rbm38	INSIDE	1.045	0.637	4526.57	2885.17	0.666	3339.60	2223.39
A_68_P21425690	chr2:91804013-91804057	NM_138306:-314	Dgkz	PROMOTER	1.045	0.607	1897.28	1151.09	0.634	1345.16	852.81
A_68_P20802626	chr1:168930754-168930798	NM_001160261:-771	Fam78b	DIVERGENT_PROMOTER	1.045	0.285	4622.37	1315.89	0.298	3650.64	1086.25
A_68_P32743940	chrX:147482252-147482296	NM_008001:499	Fgd1	INSIDE	1.044	3.471	397.38	1379.11	3.625	439.72	1593.94
A_68_P32145760	chr19:46836584-46836628	NM_001102471:508	Cnnm2	INSIDE	1.044	0.496	2655.22	1318.26	0.518	1702.01	881.86
A_68_P31863856	chr18:80560394-80560438	NM_001190373:577	Kcng2	INSIDE	1.044	2.150	909.41	1955.11	2.245	838.05	1881.65
A_68_P31425154	chr17:88197235-88197279	NM_199251:78	Kcnk12	INSIDE	1.044	0.292	1970.44	575.68	0.305	1607.53	490.33
A_68_P31306706	chr17:66869156-66869200	NM_024448:-168	Rab12	PROMOTER	1.044	0.354	2436.75	862.78	0.370	1854.29	685.54
A_68_P31117100	chr17:27692460-27692504	NM_001025427:-1036	Hmga1	PROMOTER	1.044	0.618	1841.47	1137.47	0.645	1445.91	932.39
A_68_P30585934	chr16:20097708-20097752	NM_029436:104	Klhl24	INSIDE	1.044	2.883	831.37	2396.95	3.009	646.09	1944.30
A_68_P27949791	chr11:79404266-79404312	NM_175543:-425	Rab11fip4	PROMOTER	1.044	1.580	969.43	1531.52	1.650	746.93	1232.37
A_68_P27302609	A_68_P27302609			Unknown	1.044	1.829	926.12	1694.15	1.911	700.14	1337.73
A_68_P26772684	chr9:100977961-100978005	NM_001100451:-468	Msl2	PROMOTER	1.044	0.652	4309.42	2808.43	0.680	2887.70	1964.41
A_68_P25524902	chr7:138009122-138009166	NM_133942:-279	Plekha1	PROMOTER	1.044	1.732	597.84	1035.76	1.808	526.00	951.22
A_68_P23540260	chr5:23670805-23670849	NM_153092:46	Nupl2	INSIDE	1.044	0.530	1320.41	700.33	0.554	1056.49	585.18
A_68_P23397714	chr4:148326567-148326611	NM_001159344:148088	Caszl	INSIDE	1.044	0.147	5247.25	772.95	0.154	3543.21	544.88
A_68_P32020395	chr19:23967551-23967595	NM_177034:134207	Apba1	INSIDE	1.043	2.510	1164.33	2922.39	2.618	945.19	2474.25
A_68_P30348905	chr15:76353796-76353840	NM_130893:-1259	Scrt1	PROMOTER	1.043	0.646	2808.74	1815.41	0.674	2154.59	1452.56
A_68_P29092064	chr13:63341467-63341511	NM_028079:225195	2010111I01Rik	INSIDE	1.043	2.141	3939.04	8433.08	2.233	2893.35	6461.08
A_68_P27843676	chr11:60352657-60352701	NM_172943:1494	Alkbh5	INSIDE	1.043	2.208	1290.97	2849.84	2.302	955.32	2199.48
A_68_P27623990	chr11:18918980-18919024	NM_001193271:-30	Meis1	PROMOTER	1.043	0.453	1059.42	480.30	0.473	761.84	360.29
A_68_P26887824	chr9:122076106-122076150	NM_001164572:49745	Snrk	INSIDE	1.043	1.694	2290.55	3879.77	1.766	1667.70	2945.56
A_68_P26477006	chr9:45762946-45762990	NM_172257:364	Sidt2	INSIDE	1.043	2.091	1113.08	2327.61	2.181	871.18	1899.97
A_68_P26313786	chr9:13632306-13632350	NM_176836:158	Fam76b	INSIDE	1.043	0.402	2931.39	1178.97	0.420	2155.36	904.24
A_68_P25588514	chr7:148577796-148577840	NM_011528:-240	Taldo1	PROMOTER	1.043	1.764	1182.22	2085.08	1.839	927.80	1706.38
A_68_P24056049	chr5:123958827-123958871	NM_198611:-1620	B3gnt4	PROMOTER	1.043	0.381	2624.66	1000.18	0.397	1812.46	720.10
A_68_P23541736	chr5:23931307-23931351	NM_009207:279	Slc4a2	INSIDE	1.043	0.455	2116.35	962.58	0.474	1778.63	843.52
A_68_P23437421	chr4:154624224-154624268	NM_001190445:172	2610002J02Rik	INSIDE	1.043	0.290	3543.97	1028.67	0.303	2782.09	842.27

ProbeName	Target position of probe on CpG island microarray	TargetID	GeneSymbol	CpG island Description	Ratio of relative methylation (TiO_2-NP/Vehicle)	Sham group			TiO_2-H group		
						Relative methylation (Cy5/Cy3)	Cy3 signal (Input)	Cy5 signal (MeDIP)	Relative methylation (Cy5/Cy3)	Cy3 signal (Input)	Cy5 signal (MeDIP)
A_68_P21334259	chr2:73748831-73748875	NM_175015:499	Atp5g3	INSIDE	1.043	0.194	2299.96	445.18	0.202	1625.84	328.13
A_68_P32227000	chr19:60832730-60832774	NM_178421:863	Nanos1	INSIDE	1.042	1.793	2551.15	4574.86	1.869	1819.55	3401.17
A_68_P32134051	chr19:44808722-44808771			Unknown	1.042	2.016	778.16	1569.10	2.102	494.69	1039.64
A_68_P31112724	chr17:26978218-26978262	NM_008700:270	Nkx2-5	INSIDE	1.042	0.305	2495.77	761.65	0.318	1926.59	612.51
A_68_P30575793	chr16:17804490-17804534	NM_153790:7138	Scarf2	INSIDE	1.042	0.427	1942.08	828.34	0.444	1473.27	654.81
A_68_P30546008	chr16:11066855-11066899	NM_009223:486	Snn	INSIDE	1.042	0.218	3383.84	737.86	0.227	2406.37	546.55
A_68_P30486489	chr15:100408980-100409024	NM_010127:7794	Pou6f1	INSIDE	1.042	1.869	445.77	833.00	1.946	469.97	914.71
A_68_P28529997	chr12:73171347-73171391	NM_181752:610	Gpr135	INSIDE	1.042	2.221	1758.25	3904.92	2.315	1460.06	3379.45
A_68_P27805840	chr11:53449647-53449691	NM_008355:-1464	Il13	PROMOTER	1.042	0.372	1573.75	585.33	0.388	1238.19	479.84
A_68_P27278589	chr10:79197977-79198021	NM_008226:18620	Hcn2	INSIDE	1.042	1.772	708.62	1255.58	1.846	769.34	1420.18
A_68_P26659608	chr9:78291066-78291110	NM_173386:-134	E330016A19Rik	DIVERGENT_PROMOTER	1.042	0.436	1510.15	658.25	0.454	1185.01	538.27
A_68_P26531866	chr9:55389590-55389634	NM_027397:657	Isl2	INSIDE	1.042	0.426	1586.97	675.72	0.444	1169.56	518.71
A_68_P26528578	chr9:54798480-54798524	NM_011966:-163	Psma4	PROMOTER	1.042	0.316	1835.45	580.35	0.330	1323.42	436.19
A_68_P25994476	chr8:81922795-81922839	NM_008539:551	Smad1	INSIDE	1.042	11.888	2886.25	34312.40	12.384	2821.65	34943.46
A_68_P25279939	chr7:90014474-90014518	NM_175366:-1346	Mex3b	PROMOTER	1.042	0.239	3574.28	853.88	0.249	2642.30	657.73
A_68_P23649331	chr5:44491778-44491823	NM_001163584:-5793	Prom1	PROMOTER	1.042	0.205	2695.96	553.11	0.214	1948.82	416.61
A_68_P23309920	chr4:131604337-131604381	NM_001128606:635	Epb4.1	INSIDE	1.042	0.458	2119.44	970.71	0.477	1718.81	820.05
A_68_P22185882	chr3:61963377-61963421			Unknown	1.042	2.727	756.70	2063.20	2.842	646.26	1836.66
A_68_P21113209	chr2:31939133-31939177	NM_175511:71	Fam78a	INSIDE	1.042	1.597	1657.26	2646.37	1.663	1320.37	2195.95
A_68_P20616239	chr1:133423080-133423124	NM_001081011:836	Srgap2	INSIDE	1.042	1.911	1355.10	2590.15	1.991	1007.07	2005.21
A_68_P20578314	chr1:125942313-125942357	NM_199021:-198	Dpp10	PROMOTER	1.042	0.290	1894.60	549.16	0.302	1352.07	408.38
A_68_P31108132	chr17:26206355-26206399	NM_001162868:-254	Rab11fip3	PROMOTER	1.041	1.924	1489.33	2865.41	2.002	1144.67	2291.53
A_68_P31097571	chr17:24615950-24615994	NM_019988:51	Mlst8	INSIDE	1.041	0.428	1717.88	734.41	0.445	1122.94	499.87
A_68_P29477489	chr14:26279042-26279086	NM_183208:394	Zmiz1	INSIDE	1.041	0.597	1605.46	958.01	0.621	1261.01	783.22
A_68_P28774236	chr12:118772882-118772926	NM_175930:17953	Rapgef5	INSIDE	1.041	1.743	866.48	1510.28	1.815	770.94	1399.33
A_68_P28052461	chr11:97605654-97605698	NM_054051:342	Pip4k2b	INSIDE	1.041	1.529	5683.61	8691.52	1.592	4906.11	7812.92
A_68_P27903592	chr11:70783452-70783496	NM_001083331:-10	Nup88	PROMOTER	1.041	2.950	367.83	1085.01	3.070	293.93	902.23
A_68_P27836981	chr11:59263741-59263785	NM_001205068:216	Jmjd4	INSIDE	1.041	0.355	2613.27	927.52	0.369	1689.23	623.96
A_68_P27282555	chr10:79798362-79798406	NM_001204931:5495	Reep6	INSIDE	1.041	1.915	4421.10	8466.80	1.994	3044.94	6070.69
A_68_P25542185	chr7:140900700-140900744	NM_009479:256	Uros	INSIDE	1.041	0.476	1068.94	509.24	0.496	891.04	441.87
A_68_P24864240	chr6:134871160-134871204	NM_009875:764	Cdkn1b	INSIDE	1.041	2.260	3547.91	8016.68	2.351	2813.73	6615.26
A_68_P24766250	chr6:114593146-114593190	NM_028835:26	Atg7	INSIDE	1.041	0.416	1359.39	565.72	0.433	1139.62	493.67
A_68_P23595118	chr5:34916324-34916368	NM_001024458:-16	Add1	PROMOTER	1.041	0.576	2121.98	1222.89	0.600	1416.83	850.33
A_68_P23367600	chr4:141640361-141640405	NM_145402:-163	Tmem51	PROMOTER	1.041	0.379	2510.68	951.16	0.395	1922.80	758.65
A_68_P21812069	chr2:163201531-163201575	NM_021566:22157	Jph2	INSIDE	1.041	2.619	888.46	2326.95	2.728	782.18	2133.55
A_68_P30421911	chr15:88721121-88721165	ENSMUST00000172019:63706		INSIDE	1.040	0.425	1515.89	643.78	0.442	1265.94	559.20
A_68_P28049001	chr11:97012004-97012048	NM_198100:-1000	Tbkbp1	PROMOTER	1.040	0.515	2691.09	1385.05	0.535	2051.48	1098.37
A_68_P26161392	chr8:112741955-112741999	NM_146215:117	Ftsjd1	INSIDE	1.040	0.344	3226.83	1109.47	0.357	2498.53	893.01
A_68_P25776085	chr8:35870933-35870977	NM_176933:291	Dusp4	INSIDE	1.040	0.447	1080.98	483.08	0.465	906.66	421.44
A_68_P23692827	chr5:52506449-52506493	AK076709:-2081		PROMOTER	1.040	0.258	2295.15	593.14	0.269	1790.88	481.43
A_68_P23575215	chr5:31191120-31191164	NM_174849:-82	Agbl5	PROMOTER	1.040	3.708	605.64	2245.76	3.857	441.70	1703.68
A_68_P23428418	chr4:153386065-153386109	NM_201226:175	Lrrc47	INSIDE	1.040	5.105	610.93	3118.70	5.307	554.20	2941.08
A_68_P22829977	chr4:34136995-34137039	NM_026293:26	Spaca1	INSIDE	1.040	2.128	819.28	1743.06	2.213	720.10	1593.45
A_68_P21840495	chr2:168106873-168106917	ENSMUST00000109191:-262		PROMOTER	1.040	0.540	2279.57	1229.86	0.561	1787.55	1002.56
A_68_P20012423	chr1:6373229-6373273	NM_001195732:23839	Fam150a	INSIDE	1.040	3.623	1031.14	3735.41	3.768	903.42	3404.31
A_68_P31620316	chr18:35991443-35991487	NM_133687:1993	Cxxc5	INSIDE	1.039	1.904	1281.20	2438.90	1.977	931.65	1841.97
A_68_P28748134	chr12:113382997-113383048	NM_001097621:-1396	Kif26a	PROMOTER	1.039	1.381	8606.37	11884.26	1.435	5015.70	7197.97
A_68_P27365024	chr10:94877896-94877941	NM_001168657:-1	Socs2	PROMOTER	1.039	0.409	3318.18	1357.71	0.425	2172.92	923.69
A_68_P26554632	chr9:59504864-59504908	NM_011099:472	Pkm2	INSIDE	1.039	0.187	5639.21	1052.71	0.194	4118.33	799.11
A_68_P24118746	chr5:136438629-136438673	NM_001160366:11696	Srcrb4d	INSIDE	1.039	0.403	2718.81	1096.34	0.419	2243.34	940.21
A_68_P24008162	chr5:115607960-115608004	NM_175403:203	Mlec	INSIDE	1.039	1.616	4811.70	7773.73	1.679	4410.25	7405.57
A_68_P21495965	chr2:105516343-105516387	NM_013627:-237	Pax6	DIVERGENT_PROMOTER	1.039	2.327	765.59	1781.78	2.419	635.90	1538.33
A_68_P32557243	chrX:97015613-97015657	NM_001081283:-773	Tmem28	PROMOTER	1.038	4.617	558.37	2577.83	4.791	641.94	3075.36
A_68_P32241076	chrX:7525138-7525182	NM_001130416:-145	Hdac6	PROMOTER	1.038	2.205	576.30	1270.55	2.287	764.24	1748.10
A_68_P31629941	chr18:37926527-37926571	NM_033595:1315	Pcdhga12	INSIDE	1.038	2.106	876.25	1845.60	2.187	750.87	1642.13
A_68_P29856055	chr14:102039383-102039427	NM_001033132:284	Commd6	INSIDE	1.038	3.135	2078.23	6514.56	3.253	2067.54	6726.38
A_68_P29518747	chr14:33412582-33412626	NM_001001796:-508	Prrxl1	PROMOTER	1.038	0.480	1320.66	633.77	0.498	891.96	444.43
A_68_P28189611	chr11:121121856-121121900	NM_001080932:578	Foxk2	INSIDE	1.038	0.448	1176.37	527.15	0.465	935.56	435.26
A_68_P28159846	chr11:116392786-116392830	NM_001172475:-416	Sphk1	PROMOTER	1.038	0.299	2501.99	748.72	0.311	1801.25	559.75
A_68_P26459756	chr9:42924615-42924659	NM_001034863:17	Tmem136	INSIDE	1.038	2.213	1096.79	2427.03	2.296	811.79	1864.24
A_68_P26345841	chr9:20986277-20986321	NM_183408:16141	Pde4a	INSIDE	1.038	0.477	1687.72	804.85	0.495	1241.78	614.43
A_68_P25828114	chr8:46129885-46129933	NM_001081286:94347	Fat1	INSIDE	1.038	2.521	386.40	974.14	2.618	281.54	737.04
A_68_P23293015	chr4:128444743-128444787	NM_001009819:8263	A3galt2	INSIDE	1.038	1.851	904.74	1674.96	1.922	682.43	1311.36
A_68_P30423732	chr15:89011344-89011388	NM_138749:-148	Plxnb2	PROMOTER	1.037	0.131	5159.29	674.79	0.136	3196.00	433.46
A_68_P29941246	chr14:119254241-119254285	NM_001160099:91	Cldn10	INSIDE	1.037	0.453	1610.04	728.76	0.469	1256.77	589.66

ProbeName	Target position of probe on CpG island microarray	TargetID	GeneSymbol	CpG island Description	Ratio of relative methylation (TiO_2-NP/Vehicle)	Sham group			TiO_2-H group		
						Relative methylation (Cy5/Cy3)	Cy3 signal (Input)	Cy5 signal (MeDIP)	Relative methylation (Cy5/Cy3)	Cy3 signal (Input)	Cy5 signal (MeDIP)
A_68_P28936499	chr13:34169593-34169637	NM_009450:263	Tubb2a	INSIDE	1.037	0.398	3592.32	1428.02	0.412	2779.76	1146.02
A_68_P28727040	chr12:109513867-109513911	NM_001159910:-261	Ccdc85c	PROMOTER	1.037	0.400	2880.88	1152.89	0.415	2033.99	843.91
A_68_P27835482	chr11:59022044-59022088	NM_024210:204	2310033P09Rik	INSIDE	1.037	0.529	1961.65	1037.09	0.548	1495.40	819.92
A_68_P27263123	chr10:76077247-76077291	NM_146007:8881	Col6a2	INSIDE	1.037	2.164	1905.31	4122.37	2.243	1593.12	3572.71
A_68_P26584122	chr9:64659329-64659373	NM_001162917:533	Dennd4a	INSIDE	1.037	0.192	4700.35	903.87	0.199	3223.60	643.03
A_68_P25958306	chr8:74210008-74210052	NM_001190830:9749	Jak3	INSIDE	1.037	0.432	1177.83	509.07	0.448	864.03	387.16
A_68_P25501810	chr7:133839074-133839118	NM_029420:201	Slx1b	INSIDE	1.037	0.367	2119.67	778.09	0.381	1456.26	554.14
A_68_P25022022	chr7:29244663-29244707	NM_001141921:7428	Lrfn1	INSIDE	1.037	2.642	656.02	1732.96	2.739	556.65	1524.83
A_68_P24760554	chr6:113574432-113574476	NM_009507:440	Vhl	INSIDE	1.037	0.348	2489.27	866.98	0.361	1781.44	643.23
A_68_P24054067	chr5:123582167-123582211	NM_175092:450	Rhof	INSIDE	1.037	0.385	1813.90	698.21	0.399	1228.65	490.38
A_68_P24034095	chr5:120132994-120133040	NM_011535:12339	Tbx3	INSIDE	1.037	0.455	1132.47	515.78	0.472	825.07	389.64
A_68_P22970879	chr4:63156617-63156661	NM_001008791:347	Whrn	INSIDE	1.037	0.463	2663.96	1233.64	0.480	2098.93	1008.31
A_68_P21567191	chr2:118798466-118798510	AK184532:45580		INSIDE	1.037	0.202	2353.99	474.50	0.209	1828.32	382.02
A_68_P21212082	chr2:51003647-51003691	NM_028810:963	Rnd3	INSIDE	1.037	0.571	1548.53	884.90	0.593	1181.94	700.72
A_68_P20254931	chr1:57831531-57831575	NM_144882:-152	Spats2l	PROMOTER	1.037	0.168	3201.53	539.01	0.175	2343.07	409.17
A_68_P20148454	chr1:36528308-36528352	NM_033570:-111	Cnnm4	PROMOTER	1.037	0.521	1371.41	714.09	0.540	1112.17	600.24
A_68_P20002770	chr1:3661760-3661804	NM_001011874:-203	Xkr4	PROMOTER	1.037	2.083	697.37	1452.81	2.160	636.31	1374.40
A_68_P31614612	chr18:34936814-34936858	NM_001081256:175	Kdm3b	INSIDE	1.036	0.508	2024.26	1028.61	0.526	1580.21	831.55
A_68_P30939710	chr16:87432281-87432325	NM_001081068:549	Ltn1	INSIDE	1.036	0.356	1728.32	615.59	0.369	1280.77	472.44
A_68_P30555518	chr16:13109717-13109761	NM_015769:-90	Ercc4	PROMOTER	1.036	0.448	1709.42	766.53	0.465	1267.93	589.22
A_68_P29480405	chr14:26722370-26722414	NM_207229:-13	Plac9	PROMOTER	1.036	0.500	5162.90	2583.50	0.519	3767.47	1953.46
A_68_P28873009	chr13:21560180-21560224	NM_025719:168	Nkapl	INSIDE	1.036	1.741	716.81	1247.90	1.803	576.50	1039.40
A_68_P27998250	chr11:87983215-87983259	NM_001172099:170	Cuedc1	INSIDE	1.036	1.663	778.34	1294.52	1.724	599.76	1033.81
A_68_P27804720	chr11:53244141-53244185	NM_025352:171	Uqcrq	INSIDE	1.036	0.406	2258.57	917.44	0.421	1701.75	715.90
A_68_P27278712	chr10:79218062-79218106	NM_023304:221	Fgf22	INSIDE	1.036	0.533	1869.30	997.12	0.553	1360.84	751.95
A_68_P26864772	chr9:118059149-118059193	NM_026442:144	Cmc1	INSIDE	1.036	1.618	1004.10	1624.77	1.677	788.94	1322.78
A_68_P25193392	chr7:74517038-74517082	NM_001033713:684	Mef2a	INSIDE	1.036	0.440	1332.74	586.28	0.456	1029.70	469.39
A_68_P24761384	chr6:113709003-113709047	NM_001036684:132346	Atp2b2	INSIDE	1.036	1.952	540.94	1055.85	2.022	479.06	968.76
A_68_P24760450	chr6:113555022-113555066	NM_133937:279	6720456B07Rik	INSIDE	1.036	0.440	3599.96	1583.62	0.456	2853.55	1300.84
A_68_P23281395	chr4:126355065-126355109	NM_011970:200	Psmb2	INSIDE	1.036	2.763	527.29	1456.78	2.864	382.06	1094.05
A_68_P23193626	chr4:107596160-107596204	NM_009949:12	Cpt2	INSIDE	1.036	0.394	1673.98	659.74	0.408	1334.17	544.59
A_68_P22083009	chr3:40887619-40887663	NM_027558:-672	Pgrmc2	PROMOTER	1.036	0.350	1512.75	529.43	0.362	1211.09	438.95
A_68_P20632581	chr1:136195963-136196007	NM_001144855:33521	Ppfia4	INSIDE	1.036	0.413	2514.70	1038.08	0.428	1837.58	785.76
A_68_P20353638	chr1:75379009-75379053	NM_001085370:-3154	Speg	PROMOTER	1.036	0.109	7599.68	830.71	0.113	5124.85	580.24
A_68_P31100484	chr17:25073844-25073888	NM_001163447:56	Mapk8ip3	INSIDE	1.035	1.870	998.21	1866.56	1.936	839.48	1625.31
A_68_P30991292	chr16:96367120-96367164	NM_207301:117	Wrb	INSIDE	1.035	0.158	2958.72	468.66	0.164	2172.61	356.19
A_68_P30490488	chr15:101062388-101062432	NM_019518:7773	Grasp	INSIDE	1.035	1.851	1151.13	2130.27	1.915	976.10	1869.11
A_68_P30126795	chr15:34012330-34012374	NM_026002:-121	Mtdh	PROMOTER	1.035	0.180	3569.56	641.53	0.186	2378.74	442.55
A_68_P30013961	chr15:10966774-10966818	NM_178717:927	Rxfp3	INSIDE	1.035	0.115	4582.66	526.30	0.119	3088.07	367.12
A_68_P29731239	chr14:76410408-76410452	NM_026816:242	Gtf2f2	INSIDE	1.035	0.514	2941.39	1512.27	0.532	2101.44	1118.72
A_68_P28072252	chr11:101017412-101017456	NM_134028:237	Tubg2	INSIDE	1.035	0.257	1988.59	510.42	0.266	1532.35	406.97
A_68_P26232969	chr8:124900303-124900347	NM_001029993:-191	Zc3h18	PROMOTER	1.035	0.357	1450.80	517.68	0.369	1208.30	446.31
A_68_P25728859	chr8:26864375-26864419	NM_028102:356	Ddhd2	INSIDE	1.035	0.328	3594.41	1179.04	0.339	2338.61	793.70
A_68_P24171029	chr5:147774986-147775030	NM_029581:342	Mtif3	INSIDE	1.035	0.415	5639.67	2337.86	0.429	3778.15	1621.43
A_68_P23279121	chr4:125939746-125939790	NM_013718:120	Trappc3	INSIDE	1.035	0.114	5193.90	590.87	0.118	3492.70	411.11
A_68_P22782196	chr4:23446473-23446517	ENSMUST00000118402:-907		PROMOTER	1.035	1.565	1868.30	2924.19	1.620	1557.25	2522.66
A_68_P21310282	chr2:69485168-69485213	NM_028284:-121	Bbs5	PROMOTER	1.035	0.428	1277.13	546.39	0.443	990.62	438.66
A_68_P21099288	chr2:29607027-29607071	NM_001177392:-9999	Gm13547	PROMOTER	1.035	0.458	3149.74	1441.86	0.474	2312.68	1095.21
A_68_P32361702	chrX:45245496-45245540	NM_053123:211	Smarca1	INSIDE	1.034	1.714	414.91	711.17	1.773	521.17	924.07
A_68_P32186048	chr19:54119950-54119994	NM_007417:301	Adra2a	INSIDE	1.034	2.244	1192.33	2675.42	2.320	1119.19	2596.55
A_68_P30575784	chr16:17803544-17803588	NM_153790:6192	Scarf2	INSIDE	1.034	1.805	1267.53	2288.07	1.866	1019.68	1902.39
A_68_P30572652	chr16:17233443-17233487	NM_178922:-215	Hic2	PROMOTER	1.034	0.179	3033.93	541.59	0.185	2336.85	431.48
A_68_P29639452	chr14:60216844-60216888	NM_001168535:-70	Cdadc1	PROMOTER	1.034	11.101	4014.43	44565.27	11.484	4101.86	47105.30
A_68_P28727030	chr12:109512594-109512638	NM_001159910:1011	Ccdc85c	INSIDE	1.034	0.409	1604.62	656.13	0.423	1274.49	539.00
A_68_P27780099	chr11:48630768-48630812	NM_145377:103	Trim41	INSIDE	1.034	0.649	2433.25	1578.92	0.671	1957.22	1312.86
A_68_P27284099	chr10:80039952-80039996	NM_078477:67	Klf16	INSIDE	1.034	1.421	2914.08	4140.90	1.469	2062.19	3028.85
A_68_P26134883	chr8:108138370-108138414	NR_035492:-973	Mir1966	PROMOTER	1.034	1.814	1235.10	2240.62	1.876	1010.65	1895.79
A_68_P25953303	chr8:73197017-73197061	NM_015816:-91	Lsm4	PROMOTER	1.034	0.230	3171.91	728.29	0.237	2294.07	544.54
A_68_P25269627	chr7:88137961-88138005	NM_178707:-587	Zfp592	PROMOTER	1.034	0.280	2023.04	565.89	0.289	1484.13	429.31
A_68_P25266320	chr7:87515606-87515650	NM_172903:633	Man2a2	INSIDE	1.034	0.453	1982.70	897.70	0.468	1626.52	761.64
A_68_P24785567	chr6:118147310-118147354	NM_001080780:430	Ret	INSIDE	1.034	0.498	1116.74	555.76	0.514	849.18	436.82
A_68_P24162427	chr5:145522348-145522392	NM_177735:77	Tmem130	INSIDE	1.034	0.355	1811.20	643.52	0.367	1433.43	526.77
A_68_P23442481	chr4:155484106-155484150	NM_145557:22	9430015G10Rik	INSIDE	1.034	0.350	2027.36	710.53	0.363	1845.36	668.98
A_68_P22569283	chr3:138189837-138189881	NM_007917:704	Eif4e	INSIDE	1.034	2.218	503.67	1117.02	2.293	416.35	954.67
A_68_P21710705	chr2:144993065-144993109	ENSMUST00000110007:-403		PROMOTER	1.034	0.451	1781.35	803.93	0.467	1443.20	673.26

ProbeName	Target position of probe on CpG island microarray	TargetID	GeneSymbol	CpG island Description	Ratio of relative methylation (TiO_2-NP/Vehicle)	Sham group			TiO_2-H group		
						Relative methylation (Cy5/Cy3)	Cy3 signal (Input)	Cy5 signal (MeDIP)	Relative methylation (Cy5/Cy3)	Cy3 signal (Input)	Cy5 signal (MeDIP)
A_68_P30728925	chr16:45844534-45844578	NM_153412:-65	Phldb2	PROMOTER	1.033	2.409	1505.83	3627.07	2.488	1386.70	3450.64
A_68_P29629464	chr14:58318596-58318640	NM_015771:46342	Lats2	INSIDE	1.033	5.274	1222.25	6445.74	5.447	981.44	5345.64
A_68_P26815429	chr9:108462952-108462996	NM_011830:143	Impdh2	INSIDE	1.033	0.492	3541.49	1743.29	0.508	2669.11	1357.22
A_68_P25025267	chr7:29871114-29871158	NR_035489:-2423	Mir1963	PROMOTER	1.033	3.080	917.27	2825.48	3.182	772.53	2458.15
A_68_P24993672	chr7:20094110-20094154	NM_025960:55	Trappc6a	INSIDE	1.033	0.430	1566.85	673.14	0.444	1244.10	552.20
A_68_P24974267	chr7:13620185-13620229	NM_026885:-80	Chmp2a	PROMOTER	1.033	0.352	2964.23	1042.65	0.363	2054.39	746.52
A_68_P23819789	chr5:77524030-77524074	NM_001159900:240	Hopx	INSIDE	1.033	0.418	2065.79	863.04	0.431	1523.37	657.33
A_68_P22888054	chr4:46318111-46318155	ENSMUST00000123933:38171		INSIDE	1.033	4.181	1016.54	4250.65	4.318	1116.10	4819.18
A_68_P32581184	chrX:102959900-102959944	NM_030614:107	Fgf16	INSIDE	1.032	1.785	689.59	1231.23	1.843	804.19	1482.48
A_68_P32226998	chr19:60832450-60832494	NM_178421:583	Nanos1	INSIDE	1.032	0.533	1895.38	1010.01	0.550	1625.09	894.11
A_68_P31713386	chr18:53624258-53624302	NM_001033281:81	Prdm6	INSIDE	1.032	0.211	2698.41	570.66	0.218	1952.61	425.98
A_68_P28997657	chr13:44826068-44826112	NM_001205044:-52	Jarid2	PROMOTER	1.032	2.538	3046.29	7730.32	2.619	2333.94	6111.85
A_68_P28730245	chr12:110074392-110074436	NM_181328:-328	Slc25a29	DIVERGENT_PROMOTER	1.032	0.438	1393.98	609.93	0.451	1060.88	478.99
A_68_P28514077	chr12:70457809-70457853	NM_025441:323	Nemf	INSIDE	1.032	0.566	1429.31	809.52	0.584	1243.43	726.75
A_68_P27994973	chr11:87405603-87405647	NM_133215:-94	Mtmr4	PROMOTER	1.032	0.638	4679.27	2986.55	0.659	3118.92	2054.33
A_68_P27792933	chr11:51076801-51076845	NM_007714:150	Clk4	INSIDE	1.032	0.578	2038.27	1178.68	0.597	1526.99	911.52
A_68_P25836146	chr8:47556498-47556542	NM_007981:125	Acsl1	INSIDE	1.032	2.050	937.87	1922.53	2.115	821.37	1737.40
A_68_P23591182	chr5:34125290-34125334	NM_019694:41	Letm1	INSIDE	1.032	0.455	1423.50	647.33	0.469	1100.32	516.15
A_68_P22630571	chr3:148652293-148652337	ENSMUST00000098518:-34		PROMOTER	1.032	0.400	2602.06	1040.17	0.413	1975.40	814.99
A_68_P22523145	chr3:129672979-129673023	NM_025779:124	Ccdc109b	INSIDE	1.032	2.106	861.46	1814.49	2.174	694.96	1510.95
A_68_P22378553	chr3:101816844-101816888	NM_178777:2799	Nhlh2	INSIDE	1.032	0.273	3069.80	836.80	0.281	2294.20	645.56
A_68_P21147672	chr2:38210172-38210216	NM_010710:3367	Lhx2	INSIDE	1.032	1.812	800.81	1450.92	1.870	624.95	1168.65
A_68_P20910365	chr1:188791961-188792005	NM_033077:688	D1Pas1	INSIDE	1.032	3.183	1231.21	3918.95	3.285	1014.09	3330.95
A_68_P20158329	chr1:38267215-38267259	NM_010678:454564	Aff3	INSIDE	1.032	0.463	2213.08	1025.44	0.478	1871.72	894.60
A_68_P32131186	chr19:44322737-44322781	NM_024450:44981	Scd3	DOWNSTREAM	1.031	0.534	1718.99	918.77	0.551	1288.03	709.70
A_68_P31958230	chr19:11943487-11943531	NM_001011775:2196	Olfr1419	DOWNSTREAM	1.031	2.747	946.70	2600.86	2.832	864.91	2449.17
A_68_P30351004	chr15:76709899-76709943	NM_145916:215	Zfp7	INSIDE	1.031	0.425	1121.85	477.20	0.438	899.92	394.52
A_68_P28514076	chr12:70457722-70457766	NM_025441:409	Nemf	INSIDE	1.031	0.582	2106.95	1226.20	0.600	1553.07	931.82
A_68_P28155819	chr11:115719918-115719962	NM_145438:34569	Llgl2	DOWNSTREAM	1.031	0.579	3439.97	1991.94	0.597	2390.14	1426.67
A_68_P27262525	chr10:75994355-75994399	NM_146006:5	Lss	INSIDE	1.031	1.556	1138.49	1771.27	1.604	950.21	1523.66
A_68_P24605100	chr6:85401853-85401897	NR_028081:90	1700040I03Rik	INSIDE	1.031	0.636	3396.56	2160.97	0.656	2468.67	1619.66
A_68_P24438776	chr6:50516749-50516793	NR_015581:129	5430402O13Rik	INSIDE	1.031	1.908	1519.12	2897.93	1.966	1270.26	2497.87
A_68_P24099182	chr5:132092432-132092476	NM_177047:925759	Auts2	INSIDE	1.031	0.407	1985.16	808.12	0.420	1473.61	618.66
A_68_P23251106	chr4:120078426-120078470	NM_001159630:563	Scmh1	INSIDE	1.031	0.239	2043.67	487.57	0.246	1595.42	392.33
A_68_P23111977	chr4:93002257-93002301	NM_026954:-76	Tusc1	PROMOTER	1.031	0.450	2128.74	957.21	0.463	1748.51	810.37
A_68_P22317891	chr3:89018021-89018065	NM_001161824:215	Mtx1	INSIDE	1.031	3.079	1543.54	4752.70	3.175	1123.49	3566.62
A_68_P21040932	chr2:18616488-18616532	NM_001001334:-4138	BC061194	PROMOTER	1.031	0.366	1630.88	597.58	0.378	1377.36	520.31
A_68_P20391651	chr1:82280179-82280223	NM_010570:7814	Irs1	INSIDE	1.031	0.357	5378.75	1917.79	0.368	3838.87	1411.69
A_68_P31929504	chr19:5568132-5568176	NM_001033448:81	Gm962	INSIDE	1.030	0.358	1728.08	619.01	0.369	1300.88	480.10
A_68_P30670706	chr16:35542029-35542073	NM_013661:603	Sema5b	INSIDE	1.030	0.161	10699.30	1718.03	0.165	7448.00	1231.83
A_68_P30596852	chr16:22265731-22265775	NM_009186:250	Tra2b	INSIDE	1.030	0.644	2295.50	1478.80	0.663	1698.64	1126.93
A_68_P30575402	chr16:17734359-17734403			Unknown	1.030	2.449	568.09	1391.42	2.522	464.49	1171.26
A_68_P30363894	chr15:78858490-78858534	NM_008197:-129	H1f0	PROMOTER	1.030	2.092	374.28	783.07	2.155	327.40	705.41
A_68_P28671225	chr12:99866511-99866555	NM_178914:139	Spata7	INSIDE	1.030	0.441	2007.29	885.40	0.454	1515.56	688.25
A_68_P28183237	chr11:120096983-120097028	NM_198423:2745	Bahcc1	INSIDE	1.030	3.308	266.07	880.09	3.409	185.71	633.01
A_68_P28118513	chr11:109287169-109287213	NM_025275:-69	Amz2	PROMOTER	1.030	3.563	13418.68	47814.56	3.671	9061.71	33261.51
A_68_P27284471	chr10:80097175-80097221	NM_001159591:4798	Csnk1g2	INSIDE	1.030	8.171	205.68	1680.61	8.414	181.01	1522.97
A_68_P26783903	chr9:103014881-103014925	NM_173781:499	Rab6b	INSIDE	1.030	0.404	2085.25	841.42	0.416	1707.17	709.60
A_68_P26016157	chr8:86239183-86239227	NM_197982:129	Ddx39	INSIDE	1.030	0.301	2433.19	732.23	0.310	1947.18	603.45
A_68_P25890523	chr8:59800718-59800762	NM_010402:961	Hand2	INSIDE	1.030	0.517	1661.75	859.33	0.533	1323.46	705.19
A_68_P24951683	chr7:4983086-4983130	NM_001033383:11131	Zfp865	INSIDE	1.030	2.923	1092.52	3193.90	3.010	866.67	2608.55
A_68_P24795433	chr6:119798523-119798567	NR_030776:334	3110021A11Rik	INSIDE	1.030	0.436	1708.19	745.56	0.449	1354.74	608.90
A_68_P24112564	chr5:134789737-134789781	NM_001080746:858	Gtf2i	INSIDE	1.030	0.364	2208.07	804.45	0.375	1929.98	724.21
A_68_P23297032	chr4:129190684-129190728	NM_010807:-118	Marcksl1	PROMOTER	1.030	0.489	1761.12	860.39	0.503	1095.99	551.77
A_68_P23198686	chr4:108673421-108673465	NM_146150:33	Nrd1	INSIDE	1.030	0.211	3292.36	693.38	0.217	2528.38	548.66
A_68_P22661983	chr3:153991849-153991908	NM_010713:1646	Lhx8	INSIDE	1.030	2.124	1562.26	3318.85	2.188	1150.10	2516.19
A_68_P22169031	chr3:58381260-58381314	NM_001040396:707	2810407C02Rik	INSIDE	1.030	1.957	598.31	1171.07	2.017	458.13	924.00
A_68_P20897124	chr1:186551680-186551724	NM_008250:4670	Hlx	INSIDE	1.030	0.405	1126.64	456.82	0.418	949.18	396.29
A_68_P32078821	chr19:34266845-34266890	NM_029682:108	Stambpl1	INSIDE	1.029	0.183	2606.94	477.46	0.188	1705.73	321.49
A_68_P32020401	chr19:23968179-23968223	NM_177034:134835	Apba1	INSIDE	1.029	2.270	1256.51	2852.23	2.335	1093.22	2552.56
A_68_P30580616	chr16:18588788-18588832	NM_011532:-1748	Tbx1	PROMOTER	1.029	0.345	1929.75	666.65	0.355	1380.27	490.44
A_68_P30287849	chr15:65618481-65618525	NM_133766:-100	Efr3a	PROMOTER	1.029	2.027	432.69	876.95	2.086	428.61	893.98
A_68_P29952573	chr14:121310169-121310213	NM_023579:-225	Ipo5	PROMOTER	1.029	0.465	1095.29	509.26	0.479	906.03	433.61
A_68_P29713887	chr14:73109166-73109210	NM_207636:622	Fndc3a	INSIDE	1.029	2.236	579.20	1295.37	2.302	344.29	792.64
A_68_P28788254	chr13:3477478-3477522	NR_015522:-48	2810429I04Rik	PROMOTER	1.029	0.260	3996.23	1038.29	0.267	2960.82	791.43

ProbeName	Target position of probe on CpG island microarray	TargetID	GeneSymbol	CpG island Description	Ratio of relative methylation (TiO_2-NP/Vehicle)	Sham group			TiO_2-H group		
						Relative methylation (Cy5/Cy3)	Cy3 signal (Input)	Cy5 signal (MeDIP)	Relative methylation (Cy5/Cy3)	Cy3 signal (Input)	Cy5 signal (MeDIP)
A_68_P27949795	chr11:79404844-79404888	NM_175543:153	Rab11fip4	INSIDE	1.029	2.192	721.09	1580.88	2.255	593.34	1338.04
A_68_P26544598	chr9:57787782-57787826	NM_011352:-137	Sema7a	PROMOTER	1.029	0.445	1863.88	829.98	0.458	1396.97	639.91
A_68_P26465225	chr9:43828528-43828572			Unknown	1.029	4.102	515.24	2113.60	4.221	351.07	1481.71
A_68_P25713873	chr8:24085890-24085934	NM_001110783:559	Ank1	INSIDE	1.029	1.731	1167.84	2021.41	1.782	862.93	1537.64
A_68_P25412180	chr7:117365261-117365305	NM_009302:66	Swap70	INSIDE	1.029	4.651	1084.73	5045.24	4.784	1054.36	5044.14
A_68_P25029776	chr7:30857858-30857902	NM_177889:-38	Zfp82	PROMOTER	1.029	1.711	1040.31	1780.03	1.761	809.71	1425.56
A_68_P24344492	chr6:32537469-32537513	NM_175750:702	Plxna4	INSIDE	1.029	0.544	2041.61	1111.42	0.560	1410.16	790.16
A_68_P24116584	chr5:135965213-135965257	NM_146001:55758	Hip1	INSIDE	1.029	0.460	1084.58	498.95	0.473	988.06	467.56
A_68_P23873914	chr5:89194174-89194218	NM_007832:159	Dck	INSIDE	1.029	0.345	2674.42	923.24	0.355	2175.61	772.65
A_68_P20086661	chr1:23108998-23109042	NM_025746:72	4933415F23Rik	INSIDE	1.029	1.839	646.44	1188.80	1.893	558.38	1056.77
A_68_P31759408	chr18:61885774-61885818	NM_021296:189	Grpel2	INSIDE	1.028	0.615	1846.22	1135.06	0.632	1429.94	903.43
A_68_P31622949	chr18:36440408-36440452	NM_008989:-385	Pura	PROMOTER	1.028	1.446	1410.30	2039.54	1.487	1114.08	1656.92
A_68_P29258371	chr13:99342954-99342998	AK135687:152841		INSIDE	1.028	0.490	1104.87	541.81	0.504	1040.10	524.17
A_68_P29059426	chr13:56236410-56236454	NM_001159513:479	H2afy	INSIDE	1.028	2.005	939.00	1882.42	2.061	715.18	1473.91
A_68_P29011719	chr13:47201171-47201215	NM_025900:397	Dek	INSIDE	1.028	1.754	1080.45	1894.80	1.804	869.50	1568.25
A_68_P27927584	chr11:75493253-75493297	NM_133656:463	Crk	INSIDE	1.028	0.327	3034.17	990.70	0.336	2279.47	764.91
A_68_P27308087	chr10:84849473-84849517	NM_028709:-64	Btbd11	PROMOTER	1.028	0.441	2454.27	1081.49	0.453	1978.33	896.12
A_68_P26313303	chr9:13553960-13554004	NM_023858:358	Mtmr2	INSIDE	1.028	0.456	1301.11	592.73	0.468	1103.60	516.81
A_68_P26136632	chr8:108424612-108424656	NM_173432:261	Pskh1	INSIDE	1.028	0.560	2046.90	1146.99	0.576	1555.53	896.16
A_68_P25962573	chr8:75247186-75247230	NM_001113248:22	Sin3b	INSIDE	1.028	0.240	3951.47	947.90	0.247	2925.02	721.50
A_68_P25956924	chr8:73987733-73987777	NM_022419:-198	Abhd8	DIVERGENT_PROMOTER	1.028	0.155	3741.18	580.69	0.160	2673.09	426.56
A_68_P25349937	chr7:104480730-104480774	NM_001039039:-80	Kctd21	DIVERGENT_PROMOTER	1.028	1.689	1000.12	1689.70	1.736	845.40	1467.98
A_68_P24112562	chr5:134789379-134789423	NM_001080746:1216	Gtf2i	INSIDE	1.028	1.775	967.34	1717.32	1.825	787.68	1437.66
A_68_P23222743	chr4:114659620-114659664	NM_025647:191	Cmpk1	INSIDE	1.028	0.452	1034.30	467.55	0.465	763.12	354.69
A_68_P22317133	chr3:88894786-88894830	NM_001083808:-855	Rusc1	PROMOTER	1.028	0.474	2259.42	1070.18	0.487	1672.88	814.66
A_68_P21836891	chr2:167515250-167515294	NM_009883:858	Cebpb	INSIDE	1.028	0.367	1221.59	448.21	0.377	974.73	367.58
A_68_P21747319	chr2:151874059-151874103			Unknown	1.028	0.467	1234.37	575.86	0.479	901.72	432.30
A_68_P20837648	chr1:175297264-175297308	NM_053199:540	Cadm3	INSIDE	1.028	0.287	4533.21	1302.80	0.295	3274.71	967.07
A_68_P20768944	chr1:162836948-162836992	NM_001024952:429	Rc3h1	INSIDE	1.028	1.918	490.06	940.13	1.971	438.82	865.01
A_68_P32241860	chrX:7706225-7706269	NR_029428:3727	Wdr13	INSIDE	1.027	3.001	974.44	2924.49	3.083	1222.90	3770.62
A_68_P31286845	chr17:63230423-63230467	NM_010109:222	Efna5	INSIDE	1.027	0.446	1921.93	857.28	0.458	1269.52	581.37
A_68_P30778639	chr16:55974658-55974702	NR_027965:50	2310061J03Rik	INSIDE	1.027	0.536	1226.06	657.76	0.551	1053.44	580.69
A_68_P29116252	chr13:69672648-69672692	NM_001169131:72	Papd7	INSIDE	1.027	3.092	603.18	1865.23	3.175	519.35	1648.76
A_68_P28048175	chr11:96880255-96880299	NM_031183:5394	Sp6	INSIDE	1.027	2.355	652.08	1535.57	2.418	554.57	1341.02
A_68_P27973894	chr11:83664886-83664930	NM_009330:538	Hnf1b	INSIDE	1.027	0.480	2889.18	1386.93	0.493	2159.05	1064.05
A_68_P27282448	chr10:79783216-79783260	NM_025521:55	2310011J03Rik	INSIDE	1.027	1.825	954.74	1742.14	1.873	653.04	1223.33
A_68_P27278629	chr10:79202122-79202166	NM_172551:7182	Polrmt	INSIDE	1.027	2.168	1566.03	3395.01	2.227	1193.28	2657.74
A_68_P26486126	chr9:47339043-47339087	NM_001025600:630	Cadm1	INSIDE	1.027	0.372	3008.34	1120.01	0.382	2206.02	843.78
A_68_P25228982	chr7:80562603-80562647	NM_177740:42219	Rgma	INSIDE	1.027	1.605	1375.94	2209.05	1.649	1048.42	1729.19
A_68_P25176078	chr7:71432596-71432640	NM_172742:63	Mtmr10	INSIDE	1.027	1.697	977.11	1658.48	1.743	781.19	1361.73
A_68_P25027721	chr7:30303289-30303333	ENSMUST00000141713:-913		PROMOTER	1.027	0.240	2153.16	517.39	0.247	1515.43	374.10
A_68_P23134360	chr4:97444653-97444697	NM_001122953:358	Nfia	INSIDE	1.027	0.393	1143.57	449.59	0.404	770.62	311.15
A_68_P22356952	chr3:97692829-97692873	NM_001039376:-220	Pde4dip	PROMOTER	1.027	0.441	1046.93	461.93	0.453	864.88	391.74
A_68_P21221948	chr2:52717600-52717648	NM_172409:723	Fmnl2	INSIDE	1.027	2.871	672.45	1930.59	2.950	488.15	1439.95
A_68_P21118608	chr2:32822540-32822584	NM_009076:5331	Rpl12	DOWNSTREAM	1.027	0.357	1417.95	506.13	0.367	926.68	339.68
A_68_P20412113	chr1:88330886-88330930	NM_027966:8005	1700019O17Rik	DOWNSTREAM	1.027	0.497	2049.91	1019.20	0.510	1667.06	850.95
A_68_P31145427	chr17:32533746-32533790	NR_029458:-6190	A530088E08Rik	PROMOTER	1.026	0.233	2941.05	686.64	0.240	2232.81	535.10
A_68_P30656973	chr16:33251896-33251940	NM_080557:377	Snx4	INSIDE	1.026	0.373	1588.26	591.97	0.382	1346.05	514.74
A_68_P28092301	chr11:104547023-104547067	ENSMUST00000148007:24		INSIDE	1.026	1.815	1153.70	2094.02	1.862	1035.60	1928.15
A_68_P27902564	chr11:70578153-70578197	NM_177565:226	Zfp3	INSIDE	1.026	0.466	1334.07	621.97	0.478	1000.17	478.23
A_68_P25031209	chr7:31145387-31145431	NM_175478:2383	Lrfn3	INSIDE	1.026	2.588	554.63	1435.61	2.655	443.60	1177.61
A_68_P25020499	chr7:28954819-28954863	NM_007991:74	Fbl	INSIDE	1.026	1.546	2472.54	3821.69	1.585	1855.55	2941.20
A_68_P24992917	chr7:19955431-19955475	NM_001010836:8888	Ppp1r13l	INSIDE	1.026	1.999	1811.47	3621.15	2.051	1416.63	2906.22
A_68_P24132967	chr5:139470355-139470399	NM_008808:531	Pdgfa	INSIDE	1.026	0.265	1989.14	526.38	0.271	1650.69	448.01
A_68_P23602238	chr5:36022053-36022097	NM_030127:357	Htra3	INSIDE	1.026	4.984	1983.99	9888.48	5.112	1818.93	9299.16
A_68_P23284201	chr4:126910870-126910914	NM_198618:64378	Dlgap3	INSIDE	1.026	1.870	1106.02	2067.76	1.917	859.16	1647.42
A_68_P22383909	chr3:102824881-102824925	NM_001161854:434	Csde1	INSIDE	1.026	0.334	5274.04	1762.78	0.343	3783.56	1297.93
A_68_P21006478	chr2:11423381-11423425	NM_001177752:315	Pfkfb3	INSIDE	1.026	0.585	1633.17	955.38	0.600	1060.11	636.11
A_68_P31458875	chr18:3507840-3507884	NM_026505:-92	Bambi	PROMOTER	1.025	24.747	2341.55	57946.94	25.357	2110.78	53523.43
A_68_P31253725	chr17:56218817-56218862	NM_008233:-240	Hdgfrp2	DIVERGENT_PROMOTER	1.025	0.359	7695.12	2764.73	0.368	5385.82	1982.74
A_68_P30631089	chr16:28564952-28564996	NM_010199:188314	Fgf12	INSIDE	1.025	1.779	949.60	1689.46	1.823	834.67	1521.92
A_68_P28280591	chr12:21423034-21423078	NM_011739:241	Ywhaq	INSIDE	1.025	0.615	2055.45	1264.32	0.631	1573.71	992.38
A_68_P28043747	chr11:96181632-96181676	NM_010459:2074	Hoxb4	INSIDE	1.025	1.997	781.33	1560.37	2.046	577.32	1181.40
A_68_P27234952	chr10:70810826-70810870	NM_027184:308	Ipmk	INSIDE	1.025	0.339	5226.41	1773.12	0.348	3925.12	1364.40
A_68_P26526444	chr9:54407560-54407610	NM_019686:302	Cib2	INSIDE	1.025	1.839	659.96	1213.61	1.885	615.92	1161.01

ProbeName	Target position of probe on CpG island microarray	TargetID	GeneSymbol	CpG island Description	Ratio of relative methylation (TiO_2-NP/Vehicle)	Sham group			TiO_2-H group		
						Relative methylation (Cy5/Cy3)	Cy3 signal (Input)	Cy5 signal (MeDIP)	Relative methylation (Cy5/Cy3)	Cy3 signal (Input)	Cy5 signal (MeDIP)
A_68_P26467387	chr9:44205049-44205093	NM_008063:-1188	Slc37a4	PROMOTER	1.025	0.416	1926.28	800.66	0.426	1540.69	656.35
A_68_P26304291	chr9:11277863-11277907			Unknown	1.025	2.542	278.88	708.81	2.606	305.80	796.79
A_68_P25083583	chr7:50932042-50932086	NM_145582:4664	Ctu1	INSIDE	1.025	3.405	1327.06	4518.27	3.488	977.16	3408.66
A_68_P24602908	chr6:85026279-85026323	NR_002702:166	Npm3-ps1	INSIDE	1.025	1.777	603.15	1071.64	1.822	517.50	942.89
A_68_P24381992	chr6:39155917-39155961	NM_001033430:834	Jhdm1d	INSIDE	1.025	2.372	721.82	1712.47	2.432	614.25	1494.09
A_68_P23720363	chr5:58110379-58110423	NM_018764:1141	Pcdh7	INSIDE	1.025	0.614	1834.51	1126.03	0.629	1475.00	927.98
A_68_P22973925	chr4:63678841-63678885	NM_011607:29187	Tnc	INSIDE	1.025	11.380	1460.64	16621.92	11.669	1193.38	13925.88
A_68_P21103453	chr2:30330211-30330255	NM_030244:-513	Ier5l	PROMOTER	1.025	0.514	1114.75	572.57	0.526	866.10	455.82
A_68_P20679834	chr1:145586536-145586580	NM_001038592:-139	Glrx2	PROMOTER	1.025	1.526	1382.93	2110.55	1.564	1091.06	1706.35
A_68_P20419588	chr1:89652379-89652423	NM_001205391:-185	Atg16l1	PROMOTER	1.025	1.718	867.81	1491.31	1.762	790.92	1393.41
A_68_P31156002	chr17:34973346-34973390	NM_019442:480	Stk19	INSIDE	1.024	0.540	1769.54	955.37	0.553	1402.44	775.55
A_68_P30970269	chr16:92606424-92606468	NM_001111023:91127	Runx1	INSIDE	1.024	0.539	1386.10	747.62	0.552	1039.88	574.24
A_68_P28751329	chr12:113858290-113858334	NM_007421:55	Adssl1	INSIDE	1.024	0.369	1512.73	558.14	0.378	1110.47	419.40
A_68_P28098880	chr11:105847454-105847498	NM_009598:10955	Ace	INSIDE	1.024	2.129	495.47	1054.65	2.181	429.06	935.60
A_68_P26140463	chr8:109127687-109127731	NM_009864:441	Cdh1	INSIDE	1.024	1.918	892.61	1711.96	1.963	765.80	1503.41
A_68_P25508347	chr7:135110935-135110979	NM_139149:-36	Fus	PROMOTER	1.024	1.954	1005.25	1964.16	2.002	822.08	1645.52
A_68_P25193398	chr7:74517805-74517849	NM_001033713:-82	Mef2a	PROMOTER	1.024	1.681	1558.20	2620.03	1.722	1160.82	1999.45
A_68_P25081790	chr7:50568129-50568173	NM_027264:481	Zfp715	INSIDE	1.024	0.626	2307.32	1444.52	0.641	1864.35	1195.15
A_68_P25017993	chr7:28377186-28377230	NM_001110208:630	Akt2	INSIDE	1.024	0.400	2432.02	972.15	0.409	1863.84	763.02
A_68_P24119914	chr5:136674559-136674603	NM_025774:244	Prkrip1	INSIDE	1.024	0.272	2144.34	582.67	0.278	1686.52	469.13
A_68_P23618574	chr5:38668317-38668361	NM_172709:-304	Otop1	PROMOTER	1.024	0.466	1859.43	866.96	0.478	1475.12	704.38
A_68_P21774857	chr2:156866238-156866282	NM_001164663:38741	9830001H06Rik	INSIDE	1.024	2.514	272.96	686.23	2.574	263.29	677.59
A_68_P21052783	chr2:20890077-20890121	NR_033225:-402	Gm13375	DIVERGENT_PROMOTER	1.024	0.652	2439.97	1589.78	0.667	1697.53	1132.60
A_68_P20442010	chr1:93263130-93263174	NM_173395:146	Fam132b	INSIDE	1.024	0.368	1270.54	467.78	0.377	1100.44	414.89
A_68_P20409046	chr1:87791116-87791160	NM_022417:54	Itm2c	INSIDE	1.024	0.202	4576.53	926.52	0.207	2982.64	618.35
A_68_P32128433	chr19:43827788-43827832	NM_001113562:298	Cutc	INSIDE	1.023	0.281	2553.75	717.69	0.288	1834.74	527.72
A_68_P31402642	chr17:84495931-84495975	AK043195:-259		PROMOTER	1.023	0.458	1324.21	606.54	0.469	1069.11	501.16
A_68_P31116319	chr17:27572063-27572107	NM_001013385:68165	Grm4	INSIDE	1.023	1.646	1573.72	2589.57	1.684	1125.91	1895.98
A_68_P30991545	chr16:96413628-96413672	NM_001001492:214	Lca5l	INSIDE	1.023	0.440	1238.94	545.51	0.450	1032.18	464.84
A_68_P29848865	chr14:100684720-100684764	AK021307:-1136		PROMOTER	1.023	2.171	1457.44	3163.95	2.220	1108.39	2460.45
A_68_P28036751	chr11:94979621-94979665	NM_010055:-1788	Dlx3	PROMOTER	1.023	1.824	862.53	1572.91	1.865	648.37	1209.15
A_68_P28036509	chr11:94937682-94937726	NM_013565:324	Itga3	INSIDE	1.023	0.310	1465.01	453.87	0.317	1148.49	363.86
A_68_P27787099	chr11:49988480-49988526	NM_026543:150	3010026O09Rik	INSIDE	1.023	1.498	3143.87	4710.10	1.532	2336.17	3579.79
A_68_P26715672	chr9:89949316-89949360	NM_007801:234	Ctsh	INSIDE	1.023	0.423	2527.73	1070.21	0.433	2042.20	884.82
A_68_P24816836	chr6:124705997-124706041	NM_007881:487	Atn1	INSIDE	1.023	0.600	2254.67	1351.69	0.613	1710.07	1048.33
A_68_P24424177	chr6:47601567-47601611	NR_002841:3505	Rn4.5s	DOWNSTREAM	1.023	1.352	3079.82	4162.85	1.383	2500.78	3459.18
A_68_P22592103	chr3:142364167-142364211	NM_173763:145	Ccbl2	INSIDE	1.023	0.263	3137.39	824.28	0.269	2402.30	645.60
A_68_P21779875	chr2:157741034-157741081	NM_198627:669	Vstm2l	INSIDE	1.023	2.351	1182.26	2778.93	2.404	807.80	1942.03
A_68_P21761084	chr2:154375582-154375626	NM_029305:965	1700003F12Rik	INSIDE	1.023	0.471	1472.97	694.21	0.482	1219.52	588.13
A_68_P31619440	chr18:35814836-35814880	NM_027222:-5837	2010001M09Rik	PROMOTER	1.022	0.463	1513.80	701.26	0.474	1206.24	571.32
A_68_P31166994	chr17:37188152-37188196	NM_019439:5264	Gabbr1	INSIDE	1.022	0.411	1239.74	509.28	0.420	849.05	356.37
A_68_P28094596	chr11:105026660-105026704	ENSMUST00000118770:1252		INSIDE	1.022	2.933	513.14	1505.12	2.997	438.22	1313.29
A_68_P26897299	chr9:123760705-123760749	NM_148925:56	Fyco1	INSIDE	1.022	2.599	366.86	953.32	2.655	309.76	822.46
A_68_P26811950	chr9:107838507-107838551	NM_145621:278	Camkv	INSIDE	1.022	0.268	2082.04	558.50	0.274	1726.36	473.14
A_68_P25667334	chr8:13287426-13287470	NM_172282:-564	Tmco3	PROMOTER	1.022	0.599	2882.24	1727.50	0.612	1870.48	1145.22
A_68_P24043271	chr5:121671135-121671179	ENSMUST00000119892:929		INSIDE	1.022	0.642	3333.44	2138.84	0.656	2506.12	1643.18
A_68_P23881743	chr5:90652818-90652862	NM_001033310:181	Cox18	INSIDE	1.022	4.777	671.66	3208.58	4.882	560.23	2734.83
A_68_P23222368	chr4:114598578-114598622	NM_015758:18	Foxe3	INSIDE	1.022	0.213	3127.32	665.19	0.217	2304.63	501.07
A_68_P21680284	chr2:139503094-139503138	NM_001126490:-797	Isml	PROMOTER	1.022	1.902	1345.08	2558.39	1.944	1200.01	2333.22
A_68_P21115155	chr2:32251561-32251605	NM_133783:173	Ptges2	INSIDE	1.022	0.499	3808.93	1901.07	0.510	2544.10	1297.54
A_68_P32700327	chrX:137200260-137200304	ENSMUST00000112988:672		INSIDE	1.021	2.255	599.39	1351.81	2.303	868.95	2001.40
A_68_P32127486	chr19:43675876-43675920			Unknown	1.021	0.489	2624.75	1282.20	0.499	1882.44	938.71
A_68_P31093570	chr17:23908581-23908625	NM_153791:-44	Flywch1	PROMOTER	1.021	0.294	1976.96	580.74	0.300	1446.67	433.75
A_68_P30961691	chr16:91226111-91226155	NM_016967:338	Olig2	INSIDE	1.021	0.450	1098.38	493.89	0.459	836.12	383.94
A_68_P30181641	chr15:44451230-44451274	NM_019480:66	Ebag9	INSIDE	1.021	0.344	1521.55	522.73	0.351	1316.21	461.76
A_68_P30010835	chr15:10415837-10415882	NM_011232:71	Rad1	INSIDE	1.021	0.382	4276.48	1632.67	0.390	3238.75	1262.78
A_68_P29616401	chr14:55732942-55732986	NM_177049:2808	Jph4	INSIDE	1.021	1.544	1113.71	1719.75	1.577	866.19	1366.13
A_68_P28834525	chr13:13683099-13683143	NM_010748:445	Lyst	INSIDE	1.021	0.399	1702.32	679.06	0.407	1203.19	489.82
A_68_P28724528	chr12:109155568-109155612	NM_001079883:86034	Bcl11b	INSIDE	1.021	1.643	1220.79	2005.79	1.677	917.40	1538.30
A_68_P28582807	chr12:82881694-82881738	NM_001174107:441	Map3k9	INSIDE	1.021	0.649	2690.21	1747.28	0.663	1994.23	1322.89
A_68_P26082711	chr8:97855540-97855584	NM_024467:288	Zfp319	INSIDE	1.021	4.876	2388.56	11647.24	4.977	1864.55	9278.95
A_68_P24919226	chr6:144730238-144730282	ENSMUST00000149451:179		INSIDE	1.021	2.721	752.89	2048.85	2.779	638.36	1774.23
A_68_P24127312	chr5:138182456-138182500	NM_175521:-1252	6430598A04Rik	DIVERGENT_PROMOTER	1.021	1.772	7258.33	12861.31	1.808	4209.86	7612.69
A_68_P23498118	chr5:15440585-15440629	NM_001110843:98	Cacna2d1	INSIDE	1.021	2.554	813.59	2077.92	2.607	690.34	1800.01
A_68_P23331230	chr4:135477014-135477058	NM_024243:396	Fuca1	INSIDE	1.021	0.406	2437.15	990.07	0.415	1830.81	759.47

ProbeName	Target position of probe on CpG island microarray	TargetID	GeneSymbol	CpG island Description	Ratio of relative methylation (TiO_2-NP/Vehicle)	Sham group			TiO_2-H group		
						Relative methylation (Cy5/Cy3)	Cy3 signal (Input)	Cy5 signal (MeDIP)	Relative methylation (Cy5/Cy3)	Cy3 signal (Input)	Cy5 signal (MeDIP)
A_68_P22314616	chr3:88383591-88383635	NM_025448:20	Ssr2	INSIDE	1.021	0.472	1391.90	657.02	0.482	1091.85	526.42
A_68_P21901049	chr2:179777225-179777269	NM_178750:59	Ss18l1	INSIDE	1.021	0.425	2743.77	1166.32	0.434	2102.70	912.80
A_68_P32454733	chrX:68917522-68917566	NM_010340:603	Gpr50	INSIDE	1.020	2.133	215.00	458.68	2.176	344.64	750.00
A_68_P29613437	chr14:55259966-55260010	NM_199470:213	Cdh24	INSIDE	1.020	0.344	3294.95	1134.30	0.351	2732.74	959.99
A_68_P27924619	chr11:74986101-74986145	NR_029794:-767	Mir212	DIVERGENT_PROMOTER	1.020	0.221	4252.71	940.51	0.226	2937.17	662.59
A_68_P26127081	chr8:106695023-106695067	NM_001141922:632	Bean1	INSIDE	1.020	0.488	1667.27	814.16	0.498	1361.35	678.31
A_68_P25434394	chr7:121261406-121261450	NM_025846:-133	Rras2	PROMOTER	1.020	0.327	1732.31	567.16	0.334	1408.44	470.40
A_68_P24983171	chr7:17357272-17357316	NM_009201:-9400	Slc1a5	PROMOTER	1.020	1.814	1687.13	3060.79	1.851	1236.90	2289.01
A_68_P23398855	chr4:148511571-148511616	NM_027263:116	Apitd1	INSIDE	1.020	0.408	1518.78	619.32	0.416	1096.83	456.22
A_68_P22917598	chr4:52452191-52452235	NM_008017:92	Smc2	INSIDE	1.020	0.255	2279.27	581.37	0.260	1882.65	489.95
A_68_P22889708	chr4:46633313-46633357	NM_198664:29737	Tbc1d2	INSIDE	1.020	0.595	3257.42	1938.14	0.607	2174.84	1319.90
A_68_P22356948	chr3:97692392-97692436	NM_001039376:216	Pde4dip	INSIDE	1.020	0.421	2891.90	1218.83	0.430	2232.71	959.42
A_68_P21635699	chr2:131387297-131387341	NM_013460:703	Adra1d	INSIDE	1.020	0.478	2247.21	1075.23	0.488	1692.83	825.93
A_68_P20352245	chr1:75139100-75139144	NM_026977:-180	1810031K17Rik	DIVERGENT_PROMOTER	1.020	0.599	4374.93	2620.96	0.611	3084.22	1884.51
A_68_P32203261	chr19:56896363-56896407	NM_001002238:-4314	Tdrd1	DIVERGENT_PROMOTER	1.019	0.560	1674.81	938.34	0.571	1409.71	804.64
A_68_P31627038	chr18:37303465-37303509	NM_001003672:-136	Pcdhac2	PROMOTER	1.019	0.359	1888.15	678.50	0.366	1562.96	572.16
A_68_P31347823	chr17:74058995-74059039	NM_027864:775	Galnt14	INSIDE	1.019	0.257	2636.25	677.95	0.262	2115.62	554.29
A_68_P31114358	chr17:27252888-27252932	NM_080553:58662	Itpr3	INSIDE	1.019	2.671	1110.38	2966.02	2.723	793.19	2159.82
A_68_P30350131	chr15:76541158-76541202	NM_145471:11	Lrrc14	INSIDE	1.019	0.282	2615.78	736.85	0.287	1908.44	547.97
A_68_P27922685	chr11:74643761-74643805	NM_010813:-643	Mnt	PROMOTER	1.019	2.018	805.90	1626.47	2.056	768.44	1580.02
A_68_P27815757	chr11:55283721-55283765	NM_013716:489	G3bp1	INSIDE	1.019	0.449	2063.53	925.90	0.457	1556.98	711.65
A_68_P26595469	chr9:66682493-66682537	NM_026674:-1	Aph1c	PROMOTER	1.019	0.493	1921.72	948.13	0.503	1414.38	711.13
A_68_P26346747	chr9:21172590-21172634	NM_001042708:166	Ilf3	INSIDE	1.019	0.389	1976.97	768.84	0.396	1622.07	642.75
A_68_P25997731	chr8:82581263-82581307	NM_020259:623	Hhip	INSIDE	1.019	0.446	1098.24	489.71	0.454	830.89	377.40
A_68_P25669972	chr8:13677124-13677168	NM_009025:441	Rasa3	INSIDE	1.019	0.294	2761.01	813.09	0.300	1967.04	590.17
A_68_P24951536	chr7:4960475-4960519	NM_001110330:4348	Fiz1	INSIDE	1.019	1.685	891.91	1502.88	1.717	640.23	1099.02
A_68_P24931167	chr6:146837167-146837211	NM_001170433:173	Ppfibp1	INSIDE	1.019	0.543	2461.41	1335.73	0.553	2024.70	1119.28
A_68_P24183981	chr5:150040843-150040887	AK155259:18394		DOWNSTREAM	1.019	0.378	1794.30	678.37	0.385	1494.08	575.49
A_68_P24119912	chr5:136674383-136674427	NM_025774:420	Prkrip1	INSIDE	1.019	0.540	1770.19	955.56	0.550	1336.83	735.11
A_68_P24098212	chr5:131915457-131915501	NM_177047:1102735	Auts2	INSIDE	1.019	1.753	1554.70	2725.60	1.787	1186.00	2119.39
A_68_P24002950	chr5:114724269-114724313	NM_148935:-520	Foxn4	PROMOTER	1.019	0.399	2382.85	951.52	0.407	1902.12	774.19
A_68_P21952653	chr3:13472808-13472852	NM_001163328:1176	Ralyl	INSIDE	1.019	0.456	2249.15	1025.98	0.465	1757.69	816.93
A_68_P32565870	chrX:99044973-99045017	NM_001163610:271	Nhsl2	INSIDE	1.018	2.439	602.28	1469.22	2.483	759.28	1885.52
A_68_P30979597	chr16:94347042-94347086	NM_011377:261560	Sim2	INSIDE	1.018	0.348	2559.71	891.22	0.354	1969.27	697.94
A_68_P30887977	chr16:77014158-77014202	NM_013918:-133	Usp25	PROMOTER	1.018	1.932	893.67	1726.53	1.967	831.98	1636.09
A_68_P29138729	chr13:73409301-73409345	NM_018885:11378	Irx4	DOWNSTREAM	1.018	0.493	2139.55	1054.86	0.502	1915.48	961.65
A_68_P28883194	chr13:23830758-23830802	NM_015786:105	Hist1h1c	INSIDE	1.018	2.781	494.42	1374.93	2.830	439.18	1242.98
A_68_P28107246	chr11:107330411-107330455	NM_145823:1602	Pitpnc1	INSIDE	1.018	0.290	4486.77	1299.11	0.295	3401.97	1002.91
A_68_P28072866	chr11:101115247-101115291	NM_026776:212	Vps25	INSIDE	1.018	0.441	1621.55	715.84	0.449	1195.19	537.10
A_68_P27160893	chr10:57252088-57252132	NM_019760:225	Serinc1	INSIDE	1.018	0.343	2888.03	990.22	0.349	2171.46	757.89
A_68_P26344894	chr9:20780852-20780896	NM_010333:363	S1pr2	INSIDE	1.018	20.611	3656.91	75372.51	20.977	4509.82	94601.53
A_68_P26077485	chr8:96958377-96958421	NR_015514:605	9330175E14Rik	INSIDE	1.018	2.120	2320.09	4917.83	2.157	1786.28	3853.15
A_68_P25957540	chr8:74093690-74093734	NM_011977:887	Slc27a1	INSIDE	1.018	0.467	1314.36	614.33	0.476	1045.77	497.78
A_68_P25910878	chr8:64069883-64069927	NM_001081390:311583	Palld	INSIDE	1.018	0.373	1390.60	519.32	0.380	1149.53	437.16
A_68_P25354426	chr7:105342464-105342508	NM_001081167:5499	B3gnt6	INSIDE	1.018	0.225	2126.34	478.79	0.229	1557.96	357.07
A_68_P25262394	chr7:86805544-86805588	NM_029835:485	5730590G19Rik	INSIDE	1.018	0.333	1786.40	595.68	0.340	1492.56	506.81
A_68_P24951676	chr7:4982246-4982290	NM_001033383:10291	Zfp865	INSIDE	1.018	3.149	1443.10	4544.44	3.206	1178.35	3777.53
A_68_P23443314	chr5:3100163-3100207	ENSMUST00000161709:9416		DOWNSTREAM	1.018	2.348	717.60	1684.77	2.391	721.08	1723.99
A_68_P22970308	chr4:63071362-63071406	NM_001045514:-6905	Akna	PROMOTER	1.018	0.369	1549.94	572.41	0.376	1134.12	426.28
A_68_P22964917	chr4:62180863-62180907	NM_008525:213	Alad	INSIDE	1.018	0.597	3317.38	1981.75	0.608	2415.95	1469.24
A_68_P22069518	chr3:38383415-38383459	NM_001167883:302	Ankrd50	INSIDE	1.018	0.435	1227.33	534.01	0.443	1066.25	472.27
A_68_P32109309	chr19:40588021-40588065	NM_178362:184	Sorbs1	INSIDE	1.017	0.422	1850.07	781.35	0.429	1593.16	684.14
A_68_P31932934	chr19:6140904-6140948	NM_019722:211	Arl2	INSIDE	1.017	0.426	1288.72	548.49	0.433	1099.25	475.92
A_68_P31920647	chr19:4012600-4012644	NM_133666:103	Ndufv1	INSIDE	1.017	0.552	2467.72	1362.95	0.562	1887.81	1060.78
A_68_P31475769	chr18:7004843-7004887	NM_177595:-87	Mkx	PROMOTER	1.017	2.099	861.07	1807.35	2.135	689.69	1472.40
A_68_P31110190	chr17:26568998-26569042	NM_001081656:17111	Neurl1b	INSIDE	1.017	3.030	1073.58	3253.31	3.080	835.25	2572.89
A_68_P30650845	chr16:32079162-32079206	NM_177326:175	Pak2	INSIDE	1.017	2.092	434.63	909.39	2.127	375.51	798.79
A_68_P29023970	chr13:49311714-49311758	NM_026738:-341	1110007C09Rik	PROMOTER	1.017	0.269	3136.68	845.31	0.274	2304.02	631.39
A_68_P28890408	chr13:25147877-25147921	NM_001195617:26	Dcdc2a	INSIDE	1.017	0.252	1919.78	482.88	0.256	1438.51	368.09
A_68_P27996400	chr11:87630599-87630643	NM_080420:8996	Lpo	INSIDE	1.017	2.250	2734.24	6153.07	2.288	2105.99	4818.31
A_68_P27996044	chr11:87577886-87577930	NM_172449:3866	Bzrap1	INSIDE	1.017	7.696	1250.85	9626.42	7.824	1056.45	8265.88
A_68_P27808204	chr11:53914341-53914385	NM_001136076:-63	P4ha2	PROMOTER	1.017	1.838	956.66	1758.00	1.869	793.85	1483.87
A_68_P27277827	chr10:79076635-79076679	NM_198614:107	C2cd4c	INSIDE	1.017	2.571	723.14	1859.30	2.616	607.99	1590.25
A_68_P26504864	chr9:50467840-50467884	NM_145614:23	Dlat	INSIDE	1.017	0.408	1473.54	601.51	0.415	1190.41	494.42
A_68_P25701519	chr8:18950799-18950843	NM_001113350:153	Xkr5	INSIDE	1.017	0.312	1713.18	535.27	0.318	1450.18	460.95

ProbeName	Target position of probe on CpG island microarray	TargetID	GeneSymbol	CpG island Description	Ratio of relative methylation (TiO_2-NP/Vehicle)	Sham group			TiO_2-H group		
						Relative methylation (Cy5/Cy3)	Cy3 signal (Input)	Cy5 signal (MeDIP)	Relative methylation (Cy5/Cy3)	Cy3 signal (Input)	Cy5 signal (MeDIP)
A_68_P25496224	chr7:132851162-132851206	NM_207239:18	Gtf3c1	INSIDE	1.017	0.520	1232.99	641.42	0.529	927.59	490.66
A_68_P21622285	chr2:128793081-128793125	NM_178404:-35	Zc3h6	PROMOTER	1.017	0.278	2017.69	561.53	0.283	1422.18	402.49
A_68_P20945386	chr1:194596820-194596864	NM_144881:571	Hhat	INSIDE	1.017	0.368	2237.47	823.39	0.374	1768.11	661.95
A_68_P20121779	chr1:30929974-30930018	NM_001081080:-9895	Phf3	PROMOTER	1.017	1.982	1717.79	3404.51	2.016	1334.30	2689.70
A_68_P32152399	chr19:47940223-47940267	NM_026619:-39	Gsto2	PROMOTER	1.016	2.478	941.36	2332.51	2.518	783.56	1973.21
A_68_P31614992	chr18:35000409-35000453	NM_001204914:188	Reep2	INSIDE	1.016	0.422	1279.43	540.50	0.429	988.38	424.28
A_68_P29187499	chr13:83860849-83860893	NR_015561:-6840	C130071C03Rik	PROMOTER	1.016	0.524	2173.46	1139.72	0.533	1667.33	887.95
A_68_P29020009	chr13:48641246-48641290	NR_029725:-7627	Mirlet7a-1	PROMOTER	1.016	0.354	2177.54	769.85	0.359	1693.56	608.40
A_68_P28183246	chr11:120098017-120098061	NM_198423:3778	Bahcc1	INSIDE	1.016	0.504	2488.44	1253.60	0.512	1978.68	1012.38
A_68_P27307033	chr10:84647477-84647521	NM_007771:301	Cry1	INSIDE	1.016	1.412	1729.85	2442.19	1.435	1361.26	1952.87
A_68_P26948618	chr10:12728670-12728714	NM_175102:437	Sf3b5	INSIDE	1.016	0.359	2327.17	834.39	0.364	1853.93	675.25
A_68_P26683640	chr9:83334897-83334941	NM_029434:-184	Lca5	PROMOTER	1.016	0.582	1447.56	842.04	0.591	1153.68	681.74
A_68_P24050683	chr5:122952536-122952580	NM_009722:-324	Atp2a2	PROMOTER	1.016	0.367	1532.16	562.70	0.373	1158.90	432.39
A_68_P24009588	chr5:115890672-115890717	NM_008629:11001	Msi1	INSIDE	1.016	4.203	354.62	1490.59	4.272	292.82	1250.83
A_68_P24004678	chr5:115017216-115017260	NM_001004180:-1021	BC057022	PROMOTER	1.016	3.126	543.97	1700.23	3.175	482.06	1530.77
A_68_P23893298	chr5:92706005-92706049	NM_001163687:1181	Naaa	INSIDE	1.016	2.873	1021.82	2935.51	2.919	922.46	2692.32
A_68_P23328515	chr4:134990204-134990248	NM_022980:-506	Rcan3	PROMOTER	1.016	0.485	1846.36	895.65	0.493	1464.80	722.14
A_68_P22202886	chr3:65762239-65762283	NM_019937:-113	Ccnl1	PROMOTER	1.016	0.362	2017.82	731.00	0.368	1730.89	636.80
A_68_P32142240	chr19:46211708-46211752	NM_008852:11085	Pitx3	INSIDE	1.015	0.369	1334.72	492.14	0.374	1065.24	398.83
A_68_P29337691	chr13:113717612-113717656	NM_028151:-46	Skiv2l2	DIVERGENT_PROMOTER	1.015	0.510	1719.85	877.32	0.518	1308.47	677.56
A_68_P28875601	chr13:22073468-22073512	NM_001162929:428	Pom121l2	INSIDE	1.015	1.451	1800.10	2611.98	1.473	1451.67	2138.05
A_68_P28567254	chr12:80130228-80130272	NM_181073:101	Plekhh1	INSIDE	1.015	2.071	459.98	952.65	2.103	407.59	857.01
A_68_P28101956	chr11:106362407-106362451	NR_030703:124	Snord104	DOWNSTREAM	1.015	0.523	1332.02	696.49	0.531	1127.83	598.39
A_68_P27805434	chr11:53381034-53381078	NM_008443:176	Kif3a	INSIDE	1.015	0.410	2675.09	1096.91	0.416	2223.10	924.89
A_68_P27284176	chr10:80049548-80049592	NM_145421:3516	Fam108a	INSIDE	1.015	3.120	1984.33	6190.80	3.167	1561.34	4945.20
A_68_P27261497	chr10:75807668-75807712	NM_001081419:317	Dip2a	INSIDE	1.015	2.662	416.36	1108.23	2.702	385.56	1041.79
A_68_P25957069	chr8:74009617-74009661	NM_001164679:328	Ano8	INSIDE	1.015	1.464	1593.38	2332.02	1.486	1319.98	1961.67
A_68_P25933299	chr8:69010150-69010194	NM_025465:234	1810029B16Rik	INSIDE	1.015	0.470	3182.53	1495.88	0.477	2692.53	1284.57
A_68_P25031676	chr7:31250611-31250655	NR_004443:-22	Nphs1as	PROMOTER	1.015	0.366	1880.84	687.49	0.371	1454.44	539.66
A_68_P24780080	chr6:117118449-117118493	NM_021704:-82	Cxcl12	PROMOTER	1.015	0.263	9748.86	2566.61	0.267	7221.97	1929.94
A_68_P24586697	chr6:82041942-82041986	NM_145570:50343	Fam176a	INSIDE	1.015	0.480	1333.24	640.30	0.487	1014.69	494.62
A_68_P24385313	chr6:39822756-39822801			Unknown	1.015	0.617	1936.79	1194.05	0.626	1621.90	1015.12
A_68_P23971472	chr5:108796224-108796268	ENSMUST00000100944:-2630		PROMOTER	1.015	0.353	1752.82	618.52	0.358	1135.19	406.44
A_68_P20561475	chr1:122237683-122237727	NM_207233:546	C1ql2	INSIDE	1.015	1.668	751.52	1253.65	1.693	601.28	1018.22
A_68_P32260807	chrX:11736583-11736627	NM_001168321:877	Bcor	INSIDE	1.014	2.570	236.94	608.96	2.606	267.04	695.96
A_68_P31841471	chr18:76305627-76305671	NM_145356:325817	Zbtb7c	INSIDE	1.014	3.148	654.27	2059.93	3.193	584.00	1864.86
A_68_P31829409	chr18:74224278-74224322	NM_172632:303	Mapk4	INSIDE	1.014	0.313	3044.86	954.15	0.318	1987.47	631.56
A_68_P31344184	chr17:73457546-73457590	NM_001081071:244	Lclat1	INSIDE	1.014	0.623	1831.15	1140.50	0.632	1406.80	888.54
A_68_P28974160	chr13:40823639-40823683	NM_001122948:2152	Tcfap2a	INSIDE	1.014	1.540	857.10	1319.59	1.561	824.19	1286.27
A_68_P28549764	chr12:76770450-76770494	NM_028203:52	Wdr89	INSIDE	1.014	0.247	3416.40	842.65	0.250	2411.77	603.09
A_68_P28157432	chr11:115970917-115970961	NM_001205081:611	Trim47	INSIDE	1.014	5.362	1510.42	8099.39	5.435	1099.66	5976.91
A_68_P28073805	chr11:101286632-101286676	NM_026865:-7	1700113I22Rik	PROMOTER	1.014	1.818	660.22	1200.17	1.843	549.67	1013.24
A_68_P27804993	chr11:53293588-53293632	NM_183173:87	Ankrd43	INSIDE	1.014	0.513	1417.34	726.56	0.520	1144.31	594.53
A_68_P27543828	chr10:128347300-128347344	NM_007653:401	Cd63	INSIDE	1.014	1.677	2985.38	5006.31	1.700	2331.22	3962.38
A_68_P27272486	chr10:77632469-77632513	NM_138601:23	D10Jhu81e	INSIDE	1.014	0.639	2054.65	1312.38	0.648	1706.08	1105.30
A_68_P27135751	chr10:51747176-51747220	NM_153786:4707	Vgll2	INSIDE	1.014	0.407	1792.45	728.99	0.412	1264.71	521.55
A_68_P26823387	chr9:110283734-110283778	NM_001001144:47960	Scap	INSIDE	1.014	1.682	992.98	1670.38	1.706	783.47	1336.84
A_68_P26080209	chr8:97437362-97437406	NM_001033533:4614	Ccdc102a	INSIDE	1.014	1.639	1068.42	1750.73	1.662	813.72	1352.70
A_68_P25090670	chr7:52271335-52271379	NM_001008422:263	Scaf1	INSIDE	1.014	0.408	1349.95	550.76	0.414	980.39	405.46
A_68_P24449299	chr6:52184163-52184207	NM_008263:754	Hoxa10	INSIDE	1.014	2.597	540.64	1404.07	2.634	507.17	1335.90
A_68_P23281728	chr4:126412908-126412952	NM_198960:583	Tcfap2e	INSIDE	1.014	0.495	1757.44	869.91	0.502	1420.33	712.91
A_68_P23270362	chr4:124479885-124479929	NM_008636:114	Mtf1	INSIDE	1.014	0.490	5641.00	2765.64	0.497	4219.95	2098.72
A_68_P23155321	chr4:101169179-101169223	NM_198412:-52	Dnajc6	PROMOTER	1.014	1.862	943.60	1756.84	1.887	776.17	1464.94
A_68_P22149458	chr3:54539187-54539231	NM_001163570:78	Exosc8	INSIDE	1.014	0.654	2447.94	1600.11	0.663	1808.82	1199.16
A_68_P32397416	chrX:53984482-53984526	NM_010211:-459	Fhl1	PROMOTER	1.013	2.128	576.02	1226.04	2.155	666.42	1436.46
A_68_P32240057	chrX:7339866-7339910	NM_001105196:102	Tcfe3	INSIDE	1.013	1.491	1693.36	2525.06	1.511	2302.24	3477.77
A_68_P30580561	chr16:18582686-18582730	NM_011532:4354	Tbx1	INSIDE	1.013	1.679	786.32	1320.53	1.702	691.26	1176.25
A_68_P29296005	chr13:106235083-106235127	NM_008308:1332	Htr1a	INSIDE	1.013	0.236	2444.39	577.72	0.239	1769.26	423.72
A_68_P28158193	chr11:116099596-116099640	NM_025276:-213	Evpl	PROMOTER	1.013	1.820	1032.49	1878.66	1.843	796.11	1467.07
A_68_P28151703	chr11:115025252-115025296	NM_012030:620	Slc9a3r1	INSIDE	1.013	0.589	1557.40	917.38	0.596	1173.19	699.70
A_68_P27643749	chr11:22890683-22890727	NM_009837:112	Cct4	INSIDE	1.013	0.490	1600.53	783.74	0.496	1105.62	548.67
A_68_P26897303	chr9:123761167-123761211	NM_001110253:-168	Fyco1	PROMOTER	1.013	0.197	2838.21	559.70	0.200	2202.61	439.89
A_68_P26458361	chr9:42705530-42705574	NM_175481:46902	Grik4	INSIDE	1.013	3.469	2654.27	9207.88	3.513	2427.58	8528.58
A_68_P26013519	chr8:85689979-85690023	NM_001111304:750	Tbc1d9	INSIDE	1.013	0.427	1439.12	614.17	0.432	1138.77	492.31
A_68_P25877023	chr8:55163035-55163079	NM_009506:171	Vegfc	INSIDE	1.013	0.499	2822.59	1407.19	0.505	2235.19	1128.44

ProbeName	Target position of probe on CpG island microarray	TargetID	GeneSymbol	CpG island Description	Ratio of relative methylation (TiO_2-NP/Vehicle)	Sham group			TiO_2-H group		
						Relative methylation (Cy5/Cy3)	Cy3 signal (Input)	Cy5 signal (MeDIP)	Relative methylation (Cy5/Cy3)	Cy3 signal (Input)	Cy5 signal (MeDIP)
A_68_P25285353	chr7:91040923-91040967	NM_023403:435	Mesdc2	INSIDE	1.013	0.219	3681.73	805.17	0.222	2542.50	563.32
A_68_P25184946	chr7:73007817-73007861	NM_011048:817	Pcsk6	INSIDE	1.013	0.524	1207.61	632.60	0.531	896.42	475.89
A_68_P24920736	chr6:144996969-144997013	NM_001024468:342	Bcat1	INSIDE	1.013	0.231	3401.88	786.16	0.234	2292.20	536.64
A_68_P23970433	chr5:108561687-108561731	NM_028876:-98	Tmed5	DIVERGENT_PROMOTER	1.013	0.480	1453.06	697.97	0.486	1145.65	557.30
A_68_P23440035	chr4:155068628-155068672	NM_178699:200	B930041F14Rik	INSIDE	1.013	2.393	670.38	1603.97	2.424	590.98	1432.46
A_68_P23294682	chr4:128735948-128735992	NM_029219:456	Rnf19b	INSIDE	1.013	0.475	3856.25	1830.43	0.481	2763.48	1329.24
A_68_P21069043	chr2:24817956-24818000	NM_026044:37	Wdr85	INSIDE	1.013	0.252	2378.82	600.06	0.255	1750.04	447.03
A_68_P20828236	chr1:173433692-173433736	NM_019484:106	Refbp2	INSIDE	1.013	3.937	634.65	2498.44	3.987	495.48	1975.47
A_68_P32461573	chrX:70474177-70474221	NM_007978:554	F8a	INSIDE	1.012	1.871	605.63	1133.06	1.893	728.61	1379.19
A_68_P31124214	chr17:28906962-28907006	NM_011950:723	Mapk13	INSIDE	1.012	0.596	2166.45	1290.19	0.602	1598.67	963.09
A_68_P30348856	chr15:76348296-76348340	NM_130893:4241	Scrt1	INSIDE	1.012	2.266	586.33	1328.61	2.292	553.92	1269.78
A_68_P29143668	chr13:74147651-74147695	NM_182839:806	Tppp	INSIDE	1.012	0.363	2040.99	740.45	0.367	1607.46	589.88
A_68_P28143853	chr11:113612913-113612957	NM_020006:-460	Cdc42ep4	PROMOTER	1.012	0.350	1414.62	495.67	0.355	947.01	335.74
A_68_P26290457	chr9:8004110-8004154	NM_001171147:464	Yap1	INSIDE	1.012	0.372	3277.57	1218.42	0.376	2503.39	941.44
A_68_P26170718	chr8:114400399-114400443	NM_025781:155	Tmem170	INSIDE	1.012	0.601	2923.74	1757.72	0.608	2311.07	1405.55
A_68_P24760448	chr6:113554830-113554888	NM_133937:93	6720456B07Rik	INSIDE	1.012	0.439	1367.40	599.73	0.444	1153.90	512.41
A_68_P24449225	chr6:52175521-52175565	NM_010456:1827	Hoxa9	INSIDE	1.012	0.618	2763.18	1708.53	0.626	2053.40	1284.51
A_68_P24192245	chr5:151476526-151476570	NM_175310:147	Pds5b	INSIDE	1.012	0.312	2577.40	803.34	0.315	1900.76	599.55
A_68_P24090316	chr5:130175740-130175787	NM_172462:200	Zfp11	INSIDE	1.012	0.565	2421.95	1369.31	0.572	1880.71	1076.47
A_68_P22149161	chr3:54496698-54496742	NM_019995:-306	Fam48a	PROMOTER	1.012	0.318	1744.15	553.84	0.321	1282.72	412.36
A_68_P20938880	chr1:193399417-193399461	NM_029766:-25	Dtl	DIVERGENT_PROMOTER	1.012	0.236	2294.26	540.84	0.239	1673.25	399.25
A_68_P29144942	chr13:74345862-74345906	NM_177333:264	Exoc3	INSIDE	1.011	1.811	769.63	1394.10	1.831	705.65	1291.96
A_68_P28703485	chr12:105711191-105711235	NM_010351:234	Gsc	INSIDE	1.011	2.169	850.08	1843.71	2.192	676.61	1483.32
A_68_P27924560	chr11:74979095-74979139	NM_001098203:2538	Hic1	INSIDE	1.011	2.202	422.38	930.28	2.226	380.91	847.90
A_68_P27797421	chr11:51912713-51912757	NM_019411:409	Ppp2ca	INSIDE	1.011	1.370	2596.45	3556.34	1.385	2024.59	2803.52
A_68_P26447521	chr9:40965188-40965232	NM_176860:367	Ubash3b	INSIDE	1.011	0.520	1472.65	765.96	0.526	1035.47	544.56
A_68_P25095113	chr7:53050658-53050702	NM_001005511:11364	Lmtk3	INSIDE	1.011	4.630	2296.01	10630.21	4.681	1812.44	8483.54
A_68_P25048708	chr7:36103274-36103318	NM_026181:163	Gpatch1	INSIDE	1.011	0.469	3176.12	1489.49	0.474	2541.60	1204.74
A_68_P23577314	chr5:31554107-31554151	NM_027221:50	Krtcap3	INSIDE	1.011	0.459	1799.91	826.75	0.464	1550.11	719.54
A_68_P22342939	chr3:95032854-95032898	NM_001038708:3	Cdc42se1	INSIDE	1.011	0.316	4229.53	1335.80	0.319	3109.06	992.94
A_68_P21621792	chr2:128689765-128689810	NM_024237:120	Fbln7	INSIDE	1.011	0.387	2396.72	926.70	0.391	1743.51	681.33
A_68_P21350019	chr2:76244929-76244973	NM_145525:356	Osbpl6	INSIDE	1.011	0.645	2258.97	1455.98	0.652	1906.53	1242.88
A_68_P20970770	chr2:4802809-4802853	NM_175400:221	Sephs1	INSIDE	1.011	1.881	567.20	1067.19	1.903	512.37	975.02
A_68_P20740064	chr1:157659559-157659603	NM_001024945:449	Qsox1	INSIDE	1.011	0.287	3850.38	1104.57	0.290	3060.00	887.50
A_68_P20352131	chr1:75121569-75121613	NM_029342:210	Nhej1	INSIDE	1.011	0.392	1212.69	475.76	0.396	986.55	391.11
A_68_P32672216	chrX:131010833-131010877	NM_028958:175	Taf7l	INSIDE	1.010	2.632	399.92	1052.45	2.658	572.05	1520.26
A_68_P31985662	chr19:17468432-17468476	NM_019437:-78	Rfk	PROMOTER	1.010	0.216	3786.21	819.09	0.219	2962.79	647.65
A_68_P30671438	A_68_P30671438			Unknown	1.010	1.626	1275.33	2073.27	1.642	1049.43	1723.13
A_68_P30489324	chr15:100883675-100883719	NM_011323:116995	Scn8a	DOWNSTREAM	1.010	0.501	2025.56	1015.39	0.506	1576.64	798.43
A_68_P30339170	chr15:74551517-74551561	NM_198607:-125	4930572J05Rik	PROMOTER	1.010	0.596	1459.57	869.98	0.602	1193.59	718.79
A_68_P29989753	chr15:6659068-6659112	NM_030168:710	Rictor	INSIDE	1.010	0.662	2462.66	1630.02	0.669	1760.70	1177.52
A_68_P28457453	chr12:57636766-57636810	NM_001146198:-695	Nkx2-1	PROMOTER	1.010	2.110	6444.77	13595.59	2.130	5075.26	10809.48
A_68_P28095285	chr11:105153865-105153909	NM_008626:-73	Mrc2	PROMOTER	1.010	4.489	3177.38	14263.75	4.536	2455.98	11140.10
A_68_P28072389	chr11:101035495-101035539	NM_007721:1241	Ccr10	INSIDE	1.010	0.569	2272.83	1292.12	0.574	1433.27	822.90
A_68_P27898219	chr11:69780262-69780306	NM_016887:1362	Cldn7	INSIDE	1.010	0.429	1208.24	518.41	0.433	1039.41	450.55
A_68_P27340039	chr10:90564881-90564925	NM_027078:19119	Ikbip	INSIDE	1.010	3.860	1066.31	4116.32	3.899	804.26	3135.53
A_68_P25504680	chr7:134376542-134376586	NR_030674:80	Gm4532	INSIDE	1.010	0.570	1280.90	730.35	0.576	994.93	572.73
A_68_P24325497	chr6:29222598-29222642	NR_023516:-5	2310016C08Rik	PROMOTER	1.010	0.209	2103.16	440.04	0.211	1838.71	388.74
A_68_P23374050	chr4:142802510-142802554	NM_001081355:80	Prdm2	INSIDE	1.010	0.547	1739.47	950.93	0.552	1416.03	782.13
A_68_P23183551	chr4:105989077-105989121	NM_183225:281	Usp24	INSIDE	1.010	31.884	726.69	23169.78	32.198	797.26	25670.51
A_68_P22774760	chr4:21703216-21703260	NM_152825:-178	Usp45	DIVERGENT_PROMOTER	1.010	0.437	2650.41	1158.69	0.442	2081.94	919.58
A_68_P22582960	chr3:140874546-140874590	NM_008811:751	Pdha2	INSIDE	1.010	2.026	1214.69	2460.73	2.047	1037.65	2123.79
A_68_P22029171	chr3:30494182-30494226	NM_029690:4588	Arpm1	DOWNSTREAM	1.010	0.293	1689.58	494.85	0.296	1381.33	408.44
A_68_P21874750	chr2:174153901-174153945	NM_001077510:-1667	Gnas	PROMOTER	1.010	2.010	1632.32	3280.42	2.030	1316.80	2673.09
A_68_P21835852	chr2:167329260-167329304	NM_030743:11138	Rnf114	INSIDE	1.010	0.584	1315.79	767.81	0.589	1011.02	595.77
A_68_P21328886	chr2:72818646-72818690	NM_001018042:-165	Sp3	PROMOTER	1.010	1.855	777.04	1441.67	1.873	586.13	1097.98
A_68_P31252966	chr17:56095674-56095719	NM_015766:3651	Ebi3	INSIDE	1.009	2.863	2427.28	6949.35	2.889	1853.55	5354.73
A_68_P31157364	chr17:35202217-35202261	NR_001462:857	G6b	INSIDE	1.009	0.335	2812.42	941.24	0.338	2160.99	729.97
A_68_P29508104	chr14:31638725-31638769	NM_028839:-33	Tmem110	PROMOTER	1.009	8.097	1911.74	15479.32	8.173	1743.01	14245.87
A_68_P25016925	chr7:28146852-28146896	NM_001199235:32651	Spnb4	INSIDE	1.009	0.454	1888.62	857.69	0.458	1612.29	738.65
A_68_P24104838	chr5:133017511-133017555	NM_177047:681	Auts2	INSIDE	1.009	0.565	1063.74	601.11	0.570	935.06	533.04
A_68_P23428038	chr4:153331126-153331170	NM_001163019:-197	A430005L14Rik	PROMOTER	1.009	0.465	1161.54	540.46	0.469	908.56	426.53
A_68_P23252013	chr4:120242605-120242649	NM_016748:255	Ctps	INSIDE	1.009	1.805	729.31	1316.57	1.821	692.02	1260.47
A_68_P21150016	chr2:38567183-38567227	NM_139051:2858	Nr5a1	INSIDE	1.009	0.273	1841.31	502.01	0.275	1309.77	360.41
A_68_P21113954	chr2:32069353-32069397	NM_001159634:62707	Prrc2b	INSIDE	1.009	1.537	1273.22	1956.52	1.550	1053.04	1632.72

ProbeName	Target position of probe on CpG island microarray	TargetID	GeneSymbol	CpG island Description	Ratio of relative methylation (TiO_2-NP/Vehicle)	Sham group			TiO_2-H group		
						Relative methylation (Cy5/Cy3)	Cy3 signal (Input)	Cy5 signal (MeDIP)	Relative methylation (Cy5/Cy3)	Cy3 signal (Input)	Cy5 signal (MeDIP)
A_68_P33012182	chr9_random:57481-57526	NR_027950:2811	4930526I15Rik	DOWNSTREAM	1.008	3.166	1535.95	4863.29	3.193	1052.56	3361.02
A_68_P31156012	chr17:34974444-34974488	NM_001163770:503	Dom3z	INSIDE	1.008	0.316	1770.68	559.23	0.318	1359.22	432.77
A_68_P30425919	chr15:89388600-89388644	NM_021423:58335	Shank3	INSIDE	1.008	0.453	2433.33	1101.64	0.456	1688.06	770.40
A_68_P29604236	chr14:52626490-52626534	NM_027248:13896	Zfp219	INSIDE	1.008	2.849	1105.50	3149.39	2.872	1019.27	2927.14
A_68_P29578141	chr14:47380448-47380492	NM_028222:255	Cdkn3	INSIDE	1.008	0.326	1457.87	475.26	0.329	1262.91	415.15
A_68_P29269304	chr13:101386895-101386939	NM_178410:-17	Marveld2	PROMOTER	1.008	2.036	1348.15	2744.96	2.052	1253.87	2573.39
A_68_P29041278	chr13:53075844-53075888	NM_017373:542	Nfil3	INSIDE	1.008	0.139	6009.06	836.04	0.140	3705.33	519.40
A_68_P25972614	chr8:77737372-77737416	NM_029182:-448	Rasd2	PROMOTER	1.008	0.518	1317.57	682.06	0.522	1057.62	551.67
A_68_P25954772	chr8:73416640-73416684	NM_053248:-6	Slc5a5	PROMOTER	1.008	0.335	1549.64	519.22	0.338	1235.85	417.41
A_68_P24045429	chr5:122043432-122043476	NM_009656:379	Aldh2	INSIDE	1.008	1.719	978.69	1682.42	1.733	820.05	1421.31
A_68_P24002066	chr5:114577441-114577485	NM_175016:723	Alkbh2	INSIDE	1.008	0.232	1997.09	463.33	0.234	1406.65	328.89
A_68_P23996384	chr5:113567876-113567920	NM_172884:24435	2900026A02Rik	INSIDE	1.008	0.435	1453.48	631.57	0.438	1173.62	514.20
A_68_P21717104	chr2:146048487-146048531	NM_016889:776	Insm1	INSIDE	1.008	1.907	715.68	1364.88	1.923	566.20	1088.68
A_68_P21646221	chr2:133378682-133378726	NM_007553:-230	Bmp2	PROMOTER	1.008	2.527	1939.66	4900.77	2.546	1654.18	4212.10
A_68_P20184678	chr1:42755116-42755160	NM_008900:1148	Pou3f3	INSIDE	1.008	2.910	1084.28	3155.59	2.935	913.79	2681.83
A_68_P31165741	chr17:36973900-36973945	NM_001025599:-163	Trim26	PROMOTER	1.007	0.351	4901.61	1722.91	0.354	3316.83	1173.95
A_68_P30425534	chr15:89330339-89330383	NM_021423:73	Shank3	INSIDE	1.007	0.476	919.03	437.00	0.479	763.93	365.97
A_68_P29469008	chr14:24823022-24823066	NM_010610:383	Kcnma1	INSIDE	1.007	0.270	2104.74	568.49	0.272	1541.48	419.45
A_68_P29116718	chr13:69749922-69749966	NM_175283:397	Srd5a1	INSIDE	1.007	0.477	1909.32	910.42	0.480	1611.15	774.00
A_68_P28162003	chr11:116780529-116780573	NM_172948:374	Mgat5b	INSIDE	1.007	2.844	575.34	1636.34	2.865	441.26	1264.27
A_68_P28099479	chr11:105946314-105946358	NM_011947:121	Map3k3	INSIDE	1.007	1.407	1742.12	2450.59	1.416	1471.37	2083.28
A_68_P27887970	chr11:68026508-68026552	NM_008744:173798	Ntn1	INSIDE	1.007	2.754	1383.44	3810.25	2.773	1037.88	2877.67
A_68_P27513158	chr10:122634206-122634250	NM_027604:-249	Usp15	PROMOTER	1.007	0.243	4517.56	1097.95	0.245	3293.31	805.91
A_68_P24921911	chr6:145197899-145197943	NM_021284:831	Kras	INSIDE	1.007	0.288	5302.55	1528.32	0.290	3574.03	1037.07
A_68_P24022910	chr5:118292126-118292170	ENSMUST00000142742:-4		PROMOTER	1.007	0.416	1832.99	762.42	0.419	1453.40	609.00
A_68_P23466247	chr5:8056554-8056598	NM_001080974:35	Sri	INSIDE	1.007	4.418	4355.85	19245.03	4.448	3673.96	16341.85
A_68_P22168174	chr3:58220683-58220727	NM_001081229:1094	Tsc22d2	INSIDE	1.007	0.325	1722.22	559.88	0.327	1308.40	428.31
A_68_P20970772	chr2:4803061-4803105	NM_175400:473	Sephs1	INSIDE	1.007	1.556	1200.82	1868.05	1.566	1013.27	1586.81
A_68_P20877471	chr1:182656470-182656514	NM_133225:319	Acbd3	INSIDE	1.007	0.298	2336.77	696.47	0.300	1712.18	513.65
A_68_P20644880	chr1:138157179-138157223	NM_001101516:250	Gpr25	INSIDE	1.007	2.383	553.14	1318.38	2.401	439.18	1054.28
A_68_P33007165	chr8:121970673-121970717	NM_054095:76	Necab2	INSIDE	1.006	0.668	1963.12	1311.43	0.672	1601.60	1076.07
A_68_P32142306	chr19:46221925-46221969	NM_008852:869	Pitx3	INSIDE	1.006	0.126	4783.77	601.30	0.126	3345.45	423.16
A_68_P29437783	chr14:19103060-19103104	NM_025586:418	Rpl15	INSIDE	1.006	0.315	1585.60	499.67	0.317	1267.81	401.81
A_68_P29287625	chr13:104710556-104710600	NM_001005868:16	Erbb2ip	INSIDE	1.006	2.623	644.06	1689.13	2.639	518.24	1367.38
A_68_P29269009	chr13:101322202-101322246	NM_008756:229	Ocln	INSIDE	1.006	0.640	1917.80	1227.10	0.644	1493.41	961.13
A_68_P28612727	chr12:88224458-88224502	NM_145836:1284	6430527G18Rik	INSIDE	1.006	1.669	1093.13	1824.38	1.678	892.05	1496.98
A_68_P28190288	chr11:121249326-121249370	NM_139147:217	Rab40b	INSIDE	1.006	0.504	1078.93	543.71	0.507	925.13	469.12
A_68_P27898423	chr11:69814128-69814172	NM_007888:23	Dvl2	INSIDE	1.006	0.193	2865.33	552.81	0.194	1842.74	357.73
A_68_P27795021	chr11:51449266-51449310	NM_025346:110	Rmnd5b	INSIDE	1.006	3.976	662.15	2632.55	4.001	602.45	2410.31
A_68_P26504100	chr9:50336652-50336696	NM_011220:72	Pts	INSIDE	1.006	3.180	546.21	1736.84	3.198	491.97	1573.45
A_68_P26240715	chr8:126178067-126178111	NM_133955:260	Rhou	INSIDE	1.006	0.638	2132.29	1361.30	0.642	1470.09	943.85
A_68_P26234572	chr8:125135779-125135823	NM_021502:275	Trappc2l	INSIDE	1.006	0.387	1153.02	446.24	0.389	907.91	353.41
A_68_P25957204	chr8:74031827-74031871	NM_032398:3820	Plvap	INSIDE	1.006	0.479	2071.05	992.41	0.482	1612.23	776.85
A_68_P25504738	chr7:134387769-134387813	NM_177362:-249	Zfp771	PROMOTER	1.006	0.354	3800.91	1345.04	0.356	2949.86	1050.46
A_68_P25044994	chr7:35460266-35460310	NM_175140:137442	Chst8	INSIDE	1.006	0.401	1391.58	557.53	0.403	995.97	401.37
A_68_P24540585	chr6:72184350-72184394	NM_153778:1199	Atoh8	INSIDE	1.006	0.255	2240.42	570.64	0.256	1587.63	407.00
A_68_P24423938	chr6:47545113-47545157	NM_001146689:-105	Ezh2	PROMOTER	1.006	1.860	588.89	1095.39	1.871	510.54	955.25
A_68_P24307329	chr6:25639868-25639912	NM_010338:90	Gpr37	INSIDE	1.006	0.657	2379.07	1563.17	0.661	1856.07	1226.84
A_68_P24156267	chr5:144134774-144134818	NM_133355:9210	Grid2ip	INSIDE	1.006	0.483	1079.15	521.04	0.486	874.66	424.94
A_68_P24112795	chr5:135164074-135164118	NM_010717:364	Limk1	INSIDE	1.006	0.389	5577.89	2172.14	0.392	4187.86	1639.98
A_68_P23896677	chr5:93327277-93327321	NM_001077596:275	Shroom3	INSIDE	1.006	0.532	1779.37	946.63	0.535	1492.50	799.17
A_68_P23753092	chr5:65213185-65213229	NM_008453:18445	Klf3	INSIDE	1.006	2.244	398.31	893.92	2.259	355.65	803.32
A_68_P23262156	chr4:123019628-123019672	NM_007558:396	Bmp8a	INSIDE	1.006	0.307	2209.01	677.34	0.309	1493.80	460.89
A_68_P21747682	chr2:151931567-151931611	NM_029688:123	Srxn1	INSIDE	1.006	0.479	2220.46	1064.14	0.482	1778.45	857.14
A_68_P21425684	chr2:91803378-91803422	NM_138306:320	Dgkz	INSIDE	1.006	0.330	1566.57	517.34	0.332	1323.65	439.71
A_68_P33005881	A_68_P33005881			Unknown	1.005	5.515	240.37	1325.54	5.540	180.67	1000.97
A_68_P32252893	chrX:10295189-10295233	NM_001166635:720	Mid1ip1	INSIDE	1.005	2.859	311.44	890.50	2.873	349.36	1003.65
A_68_P30642392	chr16:30550884-30550928	NM_172614:-242	Tmem44	PROMOTER	1.005	0.426	2338.55	996.13	0.428	1800.27	770.61
A_68_P30589275	chr16:20733601-20733645	NM_009893:423	Chrd	INSIDE	1.005	0.317	2007.06	636.09	0.319	1500.96	478.15
A_68_P30361556	chr15:78502582-78502626	NM_183141:45939	Elfn2	INSIDE	1.005	1.482	1587.79	2353.16	1.489	1353.76	2015.36
A_68_P29246775	chr13:97312510-97312554	NM_012048:-92	Polk	DIVERGENT_PROMOTER	1.005	0.366	1248.01	457.18	0.368	1068.91	393.38
A_68_P28547082	chr12:76277823-76277867	NM_172805:475	Kcnh5	INSIDE	1.005	4.216	961.07	4051.93	4.238	823.53	3489.80
A_68_P27804916	chr11:53280008-53280053	NM_027917:9324	Shroom1	INSIDE	1.005	0.272	2562.81	696.28	0.273	1950.58	532.77
A_68_P27212539	chr10:67011459-67011503	NM_001005419:223	Ado	INSIDE	1.005	0.293	2589.56	759.58	0.295	2037.64	600.86
A_68_P26035027	chr8:89996039-89996083	NM_019626:431	Cbln1	INSIDE	1.005	0.213	2705.22	574.93	0.214	2146.41	458.57

ProbeName	Target position of probe on CpG island microarray	TargetID	GeneSymbol	CpG island Description	Ratio of relative methylation (TiO_2-NP/Vehicle)	Sham group			TiO_2-H group		
						Relative methylation (Cy5/Cy3)	Cy3 signal (Input)	Cy5 signal (MeDIP)	Relative methylation (Cy5/Cy3)	Cy3 signal (Input)	Cy5 signal (MeDIP)
A_68_P23320948	chr4:133638061-133638105	NM_001162970:13716	Aim1l	INSIDE	1.005	0.417	1344.08	560.99	0.419	802.65	336.59
A_68_P22552062	chr3:135101824-135101868	NM_025356:124	Ube2d3	INSIDE	1.005	0.619	1820.40	1127.38	0.622	1472.43	916.57
A_68_P21727905	chr2:147870580-147870624	NM_010446:2103	Foxa2	INSIDE	1.005	0.074	8787.13	651.90	0.075	5901.05	439.87
A_68_P21477087	chr2:102290962-102291006	NM_010218:965	Fjx1	INSIDE	1.005	1.719	1286.70	2212.12	1.728	1021.27	1764.53
A_68_P21229090	chr2:54288809-54288853	NM_173030:33	Galnt13	INSIDE	1.005	0.257	2384.79	612.45	0.258	1732.47	447.36
A_68_P20599496	chr1:130141494-130141539	NM_026390:759	Ubxn4	INSIDE	1.005	0.322	1652.25	531.48	0.323	1302.20	420.86
A_68_P32233079	chrX:5659147-5659194	NM_001033211:1368	AU022751	INSIDE	1.004	2.260	435.06	983.15	2.268	567.42	1287.04
A_68_P30777939	chr16:55822496-55822540	NM_001159394:-267	Nfkbiz	PROMOTER	1.004	2.237	854.70	1911.95	2.245	875.99	1966.87
A_68_P29941714	chr14:119322290-119322334	NM_025943:77	Dzip1	INSIDE	1.004	1.768	700.11	1237.73	1.776	599.46	1064.44
A_68_P29508896	chr14:31764964-31765008	NM_011849:313	Nek4	INSIDE	1.004	0.525	1219.19	639.92	0.527	1094.02	576.71
A_68_P28925133	chr13:31902908-31902952	NM_008592:4416	Foxc1	DOWNSTREAM	1.004	0.434	1011.61	439.08	0.436	826.38	360.23
A_68_P27894856	chr11:69213335-69213379	NM_001017426:13821	Kdm6b	INSIDE	1.004	2.039	1705.78	3478.85	2.048	1598.43	3274.02
A_68_P27503061	chr10:120802012-120802056	NM_029364:-111	Gns	PROMOTER	1.004	0.555	1129.23	626.64	0.557	976.47	543.89
A_68_P27286215	chr10:80363124-80363168	NM_013895:568	Timm13	INSIDE	1.004	0.706	6090.60	4298.27	0.708	4453.43	3154.06
A_68_P27171226	chr10:59342593-59342637	NM_019965:276	Dnajb12	INSIDE	1.004	0.279	5496.14	1533.92	0.280	4060.96	1137.37
A_68_P26372311	chr9:26563473-26563520	NM_029792:4350	B3gat1	INSIDE	1.004	2.493	1356.92	3382.48	2.502	812.09	2032.05
A_68_P26136321	chr8:108378956-108379000	NM_021513:-24	Thap11	DIVERGENT_PROMOTER	1.004	0.557	2118.48	1179.04	0.559	1761.78	984.81
A_68_P25601084	chr7:150646850-150646894	NM_001161624:83	Cdkn1c	INSIDE	1.004	2.386	1321.67	3153.96	2.395	1107.09	2651.77
A_68_P25378793	chr7:109590580-109590624	NM_009103:394	Rrm1	INSIDE	1.004	0.253	6626.86	1673.47	0.254	5353.68	1357.99
A_68_P24693486	chr6:100620755-100620799	NM_181590:375	Shq1	INSIDE	1.004	0.473	1262.14	596.46	0.474	971.89	461.03
A_68_P24600269	chr6:84534408-84534452	NM_175475:9374	Cyp26b1	INSIDE	1.004	0.524	1120.07	587.18	0.527	833.57	438.88
A_68_P21744414	chr2:151368303-151368347	NM_008019:90	Fkbp1a	INSIDE	1.004	0.426	2275.89	968.91	0.427	1664.84	711.65
A_68_P21041600	chr2:18722106-18722150	AK018938:-468		PROMOTER	1.004	0.549	1133.97	622.30	0.551	929.71	512.19
A_68_P20472152	chr1:99557742-99557786	NM_133825:831	D1Ertd622e	INSIDE	1.004	1.702	952.00	1619.99	1.708	820.43	1401.00
A_68_P31862860	chr18:80389507-80389558	NM_199197:7826	Rbfa	INSIDE	1.003	2.117	673.01	1424.51	2.123	487.63	1035.14
A_68_P31412117	chr17:86087251-86087295	NM_011380:322	Six2	INSIDE	1.003	2.369	530.47	1256.59	2.375	514.30	1221.34
A_68_P31106320	chr17:25933511-25933555	NM_133719:458	Metrn	INSIDE	1.003	0.300	1574.94	472.52	0.301	1181.63	355.66
A_68_P29622464	chr14:56983521-56983565	NM_138652:-362	Atp12a	PROMOTER	1.003	0.440	1025.20	450.81	0.441	765.22	337.53
A_68_P28610310	chr12:87811236-87811280	NM_011934:48665	Esrrb	INSIDE	1.003	2.938	1144.68	3363.08	2.948	1005.37	2964.07
A_68_P27926119	chr11:75258911-75258955	NM_138950:9287	Wdr81	INSIDE	1.003	2.076	2017.37	4188.30	2.082	1623.14	3379.25
A_68_P25737278	chr8:28338295-28338339	NM_013462:1744	Adrb3	INSIDE	1.003	0.220	6115.21	1343.81	0.220	4014.50	884.79
A_68_P24948001	chr7:4103164-4103208	NM_021454:13115	Cdc42ep5	INSIDE	1.003	0.418	1456.76	609.54	0.420	1077.69	452.31
A_68_P24793354	chr6:119430660-119430704	NM_133940:997	Fbxl14	INSIDE	1.003	1.775	1341.49	2380.75	1.780	1121.95	1996.54
A_68_P23352770	chr4:139130352-139130396	NM_001205173:-260	Iffo2	PROMOTER	1.003	0.169	5400.05	910.41	0.169	3645.52	616.64
A_68_P23232777	chr4:116828074-116828118	NR_028524:-917	Snord38a	PROMOTER	1.003	0.445	1116.16	497.16	0.447	852.56	380.78
A_68_P22522353	chr3:129534614-129534673	NM_026578:-329	Gar1	DIVERGENT_PROMOTER	1.003	0.320	2292.14	732.42	0.320	1504.82	482.18
A_68_P22518746	chr3:128917465-128917509	NM_001042502:632	Pitx2	INSIDE	1.003	0.385	1492.26	575.07	0.386	1142.26	441.46
A_68_P21831917	chr2:166619198-166619242	NM_023815:-46	Trp53rk	PROMOTER	1.003	0.512	2309.32	1181.64	0.513	1718.12	882.17
A_68_P21319205	chr2:71227112-71227156	NM_026115:-182	Hat1	PROMOTER	1.003	0.492	1898.35	933.83	0.493	1733.55	854.90
A_68_P20969133	chr2:4524944-4524988	NM_001177844:44167	Frmd4a	INSIDE	1.003	1.801	802.38	1445.40	1.807	621.65	1123.09
A_68_P32767190	chrX:154036239-154036283	NM_172307:387	Mbtps2	INSIDE	1.002	1.727	405.71	700.74	1.731	622.45	1077.73
A_68_P32559512	chrX:97624640-97624684	NM_001163191:311	Otud6a	INSIDE	1.002	2.189	1582.61	3464.84	2.193	2319.96	5086.92
A_68_P32415799	chrX:58145248-58145292	NM_009237:1335	Sox3	INSIDE	1.002	1.542	1042.81	1607.85	1.544	1554.15	2400.18
A_68_P30281413	chr15:64213921-64213965	NM_010026:539	Asap1	INSIDE	1.002	0.535	1543.87	826.12	0.536	1260.27	675.63
A_68_P29616398	chr14:55732626-55732670	NM_177049:3124	Jph4	INSIDE	1.002	2.323	1050.42	2440.33	2.327	866.59	2016.45
A_68_P29047197	chr13:54151010-54151054	NM_010076:-5	Drd1a	PROMOTER	1.002	0.432	2103.86	909.10	0.433	1612.01	698.18
A_68_P28054691	chr11:98011236-98011280	NM_028149:-328	Fbxl20	PROMOTER	1.002	0.495	1936.24	958.75	0.496	1455.21	721.74
A_68_P26816016	chr9:108564748-108564792	NM_020520:342	Slc25a20	INSIDE	1.002	2.982	614.01	1830.95	2.988	550.94	1646.19
A_68_P26629704	chr9:72655004-72655048	NM_175485:-54	Prtg	PROMOTER	1.002	0.667	2082.78	1389.09	0.669	1779.31	1189.59
A_68_P26591020	chr9:65917533-65917577	NM_001025612:-16	Snx22	PROMOTER	1.002	0.574	1232.18	707.05	0.575	1090.17	626.54
A_68_P23607763	chr5:36807446-36807491	NM_024478:-365	Grpel1	PROMOTER	1.002	0.140	3433.06	479.68	0.140	2383.39	333.70
A_68_P23392000	chr4:147413032-147413076	NM_010840:-131	Mthfr	DIVERGENT_PROMOTER	1.002	0.251	2494.89	625.18	0.251	1924.69	483.03
A_68_P22630568	chr3:148652007-148652051	ENSMUST00000098518:252		INSIDE	1.002	1.865	967.58	1804.17	1.869	811.05	1515.71
A_68_P21601162	chr2:125073336-125073380	NM_023595:108	Dut	INSIDE	1.002	0.149	3798.22	564.08	0.149	2875.41	427.95
A_68_P20878147	chr1:182781059-182781103	NM_145943:-201	BC031781	PROMOTER	1.002	0.202	3252.27	658.27	0.203	2469.29	500.94
A_68_P32537858	chrX:91457905-91457949	NM_012010:64	Eif2s3x	INSIDE	1.001	0.404	1468.25	592.94	0.404	1300.14	525.78
A_68_P32319553	chrX:34185171-34185215	NM_001081956:217	Akap17b	INSIDE	1.001	2.683	412.34	1106.39	2.687	649.70	1745.84
A_68_P32121151	chr19:42507010-42507054	NM_145123:-759	Crtac1	PROMOTER	1.001	0.483	4107.03	1983.04	0.483	3207.04	1550.22
A_68_P30380962	chr15:81846721-81846765	NM_010247:-56	Xrcc6	DIVERGENT_PROMOTER	1.001	0.491	2209.26	1084.67	0.491	1637.73	804.88
A_68_P27453303	chr10:111707353-111707397	NM_001025581:-804	Kcnc2	PROMOTER	1.001	0.417	2673.07	1114.46	0.417	1995.52	832.97
A_68_P26451992	chr9:41698412-41698459			Unknown	1.001	0.458	2471.01	1132.13	0.459	1690.66	775.25
A_68_P26430869	chr9:37296739-37296783	NM_146222:-145	BC024479	PROMOTER	1.001	0.457	1684.88	770.07	0.458	1196.85	547.62
A_68_P25954144	chr8:73321416-73321460	NM_199308:-5106	Mast3	PROMOTER	1.001	0.428	3166.53	1355.46	0.429	2223.66	953.26
A_68_P25592939	chr7:149280544-149280588	NM_008748:623	Dusp8	INSIDE	1.001	0.526	2116.44	1112.68	0.526	1708.79	899.12
A_68_P24946542	chr7:3597193-3597237	NM_146176:344	Cnot3	INSIDE	1.001	0.337	1835.57	618.71	0.337	1439.78	485.72

ProbeName	Target position of probe on CpG island microarray	TargetID	GeneSymbol	CpG island Description	Ratio of relative methylation (TiO_2-NP/Vehicle)	Sham group			TiO_2-H group		
						Relative methylation (Cy5/Cy3)	Cy3 signal (Input)	Cy5 signal (MeDIP)	Relative methylation (Cy5/Cy3)	Cy3 signal (Input)	Cy5 signal (MeDIP)
A_68_P24839021	chr6:128388523-128388567	NM_010219:105	Fkbp4	INSIDE	1.001	1.568	912.24	1429.95	1.569	856.10	1343.00
A_68_P24754179	chr6:112439818-112439862	NM_001081147:-38	Oxtr	PROMOTER	1.001	0.267	1972.88	527.01	0.267	1578.23	421.91
A_68_P24224752	chr6:8159107-8159151	NM_145374:-98	Mios	PROMOTER	1.001	0.341	1811.56	617.93	0.341	1460.54	498.69
A_68_P23668848	chr5:48374910-48374954	NM_178804:539	Slit2	INSIDE	1.001	0.442	2790.41	1234.42	0.443	2022.37	895.36
A_68_P23324348	chr4:134260291-134260335	NM_207237:-107	Man1c1	PROMOTER	1.001	0.576	3228.71	1859.99	0.577	2218.31	1279.27
A_68_P22288625	chr3:83843924-83843968	NM_172681:137	D930015E06Rik	INSIDE	1.001	1.649	1191.91	1965.73	1.651	949.74	1567.63
A_68_P20354542	chr1:75501748-75501792	NM_178884:1257	Obsl1	INSIDE	1.001	0.203	2183.96	443.60	0.203	1590.64	323.49
A_68_P32082683	chr19:34953737-34953781	NM_023792:-1351	Pank1	PROMOTER	1.000	0.289	1781.93	514.28	0.289	1337.15	385.86
A_68_P30080950	chr15:25344237-25344281	NR_027376:312	Gm5468	INSIDE	1.000	2.569	530.71	1363.27	2.569	450.37	1157.16
A_68_P28748130	chr12:113382569-113382613	NM_001097621:-1828	Kif26a	PROMOTER	1.000	2.737	446.39	1221.90	2.736	346.85	949.12
A_68_P28224317	chr12:8778225-8778269	NM_011519:45	Sdc1	INSIDE	1.000	0.320	2378.23	761.40	0.320	1801.24	576.47
A_68_P28095689	chr11:105224379-105224423	NM_172568:93649	Marchf10	INSIDE	1.000	0.462	1019.28	471.31	0.462	779.62	360.35
A_68_P27854136	chr11:62272091-62272135	NM_001039536:151	Pigl	INSIDE	1.000	1.641	867.86	1424.34	1.640	764.29	1253.76
A_68_P26949006	chr10:12810713-12810757	NM_009538:141	Plagl1	INSIDE	1.000	2.749	709.34	1949.90	2.750	696.47	1915.34
A_68_P25953307	chr8:73197405-73197449	NM_015816:297	Lsm4	INSIDE	1.000	0.517	1582.25	817.48	0.517	1202.87	621.68
A_68_P25531558	chr7:139158263-139158307	NM_173410:142	Gpr26	INSIDE	1.000	0.329	1995.89	657.13	0.329	1561.98	514.02
A_68_P24444192	chr6:51382686-51382730	NM_010903:40	Nfe2l3	INSIDE	1.000	0.260	3433.02	891.86	0.260	2616.73	680.10
A_68_P23338973	chr4:136833633-136833677	NM_009523:105	Wnt4	INSIDE	1.000	3.219	592.16	1906.29	3.219	499.90	1609.32
A_68_P22313622	chr3:88214207-88214256	NM_025928:7	Pmf1	INSIDE	1.000	3.672	693.69	2547.25	3.672	622.06	2283.94
A_68_P21817618	chr2:164226131-164226175	NM_009036:-2541	Rbpjl	PROMOTER	1.000	0.550	1864.38	1025.37	0.550	1483.81	815.71
A_68_P21777899	chr2:157391883-157391927	NM_016916:193	Blcap	INSIDE	1.000	0.301	1484.91	447.01	0.301	1251.32	376.55
A_68_P21266438	chr2:61649821-61649865	NM_009322:7333	Tbr1	INSIDE	1.000	3.566	732.77	2612.71	3.566	646.52	2305.47
A_68_P32578105	chrX:101866222-101866266	NM_175358:159	Zdhhc15	INSIDE	0.999	0.601	901.94	541.74	0.600	1276.50	766.19
A_68_P31104080	chr17:25611583-25611627	NM_027951:3070	Tekt4	INSIDE	0.999	0.429	1359.50	583.80	0.429	1088.25	466.68
A_68_P29613442	chr14:55260574-55260618	NM_199470:-395	Cdh24	PROMOTER	0.999	0.562	2406.10	1351.79	0.562	1608.68	903.33
A_68_P27812595	chr11:54716247-54716291	NM_001083929:-86	Gpx3	PROMOTER	0.999	1.932	1114.92	2154.31	1.931	906.27	1749.72
A_68_P27567705	chr11:6191666-6191710	NM_010956:89	Ogdh	INSIDE	0.999	0.143	3089.09	442.31	0.143	2101.98	300.76
A_68_P27357054	chr10:93498512-93498556	NM_172538:-41	Vezt	DIVERGENT_PROMOTER	0.999	0.457	3590.51	1641.56	0.457	2513.62	1147.63
A_68_P27282472	chr10:79786332-79786376	NM_008793:5864	Pcsk4	INSIDE	0.999	0.558	3913.77	2184.92	0.558	2691.16	1500.79
A_68_P25951454	chr8:72900470-72900514	NM_016685:3046	Comp	INSIDE	0.999	0.224	2570.61	576.30	0.224	1749.78	391.99
A_68_P24990325	chr7:19479208-19479252	NM_001033314:16523	Ccdc61	INSIDE	0.999	0.597	3625.12	2163.88	0.596	2394.12	1427.23
A_68_P23536417	chr5:22940364-22940408	NM_026984:140	Mll5	INSIDE	0.999	0.118	4048.72	477.37	0.118	2828.48	333.01
A_68_P23415140	chr4:151236795-151236839	NM_001081557:-939	Camta1	PROMOTER	0.999	0.408	1180.07	481.81	0.408	869.04	354.54
A_68_P23280297	chr4:126145852-126145896	NM_153403:-209	Eif2c1	PROMOTER	0.999	2.047	417.76	855.15	2.046	390.87	799.65
A_68_P22411698	chr3:108087081-108087125	NM_019972:53	Sort1	INSIDE	0.999	2.352	3350.13	7879.60	2.350	2715.53	6380.99
A_68_P21828032	chr2:165981031-165981075	NM_028072:104	Sulf2	INSIDE	0.999	1.658	1191.87	1976.34	1.657	1005.07	1665.76
A_68_P20936389	chr1:192949970-192950014	ENSMUST00000158219:5019		DOWNSTREAM	0.999	0.231	3110.01	719.69	0.231	2171.80	502.24
A_68_P31256954	chr17:56705768-56705812	NM_001029979:18216	Safb2	INSIDE	0.998	2.355	2485.42	5852.90	2.349	1980.41	4652.53
A_68_P30473865	chr15:98087521-98087565	NM_027304:196	H1fnt	INSIDE	0.998	2.143	616.96	1322.02	2.139	591.20	1264.68
A_68_P29702035	chr14:70954292-70954336	NM_021877:452	Hr	INSIDE	0.998	0.579	1310.54	759.28	0.578	998.40	577.09
A_68_P28159660	chr11:116351347-116351391	NM_026364:292	Prpsap1	INSIDE	0.998	0.377	2020.04	760.62	0.376	1583.51	595.09
A_68_P26770339	chr9:100471468-100471512	NM_001101483:14	Tmem22	INSIDE	0.998	2.005	643.48	1290.12	2.002	532.10	1065.20
A_68_P26165985	chr8:113521608-113521652	NM_007916:93	Ddx19a	INSIDE	0.998	0.656	2035.06	1335.32	0.655	1570.83	1028.26
A_68_P24193750	chr5:151755531-151755575	NM_013823:371	Kl	INSIDE	0.998	0.335	1607.72	538.15	0.334	1205.62	402.79
A_68_P21117162	chr2:32597241-32597285	NM_013781:20671	Sh2d3c	INSIDE	0.998	0.320	1915.19	611.96	0.319	1630.94	519.97
A_68_P20600072	chr1:130256315-130256359	NM_008567:-103	Mcm6	PROMOTER	0.998	0.264	1837.94	485.52	0.264	1443.55	380.47
A_68_P31097497	chr17:24607518-24607562	NM_025954:123	Pgp	INSIDE	0.997	0.577	1872.99	1080.40	0.575	1511.88	869.49
A_68_P30495773	chr15:101904768-101904812	NM_145625:587	Eif4b	INSIDE	0.997	0.394	2254.00	887.84	0.393	1887.31	741.33
A_68_P30465076	chr15:96529677-96529721	NM_175121:431	Slc38a2	INSIDE	0.997	0.196	3920.63	769.96	0.196	2454.20	480.70
A_68_P29689073	chr14:68701763-68701807	NM_010910:-156	Nefl	PROMOTER	0.997	0.496	3785.17	1875.88	0.494	2847.33	1406.73
A_68_P27189847	chr10:62655248-62655292	NM_182992:11430	Mypn	INSIDE	0.997	2.523	352.22	888.67	2.516	317.97	799.88
A_68_P27100536	chr10:43199837-43199881	NM_199028:913	Bend3	INSIDE	0.997	1.497	1117.28	1672.72	1.493	985.48	1471.70
A_68_P26783897	chr9:103014037-103014081	NM_173781:-345	Rab6b	PROMOTER	0.997	0.554	1541.77	854.89	0.553	1260.66	697.13
A_68_P25581339	chr7:147130322-147130366	NM_009482:590	Utf1	INSIDE	0.997	0.215	3192.89	687.99	0.215	2204.12	473.51
A_68_P25370886	chr7:108147189-108147233	NM_008773:13295	P2ry2	INSIDE	0.997	2.721	1997.55	5435.94	2.713	1596.74	4331.41
A_68_P25358212	chr7:105987224-105987268	NM_009519:-108	Wnt11	PROMOTER	0.997	0.342	1417.92	484.36	0.341	1081.77	368.37
A_68_P24008563	chr5:115685673-115685717	NM_026398:-165	Pop5	PROMOTER	0.997	1.567	1326.00	2077.61	1.561	1106.29	1727.38
A_68_P23541891	chr5:23951016-23951060	NM_023229:15	Fastk	INSIDE	0.997	2.360	1088.14	2567.64	2.353	885.15	2082.83
A_68_P22006136	chr3:26230775-26230819	NM_138666:35	Nlgn1	INSIDE	0.997	0.449	2792.91	1253.42	0.447	2035.59	910.86
A_68_P21568534	chr2:119063096-119063140	NM_016907:23	Spint1	INSIDE	0.997	2.940	756.30	2223.63	2.931	526.12	1542.19
A_68_P21103105	chr2:30272089-30272133	NM_138748:541	Ppp2r4	INSIDE	0.997	0.335	4058.48	1361.23	0.334	2910.21	973.03
A_68_P28466189	chr12:59313486-59313530	NM_009216:718	Sstr1	INSIDE	0.996	2.118	1087.35	2303.06	2.110	866.74	1828.44
A_68_P28143200	chr11:113510983-113511027	NM_013581:162	Cog1	INSIDE	0.996	0.546	1499.24	818.85	0.544	1169.47	635.96
A_68_P28084674	chr11:103195207-103195251	NM_133715:10788	Arhgap27	INSIDE	0.996	0.452	1112.40	502.43	0.450	885.31	398.21
A_68_P27555762	chr11:4018372-4018416	NM_144520:338	Sec14l2	INSIDE	0.996	0.553	1420.33	785.92	0.551	1157.05	637.93

ProbeName	Target position of probe on CpG island microarray	TargetID	GeneSymbol	CpG island Description	Ratio of relative methylation (TiO_2-NP/Vehicle)	Sham group			TiO_2-H group		
						Relative methylation (Cy5/Cy3)	Cy3 signal (Input)	Cy5 signal (MeDIP)	Relative methylation (Cy5/Cy3)	Cy3 signal (Input)	Cy5 signal (MeDIP)
A_68_P27354860	chr10:93103568-93103612	NM_021320:-203	Ntn4	PROMOTER	0.996	1.674	2050.00	3432.16	1.668	1530.24	2551.83
A_68_P26872176	chr9:119312610-119312654	NM_007397:1014	Acvr2b	INSIDE	0.996	0.491	1556.61	764.08	0.489	1120.00	547.79
A_68_P26617806	chr9:70527432-70527476	NM_007399:647	Adam10	INSIDE	0.996	6.284	1007.21	6329.21	6.261	830.15	5197.49
A_68_P26595470	chr9:66682593-66682637	NM_026674:-101	Aph1c	PROMOTER	0.996	0.299	3958.45	1184.00	0.298	2934.08	873.98
A_68_P26319140	chr9:14555819-14555863	NM_010242:726	Fut4	INSIDE	0.996	0.607	2981.25	1810.81	0.605	2093.17	1266.10
A_68_P25949228	chr8:72520400-72520444	NM_001113345:-144	Gatad2a	PROMOTER	0.996	0.169	2895.36	489.93	0.168	2165.94	364.89
A_68_P25596372	chr7:149846899-149846943	NM_001122736:20	Igf2	INSIDE	0.996	0.533	2142.62	1142.75	0.531	1775.40	943.40
A_68_P25349895	chr7:104474236-104474280	NM_001177412:216	Usp35	INSIDE	0.996	1.636	840.98	1375.73	1.629	746.60	1215.88
A_68_P24085132	chr5:129288040-129288085	NM_001081388:171175	Rimbp2	INSIDE	0.996	2.009	758.25	1523.66	2.001	580.98	1162.59
A_68_P23929926	chr5:100927778-100927822	NM_001115010:-208	Lin54	PROMOTER	0.996	0.180	5492.00	987.18	0.179	3436.36	615.15
A_68_P23221707	chr4:114494191-114494235	NR_033617:-112	Gm12830	PROMOTER	0.996	0.350	2576.96	903.02	0.349	1914.64	668.09
A_68_P31961512	chr19:12670749-12670793	NR_015503:286	A330040F15Rik	INSIDE	0.995	0.249	1969.80	490.34	0.248	1356.00	335.83
A_68_P31620310	chr18:35990907-35990952	NM_133687:1458	Cxxc5	INSIDE	0.995	3.446	267.52	922.00	3.428	253.15	867.80
A_68_P31114504	chr17:27270575-27270620	NM_026063:239	2900010M23Rik	INSIDE	0.995	0.476	3275.89	1559.05	0.474	2497.61	1183.11
A_68_P30499541	chr15:102577044-102577088			Unknown	0.995	0.229	3183.00	728.11	0.228	2200.12	500.92
A_68_P27971357	chr11:83232403-83232447	NM_001013386:8851	Rasl10b	INSIDE	0.995	0.569	1374.11	781.67	0.566	1135.39	642.82
A_68_P27179601	chr10:60736090-60736134	NM_001081127:74	Adamts14	INSIDE	0.995	1.642	873.06	1433.35	1.634	779.80	1273.90
A_68_P26468507	chr9:44412404-44412448	NM_001110826:-548	Ddx6	PROMOTER	0.995	0.450	1548.45	696.67	0.448	1179.52	528.26
A_68_P25173484	chr7:70903305-70903349	NM_130880:313669	Otud7a	INSIDE	0.995	2.162	355.15	767.81	2.152	332.87	716.29
A_68_P25090464	chr7:52241287-52241331	NM_019830:394	Prmt1	INSIDE	0.995	0.573	1914.56	1097.94	0.571	1614.04	921.28
A_68_P25047600	chr7:35903087-35903131	NM_007678:-1203	Cebpa	PROMOTER	0.995	0.421	2266.84	954.25	0.419	1685.06	705.81
A_68_P23120717	chr4:94719222-94719266	NM_010591:-331	Jun	PROMOTER	0.995	0.530	1136.43	602.10	0.527	882.26	464.88
A_68_P31369867	chr17:78599714-78599759	NM_015800:149	Crim1	INSIDE	0.994	1.748	1475.80	2579.43	1.737	1075.29	1867.95
A_68_P31232584	chr17:51318828-51318872	NM_028162:-176	Tbc1d5	PROMOTER	0.994	0.392	2344.84	918.11	0.389	1699.14	661.22
A_68_P30350783	chr15:76647954-76647998	NM_001168288:624	Arhgap39	INSIDE	0.994	0.381	7678.14	2928.98	0.379	5137.18	1947.28
A_68_P29942851	chr14:119537089-119537133	NM_015820:-376	Hs6st3	PROMOTER	0.994	2.040	498.30	1016.30	2.026	434.59	880.68
A_68_P29372953	chr13:120197613-120197657	NM_008710:184	Nnt	INSIDE	0.994	0.452	1168.97	528.24	0.449	1041.43	467.91
A_68_P27921612	chr11:74452062-74452106	NM_001013784:18978	E130309D14Rik	INSIDE	0.994	2.097	780.95	1637.35	2.083	629.99	1312.49
A_68_P27127187	chr10:49502783-49502839	NM_001111268:5750	Grik2	INSIDE	0.994	3.943	430.91	1698.91	3.920	329.09	1290.16
A_68_P26885417	chr9:121672235-121672279	NM_001166644:3106	Zfp651	INSIDE	0.994	2.273	497.96	1131.66	2.258	453.81	1024.91
A_68_P25804897	chr8:41392991-41393035	NM_030110:-392	Efha2	PROMOTER	0.994	1.514	1299.91	1968.07	1.505	1089.61	1639.53
A_68_P24770500	chr6:115312177-115312222	NM_001127330:961	Pparg	INSIDE	0.994	1.464	2173.42	3181.21	1.455	1794.67	2610.64
A_68_P24155044	chr5:143874288-143874332	NM_080561:389	Rnf216	INSIDE	0.994	2.497	818.12	2043.12	2.483	673.87	1673.39
A_68_P21142144	chr2:37298887-37298931	NM_001033960:-8865	Rabgap1	PROMOTER	0.994	0.315	1540.87	485.31	0.313	885.62	277.13
A_68_P21107214	chr2:30996795-30996839	NM_001038700:712	Fnbp1	INSIDE	0.994	0.380	1887.00	717.73	0.378	1577.12	596.36
A_68_P32672206	chrX:131009632-131009676	NM_028958:1375	Taf7l	INSIDE	0.993	2.558	439.79	1125.05	2.541	683.16	1736.05
A_68_P31668412	chr18:44821284-44821328	NR_026853:-12	A930012L18Rik	PROMOTER	0.993	0.513	3037.47	1557.01	0.509	2389.28	1216.13
A_68_P31386733	chr17:81464391-81464435	NM_028138:13	Thumpd2	INSIDE	0.993	3.680	734.09	2701.69	3.653	576.92	2107.51
A_68_P29443675	chr14:20643061-20643105	NM_026528:-37	2700060E02Rik	PROMOTER	0.993	0.508	1378.53	699.93	0.504	1175.51	592.42
A_68_P28043963	chr11:96207399-96207443	NM_010458:2338	Hoxb3	INSIDE	0.993	0.414	1948.96	807.69	0.411	1528.76	628.92
A_68_P27468587	chr10:114688668-114688723	NM_025706:-146	Tbc1d15	PROMOTER	0.993	0.215	2779.10	596.63	0.213	2145.65	457.21
A_68_P26486121	chr9:47338434-47338478	NM_001025600:22	Cadm1	INSIDE	0.993	0.462	1183.87	547.48	0.459	1064.90	489.14
A_68_P26348939	chr9:21599872-21599916	NM_145611:3096	Kank2	INSIDE	0.993	3.814	467.61	1783.50	3.787	370.58	1403.47
A_68_P23409802	chr4:150431872-150431916	NM_009498:168	Vamp3	INSIDE	0.993	3.751	686.59	2575.17	3.723	573.29	2134.63
A_68_P23296404	chr4:129055173-129055217	NM_028603:78	Zbtb8a	INSIDE	0.993	0.564	1379.12	777.69	0.560	1094.12	612.58
A_68_P22356817	chr3:97671916-97671960	NM_177145:297	Pde4dip	INSIDE	0.993	0.529	1165.88	617.28	0.526	1060.07	557.61
A_68_P21419448	chr2:90725956-90726000	NM_026161:36	C1qtnf4	INSIDE	0.993	0.479	1338.71	641.62	0.476	1063.19	505.97
A_68_P21133066	chr2:35192576-35192620	NM_013515:-69	Stom	PROMOTER	0.993	3.041	2076.63	6314.64	3.019	1581.85	4775.85
A_68_P20632582	chr1:136196052-136196096	NM_001144855:33431	Ppfia4	INSIDE	0.993	0.540	1293.94	698.65	0.536	970.02	520.26
A_68_P20058164	chr1:17087723-17087767	NM_020604:226	Jph1	INSIDE	0.993	0.690	2769.18	1911.14	0.685	2226.10	1525.00
A_68_P32143068	chr19:46379305-46379349	NM_019408:-858	Nfkb2	PROMOTER	0.992	0.368	1270.91	467.95	0.365	995.05	363.43
A_68_P31159054	chr17:35496374-35496419	M19687:92581		INSIDE	0.992	0.302	1684.27	507.97	0.299	1293.48	387.13
A_68_P31003192	chr17:3114390-3114434	NM_134123:-559	Scaf8	PROMOTER	0.992	0.420	2984.32	1254.36	0.417	2262.35	943.65
A_68_P30997386	chr16:97392403-97392447	NM_031174:-82	Dscam	PROMOTER	0.992	2.198	625.35	1374.32	2.181	477.76	1042.02
A_68_P30655991	chr16:33056419-33056463	NM_001130485:-98	Rpl35a	DIVERGENT_PROMOTER	0.992	0.327	1355.66	443.23	0.324	1068.59	346.42
A_68_P30371903	chr15:80202683-80202727	NM_001044308:85037	Cacna1i	INSIDE	0.992	2.751	1078.39	2966.12	2.729	811.13	2213.66
A_68_P29702361	chr14:70999208-70999252	NM_194345:412	Fam160b2	INSIDE	0.992	0.422	2404.86	1015.18	0.419	1846.22	773.24
A_68_P29437784	chr14:19103158-19103202	NM_025586:320	Rpl15	INSIDE	0.992	0.634	1888.59	1196.44	0.629	1439.33	904.83
A_68_P28266973	chr12:16817722-16817766	NM_033270:-26	E2f6	PROMOTER	0.992	0.313	1604.36	501.55	0.310	1248.84	387.41
A_68_P26610636	chr9:69245924-69245968	NM_145618:142	Narg2	INSIDE	0.992	0.175	6351.96	1108.48	0.173	4558.66	789.04
A_68_P25502831	chr7:134028530-134028574	NM_001163774:-71	Taok2	PROMOTER	0.992	0.334	1361.09	454.33	0.331	1129.48	374.08
A_68_P24839014	chr6:128387845-128387889	NM_010219:783	Fkbp4	INSIDE	0.992	10.587	3320.10	35150.37	10.506	2630.48	27636.60
A_68_P23227293	chr4:115689981-115690025	NM_010173:505	Faah	INSIDE	0.992	0.596	3116.91	1859.02	0.592	2443.44	1446.16
A_68_P23225504	chr4:115410525-115410569	NM_172698:-131	4732418C07Rik	PROMOTER	0.992	0.667	3990.93	2660.55	0.661	3077.97	2035.20
A_68_P22406460	chr3:107261217-107261261	NM_145922:578	Kcnc4	INSIDE	0.992	0.673	2996.71	2017.81	0.668	2434.72	1626.00

ProbeName	Target position of probe on CpG island microarray	TargetID	GeneSymbol	CpG island Description	Ratio of relative methylation (TiO_2-NP/Vehicle)	Sham group			TiO_2-H group		
						Relative methylation (Cy5/Cy3)	Cy3 signal (Input)	Cy5 signal (MeDIP)	Relative methylation (Cy5/Cy3)	Cy3 signal (Input)	Cy5 signal (MeDIP)
A_68_P20956471	chr1:196446176-196446220	NM_008882:176	Plxna2	INSIDE	0.992	0.227	2247.22	510.73	0.225	1711.69	385.91
A_68_P20799110	chr1:168168534-168168578	NM_175296:319	Mael	INSIDE	0.992	2.320	1434.49	3328.25	2.302	1128.97	2598.83
A_68_P32552411	chrX:96085861-96085905	NM_052976:442	Ophn1	INSIDE	0.991	1.522	918.23	1397.60	1.508	1093.29	1648.85
A_68_P32341130	chrX:39502930-39502974	NM_021465:-925	Stag2	PROMOTER	0.991	1.986	528.51	1049.44	1.967	750.35	1475.99
A_68_P32154509	chr19:48280479-48280523	NM_025696:-14	Sorcs3	PROMOTER	0.991	0.417	3211.54	1340.49	0.414	2490.33	1029.89
A_68_P31951139	chr19:10599956-10600000	NM_001164272:245	Cpsf7	INSIDE	0.991	18.749	4658.04	87335.33	18.583	4844.25	90022.76
A_68_P30512949	chr16:4964346-4964390	NM_028301:-38	Anks3	PROMOTER	0.991	1.718	1873.88	3219.25	1.702	1273.81	2168.61
A_68_P30504522	chr15:103333324-103333368	NM_008800:-382	Pde1b	PROMOTER	0.991	0.392	6353.53	2490.54	0.388	4387.70	1703.96
A_68_P26397643	chr9:30729700-30729744	NM_001024139:315	Adamts15	INSIDE	0.991	0.323	1982.39	639.37	0.319	1539.68	491.91
A_68_P26132768	chr8:107814026-107814070	NM_177788:5950	Exoc3l	INSIDE	0.991	0.211	3281.04	693.31	0.209	2194.82	459.56
A_68_P24929401	chr6:146526297-146526341	NM_138757:39	4933424B01Rik	INSIDE	0.991	0.442	1478.87	654.16	0.438	1061.62	465.29
A_68_P24057006	chr5:124134216-124134260	NM_019765:62	Clip1	INSIDE	0.991	0.371	6284.00	2332.64	0.368	4587.32	1686.79
A_68_P24038702	chr5:120885755-120885799	NM_008499:3882	Lhx5	INSIDE	0.991	0.251	2309.22	580.42	0.249	1897.48	472.61
A_68_P23080559	chr4:86221959-86222003	NM_178376:404	Rraga	INSIDE	0.991	0.410	1332.47	546.67	0.407	1070.84	435.49
A_68_P22957030	chr4:59562123-59562167	NM_144904:92	Rod1	INSIDE	0.991	0.313	2087.12	652.34	0.310	1691.66	524.02
A_68_P22319259	chr3:89221857-89221906	NM_016904:332	Cks1b	INSIDE	0.991	0.238	2145.23	509.61	0.236	1701.16	400.65
A_68_P20354307	chr1:75471829-75471873	NM_001001565:4196	Chpf	INSIDE	0.991	2.024	550.58	1114.35	2.005	435.81	873.79
A_68_P20293012	chr1:64579202-64579246	NM_001037726:-153	Creb1	PROMOTER	0.991	0.535	1881.63	1006.66	0.530	1464.43	776.19
A_68_P30670426	chr16:35490448-35490492	NM_028295:489	Pdia5	INSIDE	0.990	0.262	2368.21	621.26	0.260	1791.55	465.45
A_68_P30337610	chr15:74348840-74348884	NM_174991:2237	Bai1	INSIDE	0.990	0.567	1773.22	1005.04	0.561	1339.31	751.47
A_68_P29730260	chr14:76244587-76244631	NM_009429:-454	Tpt1	PROMOTER	0.990	0.423	1273.98	538.43	0.419	1045.53	437.64
A_68_P29249242	chr13:97842053-97842097	NM_001100458:477	Fam169a	INSIDE	0.990	0.385	7530.65	2895.56	0.381	4992.47	1899.84
A_68_P29024920	chr13:49474941-49474985	NM_001039179:38045	Bicd2	INSIDE	0.990	1.582	1404.28	2221.24	1.566	1002.08	1568.77
A_68_P28956973	chr13:37949256-37949300	NM_001039188:30017	Rreb1	INSIDE	0.990	2.006	607.82	1219.23	1.985	543.39	1078.61
A_68_P28120302	chr11:109582605-109582649	NM_153782:944	Fam20a	INSIDE	0.990	0.560	2179.47	1220.51	0.554	1628.75	902.89
A_68_P27798798	chr11:52174210-52174254	NM_011694:-384	Vdac1	PROMOTER	0.990	0.537	1442.51	775.30	0.532	1241.09	660.48
A_68_P27479234	chr10:116500653-116500697	NM_007636:196	Cct2	INSIDE	0.990	0.525	966.27	507.47	0.520	809.81	420.95
A_68_P26685863	chr9:83728338-83728382	NM_001110265:65	Ttk	INSIDE	0.990	0.172	6252.97	1077.12	0.170	4358.65	743.05
A_68_P26658153	chr9:77955869-77955913	NM_023605:573	Fbxo9	INSIDE	0.990	0.678	2544.68	1726.07	0.672	1935.39	1299.62
A_68_P26167600	chr8:113824005-113824049	NM_146218:96	Rfwd3	INSIDE	0.990	0.364	3910.95	1422.93	0.360	2880.63	1037.41
A_68_P26056688	chr8:93593942-93593986	NM_011250:-28	Rbl2	PROMOTER	0.990	1.910	1035.60	1978.24	1.892	802.51	1518.22
A_68_P25364270	chr7:107007573-107007617	NM_001164258:-133	Xrra1	DIVERGENT_PROMOTER	0.990	2.594	898.13	2329.70	2.568	794.43	2039.98
A_68_P25354430	chr7:105342884-105342928	NM_001081167:5079	B3gnt6	INSIDE	0.990	2.865	554.25	1588.06	2.838	446.12	1266.05
A_68_P24533906	chr6:70906262-70906307	NM_001101464:-315	Foxi3	PROMOTER	0.990	0.146	3627.46	529.41	0.145	2503.42	361.86
A_68_P24141718	chr5:140981389-140981433	NM_021528:-152	Chst12	PROMOTER	0.990	0.557	1202.21	670.09	0.552	965.57	532.72
A_68_P24060290	chr5:124699281-124699325	NM_001081323:79158	Mphosph9	DOWNSTREAM	0.990	0.532	1306.41	695.41	0.527	1070.33	563.93
A_68_P21629316	chr2:130187590-130187634	NM_001110513:65938	Ebf4	INSIDE	0.990	0.350	2339.71	818.71	0.346	1700.05	588.91
A_68_P20556964	chr1:121317183-121317227	NM_008381:1621	Inhbb	INSIDE	0.990	0.529	2600.39	1375.52	0.524	1928.41	1009.92
A_68_P20198786	chr1:45852431-45852475	NM_028599:107	Wdr75	INSIDE	0.990	0.395	1269.37	501.69	0.391	972.18	380.23
A_68_P32224193	chr19:60302406-60302450	NM_029648:172	D19Ertd737e	INSIDE	0.989	0.388	1536.17	596.78	0.384	1253.23	481.32
A_68_P30381574	chr15:81958410-81958454	NM_172428:81	Ccdc134	INSIDE	0.989	0.523	2483.56	1299.53	0.517	1879.25	972.37
A_68_P30378506	chr15:81416769-81416813	NM_177821:147	Ep300	INSIDE	0.989	0.163	2701.68	439.25	0.161	2042.16	328.39
A_68_P28184671	chr11:120319473-120319517	NM_152807:52	Ccdc137	INSIDE	0.989	0.246	2383.09	586.16	0.243	1639.09	398.57
A_68_P28153582	chr11:115309493-115309537	NM_001033775:7656	4933422H20Rik	INSIDE	0.989	1.622	984.84	1597.02	1.605	728.54	1168.97
A_68_P27687432	chr11:32113340-32113384	NM_010117:8931	Rhbdf1	INSIDE	0.989	2.409	1640.62	3951.48	2.382	1238.79	2951.17
A_68_P27568687	chr11:6375581-6375625	NM_011221:318	Purb	INSIDE	0.989	2.681	677.42	1815.87	2.651	535.21	1418.87
A_68_P27497669	chr10:119912366-119912410	NM_010441:1603	Hmga2	INSIDE	0.989	0.447	1079.75	482.88	0.442	750.27	331.95
A_68_P26864197	chr9:117949927-117949971	NM_001048146:332	Azi2	INSIDE	0.989	4.595	509.25	2339.77	4.545	472.56	2147.83
A_68_P26234164	chr8:125075343-125075387	NM_001037298:-135	Fam38a	PROMOTER	0.989	0.400	1186.33	474.44	0.395	1005.39	397.62
A_68_P26227636	chr8:124114791-124114835	NM_026160:445	Map1lc3b	INSIDE	0.989	0.496	1275.73	633.06	0.491	1007.82	494.62
A_68_P23694281	chr5:52759674-52759718	NM_011435:4654	Sod3	INSIDE	0.989	1.724	1194.76	2059.78	1.706	1000.37	1706.46
A_68_P21928446	chr3:7366713-7366757	NM_008862:131	Pkia	INSIDE	0.989	1.984	1844.37	3660.02	1.962	1570.00	3080.56
A_68_P32562270	chrX:98255164-98255208	NM_031384:-208	Tex11	PROMOTER	0.988	2.323	793.02	1842.35	2.296	1106.18	2539.38
A_68_P32130261	chr19:44144105-44144149	NM_145502:50	Erlin1	INSIDE	0.988	0.461	1666.28	768.91	0.456	1458.31	664.90
A_68_P31292451	chr17:64212897-64212941	ENSMUST00000050753:222		INSIDE	0.988	0.355	2280.08	809.79	0.351	1730.10	607.23
A_68_P30601341	chr16:23108146-23108190	NR_030705:-857	Snord2	PROMOTER	0.988	0.536	1332.59	714.55	0.530	1108.31	587.09
A_68_P29613101	chr14:55196302-55196346	NM_010590:174	Jub	INSIDE	0.988	0.362	4062.01	1470.31	0.358	2991.48	1069.58
A_68_P28729566	chr12:109940197-109940241	NM_001171002:298	Degs2	INSIDE	0.988	0.423	1201.55	508.21	0.418	885.33	370.05
A_68_P27800934	chr11:52578718-52578762	NM_177059:533	Fstl4	INSIDE	0.988	0.602	4333.41	2606.59	0.594	3160.74	1878.73
A_68_P27188397	chr10:62349624-62349668	NM_027384:-6884	Tet1	PROMOTER	0.988	0.288	1584.95	455.95	0.284	1373.13	390.08
A_68_P26924748	chr10:8238264-8238308	NM_177387:337	Ust	INSIDE	0.988	0.498	2088.44	1040.16	0.492	1623.53	798.87
A_68_P26752042	chr9:97266348-97266392	NM_030219:4007	Trim42	INSIDE	0.988	2.157	1752.90	3781.11	2.130	1466.92	3125.26
A_68_P26554372	chr9:59464905-59464949	NM_001205239:-164	Parp6	PROMOTER	0.988	0.636	1627.27	1034.76	0.628	1306.18	820.39
A_68_P25950855	chr8:72807002-72807046	NM_001146153:127	Homer3	INSIDE	0.988	0.327	3313.83	1082.30	0.323	2466.64	796.27
A_68_P25351155	chr7:104728838-104728882	NM_001081267:455	Rsf1	INSIDE	0.988	0.403	2370.22	954.47	0.398	2012.74	800.73

ProbeName	Target position of probe on CpG island microarray	TargetID	GeneSymbol	CpG island Description	Ratio of relative methylation (TiO_2-NP/Vehicle)	Sham group			TiO_2-H group		
						Relative methylation (Cy5/Cy3)	Cy3 signal (Input)	Cy5 signal (MeDIP)	Relative methylation (Cy5/Cy3)	Cy3 signal (Input)	Cy5 signal (MeDIP)
A_68_P25045003	chr7:35461137-35461181	NM_175140:136572	Chst8	INSIDE	0.988	0.374	1707.25	639.02	0.370	1372.77	507.53
A_68_P23815272	chr5:76732745-76732789	NM_007715:807	Clock	INSIDE	0.988	1.765	971.64	1715.17	1.744	827.22	1442.30
A_68_P22862461	chr4:41445320-41445364	NM_001085515:4767	AI464131	INSIDE	0.988	0.503	2350.67	1182.06	0.497	1671.93	830.52
A_68_P22609446	chr3:145239414-145239458	NM_001081094:265	Znhit6	INSIDE	0.988	1.478	3622.39	5352.19	1.460	2787.50	4071.02
A_68_P20864296	chr1:180458833-180458877	NM_001161665:-401	Kif26b	PROMOTER	0.988	0.475	1401.74	666.27	0.470	1078.61	506.77
A_68_P20250405	chr1:57029997-57030046	NR_024325:1709	9130024F11Rik	INSIDE	0.988	3.842	369.19	1418.34	3.795	274.46	1041.70
A_68_P32579167	chrX:102274990-102275034	NM_026312:-101	2610029G23Rik	DIVERGENT_PROMOTER	0.987	2.990	224.98	672.70	2.951	255.84	754.91
A_68_P31929500	chr19:5567652-5567696	NM_001033448:-399	Gm962	DIVERGENT_PROMOTER	0.987	1.750	1711.33	2994.97	1.726	1320.27	2279.39
A_68_P31876071	chr18:82575755-82575799	NM_008082:393	Galr1	INSIDE	0.987	0.300	2735.73	821.46	0.296	2112.57	625.83
A_68_P31541315	chr18:20904732-20904776	NM_019737:151	B4galt6	INSIDE	0.987	0.291	3884.24	1128.80	0.287	2652.22	760.43
A_68_P30498005	chr15:102300845-102300889	NM_001103165:-196	Pcbp2	PROMOTER	0.987	0.481	1185.21	569.87	0.475	901.90	428.13
A_68_P30397290	chr15:84684091-84684135	NM_001081166:2447	Phf21b	INSIDE	0.987	1.902	1239.48	2357.30	1.876	909.82	1707.17
A_68_P29147535	chr13:74946041-74946085	NM_009817:-693	Cast	PROMOTER	0.987	1.715	3327.62	5705.86	1.693	2650.86	4488.47
A_68_P28185233	chr11:120410651-120410695	NM_001033231:369	Fam195b	INSIDE	0.987	5.152	2420.08	12467.56	5.085	2735.06	13907.61
A_68_P26785470	chr9:103267147-103267191	NM_001134427:141	Cdv3	INSIDE	0.987	0.325	2169.33	704.01	0.320	1712.76	548.34
A_68_P26699327	chr9:86358534-86358578	NM_027394:-33	Ube2cbp	DIVERGENT_PROMOTER	0.987	1.881	1406.09	2645.35	1.856	1175.44	2181.69
A_68_P26337051	chr9:18277941-18277985	NM_028935:41	Zfp558	INSIDE	0.987	0.239	4827.73	1153.34	0.236	3513.11	828.64
A_68_P26153108	chr8:111316106-111316150	NM_007496:77585	Zfhx3	INSIDE	0.987	1.864	1050.92	1958.44	1.839	825.44	1518.18
A_68_P24603555	chr6:85137374-85137418	NM_010131:-528	Emx1	PROMOTER	0.987	0.126	4959.15	626.89	0.125	3577.41	446.55
A_68_P23894277	chr5:92864276-92864320	NM_183392:-73	Nup54	PROMOTER	0.987	0.373	1934.87	722.40	0.368	1361.72	501.73
A_68_P23404230	chr4:149461292-149461336	NM_177366:-297	Gpr157	PROMOTER	0.987	0.168	6738.02	1131.12	0.166	4481.78	742.31
A_68_P23294940	chr4:128782954-128782998	NM_025452:203	Tmem54	INSIDE	0.987	0.474	2616.80	1240.73	0.468	2111.03	988.25
A_68_P22210771	chr3:67386632-67386676	NM_025813:-35	Mfsd1	PROMOTER	0.987	2.231	886.14	1977.31	2.202	637.41	1403.72
A_68_P21257458	chr2:60122109-60122153	NM_025422:345	Cd302	INSIDE	0.987	1.580	1911.25	3020.52	1.560	1529.41	2385.96
A_68_P21138488	chr2:36058729-36058773	ENSMUST00000138755:409		INSIDE	0.987	1.401	2506.05	3512.16	1.383	1916.68	2650.72
A_68_P20885895	chr1:184212870-184212914	NM_007853:-2	Degs1	PROMOTER	0.987	7.937	553.97	4396.81	7.835	469.21	3676.22
A_68_P20743649	chr1:158489100-158489144	NM_001136104:205	Abl2	INSIDE	0.987	0.461	1295.60	597.45	0.455	1055.80	480.75
A_68_P29218728	chr13:91601752-91601796	NM_024186:1073	Ssbp2	INSIDE	0.986	0.306	1755.57	537.48	0.302	1481.09	447.23
A_68_P28556955	chr12:78063502-78063546	NM_001146176:-289	Max	PROMOTER	0.986	0.234	2109.01	494.33	0.231	1594.61	368.63
A_68_P28313704	chr12:29237167-29237211	NM_001177964:-3986	Dcdc2c	PROMOTER	0.986	2.642	945.93	2498.71	2.606	821.31	2140.07
A_68_P27899471	chr11:70027749-70027793	NM_153081:359	Slc16a11	INSIDE	0.986	0.487	1251.98	609.09	0.480	901.18	432.44
A_68_P26142248	chr8:109459632-109459676	NM_009229:5	Sntb2	INSIDE	0.986	0.450	1551.10	697.70	0.443	1245.64	552.20
A_68_P25656208	chr8:11555566-11555610	NM_011919:-477	Ing1	DIVERGENT_PROMOTER	0.986	1.847	1005.91	1858.27	1.821	747.95	1362.08
A_68_P25592836	chr7:149268658-149268702	NM_008748:12509	Dusp8	INSIDE	0.986	1.672	1968.06	3291.14	1.649	1412.25	2328.85
A_68_P23435438	chr4:154358119-154358163	NM_001113360:26953	Plch2	INSIDE	0.986	2.314	830.47	1921.54	2.282	597.88	1364.27
A_68_P23323337	chr4:134090955-134090999	NM_029759:-2309	Fam54b	PROMOTER	0.986	1.655	749.81	1241.12	1.631	716.01	1168.08
A_68_P22407408	chr3:107399047-107399091	NM_007441:1120	Alx3	INSIDE	0.986	0.534	1339.37	714.87	0.526	991.94	522.10
A_68_P21604382	chr2:125548878-125548922	NM_199022:984	Shc4	INSIDE	0.986	0.293	1628.47	477.40	0.289	1242.70	359.13
A_68_P21106324	chr2:30837544-30837588	NM_144885:-105	BC005624	DIVERGENT_PROMOTER	0.986	0.160	4203.49	671.72	0.157	2591.07	408.08
A_68_P20936667	chr1:193007287-193007331	NM_007498:-96	Atf3	PROMOTER	0.986	0.318	3683.55	1170.57	0.313	2811.58	880.79
A_68_P31381746	chr17:80605842-80605886	NM_001195485:781	Srsf7	INSIDE	0.985	0.554	1522.80	844.20	0.546	1263.86	690.24
A_68_P31259473	chr17:57136900-57136944	NM_025538:161	Alkbh7	INSIDE	0.985	3.078	1037.07	3192.57	3.034	785.21	2382.16
A_68_P30230312	chr15:54921904-54921948	NM_183089:107	Dscc1	INSIDE	0.985	0.536	1084.26	581.16	0.528	1038.64	548.53
A_68_P28577471	chr12:81858975-81859019	NM_001008423:2706	Gm1568	INSIDE	0.985	2.868	465.55	1335.36	2.825	455.41	1286.44
A_68_P28107255	chr11:107331346-107331390	NM_145823:666	Pitpnc1	INSIDE	0.985	0.450	2094.22	942.42	0.443	1667.27	739.25
A_68_P28042990	chr11:96064768-96064813	NM_008267:9116	Hoxb13	DOWNSTREAM	0.985	0.198	4765.13	941.44	0.195	3120.98	607.09
A_68_P27551371	chr11:3231882-3231926	NM_178149:1171	Pik3ip1	INSIDE	0.985	0.461	1142.51	527.10	0.455	924.51	420.26
A_68_P27283562	chr10:79958458-79958502	NM_139226:830	Onecut3	INSIDE	0.985	2.025	861.50	1744.34	1.994	738.93	1473.45
A_68_P26258434	chr8:129118421-129118465	NM_001164598:-1106	Irf2bp2	PROMOTER	0.985	1.821	768.59	1399.84	1.794	633.38	1136.17
A_68_P25025138	chr7:29849498-29849542	NM_009109:60650	Ryr1	INSIDE	0.985	2.721	1836.04	4996.04	2.680	1591.59	4265.61
A_68_P24325868	chr6:29297894-29297938	NM_007594:-202	Calu	PROMOTER	0.985	0.400	1645.30	657.96	0.394	1425.29	561.30
A_68_P22859648	chr4:40895242-40895286	NM_001171739:63	Bag1	INSIDE	0.985	0.590	1236.28	729.05	0.581	1005.98	584.56
A_68_P21601163	chr2:125073424-125073468	NM_023595:196	Dut	INSIDE	0.985	0.144	3779.79	545.91	0.142	2529.56	359.98
A_68_P21067995	chr2:24618544-24618589	NM_001042528:1	Cacna1b	INSIDE	0.985	1.961	1361.18	2669.11	1.932	1132.38	2187.50
A_68_P32266423	chrX:13063488-13063532	NM_173415:18713	Nyx	INSIDE	0.984	4.832	577.25	2789.30	4.754	614.34	2920.72
A_68_P29964718	chr14:123382401-123382445	NM_028651:61	Tmtc4	INSIDE	0.984	0.484	1894.60	916.85	0.476	1750.91	833.90
A_68_P29257132	chr13:99125852-99125896	NM_008242:1675	Foxd1	INSIDE	0.984	4.770	607.78	2898.99	4.692	437.31	2051.91
A_68_P28307256	chr12:28026486-28026530	NM_009234:1075	Sox11	INSIDE	0.984	1.556	1120.84	1743.91	1.532	969.07	1484.20
A_68_P28183224	chr11:120095621-120095666	NM_198423:1383	Bahcc1	INSIDE	0.984	0.376	2594.25	974.18	0.369	1811.50	669.03
A_68_P27284472	chr10:80097247-80097291	NM_001159591:4868	Csnk1g2	INSIDE	0.984	1.850	2925.38	5410.49	1.821	2028.77	3693.81
A_68_P27176492	chr10:60215582-60215626	NM_023596:-74	Slc29a3	PROMOTER	0.984	2.348	5329.35	12513.99	2.311	3978.86	9196.00
A_68_P25837690	chr8:47826399-47826443	NM_008391:1322	Irf2	INSIDE	0.984	0.531	1924.59	1022.34	0.523	1644.00	859.42
A_68_P25022020	chr7:29244408-29244453	NM_001141921:7174	Lrfn1	INSIDE	0.984	3.119	1244.59	3881.81	3.068	976.27	2995.22
A_68_P24172984	chr5:148118315-148118359	NM_007673:489	Cdx2	INSIDE	0.984	0.561	1180.16	662.11	0.552	1044.12	576.17
A_68_P24137288	chr5:140212139-140212183	NM_174850:127	Micall2	INSIDE	0.984	0.553	1653.77	914.61	0.544	1220.65	664.15

ProbeName	Target position of probe on CpG island microarray	TargetID	GeneSymbol	CpG island Description	Ratio of relative methylation (TiO_2-NP/Vehicle)	Sham group			TiO_2-H group		
						Relative methylation (Cy5/Cy3)	Cy3 signal (Input)	Cy5 signal (MeDIP)	Relative methylation (Cy5/Cy3)	Cy3 signal (Input)	Cy5 signal (MeDIP)
A_68_P24131499	chr5:139230499-139230543	NM_030565:-514	Fam20c	PROMOTER	0.984	0.535	1620.83	867.73	0.527	1308.26	688.91
A_68_P23544555	chr5:24414575-24414619	NM_001170555:-269	Prkag2	PROMOTER	0.984	3.078	409.95	1261.78	3.027	338.05	1023.38
A_68_P21565426	chr2:118503248-118503292	NM_001145854:289	Pak6	INSIDE	0.984	0.528	2314.21	1222.38	0.520	1778.23	923.91
A_68_P21395951	chr2:84877360-84877404	NM_001136081:-225	Ssrp1	PROMOTER	0.984	0.353	4033.12	1424.60	0.348	2760.44	959.69
A_68_P30939796	chr16:87455183-87455227	NM_024258:-25	Usp16	PROMOTER	0.983	0.401	1403.37	562.81	0.394	1134.25	447.29
A_68_P29410630	chr14:13173724-13173771	NM_080433:4632	Fezf2	DOWNSTREAM	0.983	0.517	1079.98	558.01	0.508	813.73	413.12
A_68_P28189252	chr11:121066090-121066134	NM_025402:-150	1110031I02Rik	DIVERGENT_PROMOTER	0.983	0.387	3324.06	1287.27	0.381	2442.39	929.66
A_68_P27994976	chr11:87405943-87405987	NM_133215:246	Mtmr4	INSIDE	0.983	0.513	1092.94	560.46	0.504	833.30	420.14
A_68_P27805841	chr11:53449799-53449843	NM_008355:-1616	Il13	PROMOTER	0.983	0.376	1563.06	587.04	0.369	1178.04	434.78
A_68_P27177057	chr10:60293808-60293852	NM_029770:499	Unc5b	INSIDE	0.983	0.492	1244.74	612.86	0.484	962.60	466.13
A_68_P26467230	chr9:44169640-44169684	NM_027889:91	Vps11	INSIDE	0.983	0.332	2855.23	948.16	0.326	2098.93	685.18
A_68_P25953913	chr8:73292527-73292571	NM_008841:8063	Pik3r2	INSIDE	0.983	3.326	1304.02	4337.55	3.270	957.66	3131.75
A_68_P24459354	chr6:53770780-53770824	NM_025817:17	Tril	INSIDE	0.983	0.261	1939.26	506.67	0.257	1431.59	367.86
A_68_P23363266	chr4:140864579-140864623	NM_010139:7446	Epha2	INSIDE	0.983	2.953	626.66	1850.67	2.904	580.77	1686.52
A_68_P22293166	chr3:84619656-84619700	NM_001177773:180	Fbxw7	INSIDE	0.983	0.586	2468.72	1446.33	0.576	1746.50	1005.72
A_68_P22082265	chr3:40754710-40754754	NM_001040399:180	Larp1b	INSIDE	0.983	0.543	2924.82	1587.21	0.533	2494.10	1329.82
A_68_P27561410	chr11:4999411-4999455	NM_177370:-8	Rhbdd3	DIVERGENT_PROMOTER	0.982	0.537	1149.61	617.17	0.527	870.18	458.76
A_68_P27284096	chr10:80039600-80039644	NM_078477:419	Klf16	INSIDE	0.982	2.532	1551.90	3928.80	2.486	1156.86	2876.24
A_68_P27230711	chr10:70062739-70062783	NM_178621:-721	Phyhipl	PROMOTER	0.982	1.939	1214.00	2353.70	1.905	871.41	1659.69
A_68_P27186303	chr10:61949193-61949237	NM_001113355:132	Vps26a	INSIDE	0.982	19.271	902.17	17385.40	18.922	1101.64	20845.29
A_68_P25311412	chr7:97279241-97279285	NM_146194:521	Picalm	INSIDE	0.982	0.195	7380.66	1438.65	0.191	5682.40	1087.51
A_68_P24623658	chr6:88674283-88674327	NM_001166249:-101	Mgll	PROMOTER	0.982	2.002	1818.73	3641.88	1.967	1436.60	2825.92
A_68_P24155676	chr5:144014658-144014702	NM_017467:17021	Zfp316	INSIDE	0.982	0.527	3464.54	1824.31	0.517	2374.77	1228.26
A_68_P24098220	chr5:131916295-131916339	NM_177047:1101897	Auts2	INSIDE	0.982	1.909	826.33	1577.78	1.874	799.09	1497.55
A_68_P23600185	chr5:35731082-35731126	NM_010445:-661	Hmx1	PROMOTER	0.982	0.478	1853.85	885.98	0.469	1282.49	601.71
A_68_P23438947	chr4:154866365-154866409	NM_001160016:917	Gnb1	INSIDE	0.982	0.413	2673.04	1104.72	0.406	1993.24	809.21
A_68_P23397602	chr4:148312906-148312950	NM_027195:134428	Caszl	INSIDE	0.982	1.514	1739.35	2633.29	1.487	1210.76	1800.65
A_68_P23280751	chr4:126234084-126234128	NM_175554:-117	Clspn	PROMOTER	0.982	6.564	1414.28	9282.76	6.444	1182.14	7618.30
A_68_P23237282	chr4:117587476-117587521	NM_146152:106	Ipo13	INSIDE	0.982	0.230	3441.20	791.42	0.226	2373.76	536.06
A_68_P21680283	chr2:139502974-139503018	NM_001126490:-917	Isml	PROMOTER	0.982	0.250	2641.28	659.68	0.245	2044.11	501.37
A_68_P30836891	chr16:67620592-67620636	NM_001145977:539	Cadm2	INSIDE	0.981	0.303	1739.90	526.84	0.297	1290.43	383.41
A_68_P30421542	chr15:88668084-88668130	ENSMUST00000083457:2377		DOWNSTREAM	0.981	2.143	434.70	931.47	2.103	342.75	720.68
A_68_P29239505	chr13:95992702-95992746	NM_001170669:281	Pde8b	INSIDE	0.981	0.393	1291.96	507.68	0.386	950.86	366.61
A_68_P27788208	chr11:50191636-50191680	NM_021510:438	Hnrnph1	INSIDE	0.981	0.435	1305.38	567.63	0.426	1125.27	479.81
A_68_P27286931	chr10:80484539-80484583	NM_145217:3562	Diras1	INSIDE	0.981	0.626	2638.15	1650.69	0.614	2176.56	1336.54
A_68_P25960681	chr8:74842613-74842657	NM_008452:-326	Klf2	PROMOTER	0.981	0.332	2773.23	920.07	0.326	1913.55	622.98
A_68_P24993062	chr7:19979665-19979709	NM_007949:12299	Ercc2	INSIDE	0.981	2.834	283.32	802.89	2.779	249.95	694.73
A_68_P24799555	chr6:120615920-120615964	NM_001128151:-496	Cecr2	PROMOTER	0.981	0.339	4352.05	1473.92	0.332	2947.30	979.35
A_68_P24634270	chr6:90666488-90666532	NM_001134383:13	Iqsec1	INSIDE	0.981	0.466	1062.58	495.51	0.458	893.28	408.72
A_68_P23882637	chr5:90794553-90794599	NM_030886:635	Ankrd17	INSIDE	0.981	0.481	4299.72	2066.05	0.471	3289.14	1550.17
A_68_P23402407	chr4:149147586-149147630	NM_027460:768	Slc25a33	INSIDE	0.981	0.408	1311.38	535.20	0.400	1174.67	470.24
A_68_P22407409	chr3:107399162-107399206	NM_007441:1236	Alx3	INSIDE	0.981	0.253	2096.96	529.90	0.248	1497.63	371.30
A_68_P22246603	chr3:75360626-75360670	NM_019745:73	Pdcd10	INSIDE	0.981	0.339	2207.83	748.68	0.333	1756.41	584.21
A_68_P21768465	chr2:155755929-155755973	NM_018888:96	Uqcc	INSIDE	0.981	0.296	2370.49	702.42	0.291	1639.64	476.82
A_68_P21666842	chr2:136941472-136941517	NM_013822:762	Jag1	INSIDE	0.981	3.656	235.19	859.77	3.586	270.22	969.06
A_68_P21486502	chr2:103810841-103810885	NM_001142335:419	Lmo2	INSIDE	0.981	0.272	2061.16	559.91	0.266	1663.30	443.26
A_68_P20188963	chr1:43503219-43503263	NM_010879:645	Nck2	INSIDE	0.981	0.336	1974.31	662.65	0.329	1581.80	520.78
A_68_P31610612	chr18:34166849-34166893	NM_013512:-10	Epb4.1l4a	PROMOTER	0.980	0.334	1340.58	447.49	0.327	998.01	326.58
A_68_P31254141	chr17:56279185-56279230	NM_013662:-5932	Sema6b	PROMOTER	0.980	2.856	398.79	1139.13	2.799	336.14	940.73
A_68_P31120336	chr17:28217206-28217250	NM_013687:301	Tcp11	INSIDE	0.980	11.632	23862.29	277570.80	11.397	21770.90	248112.30
A_68_P30579148	chr16:18343878-18343922	NM_138583:125	D16H22S680E	INSIDE	0.980	1.636	3032.10	4961.91	1.604	2426.32	3890.88
A_68_P29620064	chr14:56444394-56444438	NM_001168346:785	Nfatc4	INSIDE	0.980	1.866	1049.85	1958.66	1.829	940.58	1719.85
A_68_P29515882	chr14:32973351-32973395	NM_019744:-5605	Ncoa4	PROMOTER	0.980	0.207	3049.95	631.97	0.203	2345.43	476.06
A_68_P29098141	chr13:64375668-64375712	NM_172587:606	Cdc14b	INSIDE	0.980	0.426	1026.65	437.46	0.418	852.11	355.91
A_68_P29055170	chr13:55565739-55565783	NM_001030296:133	Prr7	INSIDE	0.980	0.420	1241.81	521.25	0.411	901.92	370.83
A_68_P28974244	chr13:40833219-40833263	NM_011547:-4048	Tcfap2a	PROMOTER	0.980	0.349	1417.95	495.43	0.342	1449.53	496.22
A_68_P25475986	chr7:129008469-129008513	NM_011325:-134	Scnn1b	PROMOTER	0.980	0.359	3423.13	1227.66	0.352	2581.34	907.59
A_68_P24791292	chr6:119058938-119058982	NM_001159533:-753	Cacna1c	PROMOTER	0.980	0.410	1322.36	541.53	0.401	1019.84	409.38
A_68_P23971132	chr5:108741872-108741916	NM_001081234:-49	Pigg	PROMOTER	0.980	0.396	1346.16	533.44	0.388	1007.71	391.32
A_68_P22924772	chr4:53727484-53727528	NM_139309:453	Fktn	INSIDE	0.980	0.475	1559.84	741.10	0.466	1279.37	595.79
A_68_P21906907	chr2:180628640-180628684	NR_029538:-82	Mir124a-3	PROMOTER	0.980	0.575	3424.20	1968.28	0.563	2930.09	1650.72
A_68_P21150018	chr2:38567376-38567420	NM_139051:2664	Nr5a1	INSIDE	0.980	0.153	5659.54	867.68	0.150	3692.27	554.64
A_68_P20502876	chr1:107253429-107253473	NM_001160368:-163	Rnf152	PROMOTER	0.980	2.914	944.56	2752.88	2.856	705.62	2015.23
A_68_P32823846	chrX:166435801-166435845	NM_183151:118269	Mid1	DOWNSTREAM	0.979	2.367	3903.15	9240.01	2.318	3583.66	8307.91
A_68_P32389692	chrX:51278036-51278080	ENSMUST00000120892:-92		PROMOTER	0.979	1.552	1002.20	1555.60	1.519	1340.27	2035.97

ProbeName	Target position of probe on CpG island microarray	TargetID	GeneSymbol	CpG island Description	Ratio of relative methylation (TiO_2-NP/Vehicle)	Sham group			TiO_2-H group		
						Relative methylation (Cy5/Cy3)	Cy3 signal (Input)	Cy5 signal (MeDIP)	Relative methylation (Cy5/Cy3)	Cy3 signal (Input)	Cy5 signal (MeDIP)
A_68_P31936818	chr19:7015888-7015932	NM_001177360:-227	Gpr137	PROMOTER	0.979	0.518	2201.55	1141.37	0.508	1671.55	848.52
A_68_P31613233	chr18:34665967-34666011	NM_146084:489	Fam13b	INSIDE	0.979	0.617	1760.23	1086.76	0.604	1456.34	880.00
A_68_P30250406	chr15:58655527-58655571	NM_175212:-566	Tmem65	PROMOTER	0.979	1.641	1804.28	2960.60	1.607	1494.50	2401.46
A_68_P29982185	chr15:5093533-5093577	NM_001013367:-306	Prkaa1	PROMOTER	0.979	2.517	737.40	1856.30	2.465	588.62	1451.22
A_68_P28737719	chr12:111558035-111558079	AK200124:417495		INSIDE	0.979	2.407	1314.13	3162.59	2.355	1012.57	2384.52
A_68_P28099138	chr11:105881660-105881704	NM_001037712:12166	Kcnh6	INSIDE	0.979	0.174	3747.94	652.07	0.170	2697.78	459.47
A_68_P28085167	chr11:103274059-103274103	NM_183034:-102	Plekhm1	PROMOTER	0.979	3.064	783.91	2402.00	3.000	550.70	1652.25
A_68_P26883519	chr9:121363673-121363717	NM_175114:87619	Trak1	INSIDE	0.979	2.013	1054.23	2121.75	1.971	853.84	1683.10
A_68_P25954770	chr8:73416471-73416515	NM_053248:164	Slc5a5	INSIDE	0.979	1.815	1247.97	2265.10	1.776	968.23	1719.90
A_68_P25952924	chr8:73125225-73125269	NM_133772:6967	Ssbp4	INSIDE	0.979	0.569	1777.53	1010.89	0.557	1421.24	791.46
A_68_P25842219	chr8:48618727-48618771	NM_177240:76	D030016E14Rik	INSIDE	0.979	1.943	2049.63	3982.68	1.901	1688.76	3211.07
A_68_P25614882	chr8:3631030-3631074	NM_011503:-107	Stxbp2	DIVERGENT_PROMOTER	0.979	3.252	2495.51	8116.61	3.185	1902.77	6060.31
A_68_P25535522	chr7:139788929-139788973	NM_009123:2370	Nkx1-2	INSIDE	0.979	0.381	4299.73	1638.41	0.373	3258.97	1216.12
A_68_P24763851	chr6:114081597-114081641	NM_172890:384	Slc6a11	INSIDE	0.979	0.559	1331.81	744.61	0.547	1120.54	613.32
A_68_P23399809	chr4:148681348-148681392	NM_207682:437	Kif1b	INSIDE	0.979	3.856	294.73	1136.59	3.777	297.86	1124.91
A_68_P22748787	chr4:15076098-15076142	NM_178617:158	Necab1	INSIDE	0.979	0.181	4573.01	827.64	0.177	3365.00	596.05
A_68_P21152002	chr2:38921329-38921376	NM_029793:-426	Golga1	PROMOTER	0.979	2.087	516.99	1079.05	2.043	499.20	1019.71
A_68_P20350462	chr1:74829643-74829687	NM_009526:11199	Wnt6	INSIDE	0.979	1.504	1641.69	2469.17	1.473	1355.63	1997.03
A_68_P20005795	chr1:4482172-4482216	NM_011441:4300	Sox17	INSIDE	0.979	2.140	1106.13	2366.64	2.095	960.09	2011.34
A_68_P33011727	A_68_P33011727			Unknown	0.978	3.984	278.84	1110.83	3.897	163.05	635.32
A_68_P32016036	chr19:23209577-23209625	NM_010638:-6115	Klf9	PROMOTER	0.978	0.491	1520.92	746.72	0.480	1258.37	604.30
A_68_P30665016	chr16:34573818-34573862	ENSMUST00000114961:-222		PROMOTER	0.978	2.342	699.16	1637.69	2.291	501.06	1147.83
A_68_P30385944	chr15:82817919-82817963	ENSMUST00000170562:4641		INSIDE	0.978	0.381	2644.40	1006.92	0.373	1975.82	736.11
A_68_P30346615	chr15:76011854-76011898	NM_201394:14264	Plec	INSIDE	0.978	5.954	940.74	5600.75	5.824	832.44	4847.96
A_68_P27854128	chr11:62271118-62271162	NM_011308:-306	Ncor1	DIVERGENT_PROMOTER	0.978	2.252	695.96	1567.35	2.203	498.81	1099.03
A_68_P27083333	chr10:40022153-40022197	NM_009665:-180	Amd1	PROMOTER	0.978	0.443	2133.40	945.90	0.433	1698.85	736.38
A_68_P25261273	chr7:86611105-86611149	NM_017462:33	Polg	INSIDE	0.978	2.472	1184.33	2928.17	2.417	1004.32	2427.76
A_68_P24126250	chr5:137972865-137972909	NM_010312:1571	Gnb2	INSIDE	0.978	0.403	1798.77	725.36	0.394	1411.33	556.75
A_68_P23576173	chr5:31357375-31357419	NM_023525:213	Cad	INSIDE	0.978	0.568	3119.98	1771.19	0.555	2211.33	1227.16
A_68_P23006655	chr4:70196033-70196077	NM_172694:-92	Megf9	PROMOTER	0.978	0.382	1263.03	482.08	0.373	1023.32	381.93
A_68_P22311242	chr3:87803722-87803766	NM_007529:534	Bcan	INSIDE	0.978	2.111	615.51	1299.29	2.064	584.57	1206.69
A_68_P31257220	chr17:56743025-56743069	NM_001163300:18642	Safb	INSIDE	0.977	1.871	1423.76	2664.53	1.829	1164.79	2130.59
A_68_P30128579	chr15:34373192-34373236	NM_001163485:-183	Rpl30	DIVERGENT_PROMOTER	0.977	0.324	2059.38	667.51	0.317	1513.69	479.26
A_68_P29110672	chr13:68720676-68720720	NM_172480:300	Mtrr	INSIDE	0.977	0.400	1497.49	598.34	0.391	1135.32	443.36
A_68_P28156584	chr11:115836130-115836174	NM_001005608:114	Itgb4	INSIDE	0.977	0.363	1331.77	483.67	0.355	1090.21	386.76
A_68_P28039697	chr11:95448158-95448202	NM_033217:832	Ngfr	INSIDE	0.977	0.483	3585.50	1730.71	0.472	2594.19	1223.77
A_68_P27924776	chr11:75008459-75008503	NM_177708:986	Rtn4rl1	INSIDE	0.977	0.606	2650.04	1607.21	0.593	1887.17	1118.30
A_68_P26853660	chr9:116084560-116084604	NM_009371:-101	Tgfbr2	PROMOTER	0.977	0.475	1601.93	761.66	0.464	1190.40	552.86
A_68_P26254629	chr8:128435054-128435098	NM_145608:727	BC021891	INSIDE	0.977	1.794	896.32	1608.28	1.753	748.48	1311.82
A_68_P26001984	chr8:83404065-83404109	NM_021356:292	Gab1	INSIDE	0.977	0.540	2077.51	1121.30	0.527	1760.00	928.29
A_68_P25988741	chr8:80959478-80959522	NM_138944:1051	Pou4f2	INSIDE	0.977	2.507	4104.47	10288.33	2.448	3179.60	7784.32
A_68_P25581873	chr7:147222902-147222946	NM_027201:635	Zfp511	INSIDE	0.977	2.773	2115.31	5865.20	2.708	1523.08	4124.82
A_68_P25507198	chr7:134913587-134913631	NM_198424:280	Orai3	INSIDE	0.977	0.499	1395.15	696.63	0.488	1052.03	513.30
A_68_P24981713	chr7:17062092-17062136	NM_008718:15	Npas1	INSIDE	0.977	2.772	940.04	2605.42	2.706	804.01	2176.04
A_68_P24432032	chr6:49269455-49269499	NM_175098:125	Ccdc126	INSIDE	0.977	0.574	1147.58	659.20	0.561	945.78	530.97
A_68_P23145547	chr4:99322966-99323010	NM_010425:-1	Foxd3	PROMOTER	0.977	1.647	1143.62	1883.77	1.609	1022.65	1645.52
A_68_P21809532	chr2:162769099-162769143	NM_001081338:-80	L3mbtl1	PROMOTER	0.977	0.320	2302.30	736.54	0.313	1638.13	512.07
A_68_P21322068	chr2:71711196-71711241	NM_172665:-110	Pdk1	PROMOTER	0.977	1.965	1413.83	2778.28	1.921	1097.20	2107.45
A_68_P20735709	chr1:156930520-156930564	ENSMUST00000128143:-146		PROMOTER	0.977	0.352	4882.19	1720.37	0.344	3680.70	1267.72
A_68_P31619488	chr18:35821775-35821819	NM_029485:44	Spata24	INSIDE	0.976	0.387	2499.69	966.40	0.377	1917.31	723.15
A_68_P31465579	chr18:4922163-4922209	ENSMUST00000143254:463		INSIDE	0.976	0.535	1477.85	790.89	0.522	1062.24	554.76
A_68_P31220005	chr17:49071091-49071135	NM_027452:-794	Lrfn2	PROMOTER	0.976	0.284	1917.95	543.98	0.277	1398.92	387.22
A_68_P28175482	chr11:118906328-118906372	NM_013926:-4123	Cbx8	PROMOTER	0.976	2.235	853.13	1906.61	2.181	743.19	1621.21
A_68_P25503174	chr7:134093374-134093418	NM_001039645:-301	Asphd1	DIVERGENT_PROMOTER	0.976	0.459	1045.13	479.83	0.448	812.14	363.94
A_68_P25377920	chr7:109399308-109399352	NM_019566:-698	Rhog	PROMOTER	0.976	0.490	2648.75	1297.72	0.478	2041.60	976.30
A_68_P23553583	chr5:27144126-27144170	NM_010075:252	Dpp6	INSIDE	0.976	0.494	1249.38	616.80	0.482	1131.44	545.43
A_68_P23523161	chr5:20388465-20388509	NM_177601:216	Tmem60	INSIDE	0.976	0.592	1230.74	728.25	0.577	1022.97	590.52
A_68_P23329327	chr4:135129537-135129581	NM_001013756:-23	Grhl3	PROMOTER	0.976	0.454	1086.66	493.14	0.443	852.54	377.67
A_68_P23322289	chr4:133899035-133899079	NM_001039677:72	Slc30a2	INSIDE	0.976	1.682	2626.71	4419.38	1.642	2077.78	3412.48
A_68_P21823222	chr2:165151741-165151785	NM_207705:-9369	Elmo2	PROMOTER	0.976	0.331	2314.55	765.24	0.323	1850.86	597.46
A_68_P20804420	chr1:169238624-169238668	NM_001039483:-154	Tmco1	PROMOTER	0.976	0.411	1384.14	569.33	0.402	1043.89	419.27
A_68_P20456912	chr1:95582245-95582289	NM_016778:-4	Bok	PROMOTER	0.976	0.439	1174.76	515.45	0.428	1002.68	429.42
A_68_P32533270	chrX:90542781-90542825	NM_007492:10818	Arx	INSIDE	0.975	3.473	446.68	1551.54	3.387	462.79	1567.57
A_68_P31637164	chr18:39152519-39152563	NM_175164:-258	Arhgap26	PROMOTER	0.975	0.502	1273.11	638.83	0.489	1146.80	561.30
A_68_P31193443	chr17:44325522-44325566	NM_172621:24	Clic5	INSIDE	0.975	0.416	1567.81	652.81	0.406	1159.27	470.69

ProbeName	Target position of probe on CpG island microarray	TargetID	GeneSymbol	CpG island Description	Ratio of relative methylation (TiO_2-NP/Vehicle)	Sham group			TiO_2-H group		
						Relative methylation (Cy5/Cy3)	Cy3 signal (Input)	Cy5 signal (MeDIP)	Relative methylation (Cy5/Cy3)	Cy3 signal (Input)	Cy5 signal (MeDIP)
A_68_P30356786	chr15:77786446-77786490	NM_001017983:684	Foxred2	INSIDE	0.975	0.519	1400.12	726.69	0.506	992.93	502.67
A_68_P28520553	chr12:71554139-71554183	NM_028339:20	Tmx1	INSIDE	0.975	0.731	3144.58	2298.20	0.713	2377.65	1694.10
A_68_P27541278	chr10:127848946-127848990	NM_001164237:297	Rnf41	INSIDE	0.975	0.599	3589.64	2149.10	0.584	2778.07	1622.03
A_68_P27357766	chr10:93610580-93610624	NM_011629:-73	Nr2c1	PROMOTER	0.975	0.461	5685.89	2621.72	0.450	3848.71	1731.01
A_68_P26239786	chr8:125935222-125935266	NM_023279:-219	Tubb3	PROMOTER	0.975	0.387	1938.20	750.09	0.377	1524.40	575.41
A_68_P25957674	chr8:74115987-74116031	NM_025396:-74	Pgls	PROMOTER	0.975	0.541	1609.98	870.29	0.527	1047.52	552.11
A_68_P25090601	chr7:52262939-52262983	NM_001008422:8659	Scaf1	INSIDE	0.975	1.776	2523.24	4481.77	1.732	1842.63	3192.16
A_68_P24138664	chr5:140449200-140449245	NM_175522:65326	Elfn1	INSIDE	0.975	2.863	1300.98	3724.33	2.790	1009.83	2817.75
A_68_P23250422	chr4:119955245-119955289	NM_194060:4600	Foxo6	INSIDE	0.975	0.619	2131.00	1318.14	0.603	1740.46	1049.41
A_68_P22869764	chr4:43071791-43071835	NM_001081413:-43	Unc13b	PROMOTER	0.975	0.298	1554.94	464.07	0.291	1257.25	365.73
A_68_P22029170	chr3:30494111-30494155	NM_029690:4660	Arpm1	DOWNSTREAM	0.975	0.393	2479.65	974.33	0.383	1779.61	682.11
A_68_P21810975	chr2:163029586-163029630	ENSMUST00000128999:-680		PROMOTER	0.975	0.301	9098.31	2734.07	0.293	6010.83	1762.00
A_68_P31624334	chr18:36675661-36675705	NM_010415:-223	Hbegf	PROMOTER	0.974	0.350	2283.37	798.47	0.340	1487.08	506.32
A_68_P30379304	chr15:81560230-81560274	NM_001146174:97	Rangap1	INSIDE	0.974	0.181	4300.97	777.85	0.176	2937.66	517.41
A_68_P30345427	chr15:75834015-75834059	NM_001168253:5892	Fam83h	INSIDE	0.974	2.242	683.33	1532.07	2.184	627.00	1369.52
A_68_P29218723	chr13:91600897-91600941	NM_024186:217	Ssbp2	INSIDE	0.974	4.104	4254.02	17458.76	3.998	3224.82	12891.71
A_68_P28743067	chr12:112513239-112513283	NM_033603:3939	Amn	INSIDE	0.974	0.281	2425.46	680.66	0.273	1697.13	463.88
A_68_P28450015	chr12:56085848-56085892	NM_013815:1453	Baz1a	INSIDE	0.974	0.205	2720.96	557.79	0.200	2167.29	432.57
A_68_P27930792	chr11:76056879-76056923	NM_026029:301	Glod4	INSIDE	0.974	0.312	1459.58	455.57	0.304	1118.29	340.02
A_68_P27903934	chr11:70832798-70832842	NM_033562:-55	Derl2	DIVERGENT_PROMOTER	0.974	0.205	2335.58	478.46	0.199	1764.95	351.99
A_68_P27228312	chr10:69652108-69652156	NM_001111121:92264	Ccdc6	INSIDE	0.974	2.120	859.06	1820.79	2.064	712.64	1470.57
A_68_P26905361	chr10:4540405-4540449	NM_025995:-649	Fbxo5	PROMOTER	0.974	1.635	2536.94	4148.90	1.593	2196.86	3498.55
A_68_P26822033	chr9:110019921-110019966	NM_133347:-1857	Dhx30	PROMOTER	0.974	0.300	2711.25	814.67	0.293	2206.03	645.71
A_68_P25956587	chr8:73900036-73900080	NM_010150:5793	Nr2f6	INSIDE	0.974	0.376	1652.46	621.76	0.366	1260.56	461.98
A_68_P25724986	chr8:26149210-26149254	NM_001081187:202	Htra4	INSIDE	0.974	0.465	3578.72	1662.65	0.453	2711.29	1227.00
A_68_P25502656	chr7:134006226-134006271	NM_172746:763	Hirip3	INSIDE	0.974	0.272	1701.10	461.89	0.265	1282.57	339.36
A_68_P25411821	chr7:117265997-117266041	NM_009516:446	Wee1	INSIDE	0.974	0.425	1242.41	527.47	0.413	952.84	393.93
A_68_P25360312	chr7:106331247-106331291	NM_026384:-45	Dgat2	PROMOTER	0.974	1.694	837.04	1418.35	1.651	703.80	1161.87
A_68_P23984908	chr5:111714055-111714099	NM_024477:405255	Ttc28	INSIDE	0.974	1.651	1131.77	1868.97	1.609	1020.50	1642.01
A_68_P23203783	chr4:109656467-109656511	NM_172296:5859	Dmrta2	DOWNSTREAM	0.974	5.118	1654.09	8465.50	4.987	1347.31	6719.03
A_68_P21796151	chr2:160472451-160472495	NM_009408:840	Top1	INSIDE	0.974	0.585	2274.02	1330.52	0.570	1860.83	1060.65
A_68_P20639341	chr1:137326555-137326604	NM_153774:489	Ipo9	INSIDE	0.974	1.861	2588.46	4816.26	1.812	2044.19	3703.53
A_68_P33005898	A_68_P33005898			Unknown	0.973	3.916	314.20	1230.54	3.812	237.00	903.35
A_68_P32582404	chrX:103207170-103207214	NM_001190409:46	Magt1	INSIDE	0.973	1.590	1347.94	2143.82	1.548	1687.09	2610.99
A_68_P31924878	chr19:4756662-4756706	NM_025717:160	Rbm4b	INSIDE	0.973	2.359	3267.28	7705.89	2.294	2405.91	5520.08
A_68_P31868008	chr18:81171137-81171181	NM_178280:12159	Sall3	INSIDE	0.973	3.243	928.16	3009.60	3.156	848.87	2678.71
A_68_P31213522	chr17:47809817-47809861	NM_198421:42200	Usp49	INSIDE	0.973	0.422	1580.68	667.53	0.411	1078.40	442.99
A_68_P27806467	chr11:53584134-53584178	NM_001159396:182	Irf1	INSIDE	0.973	0.306	2147.95	657.93	0.298	1660.67	494.88
A_68_P26571039	chr9:62384681-62384725	NM_175484:149	Coro2b	INSIDE	0.973	13.842	14038.20	194315.00	13.474	12010.59	161832.50
A_68_P25949499	chr8:72567227-72567271	NM_027481:537	Sugp1	INSIDE	0.973	0.335	1823.82	611.27	0.326	1303.76	425.06
A_68_P25512010	chr7:135831942-135831986	NM_178641:77086	Inpp5f	INSIDE	0.973	3.137	1243.85	3901.55	3.052	1030.14	3143.78
A_68_P25471450	chr7:128125237-128125281	NM_007672:568	Cdr2	INSIDE	0.973	0.466	1557.01	725.81	0.454	1193.61	541.42
A_68_P25092989	chr7:52671296-52671340	NM_198190:2254	Ntf5	INSIDE	0.973	0.488	2260.10	1102.18	0.474	1627.83	772.24
A_68_P24153575	chr5:143582152-143582196	NM_001122730:-3108	Tnrc18	PROMOTER	0.973	0.491	1019.71	501.15	0.478	820.41	392.42
A_68_P23991447	chr5:112755530-112755574	NM_018783:164	Tfip11	INSIDE	0.973	0.282	3320.90	936.24	0.274	2301.24	631.46
A_68_P23313225	chr4:132195134-132195178	NM_010166:186	Eya3	INSIDE	0.973	0.409	3120.14	1276.66	0.398	2510.82	999.37
A_68_P21310713	chr2:69561124-69561168	NM_001081086:2	Ppig	INSIDE	0.973	0.689	2244.79	1546.19	0.670	1812.95	1214.84
A_68_P20566309	chr1:123224172-123224218	NM_133748:145	Insig2	INSIDE	0.973	0.419	1262.96	529.32	0.408	967.21	394.42
A_68_P31759172	chr18:61851786-61851830	NM_019508:4220	Il17b	INSIDE	0.972	0.164	3938.17	644.52	0.159	2667.54	424.15
A_68_P31600354	chr18:32098983-32099027	NM_144862:7844	Lims2	INSIDE	0.972	3.435	916.02	3146.59	3.337	690.93	2305.92
A_68_P30402203	chr15:85502100-85502144	AK038224:-5049		PROMOTER	0.972	2.599	398.70	1036.24	2.527	283.87	717.36
A_68_P30342959	chr15:75447008-75447052	NM_001044718:-84	Zfp41	PROMOTER	0.972	0.388	2294.12	889.81	0.377	1768.26	666.50
A_68_P29964259	chr14:123312516-123312560	NM_145466:-151	A2ld1	PROMOTER	0.972	0.195	3041.27	594.02	0.190	2153.33	408.94
A_68_P27890685	chr11:68505258-68505302	NM_175260:-136	Myh10	PROMOTER	0.972	0.184	2734.63	503.49	0.179	1858.27	332.50
A_68_P27306493	chr10:84565965-84566009	NM_001013028:615	AI597468	INSIDE	0.972	0.256	2190.40	561.41	0.249	1761.35	438.73
A_68_P27094092	chr10:41997719-41997771	NM_019740:-1196	Foxo3	PROMOTER	0.972	2.697	313.42	845.27	2.621	287.98	754.75
A_68_P27034636	chr10:29081810-29081854	NM_001110196:416	Rnf146	INSIDE	0.972	2.165	447.28	968.55	2.106	392.80	827.18
A_68_P26826442	chr9:110782965-110783009	NM_001146060:161	Als2cl	INSIDE	0.972	1.791	751.65	1346.25	1.741	662.90	1154.29
A_68_P25086957	chr7:51607437-51607481	NM_001034115:41825	Shank1	INSIDE	0.972	1.790	1099.06	1967.32	1.740	957.80	1666.47
A_68_P24951463	chr7:4946096-4946140	NM_026741:1585	Zfp579	INSIDE	0.972	2.645	742.01	1962.79	2.570	588.98	1513.62
A_68_P24053126	chr5:123402327-123402371	NM_001005866:36194	Kdm2b	INSIDE	0.972	0.306	2548.37	779.99	0.298	1991.35	592.59
A_68_P23539568	chr5:23536418-23536462	NM_053090:61	Fam126a	INSIDE	0.972	0.207	2452.30	506.78	0.201	1950.74	391.75
A_68_P21877293	chr2:174586128-174586172	NM_007903:-123	Edn3	PROMOTER	0.972	1.865	1205.57	2248.90	1.813	950.62	1723.15
A_68_P21150015	chr2:38567109-38567153	NM_139051:2932	Nr5a1	INSIDE	0.972	0.659	3245.91	2138.45	0.641	2380.39	1524.65
A_68_P21141628	chr2:37214555-37214599	NM_026176:276	Pdcl	INSIDE	0.972	0.107	6949.01	745.22	0.104	5361.45	558.73

ProbeName	Target position of probe on CpG island microarray	TargetID	GeneSymbol	CpG island Description	Ratio of relative methylation (TiO_2-NP/Vehicle)	Sham group			TiO_2-H group		
						Relative methylation (Cy5/Cy3)	Cy3 signal (Input)	Cy5 signal (MeDIP)	Relative methylation (Cy5/Cy3)	Cy3 signal (Input)	Cy5 signal (MeDIP)
A_68_P33007790	chr4_random:128337-128381	NM_001033326:18557	Dhrsx	INSIDE	0.971	2.856	7654.84	21860.49	2.773	5724.42	15875.14
A_68_P32266421	chrX:13063192-13063236	NM_173415:18417	Nyx	INSIDE	0.971	1.967	540.08	1062.52	1.911	852.37	1628.75
A_68_P30510787	chr16:4558890-4558934	NM_031182:808	Tcfap4	INSIDE	0.971	0.277	7896.37	2186.80	0.269	5231.36	1407.04
A_68_P29421305	chr14:15535746-15535790	NM_001033270:230	Slc4a7	INSIDE	0.971	4.894	1341.45	6565.38	4.750	1055.07	5011.96
A_68_P29152745	chr13:76193231-76193275	NM_175495:1192	Gpr150	INSIDE	0.971	0.497	2026.67	1008.06	0.483	1486.25	717.52
A_68_P28186435	chr11:120588296-120588340	NM_026428:277	Dcxr	INSIDE	0.971	0.317	2945.34	935.08	0.308	2078.75	640.76
A_68_P27277401	chr10:78996480-78996524	NM_015817:30	Ppap2c	INSIDE	0.971	0.242	3547.12	859.64	0.235	2597.03	611.44
A_68_P27173118	chr10:59681867-59681911	NM_016803:120	Chst3	INSIDE	0.971	0.444	1091.08	483.97	0.431	879.07	378.56
A_68_P26926722	chr10:8605525-8605569	NM_175155:322	Sash1	INSIDE	0.971	0.428	1305.11	558.61	0.416	981.34	407.97
A_68_P24120339	chr5:136751005-136751049	NM_198602:292249	Cux1	INSIDE	0.971	1.543	1668.80	2574.83	1.498	1209.37	1811.59
A_68_P23222359	chr4:114597578-114597622	NM_015758:1018	Foxe3	DOWNSTREAM	0.971	10.221	1414.73	14460.20	9.929	1238.06	12292.31
A_68_P22262791	chr3:79090407-79090451	AK032506:-885		PROMOTER	0.971	0.171	3072.80	526.98	0.167	1964.42	327.14
A_68_P22030181	chr3:30692610-30692654	NM_027016:835	Sec62	INSIDE	0.971	0.547	1239.77	678.45	0.531	982.48	522.02
A_68_P21784245	chr2:158493692-158493736	NM_001159662:844	Ppp1r16b	INSIDE	0.971	0.361	1440.40	520.44	0.351	1226.40	430.46
A_68_P21771129	chr2:156247015-156247059	NM_001003815:249	Epb4.1l1	INSIDE	0.971	0.183	3752.71	685.63	0.177	2759.75	489.64
A_68_P20596468	chr1:129574040-129574084	NM_028135:536	Tmem163	INSIDE	0.971	0.425	1578.40	671.57	0.413	1198.36	494.96
A_68_P20590468	chr1:128389485-128389529	NM_172484:-2	Nckap5	PROMOTER	0.971	0.528	4286.25	2261.68	0.512	3142.37	1609.78
A_68_P32958525	chr17:8627413-8627457	NM_009309:147	T	INSIDE	0.970	0.387	2123.86	822.82	0.376	1582.81	594.67
A_68_P32471837	chrX:72720884-72720928	ENSMUST00000117517:-44		PROMOTER	0.970	0.653	2896.32	1892.69	0.634	2780.83	1761.91
A_68_P31932937	chr19:6141281-6141325	NM_019722:-165	Arl2	PROMOTER	0.970	3.120	476.26	1486.16	3.027	420.98	1274.41
A_68_P31254158	chr17:56281426-56281470	NM_001130456:-1682	Sema6b	PROMOTER	0.970	0.205	3082.14	632.75	0.199	2216.25	441.48
A_68_P27898299	chr11:69795072-69795116	NM_018740:-357	Rai12	PROMOTER	0.970	0.471	1542.45	726.12	0.457	1291.45	590.02
A_68_P27843111	chr11:60268470-60268514	NM_021354:374	Drg2	INSIDE	0.970	0.266	1937.15	514.93	0.258	1314.72	338.83
A_68_P25087017	chr7:51615474-51615518	NM_027049:14904	1700008O03Rik	INSIDE	0.970	0.412	1470.55	605.87	0.400	1117.93	446.84
A_68_P23190570	chr4:107108505-107108549	NM_147221:1203	Glis1	INSIDE	0.970	2.011	945.66	1901.56	1.951	772.58	1507.31
A_68_P23185485	chr4:106295363-106295407	NM_177667:331	Ttc22	INSIDE	0.970	3.623	160.30	580.70	3.515	184.32	647.88
A_68_P22609957	chr3:145312789-145312833	NM_010516:139	Cyr61	INSIDE	0.970	0.460	5103.59	2345.87	0.446	3468.08	1546.66
A_68_P21722816	chr2:147019569-147019613	NM_001077632:-7452	Nkx2-2	PROMOTER	0.970	0.311	1663.11	517.57	0.302	1225.83	369.90
A_68_P20624276	chr1:134777196-134777240	NM_010732:287	Lrrn2	INSIDE	0.970	0.464	1575.55	730.52	0.450	1180.58	531.12
A_68_P20166595	chr1:39633967-39634011	NM_001033135:204	Rnf149	INSIDE	0.970	0.532	1149.86	611.40	0.516	805.26	415.30
A_68_P20076129	chr1:21069175-21069219	NM_133252:94	Tram2	INSIDE	0.970	0.550	1377.06	757.63	0.534	1084.20	578.45
A_68_P32078823	chr19:34267097-34267141	NM_029682:359	Stambpl1	INSIDE	0.969	0.617	3077.94	1897.90	0.598	2249.35	1344.38
A_68_P31378917	chr17:80113203-80113247	NM_009994:1157	Cyp1b1	INSIDE	0.969	0.437	1181.27	516.66	0.424	850.98	360.71
A_68_P30682388	chr16:37777040-37777084	NM_008047:-78	Fstl1	PROMOTER	0.969	0.421	5028.19	2119.13	0.408	3933.07	1606.19
A_68_P30337596	chr15:74347238-74347282	NM_174991:635	Bai1	INSIDE	0.969	0.463	2005.45	929.03	0.449	1526.10	685.25
A_68_P28493820	chr12:66174434-66174478	NM_013902:469	Fkbp3	INSIDE	0.969	0.711	4529.57	3219.90	0.689	3397.63	2341.54
A_68_P27846979	chr11:60988330-60988374	NM_001004143:209	Usp22	INSIDE	0.969	3.055	623.06	1903.53	2.962	570.20	1688.80
A_68_P27794865	chr11:51419667-51419711	NM_001048061:695	Hnrnpab	INSIDE	0.969	0.594	2680.75	1592.23	0.575	2096.28	1206.24
A_68_P27290578	chr10:81033661-81033705	NM_019725:-4594	Tle2	PROMOTER	0.969	0.479	1103.27	528.96	0.465	826.53	383.99
A_68_P26996375	chr10:21713739-21713783	NM_011361:-711	Sgk1	PROMOTER	0.969	3.542	515.87	1827.32	3.432	402.08	1379.98
A_68_P26540062	chr9:56920055-56920099	NM_001110350:-4106	Sin3a	PROMOTER	0.969	2.122	1077.00	2285.41	2.056	896.17	1842.24
A_68_P24197924	chr6:3151637-3151681	ENSMUST00000158475:442		DOWNSTREAM	0.969	3.177	1027.37	3264.16	3.078	880.18	2709.57
A_68_P24011284	chr5:116181838-116181882	NM_001080808:-292	Ccdc64	PROMOTER	0.969	2.042	2625.12	5360.79	1.980	2176.31	4308.05
A_68_P24008565	chr5:115685846-115685890	NM_026398:9	Pop5	INSIDE	0.969	0.525	1304.40	685.10	0.509	1068.91	543.76
A_68_P23567755	chr5:29800608-29800652	NM_019944:4380	Mnx1	INSIDE	0.969	0.504	2286.90	1151.80	0.488	1812.14	884.51
A_68_P23269837	chr4:124386139-124386183	NM_029157:-5944	Sf3a3	PROMOTER	0.969	0.375	3115.43	1167.60	0.363	2196.58	797.54
A_68_P23185613	chr4:106311998-106312042	NM_177667:16967	Ttc22	INSIDE	0.969	2.264	772.43	1748.46	2.194	695.39	1525.74
A_68_P22210126	chr3:67267629-67267673	NM_016753:179	Lxn	INSIDE	0.969	0.266	2391.58	636.98	0.258	1539.67	397.48
A_68_P21570232	chr2:119372907-119372951	NM_172857:435	Exd1	INSIDE	0.969	0.571	1397.33	797.50	0.553	1242.78	687.53
A_68_P20940735	chr1:193730717-193730761	NM_009579:79	Slc30a1	INSIDE	0.969	1.662	1055.88	1754.70	1.611	837.22	1348.79
A_68_P20877313	chr1:182627309-182627353	NM_013729:-165	Mixl1	PROMOTER	0.969	0.372	3850.26	1430.75	0.360	2995.24	1078.74
A_68_P32804787	chrX:162608456-162608500	NM_146238:67	Gemin8	INSIDE	0.968	0.460	2189.96	1007.29	0.445	2819.69	1255.98
A_68_P32148146	chr19:47253257-47253301	NM_021360:-31	Neurl1a	PROMOTER	0.968	2.350	778.65	1829.81	2.274	640.69	1457.11
A_68_P30969552	chr16:92499533-92499577	NM_172469:1163	Clic6	INSIDE	0.968	0.448	2077.59	931.67	0.434	1770.48	768.86
A_68_P30477055	chr15:98701709-98701753	NM_001033276:-116	Mll2	PROMOTER	0.968	0.318	1430.89	454.72	0.308	1118.95	344.23
A_68_P29996525	chr15:7765684-7765728	NM_010275:4696	Gdnf	INSIDE	0.968	0.519	2898.75	1503.75	0.502	2173.06	1091.31
A_68_P29476962	chr14:26197707-26197751	ENSMUST00000164287:393		INSIDE	0.968	0.178	4329.07	770.59	0.172	2746.06	473.29
A_68_P28605827	chr12:87087211-87087255	NM_145447:-256	Mfsd7c	PROMOTER	0.968	0.553	1104.26	610.51	0.535	872.69	467.16
A_68_P28184738	chr11:120328949-120328993	NM_001159328:22	Hgs	INSIDE	0.968	1.597	1792.12	2862.51	1.546	1435.11	2218.04
A_68_P28056109	chr11:98248090-98248134	NM_008890:167	Pnmt	INSIDE	0.968	0.536	1937.88	1037.90	0.518	1538.48	797.61
A_68_P27894427	chr11:69153780-69153824	NR_027827:-468	A030009H04Rik	PROMOTER	0.968	0.293	3111.23	910.46	0.283	2255.69	639.19
A_68_P25735802	chr8:28088218-28088262	NM_001101502:433	Zfp703	INSIDE	0.968	0.541	2172.30	1175.94	0.524	1729.34	905.89
A_68_P22413520	chr3:108394071-108394115	NM_181400:-103	Wdr47	PROMOTER	0.968	0.350	1800.60	630.37	0.339	1415.56	479.64
A_68_P22177975	chr3:60305139-60305183	NM_020007:-13	Mbnl1	PROMOTER	0.968	1.618	881.24	1426.17	1.567	785.99	1231.93
A_68_P22085566	chr3:41368578-41368622	NM_001130186:-201	Phf17	PROMOTER	0.968	0.440	1333.12	587.00	0.426	979.01	417.31

ProbeName	Target position of probe on CpG island microarray	TargetID	GeneSymbol	CpG island Description	Ratio of relative methylation (TiO_2-NP/Vehicle)	Sham group			TiO_2-H group		
						Relative methylation (Cy5/Cy3)	Cy3 signal (Input)	Cy5 signal (MeDIP)	Relative methylation (Cy5/Cy3)	Cy3 signal (Input)	Cy5 signal (MeDIP)
A_68_P20849637	chr1:177622396-177622440	NM_021350:-3934	Chml	PROMOTER	0.968	0.263	3383.15	889.68	0.255	2316.07	589.59
A_68_P31827569	chr18:73913791-73913835	NM_053255:321	Elac1	INSIDE	0.967	1.636	963.23	1575.81	1.581	852.18	1347.54
A_68_P31227868	chr17:50432726-50432770	NM_010021:176	Dazl	INSIDE	0.967	2.133	513.80	1095.86	2.063	444.76	917.68
A_68_P30573854	chr16:17452586-17452630	NM_007764:529	Crkl	INSIDE	0.967	0.298	1656.06	493.16	0.288	1191.40	343.01
A_68_P30447285	chr15:93333764-93333813	NM_001033217:92534	Prickle1	INSIDE	0.967	1.779	975.64	1735.35	1.720	723.27	1244.19
A_68_P28730613	chr12:110132574-110132618	NM_177602:115	Wdr25	INSIDE	0.967	0.355	1900.33	675.37	0.344	1562.96	536.90
A_68_P27993894	chr11:87171685-87171729	NM_177167:790	Ppm1e	INSIDE	0.967	0.155	3567.09	551.60	0.149	2812.99	420.50
A_68_P27383819	chr10:98376903-98376947	NM_026482:-861	Atp2b1	PROMOTER	0.967	1.509	1538.96	2323.03	1.459	1188.60	1734.10
A_68_P27262211	chr10:75931408-75931452	NM_019434:-285	Mcm3ap	PROMOTER	0.967	0.227	3543.61	805.80	0.220	2595.21	570.48
A_68_P26867069	chr9:118468837-118468881	NM_018748:53423	Golga4	INSIDE	0.967	2.120	1814.59	3847.58	2.051	1397.45	2865.98
A_68_P25587155	chr7:148354454-148354502	NM_001172101:107	Rnh1	INSIDE	0.967	1.591	1741.21	2770.68	1.540	1477.21	2274.18
A_68_P24956428	chr7:6649272-6649316	NM_011769:-151	Zim1	PROMOTER	0.967	2.177	567.25	1234.98	2.105	480.18	1010.74
A_68_P24432385	chr6:49345373-49345417	NM_029916:-208	Stk31	PROMOTER	0.967	1.570	1548.96	2431.17	1.517	1223.87	1857.07
A_68_P24159776	chr5:144934512-144934556	NM_001081109:73230	Lmtk2	INSIDE	0.967	2.618	652.01	1707.24	2.532	591.34	1497.21
A_68_P23573762	chr5:30950209-30950253	NM_175675:-81	4930471M23Rik	PROMOTER	0.967	0.481	2528.02	1214.88	0.465	1872.94	869.99
A_68_P23405235	chr4:149655313-149655357	NM_001085492:-690	Rere	PROMOTER	0.967	2.454	668.30	1639.78	2.373	603.06	1431.28
A_68_P23353413	chr4:139225072-139225116	NM_031873:15642	Tas1r2	INSIDE	0.967	2.670	825.16	2203.27	2.583	712.76	1841.14
A_68_P22964958	chr4:62186074-62186118	NM_021498:-48	Pole3	DIVERGENT_PROMOTER	0.967	0.261	1969.87	514.72	0.253	1414.78	357.65
A_68_P21874509	chr2:174124950-174124994	NM_201617:1613	Gnas	INSIDE	0.967	1.846	1444.67	2666.83	1.784	1211.41	2161.55
A_68_P32008155	chr19:21853166-21853210	NM_001033759:357	Tmem2	INSIDE	0.966	0.371	1413.40	524.92	0.359	1057.55	379.32
A_68_P30647300	chr16:31430146-31430190	NM_001122683:1330	Bdh1	INSIDE	0.966	0.522	1638.48	855.05	0.504	1157.32	583.26
A_68_P30401336	chr15:85374246-85374290	NM_001163634:34232	Wnt7b	INSIDE	0.966	1.854	788.46	1461.55	1.791	624.11	1117.88
A_68_P29517812	chr14:33278202-33278246	NM_021712:-188	Slc18a3	PROMOTER	0.966	1.901	660.80	1256.11	1.836	510.70	937.72
A_68_P29134802	chr13:72765932-72765976	NR_030701:58	D430050G20	INSIDE	0.966	0.747	3127.45	2336.94	0.722	2489.74	1798.04
A_68_P28603808	A_68_P28603808			Unknown	0.966	3.084	980.55	3023.76	2.980	824.41	2456.66
A_68_P26274326	chr9:3532514-3532558	NM_001033322:183	Gucy1a2	INSIDE	0.966	0.612	1716.64	1050.96	0.592	1281.69	758.38
A_68_P24932176	chr6:146991608-146991652	NM_145573:339	Mrps35	INSIDE	0.966	0.593	5148.56	3054.59	0.573	3732.53	2139.08
A_68_P24459355	chr6:53770847-53770891	NM_025817:-49	Tril	PROMOTER	0.966	1.705	2746.98	4684.12	1.646	2125.71	3499.69
A_68_P23138267	chr4:98062517-98062561	NM_001005784:22	Inadl	INSIDE	0.966	3.720	911.71	3391.82	3.593	881.69	3167.91
A_68_P20354341	chr1:75475955-75475999	NM_001001565:70	Chpf	INSIDE	0.966	2.246	491.36	1103.84	2.170	413.55	897.49
A_68_P30513463	chr16:5049834-5049878	NM_001079814:147	Glyr1	INSIDE	0.965	0.354	1804.18	638.62	0.342	1330.88	454.75
A_68_P29672519	chr14:65881573-65881617	NM_021458:-294	Fzd3	DIVERGENT_PROMOTER	0.965	0.488	1208.61	590.35	0.471	944.16	444.89
A_68_P29237540	chr13:95647136-95647180	NM_011021:1577	Otp	INSIDE	0.965	0.516	1308.57	675.66	0.498	1115.29	555.90
A_68_P29009462	chr13:46822720-46822764	NM_175749:476	Nup153	INSIDE	0.965	0.559	989.43	553.28	0.539	907.07	489.36
A_68_P27950228	chr11:79474495-79474539	NM_175543:69803	Rab11fip4	INSIDE	0.965	2.071	824.62	1707.78	1.998	640.50	1279.71
A_68_P27560503	chr11:4847990-4848034	NM_010904:55	Nefh	INSIDE	0.965	0.320	3183.14	1017.71	0.309	2432.52	750.51
A_68_P26875427	chr9:119883344-119883388	NM_153287:10410	Csrnp1	INSIDE	0.965	5.625	372.43	2094.74	5.429	295.13	1602.41
A_68_P26727063	chr9:92437135-92437179	NM_001142916:96	Plod2	INSIDE	0.965	0.334	1624.45	541.78	0.322	1310.89	421.79
A_68_P26605681	chr9:68502742-68502786	NM_013646:1156	Rora	INSIDE	0.965	0.245	2385.16	583.78	0.236	1643.84	388.36
A_68_P25092625	chr7:52613255-52613299	NM_029741:9113	Ppfia3	INSIDE	0.965	4.064	758.79	3083.75	3.922	663.10	2600.47
A_68_P24005520	chr5:115141122-115141166	NM_019821:-200	Gltp	PROMOTER	0.965	18.421	2270.74	41829.70	17.783	2012.01	35779.14
A_68_P23298059	chr4:129374001-129374045	NM_199305:60	Tmem39b	INSIDE	0.965	2.447	1542.56	3775.35	2.361	1201.89	2837.61
A_68_P22947349	chr4:57794962-57795006	ENSMUST00000124581:-67		PROMOTER	0.965	0.482	2601.44	1252.92	0.465	2174.46	1010.75
A_68_P20551221	chr1:120218602-120218646	NM_026472:205	Mki67ip	INSIDE	0.965	0.483	1098.52	530.21	0.466	791.48	368.59
A_68_P20454237	chr1:95132383-95132427	NM_172463:-69	Sned1	PROMOTER	0.965	2.107	887.45	1869.96	2.033	709.15	1441.47
A_68_P32139053	chr19:45619725-45619769	NM_020032:15287	Poll	DOWNSTREAM	0.964	1.752	1287.49	2255.62	1.689	973.76	1644.86
A_68_P31054931	chr17:13200874-13200921	NM_013671:193	Sod2	INSIDE	0.964	2.101	557.40	1171.38	2.027	426.94	865.32
A_68_P30370104	chr15:79913777-79913823	NR_028281:-464	Snord43	PROMOTER	0.964	0.513	1084.79	556.22	0.494	871.77	431.07
A_68_P29575955	chr14:47007582-47007626	NM_007554:2670	Bmp4	INSIDE	0.964	0.458	6264.50	2869.90	0.442	4196.06	1853.84
A_68_P29086260	chr13:60703649-60703693	NM_134062:99	Dapk1	INSIDE	0.964	0.497	2135.57	1061.42	0.479	1672.37	801.03
A_68_P29063252	chr13:56854327-56854371	AK131829:840		INSIDE	0.964	1.575	1231.46	1939.87	1.519	959.07	1457.08
A_68_P28708613	chr12:106575627-106575671	ENSMUST00000138649:446		INSIDE	0.964	0.592	2857.91	1693.01	0.571	2005.78	1145.09
A_68_P28687870	chr12:103057567-103057611	NM_011812:-323	Fbln5	PROMOTER	0.964	1.479	2796.63	4135.21	1.426	1948.47	2777.77
A_68_P28177556	chr11:119251329-119251373	NR_035453:-435	Mir1932	PROMOTER	0.964	0.373	1553.70	579.93	0.360	1321.45	475.65
A_68_P28046785	chr11:96650631-96650675	NM_007622:203	Cbx1	INSIDE	0.964	0.419	1551.88	650.53	0.404	1177.22	475.66
A_68_P27926562	chr11:75328897-75328941	NM_001004157:1876	Scarf1	INSIDE	0.964	2.410	1155.30	2784.69	2.324	1024.10	2379.81
A_68_P27682732	chr11:31271512-31271556	NM_011491:-1473	Stc2	PROMOTER	0.964	0.385	2876.53	1108.35	0.371	2185.20	811.67
A_68_P27100908	chr10:43260523-43260567	NM_026411:256	1700021F05Rik	INSIDE	0.964	0.450	1585.49	713.78	0.434	1269.69	551.24
A_68_P26612686	chr9:69609029-69609073	NM_022378:-303	Foxb1	PROMOTER	0.964	0.480	2257.90	1084.70	0.463	1691.96	783.68
A_68_P26158628	chr8:112248320-112248367	NM_029468:18356	Zfp821	INSIDE	0.964	2.943	549.86	1618.27	2.838	347.76	987.03
A_68_P25609685	chr7:152047322-152047366	NM_010202:54	Fgf4	INSIDE	0.964	0.520	1091.00	567.52	0.501	847.46	424.93
A_68_P24951823	chr7:5008286-5008330	NM_146178:-19	Ccdc106	PROMOTER	0.964	0.514	1717.77	882.16	0.495	1381.48	683.99
A_68_P24155560	chr5:143997116-143997160	NM_177681:252	Zfp12	INSIDE	0.964	0.621	1733.98	1076.64	0.599	1380.56	826.61
A_68_P23612481	chr5:37633390-37633434	NM_007765:-3618	Crmp1	PROMOTER	0.964	4.717	714.85	3372.00	4.547	546.66	2485.47
A_68_P22408448	chr3:107563100-107563144	NM_001113529:265	Csf1	INSIDE	0.964	1.745	1709.18	2981.96	1.681	1325.92	2229.05

ProbeName	Target position of probe on CpG island microarray	TargetID	GeneSymbol	CpG island Description	Ratio of relative methylation (TiO_2-NP/Vehicle)	Sham group			TiO_2-H group		
						Relative methylation (Cy5/Cy3)	Cy3 signal (Input)	Cy5 signal (MeDIP)	Relative methylation (Cy5/Cy3)	Cy3 signal (Input)	Cy5 signal (MeDIP)
A_68_P22406473	chr3:107262834-107262878	NM_145922:-1040	Kcnc4	PROMOTER	0.964	4.061	497.69	2021.25	3.914	363.38	1422.32
A_68_P21818226	chr2:164323071-164323115	NM_133779:68	Pigt	INSIDE	0.964	0.315	2220.66	698.60	0.303	1662.08	504.03
A_68_P20294265	chr1:64784540-64784584	NM_001042659:-238	Fzd5	PROMOTER	0.964	5.679	878.39	4988.28	5.472	826.30	4521.50
A_68_P20170820	chr1:40382866-40382910	NM_133193:1417	Il1rl2	INSIDE	0.964	0.440	1710.72	752.96	0.424	1338.82	568.06
A_68_P32707834	chrX:138824754-138824798	NM_001033600:302	Acsl4	INSIDE	0.963	0.372	1522.44	566.56	0.358	2209.18	791.96
A_68_P31766836	chr18:63137638-63137682	NM_028017:91	Napg	INSIDE	0.963	0.401	2091.71	838.58	0.386	1505.54	581.08
A_68_P31611596	chr18:34380712-34380756	NM_007462:97	Apc	INSIDE	0.963	0.449	1952.57	876.73	0.432	1491.94	645.24
A_68_P31600220	chr18:32070086-32070130	NM_026006:1449	Sft2d3	INSIDE	0.963	0.451	1512.92	683.02	0.435	1049.49	456.44
A_68_P30118688	chr15:32175071-32175115	NM_009154:525	Sema5a	INSIDE	0.963	0.362	1326.00	480.08	0.349	975.10	339.95
A_68_P29616217	chr14:55710378-55710422	NM_001039198:485	Zfhx2	INSIDE	0.963	0.317	3783.76	1200.76	0.305	2749.45	839.89
A_68_P29083952	chr13:60278886-60278930	NM_008086:-12	Gas1	PROMOTER	0.963	0.667	2217.52	1479.25	0.643	1832.31	1177.32
A_68_P28429503	chr12:52478533-52478577	NM_029825:-12	Scfd1	PROMOTER	0.963	0.437	2613.15	1141.23	0.421	1996.77	839.68
A_68_P27282592	chr10:79804334-79804378	NM_001113548:6835	Adamtsl5	INSIDE	0.963	0.533	2382.01	1270.07	0.513	1639.08	841.41
A_68_P26844287	chr9:114277292-114277336	NM_019922:22516	Crtap	DOWNSTREAM	0.963	1.471	2107.27	3100.48	1.417	1691.81	2398.04
A_68_P26238000	chr8:125641592-125641636	NM_170684:341	Cpne7	INSIDE	0.963	0.613	1413.93	866.07	0.590	1071.39	632.04
A_68_P25089827	chr7:52125023-52125067	NM_133949:114	Ptov1	INSIDE	0.963	2.383	1549.47	3692.20	2.295	1259.35	2890.08
A_68_P24444431	chr6:51419774-51419818	NM_016806:97	Hnrnpa2b1	INSIDE	0.963	0.469	1280.07	600.34	0.452	1023.83	462.55
A_68_P23996939	chr5:113650467-113650511	NM_001033428:-5440	Tmem211	PROMOTER	0.963	0.599	1159.06	694.77	0.577	978.08	564.74
A_68_P23195053	chr4:107890586-107890630	NM_001167936:-81	Zyg11a	PROMOTER	0.963	0.345	3960.26	1365.25	0.332	3183.40	1056.44
A_68_P20863708	chr1:180336414-180336458	NM_026626:425	Efcab2	INSIDE	0.963	0.608	1148.40	698.78	0.586	1031.56	604.30
A_68_P20353686	chr1:75384915-75384959	NM_001085370:2752	Speg	INSIDE	0.963	1.487	1581.31	2351.76	1.432	1374.82	1968.58
A_68_P20151297	chr1:36995682-36995729	NM_018872:667	Tmem131	INSIDE	0.963	0.315	2220.61	699.96	0.304	1675.05	508.55
A_68_P32385148	chrX:50341666-50341710	NM_013556:434	Hprt	INSIDE	0.962	1.617	3225.18	5213.70	1.555	4237.41	6589.26
A_68_P32044141	chr19:28086028-28086072	NM_011265:-394	Rfx3	PROMOTER	0.962	0.144	6331.25	914.86	0.139	4398.93	611.46
A_68_P31135105	chr17:30749555-30749599	NM_001113560:28	Glo1	INSIDE	0.962	0.645	1835.56	1184.72	0.621	1500.28	931.71
A_68_P28555630	chr12:77811318-77811362	NM_013675:194	Spnb1	INSIDE	0.962	0.439	1947.91	856.03	0.423	1391.01	588.23
A_68_P26998900	chr10:22365258-22365302	NM_178934:464	Slc2a12	INSIDE	0.962	0.201	3737.93	749.63	0.193	2718.25	524.21
A_68_P25616729	chr8:4238967-4239011	NM_001042557:249	Map2k7	INSIDE	0.962	0.279	2924.19	815.09	0.268	2008.27	538.50
A_68_P25375960	chr7:109044724-109044768	NM_027532:-182	3200002M19Rik	PROMOTER	0.962	0.443	1255.34	556.53	0.427	984.40	420.02
A_68_P25098817	chr7:53652411-53652455	NM_001112739:566	Kcnc1	INSIDE	0.962	0.388	2622.10	1018.58	0.374	1925.86	719.42
A_68_P23437420	chr4:154624071-154624115	NM_001190445:18	2610002J02Rik	INSIDE	0.962	0.530	1032.06	546.57	0.510	907.35	462.33
A_68_P23327089	chr4:134731774-134731818	NM_019732:55237	Runx3	INSIDE	0.962	2.693	295.21	794.92	2.590	235.10	609.02
A_68_P23317939	chr4:133054412-133054456	NM_001081156:31	Trnp1	INSIDE	0.962	0.368	2344.93	862.46	0.354	1596.41	564.69
A_68_P22385237	chr3:103084452-103084496	NM_001079830:1259	Trim33	INSIDE	0.962	0.353	2170.57	765.72	0.339	1485.46	504.18
A_68_P21905662	chr2:180436767-180436811	NM_175551:17	Dido1	INSIDE	0.962	0.385	2539.84	977.97	0.371	1965.03	728.10
A_68_P20637767	chr1:137043374-137043418	NM_026823:-14	Arl8a	PROMOTER	0.962	2.031	999.43	2030.32	1.954	836.48	1634.22
A_68_P31561530	chr18:24868456-24868500	NM_175276:533	Fhod3	INSIDE	0.961	0.519	3023.18	1568.17	0.498	2182.90	1087.74
A_68_P31020946	chr17:6079495-6079539	NM_001111017:223	Serac1	INSIDE	0.961	0.472	2780.97	1313.46	0.454	2071.39	940.11
A_68_P30966050	chr16:91931897-91931941	NM_138597:-43	Atp5o	PROMOTER	0.961	0.480	1195.32	573.51	0.461	962.40	443.93
A_68_P30605855	chr16:23983543-23983587	NM_009744:5134	Bcl6	INSIDE	0.961	0.593	2299.50	1362.84	0.569	1734.62	987.73
A_68_P28047120	chr11:96711640-96711684	NR_029562:-44	Mir152	PROMOTER	0.961	0.419	3563.32	1492.74	0.403	2621.52	1055.88
A_68_P27921011	chr11:74336607-74336651	NM_001015046:67032	Rap1gap2	INSIDE	0.961	0.348	3528.20	1227.33	0.334	2476.17	827.82
A_68_P27080350	chr10:39452886-39452930	NM_011264:943	Rev3l	INSIDE	0.961	9.714	2541.57	24689.58	9.339	1892.09	17670.98
A_68_P26628303	chr9:72379539-72379583	NM_001033536:-486	Rfx7	PROMOTER	0.961	0.178	4975.82	886.34	0.171	3289.74	563.19
A_68_P26542619	chr9:57437422-57437466	NM_027895:186	Ulk3	INSIDE	0.961	2.896	617.34	1787.81	2.784	510.58	1421.28
A_68_P26345852	chr9:20988662-20988706	NM_183408:18527	Pde4a	INSIDE	0.961	0.557	1841.66	1025.84	0.536	1427.65	764.56
A_68_P24463221	chr6:54379963-54380009	NR_033635:229	9130019P16Rik	INSIDE	0.961	0.328	1727.58	567.28	0.316	1254.28	395.99
A_68_P24163714	chr5:145725951-145725995	NM_001038627:727	Smurf1	INSIDE	0.961	0.469	3455.59	1621.53	0.451	2497.10	1125.49
A_68_P23226959	chr4:115635591-115635635	ENSMUST00000125761:54668		DOWNSTREAM	0.961	2.318	343.93	797.14	2.227	334.08	744.04
A_68_P22180928	chr3:60806900-60806944	NM_008772:206	P2ry1	INSIDE	0.961	0.343	1929.08	661.78	0.330	1474.76	486.33
A_68_P21986761	chr3:21975073-21975117	NM_030732:-479	Tbl1xr1	PROMOTER	0.961	0.359	2536.89	909.61	0.345	2175.96	750.02
A_68_P20680083	chr1:145624045-145624089	NM_013835:115	Trove2	INSIDE	0.961	1.846	827.92	1528.03	1.774	683.48	1212.72
A_68_P20597392	chr1:129765401-129765445	NM_178690:73	Rab3gap1	INSIDE	0.961	0.250	1908.44	476.27	0.240	1555.19	373.00
A_68_P32145750	chr19:46835351-46835395	NM_001102471:-726	Cnnm2	PROMOTER	0.960	2.914	1265.41	3687.26	2.797	993.45	2778.44
A_68_P31943259	chr19:8810305-8810349	NM_134139:-2	Wdr74	PROMOTER	0.960	0.549	1832.50	1006.03	0.527	1498.60	790.13
A_68_P29554792	chr14:40285336-40285380	NM_001190187:1018	Nrg3	INSIDE	0.960	0.236	5405.94	1277.25	0.227	3452.81	783.44
A_68_P29336879	chr13:113591191-113591235	NM_008903:82	Ppap2a	INSIDE	0.960	0.242	2008.69	485.63	0.232	1567.84	363.88
A_68_P29234320	chr13:95128962-95129006	NM_009680:70	Ap3b1	INSIDE	0.960	0.654	1955.81	1279.18	0.628	1576.53	989.78
A_68_P26475231	chr9:45480834-45480878	NM_001081270:242481	Dscaml1	INSIDE	0.960	2.109	1393.24	2938.76	2.024	1031.54	2088.16
A_68_P26471637	chr9:44946814-44946858	NM_001013390:-288	Scn4b	DIVERGENT_PROMOTER	0.960	0.458	1363.68	624.78	0.440	1171.38	515.06
A_68_P26224547	chr8:123632165-123632209	NM_001166482:93	Mthfsd	INSIDE	0.960	0.259	1697.55	439.40	0.249	1275.44	317.07
A_68_P25725385	chr8:26212906-26212950	NM_031257:-645	Plekha2	PROMOTER	0.960	0.399	2554.75	1020.56	0.383	1868.61	716.60
A_68_P24120141	chr5:136720302-136720346	NM_018825:449	Sh2b2	INSIDE	0.960	1.664	1363.76	2269.30	1.597	1000.27	1597.20
A_68_P23854795	chr5:84846156-84846200	NM_007937:229	Epha5	INSIDE	0.960	2.039	920.46	1876.93	1.958	743.96	1456.85
A_68_P22312508	chr3:88019276-88019320	NR_029817:-221	Mir9-1	PROMOTER	0.960	0.382	3466.01	1325.50	0.367	2417.90	887.62

ProbeName	Target position of probe on CpG island microarray	TargetID	GeneSymbol	CpG island Description	Ratio of relative methylation (TiO_2-NP/Vehicle)	Sham group			TiO_2-H group		
						Relative methylation (Cy5/Cy3)	Cy3 signal (Input)	Cy5 signal (MeDIP)	Relative methylation (Cy5/Cy3)	Cy3 signal (Input)	Cy5 signal (MeDIP)
A_68_P21909031	chr2:180921876-180921920	NM_025598:-149	Ppdpf	PROMOTER	0.960	2.258	1353.25	3055.07	2.167	1076.99	2333.48
A_68_P21573663	chr2:119979882-119979926	NM_133838:407	Ehd4	INSIDE	0.960	0.540	2321.63	1254.04	0.519	1938.34	1005.30
A_68_P21110675	chr2:31544313-31544357	NM_001112703:259	Abl1	INSIDE	0.960	0.604	1666.79	1006.22	0.580	1506.79	873.61
A_68_P20616233	chr1:133422446-133422490	NM_001081011:1470	Srgap2	INSIDE	0.960	1.575	1286.04	2024.93	1.511	1104.87	1669.63
A_68_P20066647	chr1:19204272-19204316	NM_001025305:2160	Tcfap2b	INSIDE	0.960	0.266	3343.34	889.05	0.255	2654.13	677.40
A_68_P32185221	chr19:53977782-53977826	NM_001168491:-36	Pdcd4	PROMOTER	0.959	0.571	1305.48	746.08	0.548	1033.48	566.43
A_68_P31632608	chr18:38359167-38359214	NM_029357:10226	Pcdh1	INSIDE	0.959	2.581	279.17	720.45	2.476	261.51	647.40
A_68_P31097498	chr17:24607655-24607699	NM_025954:259	Pgp	INSIDE	0.959	0.483	2862.95	1382.89	0.463	2068.23	957.77
A_68_P30641001	chr16:30283247-30283291	NM_028973:72	Lrrc15	INSIDE	0.959	0.463	1028.96	476.86	0.444	825.14	366.60
A_68_P30397289	chr15:84684008-84684052	NM_001081166:2529	Phf21b	INSIDE	0.959	0.559	2535.17	1417.25	0.536	1906.04	1022.21
A_68_P30132825	chr15:35256447-35256491	BC048602:1822		INSIDE	0.959	2.223	586.67	1303.89	2.132	545.39	1162.93
A_68_P27849837	chr11:61498300-61498344	NM_178618:411	Fam83g	INSIDE	0.959	1.714	2426.80	4160.20	1.644	1813.41	2981.16
A_68_P27288886	chr10:80787882-80787926	NM_027381:4057	2510012J08Rik	INSIDE	0.959	1.908	1168.58	2229.32	1.830	882.67	1615.37
A_68_P27093483	chr10:41917159-41917203	NM_019740:79368	Foxo3	INSIDE	0.959	1.789	1672.82	2992.41	1.716	1274.90	2187.41
A_68_P25837684	chr8:47825681-47825725	NM_008391:604	Irf2	INSIDE	0.959	0.370	1576.97	583.22	0.355	1304.25	462.64
A_68_P25666781	chr8:13200390-13200434	NM_025768:212	Grtp1	INSIDE	0.959	1.799	1287.43	2315.59	1.724	1088.79	1877.31
A_68_P25565906	chr7:144629538-144629582	NR_033611:204	Gm12669	INSIDE	0.959	0.473	1927.92	912.31	0.454	1668.07	756.84
A_68_P24819295	chr6:125141439-125141483	NR_028517:-5011	Scarna10	PROMOTER	0.959	0.253	2191.17	554.61	0.243	1593.61	387.01
A_68_P24687696	chr6:99642117-99642161	NM_008158:-534	Gpr27	PROMOTER	0.959	0.638	1668.32	1064.05	0.612	1340.40	820.09
A_68_P24161578	chr5:145306716-145306760	NM_016789:-17	Nptx2	PROMOTER	0.959	2.701	718.37	1940.37	2.590	609.03	1577.34
A_68_P23410313	chr4:150503244-150503288	NM_001081557:732611	Camta1	INSIDE	0.959	0.644	2250.49	1450.18	0.618	1564.71	966.61
A_68_P23074274	chr4:85160094-85160138	ENSMUST00000147066:-114		PROMOTER	0.959	0.581	2524.21	1467.54	0.557	1916.10	1068.17
A_68_P22531470	chr3:131005610-131005654	NM_027816:513	Cyp2u1	INSIDE	0.959	2.219	432.12	959.05	2.128	419.93	893.80
A_68_P21820020	chr2:164658912-164658956	NM_001082974:162	Neurl2	INSIDE	0.959	0.389	2320.39	903.27	0.373	1751.23	653.78
A_68_P21104695	chr2:30549015-30549059	ENSMUST00000146702:63862		DOWNSTREAM	0.959	2.931	743.27	2178.73	2.810	616.34	1732.13
A_68_P30956557	chr16:90387057-90387101	NM_015755:437	Hunk	INSIDE	0.958	0.484	2008.82	972.27	0.464	1481.86	686.88
A_68_P30726540	chr16:45408945-45408989	NM_010818:200	Cd200	INSIDE	0.958	0.629	2187.17	1374.80	0.602	1599.98	963.94
A_68_P29020720	chr13:48764426-48764470	NM_007526:6044	Barx1	DOWNSTREAM	0.958	0.252	4091.93	1031.50	0.241	2977.47	718.78
A_68_P28450023	chr12:56086848-56086892	NM_013815:453	Baz1a	INSIDE	0.958	3.119	644.61	2010.54	2.989	474.96	1419.46
A_68_P28223705	chr12:8681057-8681101	NM_030723:-408	Pum2	PROMOTER	0.958	0.385	1339.27	515.62	0.369	1046.88	386.05
A_68_P28220740	chr12:8215059-8215103	NM_001167767:148	1110057K04Rik	INSIDE	0.958	0.584	3701.00	2162.18	0.559	2781.00	1555.77
A_68_P28055768	chr11:98200119-98200163	NM_010895:-9181	Neurod2	DIVERGENT_PROMOTER	0.958	0.509	1855.03	944.02	0.487	1416.36	690.43
A_68_P27081279	chr10:39618779-39618823	NR_033628:244	AA474331	INSIDE	0.958	0.621	1853.94	1150.95	0.595	1415.84	842.11
A_68_P26629967	chr9:72704625-72704669	NM_175485:49566	Prtg	INSIDE	0.958	2.346	1456.91	3417.30	2.246	1175.50	2640.40
A_68_P25972608	chr8:77736812-77736856	NM_029182:-1008	Rasd2	PROMOTER	0.958	0.519	4043.93	2097.19	0.497	2903.04	1442.92
A_68_P25507823	chr7:135019837-135019881	NM_146259:479	Zfp668	INSIDE	0.958	0.579	1330.05	769.86	0.555	1131.56	627.67
A_68_P24837689	chr6:128150353-128150397	ENSMUST00000071101:267		INSIDE	0.958	0.399	1562.95	623.93	0.382	1354.57	517.91
A_68_P24449035	chr6:52153690-52153734	NM_010453:874	Hoxa5	INSIDE	0.958	2.187	920.82	2013.56	2.094	747.34	1564.78
A_68_P24191337	chr5:151325172-151325216	NM_009765:-3	Brca2	DIVERGENT_PROMOTER	0.958	1.860	1038.17	1931.45	1.781	893.63	1591.95
A_68_P22314314	chr3:88336898-88336942	NM_001029890:604	Mex3a	INSIDE	0.958	0.228	2244.83	512.25	0.219	1523.76	333.01
A_68_P21750610	chr2:152512595-152512639	NM_025543:-267	Mcts2	PROMOTER	0.958	1.784	1245.04	2220.74	1.709	1009.48	1725.22
A_68_P21095672	chr2:29050439-29050483	NM_133500:53100	Ntng2	INSIDE	0.958	0.549	2519.25	1384.03	0.526	2054.42	1080.80
A_68_P32537944	chrX:91480391-91480435	NM_001039059:144	Klhl15	INSIDE	0.957	1.759	1008.81	1774.68	1.683	1156.35	1946.03
A_68_P31423369	chr17:87845478-87845522	NM_007589:775	Calm2	INSIDE	0.957	1.992	528.77	1053.16	1.906	450.99	859.70
A_68_P30660865	chr16:33967049-33967093	NM_009471:19	Umps	INSIDE	0.957	0.144	3081.11	442.66	0.137	2237.25	307.58
A_68_P30470421	chr15:97535782-97535826	NM_028003:449	Rpap3	INSIDE	0.957	0.474	2985.65	1414.11	0.453	2220.24	1006.12
A_68_P28987058	chr13:43012395-43012444	NM_001005740:207470	Phactr1	INSIDE	0.957	0.405	1144.61	463.73	0.388	908.91	352.49
A_68_P25776076	chr8:35869953-35869997	NM_176933:-689	Dusp4	PROMOTER	0.957	0.384	2780.76	1067.06	0.367	2151.93	790.02
A_68_P23523567	chr5:20457129-20457173	NR_030699:344	A630072M18Rik	INSIDE	0.957	1.951	1022.27	1994.51	1.868	892.54	1667.33
A_68_P22367896	chr3:99966241-99966285	NM_175552:64	Wdr3	INSIDE	0.957	0.562	1821.97	1023.32	0.537	1413.39	759.47
A_68_P21978657	chr3:20054597-20054641	NM_013755:377	Gyg	INSIDE	0.957	1.943	826.87	1606.72	1.859	714.55	1328.61
A_68_P21957226	chr3:14534076-14534120	NM_001163579:311	Lrrcc1	INSIDE	0.957	3.152	1855.24	5847.67	3.017	1555.92	4693.90
A_68_P21952654	chr3:13472953-13472997	NM_001163328:1320	Ralyl	INSIDE	0.957	0.379	1836.40	696.45	0.363	1432.27	519.97
A_68_P32908649	chr12:89598957-89599001	NM_028105:-266	Adck1	PROMOTER	0.956	2.219	363.49	806.71	2.121	370.68	786.25
A_68_P32427549	chrX:61529891-61529935	NM_178740:259	Slitrk4	INSIDE	0.956	0.572	1378.92	789.14	0.547	2032.57	1112.24
A_68_P31425157	chr17:88197480-88197524	NM_199251:-168	Kcnk12	PROMOTER	0.956	0.155	4979.91	770.25	0.148	3811.14	563.40
A_68_P31143941	chr17:32280371-32280415	NM_008716:23405	Notch3	INSIDE	0.956	0.500	1567.43	783.79	0.478	1194.38	570.83
A_68_P30402005	chr15:85476809-85476853	AK037366:-9265		PROMOTER	0.956	0.560	1740.06	974.92	0.536	1496.65	801.68
A_68_P30397310	chr15:84686778-84686823	NM_001081166:-241	Phf21b	PROMOTER	0.956	0.378	3977.96	1503.33	0.361	2893.79	1045.55
A_68_P29052393	chr13:55057473-55057517	NM_153131:6702	Unc5a	INSIDE	0.956	1.769	2942.19	5204.26	1.690	2346.06	3965.72
A_68_P29048047	chr13:54287555-54287599	NM_008286:79	Hrh2	INSIDE	0.956	0.344	1504.07	517.89	0.329	1266.60	417.05
A_68_P28743061	chr12:112512577-112512621	NM_033603:3277	Amn	INSIDE	0.956	0.442	4130.02	1826.26	0.423	2885.90	1220.59
A_68_P27282833	chr10:79844657-79844701	NM_198615:5718	Mex3d	INSIDE	0.956	2.601	438.37	1140.41	2.486	397.10	987.15
A_68_P26511312	A_68_P26511312			Unknown	0.956	4.477	564.61	2528.03	4.279	472.23	2020.89
A_68_P26238678	chr8:125759558-125759602	NM_030176:529	Spata2L	INSIDE	0.956	0.517	1469.96	760.65	0.495	1123.44	555.65

ProbeName	Target position of probe on CpG island microarray	TargetID	GeneSymbol	CpG island Description	Ratio of relative methylation (TiO_2-NP/Vehicle)	Sham group			TiO_2-H group		
						Relative methylation (Cy5/Cy3)	Cy3 signal (Input)	Cy5 signal (MeDIP)	Relative methylation (Cy5/Cy3)	Cy3 signal (Input)	Cy5 signal (MeDIP)
A_68_P26024230	chr8:87823989-87824033	NM_001163791:480	Orc6	INSIDE	0.956	0.399	1239.38	494.53	0.382	1043.29	398.14
A_68_P24326228	chr6:29352876-29352920	NM_001201378:3973	Ccdc136	INSIDE	0.956	2.011	722.48	1453.16	1.923	585.88	1126.83
A_68_P23756860	chr5:65929084-65929128	NM_016786:607	Ube2k	INSIDE	0.956	0.564	2386.22	1344.90	0.539	1850.61	996.96
A_68_P22388053	chr3:103594655-103594699	NM_010432:522	Hipk1	INSIDE	0.956	0.385	2441.49	938.99	0.368	1703.43	626.57
A_68_P22317996	chr3:89031659-89031703	NR_030579:-560	Mir92b	DIVERGENT_PROMOTER	0.956	0.372	1695.35	630.11	0.355	1233.18	438.18
A_68_P21331222	chr2:73224458-73224502	NM_001190297:-25	Gpr155	PROMOTER	0.956	0.312	4407.91	1374.09	0.298	3352.93	998.84
A_68_P21177697	chr2:44412424-44412468	NM_172662:304696	Gtdc1	DOWNSTREAM	0.956	1.760	1764.13	3104.70	1.683	1548.95	2606.52
A_68_P32745698	chrX:147955401-147955445	NM_177201:208	Phf8	INSIDE	0.955	3.085	436.78	1347.42	2.946	605.68	1784.27
A_68_P32571131	chrX:100129101-100129145	NR_026596:440	Gm5126	INSIDE	0.955	1.759	775.10	1363.68	1.680	1080.75	1815.26
A_68_P32218817	chr19:59419763-59419807	NM_001033222:486	Pdzd8	INSIDE	0.955	0.598	2014.85	1204.94	0.571	1493.00	852.33
A_68_P31744363	chr18:58997551-58997595	NM_175506:1155	Adamts19	INSIDE	0.955	0.476	1632.03	776.87	0.455	1212.94	551.63
A_68_P31490820	chr18:10610131-10610175	NM_001081222:198	Esco1	INSIDE	0.955	1.948	571.56	1113.13	1.860	508.63	946.16
A_68_P30401337	chr15:85374375-85374419	NM_001163634:34104	Wnt7b	INSIDE	0.955	2.397	676.86	1622.57	2.290	488.91	1119.61
A_68_P29937477	chr14:118634921-118634965	NM_177753:1310	Sox21	INSIDE	0.955	2.178	641.77	1397.66	2.080	629.12	1308.30
A_68_P29024948	chr13:49478604-49478648	NM_001039179:41709	Bicd2	INSIDE	0.955	4.727	1499.40	7087.92	4.514	1338.09	6040.63
A_68_P28941007	chr13:34967553-34967597	NM_013830:212	Prpf4b	INSIDE	0.955	0.624	1477.95	922.25	0.596	1291.12	769.19
A_68_P28574824	chr12:81361474-81361518	NM_134156:-138	Actn1	PROMOTER	0.955	0.152	3194.78	486.76	0.146	2091.60	304.38
A_68_P28553607	chr12:77472331-77472375	NM_178744:1100	Zbtb1	INSIDE	0.955	1.515	1846.91	2797.26	1.446	1351.64	1954.78
A_68_P28534712	chr12:74046980-74047024	NM_011384:6071	Six6	DOWNSTREAM	0.955	2.119	831.80	1762.33	2.023	669.93	1355.25
A_68_P27278893	chr10:79243017-79243061	NM_031380:3020	Fstl3	INSIDE	0.955	0.176	3387.29	595.76	0.168	2351.26	394.79
A_68_P27254390	chr10:74524101-74524145	NM_001081412:482	Bcr	INSIDE	0.955	2.145	657.84	1411.02	2.049	501.55	1027.71
A_68_P26476240	chr9:45636562-45636606	NM_001081373:137	Cep164	INSIDE	0.955	0.342	1572.20	537.55	0.327	1260.70	411.64
A_68_P26350701	chr9:21969396-21969440	NM_001082532:8129	Pigyl	DOWNSTREAM	0.955	0.562	1214.44	682.11	0.536	975.41	523.21
A_68_P25948480	chr8:72406164-72406208	NM_026818:5105	Cilp2	INSIDE	0.955	2.802	808.56	2265.30	2.676	725.04	1939.95
A_68_P23692831	chr5:52506869-52506913	AK076709:-1661		PROMOTER	0.955	0.376	2779.84	1045.01	0.359	2295.38	824.48
A_68_P23195378	chr4:107973651-107973695	NM_001033634:23	Zyg11b	INSIDE	0.955	1.648	3896.66	6421.32	1.574	2909.28	4580.66
A_68_P21144176	chr2:37648649-37648693	NM_001163566:16902	Crb2	INSIDE	0.955	1.526	1569.46	2395.77	1.458	1202.95	1753.73
A_68_P20121785	chr1:30930713-30930757	ENSMUST00000046443:-195		PROMOTER	0.955	7.987	666.86	5326.37	7.625	733.93	5596.45
A_68_P32546912	chrX:94267952-94267996	NM_001033361:40	Pgr15l	INSIDE	0.954	2.772	240.76	667.36	2.644	283.58	749.78
A_68_P31940077	chr19:7557297-7557341	NM_053076:453	Rtn3	INSIDE	0.954	0.498	3250.53	1619.59	0.475	2216.31	1053.13
A_68_P30346598	chr15:76009831-76009878	NM_201394:16286	Plec	INSIDE	0.954	2.388	980.61	2341.62	2.277	779.60	1775.52
A_68_P30214224	chr15:51871601-51871645	NM_175503:-30	Aard	PROMOTER	0.954	0.541	1783.95	965.41	0.517	1573.34	812.69
A_68_P29977213	chr15:3976841-3976885	NM_024188:435	Oxct1	INSIDE	0.954	6.154	655.84	4035.83	5.873	510.25	2996.76
A_68_P28576811	chr12:81744792-81744836	NM_007951:34	Erh	INSIDE	0.954	4.319	4074.49	17598.06	4.121	3124.92	12877.06
A_68_P27977992	chr11:84338560-84338604	NM_008498:454	Lhx1	INSIDE	0.954	0.390	1901.83	742.08	0.372	1426.67	531.19
A_68_P27271773	chr10:77506098-77506142	NM_009646:205	Aire	INSIDE	0.954	0.736	3637.58	2676.33	0.702	2824.02	1982.40
A_68_P26055744	chr8:93433014-93433058	NM_177224:80303	Chd9	INSIDE	0.954	0.494	943.57	465.94	0.471	778.62	366.84
A_68_P24972906	chr7:13366070-13366114	NM_001017955:-5085	Zscan18	PROMOTER	0.954	2.170	990.00	2148.62	2.071	856.23	1773.12
A_68_P21212089	chr2:51004469-51004524	NM_028810:135	Rnd3	INSIDE	0.954	3.152	231.43	729.52	3.007	237.97	715.48
A_68_P20975260	chr2:5634819-5634863	NM_177343:870	Camk1d	INSIDE	0.954	0.290	2539.15	736.88	0.277	1575.43	436.31
A_68_P20163208	chr1:39044668-39044712	NM_026850:32	Pdcl3	INSIDE	0.954	3.577	2710.75	9695.03	3.412	2117.38	7225.53
A_68_P20158328	chr1:38267049-38267093	NM_010678:454730	Aff3	INSIDE	0.954	2.250	479.70	1079.47	2.146	432.92	929.10
A_68_P32128429	chr19:43827379-43827423	NM_144874:90	Cox15	INSIDE	0.953	0.422	1579.30	667.07	0.403	1182.88	476.14
A_68_P32026439	chr19:24975135-24975179	NM_008022:643	Foxd4	INSIDE	0.953	0.436	3179.01	1385.29	0.415	2543.17	1056.33
A_68_P31929908	chr19:5654240-5654284	NM_001164480:9445	Sipa1	INSIDE	0.953	2.383	1105.88	2635.50	2.271	887.52	2015.45
A_68_P31161374	chr17:36002854-36002898	NM_001146710:-196	2310014H01Rik	PROMOTER	0.953	0.414	1567.42	648.93	0.395	1103.54	435.49
A_68_P30385309	chr15:82729893-82729937	NR_027799:-1023	Tbrg3	PROMOTER	0.953	3.063	524.75	1607.10	2.919	484.63	1414.41
A_68_P28577468	chr12:81858602-81858646	NM_001008423:3078	Gm1568	INSIDE	0.953	2.543	1191.76	3030.34	2.423	1000.72	2424.96
A_68_P28277949	chr12:19888314-19888358	ENSMUST00000103822:-9494		PROMOTER	0.953	2.315	1243.44	2878.61	2.206	1088.77	2401.94
A_68_P27813412	chr11:54847029-54847073	NM_001110211:-77	Anxa6	PROMOTER	0.953	0.395	1729.17	682.24	0.376	1243.57	467.55
A_68_P27630338	chr11:20101951-20101995	NM_008996:368	Rab1	INSIDE	0.953	2.233	560.54	1251.59	2.128	452.54	962.99
A_68_P26717017	chr9:90165120-90165164	NM_194334:465	Tbc1d2b	INSIDE	0.953	0.634	2292.53	1454.47	0.605	1752.05	1059.49
A_68_P25016713	chr7:28114386-28114430	NM_001113549:4259	Ltbp4	INSIDE	0.953	0.253	1838.44	464.22	0.241	1379.45	332.05
A_68_P24820862	chr6:125444303-125444347	NM_007657:449	Cd9	INSIDE	0.953	0.428	1976.49	845.62	0.408	1502.00	612.46
A_68_P24760447	chr6:113554719-113554763	NM_133937:-25	6720456B07Rik	DIVERGENT_PROMOTER	0.953	25.109	514.86	12927.87	23.932	458.39	10970.03
A_68_P24638016	chr6:91235548-91235592			Unknown	0.953	4.944	1143.62	5654.29	4.711	868.04	4089.04
A_68_P21906908	chr2:180628801-180628845	NR_029538:78	Mir124a-3	DOWNSTREAM	0.953	2.517	469.11	1180.83	2.398	404.53	969.95
A_68_P20353582	chr1:75371730-75371774	NM_007463:-119	Speg	DIVERGENT_PROMOTER	0.953	0.245	1914.64	468.50	0.233	1356.61	316.19
A_68_P32462527	chrX:70667702-70667746	NM_009566:11283	Zfp92	INSIDE	0.952	1.933	611.50	1182.19	1.841	822.53	1513.97
A_68_P31112795	chr17:26986945-26986989	NM_008700:-8456	Nkx2-5	PROMOTER	0.952	0.391	1862.48	728.08	0.372	1457.76	542.64
A_68_P30379351	chr15:81576109-81576153	NM_001081016:853	Zc3h7b	INSIDE	0.952	0.442	1827.87	808.44	0.421	1483.37	624.37
A_68_P30371299	chr15:80117494-80117538	NM_001044308:-151	Cacna1i	PROMOTER	0.952	0.381	1829.48	696.15	0.362	1488.89	539.24
A_68_P29056641	chr13:55819458-55819502	NM_175150:3470	Txndc15	INSIDE	0.952	2.061	675.41	1391.73	1.962	549.42	1078.06
A_68_P28269097	chr12:17183053-17183097	NM_201531:620	Kcnf1	INSIDE	0.952	0.381	1727.46	658.50	0.363	1207.21	438.21
A_68_P27470615	chr10:115024818-115024862	NM_010195:-4	Lgr5	PROMOTER	0.952	0.504	1825.73	920.63	0.480	1328.38	637.91

ProbeName	Target position of probe on CpG island microarray	TargetID	GeneSymbol	CpG island Description	Ratio of relative methylation (TiO_2-NP/Vehicle)	Sham group			TiO_2-H group		
						Relative methylation (Cy5/Cy3)	Cy3 signal (Input)	Cy5 signal (MeDIP)	Relative methylation (Cy5/Cy3)	Cy3 signal (Input)	Cy5 signal (MeDIP)
A_68_P26553627	chr9:59333545-59333589	NM_019927:615	Arih1	INSIDE	0.952	0.301	1949.00	587.16	0.287	1482.23	424.92
A_68_P25848478	chr8:49641171-49641215	NM_001145937:118852	Odz3	INSIDE	0.952	0.593	2099.16	1245.06	0.565	1762.29	995.21
A_68_P25352337	chr7:104937215-104937259	ENSMUST00000172423:186		INSIDE	0.952	0.323	1769.16	572.19	0.308	1423.52	438.51
A_68_P24374361	chr6:37820805-37820849	NM_145076:16	Trim24	INSIDE	0.952	0.201	3953.74	794.11	0.191	2830.06	541.07
A_68_P23809996	chr5:75971773-75971817	NM_021099:779	Kit	INSIDE	0.952	0.600	2177.02	1307.16	0.572	1673.86	956.80
A_68_P23397203	chr4:148265241-148265285	NM_027195:86762	Casz1	INSIDE	0.952	0.223	3013.43	671.40	0.212	2024.24	429.54
A_68_P21611829	chr2:126969142-126969186	NM_001163528:-838	Itpripl1	PROMOTER	0.952	0.648	1494.33	967.99	0.616	1323.46	815.81
A_68_P21608667	chr2:126379090-126379134	NM_011978:353	Slc27a2	INSIDE	0.952	0.541	1727.79	935.13	0.515	1295.53	667.24
A_68_P32237966	chrX:6895899-6895943	ENSMUST00000132126:376		INSIDE	0.951	2.562	366.58	939.10	2.437	348.28	848.62
A_68_P32114041	chr19:41337635-41337679	NM_133352:838	Tm9sf3	INSIDE	0.951	0.623	2125.77	1325.32	0.593	1663.96	987.02
A_68_P31928722	chr19:5448027-5448071	NM_010235:351	Fosl1	INSIDE	0.951	1.610	1785.20	2873.62	1.531	1374.33	2103.63
A_68_P31084704	chr17:21146145-21146189	NM_145483:262	Zfp160	INSIDE	0.951	0.367	2962.94	1086.77	0.349	2062.42	719.30
A_68_P30366911	chr15:79342352-79342396	NM_134090:-4463	Kdelr3	DIVERGENT_PROMOTER	0.951	0.298	3891.61	1158.51	0.283	2837.14	803.10
A_68_P28729766	chr12:109996154-109996198			Unknown	0.951	0.279	3094.72	862.78	0.265	2362.15	626.48
A_68_P25958331	chr8:74213094-74213138	NM_013564:-34	Insl3	PROMOTER	0.951	1.974	991.07	1956.20	1.878	704.29	1322.60
A_68_P25269029	chr7:88005988-88006032	NM_009553:-983	Zscan2	PROMOTER	0.951	0.525	2620.57	1375.18	0.499	2003.99	1000.03
A_68_P24692854	chr6:100513607-100513651	NM_181590:107523	Shq1	DOWNSTREAM	0.951	0.450	1737.50	781.63	0.428	1383.26	591.83
A_68_P24533020	chr6:70741992-70742036	NM_009075:155	Rpia	INSIDE	0.951	1.997	1285.24	2566.69	1.900	1118.94	2125.63
A_68_P24005309	chr5:115108492-115108536	NM_022017:-84	Trpv4	PROMOTER	0.951	0.457	1616.67	738.26	0.434	1369.29	594.57
A_68_P24003587	chr5:114830039-114830083	NM_001159941:454	Kctd10	INSIDE	0.951	0.110	5608.44	615.19	0.104	3565.90	371.99
A_68_P23315908	chr4:132686610-132686654	NM_153423:85	Wasf2	INSIDE	0.951	0.459	1135.29	521.40	0.437	815.64	356.27
A_68_P22325399	chr3:90268672-90268716	NM_008727:1094	Npr1	INSIDE	0.951	0.669	2825.15	1889.82	0.636	2219.89	1412.66
A_68_P22130347	chr3:51364112-51364156	NR_033624:456	5031434O11Rik	INSIDE	0.951	0.290	1957.37	566.97	0.275	1463.83	403.04
A_68_P21628930	chr2:130109870-130109914	NM_130884:295	Idh3b	INSIDE	0.951	0.110	6816.57	750.11	0.105	4758.89	497.76
A_68_P20826008	chr1:172995959-172996003	NM_010188:-6446	Fcgr3	PROMOTER	0.951	0.148	20653.91	3047.34	0.140	16738.13	2348.72
A_68_P20088276	chr1:23389423-23389467	NM_001081079:570	Ogfrl1	INSIDE	0.951	3.453	596.67	2060.17	3.285	640.48	2103.72
A_68_P32806294	chrX:162878603-162878647	NM_177429:12	Ofd1	INSIDE	0.950	2.839	222.01	630.28	2.698	317.95	857.78
A_68_P31106579	chr17:25969550-25969594	NM_019719:734	Stub1	INSIDE	0.950	0.182	2846.91	517.75	0.173	1874.35	323.93
A_68_P31097499	chr17:24607735-24607779	NM_025954:339	Pgp	INSIDE	0.950	0.358	1523.23	545.66	0.340	1161.71	395.48
A_68_P30660864	chr16:33966979-33967023	NM_009471:89	Umps	INSIDE	0.950	0.297	1645.46	488.33	0.282	1192.98	336.33
A_68_P29116256	chr13:69673614-69673658	NM_001169131:-894	Papd7	PROMOTER	0.950	1.782	3133.57	5583.19	1.692	2276.11	3851.61
A_68_P27542074	chr10:128002848-128002892	NM_011119:120	Pa2g4	INSIDE	0.950	0.532	1648.45	876.16	0.505	1192.91	602.52
A_68_P25094127	chr7:52883891-52883935	NM_028544:1006	Rasip1	INSIDE	0.950	0.534	1234.26	659.48	0.508	1042.91	529.31
A_68_P24156001	chr5:144076400-144076444	NM_172726:617	E130309D02Rik	INSIDE	0.950	3.007	1134.56	3411.54	2.855	894.36	2553.48
A_68_P23415895	chr4:151370916-151370960	NM_172705:-650	Phf13	PROMOTER	0.950	0.443	1195.88	529.50	0.420	916.87	385.54
A_68_P23249523	chr4:119821508-119821552			Unknown	0.950	3.950	1371.78	5418.28	3.753	1144.64	4295.67
A_68_P22869479	chr4:43023268-43023312	NM_001163233:-117	Fancg	PROMOTER	0.950	0.422	3861.33	1627.82	0.401	2913.93	1167.37
A_68_P21101092	chr2:29917364-29917408	NM_023871:-129	Set	PROMOTER	0.950	0.353	2586.79	914.26	0.336	2114.54	710.18
A_68_P20826589	chr1:173126188-173126232	NM_025557:189	Pcp4l1	INSIDE	0.950	0.627	1643.54	1031.12	0.596	1370.98	817.17
A_68_P20618861	chr1:133904468-133904512	NM_007923:309	Elk4	INSIDE	0.950	0.382	2661.33	1017.47	0.363	1919.10	697.38
A_68_P20241451	chr1:55419724-55419768	NM_001113367:-195	Boll	PROMOTER	0.950	1.614	1176.00	1897.76	1.533	1052.12	1612.76
A_68_P29678557	chr14:66916156-66916200	NR_038077:-212	1700001G11Rik	PROMOTER	0.949	1.974	1068.94	2109.85	1.873	807.93	1513.15
A_68_P28221296	chr12:8305454-8305498	NM_013527:3284	Gdf7	INSIDE	0.949	2.015	1363.58	2747.18	1.912	1050.22	2008.48
A_68_P28185372	chr11:120433823-120433867	NM_011032:406	P4hb	INSIDE	0.949	0.561	1831.46	1026.93	0.532	1425.14	758.13
A_68_P25411548	chr7:117205451-117205495	NM_009281:257	Zfp143	INSIDE	0.949	3.076	322.04	990.44	2.918	317.91	927.53
A_68_P24906444	chr6:142705194-142705238	NM_009908:11	Cmas	INSIDE	0.949	4.541	1288.03	5848.82	4.309	1265.54	5453.32
A_68_P24758829	chr6:113276600-113276644	NM_010957:-347	Ogg1	PROMOTER	0.949	0.346	1668.33	577.08	0.328	1289.18	422.98
A_68_P23364321	chr4:141034275-141034319	NM_019763:60216	Spen	INSIDE	0.949	1.768	912.18	1612.28	1.677	837.06	1403.96
A_68_P23278550	chr4:125840298-125840342	NM_025856:-411	1700029G01Rik	PROMOTER	0.949	0.741	3287.32	2436.32	0.703	2414.52	1698.30
A_68_P22423108	chr3:110052868-110052912	NR_024139:1026	Prmt6	INSIDE	0.949	1.688	1577.36	2663.15	1.603	1301.78	2086.73
A_68_P21115152	chr2:32251192-32251236	NM_133783:-195	Ptges2	DIVERGENT_PROMOTER	0.949	0.562	2130.11	1196.62	0.533	1500.54	800.32
A_68_P20556979	chr1:121319156-121319200	NM_008381:-353	Inhbb	PROMOTER	0.949	0.552	1499.06	826.87	0.524	1220.99	639.38
A_68_P32044142	chr19:28086118-28086162	NM_011265:-484	Rfx3	PROMOTER	0.948	0.215	3738.35	804.02	0.204	2757.39	562.42
A_68_P29667752	chr14:65059421-65059465	NM_026322:15298	Msra	INSIDE	0.948	0.580	1203.59	698.34	0.550	1067.19	587.16
A_68_P28576810	chr12:81744684-81744728	NM_007951:142	Erh	INSIDE	0.948	0.433	3661.19	1583.62	0.410	2791.27	1144.77
A_68_P28123543	chr11:110198733-110198777	NM_147219:276	Abca5	INSIDE	0.948	0.724	4895.87	3542.61	0.686	3795.13	2603.84
A_68_P27954273	chr11:80241942-80241986	NM_178616:-152	Psmd11	PROMOTER	0.948	0.479	1909.78	915.41	0.455	1416.29	643.72
A_68_P27835013	chr11:58948172-58948216	NM_001171512:1683	Obscn	INSIDE	0.948	0.665	1514.17	1007.68	0.631	1167.29	736.82
A_68_P26884795	chr9:121578529-121578573	NM_011703:26717	Vipr1	INSIDE	0.948	0.342	2530.63	864.56	0.324	1716.93	556.11
A_68_P26574055	chr9:62986668-62986712	NM_172446:8097	Skor1	INSIDE	0.948	0.539	2253.90	1215.15	0.511	1754.68	897.14
A_68_P26246004	chr8:127044817-127044861	NM_029746:172	Cog2	INSIDE	0.948	0.312	1499.99	467.30	0.295	1131.03	333.92
A_68_P25958416	chr8:74225606-74225650	NM_028189:71	B3gnt3	INSIDE	0.948	0.635	1568.06	995.51	0.602	1155.07	695.07
A_68_P25589127	chr7:148667760-148667804	NM_053082:6644	Tspan4	INSIDE	0.948	0.355	3724.92	1320.60	0.336	2304.96	775.06
A_68_P24838519	chr6:128305511-128305555	NM_011657:337	Tulp3	INSIDE	0.948	0.354	4964.31	1758.10	0.336	3609.28	1211.74
A_68_P24141813	chr5:141000344-141000388	NM_021528:18804	Chst12	INSIDE	0.948	2.020	774.68	1564.64	1.914	571.59	1094.27

ProbeName	Target position of probe on CpG island microarray	TargetID	GeneSymbol	CpG island Description	Ratio of relative methylation (TiO_2-NP/Vehicle)	Sham group			TiO_2-H group		
						Relative methylation (Cy5/Cy3)	Cy3 signal (Input)	Cy5 signal (MeDIP)	Relative methylation (Cy5/Cy3)	Cy3 signal (Input)	Cy5 signal (MeDIP)
A_68_P23591272	chr5:34163692-34163736	ENSMUST00000168912:-590		PROMOTER	0.948	2.483	1497.85	3719.22	2.353	1241.07	2920.29
A_68_P23327085	chr4:134731250-134731294	NM_019732:54713	Runx3	INSIDE	0.948	0.504	3384.69	1704.88	0.478	2355.45	1125.10
A_68_P23314345	chr4:132399162-132399206	NM_146154:-100	Ppp1r8	PROMOTER	0.948	4.156	1912.04	7946.14	3.940	1652.28	6509.78
A_68_P23232549	chr4:116793686-116793730	NR_024078:4622	Btbd19	INSIDE	0.948	0.481	1179.81	567.71	0.456	995.59	454.36
A_68_P30981937	chr16:94748428-94748472	NM_007834:-214	Dscr3	PROMOTER	0.947	2.560	864.14	2212.10	2.425	790.72	1917.25
A_68_P30567432	chr16:15887571-15887615	NM_007679:214	Cebpd	INSIDE	0.947	0.398	3561.00	1417.19	0.377	2530.23	953.66
A_68_P30346578	chr15:76007226-76007270	NM_201394:18892	Plec	INSIDE	0.947	3.004	1762.26	5294.54	2.845	1325.51	3770.46
A_68_P29614457	chr14:55433063-55433107	ENSMUST00000095853:-2239		PROMOTER	0.947	5.294	329.96	1746.73	5.013	272.22	1364.61
A_68_P29020673	chr13:48758866-48758910	NM_007526:484	Barx1	INSIDE	0.947	0.439	1008.30	442.28	0.415	736.67	305.90
A_68_P28888883	chr13:24853418-24853464	NM_020567:366	Gmnn	INSIDE	0.947	0.616	2580.64	1590.74	0.584	1859.07	1085.58
A_68_P28606270	chr12:87166109-87166153	NM_001081423:231	Ttll5	INSIDE	0.947	2.368	928.86	2199.72	2.243	798.41	1790.77
A_68_P27623991	chr11:18919088-18919132	NM_001193271:-138	Meis1	PROMOTER	0.947	0.649	5092.37	3303.60	0.614	3184.70	1955.56
A_68_P27095620	chr10:42292871-42292915	NM_152229:10502	Nr2e1	INSIDE	0.947	2.354	397.51	935.58	2.230	312.00	695.69
A_68_P26574104	chr9:62992857-62992901	NM_172446:1909	Skor1	INSIDE	0.947	0.496	894.84	444.11	0.470	766.57	360.40
A_68_P26467841	chr9:44291334-44291378	ENSMUST00000136322:4496		DOWNSTREAM	0.947	2.071	1828.49	3786.13	1.961	1529.57	2999.83
A_68_P26258420	chr8:129116753-129116797	NM_001164598:562	Irf2bp2	INSIDE	0.947	1.586	1504.05	2386.13	1.503	1119.12	1681.74
A_68_P26204794	chr8:120325573-120325617	NM_026758:235	Mphosph6	INSIDE	0.947	0.473	1445.21	684.19	0.448	1124.42	504.14
A_68_P25954612	chr8:73392998-73393042	NM_026964:4369	Ccdc124	INSIDE	0.947	4.541	181.89	825.93	4.300	142.17	611.30
A_68_P25081793	chr7:50568397-50568441	NM_027264:213	Zfp715	INSIDE	0.947	0.329	5770.75	1895.93	0.311	4203.69	1307.21
A_68_P24951460	chr7:4945585-4945629	NM_026741:2097	Zfp579	INSIDE	0.947	2.048	2015.46	4127.87	1.939	1497.17	2903.14
A_68_P24859168	chr6:133985011-133985055	NM_007961:-692	Etv6	PROMOTER	0.947	0.302	2333.91	703.89	0.286	1662.23	474.85
A_68_P23977770	chr5:110530585-110530629	NR_027665:1029	Plcxd1	INSIDE	0.947	0.573	3175.92	1821.27	0.543	2516.49	1367.06
A_68_P21706642	chr2:144156624-144156668	NM_152947:452	Ovol2	INSIDE	0.947	0.499	2151.20	1073.68	0.473	1552.02	733.36
A_68_P21246537	chr2:58210184-58210228	NM_001111030:-37	Acvr1c	PROMOTER	0.947	0.518	978.53	506.55	0.490	754.87	370.19
A_68_P21137725	chr2:35946616-35946662	NM_001083126:12942	Lhx6	INSIDE	0.947	0.283	1769.16	500.15	0.268	1327.35	355.51
A_68_P21095253	chr2:28981070-28981114	NM_198033:581	Setx	INSIDE	0.947	0.469	1175.48	550.76	0.444	976.02	432.93
A_68_P32257884	chrX:11241665-11241709			Unknown	0.946	0.670	1423.31	954.20	0.634	1980.90	1255.84
A_68_P31792975	chr18:67801303-67801347	NM_134138:72	Psmg2	INSIDE	0.946	3.415	3821.13	13051.04	3.233	2977.62	9625.28
A_68_P31334604	chr17:71824355-71824399	NM_028887:307	Smchd1	INSIDE	0.946	0.442	3482.71	1540.47	0.418	2672.97	1118.07
A_68_P31149742	chr17:33766187-33766231	NM_026712:172	Zfp414	INSIDE	0.946	0.135	7228.92	972.80	0.127	5426.96	690.52
A_68_P31097234	chr17:24563606-24563650	NM_010023:1	Eci1	INSIDE	0.946	0.401	1155.13	463.09	0.379	897.35	340.31
A_68_P30337609	chr15:74348773-74348817	NM_174991:2169	Bai1	INSIDE	0.946	0.424	2386.32	1012.51	0.401	1776.18	712.87
A_68_P27936386	chr11:77029527-77029571	NM_177710:-378	Ssh2	PROMOTER	0.946	4.461	1642.69	7328.86	4.219	1608.98	6788.25
A_68_P27542072	chr10:128002612-128002656	NM_011119:356	Pa2g4	INSIDE	0.946	0.197	7141.55	1409.22	0.187	4701.73	877.80
A_68_P27085192	chr10:40403058-40403102	NM_177793:-7	9030224M15Rik	PROMOTER	0.946	0.488	1643.71	802.09	0.462	1240.85	573.07
A_68_P26885383	chr9:121668227-121668271	NR_033503:813	E530011L22Rik	INSIDE	0.946	0.581	2194.67	1274.37	0.549	1509.09	828.67
A_68_P25945205	chr8:71404661-71404705	NM_008509:229	Lpl	INSIDE	0.946	0.345	1435.29	495.09	0.326	1147.36	374.30
A_68_P25093840	chr7:52825839-52825883	NM_009737:128	Bcat2	INSIDE	0.946	0.213	2342.00	499.28	0.202	1812.00	365.39
A_68_P24008368	chr5:115643546-115643590	NM_013879:-7438	Cabp1	PROMOTER	0.946	0.503	2028.29	1021.18	0.476	1558.06	742.33
A_68_P24003591	chr5:114830465-114830509	NM_001159941:28	Kctd10	INSIDE	0.946	0.348	2076.98	722.41	0.329	1631.84	536.72
A_68_P23956957	chr5:106161002-106161046	NM_178701:31196	Lrrc8d	INSIDE	0.946	0.483	1058.97	511.60	0.457	967.34	442.05
A_68_P23145550	chr4:99323204-99323248	NM_010425:237	Foxd3	INSIDE	0.946	0.422	1748.76	737.14	0.399	1341.62	534.73
A_68_P22905953	chr4:49609903-49609947	NM_025944:818	2810432L12Rik	INSIDE	0.946	0.617	2400.90	1480.40	0.583	1764.98	1029.56
A_68_P21748795	chr2:152158169-152158213	NM_001083921:-29	Rbck1	PROMOTER	0.946	0.488	1523.66	742.90	0.461	1206.21	556.49
A_68_P21007903	chr2:11698976-11699020	NM_015792:156	Fbxo18	INSIDE	0.946	0.424	4018.10	1703.39	0.401	2860.23	1146.88
A_68_P33007794	chr4_random:128835-128879	NM_001033326:19055	Dhrsx	INSIDE	0.945	7.967	4445.52	35419.19	7.526	2869.74	21598.74
A_68_P32704866	chrX:138159789-138159833	NM_010572:-50	Irs4	PROMOTER	0.945	1.860	514.09	956.25	1.758	675.90	1188.28
A_68_P28976951	chr13:41345598-41345642	NM_001135577:408	BC024659	INSIDE	0.945	0.286	2701.28	772.32	0.270	1929.35	521.19
A_68_P28597424	chr12:85626225-85626269	NM_029880:-17	Ptgr2	PROMOTER	0.945	0.319	1525.25	486.63	0.301	992.96	299.23
A_68_P28170157	chr11:118109191-118109235	NM_001112699:694	Cyth1	INSIDE	0.945	0.323	2514.79	813.31	0.306	1748.97	534.58
A_68_P27661782	chr11:26110649-26110693	NR_027973:-94	5730522E02Rik	PROMOTER	0.945	0.521	1066.48	555.32	0.492	829.66	408.16
A_68_P27284102	chr10:80040405-80040449	NM_078477:-385	Klf16	PROMOTER	0.945	0.155	5362.37	829.25	0.146	3428.03	501.22
A_68_P26821062	chr9:109834457-109834501	NM_001205331:201	Mtap4	INSIDE	0.945	0.276	2516.16	695.47	0.261	1779.82	464.75
A_68_P26350217	chr9:21855419-21855463	NM_010487:1027	Elavl3	INSIDE	0.945	1.898	735.73	1396.44	1.793	623.40	1117.59
A_68_P24990794	chr7:19577916-19577960	NM_145579:1345	Mypop	INSIDE	0.945	0.442	1178.51	520.36	0.417	843.78	351.93
A_68_P24908778	chr6:143116037-143116081	NM_029250:309	Etnk1	INSIDE	0.945	3.089	895.11	2764.68	2.918	688.22	2008.45
A_68_P24336410	chr6:31166769-31166813	ENSMUST00000115107:1643		INSIDE	0.945	0.671	1735.22	1165.10	0.635	1368.28	868.65
A_68_P24111877	chr5:134660494-134660538	NM_053266:609	Gtf2ird2	INSIDE	0.945	0.667	3132.48	2090.11	0.630	2461.34	1551.51
A_68_P23317815	chr4:133036401-133036445	NM_175307:375	Fam46b	INSIDE	0.945	0.450	1765.07	794.74	0.426	1289.18	548.67
A_68_P23176715	chr4:104782702-104782746	NM_178143:-221	Prkaa2	PROMOTER	0.945	0.214	2386.08	510.77	0.202	1867.04	377.59
A_68_P21957225	chr3:14533992-14534036	NM_001163579:227	Lrrcc1	INSIDE	0.945	0.644	3110.82	2003.29	0.608	2264.95	1377.89
A_68_P21824071	chr2:165298510-165298554	NM_054055:165	Slc13a3	INSIDE	0.945	0.425	3532.93	1502.31	0.402	2631.50	1057.52
A_68_P21747512	chr2:151907346-151907390	NM_001160410:104	Scrt2	INSIDE	0.945	1.872	839.38	1571.01	1.768	673.77	1191.34
A_68_P32143179	chr19:46391989-46392033	NM_028627:9636	Psd	INSIDE	0.944	0.476	3906.90	1860.30	0.449	2950.64	1325.71
A_68_P31629837	chr18:37913139-37913183	NM_033594:-2266	Pcdhga11	PROMOTER	0.944	2.827	925.23	2615.44	2.668	711.73	1898.59

ProbeName	Target position of probe on CpG island microarray	TargetID	GeneSymbol	CpG island Description	Ratio of relative methylation (TiO_2-NP/Vehicle)	Sham group			TiO_2-H group		
						Relative methylation (Cy5/Cy3)	Cy3 signal (Input)	Cy5 signal (MeDIP)	Relative methylation (Cy5/Cy3)	Cy3 signal (Input)	Cy5 signal (MeDIP)
A_68_P31162742	chr17:36282712-36282756	NR_033517:772	2410017I17Rik	INSIDE	0.944	0.627	2925.32	1833.20	0.592	2391.98	1415.60
A_68_P31126787	chr17:29374609-29374653	NM_153166:105	Cpne5	INSIDE	0.944	0.394	1603.81	631.96	0.372	1266.82	471.15
A_68_P30786985	chr16:57549214-57549258	NM_001177871:-118	Filip1l	PROMOTER	0.944	0.510	1012.48	516.40	0.481	863.60	415.80
A_68_P30562965	chr16:14706121-14706170	NM_011415:194	Snai2	INSIDE	0.944	0.231	2688.76	620.80	0.218	1748.75	380.95
A_68_P30365306	chr15:79082860-79082904	NM_020516:2296	Slc16a8	INSIDE	0.944	0.667	3714.92	2478.77	0.630	2717.22	1711.92
A_68_P30346972	chr15:76062477-76062521	NM_001163542:-690	Plec	PROMOTER	0.944	0.442	1203.05	532.16	0.417	983.72	410.70
A_68_P29750658	chr14:79701016-79701060	NM_025427:404	1190002H23Rik	INSIDE	0.944	0.478	1085.13	518.33	0.451	714.53	322.16
A_68_P29445599	chr14:21001120-21001164	NM_021542:-138	Kcnk5	PROMOTER	0.944	0.406	1549.00	628.73	0.383	1044.01	399.90
A_68_P29440559	chr14:19725789-19725833	NM_144839:331	Ube2e2	INSIDE	0.944	0.390	1439.37	561.38	0.368	1151.77	423.94
A_68_P28941008	chr13:34967652-34967696	NM_013830:312	Prpf4b	INSIDE	0.944	0.415	1391.70	578.14	0.392	1118.45	438.60
A_68_P28778249	chr12:119437163-119437207	NM_010060:332	Dnahc11	INSIDE	0.944	0.558	1986.18	1108.51	0.527	1678.04	884.46
A_68_P28671531	chr12:99927688-99927737	NM_011877:45143	Ptpn21	INSIDE	0.944	1.820	1081.09	1967.76	1.718	933.44	1603.47
A_68_P28597122	chr12:85559358-85559402	NM_001163501:451	C130039O16Rik	INSIDE	0.944	0.638	1357.23	865.75	0.602	1152.12	693.56
A_68_P28252842	chr12:14156956-14157000	NM_029007:1866	Fam84a	INSIDE	0.944	2.617	688.60	1802.41	2.470	581.94	1437.57
A_68_P27896202	chr11:69446325-69446369	NM_011814:-126	Fxr2	PROMOTER	0.944	0.613	1962.29	1202.96	0.579	1406.21	814.18
A_68_P25481818	chr7:130113250-130113294	NM_175023:-805	Rbbp6	PROMOTER	0.944	1.689	1039.03	1754.88	1.594	956.29	1524.73
A_68_P24980794	chr7:16899349-16899393	NM_133234:4439	Bbc3	INSIDE	0.944	0.267	1681.47	448.93	0.252	1384.17	348.84
A_68_P24955007	chr7:6282885-6282929	NM_001033249:-870	Zfp583	PROMOTER	0.944	0.418	1449.78	605.61	0.394	1256.70	495.53
A_68_P24513202	chr6:65621890-65621934	NM_172399:308	A930038C07Rik	INSIDE	0.944	4.134	624.51	2581.41	3.903	477.28	1863.05
A_68_P22820135	chr4:32325268-32325312	ENSMUST00000108180:-488		PROMOTER	0.944	0.429	2141.97	918.05	0.404	1537.60	621.88
A_68_P22477034	chr3:121235139-121235183	NM_145394:102	Slc44a3	INSIDE	0.944	1.894	1043.06	1975.86	1.788	965.36	1726.50
A_68_P21112314	chr2:31806123-31806167	NM_145144:322	Aif1l	INSIDE	0.944	0.163	4328.44	707.50	0.154	2969.52	458.13
A_68_P20826030	chr1:173000636-173000680	ENSMUST00000164179:-5592		PROMOTER	0.944	0.200	5439.43	1086.40	0.189	4201.29	792.36
A_68_P29989756	chr15:6659356-6659404	NM_030168:1000	Rictor	INSIDE	0.943	4.032	500.41	2017.57	3.804	391.51	1489.21
A_68_P29033459	chr13:51740974-51741018	NM_025415:396	Cks2	INSIDE	0.943	0.404	3731.67	1506.16	0.381	2734.87	1041.25
A_68_P28833517	chr13:13485882-13485926	NM_031999:-13	Gpr137b	PROMOTER	0.943	0.323	2251.88	726.78	0.304	1899.52	578.39
A_68_P28153128	chr11:115235533-115235577	NM_175454:-6521	C630004H02Rik	DIVERGENT_PROMOTER	0.943	0.474	1373.12	650.85	0.447	1014.98	453.91
A_68_P27790975	chr11:50673295-50673343	NM_173372:9132	Grm6	INSIDE	0.943	2.272	1748.84	3973.19	2.143	1285.29	2753.78
A_68_P27536452	chr10:126971104-126971148	NM_183297:489	Nxph4	INSIDE	0.943	0.092	11811.39	1088.27	0.087	6870.62	597.22
A_68_P27360774	chr10:94151095-94151139	NR_015524:242	4932415G12Rik	INSIDE	0.943	0.332	2274.23	755.87	0.313	1726.66	540.90
A_68_P26986175	chr10:20032280-20032325	NM_001025392:28	Bclaf1	INSIDE	0.943	0.350	1638.62	574.31	0.330	1367.80	451.87
A_68_P26872302	chr9:119336590-119336634	NM_007397:24994	Acvr2b	INSIDE	0.943	1.983	1756.76	3482.89	1.869	1204.64	2251.52
A_68_P26421200	chr9:35007442-35007486	NM_001177847:26	Tirap	INSIDE	0.943	0.245	2612.03	640.60	0.231	2174.02	502.74
A_68_P25505763	chr7:134622752-134622796	NM_010183:-5960	Fbrs	PROMOTER	0.943	2.275	486.18	1105.98	2.145	391.92	840.73
A_68_P25087554	chr7:51718274-51718318	NM_198250:20440	Lrrc4b	INSIDE	0.943	0.525	1090.50	572.69	0.495	864.83	428.45
A_68_P25044864	chr7:35439779-35439823	NM_146188:-1940	Kctd15	PROMOTER	0.943	0.427	1326.04	566.55	0.403	964.07	388.35
A_68_P24772420	chr6:115626105-115626149	NM_029780:527	Raf1	INSIDE	0.943	6.116	306.97	1877.31	5.764	302.38	1742.92
A_68_P23576660	chr5:31439871-31439915	NM_021447:20908	Trim54	INSIDE	0.943	0.637	1392.70	887.26	0.601	1079.22	648.37
A_68_P21339747	chr2:74663863-74663907	NM_016804:16	Mtx2	INSIDE	0.943	2.686	417.78	1122.11	2.534	355.03	899.65
A_68_P21266387	chr2:61643809-61643853	NM_009322:1321	Tbr1	INSIDE	0.943	0.255	3761.07	960.32	0.241	3007.63	724.05
A_68_P21117858	chr2:32702735-32702779	NM_009295:-4	Stxbp1	PROMOTER	0.943	2.228	374.12	833.59	2.102	349.93	735.57
A_68_P21080072	chr2:26484180-26484224	NM_019833:226	Fam69b	INSIDE	0.943	3.436	420.30	1444.14	3.242	376.79	1221.38
A_68_P20413379	chr1:88566390-88566434	NM_010933:736	Nppc	INSIDE	0.943	0.441	1850.76	816.18	0.416	1373.40	571.23
A_68_P31411662	chr17:86008304-86008348	NR_038085:9410	Six3os1	INSIDE	0.942	0.616	2638.72	1626.02	0.580	2162.40	1254.61
A_68_P31104790	chr17:25707537-25707581	NM_011447:73	Sox8	INSIDE	0.942	0.491	1499.43	735.49	0.462	1154.09	533.12
A_68_P29152215	chr13:76080947-76080991	NM_028493:304	Rhobtb3	INSIDE	0.942	0.443	1868.80	827.27	0.417	1403.14	585.31
A_68_P28060001	chr11:98885291-98885335	NM_011623:191	Top2a	INSIDE	0.942	0.358	1500.29	536.47	0.337	1265.70	426.21
A_68_P27926120	chr11:75259006-75259050	NM_138950:9191	Wdr81	INSIDE	0.942	1.912	1409.95	2695.33	1.801	1100.05	1980.69
A_68_P27921380	chr11:74403245-74403289	NM_001015046:394	Rap1gap2	INSIDE	0.942	0.482	2563.05	1235.66	0.454	1833.42	832.90
A_68_P27311154	chr10:85391524-85391568	NM_001163614:311	Ascl4	INSIDE	0.942	3.526	387.40	1365.98	3.321	351.71	1167.99
A_68_P26766608	chr9:99770906-99770951	NM_011440:5661	Sox14	DOWNSTREAM	0.942	2.478	1035.12	2565.26	2.335	857.08	2000.85
A_68_P26168095	chr8:113917768-113917812	NM_178086:-69	Fa2h	PROMOTER	0.942	5.380	1204.05	6477.26	5.067	1207.41	6117.56
A_68_P25458543	chr7:125634738-125634782	NM_001033380:729	Itpripl2	INSIDE	0.942	0.433	1496.27	647.88	0.408	1136.66	463.84
A_68_P25272111	chr7:88599609-88599653	NM_007755:-68	Cpeb1	PROMOTER	0.942	0.372	3305.89	1230.18	0.351	2233.85	783.09
A_68_P22342538	chr3:94965011-94965055	NM_011351:-3263	Sema6c	PROMOTER	0.942	0.266	1983.28	527.05	0.250	1514.77	379.28
A_68_P22101766	chr3:45184223-45184267	NM_011043:1925	Pcdh10	INSIDE	0.942	2.118	952.55	2017.49	1.996	671.60	1340.49
A_68_P32245742	chrX:8777833-8777877	NM_173414:756	Lancl3	INSIDE	0.941	2.019	514.50	1038.70	1.899	634.71	1205.33
A_68_P31951639	chr19:10680032-10680076	NM_015735:-60	Ddb1	DIVERGENT_PROMOTER	0.941	2.128	1933.95	4115.43	2.002	1584.39	3171.86
A_68_P31213631	chr17:47825034-47825078	NM_001164729:279	Tomm6	INSIDE	0.941	0.529	864.74	457.25	0.498	765.00	380.64
A_68_P30423565	chr15:88991328-88991372	NM_001159521:12930	Plxnb2	INSIDE	0.941	2.517	352.42	887.06	2.370	330.09	782.18
A_68_P28831282	chr13:12350227-12350271	NM_001081128:19	Mtr	INSIDE	0.941	0.320	2235.55	714.33	0.301	1655.27	497.94
A_68_P28071034	chr11:100800458-100800502	NM_011486:345	Stat3	INSIDE	0.941	1.804	1181.31	2131.30	1.698	972.34	1651.34
A_68_P27215085	chr10:67442431-67442475	NM_001081346:107	Rtkn2	INSIDE	0.941	0.493	1104.42	544.72	0.464	896.31	416.13
A_68_P26224616	chr8:123641972-123642016	NM_013519:1924	Foxc2	INSIDE	0.941	0.383	1861.33	712.43	0.360	1376.04	495.48
A_68_P26169911	chr8:114255931-114255975	NM_001198839:84	Bcar1	INSIDE	0.941	0.604	3411.16	2061.84	0.569	2768.17	1574.77

ProbeName	Target position of probe on CpG island microarray	TargetID	GeneSymbol	CpG island Description	Ratio of relative methylation (TiO_2-NP/Vehicle)	Sham group			TiO_2-H group		
						Relative methylation (Cy5/Cy3)	Cy3 signal (Input)	Cy5 signal (MeDIP)	Relative methylation (Cy5/Cy3)	Cy3 signal (Input)	Cy5 signal (MeDIP)
A_68_P26021880	chr8:87308048-87308097	NM_001081981:-9804	Nfix	PROMOTER	0.941	0.306	1612.72	493.18	0.288	1251.95	360.11
A_68_P25834905	chr8:47296158-47296202	NM_007450:183	Slc25a4	INSIDE	0.941	0.190	3597.16	681.68	0.178	2533.74	451.76
A_68_P25046334	chr7:35697533-35697577	NM_008820:129	Pepd	INSIDE	0.941	0.372	1219.46	453.80	0.350	900.50	315.36
A_68_P24992780	chr7:19930438-19930482	NM_001127324:41	Ercc1	INSIDE	0.941	0.174	3308.33	575.68	0.164	2496.44	408.58
A_68_P24990614	chr7:19543086-19543130	NM_001029877:31872	Nova2	INSIDE	0.941	0.287	3528.55	1012.75	0.270	2318.66	626.04
A_68_P24835543	chr6:127837831-127837875	NM_026743:213	Tspan11	INSIDE	0.941	0.512	3199.96	1638.52	0.482	2316.61	1116.28
A_68_P24761385	chr6:113709127-113709171	NM_001036684:132222	Atp2b2	INSIDE	0.941	2.402	1061.46	2549.39	2.259	719.55	1625.61
A_68_P23442483	chr4:155484355-155484399	NM_145557:270	9430015G10Rik	INSIDE	0.941	0.313	13401.98	4194.36	0.294	8541.13	2515.25
A_68_P23416690	chr4:151500758-151500802	NM_019585:2254	Espn	INSIDE	0.941	0.321	3414.04	1095.84	0.302	2125.84	641.99
A_68_P23240955	chr4:118214466-118214510	NM_172383:1843	Tmem125	INSIDE	0.941	1.851	942.14	1744.11	1.743	810.07	1411.69
A_68_P23167115	chr4:103292261-103292306	ENSMUST00000106827:14		INSIDE	0.941	0.172	4177.26	717.96	0.162	2738.35	442.79
A_68_P22403734	chr3:106839172-106839216	NM_008418:115	Kcna3	INSIDE	0.941	0.445	7022.39	3123.80	0.419	5076.43	2125.10
A_68_P22383780	chr3:102799614-102799658	NM_025679:-26	Sike1	PROMOTER	0.941	2.165	1599.28	3462.34	2.038	1294.96	2638.79
A_68_P20941839	chr1:193961046-193961090	NR_033536:289	Gm10516	INSIDE	0.941	0.107	5357.40	572.58	0.101	3373.79	339.46
A_68_P20350234	chr1:74795399-74795445	NM_153744:107	Prkag3	INSIDE	0.941	0.697	1772.38	1235.41	0.656	1492.26	978.46
A_68_P20306026	chr1:67085213-67085257	NM_001190984:174	Lancl1	INSIDE	0.941	0.351	3151.96	1105.25	0.330	2574.88	849.66
A_68_P32143656	chr19:46470216-46470260	NM_016860:-13	Actr1a	DIVERGENT_PROMOTER	0.940	0.294	2542.96	746.76	0.276	1816.81	501.55
A_68_P32126456	chr19:43515057-43515101	NM_031396:153	Cnnm1	INSIDE	0.940	0.533	1026.48	546.70	0.501	797.96	399.60
A_68_P31330905	chr17:71167433-71167477	NM_001128180:296005	Dlgap1	INSIDE	0.940	2.466	736.28	1815.50	2.318	661.99	1534.45
A_68_P31236341	chr17:51972574-51972618	NM_009122:-624	Satb1	PROMOTER	0.940	1.562	5212.15	8141.10	1.469	3622.83	5320.40
A_68_P30684006	chr16:38089070-38089114	NM_019827:6	Gsk3b	INSIDE	0.940	0.368	2380.33	875.34	0.346	1750.54	605.13
A_68_P30478823	chr15:99055100-99055144	NM_010601:-284	Kcnh3	PROMOTER	0.940	2.005	621.51	1245.95	1.884	568.11	1070.50
A_68_P30424873	chr15:89209271-89209315	NM_001013022:393	Odf3b	INSIDE	0.940	0.430	5644.10	2424.75	0.404	4054.01	1637.09
A_68_P27992046	chr11:86806680-86806725	NM_001005341:562	Ypel2	INSIDE	0.940	0.331	2461.71	815.96	0.312	1706.21	531.83
A_68_P27170202	chr10:59165196-59165240	NM_144822:-164	Cbara1	PROMOTER	0.940	1.835	5140.79	9432.49	1.724	4291.33	7398.73
A_68_P26887821	chr9:122075732-122075776	NM_001164572:49371	Snrk	INSIDE	0.940	1.648	1033.33	1703.04	1.549	849.42	1316.13
A_68_P26080208	chr8:97437258-97437302	NM_001033533:4718	Ccdc102a	INSIDE	0.940	2.311	1770.50	4091.15	2.171	1266.52	2749.61
A_68_P26022002	chr8:87323170-87323214	NM_001081982:1047	Nfix	INSIDE	0.940	1.927	1006.30	1939.24	1.812	757.11	1371.86
A_68_P24513201	chr6:65621722-65621766	NM_172399:140	A930038C07Rik	INSIDE	0.940	0.449	3585.17	1609.87	0.422	2848.50	1202.53
A_68_P23991927	chr5:112823589-112823633	NM_028386:-2377	Asphd2	PROMOTER	0.940	0.629	1811.39	1138.57	0.591	1471.88	869.89
A_68_P21835384	chr2:167246881-167246925	NM_148929:-318	Slc9a8	PROMOTER	0.940	0.484	1285.37	622.52	0.455	1009.68	459.59
A_68_P20633021	chr1:136258633-136258677	NM_001042485:-78	Tmem183a	PROMOTER	0.940	4.757	751.02	3572.37	4.470	616.50	2755.71
A_68_P20412460	chr1:88400352-88400396			Unknown	0.940	2.588	860.28	2226.78	2.433	726.14	1766.52
A_68_P31924876	chr19:4756468-4756512	NM_025717:-34	Rbm4b	PROMOTER	0.939	3.334	629.41	2098.17	3.131	591.50	1852.12
A_68_P29695814	chr14:69955084-69955128	NM_026174:-101	Entpd4	PROMOTER	0.939	0.380	1536.16	583.71	0.357	1212.57	432.84
A_68_P26453529	chr9:41932012-41932056	NM_011436:338	Sorl1	INSIDE	0.939	3.000	782.78	2348.09	2.816	732.40	2062.31
A_68_P26349890	chr9:21792524-21792568	NM_023622:1351	Rgl3	INSIDE	0.939	0.397	1646.32	654.30	0.373	1175.45	438.67
A_68_P26290656	chr9:8042543-8042587	NM_199017:259	9230110C19Rik	INSIDE	0.939	0.378	5790.65	2189.47	0.355	3558.55	1263.22
A_68_P25594226	chr7:149558251-149558295	NM_177265:-108	6330512M04Rik	PROMOTER	0.939	3.386	525.20	1778.51	3.181	432.84	1376.88
A_68_P25194962	chr7:74790083-74790127	NM_175124:18	Lrrc28	INSIDE	0.939	0.318	1870.79	595.31	0.299	1343.38	401.38
A_68_P25092432	chr7:52582657-52582701	NM_175130:6472	Trpm4	INSIDE	0.939	2.940	1874.59	5510.81	2.762	1584.25	4375.22
A_68_P23577156	chr5:31522417-31522462	NM_008014:479	Ppm1g	INSIDE	0.939	0.693	2861.52	1982.02	0.651	2175.18	1415.33
A_68_P23333141	chr4:135846296-135846340	NM_009557:4335	Zfp46	INSIDE	0.939	0.650	3330.36	2163.16	0.610	2270.71	1384.19
A_68_P22924771	chr4:53727389-53727433	NM_139309:357	Fktn	INSIDE	0.939	0.310	3469.04	1074.25	0.291	2452.25	713.40
A_68_P21824618	chr2:165389257-165389301			Unknown	0.939	0.543	4259.69	2314.32	0.510	2843.40	1450.49
A_68_P20778062	chr1:164407645-164407689	NM_001038619:495	Dnm3	INSIDE	0.939	0.155	3839.30	596.58	0.146	2997.80	437.19
A_68_P20644876	chr1:138156784-138156828	NM_001101516:644	Gpr25	INSIDE	0.939	1.682	1521.26	2558.46	1.579	1218.97	1924.98
A_68_P20457528	chr1:95700326-95700370	NM_025454:-193	Ing5	DIVERGENT_PROMOTER	0.939	0.380	2279.61	866.00	0.357	1748.31	623.51
A_68_P32181720	chr19:53384971-53385016	NM_001008542:-2	Mxi1	PROMOTER	0.938	1.611	1763.37	2841.16	1.512	1253.72	1895.66
A_68_P30498348	chr15:102349120-102349164	NM_009319:466	Tarbp2	INSIDE	0.938	0.650	2187.50	1421.09	0.609	1696.82	1033.90
A_68_P30378088	chr15:81297114-81297159	NM_019712:391	Rbx1	INSIDE	0.938	0.115	4537.54	523.54	0.108	3302.25	357.39
A_68_P29589458	chr14:49293418-49293462	NR_029384:4478	Otx2os1	INSIDE	0.938	2.387	818.72	1954.44	2.239	630.16	1411.06
A_68_P28940998	chr13:34966207-34966251	NM_013830:-1134	Prpf4b	PROMOTER	0.938	0.174	3185.40	553.58	0.163	2434.35	396.66
A_68_P28185710	chr11:120485805-120485849	NM_153056:490	Sirt7	INSIDE	0.938	0.318	1460.23	464.73	0.299	1240.76	370.50
A_68_P28115699	chr11:108784095-108784139	NM_015732:2454	Axin2	INSIDE	0.938	0.437	1306.68	570.76	0.410	1037.14	424.97
A_68_P27992492	chr11:86899872-86899916	NM_024262:385	1200011M11Rik	INSIDE	0.938	0.562	973.39	547.30	0.528	859.15	453.33
A_68_P27911317	chr11:72225169-72225213	NM_177776:25	Smtnl2	INSIDE	0.938	0.226	2100.31	475.00	0.212	1618.32	343.24
A_68_P27535071	chr10:126726102-126726146	NM_033072:-297	Mbd6	DIVERGENT_PROMOTER	0.938	0.643	2185.22	1404.67	0.603	1714.60	1033.43
A_68_P27500770	chr10:120415220-120415264	NM_001081193:1144	Lemd3	INSIDE	0.938	0.640	1409.93	903.03	0.601	1163.88	699.44
A_68_P26888898	chr9:122261191-122261235	NM_026179:479	Abhd5	INSIDE	0.938	0.374	1501.72	562.05	0.351	1386.91	486.97
A_68_P26825042	chr9:110559281-110559325	NM_028312:296	Ccdc12	INSIDE	0.938	0.310	2454.17	759.71	0.290	1887.26	547.99
A_68_P26293545	chr9:8900606-8900650	NM_008829:796	Pgr	INSIDE	0.938	2.225	487.69	1084.99	2.087	387.55	808.95
A_68_P26228892	chr8:124308825-124308869	NM_020605:54388	Jph3	INSIDE	0.938	2.232	1280.33	2857.28	2.093	1012.31	2118.75
A_68_P25082510	chr7:50721757-50721801	NM_001164518:23615	Iglon5	DOWNSTREAM	0.938	0.235	4351.97	1021.02	0.220	3113.25	685.07
A_68_P24974124	chr7:13594887-13594931	NM_001024699:241	Zbtb45	INSIDE	0.938	0.419	6706.24	2812.28	0.394	4203.96	1654.38

ProbeName	Target position of probe on CpG island microarray	TargetID	GeneSymbol	CpG island Description	Ratio of relative methylation (TiO_2-NP/Vehicle)	Sham group			TiO_2-H group		
						Relative methylation (Cy5/Cy3)	Cy3 signal (Input)	Cy5 signal (MeDIP)	Relative methylation (Cy5/Cy3)	Cy3 signal (Input)	Cy5 signal (MeDIP)
A_68_P24424404	chr6:47763266-47763310	NR_030485:-9637	Mir704	PROMOTER	0.938	0.391	1406.91	550.22	0.367	1059.04	388.39
A_68_P24193751	chr5:151755711-151755755	NM_013823:551	Kl	INSIDE	0.938	0.395	1256.54	495.79	0.370	975.41	360.83
A_68_P21840231	chr2:168057062-168057106	NM_001160330:963	Mocs3	INSIDE	0.938	3.085	1021.61	3151.16	2.894	866.37	2506.97
A_68_P21826276	chr2:165671382-165671427	NM_027230:38784	Zmynd8	INSIDE	0.938	7.347	478.12	3512.67	6.894	579.78	3996.85
A_68_P21629726	chr2:130250214-130250258	NM_001114541:141	Fam113a	INSIDE	0.938	0.300	1599.47	479.17	0.281	1255.66	352.95
A_68_P20804316	chr1:169215214-169215258	NM_030724:22	Uck2	INSIDE	0.938	0.455	1448.10	659.42	0.427	1120.53	478.40
A_68_P20628768	chr1:135557603-135557647	NM_001166552:-162	Zbed6	PROMOTER	0.938	0.549	2149.54	1179.10	0.514	1841.30	947.34
A_68_P32051829	chr19:29326236-29326280	NM_001048177:-59	Jak2	PROMOTER	0.937	2.204	1879.54	4142.28	2.064	1462.01	3017.54
A_68_P31067733	chr17:15962847-15962891	NM_178615:682	Rgmb	INSIDE	0.937	2.024	494.55	1001.00	1.897	435.00	825.33
A_68_P30473047	chr15:97923923-97923967	NM_144851:-108	Senp1	PROMOTER	0.937	0.421	1449.72	609.74	0.394	1255.34	494.96
A_68_P30388854	chr15:83341412-83341456	NM_178869:-97	Ttll1	PROMOTER	0.937	0.182	2567.45	468.53	0.171	1800.15	307.86
A_68_P29639602	chr14:60244436-60244480	NM_145463:341	Shisa2	INSIDE	0.937	4.235	1051.70	4454.06	3.967	901.02	3574.37
A_68_P28727506	chr12:109591095-109591139	NM_010010:18526	Cyp46a1	INSIDE	0.937	0.253	1921.78	485.84	0.237	1382.26	327.60
A_68_P28724524	chr12:109155065-109155109	NM_001079883:86538	Bcl11b	INSIDE	0.937	4.023	2087.37	8397.15	3.771	1573.29	5932.95
A_68_P27539540	chr10:127534403-127534447	NM_054078:4586	Baz2a	INSIDE	0.937	0.313	2781.90	869.80	0.293	2243.72	657.60
A_68_P27277825	chr10:79076430-79076474	NM_198614:311	C2cd4c	INSIDE	0.937	0.196	2426.91	475.78	0.184	1790.55	328.87
A_68_P27185482	chr10:61803237-61803281	NM_001146100:-89	Hk1	DIVERGENT_PROMOTER	0.937	0.363	1958.87	710.81	0.340	1458.56	495.92
A_68_P26471089	chr9:44863456-44863500	NM_001093749:179	Mpzl3	INSIDE	0.937	0.468	1948.18	911.62	0.438	1459.39	639.65
A_68_P25610236	chr7:152129258-152129302	NM_007631:-3450	Ccnd1	PROMOTER	0.937	2.819	764.10	2153.76	2.640	677.85	1789.48
A_68_P25261707	chr7:86681146-86681190	ENSMUST00000141822:36166		DOWNSTREAM	0.937	0.361	2267.21	818.52	0.338	1600.98	541.31
A_68_P22881130	chr4:45120741-45120785	NM_001099675:223	Tomm5	INSIDE	0.937	0.220	2316.08	510.62	0.207	1592.44	329.06
A_68_P20998755	chr2:10002343-10002387	NM_025280:126	Kin	INSIDE	0.937	0.189	3192.68	604.64	0.177	2133.16	378.61
A_68_P20166592	chr1:39633542-39633586	NM_001033135:628	Rnf149	INSIDE	0.937	0.318	1889.66	600.09	0.298	1466.25	436.52
A_68_P20022707	chr1:9535566-9535610	NM_021511:100	Rrs1	INSIDE	0.937	0.546	1264.45	690.23	0.512	970.04	496.27
A_68_P33005896	A_68_P33005896			Unknown	0.936	3.845	231.88	891.67	3.598	188.27	677.46
A_68_P30778633	chr16:55973743-55973787	NM_173026:-152	Zbtb11	PROMOTER	0.936	0.417	1689.07	705.05	0.391	1335.11	521.66
A_68_P30484701	chr15:100058334-100058378	NM_007497:67	Atf1	INSIDE	0.936	0.467	1030.09	481.51	0.437	803.14	351.27
A_68_P29083939	chr13:60277184-60277228	NM_008086:1690	Gas1	INSIDE	0.936	0.602	2507.51	1510.22	0.564	1770.51	998.13
A_68_P28740680	chr12:112088415-112088460	NM_001199785:-51	Zfp839	DIVERGENT_PROMOTER	0.936	0.388	5199.38	2019.83	0.364	3742.39	1360.60
A_68_P28614435	chr12:88488792-88488836	NM_153415:38	Pomt2	INSIDE	0.936	0.538	1144.76	615.57	0.504	1024.25	515.73
A_68_P28330748	chr12:32035834-32035878	NM_007861:446	Dld	INSIDE	0.936	0.309	3324.19	1028.81	0.290	2430.73	704.26
A_68_P26170970	chr8:114457718-114457762	NM_001033321:-49	Tmem231	DIVERGENT_PROMOTER	0.936	0.488	1199.63	585.46	0.457	987.27	451.13
A_68_P26024223	chr8:87823249-87823293	NM_022997:126	Vps35	INSIDE	0.936	0.319	4309.55	1374.53	0.299	3070.38	916.89
A_68_P26015764	chr8:86146384-86146428	AK160924:14211		INSIDE	0.936	0.366	2665.60	975.58	0.343	1876.06	642.64
A_68_P24097670	chr5:131783250-131783294	NM_145218:120	Wbscr17	INSIDE	0.936	0.365	4602.54	1681.80	0.342	3243.21	1109.56
A_68_P23415826	chr4:151363053-151363098	NM_001145929:20	Thap3	INSIDE	0.936	2.109	602.57	1270.52	1.974	432.06	852.79
A_68_P22031194	chr3:30894990-30895034	NM_008857:320	Prkci	INSIDE	0.936	0.278	3235.16	900.29	0.260	2360.96	614.90
A_68_P21617363	chr2:127951507-127951551	NM_009754:-245	Bcl2l11	PROMOTER	0.936	0.229	3445.21	788.65	0.214	2499.16	535.58
A_68_P20416030	chr1:89051175-89051219	NM_021306:406	Ecel1	INSIDE	0.936	0.326	2165.61	705.00	0.305	1559.98	475.28
A_68_P31926383	chr19:5024065-5024109	NM_007854:81	Slc29a2	INSIDE	0.935	0.224	4836.38	1085.40	0.210	3183.28	668.21
A_68_P31542492	chr18:21160633-21160677	NM_019706:813	Rnf138	INSIDE	0.935	0.589	1888.83	1112.01	0.551	1505.72	829.19
A_68_P28174634	chr11:118770064-118770108	NM_001024931:800	Rbfox3	INSIDE	0.935	0.487	1072.28	522.28	0.455	881.00	401.02
A_68_P27902562	chr11:70577949-70577993	NM_177565:22	Zfp3	INSIDE	0.935	0.392	1523.42	597.91	0.367	1087.53	398.94
A_68_P27506870	chr10:121484975-121485019	NM_001081037:-747	Srgap1	PROMOTER	0.935	0.431	1490.65	642.06	0.403	1250.92	503.83
A_68_P27093484	chr10:41917285-41917329	NM_019740:79242	Foxo3	INSIDE	0.935	2.255	1016.61	2292.78	2.109	734.06	1548.17
A_68_P25434393	chr7:121261244-121261288	NM_025846:29	Rras2	INSIDE	0.935	0.655	1712.83	1121.79	0.613	1286.49	788.01
A_68_P24933416	chr6:147212616-147212660	NM_008970:-31	Pthlh	PROMOTER	0.935	0.583	1102.79	642.50	0.545	962.39	524.31
A_68_P24449105	chr6:52161725-52161769	NM_010454:-3123	Hoxa6	PROMOTER	0.935	2.288	599.92	1372.34	2.139	442.94	947.42
A_68_P24427217	chr6:48395684-48395728	NM_001085415:-617	Zfp467	PROMOTER	0.935	0.604	7774.35	4692.57	0.564	4832.87	2727.73
A_68_P24045149	chr5:121995337-121995381	NM_010765:543	Mapkapk5	INSIDE	0.935	2.206	634.39	1399.34	2.063	578.09	1192.62
A_68_P23537869	chr5:23181744-23181788	ENSMUST00000157734:-1567		PROMOTER	0.935	3.067	2074.87	6363.11	2.866	1739.09	4984.23
A_68_P22341854	chr3:94819230-94819274	NM_030074:-92	Zfp687	PROMOTER	0.935	0.623	2466.82	1537.22	0.583	1998.69	1164.54
A_68_P21874508	chr2:174124774-174124818	NM_201617:1437	Gnas	INSIDE	0.935	3.996	2748.98	10985.71	3.737	2314.94	8650.41
A_68_P21810970	chr2:163029056-163029100	ENSMUST00000128999:-1210		PROMOTER	0.935	0.248	5813.03	1442.17	0.232	4441.77	1030.25
A_68_P21794483	chr2:160191360-160191404	NM_010658:1419	Mafb	INSIDE	0.935	2.158	2708.29	5845.31	2.017	2121.23	4279.39
A_68_P21481084	chr2:102913796-102913840	NM_019735:-13	Apip	DIVERGENT_PROMOTER	0.935	0.680	2195.15	1493.67	0.636	1727.16	1098.70
A_68_P21339415	chr2:74601471-74601515	NM_010467:456	Hoxd1	INSIDE	0.935	0.646	2719.39	1757.24	0.604	2192.21	1324.08
A_68_P20935550	chr1:192803260-192803304	NR_027352:-107	Vash2	PROMOTER	0.935	0.604	1302.37	787.00	0.565	1032.43	583.09
A_68_P31992643	chr19:18787618-18787662	NM_177640:-85	D030056L22Rik	PROMOTER	0.934	1.887	864.44	1631.18	1.762	787.32	1387.04
A_68_P31616437	chr18:35278200-35278244	NM_009818:-343	Ctnna1	PROMOTER	0.934	0.475	1550.14	735.66	0.443	1359.56	602.89
A_68_P31553000	chr18:23198512-23198556	NM_001161483:-1370	Nol4	PROMOTER	0.934	0.574	1727.36	991.79	0.536	1249.87	669.96
A_68_P31385965	chr17:81344280-81344324	NM_026516:331	Tmem178	INSIDE	0.934	2.010	830.87	1670.39	1.878	754.81	1417.22
A_68_P31215886	chr17:48234543-48234587	NM_026625:7983	1700067P10Rik	DOWNSTREAM	0.934	0.465	1100.26	511.91	0.434	882.74	383.43
A_68_P31117526	chr17:27760252-27760296	NM_019837:123	Nudt3	INSIDE	0.934	0.578	1737.54	1004.09	0.540	1424.50	769.14
A_68_P30939715	chr16:87432766-87432810	NM_001081068:63	Ltn1	INSIDE	0.934	0.487	962.16	468.93	0.455	756.11	344.25

ProbeName	Target position of probe on CpG island microarray	TargetID	GeneSymbol	CpG island Description	Ratio of relative methylation (TiO_2-NP/Vehicle)	Sham group			TiO_2-H group		
						Relative methylation (Cy5/Cy3)	Cy3 signal (Input)	Cy5 signal (MeDIP)	Relative methylation (Cy5/Cy3)	Cy3 signal (Input)	Cy5 signal (MeDIP)
A_68_P30508110	chr16:4077547-4077591	NM_026508:242	Trap1	INSIDE	0.934	0.138	4686.36	646.77	0.129	3239.24	417.52
A_68_P30160395	chr15:40486331-40486375	NM_011766:-235	Zfpm2	PROMOTER	0.934	0.072	9937.61	713.36	0.067	6911.95	463.27
A_68_P29928909	chr14:117326153-117326197	NM_001079844:1638	Gpc6	INSIDE	0.934	0.266	2479.38	659.89	0.249	1677.27	416.85
A_68_P29753350	chr14:80170582-80170626	NM_001042726:515	Pcdh8	INSIDE	0.934	1.840	1926.70	3544.33	1.719	1472.35	2530.59
A_68_P28881184	chr13:23488366-23488410	BC160303:76510		INSIDE	0.934	0.328	1690.80	554.00	0.306	1272.45	389.56
A_68_P27638836	chr11:21893026-21893070	NM_011023:8606	Otx1	DOWNSTREAM	0.934	0.366	1948.73	713.62	0.342	1466.96	501.95
A_68_P26036156	chr8:90184417-90184461	NM_033327:299056	Zfp423	DOWNSTREAM	0.934	0.691	1761.83	1217.17	0.645	1478.12	953.61
A_68_P24086563	chr5:129526423-129526467	NM_009391:414	Ran	INSIDE	0.934	0.510	2026.76	1033.34	0.476	1472.67	701.39
A_68_P23336251	chr4:136392310-136392354	NM_010142:-482	Ephb2	PROMOTER	0.934	0.402	2070.62	832.09	0.375	1572.03	590.17
A_68_P21767850	chr2:155652193-155652237	NM_010579:447	Eif6	INSIDE	0.934	0.250	5032.92	1260.44	0.234	3548.91	830.09
A_68_P32975995	chr12:18394467-18394511	ENSMUST00000080502:-72		PROMOTER	0.933	1.898	736.80	1398.52	1.770	702.07	1242.72
A_68_P31301688	chr17:65963294-65963338	NM_013933:-421	Vapa	PROMOTER	0.933	0.604	1236.46	746.69	0.563	1135.19	639.48
A_68_P31286859	chr17:63232405-63232449	NM_010109:-1760	Efna5	PROMOTER	0.933	3.271	378.17	1236.82	3.052	330.98	1010.21
A_68_P28577473	chr12:81859415-81859459	NM_001008423:2266	Gm1568	INSIDE	0.933	3.479	362.38	1260.77	3.246	380.50	1235.29
A_68_P27932871	chr11:76384753-76384797	NM_198895:102	Abr	INSIDE	0.933	0.237	4660.51	1104.91	0.221	3186.20	705.07
A_68_P27838988	chr11:59601147-59601191	NM_183139:-9	Pld6	PROMOTER	0.933	0.236	3295.22	779.23	0.221	2543.71	561.20
A_68_P26398235	chr9:30838331-30838375	NM_001115130:124	Zbtb44	INSIDE	0.933	2.514	771.82	1940.40	2.346	682.99	1602.41
A_68_P26241996	chr8:126383242-126383286	NM_028536:845	1700054N08Rik	INSIDE	0.933	0.551	1359.16	749.38	0.514	1031.72	530.69
A_68_P25940712	chr8:70586026-70586070	ENSMUST00000150169:137		INSIDE	0.933	0.267	1991.78	531.36	0.249	1629.56	405.61
A_68_P25394239	chr7:112910735-112910779	NM_001162943:25308	Dchs1	INSIDE	0.933	1.766	994.07	1755.11	1.648	948.15	1562.58
A_68_P25285303	chr7:91032985-91033029	NM_030705:-155	Mesdc1	DIVERGENT_PROMOTER	0.933	2.405	760.23	1828.35	2.245	693.00	1555.57
A_68_P25150557	chr7:64642081-64642125	NM_008074:139	Gabrg3	INSIDE	0.933	0.609	1340.85	817.06	0.569	972.91	553.16
A_68_P25021280	chr7:29123326-29123370	NM_013676:390	Supt5h	INSIDE	0.933	0.737	3733.72	2753.16	0.688	2789.61	1918.47
A_68_P24992474	chr7:19872133-19872177	NM_001025364:-4293	Rtn2	PROMOTER	0.933	2.951	1066.69	3147.42	2.753	884.38	2434.31
A_68_P24850989	chr6:131383710-131383754			Unknown	0.933	2.373	595.54	1413.25	2.215	532.79	1180.05
A_68_P24591289	chr6:83027770-83027814	NM_197992:-591	Pcgf1	DIVERGENT_PROMOTER	0.933	3.272	388.28	1270.58	3.054	272.55	832.28
A_68_P24114684	chr5:135565665-135565709			Unknown	0.933	0.634	1967.90	1247.02	0.591	1510.71	892.84
A_68_P23618576	chr5:38668524-38668568	NM_172709:-96	Otop1	PROMOTER	0.933	0.252	2549.56	643.37	0.236	1919.01	452.00
A_68_P22393605	chr3:104667362-104667406	NM_009797:39	Capza1	INSIDE	0.933	0.566	2905.86	1643.90	0.528	2281.84	1204.97
A_68_P22142065	chr3:53282814-53282858	NM_173382:15098	2810046L04Rik	INSIDE	0.933	4.883	7085.24	34594.01	4.556	4757.00	21675.16
A_68_P21604776	chr2:125608207-125608251	NM_177608:378	Secisbp2l	INSIDE	0.933	0.585	2397.41	1402.16	0.546	1894.69	1034.14
A_68_P21339408	chr2:74600811-74600855	NM_010467:-204	Hoxd1	PROMOTER	0.933	0.475	2023.16	961.98	0.444	1444.98	641.28
A_68_P21017263	chr2:13495869-13495913	NM_011701:-47	Vim	PROMOTER	0.933	5.714	283.40	1619.48	5.330	191.05	1018.26
A_68_P20293515	chr1:64663797-64663841	NM_025964:-76	Mettl21a	PROMOTER	0.933	2.226	484.09	1077.63	2.078	421.10	874.85
A_68_P31120667	chr17:28279215-28279259	NM_001004366:-234	Scube3	PROMOTER	0.932	0.547	3137.61	1714.93	0.510	2464.25	1255.71
A_68_P27954542	chr11:80291110-80291154	NM_009871:585	Cdk5r1	INSIDE	0.932	0.225	2592.85	583.01	0.209	1627.27	340.88
A_68_P25879338	chr8:55615223-55615267	NM_029701:107	Spcs3	INSIDE	0.932	0.276	2104.73	581.13	0.257	1772.90	456.38
A_68_P25462320	chr7:126327951-126327995	NM_001024138:-84	Gpr139	PROMOTER	0.932	1.974	1076.19	2123.93	1.839	984.23	1810.11
A_68_P24754176	chr6:112439420-112439464	NM_001081147:360	Oxtr	INSIDE	0.932	2.664	977.98	2605.59	2.484	718.29	1784.19
A_68_P23333242	chr4:135866928-135866972	NM_028871:60	Hnrnpr	INSIDE	0.932	2.112	613.21	1294.80	1.968	547.62	1077.96
A_68_P23203735	chr4:109650901-109650945	NM_172296:293	Dmrta2	INSIDE	0.932	0.465	2021.92	939.76	0.433	1662.92	720.55
A_68_P23088447	chr4:87678387-87678431	NM_027326:903	Mllt3	INSIDE	0.932	0.497	2340.76	1163.46	0.463	1753.37	811.83
A_68_P21840228	chr2:168056765-168056809	NM_001160330:665	Mocs3	INSIDE	0.932	3.522	1073.39	3780.09	3.283	949.45	3116.62
A_68_P21637466	chr2:131735690-131735734	NM_011170:49	Prnp	INSIDE	0.932	0.306	1691.39	517.87	0.285	1313.53	374.92
A_68_P21436150	chr2:93483215-93483259	NM_007442:646	Alx4	INSIDE	0.932	0.382	1195.87	457.25	0.356	1065.98	379.82
A_68_P20877961	chr1:182743984-182744028	NM_008210:-272	H3f3a	PROMOTER	0.932	2.535	1625.90	4122.02	2.363	1336.51	3158.52
A_68_P31175183	A_68_P31175183			Unknown	0.931	1.656	1088.22	1802.27	1.542	894.83	1379.72
A_68_P31157812	chr17:35272083-35272127	NM_057171:-82	Bag6	DIVERGENT_PROMOTER	0.931	1.835	775.46	1422.65	1.707	640.02	1092.60
A_68_P29527054	chr14:34768675-34768719	NM_015745:1489	Rbp3	INSIDE	0.931	1.999	803.78	1606.72	1.862	639.38	1190.35
A_68_P29338087	chr13:113784480-113784524	NM_001037914:427	Gm6320	INSIDE	0.931	0.662	1694.35	1121.67	0.616	1321.81	814.60
A_68_P28072360	chr11:101031694-101031738	NM_146030:900	Plekhh3	INSIDE	0.931	1.802	1123.19	2024.14	1.678	1075.38	1804.76
A_68_P27564839	chr11:5662438-5662482	NM_001077661:-81	Urgcp	PROMOTER	0.931	0.383	1596.99	611.10	0.356	1400.96	499.24
A_68_P27563477	chr11:5344468-5344513	NM_001080924:360	Znrf3	INSIDE	0.931	1.765	1663.77	2936.37	1.643	1198.56	1969.06
A_68_P27316753	chr10:86371829-86371873	NM_138673:98837	Stab2	INSIDE	0.931	3.091	3736.47	11547.76	2.876	2877.43	8275.24
A_68_P27265099	chr10:76424280-76424324	NM_021568:390	Pcbp3	INSIDE	0.931	0.623	1264.72	787.93	0.580	1069.11	620.25
A_68_P26846738	chr9:114752895-114752940	NM_027294:353	Cmtm8	INSIDE	0.931	0.183	2535.05	464.67	0.171	1856.70	316.96
A_68_P26761641	chr9:98887034-98887078	NM_001122851:265	Faim	INSIDE	0.931	0.398	2484.70	988.62	0.371	2105.91	780.49
A_68_P26754210	chr9:97601436-97601480	NM_022319:332128	Clstn2	INSIDE	0.931	2.108	4420.29	9317.31	1.963	3771.93	7403.71
A_68_P25956773	chr8:73929899-73929944	NM_172756:11	Ankle1	INSIDE	0.931	0.569	1436.25	817.52	0.530	1116.17	591.37
A_68_P25677406	chr8:14911525-14911569	NM_001037736:-170	Arhgef10	PROMOTER	0.931	0.271	1882.85	510.43	0.253	1521.29	384.15
A_68_P25576401	chr7:146399168-146399212	NM_001162540:16	Stk32c	INSIDE	0.931	0.547	1049.69	574.55	0.510	861.29	439.02
A_68_P24725453	chr6:106749980-106750024	NM_021449:66	Crbn	INSIDE	0.931	0.668	2056.56	1374.51	0.622	1502.92	935.03
A_68_P24057005	chr5:124134134-124134178	NM_019765:144	Clip1	INSIDE	0.931	1.702	1085.45	1847.17	1.584	873.08	1382.79
A_68_P24053750	chr5:123526309-123526353	NM_001039723:47	Tmem120b	INSIDE	0.931	0.426	2008.40	855.04	0.396	1696.36	672.20
A_68_P23284269	chr4:126921347-126921391	NM_030252:341	BC003266	INSIDE	0.931	0.427	1830.37	780.66	0.397	1424.35	565.51

ProbeName	Target position of probe on CpG island microarray	TargetID	GeneSymbol	CpG island Description	Ratio of relative methylation (TiO_2-NP/Vehicle)	Sham group			TiO_2-H group		
						Relative methylation (Cy5/Cy3)	Cy3 signal (Input)	Cy5 signal (MeDIP)	Relative methylation (Cy5/Cy3)	Cy3 signal (Input)	Cy5 signal (MeDIP)
A_68_P21779874	chr2:157740919-157740963	NM_198627:552	Vstm2l	INSIDE	0.931	2.758	2564.86	7073.54	2.568	1631.25	4188.73
A_68_P21580770	chr2:121240019-121240063	NM_007952:403	Pdia3	INSIDE	0.931	0.640	1880.93	1204.68	0.596	1641.39	978.41
A_68_P21341873	chr2:74976122-74976166	ENSMUST00000143898:-289		PROMOTER	0.931	0.489	2105.27	1029.28	0.455	1681.03	764.82
A_68_P20745861	chr1:158869106-158869150	NM_001159966:390	Ralgps2	INSIDE	0.931	0.506	4867.47	2464.12	0.472	3508.02	1654.09
A_68_P20723965	chr1:154802917-154802961	NM_175460:708	Nmnat2	INSIDE	0.931	8.739	4703.28	41101.81	8.138	4293.71	34944.11
A_68_P20229135	chr1:52874565-52874609	NM_008384:-54	Inpp1	PROMOTER	0.931	0.648	1368.96	886.83	0.603	1131.18	682.14
A_68_P31982214	chr19:16854893-16854937	NM_173028:503	Vps13a	INSIDE	0.930	0.501	1102.66	552.83	0.466	910.09	424.13
A_68_P31827984	chr18:73974953-73974997	NM_145494:72	Me2	INSIDE	0.930	2.596	552.76	1434.93	2.414	456.73	1102.59
A_68_P31119196	chr17:28046558-28046602	NM_181413:296	Anks1	INSIDE	0.930	2.185	802.75	1753.90	2.032	743.31	1510.57
A_68_P28052184	chr11:97561645-97561689	NM_001163308:-704	Pcgf2	PROMOTER	0.930	1.999	1906.82	3811.88	1.860	1425.10	2650.24
A_68_P27894949	chr11:69224676-69224720	NM_001017426:2479	Kdm6b	INSIDE	0.930	0.311	6281.53	1953.29	0.289	4087.97	1182.31
A_68_P26630337	chr9:72772909-72772953	NM_028116:-526	Pygo1	PROMOTER	0.930	0.191	5042.38	963.03	0.178	3386.08	601.56
A_68_P26323067	chr9:15298474-15298518	AK146267:621		INSIDE	0.930	0.408	2762.90	1127.75	0.380	2052.04	778.88
A_68_P25528062	chr7:138514868-138514912	NM_001039534:231	Pstk	INSIDE	0.930	0.461	2246.08	1034.32	0.428	1662.48	711.65
A_68_P25186205	chr7:73224603-73224647	NM_024439:90	H47	INSIDE	0.930	2.117	1171.32	2480.04	1.969	947.93	1866.03
A_68_P24455810	chr6:53238161-53238205	NR_015553:894	9430076C15Rik	INSIDE	0.930	0.400	2178.73	872.09	0.372	1651.71	614.95
A_68_P23590297	chr5:33995249-33995293	NM_009193:-315	Slbp	PROMOTER	0.930	0.398	1216.92	484.14	0.370	991.50	366.69
A_68_P23547925	chr5:25036093-25036137	NR_027388:279	1700096K18Rik	INSIDE	0.930	0.306	1913.54	586.42	0.285	1507.47	429.56
A_68_P23312752	chr4:132089527-132089571	NM_007512:26	Atpif1	INSIDE	0.930	0.580	1317.42	764.35	0.539	1070.60	577.46
A_68_P22941964	chr4:56877971-56878015	NM_018761:91	Ctnnal1	INSIDE	0.930	0.188	4852.22	910.85	0.175	3449.06	602.42
A_68_P22359921	chr3:98186541-98186585	NM_172863:159	Zfp697	INSIDE	0.930	0.437	1804.87	788.22	0.406	1369.62	556.04
A_68_P22350683	chr3:96501427-96501471	NM_001165949:450	Pias3	INSIDE	0.930	0.287	1654.45	475.54	0.267	1392.54	372.40
A_68_P20872225	chr1:181733706-181733750	NM_026375:418	Ahctf1	INSIDE	0.930	2.175	1206.47	2623.89	2.024	959.19	1940.95
A_68_P20823477	chr1:172519538-172519582	NM_001109985:420	Nos1ap	INSIDE	0.930	0.218	2174.47	473.56	0.203	1582.25	320.53
A_68_P20293520	chr1:64664228-64664272	NM_025964:-508	Mettl21a	PROMOTER	0.930	0.381	2181.17	831.62	0.354	1656.78	587.32
A_68_P31475768	chr18:7004747-7004791	NM_177595:9	Mkx	INSIDE	0.929	0.620	1610.06	998.52	0.576	1207.92	695.63
A_68_P31099383	chr17:24890123-24890167	NM_001012402:197	Hs3st6	INSIDE	0.929	0.408	1595.34	650.12	0.379	1263.19	478.31
A_68_P30686066	chr16:38452562-38452607	NM_007414:185	Adprh	INSIDE	0.929	0.492	2540.36	1249.77	0.457	1947.00	889.54
A_68_P30586701	chr16:20241157-20241201	NM_198599:253	Map6d1	INSIDE	0.929	2.368	465.20	1101.71	2.201	401.48	883.63
A_68_P29204940	chr13:88961079-88961123	NM_001037987:24	Edil3	INSIDE	0.929	2.587	902.28	2334.17	2.403	857.94	2061.73
A_68_P26888891	chr9:122260490-122260534	NM_026179:-221	Abhd5	PROMOTER	0.929	0.607	1112.37	675.64	0.564	954.70	538.82
A_68_P26200116	chr8:119517155-119517199	NM_026572:173	Gcsh	INSIDE	0.929	0.452	2003.56	905.13	0.420	1523.94	639.41
A_68_P25725384	chr8:26212812-26212856	NM_031257:-551	Plekha2	PROMOTER	0.929	0.329	3851.07	1266.37	0.306	2797.62	855.07
A_68_P25086958	chr7:51607583-51607627	NM_001034115:41971	Shank1	INSIDE	0.929	0.444	1118.58	496.30	0.412	959.00	395.43
A_68_P24302938	chr6:24615029-24615073	NM_028459:-55	Wasl	PROMOTER	0.929	0.474	2286.34	1083.27	0.440	1971.83	867.95
A_68_P23610943	chr5:37380278-37380322	NM_011716:-79	Wfs1	PROMOTER	0.929	0.460	1493.64	687.70	0.428	1080.74	462.14
A_68_P23321455	chr4:133742851-133742895	NM_144527:128	Ccdc21	INSIDE	0.929	0.367	4034.43	1479.27	0.341	2872.10	978.56
A_68_P22325240	chr3:90237336-90237380	NM_145540:200	Ints3	INSIDE	0.929	0.714	3302.59	2357.19	0.663	2476.24	1641.53
A_68_P21580981	chr2:121275805-121275849	NM_011354:863	Serf2	INSIDE	0.929	0.226	2317.01	524.35	0.210	1658.77	348.68
A_68_P21483992	chr2:103407094-103407138	NM_178890:650	Abtb2	INSIDE	0.929	0.576	1820.49	1049.07	0.535	1555.44	832.57
A_68_P20433236	chr1:91865154-91865198	NM_139152:45976	Asb18	INSIDE	0.929	2.634	503.78	1327.00	2.447	559.66	1369.25
A_68_P31791923	chr18:67608414-67608458	NM_027130:354	Afg3l2	INSIDE	0.928	1.731	1581.28	2737.28	1.607	1267.17	2036.15
A_68_P31158975	chr17:35480111-35480155	NM_010392:855	H2-Q2	INSIDE	0.928	0.445	1312.09	583.61	0.413	944.93	390.09
A_68_P30927365	chr16:85080178-85080222	NM_001198823:93752	App	INSIDE	0.928	2.367	839.11	1986.51	2.197	722.31	1586.99
A_68_P30558815	chr16:13754812-13754856	AK044312:176		INSIDE	0.928	0.555	1832.82	1017.29	0.515	1367.66	704.34
A_68_P30421714	chr15:88692421-88692465	NM_145478:-181	Pim3	PROMOTER	0.928	0.134	3947.07	527.10	0.124	2764.79	342.47
A_68_P30368658	chr15:79634975-79635019	NM_030689:143	Nptxr	INSIDE	0.928	2.791	1438.36	4013.81	2.591	1415.95	3668.22
A_68_P28950039	chr13:36670467-36670511	AK135154:190947		INSIDE	0.928	2.460	722.70	1778.09	2.284	615.16	1404.93
A_68_P27504461	chr10:121043178-121043222	NM_001081056:20172	Xpot	INSIDE	0.928	2.398	2541.62	6093.87	2.226	1887.87	4202.02
A_68_P26936713	chr10:10408926-10408970	NM_001114333:393181	Grm1	INSIDE	0.928	0.206	2300.42	472.87	0.191	1920.73	366.30
A_68_P26876101	chr9:120019653-120019697	NM_144793:158	Slc25a38	INSIDE	0.928	0.401	1696.98	680.68	0.372	1183.94	440.54
A_68_P25091981	chr7:52489690-52489734	NM_007645:4497	Cd37	INSIDE	0.928	4.446	1551.70	6899.48	4.127	1450.00	5983.86
A_68_P25063501	chr7:38555245-38555289	NM_172385:-495	Zfp536	PROMOTER	0.928	0.444	1966.45	872.42	0.412	1547.10	636.87
A_68_P25007878	chr7:25800603-25800647	NM_008168:56764	Grik5	INSIDE	0.928	5.802	1546.37	8971.74	5.383	1026.00	5522.58
A_68_P24123991	chr5:137463053-137463097	NM_011962:186	Plod3	INSIDE	0.928	0.106	6421.78	677.74	0.098	3867.53	378.92
A_68_P23416686	chr4:151500208-151500252	NM_019585:2804	Espn	INSIDE	0.928	0.315	2051.34	647.03	0.293	1370.94	401.20
A_68_P23379973	chr4:144482635-144482679	NM_001172424:-73	Dhrs3	PROMOTER	0.928	0.428	6545.70	2804.81	0.397	4605.90	1830.80
A_68_P22201239	chr3:65462965-65463009	NM_001166659:-7163	Lekr1	PROMOTER	0.928	0.240	2111.45	505.95	0.222	1597.81	355.35
A_68_P21762928	chr2:154718164-154718208	NM_026030:456	Eif2s2	INSIDE	0.928	0.227	3805.95	862.06	0.210	2682.56	563.79
A_68_P21438176	chr2:93816807-93816851	NM_026944:34059	Alkbh3	DOWNSTREAM	0.928	6.748	1374.51	9275.29	6.266	1120.91	7023.11
A_68_P20412363	chr1:88383877-88383921			Unknown	0.928	1.973	1306.76	2578.36	1.832	1029.02	1884.72
A_68_P20091640	chr1:24106611-24106655	NM_026604:548	Fam135a	INSIDE	0.928	0.624	2495.24	1555.88	0.578	1979.18	1144.63
A_68_P31311927	chr17:67704263-67704307	NM_008984:-485	Ptprm	PROMOTER	0.927	0.298	1748.63	520.62	0.276	1384.43	382.29
A_68_P31096054	chr17:24343797-24343841	NM_001163847:-1311	Tbc1d24	PROMOTER	0.927	1.999	981.39	1961.36	1.852	763.77	1414.68
A_68_P30969342	chr16:92466123-92466167	NM_001081549:247	Rcan1	INSIDE	0.927	0.551	1835.79	1012.19	0.511	1526.34	780.46

ProbeName	Target position of probe on CpG island microarray	TargetID	GeneSymbol	CpG island Description	Ratio of relative methylation (TiO_2-NP/Vehicle)	Sham group			TiO_2-H group		
						Relative methylation (Cy5/Cy3)	Cy3 signal (Input)	Cy5 signal (MeDIP)	Relative methylation (Cy5/Cy3)	Cy3 signal (Input)	Cy5 signal (MeDIP)
A_68_P30590963	chr16:21204850-21204894	NM_010143:5	Ephb3	INSIDE	0.927	0.392	1910.87	749.26	0.364	1462.14	531.58
A_68_P29260159	chr13:99660900-99660944	NM_001048267:75	Tnpo1	INSIDE	0.927	2.105	1831.71	3855.77	1.951	1564.40	3051.56
A_68_P28601532	chr12:86337427-86337471	NM_178363:178	Ylpm1	INSIDE	0.927	4.374	171.30	749.20	4.053	150.31	609.14
A_68_P27898579	chr11:69835240-69835284	NM_001109752:3156	Dlg4	INSIDE	0.927	0.302	2530.28	765.32	0.281	1885.46	528.89
A_68_P27898214	chr11:69779823-69779867	NM_016887:922	Cldn7	INSIDE	0.927	4.501	1535.30	6910.90	4.175	1314.19	5486.10
A_68_P27095708	chr10:42303278-42303322	NM_152229:94	Nr2e1	INSIDE	0.927	2.790	441.59	1231.99	2.585	387.87	1002.66
A_68_P27034329	chr10:29033110-29033154	NM_025855:-173	Echdc1	PROMOTER	0.927	0.125	4737.67	591.02	0.116	3633.80	420.40
A_68_P26894032	chr9:123169639-123169683	NM_001002267:247	Tmem158	INSIDE	0.927	2.588	872.63	2258.25	2.399	688.52	1651.48
A_68_P25952516	chr8:73062754-73062798	NM_007924:-797	Ell	PROMOTER	0.927	0.159	8213.50	1306.73	0.147	5837.21	860.48
A_68_P24434513	chr6:49772917-49772962	NM_023456:212	Npy	INSIDE	0.927	2.609	1069.62	2790.65	2.419	906.70	2193.39
A_68_P23531277	chr5:21851025-21851070	NM_011261:-524	Reln	PROMOTER	0.927	0.564	1632.81	920.52	0.522	1146.43	598.93
A_68_P23355139	chr4:139521507-139521551	NM_173427:2413	Klhdc7a	INSIDE	0.927	2.089	886.16	1851.15	1.937	712.76	1380.81
A_68_P23232474	chr4:116782205-116782250	NM_008958:13267	Ptch2	INSIDE	0.927	2.435	977.44	2379.70	2.258	804.70	1817.07
A_68_P22405342	chr3:107082078-107082122	NM_026774:325	Hbxip	INSIDE	0.927	0.315	2199.43	692.81	0.292	1633.19	476.85
A_68_P21229087	chr2:54288431-54288477	NM_173030:-343	Galnt13	PROMOTER	0.927	0.402	2377.46	954.78	0.372	1794.59	668.07
A_68_P21073093	chr2:25433409-25433453	NM_001005424:2189	Gm996	INSIDE	0.927	0.299	1581.14	473.51	0.278	1149.54	319.11
A_68_P31769413	chr18:63545818-63545864	NM_001039485:997	Fam38b	INSIDE	0.926	2.841	928.04	2636.79	2.632	719.53	1893.47
A_68_P31110715	chr17:26645503-26645547	NM_013642:-107	Dusp1	PROMOTER	0.926	0.568	1183.01	672.06	0.526	963.33	506.91
A_68_P31093272	chr17:23863852-23863896	NM_023058:572	Pkmyt1	INSIDE	0.926	0.335	2174.57	728.23	0.310	1564.68	485.07
A_68_P31062779	chr17:15080551-15080595	NM_001083880:384	1600012H06Rik	INSIDE	0.926	0.670	1698.54	1137.99	0.620	1151.46	714.31
A_68_P30963193	chr16:91485518-91485562	NM_010508:81	Ifnar1	INSIDE	0.926	1.743	1074.61	1873.47	1.615	912.48	1473.73
A_68_P30960416	chr16:91010827-91010871	NM_001045515:492	Synj1	INSIDE	0.926	11.244	795.16	8940.51	10.413	625.75	6516.15
A_68_P30403121	chr15:85642175-85642219	NM_001164625:-69	2210021J22Rik	PROMOTER	0.926	0.164	3997.05	655.71	0.152	2921.83	443.81
A_68_P29619052	chr14:56262619-56262663	NM_028780:3	Tm9sf1	INSIDE	0.926	2.240	930.70	2084.33	2.074	775.83	1608.90
A_68_P29613302	chr14:55236499-55236543	NR_030457:-898	Mir686	PROMOTER	0.926	6.771	310.36	2101.34	6.271	259.65	1628.39
A_68_P27680407	chr11:30671725-30671769	NM_134013:-28	Psme4	PROMOTER	0.926	4.307	768.58	3310.41	3.990	563.26	2247.66
A_68_P27320008	chr10:86956714-86956759	NM_008553:-331	Ascl1	PROMOTER	0.926	2.572	533.31	1371.59	2.381	482.19	1148.19
A_68_P27278197	chr10:79138491-79138535	NM_148934:6358	Gm16517	INSIDE	0.926	4.201	1996.04	8384.62	3.891	1637.98	6373.36
A_68_P25004263	chr7:25101709-25101753	NM_001199321:-45	Zfp94	PROMOTER	0.926	0.557	2572.89	1432.32	0.516	1802.03	929.13
A_68_P24513199	chr6:65621566-65621610	NM_172399:-16	A930038C07Rik	PROMOTER	0.926	0.726	4103.93	2979.56	0.672	3101.38	2085.51
A_68_P24191782	chr5:151396383-151396427	NM_133898:696	N4bp2l1	INSIDE	0.926	0.374	1337.86	500.58	0.346	1186.50	410.88
A_68_P24053561	chr5:123479352-123479396	NM_175423:14292	Orai1	INSIDE	0.926	2.232	490.78	1095.60	2.068	441.49	912.94
A_68_P23356914	chr4:139800404-139800448	NM_198610:2300	Igsf21	INSIDE	0.926	0.452	1275.39	576.76	0.419	1078.97	451.93
A_68_P23333239	chr4:135866622-135866666	NM_028871:-246	Hnrnpr	PROMOTER	0.926	0.541	1155.19	624.38	0.500	866.59	433.65
A_68_P22362401	chr3:98945093-98945137	NM_027462:102	Wars2	INSIDE	0.926	0.366	1967.07	720.60	0.339	1586.73	538.04
A_68_P21010975	chr2:12340480-12340524	NM_024185:585	Fam188a	INSIDE	0.926	0.352	1404.30	493.97	0.326	1038.06	338.01
A_68_P31484555	chr18:9214035-9214079	NM_008058:1203	Fzd8	INSIDE	0.925	0.252	3924.00	989.12	0.233	2823.97	658.48
A_68_P30747802	chr16:49855795-49855839	NM_010581:50	Cd47	INSIDE	0.925	1.956	801.91	1568.44	1.809	600.39	1086.08
A_68_P29681978	chr14:67528942-67528986	NM_175498:-1080	Pnma2	PROMOTER	0.925	0.546	2919.45	1593.89	0.505	1998.64	1009.77
A_68_P29228407	chr13:94073846-94073890	NM_011982:-581	Homer1	PROMOTER	0.925	0.707	2281.75	1612.23	0.654	1794.29	1173.26
A_68_P29020014	chr13:48641657-48641701	NR_029725:-8037	Mirlet7a-1	PROMOTER	0.925	0.627	2242.15	1404.88	0.580	1600.51	927.66
A_68_P28183223	chr11:120095537-120095581	NM_198423:1298	Bahcc1	INSIDE	0.925	0.144	3136.97	452.89	0.134	2212.72	295.45
A_68_P28172267	chr11:118430861-118430905	NM_001024931:340004	Rbfox3	INSIDE	0.925	11.290	1671.67	18872.64	10.440	1460.79	15250.24
A_68_P27549890	chr10:129881898-129881942	NM_001103365:11030	Vmn2r86	DOWNSTREAM	0.925	2.221	519.27	1153.29	2.055	475.84	978.09
A_68_P26875482	chr9:119892235-119892279	NM_153287:1520	Csrnp1	INSIDE	0.925	0.215	3525.20	756.37	0.198	2416.20	479.39
A_68_P25503043	chr7:134072430-134072474	NM_172747:60	Kctd13	INSIDE	0.925	2.291	1997.65	4575.91	2.118	1891.18	4005.68
A_68_P25372611	chr7:108469921-108469965	NM_019990:110	Stard10	INSIDE	0.925	0.454	4521.91	2053.77	0.420	3041.84	1277.65
A_68_P25094660	chr7:52968835-52968879	NM_020011:1721	Sphk2	INSIDE	0.925	2.409	1246.73	3003.17	2.228	917.92	2044.71
A_68_P24984096	chr7:17530142-17530186	NM_001099636:134	Pnmal2	INSIDE	0.925	0.444	1195.80	531.01	0.411	858.28	352.42
A_68_P24759220	chr6:113342741-113342785	NM_133923:272	Ttll3	INSIDE	0.925	0.555	2006.04	1114.24	0.514	1616.84	830.91
A_68_P24423934	chr6:47544675-47544719	NM_001146689:333	Ezh2	INSIDE	0.925	5.988	3560.99	21324.38	5.540	2972.55	16468.96
A_68_P22629212	chr3:148482607-148482651	NM_001081298:134971	Lphn2	INSIDE	0.925	2.895	1037.25	3003.01	2.679	959.63	2570.58
A_68_P22383904	chr3:102824419-102824463	NM_001161854:-28	Csde1	PROMOTER	0.925	0.467	2137.44	998.34	0.432	1602.63	692.47
A_68_P22081524	chr3:40604311-40604355	NM_011495:460	Plk4	INSIDE	0.925	0.320	1617.73	518.14	0.296	1154.74	341.99
A_68_P21339409	chr2:74600922-74600966	NM_010467:-92	Hoxd1	PROMOTER	0.925	2.553	1111.51	2837.65	2.362	875.58	2068.02
A_68_P21330558	chr2:73115586-73115630	NM_001005343:5626	Sp9	DOWNSTREAM	0.925	2.297	559.69	1285.63	2.125	485.69	1032.30
A_68_P20936909	chr1:193048455-193048499	ENSMUST00000139056:124		INSIDE	0.925	0.416	1213.09	504.39	0.385	944.42	363.20
A_68_P20370345	chr1:78186294-78186338	NM_001159520:7395	Pax3	INSIDE	0.925	0.196	2820.04	552.29	0.181	2104.26	381.00
A_68_P20352374	chr1:75165529-75165573	NM_026846:242	Zfand2b	INSIDE	0.925	0.301	12362.20	3723.21	0.279	8448.43	2353.28
A_68_P20350878	chr1:74901948-74901992	NM_009872:368	Cdk5r2	INSIDE	0.925	0.557	1481.67	825.55	0.516	1164.94	600.55
A_68_P32473671	chrX:73211910-73211954	ENSMUST00000121090:-7625		PROMOTER	0.924	2.796	1089.36	3046.35	2.585	1344.77	3475.79
A_68_P32147459	chr19:47142283-47142327	NM_177342:67	Taf5	INSIDE	0.924	3.071	761.90	2340.06	2.838	630.65	1789.90
A_68_P31833166	chr18:74938960-74939004	NM_177470:117	Acaa2	INSIDE	0.924	0.430	1794.66	772.30	0.398	1246.68	495.89
A_68_P31418981	chr17:87152832-87152876	NM_010137:-349	Epas1	PROMOTER	0.924	0.526	5320.53	2796.56	0.486	4194.95	2037.09
A_68_P31296460	chr17:64950406-64950450	NM_008549:-560	Man2a1	PROMOTER	0.924	0.700	3667.85	2566.72	0.647	2750.91	1779.01

ProbeName	Target position of probe on CpG island microarray	TargetID	GeneSymbol	CpG island Description	Ratio of relative methylation (TiO_2-NP/Vehicle)	Sham group			TiO_2-H group		
						Relative methylation (Cy5/Cy3)	Cy3 signal (Input)	Cy5 signal (MeDIP)	Relative methylation (Cy5/Cy3)	Cy3 signal (Input)	Cy5 signal (MeDIP)
A_68_P28621835	chr12:90498748-90498792	NM_172544:306937	Nrxn3	INSIDE	0.924	2.325	1237.10	2876.75	2.148	962.97	2068.66
A_68_P28192099	chr11:121563822-121563866	NM_144797:104	Metrnl	INSIDE	0.924	0.407	2314.49	942.96	0.377	1725.45	649.76
A_68_P27281199	chr10:79599447-79599491	NM_001195268:664	Dos	INSIDE	0.924	0.476	1149.65	547.63	0.440	734.26	323.23
A_68_P26569718	chr9:62129773-62129817	NM_025721:192	Spesp1	INSIDE	0.924	2.227	575.21	1280.94	2.057	461.14	948.54
A_68_P26465869	chr9:43938100-43938144	NM_023061:-4618	Mcam	PROMOTER	0.924	0.550	1439.28	791.45	0.508	1126.56	572.44
A_68_P26445379	chr9:40608906-40608950	NM_031165:-427	Hspa8	PROMOTER	0.924	0.371	2144.34	796.12	0.343	1737.37	596.05
A_68_P26244128	chr8:126755927-126755971	NM_139272:655	Galnt2	INSIDE	0.924	0.529	1347.79	712.67	0.489	969.80	473.78
A_68_P26232725	chr8:124860203-124860247	NM_009569:54184	Zfpm1	INSIDE	0.924	0.351	3623.94	1272.01	0.324	2415.46	783.07
A_68_P25370036	chr7:108011436-108011480	NM_177073:469	Relt	INSIDE	0.924	0.441	1489.15	656.97	0.408	1227.78	500.56
A_68_P25188284	chr7:73572414-73572458	NM_053080:-73	Aldh1a3	PROMOTER	0.924	1.751	2245.48	3930.83	1.618	1811.22	2931.03
A_68_P23404233	chr4:149461609-149461653	NM_177366:19	Gpr157	INSIDE	0.924	0.185	3045.96	564.00	0.171	2338.11	399.85
A_68_P23318790	chr4:133228292-133228336	NM_001145955:248	Pigv	INSIDE	0.924	0.312	1764.01	550.55	0.288	1570.48	453.07
A_68_P21843413	chr2:168590742-168590787	NM_175303:1937	Sall4	INSIDE	0.924	0.338	1542.57	520.99	0.312	1324.02	413.20
A_68_P32462210	chrX:70599683-70599727	NM_001160229:11745	Zfp275	INSIDE	0.923	3.609	837.81	3023.28	3.332	917.61	3057.38
A_68_P29964715	chr14:123382074-123382118	NM_028651:387	Tmtc4	INSIDE	0.923	1.868	687.91	1285.26	1.724	624.66	1076.93
A_68_P29589387	chr14:49285138-49285182	NM_144841:-2613	Otx2	DIVERGENT_PROMOTER	0.923	0.483	1954.49	943.32	0.445	1360.32	605.86
A_68_P28058395	chr11:98629793-98629837	NM_145434:6742	Nr1d1	INSIDE	0.923	2.734	1208.65	3304.29	2.525	1051.00	2653.27
A_68_P27390319	chr10:99478265-99478309	NM_013598:-171	Kitl	PROMOTER	0.923	0.475	2157.94	1025.89	0.439	1778.02	780.50
A_68_P27287755	chr10:80616237-80616281	NM_021501:14207	Pias4	DOWNSTREAM	0.923	3.753	1067.72	4007.34	3.466	895.02	3101.96
A_68_P26948857	chr10:12780907-12780951	FJ425893:619		INSIDE	0.923	2.675	570.68	1526.41	2.468	541.71	1337.07
A_68_P26918726	chr10:6979506-6979550	NM_133485:863	Ppp1r14c	INSIDE	0.923	3.800	2638.35	10026.31	3.509	2137.24	7498.77
A_68_P25235045	chr7:81649413-81649459	NM_001038643:50230	Slco3a1	INSIDE	0.923	1.905	1107.70	2110.32	1.758	851.63	1497.18
A_68_P24994892	chr7:20301207-20301251	NM_001109748:-450	Tomm40	PROMOTER	0.923	0.369	1535.11	566.00	0.340	1190.19	404.93
A_68_P24868562	chr6:135682934-135682978	NM_008171:440573	Grin2b	INSIDE	0.923	2.977	314.62	936.71	2.749	233.81	642.85
A_68_P24791291	chr6:119058809-119058853	NM_001159533:-623	Cacna1c	PROMOTER	0.923	0.739	2483.98	1836.15	0.682	1980.79	1351.34
A_68_P24760947	chr6:113647724-113647768	NM_001033463:254	Tatdn2	INSIDE	0.923	0.314	1434.63	450.77	0.290	1128.51	327.22
A_68_P24362054	chr6:35489548-35489592	NM_008098:318	Mtpn	INSIDE	0.923	0.269	2263.59	609.07	0.248	1701.23	422.45
A_68_P23226011	chr4:115500811-115500855	NM_175308:136	Mobkl2c	INSIDE	0.923	0.550	1666.74	916.01	0.507	1338.71	679.34
A_68_P22319260	chr3:89222015-89222059	NM_016904:177	Cks1b	INSIDE	0.923	0.298	1978.29	588.91	0.275	1449.14	398.28
A_68_P21873198	chr2:173901908-173901952	NM_001102423:-621	Stx16	PROMOTER	0.923	0.519	2256.29	1170.15	0.479	1850.27	886.08
A_68_P20634005	chr1:136453383-136453427	NM_152895:-3350	Kdm5b	DIVERGENT_PROMOTER	0.923	0.510	1609.99	820.33	0.470	1287.10	605.58
A_68_P20347693	chr1:74331033-74331077	NM_001190444:258	Aamp	INSIDE	0.923	0.291	1893.24	551.86	0.269	1462.02	393.45
A_68_P20250397	chr1:57029081-57029125	NR_024325:790	9130024F11Rik	INSIDE	0.923	0.247	2847.01	703.67	0.228	2160.85	493.02
A_68_P32977628	A_68_P32977628			Unknown	0.922	2.689	3381.64	9094.80	2.480	1861.64	4616.41
A_68_P32567803	chrX:99446637-99446681	NM_007709:450	Cited1	INSIDE	0.922	1.755	887.51	1557.71	1.619	1101.33	1782.79
A_68_P32075616	chr19:33466245-33466289	NM_001146342:519	Rnls	INSIDE	0.922	1.836	963.77	1769.85	1.694	853.88	1446.31
A_68_P31491264	chr18:10726495-10726539	NM_144860:894	Mib1	INSIDE	0.922	1.803	1193.65	2152.74	1.663	948.00	1576.63
A_68_P31206543	chr17:46521483-46521529	NM_207671:792	Zfp318	INSIDE	0.922	3.067	344.74	1057.19	2.826	316.07	893.33
A_68_P30575738	chr16:17797523-17797567	NM_153790:170	Scarf2	INSIDE	0.922	1.565	5753.26	9004.89	1.444	4558.33	6581.19
A_68_P30486703	chr15:100445047-100445091	NM_011873:-1024	Dazap2	PROMOTER	0.922	0.714	2522.41	1801.40	0.659	1759.83	1159.36
A_68_P30403196	chr15:85652378-85652422	NM_011105:-237	Pkdrej	PROMOTER	0.922	2.221	1139.42	2530.97	2.048	1098.42	2249.15
A_68_P30402201	chr15:85501920-85501964	AK038224:-5229		PROMOTER	0.922	1.962	734.80	1441.62	1.809	651.89	1179.35
A_68_P30149668	chr15:38400702-38400746	M10062:-8338		PROMOTER	0.922	2.046	809.37	1655.73	1.886	679.02	1280.32
A_68_P28043433	chr11:96144433-96144477	NM_010461:1236	Hoxb8	INSIDE	0.922	0.586	1932.40	1132.14	0.540	1619.07	875.02
A_68_P27567707	chr11:6191869-6191913	NM_010956:291	Ogdh	INSIDE	0.922	0.118	7258.04	860.02	0.109	4810.96	525.50
A_68_P26569954	chr9:62189473-62189517	NM_009672:345	Anp32a	INSIDE	0.922	0.148	5509.97	813.62	0.136	3849.11	523.99
A_68_P26463563	chr9:43552020-43552064	NM_021424:-616	Pvrl1	PROMOTER	0.922	2.384	1945.52	4638.30	2.198	1487.28	3269.24
A_68_P26037059	chr8:90304768-90304819	NM_033327:178701	Zfp423	INSIDE	0.922	1.965	576.65	1133.34	1.813	516.49	936.40
A_68_P24830750	chr6:127059445-127059489	NM_177003:104	9630033F20Rik	INSIDE	0.922	0.514	2023.36	1039.52	0.474	1593.67	755.28
A_68_P24591236	chr6:83018754-83018798	NM_009392:1443	Tlx2	INSIDE	0.922	4.299	1007.49	4330.76	3.965	789.99	3132.39
A_68_P24288406	chr6:21899680-21899727	NM_023626:89	Ing3	INSIDE	0.922	0.170	2840.09	481.91	0.156	1848.16	289.09
A_68_P23295632	chr4:128901619-128901663	NM_001033189:24047	C77080	INSIDE	0.922	1.748	1067.99	1867.06	1.612	1026.30	1653.98
A_68_P23103758	chr4:91041293-91041337	NM_010486:-2568	Elavl2	PROMOTER	0.922	0.241	2218.92	533.75	0.222	1800.84	399.61
A_68_P21073106	chr2:25434968-25435012	NM_001005424:629	Gm996	INSIDE	0.922	0.638	1548.38	987.80	0.588	1173.16	690.37
A_68_P31412096	chr17:86084881-86084925	NM_011380:2692	Six2	INSIDE	0.921	0.511	2237.40	1143.28	0.471	1861.47	876.00
A_68_P30152565	chr15:39028705-39028749	NM_053271:-1151	Rims2	PROMOTER	0.921	0.164	3199.50	526.01	0.151	2286.51	346.36
A_68_P29152744	chr13:76193115-76193159	NM_175495:1308	Gpr150	INSIDE	0.921	0.155	5741.88	891.16	0.143	3579.37	511.54
A_68_P27851352	chr11:61770486-61770530	ENSMUST00000136971:197		INSIDE	0.921	0.499	1373.40	685.87	0.460	1065.48	489.91
A_68_P27271712	chr10:77497354-77497398	NM_001081695:-7655	Dnmt3l	PROMOTER	0.921	3.089	1302.88	4024.34	2.846	1141.51	3248.43
A_68_P26574057	chr9:62986883-62986927	NM_172446:7883	Skor1	INSIDE	0.921	10.370	552.02	5724.56	9.546	477.55	4558.74
A_68_P25953242	chr8:73183309-73183353	NM_023217:250	Pgpep1	INSIDE	0.921	1.584	3204.79	5075.32	1.458	2280.07	3325.29
A_68_P25827838	chr8:46095952-46095996	NM_001081286:60413	Fat1	INSIDE	0.921	2.211	1127.13	2491.73	2.036	924.30	1882.03
A_68_P25587560	chr7:148414430-148414474	NM_001081118:-234	Phrf1	DIVERGENT_PROMOTER	0.921	0.611	1671.37	1022.03	0.563	1154.19	649.72
A_68_P25587298	chr7:148380155-148380199	NM_153777:-865	Lrrc56	PROMOTER	0.921	0.396	1298.76	514.50	0.365	891.12	325.28
A_68_P25502255	chr7:133935924-133935968	NM_019674:39	Ppp4c	INSIDE	0.921	0.461	1301.12	599.94	0.425	1056.67	448.61

ProbeName	Target position of probe on CpG island microarray	TargetID	GeneSymbol	CpG island Description	Ratio of relative methylation (TiO_2-NP/Vehicle)	Sham group			TiO_2-H group		
						Relative methylation (Cy5/Cy3)	Cy3 signal (Input)	Cy5 signal (MeDIP)	Relative methylation (Cy5/Cy3)	Cy3 signal (Input)	Cy5 signal (MeDIP)
A_68_P25258775	chr7:86198137-86198181	NM_007424:-210	Acan	PROMOTER	0.921	0.263	2858.04	752.58	0.243	2207.19	535.36
A_68_P24981711	chr7:17061808-17061852	NM_008718:299	Npas1	INSIDE	0.921	0.542	3009.37	1631.99	0.499	1936.89	967.46
A_68_P24954139	chr7:6084115-6084159	NM_001013012:23437	Zfp787	INSIDE	0.921	3.678	1376.45	5062.26	3.387	1181.17	4000.96
A_68_P24333873	chr6:30688134-30688178	NR_029900:-3142	Mir335	PROMOTER	0.921	2.164	815.95	1765.60	1.992	719.10	1432.34
A_68_P23996383	chr5:113567701-113567745	NM_172884:24611	2900026A02Rik	INSIDE	0.921	0.520	1059.91	550.71	0.478	821.19	392.78
A_68_P23546246	chr5:24728813-24728857	NM_144908:124	Galnt11	INSIDE	0.921	2.473	558.39	1380.81	2.278	524.79	1195.72
A_68_P23278938	chr4:125911658-125911702	NM_001145970:21883	Mtap7d1	INSIDE	0.921	1.669	1539.74	2570.52	1.538	1098.80	1689.66
A_68_P22771645	chr4:20705806-20705850	NM_172987:-13	Nkain3	PROMOTER	0.921	1.544	10795.74	16665.58	1.421	7245.64	10298.85
A_68_P21096084	chr2:29108807-29108851	NR_029460:-315	6530402F18Rik	PROMOTER	0.921	1.750	1234.77	2161.13	1.611	995.94	1604.78
A_68_P20880196	chr1:183142145-183142191	NM_145514:-59	Wdr26	PROMOTER	0.921	0.638	1374.94	877.85	0.588	1027.05	603.79
A_68_P20149942	chr1:36766752-36766796	NM_146107:-4	Actr1b	PROMOTER	0.921	0.531	1666.29	884.98	0.489	1316.16	643.63
A_68_P32557245	chrX:97016277-97016321	NM_001081283:-109	Tmem28	PROMOTER	0.920	3.424	357.97	1225.79	3.152	387.02	1219.78
A_68_P30401942	chr15:85465263-85465307			Unknown	0.920	0.425	1261.99	535.76	0.390	1022.06	399.03
A_68_P30401287	chr15:85367949-85367993	NM_001163634:40530	Wnt7b	INSIDE	0.920	2.876	999.78	2875.16	2.645	939.31	2484.23
A_68_P30346581	chr15:76007523-76007567	NM_201394:18596	Plec	INSIDE	0.920	2.838	841.11	2387.06	2.612	656.84	1715.37
A_68_P29701859	chr14:70930767-70930811	NM_145219:161	Lgi3	INSIDE	0.920	0.512	1913.11	979.77	0.471	1413.05	665.72
A_68_P29676506	chr14:66572378-66572422	NM_172604:181	Scara3	INSIDE	0.920	2.651	675.85	1791.38	2.439	545.09	1329.71
A_68_P28269862	chr12:17331276-17331320	NM_001159632:238	Atp6v1c2	INSIDE	0.920	2.020	1176.04	2375.39	1.857	927.31	1722.33
A_68_P26235080	chr8:125201697-125201741	NM_001109873:357	Cbfa2t3	INSIDE	0.920	0.442	3627.75	1604.33	0.407	2880.86	1172.12
A_68_P25950464	chr8:72735989-72736033	NM_001007570:170	Slc25a42	INSIDE	0.920	0.417	4791.22	2000.22	0.384	3130.14	1202.65
A_68_P25522110	chr7:137662756-137662800	NM_001136054:266	Ate1	INSIDE	0.920	2.601	1087.26	2827.53	2.391	801.08	1915.75
A_68_P22592455	chr3:142428065-142428109	NM_145546:-124	Gtf2b	PROMOTER	0.920	0.666	1520.90	1012.88	0.613	1214.72	744.41
A_68_P22362400	chr3:98945013-98945057	NM_027462:22	Wars2	INSIDE	0.920	0.304	1561.27	475.11	0.280	1479.42	414.24
A_68_P21911110	chr2:181255507-181255551	NM_016775:319	Dnajc5	INSIDE	0.920	0.620	1893.13	1173.89	0.570	1693.05	965.64
A_68_P20354546	chr1:75502170-75502214	NM_178884:835	Obsl1	INSIDE	0.920	2.056	1460.88	3003.96	1.893	1103.31	2088.09
A_68_P32141575	chr19:46072838-46072882	NM_198296:118	9130011E15Rik	INSIDE	0.919	0.524	923.17	483.49	0.481	810.39	390.00
A_68_P32116949	chr19:41817858-41817902	NM_015748:466	Slit1	INSIDE	0.919	0.650	1415.23	919.43	0.597	1159.26	692.22
A_68_P31557924	chr18:24112862-24112906	NM_027007:-304	Zfp397	PROMOTER	0.919	0.552	1161.31	641.11	0.507	938.41	476.04
A_68_P31486450	chr18:9707680-9707724	NM_130449:57	Colec12	INSIDE	0.919	0.650	1667.33	1083.85	0.597	1284.27	767.18
A_68_P28099381	chr11:105927583-105927627	NM_027346:184	Taco1	INSIDE	0.919	0.409	2355.94	962.91	0.376	1684.46	632.58
A_68_P27327919	chr10:88349039-88349083	NM_145423:324	Slc5a8	INSIDE	0.919	0.136	4421.48	600.53	0.125	3149.57	393.26
A_68_P25007844	chr7:25795497-25795541	NM_008168:61870	Grik5	INSIDE	0.919	2.400	399.89	959.77	2.207	382.73	844.55
A_68_P24151770	chr5:142939864-142939908	NM_172725:2	C330006K01Rik	INSIDE	0.919	0.293	2949.29	863.08	0.269	1976.98	531.59
A_68_P24138256	chr5:140384605-140384649	NM_175522:730	Elfn1	INSIDE	0.919	14.575	1328.04	19355.60	13.389	1187.92	15905.01
A_68_P24043268	chr5:121670665-121670709	ENSMUST00000119892:459		INSIDE	0.919	2.513	1014.87	2550.09	2.310	846.22	1955.09
A_68_P23445681	chr5:3543833-3543877	NM_001042501:22	Fam133b	INSIDE	0.919	0.513	2413.81	1239.03	0.472	1815.71	856.75
A_68_P23093081	chr4:88584341-88584385			Unknown	0.919	2.703	404.41	1093.02	2.485	362.61	901.04
A_68_P22339050	chr3:94247442-94247486	NM_030116:207	Mrpl9	INSIDE	0.919	0.337	2698.68	909.61	0.310	2128.79	659.24
A_68_P22039667	chr3:32635840-32635884	NM_025316:-122	Ndufb5	DIVERGENT_PROMOTER	0.919	1.757	1642.21	2885.75	1.616	1385.87	2238.88
A_68_P21931967	chr3:8327343-8327387			Unknown	0.919	3.848	1815.92	6987.90	3.537	1541.42	5451.79
A_68_P21633051	chr2:130889050-130889094	NM_001163529:478	Adam33	INSIDE	0.919	4.089	677.81	2771.40	3.756	478.82	1798.44
A_68_P32750681	chrX:149204383-149204427	NM_172441:-400	Shroom2	PROMOTER	0.918	1.999	786.07	1571.67	1.835	942.20	1729.28
A_68_P32023821	chr19:24552069-24552113	NM_026520:-126	Fam122a	PROMOTER	0.918	0.411	1217.98	500.49	0.377	959.05	361.94
A_68_P31351859	chr17:74928386-74928430	NM_007566:774	Birc6	INSIDE	0.918	0.291	2619.92	762.86	0.267	1925.81	514.78
A_68_P31311921	chr17:67703645-67703689	NM_008984:133	Ptprm	INSIDE	0.918	0.567	2080.68	1179.14	0.520	1605.71	835.72
A_68_P30722300	chr16:44558693-44558737	NR_037242:-472	Mir3081	PROMOTER	0.918	0.369	6032.37	2227.28	0.339	3938.35	1334.41
A_68_P25360756	chr7:106417615-106417659	NM_001043355:781	Mtap6	INSIDE	0.918	0.437	1702.54	743.65	0.401	1317.72	528.41
A_68_P24942331	chr6:148779822-148779866	NM_001081113:145	Ipo8	INSIDE	0.918	0.469	1744.95	817.81	0.430	1418.18	610.16
A_68_P24171030	chr5:147775146-147775190	NM_029581:182	Mtif3	INSIDE	0.918	0.220	2427.47	533.74	0.202	1841.42	371.64
A_68_P24137290	chr5:140212320-140212364	NM_174850:-55	Micall2	PROMOTER	0.918	0.245	4624.44	1134.70	0.225	3417.73	769.59
A_68_P24040426	chr5:121161278-121161322	NM_008052:378	Dtx1	INSIDE	0.918	0.689	2460.54	1694.27	0.632	1728.53	1093.21
A_68_P23295723	chr4:128916179-128916223	NM_001033189:9487	C77080	INSIDE	0.918	0.251	3816.97	958.67	0.231	2839.96	654.75
A_68_P22550731	chr3:134875508-134875553	NM_173762:4	Cenpe	INSIDE	0.918	0.496	2104.05	1043.30	0.455	1533.58	697.90
A_68_P22527927	chr3:130433279-130433323	NM_001199350:-53	Rpl34-ps1	PROMOTER	0.918	0.380	1512.81	574.37	0.349	1191.69	415.38
A_68_P30925306	chr16:84735619-84735663	NM_017404:-214	Mrpl39	PROMOTER	0.917	1.615	1231.34	1988.95	1.481	1048.25	1551.96
A_68_P30385796	chr15:82793740-82793784	mmu-mir-3080:-4036		PROMOTER	0.917	2.109	1855.70	3913.56	1.934	1568.26	3032.65
A_68_P29618280	chr14:56139420-56139464	NM_008736:3586	Nrl	INSIDE	0.917	2.241	856.33	1919.22	2.054	734.25	1508.27
A_68_P29267293	chr13:100894879-100894923	NM_011420:91	Smn1	INSIDE	0.917	0.697	1401.45	976.35	0.639	1288.03	822.60
A_68_P27930796	chr11:76057294-76057338	NM_183263:79	Rnmtl1	INSIDE	0.917	0.587	2042.20	1198.73	0.538	1562.25	841.02
A_68_P27896514	chr11:69495108-69495152	NM_030702:342	Senp3	INSIDE	0.917	1.742	1434.63	2499.19	1.597	1096.83	1752.16
A_68_P27889080	chr11:68198460-68198504	NM_008744:1846	Ntn1	INSIDE	0.917	0.331	2136.14	707.26	0.304	1443.56	438.29
A_68_P27843264	chr11:60290404-60290448	NM_001103171:7586	Myo15	INSIDE	0.917	1.868	943.02	1761.09	1.712	863.09	1477.28
A_68_P26554123	chr9:59425951-59425995	NM_175235:-171	Celf6	PROMOTER	0.917	0.472	2250.10	1061.02	0.433	1759.20	761.00
A_68_P26248335	chr8:127422302-127422346	NM_001111141:539	Gm505	INSIDE	0.917	0.549	2236.29	1227.92	0.504	1561.07	786.20
A_68_P25436573	chr7:121706377-121706421	NM_177382:88	Cyp2r1	INSIDE	0.917	2.726	847.85	2311.20	2.499	801.33	2002.55

ProbeName	Target position of probe on CpG island microarray	TargetID	GeneSymbol	CpG island Description	Ratio of relative methylation (TiO_2-NP/Vehicle)	Sham group			TiO_2-H group		
						Relative methylation (Cy5/Cy3)	Cy3 signal (Input)	Cy5 signal (MeDIP)	Relative methylation (Cy5/Cy3)	Cy3 signal (Input)	Cy5 signal (MeDIP)
A_68_P24114167	chr5:135462404-135462448	NM_009902:343	Cldn3	INSIDE	0.917	2.024	1128.06	2283.09	1.856	852.10	1581.83
A_68_P24023710	chr5:118426893-118426937	NM_145564:136	Fbxo21	INSIDE	0.917	3.642	708.19	2579.06	3.339	532.96	1779.30
A_68_P23596023	chr5:35104298-35104342	NM_010414:-68	Htt	PROMOTER	0.917	0.556	1500.08	833.76	0.509	1054.64	537.32
A_68_P23361259	chr4:140517609-140517653	NM_023374:445	Sdhb	INSIDE	0.917	5.729	913.56	5233.95	5.256	776.25	4079.99
A_68_P22659607	chr3:153568738-153568782	NM_031870:342	Msh4	INSIDE	0.917	2.090	1149.80	2403.34	1.917	1005.95	1928.66
A_68_P21783932	chr2:158437350-158437394	NM_009508:879	Slc32a1	INSIDE	0.917	0.083	5879.12	489.11	0.076	3692.02	281.56
A_68_P21762363	chr2:154617145-154617189	NM_001139511:321	Raly	INSIDE	0.917	0.373	2578.37	961.81	0.342	2059.79	704.39
A_68_P30572025	chr16:17111038-17111082	NM_144954:246	Ppil2	INSIDE	0.916	0.212	2063.00	437.89	0.194	1486.84	289.02
A_68_P30126798	chr15:34012651-34012695	NM_026002:199	Mtdh	INSIDE	0.916	1.892	968.18	1832.12	1.734	854.86	1482.61
A_68_P29660137	chr14:63870430-63870474	NM_008092:-6355	Gata4	PROMOTER	0.916	0.198	2449.60	485.04	0.181	1747.98	317.04
A_68_P25954497	chr8:73376700-73376744	NM_032397:4185	Kcnn1	INSIDE	0.916	2.224	987.19	2195.33	2.038	803.94	1638.55
A_68_P25090919	chr7:52308886-52308930	NM_175022:-657	Prr12	PROMOTER	0.916	0.647	1811.59	1172.20	0.593	1503.49	891.09
A_68_P25007843	chr7:25795379-25795423	NM_008168:61988	Grik5	INSIDE	0.916	1.994	675.34	1346.83	1.826	647.79	1182.88
A_68_P24954296	chr7:6107803-6107847	NM_001013012:-251	Zfp787	PROMOTER	0.916	0.456	1260.07	574.57	0.418	1032.30	431.37
A_68_P24132976	chr5:139471493-139471537	NM_008808:-607	Pdgfa	PROMOTER	0.916	3.823	1026.68	3925.28	3.503	834.46	2922.94
A_68_P24126242	chr5:137972085-137972129	NM_010312:2351	Gnb2	INSIDE	0.916	0.450	2197.42	989.36	0.412	1604.33	661.39
A_68_P22778417	chr4:22404692-22404736	NM_008899:10564	Pou3f2	DOWNSTREAM	0.916	0.378	1923.07	726.53	0.346	1511.13	522.76
A_68_P21768604	chr2:155782604-155782648	NM_001129999:333	Cep250	INSIDE	0.916	0.448	1520.67	681.06	0.410	1441.40	591.29
A_68_P21680290	chr2:139503712-139503756	NM_001126490:-179	Ism1	PROMOTER	0.916	2.172	1094.98	2377.78	1.988	797.48	1585.69
A_68_P32364033	chrX:45694836-45694880	NM_178782:324	Bcorl1	INSIDE	0.915	2.153	470.40	1012.76	1.970	750.24	1478.09
A_68_P29448699	chr14:21553663-21553707	NM_010811:100	Ndst2	INSIDE	0.915	2.775	703.20	1951.38	2.538	674.70	1712.29
A_68_P29341545	chr13:114408648-114408692	NM_130796:102	Snx18	INSIDE	0.915	1.829	1144.67	2093.05	1.673	983.12	1644.77
A_68_P29329255	chr13:112280258-112280302	NM_001122963:-31	Gpbp1	PROMOTER	0.915	0.443	4857.42	2153.53	0.406	3698.97	1500.85
A_68_P28687313	chr12:102956276-102956320	NM_028924:-6515	Tc2n	PROMOTER	0.915	2.307	831.20	1917.23	2.111	774.60	1635.31
A_68_P28084875	chr11:103224583-103224627	NM_001205236:402	Arhgap27	INSIDE	0.915	0.630	4226.52	2661.95	0.577	3021.77	1742.11
A_68_P27386041	chr10:98726685-98726729	NM_026268:842	Dusp6	INSIDE	0.915	1.737	1984.55	3446.90	1.590	1468.49	2334.94
A_68_P26880507	chr9:120843321-120843365	NM_001165902:647	Ctnnb1	INSIDE	0.915	0.490	966.77	473.48	0.448	707.96	317.29
A_68_P26674976	chr9:81526052-81526096	NM_010482:85	Htr1b	INSIDE	0.915	0.165	6874.98	1131.64	0.151	4758.87	716.75
A_68_P26659811	chr9:78328698-78328742	NM_010106:811	Eef1a1	INSIDE	0.915	0.185	2575.79	477.67	0.170	1820.44	309.04
A_68_P26139677	chr8:108939449-108939493	NM_011764:232	Zfp90	INSIDE	0.915	0.326	1454.93	474.90	0.299	1096.31	327.55
A_68_P24156073	chr5:144090815-144090859	NM_028379:69	Zdhhc4	INSIDE	0.915	0.488	4338.92	2116.26	0.446	3440.22	1534.79
A_68_P24085131	chr5:129287972-129288016	NM_001081388:171243	Rimbp2	INSIDE	0.915	2.119	1400.27	2967.42	1.938	1142.20	2213.70
A_68_P23417747	chr4:151661148-151661192	NM_008237:4601	Hes3	INSIDE	0.915	0.256	3114.82	797.77	0.234	2380.82	558.22
A_68_P22149457	chr3:54539042-54539086	NM_001163570:222	Exosc8	INSIDE	0.915	0.624	1716.34	1070.76	0.571	1242.03	709.08
A_68_P30397622	chr15:84754271-84754315	NM_016714:435	Nup50	INSIDE	0.914	0.662	2045.22	1353.69	0.605	1557.62	942.47
A_68_P28595537	chr12:85292448-85292492	NM_023633:913	2410016O06Rik	INSIDE	0.914	0.507	1193.75	605.11	0.463	985.04	456.38
A_68_P28115689	chr11:108782814-108782858	NM_015732:1174	Axin2	INSIDE	0.914	0.717	5887.15	4221.43	0.656	3876.84	2541.81
A_68_P27280212	chr10:79451564-79451608	NM_007725:242	Cnn2	INSIDE	0.914	0.164	3560.70	583.86	0.150	2301.46	345.01
A_68_P26234573	chr8:125135855-125135899	NM_021502:351	Trappc2l	INSIDE	0.914	3.626	1894.15	6868.88	3.316	1589.10	5268.75
A_68_P26022751	chr8:87460961-87461005	NM_019945:270	Mast1	INSIDE	0.914	0.686	3269.12	2243.02	0.627	2461.62	1543.83
A_68_P25090608	chr7:52263818-52263862	NM_001008422:7779	Scaf1	INSIDE	0.914	1.739	1304.84	2269.20	1.590	1096.59	1743.68
A_68_P24133862	chr5:139626121-139626165	NM_001081265:-34	Heatr2	PROMOTER	0.914	0.569	1848.52	1051.50	0.520	1330.33	691.98
A_68_P23467735	chr5:8367240-8367284	NM_001098225:819	Adam22	INSIDE	0.914	2.227	710.17	1581.22	2.034	508.13	1033.66
A_68_P22325449	chr3:90280065-90280109	NM_026374:-36	Ilf2	PROMOTER	0.914	0.699	2203.21	1539.61	0.639	1615.30	1031.80
A_68_P21904963	chr2:180333140-180333184	NM_009936:236	Col9a3	INSIDE	0.914	0.396	2986.79	1182.31	0.362	2140.98	775.03
A_68_P21428682	chr2:92299796-92299840	NM_211358:133	Slc35c1	INSIDE	0.914	0.448	1344.97	602.43	0.409	1160.54	475.06
A_68_P20646989	chr1:138521269-138521313	NM_001160251:-187	Zfp281	PROMOTER	0.914	0.407	2097.62	853.50	0.372	1599.58	594.60
A_68_P20350233	chr1:74795245-74795297	NM_153744:259	Prkag3	INSIDE	0.914	0.570	2891.86	1647.89	0.521	2010.68	1047.75
A_68_P32107646	chr19:40345679-40345723	NM_016861:406	Pdlim1	INSIDE	0.913	0.275	2481.08	683.09	0.251	1867.51	469.31
A_68_P32030084	chr19:25580234-25580278	NM_015826:61	Dmrt1	INSIDE	0.913	0.307	2869.95	881.55	0.280	2270.98	636.91
A_68_P31863851	chr18:80559845-80559889	NM_001190373:1127	Kcng2	INSIDE	0.913	0.629	1416.38	890.30	0.574	1150.57	660.46
A_68_P31734233	chr18:57292628-57292672	NM_001001979:-93	Megf10	PROMOTER	0.913	0.224	2918.37	653.59	0.204	2173.47	444.42
A_68_P31208114	chr17:46787782-46787826	NM_025611:518	Cul7	INSIDE	0.913	1.594	3998.91	6373.54	1.455	2876.19	4184.93
A_68_P31208076	chr17:46782967-46783014	NM_025302:-206	Mrpl2	DIVERGENT_PROMOTER	0.913	0.500	935.91	467.96	0.456	751.53	342.90
A_68_P29961927	chr14:122873374-122873418	NM_009574:-1209	Zic2	DIVERGENT_PROMOTER	0.913	0.488	1312.59	640.19	0.446	1145.80	510.46
A_68_P29601195	chr14:51977843-51977887	ENSMUST00000162079:3668		DOWNSTREAM	0.913	0.615	1261.84	776.65	0.562	1021.93	574.50
A_68_P29177391	chr13:81850943-81850987	NM_028372:549	Mblac2	INSIDE	0.913	0.395	1199.40	473.84	0.361	996.54	359.51
A_68_P28143846	chr11:113612079-113612123	NM_020006:374	Cdc42ep4	INSIDE	0.913	0.577	4446.61	2563.80	0.527	3168.96	1668.79
A_68_P27973306	chr11:83567372-83567416	NM_145432:256	Heatr6	INSIDE	0.913	0.355	1472.67	522.79	0.324	1096.00	355.41
A_68_P27644168	chr11:22967930-22967974	ENSMUST00000128996:-45		DIVERGENT_PROMOTER	0.913	7.189	850.64	6114.99	6.561	802.41	5264.76
A_68_P27480858	chr10:116813406-116813450	NM_001013391:601	Cpsf6	INSIDE	0.913	0.427	2065.12	882.22	0.390	1509.81	588.86
A_68_P26886027	chr9:121766646-121766690	NM_001112668:-19	Gm9790	PROMOTER	0.913	1.999	1604.14	3206.84	1.826	1287.94	2351.21
A_68_P24769162	chr6:115085438-115085482	NM_013681:541	Syn2	INSIDE	0.913	4.537	528.74	2398.85	4.144	450.18	1865.62
A_68_P24397787	chr6:42300362-42300406	NM_011777:558	Zyx	INSIDE	0.913	0.453	3588.58	1626.35	0.414	2554.54	1056.54
A_68_P24142832	chr5:141180716-141180760	NM_172724:-238	Baat1	DIVERGENT_PROMOTER	0.913	0.240	2677.98	642.92	0.219	1930.59	423.27

ProbeName	Target position of probe on CpG island microarray	TargetID	GeneSymbol	CpG island Description	Ratio of relative methylation (TiO_2-NP/Vehicle)	Sham group			TiO_2-H group		
						Relative methylation (Cy5/Cy3)	Cy3 signal (Input)	Cy5 signal (MeDIP)	Relative methylation (Cy5/Cy3)	Cy3 signal (Input)	Cy5 signal (MeDIP)
A_68_P23361908	chr4:140616772-140616816	NM_001145958:-334	Crocc	PROMOTER	0.913	0.094	5705.84	535.29	0.086	4160.22	356.20
A_68_P23335796	chr4:136327079-136327123	NM_010142:64750	Ephb2	INSIDE	0.913	2.018	1865.98	3764.62	1.841	1384.75	2549.52
A_68_P23093077	chr4:88583855-88583899			Unknown	0.913	2.099	1264.38	2653.66	1.915	926.50	1774.62
A_68_P22863908	chr4:41660593-41660637	NM_027278:-65	2810432D09Rik	PROMOTER	0.913	0.480	1682.33	808.34	0.439	1362.50	597.59
A_68_P22775353	chr4:21806849-21806894	NM_172687:50	Coq3	INSIDE	0.913	0.406	2242.24	910.11	0.370	1633.37	605.03
A_68_P21151652	chr2:38863473-38863517	NM_028809:-164	Arpc5l	DIVERGENT_PROMOTER	0.913	0.323	1727.21	557.30	0.295	1336.34	393.88
A_68_P20658352	chr1:140516392-140516436	NM_021605:-141	Nek7	PROMOTER	0.913	0.343	1296.57	444.60	0.313	1034.95	324.12
A_68_P20621315	chr1:134314164-134314208	NM_172516:157	Dstyk	INSIDE	0.913	1.827	807.85	1475.97	1.668	652.41	1088.30
A_68_P30825270	chr16:65562578-65562622	NM_026879:342	Chmp2b	INSIDE	0.912	0.570	2234.57	1273.81	0.520	1743.03	906.44
A_68_P30381573	chr15:81958322-81958366	NM_172428:-7	Ccdc134	PROMOTER	0.912	0.628	3586.98	2253.81	0.573	2672.56	1532.11
A_68_P28516078	chr12:70825634-70825678	NM_145443:205	L2hgdh	INSIDE	0.912	2.411	2799.42	6749.88	2.198	2236.69	4917.20
A_68_P27995147	chr11:87430388-87430432	NM_001045527:-255	Hsf5	PROMOTER	0.912	5.374	1010.42	5429.56	4.898	856.32	4194.34
A_68_P27176102	chr10:60150451-60150495	NM_023370:8766	Cdh23	INSIDE	0.912	0.462	1220.07	563.68	0.421	826.71	348.38
A_68_P26375211	chr9:27107465-27107509	NM_001033323:674	Igsf9b	INSIDE	0.912	0.290	2224.09	644.66	0.264	1700.52	449.75
A_68_P25906689	chr8:63369184-63369228	NM_028299:-41	2700029M09Rik	PROMOTER	0.912	0.525	1336.37	700.94	0.478	1070.59	512.05
A_68_P23202004	chr4:109338719-109338763	NM_007671:-763	Cdkn2c	PROMOTER	0.912	0.357	1508.02	537.87	0.325	1165.22	379.14
A_68_P21621791	chr2:128689584-128689628	NM_024237:-61	Fbln7	PROMOTER	0.912	0.545	3324.98	1811.16	0.497	2343.53	1163.77
A_68_P21322071	chr2:71711579-71711623	NM_172665:272	Pdk1	INSIDE	0.912	2.591	489.84	1269.24	2.363	449.47	1062.16
A_68_P21116904	chr2:32549583-32549627	NM_010236:91	Fpgs	INSIDE	0.912	0.382	2387.04	912.59	0.349	1857.88	647.59
A_68_P20827005	chr1:173200387-173200431	NM_020579:-50	B4galt3	PROMOTER	0.912	0.449	2837.21	1274.99	0.410	2088.35	856.27
A_68_P20391715	chr1:82287292-82287336	NM_010570:700	Irs1	INSIDE	0.912	0.203	3347.48	678.61	0.185	2373.30	438.61
A_68_P32241631	chrX:7651228-7651272	NM_011514:636	Suv39h1	INSIDE	0.911	2.196	626.53	1376.01	2.000	926.80	1853.29
A_68_P32229410	chr19:61301151-61301195	NM_009970:3149	Csf2ra	INSIDE	0.911	11.255	10053.96	113161.90	10.253	8740.35	89611.32
A_68_P31791992	chr18:67624679-67624723	NM_144867:198	Slmo1	INSIDE	0.911	0.224	2328.95	521.07	0.204	1865.95	380.29
A_68_P31791362	chr18:67503417-67503461	NM_007702:221	Cidea	INSIDE	0.911	0.183	6501.19	1187.51	0.166	4250.41	707.53
A_68_P31625829	chr18:36942648-36942693	NM_008214:189	Hars	INSIDE	0.911	0.434	2194.21	951.20	0.395	1627.77	642.54
A_68_P31103787	chr17:25571314-25571358	NM_001163691:-608	Cacna1h	PROMOTER	0.911	0.339	2106.99	713.50	0.309	1626.95	502.17
A_68_P28925094	chr13:31898196-31898240	NM_008592:-296	Foxc1	PROMOTER	0.911	0.381	2136.36	813.51	0.347	1596.19	553.54
A_68_P28186834	chr11:120657869-120657913	NM_026824:-181	Dus1l	PROMOTER	0.911	0.262	2193.68	574.89	0.239	1784.17	425.92
A_68_P27278594	chr10:79198492-79198536	NM_008226:19136	Hcn2	INSIDE	0.911	2.317	961.39	2227.75	2.110	828.59	1748.25
A_68_P26809957	chr9:107514932-107514976	NM_001042779:-3382	Sema3b	PROMOTER	0.911	0.238	3053.34	726.57	0.217	2115.94	458.71
A_68_P26554627	chr9:59504327-59504371	NM_011099:-66	Pkm2	PROMOTER	0.911	0.570	2459.39	1401.52	0.519	1838.20	953.86
A_68_P26246899	chr8:127186981-127187025	NM_175149:267	2310022B05Rik	INSIDE	0.911	2.262	738.34	1670.29	2.060	542.70	1118.00
A_68_P26228656	chr8:124277409-124277453	NM_020605:22972	Jph3	INSIDE	0.911	3.691	1244.73	4593.68	3.362	1035.38	3481.04
A_68_P25458531	chr7:125633376-125633420	NM_001033380:2091	Itpripl2	INSIDE	0.911	0.671	2632.42	1765.20	0.611	1886.24	1152.28
A_68_P25406574	chr7:116318231-116318275	NM_057173:-4430	Lmo1	PROMOTER	0.911	0.645	4121.32	2659.22	0.588	2966.41	1744.32
A_68_P25093838	chr7:52825605-52825649	NM_009737:-106	Bcat2	PROMOTER	0.911	0.556	2167.10	1205.09	0.507	1670.93	846.74
A_68_P24904252	chr6:142335419-142335463	NM_001204907:167	Recql	INSIDE	0.911	0.542	1642.58	889.81	0.493	1325.68	653.92
A_68_P23957857	chr5:106305585-106305629	NM_018759:20	Zfp326	INSIDE	0.911	2.388	616.34	1471.85	2.175	506.27	1100.91
A_68_P23340748	chr4:137150166-137150210	NM_130879:85	Usp48	INSIDE	0.911	0.427	1429.78	609.90	0.389	1211.59	470.83
A_68_P23232473	chr4:116782119-116782163	NM_008958:13180	Ptch2	INSIDE	0.911	2.296	1109.65	2548.07	2.092	909.56	1902.92
A_68_P22728833	chr4:11181137-11181181	NM_178112:248	Ints8	INSIDE	0.911	0.305	2446.77	746.38	0.278	1823.84	506.81
A_68_P21639427	chr2:132088879-132088923	NM_138651:-92	Cds2	DIVERGENT_PROMOTER	0.911	6.592	594.73	3920.21	6.002	488.64	2932.80
A_68_P21418381	chr2:90519379-90519423	NM_021512:2029	Nup160	INSIDE	0.911	0.658	1287.98	847.88	0.600	1036.28	621.43
A_68_P21417955	chr2:90420030-90420074	NM_008982:752	Ptprj	INSIDE	0.911	3.546	803.45	2848.85	3.229	682.03	2202.07
A_68_P20874689	chr1:182186170-182186214	NM_001128605:239	Psen2	INSIDE	0.911	1.724	1743.20	3005.70	1.571	1523.99	2394.80
A_68_P20740948	chr1:157820278-157820322	NM_001039184:85	Cep350	INSIDE	0.911	1.898	1169.17	2219.30	1.729	940.17	1625.82
A_68_P31992642	chr19:18787440-18787484	NM_177640:-263	D030056L22Rik	PROMOTER	0.910	2.190	1629.74	3569.06	1.992	1294.63	2579.24
A_68_P31936294	chr19:6904449-6904493	NM_019924:10621	Rps6ka4	INSIDE	0.910	0.565	2105.00	1188.31	0.514	1771.88	910.71
A_68_P31621832	chr18:36227944-36227988	NM_001167891:128848	Nrg2	INSIDE	0.910	2.753	1515.05	4171.31	2.504	1225.21	3068.39
A_68_P31323980	chr17:69789181-69789225	NM_001145192:537	A330050F15Rik	INSIDE	0.910	0.421	1298.05	546.97	0.384	1011.00	387.85
A_68_P31100078	chr17:25023059-25023105	NM_011956:213	Nubp2	INSIDE	0.910	2.911	687.37	2000.85	2.650	679.75	1801.17
A_68_P29484663	chr14:27852219-27852263	NM_134437:54	Il17rd	INSIDE	0.910	0.446	1470.30	655.65	0.406	1030.80	418.48
A_68_P29338121	chr13:113789081-113789125	NM_001037914:5027	Gm6320	INSIDE	0.910	0.664	1492.45	990.87	0.604	1207.97	730.17
A_68_P28582489	chr12:82833007-82833051	NM_001174107:49129	Map3k9	INSIDE	0.910	2.593	1040.37	2697.54	2.361	828.60	1956.10
A_68_P26873359	chr9:119488345-119488389	NM_021544:-232	Scn5a	PROMOTER	0.910	1.624	1785.06	2898.58	1.477	1375.87	2032.45
A_68_P25952441	chr8:73051581-73051625	NM_001111066:-39	Fkbp8	DIVERGENT_PROMOTER	0.910	0.309	2516.09	778.54	0.282	1657.91	466.87
A_68_P25589083	chr7:148661175-148661219	NM_053082:58	Tspan4	INSIDE	0.910	0.427	2810.60	1200.52	0.389	2111.53	820.48
A_68_P23382179	chr4:144836289-144836333	NM_011610:463	Tnfrsf1b	INSIDE	0.910	0.334	2216.30	740.38	0.304	1614.20	490.56
A_68_P21909037	chr2:180922593-180922637	NM_025598:567	Ppdpf	INSIDE	0.910	0.629	1514.49	952.98	0.573	1174.05	672.25
A_68_P21717108	chr2:146049089-146049133	NM_016889:1378	Insm1	INSIDE	0.910	0.454	2350.47	1066.90	0.413	1638.76	676.58
A_68_P20240054	chr1:55084325-55084372	NM_031179:-26	Sf3b1	PROMOTER	0.910	0.385	1243.31	478.48	0.350	982.61	344.30
A_68_P32565202	chrX:98892756-98892800	ENSMUST00000073812:-5589		PROMOTER	0.909	10.772	2736.19	29474.12	9.789	3745.51	36665.85
A_68_P32287122	chrX:19637760-19637804	NM_021715:1087	Chst7	INSIDE	0.909	2.833	789.17	2235.54	2.576	990.08	2550.71
A_68_P30964662	chr16:91728888-91728932	NM_026994:137	Cryzl1	INSIDE	0.909	0.431	1432.30	617.01	0.392	888.40	348.03

ProbeName	Target position of probe on CpG island microarray	TargetID	GeneSymbol	CpG island Description	Ratio of relative methylation (TiO_2-NP/Vehicle)	Sham group			TiO_2-H group		
						Relative methylation (Cy5/Cy3)	Cy3 signal (Input)	Cy5 signal (MeDIP)	Relative methylation (Cy5/Cy3)	Cy3 signal (Input)	Cy5 signal (MeDIP)
A_68_P30639250	chr16:30008917-30008961	NR_033631:186	4632428C04Rik	INSIDE	0.909	2.487	1327.31	3300.41	2.260	1092.01	2467.94
A_68_P30546792	chr16:11203231-11203275	NM_025546:133	Rsl1d1	INSIDE	0.909	0.265	3046.35	807.39	0.241	2180.93	525.23
A_68_P29247524	chr13:97440758-97440802	NM_008255:111	Hmgcr	INSIDE	0.909	3.120	777.07	2424.16	2.836	689.26	1954.91
A_68_P27290118	chr10:80961853-80961897	NM_010102:1008	S1pr4	INSIDE	0.909	2.221	1882.00	4179.81	2.018	1416.27	2858.28
A_68_P26346111	chr9:21043277-21043321	NM_001110305:478	Keap1	INSIDE	0.909	2.164	655.79	1419.30	1.968	507.34	998.59
A_68_P26345775	chr9:20970639-20970683	NM_183408:503	Pde4a	INSIDE	0.909	2.240	719.36	1611.64	2.036	603.87	1229.21
A_68_P25090604	chr7:52263215-52263259	NM_001008422:8383	Scaf1	INSIDE	0.909	1.733	1472.65	2552.29	1.575	1064.09	1675.77
A_68_P24425523	chr6:47997406-47997450	NM_001081382:685	Zfp777	INSIDE	0.909	3.750	1929.43	7235.90	3.408	1693.45	5770.60
A_68_P23203753	chr4:109652919-109652963	NM_172296:2311	Dmrta2	INSIDE	0.909	0.511	1277.92	653.57	0.465	1095.72	509.54
A_68_P21501839	chr2:106535501-106535545	NM_029837:-235	Mpped2	PROMOTER	0.909	0.404	1794.19	725.29	0.367	1414.85	519.74
A_68_P21102299	chr2:30141747-30141791	NM_177648:106	Dolk	INSIDE	0.909	0.297	2582.41	766.36	0.270	1957.72	527.95
A_68_P20632578	chr1:136195654-136195698	NM_001144855:33829	Ppfia4	INSIDE	0.909	0.389	1384.11	538.34	0.353	1124.85	397.61
A_68_P32408596	chrX:56387279-56387323	NM_010200:313	Fgf13	INSIDE	0.908	2.323	2136.34	4961.67	2.110	2360.81	4981.23
A_68_P31731073	chr18:56722210-56722254	NM_001162989:10	Phax	INSIDE	0.908	0.473	1459.25	690.54	0.430	1058.09	454.59
A_68_P31123435	chr17:28759630-28759674	NM_016795:-253	Srpk1	PROMOTER	0.908	0.422	1556.91	656.58	0.383	1188.48	455.25
A_68_P30657579	chr16:33381453-33381497	NM_011749:614	Zfp148	INSIDE	0.908	0.278	2017.85	561.87	0.253	1571.62	397.52
A_68_P30347595	chr15:76161468-76161512	NM_010331:-233	Gpaa1	PROMOTER	0.908	0.306	1715.98	524.83	0.278	1179.41	327.39
A_68_P28578489	chr12:82047116-82047160	NM_001079694:648	Srsf5	INSIDE	0.908	4.017	669.32	2688.86	3.647	603.22	2200.11
A_68_P28529999	chr12:73171616-73171660	NM_181752:340	Gpr135	INSIDE	0.908	0.084	5695.16	479.80	0.076	3977.50	304.23
A_68_P26585375	chr9:64868889-64868933	NM_021345:614	Ptplad1	INSIDE	0.908	0.596	1855.26	1105.73	0.541	1633.70	883.67
A_68_P24112020	chr5:134697723-134697767	NM_010876:7751	Ncf1	INSIDE	0.908	0.434	2806.56	1217.84	0.394	1737.75	684.58
A_68_P23889172	chr5:91946764-91946808	NM_145562:61	Parm1	INSIDE	0.908	1.628	2333.26	3797.74	1.478	2013.95	2976.83
A_68_P23417745	chr4:151660964-151661008	NM_008237:4785	Hes3	INSIDE	0.908	0.531	1050.25	557.23	0.482	854.11	411.45
A_68_P23331723	chr4:135577209-135577253	NM_013736:334	Tceb3	INSIDE	0.908	0.670	2545.98	1705.78	0.608	1991.21	1211.42
A_68_P23234756	chr4:117167070-117167114	NM_025739:2428	Rnf220	INSIDE	0.908	7.100	1109.52	7878.01	6.448	979.24	6314.32
A_68_P23193625	chr4:107595986-107596030	NM_009949:186	Cpt2	INSIDE	0.908	0.383	1731.00	662.16	0.347	1292.61	449.00
A_68_P22360099	chr3:98232622-98232666	NM_172863:46241	Zfp697	INSIDE	0.908	0.478	1080.17	516.71	0.434	1004.75	436.55
A_68_P22262794	chr3:79090694-79090738	AK032506:-597		PROMOTER	0.908	3.003	1005.76	3020.12	2.725	928.89	2531.44
A_68_P21345222	chr2:75497220-75497264	NM_053263:-73	Hnrnpa3	PROMOTER	0.908	0.577	1377.79	795.66	0.524	1027.39	538.46
A_68_P20427304	chr1:90967161-90967205	NM_133816:146	Sh3bp4	INSIDE	0.908	0.659	1220.93	804.11	0.598	1076.61	643.93
A_68_P32383357	chrX:49965510-49965554	NM_016697:1619	Gpc3	INSIDE	0.907	2.571	676.59	1739.32	2.331	638.89	1488.95
A_68_P32132967	chr19:44637357-44637401	NM_176958:35	Hif1an	INSIDE	0.907	3.577	803.83	2875.50	3.244	659.85	2140.59
A_68_P32114044	chr19:41338030-41338074	NM_133352:442	Tm9sf3	INSIDE	0.907	2.158	896.09	1933.57	1.957	728.50	1425.39
A_68_P31405197	chr17:84911642-84911686	NM_177606:430	Plekhh2	INSIDE	0.907	0.286	1598.69	456.79	0.259	1270.44	329.39
A_68_P31381050	chr17:80461904-80461948	NM_144802:-252	Hnrpll	PROMOTER	0.907	0.469	1224.11	574.51	0.426	972.57	413.86
A_68_P30475880	chr15:98507518-98507562	NM_172612:352	Rnd1	INSIDE	0.907	0.317	2427.06	768.20	0.287	1918.54	550.77
A_68_P30382915	chr15:82176602-82176646	NM_026914:149	1500032L24Rik	INSIDE	0.907	0.453	1275.79	577.37	0.411	995.06	408.58
A_68_P30287852	chr15:65618774-65618818	NM_133766:194	Efr3a	INSIDE	0.907	2.898	309.09	895.82	2.628	270.45	710.85
A_68_P29241106	chr13:96295014-96295058	NM_007974:159	F2rl1	INSIDE	0.907	0.706	1891.28	1335.36	0.641	1442.90	924.25
A_68_P28091583	chr11:104411776-104411820	NM_145436:136	Cdc27	INSIDE	0.907	2.610	707.85	1847.66	2.369	550.52	1304.04
A_68_P27271615	chr10:77472561-77472605	NM_008826:-41	Pfkl	PROMOTER	0.907	0.368	1463.68	538.70	0.334	1139.92	380.49
A_68_P26915294	chr10:6373391-6373435	NM_001170785:54	Mthfd1l	INSIDE	0.907	0.549	2922.14	1603.89	0.498	1953.04	971.93
A_68_P26822740	chr9:110148381-110148425	NM_001166273:2116	Cspg5	INSIDE	0.907	0.229	6451.54	1474.44	0.207	4392.53	910.33
A_68_P26236468	chr8:125388849-125388893	NM_007662:16597	Cdh15	INSIDE	0.907	2.141	871.63	1866.16	1.941	807.19	1566.90
A_68_P26220856	chr8:123061767-123061811	NM_001145896:480	Gse1	INSIDE	0.907	0.694	2699.32	1873.92	0.629	2184.36	1374.67
A_68_P25962048	chr8:75072006-75072050	NM_027485:181	Med26	INSIDE	0.907	2.229	1097.50	2446.34	2.022	949.25	1919.42
A_68_P25082379	chr7:50699460-50699504	NM_026695:-30	Etfb	PROMOTER	0.907	0.625	2325.80	1453.27	0.567	1719.64	974.29
A_68_P24928968	chr6:146450992-146451036	NM_010586:-580	Itpr2	PROMOTER	0.907	0.555	1426.06	791.97	0.504	1135.93	572.40
A_68_P24122090	chr5:137042535-137042579	NM_198602:719	Cux1	INSIDE	0.907	0.394	1775.89	699.70	0.357	1463.12	523.03
A_68_P23527584	chr5:21243055-21243099	NM_028431:99	Pmpcb	INSIDE	0.907	4.587	1179.54	5410.57	4.161	911.24	3791.80
A_68_P23223183	chr4:114736145-114736189	NM_011527:4035	Tal1	INSIDE	0.907	3.337	550.65	1837.45	3.028	502.84	1522.52
A_68_P22715810	chr4:8617868-8617912	NM_001081417:-177	Chd7	PROMOTER	0.907	5.616	464.89	2610.98	5.093	471.65	2402.30
A_68_P22339047	chr3:94247097-94247141	NM_030116:-139	Mrpl9	DIVERGENT_PROMOTER	0.907	0.695	2167.92	1505.63	0.630	1688.95	1063.37
A_68_P21116979	chr2:32563681-32563725	NM_130860:4602	Cdk9	INSIDE	0.907	1.707	2329.22	3977.06	1.549	1878.21	2908.75
A_68_P21094160	chr2:28773494-28773538	NM_001164186:-1556	Barhl1	PROMOTER	0.907	2.694	278.66	750.69	2.442	328.70	802.81
A_68_P20378194	chr1:79855540-79855584	NM_009255:-322	Serpine2	PROMOTER	0.907	0.252	1954.21	491.75	0.228	1572.83	358.90
A_68_P32266422	chrX:13063296-13063340	NM_173415:18521	Nyx	INSIDE	0.906	3.800	654.70	2487.66	3.441	718.77	2473.12
A_68_P32031151	chr19:25746889-25746933	NM_145831:10	Dmrt2	INSIDE	0.906	0.369	1439.07	530.77	0.334	1080.92	361.21
A_68_P31329273	chr17:70865582-70865626	NM_001128180:-5845	Dlgap1	PROMOTER	0.906	1.723	1547.87	2666.89	1.561	1225.36	1913.01
A_68_P29961926	chr14:122873265-122873309	NM_009574:-1319	Zic2	DIVERGENT_PROMOTER	0.906	0.388	2863.90	1112.21	0.352	2137.51	751.93
A_68_P29050102	chr13:54666387-54666431	NM_133797:335	4833439L19Rik	INSIDE	0.906	0.183	3231.37	591.06	0.166	2395.32	396.92
A_68_P28939818	chr13:34744400-34744444	NM_025831:128	1300014I06Rik	INSIDE	0.906	0.424	1359.07	575.65	0.384	1075.31	412.85
A_68_P28842022	chr13:15555028-15555072	NM_008130:-505	Gli3	PROMOTER	0.906	9.851	2444.10	24076.94	8.920	1752.19	15630.02
A_68_P28832392	chr13:12553513-12553557	NM_001199043:471	Lgals8	INSIDE	0.906	0.565	1122.39	633.93	0.512	933.77	477.90
A_68_P28181983	chr11:119908193-119908237	NM_001198787:-5119	Aatk	PROMOTER	0.906	0.231	2877.44	665.94	0.210	2074.09	434.87

ProbeName	Target position of probe on CpG island microarray	TargetID	GeneSymbol	CpG island Description	Ratio of relative methylation (TiO_2-NP/Vehicle)	Sham group			TiO_2-H group		
						Relative methylation (Cy5/Cy3)	Cy3 signal (Input)	Cy5 signal (MeDIP)	Relative methylation (Cy5/Cy3)	Cy3 signal (Input)	Cy5 signal (MeDIP)
A_68_P28154358	chr11:115465110-115465154	NM_173048:88	Gga3	INSIDE	0.906	0.614	3784.30	2322.79	0.556	2764.79	1537.74
A_68_P27502796	chr10:120748000-120748044	NM_029057:223	Tbc1d30	INSIDE	0.906	0.483	1058.00	511.32	0.438	777.17	340.36
A_68_P26887823	chr9:122075943-122075987	NM_001164572:49581	Snrk	INSIDE	0.906	2.098	1427.54	2995.17	1.902	1179.64	2243.11
A_68_P25406005	chr7:116226702-116226746	NM_001038624:114	Ric3	INSIDE	0.906	2.164	1119.24	2422.51	1.961	987.08	1935.67
A_68_P24467402	chr6:55067829-55067873	NM_009953:15116	Crhr2	INSIDE	0.906	0.384	2889.88	1108.77	0.348	2123.86	738.47
A_68_P24152506	chr5:143084638-143084682	NM_175217:46	Mmd2	INSIDE	0.906	0.338	2228.20	753.18	0.306	1692.15	518.12
A_68_P23339473	chr4:136913085-136913129	NM_009861:546	Cdc42	INSIDE	0.906	2.653	1369.30	3633.43	2.404	1298.44	3121.54
A_68_P21771125	chr2:156246349-156246393	NM_001003815:-417	Epb4.1l1	PROMOTER	0.906	0.387	1236.63	479.02	0.351	869.98	305.42
A_68_P21103436	chr2:30328038-30328093	NM_030244:1654	Ier5l	DOWNSTREAM	0.906	2.558	409.89	1048.55	2.317	294.36	682.16
A_68_P20453279	chr1:94998401-94998445	NM_001110315:20	Kif1a	INSIDE	0.906	0.527	1370.27	721.62	0.477	1126.38	537.14
A_68_P20345418	chr1:73954077-73954121	NM_027884:216923	Tns1	DOWNSTREAM	0.906	5.120	999.49	5117.64	4.638	772.08	3580.90
A_68_P32362519	chrX:45387638-45387682	NM_013912:369	Apln	INSIDE	0.905	2.158	1918.86	4140.62	1.952	2189.64	4274.91
A_68_P31123768	chr17:28828786-28828830	NM_001168513:-704	Mapk14	PROMOTER	0.905	0.667	2785.62	1859.02	0.604	1939.16	1171.12
A_68_P31016356	chr17:5342041-5342085	NM_001085355:346989	Arid1b	INSIDE	0.905	2.327	1005.75	2340.64	2.106	713.89	1503.56
A_68_P30476482	chr15:98622981-98623025	NM_021279:2715	Wnt1	INSIDE	0.905	1.824	926.42	1689.34	1.651	751.40	1240.24
A_68_P30475795	chr15:98493178-98493222	NM_001080981:120	Ddx23	INSIDE	0.905	0.735	2284.86	1679.38	0.665	1723.31	1146.70
A_68_P30140501	chr15:36723049-36723093	NM_011740:1223	Ywhaz	INSIDE	0.905	2.775	1079.64	2996.25	2.511	860.74	2161.53
A_68_P27950592	chr11:79524502-79524546	NR_029579:-946	Mir193	PROMOTER	0.905	0.263	2821.41	741.09	0.238	1960.00	465.81
A_68_P27840802	chr11:59919053-59919097	NM_009021:560	Rai1	INSIDE	0.905	0.315	4809.20	1516.27	0.285	3322.08	947.62
A_68_P27280008	chr10:79422964-79423008	NM_175450:90	Wdr18	INSIDE	0.905	0.494	1870.26	923.39	0.447	1307.33	583.95
A_68_P27223320	chr10:68815779-68815823	NM_007659:-140	Cdk1	PROMOTER	0.905	0.300	2051.32	616.39	0.272	1716.59	466.67
A_68_P26844380	chr9:114294100-114294144	NM_019922:5708	Crtap	INSIDE	0.905	2.077	554.60	1152.00	1.880	490.43	921.82
A_68_P26508621	chr9:51086818-51086862	NM_001195681:-181	Gm684	PROMOTER	0.905	0.500	1624.92	812.63	0.452	1199.80	542.80
A_68_P26061534	chr8:94325346-94325390	NM_008393:-95	Irx3	PROMOTER	0.905	0.196	3741.42	731.91	0.177	2785.34	493.23
A_68_P26022611	chr8:87436417-87436461	NM_019945:24814	Mast1	INSIDE	0.905	1.782	1101.78	1963.41	1.613	772.53	1245.87
A_68_P25584911	chr7:147973663-147973707	NM_001143765:69	Syce1	INSIDE	0.905	3.135	905.11	2837.20	2.835	758.49	2150.67
A_68_P25108777	chr7:56136031-56136075	NM_001013368:359	E2f8	INSIDE	0.905	0.607	1213.75	737.33	0.550	988.08	543.13
A_68_P20354315	chr1:75472667-75472712	NM_001001565:3357	Chpf	INSIDE	0.905	3.302	709.77	2343.56	2.988	503.47	1504.24
A_68_P31501877	chr18:12802627-12802671	NM_028341:607	Ttc39c	INSIDE	0.904	0.609	2319.93	1413.88	0.551	1972.10	1086.40
A_68_P31112726	chr17:26978463-26978507	NM_008700:26	Nkx2-5	INSIDE	0.904	3.217	1222.14	3931.18	2.906	1080.85	3141.41
A_68_P30346616	chr15:76011981-76012026	NM_201394:14137	Plec	INSIDE	0.904	4.707	307.14	1445.62	4.256	234.27	997.08
A_68_P29523393	chr14:34133081-34133125	NM_153800:103094	Arhgap22	INSIDE	0.904	0.643	1809.78	1164.42	0.582	1317.49	766.64
A_68_P29330069	chr13:112476100-112476144	NM_172593:-263	Mier3	PROMOTER	0.904	0.489	1927.65	942.17	0.442	1382.74	610.63
A_68_P28939819	chr13:34744472-34744516	NM_025831:56	1300014I06Rik	INSIDE	0.904	0.480	1641.73	788.64	0.434	1417.63	615.68
A_68_P28069094	chr11:100480931-100480975	NM_019795:530	Dnajc7	INSIDE	0.904	1.619	2580.37	4176.51	1.464	1816.85	2659.84
A_68_P27282061	chr10:79727895-79727939	NM_001122604:181	Dazap1	INSIDE	0.904	3.671	1663.82	6107.27	3.319	1322.66	4390.05
A_68_P26217006	chr8:122480748-122480792	NM_009462:46019	Usp10	INSIDE	0.904	4.141	660.73	2736.08	3.742	554.75	2075.84
A_68_P25286998	chr7:91300735-91300779	NM_133722:-353	Fam108c	PROMOTER	0.904	1.677	1624.89	2725.28	1.516	1178.22	1785.62
A_68_P24764862	chr6:114232614-114232658	NM_178703:8	Slc6a1	INSIDE	0.904	27.052	3948.08	106802.50	24.468	2654.89	64959.45
A_68_P23979132	chr5:110771450-110771494	NM_001164833:582	P2rx2	INSIDE	0.904	0.435	1083.67	471.94	0.394	836.84	329.37
A_68_P22413527	chr3:108395008-108395052	NM_181400:835	Wdr47	INSIDE	0.904	0.321	1396.85	447.92	0.290	1145.09	332.04
A_68_P22148831	chr3:54437065-54437110	ENSMUST00000172420:1727		INSIDE	0.904	2.381	891.75	2123.44	2.152	674.24	1450.65
A_68_P21752416	chr2:152840886-152840930	NM_178939:211	Pdrg1	INSIDE	0.904	0.560	3403.97	1905.28	0.506	2615.25	1323.51
A_68_P21622819	chr2:128891980-128892024	NM_027192:320	Ttl	INSIDE	0.904	0.405	1122.49	454.61	0.366	985.89	361.13
A_68_P21425371	chr2:91762841-91762885	NM_007699:517	Chrm4	INSIDE	0.904	0.344	2228.94	767.86	0.311	1736.01	540.49
A_68_P21152634	chr2:39045653-39045697	NM_178778:576	Scai	INSIDE	0.904	0.360	1659.34	597.75	0.326	1228.83	400.24
A_68_P20619891	chr1:134087186-134087230	NM_001033250:-804	Lemd1	PROMOTER	0.904	0.501	977.12	489.32	0.453	793.71	359.28
A_68_P20038624	chr1:12986493-12986537	NM_172841:-5298	Slco5a1	PROMOTER	0.904	1.882	1464.86	2756.34	1.700	1042.00	1771.65
A_68_P31622630	chr18:36357100-36357144	NM_001167891:-308	Nrg2	PROMOTER	0.903	0.289	1998.45	576.87	0.261	1421.08	370.57
A_68_P31613999	chr18:34811095-34811139	NM_178347:274	Cdc23	INSIDE	0.903	0.367	5336.82	1958.22	0.331	3681.39	1220.31
A_68_P30980739	chr16:94550006-94550050	NM_133229:0	Ripply3	INSIDE	0.903	1.922	788.57	1516.01	1.735	608.89	1056.59
A_68_P30964445	chr16:91689232-91689276	NM_021720:-281	Donson	PROMOTER	0.903	2.230	1053.66	2350.02	2.013	797.97	1606.53
A_68_P30784944	chr16:57231378-57231422	NM_026254:179	Tbc1d23	INSIDE	0.903	0.391	1437.70	562.81	0.354	1096.14	387.68
A_68_P27835020	chr11:58948880-58948924	NM_001171512:975	Obscn	INSIDE	0.903	2.120	2218.01	4701.96	1.915	1855.02	3552.23
A_68_P27279615	chr10:79365943-79365988	NM_001163276:5718	Med16	INSIDE	0.903	2.740	2382.87	6529.75	2.475	1834.98	4541.85
A_68_P25448126	chr7:123587084-123587128	NM_011083:-134	Pik3c2a	PROMOTER	0.903	0.198	9770.16	1931.75	0.178	6281.99	1121.33
A_68_P25229723	chr7:80682702-80682746	NM_001081345:3908	Chd2	INSIDE	0.903	0.643	9569.04	6155.18	0.581	6731.84	3912.20
A_68_P24642399	chr6:91928996-91929040	NM_172731:-8085	Fgd5	PROMOTER	0.903	0.385	1443.38	555.50	0.348	1335.99	464.40
A_68_P24151771	chr5:142939947-142939991	NM_172725:84	C330006K01Rik	INSIDE	0.903	0.260	3314.00	862.06	0.235	2007.88	471.59
A_68_P24114164	chr5:135462079-135462123	NM_009902:17	Cldn3	INSIDE	0.903	0.211	4360.27	920.14	0.191	3213.02	612.23
A_68_P23312412	chr4:132019695-132019742	NM_026039:118	Med18	INSIDE	0.903	0.589	1143.48	673.48	0.532	969.61	515.56
A_68_P23296884	chr4:129168378-129168422	NM_001085491:-33	1700125D06Rik	DIVERGENT_PROMOTER	0.903	0.427	1226.65	523.18	0.385	998.18	384.59
A_68_P21820019	chr2:164658808-164658852	NM_001082974:266	Neurl2	INSIDE	0.903	0.335	2360.79	789.71	0.302	1846.78	557.95
A_68_P32237967	chrX:6895968-6896012	ENSMUST00000132126:306		INSIDE	0.902	2.662	319.74	851.21	2.400	451.59	1083.87
A_68_P32148597	chr19:47327774-47327818	NM_001163480:24464	Neurl1a	INSIDE	0.902	2.622	558.26	1463.83	2.365	444.48	1051.31

ProbeName	Target position of probe on CpG island microarray	TargetID	GeneSymbol	CpG island Description	Ratio of relative methylation (TiO_2-NP/Vehicle)	Sham group			TiO_2-H group		
						Relative methylation (Cy5/Cy3)	Cy3 signal (Input)	Cy5 signal (MeDIP)	Relative methylation (Cy5/Cy3)	Cy3 signal (Input)	Cy5 signal (MeDIP)
A_68_P31932932	chr19:6140654-6140698	NM_019722:461	Arl2	INSIDE	0.902	2.092	523.52	1095.22	1.887	449.18	847.82
A_68_P31876064	chr18:82575063-82575107	NM_008082:1085	Galr1	INSIDE	0.902	1.773	1570.90	2784.53	1.599	1243.14	1988.06
A_68_P31113296	chr17:27076589-27076633	NM_026948:-187	Cuta	PROMOTER	0.902	1.870	613.70	1147.62	1.686	606.52	1022.73
A_68_P28730244	chr12:110074293-110074337	NM_181328:-228	Slc25a29	DIVERGENT_PROMOTER	0.902	0.515	1903.77	980.63	0.465	1475.43	685.73
A_68_P28612730	chr12:88224807-88224851	NM_145836:936	6430527G18Rik	INSIDE	0.902	2.373	2007.47	4763.48	2.139	1535.96	3286.12
A_68_P28602202	chr12:86452173-86452217	NM_030225:412	Dlst	INSIDE	0.902	2.358	811.70	1913.90	2.126	713.07	1516.12
A_68_P27965932	chr11:82202594-82202638	NM_023438:215	Tmem132e	INSIDE	0.902	0.625	1251.69	782.35	0.564	986.50	556.35
A_68_P27543028	chr10:128184928-128184972	NM_001170869:-627	Wibg	PROMOTER	0.902	0.234	1985.71	465.40	0.211	1521.78	321.63
A_68_P27044453	chr10:31165715-31165759	NM_009413:-9	Tpd52l1	PROMOTER	0.902	1.701	1121.22	1906.95	1.534	950.26	1458.04
A_68_P26291033	chr9:8134257-8134301	NM_001045524:16	AK129341	INSIDE	0.902	2.537	684.54	1736.59	2.289	652.81	1493.96
A_68_P24591293	chr6:83028169-83028213	NM_197992:-193	Pcgf1	DIVERGENT_PROMOTER	0.902	0.527	2342.66	1235.41	0.476	1712.68	815.02
A_68_P23437238	chr4:154596264-154596308	NM_011385:358	Ski	INSIDE	0.902	0.584	1555.88	908.82	0.527	1118.80	589.16
A_68_P23057175	chr4:82151052-82151103	NM_001113209:135	Nfib	INSIDE	0.902	18.360	1851.86	33999.53	16.560	1362.18	22558.11
A_68_P22856569	chr4:40215391-40215435	NM_134097:1462	Topors	INSIDE	0.902	2.466	579.32	1428.39	2.224	543.91	1209.41
A_68_P22696143	chr4:4065948-4065992	NM_001002927:-378	Penk	PROMOTER	0.902	2.371	565.63	1340.93	2.137	511.77	1093.83
A_68_P22558746	chr3:136334203-136334247	NM_008913:491	Ppp3ca	INSIDE	0.902	0.461	6106.69	2813.98	0.416	4172.32	1734.72
A_68_P22383505	chr3:102740035-102740079	NM_011516:-33	Sycp1	PROMOTER	0.902	1.804	1111.96	2005.93	1.627	973.37	1583.60
A_68_P22069524	chr3:38384123-38384167	NM_001167883:-406	Ankrd50	PROMOTER	0.902	0.255	3571.89	912.13	0.230	2713.78	625.11
A_68_P21536547	chr2:113598045-113598089	NM_011824:739	Grem1	INSIDE	0.902	0.561	924.80	518.54	0.506	821.84	415.58
A_68_P31603478	chr18:32719306-32719350	NM_001048207:360	Gypc	INSIDE	0.901	0.554	1419.50	787.02	0.500	1113.55	556.45
A_68_P29753349	chr14:80170468-80170512	NM_001042726:629	Pcdh8	INSIDE	0.901	0.436	1362.34	593.30	0.392	1084.74	425.59
A_68_P29259035	chr13:99465145-99465189	NM_001025606:-380	Tmem171	PROMOTER	0.901	0.303	2239.74	677.76	0.273	1859.58	506.95
A_68_P28182352	chr11:119960513-119960557	NM_001110242:287	1810043H04Rik	INSIDE	0.901	0.474	1404.42	665.41	0.427	997.24	425.57
A_68_P27750957	chr11:43342796-43342840	NM_001045530:533	Ccnjl	INSIDE	0.901	0.378	2535.10	958.77	0.341	1689.26	575.44
A_68_P25589535	chr7:148725503-148725549	NM_001142681:230	Chid1	INSIDE	0.901	1.563	3784.61	5913.95	1.408	2285.40	3216.95
A_68_P25447481	chr7:123451771-123451815	ENSMUST00000106605:98		INSIDE	0.901	0.537	4346.64	2335.65	0.484	3146.16	1523.81
A_68_P25377676	chr7:109358649-109358693	NM_022979:-36	Nup98	PROMOTER	0.901	1.804	940.96	1697.87	1.625	746.08	1212.62
A_68_P25196753	chr7:75097151-75097195	NM_010513:30	Igf1r	INSIDE	0.901	5.030	1493.72	7513.88	4.531	1249.09	5659.80
A_68_P24925494	chr6:145814701-145814745	NM_024469:-862	Bhlhe41	PROMOTER	0.901	0.566	3059.35	1731.58	0.510	2116.12	1079.66
A_68_P24050684	chr5:122952627-122952671	NM_009722:-414	Atp2a2	PROMOTER	0.901	0.751	4717.03	3540.91	0.676	3554.97	2403.50
A_68_P23971131	chr5:108741769-108741813	NM_001081234:-153	Pigg	PROMOTER	0.901	0.361	1674.64	604.93	0.326	1413.53	460.17
A_68_P23746641	chr5:64040459-64040503	NM_177006:139	3110047P20Rik	INSIDE	0.901	2.965	229.51	680.41	2.672	236.64	632.28
A_68_P23351275	chr4:138908526-138908570	NM_001039200:41	C230096C10Rik	INSIDE	0.901	1.705	3380.59	5765.09	1.537	2426.12	3727.84
A_68_P21844246	chr2:168760814-168760858	NM_009564:20251	Zfp64	INSIDE	0.901	4.718	2552.60	12041.99	4.252	1973.99	8394.12
A_68_P20997496	chr2:9804242-9804286	NR_024257:1392	4930412O13Rik	INSIDE	0.901	0.434	1283.19	556.65	0.391	935.63	365.74
A_68_P20286841	chr1:63492318-63492362	NM_001177600:-137	Adam23	PROMOTER	0.901	2.227	933.53	2079.25	2.007	849.97	1706.00
A_68_P32603679	chrX:109714349-109714393	NM_177747:236	Zfp711	INSIDE	0.900	2.590	335.98	870.25	2.332	540.51	1260.66
A_68_P32132943	chr19:44629747-44629791	NM_026061:137	Ndufb8	INSIDE	0.900	0.248	6850.83	1697.90	0.223	4620.86	1030.69
A_68_P31921845	chr19:4215539-4215583	NM_019952:1169	Clcf1	INSIDE	0.900	0.180	4777.35	858.79	0.162	3469.97	561.69
A_68_P31631189	chr18:38129379-38129423	NM_175684:-15	Fchsd1	PROMOTER	0.900	0.202	2357.95	476.80	0.182	1684.26	306.41
A_68_P31259696	chr17:57170907-57170951	NM_010613:2	Khsrp	INSIDE	0.900	0.654	1413.14	924.28	0.589	1137.44	669.71
A_68_P30533736	chr16:8672646-8672705	NM_025821:-429	Carhsp1	PROMOTER	0.900	15.253	1037.43	15823.95	13.731	793.81	10900.11
A_68_P30019345	chr15:11925242-11925286	NM_011294:498	Sub1	INSIDE	0.900	0.302	2369.35	716.01	0.272	1840.46	500.59
A_68_P29625864	chr14:57676795-57676839	NM_016975:-34	Gja3	PROMOTER	0.900	2.116	1190.47	2518.51	1.903	899.59	1712.31
A_68_P27316751	chr10:86371555-86371599	NM_138673:99111	Stab2	INSIDE	0.900	1.688	1198.14	2022.71	1.519	983.74	1494.37
A_68_P27281950	chr10:79712237-79712281	NM_029272:62	Ndufs7	INSIDE	0.900	0.541	2699.04	1460.67	0.487	2146.68	1046.09
A_68_P27054668	chr10:33739164-33739208	NM_025614:236	Rwdd1	INSIDE	0.900	0.495	1085.50	537.45	0.446	974.44	434.20
A_68_P26801800	chr9:106101470-106101514	NM_198931:-222	Ppm1m	PROMOTER	0.900	2.248	518.94	1166.56	2.022	534.88	1081.59
A_68_P26779715	chr9:102255528-102255572	NM_001168296:1473	Ephb1	INSIDE	0.900	1.722	1660.14	2859.01	1.551	1372.41	2127.96
A_68_P26346391	chr9:21095271-21095315	NM_009878:361	Cdkn2d	INSIDE	0.900	0.244	3054.47	745.80	0.220	2324.08	510.54
A_68_P26234160	chr8:125074869-125074913	NM_001037298:339	Fam38a	INSIDE	0.900	3.920	936.07	3669.20	3.528	729.21	2572.53
A_68_P25186369	chr7:73254042-73254086	NM_001081163:-336	Chsy1	PROMOTER	0.900	0.609	1885.58	1148.26	0.548	1636.89	897.53
A_68_P24325223	chr6:29162126-29162170	NM_011829:123	Impdh1	INSIDE	0.900	1.949	5662.85	11038.47	1.754	3858.89	6768.95
A_68_P24053485	chr5:123465296-123465340	NM_175423:236	Orai1	INSIDE	0.900	3.951	819.11	3236.23	3.555	658.93	2342.68
A_68_P24050007	chr5:122822159-122822211	NM_026883:-212	1500011H22Rik	DIVERGENT_PROMOTER	0.900	3.186	565.97	1803.16	2.868	484.85	1390.76
A_68_P23364695	chr4:141101558-141101602	NR_030695:-496	B330016D10Rik	DIVERGENT_PROMOTER	0.900	1.987	1093.74	2172.92	1.787	858.68	1534.59
A_68_P21780068	chr2:157767229-157767273	NM_198627:26862	Vstm2l	INSIDE	0.900	0.272	2063.93	560.63	0.245	1597.72	390.79
A_68_P21761656	chr2:154482914-154482958	NM_029362:175	Chmp4b	INSIDE	0.900	2.669	1832.20	4889.34	2.402	1423.10	3418.31
A_68_P21109954	chr2:31428423-31428467	NM_001033389:200	Fubp3	INSIDE	0.900	0.504	1586.15	799.16	0.454	1355.15	614.72
A_68_P21068909	chr2:24790606-24790650	NM_001162485:173	Arrdc1	INSIDE	0.900	0.504	1985.54	1000.76	0.454	1566.36	710.51
A_68_P32536211	chrX:91077428-91077472	NM_145630:-16	Pdk3	PROMOTER	0.899	2.657	924.33	2455.56	2.389	1094.53	2614.57
A_68_P32213508	chr19:58528871-58528915	NM_010279:64	Gfra1	INSIDE	0.899	0.446	3461.05	1543.70	0.401	2573.51	1031.84
A_68_P32075619	chr19:33466689-33466733	NM_001146342:75	Rnls	INSIDE	0.899	1.629	1422.84	2317.94	1.464	1148.75	1681.67
A_68_P31927773	chr19:5272985-5273029	NM_153129:113	Pacs1	INSIDE	0.899	2.048	672.87	1378.32	1.841	613.17	1128.60
A_68_P31925522	chr19:4867768-4867812	NM_013456:10094	Actn3	INSIDE	0.899	2.190	2060.96	4513.56	1.968	1658.72	3264.48

ProbeName	Target position of probe on CpG island microarray	TargetID	GeneSymbol	CpG island Description	Ratio of relative methylation (TiO_2-NP/Vehicle)	Sham group			TiO_2-H group		
						Relative methylation (Cy5/Cy3)	Cy3 signal (Input)	Cy5 signal (MeDIP)	Relative methylation (Cy5/Cy3)	Cy3 signal (Input)	Cy5 signal (MeDIP)
A_68_P31675572	chr18:46358216-46358260	NM_001170855:234	Trim36	INSIDE	0.899	0.600	1276.32	765.38	0.539	1011.44	545.57
A_68_P29041945	chr13:53206732-53206776	NM_013846:174724	Ror2	INSIDE	0.899	3.338	814.45	2718.85	3.002	551.77	1656.40
A_68_P28158451	chr11:116144755-116144799	NM_010254:2524	Galr2	INSIDE	0.899	2.341	851.57	1993.87	2.106	670.62	1412.37
A_68_P28068130	chr11:100302472-100302516	NM_026561:-91	Nt5c3l	DIVERGENT_PROMOTER	0.899	0.259	1802.88	466.80	0.233	1317.46	306.82
A_68_P27640628	chr11:22186580-22186624	NM_153078:-761	Ehbp1	PROMOTER	0.899	0.647	2064.96	1336.01	0.582	1616.03	940.20
A_68_P27566543	chr11:5965060-5965104	NM_001174053:669	Camk2b	INSIDE	0.899	2.204	1815.71	4002.03	1.981	1536.91	3044.07
A_68_P27513153	chr10:122633430-122633474	NM_027604:527	Usp15	INSIDE	0.899	2.729	1319.56	3600.44	2.452	1102.06	2702.35
A_68_P26504618	chr9:50425465-50425510	NM_028300:62	Pih1d2	INSIDE	0.899	2.229	926.48	2065.36	2.005	849.08	1702.16
A_68_P26350662	chr9:21961189-21961233	NM_001082532:-79	Pigyl	PROMOTER	0.899	0.424	1991.76	845.07	0.381	1555.44	592.99
A_68_P24295435	chr6:23204086-23204130	NM_028462:-5844	Fezf1	PROMOTER	0.899	0.496	1488.05	738.70	0.446	1141.56	509.40
A_68_P24163719	chr5:145726566-145726610	NM_001038627:111	Smurf1	INSIDE	0.899	2.035	541.23	1101.19	1.829	508.96	930.67
A_68_P24134131	chr5:139676645-139676689	NM_024451:43	Sun1	INSIDE	0.899	0.745	5152.20	3838.23	0.670	3982.24	2668.00
A_68_P22485526	chr3:122687380-122687424	NM_133857:-37	Usp53	PROMOTER	0.899	0.337	3348.43	1128.74	0.303	2502.39	758.09
A_68_P21395067	chr2:84706557-84706601	NM_001083809:25796	Slc43a1	DOWNSTREAM	0.899	0.470	1294.21	608.83	0.423	1034.87	437.62
A_68_P21307667	chr2:69043827-69043871	NM_001199123:399	Spc25	INSIDE	0.899	0.379	1305.96	494.89	0.341	1002.56	341.62
A_68_P20262082	chr1:59176751-59176795	NM_001037812:-122	Als2cr4	PROMOTER	0.899	0.531	1784.84	947.75	0.477	1243.21	593.25
A_68_P32415791	chrX:58144223-58144267	NM_009237:2361	Sox3	DOWNSTREAM	0.898	1.892	753.58	1425.72	1.700	969.23	1647.23
A_68_P32121142	chr19:42506084-42506128	NM_145123:167	Crtac1	INSIDE	0.898	0.621	2299.12	1426.88	0.557	1772.57	987.83
A_68_P31876427	chr18:82645364-82645408	NM_001025245:872	Mbp	INSIDE	0.898	3.678	1198.97	4410.14	3.302	1008.65	3330.63
A_68_P30642670	chr16:30599886-30599930	NM_177632:100	Fam43a	INSIDE	0.898	0.302	2991.34	902.03	0.271	2125.81	575.38
A_68_P29648697	chr14:61791606-61791650	NM_172809:34335	Sacs	INSIDE	0.898	1.811	1859.28	3366.60	1.625	1512.39	2458.24
A_68_P29592280	chr14:49792675-49792719	NM_001081430:795	Naa30	INSIDE	0.898	0.430	1529.96	657.40	0.386	1138.45	439.49
A_68_P28731499	chr12:110271722-110271766	NM_001163175:34683	Begain	INSIDE	0.898	2.640	1721.58	4545.00	2.372	1587.07	3764.28
A_68_P28178432	chr11:119386132-119386176			Unknown	0.898	2.602	279.43	727.20	2.338	276.77	647.12
A_68_P28013001	chr11:90499572-90499616	NM_199008:97	Cox11	INSIDE	0.898	0.723	3171.94	2293.48	0.649	2456.30	1594.12
A_68_P27980118	chr11:84694015-84694059	NM_001077636:-249	Pigw	PROMOTER	0.898	2.649	696.56	1845.45	2.378	582.51	1385.32
A_68_P27302607	chr10:83902948-83902992	NM_001004363:246	Nuak1	INSIDE	0.898	0.644	1236.82	796.46	0.579	1009.98	584.27
A_68_P27111541	chr10:45205741-45205785	ENSMUST00000099847:-179		PROMOTER	0.898	1.807	1934.67	3496.40	1.624	1576.13	2558.85
A_68_P26785469	chr9:103266999-103267043	NM_001134427:289	Cdv3	INSIDE	0.898	0.455	1965.14	893.61	0.409	1391.33	568.42
A_68_P26629702	chr9:72654701-72654745	NM_175485:-358	Prtg	PROMOTER	0.898	0.127	3701.28	471.00	0.114	2574.05	294.16
A_68_P25806162	chr8:41596547-41596591	NM_011135:90	Cnot7	INSIDE	0.898	0.372	2504.55	931.67	0.334	1681.78	561.61
A_68_P25542608	chr7:140968333-140968377	NM_133941:132	Dhx32	INSIDE	0.898	2.713	729.73	1979.70	2.436	585.86	1427.27
A_68_P23999718	chr5:114185852-114185896	NM_001010825:84	Ficd	INSIDE	0.898	0.415	3540.42	1469.01	0.373	2720.14	1013.42
A_68_P23321772	chr4:133801543-133801587	NM_024215:-58	Zfp593	PROMOTER	0.898	0.449	1339.11	601.26	0.403	1074.50	433.40
A_68_P22695195	chr4:3866261-3866305	NM_001190322:222	Chchd7	INSIDE	0.898	0.286	4271.69	1223.09	0.257	3046.55	783.18
A_68_P22492342	chr3:124024204-124024248	NM_146140:272	Tram1l1	INSIDE	0.898	0.403	1521.47	612.55	0.361	1228.64	444.00
A_68_P22311639	chr3:87886056-87886100	NM_001033484:106	Iqgap3	INSIDE	0.898	0.598	3772.78	2257.95	0.538	2595.77	1395.52
A_68_P21752664	chr2:152880435-152880479	NM_001011732:22869	Xkr7	INSIDE	0.898	1.817	3214.14	5841.07	1.631	2388.82	3897.29
A_68_P21633705	chr2:131005466-131005510	NM_007682:260	Cenpb	INSIDE	0.898	2.411	1647.03	3971.26	2.166	1246.35	2699.97
A_68_P21432196	chr2:92885631-92885675	NM_001177536:649	Prdm11	INSIDE	0.898	0.660	3959.49	2614.93	0.593	3034.98	1800.18
A_68_P20562758	chr1:122499691-122499735	NM_010133:649	En1	INSIDE	0.898	2.766	1916.79	5301.98	2.483	1791.74	4449.62
A_68_P32266420	chrX:13063059-13063103	NM_173415:18283	Nyx	INSIDE	0.897	2.227	929.35	2069.82	1.997	992.07	1981.62
A_68_P30657576	chr16:33380868-33380912	NM_011749:30	Zfp148	INSIDE	0.897	2.242	802.81	1799.73	2.010	662.30	1331.32
A_68_P30348895	chr15:76352647-76352691	NM_130893:-109	Scrt1	PROMOTER	0.897	3.253	1150.22	3741.99	2.919	996.65	2909.62
A_68_P29018483	chr13:48357669-48357713	NM_031166:895	Id4	INSIDE	0.897	0.358	1493.05	534.36	0.321	1305.12	419.01
A_68_P28280594	chr12:21423400-21423444	NM_011739:-125	Ywhaq	PROMOTER	0.897	0.373	1842.72	687.14	0.334	1311.85	438.64
A_68_P27687370	chr11:32105281-32105325	NM_030093:-112	Snrnp25	DIVERGENT_PROMOTER	0.897	0.142	4861.60	692.50	0.128	3242.61	414.52
A_68_P27487944	chr10:118305191-118305235	NM_001014390:747	Dyrk2	INSIDE	0.897	0.374	1384.96	517.59	0.335	1142.80	382.92
A_68_P26137276	chr8:108535235-108535279	NM_028038:130	Ddx28	INSIDE	0.897	0.444	1193.08	529.96	0.399	931.87	371.47
A_68_P26084575	chr8:98202243-98202287	ENSMUST00000166358:120		INSIDE	0.897	0.456	1041.21	475.19	0.409	850.47	348.14
A_68_P25360662	chr7:106397588-106397632			Unknown	0.897	0.641	1312.02	840.85	0.575	1013.65	582.45
A_68_P25348908	chr7:104230293-104230337	NM_001162477:54	Gab2	INSIDE	0.897	3.699	533.13	1971.79	3.319	400.00	1327.47
A_68_P25048200	chr7:35989129-35989173	NM_001024707:11214	Lrp3	INSIDE	0.897	2.911	1451.16	4224.26	2.612	1253.62	3274.72
A_68_P25005091	chr7:25266519-25266563	NM_153112:-501	Cadm4	PROMOTER	0.897	0.304	10322.68	3134.82	0.273	6485.69	1767.54
A_68_P24796456	chr6:119988018-119988063	NR_030487:-3711	Mir706	PROMOTER	0.897	0.303	2515.60	761.30	0.271	1831.81	497.32
A_68_P23283635	chr4:126803524-126803568	NM_001099319:257	Gm12942	INSIDE	0.897	2.933	625.18	1833.93	2.630	480.38	1263.35
A_68_P23283137	chr4:126699570-126699614	NM_023603:1048	Sfpq	INSIDE	0.897	3.181	591.55	1881.52	2.853	471.12	1344.06
A_68_P23185680	chr4:106323776-106323820	NM_001083887:125	Pars2	INSIDE	0.897	0.257	2892.81	742.75	0.230	2275.05	523.80
A_68_P22215786	chr3:68297563-68297607	NR_036665:-530	Schip1	PROMOTER	0.897	0.308	3521.60	1083.64	0.276	2599.25	717.45
A_68_P21098343	chr2:29475430-29475474	NM_001039086:213	Rapgef1	INSIDE	0.897	2.336	638.31	1491.16	2.096	603.58	1264.91
A_68_P21037980	chr2:17969325-17969369	NM_028317:730	2810030E01Rik	INSIDE	0.897	0.407	1767.34	718.55	0.365	1386.71	505.68
A_68_P20831360	chr1:174102609-174102653	NM_153555:24485	Dcaf8	INSIDE	0.897	2.756	445.25	1227.21	2.471	308.63	762.72
A_68_P20039230	chr1:13116543-13116587	NM_001081209:680	Prdm14	INSIDE	0.897	0.267	3893.06	1040.22	0.240	2656.58	636.57
A_68_P31369876	chr17:78600703-78600747	NM_015800:1137	Criml	INSIDE	0.896	0.375	2165.03	811.45	0.336	1660.53	557.59
A_68_P31259849	chr17:57202154-57202198	NM_177638:477	Crb3	INSIDE	0.896	1.871	748.81	1401.32	1.677	650.99	1091.58

ProbeName	Target position of probe on CpG island microarray	TargetID	GeneSymbol	CpG island Description	Ratio of relative methylation (TiO_2-NP/Vehicle)	Sham group			TiO_2-H group		
						Relative methylation (Cy5/Cy3)	Cy3 signal (Input)	Cy5 signal (MeDIP)	Relative methylation (Cy5/Cy3)	Cy3 signal (Input)	Cy5 signal (MeDIP)
A_68_P30961687	chr16:91225634-91225678	NM_016967:-138	Olig2	PROMOTER	0.896	0.593	3214.78	1906.47	0.531	2219.01	1178.87
A_68_P30591270	chr16:21248808-21248852	ENSMUST00000152179:48115		INSIDE	0.896	2.421	665.42	1611.05	2.169	595.73	1292.05
A_68_P30405820	chr15:86044641-86044685	NM_145476:-226	Tbc1d22a	PROMOTER	0.896	0.237	2434.34	577.13	0.212	1726.91	366.68
A_68_P30244835	chr15:57723554-57723598	NM_024207:397	Derl1	INSIDE	0.896	0.260	2890.12	750.10	0.233	2091.49	486.56
A_68_P29511369	chr14:32149798-32149842	NM_009796:-89	Capn7	PROMOTER	0.896	2.273	1471.99	3345.75	2.037	1268.71	2584.72
A_68_P28517158	chr12:70993819-70993863	NM_178628:-251	Atl1	PROMOTER	0.896	2.891	471.67	1363.42	2.590	422.29	1093.59
A_68_P28290829	chr12:25336635-25336680	NM_178357:422	Klf11	INSIDE	0.896	0.494	2074.13	1023.76	0.442	1587.19	702.30
A_68_P28033989	chr11:94491202-94491246	NM_133807:87	Lrrc59	INSIDE	0.896	0.712	2413.95	1718.92	0.638	1873.62	1195.64
A_68_P27543732	chr10:128328704-128328748	NM_010272:48	Gdf11	INSIDE	0.896	2.554	1149.64	2936.05	2.289	911.43	2086.37
A_68_P26884586	chr9:121550854-121550898	NM_026915:-192	Lyzl4	DIVERGENT_PROMOTER	0.896	0.253	2669.17	674.08	0.226	1945.75	440.06
A_68_P26542219	chr9:57369547-57369591	NM_007747:530	Cox5a	INSIDE	0.896	2.562	1735.09	4444.64	2.294	1400.16	3212.43
A_68_P26136324	chr8:108379182-108379226	NM_021513:202	Thap11	INSIDE	0.896	0.578	1499.67	867.24	0.518	1218.05	631.16
A_68_P26044790	chr8:91567290-91567334	NM_021390:749	Sall1	INSIDE	0.896	1.871	3090.81	5783.32	1.677	2443.13	4097.39
A_68_P25951144	chr8:72854849-72854893	NM_008107:1207	Gdf1	INSIDE	0.896	4.874	574.78	2801.22	4.368	508.26	2219.89
A_68_P25021891	chr7:29220830-29220874	NM_175021:358	Samd4b	INSIDE	0.896	0.464	2010.06	933.29	0.416	1577.63	656.23
A_68_P25021279	chr7:29123215-29123259	NM_013676:502	Supt5h	INSIDE	0.896	0.342	3432.30	1174.61	0.307	2607.27	799.16
A_68_P24112565	chr5:134789851-134789895	NM_001080746:744	Gtf2i	INSIDE	0.896	0.302	2613.16	790.26	0.271	1850.05	501.03
A_68_P23539920	chr5:23606274-23606318	NM_001161800:-111	Klhl7	PROMOTER	0.896	1.529	2718.45	4155.35	1.370	2201.47	3015.20
A_68_P23419144	chr4:151852440-151852484	NM_153424:212	Nphp4	INSIDE	0.896	0.488	2745.70	1339.98	0.437	1964.89	859.01
A_68_P22202957	chr3:65778426-65778470	AK133062:4613		DOWNSTREAM	0.896	0.528	1805.07	953.84	0.474	1520.18	719.85
A_68_P21769250	chr2:155890860-155890904	NM_139151:-66	Spag4	PROMOTER	0.896	3.257	620.60	2021.27	2.920	531.59	1552.17
A_68_P21755516	chr2:153356263-153356307	NM_001134300:-577	8430427H17Rik	PROMOTER	0.896	0.547	1177.05	644.02	0.490	887.84	435.08
A_68_P20977505	chr2:6051162-6051206	NM_001159657:47	5430407P10Rik	INSIDE	0.896	2.094	9049.23	18951.61	1.876	7007.55	13146.48
A_68_P20799112	chr1:168168830-168168874	NM_175296:23	Mael	INSIDE	0.896	3.129	1079.72	3378.05	2.804	1028.60	2883.88
A_68_P20723963	chr1:154802723-154802769	NM_175460:516	Nmnat2	INSIDE	0.896	0.237	2190.77	518.15	0.212	1591.69	337.39
A_68_P20335679	chr1:72283096-72283140	ENSMUST00000123654:-7476		PROMOTER	0.896	0.564	1373.32	774.99	0.506	979.49	495.28
A_68_P32143650	chr19:46469545-46469589	NM_016860:659	Actr1a	INSIDE	0.895	3.852	2295.93	8844.68	3.450	1675.49	5779.62
A_68_P31929161	chr19:5510899-5510943	NM_001024560:-431	Snx32	PROMOTER	0.895	0.519	2277.34	1180.89	0.464	1511.46	701.76
A_68_P31792973	chr18:67800987-67801031	NM_001081073:-18	Cep76	DIVERGENT_PROMOTER	0.895	0.646	2087.93	1349.08	0.578	1533.19	886.78
A_68_P30366257	chr15:79233271-79233315	NM_172608:441	Tmem184b	INSIDE	0.895	0.549	4294.05	2356.83	0.491	3269.96	1605.88
A_68_P30332047	chr15:73526357-73526401	NM_173365:11557	Gpr20	INSIDE	0.895	3.093	5238.86	16205.93	2.768	3133.45	8674.13
A_68_P29256603	chr13:99033115-99033159	NM_023472:-60	Ankra2	DIVERGENT_PROMOTER	0.895	1.784	1006.67	1795.45	1.595	851.24	1358.10
A_68_P27629549	chr11:19921210-19921254	NM_033523:96788	Spred2	INSIDE	0.895	1.832	1768.48	3240.68	1.640	1356.65	2224.60
A_68_P25603239	chr7:151016135-151016179	NM_007856:7085	Dhcr7	INSIDE	0.895	0.367	1322.58	484.85	0.328	947.17	310.59
A_68_P25592831	chr7:149268090-149268134	NM_008748:13077	Dusp8	INSIDE	0.895	2.762	589.03	1626.77	2.473	507.00	1253.58
A_68_P25179906	chr7:72017792-72017836	NM_023239:112	Ndnl2	INSIDE	0.895	2.704	657.34	1777.62	2.421	582.01	1409.13
A_68_P25030073	chr7:30928817-30928863	NM_001081007:21874	Zfp382	DOWNSTREAM	0.895	3.079	439.37	1352.67	2.756	354.10	975.74
A_68_P23897746	chr5:93522554-93522598	NM_001009818:94	Septin11	INSIDE	0.895	0.355	7857.64	2791.56	0.318	5465.28	1738.47
A_68_P21843420	chr2:168591529-168591573	NM_175303:1151	Sall4	INSIDE	0.895	4.056	387.40	1571.11	3.631	347.43	1261.36
A_68_P21070361	chr2:25036829-25036873	NM_025980:573	Nrarp	INSIDE	0.895	0.405	2744.20	1110.80	0.362	1769.26	641.14
A_68_P20842598	chr1:176433545-176433589	NM_019445:1611	Fmn2	INSIDE	0.895	2.565	587.59	1506.98	2.296	537.55	1234.07
A_68_P20551457	chr1:120286262-120286306	NM_001081276:650	Clasp1	INSIDE	0.895	0.309	2859.05	884.85	0.277	2124.47	588.68
A_68_P28083254	chr11:102941888-102941932	NM_152813:21062	Plcd3	INSIDE	0.894	0.275	1852.58	509.46	0.246	1277.04	314.12
A_68_P27262212	chr10:75931494-75931538	NM_019434:-199	Mcm3ap	PROMOTER	0.894	0.325	1935.39	628.81	0.290	1457.38	423.32
A_68_P26659816	chr9:78329333-78329377	NM_010106:177	Eef1a1	INSIDE	0.894	2.241	818.42	1834.21	2.004	734.18	1471.10
A_68_P25947915	chr8:72298581-72298625	NM_177899:208	Zfp866	INSIDE	0.894	1.973	2189.73	4320.18	1.764	1913.94	3375.99
A_68_P25601077	chr7:150645470-150645514	NM_001161624:1463	Cdkn1c	INSIDE	0.894	2.424	322.72	782.15	2.167	336.25	728.77
A_68_P24590978	chr6:82982884-82982928	NM_010070:559	Dok1	INSIDE	0.894	1.621	5878.79	9529.39	1.449	3919.11	5676.84
A_68_P21839558	chr2:167942901-167942945			Unknown	0.894	5.614	1043.56	5858.03	5.017	2050.67	10288.04
A_68_P21812073	chr2:163201933-163201977	NM_021566:21755	Jph2	INSIDE	0.894	3.056	653.69	1997.37	2.731	516.01	1409.24
A_68_P20644881	chr1:138157260-138157304	NM_001101516:168	Gpr25	INSIDE	0.894	3.566	1416.06	5050.11	3.187	1151.25	3669.13
A_68_P32785482	chrX:158155524-158155568	NM_198409:62	Rai2	INSIDE	0.893	5.133	1178.51	6049.78	4.583	1118.06	5124.09
A_68_P31936307	chr19:6905918-6905962	NM_019924:9151	Rps6ka4	INSIDE	0.893	0.630	1464.35	923.27	0.563	1169.30	658.19
A_68_P31347824	chr17:74059066-74059113	NM_027864:702	Galnt14	INSIDE	0.893	0.303	2217.83	672.27	0.271	1398.28	378.31
A_68_P30495768	chr15:101904128-101904172	NM_145625:-53	Eif4b	PROMOTER	0.893	0.374	2164.38	808.89	0.334	1537.57	513.02
A_68_P30456842	chr15:95059493-95059537	NM_016743:299623	Nell2	INSIDE	0.893	2.031	737.44	1497.95	1.815	559.91	1016.19
A_68_P30371302	chr15:80118124-80118168	NM_001044308:479	Cacna1i	INSIDE	0.893	1.654	1542.95	2552.38	1.477	1325.80	1957.90
A_68_P27921610	chr11:74451826-74451870	NM_001013784:18742	E130309D14Rik	INSIDE	0.893	1.799	1063.85	1913.51	1.606	919.10	1476.12
A_68_P26655650	chr9:77503358-77503402	NM_172925:18842	Klhl31	INSIDE	0.893	2.047	567.05	1160.99	1.829	481.48	880.64
A_68_P26522400	chr9:53619652-53619696	NM_028060:333	Slc35f2	INSIDE	0.893	0.478	1569.40	750.34	0.427	1321.20	564.31
A_68_P26454290	chr9:42072563-42072607	NM_172769:-201	Sc5d	PROMOTER	0.893	0.361	2618.97	946.23	0.322	1814.74	585.23
A_68_P25956952	chr8:73993389-73993433	NM_025600:318	Dda1	INSIDE	0.893	0.332	2335.98	774.77	0.296	1648.60	488.23
A_68_P25954147	chr8:73321717-73321761	NM_199308:-5406	Mast3	PROMOTER	0.893	0.445	1558.25	693.34	0.397	1158.54	460.18
A_68_P25005776	chr7:25396309-25396353	NM_001102613:-16	Phldb3	PROMOTER	0.893	8.330	5473.15	45590.75	7.439	4296.48	31963.28
A_68_P23985066	chr5:111759743-111759787	NM_019640:-18	Pitpnb	PROMOTER	0.893	8.792	3509.38	30854.06	7.855	2927.39	22995.06

ProbeName	Target position of probe on CpG island microarray	TargetID	GeneSymbol	CpG island Description	Ratio of relative methylation (TiO_2-NP/Vehicle)	Sham group			TiO_2-H group		
						Relative methylation (Cy5/Cy3)	Cy3 signal (Input)	Cy5 signal (MeDIP)	Relative methylation (Cy5/Cy3)	Cy3 signal (Input)	Cy5 signal (MeDIP)
A_68_P23912412	chr5:97821337-97821381	NM_001033191:-9	Naa11	PROMOTER	0.893	3.694	1540.64	5690.87	3.297	1270.00	4187.19
A_68_P23787826	chr5:72092511-72092555	NM_008069:1278	Gabrb1	INSIDE	0.893	0.348	1733.21	604.00	0.311	1305.59	406.25
A_68_P23364201	chr4:141020235-141020279	NM_009541:19669	Zbtb17	INSIDE	0.893	2.123	1424.80	3024.38	1.896	972.78	1844.56
A_68_P23012925	chr4:71862291-71862335	NR_027923:35	C630043F03Rik	INSIDE	0.893	0.574	1344.51	771.31	0.512	1088.10	557.17
A_68_P21901052	chr2:179777532-179777576	NM_178750:367	Ss18l1	INSIDE	0.893	0.392	1240.48	486.17	0.350	787.30	275.69
A_68_P20905055	chr1:187941024-187941068	NM_146106:143	Lyplal1	INSIDE	0.893	0.314	2003.73	629.54	0.281	1467.15	411.65
A_68_P20338093	chr1:72687766-72687810	NM_176980:59350	Ankar	DOWNSTREAM	0.893	0.467	1729.03	806.68	0.417	1339.12	558.23
A_68_P32672910	chrX:131160541-131160585	ENSMUST00000137737:-279		PROMOTER	0.892	2.942	667.27	1963.27	2.624	912.96	2395.92
A_68_P31985663	chr19:17468572-17468616	NM_019437:62	Rfk	INSIDE	0.892	0.232	2373.65	551.63	0.207	1790.75	371.25
A_68_P31121752	chr17:28450748-28450792	NM_001163819:296	Fance	INSIDE	0.892	2.034	843.81	1716.62	1.814	792.74	1438.23
A_68_P29257118	chr13:99124161-99124205	NM_008242:-17	Foxd1	PROMOTER	0.892	0.451	1021.10	460.79	0.402	881.65	354.73
A_68_P28992607	chr13:43959962-43960006	ENSMUST00000158482:-8186		PROMOTER	0.892	0.337	1305.20	440.00	0.301	1027.96	309.09
A_68_P28881308	chr13:23515666-23515710	NM_013924:47	Abt1	INSIDE	0.892	0.534	1670.69	892.79	0.476	1212.29	577.58
A_68_P28048193	chr11:96882776-96882820	NM_031183:7916	Sp6	INSIDE	0.892	0.668	3386.75	2261.43	0.596	2389.32	1423.61
A_68_P27900656	chr11:70246371-70246416	NM_145429:239	Arrb2	INSIDE	0.892	0.642	1866.44	1197.87	0.572	1354.58	775.38
A_68_P27835395	chr11:59005362-59005406	NM_001159410:-4141	Guk1	PROMOTER	0.892	0.684	2197.74	1503.09	0.610	1774.57	1082.85
A_68_P26522398	chr9:53619438-53619482	NM_028060:119	Slc35f2	INSIDE	0.892	0.330	1697.66	560.85	0.295	1404.41	414.05
A_68_P26426474	chr9:36533941-36533985	NM_007691:281	Chek1	INSIDE	0.892	0.262	2922.69	765.90	0.234	2261.67	528.82
A_68_P25934945	chr8:69384119-69384163	NM_001163564:25	Naf1	INSIDE	0.892	2.119	584.76	1239.11	1.891	553.38	1046.26
A_68_P24170318	chr5:147644951-147644995	NM_019647:507	Rpl21	INSIDE	0.892	0.477	1348.78	643.01	0.425	1031.81	438.82
A_68_P24084799	chr5:129242041-129242085	NM_021311:-58	Piwil1	PROMOTER	0.892	3.540	1606.57	5687.63	3.157	1340.57	4232.13
A_68_P23819787	chr5:77523869-77523913	NM_001159900:402	Hopx	INSIDE	0.892	0.571	1530.52	874.24	0.509	1051.72	535.61
A_68_P23545737	chr5:24606038-24606082	NM_145401:400	Prkag2	INSIDE	0.892	3.274	324.87	1063.47	2.919	268.45	783.51
A_68_P23400759	chr4:148859555-148859599	NM_026963:135	Lzic	INSIDE	0.892	0.742	2181.50	1618.95	0.662	1812.89	1200.38
A_68_P23337641	chr4:136604483-136604527	NM_198248:106	Zbtb40	INSIDE	0.892	2.283	927.28	2117.06	2.036	748.68	1524.65
A_68_P23334810	chr4:136158767-136158811	NM_133872:-150	Kdm1a	DIVERGENT_PROMOTER	0.892	0.433	1002.84	434.08	0.386	886.88	342.53
A_68_P22859197	chr4:40800680-40800724	NM_022305:329	B4galt1	INSIDE	0.892	3.519	1847.64	6500.96	3.140	1524.35	4786.64
A_68_P22129521	chr3:51220268-51220312	NM_053089:353	Naa15	INSIDE	0.892	0.355	1527.27	542.58	0.317	1223.58	387.90
A_68_P21766023	chr2:155343571-155343615	NM_019811:-186	Acss2	DIVERGENT_PROMOTER	0.892	0.220	3741.29	823.82	0.196	2514.80	493.84
A_68_P32137224	chr19:45309563-45309607	NM_010691:142	Lbx1	INSIDE	0.891	0.485	964.34	467.93	0.433	781.15	337.87
A_68_P30650135	chr16:31932946-31932990	NM_172822:-968	Pigz	PROMOTER	0.891	4.000	291.54	1166.18	3.563	285.70	1017.96
A_68_P30546657	chr16:11175989-11176033	NM_145931:476	Zc3h7a	INSIDE	0.891	0.181	2719.81	492.07	0.161	1945.89	313.61
A_68_P30362719	chr15:78677947-78677991	NM_027219:4892	Cdc42ep1	INSIDE	0.891	2.038	724.99	1477.62	1.816	599.79	1089.26
A_68_P29611636	chr14:54873516-54873560	NM_001113358:66	Dad1	INSIDE	0.891	0.333	1887.81	628.21	0.297	1389.26	412.04
A_68_P29237541	chr13:95647211-95647255	NM_011021:1651	Otp	INSIDE	0.891	0.403	2388.43	963.25	0.359	1785.27	641.67
A_68_P28043964	chr11:96207537-96207581	NM_010458:2476	Hoxb3	INSIDE	0.891	0.267	2774.92	739.98	0.238	2009.92	477.42
A_68_P27950544	chr11:79517837-79517881	NR_029579:-7612	Mir193	PROMOTER	0.891	2.078	1006.20	2090.39	1.852	879.64	1628.85
A_68_P27475464	chr10:115855274-115855318	NR_028107:120	1700058G18Rik	INSIDE	0.891	6.956	271.52	1888.58	6.195	225.02	1394.03
A_68_P27093480	chr10:41916896-41916940	NM_019740:79630	Foxo3	INSIDE	0.891	2.989	910.92	2722.55	2.662	724.13	1927.78
A_68_P26816014	chr9:108564549-108564593	NM_020520:142	Slc25a20	INSIDE	0.891	0.383	1259.85	481.98	0.341	1000.81	341.27
A_68_P26458699	chr9:42752539-42752583	NM_175481:-106	Grik4	PROMOTER	0.891	3.697	425.07	1571.50	3.293	370.89	1221.52
A_68_P26142838	chr8:109572655-109572699	NM_139229:7961	Cog8	INSIDE	0.891	0.494	3866.58	1909.99	0.440	2664.09	1172.46
A_68_P25712975	chr8:23918742-23918786	NM_001122820:-2638	Ap3m2	PROMOTER	0.891	0.586	1389.51	814.20	0.522	1099.04	573.67
A_68_P25272112	chr7:88599818-88599862	NM_007755:-278	Cpeb1	PROMOTER	0.891	2.590	1053.76	2728.82	2.309	934.58	2157.50
A_68_P25262772	chr7:86866415-86866459	NM_001113471:11226	Plin1	INSIDE	0.891	1.960	601.16	1178.32	1.746	557.34	972.92
A_68_P25030459	chr7:31016969-31017013	NM_025548:58	Tbcb	INSIDE	0.891	0.717	1712.86	1228.40	0.639	1280.58	818.02
A_68_P25016990	chr7:28157258-28157302	NM_001199235:22245	Spnb4	INSIDE	0.891	3.028	947.46	2869.23	2.697	859.80	2318.93
A_68_P24816534	chr6:124662607-124662651	NM_007531:322	Phb2	INSIDE	0.891	0.551	2437.11	1343.72	0.491	1783.05	875.86
A_68_P24784005	chr6:117866930-117866974	NM_001166427:-359	Hnrnpf	PROMOTER	0.891	0.354	2004.38	709.95	0.316	1431.30	451.75
A_68_P23270091	chr4:124428867-124428911	NM_008385:9767	Inpp5b	INSIDE	0.891	0.234	5011.99	1173.28	0.209	3770.31	786.48
A_68_P21875385	chr2:174264281-174264325	NM_022325:191	Ctsz	INSIDE	0.891	0.510	1903.64	971.40	0.455	1398.56	635.69
A_68_P21111660	chr2:31701435-31701479	NM_178887:69	Fibcd1	INSIDE	0.891	3.325	1603.62	5331.92	2.963	1253.85	3715.28
A_68_P21107808	chr2:31102041-31102085	NM_019681:620	Ncs1	INSIDE	0.891	2.156	3567.74	7692.22	1.922	2660.87	5114.46
A_68_P32141576	chr19:46072976-46073020	NM_198296:-20	9130011E15Rik	DIVERGENT_PROMOTER	0.890	0.645	3825.08	2468.86	0.574	3074.14	1765.63
A_68_P30961694	chr16:91226413-91226457	NM_016967:640	Olig2	INSIDE	0.890	0.315	2966.49	935.06	0.280	2189.83	614.15
A_68_P29064233	chr13:56989277-56989325	NM_012035:7649	Trpc7	INSIDE	0.890	1.959	899.44	1761.93	1.743	596.92	1040.40
A_68_P27425024	chr10:106709059-106709103	NM_001033223:194	Lin7a	INSIDE	0.890	3.460	3804.66	13165.57	3.081	2999.43	9241.59
A_68_P27181743	chr10:61143343-61143387	NM_009120:296	Sar1a	INSIDE	0.890	0.417	2278.00	949.16	0.371	1766.44	655.13
A_68_P26801798	chr9:106101292-106101336	NM_198931:-44	Ppm1m	PROMOTER	0.890	2.077	556.91	1156.96	1.848	501.62	926.95
A_68_P26533944	chr9:55889943-55889987	NM_011992:313	Rcn2	INSIDE	0.890	2.242	572.03	1282.61	1.996	552.69	1102.94
A_68_P26467130	chr9:44152085-44152129	NM_001110251:-1642	Hmbs	PROMOTER	0.890	2.632	1431.95	3769.49	2.344	1117.13	2618.22
A_68_P24990613	chr7:19542978-19543022	NM_001029877:31764	Nova2	INSIDE	0.890	0.260	2040.96	531.11	0.232	1454.66	336.76
A_68_P24425394	chr6:47974652-47974696	NM_001081382:23439	Zfp777	INSIDE	0.890	1.939	1170.94	2270.22	1.725	842.63	1453.76
A_68_P24172796	chr5:148086210-148086254	NR_038157:-3135	2210019I11Rik	PROMOTER	0.890	0.370	2349.95	870.18	0.329	1862.00	613.47
A_68_P24045787	chr5:122110769-122110813	NM_028227:196	Brap	INSIDE	0.890	0.579	1259.90	729.22	0.515	1011.32	520.80

ProbeName	Target position of probe on CpG island microarray	TargetID	GeneSymbol	CpG island Description	Ratio of relative methylation (TiO_2-NP/Vehicle)	Sham group			TiO_2-H group		
						Relative methylation (Cy5/Cy3)	Cy3 signal (Input)	Cy5 signal (MeDIP)	Relative methylation (Cy5/Cy3)	Cy3 signal (Input)	Cy5 signal (MeDIP)
A_68_P23796834	chr5:73684070-73684114	NM_001159889:60	Ociad1	INSIDE	0.890	0.133	3849.40	513.15	0.119	2843.07	337.20
A_68_P22317611	chr3:88972711-88972761	NM_001163432:4010	Clk2	INSIDE	0.890	2.771	792.80	2196.86	2.467	607.00	1497.27
A_68_P21958958	chr3:14886343-14886387	NM_009801:-61	Car2	PROMOTER	0.890	2.200	558.87	1229.28	1.957	430.40	842.24
A_68_P21274009	chr2:63022285-63022329	NM_133207:38	Kcnh7	INSIDE	0.890	0.697	4063.64	2834.17	0.621	3195.62	1984.10
A_68_P32240895	chrX:7499312-7499356	NM_013892:2387	Pcsk1n	INSIDE	0.889	2.025	521.32	1055.87	1.801	707.13	1273.56
A_68_P31351019	chr17:74738085-74738129	NM_001162870:-220	Spast	PROMOTER	0.889	1.678	2734.73	4588.09	1.492	2046.56	3052.74
A_68_P31213681	chr17:47831661-47831705	NM_144939:-473	Frs3	DIVERGENT_PROMOTER	0.889	0.123	6492.78	801.71	0.110	4354.43	477.78
A_68_P31154762	chr17:34742611-34742655	NM_001163379:-173	Agpat1	DIVERGENT_PROMOTER	0.889	2.045	1354.02	2768.38	1.818	1191.33	2165.97
A_68_P30413823	chr15:87375395-87375439	ENSMUST00000169110:513		INSIDE	0.889	3.086	1103.10	3403.84	2.744	909.28	2495.14
A_68_P30125731	chr15:33617431-33617475	NM_001085421:186	Tspyl5	INSIDE	0.889	2.163	470.33	1017.47	1.924	464.55	893.76
A_68_P29047192	chr13:54150550-54150594	NM_010076:455	Drd1a	INSIDE	0.889	4.307	1972.76	8496.23	3.830	1506.32	5768.55
A_68_P28997649	chr13:44825063-44825107	NM_001205044:-1058	Jarid2	PROMOTER	0.889	0.359	3185.07	1141.92	0.319	2423.38	772.38
A_68_P27643752	chr11:22891041-22891085	NM_009837:470	Cct4	INSIDE	0.889	1.802	1124.07	2025.27	1.602	937.58	1502.04
A_68_P27551128	chr11:3190602-3190646	NM_019574:165	Patz1	INSIDE	0.889	0.662	1521.42	1006.97	0.589	1281.10	754.02
A_68_P26913680	chr10:6079737-6079781	NM_031185:454	Akap12	INSIDE	0.889	0.183	2970.97	543.38	0.163	2097.66	340.91
A_68_P26680226	chr9:82723911-82723955	NM_001168240:520	Irak1bp1	INSIDE	0.889	0.724	2780.20	2013.94	0.644	2038.49	1312.25
A_68_P26640622	chr9:74710098-74710142	NM_008262:393	Onecut1	INSIDE	0.889	0.361	2250.70	812.02	0.321	1655.54	530.75
A_68_P25614206	chr8:3493424-3493468	NM_080461:309	Zfp358	INSIDE	0.889	0.243	2164.46	525.54	0.216	1692.23	365.40
A_68_P25048063	chr7:35971453-35971497	NM_017394:71	Slc7a10	INSIDE	0.889	1.657	2824.25	4681.05	1.474	2089.37	3078.92
A_68_P24993776	chr7:20108843-20108887	NM_027116:4785	Nkpd1	INSIDE	0.889	0.577	1399.99	808.35	0.513	879.53	451.26
A_68_P23176987	chr4:104830931-104830975	NM_080555:1001	Ppap2b	INSIDE	0.889	1.718	1290.13	2216.71	1.527	1104.31	1686.63
A_68_P21867216	chr2:172847313-172847357	NM_019547:-68	Rbm38	PROMOTER	0.889	2.585	1545.64	3995.53	2.298	1207.90	2775.20
A_68_P31921051	chr19:4100737-4100781	NM_008851:137	Pitpnm1	INSIDE	0.888	2.843	479.07	1362.11	2.524	420.64	1061.74
A_68_P31357838	chr17:75951190-75951234	NM_133747:74	Fam98a	INSIDE	0.888	0.297	1711.50	508.05	0.264	1283.27	338.23
A_68_P30927369	chr16:85080522-85080569	NM_001198823:93407	App	INSIDE	0.888	3.220	7144.84	23003.90	2.858	4398.69	12572.60
A_68_P29117521	chr13:69878252-69878296	NM_001145162:501	Ube2ql1	INSIDE	0.888	0.710	2071.20	1470.41	0.631	1565.62	987.36
A_68_P28712493	chr12:107248661-107248705	NM_001029843:210	Vrk1	INSIDE	0.888	0.710	1743.10	1237.46	0.631	2247.92	1417.41
A_68_P28603261	chr12:86621337-86621381	NM_025421:27	Acyp1	INSIDE	0.888	2.590	1072.93	2778.40	2.299	905.47	2081.54
A_68_P28538087	chr12:74646709-74646753	NM_178715:652	Tmem30b	INSIDE	0.888	3.752	871.73	3270.34	3.331	679.37	2262.84
A_68_P28480017	chr12:62623830-62623874	NM_178714:-1766	Lrfn5	PROMOTER	0.888	0.479	1899.89	909.74	0.425	1585.68	673.95
A_68_P27286198	chr10:80360560-80360604	NM_001081688:18022	Tmprss9	INSIDE	0.888	2.387	1987.24	4742.65	2.118	1530.49	3242.21
A_68_P27271355	chr10:77441002-77441046	NM_026431:-370	1810043G02Rik	DIVERGENT_PROMOTER	0.888	0.523	1442.79	754.81	0.465	1094.56	508.66
A_68_P26397767	chr9:30750791-30750835	NM_013906:665	Adamts8	INSIDE	0.888	0.404	1251.77	505.47	0.359	1033.36	370.68
A_68_P26224549	chr8:123632350-123632394	NM_001166482:-93	Mthfsd	DIVERGENT_PROMOTER	0.888	0.363	1615.77	587.04	0.323	1188.04	383.29
A_68_P25089659	chr7:52104491-52104535	NM_001042655:-63	Tbc1d17	DIVERGENT_PROMOTER	0.888	0.482	2856.44	1376.21	0.428	1887.13	807.56
A_68_P24793341	chr6:119429106-119429150	NM_133940:-557	Fbxl14	PROMOTER	0.888	0.741	3634.16	2693.16	0.658	2566.88	1689.51
A_68_P24124229	chr5:137508063-137508107	NM_001039385:1920	Vgf	INSIDE	0.888	0.636	3099.29	1969.87	0.565	2352.98	1328.59
A_68_P23979399	chr5:110816208-110816252	NM_028596:-84	Fbrsl1	PROMOTER	0.888	0.607	2725.63	1655.45	0.540	2238.52	1207.78
A_68_P23593510	chr5:34631368-34631412	NM_001015039:-417	Zfyve28	PROMOTER	0.888	0.409	1125.75	460.77	0.364	904.95	329.08
A_68_P23283634	chr4:126803332-126803376	NM_001099319:65	Gm12942	INSIDE	0.888	0.529	2347.29	1241.24	0.470	1908.52	896.33
A_68_P23134314	chr4:97438807-97438855	NM_001122953:-5486	Nfia	PROMOTER	0.888	2.250	473.23	1064.78	1.997	398.37	795.53
A_68_P20833436	chr1:174430441-174430485	NM_178598:86	Tagln2	INSIDE	0.888	0.503	1079.33	543.23	0.447	864.73	386.49
A_68_P20293904	chr1:64737359-64737403	NR_038009:-147	2810408I11Rik	DIVERGENT_PROMOTER	0.888	2.354	1130.15	2660.83	2.091	979.93	2049.03
A_68_P32266425	chrX:13063695-13063739	NM_173415:18919	Nyx	INSIDE	0.887	3.176	661.84	2101.76	2.816	1003.45	2826.03
A_68_P32111333	chr19:40906458-40906502	NM_172839:712	Ccnj	INSIDE	0.887	2.463	734.31	1808.49	2.185	607.17	1326.50
A_68_P31923506	chr19:4510275-4510319	NM_001162946:-175	Pcx	PROMOTER	0.887	0.372	2065.94	768.79	0.330	1611.98	532.37
A_68_P31921021	chr19:4097311-4097355	NM_026373:-18	Cdk2ap2	PROMOTER	0.887	0.681	1438.79	980.40	0.605	1161.95	702.69
A_68_P31751922	chr18:60686064-60686108	NM_026302:212	Dctn4	INSIDE	0.887	0.622	1265.33	786.99	0.552	1029.60	568.17
A_68_P30010734	chr15:10399494-10399539	NM_030046:755	Dnajc21	INSIDE	0.887	3.079	574.46	1768.47	2.730	487.22	1330.07
A_68_P29702363	chr14:70999406-70999450	NM_194345:214	Fam160b2	INSIDE	0.887	0.701	1502.78	1053.71	0.622	1300.26	808.25
A_68_P28452143	chr12:56593280-56593324	NM_010907:332	Nfkbia	INSIDE	0.887	0.453	1155.12	523.81	0.402	1096.43	440.79
A_68_P28051870	chr11:97521455-97521499	NM_139311:-3249	Mllt6	PROMOTER	0.887	0.729	2655.35	1934.81	0.646	1893.78	1223.42
A_68_P27889090	chr11:68199498-68199542	NM_008744:808	Ntn1	INSIDE	0.887	0.448	1254.98	561.98	0.397	991.56	394.00
A_68_P27649725	chr11:24064408-24064452	NM_001159290:83736	Bcl11a	INSIDE	0.887	2.124	2034.52	4320.88	1.884	1457.63	2746.30
A_68_P26885918	chr9:121748458-121748502	NM_028384:99	Ccdc13	INSIDE	0.887	0.446	1450.24	647.52	0.396	1095.79	434.08
A_68_P26587154	chr9:65194762-65194806	NM_016688:910	Pdcd7	INSIDE	0.887	0.220	7548.61	1658.54	0.195	4918.42	958.32
A_68_P26579887	chr9:63908203-63908247	ENSMUST00000130862:10589		DOWNSTREAM	0.887	0.692	2472.52	1711.19	0.614	1908.42	1170.99
A_68_P25965607	chr8:75876888-75876932	NM_010687:-455	Large	PROMOTER	0.887	0.526	3964.92	2084.70	0.466	2727.18	1271.54
A_68_P25098706	chr7:53632477-53632521	NM_010866:655	Myod1	INSIDE	0.887	0.375	3788.93	1419.85	0.333	2492.38	828.86
A_68_P25035565	chr7:31993563-31993607	AK141529:209		INSIDE	0.887	0.531	1071.35	569.33	0.471	845.50	398.37
A_68_P25009651	chr7:26099846-26099890	NM_183311:8742	Tmem145	INSIDE	0.887	0.371	3389.97	1256.82	0.329	2390.64	786.05
A_68_P24385306	chr6:39822132-39822176			Unknown	0.887	0.175	3131.89	547.09	0.155	2404.98	372.80
A_68_P24059294	chr5:124545817-124545861	NM_019875:-31	Abcb9	PROMOTER	0.887	1.655	1824.72	3019.04	1.468	1566.45	2299.90
A_68_P23543722	chr5:24256716-24256760	NM_153076:6923	Crygn	DOWNSTREAM	0.887	0.520	1348.71	701.19	0.461	1107.44	510.43
A_68_P31620318	chr18:35991596-35991640	NM_133687:2147	Cxxc5	INSIDE	0.886	2.709	1707.62	4626.50	2.400	1354.87	3251.74

ProbeName	Target position of probe on CpG island microarray	TargetID	GeneSymbol	CpG island Description	Ratio of relative methylation (TiO_2-NP/Vehicle)	Sham group			TiO_2-H group		
						Relative methylation (Cy5/Cy3)	Cy3 signal (Input)	Cy5 signal (MeDIP)	Relative methylation (Cy5/Cy3)	Cy3 signal (Input)	Cy5 signal (MeDIP)
A_68_P31095207	chr17:24210070-24210114	NM_027008:360	Kctd5	INSIDE	0.886	0.647	1774.81	1148.97	0.574	1446.56	830.05
A_68_P28752828	chr12:114067098-114067142	NM_028023:480	Cdca4	INSIDE	0.886	0.438	1661.64	728.22	0.388	1206.93	468.53
A_68_P28574820	chr12:81360887-81360931	NM_134156:450	Actn1	INSIDE	0.886	2.190	1965.85	4306.13	1.941	1563.20	3034.39
A_68_P28252846	chr12:14157333-14157377	NM_029007:1490	Fam84a	INSIDE	0.886	0.400	3496.05	1400.16	0.355	2512.14	891.13
A_68_P28155137	chr11:115626646-115626697	NM_028014:-75	2310067B10Rik	PROMOTER	0.886	0.126	3461.21	436.33	0.112	2124.06	237.18
A_68_P28049717	chr11:97141114-97141159	NM_008942:754	Npepps	INSIDE	0.886	0.417	5983.53	2495.63	0.370	4194.55	1550.17
A_68_P27056138	chr10:34001761-34001805	NM_009433:-213	Tspyl1	PROMOTER	0.886	0.686	1789.52	1227.97	0.608	1415.89	860.61
A_68_P26813082	chr9:108012831-108012875	NM_007567:79862	Bsn	INSIDE	0.886	2.742	1533.83	4205.60	2.428	1340.99	3256.18
A_68_P26293556	chr9:8901995-8902039	NM_008829:2184	Pgr	INSIDE	0.886	0.499	1368.89	682.87	0.442	1169.47	517.10
A_68_P25954891	chr8:73437387-73437431	NM_173013:7536	Mtap1s	INSIDE	0.886	3.684	1948.17	7177.97	3.264	1452.46	4740.52
A_68_P25360009	chr7:106289514-106289558	NM_178635:118	Uvrag	INSIDE	0.886	0.553	1786.41	987.66	0.490	1477.78	724.26
A_68_P25045782	chr7:35597582-35597626	NM_175140:126	Chst8	INSIDE	0.886	0.116	6719.93	776.97	0.102	4333.15	444.11
A_68_P24098742	chr5:132014298-132014342	NM_177047:1003893	Auts2	INSIDE	0.886	1.709	1320.98	2258.08	1.515	1096.61	1661.29
A_68_P24044318	chr5:121847841-121847885	NM_172722:-128	Naa25	PROMOTER	0.886	0.268	1728.41	462.37	0.237	1291.24	305.92
A_68_P23354399	chr4:139377567-139377611	NM_011039:11295	Pax7	INSIDE	0.886	0.493	2595.99	1279.29	0.437	1832.13	799.83
A_68_P22793478	chr4:26273773-26273817	NM_172865:5	Manea	INSIDE	0.886	0.338	2925.44	987.36	0.299	2509.84	750.80
A_68_P21741662	chr2:150730240-150730284	NM_024465:205	Abhd12	INSIDE	0.886	0.380	2242.26	852.58	0.337	1771.92	596.90
A_68_P21680285	chr2:139503181-139503225	NM_001126490:-711	Ism1	PROMOTER	0.886	0.320	4074.41	1302.06	0.283	2894.71	819.35
A_68_P32672213	chrX:131010426-131010470	NM_028958:581	Taf7l	INSIDE	0.885	2.317	579.33	1342.46	2.052	789.68	1620.14
A_68_P31655262	chr18:42553965-42554009	NM_138945:-264	Pou4f3	PROMOTER	0.885	0.226	3322.80	749.57	0.200	2644.09	527.62
A_68_P31209392	chr17:47027759-47027803	NM_178385:211	Tbcc	INSIDE	0.885	1.880	3233.60	6078.18	1.663	2320.05	3859.32
A_68_P31097948	chr17:24670514-24670558	NM_177375:156	Rab26	INSIDE	0.885	0.409	2499.91	1023.63	0.362	1691.82	613.27
A_68_P29962216	chr14:122933648-122933692	NM_144844:121	Pcca	INSIDE	0.885	0.403	1175.67	473.63	0.356	893.19	318.29
A_68_P29644188	chr14:60997183-60997227	NM_001164705:82	Fam123a	INSIDE	0.885	0.490	1952.72	957.21	0.434	1473.91	639.06
A_68_P29626310	chr14:57752221-57752265	NM_008128:-8184	Gjb6	PROMOTER	0.885	2.726	862.63	2351.29	2.414	824.77	1990.67
A_68_P28956734	chr13:37918572-37918616	NM_001177868:-186	Rreb1	PROMOTER	0.885	0.222	4320.13	959.08	0.196	2989.10	587.31
A_68_P27897436	chr11:69651105-69651149	NM_026982:-900	1810027O10Rik	DIVERGENT_PROMOTER	0.885	0.736	3708.05	2728.27	0.652	2783.56	1813.49
A_68_P27628921	chr11:19825017-19825061	NM_033523:594	Spred2	INSIDE	0.885	0.239	4729.12	1129.53	0.211	3451.76	729.84
A_68_P27277399	chr10:78996257-78996302	NM_015817:253	Ppap2c	INSIDE	0.885	0.407	1530.94	623.01	0.360	1132.27	407.55
A_68_P26932996	chr10:9621066-9621110	NM_001081344:-250	Stxbp5	PROMOTER	0.885	3.196	517.06	1652.45	2.829	537.54	1520.91
A_68_P26471639	chr9:44947044-44947088	NM_001013390:-58	Scn4b	DIVERGENT_PROMOTER	0.885	0.514	1796.42	923.54	0.455	1356.78	617.18
A_68_P26270746	chr8:131209434-131209478	NM_010578:-97	Itgb1	PROMOTER	0.885	0.377	2317.72	873.18	0.334	1772.85	591.33
A_68_P25786792	chr8:37677018-37677062	NM_015802:-43	Dlc1	PROMOTER	0.885	1.858	2344.32	4356.53	1.644	1961.33	3224.65
A_68_P25616318	chr8:4166571-4166615	NM_001039578:26	Evi5l	INSIDE	0.885	0.473	4374.59	2067.45	0.418	3075.76	1286.94
A_68_P25458766	chr7:125676689-125676733	NM_009940:117	Coq7	INSIDE	0.885	0.619	1644.58	1018.26	0.548	1366.37	749.13
A_68_P24477532	chr6:56873994-56874038	NM_026004:-90	Nt5c3	PROMOTER	0.885	3.141	411.52	1292.45	2.779	362.96	1008.50
A_68_P24151267	chr5:142843853-142843897			Unknown	0.885	0.451	1879.48	846.87	0.399	1333.61	532.07
A_68_P23566428	chr5:29522769-29522813	NM_021470:-1755	Rnf32	PROMOTER	0.885	0.188	3452.57	650.24	0.167	2245.07	374.34
A_68_P23288223	chr4:127665242-127665286	ENSMUST00000144298:-23		PROMOTER	0.885	4.095	995.39	4076.47	3.626	835.50	3029.12
A_68_P23012916	chr4:71861284-71861328	NM_011599:647	Tle1	INSIDE	0.885	3.480	1226.29	4267.63	3.080	1086.61	3346.45
A_68_P22888785	chr4:46463681-46463725	NM_130889:-286	Anp32b	PROMOTER	0.885	1.856	1782.13	3308.49	1.644	1454.39	2390.67
A_68_P20936234	chr1:192921983-192922027	NM_030060:-288	Batf3	PROMOTER	0.885	36.510	953.01	34794.92	32.307	1035.81	33464.36
A_68_P31116850	chr17:27649440-27649484	NM_001013385:-9213	Grm4	PROMOTER	0.884	0.431	1568.00	676.17	0.381	1249.96	476.75
A_68_P30397615	chr15:84753485-84753530	NM_016714:-350	Nup50	PROMOTER	0.884	0.504	3978.87	2006.87	0.446	2844.71	1267.68
A_68_P30387346	chr15:83082276-83082320	NM_001004150:-94	A4galt	PROMOTER	0.884	2.064	801.81	1655.01	1.825	676.11	1233.66
A_68_P29681797	chr14:67487667-67487711	NM_001034881:2	Gm5464	INSIDE	0.884	3.297	292.05	962.77	2.915	309.18	901.38
A_68_P29056694	chr13:55828552-55828596	NM_028281:-154	Pcbd2	PROMOTER	0.884	2.977	681.71	2029.46	2.633	597.61	1573.37
A_68_P28974121	chr13:40818660-40818705	NM_001122948:7130	Tcfap2a	INSIDE	0.884	0.444	2484.46	1103.26	0.393	1733.45	680.81
A_68_P28194543	chr12:3310085-3310129	NM_016676:-137	Rab10	PROMOTER	0.884	0.683	1929.80	1317.40	0.604	1518.96	917.02
A_68_P27283982	chr10:80023920-80023964	NM_025852:363	Rexo1	INSIDE	0.884	0.218	2198.99	478.84	0.193	1607.91	309.64
A_68_P27217139	chr10:67739765-67739809	NM_023598:1688	Arid5b	INSIDE	0.884	0.548	2675.78	1465.82	0.484	1940.88	940.15
A_68_P24476144	chr6:56602168-56602212			Unknown	0.884	0.460	1214.43	559.01	0.407	1040.75	423.54
A_68_P24061314	chr5:124895322-124895366	NM_030241:5406	Setd8	INSIDE	0.884	4.012	753.86	3024.62	3.546	621.73	2204.63
A_68_P23694950	chr5:52862343-52862387			Unknown	0.884	0.431	1133.88	488.94	0.381	849.30	323.59
A_68_P23176984	chr4:104830529-104830573	NM_080555:599	Ppap2b	INSIDE	0.884	0.458	3628.11	1662.77	0.405	2610.24	1057.42
A_68_P22355466	chr3:97462027-97462071	NM_182997:-86	Prkab2	PROMOTER	0.884	2.550	4034.44	10289.07	2.254	3290.54	7417.68
A_68_P22010764	chr3:27270678-27270722	NM_177330:428	Ghsr	INSIDE	0.884	0.521	4475.12	2332.08	0.461	3305.04	1522.69
A_68_P21901913	chr2:179906128-179906172	NM_019822:-142	Adrm1	PROMOTER	0.884	3.224	750.80	2420.93	2.851	636.01	1812.96
A_68_P32134814	chr19:44910682-44910726	NM_011037:78821	Pax2	INSIDE	0.883	0.252	2110.25	530.75	0.222	1536.01	341.25
A_68_P31868119	chr18:81184645-81184689	NM_178280:-1349	Sall3	PROMOTER	0.883	2.566	697.09	1788.42	2.265	543.49	1230.82
A_68_P31074428	chr17:17761926-17761970	NM_172827:-495	Lnpep	PROMOTER	0.883	2.259	685.10	1547.48	1.994	612.84	1221.72
A_68_P31022096	chr17:6257118-6257162	ENSMUST00000121576:3238		DOWNSTREAM	0.883	0.689	2995.07	2063.11	0.608	2599.74	1581.80
A_68_P31015191	chr17:5189996-5190040	NM_001085355:194945	Arid1b	INSIDE	0.883	2.283	1026.39	2343.73	2.015	912.69	1839.41
A_68_P30823192	chr16:65105551-65105595	NM_008310:38	Htr1f	INSIDE	0.883	0.560	1356.60	759.23	0.494	1146.33	566.23
A_68_P29056202	chr13:55736711-55736755	NM_145975:345	Ddx46	INSIDE	0.883	0.153	5602.03	857.69	0.135	3961.85	535.84

ProbeName	Target position of probe on CpG island microarray	TargetID	GeneSymbol	CpG island Description	Ratio of relative methylation (TiO_2-NP/Vehicle)	Sham group			TiO_2-H group		
						Relative methylation (Cy5/Cy3)	Cy3 signal (Input)	Cy5 signal (MeDIP)	Relative methylation (Cy5/Cy3)	Cy3 signal (Input)	Cy5 signal (MeDIP)
A_68_P28928669	chr13:32430434-32430478	NM_146041:-43	Gmds	PROMOTER	0.883	0.651	1776.45	1156.25	0.575	1473.23	847.01
A_68_P28149093	chr11:114536801-114536845	NM_053273:41	Ttyh2	INSIDE	0.883	4.999	2368.06	11836.96	4.414	1993.57	8800.42
A_68_P28137857	chr11:112646792-112646839	NM_011448:3292	Sox9	INSIDE	0.883	2.719	348.76	948.26	2.401	295.15	708.79
A_68_P28081267	chr11:102623133-102623177	NM_001110778:402	Adam11	INSIDE	0.883	2.119	715.55	1516.41	1.871	528.03	987.85
A_68_P25951450	chr8:72900017-72900061	NM_016685:2592	Comp	INSIDE	0.883	0.625	1867.84	1166.75	0.551	1352.07	745.53
A_68_P24817707	chr6:124860198-124860242	NM_008479:1503	Lag3	INSIDE	0.883	0.599	1251.72	749.78	0.529	936.80	495.51
A_68_P24304147	chr6:24906093-24906137	NM_177013:11	Tmem229a	INSIDE	0.883	0.199	2552.54	509.14	0.176	1937.84	341.21
A_68_P24027257	chr5:119011484-119011528	NM_172424:779	Med13l	INSIDE	0.883	1.957	1129.17	2210.24	1.728	834.65	1442.48
A_68_P23407014	chr4:149944046-149944090	NM_001085492:288044	Rere	INSIDE	0.883	0.337	2775.90	935.73	0.298	2045.11	608.75
A_68_P22859776	chr4:40918121-40918165	NM_023739:167	Nfx1	INSIDE	0.883	2.751	811.86	2233.49	2.428	687.23	1668.90
A_68_P22518747	chr3:128917569-128917613	NM_001042502:736	Pitx2	INSIDE	0.883	0.243	1944.70	473.41	0.215	1443.13	310.26
A_68_P21952655	chr3:13473121-13473165	NM_001163328:1488	Ralyl	INSIDE	0.883	0.478	1301.42	622.72	0.423	1171.35	495.03
A_68_P20240345	chr1:55145369-55145413	NM_008303:399	Hspe1	INSIDE	0.883	0.420	1351.10	568.08	0.371	1131.52	419.86
A_68_P20049945	chr1:15277016-15277060			Unknown	0.883	1.983	4199.97	8328.17	1.752	3095.35	5422.23
A_68_P31383416	chr17:80882436-80882480	NM_009231:-2665	Sos1	PROMOTER	0.882	0.521	1042.04	542.84	0.460	802.61	368.90
A_68_P30593950	chr16:21694881-21694925	NM_001001881:-164	2510009E07Rik	PROMOTER	0.882	0.391	1559.01	609.67	0.345	1325.70	457.04
A_68_P30424513	chr15:89145595-89145639	NM_001081030:126	Sbf1	INSIDE	0.882	2.195	1259.22	2764.23	1.936	986.21	1909.29
A_68_P29335934	chr13:113442566-113442610	NM_001145885:-70	Ddx4	DIVERGENT_PROMOTER	0.882	3.873	1030.60	3991.92	3.417	794.10	2713.24
A_68_P29330292	chr13:112515172-112515216	NM_172593:38809	Mier3	DOWNSTREAM	0.882	1.966	1336.62	2627.22	1.734	1120.62	1942.68
A_68_P28156930	chr11:115891653-115891697	NM_172569:39	Unk	INSIDE	0.882	5.659	679.68	3846.49	4.992	527.45	2632.99
A_68_P27083332	chr10:40022071-40022115	NM_009665:-98	Amd1	PROMOTER	0.882	0.427	1568.32	669.51	0.376	1216.50	457.80
A_68_P26567939	chr9:61794038-61794082	NM_024245:546	Kif23	INSIDE	0.882	0.379	1503.64	570.25	0.334	1270.21	424.67
A_68_P26543335	chr9:57556330-57556374	NM_153799:-23	Edc3	PROMOTER	0.882	0.487	1962.09	956.25	0.430	1557.15	669.36
A_68_P26218677	chr8:122752875-122752919	ENSMUST00000127664:318156		INSIDE	0.882	7.744	1824.09	14126.68	6.832	1427.94	9756.14
A_68_P26085828	chr8:98412281-98412325	NM_010325:-37	Got2	PROMOTER	0.882	0.439	2474.46	1086.41	0.387	1853.51	717.85
A_68_P25805587	chr8:41508783-41508827	NM_178395:-364	Zdhhc2	PROMOTER	0.882	0.467	1347.08	629.51	0.412	1162.83	479.42
A_68_P24537471	chr6:71583335-71583379	NM_001038695:-457	Kdm3a	PROMOTER	0.882	0.224	2304.83	515.47	0.197	1753.50	346.03
A_68_P24119330	chr5:136559824-136559868	NM_133914:61	Rasa4	INSIDE	0.882	4.029	4735.23	19076.91	3.552	3807.60	13524.79
A_68_P23873660	chr5:89149794-89149838	NM_026735:-79	Mobkl1a	PROMOTER	0.882	2.541	1624.63	4127.68	2.242	1308.42	2932.87
A_68_P23538314	chr5:23289429-23289473	NM_178403:29	Pus7	INSIDE	0.882	0.627	2807.79	1761.05	0.553	2308.37	1276.72
A_68_P23278806	chr4:125879586-125879630	NM_146153:346	Thrap3	INSIDE	0.882	0.493	2672.31	1316.84	0.435	2349.54	1021.66
A_68_P22325408	chr3:90269752-90269796	NM_008727:14	Npr1	INSIDE	0.882	0.376	1455.66	547.08	0.332	1167.16	386.93
A_68_P22301527	chr3:86028160-86028204	NM_001077687:-429	Lrba	PROMOTER	0.882	2.261	724.72	1638.89	1.996	686.71	1370.41
A_68_P22286842	chr3:83569940-83569984	NM_009144:-280	Sfrp2	PROMOTER	0.882	2.142	2325.90	4982.61	1.890	1990.47	3762.08
A_68_P21900911	chr2:179759193-179759237	NM_177727:-477	Lsm14b	PROMOTER	0.882	0.464	1180.96	548.09	0.409	963.29	394.39
A_68_P21749132	chr2:152224537-152224581	NM_011438:-776	Sox12	PROMOTER	0.882	2.233	779.71	1741.14	1.969	655.43	1290.68
A_68_P21739398	chr2:150296010-150296054	NM_001034900:14767	Zfp345	DOWNSTREAM	0.882	4.386	268.89	1179.30	3.868	237.38	918.28
A_68_P21094999	chr2:28916017-28916061	NM_009442:256	Ttf1	INSIDE	0.882	0.474	3662.81	1737.42	0.418	2559.24	1070.41
A_68_P20456913	chr1:95582337-95582381	NM_016778:88	Bok	INSIDE	0.882	0.393	2199.83	865.21	0.347	1588.49	551.32
A_68_P20085268	chr1:22813207-22813251	NM_001012623:-665	Rims1	PROMOTER	0.882	0.273	2425.39	662.16	0.241	1863.18	448.83
A_68_P33005897	A_68_P33005897			Unknown	0.881	4.255	672.10	2860.05	3.747	540.97	2027.09
A_68_P33005868	A_68_P33005868			Unknown	0.881	8.169	560.43	4577.92	7.196	471.54	3393.08
A_68_P32048665	chr19:28754625-28754669	NM_175459:-79	Glis3	PROMOTER	0.881	3.506	1966.34	6894.40	3.090	1526.44	4716.96
A_68_P31497272	chr18:11998272-11998316	NM_001146287:512	Cables1	INSIDE	0.881	2.392	462.66	1106.83	2.108	405.19	854.18
A_68_P31256437	chr17:56615365-56615409	NM_011218:517	Ptprs	INSIDE	0.881	0.470	3171.06	1491.66	0.414	2520.88	1044.47
A_68_P30959121	chr16:90810687-90810731	NM_029497:-50	Urb1	PROMOTER	0.881	0.151	4952.04	747.51	0.133	3636.86	483.72
A_68_P30424512	chr15:89145509-89145553	NM_001081030:212	Sbf1	INSIDE	0.881	3.194	1642.63	5246.65	2.813	1293.43	3638.53
A_68_P28833742	chr13:12794705-12794749	ENSMUST00000104944:98		INSIDE	0.881	0.329	1562.46	513.86	0.290	1512.94	438.32
A_68_P28575728	chr12:81537500-81537544	NM_177267:66	Dcaf5	INSIDE	0.881	0.475	1606.23	762.46	0.418	1137.90	475.84
A_68_P28166383	chr11:117515888-117515933	NM_198022:308	Tnrc6c	INSIDE	0.881	4.649	1483.75	6898.01	4.098	1138.97	4667.19
A_68_P28153581	chr11:115309405-115309449	NM_001033775:7568	4933422H20Rik	INSIDE	0.881	5.444	1430.55	7787.86	4.798	1227.57	5890.20
A_68_P27990834	chr11:86571018-86571062	NM_001003908:-46	Cltc	PROMOTER	0.881	0.218	3786.21	823.55	0.192	2985.60	572.01
A_68_P27794866	chr11:51419856-51419900	NM_001048061:505	Hnrnpab	INSIDE	0.881	2.038	1717.19	3499.51	1.795	1345.14	2414.08
A_68_P27688949	chr11:32355960-32356004	NM_173784:611	Ubtd2	INSIDE	0.881	0.242	2722.81	659.77	0.213	2157.06	460.52
A_68_P27551860	chr11:3309041-3309085	NM_010718:176	Limk2	INSIDE	0.881	4.049	1821.19	7374.50	3.567	1509.19	5383.77
A_68_P26845718	chr9:114549214-114549258	NM_153585:82	Cnot10	INSIDE	0.881	0.457	1206.78	551.40	0.403	926.53	373.04
A_68_P26750963	chr9:97011708-97011752	NM_138756:-270	Slc25a36	PROMOTER	0.881	0.277	1735.94	480.85	0.244	1404.28	342.71
A_68_P26126979	chr8:106666779-106666826	NM_009868:41278	Cdh5	INSIDE	0.881	2.926	887.98	2597.90	2.577	659.93	1700.91
A_68_P25507031	chr7:134890071-134890115	NM_172748:-196	Fbxl19	PROMOTER	0.881	2.198	720.03	1582.38	1.936	531.20	1028.16
A_68_P25466057	chr7:127039834-127039878	NM_029610:51	Lyrm1	INSIDE	0.881	0.573	2419.19	1387.20	0.505	1962.20	991.18
A_68_P22871975	chr4:43506649-43506693	NM_001037913:434	Ccdc107	INSIDE	0.881	0.311	2942.42	914.62	0.274	2027.80	555.61
A_68_P22201289	chr3:65469993-65470037	NM_001166659:-135	Lekr1	PROMOTER	0.881	3.268	3720.46	12158.99	2.880	3080.54	8873.38
A_68_P21746101	chr2:151668709-151668753	NM_001040689:68	Rspo4	INSIDE	0.881	1.852	912.45	1689.99	1.631	682.57	1113.41
A_68_P32557249	chrX:97016733-97016777	NM_001081283:347	Tmem28	INSIDE	0.880	3.937	585.00	2303.29	3.466	653.46	2264.65
A_68_P31826517	chr18:73733495-73733539	NM_001039214:1158	Mex3c	INSIDE	0.880	0.208	2285.06	476.11	0.183	1467.85	269.26

ProbeName	Target position of probe on CpG island microarray	TargetID	GeneSymbol	CpG island Description	Ratio of relative methylation (TiO_2-NP/Vehicle)	Sham group			TiO_2-H group		
						Relative methylation (Cy5/Cy3)	Cy3 signal (Input)	Cy5 signal (MeDIP)	Relative methylation (Cy5/Cy3)	Cy3 signal (Input)	Cy5 signal (MeDIP)
A_68_P31524833	chr18:16965307-16965351	NM_007664:2230	Cdh2	INSIDE	0.880	0.401	3802.36	1524.13	0.353	2833.87	999.15
A_68_P31206983	chr17:46592824-46592868	NM_001162864:31778	Ttbk1	INSIDE	0.880	0.469	1141.92	535.84	0.413	827.65	341.75
A_68_P30426167	chr15:89422156-89422200	NM_026817:176	Rabl2	INSIDE	0.880	0.691	1869.59	1291.34	0.608	1265.32	769.41
A_68_P29531802	chr14:35582497-35582541	ENSMUST00000143244:9106		INSIDE	0.880	0.272	2613.16	710.82	0.239	2078.00	497.25
A_68_P28741429	chr12:112216865-112216909	NM_175207:345	Ankrd9	INSIDE	0.880	1.946	981.66	1910.01	1.712	761.23	1303.17
A_68_P28524377	chr12:72212495-72212539	NM_029227:102	Tomm20l	INSIDE	0.880	1.816	1772.66	3219.41	1.598	1452.03	2319.91
A_68_P28072082	chr11:100981134-100981178	NM_026501:-18	Fam134c	PROMOTER	0.880	0.423	1653.30	700.09	0.373	1043.12	388.57
A_68_P27977898	chr11:84326983-84327029	NM_019816:-3	Aatf	PROMOTER	0.880	1.748	1200.67	2098.56	1.538	1044.47	1606.31
A_68_P27896908	chr11:69571787-69571831	NM_009089:-83	Polr2a	PROMOTER	0.880	2.070	902.50	1868.40	1.822	837.42	1526.11
A_68_P26465579	chr9:43893430-43893474	NM_198091:450	Usp2	INSIDE	0.880	0.409	1125.90	460.13	0.360	821.66	295.56
A_68_P25951931	chr8:72975757-72975801	NM_001122830:25064	Klhl26	INSIDE	0.880	2.912	316.40	921.27	2.562	260.61	667.73
A_68_P25835351	chr8:47379779-47379823	NM_173789:225	Helt	INSIDE	0.880	0.704	2183.72	1537.26	0.620	1825.46	1131.36
A_68_P21736747	chr2:149656520-149656564	NM_001085521:24	Tmem90b	INSIDE	0.880	20.141	3057.47	61579.72	17.721	3011.03	53358.51
A_68_P20262083	chr1:59176826-59176870	NM_001037812:-198	Als2cr4	PROMOTER	0.880	0.242	3700.15	897.02	0.213	2622.49	559.53
A_68_P31936825	chr19:7016840-7016884	NM_001177361:78	Gpr137	INSIDE	0.879	2.964	804.91	2385.91	2.604	676.31	1761.19
A_68_P31509668	chr18:14129242-14129286	NM_145492:1978	Zfp521	INSIDE	0.879	0.593	4415.93	2618.16	0.521	3075.57	1603.40
A_68_P31418994	chr17:87154526-87154570	NM_010137:1345	Epas1	INSIDE	0.879	0.564	1248.42	704.01	0.496	989.99	490.67
A_68_P31125155	chr17:29089634-29089678	NM_025888:-504	Kctd20	PROMOTER	0.879	2.126	1616.06	3436.21	1.869	1322.32	2472.06
A_68_P28615265	chr12:88640437-88640481	NM_011479:88722	Sptlc2	DOWNSTREAM	0.879	0.324	3662.91	1187.32	0.285	2707.08	770.92
A_68_P28528480	chr12:72932514-72932558	NM_026102:472	Daam1	INSIDE	0.879	2.156	763.01	1645.04	1.896	701.55	1329.86
A_68_P28003258	chr11:88861329-88861373	NM_009546:634	Trim25	INSIDE	0.879	2.104	673.49	1416.86	1.849	645.54	1193.91
A_68_P27989204	chr11:86298222-86298266	NM_029788:86	Rnft1	INSIDE	0.879	0.484	3138.87	1520.75	0.426	2422.34	1031.38
A_68_P27928209	chr11:75609329-75609373	NM_007873:209	Doc2b	INSIDE	0.879	0.263	2723.94	715.36	0.231	1861.55	429.58
A_68_P27779981	chr11:48614053-48614098	NR_028563:-1459	Snord96a	PROMOTER	0.879	0.322	1881.44	605.02	0.283	1374.87	388.74
A_68_P27503014	chr10:120787968-120788012	ENSMUST00000078515:7663		DOWNSTREAM	0.879	4.810	4909.74	23616.89	4.227	4042.98	17090.02
A_68_P27383820	chr10:98377065-98377109	NM_026482:-699	Atp2b1	PROMOTER	0.879	0.314	7287.64	2291.52	0.276	5436.84	1502.75
A_68_P27171935	chr10:59465906-59465950	NM_025514:-68	Anapc16	DIVERGENT_PROMOTER	0.879	0.464	2815.77	1305.53	0.408	2292.05	934.03
A_68_P26983374	chr10:19570550-19570594	NM_029529:693	Slc35d3	INSIDE	0.879	1.629	1642.58	2676.04	1.431	1401.78	2006.45
A_68_P26594001	chr9:66441281-66441325	NM_144937:-515	Usp3	PROMOTER	0.879	0.558	2490.90	1390.98	0.491	1873.44	919.95
A_68_P26526553	chr9:54434458-54434502	NM_029573:163	Idh3a	INSIDE	0.879	0.548	1900.68	1042.32	0.482	1588.18	765.23
A_68_P26216697	chr8:122434504-122434548	NM_009462:-225	Usp10	PROMOTER	0.879	0.576	3046.38	1754.61	0.506	2484.53	1258.36
A_68_P25210554	chr7:77476526-77476570	AK049871:6181		INSIDE	0.879	0.127	5123.19	648.27	0.111	3559.48	395.88
A_68_P21967925	chr3:17686263-17686307	NR_033490:-3636	2610100L16Rik	PROMOTER	0.879	1.926	861.23	1658.49	1.692	816.54	1381.41
A_68_P21763712	chr2:154876566-154876610	NM_015770:37283	a	INSIDE	0.879	3.057	2628.70	8036.12	2.687	2227.38	5984.71
A_68_P20091641	chr1:24106778-24106822	NM_026604:380	Fam135a	INSIDE	0.879	2.075	1178.89	2445.62	1.824	1003.65	1830.26
A_68_P32396717	chrX:53863261-53863305	NM_172780:271	Slc9a6	INSIDE	0.878	1.768	1344.57	2376.59	1.552	1765.71	2740.06
A_68_P31125930	chr17:29232003-29232047	NM_007669:1308	Cdkn1a	INSIDE	0.878	0.202	2761.13	556.67	0.177	1933.02	342.17
A_68_P31100627	chr17:25097854-25097898	NM_198937:-308	Hn1l	PROMOTER	0.878	0.314	1413.06	443.47	0.276	1136.72	313.24
A_68_P30956060	chr16:90284235-90284280	NM_178923:413	Scaf4	INSIDE	0.878	0.355	1930.73	684.65	0.311	1526.49	475.38
A_68_P30344891	chr15:75741091-75741135	NM_178646:948	Tigd5	INSIDE	0.878	0.366	3213.06	1174.89	0.321	2101.31	674.98
A_68_P30207182	chr15:50721909-50721953	NM_032000:-343	Trps1	PROMOTER	0.878	0.199	2576.19	513.16	0.175	1958.57	342.63
A_68_P29671861	chr14:65768816-65768860	BC096449:686615		DOWNSTREAM	0.878	0.651	8167.75	5315.01	0.572	4883.14	2791.06
A_68_P29616396	chr14:55732459-55732503	NM_177049:3292	Jph4	INSIDE	0.878	2.753	1396.80	3845.05	2.416	1135.88	2743.81
A_68_P29612018	chr14:54980756-54980800	NM_026936:254	Oxa1l	INSIDE	0.878	2.367	1016.73	2406.41	2.079	860.28	1788.47
A_68_P28077571	chr11:101968496-101968540	NM_145435:572	Pyy	INSIDE	0.878	9.977	1334.32	13312.30	8.762	1208.53	10589.12
A_68_P27360776	chr10:94151341-94151385	NR_015524:-4	4932415G12Rik	DIVERGENT_PROMOTER	0.878	7.860	1187.07	9330.31	6.904	1294.04	8933.85
A_68_P27292201	chr10:81817091-81817135	DQ459435:113		INSIDE	0.878	0.236	2712.88	640.77	0.207	2011.92	417.07
A_68_P27086291	chr10:40604225-40604269	NM_031877:907	Wasf1	INSIDE	0.878	2.893	378.34	1094.44	2.541	289.69	736.05
A_68_P26430601	chr9:37237135-37237179	NM_001164767:3604	Robo3	INSIDE	0.878	2.161	1715.69	3707.55	1.897	1293.33	2453.21
A_68_P24993662	chr7:20092735-20092779	NM_177692:924	Bloc1s3	INSIDE	0.878	0.669	1313.20	878.58	0.587	1247.82	732.99
A_68_P24129161	chr5:138612875-138612919	NR_029658:-5850	Mir106b	PROMOTER	0.878	0.267	3506.97	938.07	0.235	2564.94	602.24
A_68_P24039810	chr5:121063118-121063162	NM_028041:2	Ddx54	INSIDE	0.878	3.415	833.51	2846.17	2.998	683.32	2048.33
A_68_P23441157	chr4:155244125-155244169	NM_028020:471	Cpsf3l	INSIDE	0.878	0.213	2276.81	485.23	0.187	1593.17	297.97
A_68_P22039928	chr3:32716452-32716496	NM_001013024:-73	Usp13	PROMOTER	0.878	1.997	967.85	1932.66	1.753	807.58	1416.02
A_68_P21755509	chr2:153355180-153355224	NM_001134300:505	8430427H17Rik	INSIDE	0.878	0.228	4230.14	966.31	0.201	2931.40	588.10
A_68_P21332193	chr2:73367805-73367850	NM_153138:-360	Wipf1	PROMOTER	0.878	0.417	1896.08	790.51	0.366	1481.27	542.32
A_68_P32241967	chrX:7722368-7722412	NR_033561:-162	2900002K06Rik	PROMOTER	0.877	0.586	2014.93	1181.56	0.514	2760.93	1420.22
A_68_P31644536	chr18:40379121-40379165	NM_023311:-89	Yipf5	PROMOTER	0.877	0.378	3963.85	1497.14	0.331	2875.89	952.94
A_68_P30113002	chr15:31153995-31154039	NM_146057:-123	Dap	PROMOTER	0.877	1.785	1765.68	3151.89	1.565	1155.54	1808.39
A_68_P27988569	chr11:86171411-86171455	NM_001080931:-405	Med13	PROMOTER	0.877	2.511	750.83	1884.98	2.202	588.94	1296.94
A_68_P27915806	chr11:73013237-73013281	NM_029031:275	Shpk	INSIDE	0.877	0.451	1234.38	556.72	0.396	930.39	368.18
A_68_P27553886	chr11:3661741-3661785	NM_152818:102144	Osbp2	INSIDE	0.877	0.542	1117.24	605.03	0.475	843.63	400.77
A_68_P27528049	chr10:125403615-125403659	NM_177152:362	Lrig3	INSIDE	0.877	0.524	1511.72	791.63	0.459	1333.98	612.41
A_68_P27500270	chr10:120335605-120335649	NM_177092:401	Msrb3	INSIDE	0.877	2.746	1810.92	4972.82	2.409	1476.58	3556.54
A_68_P27104595	chr10:43988364-43988408	NM_053069:223	Atg5	INSIDE	0.877	0.464	1521.50	706.03	0.407	1252.20	509.37

ProbeName	Target position of probe on CpG island microarray	TargetID	GeneSymbol	CpG island Description	Ratio of relative methylation (TiO_2-NP/Vehicle)	Sham group			TiO_2-H group		
						Relative methylation (Cy5/Cy3)	Cy3 signal (Input)	Cy5 signal (MeDIP)	Relative methylation (Cy5/Cy3)	Cy3 signal (Input)	Cy5 signal (MeDIP)
A_68_P26466890	chr9:44113631-44113675	NM_172162:102	Hinfp	INSIDE	0.877	0.393	1199.84	471.23	0.344	1025.82	353.35
A_68_P24061289	chr5:124890348-124890392	NM_030241:432	Setd8	INSIDE	0.877	0.336	5028.81	1689.25	0.295	3429.97	1010.15
A_68_P24018831	chr5:117570556-117570601	NM_001199685:441	Taok3	INSIDE	0.877	0.638	1963.16	1252.62	0.560	1573.38	880.34
A_68_P21334260	chr2:73748941-73748985	NM_175015:389	Atp5g3	INSIDE	0.877	0.305	4425.51	1351.37	0.268	3300.32	883.45
A_68_P32461570	chrX:70473796-70473840	NM_007978:174	F8a	INSIDE	0.876	3.679	426.41	1568.57	3.221	532.09	1713.76
A_68_P32288516	chrX:20002305-20002349	ENSMUST00000115384:-487		PROMOTER	0.876	2.189	588.04	1287.42	1.918	673.93	1292.64
A_68_P30656036	chr16:33062526-33062570	NM_172823:-58	Lmln	PROMOTER	0.876	3.398	315.90	1073.33	2.975	253.62	754.51
A_68_P30642386	chr16:30550244-30550288	NM_172614:398	Tmem44	INSIDE	0.876	0.505	2095.16	1059.09	0.443	1569.25	694.52
A_68_P30362677	chr15:78672776-78672820	NM_027219:-278	Cdc42ep1	PROMOTER	0.876	2.876	836.34	2405.68	2.520	689.90	1738.86
A_68_P30348872	chr15:76349910-76349954	NM_130893:2627	Scrt1	INSIDE	0.876	1.972	1236.63	2438.74	1.727	995.44	1718.84
A_68_P29605232	chr14:52816687-52816731	NM_033618:206	Supt16h	INSIDE	0.876	0.591	3918.58	2317.70	0.518	2954.25	1530.50
A_68_P28614429	chr12:88488100-88488144	NM_010363:-545	Gstz1	PROMOTER	0.876	0.564	975.00	550.00	0.494	809.87	400.09
A_68_P27798800	chr11:52174442-52174486	NM_011694:-152	Vdac1	PROMOTER	0.876	0.541	1910.18	1032.91	0.474	1413.79	669.89
A_68_P27767869	chr11:46250544-46250588	NM_001104556:104	Med7	INSIDE	0.876	2.149	1280.82	2752.43	1.882	1053.90	1983.51
A_68_P27628894	chr11:19820464-19820508	NM_033523:-3958	Spred2	PROMOTER	0.876	2.533	445.49	1128.31	2.219	413.35	917.37
A_68_P26529570	chr9:54996803-54996847	NM_180600:-351	Ube2q2	PROMOTER	0.876	2.290	1292.28	2959.05	2.006	1002.85	2011.61
A_68_P25089670	chr7:52105704-52105748	NM_026270:1130	Akt1s1	INSIDE	0.876	0.442	1237.52	547.53	0.387	939.14	363.81
A_68_P25016937	chr7:28149369-28149413	NM_001199235:30135	Spnb4	INSIDE	0.876	6.129	562.51	3447.91	5.367	422.63	2268.44
A_68_P24872958	chr6:136467356-136467400	NM_019426:7	Atf7ip	INSIDE	0.876	4.209	3107.00	13077.15	3.687	2344.69	8644.65
A_68_P23354494	chr4:139389036-139389081	NM_011039:-175	Pax7	PROMOTER	0.876	0.447	1230.14	549.68	0.391	929.62	363.82
A_68_P23333144	chr4:135846710-135846754	NM_009557:4749	Zfp46	INSIDE	0.876	0.609	1518.73	925.42	0.534	1155.99	617.37
A_68_P20166596	chr1:39634041-39634085	NM_001033135:130	Rnf149	INSIDE	0.876	2.720	1739.95	4731.89	2.381	1398.31	3330.00
A_68_P31175247	A_68_P31175247			Unknown	0.875	0.566	14885.21	8430.66	0.496	12224.19	6059.71
A_68_P30939711	chr16:87432394-87432438	NM_001081068:435	Ltn1	INSIDE	0.875	0.477	2427.67	1157.88	0.417	1874.43	781.93
A_68_P30508955	chr16:4213107-4213151	NM_001025432:276	Crebbp	INSIDE	0.875	0.566	2480.36	1403.22	0.495	1816.46	899.56
A_68_P30376708	chr15:81021101-81021145	NM_001082536:65	Mkl1	INSIDE	0.875	0.254	5212.26	1324.68	0.222	4102.77	911.87
A_68_P30347070	chr15:76077318-76077362	NM_023168:104	Grina	INSIDE	0.875	0.722	2352.81	1699.08	0.632	1853.97	1172.09
A_68_P29605618	chr14:52877257-52877301	NM_172601:21792	Rab2b	DOWNSTREAM	0.875	2.997	3067.38	9193.12	2.621	2383.77	6248.73
A_68_P28886521	chr13:24372825-24372869	NM_026825:-187	Lrrc16a	PROMOTER	0.875	1.777	1741.86	3095.10	1.554	1369.94	2129.24
A_68_P28463197	chr12:58644329-58644373	NM_008259:2758	Foxa1	INSIDE	0.875	0.594	2668.19	1586.23	0.520	2113.70	1099.55
A_68_P28048328	chr11:96902741-96902785	NM_027026:-79	Lrrc46	DIVERGENT_PROMOTER	0.875	0.291	2401.44	699.22	0.255	2150.62	547.74
A_68_P26714889	chr9:89803449-89803494	NM_001039655:-1141	Rasgrf1	PROMOTER	0.875	2.119	940.21	1992.45	1.853	712.21	1319.98
A_68_P25731023	chr8:27268359-27268403	NM_199042:-260	Thap1	PROMOTER	0.875	0.298	1499.98	446.36	0.260	1064.54	277.14
A_68_P25616527	chr8:4206553-4206597	NM_207203:10738	BC068157	DOWNSTREAM	0.875	0.577	1543.82	890.25	0.505	1081.38	545.83
A_68_P25590418	chr7:148879577-148879621	NM_023566:3338	Muc2	INSIDE	0.875	1.941	690.32	1340.13	1.699	589.75	1001.72
A_68_P25505249	chr7:134486849-134486893	NM_146202:1958	Zfp768	INSIDE	0.875	1.933	898.31	1736.00	1.690	644.12	1088.71
A_68_P25503618	chr7:134164772-134164816	NM_001102563:-69	Prrt2	PROMOTER	0.875	0.523	2839.48	1484.77	0.458	1951.50	892.94
A_68_P25328183	chr7:100229551-100229595	NM_021427:1184	Fam181b	INSIDE	0.875	1.623	2085.59	3384.50	1.419	1689.16	2397.76
A_68_P25065736	chr7:38969050-38969094	NM_028166:267	1600014C10Rik	INSIDE	0.875	0.543	1557.01	845.92	0.475	1199.73	570.35
A_68_P24754178	chr6:112439612-112439656	NM_001081147:168	Oxtr	INSIDE	0.875	0.447	1454.67	649.52	0.390	1100.34	429.66
A_68_P23147509	chr4:99768737-99768781	NM_013845:363	Ror1	INSIDE	0.875	0.482	1803.23	869.40	0.422	1287.76	543.14
A_68_P22894262	chr4:47366025-47366069	NM_009370:-130	Tgfbr1	PROMOTER	0.875	0.607	2717.42	1649.52	0.531	2357.34	1251.96
A_68_P21836026	chr2:167363834-167363878	NM_011427:130	Snai1	INSIDE	0.875	2.235	3475.97	7769.75	1.956	2661.41	5205.97
A_68_P21731903	chr2:148557781-148557825	NM_019632:354	Napb	INSIDE	0.875	0.600	1432.87	859.40	0.525	1092.40	573.31
A_68_P21061285	chr2:22924459-22924503	NM_001102437:-111	Acbd5	PROMOTER	0.875	0.386	2083.04	804.59	0.338	1419.17	479.44
A_68_P20638620	chr1:137210321-137210365	NM_011590:-28	Timm17a	PROMOTER	0.875	0.305	4596.73	1402.08	0.267	2858.11	762.97
A_68_P31937054	chr19:7048857-7048901	NM_008889:-659	Ppp1r14b	DIVERGENT_PROMOTER	0.874	0.639	2645.08	1689.43	0.558	1850.82	1033.12
A_68_P31715219	chr18:53904261-53904305	NM_178686:-81	Cep120	PROMOTER	0.874	0.677	1989.74	1347.19	0.592	1540.11	911.83
A_68_P31303252	chr17:66234323-66234367	NM_001198949:-81	Ralbp1	PROMOTER	0.874	2.862	1105.73	3165.09	2.501	862.24	2156.27
A_68_P30979306	chr16:94083777-94083821	NM_011377:-1706	Sim2	PROMOTER	0.874	0.669	1779.69	1190.91	0.585	1476.11	863.30
A_68_P30571780	chr16:17070460-17070504	NM_023249:79	Ypel1	INSIDE	0.874	2.814	691.53	1946.27	2.460	654.76	1610.96
A_68_P30481998	chr15:99556153-99556197	NM_183256:126	2310016M24Rik	INSIDE	0.874	0.300	2616.10	785.91	0.263	2022.97	531.25
A_68_P29413816	chr14:13655475-13655519	NM_001042617:97	Cadps	INSIDE	0.874	2.424	1373.00	3328.42	2.119	1104.74	2341.34
A_68_P29263981	chr13:100285080-100285124	NM_008634:1455	Mtap1b	INSIDE	0.874	0.492	2416.25	1188.57	0.430	2016.30	867.15
A_68_P28596608	chr12:85489337-85489381	NM_027438:81	Pnma1	INSIDE	0.874	0.547	3017.64	1649.96	0.478	2153.50	1029.48
A_68_P28593178	chr12:84861329-84861374	NM_030246:-64	Dcaf4	PROMOTER	0.874	0.610	1293.50	788.66	0.533	1098.21	584.98
A_68_P28031419	chr11:94072271-94072315	NM_009427:-475	Tob1	PROMOTER	0.874	0.404	1240.17	501.51	0.353	1090.80	385.43
A_68_P27590826	chr11:11362086-11362130	NM_001185153:314	Zpbp	INSIDE	0.874	6.109	686.36	4193.11	5.337	636.84	3399.12
A_68_P26802097	chr9:106149976-106150020	NM_020559:287	Alas1	INSIDE	0.874	0.556	3950.02	2195.39	0.486	2475.00	1202.15
A_68_P26469618	chr9:44607091-44607135	NM_027865:187	Tmem25	INSIDE	0.874	0.141	6082.03	860.59	0.124	4491.32	555.18
A_68_P25995583	chr8:82163277-82163321	NM_001081164:-276	Otud4	PROMOTER	0.874	0.270	1782.55	480.98	0.236	1147.75	270.77
A_68_P25618390	chr8:4678441-4678485	ENSMUST00000105524:1		INSIDE	0.874	0.659	1482.88	976.53	0.576	1203.42	692.92
A_68_P25478246	chr7:129433430-129433474	NM_008855:814	Prkcb	INSIDE	0.874	0.364	1770.22	644.10	0.318	1320.58	419.89
A_68_P25362936	chr7:106774598-106774642	NM_001195529:993	Gm4980	INSIDE	0.874	2.107	615.73	1297.03	1.841	540.57	994.99
A_68_P25241764	chr7:82926889-82926933	AK049415:-9978		PROMOTER	0.874	0.436	4630.25	2019.41	0.381	3529.15	1345.80

ProbeName	Target position of probe on CpG island microarray	TargetID	GeneSymbol	CpG island Description	Ratio of relative methylation (TiO_2-NP/Vehicle)	Sham group			TiO_2-H group		
						Relative methylation (Cy5/Cy3)	Cy3 signal (Input)	Cy5 signal (MeDIP)	Relative methylation (Cy5/Cy3)	Cy3 signal (Input)	Cy5 signal (MeDIP)
A_68_P25091030	chr7:52331941-52331985	NM_001163684:14164	Nosip	INSIDE	0.874	2.117	1090.35	2308.07	1.850	796.89	1474.08
A_68_P24735709	chr6:108733062-108733106	NM_026011:32	Arl8b	INSIDE	0.874	0.706	1744.33	1232.12	0.617	1410.75	870.48
A_68_P22508999	chr3:127201311-127201355	ENSMUST00000104965:587		DOWNSTREAM	0.874	0.666	1533.91	1022.08	0.583	1224.91	713.75
A_68_P22348135	chr3:96001508-96001552	NM_026975:-21	Bola1	PROMOTER	0.874	2.698	936.32	2526.46	2.360	734.39	1732.85
A_68_P22010765	chr3:27270773-27270817	NM_177330:522	Ghsr	INSIDE	0.874	0.231	5458.60	1258.39	0.201	3609.18	726.81
A_68_P21612308	chr2:127074016-127074060	NM_175145:328	Tmem127	INSIDE	0.874	0.208	4706.00	979.57	0.182	3509.74	638.25
A_68_P21072875	chr2:25402109-25402153	NM_009422:284	Traf2	INSIDE	0.874	0.487	5748.24	2798.69	0.425	4202.65	1787.49
A_68_P20874687	chr1:182185972-182186016	NM_011183:-9913	Psen2	PROMOTER	0.874	0.593	4774.24	2831.41	0.519	3442.83	1785.28
A_68_P20872220	chr1:181733104-181733148	NM_026375:1020	Ahctf1	INSIDE	0.874	2.731	1628.38	4446.57	2.386	1227.29	2928.32
A_68_P20555161	chr1:120950392-120950436	NM_001081125:-218	Gli2	PROMOTER	0.874	0.622	2086.85	1298.57	0.544	1598.36	868.81
A_68_P20552782	chr1:120524517-120524561	NM_023755:17	Tcfcp2l1	INSIDE	0.874	2.180	658.64	1435.95	1.907	550.37	1049.28
A_68_P20056406	chr1:16609082-16609126	NM_025773:263	Ube2w	INSIDE	0.874	0.402	1349.65	542.81	0.352	1115.90	392.32
A_68_P32975879	A_68_P32975879			Unknown	0.873	2.352	1263.05	2970.35	2.053	1074.32	2205.19
A_68_P31142314	chr17:31993086-31993130	NM_010831:-371	Sik1	PROMOTER	0.873	0.255	2094.89	533.78	0.222	1589.17	353.41
A_68_P30251032	chr15:58765375-58765419	NM_023172:32	Ndufb9	INSIDE	0.873	2.432	861.48	2095.05	2.124	760.66	1615.64
A_68_P28755050	chr12:114394591-114394636	NM_134041:-18	4930427A07Rik	PROMOTER	0.873	0.209	2548.21	533.38	0.183	1984.57	362.71
A_68_P28265956	chr12:16660397-16660441	NM_008747:143	Ntsr2	INSIDE	0.873	0.350	1399.97	489.33	0.305	1100.78	335.99
A_68_P28177060	chr11:119161422-119161466	NM_138669:-87	Eif4a3	PROMOTER	0.873	2.384	1593.46	3798.03	2.081	1365.68	2842.23
A_68_P28078749	chr11:102158447-102158491	NM_001098836:-525	Atxn7l3	PROMOTER	0.873	0.584	3895.16	2274.77	0.510	2855.80	1456.10
A_68_P27838986	chr11:59600830-59600874	NM_183139:307	Pld6	INSIDE	0.873	2.315	936.22	2167.81	2.022	846.51	1711.87
A_68_P23964039	chr5:107354837-107354881	NM_177873:51	Hfm1	INSIDE	0.873	0.508	2779.50	1413.14	0.444	1892.06	839.85
A_68_P22893293	chr4:47221194-47221238	NM_009928:333	Col15a1	INSIDE	0.873	0.359	7582.56	2722.52	0.314	5314.85	1666.48
A_68_P22342531	chr3:94964250-94964300	NM_011351:-4021	Sema6c	PROMOTER	0.873	7.055	435.86	3075.09	6.156	383.13	2358.51
A_68_P20628461	chr1:135506729-135506773	NM_009227:107	Snrpe	INSIDE	0.873	0.333	1354.63	451.04	0.291	988.71	287.48
A_68_P31097740	chr17:24643732-24643776	NM_027937:18027	Caskin1	INSIDE	0.872	0.577	1254.58	723.89	0.503	973.27	489.85
A_68_P29626123	chr14:57723264-57723308	NM_008125:253	Gjb2	INSIDE	0.872	0.733	2181.15	1599.35	0.639	1677.86	1072.75
A_68_P29056696	chr13:55828862-55828906	NM_028281:156	Pcbd2	INSIDE	0.872	2.657	753.92	2002.93	2.317	668.59	1548.87
A_68_P29038493	chr13:52626118-52626162	NM_001024474:65	Diras2	INSIDE	0.872	0.603	2566.61	1547.94	0.526	2141.06	1126.17
A_68_P28992095	chr13:43880702-43880746	NM_009856:249	Cd83	INSIDE	0.872	2.122	1575.29	3342.37	1.851	1318.78	2440.46
A_68_P28752187	chr12:113977286-113977330	NM_001024602:16924	AW555464	INSIDE	0.872	2.137	2237.70	4782.65	1.864	1884.62	3513.10
A_68_P28737539	chr12:111516074-111516118	NR_002866:182	Dio3os	INSIDE	0.872	0.474	1649.86	782.62	0.413	1277.43	528.14
A_68_P28576084	chr12:81620026-81620070	NM_001081421:72	Galntl1	INSIDE	0.872	1.713	1362.39	2333.88	1.494	1033.22	1544.12
A_68_P27790811	chr11:50641126-50641170	NM_013922:85	Zfp354c	INSIDE	0.872	0.511	2592.76	1325.28	0.446	2110.19	940.69
A_68_P27561155	chr11:4962120-4962164	NM_001190406:3188	Gas2l1	INSIDE	0.872	0.278	1894.93	526.92	0.242	1424.28	345.36
A_68_P27277828	chr10:79076720-79076764	NM_198614:21	C2cd4c	INSIDE	0.872	1.734	1265.76	2194.93	1.513	1006.42	1522.53
A_68_P25018409	chr7:28443259-28443303	NM_001081292:16337	Map3k10	INSIDE	0.872	1.982	2479.60	4914.39	1.729	1916.39	3312.91
A_68_P24133864	chr5:139626346-139626390	NM_001081265:192	Heatr2	INSIDE	0.872	3.873	2171.21	8409.42	3.376	1732.45	5849.18
A_68_P23321452	chr4:133742548-133742592	NM_144527:430	Ccdc21	INSIDE	0.872	0.115	4374.68	505.16	0.101	2263.80	227.98
A_68_P22945330	chr4:57446812-57446856	ENSMUST00000102905:-284		PROMOTER	0.872	0.502	929.31	466.26	0.438	753.06	329.65
A_68_P22543384	chr3:133208009-133208053	NM_001040400:-676	Tet2	PROMOTER	0.872	2.817	1131.72	3188.08	2.458	936.10	2300.48
A_68_P22325403	chr3:90269219-90269263	NM_008727:548	Npr1	INSIDE	0.872	0.584	4744.66	2769.80	0.509	3535.99	1799.54
A_68_P21907445	chr2:180702090-180702134	NM_001177706:108	Arfgap1	INSIDE	0.872	0.415	2353.72	977.30	0.362	1751.13	633.74
A_68_P20722814	chr1:154614048-154614092	NM_026369:399	Arpc5	INSIDE	0.872	0.679	1693.43	1149.20	0.592	1358.27	803.91
A_68_P20146390	chr1:36161035-36161084	NM_015818:35815	Hs6st1	INSIDE	0.872	2.418	534.00	1291.05	2.108	433.06	912.78
A_68_P32477695	chrX:74678912-74678956			Unknown	0.871	2.017	461.09	929.80	1.757	597.18	1049.20
A_68_P32386814	chrX:50622922-50622968	NM_001166365:38	Fam122b	INSIDE	0.871	2.077	1034.44	2148.34	1.808	1233.35	2230.50
A_68_P31766837	chr18:63137771-63137815	NM_028017:223	Napg	INSIDE	0.871	0.515	1086.71	560.01	0.449	895.61	401.97
A_68_P31503848	chr18:13132093-13132137	NM_008378:1354	Impact	INSIDE	0.871	1.697	1755.14	2978.03	1.477	1373.39	2028.52
A_68_P31475770	chr18:7004914-7004958	NM_177595:-159	Mkx	PROMOTER	0.871	2.505	1395.15	3495.13	2.181	1206.75	2632.14
A_68_P31259843	chr17:57201503-57201547	NM_177638:-1725	Crb3	DIVERGENT_PROMOTER	0.871	0.657	2608.94	1713.43	0.572	1922.68	1100.42
A_68_P30346946	chr15:76059550-76059594	NM_201385:355	Plec	INSIDE	0.871	2.825	520.45	1470.26	2.460	493.97	1215.24
A_68_P29043571	chr13:53472579-53472623	NM_009269:130	Sptlc1	INSIDE	0.871	0.502	1333.45	669.70	0.437	1062.77	464.80
A_68_P28576807	chr12:81744426-81744470	NM_007951:400	Erh	INSIDE	0.871	10.545	3146.85	33183.44	9.182	2817.72	25873.46
A_68_P27931782	chr11:76212095-76212139	NM_008750:527	Nxn	INSIDE	0.871	1.649	3027.21	4991.92	1.436	2207.36	3169.28
A_68_P27845269	chr11:60692535-60692579	NM_001168507:-16	Tmem11	PROMOTER	0.871	0.482	1297.90	625.84	0.420	1052.99	442.27
A_68_P26134825	chr8:108129199-108129243	NM_001081241:92	Fam65a	INSIDE	0.871	0.342	4926.17	1685.48	0.298	3864.60	1151.12
A_68_P26021286	chr8:87230883-87230927	NM_001081981:67364	Nfix	DOWNSTREAM	0.871	0.575	2203.14	1266.35	0.501	1623.70	812.99
A_68_P25806161	chr8:41596469-41596513	NM_011135:168	Cnot7	INSIDE	0.871	0.580	1404.10	814.55	0.505	1137.67	574.96
A_68_P25598740	chr7:150292997-150293041	NM_008434:-140	Kcnq1	PROMOTER	0.871	0.483	1379.69	666.38	0.421	1086.39	457.20
A_68_P25310386	chr7:97089350-97089394	NM_026304:323	l7Rn6	INSIDE	0.871	0.398	5405.31	2151.28	0.347	3567.61	1237.17
A_68_P22378031	chr3:101728081-101728125	NM_001039371:274	Slc22a15	INSIDE	0.871	3.261	1170.65	3817.30	2.841	920.67	2615.81
A_68_P22283295	chr3:82932608-82932652	ENSMUST00000047876:538		INSIDE	0.871	2.159	877.70	1894.59	1.881	703.21	1322.65
A_68_P21834174	chr2:167014089-167014133	NM_008420:189	Kcnb1	INSIDE	0.871	2.448	669.57	1638.84	2.132	626.72	1336.44
A_68_P21818154	chr2:164312351-164312395	NM_001048228:-4	Dbndd2	PROMOTER	0.871	0.123	4442.87	545.63	0.107	2792.92	298.63
A_68_P21483577	chr2:103324980-103325024	NM_009804:308	Cat	INSIDE	0.871	0.608	1274.34	775.27	0.530	992.00	525.88

ProbeName	Target position of probe on CpG island microarray	TargetID	GeneSymbol	CpG island Description	Ratio of relative methylation (TiO_2-NP/Vehicle)	Sham group			TiO_2-H group		
						Relative methylation (Cy5/Cy3)	Cy3 signal (Input)	Cy5 signal (MeDIP)	Relative methylation (Cy5/Cy3)	Cy3 signal (Input)	Cy5 signal (MeDIP)
A_68_P21072310	chr2:25316302-25316346	NM_153557:290	BC029214	INSIDE	0.871	0.520	3168.99	1648.31	0.453	2198.42	995.55
A_68_P20997485	chr2:9803030-9803074	NR_024257:180	4930412O13Rik	INSIDE	0.871	0.405	2363.90	958.33	0.353	1844.85	651.07
A_68_P20140407	chr1:34807751-34807795	ENSMUST00000159021:47		INSIDE	0.871	2.472	1379.62	3410.17	2.153	1237.85	2665.02
A_68_P32463052	chrX:70748623-70748667	NM_177236:220	Atp2b3	INSIDE	0.870	4.093	275.40	1127.33	3.561	382.86	1363.43
A_68_P32383371	chrX:49967421-49967465	NM_016697:-291	Gpc3	PROMOTER	0.870	0.534	1127.68	601.98	0.465	1604.11	745.24
A_68_P32119295	chr19:42222796-42222840	NM_183195:940	Marveld1	INSIDE	0.870	0.390	1234.91	482.16	0.340	958.88	325.67
A_68_P31253475	chr17:56176053-56176097	NM_013664:-75	Sh3gl1	DIVERGENT_PROMOTER	0.870	0.375	5250.68	1969.27	0.326	3466.72	1130.75
A_68_P30895371	chr16:78301854-78301898	NM_001025192:-59	Cxadr	PROMOTER	0.870	0.426	1826.36	778.78	0.371	1606.60	595.79
A_68_P28601433	chr12:86311943-86311987	NM_028632:85	Fcf1	INSIDE	0.870	0.154	2967.60	458.38	0.134	2063.93	277.24
A_68_P28185955	chr11:120522293-120522337	NM_175263:-163	Notum	PROMOTER	0.870	0.664	1201.89	798.59	0.578	978.65	565.72
A_68_P27893305	chr11:68947686-68947730	NM_011786:7830	Aloxe3	INSIDE	0.870	0.303	1605.71	485.81	0.263	1232.44	324.54
A_68_P26467229	chr9:44169555-44169599	NM_027889:177	Vps11	INSIDE	0.870	0.236	2295.12	542.67	0.206	1753.34	360.54
A_68_P25504472	chr7:134339349-134339393	NM_027353:144	Cd2bp2	INSIDE	0.870	0.455	1850.46	842.72	0.396	1319.87	522.92
A_68_P25086963	chr7:51608090-51608134	NM_001034115:42479	Shank1	INSIDE	0.870	3.537	543.53	1922.65	3.078	474.22	1459.44
A_68_P24768309	chr6:114919483-114919527	ENSMUST00000164670:-283		PROMOTER	0.870	0.683	3596.53	2456.60	0.594	2573.26	1528.68
A_68_P24438215	chr6:50406155-50406199	NM_001163645:-7	Osbpl3	PROMOTER	0.870	0.211	2466.65	521.22	0.184	1850.93	340.43
A_68_P23609442	chr5:37139552-37139596	NM_133724:344	Cno	INSIDE	0.870	0.617	2145.60	1324.13	0.537	1649.45	885.57
A_68_P23321804	chr4:133805561-133805605	NM_024215:-4076	Zfp593	PROMOTER	0.870	1.782	2267.79	4041.22	1.550	1685.37	2612.52
A_68_P22345831	chr3:95622735-95622779	NM_001081293:120	Rprd2	INSIDE	0.870	0.364	2763.10	1006.46	0.317	1898.29	601.74
A_68_P22142067	chr3:53283022-53283066	NM_173382:15306	2810046L04Rik	INSIDE	0.870	2.143	950.36	2036.98	1.865	739.62	1379.28
A_68_P21597426	chr2:124435887-124435931	NM_172537:-123	Sema6d	PROMOTER	0.870	0.514	1113.89	572.85	0.448	810.75	362.87
A_68_P20936236	chr1:192922374-192922418	NM_030060:104	Batf3	INSIDE	0.870	0.433	4576.10	1979.89	0.376	3219.02	1211.49
A_68_P20739599	chr1:157588256-157588300	NM_010712:879	Lhx4	INSIDE	0.870	0.735	2303.04	1693.41	0.639	1673.45	1069.97
A_68_P32152395	chr19:47939790-47939835	NM_030051:-222	Gsto2	PROMOTER	0.869	4.487	748.06	3356.25	3.897	592.08	2307.46
A_68_P30955584	chr16:90220772-90220816	NM_011434:-192	Sod1	PROMOTER	0.869	0.654	1639.61	1072.98	0.569	1137.24	646.69
A_68_P30378918	chr15:81494923-81494967	NM_145993:579	L3mbtl2	INSIDE	0.869	2.269	1078.02	2445.96	1.973	982.52	1938.06
A_68_P30349161	chr15:76406872-76406916	NM_172960:106	Adck5	INSIDE	0.869	0.246	2856.53	701.58	0.213	2053.82	438.17
A_68_P29644194	chr14:60997825-60997869	NM_001164705:724	Fam123a	INSIDE	0.869	2.218	1095.80	2430.18	1.926	832.04	1602.90
A_68_P29506490	chr14:31362630-31362674	NM_009388:318	Tkt	INSIDE	0.869	2.376	386.42	918.28	2.064	404.31	834.52
A_68_P29338285	chr13:113825490-113825535	NR_030452:-678	Mir449c	PROMOTER	0.869	0.372	2355.95	877.07	0.323	1842.08	595.78
A_68_P28765899	chr12:117316302-117316346	NM_009511:129	Vipr2	INSIDE	0.869	1.823	1392.15	2538.54	1.585	1242.70	1969.82
A_68_P28185092	chr11:120392138-120392182	NM_008101:120	Gcgr	INSIDE	0.869	0.272	2413.03	655.41	0.236	1700.06	401.30
A_68_P28029987	chr11:93817170-93817214	NM_001077529:-190	Nme2	PROMOTER	0.869	1.635	2565.19	4194.70	1.421	1867.62	2654.15
A_68_P27809329	chr11:54117906-54117950	NM_001033597:223	Acsl6	INSIDE	0.869	0.370	3244.01	1200.69	0.322	2676.77	861.36
A_68_P27781640	chr11:48982878-48982922	NM_172793:17691	Btnl9	INSIDE	0.869	3.877	2276.20	8825.38	3.369	2033.02	6849.97
A_68_P27675741	chr11:29593764-29593808	NM_194051:13	Rtn4	INSIDE	0.869	0.248	4014.95	995.96	0.216	2802.78	604.00
A_68_P26897433	A_68_P26897433			Unknown	0.869	0.395	2869.16	1133.29	0.343	2441.81	838.18
A_68_P25348906	chr7:104230072-104230116	NM_001162477:-166	Gab2	PROMOTER	0.869	6.325	7510.33	47499.71	5.493	5347.99	29377.52
A_68_P25021005	chr7:29079533-29079577	NM_007866:7250	Dll3	INSIDE	0.869	2.213	1227.90	2717.13	1.922	1068.58	2054.00
A_68_P24793343	chr6:119429349-119429393	NM_133940:-315	Fbxl14	PROMOTER	0.869	3.777	535.50	2022.82	3.281	453.40	1487.73
A_68_P24617814	chr6:87681107-87681152	NM_001159697:267	Ccdc48	INSIDE	0.869	2.085	1021.50	2129.40	1.812	884.56	1602.92
A_68_P24517586	chr6:66485635-66485679	NM_019499:195	Mad2l1	INSIDE	0.869	0.380	1349.52	513.15	0.330	1187.80	392.38
A_68_P24431878	chr6:49214131-49214175	NM_198102:-101	Tra2a	PROMOTER	0.869	0.455	1635.37	744.19	0.396	1312.78	519.34
A_68_P23463653	chr5:7344782-7344826	NM_001163223:-426	Zfp804b	PROMOTER	0.869	2.296	1022.60	2348.22	1.996	917.96	1832.63
A_68_P23269744	chr4:124370820-124370865	NM_026031:-44	Utp11l	DIVERGENT_PROMOTER	0.869	0.399	2552.43	1018.71	0.347	1924.37	667.74
A_68_P21818227	chr2:164323199-164323243	NM_133779:196	Pigt	INSIDE	0.869	0.278	2213.10	615.99	0.242	1578.25	381.63
A_68_P32133761	chr19:44758930-44758974	ENSMUST00000166501:10813		DOWNSTREAM	0.868	0.227	2275.12	515.68	0.197	1809.39	356.19
A_68_P31931398	chr19:5875077-5875121	NM_026169:110	Frmd8	INSIDE	0.868	1.674	2422.70	4054.40	1.452	1868.64	2713.97
A_68_P31256423	chr17:56613777-56613821	NM_011218:2105	Ptprs	INSIDE	0.868	0.242	2528.82	611.93	0.210	1860.12	390.53
A_68_P30999916	chr16:97832042-97832086	NM_015775:738	Tmprss2	INSIDE	0.868	0.472	1262.64	595.92	0.410	930.82	381.42
A_68_P30979594	chr16:94346769-94346813	NM_011377:261286	Sim2	INSIDE	0.868	0.711	2769.38	1967.69	0.617	1926.40	1187.63
A_68_P30728923	chr16:45844322-45844366	NM_153412:147	Phldb2	INSIDE	0.868	0.117	4761.62	555.48	0.101	3279.65	332.17
A_68_P30363495	chr15:78798496-78798540	NM_001039155:20365	Triobp	INSIDE	0.868	1.922	2177.40	4185.26	1.669	1605.46	2679.28
A_68_P28787940	chr13:3358038-3358084			Unknown	0.868	0.170	3278.84	557.83	0.148	2073.87	306.33
A_68_P28172264	chr11:118430533-118430577	NM_001024931:340332	Rbfox3	INSIDE	0.868	0.397	1309.43	520.26	0.345	847.48	292.14
A_68_P27977954	chr11:84333936-84333980	NM_008498:5078	Lhx1	INSIDE	0.868	2.404	609.21	1464.50	2.086	497.99	1038.64
A_68_P25584910	chr7:147973596-147973640	NM_001143765:135	Syce1	INSIDE	0.868	3.449	3344.48	11533.98	2.994	2618.11	7838.53
A_68_P21820791	chr2:164793332-164793376	NM_020333:-133	Slc12a5	PROMOTER	0.868	0.372	2309.71	859.17	0.323	1672.25	539.77
A_68_P21078053	chr2:26208046-26208090	NM_001037747:6999	Card9	INSIDE	0.868	2.100	929.95	1952.55	1.823	745.48	1358.76
A_68_P20826007	chr1:172995883-172995927	NM_010188:-6370	Fcgr3	PROMOTER	0.868	0.144	9240.49	1328.82	0.125	7229.72	902.68
A_68_P20782634	chr1:165242803-165242847	NM_011127:957	Prrx1	INSIDE	0.868	3.275	588.00	1925.51	2.843	526.07	1495.63
A_68_P20163206	chr1:39044427-39044471	NM_026850:-210	Pdcl3	PROMOTER	0.868	0.187	3160.62	591.66	0.162	2251.94	365.90
A_68_P20146150	chr1:36125563-36125607	NM_015818:340	Hs6st1	INSIDE	0.868	5.298	2820.19	14940.31	4.601	2242.03	10315.13
A_68_P31006134	chr17:3557457-3557501	NM_146074:235	Tfb1m	INSIDE	0.867	0.525	2819.21	1479.56	0.455	2078.11	945.17
A_68_P30425292	chr15:89288332-89288376	NM_021921:4013	Mapk8ip2	INSIDE	0.867	5.672	204.95	1162.52	4.917	140.49	690.82

ProbeName	Target position of probe on CpG island microarray	TargetID	GeneSymbol	CpG island Description	Ratio of relative methylation (TiO_2-NP/Vehicle)	Sham group			TiO_2-H group		
						Relative methylation (Cy5/Cy3)	Cy3 signal (Input)	Cy5 signal (MeDIP)	Relative methylation (Cy5/Cy3)	Cy3 signal (Input)	Cy5 signal (MeDIP)
A_68_P30356630	chr15:77759323-77759367	NM_019913:80	Txn2	INSIDE	0.867	0.258	1863.95	480.89	0.224	1515.06	339.03
A_68_P29622469	chr14:56983976-56984021	NM_138652:94	Atp12a	INSIDE	0.867	0.550	2834.17	1559.21	0.477	1843.70	878.94
A_68_P28051744	chr11:97490870-97490916	NM_175332:138	E130012A19Rik	INSIDE	0.867	2.706	622.54	1684.42	2.347	577.91	1356.28
A_68_P27915635	chr11:72990099-72990143	NM_001168470:390	Tmem93	INSIDE	0.867	0.418	1857.34	775.75	0.362	1485.66	538.16
A_68_P27452135	chr10:111410230-111410274	NM_178610:502	Krr1	INSIDE	0.867	0.381	2165.78	824.29	0.330	1610.31	531.37
A_68_P27138502	chr10:52411382-52411426	NM_178675:1098	Slc35f1	INSIDE	0.867	0.471	1255.24	591.14	0.408	957.59	390.91
A_68_P26924746	chr10:8238018-8238062	NM_177387:583	Ust	INSIDE	0.867	0.436	1193.17	520.16	0.378	865.20	327.13
A_68_P25190566	chr7:73985093-73985137	NM_001033877:494	Adamts17	INSIDE	0.867	2.126	1326.35	2819.66	1.843	1048.73	1932.31
A_68_P25075290	chr7:48158061-48158105	NM_021387:3435	Vstm2b	INSIDE	0.867	1.831	1276.71	2337.62	1.587	1047.98	1663.16
A_68_P24994418	chr7:20214432-20214477	NM_009046:333	Relb	INSIDE	0.867	0.348	3579.62	1246.99	0.302	2458.53	742.59
A_68_P23618838	chr5:38710862-38710906	NM_013503:137	Drd5	INSIDE	0.867	0.711	2611.70	1856.41	0.616	2104.29	1296.22
A_68_P23588824	chr5:33773360-33773404	ENSMUST00000030994:462		DOWNSTREAM	0.867	2.165	846.22	1832.28	1.878	697.58	1309.73
A_68_P22680849	chr3:157230737-157230781	NM_011196:903	Ptger3	INSIDE	0.867	0.578	1320.73	762.97	0.501	917.43	459.48
A_68_P31259250	chr17:57074995-57075039	NM_022328:-205	Mllt1	PROMOTER	0.866	0.400	1429.11	571.14	0.346	1178.40	407.61
A_68_P31201703	chr17:45682394-45682438	NM_001013749:4210	Tmem151b	INSIDE	0.866	2.193	2473.39	5425.26	1.899	2015.88	3827.33
A_68_P31123429	chr17:28758848-28758893	NM_016795:529	Srpk1	INSIDE	0.866	0.348	2519.41	876.43	0.301	1733.48	522.08
A_68_P31120664	chr17:28278724-28278768	NM_001004366:-724	Scube3	PROMOTER	0.866	1.729	1571.02	2716.56	1.498	1350.82	2023.72
A_68_P30639624	A_68_P30639624			Unknown	0.866	3.173	708.07	2246.88	2.749	519.31	1427.45
A_68_P30572149	chr16:17132425-17132469	NM_022324:30	Sdf2l1	INSIDE	0.866	0.356	3182.60	1134.41	0.309	2426.26	748.90
A_68_P30295268	chr15:66800327-66800371	NM_008681:855	Ndrg1	INSIDE	0.866	0.736	4820.62	3545.57	0.637	3820.17	2433.78
A_68_P29961943	chr14:122875333-122875377	NM_009574:749	Zic2	INSIDE	0.866	1.614	2176.74	3512.56	1.397	1782.98	2490.66
A_68_P29718153	chr14:73952777-73952821	NM_011506:206	Sucla2	INSIDE	0.866	2.414	608.22	1468.16	2.089	527.16	1101.49
A_68_P28556219	chr12:77938785-77938829	NM_145927:353	Fntb	INSIDE	0.866	0.672	1251.87	841.09	0.582	1117.33	649.77
A_68_P28323157	chr12:30687355-30687399	NM_181395:64476	Pxdn	INSIDE	0.866	1.693	1965.50	3327.29	1.466	1321.45	1937.67
A_68_P28137851	chr11:112646227-112646271	NM_011448:2725	Sox9	INSIDE	0.866	0.408	3654.72	1491.88	0.354	2700.96	955.06
A_68_P28077066	chr11:101887900-101887944	NM_007863:347	Mpp3	INSIDE	0.866	0.522	1502.30	783.61	0.452	1119.55	505.86
A_68_P27994975	chr11:87405824-87405868	NM_133215:128	Mtmr4	INSIDE	0.866	0.695	1974.91	1372.47	0.602	1480.17	890.34
A_68_P27532962	chr10:126365579-126365623			Unknown	0.866	0.609	1092.53	665.71	0.528	820.11	432.83
A_68_P26783901	chr9:103014660-103014704	NM_173781:279	Rab6b	INSIDE	0.866	0.478	3228.81	1544.87	0.415	2204.82	914.02
A_68_P25737430	chr8:28371048-28371092	NM_007918:272	Eif4ebp1	INSIDE	0.866	0.524	1689.66	884.72	0.453	1374.43	622.99
A_68_P24620369	chr6:88149762-88149806	NM_008090:1127	Gata2	INSIDE	0.866	1.900	942.69	1791.43	1.646	858.01	1412.18
A_68_P24135227	chr5:139856761-139856805	NM_001038703:-9075	Gpr146	PROMOTER	0.866	1.767	1614.57	2853.48	1.530	1257.98	1924.53
A_68_P23882635	chr5:90794338-90794382	NM_030886:851	Ankrd17	INSIDE	0.866	0.346	3135.45	1086.30	0.300	2509.42	753.20
A_68_P22820143	chr4:32326406-32326450	ENSMUST00000165661:-4279		PROMOTER	0.866	2.686	4095.52	11002.02	2.326	3195.52	7434.23
A_68_P20871446	chr1:181598547-181598591	NM_178653:207	Sccpdh	INSIDE	0.866	2.541	997.01	2533.72	2.202	791.22	1742.02
A_68_P32707835	chrX:138824834-138824878	NM_001033600:222	Acsl4	INSIDE	0.865	0.380	1355.59	515.24	0.329	1863.28	612.73
A_68_P31753941	chr18:61008559-61008603	NM_001198984:38	Tcof1	INSIDE	0.865	1.899	1048.06	1990.02	1.642	874.85	1436.59
A_68_P31259988	chr17:57226985-57227029	NM_009451:199	Tubb4	INSIDE	0.865	0.453	1201.87	544.53	0.392	1011.47	396.48
A_68_P31215014	chr17:48061036-48061080	NM_001110824:523	Foxp4	INSIDE	0.865	4.575	1320.06	6039.82	3.957	990.44	3919.15
A_68_P31098879	chr17:24806802-24806846	NM_183149:128	Zfp598	INSIDE	0.865	0.584	1993.87	1164.40	0.505	1695.39	856.72
A_68_P30476484	chr15:98623292-98623336	NM_021279:3027	Wnt1	INSIDE	0.865	6.835	969.19	6624.56	5.911	763.47	4512.51
A_68_P30374040	chr15:80542264-80542308	NM_144812:544	Tnrc6b	INSIDE	0.865	0.358	2340.18	838.03	0.310	1761.46	545.51
A_68_P30084537	chr15:25913916-25913960	NM_144523:-182	Zfp622	PROMOTER	0.865	0.394	4153.69	1636.81	0.341	3050.85	1039.72
A_68_P28683727	chr12:101963505-101963549	NM_153587:-288	Rps6ka5	PROMOTER	0.865	0.590	2028.26	1196.49	0.510	1538.45	785.01
A_68_P27915643	chr11:72991022-72991066	NM_029564:460	Tax1bp3	INSIDE	0.865	0.432	1583.92	683.64	0.373	1308.53	488.33
A_68_P26665701	chr9:79566357-79566401	NM_007730:107	Col12a1	INSIDE	0.865	1.924	2795.14	5378.20	1.665	2208.84	3678.15
A_68_P26356133	chr9:23028290-23028334	NM_028472:793	Bmper	INSIDE	0.865	0.338	5464.43	1848.13	0.293	3471.95	1016.09
A_68_P26349632	chr9:21741348-21741392	NM_144935:-1347	BC018242	PROMOTER	0.865	0.414	1138.50	471.11	0.358	867.78	310.65
A_68_P26170966	chr8:114457299-114457343	NM_001033321:371	Tmem231	INSIDE	0.865	0.567	1271.07	720.32	0.490	1055.40	517.19
A_68_P26020980	chr8:87186400-87186444	NM_010499:329	Ier2	INSIDE	0.865	0.569	1919.81	1092.67	0.493	1527.92	752.61
A_68_P25608859	chr7:151924254-151924298	NM_178642:107	Ano1	INSIDE	0.865	0.500	1682.70	841.02	0.432	1411.63	610.40
A_68_P25499770	chr7:133436200-133436244	NM_145587:20090	Sbk1	INSIDE	0.865	2.205	1038.15	2288.81	1.906	778.48	1483.82
A_68_P25351150	chr7:104728199-104728243	NM_001081267:-185	Rsf1	DIVERGENT_PROMOTER	0.865	0.111	6438.02	716.64	0.096	4181.42	402.41
A_68_P25024912	chr7:29805278-29805322	NM_009109:104870	Ryr1	INSIDE	0.865	2.152	700.97	1508.50	1.861	551.91	1026.96
A_68_P25021286	chr7:29124000-29124044	NM_013676:-284	Supt5h	PROMOTER	0.865	1.780	1143.92	2036.58	1.541	1043.94	1608.60
A_68_P24428409	chr6:48577327-48577371	NR_027236:-817	AI854703	PROMOTER	0.865	0.148	4639.25	687.06	0.128	3051.22	391.06
A_68_P24225084	chr6:8209188-8209232	NR_026632:-69	Rpa3	DIVERGENT_PROMOTER	0.865	0.359	2706.09	972.63	0.311	1498.96	466.22
A_68_P23925842	chr5:100157797-100157841	NR_030692:261	A930011G23Rik	INSIDE	0.865	2.353	1245.95	2931.96	2.035	957.21	1948.01
A_68_P23312650	chr4:132066273-132066317	NM_144907:77	Sesn2	INSIDE	0.865	0.617	1376.64	849.26	0.534	1085.58	579.58
A_68_P22321610	chr3:89577295-89577339	NM_027315:-214	Ube2q1	DIVERGENT_PROMOTER	0.865	0.591	2125.28	1256.70	0.512	1676.94	858.02
A_68_P21906887	chr2:180626371-180626415	NR_029538:-2352	Mir124a-3	PROMOTER	0.865	0.348	1580.87	550.39	0.301	1313.79	395.60
A_68_P21612307	chr2:127073935-127073979	NM_175145:246	Tmem127	INSIDE	0.865	0.106	11417.02	1214.07	0.092	8135.75	747.95
A_68_P21071165	chr2:25146200-25146244	NM_146117:792	Lrrc26	INSIDE	0.865	0.664	1724.27	1144.57	0.574	1271.86	730.49
A_68_P20939665	chr1:193542469-193542513	NM_001134829:217	Lpgat1	INSIDE	0.865	2.068	523.36	1082.54	1.790	527.41	943.89
A_68_P20645417	chr1:138242409-138242453	NM_001081360:251	Camsap1l1	INSIDE	0.865	2.409	570.17	1373.62	2.084	515.77	1074.67

ProbeName	Target position of probe on CpG island microarray	TargetID	GeneSymbol	CpG island Description	Ratio of relative methylation (TiO_2-NP/Vehicle)	Sham group			TiO_2-H group		
						Relative methylation (Cy5/Cy3)	Cy3 signal (Input)	Cy5 signal (MeDIP)	Relative methylation (Cy5/Cy3)	Cy3 signal (Input)	Cy5 signal (MeDIP)
A_68_P20618103	chr1:133763971-133764015	NM_144875:139	Rab7l1	INSIDE	0.865	0.421	1441.94	607.78	0.364	1185.95	432.26
A_68_P32463876	chrX:70882705-70882749	NM_029352:-2053	Dusp9	PROMOTER	0.864	4.189	999.08	4185.04	3.617	1202.32	4349.35
A_68_P31152225	chr17:34257765-34257809	NR_037970:-941	Brd2	PROMOTER	0.864	0.243	5408.03	1311.76	0.210	3680.31	771.34
A_68_P31052340	chr17:12700162-12700206	NM_011395:386	Slc22a3	INSIDE	0.864	0.538	2129.48	1145.14	0.465	1769.34	822.45
A_68_P30497013	chr15:102110024-102110068	NM_134100:160	Mfsd5	INSIDE	0.864	10.708	1155.14	12369.47	9.250	1169.45	10817.45
A_68_P30486140	chr15:100325173-100325217	NM_153407:476	Csrnp2	INSIDE	0.864	0.753	7005.13	5272.69	0.650	4767.95	3099.69
A_68_P30401626	chr15:85411951-85411995	NM_001163633:-813	Wnt7b	PROMOTER	0.864	1.985	1137.59	2258.40	1.715	968.68	1661.60
A_68_P29660091	chr14:63865134-63865179	NM_008092:-1059	Gata4	PROMOTER	0.864	8.338	539.23	4496.36	7.204	373.31	2689.34
A_68_P29022518	chr13:49062632-49062676	NM_001033268:543	Fam120a	INSIDE	0.864	0.551	1636.87	901.39	0.476	1367.44	650.72
A_68_P28888885	chr13:24853671-24853715	NM_020567:114	Gmnn	INSIDE	0.864	0.329	1576.11	518.55	0.284	1245.58	354.26
A_68_P28532687	chr12:73637342-73637386	NM_026327:21	1810048J11Rik	INSIDE	0.864	0.399	1147.75	458.27	0.345	1037.16	357.98
A_68_P28221326	chr12:8308667-8308711	NM_013527:72	Gdf7	INSIDE	0.864	2.801	537.47	1505.58	2.420	467.53	1131.21
A_68_P27925674	chr11:75161948-75161992	NM_001102611:36	Smyd4	INSIDE	0.864	0.432	2020.10	872.73	0.373	1668.36	622.75
A_68_P26890107	chr9:122478468-122478512	NR_015610:-3128	9530059O14Rik	PROMOTER	0.864	2.227	812.72	1809.72	1.924	708.01	1362.37
A_68_P25925202	chr8:67171298-67171342	NM_013494:517	Cpe	INSIDE	0.864	0.258	3185.36	823.21	0.223	2373.40	530.08
A_68_P25835352	chr8:47379897-47379941	NM_173789:107	Helt	INSIDE	0.864	0.320	2729.96	872.99	0.276	2280.87	630.02
A_68_P25032016	chr7:31319911-31319955	NM_178252:96	Arhgap33	INSIDE	0.864	0.450	4654.48	2092.90	0.389	3260.74	1267.30
A_68_P24061769	chr5:124981558-124981602	NM_021430:-180	Rilpl1	DIVERGENT_PROMOTER	0.864	0.476	1197.29	569.98	0.411	882.89	362.98
A_68_P23977812	chr5:110535984-110536028	NM_145147:1210	Gtpbp6	INSIDE	0.864	2.381	993.93	2366.12	2.057	773.45	1591.34
A_68_P23361405	chr4:140543030-140543074	NM_001164366:265	Atp13a2	INSIDE	0.864	0.389	3559.49	1383.08	0.336	2308.73	775.20
A_68_P22044442	chr3:33699195-33699239	NM_025978:100	Ttc14	INSIDE	0.864	3.339	244.25	815.44	2.886	206.71	596.55
A_68_P32111326	chr19:40905687-40905731	NM_172839:-60	Ccnj	PROMOTER	0.863	2.756	478.12	1317.47	2.378	420.58	1000.12
A_68_P31866355	chr18:80910611-80910655	NM_001164110:-822	Nfatc1	PROMOTER	0.863	0.485	1771.46	858.67	0.418	1354.30	566.47
A_68_P31411711	chr17:86014433-86014477	NR_038085:3282	Six3os1	INSIDE	0.863	0.265	1803.71	477.64	0.228	1449.71	331.17
A_68_P29620552	chr14:56519022-56519066	NM_001082975:25	Sdr39u1	INSIDE	0.863	0.338	3862.57	1303.79	0.291	2688.16	782.88
A_68_P29098741	chr13:64533952-64533996	NM_053180:114	Cdk20	INSIDE	0.863	2.302	896.06	2062.52	1.986	689.76	1369.97
A_68_P26139674	chr8:108939165-108939209	NM_011764:-52	Zfp90	PROMOTER	0.863	0.215	2456.64	527.84	0.185	1837.71	340.85
A_68_P25954403	chr8:73362971-73363015	NM_027560:627	Arrdc2	INSIDE	0.863	0.390	1624.98	633.02	0.336	1435.74	482.92
A_68_P25470622	chr7:127986473-127986517	NM_007908:98	Eef2k	INSIDE	0.863	0.215	2416.53	518.61	0.185	1979.61	366.76
A_68_P25370887	chr7:108147267-108147311	NM_008773:13217	P2ry2	INSIDE	0.863	2.558	2163.04	5533.16	2.207	1592.45	3514.44
A_68_P25198321	chr7:75310168-75310212	NM_010513:213048	Igf1r	INSIDE	0.863	2.068	1330.48	2751.71	1.784	1050.22	1873.78
A_68_P25025681	chr7:29946628-29946672	NM_182927:7016	Spred3	INSIDE	0.863	2.821	1968.50	5552.83	2.434	1432.23	3485.35
A_68_P24810292	chr6:122715013-122715057	ENSMUST00000171541:14941		INSIDE	0.863	0.174	3381.15	589.11	0.150	2372.02	356.59
A_68_P24318696	chr6:28085256-28085300	NM_008174:-909	Grm8	PROMOTER	0.863	0.588	875.49	514.58	0.507	759.72	385.34
A_68_P24143008	chr5:141211823-141211867	NM_173405:11764	Amz1	INSIDE	0.863	0.542	1131.76	613.46	0.468	946.35	442.88
A_68_P22500445	chr3:125641287-125641331	NM_011674:160	Ugt8a	INSIDE	0.863	0.175	3739.65	654.25	0.151	2742.75	414.00
A_68_P21579355	chr2:120997099-120997143	NM_153387:179	Tubgcp4	INSIDE	0.863	2.147	1059.58	2275.23	1.854	794.16	1472.19
A_68_P32094200	chr19:37249204-37249248	NM_198300:32557	Cpeb3	INSIDE	0.862	0.493	3900.56	1924.07	0.425	2950.21	1254.08
A_68_P31383398	chr17:80879916-80879960	NM_009231:-145	Sos1	PROMOTER	0.862	2.584	1542.57	3986.46	2.229	1276.43	2845.08
A_68_P31114981	chr17:27340635-27340679	NM_146075:727	Lemd2	INSIDE	0.862	2.372	632.76	1500.70	2.043	521.05	1064.62
A_68_P30672033	chr16:35768842-35768886	NM_153550:578	Dirc2	INSIDE	0.862	0.456	1207.69	550.25	0.393	984.01	386.54
A_68_P30478223	chr15:98927922-98927966	NM_176835:-1970	Dnajc22	DIVERGENT_PROMOTER	0.862	0.598	1199.09	716.94	0.516	934.79	482.06
A_68_P30371156	chr15:80094548-80094592	NM_175109:166	Rps19bp1	INSIDE	0.862	0.510	2612.12	1331.05	0.439	2124.86	932.97
A_68_P30370604	chr15:80003678-80003722	NM_010795:-450	Mgat3	PROMOTER	0.862	1.903	12672.80	24117.93	1.640	8240.56	13516.88
A_68_P30089769	chr15:26825262-26825306	NM_176959:35	Fbxl7	INSIDE	0.862	1.854	941.17	1745.33	1.598	780.08	1246.77
A_68_P29448698	chr14:21553543-21553587	NM_010811:220	Ndst2	INSIDE	0.862	2.819	410.15	1156.36	2.429	384.36	933.59
A_68_P28269480	chr12:17273418-17273462	NM_027959:40	Pdia6	INSIDE	0.862	0.442	2492.46	1101.59	0.381	2012.48	766.74
A_68_P27938076	chr11:77328807-77328851	NM_026185:210	Abhd15	INSIDE	0.862	5.026	1630.91	8197.67	4.335	1406.05	6094.77
A_68_P26986171	chr10:20031764-20031808	NM_001025392:-488	Bclaf1	PROMOTER	0.862	1.901	893.48	1698.64	1.638	782.21	1281.25
A_68_P26825039	chr9:110558909-110558957	NM_028312:-74	Ccdc12	DIVERGENT_PROMOTER	0.862	2.548	816.13	2079.25	2.197	738.10	1621.44
A_68_P26614836	chr9:69989309-69989353	NM_029784:34	Fam81a	INSIDE	0.862	2.847	286.12	814.51	2.455	283.10	695.08
A_68_P26228891	chr8:124308748-124308792	NM_020605:54312	Jph3	INSIDE	0.862	2.635	1357.32	3577.00	2.271	1225.16	2782.75
A_68_P25091649	chr7:52437106-52437150	NM_053256:764	Pth2	INSIDE	0.862	0.656	1307.18	857.08	0.565	970.55	548.81
A_68_P24974337	chr7:13629455-13629499	NM_145819:10637	Mzf1	INSIDE	0.862	0.332	1660.55	551.49	0.286	1375.52	393.77
A_68_P24046890	chr5:122302790-122302834	NM_175474:3776	Fam109a	INSIDE	0.862	2.311	1486.35	3435.37	1.992	1302.05	2593.15
A_68_P23250459	chr4:119960018-119960062	NM_194060:-174	Foxo6	PROMOTER	0.862	0.181	6573.35	1190.36	0.156	4159.21	648.91
A_68_P20715358	chr1:153418931-153418975	NM_022018:450	Fam129a	INSIDE	0.862	0.235	3877.14	910.30	0.202	2829.46	572.43
A_68_P20634556	chr1:136543657-136543701	NM_009307:421	Syt2	INSIDE	0.862	0.516	981.44	506.60	0.445	762.84	339.32
A_68_P31123430	chr17:28758955-28758999	NM_016795:423	Srpk1	INSIDE	0.861	0.379	4630.29	1752.65	0.326	3111.23	1013.95
A_68_P29578138	chr14:47380037-47380081	NM_028222:-157	Cdkn3	PROMOTER	0.861	1.975	1668.34	3294.47	1.700	1323.54	2250.50
A_68_P29098529	chr13:64471358-64471402	NM_009984:234	Ctsl	INSIDE	0.861	0.474	1011.46	479.67	0.408	802.94	327.82
A_68_P29055228	chr13:55573778-55573822	NM_001030296:8173	Prr7	INSIDE	0.861	0.598	898.14	537.08	0.515	786.33	404.92
A_68_P28549419	chr12:76697049-76697093	NM_012024:117	Ppp2r5e	INSIDE	0.861	0.389	5689.28	2213.47	0.335	4144.62	1388.53
A_68_P28457807	chr12:57713035-57713080	NM_008701:1214	Nkx2-9	INSIDE	0.861	3.570	788.75	2815.71	3.074	658.84	2025.41
A_68_P28140562	chr11:113063090-113063135	NR_015556:40	2610035D17Rik	INSIDE	0.861	0.372	1183.18	440.53	0.321	780.92	250.47

ProbeName	Target position of probe on CpG island microarray	TargetID	GeneSymbol	CpG island Description	Ratio of relative methylation (TiO_2-NP/Vehicle)	Sham group			TiO_2-H group		
						Relative methylation (Cy5/Cy3)	Cy3 signal (Input)	Cy5 signal (MeDIP)	Relative methylation (Cy5/Cy3)	Cy3 signal (Input)	Cy5 signal (MeDIP)
A_68_P28118144	chr11:109223851-109223895	NM_010303:-235	Gna13	PROMOTER	0.861	0.250	1887.18	472.34	0.215	1277.93	275.31
A_68_P28068816	chr11:100436337-100436381	NM_001146318:-846	Cnp	PROMOTER	0.861	3.408	916.08	3122.43	2.935	715.20	2099.10
A_68_P27850825	chr11:61668376-61668420	NM_013881:196	Ulk2	INSIDE	0.861	2.929	705.97	2067.52	2.523	566.32	1428.71
A_68_P27556419	chr11:4118075-4118119	NM_028022:-157	Gatsl3	PROMOTER	0.861	0.437	1311.49	572.57	0.376	1064.12	400.18
A_68_P26823380	chr9:110283014-110283058	NM_001001144:47240	Scap	INSIDE	0.861	2.162	1166.05	2520.48	1.862	992.63	1848.37
A_68_P26540064	chr9:56920273-56920317	NM_001110350:-3888	Sin3a	PROMOTER	0.861	2.291	844.95	1935.80	1.972	775.79	1529.87
A_68_P25962049	chr8:75072127-75072171	NM_027485:61	Med26	INSIDE	0.861	1.844	4337.40	7999.21	1.588	3446.40	5472.28
A_68_P25618724	chr8:4779472-4779516	NM_011369:40	Shcbp1	INSIDE	0.861	4.604	686.25	3159.57	3.963	584.49	2316.45
A_68_P24759117	chr6:113328299-113328343	NM_001170485:214	Arpc4	INSIDE	0.861	15.035	2381.03	35799.43	12.941	2340.29	30285.07
A_68_P23946411	chr5:103854257-103854301	NM_011204:68	Ptpn13	INSIDE	0.861	1.772	1695.28	3004.60	1.526	1312.38	2003.04
A_68_P22882285	chr4:45333452-45333496	NM_025513:1	Exosc3	INSIDE	0.861	0.415	2618.39	1085.63	0.357	1926.33	687.86
A_68_P22313617	chr3:88213719-88213763	NM_025928:498	Pmf1	INSIDE	0.861	2.020	1299.79	2625.21	1.740	1060.81	1845.51
A_68_P21935172	chr3:9004150-9004194	NM_001025263:343	Tpd52	INSIDE	0.861	0.333	3276.44	1089.75	0.286	2434.99	697.11
A_68_P21832544	chr2:166731558-166731602	NM_023565:-15	Cse1l	PROMOTER	0.861	1.936	2705.24	5237.10	1.667	1890.43	3150.48
A_68_P21133893	chr2:35347074-35347118	ENSMUST00000121664:-57		PROMOTER	0.861	3.380	778.23	2630.53	2.910	688.35	2003.26
A_68_P20778063	chr1:164407783-164407827	NM_001038619:357	Dnm3	INSIDE	0.861	2.001	2373.77	4750.57	1.722	1855.38	3195.46
A_68_P31899539	chr18:86881114-86881158	NM_172633:-1303	Cbln2	PROMOTER	0.860	0.300	4352.29	1307.63	0.258	2721.88	703.41
A_68_P31415137	chr17:86567183-86567227	NM_011104:80	Prkce	INSIDE	0.860	3.040	2630.57	7997.33	2.616	2092.84	5474.05
A_68_P31144953	chr17:32457424-32457468	NM_019774:652	Akap8	INSIDE	0.860	0.299	1574.53	471.24	0.257	1218.04	313.49
A_68_P31074433	chr17:17762462-17762506	NM_172827:-1031	Lnpep	PROMOTER	0.860	0.220	5229.38	1152.97	0.190	3661.90	694.49
A_68_P30115243	chr15:31531126-31531170	NM_007637:411	Cct5	INSIDE	0.860	0.585	923.94	540.80	0.504	803.18	404.49
A_68_P29712956	chr14:72931825-72931869	NM_207636:177964	Fndc3a	DOWNSTREAM	0.860	2.130	1962.70	4180.73	1.832	1608.05	2946.09
A_68_P29507577	chr14:31528572-31528616	NM_001166532:-206	Sfmbt1	PROMOTER	0.860	3.305	1021.42	3375.30	2.843	863.61	2455.31
A_68_P29141938	chr13:73901290-73901334	NM_011390:168	Slc12a7	INSIDE	0.860	0.352	1807.49	636.83	0.303	1371.95	415.84
A_68_P28915540	chr13:30228458-30228502	NM_153546:122	Mboat1	INSIDE	0.860	0.433	1621.30	702.15	0.373	1234.32	459.79
A_68_P28842025	chr13:15555334-15555378	NM_008130:-199	Gli3	PROMOTER	0.860	1.972	1733.47	3418.45	1.696	1413.93	2397.44
A_68_P28567993	chr12:80273243-80273287	NM_016800:181	Vti1b	INSIDE	0.860	0.463	1080.93	500.07	0.398	855.58	340.35
A_68_P28435278	chr12:53605798-53605842	AK041016:818		INSIDE	0.860	0.270	1799.10	485.56	0.232	1350.64	313.52
A_68_P28054682	chr11:98010305-98010349	NM_028149:604	Fbxl20	INSIDE	0.860	0.246	1911.08	470.56	0.212	1441.71	305.20
A_68_P27332826	chr10:89208214-89208258	NM_029166:501	Uhrf1bp1l	INSIDE	0.860	4.875	853.79	4161.99	4.190	747.49	3131.92
A_68_P26983889	chr10:19653542-19653586	NM_008580:-767	Map3k5	PROMOTER	0.860	2.288	969.70	2218.63	1.967	622.46	1224.35
A_68_P26802913	chr9:106271467-106271511	NM_153459:526	Dusp7	INSIDE	0.860	0.568	1612.13	915.13	0.488	1271.71	620.63
A_68_P26553636	chr9:59334710-59334754	NM_019927:-551	Arih1	PROMOTER	0.860	0.579	1334.08	772.68	0.498	1028.42	512.40
A_68_P26547036	chr9:58160524-58160568	NM_010729:473	Loxl1	INSIDE	0.860	0.511	1239.55	632.97	0.439	914.87	401.87
A_68_P25096455	chr7:53289295-53289339	NM_153057:251	Nomo1	INSIDE	0.860	0.459	1808.92	829.44	0.395	1384.62	546.27
A_68_P25092917	chr7:52661860-52661904	NM_010596:553	Kcna7	INSIDE	0.860	3.293	637.82	2100.07	2.831	516.12	1461.02
A_68_P24541217	chr6:72297432-72297476	NM_026696:144	0610030E20Rik	INSIDE	0.860	0.377	1405.72	530.43	0.324	1009.96	327.71
A_68_P23591968	chr5:34326440-34326484	NM_001033458:340	Gm1673	INSIDE	0.860	0.527	1623.61	855.71	0.453	1316.28	596.31
A_68_P23439842	chr4:155027173-155027217	NM_011985:299	Mmp23	INSIDE	0.860	2.007	1973.32	3960.59	1.726	1466.20	2531.16
A_68_P23360938	chr4:140462256-140462300	NM_008812:4	Padi2	INSIDE	0.860	3.934	234.81	923.81	3.382	236.71	800.58
A_68_P23300610	chr4:129815848-129815892	NM_001163027:-75	Hcrtr1	PROMOTER	0.860	4.467	795.95	3555.46	3.840	786.48	3020.46
A_68_P23199415	chr4:108790485-108790529	NM_001134791:135	Osbpl9	INSIDE	0.860	0.663	1120.79	743.21	0.570	943.37	538.19
A_68_P22601388	chr3:143861770-143861814	NM_001161770:3504	Lmo4	INSIDE	0.860	0.472	998.95	471.11	0.406	728.19	295.30
A_68_P22155914	chr3:55584133-55584177	NM_010750:-2277	Mab21l1	PROMOTER	0.860	0.648	1197.89	776.79	0.558	972.69	542.75
A_68_P21742300	chr2:150835045-150835089	NM_207204:68	Ninl	INSIDE	0.860	3.521	947.59	3336.66	3.028	795.48	2408.57
A_68_P21257003	chr2:60047554-60047598	NM_020575:-416	Marchf7	PROMOTER	0.860	3.301	1730.57	5713.30	2.838	1477.51	4192.78
A_68_P20559397	chr1:121809874-121809922	NM_175106:-153	Tmem177	PROMOTER	0.860	2.517	1153.27	2902.87	2.164	798.74	1728.20
A_68_P32206764	chr19:57435985-57436029	NM_145505:508	Fam160b1	INSIDE	0.859	1.860	926.32	1723.30	1.597	782.71	1250.27
A_68_P32094199	chr19:37249081-37249125	NM_198300:32681	Cpeb3	INSIDE	0.859	0.534	1431.33	764.13	0.458	1222.36	560.32
A_68_P31939728	chr19:7497925-7497969	NM_175381:5832	2700081O15Rik	INSIDE	0.859	1.790	1062.93	1902.45	1.538	926.97	1425.54
A_68_P31217290	A_68_P31217290			Unknown	0.859	2.380	671.86	1598.84	2.044	561.69	1148.08
A_68_P30347859	chr15:76199398-76199442	NM_021555:93	Fam203a	INSIDE	0.859	0.293	3610.79	1059.43	0.252	2687.07	677.20
A_68_P29570099	chr14:45949501-45949545	NM_025959:24	Psmc6	INSIDE	0.859	0.562	1064.75	597.99	0.482	807.13	389.33
A_68_P28989794	chr13:43460884-43460928	NM_178848:-5178	Sirt5	PROMOTER	0.859	2.921	1469.30	4291.37	2.508	1364.10	3421.61
A_68_P28744029	chr12:112667945-112667989	NM_028807:12326	1200009I06Rik	INSIDE	0.859	0.109	6972.22	760.67	0.094	4305.02	403.38
A_68_P28157439	chr11:115971727-115971771	NM_001205081:-199	Trim47	PROMOTER	0.859	0.359	1991.72	715.09	0.309	1456.93	449.54
A_68_P26891190	chr9:122656383-122656427	NM_001199736:-59	Gm9524	PROMOTER	0.859	9.202	634.65	5840.32	7.907	730.93	5779.57
A_68_P26745534	chr9:96020049-96020093	NM_177352:223	Gk5	INSIDE	0.859	0.557	2106.35	1174.27	0.479	1901.85	910.58
A_68_P26274324	chr9:3532252-3532296	NM_001033322:-79	Gucy1a2	PROMOTER	0.859	0.695	2550.65	1773.45	0.597	1953.66	1167.24
A_68_P25425957	chr7:119823141-119823185	NM_001166584:329	Tead1	INSIDE	0.859	1.879	851.43	1599.46	1.614	741.39	1196.51
A_68_P25032190	chr7:31348204-31348248	NM_025498:-23	Psenen	DIVERGENT_PROMOTER	0.859	0.443	1232.92	545.77	0.380	1008.97	383.69
A_68_P24627746	chr6:89306761-89306805	NM_008881:5825	Plxna1	INSIDE	0.859	4.162	1272.73	5297.62	3.576	990.36	3541.22
A_68_P24141810	chr5:141000020-141000064	NM_021528:18480	Chst12	INSIDE	0.859	2.436	712.02	1734.15	2.091	581.54	1216.16
A_68_P24008561	chr5:115685399-115685443	NM_026398:-439	Pop5	PROMOTER	0.859	0.489	3461.51	1692.96	0.420	2368.60	995.55
A_68_P23284224	chr4:126913454-126913498	NM_198618:66962	Dlgap3	INSIDE	0.859	1.693	1524.74	2581.74	1.455	1229.39	1788.85

ProbeName	Target position of probe on CpG island microarray	TargetID	GeneSymbol	CpG island Description	Ratio of relative methylation (TiO_2-NP/Vehicle)	Sham group			TiO_2-H group		
						Relative methylation (Cy5/Cy3)	Cy3 signal (Input)	Cy5 signal (MeDIP)	Relative methylation (Cy5/Cy3)	Cy3 signal (Input)	Cy5 signal (MeDIP)
A_68_P23244792	chr4:118967244-118967289	NM_011732:-148	Ybx1	PROMOTER	0.859	1.922	2464.12	4736.48	1.651	1689.53	2788.90
A_68_P23226518	chr4:115570734-115570778	ENSMUST00000165938:7486		INSIDE	0.859	0.457	1849.90	845.23	0.392	1375.64	539.88
A_68_P22831245	chr4:34498019-34498063	NM_001007589:177	Akirin2	INSIDE	0.859	1.945	821.26	1597.08	1.670	677.87	1131.82
A_68_P21817914	chr2:164268773-164268817	NM_011521:-106	Sdc4	PROMOTER	0.859	3.193	1269.93	4055.08	2.744	1184.47	3250.65
A_68_P21585581	chr2:122124764-122124808	NM_025777:151	Duoxa2	INSIDE	0.859	15.519	813.43	12623.45	13.327	838.87	11179.75
A_68_P21147673	chr2:38210263-38210310	NM_010710:3459	Lhx2	INSIDE	0.859	0.279	2293.17	640.62	0.240	1620.02	388.70
A_68_P32030719	chr19:25684880-25684924	NM_177360:-124	Dmrt3	PROMOTER	0.858	0.594	4314.23	2560.70	0.509	3678.45	1874.13
A_68_P30792107	chr16:58523074-58523118	NM_018784:329	St3gal6	INSIDE	0.858	0.613	1213.29	743.94	0.526	998.82	525.56
A_68_P30651713	chr16:32277691-32277735	NM_027355:166	Rnf168	INSIDE	0.858	1.747	1359.89	2376.06	1.499	1133.14	1698.14
A_68_P30588730	chr16:20651880-20651924	NM_134101:178	Psmd2	INSIDE	0.858	0.510	3122.38	1591.26	0.437	2283.82	998.29
A_68_P30567433	chr16:15887647-15887691	NM_007679:290	Cebpd	INSIDE	0.858	0.448	1608.92	721.01	0.385	1014.76	390.34
A_68_P30475041	chr15:98364232-98364276	NR_034052:-7364	Snora2b	PROMOTER	0.858	0.358	5220.14	1867.27	0.307	3429.41	1052.16
A_68_P29682516	chr14:67627743-67627787	NM_009761:-50	Bnip3l	PROMOTER	0.858	0.317	1700.53	539.03	0.272	1167.92	317.74
A_68_P29055902	chr13:55672558-55672602	NM_145382:-99	Fam193b	PROMOTER	0.858	0.525	1312.29	688.42	0.450	1013.81	456.15
A_68_P28336342	chr12:33064298-33064342	NM_001162903:667	2010109K11Rik	INSIDE	0.858	2.381	695.59	1655.94	2.042	556.00	1135.61
A_68_P28059765	chr11:98844229-98844273	NM_178596:80	Gjd3	INSIDE	0.858	0.433	3485.80	1509.65	0.372	2277.93	846.52
A_68_P27892735	chr11:68859244-68859288	NM_011496:122	Aurkb	INSIDE	0.858	0.177	4191.01	742.42	0.152	3208.50	487.92
A_68_P27788007	chr11:50139083-50139127	NM_001110499:71	Canx	INSIDE	0.858	0.464	2239.51	1038.34	0.398	1828.67	727.16
A_68_P27540018	chr10:127631429-127631473	NM_001033264:-240	Gls2	PROMOTER	0.858	0.332	2602.68	864.13	0.285	1766.25	503.12
A_68_P27184874	chr10:61693685-61693729	NM_197996:260	Tspan15	INSIDE	0.858	0.228	2652.87	604.08	0.195	1931.59	377.37
A_68_P26644654	chr9:75406902-75406946	NM_016963:540	Tmod3	INSIDE	0.858	0.454	1878.96	852.22	0.389	1553.94	604.45
A_68_P26163688	chr8:113142314-113142358	NM_146216:-201	Vac14	PROMOTER	0.858	1.922	1634.90	3142.33	1.649	1319.99	2177.28
A_68_P26126978	chr8:106666653-106666697	NM_009868:41150	Cdh5	INSIDE	0.858	4.548	936.31	4258.31	3.901	633.85	2472.89
A_68_P24425393	chr6:47974535-47974579	NM_001081382:23557	Zfp777	INSIDE	0.858	3.457	724.78	2505.33	2.965	606.01	1797.09
A_68_P22446820	chr3:115710167-115710211	NM_023214:136	Slc30a7	INSIDE	0.858	1.679	1881.90	3160.14	1.441	1313.44	1892.89
A_68_P22373269	chr3:100913808-100913852	NM_011197:259	Ptgfrn	INSIDE	0.858	5.989	675.74	4046.95	5.136	629.56	3233.57
A_68_P21973914	chr3:19088513-19088557	NM_026182:291	Mtfr1	INSIDE	0.858	0.448	1205.60	540.08	0.384	962.35	369.77
A_68_P21731577	chr2:148506945-148506989	NM_028986:111	Gzf1	INSIDE	0.858	0.496	2492.04	1236.23	0.426	1778.17	756.95
A_68_P32054913	chr19:29879873-29879917	NM_172836:605	9930021J03Rik	INSIDE	0.857	0.320	2446.25	783.95	0.275	1741.06	478.16
A_68_P31790564	chr18:67365029-67365073	NM_024190:38	Chmp1b	INSIDE	0.857	1.962	1169.34	2294.35	1.681	937.47	1575.73
A_68_P31197209	chr17:44913929-44913973	NM_178652:-169	Supt3h	PROMOTER	0.857	0.681	1433.28	976.00	0.583	1100.97	642.31
A_68_P30558910	chr16:13780799-13780843	NM_001039521:29	Rrn3	INSIDE	0.857	2.171	659.00	1430.85	1.860	600.68	1117.47
A_68_P30511519	chr16:4679291-4679335	NM_030205:408	Coro7	INSIDE	0.857	0.405	3319.96	1344.37	0.347	2423.46	840.70
A_68_P29675803	chr14:66452695-66452739	NM_028039:90	Esco2	INSIDE	0.857	0.643	1650.46	1061.45	0.551	1344.82	741.53
A_68_P29523397	chr14:34133413-34133457	NM_153800:103426	Arhgap22	INSIDE	0.857	1.873	2033.28	3808.21	1.605	1676.00	2689.18
A_68_P28889208	chr13:24923670-24923714	NM_019551:165	Tdp2	INSIDE	0.857	2.225	5723.50	12736.30	1.908	4327.07	8254.86
A_68_P28036966	chr11:95007642-95007686	NM_007867:-549	Dlx4	PROMOTER	0.857	0.497	1144.11	568.58	0.426	934.39	397.75
A_68_P27365820	chr10:95026736-95026780	NR_037212:-4803	Mir3058	PROMOTER	0.857	1.911	976.92	1867.20	1.639	918.70	1505.45
A_68_P26716008	chr9:90008880-90008924	NM_001039147:756	Morf4l1	INSIDE	0.857	0.418	5801.03	2423.09	0.358	3970.65	1421.09
A_68_P25349892	chr7:104473950-104473994	NM_001177412:502	Usp35	INSIDE	0.857	3.579	322.29	1153.53	3.066	265.47	814.01
A_68_P25026384	chr7:30074488-30074532	NM_026731:172	Ppp1r14a	INSIDE	0.857	0.527	2559.42	1349.31	0.452	1945.69	878.98
A_68_P24994890	chr7:20301020-20301066	NM_001109748:-264	Tomm40	PROMOTER	0.857	0.673	1368.60	920.40	0.577	1030.85	594.45
A_68_P24671353	chr6:97098869-97098913	NM_175313:-13	A130022J15Rik	PROMOTER	0.857	3.468	1679.45	5823.88	2.972	1431.55	4254.32
A_68_P24164622	chr5:145928149-145928193	NM_178576:78	Cpsf4	INSIDE	0.857	0.502	3459.09	1736.18	0.430	2647.64	1138.95
A_68_P24020116	chr5:117807608-117807652	NM_021539:317	Wsb2	INSIDE	0.857	2.205	965.39	2128.86	1.889	786.43	1485.60
A_68_P23985664	chr5:111847445-111847489	NM_001081235:281	Mn1	INSIDE	0.857	4.764	2056.37	9795.83	4.084	1627.58	6647.08
A_68_P23600263	chr5:35740198-35740242	NM_010445:8455	Hmx1	DOWNSTREAM	0.857	0.265	2146.76	568.48	0.227	1628.21	369.61
A_68_P23541914	chr5:23953620-23953664	NM_022418:22	Tmub1	INSIDE	0.857	0.296	2530.89	748.95	0.254	1649.36	418.30
A_68_P23185488	chr4:106295615-106295659	NM_177667:583	Ttc22	INSIDE	0.857	0.567	1026.37	581.75	0.486	840.76	408.48
A_68_P22934418	chr4:55543459-55543503	NM_010637:1867	Klf4	INSIDE	0.857	0.384	1739.02	668.37	0.329	1350.26	444.68
A_68_P22878699	chr4:44723813-44723857	NM_008782:-522	Pax5	PROMOTER	0.857	0.643	1374.02	883.12	0.551	1140.90	628.12
A_68_P22774391	chr4:21616366-21616410	NM_001080771:-3278	Prdm13	PROMOTER	0.857	4.183	2603.03	10888.59	3.584	1962.14	7032.05
A_68_P22059538	chr3:36588855-36588899	NM_019510:213	Trpc3	INSIDE	0.857	2.251	876.46	1972.81	1.929	745.93	1438.66
A_68_P21365117	chr2:78709405-78709449	NM_009454:223	Ube2e3	INSIDE	0.857	3.217	984.40	3166.60	2.757	838.85	2312.99
A_68_P21118609	chr2:32822636-32822680	NM_009076:5427	Rpl12	DOWNSTREAM	0.857	0.425	2365.55	1004.89	0.364	1687.78	614.34
A_68_P31493067	chr18:11054984-11055028	NM_010258:2499	Gata6	INSIDE	0.856	0.558	2498.67	1394.52	0.478	1853.76	885.93
A_68_P30137689	chr15:36212841-36212885	NM_013923:40	Rnf19a	INSIDE	0.856	0.588	3372.41	1982.29	0.503	2412.86	1214.73
A_68_P29454672	chr14:22570199-22570243	NM_026283:468	Samd8	INSIDE	0.856	0.292	1608.40	470.06	0.250	1237.22	309.66
A_68_P29387669	chr14:8913058-8913102	NM_025938:254	Rpp14	INSIDE	0.856	0.314	1981.41	621.38	0.269	1523.84	409.26
A_68_P28314428	chr12:29360059-29360103	NM_134052:9	Adi1	INSIDE	0.856	0.232	2670.17	619.45	0.199	2067.37	410.51
A_68_P28246268	chr12:12944044-12944088	NM_008709:4576	Mycn	INSIDE	0.856	3.060	838.23	2564.85	2.619	722.70	1893.02
A_68_P28043565	chr11:96160395-96160450	NM_008269:-62	Hoxb6	PROMOTER	0.856	0.712	1606.72	1143.47	0.609	1134.82	691.62
A_68_P27800927	chr11:52577973-52578017	NM_177059:-213	Fstl4	PROMOTER	0.856	0.710	1908.63	1355.18	0.608	1552.11	943.12
A_68_P27392327	chr10:99950250-99950294	NM_001110013:-291	Tmtc3	DIVERGENT_PROMOTER	0.856	0.620	2322.78	1440.69	0.531	1895.40	1006.14
A_68_P27055772	chr10:33927102-33927146	NM_172508:233	Dse	INSIDE	0.856	2.532	1507.09	3815.28	2.167	1355.75	2938.41

ProbeName	Target position of probe on CpG island microarray	TargetID	GeneSymbol	CpG island Description	Ratio of relative methylation (TiO_2-NP/Vehicle)	Sham group			TiO_2-H group		
						Relative methylation (Cy5/Cy3)	Cy3 signal (Input)	Cy5 signal (MeDIP)	Relative methylation (Cy5/Cy3)	Cy3 signal (Input)	Cy5 signal (MeDIP)
A_68_P26809757	chr9:107490272-107490316	NM_025903:246	Ifrd2	INSIDE	0.856	0.598	1641.41	981.38	0.512	1360.27	695.82
A_68_P26700014	chr9:86465671-86465715	NM_001145968:33	Rwdd2a	INSIDE	0.856	0.283	4944.94	1400.49	0.242	3701.37	897.25
A_68_P26154295	chr8:111474184-111474229	NM_007496:235663	Zfhx3	INSIDE	0.856	2.630	681.20	1791.70	2.252	504.85	1137.14
A_68_P26017625	chr8:86535356-86535400	NR_035431:154	Mir1199	DOWNSTREAM	0.856	3.099	391.46	1213.09	2.651	300.26	796.13
A_68_P25958333	chr8:74213329-74213373	NM_013564:200	Insl3	INSIDE	0.856	1.828	1372.90	2509.86	1.565	1108.79	1734.83
A_68_P24797810	chr6:120244069-120244113	NM_198884:487	B4galnt3	INSIDE	0.856	0.731	1649.25	1205.44	0.625	1445.33	903.90
A_68_P24624512	chr6:88792663-88792707	NM_176973:31354	Podxl2	INSIDE	0.856	0.712	2332.47	1661.57	0.610	1744.91	1064.54
A_68_P24595030	chr6:83660707-83660751	NR_002873:198	Vax2os1	INSIDE	0.856	0.265	2530.97	671.51	0.227	1771.83	402.33
A_68_P24016057	chr5:117041867-117041911	NM_026886:-62	Srrm4	PROMOTER	0.856	0.446	2778.09	1239.38	0.382	2098.25	800.85
A_68_P23352771	chr4:139130470-139130514	NM_001205173:-142	Iffo2	PROMOTER	0.856	2.002	5547.04	11103.68	1.714	4105.62	7038.15
A_68_P22350132	chr3:96402437-96402481	NM_001024851:1900	Ankrd34a	INSIDE	0.856	0.530	2337.45	1237.77	0.453	1828.77	829.12
A_68_P22081520	chr3:40603976-40604020	NM_011495:126	Plk4	INSIDE	0.856	3.305	505.09	1669.19	2.829	450.34	1273.84
A_68_P21809324	chr2:162734117-162734161	ENSMUST00000122376:479		INSIDE	0.856	4.720	1404.56	6629.11	4.038	1059.62	4278.89
A_68_P21628932	chr2:130110113-130110157	NM_130884:53	Idh3b	INSIDE	0.856	0.460	1519.53	698.75	0.393	1086.00	427.30
A_68_P20863428	chr1:180267506-180267550	NM_016805:387	Hnrnpu	INSIDE	0.856	2.505	742.11	1858.97	2.143	642.41	1376.76
A_68_P20827967	chr1:173367883-173367928	NM_172647:214	F11r	INSIDE	0.856	0.530	4856.78	2575.11	0.454	3169.42	1439.00
A_68_P20354817	chr1:75543227-75543271	NM_009208:408	Slc4a3	INSIDE	0.856	0.296	5452.05	1615.36	0.254	3519.45	892.50
A_68_P33008134	chr12:18521060-18521104	NM_001033157:-233	5730507C01Rik	PROMOTER	0.855	0.370	3016.98	1116.33	0.317	2209.66	699.38
A_68_P32141806	chr19:46119531-46119575	NM_010697:-5441	Ldb1	PROMOTER	0.855	0.495	2086.58	1032.83	0.423	1476.60	624.85
A_68_P30160399	chr15:40486877-40486921	NM_011766:311	Zfpm2	INSIDE	0.855	0.407	1221.84	497.07	0.348	947.61	329.69
A_68_P29675801	chr14:66452501-66452546	NM_028039:283	Esco2	INSIDE	0.855	0.595	1082.97	644.35	0.508	866.93	440.80
A_68_P27787452	chr11:50044549-50044593	NM_145926:5734	Mgat4b	INSIDE	0.855	1.975	1157.24	2285.38	1.688	967.33	1632.76
A_68_P26585436	chr9:64880684-64880728	NM_028906:442	Dpp8	INSIDE	0.855	0.384	1568.68	601.61	0.328	1257.64	412.51
A_68_P25262391	chr7:86805241-86805286	NM_029835:182	5730590G19Rik	INSIDE	0.855	0.385	2041.29	785.58	0.329	1579.08	519.70
A_68_P25187996	chr7:73532827-73532871	NM_146191:379	Lrrk1	INSIDE	0.855	2.930	1035.76	3034.41	2.504	1012.47	2535.14
A_68_P24904253	chr6:142335519-142335563	NM_001204907:67	Recql	INSIDE	0.855	3.584	4702.46	16854.54	3.063	3760.03	11516.51
A_68_P24829780	chr6:126889329-126889373	NM_009013:223	Rad51ap1	INSIDE	0.855	0.278	4023.74	1120.20	0.238	3038.96	723.00
A_68_P24444194	chr6:51382876-51382920	NM_010903:230	Nfe2l3	INSIDE	0.855	1.873	2459.76	4606.49	1.601	1972.79	3159.12
A_68_P23588853	chr5:33777078-33777122	ENSMUST00000030994:-3256		PROMOTER	0.855	3.661	1982.10	7256.16	3.131	1590.80	4981.12
A_68_P22749332	chr4:15193464-15193508	NM_181401:520	Tmem64	INSIDE	0.855	3.181	4124.48	13119.64	2.720	3305.47	8992.40
A_68_P22342628	chr3:94976704-94976748	NM_011351:8431	Sema6c	INSIDE	0.855	2.134	897.85	1915.57	1.824	743.58	1356.08
A_68_P20939667	chr1:193542758-193542803	NM_001134829:507	Lpgat1	INSIDE	0.855	2.115	787.30	1664.98	1.807	586.98	1060.82
A_68_P20193920	chr1:44608775-44608819	NM_028450:281	Gulp1	INSIDE	0.855	0.526	1808.79	952.11	0.450	1434.31	645.68
A_68_P31928433	chr19:5388511-5388555	NM_016882:171	Sart1	INSIDE	0.854	2.345	534.42	1253.12	2.003	480.57	962.57
A_68_P31381748	chr17:80606129-80606175	NM_001195485:493	Srsf7	INSIDE	0.854	0.333	4263.76	1420.93	0.285	2533.24	720.98
A_68_P30475796	chr15:98493283-98493327	NM_001080981:16	Ddx23	INSIDE	0.854	0.333	2209.89	736.17	0.285	1542.43	438.95
A_68_P29961929	chr14:122873515-122873559	NM_009574:-1069	Zic2	DIVERGENT_PROMOTER	0.854	0.722	2695.64	1946.98	0.617	2241.48	1383.37
A_68_P29157708	chr13:77274951-77274995	NM_001081353:176	2210408I21Rik	INSIDE	0.854	0.312	1465.85	457.09	0.266	1175.15	313.01
A_68_P28741431	chr12:112217094-112217138	NM_175207:115	Ankrd9	INSIDE	0.854	3.124	1113.32	3478.26	2.667	818.34	2182.19
A_68_P28195044	chr12:3426740-3426784	NR_030694:-15	1110002L01Rik	DIVERGENT_PROMOTER	0.854	11.380	1872.16	21304.76	9.720	1700.56	16529.16
A_68_P28033358	chr11:94361326-94361370	NM_027984:-60	Epn3	PROMOTER	0.854	1.739	1286.73	2238.17	1.485	1189.43	1766.48
A_68_P27998411	chr11:88007034-88007078	NM_001172099:23990	Cuedc1	INSIDE	0.854	2.074	863.26	1790.81	1.772	691.60	1225.72
A_68_P27840804	chr11:59919243-59919287	NM_009021:750	Rai1	INSIDE	0.854	0.467	1929.27	900.15	0.398	1465.08	583.51
A_68_P27550688	chr11:3102520-3102564	NM_023743:-43	Eif4enif1	PROMOTER	0.854	3.039	775.54	2357.03	2.597	628.75	1632.82
A_68_P26245545	chr8:126957761-126957805	NM_171824:54	Pgbd5	INSIDE	0.854	0.494	1066.46	527.34	0.422	839.98	354.51
A_68_P25089777	chr7:52119118-52119162	NM_133949:6018	Ptov1	INSIDE	0.854	2.676	2927.54	7835.48	2.286	2231.78	5101.20
A_68_P24993781	chr7:20109373-20109417	NM_027116:5315	Nkpd1	INSIDE	0.854	2.680	1659.37	4447.56	2.290	1411.02	3231.59
A_68_P23542162	chr5:23991041-23991085	NM_139153:33068	Agap3	INSIDE	0.854	1.738	1612.54	2803.21	1.485	1271.93	1889.35
A_68_P22593191	chr3:142545216-142545260	NM_178654:-270	Pkn2	PROMOTER	0.854	0.397	1807.10	718.32	0.339	1565.18	531.36
A_68_P32795516	chrX:160347102-160347146	NM_026887:33	Ap1s2	INSIDE	0.853	2.300	1050.36	2415.34	1.962	1204.83	2364.22
A_68_P32467743	chrX:71613745-71613789	NM_145405:100	Ubl4	INSIDE	0.853	1.914	1556.28	2978.92	1.633	1834.30	2995.45
A_68_P31932439	chr19:6058211-6058258	NM_001190436:268	Fau	INSIDE	0.853	0.397	1409.69	560.08	0.339	1003.72	340.04
A_68_P31927972	chr19:5307824-5307868	NM_001024717:9516	Gal3st3	INSIDE	0.853	2.211	889.62	1966.54	1.886	791.72	1492.98
A_68_P31677411	chr18:46685323-46685367	NM_173423:281	Fem1c	INSIDE	0.853	0.384	3642.64	1398.64	0.327	2495.34	817.14
A_68_P31637167	chr18:39152800-39152844	NM_175164:24	Arhgap26	INSIDE	0.853	0.241	1976.65	477.08	0.206	1574.66	324.21
A_68_P31458150	chr18:3337100-3337144	NM_001110850:465	Crem	INSIDE	0.853	2.167	1148.32	2488.72	1.850	902.33	1669.01
A_68_P30431381	chr15:90509463-90509507	NM_025815:335	Cpne8	INSIDE	0.853	0.248	2173.61	538.69	0.211	1548.70	327.48
A_68_P29639451	chr14:60216712-60216756	NM_001168535:62	Cdadc1	INSIDE	0.853	1.777	2132.96	3789.73	1.515	1725.18	2613.88
A_68_P28740913	chr12:112127116-112127160	NM_026048:217	Cinp	INSIDE	0.853	0.387	9760.05	3774.49	0.330	6761.72	2231.00
A_68_P28184669	chr11:120319314-120319358	NM_025560:41	1810049H13Rik	INSIDE	0.853	1.996	839.13	1674.54	1.703	707.53	1204.96
A_68_P27288849	chr10:80783121-80783165	NM_027381:-705	2510012J08Rik	PROMOTER	0.853	0.451	3232.31	1456.67	0.384	2221.49	854.16
A_68_P27185081	chr10:61732662-61732706	NM_001146100:70485	Hk1	INSIDE	0.853	6.146	1331.75	8185.16	5.243	1143.09	5993.45
A_68_P26915293	chr10:6373305-6373349	NM_001170785:140	Mthfd1l	INSIDE	0.853	0.233	3040.80	709.92	0.199	2370.37	471.99
A_68_P26875611	chr9:119920380-119920424	NM_011724:12314	Xirp1	DOWNSTREAM	0.853	3.922	1287.40	5049.58	3.347	1040.89	3483.43
A_68_P26644658	chr9:75407422-75407466	NM_016963:20	Tmod3	INSIDE	0.853	0.348	3664.17	1276.19	0.297	2510.79	746.00

ProbeName	Target position of probe on CpG island microarray	TargetID	GeneSymbol	CpG island Description	Ratio of relative methylation (TiO_2-NP/Vehicle)	Sham group			TiO_2-H group		
						Relative methylation (Cy5/Cy3)	Cy3 signal (Input)	Cy5 signal (MeDIP)	Relative methylation (Cy5/Cy3)	Cy3 signal (Input)	Cy5 signal (MeDIP)
A_68_P25891568	chr8:59990545-59990589	NM_008252:-73	Hmgb2	PROMOTER	0.853	0.682	1333.54	909.69	0.582	1031.58	600.24
A_68_P25261383	chr7:86640870-86640922	NR_029818:-9253	Mir9-3	PROMOTER	0.853	6.024	508.94	3065.70	5.136	443.42	2277.44
A_68_P24992476	chr7:19872340-19872384	NM_001025364:-4085	Rtn2	PROMOTER	0.853	2.175	779.76	1696.03	1.855	577.29	1070.93
A_68_P24951779	chr7:5003389-5003433	NM_026900:277	Zfp580	INSIDE	0.853	0.360	1854.92	667.52	0.307	1322.71	406.19
A_68_P24951465	chr7:4946322-4946366	NM_026741:1359	Zfp579	INSIDE	0.853	6.381	2398.94	15307.59	5.441	1632.66	8882.51
A_68_P24117365	chr5:136165147-136165191	NM_008898:85	Por	INSIDE	0.853	0.132	4196.78	553.67	0.113	3190.08	358.93
A_68_P24063260	chr5:125342106-125342150	NM_144819:463	Ccdc92	INSIDE	0.853	1.968	1310.09	2577.89	1.679	969.91	1628.57
A_68_P23999130	chr5:114064140-114064194	NM_008153:36233	Cmklr1	INSIDE	0.853	7.492	751.31	5628.64	6.392	595.42	3805.63
A_68_P23374051	chr4:142802693-142802737	NM_001081355:-102	Prdm2	PROMOTER	0.853	0.591	2105.79	1243.72	0.504	1721.36	866.76
A_68_P23312762	chr4:132091701-132091745	NM_172400:249	Dnajc8	INSIDE	0.853	0.462	960.93	443.91	0.394	925.31	364.57
A_68_P23283633	chr4:126803154-126803198	NM_001099319:-113	Gm12942	PROMOTER	0.853	2.082	641.55	1335.80	1.776	626.87	1113.17
A_68_P23222744	chr4:114659711-114659755	NM_025647:101	Cmpk1	INSIDE	0.853	0.219	2410.65	528.83	0.187	1721.18	322.19
A_68_P22803192	chr4:28742495-28742539	NM_001122889:2222	Epha7	INSIDE	0.853	0.168	3332.95	560.68	0.143	2251.48	322.95
A_68_P22202960	chr3:65778708-65778752	AK133062:4895		DOWNSTREAM	0.853	0.112	4280.99	480.32	0.096	3052.58	292.26
A_68_P21612441	chr2:127096299-127096343	NM_139308:356	Stard7	INSIDE	0.853	2.143	1030.33	2208.31	1.829	874.43	1599.45
A_68_P20411723	chr1:88255233-88255277	NR_002851:-2352	Snord82	PROMOTER	0.853	0.393	3481.07	1369.75	0.336	2527.88	848.75
A_68_P32700062	chrX:137133645-137133689	NM_001077364:1395	Tsc22d3	INSIDE	0.852	5.321	589.36	3136.03	4.533	716.09	3246.34
A_68_P31469350	chr18:5591526-5591570	NM_011546:-340	Zeb1	PROMOTER	0.852	0.630	2754.21	1736.15	0.537	2153.62	1156.82
A_68_P31421396	chr17:87534273-87534317	NM_019654:27276	Socs5	INSIDE	0.852	3.135	2438.50	7645.40	2.671	1914.06	5112.31
A_68_P31152954	chr17:34401924-34401968	NM_207105:1775	H2-Ab1	INSIDE	0.852	0.317	8382.77	2657.83	0.270	5348.99	1445.52
A_68_P31101019	chr17:25153489-25153533	NM_134126:480	Ift140	INSIDE	0.852	0.268	2287.48	613.81	0.229	1698.88	388.48
A_68_P30656037	chr16:33062602-33062646	NM_172823:18	Lmln	INSIDE	0.852	3.650	393.18	1435.03	3.109	335.90	1044.22
A_68_P30230392	chr15:54943854-54943898	NM_145470:-161	Deptor	PROMOTER	0.852	0.490	6275.62	3075.61	0.418	4151.46	1734.47
A_68_P30140494	chr15:36722668-36722712	NM_011740:1603	Ywhaz	INSIDE	0.852	2.212	2766.48	6120.67	1.885	2062.46	3888.56
A_68_P29072930	chr13:58486645-58486689	NM_027335:-80	2210016F16Rik	PROMOTER	0.852	2.959	487.54	1442.42	2.519	439.06	1106.14
A_68_P28599174	chr12:85930850-85930894	NM_007701:20071	Vsx2	INSIDE	0.852	1.967	812.00	1597.12	1.675	953.24	1596.55
A_68_P28058442	chr11:98635216-98635261	NM_145434:1318	Nr1d1	INSIDE	0.852	0.389	1864.53	726.12	0.332	1448.02	480.29
A_68_P28049249	chr11:97049576-97049622	NM_008379:-392	Kpnb1	PROMOTER	0.852	0.212	3870.79	819.51	0.180	3141.59	566.45
A_68_P26344895	chr9:20780976-20781020	NM_010333:239	S1pr2	INSIDE	0.852	0.270	2350.61	635.51	0.230	1668.55	384.23
A_68_P26276393	chr9:4309429-4309473	NM_026276:44	Aasdhppt	INSIDE	0.852	0.365	1743.57	636.94	0.311	1385.21	431.28
A_68_P25891487	chr8:59965927-59965974	NM_021788:707	Sap30	INSIDE	0.852	2.468	407.37	1005.39	2.102	352.57	741.14
A_68_P25574932	chr7:146132973-146133017	NM_028708:562	Jakmip3	INSIDE	0.852	2.131	1624.05	3460.82	1.817	1281.90	2328.68
A_68_P25354353	chr7:105326510-105326554	NM_007602:171	Capn5	INSIDE	0.852	4.839	3371.86	16314.77	4.123	2554.00	10530.23
A_68_P25111228	chr7:56501603-56501647	NM_175272:66	Nav2	INSIDE	0.852	3.517	676.06	2377.81	2.996	497.18	1489.40
A_68_P25008232	chr7:25862320-25862364	NM_175477:78	Zfp574	INSIDE	0.852	0.716	2949.71	2111.48	0.610	2181.74	1330.62
A_68_P25004857	chr7:25217107-25217151	NM_199013:13573	Irgc1	INSIDE	0.852	1.903	887.63	1689.30	1.621	738.19	1196.39
A_68_P23393403	chr4:147646187-147646231	NM_001083342:15866	Ptchd2	INSIDE	0.852	0.475	1098.61	521.56	0.404	1038.62	419.96
A_68_P23363149	chr4:140847557-140847601	NM_010139:-9576	Epha2	PROMOTER	0.852	0.402	2766.73	1111.80	0.342	2303.35	788.73
A_68_P22715108	chr4:8463392-8463436	NM_021518:624	Rab2a	INSIDE	0.852	0.483	1509.68	729.03	0.411	1218.93	501.37
A_68_P21818149	chr2:164311778-164311822	NM_026797:-154	Dbndd2	PROMOTER	0.852	0.620	1683.43	1043.78	0.528	1368.42	722.51
A_68_P21492632	chr2:104968263-104968307	NM_144783:1599	Wt1	INSIDE	0.852	0.404	1590.94	642.36	0.344	1208.62	415.59
A_68_P21151655	chr2:38863832-38863876	NM_028809:196	Arpc5l	INSIDE	0.852	0.312	4783.35	1491.63	0.266	3674.71	976.19
A_68_P32697218	chrX:136595877-136595921	NM_001081668:1209	Nup62cl	INSIDE	0.851	2.394	576.00	1379.10	2.036	593.62	1208.87
A_68_P31479231	chr18:7869684-7869728	NM_001146298:512	Wac	INSIDE	0.851	3.129	1200.37	3756.06	2.664	1049.66	2796.25
A_68_P31253472	chr17:56175685-56175729	NM_013664:293	Sh3gl1	INSIDE	0.851	3.172	813.26	2579.87	2.700	647.12	1747.02
A_68_P30931271	chr16:85802268-85802312	NM_009621:1070	Adamts1	INSIDE	0.851	0.558	1044.07	583.02	0.475	813.57	386.73
A_68_P30338797	chr15:74502578-74502622	NM_018790:400	Arc	INSIDE	0.851	1.803	1486.10	2678.92	1.534	1149.61	1763.26
A_68_P28691644	chr12:103707806-103707850	NM_013747:-291	Golga5	PROMOTER	0.851	2.766	1946.73	5385.48	2.355	1617.48	3808.93
A_68_P28182254	chr11:119947830-119947874	NM_009734:289	Azi1	INSIDE	0.851	3.023	717.42	2168.98	2.572	532.00	1368.31
A_68_P27938553	chr11:77421084-77421128	NM_144825:211	Taok1	INSIDE	0.851	14.064	1404.86	19758.38	11.965	1159.70	13876.26
A_68_P27540626	chr10:127740353-127740397	NM_133992:-16	Pan2	DIVERGENT_PROMOTER	0.851	0.409	1126.55	461.24	0.349	927.59	323.32
A_68_P27271357	chr10:77441161-77441205	NM_026431:-212	1810043G02Rik	DIVERGENT_PROMOTER	0.851	0.536	2419.15	1296.74	0.456	1935.57	883.24
A_68_P27137647	chr10:52137521-52137565	NM_030250:190	Nus1	INSIDE	0.851	0.312	6418.94	2000.22	0.265	4810.20	1276.01
A_68_P25955963	chr8:73796755-73796799	NM_001142322:164	Myo9b	INSIDE	0.851	0.253	1856.50	469.76	0.215	1386.12	298.60
A_68_P25953243	chr8:73183392-73183436	NM_023217:166	Pgpep1	INSIDE	0.851	0.344	1880.02	647.45	0.293	1280.21	375.33
A_68_P25409834	chr7:116895598-116895642	NM_020050:157	Tmem9b	INSIDE	0.851	3.345	1175.53	3932.18	2.846	1026.99	2922.48
A_68_P25190563	chr7:73984782-73984826	NM_001033877:184	Adamts17	INSIDE	0.851	0.586	2863.72	1677.94	0.498	2017.28	1005.31
A_68_P24536647	chr6:71443852-71443896	NM_009543:-13	Rnf103	PROMOTER	0.851	0.312	1462.49	455.64	0.265	1033.88	274.17
A_68_P24126341	chr5:137994693-137994737	NM_031404:-68	Actl6b	PROMOTER	0.851	0.551	1501.75	827.49	0.469	1070.19	501.62
A_68_P23392318	chr4:147462079-147462123	NM_009642:73	Agtrap	INSIDE	0.851	0.544	2041.27	1111.28	0.463	1626.39	753.69
A_68_P22522352	chr3:129534495-129534539	NM_026578:-202	Gar1	DIVERGENT_PROMOTER	0.851	0.293	1640.31	480.15	0.249	1255.24	312.56
A_68_P22129914	chr3:51288525-51288569	NM_016858:659	Rab33b	INSIDE	0.851	0.241	2510.90	604.62	0.205	2005.30	410.73
A_68_P21206006	chr2:49922023-49922067	NM_177139:63	Lypd6	INSIDE	0.851	2.449	1422.69	3483.47	2.084	1140.20	2375.87
A_68_P20834310	chr1:174562757-174562801	NM_026725:327	Dusp23	INSIDE	0.851	0.254	1788.94	453.80	0.216	1263.41	272.85
A_68_P20740066	chr1:157659774-157659818	NM_001024945:233	Qsox1	INSIDE	0.851	0.447	2117.93	945.68	0.380	1650.09	626.98

ProbeName	Target position of probe on CpG island microarray	TargetID	GeneSymbol	CpG island Description	Ratio of relative methylation (TiO_2-NP/Vehicle)	Sham group			TiO_2-H group		
						Relative methylation (Cy5/Cy3)	Cy3 signal (Input)	Cy5 signal (MeDIP)	Relative methylation (Cy5/Cy3)	Cy3 signal (Input)	Cy5 signal (MeDIP)
A_68_P32698223	chrX:136812097-136812141	ENSMUST00000130153:283		INSIDE	0.850	2.875	414.12	1190.44	2.442	561.17	1370.54
A_68_P31603480	chr18:32719536-32719580	NM_001048207:130	Gypc	INSIDE	0.850	0.223	2839.95	633.62	0.190	2070.56	392.87
A_68_P30580600	chr16:18586533-18586577	NM_011532:508	Tbx1	INSIDE	0.850	1.754	1814.87	3182.50	1.490	1421.32	2118.48
A_68_P30545644	chr16:10993293-10993337	NM_019980:-100	Litaf	PROMOTER	0.850	0.480	1971.77	946.86	0.408	1480.86	604.14
A_68_P30512109	chr16:4790382-4790426	NM_025670:-469	5730403B10Rik	PROMOTER	0.850	0.481	1017.34	489.19	0.409	800.10	327.17
A_68_P29689305	chr14:68743026-68743070	NM_008691:13	Nefm	INSIDE	0.850	0.151	5269.70	793.74	0.128	4182.58	535.50
A_68_P29232480	chr13:94827451-94827495	NM_172589:-278	Lhfpl2	PROMOTER	0.850	0.354	1402.09	496.27	0.301	1041.34	313.17
A_68_P29057251	chr13:55927242-55927286	NM_011097:5522	Pitx1	INSIDE	0.850	3.781	541.72	2048.20	3.216	412.13	1325.29
A_68_P27865436	chr11:64248735-64248779	NM_178870:-77	Hs3st3a1	PROMOTER	0.850	0.308	1710.38	526.95	0.262	1302.69	340.94
A_68_P27089727	chr10:41239601-41239645	NM_016898:317	Cd164	INSIDE	0.850	0.323	1530.09	494.13	0.274	1333.98	366.14
A_68_P26019649	chr8:86938288-86938332	NM_007578:-952	Cacna1a	PROMOTER	0.850	3.212	959.68	3082.61	2.729	817.52	2231.23
A_68_P24992475	chr7:19872268-19872312	NM_001025364:-4157	Rtn2	PROMOTER	0.850	2.375	886.21	2104.32	2.019	758.87	1531.84
A_68_P24620285	chr6:88140131-88140179	NM_008090:-8503	Gata2	PROMOTER	0.850	0.333	1995.06	664.48	0.283	1465.59	414.71
A_68_P24449040	chr6:52154168-52154212	NM_010453:396	Hoxa5	INSIDE	0.850	0.473	3373.45	1596.98	0.402	2417.09	972.22
A_68_P24326992	chr6:29457810-29457854	NM_001029985:120	Kcp	INSIDE	0.850	0.449	2982.82	1338.09	0.381	2333.70	889.96
A_68_P23542075	chr5:23980487-23980531	NM_139153:22514	Agap3	INSIDE	0.850	5.620	434.73	2443.24	4.776	341.51	1630.98
A_68_P23006658	chr4:70196288-70196332	NM_172694:-348	Megf9	PROMOTER	0.850	0.444	1415.34	628.47	0.377	1072.46	404.63
A_68_P22872576	chr4:43591441-43591485	NM_172866:-144	Rgp1	PROMOTER	0.850	3.144	619.32	1946.91	2.671	518.28	1384.12
A_68_P22774361	chr4:21612852-21612896	NM_001080771:236	Prdm13	INSIDE	0.850	0.505	2699.43	1363.85	0.430	2007.22	862.19
A_68_P22019966	chr3:28981333-28981377	NM_001167748:-144	Egfem1	PROMOTER	0.850	2.112	1354.92	2861.33	1.795	1120.93	2011.97
A_68_P21613507	chr2:127270311-127270356	NM_029629:-32	Fahd2a	PROMOTER	0.850	0.543	939.92	509.92	0.461	763.12	351.98
A_68_P20740944	chr1:157819945-157819989	NM_001039184:419	Cep350	INSIDE	0.850	2.112	1062.29	2243.24	1.795	864.30	1550.99
A_68_P20633481	chr1:136352243-136352287	NM_153128:133	Klhl12	INSIDE	0.850	3.143	1462.66	4596.99	2.672	1275.52	3408.08
A_68_P31227865	chr17:50432360-50432404	NM_010021:542	Dazl	INSIDE	0.849	3.782	360.71	1364.21	3.213	373.70	1200.54
A_68_P30953522	chr16:89885052-89885096	NM_009384:75998	Tiam1	INSIDE	0.849	2.507	1246.44	3124.45	2.127	1002.96	2133.57
A_68_P29625859	chr14:57676214-57676258	NM_016975:546	Gja3	INSIDE	0.849	0.673	1622.17	1091.82	0.571	1170.25	668.60
A_68_P29502937	chr14:30781713-30781757	NM_019979:169	Selk	INSIDE	0.849	0.406	2837.08	1150.65	0.344	2179.07	750.12
A_68_P28517159	chr12:70993954-70993998	NM_178628:-115	Atl1	PROMOTER	0.849	2.926	344.18	1007.20	2.485	294.85	732.60
A_68_P28078909	chr11:102180488-102180532	NM_011551:-100	Ubtf	PROMOTER	0.849	3.558	535.24	1904.51	3.021	434.24	1312.03
A_68_P27551119	chr11:3189697-3189741	NM_019574:-741	Patz1	PROMOTER	0.849	0.281	2079.06	584.32	0.239	1618.41	386.17
A_68_P27288262	chr10:80702920-80702964	NM_001034895:7035	Zfr2	INSIDE	0.849	2.527	818.08	2067.19	2.145	731.73	1569.44
A_68_P24636064	chr6:90937651-90937695			Unknown	0.849	1.955	1375.69	2688.81	1.659	969.87	1608.76
A_68_P24428348	chr6:48569845-48569889	NM_173429:6688	Zfp775	INSIDE	0.849	2.393	1278.21	3058.44	2.031	916.05	1860.70
A_68_P24037873	chr5:120752578-120752622			Unknown	0.849	0.356	1902.02	677.09	0.302	1438.73	434.98
A_68_P23819017	chr5:77379989-77380033	NM_025939:-425	Paics	PROMOTER	0.849	0.240	2495.17	599.04	0.204	1675.25	341.45
A_68_P23637947	chr5:42235776-42235820	NM_001081422:-244	Bod1l	PROMOTER	0.849	2.603	461.27	1200.54	2.209	491.11	1084.74
A_68_P23576448	chr5:31395845-31395889	NM_011773:34	Slc30a3	INSIDE	0.849	0.596	941.86	561.79	0.506	839.73	425.07
A_68_P23194284	chr4:107704369-107704413	NM_172874:305	Podn	INSIDE	0.849	3.620	1542.26	5583.30	3.072	1259.27	3868.38
A_68_P21067471	chr2:24534614-24534658	NM_001042528:83931	Cacna1b	INSIDE	0.849	3.640	885.61	3223.53	3.091	745.84	2305.68
A_68_P32670209	chrX:130594062-130594106	NM_133196:197	Cstf2	INSIDE	0.848	3.110	670.98	2086.64	2.636	797.97	2103.18
A_68_P31784506	chr18:66162058-66162102	NM_027400:209	Lman1	INSIDE	0.848	0.537	1101.32	591.44	0.455	825.53	375.98
A_68_P29940452	chr14:119105540-119105584	NM_001033336:-121	Abcc4	PROMOTER	0.848	2.244	1657.59	3719.84	1.903	1433.07	2726.92
A_68_P28523604	chr12:72075575-72075619	NM_011184:-13	Psma3	PROMOTER	0.848	2.926	756.17	2212.72	2.480	670.19	1662.18
A_68_P28058999	chr11:98725426-98725472	NM_197940:537	Wipf2	INSIDE	0.848	1.817	2587.69	4700.67	1.541	1752.81	2700.92
A_68_P27854122	chr11:62270378-62270422	NM_011308:434	Ncor1	INSIDE	0.848	0.697	2666.24	1857.82	0.591	1974.20	1166.52
A_68_P27286398	chr10:80392047-80392091	NM_008655:-767	Gadd45b	PROMOTER	0.848	0.574	1129.87	648.98	0.487	966.07	470.33
A_68_P27186923	chr10:62065160-62065204	NM_019553:-136	Ddx21	PROMOTER	0.848	0.415	1615.86	670.18	0.352	1290.49	453.76
A_68_P26235087	chr8:125202646-125202690	NM_001109873:-593	Cbfa2t3	PROMOTER	0.848	2.267	779.60	1767.18	1.922	730.74	1404.45
A_68_P26142247	chr8:109459545-109459589	NM_009229:-83	Sntb2	PROMOTER	0.848	2.570	734.14	1886.47	2.178	624.94	1361.03
A_68_P26011770	chr8:85388368-85388412	NM_177378:1136	Rnf150	INSIDE	0.848	3.593	1249.74	4490.47	3.047	1076.43	3279.43
A_68_P25951934	chr8:72976066-72976110	NM_001122830:24754	Klhl26	INSIDE	0.848	3.737	575.80	2151.71	3.170	540.35	1713.11
A_68_P25728012	chr8:26712805-26712849	NM_001001735:49	Whsc1l1	INSIDE	0.848	0.743	3244.94	2410.87	0.630	2493.82	1571.99
A_68_P25616821	chr8:4249374-4249418	NM_001195258:1183	Gm14378	INSIDE	0.848	0.335	4422.37	1479.44	0.284	3028.36	859.31
A_68_P25424701	chr7:119571229-119571273	NM_020606:31	Parva	INSIDE	0.848	2.005	897.02	1798.17	1.700	705.80	1199.79
A_68_P25358042	chr7:105963755-105963799			Unknown	0.848	0.114	5548.93	635.07	0.097	3530.52	342.51
A_68_P25176077	chr7:71432423-71432467	NM_172742:-111	Mtmr10	PROMOTER	0.848	3.667	323.75	1187.13	3.110	254.57	791.68
A_68_P24428257	chr6:48551937-48551981	NM_001079901:8077	Repin1	DOWNSTREAM	0.848	0.248	4592.53	1137.76	0.210	3331.14	699.96
A_68_P24056167	chr5:123974295-123974339	NM_023232:-143	Diablo	PROMOTER	0.848	0.578	2740.93	1583.40	0.490	1974.64	967.02
A_68_P23576445	chr5:31395426-31395470	NM_011773:452	Slc30a3	INSIDE	0.848	0.253	5644.37	1428.56	0.215	3762.59	807.60
A_68_P23576262	chr5:31370994-31371038	NM_023525:13833	Cad	INSIDE	0.848	1.842	1657.76	3054.04	1.562	1258.63	1965.50
A_68_P23571065	chr5:30481976-30482020	NM_145558:137	Hadhb	INSIDE	0.848	0.314	1610.92	505.76	0.266	1274.06	339.12
A_68_P23397601	chr4:148312774-148312818	NM_027195:134296	Caszl	INSIDE	0.848	2.413	2143.05	5170.91	2.046	1582.37	3238.03
A_68_P23340289	chr4:137074647-137074691	NM_008305:49951	Hspg2	INSIDE	0.848	3.907	564.07	2203.74	3.314	370.68	1228.49
A_68_P22484175	chr3:122433462-122433506	NM_153422:1409	Pde5a	INSIDE	0.848	0.398	2935.23	1167.30	0.337	2355.90	794.12
A_68_P22392368	chr3:104442587-104442631	NM_009196:18	Slc16a1	INSIDE	0.848	0.301	1948.45	585.59	0.255	1410.12	359.30

ProbeName	Target position of probe on CpG island microarray	TargetID	GeneSymbol	CpG island Description	Ratio of relative methylation (TiO_2-NP/Vehicle)	Sham group			TiO_2-H group		
						Relative methylation (Cy5/Cy3)	Cy3 signal (Input)	Cy5 signal (MeDIP)	Relative methylation (Cy5/Cy3)	Cy3 signal (Input)	Cy5 signal (MeDIP)
A_68_P21754951	chr2:153269795-153269839	NM_001001986:399	8430427H17Rik	INSIDE	0.848	2.209	1191.81	2632.40	1.872	962.84	1802.64
A_68_P31207499	chr17:46683313-46683357	NM_001081335:3	Cul9	INSIDE	0.847	0.533	1282.47	683.66	0.451	1005.06	453.75
A_68_P28161570	chr11:116704403-116704447	NM_033398:339	Jmjd6	INSIDE	0.847	0.462	2231.08	1029.81	0.391	1697.82	663.81
A_68_P27278226	chr10:79144635-79144681	NM_177613:-281	Cdc34	PROMOTER	0.847	2.252	1575.85	3549.50	1.907	1197.11	2282.80
A_68_P26736547	chr9:94438241-94438285	NM_001033145:238	1190002N15Rik	INSIDE	0.847	1.901	1350.11	2566.84	1.611	1140.92	1837.62
A_68_P26430868	chr9:37296656-37296700	NM_146222:-227	BC024479	PROMOTER	0.847	2.592	902.85	2340.36	2.196	760.51	1670.39
A_68_P26022582	chr8:87432382-87432426	NM_010062:-118	Dnase2a	PROMOTER	0.847	0.506	880.26	445.36	0.428	711.52	304.88
A_68_P25007848	chr7:25795874-25795918	NM_008168:61492	Grik5	INSIDE	0.847	3.137	2724.49	8547.57	2.658	2221.71	5906.05
A_68_P23591264	chr5:34162817-34162861	ENSMUST00000075812:-535		PROMOTER	0.847	2.903	887.24	2575.52	2.459	720.33	1770.94
A_68_P22217919	chr3:68674069-68674113	NM_001167996:583	1110032F04Rik	INSIDE	0.847	1.798	1413.49	2540.83	1.522	1180.97	1797.43
A_68_P21587250	chr2:122463614-122463658	NR_030547:-2902	Mir147	PROMOTER	0.847	0.354	1942.38	687.44	0.300	1129.61	338.52
A_68_P21569222	chr2:119177342-119177386	NM_026929:387	Chac1	INSIDE	0.847	0.392	1331.39	522.04	0.332	974.77	323.69
A_68_P21395065	chr2:84706336-84706380	NM_001083809:25576	Slc43a1	DOWNSTREAM	0.847	0.140	3784.97	529.66	0.119	2604.01	308.71
A_68_P21321484	chr2:71625261-71625305	NM_008397:143	Itga6	INSIDE	0.847	2.803	633.88	1776.58	2.373	527.56	1252.13
A_68_P21063038	chr2:23427274-23427318	NM_001165997:328	Spopl	INSIDE	0.847	0.642	3396.43	2182.05	0.544	2491.00	1356.16
A_68_P32533284	chrX:90544387-90544431	NM_007492:12424	Arx	DOWNSTREAM	0.846	2.184	808.40	1765.78	1.848	858.57	1586.32
A_68_P31549438	chr18:22503185-22503229	NM_001167777:-383	Asxl3	PROMOTER	0.846	0.719	3395.87	2443.04	0.608	2214.96	1347.34
A_68_P29940450	chr14:119105251-119105295	NM_001033336:169	Abcc4	INSIDE	0.846	0.311	2267.31	705.60	0.263	1662.69	437.74
A_68_P29937474	chr14:118634627-118634671	NM_177753:1604	Sox21	INSIDE	0.846	0.525	933.11	489.68	0.444	799.68	355.02
A_68_P28463393	chr12:58677002-58677046	NM_001037743:11923	4921506M07Rik	INSIDE	0.846	0.338	2260.75	763.58	0.286	1865.60	533.26
A_68_P27950599	chr11:79525184-79525228	NR_029579:-264	Mir193	PROMOTER	0.846	0.291	2384.09	693.22	0.246	2088.08	513.49
A_68_P27297057	chr10:82823167-82823211	NM_026579:223	D10Wsu102e	INSIDE	0.846	0.213	3202.14	681.56	0.180	2455.68	442.22
A_68_P26224602	chr8:123640403-123640447	NM_013519:354	Foxc2	INSIDE	0.846	2.610	830.10	2166.88	2.209	731.81	1616.35
A_68_P24904452	chr6:142363210-142363254	NM_145572:58397	Gys2	DOWNSTREAM	0.846	0.340	1655.20	563.53	0.288	1444.17	416.18
A_68_P23979393	chr5:110815604-110815648	NM_028596:520	Fbrsl1	INSIDE	0.846	2.707	4874.58	13196.37	2.291	3745.98	8583.86
A_68_P23613111	chr5:37727910-37727954	NM_021292:188	Evc	INSIDE	0.846	1.740	1507.84	2623.08	1.471	1136.88	1672.32
A_68_P22338872	chr3:94216808-94216852	NM_028307:-409	Tdrkh	PROMOTER	0.846	0.197	4383.23	865.07	0.167	2950.34	492.54
A_68_P21999683	chr3:25043045-25043089	AK054291:117		INSIDE	0.846	3.730	1385.41	5168.11	3.155	1221.77	3854.06
A_68_P21094090	chr2:28765327-28765371	NM_019446:6593	Barhl1	INSIDE	0.846	0.440	2161.15	950.63	0.372	1364.36	507.91
A_68_P21007915	chr2:11700413-11700457	NM_177268:1055	Ankrd16	INSIDE	0.846	0.552	2309.76	1274.79	0.467	1732.04	808.64
A_68_P20748459	chr1:159342791-159342835	NM_177644:-86	Rasal2	PROMOTER	0.846	0.315	3106.72	979.90	0.267	2081.13	555.13
A_68_P20506809	chr1:108069287-108069331	NM_133821:863	Phlpp1	INSIDE	0.846	0.341	2723.82	929.60	0.289	2090.54	603.65
A_68_P32056973	chr19:30249514-30249558	NM_138595:372	Gldc	INSIDE	0.845	0.347	1467.83	509.14	0.293	1050.68	307.92
A_68_P31928605	chr19:5424975-5425019	NM_024176:-80	Drap1	DIVERGENT_PROMOTER	0.845	0.727	4668.06	3393.97	0.614	3271.90	2009.23
A_68_P31849647	chr18:78006501-78006545	NM_146089:-3	Haus1	DIVERGENT_PROMOTER	0.845	0.384	1933.75	741.94	0.324	1631.38	529.06
A_68_P31117688	chr17:27792442-27792486	NM_011861:-162	Pacsin1	PROMOTER	0.845	4.372	808.62	3535.44	3.697	736.28	2721.69
A_68_P30486390	chr15:100381931-100381975	NM_033476:426	Tcfcp2	INSIDE	0.845	0.563	1369.62	770.49	0.475	1063.26	505.21
A_68_P30472486	chr15:97835275-97835319	NM_001113515:-141	Col2a1	PROMOTER	0.845	0.618	2248.49	1390.00	0.522	1770.48	924.75
A_68_P30146830	chr15:37890997-37891041	NM_199476:-208	Rrm2b	PROMOTER	0.845	0.690	2169.80	1496.36	0.583	1877.90	1094.86
A_68_P29080187	chr13:59658208-59658252	NM_023328:450	Agtpbp1	INSIDE	0.845	0.469	1063.19	498.24	0.396	822.49	325.73
A_68_P27903678	chr11:70796278-70796322	NM_007573:228	C1qbp	INSIDE	0.845	2.964	2279.74	6757.43	2.506	2008.50	5033.46
A_68_P26680225	chr9:82723830-82723874	NM_001168240:440	Irak1bp1	INSIDE	0.845	0.494	1678.65	828.99	0.417	1277.24	532.75
A_68_P25672217	chr8:14090101-14090145	NM_001034862:205	Erich1	INSIDE	0.845	0.445	2095.08	933.14	0.377	1623.40	611.25
A_68_P25614205	chr8:3493317-3493361	NM_080461:201	Zfp358	INSIDE	0.845	0.360	3305.20	1189.83	0.304	2605.34	792.39
A_68_P24973142	chr7:13403941-13403985	NM_026046:247	Zfp329	INSIDE	0.845	0.569	2530.80	1440.19	0.481	2044.75	983.46
A_68_P24592899	chr6:83299323-83299367	NM_175277:-133	Bola3	PROMOTER	0.845	0.520	3999.64	2080.00	0.440	3025.50	1329.99
A_68_P23433032	chr4:154009074-154009118	NM_001177995:1886	Prdm16	INSIDE	0.845	0.537	930.01	499.86	0.454	734.33	333.57
A_68_P23259663	chr4:122563383-122563428	NM_007598:-281	Cap1	PROMOTER	0.845	2.451	568.35	1392.90	2.071	490.81	1016.57
A_68_P22369560	chr3:100293076-100293120	NM_001142952:17	Fam46c	INSIDE	0.845	0.308	4676.85	1438.85	0.260	3603.16	937.14
A_68_P22314617	chr3:88383670-88383714	NM_025448:100	Ssr2	INSIDE	0.845	1.728	1746.08	3016.59	1.460	1384.49	2021.79
A_68_P22207870	chr3:66782653-66782697	NM_013665:3019	Shox2	INSIDE	0.845	0.669	2852.25	1907.25	0.565	2002.03	1130.95
A_68_P21911109	chr2:181255398-181255442	NM_016775:211	Dnajc5	INSIDE	0.845	0.283	1703.18	482.54	0.240	1077.93	258.21
A_68_P20788008	chr1:166178976-166179020	NM_054087:-187	Slc19a2	PROMOTER	0.845	0.213	7346.04	1566.82	0.180	5408.66	974.51
A_68_P20648919	chr1:138845800-138845844	NM_001159769:4385	Nr5a2	INSIDE	0.845	0.302	1864.76	563.63	0.255	1445.46	369.15
A_68_P20409050	chr1:87791484-87791528	NM_022417:422	Itm2c	INSIDE	0.845	0.307	1498.69	459.39	0.259	1091.24	282.72
A_68_P32744435	chrX:147603926-147603970	ENSMUST00000151778:-294		PROMOTER	0.844	4.144	340.01	1408.93	3.497	356.51	1246.57
A_68_P31157713	chr17:35257880-35257924	NM_009975:490	Csnk2b	INSIDE	0.844	0.229	2407.42	550.49	0.193	1607.84	310.16
A_68_P30347071	chr15:76077402-76077446	NM_023168:188	Grina	INSIDE	0.844	0.436	1630.74	711.54	0.368	1214.57	447.40
A_68_P30295267	chr15:66800261-66800306	NM_008681:920	Ndrg1	INSIDE	0.844	0.133	5833.14	776.40	0.112	4068.62	456.91
A_68_P29643581	chr14:60870353-60870397	NM_170591:-159	Nupl1	PROMOTER	0.844	1.908	1937.75	3697.23	1.610	1500.99	2417.25
A_68_P29044126	chr13:53568189-53568233	NM_013601:-61	Msx2	PROMOTER	0.844	0.493	1021.68	503.57	0.416	890.31	370.15
A_68_P28575720	chr12:81536277-81536321	NM_177267:1290	Dcaf5	INSIDE	0.844	0.513	948.77	487.11	0.433	851.26	368.78
A_68_P28030170	chr11:93858108-93858152	NM_001199203:726	Spag9	INSIDE	0.844	0.558	1913.34	1067.31	0.471	1556.34	732.55
A_68_P26039513	chr8:90724426-90724470	NM_001164497:1337	Papd5	INSIDE	0.844	0.708	4243.20	3002.43	0.597	3118.29	1861.86
A_68_P25575620	chr7:146271809-146271853	NM_011993:-69	Dpysl4	PROMOTER	0.844	0.681	4560.66	3104.31	0.575	2939.31	1688.92

ProbeName	Target position of probe on CpG island microarray	TargetID	GeneSymbol	CpG island Description	Ratio of relative methylation (TiO_2-NP/Vehicle)	Sham group			TiO_2-H group		
						Relative methylation (Cy5/Cy3)	Cy3 signal (Input)	Cy5 signal (MeDIP)	Relative methylation (Cy5/Cy3)	Cy3 signal (Input)	Cy5 signal (MeDIP)
A_68_P25326011	chr7:99817590-99817649	NM_029078:803	Pcf11	INSIDE	0.844	0.463	1223.62	567.00	0.391	1006.90	393.59
A_68_P25265479	chr7:87377398-87377442	NM_011870:82	Cib1	INSIDE	0.844	0.406	4610.47	1872.28	0.343	3500.65	1200.03
A_68_P25007800	chr7:25790566-25790610	NM_144921:326	Atp1a3	INSIDE	0.844	0.213	5769.72	1226.62	0.180	3946.96	708.55
A_68_P24600279	chr6:84535601-84535645	NM_175475:8182	Cyp26b1	INSIDE	0.844	0.576	908.62	523.39	0.486	779.73	379.22
A_68_P24449528	chr6:52210288-52210332	NM_008264:564	Hoxa13	INSIDE	0.844	12.440	604.13	7515.14	10.494	494.67	5191.08
A_68_P24141815	chr5:141000477-141000521	NM_021528:18936	Chst12	INSIDE	0.844	2.313	1143.77	2645.17	1.953	940.48	1836.62
A_68_P24138653	chr5:140448112-140448156	NM_175522:64238	Elfn1	INSIDE	0.844	2.351	2425.27	5701.53	1.984	1752.22	3475.93
A_68_P23611140	chr5:37420085-37420129	ENSMUST00000165557:12779		DOWNSTREAM	0.844	0.606	942.28	570.70	0.511	808.64	413.30
A_68_P23591878	chr5:34279071-34279115	NM_011914:-185	Whsc2	PROMOTER	0.844	0.528	1455.52	768.07	0.445	1139.39	507.45
A_68_P21224228	chr2:53051318-53051362	NM_022989:223	Arl6ip6	INSIDE	0.844	0.421	1312.20	553.08	0.356	1042.17	370.92
A_68_P21114431	chr2:32143551-32143595	NM_175190:16	2900010J23Rik	INSIDE	0.844	0.512	3248.42	1662.69	0.432	2444.78	1055.93
A_68_P20914292	chr1:189431401-189431445	ENSMUST00000127489:-1247		PROMOTER	0.844	2.717	2343.75	6368.47	2.294	1922.62	4411.38
A_68_P20825164	chr1:172815355-172815399	NM_023173:295	Dusp12	INSIDE	0.844	1.697	2149.66	3647.95	1.432	1693.50	2424.99
A_68_P31286857	chr17:63232103-63232147	NM_010109:-1458	Efna5	PROMOTER	0.843	1.857	937.07	1739.69	1.565	925.40	1448.49
A_68_P31236180	chr17:51952405-51952449	NM_001163630:-1047	Satb1	PROMOTER	0.843	2.746	772.25	2120.43	2.315	620.37	1436.19
A_68_P31054933	chr17:13200672-13200716	NM_013671:-10	Sod2	PROMOTER	0.843	0.419	1716.11	718.54	0.353	1405.09	495.77
A_68_P29713890	chr14:73109575-73109619	NM_207636:214	Fndc3a	INSIDE	0.843	0.614	1432.67	880.09	0.518	1119.16	579.53
A_68_P29050152	chr13:54676736-54676780	NM_019968:385	Arl10	INSIDE	0.843	0.580	1742.96	1010.06	0.488	1390.34	679.00
A_68_P28195048	chr12:3427213-3427257	NM_172421:351	Asxl2	INSIDE	0.843	3.706	1373.50	5089.62	3.124	1163.52	3634.48
A_68_P27677361	chr11:29924802-29924846	NM_146016:1209	Eml6	INSIDE	0.843	0.605	1292.82	781.58	0.510	1056.18	538.13
A_68_P26567942	chr9:61794410-61794454	NM_024245:174	Kif23	INSIDE	0.843	0.618	1472.61	910.44	0.521	1131.72	590.18
A_68_P26017563	chr8:86525640-86525684	NM_183097:58	1700067K01Rik	INSIDE	0.843	2.116	944.67	1999.03	1.784	826.38	1474.53
A_68_P25506523	chr7:134758122-134758166	NM_177226:-197	Zfp629	PROMOTER	0.843	0.394	1695.92	668.31	0.332	1497.81	497.42
A_68_P25265202	chr7:87331967-87332011	NM_013659:262	Sema4b	INSIDE	0.843	0.674	1158.32	781.00	0.568	983.29	558.81
A_68_P25261414	chr7:86644655-86644699	NR_029818:-5473	Mir9-3	PROMOTER	0.843	0.490	909.98	445.45	0.413	732.88	302.61
A_68_P24880088	chr6:137683751-137683795	NM_011499:170	Strap	INSIDE	0.843	3.145	656.23	2063.70	2.652	560.21	1485.69
A_68_P24828012	chr6:126594233-126594277	NM_010595:1565	Kcna1	INSIDE	0.843	0.357	3495.69	1247.38	0.301	2521.53	758.54
A_68_P24319368	chr6:28211888-28211932	NM_001081678:-309	Zfp800	PROMOTER	0.843	0.493	1520.00	748.69	0.415	1288.39	535.24
A_68_P24054126	chr5:123588945-123588989	NM_175092:-6328	Rhof	PROMOTER	0.843	2.364	853.42	2017.88	1.993	769.36	1532.97
A_68_P23524645	chr5:20692196-20692240	NM_175437:134	Pion	INSIDE	0.843	0.430	1356.89	583.96	0.363	1087.14	394.38
A_68_P22558738	chr3:136332932-136332976	NM_008913:-779	Ppp3ca	PROMOTER	0.843	0.285	1715.40	489.60	0.241	1514.56	364.36
A_68_P21839557	chr2:167942759-167942803			Unknown	0.843	3.666	714.96	2621.16	3.092	565.56	1748.75
A_68_P21706648	chr2:144157388-144157432	NM_152947:-312	Ovol2	PROMOTER	0.843	0.406	2001.56	812.10	0.342	1539.39	526.76
A_68_P21706318	chr2:144096392-144096436	NM_024225:-106	Snx5	PROMOTER	0.843	0.467	1052.60	491.47	0.394	772.24	303.95
A_68_P32593241	chrX:106208891-106208935	NM_016710:-193	Hmgn5	PROMOTER	0.842	1.834	857.43	1572.14	1.544	1176.23	1816.65
A_68_P32148029	chr19:47226204-47226248	NM_001081271:-7562	Calhm1	PROMOTER	0.842	0.483	1581.22	763.41	0.406	1211.62	492.42
A_68_P32050469	chr19:29065228-29065272	NM_025950:267	Cdc37l1	INSIDE	0.842	0.187	2500.60	467.08	0.157	1806.45	284.00
A_68_P31331866	chr17:71339942-71339986	NM_023402:-108	Myl12b	PROMOTER	0.842	0.450	2331.35	1048.26	0.378	1697.62	642.36
A_68_P30978325	chr16:93919893-93919937	NM_001165925:9898	Cldn14	INSIDE	0.842	2.986	1165.16	3478.72	2.513	967.38	2431.18
A_68_P30570063	chr16:16599690-16599734	ENSMUST00000162671:359		INSIDE	0.842	4.567	9320.89	42564.48	3.847	6985.28	26871.84
A_68_P29928896	chr14:117324667-117324711	NM_001079844:152	Gpc6	INSIDE	0.842	1.912	2270.15	4340.65	1.609	1790.98	2882.29
A_68_P28575717	chr12:81535921-81535965	NM_177267:1646	Dcaf5	INSIDE	0.842	3.108	839.94	2610.88	2.618	672.68	1761.33
A_68_P28458266	chr12:57793635-57793679	NM_011041:-2969	Pax9	PROMOTER	0.842	0.463	1458.82	675.13	0.390	1187.06	462.37
A_68_P28293505	chr12:25780980-25781025	NM_010496:-45	Id2	PROMOTER	0.842	2.478	821.46	2035.43	2.086	724.88	1511.89
A_68_P27567708	chr11:6192040-6192084	NM_010956:463	Ogdh	INSIDE	0.842	0.355	3050.86	1081.86	0.299	2480.07	740.34
A_68_P25210870	chr7:77514645-77514689	NM_183261:-3034	Nr2f2	PROMOTER	0.842	0.528	1093.91	577.96	0.445	960.68	427.44
A_68_P25017319	chr7:28232977-28233021	NM_144923:2	Blvrb	INSIDE	0.842	10.044	746.21	7495.33	8.461	605.77	5125.22
A_68_P24464807	chr6:54632035-54632079	NM_026629:291	2410066E13Rik	INSIDE	0.842	0.392	1213.54	476.16	0.330	938.66	310.11
A_68_P24156216	chr5:144126254-144126298	NM_133355:690	Grid2ip	INSIDE	0.842	0.456	2886.35	1316.29	0.384	2303.78	884.33
A_68_P23637403	chr5:42152928-42152972	NM_007524:2509	Nkx3-2	INSIDE	0.842	2.348	767.91	1803.00	1.977	582.32	1151.01
A_68_P23072596	chr4:84851915-84851959	NM_019535:577	Sh3gl2	INSIDE	0.842	0.359	1635.61	586.46	0.302	1334.95	403.04
A_68_P22888514	chr4:46402148-46402192	NM_029086:125	5830415F09Rik	INSIDE	0.842	0.497	1736.78	863.95	0.419	1309.15	548.16
A_68_P21583617	chr2:121781672-121781716	NM_008545:134	Mageb3	INSIDE	0.842	2.266	1078.26	2443.19	1.908	1001.08	1910.12
A_68_P21091465	chr2:28368543-28368587	NM_001145834:-122	Ralgds	PROMOTER	0.842	0.495	1876.13	928.28	0.417	1426.72	594.26
A_68_P21061192	chr2:22895312-22895356	NM_001077190:426	Abi1	INSIDE	0.842	0.462	1866.73	862.06	0.389	1498.76	582.87
A_68_P20559396	chr1:121809747-121809791	NM_175106:-23	Tmem177	PROMOTER	0.842	0.455	3642.11	1658.95	0.384	2959.03	1134.81
A_68_P32094198	chr19:37248988-37249032	NM_198300:32773	Cpeb3	INSIDE	0.841	0.341	3390.95	1156.22	0.287	2466.40	707.30
A_68_P31809751	chr18:70632194-70632238	NM_029019:18	Stard6	INSIDE	0.841	0.126	6403.92	805.44	0.106	4373.57	462.40
A_68_P31775663	chr18:64648900-64648944	NM_007998:-202	Fech	PROMOTER	0.841	0.330	2362.87	779.69	0.278	1590.23	441.54
A_68_P31161105	chr17:35960212-35960256	NM_008027:-67	Flot1	PROMOTER	0.841	1.937	1159.90	2246.58	1.629	972.05	1583.73
A_68_P30759541	chr16:52031831-52031875	NM_001033238:191	Cblb	INSIDE	0.841	0.488	1238.08	604.58	0.411	974.40	400.39
A_68_P29639603	chr14:60244552-60244596	NM_145463:457	Shisa2	INSIDE	0.841	2.168	1323.68	2869.66	1.823	1012.16	1845.44
A_68_P29624997	chr14:57483807-57483852	AK209334:-895		PROMOTER	0.841	2.722	926.22	2521.22	2.288	774.43	1771.97
A_68_P28149047	chr11:114530264-114530308	NM_001048058:192	Rpl38	INSIDE	0.841	0.266	1654.83	439.86	0.224	1351.16	302.06
A_68_P28079666	chr11:102317339-102317383	NM_010575:13837	Itga2b	INSIDE	0.841	0.290	2899.36	842.04	0.244	2032.76	496.42

ProbeName	Target position of probe on CpG island microarray	TargetID	GeneSymbol	CpG island Description	Ratio of relative methylation (TiO_2-NP/Vehicle)	Sham group			TiO_2-H group		
						Relative methylation (Cy5/Cy3)	Cy3 signal (Input)	Cy5 signal (MeDIP)	Relative methylation (Cy5/Cy3)	Cy3 signal (Input)	Cy5 signal (MeDIP)
A_68_P27893912	chr11:69049971-69050015	NM_008192:532	Gucy2e	INSIDE	0.841	2.875	723.87	2080.99	2.418	648.92	1569.07
A_68_P27307032	chr10:84647397-84647441	NM_007771:381	Cry1	INSIDE	0.841	0.409	1161.96	475.35	0.344	963.32	331.60
A_68_P26763584	chr9:99258801-99258845	NM_177775:127	Esyt3	INSIDE	0.841	0.616	866.76	534.23	0.518	781.93	405.38
A_68_P26261058	chr8:129588080-129588124	NM_001122850:147	Pard3	INSIDE	0.841	2.101	1219.21	2561.27	1.766	990.13	1748.42
A_68_P26171280	chr8:114515984-114516028	NM_013925:196	Adat1	INSIDE	0.841	0.430	1445.35	620.91	0.361	1191.84	430.83
A_68_P25735820	chr8:28090231-28090275	NM_001101502:2445	Zfp703	INSIDE	0.841	2.251	1750.64	3940.09	1.892	1410.55	2668.96
A_68_P24055453	chr5:123845311-123845355	NM_177582:505	Mlxip	INSIDE	0.841	0.526	1603.09	843.75	0.443	1141.56	505.55
A_68_P22311221	chr3:87801100-87801144	NM_007529:3156	Bcan	INSIDE	0.841	3.113	785.56	2445.11	2.619	616.83	1615.26
A_68_P21904883	chr2:180323917-180323961	NM_031373:-173	Ogfr	PROMOTER	0.841	0.372	1328.42	494.81	0.313	1057.91	331.56
A_68_P20557411	chr1:121401081-121401125	NM_022327:257	Ralb	INSIDE	0.841	0.335	3086.78	1033.45	0.281	2379.65	669.77
A_68_P32464062	chrX:70918709-70918753	NM_001142809:259	Slc6a8	INSIDE	0.840	2.683	925.52	2482.74	2.254	1057.91	2384.06
A_68_P31620538	chr18:36020156-36020200	NM_133687:30707	Cxxc5	INSIDE	0.840	2.021	1150.96	2325.72	1.698	936.35	1589.82
A_68_P31152214	chr17:34256524-34256568	NR_037970:299	Brd2	INSIDE	0.840	2.192	1442.33	3161.39	1.841	1133.94	2088.10
A_68_P29699886	chr14:70592016-70592060	NM_011366:15406	Sorbs3	INSIDE	0.840	0.548	1281.94	701.92	0.460	916.29	421.19
A_68_P28610312	chr12:87811484-87811528	NM_011934:48913	Esrrb	INSIDE	0.840	4.782	994.70	4756.67	4.017	797.11	3202.08
A_68_P27997243	chr11:87800391-87800435	NM_001168472:-325	Dynll2	PROMOTER	0.840	0.286	2448.88	700.28	0.240	1816.77	436.41
A_68_P27843669	chr11:60351870-60351914	NM_172943:708	Alkbh5	INSIDE	0.840	4.109	1026.06	4215.87	3.450	856.50	2955.03
A_68_P27812934	chr11:54769412-54769456	NM_001199275:190	Tnip1	INSIDE	0.840	0.611	2077.98	1268.94	0.513	1513.10	776.50
A_68_P26905370	chr10:4541439-4541483	NM_025995:385	Fbxo5	INSIDE	0.840	0.148	3995.51	589.99	0.124	2931.68	363.83
A_68_P26574056	chr9:62986746-62986790	NM_172446:8019	Skor1	INSIDE	0.840	0.331	5472.31	1812.73	0.278	3825.72	1064.95
A_68_P25010200	chr7:26180775-26180819	NM_010719:210	Lipe	INSIDE	0.840	3.219	1281.20	4124.37	2.705	1042.60	2820.40
A_68_P24431874	chr6:49213690-49213734	NM_198102:339	Tra2a	INSIDE	0.840	0.268	2730.45	732.29	0.225	2312.13	520.73
A_68_P23430517	chr4:153673893-153673937	NM_001112744:90	Arhgef16	INSIDE	0.840	0.402	2039.13	819.79	0.338	1585.14	535.07
A_68_P23308586	chr4:131393104-131393148	NM_001083119:1067	Ptpru	INSIDE	0.840	0.508	1009.36	512.61	0.426	698.28	297.78
A_68_P23134124	chr4:97412824-97412868	NM_001122952:164213	Nfia	INSIDE	0.840	0.409	1249.44	510.56	0.343	1079.21	370.28
A_68_P22881129	chr4:45120647-45120691	NM_001099675:317	Tomm5	INSIDE	0.840	0.357	3132.97	1117.08	0.300	2422.95	725.92
A_68_P22747170	chr4:14753655-14753699	NM_152812:58	Otud6b	INSIDE	0.840	2.717	466.52	1267.78	2.282	418.81	955.86
A_68_P22151288	chr3:54859609-54859653	NM_007628:-653	Ccna1	PROMOTER	0.840	2.878	1441.81	4149.65	2.416	1193.03	2882.77
A_68_P21580974	chr2:121275109-121275153	NM_011354:167	Serf2	INSIDE	0.840	0.346	2238.83	774.92	0.291	1864.96	542.38
A_68_P20897578	chr1:186634682-186634726	NM_001081361:488	Mosc1	INSIDE	0.840	0.584	1217.59	711.22	0.491	937.74	459.98
A_68_P20804312	chr1:169214668-169214712	NM_030724:568	Uck2	INSIDE	0.840	0.390	1554.16	605.36	0.327	1232.69	403.14
A_68_P20377068	chr1:79668401-79668445	NM_183027:125	Ap1s3	INSIDE	0.840	0.415	2373.61	984.41	0.348	1885.33	656.49
A_68_P31925257	chr19:4811827-4811871	NM_019869:-214	Rbm14	PROMOTER	0.839	0.342	2546.16	869.72	0.287	2141.44	614.07
A_68_P31632605	chr18:38358832-38358876	NM_029357:10562	Pcdh1	INSIDE	0.839	2.955	3957.56	11696.55	2.478	2818.48	6985.23
A_68_P31151129	chr17:34077065-34077109	NM_001185182:189	H2-Ke2	INSIDE	0.839	0.510	1992.84	1017.09	0.428	1531.21	655.37
A_68_P30639252	chr16:30009147-30009191	NR_033631:416	4632428C04Rik	INSIDE	0.839	0.424	1754.73	743.32	0.356	1419.81	504.82
A_68_P29589383	chr14:49284720-49284764	NM_144841:-2195	Otx2	DIVERGENT_PROMOTER	0.839	0.187	2521.03	471.08	0.157	1916.91	300.63
A_68_P27806466	chr11:53583987-53584031	NM_001159396:34	Irf1	INSIDE	0.839	4.299	765.81	3292.25	3.608	582.64	2102.29
A_68_P27805843	chr11:53450040-53450084	NM_008355:-1858	Il13	PROMOTER	0.839	0.708	3797.07	2687.97	0.594	2933.84	1742.14
A_68_P27288864	chr10:80785360-80785404	NM_027381:1535	2510012J08Rik	INSIDE	0.839	2.730	345.23	942.40	2.289	309.23	707.84
A_68_P25042627	chr7:34981438-34981482	NM_026549:206	Pdcd2l	INSIDE	0.839	0.612	990.27	605.63	0.513	845.60	434.07
A_68_P24444441	chr6:51420902-51420946	NM_007624:310	Cbx3	INSIDE	0.839	0.592	5066.17	3001.39	0.497	3554.20	1767.67
A_68_P24426867	chr6:48345685-48345729	NM_133922:122	Krba1	INSIDE	0.839	1.695	1886.78	3198.67	1.422	1496.69	2128.84
A_68_P24056168	chr5:123974438-123974482	NM_023232:-287	Diablo	PROMOTER	0.839	0.681	1547.41	1053.40	0.571	1231.38	703.20
A_68_P23429537	chr4:153545094-153545138	NM_001162977:295	Megf6	INSIDE	0.839	0.560	1571.09	879.88	0.470	1264.23	594.09
A_68_P23194281	chr4:107704080-107704124	NM_172874:593	Podn	INSIDE	0.839	0.389	2688.05	1045.33	0.326	1941.65	633.77
A_68_P21565427	chr2:118503318-118503362	NM_001145854:359	Pak6	INSIDE	0.839	0.596	1777.89	1059.76	0.500	1350.39	675.59
A_68_P21143643	chr2:37559408-37559452	ENSMUST00000155237:-51		PROMOTER	0.839	33.218	1660.93	55172.07	27.884	1781.52	49676.47
A_68_P21113208	chr2:31939049-31939093	NM_175511:155	Fam78a	INSIDE	0.839	1.796	1519.15	2728.57	1.508	1354.92	2042.98
A_68_P21068910	chr2:24790687-24790731	NM_001162485:93	Arrdc1	INSIDE	0.839	2.074	2244.27	4655.39	1.740	1886.11	3281.87
A_68_P20153981	chr1:37486651-37486695	NM_198006:276	6330578E17Rik	INSIDE	0.839	0.374	2579.33	964.41	0.314	1980.75	621.70
A_68_P32959962	A_68_P32959962			Unknown	0.838	4.469	266.56	1191.17	3.743	150.61	563.65
A_68_P31335318	chr17:71966149-71966193	NM_175639:616	Wdr43	INSIDE	0.838	0.265	1857.73	493.23	0.222	1472.13	327.45
A_68_P31125919	chr17:29230688-29230732	NM_007669:-6	Cdkn1a	PROMOTER	0.838	0.390	3039.00	1185.47	0.327	2191.08	716.34
A_68_P30380707	chr15:81790992-81791036	NM_013872:283	Pmm1	INSIDE	0.838	1.906	1816.44	3462.43	1.597	1137.35	1816.11
A_68_P29508895	chr14:31764856-31764900	NM_011849:205	Nek4	INSIDE	0.838	0.275	1792.32	492.27	0.230	1395.49	321.37
A_68_P29246781	chr13:97313402-97313446	NM_001164222:735	Col4a3bp	INSIDE	0.838	0.300	2698.63	809.70	0.251	2148.77	540.02
A_68_P29024660	chr13:49437384-49437428	NM_001039179:489	Bicd2	INSIDE	0.838	0.258	2285.96	589.06	0.216	1633.95	352.88
A_68_P28725020	chr12:109218453-109218497	NM_001079883:23150	Bcl11b	INSIDE	0.838	0.241	1877.08	451.68	0.202	1415.29	285.29
A_68_P28688787	chr12:103214342-103214386	NM_016856:181	Cpsf2	INSIDE	0.838	0.480	2880.18	1382.39	0.402	2173.74	874.23
A_68_P28558330	chr12:78339604-78339648	NM_016893:-381	Fut8	PROMOTER	0.838	2.024	690.95	1398.30	1.695	628.45	1065.18
A_68_P28158617	chr11:116167897-116167941	NM_001162872:134	Exoc7	INSIDE	0.838	0.304	2067.51	628.43	0.255	1476.13	375.90
A_68_P27286200	chr10:80360852-80360896	NM_001081688:18314	Tmprss9	INSIDE	0.838	0.473	2269.28	1073.02	0.396	1523.02	603.59
A_68_P26644959	chr9:75458604-75458648	NM_016711:506	Tmod2	INSIDE	0.838	0.148	3367.72	497.45	0.124	2337.71	289.40
A_68_P26135026	chr8:108159947-108159991	NM_181322:-469	Ctcf	PROMOTER	0.838	0.583	2680.82	1563.66	0.489	2193.26	1071.46

ProbeName	Target position of probe on CpG island microarray	TargetID	GeneSymbol	CpG island Description	Ratio of relative methylation (TiO_2-NP/Vehicle)	Sham group			TiO_2-H group		
						Relative methylation (Cy5/Cy3)	Cy3 signal (Input)	Cy5 signal (MeDIP)	Relative methylation (Cy5/Cy3)	Cy3 signal (Input)	Cy5 signal (MeDIP)
A_68_P25571665	chr7:145589754-145589798	NM_183289:-363	Tcergl1	PROMOTER	0.838	4.020	1252.25	5034.11	3.368	1004.77	3384.05
A_68_P25435751	chr7:121559514-121559558	NM_011055:769	Pde3b	INSIDE	0.838	5.581	987.85	5513.66	4.676	859.48	4018.94
A_68_P24817841	chr6:124881814-124881858	NM_001170341:431	Mlf2	INSIDE	0.838	0.190	3151.99	599.74	0.159	2344.03	373.56
A_68_P24171442	chr5:147840909-147840953	NM_080795:47218	Lnx2	INSIDE	0.838	4.419	726.19	3209.19	3.703	575.87	2132.63
A_68_P24053483	chr5:123464984-123465028	NM_175423:-76	Orai1	PROMOTER	0.838	0.616	1028.35	633.83	0.517	857.76	443.21
A_68_P23323338	chr4:134091096-134091140	NM_029759:-2451	Fam54b	PROMOTER	0.838	0.343	2395.03	821.86	0.288	1935.02	556.65
A_68_P22878647	chr4:44717142-44717186	NM_008782:6148	Pax5	INSIDE	0.838	1.769	1542.80	2729.64	1.482	1290.30	1912.52
A_68_P22778566	chr4:22422209-22422253	NM_008899:-6952	Pou3f2	PROMOTER	0.838	0.520	854.60	444.68	0.436	741.84	323.50
A_68_P22165656	chr3:57651792-57651836	NM_019410:-135	Pfn2	PROMOTER	0.838	0.316	3211.20	1016.18	0.265	2584.69	685.71
A_68_P21779871	chr2:157740581-157740625	NM_198627:214	Vstm2l	INSIDE	0.838	1.795	1998.74	3587.65	1.504	1476.31	2220.09
A_68_P20739568	chr1:157584694-157584738	NM_010712:4441	Lhx4	INSIDE	0.838	0.667	1698.07	1131.99	0.559	1306.86	729.94
A_68_P20370562	chr1:78214214-78214258			Unknown	0.838	0.508	950.99	483.52	0.426	734.80	312.90
A_68_P32788263	chrX:158596737-158596781	NM_001081052:964	Nhs	INSIDE	0.837	1.689	1222.31	2065.02	1.414	1496.72	2116.04
A_68_P31930176	chr19:5688332-5688376	NM_144868:554	Pcnxl3	INSIDE	0.837	0.712	2812.58	2001.39	0.596	2085.16	1241.77
A_68_P31924463	chr19:4698602-4698646	ENSMUST00000096325:-309		PROMOTER	0.837	2.773	448.28	1242.85	2.321	406.09	942.36
A_68_P29664628	chr14:64563075-64563120	NM_011446:555	Sox7	INSIDE	0.837	4.093	215.27	881.02	3.426	173.30	593.75
A_68_P29211818	chr13:90229540-90229584	NM_025335:291	Tmem167	INSIDE	0.837	0.344	2087.90	718.85	0.288	1596.72	460.03
A_68_P29110676	chr13:68721046-68721090	NM_027123:-56	Fastkd3	DIVERGENT_PROMOTER	0.837	0.633	2644.29	1674.56	0.530	2036.87	1079.89
A_68_P29033453	chr13:51740294-51740338	NM_025415:-284	Cks2	PROMOTER	0.837	0.549	1770.27	971.57	0.459	1403.96	644.89
A_68_P28691640	chr12:103707382-103707426	NM_013747:-715	Golga5	PROMOTER	0.837	7.461	412.25	3075.78	6.244	396.51	2475.63
A_68_P28269090	chr12:17182278-17182322	NM_201531:1394	Kcnf1	INSIDE	0.837	0.474	2928.64	1387.06	0.396	2067.56	819.75
A_68_P27855082	chr11:62461774-62461819	NM_198861:229	BC046404	INSIDE	0.837	4.657	647.32	3014.70	3.898	489.89	1909.67
A_68_P27534515	chr10:126627015-126627059	NM_001166413:-157	Arhgef25	PROMOTER	0.837	0.442	1579.01	698.16	0.370	1129.19	418.07
A_68_P26580972	chr9:64101263-64101307	NM_008927:128	Map2k1	INSIDE	0.837	1.833	1435.13	2630.05	1.534	1206.57	1850.33
A_68_P26260676	chr8:129494794-129494838	NM_172762:163	Rbm34	INSIDE	0.837	0.322	1996.12	642.78	0.270	1414.67	381.36
A_68_P24973956	chr7:13563487-13563531	NM_001168561:312	Zfp446	INSIDE	0.837	7.089	1109.73	7867.33	5.937	925.76	5496.45
A_68_P24925470	chr6:145811720-145811764	NM_024469:2118	Bhlhe41	INSIDE	0.837	7.140	2078.38	14839.18	5.975	1826.62	10913.55
A_68_P24609841	chr6:86338705-86338755	NM_025823:8414	Pcyox1	INSIDE	0.837	2.539	466.87	1185.34	2.126	381.11	810.13
A_68_P24540591	chr6:72184914-72184958	NM_153778:635	Atoh8	INSIDE	0.837	0.434	2435.46	1056.10	0.363	1840.25	667.88
A_68_P23909953	chr5:97426403-97426448	NM_080708:-282	Bmp2k	PROMOTER	0.837	0.576	1310.15	754.01	0.482	1127.49	542.97
A_68_P23767118	chr5:67698039-67698083	NM_178651:-135	Slc30a9	PROMOTER	0.837	0.443	2161.41	956.72	0.370	1522.94	563.99
A_68_P23294683	chr4:128736055-128736099	NM_029219:562	Rnf19b	INSIDE	0.837	0.349	1746.00	610.12	0.293	1250.87	366.06
A_68_P22933513	chr4:55363588-55363632	NM_009011:697	Rad23b	INSIDE	0.837	0.190	3709.53	703.50	0.159	2858.66	453.71
A_68_P21094978	chr2:28910349-28910393	NM_001166705:216	1700101E01Rik	INSIDE	0.837	2.393	1788.57	4279.88	2.003	1456.34	2917.02
A_68_P21078908	chr2:26321156-26321200	NM_008714:38164	Notch1	INSIDE	0.837	1.980	2128.31	4214.45	1.657	1688.29	2797.63
A_68_P32128060	chr19:43764426-43764470	NM_053103:270	Entpd7	INSIDE	0.836	0.493	1795.35	885.16	0.412	1370.09	564.59
A_68_P31929034	chr19:5490583-5490627	NM_007687:150	Cfl1	INSIDE	0.836	2.914	547.09	1594.47	2.437	467.60	1139.37
A_68_P31661237	chr18:43552558-43552602	NM_009468:405	Dpysl3	INSIDE	0.836	2.270	628.31	1426.43	1.897	606.57	1150.94
A_68_P30423406	chr15:88971030-88971074	NM_013871:81	Mapk12	INSIDE	0.836	0.206	2725.39	561.41	0.172	2031.54	349.69
A_68_P28731506	chr12:110272427-110272471	NM_001163175:33979	Begain	INSIDE	0.836	2.937	1908.31	5605.02	2.454	1490.18	3657.42
A_68_P28119820	chr11:109511689-109511733	NM_021880:-552	Prkar1a	PROMOTER	0.836	0.581	2691.90	1564.16	0.486	2232.65	1084.93
A_68_P28051740	chr11:97490459-97490503	NM_175332:550	E130012A19Rik	INSIDE	0.836	2.043	2099.63	4288.80	1.707	1699.07	2900.39
A_68_P25592942	chr7:149280795-149280840	NM_008748:372	Dusp8	INSIDE	0.836	0.730	2295.01	1676.03	0.611	1755.94	1072.32
A_68_P25578891	chr7:146767482-146767526	NR_027857:1192	Nkx6-2	INSIDE	0.836	0.311	3717.90	1156.75	0.260	2406.23	625.72
A_68_P25494915	chr7:132634916-132634960	NM_026330:118	Nsmce1	INSIDE	0.836	0.373	2678.40	998.01	0.312	2015.33	627.96
A_68_P25360010	chr7:106289656-106289700	NM_178635:-24	Uvrag	PROMOTER	0.836	2.679	1717.35	4601.04	2.239	1472.86	3297.90
A_68_P24019922	chr5:117769425-117769469	NM_001033311:172	Vsig10	INSIDE	0.836	0.608	1386.62	842.60	0.508	1078.13	547.77
A_68_P23543338	chr5:24191818-24191862	NM_016736:208	Nub1	INSIDE	0.836	2.028	983.80	1994.72	1.694	959.47	1625.58
A_68_P23403797	chr4:149383165-149383209	NM_173371:-54	H6pd	PROMOTER	0.836	9.802	2775.10	27200.30	8.196	2554.26	20935.30
A_68_P23309110	chr4:131476820-131476864	NM_001201367:-843	Tmem200b	PROMOTER	0.836	0.433	1398.40	606.15	0.362	1020.19	369.71
A_68_P21754296	chr2:153171121-153171165	NM_001039939:-732	Asxl1	PROMOTER	0.836	0.363	1607.47	582.98	0.303	1192.51	361.43
A_68_P21622287	chr2:128793258-128793302	NM_178404:143	Zc3h6	INSIDE	0.836	2.065	1645.99	3398.91	1.727	1226.79	2118.33
A_68_P20002766	chr1:3661026-3661072	NM_001011874:531	Xkr4	INSIDE	0.836	0.491	3463.39	1700.20	0.410	2502.78	1027.36
A_68_P32135962	chr19:45100671-45100715	NM_001130526:8068	Lzts2	INSIDE	0.835	4.236	1727.06	7315.78	3.538	1404.39	4968.66
A_68_P31633220	chr18:38456665-38456709	NM_001164621:228	Rnf14	INSIDE	0.835	2.630	999.77	2629.30	2.196	767.34	1685.35
A_68_P31254440	chr17:56323216-56323260	NM_080837:105	D17Wsu104e	INSIDE	0.835	2.497	1258.29	3141.81	2.086	1075.73	2243.45
A_68_P30822069	chr16:64852489-64852533	NM_178647:600	Cggbp1	INSIDE	0.835	0.409	1130.73	463.00	0.342	917.14	313.61
A_68_P30485952	chr15:100299863-100299907	NM_134093:420	Letmd1	INSIDE	0.835	0.428	2342.65	1002.28	0.357	1764.43	630.02
A_68_P30395525	chr15:84388217-84388261	NM_177630:15	Ldoc1l	INSIDE	0.835	2.115	575.76	1217.85	1.765	579.12	1022.32
A_68_P29514240	chr14:32644205-32644249	NM_001024604:-625	Ankrd28	PROMOTER	0.835	0.575	1175.82	675.71	0.480	892.42	428.30
A_68_P28748039	chr12:113368956-113369000	NR_029590:-112	Mir203	PROMOTER	0.835	3.509	314.63	1103.96	2.928	343.60	1006.20
A_68_P28601535	chr12:86337785-86337832	NM_178363:538	Ylpm1	INSIDE	0.835	0.329	2622.31	861.87	0.274	1838.65	504.63
A_68_P25082511	chr7:50721858-50721902	NM_001164518:23513	Iglon5	DOWNSTREAM	0.835	0.650	1512.58	983.38	0.543	1180.01	640.64
A_68_P24597420	chr6:84085413-84085457	NM_021469:116052	Dysf	INSIDE	0.835	1.983	1127.21	2234.72	1.656	917.70	1520.00
A_68_P24134132	chr5:139676747-139676791	NM_024451:145	Sun1	INSIDE	0.835	0.130	5550.89	723.88	0.109	3805.25	414.60

ProbeName	Target position of probe on CpG island microarray	TargetID	GeneSymbol	CpG island Description	Ratio of relative methylation (TiO_2-NP/Vehicle)	Sham group			TiO_2-H group		
						Relative methylation (Cy5/Cy3)	Cy3 signal (Input)	Cy5 signal (MeDIP)	Relative methylation (Cy5/Cy3)	Cy3 signal (Input)	Cy5 signal (MeDIP)
A_68_P24004992	chr5:115061116-115061160	NM_001004180:42879	BC057022	INSIDE	0.835	1.956	2023.07	3957.45	1.634	1437.77	2348.87
A_68_P23861881	chr5:86494186-86494230	NM_007683:400	Cenpc1	INSIDE	0.835	0.635	11948.02	7588.57	0.530	7776.67	4123.15
A_68_P23417743	chr4:151660802-151660846	NM_008237:4947	Hes3	INSIDE	0.835	0.484	1596.73	773.45	0.405	1171.89	474.12
A_68_P21761231	chr2:154395665-154395709	NM_007891:-98	E2f1	PROMOTER	0.835	0.253	2180.15	552.63	0.212	1671.17	353.81
A_68_P21118712	chr2:32837160-32837204	NM_019488:394	Slc2a8	INSIDE	0.835	2.088	863.16	1801.99	1.744	681.34	1188.02
A_68_P21037978	chr2:17969077-17969121	NM_028317:978	2810030E01Rik	INSIDE	0.835	3.419	2248.73	7688.62	2.854	1895.68	5410.29
A_68_P20416762	chr1:89161364-89161408	NM_028889:448	Efhd1	INSIDE	0.835	2.337	2447.45	5718.98	1.952	2064.82	4030.09
A_68_P32099501	chr19:38172178-38172222	NM_181748:632	O3far1	INSIDE	0.834	0.477	1820.71	867.85	0.398	1353.57	538.18
A_68_P30350023	chr15:76528479-76528523	NM_182805:1307	Gpt	INSIDE	0.834	2.370	1022.60	2423.67	1.977	879.88	1739.15
A_68_P29026629	chr13:49777484-49777528	NM_172015:8	Iars	INSIDE	0.834	0.401	1249.37	501.43	0.335	941.07	314.99
A_68_P28940412	chr13:34838589-34838633	NM_138746:7100	Fam50b	INSIDE	0.834	2.736	583.77	1596.95	2.280	493.50	1125.42
A_68_P28746794	chr12:113146363-113146407	NM_011625:-118	Ppp1r13b	PROMOTER	0.834	3.023	432.08	1306.30	2.522	414.16	1044.56
A_68_P28190285	chr11:121249100-121249144	NM_139147:443	Rab40b	INSIDE	0.834	0.420	1922.36	807.84	0.350	1408.90	493.65
A_68_P27804893	chr11:53276844-53276888	NM_027917:6160	Shroom1	INSIDE	0.834	0.624	989.14	617.55	0.521	861.81	448.79
A_68_P27181566	chr10:61111511-61111555	NM_026438:164	Ppa1	INSIDE	0.834	0.529	922.30	487.94	0.441	807.63	356.30
A_68_P27091647	chr10:41607434-41607478	NM_001013370:212	Sesn1	INSIDE	0.834	2.121	1293.34	2742.86	1.768	1170.18	2069.13
A_68_P26495381	chr9:48863943-48863987	NM_012039:279	Zw10	INSIDE	0.834	0.582	1319.79	768.17	0.485	1112.00	539.62
A_68_P25782093	chr8:36889502-36889546	NM_027998:89	Cldn23	INSIDE	0.834	0.486	2592.48	1260.29	0.405	1906.56	772.70
A_68_P25026078	chr7:30023443-30023487	NM_001110201:123	Yif1b	INSIDE	0.834	0.654	3231.69	2112.29	0.545	2378.82	1296.05
A_68_P25021121	chr7:29096863-29096907	NM_025616:181	Timm50	INSIDE	0.834	1.772	1357.86	2405.86	1.477	1184.50	1749.43
A_68_P24951824	chr7:5008363-5008407	NM_146178:57	Ccdc106	INSIDE	0.834	0.479	1509.56	723.67	0.400	1289.11	515.11
A_68_P24942902	chr6:148894983-148895027	NM_019643:-50	Fam60a	PROMOTER	0.834	0.423	1215.26	513.66	0.353	985.10	347.35
A_68_P24615402	chr6:87285175-87285219	NM_054041:573	Antxr1	INSIDE	0.834	0.657	2070.13	1359.06	0.547	1476.60	808.05
A_68_P24607364	chr6:85911255-85911299	NM_028099:385	Dusp11	INSIDE	0.834	0.295	2184.50	644.88	0.246	1464.33	360.65
A_68_P24123997	chr5:137463625-137463669	NM_027318:106	Znhit1	INSIDE	0.834	0.724	1667.56	1207.82	0.604	1329.66	803.00
A_68_P23239264	chr4:117933534-117933578	NM_011213:30446	Ptprf	INSIDE	0.834	0.464	2094.31	970.75	0.386	1553.31	600.26
A_68_P22417418	chr3:109143649-109143693	NM_020505:70	Vav3	INSIDE	0.834	2.730	461.52	1260.02	2.276	431.74	982.46
A_68_P22248625	chr3:75760605-75760649	NM_175193:127	Golim4	INSIDE	0.834	1.814	1698.35	3080.82	1.513	1298.67	1964.46
A_68_P20590467	chr1:128389351-128389395	NM_172484:132	Nckap5	INSIDE	0.834	0.527	1668.13	879.73	0.440	1196.21	525.96
A_68_P20172917	chr1:40738757-40738801	NM_001033289:222	Slc9a2	INSIDE	0.834	1.632	16006.11	26125.00	1.361	12795.21	17417.89
A_68_P31930244	chr19:5696279-5696323	NM_022012:7170	Map3k11	INSIDE	0.833	0.352	1262.62	444.82	0.293	960.34	281.81
A_68_P31929629	chr19:5602275-5602319	NM_026616:424	Rnaseh2c	INSIDE	0.833	5.286	389.23	2057.46	4.403	292.89	1289.57
A_68_P31351863	chr17:74928721-74928765	NM_007566:1108	Birc6	INSIDE	0.833	0.390	1585.64	617.69	0.324	1322.10	428.89
A_68_P29448044	chr14:21459988-21460032	NM_021508:15752	Myoz1	DOWNSTREAM	0.833	0.389	3162.57	1230.31	0.324	2330.04	754.64
A_68_P28776185	chr12:119082606-119082650	NM_146040:295	Cdca7l	INSIDE	0.833	2.834	808.16	2290.66	2.361	654.75	1545.85
A_68_P28581450	chr12:82633638-82633682	NM_025292:234	Synj2bp	INSIDE	0.833	0.671	1378.64	924.40	0.558	1039.37	580.37
A_68_P28525477	chr12:72411137-72411181	NM_001190466:288	Dact1	INSIDE	0.833	2.390	705.54	1686.24	1.992	581.81	1158.74
A_68_P27975823	chr11:83993524-83993568	ENSMUST00000137500:172		INSIDE	0.833	2.507	1682.38	4217.85	2.087	1458.50	3044.14
A_68_P27610174	chr11:16157719-16157763	NM_145967:14	Vstm2a	INSIDE	0.833	0.707	1281.12	905.89	0.589	1102.73	649.91
A_68_P26716013	chr9:90009639-90009683	NM_001039147:-2	Morf4l1	PROMOTER	0.833	0.297	2099.88	622.94	0.247	1610.46	398.20
A_68_P26466976	chr9:44127791-44127835	NM_027909:553	C2cd2l	INSIDE	0.833	0.479	1997.06	955.73	0.399	1479.75	589.80
A_68_P25537271	chr7:140044033-140044077	NM_028095:276	Mettl10	INSIDE	0.833	0.299	1553.21	465.16	0.250	1263.07	315.17
A_68_P25196741	chr7:75095612-75095656	NM_010513:-1508	Igf1r	PROMOTER	0.833	0.409	1503.95	615.12	0.341	1218.27	415.03
A_68_P24039811	chr5:121063203-121063247	NM_028041:86	Ddx54	INSIDE	0.833	0.608	1041.40	633.48	0.507	827.45	419.15
A_68_P23341301	chr4:137237373-137237417	NM_001081155:-223	Rap1gap	PROMOTER	0.833	1.929	869.19	1676.71	1.606	719.10	1154.88
A_68_P23325223	chr4:134409828-134409872	NM_025382:-590	Tmem57	PROMOTER	0.833	0.626	1001.88	627.08	0.522	865.12	451.30
A_68_P22606646	chr3:144781460-144781506	NM_001162538:-70	Odf2l	PROMOTER	0.833	0.401	2597.17	1041.59	0.334	2093.79	699.68
A_68_P22407401	chr3:107398395-107398439	NM_007441:468	Alx3	INSIDE	0.833	0.533	1467.46	782.06	0.444	1120.54	497.72
A_68_P22031195	chr3:30895073-30895117	NM_008857:402	Prkci	INSIDE	0.833	0.192	4502.95	863.65	0.160	3239.95	517.80
A_68_P21122750	chr2:33495287-33495331	NM_010725:723	Lmx1b	INSIDE	0.833	2.835	1877.62	5323.39	2.361	1577.01	3723.46
A_68_P31879126	chr18:83080440-83080484	NM_001177464:-3561	Zfp516	PROMOTER	0.832	0.497	1824.02	905.91	0.413	1293.83	534.58
A_68_P31682248	chr18:47528673-47528717	NM_018744:-172	Sema6a	PROMOTER	0.832	2.758	1458.63	4023.45	2.295	1130.48	2594.73
A_68_P30367722	chr15:79489390-79489434	NM_029407:-26	4933432B09Rik	DIVERGENT_PROMOTER	0.832	0.432	1413.17	610.85	0.360	1087.04	390.88
A_68_P30148787	chr15:38229176-38229220	NM_013692:1264	Klf10	INSIDE	0.832	3.806	492.68	1875.25	3.167	423.31	1340.74
A_68_P29614998	chr14:55531700-55531745	NM_021551:247	Slc22a17	INSIDE	0.832	0.589	1764.59	1039.75	0.490	1391.86	682.04
A_68_P29257123	chr13:99124571-99124615	NM_008242:393	Foxd1	INSIDE	0.832	2.248	563.55	1266.60	1.870	487.78	912.34
A_68_P27922701	chr11:74645689-74645733	NM_010813:1285	Mnt	INSIDE	0.832	0.665	1120.82	745.76	0.553	903.28	499.90
A_68_P27894950	chr11:69224743-69224787	NM_001017426:2413	Kdm6b	INSIDE	0.832	0.544	4209.09	2288.69	0.453	2972.93	1345.38
A_68_P27565811	chr11:5855744-5855788	NM_019661:6	Ykt6	INSIDE	0.832	2.598	617.97	1605.25	2.161	543.28	1173.79
A_68_P27553114	chr11:3548935-3548979	NR_002321:-145	Tug1	DIVERGENT_PROMOTER	0.832	0.697	2273.82	1584.29	0.580	1608.95	933.18
A_68_P26564379	chr9:61220085-61220129	NM_001083927:-66	Tle3	PROMOTER	0.832	0.539	1103.10	594.59	0.448	857.01	384.25
A_68_P26421199	chr9:35007341-35007385	NM_001177847:128	Tirap	INSIDE	0.832	0.586	4702.78	2755.54	0.487	3516.73	1713.73
A_68_P26229204	chr8:124353279-124353323	NM_145605:169	Klhdc4	INSIDE	0.832	0.513	1158.14	593.80	0.427	926.63	395.36
A_68_P26001982	chr8:83403879-83403923	NM_021356:478	Gab1	INSIDE	0.832	5.406	3439.89	18597.58	4.498	2625.72	11810.01
A_68_P25961304	chr8:74945231-74945275	NM_007944:121	Eps15l1	INSIDE	0.832	4.260	1043.40	4444.94	3.546	743.92	2638.29

ProbeName	Target position of probe on CpG island microarray	TargetID	GeneSymbol	CpG island Description	Ratio of relative methylation (TiO_2-NP/Vehicle)	Sham group			TiO_2-H group		
						Relative methylation (Cy5/Cy3)	Cy3 signal (Input)	Cy5 signal (MeDIP)	Relative methylation (Cy5/Cy3)	Cy3 signal (Input)	Cy5 signal (MeDIP)
A_68_P25958451	chr8:74233667-74233711	NM_028715:15892	Fcho1	INSIDE	0.832	3.572	2004.47	7160.04	2.972	1531.98	4553.52
A_68_P25958255	chr8:74203600-74203644	NM_001190830:3341	Jak3	INSIDE	0.832	4.161	1273.98	5300.85	3.462	992.53	3435.93
A_68_P25504741	chr7:134388254-134388298	NM_177362:237	Zfp771	INSIDE	0.832	0.390	1210.19	471.73	0.324	961.37	311.94
A_68_P25089587	chr7:52095618-52095662	NM_010215:3983	Il4i1	INSIDE	0.832	0.611	1168.24	713.40	0.508	953.26	484.61
A_68_P21836350	chr2:167423298-167423342	NM_023230:34185	Ube2v1	DOWNSTREAM	0.832	1.934	5658.53	10945.21	1.609	4142.72	6665.56
A_68_P21115068	chr2:32237650-32237694	NM_198001:-237	1110008P14Rik	PROMOTER	0.832	0.415	2648.02	1097.64	0.345	1815.28	626.34
A_68_P20897125	chr1:186551788-186551832	NM_008250:4562	Hlx	INSIDE	0.832	2.933	1230.51	3608.70	2.440	980.91	2393.91
A_68_P20614487	chr1:133142076-133142120	NM_018750:-343	Rassf5	PROMOTER	0.832	0.662	1532.69	1013.88	0.551	1242.48	684.01
A_68_P32753664	chrX:149932965-149933009	NM_018798:212	Ubqln2	INSIDE	0.831	3.515	542.88	1908.50	2.921	615.06	1796.42
A_68_P31934032	chr19:6335339-6335383	NM_001168490:322	Men1	INSIDE	0.831	0.663	1320.83	875.44	0.551	971.08	534.80
A_68_P31899537	chr18:86880867-86880911	NM_172633:-1551	Cbln2	PROMOTER	0.831	0.622	1805.08	1123.08	0.517	1410.08	728.91
A_68_P28731507	chr12:110272513-110272557	NM_001163175:33893	Begain	INSIDE	0.831	1.911	1821.55	3481.07	1.587	1375.12	2182.57
A_68_P28046742	chr11:96638698-96638742	NM_001163389:149	Snx11	INSIDE	0.831	0.274	8207.88	2250.59	0.228	5436.25	1238.00
A_68_P27848100	chr11:61191430-61191474	NM_026183:125	Slc47a1	INSIDE	0.831	2.404	858.81	2064.45	1.998	741.82	1482.10
A_68_P27765616	chr11:45869574-45869618	NM_009616:108	Adam19	INSIDE	0.831	0.583	2617.03	1525.16	0.484	2028.40	982.16
A_68_P27482567	chr10:117147762-117147806	NM_010786:-12	Mdm2	PROMOTER	0.831	2.971	1365.98	4057.99	2.468	1098.37	2711.08
A_68_P26696147	chr9:85737262-85737306	NM_001164792:826	Tpbg	INSIDE	0.831	0.477	1527.19	728.54	0.396	1070.17	424.27
A_68_P26650022	chr9:76415008-76415052	AK049628:-108		PROMOTER	0.831	0.395	1846.62	728.80	0.328	1347.18	441.62
A_68_P26595474	chr9:66683005-66683049	NM_026674:-513	Aph1c	PROMOTER	0.831	0.276	2364.64	652.80	0.229	1811.99	415.49
A_68_P26142250	chr8:109460044-109460088	NM_009229:417	Sntb2	INSIDE	0.831	0.370	1644.28	608.58	0.308	1258.56	387.15
A_68_P25448401	chr7:123648365-123648409	NM_016773:499	Nucb2	INSIDE	0.831	0.266	2079.28	553.26	0.221	1442.03	318.67
A_68_P25024597	chr7:29747515-29747559	NM_021895:-237	Actn4	PROMOTER	0.831	1.916	921.17	1764.90	1.593	787.91	1254.84
A_68_P24804130	chr6:121363049-121363093	NM_001033354:60626	Iqsec3	INSIDE	0.831	0.364	1422.63	517.23	0.302	1084.50	327.51
A_68_P24092032	chr5:130555232-130555276	NM_001130476:-101	Tpst1	PROMOTER	0.831	2.115	2408.26	5093.34	1.757	1936.67	3403.03
A_68_P22803179	chr4:28740971-28741015	NM_001122889:698	Epha7	INSIDE	0.831	2.378	772.47	1837.27	1.977	519.98	1027.88
A_68_P22338874	chr3:94217114-94217158	NM_028307:-103	Tdrkh	PROMOTER	0.831	0.328	1771.20	581.00	0.273	1320.08	359.99
A_68_P22202887	chr3:65762326-65762370	NM_019937:-201	Ccnl1	PROMOTER	0.831	0.359	1275.28	457.47	0.298	986.88	294.31
A_68_P21834173	chr2:167013977-167014021	NM_008420:301	Kcnb1	INSIDE	0.831	3.106	1844.56	5729.72	2.581	1461.41	3772.34
A_68_P21820156	chr2:164682452-164682496	NM_011125:734	Pltp	INSIDE	0.831	0.726	1645.99	1195.57	0.603	1379.61	832.46
A_68_P21777849	chr2:157385874-157385918	NM_010923:51	Nnat	INSIDE	0.831	2.024	3792.55	7675.22	1.681	2850.85	4793.32
A_68_P21754300	chr2:153171618-153171662	NM_001039939:-234	Asxl1	PROMOTER	0.831	0.584	3258.93	1904.36	0.486	2414.19	1172.51
A_68_P21556051	chr2:116946577-116946621	NM_033524:-587	Spred1	PROMOTER	0.831	0.184	3456.47	636.23	0.153	2473.59	378.17
A_68_P21152642	chr2:39046414-39046458	NM_178778:-186	Scai	PROMOTER	0.831	3.618	2197.49	7951.28	3.007	1646.07	4949.01
A_68_P20252945	chr1:57463017-57463061	NM_001081181:-353	9430016H08Rik	PROMOTER	0.831	0.236	2052.45	484.49	0.196	1597.74	313.36
A_68_P20040937	chr1:13364618-13364662	NM_001077695:-476	Ncoa2	PROMOTER	0.831	0.590	1090.44	643.75	0.491	942.19	462.23
A_68_P31849429	chr18:77952019-77952063	NM_178670:709	8030462N17Rik	INSIDE	0.830	2.462	2169.01	5339.88	2.043	1746.75	3568.66
A_68_P31757539	chr18:61559767-61559811	NM_133249:297	Ppargc1b	INSIDE	0.830	0.478	1019.58	487.87	0.397	870.25	345.60
A_68_P31209393	chr17:47027920-47027964	NM_178385:373	Tbcc	INSIDE	0.830	3.616	830.13	3002.08	3.003	675.36	2027.88
A_68_P30925872	chr16:84835744-84835788	NM_016755:53	Atp5j	INSIDE	0.830	2.338	703.08	1643.45	1.940	690.11	1338.57
A_68_P30777935	A_68_P30777935			Unknown	0.830	0.501	1431.45	717.84	0.416	1241.26	516.81
A_68_P30557333	chr16:13448464-13448508	NR_030549:-1129	Mir193b	PROMOTER	0.830	0.353	2692.38	951.70	0.293	1874.49	550.13
A_68_P30478000	chr15:98887296-98887340	NM_001163588:1714	Prph	INSIDE	0.830	0.410	1882.12	771.93	0.341	1307.95	445.50
A_68_P30476395	chr15:98606917-98606961	NM_011718:1643	Wnt10b	INSIDE	0.830	13.340	2928.94	39071.45	11.078	2320.17	25703.34
A_68_P30083648	chr15:25772800-25772844	NM_001034851:-196	Fam134b	PROMOTER	0.830	0.631	2375.26	1497.93	0.523	1983.91	1038.17
A_68_P27921609	chr11:74451666-74451710	NM_001013784:18582	E130309D14Rik	INSIDE	0.830	1.889	1035.05	1955.52	1.568	815.55	1279.14
A_68_P27628923	chr11:19825251-19825295	NM_033523:828	Spred2	INSIDE	0.830	0.341	2943.37	1004.49	0.283	2104.23	595.99
A_68_P26660235	chr9:78435636-78435680	NM_172773:176	Slc17a5	INSIDE	0.830	0.503	3259.24	1639.37	0.417	2571.57	1073.01
A_68_P25729004	chr8:26895328-26895372	NM_026121:331	Bag4	INSIDE	0.830	0.347	2227.33	773.03	0.288	1670.03	481.00
A_68_P24002949	chr5:114724178-114724222	NM_148935:-430	Foxn4	PROMOTER	0.830	0.211	2955.58	624.65	0.175	2105.38	369.41
A_68_P24001335	chr5:114443497-114443541	NM_198109:248	Ssh1	INSIDE	0.830	0.682	1297.78	885.68	0.567	1090.03	617.77
A_68_P22550730	chr3:134875375-134875419	NM_173762:-130	Cenpe	PROMOTER	0.830	0.204	2268.24	462.51	0.169	1780.55	301.41
A_68_P22336401	chr3:93248647-93248691	NM_001163098:2417	Tchh	INSIDE	0.830	0.543	1147.81	623.06	0.451	938.34	423.00
A_68_P22300151	chr3:85775250-85775294	ENSMUST00000158452:-8518		PROMOTER	0.830	2.536	760.36	1927.99	2.106	731.91	1541.19
A_68_P20893085	chr1:185857220-185857264	NM_028516:635	1700056E22Rik	INSIDE	0.830	0.524	1634.57	856.84	0.435	1222.67	532.15
A_68_P20874332	chr1:182125999-182126043	NM_001163290:-2404	Adck3	PROMOTER	0.830	0.572	1932.74	1105.85	0.475	1597.66	759.18
A_68_P20832395	chr1:174256553-174256597	NM_008429:2820	Kcnj9	INSIDE	0.830	0.376	2424.27	911.45	0.312	1991.46	621.49
A_68_P20409779	chr1:87915105-87915149	NM_027029:-3390	Spata3	PROMOTER	0.830	3.316	543.76	1803.22	2.752	454.10	1249.68
A_68_P20058158	chr1:17087056-17087100	NM_020604:892	Jph1	INSIDE	0.830	0.548	1247.95	683.69	0.455	1036.00	471.19
A_68_P31303245	chr17:66233536-66233580	NM_001198949:705	Ralbp1	INSIDE	0.829	0.639	1671.50	1068.76	0.530	1426.93	756.60
A_68_P30822066	chr16:64852004-64852048	NM_178647:116	Cggbp1	INSIDE	0.829	2.232	753.40	1681.50	1.850	598.17	1106.80
A_68_P30651587	chr16:32246570-32246614	NM_173439:519	Fbxo45	INSIDE	0.829	0.549	889.42	488.12	0.455	826.41	376.03
A_68_P30532561	chr16:8424872-8424917	NM_029070:335	Cldn26	INSIDE	0.829	0.521	2644.66	1379.01	0.433	1981.52	857.02
A_68_P30347940	chr15:76210921-76210965	NM_001162489:-9518	Heatr7a	PROMOTER	0.829	0.568	2257.50	1281.64	0.471	1785.21	840.63
A_68_P30125733	chr15:33617621-33617665	NM_001085421:-4	Tspyl5	PROMOTER	0.829	2.290	2100.15	4809.04	1.899	1719.36	3264.74
A_68_P29672921	chr14:65962760-65962804	AK028094:17615		DOWNSTREAM	0.829	0.673	3677.79	2474.98	0.558	2722.39	1518.43

ProbeName	Target position of probe on CpG island microarray	TargetID	GeneSymbol	CpG island Description	Ratio of relative methylation (TiO_2-NP/Vehicle)	Sham group			TiO_2-H group		
						Relative methylation (Cy5/Cy3)	Cy3 signal (Input)	Cy5 signal (MeDIP)	Relative methylation (Cy5/Cy3)	Cy3 signal (Input)	Cy5 signal (MeDIP)
A_68_P29265992	chr13:100670416-100670460	NM_001081493:200	Cartpt	INSIDE	0.829	0.200	3561.92	713.60	0.166	2303.29	382.67
A_68_P29142608	chr13:73984841-73984885	NM_028186:217	Nkd2	INSIDE	0.829	0.528	1457.64	770.26	0.438	1048.11	458.88
A_68_P28729569	chr12:109940441-109940485	NM_001171002:54	Degs2	INSIDE	0.829	0.479	1144.99	548.84	0.397	973.72	386.92
A_68_P27291028	chr10:81106453-81106497	NM_027480:15190	Ankrd24	INSIDE	0.829	2.321	572.83	1329.53	1.925	510.39	982.36
A_68_P27162285	chr10:57514583-57514627	NM_020561:255	Smpdl3a	INSIDE	0.829	0.500	1272.55	636.66	0.415	1092.83	453.17
A_68_P26609879	chr9:69137663-69137707	NM_013646:636076	Rora	INSIDE	0.829	0.396	1264.23	500.38	0.328	998.14	327.33
A_68_P26058232	chr8:93837095-93837139	NM_173431:5	Rpgrip1l	INSIDE	0.829	0.689	1268.16	873.44	0.571	1092.39	623.46
A_68_P25761252	chr8:33119752-33119796	ENSMUST00000080782:-193		PROMOTER	0.829	0.358	2541.40	908.64	0.296	2120.48	628.52
A_68_P25182470	chr7:72516116-72516161	NM_001163574:-8	Tjp1	PROMOTER	0.829	2.806	808.64	2269.06	2.327	653.15	1519.70
A_68_P25042529	chr7:34953233-34953277	NM_016682:294	Uba2	INSIDE	0.829	0.737	2662.98	1961.54	0.611	2122.80	1296.24
A_68_P24544189	chr6:72848961-72849005	NM_019715:991	Kcmf1	INSIDE	0.829	2.413	903.44	2179.73	2.000	793.45	1586.68
A_68_P24132969	chr5:139470681-139470725	NM_008808:205	Pdgfa	INSIDE	0.829	3.513	2063.79	7249.07	2.913	1788.89	5211.37
A_68_P23681255	chr5:50449451-50449501	NM_133911:759	Gpr125	INSIDE	0.829	0.292	1575.09	460.23	0.242	1120.27	271.20
A_68_P23238477	chr4:117807306-117807350	NM_009176:167	St3gal3	INSIDE	0.829	2.660	751.08	1998.04	2.206	605.39	1335.56
A_68_P22964623	chr4:62131950-62131994	NM_023597:-66	Wdr31	DIVERGENT_PROMOTER	0.829	2.850	681.19	1941.54	2.364	614.96	1453.87
A_68_P21629881	chr2:130276040-130276084	NM_001163688:49	Ptpra	INSIDE	0.829	0.214	5882.49	1259.84	0.178	4271.45	758.25
A_68_P21339412	chr2:74601208-74601252	NM_010467:194	Hoxd1	INSIDE	0.829	0.688	3537.62	2434.65	0.570	2713.50	1547.49
A_68_P31922246	chr19:4283254-4283298	NM_026720:-139	Ankrd13d	PROMOTER	0.828	3.084	3364.65	10375.79	2.553	2327.30	5940.79
A_68_P31157813	chr17:35272266-35272310	NM_057171:102	Bag6	INSIDE	0.828	0.457	998.65	456.46	0.378	845.40	319.90
A_68_P30577647	chr16:18128056-18128100	NM_022982:280	Rtn4r	INSIDE	0.828	0.715	3147.27	2249.90	0.592	2503.32	1481.68
A_68_P29102524	chr13:65361174-65361218	ENSMUST00000099425:350		INSIDE	0.828	6.048	857.53	5185.97	5.010	727.39	3644.35
A_68_P28959949	chr13:38437991-38438035	NM_007556:428	Bmp6	INSIDE	0.828	0.456	1377.11	627.56	0.377	868.72	327.73
A_68_P28091216	chr11:104330128-104330172	ENSMUST00000134266:-845		PROMOTER	0.828	0.210	4164.66	874.11	0.174	3328.97	578.28
A_68_P27340461	chr10:90644609-90644653	ENSMUST00000099357:-9871		PROMOTER	0.828	0.692	1309.79	906.32	0.573	1049.33	601.04
A_68_P26882494	chr9:121186351-121186395	NM_177589:-82	Ulk4	PROMOTER	0.828	0.168	4447.96	747.47	0.139	2744.31	381.86
A_68_P26166795	chr8:113669775-113669819	ENSMUST00000143719:-416		PROMOTER	0.828	2.653	779.05	2066.71	2.196	602.68	1323.45
A_68_P25447030	chr7:123380803-123380847	ENSMUST00000169410:466		INSIDE	0.828	0.326	3664.89	1194.13	0.270	2546.31	687.17
A_68_P23916493	chr5:98596245-98596289			Unknown	0.828	0.518	1504.57	779.43	0.429	1117.43	479.45
A_68_P23895411	chr5:93104067-93104111	NM_177230:65	Ccdc158	INSIDE	0.828	0.458	1711.06	783.02	0.379	1366.53	517.77
A_68_P23757987	chr5:66155002-66155046	NM_001024917:265	N4bp2	INSIDE	0.828	3.149	624.25	1965.50	2.608	522.49	1362.60
A_68_P23602808	chr5:36100579-36100623	NM_001177696:72	Ablim2	INSIDE	0.828	2.113	838.85	1772.84	1.749	712.87	1247.02
A_68_P23441979	chr4:155409215-155409259	NM_029264:15690	Ttll10	INSIDE	0.828	0.620	1280.96	793.91	0.513	1078.68	553.29
A_68_P23185482	chr4:106295106-106295150	NM_177667:75	Ttc22	INSIDE	0.828	3.101	1393.42	4321.66	2.569	1298.71	3335.91
A_68_P23103963	chr4:91067208-91067252	NM_207685:-555	Elavl2	PROMOTER	0.828	0.159	3175.18	504.81	0.132	2211.07	291.13
A_68_P22346431	chr3:95734111-95734155	NM_023210:953	Anp32e	INSIDE	0.828	2.092	766.08	1602.65	1.733	593.30	1028.12
A_68_P22343877	chr3:95238468-95238512	NM_001037737:179	Arnt	INSIDE	0.828	0.729	1956.65	1426.85	0.604	1365.08	823.96
A_68_P22319130	chr3:89199983-89200027	NM_009565:-2879	Zbtb7b	PROMOTER	0.828	0.359	1960.21	704.53	0.298	1372.54	408.37
A_68_P22059073	chr3:36512234-36512278	NM_027810:55	Bbs7	INSIDE	0.828	0.548	965.38	528.67	0.453	812.62	368.50
A_68_P21320133	chr2:71383777-71383821	NM_010054:1013	Dlx2	INSIDE	0.828	3.701	2197.73	8134.54	3.064	1660.31	5086.37
A_68_P20969130	chr2:4524621-4524665	NM_001177844:43843	Frmd4a	INSIDE	0.828	2.890	712.15	2058.36	2.393	616.01	1474.35
A_68_P20412114	chr1:88330989-88331033	NM_027966:8107	1700019O17Rik	DOWNSTREAM	0.828	0.351	3303.78	1160.47	0.291	2606.61	758.12
A_68_P32094346	chr19:37282376-37282420	NM_001164337:364	Marchf5	INSIDE	0.827	2.970	1226.78	3643.89	2.456	1089.24	2674.93
A_68_P30511523	chr16:4679735-4679779	NM_030205:-36	Coro7	DIVERGENT_PROMOTER	0.827	0.567	1809.70	1026.71	0.469	1468.87	689.36
A_68_P28597425	chr12:85626297-85626341	NM_029880:55	Ptgr2	INSIDE	0.827	0.661	2119.88	1400.75	0.546	1622.14	886.25
A_68_P28189605	chr11:121121003-121121047	NM_001080932:-276	Foxk2	PROMOTER	0.827	0.410	1067.02	437.82	0.339	857.38	290.77
A_68_P27936391	chr11:77030153-77030197	NM_177710:248	Ssh2	INSIDE	0.827	0.417	3371.84	1404.52	0.345	2396.36	825.97
A_68_P27682712	chr11:31269004-31269048	NM_011491:1035	Stc2	INSIDE	0.827	0.430	1076.93	462.78	0.355	915.92	325.49
A_68_P27271774	chr10:77506231-77506275	NM_009646:73	Aire	INSIDE	0.827	0.656	2744.24	1801.22	0.543	2113.36	1147.32
A_68_P26890130	chr9:122481560-122481604	NR_015610:-36	9530059O14Rik	PROMOTER	0.827	2.990	967.84	2893.61	2.472	774.91	1915.79
A_68_P25957933	chr8:74153614-74153658	NM_001029873:42020	Unc13a	INSIDE	0.827	0.694	1364.77	947.56	0.574	1178.61	676.90
A_68_P25365848	chr7:107270096-107270140	NM_133692:-108	Pold3	PROMOTER	0.827	0.606	1591.22	964.93	0.501	1209.60	606.28
A_68_P25354128	chr7:105293642-105293686	NM_011010:293	Omp	INSIDE	0.827	2.772	1629.65	4518.15	2.293	1265.49	2902.11
A_68_P23349653	chr4:138648926-138648970	NM_008675:-63	Nbl1	PROMOTER	0.827	0.460	1155.25	531.18	0.380	930.24	353.91
A_68_P22729537	chr4:11312715-11312759	NM_194055:1194	Esrp1	INSIDE	0.827	0.632	3619.36	2286.85	0.523	2927.31	1529.76
A_68_P22186533	chr3:62142735-62142779	NM_001081295:58	Arhgef26	INSIDE	0.827	0.549	1046.16	573.86	0.454	843.22	382.50
A_68_P21617362	chr2:127951410-127951454	NM_009754:-341	Bcl2l11	PROMOTER	0.827	0.525	2570.03	1348.15	0.434	2030.65	881.01
A_68_P21105563	chr2:30700866-30700910	NM_009116:2	Prrx2	INSIDE	0.827	0.555	962.25	533.87	0.459	802.25	368.10
A_68_P21070398	chr2:25041281-25041325	NM_025980:5025	Nrarp	DOWNSTREAM	0.827	0.539	1226.42	660.63	0.445	983.86	438.10
A_68_P20412466	chr1:88401070-88401114			Unknown	0.827	0.371	2918.17	1081.21	0.307	2290.99	702.40
A_68_P31934660	chr19:6428244-6428288	NM_001205234:9529	Nrxn2	INSIDE	0.826	4.513	723.71	3266.03	3.727	597.70	2227.84
A_68_P31619423	chr18:35812850-35812894	NM_027222:-3851	2010001M09Rik	PROMOTER	0.826	0.561	939.20	526.83	0.463	803.25	372.27
A_68_P30475794	chr15:98493006-98493053	NM_001080981:291	Ddx23	INSIDE	0.826	0.500	1767.76	884.01	0.413	1387.77	573.45
A_68_P29571975	chr14:46276736-46276780	NM_001039106:1060	Ddhd1	INSIDE	0.826	0.388	3059.95	1186.52	0.320	2198.41	703.91
A_68_P29052330	chr13:55050305-55050349	NM_153131:-466	Unc5a	PROMOTER	0.826	2.887	554.91	1601.93	2.386	516.53	1232.22
A_68_P28290828	chr12:25336571-25336615	NM_178357:357	Klf11	INSIDE	0.826	0.387	2851.70	1102.39	0.319	2075.21	662.59

ProbeName	Target position of probe on CpG island microarray	TargetID	GeneSymbol	CpG island Description	Ratio of relative methylation (TiO_2-NP/Vehicle)	Sham group			TiO_2-H group		
						Relative methylation (Cy5/Cy3)	Cy3 signal (Input)	Cy5 signal (MeDIP)	Relative methylation (Cy5/Cy3)	Cy3 signal (Input)	Cy5 signal (MeDIP)
A_68_P28158778	chr11:116196536-116196580	NM_008240:110	Foxj1	INSIDE	0.826	0.541	1944.91	1051.30	0.446	1524.43	680.54
A_68_P28091894	chr11:104469397-104469441	NM_016780:105	Itgb3	INSIDE	0.826	0.421	3646.33	1534.36	0.348	2920.23	1015.10
A_68_P27927812	chr11:75546197-75546241	NM_009536:-170	Ywhae	PROMOTER	0.826	2.216	1729.66	3833.47	1.830	1529.87	2799.59
A_68_P27798409	chr11:52096424-52096468	NM_009331:-694	Tcf7	PROMOTER	0.826	1.898	1245.14	2362.81	1.568	1102.82	1729.17
A_68_P27171639	chr10:59414574-59414618	NM_029083:-78	Ddit4	PROMOTER	0.826	0.374	2331.78	872.42	0.309	1671.66	516.85
A_68_P26616432	chr9:70269348-70269392	NM_007630:-9	Ccnb2	PROMOTER	0.826	2.182	867.66	1892.84	1.801	674.12	1214.15
A_68_P26580032	chr9:63932502-63932546	AK011166:3957		INSIDE	0.826	15.268	535.98	8183.63	12.613	518.91	6544.96
A_68_P26168091	chr8:113917437-113917481	NM_178086:263	Fa2h	INSIDE	0.826	0.655	1252.29	820.53	0.541	1083.93	586.64
A_68_P25728007	chr8:26712095-26712139	NM_001001735:-661	Whsc1l1	DIVERGENT_PROMOTER	0.826	2.741	906.05	2483.23	2.263	769.54	1741.78
A_68_P25173482	chr7:70902998-70903042	NM_130880:313363	Otud7a	INSIDE	0.826	2.934	430.21	1262.29	2.425	396.22	960.82
A_68_P25021520	chr7:29164259-29164303	NM_011756:-33	Zfp36	PROMOTER	0.826	0.425	1853.97	788.46	0.351	1517.29	532.96
A_68_P24127317	chr5:138182972-138183016	NM_175521:-1768	6430598A04Rik	DIVERGENT_PROMOTER	0.826	0.693	1342.90	931.11	0.572	1014.16	580.55
A_68_P24061232	chr5:124874999-124875043	NM_001081203:903	Sbno1	INSIDE	0.826	0.729	2807.54	2047.35	0.603	2312.87	1393.95
A_68_P23956788	chr5:106129546-106129590	NM_178701:-260	Lrrc8d	PROMOTER	0.826	0.219	2156.50	473.06	0.181	1630.58	295.56
A_68_P21840492	chr2:168106455-168106499	ENSMUST00000109191:156		INSIDE	0.826	3.974	1383.35	5496.81	3.281	1110.43	3643.52
A_68_P20262088	chr1:59177273-59177317	NM_001033449:-354	Als2cr4	PROMOTER	0.826	5.223	821.77	4292.17	4.315	648.86	2800.13
A_68_P20083589	chr1:22540314-22540358	NM_001012623:272227	Rims1	INSIDE	0.826	2.461	1520.06	3741.60	2.033	1487.59	3023.98
A_68_P31615541	chr18:35091350-35091394	NM_144866:285	Etf1	INSIDE	0.825	0.539	4664.19	2513.31	0.445	3319.79	1475.98
A_68_P30540341	chr16:9992483-9992527	NM_008170:122	Grin2a	INSIDE	0.825	1.838	1424.82	2619.03	1.517	1156.90	1755.29
A_68_P29616393	chr14:55732216-55732260	NM_177049:3534	Jph4	INSIDE	0.825	3.495	830.08	2900.70	2.884	668.07	1926.60
A_68_P29517792	chr14:33276018-33276063	NM_021712:1996	Slc18a3	INSIDE	0.825	0.331	1425.67	472.30	0.273	1179.36	322.43
A_68_P29484665	chr14:27852530-27852574	NM_134437:366	Il17rd	INSIDE	0.825	0.394	1359.38	534.98	0.325	1186.53	385.25
A_68_P28290822	chr12:25335897-25335941	NM_178357:-317	Klf11	PROMOTER	0.825	0.506	1721.58	870.66	0.417	1322.52	551.57
A_68_P28289839	chr12:25183746-25183790	NM_020614:323	Taf1b	INSIDE	0.825	0.642	2730.81	1753.28	0.530	1931.31	1023.19
A_68_P28266975	chr12:16818008-16818052	NM_033270:260	E2f6	INSIDE	0.825	2.162	1922.99	4156.75	1.784	1719.14	3067.11
A_68_P27265505	chr10:76495700-76495744	NM_031196:-234	Slc19a1	PROMOTER	0.825	0.541	886.92	480.01	0.446	753.57	336.38
A_68_P26141981	chr8:109417694-109417738	NM_011574:177	Cirh1a	INSIDE	0.825	0.262	1860.09	487.73	0.216	1329.97	287.81
A_68_P25089821	chr7:52124278-52124322	NM_133949:858	Ptov1	INSIDE	0.825	0.234	2104.73	493.38	0.193	1483.99	287.08
A_68_P25009106	chr7:26010791-26010835	NM_175436:4343	Zfp526	INSIDE	0.825	2.181	1630.40	3556.25	1.800	1269.76	2285.35
A_68_P24951575	chr7:4967227-4967271	NM_025324:139	Zfp524	INSIDE	0.825	1.977	3073.15	6074.95	1.630	2251.42	3669.79
A_68_P24947884	chr7:4089157-4089201	NM_172736:521	Leng8	INSIDE	0.825	0.551	1558.92	858.64	0.455	1233.34	560.70
A_68_P23971891	chr5:108863299-108863343	NM_007507:77	Atp5k	INSIDE	0.825	0.308	3310.75	1018.35	0.254	2504.13	635.79
A_68_P23448645	chr5:4104155-4104199	NM_020010:521	Cyp51	INSIDE	0.825	0.435	1477.54	642.80	0.359	1151.53	413.25
A_68_P23351037	chr4:138866350-138866394	NM_145384:243	Pqlc2	INSIDE	0.825	0.241	2168.26	523.32	0.199	1686.23	335.75
A_68_P23186777	chr4:106512073-106512117	ENSMUST00000151369:8431		DOWNSTREAM	0.825	0.636	3250.66	2067.52	0.525	2221.08	1165.79
A_68_P22218637	chr3:68810132-68810176	NR_029529:-3539	Mir15b	PROMOTER	0.825	0.272	2056.51	558.49	0.224	1544.36	345.83
A_68_P21566797	chr2:118728139-118728183	NM_001045523:810	Bahd1	INSIDE	0.825	0.255	3919.36	998.77	0.210	3055.99	642.10
A_68_P21565893	chr2:118572335-118572379	NR_030716:859	5430417L22Rik	INSIDE	0.825	0.654	1870.97	1223.91	0.540	1391.16	750.66
A_68_P21338805	chr2:74525959-74526003	NM_013554:-4024	Hoxd10	PROMOTER	0.825	0.664	1907.58	1266.90	0.548	1553.00	851.16
A_68_P32634534	chrX:120217171-120217215	NM_028426:25	3110007F17Rik	INSIDE	0.824	0.462	2380.39	1099.53	0.381	3361.40	1279.29
A_68_P32143271	chr19:46402975-46403019	NM_133694:323	Fbxl15	INSIDE	0.824	0.475	1954.91	929.04	0.391	1454.56	569.26
A_68_P32030722	chr19:25685283-25685327	NM_177360:278	Dmrt3	INSIDE	0.824	2.183	625.12	1364.72	1.799	585.65	1053.87
A_68_P31154934	chr17:34767881-34767925	NM_030890:1272	Prrt1	INSIDE	0.824	0.192	5171.71	992.86	0.158	4030.72	637.80
A_68_P31118681	chr17:27956946-27956990	NM_001033279:519	D17Wsu92e	INSIDE	0.824	0.386	2873.25	1110.44	0.318	2127.00	677.37
A_68_P30593952	chr16:21695060-21695104	NM_001001881:-344	2510009E07Rik	PROMOTER	0.824	1.823	1246.45	2271.84	1.502	1124.87	1689.20
A_68_P29717291	chr14:73785051-73785095	NM_008410:6	Itm2b	INSIDE	0.824	0.648	1514.32	981.87	0.534	1294.77	691.85
A_68_P28889020	chr13:24893656-24893700	NM_024473:153	BC005537	INSIDE	0.824	2.172	1049.35	2279.46	1.790	896.79	1605.12
A_68_P28198043	chr12:3981656-3981700	NM_001082483:57237	Efr3b	INSIDE	0.824	0.498	1223.07	609.54	0.410	1015.82	416.99
A_68_P27843267	chr11:60290696-60290740	NM_001103171:7878	Myo15	INSIDE	0.824	1.957	1682.16	3292.15	1.612	1259.08	2030.01
A_68_P26940975	chr10:11211781-11211825			Unknown	0.824	3.210	2011.64	6456.92	2.644	1613.08	4265.20
A_68_P26742969	chr9:95537953-95537997	NM_029620:-72	Pcolce2	PROMOTER	0.824	2.143	636.54	1363.82	1.766	559.96	988.79
A_68_P26581054	chr9:64129658-64129702	NM_025372:267	Tipin	INSIDE	0.824	3.182	4401.95	14005.06	2.622	3314.52	8691.73
A_68_P26548143	chr9:58336411-58336455	NM_176921:23	6030419C18Rik	INSIDE	0.824	2.116	1500.71	3175.57	1.743	1347.51	2348.91
A_68_P26500017	chr9:49606485-49606529	NM_001081445:668	Ncam1	INSIDE	0.824	3.123	2286.61	7141.39	2.572	1770.32	4554.08
A_68_P26476281	chr9:45647027-45647071	NM_001145947:437	Bace1	INSIDE	0.824	3.663	1634.91	5987.85	3.017	1301.61	3927.44
A_68_P26228655	chr8:124277307-124277351	NM_020605:22870	Jph3	INSIDE	0.824	6.877	821.56	5649.59	5.669	678.60	3846.77
A_68_P25952998	chr8:73135664-73135708	NM_133772:-3473	Ssbp4	DIVERGENT_PROMOTER	0.824	0.520	3707.24	1927.38	0.429	2637.33	1130.44
A_68_P24948000	chr7:4103080-4103124	NM_021454:13199	Cdc42ep5	INSIDE	0.824	0.536	2619.57	1404.24	0.442	2082.89	919.79
A_68_P24535485	chr6:71222087-71222131	NM_145568:96	Krcc1	INSIDE	0.824	13.197	1644.08	21697.39	10.879	1456.20	15841.55
A_68_P24397197	chr6:42214753-42214798	NM_007610:-262	Casp2	PROMOTER	0.824	3.072	322.01	989.29	2.532	262.74	665.14
A_68_P24132965	chr5:139470169-139470213	NM_008808:717	Pdgfa	INSIDE	0.824	0.618	2015.01	1245.87	0.509	1640.25	835.67
A_68_P23981795	chr5:111199426-111199470	NM_029337:288	Ep400	INSIDE	0.824	3.636	9664.20	35137.27	2.997	6546.03	19618.77
A_68_P23794551	chr5:73305609-73305653	NM_001113423:75	Slain2	INSIDE	0.824	0.704	1810.10	1274.56	0.580	1435.90	833.50
A_68_P23363150	chr4:140847720-140847764	NM_010139:-9412	Epha2	PROMOTER	0.824	0.280	1864.53	522.85	0.231	1359.52	313.96
A_68_P23310684	chr4:131767230-131767274	NM_145393:919	Ythdf2	INSIDE	0.824	0.381	1197.17	455.65	0.314	1023.23	320.98

ProbeName	Target position of probe on CpG island microarray	TargetID	GeneSymbol	CpG island Description	Ratio of relative methylation (TiO_2-NP/Vehicle)	Sham group			TiO_2-H group		
						Relative methylation (Cy5/Cy3)	Cy3 signal (Input)	Cy5 signal (MeDIP)	Relative methylation (Cy5/Cy3)	Cy3 signal (Input)	Cy5 signal (MeDIP)
A_68_P22791944	chr4:25726907-25726951	NM_010243:222	Fut9	INSIDE	0.824	0.446	4231.25	1887.07	0.368	3285.60	1208.04
A_68_P21780566	chr2:157854552-157854596	NM_027434:45	Rprd1b	INSIDE	0.824	0.514	2453.29	1261.27	0.423	1950.25	825.82
A_68_P20748458	chr1:159342635-159342679	NM_177644:70	Rasal2	INSIDE	0.824	2.447	823.09	2014.14	2.017	670.80	1353.13
A_68_P20611352	chr1:132613961-132614005	NM_178691:79	Yod1	INSIDE	0.824	0.713	2595.73	1850.90	0.587	2069.25	1215.41
A_68_P31102144	chr17:25321751-25321795	NM_183150:267	BC003965	INSIDE	0.823	0.625	1632.62	1020.52	0.514	1198.73	616.45
A_68_P30379099	chr15:81524550-81524594	NM_001164320:3145	Chadl	INSIDE	0.823	0.591	1316.37	778.39	0.487	1134.84	552.35
A_68_P29287628	chr13:104711178-104711222	NM_001005868:-606	Erbb2ip	PROMOTER	0.823	3.404	1482.55	5046.77	2.802	1183.20	3315.21
A_68_P28747159	chr12:113205331-113205376	NM_027360:-165	2010107E04Rik	DIVERGENT_PROMOTER	0.823	4.813	323.30	1556.04	3.960	295.31	1169.53
A_68_P28599074	chr12:85912374-85912418	NM_007701:1595	Vsx2	INSIDE	0.823	0.387	2888.91	1119.11	0.319	2353.11	749.94
A_68_P27894434	chr11:69154495-69154539	NR_027827:246	A030009H04Rik	INSIDE	0.823	1.778	1532.56	2724.97	1.464	1285.65	1881.87
A_68_P27865448	chr11:64249947-64249991	NR_028445:208	F930015N05Rik	INSIDE	0.823	0.421	1221.11	513.90	0.346	934.82	323.61
A_68_P27784457	chr11:49526433-49526477	NM_212484:-242	Cnot6	PROMOTER	0.823	0.733	1776.69	1303.13	0.604	1409.20	850.69
A_68_P26940374	chr10:11063250-11063294	NM_010146:30	Epm2a	INSIDE	0.823	3.798	366.37	1391.47	3.124	331.97	1037.17
A_68_P26159867	chr8:112472027-112472072	NM_212447:14056	Marveld3	INSIDE	0.823	4.235	183.12	775.48	3.484	247.66	862.87
A_68_P24431597	chr6:49163986-49164030	NM_023670:945	Igf2bp3	INSIDE	0.823	4.183	635.75	2659.09	3.442	619.01	2130.75
A_68_P24155792	chr5:144031402-144031446	NM_017467:277	Zfp316	INSIDE	0.823	0.476	4321.54	2058.18	0.392	3266.42	1279.77
A_68_P24040026	chr5:121098911-121098955	NM_013832:102	Rasal1	INSIDE	0.823	0.542	3257.58	1766.65	0.446	2262.82	1010.13
A_68_P23644447	chr5:43625680-43625724	NR_028417:891	Gm7854	INSIDE	0.823	3.038	1277.72	3881.30	2.500	1085.29	2713.58
A_68_P23611441	chr5:37482522-37482566	NM_178394:40449	Jakmip1	INSIDE	0.823	2.433	844.90	2055.41	2.003	748.71	1499.53
A_68_P23295050	chr4:128798908-128798952	NM_010471:-395	Hpca	PROMOTER	0.823	4.416	2247.59	9924.25	3.634	1706.91	6203.43
A_68_P23278476	chr4:125826394-125826438	NM_172145:1170	Fam176b	INSIDE	0.823	0.606	1492.50	903.83	0.499	1079.62	538.29
A_68_P22312796	chr3:88058579-88058623	NM_021375:6	Rhbg	INSIDE	0.823	0.489	1955.20	956.32	0.402	1524.46	613.47
A_68_P21565885	chr2:118571382-118571426	NR_030716:-93	5430417L22Rik	PROMOTER	0.823	2.440	1791.29	4370.12	2.008	1476.34	2964.64
A_68_P21038054	chr2:17977988-17978032	NM_010804:1113	Mllt10	INSIDE	0.823	0.469	3499.37	1640.00	0.386	2421.21	933.79
A_68_P20927663	chr1:191552534-191552578	NM_008976:410	Ptpn14	INSIDE	0.823	0.362	1354.82	489.91	0.297	971.57	288.97
A_68_P20878653	chr1:182872689-182872733	NM_144794:62	Tmem63a	INSIDE	0.823	3.459	1646.77	5696.46	2.845	1357.94	3863.72
A_68_P32471622	chrX:72661675-72661719	NM_010839:186	Mtcp1	INSIDE	0.822	4.471	1028.77	4599.31	3.677	1400.09	5148.01
A_68_P30931904	chr16:85901269-85901313	NM_011782:80	Adamts5	INSIDE	0.822	0.693	2380.09	1650.56	0.570	1821.21	1037.90
A_68_P26134298	chr8:108043017-108043061	NM_008289:393	Hsd11b2	INSIDE	0.822	2.866	322.92	925.50	2.355	365.61	860.94
A_68_P24952006	chr7:5032174-5032218	NM_010147:290	Epn1	INSIDE	0.822	0.429	1444.14	619.99	0.353	1074.30	379.23
A_68_P24864545	chr6:134933738-134933782	NM_001109914:1742	Apold1	INSIDE	0.822	2.650	962.38	2550.61	2.179	930.31	2026.93
A_68_P23560763	chr5:28445188-28445235			Unknown	0.822	0.584	1129.58	659.40	0.480	815.46	391.12
A_68_P23440849	chr4:155205840-155205884	NM_025338:108	Aurkaip1	INSIDE	0.822	0.544	2132.79	1160.03	0.447	1591.97	712.03
A_68_P23359680	chr4:140258068-140258112	NM_173867:703	Rcc2	INSIDE	0.822	2.708	1591.16	4308.98	2.226	1257.09	2797.78
A_68_P23337643	chr4:136604702-136604746	NM_198248:-114	Zbtb40	PROMOTER	0.822	0.625	2120.29	1324.71	0.514	1744.48	896.13
A_68_P23281731	chr4:126413327-126413371	NM_198960:165	Tcfap2e	INSIDE	0.822	0.472	2470.87	1166.41	0.388	1787.23	693.62
A_68_P23255467	chr4:120887341-120887385	ENSMUST00000056635:327		INSIDE	0.822	0.489	5424.29	2650.69	0.402	4066.42	1633.35
A_68_P22855811	chr4:40090528-40090572	NM_007386:253	Aco1	INSIDE	0.822	0.608	1087.76	661.88	0.500	890.34	445.32
A_68_P22834880	chr4:35172594-35172638	NM_001081343:513	3110043O21Rik	INSIDE	0.822	0.575	990.40	569.06	0.472	872.37	412.18
A_68_P22630560	chr3:148651064-148651108	ENSMUST00000098518:1194		INSIDE	0.822	0.573	2192.45	1257.26	0.471	1724.59	812.45
A_68_P22601386	chr3:143861544-143861588	NM_001161770:3730	Lmo4	INSIDE	0.822	0.659	3845.71	2534.89	0.542	2877.79	1558.53
A_68_P22318791	chr3:89154152-89154197	NM_029974:14987	Dcst1	INSIDE	0.822	3.239	670.92	2173.27	2.664	556.66	1482.95
A_68_P21844244	chr2:168760676-168760720	NM_009564:20389	Zfp64	INSIDE	0.822	4.004	860.44	3445.29	3.291	717.87	2362.24
A_68_P21715082	chr2:145686295-145686339	NM_028724:74465	Rin2	INSIDE	0.822	0.513	1446.72	741.83	0.421	1174.79	495.11
A_68_P20938879	chr1:193399292-193399336	NM_029766:99	Dtl	INSIDE	0.822	0.199	2810.71	558.20	0.163	2083.02	340.09
A_68_P20026602	chr1:10222762-10222806	NM_001102430:-33	Arfgef1	PROMOTER	0.822	1.828	1303.90	2384.13	1.503	1122.34	1686.56
A_68_P32119985	chr19:42330579-42330623	NM_027694:372	Golga7b	INSIDE	0.821	2.580	2539.53	6552.36	2.119	2058.03	4360.72
A_68_P32114045	chr19:41338252-41338296	NM_133352:220	Tm9sf3	INSIDE	0.821	2.833	1663.07	4710.97	2.325	1311.78	3049.28
A_68_P31928607	chr19:5425271-5425315	NM_134149:149	AI837181	INSIDE	0.821	0.285	6313.87	1801.80	0.234	4346.03	1018.63
A_68_P31420283	chr17:87362407-87362451	NM_145491:-22	Rhoq	PROMOTER	0.821	3.539	574.01	2031.20	2.904	448.18	1301.70
A_68_P30513996	chr16:5132766-5132810	NM_008909:-214	Ppl	PROMOTER	0.821	2.515	1139.16	2865.45	2.065	879.71	1816.32
A_68_P29977063	chr15:3945932-3945978	NM_145930:-202	AW549877	PROMOTER	0.821	2.343	871.61	2042.43	1.925	656.98	1264.66
A_68_P29863081	chr14:103380685-103380729	NM_177715:1148	Kctd12	INSIDE	0.821	0.239	2793.65	667.39	0.196	2071.75	406.13
A_68_P28187036	chr11:120685971-120686016	NM_007988:-132	Fasn	PROMOTER	0.821	0.698	1630.63	1138.31	0.573	1202.09	689.18
A_68_P28057114	chr11:98407085-98407129	NM_011771:239	Ikzf3	INSIDE	0.821	2.574	422.83	1088.29	2.114	375.53	793.96
A_68_P27750958	chr11:43342876-43342920	NM_001045530:613	Ccnjl	INSIDE	0.821	0.475	2463.17	1169.60	0.390	2019.80	787.04
A_68_P27311093	chr10:85379465-85379509	NM_181650:204	Prdm4	INSIDE	0.821	0.234	2112.50	493.41	0.192	1498.26	287.30
A_68_P27259221	chr10:75356293-75356337	NM_024440:172	Derl3	INSIDE	0.821	0.494	1593.31	786.49	0.405	1387.55	562.36
A_68_P26816890	chr9:108734418-108734462	NM_080437:5790	Celsr3	INSIDE	0.821	0.562	4521.14	2541.02	0.462	3146.40	1452.46
A_68_P26470172	chr9:44689329-44689373	NM_001081049:7	Mll1	INSIDE	0.821	2.084	1026.97	2140.48	1.711	763.51	1306.22
A_68_P26459693	chr9:42914668-42914712	NM_027144:-889	Arhgef12	PROMOTER	0.821	0.724	1490.08	1079.11	0.595	1133.71	674.43
A_68_P25715748	chr8:24367539-24367583	NM_001042484:-8	Golga7	PROMOTER	0.821	0.375	2216.85	832.30	0.308	1529.82	471.55
A_68_P25678282	chr8:15027507-15027551	NM_029116:16504	Kbtbd11	INSIDE	0.821	2.688	855.42	2299.62	2.208	675.76	1491.99
A_68_P25505272	chr7:134489178-134489222	NM_146202:-372	Zfp768	PROMOTER	0.821	2.487	636.47	1583.04	2.042	454.22	927.42
A_68_P25094672	chr7:52970422-52970466	NM_020011:133	Sphk2	INSIDE	0.821	0.471	1973.88	929.90	0.387	1429.38	552.75

ProbeName	Target position of probe on CpG island microarray	TargetID	GeneSymbol	CpG island Description	Ratio of relative methylation (TiO_2-NP/Vehicle)	Sham group			TiO_2-H group		
						Relative methylation (Cy5/Cy3)	Cy3 signal (Input)	Cy5 signal (MeDIP)	Relative methylation (Cy5/Cy3)	Cy3 signal (Input)	Cy5 signal (MeDIP)
A_68_P25030986	chr7:31107024-31107068	NM_001033140:348	Sdhaf1	INSIDE	0.821	2.818	605.74	1706.69	2.312	501.44	1159.43
A_68_P24329795	chr6:29998957-29999001	NM_001164230:991	Nrf1	INSIDE	0.821	0.570	1809.72	1031.27	0.468	1187.13	555.22
A_68_P24058536	chr5:124423825-124423869	NM_145070:210	Hip1r	INSIDE	0.821	0.464	1384.61	642.75	0.381	1050.29	400.47
A_68_P23542975	chr5:24107899-24107943	NM_025891:-100	Smarcd3	PROMOTER	0.821	4.221	366.98	1549.21	3.465	353.82	1225.91
A_68_P22325404	chr3:90269294-90269338	NM_008727:472	Npr1	INSIDE	0.821	3.462	1458.62	5049.95	2.843	1171.60	3331.24
A_68_P21037815	chr2:17950260-17950305	NR_028378:1407	LOC100034739	INSIDE	0.821	0.548	1994.85	1093.70	0.450	1369.15	616.23
A_68_P32552415	chrX:96086309-96086353	NM_052976:-6	Ophn1	PROMOTER	0.820	0.607	797.34	483.62	0.497	1154.92	574.47
A_68_P31834412	chr18:75165713-75165757	NM_001001181:181	BC031181	INSIDE	0.820	0.201	2484.77	499.86	0.165	1867.67	308.25
A_68_P31154945	chr17:34769052-34769096	NM_030890:2444	Prrt1	INSIDE	0.820	0.497	965.46	479.51	0.407	736.67	300.05
A_68_P31122729	chr17:28622707-28622751	NM_010220:329	Fkbp5	INSIDE	0.820	2.314	810.82	1876.50	1.897	724.10	1373.76
A_68_P30580553	chr16:18581703-18581747	NM_011532:5338	Tbx1	DOWNSTREAM	0.820	0.504	1486.75	749.04	0.413	1155.55	477.33
A_68_P30343814	chr15:75577843-75577887	NM_194350:488	Mafa	INSIDE	0.820	1.982	1903.16	3771.38	1.625	1507.07	2449.25
A_68_P29605728	chr14:52899342-52899386	NM_172601:-294	Rab2b	PROMOTER	0.820	0.350	3995.10	1399.25	0.287	2977.65	855.08
A_68_P28788255	chr13:3477597-3477641	NR_015522:70	2810429I04Rik	INSIDE	0.820	0.327	2159.09	706.97	0.269	1696.39	455.75
A_68_P27836132	chr11:59120062-59120106	NM_139298:-347	Wnt9a	PROMOTER	0.820	0.460	2115.54	973.98	0.377	1595.47	602.13
A_68_P26750481	chr9:96919029-96919073	NM_145134:-276	Spsb4	PROMOTER	0.820	2.928	947.07	2773.13	2.402	761.02	1827.85
A_68_P26716243	chr9:90058080-90058124	NM_001003911:287	Adamts7	INSIDE	0.820	0.368	3503.94	1289.70	0.302	2632.92	794.75
A_68_P26477291	chr9:45821396-45821440	NM_027498:516	Sik3	INSIDE	0.820	0.355	1877.40	666.73	0.291	1571.47	457.47
A_68_P25879339	chr8:55615377-55615421	NM_029701:-47	Spcs3	PROMOTER	0.820	1.989	854.80	1700.21	1.630	780.61	1272.59
A_68_P25834565	chr8:47237229-47237273	ENSMUST00000154040:126		INSIDE	0.820	2.177	691.64	1505.36	1.785	661.30	1180.74
A_68_P25475448	chr7:128877816-128877860	NM_011326:-182	Scnn1g	PROMOTER	0.820	1.922	1898.94	3649.10	1.575	1528.59	2408.14
A_68_P25092970	chr7:52669152-52669196	NM_198190:110	Ntf5	INSIDE	0.820	0.554	1169.74	647.54	0.454	924.97	419.88
A_68_P24954457	chr7:6141163-6141207	NM_001146024:17070	Zfp444	INSIDE	0.820	3.576	274.88	982.84	2.931	283.74	831.65
A_68_P24907675	chr6:142912528-142912572	NM_011374:422	St8sia1	INSIDE	0.820	1.946	1630.42	3173.60	1.597	1299.48	2074.79
A_68_P24009561	chr5:115886828-115886872	NM_008629:7157	Msi1	INSIDE	0.820	0.382	1915.47	732.27	0.313	1517.45	475.68
A_68_P24004042	chr5:114893913-114893957	NM_029956:102	Mmab	INSIDE	0.820	0.470	928.44	436.26	0.385	730.90	281.60
A_68_P22518661	chr3:128907456-128907500	NM_001042502:-9376	Pitx2	PROMOTER	0.820	3.181	2695.90	8575.74	2.610	2035.16	5311.76
A_68_P21914376	chr3:3309284-3309328			Unknown	0.820	4.611	589.97	2720.30	3.782	456.27	1725.58
A_68_P21338547	chr2:74493903-74493947	NM_007967:3552	Evx2	INSIDE	0.820	3.438	526.08	1808.58	2.819	373.84	1053.82
A_68_P32168791	chr19:50753028-50753072	NM_021377:52	Sorcs1	INSIDE	0.819	7.462	2083.54	15547.68	6.108	1611.66	9844.77
A_68_P32099500	chr19:38172076-38172120	NM_181748:530	O3far1	INSIDE	0.819	0.363	2967.92	1076.89	0.297	2223.70	660.53
A_68_P31943407	chr19:8831518-8831562	NM_016813:-52	Nxf1	PROMOTER	0.819	2.348	469.30	1101.95	1.924	424.63	816.88
A_68_P31236175	chr17:51951926-51951970	NM_001163630:-569	Satb1	PROMOTER	0.819	0.665	1855.66	1234.86	0.545	1371.38	747.54
A_68_P29223875	chr13:93196672-93196716	NM_175455:771	Ankrd34b	INSIDE	0.819	2.761	430.08	1187.42	2.260	438.84	991.86
A_68_P28160199	chr11:116442326-116442370	NM_173755:413	Ube2o	INSIDE	0.819	0.500	2320.69	1159.32	0.409	1632.95	667.92
A_68_P27188778	chr10:62442853-62442897	NM_027425:-96	Rufy2	PROMOTER	0.819	0.481	1737.98	836.15	0.394	1273.10	501.65
A_68_P26893781	chr9:123125176-123125220	NM_133974:-42	Cdcp1	PROMOTER	0.819	0.570	1745.88	995.54	0.467	1380.03	644.40
A_68_P26591325	chr9:65972739-65972783	NM_019727:-67	Snx1	DIVERGENT_PROMOTER	0.819	1.983	2977.00	5904.65	1.624	2406.85	3908.81
A_68_P26554628	chr9:59504469-59504513	NM_011099:76	Pkm2	INSIDE	0.819	0.461	4489.11	2068.71	0.378	3069.04	1158.63
A_68_P26421276	chr9:35018562-35018606	NM_172291:-74	Foxred1	DIVERGENT_PROMOTER	0.819	0.271	1680.83	454.91	0.222	1347.10	298.70
A_68_P25699252	chr8:18595573-18595617	NM_173189:422	Mcph1	INSIDE	0.819	0.585	1040.67	609.31	0.480	954.33	457.89
A_68_P25030928	chr7:31099011-31099055	NM_198027:5261	Alkbh6	INSIDE	0.819	1.868	1606.38	3000.92	1.530	1321.19	2021.82
A_68_P24785563	chr6:118146898-118146942	NM_001080780:842	Ret	INSIDE	0.819	2.556	1199.53	3065.78	2.094	1050.60	2200.03
A_68_P24631553	chr6:90259469-90259513	NM_027928:15689	Chst13	INSIDE	0.819	6.960	558.89	3890.15	5.702	527.83	3009.66
A_68_P24011285	chr5:116182026-116182070	NM_001080808:-480	Ccdc64	PROMOTER	0.819	2.399	793.37	1903.21	1.964	648.75	1273.94
A_68_P22071607	chr3:38786088-38786133	NM_183221:249	Fat4	INSIDE	0.819	2.450	564.65	1383.46	2.008	494.44	992.72
A_68_P21780568	chr2:157854743-157854787	NM_027434:235	Rprd1b	INSIDE	0.819	0.312	2535.16	791.48	0.256	2145.02	548.23
A_68_P21320134	chr2:71383919-71383963	NM_010054:871	Dlx2	INSIDE	0.819	0.273	3564.14	973.55	0.224	2623.54	587.16
A_68_P21098345	chr2:29475651-29475695	NM_001039086:433	Rapgef1	INSIDE	0.819	2.117	1397.51	2958.05	1.734	1219.33	2114.34
A_68_P32465997	chrX:71268691-71268735	NM_001177975:545	Irak1	INSIDE	0.818	2.519	426.41	1073.95	2.061	515.11	1061.40
A_68_P32463901	chrX:70885738-70885782	NM_029352:981	Dusp9	INSIDE	0.818	2.508	1118.09	2804.64	2.052	1155.03	2369.68
A_68_P31215002	chr17:48059670-48059714	NM_001110824:1889	Foxp4	INSIDE	0.818	2.580	1448.06	3735.43	2.110	1156.09	2439.25
A_68_P31098015	chr17:24687028-24687072	NM_013630:156	Pkd1	INSIDE	0.818	2.284	781.25	1784.16	1.868	669.69	1251.24
A_68_P31086418	chr17:21669673-21669717	NM_144515:-2808	Zfp52	PROMOTER	0.818	0.656	2186.25	1434.20	0.537	1716.76	921.22
A_68_P30931895	chr16:85900243-85900287	NM_011782:1106	Adamts5	INSIDE	0.818	2.644	821.77	2172.77	2.162	685.72	1482.26
A_68_P29059921	chr13:56322911-56322955	ENSMUST00000127754:22369		DOWNSTREAM	0.818	2.006	1056.35	2119.37	1.642	908.05	1491.11
A_68_P28914787	chr13:30077407-30077451	NM_010093:504	E2f3	INSIDE	0.818	0.341	2875.82	981.47	0.279	2233.22	623.17
A_68_P28175452	chr11:118902740-118902784	NM_013926:-535	Cbx8	PROMOTER	0.818	2.105	1807.80	3806.02	1.722	1395.09	2403.00
A_68_P28112590	chr11:108204958-108205002	NM_011101:222	Prkca	INSIDE	0.818	0.489	1839.05	899.33	0.400	1380.83	552.10
A_68_P27503751	chr10:120912576-120912620	NM_138956:708	Rassf3	INSIDE	0.818	0.360	2857.50	1027.69	0.294	1924.98	566.17
A_68_P27479236	chr10:116500934-116500978	NM_007636:-86	Cct2	PROMOTER	0.818	0.555	8373.25	4651.09	0.454	6288.34	2856.77
A_68_P27234635	chr10:70747809-70747853	NM_145420:180	Ube2d1	INSIDE	0.818	0.614	1637.40	1005.64	0.502	1347.95	677.13
A_68_P25232263	chr7:81157988-81158032	NM_009181:558	St8sia2	INSIDE	0.818	6.374	8195.07	52237.21	5.213	5811.94	30295.23
A_68_P24980778	chr7:16897508-16897552	NM_133234:2599	Bbc3	INSIDE	0.818	0.331	1587.00	525.65	0.271	1452.82	393.77
A_68_P24423150	chr6:47404743-47404787	NM_012042:442	Cul1	INSIDE	0.818	2.602	906.07	2358.02	2.129	698.08	1486.17

ProbeName	Target position of probe on CpG island microarray	TargetID	GeneSymbol	CpG island Description	Ratio of relative methylation (TiO_2-NP/Vehicle)	Sham group			TiO_2-H group		
						Relative methylation (Cy5/Cy3)	Cy3 signal (Input)	Cy5 signal (MeDIP)	Relative methylation (Cy5/Cy3)	Cy3 signal (Input)	Cy5 signal (MeDIP)
A_68_P23893506	chr5:92738928-92738972	NM_172713:100	Sdad1	INSIDE	0.818	0.484	1820.33	880.73	0.396	1336.86	529.29
A_68_P23437422	chr4:154624331-154624375	NM_001190445:278	2610002J02Rik	INSIDE	0.818	1.760	4997.12	8795.95	1.440	3755.76	5409.86
A_68_P21623517	chr2:129024411-129024455	NM_001159593:-76	Slc20a1	PROMOTER	0.818	0.650	1083.41	704.50	0.532	941.77	500.66
A_68_P21046081	chr2:19580665-19580709	NM_027715:998	Otud1	INSIDE	0.818	0.464	1888.91	877.29	0.380	1569.39	596.14
A_68_P33007024	A_68_P33007024			Unknown	0.817	2.135	1138.72	2431.22	1.745	1044.65	1822.43
A_68_P32579350	chrX:102316061-102316105	NM_053201:348	Magee1	INSIDE	0.817	2.520	770.55	1942.12	2.060	841.18	1732.85
A_68_P32288518	chrX:20002618-20002662	ENSMUST00000115384:-173		PROMOTER	0.817	4.673	676.83	3162.54	3.816	984.08	3754.80
A_68_P32140496	chr19:45857387-45857431	NM_023799:373	Mgea5	INSIDE	0.817	0.549	1119.02	614.11	0.449	833.44	373.82
A_68_P31378406	chr17:80014550-80014594	NM_201361:333	Fam82a1	INSIDE	0.817	3.641	1085.79	3953.58	2.975	866.79	2578.41
A_68_P31160314	chr17:35804494-35804538	NM_175137:21	Vars2	INSIDE	0.817	0.448	1126.19	503.99	0.366	871.82	318.88
A_68_P30959870	chr16:90934584-90934628	NM_026502:488	1110004E09Rik	INSIDE	0.817	0.403	5645.35	2274.99	0.329	3699.51	1217.60
A_68_P30106581	chr15:30102269-30102313	NM_008729:-57	Ctnnd2	PROMOTER	0.817	2.140	756.83	1619.62	1.749	593.09	1037.38
A_68_P28553888	chr12:77518476-77518520	NM_001163103:-87	Gm70	PROMOTER	0.817	1.919	896.26	1719.98	1.567	811.49	1271.55
A_68_P28307252	chr12:28026085-28026129	NM_009234:1477	Sox11	INSIDE	0.817	0.227	3816.12	864.47	0.185	2536.21	469.32
A_68_P28206072	chr12:5382150-5382194	NM_001164493:316	Klhl29	INSIDE	0.817	0.578	818.19	472.73	0.472	727.56	343.53
A_68_P28071221	chr11:100831940-100831984	NM_008986:-31	Ptrf	PROMOTER	0.817	0.617	2565.74	1582.37	0.504	1693.34	852.76
A_68_P27490920	chr10:118890599-118890643	NM_133442:-749	Grip1	PROMOTER	0.817	0.587	3162.55	1857.08	0.479	2277.12	1091.82
A_68_P27466130	chr10:114238213-114238257	NM_146241:192	Trhde	INSIDE	0.817	0.418	1226.45	512.63	0.341	985.93	336.50
A_68_P27453319	chr10:111708984-111709028	NM_001025581:828	Kcnc2	INSIDE	0.817	2.397	1592.75	3817.89	1.959	1262.86	2473.70
A_68_P26350219	chr9:21855638-21855682	NM_010487:807	Elavl3	INSIDE	0.817	0.130	4666.48	608.63	0.107	3023.35	321.99
A_68_P26137546	chr8:108583679-108583723	NM_010901:198	Nfatc3	INSIDE	0.817	2.591	511.96	1326.33	2.117	432.40	915.59
A_68_P25377990	chr7:109416158-109416202	NM_009287:-157	Stim1	PROMOTER	0.817	2.603	1197.02	3116.07	2.126	953.55	2026.91
A_68_P24994096	chr7:20158742-20158786	NM_027189:-72	Gemin7	DIVERGENT_PROMOTER	0.817	2.256	1322.23	2983.61	1.844	890.28	1641.47
A_68_P24944592	chr6:149302140-149302189	ENSMUST00000121073:184		INSIDE	0.817	2.331	931.61	2171.89	1.906	692.70	1320.08
A_68_P23905255	chr5:96638963-96639007	NM_001039084:-149	Mrpl1	PROMOTER	0.817	0.427	1368.31	584.32	0.349	1060.62	369.93
A_68_P23404991	chr4:149610981-149611025	NM_023119:-303	Eno1	PROMOTER	0.817	3.181	718.18	2284.77	2.599	643.40	1672.20
A_68_P23237032	chr4:117555847-117555891	NM_017377:206	B4galt2	INSIDE	0.817	0.533	1049.06	558.97	0.436	918.72	400.17
A_68_P21900914	chr2:179759500-179759544	NM_177727:-169	Lsm14b	PROMOTER	0.817	2.148	825.05	1772.60	1.755	723.67	1269.73
A_68_P20742450	chr1:158233703-158233747	NM_001134741:-245	Tdrd5	DIVERGENT_PROMOTER	0.817	3.015	1193.72	3598.49	2.463	1003.53	2471.88
A_68_P20193924	chr1:44609219-44609263	NM_028450:725	Gulp1	INSIDE	0.817	0.522	900.43	470.32	0.427	733.58	313.14
A_68_P32796351	chrX:160514199-160514243	NM_009173:-239	Siah1b	PROMOTER	0.816	2.155	713.38	1537.29	1.758	941.47	1654.85
A_68_P32107649	chr19:40345998-40346042	NM_016861:86	Pdlim1	INSIDE	0.816	0.631	2684.29	1693.30	0.515	2281.71	1173.99
A_68_P31932820	chr19:6117874-6117918	NM_133678:690	Sac3d1	INSIDE	0.816	0.632	3164.90	2000.02	0.516	2273.89	1173.00
A_68_P31625587	chr18:36904013-36904057	NM_010885:168	Ndufa2	INSIDE	0.816	0.420	2856.09	1199.12	0.343	2086.30	714.94
A_68_P31109171	chr17:26390076-26390120	NM_025881:244	Luc7l	INSIDE	0.816	0.608	2487.91	1512.98	0.496	2238.96	1110.71
A_68_P30961954	chr16:91271188-91271232	NM_016968:1197	Olig1	INSIDE	0.816	2.680	2267.00	6075.82	2.187	1782.63	3898.24
A_68_P30476479	chr15:98622678-98622722	NM_021279:2413	Wnt1	INSIDE	0.816	0.541	979.44	529.87	0.441	728.40	321.52
A_68_P29751162	chr14:79790477-79790521	NM_025832:-23	Naa16	DIVERGENT_PROMOTER	0.816	2.698	835.02	2252.58	2.203	808.07	1779.85
A_68_P29446594	chr14:21207691-21207735	NM_134081:420	Dnajc9	INSIDE	0.816	0.426	1098.94	468.53	0.348	795.75	276.72
A_68_P28813406	chr13:8870643-8870687	NM_172445:-376	Wdr37	PROMOTER	0.816	0.615	1198.75	736.77	0.501	930.25	466.35
A_68_P28450018	chr12:56086184-56086228	NM_013815:1117	Baz1a	INSIDE	0.816	0.556	3286.34	1826.08	0.453	2514.22	1139.35
A_68_P28431899	chr12:52930265-52930309	NM_144788:237	Hectd1	INSIDE	0.816	5.489	451.00	2475.51	4.478	363.60	1628.30
A_68_P28202614	chr12:4848201-4848245	NM_016863:179	Fkbp1b	INSIDE	0.816	3.222	985.92	3176.31	2.630	854.16	2246.70
A_68_P28194542	chr12:3309944-3309988	NM_016676:3	Rab10	INSIDE	0.816	1.908	1253.85	2392.86	1.557	1164.85	1814.14
A_68_P28094412	chr11:104987932-104987976	NM_172567:216	Mettl2	INSIDE	0.816	2.326	1260.59	2932.72	1.899	1113.09	2113.65
A_68_P27889089	chr11:68199419-68199463	NM_008744:888	Ntn1	INSIDE	0.816	0.371	1649.03	611.49	0.302	1251.59	378.49
A_68_P27143269	chr10:53349507-53349551	NM_027830:717	Mcm9	INSIDE	0.816	0.460	1602.18	736.94	0.375	1260.61	473.22
A_68_P26744829	chr9:95855445-95855489	NM_011916:288	Xrn1	INSIDE	0.816	2.177	1016.27	2212.06	1.777	832.90	1480.09
A_68_P26665699	chr9:79566207-79566251	NM_007730:257	Col12a1	INSIDE	0.816	0.730	1728.34	1262.22	0.596	1366.91	814.90
A_68_P26595981	chr9:66767110-66767154	NM_173413:380	Rab8b	INSIDE	0.816	0.459	4639.87	2128.20	0.374	3289.19	1231.39
A_68_P26528147	chr9:54711749-54711793	NM_022655:209	Ireb2	INSIDE	0.816	0.659	1630.27	1075.01	0.538	1409.48	758.72
A_68_P26082830	chr8:97876281-97876325	NM_008609:66	Mmp15	INSIDE	0.816	2.455	559.42	1373.36	2.002	466.31	933.60
A_68_P25265268	chr7:87343468-87343512	NM_013659:11764	Sema4b	INSIDE	0.816	5.916	1160.93	6867.76	4.827	937.00	4523.16
A_68_P25092683	chr7:52622134-52622178	NM_029741:233	Ppfia3	INSIDE	0.816	0.387	1819.09	704.55	0.316	1502.43	474.59
A_68_P25068313	chr7:46808075-46808119	AK053739:231		INSIDE	0.816	1.872	1099.76	2059.14	1.527	943.27	1440.37
A_68_P24754175	chr6:112439230-112439274	NM_001081147:550	Oxtr	INSIDE	0.816	2.629	767.41	2017.25	2.144	607.64	1302.92
A_68_P24324309	chr6:29010153-29010197	NM_008493:-46	Lep	PROMOTER	0.816	2.201	2465.86	5428.52	1.796	1775.95	3189.49
A_68_P24044663	chr5:121902036-121902080	NM_026129:425	Erp29	INSIDE	0.816	3.093	1518.34	4696.81	2.525	1213.89	3064.83
A_68_P23359685	chr4:140258885-140258929	NM_173867:1519	Rcc2	INSIDE	0.816	0.633	1247.89	789.74	0.517	966.66	499.33
A_68_P22615133	chr3:146184298-146184342	NM_027371:67	Rpf1	INSIDE	0.816	2.287	857.38	1961.25	1.867	726.50	1356.13
A_68_P20226313	chr1:52290060-52290104	NM_001081081:-6	Gls	PROMOTER	0.816	0.468	1133.76	530.55	0.382	857.27	327.33
A_68_P20047721	chr1:14745513-14745557	NM_010827:513	Msc	INSIDE	0.816	2.277	646.92	1473.13	1.858	661.04	1228.34
A_68_P32957490	chr17:8138877-8138921	NM_025789:420	Rsph3a	INSIDE	0.815	2.418	933.16	2256.01	1.969	820.07	1614.99
A_68_P32540977	chrX:92391918-92391962	ENSMUST00000113884:220		INSIDE	0.815	0.555	1184.20	657.32	0.452	1612.83	729.36
A_68_P32288523	chrX:20003140-20003184	ENSMUST00000136093:249		INSIDE	0.815	1.938	849.32	1645.86	1.579	1138.68	1797.51

ProbeName	Target position of probe on CpG island microarray	TargetID	GeneSymbol	CpG island Description	Ratio of relative methylation (TiO_2-NP/Vehicle)	Sham group			TiO_2-H group		
						Relative methylation (Cy5/Cy3)	Cy3 signal (Input)	Cy5 signal (MeDIP)	Relative methylation (Cy5/Cy3)	Cy3 signal (Input)	Cy5 signal (MeDIP)
A_68_P32147196	chr19:47089352-47089396	NM_146100:187	Ina	INSIDE	0.815	2.616	708.22	1852.42	2.131	583.30	1242.80
A_68_P32030085	chr19:25580326-25580370	NM_015826:153	Dmrt1	INSIDE	0.815	0.363	1431.17	519.12	0.296	1062.91	314.37
A_68_P31335317	chr17:71966066-71966110	NM_175639:534	Wdr43	INSIDE	0.815	0.678	2292.36	1554.96	0.553	1944.34	1074.57
A_68_P28688786	chr12:103214262-103214306	NM_016856:101	Cpsf2	INSIDE	0.815	0.563	969.83	545.61	0.459	784.80	360.03
A_68_P28341950	chr12:34114856-34114900	NM_172253:390	Twistnb	INSIDE	0.815	0.597	1052.37	627.77	0.486	874.33	425.00
A_68_P27826629	chr11:57458793-57458837	NM_134189:-129	Galnt10	PROMOTER	0.815	0.316	3921.97	1238.45	0.257	2641.89	679.79
A_68_P27536903	chr10:127031018-127031062	NM_008512:27164	Lrp1	INSIDE	0.815	1.909	1822.71	3479.30	1.555	1262.63	1963.87
A_68_P27535212	chr10:126748563-126748607	NM_001003913:258	Mars	INSIDE	0.815	0.345	1396.05	481.29	0.281	1123.33	315.60
A_68_P27383825	chr10:98377784-98377828	NM_026482:21	Atp2b1	INSIDE	0.815	3.162	794.09	2510.85	2.577	674.10	1736.86
A_68_P27303131	chr10:83997120-83997164	NM_175451:-509	Ckap4	PROMOTER	0.815	2.405	964.27	2319.34	1.960	806.83	1581.67
A_68_P27011028	chr10:24589929-24589973	NM_001166416:159	Med23	INSIDE	0.815	3.418	575.74	1968.05	2.786	549.74	1531.70
A_68_P25065279	chr7:38870220-38870264	AK181724:37896		INSIDE	0.815	0.477	1992.80	951.32	0.389	1524.66	593.00
A_68_P24816270	chr6:124613220-124613264	NM_145130:121	Lpcat3	INSIDE	0.815	0.464	2360.76	1095.15	0.378	1663.92	629.24
A_68_P24619475	chr6:87994860-87994904	ENSMUST00000113598:129		INSIDE	0.815	2.321	991.59	2301.69	1.892	928.57	1756.81
A_68_P24091119	chr5:130371741-130371785	NM_183088:170	2410018M08Rik	INSIDE	0.815	5.705	1472.05	8397.47	4.649	1245.08	5787.96
A_68_P23415960	chr4:151383595-151383639	NM_001033352:617	Klhl21	INSIDE	0.815	2.617	449.51	1176.23	2.133	378.88	808.13
A_68_P23319354	chr4:133310209-133310253	NM_001080819:-704	Arid1a	PROMOTER	0.815	1.806	3819.87	6898.66	1.473	2946.58	4338.85
A_68_P22705222	chr4:6292695-6292739	NM_001098227:-110	Sdcbp	PROMOTER	0.815	0.465	5492.71	2555.41	0.379	3832.36	1453.22
A_68_P22291209	chr3:84283512-84283556	NM_001205355:-121	Fhdc1	PROMOTER	0.815	0.265	2227.40	590.34	0.216	1657.39	357.91
A_68_P21622683	chr2:128864800-128864844	ENSMUST00000145798:16689		INSIDE	0.815	2.594	1158.04	3003.47	2.115	1010.20	2136.58
A_68_P20887293	chr1:184447465-184447509	NM_009794:128	Capn2	INSIDE	0.815	0.594	1494.65	887.70	0.484	1076.15	521.15
A_68_P20056409	chr1:16609431-16609475	NM_025773:-85	Ube2w	PROMOTER	0.815	0.360	3019.76	1087.68	0.293	2487.37	729.96
A_68_P31541307	chr18:20903913-20903957	NM_019737:971	B4galt6	INSIDE	0.814	0.483	2477.56	1196.72	0.393	1750.65	688.53
A_68_P31150605	chr17:33954480-33954524	NM_030697:7035	Kank3	INSIDE	0.814	2.738	478.49	1309.99	2.228	447.56	997.06
A_68_P30576622	chr16:17927537-17927581	NM_153150:754	Slc25a1	INSIDE	0.814	0.335	2121.23	711.45	0.273	1587.41	433.57
A_68_P30401894	chr15:85456416-85456460			Unknown	0.814	6.489	842.13	5464.71	5.285	779.38	4119.02
A_68_P29642598	chr14:60705516-60705560	NM_015803:133	Atp8a2	INSIDE	0.814	0.511	1048.09	536.05	0.417	775.84	323.17
A_68_P28204868	chr12:5216846-5216890	NM_001164493:165620	Klhl29	INSIDE	0.814	2.506	1743.85	4370.63	2.040	1327.09	2707.48
A_68_P28107295	chr11:107340618-107340662	NM_025894:-201	Psmd12	DIVERGENT_PROMOTER	0.814	0.293	2507.32	734.94	0.239	1953.79	466.38
A_68_P26463565	chr9:43552288-43552332	NM_021424:-348	Pvrl1	PROMOTER	0.814	0.605	1374.68	832.14	0.493	1019.16	502.15
A_68_P26132791	chr8:107817394-107817438	NM_177788:2582	Exoc3l	INSIDE	0.814	3.250	1168.36	3797.02	2.646	1027.79	2720.00
A_68_P25348904	chr7:104229911-104229955	NM_001162477:-328	Gab2	PROMOTER	0.814	2.428	882.23	2142.38	1.978	690.82	1366.22
A_68_P24648069	chr6:92893420-92893464	NR_015530:2867	9530026P05Rik	INSIDE	0.814	2.314	893.83	2068.16	1.883	752.81	1417.37
A_68_P24634433	chr6:90686814-90686858	NM_001134384:73281	Iqsec1	INSIDE	0.814	1.815	1869.06	3392.15	1.477	1526.61	2255.55
A_68_P24631649	chr6:90275213-90275257	NM_027928:-55	Chst13	DIVERGENT_PROMOTER	0.814	2.205	1060.80	2339.18	1.796	792.44	1422.98
A_68_P24611765	chr6:86683027-86683071	NM_011818:324	Gmcl1	INSIDE	0.814	1.927	1175.59	2265.04	1.568	966.86	1515.93
A_68_P24127559	chr5:138227476-138227520	NM_144913:431	Mepce	INSIDE	0.814	0.557	7557.53	4207.16	0.453	4644.46	2103.66
A_68_P24114331	chr5:135499854-135499898	NM_016801:435	Stx1a	INSIDE	0.814	0.630	2088.43	1316.46	0.513	1668.50	856.35
A_68_P23981857	chr5:111209299-111209343	NM_001025561:295	Pus1	INSIDE	0.814	0.605	2303.60	1393.17	0.492	1843.10	907.44
A_68_P23184553	chr4:106136681-106136725	NM_153565:228	Pcsk9	INSIDE	0.814	0.266	2578.88	687.04	0.217	1889.63	409.60
A_68_P22741774	chr4:13670169-13670215	NM_001111026:-256	Runx1t1	PROMOTER	0.814	2.535	1330.30	3372.61	2.063	937.72	1934.71
A_68_P22317585	chr3:88968583-88968627	NM_001163432:-122	Clk2	DIVERGENT_PROMOTER	0.814	2.831	1837.31	5201.40	2.305	1474.29	3398.83
A_68_P21822538	chr2:165059682-165059726	NM_174988:533	Cdh22	INSIDE	0.814	0.367	2884.35	1059.24	0.299	1956.27	584.92
A_68_P21317592	chr2:70956339-70956383	NM_028593:250	Cybrd1	INSIDE	0.814	0.276	1842.42	509.27	0.225	1436.06	323.23
A_68_P21266384	chr2:61643504-61643548	NM_009322:1017	Tbr1	INSIDE	0.814	0.659	1735.81	1144.73	0.537	1265.34	679.38
A_68_P21096534	chr2:29202663-29202707	NM_026896:329	Med27	INSIDE	0.814	0.550	2338.02	1285.28	0.447	1710.67	765.05
A_68_P20935797	chr1:192849641-192849685	NM_001081259:407	Mfsd7b	INSIDE	0.814	1.804	1786.44	3222.93	1.469	1366.85	2007.87
A_68_P32700057	chrX:137132942-137132986	NM_001077364:2097	Tsc22d3	INSIDE	0.813	1.916	963.87	1847.23	1.559	1310.06	2042.10
A_68_P32134821	chr19:44911447-44911492	NM_011037:79586	Pax2	INSIDE	0.813	1.998	3658.90	7311.07	1.624	2693.02	4374.62
A_68_P31961133	chr19:12575663-12575707	NM_172442:802	Dtx4	INSIDE	0.813	2.514	2098.02	5275.01	2.043	1602.26	3273.38
A_68_P31601524	chr18:32323271-32323316	NM_011946:551	Map3k2	INSIDE	0.813	0.337	1479.83	498.67	0.274	1125.59	308.50
A_68_P30925475	chr16:84774232-84774276	NM_023844:-113	Jam2	PROMOTER	0.813	2.095	9948.36	20841.62	1.703	7016.99	11946.83
A_68_P30502654	chr15:103012688-103012732	NM_001110216:33073	Cbx5	DOWNSTREAM	0.813	2.061	794.35	1637.25	1.676	645.37	1081.89
A_68_P30387017	chr15:83002186-83002233	NM_029787:429	Cyb5r3	INSIDE	0.813	0.298	2704.99	806.57	0.242	1704.24	413.11
A_68_P29700471	chr14:70689220-70689264	NM_008915:14	Ppp3cc	INSIDE	0.813	3.426	361.57	1238.80	2.785	352.17	980.77
A_68_P28103520	chr11:106649848-106649892	NM_007840:-62	Ddx5	DIVERGENT_PROMOTER	0.813	0.731	5391.04	3941.60	0.594	3907.66	2321.66
A_68_P27926563	chr11:75328969-75329013	NM_001004157:1948	Scarf1	INSIDE	0.813	3.107	663.78	2062.61	2.525	591.13	1492.50
A_68_P27282385	chr10:79775990-79776034	NM_011789:11448	Apc2	INSIDE	0.813	3.625	1195.42	4333.91	2.948	1131.17	3335.24
A_68_P27281294	chr10:79612495-79612539	NM_021565:1482	Midn	INSIDE	0.813	0.227	3674.75	835.50	0.185	2562.44	473.63
A_68_P27089722	chr10:41239137-41239181	NM_016898:-147	Cd164	PROMOTER	0.813	0.391	1897.04	741.25	0.318	1537.53	488.58
A_68_P26894029	chr9:123169283-123169327	NM_001002267:603	Tmem158	INSIDE	0.813	3.486	1074.46	3745.87	2.836	1009.62	2862.93
A_68_P26845378	chr9:114472828-114472872	NM_001042503:637	Trim71	INSIDE	0.813	2.535	607.88	1540.98	2.060	463.83	955.60
A_68_P25362155	chr7:106631960-106632004	NR_002172:-540	Snord15a	PROMOTER	0.813	0.347	1711.02	593.91	0.282	1364.38	385.18
A_68_P25091450	chr7:52409544-52409588	NM_029406:-201	Pih1d1	PROMOTER	0.813	0.402	2861.95	1150.39	0.327	1938.96	634.00
A_68_P25029338	chr7:30768885-30768929	NM_001033355:-67	Zfp568	PROMOTER	0.813	2.111	711.75	1502.21	1.716	600.93	1031.39

ProbeName	Target position of probe on CpG island microarray	TargetID	GeneSymbol	CpG island Description	Ratio of relative methylation (TiO_2-NP/Vehicle)	Sham group			TiO_2-H group		
						Relative methylation (Cy5/Cy3)	Cy3 signal (Input)	Cy5 signal (MeDIP)	Relative methylation (Cy5/Cy3)	Cy3 signal (Input)	Cy5 signal (MeDIP)
A_68_P24818876	chr6:125081706-125081750	NM_138747:-172	Nop2	PROMOTER	0.813	2.325	2031.38	4722.79	1.890	1686.79	3187.43
A_68_P24004046	chr5:114894276-114894320	NM_023556:-16	Mvk	DIVERGENT_PROMOTER	0.813	7.772	473.30	3678.35	6.317	381.16	2407.60
A_68_P23393397	chr4:147645490-147645534	NM_001083342:16562	Ptchd2	INSIDE	0.813	0.438	2109.40	923.43	0.356	1510.34	537.83
A_68_P23391998	chr4:147412871-147412915	NM_011929:-16	Clcn6	DIVERGENT_PROMOTER	0.813	0.544	8059.64	4381.43	0.442	5353.45	2366.74
A_68_P23264437	chr4:123395461-123395505	NM_001030274:-53	Ndufs5	PROMOTER	0.813	0.629	1279.01	803.96	0.511	1112.44	568.47
A_68_P23262655	chr4:123088845-123088889	NM_001167918:288	D830031N03Rik	INSIDE	0.813	0.238	1890.40	449.50	0.193	1460.03	282.08
A_68_P23094194	chr4:88783218-88783262	NM_024433:-33	Mtap	PROMOTER	0.813	3.940	4668.82	18397.25	3.203	3177.61	10177.18
A_68_P22663512	chr3:154259844-154259888	NM_009968:-109	Cryz	PROMOTER	0.813	0.515	903.31	464.98	0.419	678.85	284.15
A_68_P21652765	chr2:134612489-134612533	NM_001145830:611	Plcb1	INSIDE	0.813	1.947	4663.25	9080.86	1.583	3755.10	5942.89
A_68_P21576252	chr2:120435142-120435186	NM_025475:-7	Haus2	DIVERGENT_PROMOTER	0.813	2.980	683.41	2036.80	2.422	629.02	1523.29
A_68_P21338757	chr2:74520450-74520494	NM_008273:23	Hoxd11	INSIDE	0.813	2.834	1651.53	4681.09	2.305	1271.80	2930.92
A_68_P20826009	chr1:173003701-173003745	ENSMUST00000164179:-8656		PROMOTER	0.813	0.370	6463.07	2390.27	0.301	6081.83	1829.47
A_68_P31982212	chr19:16854730-16854774	NM_173028:665	Vps13a	INSIDE	0.812	0.345	1357.28	468.91	0.281	940.94	264.08
A_68_P31150844	chr17:34027540-34027584	NM_001195298:16	Kifc1	INSIDE	0.812	4.066	4870.55	19805.11	3.300	3885.41	12823.26
A_68_P30243543	chr15:57525631-57525686	NM_199449:-563	Zhx2	PROMOTER	0.812	3.100	337.52	1046.40	2.518	331.59	834.91
A_68_P29060387	chr13:56397406-56397450	NM_019568:484	Cxcl14	INSIDE	0.812	2.775	1378.24	3825.16	2.253	1056.31	2379.46
A_68_P28175417	chr11:118898832-118898876	NM_013926:3373	Cbx8	DOWNSTREAM	0.812	1.837	1931.70	3548.21	1.492	1583.96	2363.11
A_68_P27897908	chr11:69727660-69727704	NM_019726:-11	Gps2	PROMOTER	0.812	0.354	2758.04	975.69	0.287	2024.51	581.27
A_68_P27862295	chr11:63735348-63735392	NM_018805:416	Hs3st3b1	INSIDE	0.812	2.768	1732.03	4794.02	2.247	1427.07	3206.03
A_68_P27291407	chr10:81590879-81590923	NM_001163064:143	AU041133	INSIDE	0.812	0.472	4848.69	2287.77	0.383	4206.76	1612.35
A_68_P26826438	chr9:110782517-110782561	NM_146260:49	Tmie	INSIDE	0.812	0.398	2198.37	874.56	0.323	1651.69	533.73
A_68_P26812299	chr9:107888278-107888322	NM_026597:-53	6230427J02Rik	PROMOTER	0.812	78.675	897.15	70583.03	63.861	1464.12	93499.80
A_68_P26744297	chr9:95758279-95758323	NM_019864:285	Atr	INSIDE	0.812	2.500	801.90	2004.51	2.030	742.45	1506.98
A_68_P25957478	chr8:74082656-74082700	NM_001100462:92	Tmem221	INSIDE	0.812	2.333	638.07	1488.30	1.893	589.28	1115.54
A_68_P25467115	chr7:127245927-127245971	NM_145586:9	Tmem159	INSIDE	0.812	0.536	1026.77	550.50	0.435	825.99	359.41
A_68_P25368680	chr7:107755893-107755937	NM_001163663:-185	Rab6	PROMOTER	0.812	0.372	3746.66	1393.97	0.302	2751.62	831.02
A_68_P25350314	chr7:104548897-104548941	NM_024220:406	Ndufc2	INSIDE	0.812	0.317	2232.78	707.73	0.257	1809.74	465.95
A_68_P24161585	chr5:145307394-145307438	NM_016789:661	Nptx2	INSIDE	0.812	0.482	8105.61	3909.75	0.391	5189.87	2031.57
A_68_P24156268	chr5:144134918-144134962	NM_133355:9354	Grid2ip	INSIDE	0.812	0.628	2234.75	1403.80	0.510	1599.71	816.44
A_68_P23570788	chr5:30441008-30441052	NM_001167879:9295	Fam59b	INSIDE	0.812	2.619	1639.57	4293.58	2.127	1344.16	2859.53
A_68_P23248723	chr4:119705546-119705590	NM_010657:218286	Hivep3	INSIDE	0.812	3.623	1266.25	4587.75	2.943	1091.72	3213.05
A_68_P23155610	chr4:101223010-101223054	NM_001164583:-166	Dnajc6	PROMOTER	0.812	0.657	2487.75	1634.28	0.533	1953.05	1041.94
A_68_P22775355	chr4:21807011-21807055	NM_172687:211	Coq3	INSIDE	0.812	2.020	1112.10	2246.12	1.640	1023.26	1677.82
A_68_P22319356	chr3:89234182-89234226	NM_026869:-554	Pygo2	PROMOTER	0.812	0.387	1451.97	561.54	0.314	1086.97	341.51
A_68_P21904887	chr2:180324446-180324490	NM_031373:357	Ogfr	INSIDE	0.812	0.300	1862.98	559.17	0.244	1364.96	332.56
A_68_P21903063	chr2:180068623-180068667	NM_008093:740	Gata5	INSIDE	0.812	0.409	1400.33	572.48	0.332	1147.84	380.90
A_68_P21587096	chr2:122436805-122436849	NM_025961:187	Gatm	INSIDE	0.812	0.394	1287.10	506.57	0.319	1128.20	360.45
A_68_P21370052	chr2:79475902-79475946	NM_080558:343	Ssfa2	INSIDE	0.812	2.354	3417.47	8043.97	1.911	2789.99	5331.17
A_68_P32565208	chrX:98893525-98893573	ENSMUST00000073812:-6359		PROMOTER	0.811	3.392	273.90	928.96	2.752	397.13	1092.94
A_68_P32454735	chrX:68917753-68917797	NM_010340:833	Gpr50	INSIDE	0.811	3.681	752.16	2768.82	2.986	1053.37	3145.33
A_68_P31655265	chr18:42554339-42554383	NM_138945:110	Pou4f3	INSIDE	0.811	0.209	3415.42	712.38	0.169	2226.28	376.54
A_68_P31412112	chr17:86086622-86086666	NM_011380:950	Six2	INSIDE	0.811	0.362	1635.62	592.49	0.294	1151.26	338.16
A_68_P31254845	chr17:56395400-56395444	NM_010192:-793	Fem1a	PROMOTER	0.811	0.437	1885.69	824.05	0.354	1448.51	513.06
A_68_P31055442	chr17:13108620-13108664	NM_026310:315	Mrpl18	INSIDE	0.811	2.076	931.65	1934.03	1.683	768.88	1293.94
A_68_P30510888	chr16:4586584-4586628	NM_031184:-8106	Glis2	PROMOTER	0.811	0.448	1176.46	526.69	0.363	955.28	346.97
A_68_P30462677	chr15:96115709-96115753	NM_175251:-2222	Arid2	PROMOTER	0.811	1.989	956.74	1903.16	1.614	779.00	1256.93
A_68_P29585963	chr14:48740738-48740782	NM_033602:217	Peli2	INSIDE	0.811	2.085	1790.46	3733.16	1.691	1462.50	2473.20
A_68_P28073192	chr11:101163256-101163300	NM_019584:303	Becn1	INSIDE	0.811	2.409	1068.72	2574.86	1.953	740.06	1445.19
A_68_P28069997	chr11:100621341-100621385	NM_001081194:-270	Kcnh4	PROMOTER	0.811	0.476	1081.17	514.98	0.386	892.48	344.66
A_68_P27836982	chr11:59263845-59263889	NM_001205068:320	Jmjd4	INSIDE	0.811	0.642	1366.53	877.80	0.521	1140.86	594.48
A_68_P27083090	chr10:39977304-39977348	NM_026113:145	Gtf3c6	INSIDE	0.811	0.554	1992.00	1104.25	0.450	1679.49	755.31
A_68_P26846411	chr9:114690663-114690707	NM_133978:263	Cmtm7	INSIDE	0.811	0.221	3023.03	669.54	0.180	2222.23	399.25
A_68_P24124224	chr5:137507416-137507460	NM_001039385:1274	Vgf	INSIDE	0.811	11.806	2369.92	27980.36	9.575	1762.99	16880.17
A_68_P24076586	chr5:127722354-127722398	NM_175432:181	Tmem132c	INSIDE	0.811	0.460	1163.73	534.93	0.373	940.27	350.33
A_68_P23755803	chr5:65726815-65726859	NM_011258:42	Rfc1	INSIDE	0.811	0.527	1787.12	941.79	0.427	1361.35	581.51
A_68_P23579555	chr5:31999876-31999920	NM_153196:85	Rbks	INSIDE	0.811	0.676	2898.18	1959.55	0.548	2209.89	1211.45
A_68_P23547920	chr5:25035394-25035438	NR_027388:-419	1700096K18Rik	PROMOTER	0.811	0.455	3847.72	1752.45	0.369	3076.35	1136.33
A_68_P23334046	chr4:136018440-136018484	NM_024452:-7213	Luzp1	PROMOTER	0.811	3.868	666.56	2578.53	3.138	574.06	1801.42
A_68_P23328082	chr4:134908937-134908981	NM_001130477:171	Srrm1	INSIDE	0.811	0.531	2999.28	1591.40	0.430	2077.22	893.56
A_68_P23317933	chr4:133053507-133053559	NM_001081156:933	Trnp1	INSIDE	0.811	0.514	2497.13	1283.35	0.417	1714.56	714.60
A_68_P22360091	chr3:98231564-98231608	NM_172863:45183	Zfp697	INSIDE	0.811	1.763	8771.95	15465.17	1.429	5889.70	8418.37
A_68_P22321218	chr3:89519511-89519555	NM_001038587:589	Adar	INSIDE	0.811	0.528	1618.47	855.05	0.429	1206.53	517.08
A_68_P22049391	chr3:34549338-34549382	NM_011443:434	Sox2	INSIDE	0.811	5.037	633.16	3188.97	4.085	503.26	2056.00
A_68_P21076600	chr2:25996203-25996247	ENSMUST00000133808:-183		PROMOTER	0.811	9.842	913.90	8994.53	7.984	758.74	6057.40
A_68_P20827968	chr1:173368006-173368050	NM_172647:337	F11r	INSIDE	0.811	0.360	2282.97	821.73	0.292	1726.28	503.68

ProbeName	Target position of probe on CpG island microarray	TargetID	GeneSymbol	CpG island Description	Ratio of relative methylation (TiO_2-NP/Vehicle)	Sham group			TiO_2-H group		
						Relative methylation (Cy5/Cy3)	Cy3 signal (Input)	Cy5 signal (MeDIP)	Relative methylation (Cy5/Cy3)	Cy3 signal (Input)	Cy5 signal (MeDIP)
A_68_P32707352	chrX:138740429-138740473	NM_021487:291	Kcne1l	INSIDE	0.810	2.018	812.40	1639.26	1.634	1044.54	1706.59
A_68_P32054917	chr19:29880262-29880306	NM_172836:215	9930021J03Rik	INSIDE	0.810	10.198	1249.77	12745.02	8.263	1114.69	9210.34
A_68_P31919590	chr19:3852379-3852423	NM_001025566:628	Chka	INSIDE	0.810	0.344	1924.65	661.80	0.279	1447.38	403.27
A_68_P31117691	chr17:27792800-27792847	NM_011861:197	Pacsin1	INSIDE	0.810	0.390	1520.10	593.06	0.316	1086.85	343.61
A_68_P30572948	chr16:17277681-17277725	NM_177473:1310	Tmem191c	INSIDE	0.810	0.437	1935.33	845.73	0.354	1426.64	505.16
A_68_P30546660	chr16:11176366-11176410	NM_145931:98	Zc3h7a	INSIDE	0.810	0.318	1740.77	553.08	0.257	1360.44	350.21
A_68_P28098742	chr11:105829222-105829266	NM_207624:-14	Ace	PROMOTER	0.810	0.604	3592.56	2168.46	0.489	2200.07	1075.92
A_68_P27932452	chr11:76308042-76308086	NM_198018:14857	Abr	INSIDE	0.810	0.720	2051.31	1477.39	0.583	1582.51	922.80
A_68_P27371449	chr10:96079420-96079468	NM_007569:-190	Btg1	PROMOTER	0.810	0.605	1887.20	1142.49	0.491	1274.80	625.41
A_68_P26898892	chr10:3134865-3134909	NM_172546:583	Cnksr3	INSIDE	0.810	0.195	2859.70	556.46	0.158	2026.45	319.34
A_68_P26814194	chr9:108208208-108208252	NM_133986:52	Tcta	INSIDE	0.810	2.194	978.37	2146.24	1.776	822.41	1460.86
A_68_P26486124	chr9:47338813-47338857	NM_001025600:400	Cadm1	INSIDE	0.810	0.507	1950.59	988.12	0.410	1593.96	653.91
A_68_P26232175	chr8:124793420-124793464	ENSMUST00000055537:-26		PROMOTER	0.810	0.500	2128.23	1064.61	0.405	1603.08	649.17
A_68_P25370479	chr7:108079749-108079793	NM_001081116:905	Arhgef17	INSIDE	0.810	0.595	2160.15	1286.22	0.483	1646.79	794.60
A_68_P25239813	chr7:82600787-82600831	NM_029332:389	Akap13	INSIDE	0.810	0.457	2309.61	1054.40	0.370	2072.35	766.59
A_68_P25181444	chr7:72301072-72301116	NM_183087:320	Fam189a1	INSIDE	0.810	1.875	1439.03	2698.79	1.519	1250.82	1900.40
A_68_P24816527	chr6:124661976-124662020	NM_013536:198	Emg1	INSIDE	0.810	0.561	1827.88	1025.22	0.454	1325.02	602.09
A_68_P24810878	chr6:122824602-122824646	NM_026267:50	Necap1	INSIDE	0.810	0.430	1471.21	632.97	0.349	1265.10	441.11
A_68_P23429340	chr4:153516608-153516652	NM_021499:150	Wdr8	INSIDE	0.810	2.989	1986.73	5939.02	2.422	1729.62	4188.92
A_68_P23345846	chr4:138011165-138011209	NM_025451:124	Camk2n1	INSIDE	0.810	0.594	1850.10	1099.04	0.481	1217.80	586.11
A_68_P23237068	chr4:117559980-117560024	NM_033617:-68	Atp6v0b	PROMOTER	0.810	0.405	3706.82	1500.59	0.328	2754.88	903.70
A_68_P22949803	chr4:58218825-58218869	NM_022814:622	Svep1	INSIDE	0.810	4.240	604.60	2563.43	3.434	478.98	1644.74
A_68_P22412539	chr3:108218569-108218613	NM_001004177:-178	Celsr2	PROMOTER	0.810	2.277	1571.56	3579.22	1.845	1429.32	2637.54
A_68_P21796693	chr2:160556776-160556820	NM_021280:-247	Plcg1	PROMOTER	0.810	0.566	2713.65	1537.19	0.459	2057.16	943.84
A_68_P21395111	chr2:84712344-84712388	NM_199223:14483	Rtn4rl2	INSIDE	0.810	2.559	1134.03	2902.01	2.072	941.46	1950.71
A_68_P21134665	chr2:35477392-35477436	NM_001114124:-86	Dab2ip	PROMOTER	0.810	0.671	1582.41	1062.05	0.543	1137.54	618.09
A_68_P21082674	chr2:26997916-26997960	NM_175427:59	Fam163b	INSIDE	0.810	2.253	1213.61	2734.77	1.824	903.31	1648.03
A_68_P32119292	chr19:42222487-42222531	NM_183195:630	Marveld1	INSIDE	0.809	0.524	6056.17	3171.93	0.424	3936.58	1668.43
A_68_P31948839	chr19:10257741-10257785	NM_146094:385	Fads1	INSIDE	0.809	0.266	2861.35	760.73	0.215	1873.14	402.90
A_68_P31940131	chr19:7569296-7569340	NM_146091:391	Atl3	INSIDE	0.809	4.218	445.21	1878.05	3.414	364.91	1245.69
A_68_P30588851	chr16:20672864-20672908	NM_001005331:65	Eif4g1	INSIDE	0.809	0.473	2736.60	1293.84	0.383	2023.69	774.12
A_68_P30501254	chr15:102821408-102821452	NM_010466:461	Hoxc8	INSIDE	0.809	0.475	1142.47	542.28	0.384	955.28	366.86
A_68_P27987614	chr11:86014616-86014660	NM_178309:57	Brip1	INSIDE	0.809	0.571	2421.22	1383.14	0.462	1914.64	885.11
A_68_P27929274	chr11:75779968-75780012			Unknown	0.809	0.498	2463.99	1227.58	0.403	1755.45	707.66
A_68_P26610634	chr9:69245687-69245731	NM_145618:-96	Narg2	PROMOTER	0.809	5.357	441.27	2363.82	4.332	386.65	1675.00
A_68_P26365992	chr9:25060005-25060049	NM_001205367:-13	Septin7	PROMOTER	0.809	2.418	1257.38	3039.87	1.955	1150.95	2250.18
A_68_P26024229	chr8:87823886-87823930	NM_001163791:378	Orc6	INSIDE	0.809	5.749	1172.58	6741.14	4.648	954.37	4436.38
A_68_P25596358	chr7:149845220-149845264	NR_002855:-355	Igf2as	DIVERGENT_PROMOTER	0.809	4.144	1346.51	5580.30	3.352	1136.83	3810.82
A_68_P25587941	chr7:148478189-148478233	NM_007878:306	Drd4	INSIDE	0.809	0.144	3865.27	556.42	0.116	2617.54	304.94
A_68_P25027724	chr7:30303544-30303588	ENSMUST00000141713:-657		PROMOTER	0.809	2.493	1533.45	3823.18	2.018	1308.21	2639.49
A_68_P25005690	chr7:25379019-25379063	NM_001033205:-6380	Zfp575	PROMOTER	0.809	0.599	1371.62	821.02	0.484	1056.42	511.26
A_68_P22649654	chr3:151928897-151928941	NM_199465:366	Nexn	INSIDE	0.809	1.987	1532.49	3044.48	1.607	1238.49	1990.44
A_68_P22350096	chr3:96397949-96397993	NM_027241:111	Polr3gl	INSIDE	0.809	0.407	2708.28	1102.28	0.329	1956.28	643.85
A_68_P21974616	chr3:19210797-19210841	NM_001122759:504	Pde7a	INSIDE	0.809	0.542	2860.33	1549.47	0.438	2141.60	938.61
A_68_P21566801	chr2:118728573-118728617	NM_001045523:1244	Bahd1	INSIDE	0.809	0.430	1082.50	465.82	0.348	962.67	334.94
A_68_P21151178	chr2:38780768-38780812	NM_010264:1191	Nr6a1	INSIDE	0.809	0.303	3938.77	1193.86	0.245	2685.42	658.70
A_68_P20294242	chr1:64781496-64781540	NM_001042659:2806	Fzd5	INSIDE	0.809	3.501	804.94	2818.09	2.834	665.59	1886.16
A_68_P20264185	chr1:59539105-59539149	NM_008057:136	Fzd7	INSIDE	0.809	0.498	2161.22	1076.17	0.403	1633.94	658.08
A_68_P20053920	chr1:16095231-16095275	NM_133832:-710	Rdh10	DIVERGENT_PROMOTER	0.809	0.586	1435.80	841.48	0.474	1335.86	633.11
A_68_P32565204	chrX:98893057-98893101	ENSMUST00000073812:-5889		PROMOTER	0.808	11.243	4072.10	45782.95	9.084	5857.77	53213.88
A_68_P31833165	chr18:74938885-74938929	NM_177470:41	Acaa2	INSIDE	0.808	0.151	3483.59	527.20	0.122	2462.35	301.14
A_68_P31619430	chr18:35813598-35813642	NM_027222:-4599	2010001M09Rik	PROMOTER	0.808	3.300	336.25	1109.75	2.666	327.52	873.02
A_68_P30482738	chr15:99705731-99705775	NM_001113545:135	Lima1	INSIDE	0.808	8.927	2579.67	23028.31	7.210	2103.76	15167.35
A_68_P27263124	chr10:76077361-76077405	NM_146007:8767	Col6a2	INSIDE	0.808	1.943	1190.90	2313.34	1.570	985.33	1546.49
A_68_P25957036	chr8:74005119-74005163	NM_001164679:4826	Ano8	INSIDE	0.808	1.933	2077.49	4016.52	1.562	1319.76	2061.43
A_68_P25777210	chr8:36028138-36028182	NM_175091:584	Tnks	INSIDE	0.808	0.332	3665.14	1216.78	0.268	2586.68	693.57
A_68_P25090596	chr7:52262461-52262505	NM_001008422:9137	Scaf1	INSIDE	0.808	2.108	1083.34	2283.43	1.703	746.47	1271.36
A_68_P24951825	chr7:5008460-5008504	NM_146178:155	Ccdc106	INSIDE	0.808	3.080	1976.46	6087.01	2.487	1423.32	3540.29
A_68_P24838924	chr6:128374739-128374783	NM_133927:168	Itfg2	INSIDE	0.808	2.328	1002.81	2334.45	1.881	802.99	1510.70
A_68_P24834768	chr6:127718939-127718983	NM_201371:-213	Prmt8	PROMOTER	0.808	0.509	1522.21	775.28	0.411	1269.92	522.31
A_68_P24051800	chr5:123157735-123157779	NM_011026:191	P2rx4	INSIDE	0.808	0.393	1297.22	510.18	0.318	1053.08	334.62
A_68_P22317672	chr3:88981542-88981586	NM_011886:158	Scamp3	INSIDE	0.808	0.695	1231.87	855.61	0.561	1014.34	569.42
A_68_P21900709	chr2:179711291-179711335	NM_001081092:39	Taf4a	INSIDE	0.808	0.472	1069.05	504.35	0.381	833.29	317.72
A_68_P21628393	chr2:130004733-130004777	NM_009225:346	Snrpb	INSIDE	0.808	0.565	1599.24	904.36	0.457	1391.20	635.49
A_68_P32693896	chrX:135892996-135893040	ENSMUST00000122380:12		INSIDE	0.807	2.850	364.03	1037.60	2.300	481.72	1107.79

ProbeName	Target position of probe on CpG island microarray	TargetID	GeneSymbol	CpG island Description	Ratio of relative methylation (TiO_2-NP/Vehicle)	Sham group			TiO_2-H group		
						Relative methylation (Cy5/Cy3)	Cy3 signal (Input)	Cy5 signal (MeDIP)	Relative methylation (Cy5/Cy3)	Cy3 signal (Input)	Cy5 signal (MeDIP)
A_68_P32292135	chrX:20937971-20938015	NM_198633:2	Gm5124	INSIDE	0.807	0.705	1127.59	794.81	0.569	1473.05	837.76
A_68_P32141864	chr19:46131303-46131347	NM_001081214:296	Pprc1	INSIDE	0.807	0.284	3665.96	1041.79	0.229	2580.19	591.86
A_68_P31866357	chr18:80910750-80910798	NM_001164110:-964	Nfatc1	PROMOTER	0.807	0.357	2144.00	766.37	0.288	1486.35	428.52
A_68_P31784505	chr18:66161948-66161992	NM_027400:319	Lman1	INSIDE	0.807	0.593	1370.00	812.95	0.479	1186.12	567.68
A_68_P31599640	chr18:31964175-31964219	NM_001170966:486	Wdr33	INSIDE	0.807	0.399	4065.35	1623.65	0.322	3149.08	1014.92
A_68_P31559057	chr18:24363978-24364022	NM_001160404:-494	Galnt1	PROMOTER	0.807	2.207	893.72	1972.03	1.781	698.09	1243.34
A_68_P31092275	chr17:23684173-23684217	NM_001033496:16999	Zfp213	DOWNSTREAM	0.807	0.489	1507.51	737.20	0.395	1172.53	462.81
A_68_P30927978	chr16:85172880-85172924	NM_001198823:1050	App	INSIDE	0.807	4.626	1167.91	5402.60	3.731	960.86	3584.97
A_68_P29228005	chr13:93962476-93962520	NM_133905:-260	Papd4	PROMOTER	0.807	2.585	1757.50	4543.37	2.085	1568.11	3270.08
A_68_P29024152	chr13:49343166-49343210	NM_025491:344	Susd3	INSIDE	0.807	3.250	1715.51	5575.46	2.622	1416.02	3713.23
A_68_P28563529	chr12:79328194-79328238	NM_145965:575	Gphn	INSIDE	0.807	0.557	1123.74	625.38	0.449	991.48	445.21
A_68_P28140559	chr11:113062735-113062779	NR_015556:396	2610035D17Rik	INSIDE	0.807	1.971	3073.77	6058.32	1.592	2612.30	4157.56
A_68_P28068577	chr11:100388430-100388474	NM_001199296:862	Acly	INSIDE	0.807	0.568	994.04	564.93	0.459	866.36	397.23
A_68_P27096527	chr10:42481255-42481299	NM_153055:-25	Sec63	PROMOTER	0.807	0.522	1395.94	729.10	0.422	1183.91	499.20
A_68_P26804665	chr9:106588204-106588248	NM_011573:-228	Tex264	PROMOTER	0.807	0.264	3433.90	906.62	0.213	2465.94	525.31
A_68_P26078524	chr8:97126087-97126131	NM_026274:268	Rspry1	INSIDE	0.807	0.585	1537.58	899.61	0.472	1268.69	598.77
A_68_P26038241	chr8:90467953-90467997	NM_033327:15520	Zfp423	INSIDE	0.807	0.678	3031.56	2055.24	0.547	2413.72	1321.35
A_68_P25807722	chr8:41947794-41947838	NM_001044740:70	Slc7a2	INSIDE	0.807	1.969	4419.68	8702.33	1.588	3590.86	5702.63
A_68_P25475333	chr7:128850755-128850799	NM_001033173:-9	Usp31	PROMOTER	0.807	2.600	568.10	1477.12	2.098	583.62	1224.52
A_68_P25030929	chr7:31099110-31099154	NM_198027:5361	Alkbh6	INSIDE	0.807	3.151	934.80	2945.39	2.543	842.46	2142.14
A_68_P24674229	chr6:97566903-97566947	NM_145148:727	Frmd4b	INSIDE	0.807	0.696	1612.51	1122.57	0.562	1206.88	678.30
A_68_P23562832	chr5:28785049-28785093	NR_015562:-8453	9530036O11Rik	PROMOTER	0.807	2.459	1067.67	2625.75	1.985	926.20	1838.79
A_68_P23439553	chr4:154976002-154976046	NM_177186:500	Slc35e2	INSIDE	0.807	2.891	591.60	1710.44	2.334	502.10	1171.85
A_68_P23200235	chr4:108952879-108952923	NM_007943:21	Eps15	INSIDE	0.807	2.185	3227.93	7052.16	1.763	2423.69	4272.59
A_68_P22373051	chr3:100881021-100881065	NM_011197:33047	Ptgfrn	INSIDE	0.807	2.351	1395.10	3280.28	1.896	1018.92	1932.24
A_68_P21105433	chr2:30679802-30679847	NM_133346:3996	Asb6	INSIDE	0.807	2.326	2551.06	5933.00	1.876	1825.29	3424.32
A_68_P20753946	chr1:160292678-160292722	NM_001205204:266	Astn1	INSIDE	0.807	5.588	693.99	3877.69	4.507	573.80	2586.01
A_68_P32226230	chr19:60656973-60657017	NM_001172096:-68	2700078E11Rik	PROMOTER	0.806	0.512	2938.43	1505.04	0.413	2253.22	930.76
A_68_P31497270	chr18:11998017-11998061	NM_001146287:256	Cables1	INSIDE	0.806	2.460	948.29	2332.51	1.984	831.03	1648.43
A_68_P31322349	chr17:69506595-69506639	NM_013813:467	Epb4.1l3	INSIDE	0.806	3.114	1180.12	3675.38	2.511	917.02	2302.74
A_68_P30489504	chr15:100908526-100908570	AK078004:-921		PROMOTER	0.806	2.261	4102.24	9274.44	1.821	3000.82	5465.93
A_68_P29638534	chr14:60059619-60059663	NM_001081024:74	Setdb2	INSIDE	0.806	0.689	1749.03	1205.78	0.556	1407.51	782.35
A_68_P29603744	chr14:52533018-52533062	NM_001145959:123	Ndrg2	INSIDE	0.806	0.237	3148.06	745.58	0.191	2521.16	481.20
A_68_P28224326	chr12:8779101-8779145	NM_011519:921	Sdc1	INSIDE	0.806	0.439	1126.21	493.84	0.354	899.58	318.12
A_68_P28084858	chr11:103222146-103222190	NM_001205236:2838	Arhgap27	INSIDE	0.806	0.506	1235.35	625.38	0.408	899.20	367.00
A_68_P27068095	chr10:36857141-36857185	NM_008538:1570	Marcks	INSIDE	0.806	1.907	2852.31	5439.19	1.537	2418.68	3718.09
A_68_P25784753	chr8:37312019-37312063	NM_001081150:530	Lonrf1	INSIDE	0.806	2.358	547.48	1291.14	1.901	489.93	931.42
A_68_P25578896	chr7:146768160-146768204	NR_027857:514	Nkx6-2	INSIDE	0.806	0.505	1142.79	577.66	0.407	903.98	368.37
A_68_P24222134	chr6:7643189-7643233	NM_012055:-28	Asns	PROMOTER	0.806	0.250	2656.77	665.28	0.202	1975.58	398.63
A_68_P23447646	chr5:3928274-3928318	NM_194462:111	Akap9	INSIDE	0.806	3.375	1010.18	3408.87	2.719	884.05	2404.01
A_68_P22317130	chr3:88894534-88894578	NM_001083808:-603	Rusc1	PROMOTER	0.806	0.587	1957.25	1148.70	0.473	1561.99	738.88
A_68_P21099540	chr2:29658503-29658547	NM_011989:325	Slc27a4	INSIDE	0.806	0.741	2844.83	2109.22	0.597	2192.63	1310.09
A_68_P21079211	chr2:26358740-26358784	NM_008714:580	Notch1	INSIDE	0.806	0.511	1071.04	546.77	0.411	823.17	338.51
A_68_P21050002	chr2:20441328-20441372	NM_001177630:211	Etl4	INSIDE	0.806	0.634	1420.85	900.67	0.511	1132.82	578.61
A_68_P20451402	chr1:94728781-94728825	NM_016696:540	Gpc1	INSIDE	0.806	0.158	3688.53	582.34	0.127	3020.91	384.46
A_68_P32552603	chrX:96133526-96133570	NM_207633:429	Yipf6	INSIDE	0.805	4.218	609.86	2572.34	3.396	693.87	2356.69
A_68_P32481891	chrX:75829099-75829143	NM_001164275:1	Prrg1	INSIDE	0.805	2.079	621.07	1290.95	1.674	863.94	1446.01
A_68_P31623418	chr18:36508869-36508913	NM_001081365:613	0610010O12Rik	INSIDE	0.805	0.374	1610.06	602.79	0.301	1227.93	370.14
A_68_P31292460	chr17:64213972-64214016	ENSMUST00000112829:631		INSIDE	0.805	2.782	1590.04	4423.75	2.238	1333.65	2985.36
A_68_P30998363	chr16:97578192-97578236	NM_019517:-120	Bace2	PROMOTER	0.805	2.243	626.85	1406.25	1.805	577.99	1043.22
A_68_P30836894	chr16:67620955-67620999	NM_001145977:177	Cadm2	INSIDE	0.805	2.255	3635.26	8195.98	1.815	2859.45	5191.26
A_68_P29020698	chr13:48761248-48761292	NM_007526:2866	Barx1	INSIDE	0.805	0.519	1478.42	767.65	0.418	1145.07	478.72
A_68_P28553601	chr12:77471721-77471765	NM_178744:490	Zbtb1	INSIDE	0.805	0.571	920.68	526.15	0.460	801.58	368.67
A_68_P28052669	chr11:97643459-97643503	NR_028078:-400	Snora21	PROMOTER	0.805	0.426	3572.66	1521.09	0.343	2501.95	857.95
A_68_P27975235	chr11:83881690-83881734	NM_019819:147	Dusp14	INSIDE	0.805	3.283	1372.67	4505.85	2.641	1205.67	3184.57
A_68_P27489857	chr10:118677199-118677243	NM_027994:-109	Cand1	PROMOTER	0.805	0.597	1821.08	1087.49	0.481	1592.72	766.01
A_68_P27283560	chr10:79958022-79958066	NM_139226:394	Onecut3	INSIDE	0.805	0.317	3312.47	1051.66	0.255	2341.63	598.25
A_68_P25669975	chr8:13677504-13677548	NM_009025:61	Rasa3	INSIDE	0.805	0.669	2068.70	1384.65	0.539	1535.40	827.61
A_68_P25600929	chr7:150624243-150624287			Unknown	0.805	5.843	714.45	4174.28	4.703	645.49	3035.85
A_68_P24992867	chr7:19946629-19946673	NM_001010836:86	Ppp1r13l	INSIDE	0.805	1.972	2692.91	5311.67	1.587	2234.13	3545.49
A_68_P24830751	chr6:127059602-127059646	NM_177003:-54	9630033F20Rik	PROMOTER	0.805	0.427	1770.76	756.36	0.344	1372.95	471.98
A_68_P23696203	chr5:53060910-53060954	NM_172490:8	Sepsecs	INSIDE	0.805	0.656	1971.47	1292.40	0.528	1495.04	788.72
A_68_P23560504	chr5:28397844-28397888	NM_153526:-85	Insig1	PROMOTER	0.805	0.526	1337.72	704.10	0.424	995.85	422.00
A_68_P22829978	chr4:34137124-34137168	NM_026293:-104	Spaca1	PROMOTER	0.805	2.464	1530.90	3771.40	1.983	1188.91	2357.46
A_68_P22037673	chr3:32264387-32264431	NR_027966:-2	4930429B21Rik	PROMOTER	0.805	0.430	4849.61	2085.86	0.346	3654.50	1265.27

ProbeName	Target position of probe on CpG island microarray	TargetID	GeneSymbol	CpG island Description	Ratio of relative methylation (TiO_2-NP/Vehicle)	Sham group			TiO_2-H group		
						Relative methylation (Cy5/Cy3)	Cy3 signal (Input)	Cy5 signal (MeDIP)	Relative methylation (Cy5/Cy3)	Cy3 signal (Input)	Cy5 signal (MeDIP)
A_68_P21425958	chr2:91848432-91848476	NM_011957:15873	Creb3l1	INSIDE	0.805	0.414	1595.62	660.58	0.333	1185.69	395.10
A_68_P20427308	chr1:90967670-90967714	NM_133816:656	Sh3bp4	INSIDE	0.805	0.289	2857.22	826.52	0.233	2234.23	520.20
A_68_P20410046	chr1:87961433-87961477	NM_027357:261	Psmd1	INSIDE	0.805	1.917	1239.41	2375.56	1.543	1113.79	1718.74
A_68_P32048646	chr19:28752584-28752628	NM_175459:1961	Glis3	INSIDE	0.804	0.525	1847.17	969.71	0.422	1415.35	597.47
A_68_P31331958	chr17:71351874-71351918	NM_026064:-23	2900073G15Rik	PROMOTER	0.804	2.243	933.01	2092.78	1.803	875.62	1578.54
A_68_P31329271	chr17:70865401-70865445	NM_001128180:-6027	Dlgap1	PROMOTER	0.804	2.650	1884.12	4991.98	2.131	1482.93	3160.03
A_68_P30486394	chr15:100382394-100382438	NM_033476:-38	Tcfcp2	PROMOTER	0.804	0.221	2783.12	614.24	0.177	1956.99	347.31
A_68_P28517705	chr12:71087547-71087591	NM_022028:421	Sav1	INSIDE	0.804	0.439	4051.83	1779.50	0.353	2588.82	914.04
A_68_P28038651	chr11:95275101-95275145	NM_025287:-274	Spop	PROMOTER	0.804	0.407	3029.96	1234.09	0.327	2475.60	810.22
A_68_P27466138	chr10:114239189-114239233	NM_146241:-784	Trhde	PROMOTER	0.804	3.103	1197.66	3715.78	2.493	1052.64	2624.21
A_68_P27277402	chr10:78996606-78996650	NM_015817:-96	Ppap2c	PROMOTER	0.804	0.409	9249.62	3782.87	0.329	6480.61	2130.39
A_68_P26640510	chr9:74697051-74697095	ENSMUST00000160842:271		INSIDE	0.804	1.853	1562.04	2894.66	1.490	1324.56	1973.64
A_68_P26578203	chr9:63605811-63605855	NM_016769:-31	Smad3	PROMOTER	0.804	0.247	3205.89	792.61	0.199	2233.51	443.89
A_68_P25500166	chr7:133521032-133521076	NM_023712:394	Spns1	INSIDE	0.804	0.437	1020.74	446.57	0.352	871.80	306.56
A_68_P24052512	chr5:123300221-123300265	NM_030564:46	Rnf34	INSIDE	0.804	0.610	1047.41	639.38	0.491	858.85	421.27
A_68_P22081218	chr3:40549756-40549800	NM_011020:244	Hspa4l	INSIDE	0.804	4.904	992.72	4867.99	3.941	776.69	3060.72
A_68_P21902498	chr2:179992383-179992427	NM_025587:321	Rps21	INSIDE	0.804	0.557	1722.67	959.35	0.448	1374.74	615.59
A_68_P21768927	chr2:155833982-155834026	NM_025516:144	Ergic3	INSIDE	0.804	0.589	1789.49	1054.36	0.474	1457.70	690.46
A_68_P21103867	chr2:30400786-30400830	ENSMUST00000145413:10560		DOWNSTREAM	0.804	0.563	1301.36	732.87	0.453	1044.65	473.01
A_68_P20189687	chr1:43610615-43610671	NM_010879:108047	Nck2	INSIDE	0.804	4.450	306.13	1362.19	3.575	279.43	999.09
A_68_P30594366	chr16:21794327-21794371	NR_037957:-71	1300002E11Rik	DIVERGENT_PROMOTER	0.803	0.718	1783.67	1280.22	0.576	1361.07	783.97
A_68_P30575508	chr16:17759767-17759811	NM_145479:75	Klhl22	INSIDE	0.803	0.694	1153.28	800.13	0.557	1026.50	572.04
A_68_P29733543	chr14:76814437-76814481	NM_207652:-1169	Tsc22d1	PROMOTER	0.803	2.017	1657.51	3343.41	1.620	1390.50	2252.60
A_68_P28679270	chr12:101203619-101203663	NM_001164426:932	Kcnk13	INSIDE	0.803	0.629	2081.35	1309.43	0.505	1523.61	769.84
A_68_P28156837	chr11:115873664-115873708	NM_016905:347	Galk1	INSIDE	0.803	0.289	1969.34	569.29	0.232	1465.39	340.15
A_68_P27933408	chr11:76486478-76486522	NM_177182:529	Bhlha9	INSIDE	0.803	0.589	1828.71	1077.02	0.473	1440.13	681.03
A_68_P27852923	chr11:62062610-62062654	NM_007413:147	Adora2b	INSIDE	0.803	0.682	1436.92	979.32	0.547	1168.97	639.53
A_68_P27287690	chr10:80607523-80607567	NM_010731:8529	Zbtb7a	INSIDE	0.803	2.903	1705.61	4950.65	2.329	1340.15	3121.87
A_68_P27273029	chr10:77707518-77707562	NM_001081055:-153	Trappc10	PROMOTER	0.803	3.002	654.00	1963.60	2.412	638.69	1540.36
A_68_P27254396	chr10:74524768-74524812	NM_001081412:1150	Bcr	INSIDE	0.803	0.424	1243.86	527.44	0.340	989.04	336.65
A_68_P26219857	chr8:122923797-122923841	ENSMUST00000127664:489078		INSIDE	0.803	0.505	1410.24	712.40	0.406	1111.40	450.82
A_68_P25598325	chr7:150238789-150238833	NM_133655:156	Cd81	INSIDE	0.803	2.102	1347.87	2832.90	1.688	1112.90	1878.16
A_68_P24951862	chr7:5012967-5013011	NM_001205231:-756	U2af2	PROMOTER	0.803	0.496	5637.77	2793.56	0.398	3818.17	1519.82
A_68_P24830597	chr6:127030465-127030509	NM_022657:7567	Fgf23	INSIDE	0.803	0.327	3643.55	1191.78	0.263	2780.76	730.66
A_68_P24511776	chr6:65331444-65331488	NM_175524:179	C130060K24Rik	INSIDE	0.803	0.594	1510.19	896.92	0.477	1037.34	495.01
A_68_P23609897	chr5:37221748-37221792	NM_008550:118	Man2b2	INSIDE	0.803	3.634	489.35	1778.51	2.920	460.82	1345.68
A_68_P23198683	chr4:108673036-108673080	NM_146150:-351	Nrd1	PROMOTER	0.803	0.265	4685.08	1243.59	0.213	3275.79	698.04
A_68_P22878716	chr4:44725913-44725957	NM_008782:-2622	Pax5	PROMOTER	0.803	0.229	1904.19	437.01	0.184	1428.12	263.24
A_68_P22590673	chr3:142058155-142058199	NM_001190857:-3362	Pdlim5	PROMOTER	0.803	0.631	1215.82	766.68	0.506	991.66	502.17
A_68_P22407674	chr3:107434065-107434109	NM_153563:542	Fam40a	INSIDE	0.803	0.434	1707.01	740.71	0.349	1156.56	403.13
A_68_P22291852	chr3:84386166-84386211	NM_001081093:359	Arfip1	INSIDE	0.803	2.777	2083.76	5786.94	2.229	1657.32	3694.64
A_68_P31620628	chr18:36032998-36033042			Unknown	0.802	0.665	1117.71	742.99	0.533	912.09	486.41
A_68_P31256052	chr17:56568252-56568296	NM_011218:47629	Ptprs	INSIDE	0.802	2.498	754.21	1884.22	2.004	614.91	1232.44
A_68_P31108555	chr17:26275483-26275527	NM_001159598:-126	Axin1	PROMOTER	0.802	0.265	1853.53	490.70	0.212	1378.47	292.61
A_68_P30485728	chr15:100253426-100253470	NM_008732:38	Slc11a2	INSIDE	0.802	0.367	2998.42	1101.21	0.294	2120.30	624.33
A_68_P29940447	chr14:119104917-119104961	NM_001033336:503	Abcc4	INSIDE	0.802	0.322	1899.23	611.41	0.258	1423.54	367.38
A_68_P29011119	chr13:47110041-47110085	NM_175340:157	Nhlrc1	INSIDE	0.802	0.384	6252.93	2400.71	0.308	4453.79	1371.66
A_68_P28751777	chr12:113917151-113917195	NM_001100460:122	Zbtb42	INSIDE	0.802	2.333	1078.13	2515.25	1.870	913.87	1708.86
A_68_P28703312	chr12:105685478-105685522			Unknown	0.802	6.115	1481.40	9058.50	4.904	1224.62	6005.15
A_68_P28697529	chr12:104664017-104664061	NM_001159502:-48	Ddx24	PROMOTER	0.802	2.518	976.95	2460.14	2.020	792.72	1601.30
A_68_P28470862	chr12:60320683-60320727	NM_001033156:-234	Fbxo33	PROMOTER	0.802	0.586	831.80	487.09	0.470	717.99	337.20
A_68_P28042484	chr11:95985813-95985857	NM_172799:-9265	Ttll6	PROMOTER	0.802	2.912	2069.61	6027.39	2.336	1568.83	3664.96
A_68_P27980351	chr11:84729571-84729616	NM_001005223:265	Znhit3	INSIDE	0.802	0.370	2896.03	1072.60	0.297	1957.82	581.73
A_68_P27940746	chr11:77796693-77796737	NM_174852:397	Phf12	INSIDE	0.802	2.303	953.27	2195.70	1.846	841.49	1553.62
A_68_P27534325	chr10:126602946-126602990	NM_027739:82	B4galnt1	INSIDE	0.802	0.246	2329.07	572.31	0.197	1898.45	374.22
A_68_P27278456	chr10:79179900-79179944	NM_008226:544	Hcn2	INSIDE	0.802	2.086	933.67	1947.98	1.674	764.19	1279.03
A_68_P27265507	chr10:76495860-76495904	NM_031196:-74	Slc19a1	PROMOTER	0.802	14.978	3655.61	54752.41	12.009	3660.23	43955.40
A_68_P26766658	chr9:99776462-99776506	NM_011440:105	Sox14	INSIDE	0.802	0.521	1549.92	807.47	0.418	1254.53	524.23
A_68_P26348586	chr9:21528050-21528094	NM_010700:35	Ldlr	INSIDE	0.802	3.017	1110.01	3349.17	2.421	916.31	2218.54
A_68_P25958363	chr8:74217342-74217386	NM_028189:8335	B3gnt3	INSIDE	0.802	2.103	1662.38	3495.33	1.687	1391.34	2346.99
A_68_P25657379	chr8:11758150-11758194	NM_001113518:-157	Arhgef7	PROMOTER	0.802	0.285	5731.76	1635.24	0.229	4266.12	976.46
A_68_P25528246	chr7:138553835-138553879	NM_175115:136	Ikzf5	INSIDE	0.802	0.477	7204.00	3436.72	0.383	4838.75	1851.65
A_68_P25499299	chr7:133343490-133343534	NM_028816:410	Xpo6	INSIDE	0.802	0.535	3897.32	2085.70	0.429	2750.02	1180.01
A_68_P24089926	chr5:130090512-130090556	NM_011846:446	Mmp17	INSIDE	0.802	0.269	2174.38	584.98	0.216	1677.69	361.88
A_68_P23842571	chr5:82223744-82223788	NM_198702:773149	Lphn3	INSIDE	0.802	2.744	1944.02	5335.33	2.201	1362.34	2998.29

ProbeName	Target position of probe on CpG island microarray	TargetID	GeneSymbol	CpG island Description	Ratio of relative methylation (TiO_2-NP/Vehicle)	Sham group			TiO_2-H group		
						Relative methylation (Cy5/Cy3)	Cy3 signal (Input)	Cy5 signal (MeDIP)	Relative methylation (Cy5/Cy3)	Cy3 signal (Input)	Cy5 signal (MeDIP)
A_68_P23397205	chr4:148265453-148265497	NM_027195:86974	Casz1	INSIDE	0.802	0.674	1660.62	1119.23	0.540	1183.39	639.59
A_68_P22417416	chr3:109143379-109143423	NM_020505:-200	Vav3	PROMOTER	0.802	2.173	705.55	1532.82	1.742	592.52	1032.04
A_68_P22289191	chr3:83959735-83959779	NM_029797:-48	Mnd1	PROMOTER	0.802	19.064	5672.62	108140.30	15.284	5457.73	83415.73
A_68_P21833061	chr2:166821891-166821935	NM_001109905:-134	Stau1	PROMOTER	0.802	0.454	2167.36	984.01	0.364	1667.97	607.20
A_68_P21438173	chr2:93816579-93816623	NM_026944:34287	Alkbh3	DOWNSTREAM	0.802	0.432	1412.08	610.20	0.346	947.28	328.18
A_68_P20977503	chr2:6050985-6051029	NM_001159657:225	5430407P10Rik	INSIDE	0.802	0.416	2410.48	1002.43	0.333	1742.63	581.12
A_68_P20826056	chr1:173016015-173016059	BC100368:10398		DOWNSTREAM	0.802	0.629	1610.75	1013.44	0.505	1191.18	600.96
A_68_P32095546	chr19:37508728-37508772	NM_008245:-580	Hhex	PROMOTER	0.801	0.607	1012.91	614.37	0.486	921.19	447.80
A_68_P31980262	chr19:16511319-16511363	NM_008137:1184	Gna14	INSIDE	0.801	0.233	3187.74	742.78	0.187	2238.42	417.67
A_68_P30822063	chr16:64851412-64851456	NM_178647:-476	Cggbp1	PROMOTER	0.801	0.368	1694.87	624.54	0.295	1426.04	420.92
A_68_P30575819	chr16:17807668-17807712	NM_153790:10316	Scarf2	INSIDE	0.801	0.254	3132.72	795.56	0.203	2523.86	513.34
A_68_P28091896	chr11:104469667-104469711	NM_016780:375	Itgb3	INSIDE	0.801	1.909	2870.56	5479.55	1.530	2198.84	3364.08
A_68_P28043336	chr11:96132754-96132798	NM_008270:133	Hoxb9	INSIDE	0.801	2.822	1438.58	4059.60	2.259	1275.63	2881.84
A_68_P27989555	chr11:86358098-86358142	NM_001114334:189	Rps6kb1	INSIDE	0.801	0.432	1129.66	488.13	0.346	888.17	307.54
A_68_P27922135	chr11:74536870-74536914	NR_037610:468	Pafah1b1	INSIDE	0.801	1.973	2365.78	4667.06	1.580	1905.45	3010.52
A_68_P27844938	chr11:60624796-60624840	NM_009171:-51	Shmt1	PROMOTER	0.801	0.348	3890.94	1355.45	0.279	3355.92	936.04
A_68_P27564953	chr11:5688605-5688649	NM_001146308:141	Dbnl	INSIDE	0.801	0.386	2152.26	831.42	0.309	1572.10	486.32
A_68_P26866698	chr9:118416014-118416060	NM_018748:601	Golga4	INSIDE	0.801	20.556	2477.02	50918.47	16.469	2332.98	38421.09
A_68_P26761427	chr9:98856071-98856115	NM_012020:67	Foxl2	INSIDE	0.801	4.222	1331.08	5619.38	3.383	1097.78	3714.07
A_68_P26508624	chr9:51087195-51087239	NM_001195681:-557	Gm684	PROMOTER	0.801	0.499	2308.19	1152.34	0.400	1760.20	703.79
A_68_P26426681	chr9:36575026-36575070	NM_008408:115	Stt3a	INSIDE	0.801	0.400	4835.90	1932.15	0.320	3711.63	1188.31
A_68_P25736102	chr8:28134323-28134367	NM_153592:14	Erlin2	INSIDE	0.801	0.597	2472.24	1474.99	0.478	1937.01	925.43
A_68_P25661161	chr8:12385043-12385087	NR_027975:-706	Gm5607	PROMOTER	0.801	2.072	828.90	1717.47	1.660	782.54	1298.89
A_68_P24372708	chr6:37392303-37392347	NM_178661:-176	Creb3l2	PROMOTER	0.801	2.475	1040.78	2576.13	1.982	867.63	1719.26
A_68_P23523568	chr5:20457296-20457340	NM_001080977:322	Rsbn1l	INSIDE	0.801	0.456	1886.04	860.37	0.365	1558.78	569.57
A_68_P23237283	chr4:117587647-117587691	NM_146152:-64	Ipo13	PROMOTER	0.801	0.671	1786.20	1197.75	0.537	1473.47	791.43
A_68_P23186779	chr4:106512308-106512352	ENSMUST00000151369:8195		DOWNSTREAM	0.801	0.284	1735.22	492.33	0.227	1240.06	281.84
A_68_P21516956	chr2:109563795-109563839	NM_001048142:30097	Bdnf	INSIDE	0.801	2.431	1244.86	3025.81	1.946	964.34	1876.53
A_68_P20005797	chr1:4482373-4482417	NM_011441:4100	Sox17	INSIDE	0.801	1.761	1828.40	3219.32	1.410	1523.02	2147.55
A_68_P32023817	chr19:24551554-24551598	NM_026520:388	Fam122a	INSIDE	0.800	0.640	1000.52	640.08	0.512	846.85	433.55
A_68_P31204667	chr17:46168826-46168870	NM_001110266:-6736	Vegfa	PROMOTER	0.800	0.269	1793.11	483.17	0.216	1385.25	298.70
A_68_P29138631	chr13:73398065-73398109	NM_018885:142	Irx4	INSIDE	0.800	2.568	623.35	1600.83	2.055	569.85	1171.21
A_68_P28755250	chr12:114427183-114427227	NM_153776:3091	Tmem121	INSIDE	0.800	4.518	1415.09	6393.45	3.614	1165.25	4211.27
A_68_P28153959	chr11:115375052-115375096	NM_008258:610	Hn1	INSIDE	0.800	0.343	2477.28	849.44	0.274	1813.51	497.25
A_68_P28047527	chr11:96777858-96777902	NM_030248:-85	Cdk5rap3	PROMOTER	0.800	15.617	1772.33	27678.02	12.496	1917.19	23956.79
A_68_P27551127	chr11:3190518-3190562	NM_019574:81	Patz1	INSIDE	0.800	0.386	1371.31	529.28	0.309	1068.50	330.00
A_68_P27469304	chr10:114822217-114822261	NM_001033261:224	Zfc3h1	INSIDE	0.800	0.566	2177.15	1233.07	0.453	1845.10	835.81
A_68_P27282240	chr10:79755289-79755333	NM_009091:135	Rps15	INSIDE	0.800	0.451	1628.29	733.79	0.360	1199.02	432.15
A_68_P26797102	chr9:105297505-105297549	NM_172461:91	Nek11	INSIDE	0.800	0.644	1578.16	1015.58	0.515	1261.14	649.20
A_68_P26630338	chr9:72772983-72773027	NM_028116:-452	Pygo1	PROMOTER	0.800	0.287	2902.83	832.71	0.229	2210.88	507.31
A_68_P26495380	chr9:48863833-48863877	NM_012039:169	Zw10	INSIDE	0.800	0.687	2601.90	1788.60	0.550	2105.60	1158.40
A_68_P24658383	chr6:94649752-94649796	NM_008377:365	Lrig1	INSIDE	0.800	4.540	496.96	2256.22	3.632	438.93	1594.25
A_68_P23881742	chr5:90652641-90652685	NM_001033310:359	Cox18	INSIDE	0.800	3.724	1311.53	4883.78	2.977	1012.66	3015.04
A_68_P23767825	chr5:67818729-67818773	NM_001164806:288	Bend4	INSIDE	0.800	2.566	1133.00	2907.56	2.054	832.94	1710.60
A_68_P23416915	chr4:151534101-151534145	NM_008236:1147	Hes2	INSIDE	0.800	0.542	1382.25	749.23	0.434	1139.64	494.20
A_68_P23403478	chr4:149328400-149328444	NM_029035:693	Spsb1	INSIDE	0.800	0.662	1158.97	766.95	0.529	923.21	488.45
A_68_P23299680	chr4:129684126-129684170	NM_001199696:21827	Bai2	INSIDE	0.800	3.935	604.46	2378.37	3.149	508.13	1599.90
A_68_P22856643	chr4:40226344-40226388	NM_001033305:35	Ndufb6	INSIDE	0.800	0.650	1289.66	838.01	0.520	1038.65	540.02
A_68_P22716232	chr4:8679096-8679140	NM_001081417:61051	Chd7	INSIDE	0.800	1.783	2660.04	4741.98	1.426	2018.62	2879.30
A_68_P22509722	chr3:127335595-127335641	NM_009718:-444	Neurog2	PROMOTER	0.800	3.036	449.77	1365.34	2.429	487.07	1183.22
A_68_P22401915	chr3:106285595-106285639	NM_001093754:268	Dennd2d	INSIDE	0.800	0.705	2136.55	1506.06	0.564	1743.15	983.18
A_68_P22311355	chr3:87827371-87827415	NM_022031:4041	Hapln2	INSIDE	0.800	0.288	2726.23	786.22	0.231	1953.99	450.79
A_68_P21812431	chr2:163264311-163264355	NM_144891:130	Gdap1l1	INSIDE	0.800	0.525	1421.43	746.29	0.420	1171.04	492.14
A_68_P20638621	chr1:137210401-137210445	NM_011590:-108	Timm17a	PROMOTER	0.800	0.164	4684.70	769.44	0.131	3110.00	408.77
A_68_P20264182	chr1:59538765-59538809	NM_008057:-204	Fzd7	PROMOTER	0.800	0.592	1132.04	670.73	0.474	905.73	429.51
A_68_P33016565	chr7:3424475-3424519	NM_133183:-926	Cacng6	PROMOTER	0.799	0.457	1498.02	685.08	0.365	1143.54	417.70
A_68_P30566359	chr16:15637698-15637742	NM_008565:-227	Mcm4	DIVERGENT_PROMOTER	0.799	0.578	1193.20	689.53	0.462	945.75	436.59
A_68_P29531323	chr14:35487371-35487415	NM_001004436:279	Wapal	INSIDE	0.799	0.700	1484.15	1039.27	0.560	1238.84	693.20
A_68_P28831284	chr13:12350487-12350531	NM_001081128:-241	Mtr	PROMOTER	0.799	2.025	1473.20	2982.57	1.618	1245.92	2016.16
A_68_P28192101	chr11:121564092-121564136	NM_144797:374	Metrnl	INSIDE	0.799	0.619	1153.44	713.60	0.494	943.98	466.73
A_68_P28043444	chr11:96145749-96145793	NM_010461:2552	Hoxb8	INSIDE	0.799	0.230	3713.71	855.25	0.184	2793.07	514.13
A_68_P27980115	chr11:84693687-84693731	NM_027388:-11	Pigw	DIVERGENT_PROMOTER	0.799	7.759	4264.20	33084.47	6.197	3589.07	22241.39
A_68_P27910151	chr11:72020571-72020617	NM_026559:-461	Txndc17	DIVERGENT_PROMOTER	0.799	2.958	548.88	1623.79	2.365	414.23	979.58
A_68_P27897812	chr11:69715753-69715797	NM_001013414:395	Neurl4	INSIDE	0.799	0.487	1631.81	794.13	0.389	1242.87	483.15
A_68_P27835022	chr11:58949099-58949143	NM_001171512:757	Obscn	INSIDE	0.799	0.569	1054.19	599.45	0.455	772.78	351.23

ProbeName	Target position of probe on CpG island microarray	TargetID	GeneSymbol	CpG island Description	Ratio of relative methylation (TiO_2-NP/Vehicle)	Sham group			TiO_2-H group		
						Relative methylation (Cy5/Cy3)	Cy3 signal (Input)	Cy5 signal (MeDIP)	Relative methylation (Cy5/Cy3)	Cy3 signal (Input)	Cy5 signal (MeDIP)
A_68_P27288015	chr10:80655147-80655191	NM_007828:8506	Dapk3	INSIDE	0.799	2.464	698.25	1720.33	1.968	579.38	1140.23
A_68_P27099063	chr10:42894061-42894105	NM_175407:254	Sobp	INSIDE	0.799	0.232	7749.98	1794.79	0.185	5258.65	972.46
A_68_P26538539	chr9:56644557-56644601	AK155716:4490		DOWNSTREAM	0.799	2.570	4043.79	10394.50	2.054	3109.73	6388.52
A_68_P25952177	chr8:73012515-73012559	NM_182991:-1279	Tmem59l	DIVERGENT_PROMOTER	0.799	0.652	1501.66	979.29	0.521	1164.08	606.59
A_68_P25310385	chr7:97089281-97089328	NM_026304:391	I7Rn6	INSIDE	0.799	0.167	3030.28	505.59	0.133	2251.39	300.17
A_68_P25091593	chr7:52430145-52430189	NM_182993:10876	Slc17a7	INSIDE	0.799	0.432	1316.11	568.61	0.345	969.59	334.90
A_68_P24798988	chr6:120481338-120481382	NM_144815:-43	Cecr5	PROMOTER	0.799	1.806	4881.97	8818.13	1.443	3654.89	5274.65
A_68_P24053676	chr5:123507830-123507874	ENSMUST00000119611:-8398		PROMOTER	0.799	0.474	1219.16	578.11	0.379	953.21	361.28
A_68_P21580767	chr2:121239698-121239742	NM_007952:83	Pdia3	INSIDE	0.799	0.346	2078.08	720.04	0.277	1617.12	447.55
A_68_P21106325	chr2:30837610-30837654	NM_028846:-166	Usp20	DIVERGENT_PROMOTER	0.799	0.115	8427.89	969.68	0.092	5106.74	469.37
A_68_P21037814	chr2:17950160-17950204	NR_028378:1307	LOC100034739	INSIDE	0.799	0.570	1824.97	1040.76	0.455	1588.99	723.61
A_68_P20935177	chr1:192752566-192752610	NM_021421:205	Angel2	INSIDE	0.799	0.731	2240.13	1637.08	0.584	1718.12	1003.14
A_68_P20723961	chr1:154802478-154802522	NM_175460:270	Nmnat2	INSIDE	0.799	0.706	1216.79	859.38	0.565	910.18	513.86
A_68_P20351509	chr1:74997858-74997902	NM_010544:345	Ihh	INSIDE	0.799	0.151	5245.50	791.31	0.121	3768.23	454.46
A_68_P32563072	chrX:98449525-98449570	NM_018789:-319	Foxo4	PROMOTER	0.798	4.449	1230.22	5473.28	3.548	1304.69	4629.17
A_68_P31789933	chr18:67248434-67248478	NM_177137:467	Gnal	INSIDE	0.798	0.725	1937.22	1404.04	0.579	1471.46	851.25
A_68_P31412095	chr17:86084743-86084787	NM_011380:2830	Six2	INSIDE	0.798	0.485	1616.60	784.61	0.387	1262.46	488.97
A_68_P30778267	chr16:55895439-55895483	NM_001134494:-62	Fam55c	PROMOTER	0.798	0.659	2011.42	1326.26	0.526	1683.94	885.75
A_68_P30433474	chr15:90880547-90880591	NM_001109040:-189	Kif21a	PROMOTER	0.798	0.450	1276.42	574.31	0.359	991.47	355.87
A_68_P30082782	chr15:25643699-25643743	NM_019472:91416	Myo10	INSIDE	0.798	0.528	3616.44	1908.36	0.421	2653.85	1116.97
A_68_P29841642	chr14:99498547-99498591	NM_029454:-83	Pibf1	PROMOTER	0.798	0.279	1835.70	511.82	0.223	1402.75	312.13
A_68_P29624049	chr14:57287454-57287498	NM_023773:392	Mphosph8	INSIDE	0.798	0.506	3965.63	2005.42	0.403	2955.85	1192.10
A_68_P29102506	chr13:65358717-65358761	ENSMUST00000099425:-2108		PROMOTER	0.798	2.726	818.49	2231.32	2.175	708.49	1540.70
A_68_P29059430	chr13:56236999-56237043	NM_001159513:-109	H2afy	PROMOTER	0.798	4.732	742.10	3511.74	3.775	615.30	2322.56
A_68_P28871499	chr13:21272176-21272220	NM_009054:371	Trim27	INSIDE	0.798	3.462	572.71	1982.93	2.763	505.00	1395.24
A_68_P28001993	chr11:88580214-88580258	NR_038058:280	C030037D09Rik	INSIDE	0.798	2.655	1810.10	4805.47	2.119	1344.77	2849.66
A_68_P27385040	chr10:98569917-98569961	NM_015737:-830	Galnt4	PROMOTER	0.798	2.187	1204.04	2633.41	1.745	1051.01	1833.61
A_68_P27212535	chr10:67011075-67011119	NM_001005419:607	Ado	INSIDE	0.798	2.024	857.39	1735.40	1.615	711.96	1149.94
A_68_P26591019	chr9:65917414-65917458	NM_001025612:102	Snx22	INSIDE	0.798	0.400	1578.09	631.20	0.319	1255.20	400.68
A_68_P26465230	chr9:43829019-43829063			Unknown	0.798	5.989	5209.88	31200.84	4.780	3616.09	17283.22
A_68_P25599905	chr7:150482316-150482360	NR_001461:114	Kcnq1ot1	INSIDE	0.798	0.633	6590.66	4170.33	0.505	4550.56	2298.44
A_68_P25419719	chr7:118742491-118742535	NM_173739:180979	Galntl4	INSIDE	0.798	2.643	766.39	2025.93	2.109	673.96	1421.37
A_68_P24993485	chr7:20055259-20055303	ENSMUST00000083454:-8996		PROMOTER	0.798	0.736	2232.64	1644.23	0.588	1701.23	999.64
A_68_P24993424	chr7:20043542-20043586	NM_172279:279	Mark4	INSIDE	0.798	0.252	5265.06	1324.51	0.201	3981.43	799.52
A_68_P24687704	chr6:99643282-99643326	NM_008158:632	Gpr27	INSIDE	0.798	4.216	2072.40	8737.04	3.363	1662.19	5590.03
A_68_P23566425	chr5:29522442-29522486	NM_021470:-2081	Rnf32	PROMOTER	0.798	2.233	710.75	1587.34	1.782	619.16	1103.15
A_68_P23254538	chr4:120690017-120690061	NM_133716:-186	Smap2	PROMOTER	0.798	7.330	469.67	3442.46	5.849	347.86	2034.55
A_68_P23091882	chr4:88368088-88368132	NM_172871:302	Klhl9	INSIDE	0.798	0.353	2051.40	724.81	0.282	1642.45	463.11
A_68_P22346028	chr3:95659528-95659572	NM_027541:126	Prpf3	INSIDE	0.798	0.376	2608.07	979.45	0.300	1961.87	587.59
A_68_P21819739	chr2:164611647-164611691	NM_133924:148	Snx21	INSIDE	0.798	2.634	528.44	1391.78	2.101	510.63	1072.74
A_68_P21742437	chr2:150865129-150865173	NM_026086:-35	Nanp	PROMOTER	0.798	0.315	1635.99	514.56	0.251	1187.95	298.34
A_68_P21111932	chr2:31742894-31742938	NM_011836:116	Lamc3	INSIDE	0.798	0.352	2594.42	912.46	0.281	1878.61	527.20
A_68_P21078498	chr2:26264595-26264639	NM_033134:92	Inpp5e	INSIDE	0.798	0.642	916.34	588.72	0.513	831.64	426.24
A_68_P20886156	chr1:184271169-184271213	NM_175127:547	Fbxo28	INSIDE	0.798	4.037	743.73	3002.11	3.222	603.61	1944.98
A_68_P20723681	chr1:154749520-154749564	NM_001005507:234	Smg7	INSIDE	0.798	2.293	3065.52	7029.73	1.829	2750.12	5030.75
A_68_P20233542	chr1:53841734-53841778	NM_133810:303	Stk17b	INSIDE	0.798	0.316	2110.32	666.43	0.252	1740.00	438.51
A_68_P32537730	chrX:91423475-91423519			Unknown	0.797	0.273	1963.45	536.90	0.218	2776.40	605.04
A_68_P31834169	chr18:75121056-75121100	NM_010720:-161	Lipg	PROMOTER	0.797	2.158	2868.06	6188.90	1.720	2274.49	3912.31
A_68_P31731695	chr18:56867197-56867241	NM_010721:-248	Lmnb1	PROMOTER	0.797	3.738	2053.57	7677.04	2.981	1701.92	5073.42
A_68_P31654581	chr18:42435029-42435074	NM_172626:45	Rbm27	INSIDE	0.797	2.403	556.51	1337.18	1.915	477.80	915.21
A_68_P31644531	chr18:40378658-40378702	NM_023311:373	Yipf5	INSIDE	0.797	0.288	2131.09	614.14	0.230	1648.83	378.57
A_68_P31260731	A_68_P31260731			Unknown	0.797	0.346	1376.48	476.57	0.276	1319.88	364.29
A_68_P31253476	chr17:56176213-56176257	NM_013664:-235	Sh3gl1	DIVERGENT_PROMOTER	0.797	0.619	1348.77	835.10	0.494	1023.88	505.47
A_68_P30720005	chr16:44173127-44173171	NM_001029889:-361	Gm608	PROMOTER	0.797	0.526	2638.08	1388.13	0.419	2110.88	885.41
A_68_P29871957	chr14:104921836-104921880	NM_026047:25	Rnf219	INSIDE	0.797	3.830	2023.16	7747.83	3.051	1556.67	4749.83
A_68_P28683723	chr12:101962977-101963021	NM_153587:240	Rps6ka5	INSIDE	0.797	0.656	1301.02	854.05	0.523	1110.47	581.12
A_68_P28449511	chr12:55963765-55963809	NM_007688:78	Cfl2	INSIDE	0.797	0.268	2157.94	578.04	0.213	1497.65	319.64
A_68_P28109102	chr11:107655644-107655688	NM_019431:112	Cacng4	INSIDE	0.797	0.518	1940.97	1005.55	0.413	1405.62	580.12
A_68_P28099140	chr11:105881863-105881907	NM_001037712:12368	Kcnh6	INSIDE	0.797	10.247	2220.22	22750.58	8.171	2306.62	18847.48
A_68_P28011630	chr11:90279544-90279588	AK139414:-6564		PROMOTER	0.797	2.120	1333.40	2826.53	1.689	1099.86	1857.96
A_68_P27968803	chr11:82684639-82684683	NM_001164570:-414	Rffl	PROMOTER	0.797	0.616	6491.26	4001.79	0.491	5101.60	2505.61
A_68_P26986429	chr10:20067652-20067696	NM_027930:50	Fam54a	INSIDE	0.797	0.612	1015.70	621.63	0.488	833.11	406.63
A_68_P26572465	chr9:62659122-62659166	NM_010193:311	Fem1b	INSIDE	0.797	1.920	3036.35	5829.38	1.531	2411.05	3690.95
A_68_P26085826	chr8:98412091-98412135	NM_010325:153	Got2	INSIDE	0.797	0.552	1102.15	608.08	0.440	917.32	403.17
A_68_P25944440	chr8:71258901-71258945	NM_172753:119	Csgalnact1	INSIDE	0.797	0.393	2435.18	956.09	0.313	1874.75	586.91

ProbeName	Target position of probe on CpG island microarray	TargetID	GeneSymbol	CpG island Description	Ratio of relative methylation (TiO_2-NP/Vehicle)	Sham group			TiO_2-H group		
						Relative methylation (Cy5/Cy3)	Cy3 signal (Input)	Cy5 signal (MeDIP)	Relative methylation (Cy5/Cy3)	Cy3 signal (Input)	Cy5 signal (MeDIP)
A_68_P25232267	chr7:81158422-81158466	NM_009181:124	St8sia2	INSIDE	0.797	6.353	523.51	3325.70	5.061	498.76	2524.36
A_68_P25008268	chr7:25866633-25866677	NM_175477:4390	Zfp574	INSIDE	0.797	2.493	1653.60	4122.39	1.986	1347.73	2676.99
A_68_P25006993	chr7:25655058-25655102	NM_027732:5	Dmrtc2	INSIDE	0.797	0.562	1316.32	740.32	0.448	1105.60	495.78
A_68_P24993428	chr7:20044025-20044069	NM_172279:-203	Mark4	PROMOTER	0.797	0.323	1556.66	502.97	0.257	1374.21	353.81
A_68_P24868563	chr6:135683062-135683116	NM_008171:440441	Grin2b	INSIDE	0.797	2.460	1322.33	3253.52	1.960	958.18	1878.13
A_68_P24511777	chr6:65331562-65331606	NM_175524:297	C130060K24Rik	INSIDE	0.797	0.540	1661.06	896.33	0.430	1203.94	518.09
A_68_P23981859	chr5:111209498-111209542	NM_001025561:95	Pus1	INSIDE	0.797	2.518	3633.99	9150.21	2.007	2859.44	5737.49
A_68_P23618267	chr5:38611613-38611657	NM_029162:33	Zbtb49	INSIDE	0.797	0.552	1754.67	967.80	0.439	1257.42	552.62
A_68_P23297928	chr4:129349890-129349934	NM_008468:99	Kpna6	INSIDE	0.797	0.619	3148.59	1947.89	0.493	2412.82	1189.09
A_68_P23278183	chr4:125781499-125781543	NM_001145827:320	Stk40	INSIDE	0.797	0.660	1263.28	833.51	0.526	985.47	518.20
A_68_P23200903	chr4:109079476-109079520	NM_153392:-206	Ttc39a	PROMOTER	0.797	0.512	1688.30	864.32	0.408	1334.76	544.37
A_68_P22350765	chr3:96512213-96512257	NM_001162925:249	Nudt17	INSIDE	0.797	0.241	2082.14	502.32	0.192	1586.05	304.99
A_68_P22291860	chr3:84387008-84387052	NM_001081093:-483	Arfip1	PROMOTER	0.797	0.453	1976.18	895.57	0.361	1491.92	538.65
A_68_P22165657	chr3:57651863-57651907	NM_019410:-205	Pfn2	PROMOTER	0.797	0.644	2128.74	1370.26	0.513	1827.34	937.27
A_68_P21428085	chr2:92215603-92215647	NM_145122:229	Pex16	INSIDE	0.797	1.922	1674.61	3218.71	1.531	1396.82	2138.57
A_68_P21127431	chr2:34262562-34262606	ENSMUST00000147337:-118		PROMOTER	0.797	2.506	2552.72	6397.39	1.998	2089.51	4175.80
A_68_P20874334	chr1:182126153-182126197	NM_023341:-23	Adck3	PROMOTER	0.797	2.518	1158.86	2917.46	2.007	901.53	1809.59
A_68_P20601163	chr1:130488109-130488153	NM_009911:746	Cxcr4	INSIDE	0.797	0.601	1306.62	784.97	0.479	1127.24	539.45
A_68_P31632687	chr18:38369690-38369734	NM_029357:-296	Pcdh1	PROMOTER	0.796	0.535	2074.30	1109.62	0.426	1549.58	659.50
A_68_P31112796	chr17:26987048-26987092	NM_008700:-8560	Nkx2-5	PROMOTER	0.796	0.217	2486.10	538.66	0.173	1771.08	305.54
A_68_P31100626	chr17:25097776-25097820	NM_198937:-230	Hn1l	PROMOTER	0.796	0.714	2400.51	1713.18	0.568	1930.46	1096.77
A_68_P31099197	chr17:24856900-24856944	NR_027900:-12	Snhg9	DIVERGENT_PROMOTER	0.796	0.611	2848.11	1739.93	0.486	2146.65	1044.00
A_68_P30422878	chr15:88898359-88898403	NM_001002005:8225	Panx2	INSIDE	0.796	2.273	1120.60	2547.08	1.808	846.42	1530.66
A_68_P30346594	chr15:76009406-76009450	NM_201394:16712	Plec	INSIDE	0.796	4.088	1126.22	4604.54	3.253	980.62	3189.92
A_68_P29071664	chr13:58228883-58228927	NM_029872:1013	Hnrnpa0	INSIDE	0.796	4.558	2756.39	12564.54	3.627	1993.59	7231.28
A_68_P27923258	chr11:74739141-74739185	NM_001163311:138	Srr	INSIDE	0.796	0.706	2323.72	1639.47	0.562	1854.66	1041.97
A_68_P27289088	chr10:80820220-80820264	NM_028657:-72	F630110N24Rik	DIVERGENT_PROMOTER	0.796	2.724	596.90	1625.93	2.167	543.48	1177.96
A_68_P26365996	chr9:25060486-25060530	NM_001205367:469	Septin7	INSIDE	0.796	0.513	1525.01	782.31	0.409	1162.50	474.94
A_68_P25898919	chr8:61391256-61391300	NM_173411:727	BC030500	INSIDE	0.796	0.507	4777.09	2420.86	0.403	3080.07	1241.89
A_68_P25589002	chr7:148650716-148650760	NM_001025103:3746	Efcab4a	INSIDE	0.796	0.506	1763.11	892.32	0.403	1458.85	587.80
A_68_P25063517	chr7:38557044-38557088	NM_172385:-2295	Zfp536	PROMOTER	0.796	0.487	1425.70	694.94	0.388	1069.81	414.86
A_68_P25021470	chr7:29157527-29157571	NM_001083912:133	Plekhg2	INSIDE	0.796	0.336	2260.81	760.11	0.268	1787.23	478.34
A_68_P25007551	chr7:25740203-25740247			Unknown	0.796	0.214	2860.92	611.04	0.170	2252.99	382.85
A_68_P24215193	chr6:6166905-6166949	NM_001177572:247	Slc25a13	INSIDE	0.796	0.378	1220.87	461.85	0.301	993.19	299.07
A_68_P24161575	chr5:145306253-145306297	NM_016789:-481	Nptx2	PROMOTER	0.796	2.142	785.07	1681.51	1.705	646.71	1102.94
A_68_P23921377	chr5:99466430-99466474	NM_008926:-354	Prkg2	PROMOTER	0.796	0.239	3300.30	787.30	0.190	2258.20	428.60
A_68_P23246060	chr4:119212433-119212477	NM_172699:162	Foxj3	INSIDE	0.796	0.716	1286.81	920.71	0.569	1084.19	617.10
A_68_P21834185	chr2:167015628-167015672	NM_008420:-1351	Kcnb1	PROMOTER	0.796	2.053	934.05	1917.58	1.634	793.63	1296.39
A_68_P20599489	chr1:130140741-130140785	NM_026390:5	Ubxn4	INSIDE	0.796	2.171	1020.28	2214.55	1.728	857.69	1482.17
A_68_P32576287	chrX:101396366-101396410	NM_001077354:68	C77370	INSIDE	0.795	4.756	524.20	2492.99	3.779	590.38	2231.17
A_68_P32552601	chrX:96133272-96133316	NM_207633:175	Yipf6	INSIDE	0.795	2.912	937.23	2729.49	2.316	1100.35	2548.06
A_68_P32071017	chr19:32670770-32670814	NM_001201470:588	Papss2	INSIDE	0.795	0.510	1032.70	526.46	0.405	785.68	318.25
A_68_P31150132	chr17:33822462-33822506	NM_001109913:-81	Hnrnpm	PROMOTER	0.795	4.362	1889.71	8242.12	3.469	1459.37	5061.88
A_68_P31106620	chr17:25974088-25974132	NM_144816:-38	Rhbdl1	PROMOTER	0.795	3.146	2876.71	9049.68	2.502	2106.58	5270.30
A_68_P31100997	chr17:25150808-25150852	NM_020608:1345	Cramp1l	INSIDE	0.795	0.360	3818.06	1375.13	0.286	2837.01	812.04
A_68_P30386712	chr15:82952941-82952985	NM_029101:269	Rrp7a	INSIDE	0.795	0.577	1113.47	641.94	0.458	969.30	444.21
A_68_P30370814	chr15:80042595-80042639	NM_010795:38466	Mgat3	INSIDE	0.795	4.975	1278.58	6361.25	3.953	942.97	3727.84
A_68_P29681786	chr14:67486350-67486394	NM_009955:1065	Dpysl2	INSIDE	0.795	0.338	5134.39	1737.67	0.269	3611.26	971.10
A_68_P29656173	chr14:63174404-63174448	ENSMUST00000162987:-147		PROMOTER	0.795	2.363	6755.01	15965.39	1.879	4703.61	8837.53
A_68_P28751784	chr12:113917844-113917888	NM_001100460:816	Zbtb42	INSIDE	0.795	0.324	2109.41	684.42	0.258	1465.85	377.92
A_68_P28698040	chr12:104770943-104770987	NM_028980:190	Ppp4r4	INSIDE	0.795	0.277	2906.51	806.44	0.220	2327.47	513.07
A_68_P28137855	chr11:112646610-112646655	NM_011448:3109	Sox9	INSIDE	0.795	0.328	5886.03	1928.28	0.261	3689.97	961.37
A_68_P28083252	chr11:102941595-102941639	NM_152813:21356	Plcd3	INSIDE	0.795	1.977	1034.94	2046.10	1.572	829.81	1304.76
A_68_P27355934	chr10:93296766-93296810	NM_183199:2489	Usp44	INSIDE	0.795	0.526	2135.11	1123.41	0.418	1727.88	723.00
A_68_P27354277	chr10:93002683-93002727	NM_027908:74	Amdhd1	INSIDE	0.795	3.642	1118.26	4072.51	2.894	817.02	2364.54
A_68_P27188599	chr10:62409837-62409881	NM_177372:82	Dna2	INSIDE	0.795	1.997	1404.33	2804.95	1.587	1226.95	1947.05
A_68_P26783274	chr9:102910908-102910952	NM_033314:112	Slco2a1	INSIDE	0.795	0.565	1994.75	1127.83	0.449	1578.27	709.30
A_68_P26345743	chr9:20966106-20966150	NM_183408:-4029	Pde4a	PROMOTER	0.795	2.434	953.74	2321.20	1.936	803.81	1555.93
A_68_P25962056	chr8:75072887-75072931	NM_027485:-699	Med26	PROMOTER	0.795	4.319	325.79	1407.09	3.433	301.12	1033.62
A_68_P25777211	chr8:36028290-36028334	NM_175091:432	Tnks	INSIDE	0.795	4.882	830.78	4056.05	3.879	647.15	2510.28
A_68_P25581955	chr7:147234797-147234841	NM_010836:169	Msx3	INSIDE	0.795	0.482	1419.56	683.69	0.383	1115.51	427.07
A_68_P25504795	chr7:134398033-134398077	NM_177362:10015	Zfp771	INSIDE	0.795	3.258	962.41	3136.00	2.591	806.40	2089.36
A_68_P25375734	chr7:108986819-108986865	NM_010567:-102	Inppl1	PROMOTER	0.795	2.448	943.66	2309.84	1.947	841.35	1638.09
A_68_P25263207	chr7:86938148-86938192	NM_008588:306	Mesp1	INSIDE	0.795	4.224	1163.40	4913.97	3.360	1193.74	4010.38
A_68_P24382227	chr6:39206680-39206724	ENSMUST00000158797:9239		DOWNSTREAM	0.795	0.342	4726.04	1617.82	0.272	3334.60	907.77

ProbeName	Target position of probe on CpG island microarray	TargetID	GeneSymbol	CpG island Description	Ratio of relative methylation (TiO_2-NP/Vehicle)	Sham group			TiO_2-H group		
						Relative methylation (Cy5/Cy3)	Cy3 signal (Input)	Cy5 signal (MeDIP)	Relative methylation (Cy5/Cy3)	Cy3 signal (Input)	Cy5 signal (MeDIP)
A_68_P23403483	chr4:149328931-149328975	NM_029035:163	Spsb1	INSIDE	0.795	0.731	3722.42	2721.71	0.581	2785.38	1618.67
A_68_P22869409	chr4:43013406-43013450	NM_009503:-49	Vcp	PROMOTER	0.795	0.507	2165.30	1098.05	0.403	1550.44	625.42
A_68_P21094143	chr2:28771470-28771514	NM_019446:449	Barhl1	INSIDE	0.795	0.469	1994.12	935.70	0.373	1565.58	584.37
A_68_P31936816	chr19:7015638-7015682	NM_001177360:23	Gpr137	INSIDE	0.794	0.469	1352.37	633.63	0.372	1132.37	421.14
A_68_P31254846	chr17:56395500-56395544	NM_010192:-693	Fem1a	PROMOTER	0.794	0.507	2263.36	1147.58	0.402	1477.35	594.61
A_68_P30356221	chr15:77671902-77671946	NM_022410:621	Myh9	INSIDE	0.794	0.477	1663.19	793.29	0.379	1326.98	502.39
A_68_P30348812	chr15:76342113-76342157	NM_010046:114	Dgat1	INSIDE	0.794	0.422	2467.99	1040.87	0.335	1694.34	567.56
A_68_P30290901	chr15:66117980-66118024	NM_152923:-216	Kcnq3	PROMOTER	0.794	0.601	995.79	598.45	0.477	876.03	418.24
A_68_P29619205	chr14:56290728-56290772	NM_008683:-7	Nedd8	DIVERGENT_PROMOTER	0.794	0.456	1477.91	674.31	0.362	1002.05	363.00
A_68_P29134818	chr13:72768061-72768105	NM_010574:1657	Irx2	INSIDE	0.794	0.689	1268.04	873.46	0.547	1000.69	547.49
A_68_P28729768	chr12:109996385-109996429			Unknown	0.794	0.718	1661.13	1192.91	0.570	1202.23	685.69
A_68_P28577489	chr12:81861416-81861460	NM_001008423:264	Gm1568	INSIDE	0.794	3.196	1636.25	5229.48	2.539	1336.09	3392.08
A_68_P27889188	chr11:68213490-68213534	ENSMUST00000108674:813		INSIDE	0.794	2.250	1436.35	3231.53	1.786	1195.43	2135.53
A_68_P27280131	chr10:79440436-79440480	NM_001003949:6617	ORF61	INSIDE	0.794	4.545	2470.91	11231.24	3.610	1690.28	6101.34
A_68_P26022784	chr8:87469901-87469945	NM_144929:-967	Rtbdn	DIVERGENT_PROMOTER	0.794	0.650	1230.17	800.03	0.516	1032.49	532.92
A_68_P26020979	chr8:87186333-87186377	NM_010499:397	Ier2	INSIDE	0.794	0.227	1952.83	443.37	0.180	1518.57	273.79
A_68_P24993482	chr7:20054901-20054945	ENSMUST00000083454:-8638		PROMOTER	0.794	0.488	978.35	477.44	0.388	679.10	263.29
A_68_P24591487	chr6:83059326-83059370	NM_026744:247	Mrpl53	INSIDE	0.794	2.733	902.72	2466.80	2.170	690.43	1498.27
A_68_P23977819	chr5:110536642-110536688	NM_145147:552	Gtpbp6	INSIDE	0.794	0.285	7380.62	2103.78	0.226	4697.56	1062.65
A_68_P23271503	chr4:124667328-124667372	NM_138683:3677	Rspo1	INSIDE	0.794	0.177	3728.22	658.30	0.140	2387.81	334.87
A_68_P22717330	chr4:8838562-8838606			Unknown	0.794	0.187	4248.78	796.61	0.149	3127.73	465.41
A_68_P21774535	chr2:156817578-156817622	NM_013865:247	Ndrg3	INSIDE	0.794	0.604	2019.39	1220.64	0.480	1751.24	840.58
A_68_P20900640	chr1:187165153-187165197	NM_011794:9137	Bpnt1	INSIDE	0.794	2.782	663.16	1845.21	2.209	589.00	1301.20
A_68_P20353489	chr1:75357528-75357572	NM_010043:632	Des	INSIDE	0.794	0.287	1901.30	546.45	0.228	1304.21	297.58
A_68_P32134235	chr19:44833870-44833915	NM_011037:2009	Pax2	INSIDE	0.793	3.576	228.97	818.91	2.835	268.68	761.62
A_68_P31921840	chr19:4214799-4214843	NM_019952:429	Clcf1	INSIDE	0.793	2.116	950.37	2010.78	1.677	772.01	1294.54
A_68_P31678552	chr18:46901420-46901464	NM_009681:-128	Ap3s1	DIVERGENT_PROMOTER	0.793	0.686	1586.33	1087.59	0.544	1173.40	637.80
A_68_P31631085	chr18:38114509-38114553	NM_010411:112	Hdac3	INSIDE	0.793	0.240	2082.13	499.19	0.190	1200.40	228.29
A_68_P31004581	chr17:3326768-3326812	NM_001122998:218	Tiam2	INSIDE	0.793	0.326	3114.62	1015.67	0.259	2463.96	636.99
A_68_P30368650	chr15:79634238-79634282	NM_030689:879	Nptxr	INSIDE	0.793	0.592	1635.69	968.02	0.469	1274.67	598.21
A_68_P29879706	chr14:106295189-106295234	NM_011897:825	Spry2	INSIDE	0.793	0.653	989.66	646.45	0.518	852.21	441.27
A_68_P29262748	chr13:100114609-100114653	NM_026873:3	Ptcd2	INSIDE	0.793	2.156	1155.47	2491.55	1.710	981.95	1679.19
A_68_P28865537	chr13:20182539-20182583	NM_080288:185	Elmo1	INSIDE	0.793	0.154	4049.05	623.88	0.122	3011.21	367.77
A_68_P28533193	chr12:73765645-73765689	NM_025522:149	Dhrs7	INSIDE	0.793	2.424	1312.53	3182.08	1.922	996.92	1916.48
A_68_P28290824	chr12:25336101-25336145	NM_178357:-113	Klf11	PROMOTER	0.793	2.206	1071.42	2363.71	1.749	846.34	1480.50
A_68_P27889317	chr11:68245685-68245729	NM_177320:80	Pik3r5	INSIDE	0.793	2.442	606.68	1481.60	1.937	520.07	1007.50
A_68_P27542236	chr10:128027435-128027479	NM_010153:-899	Erbb3	PROMOTER	0.793	3.428	733.22	2513.28	2.718	604.25	1642.60
A_68_P27289855	chr10:80927157-80927201	NM_176954:18276	Celf5	INSIDE	0.793	0.602	3249.24	1957.35	0.478	2308.41	1102.66
A_68_P26467468	chr9:44215837-44215881	NM_024266:62	Rps25	INSIDE	0.793	0.222	3291.49	732.18	0.176	2387.42	421.34
A_68_P26000510	chr8:83135226-83135270	NM_001167898:311	Frem3	INSIDE	0.793	0.466	1899.25	885.23	0.369	1414.18	522.38
A_68_P25562262	chr7:144086069-144086113	NM_008598:-203	Mgmt	PROMOTER	0.793	0.171	10657.85	1825.31	0.136	7668.73	1041.12
A_68_P25088775	chr7:51926026-51926070	NM_028021:-5175	Myh14	PROMOTER	0.793	0.584	1397.72	816.16	0.463	1100.93	509.58
A_68_P25020502	chr7:28955130-28955174	NM_007991:386	Fbl	INSIDE	0.793	0.384	2054.51	788.36	0.304	1478.87	449.82
A_68_P24773304	chr6:115803367-115803411	NM_010774:-29	Mbd4	DIVERGENT_PROMOTER	0.793	0.306	10443.54	3192.09	0.242	6624.59	1605.92
A_68_P24006029	chr5:115223355-115223399	NM_001077360:124	Git2	INSIDE	0.793	2.319	645.64	1497.02	1.840	650.42	1196.48
A_68_P23448649	chr5:4104754-4104798	NM_020010:-79	Cyp51	PROMOTER	0.793	0.511	1524.79	779.15	0.405	1234.88	500.58
A_68_P23397278	chr4:148274906-148274950	NM_027195:96428	Caszl	INSIDE	0.793	3.582	4109.52	14722.24	2.843	3008.65	8552.34
A_68_P22558740	chr3:136333384-136333428	NM_008913:-327	Ppp3ca	PROMOTER	0.793	1.792	1889.24	3386.25	1.422	1479.79	2104.53
A_68_P22350508	chr3:96474090-96474134	NM_001081139:59	Ankrd35	INSIDE	0.793	0.339	2271.01	768.76	0.268	1831.36	491.55
A_68_P21564941	chr2:118424242-118424286	NM_009773:318	Bub1b	INSIDE	0.793	0.467	2970.05	1385.64	0.370	2183.51	807.81
A_68_P21564754	chr2:118375138-118375182	NM_138313:254	Bmf	INSIDE	0.793	0.459	7798.05	3578.58	0.364	5670.38	2063.27
A_68_P21475550	chr2:102026219-102026263	NM_178886:294	Ldlrad3	INSIDE	0.793	2.538	4125.04	10467.47	2.013	2861.62	5759.19
A_68_P20611336	chr1:132612197-132612241	NM_001162416:-1065	Pfkfb2	PROMOTER	0.793	0.444	2327.72	1034.02	0.352	1764.43	621.48
A_68_P20267734	chr1:60237319-60237363	NM_173444:-102	Nbeal1	PROMOTER	0.793	0.466	1092.47	508.61	0.369	881.04	325.43
A_68_P32019129	chr19:23762386-23762431	NM_028208:519	Ptar1	INSIDE	0.792	3.280	314.48	1031.35	2.598	249.41	647.91
A_68_P31502465	chr18:12899978-12900022	NM_001042418:137	Cabyr	INSIDE	0.792	0.603	1380.09	832.85	0.478	1070.66	511.54
A_68_P31159052	chr17:35496088-35496132	M19687:92295		INSIDE	0.792	3.460	4117.40	14247.18	2.741	3595.45	9855.90
A_68_P31117109	chr17:27693563-27693607	NM_001166476:-13	Hmga1-rs1	PROMOTER	0.792	2.061	857.28	1766.62	1.632	719.23	1173.74
A_68_P31116859	chr17:27650630-27650674	ENSMUST00000088073:-366		DIVERGENT_PROMOTER	0.792	0.468	3462.46	1619.80	0.371	2259.03	837.48
A_68_P31092954	chr17:23810547-23810591	NM_181821:-6	Hcfc1r1	PROMOTER	0.792	0.557	1250.86	697.23	0.442	935.75	413.19
A_68_P30826536	chr16:65815148-65815192	NM_028572:-707	Vgll3	PROMOTER	0.792	0.492	1299.19	639.04	0.389	1021.63	397.81
A_68_P30821643	chr16:64770368-64770412	NM_026273:375	4930453N24Rik	INSIDE	0.792	2.911	1117.82	3253.53	2.304	963.24	2219.30
A_68_P30672034	chr16:35768908-35768952	NM_153550:512	Dirc2	INSIDE	0.792	0.598	2429.40	1452.98	0.474	1863.81	883.40
A_68_P30587014	chr16:20302209-20302253	NM_001005767:205	Parl	INSIDE	0.792	0.401	1957.31	785.62	0.318	1636.68	520.05
A_68_P29700849	chr14:70750941-70750985	NM_001135152:-2722	Slc39a14	PROMOTER	0.792	0.488	2333.67	1139.62	0.387	1795.49	694.56

ProbeName	Target position of probe on CpG island microarray	TargetID	GeneSymbol	CpG island Description	Ratio of relative methylation (TiO_2-NP/Vehicle)	Sham group			TiO_2-H group		
						Relative methylation (Cy5/Cy3)	Cy3 signal (Input)	Cy5 signal (MeDIP)	Relative methylation (Cy5/Cy3)	Cy3 signal (Input)	Cy5 signal (MeDIP)
A_68_P29445595	chr14:21000753-21000797	NM_021542:230	Kcnk5	INSIDE	0.792	0.364	1361.79	495.11	0.288	1017.97	293.01
A_68_P28712204	chr12:107201277-107201321	BC156654:163438		INSIDE	0.792	0.441	1322.09	582.53	0.349	1050.91	366.56
A_68_P28698037	chr12:104770506-104770550	NM_028980:-246	Ppp4r4	PROMOTER	0.792	0.430	1616.11	694.87	0.340	1501.37	511.00
A_68_P28598114	chr12:85749779-85749823	NM_001026214:179	Entpd5	INSIDE	0.792	0.599	2724.98	1632.24	0.475	2152.30	1021.68
A_68_P28059764	chr11:98844073-98844117	NM_178596:236	Gjd3	INSIDE	0.792	0.322	1482.44	477.87	0.255	1137.59	290.37
A_68_P28051869	chr11:97521316-97521363	NM_139311:-3386	Mllt6	PROMOTER	0.792	0.239	1864.26	444.98	0.189	1227.21	232.12
A_68_P27540748	chr10:127759503-127759547	NM_019953:10	Cnpy2	INSIDE	0.792	0.731	1562.65	1141.71	0.579	1242.05	718.90
A_68_P27508735	chr10:121885914-121885958	NM_016847:382	Avpr1a	INSIDE	0.792	2.781	1554.56	4323.76	2.203	1301.08	2866.32
A_68_P27265100	chr10:76424357-76424401	NM_021568:314	Pcbp3	INSIDE	0.792	0.637	1248.76	795.34	0.505	900.55	454.47
A_68_P26135758	chr8:108282379-108282423	NM_027469:107	Gfod2	INSIDE	0.792	0.459	1384.24	634.83	0.363	1140.26	413.96
A_68_P25991119	chr8:81344708-81344752	NM_001191004:321	Lsm6	INSIDE	0.792	2.284	1320.60	3016.73	1.810	1200.89	2173.74
A_68_P25086656	chr7:51560092-51560136	NM_009131:2215	Clec11a	INSIDE	0.792	0.418	1654.52	692.13	0.332	1135.20	376.33
A_68_P24137556	chr5:140251303-140251347	NM_026748:308	Ints1	INSIDE	0.792	0.586	2365.38	1385.17	0.464	1838.27	852.33
A_68_P23190562	chr4:107107483-107107527	NM_147221:181	Glis1	INSIDE	0.792	0.641	6904.19	4428.05	0.508	5187.83	2636.08
A_68_P22291856	chr3:84386605-84386649	NM_001081093:-79	Arfip1	PROMOTER	0.792	0.538	1299.34	698.65	0.426	1068.25	454.84
A_68_P21935175	chr3:9004387-9004431	NM_001025263:107	Tpd52	INSIDE	0.792	0.559	2474.14	1383.72	0.443	1851.08	820.17
A_68_P21613745	chr2:127307805-127307849	NM_001111331:409	Kcnip3	INSIDE	0.792	0.254	2898.07	735.72	0.201	2126.18	427.64
A_68_P20596880	chr1:129671367-129671411	NM_028399:648	Ccnt2	INSIDE	0.792	0.612	1025.32	627.37	0.484	825.18	399.69
A_68_P33007791	chr4_random:128451-128495	NM_001033326:18671	Dhrsx	INSIDE	0.791	1.745	6986.69	12190.52	1.380	5133.58	7084.67
A_68_P31919591	chr19:3852552-3852596	NM_001025566:802	Chka	INSIDE	0.791	0.652	1324.91	863.86	0.516	993.14	512.24
A_68_P31207974	chr17:46766268-46766312	NM_175168:163	Ptk7	INSIDE	0.791	0.640	2119.12	1355.45	0.506	1530.95	774.45
A_68_P31108447	chr17:26260564-26260608	NM_024227:139	Mrpl28	INSIDE	0.791	2.075	1007.23	2090.00	1.642	845.14	1387.53
A_68_P30287853	chr15:65618917-65618962	NM_133766:337	Efr3a	INSIDE	0.791	0.608	958.76	582.60	0.481	873.49	419.87
A_68_P29689075	chr14:68701929-68701973	NM_010910:10	Nefl	INSIDE	0.791	0.399	2209.91	881.63	0.315	1651.91	520.96
A_68_P29628539	chr14:58161485-58161529	NM_145837:17841	Il17d	INSIDE	0.791	3.933	1281.40	5039.97	3.109	1079.44	3356.44
A_68_P27850820	chr11:61667944-61667988	NM_013881:628	Ulk2	INSIDE	0.791	3.416	1186.67	4053.93	2.701	1019.58	2754.03
A_68_P27843668	chr11:60351780-60351824	NM_172943:618	Alkbh5	INSIDE	0.791	0.337	2705.77	912.49	0.267	2090.84	557.89
A_68_P27565567	chr11:5801958-5802002	NM_010292:47622	Gck	INSIDE	0.791	0.264	1820.84	481.42	0.209	1227.54	256.72
A_68_P27234632	chr10:70747404-70747448	NM_145420:584	Ube2d1	INSIDE	0.791	6.054	2969.65	17978.37	4.787	2421.46	11591.65
A_68_P26548746	chr9:58430564-58430608	NM_009145:540	Nptn	INSIDE	0.791	0.392	4769.00	1870.81	0.310	3365.62	1043.83
A_68_P26143992	chr8:109817226-109817270	NM_018823:-121	Nfat5	PROMOTER	0.791	0.321	1360.49	436.18	0.254	1073.63	272.44
A_68_P25670892	chr8:13869760-13869804	NM_181854:142	Zfp828	INSIDE	0.791	0.342	1275.63	436.70	0.271	981.66	265.70
A_68_P24815137	chr6:124364678-124364722	NM_008995:385	Pex5	INSIDE	0.791	3.060	3565.76	10910.66	2.422	2561.33	6202.51
A_68_P24760053	chr6:113481570-113481614	NM_175101:40	Tmem111	INSIDE	0.791	0.432	1134.49	489.76	0.341	906.90	309.52
A_68_P24617829	chr6:87682986-87683030	NM_001159697:2146	Ccdc48	INSIDE	0.791	0.385	2078.40	799.95	0.305	1654.60	503.95
A_68_P24061815	chr5:124991287-124991331	NM_019770:509	Tmed2	INSIDE	0.791	4.313	998.96	4308.90	3.414	829.62	2832.24
A_68_P24061812	chr5:124990693-124990737	NM_019770:-85	Tmed2	DIVERGENT_PROMOTER	0.791	3.996	743.09	2969.62	3.163	649.50	2054.39
A_68_P24053336	chr5:123438408-123438452	NM_001005866:112	Kdm2b	INSIDE	0.791	0.661	7683.40	5080.20	0.523	5372.91	2808.68
A_68_P24038736	chr5:120890309-120890353	NM_008499:8436	Lhx5	INSIDE	0.791	0.341	1776.04	606.07	0.270	1345.07	363.21
A_68_P23778715	chr5:69983278-69983322	NM_001038015:224	Gnpda2	INSIDE	0.791	0.232	2528.97	587.04	0.184	1864.99	342.39
A_68_P23429319	chr4:153514065-153514109	NM_011642:231	Trp73	INSIDE	0.791	5.581	2041.45	11393.53	4.413	1734.88	7656.24
A_68_P23349531	chr4:138630867-138630911	NM_021358:-184	Htr6	PROMOTER	0.791	0.532	1280.07	681.20	0.421	969.94	408.43
A_68_P23260200	chr4:122673850-122673894	NM_008506:531	Mycl1	INSIDE	0.791	3.252	2673.85	8696.17	2.571	2328.14	5986.63
A_68_P22885109	chr4:45812112-45812156	NM_028270:241	Aldh1b1	INSIDE	0.791	0.320	2252.70	721.62	0.253	1630.78	413.18
A_68_P21794498	chr2:160193025-160193069	NM_010658:-245	Mafb	PROMOTER	0.791	0.405	4569.81	1848.71	0.320	3201.39	1025.03
A_68_P21775093	chr2:156904079-156904123	NM_001164663:901	9830001H06Rik	INSIDE	0.791	5.309	1442.51	7658.65	4.201	1218.05	5116.94
A_68_P21330231	chr2:73052613-73052657	NM_025942:-130	Ola1	PROMOTER	0.791	0.423	1269.11	537.45	0.335	1093.25	366.41
A_68_P20927664	chr1:191552708-191552752	NM_008976:584	Ptpn14	INSIDE	0.791	0.465	1253.63	582.37	0.368	956.37	351.57
A_68_P31207194	chr17:46624614-46624658	NM_001162864:-12	Ttbk1	DIVERGENT_PROMOTER	0.790	4.378	5503.91	24097.69	3.460	4133.96	14304.91
A_68_P30152573	chr15:39029866-39029910	NM_053271:11	Rims2	INSIDE	0.790	2.724	481.31	1311.28	2.152	404.65	870.98
A_68_P29134808	chr13:72766808-72766852	NM_010574:405	Irx2	INSIDE	0.790	0.743	3213.43	2387.56	0.587	2340.44	1373.85
A_68_P28612729	chr12:88224702-88224747	NM_145836:1040	6430527G18Rik	INSIDE	0.790	0.482	1499.72	722.17	0.381	1066.50	405.93
A_68_P28078310	chr11:102086238-102086282	NM_001077696:5226	Hdac5	INSIDE	0.790	0.541	1271.49	687.52	0.427	1131.01	483.02
A_68_P27563900	chr11:5442063-5442107	NM_134033:136	Ccdc117	INSIDE	0.790	0.506	2048.00	1035.84	0.399	1673.30	668.19
A_68_P27281467	chr10:79634488-79634532	NM_027207:-1178	1600002K03Rik	PROMOTER	0.790	5.545	4222.72	23416.67	4.381	3352.18	14686.77
A_68_P26699340	chr9:86360809-86360853	NM_177208:70	Dopey1	INSIDE	0.790	3.421	639.01	2185.91	2.702	514.26	1389.70
A_68_P26469283	chr9:44542261-44542305	NM_153537:995	Phldb1	INSIDE	0.790	0.684	1860.92	1273.31	0.540	1443.64	780.10
A_68_P26350328	A_68_P26350328			Unknown	0.790	3.080	516.29	1589.96	2.431	373.52	908.18
A_68_P26055366	chr8:93352604-93352648	NM_177224:-107	Chd9	PROMOTER	0.790	0.573	1529.35	875.68	0.452	1177.57	532.65
A_68_P25727490	chr8:26629802-26629846	NM_001079908:581	Fgfr1	INSIDE	0.790	2.544	1051.52	2675.44	2.010	883.06	1775.14
A_68_P24595041	chr6:83662285-83662329	NR_002871:-111	Vax2os2	PROMOTER	0.790	0.331	1661.63	550.59	0.262	1140.04	298.45
A_68_P24159777	chr5:144934590-144934634	NM_001081109:73308	Lmtk2	INSIDE	0.790	2.608	1308.68	3413.62	2.060	1089.85	2245.36
A_68_P24091639	chr5:130478668-130478712	NM_010368:8	Gusb	INSIDE	0.790	2.212	1633.36	3612.22	1.748	1505.43	2631.66
A_68_P21761449	chr2:154439556-154439600	NM_199304:475	Zfp341	INSIDE	0.790	0.734	1744.91	1280.17	0.579	1193.06	691.13
A_68_P21750312	chr2:152460407-152460451	NM_009047:7685	Rem1	INSIDE	0.790	2.191	1177.61	2579.56	1.731	896.75	1551.83

ProbeName	Target position of probe on CpG island microarray	TargetID	GeneSymbol	CpG island Description	Ratio of relative methylation (TiO_2-NP/Vehicle)	Sham group			TiO_2-H group		
						Relative methylation (Cy5/Cy3)	Cy3 signal (Input)	Cy5 signal (MeDIP)	Relative methylation (Cy5/Cy3)	Cy3 signal (Input)	Cy5 signal (MeDIP)
A_68_P21633706	chr2:131005544-131005588	NM_007682:182	Cenpb	INSIDE	0.790	0.619	1122.93	694.98	0.489	893.40	436.99
A_68_P20421181	chr1:89904795-89904839	NM_001033291:310	Usp40	INSIDE	0.790	2.294	965.52	2214.84	1.812	879.87	1594.34
A_68_P32369652	chrX:47013995-47014044	ENSMUST00000121891:-117		PROMOTER	0.789	4.576	515.15	2357.48	3.612	707.75	2556.13
A_68_P31625517	chr18:36894792-36894836	NM_028036:91	Tmco6	INSIDE	0.789	0.462	2126.18	982.12	0.365	1496.03	545.45
A_68_P31214873	chr17:48041518-48041563	NM_001110824:20041	Foxp4	INSIDE	0.789	5.877	2551.40	14994.77	4.638	3347.87	15528.90
A_68_P31093355	chr17:23876913-23876957	NM_023824:363	Paqr4	INSIDE	0.789	3.452	1303.85	4501.05	2.723	989.69	2694.63
A_68_P30484703	chr15:100058755-100058799	NM_007497:487	Atf1	INSIDE	0.789	4.082	207.24	845.93	3.221	197.51	636.13
A_68_P30349739	chr15:76487469-76487513	NM_019396:3001	Cyhr1	INSIDE	0.789	2.657	2065.39	5486.82	2.096	1574.47	3300.16
A_68_P28924079	chr13:31718773-31718817	NM_010225:1110	Foxf2	INSIDE	0.789	0.487	4573.06	2225.40	0.384	3277.44	1259.07
A_68_P27987617	chr11:86015036-86015080	NM_178309:-363	Brip1	PROMOTER	0.789	0.157	2974.57	466.78	0.124	2070.81	256.47
A_68_P27924764	chr11:75007008-75007052	NM_177708:-464	Rtn4rl1	DIVERGENT_PROMOTER	0.789	0.509	5721.34	2911.79	0.401	4124.17	1655.38
A_68_P27469157	chr10:114799199-114799243	NM_133683:98	Tmem19	INSIDE	0.789	0.653	1921.87	1255.76	0.516	1426.88	735.90
A_68_P26803471	chr9:106355871-106355915	NM_021567:-295	Pcbp4	PROMOTER	0.789	0.341	3619.74	1232.88	0.269	2663.85	716.23
A_68_P26744830	chr9:95855519-95855563	NM_011916:362	Xrn1	INSIDE	0.789	0.545	1107.95	604.31	0.431	1040.16	447.82
A_68_P26644962	chr9:75459020-75459064	NM_016711:90	Tmod2	INSIDE	0.789	2.172	1485.61	3227.03	1.713	1280.62	2194.20
A_68_P26199436	chr8:119387539-119387583			Unknown	0.789	0.309	1585.03	490.41	0.244	1144.58	279.39
A_68_P24982502	chr7:17200404-17200448	NM_172739:-84	Grlf1	PROMOTER	0.789	2.471	1300.71	3213.48	1.950	997.45	1944.71
A_68_P24921913	chr6:145198164-145198208	NM_021284:565	Kras	INSIDE	0.789	0.688	2020.30	1390.01	0.543	1556.15	844.99
A_68_P24622150	chr6:88396669-88396713	NM_023060:-157	Eefsec	PROMOTER	0.789	2.132	1442.95	3076.82	1.683	1207.94	2033.07
A_68_P24424918	chr6:47870647-47870691	NM_001145881:102	Zfp212	INSIDE	0.789	1.964	1192.12	2341.47	1.550	1093.55	1695.47
A_68_P24002072	chr5:114578165-114578209	NM_175016:-1	Alkbh2	DIVERGENT_PROMOTER	0.789	0.702	1543.83	1083.72	0.554	1132.60	627.25
A_68_P23072589	chr4:84851297-84851341	NM_019535:-41	Sh3gl2	PROMOTER	0.789	2.539	537.14	1363.70	2.003	473.14	947.62
A_68_P22861616	chr4:41261724-41261768	NM_026893:188	Dcaf12	INSIDE	0.789	0.414	1736.90	718.77	0.327	1395.44	455.89
A_68_P22307056	chr3:86978522-86978566	NM_001170985:125	Kirrel	INSIDE	0.789	0.283	1713.16	484.40	0.223	1329.06	296.55
A_68_P21974622	chr3:19211656-19211700	NM_001122759:-356	Pde7a	PROMOTER	0.789	0.446	998.63	445.33	0.352	781.39	275.05
A_68_P21840229	chr2:168056835-168056879	NM_001160330:735	Mocs3	INSIDE	0.789	4.046	965.93	3907.71	3.190	893.02	2849.17
A_68_P21629315	chr2:130187520-130187564	NM_001110513:65868	Ebf4	INSIDE	0.789	0.557	1386.73	771.98	0.439	1206.84	529.98
A_68_P21623514	chr2:129024077-129024121	NM_001159593:-410	Slc20a1	PROMOTER	0.789	0.243	2904.76	705.20	0.191	2080.13	398.31
A_68_P21303500	chr2:68309909-68309953	NM_016866:108	Stk39	INSIDE	0.789	0.517	1328.97	687.31	0.408	1037.87	423.26
A_68_P20936232	chr1:192921750-192921794	NM_030060:-520	Batf3	PROMOTER	0.789	0.705	2106.41	1484.09	0.556	1832.64	1019.23
A_68_P32222444	chr19:60019679-60019723	NM_001164367:-143	Rab11fip2	PROMOTER	0.788	0.643	2696.09	1734.49	0.507	2057.89	1042.82
A_68_P31425150	chr17:88196845-88196889	NM_199251:468	Kcnk12	INSIDE	0.788	2.975	856.07	2547.02	2.345	670.48	1571.99
A_68_P31257078	chr17:56723736-56723780	NM_001029979:248	Safb2	INSIDE	0.788	0.705	1599.38	1127.26	0.555	1234.88	685.93
A_68_P31062916	chr17:15097920-15097964	NM_024250:295	Phf10	INSIDE	0.788	0.504	6532.97	3294.44	0.398	5063.60	2013.09
A_68_P31053798	chr17:12962714-12962758	NM_010515:-164	Igf2r	PROMOTER	0.788	0.335	1826.77	612.78	0.264	1383.71	365.59
A_68_P30966731	chr16:92058568-92058612	NM_080456:10	Mrps6	INSIDE	0.788	0.631	2985.36	1885.22	0.498	2241.98	1115.92
A_68_P30363606	chr15:78813601-78813645	NM_001024716:137	Triobp	INSIDE	0.788	2.558	1215.26	3108.38	2.014	984.41	1983.05
A_68_P29616412	chr14:55734288-55734332	NM_177049:1462	Jph4	INSIDE	0.788	0.596	3938.26	2346.51	0.470	2807.91	1318.43
A_68_P28615264	chr12:88640362-88640406	NM_011479:88796	Sptlc2	DOWNSTREAM	0.788	0.224	3666.74	821.27	0.176	2566.01	452.81
A_68_P28599343	chr12:85958262-85958306	NM_008992:132	Abcd4	INSIDE	0.788	3.416	582.14	1988.30	2.690	497.68	1338.84
A_68_P28042073	chr11:95896364-95896408	NM_033568:156	Snf8	INSIDE	0.788	0.286	2174.09	621.42	0.225	1608.52	362.41
A_68_P27449325	chr10:110943730-110943774	NM_009344:411	Phlda1	INSIDE	0.788	2.414	914.92	2208.59	1.902	804.63	1530.71
A_68_P27282653	chr10:79812076-79812120	NM_001113548:-907	Adamtsl5	DIVERGENT_PROMOTER	0.788	0.639	877.88	561.32	0.504	794.64	400.41
A_68_P26822089	chr9:110034498-110034542	NM_009211:-7	Smarcc1	PROMOTER	0.788	13.350	17109.89	228413.60	10.516	16629.86	174878.20
A_68_P26248329	chr8:127421525-127421569	NM_198103:59	Exoc8	INSIDE	0.788	2.340	1002.12	2345.44	1.845	752.78	1389.13
A_68_P26217964	chr8:122637936-122637980	NM_001163762:-93	6430548M08Rik	PROMOTER	0.788	0.349	2811.79	982.62	0.276	1854.24	510.86
A_68_P26055738	chr8:93432239-93432283	NM_177224:79527	Chd9	INSIDE	0.788	2.574	904.21	2327.10	2.027	749.57	1519.31
A_68_P25957673	chr8:74115916-74115960	NM_025396:-144	Pgls	PROMOTER	0.788	0.265	3462.97	918.29	0.209	2662.18	556.06
A_68_P25672219	chr8:14090321-14090365	NM_001034862:-15	Erich1	DIVERGENT_PROMOTER	0.788	3.447	876.70	3021.86	2.716	674.05	1830.57
A_68_P25670364	chr8:13757610-13757654	NM_027276:-57	Cdc16	PROMOTER	0.788	0.581	2672.23	1551.87	0.457	1902.88	870.42
A_68_P25458539	chr7:125634319-125634363	NM_001033380:1149	Itpripl2	INSIDE	0.788	0.630	1658.29	1044.11	0.496	1205.99	598.15
A_68_P24938726	chr6:148160773-148160817	NR_015615:-15	4732416N19Rik	PROMOTER	0.788	0.296	5068.78	1499.10	0.233	3320.87	774.32
A_68_P24091172	chr5:130384666-130384710	NM_026916:279	4930579G22Rik	INSIDE	0.788	2.779	809.33	2249.22	2.189	701.75	1536.35
A_68_P23590587	chr5:34038039-34038083	AK172441:87326		INSIDE	0.788	2.607	1056.52	2754.31	2.053	892.87	1833.12
A_68_P23299544	chr4:129665518-129665562	NM_001199696:3219	Bai2	INSIDE	0.788	0.568	3063.71	1740.74	0.448	2284.63	1022.57
A_68_P23260764	chr4:122781955-122781999	NM_007559:-431	Bmp8b	PROMOTER	0.788	0.355	1370.84	487.08	0.280	1147.24	321.33
A_68_P20940739	chr1:193731090-193731134	NR_030669:294	1700034H15Rik	INSIDE	0.788	0.639	1665.05	1063.84	0.504	1415.74	713.23
A_68_P20456915	chr1:95582543-95582587	NM_016778:294	Bok	INSIDE	0.788	0.461	1431.04	659.24	0.363	1223.08	444.04
A_68_P32694937	chrX:136145542-136145586	NM_023270:406	Rnf128	INSIDE	0.787	2.950	466.92	1377.43	2.321	769.08	1784.71
A_68_P32119984	chr19:42330495-42330539	NM_027694:288	Golga7b	INSIDE	0.787	0.400	1554.59	622.04	0.315	1170.90	368.81
A_68_P31382676	chr17:80772525-80772569	NR_028385:336	Gm10190	INSIDE	0.787	2.483	684.96	1701.01	1.955	615.27	1202.99
A_68_P31117103	chr17:27692876-27692920	NM_001025427:-620	Hmga1	PROMOTER	0.787	2.723	657.15	1789.16	2.143	541.13	1159.60
A_68_P30884142	chr16:76372860-76372904	NM_173440:412	Nrip1	INSIDE	0.787	2.437	1048.66	2555.63	1.919	913.25	1752.51
A_68_P28614430	chr12:88488238-88488282	NM_010363:-407	Gstz1	PROMOTER	0.787	0.676	1307.28	883.61	0.532	1126.52	599.43
A_68_P28397148	chr12:45370156-45370200	NM_026164:38	Pnpla8	INSIDE	0.787	1.882	2050.55	3858.84	1.482	1701.12	2520.89

ProbeName	Target position of probe on CpG island microarray	TargetID	GeneSymbol	CpG island Description	Ratio of relative methylation (TiO_2-NP/Vehicle)	Sham group			TiO_2-H group		
						Relative methylation (Cy5/Cy3)	Cy3 signal (Input)	Cy5 signal (MeDIP)	Relative methylation (Cy5/Cy3)	Cy3 signal (Input)	Cy5 signal (MeDIP)
A_68_P27939246	chr11:77576975-77577019	ENSMUST00000164315:-2108		PROMOTER	0.787	3.256	798.48	2599.69	2.563	659.80	1691.11
A_68_P27288984	chr10:80800721-80800765	NM_148951:5269	Gipc3	INSIDE	0.787	2.551	1342.74	3425.14	2.006	1230.04	2467.85
A_68_P27286220	chr10:80363685-80363729	NM_013895:8	Timm13	INSIDE	0.787	0.300	2176.00	652.48	0.236	1669.06	393.72
A_68_P27281572	chr10:79649620-79649664	NM_007909:7416	Efna2	INSIDE	0.787	3.183	1560.88	4968.13	2.506	1192.10	2987.37
A_68_P26822090	chr9:110034578-110034622	NM_009211:73	Smarcc1	INSIDE	0.787	0.658	2120.55	1396.10	0.518	1706.40	884.43
A_68_P26660236	chr9:78435783-78435827	NM_172773:30	Slc17a5	INSIDE	0.787	0.578	893.07	516.16	0.455	784.76	357.05
A_68_P26544602	chr9:57788360-57788404	NM_011352:441	Sema7a	INSIDE	0.787	0.606	1276.74	773.08	0.476	1085.92	517.27
A_68_P24129622	chr5:138717157-138717201	NM_172412:3987	Gpc2	INSIDE	0.787	0.622	2803.00	1742.70	0.489	2015.19	985.77
A_68_P23862543	chr5:86601868-86601912	NM_172712:-122	Uba6	PROMOTER	0.787	0.420	2450.20	1029.15	0.331	1989.67	657.75
A_68_P23591267	A_68_P23591267			Unknown	0.787	6.499	326.46	2121.52	5.117	266.71	1364.74
A_68_P23223137	chr4:114730092-114730136	NM_011527:-2017	Tal1	PROMOTER	0.787	0.712	1226.39	873.08	0.561	1023.79	573.85
A_68_P21978658	chr3:20054786-20054830	NM_013755:187	Gyg	INSIDE	0.787	2.638	732.30	1931.83	2.076	626.92	1301.48
A_68_P21760474	chr2:154262689-154262733	NM_009823:491	Cbfa2t2	INSIDE	0.787	0.331	1609.30	533.35	0.261	1286.47	335.52
A_68_P21501822	chr2:106533604-106533648	NM_029837:-2131	Mpped2	PROMOTER	0.787	0.468	1100.68	515.40	0.369	920.94	339.37
A_68_P21439899	chr2:94081662-94081706	NR_029752:-74	Mir129-2	PROMOTER	0.787	0.518	2620.65	1356.82	0.407	1962.28	799.55
A_68_P20454240	chr1:95132647-95132691	NM_172463:195	Sned1	INSIDE	0.787	3.318	454.14	1506.96	2.611	384.70	1004.45
A_68_P32036095	chr19:26682223-26682267	NM_011416:2595	Smarca2	INSIDE	0.786	0.256	1951.19	498.77	0.201	1306.24	262.57
A_68_P31567781	chr18:25913080-25913124	NM_001146292:-618	Celf4	PROMOTER	0.786	0.691	1528.97	1057.13	0.543	1398.39	759.88
A_68_P31151447	chr17:34136295-34136339	NM_001001892:962	H2-K1	INSIDE	0.786	0.710	1617.78	1148.28	0.558	1271.97	709.20
A_68_P29516148	chr14:33015159-33015203	NM_011960:24	Parg	INSIDE	0.786	2.495	1488.71	3714.64	1.960	1384.11	2713.21
A_68_P28744402	chr12:112723817-112723861	NM_001033248:196	Gm266	INSIDE	0.786	2.012	1585.72	3190.79	1.581	1235.93	1954.17
A_68_P28051739	chr11:97490327-97490371	NM_175332:682	E130012A19Rik	INSIDE	0.786	5.443	440.98	2400.21	4.277	391.54	1674.44
A_68_P27901224	chr11:70353932-70353976	NM_008876:289	Pld2	INSIDE	0.786	0.257	2665.72	684.11	0.202	1897.85	382.76
A_68_P27470614	chr10:115024633-115024677	NM_010195:182	Lgr5	INSIDE	0.786	0.419	1765.02	740.28	0.330	1418.93	467.72
A_68_P27168073	chr10:58785999-58786043	NM_011030:-23	P4ha1	PROMOTER	0.786	3.479	707.02	2459.75	2.735	611.71	1673.01
A_68_P26239789	chr8:125935544-125935588	NM_023279:103	Tubb3	INSIDE	0.786	0.704	1624.54	1143.93	0.553	1330.70	736.51
A_68_P25810705	chr8:42459691-42459735	NM_019734:339	Asah1	INSIDE	0.786	0.303	2775.13	841.54	0.238	1935.32	461.36
A_68_P25420897	chr7:118923563-118923607	NM_173739:-93	Galntl4	PROMOTER	0.786	2.261	1988.32	4496.57	1.778	1852.16	3293.40
A_68_P24658386	chr6:94650061-94650105	NM_008377:57	Lrig1	INSIDE	0.786	0.656	2393.94	1570.34	0.515	1752.26	903.12
A_68_P24008367	chr5:115643468-115643512	NM_013879:-7360	Cabp1	PROMOTER	0.786	0.385	1432.49	550.98	0.302	1094.69	330.82
A_68_P23946409	chr5:103854106-103854150	NM_011204:-82	Ptpn13	PROMOTER	0.786	0.365	1877.50	686.19	0.287	1488.31	427.74
A_68_P23451683	chr5:4756856-4756900	NM_021457:1338	Fzd1	INSIDE	0.786	0.647	2352.87	1523.19	0.509	1709.09	869.22
A_68_P22934438	chr4:55546007-55546051	NM_010637:-681	Klf4	PROMOTER	0.786	0.676	964.14	651.91	0.532	880.45	468.00
A_68_P22630559	chr3:148650978-148651022	ENSMUST00000098518:1280		INSIDE	0.786	0.369	2754.15	1016.91	0.290	1951.37	566.08
A_68_P22405719	chr3:107136843-107136887	NM_001045807:-657	Rbm15	PROMOTER	0.786	0.682	1308.23	892.33	0.536	983.04	526.97
A_68_P22149940	chr3:54611394-54611438	NM_133231:297	Rfxap	INSIDE	0.786	4.379	1228.14	5378.15	3.440	1109.34	3816.21
A_68_P32668460	chrX:130222185-130222229	NM_001105245:1326	Pcdh19	INSIDE	0.785	4.303	770.22	3314.39	3.377	912.87	3082.78
A_68_P32218818	chr19:59419895-59419939	NM_001033222:354	Pdzd8	INSIDE	0.785	3.187	1105.50	3523.48	2.503	995.72	2492.12
A_68_P32147194	chr19:47089078-47089122	NM_146100:-87	Ina	PROMOTER	0.785	0.517	3828.15	1977.39	0.405	3146.89	1275.90
A_68_P32144292	chr19:46574510-46574557	NM_053100:-1604	Trim8	PROMOTER	0.785	0.443	1379.59	611.84	0.348	1156.90	402.74
A_68_P31841969	chr18:76401128-76401172	NM_010754:-428	Smad2	PROMOTER	0.785	0.385	4575.52	1759.67	0.302	3060.44	924.47
A_68_P31138857	chr17:31433598-31433642	NM_153062:1193	Slc37a1	INSIDE	0.785	2.698	1614.31	4355.77	2.117	1291.65	2734.63
A_68_P29075365	chr13:58909956-58910000	NM_001025074:785	Ntrk2	INSIDE	0.785	2.679	2169.31	5812.31	2.104	1630.37	3430.87
A_68_P28752633	chr12:114040777-114040821	NM_145450:-6387	BC022687	PROMOTER	0.785	0.724	1780.01	1288.35	0.568	1566.62	889.64
A_68_P28611535	chr12:88019970-88020014	NM_177354:343	Vash1	INSIDE	0.785	2.199	839.23	1845.33	1.726	637.86	1101.11
A_68_P28155493	chr11:115676194-115676238	NM_029557:164	Tsen54	INSIDE	0.785	1.969	2225.11	4380.72	1.545	1690.58	2611.61
A_68_P27569029	chr11:6447001-6447045	NM_001190343:133	Ccm2	INSIDE	0.785	9.356	1618.23	15139.45	7.342	1204.37	8842.75
A_68_P26936201	chr10:10277678-10277722	NM_026405:305	Rab32	INSIDE	0.785	0.488	1501.74	732.59	0.383	1199.19	459.26
A_68_P26159422	chr8:112392225-112392269	NM_001122594:-256	Phlpp2	PROMOTER	0.785	2.527	846.31	2138.58	1.983	749.51	1486.54
A_68_P24990866	chr7:19590929-19590973	NM_178757:1537	Irf2bp1	INSIDE	0.785	1.910	1399.32	2672.30	1.500	1062.03	1592.81
A_68_P24755176	chr6:112646414-112646458	NM_001167730:228	Rad18	INSIDE	0.785	0.432	1283.02	554.52	0.339	1078.07	365.68
A_68_P24430831	chr6:49024182-49024226	NM_029353:411	2410003K15Rik	INSIDE	0.785	0.301	1804.86	543.72	0.236	1470.59	347.60
A_68_P24344497	chr6:32538021-32538065	NM_175750:150	Plxna4	INSIDE	0.785	3.024	478.18	1446.25	2.373	435.01	1032.38
A_68_P23573354	chr5:30890939-30890983	NM_010608:418	Kcnk3	INSIDE	0.785	10.330	948.18	9794.37	8.106	600.90	4870.95
A_68_P23270671	chr4:124528076-124528120	NM_153566:96	Yrdc	INSIDE	0.785	2.252	575.70	1296.52	1.767	574.92	1015.84
A_68_P22826130	chr4:33397227-33397271	NM_011884:-37	Rngtt	PROMOTER	0.785	4.349	1574.78	6848.39	3.413	1393.36	4755.78
A_68_P22357697	chr3:97817451-97817495	NM_010928:12	Notch2	INSIDE	0.785	3.857	1259.88	4859.17	3.029	1085.56	3288.31
A_68_P22315263	chr3:88520957-88521001	NM_001163310:203	Rit1	INSIDE	0.785	0.177	4846.05	859.68	0.139	3547.88	494.35
A_68_P22019083	chr3:28680707-28680751	NM_177586:496	Eif5a2	INSIDE	0.785	0.566	1676.57	948.35	0.444	1274.20	565.88
A_68_P21978016	chr3:19935297-19935341	NM_001146323:-8	Hps3	PROMOTER	0.785	0.686	1375.28	943.15	0.538	1143.09	615.27
A_68_P21910958	chr2:181231832-181231876	NM_025482:-103	Tpd52l2	PROMOTER	0.785	2.419	1423.24	3443.42	1.899	1154.12	2191.35
A_68_P21780089	chr2:157770092-157770136	NM_198627:29726	Vstm2l	INSIDE	0.785	2.211	1076.89	2380.90	1.735	923.04	1601.59
A_68_P21495951	chr2:105514868-105514912	NM_013627:-1711	Pax6	DIVERGENT_PROMOTER	0.785	0.424	1057.80	448.38	0.333	823.58	274.09
A_68_P21339417	chr2:74601653-74601697	NM_010467:638	Hoxd1	INSIDE	0.785	3.377	2136.06	7212.61	2.650	1631.28	4322.45
A_68_P20644617	chr1:138112450-138112494	NM_028872:18369	5730559C18Rik	INSIDE	0.785	2.660	1580.40	4204.07	2.088	1205.26	2516.49

ProbeName	Target position of probe on CpG island microarray	TargetID	GeneSymbol	CpG island Description	Ratio of relative methylation (TiO_2-NP/Vehicle)	Sham group			TiO_2-H group		
						Relative methylation (Cy5/Cy3)	Cy3 signal (Input)	Cy5 signal (MeDIP)	Relative methylation (Cy5/Cy3)	Cy3 signal (Input)	Cy5 signal (MeDIP)
A_68_P20509920	chr1:108609243-108609287	NM_009741:1603	Bcl2	INSIDE	0.785	0.490	1387.21	680.31	0.385	1177.69	453.66
A_68_P20259678	chr1:58643163-58643207	NM_172513:-7	Fam126b	DIVERGENT_PROMOTER	0.785	0.327	1776.06	580.45	0.256	1387.78	355.89
A_68_P32147197	chr19:47089479-47089523	NM_001164363:179	Nt5c2	INSIDE	0.784	7.112	1513.17	10761.72	5.578	1325.49	7394.20
A_68_P32130739	chr19:44220905-44220949	NM_028607:10	Bloc1s2	INSIDE	0.784	0.565	1274.59	719.52	0.443	1111.88	492.39
A_68_P31862936	chr18:80403608-80403652	NM_001042408:25	Txnl4a	INSIDE	0.784	0.578	1525.84	882.47	0.453	1265.92	573.74
A_68_P30984747	chr16:95219135-95219179	NM_001025584:147	Kcnj6	INSIDE	0.784	6.409	2319.48	14865.14	5.022	1883.58	9459.60
A_68_P30650847	chr16:32079473-32079517	NM_177326:-135	Pak2	PROMOTER	0.784	0.272	1880.97	511.74	0.213	1604.76	342.51
A_68_P30295270	chr15:66800532-66800576	NM_008681:649	Ndrg1	INSIDE	0.784	0.256	1919.40	492.03	0.201	1405.01	282.49
A_68_P29773649	chr14:84846406-84846450	NM_001013753:3059	Pcdh17	INSIDE	0.784	0.611	2310.13	1411.70	0.479	1698.10	813.11
A_68_P29699882	chr14:70591597-70591641	NM_011366:15826	Sorbs3	INSIDE	0.784	0.201	4619.97	928.79	0.158	3406.96	536.97
A_68_P29235876	chr13:95365929-95365973			Unknown	0.784	2.679	1202.80	3222.55	2.101	1004.66	2110.81
A_68_P29228002	chr13:93962021-93962065	NM_133905:196	Papd4	INSIDE	0.784	5.146	1326.62	6826.91	4.033	1168.22	4711.39
A_68_P29001574	chr13:45485076-45485120	NM_153789:-12	Mylip	PROMOTER	0.784	0.464	2120.85	983.40	0.363	1842.24	669.47
A_68_P28818474	chr13:9763095-9763139	NM_001199141:1444	Zmynd11	INSIDE	0.784	0.204	2438.43	498.10	0.160	1793.89	287.45
A_68_P28696864	chr12:104563464-104563508	NM_023049:30725	Asb2	INSIDE	0.784	0.524	1397.06	732.07	0.411	1050.39	431.73
A_68_P28688689	chr12:103196183-103196227	NM_029705:249	Atxn3	INSIDE	0.784	0.687	2115.00	1453.84	0.539	1767.93	953.12
A_68_P28431157	chr12:52792863-52792907	NM_001172098:17	Strn3	INSIDE	0.784	2.892	615.19	1778.85	2.267	517.07	1172.44
A_68_P27596686	chr11:12364779-12364823	NM_172496:163	Cobl	INSIDE	0.784	2.708	1281.40	3469.42	2.122	990.55	2101.62
A_68_P27143971	chr10:53471037-53471081	NM_001081428:-343	Fam184a	PROMOTER	0.784	0.583	2490.90	1452.66	0.457	2065.23	944.27
A_68_P26931628	chr10:9394530-9394574	NM_177271:454	Samd5	INSIDE	0.784	2.947	373.50	1100.81	2.312	287.58	664.74
A_68_P26423439	chr9:35366246-35366290	NM_013932:-213	Ddx25	DIVERGENT_PROMOTER	0.784	0.602	1923.97	1159.16	0.473	1541.16	728.37
A_68_P26290459	chr9:8004389-8004433	NM_001171147:186	Yap1	INSIDE	0.784	0.547	3203.32	1753.32	0.429	2683.95	1151.86
A_68_P25652450	chr8:11008619-11008663	NM_001081212:-210	Irs2	PROMOTER	0.784	4.640	1210.18	5615.23	3.640	1052.44	3830.66
A_68_P24955196	chr7:6335606-6335650	NM_175247:-399	Zfp28	PROMOTER	0.784	0.132	4816.60	637.79	0.104	3323.94	344.99
A_68_P24630035	chr6:89785240-89785284	NM_053221:10269	Vmn1r42	DOWNSTREAM	0.784	3.025	733.89	2219.94	2.372	641.64	1521.88
A_68_P23969234	chr5:108298178-108298222	NM_007964:5926	Evi5	INSIDE	0.784	0.468	1486.07	695.79	0.367	1170.42	429.63
A_68_P23927392	chr5:100468747-100468791	NM_001163035:-244	Enoph1	DIVERGENT_PROMOTER	0.784	0.339	2629.19	890.29	0.265	1962.58	520.77
A_68_P23814579	chr5:76612836-76612880	NM_011626:-46	Tmem165	PROMOTER	0.784	2.186	1216.27	2658.44	1.713	1003.43	1719.22
A_68_P23756663	chr5:65884143-65884187	NM_133697:-90	1110003E01Rik	PROMOTER	0.784	2.533	1188.29	3010.50	1.986	992.82	1971.43
A_68_P23327086	chr4:134731348-134731392	NM_019732:54811	Runx3	INSIDE	0.784	1.912	2962.75	5665.11	1.499	2293.56	3438.34
A_68_P23197625	chr4:108453061-108453105	ENSMUST00000106658:26		INSIDE	0.784	0.730	1770.73	1292.85	0.573	1529.83	875.95
A_68_P22379761	chr3:102007586-102007630	NM_001165951:278	Vangl1	INSIDE	0.784	0.264	3537.05	934.05	0.207	2520.45	521.62
A_68_P22048260	chr3:34353370-34353414			Unknown	0.784	1.898	1363.57	2587.45	1.488	1149.64	1710.60
A_68_P22031743	chr3:30994430-30994474	NM_001039090:470	Skil	INSIDE	0.784	2.055	834.38	1714.27	1.610	718.75	1157.34
A_68_P21579905	chr2:121097022-121097066	NM_013735:78	Trp53bp1	INSIDE	0.784	0.319	2767.94	882.19	0.250	2297.16	574.33
A_68_P20149812	chr1:36748527-36748571	NM_009942:217	Cox5b	INSIDE	0.784	2.646	874.99	2314.93	2.073	772.43	1601.48
A_68_P20091644	chr1:24107112-24107156	NM_026604:46	Fam135a	INSIDE	0.784	3.439	942.13	3240.37	2.695	851.98	2296.42
A_68_P32097048	chr19:37767375-37767419	NM_001105201:7227	Cyp26c1	INSIDE	0.783	0.717	1810.55	1298.02	0.561	1419.54	796.60
A_68_P31939718	chr19:7496785-7496829	NM_175381:4692	2700081O15Rik	INSIDE	0.783	0.606	1724.18	1045.05	0.475	1450.95	688.57
A_68_P31934661	chr19:6428357-6428401	NM_001205234:9641	Nrxn2	INSIDE	0.783	4.743	811.85	3850.44	3.715	699.33	2598.20
A_68_P31208746	chr17:46889025-46889069	NM_028065:115	Cnpy3	INSIDE	0.783	3.693	2596.66	9590.02	2.893	2050.93	5934.05
A_68_P31017290	chr17:5492030-5492074	NM_146073:-547	Zdhhc14	PROMOTER	0.783	0.496	1061.37	526.27	0.388	804.36	312.10
A_68_P30497294	chr15:102162316-102162360	NM_021713:199	Mygl	INSIDE	0.783	0.141	3707.91	522.70	0.110	2719.27	299.99
A_68_P30485729	chr15:100253504-100253548	NM_008732:-40	Slc11a2	PROMOTER	0.783	0.446	1015.00	452.78	0.349	889.90	310.77
A_68_P30379303	chr15:81560107-81560151	NM_001146174:221	Rangap1	INSIDE	0.783	0.396	1879.51	745.20	0.310	1542.36	478.67
A_68_P29073060	chr13:58503550-58503594	NR_031761:-36	Rmi1	DIVERGENT_PROMOTER	0.783	0.518	1264.48	655.58	0.406	981.94	398.52
A_68_P28765898	chr12:117316198-117316242	NM_009511:25	Vipr2	INSIDE	0.783	2.267	1510.89	3424.87	1.776	1306.08	2319.39
A_68_P27892734	chr11:68859134-68859178	NM_011496:12	Aurkb	INSIDE	0.783	0.674	1932.77	1303.23	0.528	1461.26	771.53
A_68_P27278596	chr10:79198751-79198795	NM_008226:19394	Hcn2	INSIDE	0.783	3.977	1735.22	6900.79	3.115	1352.25	4212.24
A_68_P27268625	chr10:76978607-76978651	NM_133998:-70	1810008A18Rik	PROMOTER	0.783	0.661	1015.01	670.56	0.517	830.09	429.39
A_68_P26133404	chr8:107898928-107898972	NM_001081333:-330	Plekhg4	PROMOTER	0.783	4.203	1702.30	7155.20	3.292	1468.01	4832.00
A_68_P25958357	chr8:74216612-74216657	NM_028189:9065	B3gnt3	INSIDE	0.783	0.317	2589.46	822.00	0.248	1671.03	415.18
A_68_P25948482	chr8:72406463-72406507	NM_026818:4807	Cilp2	INSIDE	0.783	2.249	1216.09	2734.46	1.762	1053.78	1856.39
A_68_P24894482	chr6:140372882-140372926	NM_144920:285	Plekha5	INSIDE	0.783	2.735	891.77	2439.30	2.143	813.54	1743.15
A_68_P24591568	chr6:83072012-83072056	NM_023547:2989	Ino80b	INSIDE	0.783	0.432	2532.32	1094.49	0.339	1897.60	642.56
A_68_P24383759	chr6:39523831-39523875	NM_178873:-22	Adck2	PROMOTER	0.783	4.851	2030.07	9847.49	3.799	1604.41	6094.67
A_68_P24131505	chr5:139231244-139231288	NM_030565:232	Fam20c	INSIDE	0.783	0.501	3182.98	1596.04	0.393	2204.64	865.68
A_68_P23185129	chr4:106233508-106233552	NM_053272:-112	Dhcr24	PROMOTER	0.783	0.564	4308.71	2429.02	0.441	3130.79	1381.49
A_68_P21819504	chr2:164571506-164571550	NM_133763:14	Dnttip1	INSIDE	0.783	3.281	1315.09	4314.29	2.570	1174.12	3017.83
A_68_P21683843	chr2:140221161-140221205	NM_028387:12	Macrod2	INSIDE	0.783	0.236	2045.68	482.43	0.185	1657.92	306.20
A_68_P21601160	chr2:125073182-125073226	NM_023595:-46	Dut	PROMOTER	0.783	0.307	4899.09	1501.75	0.240	3720.26	892.39
A_68_P20910366	chr1:188792121-188792165	NM_033077:848	D1Pas1	INSIDE	0.783	2.880	1543.36	4444.28	2.256	1162.40	2622.24
A_68_P32241740	chrX:7674050-7674094	NR_003632:-140	Gm6787	DIVERGENT_PROMOTER	0.782	2.404	1004.53	2414.57	1.879	1249.79	2348.36
A_68_P32229540	A_68_P32229540			Unknown	0.782	0.654	1577.92	1031.96	0.512	1265.73	647.53
A_68_P32186047	chr19:54119811-54119855	NM_007417:161	Adra2a	INSIDE	0.782	0.531	3528.40	1872.44	0.415	2572.49	1067.89

ProbeName	Target position of probe on CpG island microarray	TargetID	GeneSymbol	CpG island Description	Ratio of relative methylation (TiO_2-NP/Vehicle)	Sham group			TiO_2-H group		
						Relative methylation (Cy5/Cy3)	Cy3 signal (Input)	Cy5 signal (MeDIP)	Relative methylation (Cy5/Cy3)	Cy3 signal (Input)	Cy5 signal (MeDIP)
A_68_P31933962	chr19:6320206-6320250	NM_001033342:13772	Cdc42bpg	INSIDE	0.782	4.522	2198.96	9944.43	3.538	1550.02	5483.76
A_68_P31921836	chr19:4214356-4214400	NM_019952:-13	Clcf1	PROMOTER	0.782	2.848	1079.00	3073.18	2.228	850.63	1895.30
A_68_P31846733	chr18:77493478-77493522	NM_013666:68915	St8sia5	INSIDE	0.782	2.227	2144.79	4776.64	1.742	1438.22	2505.30
A_68_P31418985	chr17:87153466-87153510	NM_010137:285	Epas1	INSIDE	0.782	0.177	4523.39	800.00	0.138	2933.23	405.42
A_68_P31156003	chr17:34973450-34973494	NM_019442:376	Stk19	INSIDE	0.782	0.405	1590.42	644.66	0.317	1254.25	397.43
A_68_P30979322	chr16:94086172-94086216	NM_011377:690	Sim2	INSIDE	0.782	0.639	1686.30	1077.44	0.499	1232.17	615.45
A_68_P30489319	chr15:100883076-100883120	NM_011323:116397	Scn8a	DOWNSTREAM	0.782	3.272	1564.85	5120.86	2.559	1416.22	3624.46
A_68_P30255521	chr15:59480987-59481031	NM_144549:800	Trib1	INSIDE	0.782	0.468	3261.40	1527.33	0.366	2304.33	844.00
A_68_P29879715	chr14:106296137-106296181	NM_011897:-122	Spry2	PROMOTER	0.782	4.385	195.97	859.29	3.430	192.10	658.93
A_68_P29482961	chr14:27489048-27489092	NM_178668:262	Pde12	INSIDE	0.782	0.679	1177.09	799.49	0.531	867.39	460.74
A_68_P29440562	chr14:19726350-19726394	NM_144839:-231	Ube2e2	PROMOTER	0.782	0.381	1167.68	444.73	0.298	918.13	273.29
A_68_P28836950	chr13:14155368-14155412	NM_010282:278	Ggps1	INSIDE	0.782	0.379	3051.18	1157.49	0.297	2393.52	709.98
A_68_P28152361	chr11:115119374-115119418	NM_010350:9161	Grin2c	INSIDE	0.782	2.215	1689.03	3741.23	1.733	1404.70	2433.96
A_68_P27892955	chr11:68902022-68902066	NM_009497:15	Vamp2	INSIDE	0.782	0.562	1815.56	1021.16	0.440	1610.73	708.62
A_68_P27766335	chr11:45980030-45980074	NM_172524:-191	Nipal4	PROMOTER	0.782	1.866	1673.25	3121.85	1.459	1376.00	2007.30
A_68_P27284766	chr10:80139373-80139417	NM_021462:-4673	Mknk2	PROMOTER	0.782	0.563	1804.27	1015.62	0.440	1396.91	614.65
A_68_P27254391	chr10:74524227-74524271	NM_001081412:608	Bcr	INSIDE	0.782	0.513	1556.72	799.06	0.401	1272.53	510.64
A_68_P26344985	chr9:20807480-20807524	NM_023167:322	Mrpl4	INSIDE	0.782	2.706	985.86	2667.46	2.115	779.11	1647.89
A_68_P26132792	chr8:107817484-107817528	NM_177788:2492	Exoc3l	INSIDE	0.782	2.335	1295.55	3025.04	1.826	1001.28	1828.47
A_68_P26078866	chr8:97190855-97190900	NM_024269:-128	Arl2bp	PROMOTER	0.782	2.239	1281.66	2869.49	1.752	922.00	1614.96
A_68_P24983717	chr7:17460852-17460896	NM_001081655:209	Dact3	INSIDE	0.782	0.419	1138.91	476.66	0.327	951.83	311.34
A_68_P24816769	chr6:124695789-124695833	NM_007881:10695	Atn1	INSIDE	0.782	5.405	537.34	2904.22	4.225	496.65	2098.44
A_68_P23600193	chr5:35732106-35732150	NM_010445:363	Hmx1	INSIDE	0.782	0.185	3026.07	560.80	0.145	2196.33	318.40
A_68_P23230189	chr4:116299784-116299829	NM_001081475:296	Nasp	INSIDE	0.782	2.601	1894.65	4928.30	2.035	1498.42	3049.15
A_68_P21084955	chr2:27331406-27331450	NM_001113573:-235	Brd3	PROMOTER	0.782	3.186	880.09	2804.03	2.492	707.97	1764.30
A_68_P20976551	chr2:5872361-5872405	NM_001081132:-132	Upf2	DIVERGENT_PROMOTER	0.782	0.655	1929.63	1262.98	0.512	1583.88	811.02
A_68_P20648918	chr1:138845713-138845757	NM_001159769:4473	Nr5a2	INSIDE	0.782	3.633	767.24	2787.20	2.842	730.33	2075.49
A_68_P20462986	chr1:97210270-97210314	NM_026321:88	Fam174a	INSIDE	0.782	2.699	653.22	1762.95	2.112	541.62	1143.66
A_68_P20350471	chr1:74830842-74830886	NM_009526:12399	Wnt6	INSIDE	0.782	2.747	1051.59	2889.07	2.150	846.46	1819.62
A_68_P32466328	chrX:71330831-71330875	NM_010788:80	Mecp2	INSIDE	0.781	1.807	1965.09	3550.04	1.410	2577.71	3635.38
A_68_P32378027	chrX:49034445-49034489	NM_015819:-812	Hs6st2	PROMOTER	0.781	2.851	665.45	1896.93	2.227	896.72	1996.61
A_68_P32151690	chr19:47806282-47806326	NM_026377:59	6330577E15Rik	INSIDE	0.781	0.360	1933.64	695.54	0.281	1449.37	407.25
A_68_P32109940	chr19:40686579-40686623	NM_026260:105	Tctn3	INSIDE	0.781	3.012	1284.57	3869.70	2.352	1018.80	2396.33
A_68_P32097099	chr19:37772682-37772726	NM_007811:407	Cyp26a1	INSIDE	0.781	0.528	1827.22	964.38	0.412	1337.62	551.65
A_68_P31415138	chr17:86567345-86567389	NM_011104:242	Prkce	INSIDE	0.781	0.258	1761.90	453.83	0.201	1279.85	257.54
A_68_P31259697	chr17:57171063-57171108	NM_010613:-155	Khsrp	PROMOTER	0.781	0.355	1599.87	567.29	0.277	1218.21	337.48
A_68_P31254157	chr17:56281353-56281397	NM_001130456:-1608	Sema6b	PROMOTER	0.781	0.281	3779.18	1062.63	0.220	2329.89	511.50
A_68_P31157369	chr17:35202723-35202767	NM_001033221:385	AU023871	INSIDE	0.781	0.266	2227.38	593.06	0.208	1544.84	321.06
A_68_P30979599	chr16:94347306-94347350	NM_011377:261824	Sim2	INSIDE	0.781	3.490	667.17	2328.44	2.726	505.11	1376.99
A_68_P30567430	chr16:15887377-15887421	NM_007679:20	Cebpd	INSIDE	0.781	2.685	4442.71	11928.06	2.096	3352.04	7024.82
A_68_P28938196	chr13:34436655-34436699	NM_001033167:375	Slc22a23	INSIDE	0.781	3.080	456.41	1405.77	2.406	370.24	890.77
A_68_P28054169	chr11:97914013-97914057	NM_146028:742	Stac2	INSIDE	0.781	0.329	2729.75	898.55	0.257	2015.64	518.28
A_68_P27839987	chr11:59778414-59778458	NM_009026:8	Rasd1	INSIDE	0.781	0.182	2794.24	508.27	0.142	2094.08	297.31
A_68_P27560196	chr11:4795414-4795458	NM_172438:91	Thoc5	INSIDE	0.781	0.556	2264.94	1258.39	0.434	1737.57	753.74
A_68_P26058235	chr8:93837483-93837527	NM_011936:81	Fto	INSIDE	0.781	0.151	4215.08	637.10	0.118	2899.00	342.10
A_68_P25502308	chr7:133942826-133942870	NM_007438:-80	Aldoa	PROMOTER	0.781	2.325	1601.70	3723.55	1.817	1267.59	2302.90
A_68_P25088122	chr7:51807387-51807431	NM_009473:1885	Nr1h2	INSIDE	0.781	2.551	1941.86	4954.26	1.993	1527.28	3044.39
A_68_P24973143	chr7:13404105-13404149	NM_026046:83	Zfp329	INSIDE	0.781	0.465	1147.72	533.37	0.363	1011.47	367.02
A_68_P24791601	chr6:119125187-119125231	NM_001033379:-62	Dcp1b	PROMOTER	0.781	0.377	1534.82	578.51	0.295	1229.16	362.03
A_68_P24397782	chr6:42299669-42299713	NM_011777:-136	Zyx	PROMOTER	0.781	0.498	2069.22	1030.07	0.389	1617.96	628.65
A_68_P24372703	chr6:37391889-37391933	NM_178661:238	Creb3l2	INSIDE	0.781	1.929	1715.99	3310.91	1.508	1294.03	1950.96
A_68_P24005878	chr5:115199611-115199670	NM_001077360:23860	Git2	INSIDE	0.781	3.388	500.53	1695.69	2.648	362.15	958.82
A_68_P23997297	chr5:113716248-113716292	NR_027934:-6649	Sgsm1	PROMOTER	0.781	0.220	2814.74	618.15	0.172	2262.95	388.36
A_68_P23429536	chr4:153544927-153544971	NM_001162977:127	Megf6	INSIDE	0.781	0.343	1347.08	462.40	0.268	1122.12	300.79
A_68_P23300613	chr4:129816292-129816336	NM_001163027:-519	Hcrtr1	PROMOTER	0.781	0.597	1270.73	758.48	0.466	1031.64	481.04
A_68_P21911519	chr2:181327086-181327130	NM_001164597:58	Znf512b	INSIDE	0.781	1.931	1564.46	3021.08	1.509	1356.62	2046.74
A_68_P21752059	chr2:152769043-152769087	NM_001159376:8254	Dusp15	INSIDE	0.781	2.764	542.36	1499.06	2.157	523.34	1129.10
A_68_P21346290	chr2:75670688-75670732	NM_172666:477	Agps	INSIDE	0.781	0.645	3772.99	2433.59	0.504	2679.45	1350.17
A_68_P21010978	chr2:12340776-12340820	NM_024185:289	Fam188a	INSIDE	0.781	0.355	1341.53	475.95	0.277	1027.65	284.69
A_68_P20620657	chr1:134212802-134212846	NM_001195025:123	Nuak2	INSIDE	0.781	2.507	1030.20	2582.98	1.959	902.26	1767.40
A_68_P32846303	chr1:78485277-78485321	NM_011811:123	Farsb	INSIDE	0.780	3.249	725.61	2357.54	2.535	576.87	1462.49
A_68_P32479509	chrX:75041437-75041481	NM_016979:-159	Prkx	PROMOTER	0.780	4.570	490.41	2241.38	3.565	423.18	1508.65
A_68_P32114043	chr19:41337901-41337945	NM_133352:572	Tm9sf3	INSIDE	0.780	0.640	2350.80	1504.68	0.499	1822.74	909.92
A_68_P31713139	chr18:53577609-53577653	NM_008908:31	Ppic	INSIDE	0.780	0.435	3026.79	1316.70	0.339	2228.24	756.34
A_68_P31610606	chr18:34166317-34166361	NM_013512:522	Epb4.1l4a	INSIDE	0.780	0.684	1949.73	1333.11	0.534	1565.23	835.18

ProbeName	Target position of probe on CpG island microarray	TargetID	GeneSymbol	CpG island Description	Ratio of relative methylation (TiO_2-NP/Vehicle)	Sham group			TiO_2-H group		
						Relative methylation (Cy5/Cy3)	Cy3 signal (Input)	Cy5 signal (MeDIP)	Relative methylation (Cy5/Cy3)	Cy3 signal (Input)	Cy5 signal (MeDIP)
A_68_P30955189	chr16:90144198-90144242	NR_033476:1739	Gm10789	INSIDE	0.780	7.549	1165.74	8800.69	5.886	827.83	4872.41
A_68_P30540351	chr16:9993707-9993751	NM_008170:-1102	Grin2a	PROMOTER	0.780	0.689	1848.61	1273.45	0.537	1430.73	768.33
A_68_P30132017	chr15:35085586-35085630	NM_019635:-47	Stk3	PROMOTER	0.780	0.154	5486.40	847.14	0.120	3961.97	476.93
A_68_P29322933	chr13:111185578-111185622	NM_152804:349	Plk2	INSIDE	0.780	0.182	3706.41	675.06	0.142	2454.18	348.44
A_68_P28067418	chr11:100181178-100181222	NM_011508:-109	Eif1	PROMOTER	0.780	0.210	2485.63	521.96	0.164	2057.69	337.23
A_68_P28051176	chr11:97396600-97396644	NM_018873:39818	Srcin1	INSIDE	0.780	0.612	1403.88	859.83	0.478	1148.43	548.61
A_68_P27958469	chr11:80965626-80965670	NM_007384:757	Accn1	INSIDE	0.780	0.401	2597.47	1040.88	0.313	1714.85	535.93
A_68_P27901335	chr11:70376216-70376260	NM_001045959:-144	Mink1	PROMOTER	0.780	0.676	1635.25	1104.94	0.527	1207.13	636.19
A_68_P25954890	chr8:73437278-73437322	NM_173013:7428	Mtap1s	INSIDE	0.780	3.120	1658.32	5174.21	2.433	1125.26	2737.24
A_68_P25953853	chr8:73284695-73284739	NM_183170:104	Mpv17l2	INSIDE	0.780	4.694	1297.79	6092.33	3.660	970.03	3549.97
A_68_P24991771	chr7:19742770-19742814	NM_001080815:8684	Gipr	INSIDE	0.780	1.987	1145.19	2275.94	1.550	905.96	1404.04
A_68_P24092948	chr5:130731326-130731370	NM_023248:-16	Sbds	DIVERGENT_PROMOTER	0.780	3.767	2479.34	9338.72	2.936	1906.28	5597.53
A_68_P24052807	chr5:123349777-123349821	NM_013910:561	Kdm2b	INSIDE	0.780	4.601	300.76	1383.76	3.588	247.04	886.41
A_68_P24050196	chr5:122872956-122873000	NM_019805:526	Anapc7	INSIDE	0.780	2.389	702.70	1678.54	1.863	662.56	1234.29
A_68_P23961300	chr5:106880949-106880993	NM_001005477:6215	Barhl2	DOWNSTREAM	0.780	0.385	1361.83	524.40	0.300	1092.48	328.03
A_68_P23590342	chr5:34000582-34000626	NM_026698:-123	Tmem129	DIVERGENT_PROMOTER	0.780	0.501	2068.84	1036.73	0.391	1581.11	617.67
A_68_P23467999	chr5:8423016-8423060	NM_178766:189	Slc25a40	INSIDE	0.780	3.052	501.28	1529.83	2.381	431.59	1027.44
A_68_P23260766	chr4:122782187-122782231	NM_007559:-199	Bmp8b	PROMOTER	0.780	0.721	1588.66	1145.07	0.562	1379.88	775.36
A_68_P22726931	chr4:10801456-10801500	NM_026005:-166	2610301B20Rik	PROMOTER	0.780	0.740	3060.18	2265.51	0.578	2206.58	1274.52
A_68_P22398704	chr3:105508294-105508338	NM_001163356:-59	6530418L21Rik	PROMOTER	0.780	1.979	1491.14	2951.23	1.544	1006.26	1553.54
A_68_P22014196	chr3:27837925-27837969	NM_001164056:345	Pld1	INSIDE	0.780	0.491	2049.63	1006.09	0.383	1587.36	607.92
A_68_P21428049	chr2:92210891-92210935	NM_001166633:281	Gyltl1b	INSIDE	0.780	0.309	2208.72	682.21	0.241	1581.30	381.02
A_68_P21395109	chr2:84712079-84712123	NM_199223:14749	Rtn4rl2	DOWNSTREAM	0.780	0.595	1480.16	880.88	0.464	1245.57	577.98
A_68_P21339411	chr2:74601129-74601173	NM_010467:114	Hoxd1	INSIDE	0.780	0.183	3900.64	715.04	0.143	2840.26	406.20
A_68_P21310712	chr2:69561046-69561090	NM_001081086:-76	Ppig	PROMOTER	0.780	0.499	2849.57	1421.92	0.389	2214.25	861.87
A_68_P21006734	chr2:11475492-11475536	NM_001177758:42	Pfkfb3	INSIDE	0.780	2.133	858.55	1831.14	1.663	740.30	1231.28
A_68_P20969749	chr2:4639031-4639075	NM_001190400:176	Bend7	INSIDE	0.780	2.445	1083.44	2648.94	1.907	892.42	1701.96
A_68_P20451399	chr1:94728353-94728397	NM_016696:112	Gpc1	INSIDE	0.780	10.117	1778.81	17996.28	7.893	1542.49	12174.91
A_68_P20442838	chr1:93391415-93391459	NM_028718:192	Traf3ip1	INSIDE	0.780	0.749	3502.94	2624.37	0.585	2265.67	1324.63
A_68_P32218813	chr19:59419411-59419455	NM_001033222:838	Pdzd8	INSIDE	0.779	0.529	940.91	498.02	0.412	721.69	297.51
A_68_P32030718	chr19:25684761-25684805	NM_177360:-244	Dmrt3	PROMOTER	0.779	2.550	1501.26	3828.42	1.986	1303.73	2588.82
A_68_P31207973	chr17:46766171-46766215	NM_175168:261	Ptk7	INSIDE	0.779	0.346	1483.65	513.77	0.270	1162.60	313.59
A_68_P31152221	chr17:34257414-34257458	NR_037970:-591	Brd2	PROMOTER	0.779	6.403	860.77	5511.21	4.986	768.39	3831.09
A_68_P30722301	chr16:44558812-44558856	NR_037242:-592	Mir3081	PROMOTER	0.779	0.497	1868.49	927.99	0.387	1386.81	536.80
A_68_P29110674	chr13:68720849-68720893	NM_172480:128	Mtrr	INSIDE	0.779	0.238	7504.42	1787.14	0.185	5383.69	998.63
A_68_P28105678	chr11:107050241-107050288	NM_001161329:431	Nol11	INSIDE	0.779	0.247	4023.54	994.46	0.192	2539.59	488.67
A_68_P27894945	chr11:69224208-69224252	NM_001017426:2947	Kdm6b	INSIDE	0.779	0.633	1475.72	934.28	0.493	1212.89	597.99
A_68_P27835444	chr11:59016216-59016260	NM_026035:220	Mrpl55	INSIDE	0.779	0.572	932.80	533.79	0.446	767.47	341.97
A_68_P27560708	chr11:4887125-4887169	NM_007454:239	Ap1b1	INSIDE	0.779	0.366	5216.98	1911.19	0.285	3980.83	1135.97
A_68_P27286893	chr10:80477575-80477619	NM_010319:74	Gng7	INSIDE	0.779	0.353	2369.49	837.50	0.275	1897.03	522.22
A_68_P26587298	chr9:65219738-65219782	NM_001111145:10894	Gm514	INSIDE	0.779	2.887	1421.33	4103.24	2.248	1164.54	2618.12
A_68_P25448397	chr7:123647931-123647975	NM_016773:65	Nucb2	INSIDE	0.779	0.441	2959.60	1304.75	0.343	2470.13	848.22
A_68_P25278640	chr7:89797291-89797335	NM_001159672:189	Eftud1	INSIDE	0.779	0.419	2842.60	1192.41	0.327	1975.57	645.27
A_68_P25008564	chr7:25918157-25918201	NM_001163554:-699	Pou2f2	PROMOTER	0.779	0.431	1124.82	484.72	0.336	1001.33	335.98
A_68_P24990860	chr7:19590227-19590271	NM_178757:835	Irf2bp1	INSIDE	0.779	1.926	1951.76	3759.63	1.500	1847.39	2771.04
A_68_P24595550	chr6:83745123-83745167	NM_001164187:-7	Nagk	PROMOTER	0.779	0.422	2074.74	874.64	0.329	1402.63	460.83
A_68_P24319038	chr6:28165391-28165435	ENSMUST00000143099:-142		PROMOTER	0.779	2.967	617.46	1831.76	2.312	572.11	1322.49
A_68_P24265237	chr6:17015070-17015114	NM_207176:-56	Tes	PROMOTER	0.779	3.218	857.87	2761.05	2.506	755.80	1893.91
A_68_P24182267	chr5:149740566-149740610	NM_153572:-365	Katnal1	PROMOTER	0.779	0.509	2289.09	1164.40	0.396	1749.05	693.18
A_68_P23331579	chr4:135543347-135543391	NM_025411:-209	1110049F12Rik	PROMOTER	0.779	0.501	1701.50	852.75	0.390	1446.59	564.54
A_68_P22185880	chr3:61963190-61963234			Unknown	0.779	2.082	1375.37	2863.08	1.621	1188.22	1926.03
A_68_P20852903	chr1:178204921-178204965	NM_176916:501	Pld5	INSIDE	0.779	0.327	1473.46	481.94	0.255	1161.41	295.79
A_68_P31926801	chr19:5085238-5085283	NM_001001885:217	Tmem151a	INSIDE	0.778	0.419	1232.77	516.70	0.326	1014.13	330.86
A_68_P31215267	chr17:48111395-48111439			Unknown	0.778	3.818	746.96	2851.66	2.971	617.87	1835.87
A_68_P30560746	chr16:14163433-14163477	NM_001114085:87	Nde1	INSIDE	0.778	0.648	2707.89	1754.91	0.504	2276.17	1148.22
A_68_P30464713	chr15:96472950-96472994	NM_001166456:-198	Slc38a1	PROMOTER	0.778	0.300	3064.82	920.72	0.234	1992.58	465.52
A_68_P29449128	chr14:21612623-21612667	NM_001039138:666	Camk2g	INSIDE	0.778	0.709	3725.37	2641.38	0.552	2746.88	1516.18
A_68_P28046786	chr11:96650730-96650774	NM_007622:303	Cbx1	INSIDE	0.778	0.337	1489.60	502.14	0.262	1149.14	301.46
A_68_P27910209	chr11:72028885-72028929	NM_026068:188	Med31	INSIDE	0.778	2.596	813.86	2112.65	2.020	726.88	1468.50
A_68_P26897418	A_68_P26897418			Unknown	0.778	0.446	3314.99	1477.10	0.347	2453.86	850.73
A_68_P26222126	chr8:123259917-123259961	NM_008320:-337	Irf8	PROMOTER	0.778	0.476	1746.76	832.11	0.371	1275.32	472.75
A_68_P25016727	chr7:28116145-28116189	NM_001113549:2501	Ltbp4	INSIDE	0.778	5.131	1249.79	6412.67	3.992	1072.02	4279.57
A_68_P24535482	chr6:71221514-71221558	NM_145568:-476	Krcc1	DIVERGENT_PROMOTER	0.778	6.615	2547.32	16850.43	5.148	2052.12	10564.93
A_68_P24141720	chr5:140981575-140981619	NM_021528:34	Chst12	INSIDE	0.778	0.561	2209.17	1238.69	0.436	1742.47	759.81
A_68_P23966965	chr5:107867055-107867099	NM_027599:6509	Lpcat2b	DOWNSTREAM	0.778	4.804	742.10	3565.03	3.737	701.80	2622.95

ProbeName	Target position of probe on CpG island microarray	TargetID	GeneSymbol	CpG island Description	Ratio of relative methylation (TiO_2-NP/Vehicle)	Sham group			TiO_2-H group		
						Relative methylation (Cy5/Cy3)	Cy3 signal (Input)	Cy5 signal (MeDIP)	Relative methylation (Cy5/Cy3)	Cy3 signal (Input)	Cy5 signal (MeDIP)
A_68_P23607764	chr5:36807549-36807593	NM_024478:-263	Grpel1	PROMOTER	0.778	2.218	2826.41	6270.11	1.725	2208.11	3808.92
A_68_P23299528	chr4:129663369-129663413	NM_001199696:1069	Bai2	INSIDE	0.778	0.407	4552.66	1851.40	0.316	3155.55	998.18
A_68_P22448207	chr3:115954695-115954739	NM_022427:1686	Gpr88	INSIDE	0.778	3.739	1306.20	4883.31	2.907	1225.15	3561.49
A_68_P21907946	chr2:180763743-180763787	NM_015730:10118	Chrna4	INSIDE	0.778	6.148	2965.72	18234.14	4.781	1885.54	9015.31
A_68_P21863764	chr2:172171330-172171374	NM_025912:276	2010011I20Rik	INSIDE	0.778	3.532	1181.67	4173.50	2.747	988.11	2714.39
A_68_P21817649	chr2:164230176-164230220	NM_013592:-84	Matn4	PROMOTER	0.778	0.743	3108.39	2309.35	0.578	2663.57	1540.38
A_68_P21322607	chr2:71819465-71819509	NM_001204167:215	Rapgef4	INSIDE	0.778	0.415	1469.30	609.64	0.323	1014.28	327.56
A_68_P20285211	chr1:63160025-63160069	NM_001081436:795	Ino80d	INSIDE	0.778	0.407	2015.71	819.86	0.317	1763.77	558.47
A_68_P20184654	chr1:42751615-42751659	NR_027826:34	2610017I09Rik	INSIDE	0.778	0.226	4596.33	1038.87	0.176	2994.22	526.85
A_68_P32203281	chr19:56900903-56900947	NM_001002241:-4277	Tdrd1	PROMOTER	0.777	3.678	1763.26	6485.12	2.858	1310.06	3744.00
A_68_P31774692	chr18:64500898-64500942	NM_194268:903	Onecut2	INSIDE	0.777	4.408	1753.42	7728.67	3.427	1509.86	5173.86
A_68_P31757527	chr18:61558226-61558270	NM_133249:1837	Ppargc1b	INSIDE	0.777	2.088	1592.02	3324.24	1.623	1256.34	2039.57
A_68_P30571779	chr16:17070377-17070421	NM_023249:-5	Ypel1	PROMOTER	0.777	4.708	1089.23	5127.64	3.658	902.42	3300.86
A_68_P29342440	chr13:114584633-114584677	NM_172595:-61	Arl15	PROMOTER	0.777	0.376	2478.22	930.98	0.292	1835.53	535.57
A_68_P28538325	chr12:74686617-74686661	NM_008856:611	Prkch	INSIDE	0.777	0.575	889.77	511.25	0.446	760.34	339.39
A_68_P28513753	chr12:70397631-70397675	NM_027117:-15	Klhdc2	PROMOTER	0.777	2.076	1244.74	2583.52	1.613	1099.78	1774.36
A_68_P28155818	chr11:115719846-115719890	NM_145438:34497	Llgl2	DOWNSTREAM	0.777	1.888	1655.32	3125.47	1.467	1240.26	1819.40
A_68_P27845262	chr11:60691840-60691884	NM_001168507:678	Tmem11	INSIDE	0.777	0.488	4574.96	2231.27	0.379	3609.72	1368.76
A_68_P26940376	chr10:11063434-11063478	NM_010146:214	Epm2a	INSIDE	0.777	2.644	484.77	1281.67	2.055	431.14	886.13
A_68_P24772424	chr6:115626535-115626579	NM_029780:97	Raf1	INSIDE	0.777	0.523	1265.73	661.58	0.406	998.86	405.76
A_68_P24000795	chr5:114358894-114358938	NM_011779:-201	Coro1c	PROMOTER	0.777	2.712	2460.67	6672.96	2.107	2135.95	4500.95
A_68_P22382659	chr3:102539232-102539276	NM_027533:562	Tspan2	INSIDE	0.777	0.352	3383.38	1191.34	0.274	2722.71	745.33
A_68_P22028775	chr3:30408163-30408207	NM_021442:225	Mecom	INSIDE	0.777	4.976	1907.69	9493.59	3.865	1562.76	6039.50
A_68_P21019117	chr2:13995664-13995708	NM_011484:-52	Stam	DIVERGENT_PROMOTER	0.777	0.732	2116.80	1550.40	0.569	1662.94	945.92
A_68_P20405965	chr1:84835584-84835628	NM_133975:273	Trip12	INSIDE	0.777	2.705	1723.10	4660.55	2.102	1743.09	3663.12
A_68_P31850953	chr18:78245555-78245599	NM_001101038:8431	Siglec15	INSIDE	0.776	1.915	1394.70	2671.49	1.487	1107.57	1646.93
A_68_P31144038	A_68_P31144038			Unknown	0.776	3.637	797.66	2901.11	2.821	654.75	1847.26
A_68_P30476324	chr15:98593039-98593083	NM_007478:489	Arf3	INSIDE	0.776	0.292	1580.91	461.12	0.226	1218.44	275.62
A_68_P30368655	chr15:79634703-79634747	NM_030689:415	Nptxr	INSIDE	0.776	14.002	2072.27	29016.23	10.865	1905.73	20706.05
A_68_P29308209	chr13:108681343-108681387	NM_145456:-1106	Zswim6	PROMOTER	0.776	2.117	922.89	1953.31	1.642	772.32	1267.82
A_68_P28672826	chr12:100139771-100139815	NM_001081191:-98	Eml5	PROMOTER	0.776	2.922	1243.08	3632.29	2.267	1096.29	2484.76
A_68_P27180706	chr10:60938993-60939037	NM_153542:434	Lrrc20	INSIDE	0.776	1.896	1385.51	2626.98	1.472	1109.63	1633.31
A_68_P26567740	chr9:61761969-61762013	NM_018853:327	Rplp1	INSIDE	0.776	0.295	2976.69	877.05	0.229	2039.74	466.37
A_68_P26346393	chr9:21095562-21095606	NM_009878:69	Cdkn2d	INSIDE	0.776	2.150	1438.73	3092.94	1.669	1389.58	2319.39
A_68_P25187998	chr7:73533035-73533079	NM_146191:171	Lrrk1	INSIDE	0.776	0.328	2858.00	938.44	0.255	2232.42	568.80
A_68_P25151569	chr7:64846153-64846197	NM_008071:-368	Gabrb3	PROMOTER	0.776	0.608	2367.07	1438.29	0.472	1883.23	888.33
A_68_P24943610	chr6:149049573-149049620	NM_177192:606	Dennd5b	INSIDE	0.776	0.195	2972.79	578.32	0.151	2224.90	335.87
A_68_P24697527	chr6:101327639-101327683	NM_018884:231	Pdzrn3	INSIDE	0.776	2.510	755.37	1896.28	1.948	647.94	1262.08
A_68_P24476448	chr6:56665017-56665061	NM_030235:140	Avl9	INSIDE	0.776	0.475	1167.75	554.48	0.368	1061.64	391.14
A_68_P23409800	chr4:150431562-150431606	NM_009498:478	Vamp3	INSIDE	0.776	1.913	2103.12	4023.47	1.484	1764.01	2617.15
A_68_P22765608	chr4:19497196-19497240	NM_027769:33	Cpne3	INSIDE	0.776	0.608	1797.85	1093.40	0.472	1491.53	703.77
A_68_P21108246	chr2:31170158-31170202	ENSMUST00000113532:246		INSIDE	0.776	0.349	3483.30	1214.76	0.271	2400.65	649.99
A_68_P20937887	chr1:193221193-193221237	NM_144880:-294	Ppp2r5a	PROMOTER	0.776	0.552	4995.61	2755.53	0.428	3417.09	1463.41
A_68_P20734230	chr1:156646554-156646598	AK046991:-6703		PROMOTER	0.776	5.187	821.99	4263.26	4.024	794.76	3197.73
A_68_P32823843	chrX:166435357-166435401	NM_183151:117825	Mid1	DOWNSTREAM	0.775	2.004	19191.24	38462.04	1.553	16043.32	24911.69
A_68_P32117489	chr19:41921912-41921956	NM_177603:688	Frat2	INSIDE	0.775	5.142	962.23	4947.50	3.984	748.01	2980.03
A_68_P31863476	chr18:80492765-80492809	NM_001190373:68207	Kcng2	INSIDE	0.775	2.061	922.95	1902.18	1.596	794.40	1268.10
A_68_P30651593	chr16:32247283-32247327	NM_001185162:-8	Wdr53	DIVERGENT_PROMOTER	0.775	0.707	1106.58	781.87	0.548	911.72	499.53
A_68_P30401621	chr15:85411484-85411528	NM_001163633:-347	Wnt7b	PROMOTER	0.775	2.396	744.79	1784.21	1.858	704.03	1307.83
A_68_P30246247	chr15:57966117-57966161	NM_027435:499	Atad2	INSIDE	0.775	0.586	2012.77	1179.70	0.454	1735.77	788.52
A_68_P29651207	chr14:62267059-62267103	NR_028264:34130	Dleu2	INSIDE	0.775	2.903	454.45	1319.32	2.249	377.66	849.36
A_68_P29072113	chr13:58317002-58317046	NM_026842:-10	Ubqln1	PROMOTER	0.775	2.754	681.88	1878.15	2.135	654.97	1398.28
A_68_P28469856	chr12:60113095-60113139	NM_009147:-112	Sec23a	DIVERGENT_PROMOTER	0.775	2.020	1939.77	3918.18	1.566	1389.74	2176.49
A_68_P27898022	chr11:69749285-69749329	NM_016875:-94	Ybx2	PROMOTER	0.775	0.596	1527.55	910.45	0.462	1351.21	624.51
A_68_P27542104	chr10:128007271-128007315	NM_010153:19265	Erbb3	INSIDE	0.775	2.339	528.24	1235.59	1.812	528.07	956.91
A_68_P27535080	chr10:126727505-126727549	NM_007837:-322	Ddit3	DIVERGENT_PROMOTER	0.775	0.133	6372.39	850.29	0.103	4609.80	476.42
A_68_P25508220	chr7:135079173-135079217	NM_001081374:11045	Prss36	INSIDE	0.775	0.564	2587.93	1458.92	0.437	2029.05	886.39
A_68_P25032388	chr7:31373251-31373295	NM_029274:473	Wbp7	INSIDE	0.775	0.218	2914.09	636.39	0.169	1914.17	324.13
A_68_P25016045	chr7:27980981-27981025	NM_001046637:-223	Snrpa	PROMOTER	0.775	0.671	6294.90	4222.81	0.520	4764.51	2477.33
A_68_P24541278	chr6:72305613-72305657	NM_144916:158	Tmem150a	INSIDE	0.775	0.158	3731.13	590.12	0.123	2590.83	317.74
A_68_P24469550	chr6:55401858-55401902	NM_001025372:-93	Adcyap1r1	PROMOTER	0.775	0.189	3037.93	573.43	0.146	2248.63	328.86
A_68_P23399811	chr4:148681546-148681590	NM_207682:239	Kif1b	INSIDE	0.775	3.246	596.54	1936.53	2.517	503.46	1267.37
A_68_P23332455	chr4:135728739-135728783	NM_177733:452	E2f2	INSIDE	0.775	2.456	1048.93	2576.01	1.903	927.58	1764.91
A_68_P23325516	chr4:134470628-134470672	NM_027935:181	Tmem50a	INSIDE	0.775	0.607	831.57	504.81	0.470	738.34	347.28
A_68_P23012915	chr4:71861101-71861145	NM_011599:831	Tle1	INSIDE	0.775	2.047	1336.82	2735.99	1.586	1189.23	1885.72

						Sham group			TiO_2-H group		
ProbeName	Target position of probe on CpG island microarray	TargetID	GeneSymbol	CpG island Description	Ratio of relative methylation (TiO_2-NP/Vehicle)	Relative methylation (Cy5/Cy3)	Cy3 signal (Input)	Cy5 signal (MeDIP)	Relative methylation (Cy5/Cy3)	Cy3 signal (Input)	Cy5 signal (MeDIP)
A_68_P21751499	chr2:152673945-152673989	NM_001141975:267	Tpx2	INSIDE	0.775	0.494	1314.68	650.03	0.383	1089.45	417.61
A_68_P21579354	chr2:120997006-120997050	NM_153387:87	Tubgcp4	INSIDE	0.775	0.573	949.39	544.46	0.444	809.77	359.74
A_68_P20768941	chr1:162836493-162836537	NM_001024952:-27	Rc3h1	PROMOTER	0.775	0.672	1124.44	755.81	0.521	933.04	485.95
A_68_P20304094	chr1:66747456-66747500	NM_025683:12	Rpe	INSIDE	0.775	0.509	1697.93	863.71	0.394	1389.28	547.56
A_68_P20156313	chr1:37921860-37921904	NM_207228:42	Tsga10	INSIDE	0.775	1.910	1690.75	3228.81	1.480	1367.96	2024.43
A_68_P33008827	chr4_random:110161-110205	NM_001033326:381	Dhrsx	INSIDE	0.774	2.928	2715.90	7952.93	2.268	2331.87	5288.13
A_68_P33005865	A_68_P33005865			Unknown	0.774	8.327	509.25	4240.70	6.442	445.94	2872.88
A_68_P31937110	chr19:7054912-7054956	NM_008020:-4	Fkbp2	PROMOTER	0.774	0.397	1165.53	463.15	0.308	911.08	280.18
A_68_P31140910	chr17:31773447-31773491	NM_178224:608	Cbs	INSIDE	0.774	0.356	2766.12	985.28	0.276	1881.37	518.42
A_68_P31110247	chr17:26576151-26576195	NM_001081656:24263	Neurl1b	INSIDE	0.774	3.738	1015.65	3796.18	2.892	883.04	2553.38
A_68_P31108130	chr17:26205973-26206017	NM_001162868:128	Rab11fip3	INSIDE	0.774	0.500	2136.16	1068.04	0.387	1644.95	636.98
A_68_P31106870	chr17:26005145-26005189	NM_026633:517	Fam195a	INSIDE	0.774	0.408	1247.49	508.96	0.316	936.21	295.49
A_68_P30822071	chr16:64852666-64852710	NM_178647:778	Cggbp1	INSIDE	0.774	2.172	771.30	1675.09	1.682	707.61	1190.16
A_68_P29715888	chr14:73543452-73543496	NR_033185:883	Rcbtb2	INSIDE	0.774	2.277	1750.69	3985.59	1.762	1491.57	2628.49
A_68_P28156774	chr11:115866202-115866246	NM_001005608:30186	Itgb4	INSIDE	0.774	0.694	1354.48	939.96	0.537	1072.99	576.50
A_68_P27950225	chr11:79474212-79474256	NM_175543:69521	Rab11fip4	INSIDE	0.774	0.417	2007.84	837.71	0.323	1447.10	467.39
A_68_P27281410	chr10:79627471-79627515	NM_007705:-3093	Cirbp	PROMOTER	0.774	2.346	1106.50	2595.35	1.816	829.00	1505.20
A_68_P27186312	chr10:61949993-61950037	NM_133672:-461	Vps26a	PROMOTER	0.774	0.707	3451.43	2439.02	0.547	2405.83	1316.55
A_68_P25770373	chr8:34842329-34842373	NM_001167921:-35	Gtf2e2	PROMOTER	0.774	3.206	682.70	2188.56	2.480	663.47	1645.38
A_68_P23993053	chr5:113006095-113006139	NM_019982:89	Sez6l	INSIDE	0.774	0.285	3792.95	1082.13	0.221	2841.74	627.77
A_68_P23588231	chr5:33678100-33678145	NM_021500:-98	Maea	PROMOTER	0.774	0.349	2476.77	863.43	0.270	1879.00	506.72
A_68_P23325563	chr4:134480163-134480207	NM_023665:645	D4Wsu53e	INSIDE	0.774	3.008	467.58	1406.52	2.328	408.24	950.28
A_68_P23081884	chr4:86520053-86520097	NM_139306:-243	Acer2	PROMOTER	0.774	0.507	1835.76	930.49	0.393	1407.80	552.57
A_68_P22325451	chr3:90280267-90280311	NM_026374:166	Ilf2	INSIDE	0.774	5.169	4611.90	23837.14	4.003	3714.67	14868.31
A_68_P20735710	chr1:156930629-156930673	ENSMUST00000128143:-254		PROMOTER	0.774	1.874	2276.75	4266.44	1.450	1799.08	2608.45
A_68_P31162030	chr17:36116010-36116054	NR_028516:-64	Prr3	PROMOTER	0.773	0.528	3072.53	1622.94	0.408	2268.62	925.98
A_68_P30991548	chr16:96414007-96414051	NM_001001492:-164	Lca5l	DIVERGENT_PROMOTER	0.773	0.537	1371.78	737.01	0.415	1078.08	447.68
A_68_P30991214	chr16:96349614-96349659	NM_008251:-304	Hmgn1	PROMOTER	0.773	0.566	2072.97	1172.40	0.437	1626.44	711.30
A_68_P29512056	chr14:32249107-32249151	NM_011894:91	Sh3bp5	INSIDE	0.773	2.542	406.86	1034.19	1.965	404.23	794.47
A_68_P29009046	chr13:46764820-46764864	NM_001033192:-48	C78339	PROMOTER	0.773	2.547	806.07	2053.23	1.969	613.43	1207.80
A_68_P27943642	chr11:78304280-78304324	NM_001168521:6954	Sarm1	INSIDE	0.773	0.379	1402.68	531.81	0.293	1118.59	327.76
A_68_P27797797	chr11:51981491-51981535	ENSMUST00000155549:101504		INSIDE	0.773	2.950	661.27	1950.90	2.282	511.07	1166.09
A_68_P27542079	chr10:128003411-128003455	NM_011119:-442	Pa2g4	PROMOTER	0.773	0.634	3138.58	1989.95	0.490	2224.42	1089.92
A_68_P27065106	chr10:36226802-36226846	NM_001081208:212	Hs3st5	INSIDE	0.773	1.903	1442.51	2744.53	1.470	1210.08	1778.94
A_68_P25948617	chr8:72426740-72426784	NM_023312:-305	Ndufa13	PROMOTER	0.773	0.365	1678.08	612.04	0.282	1169.66	329.57
A_68_P25279948	chr7:90015813-90015857	NM_175366:-8	Mex3b	PROMOTER	0.773	0.632	1171.95	741.09	0.489	937.83	458.67
A_68_P24464318	chr6:54545243-54545287	NM_001001335:-7876	Plekha8	PROMOTER	0.773	4.055	387.22	1570.01	3.133	381.19	1194.34
A_68_P23566427	chr5:29522649-29522693	NM_021470:-1875	Rnf32	PROMOTER	0.773	0.262	1775.74	464.74	0.202	1242.31	251.30
A_68_P23253012	chr4:120419677-120419721	NM_001081142:83	Kcnq4	INSIDE	0.773	3.709	1171.51	4345.04	2.865	1015.77	2910.32
A_68_P21682772	chr2:139996502-139996546	NM_027093:143	2310003L22Rik	INSIDE	0.773	0.623	989.93	617.09	0.482	806.47	388.76
A_68_P21152637	chr2:39045958-39046002	NM_178778:270	Scai	INSIDE	0.773	0.500	1574.20	786.60	0.386	1328.67	512.91
A_68_P31961511	chr19:12670617-12670661	NR_015503:418	A330040F15Rik	INSIDE	0.772	10.201	2142.62	21857.40	7.876	1808.03	14239.98
A_68_P31713390	chr18:53624738-53624782	NM_001033281:561	Prdm6	INSIDE	0.772	0.525	1662.06	871.98	0.405	1132.66	458.61
A_68_P31678446	chr18:46887405-46887449	NM_033037:570	Cdo1	INSIDE	0.772	0.394	1793.91	706.12	0.304	1341.46	407.50
A_68_P31601518	chr18:32322635-32322679	NM_011946:-86	Map3k2	PROMOTER	0.772	3.131	694.72	2175.06	2.416	649.77	1570.13
A_68_P30956057	chr16:90283975-90284019	NM_178923:674	Scaf4	INSIDE	0.772	2.600	733.35	1906.51	2.008	655.84	1316.91
A_68_P30478214	chr15:98926989-98927033	NM_176835:-2904	Dnajc22	DIVERGENT_PROMOTER	0.772	0.481	2140.43	1029.26	0.371	1808.44	671.72
A_68_P30404619	chr15:85863596-85863640	NM_009886:589	Celsr1	INSIDE	0.772	2.152	811.54	1746.02	1.661	683.70	1135.46
A_68_P30401334	chr15:85374054-85374098	NM_001163634:34424	Wnt7b	INSIDE	0.772	2.945	1622.09	4776.73	2.274	1407.54	3200.19
A_68_P29625000	chr14:57484103-57484147	AK209334:-600		PROMOTER	0.772	2.475	1666.85	4125.77	1.911	1122.13	2144.41
A_68_P29561105	chr14:41886163-41886207	NM_027094:-15	Dydc1	DIVERGENT_PROMOTER	0.772	0.560	1155.59	647.55	0.432	951.62	411.41
A_68_P29269007	chr13:101322013-101322057	NM_008756:419	Ocln	INSIDE	0.772	0.520	4783.74	2487.14	0.401	3380.10	1356.33
A_68_P28351020	chr12:35732452-35732496	NM_001014973:614	Snx13	INSIDE	0.772	0.627	1614.70	1012.92	0.484	1367.46	662.24
A_68_P28060080	chr11:98902347-98902391	NM_010517:-205	Igfbp4	PROMOTER	0.772	0.235	2636.89	619.70	0.181	1767.01	320.53
A_68_P27846110	chr11:60836644-60836688	ENSMUST00000089184:601		INSIDE	0.772	0.465	1024.47	476.11	0.359	834.19	299.26
A_68_P27351980	chr10:92623972-92624016	NR_035452:-1535	Mir1931	PROMOTER	0.772	0.497	2301.23	1142.58	0.383	1869.27	716.28
A_68_P26985108	chr10:19868390-19868434	NM_001198635:-313	Mtap7	PROMOTER	0.772	0.637	1057.08	673.07	0.492	919.44	452.23
A_68_P26891990	chr9:122812568-122812612	NM_001177505:15002	Zfp167	DOWNSTREAM	0.772	0.388	2200.91	854.43	0.300	1764.25	528.65
A_68_P26844413	chr9:114299730-114299774	NM_019922:78	Crtap	INSIDE	0.772	2.589	1477.50	3825.80	1.998	1301.22	2599.74
A_68_P26804413	chr9:106558146-106558190	NM_001160353:272	Grm2	INSIDE	0.772	0.368	2199.79	808.77	0.284	1707.20	484.27
A_68_P26736540	chr9:94437557-94437601	NM_001033145:922	1190002N15Rik	INSIDE	0.772	0.604	1684.36	1017.69	0.466	1302.31	607.37
A_68_P26171405	chr8:114535913-114535957	NM_020584:676	Terf2ip	INSIDE	0.772	0.514	1023.10	526.34	0.397	742.36	294.74
A_68_P25591623	chr7:149088055-149088099	NM_023764:235	Tollip	INSIDE	0.772	2.102	1095.80	2303.38	1.624	833.76	1353.65
A_68_P25283749	chr7:90780539-90780583	NM_023377:34	Stard5	INSIDE	0.772	0.699	1343.33	938.91	0.540	1114.24	601.43
A_68_P25182467	chr7:72515721-72515765	NM_001163574:388	Tjp1	INSIDE	0.772	2.461	814.27	2004.23	1.901	656.68	1248.23

ProbeName	Target position of probe on CpG island microarray	TargetID	GeneSymbol	CpG island Description	Ratio of relative methylation (TiO_2-NP/Vehicle)	Sham group			TiO_2-H group		
						Relative methylation (Cy5/Cy3)	Cy3 signal (Input)	Cy5 signal (MeDIP)	Relative methylation (Cy5/Cy3)	Cy3 signal (Input)	Cy5 signal (MeDIP)
A_68_P24624715	chr6:88824256-88824300	NM_176973:-240	Podxl2	PROMOTER	0.772	0.272	2632.89	715.98	0.210	1950.88	409.82
A_68_P23955152	chr5:105844399-105844443	NM_001033550:-373	Lrrc8b	PROMOTER	0.772	0.524	965.16	505.63	0.405	782.51	316.60
A_68_P23321241	chr4:133684793-133684837	NM_080559:-146	Sh3bgrl3	PROMOTER	0.772	4.118	2106.49	8673.58	3.178	1659.40	5273.41
A_68_P22934416	chr4:55543231-55543275	NM_010637:2095	Klf4	INSIDE	0.772	0.419	1377.92	577.30	0.323	1055.20	341.22
A_68_P21928444	chr3:7366471-7366516	NM_008862:-110	Pkia	PROMOTER	0.772	0.400	1437.75	575.61	0.309	1086.79	335.72
A_68_P20741944	chr1:158134876-158134920	NM_177838:258	Fam163a	INSIDE	0.772	2.397	1163.65	2789.17	1.851	1064.85	1970.63
A_68_P20616235	chr1:133422616-133422664	NM_001081011:1298	Srgap2	INSIDE	0.772	2.913	456.81	1330.68	2.248	386.24	868.24
A_68_P20162424	chr1:38893271-38893315	ENSMUST00000147695:-357		PROMOTER	0.772	2.100	1153.00	2420.75	1.621	1046.86	1696.65
A_68_P31614303	chr18:34879926-34879970	NM_010280:93	Gfra3	INSIDE	0.771	0.308	2382.50	734.44	0.238	2001.64	475.64
A_68_P30446387	chr15:93166777-93166821	NM_024189:568	Yaf2	INSIDE	0.771	0.608	1345.69	818.06	0.469	1033.75	484.55
A_68_P29363818	chr13:118391526-118391570	NM_010408:422	Hcn1	INSIDE	0.771	2.183	1229.72	2684.59	1.683	961.57	1618.76
A_68_P28941006	chr13:34967366-34967410	NM_013830:26	Prpf4b	INSIDE	0.771	0.361	1433.78	517.38	0.278	1088.61	302.69
A_68_P28923671	chr13:31650063-31650107	NM_008239:46	Foxq1	INSIDE	0.771	2.661	1376.84	3663.63	2.051	1182.36	2425.21
A_68_P27892698	chr11:68853700-68853744	NM_011496:-5422	Aurkb	PROMOTER	0.771	0.391	3640.00	1424.37	0.302	2483.21	749.03
A_68_P27827566	chr11:57614919-57614965	NM_001081168:-196	Sap30l	PROMOTER	0.771	0.482	1178.60	568.51	0.372	773.22	287.45
A_68_P27355933	chr10:93296604-93296648	NM_183199:2327	Usp44	INSIDE	0.771	0.400	3158.59	1264.91	0.309	2690.35	831.19
A_68_P27327692	chr10:88289255-88289299	NM_175158:283	Utp20	INSIDE	0.771	0.580	1333.29	772.88	0.447	1057.59	472.37
A_68_P27302613	chr10:83903670-83903714	NM_001004363:-476	Nuak1	PROMOTER	0.771	2.884	1082.65	3122.58	2.225	803.39	1787.53
A_68_P27280123	chr10:79439661-79439705	NM_130455:6214	Grin3b	INSIDE	0.771	2.279	1267.82	2889.57	1.758	1106.86	1946.00
A_68_P27222450	chr10:68675577-68675621	NM_001081347:194	Rhobtb1	INSIDE	0.771	3.519	4995.79	17581.44	2.713	3838.54	10415.42
A_68_P26702532	chr9:86910466-86910510	NM_001037907:117	Ripply2	INSIDE	0.771	0.410	6811.84	2791.30	0.316	5178.89	1636.34
A_68_P26397765	chr9:30750581-30750625	NM_013906:455	Adamts8	INSIDE	0.771	2.856	379.88	1085.12	2.204	399.47	880.27
A_68_P26347946	chr9:21397416-21397460	NM_001205157:-163	Yipf2	PROMOTER	0.771	0.289	2189.75	633.00	0.223	1608.95	358.67
A_68_P25261278	chr7:86611752-86611796	NM_017462:-615	Polg	PROMOTER	0.771	0.524	1153.97	604.26	0.404	909.17	366.87
A_68_P25034254	chr7:31741889-31741933	NM_011680:-88	Usf2	PROMOTER	0.771	0.249	2075.47	516.56	0.192	1519.64	291.51
A_68_P24804129	chr6:121362899-121362943	NM_001033354:60776	Iqsec3	INSIDE	0.771	0.690	1073.34	740.81	0.532	879.45	468.20
A_68_P24424176	chr6:47601491-47601535	NR_002841:3581	Rn4.5s	DOWNSTREAM	0.771	0.340	9422.32	3200.07	0.262	7099.40	1859.58
A_68_P23336932	chr4:136501941-136501985	NM_007939:10769	Epha8	INSIDE	0.771	3.220	1612.82	5192.68	2.482	1156.04	2869.01
A_68_P23145557	chr4:99323922-99323966	NM_010425:955	Foxd3	INSIDE	0.771	0.700	1354.85	948.23	0.540	1071.84	578.50
A_68_P22412465	chr3:108209988-108210032	NM_001004177:8402	Celsr2	INSIDE	0.771	2.002	2315.72	4636.37	1.543	1623.40	2504.53
A_68_P21765151	chr2:155183097-155183141	NM_001004721:42	Pigu	INSIDE	0.771	2.670	2127.37	5679.07	2.058	1833.71	3773.15
A_68_P21476820	chr2:102240650-102240694	NM_020267:385	Trim44	INSIDE	0.771	3.467	454.44	1575.48	2.673	434.33	1161.12
A_68_P21133756	chr2:35318047-35318091	NM_001145821:-1123	Ggta1	PROMOTER	0.771	0.704	1263.65	889.78	0.543	1153.99	626.72
A_68_P20639346	chr1:137327066-137327110	NM_153774:-20	Ipo9	PROMOTER	0.771	2.718	1083.56	2944.96	2.095	854.44	1789.70
A_68_P31769419	chr18:63546533-63546577	NM_001039485:283	Fam38b	INSIDE	0.770	2.730	726.53	1983.21	2.102	693.19	1457.24
A_68_P31208520	chr17:46848930-46848974	NM_145488:541	Pex6	INSIDE	0.770	2.297	2101.85	4828.09	1.768	1612.23	2851.13
A_68_P31098019	chr17:24687487-24687532	NM_013630:615	Pkd1	INSIDE	0.770	6.236	1457.86	9091.16	4.804	1210.24	5814.23
A_68_P29288686	chr13:104899839-104899883	NM_144838:-9	Sgtb	DIVERGENT_PROMOTER	0.770	0.265	3007.17	795.63	0.204	2304.51	469.37
A_68_P29117879	chr13:69948975-69949019	NM_138596:237	Med10	INSIDE	0.770	4.268	594.76	2538.51	3.287	654.06	2149.97
A_68_P28396961	chr12:45311078-45311122	NM_013760:-45	Dnajb9	PROMOTER	0.770	0.313	1977.62	618.82	0.241	1475.74	355.57
A_68_P27340135	chr10:90586440-90586484	NM_133668:246	Slc25a3	INSIDE	0.770	5.232	970.05	5075.02	4.027	857.71	3453.76
A_68_P27283984	chr10:80024121-80024165	NM_025852:163	Rexo1	INSIDE	0.770	0.540	947.13	511.58	0.416	792.34	329.39
A_68_P27165511	chr10:58276497-58276541	NM_172788:412	Sh3rf3	INSIDE	0.770	0.296	1645.41	487.80	0.228	1252.96	285.86
A_68_P26586059	chr9:64989179-64989223	NM_008988:205	Igdcc3	INSIDE	0.770	2.949	1383.13	4078.90	2.270	1238.80	2811.91
A_68_P26240284	chr8:126038626-126038670	NM_001170976:707	Dbndd1	INSIDE	0.770	0.268	2446.78	655.71	0.206	1837.27	379.19
A_68_P26129564	chr8:107154419-107154466	NM_019662:779	Rrad	INSIDE	0.770	0.447	1337.40	597.80	0.344	1190.67	409.64
A_68_P25922763	chr8:66684161-66684205	NM_009390:608	Tll1	INSIDE	0.770	3.325	539.47	1793.68	2.559	459.08	1174.62
A_68_P25430230	chr7:120513248-120513292	NM_133700:-417	Btbd10	PROMOTER	0.770	3.047	1295.24	3946.90	2.347	1014.82	2381.78
A_68_P25048709	chr7:36103373-36103417	NM_026181:65	Gpatch1	INSIDE	0.770	0.532	1398.16	743.90	0.409	1140.25	466.86
A_68_P24459340	chr6:53769060-53769104	NM_025817:1737	Tril	INSIDE	0.770	0.204	4214.44	857.95	0.157	2632.06	412.80
A_68_P24112800	chr5:135164852-135164896	NM_010717:-414	Limk1	PROMOTER	0.770	3.648	1560.54	5693.00	2.811	1198.84	3369.44
A_68_P23297752	chr4:129318175-129318219	NM_001005506:-147	Txlna	PROMOTER	0.770	0.573	932.19	534.31	0.442	832.41	367.61
A_68_P22775166	chr4:21775651-21775695	NR_028384:-77	4930528A17Rik	PROMOTER	0.770	0.500	1486.76	743.27	0.385	1201.38	462.17
A_68_P20416261	chr1:89083435-89083479	NM_027084:3389	1700027L20Rik	INSIDE	0.770	0.279	1654.45	460.86	0.215	1313.20	281.72
A_68_P20046439	chr1:14496966-14497010			Unknown	0.770	0.192	2507.69	481.11	0.148	1795.05	265.08
A_68_P33015622	chr1_random:50759-50807	NM_026866:134556	Disp1	INSIDE	0.769	2.901	822.06	2384.39	2.229	568.53	1267.47
A_68_P32364770	chrX:45807712-45807756	NM_019680:8575	Elf4	INSIDE	0.769	1.848	1614.04	2982.68	1.422	2292.72	3259.41
A_68_P32213507	chr19:58528753-58528798	NM_010279:181	Gfra1	INSIDE	0.769	0.388	1440.71	558.86	0.298	1117.94	333.43
A_68_P32065926	chr19:31739147-31739191	NM_011160:-308	Prkg1	PROMOTER	0.769	0.317	5869.38	1862.60	0.244	4045.46	986.71
A_68_P31936768	chr19:7008741-7008785	NM_008431:243	Kcnk4	INSIDE	0.769	0.328	1997.29	655.08	0.252	1559.51	393.50
A_68_P31323975	chr17:69788589-69788633	NM_001145192:-55	A330050F15Rik	PROMOTER	0.769	2.363	1085.07	2564.17	1.817	973.44	1768.52
A_68_P31145393	chr17:32529507-32529551	NM_178785:10998	Rasal3	INSIDE	0.769	2.263	824.22	1865.00	1.741	689.84	1201.11
A_68_P29612959	chr14:55172748-55172792	NM_145462:428	Haus4	INSIDE	0.769	2.079	1244.58	2587.39	1.598	1075.83	1719.01
A_68_P29346593	chr13:115249654-115249698	NM_008046:-738	Fst	PROMOTER	0.769	3.144	3758.62	11817.20	2.417	3282.03	7933.46
A_68_P28587837	chr12:83718482-83718526	NM_015812:480	Rgs6	INSIDE	0.769	5.919	5369.22	31779.44	4.551	4041.29	18393.66

ProbeName	Target position of probe on CpG island microarray	TargetID	GeneSymbol	CpG island Description	Ratio of relative methylation (TiO_2-NP/Vehicle)	Sham group			TiO_2-H group		
						Relative methylation (Cy5/Cy3)	Cy3 signal (Input)	Cy5 signal (MeDIP)	Relative methylation (Cy5/Cy3)	Cy3 signal (Input)	Cy5 signal (MeDIP)
A_68_P28291148	chr12:25393461-25393505	NM_009104:364	Rrm2	INSIDE	0.769	0.481	1627.04	783.40	0.370	1218.55	450.94
A_68_P27987931	chr11:86070791-86070835	NM_027421:258	Ints2	INSIDE	0.769	0.578	1421.60	821.09	0.444	1188.19	528.00
A_68_P27890039	chr11:68369794-68369838	NM_146004:129	Mfsd6l	INSIDE	0.769	0.665	1496.95	996.17	0.512	1211.99	620.53
A_68_P26816721	chr9:108710964-108711008	NM_030729:276	Nckipsd	INSIDE	0.769	0.559	3129.53	1748.94	0.430	2172.10	933.66
A_68_P26706400	chr9:87625023-87625067	NM_023814:1051	Tbx18	INSIDE	0.769	0.334	2024.18	675.60	0.257	1609.05	413.17
A_68_P26232279	chr8:124805350-124805394	NM_009569:-668	Zfpm1	PROMOTER	0.769	0.735	8359.78	6146.74	0.565	6001.30	3391.89
A_68_P25708992	chr8:23171208-23171252	NM_183142:39	Alg11	INSIDE	0.769	0.261	10411.69	2713.62	0.200	7136.97	1430.36
A_68_P25510694	chr7:135561980-135562024	NM_026418:-316	Rgs10	PROMOTER	0.769	2.445	746.01	1823.68	1.879	577.48	1085.01
A_68_P25416985	chr7:118225708-118225752	NM_001040131:814	Eif4g2	INSIDE	0.769	0.399	1208.46	482.49	0.307	1076.41	330.67
A_68_P24427216	chr6:48395590-48395634	NM_001085415:-523	Zfp467	PROMOTER	0.769	0.189	2935.08	554.63	0.145	2222.56	323.01
A_68_P24141202	chr5:140864898-140864942	NM_172277:281	Snx8	INSIDE	0.769	2.005	1410.75	2828.01	1.542	1215.42	1874.33
A_68_P23698176	chr5:53440601-53440645	NM_011402:31	Slc34a2	INSIDE	0.769	0.579	1234.61	714.97	0.445	890.05	396.44
A_68_P23562885	chr5:28791122-28791166	NR_015562:-2379	9530036O11Rik	PROMOTER	0.769	0.537	1002.34	538.45	0.413	915.98	378.17
A_68_P23093082	chr4:88584489-88584533			Unknown	0.769	3.500	339.69	1188.78	2.690	351.84	946.60
A_68_P32264678	chrX:12649125-12649169	NM_009481:523	Usp9x	INSIDE	0.768	3.045	1207.26	3675.60	2.339	1580.72	3697.50
A_68_P32134218	chr19:44831888-44831933	NM_011037:27	Pax2	INSIDE	0.768	0.292	3256.24	951.54	0.225	2391.18	536.83
A_68_P31958012	chr19:11893471-11893515	NM_011502:-109	Stx3	PROMOTER	0.768	2.492	1305.70	3254.11	1.914	1054.98	2019.05
A_68_P31100482	chr17:25073578-25073622	NM_001163447:322	Mapk8ip3	INSIDE	0.768	2.491	917.80	2286.59	1.913	670.50	1282.77
A_68_P30545637	chr16:10992513-10992557	NM_019980:680	Litaf	INSIDE	0.768	0.509	2741.65	1395.78	0.391	1859.76	727.49
A_68_P29605238	chr14:52817188-52817232	NM_033618:-296	Supt16h	PROMOTER	0.768	0.507	2223.85	1128.38	0.390	1804.17	703.23
A_68_P28784066	chr12:120476405-120476449	NM_177290:323	Itgb8	INSIDE	0.768	0.698	1152.14	804.69	0.537	956.66	513.38
A_68_P28166387	chr11:117516412-117516456	NM_198022:832	Tnrc6c	INSIDE	0.768	5.811	593.24	3447.51	4.462	524.42	2339.83
A_68_P28091586	chr11:104412034-104412078	NM_010858:80	Myl4	INSIDE	0.768	0.544	1365.43	742.97	0.418	1065.79	445.54
A_68_P28043344	chr11:96133745-96133789	NM_008270:1123	Hoxb9	INSIDE	0.768	3.096	687.38	2128.11	2.377	653.54	1553.24
A_68_P27280977	chr10:79565295-79565339	NM_183426:131	Sbno2	INSIDE	0.768	0.229	3573.18	817.99	0.176	2534.66	445.86
A_68_P27273028	chr10:77707431-77707475	NM_001081055:-65	Trappc10	PROMOTER	0.768	4.222	604.92	2554.17	3.245	484.87	1573.28
A_68_P26727067	chr9:92437602-92437647	NR_029474:83	B430319G15Rik	INSIDE	0.768	5.558	2538.23	14107.35	4.268	2189.48	9344.04
A_68_P26584125	chr9:64659762-64659806	NM_001162917:967	Dennd4a	INSIDE	0.768	2.231	976.13	2177.88	1.714	827.75	1418.79
A_68_P26242187	chr8:126416382-126416426	NM_009606:2232	Acta1	INSIDE	0.768	8.558	1981.89	16960.15	6.568	1730.09	11363.24
A_68_P25477105	chr7:129210400-129210444	NM_026140:155	Ears2	INSIDE	0.768	1.962	1150.70	2257.47	1.507	965.59	1455.48
A_68_P24428349	chr6:48570036-48570080	NM_173429:6880	Zfp775	INSIDE	0.768	2.309	1060.45	2448.46	1.774	789.54	1400.39
A_68_P23794320	chr5:73259625-73259669	NM_001113460:41	Tec	INSIDE	0.768	0.437	1572.48	686.81	0.336	1182.14	396.63
A_68_P23154905	chr4:101092857-101092901	NM_009647:571	Ak4	INSIDE	0.768	0.642	1354.60	869.15	0.493	1147.26	565.12
A_68_P22682766	chr3:157610204-157610248	NM_001013806:-203	Ankrd13c	PROMOTER	0.768	3.576	697.64	2494.90	2.747	615.06	1689.81
A_68_P22311349	chr3:87826614-87826658	NM_022031:4797	Hapln2	INSIDE	0.768	0.415	1309.14	543.76	0.319	1079.84	344.60
A_68_P21840494	chr2:168106782-168106826	ENSMUST00000109191:-172		PROMOTER	0.768	0.445	4430.48	1973.30	0.342	3135.55	1072.89
A_68_P21021381	chr2:14526447-14526491	NM_023116:536	Cacnb2	INSIDE	0.768	3.084	578.02	1782.65	2.369	476.61	1129.04
A_68_P20778493	chr1:164501104-164501148	NM_016796:168	Vamp4	INSIDE	0.768	0.262	3034.21	795.48	0.201	2462.32	495.69
A_68_P20768472	chr1:162722548-162722592	NM_013862:499	Rabgap1l	INSIDE	0.768	0.661	2070.29	1368.82	0.508	1890.72	960.63
A_68_P20432928	chr1:91819533-91819577	NM_010262:8197	Gbx2	DOWNSTREAM	0.768	0.452	3545.19	1601.14	0.347	2457.95	852.02
A_68_P31936612	chr19:6984205-6984249	NM_001166370:39	Trmt112	INSIDE	0.767	2.068	1713.58	3543.14	1.585	1266.92	2008.31
A_68_P31791361	chr18:67503334-67503378	NM_007702:139	Cidea	INSIDE	0.767	0.162	3379.39	547.96	0.124	2526.79	314.12
A_68_P31491261	chr18:10725960-10726004	NM_144860:360	Mib1	INSIDE	0.767	4.011	479.14	1921.66	3.077	387.13	1191.09
A_68_P31207190	chr17:46624075-46624119	NM_001162864:528	Ttbk1	INSIDE	0.767	2.697	2037.81	5496.01	2.069	1424.91	2948.21
A_68_P31157715	chr17:35258104-35258148	NM_009975:266	Csnk2b	INSIDE	0.767	0.284	2025.52	574.87	0.218	1388.18	302.06
A_68_P30630418	chr16:28446840-28446884	NM_183064:-1549	Fgf12	PROMOTER	0.767	3.003	777.28	2334.50	2.303	590.03	1358.59
A_68_P30499092	chr15:102502227-102502271	NR_030460:49	Mir688	INSIDE	0.767	0.505	948.51	478.68	0.387	766.53	296.56
A_68_P30371092	chr15:80086458-80086502	NM_009716:867	Atf4	INSIDE	0.767	0.663	1336.00	885.13	0.508	1000.87	508.72
A_68_P30347566	chr15:76158037-76158081	NR_028283:-95	LOC100302626	PROMOTER	0.767	0.556	2134.01	1186.27	0.426	1607.73	685.46
A_68_P28549766	chr12:76770694-76770738	NM_028203:-192	Wdr89	PROMOTER	0.767	3.267	366.26	1196.69	2.508	371.01	930.36
A_68_P28535267	chr12:74146949-74146993	NM_009189:729	Six1	INSIDE	0.767	0.681	1078.91	735.27	0.523	850.81	444.81
A_68_P27281213	chr10:79601565-79601609	NM_001195268:-1454	Dos	PROMOTER	0.767	0.201	2801.69	562.69	0.154	1866.65	287.45
A_68_P27095961	chr10:42351968-42352012	ENSMUST00000144806:48347		INSIDE	0.767	0.710	1279.03	908.74	0.545	991.99	540.86
A_68_P26346494	chr9:21116767-21116811	NM_001110300:-11	Ap1m2	DIVERGENT_PROMOTER	0.767	0.721	1578.66	1137.87	0.553	1337.35	739.01
A_68_P25062054	chr7:38352951-38352995	NM_172385:201799	Zfp536	INSIDE	0.767	2.277	1801.79	4103.19	1.747	1428.44	2495.17
A_68_P24800507	chr6:120786263-120786307	NM_153516:37	Bcl2l13	INSIDE	0.767	0.624	2137.25	1333.81	0.478	1612.56	771.54
A_68_P24465593	chr6:54766531-54766575	NM_199143:-357	Znrf2	PROMOTER	0.767	0.424	2359.19	1001.44	0.325	1708.71	555.98
A_68_P24098741	chr5:132014214-132014258	NM_177047:1003977	Auts2	INSIDE	0.767	2.051	1124.39	2306.27	1.573	909.50	1430.35
A_68_P24038707	chr5:120886274-120886318	NM_008499:4402	Lhx5	INSIDE	0.767	7.249	891.85	6464.94	5.558	790.10	4391.21
A_68_P23997298	chr5:113716404-113716448	NR_027934:-6805	Sgsm1	PROMOTER	0.767	0.542	813.97	441.01	0.416	728.18	302.63
A_68_P23978674	chr5:110698757-110698801	NM_001163538:140	Pgam5	INSIDE	0.767	2.663	965.31	2570.62	2.043	860.95	1758.79
A_68_P22215796	chr3:68298884-68298928	NR_036665:792	Schip1	INSIDE	0.767	0.142	5732.85	813.27	0.109	3785.62	411.71
A_68_P21680287	chr2:139503396-139503440	NM_001126490:-495	Ism1	PROMOTER	0.767	0.282	5141.36	1448.32	0.216	3526.58	762.31
A_68_P21477092	chr2:102291506-102291550	NM_010218:421	Fjx1	INSIDE	0.767	2.195	710.86	1560.13	1.684	649.85	1094.35
A_68_P21148580	chr2:38367238-38367282	NM_001159631:-135	Nek6	PROMOTER	0.767	2.416	777.21	1877.76	1.854	546.30	1012.77

ProbeName	Target position of probe on CpG island microarray	TargetID	GeneSymbol	CpG island Description	Ratio of relative methylation (TiO_2-NP/Vehicle)	Sham group			TiO_2-H group		
						Relative methylation (Cy5/Cy3)	Cy3 signal (Input)	Cy5 signal (MeDIP)	Relative methylation (Cy5/Cy3)	Cy3 signal (Input)	Cy5 signal (MeDIP)
A_68_P20737712	chr1:157264214-157264258	NM_011273:338	Xpr1	INSIDE	0.767	1.932	2547.06	4920.21	1.482	2075.47	3075.09
A_68_P31862682	chr18:80347987-80348031	NM_175028:213	Adnp2	INSIDE	0.766	0.310	7331.67	2270.54	0.237	5573.10	1321.71
A_68_P30401102	chr15:85341114-85341158	ENSMUST00000079690:-105		PROMOTER	0.766	1.919	1717.77	3295.56	1.470	1250.66	1838.28
A_68_P30378286	chr15:81353764-81353808			Unknown	0.766	2.289	1114.02	2550.37	1.754	1036.13	1816.98
A_68_P30140502	chr15:36723196-36723240	NM_011740:1075	Ywhaz	INSIDE	0.766	0.594	1242.00	737.62	0.455	1191.06	541.56
A_68_P29855894	chr14:102008105-102008149	NM_001081278:282	Tbc1d4	INSIDE	0.766	0.158	2861.06	453.33	0.121	2126.40	257.95
A_68_P27969031	chr11:82721501-82721545	NM_145431:375	Nle1	INSIDE	0.766	2.145	1927.79	4135.65	1.644	1421.47	2336.91
A_68_P27947946	chr11:79068163-79068207	NM_019653:13	Wsb1	INSIDE	0.766	2.864	2325.40	6659.77	2.193	1702.35	3733.36
A_68_P27914668	chr11:72833126-72833170	NM_018883:639	Camkk1	INSIDE	0.766	0.171	4552.32	776.46	0.131	3044.65	398.05
A_68_P27828608	chr11:57822182-57822226	NM_028451:-361	Larp1	PROMOTER	0.766	0.706	2014.53	1421.90	0.541	1600.89	865.85
A_68_P27812112	chr11:54600985-54601029	NM_178626:199	Cdc42se2	INSIDE	0.766	0.518	957.13	495.58	0.397	853.18	338.45
A_68_P26478900	chr9:46081259-46081303	NM_011752:134	Zfp259	INSIDE	0.766	0.415	1626.53	675.53	0.318	1417.39	451.03
A_68_P26024222	chr8:87823113-87823157	NM_022997:262	Vps35	INSIDE	0.766	0.157	3424.65	536.60	0.120	2186.14	262.29
A_68_P25810708	chr8:42460040-42460084	NM_019734:-11	Asah1	PROMOTER	0.766	0.638	3450.06	2200.24	0.489	2716.73	1327.73
A_68_P25766299	chr8:33994276-33994320	NM_001177589:66200	Gm3985	DOWNSTREAM	0.766	0.435	3030.48	1317.45	0.333	2265.75	754.80
A_68_P25737281	chr8:28338526-28338570	NM_013462:1512	Adrb3	INSIDE	0.766	0.311	2071.61	644.59	0.238	1480.68	353.01
A_68_P25169315	chr7:69608941-69608985	NM_013788:434	Peg12	INSIDE	0.766	4.712	494.07	2328.29	3.610	541.45	1954.51
A_68_P23993052	chr5:113005975-113006019	NM_019982:209	Sez6l	INSIDE	0.766	0.203	2244.54	456.70	0.156	1545.68	240.93
A_68_P23979135	chr5:110771856-110771900	NM_001164833:176	P2rx2	INSIDE	0.766	0.683	1501.06	1024.91	0.523	1130.68	591.54
A_68_P23749449	chr5:64550826-64550870	NM_019636:-601	Tbc1d1	PROMOTER	0.766	0.401	1949.16	780.90	0.307	1422.60	436.29
A_68_P23019857	chr4:73898113-73898157	NM_144787:352	Kdm4c	INSIDE	0.766	0.338	2062.33	697.79	0.259	1763.38	457.11
A_68_P22906126	chr4:49645116-49645160	NM_001163263:207	Rnf20	INSIDE	0.766	0.675	1297.49	875.17	0.517	888.36	459.18
A_68_P20261290	chr1:59030112-59030156	NM_172406:192	Trak2	INSIDE	0.766	0.401	1538.50	616.63	0.307	1157.67	355.34
A_68_P20147266	chr1:36326391-36326435	NM_153408:3858	Neurl3	INSIDE	0.766	3.492	1872.31	6538.89	2.674	1596.06	4268.23
A_68_P20137272	chr1:34234902-34234946	NM_133833:166255	Dst	INSIDE	0.766	3.979	641.35	2551.68	3.049	556.23	1695.75
A_68_P32226228	chr19:60656790-60656834	NM_001172096:114	2700078E11Rik	INSIDE	0.765	0.383	1381.34	529.51	0.293	1102.18	323.33
A_68_P32016080	chr19:23215457-23215501	NM_010638:-237	Klf9	PROMOTER	0.765	0.507	1251.16	634.94	0.388	1056.58	410.19
A_68_P32005497	chr19:21347432-21347476	NM_009551:687	Zfand5	INSIDE	0.765	0.571	943.49	538.54	0.437	783.84	342.39
A_68_P31982707	chr19:16947763-16947807	NM_008023:536	Foxb2	INSIDE	0.765	0.626	1515.31	948.04	0.478	1175.41	562.38
A_68_P30350975	chr15:76701912-76701956	NM_001007568:-69	Zfp251	DIVERGENT_PROMOTER	0.765	2.690	731.67	1967.83	2.058	635.78	1308.60
A_68_P30344972	chr15:75751799-75751843	NM_025412:170	Pycrl	INSIDE	0.765	0.508	1672.32	849.93	0.389	1348.39	524.14
A_68_P28332009	chr12:32243915-32243959	NM_011867:898	Slc26a4	INSIDE	0.765	2.744	835.52	2292.47	2.100	678.54	1424.78
A_68_P27921013	chr11:74336837-74336884	NM_001015046:66800	Rap1gap2	INSIDE	0.765	0.679	2737.32	1858.02	0.519	2130.45	1106.20
A_68_P26749715	chr9:96789502-96789546	NM_153420:317	Acpl2	INSIDE	0.765	0.575	11820.09	6801.86	0.440	7254.89	3193.95
A_68_P26546343	chr9:58047222-58047266	NM_001161535:2278	Islr2	INSIDE	0.765	0.557	818.72	455.81	0.426	656.15	279.58
A_68_P26486127	chr9:47339193-47339237	NM_001025600:780	Cadm1	INSIDE	0.765	3.210	609.09	1955.28	2.456	477.47	1172.63
A_68_P26321969	chr9:15083907-15083951	NM_144933:383	Med17	INSIDE	0.765	3.457	398.62	1378.20	2.643	299.30	791.12
A_68_P26234303	chr8:125100574-125100619	NM_009698:211	Aprt	INSIDE	0.765	0.501	4136.70	2073.67	0.383	3214.85	1232.46
A_68_P26132608	chr8:107792271-107792315	NM_015821:3745	Fbxl8	INSIDE	0.765	0.474	1287.03	610.67	0.363	834.57	302.74
A_68_P26068080	chr8:95351388-95351432	NM_008610:184	Mmp2	INSIDE	0.765	0.488	1007.81	491.35	0.373	854.95	318.80
A_68_P26022583	chr8:87432507-87432551	NM_010062:6	Dnase2a	INSIDE	0.765	18.684	1882.80	35177.71	14.297	1871.75	26759.77
A_68_P25905384	chr8:63111913-63111957	NM_027756:278	Mfap3l	INSIDE	0.765	0.632	2406.67	1520.89	0.484	1830.25	885.33
A_68_P25645835	chr8:9977887-9977931	NM_001081119:192	Abhd13	INSIDE	0.765	8.861	974.52	8635.59	6.782	922.30	6254.58
A_68_P24992159	chr7:19813899-19813943	NM_207525:183	Opa3	INSIDE	0.765	2.632	2874.28	7563.79	2.013	2186.09	4400.25
A_68_P24979758	chr7:16705347-16705391	NM_133727:144	Kptn	INSIDE	0.765	0.278	1687.01	469.28	0.213	1232.05	262.25
A_68_P24759850	chr6:113444920-113444964	NM_172487:6870	Prrt3	INSIDE	0.765	0.643	3831.69	2464.12	0.492	2593.07	1275.55
A_68_P24388787	chr6:40386026-40386070	NM_175528:84	E330009J07Rik	INSIDE	0.765	2.963	541.51	1604.74	2.267	464.24	1052.24
A_68_P24043140	chr5:121641038-121641082	NM_001109992:346	Ptpn11	INSIDE	0.765	3.648	1207.25	4404.60	2.791	969.56	2705.63
A_68_P23962879	chr5:107125530-107125574	NM_026856:297	Zfp644	INSIDE	0.765	0.401	1215.38	486.80	0.307	1044.01	320.06
A_68_P21611440	chr2:126896255-126896304	NM_026678:-113	Blvra	PROMOTER	0.765	0.404	2759.74	1115.32	0.309	1902.20	587.96
A_68_P21431326	chr2:92755177-92755221	NM_030725:-59	Syt13	PROMOTER	0.765	0.660	2336.30	1542.24	0.505	1711.60	864.49
A_68_P20066648	chr1:19204353-19204397	NM_001025305:2240	Tcfap2b	INSIDE	0.765	0.603	812.77	489.89	0.461	698.69	322.05
A_68_P32227241	chr19:60865885-60865929	NM_010123:690	Eif3a	INSIDE	0.764	0.469	994.42	466.13	0.358	781.18	279.80
A_68_P31934034	chr19:6335569-6335613	NM_001168490:552	Men1	INSIDE	0.764	0.272	3600.58	979.73	0.208	2276.60	473.49
A_68_P31117112	chr17:27693865-27693909	NM_001166476:289	Hmga1-rs1	INSIDE	0.764	16.490	781.83	12892.54	12.602	789.48	9949.40
A_68_P29919368	chr14:115439764-115439808	NR_029785:-3106	Mir17	PROMOTER	0.764	3.087	1082.96	3342.92	2.359	895.93	2113.45
A_68_P28737545	chr12:111517127-111517171	NM_172119:-291	Dio3	DIVERGENT_PROMOTER	0.764	0.418	2344.77	979.11	0.319	1669.03	532.23
A_68_P28514079	chr12:70458006-70458050	NM_025441:125	Nemf	INSIDE	0.764	2.883	1224.84	3530.92	2.204	1038.89	2289.41
A_68_P28089844	chr11:104092991-104093035	NM_001038609:263	Mapt	INSIDE	0.764	0.706	2570.47	1814.57	0.539	1773.30	955.95
A_68_P28051259	chr11:97408613-97408657	NM_018873:27806	Srcin1	INSIDE	0.764	0.366	2326.67	851.47	0.280	1821.15	509.41
A_68_P27902199	chr11:70513617-70513661	NM_213729:19	Inca1	INSIDE	0.764	2.371	1361.91	3228.84	1.812	1115.81	2021.91
A_68_P27894337	chr11:69140054-69140098	NM_010599:317	Kcnab3	INSIDE	0.764	0.626	4936.03	3091.12	0.478	3611.62	1727.27
A_68_P27456596	chr10:112366019-112366063	NM_001033474:42	Atxn7l3b	INSIDE	0.764	2.447	1269.04	3105.39	1.870	1154.36	2158.72
A_68_P27284379	chr10:80085336-80085380	NM_134002:-166	Csnk1g2	PROMOTER	0.764	0.393	1499.29	589.19	0.300	1071.29	321.44
A_68_P26841770	chr9:113650921-113650965	NM_001114347:352	Clasp2	INSIDE	0.764	2.706	751.81	2034.26	2.067	666.70	1377.88

ProbeName	Target position of probe on CpG island microarray	TargetID	GeneSymbol	CpG island Description	Ratio of relative methylation (TiO_2-NP/Vehicle)	Sham group			TiO_2-H group		
						Relative methylation (Cy5/Cy3)	Cy3 signal (Input)	Cy5 signal (MeDIP)	Relative methylation (Cy5/Cy3)	Cy3 signal (Input)	Cy5 signal (MeDIP)
A_68_P26469460	chr9:44575399-44575447	NM_145985:469	Arcn1	INSIDE	0.764	13.685	362.55	4961.52	10.453	306.06	3199.35
A_68_P26240287	chr8:126038926-126038970	NM_001170976:407	Dbndd1	INSIDE	0.764	0.692	2208.61	1529.38	0.529	1755.27	929.01
A_68_P25957388	chr8:74066580-74066624	NM_028617:-226	Fam125a	DIVERGENT_PROMOTER	0.764	0.410	1189.32	487.43	0.313	895.33	280.28
A_68_P25906691	chr8:63369418-63369462	NM_028299:193	2700029M09Rik	INSIDE	0.764	0.564	5748.74	3240.47	0.431	4374.78	1883.96
A_68_P25024908	chr7:29804801-29804845	NM_009109:105348	Ryr1	INSIDE	0.764	2.756	1220.02	3362.93	2.107	1176.45	2478.51
A_68_P23973156	chr5:109058413-109058457	NM_153569:324	Gak	INSIDE	0.764	0.407	4658.58	1897.28	0.311	3300.59	1026.46
A_68_P23747547	chr5:64203832-64203876	NM_029554:121	0610040J01Rik	INSIDE	0.764	3.485	574.20	2001.32	2.663	522.52	1391.68
A_68_P20880188	chr1:183140931-183140975	NM_145514:1157	Wdr26	INSIDE	0.764	0.359	3172.48	1139.67	0.275	2114.11	580.35
A_68_P20187368	chr1:43220801-43220845	NM_010212:-16	Fhl2	PROMOTER	0.764	3.196	820.66	2623.21	2.442	598.85	1462.59
A_68_P31625515	chr18:36894543-36894587	NM_028036:-159	Tmco6	DIVERGENT_PROMOTER	0.763	0.547	2386.71	1305.81	0.418	1802.45	752.54
A_68_P31614001	chr18:34811328-34811372	NM_178347:40	Cdc23	INSIDE	0.763	0.349	2394.32	836.17	0.267	1803.30	480.60
A_68_P31560666	chr18:24688433-24688477	NM_144861:251	Rprd1a	INSIDE	0.763	0.672	2576.42	1732.23	0.513	2094.33	1074.82
A_68_P31114983	chr17:27340878-27340922	NM_146075:483	Lemd2	INSIDE	0.763	2.424	680.84	1650.68	1.849	508.24	939.73
A_68_P31112707	chr17:26976313-26976357	NM_008700:2176	Nkx2-5	INSIDE	0.763	2.478	534.92	1325.35	1.890	453.43	857.05
A_68_P31063013	chr17:15115758-15115802	NM_001123367:-4	Gm3417	DIVERGENT_PROMOTER	0.763	0.477	2383.48	1136.64	0.364	2027.76	737.42
A_68_P31053796	chr17:12962471-12962515	NM_010515:80	Igf2r	INSIDE	0.763	0.488	1173.51	572.42	0.372	912.22	339.68
A_68_P30367928	chr15:79521367-79521411	NM_013818:63	Gtpbp1	INSIDE	0.763	4.196	843.81	3540.25	3.203	797.73	2555.36
A_68_P29338036	chr13:113777997-113778041	NM_001081062:9	Ccno	INSIDE	0.763	0.513	1911.45	981.05	0.391	1585.32	620.56
A_68_P28923678	chr13:31650872-31650916	NM_008239:856	Foxq1	INSIDE	0.763	0.532	1100.97	585.73	0.406	911.12	369.67
A_68_P28745653	chr12:112951441-112951485	NM_027404:5	Bag5	INSIDE	0.763	2.290	1150.36	2634.65	1.747	926.52	1618.18
A_68_P28107252	chr11:107331040-107331084	NM_145823:972	Pitpnc1	INSIDE	0.763	0.427	2714.11	1158.72	0.326	2135.32	695.93
A_68_P27992047	chr11:86806779-86806823	NM_001005341:464	Ypel2	INSIDE	0.763	0.228	5372.85	1227.32	0.174	3787.99	659.89
A_68_P26785090	chr9:103207295-103207339	NM_176979:-340	Topbp1	PROMOTER	0.763	0.154	3257.53	502.97	0.118	2351.53	276.92
A_68_P26779718	chr9:102255865-102255909	NM_001168296:1137	Ephb1	INSIDE	0.763	0.503	871.41	438.12	0.384	765.00	293.65
A_68_P26659375	chr9:78243556-78243600	NM_001191044:-5	Ddx43	PROMOTER	0.763	3.850	946.79	3645.18	2.936	860.65	2526.94
A_68_P25286562	chr7:91234889-91234933	NM_030728:105	9930013L23Rik	INSIDE	0.763	0.339	1900.88	644.53	0.259	1483.08	383.61
A_68_P25266557	chr7:87548096-87548140	NM_011046:-470	Furin	PROMOTER	0.763	0.391	1171.22	457.88	0.298	803.97	239.69
A_68_P25111232	chr7:56502025-56502069	NM_175272:488	Nav2	INSIDE	0.763	2.560	1686.21	4316.96	1.954	1339.08	2615.90
A_68_P25086723	chr7:51568193-51568237	NM_001034115:2581	Shank1	INSIDE	0.763	3.224	708.18	2282.90	2.460	576.04	1417.15
A_68_P24627788	chr6:89311893-89311937	NM_008881:693	Plxna1	INSIDE	0.763	2.171	1600.56	3474.65	1.656	1373.40	2274.72
A_68_P24449194	chr6:52171860-52171904	NM_010455:-3310	Hoxa7	PROMOTER	0.763	3.313	911.74	3020.53	2.526	751.51	1898.63
A_68_P24287934	chr6:21802232-21802276	NM_173007:261	Tspan12	INSIDE	0.763	2.554	1846.68	4717.17	1.948	1554.25	3027.42
A_68_P24253395	chr6:14851697-14851741	NM_053242:370	Foxp2	INSIDE	0.763	0.687	2276.95	1565.30	0.525	1765.65	926.33
A_68_P24172322	chr5:148001001-148001045	NM_008178:751	Gsx1	INSIDE	0.763	0.372	1673.65	622.78	0.284	1400.35	397.35
A_68_P22880599	chr4:45025284-45025328	NM_001163283:-22	Zbtb5	DIVERGENT_PROMOTER	0.763	3.192	501.78	1601.80	2.434	354.98	864.10
A_68_P22523678	chr3:129763488-129763532	NM_207209:315	Sec24b	INSIDE	0.763	3.755	377.15	1416.08	2.864	351.20	1005.69
A_68_P21840417	chr2:168094618-168094662	NM_001081134:191	Kcng1	INSIDE	0.763	2.301	1267.86	2916.76	1.756	1047.77	1840.11
A_68_P21761106	chr2:154378472-154378516	NM_001171640:983	1700007I08Rik	INSIDE	0.763	0.570	1586.11	903.59	0.435	1258.10	546.65
A_68_P21566228	chr2:118616477-118616522	NM_170593:11045	Disp2	INSIDE	0.763	0.488	1005.49	490.60	0.372	776.44	289.02
A_68_P21477091	chr2:102291430-102291474	NM_010218:497	Fjx1	INSIDE	0.763	4.325	710.88	3074.60	3.298	547.26	1804.87
A_68_P21015199	chr2:13192872-13192916	NM_009105:11	Rsu1	INSIDE	0.763	0.156	2913.89	453.66	0.119	1943.63	230.78
A_68_P32579358	chrX:102316971-102317015	NM_053201:1258	Magee1	INSIDE	0.762	3.719	721.32	2682.90	2.835	729.01	2066.65
A_68_P30356909	chr15:77801488-77801532	NR_018749:-256	Eif3d	PROMOTER	0.762	0.202	4674.02	945.65	0.154	3363.04	518.33
A_68_P30261322	chr15:60654906-60654950	NR_030696:409	9930014A18Rik	INSIDE	0.762	0.489	987.71	483.29	0.373	802.54	299.12
A_68_P29681981	chr14:67529271-67529315	NM_175498:-752	Pnma2	PROMOTER	0.762	0.428	1307.21	559.77	0.326	988.65	322.63
A_68_P29619513	chr14:56340877-56340921	NM_019519:115	Rabggta	INSIDE	0.762	0.603	12471.90	7515.84	0.459	7839.19	3600.42
A_68_P28672817	chr12:100138890-100138934	NM_001081191:782	Eml5	INSIDE	0.762	0.249	2568.06	639.07	0.190	1934.22	367.00
A_68_P28344354	chr12:34642575-34642619	NM_011658:61	Twist1	INSIDE	0.762	0.359	2483.60	891.76	0.274	2156.96	590.45
A_68_P27490918	chr10:118890399-118890443	NM_133442:-949	Grip1	PROMOTER	0.762	0.367	1282.23	470.27	0.279	1038.11	290.11
A_68_P27298384	chr10:83111351-83111395	NM_145220:37	Appl2	INSIDE	0.762	0.668	1221.83	816.10	0.509	1033.51	526.04
A_68_P27275483	chr10:78215546-78215590	NM_027630:244	Ccdc105	INSIDE	0.762	0.379	1364.22	517.67	0.289	1017.78	294.38
A_68_P26815969	chr9:108551009-108551053	NM_011790:681	Arih2	INSIDE	0.762	2.979	1081.77	3222.22	2.269	921.79	2091.69
A_68_P26782297	chr9:102737533-102737577	NM_001042607:305	Ryk	INSIDE	0.762	2.657	749.62	1991.88	2.026	687.39	1392.38
A_68_P26766653	chr9:99775810-99775854	NM_011440:757	Sox14	INSIDE	0.762	4.198	330.27	1386.65	3.201	267.97	857.76
A_68_P26337052	chr9:18278078-18278122	NM_028935:-97	Zfp558	DIVERGENT_PROMOTER	0.762	0.240	10029.36	2411.03	0.183	6714.53	1230.63
A_68_P26252592	chr8:128094354-128094398	BC072593:-720		PROMOTER	0.762	16.729	200.50	3354.20	12.740	171.72	2187.70
A_68_P25848480	chr8:49641351-49641395	NM_001145937:118672	Odz3	INSIDE	0.762	2.391	955.95	2285.64	1.821	876.05	1595.06
A_68_P25524905	chr7:138009499-138009543	NM_133942:97	Plekha1	INSIDE	0.762	3.379	5188.97	17533.75	2.576	4215.85	10858.73
A_68_P24979382	chr7:16646941-16646985			Unknown	0.762	5.572	2853.90	15902.11	4.244	2231.93	9471.49
A_68_P24952262	chr7:5077680-5077724	NM_009028:-150	Rasl2-9-ps	PROMOTER	0.762	0.597	1395.53	833.80	0.455	1349.31	614.35
A_68_P24424842	chr6:47855282-47855326	NM_146175:27751	Zfp282	INSIDE	0.762	2.151	2031.37	4368.57	1.639	1462.03	2396.35
A_68_P24112574	chr5:134790846-134790890	NM_001080746:-252	Gtf2i	PROMOTER	0.762	4.466	1292.42	5771.49	3.405	1147.07	3905.61
A_68_P23597967	chr5:35399574-35399618	NM_172708:-136	Dok7	PROMOTER	0.762	0.415	5962.77	2477.07	0.317	4322.07	1367.94
A_68_P23575729	chr5:31262539-31262583	NM_016703:174	Preb	INSIDE	0.762	0.288	2109.02	607.23	0.219	1602.30	351.65
A_68_P23527834	chr5:21290784-21290828	NM_009584:177	Dnajc2	INSIDE	0.762	0.319	3397.71	1083.26	0.243	2640.78	641.62

ProbeName	Target position of probe on CpG island microarray	TargetID	GeneSymbol	CpG island Description	Ratio of relative methylation (TiO_2-NP/Vehicle)	Sham group			TiO_2-H group		
						Relative methylation (Cy5/Cy3)	Cy3 signal (Input)	Cy5 signal (MeDIP)	Relative methylation (Cy5/Cy3)	Cy3 signal (Input)	Cy5 signal (MeDIP)
A_68_P23264562	chr4:123427164-123427208	NM_023423:356	Akirin1	INSIDE	0.762	3.025	798.32	2414.62	2.305	659.41	1520.18
A_68_P22905957	chr4:49610300-49610344	NM_025944:420	2810432L12Rik	INSIDE	0.762	2.373	1358.13	3222.96	1.807	1092.56	1974.42
A_68_P22384168	chr3:102862487-102862531	NM_010937:301	Nras	INSIDE	0.762	0.296	3124.04	923.43	0.225	2127.25	478.90
A_68_P21330518	chr2:73111208-73111252	NM_001005343:1248	Sp9	INSIDE	0.762	0.217	6144.87	1332.64	0.165	4086.41	675.32
A_68_P21081858	chr2:26883547-26883591	NM_172659:-50	Slc2a6	PROMOTER	0.762	2.964	587.80	1742.49	2.260	496.99	1123.27
A_68_P21077011	chr2:26058446-26058490	NM_001039653:3608	Lhx3	INSIDE	0.762	0.511	5019.86	2567.23	0.390	3565.99	1390.52
A_68_P20457296	chr1:95650496-95650540	NM_025920:897	Thap4	INSIDE	0.762	2.103	1112.99	2340.38	1.602	859.07	1376.03
A_68_P32464345	chrX:70961700-70961744	NM_012060:-208	Bcap31	DIVERGENT_PROMOTER	0.761	2.078	1593.95	3312.72	1.582	2287.93	3619.46
A_68_P30944968	chr16:88289921-88289969	NM_010348:559	Grik1	INSIDE	0.761	0.463	1703.42	789.47	0.353	1247.42	440.12
A_68_P30478228	chr15:98928659-98928703	NM_176835:-1234	Dnajc22	PROMOTER	0.761	2.953	1071.18	3163.24	2.247	953.20	2141.67
A_68_P30436285	chr15:91403260-91403304	NM_001033633:410	Slc2a13	INSIDE	0.761	8.746	901.21	7881.59	6.656	788.04	5245.17
A_68_P29570463	chr14:46008598-46008642	NM_019425:-149	Gnpnat1	PROMOTER	0.761	3.055	4874.42	14892.55	2.324	3742.42	8696.95
A_68_P29448497	chr14:21526842-21526886	NM_027996:91	2310021P13Rik	INSIDE	0.761	2.005	2637.82	5288.41	1.526	2086.79	3183.61
A_68_P29302881	chr13:107811807-107811851	NM_001145779:366	Kif2a	INSIDE	0.761	0.539	2169.36	1169.64	0.410	1580.26	648.31
A_68_P29233346	chr13:94944314-94944358	NM_172589:116586	Lhfpl2	INSIDE	0.761	0.472	4759.86	2245.72	0.359	3420.24	1227.35
A_68_P28781844	chr12:120081565-120081609	NM_177082:-3215	Sp8	PROMOTER	0.761	0.260	2787.14	724.59	0.198	2283.86	451.69
A_68_P28684631	chr12:102137378-102137422	NM_001177674:334	Gpr68	INSIDE	0.761	0.520	1192.05	619.70	0.395	1023.75	404.80
A_68_P28567253	chr12:80130122-80130166	NM_181073:-5	Plekhh1	PROMOTER	0.761	4.348	738.42	3210.48	3.308	693.07	2292.94
A_68_P28103446	chr11:106640661-106640705	NR_027785:147	Polg2	INSIDE	0.761	2.172	744.63	1617.68	1.653	707.82	1170.15
A_68_P28100610	chr11:106134360-106134404	NM_001130187:-96	Smarcd2	DIVERGENT_PROMOTER	0.761	0.207	5783.22	1194.66	0.157	3473.07	546.18
A_68_P27827804	chr11:57650530-57650574	NM_008213:-4903	Hand1	PROMOTER	0.761	3.345	2264.59	7574.69	2.547	1719.28	4378.60
A_68_P26520288	chr9:53192430-53192474	NM_212445:325	Kdelc2	INSIDE	0.761	0.604	1434.25	865.76	0.460	1065.29	489.55
A_68_P26228503	chr8:124254738-124254782	NM_020605:302	Jph3	INSIDE	0.761	0.351	5015.69	1758.16	0.267	3646.12	972.16
A_68_P26018982	chr8:86773469-86773513	NM_001007571:170	D8Ertd738e	INSIDE	0.761	4.683	3294.87	15428.37	3.563	3095.66	11030.17
A_68_P25933300	chr8:69010290-69010334	NM_025465:94	1810029B16Rik	INSIDE	0.761	0.470	2455.49	1154.06	0.358	1908.35	682.95
A_68_P25618383	chr8:4677621-4677665	ENSMUST00000058918:723		INSIDE	0.761	0.603	3475.95	2096.60	0.459	2492.49	1143.86
A_68_P25596355	chr7:149844923-149844967	NM_010514:-235	Igf2	DIVERGENT_PROMOTER	0.761	0.282	1655.25	467.49	0.215	1447.80	311.34
A_68_P24818880	chr6:125082083-125082127	NM_138747:204	Nop2	INSIDE	0.761	0.372	4889.08	1818.50	0.283	3872.59	1096.51
A_68_P24636899	chr6:91066454-91066498	NM_018815:344	Nup210	INSIDE	0.761	2.090	1132.17	2366.74	1.590	873.28	1388.87
A_68_P24172967	chr5:148116571-148116615	NM_007673:2233	Cdx2	INSIDE	0.761	4.824	1226.93	5918.44	3.671	1047.64	3845.79
A_68_P23979251	chr5:110792454-110792498	NM_028596:23670	Fbrsl1	INSIDE	0.761	0.676	3563.54	2407.25	0.514	2731.50	1404.70
A_68_P22872021	chr4:43512609-43512653	NM_001013377:-98	E130306D19Rik	DIVERGENT_PROMOTER	0.761	3.298	1027.73	3389.36	2.510	947.63	2378.23
A_68_P21842414	chr2:168415753-168415797	NM_001136073:-83	Nfatc2	PROMOTER	0.761	0.704	1763.98	1241.71	0.536	1346.69	721.61
A_68_P21731579	chr2:148507226-148507270	NM_028986:393	Gzf1	INSIDE	0.761	0.470	1402.59	658.76	0.357	1036.95	370.53
A_68_P21100057	chr2:29746300-29746344	NM_001177662:740	Odf2	INSIDE	0.761	0.491	951.90	466.95	0.373	771.57	288.18
A_68_P32750679	chrX:149204140-149204184	NM_172441:-158	Shroom2	PROMOTER	0.760	3.361	411.11	1381.76	2.553	545.22	1391.90
A_68_P32408599	chrX:56387764-56387808	NM_010200:-173	Fgf13	PROMOTER	0.760	2.225	406.50	904.40	1.692	607.91	1028.55
A_68_P31509663	chr18:14128717-14128761	NM_145492:2504	Zfp521	INSIDE	0.760	0.634	1667.65	1057.06	0.482	1449.76	698.45
A_68_P31488616	chr18:10181915-10181959	NM_009071:-146	Rock1	PROMOTER	0.760	0.303	2103.95	637.00	0.230	1717.45	394.98
A_68_P29856568	chr14:102128930-102128974	NM_201529:-192	Lmo7	PROMOTER	0.760	19.662	3687.43	72502.74	14.940	3416.56	51044.25
A_68_P29615710	chr14:55633787-55633831	NM_026890:-482	Ngdn	PROMOTER	0.760	0.644	1881.23	1212.07	0.490	1364.42	668.37
A_68_P28594554	chr12:85113181-85113234	NM_001205343:8624	Papln	INSIDE	0.760	4.253	308.64	1312.59	3.231	299.08	966.43
A_68_P27750513	chr11:43247484-43247528	NM_148673:274	Slu7	INSIDE	0.760	0.377	1371.17	516.70	0.286	1069.97	306.44
A_68_P27560009	chr11:4749259-4749303	NM_010898:250	Nf2	INSIDE	0.760	2.763	1380.75	3815.41	2.101	1146.65	2409.05
A_68_P27095717	chr10:42304339-42304391	NM_152229:-970	Nr2e1	PROMOTER	0.760	2.875	413.30	1188.41	2.185	420.21	917.97
A_68_P26640621	chr9:74709991-74710035	NM_008262:285	Onecut1	INSIDE	0.760	0.106	6703.86	708.65	0.080	4469.01	359.22
A_68_P26554813	chr9:59528005-59528049	NM_011099:23612	Pkm2	DOWNSTREAM	0.760	2.248	951.90	2140.00	1.709	776.57	1326.88
A_68_P25892523	chr8:60131751-60131795	NM_001167981:80	Galnt7	INSIDE	0.760	0.529	2794.58	1478.70	0.402	2048.86	824.08
A_68_P25405195	chr7:116094388-116094432	NM_025344:16482	Eif3f	DOWNSTREAM	0.760	0.636	1411.85	897.98	0.484	948.53	458.68
A_68_P25371479	chr7:108257513-108257557	NM_001146010:246	Fchsd2	INSIDE	0.760	2.040	6696.44	13659.73	1.550	4562.50	7072.84
A_68_P24620320	chr6:88144365-88144409	NM_008090:-4271	Gata2	PROMOTER	0.760	2.183	1133.51	2474.64	1.658	1073.30	1779.68
A_68_P23927386	chr5:100468112-100468156	NM_016690:107	Hnrpdl	INSIDE	0.760	2.086	2922.18	6096.60	1.586	2292.91	3636.12
A_68_P23362918	chr4:140803764-140803808	NM_172520:4988	Arhgef19	INSIDE	0.760	0.628	1294.94	813.48	0.478	1103.07	526.88
A_68_P22659975	chr3:153636613-153636657	NM_001081263:235	Slc44a5	INSIDE	0.760	2.305	1356.23	3126.05	1.752	1032.47	1808.83
A_68_P22149459	chr3:54539268-54539312	NM_001163570:-4	Exosc8	DIVERGENT_PROMOTER	0.760	0.506	2054.99	1040.29	0.385	1621.31	623.99
A_68_P31415134	chr17:86566646-86566690	NM_011104:-456	Prkce	PROMOTER	0.759	0.225	2036.13	458.73	0.171	1393.32	238.18
A_68_P31103373	chr17:25522563-25522607	NM_001163691:48144	Cacna1h	INSIDE	0.759	2.534	1843.78	4671.84	1.924	1340.32	2578.67
A_68_P30959118	chr16:90810399-90810443	NM_029497:238	Urb1	INSIDE	0.759	0.737	2099.87	1546.71	0.559	1636.26	915.25
A_68_P30647291	chr16:31429118-31429162	NM_001122683:302	Bdh1	INSIDE	0.759	0.567	1218.24	691.03	0.430	1069.32	460.19
A_68_P30484704	chr15:100058837-100058881	NM_007497:569	Atf1	INSIDE	0.759	5.045	696.00	3511.63	3.828	602.74	2306.99
A_68_P30468154	chr15:97077540-97077584	NM_172293:25	Fam113b	INSIDE	0.759	6.707	1009.85	6772.89	5.089	872.88	4442.44
A_68_P29072927	chr13:58486285-58486329	NM_027335:280	2210016F16Rik	INSIDE	0.759	2.129	1357.79	2890.74	1.616	1130.02	1825.96
A_68_P29061785	chr13:56623742-56623786	NM_178674:-104	Fbxl21	PROMOTER	0.759	0.170	5631.98	954.94	0.129	4280.63	550.93
A_68_P28558324	chr12:78338946-78338990	NM_016893:-1039	Fut8	PROMOTER	0.759	0.188	2854.91	536.90	0.143	2279.66	325.23
A_68_P28540763	chr12:75050763-75050807	NM_010431:41931	Hif1a	DOWNSTREAM	0.759	2.226	1049.01	2335.29	1.690	949.27	1604.67

ProbeName	Target position of probe on CpG island microarray	TargetID	GeneSymbol	CpG island Description	Ratio of relative methylation (TiO_2-NP/Vehicle)	Sham group			TiO_2-H group		
						Relative methylation (Cy5/Cy3)	Cy3 signal (Input)	Cy5 signal (MeDIP)	Relative methylation (Cy5/Cy3)	Cy3 signal (Input)	Cy5 signal (MeDIP)
A_68_P28183803	chr11:120166774-120166818			Unknown	0.759	0.705	2745.89	1935.37	0.535	2108.66	1127.93
A_68_P27862325	chr11:63738770-63738814	NM_018805:-3006	Hs3st3b1	PROMOTER	0.759	0.623	999.86	623.26	0.473	751.99	355.89
A_68_P27283449	chr10:79938176-79938220	ENSMUST00000158603:-898		PROMOTER	0.759	0.389	1224.22	476.08	0.295	956.25	282.35
A_68_P27054670	chr10:33739429-33739473	NM_025614:-28	Rwdd1	PROMOTER	0.759	2.816	919.78	2589.89	2.137	844.98	1805.70
A_68_P25955958	chr8:73796193-73796237	NM_001163042:275	Haus8	INSIDE	0.759	0.475	2276.90	1080.46	0.360	1558.21	561.00
A_68_P25410983	chr7:117103395-117103439	NM_021494:520	Dennd5a	INSIDE	0.759	2.762	1495.83	4131.58	2.095	1250.49	2620.29
A_68_P25374661	chr7:108812468-108812512	NM_009191:209	Clpb	INSIDE	0.759	0.569	1271.27	723.23	0.432	1124.83	486.02
A_68_P24760618	chr6:113588447-113588492	NM_001113553:9	Irak2	INSIDE	0.759	4.712	624.57	2942.94	3.576	537.51	1922.10
A_68_P24622498	chr6:88450154-88450198	NM_019685:34760	Ruvbl1	DOWNSTREAM	0.759	2.542	3306.06	8402.96	1.929	2829.23	5458.36
A_68_P24618852	chr6:87864612-87864656	NM_173737:665	8430410A17Rik	INSIDE	0.759	2.109	1459.89	3079.61	1.600	1232.19	1971.87
A_68_P23567511	chr5:29761679-29761723	NM_001033457:494	Nom1	INSIDE	0.759	0.227	1942.45	440.68	0.172	1582.07	272.49
A_68_P23513997	chr5:18733526-18733570	NM_001170746:685	Magi2	INSIDE	0.759	0.295	4476.28	1318.42	0.224	2740.30	612.97
A_68_P22774297	chr4:21605606-21605652	NM_001080771:7482	Prdm13	INSIDE	0.759	0.498	1034.96	515.33	0.378	915.00	346.01
A_68_P22482404	chr3:122123031-122123075	NM_013867:355	Bcar3	INSIDE	0.759	0.466	1967.69	916.19	0.354	1641.71	580.40
A_68_P22165653	chr3:57651276-57651320	NM_019410:381	Pfn2	INSIDE	0.759	3.087	1105.82	3414.20	2.345	968.21	2270.25
A_68_P21603042	chr2:125331829-125331873	NM_007993:324	Fbn1	INSIDE	0.759	0.492	2910.03	1432.57	0.374	2112.76	789.27
A_68_P21010260	chr2:12223375-12223419	NM_001001309:151	Itga8	INSIDE	0.759	0.703	2253.43	1584.88	0.534	1626.26	868.06
A_68_P20739612	chr1:157589907-157589951	NM_010712:-771	Lhx4	PROMOTER	0.759	2.929	1221.46	3577.26	2.223	1246.72	2770.85
A_68_P32289246	chrX:20195326-20195370	NM_001167776:464	Rbm10	INSIDE	0.758	4.359	290.23	1265.01	3.305	270.84	894.99
A_68_P31857880	chr18:79305571-79305615	NM_053099:538	Setbp1	INSIDE	0.758	0.689	1067.14	734.83	0.522	965.96	504.43
A_68_P31655273	chr18:42555254-42555298	NM_138945:1026	Pou4f3	INSIDE	0.758	2.160	2385.93	5154.04	1.637	1972.02	3229.13
A_68_P31158452	chr17:35372069-35372113	NM_010909:670	Nfkbil1	INSIDE	0.758	0.335	1466.30	491.69	0.254	1079.36	274.22
A_68_P31155202	chr17:34809736-34809780	NM_031176:2279	Tnxb	INSIDE	0.758	2.261	1657.63	3748.24	1.714	1319.46	2262.10
A_68_P31108918	chr17:26340491-26340535	NM_001081069:606	Rgs11	INSIDE	0.758	0.564	1507.81	850.36	0.428	1192.92	510.23
A_68_P30359736	chr15:78236837-78236881	NM_001162493:-283	Mpst	DIVERGENT_PROMOTER	0.758	0.380	1212.46	460.55	0.288	961.35	276.72
A_68_P29626125	chr14:57723587-57723631	NM_008125:-69	Gjb2	PROMOTER	0.758	2.368	1419.83	3361.78	1.795	1197.41	2149.42
A_68_P28918887	chr13:30842448-30842492	NM_013674:1344	Irf4	INSIDE	0.758	2.061	1448.09	2984.48	1.562	1063.43	1661.37
A_68_P28277266	chr12:19710348-19710392	BC012645:386747		INSIDE	0.758	6.971	2077.27	14480.73	5.285	1578.25	8340.98
A_68_P28192098	chr11:121563706-121563750	NM_144797:-12	Metrnl	PROMOTER	0.758	0.308	1523.45	469.16	0.233	1233.39	287.98
A_68_P28153958	chr11:115374907-115374951	NM_008258:756	Hn1	INSIDE	0.758	39.961	516.25	20629.65	30.284	501.75	15195.35
A_68_P27924763	chr11:75006899-75006943	NM_177708:-574	Rtn4rl1	DIVERGENT_PROMOTER	0.758	0.454	3467.90	1575.22	0.344	2498.88	860.81
A_68_P27687500	chr11:32122162-32122206	NM_010117:109	Rhbdf1	INSIDE	0.758	0.691	1374.93	950.33	0.524	1155.69	605.30
A_68_P27542560	chr10:128106127-128106171	NM_173733:4826	Suox	DOWNSTREAM	0.758	0.566	2361.30	1337.10	0.429	1681.89	721.79
A_68_P27101045	chr10:43298595-43298639	NM_009846:-358	Cd24a	PROMOTER	0.758	2.508	1560.14	3912.74	1.900	1203.01	2285.57
A_68_P26595206	chr9:66643069-66643113	NM_177583:140	Aph1b	INSIDE	0.758	1.982	1361.77	2699.06	1.501	1014.25	1522.81
A_68_P26351648	chr9:22193305-22193349	NM_028390:324	Anln	INSIDE	0.758	3.008	820.31	2467.81	2.281	665.81	1518.97
A_68_P24428260	chr6:48552305-48552349	NM_001079901:8445	Repin1	DOWNSTREAM	0.758	8.941	272.39	2435.36	6.779	291.48	1975.86
A_68_P23576459	chr5:31397241-31397285	NM_011773:-1362	Slc30a3	PROMOTER	0.758	0.614	1254.42	770.28	0.466	1045.60	486.94
A_68_P21901454	chr2:179838474-179838518	NM_133849:431	Hrh3	INSIDE	0.758	0.460	1449.07	666.31	0.349	1121.84	390.97
A_68_P21839352	chr2:167906373-167906417	NM_021409:-109	Pard6b	PROMOTER	0.758	6.284	387.27	2433.61	4.766	378.95	1806.21
A_68_P21019040	chr2:13976882-13976926	NM_001012396:758	Ptpla	INSIDE	0.758	0.604	2837.15	1712.64	0.458	2461.63	1126.62
A_68_P20831242	chr1:174078262-174078306	NM_153555:139	Dcaf8	INSIDE	0.758	2.199	3090.96	6796.19	1.666	2543.55	4237.33
A_68_P32130738	chr19:44220823-44220867	NM_028607:92	Bloc1s2	INSIDE	0.757	0.523	1392.45	728.04	0.396	1030.99	408.18
A_68_P32054980	chr19:29887444-29887488	NM_177721:-2	Ranbp6	PROMOTER	0.757	2.376	1416.31	3365.00	1.798	1107.46	1991.08
A_68_P31950794	chr19:10532257-10532301	NM_001111140:-341	Lrrc10b	PROMOTER	0.757	2.586	1332.32	3445.91	1.957	1035.08	2025.62
A_68_P31177905	chr17:41017051-41017095	NM_027834:435	9130008F23Rik	INSIDE	0.757	2.276	859.83	1956.74	1.723	614.54	1058.80
A_68_P31113143	chr17:27053838-27053882	NM_053173:-175	Kifc5b	PROMOTER	0.757	0.637	1485.47	946.49	0.482	979.30	472.13
A_68_P30476323	chr15:98592955-98592999	NM_007478:573	Arf3	INSIDE	0.757	0.680	5579.79	3791.71	0.515	4110.38	2115.21
A_68_P30371086	chr15:80085860-80085904	NM_009716:269	Atf4	INSIDE	0.757	0.136	6947.07	944.58	0.103	4825.52	496.88
A_68_P28908962	chr13:29043905-29043949	NM_009238:1625	Sox4	INSIDE	0.757	3.781	1710.85	6468.46	2.864	1493.92	4278.27
A_68_P28377243	chr12:40949898-40949942	NM_013562:-144	Ifrd1	PROMOTER	0.757	0.529	1877.07	992.34	0.400	1577.09	631.55
A_68_P28189253	chr11:121066168-121066212	NM_025402:-228	1110031I02Rik	DIVERGENT_PROMOTER	0.757	0.644	2994.62	1929.61	0.487	2341.37	1141.34
A_68_P28185091	chr11:120392009-120392053	NM_008101:-10	Gcgr	PROMOTER	0.757	6.281	859.90	5401.24	4.752	773.38	3675.04
A_68_P27924627	chr11:74987037-74987081	NR_029546:-125	Mir132	PROMOTER	0.757	0.390	2601.76	1015.37	0.296	2062.94	609.61
A_68_P27790976	chr11:50673440-50673484	NM_173372:9276	Grm6	INSIDE	0.757	3.589	1825.03	6549.69	2.716	1266.20	3438.97
A_68_P27674153	chr11:29274024-29274068	NM_176841:272	Ccdc88a	INSIDE	0.757	0.635	1059.54	672.47	0.480	908.33	436.15
A_68_P27297053	chr10:82822767-82822811	NM_026579:-177	D10Wsu102e	PROMOTER	0.757	0.709	7553.85	5358.89	0.537	5376.89	2886.41
A_68_P27278356	chr10:79167062-79167106	NM_001077184:-18	Bsg	PROMOTER	0.757	3.232	1040.41	3362.29	2.447	889.17	2175.39
A_68_P27033507	chr10:28867124-28867168	NM_026138:3345	6330407J23Rik	INSIDE	0.757	2.325	986.04	2292.25	1.760	771.62	1357.86
A_68_P26079854	chr8:97381360-97381404	NM_009090:33	Polr2c	INSIDE	0.757	9.832	1670.44	16423.24	7.438	1456.85	10836.49
A_68_P25846169	chr8:49314467-49314511	NM_001145937:445556	Odz3	INSIDE	0.757	4.935	806.83	3982.04	3.737	670.81	2506.99
A_68_P24804128	chr6:121362766-121362810	NM_001033354:60908	Iqsec3	INSIDE	0.757	3.715	3301.25	12264.73	2.813	2335.94	6571.69
A_68_P24164321	chr5:145875650-145875694	NM_023142:548	Arpc1b	INSIDE	0.757	0.627	1005.41	630.53	0.475	859.66	407.96
A_68_P23999131	chr5:114064213-114064257	NM_008153:36165	Cmklr1	INSIDE	0.757	5.769	1250.01	7211.52	4.364	811.02	3539.62
A_68_P23698175	chr5:53440513-53440557	NM_011402:-57	Slc34a2	PROMOTER	0.757	0.362	9239.86	3345.89	0.274	6275.87	1720.65

ProbeName	Target position of probe on CpG island microarray	TargetID	GeneSymbol	CpG island Description	Ratio of relative methylation (TiO_2-NP/Vehicle)	Sham group			TiO_2-H group		
						Relative methylation (Cy5/Cy3)	Cy3 signal (Input)	Cy5 signal (MeDIP)	Relative methylation (Cy5/Cy3)	Cy3 signal (Input)	Cy5 signal (MeDIP)
A_68_P23600260	chr5:35739942-35739986	NM_010445:8199	Hmx1	DOWNSTREAM	0.757	0.217	3390.67	736.32	0.164	2523.08	414.56
A_68_P23392750	chr4:147533918-147533962	NM_001161851:-236	Fbxo44	PROMOTER	0.757	2.722	1687.26	4593.03	2.061	1343.17	2768.69
A_68_P23270839	chr4:124558845-124558889	NM_177671:-162	Epha10	PROMOTER	0.757	0.487	2148.13	1045.68	0.368	1585.75	584.19
A_68_P22261355	chr3:78870478-78870522	NM_001099624:79297	Rapgef2	INSIDE	0.757	3.421	961.74	3290.28	2.588	807.22	2089.29
A_68_P21900711	chr2:179711603-179711647	NR_033782:67	4921531C22Rik	INSIDE	0.757	1.910	3483.20	6654.23	1.445	2818.15	4073.58
A_68_P21079215	chr2:26359143-26359187	NM_008714:178	Notch1	INSIDE	0.757	2.955	1763.61	5211.16	2.238	1358.21	3039.59
A_68_P20329006	chr1:71149346-71149390	NM_007525:178	Bard1	INSIDE	0.757	2.269	1074.10	2437.32	1.718	1042.02	1790.64
A_68_P32823841	chrX:166435161-166435205	NM_183151:117629	Mid1	DOWNSTREAM	0.756	0.465	2682.09	1246.80	0.351	2612.44	918.19
A_68_P31143790	chr17:32250687-32250731	NM_008716:53089	Notch3	DOWNSTREAM	0.756	0.529	907.88	479.93	0.399	737.90	294.72
A_68_P31029176	chr17:8995030-8995074	NM_011866:443	Pde10a	INSIDE	0.756	1.953	2221.48	4338.63	1.476	1741.43	2570.73
A_68_P30140509	chr15:36724043-36724087	NM_011740:229	Ywhaz	INSIDE	0.756	0.241	4708.08	1134.31	0.182	3150.33	573.84
A_68_P29977217	chr15:3977373-3977417	NR_028266:12	BC037032	INSIDE	0.756	0.584	894.03	522.53	0.442	797.47	352.25
A_68_P29485181	chr14:27928181-27928226	NM_134437:76017	Il17rd	DOWNSTREAM	0.756	0.465	1346.97	626.63	0.351	1049.57	368.89
A_68_P28578875	chr12:82127694-82127738	NM_001146217:-78	Smoc1	PROMOTER	0.756	0.463	1644.45	761.85	0.350	1285.47	450.15
A_68_P28048902	chr11:96999994-97000038	NM_198100:11010	Tbkbp1	INSIDE	0.756	0.653	1133.43	739.94	0.494	900.97	444.71
A_68_P26896298	chr9:123587851-123587896	NM_139142:77	Slc6a20a	INSIDE	0.756	0.226	3585.81	809.46	0.171	2433.61	415.54
A_68_P26528584	chr9:54799038-54799082	NM_145229:-299	AY074887	PROMOTER	0.756	0.598	1512.90	904.58	0.452	1151.44	520.19
A_68_P26135495	chr8:108228498-108228542	NM_198299:3548	E130303B06Rik	INSIDE	0.756	3.204	845.29	2708.09	2.423	568.63	1377.97
A_68_P26018638	chr8:86707466-86707510	NR_029821:-4628	Mir181c	PROMOTER	0.756	0.414	1736.04	718.99	0.313	1230.79	385.33
A_68_P25947916	chr8:72298647-72298691	NM_177899:142	Zfp866	INSIDE	0.756	2.630	2174.73	5718.92	1.989	1889.24	3758.00
A_68_P25190568	chr7:73985326-73985370	NM_001033877:728	Adamts17	INSIDE	0.756	0.630	1389.36	875.04	0.476	1110.22	528.91
A_68_P25017144	chr7:28189868-28189912	NM_001199236:-8318	Spnb4	PROMOTER	0.756	4.469	2504.47	11193.06	3.381	1918.10	6484.37
A_68_P24449306	chr6:52185000-52185044	NM_008263:-84	Hoxa10	PROMOTER	0.756	2.196	777.30	1707.23	1.661	680.06	1129.74
A_68_P23904973	chr5:96590810-96590854	NM_178854:-58	Cnot6l	PROMOTER	0.756	0.530	6747.31	3576.84	0.401	4394.57	1760.95
A_68_P23567512	chr5:29761751-29761795	NM_001033457:566	Noml	INSIDE	0.756	0.500	1214.94	608.00	0.379	839.60	317.80
A_68_P23396572	chr4:148178339-148178383	NM_027195:-140	Caszl	PROMOTER	0.756	2.203	1692.41	3727.61	1.666	1300.65	2166.90
A_68_P22046078	chr3:33980177-33980221	NM_001026211:50	Dnajc19	INSIDE	0.756	2.629	2178.79	5728.40	1.987	2010.53	3995.58
A_68_P21784861	chr2:158594102-158594146	NM_027975:290	Fam83d	INSIDE	0.756	0.165	3452.99	570.39	0.125	2584.06	322.53
A_68_P21100046	chr2:29745040-29745084	NM_001113214:-177	Odf2	PROMOTER	0.756	0.612	3355.27	2054.92	0.463	2412.45	1116.37
A_68_P21070954	chr2:25118703-25118747	NM_175286:607	Tprn	INSIDE	0.756	2.047	1235.77	2529.02	1.547	1062.32	1642.99
A_68_P20641220	chr1:137625770-137625814	NM_007791:19	Csrp1	INSIDE	0.756	0.587	3571.71	2094.85	0.443	2719.07	1205.71
A_68_P20075274	chr1:20925596-20925640	NM_028829:44916	Paqr8	INSIDE	0.756	2.355	939.39	2212.31	1.780	696.09	1238.78
A_68_P30497429	chr15:102186223-102186267	NM_153416:-5054	Aaas	PROMOTER	0.755	0.633	3699.61	2340.75	0.478	2845.13	1359.30
A_68_P30477113	chr15:98711795-98711839	NM_026967:29	Rhebl1	INSIDE	0.755	0.486	2566.89	1248.28	0.367	2023.24	742.57
A_68_P29661996	chr14:64162753-64162797	NM_177594:16	Mtmr9	INSIDE	0.755	2.096	2095.52	4392.41	1.583	1705.66	2700.16
A_68_P28731669	chr12:110291754-110291798	NM_001163175:14651	Begain	INSIDE	0.755	0.523	931.65	487.69	0.395	830.22	328.19
A_68_P28099477	chr11:105946015-105946059	NM_011947:-179	Map3k3	PROMOTER	0.755	2.824	981.86	2772.92	2.133	824.54	1758.50
A_68_P28089840	chr11:104092393-104092437	NM_001038609:-335	Mapt	PROMOTER	0.755	0.343	2341.83	803.16	0.259	1647.66	426.64
A_68_P28084547	chr11:103173618-103173663			Unknown	0.755	3.038	681.35	2070.22	2.295	576.94	1324.27
A_68_P28083696	chr11:103032727-103032771	NM_001077698:297	Fmnl1	INSIDE	0.755	0.618	820.25	507.08	0.467	744.82	347.67
A_68_P28060084	chr11:98902871-98902915	NM_010517:319	Igfbp4	INSIDE	0.755	2.840	1375.91	3907.44	2.144	1154.21	2474.50
A_68_P26816565	chr9:108686233-108686277	ENSMUST00000035219:128		INSIDE	0.755	0.268	2035.06	545.09	0.202	1621.23	328.06
A_68_P26813124	chr9:108017763-108017807	NM_007567:74930	Bsn	INSIDE	0.755	2.583	714.48	1845.62	1.950	527.26	1028.13
A_68_P26812600	chr9:107951503-107951547	NM_177910:-96	Gmppb	PROMOTER	0.755	3.674	389.59	1431.17	2.773	342.85	950.87
A_68_P26394906	chr9:30234770-30234814	NM_028874:-121	Snx19	PROMOTER	0.755	0.585	2956.62	1729.09	0.441	2530.55	1116.70
A_68_P26319141	chr9:14555928-14555972	NM_010242:616	Fut4	INSIDE	0.755	0.571	1107.08	631.81	0.431	792.01	341.15
A_68_P25446103	chr7:123236610-123236654	NR_029433:31	1700003G18Rik	INSIDE	0.755	0.609	2442.27	1486.36	0.460	1859.42	854.84
A_68_P25445800	chr7:123175043-123175098	NM_001025560:-509	Sox6	PROMOTER	0.755	0.445	1255.45	558.21	0.336	960.44	322.48
A_68_P24974396	chr7:13639929-13639973	NM_145819:163	Mzf1	INSIDE	0.755	0.726	1570.73	1139.78	0.548	1286.89	704.94
A_68_P24425595	A_68_P24425595			Unknown	0.755	7.107	594.27	4223.25	5.362	560.48	3005.24
A_68_P24167577	chr5:147072524-147072578	NM_153599:29300	Cdk8	INSIDE	0.755	4.138	303.83	1257.30	3.126	260.67	814.83
A_68_P24122109	chr5:137044884-137044928	NM_009986:-1605	Cux1	PROMOTER	0.755	2.215	1292.22	2862.00	1.672	1197.66	2002.44
A_68_P21567210	chr2:118801995-118802039	AK184532:42052		INSIDE	0.755	0.535	1462.52	782.71	0.404	1201.15	485.49
A_68_P21073094	chr2:25433567-25433611	NM_001005424:2031	Gm996	INSIDE	0.755	2.337	1192.46	2786.31	1.765	978.89	1727.67
A_68_P20897579	chr1:186634789-186634833	NM_001081361:382	Mosc1	INSIDE	0.755	0.568	937.96	532.44	0.428	800.09	342.76
A_68_P20877958	chr1:182743454-182743498	NM_008210:258	H3f3a	INSIDE	0.755	0.564	986.68	556.15	0.426	712.38	303.31
A_68_P20619647	chr1:134035982-134036026	NM_008795:258	Cdk18	INSIDE	0.755	3.979	1187.59	4725.19	3.003	1062.31	3190.44
A_68_P20613817	chr1:133034698-133034742	NM_145508:91	Dyrk3	INSIDE	0.755	3.065	433.80	1329.47	2.315	395.92	916.69
A_68_P32143269	chr19:46402678-46402722	NM_133694:27	Fbxl15	INSIDE	0.754	0.601	1069.12	642.44	0.453	906.52	410.63
A_68_P32139835	chr19:45734791-45734835	NM_013907:-129	Fbxw4	PROMOTER	0.754	0.628	1108.21	696.25	0.473	898.51	425.35
A_68_P31618904	chr18:35722537-35722581	NM_010771:747	Matr3	INSIDE	0.754	0.615	3062.60	1884.00	0.464	2276.79	1056.09
A_68_P31255288	chr17:56465651-56465695	NM_172132:200	Kdm4b	INSIDE	0.754	2.857	1212.80	3464.42	2.153	1126.14	2424.17
A_68_P31102749	chr17:25410196-25410240	NM_001177610:158	Ube2i	INSIDE	0.754	2.708	1329.79	3600.46	2.041	1073.85	2191.46
A_68_P30361559	chr15:78502925-78502969	NM_183141:45597	Elfn2	INSIDE	0.754	10.001	1260.74	12608.86	7.541	1060.05	7993.32
A_68_P29998440	chr15:8118886-8118930	NM_001162906:-197	2410089E03Rik	PROMOTER	0.754	0.339	1573.46	533.14	0.255	1270.01	324.25

ProbeName	Target position of probe on CpG island microarray	TargetID	GeneSymbol	CpG island Description	Ratio of relative methylation (TiO_2-NP/Vehicle)	Sham group			TiO_2-H group		
						Relative methylation (Cy5/Cy3)	Cy3 signal (Input)	Cy5 signal (MeDIP)	Relative methylation (Cy5/Cy3)	Cy3 signal (Input)	Cy5 signal (MeDIP)
A_68_P28599059	chr12:85910912-85910956	NM_007701:133	Vsx2	INSIDE	0.754	0.149	4484.90	667.63	0.112	3290.94	369.32
A_68_P27796700	chr11:51781220-51781264	NM_029976:110	Cdkn2aipnl	INSIDE	0.754	3.589	1713.27	6149.23	2.708	1475.92	3996.85
A_68_P27553116	chr11:3549120-3549164	NR_002321:-331	Tug1	DIVERGENT_PROMOTER	0.754	0.429	1918.41	822.66	0.323	1574.59	508.83
A_68_P27430002	chr10:107598906-107598950	NM_027892:-527	Ppp1r12a	PROMOTER	0.754	0.678	927.60	628.55	0.511	876.95	447.91
A_68_P26888895	chr9:122260872-122260916	NM_026179:161	Abhd5	INSIDE	0.754	4.177	1175.88	4911.17	3.149	1015.96	3199.07
A_68_P26813048	chr9:108008754-108008798	NM_007567:83938	Bsn	INSIDE	0.754	2.348	1224.25	2874.04	1.770	931.66	1649.09
A_68_P26166334	chr8:113581188-113581232	NM_028274:972	Exosc6	INSIDE	0.754	2.739	1332.19	3648.71	2.064	1064.19	2196.33
A_68_P26129051	chr8:107065181-107065225	NM_053070:496	Car7	INSIDE	0.754	0.608	2241.56	1362.67	0.458	1591.35	729.50
A_68_P26035037	chr8:89997425-89997469	NR_033430:736	Gm2694	INSIDE	0.754	4.392	480.12	2108.67	3.313	487.43	1614.67
A_68_P25954584	chr8:73389578-73389623	NM_032397:-8693	Kcnn1	PROMOTER	0.754	2.371	1610.48	3818.94	1.788	1256.26	2245.82
A_68_P25272791	chr7:88716304-88716348	NM_001004185:149	Whamm	INSIDE	0.754	2.820	698.14	1968.80	2.126	574.50	1221.68
A_68_P25115502	chr7:57167419-57167463	NM_148931:371	Slc6a5	INSIDE	0.754	0.536	1144.49	613.64	0.404	896.37	362.24
A_68_P24776189	chr6:116288297-116288341	NM_027920:178	Marchf8	INSIDE	0.754	1.887	1712.00	3231.05	1.422	1616.90	2299.49
A_68_P24639523	chr6:91465703-91465747	NM_009531:158	Xpc	INSIDE	0.754	0.566	1101.15	623.48	0.427	860.30	367.14
A_68_P23331229	chr4:135476924-135476968	NM_024243:306	Fuca1	INSIDE	0.754	0.322	2497.07	803.83	0.243	1735.44	421.02
A_68_P22752470	chr4:15872272-15872316	NM_026172:360	Decr1	INSIDE	0.754	0.464	5537.36	2569.22	0.350	3896.07	1362.20
A_68_P21570231	chr2:119372809-119372853	NM_172857:533	Exd1	INSIDE	0.754	0.403	2072.55	835.89	0.304	1476.90	449.27
A_68_P21221437	chr2:52601491-52601535	NM_019667:-329	Stam2	PROMOTER	0.754	0.521	2941.43	1533.78	0.393	2316.78	911.04
A_68_P21037798	chr2:17948361-17948405	NR_028378:-493	LOC100034739	PROMOTER	0.754	0.702	1722.51	1208.55	0.529	1293.12	684.15
A_68_P20958785	chr1:196802778-196802822	NR_033564:353	C030002c11rik	INSIDE	0.754	2.131	875.87	1866.90	1.608	714.49	1149.03
A_68_P20451933	chr1:94803719-94803763	NM_175118:175	Dusp28	INSIDE	0.754	2.223	1125.32	2502.14	1.677	867.70	1455.32
A_68_P20391720	chr1:82288083-82288127	NM_010570:-90	Irs1	PROMOTER	0.754	9.278	875.52	8123.01	6.996	701.34	4906.36
A_68_P32143669	chr19:46471505-46471549	NM_001025391:119	Sufu	INSIDE	0.753	0.169	3871.74	654.63	0.127	2812.92	358.08
A_68_P31744350	chr18:58996079-58996123	NM_175506:-317	Adamts19	PROMOTER	0.753	0.674	1628.21	1097.64	0.508	1425.36	723.95
A_68_P30895872	chr16:78377364-78377408	NM_009770:-356	Btg3	PROMOTER	0.753	0.516	864.83	446.41	0.389	750.66	291.70
A_68_P30534533	chr16:8830219-8830263	NM_001081400:48	1810013L24Rik	INSIDE	0.753	4.261	1069.47	4556.73	3.207	938.52	3010.10
A_68_P29338284	chr13:113825329-113825373	NR_030452:-840	Mir449c	PROMOTER	0.753	0.676	2569.59	1736.86	0.509	1971.54	1003.60
A_68_P28163121	chr11:116976175-116976219	NR_029459:-289	Sec14l1	PROMOTER	0.753	0.124	4760.42	589.30	0.093	3154.11	293.85
A_68_P28156585	chr11:115836281-115836325	NM_001005608:264	Itgb4	INSIDE	0.753	0.271	1873.63	507.74	0.204	1519.73	310.15
A_68_P27181451	chr10:61088741-61088785	NM_001177511:30531	Npffr1	INSIDE	0.753	0.676	1590.41	1074.40	0.509	1206.66	613.71
A_68_P25601076	chr7:150645402-150645446	NM_001161624:1531	Cdkn1c	INSIDE	0.753	3.094	1244.49	3850.14	2.331	1039.22	2422.55
A_68_P25506337	chr7:134732289-134732333	NM_172281:99	Rnf40	INSIDE	0.753	0.633	1231.87	779.69	0.476	1048.02	499.21
A_68_P25090467	chr7:52241632-52241676	NM_019830:48	Prmt1	INSIDE	0.753	0.470	2830.14	1330.50	0.354	2115.77	748.48
A_68_P24818156	chr6:124959237-124959281	NM_175557:-564	Zfp384	PROMOTER	0.753	2.860	1234.53	3531.29	2.155	1065.24	2295.10
A_68_P23692818	chr5:52505349-52505393	AK076709:-3181		PROMOTER	0.753	2.712	1178.01	3194.87	2.042	1066.90	2178.25
A_68_P23342101	chr4:137351925-137351969	NM_007431:346	Alpl	INSIDE	0.753	0.492	3065.40	1507.00	0.370	2406.43	890.49
A_68_P23318323	chr4:133140497-133140541	NM_133884:231	Gpn2	INSIDE	0.753	0.700	1831.62	1281.30	0.527	1499.49	790.18
A_68_P22531472	chr3:131005829-131005873	NM_027816:295	Cyp2u1	INSIDE	0.753	0.555	1032.85	573.49	0.418	868.35	363.01
A_68_P21839348	chr2:167905989-167906034	NM_021409:-492	Pard6b	PROMOTER	0.753	0.355	1909.78	677.54	0.267	1277.21	341.16
A_68_P21609431	chr2:126501251-126501295	NM_207669:-49	Gabpb1	PROMOTER	0.753	0.556	2405.16	1337.25	0.419	2015.16	843.74
A_68_P21095669	chr2:29050154-29050198	NM_133500:53384	Ntng2	DOWNSTREAM	0.753	2.687	1598.52	4295.21	2.023	1439.73	2911.86
A_68_P21071705	chr2:25227573-25227617	NM_001081085:-246	2010317E24Rik	DIVERGENT_PROMOTER	0.753	0.492	2086.16	1026.60	0.371	1558.49	577.43
A_68_P20233544	chr1:53842063-53842107	NM_133810:-25	Stk17b	PROMOTER	0.753	2.183	2363.91	5160.15	1.644	1945.65	3199.31
A_68_P20076127	chr1:21068999-21069043	NM_133252:270	Tram2	INSIDE	0.753	0.321	3203.74	1026.84	0.241	2444.98	589.76
A_68_P31487559	chr18:9958109-9958153	NM_153552:-47	Thoc1	PROMOTER	0.752	0.312	1497.68	467.93	0.235	1249.10	293.41
A_68_P31157497	chr17:35226598-35226642	NM_178592:385	Abhd16a	INSIDE	0.752	4.020	1732.86	6966.37	3.024	1522.71	4605.03
A_68_P31118768	chr17:27977231-27977275	NM_011432:221	Snrpc	INSIDE	0.752	7.164	536.21	3841.16	5.389	463.56	2498.20
A_68_P31102143	chr17:25321627-25321671	NM_183150:143	BC003965	INSIDE	0.752	0.382	1680.35	642.67	0.287	1377.86	396.06
A_68_P30504520	chr15:103333160-103333204	NM_008800:-546	Pde1b	PROMOTER	0.752	0.725	2210.60	1602.10	0.545	1628.71	887.50
A_68_P29512326	chr14:32307928-32307972	NM_025907:213	Mettl6	INSIDE	0.752	0.158	3910.71	618.18	0.119	3044.90	361.71
A_68_P29294056	chr13:105844725-105844769	NM_029879:264	Rgs7bp	INSIDE	0.752	0.664	1205.31	800.44	0.499	1050.90	524.72
A_68_P28853785	chr13:17896465-17896509	NM_001081058:445	Cdk13	INSIDE	0.752	2.982	1012.83	3019.94	2.243	991.97	2224.55
A_68_P28748394	chr12:113413797-113413841	NM_001097621:29400	Kif26a	INSIDE	0.752	4.385	1305.88	5725.76	3.296	1079.10	3556.83
A_68_P28094677	chr11:105043643-105043687	NM_001112705:821	Tlk2	INSIDE	0.752	2.274	764.78	1738.90	1.710	803.49	1374.20
A_68_P27688272	chr11:32247924-32247968	NM_177364:136	Sh3pxd2b	INSIDE	0.752	0.416	4171.75	1734.61	0.313	2945.06	920.43
A_68_P27638870	chr11:21897079-21897123	NM_011023:4554	Otx1	INSIDE	0.752	0.636	6431.58	4087.99	0.478	4699.01	2244.67
A_68_P26593987	chr9:66439772-66439816	NM_144937:993	Usp3	INSIDE	0.752	0.536	992.18	532.20	0.404	772.93	311.93
A_68_P25507196	chr7:134913351-134913395	NM_198424:44	Orai3	INSIDE	0.752	0.536	1067.64	571.99	0.403	859.01	346.28
A_68_P25392269	chr7:112574333-112574377	NM_007627:21	Cckbr	INSIDE	0.752	0.202	3304.08	667.31	0.152	2551.90	387.51
A_68_P25361477	chr7:106530129-106530173	NM_201352:92	Gdpd5	INSIDE	0.752	0.579	5303.57	3069.81	0.435	3957.77	1722.32
A_68_P25189684	chr7:73834910-73834954	NM_001191001:158	Lins	INSIDE	0.752	2.007	1581.84	3174.19	1.509	1269.62	1916.35
A_68_P24758531	chr6:113232209-113232253	NM_170673:-70	Cpne9	PROMOTER	0.752	0.410	1096.18	449.31	0.308	984.26	303.47
A_68_P23971471	chr5:108796094-108796138	ENSMUST00000100944:-2760		PROMOTER	0.752	0.471	1466.68	690.81	0.354	1147.95	406.44
A_68_P23909952	chr5:97426279-97426323	NM_080708:-407	Bmp2k	PROMOTER	0.752	2.369	1852.57	4388.09	1.780	1406.27	2503.38
A_68_P23410314	chr4:150503333-150503377	NM_001081557:732523	Camta1	INSIDE	0.752	0.258	2448.88	631.66	0.194	1612.68	312.82

ProbeName	Target position of probe on CpG island microarray	TargetID	GeneSymbol	CpG island Description	Ratio of relative methylation (TiO_2-NP/Vehicle)	Sham group			TiO_2-H group		
						Relative methylation (Cy5/Cy3)	Cy3 signal (Input)	Cy5 signal (MeDIP)	Relative methylation (Cy5/Cy3)	Cy3 signal (Input)	Cy5 signal (MeDIP)
A_68_P23331346	chr4:135502630-135502674	NM_008254:285	Hmgcl	INSIDE	0.752	0.354	3036.17	1073.90	0.266	2198.92	584.64
A_68_P22305775	chr3:86723959-86724003	NM_001195496:826	Dclk2	INSIDE	0.752	2.103	1253.61	2636.81	1.581	1116.42	1764.81
A_68_P20880193	chr1:183141557-183141601	NM_145514:531	Wdr26	INSIDE	0.752	4.876	822.11	4009.00	3.669	715.31	2624.78
A_68_P32127591	chr19:43689143-43689187	NM_008699:2350	Nkx2-3	INSIDE	0.751	2.431	1430.52	3476.97	1.825	1313.43	2397.18
A_68_P32008254	chr19:21867188-21867232	NM_001033759:14379	Tmem2	INSIDE	0.751	3.500	1227.57	4297.09	2.629	919.43	2417.59
A_68_P31544272	chr18:21459015-21459059	NM_001033445:-396	Fam59a	PROMOTER	0.751	0.237	2770.68	657.71	0.178	2134.93	380.51
A_68_P31375069	chr17:79419723-79419767	NM_001171004:412	Prkd3	INSIDE	0.751	2.728	1379.90	3764.64	2.050	1128.58	2313.24
A_68_P31019377	A_68_P31019377			Unknown	0.751	3.528	308.62	1088.80	2.650	245.75	651.36
A_68_P28535265	chr12:74146791-74146835	NM_009189:887	Six1	INSIDE	0.751	0.191	2789.11	533.46	0.144	2122.38	305.01
A_68_P28076811	chr11:101846175-101846219	NM_028207:-91	Dusp3	DIVERGENT_PROMOTER	0.751	0.510	1169.12	595.84	0.383	953.21	364.96
A_68_P27212542	chr10:67011815-67011859	NM_001005419:-133	Ado	PROMOTER	0.751	3.964	1001.50	3969.83	2.978	818.51	2437.62
A_68_P26660364	chr9:78463666-78463710	NM_153098:336	Cd109	INSIDE	0.751	0.361	2903.74	1049.25	0.271	2308.90	626.26
A_68_P26345299	chr9:20878051-20878095	NM_001039824:-114	Fdx1l	PROMOTER	0.751	0.396	1263.95	500.33	0.297	969.14	287.96
A_68_P26313304	chr9:13554080-13554131	NM_023858:481	Mtmr2	INSIDE	0.751	2.480	1428.72	3542.72	1.861	1212.75	2257.14
A_68_P26228504	chr8:124254834-124254878	NM_020605:398	Jph3	INSIDE	0.751	0.136	4618.90	629.81	0.102	3213.80	329.14
A_68_P26168806	chr8:114060945-114060989	NM_001168623:-1276	Znrf1	PROMOTER	0.751	2.689	1728.25	4647.26	2.019	1327.38	2680.50
A_68_P24686646	chr6:99471587-99471631	ENSMUST00001155466:145183		INSIDE	0.751	0.560	3727.76	2086.05	0.420	2830.69	1189.74
A_68_P24425029	chr6:47893194-47893238	NR_027963:43	Zfp783	INSIDE	0.751	2.793	2408.77	6728.35	2.097	1788.90	3750.69
A_68_P24091079	chr5:130362878-130362922	NM_024166:440	Chchd2	INSIDE	0.751	0.556	1891.36	1050.79	0.417	1447.40	604.08
A_68_P23892499	chr5:92566763-92566807	NM_019490:-179	Uso1	PROMOTER	0.751	1.860	3581.77	6663.17	1.398	2703.25	3778.56
A_68_P22774530	chr4:21654816-21654860	NM_001122982:-11	Ccnc	PROMOTER	0.751	0.557	864.44	481.57	0.418	675.60	282.62
A_68_P21809439	chr2:162757267-162757311	NM_026499:45	Srsf6	INSIDE	0.751	4.115	1209.81	4978.14	3.089	1138.13	3515.12
A_68_P21751498	chr2:152673870-152673914	NM_001141975:193	Tpx2	INSIDE	0.751	2.403	2661.27	6395.42	1.805	2348.76	4238.39
A_68_P32289635	chrX:20265624-20265668	NM_011049:28	Cdk16	INSIDE	0.750	3.637	544.89	1982.01	2.729	683.67	1865.65
A_68_P31863203	chr18:80453665-80453709	NM_001164423:642	Pqlc1	INSIDE	0.750	0.716	1887.89	1352.12	0.537	1491.83	801.43
A_68_P31625518	chr18:36894979-36895023	NM_028036:277	Tmco6	INSIDE	0.750	0.162	3704.18	599.76	0.122	2602.98	316.28
A_68_P30462686	chr15:96116941-96116985	NM_175251:-990	Arid2	PROMOTER	0.750	3.419	467.88	1599.92	2.566	404.32	1037.28
A_68_P29701958	chr14:70945229-70945273	NM_180588:193	Reep4	INSIDE	0.750	0.671	999.94	671.32	0.503	884.01	444.87
A_68_P29023967	chr13:49311401-49311447	NM_026738:-29	1110007C09Rik	PROMOTER	0.750	5.536	5739.10	31771.00	4.152	4639.94	19266.53
A_68_P28976807	chr13:41315714-41315758	NM_019423:36	Elovl2	INSIDE	0.750	0.609	1275.53	777.36	0.457	1060.58	484.47
A_68_P28974184	chr13:40826354-40826399	NM_001122948:-564	Tcfap2a	PROMOTER	0.750	4.547	6263.86	28484.71	3.413	4079.94	13923.00
A_68_P28184189	chr11:120239967-120240011	NM_027980:72	2310003H01Rik	INSIDE	0.750	3.020	660.19	1993.77	2.264	510.62	1156.18
A_68_P27953112	chr11:80022597-80022641	NM_001163354:62	Rhot1	INSIDE	0.750	3.552	396.36	1408.03	2.666	451.74	1204.29
A_68_P27915639	chr11:72990519-72990563	NM_001168470:-30	Tmem93	PROMOTER	0.750	3.231	1313.85	4245.31	2.424	1045.27	2533.59
A_68_P27697251	chr11:33743828-33743872	NM_001190885:-265	Kcnip1	PROMOTER	0.750	0.550	1204.94	662.32	0.412	873.15	359.75
A_68_P25954140	chr8:73321012-73321061	NM_199308:-4704	Mast3	PROMOTER	0.750	1.988	2868.59	5703.59	1.491	1894.63	2825.56
A_68_P25949908	chr8:72636524-72636568	NM_007789:8197	Ncan	INSIDE	0.750	0.558	856.01	477.64	0.419	715.42	299.50
A_68_P25655856	chr8:11497567-11497611	NM_026995:21	Carkd	INSIDE	0.750	0.525	2270.22	1192.38	0.394	1772.15	697.69
A_68_P24925471	chr6:145811847-145811891	NM_024469:1992	Bhlhe41	INSIDE	0.750	2.231	698.75	1559.16	1.673	621.52	1039.97
A_68_P23873912	chr5:89193966-89194010	NM_007832:-49	Dck	PROMOTER	0.750	0.361	6579.98	2377.62	0.271	4962.18	1344.68
A_68_P23281852	chr4:126430875-126430919	NM_133886:-117	AU040320	PROMOTER	0.750	0.279	2425.19	677.07	0.209	1856.74	388.74
A_68_P23240689	chr4:118159149-118159193	NM_011587:3284	Tie1	INSIDE	0.750	5.294	1940.20	10272.36	3.969	1592.79	6321.65
A_68_P23190566	chr4:107108029-107108073	NM_147221:727	Glis1	INSIDE	0.750	0.610	5404.44	3295.98	0.457	3745.25	1713.02
A_68_P22659976	chr3:153636770-153636814	NM_001081263:393	Slc44a5	INSIDE	0.750	0.368	1765.84	649.67	0.276	1431.27	394.87
A_68_P22388057	chr3:103595283-103595327	NM_010432:-106	Hipk1	PROMOTER	0.750	0.543	3201.79	1737.12	0.407	2452.38	998.53
A_68_P22271765	chr3:80840549-80840593	NM_019971:233	Pdgfc	INSIDE	0.750	0.340	5319.99	1811.10	0.255	4059.45	1036.56
A_68_P21931968	chr3:8327438-8327482			Unknown	0.750	3.444	1389.18	4784.26	2.584	1210.77	3128.34
A_68_P21631948	chr2:130665681-130665725	NM_029432:144	4930402H24Rik	INSIDE	0.750	2.997	1693.66	5075.43	2.247	1490.06	3348.56
A_68_P20708501	chr1:152240022-152240066	NM_133780:77	Tpr	INSIDE	0.750	0.574	1854.69	1065.07	0.431	1552.49	668.37
A_68_P20600404	chr1:130313816-130313860	NM_177445:155	Dars	INSIDE	0.750	0.265	3228.17	854.45	0.198	2236.11	443.64
A_68_P32092596	chr19:36978892-36978936			Unknown	0.749	0.649	6860.78	4453.09	0.486	4692.07	2280.30
A_68_P31615542	chr18:35091468-35091512	NM_144866:167	Etf1	INSIDE	0.749	0.557	1304.99	726.35	0.417	1108.14	461.90
A_68_P30572952	chr16:17278112-17278156	NM_177473:1742	Tmem191c	INSIDE	0.749	0.622	1546.36	962.22	0.466	1254.84	584.84
A_68_P29699779	chr14:70576599-70576643	NM_145978:859	Pdlim2	INSIDE	0.749	0.469	3393.49	1591.48	0.351	2691.29	944.81
A_68_P29694905	chr14:69808678-69808722	NM_010921:-48	Nkx3-1	PROMOTER	0.749	8.201	1364.02	11186.33	6.139	1163.61	7142.99
A_68_P29233989	chr13:95055494-95055538	NM_029153:-280	Scamp1	PROMOTER	0.749	0.466	2045.11	952.29	0.349	1578.03	550.24
A_68_P29228410	chr13:94074181-94074225	NM_011982:-247	Homer1	PROMOTER	0.749	3.043	1713.05	5212.82	2.280	1444.57	3292.96
A_68_P28202815	chr12:4881183-4881227	NM_001033488:-39	Mfsd2b	PROMOTER	0.749	4.182	1420.59	5941.24	3.134	1087.97	3409.99
A_68_P28042225	chr11:95925814-95925859	NM_172300:842	Ube2z	INSIDE	0.749	0.358	1789.64	640.07	0.268	1398.33	374.76
A_68_P26166325	chr8:113580098-113580142	NM_028274:-118	Exosc6	PROMOTER	0.749	0.570	1309.99	746.20	0.426	943.10	402.21
A_68_P26078525	chr8:97126178-97126222	NM_026274:360	Rspry1	INSIDE	0.749	0.729	1664.06	1212.38	0.545	1304.55	711.43
A_68_P25285295	chr7:91032191-91032235	NM_030705:639	Mesdc1	INSIDE	0.749	3.100	516.09	1599.98	2.322	494.53	1148.39
A_68_P24183689	chr5:149996098-149996142	NM_001013378:-15	Uspl1	DIVERGENT_PROMOTER	0.749	2.167	1102.80	2389.42	1.624	888.73	1442.99
A_68_P24114264	chr5:135485504-135485548	NR_033794:201	Abhd11	INSIDE	0.749	0.568	1267.68	719.80	0.425	1113.18	473.64
A_68_P23317937	chr4:133054043-133054087	NM_001081156:401	Trnp1	INSIDE	0.749	5.408	1160.49	6275.91	4.048	1051.79	4257.55

ProbeName	Target position of probe on CpG island microarray	TargetID	GeneSymbol	CpG island Description	Ratio of relative methylation (TiO_2-NP/Vehicle)	Sham group			TiO_2-H group		
						Relative methylation (Cy5/Cy3)	Cy3 signal (Input)	Cy5 signal (MeDIP)	Relative methylation (Cy5/Cy3)	Cy3 signal (Input)	Cy5 signal (MeDIP)
A_68_P23296723	chr4:129138984-129139028	NM_133889:84	Bsdc1	INSIDE	0.749	0.403	4961.39	1999.61	0.302	3561.43	1075.35
A_68_P23274586	chr4:125167342-125167386	NM_001081097:-710	Grik3	PROMOTER	0.749	2.813	1105.48	3109.58	2.106	817.64	1721.99
A_68_P22488130	chr3:123149844-123149888	NM_008939:36	Prss12	INSIDE	0.749	0.486	1500.39	729.92	0.364	1272.04	463.56
A_68_P22293157	chr3:84618617-84618661	NM_001177773:-860	Fbxw7	PROMOTER	0.749	0.415	3325.98	1378.91	0.311	2415.43	750.28
A_68_P22039457	chr3:32607708-32607752	NM_019673:263	Actl6a	INSIDE	0.749	0.253	2394.28	604.91	0.189	1732.92	327.71
A_68_P21839206	chr2:167875978-167876022			Unknown	0.749	2.040	1488.27	3036.19	1.528	1260.30	1925.85
A_68_P21722291	chr2:146910728-146910772	NM_023504:331	Nkx2-4	INSIDE	0.749	3.604	893.34	3219.76	2.701	745.12	2012.77
A_68_P21078051	chr2:26207828-26207873	NM_001037747:7217	Card9	INSIDE	0.749	0.677	1646.22	1114.37	0.507	1295.56	657.20
A_68_P20741945	chr1:158134987-158135031	NM_177838:148	Fam163a	INSIDE	0.749	2.026	2700.99	5472.77	1.518	2183.11	3314.90
A_68_P20620659	chr1:134213013-134213057	NM_001195025:333	Nuak2	INSIDE	0.749	0.402	1543.17	620.27	0.301	1213.66	365.44
A_68_P20165363	chr1:39424634-39424678	NM_053257:-39	Rpl31	PROMOTER	0.749	0.629	3910.50	2459.34	0.471	2826.71	1331.23
A_68_P32749010	chrX:148776690-148776734	NM_029836:315	Tspyl2	INSIDE	0.748	0.658	2374.53	1562.16	0.492	3295.13	1620.94
A_68_P32268345	chrX:13423285-13423329	NM_009806:361	Cask	INSIDE	0.748	3.226	381.49	1230.77	2.412	524.47	1265.26
A_68_P32240448	chrX:7419508-7419552	NM_138604:574	Otud5	INSIDE	0.748	2.488	701.46	1745.14	1.861	874.81	1628.42
A_68_P31253727	chr17:56219020-56219064	NM_008233:-37	Hdgfrp2	DIVERGENT_PROMOTER	0.748	3.497	1159.89	4056.60	2.616	992.35	2596.23
A_68_P30960424	chr16:91011840-91011884	NM_001164483:-309	Synj1	PROMOTER	0.748	2.299	909.07	2089.68	1.719	709.13	1218.86
A_68_P30540357	chr16:9994407-9994451	NM_008170:-1802	Grin2a	PROMOTER	0.748	0.523	1596.38	835.11	0.392	1275.14	499.28
A_68_P30404230	chr15:85809331-85809375	NM_009886:54855	Celsr1	INSIDE	0.748	2.132	1682.57	3587.05	1.595	1299.69	2072.67
A_68_P30176277	chr15:43309087-43309131	NM_025736:334	Ttc35	INSIDE	0.748	3.608	2090.87	7544.33	2.699	1873.80	5057.88
A_68_P29650241	chr14:62058563-62058607	NM_008466:200	Kpna3	INSIDE	0.748	0.279	1860.44	519.65	0.209	1457.11	304.44
A_68_P29308205	chr13:108680813-108680857	NM_145456:-576	Zswim6	PROMOTER	0.748	2.628	932.32	2449.98	1.965	737.65	1449.16
A_68_P28237422	chr12:11273354-11273398	NM_025695:685	Smc6	INSIDE	0.748	0.589	2281.33	1344.12	0.441	1609.95	709.92
A_68_P27914867	chr11:72861528-72861572	NM_025818:182	1200014J11Rik	INSIDE	0.748	0.429	5520.10	2368.94	0.321	4028.43	1292.87
A_68_P27332824	chr10:89208075-89208119	NM_029166:361	Uhrf1bp1l	INSIDE	0.748	3.511	741.70	2604.21	2.628	711.62	1870.09
A_68_P27253669	chr10:74430095-74430139	NM_010311:141	Gnaz	INSIDE	0.748	3.520	889.03	3129.39	2.632	733.27	1929.61
A_68_P26596713	chr9:66891367-66891411	NM_001164252:290	Tpm1	INSIDE	0.748	0.450	2361.54	1062.32	0.336	1914.32	643.90
A_68_P26473566	chr9:45238661-45238705	NM_001081270:307	Dscaml1	INSIDE	0.748	0.526	1987.50	1045.41	0.393	1612.75	634.11
A_68_P25307029	chr7:96487385-96487429	NM_028343:-109	Tmem135	PROMOTER	0.748	0.206	2958.43	608.03	0.154	1990.90	306.00
A_68_P25042628	chr7:34981548-34981592	NM_026549:96	Pdcd2l	INSIDE	0.748	0.507	3521.94	1785.29	0.379	2655.41	1006.36
A_68_P24908454	chr6:143048465-143048509	NM_001109688:141	5730419I09Rik	INSIDE	0.748	2.265	997.56	2259.69	1.693	817.26	1383.93
A_68_P24905346	chr6:142519942-142519986	NM_008428:-88	Kcnj8	PROMOTER	0.748	0.583	1886.91	1099.53	0.436	1369.62	596.89
A_68_P24158342	chr5:144578471-144578515	NM_013557:-168	Eif2ak1	PROMOTER	0.748	1.913	4921.01	9415.00	1.431	3876.22	5546.49
A_68_P23998432	chr5:113920097-113920141	NM_177292:652	Wscd2	INSIDE	0.748	2.008	3174.59	6373.00	1.501	2553.95	3832.86
A_68_P23880196	chr5:90312755-90312799	NM_001081401:-417	Adamts3	PROMOTER	0.748	3.404	923.38	3143.49	2.548	760.64	1938.22
A_68_P23574738	chr5:31117268-31117312	NM_133350:162	Mapre3	INSIDE	0.748	2.181	721.78	1574.55	1.631	743.11	1212.32
A_68_P23433034	chr4:154009287-154009331	NM_001177995:1674	Prdm16	INSIDE	0.748	0.311	2208.55	687.01	0.233	1709.24	397.69
A_68_P23252009	chr4:120242107-120242151	NM_016748:753	Ctps	INSIDE	0.748	0.401	2760.05	1106.17	0.300	1828.06	548.18
A_68_P23230187	chr4:116299629-116299673	NM_001081475:452	Nasp	INSIDE	0.748	2.401	806.22	1935.95	1.795	704.84	1265.38
A_68_P23156636	chr4:101390083-101390127	NM_010704:93	Lepr	INSIDE	0.748	0.456	1053.75	480.86	0.342	762.84	260.52
A_68_P22946060	chr4:57580871-57580915	NM_172868:-227	Palm2	PROMOTER	0.748	2.339	2399.49	5611.97	1.749	2196.92	3842.39
A_68_P22483661	chr3:122321647-122321691	NM_001114665:917	Fnbp1l	INSIDE	0.748	10.860	1007.91	10945.69	8.121	804.56	6533.50
A_68_P21578172	chr2:120803100-120803144	NM_175285:325	Tmem62	INSIDE	0.748	2.184	1381.87	3018.04	1.634	1158.07	1891.88
A_68_P21565424	chr2:118503074-118503118	NM_001145854:115	Pak6	INSIDE	0.748	0.330	1767.23	582.87	0.247	1452.57	358.21
A_68_P20832396	chr1:174256714-174256758	NM_008429:2658	Kcnj9	INSIDE	0.748	0.663	1132.84	751.03	0.496	920.66	456.74
A_68_P32769556	chrX:154481512-154481556	NM_177751:-492	Cnksr2	PROMOTER	0.747	3.724	507.65	1890.57	2.783	612.88	1705.95
A_68_P32769554	chrX:154481205-154481249	NM_177751:-184	Cnksr2	PROMOTER	0.747	3.488	830.02	2894.72	2.604	927.16	2414.56
A_68_P32373281	chrX:48194954-48194998	NM_133729:364	2610018G03Rik	INSIDE	0.747	2.450	702.18	1720.47	1.831	966.25	1769.09
A_68_P31982218	chr19:16855459-16855503	NM_173028:-63	Vps13a	PROMOTER	0.747	3.854	624.06	2404.92	2.879	500.86	1442.17
A_68_P31099856	chr17:24987535-24987579	NM_001159626:-55	Hagh	PROMOTER	0.747	3.750	1224.64	4592.95	2.802	990.05	2774.58
A_68_P29863085	chr14:103381169-103381213	NM_177715:664	Kctd12	INSIDE	0.747	3.628	720.10	2612.56	2.709	689.52	1867.74
A_68_P28998902	chr13:44998214-44998258	NM_001205043:171597	Jarid2	INSIDE	0.747	2.503	1420.06	3554.92	1.870	1136.46	2124.87
A_68_P28332011	chr12:32244102-32244146	NM_011867:710	Slc26a4	INSIDE	0.747	7.425	594.98	4417.73	5.543	517.00	2865.98
A_68_P27290791	chr10:81063724-81063768	NM_053254:-101	Tle6	DIVERGENT_PROMOTER	0.747	0.286	1674.08	479.29	0.214	1391.16	297.35
A_68_P26809620	chr9:107471457-107471501	NM_010489:-15	Hyal2	PROMOTER	0.747	0.648	4316.80	2799.13	0.484	3221.09	1559.98
A_68_P25779457	chr8:36439788-36439832	NM_177741:1016	Ppp1r3b	INSIDE	0.747	2.517	529.53	1332.78	1.880	462.46	869.64
A_68_P25554390	chr7:142907763-142907807	NM_001081117:278	Mki67	INSIDE	0.747	2.056	1257.35	2585.42	1.535	1135.91	1744.08
A_68_P24114959	chr5:135625601-135625645	NM_013763:42	Tbl2	INSIDE	0.747	0.667	1524.33	1017.07	0.498	1252.95	624.32
A_68_P23696527	chr5:53133072-53133116	NM_025951:282	Pi4k2b	INSIDE	0.747	3.594	760.72	2734.28	2.685	726.86	1951.46
A_68_P23542163	chr5:23991120-23991164	NM_139153:33148	Agap3	INSIDE	0.747	1.999	1403.98	2806.47	1.493	1081.36	1614.96
A_68_P23326918	chr4:134708841-134708885	NM_019732:32303	Runx3	INSIDE	0.747	0.620	2752.19	1705.55	0.463	2166.40	1002.45
A_68_P23320011	chr4:133443491-133443535	NM_009097:202	Rps6ka1	INSIDE	0.747	2.036	1177.20	2396.38	1.520	1084.10	1647.43
A_68_P23317104	chr4:132894918-132894962	NM_199306:290	Wdtc1	INSIDE	0.747	2.194	922.32	2023.14	1.639	861.37	1412.05
A_68_P22650333	chr3:152058577-152058621	NM_001080755:-375	Zzz3	PROMOTER	0.747	0.570	5083.79	2897.39	0.425	3587.26	1526.24
A_68_P22055030	chr3:35653187-35653231	NM_029570:149	Atp11b	INSIDE	0.747	0.288	2876.36	827.81	0.215	2158.67	463.92
A_68_P20834313	chr1:174563103-174563147	NM_026725:-19	Dusp23	PROMOTER	0.747	0.422	1593.05	672.32	0.315	1256.35	395.89

ProbeName	Target position of probe on CpG island microarray	TargetID	GeneSymbol	CpG island Description	Ratio of relative methylation (TiO_2-NP/Vehicle)	Sham group			TiO_2-H group		
						Relative methylation (Cy5/Cy3)	Cy3 signal (Input)	Cy5 signal (MeDIP)	Relative methylation (Cy5/Cy3)	Cy3 signal (Input)	Cy5 signal (MeDIP)
A_68_P31923503	chr19:4509996-4510046	NM_001162946:-451	Pcx	PROMOTER	0.746	2.403	790.19	1898.75	1.792	578.35	1036.31
A_68_P30497401	chr15:102180872-102180916	NM_153416:296	Aaas	INSIDE	0.746	1.904	4969.61	9459.66	1.420	3742.88	5316.29
A_68_P30426168	chr15:89422228-89422272	NM_026817:104	Rabl2	INSIDE	0.746	2.188	2242.58	4907.76	1.633	1763.90	2880.31
A_68_P30026214	chr15:13103007-13103051	NM_007666:366	Cdh6	INSIDE	0.746	4.001	3478.89	13919.16	2.987	2726.28	8142.52
A_68_P29751618	chr14:79880592-79880636	NM_007920:-386	Elf1	PROMOTER	0.746	4.563	486.66	2220.63	3.402	385.11	1310.11
A_68_P29449771	chr14:21748812-21748856	NM_009502:180	Vcl	INSIDE	0.746	2.172	1876.56	4076.16	1.620	1447.65	2344.69
A_68_P29233347	chr13:94944386-94944430	NM_172589:116658	Lhfpl2	INSIDE	0.746	0.415	2626.84	1090.62	0.310	1880.79	582.75
A_68_P29116724	chr13:69750521-69750565	NM_175283:-201	Srd5a1	DIVERGENT_PROMOTER	0.746	0.295	9339.59	2752.58	0.220	5760.78	1265.82
A_68_P29047198	chr13:54151095-54151139	NM_010076:-89	Drd1a	PROMOTER	0.746	0.502	1710.10	859.19	0.375	1314.98	492.56
A_68_P28729933	chr12:110032849-110032893	NM_009537:1350	Yy1	INSIDE	0.746	2.425	1338.42	3245.66	1.810	1131.01	2047.38
A_68_P27927429	chr11:75469879-75469923	NM_001080775:384	Myo1c	INSIDE	0.746	0.334	2423.09	808.66	0.249	1727.20	430.23
A_68_P27641463	chr11:22412449-22412493	NM_153596:185	Tmem17	INSIDE	0.746	4.436	1455.12	6454.40	3.310	1151.98	3813.44
A_68_P27288649	chr10:80755618-80755662	NM_001146687:-76	Pip5k1c	DIVERGENT_PROMOTER	0.746	3.275	948.47	3106.45	2.443	780.77	1907.19
A_68_P27262050	chr10:75905557-75905601	NM_008787:79	Pcnt	INSIDE	0.746	0.742	2287.53	1696.42	0.553	1811.11	1001.60
A_68_P25922766	chr8:66684450-66684494	NM_009390:318	Tll1	INSIDE	0.746	0.474	1326.14	628.26	0.354	1102.73	389.91
A_68_P25735815	chr8:28089687-28089731	NM_001101502:1901	Zfp703	INSIDE	0.746	0.651	3835.62	2495.91	0.485	2685.64	1303.19
A_68_P24981707	chr7:17061268-17061312	NM_008718:839	Npas1	INSIDE	0.746	5.160	656.46	3387.02	3.847	551.32	2120.94
A_68_P24325870	chr6:29298179-29298223	NM_007594:82	Calu	INSIDE	0.746	0.233	1942.42	452.75	0.174	1547.56	269.18
A_68_P23438433	chr4:154783299-154783343	NM_001166029:22	2010015L04Rik	INSIDE	0.746	0.709	1464.00	1037.75	0.529	1247.21	659.84
A_68_P23262160	chr4:123020018-123020062	NM_007558:6	Bmp8a	INSIDE	0.746	0.584	2146.98	1253.29	0.436	1548.78	674.72
A_68_P20469537	chr1:98768738-98768782	NM_172658:-12	Slco4c1	PROMOTER	0.746	0.580	1873.11	1086.04	0.433	1576.59	682.09
A_68_P32454253	chrX:68809673-68809717	NM_008253:527	Hmgb3	INSIDE	0.745	3.301	885.95	2924.77	2.460	1148.52	2825.60
A_68_P32321495	chrX:34588913-34588957	NM_173779:95	Ankrd58	INSIDE	0.745	3.327	770.06	2561.90	2.477	1084.09	2685.69
A_68_P31143794	chr17:32251060-32251105	NM_008716:52715	Notch3	DOWNSTREAM	0.745	0.404	1308.21	528.91	0.301	1004.72	302.61
A_68_P31055160	chr17:13153437-13153481	NM_009338:133	Acat2	INSIDE	0.745	0.412	1335.40	549.83	0.307	971.46	298.16
A_68_P30969546	chr16:92498878-92498922	NM_172469:509	Clic6	INSIDE	0.745	6.803	3525.59	23983.69	5.070	3113.57	15785.19
A_68_P30655032	chr16:32877777-32877821	NM_001159349:-71	Fyttd1	DIVERGENT_PROMOTER	0.745	4.713	898.55	4234.53	3.512	712.69	2502.62
A_68_P30544461	chr16:10785857-10785901	NM_009896:-249	Socs1	PROMOTER	0.745	0.355	1531.56	544.17	0.265	1168.76	309.34
A_68_P30346974	chr15:76062654-76062698	NM_001163542:-868	Plec	PROMOTER	0.745	0.321	1887.30	605.33	0.239	1431.68	341.95
A_68_P30224203	chr15:53734115-53734159	NM_177225:-145	Samd12	PROMOTER	0.745	0.668	1376.20	919.29	0.497	1105.31	549.81
A_68_P29585962	chr14:48740578-48740622	NM_033602:57	Peli2	INSIDE	0.745	3.319	651.95	2163.95	2.472	545.92	1349.38
A_68_P29026439	chr13:49747871-49747915	NM_025495:208	Cenpp	INSIDE	0.745	0.735	2061.39	1515.52	0.548	1537.87	842.19
A_68_P28909028	chr13:29051432-29051476	NM_009238:-5903	Sox4	PROMOTER	0.745	0.562	3197.39	1796.28	0.418	2426.92	1015.52
A_68_P28751330	chr12:113858462-113858506	NM_007421:227	Adssl1	INSIDE	0.745	0.635	5391.58	3425.16	0.474	4068.32	1926.70
A_68_P27129528	chr10:50312762-50312806	NM_001146089:310	Ascc3	INSIDE	0.745	0.292	4491.47	1312.14	0.218	3489.65	759.73
A_68_P26885391	chr9:121669059-121669103	NR_033503:-19	E530011L22Rik	DIVERGENT_PROMOTER	0.745	2.754	766.78	2111.88	2.052	812.15	1666.62
A_68_P26866545	A_68_P26866545			Unknown	0.745	0.648	1031.97	668.68	0.483	884.36	426.84
A_68_P26721513	chr9:91260697-91260741	NM_009573:-81	Zic1	DIVERGENT_PROMOTER	0.745	2.287	1003.23	2294.70	1.704	945.95	1612.10
A_68_P26586478	chr9:65063416-65063460	NM_177460:942	Parp16	INSIDE	0.745	0.678	1299.28	880.81	0.505	982.51	496.21
A_68_P26531898	chr9:55393314-55393358	NM_027397:4381	Isl2	INSIDE	0.745	0.463	1433.96	664.36	0.345	1075.72	371.14
A_68_P26345409	chr9:20896597-20896641	NR_038081:-166	Raver1-fdx1l	PROMOTER	0.745	0.350	1500.48	524.90	0.260	1034.51	269.44
A_68_P26289279	chr9:7764076-7764120	NM_133739:22	Tmem123	INSIDE	0.745	3.072	1542.84	4739.06	2.289	1245.56	2851.17
A_68_P26258351	chr8:129108188-129108232	NM_001164598:9126	Irf2bp2	DOWNSTREAM	0.745	0.730	1926.91	1406.19	0.544	1525.11	829.41
A_68_P26213863	chr8:121868494-121868539	NM_024219:79	Hsbp1	INSIDE	0.745	0.290	1635.68	473.81	0.216	1175.44	253.76
A_68_P24325224	chr6:29162351-29162395	NM_011829:-101	Impdh1	PROMOTER	0.745	4.195	3140.17	13172.28	3.123	2632.32	8221.13
A_68_P23439687	chr4:154998722-154998766	NM_007661:-233	Cdk11b	PROMOTER	0.745	0.476	1435.89	682.87	0.354	1155.90	409.26
A_68_P23410171	chr4:150482915-150482959	NM_001081557:752941	Camta1	INSIDE	0.745	2.559	768.38	1966.60	1.906	652.17	1243.00
A_68_P23227565	chr4:115748961-115749005	NM_153521:913	Lrrc41	INSIDE	0.745	0.638	952.20	607.97	0.476	785.87	373.72
A_68_P23190151	chr4:107040905-107040949	NM_028355:538	Tmem48	INSIDE	0.745	0.523	3612.70	1891.20	0.390	2427.04	946.73
A_68_P22379763	chr3:102007843-102007887	NM_001165951:22	Vangl1	INSIDE	0.745	0.706	2598.73	1835.93	0.526	2163.60	1138.06
A_68_P21727915	chr2:147871871-147871915	NM_010446:813	Foxa2	INSIDE	0.745	0.452	4377.20	1977.71	0.337	3352.52	1128.64
A_68_P21199928	chr2:48804507-48804551	NR_033442:259	Orc4	INSIDE	0.745	0.441	1140.45	502.83	0.328	941.46	309.07
A_68_P21147545	chr2:38194964-38195008	ENSMUST00000149664:-21		PROMOTER	0.745	0.518	1190.45	616.36	0.386	975.61	376.44
A_68_P20286429	chr1:63411957-63412001	ENSMUST00000120413:-39		PROMOTER	0.745	2.721	1130.95	3077.25	2.026	965.16	1955.79
A_68_P32051826	chr19:29325786-29325831	NM_001048177:-509	Jak2	PROMOTER	0.744	0.360	2769.63	997.24	0.268	2252.10	603.15
A_68_P31411661	chr17:86008233-86008277	NR_038085:9482	Six3os1	INSIDE	0.744	0.286	2193.81	626.71	0.213	1557.52	331.16
A_68_P31108119	chr17:26204737-26204781	NM_001162868:1364	Rab11fip3	INSIDE	0.744	0.398	2011.42	799.67	0.296	1652.42	489.03
A_68_P29510757	chr14:32064694-32064738	NM_027088:42	Bap1	INSIDE	0.744	2.247	1638.94	3682.70	1.672	1422.64	2378.33
A_68_P29210184	chr13:89879184-89879228	NM_001081249:2911	Vcan	INSIDE	0.744	3.357	606.46	2036.09	2.496	452.96	1130.72
A_68_P28170836	chr11:118216495-118216539	NM_011594:209	Timp2	INSIDE	0.744	2.770	2086.04	5778.65	2.060	1676.98	3455.00
A_68_P27542076	chr10:128003183-128003227	NM_011119:-214	Pa2g4	PROMOTER	0.744	3.360	727.45	2444.27	2.499	649.27	1622.72
A_68_P27287905	chr10:80639342-80639386	NM_007907:-11	Eef2	DIVERGENT_PROMOTER	0.744	0.554	1123.33	622.88	0.413	903.93	373.11
A_68_P27067368	chr10:36694971-36695015	NM_008229:643	Hdac2	INSIDE	0.744	0.497	1926.13	956.55	0.370	1599.60	591.13
A_68_P26983373	chr10:19570476-19570520	NM_029529:767	Slc35d3	INSIDE	0.744	6.317	2081.08	13147.09	4.702	1782.33	8380.87
A_68_P26504535	chr9:50411810-50411854	NM_025848:122	Sdhd	INSIDE	0.744	0.602	1381.60	832.00	0.448	1144.77	512.58

ProbeName	Target position of probe on CpG island microarray	TargetID	GeneSymbol	CpG island Description	Ratio of relative methylation (TiO_2-NP/Vehicle)	Sham group			TiO_2-H group		
						Relative methylation (Cy5/Cy3)	Cy3 signal (Input)	Cy5 signal (MeDIP)	Relative methylation (Cy5/Cy3)	Cy3 signal (Input)	Cy5 signal (MeDIP)
A_68_P25502554	chr7:133991255-133991299	NM_010069:210	Doc2a	INSIDE	0.744	0.585	988.80	578.03	0.435	858.43	373.46
A_68_P25393096	chr7:112730014-112730058	NM_009685:13	Apbb1	INSIDE	0.744	0.537	1828.25	982.68	0.400	1354.80	541.88
A_68_P24979386	chr7:16647485-16647529	NM_001099277:-9784	Zfp541	PROMOTER	0.744	5.620	6205.20	34870.31	4.182	4722.70	19751.79
A_68_P24602878	chr6:85019144-85019188	NM_177077:341	Exoc6b	INSIDE	0.744	2.491	1274.15	3173.68	1.854	1317.07	2441.60
A_68_P24326457	chr6:29383678-29383722	NM_001081185:548	Flnc	INSIDE	0.744	0.257	1924.09	494.12	0.191	1362.68	260.27
A_68_P24059259	chr5:124540142-124540186	NM_019875:5643	Abcb9	INSIDE	0.744	2.534	1492.48	3781.80	1.884	1208.08	2276.25
A_68_P23433055	chr4:154011802-154011846	NM_001177995:-842	Prdm16	PROMOTER	0.744	7.906	1860.60	14709.77	5.880	1697.27	9979.85
A_68_P23415137	chr4:151236473-151236517	NM_001081557:-617	Camta1	PROMOTER	0.744	0.513	1252.18	642.15	0.381	1096.75	418.31
A_68_P23397274	chr4:148274413-148274458	NM_027195:95935	Caszl	INSIDE	0.744	0.512	859.26	439.83	0.381	763.13	290.69
A_68_P22194000	chr3:63703025-63703069	ENSMUST00000159188:318		INSIDE	0.744	0.547	1168.14	639.49	0.407	923.66	376.38
A_68_P21338839	chr2:74529632-74529676	NM_013554:-350	Hoxd10	PROMOTER	0.744	0.428	1522.01	650.86	0.318	1201.60	382.37
A_68_P20350882	chr1:74902485-74902529	NM_009872:904	Cdk5r2	INSIDE	0.744	0.362	1749.30	633.56	0.270	1304.71	351.63
A_68_P32143272	chr19:46403086-46403130	NM_133694:435	Fbxl15	INSIDE	0.743	0.221	2357.09	521.36	0.164	1683.67	276.57
A_68_P31934662	chr19:6428439-6428483	NM_001205234:9723	Nrxn2	INSIDE	0.743	5.089	435.29	2215.21	3.782	313.04	1183.86
A_68_P31735610	chr18:57514422-57514466	NM_028447:58	Prrc1	INSIDE	0.743	0.376	3614.27	1360.14	0.280	2764.09	773.29
A_68_P30096385	chr15:27954397-27954441	NM_001081302:1185	Trio	INSIDE	0.743	2.049	4564.24	9353.48	1.522	3469.22	5279.68
A_68_P29624048	chr14:57287361-57287405	NM_023773:298	Mphosph8	INSIDE	0.743	0.222	3344.20	743.36	0.165	2473.49	408.69
A_68_P29150849	chr13:75845057-75845101	NM_138953:147	Ell2	INSIDE	0.743	0.524	922.68	483.67	0.390	890.64	347.11
A_68_P28925099	chr13:31899088-31899132	NM_008592:596	Foxc1	INSIDE	0.743	0.506	1147.02	580.25	0.376	959.56	360.71
A_68_P26292116	chr9:8544357-8544401	NM_013838:237	Trpc6	INSIDE	0.743	3.225	494.32	1594.25	2.395	452.50	1083.63
A_68_P26158883	chr8:112302603-112302647	NM_009677:85	Ap1g1	INSIDE	0.743	2.745	1041.43	2858.90	2.041	822.90	1679.39
A_68_P26142249	chr8:109459792-109459836	NM_009229:165	Sntb2	INSIDE	0.743	3.004	1491.09	4479.84	2.234	1252.24	2797.12
A_68_P24589799	chr6:82723709-82723753	NM_013820:718	Hk2	INSIDE	0.743	0.708	2518.32	1784.14	0.526	1884.67	992.11
A_68_P24009124	chr5:115791228-115791272	NM_026933:-5	Triap1	DIVERGENT_PROMOTER	0.743	0.534	1665.00	888.50	0.397	1237.07	490.77
A_68_P23955547	chr5:105909960-105910004	NM_001033550:65189	Lrrc8b	INSIDE	0.743	3.914	391.16	1531.18	2.909	289.92	843.46
A_68_P23588821	chr5:33773068-33773112	ENSMUST00000030994:754		DOWNSTREAM	0.743	2.549	1810.12	4613.12	1.894	1376.04	2606.88
A_68_P23440105	chr4:155079607-155079651	NM_026899:705	Ssu72	INSIDE	0.743	8.621	838.53	7228.68	6.402	909.60	5823.18
A_68_P22314313	chr3:88336733-88336777	NM_001029890:438	Mex3a	INSIDE	0.743	0.642	2337.42	1499.58	0.477	1774.11	845.62
A_68_P21901054	chr2:179777765-179777809	NM_178750:599	Ss18l1	INSIDE	0.743	3.301	1333.82	4403.15	2.452	958.80	2350.59
A_68_P21114612	chr2:32168276-32168320	NR_029811:-5681	Mir199b	PROMOTER	0.743	0.207	3252.94	672.06	0.153	2160.13	331.36
A_68_P21105306	chr2:30656954-30656998	NM_198000:251	1700001O22Rik	INSIDE	0.743	2.440	2103.29	5131.97	1.813	1742.17	3158.08
A_68_P20799884	chr1:168309567-168309611	NM_030245:291	Tada1	INSIDE	0.743	2.045	1468.63	3003.82	1.520	1188.44	1806.04
A_68_P20616243	chr1:133423463-133423507	NM_001081011:454	Srgap2	INSIDE	0.743	0.568	1752.19	994.76	0.422	1291.00	544.30
A_68_P31092987	chr17:23814347-23814391	NM_001161746:48	Tnfrsf12a	INSIDE	0.742	0.458	2521.48	1153.73	0.340	1802.14	611.93
A_68_P29619289	chr14:56300469-56300513	NM_145705:164	Tinf2	INSIDE	0.742	0.699	1168.26	816.52	0.518	994.90	515.66
A_68_P29257128	chr13:99125244-99125288	NM_008242:1067	Foxd1	INSIDE	0.742	2.556	1505.04	3847.39	1.898	1206.08	2288.60
A_68_P28836956	chr13:14156155-14156199	NM_194262:118	Arid4b	INSIDE	0.742	0.692	2212.49	1531.18	0.513	1789.76	918.76
A_68_P27288384	chr10:80720348-80720392	NM_010768:81	Matk	INSIDE	0.742	0.512	2096.46	1073.77	0.380	1664.57	632.52
A_68_P27286394	chr10:80391549-80391593	NM_008655:-1265	Gadd45b	PROMOTER	0.742	2.733	1870.65	5111.84	2.028	1436.20	2912.24
A_68_P27055773	chr10:33927208-33927252	NM_172508:127	Dse	INSIDE	0.742	0.523	4031.94	2109.20	0.388	3238.12	1256.57
A_68_P26757841	chr9:98196980-98197024	NM_144533:1	Nmnat3	INSIDE	0.742	0.663	1029.38	682.30	0.492	890.18	437.63
A_68_P26137279	chr8:108535484-108535528	NM_025518:100	Dus2l	INSIDE	0.742	0.483	3522.28	1702.41	0.359	2348.62	842.04
A_68_P26135494	chr8:108228360-108228404	NM_198299:3686	E130303B06Rik	INSIDE	0.742	2.757	1021.17	2815.39	2.047	748.11	1531.24
A_68_P26129050	chr8:107065100-107065144	NM_053070:416	Car7	INSIDE	0.742	0.531	1706.09	906.09	0.394	1438.27	567.12
A_68_P25502251	chr7:133935372-133935418	NM_019674:591	Ppp4c	INSIDE	0.742	0.521	1023.43	533.59	0.387	773.61	299.20
A_68_P25092923	chr7:52662436-52662480	NM_010596:1129	Kcna7	INSIDE	0.742	6.165	1230.07	7582.92	4.576	1123.47	5141.20
A_68_P25087380	chr7:51684809-51684853	ENSMUST00000127790:443		INSIDE	0.742	0.550	852.16	468.94	0.408	704.92	287.81
A_68_P24658382	chr6:94649670-94649714	NM_008377:447	Lrig1	INSIDE	0.742	3.439	389.98	1341.06	2.553	396.85	1013.27
A_68_P24591261	chr6:83021489-83021533	NM_009392:-1291	Tlx2	DIVERGENT_PROMOTER	0.742	0.246	2118.43	521.18	0.182	1611.10	294.02
A_68_P24428192	chr6:48544047-48544091	NM_001079901:187	Repin1	INSIDE	0.742	2.248	4919.88	11061.96	1.668	4051.12	6755.37
A_68_P24334443	chr6:30846924-30846968	NM_017478:-186	Copg2	PROMOTER	0.742	8.927	2265.68	20224.67	6.619	1903.54	12600.22
A_68_P24061764	chr5:124981000-124981044	NM_021430:378	Rilpl1	INSIDE	0.742	3.287	965.73	3174.70	2.440	856.60	2090.06
A_68_P24005517	chr5:115140712-115140756	NM_019821:210	Gltp	INSIDE	0.742	3.465	3367.32	11667.91	2.570	2565.11	6592.67
A_68_P23766886	chr5:67652442-67652486	NM_028975:574	Tmem33	INSIDE	0.742	0.346	3475.74	1203.85	0.257	2672.98	687.09
A_68_P23229786	chr4:116230170-116230214	NM_029868:-139	Gpbp1l1	DIVERGENT_PROMOTER	0.742	0.546	1387.19	757.03	0.405	1269.39	513.80
A_68_P22693141	chr4:3501940-3501984	NM_028097:-47	Tmem68	DIVERGENT_PROMOTER	0.742	0.452	974.08	440.56	0.336	693.12	232.70
A_68_P22392959	chr3:104561596-104561640	NM_183224:18847	Fam19a3	DOWNSTREAM	0.742	0.523	2338.97	1223.12	0.388	1680.66	651.79
A_68_P22006132	chr3:26230317-26230361	NM_138666:493	Nlgn1	INSIDE	0.742	2.259	1181.33	2669.17	1.676	1004.75	1683.91
A_68_P21902322	chr2:179959737-179959781	NM_001081171:806	Lama5	INSIDE	0.742	0.305	1693.93	516.03	0.226	1266.16	286.33
A_68_P21776100	chr2:157105079-157105123	NM_001039557:185	4922505G16Rik	INSIDE	0.742	2.266	1128.32	2556.34	1.680	950.57	1597.22
A_68_P20638511	chr1:137180229-137180273	NM_001159624:411	Rnpep	INSIDE	0.742	2.131	1693.75	3608.68	1.581	1377.69	2177.80
A_68_P20391719	chr1:82287804-82287848	NM_010570:188	Irs1	INSIDE	0.742	2.346	825.74	1937.06	1.741	709.29	1234.73
A_68_P20264187	chr1:59539256-59539300	NM_008057:288	Fzd7	INSIDE	0.742	0.737	1886.49	1389.65	0.547	1507.28	823.84
A_68_P32567641	chrX:99384743-99384787	NM_009094:-1054	Rps4x	PROMOTER	0.741	3.155	497.61	1569.99	2.339	589.36	1378.43
A_68_P32266424	chrX:13063560-13063604	NM_173415:18785	Nyx	INSIDE	0.741	6.183	327.10	2022.37	4.579	434.47	1989.40

ProbeName	Target position of probe on CpG island microarray	TargetID	GeneSymbol	CpG island Description	Ratio of relative methylation (TiO_2-NP/Vehicle)	Sham group			TiO_2-H group		
						Relative methylation (Cy5/Cy3)	Cy3 signal (Input)	Cy5 signal (MeDIP)	Relative methylation (Cy5/Cy3)	Cy3 signal (Input)	Cy5 signal (MeDIP)
A_68_P31336379	chr17:72131880-72131924	NM_030179:616	Clip4	INSIDE	0.741	7.555	884.12	6679.10	5.600	736.35	4123.45
A_68_P31106389	chr17:25946375-25946419	NM_001164225:367	Fbxl16	INSIDE	0.741	0.409	3042.48	1244.77	0.303	2047.78	620.57
A_68_P30861550	chr16:72663819-72663863	NM_019413:447	Robo1	INSIDE	0.741	2.368	1748.27	4140.01	1.754	1417.22	2485.82
A_68_P30447921	chr15:93426390-93426434	NM_001033217:-90	Prickle1	PROMOTER	0.741	2.394	818.77	1960.42	1.773	719.87	1276.49
A_68_P30346548	chr15:76003699-76003743	NM_201394:22420	Plec	INSIDE	0.741	2.543	1582.08	4023.51	1.885	1135.12	2139.95
A_68_P29258499	chr13:99359027-99359071	AK135687:168913		INSIDE	0.741	24.074	1385.76	33360.24	17.842	1317.67	23510.02
A_68_P29098742	chr13:64534076-64534120	NM_053180:238	Cdk20	INSIDE	0.741	0.566	845.53	478.95	0.420	662.87	278.23
A_68_P28621834	chr12:90498622-90498666	NM_172544:306811	Nrxn3	INSIDE	0.741	3.579	356.51	1275.77	2.653	280.98	745.40
A_68_P28419953	chr12:50484336-50484380	NM_008241:365	Foxg1	INSIDE	0.741	0.553	1520.54	840.21	0.409	1364.66	558.43
A_68_P28059224	chr11:98769094-98769138	NM_001025779:-86	Cdc6	PROMOTER	0.741	0.306	1446.70	442.46	0.227	1153.15	261.25
A_68_P27812448	chr11:54680388-54680432	NM_008248:471	Hint1	INSIDE	0.741	2.884	1777.81	5126.69	2.137	1844.22	3941.58
A_68_P27564654	chr11:5616764-5616808	NM_178623:24393	Urgcp	INSIDE	0.741	3.313	379.86	1258.33	2.456	312.27	766.94
A_68_P25616320	chr8:4166752-4166796	NM_001039578:208	Evi5l	INSIDE	0.741	0.587	2308.27	1354.49	0.435	1911.71	831.46
A_68_P25587306	chr7:148380913-148380957	NM_153777:-107	Lrrc56	PROMOTER	0.741	7.097	393.66	2793.86	5.259	321.07	1688.41
A_68_P25522115	chr7:137663415-137663459	NM_013799:-383	Ate1	PROMOTER	0.741	0.200	2520.57	503.51	0.148	1729.00	255.78
A_68_P24992403	chr7:19857732-19857776	NM_009499:-551	Vasp	PROMOTER	0.741	0.299	2844.76	849.61	0.221	2218.67	491.03
A_68_P24162429	chr5:145522582-145522627	NM_177735:-157	Tmem130	DIVERGENT_PROMOTER	0.741	0.357	3023.80	1080.20	0.265	2243.79	594.08
A_68_P23404995	chr4:149611450-149611494	NM_001025388:40	Gm5506	INSIDE	0.741	0.561	1020.86	573.02	0.416	763.41	317.57
A_68_P23362416	chr4:140703968-140704012	NM_172518:154	Fbxo42	INSIDE	0.741	2.642	2019.25	5335.11	1.958	1641.88	3215.60
A_68_P23195050	chr4:107890175-107890219	NM_001167936:331	Zyg11a	INSIDE	0.741	0.344	2683.58	922.54	0.255	2098.11	534.48
A_68_P23019856	chr4:73897951-73897995	NM_144787:190	Kdm4c	INSIDE	0.741	0.437	1771.77	774.12	0.324	1560.67	505.61
A_68_P22880595	chr4:45024781-45024825	NM_001163283:482	Zbtb5	INSIDE	0.741	2.082	1322.72	2753.50	1.542	1009.43	1556.30
A_68_P22039458	chr3:32607831-32607875	NM_019673:385	Actl6a	INSIDE	0.741	0.244	2248.88	548.93	0.181	1556.25	281.64
A_68_P21580973	chr2:121275014-121275058	NM_011354:73	Serf2	INSIDE	0.741	0.703	1158.68	814.58	0.521	995.30	518.28
A_68_P21082672	chr2:26997676-26997720	NM_175427:299	Fam163b	INSIDE	0.741	2.475	1568.75	3882.75	1.835	1320.88	2424.00
A_68_P20162423	chr1:38893157-38893201	ENSMUST00000147695:-243		PROMOTER	0.741	4.609	513.73	2367.68	3.414	462.14	1577.77
A_68_P32099203	chr19:38129332-38129376	NM_028293:-160	Cep55	PROMOTER	0.740	0.385	1383.42	532.61	0.285	1114.31	317.35
A_68_P31442545	chr17:91487617-91487661	NM_020252:4504	Nrxn1	INSIDE	0.740	3.756	1404.14	5273.99	2.779	1064.69	2959.02
A_68_P30576507	chr16:17911333-17911377	NM_001081633:87	Dgcr14	INSIDE	0.740	3.330	1025.60	3414.87	2.463	720.59	1774.91
A_68_P30349740	chr15:76487584-76487628	NM_019396:2885	Cyhr1	INSIDE	0.740	0.291	1705.55	496.55	0.215	1277.88	275.33
A_68_P27686041	chr11:31865736-31865780	NM_026262:126	4930524B15Rik	INSIDE	0.740	2.632	760.81	2002.52	1.948	634.72	1236.58
A_68_P25948473	chr8:72405192-72405236	NM_026818:6077	Cilp2	INSIDE	0.740	0.480	1433.71	688.78	0.356	976.14	347.23
A_68_P24946541	chr7:3597064-3597108	NM_146176:216	Cnot3	INSIDE	0.740	3.555	399.47	1420.06	2.632	347.17	913.68
A_68_P24428217	chr6:48546955-48546999	NM_001079901:3095	Repin1	INSIDE	0.740	2.405	1819.03	4374.84	1.779	1445.41	2571.97
A_68_P23981090	chr5:111082863-111082907	NM_027156:415	Ddx51	INSIDE	0.740	0.285	3043.51	866.21	0.211	2038.61	429.50
A_68_P23969365	chr5:108329935-108329979	NM_016980:410	Rpl5	INSIDE	0.740	0.618	1968.48	1217.18	0.457	1643.33	751.49
A_68_P23955160	chr5:105845255-105845299	NM_001033550:483	Lrrc8b	INSIDE	0.740	0.167	3752.09	625.98	0.123	2836.33	350.22
A_68_P23543620	chr5:24236452-24236496	NM_001081441:24	Wdr86	INSIDE	0.740	0.248	2691.05	667.23	0.184	1850.51	339.59
A_68_P23415959	chr4:151383528-151383572	NM_001033352:551	Klhl21	INSIDE	0.740	3.164	420.92	1331.93	2.340	419.65	982.03
A_68_P23270866	chr4:124563102-124563146	NM_177671:4096	Epha10	INSIDE	0.740	1.989	2852.89	5674.34	1.472	1995.30	2938.06
A_68_P22889025	chr4:46502594-46502638	NM_053179:416	Nans	INSIDE	0.740	0.629	1595.57	1003.81	0.465	1319.22	613.86
A_68_P22766411	chr4:19636348-19636392	NM_177327:-230	Wwp1	PROMOTER	0.740	2.918	846.23	2469.32	2.160	683.60	1476.53
A_68_P21394388	chr2:84583819-84583863	NR_029544:-2505	Mir130a	PROMOTER	0.740	0.544	3237.75	1762.56	0.403	2604.30	1048.48
A_68_P21117012	chr2:32568363-32568407	NR_037217:24	Mir2861	INSIDE	0.740	3.468	1114.17	3863.44	2.566	914.57	2346.42
A_68_P21113218	chr2:31940197-31940241	NM_175511:-993	Fam78a	PROMOTER	0.740	3.791	1052.46	3989.94	2.807	934.00	2621.35
A_68_P20556970	chr1:121318089-121318133	NM_008381:715	Inhbb	INSIDE	0.740	8.381	1914.28	16044.01	6.203	1485.54	9214.24
A_68_P20377652	chr1:79772594-79772638	NM_001081210:24	Mrpl44	INSIDE	0.740	0.572	1252.85	716.23	0.423	1066.88	451.40
A_68_P31924553	chr19:4712434-4712478	NM_021287:1234	Spnb3	INSIDE	0.739	0.382	1460.14	558.16	0.283	1099.75	310.84
A_68_P30338796	chr15:74502497-74502541	NM_018790:482	Arc	INSIDE	0.739	0.486	1471.42	715.23	0.359	1024.49	367.83
A_68_P30250720	chr15:58720614-58720658	NM_175226:-147	Rnf139	PROMOTER	0.739	3.499	2324.62	8133.05	2.586	1935.05	5004.48
A_68_P29904273	chr14:112075249-112075293	NM_198865:934	Slitrk5	INSIDE	0.739	2.328	661.83	1540.81	1.721	605.64	1042.17
A_68_P29642600	chr14:60705656-60705700	NM_015803:-7	Atp8a2	PROMOTER	0.739	0.702	3015.09	2115.48	0.518	2402.24	1245.29
A_68_P29631506	chr14:58695032-58695076	NM_013518:3532	Fgf9	INSIDE	0.739	0.269	3107.56	835.92	0.199	2189.00	435.42
A_68_P28448914	chr12:55757729-55757773	NM_134054:-191	1110002B05Rik	PROMOTER	0.739	2.333	644.07	1502.74	1.724	565.46	975.02
A_68_P28160636	chr11:116515196-116515240	NM_030206:409	Cygb	INSIDE	0.739	0.508	1725.45	876.75	0.376	1334.75	501.37
A_68_P27887969	chr11:68026421-68026465	NM_008744:173886	Ntn1	INSIDE	0.739	3.357	2268.13	7613.84	2.479	1735.48	4302.94
A_68_P26772688	chr9:100978469-100978513	NM_001100451:40	Msl2	INSIDE	0.739	2.221	1516.89	3368.77	1.641	1149.19	1886.40
A_68_P26665710	chr9:79567360-79567404	NM_007730:-897	Col12a1	PROMOTER	0.739	0.448	2824.03	1265.98	0.331	2050.62	679.10
A_68_P26500015	chr9:49606210-49606254	NM_001081445:942	Ncam1	INSIDE	0.739	2.936	767.37	2252.97	2.171	661.07	1435.06
A_68_P26242943	chr8:126545298-126545342	NM_133966:-111	Taf5l	DIVERGENT_PROMOTER	0.739	3.302	416.37	1374.77	2.440	371.50	906.54
A_68_P25219571	chr7:78850463-78850507			Unknown	0.739	2.000	1958.76	3917.69	1.478	1571.57	2322.73
A_68_P24658385	chr6:94649976-94650020	NM_008377:141	Lrig1	INSIDE	0.739	4.351	1583.78	6891.47	3.217	1289.19	4147.12
A_68_P24329526	chr6:29935479-29935523	ENSMUST00000165864:147		INSIDE	0.739	0.331	4257.08	1410.83	0.245	3232.72	792.19
A_68_P24117821	chr5:136253656-136253701	NM_029659:577	Styxl1	INSIDE	0.739	2.784	525.35	1462.62	2.058	479.06	985.71
A_68_P24058182	chr5:124357285-124357329	NM_026603:23	Denr	INSIDE	0.739	2.189	1684.43	3687.72	1.618	1504.60	2434.54

ProbeName	Target position of probe on CpG island microarray	TargetID	GeneSymbol	CpG island Description	Ratio of relative methylation (TiO_2-NP/Vehicle)	Sham group			TiO_2-H group		
						Relative methylation (Cy5/Cy3)	Cy3 signal (Input)	Cy5 signal (MeDIP)	Relative methylation (Cy5/Cy3)	Cy3 signal (Input)	Cy5 signal (MeDIP)
A_68_P23539922	chr5:23606508-23606552	NM_001161800:123	Klhl7	INSIDE	0.739	2.812	1039.25	2922.47	2.078	856.55	1779.63
A_68_P22611613	chr3:145587280-145587324	NM_009740:-39	Bcl10	PROMOTER	0.739	2.790	1779.59	4964.60	2.060	1507.71	3106.43
A_68_P22530128	chr3:130809594-130809638	NM_010703:-3772	Lef1	PROMOTER	0.739	2.162	973.65	2105.15	1.598	856.98	1369.52
A_68_P22028769	chr3:30407354-30407398	NM_021442:1033	Mecom	INSIDE	0.739	0.226	4534.91	1025.56	0.167	3209.18	536.30
A_68_P21812074	chr2:163202123-163202167	NM_021566:21565	Jph2	INSIDE	0.739	2.492	874.02	2177.68	1.840	753.26	1386.16
A_68_P21261144	chr2:60719799-60719843	NM_001141931:-325	Rbms1	PROMOTER	0.739	0.548	1097.17	601.35	0.405	817.45	331.11
A_68_P21070359	chr2:25036616-25036660	NM_025980:361	Nrarp	INSIDE	0.739	3.148	346.72	1091.60	2.328	323.12	752.29
A_68_P20794602	chr1:167390651-167390695	NM_028759:-78	Dcaf6	DIVERGENT_PROMOTER	0.739	1.989	1528.50	3039.95	1.471	1397.64	2055.47
A_68_P20164265	chr1:39250200-39250244	NM_008719:-894	Npas2	PROMOTER	0.739	0.445	2379.10	1059.66	0.329	1785.53	587.44
A_68_P31609276	chr18:33954463-33954507	NR_038151:-61	2410004N09Rik	PROMOTER	0.738	0.496	2533.21	1257.31	0.366	2041.79	748.14
A_68_P31497267	chr18:11997722-11997766	NM_001146287:-38	Cables1	PROMOTER	0.738	0.278	1820.38	506.55	0.205	1310.78	269.16
A_68_P31003195	chr17:3114834-3114878	NM_134123:-115	Scaf8	PROMOTER	0.738	2.409	646.08	1556.43	1.777	585.21	1040.12
A_68_P30931273	chr16:85802461-85802505	NM_009621:878	Adamts1	INSIDE	0.738	5.090	1640.90	8352.62	3.758	1298.96	4881.47
A_68_P30471849	chr15:97739034-97739078	NM_009504:-329	Vdr	PROMOTER	0.738	0.457	2020.80	923.58	0.337	1587.44	535.19
A_68_P30261334	chr15:60656407-60656451	NM_001162926:207	Fam84b	INSIDE	0.738	2.905	970.70	2820.32	2.143	867.49	1859.04
A_68_P29531803	chr14:35582622-35582666	ENSMUST00000143244:9232		INSIDE	0.738	0.313	3879.14	1212.49	0.231	2704.03	624.12
A_68_P29360869	chr13:117813423-117813467	NM_001081009:879	Parp8	INSIDE	0.738	2.741	1234.10	3382.54	2.021	1067.14	2157.15
A_68_P28613117	chr12:88289051-88289098	NM_173735:782	2310044G17Rik	INSIDE	0.738	5.616	1201.13	6745.85	4.142	955.60	3958.13
A_68_P28525541	chr12:72419505-72419549	NM_001190466:8656	Dact1	INSIDE	0.738	5.025	1150.27	5779.84	3.709	892.41	3310.16
A_68_P28447129	chr12:55414382-55414426			Unknown	0.738	11.483	607.45	6975.18	8.480	466.74	3957.76
A_68_P27894661	chr11:69182323-69182367	NM_146019:584	Chd3	INSIDE	0.738	3.699	590.00	2182.41	2.732	536.67	1465.93
A_68_P27865437	chr11:64248821-64248865	NM_178870:9	Hs3st3a1	INSIDE	0.738	2.190	1292.49	2830.05	1.617	1105.38	1787.15
A_68_P27787326	chr11:50024305-50024349	NM_011018:-34	Sqstm1	PROMOTER	0.738	3.906	624.59	2439.62	2.882	513.30	1479.30
A_68_P26567741	chr9:61762053-61762098	NM_018853:242	Rplp1	INSIDE	0.738	0.281	2661.69	748.91	0.208	1956.60	406.54
A_68_P26500012	chr9:49605941-49605986	NM_001081445:1211	Ncam1	INSIDE	0.738	0.509	1077.46	548.07	0.375	856.85	321.61
A_68_P26022161	chr8:87356199-87356243	NM_183358:40	Gadd45gip1	INSIDE	0.738	0.420	1411.23	592.33	0.310	1249.27	386.77
A_68_P25195706	chr7:74904045-74904089	NM_183312:562	Synm	INSIDE	0.738	0.380	1998.20	759.19	0.280	1614.86	452.63
A_68_P24035157	chr5:120316974-120317018	NM_011537:32325	Tbx5	INSIDE	0.738	0.238	2834.41	674.21	0.175	1952.23	342.62
A_68_P23893505	chr5:92738821-92738865	NM_172713:208	Sdad1	INSIDE	0.738	0.643	1461.76	940.62	0.475	1159.78	550.44
A_68_P23816543	chr5:76958602-76958646	NM_027270:277	Exoc1	INSIDE	0.738	0.523	1182.81	618.14	0.386	988.01	381.07
A_68_P23398856	chr4:148511654-148511698	NM_027263:33	Apitd1	INSIDE	0.738	0.689	1797.76	1239.30	0.509	1442.47	733.62
A_68_P23321770	chr4:133801322-133801366	NM_024215:162	Zfp593	INSIDE	0.738	2.527	1346.69	3402.51	1.865	1126.77	2101.86
A_68_P23188354	chr4:106739410-106739454	NM_025500:39	Mrpl37	INSIDE	0.738	3.356	1461.30	4903.55	2.476	1243.64	3079.51
A_68_P22934128	chr4:55493321-55493365			Unknown	0.738	0.625	1804.53	1127.92	0.461	1451.71	669.67
A_68_P22569091	chr3:138151998-138152042	NM_175224:326	Metap1	INSIDE	0.738	2.170	1554.60	3374.12	1.602	1340.41	2147.50
A_68_P22509665	chr3:127328307-127328351	NM_009718:-7734	Neurog2	PROMOTER	0.738	0.714	1434.16	1023.84	0.527	1221.23	642.98
A_68_P22383908	chr3:102824767-102824811	NM_001161854:320	Csde1	INSIDE	0.738	0.669	1570.62	1050.87	0.494	1175.48	580.26
A_68_P21639432	chr2:132089391-132089435	NM_138651:420	Cds2	INSIDE	0.738	0.282	2382.02	672.53	0.208	1813.72	377.76
A_68_P21578171	chr2:120803024-120803068	NM_175285:249	Tmem62	INSIDE	0.738	3.488	2063.67	7197.44	2.575	1845.51	4751.99
A_68_P21073085	chr2:25432477-25432521	NM_001005424:3121	Gm996	INSIDE	0.738	0.635	1017.26	645.81	0.468	766.90	359.26
A_68_P33012164	chr9_random:55526-55570	NM_011154:-516	Ppp2r3d	PROMOTER	0.737	2.884	1403.11	4046.39	2.125	1182.09	2511.93
A_68_P32806696	chrX:162948453-162948497	NM_001007577:-64	Tceanc	PROMOTER	0.737	2.462	646.35	1591.53	1.814	893.36	1620.23
A_68_P32287970	chrX:19868796-19868840	NM_177353:72	Slc9a7	INSIDE	0.737	2.787	742.77	2070.41	2.054	797.48	1638.36
A_68_P32039593	chr19:27291902-27291946	NM_001161420:415	Vldlr	INSIDE	0.737	0.380	1631.40	620.68	0.280	1261.85	353.64
A_68_P31260843	chr17:57415026-57415070	NM_001163815:-3474	Vav1	PROMOTER	0.737	0.638	2768.82	1766.81	0.470	2274.36	1069.70
A_68_P31215016	chr17:48061356-48061400	NM_001110824:203	Foxp4	INSIDE	0.737	3.301	636.11	2100.08	2.433	516.84	1257.69
A_68_P30593947	chr16:21694402-21694446	NM_001001881:314	2510009E07Rik	INSIDE	0.737	2.661	977.41	2600.58	1.961	832.15	1631.90
A_68_P30350780	chr15:76647637-76647681	NM_001168288:942	Arhgap39	INSIDE	0.737	2.053	1297.72	2664.14	1.513	1167.34	1765.75
A_68_P30346607	chr15:76010905-76010949	NM_201394:15214	Plec	INSIDE	0.737	9.639	2726.07	26276.82	7.103	2289.90	16265.62
A_68_P29848869	chr14:100685216-100685260	AK021307:-1632		PROMOTER	0.737	0.698	1780.42	1241.92	0.514	1359.33	698.79
A_68_P29842701	chr14:99698407-99698451	NM_009769:519	Klf5	INSIDE	0.737	2.310	689.31	1592.07	1.703	655.07	1115.80
A_68_P29266707	chr13:100785592-100785636	NM_030026:-20	Mccc2	PROMOTER	0.737	2.257	857.19	1934.50	1.663	771.44	1282.52
A_68_P28567641	chr12:80190421-80190465	NM_029988:215	Pigh	INSIDE	0.737	3.522	1680.79	5918.95	2.594	1437.86	3730.11
A_68_P28350338	chr12:35591506-35591550	AK087563:121		INSIDE	0.737	0.582	2619.39	1524.71	0.429	1926.32	825.96
A_68_P27980113	chr11:84693476-84693520	NM_027388:199	Pigw	INSIDE	0.737	3.379	1083.66	3662.21	2.491	936.71	2333.06
A_68_P27934858	chr11:76759228-76759272	NM_178645:93	Blmh	INSIDE	0.737	0.480	1293.32	621.20	0.354	937.95	331.92
A_68_P27478506	chr10:116387438-116387482	NM_001003950:-24	Rab3ip	DIVERGENT_PROMOTER	0.737	2.773	550.36	1526.24	2.045	458.81	938.24
A_68_P25957549	chr8:74094641-74094686	NM_011977:1838	Slc27a1	INSIDE	0.737	0.309	2134.23	660.37	0.228	1703.82	388.47
A_68_P25954806	chr8:73421106-73421150	NR_002901:-1373	Snora68	PROMOTER	0.737	2.211	2859.75	6323.50	1.630	2286.48	3726.48
A_68_P25016883	chr7:28140851-28140895	NM_138676:155	Shkbp1	INSIDE	0.737	0.286	3582.40	1023.93	0.211	2833.28	596.70
A_68_P24536650	chr6:71444353-71444397	NM_009543:487	Rnf103	INSIDE	0.737	2.022	2195.13	4438.51	1.490	1831.74	2729.26
A_68_P24334772	chr6:30907837-30907881	NM_001135093:1132	Klf14	INSIDE	0.737	0.373	1411.99	526.39	0.275	1024.71	281.40
A_68_P23778454	chr5:69948402-69948446	NM_172711:244	Gufl	INSIDE	0.737	14.158	505.15	7151.73	10.430	496.36	5177.10
A_68_P23763013	chr5:67009680-67009724	NM_009686:208	Apbb2	INSIDE	0.737	2.688	880.23	2365.75	1.980	690.96	1368.08
A_68_P23416103	chr4:151401853-151401897	NM_133879:-94	Zbtb48	PROMOTER	0.737	2.424	974.99	2363.03	1.787	727.99	1301.18

ProbeName	Target position of probe on CpG island microarray	TargetID	GeneSymbol	CpG island Description	Ratio of relative methylation (TiO_2-NP/Vehicle)	Sham group			TiO_2-H group		
						Relative methylation (Cy5/Cy3)	Cy3 signal (Input)	Cy5 signal (MeDIP)	Relative methylation (Cy5/Cy3)	Cy3 signal (Input)	Cy5 signal (MeDIP)
A_68_P23234767	chr4:117168730-117168774	NM_025739:768	Rnf220	INSIDE	0.737	0.540	1630.38	879.90	0.398	1293.55	514.67
A_68_P23142413	chr4:98786997-98787041	NM_026082:588	Dock7	INSIDE	0.737	2.881	782.93	2255.41	2.125	643.13	1366.35
A_68_P21834485	chr2:167066151-167066195	NM_008968:-135	Ptgis	PROMOTER	0.737	0.250	7842.50	1961.05	0.184	5410.51	997.10
A_68_P21073894	chr2:25562637-25562681	NM_023326:260	Bmyc	INSIDE	0.737	0.475	2080.23	987.47	0.350	1680.20	588.06
A_68_P20267395	chr1:60155522-60155566	NM_001199060:-200	Wdr12	PROMOTER	0.737	5.224	2216.94	11581.74	3.848	1783.43	6863.13
A_68_P31934085	chr19:6341468-6341512	NM_009006:241	Map4k2	INSIDE	0.736	0.591	1747.55	1032.49	0.435	1389.13	604.46
A_68_P31922247	chr19:4283414-4283458	NM_026720:-299	Ankrd13d	PROMOTER	0.736	5.168	668.84	3456.87	3.803	430.17	1636.04
A_68_P31150391	chr17:33897065-33897109	NM_008997:345	Rab11b	INSIDE	0.736	0.574	2985.35	1712.98	0.422	2252.33	950.99
A_68_P31099145	chr17:24844445-24844489	NM_145396:132	Tbl3	INSIDE	0.736	0.648	1233.47	798.71	0.477	891.69	425.04
A_68_P31024853	chr17:7374468-7374512	NM_011299:27	Rps6ka2	INSIDE	0.736	0.445	1515.93	674.29	0.327	1355.80	443.94
A_68_P30595257	chr16:21947236-21947280	NM_025693:367	Tmem41a	INSIDE	0.736	0.366	1535.15	561.34	0.269	1102.40	296.81
A_68_P29413817	chr14:13655575-13655619	NM_001042617:-3	Cadps	PROMOTER	0.736	0.438	1277.05	559.82	0.323	1001.96	323.42
A_68_P28470863	chr12:60320797-60320841	NM_001033156:-348	Fbxo33	PROMOTER	0.736	0.491	2144.33	1053.41	0.361	1818.35	657.07
A_68_P28071037	chr11:100800754-100800798	NM_011486:49	Stat3	INSIDE	0.736	0.408	1721.76	703.33	0.301	1226.00	368.48
A_68_P28067850	chr11:100258620-100258666	NM_010593:462	Jup	INSIDE	0.736	0.518	1674.58	867.17	0.381	1404.80	535.41
A_68_P27997848	chr11:87913317-87913362	NM_198013:692	Cuedc1	INSIDE	0.736	0.541	1475.35	797.89	0.398	1242.10	494.20
A_68_P27877797	chr11:66340283-66340327	NM_001034874:-676	Shisa6	PROMOTER	0.736	0.614	2203.06	1352.34	0.452	1721.62	777.96
A_68_P27469158	chr10:114799283-114799327	NM_133683:14	Tmem19	INSIDE	0.736	0.446	1994.15	888.60	0.328	1606.06	526.80
A_68_P27266465	chr10:76628713-76628757	NM_009929:541	Col18a1	INSIDE	0.736	0.192	4732.07	908.29	0.141	3214.13	454.27
A_68_P27098031	chr10:42741435-42741479	NM_175407:152880	Sobp	INSIDE	0.736	5.280	482.55	2547.90	3.889	397.96	1547.50
A_68_P26323068	chr9:15298594-15298638	AK146267:741		INSIDE	0.736	0.571	3088.40	1764.99	0.421	1933.89	813.36
A_68_P25951334	chr8:72877112-72877156	NM_001122829:38	Upf1	INSIDE	0.736	6.115	1953.10	11943.45	4.502	1558.89	7017.52
A_68_P25286994	chr7:91300218-91300262	NM_133722:163	Fam108c	INSIDE	0.736	4.183	1058.81	4429.08	3.081	975.63	3005.75
A_68_P24993659	chr7:20092410-20092454	NM_177692:1248	Bloc1s3	INSIDE	0.736	2.416	894.70	2161.31	1.779	807.49	1436.61
A_68_P24981704	chr7:17060818-17060862	NM_008718:1289	Npas1	INSIDE	0.736	0.584	1708.01	996.85	0.429	1335.39	573.33
A_68_P24046794	chr5:122286634-122286678	NM_008507:154	Sh2b3	INSIDE	0.736	4.089	1232.53	5040.34	3.009	994.39	2992.25
A_68_P23893507	chr5:92739062-92739106	NM_172713:-34	Sdad1	PROMOTER	0.736	0.609	958.91	583.58	0.448	779.49	349.35
A_68_P23541452	chr5:23893732-23893776	NM_001002897:4207	Atg9b	INSIDE	0.736	3.229	616.59	1990.94	2.376	583.33	1386.25
A_68_P23298638	chr4:129497304-129497348	NM_008974:-396	Ptp4a2	PROMOTER	0.736	2.889	1886.41	5449.05	2.125	1521.98	3234.86
A_68_P23250463	chr4:119960574-119960618	NM_194060:-730	Foxo6	PROMOTER	0.736	0.598	784.83	469.32	0.440	670.88	295.21
A_68_P23222249	chr4:114580688-114580732	NM_008593:793	Foxd2	INSIDE	0.736	9.868	1902.65	18774.71	7.259	1546.99	11230.03
A_68_P22698895	chr4:4719901-4719946	NM_177730:530	Impad1	INSIDE	0.736	0.383	1273.20	487.14	0.282	1079.03	303.96
A_68_P22630578	chr3:148653042-148653086	ENSMUST00000098518:-784		PROMOTER	0.736	0.698	1667.06	1163.49	0.514	1488.24	764.96
A_68_P21152640	chr2:39046217-39046261	NM_178778:12	Scai	INSIDE	0.736	3.420	905.18	3095.96	2.518	816.05	2054.75
A_68_P21054133	chr2:21127231-21127275	NM_001001297:-98	Thnsl1	DIVERGENT_PROMOTER	0.736	0.723	2660.91	1923.47	0.532	2082.72	1108.13
A_68_P20743339	chr1:158404313-158404357	NM_009230:125	Soat1	INSIDE	0.736	0.560	2623.62	1469.43	0.412	1985.43	818.51
A_68_P20455321	chr1:95298085-95298129	NM_207031:27634	Ano7	INSIDE	0.736	0.413	1685.69	696.15	0.304	1281.52	389.62
A_68_P32473672	chrX:73212006-73212050	ENSMUST00000121090:-7721		PROMOTER	0.735	2.105	1174.17	2472.10	1.547	1600.60	2476.10
A_68_P32341134	chrX:39503420-39503464	NM_021465:-435	Stag2	PROMOTER	0.735	2.892	597.11	1726.91	2.125	670.04	1423.87
A_68_P32183070	chr19:53603197-53603241	NM_001085390:-589	Dusp5	PROMOTER	0.735	0.420	1296.92	544.41	0.309	902.18	278.32
A_68_P31117120	chr17:27694784-27694828	NM_001166476:1209	Hmga1-rs1	INSIDE	0.735	2.379	1768.79	4207.25	1.748	1328.50	2322.34
A_68_P30575876	chr16:17815718-17815762	NM_153790:18366	Scarf2	DOWNSTREAM	0.735	2.621	905.95	2374.31	1.925	760.37	1463.98
A_68_P29134831	chr13:72769420-72769465	NM_010574:3017	Irx2	INSIDE	0.735	0.603	797.18	480.98	0.444	686.64	304.54
A_68_P28268127	chr12:17006562-17006606	NM_172574:340	Pqlc3	INSIDE	0.735	0.289	3994.29	1154.75	0.213	2782.01	591.50
A_68_P27673285	chr11:29073225-29073269	NM_134034:340	Smek2	INSIDE	0.735	0.583	1003.18	585.27	0.429	851.72	365.08
A_68_P26891838	chr9:122775033-122775077	NM_173364:70	Zfp445	INSIDE	0.735	0.693	1263.59	875.18	0.509	898.87	457.30
A_68_P26016334	chr8:86264880-86264924	NM_001163029:308	Cd97	INSIDE	0.735	0.437	1222.81	533.77	0.321	962.72	308.87
A_68_P25780174	chr8:36558678-36558722	NM_026067:-113	Eri1	PROMOTER	0.735	4.955	2889.57	14317.95	3.641	2506.34	9124.36
A_68_P25115908	chr7:57231147-57231191	NM_001037906:449	Nell1	INSIDE	0.735	2.883	841.46	2426.05	2.120	806.54	1710.06
A_68_P25035343	chr7:31935582-31935626	NM_027898:465	Gramd1a	INSIDE	0.735	0.281	6217.98	1746.19	0.206	4203.36	867.27
A_68_P25005236	chr7:25292275-25292319	NM_146183:191	Zfp428	INSIDE	0.735	0.380	1925.95	731.28	0.279	1590.07	443.51
A_68_P24686648	chr6:99471798-99471842	ENSMUST00000155466:144971		INSIDE	0.735	0.556	1313.03	730.19	0.409	887.27	362.53
A_68_P24591361	chr6:83037609-83037653	NM_010692:1272	Lbx2	INSIDE	0.735	2.302	703.52	1619.51	1.691	659.43	1115.20
A_68_P24534827	chr6:71094278-71094322	NM_001033929:70	Thnsl2	INSIDE	0.735	0.580	1742.50	1009.83	0.426	1456.50	620.65
A_68_P24186511	chr5:150481167-150481211	NM_001081204:357	B3galtl	INSIDE	0.735	0.343	5347.06	1831.70	0.252	3423.99	862.36
A_68_P23927128	chr5:100406693-100406737	NM_001077265:1243	Hnrnpd	INSIDE	0.735	0.444	1355.77	602.33	0.327	1160.73	379.15
A_68_P23345849	chr4:138011542-138011586	NM_025451:502	Camk2n1	INSIDE	0.735	0.516	2173.82	1122.55	0.380	1471.13	558.74
A_68_P22971148	chr4:63205777-63205821	NM_024173:0	Atp6v1g1	INSIDE	0.735	0.442	4063.89	1796.92	0.325	2906.47	944.61
A_68_P22903293	chr4:49072217-49072261	NM_178756:-95	E130309F12Rik	PROMOTER	0.735	0.171	5275.86	904.67	0.126	3536.04	445.48
A_68_P22878938	chr4:44769794-44769838	NM_138590:386	Zcchc7	INSIDE	0.735	5.449	1679.34	9151.28	4.006	1437.52	5759.06
A_68_P21093579	chr2:28696227-28696271	NM_001033294:323	Ddx31	INSIDE	0.735	0.262	3392.58	887.36	0.192	2553.35	491.09
A_68_P32589659	chrX:105011372-105011416	NM_025921:279	2610002M06Rik	INSIDE	0.734	4.027	3207.25	12916.40	2.956	4230.61	12505.00
A_68_P31738735	chr18:58038835-58038879	NM_009194:525	Slc12a2	INSIDE	0.734	3.168	922.53	2922.99	2.327	854.19	1987.36
A_68_P31484539	chr18:9212402-9212446	NM_008058:-429	Fzd8	PROMOTER	0.734	3.260	2138.74	6971.96	2.392	1908.02	4563.79
A_68_P31422103	chr17:87665170-87665214	NM_139295:95	Mcfd2	INSIDE	0.734	0.325	4504.56	1464.72	0.239	3161.41	754.67

ProbeName	Target position of probe on CpG island microarray	TargetID	GeneSymbol	CpG island Description	Ratio of relative methylation (TiO_2-NP/Vehicle)	Sham group			TiO_2-H group		
						Relative methylation (Cy5/Cy3)	Cy3 signal (Input)	Cy5 signal (MeDIP)	Relative methylation (Cy5/Cy3)	Cy3 signal (Input)	Cy5 signal (MeDIP)
A_68_P29614458	chr14:55433137-55433182	ENSMUST00000095853:-2164		PROMOTER	0.734	2.339	1257.63	2942.14	1.716	1002.10	1719.90
A_68_P29060389	chr13:56397618-56397662	NM_019568:272	Cxcl14	INSIDE	0.734	0.670	1655.64	1109.89	0.492	1228.31	604.37
A_68_P28553589	chr12:77470150-77470194	NM_001172104:279	Zbtb25	INSIDE	0.734	3.461	1017.80	3522.61	2.540	875.74	2224.64
A_68_P28538315	chr12:74685369-74685413	NM_008856:-637	Prkch	PROMOTER	0.734	4.825	1015.63	4900.63	3.540	736.26	2606.49
A_68_P28036768	chr11:94981457-94981501	NM_010055:48	Dlx3	INSIDE	0.734	0.301	7259.39	2182.70	0.221	4947.72	1092.16
A_68_P27926227	chr11:75275102-75275146	NR_029739:-2093	Mir22	PROMOTER	0.734	0.510	1571.75	801.95	0.374	1191.76	446.29
A_68_P27638905	chr11:21901185-21901229	NM_011023:448	Otx1	INSIDE	0.734	0.339	3907.61	1325.98	0.249	2584.86	644.17
A_68_P27539537	chr10:127534168-127534212	NM_054078:4352	Baz2a	INSIDE	0.734	0.585	945.67	552.92	0.429	790.28	339.08
A_68_P27385041	chr10:98569996-98570040	NM_015737:-750	Galnt4	PROMOTER	0.734	3.095	415.35	1285.32	2.271	364.23	827.22
A_68_P27262974	chr10:76059438-76059482	NM_146007:26689	Col6a2	INSIDE	0.734	2.859	2744.46	7845.44	2.099	1971.25	4138.57
A_68_P25677407	chr8:14911603-14911647	NM_001037736:-92	Arhgef10	PROMOTER	0.734	0.294	2248.33	660.35	0.216	1748.50	377.01
A_68_P25328181	chr7:100229343-100229387	NM_021427:976	Fam181b	INSIDE	0.734	0.133	4728.42	629.43	0.098	3323.34	324.61
A_68_P24924706	chr6:145695583-145695627	ENSMUST00000140966:-3486		PROMOTER	0.734	0.602	941.17	566.91	0.442	770.95	340.88
A_68_P24766865	chr6:114688621-114688665	NM_028835:95500	Atg7	INSIDE	0.734	0.577	3464.36	1997.41	0.423	2546.73	1077.34
A_68_P24424707	chr6:47827330-47827374	NM_146175:-201	Zfp282	PROMOTER	0.734	2.073	1320.57	2738.15	1.522	1210.65	1842.47
A_68_P23322288	chr4:133898952-133898996	NM_001039677:-10	Slc30a2	PROMOTER	0.734	2.582	2495.25	6443.49	1.897	2043.69	3876.00
A_68_P22931101	chr4:54957886-54957930	NM_172867:-2908	Zfp462	PROMOTER	0.734	2.779	565.21	1570.59	2.039	477.13	972.76
A_68_P22324426	chr3:90097746-90097790	AK015985:137		INSIDE	0.734	0.461	961.81	443.40	0.338	736.49	249.08
A_68_P21736745	chr2:149656284-149656328	NM_001085521:-212	Tmem90b	PROMOTER	0.734	0.663	1430.14	948.74	0.487	1033.60	503.43
A_68_P20937428	chr1:193141556-193141600	NM_025424:419	Nenf	INSIDE	0.734	0.542	1903.89	1031.84	0.398	1368.12	544.51
A_68_P20872221	chr1:181733198-181733242	NM_026375:926	Ahctf1	INSIDE	0.734	2.980	718.50	2141.32	2.187	609.22	1332.37
A_68_P20825995	chr1:172994402-172994446	NM_010188:-4890	Fcgr3	PROMOTER	0.734	0.232	27502.09	6371.71	0.170	19466.37	3309.53
A_68_P32054106	chr19:29722522-29722566	NM_001081213:366	Ermp1	INSIDE	0.733	2.171	1047.99	2275.59	1.592	977.15	1555.31
A_68_P31190365	chr17:43767352-43767396	NM_001161366:-126	Tdrd6	PROMOTER	0.733	0.657	1220.31	801.78	0.482	960.44	462.46
A_68_P30589272	chr16:20733318-20733362	NM_009893:141	Chrd	INSIDE	0.733	3.142	1075.81	3380.44	2.303	874.80	2014.40
A_68_P30207008	chr15:50701312-50701356	NM_032000:20253	Trps1	INSIDE	0.733	2.263	1679.41	3799.79	1.659	1370.80	2273.48
A_68_P28554434	chr12:77634454-77634498	NM_153804:-70	Plekhg3	PROMOTER	0.733	0.649	1779.55	1154.69	0.475	1526.48	725.81
A_68_P28331591	chr12:32183927-32183971	NM_134048:457	Cbll1	INSIDE	0.733	2.342	3592.17	8413.69	1.716	2767.86	4749.61
A_68_P28067970	chr11:100275491-100275535	NM_176830:621	Leprel4	INSIDE	0.733	2.109	992.01	2092.01	1.545	894.32	1382.11
A_68_P27997845	chr11:87913051-87913095	NM_198013:425	Cuedc1	INSIDE	0.733	0.609	1152.22	701.38	0.446	1165.29	519.85
A_68_P27897811	chr11:69715610-69715654	NM_001013414:253	Neurl4	INSIDE	0.733	2.052	1245.07	2555.51	1.505	951.55	1432.31
A_68_P27889079	chr11:68198328-68198372	NM_008744:1978	Ntn1	INSIDE	0.733	0.359	2094.75	751.77	0.263	1399.66	368.11
A_68_P27835396	chr11:59005546-59005590	NM_008193:-114	Guk1	PROMOTER	0.733	0.706	3947.64	2788.17	0.518	3234.53	1675.58
A_68_P27830383	chr11:58136875-58136919	NM_178761:-48	Zfp672	DIVERGENT_PROMOTER	0.733	0.681	1418.18	966.46	0.500	1194.71	596.93
A_68_P27190661	chr10:62801691-62801735	NM_001159590:31	Sirt1	INSIDE	0.733	2.295	1237.95	2841.69	1.683	1044.10	1756.84
A_68_P27186305	chr10:61949424-61949468	NM_001113355:-100	Vps26a	PROMOTER	0.733	0.586	2232.89	1308.91	0.430	1666.17	716.06
A_68_P26586893	chr9:65141963-65142007	NM_001044389:-117	Clpx	PROMOTER	0.733	0.683	1405.19	959.24	0.500	1075.23	538.10
A_68_P26507495	chr9:50911542-50911586	NR_029654:-349	Mir34c	PROMOTER	0.733	2.399	997.62	2393.73	1.758	911.08	1601.79
A_68_P26381828	chr9:28211224-28211268	NM_177906:612393	Opcml	INSIDE	0.733	0.296	2133.21	631.77	0.217	1729.06	375.33
A_68_P25784752	chr8:37311896-37311940	NM_001081150:652	Lonrf1	INSIDE	0.733	2.670	626.66	1672.99	1.957	622.76	1218.62
A_68_P25423373	chr7:119369629-119369673	NM_001193305:301	Mical2	INSIDE	0.733	0.492	2099.36	1033.35	0.361	1688.24	609.24
A_68_P25198318	chr7:75309835-75309879	NM_010513:212714	Igf1r	INSIDE	0.733	2.399	880.72	2112.91	1.758	732.31	1287.57
A_68_P25083582	chr7:50931965-50932009	NM_145582:4586	Ctu1	INSIDE	0.733	2.117	1640.34	3472.58	1.553	1153.18	1790.56
A_68_P25007186	chr7:25689296-25689340	NM_001130152:-46	Arhgef1	PROMOTER	0.733	0.615	1115.53	685.77	0.451	985.52	444.30
A_68_P24159038	chr5:144775460-144775504	NM_177682:240	Ccz1	INSIDE	0.733	0.717	1314.50	942.36	0.526	991.87	521.24
A_68_P23973399	chr5:109089618-109089662	NM_199011:148	Dgkq	INSIDE	0.733	2.393	907.60	2171.89	1.754	762.57	1337.45
A_68_P23916643	chr5:98614905-98614949	NM_029947:5039	Prdm8	INSIDE	0.733	2.401	663.05	1591.88	1.759	639.36	1124.79
A_68_P23316406	chr4:132776655-132776700	NM_027152:-46	Cd164l2	DIVERGENT_PROMOTER	0.733	5.280	350.77	1852.17	3.869	245.26	948.84
A_68_P22403735	chr3:106839334-106839378	NM_008418:277	Kcna3	INSIDE	0.733	0.718	1301.92	934.61	0.526	1113.12	585.66
A_68_P22182591	chr3:61169487-61169531	NM_028712:1080	Rap2b	INSIDE	0.733	0.715	3313.87	2367.88	0.524	2471.02	1294.68
A_68_P21099935	chr2:29724646-29724690	NM_207298:-345	Cercam	PROMOTER	0.733	7.517	933.62	7017.67	5.509	877.95	4836.87
A_68_P20474868	chr1:99991929-99991973	NM_013626:259	Pam	INSIDE	0.733	0.744	2920.36	2173.76	0.546	2364.06	1290.11
A_68_P20353934	chr1:75419154-75419198	NM_001085370:36992	Speg	INSIDE	0.733	0.622	4768.07	2967.32	0.456	3182.78	1451.26
A_68_P30436282	chr15:91402943-91402987	NM_001033633:728	Slc2a13	INSIDE	0.732	4.250	2051.32	8718.72	3.112	1761.16	5479.87
A_68_P30363893	chr15:78858397-78858441	NM_008197:-223	H1f0	PROMOTER	0.732	2.230	1075.75	2398.68	1.632	804.17	1312.53
A_68_P30148792	chr15:38229751-38229795	NM_013692:690	Klf10	INSIDE	0.732	2.421	1266.51	3066.73	1.773	1095.66	1942.23
A_68_P29961944	chr14:122875451-122875495	NM_009574:867	Zic2	INSIDE	0.732	0.561	2635.47	1477.41	0.410	2171.71	891.48
A_68_P29619512	chr14:56340802-56340846	NM_019519:189	Rabggta	INSIDE	0.732	0.240	3589.57	862.63	0.176	2287.21	402.26
A_68_P29071671	chr13:58229883-58229927	NM_029872:13	Hnrnpa0	INSIDE	0.732	0.620	1358.49	842.29	0.454	1207.86	548.11
A_68_P29055172	chr13:55565926-55565970	NM_001030296:321	Prr7	INSIDE	0.732	2.720	1451.04	3946.41	1.992	1164.95	2320.32
A_68_P28067856	chr11:100259165-100259209	NM_010593:-82	Jup	PROMOTER	0.732	0.559	1451.84	811.84	0.409	1162.21	475.78
A_68_P27938850	chr11:77500718-77500762	NM_001024205:1100	Nufip2	INSIDE	0.732	2.689	1003.84	2699.09	1.967	812.52	1598.25
A_68_P27841048	chr11:59954437-59954481	NM_001037764:875	Rai1	INSIDE	0.732	9.884	404.14	3994.37	7.236	400.99	2901.58
A_68_P26863467	chr9:117781995-117782039	ENSMUST00000126482:-245		PROMOTER	0.732	4.069	1068.49	4347.34	2.979	772.89	2302.53
A_68_P26232970	chr8:124900378-124900422	NM_001029993:-115	Zc3h18	PROMOTER	0.732	4.453	866.21	3857.37	3.262	724.82	2364.18

ProbeName	Target position of probe on CpG island microarray	TargetID	GeneSymbol	CpG island Description	Ratio of relative methylation (TiO_2-NP/Vehicle)	Sham group			TiO_2-H group		
						Relative methylation (Cy5/Cy3)	Cy3 signal (Input)	Cy5 signal (MeDIP)	Relative methylation (Cy5/Cy3)	Cy3 signal (Input)	Cy5 signal (MeDIP)
A_68_P25988748	chr8:80960386-80960430	NM_138944:143	Pou4f2	INSIDE	0.732	0.732	2933.63	2147.25	0.536	2065.69	1107.37
A_68_P25954902	chr8:73438710-73438754	NM_173013:8860	Mtap1s	INSIDE	0.732	3.673	1861.12	6836.70	2.688	1500.89	4035.07
A_68_P25021327	chr7:29136106-29136150	NR_028129:-430	LOC100302567	PROMOTER	0.732	2.586	5438.89	14065.28	1.894	3939.01	7459.93
A_68_P25005092	chr7:25266660-25266704	NM_153112:-359	Cadm4	PROMOTER	0.732	0.433	4840.35	2096.22	0.317	3371.87	1068.63
A_68_P24631971	chr6:90320525-90320569	NM_030260:1059	Zxdc	INSIDE	0.732	0.566	1614.25	913.71	0.414	1169.69	484.72
A_68_P24324924	chr6:29114488-29114532	NM_133925:214	Rbm28	INSIDE	0.732	0.678	3518.09	2384.82	0.496	2466.38	1223.61
A_68_P23301755	chr4:130219531-130219575	NM_001159603:279	Pum1	INSIDE	0.732	3.841	1581.87	6075.85	2.813	1134.17	3189.86
A_68_P22313144	chr3:88136817-88136861	NM_024246:1517	Tmem79	INSIDE	0.732	3.583	1150.26	4121.46	2.622	780.49	2046.60
A_68_P22009788	chr3:27081784-27081828	NM_178772:-119	Nceh1	PROMOTER	0.732	2.443	816.88	1995.88	1.788	647.06	1156.72
A_68_P21569219	chr2:119176997-119177041	NM_026929:41	Chac1	INSIDE	0.732	0.257	2259.51	580.16	0.188	1601.73	300.85
A_68_P20888736	chr1:184694036-184694080	NM_144796:-978	Susd4	PROMOTER	0.732	0.720	1478.07	1064.16	0.527	1318.09	694.32
A_68_P20332287	chr1:71699659-71699703	NM_010233:65	Fn1	INSIDE	0.732	0.262	2145.75	562.38	0.192	1541.21	295.62
A_68_P32552416	chrX:96086453-96086497	NM_052976:-150	Ophn1	PROMOTER	0.731	2.752	628.74	1730.50	2.011	600.55	1207.93
A_68_P32539665	chrX:91881517-91881561	NM_008179:131	Gspt2	INSIDE	0.731	2.708	887.47	2403.13	1.979	1158.04	2291.26
A_68_P31666413	chr18:44540296-44540340	NM_027490:165	Dcp2	INSIDE	0.731	0.309	3055.57	944.07	0.226	2309.55	521.66
A_68_P31458869	chr18:3507247-3507294	NM_026505:-684	Bambi	PROMOTER	0.731	7.813	443.35	3463.99	5.712	390.51	2230.54
A_68_P31065958	chr17:15635304-15635348	NM_011185:-86	Psmb1	DIVERGENT_PROMOTER	0.731	0.368	1211.43	445.82	0.269	880.24	236.82
A_68_P30350784	chr15:76648087-76648131	NM_001168288:492	Arhgap39	INSIDE	0.731	0.688	21740.75	14967.33	0.503	12064.36	6071.64
A_68_P30331748	chr15:73475653-73475697	ENSMUST00000150409:101		INSIDE	0.731	6.287	4383.22	27558.03	4.599	4134.32	19012.20
A_68_P30330340	chr15:73253433-73253477	NM_007982:167	Ptk2	INSIDE	0.731	1.942	1747.88	3393.85	1.419	1624.82	2304.98
A_68_P30133051	chr15:35301203-35301247	NM_177151:-76	Vps13b	PROMOTER	0.731	6.064	863.01	5233.26	4.432	814.14	3608.69
A_68_P29507209	chr14:31467715-31467759	NM_177815:176	Rft1	INSIDE	0.731	2.311	1024.96	2369.13	1.689	850.54	1436.73
A_68_P29162295	chr13:78338732-78338776	NM_010151:-511	Nr2f1	PROMOTER	0.731	3.497	1548.56	5415.60	2.558	1156.50	2957.86
A_68_P28703499	chr12:105712671-105712715	NM_010351:-1246	Gsc	PROMOTER	0.731	5.237	684.86	3586.86	3.827	561.23	2147.76
A_68_P28160197	chr11:116442143-116442187	NM_173755:597	Ube2o	INSIDE	0.731	2.234	4064.27	9077.58	1.633	3235.14	5281.88
A_68_P28149096	chr11:114537102-114537146	NM_053273:343	Ttyh2	INSIDE	0.731	0.475	1250.41	594.03	0.347	935.49	324.96
A_68_P26813992	chr9:108165850-108165894	NM_010017:195	Dag1	INSIDE	0.731	3.645	3687.20	13438.77	2.665	3181.22	8477.32
A_68_P26544601	chr9:57788261-57788305	NM_011352:341	Sema7a	INSIDE	0.731	2.349	1268.46	2979.22	1.716	967.86	1660.99
A_68_P25952346	chr8:73034117-73034161	NM_019883:128	Uba52	INSIDE	0.731	1.932	3448.43	6662.50	1.412	2569.16	3626.96
A_68_P25609546	chr7:152025123-152025167	NM_008007:628	Fgf3	INSIDE	0.731	0.417	1218.86	507.95	0.305	926.14	282.21
A_68_P25588867	chr7:148633309-148633353	NM_026020:-218	Rplp2	DIVERGENT_PROMOTER	0.731	2.266	1646.92	3732.60	1.657	1178.58	1952.57
A_68_P25508221	chr7:135079319-135079363	NM_001081374:10899	Prss36	INSIDE	0.731	3.011	3888.25	11709.03	2.202	3138.89	6912.72
A_68_P25498529	chr7:133225199-133225243	NM_001101488:705	Gsg1l	INSIDE	0.731	2.050	3066.56	6285.45	1.499	2173.60	3258.32
A_68_P25094157	chr7:52887692-52887738	NM_028544:4808	Rasip1	INSIDE	0.731	2.166	2307.15	4996.61	1.582	1733.08	2742.24
A_68_P25018371	chr7:28438749-28438793	NM_028417:-172	Ttc9b	PROMOTER	0.731	0.152	3370.09	513.14	0.111	2297.23	255.85
A_68_P24816835	chr6:124705909-124705953	NM_007881:575	Atn1	INSIDE	0.731	2.079	7110.79	14782.82	1.519	4895.54	7438.03
A_68_P24429121	chr6:48693073-48693117	NM_008376:4049	Gimap1	INSIDE	0.731	2.935	2023.18	5937.78	2.145	1595.48	3421.85
A_68_P24155692	chr5:144016313-144016357	NM_017467:15367	Zfp316	INSIDE	0.731	2.279	876.52	1997.99	1.665	748.90	1247.12
A_68_P23701553	chr5:53982267-53982311	NM_001080927:476	Rbpj	INSIDE	0.731	2.097	1879.74	3941.93	1.532	1615.07	2475.00
A_68_P22360092	chr3:98231693-98231737	NM_172863:45311	Zfp697	INSIDE	0.731	0.679	1322.34	897.78	0.496	1123.48	557.55
A_68_P22246601	chr3:75360455-75360499	NM_019745:245	Pdcd10	INSIDE	0.731	0.344	4559.53	1568.28	0.252	3020.87	759.79
A_68_P21516585	chr2:109514331-109514375	NM_007540:-504	Bdnf	PROMOTER	0.731	0.487	1916.08	933.46	0.356	1459.62	519.76
A_68_P21311215	chr2:69660694-69660738	NM_177784:290	Klhl23	INSIDE	0.731	0.411	2374.11	976.20	0.300	1739.59	522.54
A_68_P21094142	chr2:28771352-28771396	NM_019446:567	Barhl1	INSIDE	0.731	0.236	2585.30	610.15	0.172	1722.63	297.06
A_68_P20901342	chr1:187278951-187278995	NM_001033286:246	Slc30a10	INSIDE	0.731	0.294	1841.91	540.85	0.215	1432.79	307.38
A_68_P20720544	chr1:154247140-154247184	NM_177756:166	Glt25d2	INSIDE	0.731	3.453	4980.58	17200.16	2.525	3765.07	9505.50
A_68_P20502870	chr1:107252678-107252722	NM_001160368:587	Rnf152	INSIDE	0.731	0.565	2242.16	1265.71	0.413	1781.02	734.91
A_68_P32143403	chr19:46423840-46423884	NM_029186:-7507	Tmem180	PROMOTER	0.730	0.388	2683.41	1042.47	0.284	1858.10	527.18
A_68_P31296466	chr17:64951197-64951241	NM_008549:230	Man2a1	INSIDE	0.730	3.134	1025.85	3214.80	2.287	824.49	1885.66
A_68_P31260713	chr17:57386929-57386973	NM_030084:114	Gpr108	INSIDE	0.730	0.557	1377.59	767.22	0.407	1082.84	440.19
A_68_P30368847	chr15:79664630-79664674	NM_001013360:111	Npcd	INSIDE	0.730	2.085	4248.71	8856.66	1.522	3085.31	4697.08
A_68_P30146212	chr15:37721558-37721602	NM_001170866:175	Ncald	INSIDE	0.730	0.689	4353.09	2998.00	0.503	3633.68	1827.35
A_68_P30094310	chr15:27677668-27677712	NM_001081302:277913	Trio	INSIDE	0.730	2.374	1279.90	3037.99	1.733	1148.27	1989.82
A_68_P29619050	chr14:56262458-56262502	NM_028780:163	Tm9sf1	INSIDE	0.730	53.706	2168.78	116475.80	39.196	2719.83	106607.70
A_68_P28246308	chr12:12948541-12948585	NM_008709:80	Mycn	INSIDE	0.730	0.443	2164.26	959.05	0.323	1743.75	563.86
A_68_P27977997	chr11:84339091-84339135	NR_038057:-49	1500016L03Rik	DIVERGENT_PROMOTER	0.730	0.440	1099.60	483.36	0.321	760.63	243.94
A_68_P27180607	chr10:60915001-60915045	NM_010124:395	Eif4ebp2	INSIDE	0.730	2.120	1121.24	2376.47	1.546	977.73	1511.82
A_68_P26803574	chr9:106367984-106368028	NM_001159652:265	Gpr62	INSIDE	0.730	2.004	3522.75	7058.65	1.462	2507.37	3666.28
A_68_P25504796	chr7:134398154-134398198	NM_177362:10137	Zfp771	INSIDE	0.730	0.502	1992.23	999.59	0.366	1287.43	471.48
A_68_P25503172	chr7:134093232-134093276	NM_001039645:-159	Asphd1	DIVERGENT_PROMOTER	0.730	2.128	3211.09	6831.81	1.553	2407.02	3738.68
A_68_P25042532	chr7:34953588-34953632	NM_016682:-62	Uba2	PROMOTER	0.730	15.072	749.82	11301.68	11.001	738.86	8127.93
A_68_P24423144	chr6:47403969-47404013	NM_012042:-332	Cul1	PROMOTER	0.730	6.582	874.77	5757.96	4.802	802.66	3854.55
A_68_P24325039	chr6:29129114-29129158	NM_001101443:448	Prrt4	INSIDE	0.730	2.792	464.63	1297.18	2.039	485.12	989.36
A_68_P24119331	chr5:136559903-136559947	NM_133914:139	Rasa4	INSIDE	0.730	0.098	5321.55	523.09	0.072	3603.22	258.59
A_68_P23979394	chr5:110815716-110815760	NM_028596:408	Fbrsl1	INSIDE	0.730	0.742	3138.79	2329.86	0.542	2247.90	1217.81

ProbeName	Target position of probe on CpG island microarray	TargetID	GeneSymbol	CpG island Description	Ratio of relative methylation (TiO_2-NP/Vehicle)	Sham group			TiO_2-H group		
						Relative methylation (Cy5/Cy3)	Cy3 signal (Input)	Cy5 signal (MeDIP)	Relative methylation (Cy5/Cy3)	Cy3 signal (Input)	Cy5 signal (MeDIP)
A_68_P23917032	chr5:98683315-98683359	NM_010203:134	Fgf5	INSIDE	0.730	2.730	2003.23	5468.91	1.993	1573.25	3135.75
A_68_P23418022	chr4:151700088-151700132	NM_009079:124	Rpl22	INSIDE	0.730	0.592	1163.08	689.06	0.433	992.85	429.43
A_68_P23416422	chr4:151460754-151460798	ENSMUST00000118648:-190		PROMOTER	0.730	2.732	918.22	2508.53	1.995	835.58	1666.63
A_68_P23297038	chr4:129191512-129191557	NM_010807:710	Marcksl1	INSIDE	0.730	2.314	2357.12	5453.77	1.689	1863.81	3147.90
A_68_P22788125	chr4:24825516-24825560	NM_026742:309	Ndufaf4	INSIDE	0.730	2.089	2675.51	5588.17	1.524	2173.67	3313.08
A_68_P22413526	chr3:108394877-108394921	NM_181400:703	Wdr47	INSIDE	0.730	0.702	1293.84	908.02	0.513	1121.38	574.82
A_68_P22370683	chr3:100490054-100490098	NM_010763:-680	Man1a2	PROMOTER	0.730	0.504	2026.34	1020.74	0.368	1613.67	593.42
A_68_P22006133	chr3:26230419-26230463	NM_138666:391	Nlgn1	INSIDE	0.730	2.308	2518.89	5812.67	1.686	2033.91	3428.31
A_68_P21565892	chr2:118572266-118572310	NR_030716:791	5430417L22Rik	INSIDE	0.730	0.308	2218.25	683.09	0.225	1586.72	356.64
A_68_P21350014	chr2:76244191-76244236	NM_145525:-381	Osbpl6	PROMOTER	0.730	0.364	1684.10	613.17	0.266	1367.97	363.71
A_68_P21309974	chr2:69423570-69423614	NM_001081088:532	Lrp2	INSIDE	0.730	2.167	1542.41	3341.66	1.582	1319.01	2086.65
A_68_P20140950	chr1:34899440-34899484	NM_001160235:433	Fam168b	INSIDE	0.730	2.337	1022.30	2388.92	1.705	823.47	1404.14
A_68_P31846341	chr18:77424704-77424748	NM_013666:141	St8sia5	INSIDE	0.729	2.190	1578.32	3456.69	1.597	1362.48	2176.04
A_68_P31126966	chr17:29400812-29400856	NM_026845:82	Ppil1	INSIDE	0.729	0.615	922.14	567.01	0.448	778.27	348.95
A_68_P31097233	chr17:24563466-24563510	NM_010023:-139	Eci1	PROMOTER	0.729	3.139	1011.50	3174.62	2.288	811.86	1857.42
A_68_P30133050	chr15:35301092-35301136	NM_177151:-186	Vps13b	PROMOTER	0.729	2.871	455.27	1307.22	2.094	365.50	765.26
A_68_P29654920	chr14:62950855-62950899	NM_026001:-65	Rnaseh2b	PROMOTER	0.729	2.510	1981.05	4971.51	1.829	1647.05	3012.23
A_68_P29143150	chr13:74074924-74074968	NM_027182:269	Trip13	INSIDE	0.729	0.168	4185.21	702.95	0.122	2776.99	340.02
A_68_P28604201	chr12:86796427-86796471			Unknown	0.729	0.596	1771.87	1056.42	0.435	1462.20	635.80
A_68_P28585118	chr12:83270099-83270143	NM_001167983:-882	Sipa1l1	PROMOTER	0.729	0.216	2464.13	533.19	0.158	2004.13	316.09
A_68_P28430596	chr12:52694381-52694425	NM_001198835:75	Coch	INSIDE	0.729	0.670	1490.81	999.40	0.489	1284.62	627.69
A_68_P28067851	chr11:100258744-100258788	NM_010593:338	Jup	INSIDE	0.729	0.666	1726.79	1150.71	0.486	1432.88	695.75
A_68_P27212449	chr10:67001247-67001291	NM_010118:652	Egr2	INSIDE	0.729	2.274	2131.83	4848.65	1.657	1643.87	2724.48
A_68_P26954143	chr10:13684496-13684540	NM_010437:-1666	Hivep2	PROMOTER	0.729	0.417	1884.71	786.42	0.304	1416.41	430.69
A_68_P26750367	chr9:96896461-96896505	NM_145134:22292	Spsb4	INSIDE	0.729	3.447	1121.51	3866.39	2.514	829.85	2086.38
A_68_P26658191	chr9:77961162-77961206	NM_019987:140	Ick	INSIDE	0.729	0.643	2521.33	1622.03	0.469	2069.54	969.92
A_68_P26224362	chr8:123609059-123609103	NM_010426:707	Foxf1a	INSIDE	0.729	2.575	1269.28	3268.93	1.879	1096.79	2060.57
A_68_P25951052	chr8:72839724-72839768	NM_001163282:73	Gdf1	INSIDE	0.729	0.351	1357.22	476.09	0.256	967.04	247.29
A_68_P25398381	chr7:114352789-114352833	NM_029660:-148	Rbmxl2	PROMOTER	0.729	2.082	1815.39	3779.45	1.518	1602.14	2432.12
A_68_P25091650	chr7:52437190-52437234	NM_053256:848	Pth2	DOWNSTREAM	0.729	1.960	2019.86	3959.06	1.428	1574.20	2248.15
A_68_P23904971	chr5:96590602-96590646	NM_178854:150	Cnot6l	INSIDE	0.729	1.964	17652.94	34674.81	1.432	11967.55	17138.75
A_68_P23445968	chr5:3596436-3596480	NM_172991:89	C030048B08Rik	INSIDE	0.729	0.683	1111.36	758.51	0.498	953.14	474.43
A_68_P23250424	chr4:119955496-119955540	NM_194060:4348	Foxo6	INSIDE	0.729	3.610	2085.87	7530.00	2.633	1758.29	4629.31
A_68_P22774363	chr4:21613063-21613107	NM_001080771:26	Prdm13	INSIDE	0.729	0.720	3838.02	2762.16	0.525	2668.89	1400.28
A_68_P22317586	chr3:88968678-88968722	NM_001163432:-26	Clk2	DIVERGENT_PROMOTER	0.729	0.529	1098.23	580.48	0.385	938.55	361.59
A_68_P21742433	chr2:150864640-150864699	NM_026086:446	Nanp	INSIDE	0.729	3.132	556.14	1741.64	2.282	508.86	1161.35
A_68_P21345533	chr2:75542035-75542079	NM_010902:642	Nfe2l2	INSIDE	0.729	0.460	1185.38	545.30	0.336	905.43	303.79
A_68_P21309973	chr2:69423496-69423540	NM_001081088:606	Lrp2	INSIDE	0.729	2.574	869.14	2237.09	1.876	752.94	1412.69
A_68_P31731698	chr18:56867506-56867550	NM_010721:62	Lmnb1	INSIDE	0.728	3.282	1132.43	3716.12	2.389	859.37	2053.42
A_68_P31351850	chr17:74927220-74927264	NM_007566:-392	Birc6	PROMOTER	0.728	0.604	2251.52	1359.70	0.439	1631.77	717.09
A_68_P31331867	chr17:71340037-71340081	NM_023402:-202	Myl12b	PROMOTER	0.728	0.316	5779.77	1827.58	0.230	3901.39	897.79
A_68_P30980969	chr16:94592388-94592432	NM_009441:65	Ttc3	INSIDE	0.728	4.096	589.30	2413.66	2.982	551.95	1645.67
A_68_P28329063	chr12:31759041-31759085	NM_027828:230	Fam110c	INSIDE	0.728	0.656	1141.26	749.00	0.478	1014.23	484.35
A_68_P28069993	chr11:100620866-100620910	NM_001081194:204	Kcnh4	INSIDE	0.728	0.292	1690.63	492.89	0.212	1333.46	283.13
A_68_P26569949	chr9:62188962-62189006	NM_009672:-165	Anp32a	PROMOTER	0.728	0.179	3512.68	629.20	0.130	2591.46	338.09
A_68_P26247737	chr8:127318310-127318354	NM_198632:1414	Trim67	INSIDE	0.728	0.449	1381.69	620.78	0.327	1022.09	334.08
A_68_P26055742	chr8:93432780-93432824	NM_177224:80069	Chd9	INSIDE	0.728	0.408	2138.26	871.83	0.297	1852.12	549.94
A_68_P25585273	chr7:148043403-148043447	NM_053194:129	Ric8	INSIDE	0.728	0.367	1222.09	448.00	0.267	876.04	233.70
A_68_P25362940	chr7:106775025-106775069	NM_001195529:567	Gm4980	INSIDE	0.728	0.637	909.12	579.31	0.464	699.02	324.24
A_68_P24592748	chr6:83267406-83267450	NM_008638:170	Mthfd2	INSIDE	0.728	2.635	2760.11	7273.04	1.919	2012.54	3861.87
A_68_P23417946	chr4:151687386-151687430	NM_001033489:5326	Rnf207	INSIDE	0.728	0.331	1913.66	633.98	0.241	1307.46	315.29
A_68_P22614374	chr3:146068079-146068128	NM_138744:453	Ssx2ip	INSIDE	0.728	2.292	1689.60	3871.78	1.668	1288.98	2150.35
A_68_P22015873	chr3:28161770-28161814	NM_001163009:-343	Tnik	PROMOTER	0.728	5.010	262.32	1314.15	3.646	277.69	1012.54
A_68_P21638156	chr2:131855491-131855535	NM_175445:212	Rassf2	INSIDE	0.728	0.323	5927.08	1914.97	0.235	4169.72	981.27
A_68_P30396081	chr15:84493743-84493787	ENSMUST00000171460:-6285		PROMOTER	0.727	0.231	3378.03	779.74	0.168	2416.81	405.73
A_68_P30379093	chr15:81524108-81524152	NM_001164320:3587	Chadl	INSIDE	0.727	5.016	405.61	2034.41	3.645	366.49	1335.73
A_68_P29942862	chr14:119538422-119538466	NM_015820:958	Hs6st3	INSIDE	0.727	2.294	1013.41	2324.52	1.668	783.69	1306.95
A_68_P29928900	chr14:117325074-117325118	NM_001079844:560	Gpc6	INSIDE	0.727	0.523	2162.59	1130.78	0.380	1772.66	673.78
A_68_P29731792	chr14:76510870-76510914	NM_013745:195	Nufip1	INSIDE	0.727	0.631	875.42	552.79	0.459	749.00	343.98
A_68_P29041946	chr13:53206812-53206856	NM_013846:174644	Ror2	INSIDE	0.727	2.212	2976.45	6583.16	1.607	2135.78	3432.40
A_68_P28457339	chr12:57622099-57622143	AK084874:-5008		PROMOTER	0.727	0.674	1542.32	1039.10	0.490	1339.89	656.27
A_68_P28152427	chr11:115128177-115128221	NM_010350:359	Grin2c	INSIDE	0.727	0.419	2696.48	1129.19	0.304	2013.49	613.09
A_68_P27898020	chr11:69749025-69749069	NM_016875:-354	Ybx2	PROMOTER	0.727	0.321	2543.95	816.78	0.233	1900.79	443.39
A_68_P26026700	chr8:88364563-88364607	NM_028007:264	Itfg1	INSIDE	0.727	2.907	946.49	2751.85	2.113	765.88	1618.04
A_68_P25993840	chr8:81819005-81819049	NM_133823:-171	Mmaa	PROMOTER	0.727	0.436	1344.64	585.88	0.317	1071.59	339.41

ProbeName	Target position of probe on CpG island microarray	TargetID	GeneSymbol	CpG island Description	Ratio of relative methylation (TiO_2-NP/Vehicle)	Sham group			TiO_2-H group		
						Relative methylation (Cy5/Cy3)	Cy3 signal (Input)	Cy5 signal (MeDIP)	Relative methylation (Cy5/Cy3)	Cy3 signal (Input)	Cy5 signal (MeDIP)
A_68_P25728011	chr8:26712636-26712680	NM_001001735:-119	Whsc1l1	DIVERGENT_PROMOTER	0.727	3.658	418.00	1528.97	2.658	431.81	1147.73
A_68_P25525515	chr7:138080197-138080241	NM_019564:502	Htra1	INSIDE	0.727	3.248	1611.66	5235.38	2.361	1343.93	3173.40
A_68_P25354432	chr7:105343075-105343119	NM_001081167:4889	B3gnt6	INSIDE	0.727	0.336	1791.31	601.50	0.244	1448.98	353.78
A_68_P24931168	chr6:146837284-146837328	NM_001170433:291	Ppfibp1	INSIDE	0.727	0.629	1788.15	1124.45	0.457	1388.51	634.83
A_68_P24138663	chr5:140449111-140449155	NM_175522:65236	Elfn1	INSIDE	0.727	3.059	1508.71	4615.77	2.225	1333.19	2966.71
A_68_P23284225	chr4:126913544-126913588	NM_198618:67052	Dlgap3	INSIDE	0.727	0.648	1366.34	885.87	0.471	1025.92	483.51
A_68_P21368925	chr2:79297045-79297089	NM_010894:-273	Neurod1	PROMOTER	0.727	0.541	1607.27	869.14	0.393	1324.61	520.64
A_68_P21330529	chr2:73112421-73112465	NM_001005343:2460	Sp9	INSIDE	0.727	0.518	3976.32	2058.58	0.376	2810.92	1057.27
A_68_P21142145	chr2:37298992-37299036	NM_001033960:-8759	Rabgap1	PROMOTER	0.727	0.369	1935.13	713.83	0.268	1470.16	394.48
A_68_P20942041	chr1:194014550-194014594	NM_001038607:-181	Kcnh1	PROMOTER	0.727	7.911	1379.33	10911.21	5.754	1317.45	7580.18
A_68_P20008178	chr1:5008614-5008658	NM_021374:871	Rgs20	INSIDE	0.727	0.518	5033.98	2607.93	0.377	3269.53	1231.29
A_68_P32130260	chr19:44144019-44144063	NM_145502:136	Erlin1	INSIDE	0.726	1.983	3438.16	6816.67	1.440	2775.03	3995.47
A_68_P31620540	chr18:36020339-36020383	NM_133687:30889	Cxxc5	INSIDE	0.726	3.343	1218.76	4074.15	2.427	954.48	2316.95
A_68_P31425842	chr17:88374222-88374266	NM_010830:-145	Msh6	PROMOTER	0.726	2.838	1149.57	3262.13	2.059	1068.61	2200.16
A_68_P31106682	chr17:25981519-25981564	NM_145999:255	Rhot2	INSIDE	0.726	7.503	2323.56	17433.31	5.447	1841.13	10028.30
A_68_P30633249	chr16:28929086-28929130	NM_177718:676	1600021P15Rik	INSIDE	0.726	2.672	691.43	1847.60	1.940	618.77	1200.51
A_68_P30377735	chr15:81230102-81230146	NM_133726:0	St13	INSIDE	0.726	0.283	7797.37	2205.38	0.205	5352.99	1099.06
A_68_P30370943	chr15:80064789-80064834	NM_178719:302	Smcr7l	INSIDE	0.726	0.413	1950.20	806.35	0.300	1375.52	413.02
A_68_P30364183	chr15:78905793-78905837	NM_145139:162	Eif3l	INSIDE	0.726	0.535	1221.58	653.15	0.388	944.48	366.44
A_68_P30139530	chr15:36539045-36539089	NM_008774:-338	Pabpc1	PROMOTER	0.726	2.205	1754.37	3869.05	1.600	1478.32	2365.77
A_68_P29977214	chr15:3976915-3976959	NR_028266:470	BC037032	INSIDE	0.726	3.259	1594.69	5196.74	2.366	1301.25	3079.22
A_68_P29578872	chr14:47502657-47502701	NM_001037221:39	Samd4	INSIDE	0.726	2.359	2118.69	4998.96	1.712	1803.88	3088.74
A_68_P29024656	chr13:49437040-49437084	NM_001039179:145	Bicd2	INSIDE	0.726	2.644	807.13	2133.70	1.918	697.63	1338.24
A_68_P28168391	chr11:117848501-117848545	NM_133757:352	Pgs1	INSIDE	0.726	2.128	1192.41	2537.03	1.545	1073.85	1659.56
A_68_P28049250	chr11:97049684-97049728	NM_008379:-500	Kpnb1	PROMOTER	0.726	0.485	1156.95	561.62	0.353	985.99	347.68
A_68_P27966238	chr11:82248332-82248376	NM_023438:45953	Tmem132e	INSIDE	0.726	7.691	395.04	3038.19	5.581	372.24	2077.40
A_68_P27261502	chr10:75808270-75808314	NM_001081419:-285	Dip2a	PROMOTER	0.726	0.420	4274.98	1794.23	0.305	3361.89	1024.94
A_68_P27257131	chr10:75022752-75022796	NM_198860:301	AI646023	INSIDE	0.726	3.131	1291.20	4042.78	2.274	1068.53	2429.57
A_68_P26349534	chr9:21722752-21722796	NM_031874:-209	Rab3d	PROMOTER	0.726	0.307	1610.83	494.28	0.223	1247.79	278.00
A_68_P25352108	chr7:104886417-104886461	NM_175105:319	Aqp11	INSIDE	0.726	0.665	1584.67	1053.15	0.483	1150.98	555.69
A_68_P25097407	chr7:53435255-53435299	NM_011510:127	Abcc8	INSIDE	0.726	0.189	2529.87	478.40	0.137	1772.64	243.34
A_68_P25095389	chr7:53088549-53088593	NM_008172:33481	Grin2d	INSIDE	0.726	9.346	1274.43	11910.80	6.788	1025.14	6958.91
A_68_P25022017	chr7:29244098-29244142	NM_001141921:6864	Lrfn1	INSIDE	0.726	2.325	763.13	1774.08	1.688	632.37	1067.71
A_68_P24818962	chr6:125095826-125095870	NM_001039669:580	Iffo1	INSIDE	0.726	0.569	2480.20	1411.04	0.413	1837.97	759.27
A_68_P24800932	chr6:120866913-120866957	NM_007544:-96	Bid	PROMOTER	0.726	0.288	1865.43	536.50	0.209	1297.46	271.05
A_68_P24537770	chr6:71657709-71657753	NM_178608:56	Reep1	INSIDE	0.726	11.893	1140.85	13568.46	8.638	1095.82	9465.65
A_68_P24431635	chr6:49168267-49168311	NM_023670:-3335	Igf2bp3	PROMOTER	0.726	0.727	1673.15	1216.76	0.528	1337.09	705.50
A_68_P22141942	chr3:53267127-53267171	NM_172501:32	Nhlrc3	INSIDE	0.726	0.717	1731.29	1242.01	0.521	1180.94	615.40
A_68_P21587820	chr2:122564356-122564400	NM_019788:138	Pldn	INSIDE	0.726	2.979	842.54	2510.13	2.163	692.76	1498.65
A_68_P21563739	chr2:118214589-118214633	NM_001177806:-237	Eif2ak4	PROMOTER	0.726	0.455	1250.55	568.80	0.330	997.04	329.07
A_68_P21338895	chr2:74536395-74536439	NM_013555:597	Hoxd9	INSIDE	0.726	5.375	624.48	3356.86	3.903	551.06	2150.87
A_68_P21086001	chr2:27532393-27532437	NM_011305:-306	Rxra	PROMOTER	0.726	0.581	1770.14	1027.91	0.422	1267.05	534.08
A_68_P21082467	chr2:26968438-26968482	NM_175427:29537	Fam163b	INSIDE	0.726	0.635	2439.13	1547.75	0.460	1691.05	778.63
A_68_P20929010	chr1:191746024-191746068	NM_026796:121	Smyd2	INSIDE	0.726	3.083	1259.79	3883.83	2.237	1121.38	2508.96
A_68_P20354345	chr1:75476353-75476397	NM_177056:268	Tmem198	INSIDE	0.726	0.376	2805.02	1055.06	0.273	2163.99	590.53
A_68_P20350885	chr1:74902845-74902889	NM_009872:1264	Cdk5r2	INSIDE	0.726	0.364	4057.36	1477.34	0.264	2765.45	730.74
A_68_P31862937	chr18:80403704-80403748	NM_001042408:121	Txnl4a	INSIDE	0.725	0.479	3063.73	1468.22	0.347	2345.47	814.41
A_68_P31383397	chr17:80879774-80879818	NM_009231:-3	Sos1	PROMOTER	0.725	0.525	827.33	434.07	0.380	702.68	267.10
A_68_P31187137	chr17:43153945-43153989	NM_178589:463	Tnfrsf21	INSIDE	0.725	2.879	3124.23	8994.79	2.088	2492.00	5204.16
A_68_P30778635	chr16:55974040-55974084	NM_173026:146	Zbtb11	INSIDE	0.725	2.562	563.09	1442.91	1.858	467.80	869.03
A_68_P30475234	chr15:98398199-98398243	NM_009833:-153	Ccnt1	PROMOTER	0.725	0.622	2834.27	1763.50	0.451	2203.09	993.74
A_68_P30026217	chr15:13103232-13103276	NM_007666:140	Cdh6	INSIDE	0.725	0.250	4216.80	1053.54	0.181	2938.63	532.08
A_68_P29734163	chr14:76904251-76904295	NM_009366:-44	Tsc22d1	PROMOTER	0.725	5.229	521.85	2728.98	3.789	563.99	2137.10
A_68_P29509958	chr14:31951947-31951991	NM_027289:3930	Nt5dc2	INSIDE	0.725	4.118	1229.95	5064.64	2.987	893.89	2669.82
A_68_P28745892	A_68_P28745892			Unknown	0.725	6.438	328.36	2113.83	4.669	271.54	1267.88
A_68_P28480022	chr12:62624309-62624353	NM_178714:-1288	Lrfn5	PROMOTER	0.725	3.593	1894.26	6805.73	2.604	1548.93	4033.63
A_68_P28202958	chr12:4913875-4913919	NM_145441:430	Ubxn2a	INSIDE	0.725	3.011	1310.09	3944.64	2.184	1234.65	2696.30
A_68_P27279098	chr10:79281731-79281775	NM_001161747:25436	Palm	INSIDE	0.725	5.915	694.75	4109.51	4.287	675.70	2896.79
A_68_P26873355	chr9:119487746-119487790	NM_021544:366	Scn5a	INSIDE	0.725	2.923	1654.75	4837.46	2.121	1238.42	2626.30
A_68_P25087552	chr7:51718008-51718052	NM_198250:20174	Lrrc4b	INSIDE	0.725	2.196	1622.81	3563.31	1.592	1340.13	2133.97
A_68_P25009541	chr7:26082496-26082540	NM_008776:456	Pafah1b3	INSIDE	0.725	0.304	1888.04	573.74	0.220	1461.90	322.00
A_68_P24979592	chr7:16684283-16684327	NM_025898:313	Napa	INSIDE	0.725	2.220	795.77	1766.25	1.610	726.72	1170.20
A_68_P24623143	chr6:88577556-88577600	NM_001142724:-139	Kbtbd12	PROMOTER	0.725	2.065	3310.51	6837.76	1.497	2496.22	3735.82
A_68_P24591253	chr6:83020444-83020488	NM_009392:-247	Tlx2	DIVERGENT_PROMOTER	0.725	0.526	6128.07	3225.45	0.382	4483.00	1710.69
A_68_P24476445	chr6:56664725-56664769	NM_030235:-152	Avl9	PROMOTER	0.725	3.034	2515.22	7630.21	2.201	2187.64	4814.45

ProbeName	Target position of probe on CpG island microarray	TargetID	GeneSymbol	CpG island Description	Ratio of relative methylation (TiO_2-NP/Vehicle)	Sham group			TiO_2-H group		
						Relative methylation (Cy5/Cy3)	Cy3 signal (Input)	Cy5 signal (MeDIP)	Relative methylation (Cy5/Cy3)	Cy3 signal (Input)	Cy5 signal (MeDIP)
A_68_P24423149	chr6:47404611-47404655	NM_012042:310	Cul1	INSIDE	0.725	17.087	2088.52	35686.43	12.393	1958.95	24277.78
A_68_P22749326	chr4:15192772-15192816	NM_181401:-172	Tmem64	PROMOTER	0.725	2.304	1220.07	2811.26	1.670	982.19	1639.95
A_68_P22448210	chr3:115955054-115955098	NM_022427:1326	Gpr88	INSIDE	0.725	2.247	1213.00	2725.40	1.629	987.08	1607.55
A_68_P21844247	chr2:168760922-168760966	NM_009564:20143	Zfp64	INSIDE	0.725	11.525	345.11	3977.42	8.357	327.55	2737.37
A_68_P21594129	chr2:123916410-123916454	ENSMUST00000137172:728		INSIDE	0.725	0.595	2396.31	1424.85	0.431	1680.77	724.89
A_68_P21548580	chr2:115686166-115686210	NM_001159569:204169	Meis2	DOWNSTREAM	0.725	0.495	1119.66	553.89	0.359	885.07	317.36
A_68_P21147277	chr2:38142298-38142342	NM_146122:584	Dennd1a	INSIDE	0.725	0.712	3462.90	2466.67	0.516	2570.99	1327.83
A_68_P20936237	chr1:192922504-192922548	NM_030060:234	Batf3	INSIDE	0.725	0.315	2753.91	866.11	0.228	2150.17	490.59
A_68_P32260372	chrX:11668383-11668427	NM_001168321:69077	Bcor	INSIDE	0.724	0.535	1485.19	793.90	0.387	1985.80	768.47
A_68_P31868118	chr18:81184539-81184583	NM_178280:-1243	Sall3	PROMOTER	0.724	0.674	2422.56	1632.28	0.488	1961.73	957.23
A_68_P31786966	chr18:66618498-66618542	NM_021451:263	Pmaip1	INSIDE	0.724	0.390	3000.54	1170.02	0.282	2321.54	655.37
A_68_P31151613	chr17:34168502-34168546	NM_001077709:111	Slc39a7	INSIDE	0.724	0.396	1385.71	549.37	0.287	996.48	286.21
A_68_P31116857	chr17:27650341-27650385	ENSMUST00000088073:-76		DIVERGENT_PROMOTER	0.724	0.720	5347.91	3849.62	0.521	3482.23	1815.00
A_68_P30253394	chr15:59146814-59146858	NM_009270:190	Sqle	INSIDE	0.724	0.488	1347.38	656.88	0.353	1062.39	375.24
A_68_P28031043	chr11:94010730-94010774	ENSMUST00000155965:15937		INSIDE	0.724	0.690	2000.36	1380.11	0.500	1667.36	833.07
A_68_P27210043	chr10:66590012-66590056	NM_207221:29	Jmjd1c	INSIDE	0.724	3.257	922.26	3003.88	2.358	827.50	1951.07
A_68_P26920439	chr10:7445765-7445809	NM_011835:-11	Katna1	PROMOTER	0.724	3.277	1153.71	3780.27	2.373	981.93	2330.25
A_68_P25992137	chr8:81554288-81554333	NM_178267:1975	Zfp827	INSIDE	0.724	0.465	1338.06	622.77	0.337	967.90	326.32
A_68_P25009659	chr7:26100595-26100639	NM_183311:9490	Tmem145	INSIDE	0.724	2.324	1460.62	3393.97	1.683	1097.21	1846.16
A_68_P24591771	chr6:83106296-83106340	NM_023790:55	Wdr54	INSIDE	0.724	0.560	4670.26	2614.53	0.405	3263.64	1323.13
A_68_P24444443	chr6:51421130-51421174	NM_007624:538	Cbx3	INSIDE	0.724	4.422	908.60	4017.92	3.200	748.60	2395.38
A_68_P24164945	chr5:145992341-145992385	NM_001083958:-221	Zfp655	PROMOTER	0.724	0.529	1258.97	665.51	0.383	995.67	381.20
A_68_P24143544	chr5:141306207-141306251	NM_010302:157	Gna12	INSIDE	0.724	6.424	1029.62	6614.54	4.653	942.79	4386.58
A_68_P24118749	chr5:136439002-136439046	NM_001160366:11322	Srcrb4d	INSIDE	0.724	1.924	3858.36	7424.97	1.392	3066.80	4270.31
A_68_P23915348	chr5:98364470-98364514	NM_133738:95489	Antxr2	INSIDE	0.724	3.090	1490.57	4606.02	2.237	1289.77	2885.42
A_68_P22889575	chr4:46614883-46614927	NM_001164804:-103	Coro2a	PROMOTER	0.724	4.513	1702.87	7684.59	3.266	1384.13	4520.85
A_68_P21339292	chr2:74584954-74584998	NM_010468:34927	Hoxd3	INSIDE	0.724	0.486	3893.82	1890.84	0.352	3092.40	1087.27
A_68_P21239896	chr2:56954902-56954946	NM_013613:12525	Nr4a2	DOWNSTREAM	0.724	2.906	727.58	2114.19	2.103	646.51	1359.56
A_68_P20823482	chr1:172520111-172520155	NM_001109985:-152	Nos1ap	PROMOTER	0.724	0.731	1799.04	1315.02	0.529	1504.55	796.64
A_68_P31621836	chr18:36228394-36228438	NM_001167891:128398	Nrg2	INSIDE	0.723	2.954	1635.88	4832.93	2.135	1150.77	2456.39
A_68_P31516123	chr18:15309505-15309549	NM_134112:429	Kctd1	INSIDE	0.723	2.310	3704.48	8558.19	1.671	2939.15	4912.15
A_68_P31490816	chr18:10609789-10609833	NM_001081222:540	Esco1	INSIDE	0.723	7.124	1263.96	9004.09	5.149	1026.13	5284.00
A_68_P30672048	chr16:35770485-35770529	NM_175111:35	Hspbap1	INSIDE	0.723	2.592	629.05	1630.27	1.875	598.49	1122.07
A_68_P30397301	chr15:84685820-84685864	NM_001081166:717	Phf21b	INSIDE	0.723	4.442	999.52	4440.36	3.211	1034.56	3321.91
A_68_P29369780	chr13:119503164-119503208	NM_008002:-319	Fgf10	PROMOTER	0.723	2.176	1269.02	2760.83	1.573	1001.96	1575.61
A_68_P29097469	chr13:64263530-64263574	NM_019986:379	Habp4	INSIDE	0.723	2.993	525.74	1573.45	2.163	485.53	1050.36
A_68_P29050817	chr13:54789017-54789062	NM_001146025:130	Rnf44	INSIDE	0.723	2.302	2131.84	4907.23	1.665	1642.40	2734.58
A_68_P29038492	chr13:52625955-52625999	NM_001024474:229	Diras2	INSIDE	0.723	0.619	4705.04	2911.78	0.447	3549.52	1587.64
A_68_P28803334	chr13:6647782-6647826	NM_019703:166	Pfkp	INSIDE	0.723	0.472	2314.91	1092.21	0.341	1779.50	607.32
A_68_P28168286	chr11:117830189-117830233	NM_007707:470	Socs3	INSIDE	0.723	0.534	1600.39	854.47	0.386	1254.71	484.40
A_68_P27894314	chr11:69137355-69137399	NM_172560:-1	Cntrob	DIVERGENT_PROMOTER	0.723	0.727	3834.60	2788.43	0.526	2812.28	1479.24
A_68_P27856227	chr11:62693165-62693209	NM_026210:195	Fam18b	INSIDE	0.723	8.714	2015.60	17564.22	6.298	1757.76	11069.54
A_68_P27787407	chr11:50038771-50038815	NM_145926:-44	Mgat4b	PROMOTER	0.723	3.267	1060.49	3464.51	2.360	862.96	2036.93
A_68_P26885387	chr9:121668671-121668715	NR_033503:369	E530011L22Rik	INSIDE	0.723	0.457	5343.80	2440.03	0.330	3673.81	1213.49
A_68_P26044775	chr8:91565596-91565640	NM_021390:2443	Sall1	INSIDE	0.723	2.116	2421.12	5122.95	1.530	2042.14	3125.22
A_68_P25957228	chr8:74035689-74035737	NM_032398:-44	Plvap	PROMOTER	0.723	3.278	485.69	1592.08	2.368	469.43	1111.80
A_68_P24630039	chr6:89785616-89785660	NM_053221:9893	Vmn1r42	DOWNSTREAM	0.723	2.784	1274.24	3547.11	2.013	1092.83	2200.40
A_68_P24155794	chr5:144031725-144031769	NM_017467:-45	Zfp316	PROMOTER	0.723	0.346	2115.58	731.69	0.250	1706.31	426.63
A_68_P23334088	chr4:136025956-136026001	NM_024452:303	Luzp1	INSIDE	0.723	4.850	223.33	1083.25	3.506	208.49	731.04
A_68_P23279120	chr4:125939634-125939678	NM_013718:8	Trappc3	INSIDE	0.723	0.652	2289.97	1492.27	0.471	1848.88	870.59
A_68_P23072594	chr4:84851732-84851776	NM_019535:395	Sh3gl2	INSIDE	0.723	0.528	2079.17	1097.11	0.382	1579.03	602.59
A_68_P22450818	chr3:116414578-116414622	NM_144902:598	Slc35a3	INSIDE	0.723	0.289	2378.08	686.65	0.209	1811.13	378.32
A_68_P21897026	chr2:179176969-179177013	NM_009867:-192	Cdh4	PROMOTER	0.723	4.155	1034.98	4300.63	3.004	938.49	2819.65
A_68_P21814760	chr2:163679507-163679551	NM_001030292:43	Kcnk15	INSIDE	0.723	0.337	1659.00	559.57	0.244	1269.84	309.45
A_68_P21594123	chr2:123915717-123915761	ENSMUST00000137172:34		INSIDE	0.723	0.717	13070.06	9374.86	0.518	8836.19	4579.99
A_68_P21217116	chr2:51928627-51928671	NM_175238:292	Rif1	INSIDE	0.723	2.590	772.92	2001.80	1.871	656.94	1229.44
A_68_P20782630	chr1:165242350-165242396	NM_011127:1409	Prrx1	INSIDE	0.723	0.526	1231.53	647.70	0.380	1034.06	393.05
A_68_P20346580	chr1:74115002-74115046	NM_027884:55997	Tns1	INSIDE	0.723	0.586	2274.00	1333.68	0.424	1885.84	799.73
A_68_P31827982	chr18:73974763-73974807	NM_145494:262	Me2	INSIDE	0.722	0.675	1858.18	1254.18	0.487	1478.71	720.69
A_68_P31227867	chr17:50432626-50432673	NM_010021:275	Dazl	INSIDE	0.722	2.666	504.79	1345.71	1.926	422.34	813.28
A_68_P29928904	chr14:117325550-117325594	NM_001079844:1036	Gpc6	INSIDE	0.722	4.753	1761.37	8371.93	3.433	1435.32	4928.18
A_68_P28853784	chr13:17896191-17896235	NM_001081058:719	Cdk13	INSIDE	0.722	4.541	491.11	2230.28	3.277	477.19	1563.54
A_68_P28833744	chr13:12794884-12794928	ENSMUST00000104944:278		DOWNSTREAM	0.722	0.176	2856.43	503.15	0.127	2193.48	278.96
A_68_P28635716	chr12:92826987-92827031	NM_031391:1081	Gtf2a1	INSIDE	0.722	0.526	870.27	457.91	0.380	822.26	312.24
A_68_P27558539	chr11:4494769-4494813	NM_028860:28	Mtmr3	INSIDE	0.722	3.804	1089.83	4145.18	2.744	893.32	2451.66

ProbeName	Target position of probe on CpG island microarray	TargetID	GeneSymbol	CpG island Description	Ratio of relative methylation (TiO_2-NP/Vehicle)	Sham group			TiO_2-H group		
						Relative methylation (Cy5/Cy3)	Cy3 signal (Input)	Cy5 signal (MeDIP)	Relative methylation (Cy5/Cy3)	Cy3 signal (Input)	Cy5 signal (MeDIP)
A_68_P27541275	chr10:127848643-127848687	NM_001164237:-7	Rnf41	DIVERGENT_PROMOTER	0.722	8.891	1116.18	9923.99	6.423	916.12	5884.69
A_68_P27277985	chr10:79100697-79100741	NM_001024539:-55	Shc2	PROMOTER	0.722	4.479	1937.77	8678.45	3.233	1764.68	5705.67
A_68_P27003034	chr10:23070344-23070388	NM_010167:-657	Eya4	PROMOTER	0.722	2.807	1822.46	5114.77	2.026	1576.34	3193.06
A_68_P24791288	chr6:119058455-119058499	NM_001159533:-269	Cacna1c	PROMOTER	0.722	3.097	656.19	2032.02	2.235	544.03	1215.89
A_68_P23637946	chr5:42235648-42235692	NM_001081422:-116	Bod1l	PROMOTER	0.722	3.591	1240.73	4455.04	2.594	1081.78	2806.26
A_68_P23141229	chr4:98591079-98591123	NM_146144:600	Usp1	INSIDE	0.722	2.076	1610.51	3342.92	1.498	1402.97	2102.07
A_68_P21103869	chr2:30400981-30401025	ENSMUST00000145413:10754		DOWNSTREAM	0.722	3.506	393.60	1380.01	2.530	359.38	909.16
A_68_P21101319	chr2:29949323-29949367	NM_001037762:-189	Zdhhc12	PROMOTER	0.722	0.663	1030.37	683.46	0.479	860.42	412.33
A_68_P20715112	chr1:153348264-153348308	NM_011277:-333	Rnf2	PROMOTER	0.722	0.402	1294.18	520.20	0.290	1137.17	329.80
A_68_P32026207	chr19:24935981-24936025	NM_175013:330	Pgm5	INSIDE	0.721	0.507	3094.77	1570.27	0.366	2234.95	817.88
A_68_P31985666	chr19:17468871-17468915	NM_019437:360	Rfk	INSIDE	0.721	2.922	753.36	2201.43	2.106	644.85	1358.25
A_68_P31958010	chr19:11893171-11893215	NM_011502:191	Stx3	INSIDE	0.721	2.631	1058.36	2784.80	1.896	1046.36	1983.75
A_68_P31540304	chr18:20716579-20716623	NM_007883:-16	Dsg2	PROMOTER	0.721	0.612	1416.11	866.02	0.441	1224.46	539.77
A_68_P31106679	chr17:25981120-25981164	NM_145999:654	Rhot2	INSIDE	0.721	0.501	3760.86	1882.89	0.361	2541.58	917.90
A_68_P31065256	chr17:15512298-15512342	NM_007865:467	Dll1	INSIDE	0.721	0.684	3020.10	2064.30	0.493	2251.04	1108.66
A_68_P30424875	chr15:89209486-89209530	NM_001013022:177	Odf3b	INSIDE	0.721	0.465	1034.30	480.95	0.335	909.15	304.95
A_68_P30351229	A_68_P30351229			Unknown	0.721	5.889	1180.74	6953.50	4.243	1105.51	4691.02
A_68_P29712959	chr14:72932118-72932162	NM_207636:177670	Fndc3a	DOWNSTREAM	0.721	0.555	1397.33	775.31	0.400	1113.42	445.26
A_68_P29578878	chr14:47503269-47503313	NM_001037221:651	Samd4	INSIDE	0.721	0.570	1015.46	578.96	0.411	858.17	352.65
A_68_P28924067	chr13:31717371-31717415	NM_010225:-292	Foxf2	PROMOTER	0.721	2.590	3791.91	9820.42	1.867	2665.80	4977.93
A_68_P27845263	chr11:60691958-60692002	NM_001168507:560	Tmem11	INSIDE	0.721	0.131	6137.08	801.68	0.094	4802.52	452.27
A_68_P25585259	chr7:148041886-148041930	NM_018742:374	Bet1l	INSIDE	0.721	0.256	1818.24	464.77	0.184	1341.82	247.27
A_68_P24536430	chr6:71391224-71391268	NM_024288:-615	Rmnd5a	PROMOTER	0.721	0.406	1811.72	735.01	0.293	1377.66	403.15
A_68_P23576446	chr5:31395554-31395598	NM_011773:324	Slc30a3	INSIDE	0.721	0.239	2238.84	535.53	0.172	1558.52	268.82
A_68_P23201931	chr4:109330348-109330392	NM_007671:7607	Cdkn2c	DOWNSTREAM	0.721	0.209	2798.07	584.83	0.151	2305.40	347.22
A_68_P22313146	chr3:88137030-88137074	NM_024246:1303	Tmem79	INSIDE	0.721	3.404	1655.63	5635.48	2.455	1349.36	3312.97
A_68_P22071616	chr3:38786996-38787040	NM_183221:1157	Fat4	INSIDE	0.721	0.294	1527.78	448.42	0.212	1090.20	230.81
A_68_P21227529	chr2:53937298-53937342	NM_023396:643	Rprm	INSIDE	0.721	2.747	1360.14	3735.85	1.980	1173.86	2324.68
A_68_P32182248	chr19:53464624-53464668	NM_172429:417	Smndc1	INSIDE	0.720	0.656	1849.50	1213.80	0.472	1389.71	656.53
A_68_P32030091	chr19:25580881-25580925	NM_015826:707	Dmrt1	INSIDE	0.720	0.395	3784.98	1495.29	0.284	2726.90	775.23
A_68_P31776706	chr18:64820510-64820554	NM_001001488:122	Atp8b1	INSIDE	0.720	0.498	1816.80	905.08	0.359	1596.93	573.18
A_68_P30597632	chr16:22439432-22439476	NM_023794:189	Etv5	INSIDE	0.720	2.112	1317.66	2782.45	1.520	1173.95	1784.87
A_68_P30588066	chr16:20535808-20535852	NM_009679:254	Ap2m1	INSIDE	0.720	2.198	1408.47	3096.05	1.583	1202.27	1903.26
A_68_P29508107	chr14:31639042-31639086	NM_028839:285	Tmem110	INSIDE	0.720	0.406	1562.05	634.41	0.292	1236.68	361.42
A_68_P29152216	chr13:76081085-76081129	NM_028493:166	Rhobtb3	INSIDE	0.720	3.148	530.01	1668.37	2.266	490.32	1111.24
A_68_P28781890	chr12:120087139-120087183	NM_177082:2359	Sp8	INSIDE	0.720	3.257	1073.00	3495.23	2.344	871.20	2041.88
A_68_P28280373	chr12:21379873-21379917	NM_009615:-442	Adam17	PROMOTER	0.720	0.612	1147.02	702.26	0.441	1010.07	445.27
A_68_P28137823	chr11:112643047-112643091	NM_011448:-455	Sox9	PROMOTER	0.720	0.458	1085.32	496.91	0.330	822.19	271.15
A_68_P27285553	chr10:80260510-80260554	NM_023900:839	Plekhj1	INSIDE	0.720	0.462	2873.58	1328.91	0.333	2155.98	718.18
A_68_P26801603	chr9:106073078-106073122	NM_029896:-159	Wdr82	PROMOTER	0.720	3.698	981.73	3630.85	2.662	790.24	2103.75
A_68_P26626133	chr9:71959405-71959449	NM_011544:200	Tcf12	INSIDE	0.720	4.482	1049.68	4704.47	3.229	909.50	2936.65
A_68_P26554636	chr9:59505260-59505304	NM_011099:868	Pkm2	INSIDE	0.720	0.522	1307.78	682.38	0.376	1042.70	391.92
A_68_P26429013	chr9:36953392-36953436	NM_001029838:1437	Pknox2	INSIDE	0.720	0.679	2241.11	1521.39	0.489	1955.97	956.54
A_68_P26018926	chr8:86761267-86761311	NM_172503:-347	Zswim4	PROMOTER	0.720	0.626	1141.06	714.04	0.451	928.46	418.36
A_68_P24942896	chr6:148894246-148894290	NM_019643:686	Fam60a	INSIDE	0.720	2.479	527.70	1308.41	1.784	593.45	1058.79
A_68_P24632559	chr6:90412347-90412391	NM_023184:-251	Klf15	PROMOTER	0.720	0.359	1334.95	478.98	0.258	946.29	244.45
A_68_P24513207	chr6:65622605-65622649	NM_172399:1022	A930038C07Rik	INSIDE	0.720	2.165	2330.44	5045.04	1.559	1827.12	2847.70
A_68_P24118171	chr5:136328011-136328055	NM_021403:34057	Srrm3	INSIDE	0.720	6.623	1044.21	6916.20	4.772	818.00	3903.51
A_68_P23308582	chr4:131392468-131392512	NM_001083119:1703	Ptpru	INSIDE	0.720	3.684	2184.08	8045.32	2.653	1676.29	4447.33
A_68_P23222243	chr4:114580052-114580096	NM_008593:1429	Foxd2	INSIDE	0.720	4.584	1186.95	5440.96	3.300	1072.28	3538.51
A_68_P23062265	chr4:83063978-83064022	NM_029949:353	Snapc3	INSIDE	0.720	0.695	1656.05	1151.68	0.500	1308.66	654.81
A_68_P22523144	chr3:129672792-129672836	NM_025779:310	Ccdc109b	INSIDE	0.720	0.635	2159.74	1372.47	0.457	1802.22	824.22
A_68_P22467630	chr3:119485493-119485539	NM_019550:790	Ptbp2	INSIDE	0.720	0.519	1062.70	551.02	0.373	885.84	330.83
A_68_P21683844	chr2:140221275-140221319	NM_028387:126	Macrod2	INSIDE	0.720	0.248	7650.38	1900.17	0.179	4883.85	873.19
A_68_P21346289	chr2:75670532-75670576	NM_172666:321	Agps	INSIDE	0.720	0.686	1924.31	1320.02	0.494	1495.44	738.47
A_68_P21320137	chr2:71384228-71384272	NM_010054:561	Dlx2	INSIDE	0.720	0.515	2036.57	1048.80	0.371	1569.83	582.18
A_68_P21285104	chr2:65076845-65076889	NM_027225:-183	Cobll1	PROMOTER	0.720	0.258	1739.72	448.03	0.185	1346.76	249.59
A_68_P21224225	chr2:53051017-53051061	NM_022989:-79	Arl6ip6	DIVERGENT_PROMOTER	0.720	0.498	1376.79	685.67	0.359	1067.77	382.90
A_68_P21115066	chr2:32237310-32237354	NM_198001:103	1110008P14Rik	INSIDE	0.720	0.512	984.20	503.82	0.369	703.45	259.41
A_68_P20331600	chr1:71603721-71603765	NM_026195:13	Atic	INSIDE	0.720	2.757	3507.23	9670.31	1.986	2881.89	5722.97
A_68_P20262085	chr1:59176968-59177012	NM_001033449:-50	Als2cr4	PROMOTER	0.720	0.334	1865.72	623.45	0.240	1601.64	385.09
A_68_P33016566	chr7:3424393-3424437	NM_133183:-1008	Cacng6	PROMOTER	0.719	0.417	1876.62	781.94	0.300	1433.18	429.50
A_68_P31929459	chr19:5560924-5560968	NM_019935:-371	Ovol1	DIVERGENT_PROMOTER	0.719	2.115	1555.39	3289.84	1.521	1249.48	1901.07
A_68_P31694858	chr18:49992481-49992525	NM_001081371:-164	Dmxl1	PROMOTER	0.719	4.582	363.53	1665.78	3.293	335.71	1105.44
A_68_P31097237	chr17:24563905-24563949	NM_010023:299	Eci1	INSIDE	0.719	0.540	1257.26	678.57	0.388	1082.21	419.67

ProbeName	Target position of probe on CpG island microarray	TargetID	GeneSymbol	CpG island Description	Ratio of relative methylation (TiO_2-NP/Vehicle)	Sham group			TiO_2-H group		
						Relative methylation (Cy5/Cy3)	Cy3 signal (Input)	Cy5 signal (MeDIP)	Relative methylation (Cy5/Cy3)	Cy3 signal (Input)	Cy5 signal (MeDIP)
A_68_P29450508	chr14:21871849-21871893	NM_134079:16	Adk	INSIDE	0.719	0.705	2249.61	1586.99	0.507	1811.46	918.95
A_68_P28522736	chr12:71926457-71926501	NM_028127:-22	Frmd6	PROMOTER	0.719	0.706	2809.24	1982.41	0.507	2340.16	1186.66
A_68_P28356773	chr12:36883113-36883157	NM_025840:278	Bzw2	INSIDE	0.719	0.609	1141.70	695.67	0.438	946.70	414.72
A_68_P28187037	chr11:120686073-120686117	NM_007988:-233	Fasn	PROMOTER	0.719	0.455	3164.86	1440.58	0.327	2384.29	780.62
A_68_P28145843	chr11:113928475-113928519	NM_172800:-1231	Sdk2	PROMOTER	0.719	2.742	996.27	2732.08	1.971	872.24	1718.86
A_68_P26821060	chr9:109834141-109834185	NM_001205331:-115	Mtap4	PROMOTER	0.719	0.703	1228.21	862.97	0.505	1030.64	520.35
A_68_P26699343	chr9:86361138-86361182	NM_177208:400	Dopey1	INSIDE	0.719	2.779	635.67	1766.39	1.998	498.57	996.14
A_68_P26693194	chr9:85220859-85220903	NM_001160379:-123	Fam46a	PROMOTER	0.719	3.100	1211.84	3756.77	2.230	1043.37	2326.61
A_68_P25016220	chr7:28013751-28013795	NM_181593:-156	Itpkc	DIVERGENT_PROMOTER	0.719	0.307	2053.43	630.39	0.221	1482.68	327.11
A_68_P24336442	chr6:31170408-31170452	ENSMUST00000153057:-4		PROMOTER	0.719	0.622	1019.43	634.35	0.448	867.17	388.20
A_68_P24009176	chr5:115798923-115798967	NM_007748:20	Cox6a1	INSIDE	0.719	5.650	3377.16	19082.13	4.063	2551.89	10367.94
A_68_P23575086	chr5:31171940-31171984	NM_144525:-57	Tmem214	PROMOTER	0.719	4.170	1944.87	8109.36	2.999	1753.85	5260.47
A_68_P23394435	chr4:147822699-147822743	NM_020009:30	Mtor	INSIDE	0.719	7.086	2469.24	17497.70	5.092	2232.92	11369.07
A_68_P23145558	chr4:99324020-99324064	NM_010425:1053	Foxd3	INSIDE	0.719	0.652	868.52	566.51	0.469	717.01	336.30
A_68_P22142066	chr3:53282945-53282989	NM_173382:15228	2810046L04Rik	INSIDE	0.719	3.090	2311.96	7143.44	2.220	1766.15	3921.55
A_68_P21041599	chr2:18722024-18722068	AK018938:-386		PROMOTER	0.719	3.284	1204.69	3955.86	2.361	1004.82	2372.56
A_68_P20886544	chr1:184339098-184339142	NM_173378:-177	Trp53bp2	PROMOTER	0.719	2.031	1622.02	3294.03	1.460	1389.21	2027.74
A_68_P20636517	chr1:136859020-136859067	NM_026024:-110	Ube2t	DIVERGENT_PROMOTER	0.719	0.297	2029.41	602.39	0.213	1523.76	325.21
A_68_P20578315	chr1:125942392-125942436	NM_199021:-278	Dpp10	PROMOTER	0.719	0.221	3169.88	701.11	0.159	2379.28	378.44
A_68_P32385500	chrX:50410153-50410197	NR_029756:-2648	Mir322	PROMOTER	0.718	5.776	5705.80	32957.63	4.150	6592.68	27357.11
A_68_P32141594	chr19:46078185-46078229	NM_176785:239	Hps6	INSIDE	0.718	0.362	2676.33	969.52	0.260	2124.63	552.98
A_68_P31847802	chr18:77680387-77680431	NM_172834:159712	Loxhd1	INSIDE	0.718	4.567	703.57	3212.98	3.278	533.75	1749.37
A_68_P31256668	chr17:56650952-56650996	NM_011483:932	Znrf4	INSIDE	0.718	0.566	1282.38	725.42	0.406	924.91	375.54
A_68_P30084545	chr15:25914824-25914868	NM_144523:726	Zfp622	INSIDE	0.718	2.140	1476.50	3159.78	1.536	1060.00	1628.02
A_68_P29787829	chr14:87541024-87541070	NM_019670:-125	Diap3	PROMOTER	0.718	2.185	5234.16	11435.91	1.569	3886.21	6096.57
A_68_P29341546	chr13:114408820-114408864	NM_130796:-70	Snx18	PROMOTER	0.718	3.213	484.11	1555.43	2.306	428.81	988.69
A_68_P28118150	chr11:109224474-109224518	NM_010303:389	Gna13	INSIDE	0.718	0.626	2673.02	1673.59	0.450	1819.26	817.83
A_68_P28069833	chr11:100599405-100599449	NM_024456:18	Rab5c	INSIDE	0.718	0.422	4802.15	2027.81	0.303	3397.31	1029.85
A_68_P27638907	chr11:21901433-21901477	NM_011023:200	Otx1	INSIDE	0.718	0.293	1810.43	529.71	0.210	1418.80	297.96
A_68_P27532961	chr10:126365502-126365546			Unknown	0.718	0.725	2248.95	1629.72	0.520	2050.63	1066.48
A_68_P26531905	chr9:55394153-55394197	NM_027397:5219	Isl2	DOWNSTREAM	0.718	0.313	2184.39	684.63	0.225	1798.36	404.88
A_68_P26253146	chr8:128193938-128193982	NM_028908:243	4933403G14Rik	INSIDE	0.718	3.451	443.48	1530.57	2.478	415.91	1030.56
A_68_P25587939	chr7:148477922-148477966	NM_007878:40	Drd4	INSIDE	0.718	0.283	2121.06	600.37	0.203	1592.11	323.43
A_68_P25581950	chr7:147234174-147234218	NM_010836:791	Msx3	INSIDE	0.718	3.548	1347.61	4781.56	2.547	1081.29	2754.32
A_68_P25092468	chr7:52588927-52588971	NM_175130:202	Trpm4	INSIDE	0.718	0.349	2784.65	971.24	0.251	2057.89	515.65
A_68_P24783936	chr6:117857383-117857427	NM_001166428:-417	Hnrnpf	PROMOTER	0.718	0.145	8678.98	1259.06	0.104	5709.45	594.92
A_68_P24175503	chr5:148537797-148537841	NM_010228:-254	Flt1	PROMOTER	0.718	0.255	2528.78	644.62	0.183	2074.29	379.90
A_68_P24046733	chr5:122278943-122278987	NM_008507:7846	Sh2b3	INSIDE	0.718	4.372	1604.06	7012.52	3.137	1301.80	4083.66
A_68_P23611139	chr5:37419843-37419887	ENSMUST00000165557:13021		DOWNSTREAM	0.718	0.663	3284.79	2178.98	0.476	2409.62	1147.16
A_68_P23225794	chr4:115457937-115457981	NM_181040:369	Atpaf1	INSIDE	0.718	0.535	2753.60	1473.08	0.384	2222.10	853.18
A_68_P21623006	chr2:128926947-128926991	NM_009086:237	Polr1b	INSIDE	0.718	2.272	1408.48	3199.70	1.630	1171.69	1910.01
A_68_P31829416	chr18:74225103-74225147	NM_172632:-521	Mapk4	PROMOTER	0.717	0.310	1594.20	493.73	0.222	1211.64	269.02
A_68_P30650226	chr16:31948463-31948507	NR_027897:123	0610012G03Rik	INSIDE	0.717	2.431	902.52	2194.42	1.745	894.93	1561.26
A_68_P30502657	chr15:103013103-103013147	NM_001110216:32659	Cbx5	DOWNSTREAM	0.717	0.480	1374.60	660.17	0.344	1164.69	400.91
A_68_P28515343	chr12:70683664-70683708	NM_001033236:329	Mettl21d	INSIDE	0.717	1.991	1981.82	3944.96	1.428	1673.85	2390.33
A_68_P28068585	chr11:100389285-100389329	NM_001199296:8	Acly	INSIDE	0.717	0.286	1921.84	549.87	0.205	1483.60	304.31
A_68_P28057823	chr11:98544328-98544372	NM_009439:483	Psmd3	INSIDE	0.717	0.322	1780.58	573.28	0.231	1347.01	311.03
A_68_P28055838	chr11:98210489-98210533	NM_144828:459	Ppp1r1b	INSIDE	0.717	0.660	3856.10	2543.57	0.473	2904.68	1373.60
A_68_P27921007	chr11:74336152-74336196	NM_001015046:67486	Rap1gap2	INSIDE	0.717	5.376	914.77	4917.50	3.852	796.33	3067.83
A_68_P26749717	chr9:96789706-96789750	NM_153420:113	Acpl2	INSIDE	0.717	0.607	3282.30	1992.30	0.435	2335.94	1016.27
A_68_P25988753	chr8:80961280-80961324	NM_138944:-751	Pou4f2	PROMOTER	0.717	3.575	2609.08	9326.69	2.561	1801.03	4613.22
A_68_P25962574	chr8:75247287-75247331	NM_001113248:122	Sin3b	INSIDE	0.717	0.207	3445.73	713.59	0.148	2468.81	366.39
A_68_P25701518	chr8:18950728-18950772	NM_001113350:223	Xkr5	INSIDE	0.717	0.432	2217.48	958.94	0.310	1785.50	553.84
A_68_P25369179	chr7:107855638-107855683	NM_178764:445	Fam168a	INSIDE	0.717	6.163	2085.30	12850.93	4.416	1093.74	4830.19
A_68_P25230684	chr7:80885602-80885646	NM_001162532:432	Fam174b	INSIDE	0.717	0.540	2208.13	1192.91	0.387	1553.26	601.75
A_68_P25025803	chr7:29965431-29965475	NM_026545:240	Psmd8	INSIDE	0.717	2.675	956.20	2557.83	1.917	727.47	1394.52
A_68_P24817442	chr6:124813681-124813725	NM_013533:233	Gpr162	INSIDE	0.717	0.305	3047.49	928.04	0.218	2633.15	574.66
A_68_P24776190	chr6:116288445-116288489	NM_027920:326	Marchf8	INSIDE	0.717	0.496	1816.15	901.66	0.356	1447.75	515.21
A_68_P24428428	chr6:48579466-48579510	NR_027236:1323	AI854703	INSIDE	0.717	3.744	752.56	2817.78	2.685	642.84	1726.14
A_68_P24039146	chr5:120961225-120961269	NM_001177594:46	Slc24a6	INSIDE	0.717	0.201	3517.31	707.42	0.144	2707.02	390.31
A_68_P23990797	chr5:112657508-112657552	NR_003718:438	Miat	INSIDE	0.717	0.604	3252.66	1965.29	0.434	2375.35	1029.72
A_68_P22859203	chr4:40801292-40801336	NM_022305:-283	B4galt1	PROMOTER	0.717	0.305	2563.04	781.66	0.219	1962.95	429.51
A_68_P21903071	chr2:180069548-180069594	NM_008093:-186	Gata5	PROMOTER	0.717	2.573	1387.96	3571.40	1.844	1081.85	1994.93
A_68_P20451523	chr1:94745430-94745474	NR_029559:-1502	Mir149	PROMOTER	0.717	1.935	2378.45	4601.99	1.387	1876.26	2602.36
A_68_P33008888	chr12:21117412-21117456	NM_001004364:-182	Asap2	PROMOTER	0.716	0.374	2556.86	956.38	0.268	2019.60	541.24

ProbeName	Target position of probe on CpG island microarray	TargetID	GeneSymbol	CpG island Description	Ratio of relative methylation (TiO_2-NP/Vehicle)	Sham group			TiO_2-H group		
						Relative methylation (Cy5/Cy3)	Cy3 signal (Input)	Cy5 signal (MeDIP)	Relative methylation (Cy5/Cy3)	Cy3 signal (Input)	Cy5 signal (MeDIP)
A_68_P32467861	chrX:71638358-71638402	NM_025473:98	Fam3a	INSIDE	0.716	6.867	1855.72	12742.75	4.915	1970.93	9686.80
A_68_P32134817	chr19:44911075-44911119	NM_011037:79213	Pax2	INSIDE	0.716	0.260	3147.35	819.85	0.187	2164.70	403.86
A_68_P32117790	chr19:41970534-41970578	NM_199447:87	Rrp12	INSIDE	0.716	0.267	5182.73	1381.35	0.191	3138.89	599.34
A_68_P31347827	chr17:74059382-74059426	NM_027864:387	Galnt14	INSIDE	0.716	4.713	1154.71	5442.04	3.375	1027.65	3468.49
A_68_P31333824	chr17:71660558-71660602	NM_145158:-275	Emilin2	PROMOTER	0.716	0.559	3021.91	1690.17	0.400	2583.09	1034.22
A_68_P31208333	chr17:46818209-46818253	NM_010787:145	Mea1	INSIDE	0.716	0.556	1006.14	559.32	0.398	743.21	295.79
A_68_P31152609	chr17:34324546-34324590	NM_001161730:68	Tap1	INSIDE	0.716	0.284	2685.08	762.04	0.203	1999.12	406.10
A_68_P30474024	chr15:98126375-98126419	NM_173769:118	Zfp641	INSIDE	0.716	0.729	4681.42	3415.03	0.522	3400.40	1776.35
A_68_P30221114	chr15:53176756-53176800	NM_010162:960	Ext1	INSIDE	0.716	0.218	4203.70	915.63	0.156	2991.06	466.19
A_68_P29977212	chr15:3976767-3976811	NM_024188:361	Oxct1	INSIDE	0.716	0.626	1008.98	631.61	0.448	756.04	338.92
A_68_P29071678	chr13:58230674-58230718	NM_029872:-779	Hnrnpa0	PROMOTER	0.716	0.527	1900.59	1001.70	0.378	1347.92	508.84
A_68_P28771927	chr12:118392576-118392621	NR_033538:-2856	Gm10421	PROMOTER	0.716	7.974	600.99	4792.56	5.712	592.51	3384.18
A_68_P28153230	chr11:115255314-115255358	NM_001080929:12107	Cdr2l	INSIDE	0.716	2.137	1311.49	2803.16	1.529	1158.27	1771.36
A_68_P28003591	chr11:88921871-88921915	NM_019505:170	Dgke	INSIDE	0.716	5.009	974.43	4881.07	3.587	896.22	3215.07
A_68_P27998473	chr11:88017796-88017840	NM_024174:-101	Mrps23	PROMOTER	0.716	0.586	1512.87	886.70	0.420	1142.55	479.78
A_68_P27942192	chr11:78059616-78059660	NM_009143:391	Sdf2	INSIDE	0.716	0.416	1898.83	790.14	0.298	1429.55	425.83
A_68_P27093479	chr10:41916731-41916775	NM_019740:79796	Foxo3	INSIDE	0.716	2.885	516.14	1488.88	2.066	444.97	919.44
A_68_P26871809	chr9:119248880-119248924	NM_010851:256	Myd88	INSIDE	0.716	0.315	2379.52	750.59	0.226	1707.33	385.39
A_68_P26042566	chr8:91220734-91220778	NM_001128170:-170	Cyld	PROMOTER	0.716	0.392	2343.22	918.05	0.280	1613.41	452.44
A_68_P25951333	chr8:72877019-72877063	NM_001122829:132	Upf1	INSIDE	0.716	3.423	685.63	2347.03	2.451	608.94	1492.37
A_68_P25509657	chr7:135381773-135381817	NM_177001:-249	9130023H24Rik	PROMOTER	0.716	0.596	3042.26	1813.72	0.427	2208.16	942.82
A_68_P25007466	chr7:25729137-25729181			Unknown	0.716	0.598	2094.89	1252.84	0.428	1240.06	531.06
A_68_P24939590	chr6:148303285-148303329	NR_003634:129	Rps4y2	INSIDE	0.716	2.274	1731.31	3937.80	1.628	1394.71	2269.90
A_68_P24835544	chr6:127837937-127837981	NM_026743:319	Tspan11	INSIDE	0.716	0.177	3387.90	599.14	0.127	2255.42	285.45
A_68_P23332899	chr4:135804076-135804120	NM_011542:227	Tcea3	INSIDE	0.716	2.898	1446.27	4191.41	2.074	1288.96	2673.01
A_68_P21746954	chr2:151805625-151805669	NM_028666:-5936	Fam110a	PROMOTER	0.716	0.264	2023.88	533.67	0.189	1490.69	281.43
A_68_P21710713	chr2:144994120-144994164	ENSMUST00000153249:488		INSIDE	0.716	0.606	977.18	591.88	0.434	721.41	312.93
A_68_P21330589	chr2:73118929-73118979	NM_025854:31695	Cir1	DOWNSTREAM	0.716	0.221	2364.64	522.30	0.158	1605.60	253.97
A_68_P20354370	chr1:75479524-75479568	NM_177056:3440	Tmem198	INSIDE	0.716	2.324	1340.35	3115.56	1.665	924.10	1538.31
A_68_P31930681	chr19:5758063-5758107	NM_008520:17181	Ltbp3	INSIDE	0.715	5.170	906.88	4688.40	3.695	712.81	2634.10
A_68_P31774689	chr18:64500435-64500479	NM_194268:439	Onecut2	INSIDE	0.715	0.589	3266.33	1924.01	0.421	2413.34	1017.04
A_68_P31759409	chr18:61885951-61885995	NM_021296:13	Grpel2	INSIDE	0.715	0.436	3740.87	1630.37	0.312	2735.83	852.37
A_68_P31331151	chr17:71202362-71202406	NM_001164075:-1310	Tgif1	PROMOTER	0.715	2.660	1704.96	4535.25	1.902	1320.46	2511.03
A_68_P30146826	chr15:37890566-37890610	NM_199476:222	Rrm2b	INSIDE	0.715	0.187	2343.73	438.49	0.134	1758.75	235.15
A_68_P27579022	chr11:8524378-8524422	NM_001083587:40138	Tns3	INSIDE	0.715	1.986	2463.59	4892.44	1.419	1952.16	2770.82
A_68_P27279427	chr10:79337379-79337423	NM_001170935:-21	BC005764	DIVERGENT_PROMOTER	0.715	0.514	3892.01	1999.73	0.367	2705.45	994.06
A_68_P26581618	chr9:64234628-64234672	NM_172522:1218	Megf11	INSIDE	0.715	0.360	1651.88	593.95	0.257	1252.40	322.08
A_68_P26569961	chr9:62190386-62190430	NM_009672:1259	Anp32a	INSIDE	0.715	3.166	3266.14	10340.29	2.263	2575.14	5827.92
A_68_P26525029	chr9:54134177-54134221	NM_177350:-94	Gldn	PROMOTER	0.715	0.435	1705.13	741.57	0.311	1183.27	368.16
A_68_P24783633	chr6:117791390-117791434	NM_177684:153	Zfp637	INSIDE	0.715	0.645	1247.84	805.13	0.462	916.30	422.88
A_68_P24593651	chr6:83407070-83407114	NR_028382:15344	B230319C09Rik	DOWNSTREAM	0.715	2.285	1842.26	4210.10	1.635	1511.69	2471.18
A_68_P24381545	chr6:39068163-39068207	NM_172893:164	Parp12	INSIDE	0.715	3.441	411.42	1415.84	2.459	364.83	897.28
A_68_P24134134	chr5:139677035-139677079	NM_024451:433	Sun1	INSIDE	0.715	8.158	2535.17	20682.67	5.830	1900.48	11078.87
A_68_P24078665	chr5:128097485-128097529	NM_133895:256	Slc15a4	INSIDE	0.715	0.700	1160.11	812.20	0.501	951.81	476.55
A_68_P23819149	chr5:77403640-77403684	NM_025691:-63	Srp72	PROMOTER	0.715	0.501	1686.29	845.20	0.358	1318.58	472.44
A_68_P23368579	chr4:141795272-141795316	NM_001109685:22	9030409G11Rik	INSIDE	0.715	0.454	1299.37	590.01	0.325	964.34	313.01
A_68_P23364006	chr4:140981644-140981688	NM_013868:4973	Hspb7	DOWNSTREAM	0.715	0.604	3604.39	2175.65	0.432	2521.03	1088.14
A_68_P22882284	chr4:45333378-45333422	NM_025513:75	Exosc3	INSIDE	0.715	0.629	1470.48	925.10	0.450	1203.03	541.24
A_68_P22825644	chr4:33276387-33276431	NM_001034867:304	Pm20d2	INSIDE	0.715	4.045	814.01	3292.70	2.891	726.32	2100.13
A_68_P21665420	chr2:136716618-136716662	NM_028201:-313	2210009G21Rik	PROMOTER	0.715	0.229	2520.88	578.28	0.164	1857.09	304.80
A_68_P21077008	chr2:26058081-26058125	NM_001039653:3974	Lhx3	INSIDE	0.715	3.004	1526.99	4586.48	2.148	1275.97	2740.22
A_68_P20456737	chr1:95531806-95531850	NM_021537:476	Stk25	INSIDE	0.715	0.586	2436.63	1428.43	0.419	1694.93	710.51
A_68_P31625687	chr18:36919881-36919925	NM_026464:10	Wdr55	INSIDE	0.714	4.257	1806.14	7689.48	3.042	1404.43	4271.77
A_68_P31152724	chr17:34341633-34341677	NM_011530:231	Tap2	INSIDE	0.714	0.451	4071.80	1836.68	0.322	3202.18	1031.79
A_68_P31096075	chr17:24345873-24345917	NM_001163847:-3387	Tbc1d24	PROMOTER	0.714	2.955	1802.14	5326.12	2.110	1625.33	3429.46
A_68_P30369300	chr15:79763102-79763146	NM_144811:-48	Cbx7	PROMOTER	0.714	2.062	2025.69	4176.77	1.473	1639.50	2415.13
A_68_P28356776	chr12:36883470-36883514	NM_025840:-80	Bzw2	DIVERGENT_PROMOTER	0.714	0.481	3453.29	1660.52	0.343	2716.11	932.13
A_68_P28050435	chr11:97277300-97277344	ENSMUST00000121799:476		INSIDE	0.714	2.214	1318.34	2919.05	1.580	1113.84	1760.23
A_68_P28047198	chr11:96723388-96723432	NM_019877:12221	Copz2	DOWNSTREAM	0.714	0.249	2279.17	567.08	0.178	1695.44	301.16
A_68_P27594244	chr11:11925917-11925961	NM_001177629:2036	Grb10	INSIDE	0.714	6.084	2053.01	12490.43	4.343	1547.05	6718.92
A_68_P27103323	chr10:43723779-43723823	NM_172393:852	Aim1	INSIDE	0.714	2.347	1341.24	3147.36	1.675	1083.42	1814.76
A_68_P26351649	chr9:22193381-22193425	NM_028390:248	Anln	INSIDE	0.714	0.686	2548.87	1748.88	0.490	1875.82	918.98
A_68_P26214833	chr8:122099237-122099281	NM_026648:124	Lrrc50	INSIDE	0.714	0.401	2431.25	974.18	0.286	1942.62	555.65
A_68_P24979352	chr7:16638192-16638236	AK008102:4421		DOWNSTREAM	0.714	2.123	1477.82	3136.79	1.516	1233.75	1869.77
A_68_P24205541	chr6:4551256-4551300	NM_145398:213	Casd1	INSIDE	0.714	8.405	1023.29	8600.96	6.000	928.92	5573.14

ProbeName	Target position of probe on CpG island microarray	TargetID	GeneSymbol	CpG island Description	Ratio of relative methylation (TiO_2-NP/Vehicle)	Sham group			TiO_2-H group		
						Relative methylation (Cy5/Cy3)	Cy3 signal (Input)	Cy5 signal (MeDIP)	Relative methylation (Cy5/Cy3)	Cy3 signal (Input)	Cy5 signal (MeDIP)
A_68_P23527832	chr5:21290509-21290553	NM_009584:453	Dnajc2	INSIDE	0.714	0.476	1978.32	941.35	0.340	1576.14	535.71
A_68_P23417715	chr4:151657468-151657512	NM_178406:9020	Gpr153	INSIDE	0.714	3.665	1029.08	3771.56	2.617	869.98	2276.85
A_68_P23342688	chr4:137469440-137469484	NM_199307:51311	Ece1	INSIDE	0.714	0.364	1761.77	640.69	0.260	1427.15	370.65
A_68_P23322944	chr4:134024746-134024790	NM_019641:534	Stmn1	INSIDE	0.714	0.501	2371.59	1187.22	0.358	1809.81	647.27
A_68_P23230625	chr4:116393777-116393821	NM_146151:239	Tesk2	INSIDE	0.714	2.480	996.94	2472.61	1.772	852.87	1511.00
A_68_P23154636	chr4:101028607-101028651	NR_029537:-8996	Mir101a	PROMOTER	0.714	0.141	3409.13	479.23	0.100	2275.33	228.27
A_68_P22951805	chr4:58566279-58566323	NM_172989:-135	Lpar1	PROMOTER	0.714	0.547	1712.93	936.84	0.390	1317.06	514.04
A_68_P22871967	chr4:43505695-43505739	NR_001460:215	Rmrp	INSIDE	0.714	0.618	902.24	557.85	0.441	738.59	326.07
A_68_P22530174	chr3:130815152-130815196	NM_010703:1786	Lef1	INSIDE	0.714	2.930	1935.14	5670.58	2.093	1519.57	3180.19
A_68_P22503477	chr3:126299555-126299599	NM_001025438:-314	Camk2d	PROMOTER	0.714	0.600	1835.11	1100.82	0.428	1518.65	650.28
A_68_P22405344	chr3:107082299-107082343	NM_026774:545	Hbxip	INSIDE	0.714	0.397	1646.98	653.57	0.284	1324.84	375.64
A_68_P22152361	chr3:55046791-55046835	NM_001111053:365	Dclk1	INSIDE	0.714	0.652	1327.35	865.03	0.465	1145.30	532.62
A_68_P22058286	chr3:36374837-36374881	NM_028183:0	1810062G17Rik	INSIDE	0.714	0.669	2329.72	1559.66	0.478	1747.61	835.39
A_68_P21571370	chr2:119584649-119584693	NM_008523:-409	Ltk	PROMOTER	0.714	0.557	1053.57	586.50	0.397	867.20	344.67
A_68_P21339294	chr2:74585119-74585163	NM_010468:35091	Hoxd3	INSIDE	0.714	0.406	1684.50	683.78	0.290	1227.57	356.01
A_68_P20506811	chr1:108069599-108069643	NM_133821:1175	Phlpp1	INSIDE	0.714	0.358	1555.57	557.37	0.256	1289.36	329.76
A_68_P32139051	chr19:45619562-45619606	NM_020032:15449	Poll	DOWNSTREAM	0.713	0.419	1343.03	562.49	0.299	1083.83	323.53
A_68_P31602497	chr18:32537058-32537102	NM_001083334:210	Bin1	INSIDE	0.713	3.244	2423.20	7860.84	2.313	2135.03	4938.99
A_68_P30486488	chr15:100408907-100408951	NM_010127:7868	Pou6f1	INSIDE	0.713	6.424	631.18	4054.91	4.579	513.85	2352.76
A_68_P30380063	chr15:81688370-81688414	NM_020507:364	Tob2	INSIDE	0.713	0.489	2286.82	1117.62	0.349	1862.86	649.25
A_68_P29506489	chr14:31362538-31362582	NM_009388:226	Tkt	INSIDE	0.713	5.710	1555.97	8884.62	4.071	1245.17	5068.54
A_68_P28974163	chr13:40823956-40824000	NM_001122948:1834	Tcfap2a	INSIDE	0.713	0.578	3899.46	2255.65	0.412	2673.70	1102.75
A_68_P28153161	chr11:115243333-115243378	NM_001080929:126	Cdr2l	INSIDE	0.713	0.719	1562.76	1124.36	0.513	1450.72	744.57
A_68_P27891995	chr11:68714810-68714854	NM_009080:-235	Rpl26	PROMOTER	0.713	0.419	3560.55	1490.55	0.299	2674.20	798.75
A_68_P27645072	chr11:23206518-23206562	NM_001190401:-354	Usp34	PROMOTER	0.713	0.534	2243.39	1197.61	0.381	1863.59	709.12
A_68_P26529575	chr9:54997611-54997655	NM_180600:457	Ube2q2	INSIDE	0.713	2.708	589.18	1595.61	1.932	489.38	945.57
A_68_P26528579	chr9:54798597-54798641	NM_011966:-47	Psma4	PROMOTER	0.713	0.719	1306.83	939.60	0.513	1131.70	580.13
A_68_P25960691	chr8:74843873-74843917	NM_008452:934	Klf2	INSIDE	0.713	3.092	731.67	2262.29	2.205	625.69	1379.52
A_68_P25951451	chr8:72900205-72900249	NM_016685:2780	Comp	INSIDE	0.713	0.425	1345.40	571.18	0.303	975.20	295.07
A_68_P25301854	chr7:95426469-95426513	NM_009982:-112	Ctsc	PROMOTER	0.713	0.735	4407.44	3239.59	0.524	2985.25	1563.75
A_68_P24760449	chr6:113554951-113554995	NM_133937:207	6720456B07Rik	INSIDE	0.713	0.599	2149.03	1286.81	0.427	1874.51	800.65
A_68_P23976347	chr5:109986035-109986079	NM_001164735:1956	Crlf2	INSIDE	0.713	9.840	5873.91	57797.42	7.014	4680.29	32828.65
A_68_P23354400	chr4:139377667-139377712	NM_011039:11194	Pax7	INSIDE	0.713	0.614	2703.64	1660.95	0.438	2089.07	915.53
A_68_P22878645	chr4:44716965-44717009	NM_008782:6326	Pax5	INSIDE	0.713	2.055	1555.18	3195.30	1.466	1251.81	1834.63
A_68_P22142064	chr3:53282739-53282783	NM_173382:15022	2810046L04Rik	INSIDE	0.713	3.916	4891.05	19155.68	2.793	3436.76	9598.28
A_68_P21517948	chr2:109757926-109757970	NM_172671:145	Lgr4	INSIDE	0.713	2.838	1671.47	4743.94	2.024	1393.51	2819.82
A_68_P21113207	chr2:31938899-31938943	NM_175511:305	Fam78a	INSIDE	0.713	0.223	3715.54	827.06	0.159	2681.68	425.38
A_68_P21073100	chr2:25434255-25434299	NM_001005424:1343	Gm996	INSIDE	0.713	0.497	950.93	472.65	0.355	747.20	264.89
A_68_P21037916	chr2:17961721-17961765	NR_028376:-2374	A930004D18Rik	PROMOTER	0.713	0.612	1428.08	874.46	0.436	1149.53	501.70
A_68_P20354368	chr1:75479273-75479317	NM_177056:3188	Tmem198	INSIDE	0.713	0.498	2349.02	1168.84	0.355	1782.99	632.86
A_68_P20153135	chr1:37357054-37357098	NM_030266:339	Inpp4a	INSIDE	0.713	0.707	2297.76	1624.60	0.504	1867.84	941.64
A_68_P20149940	chr1:36766481-36766525	NM_146107:268	Actr1b	INSIDE	0.713	0.178	2697.35	480.31	0.127	2001.56	254.29
A_68_P31978250	chr19:16207196-16207240	NM_008139:-102	Gnaq	PROMOTER	0.712	2.513	1708.27	4292.15	1.788	1326.26	2371.72
A_68_P31731121	chr18:56732470-56732514	NM_019996:-32	Phax	PROMOTER	0.712	0.463	2278.39	1054.19	0.329	1823.50	600.41
A_68_P31258177	chr17:56896595-56896639	NM_008738:337	Nrtn	INSIDE	0.712	0.603	3376.14	2036.74	0.430	2526.11	1085.67
A_68_P31209382	chr17:47026332-47026376	NM_178385:-1215	Tbcc	PROMOTER	0.712	6.678	1146.57	7657.19	4.758	923.42	4393.83
A_68_P30981934	chr16:94748127-94748171	NM_007834:88	Dscr3	INSIDE	0.712	2.815	1213.93	3416.86	2.003	1035.47	2073.76
A_68_P30485945	chr15:100299105-100299149	NM_134093:-338	Letmd1	PROMOTER	0.712	0.695	1533.37	1065.15	0.495	1275.08	631.07
A_68_P30401623	chr15:85411668-85411712	NM_001163633:-531	Wnt7b	PROMOTER	0.712	0.615	1553.34	954.57	0.438	1104.81	483.56
A_68_P29259682	chr13:99585022-99585066	NM_172591:360	Fcho2	INSIDE	0.712	0.252	3307.03	834.24	0.180	2148.27	386.05
A_68_P29116249	chr13:69672357-69672401	NM_001169131:364	Papd7	INSIDE	0.712	2.844	628.70	1788.00	2.024	499.88	1011.70
A_68_P29071675	chr13:58230317-58230361	NM_029872:-421	Hnrnpa0	PROMOTER	0.712	0.693	1979.21	1372.10	0.493	1508.36	744.25
A_68_P29011801	chr13:47218477-47218521	NM_146042:410	Rnf144b	INSIDE	0.712	2.880	1778.90	5122.95	2.051	1485.24	3046.02
A_68_P28736904	chr12:111425727-111425771	AK044800:-37		DIVERGENT_PROMOTER	0.712	3.763	1392.01	5238.74	2.680	1198.82	3213.17
A_68_P28461536	chr12:58331561-58331605	NM_001164370:171	Mipol1	INSIDE	0.712	0.542	1814.00	982.75	0.386	1558.20	601.00
A_68_P28084677	chr11:103195536-103195580	NM_133715:10458	Arhgap27	INSIDE	0.712	0.586	3964.45	2323.18	0.417	2853.20	1190.57
A_68_P27914869	chr11:72861707-72861751	NM_025818:360	1200014J11Rik	INSIDE	0.712	0.643	5947.57	3826.86	0.458	4690.59	2150.05
A_68_P27846980	chr11:60988451-60988495	NM_001004143:89	Usp22	INSIDE	0.712	0.164	2860.43	469.25	0.117	1953.80	228.19
A_68_P27792932	chr11:51076708-51076752	NM_007714:58	Clk4	INSIDE	0.712	0.377	2285.85	861.73	0.269	1503.05	403.72
A_68_P27289569	chr10:80883025-80883071	NM_008688:6870	Nfic	INSIDE	0.712	2.664	3199.83	8524.59	1.896	2446.62	4637.75
A_68_P27288853	chr10:80783632-80783676	NM_027381:-193	2510012J08Rik	PROMOTER	0.712	2.758	1294.56	3571.02	1.964	941.09	1848.30
A_68_P26823381	chr9:110283194-110283238	NM_001001144:47420	Scap	INSIDE	0.712	2.274	2403.23	5464.58	1.620	1781.19	2884.64
A_68_P26397763	chr9:30750289-30750333	NM_013906:163	Adamts8	INSIDE	0.712	0.570	1092.70	623.32	0.406	764.37	310.61
A_68_P26021886	chr8:87308677-87308721	NM_001081982:15541	Nfix	INSIDE	0.712	5.532	3045.59	16847.04	3.936	2402.12	9455.87
A_68_P25504790	chr7:134397531-134397575	NM_177362:9513	Zfp771	INSIDE	0.712	2.726	1642.05	4475.75	1.940	1213.71	2354.13

ProbeName	Target position of probe on CpG island microarray	TargetID	GeneSymbol	CpG island Description	Ratio of relative methylation (TiO_2-NP/Vehicle)	Sham group			TiO_2-H group		
						Relative methylation (Cy5/Cy3)	Cy3 signal (Input)	Cy5 signal (MeDIP)	Relative methylation (Cy5/Cy3)	Cy3 signal (Input)	Cy5 signal (MeDIP)
A_68_P25500912	chr7:133647134-133647178	NM_183020:-340	Atxn2l	PROMOTER	0.712	0.627	874.18	548.21	0.446	763.29	340.58
A_68_P25016988	chr7:28157101-28157145	NM_001199235:22403	Spnb4	INSIDE	0.712	0.556	898.54	500.01	0.396	684.96	271.35
A_68_P24946871	chr7:3646125-3646169	NM_024168:37	Tsen34	INSIDE	0.712	2.595	1070.38	2777.63	1.847	889.38	1642.68
A_68_P24098745	chr5:132014699-132014743	NM_177047:1003493	Auts2	INSIDE	0.712	0.427	1972.68	841.93	0.304	1571.96	477.76
A_68_P24048238	chr5:122499986-122500030	NM_007804:-2174	Cux2	PROMOTER	0.712	4.862	726.91	3534.48	3.463	606.55	2100.47
A_68_P23333247	chr4:135867486-135867530	NM_028871:618	Hnrnpr	INSIDE	0.712	0.142	3526.37	502.08	0.101	2353.66	238.53
A_68_P23226467	chr4:115563355-115563399	NM_001039124:6372	Kncn	DOWNSTREAM	0.712	0.177	2633.82	465.02	0.126	1902.36	238.98
A_68_P23176985	chr4:104830723-104830767	NM_080555:793	Ppap2b	INSIDE	0.712	0.654	1188.84	777.12	0.465	950.50	442.40
A_68_P23018374	chr4:73436830-73436874	NM_001017427:-346	Rasef	PROMOTER	0.712	2.631	621.39	1635.10	1.873	560.83	1050.19
A_68_P22859652	chr4:40895697-40895741	NM_029814:133	Chmp5	INSIDE	0.712	0.422	3135.53	1324.11	0.301	2244.36	675.20
A_68_P22317588	chr3:88968981-88969026	NM_001163432:277	Clk2	INSIDE	0.712	2.619	2319.98	6076.35	1.865	1802.13	3360.59
A_68_P21977362	chr3:19795518-19795562	NR_027985:670	4632415L05Rik	INSIDE	0.712	0.506	2052.95	1038.85	0.360	1535.36	553.39
A_68_P20441216	chr1:93147363-93147407	NM_026454:489	Ube2f	INSIDE	0.712	0.683	1086.03	741.29	0.486	826.54	401.47
A_68_P20252736	chr1:57434804-57434848	NM_028546:363	1700066M21Rik	INSIDE	0.712	9.825	1555.71	15285.38	6.994	1325.46	9270.51
A_68_P32806534	chrX:162917700-162917744	NM_019773:77	Rab9	INSIDE	0.711	3.169	551.57	1747.79	2.252	720.49	1622.52
A_68_P32443538	chrX:66613886-66613930	NM_008032:403	Aff2	INSIDE	0.711	2.476	1263.96	3129.81	1.760	1764.43	3105.29
A_68_P31326351	chr17:70318541-70318585	ENSMUST00000060072:-23		PROMOTER	0.711	3.474	436.18	1515.17	2.471	370.32	915.04
A_68_P30686477	chr16:38522888-38522932	NM_024273:-163	4930455C21Rik	PROMOTER	0.711	0.271	1802.31	488.17	0.193	1312.62	252.77
A_68_P30497699	chr15:102236639-102236683	NM_013672:-86	Sp1	PROMOTER	0.711	4.050	1623.75	6575.78	2.880	1334.42	3843.43
A_68_P30374034	chr15:80541515-80541559	NM_144812:-206	Tnrc6b	PROMOTER	0.711	0.565	1018.45	575.78	0.402	763.08	306.85
A_68_P30370350	chr15:79963500-79963544	NM_025609:-61	Tab1	PROMOTER	0.711	3.074	1719.28	5285.38	2.185	1481.59	3237.34
A_68_P29501751	chr14:30535961-30536005	NM_009785:-932	Cacna2d3	PROMOTER	0.711	0.636	2007.85	1277.50	0.453	1677.87	759.39
A_68_P29501750	chr14:30535873-30535917	NM_009785:-844	Cacna2d3	PROMOTER	0.711	2.140	1201.49	2570.89	1.521	1020.13	1551.41
A_68_P29248566	chr13:97695211-97695255	NM_001166065:589	Gcnt4	INSIDE	0.711	4.697	554.32	2603.47	3.337	439.74	1467.48
A_68_P29080191	chr13:59658571-59658615	NM_023328:88	Agtpbp1	INSIDE	0.711	10.814	3916.98	42360.08	7.687	3405.85	26181.22
A_68_P27896330	chr11:69469233-69469277	NM_009235:380	Sox15	INSIDE	0.711	0.642	956.39	613.80	0.456	728.45	332.42
A_68_P27269206	chr10:77069466-77069510	NM_019929:510	Sumo3	INSIDE	0.711	0.615	906.98	557.82	0.438	787.21	344.45
A_68_P27234954	chr10:70811019-70811063	NM_027184:500	Ipmk	INSIDE	0.711	0.109	4650.88	505.34	0.077	3304.46	255.43
A_68_P27171225	chr10:59342512-59342556	NM_019965:196	Dnajb12	INSIDE	0.711	2.309	950.90	2195.74	1.642	810.90	1331.21
A_68_P27165510	chr10:58276430-58276477	NM_172788:347	Sh3rf3	INSIDE	0.711	0.288	2117.43	610.76	0.205	1468.48	301.18
A_68_P26814404	chr9:108249925-108249969	NM_011678:-215	Usp4	PROMOTER	0.711	0.467	1061.47	495.86	0.332	717.21	238.17
A_68_P26291034	chr9:8134431-8134475	NM_001045524:-158	AK129341	PROMOTER	0.711	2.852	790.64	2255.24	2.028	712.21	1444.50
A_68_P26021244	chr8:87225675-87225719	NM_008535:341	Lyl1	INSIDE	0.711	2.986	1368.89	4087.01	2.123	1166.50	2475.95
A_68_P25952317	chr8:73030680-73030724	NM_025577:-64	2810428I15Rik	PROMOTER	0.711	6.243	2281.55	14243.68	4.437	1785.80	7924.37
A_68_P25274807	chr7:89078453-89078497	NM_013886:871	Hdgfrp3	INSIDE	0.711	0.472	1550.88	731.99	0.336	1136.92	381.44
A_68_P24637120	chr6:91106803-91106847	NM_144919:16	Hdac11	INSIDE	0.711	2.189	1670.93	3657.45	1.557	1474.93	2296.14
A_68_P24541711	chr6:72389964-72390008	NM_145569:-434	Mat2a	PROMOTER	0.711	0.690	1411.43	974.00	0.491	1044.88	512.87
A_68_P24397784	chr6:42299888-42299932	NM_011777:84	Zyx	INSIDE	0.711	0.730	2339.47	1706.89	0.519	1973.14	1023.71
A_68_P24388783	chr6:40385658-40385702	NM_175528:452	E330009J07Rik	INSIDE	0.711	0.463	1559.76	722.73	0.329	1357.67	447.19
A_68_P24324980	chr6:29121309-29121353	NM_001101443:8254	Prrt4	INSIDE	0.711	0.285	2608.91	742.53	0.202	1981.80	401.07
A_68_P24209922	chr6:5248441-5248485	NM_183308:-89	Pon2	PROMOTER	0.711	0.488	3695.95	1805.14	0.347	2604.59	904.92
A_68_P24040029	chr5:121099253-121099297	NM_013832:444	Rasal1	INSIDE	0.711	0.250	1870.09	467.86	0.178	1347.90	239.74
A_68_P24009510	chr5:115880669-115880713	NM_008629:997	Msi1	INSIDE	0.711	0.421	3631.76	1527.41	0.299	2372.55	709.55
A_68_P23568207	chr5:29896110-29896154	NM_133907:351	Ube3c	INSIDE	0.711	4.114	1578.13	6491.75	2.924	1313.45	3840.24
A_68_P23498122	chr5:15441040-15441084	NM_001110843:554	Cacna2d1	INSIDE	0.711	4.568	571.19	2609.00	3.248	478.32	1553.45
A_68_P23320092	chr4:133466489-133466533	AK015234:-196		PROMOTER	0.711	0.234	3460.64	808.59	0.166	2245.67	372.91
A_68_P22367900	chr3:99966673-99966717	NM_010269:254	Gdap2	INSIDE	0.711	0.479	1280.28	612.63	0.340	1071.07	364.15
A_68_P21749134	chr2:152224754-152224798	NM_011438:-994	Sox12	PROMOTER	0.711	3.081	1368.17	4215.60	2.190	1145.48	2508.71
A_68_P21078764	chr2:26300667-26300711	NM_153125:48	Sec16a	INSIDE	0.711	4.450	2443.85	10874.00	3.164	2062.81	6527.44
A_68_P20641035	chr1:137584675-137584719	ENSMUST00000112103:-14		PROMOTER	0.711	3.480	480.82	1673.18	2.476	401.69	994.56
A_68_P20454695	chr1:95202518-95202562	NM_178051:-93	Mterfd2	PROMOTER	0.711	0.670	1033.30	691.99	0.476	898.10	427.47
A_68_P20441212	chr1:93146997-93147041	NM_026454:123	Ube2f	INSIDE	0.711	4.382	270.19	1183.95	3.116	287.06	894.48
A_68_P20156380	chr1:37932346-37932395	NM_001037918:3320	Lipt1	INSIDE	0.711	2.577	605.12	1559.33	1.832	485.06	888.73
A_68_P31320418	chr17:69191275-69191319	NM_001206661:4821	Tmem200c	INSIDE	0.710	0.730	1515.96	1106.74	0.518	1340.57	694.65
A_68_P31286841	chr17:63229963-63230007	NM_010109:682	Efna5	INSIDE	0.710	2.094	1526.93	3197.94	1.488	1263.68	1880.38
A_68_P30501636	chr15:102866066-102866110	NM_013553:1263	Hoxc4	INSIDE	0.710	0.324	1929.28	626.04	0.230	1362.07	313.77
A_68_P29117520	chr13:69878183-69878227	NM_001145162:571	Ube2ql1	INSIDE	0.710	0.283	1678.49	475.61	0.201	1180.70	237.51
A_68_P29063254	chr13:56854688-56854732	AK131829:478		INSIDE	0.710	0.338	1551.45	523.87	0.240	1175.30	281.93
A_68_P26228070	chr8:124175318-124175362	NM_080855:493	Zcchc14	INSIDE	0.710	4.231	1594.84	6747.51	3.004	1372.52	4122.66
A_68_P25922768	chr8:66684709-66684753	NM_009390:60	Tll1	INSIDE	0.710	0.583	1301.69	758.26	0.413	1074.61	444.31
A_68_P25589621	chr7:148748037-148748081	NM_007459:-20	Ap2a2	PROMOTER	0.710	3.189	692.40	2208.16	2.263	586.19	1326.62
A_68_P25229724	chr7:80682789-80682833	NM_001081345:3822	Chd2	INSIDE	0.710	1.995	4644.25	9264.05	1.416	3535.19	5004.34
A_68_P24423931	chr6:47544220-47544264	NM_001146689:787	Ezh2	INSIDE	0.710	0.249	1817.21	451.88	0.177	1454.85	256.80
A_68_P24138253	chr5:140384144-140384188	NM_175522:270	Elfn1	INSIDE	0.710	0.649	4843.96	3143.55	0.461	3431.83	1581.03
A_68_P24090500	chr5:130221446-130221490	NM_025450:66	Mrps17	INSIDE	0.710	0.574	1705.48	979.49	0.408	1366.50	557.25

ProbeName	Target position of probe on CpG island microarray	TargetID	GeneSymbol	CpG island Description	Ratio of relative methylation (TiO_2-NP/Vehicle)	Sham group			TiO_2-H group		
						Relative methylation (Cy5/Cy3)	Cy3 signal (Input)	Cy5 signal (MeDIP)	Relative methylation (Cy5/Cy3)	Cy3 signal (Input)	Cy5 signal (MeDIP)
A_68_P23355140	chr4:139521655-139521700	NM_173427:2264	Klhdc7a	INSIDE	0.710	2.067	1597.96	3303.78	1.468	1261.02	1851.10
A_68_P21969036	chr3:17955303-17955347	NM_021560:1000	Bhlhe22	INSIDE	0.710	2.153	1030.50	2219.01	1.530	1015.82	1553.77
A_68_P21875386	chr2:174264415-174264459	NM_022325:57	Ctsz	INSIDE	0.710	0.308	6088.92	1874.73	0.219	4449.02	972.76
A_68_P21820792	chr2:164793405-164793449	NM_020333:-61	Slc12a5	PROMOTER	0.710	2.975	846.77	2519.11	2.114	760.33	1607.09
A_68_P20561474	chr1:122237586-122237630	NM_207233:450	C1ql2	INSIDE	0.710	3.122	1186.67	3704.43	2.217	996.96	2210.02
A_68_P32217860	chr19:59268893-59268937			Unknown	0.709	0.616	819.71	504.84	0.437	693.10	302.70
A_68_P31930506	chr19:5731655-5731699	NM_020491:45	Sssca1	INSIDE	0.709	0.231	2933.16	676.91	0.164	2071.13	339.00
A_68_P31926693	chr19:5068431-5068475	NM_054042:375	Cd248	INSIDE	0.709	2.501	1401.14	3504.12	1.773	1106.50	1961.94
A_68_P31256045	chr17:56567492-56567536	NM_011218:48389	Ptprs	INSIDE	0.709	5.537	799.72	4427.74	3.927	624.32	2452.00
A_68_P31130474	chr17:30026534-30026578	NM_001081160:-1729	Mdga1	PROMOTER	0.709	0.477	1464.57	698.42	0.338	1109.04	374.74
A_68_P30982176	A_68_P30982176			Unknown	0.709	3.245	354.30	1149.58	2.302	303.25	698.00
A_68_P30895867	chr16:78376777-78376821	NR_002700:204	Gm7334	INSIDE	0.709	7.889	4649.04	36675.08	5.597	3912.51	21898.42
A_68_P30347069	chr15:76077165-76077209	NM_023168:-50	Grina	DIVERGENT_PROMOTER	0.709	0.608	4250.96	2586.37	0.431	3365.62	1451.95
A_68_P30021000	chr15:12251385-12251429	NM_025673:156	Golph3	INSIDE	0.709	2.904	853.30	2478.26	2.060	737.12	1518.32
A_68_P29517796	chr14:33276471-33276515	NM_021712:1544	Slc18a3	INSIDE	0.709	0.203	4267.46	865.87	0.144	2922.53	420.44
A_68_P28429306	chr12:52449562-52449606	NM_001015099:368	G2e3	INSIDE	0.709	0.581	1256.61	729.71	0.412	976.43	402.04
A_68_P28085162	chr11:103273486-103273532	NM_183034:470	Plekhm1	INSIDE	0.709	0.343	1804.92	618.47	0.243	1396.13	339.38
A_68_P28078912	chr11:102180945-102180990	NM_011551:-557	Ubtf	PROMOTER	0.709	2.087	1521.10	3174.63	1.479	1186.96	1756.05
A_68_P27921465	chr11:74424335-74424379	NM_001013784:-8750	E130309D14Rik	PROMOTER	0.709	6.950	1789.79	12439.23	4.931	1512.49	7457.68
A_68_P27281469	chr10:79634617-79634661	NM_027207:-1050	1600002K03Rik	PROMOTER	0.709	4.183	1743.44	7292.88	2.964	1405.64	4166.05
A_68_P27261499	chr10:75807838-75807882	NM_001081419:147	Dip2a	INSIDE	0.709	2.627	3523.06	9256.08	1.863	3127.94	5828.68
A_68_P26996376	chr10:21713866-21713910	NM_011361:-583	Sgk1	PROMOTER	0.709	3.064	1268.46	3886.40	2.172	1060.37	2302.81
A_68_P26469101	chr9:44516750-44516794	NM_153537:26505	Phldb1	INSIDE	0.709	2.034	4937.85	10044.63	1.441	3324.28	4791.19
A_68_P26224343	chr8:123606757-123606801	NM_010426:-1595	Foxf1a	PROMOTER	0.709	0.437	2248.40	981.88	0.310	1850.21	572.64
A_68_P25607860	chr7:151767931-151767975	NM_010175:389	Fadd	INSIDE	0.709	0.334	4355.99	1456.07	0.237	3357.86	795.97
A_68_P25005466	chr7:25332334-25332378	NM_009532:188	Xrcc1	INSIDE	0.709	2.568	982.53	2523.30	1.820	779.72	1419.36
A_68_P24984542	chr7:17612840-17612884	NM_011155:401	Ppp5c	INSIDE	0.709	2.727	1848.18	5040.74	1.933	1564.77	3024.63
A_68_P24958144	chr7:7073964-7074008	NM_172738:201	Zfp954	INSIDE	0.709	0.377	2388.33	901.04	0.267	1868.15	499.42
A_68_P24759769	chr6:113432843-113432887	NM_134159:11416	Il17rc	INSIDE	0.709	0.224	2259.45	506.69	0.159	1801.68	286.46
A_68_P23969235	chr5:108298266-108298310	NM_007964:5838	Evi5	INSIDE	0.709	1.990	1806.81	3595.66	1.411	1532.43	2162.20
A_68_P23658789	chr5:46248126-46248170	NM_172153:-357	Lcorl	PROMOTER	0.709	3.998	1398.32	5590.30	2.835	1227.23	3478.68
A_68_P23309106	chr4:131476303-131476347	NM_001201367:-1361	Tmem200b	PROMOTER	0.709	0.623	1805.27	1124.06	0.441	1436.63	633.89
A_68_P23186773	chr4:106511533-106511577	ENSMUST00000151369:8971		DOWNSTREAM	0.709	0.406	1194.82	484.68	0.288	890.07	255.96
A_68_P21601161	chr2:125073250-125073294	NM_023595:22	Dut	INSIDE	0.709	0.207	2248.63	465.07	0.147	1813.98	265.82
A_68_P21477095	chr2:102291921-102291965	NM_010218:7	Fjx1	INSIDE	0.709	2.494	720.09	1795.58	1.768	572.48	1012.20
A_68_P21250383	chr2:58999148-58999192	NM_026361:264	Pkp4	INSIDE	0.709	2.793	1038.62	2901.20	1.982	870.16	1724.29
A_68_P20977354	chr2:6022037-6022081	NM_001159657:29173	5430407P10Rik	INSIDE	0.709	3.087	526.13	1624.00	2.188	383.49	839.18
A_68_P20418214	chr1:89406592-89406636	NM_019867:323	Ngef	INSIDE	0.709	0.233	4112.56	958.73	0.165	2942.88	486.18
A_68_P20370951	chr1:78306769-78306813	NM_001004173:-130	Sgpp2	PROMOTER	0.709	2.655	3217.91	8542.01	1.882	2665.27	5016.46
A_68_P32977601	chr12:20821877-20821921	NR_015521:-258	1700030C10Rik	PROMOTER	0.708	3.903	428.98	1674.50	2.763	375.72	1038.02
A_68_P32288520	chrX:20002860-20002904	ENSMUST00000136093:-31		PROMOTER	0.708	3.662	1051.97	3852.59	2.593	1288.24	3340.99
A_68_P31425848	chr17:88374947-88374991	NM_010830:579	Msh6	INSIDE	0.708	2.346	1484.76	3483.98	1.661	1122.58	1864.68
A_68_P31166374	chr17:37079761-37079805	NM_001099632:-213	Rnf39	PROMOTER	0.708	0.266	1707.18	454.10	0.188	1232.93	232.13
A_68_P30347741	chr15:76181792-76181836	NM_001164607:91	Maf1	INSIDE	0.708	0.491	3922.74	1924.81	0.347	2544.32	884.09
A_68_P30174798	chr15:43002185-43002229	NM_172815:158	Rspo2	INSIDE	0.708	0.533	976.03	520.28	0.377	783.73	295.82
A_68_P29346590	chr13:115249262-115249306	NM_008046:-346	Fst	PROMOTER	0.708	2.212	2949.77	6523.87	1.567	2420.60	3792.77
A_68_P28683724	chr12:101963187-101963231	NM_153587:30	Rps6ka5	INSIDE	0.708	0.349	2651.77	924.35	0.247	2116.04	522.28
A_68_P28077767	chr11:102007085-102007129	NM_178053:-573	Nags	PROMOTER	0.708	0.409	1481.54	605.24	0.289	1135.95	328.42
A_68_P27941693	chr11:77978890-77978934	NM_009423:140	Traf4	INSIDE	0.708	2.716	1089.25	2958.55	1.922	957.47	1840.58
A_68_P27688942	chr11:32354979-32355023	NM_173784:-371	Ubtd2	PROMOTER	0.708	0.695	1872.99	1301.87	0.492	1513.22	745.17
A_68_P26770650	chr9:100543637-100543681	NM_009282:-383	Stag1	PROMOTER	0.708	4.668	608.00	2838.06	3.305	530.16	1752.07
A_68_P26430751	chr9:37267846-37267890			Unknown	0.708	0.237	3678.09	871.35	0.168	2593.97	434.95
A_68_P26075126	chr8:96560899-96560946	NM_026623:17	Nudt21	INSIDE	0.708	0.314	2543.22	798.51	0.222	1699.03	377.45
A_68_P25952345	chr8:73033987-73034031	NM_019883:258	Uba52	INSIDE	0.708	0.362	2136.55	772.51	0.256	1552.32	397.60
A_68_P25574425	chr7:146038687-146038731	NM_026391:640	Ppp2r2d	INSIDE	0.708	0.467	1431.26	668.11	0.330	1215.85	401.68
A_68_P25392117	chr7:112548341-112548386	NM_199009:162	Fam160a2	INSIDE	0.708	0.486	4040.10	1964.67	0.344	2775.70	955.02
A_68_P24618281	chr6:87761235-87761279	NM_133717:-1508	Rab43	PROMOTER	0.708	0.502	1428.65	717.06	0.355	1058.02	376.08
A_68_P23803969	chr5:74931765-74931809	NM_001159573:280	Fip1l1	INSIDE	0.708	0.144	5418.04	781.63	0.102	3587.27	366.57
A_68_P23324351	chr4:134260641-134260685	NM_207237:-457	Man1c1	PROMOTER	0.708	0.586	1075.81	629.89	0.414	820.07	339.79
A_68_P22340672	chr3:94590209-94590253	NM_001037711:207	Cgn	INSIDE	0.708	0.549	1651.90	906.33	0.389	1372.08	533.34
A_68_P22286848	chr3:83570629-83570673	NM_009144:408	Sfrp2	INSIDE	0.708	0.697	3260.83	2274.27	0.494	2393.49	1182.66
A_68_P22209747	chr3:67178066-67178110	NM_001039543:70	Mlf1	INSIDE	0.708	2.096	1395.73	2926.03	1.484	1146.37	1700.66
A_68_P21912147	chr2:181428245-181428289	NM_026446:363	Rgs19	INSIDE	0.708	0.452	2000.85	904.01	0.320	1615.94	516.67
A_68_P21579349	chr2:120996373-120996418	NM_178889:490	Zscan29	INSIDE	0.708	0.584	1348.93	787.13	0.413	980.65	404.91
A_68_P21089694	chr2:28061599-28061643	NM_001038612:412	Olfm1	INSIDE	0.708	3.724	1635.51	6089.85	2.638	1387.12	3659.04

ProbeName	Target position of probe on CpG island microarray	TargetID	GeneSymbol	CpG island Description	Ratio of relative methylation (TiO_2-NP/Vehicle)	Sham group			TiO_2-H group		
						Relative methylation (Cy5/Cy3)	Cy3 signal (Input)	Cy5 signal (MeDIP)	Relative methylation (Cy5/Cy3)	Cy3 signal (Input)	Cy5 signal (MeDIP)
A_68_P20007254	chr1:4798162-4798206	NM_008866:211	Lypla1	INSIDE	0.708	3.925	1446.62	5678.47	2.778	1231.01	3419.35
A_68_P33012180	chr9_random:57290-57334	NR_027950:2620	4930526I15Rik	DOWNSTREAM	0.707	0.418	1931.72	807.71	0.296	1443.36	426.84
A_68_P32244416	chrX:8469520-8469564	NM_175327:64	B630019K06Rik	INSIDE	0.707	4.119	277.24	1141.88	2.910	412.20	1199.48
A_68_P31921226	chr19:4125896-4125940	NM_001078649:-41	Tmem134	DIVERGENT_PROMOTER	0.707	2.486	2511.06	6243.67	1.759	2106.97	3706.24
A_68_P31620977	chr18:36093125-36093169			Unknown	0.707	0.468	991.72	464.51	0.331	777.40	257.56
A_68_P31467175	chr18:5163131-5163175	ENSMUST00000150337:2577		INSIDE	0.707	2.434	2648.53	6447.76	1.721	2027.70	3489.48
A_68_P31335315	chr17:71965807-71965851	NM_175639:274	Wdr43	INSIDE	0.707	4.348	1702.65	7402.79	3.075	1372.86	4221.65
A_68_P31002107	chr16:98183148-98183192	NM_001081684:616	Zfp295	INSIDE	0.707	0.446	2222.69	990.78	0.315	1832.81	577.42
A_68_P30369637	chr15:79832097-79832141	NM_011057:13120	Pdgfb	INSIDE	0.707	3.983	1890.91	7531.01	2.814	1667.20	4691.65
A_68_P29144944	chr13:74346071-74346115	NM_177333:56	Exoc3	INSIDE	0.707	0.480	3408.49	1635.90	0.339	2896.72	983.28
A_68_P27937690	chr11:77275884-77275928	NM_139128:-1508	Coro6	PROMOTER	0.707	0.552	2406.54	1327.61	0.390	1663.65	649.07
A_68_P27339935	chr10:90545393-90545437	NM_001042558:74	Apaf1	INSIDE	0.707	0.407	1275.51	518.99	0.288	1039.05	299.03
A_68_P27287963	chr10:80645829-80645873	NM_001190473:-157	Dapk3	PROMOTER	0.707	0.723	2555.10	1847.17	0.511	2033.24	1039.82
A_68_P26134295	chr8:108042675-108042719	NM_008289:51	Hsd11b2	INSIDE	0.707	0.324	2986.59	966.85	0.229	2171.71	497.38
A_68_P26074974	chr8:96535812-96535856	NM_011787:706	Amfr	INSIDE	0.707	0.380	2256.18	857.54	0.269	1790.63	481.34
A_68_P25958089	chr8:74178574-74178618	NM_001029873:17060	Unc13a	INSIDE	0.707	0.262	2064.24	540.25	0.185	1615.33	298.98
A_68_P25905383	chr8:63111766-63111810	NM_027756:132	Mfap3l	INSIDE	0.707	0.215	2559.13	550.11	0.152	2072.89	315.12
A_68_P24817706	chr6:124860067-124860111	NM_008479:1635	Lag3	INSIDE	0.707	0.509	1347.23	685.08	0.359	989.04	355.40
A_68_P24518617	chr6:66846588-66846632	NM_001177558:-274	Gng12	PROMOTER	0.707	2.382	2359.88	5620.43	1.683	1933.35	3254.36
A_68_P24117610	chr5:136209906-136209950	NM_008898:44845	Por	INSIDE	0.707	2.843	1114.89	3169.40	2.010	895.22	1798.98
A_68_P24098222	chr5:131916544-131916588	NM_177047:1101647	Auts2	INSIDE	0.707	7.333	242.26	1776.49	5.188	240.42	1247.29
A_68_P23874561	A_68_P23874561			Unknown	0.707	8.788	407.23	3578.57	6.210	345.49	2145.64
A_68_P23069985	chr4:84321596-84321640	NM_172870:-628	Bnc2	PROMOTER	0.707	2.271	1673.74	3800.66	1.606	1377.76	2212.83
A_68_P22509002	chr3:127201649-127201693	ENSMUST00000104965:249		INSIDE	0.707	0.387	1542.38	597.67	0.274	1193.99	327.17
A_68_P22384435	chr3:102906580-102906624	NM_001033303:28666	Ampd1	DOWNSTREAM	0.707	0.667	2406.12	1603.97	0.471	1895.19	893.38
A_68_P22289192	chr3:83959834-83959879	NM_029797:-148	Mnd1	PROMOTER	0.707	0.353	1581.77	557.58	0.249	1270.24	316.79
A_68_P21495956	chr2:105515369-105515413	NM_013627:-1211	Pax6	DIVERGENT_PROMOTER	0.707	0.495	1300.04	643.19	0.350	1097.22	384.06
A_68_P20644875	chr1:138156699-138156743	NM_001101516:730	Gpr25	INSIDE	0.707	0.183	2599.38	474.94	0.129	1872.57	241.90
A_68_P33005883	A_68_P33005883			Unknown	0.706	9.299	358.23	3331.05	6.562	338.20	2219.17
A_68_P32229414	A_68_P32229414			Unknown	0.706	7.616	496.60	3782.32	5.374	367.54	1975.20
A_68_P31923510	chr19:4510696-4510740	NM_001162946:247	Pcx	INSIDE	0.706	0.688	1803.56	1241.31	0.486	1439.76	699.53
A_68_P31055430	chr17:13109675-13109719	NR_028479:-6026	Snora20	PROMOTER	0.706	0.657	2115.70	1390.53	0.464	1571.71	729.36
A_68_P30491676	chr15:101242719-101242763	NM_033073:-93	Krt7	PROMOTER	0.706	2.322	2330.91	5412.96	1.641	2009.95	3297.63
A_68_P30371091	chr15:80086279-80086325	NM_009716:689	Atf4	INSIDE	0.706	5.216	1437.82	7499.20	3.682	1131.33	4165.31
A_68_P30150545	chr15:38591758-38591802	NM_025494:122	Atp6v1c1	INSIDE	0.706	0.378	2062.36	779.51	0.267	1592.55	424.77
A_68_P29976942	chr15:3929549-3929593	NM_134099:3	Fbxo4	INSIDE	0.706	3.807	1110.15	4226.16	2.688	931.47	2504.00
A_68_P29961483	chr14:122805935-122805979	NR_033326:44412	Gm5089	INSIDE	0.706	2.838	1106.81	3141.57	2.004	975.13	1953.80
A_68_P28167721	chr11:117734583-117734627	NM_027919:236	Tha1	INSIDE	0.706	2.555	715.60	1828.70	1.805	681.54	1230.01
A_68_P28159661	chr11:116351482-116351526	NM_026364:156	Prpsap1	INSIDE	0.706	3.606	6871.05	24774.95	2.546	5947.80	15141.66
A_68_P28046743	chr11:96638811-96638855	NM_001163389:37	Snx11	INSIDE	0.706	0.293	1800.47	527.95	0.207	1293.90	268.05
A_68_P27330520	chr10:88807269-88807313	ENSMUST00000168681:217		INSIDE	0.706	2.363	938.52	2217.86	1.668	742.94	1239.40
A_68_P27278593	chr10:79198358-79198402	NM_008226:19002	Hcn2	INSIDE	0.706	2.882	1035.79	2985.22	2.035	744.80	1515.59
A_68_P25950969	chr8:72826268-72826312	NM_001024922:61	Ddx49	INSIDE	0.706	0.589	2018.20	1189.02	0.416	1536.61	639.43
A_68_P25499298	chr7:133343404-133343448	NM_028816:496	Xpo6	INSIDE	0.706	0.576	3307.41	1904.60	0.406	2652.10	1077.82
A_68_P25313630	chr7:97592416-97592460	NM_145151:1148	Crebzf	INSIDE	0.706	3.991	1826.74	7290.08	2.816	1554.72	4377.55
A_68_P25228919	chr7:80554204-80554248	NM_177740:33821	Rgma	INSIDE	0.706	4.287	1656.10	7099.37	3.027	1404.98	4252.65
A_68_P25008266	chr7:25866428-25866472	NM_175477:4186	Zfp574	INSIDE	0.706	2.688	1552.11	4171.63	1.897	1192.46	2262.68
A_68_P24640496	chr6:91634068-91634112	NM_009320:30	Slc6a6	INSIDE	0.706	0.598	1219.63	729.27	0.422	901.09	380.17
A_68_P24427165	chr6:48388397-48388441	NM_001085415:6671	Zfp467	INSIDE	0.706	2.221	1077.69	2393.72	1.567	862.69	1352.00
A_68_P24183687	chr5:149995851-149995895	NR_015478:-233	5730422E09Rik	DIVERGENT_PROMOTER	0.706	0.678	3003.41	2035.67	0.479	2478.44	1186.45
A_68_P23962135	chr5:107003692-107003736	ENSMUST00000131029:20		INSIDE	0.706	3.265	1308.02	4270.77	2.304	1018.59	2346.97
A_68_P23801771	chr5:74591574-74591618	NM_026878:246	Rasl11b	INSIDE	0.706	4.167	1107.55	4614.63	2.942	940.73	2767.66
A_68_P23391999	chr4:147412940-147412984	NM_011929:-86	Clcn6	DIVERGENT_PROMOTER	0.706	0.397	1678.48	665.54	0.280	1282.58	359.05
A_68_P23059230	chr4:82505564-82505608	NM_026647:-21	Zdhhc21	PROMOTER	0.706	2.773	1222.65	3390.79	1.959	984.35	1928.36
A_68_P22953602	chr4:58925740-58925784	NM_172381:-165	AI314180	PROMOTER	0.706	0.393	1600.06	629.47	0.278	1485.66	412.81
A_68_P22775540	chr4:21858745-21858789	NM_175234:294	6230409E13Rik	INSIDE	0.706	0.254	1779.90	452.69	0.180	1333.02	239.36
A_68_P22165650	chr3:57650817-57650861	NM_019410:841	Pfn2	INSIDE	0.706	0.550	2038.34	1120.18	0.388	1642.52	637.61
A_68_P21762930	chr2:154718365-154718409	NM_026030:256	Eif2s2	INSIDE	0.706	0.395	1730.64	684.28	0.279	1307.46	364.96
A_68_P21706813	chr2:144194864-144194908	NM_181417:116	Csrp2bp	INSIDE	0.706	3.076	711.44	2188.23	2.172	556.66	1209.13
A_68_P21622817	chr2:128891746-128891790	NM_027192:86	Ttl	INSIDE	0.706	0.717	1586.23	1137.52	0.506	1234.24	624.65
A_68_P21077274	chr2:26092670-26092714	NM_153559:248	Qsox2	INSIDE	0.706	2.956	2616.22	7734.08	2.088	2188.20	4567.91
A_68_P20823718	chr1:172574573-172574617	NM_177068:-68	Olfml2b	PROMOTER	0.706	0.607	1413.68	857.50	0.428	1165.09	498.67
A_68_P32710887	chrX:139401132-139401176	NM_019496:117	Ammecr1	INSIDE	0.705	2.268	700.70	1589.38	1.599	939.93	1502.58
A_68_P32289245	chrX:20195246-20195290	NM_001167776:384	Rbm10	INSIDE	0.705	3.206	2170.34	6958.53	2.261	2957.40	6685.62
A_68_P32016045	chr19:23210547-23210591	NM_010638:-5147	Klf9	PROMOTER	0.705	3.586	750.14	2690.17	2.530	628.06	1588.76

ProbeName	Target position of probe on CpG island microarray	TargetID	GeneSymbol	CpG island Description	Ratio of relative methylation (TiO_2-NP/Vehicle)	Sham group			TiO_2-H group		
						Relative methylation (Cy5/Cy3)	Cy3 signal (Input)	Cy5 signal (MeDIP)	Relative methylation (Cy5/Cy3)	Cy3 signal (Input)	Cy5 signal (MeDIP)
A_68_P29048726	chr13:54398661-54398705	AK086128:102013		DOWNSTREAM	0.705	3.184	1745.49	5557.70	2.246	1377.67	3093.71
A_68_P29009464	chr13:46822934-46822978	NM_175749:262	Nup153	INSIDE	0.705	0.215	2052.60	441.80	0.152	1465.10	222.47
A_68_P28463221	chr12:58647081-58647125	NM_008259:6	Foxa1	INSIDE	0.705	0.665	2121.78	1410.11	0.469	1527.60	715.86
A_68_P28191918	chr11:121533998-121534047	NM_178664:443	B3gntl1	INSIDE	0.705	0.654	1348.48	882.36	0.461	896.98	413.72
A_68_P28044104	chr11:96227502-96227546	NM_008266:453	Hoxb1	INSIDE	0.705	0.484	3288.87	1593.01	0.342	2492.66	851.56
A_68_P27910207	chr11:72028699-72028750	NM_001163728:85	4930563E22Rik	INSIDE	0.705	0.583	1148.14	669.14	0.411	948.96	390.11
A_68_P27614514	chr11:17059211-17059255	NM_024459:-68	Ppp3r1	PROMOTER	0.705	3.722	1901.42	7076.31	2.623	1680.79	4408.26
A_68_P27290698	chr10:81050747-81050791	NM_019725:12492	Tle2	INSIDE	0.705	2.410	1686.01	4063.93	1.699	1352.92	2298.78
A_68_P27287865	chr10:80630602-80630646	NM_021501:-159	Pias4	DIVERGENT_PROMOTER	0.705	4.574	3059.35	13994.11	3.225	2729.46	8801.31
A_68_P27278808	chr10:79230093-79230137	NR_027505:-428	Rnf126	DIVERGENT_PROMOTER	0.705	0.386	2047.12	790.80	0.272	1622.45	441.70
A_68_P25908469	chr8:63703256-63703300	NM_021506:311	Sh3rf1	INSIDE	0.705	2.863	1582.55	4530.51	2.019	1379.85	2785.73
A_68_P25785791	chr8:37521054-37521098	ENSMUST00000036363:-134		PROMOTER	0.705	0.276	2061.67	569.50	0.195	1757.15	342.21
A_68_P25678293	chr8:15028780-15028824	NM_029116:17778	Kbtbd11	INSIDE	0.705	2.899	520.85	1509.80	2.044	424.93	868.55
A_68_P25465270	chr7:126887848-126887892	ENSMUST00000004569:4431		DOWNSTREAM	0.705	3.219	1142.56	3677.85	2.268	1027.49	2330.29
A_68_P25307422	chr7:96553151-96553195	NM_008055:297	Fzd4	INSIDE	0.705	5.877	1420.05	8346.02	4.143	1093.79	4531.45
A_68_P25111235	chr7:56502306-56502350	NM_175272:770	Nav2	INSIDE	0.705	0.252	2077.57	524.36	0.178	1461.29	259.90
A_68_P24951794	chr7:5004858-5004902	NM_026900:1747	Zfp580	INSIDE	0.705	17.389	5111.38	88884.27	12.262	4740.33	58125.23
A_68_P23416913	chr4:151533877-151533921	NM_008236:923	Hes2	INSIDE	0.705	0.719	1283.70	922.50	0.506	1067.04	540.36
A_68_P22705746	chr4:6380618-6380662	NM_010945:778	Nsmaf	INSIDE	0.705	0.475	1323.22	628.31	0.335	1232.05	412.25
A_68_P22415392	chr3:108743052-108743096	NM_001078646:-400	4921515J06Rik	PROMOTER	0.705	5.361	1478.27	7925.33	3.782	1249.25	4724.73
A_68_P22049506	chr3:34562380-34562424	NR_015580:103100	Sox2ot	INSIDE	0.705	0.516	2239.17	1155.18	0.364	1932.52	702.87
A_68_P21680297	chr2:139504517-139504561	NM_001126490:625	Ism1	INSIDE	0.705	4.759	633.16	3012.98	3.356	610.06	2047.42
A_68_P20585380	chr1:127457355-127457399	NM_028787:-216	Slc35f5	PROMOTER	0.705	0.347	1595.02	553.74	0.245	1336.78	327.36
A_68_P31292365	chr17:64199012-64199056			Unknown	0.704	2.204	2177.46	4798.92	1.551	1814.58	2814.21
A_68_P31208331	chr17:46818042-46818086	NM_010787:-21	Mea1	DIVERGENT_PROMOTER	0.704	3.394	3884.05	13182.84	2.388	3013.01	7194.81
A_68_P31207580	chr17:46692523-46692567	NM_020493:567	Srf	INSIDE	0.704	5.248	1112.37	5838.10	3.697	966.09	3571.37
A_68_P30580619	chr16:18589104-18589148	NM_011532:-2064	Tbx1	PROMOTER	0.704	0.516	2537.95	1310.63	0.363	1878.59	682.80
A_68_P29996526	chr15:7765753-7765797	NM_010275:4764	Gdnf	INSIDE	0.704	0.476	1514.29	720.99	0.335	1145.08	383.72
A_68_P29246773	chr13:97312251-97312295	NM_012048:168	Polk	INSIDE	0.704	2.551	1193.76	3045.65	1.795	1051.57	1887.96
A_68_P28708611	chr12:106575425-106575469	ENSMUST00000138649:244		INSIDE	0.704	3.795	1861.46	7064.36	2.674	1429.39	3821.61
A_68_P28307234	chr12:28023843-28023887	NM_009234:3719	Sox11	INSIDE	0.704	0.488	1323.18	645.42	0.344	1147.60	394.22
A_68_P28280595	chr12:21423493-21423537	NM_011739:-217	Ywhaq	PROMOTER	0.704	0.278	1674.33	465.90	0.196	1333.49	261.28
A_68_P28153287	chr11:115264936-115264980	NM_026729:-121	Ict1	PROMOTER	0.704	3.499	1215.73	4253.69	2.465	1013.73	2498.56
A_68_P28069090	chr11:100480471-100480515	NM_019795:990	Dnajc7	INSIDE	0.704	0.685	4534.72	3106.81	0.482	3406.39	1642.89
A_68_P27287000	chr10:80494188-80494232	NM_134135:2447	Slc39a3	INSIDE	0.704	4.425	1847.69	8176.46	3.115	1364.64	4251.00
A_68_P26020983	chr8:87186771-87186818	NM_010499:-43	Ier2	PROMOTER	0.704	2.343	1880.83	4407.51	1.650	1351.06	2228.74
A_68_P24760620	chr6:113588631-113588675	NM_001113553:192	Irak2	INSIDE	0.704	0.602	1809.40	1089.70	0.424	1378.56	584.60
A_68_P24537473	chr6:71583505-71583549	NM_001038695:-627	Kdm3a	PROMOTER	0.704	0.365	3960.21	1447.00	0.257	3051.75	784.71
A_68_P24126245	chr5:137972339-137972383	NM_010312:2097	Gnb2	INSIDE	0.704	2.149	1999.64	4296.94	1.514	1574.35	2383.29
A_68_P24012822	chr5:116474390-116474434	NM_031869:25	Prkab1	INSIDE	0.704	0.505	1725.70	872.23	0.356	1257.12	447.47
A_68_P23592737	chr5:34512192-34512236	NM_181857:-117	Poln	PROMOTER	0.704	0.090	6861.14	615.50	0.063	4311.27	272.20
A_68_P23457017	chr5:5693867-5693911	NM_001103156:189	Steap2	INSIDE	0.704	1.985	2381.46	4726.37	1.398	2045.48	2858.76
A_68_P23184551	chr4:106136489-106136533	NM_153565:420	Pcsk9	INSIDE	0.704	0.656	1464.14	961.14	0.462	1312.18	606.57
A_68_P21824257	chr2:165329532-165329576	NM_130451:77	Slc2a10	INSIDE	0.704	0.489	1558.45	761.39	0.344	1307.86	449.64
A_68_P20961508	chr2:3201225-3201269	NM_008708:-313	Nmt2	PROMOTER	0.704	0.685	2010.97	1377.61	0.482	1487.74	717.49
A_68_P20826003	chr1:172995433-172995477	NM_010188:-5920	Fcgr3	PROMOTER	0.704	0.294	22719.35	6674.13	0.207	17176.46	3552.08
A_68_P20350881	chr1:74902348-74902392	NM_009872:768	Cdk5r2	INSIDE	0.704	0.618	2438.32	1506.06	0.435	1822.03	791.99
A_68_P20293905	chr1:64737459-64737503	NR_038009:-247	2810408I11Rik	DIVERGENT_PROMOTER	0.704	6.125	747.73	4579.87	4.315	722.83	3118.86
A_68_P32145201	chr19:46728817-46728861	NM_001177813:30948	D19Wsu162e	INSIDE	0.703	3.200	800.87	2562.59	2.251	543.09	1222.37
A_68_P32116948	chr19:41817676-41817720	NM_015748:648	Slit1	INSIDE	0.703	0.367	5201.09	1910.90	0.258	3692.31	953.73
A_68_P31626863	chr18:37249680-37249724	NM_001003671:-87	Pcdhac1	PROMOTER	0.703	0.302	1521.94	459.25	0.212	1195.16	253.37
A_68_P31620305	chr18:35990289-35990333	NM_133687:839	Cxxc5	INSIDE	0.703	0.139	4352.11	602.92	0.097	3188.79	310.72
A_68_P31315916	chr17:68353564-68353617	NM_172964:-142	Arhgap28	PROMOTER	0.703	3.228	6054.54	19546.76	2.269	4156.53	9429.58
A_68_P31201715	chr17:45684089-45684133	NM_001013749:2516	Tmem151b	INSIDE	0.703	3.471	1622.18	5630.37	2.440	1058.23	2582.05
A_68_P30991920	chr16:96502337-96502381	NM_033149:-280	B3galt5	PROMOTER	0.703	0.545	834.21	454.48	0.383	678.65	260.10
A_68_P30743457	chr16:48994281-48994325	NM_172616:2	C330027C09Rik	INSIDE	0.703	4.205	508.91	2140.07	2.956	416.36	1230.55
A_68_P30377736	chr15:81230239-81230283	NM_133726:-136	St13	DIVERGENT_PROMOTER	0.703	0.482	1484.84	715.79	0.339	1230.62	417.32
A_68_P29694907	chr14:69808925-69808969	NM_010921:198	Nkx3-1	INSIDE	0.703	0.552	1726.34	953.14	0.388	1315.45	510.28
A_68_P29651216	chr14:62268043-62268087	NR_028264:33146	Dleu2	INSIDE	0.703	0.371	1525.67	566.57	0.261	1160.54	302.99
A_68_P28727037	chr12:109513497-109513541	NM_001159910:109	Ccdc85c	INSIDE	0.703	8.634	704.70	6084.23	6.067	605.75	3674.82
A_68_P28602643	chr12:86518346-86518390	NM_008827:-133	Pgf	PROMOTER	0.703	0.595	1432.58	852.20	0.418	1271.35	531.51
A_68_P28576090	chr12:81620528-81620572	NM_001081421:574	Galntl1	INSIDE	0.703	2.665	4822.99	12855.44	1.873	3664.66	6865.04
A_68_P27980110	chr11:84693182-84693228	NM_027388:493	Pigw	INSIDE	0.703	6.398	360.66	2307.33	4.498	293.08	1318.21
A_68_P27503019	chr10:120788500-120788544	ENSMUST00000078515:8195		DOWNSTREAM	0.703	0.529	1045.62	553.51	0.372	856.48	318.55
A_68_P27095714	chr10:42303999-42304043	NM_152229:-626	Nr2e1	PROMOTER	0.703	0.645	2827.41	1822.79	0.453	2031.96	921.41

ProbeName	Target position of probe on CpG island microarray	TargetID	GeneSymbol	CpG island Description	Ratio of relative methylation (TiO_2-NP/Vehicle)	Sham group			TiO_2-H group		
						Relative methylation (Cy5/Cy3)	Cy3 signal (Input)	Cy5 signal (MeDIP)	Relative methylation (Cy5/Cy3)	Cy3 signal (Input)	Cy5 signal (MeDIP)
A_68_P26920085	chr10:7382878-7382922	NR_030698:-268	A630066F11Rik	PROMOTER	0.703	0.606	1390.87	842.32	0.426	1075.69	458.23
A_68_P26135469	chr8:108225412-108225456	NM_001047435:-114	Pard6a	PROMOTER	0.703	5.619	441.70	2481.99	3.952	397.32	1570.04
A_68_P25015935	chr7:27963726-27963770	NR_037283:-2636	Mir3101	PROMOTER	0.703	0.578	2029.13	1172.06	0.406	1672.45	679.11
A_68_P24449266	chr6:52180669-52180713	NR_029912:-526	Mir196b	PROMOTER	0.703	0.501	960.29	480.77	0.352	779.96	274.34
A_68_P24397200	chr6:42215074-42215118	NM_007610:59	Casp2	INSIDE	0.703	1.934	3426.88	6628.70	1.360	2498.30	3396.52
A_68_P24161581	chr5:145306987-145307031	NM_016789:253	Nptx2	INSIDE	0.703	3.219	799.06	2572.03	2.264	730.52	1653.66
A_68_P22941965	chr4:56878080-56878124	NM_018761:-19	Ctnnal1	PROMOTER	0.703	3.011	11011.71	33153.09	2.118	8309.53	17599.19
A_68_P22282899	chr3:82859431-82859475	NM_016784:-7	Plrg1	DIVERGENT_PROMOTER	0.703	0.504	2088.22	1051.69	0.354	1665.73	589.79
A_68_P21131591	chr2:34965203-34965247	NM_012018:213	Cep110	INSIDE	0.703	0.577	4825.07	2783.66	0.406	3493.58	1417.69
A_68_P21081716	chr2:26865601-26865645	NM_029862:137	5930434B04Rik	INSIDE	0.703	0.612	3154.59	1930.43	0.430	2300.62	990.29
A_68_P20945911	chr1:194681059-194681103	NM_001177794:-3139	Sertad4	PROMOTER	0.703	4.899	1569.71	7690.41	3.444	1395.73	4807.19
A_68_P20714686	chr1:153275868-153275912	NM_026876:113	1190005F20Rik	INSIDE	0.703	0.320	2449.70	783.91	0.225	1892.76	425.93
A_68_P20421183	chr1:89905073-89905117	NM_001033291:32	Usp40	INSIDE	0.703	0.178	3904.99	695.69	0.125	2917.84	365.54
A_68_P31934086	chr19:6341582-6341626	NM_009006:355	Map4k2	INSIDE	0.702	2.465	853.82	2105.01	1.730	746.38	1291.07
A_68_P31513736	chr18:14841066-14841110	NM_001161369:335	Ss18	INSIDE	0.702	2.187	962.97	2105.59	1.535	881.23	1352.52
A_68_P30979595	chr16:94346850-94346894	NM_011377:261368	Sim2	INSIDE	0.702	0.340	2536.64	861.51	0.238	1766.49	421.12
A_68_P30392999	chr15:83998465-83998509	NM_054088:241	Pnpla3	INSIDE	0.702	2.290	1198.71	2745.27	1.608	1111.01	1786.53
A_68_P30369712	chr15:79843045-79843089	NM_011057:2172	Pdgfb	INSIDE	0.702	3.428	1139.35	3906.25	2.408	1036.18	2494.69
A_68_P28597123	chr12:85559496-85559540	NM_001163501:313	C130039O16Rik	INSIDE	0.702	0.499	972.20	485.60	0.351	799.71	280.53
A_68_P27937729	chr11:77280699-77280743	NM_139128:3306	Coro6	INSIDE	0.702	2.130	1206.93	2570.72	1.495	1033.92	1545.52
A_68_P27290416	chr10:81008062-81008106	NM_010301:-293	Gna11	PROMOTER	0.702	0.361	1678.63	605.57	0.253	1201.04	304.02
A_68_P26974631	chr10:18038563-18038607			Unknown	0.702	3.819	528.31	2017.57	2.682	473.02	1268.77
A_68_P26617801	chr9:70526697-70526741	NM_007399:-89	Adam10	PROMOTER	0.702	0.432	2340.02	1011.44	0.304	1907.69	579.14
A_68_P26270749	chr8:131209761-131209805	NM_010578:229	Itgb1	INSIDE	0.702	3.327	3483.72	11589.12	2.336	2906.30	6788.70
A_68_P26038366	chr8:90484574-90484618	NM_033327:-1102	Zfp423	PROMOTER	0.702	0.247	3052.76	752.55	0.173	2138.21	369.81
A_68_P25301153	chr7:95278950-95278994	NM_001081414:546295	Grm5	INSIDE	0.702	2.391	906.80	2168.60	1.679	754.61	1267.34
A_68_P25023454	chr7:29551526-29551579	NM_010908:-9	Nfkbib	DIVERGENT_PROMOTER	0.702	16.181	423.63	6854.85	11.366	415.75	4725.41
A_68_P24591142	chr6:83004903-83004947	NM_007517:278	Aup1	INSIDE	0.702	2.134	1331.58	2840.98	1.499	1086.45	1628.08
A_68_P23416731	chr4:151506130-151506174	NM_019585:-3118	Espn	PROMOTER	0.702	0.426	2559.33	1091.35	0.299	1823.29	545.70
A_68_P22861617	chr4:41261841-41261885	NM_026893:72	Dcaf12	INSIDE	0.702	0.607	2123.84	1288.91	0.426	1817.79	774.87
A_68_P22530159	chr3:130813231-130813275	NM_010703:-136	Lef1	PROMOTER	0.702	2.670	1001.63	2674.26	1.875	940.98	1764.11
A_68_P22388656	chr3:103718191-103718235	NM_172684:170	Rsbn1	INSIDE	0.702	5.924	982.17	5818.02	4.159	730.70	3038.86
A_68_P22342879	chr3:95021444-95021488	NM_029885:398	Gabpb2	INSIDE	0.702	0.461	1752.51	808.18	0.324	1377.57	445.75
A_68_P21538595	chr2:114027026-114027070	NM_175466:120	Zfp770	INSIDE	0.702	0.443	1878.79	832.70	0.311	1681.23	522.74
A_68_P21419477	chr2:90729763-90729807	NM_026161:3842	C1qtnf4	INSIDE	0.702	4.067	969.87	3944.29	2.855	811.44	2316.70
A_68_P20961517	chr2:3202117-3202161	NM_008708:579	Nmt2	INSIDE	0.702	2.930	1750.80	5129.72	2.056	1373.08	2823.73
A_68_P20876216	chr1:182413344-182413388	NM_001081227:269	6330403A02Rik	INSIDE	0.702	2.670	4246.13	11336.36	1.875	3259.83	6113.21
A_68_P20601161	chr1:130487885-130487929	NM_009911:970	Cxcr4	INSIDE	0.702	0.203	2699.15	547.30	0.142	1949.98	277.50
A_68_P20411440	chr1:88199681-88199725	NM_145222:-93	B3gnt7	PROMOTER	0.702	0.470	1807.05	849.22	0.330	1479.70	488.26
A_68_P20351048	chr1:74931966-74932010	NM_153111:-6	Fev	PROMOTER	0.702	0.330	2170.78	716.89	0.232	1770.65	410.22
A_68_P20026599	chr1:10222235-10222279	NM_001102430:495	Arfgef1	INSIDE	0.702	0.327	3723.24	1218.99	0.230	2653.25	609.50
A_68_P31921837	chr19:4214484-4214528	NM_019952:115	Clcf1	INSIDE	0.701	0.661	996.74	658.75	0.463	884.53	409.58
A_68_P31774685	chr18:64499703-64499747	NM_194268:-293	Onecut2	PROMOTER	0.701	0.354	2152.55	762.19	0.248	1797.00	446.09
A_68_P30478003	chr15:98887633-98887677	NM_001163588:2050	Prph	INSIDE	0.701	3.255	706.69	2300.61	2.282	638.96	1457.97
A_68_P30348898	chr15:76353129-76353173	NM_130893:-591	Scrt1	PROMOTER	0.701	0.633	1541.69	976.46	0.444	1295.83	575.18
A_68_P30018889	chr15:11834455-11834499	NM_001039181:953	Npr3	INSIDE	0.701	0.226	4426.37	1001.28	0.158	2872.09	455.13
A_68_P29701860	chr14:70930843-70930887	NM_145219:237	Lgi3	INSIDE	0.701	0.637	2980.86	1898.58	0.446	2396.76	1070.12
A_68_P28691639	chr12:103707284-103707328	NM_001199004:-37	Golga5	PROMOTER	0.701	9.341	936.59	8748.26	6.552	834.59	5468.01
A_68_P28164070	chr11:117127735-117127779	NM_001113487:197	Septin9	INSIDE	0.701	2.921	1484.36	4335.44	2.048	1154.52	2364.35
A_68_P28163661	chr11:117061039-117061083	NM_001113486:86	Septin9	INSIDE	0.701	10.414	1797.25	18716.05	7.295	1459.99	10650.73
A_68_P27365444	chr10:94964372-94964416	NM_026065:167	Mrpl42	INSIDE	0.701	2.403	1738.38	4177.21	1.684	1418.63	2388.27
A_68_P26643864	chr9:75257719-75257763	NM_027418:-574	Mapk6	PROMOTER	0.701	2.605	2937.51	7653.04	1.826	2159.38	3942.53
A_68_P26551395	chr9:58883489-58883533	NM_001042752:738	Neo1	INSIDE	0.701	0.519	2562.07	1330.60	0.364	1964.98	715.43
A_68_P25947616	chr8:72178703-72178747	NM_001177527:270	Zfp964	INSIDE	0.701	2.149	1378.27	2961.56	1.506	1193.13	1796.98
A_68_P25589538	chr7:148725824-148725868	NM_001142681:-90	Chid1	PROMOTER	0.701	2.604	2119.14	5517.90	1.826	1694.93	3094.55
A_68_P25289617	chr7:91754287-91754332	NM_010176:143	Fah	INSIDE	0.701	0.212	2286.52	485.68	0.149	1579.21	235.30
A_68_P24466826	chr6:54988021-54988065	NM_180678:48	Gars	INSIDE	0.701	2.721	1424.62	3875.74	1.906	1116.87	2129.08
A_68_P24011281	chr5:116181311-116181355	NM_001080808:236	Ccdc64	INSIDE	0.701	2.781	494.52	1375.04	1.949	479.25	934.06
A_68_P23974099	chr5:109203826-109203870	NM_026109:20534	Tmed11	DOWNSTREAM	0.701	3.260	1968.61	6417.94	2.286	1502.56	3435.33
A_68_P23573590	chr5:30925082-30925126	NM_010608:34562	Kcnk3	INSIDE	0.701	6.044	2572.75	15550.54	4.239	2017.12	8551.45
A_68_P23393398	chr4:147645613-147645662	NM_001083342:16437	Ptchd2	INSIDE	0.701	3.890	325.15	1264.66	2.727	266.30	726.25
A_68_P23345560	chr4:137951782-137951826	NM_001081672:569	Fam43b	INSIDE	0.701	4.917	820.73	4035.17	3.444	723.24	2490.90
A_68_P23324341	chr4:134259355-134259399	NM_207237:829	Man1c1	INSIDE	0.701	3.065	2036.16	6241.30	2.149	1641.57	3528.45
A_68_P22954049	chr4:59016297-59016341	NM_001033165:254	Dnajc25	INSIDE	0.701	3.392	939.96	3188.72	2.378	837.83	1992.63
A_68_P22702204	chr4:5571466-5571510	NM_173426:163	Fam110b	INSIDE	0.701	2.558	978.88	2503.81	1.793	887.52	1591.45

ProbeName	Target position of probe on CpG island microarray	TargetID	GeneSymbol	CpG island Description	Ratio of relative methylation (TiO_2-NP/Vehicle)	Sham group			TiO_2-H group		
						Relative methylation (Cy5/Cy3)	Cy3 signal (Input)	Cy5 signal (MeDIP)	Relative methylation (Cy5/Cy3)	Cy3 signal (Input)	Cy5 signal (MeDIP)
A_68_P22129174	chr3:51144832-51144876	ENSMUST00000135496:-274		PROMOTER	0.701	2.533	5281.68	13381.13	1.775	3939.63	6992.25
A_68_P21863931	chr2:172196546-172196590	NM_024199:66	Cstf1	INSIDE	0.701	0.426	2138.36	910.53	0.299	1690.20	504.72
A_68_P21750261	chr2:152453185-152453229	NM_009047:463	Rem1	INSIDE	0.701	0.274	2110.09	578.15	0.192	1629.41	312.89
A_68_P21122758	chr2:33496074-33496118	NM_010725:-65	Lmx1b	PROMOTER	0.701	0.508	1428.24	725.55	0.356	1113.39	396.47
A_68_P21101535	chr2:29989275-29989319	NM_146252:-94	Tbc1d13	DIVERGENT_PROMOTER	0.701	0.565	1150.03	649.70	0.396	951.82	377.14
A_68_P20997432	chr2:9796607-9796655	NM_008091:3597	Gata3	INSIDE	0.701	0.278	2347.82	651.57	0.194	1785.23	347.10
A_68_P20641037	chr1:137584870-137584914	ENSMUST00000112103:-210		PROMOTER	0.701	0.472	2120.10	1000.00	0.331	1602.48	529.64
A_68_P32543328	chrX:93163774-93163818			Unknown	0.700	3.297	2202.21	7259.90	2.307	3153.61	7276.83
A_68_P31752326	chr18:60756117-60756161	NM_177340:27821	Synpo	INSIDE	0.700	2.449	1527.85	3742.00	1.714	1269.77	2176.86
A_68_P31206911	chr17:46582449-46582493	NM_001162864:42154	Ttbk1	INSIDE	0.700	2.137	1997.35	4268.89	1.497	1535.09	2297.68
A_68_P30959119	chr16:90810476-90810520	NM_029497:160	Urb1	INSIDE	0.700	3.771	2160.08	8145.55	2.640	1696.04	4477.78
A_68_P30711910	chr16:42875216-42875260	ENSMUST00000142270:462		INSIDE	0.700	2.133	1673.65	3569.72	1.494	1426.53	2131.30
A_68_P29581489	chr14:47917977-47918021	NM_178684:10	Mapk1ip1l	INSIDE	0.700	0.361	1466.61	528.91	0.252	1134.21	286.25
A_68_P29057255	chr13:55927741-55927786	NM_011097:5023	Pitx1	INSIDE	0.700	0.680	1091.17	742.02	0.476	823.74	392.16
A_68_P29033456	chr13:51740655-51740699	NM_025415:76	Cks2	INSIDE	0.700	0.413	1690.89	698.52	0.289	1351.20	390.79
A_68_P28457438	chr12:57634665-57634709	NM_001146198:1407	Nkx2-1	INSIDE	0.700	4.779	2225.17	10635.16	3.344	1743.77	5830.65
A_68_P28266914	chr12:16807531-16807575	NM_015764:140	Greb1	INSIDE	0.700	2.901	810.38	2351.11	2.030	750.04	1522.57
A_68_P28051172	chr11:97396215-97396259	NM_018873:40204	Srcin1	INSIDE	0.700	0.608	1625.25	988.77	0.426	1357.49	577.83
A_68_P27643063	chr11:22760679-22760723	NM_001169114:-364	B3gnt2	PROMOTER	0.700	0.506	2421.47	1224.07	0.354	1900.37	672.12
A_68_P27281296	chr10:79612763-79612807	NM_021565:1750	Midn	INSIDE	0.700	4.321	2320.75	10027.48	3.025	2328.59	7043.76
A_68_P27080343	chr10:39452167-39452211	NM_011264:223	Rev3l	INSIDE	0.700	3.744	1700.38	6366.58	2.623	1353.46	3549.60
A_68_P25276873	chr7:89483916-89483960	NM_001190374:-265	Adamtsl3	PROMOTER	0.700	4.973	362.68	1803.74	3.484	321.18	1118.86
A_68_P25186098	chr7:73205391-73205435	NM_021336:191	Snrpa1	INSIDE	0.700	3.191	2970.05	9478.76	2.233	2477.32	5531.24
A_68_P24934669	chr6:147424894-147424938	NM_025911:524	Ccdc91	INSIDE	0.700	0.336	1347.37	452.88	0.235	1094.76	257.57
A_68_P23418114	chr4:151712427-151712471	NM_001081376:-311	Chd5	PROMOTER	0.700	3.339	1489.68	4973.58	2.336	1190.86	2781.88
A_68_P23253009	chr4:120419330-120419374	NM_001081142:429	Kcnq4	INSIDE	0.700	0.421	1627.13	685.39	0.295	1038.51	306.34
A_68_P22475542	chr3:120966000-120966044	NM_178936:212	Tmem56	INSIDE	0.700	2.318	1038.75	2407.36	1.621	931.02	1509.40
A_68_P22310058	chr3:87598739-87598783	NM_001033124:324	Ntrk1	INSIDE	0.700	0.506	2904.15	1469.70	0.354	2117.98	750.18
A_68_P21819736	chr2:164611295-164611339	NM_133924:-204	Snx21	DIVERGENT_PROMOTER	0.700	0.468	1044.70	489.18	0.328	809.32	265.27
A_68_P21736746	chr2:149656361-149656405	NM_001085521:-136	Tmem90b	PROMOTER	0.700	2.463	1270.32	3128.67	1.725	1016.41	1753.17
A_68_P20859687	chr1:179372004-179372048	NM_001012330:-2765	Zfp238	PROMOTER	0.700	0.450	1249.89	561.89	0.315	961.70	302.50
A_68_P20782631	chr1:165242518-165242562	NM_011127:1241	Prrx1	INSIDE	0.700	0.537	2592.13	1390.81	0.376	1988.13	746.77
A_68_P20411726	chr1:88255561-88255605	NR_002851:-2680	Snord82	PROMOTER	0.700	0.421	3050.70	1285.70	0.295	2189.90	646.03
A_68_P31947894	chr19:10092837-10092881	NM_144538:141	Rab3il1	INSIDE	0.699	0.711	3729.52	2652.45	0.497	2969.77	1476.84
A_68_P31371530	chr17:78817247-78817291	NM_199448:175	Fez2	INSIDE	0.699	0.653	1098.24	717.13	0.457	961.61	439.19
A_68_P31217292	chr17:48548926-48548970	NM_001110832:197	Nfya	INSIDE	0.699	0.676	3845.57	2601.50	0.473	2738.91	1294.82
A_68_P30132824	chr15:35256346-35256390	BC048602:1922		INSIDE	0.699	0.716	1512.05	1081.94	0.500	1330.85	665.22
A_68_P29625742	chr14:57655527-57655571	NM_016975:21234	Gja3	INSIDE	0.699	2.851	2535.65	7230.18	1.993	1791.99	3571.30
A_68_P28865543	chr13:20183326-20183370	NM_080288:973	Elmo1	INSIDE	0.699	2.236	1887.42	4219.99	1.563	1538.09	2404.45
A_68_P27930797	chr11:76057386-76057430	NM_183263:171	Rnmtl1	INSIDE	0.699	0.623	1075.07	669.86	0.436	791.96	344.95
A_68_P27897959	chr11:69734325-69734369	NM_001166590:-214	Eif5a	PROMOTER	0.699	0.580	1588.29	920.43	0.405	1293.01	523.46
A_68_P27892805	chr11:68874069-68874113	NM_028005:814	2310047M10Rik	INSIDE	0.699	0.619	3389.27	2099.33	0.433	2486.71	1077.40
A_68_P27624062	chr11:18927325-18927369	NM_001193271:-8374	Meis1	PROMOTER	0.699	2.834	669.78	1898.10	1.981	582.27	1153.62
A_68_P27283559	chr10:79957852-79957896	NM_139226:224	Onecut3	INSIDE	0.699	0.546	948.18	517.41	0.382	947.75	361.74
A_68_P27280129	chr10:79440293-79440337	NM_001003949:6761	ORF61	INSIDE	0.699	4.285	2639.02	11307.17	2.996	1953.12	5851.12
A_68_P27109368	chr10:44786970-44787014	NM_011156:-27	Prep	PROMOTER	0.699	2.315	1246.43	2885.28	1.619	1073.25	1737.34
A_68_P26920198	chr10:7400931-7400975	NR_033219:4	BC020402	INSIDE	0.699	3.744	1028.88	3852.45	2.616	784.77	2053.25
A_68_P26725648	chr9:92145271-92145315	NM_011636:261	Plscr1	INSIDE	0.699	5.086	4207.87	21402.74	3.557	3200.47	11383.19
A_68_P26350224	chr9:21856374-21856418	NM_010487:71	Elavl3	INSIDE	0.699	0.659	1705.24	1123.25	0.461	1395.20	642.80
A_68_P26232174	chr8:124793330-124793374	ENSMUST00000055537:-116		PROMOTER	0.699	0.463	4576.09	2116.54	0.323	3168.48	1024.16
A_68_P25842942	chr8:48759661-48759705	NM_023503:831	Ing2	INSIDE	0.699	2.540	1748.93	4442.79	1.777	1340.85	2382.26
A_68_P25410004	chr7:116925392-116925436	NM_020610:-355	Nrip3	PROMOTER	0.699	0.506	2167.56	1097.66	0.354	1781.02	630.66
A_68_P25377919	chr7:109399240-109399284	NM_019566:-630	Rhog	PROMOTER	0.699	0.610	4244.14	2588.18	0.426	3165.50	1349.85
A_68_P25352111	chr7:104886841-104886885	NM_175105:-105	Aqp11	PROMOTER	0.699	2.542	2637.54	6704.38	1.776	2109.91	3747.49
A_68_P24993786	chr7:20110039-20110083	NM_027116:5981	Nkpd1	INSIDE	0.699	0.631	1837.50	1160.09	0.441	1298.68	572.81
A_68_P24951858	chr7:5012557-5012603	NM_001205231:-1164	U2af2	PROMOTER	0.699	0.478	1184.78	566.68	0.334	914.69	305.78
A_68_P24513200	chr6:65621637-65621681	NM_172399:54	A930038C07Rik	INSIDE	0.699	0.670	4999.65	3351.52	0.469	3946.83	1850.28
A_68_P24449794	chr6:52256292-52256336	NR_038163:8512	5730457N03Rik	DOWNSTREAM	0.699	0.668	1773.50	1183.83	0.467	1425.94	665.53
A_68_P24018800	chr5:117566119-117566163	NM_001122666:-138	Suds3	DIVERGENT_PROMOTER	0.699	4.344	939.23	4080.11	3.038	760.86	2311.42
A_68_P23927393	chr5:100468878-100468922	NM_001163035:-112	Enoph1	DIVERGENT_PROMOTER	0.699	2.082	1282.23	2669.01	1.455	1160.39	1688.61
A_68_P23927139	chr5:100407949-100407993	NM_001077265:-13	Hnrnpd	PROMOTER	0.699	0.270	2769.41	747.63	0.189	2108.08	397.60
A_68_P23440102	chr4:155079253-155079297	NM_026899:351	Ssu72	INSIDE	0.699	5.078	1016.66	5162.16	3.548	849.30	3013.64
A_68_P22857437	chr4:40420294-40420338	NM_001166009:154	Tmem215	INSIDE	0.699	0.554	1565.45	867.09	0.387	1226.76	475.01
A_68_P22085571	chr3:41369106-41369150	NM_001130186:327	Phf17	INSIDE	0.699	3.561	777.00	2767.12	2.490	617.84	1538.52
A_68_P20826333	chr1:173080680-173080724	NM_025321:32	Sdhc	INSIDE	0.699	0.594	912.49	542.06	0.415	782.69	325.19

ProbeName	Target position of probe on CpG island microarray	TargetID	GeneSymbol	CpG island Description	Ratio of relative methylation (TiO_2-NP/Vehicle)	Sham group			TiO_2-H group		
						Relative methylation (Cy5/Cy3)	Cy3 signal (Input)	Cy5 signal (MeDIP)	Relative methylation (Cy5/Cy3)	Cy3 signal (Input)	Cy5 signal (MeDIP)
A_68_P20416040	chr1:89052463-89052507	NM_021306:-882	Ecel1	PROMOTER	0.699	2.351	2960.62	6959.26	1.644	2453.84	4034.07
A_68_P32467106	chrX:71500485-71500529	NM_007927:329	Emd	INSIDE	0.698	2.767	718.42	1987.89	1.932	921.66	1780.25
A_68_P32240896	chrX:7499453-7499497	NM_013892:2527	Pcsk1n	INSIDE	0.698	1.965	1511.31	2970.29	1.372	2174.74	2983.50
A_68_P32036090	chr19:26681592-26681637	NM_011416:1965	Smarca2	INSIDE	0.698	0.594	1435.12	851.95	0.415	962.49	399.10
A_68_P31565719	chr18:25645825-25645869	NM_001146292:266638	Celf4	INSIDE	0.698	2.291	4861.85	11137.46	1.598	3598.10	5750.62
A_68_P31400335	chr17:84105596-84105640	NM_001171053:116	Mta3	INSIDE	0.698	0.345	2227.11	767.40	0.240	1740.58	418.51
A_68_P30670430	chr16:35490785-35490829	NM_028295:153	Pdia5	INSIDE	0.698	2.506	13939.13	34927.18	1.748	10937.01	19116.35
A_68_P29388380	chr14:9046626-9046670	NM_027782:-13	Kctd6	PROMOTER	0.698	3.029	623.65	1889.29	2.115	509.18	1077.02
A_68_P28727296	chr12:109556884-109556928	NM_001044380:12427	Hhipl1	INSIDE	0.698	2.253	2890.11	6510.02	1.573	2116.18	3329.48
A_68_P28583196	chr12:82961089-82961133	NM_018814:94	Pcnx	INSIDE	0.698	3.272	1543.25	5049.42	2.283	1380.99	3152.93
A_68_P28307238	chr12:28024309-28024353	NM_009234:3253	Sox11	INSIDE	0.698	0.411	1377.76	566.34	0.287	1087.00	311.70
A_68_P28152749	chr11:115179633-115179677	NM_176847:3578	Ush1g	INSIDE	0.698	2.291	3543.88	8118.31	1.600	2632.49	4211.35
A_68_P27981398	chr11:84953893-84953937	NM_001029934:-457	Usp32	PROMOTER	0.698	4.303	853.79	3674.11	3.003	711.05	2135.62
A_68_P27371456	chr10:96080276-96080320	NM_007569:664	Btg1	INSIDE	0.698	0.519	1069.81	555.11	0.362	896.69	324.82
A_68_P26674972	chr9:81525625-81525671	NM_010482:511	Htr1b	INSIDE	0.698	3.457	750.44	2594.46	2.412	659.81	1591.28
A_68_P26630618	chr9:72833442-72833486	NM_001114328:107	Ccpg1	INSIDE	0.698	2.761	2154.73	5950.06	1.927	1906.35	3673.96
A_68_P26512820	chr9:51856174-51856218	NM_001104617:151	Rdx	INSIDE	0.698	2.498	959.55	2396.61	1.744	788.68	1375.24
A_68_P26044786	chr8:91566839-91566883	NM_021390:1201	Sall1	INSIDE	0.698	0.708	1844.69	1305.76	0.494	1661.39	820.95
A_68_P25377986	chr7:109415745-109415789	NM_009287:-571	Stim1	PROMOTER	0.698	0.624	2515.92	1569.10	0.435	1874.92	816.19
A_68_P25113823	chr7:56891830-56891874	NM_001005232:353	Dbx1	INSIDE	0.698	0.452	1826.83	825.95	0.315	1598.27	504.10
A_68_P24954136	chr7:6083754-6083798	NM_001013012:23797	Zfp787	INSIDE	0.698	5.799	2284.82	13248.66	4.046	1723.45	6973.04
A_68_P24016055	chr5:117041631-117041675	NM_026886:174	Srrm4	INSIDE	0.698	0.669	4020.89	2690.82	0.467	2930.68	1368.20
A_68_P22650336	chr3:152058904-152058948	NM_001080755:-47	Zzz3	PROMOTER	0.698	4.731	913.40	4321.38	3.302	786.13	2595.72
A_68_P21939653	chr3:9833472-9833516	NM_001195031:185	Pag1	INSIDE	0.698	3.715	879.13	3266.00	2.594	723.35	1876.35
A_68_P21783925	chr2:158436516-158436560	NM_009508:45	Slc32a1	INSIDE	0.698	0.322	2849.81	917.82	0.225	1957.44	440.08
A_68_P21721819	chr2:146839148-146839192	NM_011917:375	Xrn2	INSIDE	0.698	2.597	1246.41	3236.80	1.813	1066.33	1933.14
A_68_P21428541	chr2:92274385-92274429	NM_009963:-180	Cry2	PROMOTER	0.698	3.385	1287.75	4359.25	2.361	1013.92	2394.07
A_68_P20879817	chr1:183081001-183081045	NM_030131:-39	Cnih4	DIVERGENT_PROMOTER	0.698	0.660	1322.18	872.51	0.461	1144.45	527.24
A_68_P20226311	chr1:52289826-52289870	NM_001081081:228	Gls	INSIDE	0.698	4.948	825.76	4086.25	3.456	646.27	2233.26
A_68_P31925605	chr19:4878917-4878961	NM_001168517:249	Zdhhc24	INSIDE	0.697	0.594	1464.82	870.36	0.414	1168.03	483.77
A_68_P31113240	chr17:27070276-27070320	NM_009343:227	Phf1	INSIDE	0.697	3.705	1744.14	6462.25	2.581	1370.25	3536.67
A_68_P30575812	chr16:17806955-17806999	NM_153790:9602	Scarf2	INSIDE	0.697	2.686	658.24	1768.08	1.871	637.70	1193.36
A_68_P30080557	chr15:25293948-25293992	NM_027395:49549	Basp1	INSIDE	0.697	0.563	1951.92	1099.67	0.393	1596.07	626.64
A_68_P29059922	chr13:56322977-56323021	ENSMUST00000127754:22303		DOWNSTREAM	0.697	4.203	1089.83	4580.54	2.931	909.19	2665.27
A_68_P28540844	chr12:75065711-75065755	NM_178392:216	Snapc1	INSIDE	0.697	0.387	1526.58	590.13	0.269	1366.68	368.17
A_68_P27924902	chr11:75027241-75027285	NM_177708:19768	Rtn4rl1	INSIDE	0.697	0.303	2009.48	608.31	0.211	1409.78	297.28
A_68_P26880501	chr9:120842440-120842484	NM_007614:-55	Ctnnb1	PROMOTER	0.697	2.333	935.72	2183.37	1.627	809.89	1318.03
A_68_P26727062	chr9:92436930-92436974	NM_001142916:-108	Plod2	PROMOTER	0.697	0.660	3367.55	2223.58	0.460	2741.16	1260.96
A_68_P26470171	chr9:44689090-44689134	NM_001081049:245	Mll1	INSIDE	0.697	0.601	3007.98	1808.25	0.419	2408.65	1009.06
A_68_P26199699	chr8:119445245-119445289	NM_026844:70	2310061C15Rik	INSIDE	0.697	0.694	1396.03	968.77	0.484	1057.14	511.52
A_68_P25661257	chr8:12396498-12396542	NM_009233:1002	Sox1	INSIDE	0.697	0.393	2863.28	1125.20	0.274	1742.03	477.18
A_68_P24589797	chr6:82723544-82723588	NM_013820:882	Hk2	INSIDE	0.697	0.576	1498.28	862.95	0.402	1303.05	523.27
A_68_P24388781	chr6:40385401-40385445	NM_175528:710	E330009J07Rik	INSIDE	0.697	2.496	4425.43	11044.45	1.739	3203.15	5570.51
A_68_P23579558	chr5:32000236-32000280	NM_144541:-164	Bre	DIVERGENT_PROMOTER	0.697	0.644	1019.28	656.52	0.449	834.54	374.90
A_68_P23523153	chr5:20387713-20387757	NM_172992:208	Phtf2	INSIDE	0.697	2.193	1738.85	3813.08	1.527	1550.86	2368.93
A_68_P23364315	chr4:141033675-141033723	NM_019763:60814	Spen	INSIDE	0.697	0.656	1984.14	1300.80	0.457	1413.42	646.14
A_68_P21149538	chr2:38499224-38499268	NM_011187:180	Psmb7	INSIDE	0.697	2.079	4170.76	8672.91	1.449	3595.66	5211.59
A_68_P21044981	chr2:19368193-19368237	NM_018809:925	Ptf1a	INSIDE	0.697	0.600	1433.53	860.80	0.418	1051.36	439.90
A_68_P20725327	chr1:155033029-155033073	NM_008485:527	Lamc2	INSIDE	0.697	1.986	2613.01	5189.88	1.384	2127.09	2944.87
A_68_P20395058	chr1:82836029-82836073	NM_010472:-7	Agfg1	PROMOTER	0.697	3.622	600.75	2175.86	2.524	536.20	1353.44
A_68_P20372372	chr1:78655014-78655058	NM_001136226:22	Utp14b	INSIDE	0.697	0.526	2493.67	1312.38	0.367	1869.99	685.96
A_68_P31373926	chr17:79234811-79234855	NM_181649:-23	Ccdc75	DIVERGENT_PROMOTER	0.696	0.462	1641.16	758.42	0.322	1257.62	404.63
A_68_P31304342	chr17:66461159-66461203	NM_001037757:196	ORF19	INSIDE	0.696	0.622	1331.78	828.37	0.433	1023.38	443.33
A_68_P31303254	chr17:66234502-66234546	NM_001198949:-261	Ralbp1	PROMOTER	0.696	0.347	1439.83	499.71	0.241	1044.35	252.10
A_68_P31106387	chr17:25946215-25946259	NM_001164225:207	Fbxl16	INSIDE	0.696	5.457	707.19	3858.98	3.800	598.67	2275.22
A_68_P31020006	chr17:5941362-5941406	NM_001113352:105	Synj2	INSIDE	0.696	2.922	945.29	2762.39	2.033	763.25	1551.47
A_68_P30988236	chr16:95807340-95807384	ENSMUST00000121809:838		INSIDE	0.696	0.452	3848.31	1740.59	0.315	2546.45	801.70
A_68_P30655998	chr16:33057157-33057201	NM_021338:579	Rpl35a	INSIDE	0.696	0.362	4227.16	1531.35	0.252	3023.95	762.74
A_68_P30130469	chr15:34767206-34767250	NM_181317:93	Kcns2	INSIDE	0.696	3.557	3110.08	11061.32	2.476	2275.85	5634.51
A_68_P30114905	chr15:31461133-31461177	NM_172606:-362	Marchf6	PROMOTER	0.696	2.830	679.11	1921.71	1.969	633.03	1246.44
A_68_P30019878	chr15:12047227-12047275	NM_011767:-355	Zfr	PROMOTER	0.696	0.478	1334.66	637.70	0.332	1058.01	351.75
A_68_P29237510	chr13:95643777-95643821	NM_011021:-1783	Otp	PROMOTER	0.696	2.229	1208.63	2693.96	1.551	1067.97	1656.90
A_68_P29142605	chr13:73984503-73984547	NM_028186:555	Nkd2	INSIDE	0.696	0.307	1695.32	519.98	0.213	1227.90	262.02
A_68_P28577477	chr12:81859800-81859844	NM_001008423:1880	Gm1568	INSIDE	0.696	4.420	997.16	4407.01	3.076	878.24	2701.31
A_68_P28550066	chr12:76836469-76836513	NM_030750:226	Sgpp1	INSIDE	0.696	3.104	1596.06	4953.63	2.161	1275.77	2757.46

ProbeName	Target position of probe on CpG island microarray	TargetID	GeneSymbol	CpG island Description	Ratio of relative methylation (TiO_2-NP/Vehicle)	Sham group			TiO_2-H group		
						Relative methylation (Cy5/Cy3)	Cy3 signal (Input)	Cy5 signal (MeDIP)	Relative methylation (Cy5/Cy3)	Cy3 signal (Input)	Cy5 signal (MeDIP)
A_68_P28159868	chr11:116395378-116395422	NM_011451:-653	Sphk1	PROMOTER	0.696	2.097	1553.27	3256.62	1.460	1140.61	1665.00
A_68_P28055835	chr11:98210174-98210218	NM_144828:145	Ppp1r1b	INSIDE	0.696	0.379	3379.94	1281.40	0.264	2554.73	674.28
A_68_P27695167	chr11:33413838-33413882	NM_023146:-114	Ranbp17	PROMOTER	0.696	0.297	1676.15	497.72	0.207	1344.20	277.76
A_68_P27294769	chr10:82448011-82448055	NM_021439:-209	Chst11	PROMOTER	0.696	0.519	914.64	474.80	0.361	725.30	262.05
A_68_P26841766	chr9:113650462-113650506	NM_001114347:-106	Clasp2	PROMOTER	0.696	2.454	1081.36	2654.15	1.708	1022.88	1747.00
A_68_P26783272	chr9:102910605-102910649	NM_033314:-192	Slco2a1	PROMOTER	0.696	0.696	1919.50	1336.81	0.485	1592.28	772.35
A_68_P26221253	chr8:123113267-123113311	NM_178856:-313	Gins2	PROMOTER	0.696	0.723	3861.00	2792.24	0.503	2772.14	1395.26
A_68_P25520724	chr7:137409910-137409954	NM_010207:390	Fgfr2	INSIDE	0.696	3.397	857.94	2914.62	2.366	772.95	1828.76
A_68_P25414288	chr7:117758585-117758629	NM_177324:-172	Sbf2	PROMOTER	0.696	3.043	1016.15	3092.37	2.118	879.67	1863.29
A_68_P25189681	chr7:73834635-73834679	NM_001191001:-118	Lins	DIVERGENT_PROMOTER	0.696	4.534	1516.52	6875.58	3.155	1129.79	3564.01
A_68_P24780082	chr6:117118689-117118733	NM_021704:158	Cxcl12	INSIDE	0.696	2.043	2802.04	5725.22	1.423	1998.87	2843.71
A_68_P24622637	chr6:88468937-88468981	NM_016906:-164	Sec61a1	PROMOTER	0.696	0.396	2500.49	989.51	0.275	1889.13	519.99
A_68_P24620322	chr6:88144530-88144574	NM_008090:-4105	Gata2	PROMOTER	0.696	0.514	1917.75	985.92	0.358	1490.30	532.95
A_68_P24183980	chr5:150040675-150040719	AK155259:18562		DOWNSTREAM	0.696	3.227	368.97	1190.80	2.247	300.38	674.81
A_68_P24038735	chr5:120890123-120890167	NM_008499:8250	Lhx5	INSIDE	0.696	2.894	1422.27	4116.45	2.015	1063.04	2142.30
A_68_P23281727	chr4:126412759-126412803	NM_198960:733	Tcfap2e	INSIDE	0.696	3.325	6324.11	21028.92	2.314	4685.81	10842.10
A_68_P22942709	chr4:57002896-57002940	NM_001142965:345	6430704M03Rik	INSIDE	0.696	0.655	1551.95	1016.96	0.456	1155.14	526.63
A_68_P22314349	chr3:88340523-88340567	NR_035434:-240	Mir1905	PROMOTER	0.696	0.558	952.74	531.17	0.388	767.53	297.92
A_68_P21564755	chr2:118375207-118375251	NM_138313:186	Bmf	INSIDE	0.696	0.573	2025.37	1160.38	0.399	1542.99	615.33
A_68_P31939676	chr19:7491566-7491610	NM_175381:-526	2700081O15Rik	PROMOTER	0.695	2.878	728.92	2097.81	2.000	622.95	1245.97
A_68_P31669144	chr18:44971731-44971775	NM_001085373:84	Mcc	INSIDE	0.695	2.919	1843.93	5382.88	2.028	1525.52	3094.23
A_68_P30569150	chr16:16359116-16359160	NM_001025947:-15	Dnm1l	PROMOTER	0.695	9.913	441.96	4381.07	6.888	402.44	2771.94
A_68_P30542114	chr16:10411911-10411955	NM_011955:-98	Nubp1	PROMOTER	0.695	3.091	1616.84	4997.49	2.148	1207.61	2593.62
A_68_P29643651	chr14:60884174-60884218	NM_144843:132	Mtmr6	INSIDE	0.695	0.629	1035.68	651.05	0.437	785.15	342.80
A_68_P29514238	chr14:32643908-32643952	NM_001024604:-329	Ankrd28	PROMOTER	0.695	2.429	1397.66	3394.95	1.687	1170.60	1974.84
A_68_P29455222	chr14:22666790-22666836	NM_026965:1320	Comtd1	INSIDE	0.695	0.294	1661.28	489.09	0.205	1213.03	248.16
A_68_P29356808	chr13:117099881-117099929	NM_021459:-8	Isl1	PROMOTER	0.695	0.663	1444.35	957.00	0.461	1105.47	509.31
A_68_P29047196	chr13:54150905-54150949	NM_010076:101	Drd1a	INSIDE	0.695	0.223	4113.25	917.30	0.155	3006.02	465.87
A_68_P28813401	chr13:8870209-8870253	NM_172445:58	Wdr37	INSIDE	0.695	0.462	4455.16	2056.42	0.321	2960.20	950.05
A_68_P28724518	chr12:109154385-109154429	NM_001079883:87218	Bcl11b	INSIDE	0.695	3.313	3599.17	11923.69	2.301	2605.18	5995.16
A_68_P28049248	chr11:97049488-97049532	NM_008379:-304	Kpnb1	PROMOTER	0.695	0.387	3217.71	1246.27	0.269	2565.16	690.78
A_68_P27780097	chr11:48630503-48630547	NM_145377:369	Trim41	INSIDE	0.695	0.296	4281.38	1266.23	0.206	3205.52	658.80
A_68_P26913052	chr10:5959184-5959228	NM_001033466:774	Zbtb2	INSIDE	0.695	2.107	1416.25	2984.20	1.465	1220.59	1788.25
A_68_P26756439	chr9:97933339-97933383	NM_022319:226	Clstn2	INSIDE	0.695	2.776	2409.11	6688.21	1.930	1968.06	3799.15
A_68_P26743637	chr9:95650689-95650733	NM_011643:67	Trpc1	INSIDE	0.695	3.972	1897.18	7535.54	2.760	1504.59	4153.40
A_68_P26476239	chr9:45636484-45636528	NM_001081373:215	Cep164	INSIDE	0.695	0.318	4382.43	1392.33	0.221	2939.28	649.11
A_68_P26024226	chr8:87823551-87823595	NM_001163791:42	Orc6	INSIDE	0.695	0.457	1607.27	734.93	0.318	1045.35	332.24
A_68_P26002685	chr8:83538307-83538351	NM_027554:477	Usp38	INSIDE	0.695	0.533	1421.90	758.26	0.370	1065.33	394.65
A_68_P25670556	chr8:13785998-13786042	NM_025924:406	Upf3a	INSIDE	0.695	0.341	2780.85	948.03	0.237	2122.14	503.04
A_68_P25265480	chr7:87377466-87377510	NM_011870:14	Cib1	INSIDE	0.695	0.548	3958.75	2167.82	0.381	3045.61	1159.72
A_68_P24835542	chr6:127837749-127837793	NM_026743:131	Tspan11	INSIDE	0.695	2.830	3184.80	9012.80	1.967	2928.42	5759.83
A_68_P24119629	chr5:136611657-136611701	NM_027891:266	Lrwd1	INSIDE	0.695	0.261	2694.04	703.84	0.182	1890.19	343.12
A_68_P23545731	chr5:24605464-24605510	NM_145401:974	Prkag2	INSIDE	0.695	0.380	1580.94	600.66	0.264	1128.59	298.02
A_68_P23399007	chr4:148540337-148540381	NM_001081274:458	Pgd	INSIDE	0.695	0.298	3297.81	981.40	0.207	2304.45	476.40
A_68_P23353259	chr4:139198138-139198182	NM_175438:19352	Aldh4a1	INSIDE	0.695	0.392	1599.50	627.10	0.272	1113.79	303.40
A_68_P22210464	chr3:67319384-67319428	NM_001164763:39	Rarres1	INSIDE	0.695	0.239	5953.52	1424.01	0.166	4350.24	722.66
A_68_P21423816	chr2:91490456-91490500	NM_146124:-2886	Arhgap1	PROMOTER	0.695	0.233	2951.71	687.53	0.162	1759.64	285.06
A_68_P20439700	chr1:92895452-92895496	NM_001111312:171	Lrrfip1	INSIDE	0.695	0.556	1097.68	610.21	0.386	942.24	363.80
A_68_P20149053	chr1:36614863-36614907	NM_001126047:342	Sema4c	INSIDE	0.695	0.499	1804.60	899.77	0.346	1455.21	503.96
A_68_P20139530	chr1:34636521-34636565	NM_213727:5	Fam123c	INSIDE	0.695	2.681	1460.52	3916.14	1.865	1245.66	2322.82
A_68_P32140499	chr19:45857773-45857817	NM_023799:-13	Mgea5	PROMOTER	0.694	11.300	840.79	9501.38	7.845	685.74	5379.35
A_68_P32118990	chr19:42165379-42165423	NM_145501:476	Pi4k2a	INSIDE	0.694	0.218	2386.97	519.56	0.151	1759.87	265.96
A_68_P31934469	chr19:6400075-6400119	NM_011242:-486	Rasgrp2	PROMOTER	0.694	0.665	3421.29	2276.72	0.462	2530.45	1168.28
A_68_P31926489	chr19:5039460-5039504	NM_175383:657	B3gnt1	INSIDE	0.694	2.042	2330.45	4759.70	1.417	1825.87	2587.93
A_68_P31857885	chr18:79306491-79306535	NM_053099:-382	Setbp1	PROMOTER	0.694	3.920	1152.58	4517.97	2.719	1020.63	2775.09
A_68_P31400333	chr17:84105260-84105304	NM_001171053:-220	Mta3	PROMOTER	0.694	0.479	3298.39	1579.65	0.332	2524.04	838.73
A_68_P31229018	chr17:50648295-50648339	NM_013880:-555	Plcl2	PROMOTER	0.694	2.616	691.23	1808.26	1.816	674.13	1223.97
A_68_P31108363	chr17:26250594-26250638	NM_021793:356	Tmem8	INSIDE	0.694	0.473	2882.77	1363.97	0.328	2277.78	747.43
A_68_P30363283	chr15:78765483-78765527	NM_133800:142	Nol12	INSIDE	0.694	5.172	1782.01	9217.16	3.590	1513.80	5435.10
A_68_P30137684	chr15:36212197-36212241	NM_013923:684	Rnf19a	INSIDE	0.694	0.551	1500.16	826.97	0.383	1178.78	451.24
A_68_P29950628	A_68_P29950628			Unknown	0.694	2.574	1796.82	4624.34	1.787	1387.98	2479.89
A_68_P28221291	chr12:8304878-8304922	NM_013527:3860	Gdf7	INSIDE	0.694	0.209	3049.82	637.34	0.145	2147.28	311.29
A_68_P27835443	chr11:59016116-59016160	NM_026035:120	Mrpl55	INSIDE	0.694	0.506	1312.77	664.61	0.351	941.52	330.75
A_68_P27503018	chr10:120788390-120788434	ENSMUST00000078515:8085		DOWNSTREAM	0.694	3.511	496.47	1743.00	2.436	436.35	1062.84
A_68_P27271358	chr10:77441308-77441352	NM_026431:-64	1810043G02Rik	DIVERGENT_PROMOTER	0.694	2.215	1323.85	2932.13	1.538	1164.08	1790.45

ProbeName	Target position of probe on CpG island microarray	TargetID	GeneSymbol	CpG island Description	Ratio of relative methylation (TiO_2-NP/Vehicle)	Sham group			TiO_2-H group		
						Relative methylation (Cy5/Cy3)	Cy3 signal (Input)	Cy5 signal (MeDIP)	Relative methylation (Cy5/Cy3)	Cy3 signal (Input)	Cy5 signal (MeDIP)
A_68_P27189032	chr10:62486795-62486839	NM_001079824:-219	Hnrnph3	DIVERGENT_PROMOTER	0.694	3.667	1279.71	4692.71	2.545	1018.33	2591.24
A_68_P26375208	chr9:27107222-27107266	NM_001033323:432	Igsf9b	INSIDE	0.694	16.491	1493.00	24620.68	11.443	1452.90	16625.25
A_68_P26345850	chr9:20988460-20988504	NM_183408:18325	Pde4a	INSIDE	0.694	0.710	1510.15	1071.92	0.493	1199.24	590.69
A_68_P25008855	chr7:25964863-25964907	X53654:547486		INSIDE	0.694	3.689	1820.13	6714.22	2.561	1645.46	4214.65
A_68_P23796833	chr5:73683943-73683987	NM_001159889:-68	Ociad1	PROMOTER	0.694	0.361	3341.84	1207.07	0.251	2567.43	643.45
A_68_P23701543	chr5:53981055-53981099	NM_009035:-377	Rbpj	PROMOTER	0.694	0.547	1066.95	584.11	0.380	884.47	336.01
A_68_P23593815	chr5:34679727-34679773	NM_011278:712	Rnf4	INSIDE	0.694	0.467	1038.96	485.45	0.324	779.89	252.95
A_68_P23448647	chr5:4104421-4104465	NM_020010:255	Cyp51	INSIDE	0.694	2.645	1676.58	4434.73	1.837	1376.44	2528.25
A_68_P23316278	chr4:132756326-132756370	NM_153423:69801	Wasf2	DOWNSTREAM	0.694	0.532	1417.22	753.53	0.369	1075.55	396.84
A_68_P22129176	chr3:51145034-51145078	ENSMUST00000135496:-476		PROMOTER	0.694	0.413	4379.96	1807.34	0.286	3158.93	904.81
A_68_P21904290	chr2:180234531-180234575	NM_018766:-128	Ntsr1	PROMOTER	0.694	3.714	1434.24	5326.89	2.577	1213.76	3128.41
A_68_P21563665	chr2:118199048-118199092	NM_201367:85	Gpr176	INSIDE	0.694	3.405	1116.43	3801.77	2.362	932.29	2201.88
A_68_P21071434	chr2:25188169-25188213	NM_001029983:-72	Man1b1	DIVERGENT_PROMOTER	0.694	0.475	1668.86	792.52	0.330	1216.13	401.00
A_68_P20842587	chr1:176432270-176432314	NM_019445:337	Fmn2	INSIDE	0.694	0.199	4082.68	813.92	0.138	2755.89	381.42
A_68_P20641806	chr1:137715158-137715202	NM_133664:6	Lad1	INSIDE	0.694	2.945	1047.04	3083.40	2.045	902.37	1845.00
A_68_P20410595	chr1:88051237-88051281	NM_030184:-96	Armc9	PROMOTER	0.694	0.601	1101.07	661.22	0.417	861.19	358.72
A_68_P33012157	chr9_random:54677-54721	NR_027950:6	4930526I15Rik	INSIDE	0.693	2.699	616.42	1663.68	1.871	491.41	919.62
A_68_P32246203	chrX:8849653-8849697	NM_023500:-235	Xk	PROMOTER	0.693	3.007	928.86	2792.81	2.084	1113.24	2320.39
A_68_P31640473	chr18:39648559-39648603	NM_008173:-1681	Nr3c1	PROMOTER	0.693	0.385	3583.16	1380.02	0.267	2393.35	638.65
A_68_P30572026	chr16:17111205-17111249	NM_144954:80	Ppil2	INSIDE	0.693	0.474	1055.56	500.38	0.329	858.08	281.95
A_68_P30514668	chr16:5256235-5256279	NM_027446:-207	Fam86	PROMOTER	0.693	0.536	2591.22	1389.32	0.371	2069.17	768.38
A_68_P30421713	chr15:88692325-88692369	NM_145478:-277	Pim3	PROMOTER	0.693	0.747	3854.39	2880.86	0.518	2769.77	1433.80
A_68_P30284251	chr15:64752515-64752559	NM_009623:1322	Adcy8	INSIDE	0.693	5.235	1585.49	8299.27	3.627	1310.29	4752.17
A_68_P29875608	chr14:105575881-105575925	NM_134077:642	Rbm26	INSIDE	0.693	0.398	2574.21	1024.80	0.276	1930.57	532.91
A_68_P28177557	chr11:119251461-119251505	NR_035453:-303	Mir1932	PROMOTER	0.693	0.473	4301.79	2034.49	0.328	2961.75	970.79
A_68_P27980051	chr11:84683596-84683640	NM_153144:622	Ggnbp2	INSIDE	0.693	2.995	3509.15	10510.06	2.074	2506.21	5198.88
A_68_P27926121	chr11:75259177-75259221	NM_138950:9021	Wdr81	INSIDE	0.693	2.617	1926.58	5041.48	1.813	1475.33	2674.78
A_68_P27541964	chr10:127984659-127984703	NM_134003:120	Zc3h10	INSIDE	0.693	0.663	3822.66	2533.23	0.459	2839.96	1304.32
A_68_P27495879	chr10:119645822-119645866	NM_026617:-37	Tmbim4	DIVERGENT_PROMOTER	0.693	0.560	2303.83	1289.16	0.388	1669.81	647.72
A_68_P27285757	chr10:80297352-80297396	NM_001013758:9410	Lingo3	INSIDE	0.693	3.130	2221.11	6951.56	2.168	1458.02	3161.01
A_68_P26815973	chr9:108551515-108551559	NM_011790:175	Arih2	INSIDE	0.693	4.505	2225.55	10025.13	3.123	1848.93	5773.99
A_68_P26617704	chr9:70504900-70504944	NM_172772:59	Fam63b	INSIDE	0.693	3.271	954.18	3121.28	2.266	828.42	1877.50
A_68_P25948485	chr8:72406884-72406928	NM_026818:4385	Cilp2	INSIDE	0.693	0.505	1411.11	712.25	0.350	1126.30	394.10
A_68_P25089586	chr7:52095494-52095538	NM_010215:3859	Il4i1	INSIDE	0.693	0.419	2559.29	1071.26	0.290	1993.60	578.12
A_68_P24114960	chr5:135625731-135625775	NM_013763:172	Tbl2	INSIDE	0.693	5.311	1174.12	6235.39	3.683	987.03	3635.12
A_68_P24040311	chr5:121144977-121145021	NM_008052:16680	Dtx1	INSIDE	0.693	0.476	2925.56	1393.02	0.330	2107.14	695.57
A_68_P23611444	chr5:37482900-37482944	NM_178394:40827	Jakmip1	INSIDE	0.693	0.587	1471.92	863.48	0.406	1244.77	505.82
A_68_P23203786	chr4:109656795-109656839	NM_172296:6187	Dmrta2	DOWNSTREAM	0.693	11.784	7144.47	84187.91	8.169	5759.86	47053.60
A_68_P23062262	chr4:83063627-83063671	NM_029949:1	Snapc3	INSIDE	0.693	0.461	2124.44	979.69	0.320	1647.76	526.84
A_68_P22603836	chr3:144233786-144233830	NM_053102:418	Selenof	INSIDE	0.693	0.709	1983.86	1405.77	0.491	1439.08	706.56
A_68_P21901051	chr2:179777462-179777506	NM_178750:297	Ss18l1	INSIDE	0.693	2.287	3259.25	7452.52	1.584	2535.29	4016.28
A_68_P21137061	chr2:35835104-35835148	NM_028921:18	Ttll11	INSIDE	0.693	2.300	1076.76	2477.02	1.595	853.42	1361.13
A_68_P21053766	chr2:21063512-21063556	ENSMUST00000137984:47		INSIDE	0.693	0.306	4383.48	1339.83	0.212	3178.59	673.19
A_68_P31552994	chr18:23197880-23197924	NM_001161483:-738	Nol4	PROMOTER	0.692	0.615	1019.47	627.36	0.426	826.22	351.86
A_68_P31259825	chr17:57198936-57198980	NM_025877:328	Slc25a23	INSIDE	0.692	0.412	2820.76	1163.04	0.285	2124.77	606.14
A_68_P30414446	chr15:87454576-87454620	NM_134096:-1061	Fam19a5	PROMOTER	0.692	2.726	1301.36	3547.07	1.886	1099.35	2073.69
A_68_P30094309	chr15:27677579-27677623	NM_001081302:278003	Trio	INSIDE	0.692	3.780	1325.96	5012.56	2.617	980.24	2565.42
A_68_P29671620	chr14:65716836-65716880	NM_018788:85	Extl3	INSIDE	0.692	2.824	1062.10	2999.88	1.954	898.25	1755.11
A_68_P29493963	chr14:29331440-29331484	NM_009524:12804	Wnt5a	INSIDE	0.692	2.328	1095.38	2550.42	1.610	874.30	1407.82
A_68_P29477492	chr14:26279368-26279412	NM_183208:720	Zmiz1	INSIDE	0.692	0.459	1095.61	502.49	0.317	824.50	261.75
A_68_P29117527	chr13:69878894-69878938	NM_001145162:-141	Ube2ql1	PROMOTER	0.692	2.598	889.22	2309.97	1.797	707.25	1270.96
A_68_P27930557	chr11:76015854-76015898	NM_027773:319	Fam57a	INSIDE	0.692	5.179	1614.11	8359.91	3.582	1309.53	4690.64
A_68_P27551121	chr11:3189955-3189999	NM_019574:-483	Patz1	PROMOTER	0.692	0.384	2766.22	1061.37	0.265	2285.15	606.42
A_68_P27285909	chr10:80318182-80318226	NM_175195:185	3110056O03Rik	INSIDE	0.692	0.682	1929.05	1315.42	0.472	1672.84	789.36
A_68_P26540094	chr9:56924177-56924221	NM_001110350:16	Sin3a	INSIDE	0.692	2.665	2241.06	5972.87	1.844	1846.05	3404.59
A_68_P26061535	chr8:94325449-94325493	NM_008393:-197	Irx3	PROMOTER	0.692	0.530	2193.75	1163.60	0.367	1734.02	636.88
A_68_P25737017	chr8:28284572-28284616	NM_001080813:524	Rab11fip1	INSIDE	0.692	0.304	4520.82	1374.20	0.210	3706.83	780.03
A_68_P24427163	chr6:48388218-48388262	NM_001085415:6849	Zfp467	INSIDE	0.692	2.517	1896.93	4775.20	1.741	1458.89	2539.88
A_68_P23391245	chr4:147278506-147278550	NM_133201:400	Mfn2	INSIDE	0.692	3.419	1871.38	6397.76	2.365	1546.92	3658.53
A_68_P23195049	chr4:107890055-107890099	NM_001167936:451	Zyg11a	INSIDE	0.692	0.361	7797.78	2814.07	0.250	6107.91	1525.89
A_68_P22934434	chr4:55545364-55545408	NM_010637:-39	Klf4	PROMOTER	0.692	4.103	1686.79	6920.11	2.839	1458.09	4139.47
A_68_P22889892	chr4:46663146-46663190	NM_198664:-97	Tbc1d2	PROMOTER	0.692	0.730	1568.47	1145.29	0.505	1298.09	655.95
A_68_P22889234	chr4:46548936-46548980	NM_029077:58	Trim14	INSIDE	0.692	0.653	1214.19	793.01	0.452	972.03	439.43
A_68_P22058855	chr3:36470553-36470597	NM_009828:344	Ccna2	INSIDE	0.692	0.621	3981.59	2471.24	0.430	2905.12	1247.76
A_68_P21419651	chr2:90758053-90758097	NM_025576:133	Ptpmt1	INSIDE	0.692	2.317	761.86	1765.42	1.604	726.84	1165.97

ProbeName	Target position of probe on CpG island microarray	TargetID	GeneSymbol	CpG island Description	Ratio of relative methylation (TiO_2-NP/Vehicle)	Sham group			TiO_2-H group		
						Relative methylation (Cy5/Cy3)	Cy3 signal (Input)	Cy5 signal (MeDIP)	Relative methylation (Cy5/Cy3)	Cy3 signal (Input)	Cy5 signal (MeDIP)
A_68_P20598630	chr1:130000293-130000337	NM_181750:432	R3hdm1	INSIDE	0.692	4.213	542.35	2285.13	2.914	492.44	1434.97
A_68_P32239401	chrX:7215812-7215857	NM_009305:129	Syp	INSIDE	0.691	0.558	1832.72	1023.27	0.386	2424.33	935.13
A_68_P32217229	chr19:59150385-59150429	NM_001114312:153	4930506M07Rik	INSIDE	0.691	2.971	412.05	1224.09	2.053	390.90	802.54
A_68_P31791365	chr18:67503767-67503811	NR_033214:-31	B430212C06Rik	PROMOTER	0.691	0.701	1112.77	780.14	0.485	841.99	408.10
A_68_P31735661	chr18:57522721-57522765	NM_028447:8356	Prrc1	INSIDE	0.691	3.095	987.03	3054.60	2.139	837.58	1791.81
A_68_P31542075	chr18:21054261-21054305	NM_177038:297	Trappc8	INSIDE	0.691	2.376	898.34	2134.86	1.643	783.67	1287.52
A_68_P30990391	chr16:96212295-96212339	NM_019537:194	Psmg1	INSIDE	0.691	2.937	2994.71	8796.03	2.030	2438.51	4949.36
A_68_P30501630	chr15:102865341-102865385	NM_013553:537	Hoxc4	INSIDE	0.691	0.236	2064.76	486.38	0.163	1658.54	269.83
A_68_P30497696	chr15:102236237-102236289	NM_013672:-484	Sp1	PROMOTER	0.691	4.150	401.77	1667.16	2.868	371.80	1066.46
A_68_P30497693	chr15:102235932-102235976	NM_013672:-792	Sp1	PROMOTER	0.691	0.456	1727.50	787.03	0.315	1272.30	400.56
A_68_P30082178	chr15:25553210-25553254	NM_019472:928	Myo10	INSIDE	0.691	0.523	916.22	478.83	0.361	921.59	332.58
A_68_P29725073	chr14:75346827-75346871	NM_001033439:836	Lrch1	INSIDE	0.691	0.724	1595.45	1154.35	0.500	1315.30	657.81
A_68_P29223870	chr13:93196035-93196079	NM_175455:133	Ankrd34b	INSIDE	0.691	0.511	1453.56	743.19	0.353	1171.41	413.93
A_68_P27943677	chr11:78310572-78310616	NM_001168521:662	Sarm1	INSIDE	0.691	0.653	1392.75	909.40	0.452	1085.47	490.10
A_68_P27893219	chr11:68936458-68936502	NM_033041:2526	Hes7	INSIDE	0.691	6.019	3662.52	22043.21	4.157	2941.07	12224.97
A_68_P26530603	chr9:55174506-55174550	NM_172923:-227	AI118078	PROMOTER	0.691	5.320	1887.67	10042.75	3.675	1565.72	5753.86
A_68_P26023481	chr8:87584180-87584224	NM_026791:185	Fbxw9	INSIDE	0.691	0.337	1752.94	590.38	0.233	1207.46	281.05
A_68_P25101369	chr7:54051464-54051508	NM_008186:23	Gtf2h1	INSIDE	0.691	2.704	1176.40	3181.47	1.869	1007.35	1882.48
A_68_P25048763	chr7:36119695-36119739	NM_027897:461	Rhpn2	INSIDE	0.691	0.325	1972.93	641.12	0.225	1543.13	346.59
A_68_P25011333	chr7:26472908-26472952	NM_011577:910	Tgfb1	INSIDE	0.691	2.772	758.39	2102.41	1.916	646.49	1238.54
A_68_P24378363	chr6:38586630-38586674	NM_001033171:587	Klrg2	INSIDE	0.691	0.701	1513.41	1060.67	0.484	1244.40	602.62
A_68_P24126715	chr5:138070507-138070551	NM_001168652:178	Lrch4	INSIDE	0.691	0.604	1290.01	779.70	0.418	1082.01	451.77
A_68_P23401325	chr4:148960914-148960958	NM_023051:190	Clstn1	INSIDE	0.691	0.302	4396.17	1326.21	0.209	3102.33	646.90
A_68_P22728459	chr4:11118628-11118672	NM_009830:-205	Ccne2	PROMOTER	0.691	0.632	1428.76	903.64	0.437	1198.67	524.22
A_68_P22369559	chr3:100293003-100293047	NM_001142952:91	Fam46c	INSIDE	0.691	0.438	1320.98	578.53	0.303	1000.37	302.89
A_68_P21760472	chr2:154262476-154262520	NM_009823:279	Cbfa2t2	INSIDE	0.691	0.690	2741.05	1892.31	0.477	2220.46	1059.48
A_68_P21221946	chr2:52717355-52717399	NM_172409:475	Fmnl2	INSIDE	0.691	0.464	2916.82	1354.48	0.321	2333.98	748.60
A_68_P20593981	chr1:129102201-129102245	NM_145128:660	Mgat5	INSIDE	0.691	0.313	2435.16	761.64	0.216	1760.24	380.63
A_68_P31879150	chr18:83083408-83083452	NM_001177464:-593	Zfp516	PROMOTER	0.690	0.601	1447.25	869.63	0.415	1295.27	537.26
A_68_P31201909	chr17:45710173-45710217	NM_008302:16	Hsp90ab1	INSIDE	0.690	0.450	1770.91	797.37	0.311	1462.28	454.17
A_68_P30959225	chr16:90831355-90831399	NM_001199210:273	4931408A02Rik	INSIDE	0.690	3.559	1110.32	3951.96	2.457	855.26	2101.14
A_68_P30480283	chr15:99287930-99287974	NM_001001884:227	Nckap5l	INSIDE	0.690	0.642	5716.48	3670.83	0.443	4248.98	1883.33
A_68_P30139533	chr15:36539361-36539405	NM_008774:-654	Pabpc1	PROMOTER	0.690	2.447	1002.58	2452.92	1.688	856.64	1445.64
A_68_P30093915	chr15:27610984-27611028	NM_198301:247	Fam105a	INSIDE	0.690	0.669	2320.20	1552.03	0.462	1713.54	790.90
A_68_P29662309	chr14:64225736-64225780	NM_173393:392	Xkr6	INSIDE	0.690	0.635	2442.23	1551.87	0.438	1801.94	789.56
A_68_P29484378	chr14:27783198-27783242	NM_145221:517	Appl1	INSIDE	0.690	0.628	3394.18	2130.33	0.433	2539.20	1098.92
A_68_P29363819	chr13:118391652-118391696	NM_010408:548	Hcn1	INSIDE	0.690	3.210	1250.36	4013.51	2.216	1135.60	2516.22
A_68_P29102452	chr13:65343492-65343545			Unknown	0.690	2.564	3265.25	8370.86	1.770	2485.19	4398.38
A_68_P28881066	chr13:23461022-23461066	NM_001111107:27	Zfp322a	INSIDE	0.690	0.458	2895.56	1324.86	0.316	2173.87	686.52
A_68_P27938059	chr11:77326725-77326769	NM_001024920:29	Trp53i13	INSIDE	0.690	0.567	4036.41	2287.01	0.391	3040.92	1189.18
A_68_P26543685	chr9:57613651-57613695	NM_007713:-5	Clk3	PROMOTER	0.690	0.250	2708.79	676.16	0.172	2031.35	349.76
A_68_P26531860	chr9:55388975-55389019	NM_027397:41	Isl2	INSIDE	0.690	0.535	890.69	476.33	0.369	671.83	247.95
A_68_P26447522	chr9:40965293-40965337	NM_176860:263	Ubash3b	INSIDE	0.690	0.516	1095.37	564.69	0.356	863.01	307.17
A_68_P25947840	chr8:72273755-72273799	NM_001200023:85	Zfp963	INSIDE	0.690	4.567	5532.65	25268.69	3.152	4132.12	13023.66
A_68_P25786361	chr8:37612221-37612265	AK076528:2265		DOWNSTREAM	0.690	0.624	908.52	566.82	0.430	748.93	322.34
A_68_P25265266	chr7:87343315-87343359	NM_013659:11610	Sema4b	INSIDE	0.690	2.711	1207.86	3274.63	1.870	1063.23	1987.88
A_68_P24155845	chr5:144049594-144049638			Unknown	0.690	0.647	1164.36	752.79	0.446	872.85	389.42
A_68_P24140887	chr5:140797429-140797473	NM_010752:56	Mad1l1	INSIDE	0.690	2.643	814.08	2152.01	1.823	771.97	1407.61
A_68_P23595697	chr5:35003116-35003160	NM_001080743:111	Grk4	INSIDE	0.690	5.415	898.48	4864.85	3.733	741.02	2766.58
A_68_P23081886	chr4:86520283-86520327	NM_139306:-13	Acer2	PROMOTER	0.690	2.551	1582.26	4036.22	1.761	1342.64	2364.74
A_68_P22898164	chr4:48058319-48058363	NM_015743:-5779	Nr4a3	PROMOTER	0.690	0.384	2539.78	975.87	0.265	1896.39	502.97
A_68_P21794482	chr2:160191240-160191284	NM_010658:1539	Mafb	INSIDE	0.690	2.526	3443.28	8698.85	1.743	2633.49	4588.89
A_68_P21574795	chr2:120178680-120178724	NM_147153:167	Vps39	INSIDE	0.690	0.583	2155.53	1256.21	0.402	1589.63	638.96
A_68_P21283784	chr2:64860481-64860525	NM_016719:321	Grb14	INSIDE	0.690	2.320	2696.10	6255.35	1.600	2091.23	3345.49
A_68_P20930617	chr1:191994889-191994933	NM_008937:-351	Prox1	PROMOTER	0.690	2.927	806.25	2360.20	2.019	711.10	1436.04
A_68_P32700324	chrX:137199916-137199960	ENSMUST00000112988:328		INSIDE	0.689	3.894	805.73	3137.81	2.682	874.34	2344.80
A_68_P32397419	chrX:53984962-53985006	NM_001077361:-149	Fhl1	PROMOTER	0.689	3.850	1388.76	5346.52	2.653	1771.60	4700.51
A_68_P32319554	chrX:34185327-34185371	NM_001081956:61	Akap17b	INSIDE	0.689	2.331	636.10	1482.93	1.605	886.75	1423.36
A_68_P29071667	chr13:58229346-58229390	NM_029872:549	Hnrnpa0	INSIDE	0.689	2.391	1789.60	4279.83	1.648	1446.64	2384.19
A_68_P29052701	chr13:55097633-55097677	NM_153131:46862	Unc5a	INSIDE	0.689	3.070	1658.28	5091.25	2.115	1273.68	2694.29
A_68_P28707017	chr12:106270861-106270905	NM_028419:-16	Glrx5	DIVERGENT_PROMOTER	0.689	2.353	854.97	2011.83	1.622	731.71	1186.87
A_68_P27839121	chr11:59623157-59623201	NM_146018:97	Flcn	INSIDE	0.689	0.386	2406.66	929.67	0.266	1717.32	457.22
A_68_P27553983	chr11:3674675-3674719	NM_152818:89210	Osbp2	INSIDE	0.689	2.495	1302.13	3248.87	1.720	1093.49	1880.95
A_68_P27186921	chr10:62064974-62065018	NM_019553:50	Ddx21	INSIDE	0.689	0.559	922.28	515.44	0.385	787.66	303.46
A_68_P25576308	chr7:146374357-146374401	NM_021302:15	Stk32c	INSIDE	0.689	3.960	1300.83	5150.81	2.727	1075.47	2932.98

ProbeName	Target position of probe on CpG island microarray	TargetID	GeneSymbol	CpG island Description	Ratio of relative methylation (TiO_2-NP/Vehicle)	Sham group			TiO_2-H group		
						Relative methylation (Cy5/Cy3)	Cy3 signal (Input)	Cy5 signal (MeDIP)	Relative methylation (Cy5/Cy3)	Cy3 signal (Input)	Cy5 signal (MeDIP)
A_68_P25537055	chr7:140005461-140005505	NM_212473:-603	Fam53b	PROMOTER	0.689	2.612	745.14	1946.35	1.799	585.54	1053.46
A_68_P23753095	chr5:65213443-65213487	NM_008453:18703	Klf3	INSIDE	0.689	2.962	4467.87	13232.02	2.041	3369.82	6876.46
A_68_P23434596	chr4:154229972-154230016	NM_172878:-314	Ttc34	PROMOTER	0.689	4.610	225.61	1040.10	3.178	210.69	669.60
A_68_P23373724	chr4:142752116-142752160	NM_001081355:50474	Prdm2	INSIDE	0.689	7.632	1122.42	8566.44	5.262	861.36	4532.45
A_68_P23203729	chr4:109650347-109650391	NM_172296:-261	Dmrta2	PROMOTER	0.689	0.178	3121.66	556.52	0.123	2123.69	260.79
A_68_P22649021	chr3:151828589-151828633	NM_016867:254	Gipc2	INSIDE	0.689	0.508	1148.26	583.43	0.350	1000.47	350.49
A_68_P22523675	chr3:129763103-129763147	NM_207209:701	Sec24b	INSIDE	0.689	3.236	1351.84	4373.91	2.228	1376.01	3066.41
A_68_P21635701	chr2:131387590-131387634	NM_013460:409	Adra1d	INSIDE	0.689	0.189	10747.16	2031.93	0.130	6934.40	903.69
A_68_P32238972	chrX:7150718-7150762	NR_029473:15	4930524L23Rik	INSIDE	0.688	2.300	713.93	1642.39	1.583	924.92	1463.75
A_68_P32053390	chr19:29597209-29597253	NM_001081319:459	C030046E11Rik	INSIDE	0.688	0.444	2468.25	1094.78	0.305	1914.92	583.98
A_68_P32053385	chr19:29596534-29596578	NR_015567:-79	A930007I19Rik	DIVERGENT_PROMOTER	0.688	0.676	3332.49	2252.24	0.465	2655.56	1235.16
A_68_P31682231	chr18:47526609-47526653	NM_018744:1892	Sema6a	INSIDE	0.688	0.265	2330.24	616.59	0.182	1718.02	312.66
A_68_P31120663	chr17:28278567-28278611	NM_001004366:-882	Scube3	PROMOTER	0.688	0.454	1275.59	579.07	0.312	1059.24	330.64
A_68_P29940455	chr14:119105820-119105864	NM_001033336:-401	Abcc4	PROMOTER	0.688	3.459	361.61	1250.81	2.379	321.57	764.95
A_68_P29639450	chr14:60216642-60216686	NM_001168535:132	Cdadc1	INSIDE	0.688	0.633	1265.41	801.38	0.436	980.23	427.26
A_68_P29528634	chr14:35014316-35014361	NM_183209:481	Gprin2	INSIDE	0.688	0.353	4258.74	1503.62	0.243	2799.80	679.64
A_68_P29330804	chr13:112598387-112598431	NM_011945:783	Map3k1	INSIDE	0.688	3.955	2940.83	11631.27	2.721	2368.83	6445.72
A_68_P29310807	chr13:109106628-109106672	NM_178683:120	Depdc1b	INSIDE	0.688	0.651	866.48	564.03	0.448	723.06	323.98
A_68_P29237525	chr13:95645453-95645497	NM_011021:-107	Otp	PROMOTER	0.688	0.476	1539.88	733.44	0.328	1383.39	453.60
A_68_P28959953	chr13:38438458-38438503	NM_007556:896	Bmp6	INSIDE	0.688	0.211	2310.40	488.15	0.145	1731.17	251.64
A_68_P27254384	chr10:74523182-74523226	NM_001081412:-436	Bcr	PROMOTER	0.688	0.370	2637.47	976.72	0.255	2011.65	512.34
A_68_P26363022	chr9:24500423-24500467	NM_001166207:293	Dpy19l2	INSIDE	0.688	0.513	1301.14	667.06	0.353	965.21	340.60
A_68_P26078038	chr8:97056657-97056701	NM_153507:-249	Cpne2	PROMOTER	0.688	3.167	1037.59	3285.75	2.178	866.13	1886.50
A_68_P25954805	chr8:73421039-73421083	NR_002901:-1305	Snora68	PROMOTER	0.688	0.626	3878.62	2428.21	0.431	2911.33	1254.72
A_68_P24429118	chr6:48692680-48692724	NM_008376:3657	Gimap1	INSIDE	0.688	7.846	1079.96	8473.24	5.398	958.18	5172.15
A_68_P23862540	chr5:86601439-86601483	NM_172712:308	Uba6	INSIDE	0.688	0.514	2184.55	1122.55	0.353	1654.89	584.78
A_68_P22825897	chr4:33334510-33334554	NM_001033225:1230	Pnrc1	INSIDE	0.688	0.526	1457.53	767.08	0.362	1164.08	421.67
A_68_P22422580	chr3:109946888-109946938	NM_001163348:-522	Ntng1	PROMOTER	0.688	0.293	1689.03	494.64	0.201	1401.31	282.28
A_68_P21527758	chr2:112079571-112079615	NM_207206:-405	Lpcat4	PROMOTER	0.688	0.475	1189.98	565.22	0.327	1026.58	335.35
A_68_P21440995	chr2:94246447-94246491	NM_183106:378	Ttc17	INSIDE	0.688	0.513	1439.49	738.09	0.353	1228.36	433.14
A_68_P21075528	chr2:25838113-25838157	NM_001115076:668	Camsap1	INSIDE	0.688	0.439	3146.19	1381.46	0.302	2313.42	698.68
A_68_P20620522	chr1:134195663-134195707	NM_144810:482	Klhdc8a	INSIDE	0.688	5.293	1041.98	5514.99	3.643	876.32	3192.18
A_68_P32142309	chr19:46222327-46222371	NM_008852:467	Pitx3	INSIDE	0.687	2.684	2285.93	6136.55	1.845	1905.16	3515.83
A_68_P32130474	chr19:44181784-44181828	NM_001162410:161	Chuk	INSIDE	0.687	0.690	3234.65	2230.48	0.473	2430.61	1150.66
A_68_P31947675	chr19:10057125-10057169	NM_010239:-46	Fth1	PROMOTER	0.687	0.684	1326.98	908.02	0.470	1043.55	490.57
A_68_P31051120	chr17:12511344-12511388	NM_011948:160	Map3k4	INSIDE	0.687	2.077	1717.92	3567.30	1.426	1424.06	2030.06
A_68_P30496844	chr15:102078403-102078447	NM_001042727:-1641	Rarg	PROMOTER	0.687	0.239	2438.23	582.38	0.164	1702.34	279.36
A_68_P30348907	chr15:76353984-76354028	NM_130893:-1447	Scrt1	PROMOTER	0.687	0.605	1071.43	648.29	0.416	872.01	362.74
A_68_P28611534	chr12:88019825-88019869	NM_177354:197	Vash1	INSIDE	0.687	3.487	1068.17	3725.03	2.395	911.44	2183.24
A_68_P28196937	chr12:3808024-3808068	NM_007872:1067	Dnmt3a	INSIDE	0.687	0.491	4331.11	2126.87	0.337	3107.07	1048.24
A_68_P28195049	chr12:3427373-3427417	NM_172421:511	Asxl2	INSIDE	0.687	0.422	1587.83	670.72	0.290	1210.73	351.31
A_68_P28055837	chr11:98210403-98210447	NM_144828:373	Ppp1r1b	INSIDE	0.687	0.226	3941.59	891.32	0.155	2788.21	432.84
A_68_P28051868	chr11:97521238-97521282	NM_139311:-3465	Mllt6	PROMOTER	0.687	0.125	4515.95	563.45	0.086	2957.06	253.40
A_68_P27924904	chr11:75027510-75027554	NM_177708:20038	Rtn4rl1	INSIDE	0.687	0.408	1144.58	467.16	0.280	938.22	263.08
A_68_P27922356	chr11:74584498-74584542	NM_026197:156	Mettl16	INSIDE	0.687	0.424	1184.31	502.08	0.291	862.16	251.06
A_68_P27852918	chr11:62062098-62062143	NM_007413:-365	Adora2b	PROMOTER	0.687	0.520	5385.10	2801.04	0.357	3900.71	1393.85
A_68_P27294770	chr10:82448262-82448306	NM_021439:43	Chst11	INSIDE	0.687	2.081	2132.44	4437.94	1.430	1607.59	2299.16
A_68_P26773474	chr9:101153371-101153415	NM_001161362:770	Ppp2r3a	INSIDE	0.687	0.465	1390.68	647.16	0.320	1013.22	324.13
A_68_P26748152	chr9:96531410-96531454	NM_053268:490	Rasa2	INSIDE	0.687	2.733	1429.69	3906.95	1.878	1185.39	2225.80
A_68_P26464014	chr9:43611501-43611545	NM_021424:58864	Pvrl1	INSIDE	0.687	11.491	1763.19	20261.12	7.892	1061.57	8377.58
A_68_P26138375	chr8:108734930-108734974	NM_145404:-1	Prmt7	DIVERGENT_PROMOTER	0.687	2.142	2370.11	5075.94	1.472	1951.55	2873.16
A_68_P25576403	chr7:146399424-146399468	NM_001162540:-240	Stk32c	PROMOTER	0.687	0.347	2795.46	970.27	0.239	1925.90	459.33
A_68_P25034848	chr7:31852435-31852479	NM_144556:7503	Lgi4	INSIDE	0.687	4.655	945.76	4402.44	3.197	893.50	2856.48
A_68_P24658379	chr6:94649268-94649312	NM_008377:849	Lrig1	INSIDE	0.687	0.310	3427.80	1063.35	0.213	2488.18	530.38
A_68_P24449466	chr6:52203463-52203507	NR_015348:8243	Hoxa11as	DOWNSTREAM	0.687	0.341	1593.85	543.01	0.234	1128.28	263.98
A_68_P24171440	chr5:147840732-147840776	NM_080795:47394	Lnx2	INSIDE	0.687	3.434	2409.40	8274.24	2.358	2004.07	4725.62
A_68_P24127313	chr5:138182525-138182569	NM_175521:-1320	6430598A04Rik	DIVERGENT_PROMOTER	0.687	0.395	5381.90	2127.03	0.271	3531.36	958.23
A_68_P24063264	chr5:125342497-125342541	NM_144819:73	Ccdc92	INSIDE	0.687	2.878	1288.87	3709.45	1.977	1114.37	2203.42
A_68_P22680844	chr3:157230168-157230212	NM_011196:335	Ptger3	INSIDE	0.687	2.428	1077.72	2616.86	1.668	875.73	1460.97
A_68_P21838401	chr2:167757841-167757885	NM_011201:36	Ptpn1	INSIDE	0.687	0.421	1453.56	611.52	0.289	1182.14	341.73
A_68_P21395216	chr2:84726819-84726863	NM_199223:9	Rtn4rl2	INSIDE	0.687	3.173	924.69	2934.50	2.181	829.64	1809.57
A_68_P20877311	chr1:182627122-182627166	NM_013729:21	Mixl1	INSIDE	0.687	0.435	6257.63	2722.18	0.299	4662.50	1393.39
A_68_P20551186	chr1:120207831-120207875	NM_011650:-143	Tsn	PROMOTER	0.687	3.032	607.26	1841.46	2.083	609.07	1269.00
A_68_P20353690	chr1:75385333-75385377	NM_001085370:3170	Speg	INSIDE	0.687	0.272	2268.52	618.07	0.187	1480.19	277.24
A_68_P32750678	chrX:149204051-149204095	NM_172441:-68	Shroom2	PROMOTER	0.686	4.594	1764.08	8103.76	3.153	1941.47	6122.33

ProbeName	Target position of probe on CpG island microarray	TargetID	GeneSymbol	CpG island Description	Ratio of relative methylation (TiO_2-NP/Vehicle)	Sham group			TiO_2-H group		
						Relative methylation (Cy5/Cy3)	Cy3 signal (Input)	Cy5 signal (MeDIP)	Relative methylation (Cy5/Cy3)	Cy3 signal (Input)	Cy5 signal (MeDIP)
A_68_P32553139	chrX:96237904-96237948	NM_199018:7	Stard8	INSIDE	0.686	0.409	1175.33	480.91	0.281	1608.63	451.34
A_68_P32463877	chrX:70882835-70882879	NM_029352:-1923	Dusp9	PROMOTER	0.686	3.220	649.85	2092.62	2.208	802.33	1771.19
A_68_P31890103	chr18:84855123-84855167	NM_023149:-119	Cndp2	PROMOTER	0.686	2.437	7121.69	17355.88	1.672	5341.87	8931.50
A_68_P30402715	chr15:85566224-85566268	NM_011144:41	Ppara	INSIDE	0.686	0.672	2037.79	1369.34	0.461	1516.61	698.64
A_68_P30361874	chr15:78543529-78543573	NM_183141:4993	Elfn2	INSIDE	0.686	0.452	2777.49	1254.55	0.310	2108.83	653.72
A_68_P29611635	chr14:54873344-54873388	NM_001113358:238	Dad1	INSIDE	0.686	0.280	1864.79	522.17	0.192	1474.92	283.34
A_68_P28100238	chr11:106078606-106078650	NM_028074:389	Ddx42	INSIDE	0.686	0.358	2996.53	1071.32	0.245	2407.03	590.60
A_68_P27889190	chr11:68213756-68213800	ENSMUST00000108674:547		INSIDE	0.686	0.300	5095.56	1528.63	0.206	3488.25	717.65
A_68_P27804079	chr11:53113636-53113680	NM_008300:323	Hspa4	INSIDE	0.686	2.517	1601.91	4032.51	1.728	1394.81	2409.60
A_68_P27258522	chr10:75227236-75227280	NM_172549:-156	Cabin1	PROMOTER	0.686	2.047	6592.08	13492.71	1.403	5314.22	7456.50
A_68_P26977747	chr10:18564707-18564751	NM_022032:-148	Perp	PROMOTER	0.686	2.976	1928.73	5739.11	2.040	1553.69	3170.12
A_68_P24993121	chr7:19989091-19989135	NM_146182:-3907	Klc3	DIVERGENT_PROMOTER	0.686	0.584	1544.53	902.51	0.401	1236.98	495.61
A_68_P24982506	chr7:17200789-17200833	NM_172739:-468	Grlf1	PROMOTER	0.686	0.161	3388.24	545.97	0.110	2545.95	281.26
A_68_P24431877	chr6:49214057-49214101	NM_198102:-27	Tra2a	PROMOTER	0.686	0.608	1355.10	824.32	0.417	1070.83	447.01
A_68_P24119777	chr5:136646370-136646414	NM_178751:134	Orai2	INSIDE	0.686	3.952	3113.66	12303.79	2.711	2583.46	7003.59
A_68_P23720355	chr5:58109554-58109598	NM_018764:317	Pcdh7	INSIDE	0.686	0.499	1090.07	543.73	0.342	806.03	275.66
A_68_P22128531	chr3:51028825-51028869	NM_009834:478	Ccrn4l	INSIDE	0.686	3.344	1421.53	4754.07	2.295	1143.63	2624.24
A_68_P21486781	chr2:103868199-103868243	NM_020593:265	Fbxo3	INSIDE	0.686	0.451	1723.48	777.10	0.309	1403.54	434.01
A_68_P21395952	chr2:84877451-84877495	NM_001136081:-135	Ssrp1	PROMOTER	0.686	0.571	2057.31	1175.26	0.392	1677.02	657.42
A_68_P20871442	chr1:181598207-181598251	NM_178653:-133	Sccpdh	PROMOTER	0.686	4.154	1317.73	5474.43	2.849	1178.96	3358.96
A_68_P20184670	chr1:42754004-42754048	NM_008900:36	Pou3f3	INSIDE	0.686	2.237	1395.95	3122.76	1.534	1118.45	1715.38
A_68_P31323666	chr17:69732894-69732938	NM_009547:-401	Zfp161	PROMOTER	0.685	3.375	860.69	2905.09	2.312	826.10	1910.21
A_68_P31151582	chr17:34164943-34164987	NM_013543:36	H2-Ke6	INSIDE	0.685	0.721	1613.95	1162.98	0.494	1338.22	660.74
A_68_P31029178	chr17:8995181-8995225	NM_011866:593	Pde10a	INSIDE	0.685	0.377	1773.33	669.33	0.258	1306.73	337.69
A_68_P29703988	chr14:71289674-71289718	NM_008115:-240	Gfra2	PROMOTER	0.685	2.763	597.76	1651.71	1.892	479.38	907.02
A_68_P29702644	chr14:71041729-71041773	NM_008004:325	Fgf17	INSIDE	0.685	0.447	2326.27	1040.63	0.306	1818.84	557.11
A_68_P29651386	chr14:62300460-62300504	NR_028264:728	Dleu2	INSIDE	0.685	0.664	1100.83	731.41	0.455	891.62	405.51
A_68_P29130720	chr13:72100566-72100611	NM_010573:568	Irx1	INSIDE	0.685	2.450	1562.00	3827.07	1.679	1195.68	2007.08
A_68_P28924076	chr13:31718416-31718460	NM_010225:754	Foxf2	INSIDE	0.685	0.350	1340.71	469.45	0.240	982.90	235.76
A_68_P28103447	chr11:106640770-106640814	NR_027785:37	Polg2	INSIDE	0.685	3.016	1195.86	3606.49	2.065	1020.18	2107.16
A_68_P28043567	chr11:96160575-96160619	NM_008269:112	Hoxb6	INSIDE	0.685	3.144	1802.22	5666.30	2.155	1502.87	3238.83
A_68_P27896916	chr11:69572824-69572871	NM_009089:-1122	Polr2a	PROMOTER	0.685	3.858	470.71	1816.07	2.644	461.83	1221.28
A_68_P27282332	chr10:79767774-79767818	NM_011789:3232	Apc2	INSIDE	0.685	2.112	3776.56	7976.68	1.447	2584.62	3740.28
A_68_P26741563	chr9:95306199-95306243	NM_018763:1469	Chst2	INSIDE	0.685	3.031	1530.47	4638.25	2.075	1286.40	2668.90
A_68_P26674969	chr9:81525359-81525403	NM_010482:779	Htr1b	INSIDE	0.685	0.706	1094.83	773.39	0.484	846.90	409.52
A_68_P26469281	chr9:44542040-44542084	NM_153537:1215	Phldb1	INSIDE	0.685	0.404	4541.48	1833.23	0.277	3253.05	899.64
A_68_P25174411	chr7:71084399-71084443	NM_021366:-619	Klf13	PROMOTER	0.685	0.547	2369.40	1296.71	0.375	1821.75	683.05
A_68_P24983341	chr7:17396988-17397032	NM_001039878:-4227	Strn4	PROMOTER	0.685	2.008	2694.79	5411.15	1.376	2083.47	2865.92
A_68_P24872960	chr6:136467629-136467673	NM_019426:279	Atf7ip	INSIDE	0.685	0.484	2216.96	1073.68	0.332	1975.94	655.29
A_68_P23600262	chr5:35740109-35740153	NM_010445:8365	Hmx1	DOWNSTREAM	0.685	0.335	4675.61	1566.44	0.229	3411.79	782.81
A_68_P23301441	chr4:129985717-129985761	NM_010174:-283	Fabp3	PROMOTER	0.685	0.420	3368.33	1413.10	0.287	2353.31	676.37
A_68_P22592457	chr3:142428332-142428376	NM_145546:144	Gtf2b	INSIDE	0.685	2.252	1160.28	2612.69	1.543	1035.34	1597.56
A_68_P22283292	chr3:82932341-82932385	ENSMUST00000047876:806		INSIDE	0.685	2.471	1757.35	4342.68	1.694	1473.17	2495.36
A_68_P22202884	chr3:65762093-65762137	NM_019937:33	Ccnl1	INSIDE	0.685	0.286	3043.00	869.57	0.196	2327.15	455.78
A_68_P22169677	chr3:58496154-58496198	NM_009174:134	Siah2	INSIDE	0.685	0.652	1405.14	916.14	0.447	1071.69	478.73
A_68_P22164434	chr3:57379528-57379572	NM_133784:248	Wwtr1	INSIDE	0.685	0.624	2062.98	1287.77	0.428	1726.38	738.60
A_68_P21389688	chr2:83652981-83653025	NM_175514:118	Fam171b	INSIDE	0.685	2.679	884.30	2369.14	1.836	757.30	1390.74
A_68_P32029539	chr19:25484768-25484818	NM_181404:173101	Kank1	INSIDE	0.684	4.435	465.76	2065.55	3.035	308.45	936.05
A_68_P31632699	chr18:38370942-38370995	NM_029357:-1552	Pcdh1	PROMOTER	0.684	0.459	1149.96	527.84	0.314	847.51	266.00
A_68_P31465573	chr18:4921670-4921714	ENSMUST00000143254:-31		PROMOTER	0.684	0.610	1907.14	1164.14	0.418	1620.14	676.87
A_68_P31373922	chr17:79234408-79234452	NM_001081179:291	Heatr5b	INSIDE	0.684	2.779	1862.52	5176.37	1.901	1421.12	2701.48
A_68_P31311915	chr17:67703009-67703053	NM_008984:769	Ptprm	INSIDE	0.684	2.298	1331.36	3059.72	1.572	1092.13	1717.07
A_68_P30547087	chr16:11254371-11254415	NM_001130008:26	Gspt1	INSIDE	0.684	0.242	3038.83	734.24	0.165	2286.22	378.01
A_68_P30357864	chr15:77950159-77950203	NM_007583:-470	Cacng2	PROMOTER	0.684	0.620	1545.26	958.42	0.424	1337.81	567.62
A_68_P29726035	chr14:75530811-75530855	NM_027581:-142	Lrrc63	DIVERGENT_PROMOTER	0.684	0.696	2602.24	1812.43	0.476	2324.80	1106.81
A_68_P27685425	chr11:31771059-31771103	NM_026252:-1130	Cpeb4	PROMOTER	0.684	0.338	4868.43	1645.52	0.231	3467.11	801.49
A_68_P27447374	chr10:110602373-110602417	NM_001003717:537	Osbpl8	INSIDE	0.684	5.315	363.07	1929.77	3.633	281.72	1023.56
A_68_P27356288	chr10:93352563-93352607	NM_019648:1363	Metap2	INSIDE	0.684	0.541	2206.95	1194.27	0.370	1897.30	702.64
A_68_P27179603	chr10:60736419-60736463	NM_001081127:-254	Adamts14	PROMOTER	0.684	3.259	1195.75	3897.22	2.228	975.28	2173.25
A_68_P27098034	chr10:42741868-42741912	NM_175407:152446	Sobp	INSIDE	0.684	0.397	1881.65	746.92	0.272	1410.27	383.00
A_68_P26816175	chr9:108594926-108594970	NM_008924:475	Prkar2a	INSIDE	0.684	0.574	1361.70	782.13	0.393	1067.22	419.27
A_68_P26127993	chr8:106865035-106865079	NM_024217:563	Cmtm3	INSIDE	0.684	2.945	2038.29	6002.49	2.015	1642.55	3309.41
A_68_P25950463	chr8:72735887-72735931	NM_001007570:272	Slc25a42	INSIDE	0.684	0.431	1490.22	642.30	0.295	1213.19	357.90
A_68_P25047619	chr7:35905340-35905384	NM_007678:1051	Cebpa	INSIDE	0.684	0.295	1686.93	497.41	0.202	1101.94	222.16
A_68_P24816932	chr6:124719430-124719474	NM_013509:75	Eno2	INSIDE	0.684	0.289	4128.13	1191.62	0.197	2950.85	582.72

ProbeName	Target position of probe on CpG island microarray	TargetID	GeneSymbol	CpG island Description	Ratio of relative methylation (TiO_2-NP/Vehicle)	Sham group			TiO_2-H group		
						Relative methylation (Cy5/Cy3)	Cy3 signal (Input)	Cy5 signal (MeDIP)	Relative methylation (Cy5/Cy3)	Cy3 signal (Input)	Cy5 signal (MeDIP)
A_68_P24624509	chr6:88792201-88792245	NM_030251:-293	Abtb1	PROMOTER	0.684	0.463	3646.84	1689.93	0.317	2348.78	745.00
A_68_P23280028	chr4:126106770-126106814	NM_153402:-6	Eif2c3	PROMOTER	0.684	4.485	1907.34	8553.55	3.069	1539.83	4725.04
A_68_P23225791	chr4:115457470-115457514	NM_181040:-97	Atpaf1	DIVERGENT_PROMOTER	0.684	0.692	2721.60	1882.76	0.473	2167.18	1025.81
A_68_P22218623	chr3:68808767-68808811	NM_133786:-105	Smc4	DIVERGENT_PROMOTER	0.684	2.602	1131.22	2943.50	1.781	1026.23	1827.48
A_68_P21078500	chr2:26264786-26264830	NM_033134:-100	Inpp5e	PROMOTER	0.684	3.721	4724.96	17582.76	2.546	3828.92	9749.36
A_68_P20740951	chr1:157820596-157820640	NM_001039184:-233	Cep350	PROMOTER	0.684	0.414	1187.43	491.70	0.283	921.30	261.11
A_68_P20140986	chr1:34906861-34906905	NM_145516:79	Plekhb2	INSIDE	0.684	0.287	3647.75	1047.31	0.196	2627.85	515.94
A_68_P32260277	chrX:11657663-11657707	NM_029510:-5	Bcor	PROMOTER	0.683	4.932	1407.65	6943.18	3.370	1438.05	4846.19
A_68_P32245738	chrX:8777353-8777397	NM_173414:276	Lancl3	INSIDE	0.683	2.298	698.05	1604.35	1.569	1044.85	1639.83
A_68_P31116177	chr17:27552104-27552148	NM_001013385:88123	Grm4	DOWNSTREAM	0.683	24.724	1447.87	35796.68	16.878	1765.42	29797.40
A_68_P30348897	chr15:76353005-76353049	NM_130893:-467	Scrt1	PROMOTER	0.683	2.755	1025.22	2824.76	1.882	839.06	1579.30
A_68_P29241614	chr13:96388371-96388415	NM_010169:-4	F2r	PROMOTER	0.683	0.302	1983.15	598.55	0.206	1466.12	302.44
A_68_P29075352	chr13:58908396-58908440	NM_001025074:-775	Ntrk2	PROMOTER	0.683	0.500	2904.09	1452.18	0.342	2085.90	712.53
A_68_P28743066	chr12:112513158-112513202	NM_033603:3859	Amn	INSIDE	0.683	0.712	2472.92	1759.87	0.486	1006.72	489.10
A_68_P28743037	chr12:112509761-112509805	NM_033603:461	Amn	INSIDE	0.683	0.306	2437.87	746.74	0.209	1820.15	380.75
A_68_P28738219	chr12:111685115-111685159	NM_001135001:-252	Ppp2r5c	PROMOTER	0.683	4.152	1911.83	7937.30	2.837	1654.76	4695.28
A_68_P28457435	chr12:57634322-57634366	NM_001146198:1749	Nkx2-1	INSIDE	0.683	2.646	715.26	1892.30	1.808	642.19	1161.12
A_68_P27687535	chr11:32126430-32126474	NM_010822:-52	Mpg	DIVERGENT_PROMOTER	0.683	0.695	5524.58	3839.13	0.475	3748.41	1779.04
A_68_P27297646	chr10:83007010-83007054	NM_001033375:347	A230046K03Rik	INSIDE	0.683	4.086	446.50	1824.50	2.790	367.91	1026.29
A_68_P26983892	chr10:19653936-19653980	NM_008580:-373	Map3k5	PROMOTER	0.683	4.421	2106.18	9312.14	3.021	1770.73	5349.70
A_68_P26527194	chr9:54547174-54547218	NM_021422:-169	Dnaja4	PROMOTER	0.683	0.561	3222.75	1807.78	0.383	2172.97	832.71
A_68_P26505083	chr9:50507053-50507097	NM_178118:29015	Dixdc1	INSIDE	0.683	0.605	819.62	495.67	0.413	668.96	276.23
A_68_P26253145	chr8:128193794-128193838	NM_028908:99	4933403G14Rik	INSIDE	0.683	2.490	2054.63	5116.82	1.702	1686.90	2870.50
A_68_P26221509	chr8:123157870-123157914	NM_197988:390	1190005I06Rik	INSIDE	0.683	0.680	1022.58	695.77	0.465	980.61	455.75
A_68_P25327115	chr7:100023177-100023221	NM_001080995:-456	4632434I11Rik	DIVERGENT_PROMOTER	0.683	0.300	4220.49	1264.35	0.205	2794.46	571.82
A_68_P25088285	chr7:51846895-51846939	NM_008422:661	Kcnc3	INSIDE	0.683	2.420	1298.98	3143.03	1.654	1118.00	1848.68
A_68_P25027723	chr7:30303448-30303492	ENSMUST00000141713:-753		PROMOTER	0.683	0.438	1111.26	486.61	0.299	932.07	278.95
A_68_P24954291	chr7:6107085-6107129	NM_001013012:467	Zfp787	INSIDE	0.683	0.519	983.39	510.44	0.355	734.63	260.60
A_68_P24479788	chr6:57652816-57652860	NM_133737:390	Lancl2	INSIDE	0.683	2.205	3249.72	7167.08	1.506	2435.81	3667.99
A_68_P24325253	chr6:29166069-29166113	NM_011829:-3819	Impdh1	PROMOTER	0.683	2.965	336.84	998.77	2.027	388.50	787.33
A_68_P24078732	chr5:128112557-128112601	NM_177005:-53	Glt1d1	PROMOTER	0.683	0.488	2488.26	1215.30	0.334	1894.43	632.38
A_68_P23925841	chr5:100157706-100157750	NR_030692:351	A930011G23Rik	INSIDE	0.683	6.230	1564.46	9747.01	4.254	1278.96	5440.94
A_68_P23404035	chr4:149422780-149422824	ENSMUST00000134236:-319		PROMOTER	0.683	0.397	1720.73	683.97	0.272	1205.93	327.56
A_68_P22321613	chr3:89577798-89577842	NM_027315:290	Ube2q1	INSIDE	0.683	3.268	756.29	2471.64	2.233	627.33	1400.85
A_68_P21901044	chr2:179776594-179776638	NM_011969:491	Psma7	INSIDE	0.683	0.412	4320.85	1778.47	0.281	3169.41	891.40
A_68_P20617853	chr1:133724747-133724791	NM_173865:180	Slc41a1	INSIDE	0.683	0.627	1908.27	1197.10	0.428	1325.92	568.12
A_68_P20395061	chr1:82836417-82836461	NM_010472:381	Agfg1	INSIDE	0.683	0.502	2440.95	1224.95	0.343	2000.46	685.87
A_68_P31936767	chr19:7008564-7008608	NM_008431:419	Kcnk4	INSIDE	0.682	0.607	1436.77	871.62	0.414	1146.85	474.60
A_68_P31385967	chr17:81344527-81344571	NM_026516:577	Tmem178	INSIDE	0.682	3.930	930.90	3658.20	2.680	748.08	2005.05
A_68_P30568843	chr16:16302936-16302980	NM_198246:-99	Yars2	PROMOTER	0.682	0.405	1596.55	646.46	0.276	1227.22	339.09
A_68_P30221125	chr15:53177961-53178005	NM_010162:-244	Ext1	PROMOTER	0.682	2.728	1058.12	2886.57	1.862	913.54	1700.57
A_68_P28925109	chr13:31900260-31900304	NM_008592:1768	Foxc1	INSIDE	0.682	0.734	2857.68	2097.75	0.501	2299.63	1151.74
A_68_P28452661	chr12:56701135-56701179	NM_020287:1253	Insm2	INSIDE	0.682	0.221	2171.76	480.71	0.151	1605.23	242.45
A_68_P28269091	chr12:17182387-17182431	NM_201531:1286	Kcnf1	INSIDE	0.682	0.481	1116.22	536.70	0.328	878.60	288.10
A_68_P28074030	chr11:101327012-101327056	NM_012037:479	Vat1	INSIDE	0.682	5.196	3691.00	19180.05	3.545	2981.37	10568.54
A_68_P28053984	chr11:97883326-97883370	NM_001159320:-3662	Cacnb1	PROMOTER	0.682	4.056	2582.75	10475.36	2.767	2195.87	6076.69
A_68_P27869722	chr11:64975997-64976041	NM_001099288:445	Arhgap44	INSIDE	0.682	0.673	3200.98	2152.98	0.459	2725.01	1250.86
A_68_P27794864	chr11:51419555-51419599	NM_001048061:807	Hnrnpab	INSIDE	0.682	2.303	1919.46	4420.59	1.572	1517.92	2385.53
A_68_P27785188	chr11:49660391-49660435	NM_001163671:160	Mapk9	INSIDE	0.682	2.358	2116.61	4990.46	1.609	1789.92	2879.94
A_68_P27567608	chr11:6167875-6167919	NM_026538:-164	Ddx56	PROMOTER	0.682	0.333	1476.02	492.24	0.227	1213.86	276.08
A_68_P27327694	chr10:88289467-88289513	NM_175158:69	Utp20	INSIDE	0.682	0.334	6819.94	2275.46	0.227	4574.29	1040.21
A_68_P26920438	chr10:7445673-7445717	NM_011835:-103	Katna1	PROMOTER	0.682	2.534	3432.51	8697.76	1.727	2787.41	4814.39
A_68_P26913681	chr10:6079865-6079909	NM_031185:326	Akap12	INSIDE	0.682	0.645	1570.80	1012.74	0.439	1466.88	644.64
A_68_P26666122	chr9:79641306-79641350	NM_133718:-91	Tmem30a	PROMOTER	0.682	0.298	1584.81	472.40	0.203	1315.49	267.61
A_68_P26659818	chr9:78329691-78329735	NM_010106:-181	Eef1a1	PROMOTER	0.682	3.138	1063.54	3337.40	2.140	916.46	1961.57
A_68_P25587301	chr7:148380386-148380430	NM_153777:-633	Lrrc56	PROMOTER	0.682	0.744	2623.24	1951.34	0.508	1801.23	914.25
A_68_P25392337	chr7:112583863-112583907	NM_007627:9551	Cckbr	INSIDE	0.682	3.766	926.48	3488.76	2.567	808.69	2075.52
A_68_P25176629	chr7:71518985-71519029	NM_177893:-25	Fan1	PROMOTER	0.682	5.759	1629.12	9382.25	3.931	1025.43	4030.50
A_68_P25092884	chr7:52651057-52651101	NM_009224:-61	Snrnp70	PROMOTER	0.682	0.283	1904.41	539.85	0.193	1346.45	260.18
A_68_P25091297	chr7:52384491-52384535	NM_009438:-407	Rpl13a	PROMOTER	0.682	6.557	1714.51	11241.53	4.469	1402.16	6266.10
A_68_P24951795	chr7:5004972-5005016	NM_026900:1861	Zfp580	INSIDE	0.682	0.640	955.88	611.79	0.436	708.95	309.25
A_68_P23600188	chr5:35731369-35731413	NM_010445:-375	Hmx1	PROMOTER	0.682	0.703	1879.35	1321.52	0.480	1392.36	668.06
A_68_P23409805	chr4:150432256-150432300	NM_009498:-216	Vamp3	PROMOTER	0.682	6.796	4138.30	28121.86	4.635	3731.87	17296.03
A_68_P22412543	chr3:108218968-108219012	NM_001004177:-578	Celsr2	PROMOTER	0.682	4.231	791.68	3349.82	2.887	669.28	1932.28
A_68_P22151285	chr3:54859248-54859292	NM_007628:-293	Ccna1	PROMOTER	0.682	3.609	1803.78	6510.68	2.461	1562.03	3844.24

ProbeName	Target position of probe on CpG island microarray	TargetID	GeneSymbol	CpG island Description	Ratio of relative methylation (TiO_2-NP/Vehicle)	Sham group			TiO_2-H group		
						Relative methylation (Cy5/Cy3)	Cy3 signal (Input)	Cy5 signal (MeDIP)	Relative methylation (Cy5/Cy3)	Cy3 signal (Input)	Cy5 signal (MeDIP)
A_68_P21102298	chr2:30141644-30141688	NM_177648:208	Dolk	INSIDE	0.682	0.620	1902.23	1180.10	0.423	1552.63	656.64
A_68_P21044521	chr2:19293270-19293314	NM_029619:30	Msrb2	INSIDE	0.682	0.536	3712.66	1988.23	0.365	2697.98	985.09
A_68_P20639345	chr1:137326912-137326956	NM_153774:134	Ipo9	INSIDE	0.682	0.426	2634.92	1123.39	0.291	1988.87	578.48
A_68_P32750676	chrX:149203868-149203912	NM_172441:114	Shroom2	INSIDE	0.681	3.269	716.28	2341.47	2.228	948.01	2111.77
A_68_P32260574	chrX:11705655-11705699	NM_001168321:31805	Bcor	INSIDE	0.681	0.607	2829.46	1717.93	0.414	3460.65	1431.91
A_68_P32246204	chrX:8849773-8849817	NM_023500:-115	Xk	PROMOTER	0.681	6.803	258.39	1757.76	4.633	312.33	1446.96
A_68_P32183826	chr19:53751832-53751876	NM_001170847:59	Rbm20	INSIDE	0.681	8.623	2772.71	23910.24	5.874	2585.83	15188.62
A_68_P31934664	chr19:6428736-6428780	NM_001205234:10021	Nrxn2	INSIDE	0.681	0.429	1149.35	492.74	0.292	855.23	249.81
A_68_P31930917	chr19:5803602-5803646	NR_002847:-953	Malat1	PROMOTER	0.681	0.367	2579.95	947.58	0.250	1993.66	498.66
A_68_P30393148	chr15:84022873-84022917	NM_178614:232	Samm50	INSIDE	0.681	0.688	3792.15	2609.55	0.469	2929.97	1373.14
A_68_P30387966	chr15:83197892-83197936	NR_027980:-187	1700001L05Rik	PROMOTER	0.681	4.664	1762.73	8221.66	3.177	1518.64	4824.84
A_68_P30357053	chr15:77825858-77825902	NM_007583:123830	Cacng2	INSIDE	0.681	5.726	7521.08	43064.38	3.902	5929.08	23135.62
A_68_P29977061	chr15:3945633-3945677	NM_145930:98	AW549877	INSIDE	0.681	0.471	2802.34	1320.03	0.321	2275.72	730.22
A_68_P29581906	chr14:47993698-47993742	NM_001145953:186	Lgals3	INSIDE	0.681	0.642	2343.25	1505.40	0.437	1914.81	837.28
A_68_P28261128	chr12:15821694-15821738	NM_144551:1875	Trib2	INSIDE	0.681	0.405	3604.77	1459.43	0.276	2862.26	788.62
A_68_P28174632	chr11:118769905-118769949	NM_001024931:960	Rbfox3	INSIDE	0.681	3.525	1848.21	6515.16	2.401	1487.38	3571.01
A_68_P28152362	chr11:115119477-115119521	NM_010350:9059	Grin2c	INSIDE	0.681	5.973	786.42	4697.65	4.067	742.11	3018.08
A_68_P28057821	chr11:98544135-98544179	NM_009439:289	Psmd3	INSIDE	0.681	2.074	2361.53	4897.70	1.412	1633.90	2307.74
A_68_P27837109	chr11:59285613-59285657	NM_178417:342	Zfp867	INSIDE	0.681	0.545	973.95	531.12	0.371	775.51	287.79
A_68_P27532843	chr10:126338419-126338463	NM_026858:-13	Xrcc6bp1	PROMOTER	0.681	4.688	4824.72	22616.43	3.193	4151.46	13257.37
A_68_P26577166	chr9:63450424-63450468	NM_025857:-15	Aagab	DIVERGENT_PROMOTER	0.681	3.111	1121.22	3488.12	2.119	903.85	1915.05
A_68_P25093276	chr7:52715375-52715419	NM_010240:-140	Ftl1	PROMOTER	0.681	0.691	1086.70	750.44	0.471	772.46	363.48
A_68_P25019829	chr7:28834940-28834984	NM_011874:149	Psmc4	INSIDE	0.681	3.774	4524.52	17075.88	2.572	3277.49	8428.39
A_68_P24053125	chr5:123402247-123402291	NM_001005866:36274	Kdm2b	INSIDE	0.681	0.604	4750.81	2869.97	0.412	3216.27	1324.07
A_68_P24004044	chr5:114894084-114894128	NM_029956:-70	Mmab	DIVERGENT_PROMOTER	0.681	0.573	1193.70	684.40	0.391	1001.46	391.21
A_68_P23416419	chr4:151460529-151460574	ENSMUST00000118648:-415		PROMOTER	0.681	5.481	882.69	4837.67	3.730	714.16	2663.79
A_68_P22391861	chr3:104315776-104315820	NM_001025067:-19	Lrig2	PROMOTER	0.681	0.634	3697.75	2342.68	0.432	2593.36	1119.18
A_68_P22345837	chr3:95623518-95623562	NM_001081293:-664	Rprd2	PROMOTER	0.681	0.410	1896.44	776.83	0.279	1655.05	461.39
A_68_P22039461	chr3:32608077-32608121	NM_019673:631	Actl6a	INSIDE	0.681	2.801	1038.53	2908.71	1.907	882.46	1682.92
A_68_P21772343	chr2:156439879-156439923	NM_146128:460	Dlgap4	INSIDE	0.681	6.359	621.50	3952.23	4.334	563.05	2440.04
A_68_P21105204	chr2:30640432-30640476	ENSMUST00000139968:18701		DOWNSTREAM	0.681	2.633	914.56	2407.86	1.792	878.73	1575.00
A_68_P32287114	chrX:19637038-19637082	NM_021715:365	Chst7	INSIDE	0.680	2.213	812.64	1797.97	1.505	1211.10	1823.22
A_68_P31801896	chr18:69358080-69358124	AK080553:328		INSIDE	0.680	2.201	4854.69	10685.75	1.496	3498.00	5232.19
A_68_P31205409	chr17:46297933-46297979	NM_001145979:-24	Gtpbp2	DIVERGENT_PROMOTER	0.680	7.337	3423.80	25120.67	4.989	2752.84	13733.95
A_68_P30792108	chr16:58523232-58523276	NM_018784:171	St3gal6	INSIDE	0.680	0.677	1196.55	810.48	0.460	1078.39	496.51
A_68_P30719815	chr16:44140189-44140233	NM_028108:289	Naa50	INSIDE	0.680	0.429	2332.12	999.70	0.291	1726.22	503.11
A_68_P30425657	chr15:89354548-89354592	NM_021423:24283	Shank3	INSIDE	0.680	3.467	709.49	2459.86	2.359	605.43	1428.06
A_68_P29982091	chr15:5066504-5066548	NM_026069:-86	Rpl37	DIVERGENT_PROMOTER	0.680	0.591	1571.77	928.47	0.402	1294.21	519.82
A_68_P29145779	chr13:74487622-74487666	NM_023281:44	Sdha	INSIDE	0.680	2.533	4152.27	10518.15	1.722	3237.09	5574.21
A_68_P28636743	chr12:93017325-93017369	NM_175367:7530	Ston2	INSIDE	0.680	0.599	1339.86	802.79	0.408	1181.42	481.49
A_68_P28155483	chr11:115675002-115675046	NM_080643:-118	Caskin2	DIVERGENT_PROMOTER	0.680	0.584	3489.65	2037.10	0.397	2450.62	972.75
A_68_P28073804	chr11:101286517-101286561	NM_026865:109	1700113I22Rik	INSIDE	0.680	0.470	2356.29	1107.13	0.320	1788.92	571.73
A_68_P27798065	chr11:52045603-52045647	NM_011543:128	Skp1a	INSIDE	0.680	4.551	935.58	4257.37	3.096	730.61	2261.76
A_68_P27554681	chr11:3813626-3813670	NM_153142:1019	Slc35e4	INSIDE	0.680	0.345	1319.44	454.76	0.234	1031.39	241.73
A_68_P26845372	chr9:114472029-114472073	NM_001042503:1437	Trim71	INSIDE	0.680	5.248	722.14	3790.01	3.569	701.60	2504.35
A_68_P26494987	chr9:48793758-48793802	NM_175482:291	Usp28	INSIDE	0.680	0.654	1713.51	1121.07	0.445	1445.92	643.30
A_68_P26373636	chr9:26837227-26837271	NM_178027:431	Vps26b	INSIDE	0.680	2.263	1248.65	2826.14	1.539	1240.86	1909.20
A_68_P26258418	chr8:129116571-129116616	NM_001164598:743	Irf2bp2	INSIDE	0.680	3.914	495.83	1940.46	2.660	414.78	1103.39
A_68_P26134296	chr8:108042857-108042901	NM_008289:233	Hsd11b2	INSIDE	0.680	3.423	837.30	2865.71	2.329	694.47	1617.11
A_68_P25574424	chr7:146038574-146038618	NM_026391:528	Ppp2r2d	INSIDE	0.680	0.312	2001.55	624.47	0.212	1635.43	347.20
A_68_P25196761	chr7:75098172-75098216	NM_010513:1052	Igf1r	INSIDE	0.680	0.536	1810.08	970.48	0.364	1563.55	569.90
A_68_P25008233	chr7:25862458-25862502	NM_175477:216	Zfp574	INSIDE	0.680	0.455	1630.71	742.40	0.310	1348.34	417.56
A_68_P24951532	chr7:4959919-4959963	NM_001110330:4904	Fiz1	INSIDE	0.680	2.640	2711.29	7156.54	1.795	2161.32	3880.46
A_68_P24799552	chr6:120615622-120615666	NM_001128151:-794	Cecr2	PROMOTER	0.680	0.227	4011.60	912.13	0.155	3161.70	488.82
A_68_P24464806	chr6:54631922-54631966	NM_026629:179	2410066E13Rik	INSIDE	0.680	0.527	2412.50	1272.17	0.359	1901.41	682.21
A_68_P23547785	chr5:25003901-25003945	NM_001081383:679	Mll3	INSIDE	0.680	2.553	1019.56	2602.68	1.736	875.25	1519.52
A_68_P23356930	chr4:139802256-139802300	NM_198610:448	Igsf21	INSIDE	0.680	2.979	840.13	2502.53	2.025	686.93	1390.95
A_68_P23317812	chr4:133036175-133036219	NM_175307:149	Fam46b	INSIDE	0.680	0.667	1134.75	757.23	0.454	898.44	407.52
A_68_P23222201	chr4:114574854-114574898	NM_008593:6627	Foxd2	DOWNSTREAM	0.680	4.282	709.23	3037.08	2.912	637.02	1854.95
A_68_P22281784	chr3:82680685-82680738	NM_001146328:-306	Rbm46	PROMOTER	0.680	0.512	1104.74	566.09	0.349	921.02	321.06
A_68_P21836564	chr2:167457070-167457114	NM_023230:413	Ube2v1	INSIDE	0.680	2.956	1048.94	3101.12	2.010	846.94	1702.12
A_68_P21821257	chr2:164860379-164860423	NM_144892:-121	Ncoa5	PROMOTER	0.680	0.513	1960.50	1006.61	0.349	1508.45	527.05
A_68_P21494379	chr2:105239336-105239380	NM_009037:118	Rcn1	INSIDE	0.680	3.109	594.67	1848.82	2.115	482.83	1021.20
A_68_P21132195	chr2:35055732-35055776	NM_026697:886	Rab14	INSIDE	0.680	0.544	1834.97	998.53	0.370	1300.48	480.98
A_68_P21070953	chr2:25118574-25118618	NM_175286:479	Tprn	INSIDE	0.680	2.222	2787.18	6193.83	1.511	2213.57	3343.97

ProbeName	Target position of probe on CpG island microarray	TargetID	GeneSymbol	CpG island Description	Ratio of relative methylation (TiO_2-NP/Vehicle)	Sham group			TiO_2-H group		
						Relative methylation (Cy5/Cy3)	Cy3 signal (Input)	Cy5 signal (MeDIP)	Relative methylation (Cy5/Cy3)	Cy3 signal (Input)	Cy5 signal (MeDIP)
A_68_P20267137	chr1:60100051-60100095	NM_027407:-141	Ica1l	PROMOTER	0.680	7.064	581.61	4108.32	4.807	567.73	2728.92
A_68_P20010812	chr1:5906820-5906864	NM_010342:637	Npbwr1	INSIDE	0.680	0.636	2445.49	1555.15	0.432	2024.16	874.82
A_68_P32565209	chrX:98893628-98893674	ENSMUST00000073812:-6461		PROMOTER	0.679	3.925	387.59	1521.18	2.665	590.52	1573.74
A_68_P31961132	chr19:12575516-12575560	NM_172442:948	Dtx4	INSIDE	0.679	0.423	1883.32	796.88	0.287	1491.74	428.84
A_68_P31599405	chr18:31920058-31920102	NM_153515:547	Ammecr1l	INSIDE	0.679	0.492	1402.89	690.47	0.334	1184.42	395.86
A_68_P31213636	chr17:47825672-47825716	NM_001164729:-359	Tomm6	DIVERGENT_PROMOTER	0.679	0.536	1220.28	653.85	0.364	893.90	325.00
A_68_P31196995	chr17:44873188-44873232	NM_001145920:387	Runx2	INSIDE	0.679	3.997	906.63	3623.45	2.713	832.03	2257.25
A_68_P31151040	chr17:34066027-34066071	NM_001025313:9626	Tapbp	INSIDE	0.679	0.312	2256.46	703.48	0.212	1472.61	311.55
A_68_P31099379	chr17:24889503-24889547	NM_001012402:-423	Hs3st6	PROMOTER	0.679	0.581	1099.52	638.28	0.394	937.04	369.31
A_68_P30660862	chr16:33966764-33966808	NM_009471:303	Umps	INSIDE	0.679	0.303	2152.87	651.26	0.205	1649.90	338.96
A_68_P30496261	chr15:101975285-101975329	NM_008344:690	Igfbp6	INSIDE	0.679	0.616	1939.78	1194.55	0.418	1499.87	626.80
A_68_P29619800	chr14:56386757-56386801	NM_008519:1980	Ltb4r1	INSIDE	0.679	2.832	470.63	1332.71	1.922	429.78	825.95
A_68_P29580869	chr14:47809142-47809186	NM_008102:-87	Gch1	PROMOTER	0.679	0.289	2035.28	587.40	0.196	1338.95	262.49
A_68_P29154200	chr13:76521644-76521688	NM_030174:-742	Mctp1	PROMOTER	0.679	2.541	763.18	1939.23	1.724	650.96	1122.47
A_68_P28992608	chr13:43960038-43960082	ENSMUST00000158482:-8110		PROMOTER	0.679	2.772	747.65	2072.37	1.883	684.04	1287.95
A_68_P28881179	chr13:23487667-23487711	BC160303:75810		INSIDE	0.679	0.201	2598.69	522.69	0.137	1886.88	257.69
A_68_P28175796	chr11:118951645-118951689	NM_007625:-4115	Cbx4	PROMOTER	0.679	0.310	1762.21	546.50	0.211	1424.84	300.20
A_68_P27921467	chr11:74424565-74424610	NM_001013784:-8519	E130309D14Rik	PROMOTER	0.679	4.351	1484.57	6458.86	2.955	1221.90	3610.35
A_68_P27288650	chr10:80755715-80755759	NM_001146687:20	Pip5k1c	INSIDE	0.679	4.289	1374.44	5895.53	2.911	1325.08	3856.80
A_68_P26644018	chr9:75289623-75289667	NM_001039522:314	Leo1	INSIDE	0.679	0.441	1574.25	694.97	0.300	1236.90	370.72
A_68_P25946258	chr8:71612629-71612673	NM_007509:16	Atp6v1b2	INSIDE	0.679	3.185	881.62	2807.68	2.162	800.31	1730.39
A_68_P25730018	chr8:27096782-27096826	NM_029037:7789	4930444A02Rik	INSIDE	0.679	3.353	1516.94	5086.87	2.277	1116.24	2541.57
A_68_P25601117	chr7:150650897-150650941	NM_001161624:-3963	Cdkn1c	DIVERGENT_PROMOTER	0.679	3.156	629.65	1987.15	2.142	613.08	1313.45
A_68_P25504682	chr7:134376720-134376764	NR_030674:-98	Gm4532	DIVERGENT_PROMOTER	0.679	3.661	1075.71	3938.28	2.484	946.06	2350.22
A_68_P24617031	chr6:87540800-87540844	NM_021381:-127	Prokr1	PROMOTER	0.679	0.444	1686.19	749.09	0.302	1322.73	399.09
A_68_P23582143	chr5:32435937-32435982	NM_008037:-2885	Fosl2	PROMOTER	0.679	2.125	3290.26	6992.20	1.443	2267.83	3272.22
A_68_P21761082	chr2:154375340-154375384	NM_029305:723	1700003F12Rik	INSIDE	0.679	2.650	1240.27	3287.04	1.800	1050.42	1890.83
A_68_P20940737	chr1:193730916-193730960	NM_009579:279	Slc30a1	INSIDE	0.679	0.494	995.67	491.90	0.335	869.89	291.83
A_68_P20735832	chr1:156945633-156945677	NM_010500:1112	Ier5	INSIDE	0.679	0.414	1796.29	743.67	0.281	1350.47	379.61
A_68_P31866341	chr18:80909263-80909307	NM_001164111:-4372	Nfatc1	PROMOTER	0.678	0.573	845.26	484.09	0.388	767.18	297.98
A_68_P30746980	chr16:49699315-49699359	NM_028680:-70	Ift57	PROMOTER	0.678	0.508	3679.95	1868.60	0.344	2847.17	980.32
A_68_P29405446	chr14:12386981-12387025	NM_008981:936	Ptprg	INSIDE	0.678	0.159	4104.32	654.25	0.108	2824.04	305.20
A_68_P29154207	chr13:76522358-76522402	NM_030174:-28	Mctp1	PROMOTER	0.678	0.676	939.11	634.97	0.458	877.45	401.96
A_68_P29080189	chr13:59658365-59658409	NM_023328:294	Agtpbp1	INSIDE	0.678	3.901	769.31	3000.78	2.646	647.08	1712.15
A_68_P28339006	chr12:33638754-33638798	NM_013635:-33	Sypl	PROMOTER	0.678	0.302	1632.01	492.71	0.205	1358.38	277.87
A_68_P28167620	chr11:117722044-117722088	NM_001085535:1	Cldn27	INSIDE	0.678	0.589	1949.32	1147.90	0.399	1347.93	537.88
A_68_P28078649	chr11:102146096-102146140	NM_028076:-156	Tmub2	PROMOTER	0.678	0.545	1564.98	853.07	0.370	1090.92	403.17
A_68_P26983380	chr10:19571274-19571318	NM_029529:-31	Slc35d3	PROMOTER	0.678	2.079	1981.61	4118.99	1.409	1574.48	2218.99
A_68_P26644961	chr9:75458869-75458913	NM_016711:242	Tmod2	INSIDE	0.678	3.443	821.79	2829.75	2.334	639.78	1493.07
A_68_P26574097	chr9:62991929-62991973	NM_172446:2837	Skor1	INSIDE	0.678	0.497	2877.84	1429.16	0.337	1869.91	629.62
A_68_P26258436	chr8:129118597-129118641	NM_001164598:-1282	Irf2bp2	PROMOTER	0.678	0.427	1143.95	488.47	0.289	1001.45	289.91
A_68_P25716368	chr8:24522869-24522913	NM_013834:917	Sfrp1	INSIDE	0.678	2.748	2585.23	7105.40	1.865	2192.01	4087.54
A_68_P25512277	chr7:135883845-135883889	NM_145955:77	Mcmbp	INSIDE	0.678	5.210	1158.84	6037.85	3.535	1092.68	3862.45
A_68_P25285292	chr7:91031812-91031856	NM_030705:1017	Mesdc1	INSIDE	0.678	3.267	2209.20	7217.61	2.215	1878.06	4160.36
A_68_P23997296	chr5:113716088-113716132	NR_027934:-6489	Sgsm1	PROMOTER	0.678	0.188	4482.11	844.61	0.128	3317.05	423.62
A_68_P23584753	chr5:32913192-32913236	NM_001205132:-329	Yes1	PROMOTER	0.678	3.264	2816.58	9192.38	2.213	1945.06	4303.46
A_68_P23271971	chr4:124743753-124743797	NM_175246:-162	Snip1	DIVERGENT_PROMOTER	0.678	3.650	2154.56	7864.81	2.475	1872.49	4634.45
A_68_P23190560	chr4:107107097-107107141	NM_147221:-205	Glis1	PROMOTER	0.678	2.960	537.85	1591.86	2.007	512.61	1028.65
A_68_P22133745	chr3:51906640-51906685	NM_001004176:2266	Maml3	INSIDE	0.678	0.416	2328.89	967.86	0.282	1821.92	513.43
A_68_P20720547	chr1:154247487-154247531	NM_177756:512	Glt25d2	INSIDE	0.678	0.415	1185.12	491.54	0.281	830.77	233.53
A_68_P20413588	chr1:88600253-88600297	NM_001172157:-104	Dis3l2	PROMOTER	0.678	0.525	4019.66	2111.97	0.356	3011.18	1073.43
A_68_P32582485	chrX:103222578-103222622	NM_001109757:-14	Atp7a	PROMOTER	0.677	3.389	1699.08	5758.97	2.294	2109.93	4840.00
A_68_P32185502	chr19:54019941-54019985	NM_019658:597	Shoc2	INSIDE	0.677	0.671	2096.67	1406.47	0.454	1706.52	774.47
A_68_P32139052	chr19:45619644-45619688	NM_020032:15367	Poll	DOWNSTREAM	0.677	2.954	1530.19	4519.98	2.001	1201.79	2404.52
A_68_P31923332	chr19:4477049-4477093	NM_134164:73	Syt12	INSIDE	0.677	3.165	594.73	1882.10	2.144	596.36	1278.45
A_68_P31116318	chr17:27571972-27572016	NM_001013385:68255	Grm4	INSIDE	0.677	2.807	2506.13	7033.67	1.901	1760.59	3347.47
A_68_P30596851	chr16:22265603-22265647	NM_009186:378	Tra2b	INSIDE	0.677	6.487	749.00	4858.77	4.392	605.81	2660.87
A_68_P30433466	chr15:90879641-90879685	NM_001109040:717	Kif21a	INSIDE	0.677	3.475	1573.32	5466.86	2.351	1145.84	2693.75
A_68_P30363898	chr15:78858851-78858898	NM_008197:233	H1f0	INSIDE	0.677	3.021	1501.49	4536.43	2.044	1214.53	2482.97
A_68_P30348449	chr15:76287758-76287805	NM_198885:-86	Scx	PROMOTER	0.677	0.440	1867.67	822.16	0.298	1250.78	372.59
A_68_P29102526	chr13:65361405-65361450	ENSMUST00000099425:581		INSIDE	0.677	2.321	1186.28	2753.76	1.572	901.56	1417.49
A_68_P28959954	chr13:38438568-38438612	NM_007556:1006	Bmp6	INSIDE	0.677	0.410	2591.92	1063.22	0.278	2016.95	560.43
A_68_P28608855	chr12:87582934-87582978	NM_027405:129	1700020O03Rik	INSIDE	0.677	0.385	4573.65	1762.66	0.261	3657.87	954.26
A_68_P28186732	chr11:120645992-120646036	NM_145370:186	Gps1	INSIDE	0.677	2.515	735.13	1848.49	1.702	603.39	1026.99
A_68_P28158619	chr11:116168104-116168148	NM_001162872:-74	Exoc7	PROMOTER	0.677	3.768	642.74	2421.70	2.551	537.03	1370.06

ProbeName	Target position of probe on CpG island microarray	TargetID	GeneSymbol	CpG island Description	Ratio of relative methylation (TiO_2-NP/Vehicle)	Sham group			TiO_2-H group		
						Relative methylation (Cy5/Cy3)	Cy3 signal (Input)	Cy5 signal (MeDIP)	Relative methylation (Cy5/Cy3)	Cy3 signal (Input)	Cy5 signal (MeDIP)
A_68_P27283430	chr10:79934572-79934616	ENSMUST00000158603:-4502		PROMOTER	0.677	2.020	3596.53	7265.49	1.368	2600.73	3556.51
A_68_P26531859	chr9:55388856-55388900	NM_027397:-77	Isl2	PROMOTER	0.677	2.108	1536.27	3238.66	1.428	1427.67	2038.78
A_68_P26228890	chr8:124308660-124308704	NM_020605:54224	Jph3	INSIDE	0.677	2.636	980.00	2583.51	1.785	791.96	1413.36
A_68_P26129052	chr8:107065256-107065300	NM_053070:572	Car7	INSIDE	0.677	0.598	1742.51	1042.36	0.405	1223.18	495.17
A_68_P24674236	chr6:97567624-97567668	NM_145148:5	Frmd4b	INSIDE	0.677	0.568	1011.13	574.31	0.385	826.06	317.80
A_68_P24619086	chr6:87931330-87931374	NM_198622:124	H1fx	INSIDE	0.677	6.357	592.00	3763.17	4.301	543.69	2338.41
A_68_P24119633	chr5:136611987-136612031	NM_027891:-64	Lrwd1	DIVERGENT_PROMOTER	0.677	2.330	4387.99	10222.06	1.578	3189.59	5033.97
A_68_P23609096	A_68_P23609096			Unknown	0.677	5.859	245.75	1439.87	3.968	185.74	736.93
A_68_P23467740	chr5:8367848-8367892	NM_001098225:211	Adam22	INSIDE	0.677	4.740	2237.82	10608.21	3.210	1703.30	5467.28
A_68_P22169028	chr3:58380988-58381032	NM_001040396:431	2810407C02Rik	INSIDE	0.677	6.805	618.91	4211.68	4.609	511.83	2359.01
A_68_P21473128	chr2:101637014-101637058	NM_001083810:828	Prr5l	INSIDE	0.677	0.463	2699.13	1250.08	0.313	1965.65	616.20
A_68_P21265074	chr2:61431520-61431564	NM_001164071:389	Tank	INSIDE	0.677	2.351	946.65	2225.46	1.592	747.39	1190.12
A_68_P20556973	chr1:121318457-121318501	NM_008381:347	Inhbb	INSIDE	0.677	3.611	2355.31	8505.37	2.444	1872.01	4575.88
A_68_P32113657	chr19:41281416-41281460	NM_011904:-174	Tll2	PROMOTER	0.676	2.470	1921.26	4746.35	1.669	1605.83	2679.79
A_68_P32030717	chr19:25684634-25684678	NM_177360:-370	Dmrt3	PROMOTER	0.676	0.719	3632.53	2610.24	0.486	2727.22	1325.29
A_68_P31919099	chr19:3767561-3767605	NM_001167884:162	Suv420h1	INSIDE	0.676	0.327	1432.90	468.69	0.221	987.82	218.40
A_68_P31411759	chr17:86020803-86020847	NM_011381:651	Six3	INSIDE	0.676	0.733	2092.75	1534.16	0.495	1712.35	848.11
A_68_P31335038	chr17:71901756-71901800	NM_001142631:378	Spdya	INSIDE	0.676	0.693	2687.17	1862.93	0.469	2097.49	982.92
A_68_P31103786	chr17:25571232-25571276	NM_001163691:-526	Cacna1h	PROMOTER	0.676	2.971	2147.49	6380.99	2.010	1732.18	3481.53
A_68_P31103782	chr17:25570573-25570617	NM_001163691:134	Cacna1h	INSIDE	0.676	2.617	638.44	1670.56	1.770	535.74	948.14
A_68_P30364005	chr15:78873228-78873272	NM_015738:936	Galr3	INSIDE	0.676	4.113	670.07	2756.12	2.780	636.60	1769.77
A_68_P30363892	chr15:78858298-78858342	NM_008197:-321	H1f0	PROMOTER	0.676	2.283	1148.56	2622.30	1.543	977.93	1508.77
A_68_P29614999	chr14:55531847-55531891	NM_021551:101	Slc22a17	INSIDE	0.676	0.729	2118.48	1543.92	0.493	1867.94	920.79
A_68_P29581356	chr14:47896866-47896910	NM_080843:71	Socs4	INSIDE	0.676	0.311	2537.46	789.71	0.211	1933.57	407.08
A_68_P28936823	chr13:34222460-34222504	NM_023716:-259	Tubb2b	PROMOTER	0.676	0.168	8120.36	1367.36	0.114	4999.44	569.41
A_68_P28540764	chr12:75050847-75050891	NM_010431:42015	Hif1a	DOWNSTREAM	0.676	3.305	444.88	1470.12	2.233	431.44	963.54
A_68_P28195050	chr12:3427455-3427499	NM_172421:593	Asxl2	INSIDE	0.676	0.326	2827.84	922.52	0.220	2147.73	473.41
A_68_P28185950	chr11:120521651-120521695	NM_175263:479	Notum	INSIDE	0.676	0.467	1582.46	738.59	0.316	1178.56	372.06
A_68_P28043467	chr11:96148614-96148658	NM_010460:677	Hoxb7	INSIDE	0.676	0.244	3321.76	809.69	0.165	2186.13	360.48
A_68_P27901829	chr11:70460462-70460506	NM_024211:11	Slc25a11	INSIDE	0.676	2.394	1153.88	2762.08	1.618	1048.08	1695.39
A_68_P27899473	chr11:70028070-70028114	NM_153081:681	Slc16a11	INSIDE	0.676	0.557	1758.65	980.24	0.377	1440.21	542.91
A_68_P26883803	chr9:121403379-121403423	NM_031161:1407	Cck	INSIDE	0.676	0.716	2683.75	1921.03	0.484	1822.04	881.27
A_68_P26319144	chr9:14556142-14556186	NM_010242:402	Fut4	INSIDE	0.676	3.288	1548.63	5092.59	2.225	1261.16	2805.51
A_68_P25587467	chr7:148401624-148401668	NM_025886:-112	Rassf7	DIVERGENT_PROMOTER	0.676	5.017	3947.84	19807.31	3.392	3079.14	10443.64
A_68_P24994420	chr7:20214602-20214646	NM_009046:163	Relb	INSIDE	0.676	3.228	1011.12	3264.26	2.181	927.20	2022.14
A_68_P24810492	chr6:122751807-122751851	ENSMUST00000165884:-170		PROMOTER	0.676	3.195	1319.41	4216.04	2.159	989.54	2136.16
A_68_P24173494	chr5:148211545-148211589	NM_010229:499	Flt3	INSIDE	0.676	0.487	1241.49	605.21	0.330	933.31	307.79
A_68_P24124212	chr5:137506063-137506107	NM_001039385:-80	Vgf	PROMOTER	0.676	4.393	478.07	2100.28	2.972	471.38	1400.74
A_68_P23991347	chr5:112737115-112737159	NM_009419:31410	Tpst2	INSIDE	0.676	3.077	839.28	2582.74	2.079	703.07	1461.61
A_68_P23416917	chr4:151534348-151534392	NM_008236:1395	Hes2	INSIDE	0.676	0.503	1521.52	765.58	0.340	1145.20	389.61
A_68_P23271162	chr4:124614141-124614185	NM_026560:-1	Cdca8	DIVERGENT_PROMOTER	0.676	0.288	1616.19	465.85	0.195	1374.88	268.01
A_68_P23187164	chr4:106585031-106585075	NM_023672:978	Ssbp3	INSIDE	0.676	4.392	1127.47	4952.30	2.969	939.60	2789.81
A_68_P22199719	chr3:65196469-65196513	NM_026155:-15	Ssr3	PROMOTER	0.676	36.178	6109.98	221048.60	24.439	10797.88	263894.00
A_68_P21875387	chr2:174264500-174264544	NM_022325:-29	Ctsz	PROMOTER	0.676	0.427	4534.63	1935.71	0.288	3259.02	940.15
A_68_P21350020	chr2:76245001-76245045	NM_145525:428	Osbpl6	INSIDE	0.676	0.459	1196.67	549.38	0.310	955.25	296.51
A_68_P21315048	chr2:70499769-70499813	NM_027352:225	Gorasp2	INSIDE	0.676	8.953	1162.70	10409.62	6.054	925.48	5603.17
A_68_P21311406	chr2:69699566-69699610	NM_001110145:-30	Ssb	PROMOTER	0.676	2.263	1453.74	3290.09	1.529	1272.29	1945.78
A_68_P21071851	chr2:25251636-25251680	NM_009849:265	Entpd2	INSIDE	0.676	0.708	1823.96	1291.86	0.479	1577.69	755.69
A_68_P31403195	chr17:84586228-84586272	NM_001001806:1037	Zfp36l2	INSIDE	0.675	3.054	585.25	1787.34	2.060	592.11	1219.86
A_68_P31357836	A_68_P31357836			Unknown	0.675	0.714	4754.05	3392.60	0.482	3784.51	1822.52
A_68_P31335267	chr17:71948102-71948146	NR_015549:-23	Trmt61b	PROMOTER	0.675	3.107	1607.10	4993.93	2.099	1327.11	2785.16
A_68_P31157714	chr17:35257967-35258011	NM_009975:404	Csnk2b	INSIDE	0.675	0.640	1957.32	1252.60	0.432	1511.47	652.75
A_68_P30397620	chr15:84754050-84754094	NM_016714:215	Nup50	INSIDE	0.675	5.515	1271.60	7013.19	3.723	1100.12	4095.26
A_68_P30375546	chr15:80808340-80808384	NM_134091:168	Sgsm3	INSIDE	0.675	3.027	1463.10	4428.82	2.045	1334.57	2728.83
A_68_P30348904	chr15:76353697-76353741	NM_130893:-1159	Scrt1	PROMOTER	0.675	0.698	1737.04	1212.46	0.471	1269.84	598.08
A_68_P29671617	chr14:65716487-65716531	NM_018788:435	Extl3	INSIDE	0.675	0.455	1935.66	880.40	0.307	1527.33	469.22
A_68_P29619511	chr14:56340664-56340708	NM_019519:327	Rabggta	INSIDE	0.675	0.160	2950.82	472.20	0.108	2113.77	228.28
A_68_P29021091	chr13:48828074-48828118	BC167198:235103		INSIDE	0.675	2.478	1881.48	4661.37	1.673	1593.39	2665.10
A_68_P28435276	chr12:53605530-53605574	AK041016:550		INSIDE	0.675	2.056	4782.27	9830.05	1.388	3908.48	5425.55
A_68_P27901340	chr11:70376701-70376745	NM_001045959:340	Mink1	INSIDE	0.675	0.337	3688.34	1243.20	0.228	2824.64	642.68
A_68_P27677369	chr11:29925854-29925898	NM_146016:157	Eml6	INSIDE	0.675	0.377	3051.29	1150.70	0.254	2359.58	600.20
A_68_P27550009	chr10:129907495-129907539	NM_001103366:26919	Vmn2r87	DOWNSTREAM	0.675	0.624	1306.23	814.95	0.421	1131.21	476.71
A_68_P25959797	chr8:74659503-74659547	NM_001001491:334	Tpm4	INSIDE	0.675	0.392	2986.96	1170.34	0.264	2001.00	528.83
A_68_P25776083	chr8:35870669-35870713	NM_176933:27	Dusp4	INSIDE	0.675	0.518	1981.39	1025.39	0.349	1546.62	540.41
A_68_P25637624	chr8:8661448-8661492	NM_010111:-697	Efnb2	PROMOTER	0.675	2.534	803.88	2036.72	1.711	744.72	1274.05

ProbeName	Target position of probe on CpG island microarray	TargetID	GeneSymbol	CpG island Description	Ratio of relative methylation (TiO_2-NP/Vehicle)	Sham group			TiO_2-H group		
						Relative methylation (Cy5/Cy3)	Cy3 signal (Input)	Cy5 signal (MeDIP)	Relative methylation (Cy5/Cy3)	Cy3 signal (Input)	Cy5 signal (MeDIP)
A_68_P25582339	chr7:147302298-147302342	NM_053119:2	Echs1	INSIDE	0.675	2.053	2979.62	6118.05	1.387	2218.65	3077.03
A_68_P24994309	chr7:20189517-20189561	NM_016680:279	Clasrp	INSIDE	0.675	0.699	1520.53	1063.02	0.472	1215.95	573.68
A_68_P23964038	chr5:107354743-107354789	NM_177873:143	Hfm1	INSIDE	0.675	0.293	1500.37	439.32	0.198	1134.29	224.09
A_68_P23602237	chr5:36021928-36021972	NM_030127:481	Htra3	INSIDE	0.675	0.669	1050.78	702.98	0.452	878.40	396.68
A_68_P22875662	chr4:44181111-44181156	NM_175201:-723	Rnf38	PROMOTER	0.675	3.541	860.06	3045.53	2.390	732.93	1751.94
A_68_P21775428	chr2:156960888-156960932	NM_001139520:48	Samhd1	INSIDE	0.675	3.168	544.11	1723.75	2.138	413.86	884.67
A_68_P20351022	chr1:74929216-74929260	NM_153111:2744	Fev	INSIDE	0.675	2.789	1142.55	3186.78	1.882	927.14	1744.94
A_68_P31758245	chr18:61696023-61696067	NM_177828:146	Arhgef37	INSIDE	0.674	0.445	3629.85	1615.02	0.300	2612.96	783.52
A_68_P30980974	chr16:94592990-94593034	NM_001159617:-390	Pigp	PROMOTER	0.674	0.521	1111.93	579.65	0.351	1001.06	351.61
A_68_P30382137	chr15:82043157-82043202	NM_001207021:66	AI848285	INSIDE	0.674	6.705	751.67	5039.85	4.522	657.57	2973.35
A_68_P30343807	chr15:75577030-75577074	NM_194350:1300	Mafa	DOWNSTREAM	0.674	3.412	883.95	3015.78	2.298	727.68	1672.17
A_68_P29041263	chr13:53074062-53074106	NM_017373:2324	Nfil3	INSIDE	0.674	0.493	920.62	453.42	0.332	826.34	274.14
A_68_P28981522	chr13:42144875-42144919	NM_007772:-2493	Hivep1	PROMOTER	0.674	0.491	2439.51	1197.84	0.331	1798.76	595.01
A_68_P28767698	chr12:117643570-117643614	NR_028421:42	D430020J02Rik	INSIDE	0.674	0.434	1351.03	585.68	0.292	978.69	286.05
A_68_P28115688	chr11:108782730-108782774	NM_015732:1090	Axin2	INSIDE	0.674	0.732	6017.89	4407.87	0.494	4441.24	2192.52
A_68_P28051738	chr11:97490235-97490279	NM_175332:774	E130012A19Rik	INSIDE	0.674	3.474	2008.03	6975.63	2.340	1819.79	4258.83
A_68_P28034315	chr11:94538289-94538333	NM_145828:497	Xylt2	INSIDE	0.674	0.427	3285.42	1401.32	0.287	2471.19	710.21
A_68_P27977994	chr11:84338833-84338877	NM_008498:182	Lhx1	INSIDE	0.674	0.660	2227.14	1468.82	0.445	1671.44	743.04
A_68_P27055250	chr10:33847341-33847385	NM_175449:416	Fam26f	INSIDE	0.674	6.046	2675.26	16173.47	4.072	2190.60	8919.90
A_68_P26467131	chr9:44152207-44152251	NM_001110251:-1764	Hmbs	PROMOTER	0.674	2.795	1539.46	4302.65	1.883	1184.53	2229.90
A_68_P26248332	chr8:127421933-127421977	NM_001111141:169	Gm505	INSIDE	0.674	3.539	489.47	1732.05	2.385	410.91	979.89
A_68_P25174403	chr7:71083373-71083417	NM_021366:407	Klf13	INSIDE	0.674	0.547	1636.91	895.01	0.368	1337.67	492.69
A_68_P24833353	chr6:127511414-127511458			Unknown	0.674	2.752	1502.38	4134.38	1.854	1160.94	2152.83
A_68_P24120066	chr5:136707860-136707905	NM_018825:12891	Sh2b2	INSIDE	0.674	2.374	2062.73	4897.67	1.600	1433.20	2293.39
A_68_P24114957	chr5:135625410-135625454	NM_013763:-148	Tbl2	PROMOTER	0.674	0.564	1185.13	667.85	0.380	940.50	357.42
A_68_P24091288	chr5:130417664-130417708	NM_001001327:-296	Vkorc1l1	PROMOTER	0.674	0.147	9587.13	1410.05	0.099	6578.63	652.20
A_68_P24004990	chr5:115060897-115060941	NM_001004180:42659	BC057022	INSIDE	0.674	2.656	1562.92	4151.23	1.790	977.94	1750.64
A_68_P23657845	chr5:46060700-46060744	NM_019438:-441	Ncapg	PROMOTER	0.674	0.607	1450.64	880.29	0.409	1167.35	477.41
A_68_P23417944	chr4:151687118-151687162	NM_001033489:5594	Rnf207	INSIDE	0.674	0.195	2896.43	564.33	0.131	1989.89	261.21
A_68_P23345562	chr4:137952020-137952064	NM_001081672:331	Fam43b	INSIDE	0.674	3.815	973.61	3714.46	2.572	824.26	2119.67
A_68_P22407413	chr3:107399619-107399663	NM_007441:1692	Alx3	INSIDE	0.674	0.502	2194.31	1101.25	0.338	1711.09	578.52
A_68_P21958960	chr3:14886530-14886574	NM_009801:127	Car2	INSIDE	0.674	2.025	3008.43	6090.65	1.365	2472.58	3374.69
A_68_P21890603	chr2:178149107-178149151	NM_001085501:49	Ppp1r3d	INSIDE	0.674	0.555	1008.94	560.16	0.374	850.69	318.27
A_68_P21784244	chr2:158493590-158493634	NM_001159662:742	Ppp1r16b	INSIDE	0.674	2.877	896.96	2580.33	1.938	766.75	1485.81
A_68_P21257459	chr2:60122268-60122312	NM_025422:185	Cd302	INSIDE	0.674	0.556	992.28	551.57	0.374	825.69	309.19
A_68_P21116393	chr2:32468028-32468072	NM_001025310:5570	St6galnac6	INSIDE	0.674	14.138	3168.38	44796.11	9.528	2949.81	28106.42
A_68_P20613818	chr1:133034787-133034831	NM_145508:3	Dyrk3	INSIDE	0.674	3.557	719.35	2558.46	2.398	708.87	1699.60
A_68_P31623248	chr18:36479879-36479923	AK015580:1246		DOWNSTREAM	0.673	2.403	2092.11	5026.36	1.617	1737.36	2808.91
A_68_P30373741	chr15:80502565-80502609	NM_145986:310	Fam83f	INSIDE	0.673	2.645	1048.63	2774.12	1.782	812.37	1447.29
A_68_P29957984	chr14:122197004-122197048	NM_134074:-70	Dock9	PROMOTER	0.673	2.934	697.25	2045.53	1.974	590.84	1166.18
A_68_P29150850	chr13:75845171-75845215	NM_138953:261	Ell2	INSIDE	0.673	0.460	2654.27	1221.42	0.310	2015.96	623.94
A_68_P28694106	chr12:104115843-104115887	NM_172806:752	Btbd7	INSIDE	0.673	0.665	1011.29	672.84	0.448	867.00	388.25
A_68_P28192906	chr11:121691796-121691840	NM_029049:287	Ptchd3	INSIDE	0.673	5.424	1976.37	10719.00	3.650	1554.24	5672.40
A_68_P27902563	chr11:70578032-70578076	NM_177565:106	Zfp3	INSIDE	0.673	0.285	1645.31	468.44	0.192	1432.85	274.71
A_68_P27891998	chr11:68715228-68715272	NR_003373:160	Gm15772	INSIDE	0.673	2.330	1347.92	3140.07	1.569	1071.57	1681.00
A_68_P26628952	chr9:72510759-72510803	NM_010890:627	Nedd4	INSIDE	0.673	0.595	821.41	488.36	0.400	733.30	293.52
A_68_P25948481	chr8:72406321-72406365	NM_026818:4949	Cilp2	INSIDE	0.673	3.087	977.14	3016.45	2.079	966.58	2009.42
A_68_P25415073	chr7:117912172-117912216	NM_009667:-3923	Ampd3	PROMOTER	0.673	0.460	1578.18	726.08	0.310	1213.12	375.58
A_68_P25184943	chr7:73007475-73007519	NM_011048:475	Pcsk6	INSIDE	0.673	0.320	2142.84	686.42	0.215	1603.53	345.56
A_68_P24055950	chr5:123939335-123939379	NM_001033461:23	Lrrc43	INSIDE	0.673	0.688	958.29	659.38	0.463	722.88	334.71
A_68_P23947800	chr5:104084571-104084615	NM_028824:151	1700016H13Rik	INSIDE	0.673	3.119	1687.12	5262.02	2.100	1471.00	3088.50
A_68_P23787602	chr5:72048882-72048926	NM_010251:643	Gabra4	INSIDE	0.673	2.121	2799.40	5938.02	1.428	2090.62	2985.30
A_68_P23278140	chr4:125773896-125773940	NM_001163266:22	Lsm10	INSIDE	0.673	0.451	3347.52	1509.61	0.304	2470.77	750.17
A_68_P20874331	chr1:182125909-182125953	NM_001163290:-2314	Adck3	PROMOTER	0.673	2.564	723.85	1855.86	1.726	636.76	1099.05
A_68_P20261291	chr1:59030219-59030263	NM_172406:86	Trak2	INSIDE	0.673	0.298	3151.70	938.58	0.201	2181.74	437.46
A_68_P32565626	chrX:98989424-98989468	ENSMUST00000151231:-548		DIVERGENT_PROMOTER	0.672	4.394	327.56	1439.37	2.952	435.04	1284.13
A_68_P31863853	chr18:80560016-80560060	NM_001190373:955	Kcng2	INSIDE	0.672	2.865	1414.11	4051.49	1.924	1225.50	2358.45
A_68_P30464707	chr15:96472161-96472205	NM_134086:211	Slc38a1	INSIDE	0.672	0.663	1915.49	1269.07	0.445	1479.20	658.94
A_68_P28886517	chr13:24372323-24372367	NM_026825:315	Lrrc16a	INSIDE	0.672	0.352	5324.35	1874.30	0.237	3513.55	831.53
A_68_P28768146	chr12:117724807-117724852	NM_011215:637	Ptprn2	INSIDE	0.672	0.674	1258.08	848.48	0.453	962.63	436.39
A_68_P27995150	chr11:87430698-87430742	NM_001045527:55	Hsf5	INSIDE	0.672	7.127	2198.02	15665.16	4.790	1902.32	9111.32
A_68_P27895906	chr11:69395043-69395087	NM_001127233:1204	Trp53	INSIDE	0.672	3.481	1128.67	3929.10	2.338	975.28	2280.24
A_68_P27787504	chr11:50050408-50050452	NM_008521:1543	Ltc4s	INSIDE	0.672	2.215	1828.68	4050.26	1.488	1495.28	2225.15
A_68_P27297054	chr10:82822859-82822903	NM_026579:-85	D10Wsu102e	PROMOTER	0.672	0.613	4928.85	3021.09	0.412	3491.38	1437.75
A_68_P26978748	chr10:18734800-18734844	NM_001166402:-3053	Tnfaip3	PROMOTER	0.672	0.212	3575.19	758.39	0.143	2549.02	363.43

ProbeName	Target position of probe on CpG island microarray	TargetID	GeneSymbol	CpG island Description	Ratio of relative methylation (TiO_2-NP/Vehicle)	Sham group			TiO_2-H group		
						Relative methylation (Cy5/Cy3)	Cy3 signal (Input)	Cy5 signal (MeDIP)	Relative methylation (Cy5/Cy3)	Cy3 signal (Input)	Cy5 signal (MeDIP)
A_68_P26696152	chr9:85737849-85737894	NM_001164792:1413	Tpbg	INSIDE	0.672	0.394	1200.71	472.63	0.265	885.20	234.19
A_68_P26549614	chr9:58589155-58589200	NM_028598:189	2410076I21Rik	INSIDE	0.672	3.117	813.16	2534.65	2.095	706.30	1479.73
A_68_P26129290	chr8:107115528-107115572	NM_001024606:183	Pdp2	INSIDE	0.672	0.550	2787.69	1533.28	0.370	2133.60	788.52
A_68_P25195708	chr7:74904333-74904377	NM_183312:274	Synm	INSIDE	0.672	2.274	1472.92	3349.03	1.528	1229.45	1878.11
A_68_P25195707	chr7:74904146-74904190	NM_183312:460	Synm	INSIDE	0.672	3.939	1488.30	5862.17	2.645	1275.83	3375.03
A_68_P24828004	chr6:126593418-126593462	NM_010595:2379	Kcna1	INSIDE	0.672	3.064	989.72	3033.00	2.059	739.87	1523.49
A_68_P24607722	chr6:85978843-85978887	NM_012013:11680	Figla	DOWNSTREAM	0.672	0.377	1510.21	568.69	0.253	1086.74	275.20
A_68_P24382000	chr6:39157044-39157088	NM_001033430:-294	Jhdm1d	PROMOTER	0.672	18.315	7452.18	136489.00	12.299	7363.22	90563.70
A_68_P24157330	chr5:144383580-144383624	NM_011182:287	Cyth3	INSIDE	0.672	0.363	2949.12	1069.90	0.244	2379.48	580.26
A_68_P23590782	chr5:34064726-34064770	NM_001163216:-206	Fgfr3	PROMOTER	0.672	0.540	1153.60	622.60	0.362	904.29	327.79
A_68_P23541453	chr5:23893834-23893878	NM_001002897:4105	Atg9b	INSIDE	0.672	2.614	1397.49	3652.80	1.757	1125.07	1976.63
A_68_P23363728	chr4:140938466-140938511	NM_001085513:14354	Fam131c	INSIDE	0.672	0.239	2930.38	701.82	0.161	2170.88	349.42
A_68_P22715816	chr4:8618846-8618890	NM_001081417:801	Chd7	INSIDE	0.672	0.727	2116.13	1538.91	0.489	1688.90	825.51
A_68_P22567225	chr3:137806480-137806524	NM_175389:1	Rg9mtd2	INSIDE	0.672	0.187	2768.56	516.63	0.125	2018.55	253.22
A_68_P22029228	chr3:30501632-30501676	NM_030557:646	Mynn	INSIDE	0.672	0.203	2524.19	511.36	0.136	1944.53	264.72
A_68_P21110673	chr2:31544152-31544196	NM_001112703:99	Abl1	INSIDE	0.672	2.801	1414.44	3962.24	1.881	1201.08	2259.36
A_68_P20833904	chr1:174493216-174493260	NR_027644:3202	Vsig8	INSIDE	0.672	2.775	2015.85	5593.47	1.864	1630.89	3040.78
A_68_P20266463	chr1:59970586-59970630	NM_001037725:759	Fam117b	INSIDE	0.672	0.643	2872.64	1845.99	0.432	2377.14	1027.00
A_68_P32745696	chrX:147955251-147955299	NM_177201:60	Phf8	INSIDE	0.671	4.623	338.09	1562.84	3.101	434.91	1348.64
A_68_P32553702	chrX:96332024-96332068	NM_010110:578	Efnb1	INSIDE	0.671	2.951	638.86	1885.37	1.981	764.37	1514.24
A_68_P32396638	chrX:53851205-53851249	NM_146234:-130	Mmgt1	PROMOTER	0.671	3.819	1285.61	4909.91	2.562	1549.74	3971.08
A_68_P32029538	chr19:25484679-25484723	NM_181404:173009	Kank1	INSIDE	0.671	3.515	2145.72	7542.70	2.360	1528.82	3607.62
A_68_P31286855	chr17:63231908-63231952	NM_010109:-1264	Efna5	PROMOTER	0.671	0.496	1253.22	622.11	0.333	894.47	298.07
A_68_P30990392	chr16:96212465-96212509	NM_019537:24	Psmg1	INSIDE	0.671	0.550	1432.86	788.48	0.369	1122.62	414.55
A_68_P29703308	chr14:71165727-71165771	NM_023045:687	Xpo7	INSIDE	0.671	0.509	1937.16	985.35	0.341	1432.83	488.84
A_68_P29493860	chr14:29319799-29319843	NM_009524:1162	Wnt5a	INSIDE	0.671	0.126	5282.04	666.25	0.085	3901.26	330.00
A_68_P29031122	chr13:51266973-51267017	NM_029173:601	Nxnl2	INSIDE	0.671	0.236	2257.88	533.73	0.159	1671.15	265.11
A_68_P28997685	chr13:44829480-44829524	NM_001205043:2863	Jarid2	INSIDE	0.671	0.714	1405.35	1002.87	0.479	1203.77	576.14
A_68_P28974007	chr13:40804497-40804541	NM_001122948:21294	Tcfap2a	DOWNSTREAM	0.671	0.615	1562.55	960.69	0.413	1145.85	472.69
A_68_P28751776	chr12:113916958-113917002	NM_001100460:-70	Zbtb42	DIVERGENT_PROMOTER	0.671	6.027	855.92	5159.02	4.046	773.41	3129.34
A_68_P28186830	chr11:120657372-120657416	NM_026824:315	Dus1l	INSIDE	0.671	0.514	3098.12	1590.97	0.344	2117.22	729.26
A_68_P28182344	chr11:119959816-119959860	NM_183137:207	2410002I01Rik	INSIDE	0.671	0.633	1992.02	1260.93	0.425	1325.30	562.69
A_68_P27987929	chr11:86070620-86070664	NM_027421:428	Ints2	INSIDE	0.671	0.274	2461.74	674.43	0.184	1886.00	346.65
A_68_P27929276	chr11:75780169-75780213			Unknown	0.671	2.312	1476.95	3415.09	1.552	1343.61	2085.17
A_68_P26316371	chr9:14080770-14080814	NM_030261:48	Sesn3	INSIDE	0.671	0.479	2244.65	1074.22	0.321	1873.47	601.78
A_68_P25952268	chr8:73025186-73025230	NM_018827:8154	Crlf1	INSIDE	0.671	0.678	1297.31	879.84	0.455	1158.42	526.96
A_68_P25948618	chr8:72426813-72426857	NM_023312:-377	Ndufa13	PROMOTER	0.671	0.231	2635.85	607.60	0.155	1871.44	289.42
A_68_P25448400	chr7:123648290-123648334	NM_016773:425	Nucb2	INSIDE	0.671	0.159	3987.03	635.85	0.107	2616.36	279.83
A_68_P25050419	chr7:36432218-36432262	NM_019746:261	Pdcd5	INSIDE	0.671	0.422	2036.62	859.80	0.283	1536.49	435.38
A_68_P24133865	chr5:139626414-139626458	NM_001081265:260	Heatr2	INSIDE	0.671	4.393	1011.06	4441.85	2.947	903.63	2663.12
A_68_P24091170	chr5:130384460-130384504	NM_026916:73	4930579G22Rik	INSIDE	0.671	0.454	2203.86	1001.00	0.305	1876.73	572.05
A_68_P23973505	chr5:109106557-109106601	NM_174870:-1910	Slc26a1	PROMOTER	0.671	0.436	1221.38	532.44	0.292	874.22	255.58
A_68_P23956783	chr5:106128691-106128735	NM_001122768:-275	Lrrc8d	PROMOTER	0.671	2.237	1802.03	4031.93	1.501	1576.13	2365.13
A_68_P23766885	chr5:67652316-67652360	NM_028975:448	Tmem33	INSIDE	0.671	0.483	941.58	454.48	0.324	724.19	234.65
A_68_P23694283	chr5:52759912-52759956	NM_011435:4892	Sod3	INSIDE	0.671	6.281	2416.71	15178.67	4.212	2000.13	8423.64
A_68_P23409803	chr4:150432014-150432058	NM_009498:26	Vamp3	INSIDE	0.671	0.270	1847.89	498.81	0.181	1365.99	247.54
A_68_P22971251	chr4:63220017-63220061	NM_144905:-1355	6330416G13Rik	PROMOTER	0.671	0.729	1872.67	1365.84	0.489	1364.73	667.81
A_68_P22820149	chr4:32327040-32327088	ENSMUST00000165661:-3643		PROMOTER	0.671	3.098	516.92	1601.52	2.080	393.85	819.35
A_68_P21784239	chr2:158492898-158492942	NM_001159662:50	Ppp1r16b	INSIDE	0.671	4.711	612.82	2886.98	3.163	563.08	1780.94
A_68_P21311589	chr2:69735872-69735916	NM_001081548:592	Ubr3	INSIDE	0.671	3.198	2731.78	8736.89	2.147	2198.37	4719.28
A_68_P21104485	chr2:30514065-30514109	ENSMUST00000147959:11342		INSIDE	0.671	0.583	1127.09	657.18	0.391	857.81	335.65
A_68_P21101100	chr2:29918302-29918346	NM_001204875:-3669	Set	PROMOTER	0.671	3.925	2261.74	8877.48	2.635	2036.84	5367.97
A_68_P21094145	chr2:28771674-28771718	NM_019446:245	Barhl1	INSIDE	0.671	0.607	2616.67	1587.68	0.407	2031.44	827.27
A_68_P20613533	chr1:132993730-132993774	NM_008551:368	Mapkapk2	INSIDE	0.671	0.724	1958.60	1418.13	0.486	1608.70	781.05
A_68_P31757529	chr18:61558490-61558534	NM_133249:1573	Ppargc1b	INSIDE	0.670	0.359	2573.07	924.49	0.241	2128.58	512.15
A_68_P31305848	chr17:66729002-66729046	NM_001114098:70066	1110012J17Rik	INSIDE	0.670	2.937	1934.82	5681.70	1.967	1552.25	3052.67
A_68_P31186625	chr17:43013785-43013829	NM_009847:-433	Cd2ap	PROMOTER	0.670	2.683	1188.13	3187.23	1.799	1031.46	1855.20
A_68_P30578862	chr16:18289475-18289519	NM_033324:-235	Dgcr8	PROMOTER	0.670	0.438	3230.99	1414.86	0.294	2503.45	735.00
A_68_P29957982	chr14:122196774-122196818	NM_134074:160	Dock9	INSIDE	0.670	0.450	1075.79	484.17	0.302	895.43	270.05
A_68_P29560592	chr14:41780099-41780143	NM_145928:-24	Tspan14	PROMOTER	0.670	0.505	1688.71	852.98	0.338	1442.51	487.90
A_68_P28175458	chr11:118903402-118903447	NM_013926:-1197	Cbx8	PROMOTER	0.670	5.361	1318.38	7067.38	3.592	1018.95	3659.76
A_68_P28041811	chr11:95852863-95852907	NM_009951:14374	Igf2bp1	INSIDE	0.670	2.400	1638.78	3933.63	1.608	1366.71	2197.88
A_68_P27357060	chr10:93499035-93499079	NM_053072:311	Fgd6	INSIDE	0.670	0.606	1534.07	929.95	0.406	1341.76	544.75
A_68_P25511166	chr7:135667647-135667691	NM_013863:572	Bag3	INSIDE	0.670	0.628	908.08	570.00	0.421	709.11	298.37
A_68_P25011764	chr7:26539484-26539528	NM_178089:214	Hnrnpul1	INSIDE	0.670	0.577	1376.90	794.46	0.386	1026.29	396.61

ProbeName	Target position of probe on CpG island microarray	TargetID	GeneSymbol	CpG island Description	Ratio of relative methylation (TiO_2-NP/Vehicle)	Sham group: Relative methylation (Cy5/Cy3)	Sham group: Cy3 signal (Input)	Sham group: Cy5 signal (MeDIP)	TiO_2-H group: Relative methylation (Cy5/Cy3)	TiO_2-H group: Cy3 signal (Input)	TiO_2-H group: Cy5 signal (MeDIP)
A_68_P24880087	chr6:137683557-137683601	NM_011499:-24	Strap	PROMOTER	0.670	6.956	1117.39	7772.61	4.662	966.89	4507.62
A_68_P24172759	chr5:148081959-148082003	NM_008814:274	Pdx1	INSIDE	0.670	0.173	3429.37	594.67	0.116	2570.26	298.82
A_68_P23763266	chr5:67067724-67067768	NM_011670:387	Uchl1	INSIDE	0.670	6.191	1676.44	10378.11	4.145	1620.33	6715.62
A_68_P23544149	chr5:24347845-24347889	NM_053075:313	Rheb	INSIDE	0.670	3.850	1334.59	5138.40	2.580	1128.52	2911.69
A_68_P23301754	chr4:130219409-130219453	NM_001159603:157	Pum1	INSIDE	0.670	2.082	3634.49	7568.67	1.395	2814.64	3925.43
A_68_P23293641	chr4:128561860-128561904	NM_178110:499	Trim62	INSIDE	0.670	0.592	2411.14	1427.75	0.396	1889.42	749.13
A_68_P22885280	chr4:45839410-45839454	NM_018741:267	Igfbpl1	INSIDE	0.670	2.287	1911.21	4371.77	1.533	1624.11	2489.42
A_68_P21836562	chr2:167456829-167456873	NM_023230:655	Ube2v1	INSIDE	0.670	0.335	7889.07	2641.33	0.224	5325.99	1194.26
A_68_P21665423	chr2:136716895-136716939	NM_028201:-37	2210009G21Rik	PROMOTER	0.670	2.144	2553.50	5474.73	1.438	2187.50	3144.62
A_68_P21438061	chr2:93796206-93796250	NM_001145034:1029	Gm13889	INSIDE	0.670	0.290	3709.66	1074.50	0.194	2612.28	506.67
A_68_P32766767	chrX:153929659-153929703	NM_009214:298	Sms	INSIDE	0.669	6.694	655.16	4385.78	4.480	771.37	3455.64
A_68_P32553706	chrX:96332444-96332494	NM_010110:1000	Efnb1	INSIDE	0.669	2.130	1279.85	2726.09	1.425	1619.10	2307.45
A_68_P32245741	chrX:8777677-8777721	NM_173414:600	Lancl3	INSIDE	0.669	3.309	1052.47	3482.77	2.212	1326.98	2935.80
A_68_P32016034	chr19:23209424-23209468	NM_010638:-6269	Klf9	PROMOTER	0.669	0.323	4018.34	1299.41	0.216	3201.39	693.05
A_68_P31982216	chr19:16855225-16855273	NM_173028:169	Vps13a	INSIDE	0.669	3.027	529.79	1603.65	2.026	434.18	879.45
A_68_P31500013	chr18:12464044-12464088	NM_001190371:163	Ankrd29	INSIDE	0.669	0.633	1244.34	787.89	0.423	980.26	415.06
A_68_P30961948	chr16:91270536-91270580	NM_016968:545	Olig1	INSIDE	0.669	3.797	1774.11	6736.58	2.541	1355.95	3445.08
A_68_P30371089	chr15:80086079-80086123	NM_009716:487	Atf4	INSIDE	0.669	4.678	1367.85	6398.24	3.129	1280.16	4005.34
A_68_P28956736	chr13:37918899-37918943	NM_001039188:-341	Rreb1	PROMOTER	0.669	10.759	747.22	8039.54	7.195	693.57	4990.52
A_68_P27833864	chr11:58791891-58791936	NM_053168:296	Triml1	INSIDE	0.669	0.355	1327.79	471.98	0.238	1061.17	252.51
A_68_P27056939	chr10:34138119-34138163	NM_176968:194	Nt5dc1	INSIDE	0.669	0.402	1506.77	606.47	0.269	1200.32	323.24
A_68_P25898920	chr8:61391354-61391398	NM_173411:825	BC030500	INSIDE	0.669	0.316	3345.76	1058.79	0.212	2421.68	512.75
A_68_P25458765	chr7:125676599-125676643	NM_009940:207	Coq7	INSIDE	0.669	0.501	2232.37	1117.60	0.335	1567.16	525.09
A_68_P25353895	chr7:105259944-105259988	NM_008663:8037	Myo7a	INSIDE	0.669	0.421	2114.94	890.92	0.282	1546.99	436.10
A_68_P25307415	chr7:96552181-96552225	NM_008055:-673	Fzd4	PROMOTER	0.669	2.699	1607.07	4338.05	1.806	1335.78	2412.74
A_68_P24990859	chr7:19590097-19590141	NM_178757:705	Irf2bp1	INSIDE	0.669	0.504	1878.29	947.25	0.337	1404.05	473.56
A_68_P24066362	chr5:125821382-125821426	NM_001205082:60	Scarb1	INSIDE	0.669	0.188	3316.95	623.57	0.126	2486.84	312.98
A_68_P23539567	chr5:23536236-23536280	NM_053090:243	Fam126a	INSIDE	0.669	0.532	1416.83	753.61	0.356	1227.66	437.07
A_68_P22455157	chr3:117278382-117278426	NM_029425:26	4833424O15Rik	INSIDE	0.669	0.652	2378.30	1549.49	0.436	1834.26	799.44
A_68_P20005799	chr1:4482580-4482624	NM_011441:3892	Sox17	INSIDE	0.669	3.065	2166.02	6639.67	2.052	1860.33	3816.82
A_68_P31190360	chr17:43766712-43766757	NM_001161366:514	Tdrd6	INSIDE	0.668	3.573	1331.74	4758.42	2.385	1146.26	2734.31
A_68_P31097235	chr17:24563756-24563800	NM_010023:151	Eci1	INSIDE	0.668	0.445	3300.10	1469.25	0.297	2470.93	734.97
A_68_P30511262	chr16:4640285-4640329	NM_139307:362	Vasn	INSIDE	0.668	0.509	1167.83	594.20	0.340	1078.33	366.31
A_68_P29614875	chr14:55512913-55512957	NM_019402:-45	Pabpn1	PROMOTER	0.668	0.720	1717.64	1236.46	0.481	1274.68	612.77
A_68_P29455906	chr14:22808776-22808820	NM_145459:25	Zfp503	INSIDE	0.668	0.587	1313.63	771.45	0.393	1020.12	400.41
A_68_P29455887	chr14:22806463-22806507	NM_145459:2339	Zfp503	INSIDE	0.668	0.565	2521.94	1423.80	0.377	1770.32	667.92
A_68_P26814367	chr9:108242276-108242320	NM_008160:888	Gpx1	INSIDE	0.668	0.352	3183.73	1122.26	0.236	2404.90	566.67
A_68_P26803652	chr9:106379557-106379601	NM_145620:-61	Rrp9	DIVERGENT_PROMOTER	0.668	0.717	3732.92	2676.66	0.479	2910.18	1393.47
A_68_P26626137	chr9:71959791-71959835	NM_011544:-186	Tcf12	PROMOTER	0.668	5.805	2418.83	14042.19	3.876	2109.67	8176.05
A_68_P25891572	chr8:59990950-59990994	NM_008252:333	Hmgb2	INSIDE	0.668	2.594	1145.41	2970.82	1.734	930.00	1612.49
A_68_P25393401	chr7:112781612-112781656	NM_018880:379	Trim3	INSIDE	0.668	0.572	11454.72	6547.15	0.382	6528.09	2493.10
A_68_P24841052	chr6:128793077-128793121	ENSMUST00000166515:-705		PROMOTER	0.668	0.315	5330.07	1678.83	0.210	3610.05	759.45
A_68_P23966229	chr5:107717967-107718011	NM_011578:626	Tgfbr3	INSIDE	0.668	3.573	1665.82	5952.32	2.388	1497.45	3575.74
A_68_P22542153	chr3:132973212-132973256	NM_146141:155	Ppa2	INSIDE	0.668	2.462	845.84	2082.22	1.645	663.78	1091.89
A_68_P21556057	chr2:116947191-116947235	NM_033524:27	Spred1	INSIDE	0.668	5.706	365.46	2085.19	3.813	362.85	1383.42
A_68_P21370050	chr2:79475696-79475740	NM_080558:137	Ssfa2	INSIDE	0.668	5.680	1902.66	10806.59	3.792	1803.33	6838.34
A_68_P21250387	chr2:58999594-58999638	NM_026361:710	Pkp4	INSIDE	0.668	4.118	715.78	2947.84	2.753	618.77	1703.24
A_68_P32674749	chrX:131543372-131543416	NM_175446:354	Zmat1	INSIDE	0.667	4.035	435.75	1758.39	2.690	632.93	1702.58
A_68_P31655791	chr18:42671723-42671767	NM_001039474:604	Tcerg1	INSIDE	0.667	2.980	2106.68	6277.19	1.986	1697.33	3371.33
A_68_P31624569	chr18:36720617-36720661	NM_175375:382	Ankhd1	INSIDE	0.667	0.359	2365.69	849.14	0.239	1937.27	463.58
A_68_P31620715	chr18:36048154-36048198			Unknown	0.667	2.988	1103.74	3297.80	1.992	1045.86	2082.92
A_68_P31207969	chr17:46765813-46765857	NM_175168:619	Ptk7	INSIDE	0.667	0.507	1902.31	964.27	0.338	1573.95	532.18
A_68_P29657715	chr14:63456440-63456484	NM_175546:-64	Wdfy2	PROMOTER	0.667	0.718	2802.59	2011.98	0.479	2324.46	1113.59
A_68_P29072116	chr13:58317256-58317300	NM_026842:-264	Ubqln1	PROMOTER	0.667	0.516	1085.97	559.85	0.344	854.90	293.89
A_68_P28727387	chr12:109572709-109572753	NM_010010:140	Cyp46a1	INSIDE	0.667	0.689	1300.49	895.51	0.459	928.21	426.45
A_68_P28688781	chr12:103213776-103213820	NM_016856:-385	Cpsf2	PROMOTER	0.667	0.325	2568.10	835.66	0.217	1957.09	424.61
A_68_P28050945	chr11:97362080-97362124	NM_021493:50629	Arhgap23	INSIDE	0.667	5.041	780.57	3934.80	3.363	699.76	2353.62
A_68_P27941268	chr11:77893553-77893597	NM_022313:311	Eral1	INSIDE	0.667	2.272	2168.44	4926.36	1.516	1727.46	2618.36
A_68_P27937689	chr11:77275784-77275828	NM_139128:-1608	Coro6	PROMOTER	0.667	7.010	1303.26	9135.72	4.674	1178.66	5508.55
A_68_P27930555	chr11:76015561-76015605	NM_027773:25	Fam57a	INSIDE	0.667	2.799	4563.61	12775.46	1.868	3681.91	6876.88
A_68_P27924626	chr11:74986914-74986958	NR_029794:47	Mir212	INSIDE	0.667	0.410	1514.00	620.74	0.273	1233.36	337.27
A_68_P27844447	chr11:60540408-60540455	NM_022009:292	Flii	INSIDE	0.667	0.570	4557.28	2595.72	0.380	3193.15	1213.33
A_68_P27841330	chr11:59989954-59989998	NM_001037764:36393	Rai1	INSIDE	0.667	0.157	3816.12	600.27	0.105	2729.52	286.24
A_68_P27262051	chr10:75905675-75905719	NM_008787:-39	Pcnt	DIVERGENT_PROMOTER	0.667	0.353	1292.17	455.91	0.235	943.38	222.04
A_68_P26685862	chr9:83728264-83728308	NM_001110265:-9	Ttk	PROMOTER	0.667	2.834	3192.80	9047.09	1.890	2133.31	4032.65

ProbeName	Target position of probe on CpG island microarray	TargetID	GeneSymbol	CpG island Description	Ratio of relative methylation (TiO_2-NP/Vehicle)	Sham group			TiO_2-H group		
						Relative methylation (Cy5/Cy3)	Cy3 signal (Input)	Cy5 signal (MeDIP)	Relative methylation (Cy5/Cy3)	Cy3 signal (Input)	Cy5 signal (MeDIP)
A_68_P26659379	chr9:78244039-78244083	NM_001191044:477	Ddx43	INSIDE	0.667	0.513	1063.14	544.90	0.342	916.44	313.50
A_68_P26134293	chr8:108042530-108042574	NM_008289:-93	Hsd11b2	PROMOTER	0.667	0.734	2981.30	2188.27	0.490	2247.92	1101.05
A_68_P25827307	chr8:46023864-46023908	AK079043:-3373		PROMOTER	0.667	0.257	7985.42	2056.21	0.172	5223.04	896.49
A_68_P25581340	chr7:147130450-147130494	NM_009482:718	Utf1	INSIDE	0.667	2.059	3186.51	6560.63	1.374	2167.73	2978.87
A_68_P25362933	chr7:106774200-106774244	NM_001195529:1391	Gm4980	INSIDE	0.667	2.604	2277.79	5931.88	1.738	1656.00	2878.54
A_68_P24950541	chr7:4744529-4744573	NM_009081:-16	Rpl28	DIVERGENT_PROMOTER	0.667	0.390	1633.65	637.14	0.260	1273.10	331.38
A_68_P24536899	chr6:71494180-71494224	NM_025783:355	Vps24	INSIDE	0.667	0.540	1006.36	543.47	0.360	861.56	310.56
A_68_P24142218	chr5:141083418-141083462	NM_008494:146	Lfng	INSIDE	0.667	5.720	2559.80	14641.61	3.818	2143.66	8183.81
A_68_P24019918	chr5:117768976-117769020	NM_001033311:-276	Vsig10	PROMOTER	0.667	0.469	1506.18	707.04	0.313	1076.95	337.43
A_68_P23359682	chr4:140258313-140258357	NM_173867:947	Rcc2	INSIDE	0.667	0.606	1433.12	869.18	0.405	1110.52	449.23
A_68_P23309923	chr4:131604805-131604849	NM_001128606:167	Epb4.1	INSIDE	0.667	10.278	1107.86	11386.51	6.856	962.25	6597.68
A_68_P22509314	chr3:127256299-127256343	NM_138593:-53	Larp7	DIVERGENT_PROMOTER	0.667	0.242	2134.09	516.81	0.162	1674.60	270.62
A_68_P20874690	chr1:182186271-182186316	NM_001128605:138	Psen2	INSIDE	0.667	0.450	3147.82	1416.77	0.300	2458.67	738.28
A_68_P20058161	chr1:17087384-17087428	NM_020604:564	Jph1	INSIDE	0.667	0.599	3791.58	2270.69	0.400	3127.97	1250.29
A_68_P31744348	chr18:58995914-58995958	NM_175506:-481	Adamts19	PROMOTER	0.666	2.408	2790.16	6718.95	1.603	2237.18	3587.22
A_68_P31347825	chr17:74059155-74059199	NM_027864:615	Galnt14	INSIDE	0.666	0.720	7551.82	5433.89	0.479	4699.23	2251.34
A_68_P31097441	chr17:24591842-24591886	NM_007893:392	E4f1	INSIDE	0.666	0.506	871.83	441.17	0.337	699.72	235.70
A_68_P30594370	chr16:21794748-21794792	NR_037957:351	1300002E11Rik	INSIDE	0.666	2.957	2528.25	7475.71	1.968	1968.59	3874.60
A_68_P30476331	chr15:98593814-98593858	NM_007478:-287	Arf3	PROMOTER	0.666	0.219	2306.22	505.29	0.146	1666.52	243.24
A_68_P28813934	chr13:8995156-8995200	NM_027000:80	Gtpbp4	INSIDE	0.666	0.304	1830.01	555.65	0.202	1409.53	285.13
A_68_P28430594	A_68_P28430594			Unknown	0.666	2.187	1833.78	4009.62	1.456	1602.34	2333.52
A_68_P28185441	chr11:120442461-120442505	NM_133796:452	Arhgdia	INSIDE	0.666	2.890	3038.39	8781.06	1.925	2472.44	4759.83
A_68_P27869724	chr11:64976171-64976215	NM_001099288:271	Arhgap44	INSIDE	0.666	3.236	1711.84	5539.12	2.156	1463.02	3154.03
A_68_P27833862	chr11:58791684-58791728	NM_053168:89	Trim11	INSIDE	0.666	0.667	1598.81	1066.08	0.444	1188.21	527.71
A_68_P27479719	chr10:116584876-116584920	NM_177798:632	Frs2	INSIDE	0.666	0.711	1986.56	1411.91	0.473	1559.59	737.76
A_68_P26628308	chr9:72380438-72380482	NM_001033536:414	Rfx7	INSIDE	0.666	0.662	880.09	582.69	0.441	706.63	311.64
A_68_P26587800	chr9:65308975-65309019	NM_138584:253	Spg21	INSIDE	0.666	6.458	6488.78	41903.93	4.301	4694.43	20192.73
A_68_P26530601	chr9:55174289-55174334	NM_172923:-444	AI118078	PROMOTER	0.666	7.559	2176.87	16454.92	5.038	1643.95	8281.97
A_68_P25092032	chr7:52496418-52496462	NM_007645:-2231	Cd37	PROMOTER	0.666	0.559	3359.05	1877.54	0.372	2468.76	918.51
A_68_P24804559	chr6:121423187-121423231	NM_001033354:488	Iqsec3	INSIDE	0.666	2.929	686.18	2009.53	1.952	628.57	1226.79
A_68_P24798783	chr6:120443507-120443551	NM_033567:297	Cecr6	INSIDE	0.666	0.436	2314.77	1008.29	0.290	1795.16	521.01
A_68_P23861886	chr5:86494686-86494730	NM_007683:-100	Cenpc1	DIVERGENT_PROMOTER	0.666	0.643	7619.39	4899.97	0.428	5698.32	2441.10
A_68_P23441977	chr4:155409010-155409054	NM_029264:15894	Ttll10	INSIDE	0.666	0.408	1128.86	460.92	0.272	932.70	253.65
A_68_P22611968	chr3:145651227-145651271	NM_001166064:415	Syde2	INSIDE	0.666	4.458	853.73	3806.05	2.971	782.59	2324.98
A_68_P22488493	chr3:123211082-123211126	NR_028573:-120	Snora24	PROMOTER	0.666	0.582	1115.16	649.16	0.388	905.49	350.90
A_68_P21673348	chr2:138082206-138082250	NM_001025431:-91	Btbd3	PROMOTER	0.666	4.094	1744.24	7141.08	2.729	1611.60	4397.56
A_68_P21375629	chr2:80421545-80421589	NM_016965:-444	Nckap1	PROMOTER	0.666	3.131	1623.05	5082.09	2.084	1354.39	2822.61
A_68_P32576283	chrX:101395692-101395736	NM_001077354:742	C77370	INSIDE	0.665	11.288	4621.00	52161.64	7.502	4579.56	34356.70
A_68_P31961135	chr19:12575854-12575898	NM_172442:610	Dtx4	INSIDE	0.665	2.427	1302.43	3161.39	1.613	1105.36	1783.00
A_68_P31130455	chr17:30024049-30024093	NM_001081160:757	Mdga1	INSIDE	0.665	3.834	1726.56	6620.00	2.549	1531.21	3903.07
A_68_P30981932	chr16:94747888-94747932	NM_007834:326	Dscr3	INSIDE	0.665	0.506	3973.27	2008.56	0.336	2831.37	952.28
A_68_P30232957	chr15:55389253-55389297	NM_001168250:312	Mtbp	INSIDE	0.665	0.548	2715.69	1487.23	0.364	1897.42	690.55
A_68_P29612657	chr14:55098262-55098306	NM_080726:3348	Rem2	INSIDE	0.665	0.659	784.60	517.41	0.439	717.69	314.74
A_68_P28156775	chr11:115866293-115866337	NM_001005608:30276	Itgb4	INSIDE	0.665	5.231	1294.88	6773.39	3.479	1080.66	3759.61
A_68_P27915637	chr11:72990334-72990378	NM_001168470:154	Tmem93	INSIDE	0.665	2.880	1176.03	3386.91	1.915	983.77	1884.05
A_68_P27862296	chr11:63735472-63735516	NM_018805:292	Hs3st3b1	INSIDE	0.665	0.241	2218.35	534.22	0.160	1689.01	270.32
A_68_P27539432	chr10:127520351-127520395	NR_030442:-1969	Mir677	PROMOTER	0.665	0.680	1073.80	729.65	0.452	953.85	430.86
A_68_P27500776	chr10:120416131-120416176	NM_001081193:233	Lemd3	INSIDE	0.665	6.361	279.40	1777.24	4.229	266.84	1128.36
A_68_P27282838	chr10:79845116-79845160	NM_198615:5258	Mex3d	INSIDE	0.665	2.585	1483.80	3836.13	1.720	1265.13	2175.79
A_68_P26081251	chr8:97605349-97605393	NM_028805:270	Katnb1	INSIDE	0.665	0.564	4282.02	2413.21	0.375	3026.25	1133.68
A_68_P24832848	chr6:127403924-127403968	NM_181402:206	Parp11	INSIDE	0.665	2.149	1688.07	3627.60	1.430	1441.79	2061.21
A_68_P24609727	chr6:86315530-86315574	NM_025591:-124	Fam136a	PROMOTER	0.665	0.473	6645.97	3145.06	0.315	5018.17	1578.79
A_68_P24176695	chr5:148768968-148769012	NM_029920:95	Mtus2	INSIDE	0.665	0.566	1745.54	988.58	0.376	1468.09	552.62
A_68_P24154206	chr5:143722119-143722163	NM_007984:107	Fscn1	INSIDE	0.665	3.359	1763.26	5923.65	2.233	1345.15	3004.16
A_68_P23440963	chr4:155222080-155222124	NM_010091:582	Dvl1	INSIDE	0.665	2.711	758.14	2055.41	1.803	678.26	1223.21
A_68_P21723786	chr2:147191194-147191239	NM_008780:487	Pax1	INSIDE	0.665	20.511	670.62	13755.08	13.634	655.27	8933.76
A_68_P21105337	chr2:30663394-30663438	NM_170592:-80	Mettl11a	DIVERGENT_PROMOTER	0.665	0.601	7065.78	4245.08	0.400	4800.18	1919.04
A_68_P32385145	chrX:50341393-50341437	NM_013556:160	Hprt	INSIDE	0.664	7.736	907.96	7023.88	5.140	1142.58	5873.30
A_68_P32144298	chr19:46575400-46575444	NM_053100:-715	Trim8	PROMOTER	0.664	5.483	1545.33	8472.53	3.638	1234.78	4491.97
A_68_P31161191	chr17:35974038-35974082	NM_011655:1186	Tubb5	INSIDE	0.664	3.365	892.95	3004.75	2.235	786.75	1758.55
A_68_P31095792	chr17:24305949-24305993	NR_037854:370	Atp6v0c-ps2	INSIDE	0.664	0.594	10291.75	6118.01	0.395	6619.88	2612.91
A_68_P31075177	chr17:17987055-17987099	NM_008215:5076	Has1	INSIDE	0.664	2.098	2850.27	5978.69	1.394	2369.22	3301.97
A_68_P30250724	chr15:58721209-58721253	NM_175226:447	Rnf139	INSIDE	0.664	3.265	3107.94	10148.70	2.167	2402.36	5205.76
A_68_P30250398	chr15:58654714-58654758	NM_175212:246	Tmem65	INSIDE	0.664	2.611	674.70	1761.92	1.733	634.00	1098.67
A_68_P30021002	chr15:12251634-12251678	NM_025673:406	Golph3	INSIDE	0.664	0.549	1634.20	896.50	0.364	1226.02	446.60

ProbeName	Target position of probe on CpG island microarray	TargetID	GeneSymbol	CpG island Description	Ratio of relative methylation (TiO_2-NP/Vehicle)	Sham group			TiO_2-H group		
						Relative methylation (Cy5/Cy3)	Cy3 signal (Input)	Cy5 signal (MeDIP)	Relative methylation (Cy5/Cy3)	Cy3 signal (Input)	Cy5 signal (MeDIP)
A_68_P29964714	chr14:123382020-123382064	NM_028651:441	Tmtc4	INSIDE	0.664	5.492	363.50	1996.21	3.647	278.18	1014.45
A_68_P28914790	chr13:30077733-30077777	NM_010093:178	E2f3	INSIDE	0.664	0.726	2990.03	2170.27	0.482	2327.55	1122.10
A_68_P28615271	chr12:88641179-88641223	NM_011479:87980	Sptlc2	DOWNSTREAM	0.664	0.168	3968.25	665.40	0.111	2707.98	301.72
A_68_P28519206	chr12:71328237-71328281	NM_133198:412	Pygl	INSIDE	0.664	0.383	1611.91	617.39	0.254	1245.88	316.95
A_68_P28185535	chr11:120459852-120459896	NM_001038230:29	Anapc11	INSIDE	0.664	3.541	833.40	2951.25	2.352	692.77	1629.24
A_68_P28035916	chr11:94852207-94852251	NM_172261:-297	Ppp1r9b	PROMOTER	0.664	0.697	2842.56	1980.93	0.463	2087.21	966.41
A_68_P27924625	chr11:74986839-74986883	NR_029794:-29	Mir212	DIVERGENT_PROMOTER	0.664	0.458	2659.01	1216.68	0.304	2133.01	648.50
A_68_P27537442	chr10:127114141-127114185	NM_173732:42	Tmem194	INSIDE	0.664	0.212	5612.37	1189.25	0.141	3773.02	530.48
A_68_P27289568	chr10:80882945-80882989	NM_008688:6952	Nfic	INSIDE	0.664	3.658	1823.73	6671.19	2.430	1568.48	3810.97
A_68_P26883804	chr9:121403538-121403582	NM_031161:1247	Cck	INSIDE	0.664	5.614	909.60	5106.55	3.726	820.06	3055.25
A_68_P26313777	chr9:13631232-13631276	NR_037622:243	Cep57	INSIDE	0.664	0.278	3866.52	1073.51	0.184	2811.76	518.03
A_68_P26289571	chr9:7837078-7837122	NM_007465:-1845	Birc2	PROMOTER	0.664	0.507	944.11	478.50	0.337	766.08	257.79
A_68_P26248668	chr8:127473510-127473554	NM_053207:-378	Egln1	PROMOTER	0.664	4.371	661.86	2893.01	2.900	537.13	1557.90
A_68_P25051309	chr7:36588072-36588116	NM_177739:-86	Zfp507	PROMOTER	0.664	4.466	538.18	2403.49	2.963	438.71	1300.11
A_68_P25050417	chr7:36432045-36432089	NM_019746:435	Pdcd5	INSIDE	0.664	0.425	1179.48	501.46	0.282	981.05	276.98
A_68_P24984543	chr7:17612915-17612959	NM_011155:327	Ppp5c	INSIDE	0.664	3.691	1055.36	3895.75	2.452	934.96	2292.96
A_68_P24828018	chr6:126594962-126595006	NM_010595:835	Kcna1	INSIDE	0.664	0.253	2561.43	647.69	0.168	1806.70	303.50
A_68_P23974103	chr5:109204294-109204338	NM_026109:20066	Tmed11	DOWNSTREAM	0.664	5.458	353.45	1929.18	3.627	308.01	1117.04
A_68_P23574101	chr5:31013677-31013721	NM_023047:-569	Dpysl5	PROMOTER	0.664	4.037	572.68	2311.69	2.680	463.56	1242.56
A_68_P23315717	chr4:132641057-132641101	NM_146155:73658	Ahdc1	DOWNSTREAM	0.664	0.640	1064.65	681.44	0.425	853.62	362.86
A_68_P23226010	chr4:115500724-115500768	NM_175308:50	Mobkl2c	INSIDE	0.664	0.551	1112.32	613.23	0.366	917.38	335.84
A_68_P23120705	chr4:94717778-94717822	NM_010591:1113	Jun	INSIDE	0.664	3.621	1111.05	4023.42	2.404	999.06	2401.58
A_68_P23005994	chr4:70071277-70071321	NM_145990:103	Cdk5rap2	INSIDE	0.664	0.263	2240.90	589.43	0.175	1661.80	290.41
A_68_P21901458	chr2:179839037-179839081	NM_133849:-131	Hrh3	PROMOTER	0.664	0.593	7196.23	4270.60	0.394	5222.48	2059.23
A_68_P20852908	chr1:178205507-178205551	NM_176916:-85	Pld5	PROMOTER	0.664	0.437	1870.98	817.69	0.290	1543.03	447.49
A_68_P32135378	chr19:45005914-45005958	NM_001081225:328	Fam178a	INSIDE	0.663	0.576	1457.25	838.65	0.382	1105.90	422.21
A_68_P32134209	chr19:44830746-44830790	NM_011037:-1115	Pax2	PROMOTER	0.663	2.719	5207.22	14158.57	1.802	4079.18	7350.84
A_68_P31758330	chr18:61715307-61715351	NM_146087:93	Csnk1a1	INSIDE	0.663	0.528	918.07	484.56	0.350	769.28	269.14
A_68_P30478144	chr15:98917975-98918019	NM_001024702:163	C1ql4	INSIDE	0.663	14.271	711.81	10157.85	9.465	672.16	6362.29
A_68_P30224202	chr15:53734012-53734057	NM_177225:-43	Samd12	PROMOTER	0.663	0.413	1206.15	498.54	0.274	976.51	267.51
A_68_P29284641	chr13:104125004-104125048	NM_175171:-454	Mast4	PROMOTER	0.663	0.481	1658.63	797.94	0.319	1270.46	405.19
A_68_P29009048	chr13:46764994-46765038	NM_001033192:126	C78339	INSIDE	0.663	3.848	1882.86	7245.26	2.551	1612.87	4114.42
A_68_P28836964	chr13:14157071-14157115	NM_194262:1034	Arid4b	INSIDE	0.663	3.812	925.47	3527.46	2.527	791.59	2000.25
A_68_P28736915	chr12:111426863-111426907	AK044800:1099		INSIDE	0.663	4.284	848.10	3632.97	2.839	753.88	2140.25
A_68_P28577469	chr12:81858735-81858779	NM_001008423:2946	Gm1568	INSIDE	0.663	5.062	1589.35	8045.37	3.355	1239.57	4159.16
A_68_P28314928	chr12:29435138-29435182	NM_001161410:99	Ttc15	INSIDE	0.663	0.626	1705.92	1068.68	0.415	1404.47	582.95
A_68_P28154071	chr11:115396739-115396783	NM_133354:784	Sumo2	INSIDE	0.663	0.454	2167.75	984.35	0.301	1771.19	532.94
A_68_P28050112	chr11:97223519-97223563	NM_138657:-324	Socs7	PROMOTER	0.663	3.264	1124.23	3669.05	2.163	967.33	2091.97
A_68_P27804084	chr11:53114187-53114231	NM_008300:-227	Hspa4	PROMOTER	0.663	0.365	1310.43	478.09	0.242	1130.30	273.34
A_68_P27284768	chr10:80139566-80139610	NM_021462:-4867	Mknk2	PROMOTER	0.663	2.198	1743.27	3831.17	1.457	1275.95	1858.43
A_68_P26038371	chr8:90485107-90485151	NM_033327:-1634	Zfp423	PROMOTER	0.663	0.387	1552.82	600.70	0.256	1189.31	304.89
A_68_P25313627	chr7:97591983-97592027	NM_145151:714	Crebzf	INSIDE	0.663	3.686	1311.41	4833.26	2.445	1076.60	2632.20
A_68_P25088776	chr7:51926206-51926250	NM_028021:-5355	Myh14	PROMOTER	0.663	12.010	1394.28	16744.59	7.957	1172.68	9331.54
A_68_P24949868	chr7:4610619-4610663	NM_172894:-88	Ppp6r1	PROMOTER	0.663	0.643	1670.29	1074.66	0.427	1322.38	564.43
A_68_P24942897	chr6:148894399-148894443	NM_019643:534	Fam60a	INSIDE	0.663	5.557	1884.51	10472.32	3.685	2098.64	7734.40
A_68_P24819103	chr6:125115217-125115261	NM_008084:363	Gapdh	INSIDE	0.663	0.253	2902.06	733.42	0.168	2086.89	349.89
A_68_P24383895	chr6:39542839-39542883	NM_026612:279	Ndufb2	INSIDE	0.663	0.403	1163.17	468.46	0.267	789.95	210.89
A_68_P23560503	chr5:28397698-28397742	NM_153526:-231	Insig1	PROMOTER	0.663	3.833	888.29	3404.66	2.542	720.32	1831.25
A_68_P22405721	chr3:107137047-107137091	NM_001045807:-861	Rbm15	PROMOTER	0.663	3.033	838.24	2542.70	2.012	760.82	1530.88
A_68_P22342938	chr3:95032702-95032746	NM_001038708:-149	Cdc42se1	PROMOTER	0.663	2.848	1100.87	3135.17	1.888	937.60	1770.38
A_68_P21151183	chr2:38781338-38781382	NM_010264:621	Nr6a1	INSIDE	0.663	0.528	2382.09	1256.91	0.350	1833.88	641.37
A_68_P21107218	chr2:30997212-30997256	NM_001038700:294	Fnbp1	INSIDE	0.663	3.485	1159.37	4040.31	2.312	996.38	2303.18
A_68_P21107216	chr2:30997021-30997065	NM_001038700:486	Fnbp1	INSIDE	0.663	2.122	2031.14	4310.82	1.407	1620.01	2278.54
A_68_P21073104	chr2:25434737-25434781	NM_001005424:861	Gm996	INSIDE	0.663	0.568	3422.45	1944.41	0.377	2558.14	964.01
A_68_P20825163	chr1:172815285-172815329	NM_023173:365	Dusp12	INSIDE	0.663	0.560	1448.89	811.35	0.371	1216.87	452.00
A_68_P20644037	chr1:138028308-138028352	NM_001039472:353	Kif21b	INSIDE	0.663	0.350	2218.73	775.75	0.232	1671.00	387.36
A_68_P20457526	chr1:95700053-95700097	NM_025454:-467	Ing5	DIVERGENT_PROMOTER	0.663	4.810	1220.12	5868.39	3.189	1009.03	3217.41
A_68_P31925604	chr19:4878831-4878875	NM_001168517:163	Zdhhc24	INSIDE	0.662	2.627	2902.92	7625.30	1.738	2404.89	4179.64
A_68_P31841968	chr18:76400994-76401038	NM_010754:-562	Smad2	PROMOTER	0.662	3.759	790.84	2972.69	2.488	782.28	1946.28
A_68_P31147863	chr17:33103125-33103169	NM_153063:371	Zfp472	INSIDE	0.662	0.324	1577.42	511.24	0.215	1272.08	273.09
A_68_P30670423	chr16:35490181-35490225	NM_028295:757	Pdia5	INSIDE	0.662	0.162	4058.80	656.69	0.107	2935.56	314.63
A_68_P29619515	chr14:56341110-56341154	NM_019519:-119	Rabggta	PROMOTER	0.662	2.278	1268.43	2889.57	1.509	1039.85	1568.81
A_68_P28517706	chr12:71087715-71087759	NM_022028:253	Sav1	INSIDE	0.662	0.358	1483.04	530.78	0.237	1068.38	253.21
A_68_P28382007	chr12:41750810-41750854	NM_053122:156	Immp2l	INSIDE	0.662	0.446	2327.54	1038.51	0.296	1836.58	542.83
A_68_P26374351	chr9:26962634-26962678	NM_023277:350	Jam3	INSIDE	0.662	4.887	725.17	3544.05	3.238	601.20	1946.46

ProbeName	Target position of probe on CpG island microarray	TargetID	GeneSymbol	CpG island Description	Ratio of relative methylation (TiO_2-NP/Vehicle)	Sham group			TiO_2-H group		
						Relative methylation (Cy5/Cy3)	Cy3 signal (Input)	Cy5 signal (MeDIP)	Relative methylation (Cy5/Cy3)	Cy3 signal (Input)	Cy5 signal (MeDIP)
A_68_P26247724	chr8:127316847-127316891	NM_198632:-50	Trim67	PROMOTER	0.662	0.274	3558.78	973.90	0.181	2494.90	451.88
A_68_P26165834	chr8:113494088-113494132	NM_009179:50346	St3gal2	INSIDE	0.662	0.395	1300.08	514.05	0.262	1034.52	270.70
A_68_P25092990	chr7:52671388-52671432	NM_198190:2346	Ntf5	INSIDE	0.662	2.842	940.30	2672.18	1.882	753.32	1417.53
A_68_P24995084	chr7:20335086-20335130	NM_008990:-186	Pvrl2	PROMOTER	0.662	0.598	2208.44	1320.34	0.396	1521.52	602.11
A_68_P24639207	chr6:91423387-91423431	NM_133928:9	Chchd4	INSIDE	0.662	2.413	2010.49	4851.60	1.597	1619.53	2586.39
A_68_P24613524	chr6:86992684-86992728	NM_013528:-133	Gfpt1	PROMOTER	0.662	4.107	1151.38	4728.38	2.717	1014.21	2755.70
A_68_P24469549	chr6:55401776-55401820	NM_001025372:-175	Adcyap1r1	PROMOTER	0.662	0.325	2915.63	947.62	0.215	2084.09	448.69
A_68_P23991123	chr5:112705779-112705823	NM_009419:74	Tpst2	INSIDE	0.662	4.030	936.59	3774.58	2.666	801.63	2137.48
A_68_P23322026	chr4:133842693-133842737	NM_001163794:454	Pdik1l	INSIDE	0.662	3.106	1060.70	3294.43	2.056	913.87	1878.85
A_68_P23154908	chr4:101093069-101093113	NM_009647:783	Ak4	INSIDE	0.662	3.225	429.59	1385.55	2.136	363.36	776.07
A_68_P22349233	chr3:96218809-96218853	NR_001579:-74	Terc	PROMOTER	0.662	0.192	2942.58	564.18	0.127	2313.97	293.88
A_68_P21760473	chr2:154262598-154262642	NM_009823:401	Cbfa2t2	INSIDE	0.662	0.438	1125.97	492.80	0.290	1033.92	299.75
A_68_P21612035	chr2:127034187-127034231	NM_177214:69	Snrnp200	INSIDE	0.662	0.718	1313.49	943.06	0.475	1072.77	509.89
A_68_P20252732	chr1:57434304-57434348	NR_037999:62	4930558J18Rik	INSIDE	0.662	0.509	1434.72	730.56	0.337	1203.44	405.82
A_68_P20166303	chr1:39593180-39593224	NM_028043:556	D1Bwg0212e	INSIDE	0.662	3.322	693.48	2304.07	2.200	635.04	1397.08
A_68_P20157022	chr1:38055055-38055099	NM_198303:222	Eif5b	INSIDE	0.662	0.404	1567.51	632.84	0.267	1211.38	323.85
A_68_P32151687	chr19:47805901-47805945	NM_026377:-323	6330577E15Rik	PROMOTER	0.661	0.569	2178.86	1240.65	0.376	1857.58	698.66
A_68_P32143438	chr19:46431466-46431510	NM_029186:119	Tmem180	INSIDE	0.661	2.639	973.95	2570.38	1.745	734.62	1282.27
A_68_P32071925	chr19:32832040-32832084	NM_008960:-4	Pten	PROMOTER	0.661	0.521	2336.66	1217.86	0.344	1674.36	576.78
A_68_P32021817	chr19:24205542-24205588	NM_001198985:43066	Tjp2	INSIDE	0.661	3.726	1186.01	4419.04	2.462	677.04	1667.03
A_68_P31630110	chr18:37966049-37966093	NM_033582:-8662	Pcdhgc4	PROMOTER	0.661	0.488	2038.82	994.99	0.323	1607.29	518.55
A_68_P30018903	chr15:11836061-11836105	NM_001039181:-653	Npr3	PROMOTER	0.661	0.473	1950.79	922.98	0.313	1573.85	491.98
A_68_P29455871	chr14:22804612-22804656	NM_145459:4189	Zfp503	INSIDE	0.661	5.874	3121.55	18335.59	3.884	2448.48	9510.18
A_68_P28729767	chr12:109996277-109996321			Unknown	0.661	3.462	2972.15	10289.40	2.290	2118.86	4851.44
A_68_P28605005	chr12:86939999-86940043	NM_001205052:-34	Jdp2	PROMOTER	0.661	2.920	1330.16	3883.50	1.929	1103.85	2129.85
A_68_P28307255	chr12:28026369-28026413	NM_009234:1193	Sox11	INSIDE	0.661	3.332	1641.80	5470.89	2.204	1388.04	3059.62
A_68_P28195719	chr12:3572230-3572274	NM_001162465:-138	Dtnb	PROMOTER	0.661	3.739	1110.87	4153.84	2.473	948.99	2346.87
A_68_P26543337	chr9:57556573-57556620	NM_153799:221	Edc3	INSIDE	0.661	0.643	2021.04	1299.56	0.425	1478.24	628.77
A_68_P26477010	chr9:45763375-45763419	NM_172257:-64	Sidt2	PROMOTER	0.661	0.621	1049.63	651.58	0.411	795.66	326.64
A_68_P26167383	chr8:113783057-113783101	NM_009149:24	Glg1	INSIDE	0.661	13.766	2316.15	31884.71	9.096	2359.23	21458.40
A_68_P25265836	chr7:87439680-87439724	NM_145150:352	Prc1	INSIDE	0.661	0.644	1363.99	878.62	0.426	1112.17	473.71
A_68_P23657073	chr5:45911523-45911567	NM_025895:77	Med28	INSIDE	0.661	4.434	887.68	3935.81	2.931	798.95	2341.80
A_68_P23441760	chr4:155367175-155367219	NM_011341:174	Sdf4	INSIDE	0.661	0.155	3357.46	519.15	0.102	2443.54	249.87
A_68_P22869480	chr4:43023362-43023406	NM_001163233:-211	Fancg	PROMOTER	0.661	0.253	2407.11	609.76	0.167	1746.34	292.23
A_68_P22825645	chr4:33276560-33276604	NM_001034867:130	Pm20d2	INSIDE	0.661	2.684	600.64	1612.20	1.773	590.12	1046.37
A_68_P21138537	chr2:36066471-36066515	ENSMUST00000128242:201		INSIDE	0.661	2.790	718.12	2003.64	1.844	750.86	1384.53
A_68_P21084617	chr2:27281328-27281372	NM_009500:995	Vav2	INSIDE	0.661	0.630	3003.28	1893.12	0.417	2295.80	957.05
A_68_P20997431	chr2:9796515-9796559	NM_008091:3691	Gata3	INSIDE	0.661	4.341	5467.48	23734.93	2.869	4250.59	12193.23
A_68_P20946924	chr1:194862258-194862302	NM_181546:-388	Syt14	PROMOTER	0.661	0.504	5259.24	2650.57	0.333	3658.86	1219.75
A_68_P20826006	chr1:172995780-172995824	NM_010188:-6268	Fcgr3	PROMOTER	0.661	0.465	14721.57	6849.05	0.308	11937.52	3671.06
A_68_P20349427	chr1:74634546-74634590	NM_029888:34	Zfp142	INSIDE	0.661	2.463	2313.89	5699.77	1.628	1818.34	2959.53
A_68_P32203265	chr19:56896867-56896911	NM_001002238:-3810	Tdrd1	DIVERGENT_PROMOTER	0.660	0.222	2265.84	503.88	0.147	1771.15	260.15
A_68_P32186055	chr19:54120591-54120635	NM_007417:941	Adra2a	INSIDE	0.660	6.025	774.71	4667.32	3.979	627.54	2497.07
A_68_P32144304	chr19:46576472-46576516	NM_053100:357	Trim8	INSIDE	0.660	3.632	886.66	3220.62	2.398	733.32	1758.37
A_68_P32056100	chr19:30105268-30105312	NM_144873:288	Uhrf2	INSIDE	0.660	2.476	1791.43	4435.76	1.634	1433.42	2342.74
A_68_P31849787	chr18:78033303-78033347	NM_013831:36	Pstpip2	INSIDE	0.660	0.170	3874.13	658.57	0.112	2827.84	317.10
A_68_P31759276	chr18:61867012-61867056	NM_172832:255	Pcyox1l	INSIDE	0.660	0.708	3106.76	2199.06	0.467	2249.29	1051.40
A_68_P31420721	chr17:87424605-87424649	NM_008838:115	Pigf	INSIDE	0.660	3.124	1113.55	3478.42	2.060	893.77	1841.43
A_68_P30630415	chr16:28446508-28446552	NM_183064:-1217	Fgf12	PROMOTER	0.660	2.535	2411.53	6113.21	1.674	2134.53	3572.38
A_68_P29603743	chr14:52532832-52532876	NM_001145959:309	Ndrg2	INSIDE	0.660	0.278	3459.08	960.99	0.183	2385.53	437.18
A_68_P29057339	chr13:55937443-55937487	NM_011097:-4678	Pitx1	PROMOTER	0.660	0.388	1354.13	525.04	0.256	1033.48	264.53
A_68_P28143856	chr11:113613201-113613245	NM_001163346:-93	Cdc42ep4	PROMOTER	0.660	0.491	1406.11	689.81	0.324	1047.42	339.26
A_68_P28043848	chr11:96194036-96194080	NM_001079869:9619	Hoxb3	INSIDE	0.660	0.335	2594.32	870.23	0.221	1726.37	382.37
A_68_P27945543	chr11:78640074-78640118	NM_001076681:0	1810012P15Rik	INSIDE	0.660	0.388	1610.19	624.70	0.256	1487.44	380.79
A_68_P27468588	chr10:114688762-114688806	NM_025706:-235	Tbc1d15	PROMOTER	0.660	0.463	1296.89	600.39	0.305	937.61	286.37
A_68_P26665705	chr9:79566838-79566882	NM_007730:-375	Col12a1	PROMOTER	0.660	0.356	3419.79	1215.85	0.235	2331.35	546.71
A_68_P26224704	chr8:123652972-123653016	NM_008024:1410	Foxl1	INSIDE	0.660	0.401	1419.70	569.26	0.265	1115.88	295.21
A_68_P25586434	chr7:148246645-148246689	NM_177897:-506	B4galnt4	PROMOTER	0.660	0.423	4576.17	1935.85	0.279	3017.65	842.56
A_68_P25092756	chr7:52632188-52632232	NM_009224:18807	Snrnp70	INSIDE	0.660	4.607	1895.08	8731.55	3.043	1598.42	4864.31
A_68_P24776842	chr6:116410742-116410786	NM_009662:432	Alox5	INSIDE	0.660	0.585	1117.06	652.95	0.386	947.70	365.35
A_68_P24009173	chr5:115798570-115798614	NM_007748:372	Cox6a1	INSIDE	0.660	0.445	2490.70	1109.55	0.294	1901.17	558.97
A_68_P23815279	chr5:76733734-76733778	NM_007715:-183	Clock	PROMOTER	0.660	4.034	1714.98	6917.85	2.661	1345.48	3579.90
A_68_P23763606	chr5:67137204-67137248	NM_001001980:148	Limch1	INSIDE	0.660	0.608	758.18	461.00	0.402	717.05	287.90
A_68_P23317106	chr4:132895104-132895148	NM_199306:104	Wdtc1	INSIDE	0.660	6.305	11701.67	73775.12	4.158	8398.29	34920.00
A_68_P23279474	chr4:125998794-125998839	NM_133883:131	Adprhl2	INSIDE	0.660	2.803	2017.79	5656.75	1.849	1709.35	3161.38

ProbeName	Target position of probe on CpG island microarray	TargetID	GeneSymbol	CpG island Description	Ratio of relative methylation (TiO_2-NP/Vehicle)	Sham group			TiO_2-H group		
						Relative methylation (Cy5/Cy3)	Cy3 signal (Input)	Cy5 signal (MeDIP)	Relative methylation (Cy5/Cy3)	Cy3 signal (Input)	Cy5 signal (MeDIP)
A_68_P22934423	chr4:55544008-55544052	NM_010637:1317	Klf4	INSIDE	0.660	0.612	3148.06	1927.76	0.404	2298.95	929.46
A_68_P22324052	chr3:90035906-90035950	NM_206924:410	Jtb	INSIDE	0.660	2.304	1445.62	3330.58	1.522	1113.35	1693.99
A_68_P22220267	chr3:69120847-69120891	NM_178726:29	Ppm1l	INSIDE	0.660	0.358	2916.72	1043.10	0.236	2394.70	565.15
A_68_P21809984	chr2:162843268-162843312	NM_172150:83	Ift52	INSIDE	0.660	3.471	954.84	3314.14	2.291	845.26	1936.40
A_68_P21721815	chr2:146838797-146838841	NM_011917:23	Xrn2	INSIDE	0.660	2.462	2032.46	5003.02	1.626	1723.13	2801.38
A_68_P31935410	chr19:6531540-6531584	NM_001205234:112825	Nrxn2	INSIDE	0.659	0.577	1907.46	1101.02	0.381	1340.08	510.08
A_68_P31929912	chr19:5654628-5654672	NM_001164480:9057	Sipa1	INSIDE	0.659	0.593	1901.34	1128.13	0.391	1234.93	483.00
A_68_P31196991	chr17:44872774-44872818	NM_001145920:801	Runx2	INSIDE	0.659	0.727	2709.00	1968.73	0.479	2095.26	1003.58
A_68_P31139420	chr17:31523449-31523493	NM_001163748:292	Pde9a	INSIDE	0.659	0.613	1884.57	1155.80	0.404	1565.75	632.34
A_68_P30431386	chr15:90509992-90510036	NM_025815:-195	Cpne8	PROMOTER	0.659	2.418	1846.49	4465.24	1.595	1338.29	2134.04
A_68_P30221122	chr15:53177633-53177677	NM_010162:84	Ext1	INSIDE	0.659	7.075	2034.17	14390.97	4.665	1885.25	8794.00
A_68_P29516971	chr14:33135063-33135107	NM_001081130:-120	Ogdhl	PROMOTER	0.659	0.624	1007.18	628.21	0.411	913.51	375.43
A_68_P29143149	chr13:74074834-74074878	NM_027182:359	Trip13	INSIDE	0.659	0.273	4240.45	1158.11	0.180	2977.08	535.97
A_68_P29060176	chr13:56360497-56360541	NM_010896:-6994	Neurog1	PROMOTER	0.659	0.224	2315.77	518.76	0.148	1728.74	255.30
A_68_P28751775	chr12:113916856-113916901	NM_001100460:-172	Zbtb42	DIVERGENT_PROMOTER	0.659	2.423	1124.74	2724.88	1.596	911.01	1453.67
A_68_P28182256	chr11:119948049-119948093	NM_009734:71	Azi1	INSIDE	0.659	2.833	547.64	1551.37	1.868	480.92	898.17
A_68_P28155484	chr11:115675096-115675140	NM_080643:-212	Caskin2	DIVERGENT_PROMOTER	0.659	0.184	2911.22	535.03	0.121	2035.21	246.31
A_68_P27899783	chr11:70068622-70068666	NM_007440:199	Alox12	INSIDE	0.659	2.249	2840.87	6388.97	1.482	1983.29	2939.08
A_68_P27468929	chr10:114752147-114752191	NM_024454:479	Rab21	INSIDE	0.659	2.641	819.51	2164.61	1.739	645.14	1122.22
A_68_P27306491	chr10:84565795-84565839	NM_001013028:445	AI597468	INSIDE	0.659	0.481	3449.59	1659.67	0.317	2614.76	829.48
A_68_P27271929	chr10:77531813-77531858	NM_015790:-277	Icosl	PROMOTER	0.659	2.891	1246.98	3605.45	1.905	1083.22	2063.09
A_68_P25957750	chr8:74126449-74126493	NM_001166213:4926	Fam129c	INSIDE	0.659	0.635	1208.97	767.13	0.418	971.80	406.32
A_68_P25261546	chr7:86660511-86660555	ENSMUST00000150985:415		INSIDE	0.659	2.637	1628.69	4294.34	1.738	1337.09	2323.82
A_68_P25230182	chr7:80763065-80763109	AK158912:183		INSIDE	0.659	2.384	1437.83	3427.45	1.571	1290.64	2027.36
A_68_P25195709	chr7:74904507-74904551	NM_183312:100	Synm	INSIDE	0.659	0.223	3588.25	798.57	0.147	2521.00	369.91
A_68_P23927390	chr5:100468503-100468547	NM_016690:-283	Hnrpdl	DIVERGENT_PROMOTER	0.659	4.712	879.10	4142.33	3.106	757.38	2352.36
A_68_P23362371	chr4:140695718-140695762	NM_001025608:-29	D4Ertd22e	DIVERGENT_PROMOTER	0.659	3.059	1078.44	3299.01	2.016	949.33	1914.00
A_68_P23296941	chr4:129177909-129177953	NM_001085491:9497	1700125D06Rik	DOWNSTREAM	0.659	3.388	1098.70	3722.07	2.231	964.03	2150.60
A_68_P22695196	chr4:3866420-3866464	NM_001190322:382	Chchd7	INSIDE	0.659	4.869	1288.27	6272.45	3.208	989.39	3173.73
A_68_P22220265	chr3:69120566-69120610	NM_178726:-251	Ppm1l	PROMOTER	0.659	0.639	2269.43	1449.66	0.421	1675.42	704.89
A_68_P21586514	chr2:122293633-122293677	NR_027919:-121	Bambi-ps1	PROMOTER	0.659	0.383	1581.38	605.90	0.253	1257.45	317.65
A_68_P32218816	chr19:59419677-59419721	NM_001033222:572	Pdzd8	INSIDE	0.658	0.479	1183.09	566.66	0.315	975.31	307.55
A_68_P31162036	chr17:36116621-36116665	NM_145487:-230	Prr3	PROMOTER	0.658	0.506	995.46	504.02	0.333	765.90	255.13
A_68_P31092441	chr17:23713283-23713327	NM_011747:23150	Zfp13	INSIDE	0.658	3.039	1709.23	5194.00	2.001	1216.93	2434.64
A_68_P30511525	chr16:4679878-4679922	NM_030205:-180	Coro7	DIVERGENT_PROMOTER	0.658	0.524	1314.65	689.12	0.345	1077.06	371.23
A_68_P29936846	chr14:118536719-118536763	NM_021434:392	Gpr180	INSIDE	0.658	0.310	5106.93	1584.75	0.204	3041.77	620.89
A_68_P29270037	chr13:101545636-101545680	NM_021886:196	Cenph	INSIDE	0.658	0.349	2288.58	798.31	0.229	1706.39	391.51
A_68_P28493839	chr12:66176820-66176864	NM_178912:250	Fancm	INSIDE	0.658	2.915	4426.51	12903.88	1.918	3598.23	6902.67
A_68_P28103517	chr11:106649497-106649541	NR_037224:-5445	Mir3064	PROMOTER	0.658	0.618	1386.18	856.41	0.407	1113.61	452.82
A_68_P28059762	chr11:98843848-98843892	NM_178596:460	Gjd3	INSIDE	0.658	5.536	596.58	3302.56	3.642	553.31	2015.35
A_68_P28055690	chr11:98188811-98188855	NM_010895:2127	Neurod2	INSIDE	0.658	3.312	400.92	1327.88	2.178	339.98	740.57
A_68_P27556423	chr11:4118542-4118586	NM_028022:311	Gatsl3	INSIDE	0.658	0.677	1738.17	1177.53	0.446	1537.93	685.86
A_68_P27282596	chr10:79804721-79804765	NM_001113548:6449	Adamtsl5	INSIDE	0.658	0.741	3042.90	2255.07	0.488	2101.57	1024.85
A_68_P27265101	chr10:76424431-76424475	NM_021568:240	Pcbp3	INSIDE	0.658	2.946	4474.32	13182.71	1.938	3546.01	6870.54
A_68_P26540423	chr9:56978779-56978823	NM_028636:217	Man2c1	INSIDE	0.658	3.960	801.25	3172.80	2.606	753.09	1962.19
A_68_P26140461	chr8:109127454-109127498	NM_009864:209	Cdh1	INSIDE	0.658	0.609	1983.81	1207.66	0.400	1453.56	582.14
A_68_P26133220	chr8:107872806-107872850	NM_001081332:671	Slc9a5	INSIDE	0.658	2.242	1560.60	3499.31	1.475	1254.24	1849.89
A_68_P25616848	chr8:4253392-4253436	NM_133968:313	Snapc2	INSIDE	0.658	0.552	6601.73	3644.66	0.363	4322.41	1571.18
A_68_P25528245	chr7:138553702-138553746	NM_175115:268	Ikzf5	INSIDE	0.658	0.713	1720.92	1227.00	0.469	1320.08	619.57
A_68_P25481817	chr7:130113128-130113172	NM_175023:-927	Rbbp6	PROMOTER	0.658	0.491	2354.63	1157.06	0.323	1895.45	613.11
A_68_P25016714	chr7:28114472-28114516	NM_001113549:4173	Ltbp4	INSIDE	0.658	0.602	2497.06	1503.05	0.396	1878.71	743.91
A_68_P24117669	chr5:136219992-136220036	NM_172541:28	Tmem120a	INSIDE	0.658	3.980	879.92	3501.71	2.619	812.80	2129.06
A_68_P24006959	chr5:115392406-115392450	NM_028211:218	2210016L21Rik	INSIDE	0.658	2.708	3946.82	10687.72	1.782	2922.23	5208.83
A_68_P22321899	chr3:89635034-89635078	NM_172530:-235	She	PROMOTER	0.658	8.314	1545.46	12848.45	5.469	1447.86	7918.31
A_68_P21747680	chr2:151931345-151931389	NM_029688:-99	Srxn1	PROMOTER	0.658	0.402	3257.66	1309.65	0.265	2470.05	653.77
A_68_P21745020	chr2:151457975-151458019	NM_198214:273	Snph	INSIDE	0.658	6.586	472.55	3112.25	4.334	416.14	1803.38
A_68_P20632910	chr1:136241239-136241283	NM_001042485:17316	Tmem183a	DOWNSTREAM	0.658	2.305	2382.19	5492.01	1.517	1919.40	2912.31
A_68_P20454693	chr1:95202297-95202341	NM_178051:129	Mterfd2	INSIDE	0.658	0.447	3350.82	1496.37	0.294	2379.30	699.51
A_68_P32281788	chrX:17740669-17740713	NM_009483:898	Kdm6a	INSIDE	0.657	0.513	999.58	512.80	0.337	1490.43	502.24
A_68_P31829408	chr18:74224160-74224204	NM_172632:421	Mapk4	INSIDE	0.657	0.725	3255.75	2360.53	0.477	2406.03	1146.95
A_68_P30978140	chr16:93884209-93884253	NM_028083:85	Chaf1b	INSIDE	0.657	0.532	1619.71	861.76	0.350	1309.22	457.70
A_68_P30404626	chr15:85864596-85864640	NM_009886:-411	Celsr1	PROMOTER	0.657	0.581	2083.94	1210.54	0.382	1774.91	677.21
A_68_P28752835	chr12:114067766-114067810	NM_028023:-188	Cdca4	PROMOTER	0.657	0.434	2299.02	997.81	0.285	1869.73	533.55
A_68_P28344355	chr12:34642855-34642899	NM_011658:341	Twist1	INSIDE	0.657	3.850	702.80	2705.68	2.531	605.39	1532.02
A_68_P28089255	chr11:103995098-103995142	NM_007762:925	Crhr1	INSIDE	0.657	2.809	1973.33	5543.00	1.844	1706.91	3148.03

ProbeName	Target position of probe on CpG island microarray	TargetID	GeneSymbol	CpG island Description	Ratio of relative methylation (TiO_2-NP/Vehicle)	Sham group			TiO_2-H group		
						Relative methylation (Cy5/Cy3)	Cy3 signal (Input)	Cy5 signal (MeDIP)	Relative methylation (Cy5/Cy3)	Cy3 signal (Input)	Cy5 signal (MeDIP)
A_68_P28073808	chr11:101286925-101286969	NM_026865:-299	1700113I22Rik	PROMOTER	0.657	0.284	3416.50	971.91	0.187	2515.23	470.42
A_68_P27268624	chr10:76978491-76978535	NM_133998:46	1810008A18Rik	INSIDE	0.657	0.705	1600.06	1128.55	0.463	1166.57	540.55
A_68_P26540100	chr9:56924835-56924879	NM_001110350:674	Sin3a	INSIDE	0.657	0.593	937.70	556.48	0.390	857.98	334.42
A_68_P26346367	chr9:21092297-21092341	NM_145416:95	Kri1	INSIDE	0.657	0.514	911.29	468.79	0.338	805.11	271.98
A_68_P26202775	chr8:119960432-119960476	NM_028941:87385	4933407C03Rik	INSIDE	0.657	4.132	2398.43	9909.56	2.713	1553.32	4214.26
A_68_P26192688	chr8:118232099-118232143	NM_001025577:-1326	Maf	PROMOTER	0.657	0.190	2838.91	538.50	0.125	2085.80	260.09
A_68_P25586915	chr7:148317107-148317151	NM_013782:-56	Ptdss2	PROMOTER	0.657	2.616	1046.76	2738.14	1.718	952.74	1637.02
A_68_P25574426	chr7:146038756-146038800	NM_026391:710	Ppp2r2d	INSIDE	0.657	3.635	773.34	2810.95	2.388	696.51	1663.32
A_68_P23601494	chr5:35917751-35917795	NM_030208:-53	2310079F23Rik	DIVERGENT_PROMOTER	0.657	0.332	1347.69	447.55	0.218	1014.40	221.39
A_68_P23203768	chr4:109654665-109654709	NM_172296:4057	Dmrta2	INSIDE	0.657	2.954	954.12	2818.81	1.940	756.62	1468.12
A_68_P22082262	chr3:40754266-40754310	NM_001040399:-264	Larp1b	PROMOTER	0.657	3.591	391.95	1407.61	2.358	337.21	795.13
A_68_P21864924	chr2:172377062-172377106	NM_001159696:594	Tcfap2c	INSIDE	0.657	0.364	4544.19	1656.14	0.240	3087.93	739.58
A_68_P21584536	chr2:121944215-121944259	NM_145531:-114	Spg11	PROMOTER	0.657	2.137	2817.11	6019.45	1.404	2073.16	2910.78
A_68_P21339006	chr2:74550322-74550366	NM_010468:295	Hoxd3	INSIDE	0.657	3.767	1900.04	7157.20	2.473	1647.51	4074.33
A_68_P32576282	chrX:101395577-101395621	NM_001077354:858	C77370	INSIDE	0.656	5.136	1407.38	7228.93	3.371	1682.83	5672.79
A_68_P31469354	chr18:5592137-5592181	NM_011546:270	Zeb1	INSIDE	0.656	4.018	1995.10	8015.57	2.638	1563.96	4125.00
A_68_P31051121	chr17:12511550-12511594	NM_011948:-46	Map3k4	PROMOTER	0.656	2.636	1507.08	3972.04	1.730	1185.29	2050.04
A_68_P28744650	chr12:112776572-112776616	NR_033168:-2562	Snora28	PROMOTER	0.656	4.307	1024.48	4412.95	2.828	837.98	2369.67
A_68_P28538319	chr12:74685927-74685971	NM_008856:-79	Prkch	PROMOTER	0.656	6.638	437.32	2903.11	4.355	340.57	1483.18
A_68_P28293548	chr12:25785727-25785771	NM_010496:-4791	Id2	PROMOTER	0.656	2.798	2853.81	7985.62	1.835	2310.39	4239.36
A_68_P27979838	chr11:84642248-84642292	NM_177564:235	Dhrs11	INSIDE	0.656	0.553	3173.74	1753.63	0.363	2352.49	852.96
A_68_P27638837	chr11:21893202-21893246	NM_011023:8430	Otx1	DOWNSTREAM	0.656	2.965	862.32	2556.39	1.945	728.67	1417.23
A_68_P27289154	chr10:80827741-80827785	NM_001014836:803	4930404N11Rik	INSIDE	0.656	0.606	1953.13	1184.35	0.398	1435.78	570.95
A_68_P27286341	chr10:80380635-80380679	NM_010722:334	Lmnb2	INSIDE	0.656	0.425	1675.54	712.62	0.279	1245.15	347.59
A_68_P27160596	chr10:57206710-57206754	NM_008297:542	Hsf2	INSIDE	0.656	2.874	912.61	2622.59	1.885	799.26	1506.65
A_68_P26781571	chr9:102592313-102592357	AK131685:-2880		PROMOTER	0.656	0.527	1541.74	812.61	0.346	1181.68	408.45
A_68_P26254550	chr8:128421964-128422008	NM_175561:207	Pcnxl2	INSIDE	0.656	3.055	1821.17	5564.46	2.005	1508.63	3024.68
A_68_P25728674	chr8:26830692-26830736	NM_028000:195	Ppapdc1b	INSIDE	0.656	0.670	1428.07	957.13	0.439	1080.84	474.91
A_68_P25184109	chr7:72838329-72838373	NM_026795:48	Tm2d3	INSIDE	0.656	0.393	2252.22	884.61	0.258	1644.02	423.77
A_68_P25021326	chr7:29135937-29135981	NR_028129:-600	LOC100302567	PROMOTER	0.656	0.628	2196.37	1378.97	0.412	1729.26	711.70
A_68_P24046891	chr5:122302921-122302965	NM_175474:3906	Fam109a	INSIDE	0.656	3.181	1148.22	3651.93	2.087	863.35	1802.11
A_68_P23573588	chr5:30924821-30924865	NM_010608:34300	Kcnk3	INSIDE	0.656	2.609	747.65	1950.84	1.713	754.51	1292.25
A_68_P23403484	chr4:149329133-149329178	NM_029035:-40	Spsb1	PROMOTER	0.656	2.259	1446.81	3267.93	1.482	1119.36	1658.90
A_68_P23255470	chr4:120887662-120887706	ENSMUST00000056635:5		INSIDE	0.656	3.748	1725.69	6468.40	2.460	1431.85	3521.76
A_68_P22565463	chr3:137527117-137527161	NM_016750:-424	H2afz	PROMOTER	0.656	0.248	3125.98	774.95	0.163	2669.40	434.07
A_68_P22129173	chr3:51144733-51144777	ENSMUST00000135496:-174		PROMOTER	0.656	0.594	4870.35	2893.92	0.390	3686.00	1437.66
A_68_P21907361	chr2:180689354-180689398	NM_001141933:28	Nkain4	INSIDE	0.656	0.156	4928.05	766.40	0.102	3255.60	331.96
A_68_P21821252	chr2:164859772-164859816	NM_144892:485	Ncoa5	INSIDE	0.656	0.506	1147.16	580.26	0.332	859.81	285.34
A_68_P21622216	chr2:128769433-128769477	NM_020594:302	Zc3h8	INSIDE	0.656	0.655	3993.28	2617.40	0.430	3006.44	1292.54
A_68_P21114427	chr2:32143150-32143194	NM_175190:416	2900010J23Rik	INSIDE	0.656	0.485	921.08	447.14	0.318	794.97	253.15
A_68_P21105462	chr2:30683743-30683787	NM_133346:56	Asb6	INSIDE	0.656	2.121	2852.54	6050.69	1.392	2224.71	3097.74
A_68_P20910364	chr1:188791866-188791912	NM_033077:594	D1Pas1	INSIDE	0.656	9.330	1303.29	12159.80	6.124	1252.64	7671.44
A_68_P20611349	chr1:132613579-132613625	NM_178691:-301	Yod1	DIVERGENT_PROMOTER	0.656	4.171	475.98	1985.07	2.735	389.80	1066.15
A_68_P30578589	chr16:18248400-18248444	NM_011239:365	Ranbp1	INSIDE	0.655	2.574	2608.12	6714.49	1.686	2123.74	3580.20
A_68_P30503777	chr15:103196605-103196649	NM_010577:553	Itga5	INSIDE	0.655	0.605	1535.75	928.68	0.396	1311.19	519.71
A_68_P30361918	chr15:78549192-78549236	NM_183141:-671	Elfn2	PROMOTER	0.655	3.000	2785.14	8355.97	1.966	2073.22	4075.04
A_68_P28535257	chr12:74145983-74146027	NM_009189:1695	Six1	INSIDE	0.655	4.019	982.34	3947.92	2.631	851.00	2238.63
A_68_P27282604	chr10:79805724-79805768	NM_025629:5411	Adamtsl5	INSIDE	0.655	2.785	896.04	2495.57	1.825	800.91	1461.29
A_68_P27234933	chr10:70807476-70807520	NM_134007:99	Cisd1	INSIDE	0.655	3.491	1438.08	5020.46	2.288	1129.33	2584.26
A_68_P26245549	chr8:126958481-126958525	NM_171824:-666	Pgbd5	PROMOTER	0.655	7.721	1071.47	8272.96	5.057	932.52	4715.60
A_68_P26031095	chr8:89269939-89269983	NM_009172:-55	Siah1a	PROMOTER	0.655	4.399	426.05	1874.08	2.883	455.75	1313.75
A_68_P25506858	chr7:134851780-134851824	NR_030428:-198	Mir762	PROMOTER	0.655	0.576	3874.50	2231.45	0.377	2650.16	999.48
A_68_P25091980	chr7:52489623-52489667	NM_007645:4565	Cd37	INSIDE	0.655	0.555	989.64	549.53	0.364	886.38	322.47
A_68_P25017170	chr7:28193438-28193482	NM_032610:38148	Spnb4	INSIDE	0.655	5.502	1770.19	9739.16	3.603	1522.09	5484.83
A_68_P24451816	chr6:52590190-52590234	NM_145567:82	Hibadh	INSIDE	0.655	0.721	1735.81	1252.15	0.473	1489.72	704.01
A_68_P24155214	chr5:143919270-143919314	ENSMUST00000157346:-2486		PROMOTER	0.655	3.990	818.25	3265.03	2.612	882.06	2303.69
A_68_P23592026	chr5:34340043-34340087	NM_001001985:1432	Nat8l	INSIDE	0.655	3.630	413.70	1501.55	2.376	390.95	928.97
A_68_P23317755	chr4:133024124-133024168			Unknown	0.655	3.585	1925.08	6901.40	2.349	1671.44	3926.31
A_68_P22942710	chr4:57003066-57003110	NM_001142965:175	6430704M03Rik	INSIDE	0.655	4.430	734.50	3254.16	2.903	688.12	1997.39
A_68_P22310998	chr3:87774969-87775013	NM_016701:-24	Nes	PROMOTER	0.655	0.246	2865.31	705.86	0.161	2156.26	347.75
A_68_P22262798	chr3:79091123-79091167	AK032506:-169		PROMOTER	0.655	3.313	1570.54	5203.16	2.171	1306.31	2835.67
A_68_P22157981	chr3:55987567-55987611	NM_030595:35	Nbea	INSIDE	0.655	4.632	1531.89	7095.84	3.034	1285.81	3900.77
A_68_P21840497	chr2:168107059-168107103	ENSMUST00000109191:-448		PROMOTER	0.655	0.649	1456.94	945.95	0.426	1107.41	471.28
A_68_P21785050	chr2:158620772-158620816	NM_145742:239	Dhx35	INSIDE	0.655	0.481	999.00	480.27	0.315	828.79	260.84
A_68_P21350016	chr2:76244453-76244497	NM_145525:-120	Osbpl6	PROMOTER	0.655	0.424	2305.20	976.93	0.278	1736.07	482.02

ProbeName	Target position of probe on CpG island microarray	TargetID	GeneSymbol	CpG island Description	Ratio of relative methylation (TiO_2-NP/Vehicle)	Sham group			TiO_2-H group		
						Relative methylation (Cy5/Cy3)	Cy3 signal (Input)	Cy5 signal (MeDIP)	Relative methylation (Cy5/Cy3)	Cy3 signal (Input)	Cy5 signal (MeDIP)
A_68_P32383370	chrX:49967260-49967304	NM_016697:-131	Gpc3	PROMOTER	0.654	3.655	6131.96	22412.89	2.391	7696.73	18406.15
A_68_P31790105	chr18:67293085-67293129	NM_010307:-373	Gnal	PROMOTER	0.654	2.449	897.11	2197.25	1.602	834.89	1337.47
A_68_P30572558	chr16:17210568-17210612	NM_001033338:2363	Rimbp3	INSIDE	0.654	0.282	2029.93	572.50	0.185	1561.58	288.11
A_68_P30509169	chr16:4268211-4268255			Unknown	0.654	2.923	391.05	1143.08	1.912	431.38	824.62
A_68_P30344756	chr15:75724658-75724702	NM_172607:231	Naprt1	INSIDE	0.654	0.264	4065.70	1074.06	0.173	2855.64	493.66
A_68_P29073057	chr13:58503189-58503233	NR_031761:-398	Rmi1	DIVERGENT_PROMOTER	0.654	0.377	3808.08	1437.35	0.247	2955.21	729.22
A_68_P29011800	chr13:47218349-47218393	NM_146042:282	Rnf144b	INSIDE	0.654	0.579	1024.65	593.04	0.378	899.46	340.26
A_68_P28576092	chr12:81620747-81620791	NM_001081421:792	Galntl1	INSIDE	0.654	0.414	1744.47	722.82	0.271	1435.89	389.35
A_68_P28153091	chr11:115228565-115228611	NM_175454:445	C630004H02Rik	INSIDE	0.654	0.313	4449.23	1393.40	0.205	3434.93	703.82
A_68_P27932457	chr11:76308492-76308536	NM_198018:14407	Abr	INSIDE	0.654	24.286	20433.25	496239.40	15.881	18206.94	289136.50
A_68_P27792052	chr11:50873003-50873047	NM_009329:266	Zfp354a	INSIDE	0.654	6.552	3393.45	22233.00	4.287	3095.95	13273.79
A_68_P26920734	chr10:7500514-7500558	NM_145418:179	BC013529	INSIDE	0.654	2.950	1068.10	3151.26	1.930	918.84	1773.07
A_68_P26805304	chr9:106724237-106724281	NM_001015507:-48	Vprbp	PROMOTER	0.654	0.559	1581.47	883.32	0.365	1169.58	427.22
A_68_P26617706	chr9:70505115-70505159	NM_172772:-155	Fam63b	PROMOTER	0.654	4.778	1834.16	8764.04	3.124	1653.59	5165.14
A_68_P26200040	chr8:119502568-119502612	NM_027103:253	1700030J22Rik	INSIDE	0.654	0.407	7301.24	2970.77	0.266	5203.87	1384.21
A_68_P25958332	chr8:74213234-74213278	NM_013564:106	Insl3	INSIDE	0.654	3.629	521.54	1892.79	2.373	451.43	1071.42
A_68_P25731031	chr8:27269366-27269410	NM_199042:748	Thap1	INSIDE	0.654	2.719	887.41	2412.46	1.777	672.72	1195.74
A_68_P24443350	chr6:51220702-51220746	NR_029719:-815	Mir148a	PROMOTER	0.654	2.823	1500.36	4235.41	1.846	1341.47	2476.02
A_68_P23609898	chr5:37221813-37221857	NM_008550:54	Man2b2	INSIDE	0.654	2.997	412.63	1236.83	1.962	433.02	849.45
A_68_P23339476	chr4:136913526-136913570	NM_009861:104	Cdc42	INSIDE	0.654	2.377	1491.88	3546.36	1.555	1398.63	2175.23
A_68_P23331345	chr4:135502447-135502491	NM_008254:101	Hmgcl	INSIDE	0.654	0.608	1367.65	831.00	0.398	1049.18	417.24
A_68_P23284202	chr4:126910975-126911019	NM_198618:64482	Dlgap3	INSIDE	0.654	2.468	2079.05	5131.43	1.615	1657.09	2675.78
A_68_P23226097	chr4:115511687-115511731	NM_021461:-142	Mknk1	PROMOTER	0.654	0.252	2571.17	647.09	0.165	1830.83	301.43
A_68_P22039455	chr3:32607362-32607406	NM_019673:-83	Actl6a	PROMOTER	0.654	0.450	7748.96	3484.86	0.294	5767.13	1696.51
A_68_P21626036	chr2:129513877-129513921	NM_018863:1706	Pdyn	INSIDE	0.654	3.055	2358.70	7206.16	1.999	1920.54	3838.54
A_68_P21132680	chr2:35138083-35138127	NM_146120:205	Gsn	INSIDE	0.654	3.020	862.33	2604.64	1.977	698.14	1379.91
A_68_P21115865	chr2:32391002-32391046	NM_153560:146	Fam102a	INSIDE	0.654	3.058	1322.22	4043.78	2.000	1218.78	2437.91
A_68_P32246512	chrX:8905191-8905235	ENSMUST00000119858:22		INSIDE	0.653	2.756	650.97	1794.07	1.801	771.81	1389.67
A_68_P31130464	chr17:30025085-30025129	NM_001081160:-279	Mdga1	PROMOTER	0.653	9.733	2706.21	26338.31	6.352	2349.99	14926.13
A_68_P31113238	chr17:27070074-27070118	NM_009343:25	Phf1	INSIDE	0.653	0.570	1319.93	752.10	0.372	1108.21	412.32
A_68_P30490574	chr15:101076776-101076820			Unknown	0.653	0.341	1452.85	494.95	0.222	1167.30	259.71
A_68_P30486712	chr15:100446399-100446443	NM_011873:328	Dazap2	INSIDE	0.653	0.324	2710.06	877.67	0.212	1943.11	411.22
A_68_P30401591	chr15:85408071-85408115	NM_001163634:408	Wnt7b	INSIDE	0.653	2.469	814.19	2010.24	1.613	719.26	1159.83
A_68_P30382876	chr15:82171483-82171527	NM_177391:-104	Fam109b	DIVERGENT_PROMOTER	0.653	0.570	939.25	535.26	0.372	731.21	271.91
A_68_P30363284	chr15:78765610-78765654	NM_133800:270	Nol12	INSIDE	0.653	0.503	950.05	478.26	0.329	874.71	287.47
A_68_P29696921	chr14:70132430-70132474	NM_134078:-75	Chmp7	PROMOTER	0.653	2.613	2372.06	6197.19	1.706	1881.65	3209.46
A_68_P28302019	chr12:27154024-27154068	NM_020557:-33	Cmpk2	PROMOTER	0.653	0.432	2601.04	1122.67	0.282	1689.00	476.26
A_68_P28171198	chr11:118280168-118280212	NM_001025617:176	Cant1	INSIDE	0.653	0.734	2988.97	2192.62	0.479	2141.05	1025.85
A_68_P28168166	chr11:117811203-117811247			Unknown	0.653	2.317	1517.61	3516.42	1.514	1231.87	1864.94
A_68_P28086886	chr11:103610912-103610956	NM_011719:201	Wnt9b	INSIDE	0.653	0.488	1610.49	785.77	0.319	1379.32	439.56
A_68_P27813684	chr11:54911411-54911457	NM_010299:-52	Gm2a	PROMOTER	0.653	2.363	1578.93	3730.86	1.544	1369.70	2114.98
A_68_P27788006	chr11:50138948-50138992	NM_001110499:205	Canx	INSIDE	0.653	6.682	5142.45	34360.58	4.364	4329.86	18896.88
A_68_P27534326	chr10:126603018-126603062	NM_027739:154	B4galnt1	INSIDE	0.653	3.690	1179.91	4353.36	2.409	965.39	2325.89
A_68_P27189024	chr10:62485962-62486006	NM_001079824:613	Hnrnph3	INSIDE	0.653	0.502	3207.96	1610.80	0.328	2394.68	785.23
A_68_P26846253	chr9:114662207-114662251	NM_133978:28719	Cmtm7	DOWNSTREAM	0.653	0.440	1337.28	588.47	0.287	1096.59	315.15
A_68_P26581610	chr9:64233768-64233812	NM_172522:358	Megf11	INSIDE	0.653	0.398	2461.38	978.91	0.260	1940.60	503.88
A_68_P26399347	chr9:31020000-31020044	NM_001102455:-622	Aplp2	PROMOTER	0.653	0.668	2038.03	1360.81	0.436	1529.19	667.20
A_68_P25505711	chr7:134615245-134615289	NM_145589:139	Prr14	INSIDE	0.653	3.043	1226.71	3732.73	1.988	956.66	1901.68
A_68_P25415072	chr7:117911998-117912042	NM_009667:-4097	Ampd3	PROMOTER	0.653	0.226	2025.48	458.13	0.148	1631.89	241.18
A_68_P25325434	chr7:99709780-99709824	NM_001162918:144	Ccdc90b	INSIDE	0.653	0.453	1004.58	455.14	0.296	836.63	247.42
A_68_P24992859	chr7:19945635-19945679	NM_145822:-824	Cd3eap	DIVERGENT_PROMOTER	0.653	0.211	4122.28	871.61	0.138	2873.59	396.66
A_68_P24652024	chr6:93628341-93628385	NM_001083320:235322	Magi1	INSIDE	0.653	3.376	894.64	3020.03	2.204	767.35	1691.62
A_68_P24586428	chr6:81991724-81991768	NM_145570:125	Fam176a	INSIDE	0.653	0.443	1613.20	714.78	0.289	1361.48	393.64
A_68_P23342966	chr4:137514008-137514052	NM_199307:95879	Ece1	INSIDE	0.653	2.677	2940.99	7874.44	1.748	2022.63	3535.19
A_68_P22650044	chr3:152009738-152009782	NM_001076676:316	Usp33	INSIDE	0.653	5.228	786.11	4110.09	3.415	662.34	2261.62
A_68_P21908760	chr2:180869636-180869680	NM_001003824:272	Kcnq2	INSIDE	0.653	3.255	653.35	2126.76	2.124	565.28	1200.85
A_68_P21822536	chr2:165059487-165059531	NM_174988:729	Cdh22	INSIDE	0.653	0.658	1326.06	872.62	0.430	1017.23	436.94
A_68_P21104487	chr2:30514363-30514407	ENSMUST00000147959:11044		INSIDE	0.653	0.619	1173.25	726.31	0.404	882.30	356.51
A_68_P32982444	chr5:135115258-135115302	NM_033561:-82	Eif4h	PROMOTER	0.652	3.251	2202.82	7162.43	2.119	1880.78	3985.80
A_68_P32767195	chrX:154036840-154036884	NM_172307:-215	Mbtps2	PROMOTER	0.652	4.827	417.07	2013.38	3.147	622.68	1959.52
A_68_P32672454	chrX:131076324-131076368	NM_013898:-178	Timm8a1	PROMOTER	0.652	5.603	653.84	3663.71	3.656	777.20	2841.19
A_68_P31936715	chr19:7000722-7000766	NM_008431:8261	Kcnk4	INSIDE	0.652	3.800	1603.23	6091.50	2.477	1392.89	3450.48
A_68_P31620307	chr18:35990504-35990548	NM_133687:1055	Cxxc5	INSIDE	0.652	4.829	1250.28	6037.72	3.149	1022.64	3220.21
A_68_P31423372	chr17:87845921-87845965	NM_007589:333	Calm2	INSIDE	0.652	3.060	856.99	2622.03	1.995	573.44	1143.80
A_68_P30433470	chr15:90880052-90880096	NM_001109040:305	Kif21a	INSIDE	0.652	2.926	1474.02	4313.34	1.909	1154.64	2204.22

ProbeName	Target position of probe on CpG island microarray	TargetID	GeneSymbol	CpG island Description	Ratio of relative methylation (TiO_2-NP/Vehicle)	Sham group			TiO_2-H group		
						Relative methylation (Cy5/Cy3)	Cy3 signal (Input)	Cy5 signal (MeDIP)	Relative methylation (Cy5/Cy3)	Cy3 signal (Input)	Cy5 signal (MeDIP)
A_68_P30426169	chr15:89422304-89422348	NM_026817:28	Rabl2	INSIDE	0.652	4.513	1153.57	5206.58	2.941	867.27	2550.53
A_68_P27849285	chr11:61393132-61393176	NM_010148:-1	Epn2	PROMOTER	0.652	3.918	2672.87	10471.73	2.555	2060.15	5263.19
A_68_P26234304	chr8:125100653-125100697	NM_009698:133	Aprt	INSIDE	0.652	0.145	4514.90	656.57	0.095	2886.46	273.81
A_68_P26154270	chr8:111471142-111471186	NM_007496:232621	Zfhx3	INSIDE	0.652	4.715	2049.93	9666.05	3.072	1646.02	5056.85
A_68_P26135759	chr8:108282466-108282510	NM_027469:19	Gfod2	INSIDE	0.652	2.465	1592.62	3925.56	1.607	1141.70	1834.72
A_68_P25954190	chr8:73328850-73328894	NM_008353:-3475	Il12rb1	PROMOTER	0.652	0.603	3195.01	1927.03	0.393	2412.25	948.60
A_68_P25553144	chr7:142729766-142729810	NM_011212:282	Ptpre	INSIDE	0.652	0.595	2166.38	1289.60	0.388	1802.32	699.57
A_68_P25373530	chr7:108599609-108599653	NM_001143849:-135	Pde2a	PROMOTER	0.652	0.689	2865.24	1973.60	0.449	2143.41	962.23
A_68_P24992603	chr7:19894118-19894162	NM_008036:1254	Fosb	INSIDE	0.652	0.647	937.07	605.99	0.422	765.94	323.17
A_68_P24838521	chr6:128305667-128305711	NM_011657:181	Tulp3	INSIDE	0.652	0.168	7292.48	1226.23	0.110	4936.87	541.12
A_68_P24619080	chr6:87930668-87930712	NM_198622:786	H1fx	INSIDE	0.652	0.340	2472.15	840.80	0.222	1936.27	429.60
A_68_P24175497	chr5:148537110-148537154	NM_010228:432	Flt1	INSIDE	0.652	0.410	2412.06	989.49	0.267	1817.92	485.97
A_68_P23233435	chr4:116962137-116962181	NM_025739:207362	Rnf220	INSIDE	0.652	0.584	2392.16	1395.88	0.381	1861.34	708.58
A_68_P22049387	chr3:34548813-34548857	NM_011443:-92	Sox2	PROMOTER	0.652	3.744	1580.98	5919.50	2.442	1304.64	3185.36
A_68_P21129593	chr2:34609363-34609407	NM_025709:1368	Gapvd1	INSIDE	0.652	0.272	4319.16	1175.04	0.177	3142.20	557.06
A_68_P32036067	chr19:26678985-26679030	NM_011416:-642	Smarca2	PROMOTER	0.651	0.253	3581.52	906.17	0.165	2378.11	391.54
A_68_P31101000	chr17:25151186-25151230	NM_020608:967	Cramp1l	INSIDE	0.651	0.256	9098.37	2327.64	0.166	6449.60	1073.49
A_68_P30596855	chr16:22266102-22266148	NM_009186:-122	Tra2b	PROMOTER	0.651	0.377	1975.21	745.12	0.246	1456.10	357.54
A_68_P30547081	chr16:11253703-11253747	NM_001130008:694	Gspt1	INSIDE	0.651	0.216	2698.43	581.74	0.140	1876.57	263.32
A_68_P29507005	chr14:31438638-31438682	NM_011103:734	Prkcd	INSIDE	0.651	2.549	5843.10	14893.64	1.660	4852.86	8054.41
A_68_P27929565	chr11:75840897-75840941	NM_029658:366	Fam101b	INSIDE	0.651	0.600	903.27	542.41	0.391	739.44	289.27
A_68_P27480860	chr10:116813708-116813752	NM_001013391:299	Cpsf6	INSIDE	0.651	2.814	946.02	2662.14	1.831	888.50	1626.58
A_68_P27447375	chr10:110602439-110602483	NM_001003717:603	Osbpl8	INSIDE	0.651	0.467	1566.98	731.98	0.304	1219.16	370.75
A_68_P27383821	chr10:98377167-98377211	NM_026482:-597	Atp2b1	PROMOTER	0.651	2.543	857.79	2181.74	1.657	647.67	1073.14
A_68_P27091227	chr10:41530014-41530058	NM_001162908:-343	Sesn1	DIVERGENT_PROMOTER	0.651	0.503	1239.08	623.18	0.327	925.01	302.89
A_68_P26323901	chr9:15432521-15432565	NM_181816:-165	Ccdc67	PROMOTER	0.651	0.550	2745.25	1511.18	0.358	2009.38	719.62
A_68_P26079748	chr8:97362202-97362246	NM_134141:16	Ciapin1	INSIDE	0.651	4.595	471.83	2167.91	2.991	360.09	1076.88
A_68_P25467116	chr7:127246039-127246083	NM_145586:121	Tmem159	INSIDE	0.651	0.704	2527.48	1780.36	0.458	1796.40	823.40
A_68_P25018412	chr7:28443528-28443572	NM_001081292:16067	Map3k10	INSIDE	0.651	0.459	1339.91	614.72	0.299	1088.23	325.03
A_68_P24160101	chr5:144984767-144984811	NM_027410:-341	Tecpr1	PROMOTER	0.651	2.954	1297.78	3833.00	1.923	1042.01	2003.51
A_68_P24010683	chr5:116081567-116081611	NR_027810:237	1110006O24Rik	INSIDE	0.651	0.328	4497.75	1474.33	0.213	3249.13	693.26
A_68_P23658795	chr5:46248998-46249042	NM_178142:-241	Lcorl	PROMOTER	0.651	2.722	1260.23	3429.91	1.771	1078.57	1910.09
A_68_P23244791	chr4:118967175-118967219	NM_011732:-78	Ybx1	PROMOTER	0.651	0.199	7723.78	1538.23	0.130	4873.79	631.57
A_68_P22558741	chr3:136333499-136333554	NM_008913:-207	Ppp3ca	PROMOTER	0.651	3.192	459.06	1465.28	2.078	380.92	791.54
A_68_P22086571	chr3:41546266-41546310	NM_001081411:148	Sclt1	INSIDE	0.651	3.960	470.62	1863.60	2.576	412.05	1061.46
A_68_P21815513	chr2:163821038-163821082	NM_018753:128	Ywhab	INSIDE	0.651	0.367	1585.32	582.32	0.239	1377.55	329.17
A_68_P21425706	chr2:91805641-91805685	NM_138306:-1942	Dgkz	PROMOTER	0.651	2.639	777.03	2050.67	1.717	762.90	1310.17
A_68_P21075529	chr2:25838219-25838263	NM_001115076:562	Camsap1	INSIDE	0.651	0.389	2346.77	913.52	0.254	1741.88	441.75
A_68_P20091076	chr1:24012273-24012317	NM_026503:185	1110058L19Rik	INSIDE	0.651	2.787	1541.98	4297.77	1.814	1372.47	2489.53
A_68_P20024913	chr1:9934160-9934204	NR_004410:-1558	Snord87	PROMOTER	0.651	0.556	1117.95	621.59	0.362	975.05	352.84
A_68_P32241633	chrX:7651543-7651587	NM_011514:322	Suv39h1	INSIDE	0.650	4.203	772.23	3245.64	2.734	1065.32	2912.35
A_68_P32233080	chrX:5659260-5659304	NM_001033211:1256	AU022751	INSIDE	0.650	2.950	367.53	1084.39	1.918	554.38	1063.30
A_68_P32134818	chr19:44911149-44911193	NM_011037:79287	Pax2	INSIDE	0.650	0.194	6713.00	1300.47	0.126	4614.66	581.06
A_68_P32119644	chr19:42276497-42276541	NM_018780:224	Sfrp5	INSIDE	0.650	2.689	1919.50	5162.35	1.748	1653.86	2891.29
A_68_P31857881	chr18:79305640-79305684	NM_053099:468	Setbp1	INSIDE	0.650	0.451	5517.63	2487.88	0.293	3734.81	1093.80
A_68_P31839441	chr18:75980377-75980421	NM_145356:567	Zbtb7c	INSIDE	0.650	3.739	973.02	3637.92	2.430	890.25	2163.59
A_68_P31488617	chr18:10181982-10182026	NM_009071:-214	Rock1	PROMOTER	0.650	0.580	1458.84	846.38	0.377	1232.44	464.50
A_68_P30423043	chr15:88919652-88919696	NM_027905:138	1300018J18Rik	INSIDE	0.650	3.950	455.50	1799.11	2.566	366.19	939.54
A_68_P30346760	chr15:76028479-76028523	NM_201394:-2360	Plec	PROMOTER	0.650	0.613	1237.98	759.18	0.399	1012.30	403.55
A_68_P29751627	chr14:79881898-79881942	NM_018765:-845	Wbp4	PROMOTER	0.650	3.347	880.72	2948.07	2.175	926.86	2016.24
A_68_P28931385	chr13:32894778-32894822	NM_030215:902	Wrnip1	INSIDE	0.650	0.541	1334.45	721.90	0.351	990.75	348.12
A_68_P28752032	chr12:113960100-113960144	NM_001024602:-262	AW555464	PROMOTER	0.650	0.659	1456.27	959.17	0.428	1092.33	467.60
A_68_P28340238	chr12:33832153-33832197	NR_015602:277	F730043M19Rik	INSIDE	0.650	0.604	1690.78	1020.69	0.392	1206.41	473.15
A_68_P26159425	chr8:112392612-112392656	NM_001122594:132	Phlpp2	INSIDE	0.650	4.506	1708.50	7697.70	2.931	1297.91	3803.64
A_68_P26137945	chr8:108660566-108660610	NM_176838:286	Esrp2	INSIDE	0.650	0.324	2548.68	824.70	0.210	1810.20	381.01
A_68_P25809394	chr8:42219374-42219418	NM_001005863:-316	Mtus1	PROMOTER	0.650	0.680	1593.53	1083.59	0.442	1258.88	556.11
A_68_P25504835	chr7:134404064-134404108	NM_023203:95	Dctpp1	INSIDE	0.650	3.964	4662.59	18481.05	2.577	3374.00	8696.03
A_68_P25499633	chr7:133416815-133416859	NM_145587:704	Sbk1	INSIDE	0.650	0.490	1172.88	575.25	0.319	1006.54	320.79
A_68_P25090215	chr7:52185401-52185445	NM_001077264:-562	Ap2a1	PROMOTER	0.650	0.655	1801.44	1179.71	0.426	1462.38	622.33
A_68_P23839618	chr5:81758787-81758831	NM_198702:308191	Lphn3	INSIDE	0.650	2.366	1467.51	3472.65	1.538	1171.30	1801.91
A_68_P22558745	chr3:136333964-136334008	NM_008913:253	Ppp3ca	INSIDE	0.650	0.745	2676.85	1993.21	0.484	1857.76	899.35
A_68_P22375207	chr3:101255068-101255112	NM_207205:74043	Igsf3	INSIDE	0.650	2.901	1115.46	3236.07	1.886	926.56	1747.79
A_68_P21874504	chr2:174124338-174124382	NM_201617:1001	Gnas	INSIDE	0.650	2.705	1746.33	4724.44	1.759	1477.74	2598.71
A_68_P21820162	chr2:164683144-164683188	NM_011125:42	Pltp	INSIDE	0.650	0.441	1712.17	755.16	0.287	1434.13	410.95
A_68_P21799022	chr2:160934858-160934902	NM_173368:-88	Chd6	PROMOTER	0.650	0.532	866.92	460.97	0.346	647.53	223.77

ProbeName	Target position of probe on CpG island microarray	TargetID	GeneSymbol	CpG island Description	Ratio of relative methylation (TiO_2-NP/Vehicle)	Sham group			TiO_2-H group		
						Relative methylation (Cy5/Cy3)	Cy3 signal (Input)	Cy5 signal (MeDIP)	Relative methylation (Cy5/Cy3)	Cy3 signal (Input)	Cy5 signal (MeDIP)
A_68_P21117286	chr2:32612762-32612806	NM_152800:-6	Tor2a	PROMOTER	0.650	6.598	1893.50	12493.94	4.289	1584.66	6795.89
A_68_P20422165	chr1:90102210-90102254	NM_008299:91	Dnajb3	INSIDE	0.650	4.023	787.18	3166.91	2.615	642.81	1681.22
A_68_P31939554	chr19:7456968-7457012	NM_001033139:526	AI846148	INSIDE	0.649	0.587	1549.94	909.27	0.381	1349.38	513.78
A_68_P30561772	chr16:14361819-14361863	NM_008576:188	Abcc1	INSIDE	0.649	0.465	2399.02	1115.25	0.302	2018.61	608.78
A_68_P30379101	chr15:81524789-81524833	NM_001164320:2907	Chadl	INSIDE	0.649	3.052	646.63	1973.68	1.981	583.89	1156.81
A_68_P29952571	chr14:121309972-121310016	NM_023579:-421	Ipo5	PROMOTER	0.649	3.538	1798.95	6365.45	2.296	1661.44	3814.40
A_68_P29263993	chr13:100286596-100286640	NM_008634:-61	Mtap1b	PROMOTER	0.649	2.943	1197.47	3524.38	1.910	969.09	1850.65
A_68_P28873011	chr13:21560407-21560451	NM_025719:-58	Nkapl	PROMOTER	0.649	7.033	2394.40	16840.05	4.566	2088.69	9536.99
A_68_P28181194	chr11:119804412-119804456	NM_001037754:29	Baiap2	INSIDE	0.649	0.193	2727.17	525.72	0.125	1778.57	222.65
A_68_P27902925	chr11:70658259-70658303	NM_019400:16	Rabep1	INSIDE	0.649	2.421	2013.55	4873.95	1.570	1686.88	2648.14
A_68_P27898384	chr11:69809416-69809460	NM_030064:166	Phf23	INSIDE	0.649	0.584	3824.57	2234.63	0.379	2597.87	985.52
A_68_P27810583	chr11:54336349-54336393	NM_175258:22	Rapgef6	INSIDE	0.649	3.179	1124.78	3575.78	2.064	877.71	1811.57
A_68_P27541473	chr10:127896021-127896065	NM_001114096:-249	Smarcc2	PROMOTER	0.649	2.443	1830.66	4472.31	1.586	1519.34	2410.26
A_68_P27475788	chr10:115911221-115911265	NM_021452:-663	Kcnmb4	PROMOTER	0.649	4.957	1928.13	9557.83	3.218	1642.97	5287.17
A_68_P27103326	chr10:43724148-43724192	NM_172393:482	Aim1	INSIDE	0.649	3.865	1253.73	4845.24	2.508	1106.45	2774.68
A_68_P26893203	chr9:123022219-123022263	NM_026917:83	Zdhhc3	INSIDE	0.649	2.624	635.40	1667.25	1.704	724.27	1233.87
A_68_P26526554	chr9:54434551-54434595	NM_029573:255	Idh3a	INSIDE	0.649	0.277	2048.76	567.74	0.180	1613.34	290.18
A_68_P26526552	chr9:54434359-54434404	NM_029573:64	Idh3a	INSIDE	0.649	0.509	6675.40	3397.48	0.330	4601.58	1519.80
A_68_P25946260	chr8:71612907-71612951	NM_153054:193	Slc18a1	INSIDE	0.649	0.558	832.01	464.20	0.362	678.38	245.68
A_68_P25587156	chr7:148354570-148354614	NM_001172101:-7	Rnh1	PROMOTER	0.649	0.266	2206.60	585.97	0.172	1609.40	277.48
A_68_P25505266	chr7:134488622-134488666	NM_146202:184	Zfp768	INSIDE	0.649	0.217	3739.21	810.92	0.141	2431.81	342.08
A_68_P25230685	chr7:80885691-80885735	NM_001162532:520	Fam174b	INSIDE	0.649	0.686	1204.03	825.76	0.445	963.39	428.76
A_68_P24944594	chr6:149302348-149302392	ENSMUST00000121073:-22		DIVERGENT_PROMOTER	0.649	4.706	1430.22	6730.31	3.056	1103.83	3373.59
A_68_P24184313	chr5:150099222-150099276	NM_009663:22615	Alox5ap	INSIDE	0.649	0.716	1466.32	1050.60	0.465	1036.03	481.88
A_68_P23807627	chr5:75549167-75549211	NM_001083316:-3002	Pdgfra	PROMOTER	0.649	0.335	1714.13	574.87	0.218	1296.09	282.12
A_68_P23269775	chr4:124377958-124378002	NM_010213:38	Fhl3	INSIDE	0.649	0.560	3094.54	1733.12	0.364	2503.46	910.38
A_68_P23244929	chr4:118993213-118993257	NM_028677:-106	Ppih	PROMOTER	0.649	0.521	2026.80	1055.06	0.338	1542.53	521.22
A_68_P22540995	chr3:132754697-132754741	NM_026231:-14	Gstcd	DIVERGENT_PROMOTER	0.649	4.037	595.63	2404.51	2.618	473.32	1239.38
A_68_P21777116	chr2:157282885-157282929	NM_001025395:32878	Src	INSIDE	0.649	3.222	1800.13	5800.61	2.092	1413.63	2957.28
A_68_P32117863	chr19:41986389-41986433	NM_023418:50	Pgam1	INSIDE	0.648	0.197	2990.13	588.66	0.128	2024.69	258.21
A_68_P31311920	chr17:67703577-67703621	NM_008984:201	Ptprm	INSIDE	0.648	0.532	1152.25	612.48	0.344	886.52	305.37
A_68_P30647296	chr16:31429742-31429786	NM_001122683:926	Bdh1	INSIDE	0.648	0.187	4034.26	755.72	0.121	2742.05	332.80
A_68_P29072860	chr13:58460543-58460587	NM_175214:-4341	Kif27	PROMOTER	0.648	0.171	4611.61	789.67	0.111	3200.26	354.97
A_68_P28924022	chr13:31712233-31712277	NM_010225:-5430	Foxf2	PROMOTER	0.648	0.672	949.79	638.38	0.435	833.11	362.61
A_68_P28920307	chr13:31065636-31065680	NM_025588:258	Exoc2	INSIDE	0.648	3.109	3193.62	9929.31	2.016	2610.24	5262.42
A_68_P28224314	chr12:8777965-8778009	NM_011519:-215	Sdc1	PROMOTER	0.648	0.321	2549.24	817.65	0.208	1886.28	392.34
A_68_P27995858	chr11:87550476-87550520	NM_009296:-568	Supt4h1	PROMOTER	0.648	0.648	852.48	552.41	0.420	691.43	290.42
A_68_P27631992	chr11:20443745-20443789	NM_021372:511	Sertad2	INSIDE	0.648	0.676	995.72	672.69	0.438	757.99	331.66
A_68_P27284104	chr10:80040680-80040724	NM_078477:-661	Klf16	PROMOTER	0.648	0.649	2286.92	1484.04	0.420	1754.91	737.89
A_68_P26749722	chr9:96790253-96790298	NM_153420:-434	Acpl2	PROMOTER	0.648	0.366	1422.36	520.73	0.237	1075.64	255.10
A_68_P26138372	chr8:108734689-108734733	NM_001007567:123	Slc7a6os	INSIDE	0.648	4.770	1409.93	6725.36	3.093	1244.13	3847.48
A_68_P25828057	chr8:46122480-46122524	NM_001081286:86941	Fat1	INSIDE	0.648	2.457	1388.90	3413.09	1.593	1059.16	1686.75
A_68_P25656226	chr8:11557934-11557978	NM_011919:1891	Ing1	INSIDE	0.648	0.546	1033.64	564.85	0.354	897.42	317.67
A_68_P25499303	chr7:133343931-133343975	NM_028816:-30	Xpo6	PROMOTER	0.648	3.149	1643.56	5175.00	2.042	1432.71	2925.04
A_68_P25169316	chr7:69609111-69609155	NM_013788:264	Peg12	INSIDE	0.648	3.145	973.29	3060.76	2.039	897.40	1830.07
A_68_P24979593	chr7:16684397-16684441	NM_025898:427	Napa	INSIDE	0.648	0.484	1728.52	836.25	0.313	1423.04	446.05
A_68_P24796449	chr6:119987179-119987223	NR_030487:-2871	Mir706	PROMOTER	0.648	3.767	1164.46	4386.17	2.442	1011.95	2470.71
A_68_P24066624	chr5:125869903-125869947	NM_019639:463	Ubc	INSIDE	0.648	2.561	2217.68	5679.07	1.659	1820.53	3021.04
A_68_P24059257	chr5:124539964-124540008	NM_019875:5821	Abcb9	INSIDE	0.648	3.275	1372.73	4495.88	2.122	1172.09	2486.79
A_68_P23760676	chr5:66651307-66651351	NM_027602:-35	Nsun7	PROMOTER	0.648	0.510	905.12	461.50	0.330	731.09	241.61
A_68_P23415470	chr4:151308129-151308173	NM_172704:322	Dnajc11	INSIDE	0.648	3.443	526.10	1811.38	2.232	469.64	1048.00
A_68_P23404990	chr4:149610826-149610870	NM_023119:-457	Eno1	PROMOTER	0.648	0.415	1665.56	690.86	0.269	1403.49	377.46
A_68_P23308584	chr4:131392713-131392757	NM_001083119:1459	Ptpru	INSIDE	0.648	0.631	11428.85	7210.40	0.409	5778.40	2361.17
A_68_P22898162	chr4:48057954-48057998	NM_015743:-6143	Nr4a3	PROMOTER	0.648	0.237	2002.91	473.77	0.153	1487.38	227.84
A_68_P21132202	chr2:35056385-35056429	NM_026697:234	Rab14	INSIDE	0.648	3.448	3263.21	11251.83	2.236	2668.65	5966.79
A_68_P21071034	chr2:25127984-25128028	NM_175300:21	Anapc2	INSIDE	0.648	0.392	1960.60	768.65	0.254	1469.93	373.31
A_68_P20231548	chr1:53409367-53409411	NM_133728:155	Asnsd1	INSIDE	0.648	0.322	2192.29	706.62	0.209	1763.07	368.04
A_68_P32351250	chrX:42142934-42142979	NM_001190718:382	Dcaf12l1	INSIDE	0.647	2.623	1042.05	2733.41	1.698	1366.22	2320.25
A_68_P32263512	chrX:12339036-12339080	NM_001048208:41	Med14	INSIDE	0.647	12.249	703.71	8619.50	7.926	926.72	7345.63
A_68_P31757535	chr18:61559223-61559268	NM_133249:840	Ppargc1b	INSIDE	0.647	2.812	5430.66	15270.23	1.819	4172.25	7587.30
A_68_P31151553	chr17:34161608-34161652	NM_009066:-5	Ring1	PROMOTER	0.647	2.212	3117.56	6896.96	1.431	2534.09	3626.12
A_68_P30964660	chr16:91728640-91728684	NM_026994:385	Cryzl1	INSIDE	0.647	0.289	2540.49	735.24	0.187	2001.12	374.79
A_68_P30576539	chr16:17915177-17915221	NM_029469:-46	Gsc2	PROMOTER	0.647	0.469	1225.31	574.14	0.303	989.77	300.03
A_68_P29718152	chr14:73952682-73952726	NM_011506:112	Sucla2	INSIDE	0.647	9.240	1843.41	17033.81	5.981	1522.85	9108.78
A_68_P29581905	chr14:47993552-47993596	NM_001145953:40	Lgals3	INSIDE	0.647	4.124	2821.85	11637.06	2.667	2372.65	6326.72

ProbeName	Target position of probe on CpG island microarray	TargetID	GeneSymbol	CpG island Description	Ratio of relative methylation (TiO_2-NP/Vehicle)	Sham group			TiO_2-H group		
						Relative methylation (Cy5/Cy3)	Cy3 signal (Input)	Cy5 signal (MeDIP)	Relative methylation (Cy5/Cy3)	Cy3 signal (Input)	Cy5 signal (MeDIP)
A_68_P28675864	chr12:100630527-100630571	NM_183186:57736	Foxn3	INSIDE	0.647	0.263	1709.47	449.21	0.170	1345.47	228.62
A_68_P28593012	chr12:84827504-84827548	NM_058212:1132	Dpf3	INSIDE	0.647	0.179	5407.48	967.52	0.116	3580.43	414.76
A_68_P28083321	chr11:102962810-102962854	NM_152813:140	Plcd3	INSIDE	0.647	2.388	1137.71	2717.33	1.545	1007.27	1556.26
A_68_P28079748	chr11:102328467-102328511	NM_010575:2709	Itga2b	INSIDE	0.647	0.627	2262.61	1419.22	0.406	1638.68	664.68
A_68_P27430008	chr10:107599648-107599692	NM_027892:215	Ppp1r12a	INSIDE	0.647	4.061	1526.10	6197.06	2.626	1270.34	3336.52
A_68_P27180609	chr10:60915255-60915299	NM_010124:141	Eif4ebp2	INSIDE	0.647	2.238	1651.43	3696.51	1.448	1424.98	2063.05
A_68_P25835333	chr8:47377718-47377762	NM_173789:2285	Helt	INSIDE	0.647	0.388	1629.76	632.28	0.251	1314.19	329.79
A_68_P25834907	chr8:47296398-47296442	NM_007450:-57	Slc25a4	PROMOTER	0.647	0.501	1598.02	799.97	0.324	939.14	303.99
A_68_P25780168	chr8:36557978-36558022	NM_026067:587	Eri1	INSIDE	0.647	0.429	1898.39	814.57	0.278	1444.71	401.08
A_68_P24591291	chr6:83027998-83028042	NM_197992:-363	Pcgf1	DIVERGENT_PROMOTER	0.647	0.572	1747.29	999.98	0.370	1462.91	541.51
A_68_P24448786	chr6:52122729-52122773	NM_010452:40316	Hoxa3	INSIDE	0.647	5.949	2940.50	17492.75	3.850	2448.92	9427.52
A_68_P24127316	chr5:138182800-138182844	NM_175521:-1596	6430598A04Rik	DIVERGENT_PROMOTER	0.647	0.502	1555.06	781.16	0.325	1189.41	386.53
A_68_P23591967	chr5:34326256-34326300	NM_001033458:156	Gm1673	INSIDE	0.647	0.222	4414.50	981.23	0.144	3189.89	458.66
A_68_P23585886	chr5:33206293-33206337	NM_177786:-55	Depdc5	DIVERGENT_PROMOTER	0.647	2.331	1799.56	4194.97	1.509	1584.44	2390.16
A_68_P23441280	chr4:155265412-155265456	NM_001033490:437	Pusl1	INSIDE	0.647	0.664	2371.53	1574.93	0.430	1733.88	745.04
A_68_P23364649	chr4:141094339-141094383	NM_019763:152	Spen	INSIDE	0.647	3.140	2734.34	8586.71	2.031	2255.10	4580.05
A_68_P23331510	chr4:135528254-135528298	NM_011942:233	Lypla2	INSIDE	0.647	2.254	1559.64	3514.85	1.458	1320.09	1924.78
A_68_P23062645	chr4:83131826-83131870	NM_133948:446	Psip1	INSIDE	0.647	3.379	1664.25	5622.74	2.187	1451.95	3175.41
A_68_P22301214	chr3:85946197-85946241	NR_004418:-1609	Rnu73b	PROMOTER	0.647	0.433	1183.61	512.76	0.280	957.74	268.48
A_68_P21087491	chr2:27741628-27741672	NM_015734:-294	Col5a1	PROMOTER	0.647	0.529	1285.82	680.59	0.342	997.70	341.60
A_68_P21047274	chr2:19831411-19831455	ENSMUST00000114610:26		INSIDE	0.647	5.724	918.84	5259.50	3.706	769.86	2853.30
A_68_P20239573	chr1:54982711-54982755	NM_001081433:499	Ankrd44	INSIDE	0.647	0.337	2696.88	908.84	0.218	2052.25	447.36
A_68_P31930563	chr19:5742280-5742324	NM_008520:1399	Ltbp3	INSIDE	0.646	4.488	979.33	4395.29	2.900	820.07	2378.51
A_68_P31927885	chr19:5295364-5295408	NM_030109:69	Sf3b2	INSIDE	0.646	2.931	901.21	2641.50	1.893	655.96	1241.73
A_68_P31836390	chr18:75503087-75503131			Unknown	0.646	4.271	1697.10	7248.67	2.758	1505.12	4151.57
A_68_P31754305	chr18:61071638-61071682	NM_001038499:-233	Arsi	PROMOTER	0.646	0.400	2206.38	881.90	0.258	1694.14	437.22
A_68_P30345268	chr15:75812328-75812373	NM_001163518:365	2410075B13Rik	INSIDE	0.646	3.343	871.00	2911.39	2.159	684.44	1477.49
A_68_P28271087	chr12:17551396-17551440	NM_013614:-260	Odc1	PROMOTER	0.646	0.497	1751.27	870.06	0.321	1203.10	386.08
A_68_P28167619	chr11:117721965-117722009	NM_001085535:-79	Cldn27	PROMOTER	0.646	0.597	4671.79	2786.95	0.386	3294.92	1270.53
A_68_P27933172	chr11:76437300-76437344	ENSMUST00000155035:-1506		PROMOTER	0.646	0.357	2833.79	1011.26	0.231	2017.51	465.12
A_68_P27851242	chr11:61743468-61743512	NM_019921:238	Akap10	INSIDE	0.646	0.659	3257.15	2146.56	0.426	2388.34	1017.04
A_68_P27502792	chr10:120747561-120747605	NM_029057:663	Tbc1d30	INSIDE	0.646	2.864	2890.32	8277.53	1.849	2310.94	4273.08
A_68_P26770338	chr9:100471400-100471444	NM_001101483:82	Tmem22	INSIDE	0.646	2.889	867.28	2505.38	1.866	777.91	1451.59
A_68_P26017803	chr8:86566236-86566280	NM_016671:216	Il27ra	INSIDE	0.646	0.470	3994.59	1878.06	0.304	2955.02	897.99
A_68_P25910880	chr8:64070053-64070097	NM_001081390:311413	Palld	INSIDE	0.646	9.035	3129.17	28272.33	5.839	2664.18	15556.16
A_68_P25837068	chr8:47703178-47703222	NM_009810:398	Casp3	INSIDE	0.646	0.504	2655.47	1339.09	0.326	2158.43	702.81
A_68_P25597928	chr7:150154114-150154158	NM_008554:1033	Ascl2	INSIDE	0.646	0.623	730.11	455.21	0.403	656.10	264.22
A_68_P24980698	chr7:16881008-16881052	NM_001136471:-8886	Ccdc9	PROMOTER	0.646	3.116	1777.56	5539.41	2.014	1404.98	2829.57
A_68_P24160783	chr5:145118513-145118557	NM_025833:447	Baiap2l1	INSIDE	0.646	0.504	2487.14	1254.62	0.326	1963.71	640.34
A_68_P23794550	chr5:73305332-73305376	NM_001113423:-201	Slain2	PROMOTER	0.646	2.350	1936.76	4551.74	1.518	1457.36	2212.50
A_68_P23441288	chr4:155266214-155266258	NM_207223:253	Acap3	INSIDE	0.646	0.434	2115.65	917.93	0.280	1581.50	442.94
A_68_P23323200	chr4:134067066-134067110	NM_001081099:175	2610002D18Rik	INSIDE	0.646	0.349	6404.91	2237.63	0.226	4041.83	912.63
A_68_P21240022	chr2:56969332-56969376	NM_013613:-1905	Nr4a2	PROMOTER	0.646	3.098	1519.54	4708.16	2.002	1291.94	2586.20
A_68_P21102112	chr2:30112106-30112150	NM_177725:18840	Lrrc8a	INSIDE	0.646	3.503	723.21	2533.17	2.261	556.17	1257.66
A_68_P32363781	chrX:45638888-45638932	NM_028276:28800	Utp14a	DOWNSTREAM	0.645	5.807	3357.09	19493.72	3.748	3802.11	14248.69
A_68_P31731697	chr18:56867382-56867426	NM_010721:-62	Lmnb1	PROMOTER	0.645	3.522	634.40	2234.67	2.272	700.03	1590.76
A_68_P30370941	chr15:80064556-80064600	NM_178719:69	Smcr7l	INSIDE	0.645	0.343	5045.16	1728.90	0.221	3632.03	802.34
A_68_P29928902	chr14:117325279-117325323	NM_001079844:764	Gpc6	INSIDE	0.645	0.631	792.47	500.33	0.407	613.24	249.71
A_68_P29681987	chr14:67530058-67530102	NM_175498:36	Pnma2	INSIDE	0.645	0.735	2691.80	1978.50	0.474	2134.64	1011.81
A_68_P28336340	chr12:33063936-33063980	NM_001162903:305	2010109K11Rik	INSIDE	0.645	0.424	2377.78	1007.94	0.273	1819.75	497.23
A_68_P27278518	chr10:79187320-79187368	NM_008226:7966	Hcn2	INSIDE	0.645	4.968	177.15	880.01	3.205	190.86	611.75
A_68_P27167593	chr10:58684415-58684459	NM_001024910:159	Septin10	INSIDE	0.645	3.677	587.10	2158.75	2.371	411.95	976.58
A_68_P26477175	chr9:45792523-45792567	NM_008775:410	Pafah1b2	INSIDE	0.645	2.810	2243.23	6303.73	1.813	1741.72	3157.73
A_68_P26248321	chr8:127420511-127420555	NM_198103:1073	Exoc8	INSIDE	0.645	0.589	961.84	566.73	0.380	646.50	245.74
A_68_P26016847	chr8:86399178-86399222			Unknown	0.645	3.647	1453.16	5300.14	2.352	1268.38	2982.82
A_68_P25840857	chr8:48373280-48373324	NM_001114311:1454	Stox2	INSIDE	0.645	0.339	1993.07	676.63	0.219	1485.65	325.14
A_68_P25657384	chr8:11758629-11758673	NM_001113518:321	Arhgef7	INSIDE	0.645	0.727	2060.47	1497.35	0.469	1664.22	780.17
A_68_P25592835	chr7:149268541-149268585	NM_008748:12627	Dusp8	INSIDE	0.645	2.990	759.02	2269.26	1.927	544.80	1049.88
A_68_P25261270	chr7:86610733-86610777	NM_017462:405	Polg	INSIDE	0.645	3.370	2568.92	8658.13	2.174	2046.11	4448.13
A_68_P24786762	chr6:118369695-118369739	NM_194339:-281	Bms1	PROMOTER	0.645	0.627	1175.95	737.11	0.404	975.67	394.44
A_68_P24602879	chr6:85019299-85019343	NM_177077:187	Exoc6b	INSIDE	0.645	0.276	2806.97	775.79	0.178	2468.89	440.04
A_68_P24520694	chr6:67217359-67217403	NM_001113564:408	Serbp1	INSIDE	0.645	3.310	612.14	2026.20	2.135	561.04	1197.95
A_68_P24449229	chr6:52175914-52175958	NM_010456:1433	Hoxa9	INSIDE	0.645	2.952	1625.79	4799.94	1.906	1360.77	2593.18
A_68_P23417739	chr4:151660365-151660409	NM_008237:5385	Hes3	INSIDE	0.645	2.597	4746.14	12325.37	1.676	3047.20	5105.66
A_68_P23396198	chr4:148116954-148116998	AK142257:4167		DOWNSTREAM	0.645	3.842	757.28	2909.77	2.477	626.32	1551.35

ProbeName	Target position of probe on CpG island microarray	TargetID	GeneSymbol	CpG island Description	Ratio of relative methylation (TiO_2-NP/Vehicle)	Sham group			TiO_2-H group		
						Relative methylation (Cy5/Cy3)	Cy3 signal (Input)	Cy5 signal (MeDIP)	Relative methylation (Cy5/Cy3)	Cy3 signal (Input)	Cy5 signal (MeDIP)
A_68_P22871971	chr4:43506143-43506188	NM_001037913:-71	Ccdc107	DIVERGENT_PROMOTER	0.645	3.983	415.50	1654.94	2.569	357.08	917.49
A_68_P22771644	chr4:20705685-20705729	NM_172987:109	Nkain3	INSIDE	0.645	0.430	2626.42	1128.41	0.277	2023.30	561.00
A_68_P21772346	chr2:156440160-156440204	NM_146128:742	Dlgap4	INSIDE	0.645	0.373	1732.86	646.62	0.241	1273.89	306.50
A_68_P21069553	chr2:24909996-24910040	NM_001039386:120	Nelf	INSIDE	0.645	12.491	1079.55	13485.03	8.051	1149.52	9254.74
A_68_P20551183	chr1:120207585-120207629	NM_011650:103	Tsn	INSIDE	0.645	0.612	2139.88	1308.59	0.394	1646.87	649.15
A_68_P20412615	chr1:88425095-88425139	NM_008972:1806	Ptma	INSIDE	0.645	0.372	1629.33	605.92	0.240	1127.57	270.47
A_68_P20153982	chr1:37486790-37486834	NM_198006:136	6330578E17Rik	INSIDE	0.645	2.897	3632.44	10521.95	1.868	3140.86	5865.68
A_68_P31931196	chr19:5845867-5845911	NR_003513:-410	Neat1	PROMOTER	0.644	0.271	4089.18	1107.93	0.174	2804.98	489.26
A_68_P31619415	chr18:35812025-35812069	NM_027222:-3025	2010001M09Rik	PROMOTER	0.644	12.405	1414.29	17544.92	7.989	1386.57	11076.80
A_68_P31415139	chr17:86567438-86567482	NM_011104:336	Prkce	INSIDE	0.644	0.473	1427.23	674.44	0.304	1127.50	343.29
A_68_P31187140	chr17:43154341-43154385	NM_178589:859	Tnfrsf21	INSIDE	0.644	0.391	4120.10	1610.04	0.252	2750.37	691.98
A_68_P30388745	chr15:83319976-83320026	NM_178869:21337	Ttll1	INSIDE	0.644	2.992	969.10	2899.88	1.928	713.86	1376.17
A_68_P30133052	chr15:35301302-35301346	NM_177151:24	Vps13b	INSIDE	0.644	2.684	715.80	1921.11	1.728	608.95	1052.33
A_68_P29531946	chr14:35632404-35632448	NM_008166:-895	Grid1	PROMOTER	0.644	2.821	750.83	2118.01	1.817	548.21	996.24
A_68_P28729930	chr12:110032477-110032521	NM_009537:978	Yy1	INSIDE	0.644	2.855	1236.34	3529.54	1.840	1091.73	2008.52
A_68_P28087325	chr11:103671166-103671210	NM_009521:35700	Wnt3	INSIDE	0.644	4.443	1423.09	6323.34	2.861	1176.17	3365.44
A_68_P28057144	chr11:98412585-98412629	NM_001166494:196	Zpbp2	INSIDE	0.644	0.183	2926.84	535.78	0.118	2291.00	270.08
A_68_P28055911	chr11:98219606-98219650	NM_021547:-69	Stard3	PROMOTER	0.644	0.419	2385.51	998.46	0.270	1924.84	518.80
A_68_P27921510	chr11:74433466-74433510	NM_001013784:382	E130309D14Rik	INSIDE	0.644	14.844	2517.81	37374.31	9.561	2197.74	21012.85
A_68_P27835630	chr11:59041516-59041560	NM_001130408:126	Arf1	INSIDE	0.644	3.317	1943.21	6445.09	2.137	1591.19	3399.69
A_68_P27288225	chr10:80695805-80695849	NM_001034895:-81	Zfr2	DIVERGENT_PROMOTER	0.644	0.425	5747.10	2441.83	0.274	4280.50	1171.40
A_68_P27287625	chr10:80599890-80599934	NM_010731:897	Zbtb7a	INSIDE	0.644	0.482	2797.79	1349.20	0.311	2035.18	631.94
A_68_P27278360	chr10:79167439-79167483	NM_001077184:358	Bsg	INSIDE	0.644	0.526	2025.61	1066.37	0.339	1452.92	492.95
A_68_P26804663	chr9:106587992-106588036	NM_011573:-16	Tex264	PROMOTER	0.644	2.753	1150.34	3166.63	1.772	959.85	1701.16
A_68_P26766660	chr9:99776718-99776762	NM_011440:-151	Sox14	PROMOTER	0.644	0.416	1413.09	587.69	0.268	1250.45	334.78
A_68_P25995580	chr8:82162925-82162969	NM_001081164:-628	Otud4	PROMOTER	0.644	2.173	2281.28	4957.91	1.400	1862.88	2608.89
A_68_P25657382	chr8:11758359-11758403	NM_001113518:51	Arhgef7	INSIDE	0.644	0.351	1726.43	606.56	0.226	1432.94	324.29
A_68_P25503619	chr7:134164872-134164916	NM_001102563:-169	Prrt2	PROMOTER	0.644	0.471	2117.54	996.99	0.303	1571.45	476.48
A_68_P25089969	chr7:52147509-52147554	NM_029365:205	Med25	INSIDE	0.644	3.690	1616.10	5963.83	2.376	1302.29	3094.39
A_68_P25031592	chr7:31239092-31239136	NM_172898:3420	Kirrel2	INSIDE	0.644	0.534	1216.70	649.59	0.344	1045.48	359.56
A_68_P24360135	chr6:35127937-35127981	NM_027513:343	Nup205	INSIDE	0.644	0.395	1167.59	460.62	0.254	918.89	233.45
A_68_P24048831	chr5:122608072-122608116	NM_013636:-193	Ppp1cc	PROMOTER	0.644	2.715	2745.16	7453.73	1.748	2260.82	3950.81
A_68_P23315558	chr4:132617847-132617891	NM_146155:50448	Ahdc1	INSIDE	0.644	3.951	2820.22	11143.16	2.544	2120.44	5394.30
A_68_P22152358	chr3:55046521-55046565	NM_001111053:95	Dclk1	INSIDE	0.644	0.606	1276.15	772.82	0.390	1022.32	398.62
A_68_P21338756	chr2:74520344-74520390	NM_008273:-83	Hoxd11	PROMOTER	0.644	0.497	922.46	458.51	0.320	703.39	225.13
A_68_P20640400	chr1:137481754-137481798	NM_173437:156	Nav1	INSIDE	0.644	2.835	1100.91	3121.40	1.825	1033.12	1885.40
A_68_P20455651	chr1:95342033-95342077	NM_133808:33331	Hdlbp	INSIDE	0.644	2.679	1747.31	4681.35	1.724	1468.56	2532.06
A_68_P32260336	chrX:11664364-11664408	NM_029510:-6707	Bcor	PROMOTER	0.643	3.485	228.43	796.04	2.241	339.15	760.05
A_68_P31950785	chr19:10531094-10531138	NM_001111140:821	Lrrc10b	INSIDE	0.643	3.153	1144.95	3610.39	2.028	956.13	1938.94
A_68_P31937106	chr19:7054484-7054528	NM_008020:424	Fkbp2	INSIDE	0.643	0.341	4590.59	1564.47	0.219	3043.90	666.89
A_68_P31630106	chr18:37965667-37965711	NM_033581:-375	Pcdhgc3	PROMOTER	0.643	0.466	1514.64	706.44	0.300	1307.46	392.29
A_68_P30347360	chr15:76124857-76124901	NM_001195537:15	Gm10345	INSIDE	0.643	0.626	1141.46	715.00	0.403	933.41	375.72
A_68_P30330811	chr15:73342672-73342717	NM_001081066:-295	Dennd3	PROMOTER	0.643	0.729	1609.56	1172.67	0.469	1273.10	596.73
A_68_P28224331	chr12:8779590-8779634	NM_011519:1411	Sdc1	INSIDE	0.643	0.338	3446.53	1166.25	0.218	2546.27	553.94
A_68_P28071133	chr11:100820100-100820144	NM_008986:11809	Ptrf	INSIDE	0.643	5.573	2471.99	13777.32	3.586	1786.65	6406.05
A_68_P27994864	chr11:87392137-87392181	NM_011129:-2474	Septin4	PROMOTER	0.643	0.723	1534.82	1109.96	0.465	1265.72	588.28
A_68_P26658159	chr9:77956440-77956484	NM_023605:1	Fbxo9	INSIDE	0.643	5.768	1187.16	6847.55	3.707	997.51	3698.27
A_68_P26217898	chr8:122625658-122625702	NM_133967:-308	Zdhhc7	PROMOTER	0.643	0.451	1487.19	671.46	0.290	1257.00	364.67
A_68_P25948479	chr8:72406005-72406049	NM_026818:5265	Cilp2	INSIDE	0.643	3.411	625.37	2132.93	2.194	490.74	1076.68
A_68_P25507079	chr7:134895761-134895805	NM_172748:5494	Fbxl19	INSIDE	0.643	2.990	2144.60	6411.55	1.922	1698.71	3265.60
A_68_P24990865	chr7:19590861-19590905	NM_178757:1469	Irf2bp1	INSIDE	0.643	3.396	1532.94	5205.87	2.183	1355.98	2960.64
A_68_P23756355	chr5:65826987-65827031	NM_009466:73	Ugdh	INSIDE	0.643	3.100	475.49	1473.90	1.992	405.65	808.03
A_68_P23416730	chr4:151506015-151506059	NM_019585:-3002	Espn	PROMOTER	0.643	0.545	3268.94	1782.97	0.351	2509.66	880.03
A_68_P23234771	chr4:117169165-117169209	NM_025739:334	Rnf220	INSIDE	0.643	0.247	2128.46	526.55	0.159	1549.72	246.35
A_68_P23120696	chr4:94716722-94716767	NM_010591:2169	Jun	INSIDE	0.643	0.547	1318.68	720.76	0.351	1165.55	409.66
A_68_P22933503	chr4:55362594-55362638	NM_009011:-297	Rad23b	PROMOTER	0.643	3.209	1822.31	5848.06	2.064	1614.64	3332.82
A_68_P22129179	chr3:51145355-51145399	ENSMUST00000135496:-796		PROMOTER	0.643	3.929	1367.26	5372.18	2.527	1108.75	2801.27
A_68_P21769580	chr2:155937622-155937666	NM_170588:57	Cpne1	INSIDE	0.643	2.976	6531.47	19437.39	1.913	4455.40	8525.27
A_68_P21580976	chr2:121275325-121275369	NM_011354:383	Serf2	INSIDE	0.643	4.699	1353.46	6359.38	3.022	1157.58	3498.15
A_68_P32143277	chr19:46403804-46403848	NM_133694:1153	Fbxl15	INSIDE	0.642	2.993	1531.12	4582.41	1.921	1269.11	2437.81
A_68_P32131310	chr19:44341435-44341479			Unknown	0.642	0.688	1053.10	724.87	0.442	876.54	387.24
A_68_P32036092	chr19:26681843-26681887	NM_011416:2215	Smarca2	INSIDE	0.642	0.350	2413.54	845.56	0.225	1580.72	355.37
A_68_P31117527	chr17:27760515-27760559	NM_019837:-139	Nudt3	PROMOTER	0.642	2.678	704.83	1887.89	1.719	661.19	1136.69
A_68_P31058955	chr17:14417592-14417636	NM_022315:1102	Smoc2	INSIDE	0.642	2.425	1610.56	3905.96	1.556	1433.62	2230.55
A_68_P30513988	chr16:5132000-5132044	NM_008909:552	Ppl	INSIDE	0.642	0.305	2063.75	629.58	0.196	1408.36	275.65

ProbeName	Target position of probe on CpG island microarray	TargetID	GeneSymbol	CpG island Description	Ratio of relative methylation (TiO_2-NP/Vehicle)	Sham group			TiO_2-H group		
						Relative methylation (Cy5/Cy3)	Cy3 signal (Input)	Cy5 signal (MeDIP)	Relative methylation (Cy5/Cy3)	Cy3 signal (Input)	Cy5 signal (MeDIP)
A_68_P29437788	chr14:19103568-19103612	NM_023526:-65	Nkiras1	DIVERGENT_PROMOTER	0.642	0.549	804.74	441.76	0.352	639.87	225.36
A_68_P29334874	chr13:113254711-113254755	NM_010560:455	Il6st	INSIDE	0.642	0.329	1513.02	497.06	0.211	1155.11	243.72
A_68_P29240371	chr13:96145165-96145209	NM_025630:103	Aggf1	INSIDE	0.642	3.406	925.52	3152.22	2.185	817.33	1786.19
A_68_P28595367	chr12:85262475-85262519	ENSMUST00000085215:298		INSIDE	0.642	3.703	2927.26	10840.21	2.376	2424.62	5761.40
A_68_P28553591	chr12:77470382-77470426	NM_001172104:47	Zbtb25	INSIDE	0.642	3.424	1190.91	4077.48	2.199	1068.76	2349.71
A_68_P28083319	chr11:102962554-102962598	NM_152813:396	Plcd3	INSIDE	0.642	0.394	2909.55	1147.04	0.253	2109.40	533.53
A_68_P27971658	chr11:83276246-83276290	NM_080453:195	Mmp28	INSIDE	0.642	3.104	703.24	2183.16	1.994	618.52	1233.18
A_68_P27804722	chr11:53244281-53244325	NM_025352:31	Uqcrq	INSIDE	0.642	3.053	715.21	2183.24	1.959	669.01	1310.57
A_68_P27782066	chr11:49057568-49057612	NM_001110148:-102	Mgat1	PROMOTER	0.642	0.719	3484.12	2505.13	0.462	2583.02	1192.59
A_68_P27552578	chr11:3435772-3435816	NM_001159284:3501	Smtn	INSIDE	0.642	2.838	1078.60	3060.70	1.822	945.63	1722.56
A_68_P26699344	chr9:86361211-86361255	NM_177208:472	Dopey1	INSIDE	0.642	3.307	976.28	3228.41	2.124	821.56	1745.33
A_68_P26548139	chr9:58335761-58335806	NM_176921:-626	6030419C18Rik	PROMOTER	0.642	2.354	2896.69	6817.76	1.510	2250.70	3399.63
A_68_P26229920	chr8:124474514-124474558	NM_001110100:104	Banp	INSIDE	0.642	0.641	6585.00	4219.47	0.411	3923.30	1612.75
A_68_P24354189	chr6:34126897-34126941	NM_021435:136	Slc35b4	INSIDE	0.642	0.618	1721.62	1064.21	0.397	1306.18	517.99
A_68_P23985069	chr5:111760075-111760119	NM_019640:314	Pitpnb	INSIDE	0.642	4.396	1669.73	7339.69	2.820	1430.10	4033.06
A_68_P23749454	chr5:64551417-64551461	NM_019636:-11	Tbc1d1	PROMOTER	0.642	2.866	1827.38	5236.48	1.841	1511.04	2781.79
A_68_P23593955	chr5:34713045-34713089	ENSMUST00000042234:5		INSIDE	0.642	5.941	570.70	3390.34	3.812	525.02	2001.62
A_68_P23398684	chr4:148478173-148478217	NM_010044:-67	Dffa	DIVERGENT_PROMOTER	0.642	0.628	3648.23	2291.78	0.403	2619.50	1056.67
A_68_P23314346	chr4:132399235-132399279	NM_146154:-172	Ppp1r8	PROMOTER	0.642	0.722	1960.31	1415.94	0.464	1512.67	701.92
A_68_P22729159	chr4:11248834-11248879	NM_001081201:422	Dpy19l4	INSIDE	0.642	0.489	2895.40	1415.73	0.314	2216.54	695.77
A_68_P22062861	chr3:37211563-37211607	NM_001008502:109	Bbs12	INSIDE	0.642	0.595	1960.97	1166.48	0.382	1486.54	567.84
A_68_P21842488	chr2:168427130-168427174	NM_001037178:3	Nfatc2	INSIDE	0.642	4.490	4171.85	18730.44	2.881	3164.34	9115.15
A_68_P21833672	chr2:166930250-166930294	NM_008420:84027	Kcnb1	INSIDE	0.642	2.750	1746.90	4803.47	1.764	1257.15	2218.09
A_68_P21395950	chr2:84877269-84877313	NM_182990:-67	Ssrp1	DIVERGENT_PROMOTER	0.642	0.127	4495.70	571.40	0.082	2994.17	244.23
A_68_P20442008	chr1:93262992-93263036	NM_173395:8	Fam132b	INSIDE	0.642	0.443	3617.60	1601.55	0.284	2724.40	774.66
A_68_P20080973	chr1:21951507-21951551	NM_001160139:495	Kcnq5	INSIDE	0.642	3.011	1926.30	5800.88	1.932	1755.16	3390.94
A_68_P31948840	chr19:10257815-10257859	NM_146094:459	Fads1	INSIDE	0.641	0.223	3251.53	725.75	0.143	2314.52	330.98
A_68_P31838822	chr18:75856443-75856487	NM_201354:886	Ctif	INSIDE	0.641	0.313	1781.11	557.22	0.200	1411.67	283.00
A_68_P30402200	chr15:85501825-85501869	AK038224:-5325		PROMOTER	0.641	6.905	579.17	3998.93	4.429	511.26	2264.39
A_68_P29510443	chr14:32019604-32019648	NM_022656:386	Nisch	INSIDE	0.641	0.518	1688.69	874.43	0.332	1213.28	402.47
A_68_P28567643	chr12:80190635-80190679	NM_029988:1	Pigh	INSIDE	0.641	0.646	1502.42	969.96	0.414	1253.38	519.04
A_68_P28334787	chr12:32745879-32745923	NM_011158:244	Prkar2b	INSIDE	0.641	3.108	1261.42	3920.80	1.991	1166.18	2322.27
A_68_P27934410	chr11:76660169-76660213	NM_007754:320	Cpd	INSIDE	0.641	3.807	916.74	3490.16	2.441	883.87	2157.37
A_68_P27924590	chr11:74982634-74982678	NM_001098203:-1002	Hic1	PROMOTER	0.641	3.948	1060.59	4187.26	2.529	916.36	2317.76
A_68_P27899519	chr11:70034182-70034226	NM_172371:292	Slc16a13	INSIDE	0.641	0.324	1565.83	507.01	0.208	1103.68	229.02
A_68_P27288939	chr10:80795142-80795186	NM_009325:3689	Tbxa2r	INSIDE	0.641	3.761	891.23	3351.91	2.412	730.81	1763.09
A_68_P27274155	chr10:77888554-77888598	NM_007793:162	Cstb	INSIDE	0.641	4.113	1473.82	6062.23	2.635	1212.51	3195.37
A_68_P27127238	chr10:49508642-49508686	NM_001111268:-104	Grik2	PROMOTER	0.641	0.605	1115.84	675.54	0.388	881.53	342.05
A_68_P25784088	chr8:37210172-37210216	NM_172911:52313	D8Ertd82e	INSIDE	0.641	3.319	420.63	1395.89	2.127	339.78	722.82
A_68_P25427821	chr7:120097865-120097909	NM_175279:411	Rassf10	INSIDE	0.641	2.533	1643.42	4162.07	1.624	1471.94	2389.76
A_68_P24831080	chr6:127105235-127105279	NM_009829:-4190	Ccnd2	PROMOTER	0.641	0.525	1828.51	960.50	0.337	1447.77	487.82
A_68_P24055363	chr5:123823490-123823534	NM_029850:29056	Bcl7a	INSIDE	0.641	2.601	2208.81	5745.64	1.666	1665.99	2776.07
A_68_P23513996	chr5:18733426-18733470	NM_001170746:585	Magi2	INSIDE	0.641	0.715	5245.02	3749.67	0.459	3734.41	1712.63
A_68_P23155326	chr4:101169630-101169674	NM_198412:400	Dnajc6	INSIDE	0.641	0.565	2181.97	1232.96	0.362	1629.31	590.61
A_68_P22411405	chr3:108029325-108029369	NM_008596:171	Sypl2	INSIDE	0.641	0.503	4473.85	2250.19	0.322	3493.37	1126.39
A_68_P22323185	chr3:89855913-89855957	NM_028475:463	Ubap2l	INSIDE	0.641	0.473	1217.32	576.05	0.303	885.71	268.60
A_68_P20405120	chr1:84692097-84692141	NM_152915:678	Dner	INSIDE	0.641	2.937	1204.93	3538.73	1.884	976.32	1839.23
A_68_P32975877	A_68_P32975877			Unknown	0.640	2.623	2032.29	5330.76	1.679	1548.62	2599.71
A_68_P32959961	A_68_P32959961			Unknown	0.640	5.822	611.67	3561.19	3.728	470.98	1755.70
A_68_P32118991	chr19:42165475-42165519	NM_145501:572	Pi4k2a	INSIDE	0.640	0.379	3153.55	1195.75	0.243	2187.46	531.14
A_68_P31863635	chr18:80519494-80519538	NM_001190373:41477	Kcng2	INSIDE	0.640	3.831	441.64	1691.72	2.452	409.67	1004.58
A_68_P31213969	chr17:47875277-47875321	NM_001161723:419	Tcfeb	INSIDE	0.640	6.032	367.39	2216.08	3.859	322.20	1243.24
A_68_P30961686	chr16:91225569-91225613	NM_016967:-204	Olig2	PROMOTER	0.640	0.304	2629.07	800.21	0.195	1875.02	364.98
A_68_P30428912	chr15:90055009-90055053	NM_001033441:289	Alg10b	INSIDE	0.640	0.505	1377.48	695.64	0.323	1157.67	373.94
A_68_P30345384	chr15:75829239-75829283	NM_177922:5062	Mapk15	INSIDE	0.640	3.480	1737.40	6047.00	2.226	1423.99	3169.53
A_68_P29751622	chr14:79881067-79881111	NM_018765:-13	Wbp4	PROMOTER	0.640	0.505	1964.99	992.14	0.323	1592.47	514.21
A_68_P29421307	chr14:15536004-15536048	NM_001033270:488	Slc4a7	INSIDE	0.640	5.139	809.97	4162.26	3.290	747.28	2458.52
A_68_P29292645	chr13:105607251-105607295	NM_026075:-46	Srek1ip1	DIVERGENT_PROMOTER	0.640	0.386	1304.00	503.88	0.247	1120.17	277.20
A_68_P29268695	chr13:101262403-101262447	NM_022011:140	Gtf2h2	INSIDE	0.640	0.575	3647.85	2097.80	0.368	2948.48	1086.01
A_68_P29145568	chr13:74454823-74454867	NM_011051:-70	Pdcd6	PROMOTER	0.640	0.705	3326.22	2345.03	0.451	2534.64	1143.93
A_68_P28031427	chr11:94073436-94073480	NM_009427:691	Tob1	INSIDE	0.640	2.800	629.15	1761.61	1.791	671.49	1202.80
A_68_P27969032	chr11:82721609-82721653	NM_145431:267	Nle1	INSIDE	0.640	0.637	1634.68	1041.90	0.408	1275.09	519.74
A_68_P26068285	chr8:95379185-95379229	NM_173014:-42	Lpcat2	PROMOTER	0.640	10.461	2816.51	29462.88	6.694	2570.22	17204.16
A_68_P25471037	chr7:128061761-128061805	NM_001164096:525	Polr3e	INSIDE	0.640	0.712	1639.80	1167.97	0.456	1203.18	548.86
A_68_P25102013	chr7:54175369-54175413	NM_021884:-90	Tsg101	PROMOTER	0.640	2.745	991.35	2721.16	1.756	866.74	1522.17

ProbeName	Target position of probe on CpG island microarray	TargetID	GeneSymbol	CpG island Description	Ratio of relative methylation (TiO_2-NP/Vehicle)	Sham group			TiO_2-H group		
						Relative methylation (Cy5/Cy3)	Cy3 signal (Input)	Cy5 signal (MeDIP)	Relative methylation (Cy5/Cy3)	Cy3 signal (Input)	Cy5 signal (MeDIP)
A_68_P25035342	chr7:31935428-31935472	NM_027898:619	Gramd1a	INSIDE	0.640	0.534	4880.07	2604.44	0.341	3218.51	1098.69
A_68_P24691288	chr6:100237003-100237047	NM_019743:328	Rybp	INSIDE	0.640	4.719	1032.72	4872.93	3.020	924.48	2792.05
A_68_P24431605	chr6:49164853-49164897	NM_023670:79	Igf2bp3	INSIDE	0.640	2.446	1200.20	2935.23	1.564	904.04	1413.99
A_68_P24052806	chr5:123349603-123349647	NM_013910:735	Kdm2b	INSIDE	0.640	2.523	771.54	1946.25	1.615	743.18	1200.35
A_68_P24046831	chr5:122293021-122293065	NM_175474:-5994	Fam109a	DIVERGENT_PROMOTER	0.640	5.738	264.38	1517.13	3.670	240.81	883.83
A_68_P23618575	chr5:38668403-38668447	NM_172709:-218	Otop1	PROMOTER	0.640	2.865	3587.41	10278.44	1.834	2748.74	5040.53
A_68_P23325212	chr4:134408532-134408576	NM_025382:706	Tmem57	INSIDE	0.640	2.773	2056.23	5701.01	1.774	1814.54	3218.63
A_68_P23227298	chr4:115690557-115690601	NM_010173:-71	Faah	PROMOTER	0.640	3.537	687.61	2432.34	2.264	611.15	1383.39
A_68_P22301529	chr3:86028379-86028423	NM_001077687:-211	Lrba	PROMOTER	0.640	0.236	2156.75	509.86	0.151	1729.16	261.64
A_68_P22029229	chr3:30501750-30501794	NM_030557:764	Mynn	INSIDE	0.640	0.613	1888.13	1156.60	0.392	1555.51	609.73
A_68_P21646220	chr2:133378597-133378641	NM_007553:-316	Bmp2	PROMOTER	0.640	0.453	2075.40	939.62	0.290	1603.24	464.64
A_68_P20961515	chr2:3201949-3201993	NM_008708:411	Nmt2	INSIDE	0.640	0.568	1655.99	940.02	0.363	593.47	215.52
A_68_P20872224	chr1:181733633-181733677	NM_026375:492	Ahctf1	INSIDE	0.640	3.354	1185.52	3975.65	2.147	966.20	2074.22
A_68_P20723684	chr1:154750088-154750132	NM_001005507:-334	Smg7	PROMOTER	0.640	4.347	1157.82	5033.32	2.781	1103.91	3070.25
A_68_P20561486	chr1:122238913-122238957	NM_207233:1776	C1ql2	INSIDE	0.640	2.521	913.58	2302.76	1.614	780.50	1259.90
A_68_P32219569	chr19:59533670-59533714	NM_010132:513	Emx2	INSIDE	0.639	4.411	751.09	3312.96	2.820	593.46	1673.49
A_68_P32152318	chr19:47929317-47929361	NM_010362:-140	Gsto1	PROMOTER	0.639	0.463	1455.41	673.73	0.296	928.36	274.80
A_68_P32137226	chr19:45309781-45309825	NM_010691:-76	Lbx1	PROMOTER	0.639	21.413	1573.67	33696.85	13.678	1477.24	20206.26
A_68_P31862861	chr18:80389593-80389637	NM_199197:7744	Rbfa	INSIDE	0.639	4.141	703.34	2912.61	2.644	543.45	1437.11
A_68_P31150957	chr17:34053104-34053148	NM_020625:6	Zbtb22	INSIDE	0.639	7.579	10250.51	77688.67	4.842	9088.49	44010.86
A_68_P31121838	chr17:28466106-28466150	NM_011287:713	Rpl10a	INSIDE	0.639	2.559	1046.11	2676.97	1.634	836.82	1367.68
A_68_P30652424	chr16:32430803-32430847	NM_009981:-182	Pcyt1a	PROMOTER	0.639	0.580	2424.65	1405.91	0.371	1787.92	662.81
A_68_P29480407	chr14:26722558-26722602	NM_207229:-201	Plac9	PROMOTER	0.639	0.250	8892.07	2225.30	0.160	6775.33	1084.23
A_68_P29057257	chr13:55927883-55927927	NM_011097:4882	Pitx1	INSIDE	0.639	0.329	1995.88	655.69	0.210	1477.08	310.31
A_68_P28745355	chr12:112910496-112910540	NM_021273:31	Ckb	INSIDE	0.639	2.904	2004.53	5821.22	1.855	1646.20	3052.88
A_68_P28599061	chr12:85911065-85911109	NM_007701:285	Vsx2	INSIDE	0.639	0.339	1748.42	593.53	0.217	1463.05	317.31
A_68_P28336338	chr12:33063643-33063687	NM_001162903:11	2010109K11Rik	INSIDE	0.639	2.533	2129.11	5393.37	1.618	1804.96	2919.64
A_68_P28067422	chr11:100181743-100181787	NM_011508:455	Eif1	INSIDE	0.639	0.311	1870.18	581.86	0.199	1481.16	294.53
A_68_P28043442	chr11:96145448-96145492	NM_010461:2252	Hoxb8	INSIDE	0.639	4.287	2430.10	10416.96	2.740	2032.91	5569.52
A_68_P27320006	chr10:86956365-86956409	NM_008553:19	Ascl1	INSIDE	0.639	0.418	1195.03	498.99	0.267	976.15	260.52
A_68_P27272484	chr10:77632233-77632277	NM_138601:259	D10Jhu81e	INSIDE	0.639	0.435	1509.80	656.28	0.278	1271.90	353.39
A_68_P25637837	chr8:8690176-8690220	NM_176849:339	Arglu1	INSIDE	0.639	3.949	511.74	2020.94	2.523	505.08	1274.31
A_68_P25495193	chr7:132695650-132695696	NM_001008700:-123	Il4ra	PROMOTER	0.639	0.132	4626.35	610.06	0.084	3153.96	265.87
A_68_P25362045	chr7:106617271-106617315	NM_028145:2779	Klhl35	INSIDE	0.639	4.654	648.90	3019.75	2.973	490.49	1458.41
A_68_P25194961	chr7:74790016-74790060	NM_175124:84	Lrrc28	INSIDE	0.639	0.478	1833.84	877.09	0.306	1377.77	421.07
A_68_P25007548	chr7:25739804-25739848			Unknown	0.639	0.366	5331.34	1952.07	0.234	3685.77	862.21
A_68_P24983771	chr7:17470567-17470612	NM_001081655:9924	Dact3	INSIDE	0.639	3.453	835.06	2883.06	2.205	701.54	1546.76
A_68_P24172963	chr5:148116041-148116085	NM_007673:2763	Cdx2	INSIDE	0.639	3.544	872.26	3091.52	2.266	721.71	1635.18
A_68_P24063275	chr5:125343770-125343814	NM_001081750:718	Zfp664	INSIDE	0.639	4.844	2298.29	11133.67	3.096	1910.48	5914.66
A_68_P24046045	chr5:122162695-122162739	NM_009125:1099	Atxn2	INSIDE	0.639	0.688	2600.89	1789.98	0.440	1941.80	854.28
A_68_P23968426	chr5:108152356-108152400	NM_010278:985	Gfi1	INSIDE	0.639	0.178	3371.59	601.63	0.114	2289.08	261.20
A_68_P23439342	chr4:154938332-154938376	NM_001159637:431	Nadk	INSIDE	0.639	0.469	1458.15	683.82	0.300	1189.20	356.32
A_68_P23333957	chr4:135998981-135999032	NM_008309:19568	Htr1d	INSIDE	0.639	5.482	240.30	1317.21	3.504	187.29	656.28
A_68_P21817620	chr2:164226294-164226338	NM_009036:-2377	Rbpjl	PROMOTER	0.639	2.983	437.30	1304.63	1.907	433.73	827.02
A_68_P20977349	chr2:6021517-6021561	NM_001159657:29693	5430407P10Rik	INSIDE	0.639	2.809	903.63	2538.23	1.795	784.06	1407.65
A_68_P20561482	chr1:122238470-122238514	NM_207233:1334	C1ql2	INSIDE	0.639	3.274	2078.14	6803.77	2.091	1698.50	3551.38
A_68_P20153136	chr1:37357143-37357187	NM_030266:427	Inpp4a	INSIDE	0.639	0.639	1224.87	783.17	0.409	958.56	391.58
A_68_P31936369	chr19:6915237-6915281	NM_019924:-167	Rps6ka4	PROMOTER	0.638	0.407	1185.19	482.34	0.260	947.33	246.05
A_68_P31921054	chr19:4101079-4101123	NM_008851:479	Pitpnm1	INSIDE	0.638	5.453	924.78	5042.96	3.482	713.35	2483.59
A_68_P29936842	chr14:118536259-118536304	NM_021434:-67	Gpr180	DIVERGENT_PROMOTER	0.638	0.689	2485.09	1713.16	0.440	2027.47	891.64
A_68_P29531957	chr14:35633864-35633908	NM_008166:565	Grid1	INSIDE	0.638	0.315	3523.50	1108.34	0.201	2246.02	451.00
A_68_P28923670	chr13:31649941-31649985	NM_008239:-76	Foxq1	PROMOTER	0.638	0.572	1508.25	863.27	0.365	1236.15	451.09
A_68_P28744115	chr12:112680803-112680847	NM_009396:-47	Tnfaip2	PROMOTER	0.638	0.710	1804.36	1281.50	0.453	1392.86	630.78
A_68_P28036958	chr11:95006743-95006787	NM_007867:351	Dlx4	INSIDE	0.638	0.300	3997.38	1201.17	0.192	2943.26	564.37
A_68_P26560916	chr9:60642500-60642544	NM_028283:168	Uaca	INSIDE	0.638	0.621	1034.48	641.98	0.396	808.81	320.42
A_68_P26362426	chr9:24307746-24307790	NM_172920:-184	Dpy19l1	PROMOTER	0.638	4.675	232.69	1087.90	2.982	196.65	586.43
A_68_P25911981	chr8:64238686-64238730	NM_001081390:142779	Palld	INSIDE	0.638	4.294	916.27	3934.11	2.740	861.27	2360.27
A_68_P24980760	chr7:16895312-16895356	NM_133234:403	Bbc3	INSIDE	0.638	0.677	857.59	580.96	0.432	687.40	297.16
A_68_P22564417	chr3:137286650-137286694	NM_030143:37	Ddit4l	INSIDE	0.638	2.460	1423.87	3502.19	1.570	1243.61	1952.75
A_68_P22402613	chr3:106525217-106525262	NM_001039488:640	4933421E11Rik	INSIDE	0.638	0.392	3218.11	1261.44	0.250	2305.07	576.03
A_68_P21902494	chr2:179991894-179991938	NM_025587:-167	Rps21	PROMOTER	0.638	18.338	2705.41	49611.95	11.705	2314.11	27085.52
A_68_P21646236	chr2:133380456-133380500	NM_007553:1544	Bmp2	INSIDE	0.638	5.024	2068.20	10390.65	3.205	1686.06	5404.34
A_68_P21516743	chr2:109533914-109533958	NM_001048142:217	Bdnf	INSIDE	0.638	5.213	1557.85	8121.61	3.327	1736.67	5777.05
A_68_P21212091	chr2:51004685-51004729	NM_028810:-75	Rnd3	PROMOTER	0.638	5.557	283.68	1576.37	3.543	201.31	713.21
A_68_P21081268	chr2:26788702-26788746	NM_011512:307	Surf4	INSIDE	0.638	2.782	1016.38	2827.59	1.776	899.72	1597.46

ProbeName	Target position of probe on CpG island microarray	TargetID	GeneSymbol	CpG island Description	Ratio of relative methylation (TiO_2-NP/Vehicle)	Sham group			TiO_2-H group		
						Relative methylation (Cy5/Cy3)	Cy3 signal (Input)	Cy5 signal (MeDIP)	Relative methylation (Cy5/Cy3)	Cy3 signal (Input)	Cy5 signal (MeDIP)
A_68_P20226307	chr1:52289184-52289228	NM_001081081:870	Gls	INSIDE	0.638	3.774	1498.09	5653.57	2.408	1254.88	3021.53
A_68_P33005815	A_68_P33005815			Unknown	0.637	4.199	316.74	1329.88	2.675	238.74	638.67
A_68_P30512787	chr16:4939335-4939379	NR_037666:229	LOC100503704	INSIDE	0.637	0.343	6087.73	2085.69	0.218	3904.97	851.66
A_68_P29314989	chr13:109906595-109906639	NM_011056:462246	Pde4d	INSIDE	0.637	0.605	1717.73	1038.81	0.385	1351.37	520.42
A_68_P28137853	chr11:112646459-112646503	NM_011448:2957	Sox9	INSIDE	0.637	0.396	6314.11	2500.50	0.252	4360.43	1100.54
A_68_P28055696	chr11:98189596-98189640	NM_010895:1341	Neurod2	INSIDE	0.637	2.765	784.84	2170.15	1.762	617.98	1088.84
A_68_P26891892	chr9:122795954-122795998	NM_001177505:-1612	Zfp167	PROMOTER	0.637	3.025	1965.24	5944.57	1.927	1655.55	3190.15
A_68_P26551398	chr9:58883897-58883941	NM_001042752:330	Neo1	INSIDE	0.637	4.150	777.53	3226.66	2.642	703.61	1858.82
A_68_P26495379	chr9:48863756-48863800	NM_012039:93	Zw10	INSIDE	0.637	0.485	1510.54	732.02	0.309	1208.78	373.30
A_68_P26356132	chr9:23028173-23028217	NM_028472:675	Bmper	INSIDE	0.637	0.565	1220.61	690.02	0.360	996.58	358.94
A_68_P26132522	chr8:107778665-107778709	NM_178879:365	B3gnt9-ps	INSIDE	0.637	0.430	1449.09	622.79	0.274	976.80	267.36
A_68_P25954066	chr8:73311865-73311909	NM_199308:4446	Mast3	INSIDE	0.637	2.387	3158.61	7539.91	1.521	2443.16	3715.87
A_68_P25409557	chr7:116847217-116847263	NM_020616:-10	D930014E17Rik	PROMOTER	0.637	0.574	1221.63	700.80	0.366	1016.56	371.65
A_68_P25101365	chr7:54051013-54051057	NM_001005247:217	Hps5	INSIDE	0.637	0.386	3602.83	1391.76	0.246	2634.05	648.51
A_68_P25016747	chr7:28118579-28118623	NM_001113549:67	Ltbp4	INSIDE	0.637	5.449	3176.01	17304.89	3.469	2482.67	8611.15
A_68_P24995306	chr7:20382177-20382221	NM_001161844:-40	Cblc	PROMOTER	0.637	4.790	3413.87	16351.90	3.051	2815.22	8589.66
A_68_P24797812	chr6:120244301-120244345	NM_198884:255	B4galnt3	INSIDE	0.637	2.307	1710.28	3946.33	1.469	1524.83	2239.57
A_68_P23692834	chr5:52507355-52507399	AK076709:-1175		PROMOTER	0.637	3.154	2115.40	6672.50	2.008	1751.63	3517.75
A_68_P23617869	chr5:38550496-38550540	NM_010942:188	Nsg1	INSIDE	0.637	0.710	1987.09	1410.59	0.452	1487.18	672.75
A_68_P22385231	chr3:103083438-103083482	NM_001079830:245	Trim33	INSIDE	0.637	0.599	1304.98	782.08	0.382	982.78	375.33
A_68_P22045622	chr3:33919516-33919560	NM_001113188:538	Fxr1	INSIDE	0.637	2.252	2817.10	6343.72	1.434	2576.68	3695.97
A_68_P21646233	chr2:133380149-133380193	NM_007553:1236	Bmp2	INSIDE	0.637	0.683	1350.18	921.94	0.435	1236.91	538.33
A_68_P20778066	chr1:164408081-164408125	NM_001038619:59	Dnm3	INSIDE	0.637	3.284	1051.87	3454.03	2.092	903.45	1890.08
A_68_P32137213	chr19:45308119-45308163	NM_010691:1586	Lbx1	DOWNSTREAM	0.636	0.368	5457.04	2009.37	0.234	4276.70	1001.24
A_68_P32050810	chr19:29122317-29122361	NM_021299:54	Ak3	INSIDE	0.636	9.563	949.84	9083.06	6.080	738.57	4490.29
A_68_P31622955	chr18:36441203-36441247	NM_008989:409	Pura	INSIDE	0.636	0.743	3289.40	2444.45	0.473	2347.88	1109.82
A_68_P31612701	chr18:34569089-34569133	NM_001163004:34	Pkd2l2	INSIDE	0.636	0.328	1527.22	500.57	0.208	1191.08	248.16
A_68_P31065253	chr17:15511986-15512030	NM_007865:779	Dll1	INSIDE	0.636	2.992	808.76	2420.02	1.903	632.74	1204.01
A_68_P30978138	chr16:93883923-93883967	NM_028083:-201	Chaf1b	PROMOTER	0.636	4.336	2324.51	10078.63	2.757	1919.49	5292.90
A_68_P30621546	chr16:26581967-26582011	NM_134103:198	Il1rap	INSIDE	0.636	5.367	2008.65	10780.79	3.411	1704.95	5816.21
A_68_P30453204	chr15:94420006-94420050	NM_008971:227	Twf1	INSIDE	0.636	3.677	2299.31	8454.98	2.338	2057.03	4809.61
A_68_P30404613	chr15:85862950-85862994	NM_009886:1235	Celsr1	INSIDE	0.636	4.606	1615.73	7441.87	2.931	1208.20	3541.62
A_68_P30280959	chr15:64144695-64144739	NM_010026:69765	Asap1	INSIDE	0.636	2.791	580.35	1619.51	1.776	635.20	1128.04
A_68_P29449769	chr14:21748618-21748662	NM_009502:-14	Vcl	PROMOTER	0.636	0.338	4290.40	1452.10	0.215	3005.68	647.13
A_68_P29073064	chr13:58503963-58504007	NM_001168248:27	Rmi1	INSIDE	0.636	0.399	2549.66	1018.02	0.254	1919.51	487.12
A_68_P28426163	chr12:51750413-51750457	NM_008858:-224	Prkd1	PROMOTER	0.636	0.524	1861.64	974.69	0.333	1598.89	532.73
A_68_P28035927	chr11:94853399-94853443	NM_172261:895	Ppp1r9b	INSIDE	0.636	6.288	606.41	3813.08	4.002	495.49	1983.02
A_68_P27750951	chr11:43342144-43342188	NM_001045530:-119	Ccnjl	PROMOTER	0.636	0.607	1087.73	659.80	0.386	789.90	304.69
A_68_P27568544	chr11:6344567-6344611	NM_029938:-142	H2afv	PROMOTER	0.636	0.465	1341.96	624.37	0.296	1012.70	299.51
A_68_P26142251	chr8:109460182-109460226	NM_009229:555	Sntb2	INSIDE	0.636	2.321	4107.32	9532.27	1.475	3322.65	4900.66
A_68_P25614473	chr8:3537974-3538018	NM_001122818:22613	Pnpla6	INSIDE	0.636	2.270	1775.72	4031.16	1.443	1429.79	2063.06
A_68_P25354429	chr7:105342798-105342842	NM_001081167:5165	B3gnt6	INSIDE	0.636	4.631	704.81	3264.21	2.947	624.01	1839.03
A_68_P25008202	chr7:25857869-25857913	NM_008168:-502	Grik5	DIVERGENT_PROMOTER	0.636	0.636	1055.57	670.82	0.404	834.79	337.27
A_68_P24460750	chr6:53989374-53989418	NM_001163640:-529	Chn2	PROMOTER	0.636	2.797	865.28	2420.57	1.778	748.54	1331.26
A_68_P24006302	chr5:115263981-115264025	NR_027817:-17	1500011B03Rik	PROMOTER	0.636	2.862	1898.64	5433.68	1.821	1623.19	2956.13
A_68_P23374714	chr4:142940380-142940424	NM_001162983:750	Lrrc38	INSIDE	0.636	0.625	947.67	592.09	0.398	758.50	301.62
A_68_P23189866	chr4:106987036-106987080	NM_001205156:91	Yipf1	INSIDE	0.636	0.562	979.75	550.87	0.358	806.61	288.62
A_68_P22964956	chr4:62185874-62185918	NM_021498:152	Pole3	INSIDE	0.636	0.539	2503.40	1349.55	0.343	1904.11	652.39
A_68_P22953596	chr4:58924953-58924997	NM_172381:623	AI314180	INSIDE	0.636	5.438	341.12	1854.99	3.459	297.98	1030.58
A_68_P22481380	chr3:121949179-121949223	NM_008129:691	Gclm	INSIDE	0.636	0.628	854.72	536.92	0.399	693.97	277.08
A_68_P21339410	chr2:74601035-74601079	NM_010467:20	Hoxd1	INSIDE	0.636	0.650	1129.14	733.98	0.414	902.76	373.35
A_68_P20849642	chr1:177622972-177623016	NM_010098:-273	Opn3	DIVERGENT_PROMOTER	0.636	5.353	3471.86	18585.79	3.406	3086.55	10512.64
A_68_P20825998	chr1:172994760-172994804	NM_010188:-5248	Fcgr3	PROMOTER	0.636	0.206	7929.36	1637.27	0.131	6673.30	876.07
A_68_P20637315	chr1:136982242-136982286	NM_001033409:19589	Lgr6	INSIDE	0.636	3.779	485.63	1835.20	2.404	426.83	1025.91
A_68_P20442836	chr1:93391186-93391230	NM_028718:-36	Traf3ip1	PROMOTER	0.636	2.559	1105.60	2828.68	1.626	1050.47	1708.11
A_68_P31204659	chr17:46167970-46168014	NM_001110266:-5880	Vegfa	PROMOTER	0.635	0.397	2738.50	1085.98	0.252	1898.00	478.22
A_68_P31129059	chr17:29797809-29797853	NM_028791:285	Ftsjd2	INSIDE	0.635	9.117	1696.61	15468.22	5.789	1709.09	9893.73
A_68_P31108289	chr17:26232390-26232434	NM_019731:3	Nme4	INSIDE	0.635	4.440	796.63	3536.95	2.818	760.29	2142.47
A_68_P29942854	chr14:119537426-119537470	NM_015820:-38	Hs6st3	PROMOTER	0.635	4.136	1214.23	5021.90	2.627	1037.33	2724.78
A_68_P29075350	chr13:58908196-58908240	NM_001025074:-975	Ntrk2	PROMOTER	0.635	26.662	8517.08	227082.40	16.937	7482.81	126733.00
A_68_P29053525	chr13:55254569-55254613	NM_008011:412	Fgfr4	INSIDE	0.635	0.537	3946.66	2120.52	0.341	2875.49	980.71
A_68_P29032311	chr13:51504011-51504055	NM_010101:46	S1pr3	INSIDE	0.635	0.417	3748.54	1563.60	0.265	2785.80	737.42
A_68_P28185536	chr11:120459969-120460013	NM_001038230:145	Anapc11	INSIDE	0.635	0.421	1930.99	813.70	0.268	1732.35	463.83
A_68_P27830412	chr11:58144420-58144464	NM_001161338:223	Sh3bp5l	INSIDE	0.635	2.439	1305.05	3183.30	1.548	1062.66	1645.17
A_68_P27708457	chr11:35583330-35583374	NM_145962:356	Pank3	INSIDE	0.635	3.373	600.18	2024.63	2.144	558.04	1196.29

ProbeName	Target position of probe on CpG island microarray	TargetID	GeneSymbol	CpG island Description	Ratio of relative methylation (TiO_2-NP/Vehicle)	Sham group			TiO_2-H group		
						Relative methylation (Cy5/Cy3)	Cy3 signal (Input)	Cy5 signal (MeDIP)	Relative methylation (Cy5/Cy3)	Cy3 signal (Input)	Cy5 signal (MeDIP)
A_68_P27281304	chr10:79613780-79613824	NM_021565:2768	Midn	INSIDE	0.635	0.378	2704.66	1022.79	0.240	1938.29	465.59
A_68_P27183504	chr10:61441353-61441397	NM_007731:482	Col13a1	INSIDE	0.635	3.917	1082.03	4238.72	2.489	889.24	2213.29
A_68_P26142730	chr8:109555781-109555825	NM_126165:577	Vps4a	INSIDE	0.635	3.645	801.51	2921.40	2.316	627.07	1452.45
A_68_P25735811	chr8:28089322-28089366	NM_001101502:1537	Zfp703	INSIDE	0.635	3.286	723.68	2378.21	2.087	729.40	1522.42
A_68_P24795431	chr6:119798321-119798365	NR_030776:132	3110021A11Rik	INSIDE	0.635	0.680	1054.15	717.35	0.432	859.43	371.08
A_68_P24325042	chr6:29129526-29129570	NM_001101443:36	Prrt4	INSIDE	0.635	0.479	967.87	463.51	0.304	820.40	249.62
A_68_P24091776	chr5:130505164-130505208	NM_007761:11	Crcp	INSIDE	0.635	6.307	725.09	4573.17	4.003	637.70	2552.73
A_68_P24038700	chr5:120885515-120885559	NM_008499:3642	Lhx5	INSIDE	0.635	0.314	4706.35	1475.46	0.199	3504.64	697.15
A_68_P22412729	chr3:108247976-108248020	NM_001204979:179	Sars	INSIDE	0.635	0.265	1778.91	471.83	0.169	1301.67	219.36
A_68_P22394779	chr3:104855704-104855748	NM_001163332:145	Cttnbp2nl	INSIDE	0.635	0.310	1782.56	552.57	0.197	1417.95	278.91
A_68_P22063510	chr3:37318580-37318625	NM_153561:-90	Nudt6	DIVERGENT_PROMOTER	0.635	2.286	6968.69	15929.59	1.453	4674.85	6790.54
A_68_P21612309	chr2:127074111-127074155	NM_175145:422	Tmem127	INSIDE	0.635	0.209	2784.04	581.25	0.133	2224.28	294.90
A_68_P21417960	chr2:90420716-90420760	NM_008982:66	Ptprj	INSIDE	0.635	7.816	1205.37	9421.14	4.962	1031.43	5118.41
A_68_P21021376	chr2:14525863-14525907	NM_023116:-48	Cacnb2	PROMOTER	0.635	2.576	1357.19	3495.53	1.636	1199.92	1962.95
A_68_P20383169	chr1:80754785-80754829	NM_175291:322	Dock10	INSIDE	0.635	0.353	1510.44	533.37	0.224	1205.31	270.37
A_68_P32601446	chrX:109008521-109008565			Unknown	0.634	5.375	392.02	2106.98	3.408	424.32	1446.12
A_68_P32241861	chrX:7706354-7706398	NR_029428:3597	Wdr13	INSIDE	0.634	3.461	555.13	1921.05	2.193	762.62	1672.51
A_68_P30575879	chr16:17816053-17816097	NM_153790:18700	Scarf2	DOWNSTREAM	0.634	0.587	891.91	523.85	0.373	796.48	296.69
A_68_P30093919	chr15:27611395-27611439	NM_198301:-163	Fam105a	PROMOTER	0.634	13.679	2164.67	29610.62	8.669	1666.78	14449.73
A_68_P29260367	chr13:99696244-99696288	NM_178716:73	Tnpo1	INSIDE	0.634	2.741	1308.08	3586.09	1.739	1086.83	1889.51
A_68_P27687433	chr11:32113469-32113513	NM_010117:8803	Rhbdf1	INSIDE	0.634	2.817	709.74	1999.27	1.786	762.11	1361.14
A_68_P27641464	chr11:22412537-22412581	NM_153596:273	Tmem17	INSIDE	0.634	3.622	780.00	2824.76	2.298	643.08	1477.64
A_68_P27416798	chr10:105011030-105011074	NM_177368:483	Tmtc2	INSIDE	0.634	2.428	1654.31	4017.21	1.540	1382.22	2129.21
A_68_P27339932	chr10:90545143-90545187	NM_001042558:324	Apaf1	INSIDE	0.634	2.751	784.93	2159.43	1.743	601.35	1048.18
A_68_P26892620	chr9:122930391-122930435	NM_001164823:-31	Tmem42	PROMOTER	0.634	2.564	1851.59	4748.15	1.626	1515.72	2464.51
A_68_P26809495	chr9:107453757-107453801	NM_019713:-3208	Rassf1	PROMOTER	0.634	0.387	7365.54	2852.24	0.245	5014.26	1230.30
A_68_P25669976	chr8:13677580-13677624	NM_009025:-15	Rasa3	PROMOTER	0.634	4.600	1489.45	6850.75	2.915	1326.36	3866.78
A_68_P25565281	chr7:144514141-144514185	NM_001113414:-8034	Ebf3	PROMOTER	0.634	0.564	1993.40	1124.82	0.358	1677.16	600.23
A_68_P25502374	chr7:133952923-133952967	NM_026884:-7454	Fam57b	DIVERGENT_PROMOTER	0.634	0.402	1144.30	460.11	0.255	853.39	217.53
A_68_P21817647	chr2:164229981-164230025	NM_013592:112	Matn4	INSIDE	0.634	0.358	1481.17	530.91	0.227	1272.08	289.03
A_68_P20940733	chr1:193730478-193730522	NM_009579:-159	Slc30a1	PROMOTER	0.634	2.342	2407.96	5640.00	1.484	1856.91	2756.09
A_68_P31730289	chr18:56591860-56591904	NM_026240:97	Gramd3	INSIDE	0.633	2.439	1509.60	3682.31	1.544	1323.43	2043.89
A_68_P30463773	chr15:96291502-96291546	NM_028148:-250	Scaf11	PROMOTER	0.633	2.728	1397.12	3810.80	1.727	1138.18	1965.83
A_68_P30423403	chr15:88970637-88970681	NM_013871:475	Mapk12	INSIDE	0.633	0.295	1947.81	574.59	0.187	1572.17	293.36
A_68_P30366908	chr15:79341974-79342018	NM_134090:-4841	Kdelr3	DIVERGENT_PROMOTER	0.633	0.642	1622.80	1041.80	0.406	1090.42	443.23
A_68_P28554952	chr12:77707425-77707469	NM_013675:104088	Spnb1	INSIDE	0.633	4.505	1219.24	5492.35	2.851	1089.76	3106.85
A_68_P28160202	chr11:116442703-116442747	NM_173755:37	Ube2o	INSIDE	0.633	2.456	2322.71	5705.40	1.556	1934.32	3009.20
A_68_P27804897	chr11:53277337-53277381	NM_027917:6652	Shroom1	INSIDE	0.633	4.544	600.90	2730.59	2.878	591.07	1701.38
A_68_P27644767	chr11:23156427-23156471	NM_001035226:408	Xpo1	INSIDE	0.633	4.712	433.65	2043.36	2.983	346.05	1032.20
A_68_P27533648	chr10:126479103-126479147	NM_010792:137	Mettl1	INSIDE	0.633	0.627	2430.18	1524.16	0.397	1794.65	712.88
A_68_P27234347	chr10:70700994-70701038	NM_009360:-224	Tfam	PROMOTER	0.633	0.502	4368.79	2192.04	0.318	3184.49	1011.73
A_68_P27099065	chr10:42894301-42894345	NM_175407:14	Sobp	INSIDE	0.633	6.909	3896.52	26921.78	4.374	2963.48	12961.24
A_68_P26132669	chr8:107800240-107800284	NM_030152:-84	Nol3	PROMOTER	0.633	0.691	9516.51	6578.33	0.438	6741.36	2949.47
A_68_P25768631	chr8:34496720-34496764	NM_152821:-54	Purg	DIVERGENT_PROMOTER	0.633	0.213	7604.72	1620.37	0.135	5221.15	704.12
A_68_P25715449	chr8:24318844-24318888	NM_018743:59	Agpat6	INSIDE	0.633	2.909	887.82	2582.30	1.841	707.76	1303.34
A_68_P25670551	chr8:13785477-13785521	NM_025924:-116	Upf3a	PROMOTER	0.633	3.275	1463.75	4793.70	2.072	1082.54	2242.75
A_68_P25520723	chr7:137409825-137409869	NM_010207:476	Fgfr2	INSIDE	0.633	3.088	996.99	3078.95	1.955	844.51	1651.22
A_68_P25262388	chr7:86804881-86804936	NM_029835:-173	5730590G19Rik	PROMOTER	0.633	0.364	1754.72	638.28	0.230	1248.25	287.37
A_68_P24800930	chr6:120866663-120866707	NM_007544:154	Bid	INSIDE	0.633	2.207	2437.44	5379.47	1.398	1954.34	2732.17
A_68_P24537472	chr6:71583440-71583484	NM_001038695:-563	Kdm3a	PROMOTER	0.633	0.482	2058.11	992.98	0.306	1631.59	498.67
A_68_P24157328	chr5:144383397-144383441	NM_011182:103	Cyth3	INSIDE	0.633	2.907	848.95	2467.84	1.841	712.48	1311.55
A_68_P24142814	chr5:141178050-141178094	NM_028833:260	Iqce	INSIDE	0.633	0.178	3165.61	563.89	0.113	2205.24	248.58
A_68_P23392617	chr4:147514830-147514874	NM_027985:254	Mad2l2	INSIDE	0.633	0.419	1910.07	800.61	0.265	1476.80	391.99
A_68_P22888266	chr4:46358077-46358121	NM_183298:1033	Foxe1	INSIDE	0.633	4.144	2190.68	9077.89	2.624	1744.79	4577.54
A_68_P22531298	chr3:130974889-130974933	NM_008212:45	Hadh	INSIDE	0.633	0.235	3552.14	833.06	0.149	2431.21	361.16
A_68_P21270820	chr2:62502454-62502498	NM_145523:93	Gca	INSIDE	0.633	0.491	1226.25	602.09	0.311	896.10	278.42
A_68_P21007905	chr2:11699155-11699199	NM_015792:-22	Fbxo18	DIVERGENT_PROMOTER	0.633	0.132	7006.88	922.42	0.083	4577.66	381.74
A_68_P32775152	chrX:155971013-155971057	NM_001033472:435	A830080D01Rik	INSIDE	0.632	5.013	1249.98	6266.55	3.167	1311.38	4153.46
A_68_P31151041	chr17:34066176-34066220	NM_001025313:9776	Tapbp	INSIDE	0.632	0.663	1719.57	1140.76	0.419	1139.16	477.62
A_68_P30686475	chr16:38522686-38522730	NM_024273:39	4930455C21Rik	INSIDE	0.632	0.446	1451.07	647.37	0.282	1157.49	326.19
A_68_P29268696	chr13:101262505-101262549	NM_022011:38	Gtf2h2	INSIDE	0.632	0.648	1354.04	876.97	0.409	1037.52	424.46
A_68_P28039252	chr11:95374960-95375004	NM_130858:897	Nxph3	INSIDE	0.632	5.451	3174.08	17302.23	3.443	2361.14	8129.37
A_68_P28035922	chr11:94852837-94852881	NM_172261:333	Ppp1r9b	INSIDE	0.632	2.510	1136.77	2853.42	1.588	944.22	1499.01
A_68_P24994887	chr7:20300662-20300706	NM_001109748:94	Tomm40	INSIDE	0.632	0.717	10300.91	7384.59	0.453	7771.63	3522.75
A_68_P24951534	chr7:4960187-4960231	NM_001110330:4636	Fiz1	INSIDE	0.632	3.740	833.43	3117.02	2.364	842.17	1991.29

ProbeName	Target position of probe on CpG island microarray	TargetID	GeneSymbol	CpG island Description	Ratio of relative methylation (TiO_2-NP/Vehicle)	Sham group			TiO_2-H group		
						Relative methylation (Cy5/Cy3)	Cy3 signal (Input)	Cy5 signal (MeDIP)	Relative methylation (Cy5/Cy3)	Cy3 signal (Input)	Cy5 signal (MeDIP)
A_68_P24249109	chr6:13788674-13788718	NM_145066:1152	Gpr85	INSIDE	0.632	3.770	946.95	3569.63	2.384	763.62	1820.23
A_68_P24136638	chr5:140095348-140095392	AK201860:79272		INSIDE	0.632	3.188	663.90	2116.41	2.014	480.81	968.32
A_68_P23470075	chr5:8893888-8893932	NM_008830:190	Abcb4	INSIDE	0.632	2.735	1821.91	4982.46	1.729	1451.87	2510.25
A_68_P22454038	chr3:117062948-117062993	NM_177664:824	D3Bwg0562e	INSIDE	0.632	4.016	958.52	3849.34	2.537	802.13	2035.17
A_68_P21603778	chr2:125450808-125450852	NM_001081091:19	Cep152	INSIDE	0.632	0.598	6737.22	4028.77	0.378	4941.34	1866.57
A_68_P20872222	chr1:181733328-181733372	NM_026375:796	Ahctf1	INSIDE	0.632	5.733	555.31	3183.70	3.623	420.15	1522.21
A_68_P31868010	chr18:81171392-81171436	NM_178280:11903	Sall3	INSIDE	0.631	4.796	1236.88	5932.56	3.025	1065.01	3222.15
A_68_P31866306	chr18:80905336-80905380	NM_001164111:-446	Nfatc1	PROMOTER	0.631	3.247	1144.24	3715.40	2.048	973.67	1994.51
A_68_P31790570	chr18:67365551-67365595	NM_024190:560	Chmp1b	INSIDE	0.631	2.357	2289.45	5395.54	1.487	1860.02	2765.35
A_68_P31479223	chr18:7868850-7868894	NM_001146298:-322	Wac	PROMOTER	0.631	2.581	1324.18	3417.19	1.630	1161.22	1892.38
A_68_P31140484	chr17:31702098-31702142	NM_016670:403	Pknox1	INSIDE	0.631	3.692	1076.92	3975.86	2.330	878.13	2046.47
A_68_P31053607	chr17:12934828-12934873	NR_002853:674	Airn	INSIDE	0.631	3.759	729.06	2740.79	2.371	662.15	1570.01
A_68_P30480281	chr15:99287653-99287697	NM_001001884:505	Nckap5l	INSIDE	0.631	2.400	2254.69	5410.69	1.514	1503.20	2276.11
A_68_P29287623	chr13:104710322-104710366	NM_001005868:250	Erbb2ip	INSIDE	0.631	0.431	1483.04	638.91	0.272	1316.64	358.09
A_68_P29094157	chr13:63664712-63664756	NM_008957:2094	Ptch1	INSIDE	0.631	0.534	1691.12	903.48	0.337	1329.87	448.24
A_68_P28672821	chr12:100139294-100139338	NM_001081191:378	Eml5	INSIDE	0.631	2.403	1453.07	3491.18	1.515	1262.87	1913.14
A_68_P28585130	chr12:83271372-83271416	NM_001167983:392	Sipa1l1	INSIDE	0.631	0.662	1432.44	947.99	0.417	1162.02	485.02
A_68_P28086208	chr11:103510960-103511004	NM_001033212:160	Rprml	INSIDE	0.631	0.721	1681.18	1211.44	0.455	1425.68	648.19
A_68_P27930669	chr11:76030890-76030934	NM_177367:162	Gemin4	INSIDE	0.631	0.598	1083.90	648.45	0.378	962.65	363.40
A_68_P27537384	chr10:127104122-127104166	NM_001122895:-385	Nab2	DIVERGENT_PROMOTER	0.631	0.603	1096.99	661.29	0.380	956.88	363.85
A_68_P27288654	chr10:80756115-80756159	NM_001146687:420	Pip5k1c	INSIDE	0.631	0.389	1273.69	495.97	0.246	1052.36	258.67
A_68_P26745887	chr9:96096585-96096629	NM_001184706:-87	Tfdp2	PROMOTER	0.631	3.177	1415.17	4495.65	2.004	1162.14	2328.60
A_68_P26399336	chr9:31018727-31018771	NM_001102455:652	Aplp2	INSIDE	0.631	5.929	2106.35	12488.21	3.738	1822.58	6813.65
A_68_P26348964	chr9:21603129-21603173	NM_145611:-160	Kank2	PROMOTER	0.631	2.863	1128.16	3229.66	1.808	1057.41	1911.46
A_68_P25420896	chr7:118923422-118923466	NM_173739:47	Galntl4	INSIDE	0.631	0.611	1330.92	813.32	0.385	1083.76	417.61
A_68_P25288546	chr7:91558357-91558401	NM_007488:91	Arnt2	INSIDE	0.631	4.215	947.34	3993.04	2.661	886.55	2359.12
A_68_P25096865	chr7:53356200-53356244	NM_001204411:-88	Kcnj11	PROMOTER	0.631	12.183	7227.34	88051.30	7.683	6783.89	52118.43
A_68_P25009060	chr7:26004436-26004480	NM_207677:420	Dedd2	INSIDE	0.631	0.700	2854.88	1997.00	0.441	1958.50	864.23
A_68_P24127045	chr5:138125808-138125852	NM_178162:15	Agfg2	INSIDE	0.631	0.328	2796.35	916.82	0.207	2228.88	461.18
A_68_P24059369	chr5:124566689-124566733	NM_144509:594	Arl6ip4	INSIDE	0.631	0.331	1712.96	566.42	0.209	1201.77	250.93
A_68_P22882726	chr4:45421447-45421491	NM_001009949:170	Mcart1	INSIDE	0.631	3.630	1981.67	7192.47	2.289	1532.93	3509.30
A_68_P21911518	chr2:181326975-181327019	NM_001164597:170	Znf512b	INSIDE	0.631	10.607	1022.62	10846.77	6.689	992.12	6635.95
A_68_P21843263	chr2:168567207-168567251	NM_015731:72	Atp9a	INSIDE	0.631	3.113	524.70	1633.29	1.964	526.67	1034.62
A_68_P21816616	chr2:163996879-163996923	NM_008435:-51	Kcns1	PROMOTER	0.631	2.709	1230.53	3334.09	1.710	1009.44	1726.58
A_68_P21212081	chr2:51003539-51003583	NM_028810:1071	Rnd3	INSIDE	0.631	0.245	5281.23	1292.73	0.154	3451.55	532.72
A_68_P20945385	chr1:194596745-194596789	NM_144881:647	Hhat	INSIDE	0.631	2.753	3309.90	9113.81	1.737	2528.30	4391.43
A_68_P20832289	chr1:174242640-174242684	NM_080419:125	Igsf8	INSIDE	0.631	0.392	3689.98	1447.67	0.247	2802.52	693.29
A_68_P32700064	chrX:137133979-137134023	NM_001077364:1061	Tsc22d3	INSIDE	0.630	3.174	744.94	2364.09	2.000	856.32	1712.92
A_68_P31826505	chr18:73732011-73732055	NM_001039214:-326	Mex3c	PROMOTER	0.630	2.335	3205.79	7486.07	1.471	2393.53	3519.73
A_68_P30544729	chr16:10835253-10835297	NM_001162932:123	A630055G03Rik	INSIDE	0.630	3.750	529.88	1987.11	2.363	479.89	1134.18
A_68_P30503432	chr15:103144711-103144755	NM_013866:25785	Zfp385a	INSIDE	0.630	2.485	1189.61	2956.33	1.565	794.17	1242.82
A_68_P30350785	chr15:76648219-76648263	NM_001168288:360	Arhgap39	INSIDE	0.630	0.602	6059.45	3645.39	0.379	4561.18	1729.75
A_68_P30346596	chr15:76009591-76009635	NM_201394:16528	Plec	INSIDE	0.630	4.703	609.73	2867.45	2.964	445.53	1320.38
A_68_P29329254	chr13:112280052-112280096	NM_001122963:175	Gpbp1	INSIDE	0.630	0.613	6139.64	3761.00	0.386	3820.80	1473.56
A_68_P28873012	chr13:21560534-21560582	NM_025719:-188	Nkapl	PROMOTER	0.630	9.861	784.72	7738.18	6.215	673.85	4188.18
A_68_P28748141	chr12:113384025-113384069	NM_001097621:-372	Kif26a	PROMOTER	0.630	0.598	2069.50	1237.52	0.377	1770.69	666.92
A_68_P28187622	chr11:120794046-120794090	NM_027745:118	Ccdc57	INSIDE	0.630	0.525	1146.10	601.95	0.331	993.50	328.95
A_68_P27833410	chr11:58717675-58717719	NM_011758:31	Zfp39	INSIDE	0.630	0.228	4167.97	950.05	0.144	2823.16	405.16
A_68_P27796702	chr11:51781404-51781448	NM_029976:294	Cdkn2aipnl	INSIDE	0.630	0.356	1453.59	517.93	0.225	1158.01	260.14
A_68_P27503065	chr10:120802501-120802545	NM_029364:377	Gns	INSIDE	0.630	3.461	1122.84	3885.78	2.179	838.06	1826.14
A_68_P26822031	chr9:110019702-110019746	NM_133347:-1638	Dhx30	PROMOTER	0.630	2.773	1298.46	3600.71	1.748	1129.09	1973.38
A_68_P26166134	chr8:113551628-113551672	NM_001190800:20	Ddx19b	INSIDE	0.630	4.436	2211.50	9809.13	2.796	1779.54	4976.10
A_68_P25501141	chr7:133709739-133709783	NM_146200:120	Eif3c	INSIDE	0.630	0.491	3445.34	1689.94	0.309	2674.03	826.07
A_68_P25017609	chr7:28293338-28293382	NM_019412:9018	Prx	INSIDE	0.630	0.134	5077.72	678.81	0.084	3445.19	290.23
A_68_P24540594	chr6:72185394-72185438	NM_153778:155	Atoh8	INSIDE	0.630	4.633	1455.47	6743.86	2.919	1126.07	3287.49
A_68_P24157332	chr5:144383775-144383819	NM_011182:481	Cyth3	INSIDE	0.630	0.507	2427.97	1231.82	0.320	2013.63	643.50
A_68_P24119515	chr5:136592514-136592559	NM_011293:-24	Polr2j	PROMOTER	0.630	0.483	4323.18	2087.26	0.304	2616.34	795.89
A_68_P23364088	chr4:141001073-141001117	NM_009541:507	Zbtb17	INSIDE	0.630	0.727	4891.49	3554.15	0.458	3585.11	1640.41
A_68_P23205808	chr4:110024387-110024431	NM_001163397:108	Elavl4	INSIDE	0.630	0.458	1440.13	659.36	0.288	1136.72	327.63
A_68_P22481653	chr3:121996304-121996348	NR_030439:295	Mir760	DOWNSTREAM	0.630	6.174	369.96	2284.23	3.889	333.02	1295.04
A_68_P22318564	chr3:89125165-89125211	NM_010108:1613	Efna3	INSIDE	0.630	3.830	364.94	1397.75	2.412	291.88	703.94
A_68_P22207824	chr3:66777458-66777502	NM_013665:8213	Shox2	INSIDE	0.630	2.518	3490.16	8787.59	1.587	2944.89	4673.70
A_68_P21266439	chr2:61649898-61649942	NM_009322:7411	Tbr1	INSIDE	0.630	4.145	488.29	2023.74	2.612	449.65	1174.31
A_68_P33005811	A_68_P33005811			Unknown	0.629	17.765	447.45	7949.01	11.182	376.32	4207.93
A_68_P32107651	chr19:40346260-40346304	NM_016861:-176	Pdlim1	PROMOTER	0.629	0.552	882.82	487.14	0.347	730.85	253.51

ProbeName	Target position of probe on CpG island microarray	TargetID	GeneSymbol	CpG island Description	Ratio of relative methylation (TiO_2-NP/Vehicle)	Sham group			TiO_2-H group		
						Relative methylation (Cy5/Cy3)	Cy3 signal (Input)	Cy5 signal (MeDIP)	Relative methylation (Cy5/Cy3)	Cy3 signal (Input)	Cy5 signal (MeDIP)
A_68_P32030090	chr19:25580813-25580857	NM_015826:639	Dmrt1	INSIDE	0.629	0.414	4812.90	1992.83	0.260	3409.31	887.88
A_68_P31936459	chr19:6930056-6930100	NM_001081291:2623	Ccdc88b	INSIDE	0.629	0.588	1085.10	638.52	0.370	879.97	325.73
A_68_P31553001	chr18:23198592-23198636	NM_001161483:-1450	Nol4	PROMOTER	0.629	3.115	949.51	2957.68	1.958	753.86	1476.42
A_68_P29672990	chr14:65977418-65977462	NM_199029:-72	Zfp395	PROMOTER	0.629	0.393	1440.14	566.19	0.247	1207.82	298.85
A_68_P28566640	chr12:80013209-80013253	NM_013738:-5305	Plek2	PROMOTER	0.629	2.541	2776.88	7056.32	1.599	2231.34	3566.86
A_68_P28549762	chr12:76770236-76770280	NM_028203:266	Wdr89	INSIDE	0.629	0.229	2304.01	526.63	0.144	1733.11	249.37
A_68_P28549422	chr12:76697372-76697416	NM_012024:-207	Ppp2r5e	PROMOTER	0.629	0.644	4695.46	3024.70	0.405	3699.68	1499.29
A_68_P27589636	chr11:11014080-11014124	NM_177033:84	Vwc2	INSIDE	0.629	0.640	1533.69	980.99	0.402	1370.23	550.99
A_68_P27579285	chr11:8564248-8564292	NM_001083587:268	Tns3	INSIDE	0.629	2.330	2351.62	5478.42	1.465	1712.12	2507.59
A_68_P26596129	chr9:66793897-66793941	NM_026467:-6	Rps27l	PROMOTER	0.629	0.649	789.64	512.35	0.408	651.37	266.01
A_68_P26170843	chr8:114434234-114434278	NM_019950:-157	Chst5	PROMOTER	0.629	3.022	1518.32	4588.59	1.902	1230.97	2341.80
A_68_P26137064	chr8:108489850-108489894	NM_009195:67	Slc12a4	INSIDE	0.629	0.631	2083.23	1313.72	0.397	1570.95	623.13
A_68_P26038357	chr8:90483082-90483126	NM_033327:390	Zfp423	INSIDE	0.629	3.851	780.29	3005.11	2.423	678.18	1643.01
A_68_P25962045	chr8:75071657-75071701	NM_027485:531	Med26	INSIDE	0.629	4.316	3433.96	14822.61	2.717	2819.82	7661.01
A_68_P25585266	chr7:148042623-148042667	NM_018742:-362	Bet1l	DIVERGENT_PROMOTER	0.629	0.642	4076.23	2615.98	0.403	2942.13	1187.03
A_68_P25392588	chr7:112630344-112630388	NM_028444:345	Prkcdbp	INSIDE	0.629	6.082	1457.53	8864.05	3.826	1286.77	4923.07
A_68_P24539866	chr6:72047427-72047471	NM_001035228:-158	St3gal5	PROMOTER	0.629	2.538	1532.42	3889.86	1.597	1295.00	2068.25
A_68_P24120143	chr5:136720679-136720723	NM_018825:73	Sh2b2	INSIDE	0.629	2.912	1531.74	4460.46	1.831	1303.73	2387.58
A_68_P24046892	chr5:122303021-122303065	NM_175474:4006	Fam109a	INSIDE	0.629	3.215	1662.21	5344.82	2.022	1128.77	2282.66
A_68_P23792610	chr5:72971627-72971672	NM_174877:674	Zar1	INSIDE	0.629	2.495	2106.55	5256.23	1.569	1798.33	2821.68
A_68_P23417770	chr4:151664569-151664614	NM_008237:1180	Hes3	INSIDE	0.629	0.664	852.40	566.25	0.418	670.87	280.39
A_68_P23283135	chr4:126699373-126699417	NM_023603:850	Sfpq	INSIDE	0.629	10.723	6779.66	72700.71	6.747	4945.00	33364.39
A_68_P22889573	chr4:46614584-46614628	NM_001164804:195	Coro2a	INSIDE	0.629	0.499	1896.20	947.07	0.314	1537.99	483.45
A_68_P22862455	chr4:41444682-41444726	NM_001085515:5405	AI464131	INSIDE	0.629	0.592	2004.10	1187.10	0.373	1350.49	503.14
A_68_P22862454	chr4:41444579-41444623	NM_001085515:5509	AI464131	INSIDE	0.629	0.367	2117.56	777.60	0.231	1329.78	307.29
A_68_P22747701	chr4:14858053-14858097	ENSMUST00000152024:252		INSIDE	0.629	2.719	1571.04	4271.41	1.710	1315.73	2249.57
A_68_P22388171	chr3:103613470-103613514	NM_001163552:53	Ap4b1	INSIDE	0.629	5.571	844.22	4702.74	3.505	759.62	2662.26
A_68_P20765302	chr1:162125324-162125368	NM_025474:-44	Mrps14	PROMOTER	0.629	21.409	4047.51	86652.27	13.461	4112.07	55352.12
A_68_P20038584	chr1:12980774-12980818	NM_172841:420	Slco5a1	INSIDE	0.629	0.470	1005.03	472.27	0.295	901.35	266.25
A_68_P32464770	chrX:71032514-71032558	NM_009279:55	Ssr4	INSIDE	0.628	0.601	775.11	465.90	0.378	1005.70	379.91
A_68_P32134135	chr19:44821228-44821272			Unknown	0.628	2.594	942.12	2444.27	1.629	772.35	1257.99
A_68_P31609277	chr18:33954596-33954640	NR_038151:73	2410004N09Rik	INSIDE	0.628	2.170	3239.17	7027.39	1.363	2571.96	3506.32
A_68_P30350974	chr15:76701808-76701852	NM_001007568:35	Zfp251	INSIDE	0.628	3.521	1479.04	5208.16	2.212	1315.66	2910.36
A_68_P29643102	chr14:60796162-60796206	ENSMUST00000140924:192		INSIDE	0.628	2.581	1001.26	2584.45	1.621	791.87	1283.57
A_68_P29454669	chr14:22569927-22569971	NM_026283:196	Samd8	INSIDE	0.628	3.707	1125.54	4172.01	2.329	1055.85	2459.17
A_68_P28747157	chr12:113205097-113205141	NM_027360:70	2010107E04Rik	INSIDE	0.628	0.483	1002.34	484.08	0.303	747.92	226.91
A_68_P28153229	chr11:115255200-115255244	NM_001080929:11993	Cdr2l	INSIDE	0.628	3.517	668.94	2352.56	2.209	489.48	1081.14
A_68_P27279643	chr10:79371605-79371649	NM_001163276:57	Med16	INSIDE	0.628	4.140	5613.44	23242.10	2.599	4199.47	10914.67
A_68_P26400214	chr9:31193743-31193787	NM_172766:-12	Nfrkb	PROMOTER	0.628	3.245	1217.65	3950.90	2.038	1064.90	2169.98
A_68_P26346371	chr9:21092727-21092771	NM_145416:-335	Kri1	PROMOTER	0.628	0.602	3347.25	2015.55	0.378	2359.00	892.67
A_68_P25499304	chr7:133344026-133344070	NM_028816:-126	Xpo6	PROMOTER	0.628	37.151	6173.64	229358.10	23.320	6848.80	159715.30
A_68_P25005400	chr7:25318962-25319006	NM_153134:3318	Irgq	INSIDE	0.628	4.003	2042.00	8173.99	2.515	1680.68	4226.88
A_68_P24049903	chr5:122804592-122804636	NM_019780:193	Vps29	INSIDE	0.628	2.615	1950.40	5100.00	1.642	1601.18	2629.69
A_68_P23601183	chr5:35868307-35868351	NM_153107:-53	Cpz	PROMOTER	0.628	0.505	2501.29	1262.37	0.317	1756.65	556.81
A_68_P23395558	chr4:148000660-148000704	NM_001003898:423	Tardbp	INSIDE	0.628	2.963	1168.97	3463.12	1.859	954.82	1775.33
A_68_P23192848	chr4:107475278-107475322	NM_001080926:437	Lrp8	INSIDE	0.628	2.490	1142.64	2844.65	1.564	863.30	1350.61
A_68_P22382660	chr3:102539302-102539346	NM_027533:632	Tspan2	INSIDE	0.628	0.559	2367.81	1322.52	0.351	1838.22	644.70
A_68_P21831533	chr2:166539060-166539104	NM_177782:250	Prex1	INSIDE	0.628	0.685	969.04	663.77	0.430	808.08	347.51
A_68_P21722826	chr2:147020543-147020587	NM_001077632:-8426	Nkx2-2	PROMOTER	0.628	7.919	1016.60	8050.39	4.975	1029.27	5120.29
A_68_P21722727	chr2:147009567-147009611	NR_030769:-230	Nkx2-2as	PROMOTER	0.628	5.155	367.62	1895.17	3.240	341.48	1106.34
A_68_P20075374	chr1:20941893-20941937	NM_027974:208	Efhc1	INSIDE	0.628	0.376	3072.26	1154.33	0.236	2394.83	565.07
A_68_P32227328	chr19:60887531-60887575	NM_001167829:80	Fam45a	INSIDE	0.627	0.248	2126.86	527.73	0.156	1621.74	252.22
A_68_P31933640	chr19:6277337-6277381	NM_010119:463	Ehd1	INSIDE	0.627	0.523	2016.75	1054.88	0.328	1523.82	499.82
A_68_P31126967	chr17:29400949-29400993	NM_026845:-54	Ppil1	DIVERGENT_PROMOTER	0.627	3.702	1073.72	3975.37	2.322	925.55	2148.69
A_68_P30940063	chr16:87495606-87495650	NM_009840:486	Cct8	INSIDE	0.627	0.353	3684.44	1300.97	0.221	2708.06	599.48
A_68_P30641169	chr16:30309242-30309286	NM_008148:1601	Gp5	INSIDE	0.627	0.718	1508.68	1083.39	0.450	1373.54	617.99
A_68_P29582179	chr14:48037872-48037916	NM_144553:188	Dlgap5	INSIDE	0.627	0.501	3253.48	1629.81	0.314	2361.43	741.19
A_68_P29330398	chr13:112545643-112545687	NM_011945:53527	Map3k1	INSIDE	0.627	2.701	1153.51	3115.24	1.694	822.52	1392.99
A_68_P27992419	chr11:86887561-86887605	NM_025638:57	Gdpd1	INSIDE	0.627	2.928	1012.82	2965.10	1.835	740.62	1358.83
A_68_P27787321	chr11:50023806-50023850	NM_011018:464	Sqstm1	INSIDE	0.627	6.929	5593.53	38759.49	4.343	4686.81	20353.84
A_68_P27784886	chr11:49608055-49608100	NM_013529:421	Gfpt2	INSIDE	0.627	0.222	2188.53	486.49	0.139	1581.26	220.55
A_68_P26951410	chr10:13193842-13193886	NM_001195065:338	Phactr2	INSIDE	0.627	0.578	827.23	477.98	0.362	628.28	227.51
A_68_P26023270	chr8:87551092-87551137	NM_026760:383	2310036O22Rik	INSIDE	0.627	2.664	2249.34	5992.51	1.669	1834.56	3062.38
A_68_P25580590	chr7:147020054-147020098	NM_177469:4	Gpr123	INSIDE	0.627	0.705	1574.67	1110.08	0.442	1319.83	583.50
A_68_P25094544	chr7:52955351-52955395	NM_009800:36	Car11	INSIDE	0.627	0.501	2093.39	1049.23	0.314	1750.88	550.53

ProbeName	Target position of probe on CpG island microarray	TargetID	GeneSymbol	CpG island Description	Ratio of relative methylation (TiO_2-NP/Vehicle)	Sham group			TiO_2-H group		
						Relative methylation (Cy5/Cy3)	Cy3 signal (Input)	Cy5 signal (MeDIP)	Relative methylation (Cy5/Cy3)	Cy3 signal (Input)	Cy5 signal (MeDIP)
A_68_P23973160	chr5:109058811-109058855	NM_001163531:4	Tmem175	INSIDE	0.627	4.226	513.33	2169.35	2.648	405.30	1073.24
A_68_P23575088	chr5:31172158-31172203	NM_144525:161	Tmem214	INSIDE	0.627	0.259	1756.72	454.42	0.162	1055.03	171.19
A_68_P23567299	chr5:29704950-29704994	NM_020295:-42	Lmbr1	PROMOTER	0.627	0.432	1311.24	566.30	0.271	1119.30	303.32
A_68_P23081271	chr4:86394332-86394376	NM_001081014:-104	Dennd4c	PROMOTER	0.627	2.373	1574.51	3736.34	1.488	1363.29	2028.42
A_68_P21819502	chr2:164571363-164571407	NM_133763:-130	Dnttip1	DIVERGENT_PROMOTER	0.627	0.522	1533.02	799.94	0.327	1194.18	390.83
A_68_P21573666	chr2:119980254-119980299	NM_133838:35	Ehd4	INSIDE	0.627	0.395	10069.02	3976.76	0.248	6504.30	1610.84
A_68_P21114610	chr2:32168059-32168103	NR_029811:-5899	Mir199b	PROMOTER	0.627	7.410	8050.30	59652.90	4.646	7124.71	33104.31
A_68_P32401468	chrX:54756573-54756617	NM_001033360:340	Gpr101	INSIDE	0.626	2.434	745.16	1813.82	1.525	990.99	1510.80
A_68_P32143654	chr19:46469962-46470006	NM_016860:241	Actr1a	INSIDE	0.626	6.101	2936.31	17914.29	3.819	2231.25	8522.13
A_68_P31320372	chr17:69186496-69186540	NM_001206661:43	Tmem200c	INSIDE	0.626	4.099	919.72	3769.58	2.567	865.10	2221.13
A_68_P31217293	chr17:48549071-48549115	NM_001110832:53	Nfya	INSIDE	0.626	0.632	5257.62	3322.28	0.395	3696.04	1461.68
A_68_P31215872	chr17:48232748-48232792	NM_026625:6189	1700067P10Rik	DOWNSTREAM	0.626	0.465	1623.00	754.18	0.291	1300.31	377.96
A_68_P31151137	chr17:34077801-34077845	NM_020603:155	Wdr46	INSIDE	0.626	0.644	1487.98	958.75	0.403	1153.56	465.27
A_68_P30849198	A_68_P30849198			Unknown	0.626	5.230	396.89	2075.71	3.274	330.43	1081.97
A_68_P30592417	chr16:21423184-21423228	NM_001081366:16	Vps8	INSIDE	0.626	3.405	710.09	2418.06	2.133	733.28	1564.24
A_68_P30579425	chr16:18392661-18392705	NM_033474:44316	Arvcf	INSIDE	0.626	3.474	632.67	2197.84	2.174	554.37	1205.47
A_68_P30572940	chr16:17276845-17276889	NM_177473:474	Tmem191c	INSIDE	0.626	2.707	2331.16	6309.91	1.694	1990.76	3371.40
A_68_P30394506	chr15:84236917-84236961	NM_001163145:40589	1810041L15Rik	INSIDE	0.626	2.481	1544.15	3830.82	1.553	1297.94	2015.54
A_68_P29996501	chr15:7762967-7763011	NM_010275:1978	Gdnf	INSIDE	0.626	0.281	2639.98	743.13	0.176	1961.07	345.74
A_68_P29097470	chr13:64263694-64263738	NM_019986:543	Habp4	INSIDE	0.626	0.527	2107.43	1111.32	0.330	1687.00	556.80
A_68_P28566598	chr12:80007781-80007825	NM_013738:123	Plek2	INSIDE	0.626	0.459	1555.93	713.42	0.287	1208.20	346.60
A_68_P27561122	chr11:4958001-4958045	NM_145216:-108	Rasl10a	PROMOTER	0.626	3.419	1202.96	4113.24	2.141	1130.47	2420.44
A_68_P26744223	chr9:95745481-95745525	NM_001033210:196	Pls1	INSIDE	0.626	3.667	2793.23	10243.12	2.295	2477.91	5686.74
A_68_P25730817	chr8:27229449-27229493	NM_207659:226	Hook3	INSIDE	0.626	3.415	2837.07	9689.55	2.137	2199.55	4701.18
A_68_P23756665	chr5:65884378-65884423	NM_133697:-326	1110003E01Rik	PROMOTER	0.626	0.398	1498.43	597.02	0.250	1102.24	275.13
A_68_P23326916	chr4:134708430-134708475	NM_019732:31893	Runx3	INSIDE	0.626	4.864	260.86	1268.90	3.043	269.36	819.60
A_68_P23297754	chr4:129318346-129318390	NM_001199695:-59	Txlna	PROMOTER	0.626	3.668	1785.51	6549.05	2.297	1285.76	2953.12
A_68_P23294136	chr4:128639545-128639589	NM_172875:95	Adc	INSIDE	0.626	0.376	5729.19	2154.71	0.236	3816.48	898.91
A_68_P22833091	chr4:34830379-34830423	NM_013889:-203	Zfp292	PROMOTER	0.626	3.311	2242.04	7423.12	2.071	1823.68	3777.74
A_68_P22392371	chr3:104442921-104442965	NM_009196:352	Slc16a1	INSIDE	0.626	0.386	1465.46	566.38	0.242	1274.09	308.04
A_68_P22342539	chr3:94965148-94965192	NM_011351:-3125	Sema6c	PROMOTER	0.626	0.440	2816.48	1239.50	0.275	2059.45	567.24
A_68_P21745337	chr2:151528077-151528121	NM_001160363:355	5430405G05Rik	INSIDE	0.626	18.680	1901.24	35515.48	11.687	1833.46	21427.83
A_68_P21338645	chr2:74506594-74506638	NM_008275:250	Hoxd13	INSIDE	0.626	0.326	3711.37	1210.24	0.204	2726.26	556.72
A_68_P20415724	chr1:88995295-88995339	NM_001081082:2865	Alpi	INSIDE	0.626	3.404	2123.31	7228.56	2.130	1761.92	3752.38
A_68_P31962486	chr19:12869204-12869248	NR_024093:1387	U05342	INSIDE	0.625	0.377	2199.47	829.46	0.236	1807.16	425.79
A_68_P31923505	chr19:4510153-4510197	NM_001162946:-297	Pcx	PROMOTER	0.625	0.718	2297.18	1650.27	0.449	1500.34	673.19
A_68_P31915394	chr18:90679335-90679379	NM_198295:-189	Tmx3	PROMOTER	0.625	0.682	891.08	607.43	0.426	719.80	306.74
A_68_P31844550	chr18:77099205-77099249	NM_001109743:4083	Skor2	INSIDE	0.625	0.323	4990.29	1610.35	0.202	3042.20	613.93
A_68_P31655269	chr18:42554834-42554878	NM_138945:606	Pou4f3	INSIDE	0.625	0.441	2025.91	892.64	0.275	1499.75	413.05
A_68_P30753194	chr16:50732861-50732905	NR_028300:4	5330426P16Rik	INSIDE	0.625	2.320	2027.94	4705.32	1.451	1670.92	2423.74
A_68_P30665012	chr16:34573019-34573063	ENSMUST00000114961:578		INSIDE	0.625	0.458	17961.54	8219.73	0.286	12188.69	3487.19
A_68_P29240369	chr13:96144912-96144956	NM_025630:355	Aggf1	INSIDE	0.625	3.705	432.35	1601.90	2.315	417.35	966.30
A_68_P27640630	chr11:22186736-22186780	NM_153078:-917	Ehbp1	PROMOTER	0.625	3.768	1578.63	5947.50	2.354	1396.45	3287.43
A_68_P26983368	chr10:19569800-19569844	NM_029529:1443	Slc35d3	INSIDE	0.625	0.452	1701.95	769.14	0.282	1358.54	383.51
A_68_P26682076	chr9:83039410-83039454	NM_026122:782	Hmgn3	INSIDE	0.625	0.463	1642.78	760.06	0.289	1237.20	357.97
A_68_P26477292	chr9:45821526-45821572	NM_027498:646	Sik3	INSIDE	0.625	8.570	926.50	7940.16	5.357	793.63	4251.53
A_68_P26234726	chr8:125154578-125154622	NM_001109873:47475	Cbfa2t3	INSIDE	0.625	6.238	1790.95	11171.09	3.897	1539.90	6001.03
A_68_P26132621	chr8:107793840-107793884	NM_011939:89	Hsf4	INSIDE	0.625	3.372	1203.70	4058.35	2.107	941.61	1984.28
A_68_P25891489	chr8:59966195-59966239	NM_021788:441	Sap30	INSIDE	0.625	2.791	1688.06	4710.90	1.743	1439.23	2508.71
A_68_P25286563	chr7:91234999-91235044	NM_030728:-6	9930013L23Rik	PROMOTER	0.625	2.643	895.40	2366.88	1.652	751.30	1240.87
A_68_P24432388	chr6:49345736-49345780	NM_029916:156	Stk31	INSIDE	0.625	2.312	1667.02	3853.76	1.446	1308.97	1892.68
A_68_P23657737	chr5:46030747-46030791	NM_021416:-28	Fam184b	PROMOTER	0.625	3.001	1599.40	4799.13	1.875	1384.95	2596.92
A_68_P23094811	chr4:88940707-88940751	NM_009877:-205	Cdkn2a	PROMOTER	0.625	3.161	604.22	1910.18	1.975	593.11	1171.53
A_68_P22289848	chr3:84063239-84063283	ENSMUST00000147901:31		INSIDE	0.625	0.585	5914.30	3459.63	0.366	4643.77	1697.86
A_68_P20893089	chr1:185857699-185857743	NM_028516:157	1700056E22Rik	INSIDE	0.625	5.991	2528.06	15146.67	3.743	2101.24	7865.66
A_68_P20874688	chr1:182186063-182186107	NM_001128605:347	Psen2	INSIDE	0.625	0.586	4354.86	2550.75	0.366	3079.78	1127.23
A_68_P20453265	chr1:94996931-94996975	NM_001110315:1490	Kif1a	INSIDE	0.625	4.489	2277.51	10224.81	2.807	1894.47	5318.38
A_68_P20417070	chr1:89224090-89224134	NM_001110212:509	Gigyf2	INSIDE	0.625	4.583	920.16	4217.36	2.864	794.55	2275.68
A_68_P31201733	chr17:45686337-45686381	NM_001013749:268	Tmem151b	INSIDE	0.624	2.241	2075.39	4650.67	1.399	1663.84	2327.00
A_68_P29671862	chr14:65768960-65769004	BC096449:686759		DOWNSTREAM	0.624	0.541	814.61	440.79	0.337	659.08	222.39
A_68_P29668497	chr14:65206255-65206299	NR_029813:-3217	Mir124a-1	PROMOTER	0.624	4.732	6463.42	30587.16	2.952	4681.80	13821.97
A_68_P29308203	chr13:108680619-108680663	NM_145456:-382	Zswim6	PROMOTER	0.624	0.576	11774.47	6782.52	0.359	7516.87	2701.47
A_68_P29225755	chr13:93564632-93564676	NM_011582:119	Thbs4	INSIDE	0.624	0.709	2007.16	1422.38	0.442	1683.21	744.79
A_68_P28246291	chr12:12946601-12946645	NM_008709:2020	Mycn	INSIDE	0.624	0.624	2212.10	1380.89	0.389	1506.87	586.72
A_68_P27532842	chr10:126338342-126338386	NM_026858:63	Xrcc6bp1	INSIDE	0.624	6.772	3111.90	21073.50	4.227	2593.86	10962.95

ProbeName	Target position of probe on CpG island microarray	TargetID	GeneSymbol	CpG island Description	Ratio of relative methylation (TiO_2-NP/Vehicle)	Sham group			TiO_2-H group		
						Relative methylation (Cy5/Cy3)	Cy3 signal (Input)	Cy5 signal (MeDIP)	Relative methylation (Cy5/Cy3)	Cy3 signal (Input)	Cy5 signal (MeDIP)
A_68_P27279734	chr10:79383882-79383926	NM_053244:4189	Kiss1r	INSIDE	0.624	4.801	2768.17	13290.42	2.995	2320.87	6950.62
A_68_P26825597	chr9:110646599-110646643	NM_001083935:-430	Pth1r	PROMOTER	0.624	0.730	2240.81	1636.05	0.456	1744.84	795.52
A_68_P26825596	chr9:110646504-110646548	NM_001083935:-336	Pth1r	PROMOTER	0.624	0.398	1593.26	633.92	0.248	1178.52	292.71
A_68_P26580033	chr9:63932582-63932626	AK011166:4037		INSIDE	0.624	0.462	1083.26	500.62	0.288	862.26	248.51
A_68_P26352023	chr9:22280042-22280086	NM_178415:-94	Bbs9	DIVERGENT_PROMOTER	0.624	0.681	2530.15	1722.45	0.425	1873.38	795.39
A_68_P26348588	chr9:21528254-21528298	NM_010700:239	Ldlr	INSIDE	0.624	4.640	420.71	1952.05	2.895	350.96	1016.16
A_68_P25500897	chr7:133645570-133645614	NM_183020:1224	Atxn2l	INSIDE	0.624	0.637	3532.65	2251.82	0.398	2754.98	1096.48
A_68_P25496227	chr7:132851431-132851475	NM_001081022:63	D430042O09Rik	INSIDE	0.624	4.440	489.46	2173.21	2.769	466.29	1290.99
A_68_P24424708	chr6:47827424-47827468	NM_146175:-107	Zfp282	PROMOTER	0.624	3.092	728.07	2251.11	1.928	566.69	1092.83
A_68_P24195535	chr5:152036181-152036225	ENSMUST00000129088:147		INSIDE	0.624	0.489	1088.28	532.44	0.305	969.24	295.70
A_68_P23392563	chr4:147504999-147505043	NM_027426:-213	2610109H07Rik	DIVERGENT_PROMOTER	0.624	2.784	1257.54	3500.59	1.738	1112.53	1933.54
A_68_P23308588	chr4:131393415-131393459	NM_001083119:757	Ptpru	INSIDE	0.624	2.811	16370.49	46015.01	1.754	9681.65	16981.78
A_68_P23062804	chr4:83171213-83171257	NM_001081012:-214	4930473A06Rik	PROMOTER	0.624	4.196	1106.91	4644.93	2.617	959.48	2511.44
A_68_P22129479	chr3:51212850-51212894	NM_025523:5	Ndufc1	INSIDE	0.624	0.465	1382.49	643.32	0.291	1181.27	343.23
A_68_P21569912	chr2:119303420-119303464	NM_026574:-77	Ino80	PROMOTER	0.624	0.142	4321.30	611.75	0.088	2933.78	259.28
A_68_P21516744	chr2:109534011-109534055	NM_001048142:313	Bdnf	INSIDE	0.624	0.327	4866.44	1591.55	0.204	3104.13	633.11
A_68_P21491162	chr2:104689920-104689964	NM_178695:65	Prrg4	INSIDE	0.624	3.505	1523.59	5340.27	2.188	1418.23	3103.54
A_68_P21426309	chr2:91900065-91900109	ENSMUST00000123840:-560		PROMOTER	0.624	0.397	2708.90	1076.30	0.248	1914.38	474.78
A_68_P20832487	chr1:174271228-174271272	NM_001039484:-90	Kcnj10	PROMOTER	0.624	0.626	4056.32	2539.02	0.391	2832.93	1106.93
A_68_P32913428	A_68_P32913428			Unknown	0.623	4.319	701.82	3031.17	2.689	568.37	1528.45
A_68_P32707838	chrX:138825108-138825152	NM_001033600:-52	Acsl4	PROMOTER	0.623	3.089	727.07	2245.69	1.924	1147.89	2208.03
A_68_P32149065	chr19:47388402-47388446	NM_001164717:150477	Sh3pxd2a	INSIDE	0.623	3.537	400.11	1415.08	2.204	324.69	715.73
A_68_P32021818	chr19:24205720-24205764	NM_001198985:42888	Tjp2	INSIDE	0.623	5.235	2439.68	12771.47	3.263	2049.05	6686.72
A_68_P31398414	chr17:83750191-83750235	NM_001114361:-58	Eml4	PROMOTER	0.623	3.139	735.81	2309.96	1.957	615.22	1204.15
A_68_P31145776	chr17:32629460-32629505	NM_177307:39815	Cyp4f39	INSIDE	0.623	2.462	1107.20	2726.28	1.535	870.36	1336.12
A_68_P31112025	chr17:26853064-26853108	NM_029870:492	A930001N09Rik	INSIDE	0.623	0.244	2944.73	719.98	0.152	2263.71	345.07
A_68_P31097493	chr17:24606963-24607007	NM_025954:-433	Pgp	PROMOTER	0.623	15.399	2600.81	40050.20	9.594	2580.53	24757.08
A_68_P30782600	chr16:56717318-56717362	NM_019678:127	Tfg	INSIDE	0.623	2.713	1052.83	2856.10	1.690	839.37	1418.80
A_68_P30572951	chr16:17278027-17278071	NM_177473:1656	Tmem191c	INSIDE	0.623	3.377	990.52	3345.19	2.102	806.69	1696.04
A_68_P30423564	chr15:88991163-88991207	NM_001159521:13096	Plxnb2	INSIDE	0.623	4.460	833.84	3719.29	2.780	702.71	1953.79
A_68_P30367930	chr15:79521605-79521649	NM_013818:301	Gtpbp1	INSIDE	0.623	0.345	2604.32	898.00	0.215	2091.72	449.21
A_68_P30342960	chr15:75447074-75447118	NM_001044718:-18	Zfp41	PROMOTER	0.623	0.576	6231.42	3590.13	0.359	4329.92	1554.37
A_68_P29288687	chr13:104899925-104899970	NM_144838:78	Sgtb	INSIDE	0.623	0.459	3803.84	1747.20	0.286	2921.93	836.11
A_68_P28185588	chr11:120469756-120469808	NM_153288:-8	Npb	PROMOTER	0.623	0.177	2687.01	474.46	0.110	1881.60	207.09
A_68_P27910787	chr11:72128038-72128082	NM_001099688:115	Fbxo39	INSIDE	0.623	5.361	1370.43	7347.23	3.338	1159.75	3870.82
A_68_P27278520	chr10:79187527-79187571	NM_008226:8170	Hcn2	INSIDE	0.623	22.870	1189.04	27193.51	14.243	1050.53	14963.06
A_68_P26585802	chr9:64949678-64949722	NM_020043:399	Igdcc4	INSIDE	0.623	2.710	2645.64	7169.06	1.689	2158.49	3646.37
A_68_P26542152	chr9:57352020-57352064	NM_133982:134	Rpp25	INSIDE	0.623	0.410	1348.97	553.46	0.256	1025.33	262.28
A_68_P26527193	chr9:54547061-54547105	NM_021422:-283	Dnaja4	PROMOTER	0.623	0.446	6973.54	3108.14	0.278	4447.90	1234.44
A_68_P26526112	chr9:54349310-54349354	NM_172771:101	Dmxl2	INSIDE	0.623	5.160	1932.73	9972.70	3.214	1531.74	4922.86
A_68_P26016964	chr8:86423687-86423732	NM_181039:-287	Lphn1	PROMOTER	0.623	0.181	3917.41	708.75	0.113	2824.93	318.62
A_68_P25603236	chr7:151015869-151015913	NM_007856:6819	Dhcr7	INSIDE	0.623	0.607	1330.03	807.58	0.378	1006.46	380.57
A_68_P25235399	chr7:81699616-81699660	NM_001038643:28	Slco3a1	INSIDE	0.623	7.709	1065.28	8212.05	4.801	923.64	4434.64
A_68_P24383760	chr6:39523904-39523948	NM_178873:52	Adck2	INSIDE	0.623	0.439	3958.96	1738.61	0.274	2789.82	763.42
A_68_P24332082	chr6:30409887-30409931	NM_029742:58000	Klhdc10	DOWNSTREAM	0.623	0.634	1167.19	740.30	0.395	1022.73	404.42
A_68_P24330580	chr6:30123352-30123396	NR_038124:-3248	BB283400	DIVERGENT_PROMOTER	0.623	0.457	5725.42	2616.50	0.285	3971.11	1130.45
A_68_P23609093	chr5:37087355-37087399	NM_001081232:-168	D5Ertd579e	PROMOTER	0.623	5.078	3228.04	16392.35	3.162	2377.72	7517.28
A_68_P23309921	chr4:131604501-131604545	NM_001128606:471	Epb4.1	INSIDE	0.623	7.245	2333.47	16906.60	4.517	2015.94	9105.56
A_68_P22410425	chr3:107889046-107889090	NM_028779:477	Ampd2	INSIDE	0.623	3.158	3404.72	10751.44	1.967	2716.32	5342.97
A_68_P21874764	chr2:174156135-174156179	NM_001077510:567	Gnas	INSIDE	0.623	0.289	3251.12	940.13	0.180	2377.04	428.49
A_68_P21820252	chr2:164704950-164704994	NM_146129:105	Pcif1	INSIDE	0.623	0.512	3429.51	1756.11	0.319	2458.11	784.06
A_68_P21666835	chr2:136940626-136940670	NM_013822:1608	Jag1	INSIDE	0.623	0.563	1111.48	625.25	0.350	832.34	291.65
A_68_P21585601	chr2:122127327-122127371	NM_025777:2713	Duoxa2	INSIDE	0.623	0.416	4013.85	1671.38	0.259	2744.06	711.44
A_68_P21557415	chr2:117168712-117168756	NM_011246:-121	Rasgrp1	PROMOTER	0.623	0.383	1740.34	665.95	0.239	1362.08	324.93
A_68_P21476827	chr2:102241389-102241433	NM_020267:-353	Trim44	PROMOTER	0.623	0.418	1848.56	772.14	0.260	1437.23	374.26
A_68_P21086006	chr2:27533318-27533362	NM_011305:620	Rxra	INSIDE	0.623	5.439	1315.61	7155.05	3.388	1084.55	3674.72
A_68_P30501057	chr15:102796954-102796998	NM_010462:-250	Hoxc10	PROMOTER	0.622	0.627	1951.61	1223.01	0.390	1587.51	618.69
A_68_P30127461	chr15:34168406-34168450	NM_033521:648	Laptm4b	INSIDE	0.622	3.197	1526.56	4880.56	1.989	1230.31	2447.01
A_68_P29977216	chr15:3977197-3977241	NR_028266:188	BC037032	INSIDE	0.622	2.745	1179.63	3237.95	1.708	838.75	1432.27
A_68_P29239503	chr13:95992534-95992578	NM_001170669:449	Pde8b	INSIDE	0.622	8.204	1422.05	11666.24	5.106	1214.95	6203.03
A_68_P28914780	chr13:30076726-30076770	NM_010093:1184	E2f3	INSIDE	0.622	0.564	855.68	482.30	0.350	674.32	236.33
A_68_P27101048	chr10:43299118-43299162	NM_009846:166	Cd24a	INSIDE	0.622	0.233	3236.69	755.25	0.145	2227.95	323.47
A_68_P26021287	chr8:87230962-87231012	NM_001081981:67282	Nfix	DOWNSTREAM	0.622	10.542	893.18	9415.44	6.559	726.21	4763.02
A_68_P25971363	chr8:77517838-77517882	NM_178017:259	Hmgxb4	INSIDE	0.622	5.667	1304.00	7390.26	3.525	1090.59	3844.49
A_68_P25046335	chr7:35697613-35697657	NM_008820:209	Pepd	INSIDE	0.622	2.561	1639.47	4197.96	1.592	1272.31	2025.65

ProbeName	Target position of probe on CpG island microarray	TargetID	GeneSymbol	CpG island Description	Ratio of relative methylation (TiO_2-NP/Vehicle)	Sham group			TiO_2-H group		
						Relative methylation (Cy5/Cy3)	Cy3 signal (Input)	Cy5 signal (MeDIP)	Relative methylation (Cy5/Cy3)	Cy3 signal (Input)	Cy5 signal (MeDIP)
A_68_P25034849	chr7:31852601-31852645	NM_144556:7669	Lgi4	INSIDE	0.622	2.883	1235.29	3561.33	1.794	1048.81	1881.10
A_68_P24466829	chr6:54988330-54988374	NM_180678:358	Gars	INSIDE	0.622	0.265	2550.94	677.06	0.165	1923.10	317.45
A_68_P24162463	chr5:145529030-145529074	NM_001081362:-608	Trrap	DIVERGENT_PROMOTER	0.622	4.582	3665.54	16795.20	2.849	3019.60	8603.87
A_68_P24141811	chr5:141000117-141000161	NM_021528:18576	Chst12	INSIDE	0.622	2.961	3669.43	10865.00	1.842	2656.37	4892.24
A_68_P23760674	chr5:66651117-66651161	NM_027602:-225	Nsun7	PROMOTER	0.622	0.663	874.08	579.66	0.413	769.24	317.43
A_68_P23441289	chr4:155266283-155266327	NM_207223:321	Acap3	INSIDE	0.622	5.053	2175.04	10991.38	3.141	1723.05	5411.89
A_68_P22705223	chr4:6292852-6292896	NM_001098227:48	Sdcbp	INSIDE	0.622	4.126	4357.31	17976.09	2.564	3521.59	9029.89
A_68_P21767075	chr2:155517577-155517621	NM_001163452:522	Trpc4ap	INSIDE	0.622	0.097	4922.71	475.10	0.060	3575.68	214.62
A_68_P21089695	chr2:28061711-28061755	NM_001038612:524	Olfml	INSIDE	0.622	2.619	1781.67	4665.33	1.630	1436.98	2341.80
A_68_P20770398	chr1:163101115-163101159	NM_028664:28294	Ankrd45	DOWNSTREAM	0.622	0.353	2426.52	855.76	0.219	1820.75	399.46
A_68_P31807637	chr18:70212806-70212850	NM_030554:195	Rab27b	INSIDE	0.621	0.573	1332.73	763.07	0.356	1142.22	406.28
A_68_P31677409	chr18:46684937-46684981	NM_173423:667	Fem1c	INSIDE	0.621	0.254	1911.12	485.40	0.158	1233.54	194.61
A_68_P30345385	chr15:75829377-75829421	NM_177922:5200	Mapk15	INSIDE	0.621	2.862	4479.57	12821.58	1.778	3593.24	6389.98
A_68_P29863087	chr14:103381523-103381567	NM_177715:310	Kctd12	INSIDE	0.621	11.845	1906.41	22582.14	7.352	2046.13	15042.13
A_68_P29455869	chr14:22804369-22804413	NM_145459:4433	Zfp503	INSIDE	0.621	0.570	1621.00	923.93	0.354	1140.35	403.81
A_68_P28480018	chr12:62623914-62623958	NM_178714:-1682	Lrfn5	PROMOTER	0.621	0.662	1690.07	1119.24	0.411	1292.42	531.60
A_68_P28078907	chr11:102180135-102180179	NM_001044383:-1555	Ubtf	PROMOTER	0.621	0.578	1183.42	683.47	0.358	873.60	313.14
A_68_P28035919	chr11:94852484-94852528	NM_172261:-19	Ppp1r9b	PROMOTER	0.621	5.740	701.44	4026.28	3.563	655.97	2337.10
A_68_P27543981	chr10:128370721-128370765	NM_008398:-126	Itga7	PROMOTER	0.621	0.455	1960.48	892.28	0.283	1642.02	464.24
A_68_P27539153	chr10:127472665-127472709	NM_001113199:285	Naca	INSIDE	0.621	0.723	4190.81	3028.70	0.449	2684.43	1204.30
A_68_P27355937	chr10:93297003-93297047	NM_183199:2725	Usp44	INSIDE	0.621	2.803	1930.38	5410.32	1.740	1608.41	2797.89
A_68_P27282040	chr10:79723724-79723768	NM_010255:-33	Gamt	DIVERGENT_PROMOTER	0.621	0.297	3735.52	1109.67	0.184	2460.85	453.80
A_68_P26809753	chr9:107489861-107489907	NM_025903:-164	Ifrd2	PROMOTER	0.621	0.156	3321.26	518.12	0.097	2147.41	208.01
A_68_P26469197	chr9:44529171-44529218	NM_153537:14083	Phldb1	INSIDE	0.621	0.529	973.07	515.01	0.329	808.66	265.78
A_68_P26343622	chr9:20531703-20531748	NM_173777:933	Olfm2	INSIDE	0.621	0.319	3075.19	980.37	0.198	2381.44	471.48
A_68_P26134611	chr8:108090115-108090160	NM_013477:-197	Atp6v0d1	PROMOTER	0.621	0.264	2188.98	578.96	0.164	1461.52	240.24
A_68_P25477639	chr7:129303337-129303384	NM_011121:410	Plk1	INSIDE	0.621	0.630	1176.14	741.12	0.391	935.48	366.10
A_68_P24950759	chr7:4788111-4788155	NM_172737:8166	Shisa7	INSIDE	0.621	4.371	1191.87	5209.93	2.714	1008.51	2736.63
A_68_P24735989	chr6:108778919-108778963	NM_138677:306	Edem1	INSIDE	0.621	7.337	533.75	3915.99	4.555	451.21	2055.29
A_68_P23882644	chr5:90795790-90795834	NM_030886:-601	Ankrd17	PROMOTER	0.621	2.528	4075.12	10301.11	1.571	3333.51	5236.64
A_68_P23278482	chr4:125826960-125827005	NM_172145:1736	Fam176b	INSIDE	0.621	22.693	1358.59	30830.98	14.099	1663.80	23457.48
A_68_P22339768	chr3:94386146-94386190	NM_029721:456	Snx27	INSIDE	0.621	0.202	11436.09	2306.99	0.125	7841.33	982.67
A_68_P21102914	chr2:30247811-30247855	NM_020329:-103	Dolpp1	PROMOTER	0.621	0.366	4699.42	1717.82	0.227	3180.84	722.04
A_68_P20587179	chr1:127808497-127808541	NM_145100:273	Lypd1	INSIDE	0.621	2.893	876.14	2535.08	1.798	766.61	1378.38
A_68_P32096195	chr19:37625247-37625291	NM_175353:361	Exoc6	INSIDE	0.620	4.482	768.98	3446.73	2.779	836.16	2323.83
A_68_P31489152	chr18:10325147-10325191	NM_001083628:-8	Greb1l	PROMOTER	0.620	7.009	1588.16	11130.92	4.347	1481.65	6440.09
A_68_P30363604	chr15:78813243-78813287	NM_001024716:-221	Triobp	PROMOTER	0.620	0.617	3301.33	2038.24	0.383	2551.90	977.32
A_68_P30359772	chr15:78241077-78241121	NM_138670:3565	Mpst	INSIDE	0.620	0.635	1534.70	975.17	0.394	1070.64	421.61
A_68_P29644193	chr14:60997735-60997779	NM_001164705:634	Fam123a	INSIDE	0.620	5.234	1279.32	6696.27	3.244	1051.92	3412.79
A_68_P29615089	chr14:55545056-55545100	NM_010112:547	Efs	INSIDE	0.620	3.963	4140.98	16412.57	2.456	3196.81	7850.12
A_68_P29244724	chr13:96902709-96902753	NM_029210:-198	Sv2c	PROMOTER	0.620	0.296	7090.20	2097.89	0.183	5648.62	1035.85
A_68_P28957528	chr13:38022622-38022667	NM_001177869:41905	Rreb1	INSIDE	0.620	2.904	814.66	2365.55	1.799	596.37	1073.06
A_68_P28535623	chr12:74213467-74213511	NM_011382:744	Six4	INSIDE	0.620	0.277	1809.29	501.42	0.172	1015.45	174.58
A_68_P28431901	chr12:52930414-52930458	NM_144788:87	Hectd1	INSIDE	0.620	3.725	941.58	3507.00	2.308	811.98	1874.11
A_68_P28396959	chr12:45310845-45310889	NM_013760:189	Dnajb9	INSIDE	0.620	0.375	1932.72	724.84	0.233	1354.14	315.03
A_68_P28166391	chr11:117517129-117517173	NM_198022:1548	Tnrc6c	INSIDE	0.620	4.025	3118.10	12549.69	2.495	2399.34	5985.44
A_68_P27643714	chr11:22882221-22882265	NM_144514:42	Commd1	INSIDE	0.620	2.866	635.28	1820.92	1.778	571.21	1015.76
A_68_P27631991	chr11:20443663-20443707	NM_021372:429	Sertad2	INSIDE	0.620	3.967	1030.68	4089.01	2.458	875.72	2152.93
A_68_P26494144	A_68_P26494144			Unknown	0.620	2.658	1667.24	4431.47	1.647	1324.80	2181.95
A_68_P26132587	chr8:107788500-107788544	NM_015821:-25	Fbxl8	DIVERGENT_PROMOTER	0.620	6.688	818.66	5475.04	4.149	720.00	2986.96
A_68_P25228678	chr7:80521046-80521090	NM_177740:663	Rgma	INSIDE	0.620	3.324	920.53	3060.04	2.061	860.57	1773.29
A_68_P24205537	chr6:4550857-4550901	NM_145398:-187	Casd1	PROMOTER	0.620	0.429	1236.20	530.94	0.266	935.01	248.92
A_68_P24045427	chr5:122043186-122043230	NM_009656:625	Aldh2	INSIDE	0.620	0.512	1553.34	795.36	0.317	1207.96	383.37
A_68_P24010226	chr5:116009731-116009775	NM_007475:277	Rplp0	INSIDE	0.620	3.015	1157.91	3490.80	1.869	927.84	1734.05
A_68_P23296216	chr4:129012377-129012421	NM_009030:216	Rbbp4	INSIDE	0.620	2.391	1301.35	3111.39	1.482	1132.09	1678.06
A_68_P23262157	chr4:123019781-123019825	NM_007558:244	Bmp8a	INSIDE	0.620	2.786	2262.42	6303.18	1.727	1798.22	3105.00
A_68_P21138067	chr2:35992191-35992235	NM_001159635:12	Rbm18	INSIDE	0.620	0.585	1624.14	950.81	0.363	1272.33	462.00
A_68_P20737715	chr1:157264567-157264611	NM_011273:-14	Xpr1	PROMOTER	0.620	2.675	1802.36	4820.78	1.659	1416.57	2349.84
A_68_P20639343	chr1:137326748-137326792	NM_153774:298	Ipo9	INSIDE	0.620	0.209	5870.25	1225.02	0.129	4153.60	537.32
A_68_P20451396	chr1:94727977-94728021	NM_016696:-264	Gpc1	PROMOTER	0.620	0.438	3142.99	1376.76	0.272	2569.68	698.21
A_68_P20149813	chr1:36748609-36748653	NM_009942:299	Cox5b	INSIDE	0.620	0.676	1744.61	1179.52	0.419	1440.90	604.23
A_68_P32143657	chr19:46470296-46470340	NM_016860:-93	Actr1a	DIVERGENT_PROMOTER	0.619	0.266	1705.64	454.50	0.165	1316.50	217.12
A_68_P31244136	chr17:53706802-53706846	NM_020005:529	Kat2b	INSIDE	0.619	2.746	768.35	2110.09	1.700	600.24	1020.37
A_68_P30925866	chr16:84835027-84835071	NM_008065:-320	Gabpa	PROMOTER	0.619	0.299	3781.56	1129.27	0.185	2904.43	537.16
A_68_P30230393	chr15:54944017-54944061	NM_145470:1	Deptor	INSIDE	0.619	0.248	2399.95	595.03	0.153	1836.11	281.63

ProbeName	Target position of probe on CpG island microarray	TargetID	GeneSymbol	CpG island Description	Ratio of relative methylation (TiO_2-NP/Vehicle)	Sham group			TiO_2-H group		
						Relative methylation (Cy5/Cy3)	Cy3 signal (Input)	Cy5 signal (MeDIP)	Relative methylation (Cy5/Cy3)	Cy3 signal (Input)	Cy5 signal (MeDIP)
A_68_P30019884	chr15:12048005-12048049	NM_011767:421	Zfr	INSIDE	0.619	0.514	1950.82	1003.57	0.318	1385.51	441.13
A_68_P29660046	chr14:63859832-63859876	NM_008092:4243	Gata4	INSIDE	0.619	3.742	900.18	3368.69	2.315	899.96	2083.61
A_68_P28712491	chr12:107248439-107248483	NM_001029843:-12	Vrk1	PROMOTER	0.619	0.317	1521.11	482.70	0.197	1173.76	230.73
A_68_P28160474	chr11:116485631-116485675	NM_001167680:-86	Rhbdf2	PROMOTER	0.619	4.052	5239.83	21233.13	2.508	4416.02	11076.95
A_68_P28050120	chr11:97224658-97224702	NM_138657:816	Socs7	INSIDE	0.619	0.580	1565.12	908.08	0.359	1240.45	445.47
A_68_P27914870	chr11:72861801-72861845	NM_025818:454	1200014J11Rik	INSIDE	0.619	0.599	5663.69	3391.66	0.371	4153.41	1539.71
A_68_P27900566	chr11:70223756-70223800	NM_029231:-245	Pelp1	PROMOTER	0.619	0.307	2775.70	851.51	0.190	1948.56	370.09
A_68_P27561153	chr11:4961885-4961929	NM_001190406:3424	Gas2l1	INSIDE	0.619	0.677	1434.71	971.99	0.419	1094.47	459.05
A_68_P27284931	chr10:80165309-80165353	NM_027829:-107	Izumo4	DIVERGENT_PROMOTER	0.619	3.797	3907.29	14834.83	2.349	3037.74	7136.60
A_68_P27282831	chr10:79844492-79844536	NM_198615:5882	Mex3d	INSIDE	0.619	9.614	1963.34	18875.82	5.948	1617.24	9619.99
A_68_P25959792	chr8:74658995-74659039	NM_001001491:-174	Tpm4	PROMOTER	0.619	0.191	8003.40	1525.75	0.118	5199.51	613.25
A_68_P25150562	chr7:64642665-64642709	NR_033504:45	Gm9962	INSIDE	0.619	0.147	3091.74	453.42	0.091	2218.58	201.28
A_68_P25049869	chr7:36322564-36322608	NM_028034:177	Tdrd12	INSIDE	0.619	3.598	1204.97	4335.94	2.229	882.05	1965.80
A_68_P24167580	chr5:147072895-147072939	NM_153599:29666	Cdk8	INSIDE	0.619	5.660	379.19	2146.16	3.503	345.31	1209.71
A_68_P24078663	chr5:128097296-128097340	NM_133895:444	Slc15a4	INSIDE	0.619	3.087	614.40	1896.37	1.910	537.33	1026.19
A_68_P23819018	chr5:77380092-77380136	NM_025939:-321	Paics	PROMOTER	0.619	0.307	2695.99	826.54	0.190	1819.59	345.12
A_68_P23240388	chr4:118109735-118109779	NM_023223:192	Cdc20	INSIDE	0.619	0.273	5585.23	1524.87	0.169	3794.64	641.15
A_68_P21349791	chr2:76208360-76208404	NM_153405:342	Rbm45	INSIDE	0.619	0.589	1176.42	692.52	0.365	858.91	313.18
A_68_P20353935	chr1:75419232-75419276	NM_001085370:37070	Speg	INSIDE	0.619	0.457	7924.28	3617.88	0.283	5372.85	1518.28
A_68_P20351021	chr1:74929129-74929173	NM_153111:2832	Fev	INSIDE	0.619	5.870	1780.93	10454.00	3.635	1496.83	5440.77
A_68_P20165367	chr1:39425174-39425218	NM_053257:501	Rpl31	INSIDE	0.619	0.346	1655.86	572.29	0.214	1238.15	265.09
A_68_P32408601	chrX:56388072-56388116	NM_010200:-481	Fgf13	PROMOTER	0.618	3.064	965.40	2958.24	1.895	1082.71	2051.70
A_68_P32233086	chrX:5659825-5659869	NM_001033211:692	AU022751	INSIDE	0.618	2.791	432.50	1207.17	1.725	710.91	1226.30
A_68_P32217658	chr19:59240861-59240905	NM_009501:3637	Vax1	INSIDE	0.618	0.554	1146.76	635.07	0.342	1030.08	352.77
A_68_P32147605	chr19:47164876-47164920	NM_023211:217	Usmg5	INSIDE	0.618	0.700	1728.07	1209.69	0.432	1282.08	554.37
A_68_P31425147	chr17:88196405-88196449	NM_199251:908	Kcnk12	INSIDE	0.618	0.647	873.49	565.25	0.400	724.27	289.76
A_68_P30980682	chr16:94535261-94535305	ENSMUST00000163193:-112		PROMOTER	0.618	2.881	2075.65	5979.36	1.780	1590.71	2831.37
A_68_P30931894	chr16:85900162-85900206	NM_011782:1186	Adamts5	INSIDE	0.618	5.067	1408.34	7136.26	3.134	1074.45	3367.18
A_68_P30678526	chr16:37011868-37011912	NM_001159369:19	Polq	INSIDE	0.618	4.006	1317.57	5278.82	2.475	1050.69	2600.68
A_68_P30365376	chr15:79092452-79092496	NM_177580:23465	Baiap2l2	INSIDE	0.618	2.890	590.12	1705.23	1.784	524.32	935.57
A_68_P27971718	chr11:83286618-83286662	NM_027427:31	Taf15	INSIDE	0.618	11.464	1383.49	15859.68	7.083	1151.42	8155.26
A_68_P27851930	chr11:61890326-61890370	NM_001029936:-250	Specc1	PROMOTER	0.618	2.395	7250.65	17368.42	1.479	5633.34	8334.09
A_68_P27497666	chr10:119912069-119912113	NM_010441:1901	Hmga2	INSIDE	0.618	0.280	2463.22	689.41	0.173	1661.37	287.53
A_68_P27357767	chr10:93610648-93610692	NM_011629:-5	Nr2c1	PROMOTER	0.618	4.891	3680.06	17999.77	3.021	3083.36	9314.46
A_68_P27289052	chr10:80813045-80813089	NM_001163165:105	Hmg20b	INSIDE	0.618	0.549	1560.79	856.29	0.339	1217.17	412.93
A_68_P27283432	chr10:79934830-79934874	ENSMUST00000158603:-4244		PROMOTER	0.618	2.992	1721.30	5149.51	1.849	1395.52	2580.52
A_68_P27222447	chr10:68675289-68675333	NM_001081347:-94	Rhobtb1	PROMOTER	0.618	3.573	1151.97	4116.12	2.207	1052.26	2322.50
A_68_P27165512	chr10:58276626-58276670	NM_172788:542	Sh3rf3	INSIDE	0.618	4.377	979.13	4285.71	2.704	964.58	2608.64
A_68_P26528975	chr9:54874310-54874354	NM_145129:34	Chrna3	INSIDE	0.618	2.598	876.18	2276.68	1.606	811.28	1302.93
A_68_P25176744	chr7:71536910-71536954	NM_026483:190	Mphosph10	INSIDE	0.618	2.574	2038.22	5246.48	1.591	1557.27	2477.57
A_68_P24820216	chr6:125311932-125311976	NM_011609:12214	Tnfrsf1a	INSIDE	0.618	0.527	1365.02	719.29	0.325	1061.45	345.42
A_68_P23766883	chr5:67652121-67652165	NM_028975:252	Tmem33	INSIDE	0.618	0.519	1049.40	544.75	0.321	897.43	287.77
A_68_P23349526	chr4:138630332-138630376	NM_021358:350	Htr6	INSIDE	0.618	0.387	3999.32	1547.42	0.239	2661.55	636.77
A_68_P23331982	chr4:135638026-135638070	ENSMUST00000121307:-7161		PROMOTER	0.618	0.617	1295.46	799.02	0.381	1052.99	401.64
A_68_P23319594	chr4:133369212-133369256	ENSMUST00000129875:-3100		PROMOTER	0.618	0.169	4992.85	843.17	0.104	3557.13	371.42
A_68_P23186784	chr4:106512831-106512875	ENSMUST00000151369:7673		DOWNSTREAM	0.618	3.163	861.59	2724.98	1.954	798.89	1561.17
A_68_P22478235	chr3:121426699-121426743	NM_010171:266	F3	INSIDE	0.618	4.292	962.14	4129.90	2.655	857.04	2275.28
A_68_P21874393	chr2:174110277-174110321	NM_019690:478	Gnas	INSIDE	0.618	3.956	2342.57	9267.78	2.444	1863.83	4555.98
A_68_P20025490	chr1:10029865-10029909	NM_026493:1588	Cspp1	INSIDE	0.618	0.650	1250.34	812.95	0.402	939.06	377.06
A_68_P20007250	chr1:4797717-4797761	NM_008866:-235	Lypla1	PROMOTER	0.618	4.059	603.83	2451.13	2.509	499.11	1252.10
A_68_P32127607	chr19:43691200-43691244	NM_008699:4408	Nkx2-3	DOWNSTREAM	0.617	0.680	1225.57	833.76	0.420	1122.07	471.31
A_68_P32092667	chr19:36993325-36993369	NM_028263:743	Fgfbp3	INSIDE	0.617	4.072	762.42	3104.58	2.511	632.75	1588.68
A_68_P31943308	chr19:8815842-8815886	NM_001167799:-49	Stx5a	PROMOTER	0.617	0.322	3042.10	980.33	0.199	2102.17	417.85
A_68_P31939393	chr19:7416055-7416099	NM_001080388:258	Mark2	INSIDE	0.617	4.153	5950.38	24711.91	2.563	4671.16	11973.90
A_68_P31208848	chr17:46910008-46910052	NM_025966:447	2310039H08Rik	INSIDE	0.617	2.514	1145.74	2880.72	1.552	909.10	1411.01
A_68_P31092442	chr17:23713394-23713438	NM_011747:23038	Zfp13	INSIDE	0.617	2.353	1954.88	4599.91	1.452	1465.36	2127.48
A_68_P30595261	chr16:21947632-21947676	NM_025693:-29	Tmem41a	PROMOTER	0.617	2.893	1256.19	3633.66	1.786	1074.19	1918.16
A_68_P30520836	chr16:6349205-6349249	NM_021477:464341	Rbfox1	INSIDE	0.617	0.314	2729.62	856.06	0.194	2051.37	397.23
A_68_P29453022	chr14:22318726-22318770	NM_001205241:-243	Myst4	PROMOTER	0.617	0.612	2935.38	1795.20	0.377	2104.24	794.22
A_68_P29134803	chr13:72766020-72766064	NR_030701:-30	D430050G20	DIVERGENT_PROMOTER	0.617	0.506	3248.06	1644.28	0.312	2563.93	800.55
A_68_P28767699	chr12:117643707-117643751	NR_028421:-94	D430020J02Rik	DIVERGENT_PROMOTER	0.617	0.429	2037.08	874.35	0.265	1444.33	382.36
A_68_P28334082	chr12:32634899-32634943	NM_153198:480	Hbp1	INSIDE	0.617	3.210	1745.62	5603.41	1.981	1435.26	2842.98
A_68_P28238663	chr12:11462730-11462774	NM_001111118:-132	Rad51ap2	PROMOTER	0.617	3.567	3579.91	12769.26	2.200	3008.78	6618.02
A_68_P27836979	chr11:59263493-59263537	NM_001205068:-32	Jmjd4	DIVERGENT_PROMOTER	0.617	0.404	1241.18	500.96	0.249	1015.58	252.77
A_68_P27226083	chr10:69285075-69285119	NM_146005:288641	Ank3	INSIDE	0.617	2.509	2188.21	5490.55	1.549	1392.63	2157.05

ProbeName	Target position of probe on CpG island microarray	TargetID	GeneSymbol	CpG island Description	Ratio of relative methylation (TiO_2-NP/Vehicle)	Sham group			TiO_2-H group		
						Relative methylation (Cy5/Cy3)	Cy3 signal (Input)	Cy5 signal (MeDIP)	Relative methylation (Cy5/Cy3)	Cy3 signal (Input)	Cy5 signal (MeDIP)
A_68_P27172919	chr10:59649414-59649458	NM_016803:32572	Chst3	INSIDE	0.617	3.538	1321.21	4674.07	2.184	1074.13	2345.67
A_68_P26871926	chr9:119266492-119266536	NM_001033209:16	Xylb	INSIDE	0.617	0.605	1842.76	1115.11	0.374	1325.42	495.09
A_68_P26523853	chr9:53916041-53916085	NM_001033535:156	Tnfaip8l3	INSIDE	0.617	0.709	1251.44	887.81	0.438	1063.38	465.32
A_68_P26430295	chr9:37192346-37192390	NM_175189:17178	Hepacam	INSIDE	0.617	2.931	1242.31	3640.65	1.809	1003.58	1815.85
A_68_P25957808	chr8:74135004-74135048	NM_146211:104	Glt25d1	INSIDE	0.617	4.619	1507.38	6961.87	2.850	1270.62	3621.13
A_68_P25951136	chr8:72854054-72854098	NM_008107:413	Gdf1	INSIDE	0.617	2.689	870.64	2340.93	1.659	696.33	1155.04
A_68_P24424174	chr6:47601153-47601197	NR_002841:3919	Rn4.5s	DOWNSTREAM	0.617	0.681	10862.09	7397.97	0.420	7550.37	3173.78
A_68_P23763269	chr5:67068133-67068180	NM_011670:797	Uchl1	INSIDE	0.617	0.527	1662.19	875.34	0.325	1401.92	455.48
A_68_P23567513	chr5:29761841-29761885	NM_001033457:656	Noml	INSIDE	0.617	0.445	1702.54	756.86	0.274	1418.49	388.89
A_68_P23471513	chr5:9161567-9161611	NM_001110327:188	Dmtf1	INSIDE	0.617	5.639	1528.65	8619.96	3.479	1372.97	4776.48
A_68_P23416140	chr4:151406689-151406733	NM_031867:5889	Tas1r1	INSIDE	0.617	3.249	1622.87	5273.41	2.005	1058.51	2122.32
A_68_P23400514	chr4:148800490-148800534	NM_022022:228	Ube4b	INSIDE	0.617	2.289	5177.47	11852.33	1.412	4073.89	5750.92
A_68_P23190564	chr4:107107777-107107821	NM_147221:475	Glis1	INSIDE	0.617	0.601	771.05	463.15	0.371	690.12	255.97
A_68_P22951801	chr4:58565861-58565905	NM_172989:283	Lpar1	INSIDE	0.617	0.698	1198.78	837.11	0.431	906.91	391.00
A_68_P22286851	chr3:83570878-83570922	NM_009144:658	Sfrp2	INSIDE	0.617	0.366	1328.72	485.86	0.225	1055.29	237.95
A_68_P22222508	chr3:69525922-69525966	NM_133787:-32	Nmd3	PROMOTER	0.617	0.364	1555.03	565.39	0.224	1097.87	246.18
A_68_P21010259	chr2:12223211-12223256	NM_001001309:314	Itga8	INSIDE	0.617	0.568	1489.17	845.50	0.350	1228.66	430.17
A_68_P20658349	chr1:140515986-140516030	NM_021605:265	Nek7	INSIDE	0.617	3.842	1650.05	6338.98	2.372	1373.02	3256.17
A_68_P32464005	chrX:70905148-70905192	NM_001199351:23	Pnck	INSIDE	0.616	2.852	851.64	2428.53	1.757	1087.71	1910.68
A_68_P31922428	chr19:4305887-4305931	NM_130863:47	Adrbk1	INSIDE	0.616	0.555	1795.46	996.54	0.342	1528.37	522.83
A_68_P31879115	chr18:83079129-83079173	NM_183033:-1120	Zfp516	PROMOTER	0.616	0.309	1518.87	468.91	0.190	1099.75	209.03
A_68_P31215274	chr17:48112426-48112470			Unknown	0.616	2.561	899.00	2302.37	1.578	806.97	1273.02
A_68_P29453037	chr14:22320470-22320514	NM_017479:1486	Myst4	INSIDE	0.616	16.195	5806.59	94039.73	9.977	5067.49	50556.74
A_68_P28449264	chr12:55896516-55896560	NM_026998:111	Snx6	INSIDE	0.616	9.543	1283.44	12247.70	5.882	1078.64	6344.10
A_68_P28328449	chr12:31596572-31596616	NM_013709:61	Sh3yl1	INSIDE	0.616	0.466	1228.16	572.73	0.287	940.82	270.05
A_68_P28003125	chr11:88835210-88835254	NM_016706:-16	Coil	PROMOTER	0.616	4.857	1558.49	7569.01	2.993	1235.06	3696.92
A_68_P27779788	chr11:48567252-48567296	ENSMUST00000120566:-58		PROMOTER	0.616	3.773	955.28	3603.95	2.325	812.23	1888.36
A_68_P27560710	chr11:4887330-4887374	NM_007454:445	Ap1b1	INSIDE	0.616	0.286	2164.43	618.98	0.176	1565.66	275.75
A_68_P27541965	chr10:127984764-127984808	NM_134003:14	Zc3h10	INSIDE	0.616	2.392	2345.64	5610.15	1.473	1932.29	2846.34
A_68_P27278801	chr10:79229322-79229366	NM_144528:322	Rnf126	INSIDE	0.616	2.837	1463.18	4151.63	1.748	1235.59	2160.32
A_68_P26476280	chr9:45646906-45646950	NM_001145947:317	Bace1	INSIDE	0.616	0.646	1611.21	1041.16	0.398	1266.32	504.31
A_68_P25949728	chr8:72607980-72608024	NM_177900:575	Hapln4	INSIDE	0.616	0.341	2269.69	772.97	0.210	1812.79	380.31
A_68_P25533785	chr7:139508177-139508221	NM_029935:640	Chst15	INSIDE	0.616	0.467	1349.27	629.68	0.287	1094.66	314.49
A_68_P25505910	chr7:134655663-134655707	NR_024331:699	1700008J07Rik	INSIDE	0.616	0.565	1316.77	744.41	0.348	1010.59	351.99
A_68_P23591963	chr5:34325816-34325860	NM_001033458:-284	Gm1673	PROMOTER	0.616	0.721	1572.43	1133.96	0.444	1049.30	466.00
A_68_P23457020	chr5:5694251-5694295	NM_001103156:-195	Steap2	PROMOTER	0.616	0.747	3671.27	2741.46	0.460	2980.38	1371.39
A_68_P23437242	chr4:154597147-154597191	NM_011385:-524	Ski	PROMOTER	0.616	3.277	413.53	1355.19	2.020	409.16	826.46
A_68_P23164323	chr4:102787124-102787168	NM_001039081:-4848	Mier1	PROMOTER	0.616	0.396	2024.81	802.00	0.244	1560.07	380.56
A_68_P22704455	chr4:6118180-6118224	NM_026534:-49	Ubxn2b	PROMOTER	0.616	0.422	2361.50	996.79	0.260	1702.39	442.49
A_68_P22593188	chr3:142544925-142544969	NM_178654:22	Pkn2	INSIDE	0.616	4.019	508.48	2043.65	2.477	451.95	1119.41
A_68_P22522751	chr3:129604360-129604404	NM_009811:40	Casp6	INSIDE	0.616	0.536	5597.09	3002.22	0.331	4207.73	1391.42
A_68_P22056223	chr3:35899395-35899439	NM_023644:184	Mccc1	INSIDE	0.616	0.400	5125.83	2048.61	0.246	3737.33	920.19
A_68_P21586516	chr2:122293842-122293886	NR_027919:-331	Bambi-ps1	PROMOTER	0.616	0.403	1665.19	670.79	0.248	1206.52	299.55
A_68_P20457518	chr1:95698322-95698366	NM_001105667:167	Dtymk	INSIDE	0.616	0.673	1805.00	1214.25	0.415	1504.20	623.67
A_68_P32957256	chr17:8476298-8476342	NM_018819:-357	Brp44l	PROMOTER	0.615	2.902	677.40	1965.83	1.786	633.21	1130.69
A_68_P32118291	chr19:42056116-42056160	NM_145500:-114	Ubtd1	DIVERGENT_PROMOTER	0.615	0.329	3097.75	1018.03	0.202	2361.85	477.62
A_68_P31204664	chr17:46168459-46168503	NM_001110266:-6368	Vegfa	PROMOTER	0.615	6.709	2358.58	15823.53	4.124	2130.40	8786.20
A_68_P30574323	chr16:17530866-17530910	NM_026909:257	Thap7	INSIDE	0.615	0.397	1489.41	591.49	0.244	1150.18	280.77
A_68_P29662310	chr14:64225813-64225857	NM_173393:468	Xkr6	INSIDE	0.615	0.312	3067.00	955.64	0.192	2308.69	442.40
A_68_P29453027	chr14:22319300-22319344	NM_017479:316	Myst4	INSIDE	0.615	0.468	2190.32	1025.05	0.288	1686.73	485.09
A_68_P29140850	chr13:73741332-73741376	NM_146047:-95	Clptm1l	PROMOTER	0.615	2.555	1141.70	2917.23	1.572	969.04	1523.07
A_68_P29055227	chr13:55573644-55573688	NM_001030296:8039	Prr7	INSIDE	0.615	2.588	923.28	2389.15	1.592	817.01	1300.31
A_68_P28958211	chr13:38129415-38129459	NM_024242:579	Riok1	INSIDE	0.615	0.705	1971.54	1390.59	0.434	1582.57	686.78
A_68_P28679268	chr12:101203451-101203495	NM_001164426:764	Kcnk13	INSIDE	0.615	0.270	2349.47	634.17	0.166	1725.51	286.27
A_68_P28152428	chr11:115128243-115128287	NM_010350:293	Grin2c	INSIDE	0.615	3.228	1735.54	5603.06	1.985	1395.58	2769.90
A_68_P27892803	chr11:68873789-68873833	NM_028005:534	2310047M10Rik	INSIDE	0.615	2.597	2141.66	5561.58	1.596	1688.90	2695.47
A_68_P26240288	chr8:126039003-126039047	NM_001170976:331	Dbndd1	INSIDE	0.615	2.477	2080.25	5152.24	1.522	1626.01	2475.06
A_68_P25954141	chr8:73321121-73321165	NM_199308:-4810	Mast3	PROMOTER	0.615	0.607	2211.77	1341.47	0.373	1584.22	591.21
A_68_P25394242	chr7:112911135-112911179	NM_001162943:24908	Dchs1	INSIDE	0.615	2.795	2894.30	8088.42	1.719	2059.40	3539.33
A_68_P25047620	chr7:35905422-35905466	NM_007678:1133	Cebpa	INSIDE	0.615	0.512	1630.16	835.33	0.315	1265.58	398.61
A_68_P23931642	chr5:101249853-101249897	NM_172405:80	Fam175a	INSIDE	0.615	0.715	1417.59	1013.71	0.440	1061.25	467.05
A_68_P22818476	chr4:32024578-32024622	AK142691:4357		DOWNSTREAM	0.615	0.495	954.08	471.92	0.304	833.02	253.51
A_68_P22343684	chr3:95161081-95161125	NM_001163641:22	Setdb1	INSIDE	0.615	2.674	1231.55	3293.21	1.644	946.69	1556.43
A_68_P21769255	chr2:155891652-155891696	NM_139151:726	Spag4	INSIDE	0.615	0.535	1774.69	949.27	0.329	1421.86	467.60
A_68_P20739570	chr1:157584953-157584997	NM_010712:4183	Lhx4	INSIDE	0.615	0.197	9479.99	1870.50	0.121	6309.21	765.50

ProbeName	Target position of probe on CpG island microarray	TargetID	GeneSymbol	CpG island Description	Ratio of relative methylation (TiO_2-NP/Vehicle)	Sham group			TiO_2-H group		
						Relative methylation (Cy5/Cy3)	Cy3 signal (Input)	Cy5 signal (MeDIP)	Relative methylation (Cy5/Cy3)	Cy3 signal (Input)	Cy5 signal (MeDIP)
A_68_P31950560	chr19:10500789-10500833	NM_018801:37231	Syt7	INSIDE	0.614	2.397	1612.41	3865.74	1.473	1281.14	1887.21
A_68_P31351852	chr17:74927433-74927479	NM_007566:-178	Birc6	PROMOTER	0.614	0.614	950.76	583.37	0.377	720.46	271.33
A_68_P30446389	chr15:93167185-93167229	NM_024189:160	Yaf2	INSIDE	0.614	4.981	787.78	3923.84	3.058	698.95	2137.42
A_68_P29224475	chr13:93300495-93300539	NM_173392:249	Zfyve16	INSIDE	0.614	0.657	2361.86	1551.97	0.403	2126.17	857.67
A_68_P28432829	chr12:53107422-53107466	NM_029545:44	6530401N04Rik	INSIDE	0.614	2.899	2712.77	7864.76	1.780	2335.39	4157.74
A_68_P27911318	chr11:72225271-72225315	NM_177776:-77	Smtnl2	PROMOTER	0.614	0.588	2818.73	1656.50	0.361	2277.54	821.31
A_68_P27222783	chr10:68732909-68732953	NM_001081347:57526	Rhobtb1	INSIDE	0.614	2.730	1628.29	4445.35	1.676	1245.43	2086.93
A_68_P26761439	chr9:98857310-98857354	NM_012020:1307	Foxl2	INSIDE	0.614	0.233	3054.19	712.86	0.143	2387.31	342.29
A_68_P26572466	chr9:62659190-62659234	NM_010193:243	Fem1b	INSIDE	0.614	2.819	1555.36	4384.68	1.730	1209.32	2092.41
A_68_P26467075	chr9:44143629-44143673	NM_010436:853	H2afx	INSIDE	0.614	4.096	1431.25	5862.71	2.517	1093.42	2751.62
A_68_P25503044	chr7:134072503-134072547	NM_172747:132	Kctd13	INSIDE	0.614	10.313	1902.82	19624.25	6.335	1570.54	9948.65
A_68_P25352107	chr7:104886338-104886382	NM_175105:397	Aqp11	INSIDE	0.614	0.277	2201.38	610.09	0.170	1533.75	260.81
A_68_P25090529	chr7:52253176-52253220	NM_016849:169	Irf3	INSIDE	0.614	4.074	1319.91	5377.81	2.500	1173.69	2934.68
A_68_P25089584	chr7:52095285-52095329	NM_010215:3649	Il4i1	INSIDE	0.614	4.420	1944.00	8593.42	2.713	1535.92	4167.13
A_68_P25026497	chr7:30093255-30093301	NM_013874:4255	Dpf1	INSIDE	0.614	0.644	1195.12	769.71	0.395	931.55	368.34
A_68_P24051357	chr5:123064217-123064261	NM_009879:289	Ift81	INSIDE	0.614	3.796	1327.98	5041.41	2.329	993.80	2314.67
A_68_P23195767	chr4:108055814-108055858	NM_001099303:118	Fam159a	INSIDE	0.614	0.717	1519.60	1089.25	0.440	1152.63	506.88
A_68_P22900481	chr4:48486343-48486387	NM_172304:-70	Tex10	PROMOTER	0.614	4.002	582.42	2330.93	2.459	569.63	1400.81
A_68_P21328888	chr2:72818835-72818879	NM_001018042:-353	Sp3	PROMOTER	0.614	2.612	854.11	2230.59	1.605	781.34	1253.86
A_68_P21109229	chr2:31315525-31315569	ENSMUST00000142448:13292		INSIDE	0.614	0.373	4528.01	1686.93	0.229	3190.26	729.60
A_68_P20229132	chr1:52874292-52874338	NM_008384:218	Inpp1	INSIDE	0.614	2.479	1883.64	4669.43	1.522	1555.88	2367.69
A_68_P32281782	chrX:17739949-17739993	NM_009483:178	Kdm6a	INSIDE	0.613	2.405	1665.71	4006.39	1.475	1959.61	2889.88
A_68_P32109943	chr19:40686882-40686926	NM_026260:-199	Tctn3	PROMOTER	0.613	0.575	970.42	557.89	0.352	798.91	281.58
A_68_P31630116	chr18:37966805-37966849	NM_033582:-7906	Pcdhgc4	PROMOTER	0.613	0.624	937.34	585.31	0.383	813.20	311.43
A_68_P31121836	chr17:28465889-28465933	NM_011287:495	Rpl10a	INSIDE	0.613	0.687	1005.85	691.03	0.421	833.96	351.14
A_68_P31120933	chr17:28314032-28314076	NM_172617:-308	Zfp523	PROMOTER	0.613	3.594	684.82	2461.06	2.204	637.36	1405.01
A_68_P30356784	chr15:77786172-77786216	NM_001017983:958	Foxred2	INSIDE	0.613	0.414	1629.63	675.06	0.254	1148.36	291.39
A_68_P29453036	chr14:22320298-22320342	NM_017479:1314	Myst4	INSIDE	0.613	5.171	1494.11	7725.64	3.170	1339.32	4246.18
A_68_P29430092	chr14:17408235-17408279	NM_011243:-532	Rarb	PROMOTER	0.613	0.204	3111.01	636.14	0.125	2515.90	315.30
A_68_P29047306	chr13:54167631-54167675	NM_027324:439	Sfxn1	INSIDE	0.613	0.321	1832.84	589.06	0.197	1499.66	295.42
A_68_P28732704	chr12:110691154-110691198	NM_001190703:-488	Dlk1	PROMOTER	0.613	0.315	1592.39	502.08	0.193	1130.44	218.33
A_68_P27630023	chr11:20013151-20013195	NM_146243:-218	Actr2	PROMOTER	0.613	6.488	5643.42	36613.90	3.977	4722.07	18778.34
A_68_P26825589	chr9:110645592-110645636	NM_001083936:-500	Pth1r	PROMOTER	0.613	0.258	2146.88	554.64	0.158	1721.26	272.41
A_68_P26789125	chr9:103905125-103905169	NM_172460:273	Nphp3	INSIDE	0.613	0.595	2100.16	1250.59	0.365	1586.19	579.41
A_68_P26160581	chr8:112603963-112604007	NM_175480:351	Zfp612	INSIDE	0.613	0.549	2764.34	1517.47	0.337	1938.77	652.79
A_68_P26038239	chr8:90467780-90467824	NM_033327:15692	Zfp423	INSIDE	0.613	0.564	1560.94	880.73	0.346	1289.81	446.06
A_68_P25951438	chr8:72898648-72898692	NM_016685:1224	Comp	INSIDE	0.613	2.495	1300.02	3243.24	1.531	1136.70	1739.75
A_68_P25507195	chr7:134913265-134913309	NM_198424:-42	Orai3	PROMOTER	0.613	0.222	2175.93	483.65	0.136	1638.56	223.10
A_68_P24983214	chr7:17367558-17367602	NM_009201:886	Slc1a5	INSIDE	0.613	0.430	2356.48	1012.36	0.263	1749.54	460.61
A_68_P24131920	chr5:139296987-139297031	NM_001195128:975	Gm5294	INSIDE	0.613	0.494	2073.89	1024.82	0.303	1495.15	453.08
A_68_P24039145	chr5:120961107-120961151	NM_001177594:-72	Slc24a6	DIVERGENT_PROMOTER	0.613	2.581	5449.23	14066.54	1.582	3993.33	6317.74
A_68_P23301445	chr4:129986078-129986122	NM_010174:79	Fabp3	INSIDE	0.613	0.213	2396.24	509.94	0.130	1544.72	201.42
A_68_P22964786	chr4:62163240-62163284	NM_024257:-28	Hdhd3	PROMOTER	0.613	0.123	4544.34	557.85	0.075	3227.90	242.78
A_68_P22715806	chr4:8617250-8617294	NM_001081417:-795	Chd7	PROMOTER	0.613	4.086	880.35	3596.76	2.504	685.49	1716.58
A_68_P22130346	chr3:51364011-51364056	NR_033624:355	5031434O11Rik	INSIDE	0.613	4.357	3246.09	14142.83	2.669	2627.84	7013.68
A_68_P21940380	chr3:10012548-10012592	NM_010634:-35	Fabp5	PROMOTER	0.613	0.167	3118.08	522.11	0.103	2016.44	207.13
A_68_P21820797	chr2:164794239-164794283	NM_020333:773	Slc12a5	INSIDE	0.613	5.299	639.76	3390.24	3.247	496.03	1610.42
[truncated: 1,358,703 more chars]
