# Supplementary material for: Effects of Prenatal Exposure to Titanium Dioxide Nanoparticles on DNA Methylation and Gene Expression Profile in the Mouse Brain
Source: Front Toxicol. 2021 Oct 8;3:705910. doi: 10.3389/ftox.2021.705910 (PMC8915839; doi:10.3389/ftox.2021.705910)
Supplement: Supplementary file 6 [file Table9.pdf]

Supplementary Table 9

The mRNAs that expression levels were increased (A: ≥ 1.5-fold) or decreased (B: ≤ 0.67-fold) by prenatal TiO_2-NP treatment in the brain of female offspring determined by SurePrint G3 Mouse GE 8 × 60 K Microarray.

(A)

Genbank Accession	GeneSymbol	Fold change (TiO_2-H vs Sham)
NM_008029	Flt4	49.351
AK046830	Prune2	44.170
NM_181276	Tas2r136	43.956
NR_040476	4930565N06Rik	37.401
BQ554617	AI317158	31.845
NM_029499	Ms4a4c	30.232
NM_019486	Olfr71	26.909
NM_177187	D5Ertd577e	22.959
NM_146666	Olfr736	22.131
XR_001634	1700007E05Rik	22.009
NR_040317	A730082K24Rik	21.437
AK045043	Usp24	21.288
AK006214	1700029I15Rik	21.039
AK015322	4933409F18Rik	20.778
NM_199471	Prss43	20.043
AK006790	Hrasls5	19.522
NM_134253	Bnipl	18.494
NM_015767	Ttpa	17.914
NM_008941	Tmprss15	17.765
NM_018795	Abcc6	17.160
NM_147028	Olfr1124	16.235
NM_134210	Vmn1r81	16.178
NM_027031	Efcab9	15.934
NM_001163486	Hsd17b13	15.617
NM_001085529	Slc2a7	15.064
NM_134170	Vmn1r32	13.842
NM_207664	Olfr151	13.652
NM_009043	Reg2	13.623
BC044848	Dsg2	13.408
AK085162	D430047D06Rik	12.951
NM_008263	Hoxa10	12.737
XR_141471	Gm17739	12.562
BU962902	4930548J01Rik	12.553
NM_001244654	Gm9573	12.252
NM_001029893	Psg26	12.193
NM_001201390	E330021D16Rik	12.176
NM_146831	Olfr133	12.176
NM_029230	4930564B18Rik	11.794
XM_003085466	LOC100039675	11.729
NM_001001332	BC117090	11.632
NM_012008	Ddx3y	11.416
NM_009971	Csf3	11.081
NM_033616	Csprs	10.951
NM_172509	Kctd7	10.808
NM_001142539	Gm9992	10.800
NM_001081372	Ces1b	10.549
NM_147111	Olfr586	10.476
NM_008089	Gata1	10.469
NM_153052	Gzmn	10.239
NR_040493	2410088K16Rik	9.987

Genbank Accession	GeneSymbol	Fold change (TiO_2-H vs Sham)
NM_021380	Il20	9.931
NR_045157	Gm7008	9.500
NM_012011	Eif2s3y	9.448
NM_146956	Olfr525	9.363
NR_015488	A930003A15Rik	9.260
XM_001475415	Gm7972	9.221
NM_001011854	Olfr605	9.190
NM_008030	Fmo3	9.151
AK080381	9330175E14Rik	9.032
NM_001167587	Gm5916	8.889
NM_146370	Olfr47	8.889
XM_003689006	LOC100861888	8.834
NM_008040	Fpr-rs3	8.754
NM_001034100	Gm5382	8.736
NM_177243	Slc26a9	8.694
NM_178793	Ccbe1	8.682
NM_001163810	1700008P20Rik	8.568
NM_172420	Ppp1r1c	8.497
NM_146535	Olfr1370	8.282
NM_011718	Wnt10b	8.259
NM_001166030	Mylk4	8.174
NM_009123	Nkx1-2	8.157
XM_003086808	LOC100040235	8.089
NM_032394	Myo7b	8.056
NM_028025	Mageb16	7.994
NM_173395	Fam132b	7.994
NM_009484	Uty	7.983
AK044339	Esrrb	7.945
NM_177905	Piwil4	7.912
NM_146532	Olfr1170	7.895
NM_176950	Defb20	7.722
NM_175531	Mrgprb2	7.642
NM_010871	Naip6	7.621
NM_013728	Olfr154	7.418
NM_001039048	Trim63	7.408
NM_183249	1100001G20Rik	7.387
NM_008412	Ivl	7.270
NM_001001488	Atp8b1	7.235
NM_138750	Prom2	7.200
NM_001199332	LOC100041550	7.180
NM_199155	Tas2r110	7.170
NR_045343	E030044B06Rik	7.130
AK047340	Myh10	7.106
NM_001011797	Olfr782	7.032
AK039149	Plcd4	6.940
NM_028545	1700055N04Rik	6.854
NM_147037	Olfr1413	6.850
NM_203396	Fam115e	6.816
NM_025763	4933436I01Rik	6.816
NM_001005858	I830012O16Rik	6.769

Genbank Accession	GeneSymbol	Fold change (TiO_2-H vs Sham)
NR_033523	Gm11517	6.751
NR_040327	E130018N17Rik	6.690
NM_146989	Olfr1496	6.653
NM_001012434	Kctd14	6.644
NM_001167757	Ankrd7	6.644
NM_001252513	Lipi	6.575
NM_146367	Olfr976	6.561
NM_175449	Fam26f	6.539
NM_029901	Akr1c21	6.534
NM_020595	Otor	6.485
XM_003689228	LOC100862035	6.485
NM_172824	Ccdc14	6.480
NM_008361	Il1b	6.449
NM_030614	Fgf16	6.382
NM_001025585	Kcnj6	6.342
NM_205783	Chrm5	6.285
NM_019475	Olfr157	6.281
NM_177901	4933402J07Rik	6.233
NM_001005485	Olfr111	6.225
NR_028384	4930528A17Rik	6.212
NM_001166712	Vmn1r151	6.207
NM_019918	Vmn2r1	6.160
AK051627	Arvcf	6.114
AK143941	1700001D01Rik	6.101
NM_001043322	Fmn1	6.017
AK042233	A630073K07Rik	6.004
NM_146440	Olfr919	5.909
NM_138630	Arhgap4	5.905
NM_199150	BC049730	5.844
NM_010759	Mageb1	5.840
NM_146959	Olfr631	5.760
AK033147	1010001N08Rik	5.748
NM_001130412	Lpin1	5.704
AK030470	3110054G05Rik	5.700
NM_010493	Icam1	5.700
NM_001014997	Gm156	5.688
NM_001199347	Foxp3	5.684
AK146705	Filip1l	5.657
NR_045930	1700092K14Rik	5.629
NM_030711	Erap1	5.622
NM_175731	Acer1	5.598
NM_177335	D930020B18Rik	5.548
NR_045456	C330013F16Rik	5.540
NM_178780	Ric3	5.438
XM_986980	Gm8359	5.434
NM_001085503	Aadacl3	5.419
NM_172932	Nlgn3	5.374
XR_142383	LOC664934	5.359
NM_010558	Il5	5.322
NM_146543	Olfr1360	5.318
XR_104604	1700029M03Rik	5.300
NM_017371	Hpx	5.271
NM_023370	Cdh23	5.267
NM_199059	Tbpl2	5.242

Genbank Accession	GeneSymbol	Fold change (TiO_2-H vs Sham)
NM_001081211	Ptafr	5.195
NM_011667	Ube1y1	5.195
NM_001161765	Fmo5	5.191
NM_147114	Olfr575	5.152
NM_001159693	Zar1l	5.116
NM_009170	Shh	5.095
AK034192	Gm4491	4.986
NM_181579	Pof1b	4.986
AK155776	Tet2	4.959
NM_147099	Olfr616	4.938
XR_105027	A230083G16Rik	4.935
NM_172842	Lax1	4.931
NM_028529	Nipsnap3a	4.918
NM_001039889	Smok3b	4.897
NR_027806	Pea15b	4.894
NM_009220	Ssty1	4.887
NM_001042503	Trim71	4.867
NM_011294	Sub1	4.857
AK008836	2210406H18Rik	4.843
NM_001167937	D6Ertd527e	4.780
NM_201530	Sly	4.777
NM_146369	Olfr434	4.773
NM_001033541	Gm5127	4.757
NM_026105	1700093K21Rik	4.737
NM_001037748	LOC380994	4.698
NM_021517	Pdzk1	4.695
NM_010464	Hoxc13	4.643
NM_001001809	Olfr218	4.640
NM_199306	Wdtc1	4.630
NM_030237	Spz1	4.630
NR_003648	Gm5334	4.620
AK080367	A630076J17Rik	4.576
NM_001011818	Olfr316	4.573
NM_001081070	Pdia2	4.563
NM_009307	Syt2	4.560
NM_181853	Trim66	4.554
AK017398	5430435K18Rik	4.554
BY709204	Gm11266	4.541
NM_019473	Olfr155	4.538
AK037495	1810062O18Rik	4.519
NM_146925	Olfr481	4.510
NR_030763	Smok4a	4.506
NM_145383	Rho	4.485
AK052446	Pigv	4.475
NM_011426	Siglec1	4.457
NM_029264	Ttll10	4.448
NM_030192	4930562C15Rik	4.392
AK021049	C030006F08Rik	4.392
NM_001004157	Scarf1	4.386
NM_146654	Olfr237-ps1	4.386
NM_207687	Espn	4.380
AK142706	D830050J10Rik	4.377
NM_147023	Olfr385	4.368
XR_142242	Gm10610	4.341

Genbank Accession	GeneSymbol	Fold change (TiO_2-H vs Sham)
NR_033171	Ppp1r2-ps9	4.338
NM_172955	Vcan	4.323
XR_140495	Gm10846	4.317
NM_028938	Lrriq3	4.296
XM_885719	Pramel	4.275
NM_001080934	Slc16a5	4.260
NM_001011733	Olfr288	4.257
XR_002334	Lrrc31	4.222
NM_028905	4932438H23Rik	4.216
NM_028370	Pot1b	4.190
NR_045741	Gm10432	4.158
NM_008352	Il12b	4.150
NM_001081363	Cenpf	4.147
NM_181549	Clec18a	4.147
NM_030699	Ntng1	4.141
NM_026915	Lyzl4	4.135
NM_177607	4933430I17Rik	4.135
BC030499	BC030499	4.124
AK053536	E130106K03Rik	4.124
NM_177839	Tnn	4.115
NM_026806	Klk5	4.112
NM_145126	Chi3l4	4.107
AK143876	Gm10603	4.084
NR_015567	A930007I19Rik	4.061
NM_001100616	Vmn2r121	4.059
NM_146746	Olfr935	4.056
NR_033618	1700128F08Rik	4.053
XM_912668	Gm16486	4.053
NM_026296	4930548H24Rik	4.053
NM_134202	Vmn1r233	4.045
NM_147026	Olfr532	4.036
AK141472	Asxl3	4.025
NM_008092	Gata4	4.011
NM_028994	Pck2	3.994
NM_001099313	Gm11554	3.989
NM_177661	C130079G13Rik	3.964
NR_045810	4933408J17Rik	3.948
AK162913	Pigu	3.942
NM_001102665	Gm14920	3.939
NM_009509	Vil1	3.937
XR_140696	Gm3527	3.934
NM_013464	Ahr	3.926
AK006604	Gm9558	3.915
NM_010450	Hoxa11	3.885
NM_175495	Gpr150	3.880
NM_001033424	Fnd3c2	3.837
NM_153098	Cd109	3.832
AK166451	Gm10661	3.808
NM_029116	Kbtbd11	3.795
XR_106400	Gm11538	3.789
AK146348	Gm2022	3.784
AK132758	Gm10741	3.769
AK044519	Stard7	3.769
NR_045713	4930529F24Rik	3.750

Genbank Accession	GeneSymbol	Fold change (TiO_2-H vs Sham)
AK015935	Orly	3.724
NM_025751	4933425L06Rik	3.724
AK041796	Cog5	3.724
AK143754	Gm4466	3.711
AK149136	Col19a1	3.694
NM_001160142	LOC100041256	3.694
NM_030739	Vmn1r58	3.686
BC011338	Birc3	3.686
BF139390	Igkv4-53	3.673
XR_105111	Gm2788	3.665
U04807	Flt3l	3.655
NM_011146	Pparg	3.653
AK078505	Tmem220	3.650
NM_001031851	Agxt2	3.625
AK039419	A330041K01	3.623
XR_142440	LOC100048661	3.615
NM_053103	Entpd7	3.598
AK048775	C230060E24	3.585
NM_008367	Il2ra	3.580
XM_886739	Gm6310	3.575
NM_001033437	Gm889	3.568
NM_001033547	4922505E12Rik	3.526
NM_030726	Mrgprh	3.519
NM_146050	Oit1	3.519
AK155135	Dfna5	3.519
NM_027902	Tmprss6	3.506
NM_026449	Galntl5	3.502
NM_013542	Gzmb	3.497
NR_045086	AW495222	3.485
NM_026335	Lce1h	3.480
NR_036598	6030440G07Rik	3.480
NM_016775	Dnajc5	3.477
NM_011523	Synj2	3.461
NM_181849	Fgb	3.461
NR_027824	Gm13749	3.458
NM_010866	Myod1	3.446
NM_001034893	Zfp936	3.444
BC026871	Rftn2	3.441
XM_003085596	LOC100504642	3.441
NM_012010	Eif2s3x	3.439
NM_020015	Magea1	3.422
AK172643	Tmed5	3.415
NR_027915	1700120K04Rik	3.415
NR_015540	4933400F21Rik	3.406
BC080727	Gm5589	3.406
NM_010125	Elf5	3.389
BI852147	AI463170	3.373
AK016698	4933406K04Rik	3.354
NR_040547	4930509J09Rik	3.352
NM_001128103	Ano3	3.350
NM_001170935	BC005764	3.345
NM_013482	Btk	3.343
NM_001102563	Prrt2	3.343
NM_153801	Tecrl	3.322

Genbank Accession	GeneSymbol	Fold change (TiO_2-H vs Sham)
AK030628	Cwc25	3.306
NM_015743	Nr4a3	3.304
NM_001159775	Olfr391-ps	3.297
NM_030098	Rnase6	3.288
AK038843	9630028I04Rik	3.260
AK035285	Ppp1r12b	3.200
NM_008645	Mug1	3.184
NM_019544	Msgn1	3.169
NM_172578	Mis18bp1	3.158
NM_027382	Hdac8	3.134
NM_080467	Atp6v0a4	3.117
NM_001190332	Frmd7	3.078
NM_153600	Ttc26	3.053
NM_020277	Trpm5	3.050
NM_177755	Klhl38	3.031
NM_021609	Ccbp2	3.029
NM_178720	Zpld1	3.021
NM_001025353	Gm6040	3.008
NM_010502	Ifna1	3.006
AK158670	Gm10199	2.998
NM_183161	Slc17a9	2.996
NM_001039652	Oprm1	2.990
AK134268	Tmem87a	2.983
NM_001033415	Shisa3	2.975
NR_046060	4930478P22Rik	2.973
NM_001134299	Gm10220	2.953
NM_001004142	Nlrp1a	2.942
NM_008522	Ltf	2.932
AK032648	Pde4d	2.912
XM_357111	Gm5258	2.892
NM_001110320	Cd72	2.884
NM_177025	Cobll1	2.882
NM_001162945	Mtx3	2.882
NM_134195	Vmn1r227	2.880
AK041925	Svil	2.876
NR_003619	6330549D23Rik	2.872
NM_013622	Oprd1	2.870
AK031407	1700028E10Rik	2.856
NM_028051	Slc39a5	2.854
NM_181277	Col14a1	2.840
NM_138654	5033411D12Rik	2.830
NM_146618	Olfr297	2.817
NM_010410	Hcrt	2.803
XM_001481329	Spnb5	2.789
NM_198193	Raet1e	2.774
NM_001172147	Rbm41	2.772
NM_009118	Sag	2.770
NM_001081320	Cyb561d1	2.751
NM_001173553	Cdc14a	2.749
NM_011910	Uts2	2.715
NM_033325	Loxl2	2.704
NM_018738	Igtp	2.691
AK163441	Csnk1g3	2.683
AK050478	Nnt	2.661

Genbank Accession	GeneSymbol	Fold change (TiO_2-H vs Sham)
NM_007956	Esr1	2.656
AK155789	Gm10802	2.648
NM_174876	Impg2	2.646
NM_019821	Gltp	2.645
NR_045997	1700113A16Rik	2.643
NM_178258	Il22ra2	2.626
AK165082	2700007P21Rik	2.615
NM_146904	Olfr870	2.614
NM_001039188	Rreb1	2.608
XR_035409	Gm6653	2.606
NM_175526	Clec1a	2.603
NM_021409	Pard6b	2.603
NM_020576	Psors1c2	2.595
NM_177307	Cyp4f39	2.594
AK021017	B430105A11Rik	2.594
NM_145073	Hist1h3g	2.588
NM_052977	Adarb2	2.586
NM_031255	Rsph6a	2.583
NM_027089	4930579C15Rik	2.576
NM_015770	a	2.565
CB202205	AI449212	2.562
NM_001145637	Gm1661	2.558
NM_001190374	Adamtsl3	2.554
AK044320	Ocrl	2.547
NM_001193305	Mical2	2.542
AK090292	Zfp53	2.542
AK020289	9130230N09Rik	2.521
NM_013875	Pde7b	2.521
AK140520	Fam5c	2.514
AK047378	Chst10	2.509
XM_357051	Ms4a14	2.509
XR_105476	Gm9548	2.507
AK051017	Asb7	2.498
NM_001128090	Rergl	2.483
NM_009132	Scin	2.479
NM_010427	Hgf	2.469
AK080282	D10Ertd755e	2.466
BC171937	Hemt1	2.461
NM_145933	St6gal1	2.452
NM_172783	Phka2	2.449
NM_001081298	Lphn2	2.442
XM_003085861	Gm9979	2.440
NM_175681	Glp2r	2.437
AK021252	C430045I18Rik	2.432
AK031900	Adamts3	2.430
NM_030022	Grifin	2.428
NM_001163638	Ttc18	2.402
NM_009827	Cckar	2.400
NM_010469	Hoxd4	2.395
NM_152803	Hpse	2.393
XM_987873	6530409C15Rik	2.385
NM_001081428	Fam184a	2.380
AK020028	5930409G06Rik	2.380
NM_007537	Bcl2l2	2.380

Genbank Accession	GeneSymbol	Fold change (TiO_2-H vs Sham)
NM_010264	Nr6a1	2.373
XM_622784	Olfr1358	2.365
AK046843	9130004C02Rik	2.365
AK021326	D730003K21Rik	2.365
NM_008469	Krt15	2.362
AK083890	D130051D11Rik	2.357
NM_001013759	Gas2l2	2.347
NM_010059	Dmc1	2.333
NM_015796	Fbxo17	2.333
NM_026357	Ribc2	2.326
NM_001097644	Ccnyl1	2.326
NM_010551	Il16	2.323
NM_001190914	Kirrel3	2.317
NM_146359	Olfr564	2.313
NM_008008	Fgf7	2.304
NM_027717	Dydc2	2.301
NM_146235	Ercc6l	2.297
AK046639	Eif4g3	2.297
NM_011134	Pon1	2.297
NM_011485	Star	2.293
NM_028967	Batf2	2.289
AK034105	9330156P08Rik	2.286
NM_010009	Cyp27b1	2.280
AK046275	4930588A03Rik	2.278
XR_140624	Gm2401	2.270
AK028444	4631405J19Rik	2.270
XR_107893	LOC100504965	2.267
NM_008334	Ifna7	2.264
NM_207262	Abpe	2.263
NM_001110240	Slc24a2	2.258
NM_028540	1700057K13Rik	2.252
AK035822	D2Ertd295e	2.242
NM_177382	Cyp2r1	2.235
AK162820	P2rx4	2.233
AK017326	5430420F09Rik	2.230
NM_019474	Olfr156	2.227
NM_145229	AY074887	2.215
NR_033538	Gm10421	2.215
NM_022721	Fzd5	2.213
NM_146974	Olfr1262	2.208
NM_027967	1700026J04Rik	2.204
AK083592	BB217526	2.202
NM_016694	Park2	2.193
NM_001166206	Erv3	2.192
XR_104907	B230104I21Rik	2.187
NM_010024	Dct	2.178
NR_045458	4933433H22Rik	2.178
NM_001039502	Krtap1-4	2.172
NM_023380	Samsn1	2.170
AK041747	Harbi1	2.164
NM_001172123	Rbms3	2.158
NR_045047	2810011L19Rik	2.154
NM_007831	Dcc	2.151
NM_001195693	Plscr5	2.149

Genbank Accession	GeneSymbol	Fold change (TiO_2-H vs Sham)
CA463412	4933408A14Rik	2.144
AK076446	Tmem175	2.136
AK029603	Zhx3	2.136
NM_001085504	Gm436	2.136
NM_001160265	Cyp2w1	2.135
NM_028352	Pgm3	2.132
NM_001172074	3110009E18Rik	2.127
NM_001011519	Olfr1148	2.126
XR_140426	Gm10568	2.123
AK031183	3010001F23Rik	2.104
NM_007868	Dmd	2.101
NM_001164466	Dpys	2.097
NM_133984	Hemk1	2.094
NM_031389	Nlrp4c	2.089
NM_008074	Gabrg3	2.085
NR_038008	B230209K01Rik	2.083
AK170575	Arhgap6	2.082
NM_009259	Spn	2.078
NM_144515	Zfp52	2.076
NM_172622	Trerf1	2.075
NM_023631	Aox4	2.073
BG081747	AU023639	2.073
NM_001081407	Plb1	2.066
NM_009767	Chic1	2.065
NM_027649	Spats1	2.059
NM_001163502	C130039O16Rik	2.056
NM_001081050	Pard3b	2.041
AK049967	Myt1l	2.038
NM_013724	Nrk	2.035
AK033281	Cep250	2.032
NM_008961	Pter	2.029
NM_001004193	Rhox8	2.028
AK137878	A430071A18Rik	2.025
NM_183025	A430033K04Rik	2.021
NM_001113530	Csf1	2.020
NM_172874	Podn	2.015
NM_025506	Riiad1	2.007
BC100427	Gm10037	2.003
NM_181419	Zfp599	2.001
NM_001205102	Lrit3	1.993
XR_106168	A930006D01Rik	1.993
AK144790	Gxylt2	1.989
NM_008007	Fgf3	1.988
NM_009662	Alox5	1.978
NM_008308	Htr1a	1.978
NM_007860	Dio1	1.966
NR_003649	Gm5434	1.955
NM_011892	Sgcg	1.951
NM_001101510	Susd5	1.948
BC151047	Auts2	1.936
NM_033614	Pde6c	1.924
NM_008103	Gcm1	1.920
AK013575	Lias	1.895
NM_010917	Nid1	1.886

Genbank Accession	GeneSymbol	Fold change (TiO_2-H vs Sham)
NM_001163145	1810041L15Rik	1.884
NM_001111143	Cym	1.880
AK080905	Pten	1.876
NM_138584	Spg21	1.875
NM_010707	Lgals6	1.869
NM_001145978	Parp4	1.865
NM_010275	Gdnf	1.849
AK173011	Sacs	1.845
NM_013489	Cd84	1.843
AK020539	9530003O04Rik	1.842
NM_199446	Phkb	1.839
NM_146878	Olfr30	1.838
BC045142	Kcnq1	1.838
NR_040758	0610040F04Rik	1.835
AK041660	D5Ertd798e	1.826
NM_001205236	Arhgap27	1.825
NM_183151	Mid1	1.825
NM_054066	Plcz1	1.824
NM_001146080	Cntfr	1.816
NM_174846	Glyctk	1.811
NM_178738	Prss35	1.810
NM_010620	Kif15	1.808
NM_146136	Slc16a4	1.806
NM_001033380	Itpripl2	1.801
AK031795	BB070754	1.800
AK047130	B930025B16Rik	1.799
AK005011	Proz	1.799
NM_023842	Dsp	1.799
NM_001113368	Ceacam2	1.795
AA871626	Defa1	1.790
NM_021022	Abcb11	1.776
NM_009863	Cdc7	1.764
NR_015553	9430076C15Rik	1.763
NM_001033208	Myzap	1.762
NM_028053	Tmem38b	1.761
NM_133207	Kcnh7	1.761
NM_029281	Zfp820	1.758
NM_011347	Selp	1.756
NM_021050	Cftr	1.754
AF396877	Dst	1.754
NM_029416	Klf17	1.752
NM_001081160	Mdga1	1.750
AK036139	9630039A02Rik	1.742
NR_040273	9330179D12Rik	1.742
NM_019684	Srpk3	1.733
AK003710	1110014L15Rik	1.731
NM_007823	Cyp4b1	1.727
NM_001033158	Rasl12	1.725
NM_008664	Myom2	1.720
NM_009380	Thrb	1.711
NM_001039169	Eif4e2	1.697
NM_001033411	Gm826	1.696
NM_145969	Fam116a	1.689
NM_177346	Gpr149	1.688

Genbank Accession	GeneSymbol	Fold change (TiO_2-H vs Sham)
NM_029733	2010005H15Rik	1.686
NM_001037928	Gm11992	1.685
NM_027411	Ccdc99	1.685
NM_177628	Fam167a	1.684
NM_178407	Arap2	1.681
NM_031258	Chrdl1	1.681
NM_027979	Chit1	1.678
NM_001001807	Olfr279	1.677
NM_023224	Cblc	1.677
XR_142217	Gm9519	1.675
NM_175539	Dcaf12l2	1.674
NM_001159507	Grip2	1.672
NM_015820	Hs6st3	1.671
NM_001077514	Slc1a2	1.670
NM_001037916	Ccdc17	1.661
AK046043	Apol8	1.660
XR_105888	1700022N22Rik	1.660
NM_010675	Krtap8-1	1.659
NM_026922	Atp2c2	1.659
NM_011860	Nlrp5	1.651
NM_013500	Hapln1	1.649
AK036290	AU015680	1.646
NM_029324	1700018C11Rik	1.644
NM_207228	Tsga10	1.637
NR_040418	2310034G01Rik	1.631
AK047818	A730009E18Rik	1.630
AK132983	Sh3rf3	1.622
NM_145581	Siglec5	1.621
AK082713	C230094B09Rik	1.621
NM_173869	Stfa2l1	1.619
NM_175418	Mybpc1	1.611
NM_027613	Mmrn1	1.610
NM_026222	Ccdc39	1.598
AK045009	B130019D13Rik	1.595
NM_001081399	Prss33	1.593
NM_001025156	Ccdc93	1.591
NM_007876	Dpep1	1.590
AK014289	Rbm33	1.588
NR_033566	AI507597	1.577
NM_172508	Dse	1.574
NM_030560	Cwc22	1.568
BC042707	Prok1	1.566
NM_001037752	Defb45	1.558
XR_142034	LOC100862636	1.557
NM_029112	Morn3	1.557
AK049583	Mfn2	1.549
NM_177261	Kndc1	1.549
NM_016907	Spint1	1.549
XR_140816	Gm9653	1.546
NM_011681	Scgb1a1	1.542
NM_181071	Tanc2	1.542
NM_146198	Slc5a11	1.540
NM_001177951	Rpgr	1.533
NM_029714	Catsperg2	1.532

Genbank Accession	GeneSymbol	Fold change (TiO_2-H vs Sham)
NM_008184	Gstm6	1.525
NM_027706	Nbas	1.525
AK035028	Fat3	1.521
NM_009809	Casp14	1.520
NM_029025	Tmem81	1.516
NM_001042611	Cp	1.515
NM_133365	Dnahc5	1.509
NR_045120	Gm18756	1.501
NM_019809	Pdlim5	1.500
NM_028473	3110079O15Rik	1.500

(B)

Genbank Accession	GeneSymbol	Fold change (TiO_2-H vs Sham)
NM_029374	Sept12	0.005
NM_027668	4933412E24Rik	0.006
NM_011407	Slfn1	0.007
NM_029228	4930564D02Rik	0.007
AK046115	A930016O22Rik	0.008
NM_147032	Olfr705	0.008
NM_178715	Tmem30b	0.009
AK015387	4930445B16Rik	0.009
AK036917	Gm9902	0.009
NR_036452	E230016K23Rik	0.009
NM_009618	Adam2	0.009
NM_030712	Cxcr6	0.010
NM_197944	Hsh2d	0.011
NM_026090	Iqcf4	0.012
NM_009973	Csn1s2b	0.012
NM_026132	Txndc8	0.013
NM_001039047	Trim58	0.014
XR_140973	Gm10824	0.014
BU554875	2010001M06Rik	0.014
NM_001002897	Atg9b	0.014
NM_008232	Hdgfl1	0.015
AK016784	4933412A08Rik	0.016
NM_001039701	Il1rn	0.017
AK051714	Thsd7b	0.017
AF011426	Vmn2r10	0.018
AK014730	4833419O12Rik	0.019
NM_001199840	Olfr1354	0.020
XM_003689282	Fam81b	0.021
NM_177369	Myh8	0.022
CA495423	Gm15417	0.022
NM_001003670	Gm5414	0.024
NM_001038697	Gm5635	0.024
NM_001045543	Gm11435	0.024
NM_030219	Trim42	0.025
NR_033214	B430212C06Rik	0.026
AW493399	9330109K16Rik	0.026
NM_001005780	Olfr255	0.028
NM_001174099	Krt36	0.028
NM_011110	Pla2g5	0.030
NM_134203	Vmn1r73	0.030
NM_026419	Cela3b	0.032
AK076769	Gm3509	0.032
NR_040539	2410007B07Rik	0.033
NR_045920	4933404G15Rik	0.034
NM_178674	Fbxl21	0.034
NR_004853	Cdk3-ps	0.035
NM_028708	Jakmip3	0.036
AK164319	Foxp2	0.037
NM_001033332	Tmtc3	0.038
NM_207243	Muc19	0.039
NM_007704	Inadl	0.040
NM_207249	Olfr684	0.041
AF223416	Trdn	0.042

Genbank Accession	GeneSymbol	Fold change (TiO_2-H vs Sham)
NR_045168	4930447N08Rik	0.042
XM_003689102	LOC100862025	0.042
BF788177	AA408650	0.044
NM_011037	Pax2	0.044
NM_146355	Olfr692	0.044
NM_198666	Gm9758	0.044
NM_028671	Fam122c	0.045
NM_031164	F13b	0.045
NM_010340	Gpr50	0.045
NM_134163	Mbnl3	0.045
NM_001163625	Sult6b1	0.046
NM_001103157	Steap2	0.047
NM_001164289	Gm6907	0.047
AK135955	Gm3772	0.048
NM_008780	Pax1	0.048
AK011813	Slc16a10	0.049
NM_007815	Cyp2c29	0.050
NR_001570	Xist	0.050
NM_001128151	Cecr2	0.053
NM_001161855	4933416C03Rik	0.055
NM_022322	Tnmd	0.058
AK040172	Abhd15	0.058
NM_147055	Olfr649	0.058
NM_001163550	1700016H13Rik	0.059
NM_032007	Mmp1b	0.059
NM_053227	Vmn1r44	0.060
NM_001081137	Sis	0.060
NM_019461	Usp27x	0.061
NM_026822	Lce1b	0.061
NM_011465	Spna1	0.064
NM_001177482	Gm14851	0.065
NM_013793	Klra15	0.065
NM_001105160	Cyp3a59	0.065
AK143744	Gm10779	0.065
AK053725	Tcp11	0.066
XM_001472786	1700120B22Rik	0.066
NM_001005568	Olfr1281	0.066
NM_008206	H2-Oa	0.066
AK033078	Dleu2	0.066
NM_175350	Tmem146	0.067
NM_147052	Olfr589	0.067
NM_001009950	Slc38a8	0.068
AI595487	AA060545	0.069
NM_001100185	Cyp4a30b-ps	0.069
AK052948	D930001M23Rik	0.069
NM_001033290	Gpr55	0.070
NM_146630	Olfr123	0.073
NM_146456	Olfr92	0.074
NM_026594	Rpl39l	0.074
NM_007718	Ccr1l1	0.074
NM_019759	Dpt	0.074
NM_001145660	Rfx8	0.074
NM_172155	Prl3d2	0.076

Genbank Accession	GeneSymbol	Fold change (TiO_2-H vs Sham)
NM_008192	Gucy2e	0.077
NM_139222	Defb15	0.078
NM_175456	Abra	0.079
NM_010094	Lefty1	0.079
NM_001039556	Rad54b	0.081
XM_001475533	Olfr687	0.081
AK145592	Gm10204	0.082
NM_001100461	Pnma5	0.082
NM_146007	Col6a2	0.082
NM_027126	Hfe2	0.084
NM_009877	Cdkn2a	0.084
BC048590	Cep120	0.084
NM_027210	Ceacam13	0.084
AK147870	LOC627096	0.086
NM_139223	Defb13	0.088
NM_001039114	Acsbg2	0.091
NM_001252070	Dnahc7a	0.092
NM_017390	Svs2	0.094
NM_146803	Olfr906	0.095
NM_028557	Mbd3l1	0.095
AK054255	Pxdn	0.097
AK016655	4933404M09Rik	0.098
U89377	Nnmt	0.098
NM_009019	Rag1	0.100
NM_172851	Cntnap5b	0.100
NM_147005	Olfr395	0.100
NM_153588	Mkl2	0.100
NM_177230	Ccdc158	0.101
AK020766	A430105D02Rik	0.101
NM_008493	Lep	0.101
NM_022314	Tpm3	0.102
NM_028790	Acot12	0.103
NM_001126487	Heatr8	0.104
NM_009331	Tcf7	0.104
NM_007710	Ckm	0.104
NM_001081668	Nup62cl	0.105
NM_027828	Fam110c	0.105
NM_001166636	Gm4312	0.106
NM_001173550	C5ar1	0.106
NM_011709	Wap	0.106
NM_001013832	Gpr31b	0.106
NM_178886	Ldlrad3	0.107
NM_010944	Musk	0.107
XM_003086710	Gm3952	0.107
NM_008152	Gpr65	0.109
NM_130856	Krtap16-8	0.110
NM_001166631	Havcr1	0.110
AK018320	6530403M18Rik	0.111
NM_001013774	Kpna7	0.112
NM_153072	Hus1b	0.114
NR_045798	1810034E14Rik	0.114
NM_177709	Tusc5	0.115
AK139699	B020011L13Rik	0.116
NM_177083	B430306N03Rik	0.116

Genbank Accession	GeneSymbol	Fold change (TiO_2-H vs Sham)
NM_001033455	Ccdc27	0.118
AK016366	4930588G17Rik	0.120
AK041677	Gm9502	0.120
NM_001164171	Myh6	0.121
NM_001033430	Jhdm1d	0.122
NM_177779	Ccdc42	0.122
NM_022563	Ddr2	0.122
AK076724	Gm9789	0.122
NM_146738	Olfr497	0.123
BG075595	AW556556	0.123
NM_009493	Vmn2r42	0.124
NM_175501	Adamts12	0.124
AK012661	Dnahc17	0.124
NM_027651	Tmem30c	0.124
NM_001101531	Gm5538	0.125
NR_045179	Gm8633	0.126
NM_145375	Tm6sf1	0.126
BG069373	D19Ertd744e	0.126
AK139339	Spata2l	0.126
NM_173861	Csnka2ip	0.127
NM_010984	Olfr263	0.128
NM_001033333	Gm239	0.129
AK077177	Bmp2k	0.129
NM_053165	Clec2h	0.129
NM_175651	Cnpy1	0.130
NM_009265	Sprr1b	0.130
NM_023635	Rab27a	0.130
HQ713438	Clec2g	0.132
NM_001011813	Olfr93	0.132
NM_206867	Gm13280	0.133
NM_198636	Acss3	0.133
NM_018789	Foxo4	0.134
NM_001162928	Pom121l2	0.142
NM_007760	Crat	0.144
NM_008936	Prop1	0.144
NR_033493	Gm10818	0.147
NM_001003939	BC030307	0.149
XM_619430	4930488N24Rik	0.150
NR_040694	4930413E15Rik	0.150
NM_001034904	Gm5891	0.151
NM_024445	Tsnaxip1	0.152
NM_001037712	Kcnh6	0.153
NM_010821	Mpeg1	0.154
AK029744	Agbl2	0.156
NM_001081063	Prss55	0.157
NM_001102579	Vmn2r67	0.158
AK087205	9530082P21Rik	0.159
NM_009350	Adad1	0.159
XM_003085258	Pabpc1l2b-ps	0.163
NM_134199	Vmn1r235	0.165
NM_134037	Acly	0.166
NM_177638	Crb3	0.167
NM_011201	Ptpn1	0.168
NM_194335	Naif1	0.172

Genbank Accession	GeneSymbol	Fold change (TiO_2-H vs Sham)
NM_008673	Nat1	0.173
NM_001166501	Dennd1b	0.181
NM_001105055	Vmn2r58	0.183
NM_033042	Tnfrsf25	0.183
NM_024406	Fabp4	0.186
NM_007751	Cox8b	0.186
NM_181683	Defb37	0.189
XM_003084673	LOC100503615	0.191
NM_010548	Il10	0.205
NM_146953	Olfr531	0.206
NM_001177438	Aldh3b2	0.209
NM_027754	Rnf148	0.211
AK038979	Ints9	0.216
NM_177921	E230019M04Rik	0.217
AK016828	4933416A02Rik	0.219
AK138162	1700101I11Rik	0.221
NR_024323	4930426L09Rik	0.221
NM_017395	Rfx5	0.222
BY707381	1700125G22Rik	0.223
AK163481	Kdm5b	0.227
NM_207031	Ano7	0.228
NM_010505	Ifna5	0.237
NM_001045525	Cyb5d1	0.238
NM_146020	Gltpd2	0.238
NM_030141	1700061G19Rik	0.239
NM_007753	Cpa3	0.240
NM_001033493	Gpr111	0.241
NM_009202	Slc22a1	0.243
NM_198424	Orai3	0.244
NM_013599	Mmp9	0.244
AK019978	Auh	0.247
NM_172501	Nhlrc3	0.250
AK009512	2310026L22Rik	0.254
NM_001167581	Gsdmcl1	0.259
NM_001161355	Timd2	0.263
NM_001085440	Smcr8	0.265
NM_206536	AB124611	0.266
NM_206823	Olfr153	0.270
NM_010696	Lcp2	0.271
NM_001195413	Cngb1	0.274
NM_001080931	Med13	0.276
NM_144795	Pycr1	0.277
NM_008290	Hsd17b2	0.282
AK168315	Dnttip1	0.282
NM_147222	Rdh19	0.284
NM_001122953	Nfia	0.285
NM_153576	Cxcl17	0.286
NM_007758	Cr2	0.287
NM_010874	Nat2	0.290
NM_029604	1700027A23Rik	0.292
NR_038039	1700023F02Rik	0.302
NM_001045484	Mef2b	0.303
AK039088	Prkx	0.303
NM_147217	Gprc5c	0.306

Genbank Accession	GeneSymbol	Fold change (TiO_2-H vs Sham)
NM_001039198	Zfhx2	0.307
AK140648	Ptprz1	0.308
DV065341	2810427A07Rik	0.310
NM_009463	Ucp1	0.312
NM_146805	Olfr907	0.314
NM_138945	Pou4f3	0.320
NM_013684	Tbp	0.320
NM_009866	Cdh11	0.322
AK047423	Odz1	0.324
NM_001164682	Mpp4	0.327
NM_021365	Xlr4b	0.329
NM_001035531	Adrbk2	0.334
NM_008121	Gja5	0.336
AK045208	5730590G19Rik	0.348
NM_145840	Rgs9bp	0.353
NM_026422	Mrrf	0.355
NM_011838	Lynx1	0.359
NM_001139519	Zbp1	0.359
NM_007401	Adam5	0.360
NM_026563	Sdccag3	0.361
NM_019662	Rrad	0.363
NM_146749	Olfr875	0.365
XR_106015	Gm3916	0.366
AK016158	4930557B15Rik	0.367
NM_010113	Egf	0.368
NM_001111079	Uhrf1	0.368
NM_010468	Hoxd3	0.372
NM_009422	Traf2	0.372
NR_003248	Foxl2os	0.373
NM_015821	Fbxl8	0.374
NM_010196	Fga	0.375
NM_177208	Dopey1	0.378
AK041990	Ccdc64	0.379
NM_019921	Akap10	0.383
NM_054103	Stk33	0.388
NM_015809	Krtap5-4	0.391
NM_001081278	Tbc1d4	0.394
NM_027040	1700007K13Rik	0.394
NM_028751	Tjap1	0.399
AI662168	AI662168	0.402
AK078618	7330403C04Rik	0.404
NR_040511	9230102O04Rik	0.406
NM_017372	Lyz2	0.410
NM_019459	Nphs1	0.414
AK081075	Pla2g3	0.414
NM_001123362	Prdm12	0.420
NM_026596	4930591A17Rik	0.421
NM_029197	4930528F23Rik	0.426
NM_011517	Sycp3	0.427
AK077474	4930579K19Rik	0.429
AK038838	Ralgapa2	0.430
NM_001034102	Gm5800	0.434
NM_027816	Cyp2u1	0.436
NM_007392	Acta2	0.436

Genbank Accession	GeneSymbol	Fold change (TiO_2-H vs Sham)
NM_001011760	Olfr314	0.437
XR_140758	Gm10091	0.438
NM_010445	Hmx1	0.441
NM_178260	Kiss1	0.442
NR_038059	1700110I01Rik	0.442
NM_023061	Mcam	0.444
NM_023304	Fgf22	0.445
NM_028608	Glipr1	0.448
NM_175441	Mylk3	0.449
NM_183276	Nbeal2	0.451
NM_153136	Nudt18	0.452
NM_001102405	Acp5	0.455
NM_001081234	Pigg	0.458
NM_008149	Gpam	0.459
NM_172826	Dact2	0.463
NM_001164569	Rffl	0.475
NM_001039485	Fam38b	0.478
NM_144491	Dph1	0.479
NM_011432	Snrpc	0.482
NM_146567	Olfr843	0.484
XM_001471750	Exoc3l2	0.485
NM_001033769	B020031M17Rik	0.485
NM_010777	Mbp	0.485
NR_033221	BC031361	0.485
NM_175397	Sp110	0.487
NM_173770	Fam69c	0.491
NM_024438	Dusp19	0.493
NM_022434	Cyp4f14	0.493
NM_027018	Glipr1l1	0.494
NM_001082547	Gm5483	0.494
NM_172564	Tns4	0.495
NM_013524	Fut7	0.495
NM_029252	4930563D23Rik	0.496
NM_008034	Folr1	0.500
NM_145703	Kcnip2	0.500
NM_028483	Cib4	0.500
NM_021319	Pglyrp2	0.500
NM_001163614	Ascl4	0.514
NM_015783	Isg15	0.518
NM_008358	Il15ra	0.518
NM_007555	Bmp5	0.523
NM_172418	Mamstr	0.525
C78535	D7Ertd183e	0.531
NM_019732	Runx3	0.531
NM_008829	Pgr	0.536
NM_177765	Ttll13	0.540
NM_009681	Ap3s1	0.541
NM_001039220	AI429214	0.544
NM_010706	Lgals4	0.547
NR_040619	A930019D19Rik	0.549
NM_001029937	Sec14l3	0.564
NM_021274	Cxcl10	0.565
NR_045061	4921525O09Rik	0.568
NM_001013607	Vmo1	0.572
NM_001163611	Nps	0.573
NM_011582	Thbs4	0.576
NM_029107	4930417G10Rik	0.580
NM_017370	Hp	0.580
NM_177200	Svopl	0.581
NM_133193	Il1rl2	0.581
NM_013731	Sgk2	0.583
NM_001039555	Cyp2c68	0.584
AK014919	Cenpp	0.588
NM_001252569	Serpina1a	0.599
AK036480	Disp2	0.607
NR_045392	Gm10731	0.620
NR_045080	3300005D01Rik	0.621
NM_001102414	Slc2a9	0.622
NM_153510	Pilra	0.622
AK015366	4930442P07Rik	0.628
NM_018809	Ptf1a	0.631
AK170207	Zfp740	0.635
NM_009948	Cpt1b	0.637
NM_001037918	Lipt1	0.639
NM_011333	Ccl2	0.641
NM_170727	Scgb3a1	0.645
NM_144906	Sgip1	0.647
AK016268	4930571B16Rik	0.650
NM_001037822	Krtap5-5	0.652
NM_007691	Chek1	0.653
AK090034	Gm3161	0.653
NM_008631	Mt4	0.656
XM_003086124	Ms4a4a	0.660
NM_001204233	Spp1	0.662
NM_007775	Crygc	0.663
XR_105456	Gm4419	0.663
NM_001174107	Map3k9	0.663
