# Supplementary material for: Effects of Prenatal Exposure to Titanium Dioxide Nanoparticles on DNA Methylation and Gene Expression Profile in the Mouse Brain
Source: Front Toxicol. 2021 Oct 8;3:705910. doi: 10.3389/ftox.2021.705910 (PMC8915839; doi:10.3389/ftox.2021.705910)
Supplement: Supplementary file 7 [file Table6.pdf]

Supplementary Table 6. The genes of which DNA methylation were increased in the brain of female offspring prenatally exposed to TiO_2-NP.

ProbeName	Target position of probe on CpG island microarray	TargetID	GeneSymbol	CpG island Description	Ratio of relative methylation (TiO_2-NP/Vehicle)	Sham group			TiO_2-H group		
						Relative methylation (Cy5/Cy3)	Cy3 signal (Input)	Cy5 signal (MeDIP)	Relative methylation (Cy5/Cy3)	Cy3 signal (Input)	Cy5 signal (MeDIP)
A_68_P28202486	chr12:4824316-4824360	NM_025323:-75	0610009D07Rik	PROMOTER	2.066	5.998	7074.81	42438.20	12.391	5053.47	62617.15
A_68_P28183963	chr11:120210170-120210214	NR_038126:201	0610009L18Rik	INSIDE	1.932	3.213	1826.02	5866.26	6.205	1558.40	9670.64
A_68_P27646748	chr11:23533451-23533495	NM_027860:159	0610010F05Rik	INSIDE	1.632	2.999	1129.84	3388.09	4.894	941.33	4606.87
A_68_P24541220	chr6:72297774-72297818	NM_026696:486	0610030E20Rik	INSIDE	1.610	5.030	3501.90	17613.89	8.096	2811.14	22758.40
A_68_P21813623	chr2:163470481-163470525	NR_028113:-83	0610039K10Rik	PROMOTER	2.030	6.080	3175.66	19308.06	12.340	2365.83	29194.46
A_68_P28195044	chr12:3426740-3426784	NR_030694:-15	1110002L01Rik	DIVERGENT_PROMOTER	2.044	6.470	2368.60	15325.50	13.224	1639.16	21676.61
A_68_P23756659	chr5:65883667-65883711	NM_133697:386	1110003E01Rik	INSIDE	1.599	1.904	7260.24	13825.18	3.044	6186.80	18835.45
A_68_P29023967	chr13:49311401-49311447	NM_026738:-29	1110007C09Rik	PROMOTER	1.828	3.956	6255.04	24744.55	7.232	4536.90	32809.15
A_68_P24039805	chr5:121062634-121062678	NM_029096:-58	1110008J03Rik	DIVERGENT_PROMOTER	1.649	3.998	7528.67	30096.70	6.591	5424.59	35755.07
A_68_P31306319	chr17:66798578-66798622	NM_001114098:490	1110012J17Rik	INSIDE	2.195	10.809	9897.40	106984.90	23.727	6523.10	154776.90
A_68_P22217916	chr3:68673757-68673801	NM_001167996:271	1110032F04Rik	INSIDE	1.663	3.292	653.04	2149.81	5.475	646.53	3539.97
A_68_P28513315	chr12:70299066-70299110	NM_027269:328	1110034A24Rik	INSIDE	1.692	2.946	4125.79	12154.63	4.984	3535.00	17617.39
A_68_P21641570	chr2:132512792-132512837	NM_028637:-3204	1110034G24Rik	PROMOTER	1.858	6.225	4805.88	29915.80	11.569	3480.64	40266.74
A_68_P23331577	chr4:135543069-135543113	NM_025411:69	1110049F12Rik	INSIDE	1.553	3.233	1282.56	4146.47	5.022	1113.13	5590.41
A_68_P30608335	chr16:24393852-24393896	NR_037954:-133	1110054M08Rik	PROMOTER	1.556	4.325	927.24	4009.93	6.729	797.55	5366.96
A_68_P27633577	chr11:20730971-20731015	NM_173752:119	1110067D22Rik	INSIDE	1.740	5.809	1800.24	10457.74	10.107	1463.09	14786.97
A_68_P24864304	chr6:134878874-134878918	NR_037955:-213	1190002F15Rik	PROMOTER	1.710	3.687	1160.62	4279.02	6.305	851.08	5366.07
A_68_P29750657	chr14:79700949-79700993	NM_025427:472	1190002H23Rik	INSIDE	2.094	3.449	1577.46	5439.92	7.221	1229.53	8878.49
A_68_P27914868	chr11:72861634-72861678	NM_025818:288	1200014J11Rik	INSIDE	1.508	2.998	3522.26	10560.49	4.520	2793.53	12627.23
A_68_P27921682	chr11:74463075-74463119	NM_001081158:100	1300001I01Rik	INSIDE	2.088	2.414	680.04	1641.56	5.039	534.85	2695.16
A_68_P27921681	chr11:74462947-74462991	NM_001081158:-28	1300001I01Rik	PROMOTER	1.974	4.812	4348.74	20926.06	9.500	3488.37	33138.09
A_68_P30594370	chr16:21794748-21794792	NR_037957:351	1300002E11Rik	INSIDE	1.871	2.126	2580.94	5487.07	3.977	1897.25	7546.25
A_68_P24050005	chr5:122821860-122821904	NM_026883:90	1500011H22Rik	INSIDE	1.632	2.350	644.45	1514.69	3.835	582.15	2232.74
A_68_P30650943	chr16:32099956-32100000	NM_025892:91	1500031L02Rik	INSIDE	2.348	3.775	7748.81	29255.63	8.866	5745.28	50940.39
A_68_P23227949	chr4:115817024-115817068	NM_026547:175	1520402A15Rik	INSIDE	1.548	3.087	1615.03	4985.29	4.777	1317.12	6292.45
A_68_P29678557	chr14:66916156-66916200	NR_038077:-212	1700001G11Rik	PROMOTER	1.657	1.394	1033.06	1440.07	2.311	793.29	1832.91
A_68_P20134295	chr1:33727422-33727466	NR_033199:776	1700001G17Rik	INSIDE	1.808	8.194	2704.50	22161.41	14.814	1913.03	28339.87
A_68_P30387964	chr15:83197665-83197709	NR_027980:41	1700001L05Rik	INSIDE	1.652	4.523	7085.74	32046.29	7.470	5107.02	38149.22
A_68_P21105309	chr2:30657215-30657259	NM_198000:-9	1700001O22Rik	PROMOTER	1.695	3.081	983.14	3028.58	5.223	817.28	4268.44
A_68_P26541141	chr9:57109407-57109451	NM_028820:978	1700017B05Rik	INSIDE	2.493	5.134	2336.99	11998.43	12.798	1913.17	24484.04
A_68_P32050808	chr19:29122130-29122174	NR_028360:180	1700018L02Rik	INSIDE	1.797	3.071	3532.99	10850.38	5.520	2811.40	15519.09
A_68_P27293831	chr10:82275065-82275109	NR_038042:219	1700028I16Rik	INSIDE	2.437	6.388	11619.79	74232.92	15.569	8124.71	126492.50
A_68_P28450733	chr12:56225550-56225594	NM_001100116:58	1700047I17Rik2	INSIDE	1.893	4.739	1177.94	5581.73	8.969	972.67	8724.30
A_68_P28450734	chr12:56225622-56225666	NM_001100116:130	1700047I17Rik2	INSIDE	1.618	5.076	2323.24	11793.75	8.212	1827.50	15006.68
A_68_P28094671	chr11:105042686-105042730	NR_027956:39	1700052K11Rik	INSIDE	1.557	4.459	593.08	2644.78	6.941	610.23	4235.88
A_68_P20893089	chr1:185857699-185857743	NM_028516:157	1700056E22Rik	INSIDE	1.565	3.564	2094.13	7462.54	5.577	1749.00	9754.76
A_68_P20252736	chr1:57434804-57434848	NM_028546:363	1700066M21Rik	INSIDE	2.040	7.560	1416.99	10712.47	15.420	1205.70	18591.62
A_68_P21094978	chr2:28910349-28910393	NM_001166705:216	1700101E01Rik	INSIDE	1.506	1.659	1643.86	2726.81	2.498	1408.24	3517.32
A_68_P28104781	chr11:106889949-106889993	NM_001163473:434	1810010H24Rik	INSIDE	1.533	3.836	1002.22	3844.42	5.880	863.11	5074.98
A_68_P23699554	chr5:53658257-53658301	NM_001145433:-66	1810013D10Rik	PROMOTER	1.512	1.927	1747.30	3367.67	2.914	1494.68	4355.06
A_68_P30394811	chr15:84276781-84276825	NM_001163145:725	1810041L15Rik	INSIDE	1.600	3.955	1864.88	7375.82	6.327	1441.31	9119.27
A_68_P31619415	chr18:35812025-35812069	NM_027222:-3025	2010001M09Rik	PROMOTER	2.214	6.357	1547.49	9837.17	14.072	1207.65	16994.20
A_68_P24127508	chr5:138220341-138220385	NM_027242:217	2010007H12Rik	INSIDE	1.631	3.683	2150.37	7919.10	6.005	1663.78	9990.80
A_68_P29509885	chr14:31944046-31944090	NR_024069:-1952	2010107H07Rik	DIVERGENT_PROMOTER	1.757	3.885	1220.48	4741.76	6.827	944.05	6444.63
A_68_P29509872	chr14:31942367-31942412	NR_024069:-273	2010107H07Rik	DIVERGENT_PROMOTER	1.727	3.207	2485.55	7970.80	5.539	2061.63	11419.23

ProbeName	Target position of probe on CpG island microarray	TargetID	GeneSymbol	CpG island Description	Ratio of relative methylation (TiO_2-NP/Vehicle)	Sham group			TiO_2-H group		
						Relative methylation (Cy5/Cy3)	Cy3 signal (Input)	Cy5 signal (MeDIP)	Relative methylation (Cy5/Cy3)	Cy3 signal (Input)	Cy5 signal (MeDIP)
A_68_P23189066	chr4:106851077-106851121	NM_025617:-127	2210012G02Rik	DIVERGENT_PROMOTER	1.705	3.272	1742.72	5701.96	5.579	1469.17	8196.62
A_68_P24006959	chr5:115392406-115392450	NM_028211:218	2210016L21Rik	INSIDE	1.762	2.075	3762.48	7807.39	3.655	2626.23	9600.08
A_68_P25357073	chr7:105804516-105804560	NM_172280:541	2210018M11Rik	INSIDE	1.936	5.040	31781.32	160178.50	9.757	23016.13	224567.40
A_68_P21812641	chr2:163298061-163298105	NR_028122:-198	2310001K24Rik	PROMOTER	1.603	2.860	1751.00	5008.06	4.586	1461.56	6702.35
A_68_P28184189	chr11:120239967-120240011	NM_027980:72	2310003H01Rik	INSIDE	1.540	3.421	542.84	1857.08	5.269	466.14	2456.01
A_68_P31161375	chr17:36002953-36002997	NM_001146710:-98	2310014H01Rik	PROMOTER	2.081	4.776	4697.04	22432.44	9.940	3457.88	34370.91
A_68_P28613117	chr12:88289051-88289098	NM_173735:782	2310044G17Rik	INSIDE	1.794	4.048	1247.68	5050.33	7.262	971.94	7058.25
A_68_P27288028	chr10:80657209-80657253	NR_015477:-205	2310050B05Rik	PROMOTER	1.765	2.499	2529.81	6320.77	4.411	2017.03	8896.69
A_68_P28182345	chr11:119959914-119959958	NM_183137:109	2410002I01Rik	INSIDE	1.626	1.880	1012.24	1902.83	3.057	758.19	2317.78
A_68_P24430828	chr6:49023823-49023867	NM_029353:51	2410003K15Rik	INSIDE	1.662	2.935	2060.46	6047.62	4.877	1761.18	8589.71
A_68_P26631268	chr9:72951661-72951705	NM_025890:1478	2410004A20Rik	INSIDE	1.622	1.903	344.84	656.35	3.086	354.88	1095.29
A_68_P31609277	chr18:33954596-33954640	NR_038151:73	2410004N09Rik	INSIDE	1.982	1.727	3048.18	5263.10	3.423	2411.34	8253.95
A_68_P28595531	chr12:85291747-85291791	NM_023633:211	2410016O06Rik	INSIDE	2.188	2.421	371.70	899.87	5.298	458.78	2430.71
A_68_P28595535	chr12:85292140-85292184	NM_023633:605	2410016O06Rik	INSIDE	1.691	3.618	1248.07	4515.38	6.118	1021.70	6251.02
A_68_P30345268	chr15:75812328-75812373	NM_001163518:365	2410075B13Rik	INSIDE	1.504	2.082	651.05	1355.76	3.133	588.82	1844.67
A_68_P31992368	chr19:18745200-18745244	NM_026120:-47	2410127L17Rik	PROMOTER	1.970	8.119	1112.52	9032.07	15.996	768.74	12296.69
A_68_P23297489	chr4:129278119-129278163	NM_029748:190	2510006D16Rik	INSIDE	2.022	9.500	6864.88	65215.47	19.211	6025.84	115762.80
A_68_P30593952	chr16:21695060-21695104	NM_001001881:-344	2510009E07Rik	PROMOTER	1.985	1.567	1249.70	1958.13	3.110	936.20	2911.16
A_68_P30593950	chr16:21694881-21694925	NM_001001881:-164	2510009E07Rik	PROMOTER	1.503	0.434	1506.73	654.64	0.653	1233.71	805.79
A_68_P27288890	chr10:80788199-80788243	NM_027381:4373	2510012J08Rik	INSIDE	1.548	5.347	2882.86	15415.59	8.279	2017.82	16705.88
A_68_P27288853	chr10:80783632-80783676	NM_027381:-193	2510012J08Rik	PROMOTER	1.516	1.546	1103.03	1705.68	2.344	891.15	2088.80
A_68_P23323199	chr4:134066870-134066914	NM_001081099:-21	2610002D18Rik	PROMOTER	1.641	3.069	2082.85	6392.84	5.036	1674.79	8433.89
A_68_P26822984	chr9:110207818-110207862	NM_001081381:145	2610002I17Rik	INSIDE	1.574	2.186	1153.88	2522.94	3.441	942.26	3242.42
A_68_P32373277	chrX:48194552-48194596	NM_133729:-38	2610018G03Rik	PROMOTER	1.632	3.797	920.52	3495.16	6.197	695.81	4311.93
A_68_P23820887	chr5:77738314-77738358	NM_019836:775	2610024G14Rik	INSIDE	1.511	2.988	773.65	2311.49	4.515	651.71	2942.33
A_68_P30958538	chr16:90727762-90727806	NM_025642:-168	2610039C10Rik	PROMOTER	1.985	7.148	6153.67	43986.93	14.192	4023.20	57098.84
A_68_P26742258	chr9:95412445-95412489	NM_001114977:-51	2610101N10Rik	PROMOTER	2.465	9.638	28756.99	277163.20	23.754	16578.52	393802.70
A_68_P27942253	chr11:78075063-78075107	NM_001002004:-171	2610507B11Rik	PROMOTER	1.521	5.560	2221.97	12353.43	8.457	2054.15	17372.83
A_68_P25592704	chr7:149246956-149247000	NM_028308:-39	2700078K21Rik	PROMOTER	1.672	3.416	1686.03	5759.23	5.712	1471.01	8402.10
A_68_P25592703	chr7:149246867-149246911	NM_028308:51	2700078K21Rik	INSIDE	1.505	3.078	809.83	2492.47	4.632	861.56	3990.66
A_68_P31939711	chr19:7496090-7496134	NM_175381:3998	2700081O15Rik	INSIDE	1.843	5.555	2835.16	15749.15	10.237	1880.48	19250.76
A_68_P29309087	chr13:108834557-108834601	NM_001048250:-89	2810008M24Rik	PROMOTER	1.934	4.845	3948.98	19133.40	9.369	3266.40	30603.79
A_68_P20293904	chr1:64737359-64737403	NR_038009:-147	2810408I11Rik	DIVERGENT_PROMOTER	1.622	1.860	1054.52	1961.20	3.017	834.70	2518.52
A_68_P25952317	chr8:73030680-73030724	NM_025577:-64	2810428I15Rik	PROMOTER	1.683	3.799	2054.35	7803.78	6.392	1708.79	10922.38
A_68_P23545743	chr5:24606691-24606735	NR_027851:-80	2900005J15Rik	DIVERGENT_PROMOTER	1.558	2.903	729.35	2117.61	4.523	624.82	2825.84
A_68_P31114505	chr17:27270655-27270699	NM_026063:160	2900010M23Rik	INSIDE	2.157	9.667	6284.28	60751.19	20.850	4279.05	89217.88
A_68_P27787099	chr11:49988480-49988526	NM_026543:150	3010026O09Rik	INSIDE	1.742	1.554	3080.89	4787.81	2.707	2253.64	6101.51
A_68_P20417972	chr1:89371861-89371906	NM_028473:5045	3110079O15Rik	INSIDE	2.652	18.741	968.94	18158.52	49.706	756.70	37611.98
A_68_P24135766	chr5:139936328-139936372	NM_028469:138	3110082I17Rik	INSIDE	1.502	1.936	4745.40	9185.42	2.908	3667.44	10663.21
A_68_P21812328	chr2:163244957-163245001	NM_025699:228	3230401D17Rik	INSIDE	1.881	7.426	2014.28	14958.86	13.971	1569.90	21933.65
A_68_P29049835	chr13:54605357-54605401	NM_176987:213	4732471D19Rik	INSIDE	1.906	4.028	1786.81	7197.41	7.678	1384.65	10630.74
A_68_P28706885	chr12:106248338-106248382	NM_172500:-341	4831426I19Rik	PROMOTER	1.712	5.463	6987.51	38171.76	9.353	5071.87	47438.28
A_68_P31679073	chr18:47009821-47009865	NM_029008:150	4833403I15Rik	INSIDE	1.530	1.493	1261.63	1883.79	2.284	1016.66	2322.55
A_68_P29050103	chr13:54666500-54666544	NM_133797:221	4833439L19Rik	INSIDE	1.608	6.099	2522.42	15383.10	9.808	1975.41	19375.44
A_68_P30560722	chr16:14159381-14159425	NM_001081154:-35	4921513D23Rik	DIVERGENT_PROMOTER	1.575	3.794	13836.62	52494.04	5.973	8244.00	49245.55
A_68_P21770588	chr2:156137716-156137760	NM_027585:-470	4921517L17Rik	PROMOTER	1.524	2.899	1622.43	4703.88	4.418	1472.90	6507.65
A_68_P27289148	chr10:80827041-80827085	NM_001014836:1503	4930404N11Rik	INSIDE	1.964	4.488	7214.34	32377.00	8.812	5305.63	46755.12

ProbeName	Target position of probe on CpG island microarray	TargetID	GeneSymbol	CpG island Description	Ratio of relative methylation (TiO_2-NP/Vehicle)	Sham group			TiO_2-H group		
						Relative methylation (Cy5/Cy3)	Cy3 signal (Input)	Cy5 signal (MeDIP)	Relative methylation (Cy5/Cy3)	Cy3 signal (Input)	Cy5 signal (MeDIP)
A_68_P21042876	chr2:18919881-18919925	NR_024323:-43	4930426L09Rik	DIVERGENT_PROMOTER	1.977	7.301	2991.97	21843.57	14.432	2189.70	31602.67
A_68_P26018418	chr8:86671979-86672023	NM_001163752:64	4930432K21Rik	INSIDE	1.628	2.483	2729.10	6776.31	4.043	2053.68	8303.57
A_68_P30686476	chr16:38522756-38522801	NM_024273:-31	4930455C21Rik	PROMOTER	1.945	6.413	5617.05	36021.49	12.474	3776.73	47112.65
A_68_P23062804	chr4:83171213-83171257	NM_001081012:-214	4930473A06Rik	PROMOTER	1.509	2.827	912.04	2578.63	4.268	849.60	3625.74
A_68_P31122921	chr17:28660085-28660129	NM_026290:-7699	4930511I11Rik	PROMOTER	2.032	1.699	1585.67	2693.97	3.452	1228.85	4241.70
A_68_P26085413	chr8:98330883-98330927	NR_015574:175	4930513N10Rik	INSIDE	2.426	7.226	17038.37	123120.90	17.533	11905.60	208738.70
A_68_P33007146	chr9_random:49897-49941	NR_015516:-3490	4930526I15Rik	PROMOTER	3.692	6.288	3418.87	21499.06	23.215	2298.35	53355.95
A_68_P33007145	chr9_random:49764-49808	NR_015516:-3622	4930526I15Rik	PROMOTER	2.796	13.504	11845.74	159960.60	37.756	6253.91	236121.30
A_68_P33007144	chr9_random:49674-49720	NR_015516:-3712	4930526I15Rik	PROMOTER	2.238	20.949	646.83	13550.10	46.880	431.58	20232.68
A_68_P33012155	chr9_random:54509-54553	NR_027950:-162	4930526I15Rik	PROMOTER	1.961	3.299	5356.65	17673.86	6.469	3968.95	25675.00
A_68_P33012150	chr9_random:53992-54036	NR_027950:-678	4930526I15Rik	PROMOTER	1.803	7.605	24634.84	187359.00	13.714	15139.21	207618.50
A_68_P33012156	chr9_random:54584-54628	NR_027950:-86	4930526I15Rik	PROMOTER	1.535	1.664	567.58	944.36	2.554	454.38	1160.30
A_68_P22552055	chr3:135101078-135101122	NR_030689:529	4930539J05Rik	INSIDE	1.649	3.525	946.20	3335.59	5.813	843.84	4905.36
A_68_P22129414	chr3:51200267-51200311	NM_026358:181	4930583H14Rik	INSIDE	1.544	6.105	1129.35	6894.20	9.424	898.09	8463.53
A_68_P30959222	chr16:90831079-90831123	NM_001199210:-3	4931408A02Rik	PROMOTER	1.967	1.517	905.38	1373.80	2.985	747.69	2231.93
A_68_P22200394	chr3:65333128-65333172	NR_027955:186	4931440P22Rik	INSIDE	1.584	2.219	2078.16	4610.97	3.514	1826.35	6418.43
A_68_P22060448	chr3:36762071-36762115	NM_172679:65	4932438A13Rik	INSIDE	1.952	2.163	1441.07	3116.81	4.222	1152.18	4864.87
A_68_P23588493	chr5:33721117-33721161	NM_001081101:-206	4933407H18Rik	PROMOTER	1.924	5.062	8181.42	41417.93	9.741	5614.39	54690.28
A_68_P25834612	chr8:47248897-47248941	NM_025747:-5991	4933411K20Rik	PROMOTER	1.537	1.755	5189.64	9109.37	2.698	4110.67	11092.42
A_68_P27842891	chr11:60230619-60230663	NM_025757:-6	4933439F18Rik	DIVERGENT_PROMOTER	2.001	5.082	6324.86	32141.10	10.170	4611.32	46897.03
A_68_P27842896	chr11:60231205-60231249	NM_025757:580	4933439F18Rik	INSIDE	1.941	6.392	6265.28	40045.30	12.409	5241.96	65047.20
A_68_P22130346	chr3:51364011-51364056	NR_033624:355	5031434O11Rik	INSIDE	1.960	3.201	3208.33	10271.15	6.274	2445.68	15345.16
A_68_P21745337	chr2:151528077-151528121	NM_001160363:355	5430405G05Rik	INSIDE	1.617	12.534	2185.05	27386.41	20.261	1693.22	34306.73
A_68_P23929552	chr5:100858837-100858881	NR_034038:-304	5430416N02Rik	PROMOTER	1.598	4.386	2091.10	9170.96	7.010	1771.53	12417.68
A_68_P24449884	chr6:52266647-52266691	NR_038163:-1842	5730457N03Rik	PROMOTER	2.474	11.257	8241.17	92768.87	27.844	5150.16	143401.90
A_68_P20644756	chr1:138131091-138131135	NM_028872:-271	5730559C18Rik	PROMOTER	2.054	5.740	1343.02	7709.17	11.789	979.59	11548.56
A_68_P24084071	chr5:129106177-129106221	NR_015517:364	5930412G12Rik	INSIDE	1.656	2.695	2040.71	5500.05	4.464	1575.31	7032.18
A_68_P26812299	chr9:107888278-107888322	NM_026597:-53	6230427J02Rik	PROMOTER	3.211	41.034	2065.67	84762.66	131.746	820.78	108134.90
A_68_P20876216	chr1:182413344-182413388	NM_001081227:269	6330403A02Rik	INSIDE	1.559	1.478	4002.81	5914.28	2.304	3159.73	7278.97
A_68_P32151692	chr19:47806456-47806500	NM_026377:233	6330577E15Rik	INSIDE	1.543	4.098	11852.91	48570.74	6.323	7969.21	50392.81
A_68_P20153982	chr1:37486790-37486834	NM_198006:136	6330578E17Rik	INSIDE	1.695	2.028	3468.44	7034.36	3.437	2727.80	9375.07
A_68_P24127315	chr5:138182692-138182736	NM_175521:-1488	6430598A04Rik	DIVERGENT_PROMOTER	1.712	3.004	1293.54	3886.35	5.143	1008.95	5189.40
A_68_P24760447	chr6:113554719-113554763	NM_133937:-25	6720456B07Rik	DIVERGENT_PROMOTER	1.729	29.247	415.36	12148.19	50.565	408.90	20675.97
A_68_P29587993	chr14:49066241-49066285	NM_172600:236	6720456H20Rik	INSIDE	1.537	2.300	2572.13	5914.65	3.534	2101.71	7427.21
A_68_P21754954	chr2:153270097-153270141	NM_001001986:97	8430427H17Rik	INSIDE	2.028	6.050	1367.93	8276.38	12.269	1143.90	14034.44
A_68_P21755504	chr2:153354420-153354464	NM_001134300:1265	8430427H17Rik	INSIDE	1.554	4.392	2757.42	12109.24	6.824	2447.02	16699.70
A_68_P27085191	chr10:40402970-40403014	NM_177793:-95	9030224M15Rik	PROMOTER	1.686	2.249	1059.70	2383.52	3.792	947.30	3592.40
A_68_P23368580	chr4:141795400-141795444	NM_001109685:-106	9030409G11Rik	PROMOTER	1.540	4.200	12506.51	52523.82	6.467	9077.38	58703.21
A_68_P20250405	chr1:57029997-57030046	NR_024325:1709	9130024F11Rik	INSIDE	1.534	1.803	368.62	664.80	2.767	298.59	826.23
A_68_P23693365	chr5:52581836-52581880	NR_015537:-61	9230114K14Rik	DIVERGENT_PROMOTER	2.218	6.219	1084.17	6742.91	13.793	995.51	13731.22
A_68_P28513307	chr12:70298223-70298267	NR_033222:47	9330151L19Rik	INSIDE	1.565	5.402	2373.03	12819.39	8.457	1959.92	16574.74
A_68_P21782539	chr2:158187061-158187105	NR_015463:163	9430008C03Rik	INSIDE	1.922	4.489	4847.02	21757.79	8.630	4715.69	40694.87
A_68_P21782542	chr2:158187327-158187371	NR_027887:-90	9430008C03Rik	PROMOTER	1.755	4.200	1215.00	5103.27	7.372	959.59	7073.91
A_68_P31464043	chr18:4635545-4635589	NM_001081963:640	9430020K01Rik	INSIDE	1.723	2.340	2351.10	5500.43	4.031	2007.10	8090.87
A_68_P29030706	chr13:51195741-51195786	NR_029463:333	9430083A17Rik	INSIDE	1.855	7.570	295.35	2235.85	14.039	200.59	2816.15
A_68_P23562830	chr5:28784867-28784911	NR_015562:-8635	9530036O11Rik	PROMOTER	1.528	3.034	425.37	1290.71	4.636	458.45	2125.45
A_68_P26890105	chr9:122478146-122478190	NR_015610:-3450	9530059O14Rik	PROMOTER	1.828	3.077	1268.86	3904.14	5.625	1337.99	7526.78

ProbeName	Target position of probe on CpG island microarray	TargetID	GeneSymbol	CpG island Description	Ratio of relative methylation (TiO_2-NP/Vehicle)	Sham group			TiO_2-H group		
						Relative methylation (Cy5/Cy3)	Cy3 signal (Input)	Cy5 signal (MeDIP)	Relative methylation (Cy5/Cy3)	Cy3 signal (Input)	Cy5 signal (MeDIP)
A_68_P26890130	chr9:122481560-122481604	NR_015610:-36	9530059O14Rik	PROMOTER	1.742	2.246	822.11	1846.18	3.911	742.78	2905.29
A_68_P26890104	chr9:122477963-122478007	NR_015610:-3634	9530059O14Rik	PROMOTER	1.715	2.017	1416.52	2857.29	3.459	1128.93	3905.17
A_68_P21774858	chr2:156866344-156866388	NM_001164663:38635	9830001H06Rik	INSIDE	1.915	3.175	3202.56	10167.64	6.079	2541.60	15449.53
A_68_P21775093	chr2:156904079-156904123	NM_001164663:901	9830001H06Rik	INSIDE	1.552	3.069	1296.16	3978.46	4.764	1121.73	5344.35
A_68_P32054917	chr19:29880262-29880306	NM_172836:215	9930021J03Rik	INSIDE	2.013	8.406	1611.24	13543.82	16.922	1113.14	18836.77
A_68_P26344263	chr9:20672999-20673043	NM_175687:-65	A230050P20Rik	PROMOTER	1.600	11.633	911.91	10608.40	18.613	684.93	12748.62
A_68_P31961511	chr19:12670617-12670661	NR_015503:418	A330040F15Rik	INSIDE	1.811	6.470	2292.64	14834.32	11.717	1846.29	21632.64
A_68_P31145425	chr17:32533548-32533592	NR_029458:-6388	A530088E08Rik	PROMOTER	1.578	5.700	2471.14	14086.48	8.994	2301.26	20697.64
A_68_P32775148	chrX:155970504-155970548	NM_001033472:-73	A830080D01Rik	PROMOTER	1.881	4.182	1734.44	7253.34	7.867	1002.59	7887.70
A_68_P23925841	chr5:100157706-100157750	NR_030692:351	A930011G23Rik	INSIDE	2.022	3.906	1456.65	5689.62	7.898	1228.64	9704.30
A_68_P24513202	chr6:65621890-65621934	NM_172399:308	A930038C07Rik	INSIDE	1.694	3.364	496.39	1669.62	5.697	464.53	2646.23
A_68_P22921606	chr4:53172577-53172621	NM_013454:169	Abca1	INSIDE	2.437	2.829	163.57	462.78	6.896	94.45	651.32
A_68_P21072052	chr2:25283953-25283999	NM_007379:-217	Abca2	PROMOTER	1.867	6.187	1092.36	6758.71	11.554	738.63	8533.92
A_68_P28123546	chr11:110199057-110199101	NM_147219:-48	Abca5	PROMOTER	1.936	6.585	5276.42	34744.38	12.751	3677.97	46899.50
A_68_P29940452	chr14:119105540-119105584	NM_001033336:-121	Abcc4	PROMOTER	1.764	1.368	1461.86	1999.96	2.413	1301.71	3141.45
A_68_P30587610	chr16:20425965-20426009	NM_013790:481	Abcc5	INSIDE	1.505	2.189	1643.18	3597.22	3.294	1348.96	4443.74
A_68_P25097409	chr7:53435456-53435500	NM_011510:-75	Abcc8	PROMOTER	2.095	2.714	766.06	2078.86	5.685	662.42	3765.95
A_68_P24482547	chr6:58590413-58590457	NM_011920:43769	Abcg2	INSIDE	1.934	4.525	2139.50	9681.96	8.751	1636.83	14324.60
A_68_P21741665	chr2:150730549-150730593	NM_024465:-103	Abhd12	DIVERGENT_PROMOTER	1.708	3.632	2876.84	10449.71	6.204	2289.56	14203.41
A_68_P27938076	chr11:77328807-77328851	NM_026185:210	Abhd15	INSIDE	1.661	4.965	1329.88	6602.70	8.247	1178.66	9720.12
A_68_P31157497	chr17:35226598-35226642	NM_178592:385	Abhd16a	INSIDE	1.709	2.371	2002.65	4748.68	4.052	1436.97	5822.04
A_68_P20268947	chr1:60467002-60467046	NM_001198570:562	Abi2	INSIDE	2.078	4.564	2869.41	13096.93	9.483	2330.68	22101.92
A_68_P20268943	chr1:60466325-60466369	NM_001198570:-116	Abi2	PROMOTER	1.847	3.771	2029.17	7651.42	6.965	1746.26	12163.37
A_68_P20268944	chr1:60466493-60466537	NM_001198570:52	Abi2	INSIDE	1.712	4.100	1797.03	7367.11	7.017	1504.43	10555.97
A_68_P21111049	chr2:31615339-31615383	NM_009594:-104	Abl1	PROMOTER	1.603	1.565	1024.47	1603.78	2.510	855.67	2147.89
A_68_P31760609	chr18:62071314-62071358	NM_198649:142	Ablim3	INSIDE	1.729	2.646	1903.66	5036.37	4.575	1574.24	7201.53
A_68_P27932457	chr11:76308492-76308536	NM_198018:14407	Abr	INSIDE	1.921	9.569	21667.79	207339.50	18.381	18186.63	334294.90
A_68_P27932872	chr11:76384849-76384893	NM_198895:6	Abr	INSIDE	1.509	1.675	3476.87	5823.44	2.528	2873.42	7262.84
A_68_P24624506	chr6:88791877-88791921	NM_030251:31	Abtb1	INSIDE	2.158	5.442	15857.65	86291.89	11.741	10489.90	123163.10
A_68_P24624504	chr6:88791715-88791759	NM_030251:193	Abtb1	INSIDE	1.718	3.548	10382.55	36839.75	6.095	6985.27	42572.99
A_68_P21483989	chr2:103406800-103406844	NM_178890:356	Abtb2	INSIDE	1.762	2.160	513.43	1109.03	3.805	462.30	1759.28
A_68_P26373433	chr9:26807126-26807170	NM_025862:-14	Acad8	DIVERGENT_PROMOTER	2.098	5.345	17294.92	92433.87	11.212	11665.23	130790.60
A_68_P22659834	chr3:153607495-153607539	NM_007382:-120	Acadm	PROMOTER	1.924	3.919	2766.09	10839.68	7.540	2240.55	16892.71
A_68_P26521623	chr9:53418301-53418345	NM_144784:133	Acat1	INSIDE	1.541	3.585	943.56	3382.47	5.525	828.27	4576.50
A_68_P20877466	chr1:182655892-182655936	NM_133225:-259	Acbd3	PROMOTER	1.597	4.420	1550.19	6851.39	7.057	1414.09	9978.61
A_68_P30481633	chr15:99501487-99501531	NM_009597:360	Accn2	INSIDE	2.232	1.473	1396.71	2057.16	3.287	1251.13	4112.21
A_68_P28328448	chr12:31596461-31596505	NM_001110239:-5	Acp1	DIVERGENT_PROMOTER	2.003	3.807	1330.58	5065.67	7.626	1077.42	8216.80
A_68_P26749720	chr9:96790099-96790143	NM_153420:-279	Acpl2	PROMOTER	1.664	3.871	732.83	2837.06	6.444	609.12	3924.89
A_68_P32707838	chrX:138825108-138825152	NM_001033600:-52	Acsl4	PROMOTER	1.774	2.727	1079.84	2945.22	4.840	552.09	2671.95
A_68_P26242187	chr8:126416382-126416426	NM_009606:2232	Acta1	INSIDE	1.864	5.940	2048.63	12168.98	11.070	1536.20	17005.65
A_68_P24153997	chr5:143668325-143668369	NM_007393:57	Actb	INSIDE	1.581	2.044	1442.49	2948.69	3.232	1278.47	4131.59
A_68_P32143654	chr19:46469962-46470006	NM_016860:241	Actr1a	INSIDE	1.549	3.348	2979.00	9972.94	5.187	2460.95	12764.18
A_68_P27630023	chr11:20013151-20013195	NM_146243:-218	Actr2	PROMOTER	1.575	4.701	5876.49	27627.98	7.405	4547.82	33674.90
A_68_P20584830	chr1:127331799-127331843	NM_001205385:484	Actr3	INSIDE	1.500	2.916	7966.52	23227.85	4.375	6005.45	26271.98
A_68_P26617803	chr9:70527024-70527068	NM_007399:239	Adam10	INSIDE	1.859	3.341	3327.19	11116.75	6.210	2658.10	16507.02
A_68_P28081267	chr11:102623133-102623177	NM_001110778:402	Adam11	INSIDE	1.700	2.010	597.97	1201.94	3.417	526.77	1799.90
A_68_P23467734	chr5:8367164-8367208	NM_001098225:895	Adam22	INSIDE	1.589	2.734	1717.39	4695.16	4.344	1279.99	5560.36

ProbeName	Target position of probe on CpG island microarray	TargetID	GeneSymbol	CpG island Description	Ratio of relative methylation (TiO_2-NP/Vehicle)	Sham group			TiO_2-H group		
						Relative methylation (Cy5/Cy3)	Cy3 signal (Input)	Cy5 signal (MeDIP)	Relative methylation (Cy5/Cy3)	Cy3 signal (Input)	Cy5 signal (MeDIP)
A_68_P23467742	chr5:8368117-8368161	NM_001098225:-57	Adam22	PROMOTER	1.516	2.463	690.62	1701.06	3.735	631.65	2359.29
A_68_P23467741	chr5:8368019-8368063	NM_001098225:41	Adam22	INSIDE	1.570	1.732	1004.19	1738.76	2.719	921.50	2505.13
A_68_P21633051	chr2:130889050-130889094	NM_001163529:478	Adam33	INSIDE	1.542	2.455	602.88	1480.03	3.785	522.00	1975.90
A_68_P25724843	chr8:26127513-26127557	NM_007404:-140	Adam9	DIVERGENT_PROMOTER	3.403	7.316	1524.90	11155.47	24.892	1145.63	28517.06
A_68_P25724841	chr8:26127277-26127321	NM_007404:96	Adam9	INSIDE	1.608	2.741	2955.35	8101.89	4.408	2472.37	10898.08
A_68_P30931281	chr16:85803417-85803461	NM_009621:-78	Adamts1	PROMOTER	1.621	2.412	1529.85	3690.67	3.912	1277.02	4995.29
A_68_P26397640	chr9:30729401-30729445	NM_001024139:615	Adamts15	INSIDE	1.736	2.378	1314.66	3126.25	4.129	962.94	3976.27
A_68_P31744348	chr18:58995914-58995958	NM_175506:-481	Adamts19	PROMOTER	1.547	1.578	2334.17	3684.29	2.442	2112.33	5158.87
A_68_P23880187	chr5:90311691-90311735	NM_001081401:647	Adamts3	INSIDE	1.830	5.079	4130.18	20975.32	9.291	3425.03	31823.61
A_68_P23880193	chr5:90312354-90312398	NM_001081401:-17	Adamts3	PROMOTER	1.672	1.889	2641.21	4989.44	3.159	2203.61	6960.59
A_68_P23880196	chr5:90312755-90312799	NM_001081401:-417	Adamts3	PROMOTER	1.531	3.069	1012.76	3108.29	4.698	777.44	3652.78
A_68_P30931894	chr16:85900162-85900206	NM_011782:1186	Adamts5	INSIDE	1.511	4.290	1821.56	7814.98	6.483	1287.00	8343.12
A_68_P25276873	chr7:89483916-89483960	NM_001190374:-265	Adamtsl3	PROMOTER	1.957	2.496	301.10	751.49	4.885	287.88	1406.35
A_68_P25276872	chr7:89483764-89483808	NM_001190374:-417	Adamtsl3	PROMOTER	1.533	7.631	1584.93	12094.30	11.695	1347.21	15755.90
A_68_P22345154	chr3:95491735-95491779	NM_144899:25	Adamtsl4	INSIDE	1.792	1.859	2073.84	3854.71	3.330	1714.80	5711.03
A_68_P27952852	chr11:79967507-79967551	NM_172133:-135	Adap2	PROMOTER	1.668	4.869	1002.22	4879.41	8.122	801.83	6512.36
A_68_P24383759	chr6:39523831-39523875	NM_178873:-22	Adck2	PROMOTER	1.813	3.292	2014.29	6630.83	5.969	1485.03	8863.72
A_68_P29113350	chr13:69138021-69138065	NM_153534:377	Adcy2	INSIDE	1.581	2.342	1347.48	3155.18	3.703	1119.72	4146.15
A_68_P22568795	chr3:138105955-138105999	NM_007410:-151	Adh5	PROMOTER	1.639	2.652	6487.11	17202.67	4.345	5103.16	22173.81
A_68_P20633265	chr1:136312273-136312317	NM_028320:251	Adipor1	INSIDE	1.794	4.207	10396.44	43740.06	7.547	7847.36	59223.02
A_68_P20633260	chr1:136311773-136311817	NM_028320:-249	Adipor1	PROMOTER	1.638	1.899	1917.94	3642.07	3.110	1678.68	5221.19
A_68_P29450509	chr14:21871923-21871967	NM_134079:90	Adk	INSIDE	2.951	1.754	1736.13	3045.36	5.176	1477.41	7646.82
A_68_P32186048	chr19:54119950-54119994	NM_007417:301	Adra2a	INSIDE	1.514	1.870	1207.84	2258.19	2.830	1019.30	2884.49
A_68_P23599458	chr5:35621995-35622039	NM_007418:802	Adra2c	INSIDE	1.684	1.754	1240.19	2175.88	2.955	1041.02	3076.60
A_68_P23599469	chr5:35623311-35623355	NM_007418:2118	Adra2c	INSIDE	1.659	5.317	1510.31	8030.19	8.822	1307.97	11539.39
A_68_P31762242	chr18:62339733-62339777	NM_007420:-141	Adrb2	PROMOTER	1.736	3.893	1542.63	6005.45	6.760	1271.57	8595.79
A_68_P30375392	chr15:80778891-80778935	NM_009634:-36	Adsl	PROMOTER	2.048	5.773	6612.32	38176.14	11.822	4842.27	57243.28
A_68_P28751328	chr12:113858149-113858193	NM_007421:-87	Adssl1	PROMOTER	1.576	1.860	1284.81	2389.37	2.931	1070.45	3137.30
A_68_P31759815	chr18:61946490-61946534	NM_178928:-196	Afap1l1	PROMOTER	1.592	1.383	1416.45	1959.53	2.203	1122.92	2473.79
A_68_P32443538	chrX:66613886-66613930	NM_008032:403	Aff2	INSIDE	1.504	1.550	1876.72	2909.56	2.332	929.04	2166.58
A_68_P23542143	chr5:23988482-23988526	NM_139153:30510	Agap3	INSIDE	1.733	5.843	4665.00	27259.61	10.129	3513.02	35584.60
A_68_P24357881	chr6:34730747-34730791	NM_178630:337	Agbl3	INSIDE	1.543	3.147	427.26	1344.79	4.855	354.26	1720.04
A_68_P29080191	chr13:59658571-59658615	NM_023328:88	Agtpbp1	INSIDE	1.817	6.266	4305.05	26975.86	11.388	3358.48	38245.19
A_68_P20872220	chr1:181733104-181733148	NM_026375:1020	Ahctf1	INSIDE	1.602	2.264	1393.41	3155.16	3.628	1213.64	4402.86
A_68_P26530603	chr9:55174506-55174550	NM_172923:-227	AI118078	PROMOTER	1.534	3.931	1708.92	6717.38	6.028	1408.98	8493.67
A_68_P26530601	chr9:55174289-55174334	NM_172923:-444	AI118078	PROMOTER	1.532	4.214	1874.00	7897.77	6.456	1473.35	9512.37
A_68_P31212432	chr17:47605282-47605326	NM_145489:31717	AI661453	INSIDE	1.613	1.960	621.47	1218.38	3.163	511.78	1618.89
A_68_P30382137	chr15:82043157-82043202	NM_001207021:66	AI848285	INSIDE	1.580	3.256	825.33	2687.65	5.144	638.50	3284.60
A_68_P24428428	chr6:48579466-48579510	NR_027236:1323	AI854703	INSIDE	1.532	2.268	658.89	1494.33	3.475	601.02	2088.42
A_68_P27103323	chr10:43723779-43723823	NM_172393:852	Aim1	INSIDE	1.535	1.987	1204.32	2393.59	3.050	960.45	2929.32
A_68_P24158559	chr5:144670369-144670413	NM_001172146:318	Aimp2	INSIDE	1.705	4.816	2415.13	11630.91	8.213	2055.23	16879.15
A_68_P32050810	chr19:29122317-29122361	NM_021299:54	Ak3	INSIDE	1.606	6.341	972.54	6167.01	10.181	734.27	7475.93
A_68_P23154895	chr4:101091776-101091820	NM_001177602:-95	Ak4	PROMOTER	1.677	3.409	2375.71	8097.86	5.716	1900.31	10862.63
A_68_P23154908	chr4:101093069-101093113	NM_009647:783	Ak4	INSIDE	2.114	2.054	374.05	768.22	4.343	300.11	1303.25
A_68_P28002763	chr11:88726023-88726068	NM_001042541:-145	Akap1	PROMOTER	1.574	3.521	843.99	2971.51	5.541	761.65	4220.24
A_68_P31144954	chr17:32457616-32457660	NM_019774:460	Akap8	INSIDE	2.308	6.815	5331.38	36334.12	15.732	3796.01	59720.41
A_68_P31144961	chr17:32458430-32458474	NM_017476:29070	Akap8l	INSIDE	2.604	5.486	2734.95	15003.88	14.287	1952.87	27900.76

ProbeName	Target position of probe on CpG island microarray	TargetID	GeneSymbol	CpG island Description	Ratio of relative methylation (TiO_2-NP/Vehicle)	Sham group			TiO_2-H group		
						Relative methylation (Cy5/Cy3)	Cy3 signal (Input)	Cy5 signal (MeDIP)	Relative methylation (Cy5/Cy3)	Cy3 signal (Input)	Cy5 signal (MeDIP)
A_68_P23447646	chr5:3928274-3928318	NM_194462:111	Akap9	INSIDE	1.500	2.272	1022.53	2322.79	3.408	789.77	2691.39
A_68_P23264561	chr4:123427058-123427102	NM_023423:462	Akirin1	INSIDE	1.523	1.873	844.93	1582.79	2.854	666.74	1902.56
A_68_P26057102	chr8:93657583-93657627	NM_010241:1791	Aktip	INSIDE	1.541	3.702	3600.06	13328.11	5.705	2907.92	16588.87
A_68_P31731070	chr18:56721947-56721991	NM_138600:-16	Aldh7a1	DIVERGENT_PROMOTER	2.150	8.615	28263.35	243484.30	18.524	18196.29	337068.80
A_68_P31731124	chr18:56732842-56732886	NM_001127338:-271	Aldh7a1	PROMOTER	1.618	3.079	4363.12	13433.92	4.981	3711.04	18485.84
A_68_P25502305	chr7:133942445-133942489	NM_001177307:226	Aldoa	INSIDE	1.805	2.341	2242.61	5249.11	4.224	1800.05	7603.52
A_68_P25708990	chr8:23170949-23170993	NM_183142:-221	Alg11	DIVERGENT_PROMOTER	1.795	2.373	2983.00	7078.50	4.259	2521.59	10738.91
A_68_P32717145	chrX:140752482-140752526	NM_026247:-4	Alg13	PROMOTER	2.523	6.564	4083.98	26807.27	16.561	2514.84	41647.48
A_68_P31341464	chr17:72953935-72953979	NM_007439:-309	Alk	PROMOTER	1.725	3.540	6394.26	22635.29	6.108	5150.08	31456.16
A_68_P31341460	chr17:72953599-72953644	NM_007439:26	Alk	INSIDE	1.670	1.974	746.72	1473.91	3.296	648.62	2137.91
A_68_P21438176	chr2:93816807-93816851	NM_026944:34059	Alkbh3	DOWNSTREAM	1.886	5.584	1256.48	7015.68	10.528	1153.90	12148.56
A_68_P31259473	chr17:57136900-57136944	NM_025538:161	Alkbh7	INSIDE	1.735	2.983	1175.23	3506.23	5.178	897.02	4644.46
A_68_P23342100	chr4:137351824-137351868	NM_007431:446	Alpl	INSIDE	1.652	2.796	617.01	1725.19	4.619	533.23	2463.21
A_68_P26826440	chr9:110782793-110782837	NM_001146060:-11	Als2cl	PROMOTER	1.854	2.810	1878.69	5278.63	5.210	1557.45	8114.97
A_68_P20262088	chr1:59177273-59177317	NM_001033449:-354	Als2cr4	PROMOTER	1.750	4.488	745.12	3344.05	7.854	631.06	4956.38
A_68_P27083322	chr10:40020722-40020766	NM_007444:1248	Amd2	INSIDE	1.759	1.725	617.62	1065.22	3.034	556.90	1689.59
A_68_P27354277	chr10:93002683-93002727	NM_027908:74	Amdhd1	INSIDE	1.610	2.369	1000.49	2369.90	3.813	822.61	3136.64
A_68_P32710882	chrX:139400446-139400490	NM_019496:803	Ammecr1	INSIDE	1.790	3.226	976.55	3150.72	5.775	511.66	2954.91
A_68_P28743070	chr12:112513574-112513618	NM_033603:4275	Amn	INSIDE	1.565	2.620	686.38	1798.21	4.101	589.50	2417.74
A_68_P28859763	chr13:19040669-19040713	NM_175007:451	Amph	INSIDE	1.721	4.158	662.80	2756.07	7.158	559.15	4002.62
A_68_P28859761	chr13:19040366-19040410	NM_175007:149	Amph	INSIDE	1.537	2.967	5674.37	16838.28	4.562	4718.16	21522.00
A_68_P24052414	chr5:123270992-123271036	NM_001042491:334	Anapc5	INSIDE	2.546	24.473	4557.84	111543.90	62.308	2482.58	154683.20
A_68_P23447093	chr5:3802955-3802999	NM_001003909:133	Ankib1	INSIDE	2.001	4.414	2001.52	8835.30	8.832	1594.37	14080.72
A_68_P23447092	chr5:3802834-3802878	NM_001003909:253	Ankib1	INSIDE	1.703	3.584	749.53	2686.40	6.105	672.66	4106.67
A_68_P25656690	chr8:11635804-11635848	NM_133971:-72	Ankrd10	PROMOTER	1.635	2.189	2794.43	6117.45	3.578	2349.68	8407.98
A_68_P26237605	chr8:125566196-125566241	NM_001081379:-34	Ankrd11	PROMOTER	1.731	2.847	2956.92	8417.35	4.926	2522.15	12425.07
A_68_P22682770	chr3:157610734-157610778	NM_001013806:327	Ankrd13c	INSIDE	2.104	9.504	13866.58	131786.90	19.996	9715.78	194280.20
A_68_P22682769	chr3:157610620-157610664	NM_001013806:213	Ankrd13c	INSIDE	1.553	4.429	3140.67	13909.20	6.876	2744.75	18872.23
A_68_P31922246	chr19:4283254-4283298	NM_026720:-139	Ankrd13d	PROMOTER	1.555	3.090	3143.54	9713.27	4.806	2202.06	10583.65
A_68_P29514238	chr14:32643908-32643952	NM_001024604:-329	Ankrd28	PROMOTER	1.647	1.620	1147.20	1858.23	2.668	1038.20	2770.24
A_68_P25833746	chr8:47085665-47085709	NM_001039562:-482	Ankrd37	PROMOTER	1.647	2.622	3555.23	9320.60	4.318	2681.01	11575.77
A_68_P25833742	chr8:47085366-47085410	NM_001039562:-184	Ankrd37	PROMOTER	1.627	1.854	2843.21	5270.09	3.016	2169.42	6542.60
A_68_P27804984	chr11:53292506-53292550	NM_183173:1169	Ankrd43	INSIDE	1.545	2.755	606.94	1672.19	4.257	522.39	2223.93
A_68_P20239578	chr1:54983378-54983422	NM_001081433:-169	Ankrd44	PROMOTER	2.409	2.608	6346.65	16554.50	6.284	4761.34	29920.98
A_68_P22069519	chr3:38383514-38383558	NM_001167883:202	Ankrd50	INSIDE	1.621	3.267	2974.55	9716.58	5.297	2486.82	13171.62
A_68_P23897553	chr5:93473897-93473941	NM_175270:130	Ankrd56	INSIDE	1.968	1.785	1103.52	1970.25	3.513	847.35	2977.09
A_68_P32321491	chrX:34588512-34588556	NM_173779:-305	Ankrd58	PROMOTER	1.991	3.764	4900.57	18445.54	7.492	3053.16	22875.36
A_68_P31119196	chr17:28046558-28046602	NM_181413:296	Anks1	INSIDE	1.936	1.807	794.26	1435.62	3.500	648.57	2270.05
A_68_P27338390	chr10:90291759-90291803	NM_001177396:252345	Anks1b	INSIDE	2.245	4.521	1258.95	5692.26	10.151	946.42	9607.50
A_68_P27338392	chr10:90291975-90292019	NM_001177396:252561	Anks1b	INSIDE	1.702	6.325	1696.69	10730.82	10.766	1448.83	15598.30
A_68_P30459959	chr15:95620940-95620984	NM_175344:-311	Ano6	PROMOTER	1.925	3.162	2819.28	8914.46	6.086	2292.31	13950.27
A_68_P25957034	chr8:74004823-74004867	NM_001164679:5122	Ano8	INSIDE	1.543	4.355	2896.34	12613.58	6.719	2375.92	15964.47
A_68_P26569971	chr9:62191474-62191518	NM_009672:2347	Anp32a	INSIDE	1.888	1.626	2462.56	4004.47	3.070	2044.89	6278.80
A_68_P26569977	chr9:62192249-62192293	NM_009672:3121	Anp32a	INSIDE	1.503	1.406	1688.30	2373.21	2.113	1514.34	3199.71
A_68_P22888792	chr4:46464440-46464484	NM_130889:474	Anp32b	INSIDE	1.720	3.243	3492.38	11327.10	5.578	2955.22	16483.79
A_68_P27560706	chr11:4886974-4887018	NM_007454:89	Ap1b1	INSIDE	1.845	5.714	2772.77	15842.99	10.541	2186.53	23047.61
A_68_P25960292	chr8:74764393-74764437	NM_007456:384	Ap1m1	INSIDE	1.784	2.981	740.71	2207.94	5.317	583.61	3102.99

ProbeName	Target position of probe on CpG island microarray	TargetID	GeneSymbol	CpG island Description	Ratio of relative methylation (TiO_2-NP/Vehicle)	Sham group			TiO_2-H group		
						Relative methylation (Cy5/Cy3)	Cy3 signal (Input)	Cy5 signal (MeDIP)	Relative methylation (Cy5/Cy3)	Cy3 signal (Input)	Cy5 signal (MeDIP)
A_68_P30588065	chr16:20535666-20535710	NM_009679:112	Ap2m1	INSIDE	1.576	4.216	3006.62	12676.79	6.643	2140.72	14221.37
A_68_P25712956	chr8:23916111-23916155	NM_001122820:-6	Ap3m2	PROMOTER	1.652	4.125	3610.71	14895.53	6.816	2789.42	19011.75
A_68_P31678555	chr18:46901926-46901970	NM_009681:378	Ap3s1	INSIDE	2.070	5.852	3619.77	21183.18	12.116	2582.15	31286.58
A_68_P23763011	chr5:67009509-67009553	NM_009686:380	Apbb2	INSIDE	2.598	8.757	907.55	7947.11	22.748	649.96	14785.56
A_68_P32362520	chrX:45387716-45387760	NM_013912:291	Apln	INSIDE	2.111	11.695	2906.03	33987.15	24.693	1701.13	42005.98
A_68_P26399336	chr9:31018727-31018771	NM_001102455:652	Aplp2	INSIDE	1.866	3.500	1846.08	6460.53	6.530	1573.87	10276.76
A_68_P32538582	chrX:91612233-91612277	NM_026673:-194	Apoo	PROMOTER	1.639	2.182	678.97	1481.54	3.577	327.86	1172.65
A_68_P30927986	chr16:85173922-85173966	NM_001198823:8	App	INSIDE	1.810	4.246	1445.59	6137.72	7.684	1257.78	9665.34
A_68_P30481041	chr15:99414682-99414726	NM_009699:5218	Aqp2	INSIDE	1.716	3.349	1478.19	4949.91	5.747	1177.19	6764.97
A_68_P23742487	chr5:63157563-63157607	NM_178407:-168	Arap2	PROMOTER	1.654	4.428	1093.37	4841.67	7.324	873.88	6400.40
A_68_P30338802	chr15:74503171-74503215	NM_018790:-192	Arc	PROMOTER	1.692	3.131	5011.73	15690.22	5.298	3499.47	18539.99
A_68_P26469462	chr9:44575608-44575652	NM_145985:261	Arcn1	INSIDE	1.732	4.073	1190.06	4847.60	7.057	1021.52	7208.99
A_68_P26469460	chr9:44575399-44575447	NM_145985:469	Arcn1	INSIDE	1.531	7.501	660.54	4954.98	11.486	401.09	4606.84
A_68_P27835628	chr11:59041341-59041385	NM_001130408:302	Arf1	INSIDE	1.704	4.375	592.46	2591.78	7.456	507.66	3785.08
A_68_P27835630	chr11:59041516-59041560	NM_001130408:126	Arf1	INSIDE	1.592	2.033	1722.66	3502.46	3.236	1427.13	4618.53
A_68_P30476328	chr15:98593484-98593528	NM_007478:43	Arf3	INSIDE	1.594	2.018	3901.49	7874.01	3.216	3017.31	9704.51
A_68_P30476326	chr15:98593248-98593292	NM_007478:279	Arf3	INSIDE	1.808	1.391	2235.94	3111.08	2.516	1636.95	4118.23
A_68_P25983698	chr8:80041949-80041993	NM_030113:-164	Arhgap10	PROMOTER	1.754	7.007	3126.51	21906.85	12.292	2424.09	29797.49
A_68_P31315914	chr17:68353306-68353350	NM_172964:120	Arhgap28	INSIDE	1.741	3.659	2455.65	8984.22	6.368	2022.42	12879.14
A_68_P25032017	chr7:31319995-31320040	NM_178252:11	Arhgap33	INSIDE	2.650	5.817	30484.87	177343.10	15.418	21467.82	330995.40
A_68_P25032021	chr7:31320496-31320540	NM_178252:-490	Arhgap33	PROMOTER	2.197	5.889	4112.22	24218.15	12.937	2604.63	33696.82
A_68_P30350779	chr15:76647508-76647552	NM_001168288:1070	Arhgap39	INSIDE	1.516	2.606	1450.37	3779.73	3.952	1090.58	4309.65
A_68_P26295538	chr9:9239294-9239338	NM_027823:-303	Arhgap42	PROMOTER	1.569	3.162	3797.50	12008.35	4.961	2969.28	14729.70
A_68_P28185441	chr11:120442461-120442505	NM_133796:452	Arhgdia	INSIDE	1.520	1.757	2949.75	5183.66	2.671	2275.76	6078.96
A_68_P31108897	chr17:26337756-26337800	NM_008113:517	Arhgdig	INSIDE	1.657	6.939	5809.01	40307.93	11.500	4304.82	49505.05
A_68_P23430520	chr4:153674348-153674392	NM_001112744:-366	Arhgef16	PROMOTER	2.046	6.781	4768.04	32330.88	13.874	3046.91	42272.60
A_68_P23430516	chr4:153673821-153673865	NM_001112744:162	Arhgef16	INSIDE	2.012	3.473	3277.71	11382.83	6.988	2371.01	16569.62
A_68_P23362920	chr4:140804003-140804047	NM_172520:5226	Arhgef19	INSIDE	1.870	5.882	3780.71	22238.90	11.000	2681.58	29498.68
A_68_P22314836	chr3:88425692-88425736	NM_001198912:399	Arhgef2	INSIDE	1.678	6.619	2870.12	18996.50	11.103	2409.48	26752.56
A_68_P25657178	chr8:11728695-11728739	NM_001113517:612	Arhgef7	INSIDE	1.697	4.997	1742.71	8709.00	8.478	1385.60	11747.66
A_68_P23319354	chr4:133310209-133310253	NM_001080819:-704	Arid1a	PROMOTER	1.598	2.197	3613.68	7937.87	3.510	2699.47	9473.94
A_68_P31013736	chr17:4994894-4994938	NM_001085355:-157	Arid1b	PROMOTER	2.112	7.097	7741.52	54941.16	14.990	5329.90	79894.54
A_68_P28836964	chr13:14157071-14157115	NM_194262:1034	Arid4b	INSIDE	1.564	2.482	844.98	2096.93	3.880	657.33	2550.72
A_68_P29342439	chr13:114584551-114584595	NM_172595:-143	Arl15	PROMOTER	2.306	6.894	13305.65	91725.86	15.896	9055.61	143951.40
A_68_P32144718	chr19:46647649-46647693	NM_019718:-95	Arl3	DIVERGENT_PROMOTER	1.936	5.303	2710.10	14370.72	10.264	2159.84	22168.29
A_68_P20637774	chr1:137044071-137044115	NM_026823:682	Arl8a	INSIDE	2.086	7.376	14805.20	109197.40	15.389	10138.97	156028.00
A_68_P24759117	chr6:113328299-113328343	NM_001170485:214	Arpc4	INSIDE	1.632	11.956	3011.48	36006.38	19.517	2285.29	44602.84
A_68_P27900652	chr11:70246079-70246123	NM_145429:-54	Arrb2	PROMOTER	1.979	2.610	1169.34	3052.36	5.167	916.74	4736.55
A_68_P28118792	chr11:109334732-109334776	NM_028710:67	Arsg	INSIDE	1.751	1.983	2131.27	4227.17	3.474	1811.40	6292.14
A_68_P32533200	chrX:90534061-90534105	NM_007492:2098	Arx	INSIDE	3.504	13.814	6300.76	87041.16	48.399	3229.51	156305.70
A_68_P32533286	chrX:90544628-90544672	NM_007492:12666	Arx	DOWNSTREAM	2.358	4.450	2021.28	8993.94	10.492	1357.54	14243.46
A_68_P32533270	chrX:90542781-90542825	NM_007492:10818	Arx	INSIDE	2.126	3.577	832.85	2978.70	7.604	439.74	3344.00
A_68_P32533249	chrX:90540139-90540183	NM_007492:8176	Arx	INSIDE	1.592	2.504	1322.93	3312.11	3.985	969.13	3861.84
A_68_P33008885	chr12:21117800-21117844	NM_001004364:206	Asap2	INSIDE	1.629	3.874	2797.09	10835.86	6.309	2307.57	14558.86
A_68_P27681337	chr11:30854282-30854326	NM_023906:-93	Asb3	DIVERGENT_PROMOTER	2.186	6.686	9808.72	65585.14	14.615	7228.55	105644.30
A_68_P27171931	chr10:59465553-59465597	NM_026937:22	Ascc1	INSIDE	1.823	3.303	1913.76	6322.08	6.023	1652.32	9952.06
A_68_P27558730	chr11:4537928-4537972	NM_029291:155	Ascc2	INSIDE	2.265	8.303	1676.18	13917.18	18.803	1201.82	22597.63

ProbeName	Target position of probe on CpG island microarray	TargetID	GeneSymbol	CpG island Description	Ratio of relative methylation (TiO_2-NP/Vehicle)	Sham group			TiO_2-H group		
						Relative methylation (Cy5/Cy3)	Cy3 signal (Input)	Cy5 signal (MeDIP)	Relative methylation (Cy5/Cy3)	Cy3 signal (Input)	Cy5 signal (MeDIP)
A_68_P27558727	chr11:4537643-4537687	NM_029291:-131	Ascc2	DIVERGENT_PROMOTER	1.541	4.826	3891.78	18781.00	7.434	2991.62	22240.76
A_68_P27129526	chr10:50312500-50312544	NM_001146089:48	Ascc3	INSIDE	2.737	7.551	1717.70	12970.13	20.665	1310.39	27079.84
A_68_P25597935	chr7:150155005-150155050	NM_008554:142	Ascl2	INSIDE	1.527	1.424	1230.80	1753.04	2.175	972.17	2114.90
A_68_P27311153	chr10:85391455-85391499	NM_001163614:241	Ascl4	INSIDE	1.767	4.192	1634.87	6853.41	7.406	1314.91	9737.78
A_68_P27311154	chr10:85391524-85391568	NM_001163614:311	Ascl4	INSIDE	1.602	2.795	359.07	1003.47	4.477	296.83	1328.85
A_68_P20231549	chr1:53409460-53409504	NM_133728:61	Asnsd1	INSIDE	1.589	4.459	1548.31	6903.98	7.087	1276.77	9049.05
A_68_P20231550	chr1:53409540-53409584	NM_133728:-19	Asnsd1	PROMOTER	1.505	3.087	1517.71	4684.89	4.647	1377.76	6402.13
A_68_P25503171	chr7:134093152-134093196	NM_001039645:-79	Asphd1	DIVERGENT_PROMOTER	1.582	1.818	2003.95	3643.79	2.877	1578.37	4540.81
A_68_P23991928	chr5:112823693-112823737	NM_028386:-2481	Asphd2	PROMOTER	1.740	4.992	2724.62	13601.48	8.687	2226.16	19338.16
A_68_P21754297	chr2:153171289-153171333	NM_001039939:-564	Asxl1	PROMOTER	1.581	2.682	4922.89	13203.66	4.241	3681.23	15611.84
A_68_P30246249	chr15:57966345-57966389	NM_027435:271	Atad2	INSIDE	1.890	8.349	2043.61	17062.91	15.779	1656.81	26143.28
A_68_P28203004	chr12:4924079-4924123	NM_001099628:-58	Atad2b	DIVERGENT_PROMOTER	1.719	5.477	1128.71	6182.38	9.415	915.99	8624.10
A_68_P25522110	chr7:137662756-137662800	NM_001136054:266	Ate1	INSIDE	2.507	2.149	975.56	2096.60	5.388	765.52	4124.45
A_68_P30484704	chr15:100058837-100058881	NM_007497:569	Atf1	INSIDE	1.779	3.069	632.44	1941.20	5.461	547.45	2989.56
A_68_P21334154	chr2:73729891-73729935	NM_001025093:773	Atf2	INSIDE	2.330	9.939	23555.36	234127.40	23.161	14168.05	328151.80
A_68_P20936661	chr1:193006674-193006718	NM_007498:516	Atf3	INSIDE	1.738	7.410	4368.87	32372.21	12.876	3330.41	42883.92
A_68_P30371091	chr15:80086279-80086325	NM_009716:689	Atf4	INSIDE	1.948	3.218	1720.78	5537.99	6.270	1305.60	8185.62
A_68_P30371089	chr15:80086079-80086123	NM_009716:487	Atf4	INSIDE	1.583	3.049	1179.31	3596.02	4.827	964.24	4654.11
A_68_P31155038	chr17:34784397-34784441	NM_017406:294	Atf6b	INSIDE	2.986	23.608	887.30	20947.48	70.495	609.26	42949.85
A_68_P24872958	chr6:136467356-136467400	NM_019426:7	Atf7ip	INSIDE	1.785	3.282	2891.36	9490.11	5.858	2247.19	13163.42
A_68_P24766862	chr6:114688350-114688394	NM_028835:95230	Atg7	INSIDE	1.782	3.117	2619.62	8164.55	5.554	2153.76	11961.65
A_68_P24816773	chr6:124696183-124696227	NM_007881:10301	Atn1	INSIDE	2.000	4.992	1835.22	9160.55	9.981	1390.13	13875.38
A_68_P22376195	chr3:101408231-101408275	NM_144900:328	Atp1a1	INSIDE	1.933	5.227	2297.12	12008.18	10.104	1986.01	20065.78
A_68_P27914258	chr11:72775141-72775185	NM_001163336:492	Atp2a3	INSIDE	1.893	2.324	1220.11	2835.14	4.398	1120.16	4926.22
A_68_P32463053	chrX:70748921-70748965	NM_177236:518	Atp2b3	INSIDE	2.146	1.750	5188.54	9081.27	3.756	2355.67	8846.92
A_68_P32463052	chrX:70748623-70748667	NM_177236:220	Atp2b3	INSIDE	1.647	2.172	401.11	871.38	3.578	214.89	768.83
A_68_P21875562	chr2:174289603-174289647	NM_025983:-22	Atp5e	PROMOTER	1.580	3.191	1417.02	4521.49	5.041	1276.23	6434.07
A_68_P26134609	chr8:108089863-108089907	NM_013477:56	Atp6v0d1	INSIDE	1.678	1.831	3683.70	6745.62	3.072	2918.43	8965.38
A_68_P30150544	chr15:38591583-38591627	NM_025494:-54	Atp6v1c1	PROMOTER	1.727	3.206	1534.77	4919.78	5.535	1234.99	6835.11
A_68_P30150543	chr15:38591462-38591506	NM_025494:-174	Atp6v1c1	PROMOTER	1.546	2.549	521.34	1328.89	3.940	401.56	1582.16
A_68_P22971150	chr4:63206034-63206078	NM_024173:258	Atp6v1g1	INSIDE	1.732	4.331	2368.67	10258.41	7.501	2015.75	15119.91
A_68_P23770428	chr5:68238336-68238380	NM_001038999:312	Atp8a1	INSIDE	1.769	2.920	5059.10	14771.55	5.165	4019.53	20760.05
A_68_P31867752	chr18:81130655-81130699	NM_001201569:121	Atp9b	INSIDE	1.555	3.281	3663.16	12018.74	5.101	3085.38	15738.90
A_68_P21632194	chr2:130732229-130732273	NM_009730:19	Atrn	INSIDE	1.532	5.371	1345.00	7224.55	8.228	1081.03	8894.90
A_68_P29005205	chr13:46060754-46060798	NM_009124:-431	Atxn1	PROMOTER	1.754	3.197	1185.29	3789.79	5.607	956.20	5361.12
A_68_P29005211	chr13:46061364-46061408	NM_009124:-1041	Atxn1	PROMOTER	1.601	3.328	3978.55	13239.55	5.327	2945.77	15691.53
A_68_P26158736	chr8:112261757-112261801	NM_001080930:-139	Atxn1l	PROMOTER	1.951	1.963	1818.15	3569.42	3.830	1494.02	5721.52
A_68_P28078744	chr11:102157408-102157452	NM_001098836:513	Atxn7l3	INSIDE	1.982	2.125	1191.74	2532.41	4.212	930.26	3917.90
A_68_P27456596	chr10:112366019-112366063	NM_001033474:42	Atxn7l3b	INSIDE	1.872	2.055	1150.38	2364.14	3.846	889.33	3420.50
A_68_P23244381	chr4:118905195-118905239	NM_001012400:113	AU022252	INSIDE	2.333	2.690	5994.47	16124.96	6.275	4417.41	27719.49
A_68_P29040998	chr13:53024981-53025025	NM_016709:44	Auh	INSIDE	1.692	3.051	17814.71	54352.38	5.163	14317.06	73919.36
A_68_P24591144	chr6:83005100-83005144	NM_007517:476	Aup1	INSIDE	1.935	1.746	1460.28	2549.64	3.378	1153.78	3897.89
A_68_P24098738	chr5:132013832-132013876	NM_177047:1004359	Auts2	INSIDE	1.897	3.105	2416.69	7504.06	5.891	1923.10	11328.58
A_68_P24098740	chr5:132014030-132014074	NM_177047:1004161	Auts2	INSIDE	1.633	3.487	5752.28	20058.84	5.694	4561.64	25972.06
A_68_P28752186	chr12:113977208-113977252	NM_001024602:16846	AW555464	INSIDE	1.922	4.566	3478.97	15886.58	8.777	2592.08	22749.48
A_68_P28752030	chr12:113959942-113959986	NM_001024602:-420	AW555464	PROMOTER	1.833	5.367	1685.78	9047.25	9.838	1290.79	12699.32
A_68_P28752038	chr12:113960892-113960936	NM_001024602:530	AW555464	INSIDE	1.558	2.278	1280.40	2916.80	3.548	1133.46	4021.72

ProbeName	Target position of probe on CpG island microarray	TargetID	GeneSymbol	CpG island Description	Ratio of relative methylation (TiO_2-NP/Vehicle)	Sham group			TiO_2-H group		
						Relative methylation (Cy5/Cy3)	Cy3 signal (Input)	Cy5 signal (MeDIP)	Relative methylation (Cy5/Cy3)	Cy3 signal (Input)	Cy5 signal (MeDIP)
A_68_P28115686	chr11:108782547-108782591	NM_015732:906	Axin2	INSIDE	1.967	3.863	1652.35	6382.81	7.597	1331.82	10117.39
A_68_P26864195	chr9:117949694-117949738	NM_001048146:100	Azi2	INSIDE	2.577	9.490	2913.48	27648.78	24.453	2021.37	49428.26
A_68_P26864197	chr9:117949927-117949971	NM_001048146:332	Azi2	INSIDE	1.578	4.258	401.44	1709.32	6.717	385.58	2590.10
A_68_P30149946	chr15:38448361-38448405	NM_001102458:639	Azin1	INSIDE	1.768	2.411	2801.07	6754.27	4.263	2330.83	9935.58
A_68_P32573854	chrX:100810244-100810288	NR_028381:8827	B230206F22Rik	INSIDE	1.594	4.072	8298.88	33789.92	6.490	3713.15	24097.37
A_68_P32573868	chrX:100812194-100812238	NR_028381:6877	B230206F22Rik	INSIDE	1.522	1.930	590.26	1139.31	2.937	464.53	1364.45
A_68_P23364697	chr4:141101930-141101974	NR_030695:-124	B330016D10Rik	DIVERGENT_PROMOTER	1.646	2.241	420.61	942.55	3.689	394.23	1454.27
A_68_P22222029	chr3:69402614-69402658	NM_020026:147	B3galnt1	INSIDE	1.740	2.676	1545.57	4135.28	4.657	1385.46	6451.65
A_68_P27643047	chr11:22758992-22759036	NM_016888:721	B3gnt2	INSIDE	1.503	2.345	1392.02	3264.16	3.524	1171.12	4127.43
A_68_P26727067	chr9:92437602-92437647	NR_029474:83	B430319G15Rik	INSIDE	1.716	3.978	2810.97	11182.00	6.828	2049.32	13992.34
A_68_P28041452	chr11:95775809-95775854	NM_008081:354	B4galnt2	INSIDE	1.558	4.699	3119.17	14657.35	7.323	2688.16	19685.59
A_68_P24797816	chr6:120244799-120244843	NM_198884:-243	B4galnt3	PROMOTER	1.663	4.357	3431.01	14947.66	7.245	2671.21	19353.42
A_68_P22859197	chr4:40800680-40800724	NM_022305:329	B4galt1	INSIDE	1.751	3.145	1556.87	4896.93	5.509	1299.73	7159.97
A_68_P20827009	chr1:173200884-173200928	NM_020579:448	B4galt3	INSIDE	1.714	5.863	2039.79	11958.50	10.047	1518.05	15252.02
A_68_P23440042	chr4:155069270-155069314	NM_178699:842	B930041F14Rik	INSIDE	2.057	2.160	3207.52	6928.72	4.444	2257.47	10031.80
A_68_P26476281	chr9:45647027-45647071	NM_001145947:437	Bace1	INSIDE	1.538	3.408	1593.42	5430.55	5.240	1236.49	6479.45
A_68_P30337605	chr15:74348245-74348289	NM_174991:1641	Bai1	INSIDE	2.237	5.632	3019.87	17008.45	12.598	1874.84	23618.98
A_68_P30337599	chr15:74347574-74347618	NM_174991:971	Bai1	INSIDE	1.876	5.979	1825.93	10917.73	11.217	1386.24	15550.12
A_68_P23299678	chr4:129683985-129684029	NM_001199696:21685	Bai2	INSIDE	1.602	2.638	709.65	1872.38	4.226	588.49	2486.74
A_68_P20101394	chr1:25886869-25886913	NM_175642:-338	Bai3	PROMOTER	1.731	6.315	3410.04	21533.82	10.933	2400.03	26238.94
A_68_P31458875	chr18:3507840-3507884	NM_026505:-92	Bambi	PROMOTER	2.575	17.069	2820.94	48149.43	43.952	1883.09	82765.38
A_68_P21094081	chr2:28764046-28764090	NM_019446:7873	Barhl1	INSIDE	1.501	2.176	1638.99	3567.19	3.267	1373.26	4487.10
A_68_P21094161	chr2:28773677-28773726	NM_001164186:-1741	Barhl1	PROMOTER	1.575	1.669	957.36	1597.63	2.628	775.30	2037.63
A_68_P21094080	chr2:28763906-28763951	NM_019446:8013	Barhl1	INSIDE	1.759	1.881	359.60	676.37	3.309	188.20	622.75
A_68_P23961359	chr5:106887678-106887722	NM_001005477:-515	Barhl2	PROMOTER	1.660	2.219	607.95	1349.18	3.685	465.73	1716.21
A_68_P23961301	chr5:106881112-106881156	NM_001005477:6051	Barhl2	DOWNSTREAM	1.530	4.458	1187.26	5292.41	6.821	1025.21	6992.74
A_68_P24115158	chr5:135663906-135663950	NM_011714:736	Baz1b	INSIDE	1.625	4.863	843.16	4100.18	7.903	750.44	5930.37
A_68_P27539535	chr10:127533910-127533954	NM_054078:4094	Baz2a	INSIDE	1.542	1.931	1625.26	3138.52	2.978	1391.91	4145.42
A_68_P24330588	chr6:30124352-30124396	NR_038124:-2248	BB283400	DIVERGENT_PROMOTER	1.628	3.446	1069.53	3685.40	5.610	927.75	5204.62
A_68_P24980793	chr7:16899189-16899233	NM_133234:4279	Bbc3	INSIDE	1.567	1.681	1963.70	3300.92	2.634	1626.81	4285.17
A_68_P24980722	chr7:16885176-16885220	NM_133234:-9733	Bbc3	PROMOTER	1.670	1.553	819.54	1272.83	2.594	910.99	2363.33
A_68_P27448186	chr10:110735503-110735547	NM_027914:-210	Bbs10	PROMOTER	2.136	4.699	6265.44	29444.31	10.038	4580.95	45985.56
A_68_P31126996	chr17:29406043-29406087	NM_030561:332	BC004004	INSIDE	1.515	3.081	2100.11	6469.43	4.666	1938.91	9046.55
A_68_P28889015	chr13:24892904-24892948	NM_024473:-599	BC005537	PROMOTER	1.612	2.710	573.41	1553.95	4.369	537.08	2346.26
A_68_P27855082	chr11:62461774-62461819	NM_198861:229	BC046404	INSIDE	1.680	3.524	546.37	1925.28	5.918	463.03	2740.32
A_68_P24004683	chr5:115017800-115017844	NM_001004180:-437	BC057022	PROMOTER	2.324	15.416	3357.74	51764.02	35.833	2417.21	86615.99
A_68_P24004680	chr5:115017431-115017475	NM_001004180:-807	BC057022	PROMOTER	1.842	14.251	1361.17	19397.40	26.247	916.41	24053.55
A_68_P24004989	chr5:115060757-115060801	NM_001004180:42519	BC057022	INSIDE	1.798	4.701	4257.35	20015.41	8.454	3149.22	26623.86
A_68_P24004993	chr5:115061259-115061303	NM_001004180:43021	BC057022	INSIDE	1.672	4.702	9280.21	43633.32	7.863	6289.56	49452.98
A_68_P24004682	chr5:115017652-115017696	NM_001004180:-585	BC057022	PROMOTER	1.510	2.639	1484.21	3916.52	3.985	1255.60	5003.35
A_68_P25616602	chr8:4217183-4217227	NM_207203:108	BC068157	INSIDE	2.268	2.780	1157.85	3218.82	6.304	970.70	6119.43
A_68_P22311242	chr3:87803722-87803766	NM_007529:534	Bcan	INSIDE	1.584	1.717	634.64	1089.84	2.719	548.77	1492.29
A_68_P28332409	chr12:32319733-32319777	NM_001164090:-231	Bcap29	PROMOTER	2.486	9.592	2652.51	25441.57	23.849	1708.35	40742.33
A_68_P32464342	chrX:70961468-70961512	NM_012060:24	Bcap31	INSIDE	2.898	3.555	4575.87	16269.43	10.304	2149.12	22144.39
A_68_P24920738	chr6:144997199-144997243	NM_001024468:112	Bcat1	INSIDE	2.213	2.887	1538.27	4441.50	6.390	1136.41	7261.59
A_68_P27649080	chr11:23979542-23979586	NM_001159290:-1130	Bcl11a	PROMOTER	1.580	1.559	1357.49	2115.82	2.463	1197.90	2950.60
A_68_P28724517	chr12:109154270-109154314	NM_001079883:87332	Bcl11b	INSIDE	1.883	5.082	2190.88	11133.18	9.566	1718.68	16441.20

ProbeName	Target position of probe on CpG island microarray	TargetID	GeneSymbol	CpG island Description	Ratio of relative methylation (TiO_2-NP/Vehicle)	Sham group			TiO_2-H group		
						Relative methylation (Cy5/Cy3)	Cy3 signal (Input)	Cy5 signal (MeDIP)	Relative methylation (Cy5/Cy3)	Cy3 signal (Input)	Cy5 signal (MeDIP)
A_68_P28725240	chr12:109244557-109244601	NM_001079883:-2954	Bcl11b	PROMOTER	1.847	10.113	8411.21	85064.03	18.677	6212.44	116032.30
A_68_P21751445	chr2:152656855-152656899	NM_009743:542	Bcl2l1	INSIDE	1.593	2.403	439.51	1056.14	3.828	385.97	1477.30
A_68_P24995433	chr7:20408093-20408137	NM_033601:-10	Bcl3	PROMOTER	1.900	4.210	710.19	2990.18	8.001	585.96	4688.00
A_68_P30605892	chr16:23988103-23988147	NM_009744:574	Bcl6	INSIDE	1.832	5.444	3908.90	21278.28	9.974	3111.28	31031.68
A_68_P30605897	chr16:23988863-23988907	NM_009744:-186	Bcl6	PROMOTER	1.800	15.406	1820.78	28050.26	27.729	1472.85	40839.93
A_68_P32260801	chrX:11735963-11736007	NM_001168321:1497	Bcor	INSIDE	2.406	5.141	2235.03	11489.89	12.371	1508.11	18656.45
A_68_P32260816	chrX:11737647-11737691	NM_001168321:-187	Bcor	PROMOTER	2.303	3.008	999.15	3005.25	6.928	525.11	3638.03
A_68_P32260286	chrX:11658874-11658919	NM_029510:-1217	Bcor	PROMOTER	1.946	2.868	401.28	1150.87	5.582	234.46	1308.67
A_68_P32260334	chrX:11664151-11664195	NM_029510:-6493	Bcor	PROMOTER	1.808	2.229	1356.47	3024.10	4.031	815.61	3287.47
A_68_P32260277	chrX:11657663-11657707	NM_029510:-5	Bcor	PROMOTER	1.683	3.273	1448.95	4742.62	5.508	876.58	4828.15
A_68_P32260645	chrX:11714194-11714238	NM_001168321:23265	Bcor	INSIDE	2.055	1.644	1945.12	3196.98	3.377	960.88	3245.06
A_68_P32260371	chrX:11668288-11668332	NM_001168321:69171	Bcor	INSIDE	2.313	1.587	1844.59	2926.56	3.669	931.85	3419.40
A_68_P32260247	chrX:11654044-11654088	NM_029510:3613	Bcor	INSIDE	2.232	1.501	1387.28	2082.41	3.351	649.41	2176.05
A_68_P32260649	chrX:11714704-11714748	NM_001168321:22755	Bcor	INSIDE	1.548	1.375	1999.44	2748.78	2.128	960.24	2043.29
A_68_P32364063	chrX:45698568-45698612	NM_178782:4056	Bcorl1	INSIDE	1.698	3.139	2990.20	9385.67	5.330	1496.39	7975.42
A_68_P32364027	chrX:45694166-45694210	NM_178782:-346	Bcorl1	PROMOTER	1.660	4.598	1081.89	4974.40	7.632	723.01	5517.86
A_68_P32364032	chrX:45694726-45694770	NM_178782:214	Bcorl1	INSIDE	1.612	2.508	1625.91	4078.50	4.044	1095.53	4430.73
A_68_P21516743	chr2:109533914-109533958	NM_001048142:217	Bdnf	INSIDE	1.697	3.450	2201.21	7593.39	5.853	1533.04	8973.33
A_68_P28731662	chr12:110290760-110290805	NM_001163175:15645	Begain	INSIDE	1.694	2.853	1011.07	2884.77	4.833	847.23	4094.86
A_68_P28731667	chr12:110291510-110291554	NM_001163175:14895	Begain	INSIDE	1.596	2.689	2110.52	5675.94	4.291	1760.86	7556.49
A_68_P20135584	chr1:33964894-33964938	NM_177235:-450	Bend6	PROMOTER	2.037	3.894	1308.53	5095.77	7.934	871.14	6911.80
A_68_P21969040	chr3:17955766-17955810	NM_021560:1464	Bhlhe22	INSIDE	2.727	11.831	7289.10	86236.40	32.261	4657.85	150267.20
A_68_P29024653	chr13:49436665-49436709	NM_001039179:-231	Bicd2	PROMOTER	2.005	1.473	918.00	1352.30	2.953	718.95	2123.30
A_68_P31602497	chr18:32537058-32537102	NM_001083334:210	Bin1	INSIDE	1.652	1.753	1699.03	2977.56	2.895	1814.24	5252.63
A_68_P28167547	chr11:117710471-117710515	NM_001012273:-58	Birc5	PROMOTER	1.889	4.570	1254.99	5735.76	8.632	1037.63	8956.98
A_68_P25017319	chr7:28232977-28233021	NM_144923:2	Blvrb	INSIDE	1.864	5.626	831.95	4680.16	10.488	640.99	6722.65
A_68_P21646236	chr2:133380456-133380500	NM_007553:1544	Bmp2	INSIDE	1.503	3.428	1928.92	6612.98	5.151	1538.58	7925.62
A_68_P29530305	chr14:35315556-35315600	NM_009758:154	Bmpr1a	INSIDE	1.890	3.798	2069.87	7862.13	7.178	1766.58	12680.11
A_68_P24786758	chr6:118369210-118369254	NM_194339:203	Bms1	INSIDE	1.941	1.975	2909.05	5744.70	3.833	2374.51	9101.42
A_68_P31112418	chr17:26918319-26918363	NM_172149:317	Bnip1	INSIDE	2.338	3.275	5084.44	16652.05	7.656	3514.57	26906.28
A_68_P29682512	chr14:67627253-67627297	NM_009761:440	Bnip3l	INSIDE	1.739	3.096	1938.77	6002.12	5.383	2240.87	12063.71
A_68_P31152238	chr17:34259488-34259532	NM_010238:-818	Brd2	PROMOTER	2.262	8.753	1863.76	16313.88	19.798	1695.39	33564.70
A_68_P31152236	chr17:34259233-34259277	NM_010238:-562	Brd2	PROMOTER	2.106	10.329	4117.14	42527.06	21.755	2698.80	58712.71
A_68_P31152221	chr17:34257414-34257458	NR_037970:-591	Brd2	PROMOTER	1.886	4.785	791.53	3787.67	9.025	636.70	5746.45
A_68_P31152235	chr17:34259132-34259176	NM_010238:-462	Brd2	PROMOTER	1.584	2.928	654.82	1917.42	4.640	523.46	2428.63
A_68_P21084949	chr2:27330700-27330744	NM_023336:196	Brd3	INSIDE	3.270	18.917	430.60	8145.76	61.868	299.91	18554.51
A_68_P24160214	chr5:145016060-145016104	NM_018772:-11	Bri3	PROMOTER	2.046	3.409	1012.84	3452.90	6.976	891.10	6216.12
A_68_P24066928	chr5:125921960-125922004	NM_029752:45	Bri3bp	INSIDE	1.571	1.735	1634.18	2835.85	2.727	1390.05	3790.44
A_68_P24066931	chr5:125922400-125922444	NM_029752:485	Bri3bp	INSIDE	1.693	1.474	678.49	1000.22	2.495	592.40	1478.08
A_68_P31124371	chr17:28938550-28938594	NM_001081315:502	Brpf3	INSIDE	1.802	6.412	13892.16	89073.98	11.552	9383.17	108395.60
A_68_P30990999	chr16:96304407-96304451	NM_145125:-393	Brwd1	PROMOTER	1.570	2.758	5273.40	14544.89	4.331	3882.58	16817.11
A_68_P27278356	chr10:79167062-79167106	NM_001077184:-18	Bsg	PROMOTER	1.756	1.921	932.44	1791.45	3.373	697.13	2351.69
A_68_P27308088	chr10:84849580-84849624	NM_028709:44	Btbd11	INSIDE	1.577	2.863	487.57	1395.76	4.515	441.56	1993.86
A_68_P28149790	chr11:114652964-114653008	NM_028055:4220	Btbd17	INSIDE	2.356	6.334	2909.45	18427.57	14.919	2289.11	34151.24
A_68_P21673356	chr2:138082901-138082945	NM_001025431:603	Btbd3	INSIDE	1.619	1.438	3011.91	4330.58	2.328	2338.35	5442.63
A_68_P28694110	chr12:104116592-104116636	NM_172806:2	Btbd7	INSIDE	1.515	1.901	366.82	697.42	2.881	341.52	984.04
A_68_P30895871	chr16:78377255-78377299	NM_009770:-246	Btg3	PROMOTER	1.577	3.362	738.15	2481.85	5.303	628.77	3334.18

ProbeName	Target position of probe on CpG island microarray	TargetID	GeneSymbol	CpG island Description	Ratio of relative methylation (TiO_2-NP/Vehicle)	Sham group			TiO_2-H group		
						Relative methylation (Cy5/Cy3)	Cy3 signal (Input)	Cy5 signal (MeDIP)	Relative methylation (Cy5/Cy3)	Cy3 signal (Input)	Cy5 signal (MeDIP)
A_68_P28881600	chr13:23556264-23556308	NM_013483:1484	Btn1a1	INSIDE	2.014	3.623	1561.40	5656.95	7.296	1294.60	9445.96
A_68_P27781640	chr11:48982878-48982922	NM_172793:17691	Btnl9	INSIDE	1.548	4.075	2307.06	9401.42	6.310	1769.63	11166.23
A_68_P27781641	chr11:48982982-48983026	NM_172793:17587	Btnl9	INSIDE	2.360	0.624	1571.69	981.26	1.473	1783.94	2628.61
A_68_P20258663	chr1:58450520-58450564	NM_025824:563	Bzw1	INSIDE	1.599	3.266	2398.42	7833.03	5.223	2083.81	10883.14
A_68_P20411731	chr1:88256083-88256127	NR_015507:-44	C130036L24Rik	DIVERGENT_PROMOTER	1.584	5.622	3907.23	21965.87	8.906	2878.08	25631.84
A_68_P28596932	chr12:85534521-85534565	NM_001163502:423	C130039O16Rik	INSIDE	1.629	4.959	3598.27	17843.45	8.078	2828.40	22847.40
A_68_P29187496	chr13:83860499-83860543	NR_015561:-7190	C130071C03Rik	PROMOTER	1.638	4.605	1216.96	5604.25	7.543	1022.94	7716.25
A_68_P24222816	chr6:7794688-7794732	NM_052993:-513	C1galt1	PROMOTER	1.558	2.112	471.99	996.61	3.290	390.46	1284.47
A_68_P32328720	chrX:35988386-35988430	NM_021550:-88	C1galt1c1	PROMOTER	1.674	1.782	1695.13	3021.24	2.983	981.43	2927.47
A_68_P28082450	chr11:102809915-102809959	NM_011795:-2161	C1ql1	PROMOTER	1.645	1.681	2048.14	3443.75	2.766	1791.43	4955.48
A_68_P21013699	chr2:12932692-12932736	NM_153155:-223	C1ql3	PROMOTER	2.302	10.537	2128.50	22428.25	24.254	1494.20	36239.69
A_68_P30478150	chr15:98918593-98918637	NM_001024702:-455	C1ql4	PROMOTER	2.059	1.825	1640.51	2993.20	3.756	1345.67	5054.11
A_68_P30478144	chr15:98917975-98918019	NM_001024702:163	C1ql4	INSIDE	1.589	10.977	841.38	9235.98	17.442	720.72	12570.65
A_68_P21419450	chr2:90726161-90726205	NM_026161:240	C1qtnf4	INSIDE	1.985	3.211	2122.23	6815.20	6.373	1771.75	11291.25
A_68_P21419479	chr2:90729985-90730029	NM_026161:4064	C1qtnf4	INSIDE	1.707	11.448	561.15	6424.04	19.545	476.10	9305.58
A_68_P21419477	chr2:90729763-90729807	NM_026161:3842	C1qtnf4	INSIDE	1.508	2.241	777.41	1742.33	3.381	697.75	2358.80
A_68_P26536114	chr9:56265361-56265405	NM_172924:475	C230081A13Rik	INSIDE	1.612	3.543	2542.05	9007.74	5.713	1874.16	10706.98
A_68_P23351277	chr4:138908735-138908779	NM_001039200:249	C230096C10Rik	INSIDE	2.039	5.358	960.53	5146.29	10.923	813.03	8880.43
A_68_P22338534	chr3:94167626-94167670	NM_001136117:1283	C2cd4d	INSIDE	1.873	6.728	2320.94	15615.16	12.603	1545.88	19482.20
A_68_P22338541	chr3:94168285-94168329	NM_001136117:1941	C2cd4d	INSIDE	1.808	5.376	5230.63	28118.47	9.722	4155.52	40398.62
A_68_P25022916	chr7:29415707-29415751	NM_175319:306	C330005M16Rik	INSIDE	1.733	1.796	1168.65	2099.07	3.113	1029.93	3205.78
A_68_P28153096	chr11:115229081-115229125	NM_175454:-69	C630004H02Rik	PROMOTER	1.922	6.389	5115.22	32680.12	12.279	3916.94	48094.92
A_68_P23295623	chr4:128900595-128900639	NM_001033189:25071	C77080	INSIDE	1.565	2.201	993.95	2187.89	3.445	861.28	2967.49
A_68_P32576281	chrX:101395430-101395474	NM_001077354:1004	C77370	INSIDE	2.536	5.175	658.04	3405.35	13.124	385.33	5057.16
A_68_P32576283	chrX:101395692-101395736	NM_001077354:742	C77370	INSIDE	2.033	6.035	5902.41	35622.69	12.267	3428.34	42055.65
A_68_P32576286	chrX:101396172-101396216	NM_001077354:262	C77370	INSIDE	1.599	4.081	7479.24	30522.25	6.527	6051.21	39497.18
A_68_P32576287	chrX:101396366-101396410	NM_001077354:68	C77370	INSIDE	1.504	4.104	553.75	2272.55	6.172	442.53	2731.40
A_68_P29009048	chr13:46764994-46765038	NM_001033192:126	C78339	INSIDE	1.574	2.856	1542.16	4404.73	4.497	1400.30	6296.65
A_68_P29009046	chr13:46764820-46764864	NM_001033192:-48	C78339	PROMOTER	1.632	1.658	654.49	1085.11	2.705	563.16	1523.44
A_68_P27257833	chr10:75130195-75130239	NM_172549:96886	Cabin1	INSIDE	2.088	4.864	1348.50	6558.82	10.158	1195.82	12146.99
A_68_P26019650	chr8:86938371-86938415	NM_007578:-870	Cacna1a	PROMOTER	1.678	2.045	684.93	1400.45	3.431	614.58	2108.74
A_68_P24791280	chr6:119057361-119057405	NM_001159533:825	Cacna1c	INSIDE	1.775	3.638	2783.45	10125.32	6.457	2185.01	14109.12
A_68_P26808316	chr9:107302358-107302402	NM_001174047:170	Cacna2d2	INSIDE	1.933	3.324	3446.50	11455.10	6.425	2700.40	17350.81
A_68_P28053984	chr11:97883326-97883370	NM_001159320:-3662	Cacnb1	PROMOTER	1.777	3.533	2587.31	9140.65	6.279	1866.76	11721.27
A_68_P28053990	chr11:97883852-97883896	NM_001159320:-4188	Cacnb1	PROMOTER	1.526	1.645	2003.46	3296.48	2.511	1678.37	4215.21
A_68_P21221051	chr2:52531541-52531585	NM_001037099:539	Cacnb4	INSIDE	1.994	6.214	1722.92	10705.97	12.392	1153.42	14292.68
A_68_P30357053	chr15:77825858-77825902	NM_007583:123830	Cacng2	INSIDE	1.534	3.795	7182.40	27260.62	5.824	5370.61	31279.25
A_68_P30357863	chr15:77950040-77950084	NM_007583:-352	Cacng2	PROMOTER	1.517	2.852	1563.96	4460.78	4.328	1372.23	5939.16
A_68_P28109104	chr11:107655957-107656001	NM_019431:-200	Cacng4	PROMOTER	1.522	5.935	2073.53	12305.65	9.033	1748.37	15792.28
A_68_P33005168	chr7:3366552-3366596	NM_133189:29890	Cacng7	DOWNSTREAM	1.574	3.885	3043.52	11824.58	6.115	2177.56	13315.37
A_68_P26486127	chr9:47339193-47339237	NM_001025600:780	Cadm1	INSIDE	1.506	2.674	550.83	1472.74	4.026	421.57	1697.37
A_68_P26486134	chr9:47339844-47339888	NM_001025600:1432	Cadm1	INSIDE	1.559	1.847	612.83	1132.05	2.880	470.46	1354.71
A_68_P29413810	chr14:13654360-13654404	NM_001042617:1211	Cadps	INSIDE	1.750	3.642	2671.56	9730.57	6.374	2240.68	14281.93
A_68_P24298747	chr6:23789611-23789655	NM_153163:-332	Cadps2	PROMOTER	1.585	1.887	2105.53	3972.97	2.991	1721.36	5148.84
A_68_P27566544	chr11:5965149-5965193	NM_001174053:581	Camk2b	INSIDE	1.932	3.324	2280.29	7579.09	6.420	1796.50	11533.69
A_68_P23345840	chr4:138010011-138010055	NM_025451:-1030	Camk2n1	PROMOTER	1.519	2.325	1032.72	2401.05	3.531	867.53	3063.29
A_68_P24052105	chr5:123213863-123213907	NM_001199676:15535	Camkk2	INSIDE	1.545	8.285	1777.03	14721.93	12.803	1302.84	16679.92

ProbeName	Target position of probe on CpG island microarray	TargetID	GeneSymbol	CpG island Description	Ratio of relative methylation (TiO_2-NP/Vehicle)	Sham group			TiO_2-H group		
						Relative methylation (Cy5/Cy3)	Cy3 signal (Input)	Cy5 signal (MeDIP)	Relative methylation (Cy5/Cy3)	Cy3 signal (Input)	Cy5 signal (MeDIP)
A_68_P27788005	chr11:50138862-50138906	NM_001110499:291	Canx	INSIDE	1.970	3.156	5574.41	17595.39	6.220	4426.17	27529.64
A_68_P27788006	chr11:50138948-50138992	NM_001110499:205	Canx	INSIDE	1.951	4.085	5502.89	22480.34	7.970	3802.85	30309.46
A_68_P27788008	chr11:50139149-50139193	NM_001110499:5	Canx	INSIDE	1.875	2.514	5536.90	13918.62	4.712	4124.56	19436.10
A_68_P25354353	chr7:105326510-105326554	NM_007602:171	Capn5	INSIDE	2.116	4.262	3205.06	13658.50	9.017	2370.02	21370.70
A_68_P25354355	chr7:105326695-105326739	NM_007602:-13	Capn5	PROMOTER	1.889	3.757	1433.93	5386.78	7.097	1113.74	7904.33
A_68_P25354354	chr7:105326624-105326668	NM_007602:57	Capn5	INSIDE	1.746	5.034	1591.36	8011.50	8.790	1263.09	11102.05
A_68_P29511369	chr14:32149798-32149842	NM_009796:-89	Capn7	PROMOTER	1.678	1.996	1279.78	2554.88	3.350	1041.19	3487.70
A_68_P23350166	chr4:138748616-138748660	NM_009798:-207	Capzb	PROMOTER	1.967	3.798	6080.09	23094.74	7.471	4908.49	36671.62
A_68_P23350170	chr4:138749124-138749168	NM_001037761:253	Capzb	INSIDE	1.904	3.333	401.15	1336.85	6.346	334.45	2122.53
A_68_P21958957	chr3:14886237-14886281	NM_009801:-167	Car2	PROMOTER	1.622	2.677	743.86	1991.60	4.342	579.74	2517.33
A_68_P21958958	chr3:14886343-14886387	NM_009801:-61	Car2	PROMOTER	1.758	1.790	329.27	589.39	3.146	299.56	942.43
A_68_P26129049	chr8:107064933-107064977	NM_053070:248	Car7	INSIDE	1.609	4.243	3692.17	15666.86	6.830	2946.61	20123.93
A_68_P30533736	chr16:8672646-8672705	NM_025821:-429	Carhsp1	PROMOTER	1.649	9.979	1344.51	13416.96	16.451	1050.57	17282.81
A_68_P25655858	chr8:11497803-11497847	NM_026995:257	Carkd	INSIDE	1.594	2.684	1919.84	5153.21	4.279	1524.74	6524.19
A_68_P24205536	chr6:4550713-4550757	NM_145398:-331	Casd1	PROMOTER	1.915	5.905	4018.61	23729.76	11.305	3300.47	37312.12
A_68_P24205541	chr6:4551256-4551300	NM_145398:213	Casd1	INSIDE	1.553	4.127	993.11	4098.52	6.408	828.17	5306.98
A_68_P32268347	chrX:13423633-13423677	NM_009806:13	Cask	INSIDE	2.352	4.775	4033.45	19260.09	11.231	2219.51	24926.78
A_68_P32268351	chrX:13424038-13424082	NM_009806:-393	Cask	PROMOTER	1.745	2.623	3604.11	9455.09	4.577	2141.76	9802.92
A_68_P31097616	chr17:24625344-24625388	NM_027937:-361	Caskin1	DIVERGENT_PROMOTER	1.500	1.672	796.20	1330.91	2.508	669.03	1677.63
A_68_P31097620	chr17:24626114-24626158	NM_027937:409	Caskin1	INSIDE	1.821	1.379	1840.28	2537.41	2.510	1442.13	3620.09
A_68_P28155403	chr11:115664957-115665001	NM_080643:9928	Caskin2	INSIDE	2.858	7.044	4618.06	32531.25	20.130	3180.69	64028.48
A_68_P25837064	chr8:47702719-47702763	NM_009810:-62	Casp3	DIVERGENT_PROMOTER	2.255	8.065	13019.36	104998.00	18.182	9442.59	171689.80
A_68_P23397281	chr4:148275240-148275284	NM_027195:96762	Casz1	INSIDE	2.472	7.602	17403.01	132294.10	18.791	11859.44	222849.10
A_68_P23397206	chr4:148265535-148265579	NM_027195:87056	Casz1	INSIDE	2.233	5.561	2267.31	12607.68	12.418	1606.26	19945.99
A_68_P23397279	chr4:148275001-148275045	NM_027195:96522	Casz1	INSIDE	2.053	5.719	3282.49	18772.41	11.740	2431.91	28550.31
A_68_P23396578	chr4:148179082-148179126	NM_027195:604	Casz1	INSIDE	1.973	5.530	2948.56	16304.53	10.908	2126.42	23195.85
A_68_P23397204	chr4:148265329-148265373	NM_027195:86850	Casz1	INSIDE	1.513	1.413	1598.88	2258.72	2.138	1270.07	2715.47
A_68_P26132023	chr8:107694748-107694792	NM_001161456:197	Cbfb	INSIDE	1.756	4.825	1548.46	7471.14	8.475	1377.88	11677.23
A_68_P26132026	chr8:107695072-107695119	NM_001161456:522	Cbfb	INSIDE	1.921	1.830	668.55	1223.14	3.515	622.51	2188.07
A_68_P26466435	chr9:44041858-44041902	NM_007619:249	Cbl	INSIDE	2.703	8.729	4609.01	40229.81	23.595	3069.96	72435.80
A_68_P30759540	chr16:52031753-52031797	NM_001033238:113	Cblb	INSIDE	1.869	5.433	3786.69	20574.64	10.156	2974.85	30212.40
A_68_P24995306	chr7:20382177-20382221	NM_001161844:-40	Cblc	PROMOTER	1.740	2.603	3012.08	7840.71	4.530	2502.69	11336.44
A_68_P31140911	chr17:31773548-31773592	NM_178224:506	Cbs	INSIDE	1.954	3.316	7744.08	25678.94	6.479	5771.70	37394.65
A_68_P32026831	chr19:25036147-25036191	NM_146097:-62	Cbwd1	PROMOTER	2.132	6.201	3613.13	22406.37	13.219	2745.31	36290.47
A_68_P28175309	chr11:118884826-118884870	NM_007623:506	Cbx2	INSIDE	2.158	2.545	1722.26	4382.75	5.492	1449.00	7957.76
A_68_P28175799	chr11:118951917-118951961	NM_007625:-4387	Cbx4	PROMOTER	2.137	5.608	3193.53	17908.29	11.981	2453.76	29398.49
A_68_P28175418	chr11:118898955-118898999	NM_013926:3251	Cbx8	DOWNSTREAM	1.925	5.303	2342.48	12422.04	10.206	1877.46	19160.97
A_68_P28175458	chr11:118903402-118903447	NM_013926:-1197	Cbx8	PROMOTER	1.543	2.606	1629.10	4245.70	4.020	1283.32	5159.52
A_68_P28175491	chr11:118907932-118907976	NM_013926:-5727	Cbx8	PROMOTER	1.989	1.758	536.84	943.64	3.497	458.11	1601.84
A_68_P26080249	chr8:97441876-97441920	NM_001033533:100	Ccdc102a	INSIDE	1.641	1.764	2006.98	3540.67	2.895	1603.92	4644.14
A_68_P24951825	chr7:5008460-5008504	NM_146178:155	Ccdc106	INSIDE	1.771	2.209	1864.31	4118.27	3.912	1406.04	5500.30
A_68_P27169799	chr10:59079055-59079099	NM_001033259:364	Ccdc109a	INSIDE	1.507	1.574	2053.34	3232.15	2.371	1593.85	3779.80
A_68_P31676224	chr18:46471665-46471709	NM_001160399:-104	Ccdc112	PROMOTER	1.546	5.163	5877.27	30343.65	7.984	4145.92	33101.35
A_68_P27563898	chr11:5441853-5441897	NM_134033:346	Ccdc117	INSIDE	1.877	5.498	3920.15	21554.59	10.319	3139.07	32391.51
A_68_P26825039	chr9:110558909-110558957	NM_028312:-74	Ccdc12	DIVERGENT_PROMOTER	1.785	1.894	1145.22	2168.99	3.380	928.34	3138.11
A_68_P26019095	chr8:86794255-86794299	NM_026350:-17	Ccdc130	PROMOTER	1.532	2.969	3007.20	8927.37	4.548	2405.15	10938.41
A_68_P26430110	chr9:37155994-37156038	NM_001081429:-39	Ccdc15	PROMOTER	1.561	6.025	3644.25	21958.08	9.405	2772.80	26079.27

ProbeName	Target position of probe on CpG island microarray	TargetID	GeneSymbol	CpG island Description	Ratio of relative methylation (TiO_2-NP/Vehicle)	Sham group			TiO_2-H group		
						Relative methylation (Cy5/Cy3)	Cy3 signal (Input)	Cy5 signal (MeDIP)	Relative methylation (Cy5/Cy3)	Cy3 signal (Input)	Cy5 signal (MeDIP)
A_68_P20972053	chr2:5058960-5059004	NM_028804:161	Ccdc3	INSIDE	1.669	4.180	648.95	2712.90	6.979	534.03	3726.86
A_68_P24039940	chr5:121081339-121081383	NM_001195094:2884	Ccdc42b	INSIDE	2.335	2.020	661.34	1335.89	4.717	629.67	2969.92
A_68_P28112958	chr11:108286558-108286602	NM_029606:1	Ccdc46	INSIDE	1.540	1.840	1341.81	2468.67	2.833	1139.15	3226.70
A_68_P30625227	chr16:27388952-27388996	NM_001025615:-88	Ccdc50	PROMOTER	2.147	4.727	892.50	4218.57	10.148	820.82	8329.36
A_68_P27228310	chr10:69651858-69651902	NM_001111121:92012	Ccdc6	INSIDE	1.618	5.707	8650.37	49366.64	9.232	5818.45	53714.59
A_68_P26814987	chr9:108362953-108362997	NM_133744:126	Ccdc71	INSIDE	1.845	3.061	1718.26	5258.97	5.647	1503.95	8493.19
A_68_P26814990	chr9:108363250-108363294	NM_133744:424	Ccdc71	INSIDE	1.744	2.571	1714.86	4409.18	4.485	1407.04	6309.96
A_68_P31373927	chr17:79234917-79234961	NM_181649:83	Ccdc75	INSIDE	1.577	2.673	2020.61	5401.30	4.216	1599.74	6744.32
A_68_P31928777	chr19:5457059-5457103	NM_198616:469	Ccdc85b	INSIDE	1.532	2.179	1183.56	2579.21	3.338	1028.36	3432.85
A_68_P28727037	chr12:109513497-109513541	NM_001159910:109	Ccdc85c	INSIDE	1.767	4.478	592.81	2654.85	7.911	592.78	4689.72
A_68_P28727039	chr12:109513784-109513828	NM_001159910:-179	Ccdc85c	PROMOTER	1.531	3.067	3029.72	9291.40	4.694	2394.92	11242.71
A_68_P24980671	chr7:16871769-16871813	NM_172297:-1782	Ccdc9	PROMOTER	1.728	4.829	18022.74	87028.16	8.343	12430.23	103702.10
A_68_P28990871	chr13:43655098-43655142	NM_001081059:440	Ccdc90a	INSIDE	1.808	4.884	2329.94	11379.26	8.831	1930.50	17047.94
A_68_P24063264	chr5:125342497-125342541	NM_144819:73	Ccdc92	INSIDE	1.541	1.589	1082.41	1720.18	2.449	961.94	2355.74
A_68_P26883804	chr9:121403538-121403582	NM_031161:1247	Cck	INSIDE	1.634	3.579	904.53	3237.06	5.846	706.74	4131.75
A_68_P25392270	chr7:112574421-112574465	NM_007627:109	Cckbr	INSIDE	1.513	3.964	6148.54	24371.44	5.996	5077.69	30446.86
A_68_P27569029	chr11:6447001-6447045	NM_001190343:133	Ccm2	INSIDE	2.030	7.059	1906.52	13457.51	14.326	1432.75	20526.24
A_68_P25065443	chr7:38893032-38893076	NM_007633:-545	Ccne1	PROMOTER	1.913	1.928	2996.29	5777.92	3.690	2226.80	8216.66
A_68_P22728457	chr4:11118468-11118512	NM_001037134:-10	Ccne2	PROMOTER	1.767	2.141	909.54	1947.35	3.783	756.22	2860.48
A_68_P32111326	chr19:40905687-40905731	NM_172839:-60	Ccnj	PROMOTER	1.569	2.042	377.81	771.60	3.205	357.59	1146.05
A_68_P22202889	chr3:65762508-65762552	NM_019937:-383	Ccnl1	PROMOTER	2.137	1.533	854.67	1310.43	3.276	727.96	2384.86
A_68_P29338034	chr13:113777821-113777865	NM_001081062:-167	Ccno	PROMOTER	2.475	3.687	601.99	2219.36	9.124	484.42	4420.08
A_68_P29338038	chr13:113778161-113778205	NM_001081062:173	Ccno	INSIDE	1.516	1.461	3379.73	4937.96	2.215	2794.94	6190.79
A_68_P26630618	chr9:72833442-72833486	NM_001114328:107	Ccpg1	INSIDE	1.639	1.961	1956.95	3838.33	3.215	1718.91	5526.45
A_68_P20796837	chr1:167775393-167775437	NM_001113391:56603	Cd247	INSIDE	1.846	3.316	1024.24	3396.84	6.123	945.61	5790.05
A_68_P20796835	chr1:167775162-167775206	NM_001113391:56373	Cd247	INSIDE	1.656	3.749	1229.47	4609.55	6.210	1193.88	7414.07
A_68_P30747800	chr16:49855504-49855548	NM_010581:-240	Cd47	PROMOTER	2.122	2.349	1331.03	3126.76	4.984	933.56	4653.28
A_68_P30686262	chr16:38483294-38483338	NM_009855:24304	Cd80	INSIDE	1.548	3.799	3027.88	11502.80	5.880	2326.95	13681.60
A_68_P24820868	chr6:125444847-125444891	NM_007657:-95	Cd9	PROMOTER	2.390	11.281	9567.66	107930.20	26.957	5477.58	147659.80
A_68_P24820864	chr6:125444507-125444551	NM_007657:245	Cd9	INSIDE	1.633	3.222	799.10	2574.41	5.262	716.17	3768.19
A_68_P29639452	chr14:60216844-60216888	NM_001168535:-70	Cdadc1	PROMOTER	1.946	9.593	6488.31	62244.12	18.664	3827.68	71440.31
A_68_P22449315	chr3:116126619-116126663	NM_001080818:310	Cdc14a	INSIDE	1.730	3.289	1221.99	4019.51	5.690	1102.08	6270.97
A_68_P26820784	chr9:109778541-109778586	NM_007658:481	Cdc25a	INSIDE	1.836	7.655	2446.19	18725.75	14.054	1806.42	25386.49
A_68_P27278225	chr10:79144473-79144517	NM_177613:-445	Cdc34	PROMOTER	1.694	2.650	4981.62	13200.69	4.488	3951.36	17732.59
A_68_P23339473	chr4:136913085-136913129	NM_009861:546	Cdc42	INSIDE	1.525	1.562	644.29	1006.07	2.381	953.81	2270.72
A_68_P20872965	chr1:181889796-181889840	NM_001033285:-1401	Cdc42bpa	PROMOTER	1.510	1.895	2361.56	4474.53	2.861	1933.91	5532.09
A_68_P28743717	chr12:112615357-112615401	NM_183016:551	Cdc42bpb	INSIDE	1.651	11.373	1430.70	16271.87	18.774	1094.42	20546.89
A_68_P31933966	chr19:6320610-6320654	NM_001033342:14176	Cdc42bpg	INSIDE	1.866	2.451	1328.64	3256.35	4.573	993.63	4544.01
A_68_P30362680	chr15:78673148-78673192	NM_027219:94	Cdc42ep1	INSIDE	1.883	4.258	1830.06	7792.26	8.016	1529.47	12260.21
A_68_P30362677	chr15:78672776-78672820	NM_027219:-278	Cdc42ep1	PROMOTER	1.598	2.451	661.26	1620.50	3.915	598.40	2342.69
A_68_P31376853	chr17:79753911-79753955	NM_026514:499	Cdc42ep3	INSIDE	1.706	1.929	1057.53	2040.31	3.292	870.61	2866.13
A_68_P31376862	chr17:79755142-79755186	NM_026514:-733	Cdc42ep3	PROMOTER	1.516	1.712	474.69	812.66	2.596	415.37	1078.28
A_68_P28143851	chr11:113612678-113612722	NM_020006:-226	Cdc42ep4	PROMOTER	1.866	5.437	4581.48	24908.34	10.143	3566.82	36177.86
A_68_P27812111	chr11:54600916-54600960	NM_178626:267	Cdc42se2	INSIDE	1.553	2.021	2859.81	5779.07	3.138	2218.76	6963.45
A_68_P28059222	chr11:98768831-98768880	NM_001025779:-347	Cdc6	PROMOTER	1.692	7.625	1315.70	10031.63	12.899	995.61	12841.84
A_68_P28752833	chr12:114067608-114067652	NM_028023:-30	Cdca4	PROMOTER	2.176	4.177	2559.35	10690.00	9.089	1848.78	16804.37
A_68_P28752830	chr12:114067331-114067375	NM_028023:248	Cdca4	INSIDE	1.533	3.522	2449.67	8627.34	5.401	1964.09	10607.28

ProbeName	Target position of probe on CpG island microarray	TargetID	GeneSymbol	CpG island Description	Ratio of relative methylation (TiO_2-NP/Vehicle)	Sham group			TiO_2-H group		
						Relative methylation (Cy5/Cy3)	Cy3 signal (Input)	Cy5 signal (MeDIP)	Relative methylation (Cy5/Cy3)	Cy3 signal (Input)	Cy5 signal (MeDIP)
A_68_P31524836	chr18:16965652-16965696	NM_007664:1884	Cdh2	INSIDE	1.542	5.347	1273.88	6811.52	8.243	1060.08	8737.75
A_68_P31524834	chr18:16965463-16965507	NM_007664:2074	Cdh2	INSIDE	1.514	1.442	5317.30	7667.13	2.183	4181.25	9127.82
A_68_P27176099	chr10:60150136-60150180	NM_023370:9080	Cdh23	INSIDE	1.509	2.178	935.31	2037.03	3.287	895.72	2944.61
A_68_P26140014	chr8:109035535-109035580	NM_001037809:745	Cdh3	INSIDE	1.993	3.372	10881.57	36690.04	6.721	7717.80	51872.65
A_68_P21897030	chr2:179177570-179177614	NM_009867:410	Cdh4	INSIDE	1.833	3.684	2559.17	9428.91	6.755	2105.20	14221.06
A_68_P30026213	chr15:13102919-13102963	NM_007666:454	Cdh6	INSIDE	1.603	2.861	2518.02	7205.20	4.586	2042.09	9365.27
A_68_P26102845	chr8:101939884-101939928	NM_001039154:451	Cdh8	INSIDE	1.587	2.112	3141.75	6636.81	3.352	2242.95	7519.00
A_68_P26102842	chr8:101939617-101939661	NM_001039154:719	Cdh8	INSIDE	1.509	4.432	1458.56	6464.77	6.686	1284.16	8586.35
A_68_P27351976	chr10:92623453-92623497	NM_146239:-146	Cdk17	PROMOTER	1.757	4.366	815.31	3559.77	7.669	642.05	4924.18
A_68_P27351975	chr10:92623318-92623362	NM_146239:-280	Cdk17	PROMOTER	1.631	5.287	5246.48	27740.47	8.626	4202.19	36248.87
A_68_P27083521	chr10:40069136-40069180	NM_001168304:45	Cdk19	INSIDE	2.272	7.152	461.33	3299.64	16.254	351.42	5711.81
A_68_P24060833	chr5:124803880-124803924	NM_013812:735	Cdk2ap1	INSIDE	1.948	8.285	867.65	7188.25	16.135	691.74	11161.30
A_68_P28047527	chr11:96777858-96777902	NM_030248:-85	Cdk5rap3	PROMOTER	2.231	9.254	3176.78	29398.85	20.649	1753.53	36208.26
A_68_P23444287	chr5:3341583-3341627	NM_009873:-2707	Cdk6	PROMOTER	1.824	3.467	2545.92	8825.46	6.323	1807.56	11428.91
A_68_P24167580	chr5:147072895-147072939	NM_153599:29666	Cdk8	INSIDE	1.668	2.767	354.85	982.03	4.616	296.88	1370.38
A_68_P24864240	chr6:134871160-134871204	NM_009875:764	Cdkn1b	INSIDE	1.673	2.662	3361.10	8948.92	4.453	2603.18	11592.14
A_68_P24864235	chr6:134870715-134870759	NM_009875:318	Cdkn1b	INSIDE	1.707	1.405	1767.54	2483.14	2.397	1419.21	3402.40
A_68_P23094811	chr4:88940707-88940751	NM_009877:-205	Cdkn2a	PROMOTER	1.630	1.577	574.53	906.09	2.571	449.85	1156.63
A_68_P23201928	chr4:109330089-109330133	NM_007671:7867	Cdkn2c	DOWNSTREAM	1.816	2.840	954.42	2710.40	5.157	785.33	4050.01
A_68_P29578138	chr14:47380037-47380081	NM_028222:-157	Cdkn3	PROMOTER	2.191	1.390	1576.79	2191.73	3.046	1182.31	3601.48
A_68_P21639427	chr2:132088879-132088923	NM_138651:-92	Cds2	DIVERGENT_PROMOTER	1.609	4.699	469.43	2205.66	7.559	401.49	3035.01
A_68_P24172967	chr5:148116571-148116615	NM_007673:2233	Cdx2	INSIDE	1.721	4.316	1295.33	5590.72	7.427	1017.59	7557.83
A_68_P30567429	chr16:15887235-15887279	NM_007679:-122	Cebpd	PROMOTER	2.546	13.072	7367.64	96311.68	33.284	5181.40	172455.10
A_68_P30567436	chr16:15887941-15887985	NM_007679:584	Cebpd	INSIDE	1.602	4.663	696.93	3249.69	7.469	603.47	4507.38
A_68_P25047313	chr7:35841354-35841398	NM_009884:209	Cebpg	INSIDE	1.700	1.809	3138.06	5676.12	3.075	2564.46	7886.72
A_68_P24799551	chr6:120615439-120615483	NM_001128151:-978	Cecr2	PROMOTER	1.512	2.599	2655.71	6901.62	3.928	2199.66	8641.34
A_68_P24798780	chr6:120442970-120443014	NM_033567:833	Cecr6	INSIDE	1.855	9.906	4627.20	45837.77	18.374	3197.85	58756.19
A_68_P31565714	chr18:25645254-25645298	NM_001146292:267208	Celf4	INSIDE	1.775	2.149	1952.48	4194.93	3.813	1584.88	6043.79
A_68_P31565718	chr18:25645741-25645785	NM_001146292:266722	Celf4	INSIDE	1.746	1.912	1475.53	2821.49	3.339	1166.82	3896.56
A_68_P27289987	chr10:80945075-80945119	NM_176954:358	Celf5	INSIDE	2.128	8.817	8860.98	78128.69	18.767	4829.56	90637.92
A_68_P22412541	chr3:108218746-108218790	NM_001004177:-356	Celsr2	PROMOTER	2.381	3.887	1745.81	6786.45	9.257	1460.57	13520.87
A_68_P26816895	chr9:108734927-108734971	NM_080437:6298	Celsr3	INSIDE	1.600	4.517	3492.77	15777.49	7.229	2474.22	17885.33
A_68_P26816894	chr9:108734858-108734902	NM_080437:6230	Celsr3	INSIDE	1.580	3.081	4378.64	13489.38	4.868	3369.52	16404.32
A_68_P20740950	chr1:157820522-157820566	NM_001039184:-159	Cep350	PROMOTER	1.839	2.129	1799.19	3830.65	3.916	1451.01	5682.19
A_68_P32099204	chr19:38129403-38129447	NM_028293:-90	Cep55	PROMOTER	2.661	14.261	12747.94	181792.80	37.952	7793.33	295773.50
A_68_P32099202	chr19:38129262-38129306	NM_028293:-230	Cep55	PROMOTER	2.173	8.598	14671.61	126142.50	18.683	9111.35	170231.80
A_68_P27630622	chr11:20149303-20149348	NM_172260:102	Cep68	INSIDE	1.818	6.231	2082.62	12977.22	11.331	1861.11	21088.80
A_68_P29144056	chr13:74199745-74199789	NM_028959:-33	Cep72	PROMOTER	2.243	5.603	3753.23	21030.95	12.567	2544.14	31972.55
A_68_P21099935	chr2:29724646-29724690	NM_207298:-345	Cercam	PROMOTER	2.016	4.334	846.01	3666.90	8.736	713.80	6236.04
A_68_P31929035	chr19:5490695-5490739	NM_007687:262	Cfl1	INSIDE	1.782	3.659	3764.08	13772.99	6.520	2853.19	18602.65
A_68_P28449512	chr12:55963938-55963982	NM_007688:-96	Cfl2	PROMOTER	1.617	1.882	1457.03	2742.45	3.044	1200.81	3655.76
A_68_P30822064	chr16:64851589-64851633	NM_178647:-300	Cggbp1	PROMOTER	1.571	1.499	3489.85	5231.78	2.355	2884.05	6790.67
A_68_P22340674	chr3:94590393-94590437	NM_001037711:23	Cgn	INSIDE	1.781	2.740	3234.59	8862.34	4.881	2421.32	11818.44
A_68_P30379103	chr15:81524969-81525013	NM_001164320:2727	Chadl	INSIDE	1.863	5.182	1487.64	7708.88	9.656	1312.39	12672.07
A_68_P22695196	chr4:3866420-3866464	NM_001190322:382	Chchd7	INSIDE	1.561	3.322	1286.05	4272.59	5.187	980.84	5087.42
A_68_P22695192	chr4:3865915-3865959	NM_001190323:-98	Chchd7	DIVERGENT_PROMOTER	1.539	2.922	3388.26	9901.35	4.496	2546.72	11450.11
A_68_P22355191	chr3:97414110-97414154	NM_026539:-19	Chd1l	PROMOTER	1.532	4.639	481.16	2232.03	7.109	455.84	3240.45

ProbeName	Target position of probe on CpG island microarray	TargetID	GeneSymbol	CpG island Description	Ratio of relative methylation (TiO_2-NP/Vehicle)	Sham group			TiO_2-H group		
						Relative methylation (Cy5/Cy3)	Cy3 signal (Input)	Cy5 signal (MeDIP)	Relative methylation (Cy5/Cy3)	Cy3 signal (Input)	Cy5 signal (MeDIP)
A_68_P23418114	chr4:151712427-151712471	NM_001081376:-311	Chd5	PROMOTER	1.581	2.138	1253.46	2679.94	3.379	968.47	3272.88
A_68_P22715810	chr4:8617868-8617912	NM_001081417:-177	Chd7	PROMOTER	1.534	4.212	492.27	2073.62	6.460	414.93	2680.59
A_68_P26055737	chr8:93432164-93432208	NM_177224:79453	Chd9	INSIDE	1.778	5.839	1278.96	7467.43	10.384	1034.28	10739.74
A_68_P32572442	chrX:100551806-100551850	NM_009767:14	Chic1	INSIDE	1.822	3.938	3190.05	12563.39	7.174	1466.11	10517.46
A_68_P25589538	chr7:148725824-148725868	NM_001142681:-90	Chid1	PROMOTER	1.690	2.157	1913.62	4128.24	3.645	1568.84	5718.28
A_68_P31790565	chr18:67365128-67365172	NM_024190:138	Chmp1b	INSIDE	1.811	3.225	4755.01	15334.68	5.839	3568.63	20838.37
A_68_P30589274	chr16:20733484-20733528	NM_009893:307	Chrd	INSIDE	1.583	1.426	2944.10	4199.57	2.257	2340.81	5284.08
A_68_P25365140	chr7:107154438-107154482	NM_133709:-453	Chrdl2	PROMOTER	2.099	6.849	440.36	3016.19	14.378	378.06	5435.81
A_68_P20162793	chr1:38954859-38954904	NM_145142:124	Chst10	INSIDE	1.689	7.615	3082.84	23475.55	12.859	2404.60	30921.32
A_68_P24631649	chr6:90275213-90275257	NM_027928:-55	Chst13	DIVERGENT_PROMOTER	1.815	1.643	880.04	1445.53	2.982	709.33	2115.11
A_68_P21566993	chr2:118752216-118752260	NM_028117:6	Chst14	INSIDE	1.844	4.062	1825.71	7415.99	7.491	1518.00	11371.36
A_68_P21566994	chr2:118752369-118752413	NM_028117:158	Chst14	INSIDE	1.584	2.111	4274.41	9021.50	3.342	3244.99	10846.08
A_68_P26741575	chr9:95307812-95307856	NM_018763:-145	Chst2	PROMOTER	2.020	6.357	3712.67	23602.70	12.843	2845.78	36548.30
A_68_P26741563	chr9:95306199-95306243	NM_018763:1469	Chst2	INSIDE	1.521	1.952	1403.78	2740.04	2.970	1182.30	3511.08
A_68_P27173119	chr10:59681994-59682038	NM_016803:-8	Chst3	PROMOTER	1.707	1.891	3067.53	5801.21	3.228	2489.26	8036.45
A_68_P26170843	chr8:114434234-114434278	NM_019950:-157	Chst5	PROMOTER	1.643	2.517	1320.48	3323.84	4.136	1058.06	4375.93
A_68_P32287119	chrX:19637488-19637532	NM_021715:815	Chst7	INSIDE	2.452	3.851	2163.89	8333.81	9.443	1159.22	10946.07
A_68_P32287125	chrX:19638017-19638061	NM_021715:1343	Chst7	INSIDE	1.773	2.989	484.37	1447.95	5.301	264.32	1401.23
A_68_P32130475	chr19:44181890-44181934	NM_001162410:55	Chuk	INSIDE	1.727	3.622	678.44	2457.12	6.254	590.49	3693.13
A_68_P26079748	chr8:97362202-97362246	NM_134141:16	Ciapin1	INSIDE	1.892	2.871	431.89	1240.15	5.432	323.32	1756.29
A_68_P25009418	chr7:26067028-26067072	NM_001110131:-147	Cic	PROMOTER	2.126	4.923	27867.04	137201.80	10.469	18156.34	190086.60
A_68_P25009419	chr7:26067406-26067450	NM_001110131:231	Cic	INSIDE	1.714	5.002	3250.76	16259.68	8.575	2867.73	24589.71
A_68_P26141979	chr8:109417535-109417579	NM_011574:17	Cirh1a	INSIDE	1.609	5.028	1394.45	7010.82	8.089	1220.21	9870.28
A_68_P27234932	chr10:70807397-70807441	NM_134007:179	Cisd1	INSIDE	1.725	5.434	743.69	4041.59	9.376	605.45	5676.99
A_68_P32567806	chrX:99447101-99447145	NM_007709:-14	Cited1	PROMOTER	1.823	2.299	570.23	1310.75	4.190	323.08	1353.75
A_68_P26971601	chr10:17443503-17443547	NM_010828:491	Cited2	INSIDE	1.593	1.437	1021.65	1468.46	2.290	866.24	1983.53
A_68_P28745339	chr12:112908725-112908769	NM_021273:1803	Ckb	INSIDE	1.586	3.597	1932.40	6951.18	5.703	1796.13	10243.90
A_68_P22032039	chr3:31048745-31048789	NM_008770:-75	Cldn11	PROMOTER	2.122	1.605	1227.26	1969.46	3.405	969.49	3301.02
A_68_P31092997	chr17:23816594-23816638	NM_018777:285	Cldn6	INSIDE	1.846	2.792	6471.81	18068.45	5.154	4832.01	24904.13
A_68_P27898214	chr11:69779823-69779867	NM_016887:922	Cldn7	INSIDE	1.650	3.957	1568.10	6205.57	6.530	1333.22	8705.40
A_68_P30542887	chr16:10545493-10545537	NM_001204229:83	Clec16a	INSIDE	1.577	3.144	1929.46	6067.15	4.960	1642.69	8147.72
A_68_P30969546	chr16:92498878-92498922	NM_172469:509	Clic6	INSIDE	1.667	6.182	3756.63	23221.76	10.303	2929.22	30179.61
A_68_P27764455	chr11:45665845-45665889	NM_001045520:401	Clint1	INSIDE	1.572	1.508	1316.31	1985.46	2.371	1074.77	2547.83
A_68_P24057004	chr5:124133945-124133989	NM_019765:334	Clip1	INSIDE	1.911	2.160	864.01	1865.97	4.126	813.86	3358.04
A_68_P24057001	chr5:124133506-124133550	NM_019765:772	Clip1	INSIDE	1.679	2.030	4945.46	10040.83	3.408	3721.90	12684.82
A_68_P31336379	chr17:72131880-72131924	NM_030179:616	Clip4	INSIDE	1.820	4.744	765.70	3632.43	8.635	671.28	5796.46
A_68_P20258850	chr1:58480309-58480353	NM_001042634:602	Clk1	INSIDE	1.523	1.658	1405.07	2330.06	2.526	1048.58	2648.61
A_68_P22317617	chr3:88973343-88973387	NM_001163432:4638	Clk2	INSIDE	1.813	7.739	6628.02	51296.66	14.031	4272.64	59947.78
A_68_P26543678	chr9:57612898-57612942	NM_007713:747	Clk3	INSIDE	1.646	2.204	5441.20	11992.11	3.627	4191.60	15201.53
A_68_P23280751	chr4:126234084-126234128	NM_175554:-117	Clspn	PROMOTER	1.707	5.348	1230.34	6580.06	9.127	1117.06	10195.26
A_68_P26756440	chr9:97933663-97933707	NM_022319:-98	Clstn2	PROMOTER	1.876	9.816	3587.04	35211.04	18.414	3006.61	55364.42
A_68_P24906444	chr6:142705194-142705238	NM_009908:11	Cmas	INSIDE	1.615	4.227	1261.52	5332.30	6.828	1003.63	6852.26
A_68_P23999133	chr5:114064391-114064435	NM_008153:35987	Cmklr1	INSIDE	1.799	5.553	1505.92	8362.64	9.989	1033.77	10325.81
A_68_P26127993	chr8:106865035-106865079	NM_024217:563	Cmtm3	INSIDE	1.519	1.997	1888.06	3770.79	3.034	1463.37	4439.83
A_68_P26128337	chr8:106919324-106919368	NM_153582:250	Cmtm4	INSIDE	1.654	6.030	3389.72	20441.46	9.976	2593.04	25869.13
A_68_P26846741	chr9:114753280-114753324	NM_027294:-32	Cmtm8	PROMOTER	1.720	4.235	8858.42	37512.78	7.283	7283.97	53046.75
A_68_P31890103	chr18:84855123-84855167	NM_023149:-119	Cndp2	PROMOTER	1.507	2.250	7270.70	16356.27	3.391	5158.92	17494.56

ProbeName	Target position of probe on CpG island microarray	TargetID	GeneSymbol	CpG island Description	Ratio of relative methylation (TiO_2-NP/Vehicle)	Sham group			TiO_2-H group		
						Relative methylation (Cy5/Cy3)	Cy3 signal (Input)	Cy5 signal (MeDIP)	Relative methylation (Cy5/Cy3)	Cy3 signal (Input)	Cy5 signal (MeDIP)
A_68_P32769550	chrX:154480805-154480849	NM_177751:216	Cnksr2	INSIDE	1.512	2.146	1326.45	2845.95	3.244	822.04	2667.04
A_68_P20148714	chr1:36568619-36568663	NM_001039551:-80	Cnnm3	PROMOTER	2.117	8.917	14587.91	130076.10	18.878	10155.26	191711.20
A_68_P20148721	chr1:36569446-36569490	NM_001039551:748	Cnnm3	INSIDE	1.659	5.939	2091.84	12424.11	9.855	1726.52	17014.04
A_68_P20148715	chr1:36568684-36568728	NM_001039551:-14	Cnnm3	PROMOTER	1.602	2.142	3211.45	6880.19	3.431	2505.82	8597.79
A_68_P27476522	chr10:116018238-116018282	NM_001037846:307	Cnot2	INSIDE	2.636	8.518	13218.99	112595.80	22.456	8717.08	195749.60
A_68_P27476528	chr10:116018985-116019029	NM_001037846:-439	Cnot2	PROMOTER	1.657	3.208	4435.27	14229.05	5.315	3475.66	18474.40
A_68_P27476525	chr10:116018687-116018731	NM_001037846:-141	Cnot2	PROMOTER	1.501	2.634	907.22	2389.64	3.952	776.04	3067.27
A_68_P24359955	chr6:35083485-35083529	NM_001164412:231	Cnot4	INSIDE	1.889	3.638	4126.95	15015.24	6.874	3480.07	23921.77
A_68_P27784450	chr11:49525550-49525594	NM_212484:640	Cnot6	INSIDE	1.908	2.634	3975.65	10469.93	5.025	3106.29	15610.64
A_68_P27784449	chr11:49525483-49525527	NM_212484:708	Cnot6	INSIDE	1.515	4.727	1217.49	5755.20	7.160	1081.63	7744.99
A_68_P20622147	chr1:134439405-134439449	NM_177129:91	Cntn2	INSIDE	1.760	7.013	3736.09	26202.98	12.345	2879.07	35542.74
A_68_P21285107	chr2:65077163-65077207	NM_027225:-501	Cobll1	PROMOTER	1.909	4.523	6122.86	27694.30	8.633	4521.21	39031.95
A_68_P29729812	chr14:76153932-76153976	NM_177381:346	Cog3	INSIDE	1.587	1.418	1256.41	1781.25	2.249	1056.18	2375.74
A_68_P27183504	chr10:61441353-61441397	NM_007731:482	Col13a1	INSIDE	1.627	2.560	905.42	2317.85	4.164	745.87	3106.00
A_68_P23299955	chr4:129724973-129725017	NM_028266:-89	Col16a1	PROMOTER	1.606	3.786	2438.22	9230.27	6.081	2043.77	12427.41
A_68_P23299954	chr4:129724880-129724924	NM_028266:-181	Col16a1	PROMOTER	1.519	3.754	598.62	2247.47	5.703	528.13	3011.74
A_68_P22968875	chr4:62875286-62875330	NM_025685:-1137	Col27a1	PROMOTER	1.521	1.878	674.44	1266.91	2.857	542.61	1550.01
A_68_P23279343	chr4:125978444-125978488	NM_199473:14429	Col8a2	INSIDE	1.739	1.434	1429.27	2050.07	2.494	1206.55	3008.88
A_68_P21904961	chr2:180332965-180333009	NM_009936:60	Col9a3	INSIDE	1.571	2.012	1222.98	2461.18	3.162	972.24	3074.01
A_68_P29856055	chr14:102039383-102039427	NM_001033132:284	Commd6	INSIDE	1.506	3.406	2079.90	7084.80	5.129	1713.73	8790.10
A_68_P25951429	chr8:72897357-72897401	NM_016685:-68	Comp	PROMOTER	1.610	3.467	1420.43	4924.30	5.580	1152.70	6432.41
A_68_P20437267	chr1:92500071-92500115	NM_133805:93	Cops8	INSIDE	1.833	5.807	2421.48	14062.52	10.645	1968.22	20952.55
A_68_P28047199	chr11:96723478-96723522	NM_019877:12311	Copz2	DOWNSTREAM	1.769	2.368	1123.29	2659.94	4.189	762.30	3193.24
A_68_P24000798	chr5:114359230-114359275	NM_011779:-537	Coro1c	PROMOTER	1.783	5.286	11332.47	59901.40	9.423	7656.87	72153.48
A_68_P24000795	chr5:114358894-114358938	NM_011779:-201	Coro1c	PROMOTER	1.588	2.186	1922.06	4201.58	3.471	1805.60	6267.42
A_68_P26571039	chr9:62384681-62384725	NM_175484:149	Coro2b	INSIDE	2.003	9.729	15328.83	149132.30	19.488	12051.50	234861.50
A_68_P27937689	chr11:77275784-77275828	NM_139128:-1608	Coro6	PROMOTER	2.271	3.704	1483.14	5493.90	8.411	1145.14	9631.95
A_68_P27937736	chr11:77281473-77281517	NM_139128:4080	Coro6	INSIDE	1.638	1.704	1225.08	2087.69	2.791	1035.13	2889.33
A_68_P23881743	chr5:90652818-90652862	NM_001033310:181	Cox18	INSIDE	1.677	4.340	544.98	2365.02	7.278	501.88	3652.77
A_68_P26221734	chr8:123191897-123191941	NM_010926:94	Cox4nb	INSIDE	2.130	2.268	6911.12	15675.39	4.830	4809.16	23230.14
A_68_P26542219	chr9:57369547-57369591	NM_007747:530	Cox5a	INSIDE	1.507	1.737	1447.75	2514.73	2.617	1202.89	3148.03
A_68_P23644443	chr5:43625072-43625116	NM_001177379:393	Cpeb2	INSIDE	1.713	5.653	2539.23	14353.61	9.685	1936.44	18754.98
A_68_P31126789	chr17:29374780-29374824	NM_153166:-67	Cpne5	PROMOTER	2.036	2.750	5129.96	14108.92	5.599	4116.55	23049.87
A_68_P31126791	chr17:29375001-29375045	NM_153166:-287	Cpne5	PROMOTER	1.712	2.642	673.07	1778.26	4.523	569.70	2576.69
A_68_P28688783	chr12:103213937-103213981	NM_016856:-225	Cpsf2	PROMOTER	1.637	2.923	1412.05	4127.22	4.784	1184.21	5665.66
A_68_P28688785	chr12:103214150-103214194	NM_016856:-11	Cpsf2	PROMOTER	1.736	1.425	1111.50	1583.51	2.474	1079.73	2670.99
A_68_P31951139	chr19:10599956-10600000	NM_001164272:245	Cpsf7	INSIDE	2.206	15.169	6442.48	97726.43	33.464	3939.70	131838.40
A_68_P21103096	chr2:30271133-30271177	NM_007760:114	Crat	INSIDE	2.214	7.925	3581.49	28381.88	17.546	2493.99	43759.89
A_68_P21425775	chr2:91815556-91815600	NM_011957:48749	Creb3l1	DOWNSTREAM	1.651	2.114	1190.64	2516.90	3.490	1000.79	3492.82
A_68_P24372708	chr6:37392303-37392347	NM_178661:-176	Creb3l2	PROMOTER	1.764	2.324	991.77	2304.44	4.098	853.65	3498.34
A_68_P20796287	chr1:167694031-167694075	NM_011804:142	Creg1	INSIDE	1.633	2.890	2035.65	5883.48	4.720	1667.89	7872.74
A_68_P25952284	chr8:73027155-73027199	NM_018827:10122	Crlf1	INSIDE	1.751	3.433	2378.66	8165.82	6.011	1884.88	11330.54
A_68_P23976347	chr5:109986035-109986079	NM_001164735:1956	Crlf2	INSIDE	1.690	6.297	6501.58	40937.80	10.638	4205.31	44737.50
A_68_P23612481	chr5:37633390-37633434	NM_007765:-3618	Crmp1	PROMOTER	1.715	4.336	649.92	2817.97	7.435	538.48	4003.66
A_68_P23612479	chr5:37633037-37633081	NM_001136058:-260	Crmp1	PROMOTER	1.705	4.128	1558.86	6434.45	7.038	1230.62	8661.63
A_68_P23361673	chr4:140581289-140581333	NM_172122:28266	Crocc	INSIDE	1.893	5.666	2665.13	15100.28	10.727	2182.63	23412.04
A_68_P23361905	chr4:140616349-140616393	NM_172122:-6794	Crocc	PROMOTER	1.952	1.574	3797.77	5976.06	3.071	3180.60	9768.25

ProbeName	Target position of probe on CpG island microarray	TargetID	GeneSymbol	CpG island Description	Ratio of relative methylation (TiO_2-NP/Vehicle)	Sham group			TiO_2-H group		
						Relative methylation (Cy5/Cy3)	Cy3 signal (Input)	Cy5 signal (MeDIP)	Relative methylation (Cy5/Cy3)	Cy3 signal (Input)	Cy5 signal (MeDIP)
A_68_P26844412	chr9:114299556-114299600	NM_019922:252	Crtap	INSIDE	1.861	2.317	304.24	704.96	4.312	255.06	1099.81
A_68_P24850798	chr6:131338248-131338292	NM_011733:198	Csda	INSIDE	1.577	5.758	1846.89	10634.69	9.081	1644.68	14935.00
A_68_P21832544	chr2:166731558-166731602	NM_023565:-15	Cse1l	PROMOTER	1.540	2.158	2544.10	5490.79	3.323	1922.64	6388.29
A_68_P22408449	chr3:107563183-107563227	NM_001113529:183	Csf1	INSIDE	2.457	11.554	10695.95	123580.20	28.391	6189.87	175737.30
A_68_P32229410	chr19:61301151-61301195	NM_009970:3149	Csf2ra	INSIDE	1.844	8.907	12157.54	108287.80	16.425	7752.53	127333.40
A_68_P31758329	chr18:61715242-61715286	NM_146087:29	Csnk1a1	INSIDE	1.753	2.001	1909.97	3822.75	3.509	1370.39	4808.15
A_68_P26590184	chr9:65757067-65757111	NM_173185:272	Csnk1g1	INSIDE	1.657	7.170	2904.33	20822.79	11.882	2120.45	25195.40
A_68_P31715736	chr18:54022224-54022268	NM_152809:480	Csnk1g3	INSIDE	1.837	4.968	577.16	2867.29	9.127	430.82	3931.94
A_68_P21748226	chr2:152053141-152053185	NM_007788:587	Csnk2a1	INSIDE	1.652	3.954	3066.05	12123.17	6.533	2172.35	14191.76
A_68_P31157711	chr17:35257720-35257764	NM_009975:650	Csnk2b	INSIDE	1.595	1.882	2747.60	5172.15	3.002	1920.74	5766.73
A_68_P26538932	chr9:56712960-56713004	NM_139001:72	Cspg4	INSIDE	1.894	4.386	10522.96	46156.43	8.309	7877.12	65451.70
A_68_P26875426	chr9:119883221-119883265	NM_153287:10534	Csrnp1	INSIDE	1.624	3.499	1072.24	3751.45	5.682	894.90	5084.44
A_68_P31928180	chr19:5349389-5349433	NM_028623:164	Cst6	INSIDE	2.051	6.540	14012.71	91648.82	13.412	10206.67	136892.60
A_68_P23587982	chr5:33618039-33618083	NM_001198859:-407	Ctbp1	PROMOTER	1.593	2.427	696.54	1690.79	3.867	582.42	2252.40
A_68_P31864634	chr18:80666119-80666163	NM_026295:266	Ctdp1	INSIDE	1.911	4.138	713.39	2952.12	7.909	717.95	5678.08
A_68_P31838828	chr18:75857092-75857136	NM_201354:236	Ctif	INSIDE	2.291	9.102	4442.49	40434.40	20.853	3496.62	72913.37
A_68_P24568080	chr6:77929479-77929523	NM_001109764:161	Ctnna2	INSIDE	2.885	5.838	2978.31	17387.62	16.841	1751.66	29498.86
A_68_P22941962	chr4:56877715-56877759	NM_018761:347	Ctnnal1	INSIDE	1.555	6.301	881.48	5553.93	9.797	740.94	7259.07
A_68_P26880499	chr9:120842203-120842247	NM_007614:-293	Ctnnb1	PROMOTER	1.907	3.489	2486.61	8675.34	6.652	1765.98	11746.72
A_68_P25616898	chr8:4259319-4259363	NM_183315:-66	Ctxn1	PROMOTER	2.007	4.280	4968.20	21264.26	8.592	3603.90	30964.43
A_68_P27997843	chr11:87912671-87912715	NM_198013:45	Cuedc1	INSIDE	1.849	4.175	3123.06	13039.48	7.721	2763.17	21333.33
A_68_P27998411	chr11:88007034-88007078	NM_001172099:23990	Cuedc1	INSIDE	1.576	1.634	744.42	1216.08	2.574	608.68	1566.62
A_68_P24423143	chr6:47403815-47403859	NM_012042:-486	Cul1	PROMOTER	1.703	5.922	1174.27	6954.62	10.083	914.15	9217.76
A_68_P24423149	chr6:47404611-47404655	NM_012042:310	Cul1	INSIDE	1.629	10.303	2595.13	26738.51	16.788	1833.59	30782.91
A_68_P24423144	chr6:47403969-47404013	NM_012042:-332	Cul1	PROMOTER	1.552	7.853	739.60	5808.22	12.189	704.37	8585.29
A_68_P31208112	chr17:46787505-46787549	NM_025611:240	Cul7	INSIDE	1.934	4.597	1458.98	6707.18	8.893	1174.54	10445.09
A_68_P32128434	chr19:43827858-43827902	NM_001113562:368	Cutc	INSIDE	1.769	2.558	2374.32	6072.97	4.525	1895.91	8578.58
A_68_P24048238	chr5:122499986-122500030	NM_007804:-2174	Cux2	PROMOTER	1.542	2.754	614.04	1691.04	4.248	530.72	2254.36
A_68_P29060390	chr13:56397734-56397778	NM_019568:156	Cxcl14	INSIDE	3.036	3.480	3675.66	12793.06	10.568	2602.92	27507.92
A_68_P27900882	chr11:70279513-70279557	NM_023158:-6048	Cxcl16	PROMOTER	1.747	1.427	1830.22	2611.27	2.493	1401.27	3492.97
A_68_P31620539	chr18:36020271-36020315	NM_133687:30821	Cxxc5	INSIDE	1.799	4.958	1846.83	9156.90	8.922	1356.72	12104.51
A_68_P31620318	chr18:35991596-35991640	NM_133687:2147	Cxxc5	INSIDE	1.724	1.975	1603.91	3166.95	3.404	1180.80	4019.63
A_68_P30349758	chr15:76490310-76490354	NM_019396:159	Cyhr1	INSIDE	1.928	3.640	4666.59	16984.62	7.018	3324.48	23331.03
A_68_P30349739	chr15:76487469-76487513	NM_019396:3001	Cyhr1	INSIDE	1.538	2.365	2055.01	4860.62	3.637	1485.47	5402.77
A_68_P28727385	chr12:109572280-109572324	NM_010010:-288	Cyp46a1	PROMOTER	1.561	2.654	4354.11	11554.80	4.141	3150.60	13047.81
A_68_P28291023	chr12:25365989-25366033	NM_001004455:650	Cys1	INSIDE	1.929	13.581	9472.94	128648.20	26.192	5382.13	140967.50
A_68_P24157329	chr5:144383488-144383532	NM_011182:195	Cyth3	INSIDE	2.097	5.682	2773.18	15757.80	11.913	2014.79	24002.08
A_68_P20472150	chr1:99557453-99557497	NM_133825:1121	D1Ertd622e	INSIDE	1.578	3.205	1890.17	6057.91	5.058	1626.71	8228.26
A_68_P20910364	chr1:188791866-188791912	NM_033077:594	D1Pas1	INSIDE	1.629	6.821	1369.42	9340.44	11.108	1236.49	13735.20
A_68_P22454038	chr3:117062948-117062993	NM_177664:824	D3Bwg0562e	INSIDE	1.550	2.469	1291.03	3187.15	3.827	1048.07	4011.15
A_68_P23362371	chr4:140695718-140695762	NM_001025608:-29	D4Ertd22e	DIVERGENT_PROMOTER	1.674	1.754	970.90	1703.27	2.937	788.18	2314.72
A_68_P23609090	chr5:37086922-37086966	NM_001081232:264	D5Ertd579e	INSIDE	1.899	4.942	5250.33	25945.60	9.383	4159.62	39028.77
A_68_P22942460	chr4:56960340-56960384	NM_175518:-61	D730040F13Rik	PROMOTER	1.954	3.183	1863.90	5932.74	6.221	1527.94	9504.93
A_68_P22942456	chr4:56959870-56959914	NM_175518:409	D730040F13Rik	INSIDE	1.595	6.093	791.80	4824.54	9.717	707.64	6876.09
A_68_P23262658	chr4:123089142-123089186	NM_001167918:-10	D830031N03Rik	PROMOTER	2.296	9.442	1728.54	16320.16	21.680	1397.75	30303.05
A_68_P23262662	chr4:123089650-123089695	NM_001167918:-518	D830031N03Rik	PROMOTER	1.766	3.937	2775.83	10928.71	6.954	2219.30	15433.61
A_68_P25784087	chr8:37210103-37210147	NM_172911:52243	D8Ertd82e	INSIDE	1.607	4.243	5872.86	24917.49	6.816	4103.14	27967.73

ProbeName	Target position of probe on CpG island microarray	TargetID	GeneSymbol	CpG island Description	Ratio of relative methylation (TiO_2-NP/Vehicle)	Sham group			TiO_2-H group		
						Relative methylation (Cy5/Cy3)	Cy3 signal (Input)	Cy5 signal (MeDIP)	Relative methylation (Cy5/Cy3)	Cy3 signal (Input)	Cy5 signal (MeDIP)
A_68_P22288626	chr3:83844024-83844068	NM_172681:37	D930015E06Rik	INSIDE	1.520	3.459	496.73	1718.18	5.259	409.31	2152.57
A_68_P28528483	chr12:72932910-72932958	NM_026102:870	Daam1	INSIDE	1.777	7.692	781.12	6008.10	13.668	536.03	7326.51
A_68_P21134366	chr2:35438211-35438255	NM_001114125:24255	Dab2ip	INSIDE	1.951	9.744	1321.86	12879.94	19.014	1073.76	20416.14
A_68_P28525473	chr12:72410602-72410646	NM_001190466:-246	Dact1	PROMOTER	2.051	4.733	4828.66	22854.24	9.709	3756.42	36472.19
A_68_P28525474	chr12:72410830-72410874	NM_001190466:-18	Dact1	PROMOTER	1.539	2.050	5746.86	11781.79	3.155	4667.44	14727.36
A_68_P30113003	chr15:31154105-31154149	NM_146057:-13	Dap	PROMOTER	1.667	3.313	3856.43	12776.00	5.524	3116.01	17212.56
A_68_P27282058	chr10:79727411-79727455	NM_001122604:-303	Dazap1	DIVERGENT_PROMOTER	1.571	1.727	1340.24	2314.13	2.713	1046.16	2838.21
A_68_P29055349	chr13:55589582-55589626	NM_001177371:-167	Dbn1	PROMOTER	2.120	8.125	3720.33	30227.89	17.225	2832.48	48789.15
A_68_P29055348	chr13:55589381-55589425	NM_001177371:35	Dbn1	INSIDE	1.689	2.635	18468.36	48663.72	4.451	14895.89	66305.82
A_68_P27564951	chr11:5688365-5688409	NM_001146308:-99	Dbnl	PROMOTER	2.322	6.341	2538.53	16095.66	14.726	1987.15	29262.25
A_68_P22882382	chr4:45355016-45355060	NM_153167:66	Dcaf10	INSIDE	2.015	1.758	1505.23	2646.64	3.543	1371.16	4857.88
A_68_P22861615	chr4:41261631-41261675	NM_026893:282	Dcaf12	INSIDE	2.213	9.114	4804.62	43790.81	20.168	3504.05	70669.71
A_68_P32351252	chrX:42143188-42143232	NM_001190718:128	Dcaf12l1	INSIDE	2.865	7.296	3767.38	27487.72	20.901	2077.17	43415.76
A_68_P32351254	chrX:42143356-42143400	NM_178739:-4	Dcaf12l1	PROMOTER	1.772	2.845	4125.07	11736.55	5.042	2258.89	11388.94
A_68_P28082865	chr11:102878532-102878576	NM_026551:-93	Dcakd	PROMOTER	1.560	5.102	5424.26	27675.60	7.959	4348.58	34611.79
A_68_P30791337	chr16:58408386-58408430	NM_028523:-239	Dcbld2	PROMOTER	2.089	3.420	3208.27	10970.76	7.142	2683.72	19168.06
A_68_P22388169	chr3:103613288-103613332	NM_001025312:0	Dclre1b	INSIDE	1.657	8.165	1708.03	13946.79	13.526	1349.17	18249.39
A_68_P24791602	chr6:119125266-119125312	NM_001033379:18	Dcp1b	INSIDE	1.522	6.481	3512.61	22766.90	9.865	2669.19	26332.06
A_68_P25845477	chr8:49195740-49195784	NM_001161515:396	Dctd	INSIDE	3.295	10.942	2629.12	28768.68	36.052	1918.64	69170.72
A_68_P27534905	chr10:126703422-126703466	NM_001190453:127	Dctn2	INSIDE	1.938	3.315	3380.69	11207.19	6.425	2486.22	15973.56
A_68_P25772218	chr8:35171274-35171318	NM_011722:269	Dctn6	INSIDE	1.621	1.979	1786.21	3534.22	3.208	1530.23	4909.07
A_68_P25504835	chr7:134404064-134404108	NM_023203:95	Dctpp1	INSIDE	1.581	2.655	4740.09	12584.03	4.197	3408.48	14305.16
A_68_P23797913	chr5:73883146-73883190	NM_001190733:904	Dcun1d4	INSIDE	1.826	10.139	17685.04	179306.30	18.515	10597.88	196224.10
A_68_P23797912	chr5:73883055-73883099	NM_001190733:812	Dcun1d4	INSIDE	1.573	9.002	22377.90	201451.70	14.162	12948.66	183376.70
A_68_P22610589	chr3:145421770-145421814	NM_026993:137	Ddah1	INSIDE	1.654	3.326	2381.64	7920.16	5.501	1933.39	10634.74
A_68_P29571979	chr14:46277367-46277411	NM_001039106:430	Ddhd1	INSIDE	1.672	3.868	8539.33	33027.56	6.465	7021.19	45391.26
A_68_P23365645	chr4:141279495-141279539	NM_001017966:-182	Ddi2	PROMOTER	1.778	2.405	1101.33	2649.11	4.276	961.04	4109.56
A_68_P30476596	chr15:98637471-98637515	NM_001013741:864	Ddn	INSIDE	1.579	3.302	1653.03	5459.03	5.215	1322.62	6897.74
A_68_P26166134	chr8:113551628-113551672	NM_001190800:20	Ddx19b	INSIDE	1.604	2.542	2363.69	6008.76	4.078	1826.18	7447.29
A_68_P22398585	chr3:105490135-105490179	NM_017397:333	Ddx20	INSIDE	1.879	2.525	1089.30	2750.84	4.746	942.09	4471.30
A_68_P32395905	chrX:53709083-53709127	NM_172779:1089	Ddx26b	INSIDE	1.530	1.452	5384.84	7816.63	2.221	2264.74	5029.57
A_68_P32265729	chrX:12858825-12858869	NM_010028:699	Ddx3x	INSIDE	2.282	1.824	882.91	1610.03	4.162	582.92	2425.88
A_68_P32265732	chrX:12859122-12859166	NM_010028:997	Ddx3x	INSIDE	1.642	2.048	627.54	1285.32	3.363	607.86	2043.95
A_68_P32265730	chrX:12858920-12858964	NM_010028:795	Ddx3x	INSIDE	2.217	1.889	385.98	729.24	4.188	219.21	918.16
A_68_P26659378	chr9:78243891-78243935	NM_001191044:329	Ddx43	INSIDE	1.647	3.794	1438.91	5459.07	6.249	1300.98	8129.60
A_68_P28103521	chr11:106649919-106649963	NM_007840:-132	Ddx5	DIVERGENT_PROMOTER	2.625	6.666	17960.68	119723.90	17.499	11517.85	201546.70
A_68_P20645803	chr1:138311898-138311942	NM_026500:73	Ddx59	INSIDE	2.215	2.243	339.36	761.23	4.969	308.46	1532.86
A_68_P26468513	chr9:44413201-44413245	NM_001110826:248	Ddx6	INSIDE	2.063	4.714	9476.98	44677.44	9.726	6618.62	64370.16
A_68_P22752471	chr4:15872402-15872446	NM_026172:230	Decr1	INSIDE	1.727	2.505	2768.71	6935.72	4.326	2314.43	10012.17
A_68_P22752472	chr4:15872547-15872591	NM_026172:86	Decr1	INSIDE	1.699	5.267	2508.55	13212.65	8.947	2094.87	18743.45
A_68_P25009065	chr7:26004933-26004977	NM_207677:-76	Dedd2	DIVERGENT_PROMOTER	2.316	2.652	8195.44	21737.08	6.143	5505.81	33821.86
A_68_P20885895	chr1:184212870-184212914	NM_007853:-2	Degs1	PROMOTER	1.589	9.939	456.85	4540.71	15.794	377.16	5956.78
A_68_P20885892	chr1:184212460-184212504	NM_007853:408	Degs1	INSIDE	1.510	1.720	462.28	795.14	2.597	412.83	1072.19
A_68_P29011719	chr13:47201171-47201215	NM_025900:397	Dek	INSIDE	1.738	1.560	990.11	1544.24	2.711	764.35	2072.05
A_68_P25410984	chr7:117103504-117103548	NM_021494:410	Dennd5a	INSIDE	1.627	1.745	1202.04	2097.07	2.838	950.80	2698.44
A_68_P23585886	chr5:33206293-33206337	NM_177786:-55	Depdc5	DIVERGENT_PROMOTER	1.540	1.961	1701.05	3335.59	3.019	1486.86	4489.25
A_68_P30244836	chr15:57723668-57723712	NM_024207:283	Derl1	INSIDE	2.280	1.624	1458.51	2367.91	3.701	1125.77	4166.54

ProbeName	Target position of probe on CpG island microarray	TargetID	GeneSymbol	CpG island Description	Ratio of relative methylation (TiO_2-NP/Vehicle)	Sham group			TiO_2-H group		
						Relative methylation (Cy5/Cy3)	Cy3 signal (Input)	Cy5 signal (MeDIP)	Relative methylation (Cy5/Cy3)	Cy3 signal (Input)	Cy5 signal (MeDIP)
A_68_P30599077	chr16:22657267-22657311	NM_138650:16	Dgkg	INSIDE	1.502	1.609	1659.05	2669.27	2.416	1302.66	3147.24
A_68_P24371747	chr6:37249971-37250015	NM_001081206:-16	Dgki	PROMOTER	1.691	3.057	2279.23	6968.62	5.169	1966.19	10162.98
A_68_P23185134	chr4:106234075-106234119	NM_053272:454	Dhcr24	INSIDE	1.618	1.954	6180.72	12074.38	3.161	4658.67	14725.56
A_68_P23320471	chr4:133556862-133556906	NM_026144:-105	Dhdds	PROMOTER	1.911	6.695	713.05	4773.77	12.792	604.67	7734.84
A_68_P33007796	chr4_random:129068-129112	NM_001033326:19289	Dhrsx	INSIDE	2.214	6.689	9324.57	62374.43	14.813	5853.14	86702.44
A_68_P33007786	chr4_random:127770-127814	NM_001033326:17991	Dhrsx	INSIDE	2.030	7.054	14713.05	103787.70	14.321	9700.82	138923.80
A_68_P33007785	chr4_random:127689-127733	NM_001033326:17909	Dhrsx	INSIDE	2.029	9.778	14226.98	139116.50	19.840	9243.32	183387.90
A_68_P33008826	chr4_random:110027-110071	NM_001033326:247	Dhrsx	INSIDE	1.989	5.009	2018.28	10110.10	9.965	1605.78	16000.96
A_68_P33007798	chr4_random:129389-129433	NM_001033326:19609	Dhrsx	INSIDE	1.744	5.327	2192.86	11680.33	9.290	1601.36	14876.20
A_68_P33007795	chr4_random:128995-129039	NM_001033326:19215	Dhrsx	INSIDE	1.665	7.586	4871.29	36951.56	12.632	3359.62	42438.83
A_68_P27903827	chr11:70817840-70817884	NM_178367:72	Dhx33	INSIDE	1.671	2.399	725.62	1740.90	4.009	589.10	2361.84
A_68_P24980375	chr7:16807208-16807254	NM_027883:151	Dhx34	INSIDE	1.706	2.067	963.48	1991.87	3.527	747.64	2637.04
A_68_P20947409	chr1:194956122-194956166	NM_145415:301	Diexf	INSIDE	1.547	1.635	441.40	721.86	2.530	342.33	866.11
A_68_P30672037	chr16:35769289-35769333	NM_153550:132	Dirc2	INSIDE	1.707	3.802	1819.91	6918.94	6.491	1445.82	9384.94
A_68_P26249219	chr8:127578258-127578302	NM_174853:186	Disc1	INSIDE	1.745	4.120	16321.94	67241.74	7.190	12123.42	87166.19
A_68_P26505081	chr9:50506728-50506772	NM_178118:29339	Dixdc1	INSIDE	1.765	1.817	1669.25	3032.53	3.207	1162.67	3728.34
A_68_P26504861	chr9:50467491-50467535	NM_145614:373	Dlat	INSIDE	1.775	3.414	2240.05	7647.12	6.059	1937.02	11735.53
A_68_P28330749	chr12:32035950-32035994	NM_007861:330	Dld	INSIDE	1.653	4.600	1536.04	7066.43	7.607	1242.80	9453.52
A_68_P29651397	chr14:62301793-62301837	NR_028264:-604	Dleu2	PROMOTER	2.086	5.959	1659.98	9891.50	12.430	1475.76	18342.94
A_68_P29651430	chr14:62305865-62305909	NR_028264:-4676	Dleu2	PROMOTER	1.558	4.143	1151.74	4771.58	6.455	932.74	6020.70
A_68_P29651385	chr14:62300358-62300402	NR_028264:830	Dleu2	INSIDE	1.526	3.449	4014.63	13847.19	5.263	3245.48	17082.07
A_68_P29470473	chr14:25064406-25064450	NM_001163513:714	Dlg5	INSIDE	1.884	21.987	778.65	17120.00	41.423	520.80	21572.99
A_68_P29470478	chr14:25065176-25065220	NM_001163513:-56	Dlg5	PROMOTER	1.584	2.924	782.41	2287.43	4.630	663.38	3071.49
A_68_P31330059	chr17:71007053-71007097	NM_001128181:95988	Dlgap1	INSIDE	1.620	13.447	4003.88	53839.91	21.787	2580.13	56214.38
A_68_P25672261	chr8:14096967-14097011	NM_001145965:1114	Dlgap2	INSIDE	1.777	3.076	3873.43	11915.11	5.468	2879.39	15743.16
A_68_P28732711	chr12:110692025-110692069	NM_001190703:382	Dlk1	INSIDE	1.892	2.773	11271.12	31256.75	5.247	7974.42	41839.52
A_68_P31065266	chr17:15513473-15513517	NM_007865:-707	Dll1	PROMOTER	1.612	5.376	1640.73	8821.10	8.668	1157.08	10028.99
A_68_P25021007	chr7:29079751-29079795	NM_007866:7032	Dll3	INSIDE	1.529	4.149	1259.27	5225.23	6.346	1111.25	7052.15
A_68_P28036776	chr11:94982434-94982478	NM_010055:1026	Dlx3	INSIDE	2.017	2.367	3116.23	7376.19	4.774	2074.45	9904.28
A_68_P24218861	chr6:6829299-6829343	NM_010056:2748	Dlx5	INSIDE	1.699	2.436	1752.76	4269.76	4.138	1353.69	5601.47
A_68_P23226835	chr4:115616658-115616702	NM_001025567:-4149	Dmbx1	PROMOTER	1.610	3.342	3326.32	11115.32	5.381	2563.60	13794.61
A_68_P32031149	chr19:25746643-25746687	NM_145831:-236	Dmrt2	PROMOTER	2.234	3.449	1352.47	4664.11	7.702	1112.55	8569.35
A_68_P23203724	chr4:109649844-109649888	NM_172296:-763	Dmrta2	PROMOTER	3.082	29.984	17420.38	522325.10	92.404	8951.32	827137.40
A_68_P23203774	chr4:109655335-109655379	NM_172296:4727	Dmrta2	INSIDE	1.779	4.383	3173.67	13908.76	7.797	2499.63	19489.75
A_68_P23203786	chr4:109656795-109656839	NM_172296:6187	Dmrta2	DOWNSTREAM	1.621	8.757	7571.12	66301.58	14.193	5316.97	75464.36
A_68_P26526109	chr9:54349056-54349100	NM_172771:355	Dmxl2	INSIDE	1.758	3.148	2854.95	8988.13	5.534	2120.01	11732.20
A_68_P26015683	chr8:86132210-86132254	NM_018808:159	Dnajb1	INSIDE	2.012	3.343	2738.47	9154.51	6.726	2170.21	14596.54
A_68_P20422167	chr1:90102351-90102395	NM_008299:-49	Dnajb3	DIVERGENT_PROMOTER	1.628	6.145	1938.67	11913.38	10.006	1545.92	15468.64
A_68_P28396964	chr12:45311296-45311340	NM_013760:-263	Dnajb9	PROMOTER	1.813	1.965	1056.58	2076.00	3.562	892.34	3178.07
A_68_P27543292	chr10:128242453-128242497	NM_028873:-257	Dnajc14	DIVERGENT_PROMOTER	1.631	2.164	846.84	1832.34	3.529	769.79	2716.63
A_68_P30010737	chr15:10399801-10399845	NM_030046:449	Dnajc21	INSIDE	1.599	4.759	1474.51	7017.39	7.611	1290.18	9819.95
A_68_P30478229	chr15:98928779-98928823	NM_176835:-1114	Dnajc22	PROMOTER	2.194	8.321	7380.07	61409.06	18.254	5468.03	99815.07
A_68_P30478193	chr15:98924227-98924271	NM_176835:-5666	Dnajc22	DIVERGENT_PROMOTER	1.656	4.130	5541.49	22886.39	6.838	4719.82	32273.13
A_68_P26022583	chr8:87432507-87432551	NM_010062:6	Dnase2a	INSIDE	2.009	11.553	2603.56	30078.45	23.214	1599.71	37136.06
A_68_P20405120	chr1:84692097-84692141	NM_152915:678	Dner	INSIDE	1.817	2.089	1042.51	2177.44	3.794	810.47	3075.12
A_68_P20778066	chr1:164408081-164408125	NM_001038619:59	Dnm3	INSIDE	1.611	2.867	1051.30	3013.75	4.617	792.73	3660.19
A_68_P20778063	chr1:164407783-164407827	NM_001038619:357	Dnm3	INSIDE	1.503	2.197	2396.10	5263.29	3.302	1800.71	5945.11

ProbeName	Target position of probe on CpG island microarray	TargetID	GeneSymbol	CpG island Description	Ratio of relative methylation (TiO_2-NP/Vehicle)	Sham group			TiO_2-H group		
						Relative methylation (Cy5/Cy3)	Cy3 signal (Input)	Cy5 signal (MeDIP)	Relative methylation (Cy5/Cy3)	Cy3 signal (Input)	Cy5 signal (MeDIP)
A_68_P21819501	chr2:164571271-164571315	NM_133763:-222	Dnttip1	DIVERGENT_PROMOTER	1.520	2.327	7413.05	17246.82	3.535	5386.08	19041.21
A_68_P27928203	chr11:75608556-75608600	NM_007873:981	Doc2b	INSIDE	3.043	7.601	425.29	3232.70	23.131	319.99	7401.59
A_68_P32315765	chrX:33428989-33429033	NM_001009947:184	Dock11	INSIDE	1.636	4.768	664.47	3167.92	7.801	455.12	3550.48
A_68_P24590984	chr6:82983529-82983573	NM_010070:-85	Dok1	DIVERGENT_PROMOTER	2.352	3.562	1137.34	4051.68	8.379	847.92	7104.88
A_68_P21102300	chr2:30141879-30141923	NM_177648:-26	Dolk	DIVERGENT_PROMOTER	1.913	2.441	2439.73	5955.30	4.670	1879.60	8778.17
A_68_P21102918	chr2:30248319-30248363	NM_020329:405	Dolpp1	INSIDE	1.735	4.161	1997.38	8310.37	7.217	1574.57	11363.04
A_68_P27285229	chr10:80217921-80217965	NM_199322:-8	Dot1l	PROMOTER	1.659	2.909	2296.28	6680.22	4.825	1829.74	8828.72
A_68_P21840215	chr2:168055402-168055451	NM_010072:453	Dpm1	INSIDE	1.625	2.341	439.97	1029.83	3.804	363.32	1382.21
A_68_P31350938	chr17:74715809-74715853	NM_001146223:-57	Dpy30	PROMOTER	1.871	3.740	704.95	2636.74	7.000	589.60	4127.01
A_68_P31928602	chr19:5424535-5424579	NM_024176:360	Drap1	INSIDE	1.772	3.070	6270.42	19247.44	5.439	4578.67	24902.28
A_68_P29047192	chr13:54150550-54150594	NM_010076:455	Drd1a	INSIDE	1.729	3.605	1644.59	5928.09	6.233	1271.41	7925.09
A_68_P30997386	chr16:97392403-97392447	NM_031174:-82	Dscam	PROMOTER	1.644	1.595	480.90	766.97	2.622	482.13	1264.18
A_68_P21704233	chr2:143740849-143740894	NM_019771:-195	Dstn	PROMOTER	1.592	3.379	3450.55	11659.34	5.380	2818.76	15163.67
A_68_P20621314	chr1:134314077-134314121	NM_172516:69	Dstyk	INSIDE	2.025	8.533	658.52	5619.20	17.275	566.30	9782.98
A_68_P28195719	chr12:3572230-3572274	NM_001162465:-138	Dtnb	PROMOTER	1.927	2.025	1262.01	2555.32	3.901	913.73	3564.66
A_68_P31258211	chr17:56901482-56901526	NM_144858:-2669	Dus3l	DIVERGENT_PROMOTER	1.532	1.538	1244.70	1913.87	2.355	1027.37	2419.82
A_68_P24607367	chr6:85911667-85911711	NM_028099:-27	Dusp11	PROMOTER	1.716	2.947	1375.74	4054.99	5.058	1093.69	5531.42
A_68_P27975235	chr11:83881690-83881734	NM_019819:147	Dusp14	INSIDE	1.803	2.054	1290.16	2649.80	3.702	1035.86	3834.90
A_68_P20834309	chr1:174562662-174562706	NM_026725:421	Dusp23	INSIDE	1.704	4.278	19244.57	82328.57	7.290	13306.05	97004.34
A_68_P26802905	chr9:106270040-106270084	NM_153459:-900	Dusp7	PROMOTER	1.647	5.814	8987.36	52249.22	9.574	6248.62	59827.29
A_68_P32463876	chrX:70882705-70882749	NM_029352:-2053	Dusp9	PROMOTER	2.058	3.948	1322.13	5219.25	8.125	759.53	6171.13
A_68_P32463877	chrX:70882835-70882879	NM_029352:-1923	Dusp9	PROMOTER	1.602	2.572	789.86	2031.39	4.119	437.53	1802.33
A_68_P30588038	chr16:20531403-20531447	NM_007889:14288	Dvl3	INSIDE	1.524	3.268	6445.03	21060.16	4.980	4893.53	24368.66
A_68_P24213704	chr6:5955887-5955931	NM_001191023:280270	Dync1i1	INSIDE	1.827	3.203	1241.53	3976.49	5.851	992.91	5809.75
A_68_P26128587	chr8:106966661-106966705	NM_001013380:265	Dync1li2	INSIDE	1.515	3.010	3069.91	9241.24	4.561	2459.41	11216.76
A_68_P24008929	chr5:115751222-115751266	NM_019682:-245	Dynll1	PROMOTER	1.789	4.209	2244.59	9447.12	7.528	1866.35	14049.56
A_68_P27997247	chr11:87800951-87800995	NM_001168472:-885	Dynll2	PROMOTER	1.522	2.430	1692.09	4111.94	3.698	1379.73	5101.95
A_68_P25020574	chr7:28965243-28965287	NM_010092:752	Dyrk1b	INSIDE	1.615	3.240	2559.08	8290.75	5.233	2122.57	11107.96
A_68_P20613818	chr1:133034787-133034831	NM_145508:3	Dyrk3	INSIDE	1.595	2.262	671.25	1518.37	3.608	591.10	2132.62
A_68_P30743456	chr16:48994162-48994206	NM_001110017:41	Dzip3	INSIDE	1.523	3.027	2735.09	8279.27	4.610	2240.86	10331.14
A_68_P28051732	chr11:97489553-97489597	NM_175332:1456	E130012A19Rik	INSIDE	2.375	10.917	6860.96	74899.38	25.922	4341.43	112540.60
A_68_P27080340	chr10:39451597-39451641	NR_038037:195	E130307A14Rik	INSIDE	1.543	1.585	1856.03	2942.01	2.446	1386.50	3391.38
A_68_P27921611	chr11:74451961-74452005	NM_001013784:18876	E130309D14Rik	INSIDE	2.250	5.916	3541.95	20954.34	13.311	2760.39	36744.42
A_68_P27921465	chr11:74424335-74424379	NM_001013784:-8750	E130309D14Rik	PROMOTER	1.826	5.392	1636.03	8821.58	9.847	1314.62	12944.86
A_68_P27921510	chr11:74433466-74433510	NM_001013784:382	E130309D14Rik	INSIDE	1.533	8.832	2611.07	23060.27	13.542	1959.59	26536.29
A_68_P27921508	chr11:74433189-74433233	NM_001013784:104	E130309D14Rik	INSIDE	1.533	3.394	3585.16	12167.32	5.202	3056.19	15899.69
A_68_P22903296	chr4:49072520-49072564	NM_178756:209	E130309F12Rik	INSIDE	1.655	1.726	2128.61	3672.93	2.856	1666.20	4758.16
A_68_P27279294	chr10:79318023-79318067	NR_029447:-328	E130317F20Rik	PROMOTER	2.202	5.706	1545.56	8818.27	12.562	1215.69	15271.43
A_68_P28266974	chr12:16817834-16817878	NM_033270:86	E2f6	INSIDE	1.727	3.658	1329.30	4862.99	6.319	1114.54	7042.68
A_68_P27445213	chr10:110182319-110182363	NM_178609:-180	E2f7	PROMOTER	1.748	4.159	772.89	3214.54	7.272	770.36	5602.23
A_68_P25108786	chr7:56137106-56137150	NM_001013368:-717	E2f8	PROMOTER	1.688	4.136	2662.40	11010.37	6.982	2311.18	16136.32
A_68_P24388784	chr6:40385730-40385774	NM_175528:380	E330009J07Rik	INSIDE	1.842	8.401	5482.20	46054.93	15.474	4148.33	64192.08
A_68_P30253796	chr15:59205558-59205602	NM_153548:127	E430025E21Rik	INSIDE	1.715	7.918	3792.74	30030.63	13.577	2640.05	35843.98
A_68_P27756942	chr11:44432030-44432075	NM_007897:417	Ebf1	INSIDE	1.820	1.507	645.68	973.03	2.742	504.33	1383.01
A_68_P29683667	chr14:67850834-67850879	NM_010095:-1272	Ebf2	PROMOTER	2.044	7.888	448.05	3534.34	16.125	291.83	4705.88
A_68_P25565202	chr7:144505325-144505370	NM_001113414:781	Ebf3	INSIDE	1.915	5.902	4572.39	26987.78	11.301	3496.84	39517.37
A_68_P31252966	chr17:56095674-56095719	NM_015766:3651	Ebi3	INSIDE	1.548	3.023	2670.05	8070.77	4.679	1946.40	9107.33

ProbeName	Target position of probe on CpG island microarray	TargetID	GeneSymbol	CpG island Description	Ratio of relative methylation (TiO_2-NP/Vehicle)	Sham group			TiO_2-H group		
						Relative methylation (Cy5/Cy3)	Cy3 signal (Input)	Cy5 signal (MeDIP)	Relative methylation (Cy5/Cy3)	Cy3 signal (Input)	Cy5 signal (MeDIP)
A_68_P32242174	chrX:7769836-7769880	NM_007898:780	Ebp	INSIDE	2.170	1.795	3484.43	6255.21	3.896	1401.56	5460.34
A_68_P30588586	chr16:20630082-20630126	NM_139293:181	Ece2	INSIDE	1.721	2.516	2058.37	5178.35	4.328	1688.09	7306.66
A_68_P26543338	chr9:57556763-57556807	NM_153799:409	Edc3	INSIDE	1.980	7.076	3933.06	27831.33	14.009	2928.64	41028.03
A_68_P24735989	chr6:108778919-108778963	NM_138677:306	Edem1	INSIDE	1.903	3.777	394.52	1490.21	7.189	410.65	2952.03
A_68_P26659818	chr9:78329691-78329735	NM_010106:-181	Eef1a1	PROMOTER	1.735	1.761	947.75	1669.34	3.056	778.65	2379.18
A_68_P26659813	chr9:78328998-78329042	NM_010106:511	Eef1a1	INSIDE	1.607	1.741	1728.57	3008.93	2.798	1437.16	4021.22
A_68_P28962023	chr13:38751002-38751046	NM_025380:-127	Eef1e1	PROMOTER	1.575	2.491	3706.13	9230.73	3.922	2999.68	11764.15
A_68_P29631109	chr14:58617905-58617949	NM_028643:173	Efha1	INSIDE	1.610	2.344	692.10	1622.39	3.773	615.13	2321.20
A_68_P20416757	chr1:89160862-89160906	NM_028889:-54	Efhd1	PROMOTER	2.531	6.089	109823.20	668703.40	15.413	67870.99	1046093.00
A_68_P23366440	chr4:141430540-141430584	NM_025994:273	Efhd2	INSIDE	2.283	2.694	1008.85	2717.95	6.150	893.14	5493.12
A_68_P23366442	chr4:141430855-141430899	NM_025994:-41	Efhd2	PROMOTER	1.614	3.976	1143.10	4545.11	6.418	993.55	6376.66
A_68_P22318578	chr3:89126786-89126830	NM_010108:-7	Efna3	PROMOTER	1.720	1.555	6323.19	9831.44	2.675	4671.38	12494.26
A_68_P25637623	chr8:8661349-8661393	NM_010111:-597	Efnb2	PROMOTER	1.535	3.006	909.85	2734.56	4.615	760.62	3509.94
A_68_P30287852	chr15:65618774-65618818	NM_133766:194	Efr3a	INSIDE	1.753	1.928	260.85	502.80	3.380	253.86	858.03
A_68_P28198379	chr12:4038471-4038515	NM_001082483:423	Efr3b	INSIDE	1.672	4.411	3726.36	16435.80	7.373	2986.38	22017.64
A_68_P29615096	chr14:55545666-55545710	NM_010112:-63	Efs	DIVERGENT_PROMOTER	2.169	18.040	3597.05	64890.58	39.131	2404.11	94075.39
A_68_P29615089	chr14:55545056-55545100	NM_010112:547	Efs	INSIDE	1.969	2.680	3959.09	10609.07	5.277	2914.31	15377.54
A_68_P29699040	chr14:70480133-70480177	NM_018781:2903	Egr3	DOWNSTREAM	1.810	6.388	3054.13	19508.73	11.561	2484.02	28717.76
A_68_P29699037	chr14:70479901-70479945	NM_018781:2671	Egr3	INSIDE	1.583	4.276	1107.75	4736.44	6.769	861.94	5834.35
A_68_P31933641	chr19:6277445-6277489	NM_010119:571	Ehd1	INSIDE	1.863	2.535	2307.03	5847.36	4.723	1920.83	9072.38
A_68_P28067423	chr11:100181873-100181917	NM_011508:585	Eif1	INSIDE	1.570	2.429	743.63	1806.49	3.813	565.99	2158.28
A_68_P30587818	chr16:20498949-20498993	NM_172265:81	Eif2b5	INSIDE	1.626	1.709	569.66	973.48	2.778	495.87	1377.71
A_68_P23280028	chr4:126106770-126106814	NM_153402:-6	Eif2c3	PROMOTER	1.523	3.112	1596.04	4967.57	4.742	1337.85	6343.42
A_68_P21762932	chr2:154718551-154718596	NM_026030:69	Eif2s2	INSIDE	1.898	3.101	2784.28	8634.70	5.886	2108.86	12413.38
A_68_P28177063	chr11:119161761-119161805	NM_138669:-425	Eif4a3	PROMOTER	1.944	7.417	3705.01	27478.19	14.417	2789.51	40215.82
A_68_P22569284	chr3:138189919-138189963	NM_007917:786	Eif4e	INSIDE	1.665	4.216	1266.57	5340.30	7.018	1103.37	7743.61
A_68_P27897958	chr11:69734258-69734302	NM_001166590:-148	Eif5a	PROMOTER	1.595	4.279	6778.16	29002.29	6.825	5191.36	35430.35
A_68_P22019079	chr3:28680302-28680346	NM_177586:92	Eif5a2	INSIDE	1.574	5.981	1545.59	9243.98	9.412	1505.64	14171.48
A_68_P23103735	chr4:91038685-91038729	NM_010486:40	Elavl2	INSIDE	1.747	4.653	2752.25	12806.90	8.127	2275.41	18493.30
A_68_P26350216	chr9:21855292-21855336	NM_010487:1153	Elavl3	INSIDE	1.653	2.648	559.24	1480.63	4.377	446.60	1954.97
A_68_P26350213	chr9:21855003-21855047	NM_010487:1443	Elavl3	INSIDE	1.641	3.509	2235.93	7845.85	5.757	1856.32	10686.97
A_68_P22128839	chr3:51081429-51081473	NM_023502:48459	Elf2	INSIDE	1.850	5.104	1669.02	8517.86	9.439	1364.87	12883.47
A_68_P22128834	chr3:51080739-51080783	NM_023502:49149	Elf2	INSIDE	1.812	5.068	3293.52	16690.08	9.184	2567.56	23580.83
A_68_P22128832	chr3:51080406-51080450	NM_023502:49481	Elf2	INSIDE	1.552	2.100	2655.74	5575.74	3.259	2102.07	6851.41
A_68_P32364765	chrX:45806673-45806717	NM_019680:9615	Elf4	INSIDE	1.738	1.635	1048.84	1715.36	2.842	569.64	1619.14
A_68_P24138256	chr5:140384605-140384649	NM_175522:730	Elfn1	INSIDE	1.766	9.868	2008.19	19816.30	17.424	1874.43	32660.71
A_68_P24138663	chr5:140449111-140449155	NM_175522:65236	Elfn1	INSIDE	1.511	1.973	1056.98	2085.69	2.981	1263.29	3766.04
A_68_P30361559	chr15:78502925-78502969	NM_183141:45597	Elfn2	INSIDE	1.934	7.718	1295.22	9996.63	14.929	1026.00	15316.62
A_68_P30361566	chr15:78503626-78503670	NM_183141:44895	Elfn2	INSIDE	1.814	6.596	1520.94	10032.69	11.964	1350.77	16160.51
A_68_P30361565	chr15:78503546-78503590	NM_183141:44975	Elfn2	INSIDE	1.550	3.398	991.25	3368.41	5.267	733.37	3862.41
A_68_P26014555	chr8:85856129-85856173	NM_001170691:235	Elmod2	INSIDE	1.702	4.294	2829.27	12149.53	7.307	2308.53	16868.67
A_68_P28976807	chr13:41315714-41315758	NM_019423:36	Elovl2	INSIDE	1.657	0.384	1063.81	408.94	0.637	991.98	632.02
A_68_P26685702	chr9:83699787-83699831	NM_001145974:41	Elovl4	INSIDE	2.050	7.487	9488.45	71038.47	15.349	7623.95	117020.20
A_68_P21497226	chr2:105744559-105744603	NM_023876:77	Elp4	INSIDE	1.821	4.077	3355.27	13680.51	7.425	2790.48	20720.57
A_68_P31333820	chr17:71660018-71660062	NM_145158:265	Emilin2	INSIDE	1.775	3.943	1298.83	5121.79	7.000	1128.92	7902.88
A_68_P24603559	chr6:85137815-85137859	NM_010131:-88	Emx1	PROMOTER	2.198	6.948	2502.06	17385.39	15.275	1965.77	30027.50
A_68_P24603556	chr6:85137482-85137526	NM_010131:-420	Emx1	PROMOTER	1.741	1.686	1548.80	2610.51	2.934	1261.23	3700.15

ProbeName	Target position of probe on CpG island microarray	TargetID	GeneSymbol	CpG island Description	Ratio of relative methylation (TiO_2-NP/Vehicle)	Sham group			TiO_2-H group		
						Relative methylation (Cy5/Cy3)	Cy3 signal (Input)	Cy5 signal (MeDIP)	Relative methylation (Cy5/Cy3)	Cy3 signal (Input)	Cy5 signal (MeDIP)
A_68_P29250236	chr13:98010968-98011012	NM_007930:-69	Enc1	PROMOTER	1.777	7.313	1585.24	11593.63	12.995	1275.24	16571.48
A_68_P26317084	chr9:14185441-14185485	NM_028013:224	Endod1	INSIDE	1.527	1.671	826.93	1381.89	2.551	778.11	1985.00
A_68_P32573188	chrX:100689079-100689123	NR_015508:186	Enox	INSIDE	2.124	3.962	3243.02	12847.80	8.416	2143.69	18040.59
A_68_P32573186	chrX:100688845-100688889	NR_015508:-48	Enox	PROMOTER	1.921	6.156	1849.84	11386.70	11.827	1373.86	16248.66
A_68_P27010234	chr10:24431792-24431836	NM_008813:94	Enpp1	INSIDE	1.640	6.266	2456.72	15393.62	10.275	2001.34	20564.38
A_68_P21071854	chr2:25251918-25251962	NM_009849:547	Entpd2	INSIDE	1.722	4.180	2448.06	10232.39	7.196	1935.00	13923.72
A_68_P28598113	chr12:85749685-85749729	NM_001026214:273	Entpd5	INSIDE	2.269	3.661	3028.24	11086.95	8.308	2223.13	18468.75
A_68_P28598112	chr12:85749605-85749649	NM_001026214:353	Entpd5	INSIDE	1.790	3.507	2023.75	7097.12	6.279	1689.57	10608.28
A_68_P30378505	chr15:81416613-81416657	NM_177821:-9	Ep300	PROMOTER	1.634	2.357	831.78	1960.41	3.851	635.86	2448.47
A_68_P30378507	chr15:81416859-81416903	NM_177821:237	Ep300	INSIDE	1.525	1.529	2839.79	4341.31	2.331	2245.83	5234.89
A_68_P23309923	chr4:131604805-131604849	NM_001128606:167	Epb4.1	INSIDE	1.512	5.819	1030.57	5996.78	8.796	869.44	7647.47
A_68_P31610600	chr18:34165600-34165644	NM_013512:1238	Epb4.1l4a	INSIDE	1.770	4.669	7245.25	33830.49	8.266	5145.26	42531.44
A_68_P21202301	chr2:49307033-49307077	NM_172663:49	Epc2	INSIDE	1.501	2.387	1763.84	4209.42	3.583	1485.53	5322.72
A_68_P23363266	chr4:140864579-140864623	NM_010139:7446	Epha2	INSIDE	1.585	1.971	645.57	1272.51	3.125	472.81	1477.61
A_68_P22803179	chr4:28740971-28741015	NM_001122889:698	Epha7	INSIDE	1.576	2.231	745.90	1664.05	3.515	564.62	1984.76
A_68_P23336241	chr4:136391071-136391115	NM_010142:758	Ephb2	INSIDE	2.322	3.395	3359.27	11404.09	7.883	2536.99	19998.83
A_68_P30590967	chr16:21205377-21205421	NM_010143:531	Ephb3	INSIDE	2.587	10.916	1196.74	13063.33	28.244	837.42	23652.29
A_68_P26940376	chr10:11063434-11063478	NM_010146:214	Epm2a	INSIDE	1.569	2.438	362.07	882.58	3.824	380.29	1454.08
A_68_P26940374	chr10:11063250-11063294	NM_010146:30	Epm2a	INSIDE	1.543	2.782	270.82	753.53	4.294	282.19	1211.60
A_68_P28033280	chr11:94352619-94352663	NM_027984:8648	Epn3	INSIDE	1.747	14.019	1245.75	17463.81	24.492	945.71	23162.58
A_68_P29488024	chr14:28435732-28435776	NM_177814:127	Erc2	INSIDE	1.663	4.562	1555.19	7095.31	7.589	1247.87	9469.50
A_68_P24992765	chr7:19927675-19927719	NM_001127324:-2723	Ercc1	PROMOTER	1.806	8.013	991.82	7947.64	14.469	840.64	12163.27
A_68_P30555522	chr16:13110157-13110201	NM_015769:350	Ercc4	INSIDE	1.521	2.963	2035.68	6031.25	4.507	1728.16	7789.45
A_68_P20192816	chr1:44204826-44204870	NM_011729:260	Ercc5	INSIDE	1.946	6.459	1824.46	11783.62	12.569	1452.81	18260.66
A_68_P32567492	chrX:99352458-99352502	NM_146235:-50	Ercc6l	PROMOTER	1.793	4.973	810.47	4030.48	8.915	463.28	4129.97
A_68_P28576807	chr12:81744426-81744470	NM_007951:400	Erh	INSIDE	1.857	8.037	3705.65	29783.34	14.922	2743.77	40942.16
A_68_P28576811	chr12:81744792-81744836	NM_007951:34	Erh	INSIDE	1.676	3.329	3732.87	12426.91	5.578	2902.64	16192.26
A_68_P25780173	chr8:36558550-36558594	NM_026067:15	Eri1	INSIDE	1.802	2.449	747.61	1830.80	4.413	663.58	2928.45
A_68_P23235081	chr4:117222839-117222883	NM_080469:-238	Eri3	PROMOTER	1.536	1.899	1162.81	2208.21	2.917	989.53	2886.33
A_68_P25672219	chr8:14090321-14090365	NM_001034862:-15	Erich1	DIVERGENT_PROMOTER	1.518	2.067	1232.12	2546.24	3.138	955.63	2998.66
A_68_P27681336	chr11:30854073-30854117	NM_025745:37	Erlec1	INSIDE	1.849	8.253	1290.17	10647.89	15.262	994.76	15181.97
A_68_P22899537	chr4:48292195-48292239	NM_029572:245	Erp44	INSIDE	2.029	6.351	2110.38	13402.42	12.888	1564.24	20159.97
A_68_P22899538	chr4:48292337-48292381	NM_029572:103	Erp44	INSIDE	1.794	2.440	5149.30	12565.52	4.377	3986.47	17447.60
A_68_P23408691	chr4:150229048-150229092	NM_133753:-129	Errfi1	PROMOTER	1.687	3.514	1955.79	6873.54	5.929	1754.32	10401.00
A_68_P31490815	chr18:10609631-10609677	NM_001081222:696	Esco1	INSIDE	1.513	2.126	827.03	1758.24	3.216	726.99	2338.32
A_68_P29723690	chr14:75131937-75131981	NM_016903:-193	Esd	PROMOTER	1.663	4.245	5108.14	21683.61	7.059	3639.86	25692.86
A_68_P23416733	chr4:151506350-151506394	NM_019585:-3338	Espn	PROMOTER	1.863	2.714	1723.10	4676.52	5.057	1235.44	6248.09
A_68_P22729559	chr4:11315357-11315401	NM_194055:-1448	Esrp1	PROMOTER	1.596	1.674	934.79	1565.25	2.673	741.88	1982.85
A_68_P32684276	chrX:133653076-133653120	NM_007957:1767	Esx1	INSIDE	2.514	6.193	1542.14	9549.78	15.565	819.74	12759.33
A_68_P32684282	chrX:133653782-133653826	NM_007957:1061	Esx1	INSIDE	1.595	3.270	3979.99	13014.37	5.216	2354.09	12279.98
A_68_P28767111	chr12:117519591-117519635	NM_028731:-82	Esyt2	PROMOTER	1.596	2.903	1504.28	4366.28	4.632	1093.98	5067.52
A_68_P25005645	chr7:25370764-25370808	NM_023154:-1775	Ethe1	PROMOTER	1.952	5.784	9677.20	55972.42	11.292	6298.59	71125.48
A_68_P24908780	chr6:143116314-143116358	NM_029250:587	Etnk1	INSIDE	1.911	4.120	8609.83	35470.89	7.873	6271.76	49378.43
A_68_P24859175	chr6:133985925-133985969	NM_007961:222	Etv6	INSIDE	2.108	3.752	3350.18	12571.15	7.911	2729.27	21590.99
A_68_P24859179	chr6:133986298-133986342	NM_007961:596	Etv6	INSIDE	1.861	4.825	3201.66	15448.10	8.981	2185.86	19630.72
A_68_P24859169	chr6:133985084-133985128	NM_007961:-618	Etv6	PROMOTER	1.757	1.842	1887.56	3476.45	3.235	1459.11	4720.73
A_68_P23969232	chr5:108297963-108298007	NM_007964:6142	Evi5	INSIDE	1.534	3.333	2981.35	9936.38	5.114	2623.33	13416.10

ProbeName	Target position of probe on CpG island microarray	TargetID	GeneSymbol	CpG island Description	Ratio of relative methylation (TiO_2-NP/Vehicle)	Sham group			TiO_2-H group		
						Relative methylation (Cy5/Cy3)	Cy3 signal (Input)	Cy5 signal (MeDIP)	Relative methylation (Cy5/Cy3)	Cy3 signal (Input)	Cy5 signal (MeDIP)
A_68_P28728718	chr12:109792810-109792854	NM_001163394:-97	Evl	PROMOTER	1.676	3.664	11008.12	40332.18	6.141	7496.69	46040.40
A_68_P28158070	chr11:116083313-116083358	NM_025276:16070	Evpl	INSIDE	1.527	6.358	2493.61	15855.54	9.710	1964.19	19072.41
A_68_P24449862	chr6:52264017-52264061	NM_007966:547	Evx1	INSIDE	1.557	3.027	2102.31	6363.00	4.712	1730.99	8155.65
A_68_P28575798	chr12:81564728-81564777	NM_133798:671	Exd2	INSIDE	2.218	7.448	1524.74	11356.43	16.524	1070.78	17693.19
A_68_P28920307	chr13:31065636-31065680	NM_025588:258	Exoc2	INSIDE	1.651	1.876	3310.55	6212.09	3.098	2322.28	7193.72
A_68_P26132767	chr8:107813885-107813929	NM_177788:6092	Exoc3l	INSIDE	1.510	1.383	3404.14	4707.23	2.088	2623.45	5478.57
A_68_P28158619	chr11:116168104-116168148	NM_001162872:-74	Exoc7	PROMOTER	1.650	3.395	574.55	1950.42	5.602	532.83	2984.86
A_68_P26248330	chr8:127421598-127421642	NM_198103:-15	Exoc8	DIVERGENT_PROMOTER	1.850	4.396	1582.35	6956.78	8.135	1298.63	10564.61
A_68_P27003030	chr10:23069823-23069869	NM_010167:-137	Eya4	PROMOTER	1.732	3.286	434.32	1427.34	5.692	439.18	2499.66
A_68_P24423934	chr6:47544675-47544719	NM_001146689:333	Ezh2	INSIDE	2.559	4.342	3868.12	16795.29	11.113	2711.43	30131.82
A_68_P27289087	chr10:80820153-80820197	NM_028657:-140	F630110N24Rik	DIVERGENT_PROMOTER	1.569	2.472	1187.14	2934.50	3.880	956.23	3709.79
A_68_P31948062	chr19:10115784-10115828	NM_021890:-231	Fads3	PROMOTER	2.268	7.995	626.39	5008.14	18.133	509.60	9240.45
A_68_P24064187	chr5:125491813-125491857	NM_028443:7990	Fam101a	INSIDE	2.018	3.517	2606.98	9168.48	7.098	1942.22	13786.55
A_68_P21115867	chr2:32391280-32391324	NM_153560:424	Fam102a	INSIDE	1.831	2.476	1103.59	2732.88	4.533	852.12	3863.05
A_68_P21115865	chr2:32391002-32391046	NM_153560:146	Fam102a	INSIDE	1.540	2.078	1162.78	2416.42	3.200	965.79	3090.04
A_68_P22415887	chr3:108830521-108830565	NM_001163567:-17	Fam102b	PROMOTER	1.709	30.067	421.02	12658.99	51.391	305.92	15721.46
A_68_P25273848	chr7:88908491-88908535	NM_025997:674	Fam103a1	INSIDE	2.059	5.435	741.82	4031.91	11.189	588.81	6587.93
A_68_P30093919	chr15:27611395-27611439	NM_198301:-163	Fam105a	PROMOTER	1.860	6.742	2286.11	15412.02	12.541	1862.41	23356.17
A_68_P30093918	chr15:27611330-27611374	NM_198301:-99	Fam105a	PROMOTER	1.650	2.064	569.45	1175.50	3.407	609.04	2074.72
A_68_P30093655	chr15:27560477-27560521	NM_001013792:-50	Fam105b	PROMOTER	1.889	3.790	4711.05	17853.99	7.158	3397.60	24320.69
A_68_P20963945	chr2:3630790-3630834	NM_025626:83	Fam107b	INSIDE	1.973	1.721	749.08	1289.47	3.396	585.50	1988.33
A_68_P32007552	chr19:21727793-21727837	NM_146096:16	Fam108b	INSIDE	1.587	4.598	476.18	2189.70	7.296	384.95	2808.78
A_68_P24046831	chr5:122293021-122293065	NM_175474:-5994	Fam109a	DIVERGENT_PROMOTER	2.136	2.255	193.72	436.93	4.817	167.27	805.78
A_68_P22702205	chr4:5571555-5571599	NM_173426:251	Fam110b	INSIDE	1.681	6.974	1660.64	11580.84	11.726	1443.83	16930.61
A_68_P28329059	chr12:31758655-31758699	NM_027828:-156	Fam110c	PROMOTER	1.608	2.881	1404.76	4046.65	4.632	1230.34	5698.63
A_68_P23753715	chr5:65361156-65361200	NM_026667:-135	Fam114a1	DIVERGENT_PROMOTER	1.710	4.115	2530.52	10412.20	7.037	2134.05	15016.51
A_68_P20266458	chr1:59969786-59969830	NM_001037725:-41	Fam117b	PROMOTER	2.045	6.604	2387.87	15770.47	13.504	1898.02	25630.46
A_68_P26421598	chr9:35075374-35075418	NM_175411:-31	Fam118b	DIVERGENT_PROMOTER	1.752	1.543	895.51	1381.34	2.703	863.11	2333.18
A_68_P32386813	chrX:50622802-50622846	NM_001166365:158	Fam122b	INSIDE	1.789	8.506	5647.04	48031.03	15.218	2876.24	43771.99
A_68_P29644193	chr14:60997735-60997779	NM_001164705:634	Fam123a	INSIDE	1.722	3.402	1085.16	3691.29	5.858	923.00	5406.96
A_68_P21124370	chr2:33742800-33742844	NM_175184:644	Fam125b	INSIDE	1.540	2.933	4745.41	13920.28	4.516	3493.69	15778.51
A_68_P23539566	chr5:23536151-23536195	NM_053090:329	Fam126a	INSIDE	1.565	4.054	2121.20	8598.55	6.346	1830.88	11618.57
A_68_P20259677	chr1:58643036-58643080	NM_172513:119	Fam126b	INSIDE	2.034	5.228	4857.54	25394.96	10.635	3718.15	39544.05
A_68_P25957751	chr8:74126551-74126595	NM_001166213:5028	Fam129c	INSIDE	1.746	2.904	1868.37	5424.90	5.071	1476.20	7485.77
A_68_P24397660	chr6:42274774-42274818	NM_001113327:-157	Fam131b	PROMOTER	1.784	2.154	2777.70	5982.65	3.841	2513.41	9655.11
A_68_P20442011	chr1:93263227-93263272	NM_173395:243	Fam132b	INSIDE	1.744	3.148	2766.94	8709.43	5.489	2276.05	12492.45
A_68_P20012423	chr1:6373229-6373273	NM_001195732:23839	Fam150a	INSIDE	1.996	3.088	938.57	2898.62	6.165	860.84	5306.75
A_68_P25644704	chr8:9771350-9771394	NM_173446:-349	Fam155a	PROMOTER	1.907	12.194	23543.03	287086.60	23.250	14771.44	343435.70
A_68_P25644699	chr8:9770615-9770659	NM_173446:387	Fam155a	INSIDE	1.814	5.687	3346.03	19027.70	10.313	2684.47	27685.74
A_68_P25641012	chr8:9207827-9207871	NM_173446:563175	Fam155a	INSIDE	1.783	2.152	310.81	668.92	3.836	231.54	888.28
A_68_P23195768	chr4:108055890-108055934	NM_001099303:42	Fam159a	INSIDE	1.850	3.727	1717.43	6400.97	6.893	1331.16	9176.22
A_68_P25392116	chr7:112548270-112548314	NM_199009:233	Fam160a2	INSIDE	1.927	3.135	3303.29	10354.85	6.040	2514.41	15186.65
A_68_P25392118	chr7:112548478-112548522	NM_199009:25	Fam160a2	INSIDE	1.620	2.580	1482.11	3824.40	4.181	1279.22	5348.77
A_68_P20741945	chr1:158134987-158135031	NM_177838:148	Fam163a	INSIDE	2.344	0.728	1936.10	1410.28	1.708	1837.68	3138.08
A_68_P20140952	chr1:34899643-34899687	NM_001160235:231	Fam168b	INSIDE	1.568	3.941	1156.93	4558.99	6.180	1041.76	6438.55
A_68_P28079629	chr11:102308671-102308715	NM_199200:285	Fam171a2	INSIDE	1.686	1.895	1504.43	2851.23	3.195	1335.07	4265.03
A_68_P31106282	chr17:25929216-25929260	NM_145410:-9	Fam173a	PROMOTER	2.109	4.902	4593.98	22517.64	10.336	3445.66	35614.77

ProbeName	Target position of probe on CpG island microarray	TargetID	GeneSymbol	CpG island Description	Ratio of relative methylation (TiO_2-NP/Vehicle)	Sham group			TiO_2-H group		
						Relative methylation (Cy5/Cy3)	Cy3 signal (Input)	Cy5 signal (MeDIP)	Relative methylation (Cy5/Cy3)	Cy3 signal (Input)	Cy5 signal (MeDIP)
A_68_P20462986	chr1:97210270-97210314	NM_026321:88	Fam174a	INSIDE	1.572	2.255	485.23	1094.27	3.546	465.92	1651.96
A_68_P23278482	chr4:125826960-125827005	NM_172145:1736	Fam176b	INSIDE	2.373	12.234	2471.48	30237.07	29.028	1293.78	37556.21
A_68_P32135373	chr19:45005375-45005419	NM_001081225:-212	Fam178a	PROMOTER	1.623	2.807	2918.14	8191.52	4.556	2514.21	11455.31
A_68_P25328173	chr7:100228372-100228416	NM_021427:6	Fam181b	INSIDE	1.787	2.659	1172.86	3119.18	4.753	871.74	4143.13
A_68_P23657737	chr5:46030747-46030791	NM_021416:-28	Fam184b	PROMOTER	1.909	1.799	1547.18	2783.68	3.435	1287.38	4422.59
A_68_P23525900	chr5:20931065-20931109	NM_177869:366	Fam185a	INSIDE	1.501	1.522	1176.74	1790.95	2.284	1000.93	2286.17
A_68_P27856224	chr11:62692825-62692869	NM_026210:-145	Fam18b	PROMOTER	2.301	8.002	44876.32	359089.20	18.410	25812.74	475218.60
A_68_P27856227	chr11:62693165-62693209	NM_026210:195	Fam18b	INSIDE	1.741	6.334	2125.21	13461.23	11.029	1638.86	18075.66
A_68_P29055901	chr13:55672343-55672387	NM_001162881:-1028	Fam193b	PROMOTER	1.763	2.951	2122.69	6264.39	5.202	1772.94	9223.40
A_68_P25549531	chr7:142129256-142129301	NM_001143802:835	Fam196a	INSIDE	2.072	3.078	1300.45	4002.22	6.375	952.86	6074.71
A_68_P32683852	chrX:133584183-133584227	NM_146261:72	Fam199x	INSIDE	2.299	5.605	36252.91	203197.50	12.886	25460.36	328071.30
A_68_P30347864	chr15:76199968-76200012	NM_021555:663	Fam203a	INSIDE	1.590	1.972	711.02	1402.24	3.137	582.35	1826.67
A_68_P27055250	chr10:33847341-33847385	NM_175449:416	Fam26f	INSIDE	1.570	3.541	2209.17	7822.10	5.557	1786.17	9926.43
A_68_P26234161	chr8:125074980-125075024	NM_001037298:227	Fam38a	INSIDE	1.712	3.407	554.37	1888.59	5.832	500.29	2917.83
A_68_P32467861	chrX:71638358-71638402	NM_025473:98	Fam3a	INSIDE	1.682	4.438	2419.47	10737.62	7.463	1406.81	10498.39
A_68_P24329145	chr6:29866863-29866907	NM_001037740:-128	Fam40b	PROMOTER	1.720	3.229	355.25	1147.20	5.556	308.76	1715.42
A_68_P23345562	chr4:137952020-137952064	NM_001081672:331	Fam43b	INSIDE	1.512	2.773	891.39	2472.07	4.193	757.52	3175.97
A_68_P26693194	chr9:85220859-85220903	NM_001160379:-123	Fam46a	PROMOTER	2.018	2.016	961.09	1937.50	4.067	820.03	3335.24
A_68_P30278749	chr15:63829759-63829803	NM_144846:62230	Fam49b	INSIDE	2.073	3.646	2844.80	10373.26	7.558	2230.18	16854.81
A_68_P23590169	chr5:33972359-33972403	NM_178390:-96	Fam53a	PROMOTER	1.765	2.637	5515.12	14543.40	4.654	4415.98	20551.40
A_68_P23590170	chr5:33972447-33972492	NM_178390:-185	Fam53a	PROMOTER	1.690	4.543	10365.60	47091.67	7.678	7189.64	55203.47
A_68_P25537056	chr7:140005623-140005667	NM_212473:-765	Fam53b	PROMOTER	1.734	2.759	1651.86	4558.12	4.785	1316.60	6299.36
A_68_P23323339	chr4:134091201-134091245	NM_029759:-2555	Fam54b	PROMOTER	1.642	2.789	1422.20	3966.22	4.578	1228.16	5622.49
A_68_P27930557	chr11:76015854-76015898	NM_027773:319	Fam57a	INSIDE	1.904	3.875	1497.57	5802.80	7.379	1271.09	9379.09
A_68_P27930554	chr11:76015406-76015450	NM_027773:-129	Fam57a	PROMOTER	1.542	2.236	2797.45	6253.97	3.446	2477.12	8536.80
A_68_P31544271	chr18:21458929-21458973	NM_001033445:-310	Fam59a	PROMOTER	2.408	7.221	15083.00	108913.00	17.386	9309.07	161852.10
A_68_P24942904	chr6:148895207-148895251	NM_019643:-274	Fam60a	PROMOTER	1.989	4.671	2575.90	12031.24	9.289	2051.98	19060.53
A_68_P24942897	chr6:148894399-148894443	NM_019643:534	Fam60a	INSIDE	1.921	4.150	1979.80	8216.13	7.973	1511.82	12053.87
A_68_P24942894	chr6:148893997-148894041	NM_019643:936	Fam60a	INSIDE	1.544	4.314	2874.14	12399.49	6.660	2592.69	17268.14
A_68_P26617701	chr9:70504626-70504670	NM_172772:333	Fam63b	INSIDE	1.616	4.671	1854.17	8661.53	7.548	1656.06	12499.60
A_68_P27909076	chr11:71855740-71855784	NM_144526:-241	Fam64a	DIVERGENT_PROMOTER	1.620	3.912	2415.26	9447.36	6.336	2014.18	12761.04
A_68_P23314731	chr4:132478927-132478977	NM_001163792:-486	Fam76a	PROMOTER	1.821	2.349	1010.77	2374.41	4.277	696.91	2980.48
A_68_P26313788	chr9:13632555-13632599	NM_176836:406	Fam76b	INSIDE	1.600	1.733	1041.18	1804.15	2.773	816.41	2263.88
A_68_P21113215	chr2:31939833-31939877	NM_175511:-629	Fam78a	PROMOTER	2.031	5.322	1178.34	6271.46	10.808	1047.04	11316.20
A_68_P30345428	chr15:75834117-75834161	NM_001168253:5790	Fam83h	INSIDE	1.584	8.887	2371.19	21072.30	14.073	1940.59	27309.55
A_68_P30261330	chr15:60655945-60655989	NM_001162926:669	Fam84b	INSIDE	1.775	3.498	751.96	2630.19	6.210	645.31	4007.21
A_68_P30261334	chr15:60656407-60656451	NM_001162926:207	Fam84b	INSIDE	1.735	2.756	867.52	2391.32	4.784	695.50	3327.13
A_68_P21556816	chr2:117075560-117075604	NM_026620:108	Fam98b	INSIDE	1.520	2.828	1580.47	4470.05	4.299	1399.84	6017.61
A_68_P25176629	chr7:71518985-71519029	NM_177893:-25	Fan1	PROMOTER	2.306	4.347	1205.52	5239.99	10.023	908.82	9109.01
A_68_P28947125	chr13:36209443-36209487	NM_001039189:185	Fars2	INSIDE	1.673	3.957	2648.53	10479.59	6.621	2399.06	15884.25
A_68_P32846303	chr1:78485277-78485321	NM_011811:123	Farsb	INSIDE	1.701	2.498	580.73	1450.60	4.249	463.22	1968.35
A_68_P28187039	chr11:120686317-120686365	NM_007988:-479	Fasn	PROMOTER	1.682	4.348	211.73	920.69	7.315	146.38	1070.69
A_68_P29110678	chr13:68721295-68721339	NM_027123:192	Fastkd3	INSIDE	1.564	2.559	2112.04	5405.13	4.002	1553.20	6215.74
A_68_P21621794	chr2:128690006-128690050	NM_024237:361	Fbln7	INSIDE	2.332	1.496	749.56	1121.25	3.489	627.27	2188.52
A_68_P31741000	chr18:58369346-58369390	NM_010181:212	Fbn2	INSIDE	1.546	5.117	3637.60	18612.62	7.912	2582.50	20433.97
A_68_P23647569	chr5:44173172-44173216	NM_001159963:194	Fbxl5	INSIDE	1.905	5.037	4066.60	20482.39	9.597	2939.28	28208.15
A_68_P23647570	chr5:44173303-44173347	NM_001159963:64	Fbxl5	INSIDE	1.719	3.873	1869.08	7238.82	6.659	1685.32	11222.52

ProbeName	Target position of probe on CpG island microarray	TargetID	GeneSymbol	CpG island Description	Ratio of relative methylation (TiO_2-NP/Vehicle)	Sham group			TiO_2-H group		
						Relative methylation (Cy5/Cy3)	Cy3 signal (Input)	Cy5 signal (MeDIP)	Relative methylation (Cy5/Cy3)	Cy3 signal (Input)	Cy5 signal (MeDIP)
A_68_P26132587	chr8:107788500-107788544	NM_015821:-25	Fbxl8	DIVERGENT_PROMOTER	1.752	3.803	715.63	2721.64	6.664	631.31	4207.03
A_68_P25023124	chr7:29478378-29478422	NM_001163702:238	Fbxo27	INSIDE	1.599	1.525	1881.91	2870.40	2.439	1510.43	3683.48
A_68_P30246699	chr15:58046420-58046464	NM_026346:-9	Fbxo32	PROMOTER	1.802	1.596	1220.54	1947.77	2.876	1004.10	2888.22
A_68_P31764312	chr18:62708242-62708286	NM_134136:133	Fbxo38	INSIDE	2.287	2.829	9614.08	27195.70	6.469	6425.64	41568.23
A_68_P27910787	chr11:72128038-72128082	NM_001099688:115	Fbxo39	INSIDE	1.516	3.848	1223.64	4709.14	5.834	1038.74	6060.44
A_68_P23362414	chr4:140703695-140703739	NM_172518:-120	Fbxo42	DIVERGENT_PROMOTER	1.574	1.696	1041.78	1767.35	2.670	914.58	2441.71
A_68_P30137020	chr15:36094444-36094488	NM_001081253:173	Fbxo43	INSIDE	1.860	5.797	2825.11	16376.00	10.784	2325.91	25082.19
A_68_P28053223	chr11:97744951-97744995	NM_001081435:496	Fbxo47	INSIDE	2.589	10.903	9736.38	106154.20	28.224	4882.70	137808.30
A_68_P28053229	chr11:97745535-97745579	NM_001081435:-88	Fbxo47	PROMOTER	2.226	7.628	7635.22	58240.73	16.982	4804.99	81599.95
A_68_P23392700	chr4:147525826-147525870	NM_001163704:186	Fbxo6	INSIDE	1.802	5.283	4542.00	23995.44	9.522	3913.81	37267.97
A_68_P26658155	chr9:77956059-77956103	NM_023605:383	Fbxo9	INSIDE	2.294	9.040	3947.31	35683.72	20.740	3181.35	65981.75
A_68_P22293161	chr3:84619085-84619129	NM_001177773:-392	Fbxw7	PROMOTER	2.089	7.473	3528.14	26366.72	15.611	2725.81	42552.94
A_68_P20826000	chr1:172995096-172995140	NM_010188:-5584	Fcgr3	PROMOTER	1.526	2.291	17110.18	39195.13	3.495	12593.44	44016.75
A_68_P29259683	chr13:99585179-99585223	NM_172591:204	Fcho2	INSIDE	1.592	4.182	1087.69	4548.58	6.660	923.46	6149.83
A_68_P31677413	chr18:46685542-46685586	NM_173423:61	Fem1c	INSIDE	1.507	1.993	1402.64	2795.91	3.005	1532.05	4603.56
A_68_P31371532	chr17:78817452-78817496	NM_199448:-31	Fez2	PROMOTER	1.527	5.391	8891.70	47938.00	8.235	6744.37	55539.39
A_68_P24295432	chr6:23203617-23203661	NM_028462:-5374	Fezf1	PROMOTER	2.117	4.889	2303.60	11263.08	10.350	1828.01	18919.61
A_68_P29410675	chr14:13178814-13178858	NM_080433:-457	Fezf2	PROMOTER	1.726	2.747	1144.71	3144.30	4.740	883.06	4185.46
A_68_P32743933	chrX:147481461-147481505	NM_008001:-293	Fgd1	PROMOTER	1.909	4.057	3063.49	12429.23	7.745	1840.37	14252.89
A_68_P29369781	chr13:119503243-119503287	NM_008002:-241	Fgf10	PROMOTER	1.986	4.921	7424.08	36535.57	9.775	5894.22	57617.72
A_68_P30630406	chr16:28445546-28445590	NM_183064:-255	Fgf12	PROMOTER	2.053	2.897	1614.92	4677.83	5.947	1132.49	6734.62
A_68_P25609917	chr7:152082446-152082490	NM_008003:32	Fgf15	INSIDE	1.934	2.354	2711.42	6383.42	4.554	2263.15	10306.32
A_68_P25609544	chr7:152024886-152024930	NM_008007:392	Fgf3	INSIDE	1.731	2.429	1335.04	3242.95	4.204	1106.16	4650.24
A_68_P25609686	chr7:152047482-152047526	NM_010202:214	Fgf4	INSIDE	1.644	5.163	1113.15	5747.23	8.487	946.39	8032.38
A_68_P32140182	chr19:45811402-45811446	NM_001166361:5950	Fgf8	INSIDE	1.535	5.312	3067.67	16296.33	8.155	2340.21	19085.57
A_68_P29631470	chr14:58691022-58691066	NM_013518:-478	Fgf9	PROMOTER	1.882	5.806	1512.20	8780.06	10.924	1230.13	13438.37
A_68_P25727484	chr8:26629202-26629246	NM_001079908:-19	Fgfr1	PROMOTER	1.838	2.463	1456.02	3585.62	4.527	1219.57	5521.00
A_68_P29053522	chr13:55254151-55254195	NM_008011:-6	Fgfr4	PROMOTER	1.824	5.512	1658.26	9140.76	10.054	1504.20	15122.59
A_68_P20849151	chr1:177555721-177555765	NM_010209:24	Fh1	INSIDE	1.716	3.071	7119.66	21865.76	5.271	5290.52	27886.33
A_68_P20849149	chr1:177555403-177555447	NM_010209:342	Fh1	INSIDE	1.635	2.754	1911.22	5263.50	4.503	1609.59	7248.39
A_68_P29403726	chr14:11994369-11994413	NM_010210:156	Fhit	INSIDE	1.713	3.069	5639.46	17306.04	5.257	4585.36	24106.61
A_68_P20187367	chr1:43220695-43220739	NM_010212:90	Fhl2	INSIDE	1.824	6.556	1156.88	7584.06	11.958	1018.56	12180.28
A_68_P23269776	chr4:124378137-124378181	NM_010213:216	Fhl3	INSIDE	1.767	3.462	657.54	2276.40	6.116	625.75	3826.93
A_68_P31561526	chr18:24868000-24868044	NM_175276:77	Fhod3	INSIDE	1.786	4.266	1651.68	7046.32	7.617	1352.02	10298.76
A_68_P21278824	chr2:63936695-63936739	NM_021716:-647	Fign	PROMOTER	1.799	1.562	572.22	893.92	2.810	476.20	1338.03
A_68_P24951566	chr7:4966239-4966283	NM_011813:-405	Fiz1	PROMOTER	1.527	1.489	2233.90	3326.98	2.274	1677.08	3813.57
A_68_P31937108	chr19:7054737-7054781	NM_008020:172	Fkbp2	INSIDE	1.686	3.435	4409.51	15146.83	5.790	3177.15	18395.36
A_68_P31937111	chr19:7054987-7055031	NM_001166368:-57	Fkbp2	PROMOTER	1.524	1.910	1073.69	2050.39	2.910	831.21	2418.70
A_68_P24839014	chr6:128387845-128387889	NM_010219:783	Fkbp4	INSIDE	2.034	8.102	3964.90	32121.93	16.475	2576.69	42451.57
A_68_P31122728	chr17:28622553-28622597	NM_010220:483	Fkbp5	INSIDE	1.844	2.440	266.10	649.29	4.499	246.20	1107.77
A_68_P24477003	chr6:56781975-56782019	NM_012056:-56	Fkbp9	PROMOTER	2.117	2.955	752.71	2224.52	6.256	588.64	3682.38
A_68_P24326454	chr6:29383341-29383385	NM_001081185:210	Flnc	INSIDE	1.663	1.445	6320.78	9136.28	2.403	4721.24	11346.29
A_68_P31161104	chr17:35960060-35960104	NM_008027:-219	Flot1	PROMOTER	1.769	5.447	1552.32	8455.38	9.634	1231.80	11866.98
A_68_P28656384	chr12:96934105-96934150	NM_201518:3692	Flrt2	INSIDE	1.584	2.959	1264.47	3741.34	4.687	1024.72	4803.15
A_68_P32441102	chrX:65932146-65932191	NM_008031:439	Fmr1	INSIDE	2.396	4.896	1079.66	5286.01	11.733	634.73	7447.07
A_68_P21107218	chr2:30997212-30997256	NM_001038700:294	Fnbp1	INSIDE	1.689	2.193	906.60	1988.37	3.704	750.28	2778.80
A_68_P22483661	chr3:122321647-122321691	NM_001114665:917	Fnbp1l	INSIDE	1.844	6.922	969.79	6713.12	12.762	832.67	10626.89

ProbeName	Target position of probe on CpG island microarray	TargetID	GeneSymbol	CpG island Description	Ratio of relative methylation (TiO_2-NP/Vehicle)	Sham group			TiO_2-H group		
						Relative methylation (Cy5/Cy3)	Cy3 signal (Input)	Cy5 signal (MeDIP)	Relative methylation (Cy5/Cy3)	Cy3 signal (Input)	Cy5 signal (MeDIP)
A_68_P22483663	chr3:122321938-122321982	NM_001114665:625	Fnbp1l	INSIDE	1.717	5.718	894.16	5113.21	9.819	840.35	8251.31
A_68_P29713892	chr14:73109778-73109822	NM_207636:10	Fndc3a	INSIDE	1.800	3.666	15386.09	56402.37	6.598	12880.93	84987.90
A_68_P29713891	chr14:73109647-73109691	NM_207636:142	Fndc3a	INSIDE	1.641	3.333	1249.16	4163.45	5.469	1056.14	5776.46
A_68_P29713894	chr14:73110055-73110099	NM_207636:-266	Fndc3a	PROMOTER	1.613	1.503	3401.65	5113.89	2.425	2664.88	6462.15
A_68_P29713887	chr14:73109166-73109210	NM_207636:622	Fndc3a	INSIDE	1.547	1.720	371.99	639.79	2.661	324.89	864.39
A_68_P27810090	chr11:54251929-54251973	NM_173753:270	Fnip1	INSIDE	1.797	3.055	1965.54	6005.08	5.491	1626.28	8930.19
A_68_P28604394	chr12:86827035-86827079	NM_010234:12206	Fos	DOWNSTREAM	1.887	3.215	1472.75	4735.17	6.066	1107.98	6721.43
A_68_P28463190	chr12:58643591-58643635	NM_008259:3496	Foxa1	INSIDE	1.560	2.253	1457.12	3283.00	3.514	1177.59	4138.56
A_68_P29257223	chr13:99136439-99136483	NM_008242:12261	Foxd1	DOWNSTREAM	2.092	8.661	2174.59	18833.71	18.116	1799.01	32590.48
A_68_P23222249	chr4:114580688-114580732	NM_008593:793	Foxd2	INSIDE	2.427	6.487	1921.84	12467.91	15.742	1486.86	23406.71
A_68_P23222243	chr4:114580052-114580096	NM_008593:1429	Foxd2	INSIDE	1.514	3.361	1114.32	3744.93	5.088	999.09	5082.91
A_68_P23145563	chr4:99324552-99324596	NM_010425:1585	Foxd3	INSIDE	1.596	1.997	1989.00	3971.76	3.187	1744.74	5559.83
A_68_P23222359	chr4:114597578-114597622	NM_015758:1018	Foxe3	DOWNSTREAM	1.895	11.062	1484.95	16425.86	20.962	1203.20	25221.92
A_68_P23222361	chr4:114597756-114597800	NM_015758:840	Foxe3	INSIDE	1.562	2.049	646.36	1324.62	3.200	520.65	1666.31
A_68_P24002947	chr5:114723893-114723937	NM_148935:-144	Foxn4	PROMOTER	2.040	1.930	249.67	481.97	3.938	173.95	684.94
A_68_P27094083	chr10:41996329-41996375	NM_019740:196	Foxo3	INSIDE	1.730	2.798	733.02	2050.96	4.841	674.74	3266.23
A_68_P32563078	chrX:98450245-98450289	NM_018789:400	Foxo4	INSIDE	1.854	2.398	2878.19	6901.22	4.445	1681.25	7473.24
A_68_P23250319	chr4:119941839-119941885	NM_194060:18004	Foxo6	INSIDE	1.820	3.106	574.76	1785.39	5.655	460.56	2604.27
A_68_P23250457	chr4:119959679-119959723	NM_194060:166	Foxo6	INSIDE	1.669	2.359	676.94	1597.15	3.937	600.87	2365.41
A_68_P24683080	chr6:98978547-98978591	NM_001197322:-276	Foxp1	PROMOTER	1.922	1.899	1475.64	2802.18	3.650	1327.61	4846.05
A_68_P31214913	chr17:48046778-48046822	NM_001110824:14781	Foxp4	INSIDE	2.054	5.738	6145.33	35262.75	11.786	4375.44	51567.38
A_68_P32117383	chr19:41905096-41905140	NM_008043:659	Frat1	INSIDE	1.590	2.682	1948.71	5227.13	4.264	1756.91	7491.10
A_68_P32117489	chr19:41921912-41921956	NM_177603:688	Frat2	INSIDE	1.795	3.395	963.77	3271.91	6.093	735.76	4482.62
A_68_P23018909	chr4:73659645-73659689	NM_172869:160	Frmd3	INSIDE	1.623	1.485	899.52	1335.79	2.410	685.09	1650.77
A_68_P20967784	chr2:4322707-4322751	NM_001177843:707	Frmd4a	INSIDE	1.734	4.722	12366.15	58388.60	8.189	8550.72	70023.36
A_68_P21582988	chr2:121632726-121632770	NM_172673:45	Frmd5	INSIDE	2.734	8.279	6123.14	50694.51	22.637	3823.30	86547.30
A_68_P32815030	chrX:164561176-164561220	NM_001033330:453967	Frmpd4	INSIDE	1.866	1.925	4121.47	7933.14	3.592	2105.63	7564.06
A_68_P24154219	chr5:143723667-143723712	NM_007984:1656	Fscn1	INSIDE	1.784	4.276	1532.87	6554.20	7.628	1125.17	8582.46
A_68_P29346593	chr13:115249654-115249698	NM_008046:-738	Fst	PROMOTER	1.606	2.498	3716.50	9282.35	4.010	2635.06	10566.83
A_68_P21109951	chr2:31428059-31428103	NM_001033389:-164	Fubp3	PROMOTER	2.343	13.413	1326.63	17793.56	31.430	941.46	29589.95
A_68_P25266551	chr7:87547096-87547140	NM_011046:530	Furin	INSIDE	2.159	12.930	2107.76	27252.24	27.910	1267.94	35387.98
A_68_P25266552	chr7:87547168-87547212	NM_011046:458	Furin	INSIDE	1.907	4.974	1206.48	6001.21	9.487	1020.22	9678.73
A_68_P29448409	chr14:21514700-21514744	NM_028428:533	Fut11	INSIDE	1.569	1.750	974.29	1705.36	2.746	895.85	2460.26
A_68_P22045621	chr3:33919264-33919308	NM_001113188:286	Fxr1	INSIDE	1.872	5.067	1615.59	8186.84	9.487	1389.21	13179.17
A_68_P30655034	chr16:32877951-32877995	NM_001159349:103	Fyttd1	INSIDE	1.683	2.031	1813.46	3682.82	3.417	1446.40	4943.06
A_68_P28080444	chr11:102467782-102467826	NM_020510:2060	Fzd2	INSIDE	2.002	1.520	598.43	909.33	3.043	460.34	1400.67
A_68_P20294265	chr1:64784540-64784584	NM_001042659:-238	Fzd5	PROMOTER	1.987	4.693	1117.88	5245.69	9.322	838.15	7813.62
A_68_P31484539	chr18:9212402-9212446	NM_008058:-429	Fzd8	PROMOTER	1.604	2.193	2083.27	4569.12	3.518	1590.11	5594.11
A_68_P31484544	chr18:9212850-9212894	NM_008058:19	Fzd8	INSIDE	1.565	2.801	892.40	2499.57	4.384	806.66	3536.09
A_68_P23892215	chr5:92512150-92512194	NM_001080797:-229	G3bp2	PROMOTER	1.750	2.949	1937.02	5713.11	5.163	1664.45	8593.41
A_68_P23892217	chr5:92512328-92512372	NM_001080797:-407	G3bp2	PROMOTER	1.693	3.722	2491.50	9273.63	6.300	1946.07	12259.70
A_68_P26001982	chr8:83403879-83403923	NM_021356:478	Gab1	INSIDE	1.515	4.546	3277.91	14899.97	6.888	2505.41	17257.68
A_68_P25348908	chr7:104230293-104230337	NM_001162477:54	Gab2	INSIDE	1.572	2.820	412.83	1164.28	4.434	361.09	1600.99
A_68_P26171007	chr8:114465131-114465175	NM_026693:535	Gabarapl2	INSIDE	3.497	27.141	3597.79	97649.33	94.902	1892.10	179564.50
A_68_P23787605	chr5:72049192-72049236	NM_010251:333	Gabra4	INSIDE	1.882	5.032	905.66	4556.89	9.471	750.11	7104.47
A_68_P32457442	chrX:69520267-69520311	NM_017369:-228	Gabre	PROMOTER	1.789	4.370	3001.36	13115.06	7.817	2007.83	15694.92
A_68_P27286394	chr10:80391549-80391593	NM_008655:-1265	Gadd45b	PROMOTER	1.918	1.815	1699.65	3085.00	3.482	1370.18	4771.14

ProbeName	Target position of probe on CpG island microarray	TargetID	GeneSymbol	CpG island Description	Ratio of relative methylation (TiO_2-NP/Vehicle)	Sham group			TiO_2-H group		
						Relative methylation (Cy5/Cy3)	Cy3 signal (Input)	Cy5 signal (MeDIP)	Relative methylation (Cy5/Cy3)	Cy3 signal (Input)	Cy5 signal (MeDIP)
A_68_P29034741	chr13:51941420-51941464	NM_011817:-601	Gadd45g	PROMOTER	1.607	4.308	2411.71	10388.75	6.924	1861.97	12891.70
A_68_P22892659	chr4:47105046-47105090	NM_172693:244	Galnt12	INSIDE	1.570	4.111	837.62	3443.52	6.453	723.30	4667.16
A_68_P31347831	chr17:74059828-74059872	NM_027864:-59	Galnt14	PROMOTER	1.761	2.701	2475.44	6685.55	4.756	1800.05	8561.73
A_68_P27385039	chr10:98569849-98569893	NM_015737:-898	Galnt4	PROMOTER	1.754	2.881	597.56	1721.78	5.053	546.34	2760.79
A_68_P27385041	chr10:98569996-98570040	NM_015737:-750	Galnt4	PROMOTER	1.539	1.907	370.06	705.59	2.935	285.70	838.51
A_68_P25892520	chr8:60131247-60131291	NM_001167981:584	Galnt7	INSIDE	1.552	2.796	777.11	2172.51	4.339	632.87	2746.20
A_68_P23980413	chr5:110973506-110973550	NM_198306:165	Galnt9	INSIDE	2.313	2.123	903.04	1916.98	4.911	837.52	4113.03
A_68_P28576082	chr12:81619771-81619815	NM_001081421:-184	Galntl1	PROMOTER	1.562	2.629	854.62	2246.53	4.105	779.49	3200.03
A_68_P28576090	chr12:81620528-81620572	NM_001081421:574	Galntl1	INSIDE	1.536	2.152	4793.87	10316.28	3.306	3707.25	12255.35
A_68_P30364007	chr15:78873556-78873600	NM_015738:1264	Galr3	INSIDE	1.525	1.580	1550.00	2449.54	2.410	1295.18	3121.49
A_68_P27561184	chr11:4965305-4965349	NM_001190406:4	Gas2l1	INSIDE	2.125	7.670	8270.87	63438.54	16.302	5486.54	89440.16
A_68_P24620345	chr6:88147159-88147203	NM_008090:-1477	Gata2	PROMOTER	1.605	1.930	784.07	1513.35	3.097	737.04	2282.76
A_68_P24620367	chr6:88149612-88149656	NM_008090:977	Gata2	INSIDE	1.513	1.864	2081.20	3879.08	2.820	1736.45	4897.60
A_68_P24620361	chr6:88149067-88149115	NM_008090:433	Gata2	INSIDE	1.724	1.904	294.63	560.86	3.281	247.73	812.77
A_68_P20997431	chr2:9796515-9796559	NM_008091:3691	Gata3	INSIDE	1.569	3.329	5427.34	18065.56	5.224	3876.76	20251.14
A_68_P25949121	chr8:72498323-72498367	NM_001113346:-62	Gatad2a	PROMOTER	1.685	5.348	4294.38	22967.53	9.012	3393.33	30579.31
A_68_P24111498	chr5:134575433-134575477	NM_030719:-163	Gatsl2	PROMOTER	1.835	4.963	1362.28	6761.22	9.107	1124.35	10239.46
A_68_P27556422	chr11:4118442-4118486	NM_028022:211	Gatsl3	INSIDE	2.059	7.708	1310.88	10104.72	15.868	1024.76	16260.78
A_68_P30849196	chr16:70314241-70314285	NM_028803:-69	Gbe1	PROMOTER	1.701	2.303	2485.26	5724.21	3.918	1985.75	7780.62
A_68_P20432927	chr1:91819381-91819425	NM_010262:8349	Gbx2	DOWNSTREAM	2.570	8.925	4387.88	39160.32	22.935	2276.94	52221.25
A_68_P21270819	chr2:62502267-62502311	NM_145523:-95	Gca	PROMOTER	1.525	1.673	2277.55	3809.24	2.551	1855.99	4734.25
A_68_P21568184	chr2:118993532-118993576	NM_177157:31	Gchfr	INSIDE	1.586	3.148	2843.34	8949.78	4.993	2288.86	11428.31
A_68_P26656177	chr9:77602711-77602755	NM_010295:391	Gclc	INSIDE	1.572	0.426	1277.34	544.15	0.670	1078.63	722.15
A_68_P29248566	chr13:97695211-97695255	NM_001166065:589	Gcnt4	INSIDE	1.650	2.718	538.77	1464.12	4.484	429.38	1925.19
A_68_P25459669	chr7:125849289-125849333	NM_019580:-58	Gde1	DIVERGENT_PROMOTER	1.568	2.848	2463.48	7016.05	4.464	2041.48	9114.14
A_68_P25951144	chr8:72854849-72854893	NM_008107:1207	Gdf1	INSIDE	1.649	4.300	551.65	2372.07	7.090	441.95	3133.45
A_68_P29526837	chr14:34736673-34736717	NM_145741:-78	Gdf10	PROMOTER	1.742	2.152	3153.19	6786.21	3.750	2573.87	9651.14
A_68_P22722447	chr4:9771366-9771410	NM_013526:-130	Gdf6	PROMOTER	1.779	2.585	23523.29	60796.56	4.598	16395.60	75387.85
A_68_P28221329	chr12:8309070-8309114	NM_013527:-332	Gdf7	PROMOTER	1.836	2.722	5774.15	15716.10	4.998	4784.64	23914.52
A_68_P29996479	chr15:7760686-7760730	NM_010275:-302	Gdnf	PROMOTER	1.950	9.773	1415.04	13828.45	19.057	1081.20	20604.19
A_68_P29996481	chr15:7760813-7760857	NM_010275:-176	Gdnf	PROMOTER	1.625	3.802	1226.00	4661.44	6.177	960.25	5931.57
A_68_P24994096	chr7:20158742-20158786	NM_027189:-72	Gemin7	DIVERGENT_PROMOTER	1.558	1.518	990.39	1503.27	2.364	942.86	2229.39
A_68_P23968450	chr5:108155208-108155253	NM_010278:-1867	Gfi1	PROMOTER	2.050	3.755	599.09	2249.65	7.697	495.49	3813.81
A_68_P29249638	chr13:97907886-97907931	NM_177266:17	Gfm2	INSIDE	2.057	6.374	600.32	3826.62	13.115	435.69	5714.06
A_68_P32213515	chr19:58529618-58529662	NM_010279:-684	Gfra1	PROMOTER	1.748	1.741	4343.45	7562.41	3.044	2798.64	8517.84
A_68_P29703989	chr14:71289781-71289825	NM_008115:-134	Gfra2	PROMOTER	1.666	6.103	2103.42	12836.84	10.165	1664.63	16920.80
A_68_P28154359	chr11:115465196-115465240	NM_173048:2	Gga3	INSIDE	1.774	3.264	732.95	2392.19	5.789	639.72	3703.54
A_68_P27980051	chr11:84683596-84683640	NM_153144:622	Ggnbp2	INSIDE	1.514	2.029	3310.52	6716.55	3.072	2466.36	7576.01
A_68_P21765995	chr2:155340656-155340700	NM_144786:-96	Ggt7	DIVERGENT_PROMOTER	1.806	2.951	1077.94	3180.74	5.328	842.32	4487.81
A_68_P21765994	chr2:155340501-155340545	NM_144786:60	Ggt7	INSIDE	1.797	1.723	1515.62	2611.59	3.096	1202.00	3721.77
A_68_P29975444	chr15:3533365-3533409	NM_010284:-156	Ghr	PROMOTER	1.920	7.272	941.81	6848.65	13.961	869.39	12137.41
A_68_P20417070	chr1:89224090-89224134	NM_001110212:509	Gigyf2	INSIDE	1.568	2.784	842.92	2346.29	4.365	744.25	3248.54
A_68_P24429118	chr6:48692680-48692724	NM_008376:3657	Gimap1	INSIDE	1.627	6.458	1090.95	7045.70	10.506	940.90	9884.70
A_68_P26221254	chr8:123113358-123113402	NM_178856:-405	Gins2	PROMOTER	2.234	8.182	5236.56	42846.61	18.278	3565.24	65164.01
A_68_P26221250	chr8:123112916-123112960	NM_178856:37	Gins2	INSIDE	1.512	1.444	2567.51	3706.97	2.182	2080.94	4541.31
A_68_P32563826	chrX:98580104-98580148	NM_008124:7451	Gjb1	INSIDE	2.352	3.539	937.42	3317.78	8.324	424.00	3529.44
A_68_P26167383	chr8:113783057-113783101	NM_009149:24	Glg1	INSIDE	2.733	6.404	3079.88	19722.76	17.500	1937.32	33903.41

ProbeName	Target position of probe on CpG island microarray	TargetID	GeneSymbol	CpG island Description	Ratio of relative methylation (TiO_2-NP/Vehicle)	Sham group			TiO_2-H group		
						Relative methylation (Cy5/Cy3)	Cy3 signal (Input)	Cy5 signal (MeDIP)	Relative methylation (Cy5/Cy3)	Cy3 signal (Input)	Cy5 signal (MeDIP)
A_68_P20553865	chr1:120734246-120734290	NM_001081125:215928	Gli2	INSIDE	1.527	2.576	834.66	2150.49	3.933	708.23	2785.64
A_68_P28842022	chr13:15555028-15555072	NM_008130:-505	Gli3	PROMOTER	2.065	6.713	2389.98	16043.20	13.862	1884.57	26123.75
A_68_P31135104	chr17:30749389-30749433	NM_001113560:194	Glo1	INSIDE	1.571	4.088	647.44	2646.58	6.422	564.46	3624.90
A_68_P31136610	chr17:31038660-31038704	NM_021332:-129	Glp1r	PROMOTER	1.861	3.103	1597.84	4957.41	5.775	1231.07	7109.74
A_68_P22271218	chr3:80717646-80717690	NM_010298:-122	Glrb	PROMOTER	1.707	3.883	2691.97	10453.05	6.627	2172.05	14394.29
A_68_P25957808	chr8:74135004-74135048	NM_146211:104	Glt25d1	INSIDE	1.546	2.720	1477.88	4019.96	4.206	1203.59	5061.77
A_68_P20720544	chr1:154247140-154247184	NM_177756:166	Glt25d2	INSIDE	1.829	2.208	4633.39	10228.90	4.038	3755.72	15165.27
A_68_P22299774	chr3:85691661-85691705	NM_177130:-242	Glt28d2	PROMOTER	2.345	6.115	2137.83	13071.99	14.340	1643.46	23567.60
A_68_P24005520	chr5:115141122-115141166	NM_019821:-200	Gltp	PROMOTER	1.861	14.750	2824.25	41658.07	27.452	1892.70	51959.08
A_68_P24005517	chr5:115140712-115140756	NM_019821:210	Gltp	INSIDE	1.746	3.422	3875.07	13262.26	5.977	2524.78	15089.95
A_68_P28771927	chr12:118392576-118392621	NR_033538:-2856	Gm10421	PROMOTER	2.599	4.131	733.51	3030.00	10.736	537.89	5774.98
A_68_P30955189	chr16:90144198-90144242	NR_033476:1739	Gm10789	INSIDE	1.763	6.303	1076.13	6783.06	11.115	799.09	8881.84
A_68_P22737302	chr4:12833974-12834018	NM_001171801:13	Gm11818	INSIDE	2.209	5.015	1959.85	9827.78	11.076	1368.52	15158.08
A_68_P28058704	chr11:98671394-98671438	NR_033551:-13	Gm12359	PROMOTER	1.656	3.705	8276.45	30660.92	6.135	6485.89	39792.04
A_68_P21438071	chr2:93797315-93797359	NM_001145034:-79	Gm13889	PROMOTER	1.801	2.816	3220.82	9068.87	5.071	2565.75	13010.32
A_68_P21438069	chr2:93797146-93797190	NM_001145034:89	Gm13889	INSIDE	1.764	3.176	1991.48	6325.51	5.604	1596.99	8949.92
A_68_P29575892	chr14:47000105-47000149	NR_037981:928	Gm15217	INSIDE	1.787	3.607	2919.11	10529.17	6.446	2186.72	14096.10
A_68_P27845567	chr11:60745252-60745296	NR_027800:95	Gm16516	INSIDE	1.678	3.036	937.83	2847.45	5.094	736.27	3750.27
A_68_P28744403	chr12:112723947-112723992	NM_001033248:65	Gm266	INSIDE	1.784	1.381	2131.22	2942.41	2.463	1768.10	4355.31
A_68_P27813685	chr11:54911498-54911542	NM_010299:34	Gm2a	INSIDE	1.504	4.655	2755.36	12826.30	7.003	2025.41	14183.33
A_68_P25766302	chr8:33994797-33994841	NM_001177589:65680	Gm3985	DOWNSTREAM	2.287	9.404	1069.61	10058.46	21.510	750.44	16141.86
A_68_P25766300	chr8:33994475-33994519	NM_001177589:66002	Gm3985	DOWNSTREAM	1.894	2.241	608.52	1363.70	4.244	521.65	2213.84
A_68_P25362933	chr7:106774200-106774244	NM_001195529:1391	Gm4980	INSIDE	1.574	1.983	2380.55	4720.30	3.121	1714.36	5350.31
A_68_P20875118	chr1:182262286-182262330	NR_003623:-1628	Gm5069	PROMOTER	1.665	2.628	2153.41	5658.70	4.375	1699.26	7433.81
A_68_P32571132	chrX:100129205-100129249	NR_026596:336	Gm5126	INSIDE	1.927	2.774	1584.24	4393.93	5.345	859.88	4595.98
A_68_P26587299	chr9:65219825-65219869	NM_001111145:10980	Gm514	INSIDE	1.625	6.392	4269.15	27288.57	10.385	3395.63	35263.23
A_68_P29751456	chr14:79851782-79851826	NM_001034882:163	Gm5465	INSIDE	1.555	2.370	3615.36	8570.06	3.685	2881.89	10619.67
A_68_P30080950	chr15:25344237-25344281	NR_027376:312	Gm5468	INSIDE	1.575	2.606	378.57	986.49	4.105	368.66	1513.46
A_68_P25661493	chr8:12431257-12431301	NR_027975:45508	Gm5607	INSIDE	1.839	6.119	16899.96	103403.30	11.255	12229.55	137644.50
A_68_P30720009	chr16:44173533-44173577	NM_001029889:45	Gm608	INSIDE	1.621	3.061	994.91	3045.29	4.961	793.75	3937.64
A_68_P21932364	chr3:8463041-8463085	NR_033512:36	Gm6194	INSIDE	2.110	4.809	7173.47	34497.75	10.146	5470.79	55505.08
A_68_P21932363	chr3:8462950-8462994	NR_033512:126	Gm6194	INSIDE	1.712	4.894	2868.01	14035.36	8.377	2247.66	18829.32
A_68_P30333008	chr15:73670325-73670369	NM_001033365:-245	Gm628	PROMOTER	1.505	1.455	862.56	1255.19	2.189	759.49	1662.90
A_68_P21345223	chr2:75497311-75497355	NR_033513:-14	Gm6793	PROMOTER	1.817	3.214	715.56	2299.70	5.840	502.90	2936.75
A_68_P30895867	chr16:78376777-78376821	NR_002700:204	Gm7334	INSIDE	1.756	5.544	4323.13	23966.56	9.736	3610.15	35150.07
A_68_P23644448	chr5:43626040-43626084	NR_028417:531	Gm7854	INSIDE	4.107	0.491	627.14	307.93	2.017	723.63	1459.38
A_68_P31162860	chr17:36305369-36305413	NM_001081032:92	Gm8909	INSIDE	1.755	2.647	5455.52	14440.49	4.646	4867.99	22619.08
A_68_P26891190	chr9:122656383-122656427	NM_001199736:-59	Gm9524	PROMOTER	1.523	7.913	889.69	7039.99	12.054	658.89	7942.60
A_68_P26886028	chr9:121766761-121766805	NM_001112668:-133	Gm9790	PROMOTER	2.363	5.533	4191.24	23188.72	13.072	3175.11	41505.89
A_68_P22323963	chr3:90017329-90017373	NM_001190258:219	Gm9846	INSIDE	2.068	0.262	1139.06	298.86	0.543	1169.11	634.28
A_68_P24611767	chr6:86683275-86683319	NM_011818:76	Gmcl1	INSIDE	1.831	6.282	6492.21	40784.11	11.502	4673.24	53751.74
A_68_P21909702	chr2:181022305-181022349	NM_198169:345	Gmeb2	INSIDE	1.815	1.560	1875.56	2926.55	2.832	1427.15	4041.51
A_68_P22410841	chr3:107948966-107949010	NM_010306:44	Gnai3	INSIDE	1.628	3.286	970.21	3187.70	5.350	788.34	4217.64
A_68_P31790105	chr18:67293085-67293129	NM_010307:-373	Gnal	PROMOTER	1.562	2.176	769.95	1675.10	3.399	684.73	2327.47
A_68_P26073617	chr8:96334994-96335038	NM_001113384:279	Gnao1	INSIDE	1.843	6.583	10465.44	68894.13	12.130	7446.55	90329.10
A_68_P27253670	chr10:74430232-74430276	NM_010311:279	Gnaz	INSIDE	1.570	1.802	1266.85	2282.44	2.829	1006.12	2846.54
A_68_P27253669	chr10:74430095-74430139	NM_010311:141	Gnaz	INSIDE	1.529	2.357	789.78	1861.25	3.603	726.43	2617.05

ProbeName	Target position of probe on CpG island microarray	TargetID	GeneSymbol	CpG island Description	Ratio of relative methylation (TiO_2-NP/Vehicle)	Sham group			TiO_2-H group		
						Relative methylation (Cy5/Cy3)	Cy3 signal (Input)	Cy5 signal (MeDIP)	Relative methylation (Cy5/Cy3)	Cy3 signal (Input)	Cy5 signal (MeDIP)
A_68_P30580079	chr16:18499124-18499168	NM_023120:183	Gnb1l	INSIDE	2.141	2.844	1859.81	5289.01	6.089	1442.04	8781.04
A_68_P26866698	chr9:118416014-118416060	NM_018748:601	Golga4	INSIDE	2.118	11.798	3642.19	42970.34	24.992	2297.60	57421.75
A_68_P28691644	chr12:103707806-103707850	NM_013747:-291	Golga5	PROMOTER	1.726	2.151	1836.11	3949.73	3.712	1527.76	5671.67
A_68_P28691640	chr12:103707382-103707426	NM_013747:-715	Golga5	PROMOTER	1.563	6.121	432.66	2648.22	9.568	300.97	2879.67
A_68_P24904258	chr6:142336086-142336130	NM_025872:346	Golt1b	INSIDE	1.734	1.677	823.66	1381.09	2.908	721.84	2098.75
A_68_P21315049	chr2:70499932-70499976	NM_027352:389	Gorasp2	INSIDE	1.645	3.840	509.53	1956.67	6.318	440.59	2783.49
A_68_P23318268	chr4:133130953-133130997	NM_172876:315	Gpatch3	INSIDE	1.519	3.239	1150.61	3726.47	4.921	1036.16	5098.82
A_68_P28080239	chr11:102417477-102417521	NM_001159492:-26	Gpatch8	PROMOTER	2.229	6.312	7420.54	46836.99	14.069	5185.46	72952.94
A_68_P29329250	chr13:112279384-112279428	NM_001122963:843	Gpbp1	INSIDE	2.680	12.969	18361.71	238134.20	34.760	9847.22	342287.80
A_68_P20451399	chr1:94728353-94728397	NM_016696:112	Gpc1	INSIDE	2.015	7.014	1900.79	13332.30	14.136	1221.02	17260.24
A_68_P20451406	chr1:94729272-94729316	NM_016696:1032	Gpc1	INSIDE	1.884	4.338	2033.27	8819.41	8.171	1598.45	13060.72
A_68_P32383369	chrX:49967083-49967127	NM_016697:47	Gpc3	INSIDE	2.146	3.528	4704.13	16596.20	7.570	2240.78	16963.39
A_68_P32380519	chrX:49518713-49518757	NM_008150:-634	Gpc4	PROMOTER	2.000	3.934	2022.85	7957.44	7.869	1423.61	11202.84
A_68_P32380511	chrX:49517673-49517717	NM_008150:406	Gpc4	INSIDE	1.733	3.289	1340.52	4408.65	5.699	855.74	4877.06
A_68_P29928904	chr14:117325550-117325594	NM_001079844:1036	Gpc6	INSIDE	1.719	2.874	1535.27	4413.00	4.941	1284.53	6347.51
A_68_P26847149	chr9:114843024-114843068	NM_175380:59	Gpd1l	INSIDE	2.074	4.714	2036.36	9598.68	9.776	1631.58	15950.71
A_68_P32805174	chrX:162677268-162677312	NM_001177961:416	Gpm6b	INSIDE	1.765	1.759	1334.09	2347.25	3.106	749.12	2326.47
A_68_P25736450	chr8:28196606-28196651	NM_054044:316	Gpr124	INSIDE	2.545	10.594	3452.96	36580.26	26.957	2485.27	66994.82
A_68_P23681258	chr5:50449746-50449790	NM_133911:467	Gpr125	INSIDE	1.910	2.700	4056.12	10949.92	5.155	3147.75	16226.96
A_68_P23681262	chr5:50450445-50450489	NM_133911:-231	Gpr125	PROMOTER	1.757	7.745	1505.05	11656.69	13.611	1198.64	16314.77
A_68_P23681257	chr5:50449672-50449716	NM_133911:541	Gpr125	INSIDE	1.753	3.106	6380.11	19814.98	5.445	5137.68	27974.89
A_68_P31936825	chr19:7016840-7016884	NM_001177361:78	Gpr137	INSIDE	2.355	1.625	788.65	1281.49	3.826	596.15	2280.83
A_68_P29569514	chr14:45839639-45839683	NM_027518:269	Gpr137c	INSIDE	1.625	4.403	1455.02	6406.46	7.154	1082.09	7741.07
A_68_P22030511	chr3:30755072-30755116	NM_001134385:223	Gpr160	INSIDE	1.601	4.571	796.20	3639.25	7.317	677.92	4960.23
A_68_P21563665	chr2:118199048-118199092	NM_201367:85	Gpr176	INSIDE	1.661	2.525	975.61	2463.34	4.195	758.84	3183.08
A_68_P24863969	chr6:134820135-134820179	NM_001167697:17721	Gpr19	INSIDE	1.927	4.853	539.59	2618.65	9.351	411.50	3847.75
A_68_P20644877	chr1:138156885-138156929	NM_001101516:544	Gpr25	INSIDE	1.608	1.686	764.98	1289.80	2.712	648.05	1757.37
A_68_P23316365	chr4:132768551-132768595	NM_008154:-121	Gpr3	DIVERGENT_PROMOTER	1.597	4.311	3132.85	13506.40	6.884	2790.16	19208.65
A_68_P32454735	chrX:68917753-68917797	NM_010340:833	Gpr50	INSIDE	1.609	2.705	1139.03	3080.77	4.352	599.11	2607.06
A_68_P26803570	chr9:106367374-106367418	NM_001159652:875	Gpr62	INSIDE	1.735	4.565	756.92	3455.63	7.923	673.99	5340.17
A_68_P28684687	chr12:102146364-102146408	NM_001177674:-8652	Gpr68	PROMOTER	1.809	3.695	1650.80	6100.38	6.684	1533.07	10246.92
A_68_P22448215	chr3:115955466-115955511	NM_022427:914	Gpr88	INSIDE	1.726	2.313	6031.43	13952.53	3.992	4318.84	17240.68
A_68_P24864944	chr6:135015833-135015878	NM_181444:176	Gprc5a	INSIDE	2.010	8.465	1809.30	15316.48	17.014	1441.62	24527.37
A_68_P24486168	chr6:59376435-59376479	NM_183183:-172	Gprin3	PROMOTER	1.660	2.105	1615.90	3401.38	3.494	1375.65	4807.21
A_68_P21077785	chr2:26171010-26171055	NM_001199146:-20	Gpsm1	PROMOTER	1.666	9.846	2502.21	24637.89	16.405	2243.11	36797.72
A_68_P30490489	chr15:101062456-101062514	NM_019518:7847	Grasp	INSIDE	1.877	9.892	1964.82	19435.55	18.567	1397.98	25956.94
A_68_P30490425	chr15:101054602-101054646	NM_019518:-13	Grasp	PROMOTER	1.830	1.605	841.61	1350.63	2.937	635.77	1867.57
A_68_P27594327	chr11:11937393-11937437	NM_001177629:-9440	Grb10	PROMOTER	2.580	7.830	15526.51	121565.50	20.197	11291.17	228048.70
A_68_P21536555	chr2:113598914-113598958	NM_011824:-131	Grem1	PROMOTER	1.910	6.201	10172.87	63086.12	11.843	7495.12	88766.02
A_68_P24501242	chr6:63207663-63207707	NM_008167:834	Grid2	INSIDE	1.577	4.696	1727.57	8111.93	7.405	1561.71	11564.15
A_68_P24156243	chr5:144131687-144131731	NM_133355:6122	Grid2ip	INSIDE	1.624	1.924	4737.74	9114.19	3.124	4028.32	12585.02
A_68_P27127187	chr10:49502783-49502839	NM_001111268:5750	Grik2	INSIDE	1.504	2.794	331.81	927.16	4.203	255.99	1075.91
A_68_P25007878	chr7:25800603-25800647	NM_008168:56764	Grik5	INSIDE	1.608	4.820	2215.50	10677.62	7.749	1490.88	11552.75
A_68_P30540368	chr16:9995820-9995865	NM_008170:-3216	Grin2a	PROMOTER	2.085	12.926	6410.38	82858.78	26.947	4763.33	128356.60
A_68_P28152301	chr11:115111293-115111337	NM_010350:17243	Grin2c	INSIDE	1.855	4.543	821.35	3731.26	8.426	625.37	5269.14
A_68_P28152414	chr11:115126813-115126857	NM_010350:1723	Grin2c	INSIDE	1.504	1.820	7588.84	13811.37	2.738	5649.71	15466.21
A_68_P25095389	chr7:53088549-53088593	NM_008172:33481	Grin2d	INSIDE	1.835	6.204	1118.06	6936.44	11.385	875.81	9971.39

ProbeName	Target position of probe on CpG island microarray	TargetID	GeneSymbol	CpG island Description	Ratio of relative methylation (TiO_2-NP/Vehicle)	Sham group			TiO_2-H group		
						Relative methylation (Cy5/Cy3)	Cy3 signal (Input)	Cy5 signal (MeDIP)	Relative methylation (Cy5/Cy3)	Cy3 signal (Input)	Cy5 signal (MeDIP)
A_68_P26622301	chr9:71333539-71333583	NM_001164793:-795	Grinl1a	PROMOTER	1.980	2.353	1200.03	2824.26	4.660	1053.28	4908.56
A_68_P31116177	chr17:27552104-27552148	NM_001013385:88123	Grm4	DOWNSTREAM	2.139	12.359	2346.81	29005.04	26.430	1264.59	33423.64
A_68_P25301151	chr7:95278746-95278790	NM_001081414:546091	Grm5	INSIDE	1.519	2.111	1277.37	2695.95	3.206	1012.31	3245.16
A_68_P26220483	chr8:123012034-123012078	NM_198671:-709	Gse1	PROMOTER	1.762	4.405	1205.27	5309.66	7.762	902.80	7007.95
A_68_P26220480	chr8:123011650-123011694	NM_198671:-1093	Gse1	PROMOTER	1.513	2.779	968.75	2692.41	4.206	807.18	3395.13
A_68_P26220858	chr8:123061951-123061995	NM_001145896:664	Gse1	INSIDE	1.510	3.980	1269.37	5052.43	6.011	964.46	5796.91
A_68_P25498532	chr7:133225682-133225726	NM_001101488:221	Gsg1l	INSIDE	1.792	5.769	15276.74	88131.47	10.340	11739.08	121378.90
A_68_P25498529	chr7:133225199-133225243	NM_001101488:705	Gsg1l	INSIDE	1.629	1.489	2851.67	4247.28	2.426	2298.74	5577.07
A_68_P30547082	chr16:11253796-11253840	NM_001130008:600	Gspt1	INSIDE	1.838	1.881	1209.07	2273.78	3.456	1042.67	3603.49
A_68_P32539664	chrX:91881437-91881481	NM_008179:51	Gspt2	INSIDE	1.728	1.532	1092.11	1672.96	2.647	546.98	1447.80
A_68_P25770033	chr8:34764110-34764154	NM_010344:423	Gsr	INSIDE	2.372	5.357	3869.14	20727.80	12.708	3194.58	40595.16
A_68_P24757354	chr6:113026958-113027002	NR_027010:258	Gt(ROSA)26Sor	INSIDE	1.508	2.265	1639.14	3713.15	3.417	1379.95	4714.75
A_68_P24112574	chr5:134790846-134790890	NM_001080746:-252	Gtf2i	PROMOTER	1.536	2.895	1190.75	3446.85	4.446	1021.12	4539.79
A_68_P25496222	chr7:132850904-132850948	NM_207239:276	Gtf3c1	INSIDE	1.634	1.799	3778.37	6797.00	2.940	3048.35	8961.42
A_68_P21093574	chr2:28695671-28695715	NM_001166033:188	Gtf3c4	INSIDE	1.831	6.435	1088.97	7007.60	11.785	913.34	10763.79
A_68_P27083091	chr10:39977457-39977501	NM_026113:-7	Gtf3c6	PROMOTER	1.602	3.173	3397.42	10778.73	5.083	2673.04	13586.99
A_68_P30367927	chr15:79521233-79521289	NM_013818:-65	Gtpbp1	DIVERGENT_PROMOTER	1.613	3.259	2903.88	9462.94	5.255	2416.36	12697.28
A_68_P31205409	chr17:46297933-46297979	NM_001145979:-24	Gtpbp2	DIVERGENT_PROMOTER	1.761	4.260	3135.28	13356.36	7.504	2415.30	18123.83
A_68_P23977812	chr5:110535984-110536028	NM_145147:1210	Gtpbp6	INSIDE	1.567	2.103	995.06	2092.77	3.295	788.22	2597.31
A_68_P23778454	chr5:69948402-69948446	NM_172711:244	Guf1	INSIDE	1.848	11.820	651.41	7699.92	21.844	469.40	10253.48
A_68_P32507212	chrX:83022021-83022065	NM_008194:116	Gyk	INSIDE	1.620	5.178	30351.68	157156.00	8.389	20975.09	175956.90
A_68_P30363898	chr15:78858851-78858898	NM_008197:233	H1f0	INSIDE	1.637	1.917	1289.33	2471.43	3.139	1051.84	3301.42
A_68_P30363892	chr15:78858298-78858342	NM_008197:-321	H1f0	PROMOTER	1.697	1.440	972.76	1400.49	2.443	844.95	2064.48
A_68_P29059430	chr13:56236999-56237043	NM_001159513:-109	H2afy	PROMOTER	1.727	2.959	648.32	1918.32	5.110	510.41	2608.02
A_68_P27182287	chr10:61246439-61246483	NM_207000:152	H2afy2	INSIDE	1.608	3.190	2241.58	7150.70	5.131	1689.84	8670.31
A_68_P31162404	chr17:36221085-36221129	NM_008199:88	H2-Bl	INSIDE	1.904	2.502	2933.75	7341.23	4.765	2115.03	10078.02
A_68_P31158869	chr17:35457566-35457610	NM_010390:86	H2-Q1	INSIDE	2.218	3.214	2869.99	9224.70	7.128	2234.57	15927.74
A_68_P20877951	chr1:182742690-182742734	NM_008210:1022	H3f3a	INSIDE	1.879	2.919	2269.28	6623.75	5.485	1821.43	9991.04
A_68_P23403797	chr4:149383165-149383209	NM_173371:-54	H6pd	PROMOTER	1.584	6.988	3001.40	20974.56	11.069	2237.16	24763.46
A_68_P27111732	chr10:45297581-45297625	NM_172473:-32	Hace1	PROMOTER	1.900	2.556	1049.71	2683.25	4.857	871.12	4231.03
A_68_P22531299	chr3:130975017-130975061	NM_008212:-83	Hadh	PROMOTER	2.737	10.628	15469.42	164409.20	29.089	9081.46	264171.40
A_68_P27827804	chr11:57650530-57650574	NM_008213:-4903	Hand1	PROMOTER	1.522	2.081	2280.25	4744.95	3.166	1619.94	5128.94
A_68_P22311352	chr3:87826975-87827019	NM_022031:4437	Hapln2	INSIDE	1.552	1.404	4589.08	6441.50	2.178	3396.94	7399.58
A_68_P21576252	chr2:120435142-120435186	NM_025475:-7	Haus2	DIVERGENT_PROMOTER	2.082	2.017	670.15	1351.97	4.200	563.13	2365.28
A_68_P28334080	chr12:32634604-32634648	NM_153198:774	Hbp1	INSIDE	1.671	4.411	447.47	1973.84	7.371	388.89	2866.41
A_68_P28334082	chr12:32634899-32634943	NM_153198:480	Hbp1	INSIDE	1.721	1.569	1598.61	2508.49	2.700	1307.28	3530.14
A_68_P27278520	chr10:79187527-79187571	NM_008226:8170	Hcn2	INSIDE	2.081	10.178	1515.88	15429.29	21.186	1116.24	23649.08
A_68_P26550030	chr9:58670732-58670776	NM_001081192:-564	Hcn4	PROMOTER	1.738	2.314	463.53	1072.60	4.022	400.76	1611.96
A_68_P23300612	chr4:129816147-129816191	NM_001163027:-373	Hcrtr1	PROMOTER	1.503	1.713	3407.08	5835.44	2.575	2819.48	7260.01
A_68_P26648623	chr9:76171335-76171379	NM_198962:29	Hcrtr2	INSIDE	1.522	2.097	1253.70	2629.01	3.192	1031.47	3292.40
A_68_P31253727	chr17:56219020-56219064	NM_008233:-37	Hdgfrp2	DIVERGENT_PROMOTER	1.691	2.480	1095.39	2716.84	4.194	842.04	3531.18
A_68_P25274817	chr7:89079514-89079558	NM_013886:-191	Hdgfrp3	PROMOTER	2.116	6.283	3031.88	19048.71	13.295	2230.02	29648.19
A_68_P25274814	chr7:89079181-89079225	NM_013886:143	Hdgfrp3	INSIDE	1.515	3.405	2741.20	9335.16	5.161	2297.83	11858.66
A_68_P20455835	chr1:95374821-95374865	NM_133808:543	Hdlbp	INSIDE	2.286	5.679	1908.50	10839.24	12.985	1421.38	18456.94
A_68_P24133861	chr5:139626021-139626065	NM_001081265:-134	Heatr2	PROMOTER	1.923	5.203	17869.78	92974.62	10.007	11113.70	111213.60
A_68_P26039225	chr8:90661714-90661758	NM_172757:-47	Heatr3	PROMOTER	1.633	2.504	2413.44	6042.63	4.088	2631.80	10757.83
A_68_P28432636	chr12:53071968-53072012	NM_177171:318	Heatr5a	INSIDE	1.972	2.436	3384.93	8244.91	4.803	2605.10	12511.36

ProbeName	Target position of probe on CpG island microarray	TargetID	GeneSymbol	CpG island Description	Ratio of relative methylation (TiO_2-NP/Vehicle)	Sham group			TiO_2-H group		
						Relative methylation (Cy5/Cy3)	Cy3 signal (Input)	Cy5 signal (MeDIP)	Relative methylation (Cy5/Cy3)	Cy3 signal (Input)	Cy5 signal (MeDIP)
A_68_P30659187	chr16:33684576-33684620	NM_175256:47	Heg1	INSIDE	1.773	6.285	2971.27	18674.29	11.145	2361.20	26314.81
A_68_P23931499	chr5:101227260-101227304	NM_001081107:337	Helq	INSIDE	2.145	2.896	2065.63	5982.98	6.212	1669.15	10368.86
A_68_P26077217	chr8:96910495-96910539	NM_022331:117	Herpud1	INSIDE	1.689	5.907	2741.31	16193.24	9.979	2088.27	20837.91
A_68_P30639681	chr16:30070804-30070848	NM_008235:5384	Hes1	DOWNSTREAM	1.515	1.520	4721.65	7178.51	2.303	3831.89	8826.15
A_68_P23416903	chr4:151532635-151532679	NM_008236:-319	Hes2	DIVERGENT_PROMOTER	1.782	2.676	1085.29	2904.61	4.769	811.53	3870.50
A_68_P27893219	chr11:68936458-68936502	NM_033041:2526	Hes7	INSIDE	2.031	4.211	3734.43	15726.52	8.552	2629.86	22489.56
A_68_P28083428	chr11:102977758-102977802	NM_138753:142	Hexim1	INSIDE	1.594	1.775	3158.02	5605.34	2.830	2549.73	7215.73
A_68_P28083517	chr11:102994122-102994166	NM_001130516:-508	Hexim2	PROMOTER	1.615	3.255	2038.59	6635.67	5.256	1583.56	8323.43
A_68_P27041764	chr10:30562697-30562741	NM_013904:-129	Hey2	PROMOTER	1.654	2.910	517.85	1506.71	4.812	446.11	2146.52
A_68_P28184739	chr11:120329033-120329077	NM_001159328:106	Hgs	INSIDE	1.540	1.893	2172.26	4112.60	2.915	1783.07	5197.83
A_68_P32095553	chr19:37509433-37509477	NM_008245:124	Hhex	INSIDE	1.772	3.525	3556.33	12536.69	6.247	2918.16	18228.33
A_68_P22450560	chr3:116365543-116365587	NM_008246:31	Hiat1	INSIDE	2.258	4.123	1719.69	7090.76	9.309	1240.40	11546.41
A_68_P27924602	chr11:74984051-74984095	NM_010430:-315	Hic1	DIVERGENT_PROMOTER	2.102	5.021	1961.59	9850.01	10.557	1570.80	16583.45
A_68_P27924590	chr11:74982634-74982678	NM_001098203:-1002	Hic1	PROMOTER	1.631	2.257	1046.15	2361.43	3.682	817.05	3008.34
A_68_P30572653	chr16:17233627-17233671	NM_178922:-31	Hic2	PROMOTER	1.601	4.792	2127.46	10194.96	7.674	1775.80	13627.26
A_68_P28540462	chr12:75009562-75009606	NM_010431:731	Hif1a	INSIDE	1.717	3.450	1890.74	6523.81	5.924	1556.06	9217.74
A_68_P32132967	chr19:44637357-44637401	NM_176958:35	Hif1an	INSIDE	1.540	3.012	677.07	2039.17	4.638	597.35	2770.71
A_68_P27040510	chr10:30337995-30338039	NM_025798:156	Hint3	INSIDE	1.962	3.176	1065.95	3384.99	6.229	904.96	5637.29
A_68_P24116580	chr5:135964648-135964692	NM_146001:56322	Hip1	INSIDE	2.140	5.409	3084.15	16682.53	11.576	2262.33	26188.17
A_68_P21489528	chr2:104334070-104334114	NM_001145824:554	Hipk3	INSIDE	1.703	1.668	1201.67	2004.36	2.840	1086.32	3085.18
A_68_P26954148	chr10:13684931-13684975	NM_010437:-1232	Hivep2	PROMOTER	1.727	4.565	2223.13	10147.80	7.885	1782.67	14056.88
A_68_P26954155	chr10:13685730-13685774	NM_010437:-432	Hivep2	PROMOTER	1.723	4.479	4379.25	19614.59	7.715	3569.73	27541.79
A_68_P26954150	chr10:13685112-13685158	NM_010437:-1050	Hivep2	PROMOTER	1.567	2.407	1916.49	4612.93	3.773	1407.57	5310.50
A_68_P27185080	chr10:61732562-61732606	NM_001146100:70585	Hk1	INSIDE	2.269	10.646	2773.56	29527.97	24.158	2096.18	50638.62
A_68_P27185081	chr10:61732662-61732706	NM_001146100:70485	Hk1	INSIDE	1.940	4.623	1350.81	6244.16	8.967	1020.86	9154.55
A_68_P21978078	chr3:19957968-19958012	NM_144959:180	Hltf	INSIDE	1.951	4.988	7603.38	37929.07	9.733	5994.60	58346.11
A_68_P20897125	chr1:186551788-186551832	NM_008250:4562	Hlx	INSIDE	1.687	2.130	1138.59	2425.27	3.593	783.07	2813.61
A_68_P27289055	chr10:80813338-80813382	NM_001163166:-158	Hmg20b	DIVERGENT_PROMOTER	1.916	3.014	4739.46	14285.52	5.774	3859.24	22283.48
A_68_P27289029	chr10:80809838-80809882	NM_001163165:3311	Hmg20b	INSIDE	1.565	7.179	2823.24	20269.19	11.233	1862.01	20916.80
A_68_P31117105	chr17:27693117-27693161	NM_001025427:-380	Hmga1	PROMOTER	2.385	5.460	803.42	4386.29	13.019	638.81	8316.76
A_68_P31117117	chr17:27694485-27694529	NM_001166476:909	Hmga1-rs1	INSIDE	1.935	3.854	4656.86	17945.86	7.456	3396.56	25324.85
A_68_P31117120	chr17:27694784-27694828	NM_001166476:1209	Hmga1-rs1	INSIDE	1.546	1.715	1484.34	2545.31	2.651	1267.24	3358.95
A_68_P27497678	chr10:119913602-119913646	NM_010441:367	Hmga2	INSIDE	2.027	10.620	1669.67	17732.25	21.526	1108.46	23860.40
A_68_P30991207	chr16:96348653-96348697	NM_008251:658	Hmgn1	INSIDE	1.968	2.602	5404.50	14060.23	5.121	4000.73	20487.47
A_68_P25971363	chr8:77517838-77517882	NM_178017:259	Hmgxb4	INSIDE	1.504	3.776	1144.27	4320.45	5.679	1081.27	6140.09
A_68_P25528898	chr7:138685827-138685871	NM_008257:-628	Hmx3	PROMOTER	1.521	3.334	310.68	1035.88	5.071	297.74	1509.99
A_68_P25528895	chr7:138685558-138685602	NM_008257:-896	Hmx3	PROMOTER	1.515	2.685	721.58	1937.75	4.069	630.15	2564.25
A_68_P28153958	chr11:115374907-115374951	NM_008258:756	Hn1	INSIDE	2.126	17.486	989.68	17305.50	37.177	683.54	25411.86
A_68_P29071664	chr13:58228883-58228927	NM_029872:1013	Hnrnpa0	INSIDE	1.504	3.383	2280.21	7714.19	5.090	1800.45	9163.93
A_68_P29604760	chr14:52723420-52723464	NM_001170981:261	Hnrnpc	INSIDE	1.689	2.918	2786.63	8130.06	4.928	2129.02	10490.78
A_68_P23927140	chr5:100408133-100408177	NM_001077265:-197	Hnrnpd	PROMOTER	1.612	1.778	1719.81	3057.63	2.866	1408.50	4036.58
A_68_P24783935	chr6:117857266-117857310	NM_001166428:-533	Hnrnpf	PROMOTER	2.132	4.289	3396.15	14566.89	9.143	2523.63	23072.58
A_68_P24784010	chr6:117867385-117867429	NM_001166427:95	Hnrnpf	INSIDE	2.009	4.788	2320.95	11113.62	9.618	1897.26	18248.16
A_68_P24784006	chr6:117866996-117867040	NM_001166427:-293	Hnrnpf	PROMOTER	1.815	3.705	2777.20	10289.67	6.726	2018.60	13576.17
A_68_P24783941	chr6:117857819-117857863	NM_001166427:-9471	Hnrnpf	PROMOTER	1.588	6.228	909.62	5664.78	9.888	695.15	6873.69
A_68_P27788199	chr11:50190689-50190733	NM_021510:-510	Hnrnph1	PROMOTER	2.027	5.389	4503.43	24268.11	10.925	3367.63	36792.22
A_68_P27189032	chr10:62486795-62486839	NM_001079824:-219	Hnrnph3	DIVERGENT_PROMOTER	1.508	2.966	1117.74	3315.50	4.472	978.50	4376.19

ProbeName	Target position of probe on CpG island microarray	TargetID	GeneSymbol	CpG island Description	Ratio of relative methylation (TiO_2-NP/Vehicle)	Sham group			TiO_2-H group		
						Relative methylation (Cy5/Cy3)	Cy3 signal (Input)	Cy5 signal (MeDIP)	Relative methylation (Cy5/Cy3)	Cy3 signal (Input)	Cy5 signal (MeDIP)
A_68_P25023658	chr7:29594290-29594344	NM_177301:-1592	Hnrnpl	PROMOTER	1.707	4.797	1710.77	8206.30	8.186	1221.68	10000.86
A_68_P31150132	chr17:33822462-33822506	NM_001109913:-81	Hnrnpm	PROMOTER	1.528	3.972	1968.27	7818.47	6.071	1520.49	9230.34
A_68_P20863428	chr1:180267506-180267550	NM_016805:387	Hnrnpu	INSIDE	1.530	1.492	616.38	919.53	2.283	550.33	1256.38
A_68_P23927384	chr5:100467950-100467994	NM_016690:269	Hnrpdl	INSIDE	2.275	5.958	5826.38	34711.57	13.556	4005.52	54299.33
A_68_P23927386	chr5:100468112-100468156	NM_016690:107	Hnrpdl	INSIDE	1.525	1.347	2453.41	3303.61	2.053	2090.79	4292.30
A_68_P31381048	chr17:80461571-80461615	NM_144802:82	Hnrpll	INSIDE	1.615	1.950	1829.96	3569.05	3.150	1467.27	4621.90
A_68_P29614701	chr14:55482992-55483036	NM_001177705:-19	Homez	PROMOTER	1.967	3.067	3193.56	9795.99	6.033	2541.20	15329.78
A_68_P25730819	chr8:27229628-27229672	NM_207659:46	Hook3	INSIDE	1.613	2.939	1362.79	4005.67	4.742	1111.00	5268.23
A_68_P24449298	chr6:52184077-52184121	NM_008263:840	Hoxa10	INSIDE	1.716	5.558	1152.59	6405.71	9.538	928.05	8852.16
A_68_P24449403	chr6:52196333-52196377	NM_010450:-588	Hoxa11	PROMOTER	1.582	2.100	1809.28	3799.64	3.323	1412.21	4692.87
A_68_P24449533	chr6:52211079-52211123	NM_008264:-226	Hoxa13	PROMOTER	2.290	6.551	2969.10	19450.39	15.000	2271.12	34065.81
A_68_P24449528	chr6:52210288-52210332	NM_008264:564	Hoxa13	INSIDE	1.753	7.221	591.44	4270.79	12.660	478.78	6061.55
A_68_P24448786	chr6:52122729-52122773	NM_010452:40316	Hoxa3	INSIDE	1.949	3.590	2816.44	10111.45	6.999	2194.43	15358.72
A_68_P24449229	chr6:52175914-52175958	NM_010456:1433	Hoxa9	INSIDE	1.798	1.819	1546.09	2811.78	3.269	1279.83	4184.40
A_68_P28043617	chr11:96166483-96166527	NM_008268:1679	Hoxb5	INSIDE	2.377	2.543	7392.87	18798.29	6.045	5490.85	33193.03
A_68_P28043336	chr11:96132754-96132798	NM_008270:133	Hoxb9	INSIDE	1.762	2.702	1478.87	3996.34	4.761	1167.08	5556.00
A_68_P30500966	chr15:102785392-102785436	NM_001024842:458	Hoxc11	INSIDE	1.680	1.528	651.42	995.66	2.568	617.42	1585.58
A_68_P30500682	chr15:102752309-102752353	NM_010464:769	Hoxc13	INSIDE	1.950	2.090	437.20	913.94	4.076	393.05	1601.97
A_68_P21339341	chr2:74591156-74591200	NM_010467:-9858	Hoxd1	PROMOTER	1.830	4.738	3235.33	15329.05	8.673	2385.13	20685.77
A_68_P21339417	chr2:74601653-74601697	NM_010467:638	Hoxd1	INSIDE	1.542	2.014	1822.69	3670.23	3.104	1498.07	4650.35
A_68_P21339240	chr2:74578542-74578586	NM_010468:28515	Hoxd3	INSIDE	1.809	2.036	1647.74	3355.21	3.683	1258.03	4633.65
A_68_P21339006	chr2:74550322-74550366	NM_010468:295	Hoxd3	INSIDE	1.619	2.232	1636.44	3653.03	3.613	1498.00	5412.55
A_68_P21338948	chr2:74542744-74542788	NM_008276:-779	Hoxd8	PROMOTER	2.077	4.821	1280.62	6174.19	10.013	960.79	9620.80
A_68_P23344531	chr4:137772915-137772959	NM_001122897:-109	Hp1bp3	PROMOTER	2.169	16.862	7205.96	121509.40	36.582	4534.79	165892.40
A_68_P23295046	chr4:128798355-128798400	NM_010471:158	Hpca	INSIDE	1.501	5.295	891.80	4722.32	7.947	799.18	6350.88
A_68_P23295047	chr4:128798459-128798503	NM_010471:55	Hpca	INSIDE	1.541	1.416	875.12	1239.51	2.183	803.08	1753.03
A_68_P23261234	chr4:122866488-122866532	NM_174998:5764	Hpcal4	INSIDE	1.844	7.354	10025.79	73731.62	13.561	6913.90	93759.12
A_68_P23261233	chr4:122866390-122866434	NM_174998:5666	Hpcal4	INSIDE	1.647	2.404	598.81	1439.31	3.959	475.29	1881.44
A_68_P23261232	chr4:122866227-122866271	NM_174998:5502	Hpcal4	INSIDE	1.562	3.900	3399.56	13256.62	6.092	2528.50	15404.63
A_68_P32385147	chrX:50341550-50341594	NM_013556:318	Hprt	INSIDE	2.451	3.348	1957.19	6553.16	8.208	1013.19	8316.29
A_68_P32385145	chrX:50341393-50341437	NM_013556:160	Hprt	INSIDE	1.713	5.394	1232.71	6648.69	9.240	713.22	6590.19
A_68_P29702031	chr14:70953904-70953948	NM_021877:64	Hr	INSIDE	1.562	1.626	6204.00	10086.09	2.539	4768.71	12108.55
A_68_P29048046	chr13:54287380-54287425	NM_008286:-95	Hrh2	PROMOTER	1.521	2.939	1503.11	4418.07	4.470	943.46	4217.09
A_68_P24025038	chr5:118620106-118620150	NM_007545:356	Hrk	INSIDE	1.566	2.149	1563.84	3361.45	3.366	1428.17	4806.89
A_68_P25474216	chr7:128643864-128643910	NM_001081327:108077	Hs3st2	INSIDE	1.721	7.977	1898.20	15141.20	13.728	1434.35	19691.47
A_68_P27862290	chr11:63734698-63734742	NM_018805:1066	Hs3st3b1	INSIDE	2.430	7.416	3406.97	25266.90	18.019	2541.56	45797.47
A_68_P27862275	chr11:63732951-63732995	NM_018805:2814	Hs3st3b1	INSIDE	1.625	4.099	641.44	2629.45	6.661	508.06	3384.07
A_68_P27065106	chr10:36226802-36226846	NM_001081208:212	Hs3st5	INSIDE	1.660	1.708	1289.14	2202.06	2.836	1048.96	2974.51
A_68_P20146150	chr1:36125563-36125607	NM_015818:340	Hs6st1	INSIDE	1.638	4.027	2473.98	9963.62	6.599	2193.65	14474.81
A_68_P32378028	chrX:49034553-49034597	NM_015819:-920	Hs6st2	PROMOTER	1.818	2.100	1353.54	2842.92	3.817	739.84	2824.33
A_68_P27995284	chr11:87452873-87452917	NM_001045527:22229	Hsf5	INSIDE	1.658	5.403	2219.32	11991.18	8.961	1829.83	16397.01
A_68_P27995147	chr11:87430388-87430432	NM_001045527:-255	Hsf5	PROMOTER	1.519	4.025	862.35	3470.63	6.115	735.11	4494.96
A_68_P28739824	chr12:111934703-111934747	NM_010480:-119	Hsp90aa1	PROMOTER	1.917	11.944	1915.98	22884.25	22.896	1402.99	32122.28
A_68_P28553818	chr12:77505144-77505188	NM_008301:-190	Hspa2	PROMOTER	1.502	3.614	2028.44	7330.59	5.430	1623.90	8817.36
A_68_P27804074	chr11:53113101-53113145	NM_008300:859	Hspa4	INSIDE	1.982	3.316	6412.82	21267.40	6.574	5187.91	34105.70
A_68_P22081218	chr3:40549756-40549800	NM_011020:244	Hspa4l	INSIDE	1.701	3.647	627.09	2287.30	6.203	700.15	4342.90
A_68_P22081210	chr3:40548895-40548939	NM_011020:-618	Hspa4l	PROMOTER	1.674	3.368	1544.20	5200.55	5.638	1142.24	6439.66

ProbeName	Target position of probe on CpG island microarray	TargetID	GeneSymbol	CpG island Description	Ratio of relative methylation (TiO_2-NP/Vehicle)	Sham group			TiO_2-H group		
						Relative methylation (Cy5/Cy3)	Cy3 signal (Input)	Cy5 signal (MeDIP)	Relative methylation (Cy5/Cy3)	Cy3 signal (Input)	Cy5 signal (MeDIP)
A_68_P20240346	chr1:55145446-55145490	NM_008303:477	Hspe1	INSIDE	1.976	4.193	1419.21	5951.07	8.284	985.84	8167.04
A_68_P23339951	chr4:137024980-137025024	NM_008305:285	Hspg2	INSIDE	1.676	1.544	880.05	1359.16	2.588	737.14	1907.69
A_68_P25525511	chr7:138079707-138079751	NM_019564:12	Htra1	INSIDE	1.505	1.501	3874.53	5817.41	2.260	3128.33	7070.53
A_68_P23602238	chr5:36022053-36022097	NM_030127:357	Htra3	INSIDE	1.620	5.000	2167.98	10840.38	8.098	1422.78	11521.52
A_68_P26345150	chr9:20836502-20836546	NM_008319:43	Icam5	INSIDE	1.982	4.277	4438.10	18981.33	8.477	3506.13	29722.46
A_68_P26658192	chr9:77961262-77961306	NM_019987:240	Ick	INSIDE	2.003	10.295	19505.51	200801.00	20.621	11145.86	229837.30
A_68_P28153287	chr11:115264936-115264980	NM_026729:-121	Ict1	PROMOTER	1.692	2.679	1149.83	3080.74	4.535	929.72	4216.00
A_68_P28293506	chr12:25781121-25781165	NM_010496:-185	Id2	PROMOTER	1.559	2.315	4038.43	9349.15	3.609	3161.10	11406.85
A_68_P23332310	chr4:135700553-135700600	NM_008321:840	Id3	INSIDE	2.111	4.020	265.91	1068.88	8.487	194.28	1648.92
A_68_P25264893	chr7:87260180-87260224	NM_173011:34	Idh2	INSIDE	2.001	5.771	760.03	4386.16	11.547	694.19	8015.83
A_68_P21103442	chr2:30328841-30328885	NM_030244:857	Ier5l	INSIDE	2.152	11.581	3520.79	40775.03	24.925	2959.60	73769.32
A_68_P30963193	chr16:91485518-91485562	NM_010508:81	Ifnar1	INSIDE	1.633	1.748	1050.16	1835.82	2.855	791.62	2260.23
A_68_P28041915	chr11:95866931-95866976	NM_009951:305	Igf2bp1	INSIDE	2.608	8.882	6026.23	53526.77	23.168	4126.34	95599.91
A_68_P30596436	chr16:22163233-22163277	NM_183029:118	Igf2bp2	INSIDE	2.303	7.747	847.67	6567.12	17.840	768.85	13716.50
A_68_P30596432	chr16:22162719-22162763	NM_183029:632	Igf2bp2	INSIDE	1.655	5.012	979.30	4907.86	8.295	862.69	7155.78
A_68_P28060084	chr11:98902871-98902915	NM_010517:319	Igfbp4	INSIDE	1.505	1.812	1193.02	2161.77	2.726	1051.81	2867.48
A_68_P26375208	chr9:27107222-27107266	NM_001033323:432	Igsf9b	INSIDE	1.771	11.717	1501.62	17594.76	20.756	1142.28	23708.96
A_68_P20351513	chr1:74998432-74998476	NM_010544:-229	Ihh	PROMOTER	1.828	1.985	2593.05	5147.28	3.629	2305.65	8366.79
A_68_P27591949	chr11:11585605-11585649	NM_001025597:-589	Ikzf1	PROMOTER	1.971	6.461	328.40	2121.91	12.737	332.67	4237.04
A_68_P27542462	chr10:128082827-128082871	NM_011772:201	Ikzf4	INSIDE	2.014	6.594	3300.43	21761.88	13.277	2547.16	33818.33
A_68_P20170470	chr1:40323556-40323600	NM_001123382:148	Il1r1	INSIDE	2.563	9.463	1749.87	16559.53	24.254	1125.93	27308.32
A_68_P30621546	chr16:26581967-26582011	NM_134103:198	Il1rap	INSIDE	1.639	5.035	2098.47	10565.95	8.251	1560.42	12874.40
A_68_P25089584	chr7:52095285-52095329	NM_010215:3649	Il4i1	INSIDE	1.751	2.596	1830.05	4751.35	4.547	1424.35	6476.19
A_68_P25495415	chr7:132725071-132725115	NM_001008700:29297	Il4ra	DOWNSTREAM	1.745	4.300	3635.17	15632.42	7.503	2918.89	21900.68
A_68_P29334871	chr13:113254322-113254366	NM_010560:67	Il6st	INSIDE	1.652	5.429	2889.77	15688.11	8.970	2431.44	21808.93
A_68_P22325451	chr3:90280267-90280311	NM_026374:166	Ilf2	INSIDE	1.583	4.404	4144.81	18254.36	6.971	3183.87	22195.81
A_68_P23138267	chr4:98062517-98062561	NM_001005784:22	Inadl	INSIDE	1.588	4.487	849.63	3811.95	7.127	762.97	5437.57
A_68_P28751168	chr12:113838436-113838480	NM_198411:11464	Inf2	INSIDE	1.582	3.465	1310.26	4540.69	5.484	1032.55	5662.21
A_68_P25656224	chr8:11557705-11557749	NM_011919:1661	Ing1	INSIDE	1.608	5.077	2794.35	14186.40	8.162	2248.83	18355.30
A_68_P25842954	chr8:48761152-48761196	NM_023503:-661	Ing2	PROMOTER	1.618	3.119	729.58	2275.67	5.047	620.16	3130.17
A_68_P24288408	chr6:21900000-21900044	NM_023626:408	Ing3	INSIDE	1.658	3.477	6884.72	23938.05	5.766	4668.75	26919.52
A_68_P20457532	chr1:95700895-95700939	NM_025454:375	Ing5	INSIDE	2.724	6.716	2820.91	18944.70	18.295	1637.99	29967.65
A_68_P20457529	chr1:95700443-95700487	NM_025454:-77	Ing5	DIVERGENT_PROMOTER	2.133	9.915	5255.68	52111.82	21.145	2649.65	56025.88
A_68_P20457526	chr1:95700053-95700097	NM_025454:-467	Ing5	DIVERGENT_PROMOTER	1.754	3.519	1095.13	3853.92	6.171	890.34	5494.47
A_68_P20556967	chr1:121317480-121317524	NM_008381:1323	Inhbb	INSIDE	2.337	6.799	1585.18	10777.19	15.888	1233.25	19593.83
A_68_P20556970	chr1:121318089-121318133	NM_008381:715	Inhbb	INSIDE	2.159	5.335	1743.08	9299.56	11.520	1390.23	16015.89
A_68_P20556969	chr1:121317983-121318027	NM_008381:821	Inhbb	INSIDE	1.738	5.199	2087.36	10852.02	9.033	1599.33	14447.15
A_68_P21569910	chr2:119303067-119303111	NM_026574:277	Ino80	INSIDE	2.468	6.138	4882.70	29972.16	15.151	3573.68	54146.25
A_68_P21078500	chr2:26264786-26264830	NM_033134:-100	Inpp5e	PROMOTER	1.525	2.628	4230.13	11117.76	4.007	3630.11	14546.65
A_68_P21078497	chr2:26264502-26264546	NM_033134:184	Inpp5e	INSIDE	1.757	1.686	929.83	1567.59	2.963	841.93	2494.54
A_68_P28452657	chr12:56700590-56700634	NM_020287:709	Insm2	INSIDE	1.655	9.081	1368.34	12425.99	15.026	1009.74	15172.08
A_68_P22310075	chr3:87600813-87600858	NM_011832:-37	Insrr	DIVERGENT_PROMOTER	2.490	5.861	4961.28	29078.26	14.593	3144.29	45884.25
A_68_P26812405	chr9:107905152-107905196	NM_013785:196	Ip6k1	INSIDE	1.529	3.185	897.89	2859.76	4.869	759.91	3700.20
A_68_P25460625	chr7:125999193-125999237	NM_001081446:-74	Iqck	DIVERGENT_PROMOTER	1.614	2.484	2719.29	6755.40	4.010	2190.94	8785.77
A_68_P25268780	chr7:87947830-87947874	NM_016721:365	Iqgap1	INSIDE	1.551	1.594	2098.11	3343.77	2.473	1544.84	3819.74
A_68_P29243355	chr13:96661950-96661994	NM_027711:-95	Iqgap2	PROMOTER	1.865	7.954	4160.49	33091.43	14.837	3280.77	48675.82
A_68_P24804564	chr6:121423714-121423758	NM_001033354:-40	Iqsec3	PROMOTER	1.997	3.206	1771.26	5679.11	6.403	1380.83	8841.48

ProbeName	Target position of probe on CpG island microarray	TargetID	GeneSymbol	CpG island Description	Ratio of relative methylation (TiO_2-NP/Vehicle)	Sham group			TiO_2-H group		
						Relative methylation (Cy5/Cy3)	Cy3 signal (Input)	Cy5 signal (MeDIP)	Relative methylation (Cy5/Cy3)	Cy3 signal (Input)	Cy5 signal (MeDIP)
A_68_P24804128	chr6:121362766-121362810	NM_001033354:60908	Iqsec3	INSIDE	1.579	3.200	3220.17	10305.15	5.052	2257.41	11404.89
A_68_P30452934	chr15:94374073-94374117	NM_029926:4	Irak4	INSIDE	1.548	2.034	1421.64	2891.15	3.149	1041.72	3279.94
A_68_P25837693	chr8:47826718-47826762	NM_008391:1642	Irf2	INSIDE	1.521	3.072	367.43	1128.61	4.671	336.01	1569.53
A_68_P26258433	chr8:129118351-129118402	NM_001164598:-1040	Irf2bp2	PROMOTER	1.843	3.789	2286.39	8663.45	6.982	1790.20	12499.77
A_68_P26258415	chr8:129116306-129116350	NM_001164598:1008	Irf2bp2	INSIDE	1.689	2.066	5891.25	12169.15	3.488	4892.94	17068.30
A_68_P24327113	chr6:29477125-29477169	NM_012057:414	Irf5	INSIDE	2.180	6.727	2168.97	14589.91	14.666	1359.43	19936.89
A_68_P25005398	chr7:25318769-25318814	NM_153134:3125	Irgq	INSIDE	1.923	3.356	458.84	1539.90	6.454	324.51	2094.37
A_68_P25005401	chr7:25319057-25319101	NM_153134:3412	Irgq	INSIDE	1.845	7.348	962.25	7071.03	13.556	824.30	11174.33
A_68_P20391720	chr1:82288083-82288127	NM_010570:-90	Irs1	PROMOTER	1.740	6.741	738.79	4980.22	11.731	618.89	7260.25
A_68_P20391718	chr1:82287606-82287650	NM_010570:386	Irs1	INSIDE	1.668	4.812	2917.59	14038.80	8.028	2754.29	22111.30
A_68_P25652449	chr8:11008424-11008468	NM_001081212:-16	Irs2	PROMOTER	1.665	3.958	982.97	3891.03	6.591	988.57	6515.77
A_68_P29134822	chr13:72768633-72768679	NM_010574:2231	Irx2	INSIDE	1.901	2.271	4470.84	10154.53	4.319	3365.33	14533.82
A_68_P26065137	chr8:94881817-94881861	NM_018826:144	Irx5	INSIDE	1.701	3.067	1410.84	4326.52	5.216	1114.31	5812.43
A_68_P21680284	chr2:139503094-139503138	NM_001126490:-797	Ism1	PROMOTER	1.576	2.196	1282.18	2816.30	3.462	995.39	3446.44
A_68_P24618420	chr6:87788600-87788644	NM_133934:131	Isy1	INSIDE	1.579	2.686	1150.74	3090.41	4.240	1002.82	4252.19
A_68_P25952867	chr8:73118782-73118826	NM_023627:425	Isyna1	INSIDE	1.733	1.577	1487.43	2345.26	2.732	1207.58	3299.13
A_68_P21367543	chr2:79095483-79095527	NM_010576:-78	Itga4	PROMOTER	1.777	3.658	2017.49	7379.30	6.500	1621.43	10538.48
A_68_P26867372	chr9:118515925-118515969	NM_133721:120	Itga9	INSIDE	1.711	3.220	367.80	1184.40	5.510	296.16	1631.77
A_68_P26270749	chr8:131209761-131209805	NM_010578:229	Itgb1	INSIDE	1.757	2.130	3422.29	7289.03	3.743	2642.46	9891.32
A_68_P28693227	chr12:103943323-103943367	NM_172584:-265	Itpk1	PROMOTER	1.728	5.248	811.62	4259.30	9.070	709.53	6435.19
A_68_P21571253	chr2:119569096-119569140	NM_146125:1046	Itpka	INSIDE	2.303	3.874	2504.23	9700.66	8.922	1955.76	17450.11
A_68_P25016218	chr7:28013554-28013598	NM_181593:40	Itpkc	INSIDE	2.074	7.976	939.09	7490.48	16.541	687.48	11371.60
A_68_P25958256	chr8:74203690-74203734	NM_001190830:3431	Jak3	INSIDE	1.983	3.564	823.69	2935.98	7.070	656.49	4641.15
A_68_P25574929	chr7:146132486-146132530	NM_028708:76	Jakmip3	INSIDE	4.012	0.451	881.02	397.57	1.810	713.30	1291.35
A_68_P30925479	chr16:84774664-84774708	NM_023844:319	Jam2	INSIDE	1.508	3.443	838.56	2887.49	5.193	795.58	4131.20
A_68_P28997682	chr13:44829049-44829093	NM_001205043:2431	Jarid2	INSIDE	2.312	8.821	15712.06	138592.40	20.390	9201.23	187610.30
A_68_P28997663	chr13:44826717-44826761	NM_001205043:99	Jarid2	INSIDE	2.028	5.777	5348.99	30900.59	11.717	3961.04	46412.44
A_68_P28997657	chr13:44826068-44826112	NM_001205044:-52	Jarid2	PROMOTER	1.540	2.474	2897.66	7168.55	3.810	2247.25	8561.24
A_68_P28605007	chr12:86940319-86940363	NM_030887:-25	Jdp2	PROMOTER	2.462	7.922	7190.55	56964.74	19.503	5194.50	101306.80
A_68_P24381989	chr6:39155596-39155640	NM_001033430:1154	Jhdm1d	INSIDE	2.359	5.189	5116.70	26550.66	12.238	3987.22	48797.57
A_68_P24382000	chr6:39157044-39157088	NM_001033430:-294	Jhdm1d	PROMOTER	1.774	8.461	8493.65	71863.49	15.006	8119.38	121837.70
A_68_P28530101	chr12:73186989-73187033	NM_001205067:185	Jkamp	INSIDE	2.316	6.814	2167.14	14767.70	15.779	1323.51	20883.40
A_68_P30367903	chr15:79518094-79518138	NM_028792:186	Josd1	INSIDE	1.887	3.055	1146.51	3502.77	5.766	919.95	5304.26
A_68_P26228655	chr8:124277307-124277351	NM_020605:22870	Jph3	INSIDE	1.597	4.386	798.68	3502.79	7.004	595.82	4173.10
A_68_P29616407	chr14:55733626-55733670	NM_177049:2124	Jph4	INSIDE	1.505	3.567	6810.59	24294.32	5.368	4485.51	24077.71
A_68_P25953471	chr8:73223632-73223676	NM_010592:2017	Jund	INSIDE	1.654	4.674	1216.41	5685.67	7.731	1052.44	8136.16
A_68_P28067852	chr11:100258833-100258877	NM_010593:250	Jup	INSIDE	2.193	5.577	3327.06	18556.04	12.233	2328.22	28480.36
A_68_P26348962	chr9:21602957-21603001	NM_145611:12	Kank2	INSIDE	1.893	2.623	1978.53	5190.52	4.966	1544.30	7669.06
A_68_P31150606	chr17:33954625-33954670	NM_030697:7180	Kank3	INSIDE	1.539	2.105	592.68	1247.40	3.239	549.54	1780.05
A_68_P26920438	chr10:7445673-7445717	NM_011835:-103	Katna1	PROMOTER	1.654	1.716	3575.01	6133.04	2.837	2711.27	7691.58
A_68_P25678282	chr8:15027507-15027551	NM_029116:16504	Kbtbd11	INSIDE	1.651	3.122	764.09	2385.72	5.154	619.63	3193.55
A_68_P26587421	chr9:65238821-65238865	NM_028974:617	Kbtbd13	INSIDE	1.543	3.366	640.77	2157.15	5.196	558.74	2903.16
A_68_P24476912	chr6:56747694-56747738	NM_145958:91	Kbtbd2	INSIDE	1.572	1.972	1031.50	2033.69	3.100	895.96	2777.48
A_68_P26885522	chr9:121686826-121686870	NM_028202:124	Kbtbd5	INSIDE	1.570	2.948	478.89	1411.61	4.628	485.79	2248.24
A_68_P22404196	chr3:106904415-106904459	NM_008417:-48	Kcna2	PROMOTER	1.568	2.767	2265.93	6269.43	4.338	1842.84	7994.73
A_68_P25092923	chr7:52662436-52662480	NM_010596:1129	Kcna7	INSIDE	1.630	5.866	1061.24	6225.62	9.563	899.09	8597.98
A_68_P21834182	chr2:167015184-167015228	NM_008420:-907	Kcnb1	PROMOTER	1.693	5.632	3446.00	19408.40	9.534	2638.55	25156.65

ProbeName	Target position of probe on CpG island microarray	TargetID	GeneSymbol	CpG island Description	Ratio of relative methylation (TiO_2-NP/Vehicle)	Sham group			TiO_2-H group		
						Relative methylation (Cy5/Cy3)	Cy3 signal (Input)	Cy5 signal (MeDIP)	Relative methylation (Cy5/Cy3)	Cy3 signal (Input)	Cy5 signal (MeDIP)
A_68_P25099054	chr7:53683157-53683201	NM_001112739:31312	Kcnc1	INSIDE	1.626	7.224	2254.21	16284.40	11.748	1472.00	17293.73
A_68_P25098823	chr7:53653169-53653213	NM_001112739:1324	Kcnc1	INSIDE	2.122	1.801	902.15	1624.83	3.822	1053.18	4025.32
A_68_P25088286	chr7:51846961-51847005	NM_008422:727	Kcnc3	INSIDE	1.974	6.215	2829.89	17588.75	12.271	2291.82	28124.06
A_68_P25088285	chr7:51846895-51846939	NM_008422:661	Kcnc3	INSIDE	1.855	1.592	1174.97	1870.06	2.953	960.40	2835.96
A_68_P22397067	chr3:105255865-105255909	NM_019931:-5847	Kcnd3	PROMOTER	1.519	2.065	1408.52	2909.25	3.138	1170.27	3672.79
A_68_P20942041	chr1:194014550-194014594	NM_001038607:-181	Kcnh1	PROMOTER	1.857	6.657	1568.79	10443.24	12.363	1127.88	13944.47
A_68_P30478814	chr15:99054189-99054233	NM_010601:-1196	Kcnh3	PROMOTER	1.794	4.971	3105.74	15437.94	8.916	2434.66	21707.96
A_68_P30478815	chr15:99054306-99054350	NM_010601:-1078	Kcnh3	PROMOTER	1.529	2.143	1207.57	2587.92	3.278	1052.83	3450.90
A_68_P28069996	chr11:100621272-100621316	NM_001081194:-202	Kcnh4	PROMOTER	2.374	4.449	752.81	3349.60	10.562	555.22	5864.15
A_68_P28099140	chr11:105881863-105881907	NM_001037712:12368	Kcnh6	INSIDE	1.967	7.729	2711.08	20953.02	15.199	1794.72	27278.05
A_68_P27697249	chr11:33743483-33743527	NM_001190885:81	Kcnip1	INSIDE	1.699	5.080	5765.99	29288.62	8.631	4620.64	39880.89
A_68_P32140683	chr19:45890581-45890625	NM_030716:-309	Kcnip2	PROMOTER	1.823	1.932	2746.60	5307.61	3.524	2147.02	7565.05
A_68_P21613749	chr2:127308338-127308382	NM_001111331:-125	Kcnip3	PROMOTER	1.984	9.371	1665.44	15607.18	18.594	1049.56	19515.72
A_68_P25096865	chr7:53356200-53356244	NM_001204411:-88	Kcnj11	PROMOTER	1.710	7.334	10124.04	74246.41	12.539	6198.09	77718.21
A_68_P25095297	chr7:53075356-53075400	NM_145963:4739	Kcnj14	INSIDE	1.746	3.039	3221.66	9789.23	5.306	2632.50	13967.01
A_68_P30984747	chr16:95219135-95219179	NM_001025584:147	Kcnj6	INSIDE	1.505	6.088	1997.49	12160.32	9.160	1688.40	15466.36
A_68_P28670941	chr12:99812671-99812719	NM_029911:3456	Kcnk10	INSIDE	1.621	2.562	1091.94	2797.22	4.153	868.16	3605.85
A_68_P31425152	chr17:88197017-88197061	NM_199251:296	Kcnk12	INSIDE	1.678	3.655	1476.38	5396.14	6.132	1317.96	8082.18
A_68_P21814796	chr2:163684382-163684426	NM_001030292:4919	Kcnk15	INSIDE	1.766	2.761	1864.02	5147.25	4.876	1411.23	6881.10
A_68_P23573590	chr5:30925082-30925126	NM_010608:34562	Kcnk3	INSIDE	1.920	5.787	2350.06	13599.97	11.110	1736.33	19291.40
A_68_P23573354	chr5:30890939-30890983	NM_010608:418	Kcnk3	INSIDE	1.531	6.699	1037.65	6951.16	10.254	713.64	7317.35
A_68_P29445598	chr14:21001028-21001072	NM_021542:-46	Kcnk5	PROMOTER	1.833	3.715	4890.28	18167.01	6.809	3684.25	25086.54
A_68_P29469015	chr14:24824174-24824218	NM_010610:-769	Kcnma1	PROMOTER	1.772	3.849	993.01	3822.26	6.822	802.86	5476.86
A_68_P27475787	chr10:115911149-115911205	NM_021452:-597	Kcnmb4	PROMOTER	1.589	2.152	256.78	552.55	3.418	188.17	643.27
A_68_P25954496	chr8:73376577-73376621	NM_032397:4309	Kcnn1	INSIDE	1.808	5.381	1881.59	10125.04	9.727	1305.40	12698.01
A_68_P25954584	chr8:73389578-73389623	NM_032397:-8693	Kcnn1	PROMOTER	1.748	1.511	1699.52	2567.57	2.641	1171.95	3095.36
A_68_P31672402	chr18:45719605-45719649	NM_080465:-181	Kcnn2	PROMOTER	1.606	3.625	3770.40	13666.74	5.820	3126.64	18196.65
A_68_P25598742	chr7:150293216-150293260	NM_008434:80	Kcnq1	INSIDE	1.883	8.553	779.08	6663.79	16.107	603.76	9724.83
A_68_P23253010	chr4:120419437-120419481	NM_001081142:323	Kcnq4	INSIDE	1.728	2.460	1299.17	3195.38	4.251	1082.81	4602.51
A_68_P30130469	chr15:34767206-34767250	NM_181317:93	Kcns2	INSIDE	1.619	2.579	2835.18	7311.52	4.174	2259.12	9429.48
A_68_P28236792	chr12:11157416-11157460	NM_173417:-259	Kcns3	PROMOTER	1.798	12.300	3004.88	36959.76	22.110	2237.03	49461.30
A_68_P30183902	chr15:44945966-44946010	NM_026200:492	Kcnv1	INSIDE	1.925	7.547	2060.05	15548.16	14.527	1203.61	17485.23
A_68_P31515584	chr18:15221979-15222023	NM_001142731:91	Kctd1	INSIDE	1.716	3.299	1144.74	3776.04	5.660	975.32	5519.95
A_68_P29863087	chr14:103381523-103381567	NM_177715:310	Kctd12	INSIDE	1.784	8.827	2400.30	21187.50	15.751	1800.82	28365.41
A_68_P25503044	chr7:134072503-134072547	NM_172747:132	Kctd13	INSIDE	1.566	5.367	1536.35	8245.63	8.404	1331.12	11186.38
A_68_P23777458	chr5:69732840-69732884	NM_175519:86	Kctd8	INSIDE	2.354	17.034	6463.80	110101.70	40.098	4087.66	163905.10
A_68_P23334806	chr4:136158309-136158353	NM_133872:308	Kdm1a	INSIDE	2.080	7.767	4209.78	32698.79	16.158	3270.28	52841.38
A_68_P31922938	chr19:4397627-4397671	NM_001001984:-571	Kdm2a	PROMOTER	2.175	3.087	1795.80	5543.40	6.715	1491.27	10014.01
A_68_P31922932	chr19:4396607-4396651	NM_001001984:449	Kdm2a	INSIDE	1.592	2.723	3295.26	8971.97	4.335	2770.30	12010.53
A_68_P24052813	chr5:123350619-123350663	NM_013910:-281	Kdm2b	PROMOTER	1.917	2.640	545.58	1440.33	5.060	392.77	1987.24
A_68_P24052812	chr5:123350466-123350510	NM_013910:-129	Kdm2b	PROMOTER	1.667	3.445	921.33	3173.67	5.744	809.91	4651.76
A_68_P20634033	chr1:136457792-136457836	NM_152895:1060	Kdm5b	INSIDE	1.678	3.188	3541.18	11290.03	5.350	2979.49	15940.59
A_68_P32281785	chrX:17740258-17740302	NM_009483:488	Kdm6a	INSIDE	2.036	2.133	1484.78	3166.69	4.342	891.83	3872.13
A_68_P32281782	chrX:17739949-17739993	NM_009483:178	Kdm6a	INSIDE	1.915	1.913	2267.29	4337.15	3.663	1308.27	4792.41
A_68_P23812530	chr5:76374553-76374597	NM_010612:-121	Kdr	PROMOTER	1.735	4.224	6303.13	26622.36	7.328	4621.05	33863.12
A_68_P26346115	chr9:21043701-21043745	NM_001110305:54	Keap1	INSIDE	1.815	1.799	2047.85	3683.26	3.264	1688.13	5510.34
A_68_P31259695	chr17:57170806-57170850	NM_010613:102	Khsrp	INSIDE	1.597	4.699	2028.91	9532.99	7.505	1900.29	14262.48

ProbeName	Target position of probe on CpG island microarray	TargetID	GeneSymbol	CpG island Description	Ratio of relative methylation (TiO_2-NP/Vehicle)	Sham group			TiO_2-H group		
						Relative methylation (Cy5/Cy3)	Cy3 signal (Input)	Cy5 signal (MeDIP)	Relative methylation (Cy5/Cy3)	Cy3 signal (Input)	Cy5 signal (MeDIP)
A_68_P31259625	chr17:57161925-57161969	NM_010613:8984	Khsrp	INSIDE	1.510	2.764	4307.69	11905.11	4.173	3401.95	14196.21
A_68_P20646088	chr1:138363089-138363133	NM_001081258:-1424	Kif14	PROMOTER	1.504	5.511	2003.58	11041.10	8.290	1645.78	13643.44
A_68_P20453282	chr1:94998673-94998717	NM_001110315:-252	Kif1a	PROMOTER	1.678	2.859	3722.33	10641.12	4.798	2631.13	12622.95
A_68_P23399812	chr4:148681667-148681711	NM_207682:119	Kif1b	INSIDE	1.830	4.570	1413.26	6458.18	8.364	1189.26	9946.59
A_68_P23399809	chr4:148681348-148681392	NM_207682:437	Kif1b	INSIDE	1.716	3.683	258.15	950.86	6.319	229.26	1448.71
A_68_P30433473	chr15:90880476-90880520	NM_001109040:-119	Kif21a	PROMOTER	1.975	4.611	5323.48	24544.24	9.106	4245.04	38656.75
A_68_P20644107	chr1:138036961-138037006	NM_001039472:9006	Kif21b	INSIDE	2.698	10.540	4945.35	52121.62	28.439	3189.20	90698.85
A_68_P20644035	chr1:138028029-138028073	NM_001039472:73	Kif21b	INSIDE	1.532	1.639	677.67	1110.85	2.512	547.07	1374.03
A_68_P31472616	chr18:6241499-6241543	NM_008448:2	Kif5b	INSIDE	2.705	13.405	1618.85	21700.12	36.255	1036.17	37566.28
A_68_P31150844	chr17:34027540-34027584	NM_001195298:16	Kifc1	INSIDE	1.855	2.792	4744.51	13244.59	5.177	3568.63	18475.69
A_68_P30349763	chr15:76491001-76491045	NM_010630:-48	Kifc2	DIVERGENT_PROMOTER	2.034	5.654	3010.19	17019.65	11.499	2241.26	25773.16
A_68_P25031610	chr7:31242110-31242154	NM_172898:402	Kirrel2	INSIDE	2.494	15.068	2274.51	34272.62	37.583	1235.22	46423.38
A_68_P32383986	chrX:50097843-50097887	NR_003188:-94	Kis2	PROMOTER	1.618	2.686	13355.12	35865.81	4.346	6244.36	27135.25
A_68_P28745894	chr12:112997376-112997420	NM_008450:339	Klc1	INSIDE	1.732	1.607	6227.53	10010.32	2.783	4582.88	12756.15
A_68_P24993125	chr7:19989502-19989546	NM_146182:-4319	Klc3	DIVERGENT_PROMOTER	1.870	5.073	605.74	3072.82	9.488	524.06	4972.47
A_68_P26022557	chr8:87427077-87427121	NM_010635:1272	Klf1	INSIDE	1.684	1.831	1470.19	2692.40	3.084	1256.92	3876.80
A_68_P30148784	chr15:38228806-38228850	NM_013692:1634	Klf10	INSIDE	1.590	2.565	2289.00	5871.06	4.079	1935.98	7897.38
A_68_P30148791	chr15:38229661-38229705	NM_013692:780	Klf10	INSIDE	1.516	5.717	1731.12	9896.19	8.669	1387.82	12030.48
A_68_P28290823	chr12:25335979-25336023	NM_178357:-235	Klf11	PROMOTER	2.054	4.846	5228.33	25336.60	9.953	3774.20	37564.05
A_68_P25174406	chr7:71083847-71083891	NM_021366:-67	Klf13	PROMOTER	2.142	5.907	1489.42	8798.10	12.651	1164.39	14731.24
A_68_P25174396	chr7:71082541-71082585	NM_021366:1239	Klf13	INSIDE	1.663	5.204	13884.08	72255.25	8.652	11746.07	101631.50
A_68_P24632571	chr6:90413720-90413764	NM_023184:1123	Klf15	INSIDE	1.649	4.076	1932.73	7877.24	6.721	1466.57	9856.49
A_68_P22934435	chr4:55545469-55545513	NM_010637:-143	Klf4	PROMOTER	2.029	4.472	1865.43	8341.62	9.073	1581.81	14351.88
A_68_P32016047	chr19:23210802-23210846	NM_010638:-4891	Klf9	PROMOTER	1.834	3.797	3494.13	13267.89	6.963	2747.09	19127.36
A_68_P32016039	chr19:23209957-23210001	NM_010638:-5737	Klf9	PROMOTER	1.812	4.846	4436.94	21499.29	8.780	3414.58	29979.51
A_68_P20620522	chr1:134195663-134195707	NM_144810:482	Klhdc8a	INSIDE	1.606	3.422	890.52	3047.47	5.495	786.43	4321.27
A_68_P23415959	chr4:151383528-151383572	NM_001033352:551	Klhl21	INSIDE	1.506	1.829	351.42	642.62	2.754	315.28	868.14
A_68_P30585937	chr16:20098035-20098079	NM_029436:430	Klhl24	INSIDE	1.679	2.574	1667.15	4291.61	4.323	1290.36	5578.07
A_68_P30585939	chr16:20098297-20098341	NM_029436:692	Klhl24	INSIDE	1.659	2.204	1057.99	2331.82	3.656	989.42	3617.73
A_68_P25951936	chr8:72976257-72976301	NM_001122830:24564	Klhl26	INSIDE	1.688	2.504	251.71	630.21	4.226	258.31	1091.58
A_68_P23539922	chr5:23606508-23606552	NM_001161800:123	Klhl7	INSIDE	1.516	1.684	880.42	1482.98	2.553	695.55	1775.95
A_68_P31428327	chr17:88929344-88929388	NM_028658:-97	Klraq1	PROMOTER	2.326	8.666	2680.12	23227.16	20.154	1767.89	35629.85
A_68_P31428328	chr17:88929429-88929473	NM_028658:-13	Klraq1	PROMOTER	2.114	6.174	817.38	5046.29	13.049	621.87	8114.53
A_68_P28049252	chr11:97049849-97049893	NM_008379:-664	Kpnb1	PROMOTER	1.689	2.834	861.80	2442.52	4.787	836.84	4005.87
A_68_P24535485	chr6:71222087-71222131	NM_145568:96	Krcc1	INSIDE	2.134	11.063	1731.55	19155.50	23.604	1167.46	27557.14
A_68_P24535484	chr6:71221790-71221834	NM_145568:-200	Krcc1	DIVERGENT_PROMOTER	2.044	6.040	3652.61	22061.07	12.346	2689.46	33203.28
A_68_P24535482	chr6:71221514-71221558	NM_145568:-476	Krcc1	DIVERGENT_PROMOTER	1.741	4.318	2746.85	11859.56	7.516	1893.83	14233.44
A_68_P24535483	chr6:71221618-71221662	NM_145568:-372	Krcc1	DIVERGENT_PROMOTER	1.546	7.603	684.67	5205.32	11.755	621.59	7307.05
A_68_P23447098	chr5:3803414-3803458	NR_033173:256	Krit1	INSIDE	1.998	5.399	2144.76	11579.96	10.788	1737.16	18740.61
A_68_P30495553	chr15:101858609-101858653	NM_010664:-16	Krt18	PROMOTER	1.687	2.231	1396.41	3114.85	3.763	1205.16	4535.54
A_68_P28066375	chr11:100006570-100006614	NM_008471:641	Krt19	INSIDE	1.821	2.693	5241.41	14114.38	4.905	4115.85	20188.17
A_68_P23577316	chr5:31554254-31554298	NM_027221:198	Krtcap3	INSIDE	1.746	5.588	2563.92	14326.24	9.756	2030.20	19806.48
A_68_P27019249	chr10:26095302-26095346	NM_172787:-333	L3mbtl3	PROMOTER	1.683	2.585	3469.39	8967.44	4.350	2605.96	11334.74
A_68_P20042287	chr1:13650338-13650382	NM_145381:230	Lactb2	INSIDE	1.567	5.127	2361.50	12108.17	8.035	1864.14	14978.65
A_68_P21902328	chr2:179960330-179960374	NM_001081171:212	Lama5	INSIDE	2.052	2.789	706.81	1971.49	5.724	600.12	3434.97
A_68_P20726280	chr1:155180208-155180252	NM_010683:-314	Lamc1	PROMOTER	1.819	7.614	27733.70	211155.70	13.851	15482.40	214448.20
A_68_P32245740	chrX:8777550-8777594	NM_173414:474	Lancl3	INSIDE	1.856	3.553	2031.89	7220.04	6.594	1133.27	7472.63

ProbeName	Target position of probe on CpG island microarray	TargetID	GeneSymbol	CpG island Description	Ratio of relative methylation (TiO_2-NP/Vehicle)	Sham group			TiO_2-H group		
						Relative methylation (Cy5/Cy3)	Cy3 signal (Input)	Cy5 signal (MeDIP)	Relative methylation (Cy5/Cy3)	Cy3 signal (Input)	Cy5 signal (MeDIP)
A_68_P28225274	chr12:8928368-8928412	NM_008640:278	Laptm4a	INSIDE	1.621	2.539	2628.18	6673.66	4.117	2032.54	8367.79
A_68_P22082256	chr3:40753573-40753617	NM_001040399:-958	Larp1b	PROMOTER	1.793	2.331	22714.34	52937.90	4.178	16691.41	69733.62
A_68_P30483162	chr15:99803108-99803152	NM_001024526:-80	Larp4	PROMOTER	1.549	1.794	1419.86	2547.83	2.780	1207.46	3356.65
A_68_P28052725	chr11:97660923-97660967	NM_010688:-41	Lasp1	PROMOTER	1.533	5.253	3494.89	18360.31	8.055	2884.12	23231.12
A_68_P30482201	chr15:99602827-99602871	NM_028015:98	Lass5	INSIDE	1.627	3.930	4100.43	16113.09	6.392	3087.66	19737.30
A_68_P29629464	chr14:58318596-58318640	NM_015771:46342	Lats2	INSIDE	1.859	4.525	1145.35	5183.03	8.411	850.90	7156.64
A_68_P32137222	chr19:45309238-45309283	NM_010691:466	Lbx1	INSIDE	1.839	4.671	1595.40	7451.81	8.589	1245.19	10695.25
A_68_P23658789	chr5:46248126-46248170	NM_172153:-357	Lcorl	PROMOTER	1.999	2.835	1420.42	4026.53	5.666	1124.72	6372.68
A_68_P29530592	chr14:35362336-35362380	NM_001039074:39435	Ldb3	INSIDE	1.756	1.541	946.64	1459.23	2.707	874.65	2367.60
A_68_P30395524	chr15:84388079-84388123	NM_177630:153	Ldoc1l	INSIDE	1.916	7.935	2483.85	19709.22	15.205	2070.29	31478.86
A_68_P22530177	chr3:130815462-130815506	NM_010703:2096	Lef1	INSIDE	1.782	3.664	2595.49	9508.91	6.530	2057.48	13436.06
A_68_P22530159	chr3:130813231-130813275	NM_010703:-136	Lef1	PROMOTER	1.679	2.180	900.09	1962.64	3.661	805.09	2947.17
A_68_P31114987	chr17:27341486-27341531	NM_146075:-125	Lemd2	PROMOTER	1.662	1.858	956.19	1776.83	3.088	785.44	2425.69
A_68_P27500779	chr10:120416497-120416541	NM_001081193:-132	Lemd3	PROMOTER	1.790	4.313	931.72	4018.41	7.721	825.94	6376.93
A_68_P27500776	chr10:120416131-120416176	NM_001081193:233	Lemd3	INSIDE	1.634	2.904	292.41	849.04	4.744	304.63	1445.30
A_68_P24947962	chr7:4098539-4098584	NM_172736:9904	Leng8	INSIDE	1.512	4.325	1465.62	6339.23	6.541	1229.91	8045.26
A_68_P26644020	chr9:75289818-75289862	NM_001039522:510	Leo1	INSIDE	1.524	2.105	470.35	990.02	3.207	362.79	1163.57
A_68_P28067972	chr11:100275705-100275749	NM_176830:407	Leprel4	INSIDE	1.787	4.906	930.00	4562.88	8.768	870.89	7635.61
A_68_P28067969	chr11:100275377-100275421	NM_176830:735	Leprel4	INSIDE	1.527	2.451	999.41	2449.55	3.742	782.26	2927.01
A_68_P23156220	chr4:101320226-101320270	NM_175036:-139	Leprot	PROMOTER	1.638	1.858	6413.77	11913.85	3.042	4740.97	14421.47
A_68_P23591177	chr5:34124884-34124928	NM_019694:447	Letm1	INSIDE	1.696	3.111	1436.53	4468.72	5.276	1267.90	6689.45
A_68_P25727982	chr8:26707741-26707785	NM_173012:197	Letm2	INSIDE	1.597	5.315	1145.84	6090.24	8.487	988.09	8386.39
A_68_P30485948	chr15:100299486-100299530	NM_134093:44	Letmd1	INSIDE	1.660	2.013	1842.43	3709.41	3.343	1379.91	4612.61
A_68_P24142218	chr5:141083418-141083462	NM_008494:146	Lfng	INSIDE	1.738	3.087	2249.66	6945.00	5.366	1855.84	9959.10
A_68_P23695553	chr5:52957300-52957344	NM_144945:197	Lgi2	INSIDE	2.033	6.026	7794.77	46973.65	12.249	6150.40	75336.94
A_68_P20636645	chr1:136884255-136884299	NM_001033409:117577	Lgr6	INSIDE	1.892	14.177	2442.11	34620.74	26.820	1778.74	47705.97
A_68_P27977954	chr11:84333936-84333980	NM_008498:5078	Lhx1	INSIDE	1.514	2.640	571.16	1507.89	3.996	476.27	1903.32
A_68_P24038707	chr5:120886274-120886318	NM_008499:4402	Lhx5	INSIDE	1.534	5.622	734.69	4130.74	8.623	673.95	5811.19
A_68_P21137838	chr2:35960787-35960831	NM_001083126:-1228	Lhx6	PROMOTER	1.608	3.570	1602.64	5721.08	5.740	1283.41	7366.49
A_68_P22661977	chr3:153991290-153991334	NM_010713:2212	Lhx8	INSIDE	1.552	2.620	2433.89	6377.55	4.067	2210.04	8988.50
A_68_P29992340	chr15:7079603-7079647	NM_001113386:53	Lifr	INSIDE	1.545	2.541	797.88	2027.27	3.925	630.42	2474.26
A_68_P25645821	chr8:9976314-9976358	NM_176953:-13	Lig4	DIVERGENT_PROMOTER	2.332	7.229	11513.48	83233.88	16.857	7753.00	130696.00
A_68_P30482738	chr15:99705731-99705775	NM_001113545:135	Lima1	INSIDE	1.734	5.138	2702.22	13884.57	8.908	2227.72	19845.29
A_68_P23763603	chr5:67136839-67136883	NM_001001980:-218	Limch1	PROMOTER	1.888	5.879	1443.65	8487.48	11.099	1197.28	13288.10
A_68_P24112800	chr5:135164852-135164896	NM_010717:-414	Limk1	PROMOTER	1.847	3.153	1731.17	5458.42	5.825	1224.90	7134.82
A_68_P27551860	chr11:3309041-3309085	NM_010718:176	Limk2	INSIDE	1.538	3.518	1658.89	5835.56	5.410	1398.92	7568.53
A_68_P27163123	chr10:57786395-57786439	NM_026148:203	Lims1	INSIDE	1.816	4.775	3182.08	15193.93	8.672	2345.80	20343.02
A_68_P23929939	chr5:100929613-100929657	NM_001115010:-2042	Lin54	PROMOTER	1.720	3.153	2338.54	7372.86	5.423	1885.87	10226.99
A_68_P27425024	chr10:106709059-106709103	NM_001033223:194	Lin7a	INSIDE	1.571	2.971	3596.44	10684.37	4.666	2802.49	13076.50
A_68_P25092719	chr7:52625927-52625971	NM_011698:-14	Lin7b	PROMOTER	1.519	2.163	3110.79	6728.46	3.285	2368.96	7782.17
A_68_P20877047	chr1:182571834-182571878	NM_175186:392	Lin9	INSIDE	1.857	3.800	539.78	2051.03	7.055	419.29	2957.95
A_68_P26537272	chr9:56468941-56468985	NM_181074:64098	Lingo1	INSIDE	1.531	4.659	605.59	2821.38	7.135	486.37	3470.21
A_68_P25010202	chr7:26180987-26181031	NM_010719:-2	Lipe	PROMOTER	1.824	3.873	4065.25	15743.27	7.063	3161.08	22325.43
A_68_P25010077	chr7:26164977-26165021	NM_001039507:10127	Lipe	INSIDE	1.613	3.417	2557.39	8738.93	5.511	1874.40	10329.97
A_68_P31834169	chr18:75121056-75121100	NM_010720:-161	Lipg	PROMOTER	1.623	1.568	2716.88	4259.32	2.544	2197.72	5591.66
A_68_P20148355	chr1:36502065-36502109	NM_001013374:-2	Lman2l	PROMOTER	1.916	2.963	7704.43	22829.15	5.678	5809.89	32991.37
A_68_P20094994	chr1:24685459-24685503	NM_026719:98	Lmbrd1	INSIDE	2.688	20.375	4317.54	87970.49	54.775	2990.87	163823.90

ProbeName	Target position of probe on CpG island microarray	TargetID	GeneSymbol	CpG island Description	Ratio of relative methylation (TiO_2-NP/Vehicle)	Sham group			TiO_2-H group		
						Relative methylation (Cy5/Cy3)	Cy3 signal (Input)	Cy5 signal (MeDIP)	Relative methylation (Cy5/Cy3)	Cy3 signal (Input)	Cy5 signal (MeDIP)
A_68_P22601420	chr3:143865390-143865434	NM_001161769:-52	Lmo4	PROMOTER	2.081	4.028	7512.89	30265.03	8.383	5764.40	48324.41
A_68_P22601440	chr3:143867896-143867940	NM_001161769:-2558	Lmo4	PROMOTER	1.514	3.237	4765.00	15423.48	4.901	3566.87	17481.44
A_68_P29856568	chr14:102128930-102128974	NM_201529:-192	Lmo7	PROMOTER	2.621	10.226	4507.02	46087.56	26.806	2966.50	79519.01
A_68_P21122760	chr2:33496334-33496378	NM_010725:-325	Lmx1b	PROMOTER	1.517	2.550	1335.58	3406.27	3.870	1103.55	4270.81
A_68_P24171749	chr5:147887960-147888004	NM_080795:166	Lnx2	INSIDE	1.826	3.175	1701.57	5403.14	5.797	1544.24	8952.35
A_68_P24171753	chr5:147888406-147888450	NM_080795:-280	Lnx2	DIVERGENT_PROMOTER	1.707	3.199	8652.25	27677.32	5.461	6559.61	35820.20
A_68_P24171442	chr5:147840909-147840953	NM_080795:47218	Lnx2	INSIDE	1.593	2.871	522.01	1498.70	4.572	468.43	2141.74
A_68_P31708189	chr18:52688590-52688634	NM_010728:750	Lox	INSIDE	1.545	1.456	1093.75	1592.08	2.249	860.22	1934.74
A_68_P26547037	chr9:58160685-58160729	NM_010729:313	Loxl1	INSIDE	1.624	3.568	794.13	2833.46	5.793	679.29	3935.25
A_68_P22951804	chr4:58566187-58566231	NM_172989:-43	Lpar1	PROMOTER	2.313	12.789	2033.78	26009.86	29.587	1471.32	43531.41
A_68_P29140001	chr13:73605046-73605090	NM_145376:238	Lpcat1	INSIDE	1.936	4.602	5042.49	23207.43	8.909	3905.21	34793.18
A_68_P26068285	chr8:95379185-95379229	NM_173014:-42	Lpcat2	PROMOTER	1.505	6.893	3200.62	22061.99	10.374	2288.30	23738.41
A_68_P23966965	chr5:107867055-107867099	NM_027599:6509	Lpcat2b	DOWNSTREAM	1.611	5.020	584.64	2934.97	8.088	566.90	4585.04
A_68_P20939662	chr1:193541860-193541904	NM_172266:-20	Lpgat1	PROMOTER	1.640	2.460	919.65	2262.45	4.034	770.36	3107.48
A_68_P26016967	chr8:86424037-86424081	NM_181039:62	Lphn1	INSIDE	1.601	3.211	2264.65	7272.80	5.141	2047.70	10527.10
A_68_P23837522	chr5:81449591-81449635	NM_198702:-1005	Lphn3	PROMOTER	1.563	4.017	1797.47	7221.24	6.281	1431.07	8988.10
A_68_P27996402	chr11:87630757-87630801	NM_080420:8838	Lpo	INSIDE	1.874	3.124	2302.41	7193.28	5.856	1764.87	10335.15
A_68_P22301528	chr3:86028273-86028317	NM_001077687:-317	Lrba	PROMOTER	1.785	3.658	708.35	2591.21	6.529	653.27	4265.14
A_68_P32730437	chrX:143988143-143988187	NM_001081173:460	Lrch2	INSIDE	1.965	4.409	9704.97	42791.58	8.664	6149.70	53283.79
A_68_P31220008	chr17:49071618-49071662	NM_027452:-266	Lrfn2	PROMOTER	2.514	5.727	9111.94	52180.14	14.397	5782.67	83254.10
A_68_P25031213	chr7:31145836-31145880	NM_175478:1933	Lrfn3	INSIDE	1.660	6.991	3361.08	23496.42	11.602	2447.42	28394.03
A_68_P22391858	chr3:104315425-104315469	NM_001025067:333	Lrig2	INSIDE	1.760	5.016	599.41	3006.70	8.827	522.51	4612.18
A_68_P27537125	chr10:127058908-127058952	NM_008512:-726	Lrp1	PROMOTER	2.156	5.585	2639.93	14743.96	12.043	1960.62	23611.85
A_68_P27537124	chr10:127058798-127058842	NM_008512:-616	Lrp1	PROMOTER	1.712	2.092	4941.30	10335.66	3.581	4217.40	15102.89
A_68_P30156691	chr15:39775111-39775155	NM_172814:171	Lrp12	INSIDE	2.102	11.020	1141.73	12582.07	23.167	888.50	20584.27
A_68_P31950789	chr19:10531506-10531550	NM_001111140:409	Lrrc10b	INSIDE	1.695	1.584	591.01	936.18	2.684	514.45	1380.87
A_68_P28886522	chr13:24373081-24373125	NM_026825:-443	Lrrc16a	PROMOTER	1.515	5.019	1735.70	8711.35	7.604	1462.36	11119.99
A_68_P29618044	chr14:56110165-56110209	NM_001024645:257	Lrrc16b	INSIDE	1.540	2.286	3597.59	8222.54	3.520	3076.47	10829.15
A_68_P27180703	chr10:60938705-60938749	NM_153542:146	Lrrc20	INSIDE	1.762	16.348	1322.90	21626.74	28.805	1006.90	29004.18
A_68_P24322963	chr6:28783645-28783689	NM_138682:-1919	Lrrc4	PROMOTER	1.845	3.671	1049.16	3851.39	6.772	1235.47	8367.10
A_68_P23955154	chr5:105844706-105844750	NM_001033550:-65	Lrrc8b	PROMOTER	1.682	3.890	293.53	1141.87	6.542	220.20	1440.47
A_68_P21957226	chr3:14534076-14534120	NM_001163579:311	Lrrcc1	INSIDE	1.605	3.448	2014.65	6946.97	5.533	1548.92	8570.49
A_68_P26827666	chr9:111020550-111020594	NM_001164838:-42	Lrrfip2	PROMOTER	2.347	5.128	3893.76	19967.23	12.037	2948.94	35497.62
A_68_P24792500	chr6:119280593-119280637	NM_001172207:-1392	Lrtm2	PROMOTER	1.515	2.033	2029.62	4125.92	3.080	1634.56	5034.32
A_68_P27765093	chr11:45758539-45758583	NM_028185:-123	Lsm11	PROMOTER	1.928	3.590	3657.32	13130.18	6.923	2701.76	18704.49
A_68_P21900913	chr2:179759394-179759438	NM_177727:-275	Lsm14b	PROMOTER	1.891	4.621	5053.95	23356.44	8.741	3956.25	34580.53
A_68_P21900919	chr2:179760085-179760129	NM_177727:415	Lsm14b	INSIDE	1.627	4.030	1686.18	6795.38	6.555	1408.10	9230.65
A_68_P27285907	chr10:80317900-80317944	NM_025349:32	Lsm7	INSIDE	1.604	1.454	1032.35	1501.36	2.333	818.51	1909.53
A_68_P27262526	chr10:75994451-75994495	NM_146006:101	Lss	INSIDE	1.886	5.474	6782.44	37124.20	10.325	5005.00	51676.57
A_68_P29619799	chr14:56386649-56386693	NM_008519:1872	Ltb4r1	INSIDE	1.585	6.299	771.24	4857.79	9.981	638.62	6373.79
A_68_P29619756	chr14:56381232-56381276	NM_020490:990	Ltb4r2	INSIDE	1.708	1.671	743.80	1242.88	2.855	716.65	2045.84
A_68_P28600848	chr12:86217110-86217154	NM_013589:313	Ltbp2	INSIDE	1.794	1.754	853.83	1497.77	3.146	705.49	2219.75
A_68_P31930681	chr19:5758063-5758107	NM_008520:17181	Ltbp3	INSIDE	1.616	4.430	806.23	3571.76	7.160	691.28	4949.47
A_68_P25016756	chr7:28119811-28119855	NM_001113549:-1165	Ltbp4	PROMOTER	2.321	7.348	4064.11	29862.64	17.053	2879.70	49106.29
A_68_P23334085	chr4:136025641-136025685	NM_024452:-13	Luzp1	PROMOTER	1.654	5.706	10787.59	61551.52	9.440	7883.57	74420.85
A_68_P21258086	chr2:60221286-60221330	NM_013825:-20	Ly75	PROMOTER	1.526	2.902	3297.93	9570.93	4.427	2671.37	11827.18
A_68_P21204319	chr2:49643540-49643584	NM_027990:357	Lypd6b	INSIDE	2.160	4.204	1901.65	7994.55	9.081	1433.64	13018.78

ProbeName	Target position of probe on CpG island microarray	TargetID	GeneSymbol	CpG island Description	Ratio of relative methylation (TiO_2-NP/Vehicle)	Sham group			TiO_2-H group		
						Relative methylation (Cy5/Cy3)	Cy3 signal (Input)	Cy5 signal (MeDIP)	Relative methylation (Cy5/Cy3)	Cy3 signal (Input)	Cy5 signal (MeDIP)
A_68_P21204312	chr2:49642923-49642967	NM_027990:-261	Lypd6b	PROMOTER	1.915	2.123	464.44	986.20	4.067	405.43	1648.96
A_68_P20905056	chr1:187941218-187941262	NM_146106:-51	Lyplal1	PROMOTER	1.575	3.364	1318.19	4435.03	5.298	1030.23	5457.80
A_68_P27812425	chr11:54674397-54674456	NM_029327:-333	Lyrm7	DIVERGENT_PROMOTER	1.856	6.650	561.10	3731.56	12.345	393.08	4852.51
A_68_P23264212	chr4:123341892-123341936	NM_001199136:19689	Macf1	INSIDE	1.835	6.225	2756.08	17155.80	11.422	2142.75	24474.07
A_68_P23264207	chr4:123341175-123341219	NM_001199136:20407	Macf1	INSIDE	1.601	2.225	1197.98	2665.43	3.563	1057.07	3766.45
A_68_P31938321	chr19:7239801-7239845	NM_134147:108565	Macrod1	INSIDE	1.686	7.484	804.27	6019.42	12.615	664.54	8383.14
A_68_P31938323	chr19:7240213-7240257	NM_134147:108977	Macrod1	INSIDE	1.683	3.757	3541.01	13303.16	6.324	2898.79	18332.07
A_68_P21683849	chr2:140221825-140221869	NM_028387:676	Macrod2	INSIDE	1.871	6.341	1384.22	8776.69	11.861	1025.84	12167.25
A_68_P23588236	chr5:33678555-33678599	NM_021500:356	Maea	INSIDE	1.609	5.083	3030.57	15404.30	8.177	2515.92	20572.74
A_68_P30343819	chr15:75578604-75578648	NM_194350:-274	Mafa	PROMOTER	1.953	3.653	1220.29	4457.21	7.135	915.27	6530.09
A_68_P21583618	chr2:121781814-121781858	NM_008545:-8	Mageb3	DIVERGENT_PROMOTER	1.606	1.769	3483.81	6162.50	2.841	2685.15	7628.37
A_68_P32742983	chrX:147247480-147247524	NM_001199246:744	Maged2	INSIDE	2.161	4.903	2403.16	11783.38	10.596	1585.60	16800.76
A_68_P32751751	chrX:149472100-149472144	NM_023788:-16	Mageh1	PROMOTER	2.079	7.842	12021.86	94269.69	16.303	7384.41	120391.60
A_68_P24656000	chr6:94233185-94233229	NM_001029850:692	Magi1	INSIDE	1.546	1.641	4535.04	7442.85	2.537	3708.37	9406.35
A_68_P23513990	chr5:18732536-18732580	NM_001170746:-305	Magi2	PROMOTER	2.216	6.205	10538.73	65397.65	13.752	8258.67	113576.80
A_68_P23513989	chr5:18732427-18732471	NM_001170746:-415	Magi2	PROMOTER	1.729	5.351	1454.92	7784.77	9.253	1263.72	11692.73
A_68_P32582402	chrX:103206930-103206974	NM_001190409:286	Magt1	INSIDE	1.915	1.775	471.25	836.39	3.398	285.08	968.83
A_68_P31930914	chr19:5803350-5803394	NR_002847:-701	Malat1	PROMOTER	1.587	2.239	2534.95	5676.06	3.553	1910.89	6790.18
A_68_P31781218	chr18:65590541-65590585	NM_172833:-88	Malt1	PROMOTER	1.801	4.774	13185.43	62940.98	8.596	9108.70	78301.18
A_68_P31781216	chr18:65590296-65590340	NM_172833:-332	Malt1	PROMOTER	1.611	4.275	3911.10	16718.62	6.885	2564.32	17654.60
A_68_P27787812	chr11:50105519-50105563	NM_175334:298	Maml1	INSIDE	1.613	3.738	461.82	1726.44	6.031	391.72	2362.56
A_68_P27787814	chr11:50105808-50105852	NM_175334:8	Maml1	INSIDE	1.583	2.825	2008.46	5672.99	4.471	1575.17	7043.18
A_68_P22133096	chr3:51811618-51811662	NM_001004176:97288	Maml3	INSIDE	1.764	4.761	566.37	2696.29	8.396	443.15	3720.85
A_68_P23324350	chr4:134260533-134260577	NM_207237:-349	Man1c1	PROMOTER	2.820	6.404	6070.41	38876.71	18.062	3941.66	71192.51
A_68_P23609897	chr5:37221748-37221792	NM_008550:118	Man2b2	INSIDE	1.775	2.479	395.33	980.03	4.399	374.60	1648.01
A_68_P21764750	chr2:155102799-155102843	NM_025735:641	Map1lc3a	INSIDE	1.535	2.782	4218.66	11735.73	4.269	3229.90	13789.31
A_68_P29330800	chr13:112598036-112598080	NM_011945:1133	Map3k1	INSIDE	1.925	3.844	1210.59	4652.97	7.400	1025.67	7589.61
A_68_P29330804	chr13:112598387-112598431	NM_011945:783	Map3k1	INSIDE	1.523	2.477	2532.55	6272.51	3.773	2203.81	8314.87
A_68_P32777676	chrX:156426760-156426804	NM_001163085:418	Map3k15	INSIDE	1.601	3.482	3434.14	11957.67	5.574	2209.75	12316.96
A_68_P28099478	chr11:105946211-105946255	NM_011947:17	Map3k3	INSIDE	2.617	6.441	23400.69	150731.30	16.857	15983.49	269440.70
A_68_P26983892	chr10:19653936-19653980	NM_008580:-373	Map3k5	PROMOTER	1.941	2.801	2266.02	6346.81	5.436	1777.92	9664.64
A_68_P26983894	chr10:19654073-19654117	NM_008580:-237	Map3k5	PROMOTER	1.908	4.715	1137.39	5362.66	8.995	869.16	7818.51
A_68_P26983891	chr10:19653749-19653793	NM_008580:-561	Map3k5	PROMOTER	1.620	1.830	1693.83	3099.84	2.964	1458.46	4322.90
A_68_P23316515	chr4:132797012-132797056	NM_016693:302	Map3k6	INSIDE	1.601	3.752	940.36	3528.30	6.005	814.28	4889.99
A_68_P20168277	chr1:39957569-39957613	NM_008696:-167	Map4k4	PROMOTER	1.739	6.591	1829.38	12056.65	11.462	1482.10	16987.39
A_68_P30571313	chr16:16983889-16983933	NM_001038663:436	Mapk1	INSIDE	1.524	2.580	3619.05	9335.38	3.932	2928.64	11514.79
A_68_P30423478	chr15:88979773-88979817	NM_011161:242	Mapk11	INSIDE	1.578	2.423	2470.96	5987.54	3.823	2005.79	7668.84
A_68_P30345384	chr15:75829239-75829283	NM_177922:5062	Mapk15	INSIDE	1.663	2.283	1737.25	3965.36	3.796	1347.49	5115.13
A_68_P26643861	chr9:75257109-75257153	NM_027418:36	Mapk6	INSIDE	1.752	6.242	804.80	5023.46	10.938	682.80	7468.18
A_68_P21428205	chr2:92229093-92229137	NM_001202445:3726	Mapk8ip1	INSIDE	1.608	1.807	2330.81	4212.43	2.905	1815.98	5275.98
A_68_P31100482	chr17:25073578-25073622	NM_001163447:322	Mapk8ip3	INSIDE	2.063	1.495	880.29	1315.95	3.084	628.83	1939.61
A_68_P31556965	chr18:23962258-23962302	NM_153058:-190	Mapre2	PROMOTER	1.583	2.325	1721.23	4002.09	3.681	1225.12	4510.15
A_68_P28089851	chr11:104093898-104093942	NM_001038609:1171	Mapt	INSIDE	1.518	3.201	2664.13	8526.89	4.858	2303.11	11189.67
A_68_P31150250	chr17:33855758-33855802	NM_145486:-165	Marchf2	PROMOTER	1.649	2.933	2568.68	7532.69	4.837	2197.27	10627.10
A_68_P20337461	chr1:72582629-72582673	NM_001045533:488	Marchf4	INSIDE	2.205	8.769	4635.05	40642.45	19.337	3125.23	60432.55
A_68_P30114905	chr15:31461133-31461177	NM_172606:-362	Marchf6	PROMOTER	1.939	1.837	568.10	1043.48	3.561	476.87	1698.37
A_68_P30114898	chr15:31460199-31460243	NM_172606:572	Marchf6	INSIDE	1.566	1.520	2694.46	4094.33	2.380	2251.11	5357.45

ProbeName	Target position of probe on CpG island microarray	TargetID	GeneSymbol	CpG island Description	Ratio of relative methylation (TiO_2-NP/Vehicle)	Sham group			TiO_2-H group		
						Relative methylation (Cy5/Cy3)	Cy3 signal (Input)	Cy5 signal (MeDIP)	Relative methylation (Cy5/Cy3)	Cy3 signal (Input)	Cy5 signal (MeDIP)
A_68_P21257003	chr2:60047554-60047598	NM_020575:-416	Marchf7	PROMOTER	1.702	2.127	1773.27	3772.14	3.621	1423.23	5153.13
A_68_P23297033	chr4:129190774-129190818	NM_010807:-28	Marcksl1	PROMOTER	2.602	5.773	10432.74	60230.91	15.025	7036.61	105722.20
A_68_P23297034	chr4:129190844-129190888	NM_010807:42	Marcksl1	INSIDE	2.238	6.078	31663.01	192442.20	13.600	21299.62	289684.00
A_68_P31939393	chr19:7416055-7416099	NM_001080388:258	Mark2	INSIDE	1.581	2.657	5515.08	14651.84	4.199	4403.33	18491.38
A_68_P32119294	chr19:42222686-42222730	NM_183195:830	Marveld1	INSIDE	1.677	1.867	2718.81	5074.92	3.130	2118.62	6630.74
A_68_P25954148	chr8:73321826-73321870	NM_199308:-5516	Mast3	PROMOTER	1.698	3.752	5167.04	19388.07	6.371	3777.88	24067.35
A_68_P24541704	chr6:72389176-72389220	NM_145569:354	Mat2a	INSIDE	1.776	3.581	1968.78	7050.07	6.361	1773.17	11279.90
A_68_P24541702	chr6:72388860-72388904	NM_145569:670	Mat2a	INSIDE	1.602	2.735	1124.77	3076.39	4.383	983.64	4311.30
A_68_P27288382	chr10:80720130-80720174	NM_010768:-137	Matk	PROMOTER	1.787	7.778	2735.01	21273.82	13.898	2288.70	31808.01
A_68_P30127809	chr15:34236397-34236441	NM_016762:-17	Matn2	PROMOTER	1.703	2.565	3731.02	9571.39	4.369	2799.86	12232.50
A_68_P31618899	chr18:35721824-35721868	NM_010771:35	Matr3	INSIDE	2.459	1.495	1916.90	2865.17	3.676	1857.74	6829.22
A_68_P28915541	chr13:30228560-30228604	NM_153546:224	Mboat1	INSIDE	1.716	4.650	5057.74	23518.34	7.979	3984.22	31790.67
A_68_P28291793	chr12:25515677-25515721	NM_001083341:-765	Mboat2	PROMOTER	1.745	14.968	1756.93	26296.92	26.116	1229.01	32097.13
A_68_P24946816	chr7:3637239-3637283	NM_029934:7867	Mboat7	INSIDE	1.956	4.635	2593.16	12018.65	9.064	2031.67	18415.71
A_68_P24946817	chr7:3637393-3637437	NM_029934:7713	Mboat7	INSIDE	1.733	4.064	2244.83	9124.02	7.046	1661.51	11706.57
A_68_P28029575	chr11:93747831-93747875	NM_134012:320	Mbtd1	INSIDE	1.789	4.285	2190.93	9388.97	7.668	1775.23	13611.80
A_68_P26214707	chr8:122082424-122082468	NM_001167910:215	Mbtps1	INSIDE	1.636	3.018	10827.35	32676.37	4.939	9094.19	44912.50
A_68_P26465911	chr9:43943262-43943306	NM_023061:544	Mcam	INSIDE	2.490	3.450	1701.35	5869.09	8.589	1308.16	11235.64
A_68_P31668416	chr18:44821733-44821777	NM_001085374:166	Mcc	INSIDE	1.523	3.694	1617.21	5974.76	5.627	1172.44	6597.50
A_68_P29266707	chr13:100785592-100785636	NM_030026:-20	Mccc2	PROMOTER	1.618	1.916	809.86	1551.88	3.100	610.66	1893.18
A_68_P24624872	chr6:88848652-88848696	NM_008564:100	Mcm2	INSIDE	1.723	2.710	2143.08	5807.63	4.668	1669.78	7794.48
A_68_P31161218	chr17:35978447-35978494	NM_001010833:28	Mdc1	INSIDE	2.063	6.398	6929.61	44333.91	13.196	5328.21	70309.10
A_68_P31130464	chr17:30025085-30025129	NM_001081160:-279	Mdga1	PROMOTER	1.818	4.836	2874.13	13899.56	8.792	2325.88	20449.59
A_68_P31130455	chr17:30024049-30024093	NM_001081160:757	Mdga1	INSIDE	1.738	2.851	1712.55	4882.18	4.954	1318.07	6529.20
A_68_P23272064	chr4:124762396-124762440	NM_027310:28	Meaf6	INSIDE	2.105	4.439	2277.95	10112.16	9.343	1719.31	16064.02
A_68_P22028773	chr3:30407847-30407891	NM_021442:541	Mecom	INSIDE	2.882	13.646	2919.90	39844.77	39.327	2080.76	81830.92
A_68_P22028775	chr3:30408163-30408207	NM_021442:225	Mecom	INSIDE	1.615	3.398	2128.39	7231.49	5.487	1792.28	9834.61
A_68_P22028771	chr3:30407557-30407601	NM_021442:831	Mecom	INSIDE	1.619	1.748	767.78	1342.21	2.830	672.06	1901.65
A_68_P24027251	chr5:119010546-119010590	NM_172424:-159	Med13l	PROMOTER	1.689	4.857	1353.13	6571.65	8.204	1178.58	9668.76
A_68_P32263512	chrX:12339036-12339080	NM_001048208:41	Med14	INSIDE	2.302	6.479	885.86	5739.19	14.914	563.91	8410.04
A_68_P27011028	chr10:24589929-24589973	NM_001166416:159	Med23	INSIDE	1.548	4.361	469.79	2048.95	6.752	420.87	2841.82
A_68_P25962045	chr8:75071657-75071701	NM_027485:531	Med26	INSIDE	1.527	2.995	3341.21	10006.22	4.572	2633.69	12040.27
A_68_P27910209	chr11:72028885-72028929	NM_026068:188	Med31	INSIDE	1.765	1.777	736.61	1308.89	3.136	608.92	1909.51
A_68_P23006650	chr4:70195415-70195459	NM_172694:526	Megf9	INSIDE	1.975	3.715	8274.32	30737.56	7.336	6416.84	47074.16
A_68_P31350850	chr17:74695143-74695187	NM_133771:-961	Memo1	PROMOTER	5.241	2.878	450.72	1297.15	15.083	368.81	5562.66
A_68_P25285303	chr7:91032985-91033029	NM_030705:-155	Mesdc1	DIVERGENT_PROMOTER	1.623	2.693	480.29	1293.34	4.369	553.86	2419.97
A_68_P25263207	chr7:86938148-86938192	NM_008588:306	Mesp1	INSIDE	2.066	1.675	913.13	1529.48	3.460	931.29	3222.05
A_68_P31106324	chr17:25934049-25934093	NM_133719:-80	Metrn	PROMOTER	1.626	2.274	1344.63	3058.13	3.697	1092.50	4039.16
A_68_P20778372	chr1:164478561-164478605	NM_144877:-106	Mettl13	PROMOTER	1.791	2.885	7977.41	23018.06	5.169	5991.19	30966.64
A_68_P22487800	chr3:123088652-123088696	NM_201638:234	Mettl14	INSIDE	2.281	7.737	8006.62	61947.64	17.649	5831.80	102926.20
A_68_P28515346	chr12:70683995-70684039	NM_001033236:-1	Mettl21d	PROMOTER	1.574	3.709	1807.51	6704.49	5.840	1491.54	8710.42
A_68_P29605899	chr14:52924704-52924752	NM_019721:71	Mettl3	INSIDE	1.560	3.087	740.61	2286.38	4.816	590.71	2845.04
A_68_P25471703	chr7:128178649-128178693	NM_021554:712	Mettl9	INSIDE	1.607	8.648	1551.29	13416.01	13.900	1343.46	18673.87
A_68_P25279941	chr7:90014673-90014717	NM_175366:-1148	Mex3b	PROMOTER	2.224	5.070	4959.03	25142.20	11.274	3753.77	42319.18
A_68_P30497013	chr15:102110024-102110068	NM_134100:160	Mfsd5	INSIDE	1.755	10.569	1150.96	12164.70	18.546	955.38	17718.13
A_68_P20173639	chr1:40847463-40847507	NM_172499:18	Mfsd9	INSIDE	1.962	7.038	1091.71	7683.65	13.809	885.85	12232.37
A_68_P27787453	chr11:50044668-50044712	NM_145926:5854	Mgat4b	INSIDE	2.071	2.420	1573.74	3808.69	5.012	1234.71	6187.88

ProbeName	Target position of probe on CpG island microarray	TargetID	GeneSymbol	CpG island Description	Ratio of relative methylation (TiO_2-NP/Vehicle)	Sham group			TiO_2-H group		
						Relative methylation (Cy5/Cy3)	Cy3 signal (Input)	Cy5 signal (MeDIP)	Relative methylation (Cy5/Cy3)	Cy3 signal (Input)	Cy5 signal (MeDIP)
A_68_P28162003	chr11:116780529-116780573	NM_172948:374	Mgat5b	INSIDE	1.512	1.990	423.87	843.57	3.008	438.90	1320.33
A_68_P32140500	chr19:45857855-45857899	NM_023799:-95	Mgea5	PROMOTER	1.983	7.289	757.01	5518.17	14.456	564.55	8161.40
A_68_P32140499	chr19:45857773-45857817	NM_023799:-13	Mgea5	PROMOTER	1.666	6.837	801.80	5481.57	11.390	704.42	8023.62
A_68_P24623660	chr6:88674554-88674598	NM_001166250:-121	Mgll	PROMOTER	1.687	7.609	2563.93	19509.59	12.836	2246.95	28842.34
A_68_P23439858	chr4:155028864-155028908	NM_145124:14450	Mib2	INSIDE	1.576	1.782	3808.57	6788.31	2.808	2786.77	7826.64
A_68_P32823846	chrX:166435801-166435845	NM_183151:118269	Mid1	DOWNSTREAM	1.500	1.929	3218.86	6210.66	2.895	3388.99	9809.69
A_68_P27281299	chr10:79613231-79613275	NM_021565:2218	Midn	INSIDE	1.595	1.942	729.15	1415.90	3.097	609.84	1888.57
A_68_P23164325	chr4:102787321-102787365	NM_001039081:-4652	Mier1	PROMOTER	2.136	7.582	18637.64	141316.60	16.193	13089.85	211962.50
A_68_P29330297	chr13:112515612-112515656	NM_172593:39249	Mier3	DOWNSTREAM	1.948	3.136	4485.94	14066.93	6.108	3426.27	20927.12
A_68_P32070425	chr19:32560187-32560231	NM_010799:-50	Minpp1	PROMOTER	1.509	2.151	1498.34	3223.34	3.247	1254.33	4072.98
A_68_P29668497	chr14:65206255-65206299	NR_029813:-3217	Mir124a-1	PROMOTER	1.604	3.902	7366.35	28747.14	6.258	4975.08	31133.68
A_68_P21906908	chr2:180628801-180628845	NR_029538:78	Mir124a-3	DOWNSTREAM	2.003	1.584	457.25	724.51	3.173	393.78	1249.64
A_68_P26018642	chr8:86707908-86707952	NR_029821:-5070	Mir181c	PROMOTER	1.767	5.718	2600.84	14870.92	10.102	2267.63	22907.50
A_68_P26345157	chr9:20837581-20837625	NR_035438:-830	Mir1900	PROMOTER	1.532	2.271	1080.63	2454.17	3.479	904.14	3145.28
A_68_P27950601	chr11:79525364-79525408	NR_029579:-84	Mir193	PROMOTER	1.738	2.793	2055.00	5739.78	4.854	1642.00	7969.79
A_68_P26134826	chr8:108129362-108129406	NR_035492:-9981	Mir1966	PROMOTER	1.788	3.411	2337.21	7972.16	6.098	1612.29	9831.01
A_68_P21114610	chr2:32168059-32168103	NR_029811:-5899	Mir199b	PROMOTER	2.090	4.476	10196.84	45644.87	9.354	6842.97	64010.85
A_68_P21114609	chr2:32167955-32167999	NR_029811:-6003	Mir199b	PROMOTER	1.614	5.640	16942.30	95558.65	9.104	11346.08	103293.50
A_68_P28748036	chr12:113368595-113368639	NR_029590:-474	Mir203	PROMOTER	1.787	2.450	281.02	688.43	4.378	193.24	845.98
A_68_P27365819	chr10:95026630-95026674	NR_037212:-4697	Mir3058	PROMOTER	1.664	4.466	7057.96	31523.23	7.434	6155.54	45758.67
A_68_P22312502	chr3:88018703-88018747	NR_037276:-368	Mir3093	PROMOTER	1.852	2.529	1938.75	4903.30	4.684	1457.09	6825.26
A_68_P25015934	chr7:27963629-27963675	NR_037283:-2540	Mir3101	PROMOTER	1.768	3.211	11134.92	35755.86	5.678	7580.22	43038.60
A_68_P24135185	chr5:139847981-139848025	NR_029768:-2303	Mir339	DIVERGENT_PROMOTER	1.604	3.796	2433.75	9238.48	6.087	2046.61	12458.24
A_68_P32240004	chrX:7327982-7328026	NR_030454:-2210	Mir684-1	DIVERGENT_PROMOTER	2.296	3.799	1605.13	6098.03	8.723	894.64	7803.66
A_68_P29613302	chr14:55236499-55236543	NR_030457:-898	Mir686	PROMOTER	1.723	4.302	368.61	1585.89	7.412	316.57	2346.51
A_68_P22481653	chr3:121996304-121996348	NR_030439:295	Mir760	DOWNSTREAM	2.249	3.528	297.41	1049.11	7.933	238.92	1895.23
A_68_P22481656	chr3:121996594-121996638	NR_030439:5	Mir760	INSIDE	1.849	3.682	1919.95	7068.63	6.809	1611.73	10973.93
A_68_P25261466	chr7:86650289-86650333	NR_029818:161	Mir9-3	DOWNSTREAM	1.638	2.324	2376.34	5523.69	3.807	2028.31	7722.67
A_68_P25261383	chr7:86640870-86640922	NR_029818:-9253	Mir9-3	PROMOTER	1.520	4.997	818.01	4087.90	7.595	655.17	4976.12
A_68_P29020011	chr13:48641397-48641441	NR_029725:-7777	Mirlet7a-1	PROMOTER	2.016	5.034	3588.40	18065.04	10.151	2852.00	28949.53
A_68_P30556199	chr16:13256418-13256462	NM_001122667:-133	Mkl2	PROMOTER	1.806	2.014	1559.34	3140.77	3.637	1310.82	4767.44
A_68_P30556205	chr16:13257041-13257085	NM_181860:-8109	Mkl2	PROMOTER	1.574	4.312	4838.93	20866.87	6.790	3944.61	26782.12
A_68_P24337499	chr6:31349464-31349508	NM_013791:659	Mkln1	INSIDE	1.619	4.490	3001.53	13477.04	7.271	2353.24	17110.24
A_68_P24337493	chr6:31348815-31348859	NM_013791:9	Mkln1	INSIDE	1.517	7.206	1344.21	9686.86	10.934	1148.97	12562.71
A_68_P24383009	chr6:39369612-39369656	NM_018810:734	Mkrn1	INSIDE	1.773	4.571	1184.49	5414.21	8.105	871.42	7062.48
A_68_P21038125	chr2:17987169-17987213	NM_010804:10293	Mllt10	INSIDE	1.513	2.283	1455.18	3322.77	3.454	1218.79	4209.49
A_68_P28051873	chr11:97521768-97521812	NM_139311:-2935	Mllt6	PROMOTER	1.592	4.326	636.54	2753.65	6.886	596.03	4104.36
A_68_P24089922	chr5:130089988-130090034	NM_011846:-78	Mmp17	PROMOTER	2.386	10.764	5163.23	55575.58	25.684	3039.76	78072.02
A_68_P23439842	chr4:155027173-155027217	NM_011985:299	Mmp23	INSIDE	1.548	1.467	1762.94	2585.39	2.270	1420.78	3224.59
A_68_P23985683	chr5:111849467-111849511	NM_001081235:2303	Mn1	INSIDE	1.716	3.154	537.33	1694.76	5.412	481.21	2604.08
A_68_P23985664	chr5:111847445-111847489	NM_001081235:281	Mn1	INSIDE	1.589	4.139	2050.83	8487.53	6.574	1625.04	10683.59
A_68_P27922756	chr11:74651953-74651997	NM_010813:7549	Mnt	INSIDE	1.688	2.764	2511.91	6941.73	4.665	2144.68	10004.52
A_68_P27922751	chr11:74651412-74651456	NM_010813:7009	Mnt	INSIDE	1.608	4.731	2325.84	11002.83	7.608	1797.06	13671.47
A_68_P23567791	chr5:29805138-29805182	NM_019944:-150	Mnx1	PROMOTER	2.737	4.281	2107.37	9020.61	11.717	1689.85	19799.24
A_68_P23873662	chr5:89150059-89150103	NM_026735:185	Mobkl1a	INSIDE	2.246	6.208	3024.03	18774.60	13.943	2380.22	33186.33
A_68_P23873660	chr5:89149794-89149838	NM_026735:-79	Mobkl1a	PROMOTER	1.781	1.972	1492.65	2944.13	3.513	1281.13	4500.88
A_68_P24592782	chr6:83275935-83275979	NM_145571:-76	Mobkl1b	DIVERGENT_PROMOTER	1.671	2.406	1230.29	2959.67	4.021	999.48	4018.65

ProbeName	Target position of probe on CpG island microarray	TargetID	GeneSymbol	CpG island Description	Ratio of relative methylation (TiO_2-NP/Vehicle)	Sham group			TiO_2-H group		
						Relative methylation (Cy5/Cy3)	Cy3 signal (Input)	Cy5 signal (MeDIP)	Relative methylation (Cy5/Cy3)	Cy3 signal (Input)	Cy5 signal (MeDIP)
A_68_P20240462	chr1:55188131-55188175	NM_025283:64	Mobkl3	INSIDE	2.583	1.455	1093.20	1591.01	3.759	1044.28	3925.12
A_68_P26811667	chr9:107803499-107803544	NM_028369:13062	Mon1a	INSIDE	1.630	6.893	1343.62	9261.42	11.234	1132.45	12721.99
A_68_P27512350	chr10:122513515-122513559	NM_001163024:25	Mon2	INSIDE	1.881	4.934	2975.86	14683.41	9.281	2372.94	22023.12
A_68_P27512349	chr10:122513403-122513447	NM_001163024:137	Mon2	INSIDE	1.521	3.284	2131.92	7000.31	4.995	1977.70	9878.64
A_68_P31382195	chr17:80689441-80689485	NM_194269:-89	Morn2	PROMOTER	1.511	5.017	1251.41	6278.01	7.579	1032.08	7821.99
A_68_P20897790	chr1:186669470-186669514	NM_133684:234	Mosc2	INSIDE	1.840	1.853	1126.79	2088.49	3.410	827.00	2819.66
A_68_P20897789	chr1:186669350-186669394	NM_133684:354	Mosc2	INSIDE	1.567	4.109	4824.79	19825.41	6.437	4411.19	28396.76
A_68_P23051091	chr4:81088361-81088405	NM_010820:327	Mpdz	INSIDE	1.607	3.445	10469.36	36066.76	5.537	8861.19	49066.76
A_68_P31790832	chr18:67404825-67404869	NM_172630:638	Mppe1	INSIDE	1.597	2.013	1640.63	3302.46	3.214	1469.60	4723.63
A_68_P25953853	chr8:73284695-73284739	NM_183170:104	Mpv17l2	INSIDE	1.522	3.011	1073.38	3231.59	4.583	912.14	4180.48
A_68_P26764036	chr9:99336932-99336976	NM_008624:177	Mras	INSIDE	1.713	3.924	3458.26	13570.25	6.722	2687.56	18066.21
A_68_P25394537	chr7:112959576-112959620	NM_025301:3	Mrpl17	INSIDE	1.884	3.020	2285.66	6902.31	5.690	1763.25	10032.24
A_68_P26344985	chr9:20807480-20807524	NM_023167:322	Mrpl4	INSIDE	1.772	1.812	959.47	1738.23	3.210	728.81	2339.48
A_68_P20765302	chr1:162125324-162125368	NM_025474:-44	Mrps14	PROMOTER	2.468	10.418	5914.69	61622.12	25.708	3725.32	95770.00
A_68_P27564592	chr11:5607482-5607526	NM_026080:198	Mrps24	INSIDE	1.623	5.515	638.51	3521.43	8.951	525.42	4702.85
A_68_P31157235	chr17:35183322-35183366	NM_013600:207	Msh5	INSIDE	1.554	1.393	1449.87	2019.11	2.164	1177.43	2547.74
A_68_P31425843	chr17:88374364-88374408	NM_010830:-3	Msh6	PROMOTER	1.527	1.680	584.04	981.29	2.566	572.71	1469.51
A_68_P32817209	chrX:165111905-165111949	NM_010832:-92	Msl3	PROMOTER	2.457	2.364	835.90	1976.12	5.809	516.85	3002.34
A_68_P29044041	chr13:53558129-53558173	NM_013601:9999	Msx2	DOWNSTREAM	2.092	5.217	933.42	4869.21	10.914	666.32	7272.46
A_68_P25581950	chr7:147234174-147234218	NM_010836:791	Msx3	INSIDE	1.540	2.192	1382.25	3030.30	3.376	1166.43	3937.96
A_68_P25092690	chr7:52622810-52622854	NR_027802:230	Mtag2	INSIDE	1.876	3.324	2610.46	8676.44	6.236	1965.83	12259.79
A_68_P23094194	chr4:88783218-88783262	NM_024433:-33	Mtap	PROMOTER	1.809	3.549	5045.36	17904.92	6.418	3626.40	23275.54
A_68_P25954897	chr8:73438085-73438129	NM_173013:8234	Mtap1s	INSIDE	1.559	4.956	4668.80	23140.70	7.727	3349.62	25881.64
A_68_P25954894	chr8:73437692-73437736	NM_173013:7842	Mtap1s	INSIDE	1.509	2.762	422.18	1166.14	4.169	350.12	1459.49
A_68_P25360757	chr7:106417697-106417741	NM_001043355:863	Mtap6	INSIDE	2.210	4.630	2563.91	11870.65	10.232	1820.63	18628.00
A_68_P26985109	chr10:19868572-19868616	NM_001198635:-131	Mtap7	PROMOTER	1.808	5.214	1052.75	5489.10	9.426	892.38	8411.77
A_68_P22278904	chr3:82162070-82162114	NM_001081230:99	Mtap9	INSIDE	2.656	15.147	1784.01	27022.45	40.232	980.84	39461.13
A_68_P31127484	chr17:29484749-29484793	NM_019880:79	Mtch1	INSIDE	1.724	3.035	1442.67	4378.91	5.232	1135.57	5941.51
A_68_P32471622	chrX:72661675-72661719	NM_010839:186	Mtcp1	INSIDE	2.460	3.523	1439.66	5072.36	8.668	783.74	6793.80
A_68_P24592748	chr6:83267406-83267450	NM_008638:170	Mthfd2	INSIDE	1.688	1.747	2418.50	4225.33	2.950	1884.64	5558.99
A_68_P26224550	chr8:123632450-123632508	NM_001166482:-199	Mthfsd	DIVERGENT_PROMOTER	2.012	13.659	8664.67	118351.90	27.485	5760.48	158325.40
A_68_P32453217	chrX:68617827-68617871	NM_016985:-86	Mtmr1	PROMOTER	2.555	3.098	2572.42	7970.05	7.916	1433.82	11349.50
A_68_P25176077	chr7:71432423-71432467	NM_172742:-111	Mtmr10	PROMOTER	1.672	1.737	383.89	666.99	2.905	368.33	1070.00
A_68_P30020394	chr15:12134922-12134966	NM_172958:96	Mtmr12	INSIDE	1.515	2.637	924.58	2437.87	3.993	797.05	3182.95
A_68_P26659639	chr9:78296196-78296240	NM_026658:202	Mto1	INSIDE	1.960	1.795	397.62	713.77	3.518	481.25	1693.03
A_68_P23394435	chr4:147822699-147822743	NM_020009:30	Mtor	INSIDE	1.692	5.778	2393.80	13830.38	9.777	1997.31	19527.03
A_68_P24362057	chr6:35489865-35489909	NM_008098:2	Mtpn	INSIDE	1.785	3.996	2129.43	8508.36	7.134	1659.27	11837.30
A_68_P26905236	chr10:4522611-4522655	NM_175374:35	Mtrf1l	INSIDE	1.758	3.434	1637.50	5622.63	6.037	1288.35	7777.73
A_68_P26164572	chr8:113262808-113262852	NM_198625:17447	Mtss1l	INSIDE	3.486	7.191	2535.96	18235.83	25.068	1841.30	46157.41
A_68_P26164573	chr8:113262875-113262919	NM_198625:17513	Mtss1l	INSIDE	3.405	4.778	3372.57	16114.77	16.269	2472.91	40232.99
A_68_P26164568	chr8:113262460-113262504	NM_198625:17099	Mtss1l	INSIDE	2.463	10.638	6103.81	64933.75	26.205	4061.40	106430.50
A_68_P24176699	chr5:148769334-148769378	NM_029920:461	Mtus2	INSIDE	2.000	5.862	4947.09	28999.70	11.723	3794.87	44486.85
A_68_P23345719	chr4:137990637-137990681	NM_026689:72	Mul1	INSIDE	1.580	2.436	2819.27	6866.86	3.849	2247.89	8653.07
A_68_P27281785	chr10:79689246-79689290	NM_023431:-74	Mum1	PROMOTER	1.780	3.402	6975.63	23734.06	6.057	5170.10	31312.73
A_68_P28961844	chr13:38727138-38727182	NM_139063:-182	Muted	PROMOTER	1.630	1.777	971.29	1726.20	2.897	765.87	2218.59
A_68_P24004046	chr5:114894276-114894320	NM_023556:-16	Mvk	DIVERGENT_PROMOTER	1.837	4.580	511.99	2345.05	8.414	406.64	3421.63
A_68_P24611353	chr6:86619098-86619142	NM_010751:33	Mxd1	INSIDE	2.105	4.756	11879.78	56499.76	10.010	9280.46	92901.41

ProbeName	Target position of probe on CpG island microarray	TargetID	GeneSymbol	CpG island Description	Ratio of relative methylation (TiO_2-NP/Vehicle)	Sham group			TiO_2-H group		
						Relative methylation (Cy5/Cy3)	Cy3 signal (Input)	Cy5 signal (MeDIP)	Relative methylation (Cy5/Cy3)	Cy3 signal (Input)	Cy5 signal (MeDIP)
A_68_P29054388	chr13:55431309-55431353	NM_016662:-239	Mxd3	PROMOTER	1.886	4.408	1313.53	5789.64	8.315	1065.03	8855.45
A_68_P32181854	chr19:53403918-53403962	NM_001008543:-995	Mxi1	PROMOTER	1.586	2.159	1039.87	2244.67	3.423	842.54	2884.42
A_68_P20023664	chr1:9690196-9690240	NM_008651:72	Mybl1	INSIDE	2.277	6.956	950.56	6612.18	15.839	788.81	12494.10
A_68_P23260191	chr4:122672828-122672872	NM_008506:-491	Mycl1	PROMOTER	2.135	12.888	3988.63	51404.12	27.511	2697.48	74210.26
A_68_P28246269	chr12:12944121-12944165	NM_008709:4500	Mycn	INSIDE	1.884	3.332	931.52	3104.13	6.278	767.56	4818.81
A_68_P30356228	chr15:77672759-77672803	NM_022410:-235	Myh9	PROMOTER	2.233	5.791	8131.65	47090.36	12.930	5925.98	76623.80
A_68_P30356227	chr15:77672647-77672691	NM_022410:-123	Myh9	PROMOTER	1.598	4.863	5861.69	28505.42	7.773	4607.92	35815.21
A_68_P29001576	chr13:45485361-45485405	NM_181043:57	Mylip	INSIDE	1.713	1.479	1688.38	2496.84	2.533	1298.46	3288.58
A_68_P27927426	chr11:75469605-75469649	NM_001080775:110	Myo1c	INSIDE	1.501	3.042	780.85	2375.39	4.567	661.02	3019.16
A_68_P31831023	chr18:74602569-74602613	NM_201600:318	Myo5b	INSIDE	1.688	3.289	454.80	1495.78	5.552	442.03	2454.05
A_68_P25713191	chr8:23969515-23969559	NM_001081149:-474	Myst3	PROMOTER	1.757	1.557	599.52	933.45	2.735	404.86	1107.42
A_68_P29453038	chr14:22320578-22320622	NM_017479:1594	Myst4	INSIDE	1.772	10.363	8250.98	85508.03	18.364	5000.42	91827.39
A_68_P29453037	chr14:22320470-22320514	NM_017479:1486	Myst4	INSIDE	1.701	8.500	7052.63	59950.60	14.457	5362.66	77527.39
A_68_P29453036	chr14:22320298-22320342	NM_017479:1314	Myst4	INSIDE	1.676	3.472	1367.44	4747.66	5.820	1163.03	6768.46
A_68_P23757989	chr5:66155461-66155505	NM_001024917:723	N4bp2	INSIDE	1.917	2.979	1575.15	4692.02	5.709	1233.33	7041.59
A_68_P29080348	chr13:59686650-59686694	NM_030153:-21	Naa35	PROMOTER	2.207	6.748	1745.08	11775.43	14.893	1380.05	20552.67
A_68_P27569379	chr11:6505657-6505701	NM_001081652:379	Nacad	INSIDE	2.343	8.268	5339.75	44151.53	19.370	3628.25	70280.29
A_68_P30514378	chr16:5203992-5204036	NM_013796:91	Nagpa	INSIDE	2.028	3.462	3483.62	12060.51	7.023	2716.12	19074.05
A_68_P32226993	chr19:60831736-60831780	NM_178421:-131	Nanos1	PROMOTER	2.190	5.397	11809.73	63740.95	11.823	8903.57	105264.20
A_68_P32227000	chr19:60832730-60832774	NM_178421:863	Nanos1	INSIDE	1.698	1.374	2438.63	3350.63	2.333	1862.02	4344.11
A_68_P23230189	chr4:116299784-116299829	NM_001081475:296	Nasp	INSIDE	1.555	1.757	1532.01	2691.16	2.732	1330.79	3635.84
A_68_P23230191	chr4:116299963-116300007	NM_001081475:118	Nasp	INSIDE	1.849	1.862	608.42	1132.81	3.442	451.71	1554.89
A_68_P23592057	chr5:34343782-34343826	NM_001001985:5172	Nat8l	INSIDE	2.214	8.208	19505.54	160108.10	18.177	11137.08	202440.10
A_68_P22157978	chr3:55987140-55987184	NM_030595:461	Nbea	INSIDE	1.728	4.480	1138.19	5099.45	7.741	981.53	7598.19
A_68_P22752524	chr4:15885248-15885292	NM_013752:157	Nbn	INSIDE	2.141	2.779	1457.00	4048.50	5.948	1231.65	7325.64
A_68_P22752523	chr4:15885168-15885212	NM_013752:77	Nbn	INSIDE	1.526	2.495	486.75	1214.43	3.808	417.33	1589.30
A_68_P26500013	chr9:49606044-49606088	NM_001081445:1108	Ncam1	INSIDE	2.047	6.334	6147.30	38938.26	12.967	4556.04	59078.64
A_68_P26500017	chr9:49606485-49606529	NM_001081445:668	Ncam1	INSIDE	1.857	2.717	2069.79	5624.12	5.047	1608.35	8117.62
A_68_P30424721	chr15:89186360-89186404	NM_001115132:230	Ncaph2	INSIDE	1.786	4.363	3195.59	13942.59	7.793	2602.19	20277.78
A_68_P20188961	chr1:43503040-43503084	NM_010879:467	Nck2	INSIDE	1.615	2.269	1442.39	3273.18	3.665	1765.47	6470.17
A_68_P30480085	chr15:99256217-99256261	NM_001001884:31941	Nckap5l	INSIDE	1.738	4.114	1881.69	7740.46	7.150	1425.95	10195.16
A_68_P27290094	chr10:80959100-80959144	NM_134009:-14	Ncln	PROMOTER	1.662	3.944	2154.24	8495.68	6.556	1834.97	12030.15
A_68_P20040932	chr1:13363158-13363202	NM_001077695:984	Ncoa2	INSIDE	1.555	1.573	1015.20	1596.89	2.446	928.22	2270.58
A_68_P21826983	chr2:165818573-165818617	NM_008679:458	Ncoa3	INSIDE	1.704	7.206	5906.37	42563.88	12.282	4278.32	52547.31
A_68_P24064307	chr5:125507269-125507313	NM_011424:152294	Ncor2	INSIDE	1.820	5.340	9566.43	51080.29	9.717	7206.32	70024.62
A_68_P30560747	chr16:14163515-14163559	NM_001114085:169	Nde1	INSIDE	2.032	5.369	18178.12	97589.71	10.906	11694.21	127539.10
A_68_P30560744	chr16:14163261-14163305	NM_001114085:-85	Nde1	PROMOTER	1.513	1.741	1004.83	1749.37	2.633	774.54	2039.73
A_68_P27891735	chr11:68666059-68666103	NM_023668:553	Ndel1	INSIDE	1.505	1.532	1208.31	1851.00	2.306	966.35	2228.04
A_68_P25168663	chr7:69493375-69493419	NM_010882:234	Ndn	INSIDE	1.721	5.983	1564.06	9357.36	10.297	1409.83	14516.67
A_68_P30295269	chr15:66800435-66800479	NM_008681:747	Ndrg1	INSIDE	1.828	6.584	2811.46	18509.54	12.036	2244.93	27019.44
A_68_P29448698	chr14:21553543-21553587	NM_010811:220	Ndst2	INSIDE	1.542	1.813	339.59	615.81	2.797	289.37	809.26
A_68_P32322121	chrX:34731184-34731228	NM_019443:27	Ndufa1	INSIDE	1.603	1.735	1435.26	2490.36	2.782	880.76	2449.86
A_68_P25948620	chr8:72427025-72427069	NM_023312:-589	Ndufa13	PROMOTER	1.787	3.236	714.39	2311.79	5.783	683.51	3952.85
A_68_P25948621	chr8:72427093-72427137	NM_023312:-657	Ndufa13	PROMOTER	1.791	1.527	619.53	946.15	2.735	577.30	1579.17
A_68_P27536264	chr10:126948850-126948894	NM_001098789:-3122	Ndufa4l2	PROMOTER	2.359	3.890	1188.21	4622.23	9.177	968.88	8890.91
A_68_P30382971	chr15:82184657-82184701	NM_025987:43	Ndufa6	INSIDE	1.768	7.264	5277.33	38335.13	12.840	3633.10	46648.45
A_68_P27349651	chr10:92185236-92185280	NM_008682:-95	Nedd1	PROMOTER	1.968	4.104	1115.32	4576.96	8.076	934.49	7546.94

ProbeName	Target position of probe on CpG island microarray	TargetID	GeneSymbol	CpG island Description	Ratio of relative methylation (TiO_2-NP/Vehicle)	Sham group			TiO_2-H group		
						Relative methylation (Cy5/Cy3)	Cy3 signal (Input)	Cy5 signal (MeDIP)	Relative methylation (Cy5/Cy3)	Cy3 signal (Input)	Cy5 signal (MeDIP)
A_68_P31777744	chr18:65047479-65047523	NM_001114386:91	Nedd4l	INSIDE	1.843	10.346	942.70	9753.45	19.064	714.87	13628.62
A_68_P28977626	chr13:41454570-41454617	NM_017464:-223	Nedd9	PROMOTER	2.118	8.738	800.33	6993.42	18.504	665.69	12317.74
A_68_P22674484	chr3:156225009-156225062	NM_177274:113	Negr1	INSIDE	1.743	6.651	2013.48	13392.34	11.594	1409.31	16339.35
A_68_P20658349	chr1:140515986-140516030	NM_021605:265	Nek7	INSIDE	1.621	2.357	1442.96	3401.75	3.822	1214.75	4642.75
A_68_P30458578	chr15:95358757-95358801	NM_016743:359	Nell2	INSIDE	1.598	2.298	4531.86	10413.47	3.672	3655.64	13422.68
A_68_P26026035	chr8:88224110-88224154	NM_001081324:-9224	Neto2	PROMOTER	1.523	1.881	423.11	795.66	2.864	418.85	1199.59
A_68_P32148144	chr19:47253081-47253125	NM_021360:-207	Neurl1a	PROMOTER	1.772	2.016	1665.95	3358.83	3.572	1310.60	4681.33
A_68_P31110054	chr17:26552202-26552246	NM_001081656:315	Neurl1b	INSIDE	1.974	5.321	1188.55	6323.81	10.504	1036.44	10886.31
A_68_P20147266	chr1:36326391-36326435	NM_153408:3858	Neurl3	INSIDE	1.723	2.008	1892.51	3800.04	3.460	1405.54	4862.79
A_68_P28055686	chr11:98188438-98188482	NM_010895:2499	Neurod2	INSIDE	1.778	5.624	3157.40	17755.68	10.001	1993.02	19932.24
A_68_P22509722	chr3:127335595-127335641	NM_009718:-444	Neurog2	PROMOTER	1.715	2.334	539.14	1258.42	4.004	417.61	1672.11
A_68_P27560010	chr11:4749422-4749466	NM_010898:86	Nf2	INSIDE	1.596	2.351	3344.47	7862.57	3.752	2818.67	10576.64
A_68_P31866219	chr18:80894683-80894727	NM_001164111:10208	Nfatc1	INSIDE	2.561	3.778	1960.18	7405.43	9.674	1497.88	14490.19
A_68_P31866342	chr18:80909356-80909400	NM_001164111:-4466	Nfatc1	PROMOTER	2.130	3.279	1050.27	3443.57	6.983	910.41	6357.55
A_68_P26137548	chr8:108583970-108584014	NM_010901:490	Nfatc3	INSIDE	2.022	5.688	2426.16	13798.89	11.497	1782.99	20499.86
A_68_P29620076	chr14:56445768-56445812	NM_001168346:2159	Nfatc4	INSIDE	1.964	6.334	3968.26	25134.45	12.440	2785.42	34650.17
A_68_P23057175	chr4:82151052-82151103	NM_001113209:135	Nfib	INSIDE	2.186	12.701	2331.78	29615.98	27.759	1593.57	44235.74
A_68_P26021886	chr8:87308677-87308721	NM_001081982:15541	Nfix	INSIDE	2.033	3.153	2950.03	9300.15	6.408	2216.58	14204.27
A_68_P26021287	chr8:87230962-87231012	NM_001081981:67282	Nfix	DOWNSTREAM	1.743	5.485	1102.57	6047.57	9.561	909.77	8698.03
A_68_P26021284	chr8:87230641-87230685	NM_001081981:67606	Nfix	DOWNSTREAM	1.526	5.117	5399.70	27630.41	7.807	4219.66	32941.71
A_68_P26022004	chr8:87323463-87323507	NM_001081982:755	Nfix	INSIDE	1.591	1.494	801.96	1198.24	2.377	743.63	1767.40
A_68_P25023454	chr7:29551526-29551579	NM_010908:-9	Nfkbib	DIVERGENT_PROMOTER	2.257	8.477	713.98	6052.27	19.133	530.50	10149.87
A_68_P31201770	chr17:45692568-45692612	NM_008690:-74	Nfkbie	DIVERGENT_PROMOTER	2.372	9.998	12623.07	126210.50	23.715	8032.57	190492.00
A_68_P26400215	chr9:31193897-31193941	NM_172766:142	Nfrkb	INSIDE	1.566	3.163	6901.85	21828.89	4.954	5027.00	24903.77
A_68_P20352133	chr1:75121786-75121830	NM_029342:-8	Nhej1	PROMOTER	1.506	3.573	3084.75	11022.24	5.382	2114.11	11378.24
A_68_P22378552	chr3:101816741-101816785	NM_178777:2695	Nhlh2	INSIDE	1.993	4.711	4515.17	21271.37	9.388	3478.52	32656.96
A_68_P22378540	chr3:101815556-101815600	NM_178777:1511	Nhlh2	INSIDE	1.685	2.047	1560.93	3195.92	3.450	1276.07	4402.83
A_68_P32201471	chr19:56623197-56623241	NM_025811:468	Nhlrc2	INSIDE	1.723	2.753	677.65	1865.37	4.743	532.80	2526.87
A_68_P27794924	chr11:51433455-51433500	NM_026631:203	Nhp2	INSIDE	1.702	3.715	4250.56	15790.13	6.324	3209.74	20297.16
A_68_P32786683	chrX:158346162-158346206	NM_001081052:251538	Nhs	INSIDE	1.627	4.032	2483.51	10013.66	6.560	1267.66	8315.23
A_68_P32565869	chrX:99044829-99044873	NM_001163610:127	Nhsl2	INSIDE	1.771	4.869	967.20	4709.58	8.623	694.45	5988.30
A_68_P28518531	chr12:71213190-71213234	NM_008697:-300	Nin	PROMOTER	1.503	3.023	1996.26	6034.16	4.544	1812.79	8237.10
A_68_P29023779	chr13:49283004-49283048	NM_013610:111	Ninj1	INSIDE	1.968	4.358	3369.18	14682.72	8.576	2531.85	21712.21
A_68_P25144564	chr7:63274831-63274875	NM_153578:91	Nipa1	INSIDE	1.523	5.769	4245.98	24493.11	8.786	3181.43	27952.98
A_68_P25144266	chr7:63217700-63217744	NM_023647:98	Nipa2	INSIDE	1.530	1.692	1197.66	2026.59	2.589	1035.15	2680.06
A_68_P23328804	chr4:135050696-135050741	NM_028995:-299	Nipal3	DIVERGENT_PROMOTER	1.876	2.629	2957.08	7775.38	4.932	2108.10	10397.66
A_68_P30000055	chr15:8395316-8395364	NM_027707:-877	Nipbl	PROMOTER	1.807	10.334	11522.38	119074.30	18.676	6922.61	129285.00
A_68_P27050627	chr10:32609677-32609721	NM_001013411:23	Nkain2	INSIDE	2.512	7.636	4966.00	37922.42	19.182	3697.67	70929.86
A_68_P28873012	chr13:21560534-21560582	NM_025719:-188	Nkapl	PROMOTER	2.153	4.725	1181.67	5583.63	10.174	928.73	9449.34
A_68_P28873011	chr13:21560407-21560451	NM_025719:-58	Nkapl	PROMOTER	1.702	5.623	2171.94	12211.78	9.572	1787.51	17109.48
A_68_P26041379	chr8:91045377-91045421	NM_001163660:-6133	Nkd1	PROMOTER	1.941	4.503	5556.83	25021.48	8.741	4103.47	35867.01
A_68_P29142604	chr13:73984411-73984456	NM_028186:646	Nkd2	INSIDE	1.553	2.001	1418.80	2838.52	3.107	1209.81	3759.14
A_68_P28457438	chr12:57634665-57634709	NM_001146198:1407	Nkx2-1	INSIDE	1.843	2.491	2084.61	5193.37	4.591	1654.48	7596.10
A_68_P21722817	chr2:147019650-147019694	NM_001077632:-7534	Nkx2-2	PROMOTER	1.813	2.371	1751.40	4151.79	4.299	1341.88	5768.45
A_68_P21722826	chr2:147020543-147020587	NM_001077632:-8426	Nkx2-2	PROMOTER	1.749	5.439	1090.25	5929.85	9.512	881.94	8388.93
A_68_P21722727	chr2:147009567-147009611	NR_030769:-230	Nkx2-2as	PROMOTER	1.658	4.164	378.14	1574.57	6.904	306.78	2117.92
A_68_P21722283	chr2:146909704-146909748	NM_023504:1355	Nkx2-4	INSIDE	2.154	8.276	3101.29	25666.94	17.826	2213.60	39460.62

ProbeName	Target position of probe on CpG island microarray	TargetID	GeneSymbol	CpG island Description	Ratio of relative methylation (TiO_2-NP/Vehicle)	Sham group			TiO_2-H group		
						Relative methylation (Cy5/Cy3)	Cy3 signal (Input)	Cy5 signal (MeDIP)	Relative methylation (Cy5/Cy3)	Cy3 signal (Input)	Cy5 signal (MeDIP)
A_68_P31112726	chr17:26978463-26978507	NM_008700:26	Nkx2-5	INSIDE	1.875	3.055	1100.14	3361.00	5.729	941.99	5396.80
A_68_P31112722	chr17:26978019-26978063	NM_008700:470	Nkx2-5	INSIDE	1.595	2.654	1733.03	4599.01	4.234	1351.92	5723.70
A_68_P28457808	chr12:57713134-57713178	NM_008701:1115	Nkx2-9	INSIDE	1.929	4.516	1026.23	4634.92	8.713	885.78	7718.13
A_68_P28457807	chr12:57713035-57713080	NM_008701:1214	Nkx2-9	INSIDE	1.694	3.538	580.12	2052.72	5.995	556.78	3338.12
A_68_P29694905	chr14:69808678-69808722	NM_010921:-48	Nkx3-1	PROMOTER	1.542	6.019	1396.90	8407.55	9.278	1099.45	10200.87
A_68_P23936334	chr5:102093547-102093591	NM_144955:162	Nkx6-1	INSIDE	1.717	1.920	2360.88	4532.33	3.297	1938.13	6389.91
A_68_P23936340	chr5:102094274-102094318	NM_144955:-566	Nkx6-1	PROMOTER	1.601	2.012	1254.18	2523.11	3.222	1064.57	3429.80
A_68_P25578901	chr7:146768634-146768678	NR_027857:40	Nkx6-2	INSIDE	1.891	4.355	4487.96	19544.00	8.234	3456.28	28458.97
A_68_P25578895	chr7:146768050-146768094	NR_027857:624	Nkx6-2	INSIDE	1.718	3.426	1968.92	6745.40	5.887	1554.74	9153.31
A_68_P25578920	chr7:146770604-146770648	NR_027857:-1930	Nkx6-2	PROMOTER	1.676	13.760	1343.65	18488.62	23.064	1050.68	24232.72
A_68_P25715145	chr8:24268305-24268349	NM_029002:4584	Nkx6-3	INSIDE	1.947	5.658	1617.56	9152.92	11.019	1197.01	13189.99
A_68_P22222507	chr3:69525834-69525878	NM_133787:-120	Nmd3	PROMOTER	1.648	3.869	13203.19	51078.09	6.375	9180.51	58524.52
A_68_P28029989	chr11:93817362-93817406	NM_008705:-63	Nme2	PROMOTER	1.757	4.151	3025.34	12559.02	7.295	2391.06	17443.98
A_68_P20788410	chr1:166237694-166237738	NM_178071:-89	Nme7	DIVERGENT_PROMOTER	1.528	3.811	729.52	2780.46	5.824	566.98	3302.24
A_68_P20723965	chr1:154802917-154802961	NM_175460:708	Nmnat2	INSIDE	1.887	7.197	5326.04	38330.74	13.580	3487.41	47358.05
A_68_P20961517	chr2:3202117-3202161	NM_008708:579	Nmt2	INSIDE	1.633	1.432	1668.58	2389.46	2.338	1338.90	3130.71
A_68_P21777851	chr2:157386103-157386147	NM_010923:279	Nnat	INSIDE	1.695	4.656	5260.47	24494.02	7.893	3548.90	28010.42
A_68_P28004910	chr11:89163525-89163569	NM_008711:327	Nog	INSIDE	1.668	1.556	2076.56	3230.68	2.595	1813.25	4705.13
A_68_P30363283	chr15:78765483-78765527	NM_133800:142	Nol12	INSIDE	1.819	3.125	1896.06	5924.42	5.685	1496.31	8506.16
A_68_P32142001	chr19:46150433-46150477	NM_001039351:102	Nolc1	INSIDE	1.564	2.312	2268.24	5243.62	3.616	1941.60	7020.26
A_68_P24818877	chr6:125081806-125081850	NM_138747:-72	Nop2	PROMOTER	1.572	2.177	640.40	1394.03	3.421	552.07	1888.75
A_68_P23541414	chr5:23889472-23889516	NM_008713:18858	Nos3	INSIDE	1.560	1.597	1009.38	1612.02	2.491	853.11	2125.19
A_68_P25297066	chr7:94395079-94395123	NM_015760:-204	Nox4	PROMOTER	2.021	2.806	1848.01	5184.78	5.670	1421.14	8058.25
A_68_P24981713	chr7:17062092-17062136	NM_008718:15	Npas1	INSIDE	1.628	2.284	757.96	1731.04	3.718	644.47	2396.16
A_68_P28442990	chr12:54808173-54808232	NM_013780:458539	Npas3	INSIDE	1.819	5.224	899.58	4699.41	9.504	716.61	6810.66
A_68_P28445674	chr12:55169509-55169553	NM_013780:819867	Npas3	INSIDE	1.518	3.022	1322.00	3995.28	4.587	1122.49	5148.47
A_68_P31926197	chr19:4990111-4990155	NM_153553:-161	Npas4	PROMOTER	1.637	3.428	1185.76	4064.51	5.611	891.16	5000.01
A_68_P31926196	chr19:4989991-4990035	NM_153553:-41	Npas4	PROMOTER	1.696	2.125	311.58	662.19	3.605	264.17	952.35
A_68_P28185591	chr11:120470102-120470146	NM_153288:334	Npb	INSIDE	1.705	2.681	3291.21	8822.68	4.571	2345.49	10720.74
A_68_P31499629	chr18:12395086-12395130	NM_008720:-213	Npc1	PROMOTER	2.263	5.827	3711.66	21629.28	13.187	2940.31	38774.14
A_68_P28184543	chr11:120298753-120298797	NM_001195023:240	Nploc4	INSIDE	1.627	2.140	11825.52	25303.55	3.481	8744.97	30442.56
A_68_P22540268	chr3:132612964-132613008	NM_001029836:269	Npnt	INSIDE	1.527	2.202	1452.72	3198.70	3.362	1305.20	4387.93
A_68_P30018904	chr15:11836154-11836198	NM_001039181:-747	Npr3	PROMOTER	1.692	2.096	1637.38	3432.46	3.547	1416.00	5023.16
A_68_P28178599	chr11:119410964-119411008	NM_008730:-1852	Nptx1	PROMOTER	1.615	1.911	1470.34	2810.36	3.087	1225.21	3782.05
A_68_P30368655	chr15:79634703-79634747	NM_030689:415	Nptxr	INSIDE	1.767	8.180	2599.79	21265.87	14.457	2031.04	29361.75
A_68_P24434512	chr6:49772812-49772856	NM_023456:107	Npy	INSIDE	1.590	2.141	2840.99	6083.61	3.405	2464.85	8391.72
A_68_P25088136	chr7:51808933-51808977	NM_009473:339	Nr1h2	INSIDE	1.837	5.913	15841.82	93679.72	10.861	11014.63	119635.30
A_68_P25088125	chr7:51807692-51807736	NM_009473:1579	Nr1h2	INSIDE	1.772	8.216	7542.10	61962.49	14.558	5015.40	73015.23
A_68_P25088126	chr7:51807778-51807822	NM_009473:1493	Nr1h2	INSIDE	1.674	12.326	8310.29	102431.80	20.637	5463.46	112750.90
A_68_P27357767	chr10:93610648-93610692	NM_011629:-5	Nr2c1	PROMOTER	1.615	3.242	3314.61	10746.06	5.237	2772.73	14520.12
A_68_P24643143	chr6:92042164-92042208	NM_011630:775	Nr2c2	INSIDE	1.591	2.293	1246.91	2859.03	3.649	1034.35	3774.36
A_68_P27095712	chr10:42303728-42303772	NM_152229:-356	Nr2e1	PROMOTER	2.271	12.858	11284.84	145096.10	29.199	7978.87	232972.60
A_68_P27095676	chr10:42299357-42299401	NM_152229:4016	Nr2e1	INSIDE	1.589	5.157	3488.82	17991.60	8.195	2726.60	22344.39
A_68_P29162295	chr13:78338732-78338776	NM_010151:-511	Nr2f1	PROMOTER	1.522	2.715	1410.20	3828.32	4.131	1237.84	5113.65
A_68_P31640485	chr18:39650185-39650229	NM_008173:-3307	Nr3c1	PROMOTER	1.524	2.039	5516.93	11249.55	3.107	4451.15	13830.80
A_68_P21149827	chr2:38544490-38544534	NM_139051:25550	Nr5a1	DOWNSTREAM	1.746	3.993	1793.33	7161.53	6.972	1423.96	9927.68
A_68_P30346013	chr15:75920805-75920849	NM_144847:-383	Nrbp2	PROMOTER	1.880	2.482	2490.06	6180.14	4.665	1969.62	9188.98

ProbeName	Target position of probe on CpG island microarray	TargetID	GeneSymbol	CpG island Description	Ratio of relative methylation (TiO_2-NP/Vehicle)	Sham group			TiO_2-H group		
						Relative methylation (Cy5/Cy3)	Cy3 signal (Input)	Cy5 signal (MeDIP)	Relative methylation (Cy5/Cy3)	Cy3 signal (Input)	Cy5 signal (MeDIP)
A_68_P30346015	chr15:75921006-75921050	NM_144847:-585	Nrbp2	PROMOTER	1.587	2.955	3133.29	9260.38	4.690	2464.60	11559.95
A_68_P28397452	chr12:45429719-45429763	NM_001146031:-131	Nrcam	PROMOTER	1.594	3.036	974.16	2957.82	4.838	883.91	4276.75
A_68_P24329792	chr6:29998675-29998719	NM_001164230:709	Nrf1	INSIDE	1.611	1.694	1282.34	2172.15	2.730	1075.63	2936.14
A_68_P31621446	chr18:36177796-36177840	NM_001167891:178996	Nrg2	INSIDE	1.912	6.614	1194.95	7903.79	12.650	1049.81	13279.96
A_68_P31621448	chr18:36178193-36178239	NM_001167891:178598	Nrg2	INSIDE	1.845	5.845	6052.49	35379.04	10.786	4628.59	49921.89
A_68_P31621445	chr18:36177706-36177750	NM_001167891:179086	Nrg2	INSIDE	1.584	2.561	186.27	476.99	4.056	214.79	871.25
A_68_P30884140	chr16:76372608-76372652	NM_173440:664	Nrip1	INSIDE	1.673	1.856	339.43	630.12	3.106	264.27	820.80
A_68_P28950705	chr13:36816445-36816489	NM_153529:9857	Nrn1	DOWNSTREAM	1.884	8.358	4570.60	38201.00	15.746	3319.36	52265.63
A_68_P28950790	chr13:36827018-36827062	NM_153529:-717	Nrn1	PROMOTER	1.535	1.902	4299.20	8179.08	2.920	3310.23	9664.48
A_68_P20282811	chr1:62749708-62749752	NM_001077406:-160	Nrp2	PROMOTER	1.920	8.010	3164.47	25348.92	15.380	2494.48	38364.67
A_68_P31258174	chr17:56896255-56896299	NM_008738:677	Nrtn	INSIDE	1.800	9.627	1392.79	13407.73	17.330	1188.08	20589.48
A_68_P31438310	chr17:90855140-90855185	NM_020252:636980	Nrxn1	INSIDE	1.637	2.282	502.53	1146.56	3.734	388.45	1450.58
A_68_P23617871	chr5:38550739-38550783	NM_010942:-54	Nsg1	PROMOTER	1.696	3.491	4284.39	14957.57	5.920	3403.27	20146.35
A_68_P24116035	chr5:135845821-135845865	NM_145414:20	Nsun5	INSIDE	2.102	17.612	2746.94	48380.36	37.014	1782.24	65968.27
A_68_P32147197	chr19:47089479-47089523	NM_001164363:179	Nt5c2	INSIDE	1.557	5.384	1670.04	8991.04	8.381	1366.80	11454.95
A_68_P27315977	chr10:86241971-86242015	NM_175331:243	Nt5dc3	INSIDE	2.437	8.680	2362.38	20505.09	21.157	1771.77	37484.74
A_68_P30559120	chr16:13819791-13819845	NM_010946:449	Ntan1	INSIDE	1.743	2.431	941.21	2287.75	4.237	752.84	3189.89
A_68_P27887968	chr11:68026330-68026374	NM_008744:173976	Ntn1	INSIDE	2.061	8.672	4533.14	39310.19	17.872	3242.41	57949.74
A_68_P27354860	chr10:93103568-93103612	NM_021320:-203	Ntn4	PROMOTER	1.531	1.740	1786.47	3107.84	2.664	1503.59	4005.25
A_68_P25094516	chr7:52949587-52949631	NM_001033356:9825	Ntn5	INSIDE	2.535	6.663	3395.28	22623.71	16.894	2411.55	40740.20
A_68_P22422570	chr3:109945667-109945711	NM_001163348:702	Ntng1	INSIDE	2.176	1.821	2038.86	3713.60	3.963	1614.34	6397.87
A_68_P26253528	chr8:128258194-128258238	NM_025636:114	Ntpcr	INSIDE	2.043	4.405	4197.86	18493.38	8.999	3121.18	28087.87
A_68_P22310062	chr3:87599138-87599182	NM_001033124:-76	Ntrk1	DIVERGENT_PROMOTER	2.048	4.535	1048.11	4753.37	9.290	851.34	7908.49
A_68_P29075350	chr13:58908196-58908240	NM_001025074:-975	Ntrk2	PROMOTER	2.133	15.813	9970.56	157660.40	33.725	6282.78	211887.50
A_68_P27302613	chr10:83903670-83903714	NM_001004363:-476	Nuak1	PROMOTER	2.305	2.296	907.95	2084.57	5.293	794.46	4204.96
A_68_P23318161	chr4:133101757-133101801	NM_010948:164	Nudc	INSIDE	1.674	6.262	3142.55	19678.21	10.484	2430.84	25484.19
A_68_P26795688	chr9:105033875-105033919	NM_029385:239	Nudt16	INSIDE	4.477	3.082	440.46	1357.41	13.798	418.55	5775.31
A_68_P25049946	chr7:36340772-36340816	NM_033080:153	Nudt19	INSIDE	1.544	3.580	1871.68	6700.77	5.526	1707.74	9437.16
A_68_P31117527	chr17:27760515-27760559	NM_019837:-139	Nudt3	PROMOTER	1.743	1.917	633.10	1213.82	3.342	554.31	1852.33
A_68_P27938848	chr11:77500486-77500530	NM_001024205:868	Nufip2	INSIDE	1.602	2.017	1984.94	4004.32	3.232	1657.94	5359.11
A_68_P27483044	chr10:117229909-117229953	NM_134010:-169	Nup107	PROMOTER	1.724	2.587	4878.89	12620.43	4.460	3749.15	16720.20
A_68_P26242535	chr8:126473140-126473184	NM_172288:3	Nup133	INSIDE	1.970	3.959	1555.56	6157.81	7.800	1160.78	9054.05
A_68_P21418382	chr2:90519554-90519598	NM_021512:2205	Nup160	INSIDE	1.994	5.312	8159.69	43345.81	10.590	6258.95	66281.53
A_68_P21418380	chr2:90519239-90519283	NM_021512:1889	Nup160	INSIDE	1.823	5.490	7728.86	42429.44	10.009	6169.66	61751.11
A_68_P24636902	chr6:91066754-91066798	NM_018815:44	Nup210	INSIDE	1.561	4.842	904.94	4381.50	7.557	808.28	6107.96
A_68_P27931783	chr11:76212254-76212298	NM_008750:367	Nxn	INSIDE	1.686	2.497	957.35	2390.08	4.210	767.91	3232.83
A_68_P29031120	chr13:51266739-51266783	NM_029173:367	Nxnl2	INSIDE	1.600	3.920	2615.10	10251.94	6.274	2115.39	13272.40
A_68_P28039255	chr11:95375266-95375310	NM_130858:591	Nxph3	INSIDE	1.891	6.571	8238.62	54137.48	12.429	6016.37	74779.02
A_68_P28039252	chr11:95374960-95375004	NM_130858:897	Nxph3	INSIDE	1.713	3.388	3144.62	10653.15	5.802	2466.83	14312.08
A_68_P27835010	chr11:58947926-58947970	NM_001171512:1929	Obscn	INSIDE	1.685	5.288	2672.68	14132.77	8.908	1825.71	16262.78
A_68_P29269012	chr13:101322550-101322594	NM_008756:-119	Ocln	PROMOTER	1.564	2.208	944.00	2084.61	3.455	1005.10	3472.29
A_68_P22606649	chr3:144781741-144781785	NM_025714:199	Odf2l	INSIDE	1.963	4.343	4977.82	21616.42	8.526	3841.15	32748.48
A_68_P25846165	chr8:49314031-49314075	NM_001145937:445992	Odz3	INSIDE	1.660	2.794	320.07	894.25	4.638	302.78	1404.39
A_68_P25846166	chr8:49314139-49314183	NM_001145937:445884	Odz3	INSIDE	1.536	1.963	353.88	694.53	3.014	297.77	897.56
A_68_P25343512	chr7:103359060-103359104	NM_011858:-64	Odz4	PROMOTER	2.117	11.052	2664.60	29448.01	23.391	2126.61	49742.89
A_68_P24758832	chr6:113276968-113277012	NM_010957:21	Ogg1	INSIDE	1.739	4.601	10666.25	49076.78	8.002	7706.41	61667.74
A_68_P21089590	chr2:28048879-28048923	NM_001038614:288	Olfm1	INSIDE	1.610	1.554	1325.05	2058.76	2.502	1162.53	2908.28

ProbeName	Target position of probe on CpG island microarray	TargetID	GeneSymbol	CpG island Description	Ratio of relative methylation (TiO_2-NP/Vehicle)	Sham group			TiO_2-H group		
						Relative methylation (Cy5/Cy3)	Cy3 signal (Input)	Cy5 signal (MeDIP)	Relative methylation (Cy5/Cy3)	Cy3 signal (Input)	Cy5 signal (MeDIP)
A_68_P31167789	chr17:37334385-37334429	NM_001011518:505	Olfr94	INSIDE	3.185	0.553	612.40	338.39	1.760	485.62	854.62
A_68_P30961954	chr16:91271188-91271232	NM_016968:1197	Olig1	INSIDE	1.526	1.925	2176.75	4190.72	2.937	1710.79	5024.60
A_68_P30961697	chr16:91226847-91226891	NM_016967:1074	Olig2	INSIDE	1.750	3.045	5236.27	15942.01	5.329	4041.84	21540.29
A_68_P31774692	chr18:64500898-64500942	NM_194268:903	Onecut2	INSIDE	1.782	3.177	1989.72	6321.43	5.662	1432.33	8109.73
A_68_P20849641	chr1:177622831-177622875	NM_010098:-131	Opn3	DIVERGENT_PROMOTER	2.280	1.833	6986.01	12806.43	4.180	5290.64	22112.28
A_68_P24053482	chr5:123464905-123464949	NM_175423:-156	Orai1	PROMOTER	1.816	2.677	906.36	2426.00	4.861	846.86	4116.84
A_68_P24119778	chr5:136646540-136646584	NM_178751:-36	Orai2	PROMOTER	1.570	2.888	626.29	1808.83	4.534	612.18	2775.79
A_68_P20259227	chr1:58561519-58561563	NM_001025378:-3280	Orc2	PROMOTER	1.530	2.177	586.53	1276.70	3.330	452.39	1506.25
A_68_P27280126	chr10:79440000-79440044	NM_001003949:7053	ORF61	INSIDE	1.672	6.151	2393.85	14723.85	10.284	1638.63	16851.02
A_68_P27280132	chr10:79440564-79440611	NM_001003949:6488	ORF61	INSIDE	1.613	3.420	396.18	1354.95	5.517	305.43	1685.10
A_68_P27280131	chr10:79440436-79440480	NM_001003949:6617	ORF61	INSIDE	1.535	3.448	2109.25	7272.62	5.293	1727.17	9141.30
A_68_P20231213	chr1:53353942-53353986	NM_145517:26	Ormdl1	INSIDE	3.543	1.826	419.01	764.99	6.468	388.28	2511.27
A_68_P22752864	chr4:15941113-15941157	NM_145950:-110	Osgin2	PROMOTER	2.094	4.203	1844.34	7752.34	8.800	1498.32	13185.35
A_68_P27096130	chr10:42398668-42398712	NM_172416:-31	Ostm1	PROMOTER	2.043	8.153	7419.15	60484.62	16.654	5007.72	83396.75
A_68_P23618578	chr5:38668746-38668790	NM_172709:126	Otop1	INSIDE	1.845	3.000	1919.11	5756.51	5.536	1513.31	8377.17
A_68_P29237600	chr13:95653897-95653941	NM_011021:8337	Otp	DOWNSTREAM	2.471	2.116	4449.75	9414.95	5.227	3177.96	16612.19
A_68_P29237599	chr13:95653816-95653860	NM_011021:8257	Otp	DOWNSTREAM	2.133	2.276	7639.08	17383.48	4.854	5327.65	25861.44
A_68_P29237544	chr13:95647510-95647554	NM_011021:1951	Otp	INSIDE	1.900	3.442	1352.07	4653.63	6.541	951.11	6220.87
A_68_P28697278	chr12:104627062-104627106	NM_001177841:193	Otub2	INSIDE	1.615	2.049	1442.32	2955.86	3.310	1280.20	4237.09
A_68_P22347470	chr3:95908249-95908293	NM_001025613:-179	Otud7b	PROMOTER	1.539	1.937	515.73	999.13	2.982	384.89	1147.91
A_68_P27924667	chr11:74992384-74992428	NM_027136:-96	Ovca2	PROMOTER	1.889	6.221	3563.75	22170.42	11.753	2485.00	29206.57
A_68_P22180925	chr3:60806609-60806653	NM_008772:-86	P2ry1	PROMOTER	1.542	1.612	1581.70	2549.48	2.485	1392.53	3460.49
A_68_P27168078	chr10:58786437-58786481	NM_011030:415	P4ha1	INSIDE	1.840	4.803	4662.90	22393.81	8.836	3461.32	30584.71
A_68_P27168073	chr10:58785999-58786043	NM_011030:-23	P4ha1	PROMOTER	1.509	2.524	683.06	1724.28	3.809	537.46	2046.99
A_68_P30139527	chr15:36538715-36538759	NM_008774:-8	Pabpc1	PROMOTER	1.515	1.917	3127.60	5996.12	2.904	2496.02	7248.76
A_68_P30139530	chr15:36539045-36539089	NM_008774:-338	Pabpc1	PROMOTER	1.795	1.615	1468.37	2370.92	2.898	1265.04	3665.54
A_68_P32625282	chrX:117040767-117040811	NM_053114:-16	Pabpc5	PROMOTER	1.669	4.541	2248.43	10209.47	7.577	1645.15	12464.63
A_68_P29614877	chr14:55513260-55513304	NM_019402:303	Pabpn1	INSIDE	2.182	9.221	2638.58	24330.46	20.124	1966.61	39576.81
A_68_P31117838	chr17:27822326-27822370	NM_178365:150	Pacsin1	INSIDE	1.768	3.552	4612.42	16382.12	6.279	3523.84	22124.95
A_68_P31117689	chr17:27792526-27792570	NM_011861:-78	Pacsin1	PROMOTER	1.615	3.070	940.96	2889.02	4.959	739.85	3668.92
A_68_P31117688	chr17:27792442-27792486	NM_011861:-162	Pacsin1	PROMOTER	1.610	3.962	687.38	2723.32	6.378	565.99	3609.76
A_68_P21421545	chr2:91095901-91095945	NM_030880:-1047	Pacsin3	PROMOTER	1.632	4.268	1205.71	5146.03	6.967	981.70	6839.83
A_68_P25009543	chr7:26082789-26082833	NM_008776:164	Pafah1b3	INSIDE	1.626	3.401	2594.91	8824.50	5.529	2029.01	11218.18
A_68_P30650846	chr16:32079318-32079362	NM_177326:19	Pak2	INSIDE	1.975	8.408	4276.72	35957.81	16.607	3448.53	57270.10
A_68_P30650841	chr16:32078788-32078844	NM_177326:543	Pak2	INSIDE	1.572	5.599	463.67	2596.20	8.801	294.26	2589.67
A_68_P25022784	chr7:29383198-29383242	NM_027470:-17	Pak4	PROMOTER	1.853	2.382	1554.18	3702.31	4.414	1320.10	5827.10
A_68_P21565317	chr2:118489132-118489176	NM_001033254:-158	Pak6	PROMOTER	1.614	1.728	1297.75	2242.70	2.790	1069.14	2982.74
A_68_P25911981	chr8:64238686-64238730	NM_001081390:142779	Palld	INSIDE	1.978	2.275	971.13	2209.05	4.500	779.80	3508.97
A_68_P25910880	chr8:64070053-64070097	NM_001081390:311413	Palld	INSIDE	1.803	5.859	3203.60	18770.66	10.564	2389.29	25240.59
A_68_P26017663	chr8:86545319-86545363	NM_028877:-32	Palm3	DIVERGENT_PROMOTER	1.837	4.553	2619.26	11926.44	8.366	2019.89	16899.13
A_68_P24173631	chr5:148241874-148241918	NM_028291:-259	Pan3	PROMOTER	1.613	2.699	3211.48	8667.31	4.355	2514.20	10948.12
A_68_P23435277	chr4:154338248-154338292	NM_172990:29	Pank4	INSIDE	1.780	5.935	6193.36	36759.48	10.566	4696.39	49623.53
A_68_P29228002	chr13:93962021-93962065	NM_133905:196	Papd4	INSIDE	1.520	4.177	1394.45	5824.97	6.349	1073.14	6813.49
A_68_P26039507	chr8:90723531-90723575	NM_001164497:441	Papd5	INSIDE	1.748	5.336	3155.94	16841.30	9.330	2634.39	24579.68
A_68_P27648126	chr11:23795318-23795362	NM_172555:-70	Papolg	PROMOTER	1.923	3.254	5338.37	17370.64	6.258	3939.80	24656.64
A_68_P21839351	chr2:167906222-167906266	NM_021409:-259	Pard6b	PROMOTER	1.565	6.818	1258.10	8577.99	10.671	1114.90	11897.11
A_68_P26554375	chr9:59465216-59465260	NM_001205239:148	Parp6	INSIDE	1.878	4.980	2916.91	14526.13	9.350	2137.24	19983.87

ProbeName	Target position of probe on CpG island microarray	TargetID	GeneSymbol	CpG island Description	Ratio of relative methylation (TiO_2-NP/Vehicle)	Sham group			TiO_2-H group		
						Relative methylation (Cy5/Cy3)	Cy3 signal (Input)	Cy5 signal (MeDIP)	Relative methylation (Cy5/Cy3)	Cy3 signal (Input)	Cy5 signal (MeDIP)
A_68_P26554373	chr9:59464987-59465031	NM_001205239:-82	Parp6	PROMOTER	1.842	4.992	752.76	3758.08	9.197	605.13	5565.22
A_68_P29360867	chr13:117813151-117813195	NM_001081009:1151	Parp8	INSIDE	1.624	2.720	1787.53	4862.28	4.418	1510.48	6672.61
A_68_P27551118	chr11:3189560-3189605	NM_019574:-877	Patz1	PROMOTER	1.586	2.121	4010.21	8505.49	3.364	3196.90	10755.63
A_68_P32134209	chr19:44830746-44830790	NM_011037:-1115	Pax2	PROMOTER	1.621	2.469	5256.54	12977.15	4.002	3853.20	15420.81
A_68_P22878646	chr4:44717054-44717098	NM_008782:6236	Pax5	INSIDE	1.867	3.219	1464.92	4715.36	6.011	1183.08	7111.31
A_68_P27265105	chr10:76424885-76424929	NM_021568:-214	Pcbp3	PROMOTER	2.101	15.355	9942.78	152671.90	32.260	6191.69	199742.50
A_68_P26772513	chr9:100935215-100935259	NM_025835:58	Pccb	INSIDE	2.286	4.099	7042.17	28866.25	9.370	5402.54	50619.91
A_68_P31632704	chr18:38371400-38371444	NM_029357:-2006	Pcdh1	PROMOTER	1.730	3.324	1831.38	6088.28	5.751	1438.68	8273.75
A_68_P31632702	chr18:38371220-38371264	NM_029357:-1826	Pcdh1	PROMOTER	1.614	1.637	1732.59	2835.79	2.642	1331.45	3518.04
A_68_P22101775	chr3:45185176-45185220	NM_011043:2879	Pcdh10	INSIDE	2.086	3.350	1178.34	3947.95	6.990	865.17	6047.41
A_68_P29773624	chr14:84843322-84843371	NM_001013753:-23	Pcdh17	PROMOTER	1.670	2.058	854.99	1759.49	3.436	615.33	2114.28
A_68_P24591289	chr6:83027770-83027814	NM_197992:-591	Pcgf1	DIVERGENT_PROMOTER	2.204	2.692	354.10	953.35	5.934	302.72	1796.48
A_68_P23972071	chr5:108890061-108890105	NM_172716:-268	Pcgf3	PROMOTER	1.559	1.706	2087.61	3562.17	2.661	1779.09	4734.15
A_68_P23972073	chr5:108890220-108890264	NM_172716:-108	Pcgf3	PROMOTER	1.535	1.619	1040.16	1683.50	2.484	873.42	2169.90
A_68_P21820253	chr2:164705104-164705148	NM_146129:259	Pcif1	INSIDE	1.506	2.676	1462.77	3914.74	4.030	1325.28	5340.69
A_68_P25809870	chr8:42325280-42325324	NM_023662:190	Pcm1	INSIDE	2.286	10.549	531.79	5609.94	24.115	415.82	10027.66
A_68_P28583196	chr12:82961089-82961133	NM_018814:94	Pcnx	INSIDE	1.529	2.203	1583.96	3489.16	3.368	1330.25	4480.48
A_68_P31759277	chr18:61867112-61867156	NM_172832:155	Pcyox1l	INSIDE	1.517	2.206	3676.05	8109.10	3.347	2825.32	9456.43
A_68_P24164494	chr5:145900797-145900843	NM_001033313:138	Pdap1	INSIDE	1.765	2.842	4990.32	14183.28	5.016	3663.46	18375.68
A_68_P22246600	chr3:75360381-75360425	NM_019745:319	Pdcd10	INSIDE	1.539	2.256	8011.46	18072.48	3.472	5244.25	18209.34
A_68_P25435757	chr7:121559995-121560039	NM_011055:1249	Pde3b	INSIDE	1.762	3.398	1535.25	5217.14	5.987	1224.88	7333.25
A_68_P26345770	chr9:20970026-20970070	NM_183408:-109	Pde4a	PROMOTER	1.604	5.969	970.27	5791.23	9.573	837.83	8020.49
A_68_P22484168	chr3:122432681-122432725	NM_153422:627	Pde5a	INSIDE	1.670	4.891	2019.15	9875.76	8.168	1558.90	12732.31
A_68_P26142832	chr8:109571961-109572005	NM_026513:532	Pdf	INSIDE	1.539	2.131	1545.36	3293.12	3.279	1239.71	4064.46
A_68_P24132971	chr5:139470887-139470931	NM_008808:-1	Pdgfa	PROMOTER	1.916	6.049	10464.92	63300.49	11.592	10109.40	117188.10
A_68_P24132963	chr5:139469932-139469976	NM_008808:953	Pdgfa	INSIDE	1.639	2.663	4001.13	10655.82	4.365	3144.04	13724.14
A_68_P26282992	chr9:6168448-6168492	NM_027924:-141	Pdgfd	PROMOTER	1.570	2.157	1141.79	2463.16	3.387	924.44	3131.10
A_68_P23807625	chr5:75548984-75549028	NM_001083316:-3184	Pdgfra	PROMOTER	1.952	3.211	2425.96	7790.23	6.267	1751.08	10974.80
A_68_P32778409	chrX:156576281-156576325	NM_008810:-34	Pdha1	PROMOTER	3.016	7.865	716.04	5631.76	23.720	359.32	8523.27
A_68_P23322032	chr4:133843514-133843558	NM_001163794:-368	Pdik1l	PROMOTER	2.276	2.424	1209.39	2931.15	5.516	959.27	5291.41
A_68_P21322072	chr2:71711713-71711757	NM_172665:406	Pdk1	INSIDE	1.711	2.500	555.31	1388.44	4.279	440.50	1884.90
A_68_P32536210	chrX:91077357-91077401	NM_145630:56	Pdk3	INSIDE	1.538	3.659	1319.92	4829.75	5.629	988.31	5563.58
A_68_P31095624	chr17:24278101-24278145	NM_001080773:434	Pdpk1	INSIDE	1.546	2.407	3121.36	7513.71	3.722	2605.17	9697.66
A_68_P32464980	chrX:71069698-71069742	NM_001029868:588	Pdzd4	INSIDE	2.253	5.144	453.64	2333.58	11.591	243.90	2826.91
A_68_P32464984	chrX:71070091-71070135	NM_001029868:196	Pdzd4	INSIDE	1.811	4.937	765.24	3778.22	8.939	576.34	5152.02
A_68_P24697529	chr6:101327967-101328012	NM_018884:-98	Pdzrn3	PROMOTER	2.116	3.642	1129.20	4112.31	7.704	922.39	7106.53
A_68_P24956659	chr7:6682310-6682354	NM_008817:798	Peg3	INSIDE	1.748	1.520	829.29	1260.71	2.657	758.86	2016.08
A_68_P22696144	chr4:4066014-4066058	NM_001002927:-444	Penk	PROMOTER	2.009	5.062	1845.48	9341.22	10.168	1526.33	15519.67
A_68_P25046332	chr7:35697322-35697366	NM_008820:-81	Pepd	PROMOTER	1.775	4.026	3483.89	14027.35	7.147	2496.64	17842.52
A_68_P26977750	chr10:18565037-18565081	NM_022032:182	Perp	INSIDE	1.631	6.079	2807.39	17066.31	9.913	2265.96	22463.50
A_68_P23398659	chr4:148473653-148473697	NM_019781:247	Pex14	INSIDE	1.601	3.023	1074.05	3246.82	4.840	773.96	3745.89
A_68_P28803336	chr13:6648044-6648088	NM_019703:-96	Pfkp	PROMOTER	1.680	2.512	1278.91	3213.24	4.221	960.08	4052.59
A_68_P27901897	chr11:70468130-70468174	NM_011072:0	Pfn1	INSIDE	2.001	12.585	2812.51	35396.18	25.178	2091.82	52667.40
A_68_P32117861	chr19:41986212-41986256	NM_023418:-126	Pgam1	PROMOTER	1.664	2.604	2189.77	5702.93	4.335	1832.91	7945.02
A_68_P20237360	chr1:54614625-54614669	NM_001163314:-120	Pgap1	PROMOTER	1.573	1.663	1951.68	3246.19	2.616	1243.26	3252.49
A_68_P23749120	chr5:64484497-64484543	NM_025700:332	Pgm1	INSIDE	1.632	4.520	2595.29	11730.50	7.376	2168.53	15994.06
A_68_P23146552	chr4:99602526-99602570	NM_028132:493	Pgm2	INSIDE	2.610	5.642	12527.75	70679.57	14.726	8253.81	121548.50

ProbeName	Target position of probe on CpG island microarray	TargetID	GeneSymbol	CpG island Description	Ratio of relative methylation (TiO_2-NP/Vehicle)	Sham group			TiO_2-H group		
						Relative methylation (Cy5/Cy3)	Cy3 signal (Input)	Cy5 signal (MeDIP)	Relative methylation (Cy5/Cy3)	Cy3 signal (Input)	Cy5 signal (MeDIP)
A_68_P31097493	chr17:24606963-24607007	NM_025954:-433	Pgp	PROMOTER	1.951	7.982	4367.45	34859.26	15.570	2531.42	39413.34
A_68_P25953242	chr8:73183309-73183353	NM_023217:250	Pgpep1	INSIDE	1.959	1.778	3492.00	6208.53	3.483	2420.79	8431.85
A_68_P22083003	chr3:40886734-40886778	NM_027558:212	Pgrmc2	INSIDE	1.583	4.991	30520.14	152321.60	7.899	21096.52	166634.90
A_68_P26951412	chr10:13194053-13194097	NM_001195065:128	Phactr2	INSIDE	1.753	1.737	792.22	1376.34	3.045	676.01	2058.35
A_68_P21888635	chr2:177853739-177853783	NM_001177789:81	Phactr3	INSIDE	2.868	25.028	2515.99	62971.06	71.771	1619.79	116252.80
A_68_P21888793	chr2:177877242-177877286	NM_028806:627	Phactr3	INSIDE	2.033	15.233	3107.48	47336.47	30.969	1919.41	59441.49
A_68_P23415887	chr4:151369933-151369977	NM_172705:334	Phf13	INSIDE	1.522	2.338	8895.10	20795.78	3.559	7091.16	25238.78
A_68_P27796195	chr11:51671777-51671821	NM_199299:-815	Phf15	PROMOTER	1.927	3.621	11034.72	39960.52	6.977	7949.07	55458.77
A_68_P22085568	chr3:41368782-41368826	NM_001130186:3	Phf17	INSIDE	2.300	18.229	4332.09	78968.20	41.924	2483.05	104099.50
A_68_P22085575	chr3:41369640-41369684	NM_001130186:861	Phf17	INSIDE	1.805	7.734	1394.73	10786.45	13.960	1101.00	15370.41
A_68_P21130445	chr2:34769374-34769418	NM_028716:100	Phf19	INSIDE	1.696	2.328	393.02	914.86	3.947	279.14	1101.68
A_68_P21130446	chr2:34769487-34769531	NM_028716:-12	Phf19	PROMOTER	1.571	5.149	4230.44	21782.11	8.088	3388.78	27408.76
A_68_P30292506	chr15:66409382-66409426	NM_001081409:271	Phf20l1	INSIDE	1.693	2.130	765.44	1630.54	3.606	674.77	2432.95
A_68_P30292508	chr15:66409642-66409686	NM_001081409:531	Phf20l1	INSIDE	1.574	2.490	1736.18	4323.53	3.921	1286.61	5044.53
A_68_P30397291	chr15:84684245-84684289	NM_001081166:2293	Phf21b	INSIDE	1.710	4.195	3058.48	12831.61	7.173	2343.68	16811.35
A_68_P27898391	chr11:69810124-69810168	NM_030064:874	Phf23	INSIDE	1.900	6.188	16696.39	103321.70	11.758	11612.96	136541.00
A_68_P27898381	chr11:69809079-69809123	NM_030064:-172	Phf23	PROMOTER	1.704	1.792	4096.91	7341.58	3.054	2920.50	8919.54
A_68_P27898394	chr11:69810400-69810444	NM_030064:1150	Phf23	INSIDE	1.520	2.800	6398.65	17918.06	4.255	4222.08	17965.36
A_68_P27449326	chr10:110943860-110943904	NM_009344:541	Phlda1	INSIDE	1.699	4.686	709.78	3326.28	7.963	558.04	4443.61
A_68_P26469100	chr9:44516622-44516666	NM_153537:26633	Phldb1	INSIDE	2.502	21.012	5164.24	108511.90	52.574	2894.18	152159.80
A_68_P25005776	chr7:25396309-25396353	NM_001102613:-16	Phldb3	PROMOTER	1.980	6.753	6453.48	43581.39	13.373	4202.48	56201.35
A_68_P20506810	chr1:108069429-108069473	NM_133821:1005	Phlpp1	INSIDE	1.941	3.369	2493.42	8399.51	6.540	2097.84	13719.55
A_68_P20506808	chr1:108069211-108069255	NM_133821:787	Phlpp1	INSIDE	1.898	7.316	1813.92	13270.07	13.888	1509.26	20961.24
A_68_P26159425	chr8:112392612-112392656	NM_001122594:132	Phlpp2	INSIDE	1.759	3.602	1589.73	5726.16	6.337	1426.80	9041.23
A_68_P25375583	chr7:108967177-108967221	NM_008887:372	Phox2a	INSIDE	1.720	5.525	3288.44	18167.43	9.503	2745.79	26092.66
A_68_P25375611	chr7:108970306-108970350	NM_008887:3502	Phox2a	INSIDE	1.680	2.025	711.87	1441.29	3.402	593.26	2018.49
A_68_P21073067	chr2:25430182-25430226	NM_029293:187	Phpt1	INSIDE	1.554	4.301	8690.48	37378.51	6.683	6210.23	41501.24
A_68_P25587734	chr7:148445714-148445758	NM_001081118:31050	Phrf1	INSIDE	1.558	7.229	1689.14	12210.11	11.262	1175.85	13242.93
A_68_P23523151	chr5:20387300-20387344	NM_172992:620	Phtf2	INSIDE	1.700	2.310	1113.80	2573.14	3.927	1022.76	4015.91
A_68_P27230702	chr10:70061703-70061747	NM_001162846:-6164	Phyhipl	PROMOTER	2.042	3.479	2120.64	7376.67	7.102	1778.00	12627.91
A_68_P23696525	chr5:53132855-53132899	NM_025951:64	Pi4k2b	INSIDE	1.695	5.578	1428.36	7967.54	9.456	1312.72	12413.29
A_68_P26573393	chr9:62827843-62827887	NM_019663:822	Pias1	INSIDE	1.586	1.682	3374.87	5675.32	2.667	2383.82	6356.72
A_68_P27287865	chr10:80630602-80630646	NM_021501:-159	Pias4	DIVERGENT_PROMOTER	1.735	3.750	3317.59	12442.19	6.506	2416.58	15721.63
A_68_P27287763	chr10:80617060-80617104	NM_021501:13383	Pias4	INSIDE	1.503	3.958	1158.08	4583.60	5.950	877.19	5219.09
A_68_P32798239	chrX:160857687-160857731	NM_011081:-10	Piga	PROMOTER	1.846	2.501	1866.07	4667.41	4.618	951.22	4392.61
A_68_P31420721	chr17:87424605-87424649	NM_008838:115	Pigf	INSIDE	1.660	2.008	871.61	1750.16	3.333	783.45	2610.98
A_68_P31420718	chr17:87424300-87424344	NM_008838:419	Pigf	INSIDE	1.560	1.566	712.72	1116.06	2.444	619.81	1514.57
A_68_P30980968	chr16:94592282-94592326	NM_001159618:-147	Pigp	PROMOTER	1.525	3.774	2425.87	9154.33	5.756	2179.99	12547.77
A_68_P27942685	chr11:78141893-78141937	NM_201406:-9	Pigs	PROMOTER	1.662	1.612	736.88	1187.49	2.678	654.41	1752.51
A_68_P27980117	chr11:84693941-84693985	NM_001077636:-175	Pigw	PROMOTER	3.046	8.934	7846.91	70105.60	27.217	4810.06	130915.60
A_68_P27980115	chr11:84693687-84693731	NM_027388:-11	Pigw	DIVERGENT_PROMOTER	1.852	5.805	4740.80	27519.45	10.748	3448.08	37060.13
A_68_P25091451	chr7:52409634-52409678	NM_029406:-111	Pih1d1	PROMOTER	1.644	2.322	1695.68	3936.97	3.817	1344.88	5133.11
A_68_P26762516	chr9:99040770-99040814	NM_029094:-162	Pik3cb	PROMOTER	1.641	2.485	1822.34	4528.06	4.078	1394.74	5688.22
A_68_P27551374	chr11:3232269-3232313	NM_178149:1557	Pik3ip1	INSIDE	1.706	2.224	832.12	1850.51	3.793	649.12	2462.26
A_68_P25953980	chr8:73300616-73300660	NM_008841:-27	Pik3r2	DIVERGENT_PROMOTER	1.747	3.516	669.06	2352.54	6.141	614.17	3771.75
A_68_P30421719	chr15:88693018-88693062	NM_145478:417	Pim3	INSIDE	1.883	4.203	734.95	3088.77	7.913	589.31	4663.15
A_68_P29664074	chr14:64479020-64479064	NM_028228:-106	Pinx1	PROMOTER	1.575	3.862	3348.45	12932.88	6.085	2482.57	15106.29

ProbeName	Target position of probe on CpG island microarray	TargetID	GeneSymbol	CpG island Description	Ratio of relative methylation (TiO_2-NP/Vehicle)	Sham group			TiO_2-H group		
						Relative methylation (Cy5/Cy3)	Cy3 signal (Input)	Cy5 signal (MeDIP)	Relative methylation (Cy5/Cy3)	Cy3 signal (Input)	Cy5 signal (MeDIP)
A_68_P27534649	chr10:126648747-126648791	NM_054097:-90	Pip4k2c	PROMOTER	1.574	2.122	1956.33	4150.66	3.340	1693.26	5656.06
A_68_P27288650	chr10:80755715-80755759	NM_001146687:20	Pip5k1c	INSIDE	1.506	2.439	1510.25	3683.36	3.672	1267.97	4656.54
A_68_P31003040	chr17:3084893-3084937	NR_003519:-731	Pisd-ps2	PROMOTER	2.471	9.924	20698.53	205421.40	24.520	13630.62	334218.10
A_68_P23985069	chr5:111760075-111760119	NM_019640:314	Pitpnb	INSIDE	2.044	3.246	1597.28	5185.40	6.636	1287.60	8543.96
A_68_P28107262	chr11:107332069-107332113	NM_145823:-56	Pitpnc1	DIVERGENT_PROMOTER	1.915	11.985	2125.28	25472.22	22.948	1793.44	41155.20
A_68_P28107247	chr11:107330576-107330620	NM_145823:1436	Pitpnc1	INSIDE	1.562	1.920	1761.15	3381.05	2.998	1368.08	4102.19
A_68_P31921054	chr19:4101079-4101123	NM_008851:479	Pitpnm1	INSIDE	1.942	3.118	795.68	2480.76	6.056	677.00	4099.75
A_68_P29057251	chr13:55927242-55927286	NM_011097:5522	Pitx1	INSIDE	1.591	2.076	628.89	1305.28	3.303	520.93	1720.60
A_68_P22518659	chr3:128907292-128907336	NM_001042502:-9540	Pitx2	PROMOTER	1.645	3.010	1668.95	5023.52	4.951	1335.15	6610.53
A_68_P32555655	chrX:96666023-96666067	NM_001083110:30	Pja1	INSIDE	2.025	2.458	1745.67	4290.00	4.976	972.49	4838.95
A_68_P31295069	chr17:64681315-64681359	NM_001025309:-113	Pja2	PROMOTER	1.735	2.178	911.66	1985.96	3.779	794.14	3001.14
A_68_P31098019	chr17:24687487-24687532	NM_013630:615	Pkd1	INSIDE	1.785	5.306	1287.64	6831.75	9.469	1086.78	10290.64
A_68_P26016101	chr8:86222687-86222731	NM_001199593:-4748	Pkn1	PROMOTER	1.511	2.520	992.70	2501.25	3.806	882.85	3360.44
A_68_P26429021	chr9:36954305-36954349	NM_001029838:525	Pknox2	INSIDE	1.746	3.693	1007.30	3720.30	6.447	818.84	5279.12
A_68_P20642551	chr1:137815771-137815815	NM_019645:-191	Pkp1	PROMOTER	1.649	2.439	2311.39	5636.91	4.021	1869.14	7515.51
A_68_P30568393	chr16:16213292-16213336	NM_026163:-123	Pkp2	PROMOTER	1.596	2.621	1302.42	3413.60	4.184	1066.08	4460.33
A_68_P30568394	chr16:16213390-16213434	NM_026163:-25	Pkp2	PROMOTER	1.581	1.469	1701.74	2499.70	2.322	1438.16	3338.92
A_68_P21250388	chr2:58999667-58999711	NM_026361:782	Pkp4	INSIDE	2.153	7.303	2391.43	17465.64	15.726	1956.02	30760.24
A_68_P21250385	chr2:58999390-58999434	NM_026361:506	Pkp4	INSIDE	1.780	4.497	4161.06	18710.84	8.005	2857.20	22872.33
A_68_P26949005	chr10:12810608-12810652	NM_009538:37	Plagl1	INSIDE	1.519	8.094	996.87	8068.42	12.295	851.53	10469.14
A_68_P21753788	chr2:153067075-153067119	NM_018807:-2	Plagl2	DIVERGENT_PROMOTER	2.034	5.406	1530.17	8272.83	10.998	1242.41	13664.06
A_68_P24039106	chr5:120953388-120953432	NM_023625:222	Plbd2	INSIDE	1.674	3.995	959.96	3834.81	6.686	724.31	4842.55
A_68_P26203094	chr8:120022462-120022506	NM_172285:294	Plcg2	INSIDE	1.558	2.890	674.71	1949.84	4.503	610.32	2748.25
A_68_P30729955	chr16:46010463-46010507	NM_001134480:42	Plcxd2	INSIDE	1.845	4.883	876.34	4279.17	9.008	681.15	6135.69
A_68_P21587817	chr2:122564114-122564158	NM_019788:-104	Pldn	PROMOTER	1.618	4.136	1376.79	5694.31	6.692	1095.09	7328.83
A_68_P30346762	chr15:76028710-76028754	NM_201393:-93	Plec	PROMOTER	1.879	3.238	2210.87	7157.92	6.082	1649.76	10033.85
A_68_P30346633	chr15:76014154-76014198	NM_201394:11964	Plec	INSIDE	1.758	3.607	1900.53	6855.42	6.341	1385.38	8784.31
A_68_P30346599	chr15:76009927-76009971	NM_201394:16192	Plec	INSIDE	1.658	4.838	1926.06	9318.28	8.023	1489.19	11947.43
A_68_P30346619	chr15:76012289-76012333	NM_201394:13830	Plec	INSIDE	1.658	5.461	1257.05	6864.84	9.055	1034.03	9363.44
A_68_P30346592	chr15:76009110-76009154	NM_201394:17008	Plec	INSIDE	1.592	3.611	372.29	1344.43	5.748	317.36	1824.20
A_68_P30346615	chr15:76011854-76011898	NM_201394:14264	Plec	INSIDE	1.570	4.794	1004.83	4816.70	7.527	812.41	6114.67
A_68_P30346613	chr15:76011583-76011627	NM_201394:14536	Plec	INSIDE	1.805	1.900	565.89	1075.26	3.429	411.97	1412.72
A_68_P28566637	chr12:80012969-80013013	NM_013738:-5065	Plek2	PROMOTER	1.738	2.300	3127.95	7195.06	3.997	2381.41	9519.02
A_68_P28566640	chr12:80013209-80013253	NM_013738:-5305	Plek2	PROMOTER	1.503	1.504	2873.07	4322.23	2.261	2125.31	4805.89
A_68_P25524908	chr7:138009867-138009911	NM_133942:465	Plekha1	INSIDE	1.557	2.360	671.79	1585.13	3.673	567.60	2084.95
A_68_P25524905	chr7:138009499-138009543	NM_133942:97	Plekha1	INSIDE	1.523	3.165	4756.44	15053.68	4.820	3968.99	19129.86
A_68_P24894485	chr6:140373203-140373247	NM_144920:605	Plekha5	INSIDE	1.756	1.787	4206.33	7516.88	3.138	3331.26	10452.47
A_68_P26133404	chr8:107898928-107898972	NM_001081333:-330	Plekhg4	PROMOTER	1.572	3.196	1633.40	5220.99	5.026	1335.87	6714.39
A_68_P27285555	chr10:80260794-80260838	NM_023900:555	Plekhj1	INSIDE	2.029	5.818	14811.46	86178.09	11.803	10962.46	129386.30
A_68_P26588443	chr9:65427955-65427999	NM_153119:-135	Plekho2	DIVERGENT_PROMOTER	2.017	3.347	4537.00	15184.92	6.750	3697.39	24956.61
A_68_P26725648	chr9:92145271-92145315	NM_011636:261	Plscr1	INSIDE	1.735	3.586	4101.69	14709.23	6.221	3012.57	18741.24
A_68_P28053756	chr11:97847867-97847911	NM_001163608:-128	Plxdc1	PROMOTER	1.972	4.486	1026.12	4602.73	8.846	764.65	6763.79
A_68_P26818267	chr9:108998137-108998181	NM_172775:209	Plxnb1	INSIDE	1.833	3.745	978.86	3665.46	6.864	845.62	5804.39
A_68_P30423567	chr15:88991606-88991650	NM_001159521:12652	Plxnb2	INSIDE	1.556	5.958	10055.88	59914.80	9.268	6667.91	61798.74
A_68_P27362366	chr10:94407709-94407753	NM_018797:-518	Plxnc1	PROMOTER	1.676	12.375	270.07	3342.19	20.744	254.69	5283.15
A_68_P24774306	chr6:115943883-115943927	NM_026376:1119	Plxnd1	INSIDE	1.988	5.492	1597.59	8773.58	10.917	1045.67	11415.58
A_68_P24774311	chr6:115944633-115944678	NM_026376:368	Plxnd1	INSIDE	1.882	6.530	3149.31	20564.84	12.291	2421.41	29761.64

ProbeName	Target position of probe on CpG island microarray	TargetID	GeneSymbol	CpG island Description	Ratio of relative methylation (TiO_2-NP/Vehicle)	Sham group			TiO_2-H group		
						Relative methylation (Cy5/Cy3)	Cy3 signal (Input)	Cy5 signal (MeDIP)	Relative methylation (Cy5/Cy3)	Cy3 signal (Input)	Cy5 signal (MeDIP)
A_68_P22313622	chr3:88214207-88214256	NM_025928:7	Pmf1	INSIDE	1.734	3.408	876.38	2986.69	5.908	678.82	4010.58
A_68_P23527584	chr5:21243055-21243099	NM_028431:99	Pmpcb	INSIDE	1.544	3.765	910.72	3429.27	5.814	810.88	4714.20
A_68_P28470083	chr12:60168415-60168459	NM_008891:531	Pnn	INSIDE	2.123	1.820	544.02	990.33	3.864	511.63	1976.88
A_68_P27614819	chr11:17111398-17111442	NM_025443:172	Pno1	INSIDE	1.749	2.061	1394.34	2873.63	3.605	1113.56	4013.90
A_68_P24338521	chr6:31513401-31513445	NM_013723:515	Podxl	INSIDE	2.261	5.210	1194.73	6224.80	11.778	960.89	11317.24
A_68_P20800091	chr1:168339580-168339624	NM_001142948:357	Pogk	INSIDE	1.795	4.313	8825.38	38061.16	7.740	6802.34	52653.14
A_68_P22340919	chr3:94642155-94642199	NM_001165948:688	Pogz	INSIDE	1.790	7.624	1520.61	11592.56	13.646	1173.41	16012.30
A_68_P25088095	chr7:51804301-51804346	NM_011131:-138	Pold1	PROMOTER	1.691	2.746	3343.13	9180.62	4.645	2543.73	11815.50
A_68_P30678526	chr16:37011868-37011912	NM_001159369:19	Polq	INSIDE	1.889	2.244	1247.87	2800.29	4.240	1016.30	4308.64
A_68_P21623003	chr2:128926712-128926756	NM_009086:3	Polr1b	INSIDE	1.563	1.939	2491.03	4831.30	3.031	1831.52	5550.56
A_68_P21623006	chr2:128926947-128926991	NM_009086:237	Polr1b	INSIDE	1.629	1.389	1216.53	1689.37	2.262	1077.43	2436.99
A_68_P27896916	chr11:69572824-69572871	NM_009089:-1122	Polr2a	PROMOTER	1.517	1.649	653.89	1078.34	2.502	517.68	1295.20
A_68_P26079854	chr8:97381360-97381404	NM_009090:33	Polr2c	INSIDE	1.708	8.059	1623.42	13082.40	13.768	1177.99	16217.94
A_68_P30589177	chr16:20718065-20718109	NM_145632:188	Polr2h	INSIDE	1.695	2.321	1698.55	3942.10	3.934	1393.67	5482.07
A_68_P28875600	chr13:22073387-22073431	NM_001162929:346	Pom121l2	INSIDE	1.679	2.064	2503.61	5166.48	3.465	2055.02	7119.94
A_68_P24117361	chr5:136164752-136164796	NM_008898:-309	Por	PROMOTER	1.609	5.202	3240.90	16858.04	8.370	2280.86	19090.59
A_68_P20797864	chr1:167933679-167933723	NM_011137:-935	Pou2f1	PROMOTER	1.732	4.747	1600.53	7598.07	8.223	1240.42	10199.96
A_68_P23269516	chr4:124335391-124335435	NM_011141:524	Pou3f1	INSIDE	1.647	4.999	1869.29	9344.93	8.232	1390.95	11450.48
A_68_P20184680	chr1:42755426-42755470	NM_008900:1458	Pou3f3	INSIDE	2.714	6.638	5306.77	35227.30	18.016	3602.30	64898.50
A_68_P20184678	chr1:42755116-42755160	NM_008900:1148	Pou3f3	INSIDE	1.507	3.857	1035.60	3994.36	5.813	842.84	4899.23
A_68_P29871680	chr14:104872642-104872686	NM_011143:-5448	Pou4f1	PROMOTER	2.411	11.000	6367.29	70042.71	26.520	4744.22	125818.80
A_68_P30486488	chr15:100408907-100408951	NM_010127:7868	Pou6f1	INSIDE	1.685	3.988	537.02	2141.41	6.718	406.10	2728.33
A_68_P24770499	chr6:115312049-115312093	NM_001127330:832	Pparg	INSIDE	1.561	1.939	1450.39	2812.26	3.026	1089.63	3297.74
A_68_P31757526	chr18:61558105-61558149	NM_133249:1959	Ppargc1b	INSIDE	1.563	2.958	4297.15	12709.21	4.622	3216.73	14868.65
A_68_P21909031	chr2:180921876-180921920	NM_025598:-149	Ppdpf	PROMOTER	1.639	1.741	1516.43	2640.73	2.853	1252.05	3572.49
A_68_P25092565	chr7:52602361-52602407	NM_029741:20005	Ppfia3	INSIDE	2.064	16.737	4118.57	68931.68	34.541	2615.55	90342.51
A_68_P25092685	chr7:52622314-52622358	NM_029741:53	Ppfia3	INSIDE	1.790	3.630	1450.59	5264.91	6.498	1233.06	8011.86
A_68_P27568398	chr11:6316069-6316113	NM_008907:218	Ppia	INSIDE	1.776	4.454	3774.27	16809.61	7.911	3015.41	23855.94
A_68_P27568397	chr11:6315971-6316015	NM_008907:120	Ppia	INSIDE	1.632	3.616	2360.54	8534.66	5.899	1898.06	11197.31
A_68_P26590952	chr9:65908094-65908138	NM_011149:141	Ppib	INSIDE	1.871	4.387	1835.70	8054.00	8.209	1472.92	12091.40
A_68_P31126969	chr17:29401134-29401178	NM_026845:-240	Ppil1	DIVERGENT_PROMOTER	2.682	11.142	1321.08	14720.04	29.885	882.61	26376.44
A_68_P31126967	chr17:29400949-29400993	NM_026845:-54	Ppil1	DIVERGENT_PROMOTER	1.577	1.945	970.15	1886.55	3.066	846.71	2596.06
A_68_P28533645	chr12:73862367-73862411	NM_008910:191	Ppm1a	INSIDE	1.968	4.045	3217.29	13015.10	7.962	2569.35	20457.82
A_68_P27993897	chr11:87172172-87172216	NM_177167:302	Ppm1e	INSIDE	1.611	3.684	1483.62	5465.32	5.934	1280.66	7599.66
A_68_P23577160	chr5:31522948-31522992	NM_008014:-52	Ppm1g	PROMOTER	1.593	5.580	2910.39	16240.42	8.892	2461.09	21883.42
A_68_P22393109	chr3:104584285-104584329	NM_027982:333	Ppm1j	INSIDE	1.726	8.053	2584.88	20816.80	13.897	2145.43	29815.76
A_68_P23584119	chr5:32761375-32761419	NM_172707:54	Ppp1cb	INSIDE	1.713	3.796	5314.37	20175.38	6.503	4182.75	27199.38
A_68_P26918726	chr10:6979506-6979550	NM_133485:863	Ppp1r14c	INSIDE	1.631	3.344	2479.69	8292.48	5.454	1974.99	10772.07
A_68_P30349855	chr15:76502255-76502299	NM_033371:167	Ppp1r16a	INSIDE	2.037	5.148	4503.69	23185.47	10.486	3132.18	32843.45
A_68_P21784239	chr2:158492898-158492942	NM_001159662:50	Ppp1r16b	INSIDE	1.681	3.141	534.12	1677.59	5.279	476.36	2514.85
A_68_P30646578	chr16:31275334-31275378	NM_025800:7	Ppp1r2	INSIDE	1.929	3.705	2474.18	9166.92	7.148	2077.06	14846.63
A_68_P21890601	chr2:178148869-178148913	NM_001085501:287	Ppp1r3d	INSIDE	2.424	10.660	2909.91	31019.30	25.839	1951.87	50435.31
A_68_P23314345	chr4:132399162-132399206	NM_146154:-100	Ppp1r8	PROMOTER	1.626	4.182	1875.06	7842.09	6.801	1488.10	10121.29
A_68_P28035927	chr11:94853399-94853443	NM_172261:895	Ppp1r9b	INSIDE	1.509	4.641	494.07	2292.84	7.001	468.52	3280.20
A_68_P31084402	chr17:21082837-21082881	NM_016891:441	Ppp2r1a	INSIDE	1.513	1.991	424.01	844.09	3.012	391.52	1179.10
A_68_P29682917	chr14:67690619-67690663	NM_001205188:668	Ppp2r2a	INSIDE	1.791	7.070	3368.81	23816.75	12.659	2318.52	29350.15
A_68_P23610086	chr5:37259995-37260039	NM_172994:208	Ppp2r2c	INSIDE	1.617	5.030	2473.74	12442.91	8.135	1955.76	15909.70

ProbeName	Target position of probe on CpG island microarray	TargetID	GeneSymbol	CpG island Description	Ratio of relative methylation (TiO_2-NP/Vehicle)	Sham group			TiO_2-H group		
						Relative methylation (Cy5/Cy3)	Cy3 signal (Input)	Cy5 signal (MeDIP)	Relative methylation (Cy5/Cy3)	Cy3 signal (Input)	Cy5 signal (MeDIP)
A_68_P26773477	chr9:101153798-101153842	NM_001161362:342	Ppp2r3a	INSIDE	1.730	1.698	791.47	1343.88	2.937	672.35	1974.64
A_68_P31933357	chr19:6235648-6235692	NM_198168:170	Ppp2r5b	INSIDE	1.796	1.849	688.63	1273.17	3.320	592.64	1967.48
A_68_P28738217	chr12:111684829-111684873	NM_001135001:-538	Ppp2r5c	PROMOTER	1.531	2.468	2455.41	6061.07	3.780	1822.54	6888.58
A_68_P28549425	chr12:76697618-76697662	NM_012024:-453	Ppp2r5e	PROMOTER	1.582	2.537	3045.09	7725.01	4.013	2503.22	10046.44
A_68_P29700471	chr14:70689220-70689264	NM_008915:14	Ppp3cc	INSIDE	1.547	2.361	288.34	680.82	3.653	268.34	980.17
A_68_P29700473	chr14:70689417-70689461	NM_008915:-182	Ppp3cc	PROMOTER	1.847	1.470	711.29	1045.58	2.714	616.46	1673.29
A_68_P27614514	chr11:17059211-17059255	NM_024459:-68	Ppp3r1	PROMOTER	1.544	2.576	1662.81	4284.09	3.979	1513.69	6022.63
A_68_P21871010	chr2:173484993-173485037	NR_027957:26	Ppp4r1l-ps	INSIDE	1.723	4.435	2241.73	9941.73	7.641	1850.19	14137.33
A_68_P31918148	chr19:3575782-3575826	NM_001164159:-55	Ppp6r3	PROMOTER	1.673	1.972	7854.17	15489.25	3.299	5282.36	17424.43
A_68_P32239859	chrX:7305710-7305754	NM_138602:36	Praf2	INSIDE	1.949	1.561	944.87	1475.33	3.044	603.67	1837.49
A_68_P25265832	chr7:87439097-87439141	NM_145150:-232	Prc1	PROMOTER	1.748	5.867	2742.61	16091.81	10.254	2081.05	21339.65
A_68_P21431995	chr2:92855411-92855455	NM_001177536:30869	Prdm11	INSIDE	1.621	1.810	1126.49	2039.31	2.935	947.95	2782.42
A_68_P21110386	chr2:31495757-31495801	NM_001123362:222	Prdm12	INSIDE	1.676	3.384	3165.24	10710.31	5.671	2441.02	13843.20
A_68_P23433055	chr4:154011802-154011846	NM_001177995:-842	Prdm16	PROMOTER	1.599	6.269	1955.43	12259.20	10.026	1598.27	16024.05
A_68_P23430798	chr4:153715842-153715886	NM_001177995:295118	Prdm16	INSIDE	1.535	6.877	4530.81	31157.62	10.557	3110.63	32839.81
A_68_P23373724	chr4:142752116-142752160	NM_001081355:50474	Prdm2	INSIDE	1.844	3.720	941.81	3503.25	6.859	847.18	5811.11
A_68_P27311096	chr10:85379833-85379877	NM_181650:-164	Prdm4	PROMOTER	1.730	3.434	1386.39	4760.46	5.939	1135.23	6742.20
A_68_P24513815	chr6:65728982-65729026	NM_027547:49	Prdm5	INSIDE	3.419	8.570	852.11	7302.43	29.302	723.73	21206.73
A_68_P31713385	chr18:53624187-53624231	NM_001033281:9	Prdm6	INSIDE	1.891	2.311	1897.82	4386.30	4.371	1358.76	5938.95
A_68_P20029518	chr1:10983714-10983758	NM_029525:191	Prex2	INSIDE	1.684	7.473	2583.25	19305.26	12.589	1991.24	25067.06
A_68_P30447922	chr15:93426512-93426556	NM_001033217:-212	Prickle1	PROMOTER	2.106	6.137	74837.32	459288.20	12.924	48581.46	627876.40
A_68_P30447388	chr15:93349565-93349609	NM_001033217:76736	Prickle1	INSIDE	1.712	1.704	1895.94	3231.44	2.918	1539.71	4493.14
A_68_P23176712	chr4:104782284-104782328	NM_178143:197	Prkaa2	INSIDE	2.246	5.210	21364.40	111304.50	11.704	15131.89	177100.10
A_68_P23176709	chr4:104782027-104782071	NM_178143:455	Prkaa2	INSIDE	1.528	1.581	4004.06	6331.55	2.416	3082.03	7445.24
A_68_P22355467	chr3:97462102-97462146	NM_182997:-10	Prkab2	PROMOTER	1.515	4.803	8266.35	39707.11	7.277	6308.41	45903.36
A_68_P22616951	chr3:146475944-146475988	NM_011100:-56	Prkacb	PROMOTER	1.521	2.762	2101.38	5803.43	4.202	1851.47	7779.21
A_68_P23544552	chr5:24414202-24414246	NM_001170555:103	Prkag2	INSIDE	1.812	7.118	8413.99	59892.39	12.900	7495.71	96696.36
A_68_P23544553	chr5:24414393-24414437	NM_001170555:-87	Prkag2	PROMOTER	1.566	3.308	1145.83	3790.78	5.181	1015.40	5261.24
A_68_P23544551	chr5:24414128-24414172	NM_001170555:177	Prkag2	INSIDE	1.557	3.462	747.36	2587.60	5.389	642.16	3460.72
A_68_P24133762	chr5:139605565-139605609	NM_008923:120	Prkar1b	INSIDE	1.741	4.090	4595.71	18798.29	7.122	3413.53	24310.10
A_68_P26816174	chr9:108594858-108594902	NM_008924:407	Prkar2a	INSIDE	1.608	2.779	1177.52	3272.61	4.468	955.42	4269.21
A_68_P31415137	chr17:86567183-86567227	NM_011104:80	Prkce	INSIDE	2.047	2.450	2244.27	5497.38	5.015	1805.14	9053.30
A_68_P31415143	chr17:86567807-86567851	NM_011104:704	Prkce	INSIDE	1.606	2.633	1510.89	3977.79	4.228	1249.30	5282.05
A_68_P28538319	chr12:74685927-74685971	NM_008856:-79	Prkch	PROMOTER	2.273	3.609	351.00	1266.84	8.205	326.89	2682.09
A_68_P28538315	chr12:74685369-74685413	NM_008856:-637	Prkch	PROMOTER	1.603	4.059	997.12	4046.86	6.506	734.71	4779.80
A_68_P28426162	chr12:51750316-51750360	NM_008858:-128	Prkd1	PROMOTER	1.804	2.988	3996.06	11938.92	5.389	3237.18	17445.42
A_68_P25114625	chr7:57033846-57033890	NM_133740:141	Prmt3	INSIDE	1.572	1.486	840.46	1249.05	2.336	683.42	1596.68
A_68_P30577450	chr16:18088960-18089004	NM_011172:301	Prodh	INSIDE	1.719	2.615	2729.88	7138.39	4.496	2107.54	9475.10
A_68_P25736226	chr8:28152855-28152901	NM_001039077:-148	Prosc	PROMOTER	1.754	3.824	3213.83	12289.94	6.708	2627.55	17626.44
A_68_P27926352	chr11:75300162-75300206	NM_138659:-94	Prpf8	PROMOTER	1.713	2.584	2946.26	7613.67	4.425	2454.06	10860.23
A_68_P28159656	chr11:116351010-116351061	NM_026364:625	Prpsap1	INSIDE	2.269	17.758	4033.71	71630.06	40.290	2464.90	99310.50
A_68_P28159663	chr11:116351729-116351773	NM_026364:-90	Prpsap1	PROMOTER	2.107	11.663	4378.77	51068.54	24.577	3204.24	78750.14
A_68_P28159701	chr11:116359924-116359968	NM_026364:-8286	Prpsap1	PROMOTER	1.582	2.957	3257.37	9633.54	4.679	2512.02	11752.83
A_68_P25505713	chr7:134615484-134615528	NM_145589:379	Prr14	INSIDE	2.340	4.798	2228.95	10694.08	11.228	1578.60	17725.31
A_68_P25505711	chr7:134615245-134615289	NM_145589:139	Prr14	INSIDE	1.991	1.548	1093.16	1692.00	3.081	841.95	2593.98
A_68_P31162032	chr17:36116207-36116251	NR_028516:-260	Prr3	PROMOTER	1.844	4.839	2099.54	10159.79	8.921	1578.09	14077.99
A_68_P29055232	chr13:55574226-55574270	NM_001030296:8621	Prr7	INSIDE	2.293	2.788	1306.19	3641.47	6.394	1054.80	6744.14

ProbeName	Target position of probe on CpG island microarray	TargetID	GeneSymbol	CpG island Description	Ratio of relative methylation (TiO_2-NP/Vehicle)	Sham group			TiO_2-H group		
						Relative methylation (Cy5/Cy3)	Cy3 signal (Input)	Cy5 signal (MeDIP)	Relative methylation (Cy5/Cy3)	Cy3 signal (Input)	Cy5 signal (MeDIP)
A_68_P29055172	chr13:55565926-55565970	NM_001030296:321	Prr7	INSIDE	1.859	1.859	1462.78	2719.55	3.456	1167.23	4034.24
A_68_P21491162	chr2:104689920-104689964	NM_178695:65	Prrg4	INSIDE	1.547	2.682	1472.56	3950.02	4.149	1224.68	5081.32
A_68_P28875767	chr13:22097925-22097969	NM_019429:3664	Prss16	INSIDE	1.669	3.391	1172.37	3975.96	5.660	929.50	5260.78
A_68_P28875776	chr13:22098904-22098948	NM_019429:2684	Prss16	INSIDE	1.626	3.801	2569.89	9769.27	6.181	1796.94	11106.62
A_68_P25508222	chr7:135079466-135079510	NM_001081374:10751	Prss36	INSIDE	1.945	3.919	2482.62	9729.37	7.622	1728.10	13170.75
A_68_P32143181	chr19:46392296-46392341	NM_028627:9328	Psd	INSIDE	1.833	2.663	1490.81	3969.84	4.881	1320.65	6445.94
A_68_P23062645	chr4:83131826-83131870	NM_133948:446	Psip1	INSIDE	1.878	2.791	1459.70	4074.48	5.242	1255.71	6581.91
A_68_P28523569	chr12:72069499-72069543	NM_011184:-6089	Psma3	PROMOTER	1.645	2.378	192.91	458.66	3.912	164.85	644.90
A_68_P31065956	chr17:15635037-15635081	NM_011185:182	Psmb1	INSIDE	1.686	3.282	3320.49	10897.30	5.535	2703.66	14963.44
A_68_P22341205	chr3:94690730-94690774	NM_008945:128	Psmb4	INSIDE	1.559	2.185	1573.79	3438.45	3.406	1288.28	4387.92
A_68_P21149540	chr2:38499397-38499441	NM_011187:8	Psmb7	INSIDE	1.604	2.174	2497.94	5429.94	3.487	1906.18	6647.51
A_68_P21420369	chr2:90894125-90894169	NM_008948:-26	Psmc3	PROMOTER	1.827	5.506	33165.50	182611.90	10.061	22661.42	227992.10
A_68_P25019829	chr7:28834940-28834984	NM_011874:149	Psmc4	INSIDE	1.634	3.138	4381.20	13749.46	5.130	3217.72	16505.36
A_68_P30990393	chr16:96212588-96212632	NM_019537:-100	Psmg1	PROMOTER	1.549	3.381	2069.66	6997.91	5.238	1779.04	9318.84
A_68_P29624689	chr14:57396463-57396507	NM_025682:669	Pspc1	INSIDE	2.487	5.474	4853.09	26567.70	13.612	3701.11	50381.05
A_68_P29094155	chr13:63664480-63664524	NM_008957:2326	Ptch1	INSIDE	1.783	5.078	1136.97	5773.54	9.054	940.38	8514.46
A_68_P32760538	chrX:152057229-152057273	NM_001093750:620	Ptchd1	INSIDE	2.370	7.084	3332.40	23607.79	16.787	2229.53	37426.19
A_68_P23393512	chr4:147662081-147662125	NM_001083342:-28	Ptchd2	PROMOTER	1.723	2.265	1585.17	3590.51	3.903	1302.94	5085.97
A_68_P28192906	chr11:121691796-121691840	NM_029049:287	Ptchd3	INSIDE	1.968	3.225	1636.76	5277.73	6.345	1415.98	8984.10
A_68_P26015929	chr8:86192596-86192640	NM_013641:2080	Ptger1	INSIDE	1.875	5.992	6861.71	41111.95	11.236	4744.67	53309.83
A_68_P22680840	chr3:157229758-157229802	NM_011196:-75	Ptger3	PROMOTER	1.539	3.172	1382.86	4385.80	4.881	1383.01	6749.95
A_68_P30330343	chr15:73253736-73253780	NM_007982:-137	Ptk2	PROMOTER	1.556	4.901	771.75	3782.28	7.628	660.37	5037.13
A_68_P31207975	chr17:46766402-46766446	NM_175168:29	Ptk7	INSIDE	1.512	2.633	12220.97	32171.92	3.980	10248.26	40791.04
A_68_P20412593	chr1:88422665-88422709	NM_008972:-624	Ptma	PROMOTER	2.644	5.930	4838.46	28693.15	15.677	3042.74	47701.42
A_68_P20412608	chr1:88424274-88424318	NM_008972:986	Ptma	INSIDE	1.576	1.716	777.56	1334.62	2.705	670.32	1813.35
A_68_P23298636	chr4:129497126-129497170	NM_008974:-574	Ptp4a2	PROMOTER	1.610	1.810	1757.41	3181.61	2.915	1435.54	4185.16
A_68_P23298639	chr4:129497779-129497823	NM_001164745:-151	Ptp4a2	PROMOTER	1.556	2.745	1814.44	4980.11	4.270	1507.66	6437.66
A_68_P30667506	chr16:35022484-35022528	NM_023587:0	Ptplb	INSIDE	1.897	2.685	1062.16	2851.96	5.094	902.45	4597.00
A_68_P23524104	chr5:20561279-20561323	NM_011203:315	Ptpn12	INSIDE	1.620	2.868	1128.95	3237.74	4.647	1044.95	4855.85
A_68_P28671841	chr12:99975831-99975875	NM_001146199:-237	Ptpn21	DIVERGENT_PROMOTER	1.910	3.169	3504.26	11104.89	6.054	2752.12	16661.96
A_68_P22944675	chr4:57313204-57313248	NM_011207:1483	Ptpn3	INSIDE	1.531	4.074	349.23	1422.69	6.235	481.41	3001.86
A_68_P25103254	chr7:54387461-54387505	NM_001163565:486	Ptpn5	INSIDE	1.844	3.412	2019.97	6892.19	6.290	1577.30	9921.40
A_68_P23029295	chr4:76095885-76095929	NM_011211:144297	Ptprd	INSIDE	1.989	6.927	2157.13	14942.00	13.775	1517.81	20907.64
A_68_P25553143	chr7:142729512-142729556	NM_011212:28	Ptpre	INSIDE	1.511	3.674	958.74	3522.72	5.553	802.21	4454.28
A_68_P21417961	chr2:90420785-90420829	NM_008982:-2	Ptprj	PROMOTER	1.697	5.256	745.42	3917.71	8.919	603.84	5385.76
A_68_P21417960	chr2:90420716-90420760	NM_008982:66	Ptprj	INSIDE	1.650	4.856	1179.17	5726.52	8.015	976.27	7824.66
A_68_P21417962	chr2:90420971-90421015	NM_008982:-188	Ptprj	PROMOTER	1.564	4.295	2423.28	10407.62	6.717	1930.35	12966.08
A_68_P31311926	chr17:67704196-67704240	NM_008984:-419	Ptprm	PROMOTER	2.351	6.912	3804.89	26300.93	16.250	2810.62	45673.54
A_68_P31311922	chr17:67703725-67703769	NM_008984:53	Ptprm	INSIDE	1.814	7.137	1696.55	12107.99	12.949	1365.94	17687.68
A_68_P20352935	chr1:75248212-75248256	NM_008985:12549	Ptprn	INSIDE	1.578	2.927	2292.55	6709.62	4.618	1724.70	7964.70
A_68_P28768143	chr12:117724447-117724491	NM_011215:276	Ptprn2	INSIDE	1.691	7.523	3494.12	26285.46	12.721	2807.79	35716.92
A_68_P24877027	chr6:137200894-137200938	NM_001164401:97	Ptpro	INSIDE	2.079	5.823	4168.49	24271.80	12.108	3191.65	38642.87
A_68_P31256055	chr17:56568653-56568712	NM_011218:47221	Ptprs	INSIDE	1.963	9.451	507.20	4793.44	18.548	389.72	7228.65
A_68_P31256333	chr17:56601586-56601630	NM_011218:14295	Ptprs	INSIDE	2.352	1.518	961.67	1459.97	3.570	669.84	2391.35
A_68_P23308582	chr4:131392468-131392512	NM_001083119:1703	Ptpru	INSIDE	1.601	2.243	2002.36	4491.97	3.592	1510.32	5425.13
A_68_P28071133	chr11:100820100-100820144	NM_008986:11809	Ptrf	INSIDE	1.556	3.925	2274.88	8928.80	6.107	1679.27	10254.57
A_68_P28071137	chr11:100820628-100820677	NM_008986:11279	Ptrf	INSIDE	1.626	2.201	271.72	598.16	3.580	237.92	851.66

ProbeName	Target position of probe on CpG island microarray	TargetID	GeneSymbol	CpG island Description	Ratio of relative methylation (TiO_2-NP/Vehicle)	Sham group			TiO_2-H group		
						Relative methylation (Cy5/Cy3)	Cy3 signal (Input)	Cy5 signal (MeDIP)	Relative methylation (Cy5/Cy3)	Cy3 signal (Input)	Cy5 signal (MeDIP)
A_68_P23301755	chr4:130219531-130219575	NM_001159603:279	Pum1	INSIDE	1.541	2.281	1424.00	3247.45	3.515	1128.39	3965.86
A_68_P23981859	chr5:111209498-111209542	NM_001025561:95	Pus1	INSIDE	1.538	2.394	3325.28	7960.55	3.681	2575.26	9480.36
A_68_P26464014	chr9:43611501-43611545	NM_021424:58864	Pvrl1	INSIDE	1.964	6.335	2397.16	15184.99	12.442	1497.26	18628.77
A_68_P26464017	chr9:43611785-43611829	NM_021424:59148	Pvrl1	INSIDE	1.803	5.219	1554.17	8111.24	9.408	1038.88	9773.69
A_68_P26463576	chr9:43553455-43553499	NM_021424:818	Pvrl1	INSIDE	1.635	2.694	1269.60	3420.11	4.405	1012.01	4458.27
A_68_P23978770	chr5:110715093-110715137	NM_008993:73	Pxmp2	INSIDE	1.763	2.563	2154.39	5520.74	4.518	1822.63	8234.37
A_68_P23978771	chr5:110715204-110715248	NM_008993:-39	Pxmp2	DIVERGENT_PROMOTER	1.679	2.132	1477.40	3149.50	3.579	1219.87	4365.70
A_68_P21740964	chr2:150612823-150612867	NM_153781:313	Pygb	INSIDE	1.544	4.723	1686.40	7964.35	7.293	1405.35	10248.92
A_68_P26630339	chr9:72773100-72773144	NM_028116:-334	Pygo1	PROMOTER	1.536	1.416	2378.36	3368.63	2.176	2002.26	4356.21
A_68_P22319359	chr3:89234506-89234550	NM_026869:-230	Pygo2	PROMOTER	1.822	4.307	2831.88	12197.24	7.846	2355.22	18479.47
A_68_P28077571	chr11:101968496-101968540	NM_145435:572	Pyy	INSIDE	1.654	8.867	1245.68	11045.37	14.664	1014.87	14881.73
A_68_P21490977	chr2:104656727-104656771	NM_001123327:105	Qser1	INSIDE	2.622	8.015	3488.35	27959.02	21.018	2688.77	56512.11
A_68_P31107655	chr17:26122056-26122100	NM_153140:36727	Rab11fip3	DOWNSTREAM	1.712	6.619	986.65	6530.67	11.332	855.11	9690.13
A_68_P24604715	chr6:85324050-85324094	NM_001003955:556	Rab11fip5	INSIDE	2.343	8.621	4194.89	36164.41	20.197	2530.98	51118.48
A_68_P21132202	chr2:35056385-35056429	NM_026697:234	Rab14	INSIDE	1.617	2.312	2909.12	6724.51	3.738	2509.32	9380.51
A_68_P27468929	chr10:114752147-114752191	NM_024454:479	Rab21	INSIDE	1.822	1.708	878.80	1500.72	3.111	731.83	2276.69
A_68_P21871012	chr2:173485328-173485372	NM_024436:5	Rab22a	INSIDE	1.864	3.810	1753.20	6679.06	7.102	1469.63	10436.92
A_68_P22715106	chr4:8463176-8463220	NM_021518:408	Rab2a	INSIDE	1.511	2.557	1306.31	3340.23	3.864	1128.70	4361.49
A_68_P31302584	chr17:66121929-66121973	NM_133685:142	Rab31	INSIDE	1.524	5.368	2447.72	13138.28	8.182	2211.95	18098.76
A_68_P24618284	chr6:87761631-87761675	NM_133717:-1904	Rab43	PROMOTER	1.607	3.902	5101.09	19903.73	6.270	4202.66	26351.13
A_68_P27542729	chr10:128133023-128133067	NM_177411:280	Rab5b	INSIDE	1.691	2.472	2685.10	6638.46	4.180	2268.41	9481.84
A_68_P26783904	chr9:103014963-103015007	NM_173781:581	Rab6b	INSIDE	1.876	6.996	3134.89	21931.57	13.124	2261.49	29680.34
A_68_P21142183	chr2:37307736-37307780	NM_001033960:-15	Rabgap1	PROMOTER	1.596	5.228	3556.66	18595.17	8.346	2877.40	24014.49
A_68_P20768477	chr1:162723220-162723264	NM_013862:-173	Rabgap1l	PROMOTER	2.414	12.018	13257.93	159330.70	29.007	7275.12	211026.50
A_68_P30426169	chr15:89422304-89422348	NM_026817:28	Rabl2	INSIDE	1.575	3.008	939.82	2826.53	4.737	772.82	3660.98
A_68_P24156969	chr5:144288624-144288668	NM_009007:215	Rac1	INSIDE	1.514	2.574	1631.45	4199.18	3.897	1284.55	5006.06
A_68_P27840810	chr11:59919914-59919958	NM_009021:1422	Rai1	INSIDE	2.563	8.736	3024.48	26422.84	22.390	2287.83	51223.82
A_68_P27841048	chr11:59954437-59954481	NM_001037764:875	Rai1	INSIDE	1.689	8.399	414.52	3481.42	14.186	380.45	5397.20
A_68_P27840800	chr11:59918733-59918777	NM_009021:240	Rai1	INSIDE	1.656	3.280	677.65	2222.45	5.431	517.12	2808.25
A_68_P21782725	chr2:158235581-158235625	NM_177658:14	Ralgapb	INSIDE	1.795	2.817	1325.13	3733.54	5.058	1159.90	5866.99
A_68_P21091625	chr2:28389284-28389328	NM_001145835:323	Ralgds	INSIDE	1.522	1.960	879.08	1722.93	2.982	703.10	2096.69
A_68_P21762364	chr2:154617306-154617350	NM_001139511:483	Raly	INSIDE	2.443	9.092	3562.21	32388.11	22.209	2473.01	54924.23
A_68_P21762360	chr2:154616838-154616882	NM_001139511:15	Raly	INSIDE	1.600	2.771	2007.08	5561.51	4.434	1712.11	7591.07
A_68_P24086564	chr5:129526514-129526558	NM_009391:506	Ran	INSIDE	1.940	3.942	1831.96	7220.91	7.645	1437.02	10986.09
A_68_P24086565	chr5:129526623-129526667	NM_009391:614	Ran	INSIDE	1.639	2.574	1550.69	3991.92	4.220	1358.16	5731.75
A_68_P32054980	chr19:29887444-29887488	NM_177721:-2	Ranbp6	PROMOTER	1.765	2.134	1348.08	2877.14	3.766	1137.32	4283.48
A_68_P27921007	chr11:74336152-74336196	NM_001015046:67486	Rap1gap2	INSIDE	1.769	3.183	828.38	2636.47	5.632	709.37	3994.99
A_68_P22572454	chr3:138738598-138738642	NM_001040690:-457	Rap1gds1	PROMOTER	1.777	6.223	1452.24	9037.25	11.060	1227.37	13575.00
A_68_P22182581	chr3:61168376-61168420	NM_028712:-30	Rap2b	PROMOTER	1.732	3.914	4615.79	18064.14	6.779	3329.52	22571.76
A_68_P21098343	chr2:29475430-29475474	NM_001039086:213	Rapgef1	INSIDE	1.529	1.667	532.10	886.98	2.548	499.46	1272.59
A_68_P21099263	chr2:29602898-29602942	NM_001039086:127681	Rapgef1	DOWNSTREAM	1.784	1.437	1080.70	1553.22	2.563	938.01	2404.52
A_68_P28774236	chr12:118772882-118772926	NM_175930:17953	Rapgef5	INSIDE	1.573	1.763	672.93	1186.15	2.773	608.43	1687.13
A_68_P27810583	chr11:54336349-54336393	NM_175258:22	Rapgef6	INSIDE	1.627	2.089	939.96	1963.23	3.398	789.84	2684.12
A_68_P28058844	chr11:98698263-98698307	NM_001080925:186	Rapgefl1	INSIDE	1.643	2.311	1746.35	4035.94	3.797	1653.93	6279.52
A_68_P28059604	chr11:98821830-98821874	NM_001176528:68	Rara	INSIDE	2.846	9.755	2977.11	29040.29	27.760	2211.13	61381.64
A_68_P25669976	chr8:13677580-13677624	NM_009025:-15	Rasa3	PROMOTER	1.618	2.401	1590.19	3818.17	3.885	1295.35	5032.82
A_68_P24040030	chr5:121099321-121099365	NM_013832:512	Rasal1	INSIDE	1.573	2.490	6047.64	15060.88	3.917	4697.18	18400.08

ProbeName	Target position of probe on CpG island microarray	TargetID	GeneSymbol	CpG island Description	Ratio of relative methylation (TiO_2-NP/Vehicle)	Sham group			TiO_2-H group		
						Relative methylation (Cy5/Cy3)	Cy3 signal (Input)	Cy5 signal (MeDIP)	Relative methylation (Cy5/Cy3)	Cy3 signal (Input)	Cy5 signal (MeDIP)
A_68_P23018376	chr4:73436995-73437039	NM_001017427:-510	Rasef	PROMOTER	1.993	7.837	1435.05	11246.82	15.617	1145.53	17889.46
A_68_P29222297	chr13:92901976-92902020	NM_009027:-549	Rasgrf2	PROMOTER	1.551	15.440	1145.93	17693.03	23.952	845.42	20249.63
A_68_P31934467	chr19:6399578-6399622	NM_011242:-982	Rasgrp2	PROMOTER	1.756	4.373	2869.09	12545.09	7.679	2161.16	16595.64
A_68_P23801768	chr5:74591245-74591289	NM_026878:-84	Rasl11b	PROMOTER	1.543	4.567	3818.49	17438.72	7.045	3002.06	21150.25
A_68_P23801771	chr5:74591574-74591618	NM_026878:246	Rasl11b	INSIDE	1.535	2.669	1030.92	2751.48	4.097	893.49	3660.55
A_68_P27503761	chr10:120913745-120913792	NM_138956:-462	Rassf3	PROMOTER	2.197	2.114	470.03	993.45	4.644	376.06	1746.53
A_68_P20614481	chr1:133141395-133141439	NM_018750:339	Rassf5	INSIDE	2.006	1.595	980.47	1563.95	3.200	835.62	2674.01
A_68_P25587467	chr7:148401624-148401668	NM_025886:-112	Rassf7	DIVERGENT_PROMOTER	1.684	3.437	3882.06	13343.77	5.788	2929.80	16958.03
A_68_P31784091	chr18:66094837-66094881	NM_013833:3885	Rax	INSIDE	2.190	7.709	3926.57	30268.81	16.880	2989.95	50470.21
A_68_P23296217	chr4:129012516-129012560	NM_009030:76	Rbbp4	INSIDE	2.108	2.519	1338.36	3371.92	5.310	1049.62	5573.84
A_68_P23296219	chr4:129012748-129012792	NM_009030:-156	Rbbp4	DIVERGENT_PROMOTER	1.909	4.649	4131.98	19209.22	8.876	3331.75	29571.28
A_68_P23296214	chr4:129012152-129012196	NM_009030:440	Rbbp4	INSIDE	1.573	0.381	1028.53	392.14	0.600	1088.76	652.93
A_68_P31496424	chr18:11815840-11815884	NM_001081223:-488	Rbbp8	PROMOTER	2.031	5.306	1302.81	6913.15	10.775	937.39	10100.56
A_68_P31862858	chr18:80389262-80389306	NM_199197:8074	Rbfa	INSIDE	1.646	1.852	375.68	695.88	3.049	324.99	990.77
A_68_P30353507	chr15:77137504-77137548	NM_001110827:-43	Rbfox2	PROMOTER	1.715	2.280	405.10	923.60	3.911	360.13	1408.46
A_68_P28174620	chr11:118768747-118768791	NM_001024931:2118	Rbfox3	INSIDE	1.899	5.324	5970.48	31785.57	10.112	4426.10	44755.41
A_68_P28172267	chr11:118430861-118430905	NM_001024931:340004	Rbfox3	INSIDE	1.851	7.994	1742.26	13928.25	14.799	1321.84	19561.59
A_68_P28174632	chr11:118769905-118769949	NM_001024931:960	Rbfox3	INSIDE	1.555	2.369	1467.31	3476.28	3.685	1339.73	4937.01
A_68_P22405709	chr3:107135696-107135740	NM_001045807:489	Rbm15	INSIDE	1.627	3.136	3799.14	11913.80	5.103	3192.75	16291.12
A_68_P26805694	chr9:106789235-106789279	NM_175402:75	Rbm15b	INSIDE	1.609	4.059	394.04	1599.22	6.532	333.52	2178.54
A_68_P32183826	chr19:53751832-53751876	NM_001170847:59	Rbm20	INSIDE	1.813	6.095	2886.04	17589.61	11.050	2309.40	25517.88
A_68_P31654583	chr18:42435328-42435372	NM_172626:344	Rbm27	INSIDE	1.724	3.406	3192.91	10873.86	5.870	2639.01	15490.72
A_68_P21769931	chr2:156005366-156005410	NM_133242:588	Rbm39	INSIDE	1.951	4.336	1368.97	5936.39	8.462	1195.59	10116.89
A_68_P25032744	chr7:31435111-31435155	NM_133693:115	Rbm42	INSIDE	1.783	4.162	759.76	3161.83	7.420	589.95	4377.41
A_68_P26811550	chr9:107775126-107775170	NM_011251:2	Rbm6	INSIDE	1.600	4.161	612.46	2548.65	6.658	519.94	3461.97
A_68_P26492138	chr9:48303203-48303247	NM_144948:211	Rbm7	INSIDE	1.755	2.232	1857.66	4146.27	3.916	1461.89	5724.85
A_68_P21261140	chr2:60719287-60719331	NM_001141931:187	Rbms1	INSIDE	1.801	2.769	209.34	579.69	4.988	235.88	1176.47
A_68_P32365934	chrX:46048429-46048473	NM_173376:270	Rbmx2	INSIDE	2.338	12.123	3931.16	47655.81	28.346	2266.75	64254.35
A_68_P32365932	chrX:46048174-46048218	NM_173376:16	Rbmx2	INSIDE	2.307	1.483	755.30	1119.85	3.421	464.12	1587.86
A_68_P23701554	chr5:53982361-53982405	NM_001080927:570	Rbpj	INSIDE	1.943	2.359	2601.07	6135.06	4.584	2146.76	9839.79
A_68_P26588687	chr9:65478956-65479000	NM_028030:590	Rbpms2	INSIDE	2.002	7.101	1348.61	9576.98	14.215	1028.12	14614.30
A_68_P29715887	chr14:73543290-73543334	NR_033185:721	Rcbtb2	INSIDE	1.639	1.870	715.43	1337.52	3.064	603.19	1848.33
A_68_P21494379	chr2:105239336-105239380	NM_009037:118	Rcn1	INSIDE	1.638	2.339	510.90	1194.90	3.831	450.73	1726.56
A_68_P28741731	chr12:112278009-112278053	NM_198023:22	Rcor1	INSIDE	1.538	3.326	9572.13	31835.52	5.114	7748.35	39625.39
A_68_P24904253	chr6:142335519-142335563	NM_001204907:67	Recql	INSIDE	1.716	2.797	4496.67	12578.37	4.800	3780.54	18145.67
A_68_P30350128	chr15:76540931-76540975	NM_058214:37	Recql4	INSIDE	2.464	4.532	3611.54	16368.01	11.166	2597.51	29002.98
A_68_P24537770	chr6:71657709-71657753	NM_178608:56	Reep1	INSIDE	1.984	8.149	1094.29	8917.83	16.167	912.91	14759.37
A_68_P24537769	chr6:71657624-71657668	NM_178608:-28	Reep1	PROMOTER	1.729	4.365	558.91	2439.40	7.545	446.64	3369.74
A_68_P29701956	chr14:70944977-70945021	NM_180588:-59	Reep4	PROMOTER	1.739	1.515	696.61	1055.02	2.634	767.89	2022.35
A_68_P31612548	chr18:34533141-34533185	NM_007874:-93	Reep5	PROMOTER	1.764	4.572	1898.17	8677.73	8.066	1528.25	12326.48
A_68_P20828236	chr1:173433692-173433736	NM_019484:106	Refbp2	INSIDE	2.045	2.577	711.82	1834.64	5.271	588.31	3101.00
A_68_P23531274	chr5:21850512-21850556	NM_011261:-11	Reln	PROMOTER	1.710	5.008	3601.55	18035.54	8.563	3038.50	26017.57
A_68_P29612660	chr14:55098640-55098684	NM_080726:3726	Rem2	INSIDE	1.741	2.457	334.13	820.80	4.277	262.63	1123.22
A_68_P24428260	chr6:48552305-48552349	NM_001079901:8445	Repin1	DOWNSTREAM	1.920	5.777	379.35	2191.66	11.091	284.25	3152.55
A_68_P24428222	chr6:48547522-48547566	NM_001079901:3663	Repin1	INSIDE	1.605	2.155	589.84	1270.92	3.459	464.36	1606.02
A_68_P23405733	chr4:149745839-149745883	NM_001085492:89836	Rere	INSIDE	1.697	2.122	4707.06	9988.62	3.601	3562.93	12828.77
A_68_P23407012	chr4:149943746-149943790	NM_001085492:287744	Rere	INSIDE	1.599	1.670	531.33	887.33	2.670	426.21	1138.07

ProbeName	Target position of probe on CpG island microarray	TargetID	GeneSymbol	CpG island Description	Ratio of relative methylation (TiO_2-NP/Vehicle)	Sham group			TiO_2-H group		
						Relative methylation (Cy5/Cy3)	Cy3 signal (Input)	Cy5 signal (MeDIP)	Relative methylation (Cy5/Cy3)	Cy3 signal (Input)	Cy5 signal (MeDIP)
A_68_P20157818	chr1:38186099-38186143	NM_019570:387	Rev1	INSIDE	1.762	4.318	390.96	1688.19	7.608	378.46	2879.32
A_68_P27080350	chr10:39452886-39452930	NM_011264:943	Rev3l	INSIDE	1.835	9.042	2510.01	22694.51	16.596	1771.06	29392.16
A_68_P27080342	chr10:39452039-39452083	NM_011264:95	Rev3l	INSIDE	1.627	2.038	1075.82	2192.25	3.315	865.77	2869.70
A_68_P27283985	chr10:80024218-80024262	NM_025852:65	Rexo1	INSIDE	1.893	1.853	1490.13	2761.57	3.509	1217.01	4270.00
A_68_P21081484	chr2:26819762-26819806	NM_207234:122	Rexo4	INSIDE	2.111	1.454	1049.76	1526.60	3.069	779.46	2392.39
A_68_P24020335	chr5:117838986-117839030	NM_028128:24	Rfc5	INSIDE	1.641	4.483	893.29	4004.39	7.355	861.69	6337.96
A_68_P27968802	chr11:82684465-82684509	NM_001164570:-240	Rffl	PROMOTER	1.706	5.404	105512.20	570240.10	9.220	70913.33	653851.00
A_68_P26017904	chr8:86591013-86591057	NM_009055:300	Rfx1	INSIDE	2.220	4.457	1742.64	7766.47	9.895	1449.24	14340.14
A_68_P31258630	chr17:56969214-56969258	NM_009056:1195	Rfx2	INSIDE	1.925	6.313	5662.42	35747.04	12.150	4694.45	57039.64
A_68_P22341452	chr3:94758251-94758295	NM_017395:-664	Rfx5	PROMOTER	1.892	1.986	1160.58	2305.20	3.759	985.55	3704.39
A_68_P22149943	chr3:54611747-54611791	NM_133231:-55	Rfxap	PROMOTER	1.927	4.905	1112.94	5459.44	9.453	945.98	8942.71
A_68_P30778982	chr16:56037716-56037760	NM_029092:149	Rg9mtd1	INSIDE	1.742	2.409	1190.64	2868.44	4.197	1272.69	5341.78
A_68_P32567036	chrX:99266249-99266293	NM_183318:376	Rgag4	INSIDE	1.879	8.946	4538.15	40597.66	16.807	2317.83	38955.67
A_68_P31151050	chr17:34067192-34067236	NM_009059:376	Rgl2	INSIDE	1.662	1.844	5165.90	9526.14	3.065	3410.57	10453.66
A_68_P25228678	chr7:80521046-80521090	NM_177740:663	Rgma	INSIDE	4.245	0.556	663.33	368.98	2.361	644.67	1522.29
A_68_P31067776	chr17:15968184-15968228	NM_178615:-4656	Rgmb	PROMOTER	1.591	2.104	1439.15	3027.84	3.347	1305.65	4370.41
A_68_P22872576	chr4:43591441-43591485	NM_172866:-144	Rgp1	PROMOTER	1.879	2.255	663.43	1495.70	4.236	480.39	2035.06
A_68_P23597150	chr5:35292277-35292321	NM_173402:202	Rgs12	INSIDE	1.765	6.421	322.42	2070.34	11.335	287.78	3261.91
A_68_P28587837	chr12:83718482-83718526	NM_015812:480	Rgs6	INSIDE	1.865	4.469	5923.59	26471.32	8.335	4024.42	33544.94
A_68_P25050056	chr7:36369864-36369908	NM_145840:715	Rgs9bp	INSIDE	1.666	3.717	1154.63	4292.02	6.192	1035.92	6414.88
A_68_P27953576	chr11:80114526-80114570	NM_139228:135	Rhbdl3	INSIDE	2.047	5.408	2493.11	13483.69	11.071	1887.05	20891.68
A_68_P25262217	chr7:86762595-86762639	NM_019799:-73	Rhcg	PROMOTER	2.277	16.815	2412.43	40564.86	38.284	1425.80	54585.64
A_68_P26814202	chr9:108209138-108209182	NM_016802:625	Rhoa	INSIDE	2.073	11.208	7900.79	88551.97	23.233	4693.18	109038.70
A_68_P28222996	chr12:8506600-8506644	NM_007483:169	Rhob	INSIDE	3.027	29.504	8519.70	251363.70	89.305	4297.86	383821.30
A_68_P27222450	chr10:68675577-68675621	NM_001081347:194	Rhobtb1	INSIDE	1.603	3.581	5360.76	19199.15	5.739	4084.46	23442.44
A_68_P29152220	chr13:76081481-76081525	NM_028493:-230	Rhobtb3	PROMOTER	1.969	2.960	1198.54	3547.32	5.827	851.95	4963.87
A_68_P31420286	chr17:87362969-87363013	NM_145491:540	Rhoq	INSIDE	1.679	3.749	3149.75	11807.75	6.296	2640.34	16622.98
A_68_P31420283	chr17:87362407-87362451	NM_145491:-22	Rhoq	PROMOTER	2.143	2.059	433.01	891.59	4.412	401.47	1771.37
A_68_P31106682	chr17:25981519-25981564	NM_145999:255	Rhot2	INSIDE	1.566	5.770	2491.85	14378.33	9.036	1778.26	16068.59
A_68_P25585272	chr7:148043313-148043357	NM_053194:39	Ric8	INSIDE	1.548	1.798	1175.98	2113.99	2.783	980.39	2728.28
A_68_P23245805	chr4:119164931-119164975	NM_177572:251	Rimkla	INSIDE	1.970	1.959	817.12	1601.11	3.860	738.33	2849.76
A_68_P20085264	chr1:22812557-22812601	NM_001012623:-15	Rims1	PROMOTER	1.884	3.168	5138.64	16277.20	5.969	3944.43	23542.48
A_68_P20083589	chr1:22540314-22540358	NM_001012623:272227	Rims1	INSIDE	1.660	1.572	1340.67	2107.75	2.610	1166.78	3045.41
A_68_P28936016	chr13:34094703-34094748	NM_009068:-17	Ripk1	DIVERGENT_PROMOTER	2.369	5.834	3745.72	21850.67	13.820	2666.49	36851.95
A_68_P22315261	chr3:88520728-88520772	NM_001163310:-25	Rit1	PROMOTER	1.712	4.393	2291.16	10065.31	7.521	1802.82	13558.61
A_68_P26135404	chr8:108218075-108218119	NM_001033320:3291	Rltpr	INSIDE	1.520	1.873	10467.60	19607.79	2.848	8262.30	23530.51
A_68_P31929627	chr19:5602038-5602082	NM_026616:188	Rnaseh2c	INSIDE	1.822	5.312	2264.08	12027.24	9.679	1871.49	18114.83
A_68_P31929629	chr19:5602275-5602319	NM_026616:424	Rnaseh2c	INSIDE	1.733	2.818	461.91	1301.47	4.883	438.90	2143.20
A_68_P21212091	chr2:51004685-51004729	NM_028810:-75	Rnd3	PROMOTER	2.108	2.277	362.08	824.64	4.800	271.24	1301.97
A_68_P28598116	chr12:85750091-85750135	NM_025525:-8037	Rnf113a2	PROMOTER	1.952	7.688	929.08	7143.04	15.009	656.03	9846.31
A_68_P27278802	chr10:79229397-79229442	NM_144528:247	Rnf126	INSIDE	1.974	4.084	5437.70	22205.53	8.062	4054.43	32684.99
A_68_P31542491	chr18:21160534-21160578	NM_019706:715	Rnf138	INSIDE	2.507	6.232	7347.22	45784.44	15.622	4984.46	77866.60
A_68_P30250719	chr15:58720548-58720592	NM_175226:-213	Rnf139	PROMOTER	1.647	4.295	875.77	3761.37	7.074	754.23	5335.61
A_68_P29011797	chr13:47218039-47218083	NM_146042:-28	Rnf144b	PROMOTER	2.239	1.629	928.54	1512.30	3.647	772.40	2816.58
A_68_P28991174	chr13:43711396-43711440	NM_183204:253	Rnf182	INSIDE	1.552	4.911	1583.61	7776.79	7.619	1313.01	10004.08
A_68_P30137691	chr15:36213148-36213192	NM_013923:-268	Rnf19a	PROMOTER	2.895	7.973	1368.64	10911.67	23.081	795.84	18368.76
A_68_P23234760	chr4:117167587-117167631	NM_025739:1912	Rnf220	INSIDE	1.738	3.133	2488.25	7795.60	5.446	2043.22	11127.02

ProbeName	Target position of probe on CpG island microarray	TargetID	GeneSymbol	CpG island Description	Ratio of relative methylation (TiO_2-NP/Vehicle)	Sham group			TiO_2-H group		
						Relative methylation (Cy5/Cy3)	Cy3 signal (Input)	Cy5 signal (MeDIP)	Relative methylation (Cy5/Cy3)	Cy3 signal (Input)	Cy5 signal (MeDIP)
A_68_P23234756	chr4:117167070-117167114	NM_025739:2428	Rnf220	INSIDE	1.628	6.644	1123.56	7465.08	10.820	864.45	9353.10
A_68_P27541275	chr10:127848643-127848687	NM_001164237:-7	Rnf41	DIVERGENT_PROMOTER	1.883	5.832	1251.46	7299.10	10.982	997.40	10953.87
A_68_P22826133	chr4:33397493-33397537	NM_011884:229	Rngtt	INSIDE	1.659	2.930	1834.42	5374.19	4.859	1525.29	7411.58
A_68_P26430635	chr9:37240928-37240972	NM_001164767:-190	Robo3	PROMOTER	1.986	5.036	3993.54	20110.91	10.002	2951.93	29524.69
A_68_P31488614	chr18:10181658-10181702	NM_009071:110	Rock1	INSIDE	1.758	5.975	3034.69	18133.36	10.504	2295.97	24116.75
A_68_P30513218	chr16:5013781-5013827	NM_133185:-158	Rogdi	PROMOTER	1.634	3.783	2479.14	9378.26	6.183	1863.86	11524.08
A_68_P26352003	chr9:22272492-22272536	NM_018739:286	Rp9	INSIDE	1.937	6.711	2403.58	16130.31	13.000	1977.00	25701.63
A_68_P31121831	chr17:28465275-28465320	NM_011287:-118	Rpl10a	PROMOTER	1.891	8.952	1034.42	9260.53	16.925	734.71	12435.11
A_68_P31121837	chr17:28465985-28466029	NM_011287:591	Rpl10a	INSIDE	1.585	2.638	2942.12	7761.29	4.181	2365.40	9888.84
A_68_P25091297	chr7:52384491-52384535	NM_009438:-407	Rpl13a	PROMOTER	1.705	6.002	1607.98	9650.27	10.230	1214.67	12426.62
A_68_P26878665	chr9:120486576-120486620	NM_025974:5965	Rpl14	DOWNSTREAM	1.684	1.848	1419.03	2622.10	3.112	1187.42	3695.48
A_68_P26878663	chr9:120486371-120486416	NM_025974:5760	Rpl14	DOWNSTREAM	2.712	1.510	1244.63	1879.28	4.095	974.70	3991.69
A_68_P23418024	chr4:151700327-151700371	NM_009079:362	Rpl22	INSIDE	2.437	4.492	1567.13	7039.64	10.946	1298.21	14210.70
A_68_P30128581	chr15:34373372-34373416	NM_001163485:-363	Rpl30	DIVERGENT_PROMOTER	1.902	2.460	2857.63	7030.14	4.679	2030.46	9500.08
A_68_P20165362	chr1:39424508-39424553	NM_053257:-165	Rpl31	PROMOTER	1.737	3.441	688.17	2368.01	5.977	527.67	3153.77
A_68_P21151631	chr2:38860577-38860621	NM_025592:53	Rpl35	INSIDE	1.763	2.925	2620.19	7663.81	5.157	2061.53	10630.55
A_68_P25588869	chr7:148633509-148633553	NM_026020:-18	Rplp2	DIVERGENT_PROMOTER	2.036	1.443	1519.74	2192.72	2.938	1093.90	3214.10
A_68_P24619664	chr6:88035091-88035135	NM_133933:646	Rpn1	INSIDE	2.023	2.406	687.90	1655.17	4.867	583.12	2838.15
A_68_P31163327	chr17:36394876-36394920	NM_026308:-107	Rpp21	PROMOTER	1.952	6.137	1187.76	7289.19	11.981	1029.28	12331.59
A_68_P21780563	chr2:157854132-157854176	NM_027434:-375	Rprd1b	PROMOTER	2.174	3.021	1078.36	3258.04	6.568	867.01	5694.82
A_68_P21780565	chr2:157854409-157854453	NM_027434:-99	Rprd1b	PROMOTER	1.849	2.117	3552.85	7520.53	3.913	2754.84	10779.73
A_68_P25456593	chr7:125259495-125259539	NM_170669:145	Rps15a	INSIDE	2.426	5.742	1683.11	9663.99	13.927	1116.17	15544.89
A_68_P25007073	chr7:25669913-25669957	NM_023133:202	Rps19	INSIDE	1.597	3.412	1484.11	5063.19	5.450	1292.39	7043.04
A_68_P21902494	chr2:179991894-179991938	NM_025587:-167	Rps21	PROMOTER	1.713	9.493	3506.19	33283.25	16.258	2557.31	41577.61
A_68_P26596131	chr9:66794074-66794118	NM_026467:172	Rps27l	INSIDE	1.506	2.303	551.36	1269.99	3.468	380.12	1318.23
A_68_P32567641	chrX:99384743-99384787	NM_009094:-1054	Rps4x	PROMOTER	1.622	2.279	620.42	1413.98	3.696	430.02	1589.15
A_68_P24973639	chr7:13507675-13507719	NM_009095:37	Rps5	INSIDE	1.834	2.127	320.23	681.01	3.899	316.64	1234.64
A_68_P28314231	chr12:29320191-29320235	NM_011300:606	Rps7	INSIDE	1.539	1.793	3656.30	6553.99	2.759	3029.59	8358.80
A_68_P28956729	chr13:37918014-37918058	NM_001177868:-744	Rreb1	PROMOTER	1.641	2.736	2349.87	6428.20	4.490	1779.07	7988.06
A_68_P31143302	chr17:32173587-32173631	NM_028244:502	Rrp1b	INSIDE	2.195	6.110	3200.19	19554.71	13.412	2348.29	31494.87
A_68_P31208271	chr17:46811147-46811191	NM_144857:36	Rrp36	INSIDE	1.656	2.295	5152.14	11824.65	3.800	3977.79	15116.76
A_68_P22388656	chr3:103718191-103718235	NM_172684:170	Rsbn1	INSIDE	1.704	3.997	845.40	3378.85	6.812	691.87	4712.77
A_68_P25351157	chr7:104729030-104729074	NM_001081267:647	Rsf1	INSIDE	1.506	2.725	802.51	2186.66	4.104	732.60	3006.58
A_68_P24991219	chr7:19650979-19651023	NM_001159671:10965	Rsph6a	INSIDE	1.562	5.179	3800.91	19684.78	8.090	2925.80	23668.66
A_68_P23271474	chr4:124663490-124663534	NM_138683:-161	Rspo1	PROMOTER	2.078	4.498	1224.00	5505.66	9.348	917.28	8574.29
A_68_P27215083	chr10:67442230-67442274	NM_001081346:-93	Rtkn2	PROMOTER	1.616	3.542	8566.13	30338.77	5.723	5946.50	34030.73
A_68_P21395110	chr2:84712227-84712271	NM_199223:14601	Rtn4rl2	INSIDE	1.979	6.057	2764.73	16746.15	11.988	2317.19	27778.80
A_68_P28073802	chr11:101286306-101286350	NM_172566:-70	Rundc1	PROMOTER	2.090	4.644	5587.40	25946.09	9.706	3826.56	37141.60
A_68_P23468796	chr5:8622618-8622662	NM_198620:312	Rundc3b	INSIDE	2.023	2.921	1876.82	5481.80	5.908	1531.02	9044.67
A_68_P23468797	chr5:8622724-8622768	NM_198620:206	Rundc3b	INSIDE	1.884	1.793	6375.10	11431.71	3.379	5084.04	17177.17
A_68_P31196995	chr17:44873188-44873232	NM_001145920:387	Runx2	INSIDE	1.757	2.269	882.30	2001.78	3.986	705.48	2812.18
A_68_P23326917	chr4:134708685-134708729	NM_019732:32147	Runx3	INSIDE	1.612	3.639	3739.75	13608.80	5.865	2960.41	17362.93
A_68_P22317129	chr3:88894411-88894455	NM_001083808:-479	Rusc1	PROMOTER	1.816	3.505	2746.83	9627.80	6.364	2121.72	13503.65
A_68_P30013962	chr15:10966866-10966910	NM_178717:835	Rxfp3	INSIDE	1.734	6.001	3825.75	22960.00	10.408	2752.13	28644.35
A_68_P21086006	chr2:27533318-27533362	NM_011305:620	Rxra	INSIDE	1.638	3.240	1257.40	4074.37	5.307	1004.52	5331.24
A_68_P31151615	chr17:34168722-34168766	NM_001205214:-12	Rxrb	DIVERGENT_PROMOTER	2.525	2.326	584.39	1359.19	5.873	474.20	2785.18
A_68_P24691294	chr6:100237868-100237912	NM_019743:-538	Rybp	PROMOTER	1.656	3.440	658.40	2264.60	5.697	567.63	3233.73

ProbeName	Target position of probe on CpG island microarray	TargetID	GeneSymbol	CpG island Description	Ratio of relative methylation (TiO_2-NP/Vehicle)	Sham group			TiO_2-H group		
						Relative methylation (Cy5/Cy3)	Cy3 signal (Input)	Cy5 signal (MeDIP)	Relative methylation (Cy5/Cy3)	Cy3 signal (Input)	Cy5 signal (MeDIP)
A_68_P24691293	chr6:100237792-100237836	NM_019743:-462	Rybp	PROMOTER	1.600	3.966	2131.78	8453.78	6.345	1704.68	10815.92
A_68_P26344894	chr9:20780852-20780896	NM_010333:363	S1pr2	INSIDE	1.822	15.703	5257.87	82562.47	28.611	3131.07	89583.18
A_68_P26344891	chr9:20780545-20780589	NM_010333:671	S1pr2	INSIDE	2.748	1.576	1964.61	3095.35	4.330	1484.57	6427.85
A_68_P29032310	chr13:51503858-51503902	NM_010101:-106	S1pr3	PROMOTER	2.347	4.881	1406.71	6866.52	11.458	969.33	11106.33
A_68_P29648696	chr14:61791453-61791497	NM_172809:34181	Sacs	INSIDE	1.659	3.057	914.38	2795.69	5.073	777.56	3944.66
A_68_P29648697	chr14:61791606-61791650	NM_172809:34335	Sacs	INSIDE	1.517	1.545	1833.51	2833.14	2.345	1362.27	3193.90
A_68_P24981209	chr7:16973109-16973153	NM_019748:4	Sae1	INSIDE	1.745	2.708	1486.23	4024.38	4.726	1180.87	5580.70
A_68_P26044774	chr8:91565521-91565568	NM_021390:2517	Sall1	INSIDE	1.582	4.277	385.41	1648.29	6.766	343.98	2327.19
A_68_P26044646	chr8:91549828-91549872	NM_021390:18211	Sall1	DOWNSTREAM	1.826	1.816	777.09	1411.38	3.317	681.97	2261.82
A_68_P31868107	chr18:81182856-81182900	NM_178280:439	Sall3	INSIDE	1.600	3.902	3523.83	13749.52	6.244	2824.25	17633.53
A_68_P21843420	chr2:168591529-168591573	NM_175303:1151	Sall4	INSIDE	1.944	2.326	400.24	930.99	4.522	339.93	1537.30
A_68_P28036074	chr11:94871790-94871834	NM_146025:620	Samd14	INSIDE	1.502	2.501	2221.81	5557.73	3.757	1714.51	6441.51
A_68_P25021894	chr7:29221444-29221488	NM_175021:-256	Samd4b	DIVERGENT_PROMOTER	1.648	4.085	2074.50	8475.26	6.732	1684.39	11340.10
A_68_P26931630	chr10:9394840-9394884	NM_177271:144	Samd5	INSIDE	1.867	2.575	252.30	649.59	4.807	193.73	931.21
A_68_P23999965	chr5:114221529-114221573	NM_016926:108	Sart3	INSIDE	1.607	2.436	1029.35	2507.68	3.916	916.10	3587.48
A_68_P26926726	chr10:8605914-8605958	NM_175155:-68	Sash1	PROMOTER	1.736	2.375	1853.06	4400.32	4.123	1678.72	6920.83
A_68_P31236178	chr17:51952223-51952267	NM_001163630:-865	Satb1	PROMOTER	1.934	4.659	2674.30	12459.88	9.009	2114.51	19049.22
A_68_P24092948	chr5:130731326-130731370	NM_023248:-16	Sbds	DIVERGENT_PROMOTER	2.697	2.449	2368.30	5800.12	6.606	1776.61	11735.43
A_68_P30463766	chr15:96290439-96290483	NM_028148:814	Scaf11	INSIDE	1.881	1.929	1477.99	2851.50	3.629	1168.09	4239.56
A_68_P21152636	chr2:39045852-39045896	NM_178778:376	Scai	INSIDE	2.003	6.019	21465.77	129193.80	12.053	15269.99	184042.30
A_68_P21152640	chr2:39046217-39046261	NM_178778:12	Scai	INSIDE	1.500	2.913	813.18	2369.11	4.370	698.70	3053.46
A_68_P26542457	chr9:57408653-57408697	NM_022813:-76	Scamp2	DIVERGENT_PROMOTER	1.862	8.524	2860.02	24377.60	15.873	2166.72	34391.44
A_68_P27284238	chr10:80065633-80065677	NM_019575:28	Scamp4	INSIDE	1.557	3.207	2217.98	7114.18	4.993	1596.30	7969.72
A_68_P26533458	chr9:55785595-55785639	NM_001081341:306	Scaper	INSIDE	1.591	1.692	6215.45	10515.26	2.692	5095.27	13718.22
A_68_P22215798	chr3:68299063-68299107	NR_036665:970	Schip1	INSIDE	2.061	4.193	1799.08	7543.33	8.640	1428.43	12341.43
A_68_P28376737	chr12:40860793-40860837	NM_001146196:1	Scin	INSIDE	1.874	8.543	1868.55	15962.97	16.011	1151.05	18429.85
A_68_P20441527	chr1:93194765-93194809	NM_016717:-128	Scly	PROMOTER	1.586	4.107	3339.04	13712.54	6.514	2809.46	18299.80
A_68_P26873355	chr9:119487746-119487790	NM_021544:366	Scn5a	INSIDE	1.532	1.767	1439.43	2543.43	2.708	1209.86	3275.78
A_68_P30489270	chr15:100877044-100877088	NM_011323:110365	Scn8a	DOWNSTREAM	1.704	1.428	2703.94	3861.00	2.433	2254.44	5485.81
A_68_P30345853	chr15:75900100-75900144	NM_134089:38	Scrib	INSIDE	1.923	4.663	3776.94	17610.21	8.966	3372.04	30235.25
A_68_P30348856	chr15:76348296-76348340	NM_130893:4241	Scrt1	INSIDE	1.861	1.663	646.71	1075.76	3.096	568.99	1761.59
A_68_P21747518	chr2:151908021-151908065	NM_001160410:778	Scrt2	INSIDE	2.015	5.702	6790.30	38718.10	11.488	4305.90	49465.33
A_68_P21747609	chr2:151919690-151919734	NM_001160410:12448	Scrt2	INSIDE	1.522	7.979	1606.17	12814.85	12.144	1057.18	12838.79
A_68_P30390278	chr15:83555204-83555248	NM_022723:225	Scube1	INSIDE	1.927	4.462	1759.53	7850.47	8.596	1610.50	13843.26
A_68_P22705223	chr4:6292852-6292896	NM_001098227:48	Sdcbp	INSIDE	1.618	2.569	3970.18	10197.67	4.156	3264.23	13567.49
A_68_P30572142	chr16:17131635-17131679	NM_022324:820	Sdf2l1	INSIDE	1.586	2.415	2158.71	5212.22	3.830	1737.14	6652.61
A_68_P21078770	chr2:26301305-26301349	NM_153125:-590	Sec16a	PROMOTER	1.731	2.186	792.56	1732.82	3.784	665.25	2517.04
A_68_P21078768	chr2:26301082-26301126	NM_153125:-368	Sec16a	PROMOTER	1.588	5.643	10762.58	60732.44	8.962	7729.46	69269.22
A_68_P30669634	chr16:35363730-35363774	NM_133704:252	Sec22a	INSIDE	1.544	2.387	1288.67	3075.84	3.686	1150.47	4240.27
A_68_P29033511	chr13:51746883-51746927	NM_029279:-178	Secisbp2	PROMOTER	1.517	2.454	3102.77	7613.38	3.723	2647.15	9854.78
A_68_P27552432	chr11:3417267-3417311	NM_053267:2584	Selm	INSIDE	1.612	2.862	653.75	1870.73	4.613	526.96	2431.08
A_68_P20149055	chr1:36615091-36615135	NM_001126047:114	Sema4c	INSIDE	2.308	2.850	1328.56	3786.16	6.578	972.00	6393.64
A_68_P20149049	chr1:36614326-36614370	NM_001126047:878	Sema4c	INSIDE	1.764	7.297	6518.75	47565.21	12.870	4549.54	58550.61
A_68_P22342541	chr3:94965423-94965467	NM_011351:-2851	Sema6c	PROMOTER	1.675	3.265	2048.94	6690.65	5.471	1637.88	8960.23
A_68_P30779064	chr16:56075886-56075930	NM_001003972:387	Senp7	INSIDE	1.763	6.223	2092.73	13022.48	10.970	1717.71	18843.10
A_68_P26555222	chr9:59598670-59598714	NM_001172068:-236	Senp8	DIVERGENT_PROMOTER	1.739	3.056	1609.98	4920.90	5.317	1312.18	6976.36
A_68_P27167594	chr10:58684574-58684618	NM_001024910:-1	Sept10	DIVERGENT_PROMOTER	1.874	6.097	4520.24	27558.99	11.425	3322.33	37957.26

ProbeName	Target position of probe on CpG island microarray	TargetID	GeneSymbol	CpG island Description	Ratio of relative methylation (TiO_2-NP/Vehicle)	Sham group			TiO_2-H group		
						Relative methylation (Cy5/Cy3)	Cy3 signal (Input)	Cy5 signal (MeDIP)	Relative methylation (Cy5/Cy3)	Cy3 signal (Input)	Cy5 signal (MeDIP)
A_68_P26365989	chr9:25059719-25059763	NM_001205367:-299	Sept7	PROMOTER	1.582	3.061	4140.89	12675.54	4.843	3365.70	16299.80
A_68_P28164254	chr11:117151837-117151881	NM_001113487:24299	Sept9	INSIDE	1.918	4.704	4337.00	20399.80	9.019	3424.73	30888.83
A_68_P28163660	chr11:117060956-117061000	NM_001113486:4	Sept9	INSIDE	1.579	3.981	1641.20	6532.99	6.285	1352.89	8503.57
A_68_P31020947	chr17:6079672-6079716	NM_001111017:45	Serac1	INSIDE	2.142	2.556	2521.73	6444.82	5.475	1977.12	10824.66
A_68_P24520694	chr6:67217359-67217403	NM_001113564:408	Serbp1	INSIDE	1.510	1.793	659.14	1181.96	2.707	523.12	1416.16
A_68_P21580978	chr2:121275537-121275581	NM_011354:595	Serf2	INSIDE	1.801	2.582	3099.68	8003.43	4.651	2734.38	12717.10
A_68_P21580976	chr2:121275325-121275369	NM_011354:383	Serf2	INSIDE	1.783	2.627	1183.49	3109.35	4.685	1067.15	4999.11
A_68_P28936013	chr13:34094245-34094289	NR_028309:352	Serpinb6a	INSIDE	1.667	2.063	1185.68	2445.85	3.438	1038.65	3571.02
A_68_P25017511	chr7:28271855-28271899	NM_018820:-95	Sertad1	PROMOTER	1.637	3.640	3792.42	13803.19	5.956	2868.55	17086.23
A_68_P27632484	chr11:20531833-20531877	NM_001038625:-125	Sertad2	PROMOTER	1.614	3.475	6104.15	21214.46	5.608	4460.01	25012.99
A_68_P27631993	chr11:20443834-20443878	NM_021372:601	Sertad2	INSIDE	1.613	10.061	10624.97	106902.70	16.231	7456.42	121021.70
A_68_P27631991	chr11:20443663-20443707	NM_021372:429	Sertad2	INSIDE	1.740	1.563	615.95	962.46	2.718	770.09	2093.18
A_68_P27091229	chr10:41530226-41530270	NM_001162908:-131	Sesn1	DIVERGENT_PROMOTER	1.930	4.951	3065.18	15175.30	9.557	2488.78	23786.35
A_68_P27091644	chr10:41607176-41607220	NM_001013370:-46	Sesn1	PROMOTER	1.903	6.522	2943.93	19199.99	12.412	2476.28	30734.88
A_68_P31857885	chr18:79306491-79306535	NM_053099:-382	Setbp1	PROMOTER	1.642	3.094	1006.69	3114.38	5.081	841.53	4275.84
A_68_P25507242	chr7:134920047-134920091	NM_178029:-834	Setd1a	PROMOTER	1.997	4.883	785.76	3837.16	9.754	536.84	5236.52
A_68_P28726319	chr12:109416700-109416744	NM_028262:772	Setd3	INSIDE	1.939	4.260	1042.01	4439.33	8.260	798.11	6592.16
A_68_P28726327	chr12:109417619-109417664	NM_028262:-147	Setd3	DIVERGENT_PROMOTER	1.908	4.603	1903.06	8759.66	8.782	1566.38	13755.89
A_68_P28726321	chr12:109416856-109416900	NM_028262:616	Setd3	INSIDE	1.536	2.422	1209.78	2930.42	3.721	910.50	3388.06
A_68_P30976720	chr16:93603886-93603930	NM_145482:152	Setd4	INSIDE	1.603	4.985	4901.08	24430.88	7.990	3852.54	30782.88
A_68_P22130348	chr3:51364282-51364326	NM_080793:441	Setd7	INSIDE	1.614	4.461	2334.51	10415.15	7.201	2072.84	14926.78
A_68_P24061285	chr5:124889815-124889859	NM_030241:-102	Setd8	PROMOTER	1.772	6.736	706.20	4756.98	11.934	574.76	6858.93
A_68_P31934224	chr19:6364358-6364402	NM_001110791:691	Sf1	INSIDE	2.459	12.680	1791.20	22713.16	31.175	1325.53	41323.44
A_68_P31934221	chr19:6363781-6363825	NM_001110791:113	Sf1	INSIDE	1.699	2.366	508.77	1203.74	4.020	393.59	1582.14
A_68_P29507580	chr14:31529106-31529150	NM_001166532:328	Sfmbt1	INSIDE	1.522	4.440	1023.75	4545.37	6.758	860.17	5813.21
A_68_P23318413	chr4:133155649-133155693	NM_018754:2413	Sfn	DOWNSTREAM	1.644	1.623	1136.06	1843.39	2.668	943.40	2516.70
A_68_P23283131	chr4:126698502-126698546	NM_023603:-20	Sfpq	PROMOTER	1.510	10.628	252.76	2686.37	16.045	227.79	3654.84
A_68_P25716360	chr8:24521956-24522000	NM_013834:5	Sfrp1	INSIDE	2.193	9.499	2326.53	22099.39	20.830	1818.84	37887.13
A_68_P31600224	chr18:32070540-32070584	NM_026006:995	Sft2d3	INSIDE	1.832	5.139	2574.66	13231.95	9.415	2098.93	19762.36
A_68_P25797392	chr8:39723329-39723373	NM_145841:1212	Sgcz	INSIDE	2.063	33.866	2213.30	74955.73	69.872	1292.88	90336.10
A_68_P26996375	chr10:21713739-21713783	NM_011361:-711	Sgk1	PROMOTER	1.566	3.211	605.84	1945.46	5.029	456.14	2293.94
A_68_P27178875	chr10:60609327-60609371	NM_009163:1065	Sgpl1	INSIDE	1.598	1.769	2592.83	4587.47	2.828	2049.41	5795.72
A_68_P23997432	chr5:113739494-113739538	NM_172718:290	Sgsm1	INSIDE	1.661	4.145	4114.39	17054.40	6.886	3205.79	22073.60
A_68_P24046730	chr5:122278610-122278654	NM_008507:8178	Sh2b3	INSIDE	1.613	4.679	832.36	3894.34	7.546	731.05	5516.12
A_68_P23321241	chr4:133684793-133684837	NM_080559:-146	Sh3bgrl3	PROMOTER	1.695	3.600	2045.37	7362.38	6.101	1544.79	9425.19
A_68_P23594805	chr5:34868409-34868453	NM_011893:-2	Sh3bp2	PROMOTER	2.402	20.833	5614.36	116962.20	50.042	3130.32	156648.20
A_68_P23594804	chr5:34868325-34868369	NM_011893:-86	Sh3bp2	PROMOTER	1.522	3.182	1059.93	3372.88	4.843	1020.92	4944.01
A_68_P31253474	chr17:56175980-56176024	NM_013664:-3	Sh3gl1	DIVERGENT_PROMOTER	2.295	5.057	8492.46	42943.44	11.605	6803.83	78960.70
A_68_P23072589	chr4:84851297-84851341	NM_019535:-41	Sh3gl2	PROMOTER	1.598	1.967	485.40	954.70	3.142	462.98	1454.73
A_68_P27165498	chr10:58275060-58275104	NM_172788:-1024	Sh3rf3	PROMOTER	1.772	1.997	6835.60	13649.97	3.539	5808.04	20551.85
A_68_P25086855	chr7:51591550-51591594	NM_001034115:25939	Shank1	INSIDE	1.558	3.042	2355.54	7164.91	4.738	1933.02	9158.01
A_68_P25086960	chr7:51607818-51607862	NM_001034115:42207	Shank1	INSIDE	1.516	5.044	7768.35	39184.21	7.649	5667.39	43348.23
A_68_P25086963	chr7:51608090-51608134	NM_001034115:42479	Shank1	INSIDE	1.934	1.856	483.92	897.95	3.589	416.06	1493.20
A_68_P22883653	chr4:45543529-45543573	NM_001033306:150	Shb	INSIDE	1.658	3.819	859.99	3284.17	6.333	816.57	5171.07
A_68_P22883647	chr4:45542639-45542683	NM_001033306:1040	Shb	INSIDE	1.551	3.693	825.96	3050.21	5.728	737.06	4222.19
A_68_P27277985	chr10:79100697-79100741	NM_001024539:-55	Shc2	PROMOTER	1.790	3.240	1741.42	5642.11	5.799	1450.86	8414.19
A_68_P22321899	chr3:89635034-89635078	NM_172530:-235	She	PROMOTER	2.040	5.853	1470.83	8608.54	11.942	1263.72	15091.48

ProbeName	Target position of probe on CpG island microarray	TargetID	GeneSymbol	CpG island Description	Ratio of relative methylation (TiO_2-NP/Vehicle)	Sham group			TiO_2-H group		
						Relative methylation (Cy5/Cy3)	Cy3 signal (Input)	Cy5 signal (MeDIP)	Relative methylation (Cy5/Cy3)	Cy3 signal (Input)	Cy5 signal (MeDIP)
A_68_P29639602	chr14:60244436-60244480	NM_145463:341	Shisa2	INSIDE	1.576	3.893	992.08	3861.92	6.133	779.98	4783.79
A_68_P20638994	chr1:137271658-137271702	NM_175259:-40	Shisa4	PROMOTER	1.525	1.713	767.81	1315.14	2.612	633.58	1654.92
A_68_P24950760	chr7:4788206-4788250	NM_172737:8070	Shisa7	INSIDE	1.567	4.057	712.16	2889.21	6.357	591.98	3763.03
A_68_P30550104	chr16:11984103-11984147	NM_001174086:-81	Shisa9	PROMOTER	2.405	2.187	9712.97	21244.19	5.261	6807.54	35811.66
A_68_P32185492	chr19:54018463-54018507	NM_001168505:-311	Shoc2	PROMOTER	1.873	3.317	398.42	1321.74	6.212	340.43	2114.92
A_68_P22207824	chr3:66777458-66777502	NM_013665:8213	Shox2	INSIDE	1.541	1.628	3214.15	5233.73	2.510	2755.27	6914.40
A_68_P32750678	chrX:149204051-149204095	NM_172441:-68	Shroom2	PROMOTER	1.622	2.671	2098.76	5606.54	4.334	1352.04	5859.93
A_68_P23896971	chr5:93371578-93371622	NM_001077596:44577	Shroom3	INSIDE	1.638	2.786	519.73	1447.84	4.563	449.18	2049.51
A_68_P23896964	chr5:93370721-93370767	NM_001077596:43721	Shroom3	INSIDE	1.653	2.044	393.57	804.42	3.378	328.44	1109.39
A_68_P32234207	chrX:5977686-5977730	NM_001040459:446	Shroom4	INSIDE	1.898	3.037	992.20	3013.76	5.764	637.73	3675.91
A_68_P32234202	chrX:5977172-5977216	NM_001040459:-68	Shroom4	PROMOTER	1.512	2.645	1691.44	4473.07	3.997	1083.40	4330.82
A_68_P32796350	chrX:160514136-160514180	NM_009173:-177	Siah1b	PROMOTER	2.621	3.643	1852.28	6747.79	9.547	1061.03	10129.19
A_68_P26477292	chr9:45821526-45821572	NM_027498:646	Sik3	INSIDE	1.526	3.393	1087.02	3688.45	5.178	884.56	4579.83
A_68_P22383780	chr3:102799614-102799658	NM_025679:-26	Sike1	PROMOTER	1.796	1.652	1622.33	2680.09	2.967	1345.94	3993.37
A_68_P26540094	chr9:56924177-56924221	NM_001110350:16	Sin3a	INSIDE	1.510	1.692	1975.15	3341.37	2.555	1590.03	4062.80
A_68_P28585119	chr12:83270170-83270214	NM_001167983:-810	Sipa1l1	PROMOTER	2.427	9.389	1250.51	11740.61	22.786	826.90	18841.71
A_68_P21625558	chr2:129418959-129419003	NM_001177647:50	Sirpa	INSIDE	2.259	2.888	1133.84	3275.07	6.526	933.94	6095.30
A_68_P28989794	chr13:43460884-43460928	NM_178848:-5178	Sirt5	PROMOTER	2.332	3.269	1508.13	4930.73	7.626	1101.35	8398.55
A_68_P31412114	chr17:86086928-86086972	NM_011380:644	Six2	INSIDE	1.817	4.305	1547.01	6659.82	7.824	1263.46	9885.38
A_68_P31412120	chr17:86087935-86087979	NM_011380:-362	Six2	PROMOTER	1.784	7.698	5465.70	42077.02	13.734	4681.46	64296.65
A_68_P31412116	chr17:86087149-86087193	NM_011380:424	Six2	INSIDE	1.667	2.596	711.32	1846.23	4.328	623.31	2697.39
A_68_P31411779	chr17:86022957-86023001	NM_011381:2805	Six3	INSIDE	2.050	2.885	1519.99	4385.06	5.913	1232.54	7288.03
A_68_P31411712	chr17:86014601-86014645	NR_038085:3114	Six3os1	INSIDE	1.984	4.684	10986.84	51460.60	9.291	7340.72	68205.52
A_68_P31156111	chr17:34987166-34987210	NM_021337:-39	Skiv2l	DIVERGENT_PROMOTER	2.007	5.126	962.58	4934.17	10.287	766.37	7883.27
A_68_P26574053	chr9:62986483-62986527	NM_172446:8283	Skor1	INSIDE	1.844	6.491	392.52	2547.85	11.969	308.45	3691.74
A_68_P26574057	chr9:62986883-62986927	NM_172446:7883	Skor1	INSIDE	1.735	6.931	588.37	4077.83	12.028	469.55	5647.64
A_68_P27798065	chr11:52045603-52045647	NM_011543:128	Skp1a	INSIDE	1.531	2.037	756.45	1540.63	3.117	659.84	2056.86
A_68_P23590298	chr5:33995400-33995444	NM_009193:-467	Slbp	PROMOTER	1.836	2.597	4361.04	11323.66	4.766	3399.33	16202.53
A_68_P21820794	chr2:164793741-164793785	NM_020333:275	Slc12a5	INSIDE	2.299	8.368	8253.59	69064.27	19.241	5190.38	99869.07
A_68_P21820992	chr2:164823190-164823234	NM_020333:29725	Slc12a5	INSIDE	1.863	4.280	1560.22	6677.49	7.972	1157.02	9223.73
A_68_P21820797	chr2:164794239-164794283	NM_020333:773	Slc12a5	INSIDE	1.843	2.128	874.21	1860.62	3.922	698.46	2739.65
A_68_P21527966	chr2:112105941-112105985	NM_133649:-508	Slc12a6	PROMOTER	1.715	1.556	781.59	1216.41	2.669	608.90	1625.27
A_68_P27228617	chr10:69707740-69707784	NM_025807:-261	Slc16a9	PROMOTER	1.825	3.012	3026.06	9114.75	5.497	2482.91	13648.79
A_68_P27228619	chr10:69707994-69708038	NM_025807:-7	Slc16a9	PROMOTER	1.620	7.744	1500.53	11620.31	12.543	1322.87	16592.67
A_68_P25091507	chr7:52417775-52417819	NM_182993:-1494	Slc17a7	PROMOTER	1.783	4.040	3391.39	13699.88	7.203	2543.47	18320.64
A_68_P27265507	chr10:76495860-76495904	NM_031196:-74	Slc19a1	PROMOTER	2.499	9.071	5096.34	46231.10	22.666	3101.46	70297.76
A_68_P21478262	chr2:102499332-102499376	NM_001077514:515	Slc1a2	INSIDE	1.869	1.470	1996.54	2934.82	2.748	1860.15	5111.37
A_68_P28938191	chr13:34436110-34436154	NM_001033167:919	Slc22a23	INSIDE	1.574	3.253	3942.01	12824.17	5.122	2927.73	14995.06
A_68_P28938196	chr13:34436655-34436699	NM_001033167:375	Slc22a23	INSIDE	1.520	1.966	319.47	628.20	2.988	317.30	948.12
A_68_P27901829	chr11:70460462-70460506	NM_024211:11	Slc25a11	INSIDE	2.215	1.633	1156.52	1888.88	3.617	951.49	3441.89
A_68_P32127978	chr19:43749225-43749269	NM_145156:125	Slc25a28	INSIDE	1.860	3.834	4546.31	17431.31	7.130	3722.09	26537.24
A_68_P27340135	chr10:90586440-90586484	NM_133668:246	Slc25a3	INSIDE	2.048	3.639	885.81	3223.11	7.451	782.32	5828.85
A_68_P23402414	chr4:149148446-149148491	NM_027460:-92	Slc25a33	PROMOTER	1.732	6.311	5319.73	33572.15	10.928	4115.00	44966.77
A_68_P31598791	chr18:31769247-31769291	NM_026165:288	Slc25a46	INSIDE	1.552	3.700	928.88	3437.12	5.743	845.62	4856.30
A_68_P28177386	chr11:119217235-119217283	NM_178743:388	Slc26a11	INSIDE	1.744	3.869	1785.95	6909.52	6.749	1449.59	9782.59
A_68_P28332010	chr12:32244021-32244065	NM_011867:792	Slc26a4	INSIDE	1.552	2.591	663.55	1719.32	4.020	565.21	2272.30
A_68_P25957541	chr8:74093810-74093856	NM_011977:1007	Slc27a1	INSIDE	1.677	1.544	2369.95	3658.60	2.589	1777.86	4603.28

ProbeName	Target position of probe on CpG island microarray	TargetID	GeneSymbol	CpG island Description	Ratio of relative methylation (TiO_2-NP/Vehicle)	Sham group			TiO_2-H group		
						Relative methylation (Cy5/Cy3)	Cy3 signal (Input)	Cy5 signal (MeDIP)	Relative methylation (Cy5/Cy3)	Cy3 signal (Input)	Cy5 signal (MeDIP)
A_68_P30436287	chr15:91403612-91403656	NM_001033633:58	Slc2a13	INSIDE	2.026	8.109	4830.94	39175.08	16.427	3504.49	57568.49
A_68_P30436282	chr15:91402943-91402987	NM_001033633:728	Slc2a13	INSIDE	1.767	3.345	1912.48	6397.63	5.912	1714.23	10134.76
A_68_P30436288	chr15:91403683-91403727	NM_001033633:-12	Slc2a13	PROMOTER	1.642	1.669	457.19	762.82	2.740	420.44	1152.03
A_68_P23576447	chr5:31395717-31395761	NM_011773:162	Slc30a3	INSIDE	1.669	3.772	1644.43	6202.22	6.295	1250.89	7873.75
A_68_P21587592	chr2:122528587-122528631	NM_011774:-209	Slc30a4	PROMOTER	2.167	4.479	2103.53	9422.12	9.705	1687.24	16374.21
A_68_P29270336	chr13:101602642-101602692	NM_022885:716	Slc30a5	INSIDE	2.064	4.883	1259.48	6149.51	10.078	939.30	9466.16
A_68_P22963537	chr4:61947638-61947682	NM_025286:182	Slc31a2	INSIDE	1.575	2.257	4243.51	9576.03	3.553	3512.67	12481.00
A_68_P21783928	chr2:158436941-158436985	NM_009508:469	Slc32a1	INSIDE	1.973	5.667	21029.26	119167.00	11.180	15800.81	176658.30
A_68_P28963697	chr13:39052368-39052412	NM_001170430:16	Slc35b3	INSIDE	1.563	1.824	2096.03	3823.89	2.851	1602.74	4569.52
A_68_P26983377	chr10:19570989-19571033	NM_029529:255	Slc35d3	INSIDE	1.762	1.437	1770.66	2545.16	2.533	1354.11	3430.35
A_68_P23439553	chr4:154976002-154976046	NM_177186:500	Slc35e2	INSIDE	1.540	2.236	771.25	1724.72	3.443	614.18	2114.67
A_68_P27138494	chr10:52410512-52410556	NM_178675:228	Slc35f1	INSIDE	2.495	3.924	618.16	2425.63	9.792	614.22	6014.29
A_68_P20585375	chr1:127456906-127456950	NM_028787:-664	Slc35f5	PROMOTER	2.071	5.325	2201.21	11722.48	11.029	1528.85	16862.37
A_68_P20585382	chr1:127457522-127457566	NM_028787:-48	Slc35f5	PROMOTER	1.587	3.073	987.60	3034.69	4.876	876.38	4272.84
A_68_P21420493	chr2:90910689-90910733	NM_026721:-332	Slc39a13	PROMOTER	1.802	3.480	3319.90	11553.43	6.269	2783.38	17450.22
A_68_P21420490	chr2:90910289-90910333	NM_026721:68	Slc39a13	INSIDE	1.576	1.606	2381.15	3824.63	2.532	1770.20	4482.07
A_68_P31560905	chr18:24761962-24762006	NM_139143:334	Slc39a6	INSIDE	1.868	2.161	1272.21	2749.55	4.037	1035.29	4179.32
A_68_P31560903	chr18:24761642-24761686	NM_139143:654	Slc39a6	INSIDE	1.834	2.435	2444.17	5951.24	4.466	2013.51	8992.61
A_68_P31943158	chr19:8788397-8788441	NM_008577:-31	Slc3a2	PROMOTER	1.514	3.055	1784.84	5452.80	4.624	1356.57	6272.64
A_68_P22923084	chr4:53453754-53453798	NM_001159633:492	Slc44a1	INSIDE	1.758	4.226	3284.90	13883.52	7.429	2686.04	19955.22
A_68_P23407530	chr4:150026271-150026315	NM_173774:-9	Slc45a1	PROMOTER	1.691	6.326	669.12	4233.06	10.699	664.88	7113.70
A_68_P27943494	chr11:78279579-78279623	NM_026740:398	Slc46a1	INSIDE	2.212	5.310	7800.47	41419.02	11.745	5159.24	60594.41
A_68_P21631241	chr2:130523070-130523114	NM_001081162:163	Slc4a11	INSIDE	1.784	2.805	856.46	2402.29	5.003	680.46	3404.16
A_68_P20354818	chr1:75543316-75543360	NM_009208:498	Slc4a3	INSIDE	2.116	4.675	6516.45	30464.92	9.891	4369.35	43216.35
A_68_P27327917	chr10:88348711-88348755	NM_145423:-4	Slc5a8	PROMOTER	1.759	5.743	1545.80	8878.18	10.104	1367.68	13818.54
A_68_P24764862	chr6:114232614-114232658	NM_178703:8	Slc6a1	INSIDE	2.549	17.190	6602.31	113494.20	43.825	3404.89	149218.90
A_68_P24764867	chr6:114233070-114233114	NM_178703:464	Slc6a1	INSIDE	1.815	2.185	1964.99	4292.57	3.964	1703.19	6751.78
A_68_P26068970	chr8:95484812-95484856	NM_009209:-111	Slc6a2	PROMOTER	2.056	2.600	328.68	854.73	5.348	259.54	1387.98
A_68_P27935204	chr11:76811878-76811922	NM_010484:-198	Slc6a4	PROMOTER	1.732	2.191	1262.92	2767.27	3.795	1066.19	4046.40
A_68_P32464061	chrX:70918592-70918636	NM_001142809:143	Slc6a8	INSIDE	1.948	4.473	1953.12	8735.75	8.713	1190.32	10371.57
A_68_P23236664	chr4:117508530-117508574	NM_008135:690	Slc6a9	INSIDE	1.748	2.240	1988.17	4452.72	3.914	1745.17	6830.68
A_68_P23236670	chr4:117509289-117509333	NM_008135:1448	Slc6a9	INSIDE	1.719	3.621	3065.86	11101.40	6.224	2295.46	14288.00
A_68_P25807719	chr8:41947388-41947432	NM_007514:-310	Slc7a2	PROMOTER	1.982	6.030	7612.40	45905.08	11.954	5257.46	62847.22
A_68_P30574556	chr16:17576756-17576800	NM_144852:-14	Slc7a4	PROMOTER	1.675	2.076	1704.75	3539.73	3.478	1349.50	4693.46
A_68_P26229674	chr8:124431610-124431654	NM_011404:-46	Slc7a5	DIVERGENT_PROMOTER	1.515	2.080	3412.27	7097.53	3.152	2669.32	8414.32
A_68_P26138122	chr8:108692733-108692777	NM_178798:-20	Slc7a6	PROMOTER	2.133	9.459	3267.93	30911.10	20.172	2009.26	40530.44
A_68_P26138372	chr8:108734689-108734733	NM_001007567:123	Slc7a6os	INSIDE	1.521	2.938	1210.21	3555.44	4.469	1091.81	4879.33
A_68_P32396713	chrX:53862851-53862895	NM_172780:-139	Slc9a6	PROMOTER	2.649	5.416	1489.93	8069.17	14.349	796.83	11433.70
A_68_P22236489	chr3:72861536-72861580	NM_198864:-693	Slitrk3	PROMOTER	1.653	2.647	8475.59	22433.80	4.376	6429.11	28135.05
A_68_P22236487	chr3:72861315-72861359	NM_198864:-471	Slitrk3	PROMOTER	1.556	1.938	2102.13	4073.97	3.015	1678.91	5062.21
A_68_P32150744	chr19:47653811-47653855	NM_001164639:-676	Slk	PROMOTER	1.806	1.474	1101.74	1624.42	2.663	969.80	2583.01
A_68_P31791990	chr18:67624448-67624492	NM_144867:-32	Slmo1	PROMOTER	1.776	1.828	573.52	1048.66	3.248	617.54	2005.65
A_68_P25994476	chr8:81922795-81922839	NM_008539:551	Smad1	INSIDE	1.795	10.589	3319.82	35154.77	19.006	2493.42	47388.88
A_68_P29062977	chr13:56805003-56805048	NM_001164042:-3	Smad5	PROMOTER	1.663	6.202	2821.79	17499.50	10.311	2385.19	24592.75
A_68_P26579683	chr9:63870481-63870525	NM_008542:-636	Smad6	PROMOTER	1.662	2.949	2979.18	8784.45	4.901	2298.79	11266.06
A_68_P30486834	chr15:100467426-100467470	NM_001033872:-152	Smagp	PROMOTER	1.684	1.671	1685.07	2816.45	2.815	1373.73	3866.96
A_68_P23254538	chr4:120690017-120690061	NM_133716:-186	Smap2	PROMOTER	1.714	4.098	636.02	2606.32	7.024	524.34	3683.03

ProbeName	Target position of probe on CpG island microarray	TargetID	GeneSymbol	CpG island Description	Ratio of relative methylation (TiO_2-NP/Vehicle)	Sham group			TiO_2-H group		
						Relative methylation (Cy5/Cy3)	Cy3 signal (Input)	Cy5 signal (MeDIP)	Relative methylation (Cy5/Cy3)	Cy3 signal (Input)	Cy5 signal (MeDIP)
A_68_P32036088	chr19:26681356-26681400	NM_011416:1729	Smarca2	INSIDE	1.581	3.893	616.82	2401.14	6.156	548.04	3373.76
A_68_P32036079	chr19:26680265-26680310	NM_011416:638	Smarca2	INSIDE	1.551	1.658	1913.20	3172.21	2.571	1502.58	3863.03
A_68_P26822089	chr9:110034498-110034542	NM_009211:-7	Smarcc1	PROMOTER	1.586	6.946	17001.44	118093.90	11.014	16425.11	180909.50
A_68_P23542998	chr5:24110558-24110602	NM_025891:-2760	Smarcd3	PROMOTER	1.631	8.477	7541.98	63933.24	13.824	5614.49	77617.03
A_68_P28061204	chr11:99091669-99091713	NM_020618:641	Smarce1	INSIDE	1.700	1.808	1456.70	2633.66	3.073	1302.10	4000.92
A_68_P28237421	chr12:11273151-11273195	NM_025695:481	Smc6	INSIDE	1.577	2.663	948.41	2526.00	4.199	774.69	3253.12
A_68_P28685879	chr12:102321594-102321638	NM_001160214:296	Smek1	INSIDE	1.596	2.273	1733.74	3940.91	3.627	1557.98	5650.62
A_68_P25457391	chr7:125387085-125387129	NM_001031814:45	Smg1	INSIDE	2.106	4.551	16026.86	72944.30	9.587	11915.57	114231.50
A_68_P24328142	chr6:29685305-29685349	NM_176996:-170	Smo	PROMOTER	2.031	6.080	4339.25	26382.27	12.347	3096.61	38233.21
A_68_P24328146	chr6:29685667-29685711	NM_176996:192	Smo	INSIDE	1.511	4.847	1094.66	5305.59	7.325	874.54	6405.71
A_68_P21635209	chr2:131317684-131317728	NM_001177833:109	Smox	INSIDE	1.607	7.670	1752.26	13439.82	12.323	1384.68	17062.93
A_68_P30574719	chr16:17619415-17619459	NM_001164609:-10	Smpd4	PROMOTER	1.740	3.457	1114.97	3854.16	6.013	928.41	5582.68
A_68_P32766765	chrX:153929388-153929432	NM_009214:568	Sms	INSIDE	2.423	5.494	1673.83	9195.71	13.311	1054.97	14042.84
A_68_P32766764	chrX:153929309-153929353	NM_009214:648	Sms	INSIDE	1.997	4.112	1440.88	5924.68	8.211	837.57	6877.17
A_68_P32766772	chrX:153930279-153930323	NM_009214:-322	Sms	PROMOTER	1.698	4.058	1323.76	5372.22	6.890	777.52	5357.23
A_68_P20929010	chr1:191746024-191746068	NM_026796:121	Smyd2	INSIDE	1.856	1.637	1106.26	1810.60	3.039	961.74	2922.27
A_68_P27925675	chr11:75162047-75162091	NM_001102611:134	Smyd4	INSIDE	1.621	1.499	641.77	962.31	2.431	578.57	1406.70
A_68_P21836025	chr2:167363730-167363774	NM_011427:26	Snai1	INSIDE	1.711	1.602	1591.40	2549.53	2.740	1316.52	3607.71
A_68_P21078285	chr2:26236006-26236050	NM_172339:145	Snapc4	INSIDE	1.709	3.792	4307.71	16333.25	6.478	3441.90	22297.41
A_68_P29051301	chr13:54867120-54867164	NM_033610:659	Sncb	INSIDE	1.752	3.620	2927.26	10596.65	6.343	2313.86	14677.38
A_68_P30546005	chr16:11066391-11066435	NM_009223:22	Snn	INSIDE	1.563	1.775	1499.49	2661.18	2.774	1273.43	3531.86
A_68_P25588870	chr7:148633634-148633678	NR_034049:-1075	Snora52	PROMOTER	1.789	2.259	6061.79	13694.99	4.041	4450.07	17984.60
A_68_P25954806	chr8:73421106-73421150	NR_002901:-1373	Snora68	PROMOTER	1.562	1.778	2759.35	4907.28	2.777	2115.56	5875.71
A_68_P23232782	chr4:116828713-116828757	NR_030704:-281	Snord55	PROMOTER	2.140	3.084	637.06	1964.47	6.599	492.18	3247.96
A_68_P20411724	chr1:88255322-88255366	NR_002851:-2442	Snord82	PROMOTER	1.827	5.464	1772.42	9685.24	9.982	1286.89	12845.70
A_68_P21745020	chr2:151457975-151458019	NM_198214:273	Snph	INSIDE	1.503	4.184	431.24	1804.12	6.287	374.44	2354.14
A_68_P26887498	chr9:122026335-122026379	NM_001164572:-27	Snrk	PROMOTER	1.574	3.457	2955.78	10218.80	5.442	2375.99	12929.20
A_68_P31118768	chr17:27977231-27977275	NM_011432:221	Snrpc	INSIDE	1.763	5.799	496.83	2881.28	10.226	367.40	3757.01
A_68_P20022045	chr1:9289343-9289387	NM_027671:447	Sntg1	INSIDE	1.752	5.857	3617.91	21188.75	10.260	2628.46	26966.77
A_68_P26591323	chr9:65972506-65972550	NM_019727:165	Snx1	INSIDE	1.782	2.993	1149.29	3439.90	5.332	971.18	5178.63
A_68_P24445013	chr6:51542631-51542675	NM_001127348:48131	Snx10	DOWNSTREAM	1.513	1.619	5835.38	9446.43	2.449	4234.39	10370.79
A_68_P28351016	chr12:35731926-35731970	NM_001014973:88	Snx13	INSIDE	1.793	5.061	1661.88	8411.34	9.077	1424.16	12927.00
A_68_P26394905	chr9:30234659-30234703	NM_028874:-233	Snx19	PROMOTER	1.657	1.766	790.61	1396.01	2.926	755.39	2210.60
A_68_P26591015	chr9:65917041-65917085	NM_001025612:476	Snx22	INSIDE	1.933	3.673	1247.31	4581.80	7.101	1043.81	7412.32
A_68_P27095209	chr10:42222002-42222046	NM_017472:165	Snx3	INSIDE	2.314	4.285	1119.07	4795.47	9.918	862.62	8555.08
A_68_P30656969	chr16:33251415-33251459	NM_080557:-105	Snx4	PROMOTER	2.486	10.407	3440.30	35804.83	25.873	2337.93	60489.97
A_68_P28449264	chr12:55896516-55896560	NM_026998:111	Snx6	INSIDE	1.818	4.739	1566.84	7425.23	8.616	1292.67	11137.82
A_68_P24141205	chr5:140865282-140865326	NM_172277:-103	Snx8	PROMOTER	2.512	5.782	13712.53	79285.45	14.522	8932.21	129716.30
A_68_P30544459	chr16:10785615-10785659	NM_009896:-7	Socs1	PROMOTER	1.940	4.714	4002.87	18868.14	9.143	3241.30	29633.88
A_68_P27365029	chr10:94878409-94878453	NM_001168656:-312	Socs2	PROMOTER	1.957	3.650	1261.25	4603.74	7.144	1029.49	7354.78
A_68_P31054932	chr17:13200778-13200822	NM_013671:96	Sod2	INSIDE	1.676	3.293	1214.17	3997.88	5.520	988.75	5457.47
A_68_P23694283	chr5:52759912-52759956	NM_011435:4892	Sod3	INSIDE	1.595	4.202	2347.88	9864.82	6.699	1963.26	13152.84
A_68_P29699987	chr14:70606016-70606060	NM_011366:1406	Sorbs3	INSIDE	1.837	4.285	2648.09	11346.76	7.872	2086.17	16422.34
A_68_P32168785	chr19:50752526-50752570	NM_021377:554	Sorcs1	INSIDE	2.133	7.067	4953.93	35007.53	15.071	3490.88	52610.12
A_68_P32168791	chr19:50753028-50753072	NM_021377:52	Sorcs1	INSIDE	1.765	5.090	1771.47	9017.51	8.987	1696.90	15249.77
A_68_P23607369	chr5:36740782-36740826	NM_030889:-16	Sorcs2	PROMOTER	1.758	6.372	4995.22	31827.52	11.198	4038.19	45221.17
A_68_P22411695	chr3:108086638-108086682	NM_019972:-389	Sort1	PROMOTER	1.860	12.153	4225.97	51358.98	22.603	3331.36	75298.24

ProbeName	Target position of probe on CpG island microarray	TargetID	GeneSymbol	CpG island Description	Ratio of relative methylation (TiO_2-NP/Vehicle)	Sham group			TiO_2-H group		
						Relative methylation (Cy5/Cy3)	Cy3 signal (Input)	Cy5 signal (MeDIP)	Relative methylation (Cy5/Cy3)	Cy3 signal (Input)	Cy5 signal (MeDIP)
A_68_P28515845	chr12:70782400-70782444	NM_001135559:417	Sos2	INSIDE	1.969	2.541	1958.50	4975.69	5.001	1528.15	7642.91
A_68_P28307263	chr12:28027320-28027364	NM_009234:241	Sox11	INSIDE	2.795	16.492	4774.96	78748.73	46.092	3149.38	145159.90
A_68_P28307270	chr12:28028339-28028383	NM_009234:-777	Sox11	PROMOTER	1.666	2.492	5072.19	12642.29	4.152	3722.72	15456.05
A_68_P26766653	chr9:99775810-99775854	NM_011440:757	Sox14	INSIDE	1.624	2.131	357.97	762.89	3.461	336.16	1163.37
A_68_P20005810	chr1:4483711-4483755	NM_011441:2762	Sox17	INSIDE	1.927	4.121	693.64	2858.49	7.941	700.21	5560.40
A_68_P20005794	chr1:4482093-4482137	NM_011441:4380	Sox17	INSIDE	1.589	1.584	758.20	1201.34	2.517	626.08	1575.97
A_68_P29937469	chr14:118633887-118633936	NM_177753:2341	Sox21	INSIDE	1.566	2.106	666.97	1404.33	3.298	548.73	1809.84
A_68_P28909030	chr13:29051600-29051644	NM_009238:-6071	Sox4	PROMOTER	2.248	3.638	3625.39	13187.66	8.177	2539.05	20760.84
A_68_P25445817	chr7:123177303-123177347	NM_001025560:-2763	Sox6	PROMOTER	1.917	3.509	3173.69	11135.04	6.726	2390.20	16075.83
A_68_P29664626	chr14:64562881-64562925	NM_011446:360	Sox7	INSIDE	1.647	2.929	1685.38	4936.40	4.824	1370.78	6612.59
A_68_P31104792	chr17:25707704-25707748	NM_011447:-95	Sox8	DIVERGENT_PROMOTER	1.535	5.972	1910.27	11407.68	9.169	1274.07	11682.41
A_68_P28137828	chr11:112643707-112643751	NM_011448:205	Sox9	INSIDE	1.701	3.547	3362.01	11926.44	6.035	2633.99	15895.53
A_68_P28137825	chr11:112643344-112643388	NM_011448:-157	Sox9	PROMOTER	1.664	1.701	4828.16	8213.36	2.830	3355.10	9494.71
A_68_P30497696	chr15:102236237-102236289	NM_013672:-484	Sp1	PROMOTER	1.690	2.365	620.64	1467.90	3.996	432.04	1726.52
A_68_P30497699	chr15:102236639-102236683	NM_013672:-86	Sp1	PROMOTER	1.634	2.607	1459.41	3804.34	4.259	1211.32	5158.72
A_68_P28778754	chr12:119539626-119539670	NM_001166385:265	Sp4	INSIDE	1.862	2.643	5538.52	14636.78	4.922	3708.25	18250.32
A_68_P28048173	chr11:96879906-96879950	NM_031183:5046	Sp6	INSIDE	2.171	8.587	6504.89	55854.27	18.644	4870.51	90804.01
A_68_P28781893	chr12:120087514-120087558	NM_177082:2735	Sp8	INSIDE	1.855	6.228	2260.85	14079.59	11.549	1813.01	20938.56
A_68_P28781792	chr12:120075230-120075274	NM_177082:-9549	Sp8	PROMOTER	1.718	2.608	3721.05	9704.84	4.481	2814.29	12609.79
A_68_P21330556	chr2:73115402-73115446	NM_001005343:5442	Sp9	DOWNSTREAM	1.969	4.836	4620.55	22345.30	9.521	3436.51	32718.53
A_68_P27942521	chr11:78115275-78115319	NM_017407:204	Spag5	INSIDE	1.642	5.783	1791.57	10361.45	9.494	1481.68	14067.16
A_68_P31351023	chr17:74738587-74738631	NM_001162870:282	Spast	INSIDE	1.515	3.242	614.24	1991.24	4.912	530.71	2606.66
A_68_P22614971	chr3:146162605-146162649	NM_027617:91	Spata1	INSIDE	1.599	3.443	830.28	2858.85	5.504	725.21	3991.58
A_68_P22063513	chr3:37319180-37319224	NM_001163511:1	Spata5	INSIDE	1.686	1.914	702.42	1344.19	3.226	643.62	2076.33
A_68_P21307670	chr2:69044152-69044196	NM_001199123:73	Spc25	INSIDE	1.852	3.706	1585.79	5877.44	6.865	1353.50	9291.82
A_68_P21633642	chr2:130997305-130997349	NM_027641:3220	Spef1	INSIDE	1.534	2.060	1345.19	2770.51	3.159	1104.99	3490.29
A_68_P26587800	chr9:65308975-65309019	NM_138584:253	Spg21	INSIDE	1.582	4.852	7065.33	34279.80	7.673	4943.68	37934.16
A_68_P21568536	chr2:119063328-119063372	NM_016907:255	Spint1	INSIDE	1.718	2.031	3187.21	6473.42	3.490	2311.72	8067.09
A_68_P21100500	chr2:29821292-29821336	NM_001076554:235	Spna2	INSIDE	2.415	5.503	3190.19	17554.30	13.290	2465.20	32762.30
A_68_P21100497	chr2:29820983-29821027	NM_001076554:-75	Spna2	PROMOTER	1.655	1.873	8262.90	15476.18	3.100	6187.29	19181.71
A_68_P21100496	chr2:29820843-29820887	NM_001076554:-215	Spna2	PROMOTER	1.585	2.106	2808.10	5914.80	3.338	2344.22	7824.94
A_68_P25017170	chr7:28193438-28193482	NM_032610:38148	Spnb4	INSIDE	1.837	3.254	1617.69	5263.61	5.977	1239.79	7410.37
A_68_P25017009	chr7:28160497-28160541	NM_001199235:19007	Spnb4	INSIDE	1.642	2.209	3530.92	7801.52	3.628	3013.58	10934.32
A_68_P21556049	chr2:116946411-116946455	NM_033524:-753	Spred1	PROMOTER	2.559	7.004	7406.18	51873.25	17.920	5638.84	101048.30
A_68_P21556050	chr2:116946481-116946525	NM_033524:-683	Spred1	PROMOTER	1.842	3.294	4864.40	16025.06	6.069	4099.83	24880.16
A_68_P27628926	chr11:19825568-19825612	NM_033523:1146	Spred2	INSIDE	1.848	5.940	1757.69	10440.87	10.975	1358.52	14909.89
A_68_P27629548	chr11:19921114-19921161	NM_033523:96693	Spred2	INSIDE	1.678	2.842	623.29	1771.16	4.768	471.80	2249.64
A_68_P30496205	chr15:101966489-101966533	NM_001033277:136	Spryd3	INSIDE	1.745	2.590	3279.72	8492.93	4.519	2677.66	12099.03
A_68_P28615786	chr12:88729140-88729184	NM_011479:18	Sptlc2	INSIDE	1.965	7.386	683.93	5051.85	14.516	646.96	9391.54
A_68_P28615267	chr12:88640639-88640683	NM_011479:88520	Sptlc2	DOWNSTREAM	1.638	3.014	654.26	1971.91	4.938	576.68	2847.47
A_68_P28615783	chr12:88728757-88728801	NM_011479:402	Sptlc2	INSIDE	1.534	2.160	1028.58	2221.43	3.312	828.94	2745.68
A_68_P25102500	chr7:54263669-54263713	NM_175318:94	Spty2d1	INSIDE	1.742	3.016	3244.63	9784.45	5.252	2553.17	13408.54
A_68_P21587981	chr2:122591010-122591054	NM_001162503:-62	Sqrdl	PROMOTER	2.501	7.553	2476.02	18700.67	18.891	1784.22	33705.88
A_68_P27787321	chr11:50023806-50023850	NM_011018:464	Sqstm1	INSIDE	1.738	4.742	6644.51	31510.94	8.244	4503.50	37125.14
A_68_P21777116	chr2:157282885-157282929	NM_001025395:32878	Src	INSIDE	1.624	1.761	1431.74	2521.94	2.861	1201.96	3438.40
A_68_P24118747	chr5:136438697-136438741	NM_001160366:11628	Srcrb4d	INSIDE	2.078	3.036	1843.78	5597.98	6.308	1464.81	9240.73
A_68_P23814358	chr5:76569347-76569391	NM_020611:71	Srd5a3	INSIDE	1.913	3.279	1314.92	4311.92	6.273	972.42	6100.40

ProbeName	Target position of probe on CpG island microarray	TargetID	GeneSymbol	CpG island Description	Ratio of relative methylation (TiO_2-NP/Vehicle)	Sham group			TiO_2-H group		
						Relative methylation (Cy5/Cy3)	Cy3 signal (Input)	Cy5 signal (MeDIP)	Relative methylation (Cy5/Cy3)	Cy3 signal (Input)	Cy5 signal (MeDIP)
A_68_P27841688	chr11:60034191-60034235	NM_011480:-106	Srebf1	PROMOTER	1.635	1.775	1170.73	2077.55	2.902	1024.89	2974.20
A_68_P30381701	chr15:81977690-81977734	NM_033218:14	Srebf2	INSIDE	1.641	2.567	7370.16	18917.36	4.213	5529.92	23299.25
A_68_P31207579	chr17:46692454-46692498	NM_020493:635	Srf	INSIDE	1.535	2.991	2127.40	6364.07	4.593	1763.88	8101.18
A_68_P31612299	chr18:34490607-34490651	NM_025527:-170	Srp19	PROMOTER	1.509	2.820	1687.65	4759.55	4.255	1369.71	5827.73
A_68_P23819150	chr5:77403737-77403781	NM_025691:33	Srp72	INSIDE	1.827	5.143	3782.81	19453.76	9.396	2820.82	26505.70
A_68_P23991577	chr5:112772027-112772071	NM_027323:12	Srrd	INSIDE	1.788	3.757	18048.04	67814.54	6.717	14956.85	100468.50
A_68_P31093720	chr17:23940883-23940927	NM_175229:751	Srrm2	INSIDE	2.212	2.483	1982.45	4923.18	5.494	1454.97	7994.04
A_68_P22825736	chr4:33296596-33296640	NM_177774:653	Srsf12	INSIDE	1.527	3.172	5337.83	16931.31	4.845	3663.72	17751.12
A_68_P28161643	chr11:116713965-116714009	NM_011358:422	Srsf2	INSIDE	1.515	2.553	535.98	1368.48	3.867	467.60	1808.41
A_68_P23308821	chr4:131429974-131430018	NM_020587:443	Srsf4	INSIDE	1.775	1.555	884.71	1375.62	2.760	827.18	2283.21
A_68_P28578489	chr12:82047116-82047160	NM_001079694:648	Srsf5	INSIDE	2.113	3.899	533.87	2081.51	8.237	451.24	3716.87
A_68_P24009031	chr5:115777029-115777073	NM_025573:-135	Srsf9	PROMOTER	1.556	4.084	2154.06	8797.49	6.357	1708.11	10857.85
A_68_P31513737	chr18:14841164-14841208	NM_001161369:237	Ss18	INSIDE	1.623	5.982	1313.71	7859.24	9.711	1187.27	11529.15
A_68_P31513736	chr18:14841066-14841110	NM_001161369:335	Ss18	INSIDE	1.730	1.539	893.89	1376.05	2.663	779.30	2075.10
A_68_P29218716	chr13:91599885-91599929	NM_024186:-795	Ssbp2	PROMOTER	2.476	16.971	563.62	9565.23	42.025	488.52	20530.01
A_68_P29218720	chr13:91600319-91600363	NM_024186:-361	Ssbp2	PROMOTER	1.588	4.755	2770.98	13176.69	7.551	2307.64	17424.88
A_68_P29218723	chr13:91600897-91600941	NM_024186:217	Ssbp2	INSIDE	1.517	4.122	4099.33	16896.23	6.251	3046.95	19047.37
A_68_P27937051	chr11:77161599-77161643	NM_177710:131694	Ssh2	INSIDE	1.533	4.004	1450.50	5807.99	6.140	1278.99	7852.84
A_68_P21071033	chr2:25127873-25127917	NM_023464:44	Ssna1	INSIDE	1.645	2.674	2235.25	5976.17	4.398	1902.58	8367.84
A_68_P22199719	chr3:65196469-65196513	NM_026155:-15	Ssr3	PROMOTER	2.746	17.796	15091.40	268560.50	48.865	7125.09	348164.30
A_68_P28466190	chr12:59313609-59313653	NM_009216:840	Sstr1	INSIDE	1.619	5.454	1300.46	7092.60	8.829	990.04	8741.09
A_68_P23440100	chr4:155079091-155079135	NM_026899:189	Ssu72	INSIDE	2.023	4.457	9756.79	43489.91	9.016	7677.19	69218.56
A_68_P23440105	chr4:155079607-155079651	NM_026899:705	Ssu72	INSIDE	2.020	6.071	1128.51	6850.95	12.263	891.79	10935.81
A_68_P22614372	chr3:146067861-146067905	NM_138744:232	Ssx2ip	INSIDE	1.658	6.185	795.13	4917.62	10.254	736.50	7552.35
A_68_P26398844	chr9:30939486-30939530	NM_011176:-124	St14	PROMOTER	2.197	4.125	7544.95	31124.72	9.064	5133.42	46531.13
A_68_P26165552	chr8:113443992-113444036	NM_009179:250	St3gal2	INSIDE	1.692	4.347	2103.08	9142.78	7.357	1692.26	12450.05
A_68_P26165551	chr8:113443849-113443893	NM_009179:106	St3gal2	INSIDE	1.585	1.358	1611.73	2188.02	2.152	1539.91	3313.18
A_68_P30601918	chr16:23225193-23225237	NM_145933:380	St6gal1	INSIDE	1.904	2.661	1893.74	5038.34	5.066	1478.16	7487.62
A_68_P30601919	chr16:23225265-23225309	NM_145933:452	St6gal1	INSIDE	1.641	2.234	1424.73	3183.27	3.666	1177.58	4317.57
A_68_P31251161	chr17:55585081-55585126	NM_172829:89	St6gal2	INSIDE	1.508	1.632	618.68	1009.67	2.461	567.75	1397.19
A_68_P22658557	chr3:153388050-153388094	NM_011372:25	St6galnac3	INSIDE	1.583	2.667	3430.94	9151.06	4.222	2955.87	12479.41
A_68_P21116393	chr2:32468028-32468072	NM_001025310:5570	St6galnac6	INSIDE	1.925	8.406	3661.88	30782.19	16.185	2404.68	38920.51
A_68_P25232263	chr7:81157988-81158032	NM_009181:558	St8sia2	INSIDE	2.023	4.807	8465.95	40693.13	9.725	6151.28	59819.75
A_68_P25232267	chr7:81158422-81158466	NM_009181:124	St8sia2	INSIDE	1.639	4.897	579.54	2837.93	8.027	450.86	3618.93
A_68_P28054168	chr11:97913868-97913912	NM_146028:886	Stac2	INSIDE	1.654	2.484	8806.47	21877.27	4.109	6437.64	26455.20
A_68_P25372610	chr7:108469852-108469896	NM_019990:42	Stard10	INSIDE	2.367	5.981	15543.06	92958.13	14.156	11160.96	157990.00
A_68_P28862223	chr13:19487305-19487349	NM_024270:295	Stard3nl	INSIDE	1.660	1.728	1020.53	1763.43	2.869	858.88	2464.22
A_68_P32553143	chrX:96238297-96238345	NM_199018:401	Stard8	INSIDE	2.484	6.859	445.03	3052.42	17.039	246.79	4205.01
A_68_P32553141	chrX:96238064-96238108	NM_199018:167	Stard8	INSIDE	1.904	4.549	2633.26	11979.79	8.662	1594.17	13808.89
A_68_P23457345	chr5:5749151-5749195	NM_027399:145	Steap1	INSIDE	1.814	5.963	5147.22	30691.20	10.818	4433.72	47962.54
A_68_P25377989	chr7:109416038-109416082	NM_009287:-277	Stim1	PROMOTER	1.661	5.566	1377.22	7666.18	9.247	1143.42	10572.66
A_68_P25377988	chr7:109415960-109416004	NM_009287:-355	Stim1	PROMOTER	1.588	3.944	1503.95	5931.63	6.262	1256.37	7866.97
A_68_P23703749	chr5:54390129-54390173	NM_001081103:389	Stim2	INSIDE	1.572	4.884	1311.45	6404.52	7.676	1102.18	8460.46
A_68_P20456742	chr1:95532281-95532325	NM_021537:2	Stk25	INSIDE	1.901	5.375	5391.60	28979.31	10.220	4104.03	41941.65
A_68_P24432387	chr6:49345594-49345638	NM_029916:14	Stk31	INSIDE	1.731	6.766	7487.28	50658.23	11.713	5534.60	64827.12
A_68_P31659996	chr18:43367102-43367146	NM_178749:-226	Stk32a	PROMOTER	1.899	6.224	3351.87	20860.38	11.820	2474.30	29245.91
A_68_P25576307	chr7:146374240-146374284	NM_021302:131	Stk32c	INSIDE	1.803	4.492	1127.51	5065.01	8.099	961.82	7790.11

ProbeName	Target position of probe on CpG island microarray	TargetID	GeneSymbol	CpG island Description	Ratio of relative methylation (TiO_2-NP/Vehicle)	Sham group			TiO_2-H group		
						Relative methylation (Cy5/Cy3)	Cy3 signal (Input)	Cy5 signal (MeDIP)	Relative methylation (Cy5/Cy3)	Cy3 signal (Input)	Cy5 signal (MeDIP)
A_68_P25408016	chr7:116582357-116582401	NM_054103:189	Stk33	INSIDE	1.933	3.287	1844.54	6063.05	6.353	1448.65	9202.64
A_68_P28635993	chr12:92886575-92886620	NM_175367:138279	Ston2	INSIDE	1.516	2.163	294.02	636.00	3.280	230.66	756.53
A_68_P27187531	chr10:62188832-62188876	NM_001033260:-7	Stox1	PROMOTER	1.517	3.109	1793.62	5576.70	4.717	1443.07	6807.33
A_68_P24880087	chr6:137683557-137683601	NM_011499:-24	Strap	PROMOTER	1.675	4.583	993.38	4552.59	7.675	898.68	6897.24
A_68_P31373358	chr17:79136029-79136073	NM_011500:-150	Strn	PROMOTER	2.327	2.976	1194.14	3553.45	6.925	955.41	6616.44
A_68_P28431157	chr12:52792863-52792907	NM_001172098:17	Strn3	INSIDE	1.570	1.974	635.80	1255.32	3.100	541.32	1678.28
A_68_P24114435	chr5:135518130-135518174	NM_016801:18711	Stx1a	INSIDE	1.518	10.296	11814.23	121639.30	15.628	7533.59	117737.10
A_68_P25507562	chr7:134967999-134968043	NM_024414:25	Stx1b	INSIDE	1.656	2.692	6088.86	16388.52	4.459	4698.34	20947.61
A_68_P26932995	chr10:9620910-9620954	NM_001081344:-94	Stxbp5	PROMOTER	1.559	2.555	589.32	1505.86	3.985	591.01	2355.10
A_68_P26932993	chr10:9620586-9620630	NM_001081344:230	Stxbp5	INSIDE	1.538	17.246	1505.89	25970.25	26.529	1250.90	33185.50
A_68_P29718152	chr14:73952682-73952726	NM_011506:112	Sucla2	INSIDE	1.843	4.831	1652.03	7980.50	8.905	1393.34	12407.05
A_68_P24018800	chr5:117566119-117566163	NM_001122666:-138	Suds3	DIVERGENT_PROMOTER	1.578	3.103	670.81	2081.47	4.896	648.91	3176.91
A_68_P29752204	chr14:79987400-79987444	NM_026474:-75	Sugt1	PROMOTER	1.924	4.763	3302.53	15728.79	9.163	2623.06	24035.36
A_68_P28154074	chr11:115397049-115397093	NM_133354:474	Sumo2	INSIDE	1.984	7.154	14395.30	102980.70	14.191	11130.77	157957.40
A_68_P24134134	chr5:139677035-139677079	NM_024451:433	Sun1	INSIDE	2.181	5.783	2783.77	16099.74	12.615	1839.83	23209.33
A_68_P24134133	chr5:139676870-139676914	NM_024451:269	Sun1	INSIDE	1.817	1.607	767.61	1233.68	2.920	1083.62	3164.28
A_68_P27542559	chr10:128105989-128106033	NM_173733:4964	Suox	DOWNSTREAM	1.765	4.909	11211.50	55040.77	8.663	7886.81	68326.57
A_68_P29605230	chr14:52816435-52816479	NM_033618:458	Supt16h	INSIDE	1.940	4.812	2932.18	14109.05	9.334	2379.69	22211.42
A_68_P29605237	chr14:52817103-52817147	NM_033618:-210	Supt16h	PROMOTER	1.755	1.527	2216.53	3384.63	2.681	1820.08	4878.78
A_68_P31197210	chr17:44914016-44914060	NM_178652:-81	Supt3h	PROMOTER	1.782	4.329	2694.77	11664.92	7.713	2128.74	16418.48
A_68_P27995861	chr11:87550950-87550994	NM_009296:-94	Supt4h1	PROMOTER	2.114	6.564	2326.72	15273.06	13.877	1692.93	23493.62
A_68_P29024152	chr13:49343166-49343210	NM_025491:344	Susd3	INSIDE	1.817	1.837	1441.29	2648.13	3.338	1207.12	4029.69
A_68_P25239159	chr7:82453955-82453999	NM_001109753:-704	Sv2b	PROMOTER	1.514	4.067	2346.69	9543.51	6.158	1944.04	11971.56
A_68_P29244723	chr13:96902535-96902579	NM_029210:-24	Sv2c	PROMOTER	1.517	1.877	1202.09	2255.91	2.847	970.77	2764.28
A_68_P25126430	chr7:59261254-59261298	NR_027704:-192	Svip	PROMOTER	1.626	3.084	7032.53	21687.90	5.015	5516.22	27665.59
A_68_P24001851	chr5:114541342-114541386	NM_026805:25	Svop	INSIDE	1.753	5.740	2988.43	17153.90	10.061	2550.77	25662.82
A_68_P25412183	chr7:117365487-117365531	NM_009302:292	Swap70	INSIDE	1.972	5.336	5679.82	30307.86	10.520	4381.06	46089.90
A_68_P25412180	chr7:117365261-117365305	NM_009302:66	Swap70	INSIDE	1.550	5.265	1061.01	5585.94	8.159	899.62	7339.83
A_68_P22383504	chr3:102739949-102739993	NM_011516:53	Sycp1	INSIDE	1.555	3.031	522.26	1582.80	4.713	383.30	1806.57
A_68_P21890548	chr2:178142227-178142271	NM_177191:115	Sycp2	INSIDE	1.673	4.488	7874.68	35339.82	7.508	6281.03	47156.69
A_68_P21890546	chr2:178142021-178142065	NM_177191:321	Sycp2	INSIDE	1.616	3.732	5809.31	21681.31	6.031	4181.96	25221.88
A_68_P27325555	chr10:87922666-87922710	NM_011517:357	Sycp3	INSIDE	1.532	7.924	5644.23	44726.54	12.140	3909.36	47458.33
A_68_P23325610	chr4:134487232-134487276	NM_026780:360	Syf2	INSIDE	2.288	21.890	11179.55	244721.20	50.094	7846.17	393047.30
A_68_P24769157	chr6:115084895-115084939	NM_013681:-3	Syn2	PROMOTER	2.043	2.768	1816.41	5028.12	5.654	1390.76	7863.42
A_68_P26710370	chr9:88377975-88378019	NM_019796:-761	Syncrip	PROMOTER	2.138	5.150	1514.87	7801.64	11.009	1191.15	13113.61
A_68_P28167342	chr11:117671257-117671301	NM_009304:298	Syngr2	INSIDE	1.550	2.821	975.53	2752.34	4.374	772.61	3379.42
A_68_P31099001	chr17:24824425-24824469	NM_011522:2448	Syngr3	INSIDE	2.091	8.282	15324.02	126913.40	17.318	9704.87	168065.90
A_68_P30960416	chr16:91010827-91010871	NM_001045515:492	Synj1	INSIDE	2.196	7.714	957.87	7388.76	16.937	792.63	13424.86
A_68_P25195707	chr7:74904146-74904190	NM_183312:460	Synm	INSIDE	1.606	1.807	1191.57	2153.03	2.902	1015.20	2946.60
A_68_P25195705	chr7:74903906-74903950	NM_183312:700	Synm	INSIDE	1.543	2.131	1661.38	3540.87	3.288	1378.62	4533.34
A_68_P27974634	chr11:83777899-83777943	NM_001115009:-9	Synrg	PROMOTER	1.523	2.208	376.20	830.79	3.364	416.91	1402.61
A_68_P20946920	chr1:194861857-194861901	NM_181546:14	Syt14	INSIDE	1.758	3.505	904.97	3171.63	6.162	732.36	4513.01
A_68_P25458299	chr7:125591289-125591333	NM_138649:-4244	Syt17	PROMOTER	1.683	5.072	2430.45	12326.97	8.537	2032.42	17351.21
A_68_P25458297	chr7:125591038-125591082	NM_138649:-3994	Syt17	PROMOTER	1.649	2.016	354.57	714.92	3.324	307.88	1023.45
A_68_P20634933	chr1:136605811-136605855	NM_009307:62575	Syt2	INSIDE	1.509	2.023	1859.43	3762.50	3.053	1570.85	4795.85
A_68_P32506209	chrX:82819101-82819145	NM_025729:-238	Tab3	PROMOTER	2.228	5.900	2141.79	12636.44	13.145	1254.89	16495.16
A_68_P24759109	chr6:113327446-113327490	NM_133932:46	Tada3	INSIDE	1.691	3.894	1254.60	4885.71	6.584	1005.79	6621.83

ProbeName	Target position of probe on CpG island microarray	TargetID	GeneSymbol	CpG island Description	Ratio of relative methylation (TiO_2-NP/Vehicle)	Sham group			TiO_2-H group		
						Relative methylation (Cy5/Cy3)	Cy3 signal (Input)	Cy5 signal (MeDIP)	Relative methylation (Cy5/Cy3)	Cy3 signal (Input)	Cy5 signal (MeDIP)
A_68_P23311001	chr4:131830273-131830317	NM_025579:5	Taf12	INSIDE	1.613	11.344	3315.90	37616.64	18.297	2386.77	43669.74
A_68_P30230212	chr15:54903756-54903800	NM_001081288:-71	Taf2	PROMOTER	1.901	4.632	1981.73	9179.20	8.804	1705.00	15010.15
A_68_P20998559	chr2:9970073-9970117	NM_027748:142	Taf3	INSIDE	1.999	3.882	2680.44	10404.16	7.759	2242.04	17396.16
A_68_P26242940	chr8:126544834-126544878	NM_133966:353	Taf5l	INSIDE	1.763	4.722	1079.06	5094.78	8.322	864.28	7192.74
A_68_P26242939	chr8:126544720-126544764	NM_133966:467	Taf5l	INSIDE	1.731	7.775	676.84	5262.41	13.462	525.48	7074.04
A_68_P32672214	chrX:131010586-131010630	NM_028958:421	Taf7l	INSIDE	2.192	4.631	1820.18	8429.86	10.151	797.38	8094.39
A_68_P32583453	chrX:103415136-103415180	NM_001001176:23	Taf9b	INSIDE	2.223	6.738	958.05	6455.48	14.979	638.42	9563.02
A_68_P23223131	chr4:114729380-114729424	NM_011527:-2729	Tal1	PROMOTER	1.528	1.748	4063.28	7104.22	2.671	3092.87	8261.82
A_68_P25588514	chr7:148577796-148577840	NM_011528:-240	Taldo1	PROMOTER	1.699	1.899	1080.94	2052.65	3.227	874.05	2820.31
A_68_P27938553	chr11:77421084-77421128	NM_144825:211	Taok1	INSIDE	2.421	10.195	1625.76	16575.32	24.688	1096.06	27059.37
A_68_P20936016	chr1:192886793-192886837	NM_001163421:-3	Tatdn3	DIVERGENT_PROMOTER	1.528	2.584	13552.33	35014.66	3.948	11020.49	43506.03
A_68_P24452215	chr6:52664021-52664065	NM_025816:320	Tax1bp1	INSIDE	1.697	6.629	2894.54	19187.84	11.248	2434.57	27383.92
A_68_P23749456	chr5:64551627-64551671	NM_019636:199	Tbc1d1	INSIDE	2.251	6.568	13603.88	89353.73	14.786	9241.51	136648.90
A_68_P23749454	chr5:64551417-64551461	NM_019636:-11	Tbc1d1	PROMOTER	1.529	1.732	1641.37	2843.35	2.650	1396.68	3700.54
A_68_P23749879	chr5:64621748-64621793	NM_019636:70321	Tbc1d1	INSIDE	1.527	3.790	7787.71	29513.45	5.786	6371.74	36869.30
A_68_P23608585	chr5:36924742-36924786	NM_133910:282	Tbc1d14	INSIDE	1.901	2.812	13547.19	38091.92	5.346	9749.13	52114.66
A_68_P21748557	chr2:152119766-152119810	NM_024196:181	Tbc1d20	INSIDE	1.548	5.502	3289.69	18098.61	8.515	2765.79	23551.72
A_68_P21748558	chr2:152119855-152119899	NM_024196:269	Tbc1d20	INSIDE	1.510	3.767	2842.08	10706.49	5.687	2383.82	13556.14
A_68_P31232583	chr17:51318678-51318722	NM_028162:-26	Tbc1d5	PROMOTER	1.627	2.670	1622.53	4332.51	4.344	1220.79	5303.73
A_68_P29236942	chr13:95558754-95558798	NM_009321:-121	Tbca	PROMOTER	2.017	4.511	2758.98	12446.79	9.101	2323.29	21144.94
A_68_P31209382	chr17:47026332-47026376	NM_178385:-1215	Tbcc	PROMOTER	1.733	4.014	1076.92	4322.30	6.957	914.68	6363.05
A_68_P27504343	chr10:121023890-121023934	NM_019786:-62	Tbk1	PROMOTER	1.835	6.046	1165.23	7045.24	11.095	1100.61	12211.23
A_68_P28048904	chr11:97000172-97000216	NM_198100:10832	Tbkbp1	INSIDE	1.662	4.015	955.93	3838.25	6.672	792.12	5284.78
A_68_P24114960	chr5:135625731-135625775	NM_013763:172	Tbl2	INSIDE	1.670	3.290	1092.96	3595.91	5.494	907.58	4986.45
A_68_P21266439	chr2:61649898-61649942	NM_009322:7411	Tbr1	INSIDE	1.537	2.571	364.72	937.82	3.952	383.31	1514.93
A_68_P21266440	chr2:61650101-61650145	NM_009322:7613	Tbr1	INSIDE	1.879	2.100	424.79	892.06	3.945	412.76	1628.36
A_68_P30580602	chr16:18586865-18586909	NM_011532:176	Tbx1	INSIDE	1.611	3.668	2181.79	8002.39	5.908	1820.13	10753.60
A_68_P27985587	chr11:85651691-85651735	NM_009324:5596	Tbx2	INSIDE	1.595	1.611	1802.26	2903.35	2.570	1401.10	3600.16
A_68_P23332899	chr4:135804076-135804120	NM_011542:227	Tcea3	INSIDE	1.586	2.093	1284.42	2687.88	3.319	1047.49	3476.56
A_68_P23331724	chr4:135577368-135577412	NM_013736:174	Tceb3	INSIDE	1.724	5.009	4526.26	22670.37	8.633	3689.98	31854.49
A_68_P31655789	chr18:42671379-42671423	NM_001039474:260	Tcerg1	INSIDE	1.706	7.326	13803.96	101132.70	12.500	9150.65	114384.80
A_68_P31655791	chr18:42671723-42671767	NM_001039474:604	Tcerg1	INSIDE	1.537	1.628	1908.82	3108.31	2.502	1457.80	3647.64
A_68_P25571662	chr7:145589207-145589251	NM_183289:185	Tcerg1l	INSIDE	2.863	9.562	4573.94	43735.11	27.371	2937.71	80409.07
A_68_P26626137	chr9:71959791-71959835	NM_011544:-186	Tcf12	PROMOTER	1.508	4.049	2317.19	9381.37	6.106	2028.59	12386.53
A_68_P31159487	chr17:35653700-35653744	NM_001163763:47	Tcf19	INSIDE	1.620	2.076	1916.59	3979.58	3.364	1478.90	4975.61
A_68_P26239531	chr8:125898300-125898344	NM_001037877:588	Tcf25	INSIDE	1.637	4.492	13008.45	58440.41	7.353	8218.25	60426.39
A_68_P27798408	chr11:52096304-52096348	NM_009331:-574	Tcf7	PROMOTER	1.611	1.663	2733.97	4546.98	2.679	2168.01	5807.09
A_68_P32197338	chr19:55969833-55969892	NM_001142924:52	Tcf7l2	INSIDE	1.968	5.017	167.54	840.57	9.872	114.89	1134.25
A_68_P28974187	chr13:40826726-40826770	NM_001122948:-936	Tcfap2a	PROMOTER	1.725	2.982	3597.64	10726.98	5.142	2801.51	14405.41
A_68_P28974164	chr13:40824040-40824084	NM_001122948:1750	Tcfap2a	INSIDE	1.709	4.053	3801.80	15408.83	6.928	3002.30	20798.76
A_68_P22336399	chr3:93248419-93248463	NM_001163098:2189	Tchh	INSIDE	2.183	41.121	2242.23	92201.51	89.767	1225.81	110037.50
A_68_P31120338	chr17:28217573-28217617	NM_001085555:-10	Tcp11	PROMOTER	2.106	4.187	3641.41	15248.33	8.819	2657.60	23437.56
A_68_P31120336	chr17:28217206-28217250	NM_013687:301	Tcp11	INSIDE	1.703	8.975	36936.59	331494.10	15.286	22975.57	351212.70
A_68_P20742449	chr1:158233625-158233669	NM_001134741:-167	Tdrd5	DIVERGENT_PROMOTER	2.212	9.538	2976.99	28395.71	21.102	2346.43	49515.32
A_68_P25091867	chr7:52471545-52471589	NM_011565:444	Tead2	INSIDE	2.215	15.992	4898.00	78330.04	35.420	3007.66	106531.60
A_68_P31121999	chr17:28487626-28487670	NM_001098226:-103	Tead3	PROMOTER	1.576	1.906	4153.46	7917.25	3.004	3102.91	9321.84
A_68_P24160101	chr5:144984767-144984811	NM_027410:-341	Tecpr1	PROMOTER	1.827	1.830	1178.84	2157.15	3.343	846.77	2831.13

ProbeName	Target position of probe on CpG island microarray	TargetID	GeneSymbol	CpG island Description	Ratio of relative methylation (TiO_2-NP/Vehicle)	Sham group			TiO_2-H group		
						Relative methylation (Cy5/Cy3)	Cy3 signal (Input)	Cy5 signal (MeDIP)	Relative methylation (Cy5/Cy3)	Cy3 signal (Input)	Cy5 signal (MeDIP)
A_68_P26015636	chr8:86118488-86118532	NM_027179:-120	Tecr	PROMOTER	1.682	3.147	1544.48	4861.07	5.293	1080.67	5720.53
A_68_P30379756	chr15:81641379-81641423	NM_017376:-443	Tef	PROMOTER	1.608	3.952	12834.27	50717.44	6.355	10155.86	64536.56
A_68_P30379760	chr15:81641863-81641907	NM_017376:41	Tef	INSIDE	1.573	2.878	884.23	2544.59	4.526	802.40	3631.53
A_68_P26143096	chr8:109620241-109620285	NM_009353:183	Terf2	INSIDE	1.842	7.143	4769.95	34073.04	13.156	3569.45	46958.07
A_68_P24024083	chr5:118478594-118478638	NM_021344:784	Tesc	INSIDE	1.625	4.891	4373.06	21388.98	7.949	3586.56	28509.15
A_68_P26804662	chr9:106587823-106587867	NM_011573:154	Tex264	INSIDE	1.815	2.861	632.52	1809.56	5.192	463.35	2405.94
A_68_P26804666	chr9:106588275-106588319	NM_001081654:-17	Tex264	PROMOTER	1.765	2.185	1727.76	3774.92	3.855	1342.99	5177.58
A_68_P30782600	chr16:56717318-56717362	NM_019678:127	Tfg	INSIDE	1.773	2.471	835.50	2064.67	4.382	706.35	3094.87
A_68_P24608765	chr6:86145281-86145325	NM_031199:58	Tgfa	INSIDE	2.011	4.196	265.89	1115.69	8.439	229.08	1933.18
A_68_P25011329	chr7:26472470-26472514	NM_011577:472	Tgfb1	INSIDE	1.819	2.718	5512.94	14984.39	4.943	4060.73	20072.39
A_68_P25011349	chr7:26474463-26474507	NM_011577:2464	Tgfb1	INSIDE	1.654	4.639	1133.34	5257.87	7.675	964.32	7401.22
A_68_P25011324	chr7:26471878-26471922	NM_011577:-120	Tgfb1	PROMOTER	1.568	2.535	2686.22	6810.85	3.977	2203.33	8762.21
A_68_P29062337	chr13:56710941-56710985	NM_009369:-1	Tgfbi	PROMOTER	1.526	3.737	5366.24	20054.80	5.704	4386.93	25022.32
A_68_P22894261	chr4:47365847-47365891	NM_009370:-308	Tgfbr1	PROMOTER	2.169	5.306	1036.17	5498.30	11.508	847.24	9749.72
A_68_P21773626	chr2:156665615-156665659	NM_173396:-176	Tgif2	PROMOTER	1.629	4.810	1506.49	7246.49	7.837	1272.30	9971.61
A_68_P23415826	chr4:151363053-151363098	NM_001145929:20	Thap3	INSIDE	1.604	1.650	491.30	810.45	2.646	399.61	1057.32
A_68_P20457301	chr1:95651221-95651265	NM_025920:173	Thap4	INSIDE	1.699	10.560	1017.56	10745.42	17.936	928.45	16652.85
A_68_P29418922	chr14:14794169-14794213	NM_025435:-451	Thoc7	PROMOTER	1.598	2.309	2178.68	5030.69	3.690	1673.07	6174.15
A_68_P31931517	chr19:5894108-5894152	NM_198634:-23	Tigd3	PROMOTER	1.502	1.820	1100.87	2003.78	2.734	879.37	2403.95
A_68_P30344883	chr15:75740087-75740131	NM_178646:-56	Tigd5	DIVERGENT_PROMOTER	1.717	4.105	2199.15	9027.46	7.048	1901.13	13399.69
A_68_P32672454	chrX:131076324-131076368	NM_013898:-178	Timm8a1	PROMOTER	1.633	3.393	810.91	2751.17	5.540	428.52	2374.03
A_68_P26581054	chr9:64129658-64129702	NM_025372:267	Tipin	INSIDE	1.784	1.772	3811.93	6754.17	3.161	3116.88	9853.57
A_68_P27290790	chr10:81063642-81063689	NM_053254:-20	Tle6	DIVERGENT_PROMOTER	2.176	4.811	8247.25	39678.77	10.468	6013.28	62949.87
A_68_P25922763	chr8:66684161-66684205	NM_009390:608	Tll1	INSIDE	1.504	2.938	650.04	1910.15	4.418	499.30	2205.96
A_68_P24591236	chr6:83018754-83018798	NM_009392:1443	Tlx2	INSIDE	1.532	2.775	421.84	1170.68	4.251	650.07	2763.26
A_68_P27693341	chr11:33101001-33101045	NM_019916:2566	Tlx3	INSIDE	3.266	11.424	11521.46	131618.90	37.311	7480.28	279095.00
A_68_P27693355	chr11:33102836-33102880	NM_019916:730	Tlx3	INSIDE	2.123	3.391	1478.25	5012.70	7.199	1172.83	8443.77
A_68_P27693347	chr11:33101808-33101852	NM_019916:1758	Tlx3	INSIDE	1.876	2.938	673.36	1978.25	5.511	549.92	3030.69
A_68_P27693346	chr11:33101716-33101763	NM_019916:1849	Tlx3	INSIDE	1.717	4.968	736.97	3661.61	8.529	543.56	4636.17
A_68_P25724844	chr8:26127584-26127628	NM_027194:-76	Tm2d2	DIVERGENT_PROMOTER	1.585	3.211	1452.58	4664.16	5.088	1112.65	5661.61
A_68_P29619050	chr14:56262458-56262502	NM_028780:163	Tm9sf1	INSIDE	3.144	21.737	4434.65	96395.99	68.347	2167.49	148140.80
A_68_P25459064	chr7:125728226-125728270	NM_172476:-48	Tmc7	PROMOTER	1.534	3.283	1856.92	6095.49	5.036	1642.42	8271.82
A_68_P26713768	chr9:89599588-89599632	NM_025360:271	Tmed3	INSIDE	1.529	1.959	612.80	1200.66	2.995	523.72	1568.54
A_68_P28614745	chr12:88540669-88540713	NM_001033475:489	Tmed8	INSIDE	1.596	5.480	3474.84	19041.55	8.745	2597.15	22713.11
A_68_P26787844	chr9:103663476-103663520	NM_178638:669	Tmem108	INSIDE	1.680	6.287	1990.88	12516.72	10.564	1533.51	16200.24
A_68_P29508104	chr14:31638725-31638769	NM_028839:-33	Tmem110	PROMOTER	1.630	7.519	1861.83	13998.85	12.256	1443.88	17696.25
A_68_P30453398	chr15:94459261-94459305	NM_178789:-333	Tmem117	PROMOTER	1.751	4.555	9344.40	42566.47	7.978	6963.78	55556.36
A_68_P26289277	chr9:7763854-7763898	NM_133739:-200	Tmem123	PROMOTER	1.557	2.817	10958.54	30867.54	4.385	7497.56	32880.13
A_68_P20151305	chr1:36996651-36996695	NM_018872:-300	Tmem131	PROMOTER	1.596	2.608	1176.45	3067.66	4.162	972.02	4045.50
A_68_P27965917	chr11:82200919-82200963	NM_023438:-1461	Tmem132e	PROMOTER	2.280	7.265	2269.87	16491.30	16.564	1718.62	28467.81
A_68_P27965931	chr11:82202507-82202551	NM_023438:127	Tmem132e	INSIDE	1.807	5.438	5787.76	31475.98	9.825	4556.78	44770.51
A_68_P27966238	chr11:82248332-82248376	NM_023438:45953	Tmem132e	INSIDE	1.666	6.280	392.39	2464.03	10.459	334.54	3499.15
A_68_P31921226	chr19:4125896-4125940	NM_001078649:-41	Tmem134	DIVERGENT_PROMOTER	1.535	1.599	2143.89	3428.02	2.454	1865.14	4577.64
A_68_P31201735	chr17:45686486-45686530	NM_001013749:118	Tmem151b	INSIDE	1.921	4.523	4033.96	18243.61	8.688	2866.15	24900.98
A_68_P26894035	chr9:123169998-123170042	NM_001002267:-113	Tmem158	PROMOTER	1.570	5.235	1322.39	6923.00	8.219	1293.02	10627.22
A_68_P26894026	chr9:123168701-123168745	NM_001002267:1185	Tmem158	INSIDE	1.561	2.872	2065.30	5931.81	4.485	1627.76	7300.04
A_68_P32709174	chrX:139116023-139116067	NM_001199360:-224	Tmem164	PROMOTER	1.899	4.862	2349.83	11425.20	9.232	1450.14	13387.17

ProbeName	Target position of probe on CpG island microarray	TargetID	GeneSymbol	CpG island Description	Ratio of relative methylation (TiO_2-NP/Vehicle)	Sham group			TiO_2-H group		
						Relative methylation (Cy5/Cy3)	Cy3 signal (Input)	Cy5 signal (MeDIP)	Relative methylation (Cy5/Cy3)	Cy3 signal (Input)	Cy5 signal (MeDIP)
A_68_P22413373	chr3:108365105-108365149	NM_026198:258	Tmem167b	INSIDE	2.014	5.899	11035.30	65100.15	11.883	7806.95	92767.71
A_68_P31385967	chr17:81344527-81344571	NM_026516:577	Tmem178	INSIDE	1.739	2.003	790.27	1582.55	3.483	671.56	2339.01
A_68_P20632910	chr1:136241239-136241283	NM_001042485:17316	Tmem183a	DOWNSTREAM	1.507	1.589	1960.62	3116.15	2.395	1775.76	4253.73
A_68_P30366260	chr15:79233510-79233554	NM_172608:201	Tmem184b	INSIDE	1.696	2.289	1282.09	2934.90	3.882	1107.15	4297.46
A_68_P30572936	chr16:17276365-17276409	NM_177473:-6	Tmem191c	PROMOTER	2.406	10.155	1116.24	11334.84	24.427	920.55	22486.71
A_68_P25926494	chr8:67471391-67471435	NM_001163747:329	Tmem192	INSIDE	2.049	1.823	3191.17	5815.94	3.734	2491.95	9305.58
A_68_P32008156	chr19:21853273-21853317	NM_001033759:463	Tmem2	INSIDE	2.079	7.297	4595.47	33532.42	15.169	3279.14	49740.42
A_68_P23402215	chr4:149111795-149111839	NM_001025106:327	Tmem201	INSIDE	1.750	6.996	1464.17	10243.76	12.245	1030.76	12621.58
A_68_P26133052	chr8:107850532-107850576	NM_025486:291	Tmem208	INSIDE	1.617	2.535	7908.84	20050.59	4.099	6297.19	25813.56
A_68_P24304143	chr6:24905716-24905760	NM_177013:387	Tmem229a	INSIDE	1.538	2.687	1431.27	3845.77	4.133	1315.59	5436.84
A_68_P28567140	chr12:80108151-80108195	NM_178745:92	Tmem229b	INSIDE	2.358	6.724	9582.42	64433.40	15.858	7192.83	114067.00
A_68_P32557243	chrX:97015613-97015657	NM_001081283:-773	Tmem28	PROMOTER	1.986	2.695	738.08	1989.24	5.353	374.47	2004.59
A_68_P25962148	chr8:75095909-75095953	NM_144534:-71	Tmem38a	DIVERGENT_PROMOTER	2.027	3.910	3988.38	15596.27	7.928	3405.73	27001.85
A_68_P25962147	chr8:75095836-75095880	NM_144534:-143	Tmem38a	DIVERGENT_PROMOTER	1.542	2.800	1226.51	3434.52	4.317	1052.87	4545.69
A_68_P23298058	chr4:129373895-129373939	NM_199305:166	Tmem39b	INSIDE	1.738	2.740	2790.29	7644.16	4.761	2354.98	11212.01
A_68_P23298057	chr4:129373770-129373814	NM_199305:290	Tmem39b	INSIDE	1.624	1.711	1592.30	2724.61	2.779	1302.78	3620.70
A_68_P25411125	chr7:117129431-117129475	NM_153525:292	Tmem41b	INSIDE	1.594	2.631	4442.00	11687.76	4.195	3120.46	13091.02
A_68_P30642387	chr16:30550318-30550362	NM_172614:324	Tmem44	INSIDE	1.547	1.662	2144.39	3563.03	2.571	1758.73	4521.84
A_68_P32488667	chrX:78316560-78316604	NM_138751:600	Tmem47	INSIDE	1.926	4.390	5758.30	25281.21	8.457	3130.92	26478.88
A_68_P32488661	chrX:78315918-78315962	NM_138751:-42	Tmem47	PROMOTER	1.573	2.978	1549.11	4612.70	4.684	1104.22	5171.91
A_68_P23190148	chr4:107040619-107040663	NM_028355:252	Tmem48	INSIDE	1.913	8.198	1328.94	10894.72	15.684	1001.80	15712.00
A_68_P27507134	chr10:121533967-121534011	NM_153059:170	Tmem5	INSIDE	1.756	2.901	22532.79	65360.53	5.093	17904.44	91188.08
A_68_P23294938	chr4:128782726-128782770	NM_025452:-25	Tmem54	PROMOTER	1.536	4.533	3146.01	14260.84	6.964	2437.77	16975.62
A_68_P23294939	chr4:128782828-128782872	NM_025452:77	Tmem54	INSIDE	1.525	2.730	3022.03	8250.80	4.163	2406.60	10019.09
A_68_P29599187	chr14:51550282-51550326	NM_001033271:220	Tmem55b	INSIDE	1.740	8.543	1131.21	9664.49	14.870	707.37	10518.48
A_68_P29599189	chr14:51550570-51550614	NM_001033271:-68	Tmem55b	PROMOTER	1.728	6.256	6967.38	43587.79	10.809	5207.44	56284.93
A_68_P22475542	chr3:120966000-120966044	NM_178936:212	Tmem56	INSIDE	1.675	2.106	842.69	1774.56	3.527	788.56	2781.43
A_68_P22475538	chr3:120965447-120965491	NM_178936:766	Tmem56	INSIDE	1.629	3.110	1198.08	3726.36	5.068	1119.74	5674.32
A_68_P25588178	chr7:148514084-148514128	NR_024509:78	Tmem80	INSIDE	1.587	2.664	2355.48	6275.28	4.228	1974.60	8349.00
A_68_P21736747	chr2:149656520-149656564	NM_001085521:24	Tmem90b	INSIDE	2.019	14.996	3615.40	54215.89	30.269	2609.57	78989.27
A_68_P22886521	chr4:46051942-46051986	NM_021883:-129	Tmod1	PROMOTER	1.530	2.741	2565.11	7031.71	4.195	2101.39	8815.76
A_68_P29964713	chr14:123381923-123381967	NM_028651:539	Tmtc4	INSIDE	1.561	5.108	1203.79	6149.55	7.972	922.30	7352.89
A_68_P29964714	chr14:123382020-123382064	NM_028651:441	Tmtc4	INSIDE	1.543	3.639	289.34	1053.04	5.616	247.28	1388.75
A_68_P23541913	chr5:23953475-23953519	NM_022418:168	Tmub1	INSIDE	1.550	2.442	1120.23	2736.03	3.785	956.45	3620.14
A_68_P28520555	chr12:71554358-71554402	NM_028339:240	Tmx1	INSIDE	1.517	1.462	1187.01	1735.79	2.218	1077.70	2390.47
A_68_P22973925	chr4:63678841-63678885	NM_011607:29187	Tnc	INSIDE	2.532	7.689	1740.85	13385.58	19.465	1214.65	23643.61
A_68_P31187135	chr17:43153738-43153782	NM_178589:257	Tnfrsf21	INSIDE	1.628	4.557	2213.00	10085.59	7.421	1633.92	12124.77
A_68_P29745018	chr14:78707731-78707775	NM_011613:98	Tnfsf11	INSIDE	1.552	3.653	2008.53	7338.06	5.671	1580.84	8964.18
A_68_P22015873	chr3:28161770-28161814	NM_001163009:-343	Tnik	PROMOTER	1.744	3.559	324.25	1153.94	6.206	261.68	1623.89
A_68_P25777211	chr8:36028290-36028334	NM_175091:432	Tnks	INSIDE	1.503	3.928	823.33	3233.88	5.903	639.69	3775.94
A_68_P24153558	chr5:143580250-143580294	NM_001122730:-1206	Tnrc18	PROMOTER	1.865	3.080	1012.96	3120.38	5.746	755.98	4343.72
A_68_P30374043	chr15:80542551-80542595	NM_144812:830	Tnrc6b	INSIDE	2.901	10.575	3462.10	36612.34	30.679	2420.22	74249.83
A_68_P28166383	chr11:117515888-117515933	NM_198022:308	Tnrc6c	INSIDE	1.761	3.753	1659.74	6229.66	6.610	1320.71	8729.71
A_68_P20345418	chr1:73954077-73954121	NM_027884:216923	Tns1	DOWNSTREAM	1.662	4.095	1102.23	4514.15	6.807	870.43	5925.32
A_68_P27579284	chr11:8564125-8564169	NM_001083587:392	Tns3	INSIDE	2.088	6.420	1932.44	12405.84	13.402	1474.21	19757.56
A_68_P30380067	chr15:81688792-81688836	NM_020507:-58	Tob2	PROMOTER	1.902	4.656	2525.26	11757.26	8.854	1887.14	16709.04
A_68_P30784285	chr16:57122018-57122062	NM_138599:214	Tomm70a	INSIDE	2.470	9.905	4797.46	47520.59	24.470	3311.14	81023.28

ProbeName	Target position of probe on CpG island microarray	TargetID	GeneSymbol	CpG island Description	Ratio of relative methylation (TiO_2-NP/Vehicle)	Sham group			TiO_2-H group		
						Relative methylation (Cy5/Cy3)	Cy3 signal (Input)	Cy5 signal (MeDIP)	Relative methylation (Cy5/Cy3)	Cy3 signal (Input)	Cy5 signal (MeDIP)
A_68_P30349539	chr15:76462358-76462402	NM_183091:7979	Tonsl	INSIDE	1.799	6.542	8551.76	55942.93	11.767	6017.12	70801.82
A_68_P29428731	chr14:17197979-17198023	NM_009409:281	Top2b	INSIDE	1.749	2.011	1529.45	3075.09	3.516	1246.60	4382.69
A_68_P21117289	chr2:32612990-32613034	NM_152800:222	Tor2a	INSIDE	1.896	6.493	1602.39	10403.93	12.309	1213.79	14940.74
A_68_P21117286	chr2:32612762-32612806	NM_152800:-6	Tor2a	PROMOTER	1.855	3.906	1786.12	6976.17	7.247	1455.65	10548.40
A_68_P22708703	chr4:6917953-6917997	NM_145711:-104	Tox	PROMOTER	1.725	3.353	5320.50	17841.79	5.786	4525.23	26181.49
A_68_P21910960	chr2:181231996-181232040	NM_025482:61	Tpd52l2	INSIDE	1.588	2.405	810.93	1950.67	3.820	706.07	2696.99
A_68_P22872185	chr4:43536399-43536443	NM_009416:-160	Tpm2	PROMOTER	1.600	2.434	1937.75	4715.58	3.894	1520.65	5921.09
A_68_P20191548	chr1:43991025-43991069	NM_009418:195	Tpp2	INSIDE	1.567	4.232	826.31	3496.70	6.632	684.81	4541.32
A_68_P29143670	chr13:74147870-74147914	NM_182839:1026	Tppp	INSIDE	1.560	3.518	1149.79	4045.47	5.489	940.34	5161.41
A_68_P26134000	chr8:107995392-107995436	NM_026481:-92	Tppp3	PROMOTER	1.666	4.999	3639.91	18195.52	8.330	2948.59	24560.81
A_68_P30596851	chr16:22265603-22265647	NM_009186:378	Tra2b	INSIDE	2.031	3.657	723.92	2647.65	7.430	568.60	4224.62
A_68_P22492339	chr3:124023918-124023962	NM_146140:-14	Tram1l1	PROMOTER	1.550	1.883	1710.74	3222.01	2.919	1366.00	3986.99
A_68_P26234573	chr8:125135855-125135899	NM_021502:351	Trappc2l	INSIDE	1.589	2.915	1854.83	5407.17	4.632	1334.71	6182.19
A_68_P30255506	chr15:59479250-59479294	NM_144549:-936	Trib1	PROMOTER	2.036	6.079	9403.85	57161.85	12.376	6351.70	78609.39
A_68_P30255510	chr15:59479785-59479829	NM_144549:-402	Trib1	PROMOTER	1.521	2.815	1344.26	3784.38	4.282	1178.22	5044.74
A_68_P30255512	chr15:59479961-59480005	NM_144549:-226	Trib1	PROMOTER	1.502	2.370	2851.21	6758.58	3.560	2415.27	8599.13
A_68_P24459338	chr6:53768811-53768855	NM_025817:1987	Tril	INSIDE	1.951	6.346	4390.67	27862.05	12.382	3566.54	44159.98
A_68_P28871505	chr13:21272743-21272787	NM_009054:937	Trim27	INSIDE	1.862	3.993	1642.00	6556.20	7.435	1345.09	10000.14
A_68_P28871497	chr13:21271956-21272000	NM_009054:151	Trim27	INSIDE	1.509	1.526	771.34	1177.15	2.304	742.76	1710.95
A_68_P31675573	chr18:46358367-46358411	NM_001170855:84	Trim36	INSIDE	1.795	2.018	1190.25	2402.41	3.623	1052.42	3813.30
A_68_P31675568	chr18:46357840-46357884	NM_001170855:610	Trim36	INSIDE	1.561	2.855	486.55	1389.25	4.458	453.71	2022.68
A_68_P28157432	chr11:115970917-115970961	NM_001205081:611	Trim47	INSIDE	1.682	5.953	1398.78	8326.68	10.014	1042.65	10441.36
A_68_P28157433	chr11:115971055-115971099	NM_001205081:473	Trim47	INSIDE	1.574	7.514	4631.18	34796.67	11.828	3674.12	43458.28
A_68_P26247734	chr8:127317918-127317962	NM_198632:1022	Trim67	INSIDE	2.219	5.090	4707.86	23962.96	11.297	3622.37	40921.94
A_68_P26845381	chr9:114473232-114473276	NM_001042503:233	Trim71	INSIDE	2.604	6.462	664.84	4295.91	16.825	520.90	8764.06
A_68_P30096385	chr15:27954397-27954441	NM_001081302:1185	Trio	INSIDE	1.750	1.463	4319.80	6321.36	2.561	3326.99	8521.62
A_68_P30363493	chr15:78798329-78798373	NM_001039155:20197	Triobp	INSIDE	1.982	11.133	1510.57	16816.93	22.062	976.39	21540.76
A_68_P30363492	chr15:78798174-78798218	NM_001039155:20043	Triobp	INSIDE	1.531	4.304	3043.35	13098.90	6.588	2143.46	14121.76
A_68_P28688419	chr12:103150855-103150899	NM_028446:505	Trip11	INSIDE	2.414	4.319	6889.77	29759.31	10.425	5098.42	53151.19
A_68_P23429319	chr4:153514065-153514109	NM_011642:231	Trp73	INSIDE	1.625	4.438	1760.55	7813.59	7.211	1637.29	11805.84
A_68_P21767078	chr2:155518080-155518124	NM_001163452:18	Trpc4ap	INSIDE	2.112	3.247	805.77	2616.47	6.857	678.22	4650.51
A_68_P26292118	chr9:8544562-8544606	NM_013838:443	Trpc6	INSIDE	2.466	3.342	924.53	3089.86	8.241	744.87	6138.87
A_68_P32010020	chr19:22213374-22213418	NM_001035239:-210	Trpm3	PROMOTER	1.597	2.658	1036.70	2755.98	4.245	859.73	3649.29
A_68_P24162469	chr5:145529670-145529714	NM_001081362:32	Trrap	INSIDE	1.637	2.912	5153.75	15010.24	4.768	3947.39	18822.66
A_68_P31098616	chr17:24769435-24769479	NM_001039363:118	Tsc2	INSIDE	1.589	1.349	4463.30	6019.95	2.143	3751.78	8041.93
A_68_P29733553	chr14:76816095-76816139	NM_207652:489	Tsc22d1	INSIDE	1.725	3.435	3654.98	12555.67	5.926	2628.47	15576.82
A_68_P32700067	chrX:137134400-137134444	NM_001077364:639	Tsc22d3	INSIDE	1.525	2.951	4716.72	13920.73	4.500	2959.65	13317.83
A_68_P24771681	chr6:115494875-115494919	NM_199033:175	Tsen2	INSIDE	1.820	7.577	2438.08	18473.32	13.787	1654.35	22808.57
A_68_P25102011	chr7:54175094-54175138	NM_021884:184	Tsg101	INSIDE	1.710	3.474	1126.98	3915.36	5.942	917.98	5454.39
A_68_P20156316	chr1:37922113-37922157	NM_207228:-210	Tsga10	DIVERGENT_PROMOTER	1.586	1.464	728.86	1066.79	2.321	599.54	1391.72
A_68_P25056449	chr7:37481732-37481776	NM_172298:-1382	Tshz3	PROMOTER	2.179	4.522	6942.59	31394.05	9.853	5384.81	53058.30
A_68_P25056451	chr7:37481970-37482014	NM_172298:-1144	Tshz3	PROMOTER	1.556	2.088	1067.78	2229.64	3.249	934.35	3035.37
A_68_P29560590	chr14:41779806-41779850	NM_145928:268	Tspan14	INSIDE	1.852	3.937	6353.90	25015.85	7.292	5038.57	36742.06
A_68_P29051405	chr13:54890823-54890867	NM_028841:79	Tspan17	INSIDE	1.881	3.452	458.95	1584.22	6.494	329.33	2138.76
A_68_P22382656	chr3:102538778-102538822	NM_027533:108	Tspan2	INSIDE	1.897	4.171	9887.21	41239.90	7.911	7434.75	58815.90
A_68_P22570418	chr3:138405498-138405542	NM_019571:362	Tspan5	INSIDE	1.680	4.376	1624.13	7106.38	7.351	1326.85	9753.60
A_68_P32749012	chrX:148777026-148777070	NM_029836:-21	Tspyl2	PROMOTER	2.020	6.415	31502.22	202084.40	12.961	19139.72	248069.50

ProbeName	Target position of probe on CpG island microarray	TargetID	GeneSymbol	CpG island Description	Ratio of relative methylation (TiO_2-NP/Vehicle)	Sham group			TiO_2-H group		
						Relative methylation (Cy5/Cy3)	Cy3 signal (Input)	Cy5 signal (MeDIP)	Relative methylation (Cy5/Cy3)	Cy3 signal (Input)	Cy5 signal (MeDIP)
A_68_P23982393	chr5:111308521-111308565	NM_024477:-279	Ttc28	PROMOTER	1.724	6.433	286.30	1841.66	11.088	176.30	1954.72
A_68_P23982396	chr5:111309011-111309055	NM_024477:211	Ttc28	INSIDE	1.676	4.893	6002.93	29374.08	8.200	4758.82	39022.67
A_68_P30176273	chr15:43308698-43308742	NM_025736:-54	Ttc35	PROMOTER	1.857	2.199	1903.70	4185.70	4.083	1443.41	5892.75
A_68_P30176277	chr15:43309087-43309131	NM_025736:334	Ttc35	INSIDE	1.552	2.774	2444.85	6782.67	4.306	2147.63	9247.83
A_68_P31422209	chr17:87682307-87682351	NM_028639:103	Ttc7	INSIDE	2.100	3.330	695.26	2315.04	6.993	552.07	3860.37
A_68_P21622816	chr2:128891587-128891631	NM_027192:-74	Ttl	PROMOTER	1.596	4.591	2434.91	11177.48	7.325	1775.89	13008.52
A_68_P28606270	chr12:87166109-87166153	NM_001081423:231	Ttll5	INSIDE	2.203	1.934	783.80	1515.98	4.262	601.72	2564.47
A_68_P28149093	chr11:114536801-114536845	NM_053273:41	Ttyh2	INSIDE	1.586	5.212	2080.39	10842.37	8.265	1728.79	14288.31
A_68_P21070656	chr2:25080752-25080796	NM_146116:-552	Tubb2c	PROMOTER	1.976	4.220	2765.86	11672.93	8.339	2234.63	18633.69
A_68_P31791603	chr18:67550248-67550292	NM_026473:-114	Tubb6	PROMOTER	1.833	2.112	1364.48	2881.84	3.872	1120.72	4339.74
A_68_P25798746	chr8:40068990-40069034	NM_030254:92	Tusc3	INSIDE	1.691	3.748	1215.15	4554.28	6.337	912.00	5779.74
A_68_P28344356	chr12:34642925-34642969	NM_011658:411	Twist1	INSIDE	1.540	1.644	423.44	696.13	2.532	392.98	995.14
A_68_P23297754	chr4:129318346-129318390	NM_001199695:-59	Txlna	PROMOTER	1.557	2.710	1619.38	4388.83	4.220	1351.27	5701.81
A_68_P22948244	chr4:57969284-57969328	NM_011660:-23	Txn1	PROMOTER	1.595	3.002	664.99	1996.03	4.788	619.35	2965.60
A_68_P21571594	chr2:119625143-119625187	NM_019392:-85	Tyro3	PROMOTER	2.071	8.860	3983.38	35291.72	18.347	3170.68	58172.74
A_68_P27181843	chr10:61158699-61158743	NM_027912:459	Tysnd1	INSIDE	1.616	3.373	978.98	3301.84	5.452	761.99	4154.37
A_68_P24951859	chr7:5012645-5012689	NM_001205231:-1078	U2af2	PROMOTER	1.555	1.754	3975.91	6972.82	2.727	2829.09	7714.78
A_68_P25042532	chr7:34953588-34953632	NM_016682:-62	Uba2	PROMOTER	1.664	13.458	665.13	8951.22	22.398	588.62	13184.03
A_68_P24671729	chr6:97155626-97155670	NM_001111106:-12	Uba3	DIVERGENT_PROMOTER	1.837	4.483	7712.25	34570.24	8.234	5398.68	44452.74
A_68_P21075771	chr2:25877080-25877124	NM_133835:178	Ubac1	INSIDE	1.718	3.692	611.91	2259.37	6.342	595.33	3775.64
A_68_P29958338	chr14:122278102-122278146	NM_026861:297	Ubac2	INSIDE	2.334	4.232	2780.86	11768.46	9.877	2012.80	19879.47
A_68_P29958335	chr14:122277673-122277717	NM_026861:-133	Ubac2	PROMOTER	2.099	1.758	1369.19	2407.09	3.691	1136.02	4192.80
A_68_P22323188	chr3:89856392-89856436	NM_028475:-17	Ubap2l	PROMOTER	1.626	1.802	9446.78	17020.32	2.929	6798.45	19913.72
A_68_P32320545	chrX:34415031-34415075	NM_019668:692	Ube2a	INSIDE	2.025	3.110	1464.24	4554.02	6.297	822.03	5176.49
A_68_P21819631	chr2:164595113-164595157	NM_026785:-294	Ube2c	PROMOTER	1.949	1.547	2320.10	3588.52	3.015	1901.78	5734.30
A_68_P27234632	chr10:70747404-70747448	NM_145420:584	Ube2d1	INSIDE	1.723	4.515	3345.42	15106.07	7.781	2432.05	18923.28
A_68_P27912376	chr11:72420588-72420632	NM_025985:-152	Ube2g1	PROMOTER	1.630	3.721	980.28	3647.77	6.065	904.63	5486.35
A_68_P28159934	chr11:116403055-116403099	NM_173755:39685	Ube2o	INSIDE	1.780	3.415	1066.32	3641.22	6.080	893.63	5433.21
A_68_P28160203	chr11:116442887-116442931	NM_173755:-147	Ube2o	PROMOTER	1.575	2.804	3375.46	9466.43	4.418	2808.59	12408.08
A_68_P22321608	chr3:89577074-89577118	NM_027315:-434	Ube2q1	DIVERGENT_PROMOTER	1.517	1.749	2102.20	3677.00	2.653	1641.16	4353.86
A_68_P26529572	chr9:54997117-54997161	NM_180600:-37	Ube2q2	PROMOTER	2.656	11.264	2664.79	30016.69	29.913	1875.82	56110.90
A_68_P26529571	chr9:54996925-54996969	NM_180600:-229	Ube2q2	PROMOTER	1.914	7.210	1010.45	7285.18	13.799	790.60	10909.70
A_68_P29117519	chr13:69878102-69878146	NM_001145162:651	Ube2ql1	INSIDE	1.721	3.906	4725.31	18459.07	6.724	3879.84	26087.56
A_68_P29117527	chr13:69878894-69878938	NM_001145162:-141	Ube2ql1	PROMOTER	1.831	1.454	759.42	1103.94	2.661	644.53	1715.34
A_68_P22860680	chr4:41082241-41082285	NM_026275:-791	Ube2r2	PROMOTER	1.629	1.364	2700.44	3684.57	2.222	2131.24	4735.93
A_68_P21836350	chr2:167423298-167423342	NM_023230:34185	Ube2v1	DOWNSTREAM	2.559	1.736	6057.44	10517.36	4.443	3948.40	17542.62
A_68_P21836569	chr2:167457610-167457654	NM_023230:-127	Ube2v1	PROMOTER	1.633	3.680	822.83	3028.11	6.008	636.44	3824.00
A_68_P23568207	chr5:29896110-29896154	NM_133907:351	Ube3c	INSIDE	1.512	2.739	1584.16	4339.25	4.140	1228.08	5084.59
A_68_P25477108	chr7:129210764-129210808	NM_138589:75	Ubfd1	INSIDE	1.622	5.308	1922.64	10205.63	8.608	1580.94	13609.45
A_68_P32467744	chrX:71613927-71613971	NM_145405:-82	Ubl4	PROMOTER	1.663	3.501	1792.31	6275.29	5.822	1302.37	7581.90
A_68_P21311589	chr2:69735872-69735916	NM_001081548:592	Ubr3	INSIDE	1.552	2.467	2694.52	6646.05	3.829	2134.09	8170.82
A_68_P32118295	chr19:42056730-42056774	NM_145500:500	Ubtd1	INSIDE	1.886	6.127	949.72	5818.85	11.553	816.00	9427.02
A_68_P27688946	chr11:32355594-32355638	NM_173784:245	Ubtd2	INSIDE	2.445	5.514	5760.64	31763.89	13.479	4170.34	56213.19
A_68_P28078909	chr11:102180488-102180532	NM_011551:-100	Ubtf	PROMOTER	1.826	2.132	552.33	1177.68	3.893	399.39	1554.76
A_68_P20599489	chr1:130140741-130140785	NM_026390:5	Ubxn4	INSIDE	2.077	1.596	867.00	1383.46	3.314	766.74	2541.27
A_68_P20821183	chr1:172145120-172145164	NM_010633:382	Uhmk1	INSIDE	1.643	1.951	1817.60	3545.33	3.204	1568.10	5024.87
A_68_P27332821	chr10:89207726-89207770	NM_029166:13	Uhrf1bp1l	INSIDE	2.154	3.424	2654.20	9088.83	7.375	2280.54	16819.81

ProbeName	Target position of probe on CpG island microarray	TargetID	GeneSymbol	CpG island Description	Ratio of relative methylation (TiO_2-NP/Vehicle)	Sham group			TiO_2-H group		
						Relative methylation (Cy5/Cy3)	Cy3 signal (Input)	Cy5 signal (MeDIP)	Relative methylation (Cy5/Cy3)	Cy3 signal (Input)	Cy5 signal (MeDIP)
A_68_P32056099	chr19:30105106-30105150	NM_144873:126	Uhrf2	INSIDE	2.097	6.089	1920.53	11693.79	12.766	1554.80	19847.81
A_68_P27850822	chr11:61668093-61668137	NM_013881:480	Ulk2	INSIDE	2.241	12.017	5428.39	65234.44	26.936	3559.78	95885.15
A_68_P27850828	chr11:61668703-61668747	NM_013881:-130	Ulk2	PROMOTER	1.866	9.965	2530.43	25216.61	18.596	1822.22	33886.04
A_68_P27942762	chr11:78157060-78157104	NM_011676:59	Unc119	INSIDE	1.644	4.324	5863.68	25357.28	7.109	4592.37	32645.92
A_68_P25958205	chr8:74195695-74195739	NM_001029873:-60	Unc13a	DIVERGENT_PROMOTER	1.748	3.016	8338.50	25152.77	5.272	6293.49	33177.18
A_68_P27177056	chr10:60293675-60293719	NM_029770:633	Unc5b	INSIDE	1.989	3.673	1451.34	5330.04	7.303	1228.29	8970.39
A_68_P27177053	chr10:60293340-60293384	NM_029770:967	Unc5b	INSIDE	1.681	2.249	1672.25	3760.27	3.780	1433.56	5418.84
A_68_P25747455	chr8:30329725-30329769	NM_153135:362	Unc5d	INSIDE	2.094	7.058	8318.20	58706.24	14.778	6527.83	96467.42
A_68_P20303908	chr1:66724124-66724168	NM_175510:209126	Unc80	INSIDE	2.192	12.767	15968.88	203869.80	27.990	10327.29	289058.50
A_68_P24136157	chr5:140020297-140020341	NM_013702:467	Uncx	INSIDE	1.526	3.136	2512.84	7880.53	4.786	2233.60	10689.77
A_68_P28156930	chr11:115891653-115891697	NM_172569:39	Unk	INSIDE	1.982	3.224	656.27	2115.97	6.391	542.34	3466.17
A_68_P25951334	chr8:72877112-72877156	NM_001122829:38	Upf1	INSIDE	1.535	4.103	1615.14	6627.05	6.300	1315.38	8287.26
A_68_P25670554	chr8:13785750-13785794	NM_025924:158	Upf3a	INSIDE	1.571	3.934	1989.76	7828.20	6.182	1622.32	10028.85
A_68_P25670559	chr8:13786249-13786293	NM_025924:656	Upf3a	INSIDE	1.531	1.530	2669.49	4083.41	2.342	2133.74	4998.14
A_68_P25670551	chr8:13785477-13785521	NM_025924:-116	Upf3a	PROMOTER	2.006	1.435	1349.51	1936.78	2.880	977.43	2814.58
A_68_P21768463	chr2:155755683-155755727	NM_018888:342	Uqcc	INSIDE	1.743	3.108	3340.04	10381.87	5.418	2243.48	12155.32
A_68_P26817224	chr9:108838919-108838963	NM_025407:280	Uqcrc1	INSIDE	1.634	3.523	5287.92	18628.69	5.757	4049.89	23316.55
A_68_P27804722	chr11:53244281-53244325	NM_025352:31	Uqcrq	INSIDE	1.769	1.652	602.65	995.78	2.923	574.97	1680.60
A_68_P30959119	chr16:90810476-90810520	NM_029497:160	Urb1	INSIDE	2.136	3.049	1905.85	5811.50	6.515	1616.24	10529.11
A_68_P27564785	chr11:5641343-5641387	NM_178623:-185	Urgcp	PROMOTER	2.009	0.300	1036.82	310.68	0.602	1175.91	707.96
A_68_P25034247	chr7:31740799-31740843	NM_011680:1002	Usf2	INSIDE	1.512	2.427	822.98	1997.11	3.670	742.19	2723.71
A_68_P28152781	chr11:115183238-115183282	NM_176847:-28	Ush1g	PROMOTER	1.970	9.441	10000.64	94413.83	18.600	6015.03	111879.70
A_68_P26217005	chr8:122480667-122480711	NM_009462:45937	Usp10	INSIDE	2.097	5.459	7807.56	42622.45	11.448	5530.16	63309.99
A_68_P32975180	chr9:108393143-108393187	NM_027804:158	Usp19	INSIDE	1.610	2.831	1154.50	3268.25	4.559	1078.28	4915.64
A_68_P27846979	chr11:60988330-60988374	NM_001004143:209	Usp22	INSIDE	1.658	2.382	587.13	1398.64	3.949	512.90	2025.31
A_68_P22650044	chr3:152009738-152009782	NM_001076676:316	Usp33	INSIDE	1.582	2.830	733.50	2075.90	4.476	652.95	2922.67
A_68_P25349892	chr7:104473950-104473994	NM_001177412:502	Usp35	INSIDE	2.080	2.019	288.84	583.25	4.200	214.06	899.00
A_68_P26814405	chr9:108250089-108250133	NM_011678:-51	Usp4	PROMOTER	1.604	2.988	1313.14	3923.16	4.793	1096.62	5256.58
A_68_P20421182	chr1:89904977-89905021	NM_001033291:128	Usp40	INSIDE	1.787	3.697	4393.92	16244.86	6.605	3280.38	21667.59
A_68_P31213521	chr17:47809690-47809734	NM_198421:42074	Usp49	INSIDE	1.612	2.789	2117.42	5904.90	4.496	1779.78	8002.52
A_68_P20372363	chr1:78654178-78654222	NM_001001981:-199	Utp14b	PROMOTER	1.779	2.805	1095.37	3072.37	4.989	992.11	4949.18
A_68_P25360010	chr7:106289656-106289700	NM_178635:-24	Uvrag	PROMOTER	1.703	2.507	1624.33	4071.82	4.270	1259.73	5378.94
A_68_P27892954	chr11:68901870-68901914	NM_009497:-137	Vamp2	PROMOTER	1.760	1.492	1659.78	2477.16	2.626	1196.24	3141.60
A_68_P23409805	chr4:150432256-150432300	NM_009498:-216	Vamp3	PROMOTER	1.723	5.061	4467.10	22606.50	8.719	3000.37	26159.48
A_68_P20935551	chr1:192803342-192803386	NR_027352:-189	Vash2	PROMOTER	1.515	2.830	1064.05	3011.52	4.287	847.11	3631.88
A_68_P24992404	chr7:19857862-19857906	NM_009499:-681	Vasp	PROMOTER	1.643	1.942	3920.49	7612.56	3.190	3319.71	10591.52
A_68_P28074030	chr11:101327012-101327056	NM_012037:479	Vat1	INSIDE	1.577	3.290	3016.42	9925.08	5.188	2617.11	13577.53
A_68_P21084621	chr2:27281926-27281970	NM_009500:397	Vav2	INSIDE	1.547	2.812	1136.36	3195.93	4.352	994.41	4327.32
A_68_P32217704	chr19:59246215-59246259	NM_009501:-1717	Vax1	PROMOTER	1.684	2.583	3160.67	8165.50	4.349	2353.74	10237.25
A_68_P29210184	chr13:89879184-89879228	NM_001081249:2911	Vcan	INSIDE	1.606	2.401	503.61	1209.38	3.856	446.94	1723.49
A_68_P29449770	chr14:21748700-21748744	NM_009502:68	Vcl	INSIDE	1.969	3.193	4035.96	12886.43	6.286	3134.26	19703.38
A_68_P29449771	chr14:21748812-21748856	NM_009502:180	Vcl	INSIDE	2.043	1.657	1740.03	2882.66	3.385	1324.16	4482.62
A_68_P31204664	chr17:46168459-46168503	NM_001110266:-6368	Vegfa	PROMOTER	1.868	3.938	2692.61	10604.08	7.358	1954.89	14383.83
A_68_P31937159	chr19:7062178-7062222	NM_001185164:-59	Vegfb	PROMOTER	1.636	4.237	3920.15	16608.12	6.932	3142.11	21780.69
A_68_P24124224	chr5:137507416-137507460	NM_001039385:1274	Vgf	INSIDE	1.972	7.730	2393.17	18499.10	15.247	1785.80	27227.51
A_68_P24124212	chr5:137506063-137506107	NM_001039385:-80	Vgf	PROMOTER	1.663	2.955	535.21	1581.64	4.915	464.93	2285.03
A_68_P24124227	chr5:137507750-137507794	NM_001039385:1608	Vgf	INSIDE	1.591	6.809	422.38	2876.05	10.831	459.61	4978.05

ProbeName	Target position of probe on CpG island microarray	TargetID	GeneSymbol	CpG island Description	Ratio of relative methylation (TiO_2-NP/Vehicle)	Sham group			TiO_2-H group		
						Relative methylation (Cy5/Cy3)	Cy3 signal (Input)	Cy5 signal (MeDIP)	Relative methylation (Cy5/Cy3)	Cy3 signal (Input)	Cy5 signal (MeDIP)
A_68_P21017263	chr2:13495869-13495913	NM_011701:-47	Vim	PROMOTER	2.359	3.510	257.85	905.15	8.280	190.22	1574.90
A_68_P21017264	chr2:13495981-13496025	NM_011701:65	Vim	INSIDE	1.832	3.783	3082.57	11659.97	6.931	2498.75	17319.85
A_68_P28765899	chr12:117316302-117316346	NM_009511:129	Vipr2	INSIDE	1.659	1.819	1316.78	2395.76	3.018	1029.57	3107.40
A_68_P24091293	chr5:130418207-130418251	NM_001001327:246	Vkorc1l1	INSIDE	1.717	6.268	943.56	5914.16	10.764	1054.67	11352.37
A_68_P31257913	chr17:56856203-56856247	NM_178926:120	Vmac	INSIDE	1.560	1.548	592.48	917.36	2.415	522.90	1262.62
A_68_P31982219	chr19:16855558-16855602	NM_173028:-163	Vps13a	PROMOTER	1.656	3.918	1634.12	6403.20	6.488	1341.32	8703.07
A_68_P31982216	chr19:16855225-16855273	NM_173028:169	Vps13a	INSIDE	1.537	1.643	672.73	1105.45	2.526	541.01	1366.40
A_68_P30133051	chr15:35301203-35301247	NM_177151:-76	Vps13b	PROMOTER	1.547	3.342	818.23	2734.90	5.169	728.49	3765.74
A_68_P23381901	chr4:144785001-144785047	NM_001128198:-4529	Vps13d	PROMOTER	1.969	2.303	958.14	2206.35	4.534	812.58	3684.09
A_68_P23381898	chr4:144784634-144784678	NM_001128198:-4161	Vps13d	PROMOTER	1.523	2.268	1021.53	2316.37	3.453	893.16	3083.71
A_68_P24536895	chr6:71493871-71493915	NM_025783:45	Vps24	INSIDE	2.044	6.284	3257.70	20471.36	12.844	2453.21	31509.98
A_68_P27186303	chr10:61949193-61949237	NM_001113355:132	Vps26a	INSIDE	2.075	17.294	1212.62	20971.44	35.885	866.21	31083.98
A_68_P22347225	chr3:95861928-95861972	NM_013841:428	Vps45	INSIDE	1.773	1.463	941.12	1376.55	2.593	861.27	2233.70
A_68_P27635752	chr11:21139299-21139343	NM_139061:429	Vps54	INSIDE	1.804	4.071	1720.94	7005.72	7.344	1344.29	9872.08
A_68_P24019920	chr5:117769201-117769245	NM_001033311:-52	Vsig10	PROMOTER	1.898	1.432	926.32	1326.26	2.717	833.12	2263.95
A_68_P32203587	chr19:56948670-56948714	NM_172840:-213	Vwa2	PROMOTER	1.857	2.149	2555.17	5491.18	3.992	1976.20	7888.36
A_68_P30588341	chr16:20591588-20591632	NM_182636:1956	Vwa5b2	INSIDE	2.020	2.519	1889.99	4761.39	5.088	1498.07	7621.73
A_68_P30588338	chr16:20591336-20591380	NM_182636:1704	Vwa5b2	INSIDE	1.582	3.605	2568.88	9259.68	5.704	1877.16	10707.37
A_68_P27589635	chr11:11013930-11013974	NM_177033:-66	Vwc2	PROMOTER	1.594	2.232	460.96	1029.09	3.559	447.80	1593.57
A_68_P31479225	chr18:7869058-7869102	NM_001146298:-114	Wac	PROMOTER	1.974	4.017	3388.04	13608.31	7.928	2757.80	21863.00
A_68_P31479231	chr18:7869684-7869728	NM_001146298:512	Wac	INSIDE	1.656	2.321	1157.71	2686.65	3.844	944.48	3630.44
A_68_P31479228	chr18:7869362-7869406	NM_001146298:190	Wac	INSIDE	1.645	2.198	2696.63	5927.22	3.616	2065.93	7469.93
A_68_P29751622	chr14:79881067-79881111	NM_018765:-13	Wbp4	PROMOTER	1.876	0.357	1577.79	563.98	0.670	1430.02	958.78
A_68_P23938671	chr5:102499208-102499252	NM_172882:-290	Wdfy3	PROMOTER	1.658	3.288	855.64	2813.62	5.451	689.08	3756.44
A_68_P25516578	chr7:136735619-136735663	NM_172255:264	Wdr11	INSIDE	2.632	0.212	1316.65	279.76	0.559	1149.78	642.91
A_68_P20267395	chr1:60155522-60155566	NM_001199060:-200	Wdr12	PROMOTER	1.939	3.578	1891.30	6767.76	6.938	1500.30	10409.78
A_68_P28739967	chr12:111976055-111976099	NM_027149:-82	Wdr20a	PROMOTER	1.544	3.066	1411.64	4327.86	4.735	1260.98	5970.71
A_68_P20880195	chr1:183141802-183141846	NM_145514:285	Wdr26	INSIDE	1.779	4.212	2162.58	9109.20	7.493	1734.85	12999.94
A_68_P28225578	chr12:8980743-8980787	NM_001159527:-42	Wdr35	PROMOTER	1.921	2.640	7446.62	19660.79	5.071	5556.74	28180.03
A_68_P31335313	chr17:71965569-71965613	NM_175639:36	Wdr43	INSIDE	1.523	1.559	551.97	860.51	2.374	500.10	1187.19
A_68_P28190095	chr11:121215875-121215919	NM_025793:-135	Wdr45l	PROMOTER	1.630	9.024	2659.39	23999.48	14.714	1804.12	26545.49
A_68_P31625687	chr18:36919881-36919925	NM_026464:10	Wdr55	INSIDE	1.574	3.041	1928.33	5863.74	4.786	1532.31	7333.66
A_68_P27926183	chr11:75267995-75268039	NM_138950:203	Wdr81	INSIDE	1.612	1.934	384.97	744.61	3.117	383.70	1196.10
A_68_P24358614	chr6:34860895-34860939	NM_001013366:-85	Wdr91	PROMOTER	1.649	1.524	6329.07	9643.26	2.512	4657.72	11699.40
A_68_P32152243	chr19:47911627-47911671	NM_027559:203	Wdr96	INSIDE	1.512	1.971	1964.46	3872.11	2.981	1536.97	4581.51
A_68_P23317106	chr4:132895104-132895148	NM_199306:104	Wdtc1	INSIDE	1.708	4.498	11435.70	51436.61	7.681	8078.16	62051.66
A_68_P25411826	chr7:117266404-117266448	NM_009516:854	Wee1	INSIDE	1.538	2.467	904.96	2232.32	3.794	799.90	3034.79
A_68_P25411827	chr7:117266477-117266521	NM_009516:926	Wee1	INSIDE	1.830	1.663	628.36	1044.80	3.043	619.02	1883.92
A_68_P27543031	chr10:128185237-128185281	NM_001170869:-319	Wibg	PROMOTER	1.949	1.732	406.22	703.63	3.376	336.20	1134.90
A_68_P28119629	chr11:109472837-109472881	NM_145940:-155	Wipi1	PROMOTER	1.945	2.160	5773.50	12469.26	4.201	4146.90	17421.64
A_68_P28119631	chr11:109473059-109473103	NM_145940:-377	Wipi1	PROMOTER	1.670	3.869	13921.71	53858.78	6.460	10681.12	68996.43
A_68_P24152591	chr5:143105523-143105567	NM_178398:7	Wipi2	INSIDE	1.820	3.101	1127.27	3495.87	5.644	958.11	5407.37
A_68_P24152597	chr5:143106025-143106069	NM_178398:509	Wipi2	INSIDE	1.725	2.031	1971.87	4004.07	3.502	1589.00	5564.96
A_68_P20350627	chr1:74849814-74849858	NM_009518:11244	Wnt10a	INSIDE	1.725	3.465	3093.07	10717.89	5.977	2626.35	15696.38
A_68_P20350529	chr1:74838384-74838428	NM_009518:-186	Wnt10a	PROMOTER	1.613	4.124	4948.69	20405.98	6.653	3730.25	24816.45
A_68_P30476395	chr15:98606917-98606961	NM_011718:1643	Wnt10b	INSIDE	1.706	9.467	3105.78	29401.01	16.152	2449.64	39567.60
A_68_P30476410	chr15:98608643-98608687	NM_011718:-83	Wnt10b	PROMOTER	1.604	1.353	4751.71	6428.46	2.170	3765.60	8171.33

ProbeName	Target position of probe on CpG island microarray	TargetID	GeneSymbol	CpG island Description	Ratio of relative methylation (TiO_2-NP/Vehicle)	Sham group			TiO_2-H group		
						Relative methylation (Cy5/Cy3)	Cy3 signal (Input)	Cy5 signal (MeDIP)	Relative methylation (Cy5/Cy3)	Cy3 signal (Input)	Cy5 signal (MeDIP)
A_68_P22394252	chr3:104770874-104770918	NM_009520:-6269	Wnt2b	PROMOTER	1.531	2.252	1357.05	3055.94	3.448	1114.67	3843.22
A_68_P27836028	chr11:59103858-59103902	NM_009522:373	Wnt3a	INSIDE	1.883	2.885	1333.86	3847.79	5.431	1095.47	5949.46
A_68_P27835858	chr11:59077515-59077559	NM_009522:26717	Wnt3a	INSIDE	1.500	2.344	2131.54	4997.33	3.518	1898.80	6679.52
A_68_P24638868	chr6:91361534-91361578	NM_009527:-193	Wnt7a	PROMOTER	3.112	4.262	1684.23	7177.50	13.263	1343.70	17822.06
A_68_P24638863	chr6:91360911-91360955	NM_009527:431	Wnt7a	INSIDE	1.557	2.287	1572.05	3594.67	3.560	1198.48	4266.50
A_68_P28086889	chr11:103611261-103611305	NM_011719:-147	Wnt9b	PROMOTER	1.839	4.097	1081.37	4430.58	7.536	805.24	6068.44
A_68_P28931383	chr13:32894479-32894523	NM_030215:602	Wrnip1	INSIDE	1.502	3.982	1699.99	6769.26	5.980	1708.82	10218.32
A_68_P27907296	chr11:71564250-71564294	NM_177618:68	Wscd1	INSIDE	1.860	4.496	4666.83	20981.39	8.363	3397.05	28410.13
A_68_P27907301	chr11:71564787-71564831	NM_177618:604	Wscd1	INSIDE	1.681	2.114	1199.61	2536.24	3.555	939.08	3338.21
A_68_P21492621	chr2:104966817-104966861	NM_144783:153	Wt1	INSIDE	2.243	4.140	9624.40	39847.81	9.288	6641.61	61684.67
A_68_P25844867	chr8:49075341-49075385	NM_133791:543	Wwc2	INSIDE	2.049	5.154	5036.50	25958.43	10.560	3628.59	38319.47
A_68_P25844872	chr8:49076065-49076109	NM_133791:-181	Wwc2	PROMOTER	1.558	3.399	3388.99	11519.48	5.297	3233.27	17126.42
A_68_P22766408	chr4:19636033-19636077	NM_177327:86	Wwp1	INSIDE	1.633	3.436	1455.50	5000.53	5.611	1334.52	7487.54
A_68_P32340723	chrX:39421307-39421351	NM_009688:316	Xiap	INSIDE	2.308	3.837	2381.77	9138.39	8.854	1191.40	10548.87
A_68_P32340725	chrX:39421489-39421533	NM_009688:498	Xiap	INSIDE	1.543	3.144	351.26	1104.43	4.851	150.06	727.98
A_68_P26875613	chr9:119920587-119920631	NM_011724:12108	Xirp1	DOWNSTREAM	1.754	2.196	2506.01	5503.35	3.852	2088.07	8042.98
A_68_P32573117	chrX:100677336-100677380	NR_001463:1214	Xist	INSIDE	1.721	2.053	4041.71	8298.95	3.533	1728.31	6105.93
A_68_P32573118	chrX:100677473-100677517	NR_001463:1078	Xist	INSIDE	1.547	3.534	3844.65	13587.88	5.467	1405.29	7682.44
A_68_P21752520	chr2:152857602-152857646	NM_001011732:37	Xkr7	INSIDE	1.966	7.358	1460.41	10745.14	14.466	1068.59	15458.44
A_68_P21752521	chr2:152857720-152857764	NM_001011732:155	Xkr7	INSIDE	1.930	4.795	3267.73	15669.96	9.254	2596.29	24027.08
A_68_P21752666	chr2:152880620-152880664	NM_001011732:23055	Xkr7	INSIDE	1.679	4.032	1917.47	7731.35	6.769	1550.20	10493.70
A_68_P25499304	chr7:133344026-133344070	NM_028816:-126	Xpo6	PROMOTER	2.285	19.109	8771.90	167619.40	43.660	5610.62	244958.70
A_68_P20737714	chr1:157264468-157264512	NM_011273:84	Xpr1	INSIDE	1.558	3.906	11830.67	46212.57	6.084	9122.39	55504.65
A_68_P25005464	chr7:25332066-25332110	NM_009532:-80	Xrcc1	DIVERGENT_PROMOTER	1.719	2.836	1884.27	5344.33	4.875	1417.59	6910.93
A_68_P27532842	chr10:126338342-126338386	NM_026858:63	Xrcc6bp1	INSIDE	2.017	4.400	3059.98	13463.66	8.873	2300.66	20414.17
A_68_P25364270	chr7:107007573-107007617	NM_001164258:-133	Xrra1	DIVERGENT_PROMOTER	1.512	3.183	829.70	2640.68	4.811	747.86	3598.25
A_68_P26290461	chr9:8004643-8004687	NM_001171147:-68	Yap1	PROMOTER	1.878	5.407	3903.80	21106.63	10.155	3228.67	32788.72
A_68_P23584753	chr5:32913192-32913236	NM_001205132:-329	Yes1	PROMOTER	1.611	2.569	2853.62	7330.54	4.138	2033.11	8412.94
A_68_P32552603	chrX:96133526-96133570	NM_207633:429	Yipf6	INSIDE	1.698	2.647	821.91	2175.68	4.496	459.10	2064.03
A_68_P27565811	chr11:5855744-5855788	NM_019661:6	Ykt6	INSIDE	1.592	1.660	494.53	820.84	2.643	392.71	1037.95
A_68_P28601528	chr12:86337027-86337071	NM_178363:-222	Ylpm1	PROMOTER	1.686	2.102	574.60	1208.09	3.545	463.35	1642.77
A_68_P20611349	chr1:132613579-132613625	NM_178691:-301	Yod1	DIVERGENT_PROMOTER	1.830	2.158	504.55	1088.79	3.949	468.83	1851.55
A_68_P23865427	chr5:87233816-87233860	NM_177680:324	Ythdc1	INSIDE	1.725	1.739	413.95	720.01	3.000	361.58	1084.78
A_68_P23310689	chr4:131767916-131767960	NM_145393:233	Ythdf2	INSIDE	1.732	4.831	8487.61	41002.43	8.366	6485.99	54258.90
A_68_P24118628	chr5:136410394-136410438	NM_018871:95	Ywhag	INSIDE	1.695	1.624	1284.33	2086.11	2.752	987.55	2718.20
A_68_P30140505	chr15:36723538-36723582	NM_011740:733	Ywhaz	INSIDE	1.623	2.586	1808.20	4675.67	4.198	1640.94	6888.50
A_68_P28729933	chr12:110032849-110032893	NM_009537:1350	Yy1	INSIDE	1.634	2.528	1099.01	2778.47	4.132	930.27	3843.93
A_68_P21936419	chr3:9251754-9251798	NM_177660:1210	Zbtb10	INSIDE	1.503	2.892	5521.23	15969.96	4.346	4422.08	19220.12
A_68_P26494143	chr9:48643240-48643284	NM_001033324:788	Zbtb16	INSIDE	1.836	5.724	2309.58	13219.75	10.510	1649.43	17335.96
A_68_P31150957	chr17:34053104-34053148	NM_020625:6	Zbtb22	INSIDE	1.779	4.398	11456.48	50390.67	7.827	8771.91	68655.84
A_68_P28553589	chr12:77470150-77470194	NM_001172104:279	Zbtb25	INSIDE	1.578	2.047	870.21	1781.03	3.230	770.10	2487.54
A_68_P32326603	chrX:35543022-35543066	NM_001079513:75	Zbtb33	INSIDE	2.011	4.310	831.73	3584.55	8.668	491.76	4262.69
A_68_P27896951	chr11:69579684-69579728	NM_029348:293	Zbtb4	INSIDE	1.927	6.578	853.34	5613.07	12.676	665.38	8434.45
A_68_P28751776	chr12:113916958-113917002	NM_001100460:-70	Zbtb42	DIVERGENT_PROMOTER	2.209	2.980	806.11	2402.03	6.582	700.69	4611.84
A_68_P21121774	chr2:33323833-33323877	NM_001025594:198	Zbtb43	INSIDE	1.528	2.141	1900.30	4068.74	3.271	1754.03	5738.29
A_68_P26398232	chr9:30838076-30838120	NM_001115130:-130	Zbtb44	PROMOTER	1.584	5.966	5734.07	34207.50	9.452	4537.35	42885.20
A_68_P22880598	chr4:45025167-45025211	NM_001163283:96	Zbtb5	INSIDE	1.503	4.230	645.60	2730.70	6.358	540.93	3439.40

ProbeName	Target position of probe on CpG island microarray	TargetID	GeneSymbol	CpG island Description	Ratio of relative methylation (TiO_2-NP/Vehicle)	Sham group			TiO_2-H group		
						Relative methylation (Cy5/Cy3)	Cy3 signal (Input)	Cy5 signal (MeDIP)	Relative methylation (Cy5/Cy3)	Cy3 signal (Input)	Cy5 signal (MeDIP)
A_68_P27287617	chr10:80598897-80598941	NM_010731:-97	Zbtb7a	PROMOTER	1.721	3.469	1436.35	4982.19	5.968	1224.08	7305.29
A_68_P27287712	chr10:80611089-80611133	NM_010731:12095	Zbtb7a	INSIDE	1.568	3.451	1624.44	5606.41	5.410	1331.74	7204.85
A_68_P27287631	chr10:80600637-80600681	NM_010731:1643	Zbtb7a	INSIDE	1.509	2.562	2040.84	5227.85	3.864	1790.23	6917.92
A_68_P27287623	chr10:80599677-80599721	NM_010731:683	Zbtb7a	INSIDE	1.663	1.563	1306.00	2041.31	2.600	1135.28	2951.57
A_68_P22319131	chr3:89200123-89200167	NM_009565:-3019	Zbtb7b	PROMOTER	2.104	1.910	3518.56	6721.41	4.019	2746.25	11038.09
A_68_P22319097	chr3:89196146-89196190	NM_009565:957	Zbtb7b	INSIDE	1.989	0.704	1297.39	913.61	1.400	1152.06	1613.43
A_68_P31839435	chr18:75979484-75979528	NM_145356:-325	Zbtb7c	PROMOTER	2.041	4.500	2203.19	9914.56	9.184	1824.68	16758.33
A_68_P23272336	chr4:124804630-124804674	NM_153159:473	Zc3h12a	INSIDE	2.534	8.868	9988.09	88569.59	22.468	6409.08	143996.90
A_68_P29726978	chr14:75684076-75684120	NM_026083:-81	Zc3h13	PROMOTER	2.120	8.539	2031.43	17345.71	18.101	1558.55	28211.02
A_68_P26232970	chr8:124900378-124900422	NM_001029993:-115	Zc3h18	PROMOTER	1.815	4.101	833.27	3417.54	7.443	688.30	5123.13
A_68_P21622283	chr2:128792891-128792935	NM_178404:-225	Zc3h6	PROMOTER	1.942	7.387	16791.77	124034.50	14.346	11192.29	160559.10
A_68_P27804178	chr11:53138130-53138174	NM_026479:-38	Zcchc10	PROMOTER	2.182	3.232	3439.26	11114.35	7.051	2390.08	16853.17
A_68_P23196094	chr4:108132526-108132570	NM_175472:518	Zcchc11	INSIDE	1.817	10.177	5197.71	52899.47	18.496	3681.07	68086.84
A_68_P20505897	chr1:107890521-107890565	NM_001122675:3560	Zcchc2	INSIDE	1.734	3.021	2180.83	6589.31	5.239	1749.16	9164.54
A_68_P21749221	chr2:152240295-152240339	NM_175126:464	Zcchc3	INSIDE	1.589	3.004	2569.64	7720.35	4.775	2282.28	10898.69
A_68_P23696773	chr5:53174399-53174443	NM_030185:115	Zcchc4	INSIDE	1.511	2.632	2596.84	6834.23	3.977	2118.43	8424.77
A_68_P22878938	chr4:44769794-44769838	NM_138590:386	Zcchc7	INSIDE	2.063	3.570	1474.78	5264.24	7.363	1251.77	9216.30
A_68_P32578106	chrX:101866324-101866368	NM_175358:57	Zdhhc15	INSIDE	2.357	1.895	739.72	1402.08	4.467	457.94	2045.51
A_68_P32578104	chrX:101866109-101866153	NM_175358:273	Zdhhc15	INSIDE	1.899	3.304	4590.08	15164.50	6.274	2668.97	16744.35
A_68_P27446660	chr10:110446716-110446760	NM_172554:384	Zdhhc17	INSIDE	1.608	1.530	3567.01	5458.66	2.461	2850.16	7014.34
A_68_P23059231	chr4:82505707-82505751	NM_026647:-163	Zdhhc21	PROMOTER	1.617	2.859	3478.55	9944.80	4.624	2488.71	11508.13
A_68_P28613409	chr12:88331117-88331161	NM_001080943:-1512	Zdhhc22	PROMOTER	1.571	3.192	1058.11	3377.73	5.015	913.34	4579.94
A_68_P21394209	chr2:84555435-84555479	NM_144887:-135	Zdhhc5	PROMOTER	2.041	7.232	8025.09	58033.93	14.762	6272.09	92586.57
A_68_P26217897	chr8:122625576-122625620	NM_133967:-226	Zdhhc7	PROMOTER	1.721	1.447	1487.66	2151.96	2.490	1206.32	3003.91
A_68_P30578494	chr16:18234940-18234984	NM_172151:267	Zdhhc8	INSIDE	2.017	3.442	5731.05	19726.11	6.943	4192.76	29109.48
A_68_P30578495	chr16:18235059-18235103	NM_172151:149	Zdhhc8	INSIDE	1.591	2.521	1763.98	4446.77	4.011	1455.75	5839.54
A_68_P30578497	chr16:18235357-18235401	NM_172151:-149	Zdhhc8	PROMOTER	1.577	2.553	2395.00	6115.41	4.026	1963.17	7903.27
A_68_P31469349	chr18:5591373-5591417	NM_011546:-494	Zeb1	PROMOTER	1.630	2.036	1186.01	2415.11	3.319	998.95	3315.18
A_68_P26153173	chr8:111324170-111324214	NM_007496:85649	Zfhx3	INSIDE	1.631	2.018	591.48	1193.66	3.292	440.15	1448.85
A_68_P26169432	chr8:114167808-114167852	NM_001037665:488	Zfp1	INSIDE	2.202	5.107	2651.81	13541.72	11.244	1977.35	22233.90
A_68_P31092441	chr17:23713283-23713327	NM_011747:23150	Zfp13	INSIDE	2.092	1.581	1651.05	2609.73	3.307	1243.51	4112.10
A_68_P31323668	chr17:69733260-69733304	NM_009547:-35	Zfp161	PROMOTER	1.505	4.474	2569.08	11495.08	6.736	2114.62	14243.16
A_68_P21851410	chr2:169955855-169955909	NM_001033299:838	Zfp217	INSIDE	1.663	1.431	1120.11	1603.07	2.379	944.80	2248.13
A_68_P24786877	chr6:118405159-118405203	NM_028335:344	Zfp248	INSIDE	1.560	2.221	2692.98	5980.02	3.464	2110.48	7311.15
A_68_P26342514	chr9:20264309-20264353	NM_011753:274	Zfp26	INSIDE	2.597	5.833	12946.13	75517.71	15.147	9104.24	137900.20
A_68_P32462210	chrX:70599683-70599727	NM_001160229:11745	Zfp275	INSIDE	1.580	3.811	1027.01	3913.44	6.020	642.16	3866.00
A_68_P26238816	chr8:125778247-125778291	NM_020497:174	Zfp276	INSIDE	1.951	7.620	4136.00	31517.69	14.868	3031.78	45076.72
A_68_P32365466	chrX:45947510-45947554	NM_153532:-2222	Zfp280c	PROMOTER	2.676	5.021	1811.17	9094.51	13.439	945.31	12704.10
A_68_P24973879	chr7:13551564-13551609	NM_178732:374	Zfp324	INSIDE	2.727	10.119	12804.14	129564.20	27.590	6837.10	188638.10
A_68_P27792052	chr11:50873003-50873047	NM_009329:266	Zfp354a	INSIDE	1.620	4.509	3530.93	15919.26	7.304	2608.28	19049.86
A_68_P21359595	chr2:77654503-77654547	NM_178723:349	Zfp385b	INSIDE	2.000	4.636	925.18	4289.04	9.270	697.33	6464.28
A_68_P27833411	chr11:58717772-58717816	NM_011758:-67	Zfp39	PROMOTER	1.812	5.846	8399.74	49106.03	10.592	6079.29	64392.64
A_68_P29672987	chr14:65977066-65977114	NM_199029:-422	Zfp395	PROMOTER	1.711	3.926	498.77	1958.26	6.719	434.67	2920.36
A_68_P29672994	chr14:65977929-65977973	NM_199029:438	Zfp395	INSIDE	1.551	0.410	847.18	347.45	0.636	967.85	615.83
A_68_P26036153	chr8:90184081-90184125	NM_033327:299392	Zfp423	DOWNSTREAM	1.860	2.586	2470.14	6388.56	4.811	1808.14	8699.09
A_68_P26038373	chr8:90485269-90485313	NM_033327:-1796	Zfp423	PROMOTER	1.672	9.737	873.67	8506.77	16.277	734.07	11948.65
A_68_P26038355	chr8:90482696-90482740	NM_033327:776	Zfp423	INSIDE	1.523	2.485	1179.34	2930.14	3.784	1066.12	4034.58

ProbeName	Target position of probe on CpG island microarray	TargetID	GeneSymbol	CpG island Description	Ratio of relative methylation (TiO_2-NP/Vehicle)	Sham group			TiO_2-H group		
						Relative methylation (Cy5/Cy3)	Cy3 signal (Input)	Cy5 signal (MeDIP)	Relative methylation (Cy5/Cy3)	Cy3 signal (Input)	Cy5 signal (MeDIP)
A_68_P25005234	chr7:25292077-25292122	NM_146183:-6	Zfp428	PROMOTER	1.664	1.725	2355.04	4063.24	2.871	1775.29	5097.13
A_68_P24954457	chr7:6141163-6141207	NM_001146024:17070	Zfp444	INSIDE	1.633	2.299	250.55	575.91	3.754	227.18	852.87
A_68_P24973956	chr7:13563487-13563531	NM_001168561:312	Zfp446	INSIDE	1.927	4.269	926.05	3952.94	8.225	813.97	6694.80
A_68_P22931104	chr4:54958261-54958305	NM_172867:-2534	Zfp462	PROMOTER	1.701	2.761	1914.64	5286.90	4.698	1621.76	7619.02
A_68_P29455874	chr14:22804989-22805033	NM_145459:3813	Zfp503	INSIDE	6.806	12.105	640.93	7758.46	82.389	599.62	49402.73
A_68_P29455876	chr14:22805255-22805299	NM_145459:3547	Zfp503	INSIDE	2.388	4.903	8384.65	41113.51	11.710	5045.05	59078.81
A_68_P29455871	chr14:22804612-22804656	NM_145459:4189	Zfp503	INSIDE	2.022	3.937	2979.74	11729.99	7.960	2329.53	18543.92
A_68_P29455867	chr14:22804205-22804249	NM_145459:4597	Zfp503	INSIDE	1.705	2.108	6999.60	14757.56	3.594	5362.43	19273.32
A_68_P25051309	chr7:36588072-36588116	NM_177739:-86	Zfp507	PROMOTER	1.670	3.097	459.55	1423.12	5.172	360.31	1863.39
A_68_P25581873	chr7:147222902-147222946	NM_027201:635	Zfp511	INSIDE	1.804	2.157	2142.84	4623.02	3.891	1510.89	5879.04
A_68_P31879479	chr18:83126499-83126543	NM_001177464:42497	Zfp516	INSIDE	2.098	9.025	2560.18	23104.84	18.937	2102.69	39819.04
A_68_P31086417	chr17:21669593-21669637	NM_144515:-2888	Zfp52	PROMOTER	1.557	2.758	2058.15	5676.25	4.293	1665.77	7151.85
A_68_P31120939	chr17:28314640-28314684	NM_172617:300	Zfp523	INSIDE	1.524	2.620	5376.46	14087.07	3.993	3975.25	15871.32
A_68_P31086236	chr17:21626256-21626300	NM_013843:327	Zfp53	INSIDE	1.652	1.868	1741.74	3253.80	3.086	1520.40	4691.44
A_68_P31782005	chr18:65740650-65740694	NM_207255:789	Zfp532	INSIDE	1.804	4.365	4337.80	18933.93	7.874	3516.66	27688.88
A_68_P25504683	chr7:134376847-134376891	NM_146201:-88	Zfp553	DIVERGENT_PROMOTER	1.562	6.493	500.43	3249.53	10.143	454.10	4605.86
A_68_P25008269	chr7:25866719-25866763	NM_175477:4476	Zfp574	INSIDE	1.779	4.221	4048.08	17085.21	7.509	3275.89	24598.20
A_68_P24951794	chr7:5004858-5004902	NM_026900:1747	Zfp580	INSIDE	1.953	11.439	6702.51	76672.71	22.347	4388.05	98058.91
A_68_P30084544	chr15:25914715-25914759	NM_144523:616	Zfp622	INSIDE	1.615	2.547	2462.55	6272.56	4.112	2001.95	8232.98
A_68_P21844247	chr2:168760922-168760966	NM_009564:20143	Zfp64	INSIDE	1.844	7.758	353.32	2740.99	14.308	325.64	4659.13
A_68_P23962878	chr5:107125443-107125487	NM_026856:385	Zfp644	INSIDE	1.982	5.364	4973.92	26679.75	10.633	4228.15	44958.50
A_68_P26350251	chr9:21860554-21860598	NM_177318:15213	Zfp653	INSIDE	1.500	2.831	1323.23	3746.33	4.247	1055.78	4483.80
A_68_P24063275	chr5:125343770-125343814	NM_001081750:718	Zfp664	INSIDE	1.643	3.056	2191.66	6698.03	5.021	1670.90	8389.72
A_68_P27830380	chr11:58136423-58136467	NM_178761:404	Zfp672	INSIDE	1.629	4.207	4136.22	17401.81	6.852	3392.02	23243.79
A_68_P22341853	chr3:94819143-94819187	NM_030074:-4	Zfp687	PROMOTER	1.962	4.849	808.04	3917.86	9.511	672.38	6395.29
A_68_P25505623	chr7:134587980-134588024	NM_175163:4670	Zfp689	INSIDE	2.195	6.386	1233.58	7878.17	14.018	1140.67	15989.94
A_68_P32603687	chrX:109715096-109715140	NM_177747:984	Zfp711	INSIDE	2.086	6.119	1571.52	9616.70	12.767	999.78	12764.45
A_68_P32603677	chrX:109714158-109714202	NM_177747:46	Zfp711	INSIDE	1.557	2.212	329.82	729.70	3.444	225.13	775.44
A_68_P25505272	chr7:134489178-134489222	NM_146202:-372	Zfp768	PROMOTER	2.021	1.770	593.28	1049.86	3.577	545.91	1952.67
A_68_P25504790	chr7:134397531-134397575	NM_177362:9513	Zfp771	INSIDE	1.532	1.867	1286.01	2400.51	2.860	1137.32	3252.78
A_68_P24425393	chr6:47974535-47974579	NM_001081382:23557	Zfp777	INSIDE	1.551	3.239	550.43	1782.89	5.025	529.21	2659.20
A_68_P24954294	chr7:6107601-6107645	NM_001013012:-49	Zfp787	PROMOTER	1.967	4.333	5409.37	23436.41	8.522	4239.71	36128.89
A_68_P24954136	chr7:6083754-6083798	NM_001013012:23797	Zfp787	INSIDE	1.711	3.719	1970.48	7328.70	6.364	1617.93	10296.73
A_68_P24954292	chr7:6107153-6107197	NM_001013012:399	Zfp787	INSIDE	1.622	1.848	928.31	1715.63	2.997	1187.74	3560.21
A_68_P24954289	chr7:6106868-6106913	NM_001013012:683	Zfp787	INSIDE	1.579	3.682	2148.52	7910.72	5.812	1736.73	10094.57
A_68_P31147212	chr17:32966976-32967020	NM_177359:195	Zfp799	INSIDE	2.230	7.205	2501.50	18022.65	16.067	1776.93	28549.85
A_68_P31148785	chr17:33495528-33495572	NM_207541:273	Zfp81	INSIDE	1.787	2.112	3403.81	7188.31	3.773	2674.22	10090.12
A_68_P25029775	chr7:30857776-30857820	NM_177889:44	Zfp82	INSIDE	1.702	2.524	1477.15	3728.99	4.296	1209.50	5196.36
A_68_P25992690	chr8:81636588-81636632	NM_178267:84275	Zfp827	INSIDE	2.710	18.337	18328.51	336097.60	49.688	10239.56	508780.30
A_68_P25992689	chr8:81636479-81636523	NM_178267:84165	Zfp827	INSIDE	2.072	12.786	10404.52	133037.20	26.490	6169.84	163436.70
A_68_P25992691	chr8:81636666-81636710	NM_178267:84353	Zfp827	INSIDE	1.545	9.692	5183.46	50237.54	14.970	3215.13	48129.18
A_68_P25670894	chr8:13870055-13870099	NM_181854:436	Zfp828	INSIDE	1.587	1.726	1360.09	2347.50	2.740	1066.27	2921.49
A_68_P24951673	chr7:4981991-4982035	NM_001033383:10035	Zfp865	INSIDE	1.668	3.797	2167.70	8231.60	6.333	1920.71	12163.49
A_68_P31147009	chr17:32924787-32924831	NM_172458:424	Zfp871	INSIDE	1.948	4.509	5798.67	26143.80	8.782	5006.12	43964.92
A_68_P31087861	chr17:22058135-22058179	NM_001199048:41275	Zfp942	DOWNSTREAM	1.693	6.043	8521.82	51496.41	10.228	6647.56	67992.19
A_68_P31090923	chr17:23013560-23013604	NM_001110254:-9481	Zfp945	PROMOTER	1.524	3.867	1295.52	5010.37	5.892	1139.64	6715.07
A_68_P25947840	chr8:72273755-72273799	NM_001200023:85	Zfp963	INSIDE	1.510	2.939	4922.90	14468.46	4.439	3866.13	17161.24

ProbeName	Target position of probe on CpG island microarray	TargetID	GeneSymbol	CpG island Description	Ratio of relative methylation (TiO_2-NP/Vehicle)	Sham group			TiO_2-H group		
						Relative methylation (Cy5/Cy3)	Cy3 signal (Input)	Cy5 signal (MeDIP)	Relative methylation (Cy5/Cy3)	Cy3 signal (Input)	Cy5 signal (MeDIP)
A_68_P25947613	chr8:72178336-72178380	NM_001177527:-96	Zfp964	PROMOTER	1.934	5.353	2590.06	13863.49	10.354	1969.80	20395.55
A_68_P30160398	chr15:40486792-40486836	NM_011766:227	Zfpm2	INSIDE	1.934	4.138	7704.09	31875.86	8.003	5311.51	42509.08
A_68_P32537531	chrX:91368428-91368472	NM_001044386:296	Zfx	INSIDE	1.616	5.107	1044.72	5335.77	8.253	681.29	5622.91
A_68_P32537532	chrX:91368506-91368550	NM_001044386:218	Zfx	INSIDE	1.572	4.782	3575.38	17099.25	7.520	2303.95	17325.96
A_68_P30243544	chr15:57525714-57525758	NM_199449:-485	Zhx2	PROMOTER	1.907	2.755	1625.86	4478.68	5.253	1339.99	7039.37
A_68_P30243549	chr15:57526400-57526444	NM_199449:201	Zhx2	INSIDE	1.578	5.586	1314.57	7343.29	8.817	1022.93	9019.07
A_68_P26721663	chr9:91278946-91278990	NM_009576:15159	Zic4	INSIDE	1.721	2.922	909.30	2656.60	5.028	697.42	3506.49
A_68_P32674751	chrX:131543547-131543591	NM_175446:180	Zmat1	INSIDE	2.422	19.742	5109.23	100864.00	47.809	3031.64	144939.50
A_68_P32564024	chrX:98615209-98615253	NM_001177985:23	Zmym3	INSIDE	1.891	2.856	582.27	1662.67	5.400	372.25	2010.15
A_68_P23282958	chr4:126644783-126644827	NM_001114399:363	Zmym4	INSIDE	1.734	6.315	4207.22	26569.82	10.948	3446.79	37736.43
A_68_P21826276	chr2:165671382-165671427	NM_027230:38784	Zmynd8	INSIDE	1.699	7.060	678.38	4789.61	11.993	547.10	6561.33
A_68_P21911521	chr2:181327466-181327510	NM_001164597:-322	Znf512b	PROMOTER	1.765	3.573	1536.04	5488.81	6.305	1321.95	8335.16
A_68_P21911518	chr2:181326975-181327019	NM_001164597:170	Znf512b	INSIDE	1.689	8.697	1229.90	10697.01	14.691	829.14	12180.90
A_68_P31932456	chr19:6060898-6060957	NM_013859:-279	Znhit2-ps	PROMOTER	1.863	9.177	22684.11	208180.60	17.096	12764.16	218213.80
A_68_P27563479	chr11:5344842-5344886	NM_001080924:-14	Znrf3	PROMOTER	1.574	1.826	2713.11	4953.86	2.874	2271.20	6527.31
A_68_P28057142	chr11:98412381-98412425	NM_001166494:-8	Zpbp2	DIVERGENT_PROMOTER	2.100	7.127	4869.52	34703.67	14.967	3233.09	48389.12
A_68_P32795839	chrX:160396590-160396634	NM_009453:-14	Zrsr2	PROMOTER	1.750	2.246	2229.98	5007.44	3.929	1201.96	4722.33
A_68_P23231372	chr4:116550094-116550138	NM_001029912:110	Zswim5	INSIDE	1.866	3.471	2852.81	9901.03	6.476	2279.39	14761.92
A_68_P29308210	chr13:108681440-108681484	NM_145456:-1204	Zswim6	PROMOTER	1.530	3.652	1260.57	4603.49	5.589	1054.22	5891.68
A_68_P29308205	chr13:108680813-108680857	NM_145456:-576	Zswim6	PROMOTER	2.259	1.450	1072.99	1555.79	3.276	830.04	2719.13
A_68_P24631961	chr6:90319416-90319460	NM_030260:-49	Zxdc	PROMOTER	1.853	2.577	1348.62	3475.42	4.775	1139.85	5442.93
A_68_P23195047	chr4:107889859-107889903	NM_001167936:647	Zyg11a	INSIDE	1.926	5.752	19495.88	112147.10	11.079	13845.63	153397.70
A_68_P22650336	chr3:152058904-152058948	NM_001080755:-47	Zzz3	PROMOTER	1.639	2.012	590.54	1188.06	3.298	679.08	2239.34
