# Supplementary material for: Effects of Prenatal Exposure to Titanium Dioxide Nanoparticles on DNA Methylation and Gene Expression Profile in the Mouse Brain
Source: Front Toxicol. 2021 Oct 8;3:705910. doi: 10.3389/ftox.2021.705910 (PMC8915839; doi:10.3389/ftox.2021.705910)
Supplement: Supplementary file 8 [file Table3.pdf]

Supplementary Table 3. Alteration of DNA methylation state of the brain of female offspring prenatally exposed to TiO_2-NP.

The relative DNA methylation was calculated by dividing Cy5 signal by Cy3 signal obtained from CpG island microarray and then compared between Sham and TiO_2-NP groups.

ProbeName	Target position of probe on CpG island microarray	TargetID	GeneSymbol	CpG island Description	Ratio of relative methylation (TiO_2-NP/Vehicle)	Sham group			TiO_2-H group		
						Relative methylation (Cy5/Cy3)	Cy3 signal (Input)	Cy5 signal (MeDIP)	Relative methylation (Cy5/Cy3)	Cy3 signal (Input)	Cy5 signal (MeDIP)
A_68_P29455874	chr14:22804989-22805033	NM_145459:3813	Zfp503	INSIDE	6.806	12.105	640.93	7758.46	82.389	599.62	49402.73
A_68_P31350850	chr17:74695143-74695187	NM_133771:-961	Memo1	PROMOTER	5.241	2.878	450.72	1297.15	15.083	368.81	5562.66
A_68_P26795688	chr9:105033875-105033919	NM_029385:239	Nudt16	INSIDE	4.477	3.082	440.46	1357.41	13.798	418.55	5775.31
A_68_P25228678	chr7:80521046-80521090	NM_177740:663	Rgma	INSIDE	4.245	0.556	663.33	368.98	2.361	644.67	1522.29
A_68_P23644448	chr5:43626040-43626084	NR_028417:531	Gm7854	INSIDE	4.107	0.491	627.14	307.93	2.017	723.63	1459.38
A_68_P25574929	chr7:146132486-146132530	NM_028708:76	Jakmip3	INSIDE	4.012	0.451	881.02	397.57	1.810	713.30	1291.35
A_68_P33007146	chr9_random:49897-49941	NR_015516:-3490	4930526I15Rik	PROMOTER	3.692	6.288	3418.87	21499.06	23.215	2298.35	53355.95
A_68_P20231213	chr1:53353942-53353986	NM_145517:26	Ormdl1	INSIDE	3.543	1.826	419.01	764.99	6.468	388.28	2511.27
A_68_P32533200	chrX:90534061-90534105	NM_007492:2098	Arx	INSIDE	3.504	13.814	6300.76	87041.16	48.399	3229.51	156305.70
A_68_P26171007	chr8:114465131-114465175	NM_026693:535	Gabarapl2	INSIDE	3.497	27.141	3597.79	97649.33	94.902	1892.10	179564.50
A_68_P26164572	chr8:113262808-113262852	NM_198625:17447	Mtss1l	INSIDE	3.486	7.191	2535.96	18235.83	25.068	1841.30	46157.41
A_68_P24513815	chr6:65728982-65729026	NM_027547:49	Prdm5	INSIDE	3.419	8.570	852.11	7302.43	29.302	723.73	21206.73
A_68_P26164573	chr8:113262875-113262919	NM_198625:17513	Mtss1l	INSIDE	3.405	4.778	3372.57	16114.77	16.269	2472.91	40232.99
A_68_P25724843	chr8:26127513-26127557	NM_007404:-140	Adam9	DIVERGENT_PROMOTER	3.403	7.316	1524.90	11155.47	24.892	1145.63	28517.06
A_68_P25845477	chr8:49195740-49195784	NM_001161515:396	Dctd	INSIDE	3.295	10.942	2629.12	28768.68	36.052	1918.64	69170.72
A_68_P21084949	chr2:27330700-27330744	NM_023336:196	Brd3	INSIDE	3.270	18.917	430.60	8145.76	61.868	299.91	18554.51
A_68_P27693341	chr11:33101001-33101045	NM_019916:2566	Tlx3	INSIDE	3.266	11.424	11521.46	131618.90	37.311	7480.28	279095.00
A_68_P26812299	chr9:107888278-107888322	NM_026597:-53	6230427J02Rik	PROMOTER	3.211	41.034	2065.67	84762.66	131.746	820.78	108134.90
A_68_P31167789	chr17:37334385-37334429	NM_001011518:505	Olfr94	INSIDE	3.185	0.553	612.40	338.39	1.760	485.62	854.62
A_68_P29619050	chr14:56262458-56262502	NM_028780:163	Tm9sf1	INSIDE	3.144	21.737	4434.65	96395.99	68.347	2167.49	148140.80
A_68_P24638868	chr6:91361534-91361578	NM_009527:-193	Wnt7a	PROMOTER	3.112	4.262	1684.23	7177.50	13.263	1343.70	17822.06
A_68_P23203724	chr4:109649844-109649888	NM_172296:-763	Dmrta2	PROMOTER	3.082	29.984	17420.38	522325.10	92.404	8951.32	827137.40
A_68_P27980117	chr11:84693941-84693985	NM_001077636:-175	Pigw	PROMOTER	3.046	8.934	7846.91	70105.60	27.217	4810.06	130915.60
A_68_P27928203	chr11:75608556-75608600	NM_007873:981	Doc2b	INSIDE	3.043	7.601	425.29	3232.70	23.131	319.99	7401.59
A_68_P29060390	chr13:56397734-56397778	NM_019568:156	Cxcl14	INSIDE	3.036	3.480	3675.66	12793.06	10.568	2602.92	27507.92
A_68_P28222996	chr12:8506600-8506644	NM_007483:169	Rhob	INSIDE	3.027	29.504	8519.70	251363.70	89.305	4297.86	383821.30
A_68_P32778409	chrX:156576281-156576325	NM_008810:-34	Pdha1	PROMOTER	3.016	7.865	716.04	5631.76	23.720	359.32	8523.27
A_68_P31155038	chr17:34784397-34784441	NM_017406:294	Atf6b	INSIDE	2.986	23.608	887.30	20947.48	70.495	609.26	42949.85
A_68_P32536642	chrX:91168388-91168432	ENSMUST00000117999:-1109		DIVERGENT_PROMOTER	2.977	7.201	1376.55	9912.26	21.433	728.70	15618.38
A_68_P29450509	chr14:21871923-21871967	NM_134079:90	Adk	INSIDE	2.951	1.754	1736.13	3045.36	5.176	1477.41	7646.82
A_68_P30374043	chr15:80542551-80542595	NM_144812:830	Tnrc6b	INSIDE	2.901	10.575	3462.10	36612.34	30.679	2420.22	74249.83
A_68_P32464342	chrX:70961468-70961512	NM_012060:24	Bcap31	INSIDE	2.898	3.555	4575.87	16269.43	10.304	2149.12	22144.39
A_68_P30137691	chr15:36213148-36213192	NM_013923:-268	Rnf19a	PROMOTER	2.895	7.973	1368.64	10911.67	23.081	795.84	18368.76
A_68_P25703829	chr8:19535136-19535180	ENSMUST00000118143:-745		PROMOTER	2.887	8.233	5523.50	45473.70	23.771	3532.43	83970.59
A_68_P24568080	chr6:77929479-77929523	NM_001109764:161	Ctnna2	INSIDE	2.885	5.838	2978.31	17387.62	16.841	1751.66	29498.86
A_68_P22028773	chr3:30407847-30407891	NM_021442:541	Mecom	INSIDE	2.882	13.646	2919.90	39844.77	39.327	2080.76	81830.92
A_68_P21888635	chr2:177853739-177853783	NM_001177789:81	Phactr3	INSIDE	2.868	25.028	2515.99	62971.06	71.771	1619.79	116252.80
A_68_P32351252	chrX:42143188-42143232	NM_001190718:128	Dcaf12l1	INSIDE	2.865	7.296	3767.38	27487.72	20.901	2077.17	43415.76
A_68_P25571662	chr7:145589207-145589251	NM_183289:185	Tcerg1l	INSIDE	2.863	9.562	4573.94	43735.11	27.371	2937.71	80409.07
A_68_P28155403	chr11:115664957-115665001	NM_080643:9928	Caskin2	INSIDE	2.858	7.044	4618.06	32531.25	20.130	3180.69	64028.48
A_68_P28059604	chr11:98821830-98821874	NM_001176528:68	Rara	INSIDE	2.846	9.755	2977.11	29040.29	27.760	2211.13	61381.64
A_68_P23324350	chr4:134260533-134260577	NM_207237:-349	Man1c1	PROMOTER	2.820	6.404	6070.41	38876.71	18.062	3941.66	71192.51
A_68_P33007145	chr9_random:49764-49808	NR_015516:-3622	4930526I15Rik	PROMOTER	2.796	13.504	11845.74	159960.60	37.756	6253.91	236121.30
A_68_P28307263	chr12:28027320-28027364	NM_009234:241	Sox11	INSIDE	2.795	16.492	4774.96	78748.73	46.092	3149.38	145159.90
A_68_P25196205	chr7:74994369-74994413			Unknown	2.771	7.120	6927.70	49326.62	19.730	5073.07	100093.90
A_68_P26344891	chr9:20780545-20780589	NM_010333:671	S1pr2	INSIDE	2.748	1.576	1964.61	3095.35	4.330	1484.57	6427.85
A_68_P22199719	chr3:65196469-65196513	NM_026155:-15	Ssr3	PROMOTER	2.746	17.796	15091.40	268560.50	48.865	7125.09	348164.30

ProbeName	Target position of probe on CpG island microarray	TargetID	GeneSymbol	CpG island Description	Ratio of relative methylation (TiO_2-NP/Vehicle)	Sham group			TiO_2-H group		
						Relative methylation (Cy5/Cy3)	Cy3 signal (Input)	Cy5 signal (MeDIP)	Relative methylation (Cy5/Cy3)	Cy3 signal (Input)	Cy5 signal (MeDIP)
A_68_P27129526	chr10:50312500-50312544	NM_001146089:48	Ascc3	INSIDE	2.737	7.551	1717.70	12970.13	20.665	1310.39	27079.84
A_68_P22531299	chr3:130975017-130975061	NM_008212:-83	Hadh	PROMOTER	2.737	10.628	15469.42	164409.20	29.089	9081.46	264171.40
A_68_P23567791	chr5:29805138-29805182	NM_019944:-150	Mnx1	PROMOTER	2.737	4.281	2107.37	9020.61	11.717	1689.85	19799.24
A_68_P20412166	chr1:88338644-88338688	AK089549:7401		DOWNSTREAM	2.737	6.103	1274.33	7777.15	16.703	910.84	15214.20
A_68_P21582988	chr2:121632726-121632770	NM_172673:45	Frmd5	INSIDE	2.734	8.279	6123.14	50694.51	22.637	3823.30	86547.30
A_68_P26167383	chr8:113783057-113783101	NM_009149:24	Glg1	INSIDE	2.733	6.404	3079.88	19722.76	17.500	1937.32	33903.41
A_68_P24686653	chr6:99472198-99472242	ENSMUST00000155466:144571		INSIDE	2.730	8.343	2538.43	21179.35	22.775	1588.22	36171.95
A_68_P21969040	chr3:17955766-17955810	NM_021560:1464	Bhlhe22	INSIDE	2.727	11.831	7289.10	86236.40	32.261	4657.85	150267.20
A_68_P24973879	chr7:13551564-13551609	NM_178732:374	Zfp324	INSIDE	2.727	10.119	12804.14	129564.20	27.590	6837.10	188638.10
A_68_P20457532	chr1:95700895-95700939	NM_025454:375	Ing5	INSIDE	2.724	6.716	2820.91	18944.70	18.295	1637.99	29967.65
A_68_P20184680	chr1:42755426-42755470	NM_008900:1458	Pou3f3	INSIDE	2.714	6.638	5306.77	35227.30	18.016	3602.30	64898.50
A_68_P26878663	chr9:120486371-120486416	NM_025974:5760	Rpl14	DOWNSTREAM	2.712	1.510	1244.63	1879.28	4.095	974.70	3991.69
A_68_P25992690	chr8:81636588-81636632	NM_178267:84275	Zfp827	INSIDE	2.710	18.337	18328.51	336097.60	49.688	10239.56	508780.30
A_68_P31472616	chr18:6241499-6241543	NM_008448:2	Kif5b	INSIDE	2.705	13.405	1618.85	21700.12	36.255	1036.17	37566.28
A_68_P26466435	chr9:44041858-44041902	NM_007619:249	Cbl	INSIDE	2.703	8.729	4609.01	40229.81	23.595	3069.96	72435.80
A_68_P20644107	chr1:138036961-138037006	NM_001039472:9006	Kif21b	INSIDE	2.698	10.540	4945.35	52121.62	28.439	3189.20	90698.85
A_68_P24092948	chr5:130731326-130731370	NM_023248:-16	Sbds	DIVERGENT_PROMOTER	2.697	2.449	2368.30	5800.12	6.606	1776.61	11735.43
A_68_P20094994	chr1:24685459-24685503	NM_026719:98	Lmbrd1	INSIDE	2.688	20.375	4317.54	87970.49	54.775	2990.87	163823.90
A_68_P31126969	chr17:29401134-29401178	NM_026845:-240	Ppil1	DIVERGENT_PROMOTER	2.682	11.142	1321.08	14720.04	29.885	882.61	26376.44
A_68_P29329250	chr13:112279384-112279428	NM_001122963:843	Gpbp1	INSIDE	2.680	12.969	18361.71	238134.20	34.760	9847.22	342287.80
A_68_P32365466	chrX:45947510-45947554	NM_153532:-2222	Zfp280c	PROMOTER	2.676	5.021	1811.17	9094.51	13.439	945.31	12704.10
A_68_P32099204	chr19:38129403-38129447	NM_028293:-90	Cep55	PROMOTER	2.661	14.261	12747.94	181792.80	37.952	7793.33	295773.50
A_68_P21426271	chr2:91895625-91895669	ENSMUST00000123840:3880		INSIDE	2.658	8.286	5247.23	43479.63	22.024	3647.86	80341.66
A_68_P22278904	chr3:82162070-82162114	NM_001081230:99	Mtap9	INSIDE	2.656	15.147	1784.01	27022.45	40.232	980.84	39461.13
A_68_P26529572	chr9:54997117-54997161	NM_180600:-37	Ube2q2	PROMOTER	2.656	11.264	2664.79	30016.69	29.913	1875.82	56110.90
A_68_P20417972	chr1:89371861-89371906	NM_028473:5045	3110079O15Rik	INSIDE	2.652	18.741	968.94	18158.52	49.706	756.70	37611.98
A_68_P25032017	chr7:31319995-31320040	NM_178252:11	Arhgap33	INSIDE	2.650	5.817	30484.87	177343.10	15.418	21467.82	330995.40
A_68_P32396713	chrX:53862851-53862895	NM_172780:-139	Slc9a6	PROMOTER	2.649	5.416	1489.93	8069.17	14.349	796.83	11433.70
A_68_P31322348	A_68_P31322348			Unknown	2.647	7.240	1352.79	9794.43	19.167	1045.04	20030.55
A_68_P24922453	chr6:145289480-145289524			Unknown	2.645	0.564	817.78	461.17	1.492	706.91	1054.43
A_68_P20412593	chr1:88422665-88422709	NM_008972:-624	Ptma	PROMOTER	2.644	5.930	4838.46	28693.15	15.677	3042.74	47701.42
A_68_P27476522	chr10:116018238-116018282	NM_001037846:307	Cnot2	INSIDE	2.636	8.518	13218.99	112595.80	22.456	8717.08	195749.60
A_68_P25516578	chr7:136735619-136735663	NM_172255:264	Wdr11	INSIDE	2.632	0.212	1316.65	279.76	0.559	1149.78	642.91
A_68_P28103521	chr11:106649919-106649963	NM_007840:-132	Ddx5	DIVERGENT_PROMOTER	2.625	6.666	17960.68	119723.90	17.499	11517.85	201546.70
A_68_P21490977	chr2:104656727-104656771	NM_001123327:105	Qser1	INSIDE	2.622	8.015	3488.35	27959.02	21.018	2688.77	56512.11
A_68_P29856568	chr14:102128930-102128974	NM_201529:-192	Lmo7	PROMOTER	2.621	10.226	4507.02	46087.56	26.806	2966.50	79519.01
A_68_P32796350	chrX:160514136-160514180	NM_009173:-177	Siah1b	PROMOTER	2.621	3.643	1852.28	6747.79	9.547	1061.03	10129.19
A_68_P28099478	chr11:105946211-105946255	NM_011947:17	Map3k3	INSIDE	2.617	6.441	23400.69	150731.30	16.857	15983.49	269440.70
A_68_P23146552	chr4:99602526-99602570	NM_028132:493	Pgm2	INSIDE	2.610	5.642	12527.75	70679.57	14.726	8253.81	121548.50
A_68_P21076600	chr2:25996203-25996247	ENSMUST00000133808:-183		PROMOTER	2.610	5.557	1035.44	5754.15	14.505	736.72	10685.75
A_68_P28041915	chr11:95866931-95866976	NM_009951:305	Igf2bp1	INSIDE	2.608	8.882	6026.23	53526.77	23.168	4126.34	95599.91
A_68_P31144961	chr17:32458430-32458474	NM_017476:29070	Akap8l	INSIDE	2.604	5.486	2734.95	15003.88	14.287	1952.87	27900.76
A_68_P26845381	chr9:114473232-114473276	NM_001042503:233	Trim71	INSIDE	2.604	6.462	664.84	4295.91	16.825	520.90	8764.06
A_68_P23297033	chr4:129190774-129190818	NM_010807:-28	Marcksl1	PROMOTER	2.602	5.773	10432.74	60230.91	15.025	7036.61	105722.20
A_68_P28771927	chr12:118392576-118392621	NR_033538:-2856	Gm10421	PROMOTER	2.599	4.131	733.51	3030.00	10.736	537.89	5774.98
A_68_P23763011	chr5:67009509-67009553	NM_009686:380	Apbb2	INSIDE	2.598	8.757	907.55	7947.11	22.748	649.96	14785.56
A_68_P26342514	chr9:20264309-20264353	NM_011753:274	Zfp26	INSIDE	2.597	5.833	12946.13	75517.71	15.147	9104.24	137900.20
A_68_P28053223	chr11:97744951-97744995	NM_001081435:496	Fbxo47	INSIDE	2.589	10.903	9736.38	106154.20	28.224	4882.70	137808.30
A_68_P30590967	chr16:21205377-21205421	NM_010143:531	Ephb3	INSIDE	2.587	10.916	1196.74	13063.33	28.244	837.42	23652.29
A_68_P20240462	chr1:55188131-55188175	NM_025283:64	Mobkl3	INSIDE	2.583	1.455	1093.20	1591.01	3.759	1044.28	3925.12
A_68_P27594327	chr11:11937393-11937437	NM_001177629:-9440	Grb10	PROMOTER	2.580	7.830	15526.51	121565.50	20.197	11291.17	228048.70

ProbeName	Target position of probe on CpG island microarray	TargetID	GeneSymbol	CpG island Description	Ratio of relative methylation (TiO_2-NP/Vehicle)	Sham group			TiO_2-H group		
						Relative methylation (Cy5/Cy3)	Cy3 signal (Input)	Cy5 signal (MeDIP)	Relative methylation (Cy5/Cy3)	Cy3 signal (Input)	Cy5 signal (MeDIP)
A_68_P26864195	chr9:117949694-117949738	NM_001048146:100	Azi2	INSIDE	2.577	9.490	2913.48	27648.78	24.453	2021.37	49428.26
A_68_P31458875	chr18:3507840-3507884	NM_026505:-92	Bambi	PROMOTER	2.575	17.069	2820.94	48149.43	43.952	1883.09	82765.38
A_68_P20432927	chr1:91819381-91819425	NM_010262:8349	Gbx2	DOWNSTREAM	2.570	8.925	4387.88	39160.32	22.935	2276.94	52221.25
A_68_P20170470	chr1:40323556-40323600	NM_001123382:148	Il1rl	INSIDE	2.563	9.463	1749.87	16559.53	24.254	1125.93	27308.32
A_68_P27840810	chr11:59919914-59919958	NM_009021:1422	Rai1	INSIDE	2.563	8.736	3024.48	26422.84	22.390	2287.83	51223.82
A_68_P31866219	chr18:80894683-80894727	NM_001164111:10208	Nfatc1	INSIDE	2.561	3.778	1960.18	7405.43	9.674	1497.88	14490.19
A_68_P24423934	chr6:47544675-47544719	NM_001146689:333	Ezh2	INSIDE	2.559	4.342	3868.12	16795.29	11.113	2711.43	30131.82
A_68_P21556049	chr2:116946411-116946455	NM_033524:-753	Spred1	PROMOTER	2.559	7.004	7406.18	51873.25	17.920	5638.84	101048.30
A_68_P21836350	chr2:167423298-167423342	NM_023230:34185	Ube2v1	DOWNSTREAM	2.559	1.736	6057.44	10517.36	4.443	3948.40	17542.62
A_68_P32453217	chrX:68617827-68617871	NM_016985:-86	Mtmr1	PROMOTER	2.555	3.098	2572.42	7970.05	7.916	1433.82	11349.50
A_68_P33007166	A_68_P33007166			Unknown	2.552	11.771	2314.31	27242.83	30.035	1471.56	44198.88
A_68_P24764862	chr6:114232614-114232658	NM_178703:8	Slc6a1	INSIDE	2.549	17.190	6602.31	113494.20	43.825	3404.89	149218.90
A_68_P24052414	chr5:123270992-123271036	NM_001042491:334	Anapc5	INSIDE	2.546	24.473	4557.84	111543.90	62.308	2482.58	154683.20
A_68_P30567429	chr16:15887235-15887279	NM_007679:-122	Cebpd	PROMOTER	2.546	13.072	7367.64	96311.68	33.284	5181.40	172455.10
A_68_P25736450	chr8:28196606-28196651	NM_054044:316	Gpr124	INSIDE	2.545	10.594	3452.96	36580.26	26.957	2485.27	66994.82
A_68_P27358653	chr10:93774633-93774677	ENSMUST00000147869:-39		PROMOTER	2.542	4.982	10801.78	53819.71	12.664	7829.95	99155.27
A_68_P32576281	chrX:101395430-101395474	NM_001077354:1004	C77370	INSIDE	2.536	5.175	658.04	3405.35	13.124	385.33	5057.16
A_68_P25094516	chr7:52949587-52949631	NM_001033356:9825	Ntn5	INSIDE	2.535	6.663	3395.28	22623.71	16.894	2411.55	40740.20
A_68_P23272336	chr4:124804630-124804674	NM_153159:473	Zc3h12a	INSIDE	2.534	8.868	9988.09	88569.59	22.468	6409.08	143996.90
A_68_P22973925	chr4:63678841-63678885	NM_011607:29187	Tnc	INSIDE	2.532	7.689	1740.85	13385.58	19.465	1214.65	23643.61
A_68_P20416757	chr1:89160862-89160906	NM_028889:-54	Efhd1	PROMOTER	2.531	6.089	109823.20	668703.40	15.413	67870.99	1046093.00
A_68_P31151615	chr17:34168722-34168766	NM_001205214:-12	Rxrb	DIVERGENT_PROMOTER	2.525	2.326	584.39	1359.19	5.873	474.20	2785.18
A_68_P32717145	chrX:140752482-140752526	NM_026247:-4	Alg13	PROMOTER	2.523	6.564	4083.98	26807.27	16.561	2514.84	41647.48
A_68_P32684276	chrX:133653076-133653120	NM_007957:1767	Esx1	INSIDE	2.514	6.193	1542.14	9549.78	15.565	819.74	12759.33
A_68_P31220008	chr17:49071618-49071662	NM_027452:-266	Lrfn2	PROMOTER	2.514	5.727	9111.94	52180.14	14.397	5782.67	83254.10
A_68_P27050627	chr10:32609677-32609721	NM_001013411:23	Nkain2	INSIDE	2.512	7.636	4966.00	37922.42	19.182	3697.67	70929.86
A_68_P24141205	chr5:140865282-140865326	NM_172277:-103	Snx8	PROMOTER	2.512	5.782	13712.53	79285.45	14.522	8932.21	129716.30
A_68_P25522110	chr7:137662756-137662800	NM_001136054:266	Ate1	INSIDE	2.507	2.149	975.56	2096.60	5.388	765.52	4124.45
A_68_P31542491	chr18:21160534-21160578	NM_019706:715	Rnf138	INSIDE	2.507	6.232	7347.22	45784.44	15.622	4984.46	77866.60
A_68_P26469100	chr9:44516622-44516666	NM_153537:26633	Phldb1	INSIDE	2.502	21.012	5164.24	108511.90	52.574	2894.18	152159.80
A_68_P21587981	chr2:122591010-122591054	NM_001162503:-62	Sqrdl	PROMOTER	2.501	7.553	2476.02	18700.67	18.891	1784.22	33705.88
A_68_P27265507	chr10:76495860-76495904	NM_031196:-74	Slc19a1	PROMOTER	2.499	9.071	5096.34	46231.10	22.666	3101.46	70297.76
A_68_P27138494	chr10:52410512-52410556	NM_178675:228	Slc35f1	INSIDE	2.495	3.924	618.16	2425.63	9.792	614.22	6014.29
A_68_P25031610	chr7:31242110-31242154	NM_172898:402	Kirrel2	INSIDE	2.494	15.068	2274.51	34272.62	37.583	1235.22	46423.38
A_68_P26541141	chr9:57109407-57109451	NM_028820:978	1700017B05Rik	INSIDE	2.493	5.134	2336.99	11998.43	12.798	1913.17	24484.04
A_68_P22310075	chr3:87600813-87600858	NM_011832:-37	Insrr	DIVERGENT_PROMOTER	2.490	5.861	4961.28	29078.26	14.593	3144.29	45884.25
A_68_P26465911	chr9:43943262-43943306	NM_023061:544	Mcam	INSIDE	2.490	3.450	1701.35	5869.09	8.589	1308.16	11235.64
A_68_P29624689	chr14:57396463-57396507	NM_025682:669	Pspc1	INSIDE	2.487	5.474	4853.09	26567.70	13.612	3701.11	50381.05
A_68_P28332409	chr12:32319733-32319777	NM_001164090:-231	Bcap29	PROMOTER	2.486	9.592	2652.51	25441.57	23.849	1708.35	40742.33
A_68_P30656969	chr16:33251415-33251459	NM_080557:-105	Snx4	PROMOTER	2.486	10.407	3440.30	35804.83	25.873	2337.93	60489.97
A_68_P32553143	chrX:96238297-96238345	NM_199018:401	Stard8	INSIDE	2.484	6.859	445.03	3052.42	17.039	246.79	4205.01
A_68_P29218716	chr13:91599885-91599929	NM_024186:-795	Ssbp2	PROMOTER	2.476	16.971	563.62	9565.23	42.025	488.52	20530.01
A_68_P29338034	chr13:113777821-113777865	NM_001081062:-167	Ccno	PROMOTER	2.475	3.687	601.99	2219.36	9.124	484.42	4420.08
A_68_P24449884	chr6:52266647-52266691	NR_038163:-1842	5730457N03Rik	PROMOTER	2.474	11.257	8241.17	92768.87	27.844	5150.16	143401.90
A_68_P29643100	chr14:60795943-60795987	ENSMUST00000140924:412		INSIDE	2.473	5.834	4663.00	27202.57	14.424	3814.13	55015.58
A_68_P23397281	chr4:148275240-148275284	NM_027195:96762	Casz1	INSIDE	2.472	7.602	17403.01	132294.10	18.791	11859.44	222849.10
A_68_P29237600	chr13:95653897-95653941	NM_011021:8337	Otp	DOWNSTREAM	2.471	2.116	4449.75	9414.95	5.227	3177.96	16612.19
A_68_P31003040	chr17:3084893-3084937	NR_003519:-731	Pisd-ps2	PROMOTER	2.471	9.924	20698.53	205421.40	24.520	13630.62	334218.10
A_68_P30784285	chr16:57122018-57122062	NM_138599:214	Tomm70a	INSIDE	2.470	9.905	4797.46	47520.59	24.470	3311.14	81023.28
A_68_P21569910	chr2:119303067-119303111	NM_026574:277	Ino80	INSIDE	2.468	6.138	4882.70	29972.16	15.151	3573.68	54146.25
A_68_P20765302	chr1:162125324-162125368	NM_025474:-44	Mrps14	PROMOTER	2.468	10.418	5914.69	61622.12	25.708	3725.32	95770.00

ProbeName	Target position of probe on CpG island microarray	TargetID	GeneSymbol	CpG island Description	Ratio of relative methylation (TiO_2-NP/Vehicle)	Sham group			TiO_2-H group		
						Relative methylation (Cy5/Cy3)	Cy3 signal (Input)	Cy5 signal (MeDIP)	Relative methylation (Cy5/Cy3)	Cy3 signal (Input)	Cy5 signal (MeDIP)
A_68_P26292118	chr9:8544562-8544606	NM_013838:443	Trpc6	INSIDE	2.466	3.342	924.53	3089.86	8.241	744.87	6138.87
A_68_P26742258	chr9:95412445-95412489	NM_001114977:-51	2610101N10Rik	PROMOTER	2.465	9.638	28756.99	277163.20	23.754	16578.52	393802.70
A_68_P30350128	chr15:76540931-76540975	NM_058214:37	Recql4	INSIDE	2.464	4.532	3611.54	16368.01	11.166	2597.51	29002.98
A_68_P26164568	chr8:113262460-113262504	NM_198625:17099	Mtss1l	INSIDE	2.463	10.638	6103.81	64933.75	26.205	4061.40	106430.50
A_68_P28605007	chr12:86940319-86940363	NM_030887:-25	Jdp2	PROMOTER	2.462	7.922	7190.55	56964.74	19.503	5194.50	101306.80
A_68_P32471622	chrX:72661675-72661719	NM_010839:186	Mtcp1	INSIDE	2.460	3.523	1439.66	5072.36	8.668	783.74	6793.80
A_68_P31618899	chr18:35721824-35721868	NM_010771:35	Matr3	INSIDE	2.459	1.495	1916.90	2865.17	3.676	1857.74	6829.22
A_68_P31934224	chr19:6364358-6364402	NM_001110791:691	Sf1	INSIDE	2.459	12.680	1791.20	22713.16	31.175	1325.53	41323.44
A_68_P22408449	chr3:107563183-107563227	NM_001113529:183	Csf1	INSIDE	2.457	11.554	10695.95	123580.20	28.391	6189.87	175737.30
A_68_P32817209	chrX:165111905-165111949	NM_010832:-92	Msl3	PROMOTER	2.457	2.364	835.90	1976.12	5.809	516.85	3002.34
A_68_P32287119	chrX:19637488-19637532	NM_021715:815	Chst7	INSIDE	2.452	3.851	2163.89	8333.81	9.443	1159.22	10946.07
A_68_P32385147	chrX:50341550-50341594	NM_013556:318	Hprt	INSIDE	2.451	3.348	1957.19	6553.16	8.208	1013.19	8316.29
A_68_P30575400	chr16:17734120-17734164			Unknown	2.447	6.556	12299.05	80630.62	16.041	9298.61	149159.30
A_68_P22888054	chr4:46318111-46318155	ENSMUST00000123933:38171		INSIDE	2.447	4.268	1176.07	5019.52	10.445	855.56	8936.54
A_68_P27688946	chr11:32355594-32355638	NM_173784:245	Ubtd2	INSIDE	2.445	5.514	5760.64	31763.89	13.479	4170.34	56213.19
A_68_P21762364	chr2:154617306-154617350	NM_001139511:483	Raly	INSIDE	2.443	9.092	3562.21	32388.11	22.209	2473.01	54924.23
A_68_P27293831	chr10:82275065-82275109	NR_038042:219	1700028I16Rik	INSIDE	2.437	6.388	11619.79	74232.92	15.569	8124.71	126492.50
A_68_P22921606	chr4:53172577-53172621	NM_013454:169	Abca1	INSIDE	2.437	2.829	163.57	462.78	6.896	94.45	651.32
A_68_P27315977	chr10:86241971-86242015	NM_175331:243	Nt5dc3	INSIDE	2.437	8.680	2362.38	20505.09	21.157	1771.77	37484.74
A_68_P23418024	chr4:151700327-151700371	NM_009079:362	Rpl22	INSIDE	2.437	4.492	1567.13	7039.64	10.946	1298.21	14210.70
A_68_P31427919	A_68_P31427919			Unknown	2.436	9.712	763.42	7414.61	23.661	575.48	13616.52
A_68_P27862290	chr11:63734698-63734742	NM_018805:1066	Hs3st3b1	INSIDE	2.430	7.416	3406.97	25266.90	18.019	2541.56	45797.47
A_68_P23222249	chr4:114580688-114580732	NM_008593:793	Foxd2	INSIDE	2.427	6.487	1921.84	12467.91	15.742	1486.86	23406.71
A_68_P28585119	chr12:83270170-83270214	NM_001167983:-810	Sipa1l1	PROMOTER	2.427	9.389	1250.51	11740.61	22.786	826.90	18841.71
A_68_P26085413	chr8:98330883-98330927	NR_015574:175	4930513N10Rik	INSIDE	2.426	7.226	17038.37	123120.90	17.533	11905.60	208738.70
A_68_P25456593	chr7:125259495-125259539	NM_170669:145	Rps15a	INSIDE	2.426	5.742	1683.11	9663.99	13.927	1116.17	15544.89
A_68_P27951947	chr11:79787924-79787968			Unknown	2.425	2.515	1153.67	2901.97	6.099	1034.23	6307.86
A_68_P21890601	chr2:178148869-178148913	NM_001085501:287	Ppp1r3d	INSIDE	2.424	10.660	2909.91	31019.30	25.839	1951.87	50435.31
A_68_P32766765	chrX:153929388-153929432	NM_009214:568	Sms	INSIDE	2.423	5.494	1673.83	9195.71	13.311	1054.97	14042.84
A_68_P32674751	chrX:131543547-131543591	NM_175446:180	Zmat1	INSIDE	2.422	19.742	5109.23	100864.00	47.809	3031.64	144939.50
A_68_P21143643	chr2:37559408-37559452	ENSMUST00000155237:-51		PROMOTER	2.422	21.024	2553.66	53687.95	50.919	1577.89	80345.03
A_68_P27938553	chr11:77421084-77421128	NM_144825:211	Taok1	INSIDE	2.421	10.195	1625.76	16575.32	24.688	1096.06	27059.37
A_68_P21100500	chr2:29821292-29821336	NM_001076554:235	Spna2	INSIDE	2.415	5.503	3190.19	17554.30	13.290	2465.20	32762.30
A_68_P20768477	chr1:162723220-162723264	NM_013862:-173	Rabgap1l	PROMOTER	2.414	12.018	13257.93	159330.70	29.007	7275.12	211026.50
A_68_P28688419	chr12:103150855-103150899	NM_028446:505	Trip11	INSIDE	2.414	4.319	6889.77	29759.31	10.425	5098.42	53151.19
A_68_P23288229	chr4:127665953-127665997	ENSMUST00000144298:687		INSIDE	2.412	4.500	4613.46	20761.36	10.856	3159.82	34303.98
A_68_P29871680	chr14:104872642-104872686	NM_011143:-5448	Pou4f1	PROMOTER	2.411	11.000	6367.29	70042.71	26.520	4744.22	125818.80
A_68_P20239578	chr1:54983378-54983422	NM_001081433:-169	Ankrd44	PROMOTER	2.409	2.608	6346.65	16554.50	6.284	4761.34	29920.98
A_68_P31544271	chr18:21458929-21458973	NM_001033445:-310	Fam59a	PROMOTER	2.408	7.221	15083.00	108913.00	17.386	9309.07	161852.10
A_68_P32260801	chrX:11735963-11736007	NM_001168321:1497	Bcor	INSIDE	2.406	5.141	2235.03	11489.89	12.371	1508.11	18656.45
A_68_P30572936	chr16:17276365-17276409	NM_177473:-6	Tmem191c	PROMOTER	2.406	10.155	1116.24	11334.84	24.427	920.55	22486.71
A_68_P30550104	chr16:11984103-11984147	NM_001174086:-81	Shisa9	PROMOTER	2.405	2.187	9712.97	21244.19	5.261	6807.54	35811.66
A_68_P27282935	A_68_P27282935			Unknown	2.403	14.380	970.24	13952.13	34.556	611.57	21133.49
A_68_P23594805	chr5:34868409-34868453	NM_011893:-2	Sh3bp2	PROMOTER	2.402	20.833	5614.36	116962.20	50.042	3130.32	156648.20
A_68_P32441102	chrX:65932146-65932191	NM_008031:439	Fmr1	INSIDE	2.396	4.896	1079.66	5286.01	11.733	634.73	7447.07
A_68_P24820868	chr6:125444847-125444891	NM_007657:-95	Cd9	PROMOTER	2.390	11.281	9567.66	107930.20	26.957	5477.58	147659.80
A_68_P29455876	chr14:22805255-22805299	NM_145459:3547	Zfp503	INSIDE	2.388	4.903	8384.65	41113.51	11.710	5045.05	59078.81
A_68_P24089922	chr5:130089988-130090034	NM_011846:-78	Mmp17	PROMOTER	2.386	10.764	5163.23	55575.58	25.684	3039.76	78072.02
A_68_P31117105	chr17:27693117-27693161	NM_001025427:-380	Hmga1	PROMOTER	2.385	5.460	803.42	4386.29	13.019	638.81	8316.76
A_68_P22412541	chr3:108218746-108218790	NM_001004177:-356	Celsr2	PROMOTER	2.381	3.887	1745.81	6786.45	9.257	1460.57	13520.87
A_68_P28043617	chr11:96166483-96166527	NM_008268:1679	Hoxb5	INSIDE	2.377	2.543	7392.87	18798.29	6.045	5490.85	33193.03

ProbeName	Target position of probe on CpG island microarray	TargetID	GeneSymbol	CpG island Description	Ratio of relative methylation (TiO_2-NP/Vehicle)	Sham group			TiO_2-H group		
						Relative methylation (Cy5/Cy3)	Cy3 signal (Input)	Cy5 signal (MeDIP)	Relative methylation (Cy5/Cy3)	Cy3 signal (Input)	Cy5 signal (MeDIP)
A_68_P28051732	chr11:97489553-97489597	NM_175332:1456	E130012A19Rik	INSIDE	2.375	10.917	6860.96	74899.38	25.922	4341.43	112540.60
A_68_P28069996	chr11:100621272-100621316	NM_001081194:-202	Kcnh4	PROMOTER	2.374	4.449	752.81	3349.60	10.562	555.22	5864.15
A_68_P23278482	chr4:125826960-125827005	NM_172145:1736	Fam176b	INSIDE	2.373	12.234	2471.48	30237.07	29.028	1293.78	37556.21
A_68_P25770033	chr8:34764110-34764154	NM_010344:423	Gsr	INSIDE	2.372	5.357	3869.14	20727.80	12.708	3194.58	40595.16
A_68_P31201770	chr17:45692568-45692612	NM_008690:-74	Nfkbie	DIVERGENT_PROMOTER	2.372	9.998	12623.07	126210.50	23.715	8032.57	190492.00
A_68_P22532055	chr3:131153686-131153730			Unknown	2.371	10.441	2576.84	26904.95	24.751	1612.79	39917.49
A_68_P32760538	chrX:152057229-152057273	NM_001093750:620	Ptchd1	INSIDE	2.370	7.084	3332.40	23607.79	16.787	2229.53	37426.19
A_68_P28936016	chr13:34094703-34094748	NM_009068:-17	Ripk1	DIVERGENT_PROMOTER	2.369	5.834	3745.72	21850.67	13.820	2666.49	36851.95
A_68_P25372610	chr7:108469852-108469896	NM_019990:42	Stard10	INSIDE	2.367	5.981	15543.06	92958.13	14.156	11160.96	157990.00
A_68_P26886028	chr9:121766761-121766805	NM_001112668:-133	Gm9790	PROMOTER	2.363	5.533	4191.24	23188.72	13.072	3175.11	41505.89
A_68_P27781641	chr11:48982982-48983026	NM_172793:17587	Btnl9	INSIDE	2.360	0.624	1571.69	981.26	1.473	1783.94	2628.61
A_68_P24381989	chr6:39155596-39155640	NM_001033430:1154	Jhdm1d	INSIDE	2.359	5.189	5116.70	26550.66	12.238	3987.22	48797.57
A_68_P27536264	chr10:126948850-126948894	NM_001098789:-3122	Ndufa4l2	PROMOTER	2.359	3.890	1188.21	4622.23	9.177	968.88	8890.91
A_68_P21017263	chr2:13495869-13495913	NM_011701:-47	Vim	PROMOTER	2.359	3.510	257.85	905.15	8.280	190.22	1574.90
A_68_P32533286	chrX:90544628-90544672	NM_007492:12666	Arx	DOWNSTREAM	2.358	4.450	2021.28	8993.94	10.492	1357.54	14243.46
A_68_P28567140	chr12:80108151-80108195	NM_178745:92	Tmem229b	INSIDE	2.358	6.724	9582.42	64433.40	15.858	7192.83	114067.00
A_68_P32578106	chrX:101866324-101866368	NM_175358:57	Zdhhc15	INSIDE	2.357	1.895	739.72	1402.08	4.467	457.94	2045.51
A_68_P28149790	chr11:114652964-114653008	NM_028055:4220	Btbd17	INSIDE	2.356	6.334	2909.45	18427.57	14.919	2289.11	34151.24
A_68_P31936825	chr19:7016840-7016884	NM_001177361:78	Gpr137	INSIDE	2.355	1.625	788.65	1281.49	3.826	596.15	2280.83
A_68_P23777458	chr5:69732840-69732884	NM_175519:86	Kctd8	INSIDE	2.354	17.034	6463.80	110101.70	40.098	4087.66	163905.10
A_68_P32268347	chrX:13423633-13423677	NM_009806:13	Cask	INSIDE	2.352	4.775	4033.45	19260.09	11.231	2219.51	24926.78
A_68_P24590984	chr6:82983529-82983573	NM_010070:-85	Dok1	DIVERGENT_PROMOTER	2.352	3.562	1137.34	4051.68	8.379	847.92	7104.88
A_68_P32563826	chrX:98580104-98580148	NM_008124:7451	Gjb1	INSIDE	2.352	3.539	937.42	3317.78	8.324	424.00	3529.44
A_68_P31256333	chr17:56601586-56601630	NM_011218:14295	Ptprs	INSIDE	2.352	1.518	961.67	1459.97	3.570	669.84	2391.35
A_68_P31311926	chr17:67704196-67704240	NM_008984:-419	Ptprm	PROMOTER	2.351	6.912	3804.89	26300.93	16.250	2810.62	45673.54
A_68_P30650943	chr16:32099956-32100000	NM_025892:91	1500031L02Rik	INSIDE	2.348	3.775	7748.81	29255.63	8.866	5745.28	50940.39
A_68_P26827666	chr9:111020550-111020594	NM_001164838:-42	Lrrfip2	PROMOTER	2.347	5.128	3893.76	19967.23	12.037	2948.94	35497.62
A_68_P29032310	chr13:51503858-51503902	NM_010101:-106	S1pr3	PROMOTER	2.347	4.881	1406.71	6866.52	11.458	969.33	11106.33
A_68_P22299774	chr3:85691661-85691705	NM_177130:-242	Glt28d2	PROMOTER	2.345	6.115	2137.83	13071.99	14.340	1643.46	23567.60
A_68_P20741945	chr1:158134987-158135031	NM_177838:148	Fam163a	INSIDE	2.344	0.728	1936.10	1410.28	1.708	1837.68	3138.08
A_68_P21109951	chr2:31428059-31428103	NM_001033389:-164	Fubp3	PROMOTER	2.343	13.413	1326.63	17793.56	31.430	941.46	29589.95
A_68_P27569379	chr11:6505657-6505701	NM_001081652:379	Nacad	INSIDE	2.343	8.268	5339.75	44151.53	19.370	3628.25	70280.29
A_68_P24604715	chr6:85324050-85324094	NM_001003955:556	Rab11fip5	INSIDE	2.343	8.621	4194.89	36164.41	20.197	2530.98	51118.48
A_68_P25505713	chr7:134615484-134615528	NM_145589:379	Prr14	INSIDE	2.340	4.798	2228.95	10694.08	11.228	1578.60	17725.31
A_68_P31112418	chr17:26918319-26918363	NM_172149:317	Bnip1	INSIDE	2.338	3.275	5084.44	16652.05	7.656	3514.57	26906.28
A_68_P32365934	chrX:46048429-46048473	NM_173376:270	Rbmx2	INSIDE	2.338	12.123	3931.16	47655.81	28.346	2266.75	64254.35
A_68_P20556967	chr1:121317480-121317524	NM_008381:1323	Inhbb	INSIDE	2.337	6.799	1585.18	10777.19	15.888	1233.25	19593.83
A_68_P24039940	chr5:121081339-121081383	NM_001195094:2884	Ccdc42b	INSIDE	2.335	2.020	661.34	1335.89	4.717	629.67	2969.92
A_68_P29958338	chr14:122278102-122278146	NM_026861:297	Ubac2	INSIDE	2.334	4.232	2780.86	11768.46	9.877	2012.80	19879.47
A_68_P23244381	chr4:118905195-118905239	NM_001012400:113	AU022252	INSIDE	2.333	2.690	5994.47	16124.96	6.275	4417.41	27719.49
A_68_P21621794	chr2:128690006-128690050	NM_024237:361	Fbln7	INSIDE	2.332	1.496	749.56	1121.25	3.489	627.27	2188.52
A_68_P25645821	chr8:9976314-9976358	NM_176953:-13	Lig4	DIVERGENT_PROMOTER	2.332	7.229	11513.48	83233.88	16.857	7753.00	130696.00
A_68_P28989794	chr13:43460884-43460928	NM_178848:-5178	Sirt5	PROMOTER	2.332	3.269	1508.13	4930.73	7.626	1101.35	8398.55
A_68_P21334154	chr2:73729891-73729935	NM_001025093:773	Atf2	INSIDE	2.330	9.939	23555.36	234127.40	23.161	14168.05	328151.80
A_68_P31373358	chr17:79136029-79136073	NM_011500:-150	Strn	PROMOTER	2.327	2.976	1194.14	3553.45	6.925	955.41	6616.44
A_68_P31428327	chr17:88929344-88929388	NM_028658:-97	Klraq1	PROMOTER	2.326	8.666	2680.12	23227.16	20.154	1767.89	35629.85
A_68_P24004683	chr5:115017800-115017844	NM_001004180:-437	BC057022	PROMOTER	2.324	15.416	3357.74	51764.02	35.833	2417.21	86615.99
A_68_P27564951	chr11:5688365-5688409	NM_001146308:-99	Dbnl	PROMOTER	2.322	6.341	2538.53	16095.66	14.726	1987.15	29262.25
A_68_P23336241	chr4:136391071-136391115	NM_010142:758	Ephb2	INSIDE	2.322	3.395	3359.27	11404.09	7.883	2536.99	19998.83
A_68_P25016756	chr7:28119811-28119855	NM_001113549:-1165	Ltbp4	PROMOTER	2.321	7.348	4064.11	29862.64	17.053	2879.70	49106.29
A_68_P25009065	chr7:26004933-26004977	NM_207677:-76	Dedd2	DIVERGENT_PROMOTER	2.316	2.652	8195.44	21737.08	6.143	5505.81	33821.86

ProbeName	Target position of probe on CpG island microarray	TargetID	GeneSymbol	CpG island Description	Ratio of relative methylation (TiO_2-NP/Vehicle)	Sham group			TiO_2-H group		
						Relative methylation (Cy5/Cy3)	Cy3 signal (Input)	Cy5 signal (MeDIP)	Relative methylation (Cy5/Cy3)	Cy3 signal (Input)	Cy5 signal (MeDIP)
A_68_P28530101	chr12:73186989-73187033	NM_001205067:185	Jkamp	INSIDE	2.316	6.814	2167.14	14767.70	15.779	1323.51	20883.40
A_68_P27095209	chr10:42222002-42222046	NM_017472:165	Snx3	INSIDE	2.314	4.285	1119.07	4795.47	9.918	862.62	8555.08
A_68_P32260371	chrX:11668288-11668332	NM_001168321:69171	Bcor	INSIDE	2.313	1.587	1844.59	2926.56	3.669	931.85	3419.40
A_68_P23980413	chr5:110973506-110973550	NM_198306:165	Galnt9	INSIDE	2.313	2.123	903.04	1916.98	4.911	837.52	4113.03
A_68_P22951804	chr4:58566187-58566231	NM_172989:-43	Lpar1	PROMOTER	2.313	12.789	2033.78	26009.86	29.587	1471.32	43531.41
A_68_P28997682	chr13:44829049-44829093	NM_001205043:2431	Jarid2	INSIDE	2.312	8.821	15712.06	138592.40	20.390	9201.23	187610.30
A_68_P31144954	chr17:32457616-32457660	NM_019774:460	Akap8	INSIDE	2.308	6.815	5331.38	36334.12	15.732	3796.01	59720.41
A_68_P20149055	chr1:36615091-36615135	NM_001126047:114	Sema4c	INSIDE	2.308	2.850	1328.56	3786.16	6.578	972.00	6393.64
A_68_P32340723	chrX:39421307-39421351	NM_009688:316	Xiap	INSIDE	2.308	3.837	2381.77	9138.39	8.854	1191.40	10548.87
A_68_P32365932	chrX:46048174-46048218	NM_173376:16	Rbmx2	INSIDE	2.307	1.483	755.30	1119.85	3.421	464.12	1587.86
A_68_P29342439	chr13:114584551-114584595	NM_172595:-143	Arl15	PROMOTER	2.306	6.894	13305.65	91725.86	15.896	9055.61	143951.40
A_68_P25176629	chr7:71518985-71519029	NM_177893:-25	Fan1	PROMOTER	2.306	4.347	1205.52	5239.99	10.023	908.82	9109.01
A_68_P27302613	chr10:83903670-83903714	NM_001004363:-476	Nuak1	PROMOTER	2.305	2.296	907.95	2084.57	5.293	794.46	4204.96
A_68_P32260816	chrX:11737647-11737691	NM_001168321:-187	Bcor	PROMOTER	2.303	3.008	999.15	3005.25	6.928	525.11	3638.03
A_68_P30596436	chr16:22163233-22163277	NM_183029:118	Igf2bp2	INSIDE	2.303	7.747	847.67	6567.12	17.840	768.85	13716.50
A_68_P21571253	chr2:119569096-119569140	NM_146125:1046	Itpka	INSIDE	2.303	3.874	2504.23	9700.66	8.922	1955.76	17450.11
A_68_P21013699	chr2:12932692-12932736	NM_153155:-223	C1ql3	PROMOTER	2.302	10.537	2128.50	22428.25	24.254	1494.20	36239.69
A_68_P32263512	chrX:12339036-12339080	NM_001048208:41	Med14	INSIDE	2.302	6.479	885.86	5739.19	14.914	563.91	8410.04
A_68_P27856224	chr11:62692825-62692869	NM_026210:-145	Fam18b	PROMOTER	2.301	8.002	44876.32	359089.20	18.410	25812.74	475218.60
A_68_P22085568	chr3:41368782-41368826	NM_001130186:3	Phf17	INSIDE	2.300	18.229	4332.09	78968.20	41.924	2483.05	104099.50
A_68_P32683852	chrX:133584183-133584227	NM_146261:72	Fam199x	INSIDE	2.299	5.605	36252.91	203197.50	12.886	25460.36	328071.30
A_68_P21820794	chr2:164793741-164793785	NM_020333:275	Slc12a5	INSIDE	2.299	8.368	8253.59	69064.27	19.241	5190.38	99869.07
A_68_P28183801	chr11:120166516-120166560			Unknown	2.298	12.608	2249.12	28357.44	28.973	1397.42	40487.78
A_68_P23262658	chr4:123089142-123089186	NM_001167918:-10	D830031N03Rik	PROMOTER	2.296	9.442	1728.54	16320.16	21.680	1397.75	30303.05
A_68_P32240004	chrX:7327982-7328026	NR_030454:-2210	Mir684-1	DIVERGENT_PROMOTER	2.296	3.799	1605.13	6098.03	8.723	894.64	7803.66
A_68_P31253474	chr17:56175980-56176024	NM_013664:-3	Sh3gl1	DIVERGENT_PROMOTER	2.295	5.057	8492.46	42943.44	11.605	6803.83	78960.70
A_68_P26658155	chr9:77956059-77956103	NM_023605:383	Fbxo9	INSIDE	2.294	9.040	3947.31	35683.72	20.740	3181.35	65981.75
A_68_P29055232	chr13:55574226-55574270	NM_001030296:8621	Prr7	INSIDE	2.293	2.788	1306.19	3641.47	6.394	1054.80	6744.14
A_68_P31838828	chr18:75857092-75857136	NM_201354:236	Ctif	INSIDE	2.291	9.102	4442.49	40434.40	20.853	3496.62	72913.37
A_68_P24449533	chr6:52211079-52211123	NM_008264:-226	Hoxa13	PROMOTER	2.290	6.551	2969.10	19450.39	15.000	2271.12	34065.81
A_68_P23325610	chr4:134487232-134487276	NM_026780:360	Syf2	INSIDE	2.288	21.890	11179.55	244721.20	50.094	7846.17	393047.30
A_68_P31764312	chr18:62708242-62708286	NM_134136:133	Fbxo38	INSIDE	2.287	2.829	9614.08	27195.70	6.469	6425.64	41568.23
A_68_P25766302	chr8:33994797-33994841	NM_001177589:65680	Gm3985	DOWNSTREAM	2.287	9.404	1069.61	10058.46	21.510	750.44	16141.86
A_68_P20455835	chr1:95374821-95374865	NM_133808:543	Hdlbp	INSIDE	2.286	5.679	1908.50	10839.24	12.985	1421.38	18456.94
A_68_P26772513	chr9:100935215-100935259	NM_025835:58	Pccb	INSIDE	2.286	4.099	7042.17	28866.25	9.370	5402.54	50619.91
A_68_P25809870	chr8:42325280-42325324	NM_023662:190	Pcm1	INSIDE	2.286	10.549	531.79	5609.94	24.115	415.82	10027.66
A_68_P25499304	chr7:133344026-133344070	NM_028816:-126	Xpo6	PROMOTER	2.285	19.109	8771.90	167619.40	43.660	5610.62	244958.70
A_68_P23366440	chr4:141430540-141430584	NM_025994:273	Efhd2	INSIDE	2.283	2.694	1008.85	2717.95	6.150	893.14	5493.12
A_68_P32265729	chrX:12858825-12858869	NM_010028:699	Ddx3x	INSIDE	2.282	1.824	882.91	1610.03	4.162	582.92	2425.88
A_68_P22487800	chr3:123088652-123088696	NM_201638:234	Mettl14	INSIDE	2.281	7.737	8006.62	61947.64	17.649	5831.80	102926.20
A_68_P30244836	chr15:57723668-57723712	NM_024207:283	Derl1	INSIDE	2.280	1.624	1458.51	2367.91	3.701	1125.77	4166.54
A_68_P20849641	chr1:177622831-177622875	NM_010098:-131	Opn3	DIVERGENT_PROMOTER	2.280	1.833	6986.01	12806.43	4.180	5290.64	22112.28
A_68_P27965917	chr11:82200919-82200963	NM_023438:-1461	Tmem132e	PROMOTER	2.280	7.265	2269.87	16491.30	16.564	1718.62	28467.81
A_68_P20023664	chr1:9690196-9690240	NM_008651:72	Mybl1	INSIDE	2.277	6.956	950.56	6612.18	15.839	788.81	12494.10
A_68_P25262217	chr7:86762595-86762639	NM_019799:-73	Rhcg	PROMOTER	2.277	16.815	2412.43	40564.86	38.284	1425.80	54585.64
A_68_P23322032	chr4:133843514-133843558	NM_001163794:-368	Pdik1l	PROMOTER	2.276	2.424	1209.39	2931.15	5.516	959.27	5291.41
A_68_P23927384	chr5:100467950-100467994	NM_016690:269	Hnrpdl	INSIDE	2.275	5.958	5826.38	34711.57	13.556	4005.52	54299.33
A_68_P28538319	chr12:74685927-74685971	NM_008856:-79	Prkch	PROMOTER	2.273	3.609	351.00	1266.84	8.205	326.89	2682.09
A_68_P27083521	chr10:40069136-40069180	NM_001168304:45	Cdk19	INSIDE	2.272	7.152	461.33	3299.64	16.254	351.42	5711.81
A_68_P27937689	chr11:77275784-77275828	NM_139128:-1608	Coro6	PROMOTER	2.271	3.704	1483.14	5493.90	8.411	1145.14	9631.95
A_68_P27095712	chr10:42303728-42303772	NM_152229:-356	Nr2e1	PROMOTER	2.271	12.858	11284.84	145096.10	29.199	7978.87	232972.60

ProbeName	Target position of probe on CpG island microarray	TargetID	GeneSymbol	CpG island Description	Ratio of relative methylation (TiO_2-NP/Vehicle)	Sham group			TiO_2-H group		
						Relative methylation (Cy5/Cy3)	Cy3 signal (Input)	Cy5 signal (MeDIP)	Relative methylation (Cy5/Cy3)	Cy3 signal (Input)	Cy5 signal (MeDIP)
A_68_P28598113	chr12:85749685-85749729	NM_001026214:273	Entpd5	INSIDE	2.269	3.661	3028.24	11086.95	8.308	2223.13	18468.75
A_68_P27185080	chr10:61732562-61732606	NM_001146100:70585	Hk1	INSIDE	2.269	10.646	2773.56	29527.97	24.158	2096.18	50638.62
A_68_P28159656	chr11:116351010-116351061	NM_026364:625	Prpsap1	INSIDE	2.269	17.758	4033.71	71630.06	40.290	2464.90	99310.50
A_68_P25616602	chr8:4217183-4217227	NM_207203:108	BC068157	INSIDE	2.268	2.780	1157.85	3218.82	6.304	970.70	6119.43
A_68_P31948062	chr19:10115784-10115828	NM_021890:-231	Fads3	PROMOTER	2.268	7.995	626.39	5008.14	18.133	509.60	9240.45
A_68_P27558730	chr11:4537928-4537972	NM_029291:155	Ascc2	INSIDE	2.265	8.303	1676.18	13917.18	18.803	1201.82	22597.63
A_68_P31499629	chr18:12395086-12395130	NM_008720:-213	Npc1	PROMOTER	2.263	5.827	3711.66	21629.28	13.187	2940.31	38774.14
A_68_P20088280	A_68_P20088280			Unknown	2.263	6.286	1897.88	11930.69	14.227	1381.63	19656.91
A_68_P31152238	chr17:34259488-34259532	NM_010238:-818	Brd2	PROMOTER	2.262	8.753	1863.76	16313.88	19.798	1695.39	33564.70
A_68_P24338521	chr6:31513401-31513445	NM_013723:515	Podxl	INSIDE	2.261	5.210	1194.73	6224.80	11.778	960.89	11317.24
A_68_P21625558	chr2:129418959-129419003	NM_001177647:50	Sirpa	INSIDE	2.259	2.888	1133.84	3275.07	6.526	933.94	6095.30
A_68_P29308205	chr13:108680813-108680857	NM_145456:-576	Zswim6	PROMOTER	2.259	1.450	1072.99	1555.79	3.276	830.04	2719.13
A_68_P22450560	chr3:116365543-116365587	NM_008246:31	Hiat1	INSIDE	2.258	4.123	1719.69	7090.76	9.309	1240.40	11546.41
A_68_P25023454	chr7:29551526-29551579	NM_010908:-9	Nfkbib	DIVERGENT_PROMOTER	2.257	8.477	713.98	6052.27	19.133	530.50	10149.87
A_68_P25837064	chr8:47702719-47702763	NM_009810:-62	Casp3	DIVERGENT_PROMOTER	2.255	8.065	13019.36	104998.00	18.182	9442.59	171689.80
A_68_P32464980	chrX:71069698-71069742	NM_001029868:588	Pdzd4	INSIDE	2.253	5.144	453.64	2333.58	11.591	243.90	2826.91
A_68_P23749456	chr5:64551627-64551671	NM_019636:199	Tbc1d1	INSIDE	2.251	6.568	13603.88	89353.73	14.786	9241.51	136648.90
A_68_P27921611	chr11:74451961-74452005	NM_001013784:18876	E130309D14Rik	INSIDE	2.250	5.916	3541.95	20954.34	13.311	2760.39	36744.42
A_68_P22481653	chr3:121996304-121996348	NR_030439:295	Mir760	DOWNSTREAM	2.249	3.528	297.41	1049.11	7.933	238.92	1895.23
A_68_P28909030	chr13:29051600-29051644	NM_009238:-6071	Sox4	PROMOTER	2.248	3.638	3625.39	13187.66	8.177	2539.05	20760.84
A_68_P25761256	chr8:33120256-33120300	ENSMUST00000080782:-697		PROMOTER	2.247	4.259	4672.03	19896.15	9.570	3228.37	30896.25
A_68_P23873662	chr5:89150059-89150103	NM_026735:185	Mobkl1a	INSIDE	2.246	6.208	3024.03	18774.60	13.943	2380.22	33186.33
A_68_P23176712	chr4:104782284-104782328	NM_178143:197	Prkaa2	INSIDE	2.246	5.210	21364.40	111304.50	11.704	15131.89	177100.10
A_68_P27338390	chr10:90291759-90291803	NM_001177396:252345	Anks1b	INSIDE	2.245	4.521	1258.95	5692.26	10.151	946.42	9607.50
A_68_P29144056	chr13:74199745-74199789	NM_028959:-33	Cep72	PROMOTER	2.243	5.603	3753.23	21030.95	12.567	2544.14	31972.55
A_68_P21492621	chr2:104966817-104966861	NM_144783:153	Wt1	INSIDE	2.243	4.140	9624.40	39847.81	9.288	6641.61	61684.67
A_68_P27850822	chr11:61668093-61668137	NM_013881:480	Ulk2	INSIDE	2.241	12.017	5428.39	65234.44	26.936	3559.78	95885.15
A_68_P29011797	chr13:47218039-47218083	NM_146042:-28	Rnf144b	PROMOTER	2.239	1.629	928.54	1512.30	3.647	772.40	2816.58
A_68_P33007144	chr9_random:49674-49720	NR_015516:-3712	4930526I15Rik	PROMOTER	2.238	20.949	646.83	13550.10	46.880	431.58	20232.68
A_68_P23297034	chr4:129190844-129190888	NM_010807:42	Marcksl1	INSIDE	2.238	6.078	31663.01	192442.20	13.600	21299.62	289684.00
A_68_P30337605	chr15:74348245-74348289	NM_174991:1641	Bai1	INSIDE	2.237	5.632	3019.87	17008.45	12.598	1874.84	23618.98
A_68_P32031149	chr19:25746643-25746687	NM_145831:-236	Dmrt2	PROMOTER	2.234	3.449	1352.47	4664.11	7.702	1112.55	8569.35
A_68_P26221254	chr8:123113358-123113402	NM_178856:-405	Gins2	PROMOTER	2.234	8.182	5236.56	42846.61	18.278	3565.24	65164.01
A_68_P23397206	chr4:148265535-148265579	NM_027195:87056	Casz1	INSIDE	2.233	5.561	2267.31	12607.68	12.418	1606.26	19945.99
A_68_P30356228	chr15:77672759-77672803	NM_022410:-235	Myh9	PROMOTER	2.233	5.791	8131.65	47090.36	12.930	5925.98	76623.80
A_68_P30481633	chr15:99501487-99501531	NM_009597:360	Accn2	INSIDE	2.232	1.473	1396.71	2057.16	3.287	1251.13	4112.21
A_68_P32260247	chrX:11654044-11654088	NM_029510:3613	Bcor	INSIDE	2.232	1.501	1387.28	2082.41	3.351	649.41	2176.05
A_68_P28047527	chr11:96777858-96777902	NM_030248:-85	Cdk5rap3	PROMOTER	2.231	9.254	3176.78	29398.85	20.649	1753.53	36208.26
A_68_P31147212	chr17:32966976-32967020	NM_177359:195	Zfp799	INSIDE	2.230	7.205	2501.50	18022.65	16.067	1776.93	28549.85
A_68_P28080239	chr11:102417477-102417521	NM_001159492:-26	Gpatch8	PROMOTER	2.229	6.312	7420.54	46836.99	14.069	5185.46	72952.94
A_68_P32506209	chrX:82819101-82819145	NM_025729:-238	Tab3	PROMOTER	2.228	5.900	2141.79	12636.44	13.145	1254.89	16495.16
A_68_P28053229	chr11:97745535-97745579	NM_001081435:-88	Fbxo47	PROMOTER	2.226	7.628	7635.22	58240.73	16.982	4804.99	81599.95
A_68_P25279941	chr7:90014673-90014717	NM_175366:-1148	Mex3b	PROMOTER	2.224	5.070	4959.03	25142.20	11.274	3753.77	42319.18
A_68_P32583453	chrX:103415136-103415180	NM_001001176:23	Taf9b	INSIDE	2.223	6.738	958.05	6455.48	14.979	638.42	9563.02
A_68_P26017904	chr8:86591013-86591057	NM_009055:300	Rfx1	INSIDE	2.220	4.457	1742.64	7766.47	9.895	1449.24	14340.14
A_68_P26247734	chr8:127317918-127317962	NM_198632:1022	Trim67	INSIDE	2.219	5.090	4707.86	23962.96	11.297	3622.37	40921.94
A_68_P23693365	chr5:52581836-52581880	NR_015537:-61	9230114K14Rik	DIVERGENT_PROMOTER	2.218	6.219	1084.17	6742.91	13.793	995.51	13731.22
A_68_P28575798	chr12:81564728-81564777	NM_133798:671	Exd2	INSIDE	2.218	7.448	1524.74	11356.43	16.524	1070.78	17693.19
A_68_P31158869	chr17:35457566-35457610	NM_010390:86	H2-Q1	INSIDE	2.218	3.214	2869.99	9224.70	7.128	2234.57	15927.74
A_68_P32265730	chrX:12858920-12858964	NM_010028:795	Ddx3x	INSIDE	2.217	1.889	385.98	729.24	4.188	219.21	918.16
A_68_P23513990	chr5:18732536-18732580	NM_001170746:-305	Magi2	PROMOTER	2.216	6.205	10538.73	65397.65	13.752	8258.67	113576.80

ProbeName	Target position of probe on CpG island microarray	TargetID	GeneSymbol	CpG island Description	Ratio of relative methylation (TiO_2-NP/Vehicle)	Sham group			TiO_2-H group		
						Relative methylation (Cy5/Cy3)	Cy3 signal (Input)	Cy5 signal (MeDIP)	Relative methylation (Cy5/Cy3)	Cy3 signal (Input)	Cy5 signal (MeDIP)
A_68_P32700325	chrX:137200051-137200095	ENSMUST00000112988:462		INSIDE	2.216	6.332	1007.94	6382.27	14.033	816.09	11452.23
A_68_P20645803	chr1:138311898-138311942	NM_026500:73	Ddx59	INSIDE	2.215	2.243	339.36	761.23	4.969	308.46	1532.86
A_68_P27901829	chr11:70460462-70460506	NM_024211:11	Slc25a11	INSIDE	2.215	1.633	1156.52	1888.88	3.617	951.49	3441.89
A_68_P25091867	chr7:52471545-52471589	NM_011565:444	Tead2	INSIDE	2.215	15.992	4898.00	78330.04	35.420	3007.66	106531.60
A_68_P31619415	chr18:35812025-35812069	NM_027222:-3025	2010001M09Rik	PROMOTER	2.214	6.357	1547.49	9837.17	14.072	1207.65	16994.20
A_68_P21103096	chr2:30271133-30271177	NM_007760:114	Crat	INSIDE	2.214	7.925	3581.49	28381.88	17.546	2493.99	43759.89
A_68_P33007796	chr4_random:129068-129112	NM_001033326:19289	Dhrsx	INSIDE	2.214	6.689	9324.57	62374.43	14.813	5853.14	86702.44
A_68_P23592057	chr5:34343782-34343826	NM_001001985:5172	Nat8l	INSIDE	2.214	8.208	19505.54	160108.10	18.177	11137.08	202440.10
A_68_P24920738	chr6:144997199-144997243	NM_001024468:112	Bcat1	INSIDE	2.213	2.887	1538.27	4441.50	6.390	1136.41	7261.59
A_68_P22861615	chr4:41261631-41261675	NM_026893:282	Dcaf12	INSIDE	2.213	9.114	4804.62	43790.81	20.168	3504.05	70669.71
A_68_P27943494	chr11:78279579-78279623	NM_026740:398	Slc46a1	INSIDE	2.212	5.310	7800.47	41419.02	11.745	5159.24	60594.41
A_68_P31093720	chr17:23940883-23940927	NM_175229:751	Srrm2	INSIDE	2.212	2.483	1982.45	4923.18	5.494	1454.97	7994.04
A_68_P20742449	chr1:158233625-158233669	NM_001134741:-167	Tdrd5	DIVERGENT_PROMOTER	2.212	9.538	2976.99	28395.71	21.102	2346.43	49515.32
A_68_P25360757	chr7:106417697-106417741	NM_001043355:863	Mtap6	INSIDE	2.210	4.630	2563.91	11870.65	10.232	1820.63	18628.00
A_68_P22737302	chr4:12833974-12834018	NM_001171801:13	Gm11818	INSIDE	2.209	5.015	1959.85	9827.78	11.076	1368.52	15158.08
A_68_P28751776	chr12:113916958-113917002	NM_001100460:-70	Zbtb42	DIVERGENT_PROMOTER	2.209	2.980	806.11	2402.03	6.582	700.69	4611.84
A_68_P29080348	chr13:59686650-59686694	NM_030153:-21	Naa35	PROMOTER	2.207	6.748	1745.08	11775.43	14.893	1380.05	20552.67
A_68_P31951139	chr19:10599956-10600000	NM_001164272:245	Cpsf7	INSIDE	2.206	15.169	6442.48	97726.43	33.464	3939.70	131838.40
A_68_P20337461	chr1:72582629-72582673	NM_001045533:488	Marchf4	INSIDE	2.205	8.769	4635.05	40642.45	19.337	3125.23	60432.55
A_68_P20335713	chr1:72290528-72290572	ENSMUST00000123654:-44		PROMOTER	2.205	3.019	1186.36	3581.85	6.658	844.95	5625.61
A_68_P24591289	chr6:83027770-83027814	NM_197992:-591	Pcgf1	DIVERGENT_PROMOTER	2.204	2.692	354.10	953.35	5.934	302.72	1796.48
A_68_P28606270	chr12:87166109-87166153	NM_001081423:231	Ttll5	INSIDE	2.203	1.934	783.80	1515.98	4.262	601.72	2564.47
A_68_P28712200	chr12:107200958-107201002	BC156654:163120		INSIDE	2.203	8.056	7461.56	60109.60	17.745	5395.10	95735.46
A_68_P27279294	chr10:79318023-79318067	NR_029447:-328	E130317F20Rik	PROMOTER	2.202	5.706	1545.56	8818.27	12.562	1215.69	15271.43
A_68_P26169432	chr8:114167808-114167852	NM_001037665:488	Zfp1	INSIDE	2.202	5.107	2651.81	13541.72	11.244	1977.35	22233.90
A_68_P24603559	chr6:85137815-85137859	NM_010131:-88	Emx1	PROMOTER	2.198	6.948	2502.06	17385.39	15.275	1965.77	30027.50
A_68_P25032021	chr7:31320496-31320540	NM_178252:-490	Arhgap33	PROMOTER	2.197	5.889	4112.22	24218.15	12.937	2604.63	33696.82
A_68_P27503761	chr10:120913745-120913792	NM_138956:-462	Rassf3	PROMOTER	2.197	2.114	470.03	993.45	4.644	376.06	1746.53
A_68_P26398844	chr9:30939486-30939530	NM_011176:-124	St14	PROMOTER	2.197	4.125	7544.95	31124.72	9.064	5133.42	46531.13
A_68_P30960416	chr16:91010827-91010871	NM_001045515:492	Synj1	INSIDE	2.196	7.714	957.87	7388.76	16.937	792.63	13424.86
A_68_P31306319	chr17:66798578-66798622	NM_001114098:490	1110012J17Rik	INSIDE	2.195	10.809	9897.40	106984.90	23.727	6523.10	154776.90
A_68_P31143302	chr17:32173587-32173631	NM_028244:502	Rrp1b	INSIDE	2.195	6.110	3200.19	19554.71	13.412	2348.29	31494.87
A_68_P25505623	chr7:134587980-134588024	NM_175163:4670	Zfp689	INSIDE	2.195	6.386	1233.58	7878.17	14.018	1140.67	15989.94
A_68_P30478229	chr15:98928779-98928823	NM_176835:-1114	Dnajc22	PROMOTER	2.194	8.321	7380.07	61409.06	18.254	5468.03	99815.07
A_68_P28067852	chr11:100258833-100258877	NM_010593:250	Jup	INSIDE	2.193	5.577	3327.06	18556.04	12.233	2328.22	28480.36
A_68_P25716360	chr8:24521956-24522000	NM_013834:5	Sfrp1	INSIDE	2.193	9.499	2326.53	22099.39	20.830	1818.84	37887.13
A_68_P30668269	A_68_P30668269			Unknown	2.193	10.012	1958.33	19606.21	21.951	1317.28	28916.10
A_68_P32672214	chrX:131010586-131010630	NM_028958:421	Taf7l	INSIDE	2.192	4.631	1820.18	8429.86	10.151	797.38	8094.39
A_68_P20303908	chr1:66724124-66724168	NM_175510:209126	Unc80	INSIDE	2.192	12.767	15968.88	203869.80	27.990	10327.29	289058.50
A_68_P29578138	chr14:47380037-47380081	NM_028222:-157	Cdkn3	PROMOTER	2.191	1.390	1576.79	2191.73	3.046	1182.31	3601.48
A_68_P32226993	chr19:60831736-60831780	NM_178421:-131	Nanos1	PROMOTER	2.190	5.397	11809.73	63740.95	11.823	8903.57	105264.20
A_68_P31784091	chr18:66094837-66094881	NM_013833:3885	Rax	INSIDE	2.190	7.709	3926.57	30268.81	16.880	2989.95	50470.21
A_68_P28595531	chr12:85291747-85291791	NM_023633:211	2410016O06Rik	INSIDE	2.188	2.421	371.70	899.87	5.298	458.78	2430.71
A_68_P27681337	chr11:30854282-30854326	NM_023906:-93	Asb3	DIVERGENT_PROMOTER	2.186	6.686	9808.72	65585.14	14.615	7228.55	105644.30
A_68_P23057175	chr4:82151052-82151103	NM_001113209:135	Nfib	INSIDE	2.186	12.701	2331.78	29615.98	27.759	1593.57	44235.74
A_68_P22336399	chr3:93248419-93248463	NM_001163098:2189	Tchh	INSIDE	2.183	41.121	2242.23	92201.51	89.767	1225.81	110037.50
A_68_P29614877	chr14:55513260-55513304	NM_019402:303	Pabpn1	INSIDE	2.182	9.221	2638.58	24330.46	20.124	1966.61	39576.81
A_68_P27804178	chr11:53138130-53138174	NM_026479:-38	Zcchc10	PROMOTER	2.182	3.232	3439.26	11114.35	7.051	2390.08	16853.17
A_68_P24134134	chr5:139677035-139677079	NM_024451:433	Sun1	INSIDE	2.181	5.783	2783.77	16099.74	12.615	1839.83	23209.33
A_68_P24327113	chr6:29477125-29477169	NM_012057:414	Irf5	INSIDE	2.180	6.727	2168.97	14589.91	14.666	1359.43	19936.89
A_68_P25056449	chr7:37481732-37481776	NM_172298:-1382	Tshz3	PROMOTER	2.179	4.522	6942.59	31394.05	9.853	5384.81	53058.30

ProbeName	Target position of probe on CpG island microarray	TargetID	GeneSymbol	CpG island Description	Ratio of relative methylation (TiO_2-NP/Vehicle)	Sham group			TiO_2-H group		
						Relative methylation (Cy5/Cy3)	Cy3 signal (Input)	Cy5 signal (MeDIP)	Relative methylation (Cy5/Cy3)	Cy3 signal (Input)	Cy5 signal (MeDIP)
A_68_P28752833	chr12:114067608-114067652	NM_028023:-30	Cdca4	PROMOTER	2.176	4.177	2559.35	10690.00	9.089	1848.78	16804.37
A_68_P22422570	chr3:109945667-109945711	NM_001163348:702	Ntng1	INSIDE	2.176	1.821	2038.86	3713.60	3.963	1614.34	6397.87
A_68_P27290790	chr10:81063642-81063689	NM_053254:-20	Tle6	DIVERGENT_PROMOTER	2.176	4.811	8247.25	39678.77	10.468	6013.28	62949.87
A_68_P31922938	chr19:4397627-4397671	NM_001001984:-571	Kdm2a	PROMOTER	2.175	3.087	1795.80	5543.40	6.715	1491.27	10014.01
A_68_P21780563	chr2:157854132-157854176	NM_027434:-375	Rprd1b	PROMOTER	2.174	3.021	1078.36	3258.04	6.568	867.01	5694.82
A_68_P32099202	chr19:38129262-38129306	NM_028293:-230	Cep55	PROMOTER	2.173	8.598	14671.61	126142.50	18.683	9111.35	170231.80
A_68_P28048173	chr11:96879906-96879950	NM_031183:5046	Sp6	INSIDE	2.171	8.587	6504.89	55854.27	18.644	4870.51	90804.01
A_68_P32242174	chrX:7769836-7769880	NM_007898:780	Ebp	INSIDE	2.170	1.795	3484.43	6255.21	3.896	1401.56	5460.34
A_68_P29615096	chr14:55545666-55545710	NM_010112:-63	Efs	DIVERGENT_PROMOTER	2.169	18.040	3597.05	64890.58	39.131	2404.11	94075.39
A_68_P23344531	chr4:137772915-137772959	NM_001122897:-109	Hp1bp3	PROMOTER	2.169	16.862	7205.96	121509.40	36.582	4534.79	165892.40
A_68_P22894261	chr4:47365847-47365891	NM_009370:-308	Tgfbr1	PROMOTER	2.169	5.306	1036.17	5498.30	11.508	847.24	9749.72
A_68_P21587592	chr2:122528587-122528631	NM_011774:-209	Slc30a4	PROMOTER	2.167	4.479	2103.53	9422.12	9.705	1687.24	16374.21
A_68_P32742983	chrX:147247480-147247524	NM_001199246:744	Maged2	INSIDE	2.161	4.903	2403.16	11783.38	10.596	1585.60	16800.76
A_68_P21204319	chr2:49643540-49643584	NM_027990:357	Lypd6b	INSIDE	2.160	4.204	1901.65	7994.55	9.081	1433.64	13018.78
A_68_P25266551	chr7:87547096-87547140	NM_011046:530	Furin	INSIDE	2.159	12.930	2107.76	27252.24	27.910	1267.94	35387.98
A_68_P20556970	chr1:121318089-121318133	NM_008381:715	Inhbb	INSIDE	2.159	5.335	1743.08	9299.56	11.520	1390.23	16015.89
A_68_P27846103	chr11:60835840-60835884	ENSMUST00000089184:-203		PROMOTER	2.159	4.342	8041.17	34914.08	9.374	6184.87	57977.20
A_68_P24624506	chr6:88791877-88791921	NM_030251:31	Abtb1	INSIDE	2.158	5.442	15857.65	86291.89	11.741	10489.90	123163.10
A_68_P28175309	chr11:118884826-118884870	NM_007623:506	Cbx2	INSIDE	2.158	2.545	1722.26	4382.75	5.492	1449.00	7957.76
A_68_P31114505	chr17:27270655-27270699	NM_026063:160	2900010M23Rik	INSIDE	2.157	9.667	6284.28	60751.19	20.850	4279.05	89217.88
A_68_P27537125	chr10:127058908-127058952	NM_008512:-726	Lrp1	PROMOTER	2.156	5.585	2639.93	14743.96	12.043	1960.62	23611.85
A_68_P21722283	chr2:146909704-146909748	NM_023504:1355	Nkx2-4	INSIDE	2.154	8.276	3101.29	25666.94	17.826	2213.60	39460.62
A_68_P27332821	chr10:89207726-89207770	NM_029166:13	Uhrf1bp1l	INSIDE	2.154	3.424	2654.20	9088.83	7.375	2280.54	16819.81
A_68_P28873012	chr13:21560534-21560582	NM_025719:-188	Nkapl	PROMOTER	2.153	4.725	1181.67	5583.63	10.174	928.73	9449.34
A_68_P21250388	chr2:58999667-58999711	NM_026361:782	Pkp4	INSIDE	2.153	7.303	2391.43	17465.64	15.726	1956.02	30760.24
A_68_P21103442	chr2:30328841-30328885	NM_030244:857	Ier5l	INSIDE	2.152	11.581	3520.79	40775.03	24.925	2959.60	73769.32
A_68_P31731070	chr18:56721947-56721991	NM_138600:-16	Aldh7a1	DIVERGENT_PROMOTER	2.150	8.615	28263.35	243484.30	18.524	18196.29	337068.80
A_68_P30625227	chr16:27388952-27388996	NM_001025615:-88	Ccdc50	PROMOTER	2.147	4.727	892.50	4218.57	10.148	820.82	8329.36
A_68_P32463053	chrX:70748921-70748965	NM_177236:518	Atp2b3	INSIDE	2.146	1.750	5188.54	9081.27	3.756	2355.67	8846.92
A_68_P32383369	chrX:49967083-49967127	NM_016697:47	Gpc3	INSIDE	2.146	3.528	4704.13	16596.20	7.570	2240.78	16963.39
A_68_P23931499	chr5:101227260-101227304	NM_001081107:337	Helq	INSIDE	2.145	2.896	2065.63	5982.98	6.212	1669.15	10368.86
A_68_P31420283	chr17:87362407-87362451	NM_145491:-22	Rhoq	PROMOTER	2.143	2.059	433.01	891.59	4.412	401.47	1771.37
A_68_P25174406	chr7:71083847-71083891	NM_021366:-67	Klf13	PROMOTER	2.142	5.907	1489.42	8798.10	12.651	1164.39	14731.24
A_68_P31020947	chr17:6079672-6079716	NM_001111017:45	Serac1	INSIDE	2.142	2.556	2521.73	6444.82	5.475	1977.12	10824.66
A_68_P30580079	chr16:18499124-18499168	NM_023120:183	Gnb1l	INSIDE	2.141	2.844	1859.81	5289.01	6.089	1442.04	8781.04
A_68_P22752524	chr4:15885248-15885292	NM_013752:157	Nbn	INSIDE	2.141	2.779	1457.00	4048.50	5.948	1231.65	7325.64
A_68_P24116580	chr5:135964648-135964692	NM_146001:56322	Hip1	INSIDE	2.140	5.409	3084.15	16682.53	11.576	2262.33	26188.17
A_68_P23232782	chr4:116828713-116828757	NR_030704:-281	Snord55	PROMOTER	2.140	3.084	637.06	1964.47	6.599	492.18	3247.96
A_68_P31116177	chr17:27552104-27552148	NM_001013385:88123	Grm4	DOWNSTREAM	2.139	12.359	2346.81	29005.04	26.430	1264.59	33423.64
A_68_P27776272	chr11:47802609-47802653	ENSMUST00000128558:-159		PROMOTER	2.139	4.332	1174.70	5089.32	9.265	917.71	8502.75
A_68_P26710370	chr9:88377975-88378019	NM_019796:-761	Syncrip	PROMOTER	2.138	5.150	1514.87	7801.64	11.009	1191.15	13113.61
A_68_P26405232	chr9:32217116-32217162	AK146406:436717		INSIDE	2.138	3.197	3016.19	9643.67	6.836	2325.43	15897.00
A_68_P28175799	chr11:118951917-118951961	NM_007625:-4387	Cbx4	PROMOTER	2.137	5.608	3193.53	17908.29	11.981	2453.76	29398.49
A_68_P22202889	chr3:65762508-65762552	NM_019937:-383	Ccnl1	PROMOTER	2.137	1.533	854.67	1310.43	3.276	727.96	2384.86
A_68_P27448186	chr10:110735503-110735547	NM_027914:-210	Bbs10	PROMOTER	2.136	4.699	6265.44	29444.31	10.038	4580.95	45985.56
A_68_P24046831	chr5:122293021-122293065	NM_175474:-5994	Fam109a	DIVERGENT_PROMOTER	2.136	2.255	193.72	436.93	4.817	167.27	805.78
A_68_P23164325	chr4:102787321-102787365	NM_001039081:-4652	Mier1	PROMOTER	2.136	7.582	18637.64	141316.60	16.193	13089.85	211962.50
A_68_P30959119	chr16:90810476-90810520	NM_029497:160	Urb1	INSIDE	2.136	3.049	1905.85	5811.50	6.515	1616.24	10529.11
A_68_P23260191	chr4:122672828-122672872	NM_008506:-491	Mycl1	PROMOTER	2.135	12.888	3988.63	51404.12	27.511	2697.48	74210.26
A_68_P24535485	chr6:71222087-71222131	NM_145568:96	Krcc1	INSIDE	2.134	11.063	1731.55	19155.50	23.604	1167.46	27557.14
A_68_P20457529	chr1:95700443-95700487	NM_025454:-77	Ing5	DIVERGENT_PROMOTER	2.133	9.915	5255.68	52111.82	21.145	2649.65	56025.88

ProbeName	Target position of probe on CpG island microarray	TargetID	GeneSymbol	CpG island Description	Ratio of relative methylation (TiO_2-NP/Vehicle)	Sham group			TiO_2-H group		
						Relative methylation (Cy5/Cy3)	Cy3 signal (Input)	Cy5 signal (MeDIP)	Relative methylation (Cy5/Cy3)	Cy3 signal (Input)	Cy5 signal (MeDIP)
A_68_P29075350	chr13:58908196-58908240	NM_001025074:-975	Ntrk2	PROMOTER	2.133	15.813	9970.56	157660.40	33.725	6282.78	211887.50
A_68_P29237599	chr13:95653816-95653860	NM_011021:8257	Otp	DOWNSTREAM	2.133	2.276	7639.08	17383.48	4.854	5327.65	25861.44
A_68_P26138122	chr8:108692733-108692777	NM_178798:-20	Slc7a6	PROMOTER	2.133	9.459	3267.93	30911.10	20.172	2009.26	40530.44
A_68_P32168785	chr19:50752526-50752570	NM_021377:554	Sorcs1	INSIDE	2.133	7.067	4953.93	35007.53	15.071	3490.88	52610.12
A_68_P32026831	chr19:25036147-25036191	NM_146097:-62	Cbwd1	PROMOTER	2.132	6.201	3613.13	22406.37	13.219	2745.31	36290.47
A_68_P24783935	chr6:117857266-117857310	NM_001166428:-533	Hnrnpf	PROMOTER	2.132	4.289	3396.15	14566.89	9.143	2523.63	23072.58
A_68_P26221734	chr8:123191897-123191941	NM_010926:94	Cox4nb	INSIDE	2.130	2.268	6911.12	15675.39	4.830	4809.16	23230.14
A_68_P31866342	chr18:80909356-80909400	NM_001164111:-4466	Nfatc1	PROMOTER	2.130	3.279	1050.27	3443.57	6.983	910.41	6357.55
A_68_P27289987	chr10:80945075-80945119	NM_176954:358	Celf5	INSIDE	2.128	8.817	8860.98	78128.69	18.767	4829.56	90637.92
A_68_P25850572	chr8:49928690-49928734	AK013771:-216		PROMOTER	2.127	7.939	5676.85	45068.92	16.886	4392.61	74172.80
A_68_P32533270	chrX:90542781-90542825	NM_007492:10818	Arx	INSIDE	2.126	3.577	832.85	2978.70	7.604	439.74	3344.00
A_68_P25009418	chr7:26067028-26067072	NM_001110131:-147	Cic	PROMOTER	2.126	4.923	27867.04	137201.80	10.469	18156.34	190086.60
A_68_P28153958	chr11:115374907-115374951	NM_008258:756	Hn1	INSIDE	2.126	17.486	989.68	17305.50	37.177	683.54	25411.86
A_68_P27561184	chr11:4965305-4965349	NM_001190406:4	Gas2l1	INSIDE	2.125	7.670	8270.87	63438.54	16.302	5486.54	89440.16
A_68_P32573188	chrX:100689079-100689123	NR_015508:186	Enox	INSIDE	2.124	3.962	3243.02	12847.80	8.416	2143.69	18040.59
A_68_P28470083	chr12:60168415-60168459	NM_008891:531	Pnn	INSIDE	2.123	1.820	544.02	990.33	3.864	511.63	1976.88
A_68_P27693355	chr11:33102836-33102880	NM_019916:730	Tlx3	INSIDE	2.123	3.391	1478.25	5012.70	7.199	1172.83	8443.77
A_68_P30747800	chr16:49855504-49855548	NM_010581:-240	Cd47	PROMOTER	2.122	2.349	1331.03	3126.76	4.984	933.56	4653.28
A_68_P22032039	chr3:31048745-31048789	NM_008770:-75	Cldn11	PROMOTER	2.122	1.605	1227.26	1969.46	3.405	969.49	3301.02
A_68_P25098823	chr7:53653169-53653213	NM_001112739:1324	Kcnc1	INSIDE	2.122	1.801	902.15	1624.83	3.822	1053.18	4025.32
A_68_P29055349	chr13:55589582-55589626	NM_001177371:-167	Dbn1	PROMOTER	2.120	8.125	3720.33	30227.89	17.225	2832.48	48789.15
A_68_P29726978	chr14:75684076-75684120	NM_026083:-81	Zc3h13	PROMOTER	2.120	8.539	2031.43	17345.71	18.101	1558.55	28211.02
A_68_P27939273	chr11:77580346-77580390	ENSMUST00000102488:-5284		PROMOTER	2.119	6.643	2128.84	14141.13	14.075	1689.26	23776.24
A_68_P26866698	chr9:118416014-118416060	NM_018748:601	Golga4	INSIDE	2.118	11.798	3642.19	42970.34	24.992	2297.60	57421.75
A_68_P28977626	chr13:41454570-41454617	NM_017464:-223	Nedd9	PROMOTER	2.118	8.738	800.33	6993.42	18.504	665.69	12317.74
A_68_P20148714	chr1:36568619-36568663	NM_001039551:-80	Cnnm3	PROMOTER	2.117	8.917	14587.91	130076.10	18.878	10155.26	191711.20
A_68_P24295432	chr6:23203617-23203661	NM_028462:-5374	Fezf1	PROMOTER	2.117	4.889	2303.60	11263.08	10.350	1828.01	18919.61
A_68_P24477003	chr6:56781975-56782019	NM_012056:-56	Fkbp9	PROMOTER	2.117	2.955	752.71	2224.52	6.256	588.64	3682.38
A_68_P25343512	chr7:103359060-103359104	NM_011858:-64	Odz4	PROMOTER	2.117	11.052	2664.60	29448.01	23.391	2126.61	49742.89
A_68_P25354353	chr7:105326510-105326554	NM_007602:171	Capn5	INSIDE	2.116	4.262	3205.06	13658.50	9.017	2370.02	21370.70
A_68_P25274817	chr7:89079514-89079558	NM_013886:-191	Hdgfrp3	PROMOTER	2.116	6.283	3031.88	19048.71	13.295	2230.02	29648.19
A_68_P24697529	chr6:101327967-101328012	NM_018884:-98	Pdzrn3	PROMOTER	2.116	3.642	1129.20	4112.31	7.704	922.39	7106.53
A_68_P20354818	chr1:75543316-75543360	NM_009208:498	Slc4a3	INSIDE	2.116	4.675	6516.45	30464.92	9.891	4369.35	43216.35
A_68_P23154908	chr4:101093069-101093113	NM_009647:783	Ak4	INSIDE	2.114	2.054	374.05	768.22	4.343	300.11	1303.25
A_68_P31428328	chr17:88929429-88929473	NM_028658:-13	Klraq1	PROMOTER	2.114	6.174	817.38	5046.29	13.049	621.87	8114.53
A_68_P27995861	chr11:87550950-87550994	NM_009296:-94	Supt4h1	PROMOTER	2.114	6.564	2326.72	15273.06	13.877	1692.93	23493.62
A_68_P28578489	chr12:82047116-82047160	NM_001079694:648	Srsf5	INSIDE	2.113	3.899	533.87	2081.51	8.237	451.24	3716.87
A_68_P31013736	chr17:4994894-4994938	NM_001085355:-157	Arid1b	PROMOTER	2.112	7.097	7741.52	54941.16	14.990	5329.90	79894.54
A_68_P21767078	chr2:155518080-155518124	NM_001163452:18	Trpc4ap	INSIDE	2.112	3.247	805.77	2616.47	6.857	678.22	4650.51
A_68_P32362520	chrX:45387716-45387760	NM_013912:291	Apln	INSIDE	2.111	11.695	2906.03	33987.15	24.693	1701.13	42005.98
A_68_P23332310	chr4:135700553-135700600	NM_008321:840	Id3	INSIDE	2.111	4.020	265.91	1068.88	8.487	194.28	1648.92
A_68_P21081484	chr2:26819762-26819806	NM_207234:122	Rexo4	INSIDE	2.111	1.454	1049.76	1526.60	3.069	779.46	2392.39
A_68_P21932364	chr3:8463041-8463085	NR_033512:36	Gm6194	INSIDE	2.110	4.809	7173.47	34497.75	10.146	5470.79	55505.08
A_68_P26580032	chr9:63932502-63932546	AK011166:3957		INSIDE	2.110	8.689	923.42	8023.56	18.334	876.07	16061.57
A_68_P31106282	chr17:25929216-25929260	NM_145410:-9	Fam173a	PROMOTER	2.109	4.902	4593.98	22517.64	10.336	3445.66	35614.77
A_68_P24859175	chr6:133985925-133985969	NM_007961:222	Etv6	INSIDE	2.108	3.752	3350.18	12571.15	7.911	2729.27	21590.99
A_68_P23296217	chr4:129012516-129012560	NM_009030:76	Rbbp4	INSIDE	2.108	2.519	1338.36	3371.92	5.310	1049.62	5573.84
A_68_P21212091	chr2:51004685-51004729	NM_028810:-75	Rnd3	PROMOTER	2.108	2.277	362.08	824.64	4.800	271.24	1301.97
A_68_P28159663	chr11:116351729-116351773	NM_026364:-90	Prpsap1	PROMOTER	2.107	11.663	4378.77	51068.54	24.577	3204.24	78750.14
A_68_P31152236	chr17:34259233-34259277	NM_010238:-562	Brd2	PROMOTER	2.106	10.329	4117.14	42527.06	21.755	2698.80	58712.71
A_68_P30447922	chr15:93426512-93426556	NM_001033217:-212	Prickle1	PROMOTER	2.106	6.137	74837.32	459288.20	12.924	48581.46	627876.40

ProbeName	Target position of probe on CpG island microarray	TargetID	GeneSymbol	CpG island Description	Ratio of relative methylation (TiO_2-NP/Vehicle)	Sham group			TiO_2-H group		
						Relative methylation (Cy5/Cy3)	Cy3 signal (Input)	Cy5 signal (MeDIP)	Relative methylation (Cy5/Cy3)	Cy3 signal (Input)	Cy5 signal (MeDIP)
A_68_P25457391	chr7:125387085-125387129	NM_001031814:45	Smg1	INSIDE	2.106	4.551	16026.86	72944.30	9.587	11915.57	114231.50
A_68_P31120338	chr17:28217573-28217617	NM_001085555:-10	Tcp11	PROMOTER	2.106	4.187	3641.41	15248.33	8.819	2657.60	23437.56
A_68_P23272064	chr4:124762396-124762440	NM_027310:28	Meaf6	INSIDE	2.105	4.439	2277.95	10112.16	9.343	1719.31	16064.02
A_68_P24611353	chr6:86619098-86619142	NM_010751:33	Mxd1	INSIDE	2.105	4.756	11879.78	56499.76	10.010	9280.46	92901.41
A_68_P22682770	chr3:157610734-157610778	NM_001013806:327	Ankrd13c	INSIDE	2.104	9.504	13866.58	131786.90	19.996	9715.78	194280.20
A_68_P22319131	chr3:89200123-89200167	NM_009565:-3019	Zbtb7b	PROMOTER	2.104	1.910	3518.56	6721.41	4.019	2746.25	11038.09
A_68_P21108244	chr2:31169965-31170009	ENSMUST00000113532:52		INSIDE	2.104	8.863	1254.86	11121.21	18.649	932.21	17385.19
A_68_P27924602	chr11:74984051-74984095	NM_010430:-315	Hic1	DIVERGENT_PROMOTER	2.102	5.021	1961.59	9850.01	10.557	1570.80	16583.45
A_68_P30156691	chr15:39775111-39775155	NM_172814:171	Lrp12	INSIDE	2.102	11.020	1141.73	12582.07	23.167	888.50	20584.27
A_68_P24116035	chr5:135845821-135845865	NM_145414:20	Nsun5	INSIDE	2.102	17.612	2746.94	48380.36	37.014	1782.24	65968.27
A_68_P27265105	chr10:76424885-76424929	NM_021568:-214	Pcbp3	PROMOTER	2.101	15.355	9942.78	152671.90	32.260	6191.69	199742.50
A_68_P31422209	chr17:87682307-87682351	NM_028639:103	Ttc7	INSIDE	2.100	3.330	695.26	2315.04	6.993	552.07	3860.37
A_68_P28057142	chr11:98412381-98412425	NM_001166494:-8	Zpbp2	DIVERGENT_PROMOTER	2.100	7.127	4869.52	34703.67	14.967	3233.09	48389.12
A_68_P25365140	chr7:107154438-107154482	NM_133709:-453	Chrdl2	PROMOTER	2.099	6.849	440.36	3016.19	14.378	378.06	5435.81
A_68_P29958335	chr14:122277673-122277717	NM_026861:-133	Ubac2	PROMOTER	2.099	1.758	1369.19	2407.09	3.691	1136.02	4192.80
A_68_P26373433	chr9:26807126-26807170	NM_025862:-14	Acad8	DIVERGENT_PROMOTER	2.098	5.345	17294.92	92433.87	11.212	11665.23	130790.60
A_68_P31879479	chr18:83126499-83126543	NM_001177464:42497	Zfp516	INSIDE	2.098	9.025	2560.18	23104.84	18.937	2102.69	39819.04
A_68_P22589086	chr3:141832410-141832454	ENSMUST00000106230:-43		PROMOTER	2.098	5.085	1374.40	6989.17	10.667	1016.87	10846.50
A_68_P31465581	chr18:4922333-4922377	ENSMUST00000143254:631		INSIDE	2.098	4.943	1041.55	5148.69	10.369	737.86	7651.00
A_68_P24157329	chr5:144383488-144383532	NM_011182:195	Cyth3	INSIDE	2.097	5.682	2773.18	15757.80	11.913	2014.79	24002.08
A_68_P32056099	chr19:30105106-30105150	NM_144873:126	Uhrf2	INSIDE	2.097	6.089	1920.53	11693.79	12.766	1554.80	19847.81
A_68_P26217005	chr8:122480667-122480711	NM_009462:45937	Usp10	INSIDE	2.097	5.459	7807.56	42622.45	11.448	5530.16	63309.99
A_68_P25097409	chr7:53435456-53435500	NM_011510:-75	Abcc8	PROMOTER	2.095	2.714	766.06	2078.86	5.685	662.42	3765.95
A_68_P29750657	chr14:79700949-79700993	NM_025427:472	1190002H23Rik	INSIDE	2.094	3.449	1577.46	5439.92	7.221	1229.53	8878.49
A_68_P22752864	chr4:15941113-15941157	NM_145950:-110	Osgin2	PROMOTER	2.094	4.203	1844.34	7752.34	8.800	1498.32	13185.35
A_68_P25747455	chr8:30329725-30329769	NM_153135:362	Unc5d	INSIDE	2.094	7.058	8318.20	58706.24	14.778	6527.83	96467.42
A_68_P30141373	chr15:36868452-36868496			Unknown	2.093	6.700	1134.05	7597.84	14.025	878.71	12323.46
A_68_P29257223	chr13:99136439-99136483	NM_008242:12261	Foxd1	DOWNSTREAM	2.092	8.661	2174.59	18833.71	18.116	1799.01	32590.48
A_68_P29044041	chr13:53558129-53558173	NM_013601:9999	Msx2	DOWNSTREAM	2.092	5.217	933.42	4869.21	10.914	666.32	7272.46
A_68_P31092441	chr17:23713283-23713327	NM_011747:23150	Zfp13	INSIDE	2.092	1.581	1651.05	2609.73	3.307	1243.51	4112.10
A_68_P31099001	chr17:24824425-24824469	NM_011522:2448	Syngr3	INSIDE	2.091	8.282	15324.02	126913.40	17.318	9704.87	168065.90
A_68_P21114610	chr2:32168059-32168103	NR_029811:-5899	Mir199b	PROMOTER	2.090	4.476	10196.84	45644.87	9.354	6842.97	64010.85
A_68_P28073802	chr11:101286306-101286350	NM_172566:-70	Rundc1	PROMOTER	2.090	4.644	5587.40	25946.09	9.706	3826.56	37141.60
A_68_P30791337	chr16:58408386-58408430	NM_028523:-239	Dcbld2	PROMOTER	2.089	3.420	3208.27	10970.76	7.142	2683.72	19168.06
A_68_P22293161	chr3:84619085-84619129	NM_001177773:-392	Fbxw7	PROMOTER	2.089	7.473	3528.14	26366.72	15.611	2725.81	42552.94
A_68_P27921682	chr11:74463075-74463119	NM_001081158:100	1300001I01Rik	INSIDE	2.088	2.414	680.04	1641.56	5.039	534.85	2695.16
A_68_P27257833	chr10:75130195-75130239	NM_172549:96886	Cabin1	INSIDE	2.088	4.864	1348.50	6558.82	10.158	1195.82	12146.99
A_68_P27579284	chr11:8564125-8564169	NM_001083587:392	Tns3	INSIDE	2.088	6.420	1932.44	12405.84	13.402	1474.21	19757.56
A_68_P20637774	chr1:137044071-137044115	NM_026823:682	Arl8a	INSIDE	2.086	7.376	14805.20	109197.40	15.389	10138.97	156028.00
A_68_P29651397	chr14:62301793-62301837	NR_028264:-604	Dleu2	PROMOTER	2.086	5.959	1659.98	9891.50	12.430	1475.76	18342.94
A_68_P22101775	chr3:45185176-45185220	NM_011043:2879	Pcdh10	INSIDE	2.086	3.350	1178.34	3947.95	6.990	865.17	6047.41
A_68_P32603687	chrX:109715096-109715140	NM_177747:984	Zfp711	INSIDE	2.086	6.119	1571.52	9616.70	12.767	999.78	12764.45
A_68_P30540368	chr16:9995820-9995865	NM_008170:-3216	Grin2a	PROMOTER	2.085	12.926	6410.38	82858.78	26.947	4763.33	128356.60
A_68_P28956451	chr13:37870397-37870441	ENSMUST00000122842:150		INSIDE	2.085	7.454	6420.13	47855.92	15.540	4503.02	69975.92
A_68_P32957122	A_68_P32957122			Unknown	2.083	2.877	2800.99	8058.38	5.992	2104.57	12611.20
A_68_P21576252	chr2:120435142-120435186	NM_025475:-7	Haus2	DIVERGENT_PROMOTER	2.082	2.017	670.15	1351.97	4.200	563.13	2365.28
A_68_P31161375	chr17:36002953-36002997	NM_001146710:-98	2310014H01Rik	PROMOTER	2.081	4.776	4697.04	22432.44	9.940	3457.88	34370.91
A_68_P27278520	chr10:79187527-79187571	NM_008226:8170	Hcn2	INSIDE	2.081	10.178	1515.88	15429.29	21.186	1116.24	23649.08
A_68_P22601420	chr3:143865390-143865434	NM_001161769:-52	Lmo4	PROMOTER	2.081	4.028	7512.89	30265.03	8.383	5764.40	48324.41
A_68_P23334806	chr4:136158309-136158353	NM_133872:308	Kdm1a	INSIDE	2.080	7.767	4209.78	32698.79	16.158	3270.28	52841.38
A_68_P25349892	chr7:104473950-104473994	NM_001177412:502	Usp35	INSIDE	2.080	2.019	288.84	583.25	4.200	214.06	899.00

ProbeName	Target position of probe on CpG island microarray	TargetID	GeneSymbol	CpG island Description	Ratio of relative methylation (TiO_2-NP/Vehicle)	Sham group			TiO_2-H group		
						Relative methylation (Cy5/Cy3)	Cy3 signal (Input)	Cy5 signal (MeDIP)	Relative methylation (Cy5/Cy3)	Cy3 signal (Input)	Cy5 signal (MeDIP)
A_68_P32751751	chrX:149472100-149472144	NM_023788:-16	Mageh1	PROMOTER	2.079	7.842	12021.86	94269.69	16.303	7384.41	120391.60
A_68_P24877027	chr6:137200894-137200938	NM_001164401:97	Ptpro	INSIDE	2.079	5.823	4168.49	24271.80	12.108	3191.65	38642.87
A_68_P32008156	chr19:21853273-21853317	NM_001033759:463	Tmem2	INSIDE	2.079	7.297	4595.47	33532.42	15.169	3279.14	49740.42
A_68_P20268947	chr1:60467002-60467046	NM_001198570:562	Abi2	INSIDE	2.078	4.564	2869.41	13096.93	9.483	2330.68	22101.92
A_68_P23271474	chr4:124663490-124663534	NM_138683:-161	Rspo1	PROMOTER	2.078	4.498	1224.00	5505.66	9.348	917.28	8574.29
A_68_P24118747	chr5:136438697-136438741	NM_001160366:11628	Srcrb4d	INSIDE	2.078	3.036	1843.78	5597.98	6.308	1464.81	9240.73
A_68_P21338948	chr2:74542744-74542788	NM_008276:-779	Hoxd8	PROMOTER	2.077	4.821	1280.62	6174.19	10.013	960.79	9620.80
A_68_P20599489	chr1:130140741-130140785	NM_026390:5	Ubxn4	INSIDE	2.077	1.596	867.00	1383.46	3.314	766.74	2541.27
A_68_P27186303	chr10:61949193-61949237	NM_001113355:132	Vps26a	INSIDE	2.075	17.294	1212.62	20971.44	35.885	866.21	31083.98
A_68_P26847149	chr9:114843024-114843068	NM_175380:59	Gpd1l	INSIDE	2.074	4.714	2036.36	9598.68	9.776	1631.58	15950.71
A_68_P25016218	chr7:28013554-28013598	NM_181593:40	Itpkc	INSIDE	2.074	7.976	939.09	7490.48	16.541	687.48	11371.60
A_68_P30278749	chr15:63829759-63829803	NM_144846:62230	Fam49b	INSIDE	2.073	3.646	2844.80	10373.26	7.558	2230.18	16854.81
A_68_P26814202	chr9:108209138-108209182	NM_016802:625	Rhoa	INSIDE	2.073	11.208	7900.79	88551.97	23.233	4693.18	109038.70
A_68_P25549531	chr7:142129256-142129301	NM_001143802:835	Fam196a	INSIDE	2.072	3.078	1300.45	4002.22	6.375	952.86	6074.71
A_68_P25992689	chr8:81636479-81636523	NM_178267:84165	Zfp827	INSIDE	2.072	12.786	10404.52	133037.20	26.490	6169.84	163436.70
A_68_P27787453	chr11:50044668-50044712	NM_145926:5854	Mgat4b	INSIDE	2.071	2.420	1573.74	3808.69	5.012	1234.71	6187.88
A_68_P20585375	chr1:127456906-127456950	NM_028787:-664	Slc35f5	PROMOTER	2.071	5.325	2201.21	11722.48	11.029	1528.85	16862.37
A_68_P21571594	chr2:119625143-119625187	NM_019392:-85	Tyro3	PROMOTER	2.071	8.860	3983.38	35291.72	18.347	3170.68	58172.74
A_68_P31678555	chr18:46901926-46901970	NM_009681:378	Ap3s1	INSIDE	2.070	5.852	3619.77	21183.18	12.116	2582.15	31286.58
A_68_P22323963	chr3:90017329-90017373	NM_001190258:219	Gm9846	INSIDE	2.068	0.262	1139.06	298.86	0.543	1169.11	634.28
A_68_P28202486	chr12:4824316-4824360	NM_025323:-75	0610009D07Rik	PROMOTER	2.066	5.998	7074.81	42438.20	12.391	5053.47	62617.15
A_68_P25263207	chr7:86938148-86938192	NM_008588:306	Mesp1	INSIDE	2.066	1.675	913.13	1529.48	3.460	931.29	3222.05
A_68_P23962136	chr5:107003898-107003942	ENSMUST00000124394:-185		PROMOTER	2.066	9.107	6108.38	55630.31	18.816	4224.86	79494.57
A_68_P28842022	chr13:15555028-15555072	NM_008130:-505	Gli3	PROMOTER	2.065	6.713	2389.98	16043.20	13.862	1884.57	26123.75
A_68_P25092565	chr7:52602361-52602407	NM_029741:20005	Ppfia3	INSIDE	2.064	16.737	4118.57	68931.68	34.541	2615.55	90342.51
A_68_P29270336	chr13:101602642-101602692	NM_022885:716	Slc30a5	INSIDE	2.064	4.883	1259.48	6149.51	10.078	939.30	9466.16
A_68_P26468513	chr9:44413201-44413245	NM_001110826:248	Ddx6	INSIDE	2.063	4.714	9476.98	44677.44	9.726	6618.62	64370.16
A_68_P31100482	chr17:25073578-25073622	NM_001163447:322	Mapk8ip3	INSIDE	2.063	1.495	880.29	1315.95	3.084	628.83	1939.61
A_68_P31161218	chr17:35978447-35978494	NM_001010833:28	Mdc1	INSIDE	2.063	6.398	6929.61	44333.91	13.196	5328.21	70309.10
A_68_P25797392	chr8:39723329-39723373	NM_145841:1212	Sgcz	INSIDE	2.063	33.866	2213.30	74955.73	69.872	1292.88	90336.10
A_68_P22878938	chr4:44769794-44769838	NM_138590:386	Zcchc7	INSIDE	2.063	3.570	1474.78	5264.24	7.363	1251.77	9216.30
A_68_P27887968	chr11:68026330-68026374	NM_008744:173976	Ntn1	INSIDE	2.061	8.672	4533.14	39310.19	17.872	3242.41	57949.74
A_68_P22215798	chr3:68299063-68299107	NR_036665:970	Schip1	INSIDE	2.061	4.193	1799.08	7543.33	8.640	1428.43	12341.43
A_68_P30478150	chr15:98918593-98918637	NM_001024702:-455	C1ql4	PROMOTER	2.059	1.825	1640.51	2993.20	3.756	1345.67	5054.11
A_68_P25273848	chr7:88908491-88908535	NM_025997:674	Fam103a1	INSIDE	2.059	5.435	741.82	4031.91	11.189	588.81	6587.93
A_68_P27556422	chr11:4118442-4118486	NM_028022:211	Gatsl3	INSIDE	2.059	7.708	1310.88	10104.72	15.868	1024.76	16260.78
A_68_P32463876	chrX:70882705-70882749	NM_029352:-2053	Dusp9	PROMOTER	2.058	3.948	1322.13	5219.25	8.125	759.53	6171.13
A_68_P23440042	chr4:155069270-155069314	NM_178699:842	B930041F14Rik	INSIDE	2.057	2.160	3207.52	6928.72	4.444	2257.47	10031.80
A_68_P29249638	chr13:97907886-97907931	NM_177266:17	Gfm2	INSIDE	2.057	6.374	600.32	3826.62	13.115	435.69	5714.06
A_68_P25008850	chr7:25963997-25964041	X53654:548352		INSIDE	2.057	5.198	4666.59	24258.15	10.691	3443.42	36814.72
A_68_P26068970	chr8:95484812-95484856	NM_009209:-111	Slc6a2	PROMOTER	2.056	2.600	328.68	854.73	5.348	259.54	1387.98
A_68_P31385961	A_68_P31385961			Unknown	2.056	9.617	10513.88	101112.50	19.776	6821.81	134906.90
A_68_P32260645	chrX:11714194-11714238	NM_001168321:23265	Bcor	INSIDE	2.055	1.644	1945.12	3196.98	3.377	960.88	3245.06
A_68_P20644756	chr1:138131091-138131135	NM_028872:-271	5730559C18Rik	PROMOTER	2.054	5.740	1343.02	7709.17	11.789	979.59	11548.56
A_68_P31214913	chr17:48046778-48046822	NM_001110824:14781	Foxp4	INSIDE	2.054	5.738	6145.33	35262.75	11.786	4375.44	51567.38
A_68_P28290823	chr12:25335979-25336023	NM_178357:-235	Klf11	PROMOTER	2.054	4.846	5228.33	25336.60	9.953	3774.20	37564.05
A_68_P23397279	chr4:148275001-148275045	NM_027195:96522	Casz1	INSIDE	2.053	5.719	3282.49	18772.41	11.740	2431.91	28550.31
A_68_P30630406	chr16:28445546-28445590	NM_183064:-255	Fgf12	PROMOTER	2.053	2.897	1614.92	4677.83	5.947	1132.49	6734.62
A_68_P21902328	chr2:179960330-179960374	NM_001081171:212	Lama5	INSIDE	2.052	2.789	706.81	1971.49	5.724	600.12	3434.97
A_68_P31928180	chr19:5349389-5349433	NM_028623:164	Cst6	INSIDE	2.051	6.540	14012.71	91648.82	13.412	10206.67	136892.60
A_68_P28525473	chr12:72410602-72410646	NM_001190466:-246	Dact1	PROMOTER	2.051	4.733	4828.66	22854.24	9.709	3756.42	36472.19

ProbeName	Target position of probe on CpG island microarray	TargetID	GeneSymbol	CpG island Description	Ratio of relative methylation (TiO_2-NP/Vehicle)	Sham group			TiO_2-H group		
						Relative methylation (Cy5/Cy3)	Cy3 signal (Input)	Cy5 signal (MeDIP)	Relative methylation (Cy5/Cy3)	Cy3 signal (Input)	Cy5 signal (MeDIP)
A_68_P24382228	chr6:39206775-39206819	ENSMUST00000158797:9145		DOWNSTREAM	2.051	8.436	13139.72	110850.80	17.307	8633.86	149423.40
A_68_P26685702	chr9:83699787-83699831	NM_001145974:41	Elovl4	INSIDE	2.050	7.487	9488.45	71038.47	15.349	7623.95	117020.20
A_68_P23968450	chr5:108155208-108155253	NM_010278:-1867	Gfi1	PROMOTER	2.050	3.755	599.09	2249.65	7.697	495.49	3813.81
A_68_P31411779	chr17:86022957-86023001	NM_011381:2805	Six3	INSIDE	2.050	2.885	1519.99	4385.06	5.913	1232.54	7288.03
A_68_P25926494	chr8:67471391-67471435	NM_001163747:329	Tmem192	INSIDE	2.049	1.823	3191.17	5815.94	3.734	2491.95	9305.58
A_68_P25844867	chr8:49075341-49075385	NM_133791:543	Wwc2	INSIDE	2.049	5.154	5036.50	25958.43	10.560	3628.59	38319.47
A_68_P32698227	chrX:136812773-136812817	ENSMUST00000130153:-393		PROMOTER	2.049	4.073	7538.47	30704.72	8.344	4410.05	36799.63
A_68_P30375392	chr15:80778891-80778935	NM_009634:-36	Adsl	PROMOTER	2.048	5.773	6612.32	38176.14	11.822	4842.27	57243.28
A_68_P22310062	chr3:87599138-87599182	NM_001033124:-76	Ntrk1	DIVERGENT_PROMOTER	2.048	4.535	1048.11	4753.37	9.290	851.34	7908.49
A_68_P27340135	chr10:90586440-90586484	NM_133668:246	Slc25a3	INSIDE	2.048	3.639	885.81	3223.11	7.451	782.32	5828.85
A_68_P26500013	chr9:49606044-49606088	NM_001081445:1108	Ncam1	INSIDE	2.047	6.334	6147.30	38938.26	12.967	4556.04	59078.64
A_68_P31415137	chr17:86567183-86567227	NM_011104:80	Prkce	INSIDE	2.047	2.450	2244.27	5497.38	5.015	1805.14	9053.30
A_68_P27953576	chr11:80114526-80114570	NM_139228:135	Rhbdl3	INSIDE	2.047	5.408	2493.11	13483.69	11.071	1887.05	20891.68
A_68_P23430520	chr4:153674348-153674392	NM_001112744:-366	Arhgef16	PROMOTER	2.046	6.781	4768.04	32330.88	13.874	3046.91	42272.60
A_68_P24160214	chr5:145016060-145016104	NM_018772:-11	Bri3	PROMOTER	2.046	3.409	1012.84	3452.90	6.976	891.10	6216.12
A_68_P20266458	chr1:59969786-59969830	NM_001037725:-41	Fam117b	PROMOTER	2.045	6.604	2387.87	15770.47	13.504	1898.02	25630.46
A_68_P20828236	chr1:173433692-173433736	NM_019484:106	Refbp2	INSIDE	2.045	2.577	711.82	1834.64	5.271	588.31	3101.00
A_68_P28195044	chr12:3426740-3426784	NR_030694:-15	1110002L01Rik	DIVERGENT_PROMOTER	2.044	6.470	2368.60	15325.50	13.224	1639.16	21676.61
A_68_P29683667	chr14:67850834-67850879	NM_010095:-1272	Ebf2	PROMOTER	2.044	7.888	448.05	3534.34	16.125	291.83	4705.88
A_68_P24535484	chr6:71221790-71221834	NM_145568:-200	Krcc1	DIVERGENT_PROMOTER	2.044	6.040	3652.61	22061.07	12.346	2689.46	33203.28
A_68_P23985069	chr5:111760075-111760119	NM_019640:314	Pitpnb	INSIDE	2.044	3.246	1597.28	5185.40	6.636	1287.60	8543.96
A_68_P24536895	chr6:71493871-71493915	NM_025783:45	Vps24	INSIDE	2.044	6.284	3257.70	20471.36	12.844	2453.21	31509.98
A_68_P26253528	chr8:128258194-128258238	NM_025636:114	Ntpcr	INSIDE	2.043	4.405	4197.86	18493.38	8.999	3121.18	28087.87
A_68_P27096130	chr10:42398668-42398712	NM_172416:-31	Ostm1	PROMOTER	2.043	8.153	7419.15	60484.62	16.654	5007.72	83396.75
A_68_P24769157	chr6:115084895-115084939	NM_013681:-3	Syn2	PROMOTER	2.043	2.768	1816.41	5028.12	5.654	1390.76	7863.42
A_68_P29449771	chr14:21748812-21748856	NM_009502:180	Vcl	INSIDE	2.043	1.657	1740.03	2882.66	3.385	1324.16	4482.62
A_68_P22947351	chr4:57795187-57795231	ENSMUST00000124581:157		INSIDE	2.043	4.310	5761.20	24831.12	8.805	3955.52	34829.01
A_68_P27230702	chr10:70061703-70061747	NM_001162846:-6164	Phyhipl	PROMOTER	2.042	3.479	2120.64	7376.67	7.102	1778.00	12627.91
A_68_P31839435	chr18:75979484-75979528	NM_145356:-325	Zbtb7c	PROMOTER	2.041	4.500	2203.19	9914.56	9.184	1824.68	16758.33
A_68_P21394209	chr2:84555435-84555479	NM_144887:-135	Zdhhc5	PROMOTER	2.041	7.232	8025.09	58033.93	14.762	6272.09	92586.57
A_68_P20252736	chr1:57434804-57434848	NM_028546:363	1700066M21Rik	INSIDE	2.040	7.560	1416.99	10712.47	15.420	1205.70	18591.62
A_68_P24002947	chr5:114723893-114723937	NM_148935:-144	Foxn4	PROMOTER	2.040	1.930	249.67	481.97	3.938	173.95	684.94
A_68_P22321899	chr3:89635034-89635078	NM_172530:-235	She	PROMOTER	2.040	5.853	1470.83	8608.54	11.942	1263.72	15091.48
A_68_P23351277	chr4:138908735-138908779	NM_001039200:249	C230096C10Rik	INSIDE	2.039	5.358	960.53	5146.29	10.923	813.03	8880.43
A_68_P20135584	chr1:33964894-33964938	NM_177235:-450	Bend6	PROMOTER	2.037	3.894	1308.53	5095.77	7.934	871.14	6911.80
A_68_P30349855	chr15:76502255-76502299	NM_033371:167	Ppp1r16a	INSIDE	2.037	5.148	4503.69	23185.47	10.486	3132.18	32843.45
A_68_P31126789	chr17:29374780-29374824	NM_153166:-67	Cpne5	PROMOTER	2.036	2.750	5129.96	14108.92	5.599	4116.55	23049.87
A_68_P32281785	chrX:17740258-17740302	NM_009483:488	Kdm6a	INSIDE	2.036	2.133	1484.78	3166.69	4.342	891.83	3872.13
A_68_P25588869	chr7:148633509-148633553	NM_026020:-18	Rplp2	DIVERGENT_PROMOTER	2.036	1.443	1519.74	2192.72	2.938	1093.90	3214.10
A_68_P30255506	chr15:59479250-59479294	NM_144549:-936	Trib1	PROMOTER	2.036	6.079	9403.85	57161.85	12.376	6351.70	78609.39
A_68_P20259677	chr1:58643036-58643080	NM_172513:119	Fam126b	INSIDE	2.034	5.228	4857.54	25394.96	10.635	3718.15	39544.05
A_68_P24839014	chr6:128387845-128387889	NM_010219:783	Fkbp4	INSIDE	2.034	8.102	3964.90	32121.93	16.475	2576.69	42451.57
A_68_P30349763	chr15:76491001-76491045	NM_010630:-48	Kifc2	DIVERGENT_PROMOTER	2.034	5.654	3010.19	17019.65	11.499	2241.26	25773.16
A_68_P21753788	chr2:153067075-153067119	NM_018807:-2	Plagl2	DIVERGENT_PROMOTER	2.034	5.406	1530.17	8272.83	10.998	1242.41	13664.06
A_68_P32576283	chrX:101395692-101395736	NM_001077354:742	C77370	INSIDE	2.033	6.035	5902.41	35622.69	12.267	3428.34	42055.65
A_68_P23695553	chr5:52957300-52957344	NM_144945:197	Lgi2	INSIDE	2.033	6.026	7794.77	46973.65	12.249	6150.40	75336.94
A_68_P26021886	chr8:87308677-87308721	NM_001081982:15541	Nfix	INSIDE	2.033	3.153	2950.03	9300.15	6.408	2216.58	14204.27
A_68_P21888793	chr2:177877242-177877286	NM_028806:627	Phactr3	INSIDE	2.033	15.233	3107.48	47336.47	30.969	1919.41	59441.49
A_68_P31122921	chr17:28660085-28660129	NM_026290:-7699	4930511I11Rik	PROMOTER	2.032	1.699	1585.67	2693.97	3.452	1228.85	4241.70
A_68_P30560747	chr16:14163515-14163559	NM_001114085:169	Nde1	INSIDE	2.032	5.369	18178.12	97589.71	10.906	11694.21	127539.10
A_68_P31013265	chr17:4907805-4907849	AK186662:128019		INSIDE	2.032	5.461	2831.22	15461.30	11.095	2051.46	22761.46

ProbeName	Target position of probe on CpG island microarray	TargetID	GeneSymbol	CpG island Description	Ratio of relative methylation (TiO_2-NP/Vehicle)	Sham group			TiO_2-H group		
						Relative methylation (Cy5/Cy3)	Cy3 signal (Input)	Cy5 signal (MeDIP)	Relative methylation (Cy5/Cy3)	Cy3 signal (Input)	Cy5 signal (MeDIP)
A_68_P21113215	chr2:31939833-31939877	NM_175511:-629	Fam78a	PROMOTER	2.031	5.322	1178.34	6271.46	10.808	1047.04	11316.20
A_68_P27893219	chr11:68936458-68936502	NM_033041:2526	Hes7	INSIDE	2.031	4.211	3734.43	15726.52	8.552	2629.86	22489.56
A_68_P31496424	chr18:11815840-11815884	NM_001081223:-488	Rbbp8	PROMOTER	2.031	5.306	1302.81	6913.15	10.775	937.39	10100.56
A_68_P24328142	chr6:29685305-29685349	NM_176996:-170	Smo	PROMOTER	2.031	6.080	4339.25	26382.27	12.347	3096.61	38233.21
A_68_P30596851	chr16:22265603-22265647	NM_009186:378	Tra2b	INSIDE	2.031	3.657	723.92	2647.65	7.430	568.60	4224.62
A_68_P21813623	chr2:163470481-163470525	NR_028113:-83	0610039K10Rik	PROMOTER	2.030	6.080	3175.66	19308.06	12.340	2365.83	29194.46
A_68_P27569029	chr11:6447001-6447045	NM_001190343:133	Ccm2	INSIDE	2.030	7.059	1906.52	13457.51	14.326	1432.75	20526.24
A_68_P33007786	chr4_random:127770-127814	NM_001033326:17991	Dhrsx	INSIDE	2.030	7.054	14713.05	103787.70	14.321	9700.82	138923.80
A_68_P33007785	chr4_random:127689-127733	NM_001033326:17909	Dhrsx	INSIDE	2.029	9.778	14226.98	139116.50	19.840	9243.32	183387.90
A_68_P22899537	chr4:48292195-48292239	NM_029572:245	Erp44	INSIDE	2.029	6.351	2110.38	13402.42	12.888	1564.24	20159.97
A_68_P22934435	chr4:55545469-55545513	NM_010637:-143	Klf4	PROMOTER	2.029	4.472	1865.43	8341.62	9.073	1581.81	14351.88
A_68_P27285555	chr10:80260794-80260838	NM_023900:555	Plekhj1	INSIDE	2.029	5.818	14811.46	86178.09	11.803	10962.46	129386.30
A_68_P21754954	chr2:153270097-153270141	NM_001001986:97	8430427H17Rik	INSIDE	2.028	6.050	1367.93	8276.38	12.269	1143.90	14034.44
A_68_P28997663	chr13:44826717-44826761	NM_001205043:99	Jarid2	INSIDE	2.028	5.777	5348.99	30900.59	11.717	3961.04	46412.44
A_68_P30514378	chr16:5203992-5204036	NM_013796:91	Nagpa	INSIDE	2.028	3.462	3483.62	12060.51	7.023	2716.12	19074.05
A_68_P27497678	chr10:119913602-119913646	NM_010441:367	Hmga2	INSIDE	2.027	10.620	1669.67	17732.25	21.526	1108.46	23860.40
A_68_P27788199	chr11:50190689-50190733	NM_021510:-510	Hnrnph1	PROMOTER	2.027	5.389	4503.43	24268.11	10.925	3367.63	36792.22
A_68_P25962148	chr8:75095909-75095953	NM_144534:-71	Tmem38a	DIVERGENT_PROMOTER	2.027	3.910	3988.38	15596.27	7.928	3405.73	27001.85
A_68_P30436287	chr15:91403612-91403656	NM_001033633:58	Slc2a13	INSIDE	2.026	8.109	4830.94	39175.08	16.427	3504.49	57568.49
A_68_P20621314	chr1:134314077-134314121	NM_172516:69	Dstyk	INSIDE	2.025	8.533	658.52	5619.20	17.275	566.30	9782.98
A_68_P32555655	chrX:96666023-96666067	NM_001083110:30	Pja1	INSIDE	2.025	2.458	1745.67	4290.00	4.976	972.49	4838.95
A_68_P32320545	chrX:34415031-34415075	NM_019668:692	Ube2a	INSIDE	2.025	3.110	1464.24	4554.02	6.297	822.03	5176.49
A_68_P24619664	chr6:88035091-88035135	NM_133933:646	Rpn1	INSIDE	2.023	2.406	687.90	1655.17	4.867	583.12	2838.15
A_68_P23468796	chr5:8622618-8622662	NM_198620:312	Rundc3b	INSIDE	2.023	2.921	1876.82	5481.80	5.908	1531.02	9044.67
A_68_P23440100	chr4:155079091-155079135	NM_026899:189	Ssu72	INSIDE	2.023	4.457	9756.79	43489.91	9.016	7677.19	69218.56
A_68_P25232263	chr7:81157988-81158032	NM_009181:558	St8sia2	INSIDE	2.023	4.807	8465.95	40693.13	9.725	6151.28	59819.75
A_68_P32258635	chrX:11384158-11384202			Unknown	2.023	3.830	857.33	3283.38	7.748	470.95	3649.06
A_68_P23297489	chr4:129278119-129278163	NM_029748:190	2510006D16Rik	INSIDE	2.022	9.500	6864.88	65215.47	19.211	6025.84	115762.80
A_68_P23925841	chr5:100157706-100157750	NR_030692:351	A930011G23Rik	INSIDE	2.022	3.906	1456.65	5689.62	7.898	1228.64	9704.30
A_68_P26137548	chr8:108583970-108584014	NM_010901:490	Nfatc3	INSIDE	2.022	5.688	2426.16	13798.89	11.497	1782.99	20499.86
A_68_P29455871	chr14:22804612-22804656	NM_145459:4189	Zfp503	INSIDE	2.022	3.937	2979.74	11729.99	7.960	2329.53	18543.92
A_68_P25297066	chr7:94395079-94395123	NM_015760:-204	Nox4	PROMOTER	2.021	2.806	1848.01	5184.78	5.670	1421.14	8058.25
A_68_P25505272	chr7:134489178-134489222	NM_146202:-372	Zfp768	PROMOTER	2.021	1.770	593.28	1049.86	3.577	545.91	1952.67
A_68_P29018253	chr13:48323422-48323466	ENSMUST00000143259:34548		INSIDE	2.021	6.626	8014.06	53098.56	13.390	6186.98	82841.30
A_68_P26741575	chr9:95307812-95307856	NM_018763:-145	Chst2	PROMOTER	2.020	6.357	3712.67	23602.70	12.843	2845.78	36548.30
A_68_P23440105	chr4:155079607-155079651	NM_026899:705	Ssu72	INSIDE	2.020	6.071	1128.51	6850.95	12.263	891.79	10935.81
A_68_P32749012	chrX:148777026-148777070	NM_029836:-21	Tspyl2	PROMOTER	2.020	6.415	31502.22	202084.40	12.961	19139.72	248069.50
A_68_P30588341	chr16:20591588-20591632	NM_182636:1956	Vwa5b2	INSIDE	2.020	2.519	1889.99	4761.39	5.088	1498.07	7621.73
A_68_P21736747	chr2:149656520-149656564	NM_001085521:24	Tmem90b	INSIDE	2.019	14.996	3615.40	54215.89	30.269	2609.57	78989.27
A_68_P24064187	chr5:125491813-125491857	NM_028443:7990	Fam101a	INSIDE	2.018	3.517	2606.98	9168.48	7.098	1942.22	13786.55
A_68_P26693194	chr9:85220859-85220903	NM_001160379:-123	Fam46a	PROMOTER	2.018	2.016	961.09	1937.50	4.067	820.03	3335.24
A_68_P28036776	chr11:94982434-94982478	NM_010055:1026	Dlx3	INSIDE	2.017	2.367	3116.23	7376.19	4.774	2074.45	9904.28
A_68_P26588443	chr9:65427955-65427999	NM_153119:-135	Plekho2	DIVERGENT_PROMOTER	2.017	3.347	4537.00	15184.92	6.750	3697.39	24956.61
A_68_P29236942	chr13:95558754-95558798	NM_009321:-121	Tbca	PROMOTER	2.017	4.511	2758.98	12446.79	9.101	2323.29	21144.94
A_68_P27532842	chr10:126338342-126338386	NM_026858:63	Xrcc6bp1	INSIDE	2.017	4.400	3059.98	13463.66	8.873	2300.66	20414.17
A_68_P30578494	chr16:18234940-18234984	NM_172151:267	Zdhhc8	INSIDE	2.017	3.442	5731.05	19726.11	6.943	4192.76	29109.48
A_68_P21099935	chr2:29724646-29724690	NM_207298:-345	Cercam	PROMOTER	2.016	4.334	846.01	3666.90	8.736	713.80	6236.04
A_68_P29020011	chr13:48641397-48641441	NR_029725:-7777	Mirlet7a-1	PROMOTER	2.016	5.034	3588.40	18065.04	10.151	2852.00	28949.53
A_68_P32540976	chrX:92391835-92391879	ENSMUST00000113884:304		INSIDE	2.016	7.714	9301.38	71751.77	15.552	6043.94	93993.33
A_68_P22882382	chr4:45355016-45355060	NM_153167:66	Dcaf10	INSIDE	2.015	1.758	1505.23	2646.64	3.543	1371.16	4857.88
A_68_P20451399	chr1:94728353-94728397	NM_016696:112	Gpc1	INSIDE	2.015	7.014	1900.79	13332.30	14.136	1221.02	17260.24

ProbeName	Target position of probe on CpG island microarray	TargetID	GeneSymbol	CpG island Description	Ratio of relative methylation (TiO_2-NP/Vehicle)	Sham group			TiO_2-H group		
						Relative methylation (Cy5/Cy3)	Cy3 signal (Input)	Cy5 signal (MeDIP)	Relative methylation (Cy5/Cy3)	Cy3 signal (Input)	Cy5 signal (MeDIP)
A_68_P21747518	chr2:151908021-151908065	NM_001160410:778	Scrt2	INSIDE	2.015	5.702	6790.30	38718.10	11.488	4305.90	49465.33
A_68_P28881600	chr13:23556264-23556308	NM_013483:1484	Btn1a1	INSIDE	2.014	3.623	1561.40	5656.95	7.296	1294.60	9445.96
A_68_P27542462	chr10:128082827-128082871	NM_011772:201	Ikzf4	INSIDE	2.014	6.594	3300.43	21761.88	13.277	2547.16	33818.33
A_68_P22413373	chr3:108365105-108365149	NM_026198:258	Tmem167b	INSIDE	2.014	5.899	11035.30	65100.15	11.883	7806.95	92767.71
A_68_P32054917	chr19:29880262-29880306	NM_172836:215	9930021J03Rik	INSIDE	2.013	8.406	1611.24	13543.82	16.922	1113.14	18836.77
A_68_P23430516	chr4:153673821-153673865	NM_001112744:162	Arhgef16	INSIDE	2.012	3.473	3277.71	11382.83	6.988	2371.01	16569.62
A_68_P26015683	chr8:86132210-86132254	NM_018808:159	Dnajb1	INSIDE	2.012	3.343	2738.47	9154.51	6.726	2170.21	14596.54
A_68_P26224550	chr8:123632450-123632508	NM_001166482:-199	Mthfsd	DIVERGENT_PROMOTER	2.012	13.659	8664.67	118351.90	27.485	5760.48	158325.40
A_68_P29596330	chr14:50682188-50682232	AK041390:1176		INSIDE	2.012	2.046	949.94	1944.02	4.118	747.94	3080.16
A_68_P24608765	chr6:86145281-86145325	NM_031199:58	Tgfa	INSIDE	2.011	4.196	265.89	1115.69	8.439	229.08	1933.18
A_68_P32326603	chrX:35543022-35543066	NM_001079513:75	Zbtb33	INSIDE	2.011	4.310	831.73	3584.55	8.668	491.76	4262.69
A_68_P24864944	chr6:135015833-135015878	NM_181444:176	Gprc5a	INSIDE	2.010	8.465	1809.30	15316.48	17.014	1441.62	24527.37
A_68_P33005811	A_68_P33005811			Unknown	2.010	7.835	405.82	3179.55	15.748	365.25	5751.97
A_68_P26022583	chr8:87432507-87432551	NM_010062:6	Dnase2a	INSIDE	2.009	11.553	2603.56	30078.45	23.214	1599.71	37136.06
A_68_P24784010	chr6:117867385-117867429	NM_001166427:95	Hnrnpf	INSIDE	2.009	4.788	2320.95	11113.62	9.618	1897.26	18248.16
A_68_P22696144	chr4:4066014-4066058	NM_001002927:-444	Penk	PROMOTER	2.009	5.062	1845.48	9341.22	10.168	1526.33	15519.67
A_68_P27564785	chr11:5641343-5641387	NM_178623:-185	Urgcp	PROMOTER	2.009	0.300	1036.82	310.68	0.602	1175.91	707.96
A_68_P25616898	chr8:4259319-4259363	NM_183315:-66	Ctxn1	PROMOTER	2.007	4.280	4968.20	21264.26	8.592	3603.90	30964.43
A_68_P31156111	chr17:34987166-34987210	NM_021337:-39	Skiv2l	DIVERGENT_PROMOTER	2.007	5.126	962.58	4934.17	10.287	766.37	7883.27
A_68_P20614481	chr1:133141395-133141439	NM_018750:339	Rassf5	INSIDE	2.006	1.595	980.47	1563.95	3.200	835.62	2674.01
A_68_P25670551	chr8:13785477-13785521	NM_025924:-116	Upf3a	PROMOTER	2.006	1.435	1349.51	1936.78	2.880	977.43	2814.58
A_68_P29024653	chr13:49436665-49436709	NM_001039179:-231	Bicd2	PROMOTER	2.005	1.473	918.00	1352.30	2.953	718.95	2123.30
A_68_P31836381	chr18:75502057-75502101			Unknown	2.005	5.647	7194.94	40631.71	11.322	5950.74	67373.66
A_68_P28328448	chr12:31596461-31596505	NM_001110239:-5	Acp1	DIVERGENT_PROMOTER	2.003	3.807	1330.58	5065.67	7.626	1077.42	8216.80
A_68_P26571039	chr9:62384681-62384725	NM_175484:149	Coro2b	INSIDE	2.003	9.729	15328.83	149132.30	19.488	12051.50	234861.50
A_68_P26658192	chr9:77961262-77961306	NM_019987:240	Ick	INSIDE	2.003	10.295	19505.51	200801.00	20.621	11145.86	229837.30
A_68_P21906908	chr2:180628801-180628845	NR_029538:78	Mir124a-3	DOWNSTREAM	2.003	1.584	457.25	724.51	3.173	393.78	1249.64
A_68_P21152636	chr2:39045852-39045896	NM_178778:376	Scai	INSIDE	2.003	6.019	21465.77	129193.80	12.053	15269.99	184042.30
A_68_P28080444	chr11:102467782-102467826	NM_020510:2060	Fzd2	INSIDE	2.002	1.520	598.43	909.33	3.043	460.34	1400.67
A_68_P26588687	chr9:65478956-65479000	NM_028030:590	Rbpms2	INSIDE	2.002	7.101	1348.61	9576.98	14.215	1028.12	14614.30
A_68_P27842891	chr11:60230619-60230663	NM_025757:-6	4933439F18Rik	DIVERGENT_PROMOTER	2.001	5.082	6324.86	32141.10	10.170	4611.32	46897.03
A_68_P23447093	chr5:3802955-3802999	NM_001003909:133	Ankib1	INSIDE	2.001	4.414	2001.52	8835.30	8.832	1594.37	14080.72
A_68_P25264893	chr7:87260180-87260224	NM_173011:34	Idh2	INSIDE	2.001	5.771	760.03	4386.16	11.547	694.19	8015.83
A_68_P27901897	chr11:70468130-70468174	NM_011072:0	Pfn1	INSIDE	2.001	12.585	2812.51	35396.18	25.178	2091.82	52667.40
A_68_P24816773	chr6:124696183-124696227	NM_007881:10301	Atn1	INSIDE	2.000	4.992	1835.22	9160.55	9.981	1390.13	13875.38
A_68_P32380519	chrX:49518713-49518757	NM_008150:-634	Gpc4	PROMOTER	2.000	3.934	2022.85	7957.44	7.869	1423.61	11202.84
A_68_P24176699	chr5:148769334-148769378	NM_029920:461	Mtus2	INSIDE	2.000	5.862	4947.09	28999.70	11.723	3794.87	44486.85
A_68_P21359595	chr2:77654503-77654547	NM_178723:349	Zfp385b	INSIDE	2.000	4.636	925.18	4289.04	9.270	697.33	6464.28
A_68_P23658789	chr5:46248126-46248170	NM_172153:-357	Lcorl	PROMOTER	1.999	2.835	1420.42	4026.53	5.666	1124.72	6372.68
A_68_P20998559	chr2:9970073-9970117	NM_027748:142	Taf3	INSIDE	1.999	3.882	2680.44	10404.16	7.759	2242.04	17396.16
A_68_P23447098	chr5:3803414-3803458	NR_033173:256	Krit1	INSIDE	1.998	5.399	2144.76	11579.96	10.788	1737.16	18740.61
A_68_P24804564	chr6:121423714-121423758	NM_001033354:-40	Iqsec3	PROMOTER	1.997	3.206	1771.26	5679.11	6.403	1380.83	8841.48
A_68_P25507242	chr7:134920047-134920091	NM_178029:-834	Setd1a	PROMOTER	1.997	4.883	785.76	3837.16	9.754	536.84	5236.52
A_68_P32766764	chrX:153929309-153929353	NM_009214:648	Sms	INSIDE	1.997	4.112	1440.88	5924.68	8.211	837.57	6877.17
A_68_P20012423	chr1:6373229-6373273	NM_001195732:23839	Fam150a	INSIDE	1.996	3.088	938.57	2898.62	6.165	860.84	5306.75
A_68_P21221051	chr2:52531541-52531585	NM_001037099:539	Cacnb4	INSIDE	1.994	6.214	1722.92	10705.97	12.392	1153.42	14292.68
A_68_P21418382	chr2:90519554-90519598	NM_021512:2205	Nup160	INSIDE	1.994	5.312	8159.69	43345.81	10.590	6258.95	66281.53
A_68_P26140014	chr8:109035535-109035580	NM_001037809:745	Cdh3	INSIDE	1.993	3.372	10881.57	36690.04	6.721	7717.80	51872.65
A_68_P22378552	chr3:101816741-101816785	NM_178777:2695	Nhlh2	INSIDE	1.993	4.711	4515.17	21271.37	9.388	3478.52	32656.96
A_68_P23018376	chr4:73436995-73437039	NM_001017427:-510	Rasef	PROMOTER	1.993	7.837	1435.05	11246.82	15.617	1145.53	17889.46
A_68_P20877753	chr1:182708067-182708111	AK013590:116		INSIDE	1.993	2.064	359.44	741.95	4.115	393.88	1620.70

ProbeName	Target position of probe on CpG island microarray	TargetID	GeneSymbol	CpG island Description	Ratio of relative methylation (TiO_2-NP/Vehicle)	Sham group			TiO_2-H group		
						Relative methylation (Cy5/Cy3)	Cy3 signal (Input)	Cy5 signal (MeDIP)	Relative methylation (Cy5/Cy3)	Cy3 signal (Input)	Cy5 signal (MeDIP)
A_68_P32321491	chrX:34588512-34588556	NM_173779:-305	Ankrd58	PROMOTER	1.991	3.764	4900.57	18445.54	7.492	3053.16	22875.36
A_68_P25505711	chr7:134615245-134615289	NM_145589:139	Prr14	INSIDE	1.991	1.548	1093.16	1692.00	3.081	841.95	2593.98
A_68_P28175491	chr11:118907932-118907976	NM_013926:-5727	Cbx8	PROMOTER	1.989	1.758	536.84	943.64	3.497	458.11	1601.84
A_68_P33008826	chr4_random:110027-110071	NM_001033326:247	Dhrsx	INSIDE	1.989	5.009	2018.28	10110.10	9.965	1605.78	16000.96
A_68_P24942904	chr6:148895207-148895251	NM_019643:-274	Fam60a	PROMOTER	1.989	4.671	2575.90	12031.24	9.289	2051.98	19060.53
A_68_P23029295	chr4:76095885-76095929	NM_011211:144297	Ptprd	INSIDE	1.989	6.927	2157.13	14942.00	13.775	1517.81	20907.64
A_68_P27177056	chr10:60293675-60293719	NM_029770:633	Unc5b	INSIDE	1.989	3.673	1451.34	5330.04	7.303	1228.29	8970.39
A_68_P22319097	chr3:89196146-89196190	NM_009565:957	Zbtb7b	INSIDE	1.989	0.704	1297.39	913.61	1.400	1152.06	1613.43
A_68_P24774306	chr6:115943883-115943927	NM_026376:1119	Plxnd1	INSIDE	1.988	5.492	1597.59	8773.58	10.917	1045.67	11415.58
A_68_P20294265	chr1:64784540-64784584	NM_001042659:-238	Fzd5	PROMOTER	1.987	4.693	1117.88	5245.69	9.322	838.15	7813.62
A_68_P29369781	chr13:119503243-119503287	NM_008002:-241	Fgf10	PROMOTER	1.986	4.921	7424.08	36535.57	9.775	5894.22	57617.72
A_68_P26430635	chr9:37240928-37240972	NM_001164767:-190	Robo3	PROMOTER	1.986	5.036	3993.54	20110.91	10.002	2951.93	29524.69
A_68_P32557243	chrX:97015613-97015657	NM_001081283:-773	Tmem28	PROMOTER	1.986	2.695	738.08	1989.24	5.353	374.47	2004.59
A_68_P30593952	chr16:21695060-21695104	NM_001001881:-344	2510009E07Rik	PROMOTER	1.985	1.567	1249.70	1958.13	3.110	936.20	2911.16
A_68_P30958538	chr16:90727762-90727806	NM_025642:-168	2610039C10Rik	PROMOTER	1.985	7.148	6153.67	43986.93	14.192	4023.20	57098.84
A_68_P21419450	chr2:90726161-90726205	NM_026161:240	C1qtnf4	INSIDE	1.985	3.211	2122.23	6815.20	6.373	1771.75	11291.25
A_68_P21613749	chr2:127308338-127308382	NM_001111331:-125	Kcnip3	PROMOTER	1.984	9.371	1665.44	15607.18	18.594	1049.56	19515.72
A_68_P24537770	chr6:71657709-71657753	NM_178608:56	Reep1	INSIDE	1.984	8.149	1094.29	8917.83	16.167	912.91	14759.37
A_68_P31411712	chr17:86014601-86014645	NR_038085:3114	Six3os1	INSIDE	1.984	4.684	10986.84	51460.60	9.291	7340.72	68205.52
A_68_P28154074	chr11:115397049-115397093	NM_133354:474	Sumo2	INSIDE	1.984	7.154	14395.30	102980.70	14.191	11130.77	157957.40
A_68_P25958256	chr8:74203690-74203734	NM_001190830:3431	Jak3	INSIDE	1.983	3.564	823.69	2935.98	7.070	656.49	4641.15
A_68_P32140500	chr19:45857855-45857899	NM_023799:-95	Mgea5	PROMOTER	1.983	7.289	757.01	5518.17	14.456	564.55	8161.40
A_68_P31609277	chr18:33954596-33954640	NR_038151:73	2410004N09Rik	INSIDE	1.982	1.727	3048.18	5263.10	3.423	2411.34	8253.95
A_68_P28078744	chr11:102157408-102157452	NM_001098836:513	Atxn7l3	INSIDE	1.982	2.125	1191.74	2532.41	4.212	930.26	3917.90
A_68_P27804074	chr11:53113101-53113145	NM_008300:859	Hspa4	INSIDE	1.982	3.316	6412.82	21267.40	6.574	5187.91	34105.70
A_68_P26345150	chr9:20836502-20836546	NM_008319:43	Icam5	INSIDE	1.982	4.277	4438.10	18981.33	8.477	3506.13	29722.46
A_68_P25807719	chr8:41947388-41947432	NM_007514:-310	Slc7a2	PROMOTER	1.982	6.030	7612.40	45905.08	11.954	5257.46	62847.22
A_68_P30363493	chr15:78798329-78798373	NM_001039155:20197	Triobp	INSIDE	1.982	11.133	1510.57	16816.93	22.062	976.39	21540.76
A_68_P28156930	chr11:115891653-115891697	NM_172569:39	Unk	INSIDE	1.982	3.224	656.27	2115.97	6.391	542.34	3466.17
A_68_P23962878	chr5:107125443-107125487	NM_026856:385	Zfp644	INSIDE	1.982	5.364	4973.92	26679.75	10.633	4228.15	44958.50
A_68_P26543338	chr9:57556763-57556807	NM_153799:409	Edc3	INSIDE	1.980	7.076	3933.06	27831.33	14.009	2928.64	41028.03
A_68_P26622301	chr9:71333539-71333583	NM_001164793:-795	Grinl1a	PROMOTER	1.980	2.353	1200.03	2824.26	4.660	1053.28	4908.56
A_68_P25005776	chr7:25396309-25396353	NM_001102613:-16	Phldb3	PROMOTER	1.980	6.753	6453.48	43581.39	13.373	4202.48	56201.35
A_68_P28595369	chr12:85262700-85262744	ENSMUST00000085215:72		INSIDE	1.980	3.043	16786.27	51079.68	6.026	12030.00	72496.26
A_68_P27900652	chr11:70246079-70246123	NM_145429:-54	Arrb2	PROMOTER	1.979	2.610	1169.34	3052.36	5.167	916.74	4736.55
A_68_P21395110	chr2:84712227-84712271	NM_199223:14601	Rtn4rl2	INSIDE	1.979	6.057	2764.73	16746.15	11.988	2317.19	27778.80
A_68_P25370744	chr7:108123196-108123240			Unknown	1.979	2.486	1180.31	2934.71	4.921	930.79	4580.85
A_68_P25911981	chr8:64238686-64238730	NM_001081390:142779	Palld	INSIDE	1.978	2.275	971.13	2209.05	4.500	779.80	3508.97
A_68_P21042876	chr2:18919881-18919925	NR_024323:-43	4930426L09Rik	DIVERGENT_PROMOTER	1.977	7.301	2991.97	21843.57	14.432	2189.70	31602.67
A_68_P20016449	chr1:7388374-7388418	ENSMUST00000138711:-514		PROMOTER	1.977	4.924	4200.99	20683.63	9.733	3349.27	32597.71
A_68_P20240346	chr1:55145446-55145490	NM_008303:477	Hspe1	INSIDE	1.976	4.193	1419.21	5951.07	8.284	985.84	8167.04
A_68_P21070656	chr2:25080752-25080796	NM_146116:-552	Tubb2c	PROMOTER	1.976	4.220	2765.86	11672.93	8.339	2234.63	18633.69
A_68_P30433473	chr15:90880476-90880520	NM_001109040:-119	Kif21a	PROMOTER	1.975	4.611	5323.48	24544.24	9.106	4245.04	38656.75
A_68_P23006650	chr4:70195415-70195459	NM_172694:526	Megf9	INSIDE	1.975	3.715	8274.32	30737.56	7.336	6416.84	47074.16
A_68_P30650846	chr16:32079318-32079362	NM_177326:19	Pak2	INSIDE	1.975	8.408	4276.72	35957.81	16.607	3448.53	57270.10
A_68_P27921681	chr11:74462947-74462991	NM_001081158:-28	1300001I01Rik	PROMOTER	1.974	4.812	4348.74	20926.06	9.500	3488.37	33138.09
A_68_P25088286	chr7:51846961-51847005	NM_008422:727	Kcnc3	INSIDE	1.974	6.215	2829.89	17588.75	12.271	2291.82	28124.06
A_68_P31110054	chr17:26552202-26552246	NM_001081656:315	Neurl1b	INSIDE	1.974	5.321	1188.55	6323.81	10.504	1036.44	10886.31
A_68_P27278802	chr10:79229397-79229442	NM_144528:247	Rnf126	INSIDE	1.974	4.084	5437.70	22205.53	8.062	4054.43	32684.99
A_68_P31479225	chr18:7869058-7869102	NM_001146298:-114	Wac	PROMOTER	1.974	4.017	3388.04	13608.31	7.928	2757.80	21863.00
A_68_P23396578	chr4:148179082-148179126	NM_027195:604	Casz1	INSIDE	1.973	5.530	2948.56	16304.53	10.908	2126.42	23195.85

ProbeName	Target position of probe on CpG island microarray	TargetID	GeneSymbol	CpG island Description	Ratio of relative methylation (TiO_2-NP/Vehicle)	Sham group			TiO_2-H group		
						Relative methylation (Cy5/Cy3)	Cy3 signal (Input)	Cy5 signal (MeDIP)	Relative methylation (Cy5/Cy3)	Cy3 signal (Input)	Cy5 signal (MeDIP)
A_68_P20963945	chr2:3630790-3630834	NM_025626:83	Fam107b	INSIDE	1.973	1.721	749.08	1289.47	3.396	585.50	1988.33
A_68_P21783928	chr2:158436941-158436985	NM_009508:469	Slc32a1	INSIDE	1.973	5.667	21029.26	119167.00	11.180	15800.81	176658.30
A_68_P22630556	chr3:148650621-148650665	ENSMUST00000098518:1638		INSIDE	1.973	2.845	6332.89	18017.74	5.614	4669.41	26214.69
A_68_P28432636	chr12:53071968-53072012	NM_177171:318	Heatr5a	INSIDE	1.972	2.436	3384.93	8244.91	4.803	2605.10	12511.36
A_68_P28053756	chr11:97847867-97847911	NM_001163608:-128	Plxdc1	PROMOTER	1.972	4.486	1026.12	4602.73	8.846	764.65	6763.79
A_68_P25412183	chr7:117365487-117365531	NM_009302:292	Swap70	INSIDE	1.972	5.336	5679.82	30307.86	10.520	4381.06	46089.90
A_68_P24124224	chr5:137507416-137507460	NM_001039385:1274	Vgf	INSIDE	1.972	7.730	2393.17	18499.10	15.247	1785.80	27227.51
A_68_P27591949	chr11:11585605-11585649	NM_001025597:-589	Ikzf1	PROMOTER	1.971	6.461	328.40	2121.91	12.737	332.67	4237.04
A_68_P31992368	chr19:18745200-18745244	NM_026120:-47	2410127L17Rik	PROMOTER	1.970	8.119	1112.52	9032.07	15.996	768.74	12296.69
A_68_P27788005	chr11:50138862-50138906	NM_001110499:291	Canx	INSIDE	1.970	3.156	5574.41	17595.39	6.220	4426.17	27529.64
A_68_P26242535	chr8:126473140-126473184	NM_172288:3	Nup133	INSIDE	1.970	3.959	1555.56	6157.81	7.800	1160.78	9054.05
A_68_P23245805	chr4:119164931-119164975	NM_177572:251	Rimkla	INSIDE	1.970	1.959	817.12	1601.11	3.860	738.33	2849.76
A_68_P28152781	chr11:115183238-115183282	NM_176847:-28	Ush1g	PROMOTER	1.970	9.441	10000.64	94413.83	18.600	6015.03	111879.70
A_68_P29615089	chr14:55545056-55545100	NM_010112:547	Efs	INSIDE	1.969	2.680	3959.09	10609.07	5.277	2914.31	15377.54
A_68_P29152220	chr13:76081481-76081525	NM_028493:-230	Rhobtb3	PROMOTER	1.969	2.960	1198.54	3547.32	5.827	851.95	4963.87
A_68_P28515845	chr12:70782400-70782444	NM_001135559:417	Sos2	INSIDE	1.969	2.541	1958.50	4975.69	5.001	1528.15	7642.91
A_68_P21330556	chr2:73115402-73115446	NM_001005343:5442	Sp9	DOWNSTREAM	1.969	4.836	4620.55	22345.30	9.521	3436.51	32718.53
A_68_P29449770	chr14:21748700-21748744	NM_009502:68	Vcl	INSIDE	1.969	3.193	4035.96	12886.43	6.286	3134.26	19703.38
A_68_P23381901	chr4:144785001-144785047	NM_001128198:-4529	Vps13d	PROMOTER	1.969	2.303	958.14	2206.35	4.534	812.58	3684.09
A_68_P23897553	chr5:93473897-93473941	NM_175270:130	Ankrd56	INSIDE	1.968	1.785	1103.52	1970.25	3.513	847.35	2977.09
A_68_P30991207	chr16:96348653-96348697	NM_008251:658	Hmgn1	INSIDE	1.968	2.602	5404.50	14060.23	5.121	4000.73	20487.47
A_68_P27349651	chr10:92185236-92185280	NM_008682:-95	Nedd1	PROMOTER	1.968	4.104	1115.32	4576.96	8.076	934.49	7546.94
A_68_P29023779	chr13:49283004-49283048	NM_013610:111	Ninj1	INSIDE	1.968	4.358	3369.18	14682.72	8.576	2531.85	21712.21
A_68_P28533645	chr12:73862367-73862411	NM_008910:191	Ppm1a	INSIDE	1.968	4.045	3217.29	13015.10	7.962	2569.35	20457.82
A_68_P28192906	chr11:121691796-121691840	NM_029049:287	Ptchd3	INSIDE	1.968	3.225	1636.76	5277.73	6.345	1415.98	8984.10
A_68_P32197338	chr19:55969833-55969892	NM_001142924:52	Tcf7l2	INSIDE	1.968	5.017	167.54	840.57	9.872	114.89	1134.25
A_68_P30959222	chr16:90831079-90831123	NM_001199210:-3	4931408A02Rik	PROMOTER	1.967	1.517	905.38	1373.80	2.985	747.69	2231.93
A_68_P28115686	chr11:108782547-108782591	NM_015732:906	Axin2	INSIDE	1.967	3.863	1652.35	6382.81	7.597	1331.82	10117.39
A_68_P23350166	chr4:138748616-138748660	NM_009798:-207	Capzb	PROMOTER	1.967	3.798	6080.09	23094.74	7.471	4908.49	36671.62
A_68_P29614701	chr14:55482992-55483036	NM_001177705:-19	Homez	PROMOTER	1.967	3.067	3193.56	9795.99	6.033	2541.20	15329.78
A_68_P28099140	chr11:105881863-105881907	NM_001037712:12368	Kcnh6	INSIDE	1.967	7.729	2711.08	20953.02	15.199	1794.72	27278.05
A_68_P24954294	chr7:6107601-6107645	NM_001013012:-49	Zfp787	PROMOTER	1.967	4.333	5409.37	23436.41	8.522	4239.71	36128.89
A_68_P32477697	chrX:74679072-74679116			Unknown	1.967	4.351	10388.98	45204.71	8.559	7505.05	64237.52
A_68_P21752520	chr2:152857602-152857646	NM_001011732:37	Xkr7	INSIDE	1.966	7.358	1460.41	10745.14	14.466	1068.59	15458.44
A_68_P32730437	chrX:143988143-143988187	NM_001081173:460	Lrch2	INSIDE	1.965	4.409	9704.97	42791.58	8.664	6149.70	53283.79
A_68_P28615786	chr12:88729140-88729184	NM_011479:18	Sptlc2	INSIDE	1.965	7.386	683.93	5051.85	14.516	646.96	9391.54
A_68_P27644168	chr11:22967930-22967974	ENSMUST00000128996:-45		DIVERGENT_PROMOTER	1.965	6.515	903.14	5884.30	12.805	735.16	9413.94
A_68_P27289148	chr10:80827041-80827085	NM_001014836:1503	4930404N11Rik	INSIDE	1.964	4.488	7214.34	32377.00	8.812	5305.63	46755.12
A_68_P29620076	chr14:56445768-56445812	NM_001168346:2159	Nfatc4	INSIDE	1.964	6.334	3968.26	25134.45	12.440	2785.42	34650.17
A_68_P26464014	chr9:43611501-43611545	NM_021424:58864	Pvrl1	INSIDE	1.964	6.335	2397.16	15184.99	12.442	1497.26	18628.77
A_68_P22606649	chr3:144781741-144781785	NM_025714:199	Odf2l	INSIDE	1.963	4.343	4977.82	21616.42	8.526	3841.15	32748.48
A_68_P31256055	chr17:56568653-56568712	NM_011218:47221	Ptprs	INSIDE	1.963	9.451	507.20	4793.44	18.548	389.72	7228.65
A_68_P27040510	chr10:30337995-30338039	NM_025798:156	Hint3	INSIDE	1.962	3.176	1065.95	3384.99	6.229	904.96	5637.29
A_68_P20173639	chr1:40847463-40847507	NM_172499:18	Mfsd9	INSIDE	1.962	7.038	1091.71	7683.65	13.809	885.85	12232.37
A_68_P22341853	chr3:94819143-94819187	NM_030074:-4	Zfp687	PROMOTER	1.962	4.849	808.04	3917.86	9.511	672.38	6395.29
A_68_P33012155	chr9_random:54509-54553	NR_027950:-162	4930526I15Rik	PROMOTER	1.961	3.299	5356.65	17673.86	6.469	3968.95	25675.00
A_68_P22130346	chr3:51364011-51364056	NR_033624:355	5031434O11Rik	INSIDE	1.960	3.201	3208.33	10271.15	6.274	2445.68	15345.16
A_68_P26659639	chr9:78296196-78296240	NM_026658:202	Mto1	INSIDE	1.960	1.795	397.62	713.77	3.518	481.25	1693.03
A_68_P24850990	chr6:131383791-131383835			Unknown	1.960	3.368	888.30	2991.42	6.601	808.91	5339.71
A_68_P25953242	chr8:73183309-73183353	NM_023217:250	Pgpep1	INSIDE	1.959	1.778	3492.00	6208.53	3.483	2420.79	8431.85
A_68_P25276873	chr7:89483916-89483960	NM_001190374:-265	Adamtsl3	PROMOTER	1.957	2.496	301.10	751.49	4.885	287.88	1406.35

ProbeName	Target position of probe on CpG island microarray	TargetID	GeneSymbol	CpG island Description	Ratio of relative methylation (TiO_2-NP/Vehicle)	Sham group			TiO_2-H group		
						Relative methylation (Cy5/Cy3)	Cy3 signal (Input)	Cy5 signal (MeDIP)	Relative methylation (Cy5/Cy3)	Cy3 signal (Input)	Cy5 signal (MeDIP)
A_68_P27365029	chr10:94878409-94878453	NM_001168656:-312	Socs2	PROMOTER	1.957	3.650	1261.25	4603.74	7.144	1029.49	7354.78
A_68_P24946816	chr7:3637239-3637283	NM_029934:7867	Mboat7	INSIDE	1.956	4.635	2593.16	12018.65	9.064	2031.67	18415.71
A_68_P31140911	chr17:31773548-31773592	NM_178224:506	Cbs	INSIDE	1.954	3.316	7744.08	25678.94	6.479	5771.70	37394.65
A_68_P22942460	chr4:56960340-56960384	NM_175518:-61	D730040F13Rik	PROMOTER	1.954	3.183	1863.90	5932.74	6.221	1527.94	9504.93
A_68_P30343819	chr15:75578604-75578648	NM_194350:-274	Mafa	PROMOTER	1.953	3.653	1220.29	4457.21	7.135	915.27	6530.09
A_68_P24951794	chr7:5004858-5004902	NM_026900:1747	Zfp580	INSIDE	1.953	11.439	6702.51	76672.71	22.347	4388.05	98058.91
A_68_P22060448	chr3:36762071-36762115	NM_172679:65	4932438A13Rik	INSIDE	1.952	2.163	1441.07	3116.81	4.222	1152.18	4864.87
A_68_P23361905	chr4:140616349-140616393	NM_172122:-6794	Crocc	PROMOTER	1.952	1.574	3797.77	5976.06	3.071	3180.60	9768.25
A_68_P25005645	chr7:25370764-25370808	NM_023154:-1775	Ethe1	PROMOTER	1.952	5.784	9677.20	55972.42	11.292	6298.59	71125.48
A_68_P23807625	chr5:75548984-75549028	NM_001083316:-3184	Pdgfra	PROMOTER	1.952	3.211	2425.96	7790.23	6.267	1751.08	10974.80
A_68_P28598116	chr12:85750091-85750135	NM_025525:-8037	Rnf113a2	PROMOTER	1.952	7.688	929.08	7143.04	15.009	656.03	9846.31
A_68_P31163327	chr17:36394876-36394920	NM_026308:-107	Rpp21	PROMOTER	1.952	6.137	1187.76	7289.19	11.981	1029.28	12331.59
A_68_P26158736	chr8:112261757-112261801	NM_001080930:-139	Atxn1l	PROMOTER	1.951	1.963	1818.15	3569.42	3.830	1494.02	5721.52
A_68_P27788006	chr11:50138948-50138992	NM_001110499:205	Canx	INSIDE	1.951	4.085	5502.89	22480.34	7.970	3802.85	30309.46
A_68_P21134366	chr2:35438211-35438255	NM_001114125:24255	Dab2ip	INSIDE	1.951	9.744	1321.86	12879.94	19.014	1073.76	20416.14
A_68_P21978078	chr3:19957968-19958012	NM_144959:180	Hltf	INSIDE	1.951	4.988	7603.38	37929.07	9.733	5994.60	58346.11
A_68_P31097493	chr17:24606963-24607007	NM_025954:-433	Pgp	PROMOTER	1.951	7.982	4367.45	34859.26	15.570	2531.42	39413.34
A_68_P21769931	chr2:156005366-156005410	NM_133242:588	Rbm39	INSIDE	1.951	4.336	1368.97	5936.39	8.462	1195.59	10116.89
A_68_P24459338	chr6:53768811-53768855	NM_025817:1987	Tril	INSIDE	1.951	6.346	4390.67	27862.05	12.382	3566.54	44159.98
A_68_P26238816	chr8:125778247-125778291	NM_020497:174	Zfp276	INSIDE	1.951	7.620	4136.00	31517.69	14.868	3031.78	45076.72
A_68_P29996479	chr15:7760686-7760730	NM_010275:-302	Gdnf	PROMOTER	1.950	9.773	1415.04	13828.45	19.057	1081.20	20604.19
A_68_P30500682	chr15:102752309-102752353	NM_010464:769	Hoxc13	INSIDE	1.950	2.090	437.20	913.94	4.076	393.05	1601.97
A_68_P24448786	chr6:52122729-52122773	NM_010452:40316	Hoxa3	INSIDE	1.949	3.590	2816.44	10111.45	6.999	2194.43	15358.72
A_68_P32239859	chrX:7305710-7305754	NM_138602:36	Praf2	INSIDE	1.949	1.561	944.87	1475.33	3.044	603.67	1837.49
A_68_P21819631	chr2:164595113-164595157	NM_026785:-294	Ube2c	PROMOTER	1.949	1.547	2320.10	3588.52	3.015	1901.78	5734.30
A_68_P27543031	chr10:128185237-128185281	NM_001170869:-319	Wibg	PROMOTER	1.949	1.732	406.22	703.63	3.376	336.20	1134.90
A_68_P30371091	chr15:80086279-80086325	NM_009716:689	Atf4	INSIDE	1.948	3.218	1720.78	5537.99	6.270	1305.60	8185.62
A_68_P24060833	chr5:124803880-124803924	NM_013812:735	Cdk2ap1	INSIDE	1.948	8.285	867.65	7188.25	16.135	691.74	11161.30
A_68_P29330297	chr13:112515612-112515656	NM_172593:39249	Mier3	DOWNSTREAM	1.948	3.136	4485.94	14066.93	6.108	3426.27	20927.12
A_68_P32464061	chrX:70918592-70918636	NM_001142809:143	Slc6a8	INSIDE	1.948	4.473	1953.12	8735.75	8.713	1190.32	10371.57
A_68_P31147009	chr17:32924787-32924831	NM_172458:424	Zfp871	INSIDE	1.948	4.509	5798.67	26143.80	8.782	5006.12	43964.92
A_68_P25715145	chr8:24268305-24268349	NM_029002:4584	Nkx6-3	INSIDE	1.947	5.658	1617.56	9152.92	11.019	1197.01	13189.99
A_68_P32260286	chrX:11658874-11658919	NM_029510:-1217	Bcor	PROMOTER	1.946	2.868	401.28	1150.87	5.582	234.46	1308.67
A_68_P29639452	chr14:60216844-60216888	NM_001168535:-70	Cdadc1	PROMOTER	1.946	9.593	6488.31	62244.12	18.664	3827.68	71440.31
A_68_P20192816	chr1:44204826-44204870	NM_011729:260	Ercc5	INSIDE	1.946	6.459	1824.46	11783.62	12.569	1452.81	18260.66
A_68_P30686476	chr16:38522756-38522801	NM_024273:-31	4930455C21Rik	PROMOTER	1.945	6.413	5617.05	36021.49	12.474	3776.73	47112.65
A_68_P25508222	chr7:135079466-135079510	NM_001081374:10751	Prss36	INSIDE	1.945	3.919	2482.62	9729.37	7.622	1728.10	13170.75
A_68_P28119629	chr11:109472837-109472881	NM_145940:-155	Wipi1	PROMOTER	1.945	2.160	5773.50	12469.26	4.201	4146.90	17421.64
A_68_P28177063	chr11:119161761-119161805	NM_138669:-425	Eif4a3	PROMOTER	1.944	7.417	3705.01	27478.19	14.417	2789.51	40215.82
A_68_P21843420	chr2:168591529-168591573	NM_175303:1151	Sall4	INSIDE	1.944	2.326	400.24	930.99	4.522	339.93	1537.30
A_68_P23701554	chr5:53982361-53982405	NM_001080927:570	Rbpj	INSIDE	1.943	2.359	2601.07	6135.06	4.584	2146.76	9839.79
A_68_P31921054	chr19:4101079-4101123	NM_008851:479	Pitpnm1	INSIDE	1.942	3.118	795.68	2480.76	6.056	677.00	4099.75
A_68_P21622283	chr2:128792891-128792935	NM_178404:-225	Zc3h6	PROMOTER	1.942	7.387	16791.77	124034.50	14.346	11192.29	160559.10
A_68_P27842896	chr11:60231205-60231249	NM_025757:580	4933439F18Rik	INSIDE	1.941	6.392	6265.28	40045.30	12.409	5241.96	65047.20
A_68_P24786758	chr6:118369210-118369254	NM_194339:203	Bms1	INSIDE	1.941	1.975	2909.05	5744.70	3.833	2374.51	9101.42
A_68_P26983892	chr10:19653936-19653980	NM_008580:-373	Map3k5	PROMOTER	1.941	2.801	2266.02	6346.81	5.436	1777.92	9664.64
A_68_P26041379	chr8:91045377-91045421	NM_001163660:-6133	Nkd1	PROMOTER	1.941	4.503	5556.83	25021.48	8.741	4103.47	35867.01
A_68_P20506810	chr1:108069429-108069473	NM_133821:1005	Phlpp1	INSIDE	1.941	3.369	2493.42	8399.51	6.540	2097.84	13719.55
A_68_P27185081	chr10:61732662-61732706	NM_001146100:70485	Hk1	INSIDE	1.940	4.623	1350.81	6244.16	8.967	1020.86	9154.55
A_68_P24086564	chr5:129526514-129526558	NM_009391:506	Ran	INSIDE	1.940	3.942	1831.96	7220.91	7.645	1437.02	10986.09
A_68_P30544459	chr16:10785615-10785659	NM_009896:-7	Socs1	PROMOTER	1.940	4.714	4002.87	18868.14	9.143	3241.30	29633.88

ProbeName	Target position of probe on CpG island microarray	TargetID	GeneSymbol	CpG island Description	Ratio of relative methylation (TiO_2-NP/Vehicle)	Sham group			TiO_2-H group		
						Relative methylation (Cy5/Cy3)	Cy3 signal (Input)	Cy5 signal (MeDIP)	Relative methylation (Cy5/Cy3)	Cy3 signal (Input)	Cy5 signal (MeDIP)
A_68_P29605230	chr14:52816435-52816479	NM_033618:458	Supt16h	INSIDE	1.940	4.812	2932.18	14109.05	9.334	2379.69	22211.42
A_68_P30114905	chr15:31461133-31461177	NM_172606:-362	Marchf6	PROMOTER	1.939	1.837	568.10	1043.48	3.561	476.87	1698.37
A_68_P28726319	chr12:109416700-109416744	NM_028262:772	Setd3	INSIDE	1.939	4.260	1042.01	4439.33	8.260	798.11	6592.16
A_68_P20267395	chr1:60155522-60155566	NM_001199060:-200	Wdr12	PROMOTER	1.939	3.578	1891.30	6767.76	6.938	1500.30	10409.78
A_68_P27534905	chr10:126703422-126703466	NM_001190453:127	Dctn2	INSIDE	1.938	3.315	3380.69	11207.19	6.425	2486.22	15973.56
A_68_P26352003	chr9:22272492-22272536	NM_018739:286	Rp9	INSIDE	1.937	6.711	2403.58	16130.31	13.000	1977.00	25701.63
A_68_P25357073	chr7:105804516-105804560	NM_172280:541	2210018M11Rik	INSIDE	1.936	5.040	31781.32	160178.50	9.757	23016.13	224567.40
A_68_P28123546	chr11:110199057-110199101	NM_147219:-48	Abca5	PROMOTER	1.936	6.585	5276.42	34744.38	12.751	3677.97	46899.50
A_68_P31119196	chr17:28046558-28046602	NM_181413:296	Anks1	INSIDE	1.936	1.807	794.26	1435.62	3.500	648.57	2270.05
A_68_P32144718	chr19:46647649-46647693	NM_019718:-95	Arl3	DIVERGENT_PROMOTER	1.936	5.303	2710.10	14370.72	10.264	2159.84	22168.29
A_68_P29140001	chr13:73605046-73605090	NM_145376:238	Lpcat1	INSIDE	1.936	4.602	5042.49	23207.43	8.909	3905.21	34793.18
A_68_P24591144	chr6:83005100-83005144	NM_007517:476	Aup1	INSIDE	1.935	1.746	1460.28	2549.64	3.378	1153.78	3897.89
A_68_P31117117	chr17:27694485-27694529	NM_001166476:909	Hmga1-rs1	INSIDE	1.935	3.854	4656.86	17945.86	7.456	3396.56	25324.85
A_68_P29309087	chr13:108834557-108834601	NM_001048250:-89	2810008M24Rik	PROMOTER	1.934	4.845	3948.98	19133.40	9.369	3266.40	30603.79
A_68_P24482547	chr6:58590413-58590457	NM_011920:43769	Abcg2	INSIDE	1.934	4.525	2139.50	9681.96	8.751	1636.83	14324.60
A_68_P31208112	chr17:46787505-46787549	NM_025611:240	Cul7	INSIDE	1.934	4.597	1458.98	6707.18	8.893	1174.54	10445.09
A_68_P30361559	chr15:78502925-78502969	NM_183141:45597	Elfn2	INSIDE	1.934	7.718	1295.22	9996.63	14.929	1026.00	15316.62
A_68_P25609917	chr7:152082446-152082490	NM_008003:32	Fgf15	INSIDE	1.934	2.354	2711.42	6383.42	4.554	2263.15	10306.32
A_68_P31236178	chr17:51952223-51952267	NM_001163630:-865	Satb1	PROMOTER	1.934	4.659	2674.30	12459.88	9.009	2114.51	19049.22
A_68_P25086963	chr7:51608090-51608134	NM_001034115:42479	Shank1	INSIDE	1.934	1.856	483.92	897.95	3.589	416.06	1493.20
A_68_P25947613	chr8:72178336-72178380	NM_001177527:-96	Zfp964	PROMOTER	1.934	5.353	2590.06	13863.49	10.354	1969.80	20395.55
A_68_P30160398	chr15:40486792-40486836	NM_011766:227	Zfpm2	INSIDE	1.934	4.138	7704.09	31875.86	8.003	5311.51	42509.08
A_68_P22376195	chr3:101408231-101408275	NM_144900:328	Atp1a1	INSIDE	1.933	5.227	2297.12	12008.18	10.104	1986.01	20065.78
A_68_P26808316	chr9:107302358-107302402	NM_001174047:170	Cacna2d2	INSIDE	1.933	3.324	3446.50	11455.10	6.425	2700.40	17350.81
A_68_P26591015	chr9:65917041-65917085	NM_001025612:476	Snx22	INSIDE	1.933	3.673	1247.31	4581.80	7.101	1043.81	7412.32
A_68_P25408016	chr7:116582357-116582401	NM_054103:189	Stk33	INSIDE	1.933	3.287	1844.54	6063.05	6.353	1448.65	9202.64
A_68_P28183963	chr11:120210170-120210214	NR_038126:201	0610009L18Rik	INSIDE	1.932	3.213	1826.02	5866.26	6.205	1558.40	9670.64
A_68_P27566544	chr11:5965149-5965193	NM_001174053:581	Camk2b	INSIDE	1.932	3.324	2280.29	7579.09	6.420	1796.50	11533.69
A_68_P27091229	chr10:41530226-41530270	NM_001162908:-131	Sesn1	DIVERGENT_PROMOTER	1.930	4.951	3065.18	15175.30	9.557	2488.78	23786.35
A_68_P21752521	chr2:152857720-152857764	NM_001011732:155	Xkr7	INSIDE	1.930	4.795	3267.73	15669.96	9.254	2596.29	24027.08
A_68_P28291023	chr12:25365989-25366033	NM_001004455:650	Cys1	INSIDE	1.929	13.581	9472.94	128648.20	26.192	5382.13	140967.50
A_68_P28457808	chr12:57713134-57713178	NM_008701:1115	Nkx2-9	INSIDE	1.929	4.516	1026.23	4634.92	8.713	885.78	7718.13
A_68_P30646578	chr16:31275334-31275378	NM_025800:7	Ppp1r2	INSIDE	1.929	3.705	2474.18	9166.92	7.148	2077.06	14846.63
A_68_P30349758	chr15:76490310-76490354	NM_019396:159	Cyhr1	INSIDE	1.928	3.640	4666.59	16984.62	7.018	3324.48	23331.03
A_68_P27765093	chr11:45758539-45758583	NM_028185:-123	Lsm11	PROMOTER	1.928	3.590	3657.32	13130.18	6.923	2701.76	18704.49
A_68_P28195719	chr12:3572230-3572274	NM_001162465:-138	Dtnb	PROMOTER	1.927	2.025	1262.01	2555.32	3.901	913.73	3564.66
A_68_P25392116	chr7:112548270-112548314	NM_199009:233	Fam160a2	INSIDE	1.927	3.135	3303.29	10354.85	6.040	2514.41	15186.65
A_68_P32571132	chrX:100129205-100129249	NR_026596:336	Gm5126	INSIDE	1.927	2.774	1584.24	4393.93	5.345	859.88	4595.98
A_68_P24863969	chr6:134820135-134820179	NM_001167697:17721	Gpr19	INSIDE	1.927	4.853	539.59	2618.65	9.351	411.50	3847.75
A_68_P27796195	chr11:51671777-51671821	NM_199299:-815	Phf15	PROMOTER	1.927	3.621	11034.72	39960.52	6.977	7949.07	55458.77
A_68_P22149943	chr3:54611747-54611791	NM_133231:-55	Rfxap	PROMOTER	1.927	4.905	1112.94	5459.44	9.453	945.98	8942.71
A_68_P30390278	chr15:83555204-83555248	NM_022723:225	Scube1	INSIDE	1.927	4.462	1759.53	7850.47	8.596	1610.50	13843.26
A_68_P20005810	chr1:4483711-4483755	NM_011441:2762	Sox17	INSIDE	1.927	4.121	693.64	2858.49	7.941	700.21	5560.40
A_68_P27896951	chr11:69579684-69579728	NM_029348:293	Zbtb4	INSIDE	1.927	6.578	853.34	5613.07	12.676	665.38	8434.45
A_68_P24973956	chr7:13563487-13563531	NM_001168561:312	Zfp446	INSIDE	1.927	4.269	926.05	3952.94	8.225	813.97	6694.80
A_68_P32488667	chrX:78316560-78316604	NM_138751:600	Tmem47	INSIDE	1.926	4.390	5758.30	25281.21	8.457	3130.92	26478.88
A_68_P23195047	chr4:107889859-107889903	NM_001167936:647	Zyg11a	INSIDE	1.926	5.752	19495.88	112147.10	11.079	13845.63	153397.70
A_68_P30459959	chr15:95620940-95620984	NM_175344:-311	Ano6	PROMOTER	1.925	3.162	2819.28	8914.46	6.086	2292.31	13950.27
A_68_P28175418	chr11:118898955-118898999	NM_013926:3251	Cbx8	DOWNSTREAM	1.925	5.303	2342.48	12422.04	10.206	1877.46	19160.97
A_68_P30183902	chr15:44945966-44946010	NM_026200:492	Kcnv1	INSIDE	1.925	7.547	2060.05	15548.16	14.527	1203.61	17485.23
A_68_P29330800	chr13:112598036-112598080	NM_011945:1133	Map3k1	INSIDE	1.925	3.844	1210.59	4652.97	7.400	1025.67	7589.61

ProbeName	Target position of probe on CpG island microarray	TargetID	GeneSymbol	CpG island Description	Ratio of relative methylation (TiO_2-NP/Vehicle)	Sham group			TiO_2-H group		
						Relative methylation (Cy5/Cy3)	Cy3 signal (Input)	Cy5 signal (MeDIP)	Relative methylation (Cy5/Cy3)	Cy3 signal (Input)	Cy5 signal (MeDIP)
A_68_P31258630	chr17:56969214-56969258	NM_009056:1195	Rfx2	INSIDE	1.925	6.313	5662.42	35747.04	12.150	4694.45	57039.64
A_68_P21116393	chr2:32468028-32468072	NM_001025310:5570	St6galnac6	INSIDE	1.925	8.406	3661.88	30782.19	16.185	2404.68	38920.51
A_68_P23588493	chr5:33721117-33721161	NM_001081101:-206	4933407H18Rik	PROMOTER	1.924	5.062	8181.42	41417.93	9.741	5614.39	54690.28
A_68_P22659834	chr3:153607495-153607539	NM_007382:-120	Acadm	PROMOTER	1.924	3.919	2766.09	10839.68	7.540	2240.55	16892.71
A_68_P29752204	chr14:79987400-79987444	NM_026474:-75	Sugt1	PROMOTER	1.924	4.763	3302.53	15728.79	9.163	2623.06	24035.36
A_68_P24133861	chr5:139626021-139626065	NM_001081265:-134	Heatr2	PROMOTER	1.923	5.203	17869.78	92974.62	10.007	11113.70	111213.60
A_68_P25005398	chr7:25318769-25318814	NM_153134:3125	Irgq	INSIDE	1.923	3.356	458.84	1539.90	6.454	324.51	2094.37
A_68_P27648126	chr11:23795318-23795362	NM_172555:-70	Papolg	PROMOTER	1.923	3.254	5338.37	17370.64	6.258	3939.80	24656.64
A_68_P30345853	chr15:75900100-75900144	NM_134089:38	Scrib	INSIDE	1.923	4.663	3776.94	17610.21	8.966	3372.04	30235.25
A_68_P21782539	chr2:158187061-158187105	NR_015463:163	9430008C03Rik	INSIDE	1.922	4.489	4847.02	21757.79	8.630	4715.69	40694.87
A_68_P28752186	chr12:113977208-113977252	NM_001024602:16846	AW555464	INSIDE	1.922	4.566	3478.97	15886.58	8.777	2592.08	22749.48
A_68_P28153096	chr11:115229081-115229125	NM_175454:-69	C630004H02Rik	PROMOTER	1.922	6.389	5115.22	32680.12	12.279	3916.94	48094.92
A_68_P24683080	chr6:98978547-98978591	NM_001197322:-276	Foxp1	PROMOTER	1.922	1.899	1475.64	2802.18	3.650	1327.61	4846.05
A_68_P27932457	chr11:76308492-76308536	NM_198018:14407	Abr	INSIDE	1.921	9.569	21667.79	207339.50	18.381	18186.63	334294.90
A_68_P26132026	chr8:107695072-107695119	NM_001161456:522	Cbfb	INSIDE	1.921	1.830	668.55	1223.14	3.515	622.51	2188.07
A_68_P32573186	chrX:100688845-100688889	NR_015508:-48	Enox	PROMOTER	1.921	6.156	1849.84	11386.70	11.827	1373.86	16248.66
A_68_P24942897	chr6:148894399-148894443	NM_019643:534	Fam60a	INSIDE	1.921	4.150	1979.80	8216.13	7.973	1511.82	12053.87
A_68_P31201735	chr17:45686486-45686530	NM_001013749:118	Tmem151b	INSIDE	1.921	4.523	4033.96	18243.61	8.688	2866.15	24900.98
A_68_P28225578	chr12:8980743-8980787	NM_001159527:-42	Wdr35	PROMOTER	1.921	2.640	7446.62	19660.79	5.071	5556.74	28180.03
A_68_P29975444	chr15:3533365-3533409	NM_010284:-156	Ghr	PROMOTER	1.920	7.272	941.81	6848.65	13.961	869.39	12137.41
A_68_P23573590	chr5:30925082-30925126	NM_010608:34562	Kcnk3	INSIDE	1.920	5.787	2350.06	13599.97	11.110	1736.33	19291.40
A_68_P20282811	chr1:62749708-62749752	NM_001077406:-160	Nrp2	PROMOTER	1.920	8.010	3164.47	25348.92	15.380	2494.48	38364.67
A_68_P24428260	chr6:48552305-48552349	NM_001079901:8445	Repin1	DOWNSTREAM	1.920	5.777	379.35	2191.66	11.091	284.25	3152.55
A_68_P27286394	chr10:80391549-80391593	NM_008655:-1265	Gadd45b	PROMOTER	1.918	1.815	1699.65	3085.00	3.482	1370.18	4771.14
A_68_P28164254	chr11:117151837-117151881	NM_001113487:24299	Septin9	INSIDE	1.918	4.704	4337.00	20399.80	9.019	3424.73	30888.83
A_68_P28739824	chr12:111934703-111934747	NM_010480:-119	Hsp90aa1	PROMOTER	1.917	11.944	1915.98	22884.25	22.896	1402.99	32122.28
A_68_P24052813	chr5:123350619-123350663	NM_013910:-281	Kdm2b	PROMOTER	1.917	2.640	545.58	1440.33	5.060	392.77	1987.24
A_68_P23757989	chr5:66155461-66155505	NM_001024917:723	N4bp2	INSIDE	1.917	2.979	1575.15	4692.02	5.709	1233.33	7041.59
A_68_P25445817	chr7:123177303-123177347	NM_001025560:-2763	Sox6	PROMOTER	1.917	3.509	3173.69	11135.04	6.726	2390.20	16075.83
A_68_P27289055	chr10:80813338-80813382	NM_001163166:-158	Hmg20b	DIVERGENT_PROMOTER	1.916	3.014	4739.46	14285.52	5.774	3859.24	22283.48
A_68_P30395524	chr15:84388079-84388123	NM_177630:153	Ldoc1l	INSIDE	1.916	7.935	2483.85	19709.22	15.205	2070.29	31478.86
A_68_P20148355	chr1:36502065-36502109	NM_001013374:-2	Lman2l	PROMOTER	1.916	2.963	7704.43	22829.15	5.678	5809.89	32991.37
A_68_P24132971	chr5:139470887-139470931	NM_008808:-1	Pdgfa	PROMOTER	1.916	6.049	10464.92	63300.49	11.592	10109.40	117188.10
A_68_P21774858	chr2:156866344-156866388	NM_001164663:38635	9830001H06Rik	INSIDE	1.915	3.175	3202.56	10167.64	6.079	2541.60	15449.53
A_68_P24205536	chr6:4550713-4550757	NM_145398:-331	Casd1	PROMOTER	1.915	5.905	4018.61	23729.76	11.305	3300.47	37312.12
A_68_P25565202	chr7:144505325-144505370	NM_001113414:781	Ebf3	INSIDE	1.915	5.902	4572.39	26987.78	11.301	3496.84	39517.37
A_68_P32281782	chrX:17739949-17739993	NM_009483:178	Kdm6a	INSIDE	1.915	1.913	2267.29	4337.15	3.663	1308.27	4792.41
A_68_P21204312	chr2:49642923-49642967	NM_027990:-261	Lypd6b	PROMOTER	1.915	2.123	464.44	986.20	4.067	405.43	1648.96
A_68_P32582402	chrX:103206930-103206974	NM_001190409:286	Magt1	INSIDE	1.915	1.775	471.25	836.39	3.398	285.08	968.83
A_68_P28107262	chr11:107332069-107332113	NM_145823:-56	Pitpnc1	DIVERGENT_PROMOTER	1.915	11.985	2125.28	25472.22	22.948	1793.44	41155.20
A_68_P26529571	chr9:54996925-54996969	NM_180600:-229	Ube2q2	PROMOTER	1.914	7.210	1010.45	7285.18	13.799	790.60	10909.70
A_68_P25065443	chr7:38893032-38893076	NM_007633:-545	Ccne1	PROMOTER	1.913	1.928	2996.29	5777.92	3.690	2226.80	8216.66
A_68_P21102300	chr2:30141879-30141923	NM_177648:-26	Dolk	DIVERGENT_PROMOTER	1.913	2.441	2439.73	5955.30	4.670	1879.60	8778.17
A_68_P23814358	chr5:76569347-76569391	NM_020611:71	Srd5a3	INSIDE	1.913	3.279	1314.92	4311.92	6.273	972.42	6100.40
A_68_P23190148	chr4:107040619-107040663	NM_028355:252	Tmem48	INSIDE	1.913	8.198	1328.94	10894.72	15.684	1001.80	15712.00
A_68_P31621446	chr18:36177796-36177840	NM_001167891:178996	Nrg2	INSIDE	1.912	6.614	1194.95	7903.79	12.650	1049.81	13279.96
A_68_P24057004	chr5:124133945-124133989	NM_019765:334	Clip1	INSIDE	1.911	2.160	864.01	1865.97	4.126	813.86	3358.04
A_68_P31864634	chr18:80666119-80666163	NM_026295:266	Ctdp1	INSIDE	1.911	4.138	713.39	2952.12	7.909	717.95	5678.08
A_68_P23320471	chr4:133556862-133556906	NM_026144:-105	Dhdds	PROMOTER	1.911	6.695	713.05	4773.77	12.792	604.67	7734.84
A_68_P24908780	chr6:143116314-143116358	NM_029250:587	Etnk1	INSIDE	1.911	4.120	8609.83	35470.89	7.873	6271.76	49378.43
A_68_P23681258	chr5:50449746-50449790	NM_133911:467	Gpr125	INSIDE	1.910	2.700	4056.12	10949.92	5.155	3147.75	16226.96

ProbeName	Target position of probe on CpG island microarray	TargetID	GeneSymbol	CpG island Description	Ratio of relative methylation (TiO_2-NP/Vehicle)	Sham group			TiO_2-H group		
						Relative methylation (Cy5/Cy3)	Cy3 signal (Input)	Cy5 signal (MeDIP)	Relative methylation (Cy5/Cy3)	Cy3 signal (Input)	Cy5 signal (MeDIP)
A_68_P21536555	chr2:113598914-113598958	NM_011824:-131	Grem1	PROMOTER	1.910	6.201	10172.87	63086.12	11.843	7495.12	88766.02
A_68_P28671841	chr12:99975831-99975875	NM_001146199:-237	Ptpn21	DIVERGENT_PROMOTER	1.910	3.169	3504.26	11104.89	6.054	2752.12	16661.96
A_68_P21285107	chr2:65077163-65077207	NM_027225:-501	Cobll1	PROMOTER	1.909	4.523	6122.86	27694.30	8.633	4521.21	39031.95
A_68_P23657737	chr5:46030747-46030791	NM_021416:-28	Fam184b	PROMOTER	1.909	1.799	1547.18	2783.68	3.435	1287.38	4422.59
A_68_P32743933	chrX:147481461-147481505	NM_008001:-293	Fgd1	PROMOTER	1.909	4.057	3063.49	12429.23	7.745	1840.37	14252.89
A_68_P23296219	chr4:129012748-129012792	NM_009030:-156	Rbbp4	DIVERGENT_PROMOTER	1.909	4.649	4131.98	19209.22	8.876	3331.75	29571.28
A_68_P30499546	chr15:102577711-102577755			Unknown	1.909	1.480	2355.57	3485.49	2.824	1811.15	5114.82
A_68_P27784450	chr11:49525550-49525594	NM_212484:640	Cnot6	INSIDE	1.908	2.634	3975.65	10469.93	5.025	3106.29	15610.64
A_68_P26983894	chr10:19654073-19654117	NM_008580:-237	Map3k5	PROMOTER	1.908	4.715	1137.39	5362.66	8.995	869.16	7818.51
A_68_P28726327	chr12:109417619-109417664	NM_028262:-147	Setd3	DIVERGENT_PROMOTER	1.908	4.603	1903.06	8759.66	8.782	1566.38	13755.89
A_68_P26880499	chr9:120842203-120842247	NM_007614:-293	Ctnnb1	PROMOTER	1.907	3.489	2486.61	8675.34	6.652	1765.98	11746.72
A_68_P25644704	chr8:9771350-9771394	NM_173446:-349	Fam155a	PROMOTER	1.907	12.194	23543.03	287086.60	23.250	14771.44	343435.70
A_68_P25266552	chr7:87547168-87547212	NM_011046:458	Furin	INSIDE	1.907	4.974	1206.48	6001.21	9.487	1020.22	9678.73
A_68_P30243544	chr15:57525714-57525758	NM_199449:-485	Zhx2	PROMOTER	1.907	2.755	1625.86	4478.68	5.253	1339.99	7039.37
A_68_P29049835	chr13:54605357-54605401	NM_176987:213	4732471D19Rik	INSIDE	1.906	4.028	1786.81	7197.41	7.678	1384.65	10630.74
A_68_P23647569	chr5:44173172-44173216	NM_001159963:194	Fbxl5	INSIDE	1.905	5.037	4066.60	20482.39	9.597	2939.28	28208.15
A_68_P23350170	chr4:138749124-138749168	NM_001037761:253	Capzb	INSIDE	1.904	3.333	401.15	1336.85	6.346	334.45	2122.53
A_68_P27930557	chr11:76015854-76015898	NM_027773:319	Fam57a	INSIDE	1.904	3.875	1497.57	5802.80	7.379	1271.09	9379.09
A_68_P31162404	chr17:36221085-36221129	NM_008199:88	H2-Bl	INSIDE	1.904	2.502	2933.75	7341.23	4.765	2115.03	10078.02
A_68_P30601918	chr16:23225193-23225237	NM_145933:380	St6gal1	INSIDE	1.904	2.661	1893.74	5038.34	5.066	1478.16	7487.62
A_68_P32553141	chrX:96238064-96238108	NM_199018:167	Stard8	INSIDE	1.904	4.549	2633.26	11979.79	8.662	1594.17	13808.89
A_68_P24735989	chr6:108778919-108778963	NM_138677:306	Edem1	INSIDE	1.903	3.777	394.52	1490.21	7.189	410.65	2952.03
A_68_P27091644	chr10:41607176-41607220	NM_001013370:-46	Sesn1	PROMOTER	1.903	6.522	2943.93	19199.99	12.412	2476.28	30734.88
A_68_P30128581	chr15:34373372-34373416	NM_001163485:-363	Rpl30	DIVERGENT_PROMOTER	1.902	2.460	2857.63	7030.14	4.679	2030.46	9500.08
A_68_P30380067	chr15:81688792-81688836	NM_020507:-58	Tob2	PROMOTER	1.902	4.656	2525.26	11757.26	8.854	1887.14	16709.04
A_68_P29134822	chr13:72768633-72768679	NM_010574:2231	Irx2	INSIDE	1.901	2.271	4470.84	10154.53	4.319	3365.33	14533.82
A_68_P20456742	chr1:95532281-95532325	NM_021537:2	Stk25	INSIDE	1.901	5.375	5391.60	28979.31	10.220	4104.03	41941.65
A_68_P30230212	chr15:54903756-54903800	NM_001081288:-71	Taf2	PROMOTER	1.901	4.632	1981.73	9179.20	8.804	1705.00	15010.15
A_68_P23608585	chr5:36924742-36924786	NM_133910:282	Tbc1d14	INSIDE	1.901	2.812	13547.19	38091.92	5.346	9749.13	52114.66
A_68_P32258637	chrX:11384409-11384453			Unknown	1.901	1.417	1543.52	2187.58	2.694	810.68	2183.79
A_68_P24995433	chr7:20408093-20408137	NM_033601:-10	Bcl3	PROMOTER	1.900	4.210	710.19	2990.18	8.001	585.96	4688.00
A_68_P27111732	chr10:45297581-45297625	NM_172473:-32	Hace1	PROMOTER	1.900	2.556	1049.71	2683.25	4.857	871.12	4231.03
A_68_P29237544	chr13:95647510-95647554	NM_011021:1951	Otp	INSIDE	1.900	3.442	1352.07	4653.63	6.541	951.11	6220.87
A_68_P27898391	chr11:69810124-69810168	NM_030064:874	Phf23	INSIDE	1.900	6.188	16696.39	103321.70	11.758	11612.96	136541.00
A_68_P23609090	chr5:37086922-37086966	NM_001081232:264	D5Ertd579e	INSIDE	1.899	4.942	5250.33	25945.60	9.383	4159.62	39028.77
A_68_P28174620	chr11:118768747-118768791	NM_001024931:2118	Rbfox3	INSIDE	1.899	5.324	5970.48	31785.57	10.112	4426.10	44755.41
A_68_P31659996	chr18:43367102-43367146	NM_178749:-226	Stk32a	PROMOTER	1.899	6.224	3351.87	20860.38	11.820	2474.30	29245.91
A_68_P32709174	chrX:139116023-139116067	NM_001199360:-224	Tmem164	PROMOTER	1.899	4.862	2349.83	11425.20	9.232	1450.14	13387.17
A_68_P32578104	chrX:101866109-101866153	NM_175358:273	Zdhhc15	INSIDE	1.899	3.304	4590.08	15164.50	6.274	2668.97	16744.35
A_68_P21762932	chr2:154718551-154718596	NM_026030:69	Eif2s2	INSIDE	1.898	3.101	2784.28	8634.70	5.886	2108.86	12413.38
A_68_P20506808	chr1:108069211-108069255	NM_133821:787	Phlpp1	INSIDE	1.898	7.316	1813.92	13270.07	13.888	1509.26	20961.24
A_68_P32234207	chrX:5977686-5977730	NM_001040459:446	Shroom4	INSIDE	1.898	3.037	992.20	3013.76	5.764	637.73	3675.91
A_68_P24019920	chr5:117769201-117769245	NM_001033311:-52	Vsig10	PROMOTER	1.898	1.432	926.32	1326.26	2.717	833.12	2263.95
A_68_P24098738	chr5:132013832-132013876	NM_177047:1004359	Auts2	INSIDE	1.897	3.105	2416.69	7504.06	5.891	1923.10	11328.58
A_68_P30667506	chr16:35022484-35022528	NM_023587:0	Ptplb	INSIDE	1.897	2.685	1062.16	2851.96	5.094	902.45	4597.00
A_68_P22382656	chr3:102538778-102538822	NM_027533:108	Tspan2	INSIDE	1.897	4.171	9887.21	41239.90	7.911	7434.75	58815.90
A_68_P21117289	chr2:32612990-32613034	NM_152800:222	Tor2a	INSIDE	1.896	6.493	1602.39	10403.93	12.309	1213.79	14940.74
A_68_P32700327	chrX:137200260-137200304	ENSMUST00000112988:672		INSIDE	1.896	2.665	1016.59	2709.60	5.053	477.49	2412.85
A_68_P23222359	chr4:114597578-114597622	NM_015758:1018	Foxe3	DOWNSTREAM	1.895	11.062	1484.95	16425.86	20.962	1203.20	25221.92
A_68_P26538932	chr9:56712960-56713004	NM_139001:72	Cspg4	INSIDE	1.894	4.386	10522.96	46156.43	8.309	7877.12	65451.70
A_68_P25766300	chr8:33994475-33994519	NM_001177589:66002	Gm3985	DOWNSTREAM	1.894	2.241	608.52	1363.70	4.244	521.65	2213.84

ProbeName	Target position of probe on CpG island microarray	TargetID	GeneSymbol	CpG island Description	Ratio of relative methylation (TiO_2-NP/Vehicle)	Sham group			TiO_2-H group		
						Relative methylation (Cy5/Cy3)	Cy3 signal (Input)	Cy5 signal (MeDIP)	Relative methylation (Cy5/Cy3)	Cy3 signal (Input)	Cy5 signal (MeDIP)
A_68_P28450733	chr12:56225550-56225594	NM_001100116:58	1700047I17Rik2	INSIDE	1.893	4.739	1177.94	5581.73	8.969	972.67	8724.30
A_68_P27914258	chr11:72775141-72775185	NM_001163336:492	Atp2a3	INSIDE	1.893	2.324	1220.11	2835.14	4.398	1120.16	4926.22
A_68_P23361673	chr4:140581289-140581333	NM_172122:28266	Crocc	INSIDE	1.893	5.666	2665.13	15100.28	10.727	2182.63	23412.04
A_68_P26348962	chr9:21602957-21603001	NM_145611:12	Kank2	INSIDE	1.893	2.623	1978.53	5190.52	4.966	1544.30	7669.06
A_68_P27283985	chr10:80024218-80024262	NM_025852:65	Rexo1	INSIDE	1.893	1.853	1490.13	2761.57	3.509	1217.01	4270.00
A_68_P26079748	chr8:97362202-97362246	NM_134141:16	Ciapin1	INSIDE	1.892	2.871	431.89	1240.15	5.432	323.32	1756.29
A_68_P28732711	chr12:110692025-110692069	NM_001190703:382	Dlk1	INSIDE	1.892	2.773	11271.12	31256.75	5.247	7974.42	41839.52
A_68_P20636645	chr1:136884255-136884299	NM_001033409:117577	Lgr6	INSIDE	1.892	14.177	2442.11	34620.74	26.820	1778.74	47705.97
A_68_P22341452	chr3:94758251-94758295	NM_017395:-664	Rfx5	PROMOTER	1.892	1.986	1160.58	2305.20	3.759	985.55	3704.39
A_68_P23442283	chr4:155452135-155452179			Unknown	1.892	3.276	2422.93	7938.13	6.200	1916.25	11880.43
A_68_P21900913	chr2:179759394-179759438	NM_177727:-275	Lsm14b	PROMOTER	1.891	4.621	5053.95	23356.44	8.741	3956.25	34580.53
A_68_P25578901	chr7:146768634-146768678	NR_027857:40	Nkx6-2	INSIDE	1.891	4.355	4487.96	19544.00	8.234	3456.28	28458.97
A_68_P28039255	chr11:95375266-95375310	NM_130858:591	Nxph3	INSIDE	1.891	6.571	8238.62	54137.48	12.429	6016.37	74779.02
A_68_P31713385	chr18:53624187-53624231	NM_001033281:9	Prdm6	INSIDE	1.891	2.311	1897.82	4386.30	4.371	1358.76	5938.95
A_68_P31121831	chr17:28465275-28465320	NM_011287:-118	Rpl10a	PROMOTER	1.891	8.952	1034.42	9260.53	16.925	734.71	12435.11
A_68_P32564024	chrX:98615209-98615253	NM_001177985:23	Zmym3	INSIDE	1.891	2.856	582.27	1662.67	5.400	372.25	2010.15
A_68_P30246249	chr15:57966345-57966389	NM_027435:271	Atad2	INSIDE	1.890	8.349	2043.61	17062.91	15.779	1656.81	26143.28
A_68_P29530305	chr14:35315556-35315600	NM_009758:154	Bmpr1a	INSIDE	1.890	3.798	2069.87	7862.13	7.178	1766.58	12680.11
A_68_P28167547	chr11:117710471-117710515	NM_001012273:-58	Birc5	PROMOTER	1.889	4.570	1254.99	5735.76	8.632	1037.63	8956.98
A_68_P25354355	chr7:105326695-105326739	NM_007602:-13	Capn5	PROMOTER	1.889	3.757	1433.93	5386.78	7.097	1113.74	7904.33
A_68_P24359955	chr6:35083485-35083529	NM_001164412:231	Cnot4	INSIDE	1.889	3.638	4126.95	15015.24	6.874	3480.07	23921.77
A_68_P30093655	chr15:27560477-27560521	NM_001013792:-50	Fam105b	PROMOTER	1.889	3.790	4711.05	17853.99	7.158	3397.60	24320.69
A_68_P27924667	chr11:74992384-74992428	NM_027136:-96	Ovca2	PROMOTER	1.889	6.221	3563.75	22170.42	11.753	2485.00	29206.57
A_68_P30678526	chr16:37011868-37011912	NM_001159369:19	Polq	INSIDE	1.889	2.244	1247.87	2800.29	4.240	1016.30	4308.64
A_68_P26569971	chr9:62191474-62191518	NM_009672:2347	Anp32a	INSIDE	1.888	1.626	2462.56	4004.47	3.070	2044.89	6278.80
A_68_P23763603	chr5:67136839-67136883	NM_001001980:-218	Limch1	PROMOTER	1.888	5.879	1443.65	8487.48	11.099	1197.28	13288.10
A_68_P28604394	chr12:86827035-86827079	NM_010234:12206	Fos	DOWNSTREAM	1.887	3.215	1472.75	4735.17	6.066	1107.98	6721.43
A_68_P30367903	chr15:79518094-79518138	NM_028792:186	Josd1	INSIDE	1.887	3.055	1146.51	3502.77	5.766	919.95	5304.26
A_68_P20723965	chr1:154802917-154802961	NM_175460:708	Nmnat2	INSIDE	1.887	7.197	5326.04	38330.74	13.580	3487.41	47358.05
A_68_P29624997	chr14:57483807-57483852	AK209334:-895		PROMOTER	1.887	1.472	811.40	1194.05	2.778	730.58	2029.25
A_68_P21438176	chr2:93816807-93816851	NM_026944:34059	Alkbh3	DOWNSTREAM	1.886	5.584	1256.48	7015.68	10.528	1153.90	12148.56
A_68_P31152221	chr17:34257414-34257458	NR_037970:-591	Brd2	PROMOTER	1.886	4.785	791.53	3787.67	9.025	636.70	5746.45
A_68_P27262526	chr10:75994451-75994495	NM_146006:101	Lss	INSIDE	1.886	5.474	6782.44	37124.20	10.325	5005.00	51676.57
A_68_P29054388	chr13:55431309-55431353	NM_016662:-239	Mxd3	PROMOTER	1.886	4.408	1313.53	5789.64	8.315	1065.03	8855.45
A_68_P32118295	chr19:42056730-42056774	NM_145500:500	Ubtd1	INSIDE	1.886	6.127	949.72	5818.85	11.553	816.00	9427.02
A_68_P25734548	chr8:27891120-27891164			Unknown	1.886	2.789	931.33	2597.80	5.262	886.53	4664.93
A_68_P29470473	chr14:25064406-25064450	NM_001163513:714	Dlg5	INSIDE	1.884	21.987	778.65	17120.00	41.423	520.80	21572.99
A_68_P20451406	chr1:94729272-94729316	NM_016696:1032	Gpc1	INSIDE	1.884	4.338	2033.27	8819.41	8.171	1598.45	13060.72
A_68_P25394537	chr7:112959576-112959620	NM_025301:3	Mrpl17	INSIDE	1.884	3.020	2285.66	6902.31	5.690	1763.25	10032.24
A_68_P28246269	chr12:12944121-12944165	NM_008709:4500	Mycn	INSIDE	1.884	3.332	931.52	3104.13	6.278	767.56	4818.81
A_68_P28950705	chr13:36816445-36816489	NM_153529:9857	Nrn1	DOWNSTREAM	1.884	8.358	4570.60	38201.00	15.746	3319.36	52265.63
A_68_P20085264	chr1:22812557-22812601	NM_001012623:-15	Rims1	PROMOTER	1.884	3.168	5138.64	16277.20	5.969	3944.43	23542.48
A_68_P23468797	chr5:8622724-8622768	NM_198620:206	Rundc3b	INSIDE	1.884	1.793	6375.10	11431.71	3.379	5084.04	17177.17
A_68_P28724517	chr12:109154270-109154314	NM_001079883:87332	Bcl11b	INSIDE	1.883	5.082	2190.88	11133.18	9.566	1718.68	16441.20
A_68_P30362680	chr15:78673148-78673192	NM_027219:94	Cdc42ep1	INSIDE	1.883	4.258	1830.06	7792.26	8.016	1529.47	12260.21
A_68_P25598742	chr7:150293216-150293260	NM_008434:80	Kcnq1	INSIDE	1.883	8.553	779.08	6663.79	16.107	603.76	9724.83
A_68_P30421719	chr15:88693018-88693062	NM_145478:417	Pim3	INSIDE	1.883	4.203	734.95	3088.77	7.913	589.31	4663.15
A_68_P27541275	chr10:127848643-127848687	NM_001164237:-7	Rnf41	DIVERGENT_PROMOTER	1.883	5.832	1251.46	7299.10	10.982	997.40	10953.87
A_68_P27836028	chr11:59103858-59103902	NM_009522:373	Wnt3a	INSIDE	1.883	2.885	1333.86	3847.79	5.431	1095.47	5949.46
A_68_P29631470	chr14:58691022-58691066	NM_013518:-478	Fgf9	PROMOTER	1.882	5.806	1512.20	8780.06	10.924	1230.13	13438.37
A_68_P23787605	chr5:72049192-72049236	NM_010251:333	Gabra4	INSIDE	1.882	5.032	905.66	4556.89	9.471	750.11	7104.47

ProbeName	Target position of probe on CpG island microarray	TargetID	GeneSymbol	CpG island Description	Ratio of relative methylation (TiO_2-NP/Vehicle)	Sham group			TiO_2-H group		
						Relative methylation (Cy5/Cy3)	Cy3 signal (Input)	Cy5 signal (MeDIP)	Relative methylation (Cy5/Cy3)	Cy3 signal (Input)	Cy5 signal (MeDIP)
A_68_P24774311	chr6:115944633-115944678	NM_026376:368	Plxnd1	INSIDE	1.882	6.530	3149.31	20564.84	12.291	2421.41	29761.64
A_68_P21812328	chr2:163244957-163245001	NM_025699:228	3230401D17Rik	INSIDE	1.881	7.426	2014.28	14958.86	13.971	1569.90	21933.65
A_68_P32775148	chrX:155970504-155970548	NM_001033472:-73	A830080D01Rik	PROMOTER	1.881	4.182	1734.44	7253.34	7.867	1002.59	7887.70
A_68_P27512350	chr10:122513515-122513559	NM_001163024:25	Mon2	INSIDE	1.881	4.934	2975.86	14683.41	9.281	2372.94	22023.12
A_68_P30463766	chr15:96290439-96290483	NM_028148:814	Scaf11	INSIDE	1.881	1.929	1477.99	2851.50	3.629	1168.09	4239.56
A_68_P29051405	chr13:54890823-54890867	NM_028841:79	Tspan17	INSIDE	1.881	3.452	458.95	1584.22	6.494	329.33	2138.76
A_68_P30346013	chr15:75920805-75920849	NM_144847:-383	Nrbp2	PROMOTER	1.880	2.482	2490.06	6180.14	4.665	1969.62	9188.98
A_68_P22398585	chr3:105490135-105490179	NM_017397:333	Ddx20	INSIDE	1.879	2.525	1089.30	2750.84	4.746	942.09	4471.30
A_68_P20877951	chr1:182742690-182742734	NM_008210:1022	H3f3a	INSIDE	1.879	2.919	2269.28	6623.75	5.485	1821.43	9991.04
A_68_P30346762	chr15:76028710-76028754	NM_201393:-93	Plec	PROMOTER	1.879	3.238	2210.87	7157.92	6.082	1649.76	10033.85
A_68_P32567036	chrX:99266249-99266293	NM_183318:376	Rgag4	INSIDE	1.879	8.946	4538.15	40597.66	16.807	2317.83	38955.67
A_68_P22872576	chr4:43591441-43591485	NM_172866:-144	Rgp1	PROMOTER	1.879	2.255	663.43	1495.70	4.236	480.39	2035.06
A_68_P21266440	chr2:61650101-61650145	NM_009322:7613	Tbr1	INSIDE	1.879	2.100	424.79	892.06	3.945	412.76	1628.36
A_68_P26554375	chr9:59465216-59465260	NM_001205239:148	Parp6	INSIDE	1.878	4.980	2916.91	14526.13	9.350	2137.24	19983.87
A_68_P23062645	chr4:83131826-83131870	NM_133948:446	Psip1	INSIDE	1.878	2.791	1459.70	4074.48	5.242	1255.71	6581.91
A_68_P26290461	chr9:8004643-8004687	NM_001171147:-68	Yap1	PROMOTER	1.878	5.407	3903.80	21106.63	10.155	3228.67	32788.72
A_68_P32565199	chrX:98892409-98892453	ENSMUST00000073812:-5241		PROMOTER	1.878	6.112	8917.51	54505.77	11.481	3674.32	42184.32
A_68_P27563898	chr11:5441853-5441897	NM_134033:346	Ccdc117	INSIDE	1.877	5.498	3920.15	21554.59	10.319	3139.07	32391.51
A_68_P30490489	chr15:101062456-101062514	NM_019518:7847	Grasp	INSIDE	1.877	9.892	1964.82	19435.55	18.567	1397.98	25956.94
A_68_P30337599	chr15:74347574-74347618	NM_174991:971	Bai1	INSIDE	1.876	5.979	1825.93	10917.73	11.217	1386.24	15550.12
A_68_P26756440	chr9:97933663-97933707	NM_022319:-98	Clstn2	PROMOTER	1.876	9.816	3587.04	35211.04	18.414	3006.61	55364.42
A_68_P25092690	chr7:52622810-52622854	NR_027802:230	Mtag2	INSIDE	1.876	3.324	2610.46	8676.44	6.236	1965.83	12259.79
A_68_P23328804	chr4:135050696-135050741	NM_028995:-299	Nipal3	DIVERGENT_PROMOTER	1.876	2.629	2957.08	7775.38	4.932	2108.10	10397.66
A_68_P26783904	chr9:103014963-103015007	NM_173781:581	Rab6b	INSIDE	1.876	6.996	3134.89	21931.57	13.124	2261.49	29680.34
A_68_P27693347	chr11:33101808-33101852	NM_019916:1758	Tlx3	INSIDE	1.876	2.938	673.36	1978.25	5.511	549.92	3030.69
A_68_P29751622	chr14:79881067-79881111	NM_018765:-13	Wbp4	PROMOTER	1.876	0.357	1577.79	563.98	0.670	1430.02	958.78
A_68_P27788008	chr11:50139149-50139193	NM_001110499:5	Canx	INSIDE	1.875	2.514	5536.90	13918.62	4.712	4124.56	19436.10
A_68_P31112726	chr17:26978463-26978507	NM_008700:26	Nkx2-5	INSIDE	1.875	3.055	1100.14	3361.00	5.729	941.99	5396.80
A_68_P26015929	chr8:86192596-86192640	NM_013641:2080	Ptger1	INSIDE	1.875	5.992	6861.71	41111.95	11.236	4744.67	53309.83
A_68_P27996402	chr11:87630757-87630801	NM_080420:8838	Lpo	INSIDE	1.874	3.124	2302.41	7193.28	5.856	1764.87	10335.15
A_68_P28376737	chr12:40860793-40860837	NM_001146196:1	Scin	INSIDE	1.874	8.543	1868.55	15962.97	16.011	1151.05	18429.85
A_68_P27167594	chr10:58684574-58684618	NM_001024910:-1	Septin10	DIVERGENT_PROMOTER	1.874	6.097	4520.24	27558.99	11.425	3322.33	37957.26
A_68_P22338534	chr3:94167626-94167670	NM_001136117:1283	C2cd4d	INSIDE	1.873	6.728	2320.94	15615.16	12.603	1545.88	19482.20
A_68_P32185492	chr19:54018463-54018507	NM_001168505:-311	Shoc2	PROMOTER	1.873	3.317	398.42	1321.74	6.212	340.43	2114.92
A_68_P27456596	chr10:112366019-112366063	NM_001033474:42	Atxn7l3b	INSIDE	1.872	2.055	1150.38	2364.14	3.846	889.33	3420.50
A_68_P22045621	chr3:33919264-33919308	NM_001113188:286	Fxr1	INSIDE	1.872	5.067	1615.59	8186.84	9.487	1389.21	13179.17
A_68_P26974637	chr10:18039197-18039241			Unknown	1.872	4.510	1580.08	7125.83	8.444	1398.48	11808.21
A_68_P20453775	chr1:95073064-95073108			Unknown	1.872	4.538	4204.56	19080.88	8.496	3052.39	25933.24
A_68_P30594370	chr16:21794748-21794792	NR_037957:351	1300002E11Rik	INSIDE	1.871	2.126	2580.94	5487.07	3.977	1897.25	7546.25
A_68_P31350938	chr17:74715809-74715853	NM_001146223:-57	Dpy30	PROMOTER	1.871	3.740	704.95	2636.74	7.000	589.60	4127.01
A_68_P21683849	chr2:140221825-140221869	NM_028387:676	Macrod2	INSIDE	1.871	6.341	1384.22	8776.69	11.861	1025.84	12167.25
A_68_P26590952	chr9:65908094-65908138	NM_011149:141	Ppib	INSIDE	1.871	4.387	1835.70	8054.00	8.209	1472.92	12091.40
A_68_P23362920	chr4:140804003-140804047	NM_172520:5226	Arhgef19	INSIDE	1.870	5.882	3780.71	22238.90	11.000	2681.58	29498.68
A_68_P24993125	chr7:19989502-19989546	NM_146182:-4319	Klc3	DIVERGENT_PROMOTER	1.870	5.073	605.74	3072.82	9.488	524.06	4972.47
A_68_P30759540	chr16:52031753-52031797	NM_001033238:113	Cblb	INSIDE	1.869	5.433	3786.69	20574.64	10.156	2974.85	30212.40
A_68_P21478262	chr2:102499332-102499376	NM_001077514:515	Slc1a2	INSIDE	1.869	1.470	1996.54	2934.82	2.748	1860.15	5111.37
A_68_P31560905	chr18:24761962-24762006	NM_139143:334	Slc39a6	INSIDE	1.868	2.161	1272.21	2749.55	4.037	1035.29	4179.32
A_68_P31204664	chr17:46168459-46168503	NM_001110266:-6368	Vegfa	PROMOTER	1.868	3.938	2692.61	10604.08	7.358	1954.89	14383.83
A_68_P21072052	chr2:25283953-25283999	NM_007379:-217	Abca2	PROMOTER	1.867	6.187	1092.36	6758.71	11.554	738.63	8533.92
A_68_P22878646	chr4:44717054-44717098	NM_008782:6236	Pax5	INSIDE	1.867	3.219	1464.92	4715.36	6.011	1183.08	7111.31
A_68_P26931630	chr10:9394840-9394884	NM_177271:144	Samd5	INSIDE	1.867	2.575	252.30	649.59	4.807	193.73	931.21

ProbeName	Target position of probe on CpG island microarray	TargetID	GeneSymbol	CpG island Description	Ratio of relative methylation (TiO_2-NP/Vehicle)	Sham group			TiO_2-H group		
						Relative methylation (Cy5/Cy3)	Cy3 signal (Input)	Cy5 signal (MeDIP)	Relative methylation (Cy5/Cy3)	Cy3 signal (Input)	Cy5 signal (MeDIP)
A_68_P26399336	chr9:31018727-31018771	NM_001102455:652	Aplp2	INSIDE	1.866	3.500	1846.08	6460.53	6.530	1573.87	10276.76
A_68_P31933966	chr19:6320610-6320654	NM_001033342:14176	Cdc42bpg	INSIDE	1.866	2.451	1328.64	3256.35	4.573	993.63	4544.01
A_68_P28143851	chr11:113612678-113612722	NM_020006:-226	Cdc42ep4	PROMOTER	1.866	5.437	4581.48	24908.34	10.143	3566.82	36177.86
A_68_P32815030	chrX:164561176-164561220	NM_001033330:453967	Frmpd4	INSIDE	1.866	1.925	4121.47	7933.14	3.592	2105.63	7564.06
A_68_P27850828	chr11:61668703-61668747	NM_013881:-130	Ulk2	PROMOTER	1.866	9.965	2530.43	25216.61	18.596	1822.22	33886.04
A_68_P23231372	chr4:116550094-116550138	NM_001029912:110	Zswim5	INSIDE	1.866	3.471	2852.81	9901.03	6.476	2279.39	14761.92
A_68_P29243355	chr13:96661950-96661994	NM_027711:-95	Iqgap2	PROMOTER	1.865	7.954	4160.49	33091.43	14.837	3280.77	48675.82
A_68_P28587837	chr12:83718482-83718526	NM_015812:480	Rgs6	INSIDE	1.865	4.469	5923.59	26471.32	8.335	4024.42	33544.94
A_68_P24153558	chr5:143580250-143580294	NM_001122730:-1206	Tnrc18	PROMOTER	1.865	3.080	1012.96	3120.38	5.746	755.98	4343.72
A_68_P26242187	chr8:126416382-126416426	NM_009606:2232	Acta1	INSIDE	1.864	5.940	2048.63	12168.98	11.070	1536.20	17005.65
A_68_P25017319	chr7:28232977-28233021	NM_144923:2	Blvrb	INSIDE	1.864	5.626	831.95	4680.16	10.488	640.99	6722.65
A_68_P21871012	chr2:173485328-173485372	NM_024436:5	Rab22a	INSIDE	1.864	3.810	1753.20	6679.06	7.102	1469.63	10436.92
A_68_P30379103	chr15:81524969-81525013	NM_001164320:2727	Chadl	INSIDE	1.863	5.182	1487.64	7708.88	9.656	1312.39	12672.07
A_68_P31933641	chr19:6277445-6277489	NM_010119:571	Ehd1	INSIDE	1.863	2.535	2307.03	5847.36	4.723	1920.83	9072.38
A_68_P23416733	chr4:151506350-151506394	NM_019585:-3338	Espn	PROMOTER	1.863	2.714	1723.10	4676.52	5.057	1235.44	6248.09
A_68_P21820992	chr2:164823190-164823234	NM_020333:29725	Slc12a5	INSIDE	1.863	4.280	1560.22	6677.49	7.972	1157.02	9223.73
A_68_P31932456	chr19:6060898-6060957	NM_013859:-279	Znhit2-ps	PROMOTER	1.863	9.177	22684.11	208180.60	17.096	12764.16	218213.80
A_68_P26542457	chr9:57408653-57408697	NM_022813:-76	Scamp2	DIVERGENT_PROMOTER	1.862	8.524	2860.02	24377.60	15.873	2166.72	34391.44
A_68_P28778754	chr12:119539626-119539670	NM_001166385:265	Sp4	INSIDE	1.862	2.643	5538.52	14636.78	4.922	3708.25	18250.32
A_68_P28871505	chr13:21272743-21272787	NM_009054:937	Trim27	INSIDE	1.862	3.993	1642.00	6556.20	7.435	1345.09	10000.14
A_68_P26844412	chr9:114299556-114299600	NM_019922:252	Crtap	INSIDE	1.861	2.317	304.24	704.96	4.312	255.06	1099.81
A_68_P24859179	chr6:133986298-133986342	NM_007961:596	Etv6	INSIDE	1.861	4.825	3201.66	15448.10	8.981	2185.86	19630.72
A_68_P31136610	chr17:31038660-31038704	NM_021332:-129	Glp1r	PROMOTER	1.861	3.103	1597.84	4957.41	5.775	1231.07	7109.74
A_68_P24005520	chr5:115141122-115141166	NM_019821:-200	Gltp	PROMOTER	1.861	14.750	2824.25	41658.07	27.452	1892.70	51959.08
A_68_P30348856	chr15:76348296-76348340	NM_130893:4241	Scrt1	INSIDE	1.861	1.663	646.71	1075.76	3.096	568.99	1761.59
A_68_P33005878	A_68_P33005878			Unknown	1.861	2.483	1273.31	3161.72	4.622	1150.16	5316.27
A_68_P32288523	chrX:20003140-20003184	ENSMUST00000136093:249		INSIDE	1.861	2.307	1224.44	2824.45	4.292	634.04	2721.51
A_68_P30093919	chr15:27611395-27611439	NM_198301:-163	Fam105a	PROMOTER	1.860	6.742	2286.11	15412.02	12.541	1862.41	23356.17
A_68_P30137020	chr15:36094444-36094488	NM_001081253:173	Fbxo43	INSIDE	1.860	5.797	2825.11	16376.00	10.784	2325.91	25082.19
A_68_P32127978	chr19:43749225-43749269	NM_145156:125	Slc25a28	INSIDE	1.860	3.834	4546.31	17431.31	7.130	3722.09	26537.24
A_68_P22411695	chr3:108086638-108086682	NM_019972:-389	Sort1	PROMOTER	1.860	12.153	4225.97	51358.98	22.603	3331.36	75298.24
A_68_P27907296	chr11:71564250-71564294	NM_177618:68	Wscd1	INSIDE	1.860	4.496	4666.83	20981.39	8.363	3397.05	28410.13
A_68_P26036153	chr8:90184081-90184125	NM_033327:299392	Zfp423	DOWNSTREAM	1.860	2.586	2470.14	6388.56	4.811	1808.14	8699.09
A_68_P26617803	chr9:70527024-70527068	NM_007399:239	Adam10	INSIDE	1.859	3.341	3327.19	11116.75	6.210	2658.10	16507.02
A_68_P29629464	chr14:58318596-58318640	NM_015771:46342	Lats2	INSIDE	1.859	4.525	1145.35	5183.03	8.411	850.90	7156.64
A_68_P29055172	chr13:55565926-55565970	NM_001030296:321	Prr7	INSIDE	1.859	1.859	1462.78	2719.55	3.456	1167.23	4034.24
A_68_P21641570	chr2:132512792-132512837	NM_028637:-3204	1110034G24Rik	PROMOTER	1.858	6.225	4805.88	29915.80	11.569	3480.64	40266.74
A_68_P28576807	chr12:81744426-81744470	NM_007951:400	Erh	INSIDE	1.857	8.037	3705.65	29783.34	14.922	2743.77	40942.16
A_68_P20942041	chr1:194014550-194014594	NM_001038607:-181	Kcnh1	PROMOTER	1.857	6.657	1568.79	10443.24	12.363	1127.88	13944.47
A_68_P20877047	chr1:182571834-182571878	NM_175186:392	Lin9	INSIDE	1.857	3.800	539.78	2051.03	7.055	419.29	2957.95
A_68_P26500017	chr9:49606485-49606529	NM_001081445:668	Ncam1	INSIDE	1.857	2.717	2069.79	5624.12	5.047	1608.35	8117.62
A_68_P30176273	chr15:43308698-43308742	NM_025736:-54	Ttc35	PROMOTER	1.857	2.199	1903.70	4185.70	4.083	1443.41	5892.75
A_68_P32203587	chr19:56948670-56948714	NM_172840:-213	Vwa2	PROMOTER	1.857	2.149	2555.17	5491.18	3.992	1976.20	7888.36
A_68_P32245740	chrX:8777550-8777594	NM_173414:474	Lancl3	INSIDE	1.856	3.553	2031.89	7220.04	6.594	1133.27	7472.63
A_68_P27812425	chr11:54674397-54674456	NM_029327:-333	Lyrm7	DIVERGENT_PROMOTER	1.856	6.650	561.10	3731.56	12.345	393.08	4852.51
A_68_P20929010	chr1:191746024-191746068	NM_026796:121	Smyd2	INSIDE	1.856	1.637	1106.26	1810.60	3.039	961.74	2922.27
A_68_P29030706	chr13:51195741-51195786	NR_029463:333	9430083A17Rik	INSIDE	1.855	7.570	295.35	2235.85	14.039	200.59	2816.15
A_68_P24798780	chr6:120442970-120443014	NM_033567:833	Cecr6	INSIDE	1.855	9.906	4627.20	45837.77	18.374	3197.85	58756.19
A_68_P28152301	chr11:115111293-115111337	NM_010350:17243	Grin2c	INSIDE	1.855	4.543	821.35	3731.26	8.426	625.37	5269.14
A_68_P25088285	chr7:51846895-51846939	NM_008422:661	Kcnc3	INSIDE	1.855	1.592	1174.97	1870.06	2.953	960.40	2835.96
A_68_P31150844	chr17:34027540-34027584	NM_001195298:16	Kifc1	INSIDE	1.855	2.792	4744.51	13244.59	5.177	3568.63	18475.69

ProbeName	Target position of probe on CpG island microarray	TargetID	GeneSymbol	CpG island Description	Ratio of relative methylation (TiO_2-NP/Vehicle)	Sham group			TiO_2-H group		
						Relative methylation (Cy5/Cy3)	Cy3 signal (Input)	Cy5 signal (MeDIP)	Relative methylation (Cy5/Cy3)	Cy3 signal (Input)	Cy5 signal (MeDIP)
A_68_P28781893	chr12:120087514-120087558	NM_177082:2735	Sp8	INSIDE	1.855	6.228	2260.85	14079.59	11.549	1813.01	20938.56
A_68_P21117286	chr2:32612762-32612806	NM_152800:-6	Tor2a	PROMOTER	1.855	3.906	1786.12	6976.17	7.247	1455.65	10548.40
A_68_P26826440	chr9:110782793-110782837	NM_001146060:-11	Als2cl	PROMOTER	1.854	2.810	1878.69	5278.63	5.210	1557.45	8114.97
A_68_P32563078	chrX:98450245-98450289	NM_018789:400	Foxo4	INSIDE	1.854	2.398	2878.19	6901.22	4.445	1681.25	7473.24
A_68_P25022784	chr7:29383198-29383242	NM_027470:-17	Pak4	PROMOTER	1.853	2.382	1554.18	3702.31	4.414	1320.10	5827.10
A_68_P24631961	chr6:90319416-90319460	NM_030260:-49	Zxdc	PROMOTER	1.853	2.577	1348.62	3475.42	4.775	1139.85	5442.93
A_68_P26897431	A_68_P26897431			Unknown	1.853	3.957	2107.03	8337.27	7.333	1705.38	12505.80
A_68_P22312502	chr3:88018703-88018747	NR_037276:-368	Mir3093	PROMOTER	1.852	2.529	1938.75	4903.30	4.684	1457.09	6825.26
A_68_P27980115	chr11:84693687-84693731	NM_027388:-11	Pigw	DIVERGENT_PROMOTER	1.852	5.805	4740.80	27519.45	10.748	3448.08	37060.13
A_68_P21307670	chr2:69044152-69044196	NM_001199123:73	Spc25	INSIDE	1.852	3.706	1585.79	5877.44	6.865	1353.50	9291.82
A_68_P29560590	chr14:41779806-41779850	NM_145928:268	Tspan14	INSIDE	1.852	3.937	6353.90	25015.85	7.292	5038.57	36742.06
A_68_P28172267	chr11:118430861-118430905	NM_001024931:340004	Rbfox3	INSIDE	1.851	7.994	1742.26	13928.25	14.799	1321.84	19561.59
A_68_P23588840	chr5:33775538-33775582	ENSMUST00000030994:-1716		PROMOTER	1.851	3.157	943.98	2980.57	5.843	721.78	4217.52
A_68_P22128839	chr3:51081429-51081473	NM_023502:48459	Elf2	INSIDE	1.850	5.104	1669.02	8517.86	9.439	1364.87	12883.47
A_68_P26248330	chr8:127421598-127421642	NM_198103:-15	Exoc8	DIVERGENT_PROMOTER	1.850	4.396	1582.35	6956.78	8.135	1298.63	10564.61
A_68_P23195768	chr4:108055890-108055934	NM_001099303:42	Fam159a	INSIDE	1.850	3.727	1717.43	6400.97	6.893	1331.16	9176.22
A_68_P27997843	chr11:87912671-87912715	NM_198013:45	Cuedc1	INSIDE	1.849	4.175	3123.06	13039.48	7.721	2763.17	21333.33
A_68_P27681336	chr11:30854073-30854117	NM_025745:37	Erlec1	INSIDE	1.849	8.253	1290.17	10647.89	15.262	994.76	15181.97
A_68_P22481656	chr3:121996594-121996638	NR_030439:5	Mir760	INSIDE	1.849	3.682	1919.95	7068.63	6.809	1611.73	10973.93
A_68_P23230191	chr4:116299963-116300007	NM_001081475:118	Nasp	INSIDE	1.849	1.862	608.42	1132.81	3.442	451.71	1554.89
A_68_P21780565	chr2:157854409-157854453	NM_027434:-99	Rprd1b	PROMOTER	1.849	2.117	3552.85	7520.53	3.913	2754.84	10779.73
A_68_P23778454	chr5:69948402-69948446	NM_172711:244	Guf1	INSIDE	1.848	11.820	651.41	7699.92	21.844	469.40	10253.48
A_68_P27628926	chr11:19825568-19825612	NM_033523:1146	Spred2	INSIDE	1.848	5.940	1757.69	10440.87	10.975	1358.52	14909.89
A_68_P20268943	chr1:60466325-60466369	NM_001198570:-116	Abi2	PROMOTER	1.847	3.771	2029.17	7651.42	6.965	1746.26	12163.37
A_68_P28725240	chr12:109244557-109244601	NM_001079883:-2954	Bcl11b	PROMOTER	1.847	10.113	8411.21	85064.03	18.677	6212.44	116032.30
A_68_P24112800	chr5:135164852-135164896	NM_010717:-414	Limk1	PROMOTER	1.847	3.153	1731.17	5458.42	5.825	1224.90	7134.82
A_68_P29700473	chr14:70689417-70689461	NM_008915:-182	Ppp3cc	PROMOTER	1.847	1.470	711.29	1045.58	2.714	616.46	1673.29
A_68_P20796837	chr1:167775393-167775437	NM_001113391:56603	Cd247	INSIDE	1.846	3.316	1024.24	3396.84	6.123	945.61	5790.05
A_68_P31092997	chr17:23816594-23816638	NM_018777:285	Cldn6	INSIDE	1.846	2.792	6471.81	18068.45	5.154	4832.01	24904.13
A_68_P32798239	chrX:160857687-160857731	NM_011081:-10	Piga	PROMOTER	1.846	2.501	1866.07	4667.41	4.618	951.22	4392.61
A_68_P27560706	chr11:4886974-4887018	NM_007454:89	Ap1b1	INSIDE	1.845	5.714	2772.77	15842.99	10.541	2186.53	23047.61
A_68_P26814987	chr9:108362953-108362997	NM_133744:126	Ccdc71	INSIDE	1.845	3.061	1718.26	5258.97	5.647	1503.95	8493.19
A_68_P25005401	chr7:25319057-25319101	NM_153134:3412	Irgq	INSIDE	1.845	7.348	962.25	7071.03	13.556	824.30	11174.33
A_68_P24322963	chr6:28783645-28783689	NM_138682:-1919	Lrrc4	PROMOTER	1.845	3.671	1049.16	3851.39	6.772	1235.47	8367.10
A_68_P31621448	chr18:36178193-36178239	NM_001167891:178598	Nrg2	INSIDE	1.845	5.845	6052.49	35379.04	10.786	4628.59	49921.89
A_68_P23618578	chr5:38668746-38668790	NM_172709:126	Otop1	INSIDE	1.845	3.000	1919.11	5756.51	5.536	1513.31	8377.17
A_68_P30729955	chr16:46010463-46010507	NM_001134480:42	Plcxd2	INSIDE	1.845	4.883	876.34	4279.17	9.008	681.15	6135.69
A_68_P21566993	chr2:118752216-118752260	NM_028117:6	Chst14	INSIDE	1.844	4.062	1825.71	7415.99	7.491	1518.00	11371.36
A_68_P32229410	chr19:61301151-61301195	NM_009970:3149	Csf2ra	INSIDE	1.844	8.907	12157.54	108287.80	16.425	7752.53	127333.40
A_68_P31122728	chr17:28622553-28622597	NM_010220:483	Fkbp5	INSIDE	1.844	2.440	266.10	649.29	4.499	246.20	1107.77
A_68_P22483661	chr3:122321647-122321691	NM_001114665:917	Fnbp1l	INSIDE	1.844	6.922	969.79	6713.12	12.762	832.67	10626.89
A_68_P23261234	chr4:122866488-122866532	NM_174998:5764	Hpcal4	INSIDE	1.844	7.354	10025.79	73731.62	13.561	6913.90	93759.12
A_68_P23373724	chr4:142752116-142752160	NM_001081355:50474	Prdm2	INSIDE	1.844	3.720	941.81	3503.25	6.859	847.18	5811.11
A_68_P31162032	chr17:36116207-36116251	NR_028516:-260	Prr3	PROMOTER	1.844	4.839	2099.54	10159.79	8.921	1578.09	14077.99
A_68_P25103254	chr7:54387461-54387505	NM_001163565:486	Ptpn5	INSIDE	1.844	3.412	2019.97	6892.19	6.290	1577.30	9921.40
A_68_P26574053	chr9:62986483-62986527	NM_172446:8283	Skor1	INSIDE	1.844	6.491	392.52	2547.85	11.969	308.45	3691.74
A_68_P21844247	chr2:168760922-168760966	NM_009564:20143	Zfp64	INSIDE	1.844	7.758	353.32	2740.99	14.308	325.64	4659.13
A_68_P32565200	chrX:98892529-98892573	ENSMUST00000073812:-5361		PROMOTER	1.844	7.736	2925.10	22628.70	14.269	1541.65	21997.32
A_68_P31939711	chr19:7496090-7496134	NM_175381:3998	2700081O15Rik	INSIDE	1.843	5.555	2835.16	15749.15	10.237	1880.48	19250.76
A_68_P26073617	chr8:96334994-96335038	NM_001113384:279	Gnao1	INSIDE	1.843	6.583	10465.44	68894.13	12.130	7446.55	90329.10
A_68_P26258433	chr8:129118351-129118402	NM_001164598:-1040	Irf2bp2	PROMOTER	1.843	3.789	2286.39	8663.45	6.982	1790.20	12499.77

ProbeName	Target position of probe on CpG island microarray	TargetID	GeneSymbol	CpG island Description	Ratio of relative methylation (TiO_2-NP/Vehicle)	Sham group			TiO_2-H group		
						Relative methylation (Cy5/Cy3)	Cy3 signal (Input)	Cy5 signal (MeDIP)	Relative methylation (Cy5/Cy3)	Cy3 signal (Input)	Cy5 signal (MeDIP)
A_68_P31777744	chr18:65047479-65047523	NM_001114386:91	Nedd4l	INSIDE	1.843	10.346	942.70	9753.45	19.064	714.87	13628.62
A_68_P28457438	chr12:57634665-57634709	NM_001146198:1407	Nkx2-1	INSIDE	1.843	2.491	2084.61	5193.37	4.591	1654.48	7596.10
A_68_P21820797	chr2:164794239-164794283	NM_020333:773	Slc12a5	INSIDE	1.843	2.128	874.21	1860.62	3.922	698.46	2739.65
A_68_P29718152	chr14:73952682-73952726	NM_011506:112	Sucla2	INSIDE	1.843	4.831	1652.03	7980.50	8.905	1393.34	12407.05
A_68_P32906197	A_68_P32906197			Unknown	1.843	5.241	235.53	1234.33	9.660	181.13	1749.76
A_68_P23093082	chr4:88584489-88584533			Unknown	1.843	2.136	260.76	556.96	3.937	258.24	1016.83
A_68_P23253636	chr4:120527346-120527390	ENSMUST00000132895:-52		PROMOTER	1.843	5.879	13179.14	77479.07	10.837	9680.76	104913.40
A_68_P24004680	chr5:115017431-115017475	NM_001004180:-807	BC057022	PROMOTER	1.842	14.251	1361.17	19397.40	26.247	916.41	24053.55
A_68_P24388784	chr6:40385730-40385774	NM_175528:380	E330009J07Rik	INSIDE	1.842	8.401	5482.20	46054.93	15.474	4148.33	64192.08
A_68_P26554373	chr9:59464987-59465031	NM_001205239:-82	Parp6	PROMOTER	1.842	4.992	752.76	3758.08	9.197	605.13	5565.22
A_68_P21556050	chr2:116946481-116946525	NM_033524:-683	Spred1	PROMOTER	1.842	3.294	4864.40	16025.06	6.069	4099.83	24880.16
A_68_P26143096	chr8:109620241-109620285	NM_009353:183	Terf2	INSIDE	1.842	7.143	4769.95	34073.04	13.156	3569.45	46958.07
A_68_P29385917	chr14:8624251-8624295	ENSMUST00000081331:-107		DIVERGENT_PROMOTER	1.842	2.171	1122.88	2438.04	4.000	1068.56	4274.30
A_68_P26863467	chr9:117781995-117782039	ENSMUST00000126482:-245		PROMOTER	1.841	2.890	892.04	2577.83	5.319	690.12	3670.96
A_68_P20897790	chr1:186669470-186669514	NM_133684:234	Mosc2	INSIDE	1.840	1.853	1126.79	2088.49	3.410	827.00	2819.66
A_68_P27168078	chr10:58786437-58786481	NM_011030:415	P4ha1	INSIDE	1.840	4.803	4662.90	22393.81	8.836	3461.32	30584.71
A_68_P20740950	chr1:157820522-157820566	NM_001039184:-159	Cep350	PROMOTER	1.839	2.129	1799.19	3830.65	3.916	1451.01	5682.19
A_68_P25661493	chr8:12431257-12431301	NR_027975:45508	Gm5607	INSIDE	1.839	6.119	16899.96	103403.30	11.255	12229.55	137644.50
A_68_P32137222	chr19:45309238-45309283	NM_010691:466	Lbx1	INSIDE	1.839	4.671	1595.40	7451.81	8.589	1245.19	10695.25
A_68_P28086889	chr11:103611261-103611305	NM_011719:-147	Wnt9b	PROMOTER	1.839	4.097	1081.37	4430.58	7.536	805.24	6068.44
A_68_P25727484	chr8:26629202-26629246	NM_001079908:-19	Fgfr1	PROMOTER	1.838	2.463	1456.02	3585.62	4.527	1219.57	5521.00
A_68_P30547082	chr16:11253796-11253840	NM_001130008:600	Gspt1	INSIDE	1.838	1.881	1209.07	2273.78	3.456	1042.67	3603.49
A_68_P31715736	chr18:54022224-54022268	NM_152809:480	Csnk1g3	INSIDE	1.837	4.968	577.16	2867.29	9.127	430.82	3931.94
A_68_P24004046	chr5:114894276-114894320	NM_023556:-16	Mvk	DIVERGENT_PROMOTER	1.837	4.580	511.99	2345.05	8.414	406.64	3421.63
A_68_P25088136	chr7:51808933-51808977	NM_009473:339	Nr1h2	INSIDE	1.837	5.913	15841.82	93679.72	10.861	11014.63	119635.30
A_68_P26017663	chr8:86545319-86545363	NM_028877:-32	Palm3	DIVERGENT_PROMOTER	1.837	4.553	2619.26	11926.44	8.366	2019.89	16899.13
A_68_P29699987	chr14:70606016-70606060	NM_011366:1406	Sorbs3	INSIDE	1.837	4.285	2648.09	11346.76	7.872	2086.17	16422.34
A_68_P25017170	chr7:28193438-28193482	NM_032610:38148	Spnb4	INSIDE	1.837	3.254	1617.69	5263.61	5.977	1239.79	7410.37
A_68_P24671729	chr6:97155626-97155670	NM_001111106:-12	Uba3	DIVERGENT_PROMOTER	1.837	4.483	7712.25	34570.24	8.234	5398.68	44452.74
A_68_P21664157	chr2:136504412-136504456			Unknown	1.837	7.495	4216.07	31601.16	13.766	2972.39	40917.96
A_68_P20087502	chr1:23263626-23263674	ENSMUST00000097807:-405		PROMOTER	1.837	2.686	714.35	1918.87	4.933	620.39	3060.58
A_68_P26820784	chr9:109778541-109778586	NM_007658:481	Cdc25a	INSIDE	1.836	7.655	2446.19	18725.75	14.054	1806.42	25386.49
A_68_P28221329	chr12:8309070-8309114	NM_013527:-332	Gdf7	PROMOTER	1.836	2.722	5774.15	15716.10	4.998	4784.64	23914.52
A_68_P23590298	chr5:33995400-33995444	NM_009193:-467	Slbp	PROMOTER	1.836	2.597	4361.04	11323.66	4.766	3399.33	16202.53
A_68_P26494143	chr9:48643240-48643284	NM_001033324:788	Zbtb16	INSIDE	1.836	5.724	2309.58	13219.75	10.510	1649.43	17335.96
A_68_P24111498	chr5:134575433-134575477	NM_030719:-163	Gatsl2	PROMOTER	1.835	4.963	1362.28	6761.22	9.107	1124.35	10239.46
A_68_P25095389	chr7:53088549-53088593	NM_008172:33481	Grin2d	INSIDE	1.835	6.204	1118.06	6936.44	11.385	875.81	9971.39
A_68_P23264212	chr4:123341892-123341936	NM_001199136:19689	Macf1	INSIDE	1.835	6.225	2756.08	17155.80	11.422	2142.75	24474.07
A_68_P27080350	chr10:39452886-39452930	NM_011264:943	Rev3l	INSIDE	1.835	9.042	2510.01	22694.51	16.596	1771.06	29392.16
A_68_P27504343	chr10:121023890-121023934	NM_019786:-62	Tbk1	PROMOTER	1.835	6.046	1165.23	7045.24	11.095	1100.61	12211.23
A_68_P32016047	chr19:23210802-23210846	NM_010638:-4891	Klf9	PROMOTER	1.834	3.797	3494.13	13267.89	6.963	2747.09	19127.36
A_68_P24973639	chr7:13507675-13507719	NM_009095:37	Rps5	INSIDE	1.834	2.127	320.23	681.01	3.899	316.64	1234.64
A_68_P31560903	chr18:24761642-24761686	NM_139143:654	Slc39a6	INSIDE	1.834	2.435	2444.17	5951.24	4.466	2013.51	8992.61
A_68_P28752030	chr12:113959942-113959986	NM_001024602:-420	AW555464	PROMOTER	1.833	5.367	1685.78	9047.25	9.838	1290.79	12699.32
A_68_P21897030	chr2:179177570-179177614	NM_009867:410	Cdh4	INSIDE	1.833	3.684	2559.17	9428.91	6.755	2105.20	14221.06
A_68_P20437267	chr1:92500071-92500115	NM_133805:93	Cops8	INSIDE	1.833	5.807	2421.48	14062.52	10.645	1968.22	20952.55
A_68_P29445598	chr14:21001028-21001072	NM_021542:-46	Kcnk5	PROMOTER	1.833	3.715	4890.28	18167.01	6.809	3684.25	25086.54
A_68_P26818267	chr9:108998137-108998181	NM_172775:209	Plxnb1	INSIDE	1.833	3.745	978.86	3665.46	6.864	845.62	5804.39
A_68_P32143181	chr19:46392296-46392341	NM_028627:9328	Psd	INSIDE	1.833	2.663	1490.81	3969.84	4.881	1320.65	6445.94
A_68_P31791603	chr18:67550248-67550292	NM_026473:-114	Tubb6	PROMOTER	1.833	2.112	1364.48	2881.84	3.872	1120.72	4339.74
A_68_P30605892	chr16:23988103-23988147	NM_009744:574	Bcl6	INSIDE	1.832	5.444	3908.90	21278.28	9.974	3111.28	31031.68

ProbeName	Target position of probe on CpG island microarray	TargetID	GeneSymbol	CpG island Description	Ratio of relative methylation (TiO_2-NP/Vehicle)	Sham group			TiO_2-H group		
						Relative methylation (Cy5/Cy3)	Cy3 signal (Input)	Cy5 signal (MeDIP)	Relative methylation (Cy5/Cy3)	Cy3 signal (Input)	Cy5 signal (MeDIP)
A_68_P31600224	chr18:32070540-32070584	NM_026006:995	Sft2d3	INSIDE	1.832	5.139	2574.66	13231.95	9.415	2098.93	19762.36
A_68_P21017264	chr2:13495981-13496025	NM_011701:65	Vim	INSIDE	1.832	3.783	3082.57	11659.97	6.931	2498.75	17319.85
A_68_P31022093	chr17:6256852-6256896	ENSMUST00000121576:2972		DOWNSTREAM	1.832	3.733	1974.82	7371.73	6.840	1596.63	10920.47
A_68_P21115867	chr2:32391280-32391324	NM_153560:424	Fam102a	INSIDE	1.831	2.476	1103.59	2732.88	4.533	852.12	3863.05
A_68_P24611767	chr6:86683275-86683319	NM_011818:76	Gmcl1	INSIDE	1.831	6.282	6492.21	40784.11	11.502	4673.24	53751.74
A_68_P21093574	chr2:28695671-28695715	NM_001166033:188	Gtf3c4	INSIDE	1.831	6.435	1088.97	7007.60	11.785	913.34	10763.79
A_68_P29117527	chr13:69878894-69878938	NM_001145162:-141	Ube2ql1	PROMOTER	1.831	1.454	759.42	1103.94	2.661	644.53	1715.34
A_68_P23880187	chr5:90311691-90311735	NM_001081401:647	Adamts3	INSIDE	1.830	5.079	4130.18	20975.32	9.291	3425.03	31823.61
A_68_P30490425	chr15:101054602-101054646	NM_019518:-13	Grasp	PROMOTER	1.830	1.605	841.61	1350.63	2.937	635.77	1867.57
A_68_P21339341	chr2:74591156-74591200	NM_010467:-9858	Hoxd1	PROMOTER	1.830	4.738	3235.33	15329.05	8.673	2385.13	20685.77
A_68_P23399812	chr4:148681667-148681711	NM_207682:119	Kif1b	INSIDE	1.830	4.570	1413.26	6458.18	8.364	1189.26	9946.59
A_68_P25411827	chr7:117266477-117266521	NM_009516:926	Wee1	INSIDE	1.830	1.663	628.36	1044.80	3.043	619.02	1883.92
A_68_P20611349	chr1:132613579-132613625	NM_178691:-301	Yod1	DIVERGENT_PROMOTER	1.830	2.158	504.55	1088.79	3.949	468.83	1851.55
A_68_P20720544	chr1:154247140-154247184	NM_177756:166	Glt25d2	INSIDE	1.829	2.208	4633.39	10228.90	4.038	3755.72	15165.27
A_68_P29023967	chr13:49311401-49311447	NM_026738:-29	1110007C09Rik	PROMOTER	1.828	3.956	6255.04	24744.55	7.232	4536.90	32809.15
A_68_P26890105	chr9:122478146-122478190	NR_015610:-3450	9530059O14Rik	PROMOTER	1.828	3.077	1268.86	3904.14	5.625	1337.99	7526.78
A_68_P20351513	chr1:74998432-74998476	NM_010544:-229	Ihh	PROMOTER	1.828	1.985	2593.05	5147.28	3.629	2305.65	8366.79
A_68_P30295269	chr15:66800435-66800479	NM_008681:747	Ndrg1	INSIDE	1.828	6.584	2811.46	18509.54	12.036	2244.93	27019.44
A_68_P24213704	chr6:5955887-5955931	NM_001191023:280270	Dync1i1	INSIDE	1.827	3.203	1241.53	3976.49	5.851	992.91	5809.75
A_68_P21420369	chr2:90894125-90894169	NM_008948:-26	Psmc3	PROMOTER	1.827	5.506	33165.50	182611.90	10.061	22661.42	227992.10
A_68_P20411724	chr1:88255322-88255366	NR_002851:-2442	Snord82	PROMOTER	1.827	5.464	1772.42	9685.24	9.982	1286.89	12845.70
A_68_P23819150	chr5:77403737-77403781	NM_025691:33	Srp72	INSIDE	1.827	5.143	3782.81	19453.76	9.396	2820.82	26505.70
A_68_P24160101	chr5:144984767-144984811	NM_027410:-341	Tecpr1	PROMOTER	1.827	1.830	1178.84	2157.15	3.343	846.77	2831.13
A_68_P24979381	chr7:16646861-16646906			Unknown	1.827	4.435	3850.42	17074.98	8.102	2834.25	22963.00
A_68_P23797913	chr5:73883146-73883190	NM_001190733:904	Dcun1d4	INSIDE	1.826	10.139	17685.04	179306.30	18.515	10597.88	196224.10
A_68_P27921465	chr11:74424335-74424379	NM_001013784:-8750	E130309D14Rik	PROMOTER	1.826	5.392	1636.03	8821.58	9.847	1314.62	12944.86
A_68_P24171749	chr5:147887960-147888004	NM_080795:166	Lnx2	INSIDE	1.826	3.175	1701.57	5403.14	5.797	1544.24	8952.35
A_68_P26044646	chr8:91549828-91549872	NM_021390:18211	Sall1	DOWNSTREAM	1.826	1.816	777.09	1411.38	3.317	681.97	2261.82
A_68_P28078909	chr11:102180488-102180532	NM_011551:-100	Ubtf	PROMOTER	1.826	2.132	552.33	1177.68	3.893	399.39	1554.76
A_68_P27228617	chr10:69707740-69707784	NM_025807:-261	Slc16a9	PROMOTER	1.825	3.012	3026.06	9114.75	5.497	2482.91	13648.79
A_68_P23444287	chr5:3341583-3341627	NM_009873:-2707	Cdk6	PROMOTER	1.824	3.467	2545.92	8825.46	6.323	1807.56	11428.91
A_68_P29053522	chr13:55254151-55254195	NM_008011:-6	Fgfr4	PROMOTER	1.824	5.512	1658.26	9140.76	10.054	1504.20	15122.59
A_68_P20187367	chr1:43220695-43220739	NM_010212:90	Fhl2	INSIDE	1.824	6.556	1156.88	7584.06	11.958	1018.56	12180.28
A_68_P25010202	chr7:26180987-26181031	NM_010719:-2	Lipe	PROMOTER	1.824	3.873	4065.25	15743.27	7.063	3161.08	22325.43
A_68_P27171931	chr10:59465553-59465597	NM_026937:22	Ascc1	INSIDE	1.823	3.303	1913.76	6322.08	6.023	1652.32	9952.06
A_68_P32567806	chrX:99447101-99447145	NM_007709:-14	Cited1	PROMOTER	1.823	2.299	570.23	1310.75	4.190	323.08	1353.75
A_68_P32140683	chr19:45890581-45890625	NM_030716:-309	Kcnip2	PROMOTER	1.823	1.932	2746.60	5307.61	3.524	2147.02	7565.05
A_68_P21418380	chr2:90519239-90519283	NM_021512:1889	Nup160	INSIDE	1.823	5.490	7728.86	42429.44	10.009	6169.66	61751.11
A_68_P30489504	chr15:100908526-100908570	AK078004:-921		PROMOTER	1.823	1.626	3698.21	6011.74	2.964	2761.17	8183.88
A_68_P32572442	chrX:100551806-100551850	NM_009767:14	Chic1	INSIDE	1.822	3.938	3190.05	12563.39	7.174	1466.11	10517.46
A_68_P22319359	chr3:89234506-89234550	NM_026869:-230	Pygo2	PROMOTER	1.822	4.307	2831.88	12197.24	7.846	2355.22	18479.47
A_68_P27468929	chr10:114752147-114752191	NM_024454:479	Rab21	INSIDE	1.822	1.708	878.80	1500.72	3.111	731.83	2276.69
A_68_P31929627	chr19:5602038-5602082	NM_026616:188	Rnaseh2c	INSIDE	1.822	5.312	2264.08	12027.24	9.679	1871.49	18114.83
A_68_P26344894	chr9:20780852-20780896	NM_010333:363	S1pr2	INSIDE	1.822	15.703	5257.87	82562.47	28.611	3131.07	89583.18
A_68_P31097620	chr17:24626114-24626158	NM_027937:409	Caskin1	INSIDE	1.821	1.379	1840.28	2537.41	2.510	1442.13	3620.09
A_68_P21497226	chr2:105744559-105744603	NM_023876:77	Elp4	INSIDE	1.821	4.077	3355.27	13680.51	7.425	2790.48	20720.57
A_68_P23314731	chr4:132478927-132478977	NM_001163792:-486	Fam76a	PROMOTER	1.821	2.349	1010.77	2374.41	4.277	696.91	2980.48
A_68_P28066375	chr11:100006570-100006614	NM_008471:641	Krt19	INSIDE	1.821	2.693	5241.41	14114.38	4.905	4115.85	20188.17
A_68_P31336379	chr17:72131880-72131924	NM_030179:616	Clip4	INSIDE	1.820	4.744	765.70	3632.43	8.635	671.28	5796.46
A_68_P27756942	chr11:44432030-44432075	NM_007897:417	Ebf1	INSIDE	1.820	1.507	645.68	973.03	2.742	504.33	1383.01
A_68_P23250319	chr4:119941839-119941885	NM_194060:18004	Foxo6	INSIDE	1.820	3.106	574.76	1785.39	5.655	460.56	2604.27

ProbeName	Target position of probe on CpG island microarray	TargetID	GeneSymbol	CpG island Description	Ratio of relative methylation (TiO_2-NP/Vehicle)	Sham group			TiO_2-H group		
						Relative methylation (Cy5/Cy3)	Cy3 signal (Input)	Cy5 signal (MeDIP)	Relative methylation (Cy5/Cy3)	Cy3 signal (Input)	Cy5 signal (MeDIP)
A_68_P24064307	chr5:125507269-125507313	NM_011424:152294	Ncor2	INSIDE	1.820	5.340	9566.43	51080.29	9.717	7206.32	70024.62
A_68_P24771681	chr6:115494875-115494919	NM_199033:175	Tsen2	INSIDE	1.820	7.577	2438.08	18473.32	13.787	1654.35	22808.57
A_68_P24152591	chr5:143105523-143105567	NM_178398:7	Wipi2	INSIDE	1.820	3.101	1127.27	3495.87	5.644	958.11	5407.37
A_68_P22630575	chr3:148652818-148652862	ENSMUST00000098518:-560		PROMOTER	1.820	9.814	1470.88	14435.63	17.863	1052.46	18800.46
A_68_P20726280	chr1:155180208-155180252	NM_010683:-314	Lamc1	PROMOTER	1.819	7.614	27733.70	211155.70	13.851	15482.40	214448.20
A_68_P30363283	chr15:78765483-78765527	NM_133800:142	Nol12	INSIDE	1.819	3.125	1896.06	5924.42	5.685	1496.31	8506.16
A_68_P28442990	chr12:54808173-54808232	NM_013780:458539	Npas3	INSIDE	1.819	5.224	899.58	4699.41	9.504	716.61	6810.66
A_68_P25011329	chr7:26472470-26472514	NM_011577:472	Tgfb1	INSIDE	1.819	2.718	5512.94	14984.39	4.943	4060.73	20072.39
A_68_P27630622	chr11:20149303-20149348	NM_172260:102	Cep68	INSIDE	1.818	6.231	2082.62	12977.22	11.331	1861.11	21088.80
A_68_P32378028	chrX:49034553-49034597	NM_015819:-920	Hs6st2	PROMOTER	1.818	2.100	1353.54	2842.92	3.817	739.84	2824.33
A_68_P31130464	chr17:30025085-30025129	NM_001081160:-279	Mdga1	PROMOTER	1.818	4.836	2874.13	13899.56	8.792	2325.88	20449.59
A_68_P28449264	chr12:55896516-55896560	NM_026998:111	Snx6	INSIDE	1.818	4.739	1566.84	7425.23	8.616	1292.67	11137.82
A_68_P29080191	chr13:59658571-59658615	NM_023328:88	Agtpbp1	INSIDE	1.817	6.266	4305.05	26975.86	11.388	3358.48	38245.19
A_68_P20405120	chr1:84692097-84692141	NM_152915:678	Dner	INSIDE	1.817	2.089	1042.51	2177.44	3.794	810.47	3075.12
A_68_P21345223	chr2:75497311-75497355	NR_033513:-14	Gm6793	PROMOTER	1.817	3.214	715.56	2299.70	5.840	502.90	2936.75
A_68_P31412114	chr17:86086928-86086972	NM_011380:644	Six2	INSIDE	1.817	4.305	1547.01	6659.82	7.824	1263.46	9885.38
A_68_P24134133	chr5:139676870-139676914	NM_024451:269	Sun1	INSIDE	1.817	1.607	767.61	1233.68	2.920	1083.62	3164.28
A_68_P29024152	chr13:49343166-49343210	NM_025491:344	Susd3	INSIDE	1.817	1.837	1441.29	2648.13	3.338	1207.12	4029.69
A_68_P23196094	chr4:108132526-108132570	NM_175472:518	Zcchc11	INSIDE	1.817	10.177	5197.71	52899.47	18.496	3681.07	68086.84
A_68_P23201928	chr4:109330089-109330133	NM_007671:7867	Cdkn2c	DOWNSTREAM	1.816	2.840	954.42	2710.40	5.157	785.33	4050.01
A_68_P27163123	chr10:57786395-57786439	NM_026148:203	Lims1	INSIDE	1.816	4.775	3182.08	15193.93	8.672	2345.80	20343.02
A_68_P24053482	chr5:123464905-123464949	NM_175423:-156	Orai1	PROMOTER	1.816	2.677	906.36	2426.00	4.861	846.86	4116.84
A_68_P22317129	chr3:88894411-88894455	NM_001083808:-479	Rusc1	PROMOTER	1.816	3.505	2746.83	9627.80	6.364	2121.72	13503.65
A_68_P24631649	chr6:90275213-90275257	NM_027928:-55	Chst13	DIVERGENT_PROMOTER	1.815	1.643	880.04	1445.53	2.982	709.33	2115.11
A_68_P21909702	chr2:181022305-181022349	NM_198169:345	Gmeb2	INSIDE	1.815	1.560	1875.56	2926.55	2.832	1427.15	4041.51
A_68_P24784006	chr6:117866996-117867040	NM_001166427:-293	Hnrnpf	PROMOTER	1.815	3.705	2777.20	10289.67	6.726	2018.60	13576.17
A_68_P26346115	chr9:21043701-21043745	NM_001110305:54	Keap1	INSIDE	1.815	1.799	2047.85	3683.26	3.264	1688.13	5510.34
A_68_P24764867	chr6:114233070-114233114	NM_178703:464	Slc6a1	INSIDE	1.815	2.185	1964.99	4292.57	3.964	1703.19	6751.78
A_68_P26804662	chr9:106587823-106587867	NM_011573:154	Tex264	INSIDE	1.815	2.861	632.52	1809.56	5.192	463.35	2405.94
A_68_P26232970	chr8:124900378-124900422	NM_001029993:-115	Zc3h18	PROMOTER	1.815	4.101	833.27	3417.54	7.443	688.30	5123.13
A_68_P28050431	chr11:97276806-97276850	ENSMUST00000121799:-18		PROMOTER	1.815	3.825	2894.65	11072.80	6.942	2139.59	14852.50
A_68_P30361566	chr15:78503626-78503670	NM_183141:44895	Elfn2	INSIDE	1.814	6.596	1520.94	10032.69	11.964	1350.77	16160.51
A_68_P25644699	chr8:9770615-9770659	NM_173446:387	Fam155a	INSIDE	1.814	5.687	3346.03	19027.70	10.313	2684.47	27685.74
A_68_P31311922	chr17:67703725-67703769	NM_008984:53	Ptprm	INSIDE	1.814	7.137	1696.55	12107.99	12.949	1365.94	17687.68
A_68_P23457345	chr5:5749151-5749195	NM_027399:145	Steap1	INSIDE	1.814	5.963	5147.22	30691.20	10.818	4433.72	47962.54
A_68_P24383759	chr6:39523831-39523875	NM_178873:-22	Adck2	PROMOTER	1.813	3.292	2014.29	6630.83	5.969	1485.03	8863.72
A_68_P22317617	chr3:88973343-88973387	NM_001163432:4638	Clk2	INSIDE	1.813	7.739	6628.02	51296.66	14.031	4272.64	59947.78
A_68_P28396964	chr12:45311296-45311340	NM_013760:-263	Dnajb9	PROMOTER	1.813	1.965	1056.58	2076.00	3.562	892.34	3178.07
A_68_P21722817	chr2:147019650-147019694	NM_001077632:-7534	Nkx2-2	PROMOTER	1.813	2.371	1751.40	4151.79	4.299	1341.88	5768.45
A_68_P32183826	chr19:53751832-53751876	NM_001170847:59	Rbm20	INSIDE	1.813	6.095	2886.04	17589.61	11.050	2309.40	25517.88
A_68_P22128834	chr3:51080739-51080783	NM_023502:49149	Elf2	INSIDE	1.812	5.068	3293.52	16690.08	9.184	2567.56	23580.83
A_68_P32016039	chr19:23209957-23210001	NM_010638:-5737	Klf9	PROMOTER	1.812	4.846	4436.94	21499.29	8.780	3414.58	29979.51
A_68_P23544552	chr5:24414202-24414246	NM_001170555:103	Prkag2	INSIDE	1.812	7.118	8413.99	59892.39	12.900	7495.71	96696.36
A_68_P27833411	chr11:58717772-58717816	NM_011758:-67	Zfp39	PROMOTER	1.812	5.846	8399.74	49106.03	10.592	6079.29	64392.64
A_68_P29703311	A_68_P29703311			Unknown	1.812	7.959	3332.17	26519.53	14.422	2490.01	35910.79
A_68_P31019376	A_68_P31019376			Unknown	1.812	3.074	1463.58	4499.53	5.571	1156.81	6444.19
A_68_P31961511	chr19:12670617-12670661	NR_015503:418	A330040F15Rik	INSIDE	1.811	6.470	2292.64	14834.32	11.717	1846.29	21632.64
A_68_P31790565	chr18:67365128-67365172	NM_024190:138	Chmp1b	INSIDE	1.811	3.225	4755.01	15334.68	5.839	3568.63	20838.37
A_68_P32464984	chrX:71070091-71070135	NM_001029868:196	Pdzd4	INSIDE	1.811	4.937	765.24	3778.22	8.939	576.34	5152.02
A_68_P30927986	chr16:85173922-85173966	NM_001198823:8	App	INSIDE	1.810	4.246	1445.59	6137.72	7.684	1257.78	9665.34
A_68_P29699040	chr14:70480133-70480177	NM_018781:2903	Egr3	DOWNSTREAM	1.810	6.388	3054.13	19508.73	11.561	2484.02	28717.76

ProbeName	Target position of probe on CpG island microarray	TargetID	GeneSymbol	CpG island Description	Ratio of relative methylation (TiO_2-NP/Vehicle)	Sham group			TiO_2-H group		
						Relative methylation (Cy5/Cy3)	Cy3 signal (Input)	Cy5 signal (MeDIP)	Relative methylation (Cy5/Cy3)	Cy3 signal (Input)	Cy5 signal (MeDIP)
A_68_P27274519	chr10:77945016-77945060	ENSMUST00000069431:5549		DOWNSTREAM	1.810	1.673	1145.55	1916.89	3.028	1011.16	3061.94
A_68_P28684687	chr12:102146364-102146408	NM_001177674:-8652	Gpr68	PROMOTER	1.809	3.695	1650.80	6100.38	6.684	1533.07	10246.92
A_68_P21339240	chr2:74578542-74578586	NM_010468:28515	Hoxd3	INSIDE	1.809	2.036	1647.74	3355.21	3.683	1258.03	4633.65
A_68_P23094194	chr4:88783218-88783262	NM_024433:-33	Mtap	PROMOTER	1.809	3.549	5045.36	17904.92	6.418	3626.40	23275.54
A_68_P20134295	chr1:33727422-33727466	NR_033199:776	1700001G17Rik	INSIDE	1.808	8.194	2704.50	22161.41	14.814	1913.03	28339.87
A_68_P30476326	chr15:98593248-98593292	NM_007478:279	Arf3	INSIDE	1.808	1.391	2235.94	3111.08	2.516	1636.95	4118.23
A_68_P32260334	chrX:11664151-11664195	NM_029510:-6493	Bcor	PROMOTER	1.808	2.229	1356.47	3024.10	4.031	815.61	3287.47
A_68_P22338541	chr3:94168285-94168329	NM_001136117:1941	C2cd4d	INSIDE	1.808	5.376	5230.63	28118.47	9.722	4155.52	40398.62
A_68_P28990871	chr13:43655098-43655142	NM_001081059:440	Ccdc90a	INSIDE	1.808	4.884	2329.94	11379.26	8.831	1930.50	17047.94
A_68_P25954496	chr8:73376577-73376621	NM_032397:4309	Kcnn1	INSIDE	1.808	5.381	1881.59	10125.04	9.727	1305.40	12698.01
A_68_P26985109	chr10:19868572-19868616	NM_001198635:-131	Mtap7	PROMOTER	1.808	5.214	1052.75	5489.10	9.426	892.38	8411.77
A_68_P30000055	chr15:8395316-8395364	NM_027707:-877	Nipbl	PROMOTER	1.807	10.334	11522.38	119074.30	18.676	6922.61	129285.00
A_68_P27965931	chr11:82202507-82202551	NM_023438:127	Tmem132e	INSIDE	1.807	5.438	5787.76	31475.98	9.825	4556.78	44770.51
A_68_P24638016	chr6:91235548-91235592			Unknown	1.807	3.859	1017.61	3927.24	6.973	821.14	5725.42
A_68_P24992765	chr7:19927675-19927719	NM_001127324:-2723	Ercc1	PROMOTER	1.806	8.013	991.82	7947.64	14.469	840.64	12163.27
A_68_P21765995	chr2:155340656-155340700	NM_144786:-96	Ggt7	DIVERGENT_PROMOTER	1.806	2.951	1077.94	3180.74	5.328	842.32	4487.81
A_68_P30556199	chr16:13256418-13256462	NM_001122667:-133	Mkl2	PROMOTER	1.806	2.014	1559.34	3140.77	3.637	1310.82	4767.44
A_68_P32150744	chr19:47653811-47653855	NM_001164639:-676	Slk	PROMOTER	1.806	1.474	1101.74	1624.42	2.663	969.80	2583.01
A_68_P25502305	chr7:133942445-133942489	NM_001177307:226	Aldoa	INSIDE	1.805	2.341	2242.61	5249.11	4.224	1800.05	7603.52
A_68_P22085575	chr3:41369640-41369684	NM_001130186:861	Phf17	INSIDE	1.805	7.734	1394.73	10786.45	13.960	1101.00	15370.41
A_68_P30346613	chr15:76011583-76011627	NM_201394:14536	Plec	INSIDE	1.805	1.900	565.89	1075.26	3.429	411.97	1412.72
A_68_P28426162	chr12:51750316-51750360	NM_008858:-128	Prkd1	PROMOTER	1.804	2.988	3996.06	11938.92	5.389	3237.18	17445.42
A_68_P27635752	chr11:21139299-21139343	NM_139061:429	Vps54	INSIDE	1.804	4.071	1720.94	7005.72	7.344	1344.29	9872.08
A_68_P25581873	chr7:147222902-147222946	NM_027201:635	Zfp511	INSIDE	1.804	2.157	2142.84	4623.02	3.891	1510.89	5879.04
A_68_P31782005	chr18:65740650-65740694	NM_207255:789	Zfp532	INSIDE	1.804	4.365	4337.80	18933.93	7.874	3516.66	27688.88
A_68_P33012150	chr9_random:53992-54036	NR_027950:-678	4930526I15Rik	PROMOTER	1.803	7.605	24634.84	187359.00	13.714	15139.21	207618.50
A_68_P27975235	chr11:83881690-83881734	NM_019819:147	Dusp14	INSIDE	1.803	2.054	1290.16	2649.80	3.702	1035.86	3834.90
A_68_P25910880	chr8:64070053-64070097	NM_001081390:311413	Palld	INSIDE	1.803	5.859	3203.60	18770.66	10.564	2389.29	25240.59
A_68_P26464017	chr9:43611785-43611829	NM_021424:59148	Pvrl1	INSIDE	1.803	5.219	1554.17	8111.24	9.408	1038.88	9773.69
A_68_P25576307	chr7:146374240-146374284	NM_021302:131	Stk32c	INSIDE	1.803	4.492	1127.51	5065.01	8.099	961.82	7790.11
A_68_P31124371	chr17:28938550-28938594	NM_001081315:502	Brpf3	INSIDE	1.802	6.412	13892.16	89073.98	11.552	9383.17	108395.60
A_68_P25780173	chr8:36558550-36558594	NM_026067:15	Eri1	INSIDE	1.802	2.449	747.61	1830.80	4.413	663.58	2928.45
A_68_P30246699	chr15:58046420-58046464	NM_026346:-9	Fbxo32	PROMOTER	1.802	1.596	1220.54	1947.77	2.876	1004.10	2888.22
A_68_P23392700	chr4:147525826-147525870	NM_001163704:186	Fbxo6	INSIDE	1.802	5.283	4542.00	23995.44	9.522	3913.81	37267.97
A_68_P21420493	chr2:90910689-90910733	NM_026721:-332	Slc39a13	PROMOTER	1.802	3.480	3319.90	11553.43	6.269	2783.38	17450.22
A_68_P21438071	chr2:93797315-93797359	NM_001145034:-79	Gm13889	PROMOTER	1.801	2.816	3220.82	9068.87	5.071	2565.75	13010.32
A_68_P31781218	chr18:65590541-65590585	NM_172833:-88	Malt1	PROMOTER	1.801	4.774	13185.43	62940.98	8.596	9108.70	78301.18
A_68_P21261140	chr2:60719287-60719331	NM_001141931:187	Rbms1	INSIDE	1.801	2.769	209.34	579.69	4.988	235.88	1176.47
A_68_P21580978	chr2:121275537-121275581	NM_011354:595	Serf2	INSIDE	1.801	2.582	3099.68	8003.43	4.651	2734.38	12717.10
A_68_P30605897	chr16:23988863-23988907	NM_009744:-186	Bcl6	PROMOTER	1.800	15.406	1820.78	28050.26	27.729	1472.85	40839.93
A_68_P29713892	chr14:73109778-73109822	NM_207636:10	Fndc3a	INSIDE	1.800	3.666	15386.09	56402.37	6.598	12880.93	84987.90
A_68_P31258174	chr17:56896255-56896299	NM_008738:677	Nrtn	INSIDE	1.800	9.627	1392.79	13407.73	17.330	1188.08	20589.48
A_68_P23999133	chr5:114064391-114064435	NM_008153:35987	Cmklr1	INSIDE	1.799	5.553	1505.92	8362.64	9.989	1033.77	10325.81
A_68_P31620539	chr18:36020271-36020315	NM_133687:30821	Cxxc5	INSIDE	1.799	4.958	1846.83	9156.90	8.922	1356.72	12104.51
A_68_P21278824	chr2:63936695-63936739	NM_021716:-647	Fign	PROMOTER	1.799	1.562	572.22	893.92	2.810	476.20	1338.03
A_68_P30349539	chr15:76462358-76462402	NM_183091:7979	Tonsl	INSIDE	1.799	6.542	8551.76	55942.93	11.767	6017.12	70801.82
A_68_P29257361	chr13:99161507-99161552	BC059100:739690		INSIDE	1.799	5.081	3273.53	16631.91	9.141	2534.57	23167.47
A_68_P24004989	chr5:115060757-115060801	NM_001004180:42519	BC057022	INSIDE	1.798	4.701	4257.35	20015.41	8.454	3149.22	26623.86
A_68_P24449229	chr6:52175914-52175958	NM_010456:1433	Hoxa9	INSIDE	1.798	1.819	1546.09	2811.78	3.269	1279.83	4184.40
A_68_P28236792	chr12:11157416-11157460	NM_173417:-259	Kcns3	PROMOTER	1.798	12.300	3004.88	36959.76	22.110	2237.03	49461.30
A_68_P32050808	chr19:29122130-29122174	NR_028360:180	1700018L02Rik	INSIDE	1.797	3.071	3532.99	10850.38	5.520	2811.40	15519.09

ProbeName	Target position of probe on CpG island microarray	TargetID	GeneSymbol	CpG island Description	Ratio of relative methylation (TiO_2-NP/Vehicle)	Sham group			TiO_2-H group		
						Relative methylation (Cy5/Cy3)	Cy3 signal (Input)	Cy5 signal (MeDIP)	Relative methylation (Cy5/Cy3)	Cy3 signal (Input)	Cy5 signal (MeDIP)
A_68_P27810090	chr11:54251929-54251973	NM_173753:270	Fnip1	INSIDE	1.797	3.055	1965.54	6005.08	5.491	1626.28	8930.19
A_68_P21765994	chr2:155340501-155340545	NM_144786:60	Ggt7	INSIDE	1.797	1.723	1515.62	2611.59	3.096	1202.00	3721.77
A_68_P31933357	chr19:6235648-6235692	NM_198168:170	Ppp2r5b	INSIDE	1.796	1.849	688.63	1273.17	3.320	592.64	1967.48
A_68_P22383780	chr3:102799614-102799658	NM_025679:-26	Sike1	PROMOTER	1.796	1.652	1622.33	2680.09	2.967	1345.94	3993.37
A_68_P21147548	chr2:38195289-38195333	ENSMUST00000133661:-1301		PROMOTER	1.796	2.717	1425.62	3873.62	4.880	1225.15	5978.96
A_68_P29656173	chr14:63174404-63174448	ENSMUST00000162987:-147		PROMOTER	1.796	1.704	6632.57	11303.29	3.060	4694.28	14364.54
A_68_P25708990	chr8:23170949-23170993	NM_183142:-221	Alg11	DIVERGENT_PROMOTER	1.795	2.373	2983.00	7078.50	4.259	2521.59	10738.91
A_68_P32117489	chr19:41921912-41921956	NM_177603:688	Frat2	INSIDE	1.795	3.395	963.77	3271.91	6.093	735.76	4482.62
A_68_P30139530	chr15:36539045-36539089	NM_008774:-338	Pabpc1	PROMOTER	1.795	1.615	1468.37	2370.92	2.898	1265.04	3665.54
A_68_P20800091	chr1:168339580-168339624	NM_001142948:357	Pogk	INSIDE	1.795	4.313	8825.38	38061.16	7.740	6802.34	52653.14
A_68_P21782725	chr2:158235581-158235625	NM_177658:14	Ralgapb	INSIDE	1.795	2.817	1325.13	3733.54	5.058	1159.90	5866.99
A_68_P25994476	chr8:81922795-81922839	NM_008539:551	Smad1	INSIDE	1.795	10.589	3319.82	35154.77	19.006	2493.42	47388.88
A_68_P31675573	chr18:46358367-46358411	NM_001170855:84	Trim36	INSIDE	1.795	2.018	1190.25	2402.41	3.623	1052.42	3813.30
A_68_P28613117	chr12:88289051-88289098	NM_173735:782	2310044G17Rik	INSIDE	1.794	4.048	1247.68	5050.33	7.262	971.94	7058.25
A_68_P20633265	chr1:136312273-136312317	NM_028320:251	Adipor1	INSIDE	1.794	4.207	10396.44	43740.06	7.547	7847.36	59223.02
A_68_P22899538	chr4:48292337-48292381	NM_029572:103	Erp44	INSIDE	1.794	2.440	5149.30	12565.52	4.377	3986.47	17447.60
A_68_P30478814	chr15:99054189-99054233	NM_010601:-1196	Kcnh3	PROMOTER	1.794	4.971	3105.74	15437.94	8.916	2434.66	21707.96
A_68_P28600848	chr12:86217110-86217154	NM_013589:313	Ltbp2	INSIDE	1.794	1.754	853.83	1497.77	3.146	705.49	2219.75
A_68_P32567492	chrX:99352458-99352502	NM_146235:-50	Ercc6l	PROMOTER	1.793	4.973	810.47	4030.48	8.915	463.28	4129.97
A_68_P22082256	chr3:40753573-40753617	NM_001040399:-958	Larp1b	PROMOTER	1.793	2.331	22714.34	52937.90	4.178	16691.41	69733.62
A_68_P28351016	chr12:35731926-35731970	NM_001014973:88	Snx13	INSIDE	1.793	5.061	1661.88	8411.34	9.077	1424.16	12927.00
A_68_P22345154	chr3:95491735-95491779	NM_144899:25	Adamtsl4	INSIDE	1.792	1.859	2073.84	3854.71	3.330	1714.80	5711.03
A_68_P25498532	chr7:133225682-133225726	NM_001101488:221	Gsg1l	INSIDE	1.792	5.769	15276.74	88131.47	10.340	11739.08	121378.90
A_68_P20778372	chr1:164478561-164478605	NM_144877:-106	Mettl13	PROMOTER	1.791	2.885	7977.41	23018.06	5.169	5991.19	30966.64
A_68_P25948621	chr8:72427093-72427137	NM_023312:-657	Ndufa13	PROMOTER	1.791	1.527	619.53	946.15	2.735	577.30	1579.17
A_68_P29682917	chr14:67690619-67690663	NM_001205188:668	Ppp2r2a	INSIDE	1.791	7.070	3368.81	23816.75	12.659	2318.52	29350.15
A_68_P32710882	chrX:139400446-139400490	NM_019496:803	Ammecr1	INSIDE	1.790	3.226	976.55	3150.72	5.775	511.66	2954.91
A_68_P28598112	chr12:85749605-85749649	NM_001026214:353	Entpd5	INSIDE	1.790	3.507	2023.75	7097.12	6.279	1689.57	10608.28
A_68_P27500779	chr10:120416497-120416541	NM_001081193:-132	Lemd3	PROMOTER	1.790	4.313	931.72	4018.41	7.721	825.94	6376.93
A_68_P22340919	chr3:94642155-94642199	NM_001165948:688	Pogz	INSIDE	1.790	7.624	1520.61	11592.56	13.646	1173.41	16012.30
A_68_P25092685	chr7:52622314-52622358	NM_029741:53	Ppfia3	INSIDE	1.790	3.630	1450.59	5264.91	6.498	1233.06	8011.86
A_68_P27277985	chr10:79100697-79100741	NM_001024539:-55	Shc2	PROMOTER	1.790	3.240	1741.42	5642.11	5.799	1450.86	8414.19
A_68_P24008929	chr5:115751222-115751266	NM_019682:-245	Dynll1	PROMOTER	1.789	4.209	2244.59	9447.12	7.528	1866.35	14049.56
A_68_P32386813	chrX:50622802-50622846	NM_001166365:158	Fam122b	INSIDE	1.789	8.506	5647.04	48031.03	15.218	2876.24	43771.99
A_68_P32457442	chrX:69520267-69520311	NM_017369:-228	Gabre	PROMOTER	1.789	4.370	3001.36	13115.06	7.817	2007.83	15694.92
A_68_P28029575	chr11:93747831-93747875	NM_134012:320	Mbtd1	INSIDE	1.789	4.285	2190.93	9388.97	7.668	1775.23	13611.80
A_68_P25588870	chr7:148633634-148633678	NR_034049:-1075	Snora52	PROMOTER	1.789	2.259	6061.79	13694.99	4.041	4450.07	17984.60
A_68_P26134826	chr8:108129362-108129406	NR_035492:-9981	Mir1966	PROMOTER	1.788	3.411	2337.21	7972.16	6.098	1612.29	9831.01
A_68_P23991577	chr5:112772027-112772071	NM_027323:12	Srrd	INSIDE	1.788	3.757	18048.04	67814.54	6.717	14956.85	100468.50
A_68_P25328173	chr7:100228372-100228416	NM_021427:6	Fam181b	INSIDE	1.787	2.659	1172.86	3119.18	4.753	871.74	4143.13
A_68_P29575892	chr14:47000105-47000149	NR_037981:928	Gm15217	INSIDE	1.787	3.607	2919.11	10529.17	6.446	2186.72	14096.10
A_68_P28067972	chr11:100275705-100275749	NM_176830:407	Leprel4	INSIDE	1.787	4.906	930.00	4562.88	8.768	870.89	7635.61
A_68_P27288382	chr10:80720130-80720174	NM_010768:-137	Matk	PROMOTER	1.787	7.778	2735.01	21273.82	13.898	2288.70	31808.01
A_68_P28748036	chr12:113368595-113368639	NR_029590:-474	Mir203	PROMOTER	1.787	2.450	281.02	688.43	4.378	193.24	845.98
A_68_P25948620	chr8:72427025-72427069	NM_023312:-589	Ndufa13	PROMOTER	1.787	3.236	714.39	2311.79	5.783	683.51	3952.85
A_68_P20421182	chr1:89904977-89905021	NM_001033291:128	Usp40	INSIDE	1.787	3.697	4393.92	16244.86	6.605	3280.38	21667.59
A_68_P31148785	chr17:33495528-33495572	NM_207541:273	Zfp81	INSIDE	1.787	2.112	3403.81	7188.31	3.773	2674.22	10090.12
A_68_P31561526	chr18:24868000-24868044	NM_175276:77	Fhod3	INSIDE	1.786	4.266	1651.68	7046.32	7.617	1352.02	10298.76
A_68_P30424721	chr15:89186360-89186404	NM_001115132:230	Ncaph2	INSIDE	1.786	4.363	3195.59	13942.59	7.793	2602.19	20277.78
A_68_P24872958	chr6:136467356-136467400	NM_019426:7	Atf7ip	INSIDE	1.785	3.282	2891.36	9490.11	5.858	2247.19	13163.42
A_68_P26825039	chr9:110558909-110558957	NM_028312:-74	Ccdc12	DIVERGENT_PROMOTER	1.785	1.894	1145.22	2168.99	3.380	928.34	3138.11

ProbeName	Target position of probe on CpG island microarray	TargetID	GeneSymbol	CpG island Description	Ratio of relative methylation (TiO_2-NP/Vehicle)	Sham group			TiO_2-H group		
						Relative methylation (Cy5/Cy3)	Cy3 signal (Input)	Cy5 signal (MeDIP)	Relative methylation (Cy5/Cy3)	Cy3 signal (Input)	Cy5 signal (MeDIP)
A_68_P22301528	chr3:86028273-86028317	NM_001077687:-317	Lrba	PROMOTER	1.785	3.658	708.35	2591.21	6.529	653.27	4265.14
A_68_P24362057	chr6:35489865-35489909	NM_008098:2	Mtpn	INSIDE	1.785	3.996	2129.43	8508.36	7.134	1659.27	11837.30
A_68_P31098019	chr17:24687487-24687532	NM_013630:615	Pkd1	INSIDE	1.785	5.306	1287.64	6831.75	9.469	1086.78	10290.64
A_68_P25960292	chr8:74764393-74764437	NM_007456:384	Ap1m1	INSIDE	1.784	2.981	740.71	2207.94	5.317	583.61	3102.99
A_68_P24397660	chr6:42274774-42274818	NM_001113327:-157	Fam131b	PROMOTER	1.784	2.154	2777.70	5982.65	3.841	2513.41	9655.11
A_68_P24154219	chr5:143723667-143723712	NM_007984:1656	Fscn1	INSIDE	1.784	4.276	1532.87	6554.20	7.628	1125.17	8582.46
A_68_P28744403	chr12:112723947-112723992	NM_001033248:65	Gm266	INSIDE	1.784	1.381	2131.22	2942.41	2.463	1768.10	4355.31
A_68_P29863087	chr14:103381523-103381567	NM_177715:310	Kctd12	INSIDE	1.784	8.827	2400.30	21187.50	15.751	1800.82	28365.41
A_68_P21099263	chr2:29602898-29602942	NM_001039086:127681	Rapgef1	DOWNSTREAM	1.784	1.437	1080.70	1553.22	2.563	938.01	2404.52
A_68_P31412120	chr17:86087935-86087979	NM_011380:-362	Six2	PROMOTER	1.784	7.698	5465.70	42077.02	13.734	4681.46	64296.65
A_68_P21631241	chr2:130523070-130523114	NM_001081162:163	Slc4a11	INSIDE	1.784	2.805	856.46	2402.29	5.003	680.46	3404.16
A_68_P26581054	chr9:64129658-64129702	NM_025372:267	Tipin	INSIDE	1.784	1.772	3811.93	6754.17	3.161	3116.88	9853.57
A_68_P25940715	chr8:70586499-70586543	ENSMUST00000150169:-335		PROMOTER	1.784	5.329	693.49	3695.53	9.509	526.22	5003.90
A_68_P24000798	chr5:114359230-114359275	NM_011779:-537	Coro1c	PROMOTER	1.783	5.286	11332.47	59901.40	9.423	7656.87	72153.48
A_68_P25641012	chr8:9207827-9207871	NM_173446:563175	Fam155a	INSIDE	1.783	2.152	310.81	668.92	3.836	231.54	888.28
A_68_P29094155	chr13:63664480-63664524	NM_008957:2326	Ptch1	INSIDE	1.783	5.078	1136.97	5773.54	9.054	940.38	8514.46
A_68_P25032744	chr7:31435111-31435155	NM_133693:115	Rbm42	INSIDE	1.783	4.162	759.76	3161.83	7.420	589.95	4377.41
A_68_P21580976	chr2:121275325-121275369	NM_011354:383	Serf2	INSIDE	1.783	2.627	1183.49	3109.35	4.685	1067.15	4999.11
A_68_P25091507	chr7:52417775-52417819	NM_182993:-1494	Slc17a7	PROMOTER	1.783	4.040	3391.39	13699.88	7.203	2543.47	18320.64
A_68_P24766862	chr6:114688350-114688394	NM_028835:95230	Atg7	INSIDE	1.782	3.117	2619.62	8164.55	5.554	2153.76	11961.65
A_68_P31929035	chr19:5490695-5490739	NM_007687:262	Cfl1	INSIDE	1.782	3.659	3764.08	13772.99	6.520	2853.19	18602.65
A_68_P23416903	chr4:151532635-151532679	NM_008236:-319	Hes2	DIVERGENT_PROMOTER	1.782	2.676	1085.29	2904.61	4.769	811.53	3870.50
A_68_P22530177	chr3:130815462-130815506	NM_010703:2096	Lef1	INSIDE	1.782	3.664	2595.49	9508.91	6.530	2057.48	13436.06
A_68_P31774692	chr18:64500898-64500942	NM_194268:903	Onecut2	INSIDE	1.782	3.177	1989.72	6321.43	5.662	1432.33	8109.73
A_68_P26591323	chr9:65972506-65972550	NM_019727:165	Snx1	INSIDE	1.782	2.993	1149.29	3439.90	5.332	971.18	5178.63
A_68_P31197210	chr17:44914016-44914060	NM_178652:-81	Supt3h	PROMOTER	1.782	4.329	2694.77	11664.92	7.713	2128.74	16418.48
A_68_P27269640	chr10:77140522-77140570			Unknown	1.782	2.558	3658.89	9360.00	4.557	2742.95	12500.90
A_68_P22340674	chr3:94590393-94590437	NM_001037711:23	Cgn	INSIDE	1.781	2.740	3234.59	8862.34	4.881	2421.32	11818.44
A_68_P23873660	chr5:89149794-89149838	NM_026735:-79	Mobkl1a	PROMOTER	1.781	1.972	1492.65	2944.13	3.513	1281.13	4500.88
A_68_P27281785	chr10:79689246-79689290	NM_023431:-74	Mum1	PROMOTER	1.780	3.402	6975.63	23734.06	6.057	5170.10	31312.73
A_68_P23435277	chr4:154338248-154338292	NM_172990:29	Pank4	INSIDE	1.780	5.935	6193.36	36759.48	10.566	4696.39	49623.53
A_68_P21250385	chr2:58999390-58999434	NM_026361:506	Pkp4	INSIDE	1.780	4.497	4161.06	18710.84	8.005	2857.20	22872.33
A_68_P28159934	chr11:116403055-116403099	NM_173755:39685	Ube2o	INSIDE	1.780	3.415	1066.32	3641.22	6.080	893.63	5433.21
A_68_P30484704	chr15:100058837-100058881	NM_007497:569	Atf1	INSIDE	1.779	3.069	632.44	1941.20	5.461	547.45	2989.56
A_68_P23203774	chr4:109655335-109655379	NM_172296:4727	Dmrta2	INSIDE	1.779	4.383	3173.67	13908.76	7.797	2499.63	19489.75
A_68_P22722447	chr4:9771366-9771410	NM_013526:-130	Gdf6	PROMOTER	1.779	2.585	23523.29	60796.56	4.598	16395.60	75387.85
A_68_P20372363	chr1:78654178-78654222	NM_001001981:-199	Utp14b	PROMOTER	1.779	2.805	1095.37	3072.37	4.989	992.11	4949.18
A_68_P20880195	chr1:183141802-183141846	NM_145514:285	Wdr26	INSIDE	1.779	4.212	2162.58	9109.20	7.493	1734.85	12999.94
A_68_P31150957	chr17:34053104-34053148	NM_020625:6	Zbtb22	INSIDE	1.779	4.398	11456.48	50390.67	7.827	8771.91	68655.84
A_68_P25008269	chr7:25866719-25866763	NM_175477:4476	Zfp574	INSIDE	1.779	4.221	4048.08	17085.21	7.509	3275.89	24598.20
A_68_P26055737	chr8:93432164-93432208	NM_177224:79453	Chd9	INSIDE	1.778	5.839	1278.96	7467.43	10.384	1034.28	10739.74
A_68_P23365645	chr4:141279495-141279539	NM_001017966:-182	Ddi2	PROMOTER	1.778	2.405	1101.33	2649.11	4.276	961.04	4109.56
A_68_P28055686	chr11:98188438-98188482	NM_010895:2499	Neurod2	INSIDE	1.778	5.624	3157.40	17755.68	10.001	1993.02	19932.24
A_68_P28053984	chr11:97883326-97883370	NM_001159320:-3662	Cacnb1	PROMOTER	1.777	3.533	2587.31	9140.65	6.279	1866.76	11721.27
A_68_P28528483	chr12:72932910-72932958	NM_026102:870	Daam1	INSIDE	1.777	7.692	781.12	6008.10	13.668	536.03	7326.51
A_68_P25672261	chr8:14096967-14097011	NM_001145965:1114	Dlgap2	INSIDE	1.777	3.076	3873.43	11915.11	5.468	2879.39	15743.16
A_68_P29250236	chr13:98010968-98011012	NM_007930:-69	Enc1	PROMOTER	1.777	7.313	1585.24	11593.63	12.995	1275.24	16571.48
A_68_P21367543	chr2:79095483-79095527	NM_010576:-78	Itga4	PROMOTER	1.777	3.658	2017.49	7379.30	6.500	1621.43	10538.48
A_68_P22572454	chr3:138738598-138738642	NM_001040690:-457	Rap1gds1	PROMOTER	1.777	6.223	1452.24	9037.25	11.060	1227.37	13575.00
A_68_P24541704	chr6:72389176-72389220	NM_145569:354	Mat2a	INSIDE	1.776	3.581	1968.78	7050.07	6.361	1773.17	11279.90
A_68_P27568398	chr11:6316069-6316113	NM_008907:218	Ppia	INSIDE	1.776	4.454	3774.27	16809.61	7.911	3015.41	23855.94

ProbeName	Target position of probe on CpG island microarray	TargetID	GeneSymbol	CpG island Description	Ratio of relative methylation (TiO_2-NP/Vehicle)	Sham group			TiO_2-H group		
						Relative methylation (Cy5/Cy3)	Cy3 signal (Input)	Cy5 signal (MeDIP)	Relative methylation (Cy5/Cy3)	Cy3 signal (Input)	Cy5 signal (MeDIP)
A_68_P31791990	chr18:67624448-67624492	NM_144867:-32	Slmo1	PROMOTER	1.776	1.828	573.52	1048.66	3.248	617.54	2005.65
A_68_P20863553	chr1:180299131-180299175			Unknown	1.776	4.221	3754.06	15846.01	7.498	3098.90	23236.86
A_68_P24791280	chr6:119057361-119057405	NM_001159533:825	Cacna1c	INSIDE	1.775	3.638	2783.45	10125.32	6.457	2185.01	14109.12
A_68_P31565714	chr18:25645254-25645298	NM_001146292:267208	Celf4	INSIDE	1.775	2.149	1952.48	4194.93	3.813	1584.88	6043.79
A_68_P26504861	chr9:50467491-50467535	NM_145614:373	Dlat	INSIDE	1.775	3.414	2240.05	7647.12	6.059	1937.02	11735.53
A_68_P31333820	chr17:71660018-71660062	NM_145158:265	Emilin2	INSIDE	1.775	3.943	1298.83	5121.79	7.000	1128.92	7902.88
A_68_P30261330	chr15:60655945-60655989	NM_001162926:669	Fam84b	INSIDE	1.775	3.498	751.96	2630.19	6.210	645.31	4007.21
A_68_P23609897	chr5:37221748-37221792	NM_008550:118	Man2b2	INSIDE	1.775	2.479	395.33	980.03	4.399	374.60	1648.01
A_68_P25046332	chr7:35697322-35697366	NM_008820:-81	Pepd	PROMOTER	1.775	4.026	3483.89	14027.35	7.147	2496.64	17842.52
A_68_P23308821	chr4:131429974-131430018	NM_020587:443	Srsf4	INSIDE	1.775	1.555	884.71	1375.62	2.760	827.18	2283.21
A_68_P32707838	chrX:138825108-138825152	NM_001033600:-52	Acsl4	PROMOTER	1.774	2.727	1079.84	2945.22	4.840	552.09	2671.95
A_68_P28154359	chr11:115465196-115465240	NM_173048:2	Gga3	INSIDE	1.774	3.264	732.95	2392.19	5.789	639.72	3703.54
A_68_P24382000	chr6:39157044-39157088	NM_001033430:-294	Jhdm1d	PROMOTER	1.774	8.461	8493.65	71863.49	15.006	8119.38	121837.70
A_68_P32579463	chrX:102347552-102347596	AK136781:-2464		PROMOTER	1.774	2.645	1412.04	3734.71	4.692	791.80	3715.44
A_68_P32287125	chrX:19638017-19638061	NM_021715:1343	Chst7	INSIDE	1.773	2.989	484.37	1447.95	5.301	264.32	1401.23
A_68_P30659187	chr16:33684576-33684620	NM_175256:47	Heg1	INSIDE	1.773	6.285	2971.27	18674.29	11.145	2361.20	26314.81
A_68_P24383009	chr6:39369612-39369656	NM_018810:734	Mkrn1	INSIDE	1.773	4.571	1184.49	5414.21	8.105	871.42	7062.48
A_68_P30782600	chr16:56717318-56717362	NM_019678:127	Tfg	INSIDE	1.773	2.471	835.50	2064.67	4.382	706.35	3094.87
A_68_P22347225	chr3:95861928-95861972	NM_013841:428	Vps45	INSIDE	1.773	1.463	941.12	1376.55	2.593	861.27	2233.70
A_68_P32351254	chrX:42143356-42143400	NM_178739:-4	Dcaf12l1	PROMOTER	1.772	2.845	4125.07	11736.55	5.042	2258.89	11388.94
A_68_P31928602	chr19:5424535-5424579	NM_024176:360	Drap1	INSIDE	1.772	3.070	6270.42	19247.44	5.439	4578.67	24902.28
A_68_P32095553	chr19:37509433-37509477	NM_008245:124	Hhex	INSIDE	1.772	3.525	3556.33	12536.69	6.247	2918.16	18228.33
A_68_P29469015	chr14:24824174-24824218	NM_010610:-769	Kcnma1	PROMOTER	1.772	3.849	993.01	3822.26	6.822	802.86	5476.86
A_68_P26344985	chr9:20807480-20807524	NM_023167:322	Mrpl4	INSIDE	1.772	1.812	959.47	1738.23	3.210	728.81	2339.48
A_68_P29453038	chr14:22320578-22320622	NM_017479:1594	Myst4	INSIDE	1.772	10.363	8250.98	85508.03	18.364	5000.42	91827.39
A_68_P32148144	chr19:47253081-47253125	NM_021360:-207	Neurl1a	PROMOTER	1.772	2.016	1665.95	3358.83	3.572	1310.60	4681.33
A_68_P25088125	chr7:51807692-51807736	NM_009473:1579	Nr1h2	INSIDE	1.772	8.216	7542.10	61962.49	14.558	5015.40	73015.23
A_68_P24061285	chr5:124889815-124889859	NM_030241:-102	Setd8	PROMOTER	1.772	6.736	706.20	4756.98	11.934	574.76	6858.93
A_68_P27165498	chr10:58275060-58275104	NM_172788:-1024	Sh3rf3	PROMOTER	1.772	1.997	6835.60	13649.97	3.539	5808.04	20551.85
A_68_P24951825	chr7:5008460-5008504	NM_146178:155	Ccdc106	INSIDE	1.771	2.209	1864.31	4118.27	3.912	1406.04	5500.30
A_68_P26375208	chr9:27107222-27107266	NM_001033323:432	Igsf9b	INSIDE	1.771	11.717	1501.62	17594.76	20.756	1142.28	23708.96
A_68_P32565869	chrX:99044829-99044873	NM_001163610:127	Nhsl2	INSIDE	1.771	4.869	967.20	4709.58	8.623	694.45	5988.30
A_68_P29258499	chr13:99359027-99359071	AK135687:168913		INSIDE	1.771	14.231	1753.95	24959.88	25.208	1094.15	27581.51
A_68_P31610600	chr18:34165600-34165644	NM_013512:1238	Epb4.1l4a	INSIDE	1.770	4.669	7245.25	33830.49	8.266	5145.26	42531.44
A_68_P31873837	chr18:82113134-82113178	AK046168:311		INSIDE	1.770	1.694	1350.04	2286.41	2.998	1131.40	3392.14
A_68_P23770428	chr5:68238336-68238380	NM_001038999:312	Atp8a1	INSIDE	1.769	2.920	5059.10	14771.55	5.165	4019.53	20760.05
A_68_P28047199	chr11:96723478-96723522	NM_019877:12311	Copz2	DOWNSTREAM	1.769	2.368	1123.29	2659.94	4.189	762.30	3193.24
A_68_P32128434	chr19:43827858-43827902	NM_001113562:368	Cutc	INSIDE	1.769	2.558	2374.32	6072.97	4.525	1895.91	8578.58
A_68_P31161104	chr17:35960060-35960104	NM_008027:-219	Flot1	PROMOTER	1.769	5.447	1552.32	8455.38	9.634	1231.80	11866.98
A_68_P27921007	chr11:74336152-74336196	NM_001015046:67486	Rap1gap2	INSIDE	1.769	3.183	828.38	2636.47	5.632	709.37	3994.99
A_68_P27804722	chr11:53244281-53244325	NM_025352:31	Uqcrq	INSIDE	1.769	1.652	602.65	995.78	2.923	574.97	1680.60
A_68_P30149946	chr15:38448361-38448405	NM_001102458:639	Azin1	INSIDE	1.768	2.411	2801.07	6754.27	4.263	2330.83	9935.58
A_68_P25015934	chr7:27963629-27963675	NR_037283:-2540	Mir3101	PROMOTER	1.768	3.211	11134.92	35755.86	5.678	7580.22	43038.60
A_68_P30382971	chr15:82184657-82184701	NM_025987:43	Ndufa6	INSIDE	1.768	7.264	5277.33	38335.13	12.840	3633.10	46648.45
A_68_P31117838	chr17:27822326-27822370	NM_178365:150	Pacsin1	INSIDE	1.768	3.552	4612.42	16382.12	6.279	3523.84	22124.95
A_68_P27311153	chr10:85391455-85391499	NM_001163614:241	Ascl4	INSIDE	1.767	4.192	1634.87	6853.41	7.406	1314.91	9737.78
A_68_P28727037	chr12:109513497-109513541	NM_001159910:109	Ccdc85c	INSIDE	1.767	4.478	592.81	2654.85	7.911	592.78	4689.72
A_68_P22728457	chr4:11118468-11118512	NM_001037134:-10	Ccne2	PROMOTER	1.767	2.141	909.54	1947.35	3.783	756.22	2860.48
A_68_P23269776	chr4:124378137-124378181	NM_010213:216	Fhl3	INSIDE	1.767	3.462	657.54	2276.40	6.116	625.75	3826.93
A_68_P26018642	chr8:86707908-86707952	NR_029821:-5070	Mir181c	PROMOTER	1.767	5.718	2600.84	14870.92	10.102	2267.63	22907.50
A_68_P30368655	chr15:79634703-79634747	NM_030689:415	Nptxr	INSIDE	1.767	8.180	2599.79	21265.87	14.457	2031.04	29361.75

ProbeName	Target position of probe on CpG island microarray	TargetID	GeneSymbol	CpG island Description	Ratio of relative methylation (TiO_2-NP/Vehicle)	Sham group			TiO_2-H group		
						Relative methylation (Cy5/Cy3)	Cy3 signal (Input)	Cy5 signal (MeDIP)	Relative methylation (Cy5/Cy3)	Cy3 signal (Input)	Cy5 signal (MeDIP)
A_68_P30436282	chr15:91402943-91402987	NM_001033633:728	Slc2a13	INSIDE	1.767	3.345	1912.48	6397.63	5.912	1714.23	10134.76
A_68_P32393600	chrX:52896641-52896685	ENSMUST00000163353:91930		INSIDE	1.767	3.118	1362.56	4247.89	5.508	790.77	4355.57
A_68_P23262662	chr4:123089650-123089695	NM_001167918:-518	D830031N03Rik	PROMOTER	1.766	3.937	2775.83	10928.71	6.954	2219.30	15433.61
A_68_P24138256	chr5:140384605-140384649	NM_175522:730	Elfn1	INSIDE	1.766	9.868	2008.19	19816.30	17.424	1874.43	32660.71
A_68_P21814796	chr2:163684382-163684426	NM_001030292:4919	Kcnk15	INSIDE	1.766	2.761	1864.02	5147.25	4.876	1411.23	6881.10
A_68_P25230185	chr7:80763469-80763513	AK158912:-221		PROMOTER	1.766	7.062	5298.66	37420.68	12.469	4235.27	52810.32
A_68_P27288028	chr10:80657209-80657253	NR_015477:-205	2310050B05Rik	PROMOTER	1.765	2.499	2529.81	6320.77	4.411	2017.03	8896.69
A_68_P26505081	chr9:50506728-50506772	NM_178118:29339	Dixdc1	INSIDE	1.765	1.817	1669.25	3032.53	3.207	1162.67	3728.34
A_68_P23590169	chr5:33972359-33972403	NM_178390:-96	Fam53a	PROMOTER	1.765	2.637	5515.12	14543.40	4.654	4415.98	20551.40
A_68_P32805174	chrX:162677268-162677312	NM_001177961:416	Gpm6b	INSIDE	1.765	1.759	1334.09	2347.25	3.106	749.12	2326.47
A_68_P27910209	chr11:72028885-72028929	NM_026068:188	Med31	INSIDE	1.765	1.777	736.61	1308.89	3.136	608.92	1909.51
A_68_P24164494	chr5:145900797-145900843	NM_001033313:138	Pdap1	INSIDE	1.765	2.842	4990.32	14183.28	5.016	3663.46	18375.68
A_68_P32054980	chr19:29887444-29887488	NM_177721:-2	Ranbp6	PROMOTER	1.765	2.134	1348.08	2877.14	3.766	1137.32	4283.48
A_68_P23597150	chr5:35292277-35292321	NM_173402:202	Rgs12	INSIDE	1.765	6.421	322.42	2070.34	11.335	287.78	3261.91
A_68_P32168791	chr19:50753028-50753072	NM_021377:52	Sorcs1	INSIDE	1.765	5.090	1771.47	9017.51	8.987	1696.90	15249.77
A_68_P27542559	chr10:128105989-128106033	NM_173733:4964	Suox	DOWNSTREAM	1.765	4.909	11211.50	55040.77	8.663	7886.81	68326.57
A_68_P26804666	chr9:106588275-106588319	NM_001081654:-17	Tex264	PROMOTER	1.765	2.185	1727.76	3774.92	3.855	1342.99	5177.58
A_68_P21911521	chr2:181327466-181327510	NM_001164597:-322	Znf512b	PROMOTER	1.765	3.573	1536.04	5488.81	6.305	1321.95	8335.16
A_68_P29940452	chr14:119105540-119105584	NM_001033336:-121	Abcc4	PROMOTER	1.764	1.368	1461.86	1999.96	2.413	1301.71	3141.45
A_68_P24372708	chr6:37392303-37392347	NM_178661:-176	Creb3l2	PROMOTER	1.764	2.324	991.77	2304.44	4.098	853.65	3498.34
A_68_P21438069	chr2:93797146-93797190	NM_001145034:89	Gm13889	INSIDE	1.764	3.176	1991.48	6325.51	5.604	1596.99	8949.92
A_68_P22133096	chr3:51811618-51811662	NM_001004176:97288	Maml3	INSIDE	1.764	4.761	566.37	2696.29	8.396	443.15	3720.85
A_68_P31612548	chr18:34533141-34533185	NM_007874:-93	Reep5	PROMOTER	1.764	4.572	1898.17	8677.73	8.066	1528.25	12326.48
A_68_P20149049	chr1:36614326-36614370	NM_001126047:878	Sema4c	INSIDE	1.764	7.297	6518.75	47565.21	12.870	4549.54	58550.61
A_68_P29055901	chr13:55672343-55672387	NM_001162881:-1028	Fam193b	PROMOTER	1.763	2.951	2122.69	6264.39	5.202	1772.94	9223.40
A_68_P30955189	chr16:90144198-90144242	NR_033476:1739	Gm10789	INSIDE	1.763	6.303	1076.13	6783.06	11.115	799.09	8881.84
A_68_P23978770	chr5:110715093-110715137	NM_008993:73	Pxmp2	INSIDE	1.763	2.563	2154.39	5520.74	4.518	1822.63	8234.37
A_68_P21151631	chr2:38860577-38860621	NM_025592:53	Rpl35	INSIDE	1.763	2.925	2620.19	7663.81	5.157	2061.53	10630.55
A_68_P30779064	chr16:56075886-56075930	NM_001003972:387	Senp7	INSIDE	1.763	6.223	2092.73	13022.48	10.970	1717.71	18843.10
A_68_P31118768	chr17:27977231-27977275	NM_011432:221	Snrpc	INSIDE	1.763	5.799	496.83	2881.28	10.226	367.40	3757.01
A_68_P26242940	chr8:126544834-126544878	NM_133966:353	Taf5l	INSIDE	1.763	4.722	1079.06	5094.78	8.322	864.28	7192.74
A_68_P24006959	chr5:115392406-115392450	NM_028211:218	2210016L21Rik	INSIDE	1.762	2.075	3762.48	7807.39	3.655	2626.23	9600.08
A_68_P21483989	chr2:103406800-103406844	NM_178890:356	Abtb2	INSIDE	1.762	2.160	513.43	1109.03	3.805	462.30	1759.28
A_68_P26220483	chr8:123012034-123012078	NM_198671:-709	Gse1	PROMOTER	1.762	4.405	1205.27	5309.66	7.762	902.80	7007.95
A_68_P28043336	chr11:96132754-96132798	NM_008270:133	Hoxb9	INSIDE	1.762	2.702	1478.87	3996.34	4.761	1167.08	5556.00
A_68_P27180703	chr10:60938705-60938749	NM_153542:146	Lrrc20	INSIDE	1.762	16.348	1322.90	21626.74	28.805	1006.90	29004.18
A_68_P25435757	chr7:121559995-121560039	NM_011055:1249	Pde3b	INSIDE	1.762	3.398	1535.25	5217.14	5.987	1224.88	7333.25
A_68_P20157818	chr1:38186099-38186143	NM_019570:387	Rev1	INSIDE	1.762	4.318	390.96	1688.19	7.608	378.46	2879.32
A_68_P26983377	chr10:19570989-19571033	NM_029529:255	Slc35d3	INSIDE	1.762	1.437	1770.66	2545.16	2.533	1354.11	3430.35
A_68_P31347831	chr17:74059828-74059872	NM_027864:-59	Galnt14	PROMOTER	1.761	2.701	2475.44	6685.55	4.756	1800.05	8561.73
A_68_P31205409	chr17:46297933-46297979	NM_001145979:-24	Gtpbp2	DIVERGENT_PROMOTER	1.761	4.260	3135.28	13356.36	7.504	2415.30	18123.83
A_68_P28166383	chr11:117515888-117515933	NM_198022:308	Tnrc6c	INSIDE	1.761	3.753	1659.74	6229.66	6.610	1320.71	8729.71
A_68_P20622147	chr1:134439405-134439449	NM_177129:91	Cntn2	INSIDE	1.760	7.013	3736.09	26202.98	12.345	2879.07	35542.74
A_68_P22391858	chr3:104315425-104315469	NM_001025067:333	Lrig2	INSIDE	1.760	5.016	599.41	3006.70	8.827	522.51	4612.18
A_68_P27892954	chr11:68901870-68901914	NM_009497:-137	Vamp2	PROMOTER	1.760	1.492	1659.78	2477.16	2.626	1196.24	3141.60
A_68_P27083322	chr10:40020722-40020766	NM_007444:1248	Amd2	INSIDE	1.759	1.725	617.62	1065.22	3.034	556.90	1689.59
A_68_P21094080	chr2:28763906-28763951	NM_019446:8013	Barhl1	INSIDE	1.759	1.881	359.60	676.37	3.309	188.20	622.75
A_68_P26159425	chr8:112392612-112392656	NM_001122594:132	Phlpp2	INSIDE	1.759	3.602	1589.73	5726.16	6.337	1426.80	9041.23
A_68_P27327917	chr10:88348711-88348755	NM_145423:-4	Slc5a8	PROMOTER	1.759	5.743	1545.80	8878.18	10.104	1367.68	13818.54
A_68_P26218688	chr8:122754127-122754171	ENSMUST00000127664:319408		INSIDE	1.759	4.700	1631.72	7669.08	8.267	1341.60	11090.44
A_68_P21958958	chr3:14886343-14886387	NM_009801:-61	Car2	PROMOTER	1.758	1.790	329.27	589.39	3.146	299.56	942.43

ProbeName	Target position of probe on CpG island microarray	TargetID	GeneSymbol	CpG island Description	Ratio of relative methylation (TiO_2-NP/Vehicle)	Sham group			TiO_2-H group		
						Relative methylation (Cy5/Cy3)	Cy3 signal (Input)	Cy5 signal (MeDIP)	Relative methylation (Cy5/Cy3)	Cy3 signal (Input)	Cy5 signal (MeDIP)
A_68_P26526109	chr9:54349056-54349100	NM_172771:355	Dmxl2	INSIDE	1.758	3.148	2854.95	8988.13	5.534	2120.01	11732.20
A_68_P26905236	chr10:4522611-4522655	NM_175374:35	Mtrf1l	INSIDE	1.758	3.434	1637.50	5622.63	6.037	1288.35	7777.73
A_68_P30346633	chr15:76014154-76014198	NM_201394:11964	Plec	INSIDE	1.758	3.607	1900.53	6855.42	6.341	1385.38	8784.31
A_68_P31488614	chr18:10181658-10181702	NM_009071:110	Rock1	INSIDE	1.758	5.975	3034.69	18133.36	10.504	2295.97	24116.75
A_68_P22923084	chr4:53453754-53453798	NM_001159633:492	Slc44a1	INSIDE	1.758	4.226	3284.90	13883.52	7.429	2686.04	19955.22
A_68_P23607369	chr5:36740782-36740826	NM_030889:-16	Sorcs2	PROMOTER	1.758	6.372	4995.22	31827.52	11.198	4038.19	45221.17
A_68_P20946920	chr1:194861857-194861901	NM_181546:14	Syt14	INSIDE	1.758	3.505	904.97	3171.63	6.162	732.36	4513.01
A_68_P29509885	chr14:31944046-31944090	NR_024069:-1952	2010107H07Rik	DIVERGENT_PROMOTER	1.757	3.885	1220.48	4741.76	6.827	944.05	6444.63
A_68_P27351976	chr10:92623453-92623497	NM_146239:-146	Cdk17	PROMOTER	1.757	4.366	815.31	3559.77	7.669	642.05	4924.18
A_68_P24859169	chr6:133985084-133985128	NM_007961:-618	Etv6	PROMOTER	1.757	1.842	1887.56	3476.45	3.235	1459.11	4720.73
A_68_P23681262	chr5:50450445-50450489	NM_133911:-231	Gpr125	PROMOTER	1.757	7.745	1505.05	11656.69	13.611	1198.64	16314.77
A_68_P21078497	chr2:26264502-26264546	NM_033134:184	Inpp5e	INSIDE	1.757	1.686	929.83	1567.59	2.963	841.93	2494.54
A_68_P26270749	chr8:131209761-131209805	NM_010578:229	Itgb1	INSIDE	1.757	2.130	3422.29	7289.03	3.743	2642.46	9891.32
A_68_P25713191	chr8:23969515-23969559	NM_001081149:-474	Myst3	PROMOTER	1.757	1.557	599.52	933.45	2.735	404.86	1107.42
A_68_P28029989	chr11:93817362-93817406	NM_008705:-63	Nme2	PROMOTER	1.757	4.151	3025.34	12559.02	7.295	2391.06	17443.98
A_68_P31196995	chr17:44873188-44873232	NM_001145920:387	Runx2	INSIDE	1.757	2.269	882.30	2001.78	3.986	705.48	2812.18
A_68_P27278356	chr10:79167062-79167106	NM_001077184:-18	Bsg	PROMOTER	1.756	1.921	932.44	1791.45	3.373	697.13	2351.69
A_68_P26132023	chr8:107694748-107694792	NM_001161456:197	Cbfb	INSIDE	1.756	4.825	1548.46	7471.14	8.475	1377.88	11677.23
A_68_P30895867	chr16:78376777-78376821	NR_002700:204	Gm7334	INSIDE	1.756	5.544	4323.13	23966.56	9.736	3610.15	35150.07
A_68_P29530592	chr14:35362336-35362380	NM_001039074:39435	Ldb3	INSIDE	1.756	1.541	946.64	1459.23	2.707	874.65	2367.60
A_68_P24894485	chr6:140373203-140373247	NM_144920:605	Plekha5	INSIDE	1.756	1.787	4206.33	7516.88	3.138	3331.26	10452.47
A_68_P31934467	chr19:6399578-6399622	NM_011242:-982	Rasgrp2	PROMOTER	1.756	4.373	2869.09	12545.09	7.679	2161.16	16595.64
A_68_P27507134	chr10:121533967-121534011	NM_153059:170	Tmem5	INSIDE	1.756	2.901	22532.79	65360.53	5.093	17904.44	91188.08
A_68_P21782542	chr2:158187327-158187371	NR_027887:-90	9430008C03Rik	PROMOTER	1.755	4.200	1215.00	5103.27	7.372	959.59	7073.91
A_68_P31162860	chr17:36305369-36305413	NM_001081032:92	Gm8909	INSIDE	1.755	2.647	5455.52	14440.49	4.646	4867.99	22619.08
A_68_P30497013	chr15:102110024-102110068	NM_134100:160	Mfsd5	INSIDE	1.755	10.569	1150.96	12164.70	18.546	955.38	17718.13
A_68_P26492138	chr9:48303203-48303247	NM_144948:211	Rbm7	INSIDE	1.755	2.232	1857.66	4146.27	3.916	1461.89	5724.85
A_68_P29605237	chr14:52817103-52817147	NM_033618:-210	Supt16h	PROMOTER	1.755	1.527	2216.53	3384.63	2.681	1820.08	4878.78
A_68_P25983698	chr8:80041949-80041993	NM_030113:-164	Arhgap10	PROMOTER	1.754	7.007	3126.51	21906.85	12.292	2424.09	29797.49
A_68_P29005205	chr13:46060754-46060798	NM_009124:-431	Atxn1	PROMOTER	1.754	3.197	1185.29	3789.79	5.607	956.20	5361.12
A_68_P27385039	chr10:98569849-98569893	NM_015737:-898	Galnt4	PROMOTER	1.754	2.881	597.56	1721.78	5.053	546.34	2760.79
A_68_P20457526	chr1:95700053-95700097	NM_025454:-467	Ing5	DIVERGENT_PROMOTER	1.754	3.519	1095.13	3853.92	6.171	890.34	5494.47
A_68_P25736226	chr8:28152855-28152901	NM_001039077:-148	Prosc	PROMOTER	1.754	3.824	3213.83	12289.94	6.708	2627.55	17626.44
A_68_P26875613	chr9:119920587-119920631	NM_011724:12108	Xirp1	DOWNSTREAM	1.754	2.196	2506.01	5503.35	3.852	2088.07	8042.98
A_68_P31758329	chr18:61715242-61715286	NM_146087:29	Csnk1a1	INSIDE	1.753	2.001	1909.97	3822.75	3.509	1370.39	4808.15
A_68_P30287852	chr15:65618774-65618818	NM_133766:194	Efr3a	INSIDE	1.753	1.928	260.85	502.80	3.380	253.86	858.03
A_68_P23681257	chr5:50449672-50449716	NM_133911:541	Gpr125	INSIDE	1.753	3.106	6380.11	19814.98	5.445	5137.68	27974.89
A_68_P24449528	chr6:52210288-52210332	NM_008264:564	Hoxa13	INSIDE	1.753	7.221	591.44	4270.79	12.660	478.78	6061.55
A_68_P26951412	chr10:13194053-13194097	NM_001195065:128	Phactr2	INSIDE	1.753	1.737	792.22	1376.34	3.045	676.01	2058.35
A_68_P24001851	chr5:114541342-114541386	NM_026805:25	Svop	INSIDE	1.753	5.740	2988.43	17153.90	10.061	2550.77	25662.82
A_68_P26421598	chr9:35075374-35075418	NM_175411:-31	Fam118b	DIVERGENT_PROMOTER	1.752	1.543	895.51	1381.34	2.703	863.11	2333.18
A_68_P26132587	chr8:107788500-107788544	NM_015821:-25	Fbxl8	DIVERGENT_PROMOTER	1.752	3.803	715.63	2721.64	6.664	631.31	4207.03
A_68_P26643861	chr9:75257109-75257153	NM_027418:36	Mapk6	INSIDE	1.752	6.242	804.80	5023.46	10.938	682.80	7468.18
A_68_P29051301	chr13:54867120-54867164	NM_033610:659	Sncb	INSIDE	1.752	3.620	2927.26	10596.65	6.343	2313.86	14677.38
A_68_P20022045	chr1:9289343-9289387	NM_027671:447	Sntg1	INSIDE	1.752	5.857	3617.91	21188.75	10.260	2628.46	26966.77
A_68_P28118792	chr11:109334732-109334776	NM_028710:67	Arsg	INSIDE	1.751	1.983	2131.27	4227.17	3.474	1811.40	6292.14
A_68_P22859197	chr4:40800680-40800724	NM_022305:329	B4galt1	INSIDE	1.751	3.145	1556.87	4896.93	5.509	1299.73	7159.97
A_68_P25952284	chr8:73027155-73027199	NM_018827:10122	Crlf1	INSIDE	1.751	3.433	2378.66	8165.82	6.011	1884.88	11330.54
A_68_P25089584	chr7:52095285-52095329	NM_010215:3649	Il4i1	INSIDE	1.751	2.596	1830.05	4751.35	4.547	1424.35	6476.19
A_68_P30453398	chr15:94459261-94459305	NM_178789:-333	Tmem117	PROMOTER	1.751	4.555	9344.40	42566.47	7.978	6963.78	55556.36
A_68_P20262088	chr1:59177273-59177317	NM_001033449:-354	Als2cr4	PROMOTER	1.750	4.488	745.12	3344.05	7.854	631.06	4956.38

ProbeName	Target position of probe on CpG island microarray	TargetID	GeneSymbol	CpG island Description	Ratio of relative methylation (TiO_2-NP/Vehicle)	Sham group			TiO_2-H group		
						Relative methylation (Cy5/Cy3)	Cy3 signal (Input)	Cy5 signal (MeDIP)	Relative methylation (Cy5/Cy3)	Cy3 signal (Input)	Cy5 signal (MeDIP)
A_68_P29413810	chr14:13654360-13654404	NM_001042617:1211	Cadps	INSIDE	1.750	3.642	2671.56	9730.57	6.374	2240.68	14281.93
A_68_P23892215	chr5:92512150-92512194	NM_001080797:-229	G3bp2	PROMOTER	1.750	2.949	1937.02	5713.11	5.163	1664.45	8593.41
A_68_P30961697	chr16:91226847-91226891	NM_016967:1074	Olig2	INSIDE	1.750	3.045	5236.27	15942.01	5.329	4041.84	21540.29
A_68_P23402215	chr4:149111795-149111839	NM_001025106:327	Tmem201	INSIDE	1.750	6.996	1464.17	10243.76	12.245	1030.76	12621.58
A_68_P30096385	chr15:27954397-27954441	NM_001081302:1185	Trio	INSIDE	1.750	1.463	4319.80	6321.36	2.561	3326.99	8521.62
A_68_P32795839	chrX:160396590-160396634	NM_009453:-14	Zrsr2	PROMOTER	1.750	2.246	2229.98	5007.44	3.929	1201.96	4722.33
A_68_P21722826	chr2:147020543-147020587	NM_001077632:-8426	Nkx2-2	PROMOTER	1.749	5.439	1090.25	5929.85	9.512	881.94	8388.93
A_68_P27614819	chr11:17111398-17111442	NM_025443:172	Pno1	INSIDE	1.749	2.061	1394.34	2873.63	3.605	1113.56	4013.90
A_68_P29428731	chr14:17197979-17198023	NM_009409:281	Top2b	INSIDE	1.749	2.011	1529.45	3075.09	3.516	1246.60	4382.69
A_68_P27445213	chr10:110182319-110182363	NM_178609:-180	E2f7	PROMOTER	1.748	4.159	772.89	3214.54	7.272	770.36	5602.23
A_68_P32213515	chr19:58529618-58529662	NM_010279:-684	Gfra1	PROMOTER	1.748	1.741	4343.45	7562.41	3.044	2798.64	8517.84
A_68_P25954584	chr8:73389578-73389623	NM_032397:-8693	Kcnn1	PROMOTER	1.748	1.511	1699.52	2567.57	2.641	1171.95	3095.36
A_68_P26039507	chr8:90723531-90723575	NM_001164497:441	Papd5	INSIDE	1.748	5.336	3155.94	16841.30	9.330	2634.39	24579.68
A_68_P24956659	chr7:6682310-6682354	NM_008817:798	Peg3	INSIDE	1.748	1.520	829.29	1260.71	2.657	758.86	2016.08
A_68_P25265832	chr7:87439097-87439141	NM_145150:-232	Prc1	PROMOTER	1.748	5.867	2742.61	16091.81	10.254	2081.05	21339.65
A_68_P23236664	chr4:117508530-117508574	NM_008135:690	Slc6a9	INSIDE	1.748	2.240	1988.17	4452.72	3.914	1745.17	6830.68
A_68_P25958205	chr8:74195695-74195739	NM_001029873:-60	Unc13a	DIVERGENT_PROMOTER	1.748	3.016	8338.50	25152.77	5.272	6293.49	33177.18
A_68_P32477696	chrX:74678992-74679036			Unknown	1.748	4.677	553.98	2590.80	8.177	333.85	2729.94
A_68_P27900882	chr11:70279513-70279557	NM_023158:-6048	Cxcl16	PROMOTER	1.747	1.427	1830.22	2611.27	2.493	1401.27	3492.97
A_68_P23103735	chr4:91038685-91038729	NM_010486:40	Elavl2	INSIDE	1.747	4.653	2752.25	12806.90	8.127	2275.41	18493.30
A_68_P28033280	chr11:94352619-94352663	NM_027984:8648	Epn3	INSIDE	1.747	14.019	1245.75	17463.81	24.492	945.71	23162.58
A_68_P25953980	chr8:73300616-73300660	NM_008841:-27	Pik3r2	DIVERGENT_PROMOTER	1.747	3.516	669.06	2352.54	6.141	614.17	3771.75
A_68_P25354354	chr7:105326624-105326668	NM_007602:57	Capn5	INSIDE	1.746	5.034	1591.36	8011.50	8.790	1263.09	11102.05
A_68_P31565718	chr18:25645741-25645785	NM_001146292:266722	Celf4	INSIDE	1.746	1.912	1475.53	2821.49	3.339	1166.82	3896.56
A_68_P25957751	chr8:74126551-74126595	NM_001166213:5028	Fam129c	INSIDE	1.746	2.904	1868.37	5424.90	5.071	1476.20	7485.77
A_68_P24005517	chr5:115140712-115140756	NM_019821:210	Gltp	INSIDE	1.746	3.422	3875.07	13262.26	5.977	2524.78	15089.95
A_68_P25095297	chr7:53075356-53075400	NM_145963:4739	Kcnj14	INSIDE	1.746	3.039	3221.66	9789.23	5.306	2632.50	13967.01
A_68_P23577316	chr5:31554254-31554298	NM_027221:198	Krtcap3	INSIDE	1.746	5.588	2563.92	14326.24	9.756	2030.20	19806.48
A_68_P21149827	chr2:38544490-38544534	NM_139051:25550	Nr5a1	DOWNSTREAM	1.746	3.993	1793.33	7161.53	6.972	1423.96	9927.68
A_68_P26429021	chr9:36954305-36954349	NM_001029838:525	Pknox2	INSIDE	1.746	3.693	1007.30	3720.30	6.447	818.84	5279.12
A_68_P32268351	chrX:13424038-13424082	NM_009806:-393	Cask	PROMOTER	1.745	2.623	3604.11	9455.09	4.577	2141.76	9802.92
A_68_P26249219	chr8:127578258-127578302	NM_174853:186	Disc1	INSIDE	1.745	4.120	16321.94	67241.74	7.190	12123.42	87166.19
A_68_P25495415	chr7:132725071-132725115	NM_001008700:29297	Il4ra	DOWNSTREAM	1.745	4.300	3635.17	15632.42	7.503	2918.89	21900.68
A_68_P28291793	chr12:25515677-25515721	NM_001083341:-765	Mboat2	PROMOTER	1.745	14.968	1756.93	26296.92	26.116	1229.01	32097.13
A_68_P24981209	chr7:16973109-16973153	NM_019748:4	Sae1	INSIDE	1.745	2.708	1486.23	4024.38	4.726	1180.87	5580.70
A_68_P30496205	chr15:101966489-101966533	NM_001033277:136	Spryd3	INSIDE	1.745	2.590	3279.72	8492.93	4.519	2677.66	12099.03
A_68_P31326352	chr17:70318650-70318694	ENSMUST00000155823:-35		PROMOTER	1.745	2.085	673.37	1403.79	3.638	630.45	2293.40
A_68_P26814990	chr9:108363250-108363294	NM_133744:424	Ccdc71	INSIDE	1.744	2.571	1714.86	4409.18	4.485	1407.04	6309.96
A_68_P33007798	chr4_random:129389-129433	NM_001033326:19609	Dhrsx	INSIDE	1.744	5.327	2192.86	11680.33	9.290	1601.36	14876.20
A_68_P20442011	chr1:93263227-93263272	NM_173395:243	Fam132b	INSIDE	1.744	3.148	2766.94	8709.43	5.489	2276.05	12492.45
A_68_P28177386	chr11:119217235-119217283	NM_178743:388	Slc26a11	INSIDE	1.744	3.869	1785.95	6909.52	6.749	1449.59	9782.59
A_68_P22015873	chr3:28161770-28161814	NM_001163009:-343	Tnik	PROMOTER	1.744	3.559	324.25	1153.94	6.206	261.68	1623.89
A_68_P22674484	chr3:156225009-156225062	NM_177274:113	Negr1	INSIDE	1.743	6.651	2013.48	13392.34	11.594	1409.31	16339.35
A_68_P26021287	chr8:87230962-87231012	NM_001081981:67282	Nfix	DOWNSTREAM	1.743	5.485	1102.57	6047.57	9.561	909.77	8698.03
A_68_P30559120	chr16:13819791-13819845	NM_010946:449	Ntan1	INSIDE	1.743	2.431	941.21	2287.75	4.237	752.84	3189.89
A_68_P31117527	chr17:27760515-27760559	NM_019837:-139	Nudt3	PROMOTER	1.743	1.917	633.10	1213.82	3.342	554.31	1852.33
A_68_P21768463	chr2:155755683-155755727	NM_018888:342	Uqcc	INSIDE	1.743	3.108	3340.04	10381.87	5.418	2243.48	12155.32
A_68_P27787099	chr11:49988480-49988526	NM_026543:150	3010026O09Rik	INSIDE	1.742	1.554	3080.89	4787.81	2.707	2253.64	6101.51
A_68_P26890130	chr9:122481560-122481604	NR_015610:-36	9530059O14Rik	PROMOTER	1.742	2.246	822.11	1846.18	3.911	742.78	2905.29
A_68_P29526837	chr14:34736673-34736717	NM_145741:-78	Gdf10	PROMOTER	1.742	2.152	3153.19	6786.21	3.750	2573.87	9651.14
A_68_P30778982	chr16:56037716-56037760	NM_029092:149	Rg9mtd1	INSIDE	1.742	2.409	1190.64	2868.44	4.197	1272.69	5341.78

ProbeName	Target position of probe on CpG island microarray	TargetID	GeneSymbol	CpG island Description	Ratio of relative methylation (TiO_2-NP/Vehicle)	Sham group			TiO_2-H group		
						Relative methylation (Cy5/Cy3)	Cy3 signal (Input)	Cy5 signal (MeDIP)	Relative methylation (Cy5/Cy3)	Cy3 signal (Input)	Cy5 signal (MeDIP)
A_68_P25102500	chr7:54263669-54263713	NM_175318:94	Spty2d1	INSIDE	1.742	3.016	3244.63	9784.45	5.252	2553.17	13408.54
A_68_P31315914	chr17:68353306-68353350	NM_172964:120	Arhgap28	INSIDE	1.741	3.659	2455.65	8984.22	6.368	2022.42	12879.14
A_68_P24603556	chr6:85137482-85137526	NM_010131:-420	Emx1	PROMOTER	1.741	1.686	1548.80	2610.51	2.934	1261.23	3700.15
A_68_P27856227	chr11:62693165-62693209	NM_026210:195	Fam18b	INSIDE	1.741	6.334	2125.21	13461.23	11.029	1638.86	18075.66
A_68_P24535482	chr6:71221514-71221558	NM_145568:-476	Krcc1	DIVERGENT_PROMOTER	1.741	4.318	2746.85	11859.56	7.516	1893.83	14233.44
A_68_P24133762	chr5:139605565-139605609	NM_008923:120	Prkar1b	INSIDE	1.741	4.090	4595.71	18798.29	7.122	3413.53	24310.10
A_68_P29612660	chr14:55098640-55098684	NM_080726:3726	Rem2	INSIDE	1.741	2.457	334.13	820.80	4.277	262.63	1123.22
A_68_P27633577	chr11:20730971-20731015	NM_173752:119	1110067D22Rik	INSIDE	1.740	5.809	1800.24	10457.74	10.107	1463.09	14786.97
A_68_P23991928	chr5:112823693-112823737	NM_028386:-2481	Asphd2	PROMOTER	1.740	4.992	2724.62	13601.48	8.687	2226.16	19338.16
A_68_P22222029	chr3:69402614-69402658	NM_020026:147	B3galnt1	INSIDE	1.740	2.676	1545.57	4135.28	4.657	1385.46	6451.65
A_68_P24995306	chr7:20382177-20382221	NM_001161844:-40	Cblc	PROMOTER	1.740	2.603	3012.08	7840.71	4.530	2502.69	11336.44
A_68_P20391720	chr1:82288083-82288127	NM_010570:-90	Irs1	PROMOTER	1.740	6.741	738.79	4980.22	11.731	618.89	7260.25
A_68_P27631991	chr11:20443663-20443707	NM_021372:429	Sertad2	INSIDE	1.740	1.563	615.95	962.46	2.718	770.09	2093.18
A_68_P30574719	chr16:17619415-17619459	NM_001164609:-10	Smpd4	PROMOTER	1.740	3.457	1114.97	3854.16	6.013	928.41	5582.68
A_68_P29599187	chr14:51550282-51550326	NM_001033271:220	Tmem55b	INSIDE	1.740	8.543	1131.21	9664.49	14.870	707.37	10518.48
A_68_P29682512	chr14:67627253-67627297	NM_009761:440	Bnip3l	INSIDE	1.739	3.096	1938.77	6002.12	5.383	2240.87	12063.71
A_68_P23279343	chr4:125978444-125978488	NM_199473:14429	Col8a2	INSIDE	1.739	1.434	1429.27	2050.07	2.494	1206.55	3008.88
A_68_P20168277	chr1:39957569-39957613	NM_008696:-167	Map4k4	PROMOTER	1.739	6.591	1829.38	12056.65	11.462	1482.10	16987.39
A_68_P24758832	chr6:113276968-113277012	NM_010957:21	Ogg1	INSIDE	1.739	4.601	10666.25	49076.78	8.002	7706.41	61667.74
A_68_P29701956	chr14:70944977-70945021	NM_180588:-59	Reep4	PROMOTER	1.739	1.515	696.61	1055.02	2.634	767.89	2022.35
A_68_P26555222	chr9:59598670-59598714	NM_001172068:-236	Senp8	DIVERGENT_PROMOTER	1.739	3.056	1609.98	4920.90	5.317	1312.18	6976.36
A_68_P31385967	chr17:81344527-81344571	NM_026516:577	Tmem178	INSIDE	1.739	2.003	790.27	1582.55	3.483	671.56	2339.01
A_68_P20936661	chr1:193006674-193006718	NM_007498:516	Atf3	INSIDE	1.738	7.410	4368.87	32372.21	12.876	3330.41	42883.92
A_68_P29011719	chr13:47201171-47201215	NM_025900:397	Dek	INSIDE	1.738	1.560	990.11	1544.24	2.711	764.35	2072.05
A_68_P32364765	chrX:45806673-45806717	NM_019680:9615	Elf4	INSIDE	1.738	1.635	1048.84	1715.36	2.842	569.64	1619.14
A_68_P26550030	chr9:58670732-58670776	NM_001081192:-564	Hcn4	PROMOTER	1.738	2.314	463.53	1072.60	4.022	400.76	1611.96
A_68_P20556969	chr1:121317983-121318027	NM_008381:821	Inhbb	INSIDE	1.738	5.199	2087.36	10852.02	9.033	1599.33	14447.15
A_68_P24142218	chr5:141083418-141083462	NM_008494:146	Lfng	INSIDE	1.738	3.087	2249.66	6945.00	5.366	1855.84	9959.10
A_68_P31130455	chr17:30024049-30024093	NM_001081160:757	Mdga1	INSIDE	1.738	2.851	1712.55	4882.18	4.954	1318.07	6529.20
A_68_P27950601	chr11:79525364-79525408	NR_029579:-84	Mir193	PROMOTER	1.738	2.793	2055.00	5739.78	4.854	1642.00	7969.79
A_68_P30480085	chr15:99256217-99256261	NM_001001884:31941	Nckap5l	INSIDE	1.738	4.114	1881.69	7740.46	7.150	1425.95	10195.16
A_68_P28566637	chr12:80012969-80013013	NM_013738:-5065	Plek2	PROMOTER	1.738	2.300	3127.95	7195.06	3.997	2381.41	9519.02
A_68_P23234760	chr4:117167587-117167631	NM_025739:1912	Rnf220	INSIDE	1.738	3.133	2488.25	7795.60	5.446	2043.22	11127.02
A_68_P27787321	chr11:50023806-50023850	NM_011018:464	Sqstm1	INSIDE	1.738	4.742	6644.51	31510.94	8.244	4503.50	37125.14
A_68_P23298058	chr4:129373895-129373939	NM_199305:166	Tmem39b	INSIDE	1.738	2.740	2790.29	7644.16	4.761	2354.98	11212.01
A_68_P26871705	chr9:119231516-119231560	ENSMUST00000128880:-98		PROMOTER	1.738	5.111	4707.16	24057.97	8.881	3557.34	31594.19
A_68_P20165362	chr1:39424508-39424553	NM_053257:-165	Rpl31	PROMOTER	1.737	3.441	688.17	2368.01	5.977	527.67	3153.77
A_68_P26397640	chr9:30729401-30729445	NM_001024139:615	Adamts15	INSIDE	1.736	2.378	1314.66	3126.25	4.129	962.94	3976.27
A_68_P31762242	chr18:62339733-62339777	NM_007420:-141	Adrb2	PROMOTER	1.736	3.893	1542.63	6005.45	6.760	1271.57	8595.79
A_68_P28688785	chr12:103214150-103214194	NM_016856:-11	Cpsf2	PROMOTER	1.736	1.425	1111.50	1583.51	2.474	1079.73	2670.99
A_68_P26926726	chr10:8605914-8605958	NM_175155:-68	Sash1	PROMOTER	1.736	2.375	1853.06	4400.32	4.123	1678.72	6920.83
A_68_P20453778	chr1:95073519-95073563			Unknown	1.736	7.128	2346.40	16725.72	12.378	1903.78	23564.98
A_68_P32127475	chr19:43674323-43674367			Unknown	1.736	3.029	4914.69	14886.60	5.260	3642.82	19160.25
A_68_P31259473	chr17:57136900-57136944	NM_025538:161	Alkbh7	INSIDE	1.735	2.983	1175.23	3506.23	5.178	897.02	4644.46
A_68_P21102918	chr2:30248319-30248363	NM_020329:405	Dolpp1	INSIDE	1.735	4.161	1997.38	8310.37	7.217	1574.57	11363.04
A_68_P26659818	chr9:78329691-78329735	NM_010106:-181	Eef1a1	PROMOTER	1.735	1.761	947.75	1669.34	3.056	778.65	2379.18
A_68_P30261334	chr15:60656407-60656451	NM_001162926:207	Fam84b	INSIDE	1.735	2.756	867.52	2391.32	4.784	695.50	3327.13
A_68_P26803570	chr9:106367374-106367418	NM_001159652:875	Gpr62	INSIDE	1.735	4.565	756.92	3455.63	7.923	673.99	5340.17
A_68_P23812530	chr5:76374553-76374597	NM_010612:-121	Kdr	PROMOTER	1.735	4.224	6303.13	26622.36	7.328	4621.05	33863.12
A_68_P27287865	chr10:80630602-80630646	NM_021501:-159	Pias4	DIVERGENT_PROMOTER	1.735	3.750	3317.59	12442.19	6.506	2416.58	15721.63
A_68_P31295069	chr17:64681315-64681359	NM_001025309:-113	Pja2	PROMOTER	1.735	2.178	911.66	1985.96	3.779	794.14	3001.14

ProbeName	Target position of probe on CpG island microarray	TargetID	GeneSymbol	CpG island Description	Ratio of relative methylation (TiO_2-NP/Vehicle)	Sham group			TiO_2-H group		
						Relative methylation (Cy5/Cy3)	Cy3 signal (Input)	Cy5 signal (MeDIP)	Relative methylation (Cy5/Cy3)	Cy3 signal (Input)	Cy5 signal (MeDIP)
A_68_P26725648	chr9:92145271-92145315	NM_011636:261	Plscr1	INSIDE	1.735	3.586	4101.69	14709.23	6.221	3012.57	18741.24
A_68_P26574057	chr9:62986883-62986927	NM_172446:7883	Skor1	INSIDE	1.735	6.931	588.37	4077.83	12.028	469.55	5647.64
A_68_P25537056	chr7:140005623-140005667	NM_212473:-765	Fam53b	PROMOTER	1.734	2.759	1651.86	4558.12	4.785	1316.60	6299.36
A_68_P20967784	chr2:4322707-4322751	NM_001177843:707	Frmd4a	INSIDE	1.734	4.722	12366.15	58388.60	8.189	8550.72	70023.36
A_68_P24904258	chr6:142336086-142336130	NM_025872:346	Golt1b	INSIDE	1.734	1.677	823.66	1381.09	2.908	721.84	2098.75
A_68_P30482738	chr15:99705731-99705775	NM_001113545:135	Lima1	INSIDE	1.734	5.138	2702.22	13884.57	8.908	2227.72	19845.29
A_68_P22313622	chr3:88214207-88214256	NM_025928:7	Pmf1	INSIDE	1.734	3.408	876.38	2986.69	5.908	678.82	4010.58
A_68_P30013962	chr15:10966866-10966910	NM_178717:835	Rxfp3	INSIDE	1.734	6.001	3825.75	22960.00	10.408	2752.13	28644.35
A_68_P20505897	chr1:107890521-107890565	NM_001122675:3560	Zcchc2	INSIDE	1.734	3.021	2180.83	6589.31	5.239	1749.16	9164.54
A_68_P23282958	chr4:126644783-126644827	NM_001114399:363	Zmym4	INSIDE	1.734	6.315	4207.22	26569.82	10.948	3446.79	37736.43
A_68_P23542143	chr5:23988482-23988526	NM_139153:30510	Agap3	INSIDE	1.733	5.843	4665.00	27259.61	10.129	3513.02	35584.60
A_68_P25022916	chr7:29415707-29415751	NM_175319:306	C330005M16Rik	INSIDE	1.733	1.796	1168.65	2099.07	3.113	1029.93	3205.78
A_68_P32380511	chrX:49517673-49517717	NM_008150:406	Gpc4	INSIDE	1.733	3.289	1340.52	4408.65	5.699	855.74	4877.06
A_68_P25952867	chr8:73118782-73118826	NM_023627:425	Isyna1	INSIDE	1.733	1.577	1487.43	2345.26	2.732	1207.58	3299.13
A_68_P24946817	chr7:3637393-3637437	NM_029934:7713	Mboat7	INSIDE	1.733	4.064	2244.83	9124.02	7.046	1661.51	11706.57
A_68_P31929629	chr19:5602275-5602319	NM_026616:424	Rnaseh2c	INSIDE	1.733	2.818	461.91	1301.47	4.883	438.90	2143.20
A_68_P31209382	chr17:47026332-47026376	NM_178385:-1215	Tbcc	PROMOTER	1.733	4.014	1076.92	4322.30	6.957	914.68	6363.05
A_68_P33005868	A_68_P33005868			Unknown	1.733	6.745	406.82	2743.92	11.687	336.19	3929.21
A_68_P26469462	chr9:44575608-44575652	NM_145985:261	Arcn1	INSIDE	1.732	4.073	1190.06	4847.60	7.057	1021.52	7208.99
A_68_P22971150	chr4:63206034-63206078	NM_024173:258	Atp6v1g1	INSIDE	1.732	4.331	2368.67	10258.41	7.501	2015.75	15119.91
A_68_P27003030	chr10:23069823-23069869	NM_010167:-137	Eya4	PROMOTER	1.732	3.286	434.32	1427.34	5.692	439.18	2499.66
A_68_P28745894	chr12:112997376-112997420	NM_008450:339	Klc1	INSIDE	1.732	1.607	6227.53	10010.32	2.783	4582.88	12756.15
A_68_P20797864	chr1:167933679-167933723	NM_011137:-935	Pou2f1	PROMOTER	1.732	4.747	1600.53	7598.07	8.223	1240.42	10199.96
A_68_P22182581	chr3:61168376-61168420	NM_028712:-30	Rap2b	PROMOTER	1.732	3.914	4615.79	18064.14	6.779	3329.52	22571.76
A_68_P23402414	chr4:149148446-149148491	NM_027460:-92	Slc25a33	PROMOTER	1.732	6.311	5319.73	33572.15	10.928	4115.00	44966.77
A_68_P27935204	chr11:76811878-76811922	NM_010484:-198	Slc6a4	PROMOTER	1.732	2.191	1262.92	2767.27	3.795	1066.19	4046.40
A_68_P23310689	chr4:131767916-131767960	NM_145393:233	Ythdf2	INSIDE	1.732	4.831	8487.61	41002.43	8.366	6485.99	54258.90
A_68_P26237605	chr8:125566196-125566241	NM_001081379:-34	Ankrd11	PROMOTER	1.731	2.847	2956.92	8417.35	4.926	2522.15	12425.07
A_68_P20101394	chr1:25886869-25886913	NM_175642:-338	Bai3	PROMOTER	1.731	6.315	3410.04	21533.82	10.933	2400.03	26238.94
A_68_P25609544	chr7:152024886-152024930	NM_008007:392	Fgf3	INSIDE	1.731	2.429	1335.04	3242.95	4.204	1106.16	4650.24
A_68_P21078770	chr2:26301305-26301349	NM_153125:-590	Sec16a	PROMOTER	1.731	2.186	792.56	1732.82	3.784	665.25	2517.04
A_68_P24432387	chr6:49345594-49345638	NM_029916:14	Stk31	INSIDE	1.731	6.766	7487.28	50658.23	11.713	5534.60	64827.12
A_68_P26242939	chr8:126544720-126544764	NM_133966:467	Taf5l	INSIDE	1.731	7.775	676.84	5262.41	13.462	525.48	7074.04
A_68_P22449315	chr3:116126619-116126663	NM_001080818:310	Cdc14a	INSIDE	1.730	3.289	1221.99	4019.51	5.690	1102.08	6270.97
A_68_P27094083	chr10:41996329-41996375	NM_019740:196	Foxo3	INSIDE	1.730	2.798	733.02	2050.96	4.841	674.74	3266.23
A_68_P31632704	chr18:38371400-38371444	NM_029357:-2006	Pcdh1	PROMOTER	1.730	3.324	1831.38	6088.28	5.751	1438.68	8273.75
A_68_P26773477	chr9:101153798-101153842	NM_001161362:342	Ppp2r3a	INSIDE	1.730	1.698	791.47	1343.88	2.937	672.35	1974.64
A_68_P27311096	chr10:85379833-85379877	NM_181650:-164	Prdm4	PROMOTER	1.730	3.434	1386.39	4760.46	5.939	1135.23	6742.20
A_68_P31513736	chr18:14841066-14841110	NM_001161369:335	Ss18	INSIDE	1.730	1.539	893.89	1376.05	2.663	779.30	2075.10
A_68_P24760447	chr6:113554719-113554763	NM_133937:-25	6720456B07Rik	DIVERGENT_PROMOTER	1.729	29.247	415.36	12148.19	50.565	408.90	20675.97
A_68_P31760609	chr18:62071314-62071358	NM_198649:142	Ablim3	INSIDE	1.729	2.646	1903.66	5036.37	4.575	1574.24	7201.53
A_68_P29047192	chr13:54150550-54150594	NM_010076:455	Drd1a	INSIDE	1.729	3.605	1644.59	5928.09	6.233	1271.41	7925.09
A_68_P23513989	chr5:18732427-18732471	NM_001170746:-415	Magi2	PROMOTER	1.729	5.351	1454.92	7784.77	9.253	1263.72	11692.73
A_68_P24537769	chr6:71657624-71657668	NM_178608:-28	Reep1	PROMOTER	1.729	4.365	558.91	2439.40	7.545	446.64	3369.74
A_68_P32906199	A_68_P32906199			Unknown	1.729	8.050	1059.06	8524.89	13.918	818.63	11394.02
A_68_P24980671	chr7:16871769-16871813	NM_172297:-1782	Ccdc9	PROMOTER	1.728	4.829	18022.74	87028.16	8.343	12430.23	103702.10
A_68_P32539664	chrX:91881437-91881481	NM_008179:51	Gspt2	INSIDE	1.728	1.532	1092.11	1672.96	2.647	546.98	1447.80
A_68_P28693227	chr12:103943323-103943367	NM_172584:-265	Itpk1	PROMOTER	1.728	5.248	811.62	4259.30	9.070	709.53	6435.19
A_68_P23253010	chr4:120419437-120419481	NM_001081142:323	Kcnq4	INSIDE	1.728	2.460	1299.17	3195.38	4.251	1082.81	4602.51
A_68_P22157978	chr3:55987140-55987184	NM_030595:461	Nbea	INSIDE	1.728	4.480	1138.19	5099.45	7.741	981.53	7598.19
A_68_P29599189	chr14:51550570-51550614	NM_001033271:-68	Tmem55b	PROMOTER	1.728	6.256	6967.38	43587.79	10.809	5207.44	56284.93

ProbeName	Target position of probe on CpG island microarray	TargetID	GeneSymbol	CpG island Description	Ratio of relative methylation (TiO_2-NP/Vehicle)	Sham group			TiO_2-H group		
						Relative methylation (Cy5/Cy3)	Cy3 signal (Input)	Cy5 signal (MeDIP)	Relative methylation (Cy5/Cy3)	Cy3 signal (Input)	Cy5 signal (MeDIP)
A_68_P29509872	chr14:31942367-31942412	NR_024069:-273	2010107H07Rik	DIVERGENT_PROMOTER	1.727	3.207	2485.55	7970.80	5.539	2061.63	11419.23
A_68_P30150544	chr15:38591583-38591627	NM_025494:-54	Atp6v1c1	PROMOTER	1.727	3.206	1534.77	4919.78	5.535	1234.99	6835.11
A_68_P32130475	chr19:44181890-44181934	NM_001162410:55	Chuk	INSIDE	1.727	3.622	678.44	2457.12	6.254	590.49	3693.13
A_68_P22752471	chr4:15872402-15872446	NM_026172:230	Decr1	INSIDE	1.727	2.505	2768.71	6935.72	4.326	2314.43	10012.17
A_68_P28266974	chr12:16817834-16817878	NM_033270:86	E2f6	INSIDE	1.727	3.658	1329.30	4862.99	6.319	1114.54	7042.68
A_68_P29059430	chr13:56236999-56237043	NM_001159513:-109	H2afy	PROMOTER	1.727	2.959	648.32	1918.32	5.110	510.41	2608.02
A_68_P26954148	chr10:13684931-13684975	NM_010437:-1232	Hivep2	PROMOTER	1.727	4.565	2223.13	10147.80	7.885	1782.67	14056.88
A_68_P23288232	chr4:127666279-127666323	ENSMUST00000144298:1013		INSIDE	1.727	2.333	1772.54	4135.39	4.030	1329.21	5356.51
A_68_P29410675	chr14:13178814-13178858	NM_080433:-457	Fezf2	PROMOTER	1.726	2.747	1144.71	3144.30	4.740	883.06	4185.46
A_68_P28691644	chr12:103707806-103707850	NM_013747:-291	Golga5	PROMOTER	1.726	2.151	1836.11	3949.73	3.712	1527.76	5671.67
A_68_P22448215	chr3:115955466-115955511	NM_022427:914	Gpr88	INSIDE	1.726	2.313	6031.43	13952.53	3.992	4318.84	17240.68
A_68_P22393109	chr3:104584285-104584329	NM_027982:333	Ppm1j	INSIDE	1.726	8.053	2584.88	20816.80	13.897	2145.43	29815.76
A_68_P31341464	chr17:72953935-72953979	NM_007439:-309	Alk	PROMOTER	1.725	3.540	6394.26	22635.29	6.108	5150.08	31456.16
A_68_P27234932	chr10:70807397-70807441	NM_134007:179	Cisd1	INSIDE	1.725	5.434	743.69	4041.59	9.376	605.45	5676.99
A_68_P28974187	chr13:40826726-40826770	NM_001122948:-936	Tcfap2a	PROMOTER	1.725	2.982	3597.64	10726.98	5.142	2801.51	14405.41
A_68_P22708703	chr4:6917953-6917997	NM_145711:-104	Tox	PROMOTER	1.725	3.353	5320.50	17841.79	5.786	4525.23	26181.49
A_68_P29733553	chr14:76816095-76816139	NM_207652:489	Tsc22d1	INSIDE	1.725	3.435	3654.98	12555.67	5.926	2628.47	15576.82
A_68_P24152597	chr5:143106025-143106069	NM_178398:509	Wipi2	INSIDE	1.725	2.031	1971.87	4004.07	3.502	1589.00	5564.96
A_68_P20350627	chr1:74849814-74849858	NM_009518:11244	Wnt10a	INSIDE	1.725	3.465	3093.07	10717.89	5.977	2626.35	15696.38
A_68_P23865427	chr5:87233816-87233860	NM_177680:324	Ythdc1	INSIDE	1.725	1.739	413.95	720.01	3.000	361.58	1084.78
A_68_P31620318	chr18:35991596-35991640	NM_133687:2147	Cxxc5	INSIDE	1.724	1.975	1603.91	3166.95	3.404	1180.80	4019.63
A_68_P24620361	chr6:88149067-88149115	NM_008090:433	Gata2	INSIDE	1.724	1.904	294.63	560.86	3.281	247.73	812.77
A_68_P31127484	chr17:29484749-29484793	NM_019880:79	Mtch1	INSIDE	1.724	3.035	1442.67	4378.91	5.232	1135.57	5941.51
A_68_P27483044	chr10:117229909-117229953	NM_134010:-169	Nup107	PROMOTER	1.724	2.587	4878.89	12620.43	4.460	3749.15	16720.20
A_68_P31654583	chr18:42435328-42435372	NM_172626:344	Rbm27	INSIDE	1.724	3.406	3192.91	10873.86	5.870	2639.01	15490.72
A_68_P23331724	chr4:135577368-135577412	NM_013736:174	Tceb3	INSIDE	1.724	5.009	4526.26	22670.37	8.633	3689.98	31854.49
A_68_P23982393	chr5:111308521-111308565	NM_024477:-279	Ttc28	PROMOTER	1.724	6.433	286.30	1841.66	11.088	176.30	1954.72
A_68_P31464043	chr18:4635545-4635589	NM_001081963:640	9430020K01Rik	INSIDE	1.723	2.340	2351.10	5500.43	4.031	2007.10	8090.87
A_68_P26954155	chr10:13685730-13685774	NM_010437:-432	Hivep2	PROMOTER	1.723	4.479	4379.25	19614.59	7.715	3569.73	27541.79
A_68_P24624872	chr6:88848652-88848696	NM_008564:100	Mcm2	INSIDE	1.723	2.710	2143.08	5807.63	4.668	1669.78	7794.48
A_68_P29613302	chr14:55236499-55236543	NR_030457:-898	Mir686	PROMOTER	1.723	4.302	368.61	1585.89	7.412	316.57	2346.51
A_68_P20147266	chr1:36326391-36326435	NM_153408:3858	Neurl3	INSIDE	1.723	2.008	1892.51	3800.04	3.460	1405.54	4862.79
A_68_P32201471	chr19:56623197-56623241	NM_025811:468	Nhlrc2	INSIDE	1.723	2.753	677.65	1865.37	4.743	532.80	2526.87
A_68_P21871010	chr2:173484993-173485037	NR_027957:26	Ppp4r1l-ps	INSIDE	1.723	4.435	2241.73	9941.73	7.641	1850.19	14137.33
A_68_P23393512	chr4:147662081-147662125	NM_001083342:-28	Ptchd2	PROMOTER	1.723	2.265	1585.17	3590.51	3.903	1302.94	5085.97
A_68_P27234632	chr10:70747404-70747448	NM_145420:584	Ube2d1	INSIDE	1.723	4.515	3345.42	15106.07	7.781	2432.05	18923.28
A_68_P23409805	chr4:150432256-150432300	NM_009498:-216	Vamp3	PROMOTER	1.723	5.061	4467.10	22606.50	8.719	3000.37	26159.48
A_68_P31154661	A_68_P31154661			Unknown	1.723	5.020	1377.34	6913.80	8.649	935.76	8093.31
A_68_P21071854	chr2:25251918-25251962	NM_009849:547	Entpd2	INSIDE	1.722	4.180	2448.06	10232.39	7.196	1935.00	13923.72
A_68_P29644193	chr14:60997735-60997779	NM_001164705:634	Fam123a	INSIDE	1.722	3.402	1085.16	3691.29	5.858	923.00	5406.96
A_68_P28084547	chr11:103173618-103173663			Unknown	1.722	1.532	679.97	1041.72	2.638	547.69	1444.83
A_68_P28859763	chr13:19040669-19040713	NM_175007:451	Amph	INSIDE	1.721	4.158	662.80	2756.07	7.158	559.15	4002.62
A_68_P24172967	chr5:148116571-148116615	NM_007673:2233	Cdx2	INSIDE	1.721	4.316	1295.33	5590.72	7.427	1017.59	7557.83
A_68_P30588586	chr16:20630082-20630126	NM_139293:181	Ece2	INSIDE	1.721	2.516	2058.37	5178.35	4.328	1688.09	7306.66
A_68_P28334082	chr12:32634899-32634943	NM_153198:480	Hbp1	INSIDE	1.721	1.569	1598.61	2508.49	2.700	1307.28	3530.14
A_68_P25474216	chr7:128643864-128643910	NM_001081327:108077	Hs3st2	INSIDE	1.721	7.977	1898.20	15141.20	13.728	1434.35	19691.47
A_68_P25168663	chr7:69493375-69493419	NM_010882:234	Ndn	INSIDE	1.721	5.983	1564.06	9357.36	10.297	1409.83	14516.67
A_68_P29117519	chr13:69878102-69878146	NM_001145162:651	Ube2ql1	INSIDE	1.721	3.906	4725.31	18459.07	6.724	3879.84	26087.56
A_68_P32573117	chrX:100677336-100677380	NR_001463:1214	Xist	INSIDE	1.721	2.053	4041.71	8298.95	3.533	1728.31	6105.93
A_68_P27287617	chr10:80598897-80598941	NM_010731:-97	Zbtb7a	PROMOTER	1.721	3.469	1436.35	4982.19	5.968	1224.08	7305.29
A_68_P26217897	chr8:122625576-122625620	NM_133967:-226	Zdhhc7	PROMOTER	1.721	1.447	1487.66	2151.96	2.490	1206.32	3003.91

ProbeName	Target position of probe on CpG island microarray	TargetID	GeneSymbol	CpG island Description	Ratio of relative methylation (TiO_2-NP/Vehicle)	Sham group			TiO_2-H group		
						Relative methylation (Cy5/Cy3)	Cy3 signal (Input)	Cy5 signal (MeDIP)	Relative methylation (Cy5/Cy3)	Cy3 signal (Input)	Cy5 signal (MeDIP)
A_68_P26721663	chr9:91278946-91278990	NM_009576:15159	Zic4	INSIDE	1.721	2.922	909.30	2656.60	5.028	697.42	3506.49
A_68_P30534202	A_68_P30534202			Unknown	1.721	3.265	1026.16	3350.15	5.619	897.05	5040.94
A_68_P28736910	chr12:111426306-111426350	AK044800:543		INSIDE	1.721	12.342	2821.20	34819.40	21.240	1891.38	40173.25
A_68_P22888792	chr4:46464440-46464484	NM_130889:474	Anp32b	INSIDE	1.720	3.243	3492.38	11327.10	5.578	2955.22	16483.79
A_68_P26846741	chr9:114753280-114753324	NM_027294:-32	Cmtm8	PROMOTER	1.720	4.235	8858.42	37512.78	7.283	7283.97	53046.75
A_68_P22318578	chr3:89126786-89126830	NM_010108:-7	Efna3	PROMOTER	1.720	1.555	6323.19	9831.44	2.675	4671.38	12494.26
A_68_P24329145	chr6:29866863-29866907	NM_001037740:-128	Fam40b	PROMOTER	1.720	3.229	355.25	1147.20	5.556	308.76	1715.42
A_68_P23929939	chr5:100929613-100929657	NM_001115010:-2042	Lin54	PROMOTER	1.720	3.153	2338.54	7372.86	5.423	1885.87	10226.99
A_68_P25375583	chr7:108967177-108967221	NM_008887:372	Phox2a	INSIDE	1.720	5.525	3288.44	18167.43	9.503	2745.79	26092.66
A_68_P28203004	chr12:4924079-4924123	NM_001099628:-58	Atad2b	DIVERGENT_PROMOTER	1.719	5.477	1128.71	6182.38	9.415	915.99	8624.10
A_68_P23647570	chr5:44173303-44173347	NM_001159963:64	Fbxl5	INSIDE	1.719	3.873	1869.08	7238.82	6.659	1685.32	11222.52
A_68_P29928904	chr14:117325550-117325594	NM_001079844:1036	Gpc6	INSIDE	1.719	2.874	1535.27	4413.00	4.941	1284.53	6347.51
A_68_P30577450	chr16:18088960-18089004	NM_011172:301	Prodh	INSIDE	1.719	2.615	2729.88	7138.39	4.496	2107.54	9475.10
A_68_P23236670	chr4:117509289-117509333	NM_008135:1448	Slc6a9	INSIDE	1.719	3.621	3065.86	11101.40	6.224	2295.46	14288.00
A_68_P25005464	chr7:25332066-25332110	NM_009532:-80	Xrcc1	DIVERGENT_PROMOTER	1.719	2.836	1884.27	5344.33	4.875	1417.59	6910.93
A_68_P24624504	chr6:88791715-88791759	NM_030251:193	Abtb1	INSIDE	1.718	3.548	10382.55	36839.75	6.095	6985.27	42572.99
A_68_P25578895	chr7:146768050-146768094	NR_027857:624	Nkx6-2	INSIDE	1.718	3.426	1968.92	6745.40	5.887	1554.74	9153.31
A_68_P28781792	chr12:120075230-120075274	NM_177082:-9549	Sp8	PROMOTER	1.718	2.608	3721.05	9704.84	4.481	2814.29	12609.79
A_68_P21568536	chr2:119063328-119063372	NM_016907:255	Spint1	INSIDE	1.718	2.031	3187.21	6473.42	3.490	2311.72	8067.09
A_68_P21075771	chr3:25877080-25877124	NM_133835:178	Ubac1	INSIDE	1.718	3.692	611.91	2259.37	6.342	595.33	3775.64
A_68_P22483663	chr3:122321938-122321982	NM_001114665:625	Fnbp1l	INSIDE	1.717	5.718	894.16	5113.21	9.819	840.35	8251.31
A_68_P28540462	chr12:75009562-75009606	NM_010431:731	Hif1a	INSIDE	1.717	3.450	1890.74	6523.81	5.924	1556.06	9217.74
A_68_P23936334	chr5:102093547-102093591	NM_144955:162	Nkx6-1	INSIDE	1.717	1.920	2360.88	4532.33	3.297	1938.13	6389.91
A_68_P30344883	chr15:75740087-75740131	NM_178646:-56	Tigd5	DIVERGENT_PROMOTER	1.717	4.105	2199.15	9027.46	7.048	1901.13	13399.69
A_68_P27693346	chr11:33101716-33101763	NM_019916:1849	Tlx3	INSIDE	1.717	4.968	736.97	3661.61	8.529	543.56	4636.17
A_68_P24091293	chr5:130418207-130418251	NM_001001327:246	Vkorc1l1	INSIDE	1.717	6.268	943.56	5914.16	10.764	1054.67	11352.37
A_68_P30481041	chr15:99414682-99414726	NM_009699:5218	Aqp2	INSIDE	1.716	3.349	1478.19	4949.91	5.747	1177.19	6764.97
A_68_P26727067	chr9:92437602-92437647	NR_029474:83	B430319G15Rik	INSIDE	1.716	3.978	2810.97	11182.00	6.828	2049.32	13992.34
A_68_P24607367	chr6:85911667-85911711	NM_028099:-27	Dusp11	PROMOTER	1.716	2.947	1375.74	4054.99	5.058	1093.69	5531.42
A_68_P20849151	chr1:177555721-177555765	NM_010209:24	Fh1	INSIDE	1.716	3.071	7119.66	21865.76	5.271	5290.52	27886.33
A_68_P24449298	chr6:52184077-52184121	NM_008263:840	Hoxa10	INSIDE	1.716	5.558	1152.59	6405.71	9.538	928.05	8852.16
A_68_P31515584	chr18:15221979-15222023	NM_001142731:91	Kctd1	INSIDE	1.716	3.299	1144.74	3776.04	5.660	975.32	5519.95
A_68_P23399809	chr4:148681348-148681392	NM_207682:437	Kif1b	INSIDE	1.716	3.683	258.15	950.86	6.319	229.26	1448.71
A_68_P28915541	chr13:30228560-30228604	NM_153546:224	Mboat1	INSIDE	1.716	4.650	5057.74	23518.34	7.979	3984.22	31790.67
A_68_P23985683	chr5:111849467-111849511	NM_001081235:2303	Mn1	INSIDE	1.716	3.154	537.33	1694.76	5.412	481.21	2604.08
A_68_P24904253	chr6:142335519-142335563	NM_001204907:67	Recql	INSIDE	1.716	2.797	4496.67	12578.37	4.800	3780.54	18145.67
A_68_P26890104	chr9:122477963-122478007	NR_015610:-3634	9530059O14Rik	PROMOTER	1.715	2.017	1416.52	2857.29	3.459	1128.93	3905.17
A_68_P23612481	chr5:37633390-37633434	NM_007765:-3618	Crmp1	PROMOTER	1.715	4.336	649.92	2817.97	7.435	538.48	4003.66
A_68_P30253796	chr15:59205558-59205602	NM_153548:127	E430025E21Rik	INSIDE	1.715	7.918	3792.74	30030.63	13.577	2640.05	35843.98
A_68_P22509722	chr3:127335595-127335641	NM_009718:-444	Neurog2	PROMOTER	1.715	2.334	539.14	1258.42	4.004	417.61	1672.11
A_68_P30353507	chr15:77137504-77137548	NM_001110827:-43	Rbfox2	PROMOTER	1.715	2.280	405.10	923.60	3.911	360.13	1408.46
A_68_P21527966	chr2:112105941-112105985	NM_133649:-508	Slc12a6	PROMOTER	1.715	1.556	781.59	1216.41	2.669	608.90	1625.27
A_68_P25261701	chr7:86680448-86680492	ENSMUST00000141822:35468		DOWNSTREAM	1.715	3.255	1707.77	5559.10	5.582	1369.99	7646.90
A_68_P20827009	chr1:173200884-173200928	NM_020579:448	B4galt3	INSIDE	1.714	5.863	2039.79	11958.50	10.047	1518.05	15252.02
A_68_P25009419	chr7:26067406-26067450	NM_001110131:231	Cic	INSIDE	1.714	5.002	3250.76	16259.68	8.575	2867.73	24589.71
A_68_P23254538	chr4:120690017-120690061	NM_133716:-186	Smap2	PROMOTER	1.714	4.098	636.02	2606.32	7.024	524.34	3683.03
A_68_P23817348	chr5:77086896-77086940	ENSMUST00000125922:173		INSIDE	1.714	2.665	1380.62	3679.33	4.568	1126.22	5144.39
A_68_P23644443	chr5:43625072-43625116	NM_001177379:393	Cpeb2	INSIDE	1.713	5.653	2539.23	14353.61	9.685	1936.44	18754.98
A_68_P29403726	chr14:11994369-11994413	NM_010210:156	Fhit	INSIDE	1.713	3.069	5639.46	17306.04	5.257	4585.36	24106.61
A_68_P32385145	chrX:50341393-50341437	NM_013556:160	Hprt	INSIDE	1.713	5.394	1232.71	6648.69	9.240	713.22	6590.19
A_68_P26764036	chr9:99336932-99336976	NM_008624:177	Mras	INSIDE	1.713	3.924	3458.26	13570.25	6.722	2687.56	18066.21

ProbeName	Target position of probe on CpG island microarray	TargetID	GeneSymbol	CpG island Description	Ratio of relative methylation (TiO_2-NP/Vehicle)	Sham group			TiO_2-H group		
						Relative methylation (Cy5/Cy3)	Cy3 signal (Input)	Cy5 signal (MeDIP)	Relative methylation (Cy5/Cy3)	Cy3 signal (Input)	Cy5 signal (MeDIP)
A_68_P29001576	chr13:45485361-45485405	NM_181043:57	Mylip	INSIDE	1.713	1.479	1688.38	2496.84	2.533	1298.46	3288.58
A_68_P28039252	chr11:95374960-95375004	NM_130858:897	Nxph3	INSIDE	1.713	3.388	3144.62	10653.15	5.802	2466.83	14312.08
A_68_P23584119	chr5:32761375-32761419	NM_172707:54	Ppp1cb	INSIDE	1.713	3.796	5314.37	20175.38	6.503	4182.75	27199.38
A_68_P27926352	chr11:75300162-75300206	NM_138659:-94	Prpf8	PROMOTER	1.713	2.584	2946.26	7613.67	4.425	2454.06	10860.23
A_68_P21902494	chr2:179991894-179991938	NM_025587:-167	Rps21	PROMOTER	1.713	9.493	3506.19	33283.25	16.258	2557.31	41577.61
A_68_P26847329	chr9:114887053-114887097	CCDS52952.1:4751		DOWNSTREAM	1.713	7.635	2948.35	22510.50	13.079	1995.00	26092.04
A_68_P28706885	chr12:106248338-106248382	NM_172500:-341	4831426I19Rik	PROMOTER	1.712	5.463	6987.51	38171.76	9.353	5071.87	47438.28
A_68_P24127315	chr5:138182692-138182736	NM_175521:-1488	6430598A04Rik	DIVERGENT_PROMOTER	1.712	3.004	1293.54	3886.35	5.143	1008.95	5189.40
A_68_P20268944	chr1:60466493-60466537	NM_001198570:52	Abi2	INSIDE	1.712	4.100	1797.03	7367.11	7.017	1504.43	10555.97
A_68_P31126791	chr17:29375001-29375045	NM_153166:-287	Cpne5	PROMOTER	1.712	2.642	673.07	1778.26	4.523	569.70	2576.69
A_68_P26234161	chr8:125074980-125075024	NM_001037298:227	Fam38a	INSIDE	1.712	3.407	554.37	1888.59	5.832	500.29	2917.83
A_68_P21932363	chr3:8462950-8462994	NR_033512:126	Gm6194	INSIDE	1.712	4.894	2868.01	14035.36	8.377	2247.66	18829.32
A_68_P27537124	chr10:127058798-127058842	NM_008512:-616	Lrp1	PROMOTER	1.712	2.092	4941.30	10335.66	3.581	4217.40	15102.89
A_68_P30447388	chr15:93349565-93349609	NM_001033217:76736	Prickle1	INSIDE	1.712	1.704	1895.94	3231.44	2.918	1539.71	4493.14
A_68_P31107655	chr17:26122056-26122100	NM_153140:36727	Rab11fip3	DOWNSTREAM	1.712	6.619	986.65	6530.67	11.332	855.11	9690.13
A_68_P22315261	chr3:88520728-88520772	NM_001163310:-25	Rit1	PROMOTER	1.712	4.393	2291.16	10065.31	7.521	1802.82	13558.61
A_68_P28277266	chr12:19710348-19710392	BC012645:386747		INSIDE	1.712	5.328	2009.26	10704.84	9.122	1558.95	14220.18
A_68_P23678475	chr5:49916199-49916243	ENSMUST00000087395:-280		PROMOTER	1.712	1.441	1179.39	1699.97	2.468	1102.21	2719.84
A_68_P26867372	chr9:118515925-118515969	NM_133721:120	Itga9	INSIDE	1.711	3.220	367.80	1184.40	5.510	296.16	1631.77
A_68_P21322072	chr2:71711713-71711757	NM_172665:406	Pdk1	INSIDE	1.711	2.500	555.31	1388.44	4.279	440.50	1884.90
A_68_P21836025	chr2:167363730-167363774	NM_011427:26	Snai1	INSIDE	1.711	1.602	1591.40	2549.53	2.740	1316.52	3607.71
A_68_P29672987	chr14:65977066-65977114	NM_199029:-422	Zfp395	PROMOTER	1.711	3.926	498.77	1958.26	6.719	434.67	2920.36
A_68_P24954136	chr7:6083754-6083798	NM_001013012:23797	Zfp787	INSIDE	1.711	3.719	1970.48	7328.70	6.364	1617.93	10296.73
A_68_P24864304	chr6:134878874-134878918	NR_037955:-213	1190002F15Rik	PROMOTER	1.710	3.687	1160.62	4279.02	6.305	851.08	5366.07
A_68_P23753715	chr5:65361156-65361200	NM_026667:-135	Fam114a1	DIVERGENT_PROMOTER	1.710	4.115	2530.52	10412.20	7.037	2134.05	15016.51
A_68_P25096865	chr7:53356200-53356244	NM_001204411:-88	Kcnj11	PROMOTER	1.710	7.334	10124.04	74246.41	12.539	6198.09	77718.21
A_68_P30397291	chr15:84684245-84684289	NM_001081166:2293	Phf21b	INSIDE	1.710	4.195	3058.48	12831.61	7.173	2343.68	16811.35
A_68_P23531274	chr5:21850512-21850556	NM_011261:-11	Reln	PROMOTER	1.710	5.008	3601.55	18035.54	8.563	3038.50	26017.57
A_68_P25102011	chr7:54175094-54175138	NM_021884:184	Tsg101	INSIDE	1.710	3.474	1126.98	3915.36	5.942	917.98	5454.39
A_68_P23317755	chr4:133024124-133024168			Unknown	1.710	2.913	1711.16	4983.75	4.982	1437.64	7161.96
A_68_P31157497	chr17:35226598-35226642	NM_178592:385	Abhd16a	INSIDE	1.709	2.371	2002.65	4748.68	4.052	1436.97	5822.04
A_68_P22415887	chr3:108830521-108830565	NM_001163567:-17	Fam102b	PROMOTER	1.709	30.067	421.02	12658.99	51.391	305.92	15721.46
A_68_P21078285	chr2:26236006-26236050	NM_172339:145	Snapc4	INSIDE	1.709	3.792	4307.71	16333.25	6.478	3441.90	22297.41
A_68_P28974164	chr13:40824040-40824084	NM_001122948:1750	Tcfap2a	INSIDE	1.709	4.053	3801.80	15408.83	6.928	3002.30	20798.76
A_68_P21741665	chr2:150730549-150730593	NM_024465:-103	Abhd12	DIVERGENT_PROMOTER	1.708	3.632	2876.84	10449.71	6.204	2289.56	14203.41
A_68_P29619756	chr14:56381232-56381276	NM_020490:990	Ltb4r2	INSIDE	1.708	1.671	743.80	1242.88	2.855	716.65	2045.84
A_68_P26079854	chr8:97381360-97381404	NM_009090:33	Polr2c	INSIDE	1.708	8.059	1623.42	13082.40	13.768	1177.99	16217.94
A_68_P23317106	chr4:132895104-132895148	NM_199306:104	Wdtc1	INSIDE	1.708	4.498	11435.70	51436.61	7.681	8078.16	62051.66
A_68_P21419479	chr2:90729985-90730029	NM_026161:4064	C1qtnf4	INSIDE	1.707	11.448	561.15	6424.04	19.545	476.10	9305.58
A_68_P24864235	chr6:134870715-134870759	NM_009875:318	Cdkn1b	INSIDE	1.707	1.405	1767.54	2483.14	2.397	1419.21	3402.40
A_68_P27173119	chr10:59681994-59682038	NM_016803:-8	Chst3	PROMOTER	1.707	1.891	3067.53	5801.21	3.228	2489.26	8036.45
A_68_P23280751	chr4:126234084-126234128	NM_175554:-117	Clspn	PROMOTER	1.707	5.348	1230.34	6580.06	9.127	1117.06	10195.26
A_68_P30672037	chr16:35769289-35769333	NM_153550:132	Dirc2	INSIDE	1.707	3.802	1819.91	6918.94	6.491	1445.82	9384.94
A_68_P22271218	chr3:80717646-80717690	NM_010298:-122	Glrb	PROMOTER	1.707	3.883	2691.97	10453.05	6.627	2172.05	14394.29
A_68_P25023658	chr7:29594290-29594344	NM_177301:-1592	Hnrnpl	PROMOTER	1.707	4.797	1710.77	8206.30	8.186	1221.68	10000.86
A_68_P24171753	chr5:147888406-147888450	NM_080795:-280	Lnx2	DIVERGENT_PROMOTER	1.707	3.199	8652.25	27677.32	5.461	6559.61	35820.20
A_68_P23609096	A_68_P23609096			Unknown	1.707	2.868	253.04	725.71	4.895	223.66	1094.77
A_68_P31376853	chr17:79753911-79753955	NM_026514:499	Cdc42ep3	INSIDE	1.706	1.929	1057.53	2040.31	3.292	870.61	2866.13
A_68_P24980375	chr7:16807208-16807254	NM_027883:151	Dhx34	INSIDE	1.706	2.067	963.48	1991.87	3.527	747.64	2637.04
A_68_P27551374	chr11:3232269-3232313	NM_178149:1557	Pik3ip1	INSIDE	1.706	2.224	832.12	1850.51	3.793	649.12	2462.26
A_68_P27968802	chr11:82684465-82684509	NM_001164570:-240	Rffl	PROMOTER	1.706	5.404	105512.20	570240.10	9.220	70913.33	653851.00

ProbeName	Target position of probe on CpG island microarray	TargetID	GeneSymbol	CpG island Description	Ratio of relative methylation (TiO_2-NP/Vehicle)	Sham group			TiO_2-H group		
						Relative methylation (Cy5/Cy3)	Cy3 signal (Input)	Cy5 signal (MeDIP)	Relative methylation (Cy5/Cy3)	Cy3 signal (Input)	Cy5 signal (MeDIP)
A_68_P31655789	chr18:42671379-42671423	NM_001039474:260	Tcerg1	INSIDE	1.706	7.326	13803.96	101132.70	12.500	9150.65	114384.80
A_68_P30476395	chr15:98606917-98606961	NM_011718:1643	Wnt10b	INSIDE	1.706	9.467	3105.78	29401.01	16.152	2449.64	39567.60
A_68_P23189066	chr4:106851077-106851121	NM_025617:-127	2210012G02Rik	DIVERGENT_PROMOTER	1.705	3.272	1742.72	5701.96	5.579	1469.17	8196.62
A_68_P24158559	chr5:144670369-144670413	NM_001172146:318	Aimp2	INSIDE	1.705	4.816	2415.13	11630.91	8.213	2055.23	16879.15
A_68_P23612479	chr5:37633037-37633081	NM_001136058:-260	Crmp1	PROMOTER	1.705	4.128	1558.86	6434.45	7.038	1230.62	8661.63
A_68_P28185591	chr11:120470102-120470146	NM_153288:334	Npb	INSIDE	1.705	2.681	3291.21	8822.68	4.571	2345.49	10720.74
A_68_P25091297	chr7:52384491-52384535	NM_009438:-407	Rpl13a	PROMOTER	1.705	6.002	1607.98	9650.27	10.230	1214.67	12426.62
A_68_P29455867	chr14:22804205-22804249	NM_145459:4597	Zfp503	INSIDE	1.705	2.108	6999.60	14757.56	3.594	5362.43	19273.32
A_68_P27835628	chr11:59041341-59041385	NM_001130408:302	Arf1	INSIDE	1.704	4.375	592.46	2591.78	7.456	507.66	3785.08
A_68_P20834309	chr1:174562662-174562706	NM_026725:421	Dusp23	INSIDE	1.704	4.278	19244.57	82328.57	7.290	13306.05	97004.34
A_68_P21826983	chr2:165818573-165818617	NM_008679:458	Ncoa3	INSIDE	1.704	7.206	5906.37	42563.88	12.282	4278.32	52547.31
A_68_P27898381	chr11:69809079-69809123	NM_030064:-172	Phf23	PROMOTER	1.704	1.792	4096.91	7341.58	3.054	2920.50	8919.54
A_68_P22388656	chr3:103718191-103718235	NM_172684:170	Rsbn1	INSIDE	1.704	3.997	845.40	3378.85	6.812	691.87	4712.77
A_68_P30489270	chr15:100877044-100877088	NM_011323:110365	Scn8a	DOWNSTREAM	1.704	1.428	2703.94	3861.00	2.433	2254.44	5485.81
A_68_P23447092	chr5:3802834-3802878	NM_001003909:253	Ankib1	INSIDE	1.703	3.584	749.53	2686.40	6.105	672.66	4106.67
A_68_P24423143	chr6:47403815-47403859	NM_012042:-486	Cul1	PROMOTER	1.703	5.922	1174.27	6954.62	10.083	914.15	9217.76
A_68_P21489528	chr2:104334070-104334114	NM_001145824:554	Hipk3	INSIDE	1.703	1.668	1201.67	2004.36	2.840	1086.32	3085.18
A_68_P30127809	chr15:34236397-34236441	NM_016762:-17	Matn2	PROMOTER	1.703	2.565	3731.02	9571.39	4.369	2799.86	12232.50
A_68_P31120336	chr17:28217206-28217250	NM_013687:301	Tcp11	INSIDE	1.703	8.975	36936.59	331494.10	15.286	22975.57	351212.70
A_68_P25360010	chr7:106289656-106289700	NM_178635:-24	Uvrag	PROMOTER	1.703	2.507	1624.33	4071.82	4.270	1259.73	5378.94
A_68_P27338392	chr10:90291975-90292019	NM_001177396:252561	Anks1b	INSIDE	1.702	6.325	1696.69	10730.82	10.766	1448.83	15598.30
A_68_P26014555	chr8:85856129-85856173	NM_001170691:235	Elmod2	INSIDE	1.702	4.294	2829.27	12149.53	7.307	2308.53	16868.67
A_68_P21257003	chr2:60047554-60047598	NM_020575:-416	Marchf7	PROMOTER	1.702	2.127	1773.27	3772.14	3.621	1423.23	5153.13
A_68_P27794924	chr11:51433455-51433500	NM_026631:203	Nhp2	INSIDE	1.702	3.715	4250.56	15790.13	6.324	3209.74	20297.16
A_68_P28873011	chr13:21560407-21560451	NM_025719:-58	Nkapl	PROMOTER	1.702	5.623	2171.94	12211.78	9.572	1787.51	17109.48
A_68_P25029775	chr7:30857776-30857820	NM_177889:44	Zfp82	INSIDE	1.702	2.524	1477.15	3728.99	4.296	1209.50	5196.36
A_68_P32846303	chr1:78485277-78485321	NM_011811:123	Farsb	INSIDE	1.701	2.498	580.73	1450.60	4.249	463.22	1968.35
A_68_P30849196	chr16:70314241-70314285	NM_028803:-69	Gbe1	PROMOTER	1.701	2.303	2485.26	5724.21	3.918	1985.75	7780.62
A_68_P22081218	chr3:40549756-40549800	NM_011020:244	Hspa4l	INSIDE	1.701	3.647	627.09	2287.30	6.203	700.15	4342.90
A_68_P26065137	chr8:94881817-94881861	NM_018826:144	Irx5	INSIDE	1.701	3.067	1410.84	4326.52	5.216	1114.31	5812.43
A_68_P29453037	chr14:22320470-22320514	NM_017479:1486	Myst4	INSIDE	1.701	8.500	7052.63	59950.60	14.457	5362.66	77527.39
A_68_P28137828	chr11:112643707-112643751	NM_011448:205	Sox9	INSIDE	1.701	3.547	3362.01	11926.44	6.035	2633.99	15895.53
A_68_P22931104	chr4:54958261-54958305	NM_172867:-2534	Zfp462	PROMOTER	1.701	2.761	1914.64	5286.90	4.698	1621.76	7619.02
A_68_P28081267	chr11:102623133-102623177	NM_001110778:402	Adam11	INSIDE	1.700	2.010	597.97	1201.94	3.417	526.77	1799.90
A_68_P25047313	chr7:35841354-35841398	NM_009884:209	Cebpg	INSIDE	1.700	1.809	3138.06	5676.12	3.075	2564.46	7886.72
A_68_P23523151	chr5:20387300-20387344	NM_172992:620	Phtf2	INSIDE	1.700	2.310	1113.80	2573.14	3.927	1022.76	4015.91
A_68_P28061204	chr11:99091669-99091713	NM_020618:641	Smarce1	INSIDE	1.700	1.808	1456.70	2633.66	3.073	1302.10	4000.92
A_68_P22752472	chr4:15872547-15872591	NM_026172:86	Decr1	INSIDE	1.699	5.267	2508.55	13212.65	8.947	2094.87	18743.45
A_68_P24218861	chr6:6829299-6829343	NM_010056:2748	Dlx5	INSIDE	1.699	2.436	1752.76	4269.76	4.138	1353.69	5601.47
A_68_P27697249	chr11:33743483-33743527	NM_001190885:81	Kcnip1	INSIDE	1.699	5.080	5765.99	29288.62	8.631	4620.64	39880.89
A_68_P27449326	chr10:110943860-110943904	NM_009344:541	Phlda1	INSIDE	1.699	4.686	709.78	3326.28	7.963	558.04	4443.61
A_68_P31934221	chr19:6363781-6363825	NM_001110791:113	Sf1	INSIDE	1.699	2.366	508.77	1203.74	4.020	393.59	1582.14
A_68_P25588514	chr7:148577796-148577840	NM_011528:-240	Taldo1	PROMOTER	1.699	1.899	1080.94	2052.65	3.227	874.05	2820.31
A_68_P20457301	chr1:95651221-95651265	NM_025920:173	Thap4	INSIDE	1.699	10.560	1017.56	10745.42	17.936	928.45	16652.85
A_68_P21826276	chr2:165671382-165671427	NM_027230:38784	Zmynd8	INSIDE	1.699	7.060	678.38	4789.61	11.993	547.10	6561.33
A_68_P32364063	chrX:45698568-45698612	NM_178782:4056	Bcorl1	INSIDE	1.698	3.139	2990.20	9385.67	5.330	1496.39	7975.42
A_68_P25954148	chr8:73321826-73321870	NM_199308:-5516	Mast3	PROMOTER	1.698	3.752	5167.04	19388.07	6.371	3777.88	24067.35
A_68_P32227000	chr19:60832730-60832774	NM_178421:863	Nanos1	INSIDE	1.698	1.374	2438.63	3350.63	2.333	1862.02	4344.11
A_68_P32766772	chrX:153930279-153930323	NM_009214:-322	Sms	PROMOTER	1.698	4.058	1323.76	5372.22	6.890	777.52	5357.23
A_68_P32552603	chrX:96133526-96133570	NM_207633:429	Yipf6	INSIDE	1.698	2.647	821.91	2175.68	4.496	459.10	2064.03
A_68_P25657178	chr8:11728695-11728739	NM_001113517:612	Arhgef7	INSIDE	1.697	4.997	1742.71	8709.00	8.478	1385.60	11747.66

ProbeName	Target position of probe on CpG island microarray	TargetID	GeneSymbol	CpG island Description	Ratio of relative methylation (TiO_2-NP/Vehicle)	Sham group			TiO_2-H group		
						Relative methylation (Cy5/Cy3)	Cy3 signal (Input)	Cy5 signal (MeDIP)	Relative methylation (Cy5/Cy3)	Cy3 signal (Input)	Cy5 signal (MeDIP)
A_68_P21516743	chr2:109533914-109533958	NM_001048142:217	Bdnf	INSIDE	1.697	3.450	2201.21	7593.39	5.853	1533.04	8973.33
A_68_P30363892	chr15:78858298-78858342	NM_008197:-321	H1f0	PROMOTER	1.697	1.440	972.76	1400.49	2.443	844.95	2064.48
A_68_P21417961	chr2:90420785-90420829	NM_008982:-2	Ptprj	PROMOTER	1.697	5.256	745.42	3917.71	8.919	603.84	5385.76
A_68_P23405733	chr4:149745839-149745883	NM_001085492:89836	Rere	INSIDE	1.697	2.122	4707.06	9988.62	3.601	3562.93	12828.77
A_68_P24452215	chr6:52664021-52664065	NM_025816:320	Tax1bp1	INSIDE	1.697	6.629	2894.54	19187.84	11.248	2434.57	27383.92
A_68_P23591177	chr5:34124884-34124928	NM_019694:447	Letm1	INSIDE	1.696	3.111	1436.53	4468.72	5.276	1267.90	6689.45
A_68_P31926196	chr19:4989991-4990035	NM_153553:-41	Npas4	PROMOTER	1.696	2.125	311.58	662.19	3.605	264.17	952.35
A_68_P23617871	chr5:38550739-38550783	NM_010942:-54	Nsg1	PROMOTER	1.696	3.491	4284.39	14957.57	5.920	3403.27	20146.35
A_68_P21130445	chr2:34769374-34769418	NM_028716:100	Phf19	INSIDE	1.696	2.328	393.02	914.86	3.947	279.14	1101.68
A_68_P30366260	chr15:79233510-79233554	NM_172608:201	Tmem184b	INSIDE	1.696	2.289	1282.09	2934.90	3.882	1107.15	4297.46
A_68_P21105309	chr2:30657215-30657259	NM_198000:-9	1700001O22Rik	PROMOTER	1.695	3.081	983.14	3028.58	5.223	817.28	4268.44
A_68_P20153982	chr1:37486790-37486834	NM_198006:136	6330578E17Rik	INSIDE	1.695	2.028	3468.44	7034.36	3.437	2727.80	9375.07
A_68_P31950789	chr19:10531506-10531550	NM_001111140:409	Lrrc10b	INSIDE	1.695	1.584	591.01	936.18	2.684	514.45	1380.87
A_68_P21777851	chr2:157386103-157386147	NM_010923:279	Nnat	INSIDE	1.695	4.656	5260.47	24494.02	7.893	3548.90	28010.42
A_68_P23696525	chr5:53132855-53132899	NM_025951:64	Pi4k2b	INSIDE	1.695	5.578	1428.36	7967.54	9.456	1312.72	12413.29
A_68_P30589177	chr16:20718065-20718109	NM_145632:188	Polr2h	INSIDE	1.695	2.321	1698.55	3942.10	3.934	1393.67	5482.07
A_68_P23321241	chr4:133684793-133684837	NM_080559:-146	Sh3bgrl3	PROMOTER	1.695	3.600	2045.37	7362.38	6.101	1544.79	9425.19
A_68_P24118628	chr5:136410394-136410438	NM_018871:95	Ywhag	INSIDE	1.695	1.624	1284.33	2086.11	2.752	987.55	2718.20
A_68_P24513202	chr6:65621890-65621934	NM_172399:308	A930038C07Rik	INSIDE	1.694	3.364	496.39	1669.62	5.697	464.53	2646.23
A_68_P28731662	chr12:110290760-110290805	NM_001163175:15645	Begain	INSIDE	1.694	2.853	1011.07	2884.77	4.833	847.23	4094.86
A_68_P27278225	chr10:79144473-79144517	NM_177613:-445	Cdc34	PROMOTER	1.694	2.650	4981.62	13200.69	4.488	3951.36	17732.59
A_68_P28457807	chr12:57713035-57713080	NM_008701:1214	Nkx2-9	INSIDE	1.694	3.538	580.12	2052.72	5.995	556.78	3338.12
A_68_P24066931	chr5:125922400-125922444	NM_029752:485	Bri3bp	INSIDE	1.693	1.474	678.49	1000.22	2.495	592.40	1478.08
A_68_P23892217	chr5:92512328-92512372	NM_001080797:-407	G3bp2	PROMOTER	1.693	3.722	2491.50	9273.63	6.300	1946.07	12259.70
A_68_P21834182	chr2:167015184-167015228	NM_008420:-907	Kcnb1	PROMOTER	1.693	5.632	3446.00	19408.40	9.534	2638.55	25156.65
A_68_P30292506	chr15:66409382-66409426	NM_001081409:271	Phf20l1	INSIDE	1.693	2.130	765.44	1630.54	3.606	674.77	2432.95
A_68_P31087861	chr17:22058135-22058179	NM_001199048:41275	Zfp942	DOWNSTREAM	1.693	6.043	8521.82	51496.41	10.228	6647.56	67992.19
A_68_P28513315	chr12:70299066-70299110	NM_027269:328	1110034A24Rik	INSIDE	1.692	2.946	4125.79	12154.63	4.984	3535.00	17617.39
A_68_P30338802	chr15:74503171-74503215	NM_018790:-192	Arc	PROMOTER	1.692	3.131	5011.73	15690.22	5.298	3499.47	18539.99
A_68_P29040998	chr13:53024981-53025025	NM_016709:44	Auh	INSIDE	1.692	3.051	17814.71	54352.38	5.163	14317.06	73919.36
A_68_P28059222	chr11:98768831-98768880	NM_001025779:-347	Cdc6	PROMOTER	1.692	7.625	1315.70	10031.63	12.899	995.61	12841.84
A_68_P28153287	chr11:115264936-115264980	NM_026729:-121	Ict1	PROMOTER	1.692	2.679	1149.83	3080.74	4.535	929.72	4216.00
A_68_P23394435	chr4:147822699-147822743	NM_020009:30	Mtor	INSIDE	1.692	5.778	2393.80	13830.38	9.777	1997.31	19527.03
A_68_P30018904	chr15:11836154-11836198	NM_001039181:-747	Npr3	PROMOTER	1.692	2.096	1637.38	3432.46	3.547	1416.00	5023.16
A_68_P26165552	chr8:113443992-113444036	NM_009179:250	St3gal2	INSIDE	1.692	4.347	2103.08	9142.78	7.357	1692.26	12450.05
A_68_P28595535	chr12:85292140-85292184	NM_023633:605	2410016O06Rik	INSIDE	1.691	3.618	1248.07	4515.38	6.118	1021.70	6251.02
A_68_P24371747	chr6:37249971-37250015	NM_001081206:-16	Dgki	PROMOTER	1.691	3.057	2279.23	6968.62	5.169	1966.19	10162.98
A_68_P31253727	chr17:56219020-56219064	NM_008233:-37	Hdgfrp2	DIVERGENT_PROMOTER	1.691	2.480	1095.39	2716.84	4.194	842.04	3531.18
A_68_P25088095	chr7:51804301-51804346	NM_011131:-138	Pold1	PROMOTER	1.691	2.746	3343.13	9180.62	4.645	2543.73	11815.50
A_68_P28768143	chr12:117724447-117724491	NM_011215:276	Ptprn2	INSIDE	1.691	7.523	3494.12	26285.46	12.721	2807.79	35716.92
A_68_P27542729	chr10:128133023-128133067	NM_177411:280	Rab5b	INSIDE	1.691	2.472	2685.10	6638.46	4.180	2268.41	9481.84
A_68_P23407530	chr4:150026271-150026315	NM_173774:-9	Slc45a1	PROMOTER	1.691	6.326	669.12	4233.06	10.699	664.88	7113.70
A_68_P24759109	chr6:113327446-113327490	NM_133932:46	Tada3	INSIDE	1.691	3.894	1254.60	4885.71	6.584	1005.79	6621.83
A_68_P25798746	chr8:40068990-40069034	NM_030254:92	Tusc3	INSIDE	1.691	3.748	1215.15	4554.28	6.337	912.00	5779.74
A_68_P25589538	chr7:148725824-148725868	NM_001142681:-90	Chid1	PROMOTER	1.690	2.157	1913.62	4128.24	3.645	1568.84	5718.28
A_68_P23976347	chr5:109986035-109986079	NM_001164735:1956	Crlf2	INSIDE	1.690	6.297	6501.58	40937.80	10.638	4205.31	44737.50
A_68_P23590170	chr5:33972447-33972492	NM_178390:-185	Fam53a	PROMOTER	1.690	4.543	10365.60	47091.67	7.678	7189.64	55203.47
A_68_P30497696	chr15:102236237-102236289	NM_013672:-484	Sp1	PROMOTER	1.690	2.365	620.64	1467.90	3.996	432.04	1726.52
A_68_P20162793	chr1:38954859-38954904	NM_145142:124	Chst10	INSIDE	1.689	7.615	3082.84	23475.55	12.859	2404.60	30921.32
A_68_P29055348	chr13:55589381-55589425	NM_001177371:35	Dbn1	INSIDE	1.689	2.635	18468.36	48663.72	4.451	14895.89	66305.82
A_68_P21107218	chr2:30997212-30997256	NM_001038700:294	Fnbp1	INSIDE	1.689	2.193	906.60	1988.37	3.704	750.28	2778.80

ProbeName	Target position of probe on CpG island microarray	TargetID	GeneSymbol	CpG island Description	Ratio of relative methylation (TiO_2-NP/Vehicle)	Sham group			TiO_2-H group		
						Relative methylation (Cy5/Cy3)	Cy3 signal (Input)	Cy5 signal (MeDIP)	Relative methylation (Cy5/Cy3)	Cy3 signal (Input)	Cy5 signal (MeDIP)
A_68_P26077217	chr8:96910495-96910539	NM_022331:117	Herpud1	INSIDE	1.689	5.907	2741.31	16193.24	9.979	2088.27	20837.91
A_68_P29604760	chr14:52723420-52723464	NM_001170981:261	Hnrnpc	INSIDE	1.689	2.918	2786.63	8130.06	4.928	2129.02	10490.78
A_68_P26258415	chr8:129116306-129116350	NM_001164598:1008	Irf2bp2	INSIDE	1.689	2.066	5891.25	12169.15	3.488	4892.94	17068.30
A_68_P28049252	chr11:97049849-97049893	NM_008379:-664	Kpnb1	PROMOTER	1.689	2.834	861.80	2442.52	4.787	836.84	4005.87
A_68_P24027251	chr5:119010546-119010590	NM_172424:-159	Med13l	PROMOTER	1.689	4.857	1353.13	6571.65	8.204	1178.58	9668.76
A_68_P27841048	chr11:59954437-59954481	NM_001037764:875	Rai1	INSIDE	1.689	8.399	414.52	3481.42	14.186	380.45	5397.20
A_68_P21911518	chr2:181326975-181327019	NM_001164597:170	Znf512b	INSIDE	1.689	8.697	1229.90	10697.01	14.691	829.14	12180.90
A_68_P25108786	chr7:56137106-56137150	NM_001013368:-717	E2f8	PROMOTER	1.688	4.136	2662.40	11010.37	6.982	2311.18	16136.32
A_68_P25951936	chr8:72976257-72976301	NM_001122830:24564	Klhl26	INSIDE	1.688	2.504	251.71	630.21	4.226	258.31	1091.58
A_68_P27922756	chr11:74651953-74651997	NM_010813:7549	Mnt	INSIDE	1.688	2.764	2511.91	6941.73	4.665	2144.68	10004.52
A_68_P24592748	chr6:83267406-83267450	NM_008638:170	Mthfd2	INSIDE	1.688	1.747	2418.50	4225.33	2.950	1884.64	5558.99
A_68_P31831023	chr18:74602569-74602613	NM_201600:318	Myo5b	INSIDE	1.688	3.289	454.80	1495.78	5.552	442.03	2454.05
A_68_P27503014	chr10:120787968-120788012	ENSMUST00000078515:7663		DOWNSTREAM	1.688	3.951	4977.57	19668.80	6.670	3764.39	25109.76
A_68_P23408691	chr4:150229048-150229092	NM_133753:-129	Errfi1	PROMOTER	1.687	3.514	1955.79	6873.54	5.929	1754.32	10401.00
A_68_P20897125	chr1:186551788-186551832	NM_008250:4562	Hlx	INSIDE	1.687	2.130	1138.59	2425.27	3.593	783.07	2813.61
A_68_P30495553	chr15:101858609-101858653	NM_010664:-16	Krt18	PROMOTER	1.687	2.231	1396.41	3114.85	3.763	1205.16	4535.54
A_68_P24623660	chr6:88674554-88674598	NM_001166250:-121	Mgll	PROMOTER	1.687	7.609	2563.93	19509.59	12.836	2246.95	28842.34
A_68_P32579464	chrX:102347631-102347675	AK136781:-2542		PROMOTER	1.687	3.600	31139.15	112101.40	6.072	23137.23	140486.60
A_68_P27085191	chr10:40402970-40403014	NM_177793:-95	9030224M15Rik	PROMOTER	1.686	2.249	1059.70	2383.52	3.792	947.30	3592.40
A_68_P28079629	chr11:102308671-102308715	NM_199200:285	Fam171a2	INSIDE	1.686	1.895	1504.43	2851.23	3.195	1335.07	4265.03
A_68_P31937108	chr19:7054737-7054781	NM_008020:172	Fkbp2	INSIDE	1.686	3.435	4409.51	15146.83	5.790	3177.15	18395.36
A_68_P31938321	chr19:7239801-7239845	NM_134147:108565	Macrod1	INSIDE	1.686	7.484	804.27	6019.42	12.615	664.54	8383.14
A_68_P27931783	chr11:76212254-76212298	NM_008750:367	Nxn	INSIDE	1.686	2.497	957.35	2390.08	4.210	767.91	3232.83
A_68_P31065956	chr17:15635037-15635081	NM_011185:182	Psmb1	INSIDE	1.686	3.282	3320.49	10897.30	5.535	2703.66	14963.44
A_68_P22063513	chr3:37319180-37319224	NM_001163511:1	Spata5	INSIDE	1.686	1.914	702.42	1344.19	3.226	643.62	2076.33
A_68_P28601528	chr12:86337027-86337071	NM_178363:-222	Ylpm1	PROMOTER	1.686	2.102	574.60	1208.09	3.545	463.35	1642.77
A_68_P25949121	chr8:72498323-72498367	NM_001113346:-62	Gatad2a	PROMOTER	1.685	5.348	4294.38	22967.53	9.012	3393.33	30579.31
A_68_P22378540	chr3:101815556-101815600	NM_178777:1511	Nhlh2	INSIDE	1.685	2.047	1560.93	3195.92	3.450	1276.07	4402.83
A_68_P27835010	chr11:58947926-58947970	NM_001171512:1929	Obscn	INSIDE	1.685	5.288	2672.68	14132.77	8.908	1825.71	16262.78
A_68_P30486488	chr15:100408907-100408951	NM_010127:7868	Pou6f1	INSIDE	1.685	3.988	537.02	2141.41	6.718	406.10	2728.33
A_68_P23599458	chr5:35621995-35622039	NM_007418:802	Adra2c	INSIDE	1.684	1.754	1240.19	2175.88	2.955	1041.02	3076.60
A_68_P26022557	chr8:87427077-87427121	NM_010635:1272	Klf1	INSIDE	1.684	1.831	1470.19	2692.40	3.084	1256.92	3876.80
A_68_P20029518	chr1:10983714-10983758	NM_029525:191	Prex2	INSIDE	1.684	7.473	2583.25	19305.26	12.589	1991.24	25067.06
A_68_P25587467	chr7:148401624-148401668	NM_025886:-112	Rassf7	DIVERGENT_PROMOTER	1.684	3.437	3882.06	13343.77	5.788	2929.80	16958.03
A_68_P26878665	chr9:120486576-120486620	NM_025974:5965	Rpl14	DOWNSTREAM	1.684	1.848	1419.03	2622.10	3.112	1187.42	3695.48
A_68_P30486834	chr15:100467426-100467470	NM_001033872:-152	Smagp	PROMOTER	1.684	1.671	1685.07	2816.45	2.815	1373.73	3866.96
A_68_P32217704	chr19:59246215-59246259	NM_009501:-1717	Vax1	PROMOTER	1.684	2.583	3160.67	8165.50	4.349	2353.74	10237.25
A_68_P25952317	chr8:73030680-73030724	NM_025577:-64	2810428I15Rik	PROMOTER	1.683	3.799	2054.35	7803.78	6.392	1708.79	10922.38
A_68_P32260277	chrX:11657663-11657707	NM_029510:-5	Bcor	PROMOTER	1.683	3.273	1448.95	4742.62	5.508	876.58	4828.15
A_68_P30655034	chr16:32877951-32877995	NM_001159349:103	Fyttd1	INSIDE	1.683	2.031	1813.46	3682.82	3.417	1446.40	4943.06
A_68_P27019249	chr10:26095302-26095346	NM_172787:-333	L3mbtl3	PROMOTER	1.683	2.585	3469.39	8967.44	4.350	2605.96	11334.74
A_68_P31938323	chr19:7240213-7240257	NM_134147:108977	Macrod1	INSIDE	1.683	3.757	3541.01	13303.16	6.324	2898.79	18332.07
A_68_P25458299	chr7:125591289-125591333	NM_138649:-4244	Syt17	PROMOTER	1.683	5.072	2430.45	12326.97	8.537	2032.42	17351.21
A_68_P31713387	A_68_P31713387			Unknown	1.683	3.053	751.00	2292.45	5.137	499.62	2566.44
A_68_P32467861	chrX:71638358-71638402	NM_025473:98	Fam3a	INSIDE	1.682	4.438	2419.47	10737.62	7.463	1406.81	10498.39
A_68_P28187039	chr11:120686317-120686365	NM_007988:-479	Fasn	PROMOTER	1.682	4.348	211.73	920.69	7.315	146.38	1070.69
A_68_P23955154	chr5:105844706-105844750	NM_001033550:-65	Lrrc8b	PROMOTER	1.682	3.890	293.53	1141.87	6.542	220.20	1440.47
A_68_P26015636	chr8:86118488-86118532	NM_027179:-120	Tecr	PROMOTER	1.682	3.147	1544.48	4861.07	5.293	1080.67	5720.53
A_68_P28157432	chr11:115970917-115970961	NM_001205081:611	Trim47	INSIDE	1.682	5.953	1398.78	8326.68	10.014	1042.65	10441.36
A_68_P22702205	chr4:5571555-5571599	NM_173426:251	Fam110b	INSIDE	1.681	6.974	1660.64	11580.84	11.726	1443.83	16930.61
A_68_P21784239	chr2:158492898-158492942	NM_001159662:50	Ppp1r16b	INSIDE	1.681	3.141	534.12	1677.59	5.279	476.36	2514.85

ProbeName	Target position of probe on CpG island microarray	TargetID	GeneSymbol	CpG island Description	Ratio of relative methylation (TiO_2-NP/Vehicle)	Sham group			TiO_2-H group		
						Relative methylation (Cy5/Cy3)	Cy3 signal (Input)	Cy5 signal (MeDIP)	Relative methylation (Cy5/Cy3)	Cy3 signal (Input)	Cy5 signal (MeDIP)
A_68_P27177053	chr10:60293340-60293384	NM_029770:967	Unc5b	INSIDE	1.681	2.249	1672.25	3760.27	3.780	1433.56	5418.84
A_68_P27907301	chr11:71564787-71564831	NM_177618:604	Wscd1	INSIDE	1.681	2.114	1199.61	2536.24	3.555	939.08	3338.21
A_68_P26600189	chr9:67480966-67481010			Unknown	1.681	1.691	3911.43	6613.30	2.842	3124.26	8879.43
A_68_P29643102	chr14:60796162-60796206	ENSMUST00000140924:192		INSIDE	1.681	1.602	871.83	1396.31	2.692	731.71	1969.76
A_68_P27855082	chr11:62461774-62461819	NM_198861:229	BC046404	INSIDE	1.680	3.524	546.37	1925.28	5.918	463.03	2740.32
A_68_P30500966	chr15:102785392-102785436	NM_001024842:458	Hoxc11	INSIDE	1.680	1.528	651.42	995.66	2.568	617.42	1585.58
A_68_P28803336	chr13:6648044-6648088	NM_019703:-96	Pfkp	PROMOTER	1.680	2.512	1278.91	3213.24	4.221	960.08	4052.59
A_68_P25375611	chr7:108970306-108970350	NM_008887:3502	Phox2a	INSIDE	1.680	2.025	711.87	1441.29	3.402	593.26	2018.49
A_68_P26787844	chr9:103663476-103663520	NM_178638:669	Tmem108	INSIDE	1.680	6.287	1990.88	12516.72	10.564	1533.51	16200.24
A_68_P22570418	chr3:138405498-138405542	NM_019571:362	Tspan5	INSIDE	1.680	4.376	1624.13	7106.38	7.351	1326.85	9753.60
A_68_P25219571	chr7:78850463-78850507			Unknown	1.680	1.438	1733.56	2493.05	2.415	1453.41	3510.59
A_68_P21109230	chr2:31315614-31315658	ENSMUST00000142448:13382		INSIDE	1.680	4.368	3026.10	13217.90	7.338	2593.91	19033.59
A_68_P24057001	chr5:124133506-124133550	NM_019765:772	Clip1	INSIDE	1.679	2.030	4945.46	10040.83	3.408	3721.90	12684.82
A_68_P30585937	chr16:20098035-20098079	NM_029436:430	Klhl24	INSIDE	1.679	2.574	1667.15	4291.61	4.323	1290.36	5578.07
A_68_P22530159	chr3:130813231-130813275	NM_010703:-136	Lef1	PROMOTER	1.679	2.180	900.09	1962.64	3.661	805.09	2947.17
A_68_P28875600	chr13:22073387-22073431	NM_001162929:346	Pom121l2	INSIDE	1.679	2.064	2503.61	5166.48	3.465	2055.02	7119.94
A_68_P23978771	chr5:110715204-110715248	NM_008993:-39	Pxmp2	DIVERGENT_PROMOTER	1.679	2.132	1477.40	3149.50	3.579	1219.87	4365.70
A_68_P31420286	chr17:87362969-87363013	NM_145491:540	Rhoq	INSIDE	1.679	3.749	3149.75	11807.75	6.296	2640.34	16622.98
A_68_P21752666	chr2:152880620-152880664	NM_001011732:23055	Xkr7	INSIDE	1.679	4.032	1917.47	7731.35	6.769	1550.20	10493.70
A_68_P32369653	chrX:47014094-47014138	ENSMUST00000121891:-20		PROMOTER	1.679	3.462	1160.79	4018.34	5.812	565.03	3283.98
A_68_P22314836	chr3:88425692-88425736	NM_001198912:399	Arhgef2	INSIDE	1.678	6.619	2870.12	18996.50	11.103	2409.48	26752.56
A_68_P26134609	chr8:108089863-108089907	NM_013477:56	Atp6v0d1	INSIDE	1.678	1.831	3683.70	6745.62	3.072	2918.43	8965.38
A_68_P26019650	chr8:86938371-86938415	NM_007578:-870	Cacna1a	PROMOTER	1.678	2.045	684.93	1400.45	3.431	614.58	2108.74
A_68_P29511369	chr14:32149798-32149842	NM_009796:-89	Capn7	PROMOTER	1.678	1.996	1279.78	2554.88	3.350	1041.19	3487.70
A_68_P27845567	chr11:60745252-60745296	NR_027800:95	Gm16516	INSIDE	1.678	3.036	937.83	2847.45	5.094	736.27	3750.27
A_68_P31425152	chr17:88197017-88197061	NM_199251:296	Kcnk12	INSIDE	1.678	3.655	1476.38	5396.14	6.132	1317.96	8082.18
A_68_P20634033	chr1:136457792-136457836	NM_152895:1060	Kdm5b	INSIDE	1.678	3.188	3541.18	11290.03	5.350	2979.49	15940.59
A_68_P20453282	chr1:94998673-94998717	NM_001110315:-252	Kif1a	PROMOTER	1.678	2.859	3722.33	10641.12	4.798	2631.13	12622.95
A_68_P27629548	chr11:19921114-19921161	NM_033523:96693	Spred2	INSIDE	1.678	2.842	623.29	1771.16	4.768	471.80	2249.64
A_68_P26974633	chr10:18038772-18038816			Unknown	1.678	3.825	2132.84	8158.55	6.421	1708.52	10969.63
A_68_P23154895	chr4:101091776-101091820	NM_001177602:-95	Ak4	PROMOTER	1.677	3.409	2375.71	8097.86	5.716	1900.31	10862.63
A_68_P23881743	chr5:90652818-90652862	NM_001033310:181	Cox18	INSIDE	1.677	4.340	544.98	2365.02	7.278	501.88	3652.77
A_68_P32119294	chr19:42222686-42222730	NM_183195:830	Marveld1	INSIDE	1.677	1.867	2718.81	5074.92	3.130	2118.62	6630.74
A_68_P25957541	chr8:74093810-74093856	NM_011977:1007	Slc27a1	INSIDE	1.677	1.544	2369.95	3658.60	2.589	1777.86	4603.28
A_68_P28447129	chr12:55414382-55414426			Unknown	1.677	5.392	728.27	3926.73	9.043	644.52	5828.41
A_68_P28576811	chr12:81744792-81744836	NM_007951:34	Erh	INSIDE	1.676	3.329	3732.87	12426.91	5.578	2902.64	16192.26
A_68_P28728718	chr12:109792810-109792854	NM_001163394:-97	Evl	PROMOTER	1.676	3.664	11008.12	40332.18	6.141	7496.69	46040.40
A_68_P23339951	chr4:137024980-137025024	NM_008305:285	Hspg2	INSIDE	1.676	1.544	880.05	1359.16	2.588	737.14	1907.69
A_68_P29453036	chr14:22320298-22320342	NM_017479:1314	Myst4	INSIDE	1.676	3.472	1367.44	4747.66	5.820	1163.03	6768.46
A_68_P25578920	chr7:146770604-146770648	NR_027857:-1930	Nkx6-2	PROMOTER	1.676	13.760	1343.65	18488.62	23.064	1050.68	24232.72
A_68_P27362366	chr10:94407709-94407753	NM_018797:-518	Plxnc1	PROMOTER	1.676	12.375	270.07	3342.19	20.744	254.69	5283.15
A_68_P21110386	chr2:31495757-31495801	NM_001123362:222	Prdm12	INSIDE	1.676	3.384	3165.24	10710.31	5.671	2441.02	13843.20
A_68_P31054932	chr17:13200778-13200822	NM_013671:96	Sod2	INSIDE	1.676	3.293	1214.17	3997.88	5.520	988.75	5457.47
A_68_P23982396	chr5:111309011-111309055	NM_024477:211	Ttc28	INSIDE	1.676	4.893	6002.93	29374.08	8.200	4758.82	39022.67
A_68_P25023459	A_68_P25023459			Unknown	1.676	3.074	1800.55	5535.34	5.153	1467.75	7563.92
A_68_P26538539	chr9:56644557-56644601	AK155716:4490		DOWNSTREAM	1.676	2.512	4014.41	10083.20	4.210	3008.83	12668.33
A_68_P22342541	chr3:94965423-94965467	NM_011351:-2851	Sema6c	PROMOTER	1.675	3.265	2048.94	6690.65	5.471	1637.88	8960.23
A_68_P30574556	chr16:17576756-17576800	NM_144852:-14	Slc7a4	PROMOTER	1.675	2.076	1704.75	3539.73	3.478	1349.50	4693.46
A_68_P24880087	chr6:137683557-137683601	NM_011499:-24	Strap	PROMOTER	1.675	4.583	993.38	4552.59	7.675	898.68	6897.24
A_68_P22475542	chr3:120966000-120966044	NM_178936:212	Tmem56	INSIDE	1.675	2.106	842.69	1774.56	3.527	788.56	2781.43
A_68_P32328720	chrX:35988386-35988430	NM_021550:-88	C1galt1c1	PROMOTER	1.674	1.782	1695.13	3021.24	2.983	981.43	2927.47

ProbeName	Target position of probe on CpG island microarray	TargetID	GeneSymbol	CpG island Description	Ratio of relative methylation (TiO_2-NP/Vehicle)	Sham group			TiO_2-H group		
						Relative methylation (Cy5/Cy3)	Cy3 signal (Input)	Cy5 signal (MeDIP)	Relative methylation (Cy5/Cy3)	Cy3 signal (Input)	Cy5 signal (MeDIP)
A_68_P23362371	chr4:140695718-140695762	NM_001025608:-29	D4Ertd22e	DIVERGENT_PROMOTER	1.674	1.754	970.90	1703.27	2.937	788.18	2314.72
A_68_P22081210	chr3:40548895-40548939	NM_011020:-618	Hspa4l	PROMOTER	1.674	3.368	1544.20	5200.55	5.638	1142.24	6439.66
A_68_P25088126	chr7:51807778-51807822	NM_009473:1493	Nr1h2	INSIDE	1.674	12.326	8310.29	102431.80	20.637	5463.46	112750.90
A_68_P23318161	chr4:133101757-133101801	NM_010948:164	Nudc	INSIDE	1.674	6.262	3142.55	19678.21	10.484	2430.84	25484.19
A_68_P24039106	chr5:120953388-120953432	NM_023625:222	Plbd2	INSIDE	1.674	3.995	959.96	3834.81	6.686	724.31	4842.55
A_68_P24864240	chr6:134871160-134871204	NM_009875:764	Cdkn1b	INSIDE	1.673	2.662	3361.10	8948.92	4.453	2603.18	11592.14
A_68_P28947125	chr13:36209443-36209487	NM_001039189:185	Fars2	INSIDE	1.673	3.957	2648.53	10479.59	6.621	2399.06	15884.25
A_68_P30884140	chr16:76372608-76372652	NM_173440:664	Nrip1	INSIDE	1.673	1.856	339.43	630.12	3.106	264.27	820.80
A_68_P31918148	chr19:3575782-3575826	NM_001164159:-55	Ppp6r3	PROMOTER	1.673	1.972	7854.17	15489.25	3.299	5282.36	17424.43
A_68_P21890548	chr2:178142227-178142271	NM_177191:115	Sycp2	INSIDE	1.673	4.488	7874.68	35339.82	7.508	6281.03	47156.69
A_68_P24810492	chr6:122751807-122751851	ENSMUST00000165884:-170		PROMOTER	1.673	2.515	1274.24	3204.41	4.207	950.85	3999.78
A_68_P25592704	chr7:149246956-149247000	NM_028308:-39	2700078K21Rik	PROMOTER	1.672	3.416	1686.03	5759.23	5.712	1471.01	8402.10
A_68_P23880193	chr5:90312354-90312398	NM_001081401:-17	Adamts3	PROMOTER	1.672	1.889	2641.21	4989.44	3.159	2203.61	6960.59
A_68_P24004993	chr5:115061259-115061303	NM_001004180:43021	BC057022	INSIDE	1.672	4.702	9280.21	43633.32	7.863	6289.56	49452.98
A_68_P29571979	chr14:46277367-46277411	NM_001039106:430	Ddhd1	INSIDE	1.672	3.868	8539.33	33027.56	6.465	7021.19	45391.26
A_68_P28198379	chr12:4038471-4038515	NM_001082483:423	Efr3b	INSIDE	1.672	4.411	3726.36	16435.80	7.373	2986.38	22017.64
A_68_P25176077	chr7:71432423-71432467	NM_172742:-111	Mtmr10	PROMOTER	1.672	1.737	383.89	666.99	2.905	368.33	1070.00
A_68_P27280126	chr10:79440000-79440044	NM_001003949:7053	ORF61	INSIDE	1.672	6.151	2393.85	14723.85	10.284	1638.63	16851.02
A_68_P26038373	chr8:90485269-90485313	NM_033327:-1796	Zfp423	PROMOTER	1.672	9.737	873.67	8506.77	16.277	734.07	11948.65
A_68_P27903827	chr11:70817840-70817884	NM_178367:72	Dhx33	INSIDE	1.671	2.399	725.62	1740.90	4.009	589.10	2361.84
A_68_P28334080	chr12:32634604-32634648	NM_153198:774	Hbp1	INSIDE	1.671	4.411	447.47	1973.84	7.371	388.89	2866.41
A_68_P24592782	chr6:83275935-83275979	NM_145571:-76	Mobkl1b	DIVERGENT_PROMOTER	1.671	2.406	1230.29	2959.67	4.021	999.48	4018.65
A_68_P33005865	A_68_P33005865			Unknown	1.671	5.393	410.63	2214.72	9.013	385.94	3478.49
A_68_P23593951	chr5:34712334-34712378	ENSMUST00000042234:-705		PROMOTER	1.671	1.855	3498.72	6491.48	3.100	2620.62	8123.74
A_68_P31341460	chr17:72953599-72953644	NM_007439:26	Alk	INSIDE	1.670	1.974	746.72	1473.91	3.296	648.62	2137.91
A_68_P24980722	chr7:16885176-16885220	NM_133234:-9733	Bbc3	PROMOTER	1.670	1.553	819.54	1272.83	2.594	910.99	2363.33
A_68_P29773624	chr14:84843322-84843371	NM_001013753:-23	Pcdh17	PROMOTER	1.670	2.058	854.99	1759.49	3.436	615.33	2114.28
A_68_P22484168	chr3:122432681-122432725	NM_153422:627	Pde5a	INSIDE	1.670	4.891	2019.15	9875.76	8.168	1558.90	12732.31
A_68_P24114960	chr5:135625731-135625775	NM_013763:172	Tbl2	INSIDE	1.670	3.290	1092.96	3595.91	5.494	907.58	4986.45
A_68_P28119631	chr11:109473059-109473103	NM_145940:-377	Wipi1	PROMOTER	1.670	3.869	13921.71	53858.78	6.460	10681.12	68996.43
A_68_P25051309	chr7:36588072-36588116	NM_177739:-86	Zfp507	PROMOTER	1.670	3.097	459.55	1423.12	5.172	360.31	1863.39
A_68_P29672919	chr14:65962555-65962599	AK028094:17409		DOWNSTREAM	1.670	3.682	6108.28	22488.65	6.147	4492.65	27616.55
A_68_P20972053	chr2:5058960-5059004	NM_028804:161	Ccdc3	INSIDE	1.669	4.180	648.95	2712.90	6.979	534.03	3726.86
A_68_P23250457	chr4:119959679-119959723	NM_194060:166	Foxo6	INSIDE	1.669	2.359	676.94	1597.15	3.937	600.87	2365.41
A_68_P32625282	chrX:117040767-117040811	NM_053114:-16	Pabpc5	PROMOTER	1.669	4.541	2248.43	10209.47	7.577	1645.15	12464.63
A_68_P28875767	chr13:22097925-22097969	NM_019429:3664	Prss16	INSIDE	1.669	3.391	1172.37	3975.96	5.660	929.50	5260.78
A_68_P23576447	chr5:31395717-31395761	NM_011773:162	Slc30a3	INSIDE	1.669	3.772	1644.43	6202.22	6.295	1250.89	7873.75
A_68_P27952852	chr11:79967507-79967551	NM_172133:-135	Adap2	PROMOTER	1.668	4.869	1002.22	4879.41	8.122	801.83	6512.36
A_68_P24167580	chr5:147072895-147072939	NM_153599:29666	Cdk8	INSIDE	1.668	2.767	354.85	982.03	4.616	296.88	1370.38
A_68_P20391718	chr1:82287606-82287650	NM_010570:386	Irs1	INSIDE	1.668	4.812	2917.59	14038.80	8.028	2754.29	22111.30
A_68_P28004910	chr11:89163525-89163569	NM_008711:327	Nog	INSIDE	1.668	1.556	2076.56	3230.68	2.595	1813.25	4705.13
A_68_P24951673	chr7:4981991-4982035	NM_001033383:10035	Zfp865	INSIDE	1.668	3.797	2167.70	8231.60	6.333	1920.71	12163.49
A_68_P33005883	A_68_P33005883			Unknown	1.668	6.752	372.39	2514.24	11.259	306.52	3451.06
A_68_P30969546	chr16:92498878-92498922	NM_172469:509	Clic6	INSIDE	1.667	6.182	3756.63	23221.76	10.303	2929.22	30179.61
A_68_P30113003	chr15:31154105-31154149	NM_146057:-13	Dap	PROMOTER	1.667	3.313	3856.43	12776.00	5.524	3116.01	17212.56
A_68_P24052812	chr5:123350466-123350510	NM_013910:-129	Kdm2b	PROMOTER	1.667	3.445	921.33	3173.67	5.744	809.91	4651.76
A_68_P28936013	chr13:34094245-34094289	NR_028309:352	Serpinb6a	INSIDE	1.667	2.063	1185.68	2445.85	3.438	1038.65	3571.02
A_68_P31412116	chr17:86087149-86087193	NM_011380:424	Six2	INSIDE	1.667	2.596	711.32	1846.23	4.328	623.31	2697.39
A_68_P29703989	chr14:71289781-71289825	NM_008115:-134	Gfra2	PROMOTER	1.666	6.103	2103.42	12836.84	10.165	1664.63	16920.80
A_68_P21077785	chr2:26171010-26171055	NM_001199146:-20	Gpsm1	PROMOTER	1.666	9.846	2502.21	24637.89	16.405	2243.11	36797.72
A_68_P32140499	chr19:45857773-45857817	NM_023799:-13	Mgea5	PROMOTER	1.666	6.837	801.80	5481.57	11.390	704.42	8023.62

ProbeName	Target position of probe on CpG island microarray	TargetID	GeneSymbol	CpG island Description	Ratio of relative methylation (TiO_2-NP/Vehicle)	Sham group			TiO_2-H group		
						Relative methylation (Cy5/Cy3)	Cy3 signal (Input)	Cy5 signal (MeDIP)	Relative methylation (Cy5/Cy3)	Cy3 signal (Input)	Cy5 signal (MeDIP)
A_68_P25050056	chr7:36369864-36369908	NM_145840:715	Rgs9bp	INSIDE	1.666	3.717	1154.63	4292.02	6.192	1035.92	6414.88
A_68_P28307270	chr12:28028339-28028383	NM_009234:-777	Sox11	PROMOTER	1.666	2.492	5072.19	12642.29	4.152	3722.72	15456.05
A_68_P27966238	chr11:82248332-82248376	NM_023438:45953	Tmem132e	INSIDE	1.666	6.280	392.39	2464.03	10.459	334.54	3499.15
A_68_P26134000	chr8:107995392-107995436	NM_026481:-92	Tppp3	PROMOTER	1.666	4.999	3639.91	18195.52	8.330	2948.59	24560.81
A_68_P28973959	chr13:40799401-40799445	ENSMUST00000069457:49		INSIDE	1.666	2.489	2892.03	7197.48	4.146	2339.63	9699.25
A_68_P33007795	chr4_random:128995-129039	NM_001033326:19215	Dhrsx	INSIDE	1.665	7.586	4871.29	36951.56	12.632	3359.62	42438.83
A_68_P22569284	chr3:138189919-138189963	NM_007917:786	Eif4e	INSIDE	1.665	4.216	1266.57	5340.30	7.018	1103.37	7743.61
A_68_P20875118	chr1:182262286-182262330	NR_003623:-1628	Gm5069	PROMOTER	1.665	2.628	2153.41	5658.70	4.375	1699.26	7433.81
A_68_P25652449	chr8:11008424-11008468	NM_001081212:-16	Irs2	PROMOTER	1.665	3.958	982.97	3891.03	6.591	988.57	6515.77
A_68_P23591267	A_68_P23591267			Unknown	1.665	4.040	288.57	1165.95	6.729	219.26	1475.35
A_68_P24636070	chr6:90938255-90938299			Unknown	1.665	1.851	336.69	623.25	3.082	268.57	827.71
A_68_P26749720	chr9:96790099-96790143	NM_153420:-279	Acpl2	PROMOTER	1.664	3.871	732.83	2837.06	6.444	609.12	3924.89
A_68_P27365819	chr10:95026630-95026674	NR_037212:-4697	Mir3058	PROMOTER	1.664	4.466	7057.96	31523.23	7.434	6155.54	45758.67
A_68_P32117861	chr19:41986212-41986256	NM_023418:-126	Pgam1	PROMOTER	1.664	2.604	2189.77	5702.93	4.335	1832.91	7945.02
A_68_P28137825	chr11:112643344-112643388	NM_011448:-157	Sox9	PROMOTER	1.664	1.701	4828.16	8213.36	2.830	3355.10	9494.71
A_68_P25042532	chr7:34953588-34953632	NM_016682:-62	Uba2	PROMOTER	1.664	13.458	665.13	8951.22	22.398	588.62	13184.03
A_68_P25005234	chr7:25292077-25292122	NM_146183:-6	Zfp428	PROMOTER	1.664	1.725	2355.04	4063.24	2.871	1775.29	5097.13
A_68_P22217916	chr3:68673757-68673801	NM_001167996:271	1110032F04Rik	INSIDE	1.663	3.292	653.04	2149.81	5.475	646.53	3539.97
A_68_P24797816	chr6:120244799-120244843	NM_198884:-243	B4galnt3	PROMOTER	1.663	4.357	3431.01	14947.66	7.245	2671.21	19353.42
A_68_P29488024	chr14:28435732-28435776	NM_177814:127	Erc2	INSIDE	1.663	4.562	1555.19	7095.31	7.589	1247.87	9469.50
A_68_P29723690	chr14:75131937-75131981	NM_016903:-193	Esd	PROMOTER	1.663	4.245	5108.14	21683.61	7.059	3639.86	25692.86
A_68_P24326454	chr6:29383341-29383385	NM_001081185:210	Flnc	INSIDE	1.663	1.445	6320.78	9136.28	2.403	4721.24	11346.29
A_68_P25174396	chr7:71082541-71082585	NM_021366:1239	Klf13	INSIDE	1.663	5.204	13884.08	72255.25	8.652	11746.07	101631.50
A_68_P30345384	chr15:75829239-75829283	NM_177922:5062	Mapk15	INSIDE	1.663	2.283	1737.25	3965.36	3.796	1347.49	5115.13
A_68_P29062977	chr13:56805003-56805048	NM_001164042:-3	Smad5	PROMOTER	1.663	6.202	2821.79	17499.50	10.311	2385.19	24592.75
A_68_P32467744	chrX:71613927-71613971	NM_145405:-82	Ubl4	PROMOTER	1.663	3.501	1792.31	6275.29	5.822	1302.37	7581.90
A_68_P24124212	chr5:137506063-137506107	NM_001039385:-80	Vgf	PROMOTER	1.663	2.955	535.21	1581.64	4.915	464.93	2285.03
A_68_P27287623	chr10:80599677-80599721	NM_010731:683	Zbtb7a	INSIDE	1.663	1.563	1306.00	2041.31	2.600	1135.28	2951.57
A_68_P21851410	chr2:169955855-169955909	NM_001033299:838	Zfp217	INSIDE	1.663	1.431	1120.11	1603.07	2.379	944.80	2248.13
A_68_P24430828	chr6:49023823-49023867	NM_029353:51	2410003K15Rik	INSIDE	1.662	2.935	2060.46	6047.62	4.877	1761.18	8589.71
A_68_P31114987	chr17:27341486-27341531	NM_146075:-125	Lemd2	PROMOTER	1.662	1.858	956.19	1776.83	3.088	785.44	2425.69
A_68_P27290094	chr10:80959100-80959144	NM_134009:-14	Ncln	PROMOTER	1.662	3.944	2154.24	8495.68	6.556	1834.97	12030.15
A_68_P27942685	chr11:78141893-78141937	NM_201406:-9	Pigs	PROMOTER	1.662	1.612	736.88	1187.49	2.678	654.41	1752.51
A_68_P31151050	chr17:34067192-34067236	NM_009059:376	Rgl2	INSIDE	1.662	1.844	5165.90	9526.14	3.065	3410.57	10453.66
A_68_P26579683	chr9:63870481-63870525	NM_008542:-636	Smad6	PROMOTER	1.662	2.949	2979.18	8784.45	4.901	2298.79	11266.06
A_68_P28048904	chr11:97000172-97000216	NM_198100:10832	Tbkbp1	INSIDE	1.662	4.015	955.93	3838.25	6.672	792.12	5284.78
A_68_P20345418	chr1:73954077-73954121	NM_027884:216923	Tns1	DOWNSTREAM	1.662	4.095	1102.23	4514.15	6.807	870.43	5925.32
A_68_P27938076	chr11:77328807-77328851	NM_026185:210	Abhd15	INSIDE	1.661	4.965	1329.88	6602.70	8.247	1178.66	9720.12
A_68_P21563665	chr2:118199048-118199092	NM_201367:85	Gpr176	INSIDE	1.661	2.525	975.61	2463.34	4.195	758.84	3183.08
A_68_P23997432	chr5:113739494-113739538	NM_172718:290	Sgsm1	INSIDE	1.661	4.145	4114.39	17054.40	6.886	3205.79	22073.60
A_68_P25377989	chr7:109416038-109416082	NM_009287:-277	Stim1	PROMOTER	1.661	5.566	1377.22	7666.18	9.247	1143.42	10572.66
A_68_P31326355	chr17:70318978-70319022	ENSMUST00000130971:240		INSIDE	1.661	1.412	1882.05	2656.86	2.345	1674.32	3925.97
A_68_P23961359	chr5:106887678-106887722	NM_001005477:-515	Barhl2	PROMOTER	1.660	2.219	607.95	1349.18	3.685	465.73	1716.21
A_68_P32364027	chrX:45694166-45694210	NM_178782:-346	Bcorl1	PROMOTER	1.660	4.598	1081.89	4974.40	7.632	723.01	5517.86
A_68_P24486168	chr6:59376435-59376479	NM_183183:-172	Gprin3	PROMOTER	1.660	2.105	1615.90	3401.38	3.494	1375.65	4807.21
A_68_P27065106	chr10:36226802-36226846	NM_001081208:212	Hs3st5	INSIDE	1.660	1.708	1289.14	2202.06	2.836	1048.96	2974.51
A_68_P30485948	chr15:100299486-100299530	NM_134093:44	Letmd1	INSIDE	1.660	2.013	1842.43	3709.41	3.343	1379.91	4612.61
A_68_P25031213	chr7:31145836-31145880	NM_175478:1933	Lrfn3	INSIDE	1.660	6.991	3361.08	23496.42	11.602	2447.42	28394.03
A_68_P25846165	chr8:49314031-49314075	NM_001145937:445992	Odz3	INSIDE	1.660	2.794	320.07	894.25	4.638	302.78	1404.39
A_68_P31420721	chr17:87424605-87424649	NM_008838:115	Pigf	INSIDE	1.660	2.008	871.61	1750.16	3.333	783.45	2610.98
A_68_P20083589	chr1:22540314-22540358	NM_001012623:272227	Rims1	INSIDE	1.660	1.572	1340.67	2107.75	2.610	1166.78	3045.41

ProbeName	Target position of probe on CpG island microarray	TargetID	GeneSymbol	CpG island Description	Ratio of relative methylation (TiO_2-NP/Vehicle)	Sham group			TiO_2-H group		
						Relative methylation (Cy5/Cy3)	Cy3 signal (Input)	Cy5 signal (MeDIP)	Relative methylation (Cy5/Cy3)	Cy3 signal (Input)	Cy5 signal (MeDIP)
A_68_P28862223	chr13:19487305-19487349	NM_024270:295	Stard3nl	INSIDE	1.660	1.728	1020.53	1763.43	2.869	858.88	2464.22
A_68_P23599469	chr5:35623311-35623355	NM_007418:2118	Adra2c	INSIDE	1.659	5.317	1510.31	8030.19	8.822	1307.97	11539.39
A_68_P20148721	chr1:36569446-36569490	NM_001039551:748	Cnnm3	INSIDE	1.659	5.939	2091.84	12424.11	9.855	1726.52	17014.04
A_68_P27285229	chr10:80217921-80217965	NM_199322:-8	Dot1l	PROMOTER	1.659	2.909	2296.28	6680.22	4.825	1829.74	8828.72
A_68_P30585939	chr16:20098297-20098341	NM_029436:692	Klhl24	INSIDE	1.659	2.204	1057.99	2331.82	3.656	989.42	3617.73
A_68_P22826133	chr4:33397493-33397537	NM_011884:229	Rngtt	INSIDE	1.659	2.930	1834.42	5374.19	4.859	1525.29	7411.58
A_68_P29648696	chr14:61791453-61791497	NM_172809:34181	Sacs	INSIDE	1.659	3.057	914.38	2795.69	5.073	777.56	3944.66
A_68_P28765899	chr12:117316302-117316346	NM_009511:129	Vipr2	INSIDE	1.659	1.819	1316.78	2395.76	3.018	1029.57	3107.40
A_68_P27995284	chr11:87452873-87452917	NM_001045527:22229	Hsf5	INSIDE	1.658	5.403	2219.32	11991.18	8.961	1829.83	16397.01
A_68_P24288408	chr6:21900000-21900044	NM_023626:408	Ing3	INSIDE	1.658	3.477	6884.72	23938.05	5.766	4668.75	26919.52
A_68_P21722727	chr2:147009567-147009611	NR_030769:-230	Nkx2-2as	PROMOTER	1.658	4.164	378.14	1574.57	6.904	306.78	2117.92
A_68_P30346619	chr15:76012289-76012333	NM_201394:13830	Plec	INSIDE	1.658	5.461	1257.05	6864.84	9.055	1034.03	9363.44
A_68_P30346599	chr15:76009927-76009971	NM_201394:16192	Plec	INSIDE	1.658	4.838	1926.06	9318.28	8.023	1489.19	11947.43
A_68_P22883653	chr4:45543529-45543573	NM_001033306:150	Shb	INSIDE	1.658	3.819	859.99	3284.17	6.333	816.57	5171.07
A_68_P22614372	chr3:146067861-146067905	NM_138744:232	Ssx2ip	INSIDE	1.658	6.185	795.13	4917.62	10.254	736.50	7552.35
A_68_P27846979	chr11:60988330-60988374	NM_001004143:209	Usp22	INSIDE	1.658	2.382	587.13	1398.64	3.949	512.90	2025.31
A_68_P23938671	chr5:102499208-102499252	NM_172882:-290	Wdfy3	PROMOTER	1.658	3.288	855.64	2813.62	5.451	689.08	3756.44
A_68_P29678557	chr14:66916156-66916200	NR_038077:-212	1700001G11Rik	PROMOTER	1.657	1.394	1033.06	1440.07	2.311	793.29	1832.91
A_68_P31108897	chr17:26337756-26337800	NM_008113:517	Arhgdig	INSIDE	1.657	6.939	5809.01	40307.93	11.500	4304.82	49505.05
A_68_P27476528	chr10:116018985-116019029	NM_001037846:-439	Cnot2	PROMOTER	1.657	3.208	4435.27	14229.05	5.315	3475.66	18474.40
A_68_P26590184	chr9:65757067-65757111	NM_173185:272	Csnk1g1	INSIDE	1.657	7.170	2904.33	20822.79	11.882	2120.45	25195.40
A_68_P22388169	chr3:103613288-103613332	NM_001025312:0	Dclre1b	INSIDE	1.657	8.165	1708.03	13946.79	13.526	1349.17	18249.39
A_68_P28976807	chr13:41315714-41315758	NM_019423:36	Elovl2	INSIDE	1.657	0.384	1063.81	408.94	0.637	991.98	632.02
A_68_P26394905	chr9:30234659-30234703	NM_028874:-233	Snx19	PROMOTER	1.657	1.766	790.61	1396.01	2.926	755.39	2210.60
A_68_P23320096	chr4:133467057-133467101	AK015234:372		INSIDE	1.657	1.740	1508.28	2624.10	2.883	1292.84	3727.63
A_68_P29102524	chr13:65361174-65361218	ENSMUST00000099425:350		INSIDE	1.657	3.466	742.09	2572.19	5.744	727.94	4181.38
A_68_P24084071	chr5:129106177-129106221	NR_015517:364	5930412G12Rik	INSIDE	1.656	2.695	2040.71	5500.05	4.464	1575.31	7032.18
A_68_P20796835	chr1:167775162-167775206	NM_001113391:56373	Cd247	INSIDE	1.656	3.749	1229.47	4609.55	6.210	1193.88	7414.07
A_68_P30478193	chr15:98924227-98924271	NM_176835:-5666	Dnajc22	DIVERGENT_PROMOTER	1.656	4.130	5541.49	22886.39	6.838	4719.82	32273.13
A_68_P28058704	chr11:98671394-98671438	NR_033551:-13	Gm12359	PROMOTER	1.656	3.705	8276.45	30660.92	6.135	6485.89	39792.04
A_68_P27840800	chr11:59918733-59918777	NM_009021:240	Rai1	INSIDE	1.656	3.280	677.65	2222.45	5.431	517.12	2808.25
A_68_P31208271	chr17:46811147-46811191	NM_144857:36	Rrp36	INSIDE	1.656	2.295	5152.14	11824.65	3.800	3977.79	15116.76
A_68_P24691294	chr6:100237868-100237912	NM_019743:-538	Rybp	PROMOTER	1.656	3.440	658.40	2264.60	5.697	567.63	3233.73
A_68_P25507562	chr7:134967999-134968043	NM_024414:25	Stx1b	INSIDE	1.656	2.692	6088.86	16388.52	4.459	4698.34	20947.61
A_68_P31982219	chr19:16855558-16855602	NM_173028:-163	Vps13a	PROMOTER	1.656	3.918	1634.12	6403.20	6.488	1341.32	8703.07
A_68_P31479231	chr18:7869684-7869728	NM_001146298:512	Wac	INSIDE	1.656	2.321	1157.71	2686.65	3.844	944.48	3630.44
A_68_P22903296	chr4:49072520-49072564	NM_178756:209	E130309F12Rik	INSIDE	1.655	1.726	2128.61	3672.93	2.856	1666.20	4758.16
A_68_P30596432	chr16:22162719-22162763	NM_183029:632	Igf2bp2	INSIDE	1.655	5.012	979.30	4907.86	8.295	862.69	7155.78
A_68_P28452657	chr12:56700590-56700634	NM_020287:709	Insm2	INSIDE	1.655	9.081	1368.34	12425.99	15.026	1009.74	15172.08
A_68_P21100497	chr2:29820983-29821027	NM_001076554:-75	Spna2	PROMOTER	1.655	1.873	8262.90	15476.18	3.100	6187.29	19181.71
A_68_P23742487	chr5:63157563-63157607	NM_178407:-168	Arap2	PROMOTER	1.654	4.428	1093.37	4841.67	7.324	873.88	6400.40
A_68_P26128337	chr8:106919324-106919368	NM_153582:250	Cmtm4	INSIDE	1.654	6.030	3389.72	20441.46	9.976	2593.04	25869.13
A_68_P22610589	chr3:145421770-145421814	NM_026993:137	Ddah1	INSIDE	1.654	3.326	2381.64	7920.16	5.501	1933.39	10634.74
A_68_P27041764	chr10:30562697-30562741	NM_013904:-129	Hey2	PROMOTER	1.654	2.910	517.85	1506.71	4.812	446.11	2146.52
A_68_P25953471	chr8:73223632-73223676	NM_010592:2017	Jund	INSIDE	1.654	4.674	1216.41	5685.67	7.731	1052.44	8136.16
A_68_P26920438	chr10:7445673-7445717	NM_011835:-103	Katna1	PROMOTER	1.654	1.716	3575.01	6133.04	2.837	2711.27	7691.58
A_68_P23334085	chr4:136025641-136025685	NM_024452:-13	Luzp1	PROMOTER	1.654	5.706	10787.59	61551.52	9.440	7883.57	74420.85
A_68_P28077571	chr11:101968496-101968540	NM_145435:572	Pyy	INSIDE	1.654	8.867	1245.68	11045.37	14.664	1014.87	14881.73
A_68_P28054168	chr11:97913868-97913912	NM_146028:886	Stac2	INSIDE	1.654	2.484	8806.47	21877.27	4.109	6437.64	26455.20
A_68_P25011349	chr7:26474463-26474507	NM_011577:2464	Tgfb1	INSIDE	1.654	4.639	1133.34	5257.87	7.675	964.32	7401.22
A_68_P20936911	chr1:193048707-193048751	ENSMUST00000139056:376		INSIDE	1.654	1.704	1637.26	2789.55	2.819	1242.74	3502.78

ProbeName	Target position of probe on CpG island microarray	TargetID	GeneSymbol	CpG island Description	Ratio of relative methylation (TiO_2-NP/Vehicle)	Sham group			TiO_2-H group		
						Relative methylation (Cy5/Cy3)	Cy3 signal (Input)	Cy5 signal (MeDIP)	Relative methylation (Cy5/Cy3)	Cy3 signal (Input)	Cy5 signal (MeDIP)
A_68_P28330749	chr12:32035950-32035994	NM_007861:330	Dld	INSIDE	1.653	4.600	1536.04	7066.43	7.607	1242.80	9453.52
A_68_P26350216	chr9:21855292-21855336	NM_010487:1153	Elavl3	INSIDE	1.653	2.648	559.24	1480.63	4.377	446.60	1954.97
A_68_P23896964	chr5:93370721-93370767	NM_001077596:43721	Shroom3	INSIDE	1.653	2.044	393.57	804.42	3.378	328.44	1109.39
A_68_P22236489	chr3:72861536-72861580	NM_198864:-693	Slitrk3	PROMOTER	1.653	2.647	8475.59	22433.80	4.376	6429.11	28135.05
A_68_P22185881	chr3:61963288-61963332			Unknown	1.653	6.500	1617.67	10514.98	10.745	1247.80	13407.95
A_68_P30387964	chr15:83197665-83197709	NR_027980:41	1700001L05Rik	INSIDE	1.652	4.523	7085.74	32046.29	7.470	5107.02	38149.22
A_68_P23342100	chr4:137351824-137351868	NM_007431:446	Alpl	INSIDE	1.652	2.796	617.01	1725.19	4.619	533.23	2463.21
A_68_P25712956	chr8:23916111-23916155	NM_001122820:-6	Ap3m2	PROMOTER	1.652	4.125	3610.71	14895.53	6.816	2789.42	19011.75
A_68_P31602497	chr18:32537058-32537102	NM_001083334:210	Bin1	INSIDE	1.652	1.753	1699.03	2977.56	2.895	1814.24	5252.63
A_68_P21748226	chr2:152053141-152053185	NM_007788:587	Csnk2a1	INSIDE	1.652	3.954	3066.05	12123.17	6.533	2172.35	14191.76
A_68_P29334871	chr13:113254322-113254366	NM_010560:67	Il6st	INSIDE	1.652	5.429	2889.77	15688.11	8.970	2431.44	21808.93
A_68_P31086236	chr17:21626256-21626300	NM_013843:327	Zfp53	INSIDE	1.652	1.868	1741.74	3253.80	3.086	1520.40	4691.44
A_68_P28743717	chr12:112615357-112615401	NM_183016:551	Cdc42bpb	INSIDE	1.651	11.373	1430.70	16271.87	18.774	1094.42	20546.89
A_68_P21425775	chr2:91815556-91815600	NM_011957:48749	Creb3l1	DOWNSTREAM	1.651	2.114	1190.64	2516.90	3.490	1000.79	3492.82
A_68_P28920307	chr13:31065636-31065680	NM_025588:258	Exoc2	INSIDE	1.651	1.876	3310.55	6212.09	3.098	2322.28	7193.72
A_68_P25678282	chr8:15027507-15027551	NM_029116:16504	Kbtbd11	INSIDE	1.651	3.122	764.09	2385.72	5.154	619.63	3193.55
A_68_P27898214	chr11:69779823-69779867	NM_016887:922	Cldn7	INSIDE	1.650	3.957	1568.10	6205.57	6.530	1333.22	8705.40
A_68_P28158619	chr11:116168104-116168148	NM_001162872:-74	Exoc7	PROMOTER	1.650	3.395	574.55	1950.42	5.602	532.83	2984.86
A_68_P30093918	chr15:27611330-27611374	NM_198301:-99	Fam105a	PROMOTER	1.650	2.064	569.45	1175.50	3.407	609.04	2074.72
A_68_P29248566	chr13:97695211-97695255	NM_001166065:589	Gcnt4	INSIDE	1.650	2.718	538.77	1464.12	4.484	429.38	1925.19
A_68_P21417960	chr2:90420716-90420760	NM_008982:66	Ptprj	INSIDE	1.650	4.856	1179.17	5726.52	8.015	976.27	7824.66
A_68_P24039805	chr5:121062634-121062678	NM_029096:-58	1110008J03Rik	DIVERGENT_PROMOTER	1.649	3.998	7528.67	30096.70	6.591	5424.59	35755.07
A_68_P22552055	chr3:135101078-135101122	NR_030689:529	4930539J05Rik	INSIDE	1.649	3.525	946.20	3335.59	5.813	843.84	4905.36
A_68_P30533736	chr16:8672646-8672705	NM_025821:-429	Carhsp1	PROMOTER	1.649	9.979	1344.51	13416.96	16.451	1050.57	17282.81
A_68_P25951144	chr8:72854849-72854893	NM_008107:1207	Gdf1	INSIDE	1.649	4.300	551.65	2372.07	7.090	441.95	3133.45
A_68_P24632571	chr6:90413720-90413764	NM_023184:1123	Klf15	INSIDE	1.649	4.076	1932.73	7877.24	6.721	1466.57	9856.49
A_68_P31150250	chr17:33855758-33855802	NM_145486:-165	Marchf2	PROMOTER	1.649	2.933	2568.68	7532.69	4.837	2197.27	10627.10
A_68_P20642551	chr1:137815771-137815815	NM_019645:-191	Pkp1	PROMOTER	1.649	2.439	2311.39	5636.91	4.021	1869.14	7515.51
A_68_P25458297	chr7:125591038-125591082	NM_138649:-3994	Syt17	PROMOTER	1.649	2.016	354.57	714.92	3.324	307.88	1023.45
A_68_P24358614	chr6:34860895-34860939	NM_001013366:-85	Wdr91	PROMOTER	1.649	1.524	6329.07	9643.26	2.512	4657.72	11699.40
A_68_P22222507	chr3:69525834-69525878	NM_133787:-120	Nmd3	PROMOTER	1.648	3.869	13203.19	51078.09	6.375	9180.51	58524.52
A_68_P25021894	chr7:29221444-29221488	NM_175021:-256	Samd4b	DIVERGENT_PROMOTER	1.648	4.085	2074.50	8475.26	6.732	1684.39	11340.10
A_68_P29514238	chr14:32643908-32643952	NM_001024604:-329	Ankrd28	PROMOTER	1.647	1.620	1147.20	1858.23	2.668	1038.20	2770.24
A_68_P25833746	chr8:47085665-47085709	NM_001039562:-482	Ankrd37	PROMOTER	1.647	2.622	3555.23	9320.60	4.318	2681.01	11575.77
A_68_P32463052	chrX:70748623-70748667	NM_177236:220	Atp2b3	INSIDE	1.647	2.172	401.11	871.38	3.578	214.89	768.83
A_68_P26659378	chr9:78243891-78243935	NM_001191044:329	Ddx43	INSIDE	1.647	3.794	1438.91	5459.07	6.249	1300.98	8129.60
A_68_P26802905	chr9:106270040-106270084	NM_153459:-900	Dusp7	PROMOTER	1.647	5.814	8987.36	52249.22	9.574	6248.62	59827.29
A_68_P23261233	chr4:122866390-122866434	NM_174998:5666	Hpcal4	INSIDE	1.647	2.404	598.81	1439.31	3.959	475.29	1881.44
A_68_P23269516	chr4:124335391-124335435	NM_011141:524	Pou3f1	INSIDE	1.647	4.999	1869.29	9344.93	8.232	1390.95	11450.48
A_68_P30250719	chr15:58720548-58720592	NM_175226:-213	Rnf139	PROMOTER	1.647	4.295	875.77	3761.37	7.074	754.23	5335.61
A_68_P29664626	chr14:64562881-64562925	NM_011446:360	Sox7	INSIDE	1.647	2.929	1685.38	4936.40	4.824	1370.78	6612.59
A_68_P23364697	chr4:141101930-141101974	NR_030695:-124	B330016D10Rik	DIVERGENT_PROMOTER	1.646	2.241	420.61	942.55	3.689	394.23	1454.27
A_68_P26543678	chr9:57612898-57612942	NM_007713:747	Clk3	INSIDE	1.646	2.204	5441.20	11992.11	3.627	4191.60	15201.53
A_68_P31862858	chr18:80389262-80389306	NM_199197:8074	Rbfa	INSIDE	1.646	1.852	375.68	695.88	3.049	324.99	990.77
A_68_P28082450	chr11:102809915-102809959	NM_011795:-2161	C1ql1	PROMOTER	1.645	1.681	2048.14	3443.75	2.766	1791.43	4955.48
A_68_P21315049	chr2:70499932-70499976	NM_027352:389	Gorasp2	INSIDE	1.645	3.840	509.53	1956.67	6.318	440.59	2783.49
A_68_P22518659	chr3:128907292-128907336	NM_001042502:-9540	Pitx2	PROMOTER	1.645	3.010	1668.95	5023.52	4.951	1335.15	6610.53
A_68_P28523569	chr12:72069499-72069543	NM_011184:-6089	Psma3	PROMOTER	1.645	2.378	192.91	458.66	3.912	164.85	644.90
A_68_P21071033	chr2:25127873-25127917	NM_023464:44	Ssna1	INSIDE	1.645	2.674	2235.25	5976.17	4.398	1902.58	8367.84
A_68_P31479228	chr18:7869362-7869406	NM_001146298:190	Wac	INSIDE	1.645	2.198	2696.63	5927.22	3.616	2065.93	7469.93
A_68_P29657349	A_68_P29657349			Unknown	1.645	3.129	1783.91	5581.20	5.148	1297.42	6679.07

ProbeName	Target position of probe on CpG island microarray	TargetID	GeneSymbol	CpG island Description	Ratio of relative methylation (TiO_2-NP/Vehicle)	Sham group			TiO_2-H group		
						Relative methylation (Cy5/Cy3)	Cy3 signal (Input)	Cy5 signal (MeDIP)	Relative methylation (Cy5/Cy3)	Cy3 signal (Input)	Cy5 signal (MeDIP)
A_68_P30997386	chr16:97392403-97392447	NM_031174:-82	Dscam	PROMOTER	1.644	1.595	480.90	766.97	2.622	482.13	1264.18
A_68_P25609686	chr7:152047482-152047526	NM_010202:214	Fgf4	INSIDE	1.644	5.163	1113.15	5747.23	8.487	946.39	8032.38
A_68_P25091451	chr7:52409634-52409678	NM_029406:-111	Pih1d1	PROMOTER	1.644	2.322	1695.68	3936.97	3.817	1344.88	5133.11
A_68_P23318413	chr4:133155649-133155693	NM_018754:2413	Sfn	DOWNSTREAM	1.644	1.623	1136.06	1843.39	2.668	943.40	2516.70
A_68_P27942762	chr11:78157060-78157104	NM_011676:59	Unc119	INSIDE	1.644	4.324	5863.68	25357.28	7.109	4592.37	32645.92
A_68_P23249523	chr4:119821508-119821552			Unknown	1.644	2.927	1297.96	3798.79	4.811	1073.75	5165.37
A_68_P26170843	chr8:114434234-114434278	NM_019950:-157	Chst5	PROMOTER	1.643	2.517	1320.48	3323.84	4.136	1058.06	4375.93
A_68_P28058844	chr11:98698263-98698307	NM_001080925:186	Rapgefl1	INSIDE	1.643	2.311	1746.35	4035.94	3.797	1653.93	6279.52
A_68_P20821183	chr1:172145120-172145164	NM_010633:382	Uhmk1	INSIDE	1.643	1.951	1817.60	3545.33	3.204	1568.10	5024.87
A_68_P24992404	chr7:19857862-19857906	NM_009499:-681	Vasp	PROMOTER	1.643	1.942	3920.49	7612.56	3.190	3319.71	10591.52
A_68_P24063275	chr5:125343770-125343814	NM_001081750:718	Zfp664	INSIDE	1.643	3.056	2191.66	6698.03	5.021	1670.90	8389.72
A_68_P32265732	chrX:12859122-12859166	NM_010028:997	Ddx3x	INSIDE	1.642	2.048	627.54	1285.32	3.363	607.86	2043.95
A_68_P23323339	chr4:134091201-134091245	NM_029759:-2555	Fam54b	PROMOTER	1.642	2.789	1422.20	3966.22	4.578	1228.16	5622.49
A_68_P31857885	chr18:79306491-79306535	NM_053099:-382	Setbp1	PROMOTER	1.642	3.094	1006.69	3114.38	5.081	841.53	4275.84
A_68_P30436288	chr15:91403683-91403727	NM_001033633:-12	Slc2a13	PROMOTER	1.642	1.669	457.19	762.82	2.740	420.44	1152.03
A_68_P27942521	chr11:78115275-78115319	NM_017407:204	Spag5	INSIDE	1.642	5.783	1791.57	10361.45	9.494	1481.68	14067.16
A_68_P25017009	chr7:28160497-28160541	NM_001199235:19007	Spnb4	INSIDE	1.642	2.209	3530.92	7801.52	3.628	3013.58	10934.32
A_68_P30378284	chr15:81353498-81353542			Unknown	1.642	1.558	675.50	1052.36	2.557	654.93	1674.93
A_68_P23323199	chr4:134066870-134066914	NM_001081099:-21	2610002D18Rik	PROMOTER	1.641	3.069	2082.85	6392.84	5.036	1674.79	8433.89
A_68_P26080249	chr8:97441876-97441920	NM_001033533:100	Ccdc102a	INSIDE	1.641	1.764	2006.98	3540.67	2.895	1603.92	4644.14
A_68_P26350213	chr9:21855003-21855047	NM_010487:1443	Elavl3	INSIDE	1.641	3.509	2235.93	7845.85	5.757	1856.32	10686.97
A_68_P29713891	chr14:73109647-73109691	NM_207636:142	Fndc3a	INSIDE	1.641	3.333	1249.16	4163.45	5.469	1056.14	5776.46
A_68_P26762516	chr9:99040770-99040814	NM_029094:-162	Pik3cb	PROMOTER	1.641	2.485	1822.34	4528.06	4.078	1394.74	5688.22
A_68_P24020335	chr5:117838986-117839030	NM_028128:24	Rfc5	INSIDE	1.641	4.483	893.29	4004.39	7.355	861.69	6337.96
A_68_P28956729	chr13:37918014-37918058	NM_001177868:-744	Rreb1	PROMOTER	1.641	2.736	2349.87	6428.20	4.490	1779.07	7988.06
A_68_P30381701	chr15:81977690-81977734	NM_033218:14	Srebf2	INSIDE	1.641	2.567	7370.16	18917.36	4.213	5529.92	23299.25
A_68_P30601919	chr16:23225265-23225309	NM_145933:452	St6gal1	INSIDE	1.641	2.234	1424.73	3183.27	3.666	1177.58	4317.57
A_68_P21485725	chr2:103659783-103659827	ENSMUST00000167989:-171		PROMOTER	1.641	5.057	2260.32	11430.58	8.297	1657.10	13748.53
A_68_P27010234	chr10:24431792-24431836	NM_008813:94	Enpp1	INSIDE	1.640	6.266	2456.72	15393.62	10.275	2001.34	20564.38
A_68_P20939662	chr1:193541860-193541904	NM_172266:-20	Lpgat1	PROMOTER	1.640	2.460	919.65	2262.45	4.034	770.36	3107.48
A_68_P22568795	chr3:138105955-138105999	NM_007410:-151	Adh5	PROMOTER	1.639	2.652	6487.11	17202.67	4.345	5103.16	22173.81
A_68_P32538582	chrX:91612233-91612277	NM_026673:-194	Apoo	PROMOTER	1.639	2.182	678.97	1481.54	3.577	327.86	1172.65
A_68_P26630618	chr9:72833442-72833486	NM_001114328:107	Ccpg1	INSIDE	1.639	1.961	1956.95	3838.33	3.215	1718.91	5526.45
A_68_P30621546	chr16:26581967-26582011	NM_134103:198	Il1rap	INSIDE	1.639	5.035	2098.47	10565.95	8.251	1560.42	12874.40
A_68_P24132963	chr5:139469932-139469976	NM_008808:953	Pdgfa	INSIDE	1.639	2.663	4001.13	10655.82	4.365	3144.04	13724.14
A_68_P21909031	chr2:180921876-180921920	NM_025598:-149	Ppdpf	PROMOTER	1.639	1.741	1516.43	2640.73	2.853	1252.05	3572.49
A_68_P24086565	chr5:129526623-129526667	NM_009391:614	Ran	INSIDE	1.639	2.574	1550.69	3991.92	4.220	1358.16	5731.75
A_68_P29715887	chr14:73543290-73543334	NR_033185:721	Rcbtb2	INSIDE	1.639	1.870	715.43	1337.52	3.064	603.19	1848.33
A_68_P25232267	chr7:81158422-81158466	NM_009181:124	St8sia2	INSIDE	1.639	4.897	579.54	2837.93	8.027	450.86	3618.93
A_68_P22650336	chr3:152058904-152058948	NM_001080755:-47	Zzz3	PROMOTER	1.639	2.012	590.54	1188.06	3.298	679.08	2239.34
A_68_P21747321	chr2:151874396-151874440			Unknown	1.639	8.573	1419.06	12165.45	14.053	1112.80	15637.85
A_68_P20633260	chr1:136311773-136311817	NM_028320:-249	Adipor1	PROMOTER	1.638	1.899	1917.94	3642.07	3.110	1678.68	5221.19
A_68_P29187496	chr13:83860499-83860543	NR_015561:-7190	C130071C03Rik	PROMOTER	1.638	4.605	1216.96	5604.25	7.543	1022.94	7716.25
A_68_P27937736	chr11:77281473-77281517	NM_139128:4080	Coro6	INSIDE	1.638	1.704	1225.08	2087.69	2.791	1035.13	2889.33
A_68_P20146150	chr1:36125563-36125607	NM_015818:340	Hs6st1	INSIDE	1.638	4.027	2473.98	9963.62	6.599	2193.65	14474.81
A_68_P23156220	chr4:101320226-101320270	NM_175036:-139	Leprot	PROMOTER	1.638	1.858	6413.77	11913.85	3.042	4740.97	14421.47
A_68_P25261466	chr7:86650289-86650333	NR_029818:161	Mir9-3	DOWNSTREAM	1.638	2.324	2376.34	5523.69	3.807	2028.31	7722.67
A_68_P21494379	chr2:105239336-105239380	NM_009037:118	Rcn1	INSIDE	1.638	2.339	510.90	1194.90	3.831	450.73	1726.56
A_68_P21086006	chr2:27533318-27533362	NM_011305:620	Rxra	INSIDE	1.638	3.240	1257.40	4074.37	5.307	1004.52	5331.24
A_68_P23896971	chr5:93371578-93371622	NM_001077596:44577	Shroom3	INSIDE	1.638	2.786	519.73	1447.84	4.563	449.18	2049.51
A_68_P28615267	chr12:88640639-88640683	NM_011479:88520	Sptlc2	DOWNSTREAM	1.638	3.014	654.26	1971.91	4.938	576.68	2847.47

ProbeName	Target position of probe on CpG island microarray	TargetID	GeneSymbol	CpG island Description	Ratio of relative methylation (TiO_2-NP/Vehicle)	Sham group			TiO_2-H group		
						Relative methylation (Cy5/Cy3)	Cy3 signal (Input)	Cy5 signal (MeDIP)	Relative methylation (Cy5/Cy3)	Cy3 signal (Input)	Cy5 signal (MeDIP)
A_68_P28688783	chr12:103213937-103213981	NM_016856:-225	Cpsf2	PROMOTER	1.637	2.923	1412.05	4127.22	4.784	1184.21	5665.66
A_68_P30363898	chr15:78858851-78858898	NM_008197:233	H1f0	INSIDE	1.637	1.917	1289.33	2471.43	3.139	1051.84	3301.42
A_68_P31926197	chr19:4990111-4990155	NM_153553:-161	Npas4	PROMOTER	1.637	3.428	1185.76	4064.51	5.611	891.16	5000.01
A_68_P31438310	chr17:90855140-90855185	NM_020252:636980	Nrxn1	INSIDE	1.637	2.282	502.53	1146.56	3.734	388.45	1450.58
A_68_P25017511	chr7:28271855-28271899	NM_018820:-95	Sertad1	PROMOTER	1.637	3.640	3792.42	13803.19	5.956	2868.55	17086.23
A_68_P26239531	chr8:125898300-125898344	NM_001037877:588	Tcf25	INSIDE	1.637	4.492	13008.45	58440.41	7.353	8218.25	60426.39
A_68_P24162469	chr5:145529670-145529714	NM_001081362:32	Trrap	INSIDE	1.637	2.912	5153.75	15010.24	4.768	3947.39	18822.66
A_68_P32315765	chrX:33428989-33429033	NM_001009947:184	Dock11	INSIDE	1.636	4.768	664.47	3167.92	7.801	455.12	3550.48
A_68_P26214707	chr8:122082424-122082468	NM_001167910:215	Mbtps1	INSIDE	1.636	3.018	10827.35	32676.37	4.939	9094.19	44912.50
A_68_P31937159	chr19:7062178-7062222	NM_001185164:-59	Vegfb	PROMOTER	1.636	4.237	3920.15	16608.12	6.932	3142.11	21780.69
A_68_P25656690	chr8:11635804-11635848	NM_133971:-72	Ankrd10	PROMOTER	1.635	2.189	2794.43	6117.45	3.578	2349.68	8407.98
A_68_P20849149	chr1:177555403-177555447	NM_010209:342	Fh1	INSIDE	1.635	2.754	1911.22	5263.50	4.503	1609.59	7248.39
A_68_P26463576	chr9:43553455-43553499	NM_021424:818	Pvrl1	INSIDE	1.635	2.694	1269.60	3420.11	4.405	1012.01	4458.27
A_68_P27841688	chr11:60034191-60034235	NM_011480:-106	Srebf1	PROMOTER	1.635	1.775	1170.73	2077.55	2.902	1024.89	2974.20
A_68_P26883804	chr9:121403538-121403582	NM_031161:1247	Cck	INSIDE	1.634	3.579	904.53	3237.06	5.846	706.74	4131.75
A_68_P30378505	chr15:81416613-81416657	NM_177821:-9	Ep300	PROMOTER	1.634	2.357	831.78	1960.41	3.851	635.86	2448.47
A_68_P25496222	chr7:132850904-132850948	NM_207239:276	Gtf3c1	INSIDE	1.634	1.799	3778.37	6797.00	2.940	3048.35	8961.42
A_68_P27500776	chr10:120416131-120416176	NM_001081193:233	Lemd3	INSIDE	1.634	2.904	292.41	849.04	4.744	304.63	1445.30
A_68_P25019829	chr7:28834940-28834984	NM_011874:149	Psmc4	INSIDE	1.634	3.138	4381.20	13749.46	5.130	3217.72	16505.36
A_68_P30513218	chr16:5013781-5013827	NM_133185:-158	Rogdi	PROMOTER	1.634	3.783	2479.14	9378.26	6.183	1863.86	11524.08
A_68_P30497699	chr15:102236639-102236683	NM_013672:-86	Sp1	PROMOTER	1.634	2.607	1459.41	3804.34	4.259	1211.32	5158.72
A_68_P26817224	chr9:108838919-108838963	NM_025407:280	Uqcrc1	INSIDE	1.634	3.523	5287.92	18628.69	5.757	4049.89	23316.55
A_68_P28729933	chr12:110032849-110032893	NM_009537:1350	Yy1	INSIDE	1.634	2.528	1099.01	2778.47	4.132	930.27	3843.93
A_68_P25210201	chr7:77425441-77425485	AK148659:-817		PROMOTER	1.634	2.457	4533.42	11136.61	4.014	3412.47	13696.92
A_68_P24098740	chr5:132014030-132014074	NM_177047:1004161	Auts2	INSIDE	1.633	3.487	5752.28	20058.84	5.694	4561.64	25972.06
A_68_P24820864	chr6:125444507-125444551	NM_007657:245	Cd9	INSIDE	1.633	3.222	799.10	2574.41	5.262	716.17	3768.19
A_68_P20796287	chr1:167694031-167694075	NM_011804:142	Creg1	INSIDE	1.633	2.890	2035.65	5883.48	4.720	1667.89	7872.74
A_68_P26039225	chr8:90661714-90661758	NM_172757:-47	Heatr3	PROMOTER	1.633	2.504	2413.44	6042.63	4.088	2631.80	10757.83
A_68_P30963193	chr16:91485518-91485562	NM_010508:81	Ifnar1	INSIDE	1.633	1.748	1050.16	1835.82	2.855	791.62	2260.23
A_68_P20961517	chr2:3202117-3202161	NM_008708:579	Nmt2	INSIDE	1.633	1.432	1668.58	2389.46	2.338	1338.90	3130.71
A_68_P32672454	chrX:131076324-131076368	NM_013898:-178	Timm8a1	PROMOTER	1.633	3.393	810.91	2751.17	5.540	428.52	2374.03
A_68_P21836569	chr2:167457610-167457654	NM_023230:-127	Ube2v1	PROMOTER	1.633	3.680	822.83	3028.11	6.008	636.44	3824.00
A_68_P22766408	chr4:19636033-19636077	NM_177327:86	Wwp1	INSIDE	1.633	3.436	1455.50	5000.53	5.611	1334.52	7487.54
A_68_P24954457	chr7:6141163-6141207	NM_001146024:17070	Zfp444	INSIDE	1.633	2.299	250.55	575.91	3.754	227.18	852.87
A_68_P23591268	A_68_P23591268			Unknown	1.633	3.481	806.43	2807.22	5.686	553.20	3145.63
A_68_P33011727	A_68_P33011727			Unknown	1.633	2.385	193.78	462.11	3.894	160.14	623.51
A_68_P20914292	chr1:189431401-189431445	ENSMUST00000127489:-1247		PROMOTER	1.633	3.202	2279.69	7300.11	5.230	1679.29	8782.37
A_68_P27646748	chr11:23533451-23533495	NM_027860:159	0610010F05Rik	INSIDE	1.632	2.999	1129.84	3388.09	4.894	941.33	4606.87
A_68_P24050005	chr5:122821860-122821904	NM_026883:90	1500011H22Rik	INSIDE	1.632	2.350	644.45	1514.69	3.835	582.15	2232.74
A_68_P32373277	chrX:48194552-48194596	NM_133729:-38	2610018G03Rik	PROMOTER	1.632	3.797	920.52	3495.16	6.197	695.81	4311.93
A_68_P24759117	chr6:113328299-113328343	NM_001170485:214	Arpc4	INSIDE	1.632	11.956	3011.48	36006.38	19.517	2285.29	44602.84
A_68_P29009046	chr13:46764820-46764864	NM_001033192:-48	C78339	PROMOTER	1.632	1.658	654.49	1085.11	2.705	563.16	1523.44
A_68_P21421545	chr2:91095901-91095945	NM_030880:-1047	Pacsin3	PROMOTER	1.632	4.268	1205.71	5146.03	6.967	981.70	6839.83
A_68_P23749120	chr5:64484497-64484543	NM_025700:332	Pgm1	INSIDE	1.632	4.520	2595.29	11730.50	7.376	2168.53	15994.06
A_68_P27568397	chr11:6315971-6316015	NM_008907:120	Ppia	INSIDE	1.632	3.616	2360.54	8534.66	5.899	1898.06	11197.31
A_68_P24127508	chr5:138220341-138220385	NM_027242:217	2010007H12Rik	INSIDE	1.631	3.683	2150.37	7919.10	6.005	1663.78	9990.80
A_68_P27351975	chr10:92623318-92623362	NM_146239:-280	Cdk17	PROMOTER	1.631	5.287	5246.48	27740.47	8.626	4202.19	36248.87
A_68_P27543292	chr10:128242453-128242497	NM_028873:-257	Dnajc14	DIVERGENT_PROMOTER	1.631	2.164	846.84	1832.34	3.529	769.79	2716.63
A_68_P27924590	chr11:74982634-74982678	NM_001098203:-1002	Hic1	PROMOTER	1.631	2.257	1046.15	2361.43	3.682	817.05	3008.34
A_68_P26977750	chr10:18565037-18565081	NM_022032:182	Perp	INSIDE	1.631	6.079	2807.39	17066.31	9.913	2265.96	22463.50
A_68_P26918726	chr10:6979506-6979550	NM_133485:863	Ppp1r14c	INSIDE	1.631	3.344	2479.69	8292.48	5.454	1974.99	10772.07

ProbeName	Target position of probe on CpG island microarray	TargetID	GeneSymbol	CpG island Description	Ratio of relative methylation (TiO_2-NP/Vehicle)	Sham group			TiO_2-H group		
						Relative methylation (Cy5/Cy3)	Cy3 signal (Input)	Cy5 signal (MeDIP)	Relative methylation (Cy5/Cy3)	Cy3 signal (Input)	Cy5 signal (MeDIP)
A_68_P23542998	chr5:24110558-24110602	NM_025891:-2760	Smarcd3	PROMOTER	1.631	8.477	7541.98	63933.24	13.824	5614.49	77617.03
A_68_P26153173	chr8:111324170-111324214	NM_007496:85649	Zfhx3	INSIDE	1.631	2.018	591.48	1193.66	3.292	440.15	1448.85
A_68_P23094811	chr4:88940707-88940751	NM_009877:-205	Cdkn2a	PROMOTER	1.630	1.577	574.53	906.09	2.571	449.85	1156.63
A_68_P25092923	chr7:52662436-52662480	NM_010596:1129	Kcna7	INSIDE	1.630	5.866	1061.24	6225.62	9.563	899.09	8597.98
A_68_P26811667	chr9:107803499-107803544	NM_028369:13062	Mon1a	INSIDE	1.630	6.893	1343.62	9261.42	11.234	1132.45	12721.99
A_68_P28961844	chr13:38727138-38727182	NM_139063:-182	Muted	PROMOTER	1.630	1.777	971.29	1726.20	2.897	765.87	2218.59
A_68_P29508104	chr14:31638725-31638769	NM_028839:-33	Tmem110	PROMOTER	1.630	7.519	1861.83	13998.85	12.256	1443.88	17696.25
A_68_P27912376	chr11:72420588-72420632	NM_025985:-152	Ube2g1	PROMOTER	1.630	3.721	980.28	3647.77	6.065	904.63	5486.35
A_68_P28190095	chr11:121215875-121215919	NM_025793:-135	Wdr45l	PROMOTER	1.630	9.024	2659.39	23999.48	14.714	1804.12	26545.49
A_68_P31469349	chr18:5591373-5591417	NM_011546:-494	Zeb1	PROMOTER	1.630	2.036	1186.01	2415.11	3.319	998.95	3315.18
A_68_P21105115	chr2:30627096-30627141			Unknown	1.630	2.805	2376.46	6665.33	4.572	1891.60	8648.49
A_68_P33008885	chr12:21117800-21117844	NM_001004364:206	Asap2	INSIDE	1.629	3.874	2797.09	10835.86	6.309	2307.57	14558.86
A_68_P28596932	chr12:85534521-85534565	NM_001163502:423	C130039O16Rik	INSIDE	1.629	4.959	3598.27	17843.45	8.078	2828.40	22847.40
A_68_P24423149	chr6:47404611-47404655	NM_012042:310	Cul1	INSIDE	1.629	10.303	2595.13	26738.51	16.788	1833.59	30782.91
A_68_P20910364	chr1:188791866-188791912	NM_033077:594	D1Pas1	INSIDE	1.629	6.821	1369.42	9340.44	11.108	1236.49	13735.20
A_68_P25498529	chr7:133225199-133225243	NM_001101488:705	Gsg1l	INSIDE	1.629	1.489	2851.67	4247.28	2.426	2298.74	5577.07
A_68_P21623006	chr2:128926947-128926991	NM_009086:237	Polr1b	INSIDE	1.629	1.389	1216.53	1689.37	2.262	1077.43	2436.99
A_68_P21773626	chr2:156665615-156665659	NM_173396:-176	Tgif2	PROMOTER	1.629	4.810	1506.49	7246.49	7.837	1272.30	9971.61
A_68_P22475538	chr3:120965447-120965491	NM_178936:766	Tmem56	INSIDE	1.629	3.110	1198.08	3726.36	5.068	1119.74	5674.32
A_68_P22860680	chr4:41082241-41082285	NM_026275:-791	Ube2r2	PROMOTER	1.629	1.364	2700.44	3684.57	2.222	2131.24	4735.93
A_68_P27830380	chr11:58136423-58136467	NM_178761:404	Zfp672	INSIDE	1.629	4.207	4136.22	17401.81	6.852	3392.02	23243.79
A_68_P20412463	chr1:88400704-88400748			Unknown	1.629	4.268	5210.04	22236.22	6.951	4080.44	28361.68
A_68_P26018418	chr8:86671979-86672023	NM_001163752:64	4930432K21Rik	INSIDE	1.628	2.483	2729.10	6776.31	4.043	2053.68	8303.57
A_68_P24330588	chr6:30124352-30124396	NR_038124:-2248	BB283400	DIVERGENT_PROMOTER	1.628	3.446	1069.53	3685.40	5.610	927.75	5204.62
A_68_P20422167	chr1:90102351-90102395	NM_008299:-49	Dnajb3	DIVERGENT_PROMOTER	1.628	6.145	1938.67	11913.38	10.006	1545.92	15468.64
A_68_P22410841	chr3:107948966-107949010	NM_010306:44	Gnai3	INSIDE	1.628	3.286	970.21	3187.70	5.350	788.34	4217.64
A_68_P24981713	chr7:17062092-17062136	NM_008718:15	Npas1	INSIDE	1.628	2.284	757.96	1731.04	3.718	644.47	2396.16
A_68_P23234756	chr4:117167070-117167114	NM_025739:2428	Rnf220	INSIDE	1.628	6.644	1123.56	7465.08	10.820	864.45	9353.10
A_68_P31187135	chr17:43153738-43153782	NM_178589:257	Tnfrsf21	INSIDE	1.628	4.557	2213.00	10085.59	7.421	1633.92	12124.77
A_68_P32565197	chrX:98892164-98892208	ENSMUST00000073812:-4997		PROMOTER	1.628	5.281	10261.67	54192.20	8.596	3845.96	33060.00
A_68_P25833742	chr8:47085366-47085410	NM_001039562:-184	Ankrd37	PROMOTER	1.627	1.854	2843.21	5270.09	3.016	2169.42	6542.60
A_68_P27183504	chr10:61441353-61441397	NM_007731:482	Col13a1	INSIDE	1.627	2.560	905.42	2317.85	4.164	745.87	3106.00
A_68_P25410984	chr7:117103504-117103548	NM_021494:410	Dennd5a	INSIDE	1.627	1.745	1202.04	2097.07	2.838	950.80	2698.44
A_68_P24429118	chr6:48692680-48692724	NM_008376:3657	Gimap1	INSIDE	1.627	6.458	1090.95	7045.70	10.506	940.90	9884.70
A_68_P30482201	chr15:99602827-99602871	NM_028015:98	Lass5	INSIDE	1.627	3.930	4100.43	16113.09	6.392	3087.66	19737.30
A_68_P21900919	chr2:179760085-179760129	NM_177727:415	Lsm14b	INSIDE	1.627	4.030	1686.18	6795.38	6.555	1408.10	9230.65
A_68_P32786683	chrX:158346162-158346206	NM_001081052:251538	Nhs	INSIDE	1.627	4.032	2483.51	10013.66	6.560	1267.66	8315.23
A_68_P28184543	chr11:120298753-120298797	NM_001195023:240	Nploc4	INSIDE	1.627	2.140	11825.52	25303.55	3.481	8744.97	30442.56
A_68_P27810583	chr11:54336349-54336393	NM_175258:22	Rapgef6	INSIDE	1.627	2.089	939.96	1963.23	3.398	789.84	2684.12
A_68_P22405709	chr3:107135696-107135740	NM_001045807:489	Rbm15	INSIDE	1.627	3.136	3799.14	11913.80	5.103	3192.75	16291.12
A_68_P27080342	chr10:39452039-39452083	NM_011264:95	Rev3l	INSIDE	1.627	2.038	1075.82	2192.25	3.315	865.77	2869.70
A_68_P31232583	chr17:51318678-51318722	NM_028162:-26	Tbc1d5	PROMOTER	1.627	2.670	1622.53	4332.51	4.344	1220.79	5303.73
A_68_P28182345	chr11:119959914-119959958	NM_183137:109	2410002I01Rik	INSIDE	1.626	1.880	1012.24	1902.83	3.057	758.19	2317.78
A_68_P30587818	chr16:20498949-20498993	NM_172265:81	Eif2b5	INSIDE	1.626	1.709	569.66	973.48	2.778	495.87	1377.71
A_68_P25099054	chr7:53683157-53683201	NM_001112739:31312	Kcnc1	INSIDE	1.626	7.224	2254.21	16284.40	11.748	1472.00	17293.73
A_68_P31106324	chr17:25934049-25934093	NM_133719:-80	Metrn	PROMOTER	1.626	2.274	1344.63	3058.13	3.697	1092.50	4039.16
A_68_P25009543	chr7:26082789-26082833	NM_008776:164	Pafah1b3	INSIDE	1.626	3.401	2594.91	8824.50	5.529	2029.01	11218.18
A_68_P23314345	chr4:132399162-132399206	NM_146154:-100	Ppp1r8	PROMOTER	1.626	4.182	1875.06	7842.09	6.801	1488.10	10121.29
A_68_P28875776	chr13:22098904-22098948	NM_019429:2684	Prss16	INSIDE	1.626	3.801	2569.89	9769.27	6.181	1796.94	11106.62
A_68_P28071137	chr11:100820628-100820677	NM_008986:11279	Ptrf	INSIDE	1.626	2.201	271.72	598.16	3.580	237.92	851.66
A_68_P25126430	chr7:59261254-59261298	NR_027704:-192	Svip	PROMOTER	1.626	3.084	7032.53	21687.90	5.015	5516.22	27665.59

ProbeName	Target position of probe on CpG island microarray	TargetID	GeneSymbol	CpG island Description	Ratio of relative methylation (TiO_2-NP/Vehicle)	Sham group			TiO_2-H group		
						Relative methylation (Cy5/Cy3)	Cy3 signal (Input)	Cy5 signal (MeDIP)	Relative methylation (Cy5/Cy3)	Cy3 signal (Input)	Cy5 signal (MeDIP)
A_68_P22323188	chr3:89856392-89856436	NM_028475:-17	Ubap2l	PROMOTER	1.626	1.802	9446.78	17020.32	2.929	6798.45	19913.72
A_68_P24115158	chr5:135663906-135663950	NM_011714:736	Baz1b	INSIDE	1.625	4.863	843.16	4100.18	7.903	750.44	5930.37
A_68_P21840215	chr2:168055402-168055451	NM_010072:453	Dpm1	INSIDE	1.625	2.341	439.97	1029.83	3.804	363.32	1382.21
A_68_P29996481	chr15:7760813-7760857	NM_010275:-176	Gdnf	PROMOTER	1.625	3.802	1226.00	4661.44	6.177	960.25	5931.57
A_68_P26587299	chr9:65219825-65219869	NM_001111145:10980	Gm514	INSIDE	1.625	6.392	4269.15	27288.57	10.385	3395.63	35263.23
A_68_P29569514	chr14:45839639-45839683	NM_027518:269	Gpr137c	INSIDE	1.625	4.403	1455.02	6406.46	7.154	1082.09	7741.07
A_68_P27862275	chr11:63732951-63732995	NM_018805:2814	Hs3st3b1	INSIDE	1.625	4.099	641.44	2629.45	6.661	508.06	3384.07
A_68_P24024083	chr5:118478594-118478638	NM_021344:784	Tesc	INSIDE	1.625	4.891	4373.06	21388.98	7.949	3586.56	28509.15
A_68_P23429319	chr4:153514065-153514109	NM_011642:231	Trp73	INSIDE	1.625	4.438	1760.55	7813.59	7.211	1637.29	11805.84
A_68_P26875426	chr9:119883221-119883265	NM_153287:10534	Csrnp1	INSIDE	1.624	3.499	1072.24	3751.45	5.682	894.90	5084.44
A_68_P24156243	chr5:144131687-144131731	NM_133355:6122	Grid2ip	INSIDE	1.624	1.924	4737.74	9114.19	3.124	4028.32	12585.02
A_68_P26547037	chr9:58160685-58160729	NM_010729:313	Loxl1	INSIDE	1.624	3.568	794.13	2833.46	5.793	679.29	3935.25
A_68_P29360867	chr13:117813151-117813195	NM_001081009:1151	Parp8	INSIDE	1.624	2.720	1787.53	4862.28	4.418	1510.48	6672.61
A_68_P26766653	chr9:99775810-99775854	NM_011440:757	Sox14	INSIDE	1.624	2.131	357.97	762.89	3.461	336.16	1163.37
A_68_P21777116	chr2:157282885-157282929	NM_001025395:32878	Src	INSIDE	1.624	1.761	1431.74	2521.94	2.861	1201.96	3438.40
A_68_P23298057	chr4:129373770-129373814	NM_199305:290	Tmem39b	INSIDE	1.624	1.711	1592.30	2724.61	2.779	1302.78	3620.70
A_68_P26948857	chr10:12780907-12780951	FJ425893:619		INSIDE	1.624	2.131	456.21	972.32	3.462	445.93	1543.60
A_68_P32135373	chr19:45005375-45005419	NM_001081225:-212	Fam178a	PROMOTER	1.623	2.807	2918.14	8191.52	4.556	2514.21	11455.31
A_68_P23018909	chr4:73659645-73659689	NM_172869:160	Frmd3	INSIDE	1.623	1.485	899.52	1335.79	2.410	685.09	1650.77
A_68_P31834169	chr18:75121056-75121100	NM_010720:-161	Lipg	PROMOTER	1.623	1.568	2716.88	4259.32	2.544	2197.72	5591.66
A_68_P25285303	chr7:91032985-91033029	NM_030705:-155	Mesdc1	DIVERGENT_PROMOTER	1.623	2.693	480.29	1293.34	4.369	553.86	2419.97
A_68_P27564592	chr11:5607482-5607526	NM_026080:198	Mrps24	INSIDE	1.623	5.515	638.51	3521.43	8.951	525.42	4702.85
A_68_P31513737	chr18:14841164-14841208	NM_001161369:237	Ss18	INSIDE	1.623	5.982	1313.71	7859.24	9.711	1187.27	11529.15
A_68_P30140505	chr15:36723538-36723582	NM_011740:733	Ywhaz	INSIDE	1.623	2.586	1808.20	4675.67	4.198	1640.94	6888.50
A_68_P32410977	chrX:56820526-56820570	ENSMUST00000124402:101		INSIDE	1.623	3.590	723.06	2595.98	5.828	472.85	2755.59
A_68_P26631268	chr9:72951661-72951705	NM_025890:1478	2410004A20Rik	INSIDE	1.622	1.903	344.84	656.35	3.086	354.88	1095.29
A_68_P20293904	chr1:64737359-64737403	NR_038009:-147	2810408I11Rik	DIVERGENT_PROMOTER	1.622	1.860	1054.52	1961.20	3.017	834.70	2518.52
A_68_P21958957	chr3:14886237-14886281	NM_009801:-167	Car2	PROMOTER	1.622	2.677	743.86	1991.60	4.342	579.74	2517.33
A_68_P32567641	chrX:99384743-99384787	NM_009094:-1054	Rps4x	PROMOTER	1.622	2.279	620.42	1413.98	3.696	430.02	1589.15
A_68_P32750678	chrX:149204051-149204095	NM_172441:-68	Shroom2	PROMOTER	1.622	2.671	2098.76	5606.54	4.334	1352.04	5859.93
A_68_P25477108	chr7:129210764-129210808	NM_138589:75	Ubfd1	INSIDE	1.622	5.308	1922.64	10205.63	8.608	1580.94	13609.45
A_68_P24954292	chr7:6107153-6107197	NM_001013012:399	Zfp787	INSIDE	1.622	1.848	928.31	1715.63	2.997	1187.74	3560.21
A_68_P30931281	chr16:85803417-85803461	NM_009621:-78	Adamts1	PROMOTER	1.621	2.412	1529.85	3690.67	3.912	1277.02	4995.29
A_68_P22069519	chr3:38383514-38383558	NM_001167883:202	Ankrd50	INSIDE	1.621	3.267	2974.55	9716.58	5.297	2486.82	13171.62
A_68_P25772218	chr8:35171274-35171318	NM_011722:269	Dctn6	INSIDE	1.621	1.979	1786.21	3534.22	3.208	1530.23	4909.07
A_68_P23203786	chr4:109656795-109656839	NM_172296:6187	Dmrta2	DOWNSTREAM	1.621	8.757	7571.12	66301.58	14.193	5316.97	75464.36
A_68_P30720009	chr16:44173533-44173577	NM_001029889:45	Gm608	INSIDE	1.621	3.061	994.91	3045.29	4.961	793.75	3937.64
A_68_P28670941	chr12:99812671-99812719	NM_029911:3456	Kcnk10	INSIDE	1.621	2.562	1091.94	2797.22	4.153	868.16	3605.85
A_68_P28225274	chr12:8928368-8928412	NM_008640:278	Laptm4a	INSIDE	1.621	2.539	2628.18	6673.66	4.117	2032.54	8367.79
A_68_P20658349	chr1:140515986-140516030	NM_021605:265	Nek7	INSIDE	1.621	2.357	1442.96	3401.75	3.822	1214.75	4642.75
A_68_P32134209	chr19:44830746-44830790	NM_011037:-1115	Pax2	PROMOTER	1.621	2.469	5256.54	12977.15	4.002	3853.20	15420.81
A_68_P21431995	chr2:92855411-92855455	NM_001177536:30869	Prdm11	INSIDE	1.621	1.810	1126.49	2039.31	2.935	947.95	2782.42
A_68_P27925675	chr11:75162047-75162091	NM_001102611:134	Smyd4	INSIDE	1.621	1.499	641.77	962.31	2.431	578.57	1406.70
A_68_P31330059	chr17:71007053-71007097	NM_001128181:95988	Dlgap1	INSIDE	1.620	13.447	4003.88	53839.91	21.787	2580.13	56214.38
A_68_P25392118	chr7:112548478-112548522	NM_199009:25	Fam160a2	INSIDE	1.620	2.580	1482.11	3824.40	4.181	1279.22	5348.77
A_68_P27909076	chr11:71855740-71855784	NM_144526:-241	Fam64a	DIVERGENT_PROMOTER	1.620	3.912	2415.26	9447.36	6.336	2014.18	12761.04
A_68_P32507212	chrX:83022021-83022065	NM_008194:116	Gyk	INSIDE	1.620	5.178	30351.68	157156.00	8.389	20975.09	175956.90
A_68_P23602238	chr5:36022053-36022097	NM_030127:357	Htra3	INSIDE	1.620	5.000	2167.98	10840.38	8.098	1422.78	11521.52
A_68_P26983891	chr10:19653749-19653793	NM_008580:-561	Map3k5	PROMOTER	1.620	1.830	1693.83	3099.84	2.964	1458.46	4322.90
A_68_P23524104	chr5:20561279-20561323	NM_011203:315	Ptpn12	INSIDE	1.620	2.868	1128.95	3237.74	4.647	1044.95	4855.85
A_68_P27228619	chr10:69707994-69708038	NM_025807:-7	Slc16a9	PROMOTER	1.620	7.744	1500.53	11620.31	12.543	1322.87	16592.67

ProbeName	Target position of probe on CpG island microarray	TargetID	GeneSymbol	CpG island Description	Ratio of relative methylation (TiO_2-NP/Vehicle)	Sham group			TiO_2-H group		
						Relative methylation (Cy5/Cy3)	Cy3 signal (Input)	Cy5 signal (MeDIP)	Relative methylation (Cy5/Cy3)	Cy3 signal (Input)	Cy5 signal (MeDIP)
A_68_P31159487	chr17:35653700-35653744	NM_001163763:47	Tcf19	INSIDE	1.620	2.076	1916.59	3979.58	3.364	1478.90	4975.61
A_68_P27792052	chr11:50873003-50873047	NM_009329:266	Zfp354a	INSIDE	1.620	4.509	3530.93	15919.26	7.304	2608.28	19049.86
A_68_P32257887	chrX:11242111-11242155			Unknown	1.620	2.006	2657.86	5331.53	3.249	1402.51	4556.80
A_68_P21673356	chr2:138082901-138082945	NM_001025431:603	Btbd3	INSIDE	1.619	1.438	3011.91	4330.58	2.328	2338.35	5442.63
A_68_P21339006	chr2:74550322-74550366	NM_010468:295	Hoxd3	INSIDE	1.619	2.232	1636.44	3653.03	3.613	1498.00	5412.55
A_68_P30130469	chr15:34767206-34767250	NM_181317:93	Kcns2	INSIDE	1.619	2.579	2835.18	7311.52	4.174	2259.12	9429.48
A_68_P22028771	chr3:30407557-30407601	NM_021442:831	Mecom	INSIDE	1.619	1.748	767.78	1342.21	2.830	672.06	1901.65
A_68_P24337499	chr6:31349464-31349508	NM_013791:659	Mkln1	INSIDE	1.619	4.490	3001.53	13477.04	7.271	2353.24	17110.24
A_68_P28466190	chr12:59313609-59313653	NM_009216:840	Sstr1	INSIDE	1.619	5.454	1300.46	7092.60	8.829	990.04	8741.09
A_68_P28450734	chr12:56225622-56225666	NM_001100116:130	1700047I17Rik2	INSIDE	1.618	5.076	2323.24	11793.75	8.212	1827.50	15006.68
A_68_P31731124	chr18:56732842-56732886	NM_001127338:-271	Aldh7a1	PROMOTER	1.618	3.079	4363.12	13433.92	4.981	3711.04	18485.84
A_68_P27228310	chr10:69651858-69651902	NM_001111121:92012	Ccdc6	INSIDE	1.618	5.707	8650.37	49366.64	9.232	5818.45	53714.59
A_68_P23185134	chr4:106234075-106234119	NM_053272:454	Dhcr24	INSIDE	1.618	1.954	6180.72	12074.38	3.161	4658.67	14725.56
A_68_P25842954	chr8:48761152-48761196	NM_023503:-661	Ing2	PROMOTER	1.618	3.119	729.58	2275.67	5.047	620.16	3130.17
A_68_P32383986	chrX:50097843-50097887	NR_003188:-94	Kis2	PROMOTER	1.618	2.686	13355.12	35865.81	4.346	6244.36	27135.25
A_68_P29266707	chr13:100785592-100785636	NM_030026:-20	Mccc2	PROMOTER	1.618	1.916	809.86	1551.88	3.100	610.66	1893.18
A_68_P21587817	chr2:122564114-122564158	NM_019788:-104	Pldn	PROMOTER	1.618	4.136	1376.79	5694.31	6.692	1095.09	7328.83
A_68_P25669976	chr8:13677580-13677624	NM_009025:-15	Rasa3	PROMOTER	1.618	2.401	1590.19	3818.17	3.885	1295.35	5032.82
A_68_P22705223	chr4:6292852-6292896	NM_001098227:48	Sdcbp	INSIDE	1.618	2.569	3970.18	10197.67	4.156	3264.23	13567.49
A_68_P27929278	chr11:75780405-75780449			Unknown	1.618	2.631	267.46	703.76	4.257	283.23	1205.58
A_68_P21745337	chr2:151528077-151528121	NM_001160363:355	5430405G05Rik	INSIDE	1.617	12.534	2185.05	27386.41	20.261	1693.22	34306.73
A_68_P28449512	chr12:55963938-55963982	NM_007688:-96	Cfl2	PROMOTER	1.617	1.882	1457.03	2742.45	3.044	1200.81	3655.76
A_68_P23610086	chr5:37259995-37260039	NM_172994:208	Ppp2r2c	INSIDE	1.617	5.030	2473.74	12442.91	8.135	1955.76	15909.70
A_68_P21132202	chr2:35056385-35056429	NM_026697:234	Rab14	INSIDE	1.617	2.312	2909.12	6724.51	3.738	2509.32	9380.51
A_68_P26133052	chr8:107850532-107850576	NM_025486:291	Tmem208	INSIDE	1.617	2.535	7908.84	20050.59	4.099	6297.19	25813.56
A_68_P23059231	chr4:82505707-82505751	NM_026647:-163	Zdhhc21	PROMOTER	1.617	2.859	3478.55	9944.80	4.624	2488.71	11508.13
A_68_P26617701	chr9:70504626-70504670	NM_172772:333	Fam63b	INSIDE	1.616	4.671	1854.17	8661.53	7.548	1656.06	12499.60
A_68_P31930681	chr19:5758063-5758107	NM_008520:17181	Ltbp3	INSIDE	1.616	4.430	806.23	3571.76	7.160	691.28	4949.47
A_68_P27215083	chr10:67442230-67442274	NM_001081346:-93	Rtkn2	PROMOTER	1.616	3.542	8566.13	30338.77	5.723	5946.50	34030.73
A_68_P21890546	chr2:178142021-178142065	NM_177191:321	Sycp2	INSIDE	1.616	3.732	5809.31	21681.31	6.031	4181.96	25221.88
A_68_P27181843	chr10:61158699-61158743	NM_027912:459	Tysnd1	INSIDE	1.616	3.373	978.98	3301.84	5.452	761.99	4154.37
A_68_P32537531	chrX:91368428-91368472	NM_001044386:296	Zfx	INSIDE	1.616	5.107	1044.72	5335.77	8.253	681.29	5622.91
A_68_P24906444	chr6:142705194-142705238	NM_009908:11	Cmas	INSIDE	1.615	4.227	1261.52	5332.30	6.828	1003.63	6852.26
A_68_P25020574	chr7:28965243-28965287	NM_010092:752	Dyrk1b	INSIDE	1.615	3.240	2559.08	8290.75	5.233	2122.57	11107.96
A_68_P28083517	chr11:102994122-102994166	NM_001130516:-508	Hexim2	PROMOTER	1.615	3.255	2038.59	6635.67	5.256	1583.56	8323.43
A_68_P31381048	chr17:80461571-80461615	NM_144802:82	Hnrpll	INSIDE	1.615	1.950	1829.96	3569.05	3.150	1467.27	4621.90
A_68_P22028775	chr3:30408163-30408207	NM_021442:225	Mecom	INSIDE	1.615	3.398	2128.39	7231.49	5.487	1792.28	9834.61
A_68_P20188961	chr1:43503040-43503084	NM_010879:467	Nck2	INSIDE	1.615	2.269	1442.39	3273.18	3.665	1765.47	6470.17
A_68_P28178599	chr11:119410964-119411008	NM_008730:-1852	Nptx1	PROMOTER	1.615	1.911	1470.34	2810.36	3.087	1225.21	3782.05
A_68_P27357767	chr10:93610648-93610692	NM_011629:-5	Nr2c1	PROMOTER	1.615	3.242	3314.61	10746.06	5.237	2772.73	14520.12
A_68_P28697278	chr12:104627062-104627106	NM_001177841:193	Otub2	INSIDE	1.615	2.049	1442.32	2955.86	3.310	1280.20	4237.09
A_68_P31117689	chr17:27792526-27792570	NM_011861:-78	Pacsin1	PROMOTER	1.615	3.070	940.96	2889.02	4.959	739.85	3668.92
A_68_P30084544	chr15:25914715-25914759	NM_144523:616	Zfp622	INSIDE	1.615	2.547	2462.55	6272.56	4.112	2001.95	8232.98
A_68_P23366442	chr4:141430855-141430899	NM_025994:-41	Efhd2	PROMOTER	1.614	3.976	1143.10	4545.11	6.418	993.55	6376.66
A_68_P25460625	chr7:125999193-125999237	NM_001081446:-74	Iqck	DIVERGENT_PROMOTER	1.614	2.484	2719.29	6755.40	4.010	2190.94	8785.77
A_68_P21114609	chr2:32167955-32167999	NR_029811:-6003	Mir199b	PROMOTER	1.614	5.640	16942.30	95558.65	9.104	11346.08	103293.50
A_68_P21565317	chr2:118489132-118489176	NM_001033254:-158	Pak6	PROMOTER	1.614	1.728	1297.75	2242.70	2.790	1069.14	2982.74
A_68_P31632702	chr18:38371220-38371264	NM_029357:-1826	Pcdh1	PROMOTER	1.614	1.637	1732.59	2835.79	2.642	1331.45	3518.04
A_68_P27632484	chr11:20531833-20531877	NM_001038625:-125	Sertad2	PROMOTER	1.614	3.475	6104.15	21214.46	5.608	4460.01	25012.99
A_68_P22130348	chr3:51364282-51364326	NM_080793:441	Setd7	INSIDE	1.614	4.461	2334.51	10415.15	7.201	2072.84	14926.78
A_68_P31212432	chr17:47605282-47605326	NM_145489:31717	AI661453	INSIDE	1.613	1.960	621.47	1218.38	3.163	511.78	1618.89

ProbeName	Target position of probe on CpG island microarray	TargetID	GeneSymbol	CpG island Description	Ratio of relative methylation (TiO_2-NP/Vehicle)	Sham group			TiO_2-H group		
						Relative methylation (Cy5/Cy3)	Cy3 signal (Input)	Cy5 signal (MeDIP)	Relative methylation (Cy5/Cy3)	Cy3 signal (Input)	Cy5 signal (MeDIP)
A_68_P29713894	chr14:73110055-73110099	NM_207636:-266	Fndc3a	PROMOTER	1.613	1.503	3401.65	5113.89	2.425	2664.88	6462.15
A_68_P30367927	chr15:79521233-79521289	NM_013818:-65	Gtpbp1	DIVERGENT_PROMOTER	1.613	3.259	2903.88	9462.94	5.255	2416.36	12697.28
A_68_P25730819	chr8:27229628-27229672	NM_207659:46	Hook3	INSIDE	1.613	2.939	1362.79	4005.67	4.742	1111.00	5268.23
A_68_P25010077	chr7:26164977-26165021	NM_001039507:10127	Lipe	INSIDE	1.613	3.417	2557.39	8738.93	5.511	1874.40	10329.97
A_68_P27787812	chr11:50105519-50105563	NM_175334:298	Maml1	INSIDE	1.613	3.738	461.82	1726.44	6.031	391.72	2362.56
A_68_P27280132	chr10:79440564-79440611	NM_001003949:6488	ORF61	INSIDE	1.613	3.420	396.18	1354.95	5.517	305.43	1685.10
A_68_P24173631	chr5:148241874-148241918	NM_028291:-259	Pan3	PROMOTER	1.613	2.699	3211.48	8667.31	4.355	2514.20	10948.12
A_68_P27631993	chr11:20443834-20443878	NM_021372:601	Sertad2	INSIDE	1.613	10.061	10624.97	106902.70	16.231	7456.42	121021.70
A_68_P24046730	chr5:122278610-122278654	NM_008507:8178	Sh2b3	INSIDE	1.613	4.679	832.36	3894.34	7.546	731.05	5516.12
A_68_P23311001	chr4:131830273-131830317	NM_025579:5	Taf12	INSIDE	1.613	11.344	3315.90	37616.64	18.297	2386.77	43669.74
A_68_P20350529	chr1:74838384-74838428	NM_009518:-186	Wnt10a	PROMOTER	1.613	4.124	4948.69	20405.98	6.653	3730.25	24816.45
A_68_P30499545	chr15:102577606-102577650			Unknown	1.613	2.317	1696.36	3931.06	3.738	1417.87	5299.73
A_68_P28889015	chr13:24892904-24892948	NM_024473:-599	BC005537	PROMOTER	1.612	2.710	573.41	1553.95	4.369	537.08	2346.26
A_68_P32364032	chrX:45694726-45694770	NM_178782:214	Bcorl1	INSIDE	1.612	2.508	1625.91	4078.50	4.044	1095.53	4430.73
A_68_P26536114	chr9:56265361-56265405	NM_172924:475	C230081A13Rik	INSIDE	1.612	3.543	2542.05	9007.74	5.713	1874.16	10706.98
A_68_P31065266	chr17:15513473-15513517	NM_007865:-707	Dll1	PROMOTER	1.612	5.376	1640.73	8821.10	8.668	1157.08	10028.99
A_68_P23927140	chr5:100408133-100408177	NM_001077265:-197	Hnrnpd	PROMOTER	1.612	1.778	1719.81	3057.63	2.866	1408.50	4036.58
A_68_P23326917	chr4:134708685-134708729	NM_019732:32147	Runx3	INSIDE	1.612	3.639	3739.75	13608.80	5.865	2960.41	17362.93
A_68_P27552432	chr11:3417267-3417311	NM_053267:2584	Selm	INSIDE	1.612	2.862	653.75	1870.73	4.613	526.96	2431.08
A_68_P31213521	chr17:47809690-47809734	NM_198421:42074	Usp49	INSIDE	1.612	2.789	2117.42	5904.90	4.496	1779.78	8002.52
A_68_P27926183	chr11:75267995-75268039	NM_138950:203	Wdr81	INSIDE	1.612	1.934	384.97	744.61	3.117	383.70	1196.10
A_68_P21103868	chr2:30400904-30400948	ENSMUST00000145413:10678		DOWNSTREAM	1.612	2.044	2240.78	4580.20	3.295	1770.68	5834.67
A_68_P20778066	chr1:164408081-164408125	NM_001038619:59	Dnm3	INSIDE	1.611	2.867	1051.30	3013.75	4.617	792.73	3660.19
A_68_P23966965	chr5:107867055-107867099	NM_027599:6509	Lpcat2b	DOWNSTREAM	1.611	5.020	584.64	2934.97	8.088	566.90	4585.04
A_68_P31781216	chr18:65590296-65590340	NM_172833:-332	Malt1	PROMOTER	1.611	4.275	3911.10	16718.62	6.885	2564.32	17654.60
A_68_P24329792	chr6:29998675-29998719	NM_001164230:709	Nrf1	INSIDE	1.611	1.694	1282.34	2172.15	2.730	1075.63	2936.14
A_68_P27993897	chr11:87172172-87172216	NM_177167:302	Ppm1e	INSIDE	1.611	3.684	1483.62	5465.32	5.934	1280.66	7599.66
A_68_P30580602	chr16:18586865-18586909	NM_011532:176	Tbx1	INSIDE	1.611	3.668	2181.79	8002.39	5.908	1820.13	10753.60
A_68_P27798408	chr11:52096304-52096348	NM_009331:-574	Tcf7	PROMOTER	1.611	1.663	2733.97	4546.98	2.679	2168.01	5807.09
A_68_P23584753	chr5:32913192-32913236	NM_001205132:-329	Yes1	PROMOTER	1.611	2.569	2853.62	7330.54	4.138	2033.11	8412.94
A_68_P24541220	chr6:72297774-72297818	NM_026696:486	0610030E20Rik	INSIDE	1.610	5.030	3501.90	17613.89	8.096	2811.14	22758.40
A_68_P27354277	chr10:93002683-93002727	NM_027908:74	Amdhd1	INSIDE	1.610	2.369	1000.49	2369.90	3.813	822.61	3136.64
A_68_P25951429	chr8:72897357-72897401	NM_016685:-68	Comp	PROMOTER	1.610	3.467	1420.43	4924.30	5.580	1152.70	6432.41
A_68_P23226835	chr4:115616658-115616702	NM_001025567:-4149	Dmbx1	PROMOTER	1.610	3.342	3326.32	11115.32	5.381	2563.60	13794.61
A_68_P29631109	chr14:58617905-58617949	NM_028643:173	Efha1	INSIDE	1.610	2.344	692.10	1622.39	3.773	615.13	2321.20
A_68_P21089590	chr2:28048879-28048923	NM_001038614:288	Olfm1	INSIDE	1.610	1.554	1325.05	2058.76	2.502	1162.53	2908.28
A_68_P31117688	chr17:27792442-27792486	NM_011861:-162	Pacsin1	PROMOTER	1.610	3.962	687.38	2723.32	6.378	565.99	3609.76
A_68_P23298636	chr4:129497126-129497170	NM_008974:-574	Ptp4a2	PROMOTER	1.610	1.810	1757.41	3181.61	2.915	1435.54	4185.16
A_68_P32975180	chr9:108393143-108393187	NM_027804:158	Usp19	INSIDE	1.610	2.831	1154.50	3268.25	4.559	1078.28	4915.64
A_68_P26129049	chr8:107064933-107064977	NM_053070:248	Car7	INSIDE	1.609	4.243	3692.17	15666.86	6.830	2946.61	20123.93
A_68_P21639427	chr2:132088879-132088923	NM_138651:-92	Cds2	DIVERGENT_PROMOTER	1.609	4.699	469.43	2205.66	7.559	401.49	3035.01
A_68_P26141979	chr8:109417535-109417579	NM_011574:17	Cirh1a	INSIDE	1.609	5.028	1394.45	7010.82	8.089	1220.21	9870.28
A_68_P32454735	chrX:68917753-68917797	NM_010340:833	Gpr50	INSIDE	1.609	2.705	1139.03	3080.77	4.352	599.11	2607.06
A_68_P23588236	chr5:33678555-33678599	NM_021500:356	Maea	INSIDE	1.609	5.083	3030.57	15404.30	8.177	2515.92	20572.74
A_68_P24117361	chr5:136164752-136164796	NM_008898:-309	Por	PROMOTER	1.609	5.202	3240.90	16858.04	8.370	2280.86	19090.59
A_68_P26805694	chr9:106789235-106789279	NM_175402:75	Rbm15b	INSIDE	1.609	4.059	394.04	1599.22	6.532	333.52	2178.54
A_68_P23158199	chr4:101760754-101760798	ENSMUST00000106911:629		INSIDE	1.609	4.814	2102.05	10119.75	7.746	2023.87	15677.45
A_68_P29050103	chr13:54666500-54666544	NM_133797:221	4833439L19Rik	INSIDE	1.608	6.099	2522.42	15383.10	9.808	1975.41	19375.44
A_68_P25724841	chr8:26127277-26127321	NM_007404:96	Adam9	INSIDE	1.608	2.741	2955.35	8101.89	4.408	2472.37	10898.08
A_68_P28329059	chr12:31758655-31758699	NM_027828:-156	Fam110c	PROMOTER	1.608	2.881	1404.76	4046.65	4.632	1230.34	5698.63
A_68_P20644877	chr1:138156885-138156929	NM_001101516:544	Gpr25	INSIDE	1.608	1.686	764.98	1289.80	2.712	648.05	1757.37

ProbeName	Target position of probe on CpG island microarray	TargetID	GeneSymbol	CpG island Description	Ratio of relative methylation (TiO_2-NP/Vehicle)	Sham group			TiO_2-H group		
						Relative methylation (Cy5/Cy3)	Cy3 signal (Input)	Cy5 signal (MeDIP)	Relative methylation (Cy5/Cy3)	Cy3 signal (Input)	Cy5 signal (MeDIP)
A_68_P25007878	chr7:25800603-25800647	NM_008168:56764	Grik5	INSIDE	1.608	4.820	2215.50	10677.62	7.749	1490.88	11552.75
A_68_P27182287	chr10:61246439-61246483	NM_207000:152	H2afy2	INSIDE	1.608	3.190	2241.58	7150.70	5.131	1689.84	8670.31
A_68_P25656224	chr8:11557705-11557749	NM_011919:1661	Ing1	INSIDE	1.608	5.077	2794.35	14186.40	8.162	2248.83	18355.30
A_68_P21137838	chr2:35960787-35960831	NM_001083126:-1228	Lhx6	PROMOTER	1.608	3.570	1602.64	5721.08	5.740	1283.41	7366.49
A_68_P21428205	chr2:92229093-92229137	NM_001202445:3726	Mapk8ip1	INSIDE	1.608	1.807	2330.81	4212.43	2.905	1815.98	5275.98
A_68_P27922751	chr11:74651412-74651456	NM_010813:7009	Mnt	INSIDE	1.608	4.731	2325.84	11002.83	7.608	1797.06	13671.47
A_68_P26816174	chr9:108594858-108594902	NM_008924:407	Prkar2a	INSIDE	1.608	2.779	1177.52	3272.61	4.468	955.42	4269.21
A_68_P30379756	chr15:81641379-81641423	NM_017376:-443	Tef	PROMOTER	1.608	3.952	12834.27	50717.44	6.355	10155.86	64536.56
A_68_P27446660	chr10:110446716-110446760	NM_172554:384	Zdhhc17	INSIDE	1.608	1.530	3567.01	5458.66	2.461	2850.16	7014.34
A_68_P25784087	chr8:37210103-37210147	NM_172911:52243	D8Ertd82e	INSIDE	1.607	4.243	5872.86	24917.49	6.816	4103.14	27967.73
A_68_P26659813	chr9:78328998-78329042	NM_010106:511	Eef1a1	INSIDE	1.607	1.741	1728.57	3008.93	2.798	1437.16	4021.22
A_68_P29034741	chr13:51941420-51941464	NM_011817:-601	Gadd45g	PROMOTER	1.607	4.308	2411.71	10388.75	6.924	1861.97	12891.70
A_68_P25471703	chr7:128178649-128178693	NM_021554:712	Mettl9	INSIDE	1.607	8.648	1551.29	13416.01	13.900	1343.46	18673.87
A_68_P23051091	chr4:81088361-81088405	NM_010820:327	Mpdz	INSIDE	1.607	3.445	10469.36	36066.76	5.537	8861.19	49066.76
A_68_P24618284	chr6:87761631-87761675	NM_133717:-1904	Rab43	PROMOTER	1.607	3.902	5101.09	19903.73	6.270	4202.66	26351.13
A_68_P23999965	chr5:114221529-114221573	NM_016926:108	Sart3	INSIDE	1.607	2.436	1029.35	2507.68	3.916	916.10	3587.48
A_68_P21635209	chr2:131317684-131317728	NM_001177833:109	Smox	INSIDE	1.607	7.670	1752.26	13439.82	12.323	1384.68	17062.93
A_68_P32050810	chr19:29122317-29122361	NM_021299:54	Ak3	INSIDE	1.606	6.341	972.54	6167.01	10.181	734.27	7475.93
A_68_P23299955	chr4:129724973-129725017	NM_028266:-89	Col16a1	PROMOTER	1.606	3.786	2438.22	9230.27	6.081	2043.77	12427.41
A_68_P29346593	chr13:115249654-115249698	NM_008046:-738	Fst	PROMOTER	1.606	2.498	3716.50	9282.35	4.010	2635.06	10566.83
A_68_P31672402	chr18:45719605-45719649	NM_080465:-181	Kcnn2	PROMOTER	1.606	3.625	3770.40	13666.74	5.820	3126.64	18196.65
A_68_P20620522	chr1:134195663-134195707	NM_144810:482	Klhdc8a	INSIDE	1.606	3.422	890.52	3047.47	5.495	786.43	4321.27
A_68_P21583618	chr2:121781814-121781858	NM_008545:-8	Mageb3	DIVERGENT_PROMOTER	1.606	1.769	3483.81	6162.50	2.841	2685.15	7628.37
A_68_P31415143	chr17:86567807-86567851	NM_011104:704	Prkce	INSIDE	1.606	2.633	1510.89	3977.79	4.228	1249.30	5282.05
A_68_P25195707	chr7:74904146-74904190	NM_183312:460	Synm	INSIDE	1.606	1.807	1191.57	2153.03	2.902	1015.20	2946.60
A_68_P29210184	chr13:89879184-89879228	NM_001081249:2911	Vcan	INSIDE	1.606	2.401	503.61	1209.38	3.856	446.94	1723.49
A_68_P24620345	chr6:88147159-88147203	NM_008090:-1477	Gata2	PROMOTER	1.605	1.930	784.07	1513.35	3.097	737.04	2282.76
A_68_P21957226	chr3:14534076-14534120	NM_001163579:311	Lrrcc1	INSIDE	1.605	3.448	2014.65	6946.97	5.533	1548.92	8570.49
A_68_P24428222	chr6:48547522-48547566	NM_001079901:3663	Repin1	INSIDE	1.605	2.155	589.84	1270.92	3.459	464.36	1606.02
A_68_P32601446	chrX:109008521-109008565			Unknown	1.605	2.464	449.98	1108.76	3.955	314.96	1245.81
A_68_P31620631	chr18:36033282-36033326			Unknown	1.605	3.000	1800.41	5401.85	4.815	1442.62	6946.38
A_68_P26166134	chr8:113551628-113551672	NM_001190800:20	Ddx19b	INSIDE	1.604	2.542	2363.69	6008.76	4.078	1826.18	7447.29
A_68_P31484539	chr18:9212402-9212446	NM_008058:-429	Fzd8	PROMOTER	1.604	2.193	2083.27	4569.12	3.518	1590.11	5594.11
A_68_P27285907	chr10:80317900-80317944	NM_025349:32	Lsm7	INSIDE	1.604	1.454	1032.35	1501.36	2.333	818.51	1909.53
A_68_P29668497	chr14:65206255-65206299	NR_029813:-3217	Mir124a-1	PROMOTER	1.604	3.902	7366.35	28747.14	6.258	4975.08	31133.68
A_68_P24135185	chr5:139847981-139848025	NR_029768:-2303	Mir339	DIVERGENT_PROMOTER	1.604	3.796	2433.75	9238.48	6.087	2046.61	12458.24
A_68_P26345770	chr9:20970026-20970070	NM_183408:-109	Pde4a	PROMOTER	1.604	5.969	970.27	5791.23	9.573	837.83	8020.49
A_68_P21149540	chr2:38499397-38499441	NM_011187:8	Psmb7	INSIDE	1.604	2.174	2497.94	5429.94	3.487	1906.18	6647.51
A_68_P23415826	chr4:151363053-151363098	NM_001145929:20	Thap3	INSIDE	1.604	1.650	491.30	810.45	2.646	399.61	1057.32
A_68_P26814405	chr9:108250089-108250133	NM_011678:-51	Usp4	PROMOTER	1.604	2.988	1313.14	3923.16	4.793	1096.62	5256.58
A_68_P30476410	chr15:98608643-98608687	NM_011718:-83	Wnt10b	PROMOTER	1.604	1.353	4751.71	6428.46	2.170	3765.60	8171.33
A_68_P21812641	chr2:163298061-163298105	NR_028122:-198	2310001K24Rik	PROMOTER	1.603	2.860	1751.00	5008.06	4.586	1461.56	6702.35
A_68_P21111049	chr2:31615339-31615383	NM_009594:-104	Abl1	PROMOTER	1.603	1.565	1024.47	1603.78	2.510	855.67	2147.89
A_68_P30026213	chr15:13102919-13102963	NM_007666:454	Cdh6	INSIDE	1.603	2.861	2518.02	7205.20	4.586	2042.09	9365.27
A_68_P32322121	chrX:34731184-34731228	NM_019443:27	Ndufa1	INSIDE	1.603	1.735	1435.26	2490.36	2.782	880.76	2449.86
A_68_P28538315	chr12:74685369-74685413	NM_008856:-637	Prkch	PROMOTER	1.603	4.059	997.12	4046.86	6.506	734.71	4779.80
A_68_P27222450	chr10:68675577-68675621	NM_001081347:194	Rhobtb1	INSIDE	1.603	3.581	5360.76	19199.15	5.739	4084.46	23442.44
A_68_P30976720	chr16:93603886-93603930	NM_145482:152	Setd4	INSIDE	1.603	4.985	4901.08	24430.88	7.990	3852.54	30782.88
A_68_P20872220	chr1:181733104-181733148	NM_026375:1020	Ahctf1	INSIDE	1.602	2.264	1393.41	3155.16	3.628	1213.64	4402.86
A_68_P27311154	chr10:85391524-85391568	NM_001163614:311	Ascl4	INSIDE	1.602	2.795	359.07	1003.47	4.477	296.83	1328.85
A_68_P23299678	chr4:129683985-129684029	NM_001199696:21685	Bai2	INSIDE	1.602	2.638	709.65	1872.38	4.226	588.49	2486.74

ProbeName	Target position of probe on CpG island microarray	TargetID	GeneSymbol	CpG island Description	Ratio of relative methylation (TiO_2-NP/Vehicle)	Sham group			TiO_2-H group		
						Relative methylation (Cy5/Cy3)	Cy3 signal (Input)	Cy5 signal (MeDIP)	Relative methylation (Cy5/Cy3)	Cy3 signal (Input)	Cy5 signal (MeDIP)
A_68_P30567436	chr16:15887941-15887985	NM_007679:584	Cebpd	INSIDE	1.602	4.663	696.93	3249.69	7.469	603.47	4507.38
A_68_P20148715	chr1:36568684-36568728	NM_001039551:-14	Cnnm3	PROMOTER	1.602	2.142	3211.45	6880.19	3.431	2505.82	8597.79
A_68_P32463877	chrX:70882835-70882879	NM_029352:-1923	Dusp9	PROMOTER	1.602	2.572	789.86	2031.39	4.119	437.53	1802.33
A_68_P27083091	chr10:39977457-39977501	NM_026113:-7	Gtf3c6	PROMOTER	1.602	3.173	3397.42	10778.73	5.083	2673.04	13586.99
A_68_P24541702	chr6:72388860-72388904	NM_145569:670	Mat2a	INSIDE	1.602	2.735	1124.77	3076.39	4.383	983.64	4311.30
A_68_P27938848	chr11:77500486-77500530	NM_001024205:868	Nufip2	INSIDE	1.602	2.017	1984.94	4004.32	3.232	1657.94	5359.11
A_68_P24323855	chr6:28931080-28931124	ENSMUST00000164104:-531		PROMOTER	1.602	1.361	1615.01	2197.83	2.181	1235.55	2694.25
A_68_P29005211	chr13:46061364-46061408	NM_009124:-1041	Atxn1	PROMOTER	1.601	3.328	3978.55	13239.55	5.327	2945.77	15691.53
A_68_P22030511	chr3:30755072-30755116	NM_001134385:223	Gpr160	INSIDE	1.601	4.571	796.20	3639.25	7.317	677.92	4960.23
A_68_P30572653	chr16:17233627-17233671	NM_178922:-31	Hic2	PROMOTER	1.601	4.792	2127.46	10194.96	7.674	1775.80	13627.26
A_68_P26016967	chr8:86424037-86424081	NM_181039:62	Lphn1	INSIDE	1.601	3.211	2264.65	7272.80	5.141	2047.70	10527.10
A_68_P23264207	chr4:123341175-123341219	NM_001199136:20407	Macf1	INSIDE	1.601	2.225	1197.98	2665.43	3.563	1057.07	3766.45
A_68_P32777676	chrX:156426760-156426804	NM_001163085:418	Map3k15	INSIDE	1.601	3.482	3434.14	11957.67	5.574	2209.75	12316.96
A_68_P23316515	chr4:132797012-132797056	NM_016693:302	Map3k6	INSIDE	1.601	3.752	940.36	3528.30	6.005	814.28	4889.99
A_68_P23936340	chr5:102094274-102094318	NM_144955:-566	Nkx6-1	PROMOTER	1.601	2.012	1254.18	2523.11	3.222	1064.57	3429.80
A_68_P23398659	chr4:148473653-148473697	NM_019781:247	Pex14	INSIDE	1.601	3.023	1074.05	3246.82	4.840	773.96	3745.89
A_68_P23308582	chr4:131392468-131392512	NM_001083119:1703	Ptpru	INSIDE	1.601	2.243	2002.36	4491.97	3.592	1510.32	5425.13
A_68_P30394811	chr15:84276781-84276825	NM_001163145:725	1810041L15Rik	INSIDE	1.600	3.955	1864.88	7375.82	6.327	1441.31	9119.27
A_68_P26344263	chr9:20672999-20673043	NM_175687:-65	A230050P20Rik	PROMOTER	1.600	11.633	911.91	10608.40	18.613	684.93	12748.62
A_68_P26816895	chr9:108734927-108734971	NM_080437:6298	Celsr3	INSIDE	1.600	4.517	3492.77	15777.49	7.229	2474.22	17885.33
A_68_P26313788	chr9:13632555-13632599	NM_176836:406	Fam76b	INSIDE	1.600	1.733	1041.18	1804.15	2.773	816.41	2263.88
A_68_P29031120	chr13:51266739-51266783	NM_029173:367	Nxnl2	INSIDE	1.600	3.920	2615.10	10251.94	6.274	2115.39	13272.40
A_68_P21762360	chr2:154616838-154616882	NM_001139511:15	Raly	INSIDE	1.600	2.771	2007.08	5561.51	4.434	1712.11	7591.07
A_68_P26811550	chr9:107775126-107775170	NM_011251:2	Rbm6	INSIDE	1.600	4.161	612.46	2548.65	6.658	519.94	3461.97
A_68_P24691293	chr6:100237792-100237836	NM_019743:-462	Rybp	PROMOTER	1.600	3.966	2131.78	8453.78	6.345	1704.68	10815.92
A_68_P31868107	chr18:81182856-81182900	NM_178280:439	Sall3	INSIDE	1.600	3.902	3523.83	13749.52	6.244	2824.25	17633.53
A_68_P22872185	chr4:43536399-43536443	NM_009416:-160	Tpm2	PROMOTER	1.600	2.434	1937.75	4715.58	3.894	1520.65	5921.09
A_68_P23756659	chr5:65883667-65883711	NM_133697:386	1110003E01Rik	INSIDE	1.599	1.904	7260.24	13825.18	3.044	6186.80	18835.45
A_68_P20258663	chr1:58450520-58450564	NM_025824:563	Bzw1	INSIDE	1.599	3.266	2398.42	7833.03	5.223	2083.81	10883.14
A_68_P32576286	chrX:101396172-101396216	NM_001077354:262	C77370	INSIDE	1.599	4.081	7479.24	30522.25	6.527	6051.21	39497.18
A_68_P30010737	chr15:10399801-10399845	NM_030046:449	Dnajc21	INSIDE	1.599	4.759	1474.51	7017.39	7.611	1290.18	9819.95
A_68_P25023124	chr7:29478378-29478422	NM_001163702:238	Fbxo27	INSIDE	1.599	1.525	1881.91	2870.40	2.439	1510.43	3683.48
A_68_P23433055	chr4:154011802-154011846	NM_001177995:-842	Prdm16	PROMOTER	1.599	6.269	1955.43	12259.20	10.026	1598.27	16024.05
A_68_P23407012	chr4:149943746-149943790	NM_001085492:287744	Rere	INSIDE	1.599	1.670	531.33	887.33	2.670	426.21	1138.07
A_68_P22614971	chr3:146162605-146162649	NM_027617:91	Spata1	INSIDE	1.599	3.443	830.28	2858.85	5.504	725.21	3991.58
A_68_P23929552	chr5:100858837-100858881	NR_034038:-304	5430416N02Rik	PROMOTER	1.598	4.386	2091.10	9170.96	7.010	1771.53	12417.68
A_68_P23319354	chr4:133310209-133310253	NM_001080819:-704	Arid1a	PROMOTER	1.598	2.197	3613.68	7937.87	3.510	2699.47	9473.94
A_68_P30362677	chr15:78672776-78672820	NM_027219:-278	Cdc42ep1	PROMOTER	1.598	2.451	661.26	1620.50	3.915	598.40	2342.69
A_68_P30356227	chr15:77672647-77672691	NM_022410:-123	Myh9	PROMOTER	1.598	4.863	5861.69	28505.42	7.773	4607.92	35815.21
A_68_P30458578	chr15:95358757-95358801	NM_016743:359	Nell2	INSIDE	1.598	2.298	4531.86	10413.47	3.672	3655.64	13422.68
A_68_P27178875	chr10:60609327-60609371	NM_009163:1065	Sgpl1	INSIDE	1.598	1.769	2592.83	4587.47	2.828	2049.41	5795.72
A_68_P23072589	chr4:84851297-84851341	NM_019535:-41	Sh3gl2	PROMOTER	1.598	1.967	485.40	954.70	3.142	462.98	1454.73
A_68_P29418922	chr14:14794169-14794213	NM_025435:-451	Thoc7	PROMOTER	1.598	2.309	2178.68	5030.69	3.690	1673.07	6174.15
A_68_P22875967	chr4:44246168-44246212	ENSMUST00000123844:-5006		PROMOTER	1.598	5.630	5663.68	31884.31	8.996	4334.87	38996.60
A_68_P20877466	chr1:182655892-182655936	NM_133225:-259	Acbd3	PROMOTER	1.597	4.420	1550.19	6851.39	7.057	1414.09	9978.61
A_68_P23316365	chr4:132768551-132768595	NM_008154:-121	Gpr3	DIVERGENT_PROMOTER	1.597	4.311	3132.85	13506.40	6.884	2790.16	19208.65
A_68_P26228655	chr8:124277307-124277351	NM_020605:22870	Jph3	INSIDE	1.597	4.386	798.68	3502.79	7.004	595.82	4173.10
A_68_P31259695	chr17:57170806-57170850	NM_010613:102	Khsrp	INSIDE	1.597	4.699	2028.91	9532.99	7.505	1900.29	14262.48
A_68_P25727982	chr8:26707741-26707785	NM_173012:197	Letm2	INSIDE	1.597	5.315	1145.84	6090.24	8.487	988.09	8386.39
A_68_P31790832	chr18:67404825-67404869	NM_172630:638	Mppe1	INSIDE	1.597	2.013	1640.63	3302.46	3.214	1469.60	4723.63
A_68_P25007073	chr7:25669913-25669957	NM_023133:202	Rps19	INSIDE	1.597	3.412	1484.11	5063.19	5.450	1292.39	7043.04

ProbeName	Target position of probe on CpG island microarray	TargetID	GeneSymbol	CpG island Description	Ratio of relative methylation (TiO_2-NP/Vehicle)	Sham group			TiO_2-H group		
						Relative methylation (Cy5/Cy3)	Cy3 signal (Input)	Cy5 signal (MeDIP)	Relative methylation (Cy5/Cy3)	Cy3 signal (Input)	Cy5 signal (MeDIP)
A_68_P32010020	chr19:22213374-22213418	NM_001035239:-210	Trpm3	PROMOTER	1.597	2.658	1036.70	2755.98	4.245	859.73	3649.29
A_68_P28731667	chr12:110291510-110291554	NM_001163175:14895	Begain	INSIDE	1.596	2.689	2110.52	5675.94	4.291	1760.86	7556.49
A_68_P22729559	chr4:11315357-11315401	NM_194055:-1448	Esrp1	PROMOTER	1.596	1.674	934.79	1565.25	2.673	741.88	1982.85
A_68_P28767111	chr12:117519591-117519635	NM_028731:-82	Esyt2	PROMOTER	1.596	2.903	1504.28	4366.28	4.632	1093.98	5067.52
A_68_P23145563	chr4:99324552-99324596	NM_010425:1585	Foxd3	INSIDE	1.596	1.997	1989.00	3971.76	3.187	1744.74	5559.83
A_68_P27560010	chr11:4749422-4749466	NM_010898:86	Nf2	INSIDE	1.596	2.351	3344.47	7862.57	3.752	2818.67	10576.64
A_68_P30568393	chr16:16213292-16213336	NM_026163:-123	Pkp2	PROMOTER	1.596	2.621	1302.42	3413.60	4.184	1066.08	4460.33
A_68_P21142183	chr2:37307736-37307780	NM_001033960:-15	Rabgap1	PROMOTER	1.596	5.228	3556.66	18595.17	8.346	2877.40	24014.49
A_68_P28685879	chr12:102321594-102321638	NM_001160214:296	Smek1	INSIDE	1.596	2.273	1733.74	3940.91	3.627	1557.98	5650.62
A_68_P28614745	chr12:88540669-88540713	NM_001033475:489	Tmed8	INSIDE	1.596	5.480	3474.84	19041.55	8.745	2597.15	22713.11
A_68_P20151305	chr1:36996651-36996695	NM_018872:-300	Tmem131	PROMOTER	1.596	2.608	1176.45	3067.66	4.162	972.02	4045.50
A_68_P21622816	chr2:128891587-128891631	NM_027192:-74	Ttl	PROMOTER	1.596	4.591	2434.91	11177.48	7.325	1775.89	13008.52
A_68_P26276227	chr9:4260687-4260731			Unknown	1.596	3.594	1726.01	6204.06	5.737	1444.73	8288.80
A_68_P31326351	chr17:70318541-70318585	ENSMUST00000060072:-23		PROMOTER	1.596	2.290	406.10	929.83	3.655	342.56	1252.03
A_68_P23817349	chr5:77087036-77087080	ENSMUST00000125922:313		INSIDE	1.596	3.109	1045.08	3248.75	4.961	938.76	4656.77
A_68_P31157711	chr17:35257720-35257764	NM_009975:650	Csnk2b	INSIDE	1.595	1.882	2747.60	5172.15	3.002	1920.74	5766.73
A_68_P22942456	chr4:56959870-56959914	NM_175518:409	D730040F13Rik	INSIDE	1.595	6.093	791.80	4824.54	9.717	707.64	6876.09
A_68_P20613818	chr1:133034787-133034831	NM_145508:3	Dyrk3	INSIDE	1.595	2.262	671.25	1518.37	3.608	591.10	2132.62
A_68_P27897958	chr11:69734258-69734302	NM_001166590:-148	Eif5a	PROMOTER	1.595	4.279	6778.16	29002.29	6.825	5191.36	35430.35
A_68_P32684282	chrX:133653782-133653826	NM_007957:1061	Esx1	INSIDE	1.595	3.270	3979.99	13014.37	5.216	2354.09	12279.98
A_68_P27281299	chr10:79613231-79613275	NM_021565:2218	Midn	INSIDE	1.595	1.942	729.15	1415.90	3.097	609.84	1888.57
A_68_P31112722	chr17:26978019-26978063	NM_008700:470	Nkx2-5	INSIDE	1.595	2.654	1733.03	4599.01	4.234	1351.92	5723.70
A_68_P23694283	chr5:52759912-52759956	NM_011435:4892	Sod3	INSIDE	1.595	4.202	2347.88	9864.82	6.699	1963.26	13152.84
A_68_P27985587	chr11:85651691-85651735	NM_009324:5596	Tbx2	INSIDE	1.595	1.611	1802.26	2903.35	2.570	1401.10	3600.16
A_68_P22948244	chr4:57969284-57969328	NM_011660:-23	Txn1	PROMOTER	1.595	3.002	664.99	1996.03	4.788	619.35	2965.60
A_68_P30476328	chr15:98593484-98593528	NM_007478:43	Arf3	INSIDE	1.594	2.018	3901.49	7874.01	3.216	3017.31	9704.51
A_68_P32573854	chrX:100810244-100810288	NR_028381:8827	B230206F22Rik	INSIDE	1.594	4.072	8298.88	33789.92	6.490	3713.15	24097.37
A_68_P25655858	chr8:11497803-11497847	NM_026995:257	Carkd	INSIDE	1.594	2.684	1919.84	5153.21	4.279	1524.74	6524.19
A_68_P28083428	chr11:102977758-102977802	NM_138753:142	Hexim1	INSIDE	1.594	1.775	3158.02	5605.34	2.830	2549.73	7215.73
A_68_P28397452	chr12:45429719-45429763	NM_001146031:-131	Nrcam	PROMOTER	1.594	3.036	974.16	2957.82	4.838	883.91	4276.75
A_68_P25411125	chr7:117129431-117129475	NM_153525:292	Tmem41b	INSIDE	1.594	2.631	4442.00	11687.76	4.195	3120.46	13091.02
A_68_P27589635	chr11:11013930-11013974	NM_177033:-66	Vwc2	PROMOTER	1.594	2.232	460.96	1029.09	3.559	447.80	1593.57
A_68_P21751445	chr2:152656855-152656899	NM_009743:542	Bcl2l1	INSIDE	1.593	2.403	439.51	1056.14	3.828	385.97	1477.30
A_68_P26971601	chr10:17443503-17443547	NM_010828:491	Cited2	INSIDE	1.593	1.437	1021.65	1468.46	2.290	866.24	1983.53
A_68_P23587982	chr5:33618039-33618083	NM_001198859:-407	Ctbp1	PROMOTER	1.593	2.427	696.54	1690.79	3.867	582.42	2252.40
A_68_P24171442	chr5:147840909-147840953	NM_080795:47218	Lnx2	INSIDE	1.593	2.871	522.01	1498.70	4.572	468.43	2141.74
A_68_P23577160	chr5:31522948-31522992	NM_008014:-52	Ppm1g	PROMOTER	1.593	5.580	2910.39	16240.42	8.892	2461.09	21883.42
A_68_P31759815	chr18:61946490-61946534	NM_178928:-196	Afap1l1	PROMOTER	1.592	1.383	1416.45	1959.53	2.203	1122.92	2473.79
A_68_P27835630	chr11:59041516-59041560	NM_001130408:126	Arf1	INSIDE	1.592	2.033	1722.66	3502.46	3.236	1427.13	4618.53
A_68_P32533249	chrX:90540139-90540183	NM_007492:8176	Arx	INSIDE	1.592	2.504	1322.93	3312.11	3.985	969.13	3861.84
A_68_P21704233	chr2:143740849-143740894	NM_019771:-195	Dstn	PROMOTER	1.592	3.379	3450.55	11659.34	5.380	2818.76	15163.67
A_68_P29259683	chr13:99585179-99585223	NM_172591:204	Fcho2	INSIDE	1.592	4.182	1087.69	4548.58	6.660	923.46	6149.83
A_68_P31922932	chr19:4396607-4396651	NM_001001984:449	Kdm2a	INSIDE	1.592	2.723	3295.26	8971.97	4.335	2770.30	12010.53
A_68_P28051873	chr11:97521768-97521812	NM_139311:-2935	Mllt6	PROMOTER	1.592	4.326	636.54	2753.65	6.886	596.03	4104.36
A_68_P30346592	chr15:76009110-76009154	NM_201394:17008	Plec	INSIDE	1.592	3.611	372.29	1344.43	5.748	317.36	1824.20
A_68_P27565811	chr11:5855744-5855788	NM_019661:6	Ykt6	INSIDE	1.592	1.660	494.53	820.84	2.643	392.71	1037.95
A_68_P22129179	chr3:51145355-51145399	ENSMUST00000135496:-796		PROMOTER	1.592	2.407	1351.50	3253.62	3.832	1112.68	4263.75
A_68_P26022004	chr8:87323463-87323507	NM_001081982:755	Nfix	INSIDE	1.591	1.494	801.96	1198.24	2.377	743.63	1767.40
A_68_P24643143	chr6:92042164-92042208	NM_011630:775	Nr2c2	INSIDE	1.591	2.293	1246.91	2859.03	3.649	1034.35	3774.36
A_68_P29057251	chr13:55927242-55927286	NM_011097:5522	Pitx1	INSIDE	1.591	2.076	628.89	1305.28	3.303	520.93	1720.60
A_68_P31067776	chr17:15968184-15968228	NM_178615:-4656	Rgmb	PROMOTER	1.591	2.104	1439.15	3027.84	3.347	1305.65	4370.41

ProbeName	Target position of probe on CpG island microarray	TargetID	GeneSymbol	CpG island Description	Ratio of relative methylation (TiO_2-NP/Vehicle)	Sham group			TiO_2-H group		
						Relative methylation (Cy5/Cy3)	Cy3 signal (Input)	Cy5 signal (MeDIP)	Relative methylation (Cy5/Cy3)	Cy3 signal (Input)	Cy5 signal (MeDIP)
A_68_P26533458	chr9:55785595-55785639	NM_001081341:306	Scaper	INSIDE	1.591	1.692	6215.45	10515.26	2.692	5095.27	13718.22
A_68_P24124227	chr5:137507750-137507794	NM_001039385:1608	Vgf	INSIDE	1.591	6.809	422.38	2876.05	10.831	459.61	4978.05
A_68_P30578495	chr16:18235059-18235103	NM_172151:149	Zdhhc8	INSIDE	1.591	2.521	1763.98	4446.77	4.011	1455.75	5839.54
A_68_P30347864	chr15:76199968-76200012	NM_021555:663	Fam203a	INSIDE	1.590	1.972	711.02	1402.24	3.137	582.35	1826.67
A_68_P32117383	chr19:41905096-41905140	NM_008043:659	Frat1	INSIDE	1.590	2.682	1948.71	5227.13	4.264	1756.91	7491.10
A_68_P30148784	chr15:38228806-38228850	NM_013692:1634	Klf10	INSIDE	1.590	2.565	2289.00	5871.06	4.079	1935.98	7897.38
A_68_P24434512	chr6:49772812-49772856	NM_023456:107	Npy	INSIDE	1.590	2.141	2840.99	6083.61	3.405	2464.85	8391.72
A_68_P33008139	A_68_P33008139			Unknown	1.590	3.923	9272.31	36375.59	6.238	6729.01	41976.06
A_68_P23467734	chr5:8367164-8367208	NM_001098225:895	Adam22	INSIDE	1.589	2.734	1717.39	4695.16	4.344	1279.99	5560.36
A_68_P20231549	chr1:53409460-53409504	NM_133728:61	Asnsd1	INSIDE	1.589	4.459	1548.31	6903.98	7.087	1276.77	9049.05
A_68_P30478144	chr15:98917975-98918019	NM_001024702:163	C1ql4	INSIDE	1.589	10.977	841.38	9235.98	17.442	720.72	12570.65
A_68_P20885895	chr1:184212870-184212914	NM_007853:-2	Degs1	PROMOTER	1.589	9.939	456.85	4540.71	15.794	377.16	5956.78
A_68_P27475787	chr10:115911149-115911205	NM_021452:-597	Kcnmb4	PROMOTER	1.589	2.152	256.78	552.55	3.418	188.17	643.27
A_68_P23985664	chr5:111847445-111847489	NM_001081235:281	Mn1	INSIDE	1.589	4.139	2050.83	8487.53	6.574	1625.04	10683.59
A_68_P27095676	chr10:42299357-42299401	NM_152229:4016	Nr2e1	INSIDE	1.589	5.157	3488.82	17991.60	8.195	2726.60	22344.39
A_68_P20005794	chr1:4482093-4482137	NM_011441:4380	Sox17	INSIDE	1.589	1.584	758.20	1201.34	2.517	626.08	1575.97
A_68_P26234573	chr8:125135855-125135899	NM_021502:351	Trappc2l	INSIDE	1.589	2.915	1854.83	5407.17	4.632	1334.71	6182.19
A_68_P31098616	chr17:24769435-24769479	NM_001039363:118	Tsc2	INSIDE	1.589	1.349	4463.30	6019.95	2.143	3751.78	8041.93
A_68_P21749221	chr2:152240295-152240339	NM_175126:464	Zcchc3	INSIDE	1.589	3.004	2569.64	7720.35	4.775	2282.28	10898.69
A_68_P24000795	chr5:114358894-114358938	NM_011779:-201	Coro1c	PROMOTER	1.588	2.186	1922.06	4201.58	3.471	1805.60	6267.42
A_68_P24783941	chr6:117857819-117857863	NM_001166427:-9471	Hnrnpf	PROMOTER	1.588	6.228	909.62	5664.78	9.888	695.15	6873.69
A_68_P23138267	chr4:98062517-98062561	NM_001005784:22	Inadl	INSIDE	1.588	4.487	849.63	3811.95	7.127	762.97	5437.57
A_68_P21078768	chr2:26301082-26301126	NM_153125:-368	Sec16a	PROMOTER	1.588	5.643	10762.58	60732.44	8.962	7729.46	69269.22
A_68_P29218720	chr13:91600319-91600363	NM_024186:-361	Ssbp2	PROMOTER	1.588	4.755	2770.98	13176.69	7.551	2307.64	17424.88
A_68_P25377988	chr7:109415960-109416004	NM_009287:-355	Stim1	PROMOTER	1.588	3.944	1503.95	5931.63	6.262	1256.37	7866.97
A_68_P21910960	chr2:181231996-181232040	NM_025482:61	Tpd52l2	INSIDE	1.588	2.405	810.93	1950.67	3.820	706.07	2696.99
A_68_P26974632	chr10:18038691-18038735			Unknown	1.588	2.681	1100.73	2951.11	4.258	938.92	3997.93
A_68_P26102845	chr8:101939884-101939928	NM_001039154:451	Cdh8	INSIDE	1.587	2.112	3141.75	6636.81	3.352	2242.95	7519.00
A_68_P29729812	chr14:76153932-76153976	NM_177381:346	Cog3	INSIDE	1.587	1.418	1256.41	1781.25	2.249	1056.18	2375.74
A_68_P32007552	chr19:21727793-21727837	NM_146096:16	Fam108b	INSIDE	1.587	4.598	476.18	2189.70	7.296	384.95	2808.78
A_68_P31930914	chr19:5803350-5803394	NR_002847:-701	Malat1	PROMOTER	1.587	2.239	2534.95	5676.06	3.553	1910.89	6790.18
A_68_P30346015	chr15:75921006-75921050	NM_144847:-585	Nrbp2	PROMOTER	1.587	2.955	3133.29	9260.38	4.690	2464.60	11559.95
A_68_P20585382	chr1:127457522-127457566	NM_028787:-48	Slc35f5	PROMOTER	1.587	3.073	987.60	3034.69	4.876	876.38	4272.84
A_68_P25588178	chr7:148514084-148514128	NR_024509:78	Tmem80	INSIDE	1.587	2.664	2355.48	6275.28	4.228	1974.60	8349.00
A_68_P25670894	chr8:13870055-13870099	NM_181854:436	Zfp828	INSIDE	1.587	1.726	1360.09	2347.50	2.740	1066.27	2921.49
A_68_P28745339	chr12:112908725-112908769	NM_021273:1803	Ckb	INSIDE	1.586	3.597	1932.40	6951.18	5.703	1796.13	10243.90
A_68_P21568184	chr2:118993532-118993576	NM_177157:31	Gchfr	INSIDE	1.586	3.148	2843.34	8949.78	4.993	2288.86	11428.31
A_68_P32181854	chr19:53403918-53403962	NM_001008543:-995	Mxi1	PROMOTER	1.586	2.159	1039.87	2244.67	3.423	842.54	2884.42
A_68_P27551118	chr11:3189560-3189605	NM_019574:-877	Patz1	PROMOTER	1.586	2.121	4010.21	8505.49	3.364	3196.90	10755.63
A_68_P26573393	chr9:62827843-62827887	NM_019663:822	Pias1	INSIDE	1.586	1.682	3374.87	5675.32	2.667	2383.82	6356.72
A_68_P20441527	chr1:93194765-93194809	NM_016717:-128	Scly	PROMOTER	1.586	4.107	3339.04	13712.54	6.514	2809.46	18299.80
A_68_P30572142	chr16:17131635-17131679	NM_022324:820	Sdf2l1	INSIDE	1.586	2.415	2158.71	5212.22	3.830	1737.14	6652.61
A_68_P26822089	chr9:110034498-110034542	NM_009211:-7	Smarcc1	PROMOTER	1.586	6.946	17001.44	118093.90	11.014	16425.11	180909.50
A_68_P23332899	chr4:135804076-135804120	NM_011542:227	Tcea3	INSIDE	1.586	2.093	1284.42	2687.88	3.319	1047.49	3476.56
A_68_P20156316	chr1:37922113-37922157	NM_207228:-210	Tsga10	DIVERGENT_PROMOTER	1.586	1.464	728.86	1066.79	2.321	599.54	1391.72
A_68_P28149093	chr11:114536801-114536845	NM_053273:41	Ttyh2	INSIDE	1.586	5.212	2080.39	10842.37	8.265	1728.79	14288.31
A_68_P24298747	chr6:23789611-23789655	NM_153163:-332	Cadps2	PROMOTER	1.585	1.887	2105.53	3972.97	2.991	1721.36	5148.84
A_68_P23363266	chr4:140864579-140864623	NM_010139:7446	Epha2	INSIDE	1.585	1.971	645.57	1272.51	3.125	472.81	1477.61
A_68_P29619799	chr14:56386649-56386693	NM_008519:1872	Ltb4r1	INSIDE	1.585	6.299	771.24	4857.79	9.981	638.62	6373.79
A_68_P31121837	chr17:28465985-28466029	NM_011287:591	Rpl10a	INSIDE	1.585	2.638	2942.12	7761.29	4.181	2365.40	9888.84
A_68_P21100496	chr2:29820843-29820887	NM_001076554:-215	Spna2	PROMOTER	1.585	2.106	2808.10	5914.80	3.338	2344.22	7824.94

ProbeName	Target position of probe on CpG island microarray	TargetID	GeneSymbol	CpG island Description	Ratio of relative methylation (TiO_2-NP/Vehicle)	Sham group			TiO_2-H group		
						Relative methylation (Cy5/Cy3)	Cy3 signal (Input)	Cy5 signal (MeDIP)	Relative methylation (Cy5/Cy3)	Cy3 signal (Input)	Cy5 signal (MeDIP)
A_68_P26165551	chr8:113443849-113443893	NM_009179:106	St3gal2	INSIDE	1.585	1.358	1611.73	2188.02	2.152	1539.91	3313.18
A_68_P25724844	chr8:26127584-26127628	NM_027194:-76	Tm2d2	DIVERGENT_PROMOTER	1.585	3.211	1452.58	4664.16	5.088	1112.65	5661.61
A_68_P22200394	chr3:65333128-65333172	NR_027955:186	4931440P22Rik	INSIDE	1.584	2.219	2078.16	4610.97	3.514	1826.35	6418.43
A_68_P22311242	chr3:87803722-87803766	NM_007529:534	Bcan	INSIDE	1.584	1.717	634.64	1089.84	2.719	548.77	1492.29
A_68_P31152235	chr17:34259132-34259176	NM_010238:-462	Brd2	PROMOTER	1.584	2.928	654.82	1917.42	4.640	523.46	2428.63
A_68_P20411731	chr1:88256083-88256127	NR_015507:-44	C130036L24Rik	DIVERGENT_PROMOTER	1.584	5.622	3907.23	21965.87	8.906	2878.08	25631.84
A_68_P21566994	chr2:118752369-118752413	NM_028117:158	Chst14	INSIDE	1.584	2.111	4274.41	9021.50	3.342	3244.99	10846.08
A_68_P29470478	chr14:25065176-25065220	NM_001163513:-56	Dlg5	PROMOTER	1.584	2.924	782.41	2287.43	4.630	663.38	3071.49
A_68_P30345428	chr15:75834117-75834161	NM_001168253:5790	Fam83h	INSIDE	1.584	8.887	2371.19	21072.30	14.073	1940.59	27309.55
A_68_P28656384	chr12:96934105-96934150	NM_201518:3692	Flrt2	INSIDE	1.584	2.959	1264.47	3741.34	4.687	1024.72	4803.15
A_68_P23403797	chr4:149383165-149383209	NM_173371:-54	H6pd	PROMOTER	1.584	6.988	3001.40	20974.56	11.069	2237.16	24763.46
A_68_P31621445	chr18:36177706-36177750	NM_001167891:179086	Nrg2	INSIDE	1.584	2.561	186.27	476.99	4.056	214.79	871.25
A_68_P26398232	chr9:30838076-30838120	NM_001115130:-130	Zbtb44	PROMOTER	1.584	5.966	5734.07	34207.50	9.452	4537.35	42885.20
A_68_P30371089	chr15:80086079-80086123	NM_009716:487	Atf4	INSIDE	1.583	3.049	1179.31	3596.02	4.827	964.24	4654.11
A_68_P30589274	chr16:20733484-20733528	NM_009893:307	Chrd	INSIDE	1.583	1.426	2944.10	4199.57	2.257	2340.81	5284.08
A_68_P29699037	chr14:70479901-70479945	NM_018781:2671	Egr3	INSIDE	1.583	4.276	1107.75	4736.44	6.769	861.94	5834.35
A_68_P22325451	chr3:90280267-90280311	NM_026374:166	Ilf2	INSIDE	1.583	4.404	4144.81	18254.36	6.971	3183.87	22195.81
A_68_P27787814	chr11:50105808-50105852	NM_175334:8	Maml1	INSIDE	1.583	2.825	2008.46	5672.99	4.471	1575.17	7043.18
A_68_P31556965	chr18:23962258-23962302	NM_153058:-190	Mapre2	PROMOTER	1.583	2.325	1721.23	4002.09	3.681	1225.12	4510.15
A_68_P22083003	chr3:40886734-40886778	NM_027558:212	Pgrmc2	INSIDE	1.583	4.991	30520.14	152321.60	7.899	21096.52	166634.90
A_68_P22658557	chr3:153388050-153388094	NM_011372:25	St6galnac3	INSIDE	1.583	2.667	3430.94	9151.06	4.222	2955.87	12479.41
A_68_P25503171	chr7:134093152-134093196	NM_001039645:-79	Asphd1	DIVERGENT_PROMOTER	1.582	1.818	2003.95	3643.79	2.877	1578.37	4540.81
A_68_P24449403	chr6:52196333-52196377	NM_010450:-588	Hoxa11	PROMOTER	1.582	2.100	1809.28	3799.64	3.323	1412.21	4692.87
A_68_P28751168	chr12:113838436-113838480	NM_198411:11464	Inf2	INSIDE	1.582	3.465	1310.26	4540.69	5.484	1032.55	5662.21
A_68_P28549425	chr12:76697618-76697662	NM_012024:-453	Ppp2r5e	PROMOTER	1.582	2.537	3045.09	7725.01	4.013	2503.22	10046.44
A_68_P28159701	chr11:116359924-116359968	NM_026364:-8286	Prpsap1	PROMOTER	1.582	2.957	3257.37	9633.54	4.679	2512.02	11752.83
A_68_P26044774	chr8:91565521-91565568	NM_021390:2517	Sall1	INSIDE	1.582	4.277	385.41	1648.29	6.766	343.98	2327.19
A_68_P26365989	chr9:25059719-25059763	NM_001205367:-299	Septin7	PROMOTER	1.582	3.061	4140.89	12675.54	4.843	3365.70	16299.80
A_68_P26587800	chr9:65308975-65309019	NM_138584:253	Spg21	INSIDE	1.582	4.852	7065.33	34279.80	7.673	4943.68	37934.16
A_68_P22650044	chr3:152009738-152009782	NM_001076676:316	Usp33	INSIDE	1.582	2.830	733.50	2075.90	4.476	652.95	2922.67
A_68_P30588338	chr16:20591336-20591380	NM_182636:1704	Vwa5b2	INSIDE	1.582	3.605	2568.88	9259.68	5.704	1877.16	10707.37
A_68_P24153997	chr5:143668325-143668369	NM_007393:57	Actb	INSIDE	1.581	2.044	1442.49	2948.69	3.232	1278.47	4131.59
A_68_P29113350	chr13:69138021-69138065	NM_153534:377	Adcy2	INSIDE	1.581	2.342	1347.48	3155.18	3.703	1119.72	4146.15
A_68_P21754297	chr2:153171289-153171333	NM_001039939:-564	Asxl1	PROMOTER	1.581	2.682	4922.89	13203.66	4.241	3681.23	15611.84
A_68_P23418114	chr4:151712427-151712471	NM_001081376:-311	Chd5	PROMOTER	1.581	2.138	1253.46	2679.94	3.379	968.47	3272.88
A_68_P25504835	chr7:134404064-134404108	NM_023203:95	Dctpp1	INSIDE	1.581	2.655	4740.09	12584.03	4.197	3408.48	14305.16
A_68_P31939393	chr19:7416055-7416099	NM_001080388:258	Mark2	INSIDE	1.581	2.657	5515.08	14651.84	4.199	4403.33	18491.38
A_68_P30568394	chr16:16213390-16213434	NM_026163:-25	Pkp2	PROMOTER	1.581	1.469	1701.74	2499.70	2.322	1438.16	3338.92
A_68_P32036088	chr19:26681356-26681400	NM_011416:1729	Smarca2	INSIDE	1.581	3.893	616.82	2401.14	6.156	548.04	3373.76
A_68_P24595943	chr6:83817210-83817254	ENSMUST00000113842:8189		DOWNSTREAM	1.581	1.572	847.83	1332.65	2.486	720.15	1790.19
A_68_P30382137	chr15:82043157-82043202	NM_001207021:66	AI848285	INSIDE	1.580	3.256	825.33	2687.65	5.144	638.50	3284.60
A_68_P21875562	chr2:174289603-174289647	NM_025983:-22	Atp5e	PROMOTER	1.580	3.191	1417.02	4521.49	5.041	1276.23	6434.07
A_68_P27649080	chr11:23979542-23979586	NM_001159290:-1130	Bcl11a	PROMOTER	1.580	1.559	1357.49	2115.82	2.463	1197.90	2950.60
A_68_P26816894	chr9:108734858-108734902	NM_080437:6230	Celsr3	INSIDE	1.580	3.081	4378.64	13489.38	4.868	3369.52	16404.32
A_68_P23345719	chr4:137990637-137990681	NM_026689:72	Mul1	INSIDE	1.580	2.436	2819.27	6866.86	3.849	2247.89	8653.07
A_68_P32462210	chrX:70599683-70599727	NM_001160229:11745	Zfp275	INSIDE	1.580	3.811	1027.01	3913.44	6.020	642.16	3866.00
A_68_P30356303	chr15:77688418-77688462			Unknown	1.580	1.913	1944.48	3720.65	3.023	1658.89	5014.82
A_68_P28458172	chr12:57782364-57782408	ENSMUST00000159874:-8518		PROMOTER	1.580	3.846	1132.52	4355.31	6.075	886.88	5387.73
A_68_P26342888	chr9:20348946-20348990	ENSMUST00000171046:111765		INSIDE	1.580	1.813	1457.75	2642.66	2.864	1294.14	3706.00
A_68_P30476596	chr15:98637471-98637515	NM_001013741:864	Ddn	INSIDE	1.579	3.302	1653.03	5459.03	5.215	1322.62	6897.74
A_68_P24804128	chr6:121362766-121362810	NM_001033354:60908	Iqsec3	INSIDE	1.579	3.200	3220.17	10305.15	5.052	2257.41	11404.89

ProbeName	Target position of probe on CpG island microarray	TargetID	GeneSymbol	CpG island Description	Ratio of relative methylation (TiO_2-NP/Vehicle)	Sham group			TiO_2-H group		
						Relative methylation (Cy5/Cy3)	Cy3 signal (Input)	Cy5 signal (MeDIP)	Relative methylation (Cy5/Cy3)	Cy3 signal (Input)	Cy5 signal (MeDIP)
A_68_P24618420	chr6:87788600-87788644	NM_133934:131	Isy1	INSIDE	1.579	2.686	1150.74	3090.41	4.240	1002.82	4252.19
A_68_P28163660	chr11:117060956-117061000	NM_001113486:4	Septin9	INSIDE	1.579	3.981	1641.20	6532.99	6.285	1352.89	8503.57
A_68_P24954289	chr7:6106868-6106913	NM_001013012:683	Zfp787	INSIDE	1.579	3.682	2148.52	7910.72	5.812	1736.73	10094.57
A_68_P31145425	chr17:32533548-32533592	NR_029458:-6388	A530088E08Rik	PROMOTER	1.578	5.700	2471.14	14086.48	8.994	2301.26	20697.64
A_68_P26864197	chr9:117949927-117949971	NM_001048146:332	Azi2	INSIDE	1.578	4.258	401.44	1709.32	6.717	385.58	2590.10
A_68_P20472150	chr1:99557453-99557497	NM_133825:1121	D1Ertd622e	INSIDE	1.578	3.205	1890.17	6057.91	5.058	1626.71	8228.26
A_68_P30423478	chr15:88979773-88979817	NM_011161:242	Mapk11	INSIDE	1.578	2.423	2470.96	5987.54	3.823	2005.79	7668.84
A_68_P20352935	chr1:75248212-75248256	NM_008985:12549	Ptprn	INSIDE	1.578	2.927	2292.55	6709.62	4.618	1724.70	7964.70
A_68_P24018800	chr5:117566119-117566163	NM_001122666:-138	Suds3	DIVERGENT_PROMOTER	1.578	3.103	670.81	2081.47	4.896	648.91	3176.91
A_68_P28553589	chr12:77470150-77470194	NM_001172104:279	Zbtb25	INSIDE	1.578	2.047	870.21	1781.03	3.230	770.10	2487.54
A_68_P30243549	chr15:57526400-57526444	NM_199449:201	Zhx2	INSIDE	1.578	5.586	1314.57	7343.29	8.817	1022.93	9019.07
A_68_P27308088	chr10:84849580-84849624	NM_028709:44	Btbd11	INSIDE	1.577	2.863	487.57	1395.76	4.515	441.56	1993.86
A_68_P30895871	chr16:78377255-78377299	NM_009770:-246	Btg3	PROMOTER	1.577	3.362	738.15	2481.85	5.303	628.77	3334.18
A_68_P31373927	chr17:79234917-79234961	NM_181649:83	Ccdc75	INSIDE	1.577	2.673	2020.61	5401.30	4.216	1599.74	6744.32
A_68_P30542887	chr16:10545493-10545537	NM_001204229:83	Clec16a	INSIDE	1.577	3.144	1929.46	6067.15	4.960	1642.69	8147.72
A_68_P24850798	chr6:131338248-131338292	NM_011733:198	Csda	INSIDE	1.577	5.758	1846.89	10634.69	9.081	1644.68	14935.00
A_68_P24501242	chr6:63207663-63207707	NM_008167:834	Grid2	INSIDE	1.577	4.696	1727.57	8111.93	7.405	1561.71	11564.15
A_68_P31126967	chr17:29400949-29400993	NM_026845:-54	Ppil1	DIVERGENT_PROMOTER	1.577	1.945	970.15	1886.55	3.066	846.71	2596.06
A_68_P28237421	chr12:11273151-11273195	NM_025695:481	Smc6	INSIDE	1.577	2.663	948.41	2526.00	4.199	774.69	3253.12
A_68_P28074030	chr11:101327012-101327056	NM_012037:479	Vat1	INSIDE	1.577	3.290	3016.42	9925.08	5.188	2617.11	13577.53
A_68_P30578497	chr16:18235357-18235401	NM_172151:-149	Zdhhc8	PROMOTER	1.577	2.553	2395.00	6115.41	4.026	1963.17	7903.27
A_68_P28751328	chr12:113858149-113858193	NM_007421:-87	Adssl1	PROMOTER	1.576	1.860	1284.81	2389.37	2.931	1070.45	3137.30
A_68_P30588065	chr16:20535666-20535710	NM_009679:112	Ap2m1	INSIDE	1.576	4.216	3006.62	12676.79	6.643	2140.72	14221.37
A_68_P27998411	chr11:88007034-88007078	NM_001172099:23990	Cuedc1	INSIDE	1.576	1.634	744.42	1216.08	2.574	608.68	1566.62
A_68_P22803179	chr4:28740971-28741015	NM_001122889:698	Epha7	INSIDE	1.576	2.231	745.90	1664.05	3.515	564.62	1984.76
A_68_P21680284	chr2:139503094-139503138	NM_001126490:-797	Ism1	PROMOTER	1.576	2.196	1282.18	2816.30	3.462	995.39	3446.44
A_68_P23439858	chr4:155028864-155028908	NM_145124:14450	Mib2	INSIDE	1.576	1.782	3808.57	6788.31	2.808	2786.77	7826.64
A_68_P20412608	chr1:88424274-88424318	NM_008972:986	Ptma	INSIDE	1.576	1.716	777.56	1334.62	2.705	670.32	1813.35
A_68_P29639602	chr14:60244436-60244480	NM_145463:341	Shisa2	INSIDE	1.576	3.893	992.08	3861.92	6.133	779.98	4783.79
A_68_P21420490	chr2:90910289-90910333	NM_026721:68	Slc39a13	INSIDE	1.576	1.606	2381.15	3824.63	2.532	1770.20	4482.07
A_68_P31121999	chr17:28487626-28487670	NM_001098226:-103	Tead3	PROMOTER	1.576	1.906	4153.46	7917.25	3.004	3102.91	9321.84
A_68_P30560722	chr16:14159381-14159425	NM_001081154:-35	4921513D23Rik	DIVERGENT_PROMOTER	1.575	3.794	13836.62	52494.04	5.973	8244.00	49245.55
A_68_P27630023	chr11:20013151-20013195	NM_146243:-218	Actr2	PROMOTER	1.575	4.701	5876.49	27627.98	7.405	4547.82	33674.90
A_68_P21094161	chr2:28773677-28773726	NM_001164186:-1741	Barhl1	PROMOTER	1.575	1.669	957.36	1597.63	2.628	775.30	2037.63
A_68_P28962023	chr13:38751002-38751046	NM_025380:-127	Eef1e1	PROMOTER	1.575	2.491	3706.13	9230.73	3.922	2999.68	11764.15
A_68_P30080950	chr15:25344237-25344281	NR_027376:312	Gm5468	INSIDE	1.575	2.606	378.57	986.49	4.105	368.66	1513.46
A_68_P20905056	chr1:187941218-187941262	NM_146106:-51	Lyplal1	PROMOTER	1.575	3.364	1318.19	4435.03	5.298	1030.23	5457.80
A_68_P29664074	chr14:64479020-64479064	NM_028228:-106	Pinx1	PROMOTER	1.575	3.862	3348.45	12932.88	6.085	2482.57	15106.29
A_68_P30426169	chr15:89422304-89422348	NM_026817:28	Rabl2	INSIDE	1.575	3.008	939.82	2826.53	4.737	772.82	3660.98
A_68_P22963537	chr4:61947638-61947682	NM_025286:182	Slc31a2	INSIDE	1.575	2.257	4243.51	9576.03	3.553	3512.67	12481.00
A_68_P28160203	chr11:116442887-116442931	NM_173755:-147	Ube2o	PROMOTER	1.575	2.804	3375.46	9466.43	4.418	2808.59	12408.08
A_68_P26822984	chr9:110207818-110207862	NM_001081381:145	2610002I17Rik	INSIDE	1.574	2.186	1153.88	2522.94	3.441	942.26	3242.42
A_68_P28002763	chr11:88726023-88726068	NM_001042541:-145	Akap1	PROMOTER	1.574	3.521	843.99	2971.51	5.541	761.65	4220.24
A_68_P29009048	chr13:46764994-46765038	NM_001033192:126	C78339	INSIDE	1.574	2.856	1542.16	4404.73	4.497	1400.30	6296.65
A_68_P33005168	chr7:3366552-3366596	NM_133189:29890	Cacng7	DOWNSTREAM	1.574	3.885	3043.52	11824.58	6.115	2177.56	13315.37
A_68_P22019079	chr3:28680302-28680346	NM_177586:92	Eif5a2	INSIDE	1.574	5.981	1545.59	9243.98	9.412	1505.64	14171.48
A_68_P23362414	chr4:140703695-140703739	NM_172518:-120	Fbxo42	DIVERGENT_PROMOTER	1.574	1.696	1041.78	1767.35	2.670	914.58	2441.71
A_68_P25362933	chr7:106774200-106774244	NM_001195529:1391	Gm4980	INSIDE	1.574	1.983	2380.55	4720.30	3.121	1714.36	5350.31
A_68_P28515346	chr12:70683995-70684039	NM_001033236:-1	Mettl21d	PROMOTER	1.574	3.709	1807.51	6704.49	5.840	1491.54	8710.42
A_68_P30556205	chr16:13257041-13257085	NM_181860:-8109	Mkl2	PROMOTER	1.574	4.312	4838.93	20866.87	6.790	3944.61	26782.12
A_68_P30292508	chr15:66409642-66409686	NM_001081409:531	Phf20l1	INSIDE	1.574	2.490	1736.18	4323.53	3.921	1286.61	5044.53

ProbeName	Target position of probe on CpG island microarray	TargetID	GeneSymbol	CpG island Description	Ratio of relative methylation (TiO_2-NP/Vehicle)	Sham group			TiO_2-H group		
						Relative methylation (Cy5/Cy3)	Cy3 signal (Input)	Cy5 signal (MeDIP)	Relative methylation (Cy5/Cy3)	Cy3 signal (Input)	Cy5 signal (MeDIP)
A_68_P27534649	chr10:126648747-126648791	NM_054097:-90	Pip4k2c	PROMOTER	1.574	2.122	1956.33	4150.66	3.340	1693.26	5656.06
A_68_P28938191	chr13:34436110-34436154	NM_001033167:919	Slc22a23	INSIDE	1.574	3.253	3942.01	12824.17	5.122	2927.73	14995.06
A_68_P26887498	chr9:122026335-122026379	NM_001164572:-27	Snrk	PROMOTER	1.574	3.457	2955.78	10218.80	5.442	2375.99	12929.20
A_68_P28157433	chr11:115971055-115971099	NM_001205081:473	Trim47	INSIDE	1.574	7.514	4631.18	34796.67	11.828	3674.12	43458.28
A_68_P31625687	chr18:36919881-36919925	NM_026464:10	Wdr55	INSIDE	1.574	3.041	1928.33	5863.74	4.786	1532.31	7333.66
A_68_P27563479	chr11:5344842-5344886	NM_001080924:-14	Znrf3	PROMOTER	1.574	1.826	2713.11	4953.86	2.874	2271.20	6527.31
A_68_P23797912	chr5:73883055-73883099	NM_001190733:812	Dcun1d4	INSIDE	1.573	9.002	22377.90	201451.70	14.162	12948.66	183376.70
A_68_P20237360	chr1:54614625-54614669	NM_001163314:-120	Pgap1	PROMOTER	1.573	1.663	1951.68	3246.19	2.616	1243.26	3252.49
A_68_P28774236	chr12:118772882-118772926	NM_175930:17953	Rapgef5	INSIDE	1.573	1.763	672.93	1186.15	2.773	608.43	1687.13
A_68_P24040030	chr5:121099321-121099365	NM_013832:512	Rasal1	INSIDE	1.573	2.490	6047.64	15060.88	3.917	4697.18	18400.08
A_68_P23296214	chr4:129012152-129012196	NM_009030:440	Rbbp4	INSIDE	1.573	0.381	1028.53	392.14	0.600	1088.76	652.93
A_68_P30379760	chr15:81641863-81641907	NM_017376:41	Tef	INSIDE	1.573	2.878	884.23	2544.59	4.526	802.40	3631.53
A_68_P32488661	chrX:78315918-78315962	NM_138751:-42	Tmem47	PROMOTER	1.573	2.978	1549.11	4612.70	4.684	1104.22	5171.91
A_68_P23246908	chr4:119406642-119406686	ENSMUST00000123698:219		INSIDE	1.573	2.387	438.30	1046.35	3.756	363.98	1367.23
A_68_P22129174	chr3:51144832-51144876	ENSMUST00000135496:-274		PROMOTER	1.573	2.065	4796.61	9906.33	3.248	3599.83	11691.13
A_68_P27764455	chr11:45665845-45665889	NM_001045520:401	Clint1	INSIDE	1.572	1.508	1316.31	1985.46	2.371	1074.77	2547.83
A_68_P20462986	chr1:97210270-97210314	NM_026321:88	Fam174a	INSIDE	1.572	2.255	485.23	1094.27	3.546	465.92	1651.96
A_68_P25348908	chr7:104230293-104230337	NM_001162477:54	Gab2	INSIDE	1.572	2.820	412.83	1164.28	4.434	361.09	1600.99
A_68_P26656177	chr9:77602711-77602755	NM_010295:391	Gclc	INSIDE	1.572	0.426	1277.34	544.15	0.670	1078.63	722.15
A_68_P24476912	chr6:56747694-56747738	NM_145958:91	Kbtbd2	INSIDE	1.572	1.972	1031.50	2033.69	3.100	895.96	2777.48
A_68_P24818877	chr6:125081806-125081850	NM_138747:-72	Nop2	PROMOTER	1.572	2.177	640.40	1394.03	3.421	552.07	1888.75
A_68_P30650841	chr16:32078788-32078844	NM_177326:543	Pak2	INSIDE	1.572	5.599	463.67	2596.20	8.801	294.26	2589.67
A_68_P26133404	chr8:107898928-107898972	NM_001081333:-330	Plekhg4	PROMOTER	1.572	3.196	1633.40	5220.99	5.026	1335.87	6714.39
A_68_P25114625	chr7:57033846-57033890	NM_133740:141	Prmt3	INSIDE	1.572	1.486	840.46	1249.05	2.336	683.42	1596.68
A_68_P23703749	chr5:54390129-54390173	NM_001081103:389	Stim2	INSIDE	1.572	4.884	1311.45	6404.52	7.676	1102.18	8460.46
A_68_P32537532	chrX:91368506-91368550	NM_001044386:218	Zfx	INSIDE	1.572	4.782	3575.38	17099.25	7.520	2303.95	17325.96
A_68_P24066928	chr5:125921960-125922004	NM_029752:45	Bri3bp	INSIDE	1.571	1.735	1634.18	2835.85	2.727	1390.05	3790.44
A_68_P30822064	chr16:64851589-64851633	NM_178647:-300	Cggbp1	PROMOTER	1.571	1.499	3489.85	5231.78	2.355	2884.05	6790.67
A_68_P21904961	chr2:180332965-180333009	NM_009936:60	Col9a3	INSIDE	1.571	2.012	1222.98	2461.18	3.162	972.24	3074.01
A_68_P27282058	chr10:79727411-79727455	NM_001122604:-303	Dazap1	DIVERGENT_PROMOTER	1.571	1.727	1340.24	2314.13	2.713	1046.16	2838.21
A_68_P31135104	chr17:30749389-30749433	NM_001113560:194	Glo1	INSIDE	1.571	4.088	647.44	2646.58	6.422	564.46	3624.90
A_68_P27425024	chr10:106709059-106709103	NM_001033223:194	Lin7a	INSIDE	1.571	2.971	3596.44	10684.37	4.666	2802.49	13076.50
A_68_P21130446	chr2:34769487-34769531	NM_028716:-12	Phf19	PROMOTER	1.571	5.149	4230.44	21782.11	8.088	3388.78	27408.76
A_68_P25670554	chr8:13785750-13785794	NM_025924:158	Upf3a	INSIDE	1.571	3.934	1989.76	7828.20	6.182	1622.32	10028.85
A_68_P28613409	chr12:88331117-88331161	NM_001080943:-1512	Zdhhc22	PROMOTER	1.571	3.192	1058.11	3377.73	5.015	913.34	4579.94
A_68_P23467741	chr5:8368019-8368063	NM_001098225:41	Adam22	INSIDE	1.570	1.732	1004.19	1738.76	2.719	921.50	2505.13
A_68_P30990999	chr16:96304407-96304451	NM_145125:-393	Brwd1	PROMOTER	1.570	2.758	5273.40	14544.89	4.331	3882.58	16817.11
A_68_P28067423	chr11:100181873-100181917	NM_011508:585	Eif1	INSIDE	1.570	2.429	743.63	1806.49	3.813	565.99	2158.28
A_68_P27055250	chr10:33847341-33847385	NM_175449:416	Fam26f	INSIDE	1.570	3.541	2209.17	7822.10	5.557	1786.17	9926.43
A_68_P22892659	chr4:47105046-47105090	NM_172693:244	Galnt12	INSIDE	1.570	4.111	837.62	3443.52	6.453	723.30	4667.16
A_68_P27253670	chr10:74430232-74430276	NM_010311:279	Gnaz	INSIDE	1.570	1.802	1266.85	2282.44	2.829	1006.12	2846.54
A_68_P26885522	chr9:121686826-121686870	NM_028202:124	Kbtbd5	INSIDE	1.570	2.948	478.89	1411.61	4.628	485.79	2248.24
A_68_P24119778	chr5:136646540-136646584	NM_178751:-36	Orai2	PROMOTER	1.570	2.888	626.29	1808.83	4.534	612.18	2775.79
A_68_P26282992	chr9:6168448-6168492	NM_027924:-141	Pdgfd	PROMOTER	1.570	2.157	1141.79	2463.16	3.387	924.44	3131.10
A_68_P30346615	chr15:76011854-76011898	NM_201394:14264	Plec	INSIDE	1.570	4.794	1004.83	4816.70	7.527	812.41	6114.67
A_68_P28431157	chr12:52792863-52792907	NM_001172098:17	Strn3	INSIDE	1.570	1.974	635.80	1255.32	3.100	541.32	1678.28
A_68_P26894035	chr9:123169998-123170042	NM_001002267:-113	Tmem158	PROMOTER	1.570	5.235	1322.39	6923.00	8.219	1293.02	10627.22
A_68_P26295538	chr9:9239294-9239338	NM_027823:-303	Arhgap42	PROMOTER	1.569	3.162	3797.50	12008.35	4.961	2969.28	14729.70
A_68_P32111326	chr19:40905687-40905731	NM_172839:-60	Ccnj	PROMOTER	1.569	2.042	377.81	771.60	3.205	357.59	1146.05
A_68_P26940376	chr10:11063434-11063478	NM_010146:214	Epm2a	INSIDE	1.569	2.438	362.07	882.58	3.824	380.29	1454.08
A_68_P27289087	chr10:80820153-80820197	NM_028657:-140	F630110N24Rik	DIVERGENT_PROMOTER	1.569	2.472	1187.14	2934.50	3.880	956.23	3709.79

ProbeName	Target position of probe on CpG island microarray	TargetID	GeneSymbol	CpG island Description	Ratio of relative methylation (TiO_2-NP/Vehicle)	Sham group			TiO_2-H group		
						Relative methylation (Cy5/Cy3)	Cy3 signal (Input)	Cy5 signal (MeDIP)	Relative methylation (Cy5/Cy3)	Cy3 signal (Input)	Cy5 signal (MeDIP)
A_68_P29448409	chr14:21514700-21514744	NM_028428:533	Fut11	INSIDE	1.569	1.750	974.29	1705.36	2.746	895.85	2460.26
A_68_P20997431	chr2:9796515-9796559	NM_008091:3691	Gata3	INSIDE	1.569	3.329	5427.34	18065.56	5.224	3876.76	20251.14
A_68_P26003887	chr8:83866396-83866440	ENSMUST00000172167:-36		PROMOTER	1.569	2.225	3413.89	7597.57	3.491	2846.17	9936.75
A_68_P20140952	chr1:34899643-34899687	NM_001160235:231	Fam168b	INSIDE	1.568	3.941	1156.93	4558.99	6.180	1041.76	6438.55
A_68_P25459669	chr7:125849289-125849333	NM_019580:-58	Gde1	DIVERGENT_PROMOTER	1.568	2.848	2463.48	7016.05	4.464	2041.48	9114.14
A_68_P20417070	chr1:89224090-89224134	NM_001110212:509	Gigyf2	INSIDE	1.568	2.784	842.92	2346.29	4.365	744.25	3248.54
A_68_P22404196	chr3:106904415-106904459	NM_008417:-48	Kcna2	PROMOTER	1.568	2.767	2265.93	6269.43	4.338	1842.84	7994.73
A_68_P25011324	chr7:26471878-26471922	NM_011577:-120	Tgfb1	PROMOTER	1.568	2.535	2686.22	6810.85	3.977	2203.33	8762.21
A_68_P27287712	chr10:80611089-80611133	NM_010731:12095	Zbtb7a	INSIDE	1.568	3.451	1624.44	5606.41	5.410	1331.74	7204.85
A_68_P25230180	chr7:80762845-80762889	AK158912:403		INSIDE	1.568	1.546	841.61	1301.17	2.425	793.56	1924.14
A_68_P23396096	chr4:148098703-148098747	ENSMUST00000139895:10913		DOWNSTREAM	1.568	2.054	3592.36	7376.99	3.220	3195.88	10289.84
A_68_P22820149	chr4:32327040-32327088	ENSMUST00000165661:-3643		PROMOTER	1.568	2.459	817.83	2011.16	3.856	654.28	2522.89
A_68_P24980793	chr7:16899189-16899233	NM_133234:4279	Bbc3	INSIDE	1.567	1.681	1963.70	3300.92	2.634	1626.81	4285.17
A_68_P23977812	chr5:110535984-110536028	NM_145147:1210	Gtpbp6	INSIDE	1.567	2.103	995.06	2092.77	3.295	788.22	2597.31
A_68_P26954150	chr10:13685112-13685158	NM_010437:-1050	Hivep2	PROMOTER	1.567	2.407	1916.49	4612.93	3.773	1407.57	5310.50
A_68_P20042287	chr1:13650338-13650382	NM_145381:230	Lactb2	INSIDE	1.567	5.127	2361.50	12108.17	8.035	1864.14	14978.65
A_68_P20897789	chr1:186669350-186669394	NM_133684:354	Mosc2	INSIDE	1.567	4.109	4824.79	19825.41	6.437	4411.19	28396.76
A_68_P24950760	chr7:4788206-4788250	NM_172737:8070	Shisa7	INSIDE	1.567	4.057	712.16	2889.21	6.357	591.98	3763.03
A_68_P20191548	chr1:43991025-43991069	NM_009418:195	Tpp2	INSIDE	1.567	4.232	826.31	3496.70	6.632	684.81	4541.32
A_68_P24025038	chr5:118620106-118620150	NM_007545:356	Hrk	INSIDE	1.566	2.149	1563.84	3361.45	3.366	1428.17	4806.89
A_68_P25503044	chr7:134072503-134072547	NM_172747:132	Kctd13	INSIDE	1.566	5.367	1536.35	8245.63	8.404	1331.12	11186.38
A_68_P30114898	chr15:31460199-31460243	NM_172606:572	Marchf6	INSIDE	1.566	1.520	2694.46	4094.33	2.380	2251.11	5357.45
A_68_P26400215	chr9:31193897-31193941	NM_172766:142	Nfrkb	INSIDE	1.566	3.163	6901.85	21828.89	4.954	5027.00	24903.77
A_68_P23544553	chr5:24414393-24414437	NM_001170555:-87	Prkag2	PROMOTER	1.566	3.308	1145.83	3790.78	5.181	1015.40	5261.24
A_68_P31106682	chr17:25981519-25981564	NM_145999:255	Rhot2	INSIDE	1.566	5.770	2491.85	14378.33	9.036	1778.26	16068.59
A_68_P26996375	chr10:21713739-21713783	NM_011361:-711	Sgk1	PROMOTER	1.566	3.211	605.84	1945.46	5.029	456.14	2293.94
A_68_P29937469	chr14:118633887-118633936	NM_177753:2341	Sox21	INSIDE	1.566	2.106	666.97	1404.33	3.298	548.73	1809.84
A_68_P24425595	A_68_P24425595			Unknown	1.566	4.863	796.97	3875.36	7.616	735.68	5602.61
A_68_P27644170	chr11:22968078-22968122	ENSMUST00000128996:103		INSIDE	1.566	3.114	326.32	1016.16	4.878	341.56	1666.04
A_68_P20893089	chr1:185857699-185857743	NM_028516:157	1700056E22Rik	INSIDE	1.565	3.564	2094.13	7462.54	5.577	1749.00	9754.76
A_68_P28513307	chr12:70298223-70298267	NR_033222:47	9330151L19Rik	INSIDE	1.565	5.402	2373.03	12819.39	8.457	1959.92	16574.74
A_68_P28743070	chr12:112513574-112513618	NM_033603:4275	Amn	INSIDE	1.565	2.620	686.38	1798.21	4.101	589.50	2417.74
A_68_P23295623	chr4:128900595-128900639	NM_001033189:25071	C77080	INSIDE	1.565	2.201	993.95	2187.89	3.445	861.28	2967.49
A_68_P23539566	chr5:23536151-23536195	NM_053090:329	Fam126a	INSIDE	1.565	4.054	2121.20	8598.55	6.346	1830.88	11618.57
A_68_P31484544	chr18:9212850-9212894	NM_008058:19	Fzd8	INSIDE	1.565	2.801	892.40	2499.57	4.384	806.66	3536.09
A_68_P27289029	chr10:80809838-80809882	NM_001163165:3311	Hmg20b	INSIDE	1.565	7.179	2823.24	20269.19	11.233	1862.01	20916.80
A_68_P21839351	chr2:167906222-167906266	NM_021409:-259	Pard6b	PROMOTER	1.565	6.818	1258.10	8577.99	10.671	1114.90	11897.11
A_68_P24979352	chr7:16638192-16638236	AK008102:4421		DOWNSTREAM	1.565	1.509	1254.00	1892.14	2.361	1022.36	2413.86
A_68_P28836964	chr13:14157071-14157115	NM_194262:1034	Arid4b	INSIDE	1.564	2.482	844.98	2096.93	3.880	657.33	2550.72
A_68_P29110678	chr13:68721295-68721339	NM_027123:192	Fastkd3	INSIDE	1.564	2.559	2112.04	5405.13	4.002	1553.20	6215.74
A_68_P32142001	chr19:46150433-46150477	NM_001039351:102	Nolc1	INSIDE	1.564	2.312	2268.24	5243.62	3.616	1941.60	7020.26
A_68_P29269012	chr13:101322550-101322594	NM_008756:-119	Ocln	PROMOTER	1.564	2.208	944.00	2084.61	3.455	1005.10	3472.29
A_68_P21417962	chr2:90420971-90421015	NM_008982:-188	Ptprj	PROMOTER	1.564	4.295	2423.28	10407.62	6.717	1930.35	12966.08
A_68_P28595365	chr12:85262189-85262233	ENSMUST00000085215:584		INSIDE	1.564	2.328	3510.08	8172.80	3.641	2840.92	10345.21
A_68_P28691640	chr12:103707382-103707426	NM_013747:-715	Golga5	PROMOTER	1.563	6.121	432.66	2648.22	9.568	300.97	2879.67
A_68_P23837522	chr5:81449591-81449635	NM_198702:-1005	Lphn3	PROMOTER	1.563	4.017	1797.47	7221.24	6.281	1431.07	8988.10
A_68_P21623003	chr2:128926712-128926756	NM_009086:3	Polr1b	INSIDE	1.563	1.939	2491.03	4831.30	3.031	1831.52	5550.56
A_68_P31757526	chr18:61558105-61558149	NM_133249:1959	Ppargc1b	INSIDE	1.563	2.958	4297.15	12709.21	4.622	3216.73	14868.65
A_68_P28963697	chr13:39052368-39052412	NM_001170430:16	Slc35b3	INSIDE	1.563	1.824	2096.03	3823.89	2.851	1602.74	4569.52
A_68_P30546005	chr16:11066391-11066435	NM_009223:22	Snn	INSIDE	1.563	1.775	1499.49	2661.18	2.774	1273.43	3531.86
A_68_P23222361	chr4:114597756-114597800	NM_015758:840	Foxe3	INSIDE	1.562	2.049	646.36	1324.62	3.200	520.65	1666.31

						Sham group			TiO_2-H group		
ProbeName	Target position of probe on CpG island microarray	TargetID	GeneSymbol	CpG island Description	Ratio of relative methylation (TiO_2-NP/Vehicle)	Relative methylation (Cy5/Cy3)	Cy3 signal (Input)	Cy5 signal (MeDIP)	Relative methylation (Cy5/Cy3)	Cy3 signal (Input)	Cy5 signal (MeDIP)
A_68_P28576082	chr12:81619771-81619815	NM_001081421:-184	Galntl1	PROMOTER	1.562	2.629	854.62	2246.53	4.105	779.49	3200.03
A_68_P31790105	chr18:67293085-67293129	NM_010307:-373	Gnal	PROMOTER	1.562	2.176	769.95	1675.10	3.399	684.73	2327.47
A_68_P23261232	chr4:122866227-122866271	NM_174998:5502	Hpcal4	INSIDE	1.562	3.900	3399.56	13256.62	6.092	2528.50	15404.63
A_68_P29702031	chr14:70953904-70953948	NM_021877:64	Hr	INSIDE	1.562	1.626	6204.00	10086.09	2.539	4768.71	12108.55
A_68_P28107247	chr11:107330576-107330620	NM_145823:1436	Pitpnc1	INSIDE	1.562	1.920	1761.15	3381.05	2.998	1368.08	4102.19
A_68_P24991219	chr7:19650979-19651023	NM_001159671:10965	Rsph6a	INSIDE	1.562	5.179	3800.91	19684.78	8.090	2925.80	23668.66
A_68_P25954806	chr8:73421106-73421150	NR_002901:-1373	Snora68	PROMOTER	1.562	1.778	2759.35	4907.28	2.777	2115.56	5875.71
A_68_P25504683	chr7:134376847-134376891	NM_146201:-88	Zfp553	DIVERGENT_PROMOTER	1.562	6.493	500.43	3249.53	10.143	454.10	4605.86
A_68_P26430110	chr9:37155994-37156038	NM_001081429:-39	Ccdc15	PROMOTER	1.561	6.025	3644.25	21958.08	9.405	2772.80	26079.27
A_68_P22695196	chr4:3866420-3866464	NM_001190322:382	Chchd7	INSIDE	1.561	3.322	1286.05	4272.59	5.187	980.84	5087.42
A_68_P28727385	chr12:109572280-109572324	NM_010010:-288	Cyp46a1	PROMOTER	1.561	2.654	4354.11	11554.80	4.141	3150.60	13047.81
A_68_P24636902	chr6:91066754-91066798	NM_018815:44	Nup210	INSIDE	1.561	4.842	904.94	4381.50	7.557	808.28	6107.96
A_68_P24770499	chr6:115312049-115312093	NM_001127330:832	Pparg	INSIDE	1.561	1.939	1450.39	2812.26	3.026	1089.63	3297.74
A_68_P26894026	chr9:123168701-123168745	NM_001002267:1185	Tmem158	INSIDE	1.561	2.872	2065.30	5931.81	4.485	1627.76	7300.04
A_68_P29964713	chr14:123381923-123381967	NM_028651:539	Tmtc4	INSIDE	1.561	5.108	1203.79	6149.55	7.972	922.30	7352.89
A_68_P31675568	chr18:46357840-46357884	NM_001170855:610	Trim36	INSIDE	1.561	2.855	486.55	1389.25	4.458	453.71	2022.68
A_68_P28082865	chr11:102878532-102878576	NM_026551:-93	Dcakd	PROMOTER	1.560	5.102	5424.26	27675.60	7.959	4348.58	34611.79
A_68_P28463190	chr12:58643591-58643635	NM_008259:3496	Foxa1	INSIDE	1.560	2.253	1457.12	3283.00	3.514	1177.59	4138.56
A_68_P29605899	chr14:52924704-52924752	NM_019721:71	Mettl3	INSIDE	1.560	3.087	740.61	2286.38	4.816	590.71	2845.04
A_68_P23541414	chr5:23889472-23889516	NM_008713:18858	Nos3	INSIDE	1.560	1.597	1009.38	1612.02	2.491	853.11	2125.19
A_68_P31420718	chr17:87424300-87424344	NM_008838:419	Pigf	INSIDE	1.560	1.566	712.72	1116.06	2.444	619.81	1514.57
A_68_P29143670	chr13:74147870-74147914	NM_182839:1026	Tppp	INSIDE	1.560	3.518	1149.79	4045.47	5.489	940.34	5161.41
A_68_P31257913	chr17:56856203-56856247	NM_178926:120	Vmac	INSIDE	1.560	1.548	592.48	917.36	2.415	522.90	1262.62
A_68_P24786877	chr6:118405159-118405203	NM_028335:344	Zfp248	INSIDE	1.560	2.221	2692.98	5980.02	3.464	2110.48	7311.15
A_68_P32565205	chrX:98893158-98893202	ENSMUST00000073812:-5991		PROMOTER	1.560	6.701	3873.46	25954.72	10.456	1949.34	20382.57
A_68_P20876216	chr1:182413344-182413388	NM_001081227:269	6330403A02Rik	INSIDE	1.559	1.478	4002.81	5914.28	2.304	3159.73	7278.97
A_68_P26486134	chr9:47339844-47339888	NM_001025600:1432	Cadm1	INSIDE	1.559	1.847	612.83	1132.05	2.880	470.46	1354.71
A_68_P28293506	chr12:25781121-25781165	NM_010496:-185	Id2	PROMOTER	1.559	2.315	4038.43	9349.15	3.609	3161.10	11406.85
A_68_P25954897	chr8:73438085-73438129	NM_173013:8234	Mtap1s	INSIDE	1.559	4.956	4668.80	23140.70	7.727	3349.62	25881.64
A_68_P23972071	chr5:108890061-108890105	NM_172716:-268	Pcgf3	PROMOTER	1.559	1.706	2087.61	3562.17	2.661	1779.09	4734.15
A_68_P22341205	chr3:94690730-94690774	NM_008945:128	Psmb4	INSIDE	1.559	2.185	1573.79	3438.45	3.406	1288.28	4387.92
A_68_P26932995	chr10:9620910-9620954	NM_001081344:-94	Stxbp5	PROMOTER	1.559	2.555	589.32	1505.86	3.985	591.01	2355.10
A_68_P23545743	chr5:24606691-24606735	NR_027851:-80	2900005J15Rik	DIVERGENT_PROMOTER	1.558	2.903	729.35	2117.61	4.523	624.82	2825.84
A_68_P28752038	chr12:113960892-113960936	NM_001024602:530	AW555464	INSIDE	1.558	2.278	1280.40	2916.80	3.548	1133.46	4021.72
A_68_P28041452	chr11:95775809-95775854	NM_008081:354	B4galnt2	INSIDE	1.558	4.699	3119.17	14657.35	7.323	2688.16	19685.59
A_68_P24222816	chr6:7794688-7794732	NM_052993:-513	C1galt1	PROMOTER	1.558	2.112	471.99	996.61	3.290	390.46	1284.47
A_68_P29651430	chr14:62305865-62305909	NR_028264:-4676	Dleu2	PROMOTER	1.558	4.143	1151.74	4771.58	6.455	932.74	6020.70
A_68_P24994096	chr7:20158742-20158786	NM_027189:-72	Gemin7	DIVERGENT_PROMOTER	1.558	1.518	990.39	1503.27	2.364	942.86	2229.39
A_68_P25587734	chr7:148445714-148445758	NM_001081118:31050	Phrf1	INSIDE	1.558	7.229	1689.14	12210.11	11.262	1175.85	13242.93
A_68_P26203094	chr8:120022462-120022506	NM_172285:294	Plcg2	INSIDE	1.558	2.890	674.71	1949.84	4.503	610.32	2748.25
A_68_P25086855	chr7:51591550-51591594	NM_001034115:25939	Shank1	INSIDE	1.558	3.042	2355.54	7164.91	4.738	1933.02	9158.01
A_68_P25844872	chr8:49076065-49076109	NM_133791:-181	Wwc2	PROMOTER	1.558	3.399	3388.99	11519.48	5.297	3233.27	17126.42
A_68_P20737714	chr1:157264468-157264512	NM_011273:84	Xpr1	INSIDE	1.558	3.906	11830.67	46212.57	6.084	9122.39	55504.65
A_68_P27274520	chr10:77945131-77945175	ENSMUST00000069431:5435		DOWNSTREAM	1.558	2.753	1617.45	4452.65	4.290	1515.23	6500.21
A_68_P28094671	chr11:105042686-105042730	NR_027956:39	1700052K11Rik	INSIDE	1.557	4.459	593.08	2644.78	6.941	610.23	4235.88
A_68_P24449862	chr6:52264017-52264061	NM_007966:547	Evx1	INSIDE	1.557	3.027	2102.31	6363.00	4.712	1730.99	8155.65
A_68_P32147197	chr19:47089479-47089523	NM_001164363:179	Nt5c2	INSIDE	1.557	5.384	1670.04	8991.04	8.381	1366.80	11454.95
A_68_P25524908	chr7:138009867-138009911	NM_133942:465	Plekha1	INSIDE	1.557	2.360	671.79	1585.13	3.673	567.60	2084.95
A_68_P23544551	chr5:24414128-24414172	NM_001170555:177	Prkag2	INSIDE	1.557	3.462	747.36	2587.60	5.389	642.16	3460.72
A_68_P27284238	chr10:80065633-80065677	NM_019575:28	Scamp4	INSIDE	1.557	3.207	2217.98	7114.18	4.993	1596.30	7969.72
A_68_P26289277	chr9:7763854-7763898	NM_133739:-200	Tmem123	PROMOTER	1.557	2.817	10958.54	30867.54	4.385	7497.56	32880.13

ProbeName	Target position of probe on CpG island microarray	TargetID	GeneSymbol	CpG island Description	Ratio of relative methylation (TiO_2-NP/Vehicle)	Sham group			TiO_2-H group		
						Relative methylation (Cy5/Cy3)	Cy3 signal (Input)	Cy5 signal (MeDIP)	Relative methylation (Cy5/Cy3)	Cy3 signal (Input)	Cy5 signal (MeDIP)
A_68_P23297754	chr4:129318346-129318390	NM_001199695:-59	Txlna	PROMOTER	1.557	2.710	1619.38	4388.83	4.220	1351.27	5701.81
A_68_P24638863	chr6:91360911-91360955	NM_009527:431	Wnt7a	INSIDE	1.557	2.287	1572.05	3594.67	3.560	1198.48	4266.50
A_68_P31086417	chr17:21669593-21669637	NM_144515:-2888	Zfp52	PROMOTER	1.557	2.758	2058.15	5676.25	4.293	1665.77	7151.85
A_68_P32603677	chrX:109714158-109714202	NM_177747:46	Zfp711	INSIDE	1.557	2.212	329.82	729.70	3.444	225.13	775.44
A_68_P22312382	chr3:88004398-88004442	ENSMUST00000167043:-124		PROMOTER	1.557	2.729	3170.52	8651.45	4.248	2563.81	10891.21
A_68_P30608335	chr16:24393852-24393896	NR_037954:-133	1110054M08Rik	PROMOTER	1.556	4.325	927.24	4009.93	6.729	797.55	5366.96
A_68_P30423567	chr15:88991606-88991650	NM_001159521:12652	Plxnb2	INSIDE	1.556	5.958	10055.88	59914.80	9.268	6667.91	61798.74
A_68_P30330343	chr15:73253736-73253780	NM_007982:-137	Ptk2	PROMOTER	1.556	4.901	771.75	3782.28	7.628	660.37	5037.13
A_68_P23298639	chr4:129497779-129497823	NM_001164745:-151	Ptp4a2	PROMOTER	1.556	2.745	1814.44	4980.11	4.270	1507.66	6437.66
A_68_P28071133	chr11:100820100-100820144	NM_008986:11809	Ptrf	INSIDE	1.556	3.925	2274.88	8928.80	6.107	1679.27	10254.57
A_68_P22236487	chr3:72861315-72861359	NM_198864:-471	Slitrk3	PROMOTER	1.556	1.938	2102.13	4073.97	3.015	1678.91	5062.21
A_68_P24009031	chr5:115777029-115777073	NM_025573:-135	Srsf9	PROMOTER	1.556	4.084	2154.06	8797.49	6.357	1708.11	10857.85
A_68_P25056451	chr7:37481970-37482014	NM_172298:-1144	Tshz3	PROMOTER	1.556	2.088	1067.78	2229.64	3.249	934.35	3035.37
A_68_P31922246	chr19:4283254-4283298	NM_026720:-139	Ankrd13d	PROMOTER	1.555	3.090	3143.54	9713.27	4.806	2202.06	10583.65
A_68_P31867752	chr18:81130655-81130699	NM_001201569:121	Atp9b	INSIDE	1.555	3.281	3663.16	12018.74	5.101	3085.38	15738.90
A_68_P22941962	chr4:56877715-56877759	NM_018761:347	Ctnnal1	INSIDE	1.555	6.301	881.48	5553.93	9.797	740.94	7259.07
A_68_P29751456	chr14:79851782-79851826	NM_001034882:163	Gm5465	INSIDE	1.555	2.370	3615.36	8570.06	3.685	2881.89	10619.67
A_68_P23230189	chr4:116299784-116299829	NM_001081475:296	Nasp	INSIDE	1.555	1.757	1532.01	2691.16	2.732	1330.79	3635.84
A_68_P20040932	chr1:13363158-13363202	NM_001077695:984	Ncoa2	INSIDE	1.555	1.573	1015.20	1596.89	2.446	928.22	2270.58
A_68_P28174632	chr11:118769905-118769949	NM_001024931:960	Rbfox3	INSIDE	1.555	2.369	1467.31	3476.28	3.685	1339.73	4937.01
A_68_P22383504	chr3:102739949-102739993	NM_011516:53	Sycp1	INSIDE	1.555	3.031	522.26	1582.80	4.713	383.30	1806.57
A_68_P24951859	chr7:5012645-5012689	NM_001205231:-1078	U2af2	PROMOTER	1.555	1.754	3975.91	6972.82	2.727	2829.09	7714.78
A_68_P24979382	chr7:16646941-16646985			Unknown	1.555	4.452	3071.18	13673.68	6.922	2294.18	15879.60
A_68_P21755504	chr2:153354420-153354464	NM_001134300:1265	8430427H17Rik	INSIDE	1.554	4.392	2757.42	12109.24	6.824	2447.02	16699.70
A_68_P31157235	chr17:35183322-35183366	NM_013600:207	Msh5	INSIDE	1.554	1.393	1449.87	2019.11	2.164	1177.43	2547.74
A_68_P21073067	chr2:25430182-25430226	NM_029293:187	Phpt1	INSIDE	1.554	4.301	8690.48	37378.51	6.683	6210.23	41501.24
A_68_P23331577	chr4:135543069-135543113	NM_025411:69	1110049F12Rik	INSIDE	1.553	3.233	1282.56	4146.47	5.022	1113.13	5590.41
A_68_P22682769	chr3:157610620-157610664	NM_001013806:213	Ankrd13c	INSIDE	1.553	4.429	3140.67	13909.20	6.876	2744.75	18872.23
A_68_P24205541	chr6:4551256-4551300	NM_145398:213	Casd1	INSIDE	1.553	4.127	993.11	4098.52	6.408	828.17	5306.98
A_68_P27812111	chr11:54600916-54600960	NM_178626:267	Cdc42se2	INSIDE	1.553	2.021	2859.81	5779.07	3.138	2218.76	6963.45
A_68_P29142604	chr13:73984411-73984456	NM_028186:646	Nkd2	INSIDE	1.553	2.001	1418.80	2838.52	3.107	1209.81	3759.14
A_68_P21775093	chr2:156904079-156904123	NM_001164663:901	9830001H06Rik	INSIDE	1.552	3.069	1296.16	3978.46	4.764	1121.73	5344.35
A_68_P24423144	chr6:47403969-47404013	NM_012042:-332	Cul1	PROMOTER	1.552	7.853	739.60	5808.22	12.189	704.37	8585.29
A_68_P22128832	chr3:51080406-51080450	NM_023502:49481	Elf2	INSIDE	1.552	2.100	2655.74	5575.74	3.259	2102.07	6851.41
A_68_P25892520	chr8:60131247-60131291	NM_001167981:584	Galnt7	INSIDE	1.552	2.796	777.11	2172.51	4.339	632.87	2746.20
A_68_P22311352	chr3:87826975-87827019	NM_022031:4437	Hapln2	INSIDE	1.552	1.404	4589.08	6441.50	2.178	3396.94	7399.58
A_68_P22661977	chr3:153991290-153991334	NM_010713:2212	Lhx8	INSIDE	1.552	2.620	2433.89	6377.55	4.067	2210.04	8988.50
A_68_P28991174	chr13:43711396-43711440	NM_183204:253	Rnf182	INSIDE	1.552	4.911	1583.61	7776.79	7.619	1313.01	10004.08
A_68_P31598791	chr18:31769247-31769291	NM_026165:288	Slc25a46	INSIDE	1.552	3.700	928.88	3437.12	5.743	845.62	4856.30
A_68_P28332010	chr12:32244021-32244065	NM_011867:792	Slc26a4	INSIDE	1.552	2.591	663.55	1719.32	4.020	565.21	2272.30
A_68_P29745018	chr14:78707731-78707775	NM_011613:98	Tnfsf11	INSIDE	1.552	3.653	2008.53	7338.06	5.671	1580.84	8964.18
A_68_P30176277	chr15:43309087-43309131	NM_025736:334	Ttc35	INSIDE	1.552	2.774	2444.85	6782.67	4.306	2147.63	9247.83
A_68_P21311589	chr2:69735872-69735916	NM_001081548:592	Ubr3	INSIDE	1.552	2.467	2694.52	6646.05	3.829	2134.09	8170.82
A_68_P25268780	chr7:87947830-87947874	NM_016721:365	Iqgap1	INSIDE	1.551	1.594	2098.11	3343.77	2.473	1544.84	3819.74
A_68_P29222297	chr13:92901976-92902020	NM_009027:-549	Rasgrf2	PROMOTER	1.551	15.440	1145.93	17693.03	23.952	845.42	20249.63
A_68_P22883647	chr4:45542639-45542683	NM_001033306:1040	Shb	INSIDE	1.551	3.693	825.96	3050.21	5.728	737.06	4222.19
A_68_P32036079	chr19:26680265-26680310	NM_011416:638	Smarca2	INSIDE	1.551	1.658	1913.20	3172.21	2.571	1502.58	3863.03
A_68_P29672994	chr14:65977929-65977973	NM_199029:438	Zfp395	INSIDE	1.551	0.410	847.18	347.45	0.636	967.85	615.83
A_68_P24425393	chr6:47974535-47974579	NM_001081382:23557	Zfp777	INSIDE	1.551	3.239	550.43	1782.89	5.025	529.21	2659.20
A_68_P22454038	chr3:117062948-117062993	NM_177664:824	D3Bwg0562e	INSIDE	1.550	2.469	1291.03	3187.15	3.827	1048.07	4011.15
A_68_P30361565	chr15:78503546-78503590	NM_183141:44975	Elfn2	INSIDE	1.550	3.398	991.25	3368.41	5.267	733.37	3862.41

ProbeName	Target position of probe on CpG island microarray	TargetID	GeneSymbol	CpG island Description	Ratio of relative methylation (TiO_2-NP/Vehicle)	Sham group			TiO_2-H group		
						Relative methylation (Cy5/Cy3)	Cy3 signal (Input)	Cy5 signal (MeDIP)	Relative methylation (Cy5/Cy3)	Cy3 signal (Input)	Cy5 signal (MeDIP)
A_68_P25412180	chr7:117365261-117365305	NM_009302:66	Swap70	INSIDE	1.550	5.265	1061.01	5585.94	8.159	899.62	7339.83
A_68_P28167342	chr11:117671257-117671301	NM_009304:298	Syngr2	INSIDE	1.550	2.821	975.53	2752.34	4.374	772.61	3379.42
A_68_P23541913	chr5:23953475-23953519	NM_022418:168	Tmub1	INSIDE	1.550	2.442	1120.23	2736.03	3.785	956.45	3620.14
A_68_P22492339	chr3:124023918-124023962	NM_146140:-14	Tram1l1	PROMOTER	1.550	1.883	1710.74	3222.01	2.919	1366.00	3986.99
A_68_P31301686	A_68_P31301686			Unknown	1.550	2.978	386.38	1150.55	4.615	316.07	1458.75
A_68_P29055344	A_68_P29055344			Unknown	1.550	2.883	1046.86	3018.37	4.468	814.21	3637.95
A_68_P32143654	chr19:46469962-46470006	NM_016860:241	Actr1a	INSIDE	1.549	3.348	2979.00	9972.94	5.187	2460.95	12764.18
A_68_P30483162	chr15:99803108-99803152	NM_001024526:-80	Larp4	PROMOTER	1.549	1.794	1419.86	2547.83	2.780	1207.46	3356.65
A_68_P30990393	chr16:96212588-96212632	NM_019537:-100	Psmg1	PROMOTER	1.549	3.381	2069.66	6997.91	5.238	1779.04	9318.84
A_68_P23227949	chr4:115817024-115817068	NM_026547:175	1520402A15Rik	INSIDE	1.548	3.087	1615.03	4985.29	4.777	1317.12	6292.45
A_68_P27288890	chr10:80788199-80788243	NM_027381:4373	2510012J08Rik	INSIDE	1.548	5.347	2882.86	15415.59	8.279	2017.82	16705.88
A_68_P32260649	chrX:11714704-11714748	NM_001168321:22755	Bcor	INSIDE	1.548	1.375	1999.44	2748.78	2.128	960.24	2043.29
A_68_P27781640	chr11:48982878-48982922	NM_172793:17691	Btnl9	INSIDE	1.548	4.075	2307.06	9401.42	6.310	1769.63	11166.23
A_68_P30686262	chr16:38483294-38483338	NM_009855:24304	Cd80	INSIDE	1.548	3.799	3027.88	11502.80	5.880	2326.95	13681.60
A_68_P31252966	chr17:56095674-56095719	NM_015766:3651	Ebi3	INSIDE	1.548	3.023	2670.05	8070.77	4.679	1946.40	9107.33
A_68_P30452934	chr15:94374073-94374117	NM_029926:4	Irak4	INSIDE	1.548	2.034	1421.64	2891.15	3.149	1041.72	3279.94
A_68_P27011028	chr10:24589929-24589973	NM_001166416:159	Med23	INSIDE	1.548	4.361	469.79	2048.95	6.752	420.87	2841.82
A_68_P23439842	chr4:155027173-155027217	NM_011985:299	Mmp23	INSIDE	1.548	1.467	1762.94	2585.39	2.270	1420.78	3224.59
A_68_P25585272	chr7:148043313-148043357	NM_053194:39	Ric8	INSIDE	1.548	1.798	1175.98	2113.99	2.783	980.39	2728.28
A_68_P21748557	chr2:152119766-152119810	NM_024196:181	Tbc1d20	INSIDE	1.548	5.502	3289.69	18098.61	8.515	2765.79	23551.72
A_68_P31744348	chr18:58995914-58995958	NM_175506:-481	Adamts19	PROMOTER	1.547	1.578	2334.17	3684.29	2.442	2112.33	5158.87
A_68_P20947409	chr1:194956122-194956166	NM_145415:301	Diexf	INSIDE	1.547	1.635	441.40	721.86	2.530	342.33	866.11
A_68_P29713887	chr14:73109166-73109210	NM_207636:622	Fndc3a	INSIDE	1.547	1.720	371.99	639.79	2.661	324.89	864.39
A_68_P29700471	chr14:70689220-70689264	NM_008915:14	Ppp3cc	INSIDE	1.547	2.361	288.34	680.82	3.653	268.34	980.17
A_68_P21491162	chr2:104689920-104689964	NM_178695:65	Prrg4	INSIDE	1.547	2.682	1472.56	3950.02	4.149	1224.68	5081.32
A_68_P30642387	chr16:30550318-30550362	NM_172614:324	Tmem44	INSIDE	1.547	1.662	2144.39	3563.03	2.571	1758.73	4521.84
A_68_P21084621	chr2:27281926-27281970	NM_009500:397	Vav2	INSIDE	1.547	2.812	1136.36	3195.93	4.352	994.41	4327.32
A_68_P30133051	chr15:35301203-35301247	NM_177151:-76	Vps13b	PROMOTER	1.547	3.342	818.23	2734.90	5.169	728.49	3765.74
A_68_P32573118	chrX:100677473-100677517	NR_001463:1078	Xist	INSIDE	1.547	3.534	3844.65	13587.88	5.467	1405.29	7682.44
A_68_P30150543	chr15:38591462-38591506	NM_025494:-174	Atp6v1c1	PROMOTER	1.546	2.549	521.34	1328.89	3.940	401.56	1582.16
A_68_P31676224	chr18:46471665-46471709	NM_001160399:-104	Ccdc112	PROMOTER	1.546	5.163	5877.27	30343.65	7.984	4145.92	33101.35
A_68_P31741000	chr18:58369346-58369390	NM_010181:212	Fbn2	INSIDE	1.546	5.117	3637.60	18612.62	7.912	2582.50	20433.97
A_68_P25957808	chr8:74135004-74135048	NM_146211:104	Glt25d1	INSIDE	1.546	2.720	1477.88	4019.96	4.206	1203.59	5061.77
A_68_P31117120	chr17:27694784-27694828	NM_001166476:1209	Hmga1-rs1	INSIDE	1.546	1.715	1484.34	2545.31	2.651	1267.24	3358.95
A_68_P24535483	chr6:71221618-71221662	NM_145568:-372	Krcc1	DIVERGENT_PROMOTER	1.546	7.603	684.67	5205.32	11.755	621.59	7307.05
A_68_P24656000	chr6:94233185-94233229	NM_001029850:692	Magi1	INSIDE	1.546	1.641	4535.04	7442.85	2.537	3708.37	9406.35
A_68_P31095624	chr17:24278101-24278145	NM_001080773:434	Pdpk1	INSIDE	1.546	2.407	3121.36	7513.71	3.722	2605.17	9697.66
A_68_P27804984	chr11:53292506-53292550	NM_183173:1169	Ankrd43	INSIDE	1.545	2.755	606.94	1672.19	4.257	522.39	2223.93
A_68_P24052105	chr5:123213863-123213907	NM_001199676:15535	Camkk2	INSIDE	1.545	8.285	1777.03	14721.93	12.803	1302.84	16679.92
A_68_P29992340	chr15:7079603-7079647	NM_001113386:53	Lifr	INSIDE	1.545	2.541	797.88	2027.27	3.925	630.42	2474.26
A_68_P31708189	chr18:52688590-52688634	NM_010728:750	Lox	INSIDE	1.545	1.456	1093.75	1592.08	2.249	860.22	1934.74
A_68_P25992691	chr8:81636666-81636710	NM_178267:84353	Zfp827	INSIDE	1.545	9.692	5183.46	50237.54	14.970	3215.13	48129.18
A_68_P22129414	chr3:51200267-51200311	NM_026358:181	4930583H14Rik	INSIDE	1.544	6.105	1129.35	6894.20	9.424	898.09	8463.53
A_68_P24942894	chr6:148893997-148894041	NM_019643:936	Fam60a	INSIDE	1.544	4.314	2874.14	12399.49	6.660	2592.69	17268.14
A_68_P25049946	chr7:36340772-36340816	NM_033080:153	Nudt19	INSIDE	1.544	3.580	1871.68	6700.77	5.526	1707.74	9437.16
A_68_P23527584	chr5:21243055-21243099	NM_028431:99	Pmpcb	INSIDE	1.544	3.765	910.72	3429.27	5.814	810.88	4714.20
A_68_P27614514	chr11:17059211-17059255	NM_024459:-68	Ppp3r1	PROMOTER	1.544	2.576	1662.81	4284.09	3.979	1513.69	6022.63
A_68_P21740964	chr2:150612823-150612867	NM_153781:313	Pygb	INSIDE	1.544	4.723	1686.40	7964.35	7.293	1405.35	10248.92
A_68_P30669634	chr16:35363730-35363774	NM_133704:252	Sec22a	INSIDE	1.544	2.387	1288.67	3075.84	3.686	1150.47	4240.27
A_68_P28739967	chr12:111976055-111976099	NM_027149:-82	Wdr20a	PROMOTER	1.544	3.066	1411.64	4327.86	4.735	1260.98	5970.71
A_68_P32151692	chr19:47806456-47806500	NM_026377:233	6330577E15Rik	INSIDE	1.543	4.098	11852.91	48570.74	6.323	7969.21	50392.81

ProbeName	Target position of probe on CpG island microarray	TargetID	GeneSymbol	CpG island Description	Ratio of relative methylation (TiO_2-NP/Vehicle)	Sham group			TiO_2-H group		
						Relative methylation (Cy5/Cy3)	Cy3 signal (Input)	Cy5 signal (MeDIP)	Relative methylation (Cy5/Cy3)	Cy3 signal (Input)	Cy5 signal (MeDIP)
A_68_P24357881	chr6:34730747-34730791	NM_178630:337	Agbl3	INSIDE	1.543	3.147	427.26	1344.79	4.855	354.26	1720.04
A_68_P25957034	chr8:74004823-74004867	NM_001164679:5122	Ano8	INSIDE	1.543	4.355	2896.34	12613.58	6.719	2375.92	15964.47
A_68_P28175458	chr11:118903402-118903447	NM_013926:-1197	Cbx8	PROMOTER	1.543	2.606	1629.10	4245.70	4.020	1283.32	5159.52
A_68_P27080340	chr10:39451597-39451641	NR_038037:195	E130307A14Rik	INSIDE	1.543	1.585	1856.03	2942.01	2.446	1386.50	3391.38
A_68_P26940374	chr10:11063250-11063294	NM_010146:30	Epm2a	INSIDE	1.543	2.782	270.82	753.53	4.294	282.19	1211.60
A_68_P26587421	chr9:65238821-65238865	NM_028974:617	Kbtbd13	INSIDE	1.543	3.366	640.77	2157.15	5.196	558.74	2903.16
A_68_P23801768	chr5:74591245-74591289	NM_026878:-84	Rasl11b	PROMOTER	1.543	4.567	3818.49	17438.72	7.045	3002.06	21150.25
A_68_P25195705	chr7:74903906-74903950	NM_183312:700	Synm	INSIDE	1.543	2.131	1661.38	3540.87	3.288	1378.62	4533.34
A_68_P29964714	chr14:123382020-123382064	NM_028651:441	Tmtc4	INSIDE	1.543	3.639	289.34	1053.04	5.616	247.28	1388.75
A_68_P32340725	chrX:39421489-39421533	NM_009688:498	Xiap	INSIDE	1.543	3.144	351.26	1104.43	4.851	150.06	727.98
A_68_P24688740	chr6:99828403-99828447	ENSMUST00000121046:-96		PROMOTER	1.543	3.656	1559.34	5700.35	5.642	1234.95	6967.17
A_68_P21633051	chr2:130889050-130889094	NM_001163529:478	Adam33	INSIDE	1.542	2.455	602.88	1480.03	3.785	522.00	1975.90
A_68_P27539535	chr10:127533910-127533954	NM_054078:4094	Baz2a	INSIDE	1.542	1.931	1625.26	3138.52	2.978	1391.91	4145.42
A_68_P31524836	chr18:16965652-16965696	NM_007664:1884	Cdh2	INSIDE	1.542	5.347	1273.88	6811.52	8.243	1060.08	8737.75
A_68_P24048238	chr5:122499986-122500030	NM_007804:-2174	Cux2	PROMOTER	1.542	2.754	614.04	1691.04	4.248	530.72	2254.36
A_68_P27930554	chr11:76015406-76015450	NM_027773:-129	Fam57a	PROMOTER	1.542	2.236	2797.45	6253.97	3.446	2477.12	8536.80
A_68_P21339417	chr2:74601653-74601697	NM_010467:638	Hoxd1	INSIDE	1.542	2.014	1822.69	3670.23	3.104	1498.07	4650.35
A_68_P29448698	chr14:21553543-21553587	NM_010811:220	Ndst2	INSIDE	1.542	1.813	339.59	615.81	2.797	289.37	809.26
A_68_P29694905	chr14:69808678-69808722	NM_010921:-48	Nkx3-1	PROMOTER	1.542	6.019	1396.90	8407.55	9.278	1099.45	10200.87
A_68_P22180925	chr3:60806609-60806653	NM_008772:-86	P2ry1	PROMOTER	1.542	1.612	1581.70	2549.48	2.485	1392.53	3460.49
A_68_P25962147	chr8:75095836-75095880	NM_144534:-143	Tmem38a	DIVERGENT_PROMOTER	1.542	2.800	1226.51	3434.52	4.317	1052.87	4545.69
A_68_P26521623	chr9:53418301-53418345	NM_144784:133	Acat1	INSIDE	1.541	3.585	943.56	3382.47	5.525	828.27	4576.50
A_68_P26057102	chr8:93657583-93657627	NM_010241:1791	Aktip	INSIDE	1.541	3.702	3600.06	13328.11	5.705	2907.92	16588.87
A_68_P27558727	chr11:4537643-4537687	NM_029291:-131	Ascc2	DIVERGENT_PROMOTER	1.541	4.826	3891.78	18781.00	7.434	2991.62	22240.76
A_68_P24063264	chr5:125342497-125342541	NM_144819:73	Ccdc92	INSIDE	1.541	1.589	1082.41	1720.18	2.449	961.94	2355.74
A_68_P23295047	chr4:128798459-128798503	NM_010471:55	Hpca	INSIDE	1.541	1.416	875.12	1239.51	2.183	803.08	1753.03
A_68_P23301755	chr4:130219531-130219575	NM_001159603:279	Pum1	INSIDE	1.541	2.281	1424.00	3247.45	3.515	1128.39	3965.86
A_68_P22207824	chr3:66777458-66777502	NM_013665:8213	Shox2	INSIDE	1.541	1.628	3214.15	5233.73	2.510	2755.27	6914.40
A_68_P28184189	chr11:120239967-120240011	NM_027980:72	2310003H01Rik	INSIDE	1.540	3.421	542.84	1857.08	5.269	466.14	2456.01
A_68_P23368580	chr4:141795400-141795444	NM_001109685:-106	9030409G11Rik	PROMOTER	1.540	4.200	12506.51	52523.82	6.467	9077.38	58703.21
A_68_P28112958	chr11:108286558-108286602	NM_029606:1	Ccdc46	INSIDE	1.540	1.840	1341.81	2468.67	2.833	1139.15	3226.70
A_68_P21832544	chr2:166731558-166731602	NM_023565:-15	Cse1l	PROMOTER	1.540	2.158	2544.10	5490.79	3.323	1922.64	6388.29
A_68_P23585886	chr5:33206293-33206337	NM_177786:-55	Depdc5	DIVERGENT_PROMOTER	1.540	1.961	1701.05	3335.59	3.019	1486.86	4489.25
A_68_P21115865	chr2:32391002-32391046	NM_153560:146	Fam102a	INSIDE	1.540	2.078	1162.78	2416.42	3.200	965.79	3090.04
A_68_P21124370	chr2:33742800-33742844	NM_175184:644	Fam125b	INSIDE	1.540	2.933	4745.41	13920.28	4.516	3493.69	15778.51
A_68_P28184739	chr11:120329033-120329077	NM_001159328:106	Hgs	INSIDE	1.540	1.893	2172.26	4112.60	2.915	1783.07	5197.83
A_68_P32132967	chr19:44637357-44637401	NM_176958:35	Hif1an	INSIDE	1.540	3.012	677.07	2039.17	4.638	597.35	2770.71
A_68_P28997657	chr13:44826068-44826112	NM_001205044:-52	Jarid2	PROMOTER	1.540	2.474	2897.66	7168.55	3.810	2247.25	8561.24
A_68_P29618044	chr14:56110165-56110209	NM_001024645:257	Lrrc16b	INSIDE	1.540	2.286	3597.59	8222.54	3.520	3076.47	10829.15
A_68_P25581950	chr7:147234174-147234218	NM_010836:791	Msx3	INSIDE	1.540	2.192	1382.25	3030.30	3.376	1166.43	3937.96
A_68_P23439553	chr4:154976002-154976046	NM_177186:500	Slc35e2	INSIDE	1.540	2.236	771.25	1724.72	3.443	614.18	2114.67
A_68_P28344356	chr12:34642925-34642969	NM_011658:411	Twist1	INSIDE	1.540	1.644	423.44	696.13	2.532	392.98	995.14
A_68_P22695192	chr4:3865915-3865959	NM_001190323:-98	Chchd7	DIVERGENT_PROMOTER	1.539	2.922	3388.26	9901.35	4.496	2546.72	11450.11
A_68_P28525474	chr12:72410830-72410874	NM_001190466:-18	Dact1	PROMOTER	1.539	2.050	5746.86	11781.79	3.155	4667.44	14727.36
A_68_P27385041	chr10:98569996-98570040	NM_015737:-750	Galnt4	PROMOTER	1.539	1.907	370.06	705.59	2.935	285.70	838.51
A_68_P31150606	chr17:33954625-33954670	NM_030697:7180	Kank3	INSIDE	1.539	2.105	592.68	1247.40	3.239	549.54	1780.05
A_68_P22347470	chr3:95908249-95908293	NM_001025613:-179	Otud7b	PROMOTER	1.539	1.937	515.73	999.13	2.982	384.89	1147.91
A_68_P22246600	chr3:75360381-75360425	NM_019745:319	Pdcd10	INSIDE	1.539	2.256	8011.46	18072.48	3.472	5244.25	18209.34
A_68_P26142832	chr8:109571961-109572005	NM_026513:532	Pdf	INSIDE	1.539	2.131	1545.36	3293.12	3.279	1239.71	4064.46
A_68_P22680840	chr3:157229758-157229802	NM_011196:-75	Ptger3	PROMOTER	1.539	3.172	1382.86	4385.80	4.881	1383.01	6749.95
A_68_P28314231	chr12:29320191-29320235	NM_011300:606	Rps7	INSIDE	1.539	1.793	3656.30	6553.99	2.759	3029.59	8358.80

ProbeName	Target position of probe on CpG island microarray	TargetID	GeneSymbol	CpG island Description	Ratio of relative methylation (TiO_2-NP/Vehicle)	Sham group			TiO_2-H group		
						Relative methylation (Cy5/Cy3)	Cy3 signal (Input)	Cy5 signal (MeDIP)	Relative methylation (Cy5/Cy3)	Cy3 signal (Input)	Cy5 signal (MeDIP)
A_68_P33007029	A_68_P33007029			Unknown	1.539	3.724	3393.02	12634.27	5.729	2908.95	16665.78
A_68_P20121782	chr1:30930313-30930357	ENSMUST00000046443:205		INSIDE	1.539	1.825	3153.60	5755.99	2.809	2895.33	8133.96
A_68_P26476281	chr9:45647027-45647071	NM_001145947:437	Bace1	INSIDE	1.538	3.408	1593.42	5430.55	5.240	1236.49	6479.45
A_68_P30349739	chr15:76487469-76487513	NM_019396:3001	Cyhr1	INSIDE	1.538	2.365	2055.01	4860.62	3.637	1485.47	5402.77
A_68_P27551860	chr11:3309041-3309085	NM_010718:176	Limk2	INSIDE	1.538	3.518	1658.89	5835.56	5.410	1398.92	7568.53
A_68_P32536210	chrX:91077357-91077401	NM_145630:56	Pdk3	INSIDE	1.538	3.659	1319.92	4829.75	5.629	988.31	5563.58
A_68_P23981859	chr5:111209498-111209542	NM_001025561:95	Pus1	INSIDE	1.538	2.394	3325.28	7960.55	3.681	2575.26	9480.36
A_68_P28741731	chr12:112278009-112278053	NM_198023:22	Rcor1	INSIDE	1.538	3.326	9572.13	31835.52	5.114	7748.35	39625.39
A_68_P26932993	chr10:9620586-9620630	NM_001081344:230	Stxbp5	INSIDE	1.538	17.246	1505.89	25970.25	26.529	1250.90	33185.50
A_68_P24304143	chr6:24905716-24905760	NM_177013:387	Tmem229a	INSIDE	1.538	2.687	1431.27	3845.77	4.133	1315.59	5436.84
A_68_P25411826	chr7:117266404-117266448	NM_009516:854	Wee1	INSIDE	1.538	2.467	904.96	2232.32	3.794	799.90	3034.79
A_68_P26252592	chr8:128094354-128094398	BC072593:-720		PROMOTER	1.538	8.899	274.41	2442.03	13.684	230.31	3151.62
A_68_P25834612	chr8:47248897-47248941	NM_025747:-5991	4933411K20Rik	PROMOTER	1.537	1.755	5189.64	9109.37	2.698	4110.67	11092.42
A_68_P29587993	chr14:49066241-49066285	NM_172600:236	6720456H20Rik	INSIDE	1.537	2.300	2572.13	5914.65	3.534	2101.71	7427.21
A_68_P28859761	chr13:19040366-19040410	NM_175007:149	Amph	INSIDE	1.537	2.967	5674.37	16838.28	4.562	4718.16	21522.00
A_68_P21266439	chr2:61649898-61649942	NM_009322:7411	Tbr1	INSIDE	1.537	2.571	364.72	937.82	3.952	383.31	1514.93
A_68_P31655791	chr18:42671723-42671767	NM_001039474:604	Tcerg1	INSIDE	1.537	1.628	1908.82	3108.31	2.502	1457.80	3647.64
A_68_P31982216	chr19:16855225-16855273	NM_173028:169	Vps13a	INSIDE	1.537	1.643	672.73	1105.45	2.526	541.01	1366.40
A_68_P29102522	chr13:65360990-65361034	ENSMUST00000099425:166		INSIDE	1.537	5.390	861.37	4642.76	8.282	759.18	6287.68
A_68_P23235081	chr4:117222839-117222883	NM_080469:-238	Eri3	PROMOTER	1.536	1.899	1162.81	2208.21	2.917	989.53	2886.33
A_68_P28576090	chr12:81620528-81620572	NM_001081421:574	Galntl1	INSIDE	1.536	2.152	4793.87	10316.28	3.306	3707.25	12255.35
A_68_P24112574	chr5:134790846-134790890	NM_001080746:-252	Gtf2i	PROMOTER	1.536	2.895	1190.75	3446.85	4.446	1021.12	4539.79
A_68_P25846166	chr8:49314139-49314183	NM_001145937:445884	Odz3	INSIDE	1.536	1.963	353.88	694.53	3.014	297.77	897.56
A_68_P26630339	chr9:72773100-72773144	NM_028116:-334	Pygo1	PROMOTER	1.536	1.416	2378.36	3368.63	2.176	2002.26	4356.21
A_68_P28726321	chr12:109416856-109416900	NM_028262:616	Setd3	INSIDE	1.536	2.422	1209.78	2930.42	3.721	910.50	3388.06
A_68_P23294938	chr4:128782726-128782770	NM_025452:-25	Tmem54	PROMOTER	1.536	4.533	3146.01	14260.84	6.964	2437.77	16975.62
A_68_P33012156	chr9_random:54584-54628	NR_027950:-86	4930526I15Rik	PROMOTER	1.535	1.664	567.58	944.36	2.554	454.38	1160.30
A_68_P27103323	chr10:43723779-43723823	NM_172393:852	Aim1	INSIDE	1.535	1.987	1204.32	2393.59	3.050	960.45	2929.32
A_68_P25637623	chr8:8661349-8661393	NM_010111:-597	Efnb2	PROMOTER	1.535	3.006	909.85	2734.56	4.615	760.62	3509.94
A_68_P32140182	chr19:45811402-45811446	NM_001166361:5950	Fgf8	INSIDE	1.535	5.312	3067.67	16296.33	8.155	2340.21	19085.57
A_68_P21764750	chr2:155102799-155102843	NM_025735:641	Map1lc3a	INSIDE	1.535	2.782	4218.66	11735.73	4.269	3229.90	13789.31
A_68_P28950790	chr13:36827018-36827062	NM_153529:-717	Nrn1	PROMOTER	1.535	1.902	4299.20	8179.08	2.920	3310.23	9664.48
A_68_P27280131	chr10:79440436-79440480	NM_001003949:6617	ORF61	INSIDE	1.535	3.448	2109.25	7272.62	5.293	1727.17	9141.30
A_68_P23972073	chr5:108890220-108890264	NM_172716:-108	Pcgf3	PROMOTER	1.535	1.619	1040.16	1683.50	2.484	873.42	2169.90
A_68_P23430798	chr4:153715842-153715886	NM_001177995:295118	Prdm16	INSIDE	1.535	6.877	4530.81	31157.62	10.557	3110.63	32839.81
A_68_P23801771	chr5:74591574-74591618	NM_026878:246	Rasl11b	INSIDE	1.535	2.669	1030.92	2751.48	4.097	893.49	3660.55
A_68_P31104792	chr17:25707704-25707748	NM_011447:-95	Sox8	DIVERGENT_PROMOTER	1.535	5.972	1910.27	11407.68	9.169	1274.07	11682.41
A_68_P31207579	chr17:46692454-46692498	NM_020493:635	Srf	INSIDE	1.535	2.991	2127.40	6364.07	4.593	1763.88	8101.18
A_68_P31921226	chr19:4125896-4125940	NM_001078649:-41	Tmem134	DIVERGENT_PROMOTER	1.535	1.599	2143.89	3428.02	2.454	1865.14	4577.64
A_68_P25951334	chr8:72877112-72877156	NM_001122829:38	Upf1	INSIDE	1.535	4.103	1615.14	6627.05	6.300	1315.38	8287.26
A_68_P27939271	chr11:77580034-77580078	ENSMUST00000108376:-181		PROMOTER	1.535	3.753	812.30	3048.39	5.762	671.76	3870.66
A_68_P20250405	chr1:57029997-57030046	NR_024325:1709	9130024F11Rik	INSIDE	1.534	1.803	368.62	664.80	2.767	298.59	826.23
A_68_P26530603	chr9:55174506-55174550	NM_172923:-227	AI118078	PROMOTER	1.534	3.931	1708.92	6717.38	6.028	1408.98	8493.67
A_68_P30357053	chr15:77825858-77825902	NM_007583:123830	Cacng2	INSIDE	1.534	3.795	7182.40	27260.62	5.824	5370.61	31279.25
A_68_P22715810	chr4:8617868-8617912	NM_001081417:-177	Chd7	PROMOTER	1.534	4.212	492.27	2073.62	6.460	414.93	2680.59
A_68_P23969232	chr5:108297963-108298007	NM_007964:6142	Evi5	INSIDE	1.534	3.333	2981.35	9936.38	5.114	2623.33	13416.10
A_68_P24038707	chr5:120886274-120886318	NM_008499:4402	Lhx5	INSIDE	1.534	5.622	734.69	4130.74	8.623	673.95	5811.19
A_68_P21633642	chr2:130997305-130997349	NM_027641:3220	Spef1	INSIDE	1.534	2.060	1345.19	2770.51	3.159	1104.99	3490.29
A_68_P28615783	chr12:88728757-88728801	NM_011479:402	Sptlc2	INSIDE	1.534	2.160	1028.58	2221.43	3.312	828.94	2745.68
A_68_P25459064	chr7:125728226-125728270	NM_172476:-48	Tmc7	PROMOTER	1.534	3.283	1856.92	6095.49	5.036	1642.42	8271.82
A_68_P20087498	chr1:23262935-23262979	ENSMUST00000097807:289		INSIDE	1.534	1.963	1047.48	2056.14	3.011	901.14	2713.14

ProbeName	Target position of probe on CpG island microarray	TargetID	GeneSymbol	CpG island Description	Ratio of relative methylation (TiO_2-NP/Vehicle)	Sham group			TiO_2-H group		
						Relative methylation (Cy5/Cy3)	Cy3 signal (Input)	Cy5 signal (MeDIP)	Relative methylation (Cy5/Cy3)	Cy3 signal (Input)	Cy5 signal (MeDIP)
A_68_P28104781	chr11:106889949-106889993	NM_001163473:434	1810010H24Rik	INSIDE	1.533	3.836	1002.22	3844.42	5.880	863.11	5074.98
A_68_P25276872	chr7:89483764-89483808	NM_001190374:-417	Adamtsl3	PROMOTER	1.533	7.631	1584.93	12094.30	11.695	1347.21	15755.90
A_68_P28752830	chr12:114067331-114067375	NM_028023:248	Cdca4	INSIDE	1.533	3.522	2449.67	8627.34	5.401	1964.09	10607.28
A_68_P27921508	chr11:74433189-74433233	NM_001013784:104	E130309D14Rik	INSIDE	1.533	3.394	3585.16	12167.32	5.202	3056.19	15899.69
A_68_P27921510	chr11:74433466-74433510	NM_001013784:382	E130309D14Rik	INSIDE	1.533	8.832	2611.07	23060.27	13.542	1959.59	26536.29
A_68_P28052725	chr11:97660923-97660967	NM_010688:-41	Lasp1	PROMOTER	1.533	5.253	3494.89	18360.31	8.055	2884.12	23231.12
A_68_P27937051	chr11:77161599-77161643	NM_177710:131694	Ssh2	INSIDE	1.533	4.004	1450.50	5807.99	6.140	1278.99	7852.84
A_68_P26530601	chr9:55174289-55174334	NM_172923:-444	AI118078	PROMOTER	1.532	4.214	1874.00	7897.77	6.456	1473.35	9512.37
A_68_P24428428	chr6:48579466-48579510	NR_027236:1323	AI854703	INSIDE	1.532	2.268	658.89	1494.33	3.475	601.02	2088.42
A_68_P21632194	chr2:130732229-130732273	NM_009730:19	Atrn	INSIDE	1.532	5.371	1345.00	7224.55	8.228	1081.03	8894.90
A_68_P26019095	chr8:86794255-86794299	NM_026350:-17	Ccdc130	PROMOTER	1.532	2.969	3007.20	8927.37	4.548	2405.15	10938.41
A_68_P31928777	chr19:5457059-5457103	NM_198616:469	Ccdc85b	INSIDE	1.532	2.179	1183.56	2579.21	3.338	1028.36	3432.85
A_68_P22355191	chr3:97414110-97414154	NM_026539:-19	Chd1l	PROMOTER	1.532	4.639	481.16	2232.03	7.109	455.84	3240.45
A_68_P31258211	chr17:56901482-56901526	NM_144858:-2669	Dus3l	DIVERGENT_PROMOTER	1.532	1.538	1244.70	1913.87	2.355	1027.37	2419.82
A_68_P20644035	chr1:138028029-138028073	NM_001039472:73	Kif21b	INSIDE	1.532	1.639	677.67	1110.85	2.512	547.07	1374.03
A_68_P26345157	chr9:20837581-20837625	NR_035438:-830	Mir1900	PROMOTER	1.532	2.271	1080.63	2454.17	3.479	904.14	3145.28
A_68_P26873355	chr9:119487746-119487790	NM_021544:366	Scn5a	INSIDE	1.532	1.767	1439.43	2543.43	2.708	1209.86	3275.78
A_68_P27325555	chr10:87922666-87922710	NM_011517:357	Sycp3	INSIDE	1.532	7.924	5644.23	44726.54	12.140	3909.36	47458.33
A_68_P24591236	chr6:83018754-83018798	NM_009392:1443	Tlx2	INSIDE	1.532	2.775	421.84	1170.68	4.251	650.07	2763.26
A_68_P25504790	chr7:134397531-134397575	NM_177362:9513	Zfp771	INSIDE	1.532	1.867	1286.01	2400.51	2.860	1137.32	3252.78
A_68_P23880196	chr5:90312755-90312799	NM_001081401:-417	Adamts3	PROMOTER	1.531	3.069	1012.76	3108.29	4.698	777.44	3652.78
A_68_P26469460	chr9:44575399-44575447	NM_145985:469	Arcn1	INSIDE	1.531	7.501	660.54	4954.98	11.486	401.09	4606.84
A_68_P28727039	chr12:109513784-109513828	NM_001159910:-179	Ccdc85c	PROMOTER	1.531	3.067	3029.72	9291.40	4.694	2394.92	11242.71
A_68_P23573354	chr5:30890939-30890983	NM_010608:418	Kcnk3	INSIDE	1.531	6.699	1037.65	6951.16	10.254	713.64	7317.35
A_68_P26537272	chr9:56468941-56468985	NM_181074:64098	Lingo1	INSIDE	1.531	4.659	605.59	2821.38	7.135	486.37	3470.21
A_68_P27354860	chr10:93103568-93103612	NM_021320:-203	Ntn4	PROMOTER	1.531	1.740	1786.47	3107.84	2.664	1503.59	4005.25
A_68_P28738217	chr12:111684829-111684873	NM_001135001:-538	Ppp2r5c	PROMOTER	1.531	2.468	2455.41	6061.07	3.780	1822.54	6888.58
A_68_P22944675	chr4:57313204-57313248	NM_011207:1483	Ptpn3	INSIDE	1.531	4.074	349.23	1422.69	6.235	481.41	3001.86
A_68_P27798065	chr11:52045603-52045647	NM_011543:128	Skp1a	INSIDE	1.531	2.037	756.45	1540.63	3.117	659.84	2056.86
A_68_P30363492	chr15:78798174-78798218	NM_001039155:20043	Triobp	INSIDE	1.531	4.304	3043.35	13098.90	6.588	2143.46	14121.76
A_68_P25670559	chr8:13786249-13786293	NM_025924:656	Upf3a	INSIDE	1.531	1.530	2669.49	4083.41	2.342	2133.74	4998.14
A_68_P22394252	chr3:104770874-104770918	NM_009520:-6269	Wnt2b	PROMOTER	1.531	2.252	1357.05	3055.94	3.448	1114.67	3843.22
A_68_P24086494	A_68_P24086494			Unknown	1.531	2.899	569.27	1650.39	4.439	505.79	2244.97
A_68_P25352334	chr7:104936888-104936932	ENSMUST00000172423:-140		PROMOTER	1.531	1.373	1404.07	1927.65	2.103	1176.25	2473.09
A_68_P31679073	chr18:47009821-47009865	NM_029008:150	4833403I15Rik	INSIDE	1.530	1.493	1261.63	1883.79	2.284	1016.66	2322.55
A_68_P23961301	chr5:106881112-106881156	NM_001005477:6051	Barhl2	DOWNSTREAM	1.530	4.458	1187.26	5292.41	6.821	1025.21	6992.74
A_68_P32395905	chrX:53709083-53709127	NM_172779:1089	Ddx26b	INSIDE	1.530	1.452	5384.84	7816.63	2.221	2264.74	5029.57
A_68_P20863428	chr1:180267506-180267550	NM_016805:387	Hnrnpu	INSIDE	1.530	1.492	616.38	919.53	2.283	550.33	1256.38
A_68_P25144266	chr7:63217700-63217744	NM_023647:98	Nipa2	INSIDE	1.530	1.692	1197.66	2026.59	2.589	1035.15	2680.06
A_68_P20259227	chr1:58561519-58561563	NM_001025378:-3280	Orc2	PROMOTER	1.530	2.177	586.53	1276.70	3.330	452.39	1506.25
A_68_P22886521	chr4:46051942-46051986	NM_021883:-129	Tmod1	PROMOTER	1.530	2.741	2565.11	7031.71	4.195	2101.39	8815.76
A_68_P29308210	chr13:108681440-108681484	NM_145456:-1204	Zswim6	PROMOTER	1.530	3.652	1260.57	4603.49	5.589	1054.22	5891.68
A_68_P24086560	A_68_P24086560			Unknown	1.530	4.553	524.04	2385.92	6.964	363.09	2528.68
A_68_P25021007	chr7:29079751-29079795	NM_007866:7032	Dll3	INSIDE	1.529	4.149	1259.27	5225.23	6.346	1111.25	7052.15
A_68_P27253669	chr10:74430095-74430139	NM_010311:141	Gnaz	INSIDE	1.529	2.357	789.78	1861.25	3.603	726.43	2617.05
A_68_P26812405	chr9:107905152-107905196	NM_013785:196	Ip6k1	INSIDE	1.529	3.185	897.89	2859.76	4.869	759.91	3700.20
A_68_P30478815	chr15:99054306-99054350	NM_010601:-1078	Kcnh3	PROMOTER	1.529	2.143	1207.57	2587.92	3.278	1052.83	3450.90
A_68_P28583196	chr12:82961089-82961133	NM_018814:94	Pcnx	INSIDE	1.529	2.203	1583.96	3489.16	3.368	1330.25	4480.48
A_68_P21098343	chr2:29475430-29475474	NM_001039086:213	Rapgef1	INSIDE	1.529	1.667	532.10	886.98	2.548	499.46	1272.59
A_68_P23749454	chr5:64551417-64551461	NM_019636:-11	Tbc1d1	PROMOTER	1.529	1.732	1641.37	2843.35	2.650	1396.68	3700.54
A_68_P26713768	chr9:89599588-89599632	NM_025360:271	Tmed3	INSIDE	1.529	1.959	612.80	1200.66	2.995	523.72	1568.54

ProbeName	Target position of probe on CpG island microarray	TargetID	GeneSymbol	CpG island Description	Ratio of relative methylation (TiO_2-NP/Vehicle)	Sham group			TiO_2-H group		
						Relative methylation (Cy5/Cy3)	Cy3 signal (Input)	Cy5 signal (MeDIP)	Relative methylation (Cy5/Cy3)	Cy3 signal (Input)	Cy5 signal (MeDIP)
A_68_P29286853	chr13:104564644-104564688	ENSMUST00000109322:-127		PROMOTER	1.529	2.288	823.90	1884.69	3.498	732.31	2561.91
A_68_P21809324	chr2:162734117-162734161	ENSMUST00000122376:479		INSIDE	1.529	4.797	1120.76	5376.75	7.337	932.71	6843.66
A_68_P23562830	chr5:28784867-28784911	NR_015562:-8635	9530036O11Rik	PROMOTER	1.528	3.034	425.37	1290.71	4.636	458.45	2125.45
A_68_P31150132	chr17:33822462-33822506	NM_001109913:-81	Hnrnpm	PROMOTER	1.528	3.972	1968.27	7818.47	6.071	1520.49	9230.34
A_68_P20788410	chr1:166237694-166237738	NM_178071:-89	Nme7	DIVERGENT_PROMOTER	1.528	3.811	729.52	2780.46	5.824	566.98	3302.24
A_68_P23176709	chr4:104782027-104782071	NM_178143:455	Prkaa2	INSIDE	1.528	1.581	4004.06	6331.55	2.416	3082.03	7445.24
A_68_P23223131	chr4:114729380-114729424	NM_011527:-2729	Tal1	PROMOTER	1.528	1.748	4063.28	7104.22	2.671	3092.87	8261.82
A_68_P20936016	chr1:192886793-192886837	NM_001163421:-3	Tatdn3	DIVERGENT_PROMOTER	1.528	2.584	13552.33	35014.66	3.948	11020.49	43506.03
A_68_P21121774	chr2:33323833-33323877	NM_001025594:198	Zbtb43	INSIDE	1.528	2.141	1900.30	4068.74	3.271	1754.03	5738.29
A_68_P32288518	chrX:20002618-20002662	ENSMUST00000115384:-173		PROMOTER	1.528	3.462	1052.72	3644.25	5.289	598.35	3164.43
A_68_P25597935	chr7:150155005-150155050	NM_008554:142	Ascl2	INSIDE	1.527	1.424	1230.80	1753.04	2.175	972.17	2114.90
A_68_P26317084	chr9:14185441-14185485	NM_028013:224	Endod1	INSIDE	1.527	1.671	826.93	1381.89	2.551	778.11	1985.00
A_68_P28158070	chr11:116083313-116083358	NM_025276:16070	Evpl	INSIDE	1.527	6.358	2493.61	15855.54	9.710	1964.19	19072.41
A_68_P31371532	chr17:78817452-78817496	NM_199448:-31	Fez2	PROMOTER	1.527	5.391	8891.70	47938.00	8.235	6744.37	55539.39
A_68_P24951566	chr7:4966239-4966283	NM_011813:-405	Fiz1	PROMOTER	1.527	1.489	2233.90	3326.98	2.274	1677.08	3813.57
A_68_P20553865	chr1:120734246-120734290	NM_001081125:215928	Gli2	INSIDE	1.527	2.576	834.66	2150.49	3.933	708.23	2785.64
A_68_P28067969	chr11:100275377-100275421	NM_176830:735	Leprel4	INSIDE	1.527	2.451	999.41	2449.55	3.742	782.26	2927.01
A_68_P25962045	chr8:75071657-75071701	NM_027485:531	Med26	INSIDE	1.527	2.995	3341.21	10006.22	4.572	2633.69	12040.27
A_68_P31425843	chr17:88374364-88374408	NM_010830:-3	Msh6	PROMOTER	1.527	1.680	584.04	981.29	2.566	572.71	1469.51
A_68_P22540268	chr3:132612964-132613008	NM_001029836:269	Npnt	INSIDE	1.527	2.202	1452.72	3198.70	3.362	1305.20	4387.93
A_68_P22825736	chr4:33296596-33296640	NM_177774:653	Srsf12	INSIDE	1.527	3.172	5337.83	16931.31	4.845	3663.72	17751.12
A_68_P23749879	chr5:64621748-64621793	NM_019636:70321	Tbc1d1	INSIDE	1.527	3.790	7787.71	29513.45	5.786	6371.74	36869.30
A_68_P28053990	chr11:97883852-97883896	NM_001159320:-4188	Cacnb1	PROMOTER	1.526	1.645	2003.46	3296.48	2.511	1678.37	4215.21
A_68_P29651385	chr14:62300358-62300402	NR_028264:830	Dleu2	INSIDE	1.526	3.449	4014.63	13847.19	5.263	3245.48	17082.07
A_68_P20826000	chr1:172995096-172995140	NM_010188:-5584	Fcgr3	PROMOTER	1.526	2.291	17110.18	39195.13	3.495	12593.44	44016.75
A_68_P21258086	chr2:60221286-60221330	NM_013825:-20	Ly75	PROMOTER	1.526	2.902	3297.93	9570.93	4.427	2671.37	11827.18
A_68_P22752523	chr4:15885168-15885212	NM_013752:77	Nbn	INSIDE	1.526	2.495	486.75	1214.43	3.808	417.33	1589.30
A_68_P26021284	chr8:87230641-87230685	NM_001081981:67606	Nfix	DOWNSTREAM	1.526	5.117	5399.70	27630.41	7.807	4219.66	32941.71
A_68_P30961954	chr16:91271188-91271232	NM_016968:1197	Olig1	INSIDE	1.526	1.925	2176.75	4190.72	2.937	1710.79	5024.60
A_68_P26477292	chr9:45821526-45821572	NM_027498:646	Sik3	INSIDE	1.526	3.393	1087.02	3688.45	5.178	884.56	4579.83
A_68_P29062337	chr13:56710941-56710985	NM_009369:-1	Tgfbi	PROMOTER	1.526	3.737	5366.24	20054.80	5.704	4386.93	25022.32
A_68_P24136157	chr5:140020297-140020341	NM_013702:467	Uncx	INSIDE	1.526	3.136	2512.84	7880.53	4.786	2233.60	10689.77
A_68_P32410980	chrX:56820824-56820868	ENSMUST00000124402:-197		PROMOTER	1.526	2.019	554.33	1119.11	3.080	325.16	1001.56
A_68_P24189032	chr5:150921298-150921342	ENSMUST00000165030:79		INSIDE	1.526	1.614	1184.67	1911.70	2.462	1095.03	2696.21
A_68_P23339473	chr4:136913085-136913129	NM_009861:546	Cdc42	INSIDE	1.525	1.562	644.29	1006.07	2.381	953.81	2270.72
A_68_P30378507	chr15:81416859-81416903	NM_177821:237	Ep300	INSIDE	1.525	1.529	2839.79	4341.31	2.331	2245.83	5234.89
A_68_P30364007	chr15:78873556-78873600	NM_015738:1264	Galr3	INSIDE	1.525	1.580	1550.00	2449.54	2.410	1295.18	3121.49
A_68_P21270819	chr2:62502267-62502311	NM_145523:-95	Gca	PROMOTER	1.525	1.673	2277.55	3809.24	2.551	1855.99	4734.25
A_68_P23927386	chr5:100468112-100468156	NM_016690:107	Hnrpdl	INSIDE	1.525	1.347	2453.41	3303.61	2.053	2090.79	4292.30
A_68_P21078500	chr2:26264786-26264830	NM_033134:-100	Inpp5e	PROMOTER	1.525	2.628	4230.13	11117.76	4.007	3630.11	14546.65
A_68_P30980968	chr16:94592282-94592326	NM_001159618:-147	Pigp	PROMOTER	1.525	3.774	2425.87	9154.33	5.756	2179.99	12547.77
A_68_P20638994	chr1:137271658-137271702	NM_175259:-40	Shisa4	PROMOTER	1.525	1.713	767.81	1315.14	2.612	633.58	1654.92
A_68_P23294939	chr4:128782828-128782872	NM_025452:77	Tmem54	INSIDE	1.525	2.730	3022.03	8250.80	4.163	2406.60	10019.09
A_68_P32700067	chrX:137134400-137134444	NM_001077364:639	Tsc22d3	INSIDE	1.525	2.951	4716.72	13920.73	4.500	2959.65	13317.83
A_68_P21770588	chr2:156137716-156137760	NM_027585:-470	4921517L17Rik	PROMOTER	1.524	2.899	1622.43	4703.88	4.418	1472.90	6507.65
A_68_P30588038	chr16:20531403-20531447	NM_007889:14288	Dvl3	INSIDE	1.524	3.268	6445.03	21060.16	4.980	4893.53	24368.66
A_68_P31937111	chr19:7054987-7055031	NM_001166368:-57	Fkbp2	PROMOTER	1.524	1.910	1073.69	2050.39	2.910	831.21	2418.70
A_68_P26644020	chr9:75289818-75289862	NM_001039522:510	Leo1	INSIDE	1.524	2.105	470.35	990.02	3.207	362.79	1163.57
A_68_P30571313	chr16:16983889-16983933	NM_001038663:436	Mapk1	INSIDE	1.524	2.580	3619.05	9335.38	3.932	2928.64	11514.79
A_68_P31640485	chr18:39650185-39650229	NM_008173:-3307	Nr3c1	PROMOTER	1.524	2.039	5516.93	11249.55	3.107	4451.15	13830.80
A_68_P31302584	chr17:66121929-66121973	NM_133685:142	Rab31	INSIDE	1.524	5.368	2447.72	13138.28	8.182	2211.95	18098.76

ProbeName	Target position of probe on CpG island microarray	TargetID	GeneSymbol	CpG island Description	Ratio of relative methylation (TiO_2-NP/Vehicle)	Sham group			TiO_2-H group		
						Relative methylation (Cy5/Cy3)	Cy3 signal (Input)	Cy5 signal (MeDIP)	Relative methylation (Cy5/Cy3)	Cy3 signal (Input)	Cy5 signal (MeDIP)
A_68_P31120939	chr17:28314640-28314684	NM_172617:300	Zfp523	INSIDE	1.524	2.620	5376.46	14087.07	3.993	3975.25	15871.32
A_68_P31090923	chr17:23013560-23013604	NM_001110254:-9481	Zfp945	PROMOTER	1.524	3.867	1295.52	5010.37	5.892	1139.64	6715.07
A_68_P30591825	chr16:21333336-21333380	AK018285:71		INSIDE	1.524	1.848	1711.65	3162.98	2.817	1599.45	4505.55
A_68_P31127961	chr17:29574415-29574459	AK153778:-2035		PROMOTER	1.524	2.268	647.74	1469.09	3.456	595.62	2058.33
A_68_P23264561	chr4:123427058-123427102	NM_023423:462	Akirin1	INSIDE	1.523	1.873	844.93	1582.79	2.854	666.74	1902.56
A_68_P20258850	chr1:58480309-58480353	NM_001042634:602	Clk1	INSIDE	1.523	1.658	1405.07	2330.06	2.526	1048.58	2648.61
A_68_P30743456	chr16:48994162-48994206	NM_001110017:41	Dzip3	INSIDE	1.523	3.027	2735.09	8279.27	4.610	2240.86	10331.14
A_68_P23280028	chr4:126106770-126106814	NM_153402:-6	Eif2c3	PROMOTER	1.523	3.112	1596.04	4967.57	4.742	1337.85	6343.42
A_68_P26891190	chr9:122656383-122656427	NM_001199736:-59	Gm9524	PROMOTER	1.523	7.913	889.69	7039.99	12.054	658.89	7942.60
A_68_P29330804	chr13:112598387-112598431	NM_011945:783	Map3k1	INSIDE	1.523	2.477	2532.55	6272.51	3.773	2203.81	8314.87
A_68_P31668416	chr18:44821733-44821777	NM_001085374:166	Mcc	INSIDE	1.523	3.694	1617.21	5974.76	5.627	1172.44	6597.50
A_68_P26026035	chr8:88224110-88224154	NM_001081324:-9224	Neto2	PROMOTER	1.523	1.881	423.11	795.66	2.864	418.85	1199.59
A_68_P25144564	chr7:63274831-63274875	NM_153578:91	Nipa1	INSIDE	1.523	5.769	4245.98	24493.11	8.786	3181.43	27952.98
A_68_P25524905	chr7:138009499-138009543	NM_133942:97	Plekha1	INSIDE	1.523	3.165	4756.44	15053.68	4.820	3968.99	19129.86
A_68_P27974634	chr11:83777899-83777943	NM_001115009:-9	Synrg	PROMOTER	1.523	2.208	376.20	830.79	3.364	416.91	1402.61
A_68_P23381898	chr4:144784634-144784678	NM_001128198:-4161	Vps13d	PROMOTER	1.523	2.268	1021.53	2316.37	3.453	893.16	3083.71
A_68_P31335313	chr17:71965569-71965613	NM_175639:36	Wdr43	INSIDE	1.523	1.559	551.97	860.51	2.374	500.10	1187.19
A_68_P26038355	chr8:90482696-90482740	NM_033327:776	Zfp423	INSIDE	1.523	2.485	1179.34	2930.14	3.784	1066.12	4034.58
A_68_P32573868	chrX:100812194-100812238	NR_028381:6877	B230206F22Rik	INSIDE	1.522	1.930	590.26	1139.31	2.937	464.53	1364.45
A_68_P28109104	chr11:107655957-107656001	NM_019431:-200	Cacng4	PROMOTER	1.522	5.935	2073.53	12305.65	9.033	1748.37	15792.28
A_68_P24791602	chr6:119125266-119125312	NM_001033379:18	Dcp1b	INSIDE	1.522	6.481	3512.61	22766.90	9.865	2669.19	26332.06
A_68_P27997247	chr11:87800951-87800995	NM_001168472:-885	Dynll2	PROMOTER	1.522	2.430	1692.09	4111.94	3.698	1379.73	5101.95
A_68_P27827804	chr11:57650530-57650574	NM_008213:-4903	Hand1	PROMOTER	1.522	2.081	2280.25	4744.95	3.166	1619.94	5128.94
A_68_P26648623	chr9:76171335-76171379	NM_198962:29	Hcrtr2	INSIDE	1.522	2.097	1253.70	2629.01	3.192	1031.47	3292.40
A_68_P25953853	chr8:73284695-73284739	NM_183170:104	Mpv17l2	INSIDE	1.522	3.011	1073.38	3231.59	4.583	912.14	4180.48
A_68_P29162295	chr13:78338732-78338776	NM_010151:-511	Nr2f1	PROMOTER	1.522	2.715	1410.20	3828.32	4.131	1237.84	5113.65
A_68_P23415887	chr4:151369933-151369977	NM_172705:334	Phf13	INSIDE	1.522	2.338	8895.10	20795.78	3.559	7091.16	25238.78
A_68_P21091625	chr2:28389284-28389328	NM_001145835:323	Ralgds	INSIDE	1.522	1.960	879.08	1722.93	2.982	703.10	2096.69
A_68_P21747609	chr2:151919690-151919734	NM_001160410:12448	Scrt2	INSIDE	1.522	7.979	1606.17	12814.85	12.144	1057.18	12838.79
A_68_P29507580	chr14:31529106-31529150	NM_001166532:328	Sfmbt1	INSIDE	1.522	4.440	1023.75	4545.37	6.758	860.17	5813.21
A_68_P23594804	chr5:34868325-34868369	NM_011893:-86	Sh3bp2	PROMOTER	1.522	3.182	1059.93	3372.88	4.843	1020.92	4944.01
A_68_P21840492	chr2:168106455-168106499	ENSMUST00000109191:156		INSIDE	1.522	3.036	1075.46	3265.51	4.621	990.43	4576.44
A_68_P27942253	chr11:78075063-78075107	NM_001002004:-171	2610507B11Rik	PROMOTER	1.521	5.560	2221.97	12353.43	8.457	2054.15	17372.83
A_68_P26741563	chr9:95306199-95306243	NM_018763:1469	Chst2	INSIDE	1.521	1.952	1403.78	2740.04	2.970	1182.30	3511.08
A_68_P22968875	chr4:62875286-62875330	NM_025685:-1137	Col27a1	PROMOTER	1.521	1.878	674.44	1266.91	2.857	542.61	1550.01
A_68_P30555522	chr16:13110157-13110201	NM_015769:350	Ercc4	INSIDE	1.521	2.963	2035.68	6031.25	4.507	1728.16	7789.45
A_68_P25528898	chr7:138685827-138685871	NM_008257:-628	Hmx3	PROMOTER	1.521	3.334	310.68	1035.88	5.071	297.74	1509.99
A_68_P29048046	chr13:54287380-54287425	NM_008286:-95	Hrh2	PROMOTER	1.521	2.939	1503.11	4418.07	4.470	943.46	4217.09
A_68_P25837693	chr8:47826718-47826762	NM_008391:1642	Irf2	INSIDE	1.521	3.072	367.43	1128.61	4.671	336.01	1569.53
A_68_P27512349	chr10:122513403-122513447	NM_001163024:137	Mon2	INSIDE	1.521	3.284	2131.92	7000.31	4.995	1977.70	9878.64
A_68_P22616951	chr3:146475944-146475988	NM_011100:-56	Prkacb	PROMOTER	1.521	2.762	2101.38	5803.43	4.202	1851.47	7779.21
A_68_P26138372	chr8:108734689-108734733	NM_001007567:123	Slc7a6os	INSIDE	1.521	2.938	1210.21	3555.44	4.469	1091.81	4879.33
A_68_P30255510	chr15:59479785-59479829	NM_144549:-402	Trib1	PROMOTER	1.521	2.815	1344.26	3784.38	4.282	1178.22	5044.74
A_68_P28185441	chr11:120442461-120442505	NM_133796:452	Arhgdia	INSIDE	1.520	1.757	2949.75	5183.66	2.671	2275.76	6078.96
A_68_P22288626	chr3:83844024-83844068	NM_172681:37	D930015E06Rik	INSIDE	1.520	3.459	496.73	1718.18	5.259	409.31	2152.57
A_68_P21819501	chr2:164571271-164571315	NM_133763:-222	Dnttip1	DIVERGENT_PROMOTER	1.520	2.327	7413.05	17246.82	3.535	5386.08	19041.21
A_68_P21556816	chr2:117075560-117075604	NM_026620:108	Fam98b	INSIDE	1.520	2.828	1580.47	4470.05	4.299	1399.84	6017.61
A_68_P25261383	chr7:86640870-86640922	NR_029818:-9253	Mir9-3	PROMOTER	1.520	4.997	818.01	4087.90	7.595	655.17	4976.12
A_68_P29228002	chr13:93962021-93962065	NM_133905:196	Papd4	INSIDE	1.520	4.177	1394.45	5824.97	6.349	1073.14	6813.49
A_68_P27898394	chr11:69810400-69810444	NM_030064:1150	Phf23	INSIDE	1.520	2.800	6398.65	17918.06	4.255	4222.08	17965.36
A_68_P26135404	chr8:108218075-108218119	NM_001033320:3291	Rltpr	INSIDE	1.520	1.873	10467.60	19607.79	2.848	8262.30	23530.51

ProbeName	Target position of probe on CpG island microarray	TargetID	GeneSymbol	CpG island Description	Ratio of relative methylation (TiO_2-NP/Vehicle)	Sham group			TiO_2-H group		
						Relative methylation (Cy5/Cy3)	Cy3 signal (Input)	Cy5 signal (MeDIP)	Relative methylation (Cy5/Cy3)	Cy3 signal (Input)	Cy5 signal (MeDIP)
A_68_P28938196	chr13:34436655-34436699	NM_001033167:375	Slc22a23	INSIDE	1.520	1.966	319.47	628.20	2.988	317.30	948.12
A_68_P23416421	chr4:151460684-151460728	ENSMUST00000118648:-260		PROMOTER	1.520	7.398	551.53	4080.29	11.246	515.12	5792.83
A_68_P23345840	chr4:138010011-138010055	NM_025451:-1030	Camk2n1	PROMOTER	1.519	2.325	1032.72	2401.05	3.531	867.53	3063.29
A_68_P26127993	chr8:106865035-106865079	NM_024217:563	Cmtm3	INSIDE	1.519	1.997	1888.06	3770.79	3.034	1463.37	4439.83
A_68_P23299954	chr4:129724880-129724924	NM_028266:-181	Col16a1	PROMOTER	1.519	3.754	598.62	2247.47	5.703	528.13	3011.74
A_68_P23318268	chr4:133130953-133130997	NM_172876:315	Gpatch3	INSIDE	1.519	3.239	1150.61	3726.47	4.921	1036.16	5098.82
A_68_P25301151	chr7:95278746-95278790	NM_001081414:546091	Grm5	INSIDE	1.519	2.111	1277.37	2695.95	3.206	1012.31	3245.16
A_68_P27995147	chr11:87430388-87430432	NM_001045527:-255	Hsf5	PROMOTER	1.519	4.025	862.35	3470.63	6.115	735.11	4494.96
A_68_P22397067	chr3:105255865-105255909	NM_019931:-5847	Kcnd3	PROMOTER	1.519	2.065	1408.52	2909.25	3.138	1170.27	3672.79
A_68_P25092719	chr7:52625927-52625971	NM_011698:-14	Lin7b	PROMOTER	1.519	2.163	3110.79	6728.46	3.285	2368.96	7782.17
A_68_P26949005	chr10:12810608-12810652	NM_009538:37	Plagl1	INSIDE	1.519	8.094	996.87	8068.42	12.295	851.53	10469.14
A_68_P25672219	chr8:14090321-14090365	NM_001034862:-15	Erich1	DIVERGENT_PROMOTER	1.518	2.067	1232.12	2546.24	3.138	955.63	2998.66
A_68_P28089851	chr11:104093898-104093942	NM_001038609:1171	Mapt	INSIDE	1.518	3.201	2664.13	8526.89	4.858	2303.11	11189.67
A_68_P28445674	chr12:55169509-55169553	NM_013780:819867	Npas3	INSIDE	1.518	3.022	1322.00	3995.28	4.587	1122.49	5148.47
A_68_P24114435	chr5:135518130-135518174	NM_016801:18711	Stx1a	INSIDE	1.518	10.296	11814.23	121639.30	15.628	7533.59	117737.10
A_68_P24151265	chr5:142843572-142843616			Unknown	1.518	1.665	1769.80	2946.27	2.527	1501.31	3794.47
A_68_P24182785	chr5:149826986-149827030	ENSMUST00000133243:381		INSIDE	1.518	3.325	7588.27	25228.93	5.047	4600.37	23216.64
A_68_P30357863	chr15:77950040-77950084	NM_007583:-352	Cacng2	PROMOTER	1.517	2.852	1563.96	4460.78	4.328	1372.23	5939.16
A_68_P21122760	chr2:33496334-33496378	NM_010725:-325	Lmx1b	PROMOTER	1.517	2.550	1335.58	3406.27	3.870	1103.55	4270.81
A_68_P24337493	chr6:31348815-31348859	NM_013791:9	Mkln1	INSIDE	1.517	7.206	1344.21	9686.86	10.934	1148.97	12562.71
A_68_P31759277	chr18:61867112-61867156	NM_172832:155	Pcyox1l	INSIDE	1.517	2.206	3676.05	8109.10	3.347	2825.32	9456.43
A_68_P27896916	chr11:69572824-69572871	NM_009089:-1122	Polr2a	PROMOTER	1.517	1.649	653.89	1078.34	2.502	517.68	1295.20
A_68_P29648697	chr14:61791606-61791650	NM_172809:34335	Sacs	INSIDE	1.517	1.545	1833.51	2833.14	2.345	1362.27	3193.90
A_68_P29033511	chr13:51746883-51746927	NM_029279:-178	Secisbp2	PROMOTER	1.517	2.454	3102.77	7613.38	3.723	2647.15	9854.78
A_68_P29218723	chr13:91600897-91600941	NM_024186:217	Ssbp2	INSIDE	1.517	4.122	4099.33	16896.23	6.251	3046.95	19047.37
A_68_P27187531	chr10:62188832-62188876	NM_001033260:-7	Stox1	PROMOTER	1.517	3.109	1793.62	5576.70	4.717	1443.07	6807.33
A_68_P29244723	chr13:96902535-96902579	NM_029210:-24	Sv2c	PROMOTER	1.517	1.877	1202.09	2255.91	2.847	970.77	2764.28
A_68_P28520555	chr12:71554358-71554402	NM_028339:240	Tmx1	INSIDE	1.517	1.462	1187.01	1735.79	2.218	1077.70	2390.47
A_68_P22321608	chr3:89577074-89577118	NM_027315:-434	Ube2q1	DIVERGENT_PROMOTER	1.517	1.749	2102.20	3677.00	2.653	1641.16	4353.86
A_68_P26350328	A_68_P26350328			Unknown	1.517	2.943	449.87	1324.02	4.464	345.01	1540.05
A_68_P27288853	chr10:80783632-80783676	NM_027381:-193	2510012J08Rik	PROMOTER	1.516	1.546	1103.03	1705.68	2.344	891.15	2088.80
A_68_P23467742	chr5:8368117-8368161	NM_001098225:-57	Adam22	PROMOTER	1.516	2.463	690.62	1701.06	3.735	631.65	2359.29
A_68_P30350779	chr15:76647508-76647552	NM_001168288:1070	Arhgap39	INSIDE	1.516	2.606	1450.37	3779.73	3.952	1090.58	4309.65
A_68_P29338038	chr13:113778161-113778205	NM_001081062:173	Ccno	INSIDE	1.516	1.461	3379.73	4937.96	2.215	2794.94	6190.79
A_68_P31376862	chr17:79755142-79755186	NM_026514:-733	Cdc42ep3	PROMOTER	1.516	1.712	474.69	812.66	2.596	415.37	1078.28
A_68_P27910787	chr11:72128038-72128082	NM_001099688:115	Fbxo39	INSIDE	1.516	3.848	1223.64	4709.14	5.834	1038.74	6060.44
A_68_P30148791	chr15:38229661-38229705	NM_013692:780	Klf10	INSIDE	1.516	5.717	1731.12	9896.19	8.669	1387.82	12030.48
A_68_P23539922	chr5:23606508-23606552	NM_001161800:123	Klhl7	INSIDE	1.516	1.684	880.42	1482.98	2.553	695.55	1775.95
A_68_P25086960	chr7:51607818-51607862	NM_001034115:42207	Shank1	INSIDE	1.516	5.044	7768.35	39184.21	7.649	5667.39	43348.23
A_68_P28635993	chr12:92886575-92886620	NM_175367:138279	Ston2	INSIDE	1.516	2.163	294.02	636.00	3.280	230.66	756.53
A_68_P30980679	chr16:94534837-94534881	ENSMUST00000163193:312		INSIDE	1.516	4.109	565.54	2323.63	6.228	554.91	3456.16
A_68_P31126996	chr17:29406043-29406087	NM_030561:332	BC004004	INSIDE	1.515	3.081	2100.11	6469.43	4.666	1938.91	9046.55
A_68_P28694110	chr12:104116592-104116636	NM_172806:2	Btbd7	INSIDE	1.515	1.901	366.82	697.42	2.881	341.52	984.04
A_68_P27784449	chr11:49525483-49525527	NM_212484:708	Cnot6	INSIDE	1.515	4.727	1217.49	5755.20	7.160	1081.63	7744.99
A_68_P26128587	chr8:106966661-106966705	NM_001013380:265	Dync1li2	INSIDE	1.515	3.010	3069.91	9241.24	4.561	2459.41	11216.76
A_68_P26001982	chr8:83403879-83403923	NM_021356:478	Gab1	INSIDE	1.515	4.546	3277.91	14899.97	6.888	2505.41	17257.68
A_68_P25274814	chr7:89079181-89079225	NM_013886:143	Hdgfrp3	INSIDE	1.515	3.405	2741.20	9335.16	5.161	2297.83	11858.66
A_68_P30639681	chr16:30070804-30070848	NM_008235:5384	Hes1	DOWNSTREAM	1.515	1.520	4721.65	7178.51	2.303	3831.89	8826.15
A_68_P25528895	chr7:138685558-138685602	NM_008257:-896	Hmx3	PROMOTER	1.515	2.685	721.58	1937.75	4.069	630.15	2564.25
A_68_P28886522	chr13:24373081-24373125	NM_026825:-443	Lrrc16a	PROMOTER	1.515	5.019	1735.70	8711.35	7.604	1462.36	11119.99
A_68_P24792500	chr6:119280593-119280637	NM_001172207:-1392	Lrtm2	PROMOTER	1.515	2.033	2029.62	4125.92	3.080	1634.56	5034.32

ProbeName	Target position of probe on CpG island microarray	TargetID	GeneSymbol	CpG island Description	Ratio of relative methylation (TiO_2-NP/Vehicle)	Sham group			TiO_2-H group		
						Relative methylation (Cy5/Cy3)	Cy3 signal (Input)	Cy5 signal (MeDIP)	Relative methylation (Cy5/Cy3)	Cy3 signal (Input)	Cy5 signal (MeDIP)
A_68_P30020394	chr15:12134922-12134966	NM_172958:96	Mtmr12	INSIDE	1.515	2.637	924.58	2437.87	3.993	797.05	3182.95
A_68_P30139527	chr15:36538715-36538759	NM_008774:-8	Pabpc1	PROMOTER	1.515	1.917	3127.60	5996.12	2.904	2496.02	7248.76
A_68_P22355467	chr3:97462102-97462146	NM_182997:-10	Prkab2	PROMOTER	1.515	4.803	8266.35	39707.11	7.277	6308.41	45903.36
A_68_P26229674	chr8:124431610-124431654	NM_011404:-46	Slc7a5	DIVERGENT_PROMOTER	1.515	2.080	3412.27	7097.53	3.152	2669.32	8414.32
A_68_P31351023	chr17:74738587-74738631	NM_001162870:282	Spast	INSIDE	1.515	3.242	614.24	1991.24	4.912	530.71	2606.66
A_68_P28161643	chr11:116713965-116714009	NM_011358:422	Srsf2	INSIDE	1.515	2.553	535.98	1368.48	3.867	467.60	1808.41
A_68_P20935551	chr1:192803342-192803386	NR_027352:-189	Vash2	PROMOTER	1.515	2.830	1064.05	3011.52	4.287	847.11	3631.88
A_68_P32186048	chr19:54119950-54119994	NM_007417:301	Adra2a	INSIDE	1.514	1.870	1207.84	2258.19	2.830	1019.30	2884.49
A_68_P31524834	chr18:16965463-16965507	NM_007664:2074	Cdh2	INSIDE	1.514	1.442	5317.30	7667.13	2.183	4181.25	9127.82
A_68_P23222243	chr4:114580052-114580096	NM_008593:1429	Foxd2	INSIDE	1.514	3.361	1114.32	3744.93	5.088	999.09	5082.91
A_68_P27980051	chr11:84683596-84683640	NM_153144:622	Ggnbp2	INSIDE	1.514	2.029	3310.52	6716.55	3.072	2466.36	7576.01
A_68_P27977954	chr11:84333936-84333980	NM_008498:5078	Lhx1	INSIDE	1.514	2.640	571.16	1507.89	3.996	476.27	1903.32
A_68_P22601440	chr3:143867896-143867940	NM_001161769:-2558	Lmo4	PROMOTER	1.514	3.237	4765.00	15423.48	4.901	3566.87	17481.44
A_68_P24156969	chr5:144288624-144288668	NM_009007:215	Rac1	INSIDE	1.514	2.574	1631.45	4199.18	3.897	1284.55	5006.06
A_68_P31943158	chr19:8788397-8788441	NM_008577:-31	Slc3a2	PROMOTER	1.514	3.055	1784.84	5452.80	4.624	1356.57	6272.64
A_68_P25239159	chr7:82453955-82453999	NM_001109753:-704	Sv2b	PROMOTER	1.514	4.067	2346.69	9543.51	6.158	1944.04	11971.56
A_68_P23397204	chr4:148265329-148265373	NM_027195:86850	Casz1	INSIDE	1.513	1.413	1598.88	2258.72	2.138	1270.07	2715.47
A_68_P25392270	chr7:112574421-112574465	NM_007627:109	Cckbr	INSIDE	1.513	3.964	6148.54	24371.44	5.996	5077.69	30446.86
A_68_P31490815	chr18:10609631-10609677	NM_001081222:696	Esco1	INSIDE	1.513	2.126	827.03	1758.24	3.216	726.99	2338.32
A_68_P24620367	chr6:88149612-88149656	NM_008090:977	Gata2	INSIDE	1.513	1.864	2081.20	3879.08	2.820	1736.45	4897.60
A_68_P26220480	chr8:123011650-123011694	NM_198671:-1093	Gse1	PROMOTER	1.513	2.779	968.75	2692.41	4.206	807.18	3395.13
A_68_P21038125	chr2:17987169-17987213	NM_010804:10293	Mllt10	INSIDE	1.513	2.283	1455.18	3322.77	3.454	1218.79	4209.49
A_68_P30560744	chr16:14163261-14163305	NM_001114085:-85	Nde1	PROMOTER	1.513	1.741	1004.83	1749.37	2.633	774.54	2039.73
A_68_P31084402	chr17:21082837-21082881	NM_016891:441	Ppp2r1a	INSIDE	1.513	1.991	424.01	844.09	3.012	391.52	1179.10
A_68_P24445013	chr6:51542631-51542675	NM_001127348:48131	Snx10	DOWNSTREAM	1.513	1.619	5835.38	9446.43	2.449	4234.39	10370.79
A_68_P30401894	chr15:85456416-85456460			Unknown	1.513	4.687	777.98	3646.20	7.091	665.73	4720.50
A_68_P23699554	chr5:53658257-53658301	NM_001145433:-66	1810013D10Rik	PROMOTER	1.512	1.927	1747.30	3367.67	2.914	1494.68	4355.06
A_68_P24799551	chr6:120615439-120615483	NM_001128151:-978	Cecr2	PROMOTER	1.512	2.599	2655.71	6901.62	3.928	2199.66	8641.34
A_68_P32769550	chrX:154480805-154480849	NM_177751:216	Cnksr2	INSIDE	1.512	2.146	1326.45	2845.95	3.244	822.04	2667.04
A_68_P23309923	chr4:131604805-131604849	NM_001128606:167	Epb4.1	INSIDE	1.512	5.819	1030.57	5996.78	8.796	869.44	7647.47
A_68_P23345562	chr4:137952020-137952064	NM_001081672:331	Fam43b	INSIDE	1.512	2.773	891.39	2472.07	4.193	757.52	3175.97
A_68_P26221250	chr8:123112916-123112960	NM_178856:37	Gins2	INSIDE	1.512	1.444	2567.51	3706.97	2.182	2080.94	4541.31
A_68_P24947962	chr7:4098539-4098584	NM_172736:9904	Leng8	INSIDE	1.512	4.325	1465.62	6339.23	6.541	1229.91	8045.26
A_68_P28162003	chr11:116780529-116780573	NM_172948:374	Mgat5b	INSIDE	1.512	1.990	423.87	843.57	3.008	438.90	1320.33
A_68_P31207975	chr17:46766402-46766446	NM_175168:29	Ptk7	INSIDE	1.512	2.633	12220.97	32171.92	3.980	10248.26	40791.04
A_68_P32234202	chrX:5977172-5977216	NM_001040459:-68	Shroom4	PROMOTER	1.512	2.645	1691.44	4473.07	3.997	1083.40	4330.82
A_68_P23568207	chr5:29896110-29896154	NM_133907:351	Ube3c	INSIDE	1.512	2.739	1584.16	4339.25	4.140	1228.08	5084.59
A_68_P25034247	chr7:31740799-31740843	NM_011680:1002	Usf2	INSIDE	1.512	2.427	822.98	1997.11	3.670	742.19	2723.71
A_68_P32152243	chr19:47911627-47911671	NM_027559:203	Wdr96	INSIDE	1.512	1.971	1964.46	3872.11	2.981	1536.97	4581.51
A_68_P25364270	chr7:107007573-107007617	NM_001164258:-133	Xrra1	DIVERGENT_PROMOTER	1.512	3.183	829.70	2640.68	4.811	747.86	3598.25
A_68_P23820887	chr5:77738314-77738358	NM_019836:775	2610024G14Rik	INSIDE	1.511	2.988	773.65	2311.49	4.515	651.71	2942.33
A_68_P30931894	chr16:85900162-85900206	NM_011782:1186	Adamts5	INSIDE	1.511	4.290	1821.56	7814.98	6.483	1287.00	8343.12
A_68_P24138663	chr5:140449111-140449155	NM_175522:65236	Elfn1	INSIDE	1.511	1.973	1056.98	2085.69	2.981	1263.29	3766.04
A_68_P31382195	chr17:80689441-80689485	NM_194269:-89	Morn2	PROMOTER	1.511	5.017	1251.41	6278.01	7.579	1032.08	7821.99
A_68_P26016101	chr8:86222687-86222731	NM_001199593:-4748	Pkn1	PROMOTER	1.511	2.520	992.70	2501.25	3.806	882.85	3360.44
A_68_P25553143	chr7:142729512-142729556	NM_011212:28	Ptpre	INSIDE	1.511	3.674	958.74	3522.72	5.553	802.21	4454.28
A_68_P22715106	chr4:8463176-8463220	NM_021518:408	Rab2a	INSIDE	1.511	2.557	1306.31	3340.23	3.864	1128.70	4361.49
A_68_P24328146	chr6:29685667-29685711	NM_176996:192	Smo	INSIDE	1.511	4.847	1094.66	5305.59	7.325	874.54	6405.71
A_68_P23696773	chr5:53174399-53174443	NM_030185:115	Zcchc4	INSIDE	1.511	2.632	2596.84	6834.23	3.977	2118.43	8424.77
A_68_P24004682	chr5:115017652-115017696	NM_001004180:-585	BC057022	PROMOTER	1.510	2.639	1484.21	3916.52	3.985	1255.60	5003.35
A_68_P20872965	chr1:181889796-181889840	NM_001033285:-1401	Cdc42bpa	PROMOTER	1.510	1.895	2361.56	4474.53	2.861	1933.91	5532.09

ProbeName	Target position of probe on CpG island microarray	TargetID	GeneSymbol	CpG island Description	Ratio of relative methylation (TiO_2-NP/Vehicle)	Sham group			TiO_2-H group		
						Relative methylation (Cy5/Cy3)	Cy3 signal (Input)	Cy5 signal (MeDIP)	Relative methylation (Cy5/Cy3)	Cy3 signal (Input)	Cy5 signal (MeDIP)
A_68_P20885892	chr1:184212460-184212504	NM_007853:408	Degs1	INSIDE	1.510	1.720	462.28	795.14	2.597	412.83	1072.19
A_68_P26132767	chr8:107813885-107813929	NM_177788:6092	Exoc3l	INSIDE	1.510	1.383	3404.14	4707.23	2.088	2623.45	5478.57
A_68_P26220858	chr8:123061951-123061995	NM_001145896:664	Gse1	INSIDE	1.510	3.980	1269.37	5052.43	6.011	964.46	5796.91
A_68_P31259625	chr17:57161925-57161969	NM_010613:8984	Khsrp	INSIDE	1.510	2.764	4307.69	11905.11	4.173	3401.95	14196.21
A_68_P24520694	chr6:67217359-67217403	NM_001113564:408	Serbp1	INSIDE	1.510	1.793	659.14	1181.96	2.707	523.12	1416.16
A_68_P23283131	chr4:126698502-126698546	NM_023603:-20	Sfpq	PROMOTER	1.510	10.628	252.76	2686.37	16.045	227.79	3654.84
A_68_P26540094	chr9:56924177-56924221	NM_001110350:16	Sin3a	INSIDE	1.510	1.692	1975.15	3341.37	2.555	1590.03	4062.80
A_68_P21748558	chr2:152119855-152119899	NM_024196:269	Tbc1d20	INSIDE	1.510	3.767	2842.08	10706.49	5.687	2383.82	13556.14
A_68_P25947840	chr8:72273755-72273799	NM_001200023:85	Zfp963	INSIDE	1.510	2.939	4922.90	14468.46	4.439	3866.13	17161.24
A_68_P21070248	chr2:25023708-25023752			Unknown	1.510	1.986	1032.55	2050.38	2.999	889.93	2668.77
A_68_P23062804	chr4:83171213-83171257	NM_001081012:-214	4930473A06Rik	PROMOTER	1.509	2.827	912.04	2578.63	4.268	849.60	3625.74
A_68_P27932872	chr11:76384849-76384893	NM_198895:6	Abr	INSIDE	1.509	1.675	3476.87	5823.44	2.528	2873.42	7262.84
A_68_P27176099	chr10:60150136-60150180	NM_023370:9080	Cdh23	INSIDE	1.509	2.178	935.31	2037.03	3.287	895.72	2944.61
A_68_P26102842	chr8:101939617-101939661	NM_001039154:719	Cdh8	INSIDE	1.509	4.432	1458.56	6464.77	6.686	1284.16	8586.35
A_68_P32070425	chr19:32560187-32560231	NM_010799:-50	Minpp1	PROMOTER	1.509	2.151	1498.34	3223.34	3.247	1254.33	4072.98
A_68_P25954894	chr8:73437692-73437736	NM_173013:7842	Mtap1s	INSIDE	1.509	2.762	422.18	1166.14	4.169	350.12	1459.49
A_68_P27168073	chr10:58785999-58786043	NM_011030:-23	P4ha1	PROMOTER	1.509	2.524	683.06	1724.28	3.809	537.46	2046.99
A_68_P28035927	chr11:94853399-94853443	NM_172261:895	Ppp1r9b	INSIDE	1.509	4.641	494.07	2292.84	7.001	468.52	3280.20
A_68_P31612299	chr18:34490607-34490651	NM_025527:-170	Srp19	PROMOTER	1.509	2.820	1687.65	4759.55	4.255	1369.71	5827.73
A_68_P20634933	chr1:136605811-136605855	NM_009307:62575	Syt2	INSIDE	1.509	2.023	1859.43	3762.50	3.053	1570.85	4795.85
A_68_P28871497	chr13:21271956-21272000	NM_009054:151	Trim27	INSIDE	1.509	1.526	771.34	1177.15	2.304	742.76	1710.95
A_68_P27287631	chr10:80600637-80600681	NM_010731:1643	Zbtb7a	INSIDE	1.509	2.562	2040.84	5227.85	3.864	1790.23	6917.92
A_68_P27914868	chr11:72861634-72861678	NM_025818:288	1200014J11Rik	INSIDE	1.508	2.998	3522.26	10560.49	4.520	2793.53	12627.23
A_68_P21419477	chr2:90729763-90729807	NM_026161:3842	C1qtnf4	INSIDE	1.508	2.241	777.41	1742.33	3.381	697.75	2358.80
A_68_P24757354	chr6:113026958-113027002	NR_027010:258	Gt(ROSA)26Sor	INSIDE	1.508	2.265	1639.14	3713.15	3.417	1379.95	4714.75
A_68_P27189032	chr10:62486795-62486839	NM_001079824:-219	Hnrnph3	DIVERGENT_PROMOTER	1.508	2.966	1117.74	3315.50	4.472	978.50	4376.19
A_68_P30925479	chr16:84774664-84774708	NM_023844:319	Jam2	INSIDE	1.508	3.443	838.56	2887.49	5.193	795.58	4131.20
A_68_P31251161	chr17:55585081-55585126	NM_172829:89	St6gal2	INSIDE	1.508	1.632	618.68	1009.67	2.461	567.75	1397.19
A_68_P26626137	chr9:71959791-71959835	NM_011544:-186	Tcf12	PROMOTER	1.508	4.049	2317.19	9381.37	6.106	2028.59	12386.53
A_68_P22630580	chr3:148653297-148653341	ENSMUST00000098518:-1038		PROMOTER	1.508	1.703	1888.29	3215.15	2.567	1552.98	3987.28
A_68_P27169799	chr10:59079055-59079099	NM_001033259:364	Ccdc109a	INSIDE	1.507	1.574	2053.34	3232.15	2.371	1593.85	3779.80
A_68_P31890103	chr18:84855123-84855167	NM_023149:-119	Cndp2	PROMOTER	1.507	2.250	7270.70	16356.27	3.391	5158.92	17494.56
A_68_P26542219	chr9:57369547-57369591	NM_007747:530	Cox5a	INSIDE	1.507	1.737	1447.75	2514.73	2.617	1202.89	3148.03
A_68_P31677413	chr18:46685542-46685586	NM_173423:61	Fem1c	INSIDE	1.507	1.993	1402.64	2795.91	3.005	1532.05	4603.56
A_68_P20184678	chr1:42755116-42755160	NM_008900:1148	Pou3f3	INSIDE	1.507	3.857	1035.60	3994.36	5.813	842.84	4899.23
A_68_P20632910	chr1:136241239-136241283	NM_001042485:17316	Tmem183a	DOWNSTREAM	1.507	1.589	1960.62	3116.15	2.395	1775.76	4253.73
A_68_P21094978	chr2:28910349-28910393	NM_001166705:216	1700101E01Rik	INSIDE	1.506	1.659	1643.86	2726.81	2.498	1408.24	3517.32
A_68_P26486127	chr9:47339193-47339237	NM_001025600:780	Cadm1	INSIDE	1.506	2.674	550.83	1472.74	4.026	421.57	1697.37
A_68_P29856055	chr14:102039383-102039427	NM_001033132:284	Commd6	INSIDE	1.506	3.406	2079.90	7084.80	5.129	1713.73	8790.10
A_68_P23415959	chr4:151383528-151383572	NM_001033352:551	Klhl21	INSIDE	1.506	1.829	351.42	642.62	2.754	315.28	868.14
A_68_P20352133	chr1:75121786-75121830	NM_029342:-8	Nhej1	PROMOTER	1.506	3.573	3084.75	11022.24	5.382	2114.11	11378.24
A_68_P21820253	chr2:164705104-164705148	NM_146129:259	Pcif1	INSIDE	1.506	2.676	1462.77	3914.74	4.030	1325.28	5340.69
A_68_P27288650	chr10:80755715-80755759	NM_001146687:20	Pip5k1c	INSIDE	1.506	2.439	1510.25	3683.36	3.672	1267.97	4656.54
A_68_P26596131	chr9:66794074-66794118	NM_026467:172	Rps27l	INSIDE	1.506	2.303	551.36	1269.99	3.468	380.12	1318.23
A_68_P25351157	chr7:104729030-104729074	NM_001081267:647	Rsf1	INSIDE	1.506	2.725	802.51	2186.66	4.104	732.60	3006.58
A_68_P25592703	chr7:149246867-149246911	NM_028308:51	2700078K21Rik	INSIDE	1.505	3.078	809.83	2492.47	4.632	861.56	3990.66
A_68_P30587610	chr16:20425965-20426009	NM_013790:481	Abcc5	INSIDE	1.505	2.189	1643.18	3597.22	3.294	1348.96	4443.74
A_68_P20231550	chr1:53409540-53409584	NM_133728:-19	Asnsd1	PROMOTER	1.505	3.087	1517.71	4684.89	4.647	1377.76	6402.13
A_68_P30333008	chr15:73670325-73670369	NM_001033365:-245	Gm628	PROMOTER	1.505	1.455	862.56	1255.19	2.189	759.49	1662.90
A_68_P25525511	chr7:138079707-138079751	NM_019564:12	Htra1	INSIDE	1.505	1.501	3874.53	5817.41	2.260	3128.33	7070.53
A_68_P28060084	chr11:98902871-98902915	NM_010517:319	Igfbp4	INSIDE	1.505	1.812	1193.02	2161.77	2.726	1051.81	2867.48

ProbeName	Target position of probe on CpG island microarray	TargetID	GeneSymbol	CpG island Description	Ratio of relative methylation (TiO_2-NP/Vehicle)	Sham group			TiO_2-H group		
						Relative methylation (Cy5/Cy3)	Cy3 signal (Input)	Cy5 signal (MeDIP)	Relative methylation (Cy5/Cy3)	Cy3 signal (Input)	Cy5 signal (MeDIP)
A_68_P29616407	chr14:55733626-55733670	NM_177049:2124	Jph4	INSIDE	1.505	3.567	6810.59	24294.32	5.368	4485.51	24077.71
A_68_P30984747	chr16:95219135-95219179	NM_001025584:147	Kcnj6	INSIDE	1.505	6.088	1997.49	12160.32	9.160	1688.40	15466.36
A_68_P26068285	chr8:95379185-95379229	NM_173014:-42	Lpcat2	PROMOTER	1.505	6.893	3200.62	22061.99	10.374	2288.30	23738.41
A_68_P27891735	chr11:68666059-68666103	NM_023668:553	Ndel1	INSIDE	1.505	1.532	1208.31	1851.00	2.306	966.35	2228.04
A_68_P31323668	chr17:69733260-69733304	NM_009547:-35	Zfp161	PROMOTER	1.505	4.474	2569.08	11495.08	6.736	2114.62	14243.16
A_68_P32288519	chrX:20002775-20002819	ENSMUST00000115384:-17		PROMOTER	1.505	2.230	1112.18	2480.71	3.356	672.13	2255.95
A_68_P30345268	chr15:75812328-75812373	NM_001163518:365	2410075B13Rik	INSIDE	1.504	2.082	651.05	1355.76	3.133	588.82	1844.67
A_68_P32443538	chrX:66613886-66613930	NM_008032:403	Aff2	INSIDE	1.504	1.550	1876.72	2909.56	2.332	929.04	2166.58
A_68_P32576287	chrX:101396366-101396410	NM_001077354:68	C77370	INSIDE	1.504	4.104	553.75	2272.55	6.172	442.53	2731.40
A_68_P27813685	chr11:54911498-54911542	NM_010299:34	Gm2a	INSIDE	1.504	4.655	2755.36	12826.30	7.003	2025.41	14183.33
A_68_P27127187	chr10:49502783-49502839	NM_001111268:5750	Grik2	INSIDE	1.504	2.794	331.81	927.16	4.203	255.99	1075.91
A_68_P28152414	chr11:115126813-115126857	NM_010350:1723	Grin2c	INSIDE	1.504	1.820	7588.84	13811.37	2.738	5649.71	15466.21
A_68_P25971363	chr8:77517838-77517882	NM_178017:259	Hmgxb4	INSIDE	1.504	3.776	1144.27	4320.45	5.679	1081.27	6140.09
A_68_P29071664	chr13:58228883-58228927	NM_029872:1013	Hnrnpa0	INSIDE	1.504	3.383	2280.21	7714.19	5.090	1800.45	9163.93
A_68_P20646088	chr1:138363089-138363133	NM_001081258:-1424	Kif14	PROMOTER	1.504	5.511	2003.58	11041.10	8.290	1645.78	13643.44
A_68_P25922763	chr8:66684161-66684205	NM_009390:608	Tll1	INSIDE	1.504	2.938	650.04	1910.15	4.418	499.30	2205.96
A_68_P30593950	chr16:21694881-21694925	NM_001001881:-164	2510009E07Rik	PROMOTER	1.503	0.434	1506.73	654.64	0.653	1233.71	805.79
A_68_P26569977	chr9:62192249-62192293	NM_009672:3121	Anp32a	INSIDE	1.503	1.406	1688.30	2373.21	2.113	1514.34	3199.71
A_68_P27643047	chr11:22758992-22759036	NM_016888:721	B3gnt2	INSIDE	1.503	2.345	1392.02	3264.16	3.524	1171.12	4127.43
A_68_P21646236	chr2:133380456-133380500	NM_007553:1544	Bmp2	INSIDE	1.503	3.428	1928.92	6612.98	5.151	1538.58	7925.62
A_68_P20778063	chr1:164407783-164407827	NM_001038619:357	Dnm3	INSIDE	1.503	2.197	2396.10	5263.29	3.302	1800.71	5945.11
A_68_P23300612	chr4:129816147-129816191	NM_001163027:-373	Hcrtr1	PROMOTER	1.503	1.713	3407.08	5835.44	2.575	2819.48	7260.01
A_68_P28518531	chr12:71213190-71213234	NM_008697:-300	Nin	PROMOTER	1.503	3.023	1996.26	6034.16	4.544	1812.79	8237.10
A_68_P27287763	chr10:80617060-80617104	NM_021501:13383	Pias4	INSIDE	1.503	3.958	1158.08	4583.60	5.950	877.19	5219.09
A_68_P28566640	chr12:80013209-80013253	NM_013738:-5305	Plek2	PROMOTER	1.503	1.504	2873.07	4322.23	2.261	2125.31	4805.89
A_68_P21745020	chr2:151457975-151458019	NM_198214:273	Snph	INSIDE	1.503	4.184	431.24	1804.12	6.287	374.44	2354.14
A_68_P25777211	chr8:36028290-36028334	NM_175091:432	Tnks	INSIDE	1.503	3.928	823.33	3233.88	5.903	639.69	3775.94
A_68_P21936419	chr3:9251754-9251798	NM_177660:1210	Zbtb10	INSIDE	1.503	2.892	5521.23	15969.96	4.346	4422.08	19220.12
A_68_P22880598	chr4:45025167-45025211	NM_001163283:96	Zbtb5	INSIDE	1.503	4.230	645.60	2730.70	6.358	540.93	3439.40
A_68_P26218677	chr8:122752875-122752919	ENSMUST00000127664:318156		INSIDE	1.503	7.820	1854.22	14499.10	11.750	1420.25	16687.41
A_68_P24135766	chr5:139936328-139936372	NM_028469:138	3110082I17Rik	INSIDE	1.502	1.936	4745.40	9185.42	2.908	3667.44	10663.21
A_68_P30599077	chr16:22657267-22657311	NM_138650:16	Dgkg	INSIDE	1.502	1.609	1659.05	2669.27	2.416	1302.66	3147.24
A_68_P28553818	chr12:77505144-77505188	NM_008301:-190	Hspa2	PROMOTER	1.502	3.614	2028.44	7330.59	5.430	1623.90	8817.36
A_68_P28036074	chr11:94871790-94871834	NM_146025:620	Samd14	INSIDE	1.502	2.501	2221.81	5557.73	3.757	1714.51	6441.51
A_68_P31931517	chr19:5894108-5894152	NM_198634:-23	Tigd3	PROMOTER	1.502	1.820	1100.87	2003.78	2.734	879.37	2403.95
A_68_P30255512	chr15:59479961-59480005	NM_144549:-226	Trib1	PROMOTER	1.502	2.370	2851.21	6758.58	3.560	2415.27	8599.13
A_68_P28931383	chr13:32894479-32894523	NM_030215:602	Wrnip1	INSIDE	1.502	3.982	1699.99	6769.26	5.980	1708.82	10218.32
A_68_P29063253	chr13:56854488-56854532	AK131829:678		INSIDE	1.502	4.787	2312.37	11068.22	7.188	1936.18	13917.98
A_68_P21094081	chr2:28764046-28764090	NM_019446:7873	Barhl1	INSIDE	1.501	2.176	1638.99	3567.19	3.267	1373.26	4487.10
A_68_P27476525	chr10:116018687-116018731	NM_001037846:-141	Cnot2	PROMOTER	1.501	2.634	907.22	2389.64	3.952	776.04	3067.27
A_68_P21202301	chr2:49307033-49307077	NM_172663:49	Epc2	INSIDE	1.501	2.387	1763.84	4209.42	3.583	1485.53	5322.72
A_68_P23525900	chr5:20931065-20931109	NM_177869:366	Fam185a	INSIDE	1.501	1.522	1176.74	1790.95	2.284	1000.93	2286.17
A_68_P23295046	chr4:128798355-128798400	NM_010471:158	Hpca	INSIDE	1.501	5.295	891.80	4722.32	7.947	799.18	6350.88
A_68_P27927426	chr11:75469605-75469649	NM_001080775:110	Myo1c	INSIDE	1.501	3.042	780.85	2375.39	4.567	661.02	3019.16
A_68_P27678665	chr11:30167644-30167688	ENSMUST00000146985:203		INSIDE	1.501	7.255	3141.26	22790.57	10.889	2975.88	32405.32
A_68_P22148834	chr3:54437446-54437490	ENSMUST00000172420:2108		INSIDE	1.501	4.213	925.25	3898.13	6.324	727.00	4597.46
A_68_P20584830	chr1:127331799-127331843	NM_001205385:484	Actr3	INSIDE	1.500	2.916	7966.52	23227.85	4.375	6005.45	26271.98
A_68_P23447646	chr5:3928274-3928318	NM_194462:111	Akap9	INSIDE	1.500	2.272	1022.53	2322.79	3.408	789.77	2691.39
A_68_P24186507	chr5:150480724-150480768	NM_001081204:-85	B3galtl	PROMOTER	1.500	1.836	485.61	891.45	2.753	476.66	1312.40
A_68_P31097616	chr17:24625344-24625388	NM_027937:-361	Caskin1	DIVERGENT_PROMOTER	1.500	1.672	796.20	1330.91	2.508	669.03	1677.63
A_68_P23571444	chr5:30559486-30559531	NM_027652:351	Ept1	INSIDE	1.500	1.791	1159.99	2077.24	2.686	1065.22	2860.95

ProbeName	Target position of probe on CpG island microarray	TargetID	GeneSymbol	CpG island Description	Ratio of relative methylation (TiO_2-NP/Vehicle)	Sham group			TiO_2-H group		
						Relative methylation (Cy5/Cy3)	Cy3 signal (Input)	Cy5 signal (MeDIP)	Relative methylation (Cy5/Cy3)	Cy3 signal (Input)	Cy5 signal (MeDIP)
A_68_P32823846	chrX:166435801-166435845	NM_183151:118269	Mid1	DOWNSTREAM	1.500	1.929	3218.86	6210.66	2.895	3388.99	9809.69
A_68_P21152640	chr2:39046217-39046261	NM_178778:12	Scai	INSIDE	1.500	2.913	813.18	2369.11	4.370	698.70	3053.46
A_68_P24141204	chr5:140865179-140865223	NM_172277:1	Snx8	INSIDE	1.500	1.386	1327.27	1839.93	2.079	1159.03	2409.47
A_68_P27835858	chr11:59077515-59077559	NM_009522:26717	Wnt3a	INSIDE	1.500	2.344	2131.54	4997.33	3.518	1898.80	6679.52
A_68_P26350251	chr9:21860554-21860598	NM_177318:15213	Zfp653	INSIDE	1.500	2.831	1323.23	3746.33	4.247	1055.78	4483.80
A_68_P32977632	A_68_P32977632			Unknown	1.500	4.971	1611.43	8011.05	7.456	1167.80	8707.48
A_68_P30402200	chr15:85501825-85501869	AK038224:-5325		PROMOTER	1.500	3.951	537.13	2122.34	5.926	487.27	2887.43
A_68_P25486797	chr7:131127028-131127072	ENSMUST00000106437:668		INSIDE	1.500	1.926	661.20	1273.48	2.889	547.21	1580.74
A_68_P32410978	chrX:56820599-56820643	ENSMUST00000124402:29		INSIDE	1.500	3.436	446.12	1532.94	5.153	273.20	1407.78
A_68_P22540995	chr3:132754697-132754741	NM_026231:-14	Gstcd	DIVERGENT_PROMOTER	1.499	2.324	672.14	1561.92	3.483	565.10	1968.07
A_68_P27922692	chr11:74644684-74644728	NM_010813:281	Mnt	INSIDE	1.499	2.830	1929.46	5460.65	4.243	1709.52	7252.82
A_68_P29977213	chr15:3976841-3976885	NM_024188:435	Oxct1	INSIDE	1.499	3.650	544.74	1988.15	5.470	451.02	2467.31
A_68_P28194540	chr12:3309680-3309724	NM_016676:267	Rab10	INSIDE	1.499	4.003	1635.64	6547.04	6.001	1438.90	8635.07
A_68_P23283135	chr4:126699373-126699417	NM_023603:850	Sfpq	INSIDE	1.499	8.155	8727.66	71176.42	12.221	6037.34	73783.94
A_68_P28938194	chr13:34436403-34436447	NM_001033167:627	Slc22a23	INSIDE	1.499	1.665	1049.25	1747.35	2.496	979.35	2444.71
A_68_P30387965	chr15:83197791-83197835	NR_027980:-85	1700001L05Rik	PROMOTER	1.498	3.254	10728.36	34910.23	4.876	7471.87	36432.60
A_68_P21436146	chr2:93482709-93482753	NM_007442:140	Alx4	INSIDE	1.498	3.971	1582.58	6284.85	5.950	1249.96	7437.44
A_68_P21630555	chr2:130406725-130406769	NM_009732:1531	Avp	INSIDE	1.498	1.378	5629.21	7758.02	2.064	4446.85	9178.68
A_68_P31921521	chr19:4169457-4169501	NM_134148:6001	Carns1	INSIDE	1.498	1.811	5127.86	9285.25	2.712	3896.91	10569.98
A_68_P28098603	chr11:105805256-105805300	NM_007805:183	Cyb561	INSIDE	1.498	3.582	1476.04	5286.87	5.364	1148.77	6161.80
A_68_P25554390	chr7:142907763-142907807	NM_001081117:278	Mki67	INSIDE	1.498	1.812	1042.27	1888.86	2.715	958.07	2601.06
A_68_P29753346	chr14:80170179-80170223	NM_001042726:919	Pcdh8	INSIDE	1.498	1.635	727.63	1189.84	2.450	621.18	1521.77
A_68_P22889129	chr4:46520101-46520145	NM_029077:28894	Trim14	INSIDE	1.498	2.217	1000.12	2216.93	3.321	989.66	3286.24
A_68_P26601300	chr9:67688155-67688199	NM_177184:-26	Vps13c	DIVERGENT_PROMOTER	1.498	5.144	3237.18	16652.92	7.705	2570.25	19803.03
A_68_P28596933	chr12:85534608-85534652	NM_001163502:335	C130039O16Rik	INSIDE	1.497	5.387	1169.98	6302.50	8.063	1274.29	10274.97
A_68_P26762950	chr9:99144040-99144084	NM_023873:176	Cep70	INSIDE	1.497	6.111	1295.82	7919.20	9.151	1096.40	10033.53
A_68_P20794600	chr1:167390414-167390458	NM_028759:158	Dcaf6	INSIDE	1.497	2.139	2568.30	5493.40	3.201	2066.97	6616.97
A_68_P29751618	chr14:79880592-79880636	NM_007920:-386	Elf1	PROMOTER	1.497	2.751	378.29	1040.82	4.118	390.19	1606.65
A_68_P30363895	chr15:78858579-78858623	NM_008197:-41	H1f0	PROMOTER	1.497	2.021	385.83	779.59	3.024	312.88	946.11
A_68_P31936765	chr19:7008355-7008399	NM_008431:629	Kcnk4	INSIDE	1.497	1.604	5143.15	8248.42	2.400	3717.11	8922.84
A_68_P21101981	chr2:30093673-30093717	NM_177725:406	Lrrc8a	INSIDE	1.497	1.509	1338.03	2019.61	2.260	1058.68	2392.53
A_68_P30252145	chr15:58913581-58913625	NM_001146180:-21	Mtss1	PROMOTER	1.497	3.797	3359.57	12755.92	5.685	2750.82	15637.27
A_68_P26744225	chr9:95745638-95745682	NM_001033210:38	Pls1	INSIDE	1.497	2.484	3334.46	8281.80	3.717	2629.21	9772.42
A_68_P24152262	chr5:143027306-143027350	NM_178702:-297	Radil	PROMOTER	1.497	1.658	2003.76	3323.21	2.483	1539.05	3821.45
A_68_P28051255	chr11:97407967-97408011	NM_018873:28452	Srcin1	INSIDE	1.497	3.347	1094.35	3663.05	5.011	871.73	4368.59
A_68_P27981398	chr11:84953893-84953937	NM_001029934:-457	Usp32	PROMOTER	1.497	2.203	743.47	1638.01	3.298	590.65	1948.14
A_68_P26801603	chr9:106073078-106073122	NM_029896:-159	Wdr82	PROMOTER	1.497	2.599	798.22	2074.18	3.889	757.80	2947.20
A_68_P27797797	chr11:51981491-51981535	ENSMUST00000155549:101504		INSIDE	1.497	2.585	570.77	1475.40	3.870	508.48	1967.72
A_68_P24671353	chr6:97098869-97098913	NM_175313:-13	A130022J15Rik	PROMOTER	1.496	2.483	1343.44	3335.11	3.713	1158.58	4302.05
A_68_P20804630	chr1:169280208-169280252	NM_019993:109	Aldh9a1	INSIDE	1.496	2.695	2883.49	7770.36	4.031	2382.36	9604.02
A_68_P24098215	chr5:131915714-131915758	NM_177047:1102477	Auts2	INSIDE	1.496	4.855	1835.06	8909.31	7.265	1385.90	10069.19
A_68_P26018982	chr8:86773469-86773513	NM_001007571:170	D8Ertd738e	INSIDE	1.496	4.477	3650.41	16344.52	6.698	2731.43	18295.77
A_68_P28291796	chr12:25516059-25516103	NM_001083341:-383	Mboat2	PROMOTER	1.496	2.432	781.87	1901.35	3.639	595.30	2166.22
A_68_P28457433	chr12:57634159-57634203	NM_001146198:1913	Nkx2-1	INSIDE	1.496	4.586	2023.54	9279.60	6.859	1756.49	12048.05
A_68_P30368658	chr15:79634975-79635019	NM_030689:143	Nptxr	INSIDE	1.496	1.954	1162.70	2271.90	2.922	1261.40	3686.23
A_68_P32415817	chrX:58147657-58147701	NM_009237:-1073	Sox3	PROMOTER	1.496	4.941	926.43	4577.92	7.391	691.04	5107.77
A_68_P20737712	chr1:157264214-157264258	NM_011273:338	Xpr1	INSIDE	1.496	1.619	2493.29	4035.43	2.421	1928.05	4667.12
A_68_P31620951	chr18:36090196-36090240			Unknown	1.496	3.298	274.83	906.53	4.936	237.68	1173.16
A_68_P28175452	chr11:118902740-118902784	NM_013926:-535	Cbx8	PROMOTER	1.495	1.563	1619.67	2532.11	2.337	1329.59	3107.83
A_68_P28051744	chr11:97490870-97490916	NM_175332:138	E130012A19Rik	INSIDE	1.495	1.730	541.17	936.05	2.586	478.99	1238.69
A_68_P24613524	chr6:86992684-86992728	NM_013528:-133	Gfpt1	PROMOTER	1.495	2.284	888.58	2029.77	3.416	884.07	3020.06

ProbeName	Target position of probe on CpG island microarray	TargetID	GeneSymbol	CpG island Description	Ratio of relative methylation (TiO_2-NP/Vehicle)	Sham group			TiO_2-H group		
						Relative methylation (Cy5/Cy3)	Cy3 signal (Input)	Cy5 signal (MeDIP)	Relative methylation (Cy5/Cy3)	Cy3 signal (Input)	Cy5 signal (MeDIP)
A_68_P30639626	chr16:30064558-30064602	NM_008235:-862	Hes1	PROMOTER	1.495	4.123	2347.01	9677.81	6.166	1933.93	11924.39
A_68_P28192109	chr11:121564851-121564895	NM_144797:1132	Metrnl	INSIDE	1.495	1.942	2716.65	5276.61	2.904	1976.74	5740.69
A_68_P21495965	chr2:105516343-105516387	NM_013627:-237	Pax6	DIVERGENT_PROMOTER	1.495	1.911	694.64	1327.46	2.857	606.61	1732.91
A_68_P20022046	chr1:9289451-9289495	NM_027671:339	Sntg1	INSIDE	1.495	1.833	443.34	812.55	2.740	419.04	1148.14
A_68_P24002091	chr5:114581110-114581154	NM_011677:-32	Ung	PROMOTER	1.495	2.127	2054.94	4371.54	3.181	1704.40	5421.67
A_68_P24596101	chr6:83864271-83864315	NM_001166371:-54	Zfml	PROMOTER	1.495	3.231	532.77	1721.39	4.831	496.47	2398.62
A_68_P30849195	chr16:70314120-70314164	NM_028803:-189	Gbe1	PROMOTER	1.494	2.616	1831.98	4791.95	3.907	1550.72	6058.97
A_68_P23322029	chr4:133843071-133843115	NM_001163794:76	Pdik1l	INSIDE	1.494	2.372	10225.48	24258.79	3.545	8594.51	30466.36
A_68_P30423622	chr15:88998029-88998073	NM_001159521:6230	Plxnb2	INSIDE	1.494	1.877	524.77	984.97	2.804	429.82	1205.04
A_68_P25410469	chr7:117008961-117009005	NM_020052:211	Scube2	INSIDE	1.494	5.181	868.74	4501.01	7.739	825.26	6386.80
A_68_P28744649	chr12:112776399-112776444	NR_033168:-2735	Snora28	PROMOTER	1.494	3.047	9826.32	29943.34	4.552	7671.96	34924.14
A_68_P27985536	chr11:85645744-85645788	NM_009324:-350	Tbx2	PROMOTER	1.494	1.835	2705.43	4964.16	2.741	2125.14	5824.30
A_68_P27466136	chr10:114238907-114238951	NM_146241:-502	Trhde	PROMOTER	1.494	1.663	869.87	1446.85	2.485	737.16	1832.11
A_68_P20421181	chr1:89904795-89904839	NM_001033291:310	Usp40	INSIDE	1.494	1.493	776.15	1158.90	2.231	660.26	1473.15
A_68_P30351229	A_68_P30351229			Unknown	1.494	4.281	1145.52	4904.46	6.397	1071.97	6857.53
A_68_P22262798	chr3:79091123-79091167	AK032506:-169		PROMOTER	1.494	2.337	1452.04	3392.81	3.492	1172.92	4095.33
A_68_P31161581	chr17:36034296-36034340	NM_001033630:5	2310061I04Rik	INSIDE	1.493	3.516	1094.62	3848.68	5.249	932.82	4895.98
A_68_P21754951	chr2:153269795-153269839	NM_001001986:399	8430427H17Rik	INSIDE	1.493	1.796	1084.60	1948.13	2.682	930.08	2494.60
A_68_P26890107	chr9:122478468-122478512	NR_015610:-3128	9530059O14Rik	PROMOTER	1.493	1.625	802.64	1303.97	2.425	628.18	1523.47
A_68_P28098880	chr11:105847454-105847498	NM_009598:10955	Ace	INSIDE	1.493	1.549	581.37	900.55	2.313	444.02	1026.80
A_68_P25093839	chr7:52825720-52825764	NM_009737:10	Bcat2	INSIDE	1.493	2.461	1979.07	4869.89	3.675	1656.09	6086.18
A_68_P30433470	chr15:90880052-90880096	NM_001109040:305	Kif21a	INSIDE	1.493	1.872	1133.72	2122.14	2.794	984.50	2750.42
A_68_P32266422	chrX:13063296-13063340	NM_173415:18521	Nyx	INSIDE	1.493	2.556	715.31	1828.01	3.816	512.63	1955.93
A_68_P26349531	chr9:21722321-21722365	NM_031874:223	Rab3d	INSIDE	1.493	2.793	847.44	2367.19	4.170	739.45	3083.42
A_68_P21851412	chr2:169956064-169956108	NM_001033299:634	Zfp217	INSIDE	1.493	2.203	383.89	845.52	3.289	346.43	1139.56
A_68_P27830382	chr11:58136668-58136712	NM_178761:158	Zfp672	INSIDE	1.493	1.613	493.52	796.12	2.408	499.73	1203.21
A_68_P24336409	chr6:31166646-31166691	ENSMUST00000115107:1765		INSIDE	1.493	2.097	1806.75	3789.15	3.132	1653.24	5178.09
A_68_P23425243	chr4:152857051-152857095	NM_001099299:-133	Ajap1	PROMOTER	1.492	4.288	1661.98	7127.39	6.399	1493.27	9555.03
A_68_P22941965	chr4:56878080-56878124	NM_018761:-19	Ctnnal1	PROMOTER	1.492	2.293	10764.22	24683.44	3.420	7836.12	26802.11
A_68_P25010200	chr7:26180775-26180819	NM_010719:210	Lipe	INSIDE	1.492	2.450	1203.56	2948.31	3.655	957.61	3500.53
A_68_P21742434	chr2:150864733-150864777	NM_026086:361	Nanp	INSIDE	1.492	3.383	725.58	2454.59	5.047	652.76	3294.63
A_68_P27551122	chr11:3190063-3190107	NM_019574:-375	Patz1	PROMOTER	1.492	3.884	2049.99	7961.96	5.796	1553.45	9004.29
A_68_P21351543	chr2:76486449-76486493	NM_011871:-419	Prkra	DIVERGENT_PROMOTER	1.492	1.899	4446.39	8444.13	2.833	3396.36	9622.74
A_68_P25571665	chr7:145589754-145589798	NM_183289:-363	Tcerg1l	PROMOTER	1.492	3.210	1121.24	3599.02	4.789	938.78	4496.17
A_68_P32196169	chr19:55816232-55816276	NM_001142918:-45	Tcf7l2	PROMOTER	1.492	3.108	5003.32	15550.53	4.637	3907.26	18118.76
A_68_P23183551	chr4:105989077-105989121	NM_183225:281	Usp24	INSIDE	1.492	30.482	1090.71	33247.34	45.472	799.65	36361.55
A_68_P27663517	chr11:26493581-26493625	NM_027260:318	Vrk2	INSIDE	1.492	1.570	1306.37	2050.94	2.343	1375.56	3222.57
A_68_P28739968	chr12:111976134-111976178	NM_027149:-2	Wdr20a	PROMOTER	1.492	1.771	1358.99	2406.85	2.642	1134.91	2998.01
A_68_P21713971	chr2:145500669-145500713	ENSMUST00000144661:-412		PROMOTER	1.492	3.041	8805.83	26779.05	4.538	6759.79	30676.79
A_68_P32186055	chr19:54120591-54120635	NM_007417:941	Adra2a	INSIDE	1.491	3.574	745.23	2663.79	5.331	602.49	3211.68
A_68_P20429509	chr1:91351220-91351264	NM_001037136:-143	Agap1	PROMOTER	1.491	2.172	1218.02	2645.15	3.237	1092.67	3536.90
A_68_P20258853	chr1:58480687-58480731	NM_001042634:224	Clk1	INSIDE	1.491	2.517	965.55	2430.58	3.754	821.43	3083.74
A_68_P30213289	chr15:51697145-51697189	NM_080635:-159	Eif3h	PROMOTER	1.491	4.118	811.57	3341.85	6.140	689.75	4235.10
A_68_P26248326	chr8:127421066-127421110	NM_198103:517	Exoc8	INSIDE	1.491	2.689	1459.41	3923.64	4.010	1160.08	4651.42
A_68_P27556420	chr11:4118164-4118208	NM_028022:-67	Gatsl3	PROMOTER	1.491	3.351	3994.43	13386.75	4.999	3124.31	15616.92
A_68_P20417068	chr1:89223852-89223896	NM_001110212:271	Gigyf2	INSIDE	1.491	5.680	1362.29	7738.35	8.472	1230.82	10427.60
A_68_P23627551	chr5:40035622-40035667	NM_010474:226	Hs3st1	INSIDE	1.491	2.146	4082.80	8763.09	3.201	3300.09	10564.46
A_68_P24325224	chr6:29162351-29162395	NM_011829:-101	Impdh1	PROMOTER	1.491	3.611	2888.66	10432.29	5.386	2362.75	12725.29
A_68_P22530123	chr3:130809073-130809117	NM_010703:-4294	Lef1	PROMOTER	1.491	13.626	995.20	13560.91	20.319	868.75	17651.82
A_68_P32565868	chrX:99044658-99044702	NM_001163610:-43	Nhsl2	PROMOTER	1.491	3.614	1420.71	5134.02	5.386	897.42	4833.88
A_68_P31067738	chr17:15963543-15963587	NM_178615:-14	Rgmb	PROMOTER	1.491	2.517	8925.03	22468.51	3.753	6475.95	24302.21

ProbeName	Target position of probe on CpG island microarray	TargetID	GeneSymbol	CpG island Description	Ratio of relative methylation (TiO_2-NP/Vehicle)	Sham group			TiO_2-H group		
						Relative methylation (Cy5/Cy3)	Cy3 signal (Input)	Cy5 signal (MeDIP)	Relative methylation (Cy5/Cy3)	Cy3 signal (Input)	Cy5 signal (MeDIP)
A_68_P24127573	chr5:138228997-138229041	NM_001005426:-11	Zcwpw1	DIVERGENT_PROMOTER	1.491	1.562	697.44	1089.17	2.329	617.84	1438.97
A_68_P25959077	chr8:74446861-74446905	NM_133358:159	Zfp617	INSIDE	1.491	5.588	2263.07	12647.16	8.330	1860.47	15498.50
A_68_P31256671	chr17:56651263-56651307	NM_011483:622	Znrf4	INSIDE	1.491	3.365	1288.91	4337.82	5.019	1098.38	5513.26
A_68_P29527055	A_68_P29527055			Unknown	1.491	3.497	196.73	687.87	5.214	153.74	801.59
A_68_P28743060	chr12:112512501-112512545	NM_033603:3201	Amn	INSIDE	1.490	1.423	7033.05	10010.01	2.121	5389.09	11430.75
A_68_P22860481	chr4:41045310-41045354	NM_016689:-116	Aqp3	PROMOTER	1.490	4.660	1305.12	6081.64	6.945	1101.64	7650.92
A_68_P27849285	chr11:61393132-61393176	NM_010148:-1	Epn2	PROMOTER	1.490	2.945	2245.53	6613.71	4.388	1779.36	7807.81
A_68_P25616728	chr8:4238821-4238865	NM_001042557:103	Map2k7	INSIDE	1.490	1.822	1137.79	2072.92	2.715	1027.75	2790.18
A_68_P24536650	chr6:71444353-71444397	NM_009543:487	Rnf103	INSIDE	1.490	1.806	1872.30	3381.13	2.691	1641.75	4418.32
A_68_P22342531	chr3:94964250-94964300	NM_011351:-4021	Sema6c	PROMOTER	1.490	4.451	357.82	1592.58	6.634	354.91	2354.44
A_68_P26596714	chr9:66891475-66891519	NM_001164252:182	Tpm1	INSIDE	1.490	2.510	900.44	2260.05	3.741	720.21	2694.31
A_68_P30826541	chr16:65815730-65815774	NM_028572:-125	Vgll3	PROMOTER	1.490	3.059	712.43	2179.14	4.557	617.08	2811.93
A_68_P24850992	chr6:131383975-131384019			Unknown	1.490	2.161	1306.32	2823.15	3.221	1119.61	3605.74
A_68_P22630569	chr3:148652119-148652163	ENSMUST00000098518:140		INSIDE	1.490	1.650	1457.29	2405.23	2.459	1295.15	3185.08
A_68_P25027724	chr7:30303544-30303588	ENSMUST00000141713:-657		PROMOTER	1.490	1.774	1239.78	2198.95	2.644	1094.62	2893.71
A_68_P30558454	chr16:13672209-13672253	NM_025653:118	3110001I22Rik	INSIDE	1.489	2.198	1853.67	4074.40	3.272	1443.14	4721.77
A_68_P25525515	chr7:138080197-138080241	NM_019564:502	Htra1	INSIDE	1.489	1.895	1370.52	2597.52	2.823	1195.46	3374.46
A_68_P30585934	chr16:20097708-20097752	NM_029436:104	Klhl24	INSIDE	1.489	2.283	662.18	1512.05	3.399	645.27	2193.26
A_68_P25018407	chr7:28442995-28443039	NM_001081292:16601	Map3k10	INSIDE	1.489	5.153	1305.66	6727.86	7.673	1031.07	7911.18
A_68_P23331869	chr4:135609312-135609356	NM_025919:-48	Rpl11	PROMOTER	1.489	3.002	1260.15	3783.48	4.469	1027.83	4593.88
A_68_P24158560	chr5:144670456-144670500	NM_001172146:230	Aimp2	INSIDE	1.488	5.856	2490.98	14587.44	8.716	2174.48	18953.42
A_68_P23818500	chr5:77286506-77286550	NM_001163793:16905	C530008M17Rik	INSIDE	1.488	6.205	7896.04	48992.45	9.233	4990.86	46079.35
A_68_P22524273	chr3:129883661-129883705	NM_029838:-113	Col25a1	PROMOTER	1.488	6.654	699.40	4653.89	9.898	635.72	6292.63
A_68_P21428537	chr2:92273951-92273995	NM_009963:254	Cry2	INSIDE	1.488	1.459	782.43	1141.89	2.172	670.94	1457.25
A_68_P32383357	chrX:49965510-49965554	NM_016697:1619	Gpc3	INSIDE	1.488	2.556	704.94	1801.58	3.802	445.28	1693.02
A_68_P27271929	chr10:77531813-77531858	NM_015790:-277	Icosl	PROMOTER	1.488	2.482	1296.29	3217.58	3.694	1047.40	3868.77
A_68_P20639346	chr1:137327066-137327110	NM_153774:-20	Ipo9	PROMOTER	1.488	1.519	815.68	1238.76	2.259	767.03	1732.96
A_68_P26531872	chr9:55390256-55390300	NM_027397:1323	Isl2	INSIDE	1.488	4.671	1085.66	5070.71	6.951	978.25	6799.90
A_68_P25948235	chr8:72356375-72356419	NM_001024954:-206	Pbx4	PROMOTER	1.488	1.638	680.46	1114.66	2.438	581.17	1416.90
A_68_P26011770	chr8:85388368-85388412	NM_177378:1136	Rnf150	INSIDE	1.488	3.080	1079.30	3324.26	4.583	970.72	4448.36
A_68_P24769162	chr6:115085438-115085482	NM_013681:541	Syn2	INSIDE	1.488	4.224	615.78	2600.91	6.283	470.27	2954.76
A_68_P24760941	chr6:113647011-113647055	NM_001033463:-460	Tatdn2	PROMOTER	1.488	2.222	1072.54	2383.67	3.307	953.60	3153.27
A_68_P27886201	chr11:67735651-67735695	NM_173754:-17	Usp43	PROMOTER	1.488	2.241	486.73	1090.62	3.334	360.25	1201.16
A_68_P20737716	chr1:157264675-157264719	NM_011273:-122	Xpr1	PROMOTER	1.488	2.422	1335.19	3233.80	3.603	1135.65	4091.66
A_68_P25614202	chr8:3492951-3492995	NM_080461:-165	Zfp358	PROMOTER	1.488	1.596	6033.02	9628.47	2.375	4331.43	10288.39
A_68_P26349677	chr9:21748879-21748923	NM_144935:6183	BC018242	INSIDE	1.487	3.028	3130.24	9478.02	4.502	2573.97	11588.81
A_68_P25601080	chr7:150646296-150646340	NM_001161624:637	Cdkn1c	INSIDE	1.487	4.382	1274.18	5583.61	6.515	1004.83	6546.13
A_68_P27849286	chr11:61393305-61393349	NM_010148:-173	Epn2	PROMOTER	1.487	4.727	4819.02	22780.15	7.029	3257.06	22894.05
A_68_P23356930	chr4:139802256-139802300	NM_198610:448	Igsf21	INSIDE	1.487	2.045	786.67	1608.36	3.041	607.76	1848.19
A_68_P21389130	chr2:83564617-83564661	NM_008402:85	Itgav	INSIDE	1.487	2.157	1205.50	2600.52	3.207	1076.10	3451.24
A_68_P31120666	chr17:28279127-28279172	NM_001004366:-321	Scube3	PROMOTER	1.487	1.332	5679.82	7564.81	1.981	3576.07	7084.14
A_68_P22383782	chr3:102799932-102799976	NM_025679:292	Sike1	INSIDE	1.487	2.904	3165.15	9192.18	4.317	2396.96	10348.20
A_68_P31301690	chr17:65963450-65963494	NM_013933:-577	Vapa	PROMOTER	1.487	8.798	1010.79	8893.14	13.087	685.67	8973.47
A_68_P23296941	chr4:129177909-129177953	NM_001085491:9497	1700125D06Rik	DOWNSTREAM	1.486	2.494	959.72	2393.20	3.705	822.00	3045.18
A_68_P23956783	chr5:106128691-106128735	NM_001122768:-275	Lrrc8d	PROMOTER	1.486	1.842	1832.21	3375.58	2.737	1481.10	4053.61
A_68_P20640400	chr1:137481754-137481798	NM_173437:156	Nav1	INSIDE	1.486	1.516	848.35	1286.26	2.253	795.55	1792.57
A_68_P31112710	chr17:26976655-26976699	NM_008700:1834	Nkx2-5	INSIDE	1.486	2.074	1893.57	3926.91	3.082	1586.57	4890.57
A_68_P26135469	chr8:108225412-108225456	NM_001047435:-114	Pard6a	PROMOTER	1.486	3.119	449.62	1402.26	4.634	385.94	1788.30
A_68_P24338524	chr6:31513799-31513843	NM_013723:117	Podxl	INSIDE	1.486	1.746	769.41	1343.69	2.595	715.71	1856.99
A_68_P31102167	chr17:25325180-25325224	NM_001197024:-142	Unkl	PROMOTER	1.486	2.798	921.18	2577.58	4.159	811.41	3374.68
A_68_P28746261	chr12:113052815-113052859	NM_026752:456	Zfyve21	INSIDE	1.486	3.714	1302.12	4836.52	5.520	1086.85	5999.50

ProbeName	Target position of probe on CpG island microarray	TargetID	GeneSymbol	CpG island Description	Ratio of relative methylation (TiO_2-NP/Vehicle)	Sham group			TiO_2-H group		
						Relative methylation (Cy5/Cy3)	Cy3 signal (Input)	Cy5 signal (MeDIP)	Relative methylation (Cy5/Cy3)	Cy3 signal (Input)	Cy5 signal (MeDIP)
A_68_P28168166	chr11:117811203-117811247			Unknown	1.486	1.853	1410.42	2613.60	2.754	1231.53	3391.73
A_68_P27633195	chr11:20640941-20640985	NM_181411:525	Aftph	INSIDE	1.485	6.232	1309.64	8162.06	9.254	1017.41	9415.47
A_68_P25188282	chr7:73572179-73572223	NM_053080:163	Aldh1a3	INSIDE	1.485	1.775	482.69	856.84	2.636	411.20	1083.82
A_68_P32244416	chrX:8469520-8469564	NM_175327:64	B630019K06Rik	INSIDE	1.485	3.190	515.88	1645.62	4.736	267.16	1265.36
A_68_P31756138	chr18:61336272-61336316	NM_178277:409	Hmgxb3	INSIDE	1.485	1.870	2277.30	4258.67	2.777	1653.25	4591.76
A_68_P28041916	chr11:95867049-95867093	NM_009951:188	Igf2bp1	INSIDE	1.485	2.030	1304.20	2646.88	3.014	1157.60	3488.96
A_68_P22509730	chr3:127336734-127336778	NM_009718:694	Neurog2	INSIDE	1.485	5.671	1068.27	6058.09	8.419	859.81	7239.13
A_68_P23361259	chr4:140517609-140517653	NM_023374:445	Sdhb	INSIDE	1.485	5.325	986.50	5253.58	7.911	849.33	6718.97
A_68_P26932996	chr10:9621066-9621110	NM_001081344:-250	Stxbp5	PROMOTER	1.485	2.612	314.38	821.25	3.879	442.32	1715.69
A_68_P28078912	chr11:102180945-102180990	NM_011551:-557	Ubtf	PROMOTER	1.485	1.841	1467.58	2701.44	2.733	1097.83	3000.03
A_68_P31839441	chr18:75980377-75980421	NM_145356:567	Zbtb7c	INSIDE	1.485	1.935	940.22	1819.10	2.874	811.86	2333.14
A_68_P30331748	chr15:73475653-73475697	ENSMUST00000150409:101		INSIDE	1.485	5.345	5095.75	27234.62	7.939	3654.55	29013.70
A_68_P22789806	chr4:25208898-25208942	NM_026194:48	1810074P20Rik	INSIDE	1.484	1.911	1480.86	2829.19	2.836	1269.09	3598.53
A_68_P23607908	chr5:36827600-36827644	NM_025725:386	Ccdc96	INSIDE	1.484	5.235	2489.82	13033.21	7.770	1941.17	15083.03
A_68_P26168095	chr8:113917768-113917812	NM_178086:-69	Fa2h	PROMOTER	1.484	6.080	1257.89	7648.54	9.026	1008.97	9106.88
A_68_P27497681	chr10:119914095-119914139	NM_010441:-125	Hmga2	PROMOTER	1.484	2.135	2093.43	4468.85	3.168	1657.10	5250.27
A_68_P30267513	chr15:61816737-61816781	NM_001177352:-137	Myc	PROMOTER	1.484	3.242	3790.60	12290.28	4.810	2991.79	14391.14
A_68_P26024229	chr8:87823886-87823930	NM_001163791:378	Orc6	INSIDE	1.484	5.626	1217.96	6852.12	8.349	978.99	8173.95
A_68_P30139531	chr15:36539130-36539174	NM_008774:-424	Pabpc1	PROMOTER	1.484	2.124	1105.00	2347.11	3.151	925.10	2915.31
A_68_P22129906	chr3:51287666-51287710	NM_016858:-199	Rab33b	PROMOTER	1.484	3.575	3179.94	11369.76	5.304	2674.89	14188.17
A_68_P28051170	chr11:97396005-97396049	NM_018873:40414	Srcin1	INSIDE	1.484	2.729	1143.80	3121.76	4.052	991.18	4015.84
A_68_P22411406	chr3:108029457-108029501	NM_008596:39	Sypl2	INSIDE	1.484	2.930	2603.75	7628.82	4.348	1948.88	8474.21
A_68_P30497401	chr15:102180872-102180916	NM_153416:296	Aaas	INSIDE	1.483	1.718	4393.93	7547.58	2.548	3456.38	8806.91
A_68_P27255925	chr10:74779618-74779662	NM_009630:-47	Adora2a	PROMOTER	1.483	2.081	3518.28	7321.57	3.086	2975.00	9180.44
A_68_P28533193	chr12:73765645-73765689	NM_025522:149	Dhrs7	INSIDE	1.483	1.684	1131.18	1904.55	2.498	939.62	2346.87
A_68_P28669125	chr12:99497683-99497727	NM_008079:-157	Galc	DIVERGENT_PROMOTER	1.483	2.241	1222.21	2738.64	3.322	1049.95	3488.01
A_68_P23756018	chr5:65771113-65771157	NM_031180:31485	Klb	INSIDE	1.483	4.473	1280.38	5727.59	6.635	1092.86	7251.53
A_68_P31731698	chr18:56867506-56867550	NM_010721:62	Lmnb1	INSIDE	1.483	1.891	1001.30	1893.37	2.804	868.82	2436.10
A_68_P26991379	chr10:20879243-20879287	NM_001198914:1526	Myb	INSIDE	1.483	4.136	1333.68	5516.51	6.135	1248.56	7659.30
A_68_P22325410	chr3:90270013-90270057	NM_008727:-246	Npr1	PROMOTER	1.483	2.568	1376.39	3535.17	3.809	1090.25	4152.71
A_68_P20876659	chr1:182498959-182499003	NM_007415:-125	Parp1	PROMOTER	1.483	5.070	2443.18	12386.49	7.518	1978.81	14877.19
A_68_P27838986	chr11:59600830-59600874	NM_183139:307	Pld6	INSIDE	1.483	2.031	820.37	1665.86	3.011	692.96	2086.64
A_68_P29419901	chr14:14953551-14953595	NM_025550:-154	Psmd6	PROMOTER	1.483	5.375	1361.25	7316.32	7.971	1043.09	8314.32
A_68_P27554683	chr11:3813912-3813956	NM_153142:733	Slc35e4	INSIDE	1.483	3.181	5584.46	17766.24	4.718	4111.19	19396.84
A_68_P28183961	chr11:120209795-120209839	NM_009609:-18	Actg1	DIVERGENT_PROMOTER	1.482	4.193	1639.68	6875.37	6.213	1414.71	8790.24
A_68_P30284258	chr15:64753242-64753286	NM_009623:594	Adcy8	INSIDE	1.482	2.953	6285.38	18562.99	4.376	4889.33	21396.26
A_68_P24950090	chr7:4658912-4658956	NM_001003920:16405	Brsk1	INSIDE	1.482	2.699	4374.20	11806.01	4.000	3848.05	15392.56
A_68_P27227733	chr10:69559705-69559749	NM_001111121:-142	Ccdc6	PROMOTER	1.482	5.110	3870.06	19777.68	7.576	2600.34	19699.88
A_68_P23980414	chr5:110973587-110973631	NM_198306:245	Galnt9	INSIDE	1.482	4.093	1922.29	7867.08	6.063	1572.34	9533.56
A_68_P22301527	chr3:86028160-86028204	NM_001077687:-429	Lrba	PROMOTER	1.482	1.595	746.77	1190.92	2.363	658.90	1556.83
A_68_P25995588	chr8:82163767-82163811	NM_001081164:214	Otud4	INSIDE	1.482	3.426	421.02	1442.45	5.079	366.04	1859.13
A_68_P28238663	chr12:11462730-11462774	NM_001111118:-132	Rad51ap2	PROMOTER	1.482	2.658	3232.76	8594.13	3.940	2793.60	11007.71
A_68_P20233544	chr1:53842063-53842107	NM_133810:-25	Stk17b	PROMOTER	1.482	1.878	2289.72	4300.75	2.784	1885.34	5248.64
A_68_P24951873	chr7:5014151-5014195	NM_001205231:428	U2af2	INSIDE	1.482	3.304	2306.57	7620.07	4.897	1919.29	9399.65
A_68_P26529570	chr9:54996803-54996847	NM_180600:-351	Ube2q2	PROMOTER	1.482	2.667	1031.63	2750.92	3.953	925.31	3657.95
A_68_P27475464	chr10:115855274-115855318	NR_028107:120	1700058G18Rik	INSIDE	1.481	4.934	292.58	1443.64	7.307	244.85	1789.09
A_68_P27958482	chr11:80967088-80967132	NM_007384:-705	Accn1	PROMOTER	1.481	4.388	962.52	4223.79	6.500	813.40	5287.31
A_68_P20753949	chr1:160293080-160293124	NM_001205204:668	Astn1	INSIDE	1.481	1.918	5761.52	11049.78	2.840	4203.78	11939.94
A_68_P25272112	chr7:88599818-88599862	NM_007755:-278	Cpeb1	PROMOTER	1.481	2.359	1025.11	2418.14	3.493	831.90	2905.84
A_68_P32219567	chr19:59533292-59533336	NM_010132:135	Emx2	INSIDE	1.481	1.925	451.55	869.14	2.851	413.29	1178.37
A_68_P32383368	chrX:49966913-49966957	NM_016697:217	Gpc3	INSIDE	1.481	2.480	1551.13	3847.02	3.674	1061.86	3901.58

ProbeName	Target position of probe on CpG island microarray	TargetID	GeneSymbol	CpG island Description	Ratio of relative methylation (TiO_2-NP/Vehicle)	Sham group			TiO_2-H group		
						Relative methylation (Cy5/Cy3)	Cy3 signal (Input)	Cy5 signal (MeDIP)	Relative methylation (Cy5/Cy3)	Cy3 signal (Input)	Cy5 signal (MeDIP)
A_68_P23361559	chr4:140566637-140566681	NM_008546:107	Mfap2	INSIDE	1.481	1.545	1918.29	2964.27	2.289	1684.65	3855.41
A_68_P24132976	chr5:139471493-139471537	NM_008808:-607	Pdgfa	PROMOTER	1.481	4.271	882.01	3766.82	6.325	815.04	5155.40
A_68_P29578866	chr14:47501811-47501855	NM_001037221:-807	Samd4	PROMOTER	1.481	3.095	2088.77	6465.44	4.585	1551.32	7112.98
A_68_P22413523	chr3:108394473-108394517	NM_181400:299	Wdr47	INSIDE	1.481	1.823	830.50	1513.68	2.700	719.16	1941.53
A_68_P30387966	chr15:83197892-83197936	NR_027980:-187	1700001L05Rik	PROMOTER	1.480	3.809	1597.10	6083.53	5.638	1346.38	7590.37
A_68_P27945542	chr11:78639981-78640025	NM_001076681:-94	1810012P15Rik	PROMOTER	1.480	1.923	2346.71	4511.92	2.846	2058.79	5859.47
A_68_P25952417	chr8:73047003-73047047	NM_029366:55	2810422J05Rik	INSIDE	1.480	1.630	1050.66	1712.07	2.411	849.65	2048.77
A_68_P31776707	chr18:64820614-64820658	NM_001001488:18	Atp8b1	INSIDE	1.480	2.527	709.08	1791.99	3.740	629.48	2354.57
A_68_P29005199	chr13:46059825-46059869	NM_009124:499	Atxn1	INSIDE	1.480	4.210	1091.25	4593.78	6.230	913.47	5690.97
A_68_P24476446	chr6:56664815-56664859	NM_030235:-62	Avl9	PROMOTER	1.480	3.367	2391.78	8053.22	4.982	1936.96	9649.44
A_68_P26119098	chr8:105308180-105308224	NM_009866:809	Cdh11	INSIDE	1.480	2.061	8473.02	17463.92	3.049	6293.29	19191.28
A_68_P26898893	chr10:3134939-3134983	NM_172546:657	Cnksr3	INSIDE	1.480	2.790	3564.58	9944.12	4.129	2849.51	11766.46
A_68_P21769580	chr2:155937622-155937666	NM_170588:57	Cpne1	INSIDE	1.480	2.533	6122.06	15507.23	3.748	4655.04	17448.24
A_68_P22318791	chr3:89154152-89154197	NM_029974:14987	Dcst1	INSIDE	1.480	2.249	710.24	1597.59	3.330	619.90	2064.17
A_68_P20140950	chr1:34899440-34899484	NM_001160235:433	Fam168b	INSIDE	1.480	2.391	1108.36	2650.23	3.539	877.79	3106.40
A_68_P24784008	chr6:117867198-117867242	NM_001166427:-91	Hnrnpf	PROMOTER	1.480	2.749	2925.73	8044.26	4.068	2294.90	9336.79
A_68_P29616398	chr14:55732626-55732670	NM_177049:3124	Jph4	INSIDE	1.480	1.870	926.03	1731.65	2.767	721.71	1996.85
A_68_P31552995	chr18:23197987-23198031	NM_001161483:-844	Nol4	PROMOTER	1.480	2.708	3982.48	10782.62	4.006	3148.81	12614.76
A_68_P28151868	chr11:115048748-115048792	NM_001033393:-30	Tmem104	DIVERGENT_PROMOTER	1.480	3.488	3588.34	12515.17	5.162	3236.22	16706.76
A_68_P30366258	chr15:79233359-79233403	NM_172608:353	Tmem184b	INSIDE	1.480	2.133	1972.86	4207.76	3.156	1659.57	5236.82
A_68_P20405965	chr1:84835584-84835628	NM_133975:273	Trip12	INSIDE	1.480	2.436	1733.53	4222.22	3.606	1450.76	5230.82
A_68_P25355272	chr7:105509671-105509715	NM_001024619:106	Tsku	INSIDE	1.480	1.578	789.68	1245.92	2.335	720.02	1681.44
A_68_P27910155	chr11:72021079-72021123	NM_026559:45	Txndc17	INSIDE	1.480	1.464	1165.15	1705.59	2.167	977.40	2117.86
A_68_P27554559	chr11:3794810-3794858	NM_026150:295	4921536K21Rik	INSIDE	1.479	1.995	823.65	1642.99	2.950	659.99	1946.71
A_68_P25645835	chr8:9977887-9977931	NM_001081119:192	Abhd13	INSIDE	1.479	8.814	857.51	7558.11	13.035	763.22	9948.28
A_68_P31759814	chr18:61946393-61946437	NM_178928:-98	Afap1l1	PROMOTER	1.479	2.041	3425.45	6990.74	3.018	3032.52	9153.63
A_68_P26818180	chr9:108984801-108984845	NM_183250:73	Ccdc72	INSIDE	1.479	1.486	6981.24	10376.87	2.199	5026.10	11050.19
A_68_P28914111	chr13:29947064-29947108	NM_144536:371	Cdkal1	INSIDE	1.479	3.371	944.34	3183.75	4.986	929.45	4633.90
A_68_P31715218	chr18:53904131-53904175	NM_178686:49	Cep120	INSIDE	1.479	1.731	2423.12	4193.44	2.560	1996.83	5112.27
A_68_P31101001	chr17:25151361-25151405	NM_020608:793	Cramp1l	INSIDE	1.479	2.667	3775.30	10067.56	3.943	3133.13	12353.35
A_68_P24620316	chr6:88143988-88144032	NM_008090:-4647	Gata2	PROMOTER	1.479	3.030	1870.87	5668.13	4.480	1565.73	7013.72
A_68_P24862663	chr6:134582475-134582519	NM_026345:10	Mansc1	INSIDE	1.479	3.308	1719.31	5688.01	4.892	1484.89	7264.64
A_68_P24894482	chr6:140372882-140372926	NM_144920:285	Plekha5	INSIDE	1.479	1.478	786.61	1162.51	2.186	680.70	1488.20
A_68_P22321898	chr3:89634943-89634987	NM_172530:-327	She	PROMOTER	1.479	2.768	1127.27	3120.33	4.094	1072.50	4390.86
A_68_P28118794	chr11:109334955-109334999	NM_001029842:-66	Slc16a6	PROMOTER	1.479	1.583	2491.68	3944.60	2.342	1947.22	4560.78
A_68_P20734232	chr1:156646813-156646857	AK046991:-6961		PROMOTER	1.479	3.585	816.76	2928.19	5.304	648.34	3438.64
A_68_P31292460	chr17:64213972-64214016	ENSMUST00000112829:631		INSIDE	1.479	1.881	1392.48	2618.96	2.781	1147.32	3191.23
A_68_P31398060	chr17:83675697-83675741	ENSMUST00000157785:6408		DOWNSTREAM	1.479	2.246	610.82	1371.64	3.321	602.51	2000.92
A_68_P24115156	chr5:135663662-135663706	NM_011714:492	Baz1b	INSIDE	1.478	1.392	1457.67	2028.70	2.057	1309.96	2694.99
A_68_P32982437	chr5:135114511-135114555	NM_033561:666	Eif4h	INSIDE	1.478	1.455	1737.39	2528.45	2.151	1363.07	2931.61
A_68_P32092668	chr19:36993413-36993457	NM_028263:655	Fgfbp3	INSIDE	1.478	3.750	1172.92	4398.74	5.545	945.62	5243.20
A_68_P23201329	chr4:109148711-109148755	NM_013876:378	Rnf11	INSIDE	1.478	2.590	15012.97	38881.78	3.829	12506.99	47883.74
A_68_P29233990	chr13:95055582-95055626	NM_029153:-368	Scamp1	PROMOTER	1.478	2.340	3444.93	8061.13	3.458	2510.77	8681.86
A_68_P24951790	chr7:5004491-5004536	NM_026900:1380	Zfp580	INSIDE	1.478	1.663	883.25	1468.74	2.457	733.71	1802.93
A_68_P29145780	chr13:74487726-74487770	NM_001168659:-16	Ccdc127	DIVERGENT_PROMOTER	1.477	1.989	1765.25	3510.96	2.938	1523.36	4475.30
A_68_P29631499	chr14:58694324-58694368	NM_013518:2824	Fgf9	INSIDE	1.477	2.331	3091.01	7204.39	3.443	2442.41	8408.05
A_68_P28043342	chr11:96133436-96133480	NM_008270:815	Hoxb9	INSIDE	1.477	3.738	3533.47	13209.48	5.520	2622.75	14478.89
A_68_P25089581	chr7:52094932-52094976	NM_010215:3297	Il4i1	INSIDE	1.477	3.776	2505.62	9461.75	5.578	1728.23	9639.62
A_68_P30290903	chr15:66118372-66118416	NM_152923:-608	Kcnq3	PROMOTER	1.477	2.368	814.02	1927.72	3.499	706.35	2471.19
A_68_P32299549	chrX:22942071-22942115	NM_026167:87	Klhl13	INSIDE	1.477	1.778	456.55	811.58	2.625	245.96	645.62
A_68_P23657073	chr5:45911523-45911567	NM_025895:77	Med28	INSIDE	1.477	2.740	677.29	1855.49	4.047	663.80	2686.45

ProbeName	Target position of probe on CpG island microarray	TargetID	GeneSymbol	CpG island Description	Ratio of relative methylation (TiO_2-NP/Vehicle)	Sham group			TiO_2-H group		
						Relative methylation (Cy5/Cy3)	Cy3 signal (Input)	Cy5 signal (MeDIP)	Relative methylation (Cy5/Cy3)	Cy3 signal (Input)	Cy5 signal (MeDIP)
A_68_P28202815	chr12:4881183-4881227	NM_001033488:-39	Mfsd2b	PROMOTER	1.477	3.023	1201.75	3632.72	4.464	1000.10	4464.73
A_68_P31621449	chr18:36178281-36178325	NM_001167891:178512	Nrg2	INSIDE	1.477	1.702	3373.35	5741.23	2.514	2787.93	7007.95
A_68_P31844540	chr18:77098052-77098096	NM_001109743:2931	Skor2	INSIDE	1.477	3.276	1955.84	6407.97	4.840	1593.65	7712.69
A_68_P21303493	chr2:68309091-68309135	NM_016866:926	Stk39	INSIDE	1.477	1.631	1186.56	1935.83	2.409	940.06	2264.78
A_68_P21763712	chr2:154876566-154876610	NM_015770:37283	a	INSIDE	1.476	2.976	2522.97	7508.93	4.393	2039.92	8962.22
A_68_P21068908	chr2:24790503-24790547	NM_001162485:277	Arrdc1	INSIDE	1.476	2.759	3219.05	8881.57	4.072	2742.21	11166.22
A_68_P28175493	chr11:118908126-118908170	NM_013926:-5921	Cbx8	PROMOTER	1.476	3.311	1112.60	3683.47	4.887	1022.16	4995.76
A_68_P29961483	chr14:122805935-122805979	NR_033326:44412	Gm5089	INSIDE	1.476	2.444	1001.95	2448.52	3.607	904.65	3263.19
A_68_P24687704	chr6:99643282-99643326	NM_008158:632	Gpr27	INSIDE	1.476	2.762	1794.15	4955.86	4.077	1534.99	6257.79
A_68_P30490486	chr15:101062223-101062267	NM_019518:7607	Grasp	INSIDE	1.476	3.723	1411.87	5256.42	5.494	1174.75	6454.27
A_68_P28540459	chr12:75009270-75009315	NM_010431:439	Hif1a	INSIDE	1.476	1.586	647.19	1026.57	2.341	554.41	1297.97
A_68_P25842937	chr8:48759109-48759153	NM_023503:1383	Ing2	INSIDE	1.476	4.309	13402.38	57746.43	6.362	10088.99	64183.56
A_68_P25095113	chr7:53050658-53050702	NM_001005511:11364	Lmtk3	INSIDE	1.476	3.613	2223.54	8034.61	5.333	1761.72	9394.45
A_68_P31937501	chr19:7131176-7131220	NM_134147:-59	Macrod1	PROMOTER	1.476	1.539	564.55	868.61	2.271	522.48	1186.46
A_68_P31108447	chr17:26260564-26260608	NM_024227:139	Mrpl28	INSIDE	1.476	2.097	887.71	1861.50	3.096	711.74	2203.30
A_68_P27647504	chr11:23671033-23671077	NM_009044:-84	Rel	PROMOTER	1.476	3.631	2050.13	7443.76	5.360	1691.62	9067.89
A_68_P30424512	chr15:89145509-89145553	NM_001081030:212	Sbf1	INSIDE	1.476	2.299	1415.78	3255.00	3.394	1200.13	4072.88
A_68_P31927885	chr19:5295364-5295408	NM_030109:69	Sf3b2	INSIDE	1.476	1.915	760.31	1456.16	2.828	580.86	1642.40
A_68_P25516577	chr7:136735544-136735588	NM_172255:190	Wdr11	INSIDE	1.476	2.364	1435.09	3393.22	3.491	1131.32	3949.42
A_68_P28729932	chr12:110032712-110032756	NM_009537:1214	Yy1	INSIDE	1.476	2.304	672.44	1549.27	3.400	504.55	1715.50
A_68_P24934667	chr6:147424670-147424714	NM_025911:300	Ccdc91	INSIDE	1.475	1.446	1335.79	1931.52	2.133	1180.74	2518.27
A_68_P31208746	chr17:46889025-46889069	NM_028065:115	Cnpy3	INSIDE	1.475	2.679	2370.77	6352.00	3.953	2092.09	8270.42
A_68_P22564417	chr3:137286650-137286694	NM_030143:37	Ddit4l	INSIDE	1.475	1.539	1359.15	2091.21	2.269	1223.36	2775.50
A_68_P25005644	chr7:25370658-25370702	NM_023154:-1881	Ethe1	PROMOTER	1.475	3.742	7705.46	28832.32	5.518	5065.39	27952.41
A_68_P31213683	chr17:47831917-47831961	NM_144939:-217	Frs3	DIVERGENT_PROMOTER	1.475	3.687	4050.49	14933.40	5.437	3058.19	16628.36
A_68_P28558333	chr12:78339907-78339951	NM_016893:-79	Fut8	PROMOTER	1.475	4.612	1535.76	7083.22	6.801	1349.59	9179.20
A_68_P31256045	chr17:56567492-56567536	NM_011218:48389	Ptprs	INSIDE	1.475	3.272	739.29	2419.22	4.827	634.65	3063.46
A_68_P30980682	chr16:94535261-94535305	ENSMUST00000163193:-112		PROMOTER	1.475	1.966	1953.55	3840.35	2.899	1530.36	4436.57
A_68_P22410430	chr3:107889618-107889662	NM_028779:-95	Ampd2	DIVERGENT_PROMOTER	1.474	2.476	2023.84	5010.93	3.650	1662.24	6066.48
A_68_P21105464	chr2:30683897-30683941	NM_133346:-98	Asb6	PROMOTER	1.474	2.095	2429.69	5091.41	3.090	1863.61	5758.05
A_68_P28731499	chr12:110271722-110271766	NM_001163175:34683	Begain	INSIDE	1.474	1.755	1061.50	1862.57	2.587	1268.33	3280.56
A_68_P22342938	chr3:95032702-95032746	NM_001038708:-149	Cdc42se1	PROMOTER	1.474	1.703	954.06	1624.94	2.511	824.34	2070.21
A_68_P24290981	chr6:22306235-22306279	NM_138587:-175	Fam3c	PROMOTER	1.474	2.029	914.58	1855.65	2.991	785.68	2350.17
A_68_P26676070	chr9:81757205-81757249	NM_175213:-124	Mei4	PROMOTER	1.474	5.220	1402.00	7318.95	7.693	1110.60	8544.04
A_68_P25090468	chr7:52241783-52241827	NM_019830:-102	Prmt1	PROMOTER	1.474	1.607	2363.69	3798.86	2.369	1816.10	4303.19
A_68_P24001336	chr5:114443642-114443686	NM_198109:102	Ssh1	INSIDE	1.474	4.300	884.83	3805.16	6.338	760.01	4816.69
A_68_P24425029	chr6:47893194-47893238	NR_027963:43	Zfp783	INSIDE	1.474	2.346	1905.66	4470.50	3.459	1740.08	6018.63
A_68_P32913428	A_68_P32913428			Unknown	1.474	2.135	546.97	1168.06	3.147	484.41	1524.41
A_68_P31936888	chr19:7026555-7026600	NM_007522:10233	Bad	DOWNSTREAM	1.473	1.814	420.70	763.12	2.671	360.42	962.84
A_68_P23327642	chr4:134829090-134829134	NM_013885:-437	Clic4	PROMOTER	1.473	2.877	1202.13	3458.33	4.238	1110.86	4707.56
A_68_P25307422	chr7:96553151-96553195	NM_008055:297	Fzd4	INSIDE	1.473	4.265	1247.89	5321.97	6.281	967.76	6078.42
A_68_P24991771	chr7:19742770-19742814	NM_001080815:8684	Gipr	INSIDE	1.473	1.415	964.19	1364.02	2.084	816.47	1701.45
A_68_P27279734	chr10:79383882-79383926	NM_053244:4189	Kiss1r	INSIDE	1.473	2.818	2528.95	7125.36	4.150	2110.00	8756.97
A_68_P22882678	chr4:45412906-45412950	NM_001009949:8710	Mcart1	INSIDE	1.473	3.680	1682.66	6191.40	5.420	1213.04	6574.76
A_68_P31947893	chr19:10092733-10092777	NM_144538:37	Rab3il1	INSIDE	1.473	2.026	2171.86	4400.28	2.985	1800.08	5373.28
A_68_P25434388	chr7:121260597-121260641	NM_025846:677	Rras2	INSIDE	1.473	2.462	1960.46	4826.78	3.626	1799.97	6525.85
A_68_P27942519	chr11:78115075-78115119	NM_017407:4	Spag5	INSIDE	1.473	1.878	1066.65	2003.56	2.767	949.08	2625.70
A_68_P28940415	chr13:34838970-34839014	NM_138746:7482	Fam50b	INSIDE	1.472	4.375	983.48	4303.21	6.443	877.56	5653.70
A_68_P24658385	chr6:94649976-94650020	NM_008377:141	Lrig1	INSIDE	1.472	2.625	1485.04	3898.55	3.863	1194.57	4614.70
A_68_P25957682	chr8:74116758-74116804	NM_025396:698	Pgls	INSIDE	1.472	3.782	238.51	902.11	5.567	196.15	1092.01
A_68_P32144302	chr19:46576160-46576204	NM_053100:45	Trim8	INSIDE	1.472	7.776	169.31	1316.65	11.445	147.21	1684.80

ProbeName	Target position of probe on CpG island microarray	TargetID	GeneSymbol	CpG island Description	Ratio of relative methylation (TiO_2-NP/Vehicle)	Sham group			TiO_2-H group		
						Relative methylation (Cy5/Cy3)	Cy3 signal (Input)	Cy5 signal (MeDIP)	Relative methylation (Cy5/Cy3)	Cy3 signal (Input)	Cy5 signal (MeDIP)
A_68_P28606269	chr12:87165967-87166011	NM_001081423:89	Ttll5	INSIDE	1.472	2.014	2091.25	4211.80	2.964	1633.16	4840.56
A_68_P27179603	chr10:60736419-60736463	NM_001081127:-254	Adamts14	PROMOTER	1.471	2.066	1282.18	2648.46	3.039	949.89	2886.88
A_68_P30221123	chr15:53177744-53177788	NM_010162:-28	Ext1	PROMOTER	1.471	2.194	858.05	1882.41	3.228	768.85	2481.85
A_68_P30278744	chr15:63829220-63829264	NM_144846:62768	Fam49b	INSIDE	1.471	2.677	3820.71	10227.82	3.938	3260.43	12839.04
A_68_P28470857	chr12:60320041-60320085	NM_001033156:408	Fbxo33	INSIDE	1.471	2.157	1060.35	2287.30	3.173	764.70	2426.15
A_68_P29631503	chr14:58694761-58694806	NM_013518:3261	Fgf9	INSIDE	1.471	2.692	4216.43	11351.25	3.960	2938.61	11637.08
A_68_P27946741	chr11:78858490-78858534	NM_013571:101344	Ksr1	INSIDE	1.471	5.982	3427.78	20503.67	8.798	2564.13	22559.04
A_68_P27284853	chr10:80153805-80153849	NM_172457:10739	Mobkl2a	INSIDE	1.471	1.689	400.71	676.92	2.485	310.65	771.97
A_68_P23364089	chr4:141001208-141001252	NM_009541:643	Zbtb17	INSIDE	1.471	1.663	1350.23	2246.10	2.446	1220.67	2986.11
A_68_P20148961	chr1:36604157-36604208	NM_026241:-136	Ankrd39	PROMOTER	1.470	2.684	1725.21	4631.27	3.947	1246.17	4918.90
A_68_P31458869	chr18:3507247-3507294	NM_026505:-684	Bambi	PROMOTER	1.470	5.395	495.23	2671.77	7.929	382.40	3032.14
A_68_P20561486	chr1:122238913-122238957	NM_207233:1776	C1ql2	INSIDE	1.470	1.678	855.66	1436.04	2.466	656.37	1618.88
A_68_P25610236	chr7:152129258-152129302	NM_007631:-3450	Ccnd1	PROMOTER	1.470	1.564	751.82	1175.58	2.299	574.03	1319.74
A_68_P31864637	chr18:80666392-80666436	NM_026295:-8	Ctdp1	PROMOTER	1.470	3.845	1779.09	6839.92	5.651	1596.78	9024.09
A_68_P31933635	chr19:6276972-6277016	NM_010119:99	Ehd1	INSIDE	1.470	1.513	1146.59	1734.21	2.224	911.20	2026.38
A_68_P26220864	chr8:123062616-123062660	NM_001145896:1330	Gse1	INSIDE	1.470	2.649	773.57	2049.13	3.893	694.67	2704.14
A_68_P24619086	chr6:87931330-87931374	NM_198622:124	H1fx	INSIDE	1.470	4.319	530.03	2289.23	6.347	452.06	2869.37
A_68_P30823193	chr16:65105676-65105720	NM_008310:-88	Htr1f	PROMOTER	1.470	3.713	536.70	1992.53	5.458	444.92	2428.27
A_68_P24759667	chr6:113419865-113419909	NM_001034029:10998	Il17re	INSIDE	1.470	4.822	1404.21	6771.30	7.088	1016.82	7207.65
A_68_P31029172	chr17:8994701-8994745	NM_011866:113	Pde10a	INSIDE	1.470	2.005	3853.22	7725.23	2.948	3130.87	9228.68
A_68_P32183825	chr19:53751761-53751805	NM_001170847:-13	Rbm20	PROMOTER	1.470	2.473	1485.31	3673.77	3.636	1185.94	4311.76
A_68_P23466247	chr5:8056554-8056598	NM_001080974:35	Sri	INSIDE	1.470	4.638	4087.16	18956.12	6.819	3246.30	22137.09
A_68_P25614882	chr8:3631030-3631074	NM_011503:-107	Stxbp2	DIVERGENT_PROMOTER	1.470	2.653	1940.51	5147.96	3.901	1699.86	6630.50
A_68_P31922247	chr19:4283414-4283458	NM_026720:-299	Ankrd13d	PROMOTER	1.469	3.237	851.81	2757.27	4.755	713.16	3391.01
A_68_P20347398	chr1:74283332-74283376	NM_029711:231	Arpc2	INSIDE	1.469	2.067	4621.98	9555.06	3.037	3605.94	10952.21
A_68_P26624019	chr9:71619304-71619348	NM_026599:83	Cgnl1	INSIDE	1.469	2.364	1632.58	3859.07	3.474	1414.90	4914.66
A_68_P24613526	chr6:86992962-86993006	NM_013528:145	Gfpt1	INSIDE	1.469	1.416	2116.35	2995.80	2.079	1656.41	3444.36
A_68_P27127237	chr10:49508470-49508514	NM_001111268:68	Grik2	INSIDE	1.469	2.389	940.44	2246.97	3.510	827.91	2906.39
A_68_P26954164	chr10:13687012-13687056	NM_010437:850	Hivep2	INSIDE	1.469	4.341	882.92	3832.82	6.377	692.35	4415.08
A_68_P29130726	chr13:72101391-72101435	NM_010573:-241	Irx1	PROMOTER	1.469	2.409	1015.37	2445.87	3.539	1123.70	3976.49
A_68_P27989203	chr11:86298068-86298112	NM_029788:-68	Rnft1	PROMOTER	1.469	3.318	3448.05	11440.91	4.873	2688.37	13099.53
A_68_P24590571	chr6:82889627-82889671	NM_011350:96	Sema4f	INSIDE	1.469	4.504	1390.53	6262.53	6.617	1245.93	8244.76
A_68_P21440134	chr2:94113837-94113881	ENSMUST00000167726:-8943		PROMOTER	1.469	2.315	5022.32	11624.23	3.399	4033.70	13711.46
A_68_P32704868	chrX:138159961-138160005	NM_010572:-222	Irs4	PROMOTER	1.468	1.836	1144.09	2100.11	2.695	660.25	1779.33
A_68_P25098813	chr7:53652081-53652125	NM_001112739:236	Kcnc1	INSIDE	1.468	4.084	1056.70	4316.07	5.996	1000.33	5997.67
A_68_P23331510	chr4:135528254-135528298	NM_011942:233	Lypla2	INSIDE	1.468	1.701	1471.16	2502.09	2.496	1180.83	2947.92
A_68_P28154417	chr11:115474024-115474068	NM_027162:-229	Mif4gd	PROMOTER	1.468	1.814	4942.93	8967.52	2.663	3768.32	10035.51
A_68_P31095629	chr17:24278798-24278842	NM_011062:-259	Pdpk1	PROMOTER	1.468	2.422	1361.05	3296.96	3.557	993.71	3534.40
A_68_P30348897	chr15:76353005-76353049	NM_130893:-467	Scrt1	PROMOTER	1.468	1.878	942.14	1769.61	2.757	740.71	2042.07
A_68_P22873079	chr4:43682099-43682143	NM_001085508:278	Tmem8b	INSIDE	1.468	3.001	2359.24	7081.21	4.405	1952.29	8600.32
A_68_P28166391	chr11:117517129-117517173	NM_198022:1548	Tnrc6c	INSIDE	1.468	2.606	2960.80	7715.59	3.825	2299.18	8794.43
A_68_P23576671	chr5:31441022-31441066	NM_021290:224	Ucn	INSIDE	1.468	3.501	2317.10	8112.83	5.142	1802.53	9267.79
A_68_P23272342	chr4:124805193-124805237	NM_153159:-89	Zc3h12a	PROMOTER	1.468	3.508	1523.15	5342.63	5.148	1237.05	6368.43
A_68_P23441289	chr4:155266283-155266327	NM_207223:321	Acap3	INSIDE	1.467	3.063	1838.00	5630.53	4.495	1604.68	7213.71
A_68_P32769554	chrX:154481205-154481249	NM_177751:-184	Cnksr2	PROMOTER	1.467	1.785	1035.89	1848.56	2.618	662.20	1733.68
A_68_P24009176	chr5:115798923-115798967	NM_007748:20	Cox6a1	INSIDE	1.467	3.389	3166.66	10730.30	4.969	2503.40	12440.52
A_68_P31347827	chr17:74059382-74059426	NM_027864:387	Galnt14	INSIDE	1.467	3.466	987.63	3423.01	5.085	958.75	4875.38
A_68_P31493066	chr18:11054906-11054950	NM_010258:2421	Gata6	INSIDE	1.467	4.748	2399.43	11393.63	6.966	2124.96	14802.92
A_68_P29581905	chr14:47993552-47993596	NM_001145953:40	Lgals3	INSIDE	1.467	2.547	2541.06	6470.88	3.736	2078.11	7763.12
A_68_P20806523	chr1:169619018-169619062	NM_033652:-648	Lmx1a	PROMOTER	1.467	3.092	1588.97	4913.37	4.536	1242.36	5635.68
A_68_P25934950	chr8:69384793-69384837	NM_001163564:699	Naf1	INSIDE	1.467	1.903	1890.51	3598.40	2.793	1778.06	4966.22

ProbeName	Target position of probe on CpG island microarray	TargetID	GeneSymbol	CpG island Description	Ratio of relative methylation (TiO_2-NP/Vehicle)	Sham group			TiO_2-H group		
						Relative methylation (Cy5/Cy3)	Cy3 signal (Input)	Cy5 signal (MeDIP)	Relative methylation (Cy5/Cy3)	Cy3 signal (Input)	Cy5 signal (MeDIP)
A_68_P31093355	chr17:23876913-23876957	NM_023824:363	Paqr4	INSIDE	1.467	1.879	1257.55	2362.82	2.756	997.12	2748.02
A_68_P31940079	chr19:7557572-7557616	NM_053076:177	Rtn3	INSIDE	1.467	1.906	2243.05	4274.38	2.795	1833.22	5124.16
A_68_P26892104	chr9:122832365-122832409	NM_009544:191	Zfp105	INSIDE	1.467	3.399	1751.18	5952.06	4.986	1650.02	8227.28
A_68_P25600927	chr7:150623918-150623962			Unknown	1.467	2.640	1461.25	3857.25	3.872	1226.22	4748.16
A_68_P32565204	chrX:98893057-98893101	ENSMUST00000073812:-5889		PROMOTER	1.467	6.825	7499.20	51181.65	10.011	3237.87	32412.92
A_68_P21105459	chr2:30683485-30683529	NM_133346:314	Asb6	INSIDE	1.466	1.854	831.64	1541.56	2.718	687.12	1867.83
A_68_P26235085	chr8:125202360-125202404	NM_001109873:-307	Cbfa2t3	PROMOTER	1.466	5.797	1592.00	9228.23	8.499	1563.32	13286.47
A_68_P25082512	chr7:50721931-50721975	NM_001164518:23441	Iglon5	DOWNSTREAM	1.466	6.673	1298.78	8666.90	9.782	1293.35	12651.86
A_68_P23560503	chr5:28397698-28397742	NM_153526:-231	Insig1	PROMOTER	1.466	2.699	830.23	2240.95	3.956	742.87	2938.95
A_68_P24838924	chr6:128374739-128374783	NM_133927:168	Itfg2	INSIDE	1.466	2.138	879.52	1880.20	3.135	701.85	2200.15
A_68_P21305453	chr2:68700233-68700278	NM_172856:642	Lass6	INSIDE	1.466	3.774	2495.47	9417.92	5.533	2252.29	12461.69
A_68_P32137226	chr19:45309781-45309825	NM_010691:-76	Lbx1	PROMOTER	1.466	11.283	1551.16	17502.06	16.539	1307.13	21618.77
A_68_P23260196	chr4:122673426-122673470	NM_008506:107	Mycl1	INSIDE	1.466	3.773	3472.25	13102.03	5.531	2920.99	16155.65
A_68_P23298638	chr4:129497304-129497348	NM_008974:-396	Ptp4a2	PROMOTER	1.466	2.069	1542.71	3191.27	3.033	1412.18	4283.62
A_68_P27949795	chr11:79404844-79404888	NM_175543:153	Rab11fip4	INSIDE	1.466	1.779	517.95	921.50	2.609	505.92	1319.93
A_68_P26569961	chr9:62190386-62190430	NM_009672:1259	Anp32a	INSIDE	1.465	2.912	3661.25	10661.31	4.265	2424.99	10342.80
A_68_P26135515	chr8:108231965-108232009	NM_198299:82	E130303B06Rik	INSIDE	1.465	2.072	5535.37	11468.49	3.036	4238.37	12868.85
A_68_P21940379	chr3:10012439-10012483	NM_010634:-145	Fabp5	PROMOTER	1.465	3.164	4173.05	13202.55	4.634	3534.74	16381.28
A_68_P21338643	chr2:74506367-74506411	NM_008275:22	Hoxd13	INSIDE	1.465	1.856	2823.18	5239.55	2.719	2470.82	6719.12
A_68_P25016216	chr7:28013358-28013402	NM_181593:236	Itpkc	INSIDE	1.465	1.916	810.49	1553.14	2.808	728.28	2045.11
A_68_P28290827	chr12:25336485-25336529	NM_178357:271	Klf11	INSIDE	1.465	1.528	4868.62	7437.65	2.239	3523.30	7887.89
A_68_P25617732	chr8:4493215-4493259	NM_026058:-168	Lass4	PROMOTER	1.465	2.568	11509.06	29554.92	3.763	7642.87	28758.22
A_68_P24159372	chr5:144861058-144861106	NM_001081109:-222	Lmtk2	PROMOTER	1.465	2.419	1587.58	3840.24	3.544	1278.35	4530.73
A_68_P31296466	chr17:64951197-64951241	NM_008549:230	Man2a1	INSIDE	1.465	1.975	798.17	1576.45	2.894	699.34	2024.02
A_68_P32384750	chrX:50265645-50265689	NM_027642:224	Phf6	INSIDE	1.465	2.584	518.16	1338.87	3.786	317.74	1202.89
A_68_P28103448	chr11:106640841-106640885	NM_015810:-11	Polg2	PROMOTER	1.465	2.977	1175.26	3498.35	4.361	987.01	4303.98
A_68_P31303252	chr17:66234323-66234367	NM_001198949:-81	Ralbp1	PROMOTER	1.465	2.500	1059.86	2650.05	3.662	858.46	3143.61
A_68_P25050063	chr7:36370599-36370643	NM_145840:-19	Rgs9bp	DIVERGENT_PROMOTER	1.465	2.928	2932.41	8585.37	4.289	2328.77	9987.40
A_68_P22049391	chr3:34549338-34549382	NM_011443:434	Sox2	INSIDE	1.465	2.631	565.14	1486.72	3.854	461.29	1777.59
A_68_P31210123	chr17:47147224-47147268	NM_001177374:235	Ubr2	INSIDE	1.465	2.951	1438.47	4244.75	4.324	1223.15	5288.34
A_68_P20737709	chr1:157263929-157263973	NM_011273:624	Xpr1	INSIDE	1.465	1.630	3904.60	6363.37	2.387	3173.16	7575.19
A_68_P29478601	chr14:26427680-26427724	NM_183208:149032	Zmiz1	INSIDE	1.465	3.821	2002.18	7651.11	5.600	1832.49	10261.89
A_68_P24044663	chr5:121902036-121902080	NM_026129:425	Erp29	INSIDE	1.464	2.054	1586.70	3258.30	3.006	1213.06	3646.79
A_68_P24397652	chr6:42273788-42273832	NM_001113327:829	Fam131b	INSIDE	1.464	1.928	4011.99	7734.52	2.823	2915.36	8228.84
A_68_P27087305	chr10:40792189-40792233	NM_199058:-820	Gpr6	PROMOTER	1.464	2.788	1679.17	4680.85	4.082	1357.03	5538.88
A_68_P24249109	chr6:13788674-13788718	NM_145066:1152	Gpr85	INSIDE	1.464	2.339	811.08	1896.88	3.423	698.33	2390.38
A_68_P29186150	chr13:83662891-83662935	NM_001170537:19880	Mef2c	INSIDE	1.464	2.933	282.49	828.67	4.294	332.88	1429.43
A_68_P26977748	chr10:18564800-18564844	NM_022032:-54	Perp	PROMOTER	1.464	4.788	899.74	4307.54	7.009	868.93	6090.44
A_68_P25262772	chr7:86866415-86866459	NM_001113471:11226	Plin1	INSIDE	1.464	1.697	461.93	784.11	2.484	394.86	981.02
A_68_P29033142	chr13:51662332-51662377	NM_009167:99	Shc3	INSIDE	1.464	2.474	2443.96	6045.47	3.622	2096.84	7594.48
A_68_P27915805	chr11:73013090-73013134	NM_029031:129	Shpk	INSIDE	1.464	4.681	893.22	4181.24	6.852	813.06	5571.09
A_68_P32341125	chrX:39502207-39502251	NM_001077712:-360	Stag2	PROMOTER	1.464	3.496	726.62	2540.10	5.118	473.46	2423.23
A_68_P20186939	chr1:43149198-43149242	NM_001013025:6247	Tgfbrap1	INSIDE	1.464	1.582	1869.01	2956.34	2.316	1806.50	4183.12
A_68_P26349635	chr9:21741759-21741803	NM_144935:-937	BC018242	PROMOTER	1.463	2.870	814.01	2336.54	4.200	613.10	2574.99
A_68_P23895412	chr5:93104159-93104203	NM_177230:-27	Ccdc158	DIVERGENT_PROMOTER	1.463	2.184	2971.54	6488.40	3.195	2388.53	7632.27
A_68_P28727032	chr12:109512870-109512914	NM_001159910:735	Ccdc85c	INSIDE	1.463	3.637	1292.84	4702.43	5.322	989.31	5264.62
A_68_P27495835	chr10:119638550-119638594	NM_028679:21	Irak3	INSIDE	1.463	1.455	1018.19	1481.09	2.128	954.18	2030.95
A_68_P24623141	chr6:88577383-88577427	NM_001142724:35	Kbtbd12	INSIDE	1.463	1.486	1134.15	1685.45	2.174	954.46	2075.22
A_68_P30363285	chr15:78765693-78765737	NM_133800:352	Nol12	INSIDE	1.463	2.228	927.44	2066.66	3.260	731.54	2384.58
A_68_P30167378	chr15:41620567-41620611	NM_001130163:-472	Oxr1	PROMOTER	1.463	5.835	1351.56	7886.92	8.537	1228.20	10485.06
A_68_P20015305	chr1:7078905-7078949	NM_183028:-74	Pcmtd1	PROMOTER	1.463	1.868	1307.36	2442.13	2.733	1061.13	2900.19

ProbeName	Target position of probe on CpG island microarray	TargetID	GeneSymbol	CpG island Description	Ratio of relative methylation (TiO_2-NP/Vehicle)	Sham group			TiO_2-H group		
						Relative methylation (Cy5/Cy3)	Cy3 signal (Input)	Cy5 signal (MeDIP)	Relative methylation (Cy5/Cy3)	Cy3 signal (Input)	Cy5 signal (MeDIP)
A_68_P25435751	chr7:121559514-121559558	NM_011055:769	Pde3b	INSIDE	1.463	5.170	900.72	4656.88	7.565	729.69	5520.09
A_68_P25504895	chr7:134417127-134417171	NM_009266:425	Sephs2	INSIDE	1.463	2.790	990.62	2763.79	4.081	1003.94	4097.31
A_68_P25482572	chr7:130267793-130267837	NM_144925:416	Tnrc6a	INSIDE	1.463	2.144	694.03	1488.05	3.136	563.02	1765.67
A_68_P33008823	chr4_random:109636-109680	NM_001033326:-143	Dhrsx	PROMOTER	1.462	5.931	7787.85	46191.47	8.670	5024.52	43561.95
A_68_P24218878	chr6:6831381-6831425	NM_010056:666	Dlx5	INSIDE	1.462	1.541	1552.47	2392.93	2.253	1183.51	2666.48
A_68_P32263511	chrX:12338951-12338995	NM_001048208:127	Med14	INSIDE	1.462	2.879	1207.83	3477.80	4.208	984.15	4141.68
A_68_P23618575	chr5:38668403-38668447	NM_172709:-218	Otop1	PROMOTER	1.462	1.997	3392.73	6775.24	2.920	2565.88	7491.60
A_68_P21417957	chr2:90420274-90420318	NM_008982:508	Ptprj	INSIDE	1.462	3.067	1620.25	4968.54	4.483	1335.27	5985.98
A_68_P29605618	chr14:52877257-52877301	NM_172601:21792	Rab2b	DOWNSTREAM	1.462	2.734	2916.60	7973.69	3.998	2339.42	9352.89
A_68_P31302582	chr17:66121758-66121802	NM_133685:312	Rab31	INSIDE	1.462	2.303	5249.27	12088.18	3.368	4134.74	13924.93
A_68_P31924143	chr19:4625643-4625687	NM_023131:-47	Rce1	PROMOTER	1.462	1.354	1807.90	2447.64	1.980	1502.27	2973.96
A_68_P21568741	chr2:119096977-119097021	NM_145530:-36	Rhov	PROMOTER	1.462	2.931	2635.94	7726.37	4.286	2156.95	9244.83
A_68_P21911578	chr2:181333602-181333646	NM_172676:228	Samd10	INSIDE	1.462	1.779	1637.73	2913.00	2.601	1257.33	3269.68
A_68_P31929910	chr19:5654473-5654517	NM_001164480:9213	Sipa1	INSIDE	1.462	1.743	4619.05	8051.53	2.549	3528.01	8992.71
A_68_P22611968	chr3:145651227-145651271	NM_001166064:415	Syde2	INSIDE	1.462	2.559	707.01	1809.58	3.743	622.20	2329.01
A_68_P25581340	chr7:147130450-147130494	NM_009482:718	Utf1	INSIDE	1.462	1.579	3203.71	5057.77	2.308	2204.81	5089.26
A_68_P29455108	chr14:22650786-22650830	NM_011695:26	Vdac2	INSIDE	1.462	8.363	669.25	5597.08	12.226	576.83	7052.33
A_68_P21775097	chr2:156904527-156904571	NM_001164663:453	9830001H06Rik	INSIDE	1.461	6.751	1401.95	9464.94	9.864	1289.26	12716.90
A_68_P27339932	chr10:90545143-90545187	NM_001042558:324	Apaf1	INSIDE	1.461	1.626	581.25	944.94	2.375	514.37	1221.49
A_68_P28525541	chr12:72419505-72419549	NM_001190466:8656	Dact1	INSIDE	1.461	3.271	948.47	3102.20	4.779	740.23	3537.29
A_68_P27540020	chr10:127631671-127631715	NM_001033264:2	Gls2	INSIDE	1.461	2.403	1119.85	2691.21	3.510	1006.38	3532.25
A_68_P22028772	chr3:30407701-30407745	NM_021442:687	Mecom	INSIDE	1.461	2.306	973.83	2245.24	3.368	814.47	2742.77
A_68_P32148151	chr19:47253878-47253922	NM_021360:591	Neurl1a	INSIDE	1.461	3.015	2347.76	7079.64	4.406	2046.66	9017.99
A_68_P20641449	chr1:137662705-137662749	NM_013750:65	Phlda3	INSIDE	1.461	3.491	4817.35	16815.45	5.101	4035.59	20586.57
A_68_P27163773	chr10:57909758-57909803	NM_011240:181	Ranbp2	INSIDE	1.461	2.070	1044.40	2161.81	3.024	955.85	2890.17
A_68_P21494381	chr2:105239586-105239630	NM_009037:-132	Rcn1	PROMOTER	1.461	2.834	960.25	2720.91	4.141	859.45	3559.00
A_68_P28031422	chr11:94072689-94072733	NM_009427:-57	Tob1	PROMOTER	1.461	2.069	3383.56	6999.79	3.022	2629.56	7945.46
A_68_P20451933	chr1:94803719-94803763	NM_175118:175	Dusp28	INSIDE	1.460	1.836	1000.17	1836.32	2.680	851.65	2282.69
A_68_P31424359	chr17:88039798-88039842	NM_008532:4502	Epcam	INSIDE	1.460	2.523	659.90	1665.23	3.684	541.12	1993.23
A_68_P25018411	chr7:28443445-28443489	NM_001081292:16151	Map3k10	INSIDE	1.460	2.763	3810.58	10527.58	4.033	2767.38	11161.07
A_68_P26539664	chr9:56843551-56843595	NM_019651:798	Ptpn9	INSIDE	1.460	2.050	548.75	1125.07	2.993	444.66	1330.79
A_68_P32099689	chr19:38199320-38199364	NM_011255:-41	Rbp4	PROMOTER	1.460	2.293	652.67	1496.39	3.348	581.81	1947.91
A_68_P20833904	chr1:174493216-174493260	NR_027644:3202	Vsig8	INSIDE	1.460	1.492	1794.63	2677.71	2.178	1418.87	3090.24
A_68_P31468065	chr18:5334203-5334247	NM_178722:213	Zfp438	INSIDE	1.460	3.748	1186.50	4446.93	5.472	1077.59	5896.12
A_68_P24438776	chr6:50516749-50516793	NR_015581:129	5430402O13Rik	INSIDE	1.459	1.688	1333.34	2251.10	2.464	1109.36	2733.16
A_68_P28553305	chr12:77425730-77425774	NM_001101471:-125	Akap5	PROMOTER	1.459	5.759	2747.26	15821.81	8.404	2184.46	18357.78
A_68_P27080339	chr10:39451377-39451421	NR_038037:415	E130307A14Rik	INSIDE	1.459	2.576	3212.43	8274.16	3.759	2879.49	10823.71
A_68_P21107216	chr2:30997021-30997065	NM_001038700:486	Fnbp1	INSIDE	1.459	1.721	1615.84	2780.84	2.510	1425.03	3576.91
A_68_P23510646	chr5:17866146-17866190	NM_010305:63	Gnai1	INSIDE	1.459	2.612	1092.96	2855.21	3.812	932.74	3555.40
A_68_P31381047	chr17:80461465-80461509	NM_144802:188	Hnrpll	INSIDE	1.459	5.020	1241.74	6233.03	7.323	1099.87	8054.72
A_68_P31826505	chr18:73732011-73732055	NM_001039214:-326	Mex3c	PROMOTER	1.459	1.754	2862.22	5019.82	2.558	2182.65	5583.74
A_68_P21839356	chr2:167906923-167906967	NM_021409:441	Pard6b	INSIDE	1.459	1.612	1921.41	3097.64	2.352	1712.76	4027.98
A_68_P22874082	chr4:43888324-43888368	NM_016678:-55	Reck	PROMOTER	1.459	4.573	1315.45	6015.70	6.671	1076.68	7182.01
A_68_P23325608	chr4:134487062-134487106	NM_026780:190	Syf2	INSIDE	1.459	1.768	617.65	1092.25	2.580	512.56	1322.59
A_68_P30476484	chr15:98623292-98623336	NM_021279:3027	Wnt1	INSIDE	1.459	5.005	944.81	4728.35	7.304	680.07	4966.93
A_68_P23244790	chr4:118967092-118967136	NM_011732:4	Ybx1	INSIDE	1.459	1.877	4211.74	7907.02	2.739	2896.16	7932.24
A_68_P27873632	chr11:65621027-65621071	NM_026107:303	Zkscan6	INSIDE	1.459	3.212	942.34	3027.10	4.688	847.63	3973.56
A_68_P21101977	chr2:30093187-30093231	NR_028299:-57	1700084E18Rik	DIVERGENT_PROMOTER	1.458	2.115	2810.65	5945.31	3.085	2342.34	7225.85
A_68_P25933298	chr8:69010075-69010123	NM_025465:308	1810029B16Rik	INSIDE	1.458	2.297	420.54	965.79	3.349	366.75	1228.11
A_68_P26273049	chr9:3199691-3199735	NR_027988:101	4930433N12Rik	INSIDE	1.458	9.650	3965.00	38263.77	14.072	2614.39	36790.53
A_68_P28002759	chr11:88725539-88725583	NM_001042541:340	Akap1	INSIDE	1.458	1.390	1362.89	1894.77	2.028	1133.86	2298.98

ProbeName	Target position of probe on CpG island microarray	TargetID	GeneSymbol	CpG island Description	Ratio of relative methylation (TiO_2-NP/Vehicle)	Sham group			TiO_2-H group		
						Relative methylation (Cy5/Cy3)	Cy3 signal (Input)	Cy5 signal (MeDIP)	Relative methylation (Cy5/Cy3)	Cy3 signal (Input)	Cy5 signal (MeDIP)
A_68_P21023862	chr2:14976924-14976968	NM_029466:-42	Arl5b	DIVERGENT_PROMOTER	1.458	1.951	1371.22	2675.65	2.845	1248.84	3552.80
A_68_P27954537	chr11:80290536-80290580	NM_009871:11	Cdk5r1	INSIDE	1.458	3.634	10555.96	38360.57	5.299	9525.91	50479.30
A_68_P28202614	chr12:4848201-4848245	NM_016863:179	Fkbp1b	INSIDE	1.458	3.457	890.42	3078.30	5.040	753.94	3799.81
A_68_P29542521	chr14:37781798-37781842	NM_027045:130	Gcap14	INSIDE	1.458	1.822	1608.46	2930.62	2.657	1283.32	3409.15
A_68_P25092916	chr7:52661724-52661768	NM_010596:417	Kcna7	INSIDE	1.458	3.884	1484.75	5766.07	5.663	1337.18	7572.44
A_68_P22393168	chr3:104591684-104591728	NM_007484:-245	Rhoc	PROMOTER	1.458	3.384	2910.92	9851.91	4.934	2172.18	10716.60
A_68_P24939589	chr6:148303183-148303227	NR_003634:27	Rps4y2	INSIDE	1.458	2.311	403.52	932.55	3.369	366.69	1235.46
A_68_P22324942	chr3:90193856-90193900	NM_011988:-29	Slc27a3	PROMOTER	1.458	2.853	781.53	2229.86	4.159	719.70	2993.24
A_68_P21328883	chr2:72818359-72818403	NM_001098425:-1042	Sp3	PROMOTER	1.458	3.094	36100.64	111680.40	4.510	29854.51	134653.00
A_68_P26875611	chr9:119920380-119920424	NM_011724:12314	Xirp1	DOWNSTREAM	1.458	2.503	1178.16	2949.18	3.649	919.22	3354.32
A_68_P21575995	chr2:120389596-120389640	NM_011743:-51	Zfp106	DIVERGENT_PROMOTER	1.458	2.231	4259.53	9503.35	3.253	3837.58	12482.20
A_68_P21863763	chr2:172171241-172171285	NM_025912:186	2010011I20Rik	INSIDE	1.457	2.311	1062.68	2455.68	3.367	931.57	3136.61
A_68_P27843669	chr11:60351870-60351914	NM_172943:708	Alkbh5	INSIDE	1.457	3.672	929.96	3414.82	5.351	763.55	4085.77
A_68_P25957072	chr8:74009934-74009979	NM_001164679:10	Ano8	INSIDE	1.457	1.346	2736.69	3683.83	1.961	2053.58	4026.41
A_68_P28195048	chr12:3427213-3427257	NM_172421:351	Asxl2	INSIDE	1.457	3.449	1549.18	5343.48	5.026	1221.20	6137.28
A_68_P28731660	chr12:110290621-110290665	NM_001163175:15785	Begain	INSIDE	1.457	3.320	520.84	1728.97	4.836	521.48	2521.94
A_68_P28082431	chr11:102807578-102807622	NM_011795:175	C1ql1	INSIDE	1.457	6.794	514.32	3494.08	9.896	465.73	4609.07
A_68_P31022586	chr17:6987747-6987791	NM_009510:-639	Ezr	PROMOTER	1.457	1.571	752.56	1182.48	2.289	639.94	1464.77
A_68_P22483671	chr3:122323019-122323074	NM_001114665:-461	Fnbp1l	PROMOTER	1.457	3.387	944.45	3198.37	4.933	826.14	4075.32
A_68_P21052792	chr2:20891136-20891180	NR_033225:658	Gm13375	INSIDE	1.457	4.676	1729.00	8084.67	6.813	1409.02	9600.07
A_68_P25842949	chr8:48760597-48760641	NM_023503:-105	Ing2	PROMOTER	1.457	2.518	1788.88	4503.57	3.668	1504.15	5517.81
A_68_P20166596	chr1:39634041-39634085	NM_001033135:130	Rnf149	INSIDE	1.457	2.325	1362.29	3166.74	3.386	1243.87	4211.82
A_68_P20638511	chr1:137180229-137180273	NM_001159624:411	Rnpep	INSIDE	1.457	2.202	1517.55	3341.03	3.207	1262.64	4049.56
A_68_P30970962	chr16:92698887-92698931	NM_001111023:-1335	Runx1	PROMOTER	1.457	1.835	641.32	1176.91	2.675	530.30	1418.40
A_68_P24803059	chr6:121195955-121195999	NM_011909:53	Usp18	INSIDE	1.457	2.204	926.39	2041.83	3.212	1000.99	3215.04
A_68_P26815586	chr9:108481063-108481107	NM_031392:-83	Wdr6	PROMOTER	1.457	2.069	1765.90	3654.08	3.015	1426.63	4301.72
A_68_P23575215	chr5:31191120-31191164	NM_174849:-82	Agbl5	PROMOTER	1.456	2.933	607.02	1780.58	4.272	413.55	1766.63
A_68_P33005169	chr7:3366442-3366487	NM_133189:29780	Cacng7	INSIDE	1.456	1.529	1339.66	2048.53	2.227	991.30	2207.71
A_68_P23792211	chr5:72895109-72895153	NM_016869:317	Corin	INSIDE	1.456	1.865	1598.60	2981.29	2.716	1215.77	3302.39
A_68_P23416728	chr4:151505797-151505841	NM_019585:-2784	Espn	PROMOTER	1.456	1.612	1539.60	2481.95	2.346	1367.83	3209.46
A_68_P30630418	chr16:28446840-28446884	NM_183064:-1549	Fgf12	PROMOTER	1.456	2.036	569.25	1158.74	2.964	537.05	1591.64
A_68_P29388379	chr14:9046505-9046549	NM_027782:-135	Kctd6	PROMOTER	1.456	6.924	4007.28	27747.18	10.079	3170.03	31951.83
A_68_P24325043	chr6:29129618-29129662	NM_001101443:-56	Prrt4	PROMOTER	1.456	7.255	8481.06	61526.61	10.564	6185.29	65343.71
A_68_P31102749	chr17:25410196-25410240	NM_001177610:158	Ube2i	INSIDE	1.456	2.075	1046.40	2171.19	3.020	923.66	2789.66
A_68_P27997644	chr11:87881461-87881505	NM_016686:-360	Vezf1	PROMOTER	1.456	1.830	2438.54	4462.54	2.665	2358.99	6286.77
A_68_P23957857	chr5:106305585-106305629	NM_018759:20	Zfp326	INSIDE	1.456	2.246	415.39	932.83	3.269	423.88	1385.76
A_68_P31134871	chr17:30713349-30713393	NM_027060:-138	Btbd9	PROMOTER	1.455	2.181	1117.76	2438.28	3.173	1010.81	3207.63
A_68_P24993219	chr7:20006500-20006544	NM_007710:10080	Ckm	INSIDE	1.455	2.148	1288.84	2768.37	3.125	1070.58	3345.70
A_68_P23345560	chr4:137951782-137951826	NM_001081672:569	Fam43b	INSIDE	1.455	3.959	706.97	2799.21	5.763	601.77	3467.91
A_68_P23582152	chr5:32437568-32437612	NM_008037:-1254	Fosl2	PROMOTER	1.455	4.137	641.42	2653.38	6.018	616.21	3708.17
A_68_P31515586	chr18:15222359-15222403	NM_001142731:-289	Kctd1	PROMOTER	1.455	1.733	1386.30	2403.07	2.523	1097.60	2769.12
A_68_P27785188	chr11:49660391-49660435	NM_001163671:160	Mapk9	INSIDE	1.455	1.566	1886.97	2955.07	2.278	1586.85	3614.59
A_68_P25981540	chr8:79709769-79709813	NM_001083906:283384	Nr3c2	INSIDE	1.455	3.288	943.76	3102.85	4.785	725.24	3470.35
A_68_P20163208	chr1:39044668-39044712	NM_026850:32	Pdcl3	INSIDE	1.455	3.480	2919.10	10159.42	5.065	2028.94	10275.96
A_68_P27430008	chr10:107599648-107599692	NM_027892:215	Ppp1r12a	INSIDE	1.455	2.288	1378.25	3153.33	3.330	1209.49	4027.44
A_68_P24691288	chr6:100237003-100237047	NM_019743:328	Rybp	INSIDE	1.455	3.187	1046.04	3333.77	4.638	817.40	3791.10
A_68_P22286842	chr3:83569940-83569984	NM_009144:-280	Sfrp2	PROMOTER	1.455	1.712	2189.69	3748.71	2.492	1691.78	4215.27
A_68_P29052701	chr13:55097633-55097677	NM_153131:46862	Unc5a	INSIDE	1.455	2.295	1681.83	3859.82	3.339	1344.53	4489.11
A_68_P24979386	chr7:16647485-16647529	NM_001099277:-9784	Zfp541	PROMOTER	1.455	3.901	6432.66	25096.37	5.677	4589.99	26058.32
A_68_P30379093	chr15:81524108-81524152	NM_001164320:3587	Chadl	INSIDE	1.454	3.450	292.29	1008.53	5.017	330.10	1656.11
A_68_P24818548	chr6:125031803-125031847	NM_001163268:10530	Lpar5	INSIDE	1.454	1.700	1581.55	2689.12	2.473	1399.54	3460.97

ProbeName	Target position of probe on CpG island microarray	TargetID	GeneSymbol	CpG island Description	Ratio of relative methylation (TiO_2-NP/Vehicle)	Sham group			TiO_2-H group		
						Relative methylation (Cy5/Cy3)	Cy3 signal (Input)	Cy5 signal (MeDIP)	Relative methylation (Cy5/Cy3)	Cy3 signal (Input)	Cy5 signal (MeDIP)
A_68_P23227558	chr4:115748182-115748226	NM_153521:135	Lrrc41	INSIDE	1.454	3.338	1274.86	4255.23	4.853	1149.75	5579.65
A_68_P21780569	chr2:157854821-157854865	NM_027434:313	Rprd1b	INSIDE	1.454	1.517	1485.79	2254.60	2.206	1097.49	2420.89
A_68_P31331151	chr17:71202362-71202406	NM_001164075:-1310	Tgif1	PROMOTER	1.454	2.468	1761.00	4345.77	3.589	1299.47	4663.50
A_68_P25850571	chr8:49928417-49928461	AK013771:58		INSIDE	1.454	2.771	1314.67	3642.66	4.028	1193.16	4806.45
A_68_P32217229	chr19:59150385-59150429	NM_001114312:153	4930506M07Rik	INSIDE	1.453	1.820	399.24	726.66	2.645	346.26	915.90
A_68_P31053606	chr17:12934729-12934773	NR_002853:574	Airn	INSIDE	1.453	3.100	634.08	1965.72	4.503	625.99	2819.07
A_68_P28925107	chr13:31900007-31900051	NM_008592:1514	Foxc1	INSIDE	1.453	2.368	1067.03	2527.10	3.441	888.61	3058.02
A_68_P29660091	chr14:63865134-63865179	NM_008092:-1059	Gata4	PROMOTER	1.453	7.098	511.45	3630.21	10.310	404.51	4170.34
A_68_P22898182	chr4:48060289-48060333	NM_015743:-3809	Nr4a3	PROMOTER	1.453	2.764	766.56	2118.88	4.017	589.66	2368.92
A_68_P26574084	chr9:62990320-62990364	NM_172446:4445	Skor1	INSIDE	1.453	2.763	1499.36	4143.06	4.015	1218.84	4894.05
A_68_P28938195	chr13:34436488-34436532	NM_001033167:541	Slc22a23	INSIDE	1.453	2.762	403.59	1114.57	4.014	358.05	1437.23
A_68_P24118171	chr5:136328011-136328055	NM_021403:34057	Srrm3	INSIDE	1.453	3.840	1032.86	3965.69	5.579	906.14	5055.25
A_68_P21751498	chr2:152673870-152673914	NM_001141975:193	Tpx2	INSIDE	1.453	2.514	2648.33	6656.99	3.651	2122.21	7748.70
A_68_P24979385	chr7:16647414-16647458	NM_001099277:-9854	Zfp541	PROMOTER	1.453	4.543	2956.79	13432.03	6.600	2184.19	14415.65
A_68_P25174399	chr7:71082777-71082821	NM_021366:1003	Klf13	INSIDE	1.452	4.175	3133.31	13082.74	6.062	2360.67	14310.05
A_68_P22887154	chr4:46151513-46151557	NM_001033201:152	Ncbp1	INSIDE	1.452	1.423	840.75	1196.44	2.066	754.96	1559.62
A_68_P27230706	chr10:70062237-70062281	NM_178621:-219	Phyhipl	PROMOTER	1.452	2.073	7987.04	16559.84	3.011	6789.13	20438.82
A_68_P32785482	chrX:158155524-158155568	NM_198409:62	Rai2	INSIDE	1.452	4.180	1229.62	5140.23	6.068	905.40	5493.85
A_68_P20157823	chr1:38186758-38186802	NM_019570:-273	Rev1	PROMOTER	1.452	2.296	1303.21	2992.19	3.335	1132.40	3776.31
A_68_P22753435	chr4:16090304-16090348	NM_138952:319	Ripk2	INSIDE	1.452	2.680	1070.14	2867.87	3.891	950.19	3697.39
A_68_P20885441	chr1:184054789-184054833	NM_012058:-57	Srp9	PROMOTER	1.452	2.437	1536.69	3744.66	3.539	1275.05	4512.09
A_68_P24954139	chr7:6084115-6084159	NM_001013012:23437	Zfp787	INSIDE	1.452	2.746	1342.01	3685.47	3.988	1257.91	5016.35
A_68_P30284251	chr15:64752515-64752559	NM_009623:1322	Adcy8	INSIDE	1.451	3.100	1343.62	4165.22	4.499	1107.10	4981.16
A_68_P20886156	chr1:184271169-184271213	NM_175127:547	Fbxo28	INSIDE	1.451	2.477	645.07	1597.68	3.593	543.63	1953.12
A_68_P21840388	chr2:168088389-168088433	NM_001081134:6421	Kcng1	INSIDE	1.451	1.671	942.82	1575.61	2.425	706.71	1713.57
A_68_P25176078	chr7:71432596-71432640	NM_172742:63	Mtmr10	INSIDE	1.451	1.816	781.96	1419.94	2.635	718.56	1893.58
A_68_P23239263	chr4:117933446-117933490	NM_011213:30534	Ptprf	INSIDE	1.451	6.603	3880.11	25620.73	9.581	3001.41	28755.82
A_68_P21476820	chr2:102240650-102240694	NM_020267:385	Trim44	INSIDE	1.451	2.474	373.14	923.15	3.591	352.20	1264.71
A_68_P30570063	chr16:16599690-16599734	ENSMUST00000162671:359		INSIDE	1.451	4.347	10461.80	45473.02	6.307	7454.08	47015.58
A_68_P24816769	chr6:124695789-124695833	NM_007881:10695	Atn1	INSIDE	1.450	6.660	552.01	3676.21	9.657	451.90	4364.14
A_68_P26600872	chr9:67607645-67607689	NM_001081314:423	C2cd4b	INSIDE	1.450	3.347	1542.11	5161.61	4.852	1305.26	6332.76
A_68_P25954643	chr8:73397351-73397395	NM_026964:17	Ccdc124	INSIDE	1.450	1.547	1591.20	2461.74	2.243	1363.71	3058.24
A_68_P24334443	chr6:30846924-30846968	NM_017478:-186	Copg2	PROMOTER	1.450	6.673	2212.72	14766.05	9.679	1733.39	16776.79
A_68_P32092666	chr19:36993233-36993277	NM_028263:835	Fgfbp3	INSIDE	1.450	6.685	1885.91	12606.49	9.692	1657.17	16061.23
A_68_P24610513	chr6:86475289-86475333	NM_011865:849	Pcbp1	INSIDE	1.450	3.509	1375.79	4827.22	5.088	1197.78	6094.21
A_68_P25092562	chr7:52601979-52602025	NM_029741:20387	Ppfia3	INSIDE	1.450	7.101	288.86	2051.21	10.300	297.84	3067.79
A_68_P24090826	chr5:130293163-130293207	NM_133900:-55	Psph	DIVERGENT_PROMOTER	1.450	3.228	1767.44	5705.91	4.680	1507.77	7055.94
A_68_P27325556	chr10:87922749-87922793	NM_011517:439	Sycp3	INSIDE	1.450	9.760	7040.68	68715.96	14.156	4470.72	63288.14
A_68_P25011335	chr7:26473118-26473162	NM_011577:1120	Tgfb1	INSIDE	1.450	4.692	406.37	1906.80	6.804	351.15	2389.26
A_68_P20302822	chr1:66514910-66514954	NM_175510:-88	Unc80	PROMOTER	1.450	1.796	1118.10	2008.23	2.605	909.12	2368.43
A_68_P26084570	chr8:98201548-98201592	ENSMUST00000041318:-414		PROMOTER	1.450	1.955	3405.68	6657.29	2.835	2737.00	7759.89
A_68_P23328301	chr4:134954162-134954206	NM_001168500:-42	A330049M08Rik	PROMOTER	1.449	2.446	11938.32	29200.64	3.544	9050.21	32075.46
A_68_P23264562	chr4:123427164-123427208	NM_023423:356	Akirin1	INSIDE	1.449	3.460	686.55	2375.57	5.014	603.99	3028.44
A_68_P24120341	chr5:136751221-136751265	NM_198602:292033	Cux1	INSIDE	1.449	2.086	434.49	906.44	3.023	389.30	1176.94
A_68_P28072079	chr11:100980744-100980788	NM_026501:372	Fam134c	INSIDE	1.449	2.544	5815.49	14794.36	3.687	3975.99	14659.23
A_68_P27787811	chr11:50105373-50105417	NM_175334:444	Maml1	INSIDE	1.449	1.874	448.97	841.25	2.716	406.52	1103.98
A_68_P31296462	chr17:64950677-64950721	NM_008549:-290	Man2a1	PROMOTER	1.449	3.363	771.63	2595.34	4.873	652.61	3180.49
A_68_P23318794	chr4:133228762-133228806	NM_001145955:-222	Pigv	PROMOTER	1.449	2.193	1112.08	2438.55	3.177	1046.86	3326.16
A_68_P28956736	chr13:37918899-37918943	NM_001039188:-341	Rreb1	PROMOTER	1.449	7.469	673.49	5030.59	10.824	647.42	7007.82
A_68_P21101100	chr2:29918302-29918346	NM_001204875:-3669	Set	PROMOTER	1.449	3.154	2323.74	7328.64	4.570	1936.20	8849.05
A_68_P25618724	chr8:4779472-4779516	NM_011369:40	Shcbp1	INSIDE	1.449	2.534	676.91	1715.14	3.672	595.88	2188.15

ProbeName	Target position of probe on CpG island microarray	TargetID	GeneSymbol	CpG island Description	Ratio of relative methylation (TiO_2-NP/Vehicle)	Sham group			TiO_2-H group		
						Relative methylation (Cy5/Cy3)	Cy3 signal (Input)	Cy5 signal (MeDIP)	Relative methylation (Cy5/Cy3)	Cy3 signal (Input)	Cy5 signal (MeDIP)
A_68_P31259834	chr17:57199854-57199898	NM_025877:-590	Slc25a23	DIVERGENT_PROMOTER	1.449	2.701	1618.44	4371.00	3.912	1334.36	5220.64
A_68_P26828805	chr9:111214091-111214135	NM_001164659:-130	Trank1	PROMOTER	1.449	1.976	1514.65	2993.04	2.863	1318.17	3774.06
A_68_P23195055	chr4:107890802-107890861	NM_001167936:-304	Zyg11a	PROMOTER	1.449	1.839	675.01	1241.37	2.664	614.10	1636.04
A_68_P26016847	chr8:86399178-86399222			Unknown	1.449	2.692	1231.64	3315.53	3.901	1043.50	4070.90
A_68_P21040878	chr2:18610287-18610336	NM_007552:11667	Bmi1	DOWNSTREAM	1.448	3.632	294.61	1069.87	5.260	290.08	1525.82
A_68_P28068816	chr11:100436337-100436381	NM_001146318:-846	Cnp	PROMOTER	1.448	2.750	627.90	1726.48	3.980	661.69	2633.81
A_68_P27480860	chr10:116813708-116813752	NM_001013391:299	Cpsf6	INSIDE	1.448	1.714	752.02	1289.24	2.483	783.49	1945.15
A_68_P25470625	chr7:127986822-127986866	NM_007908:448	Eef2k	INSIDE	1.448	2.070	1084.98	2245.87	2.996	836.11	2505.35
A_68_P23250309	chr4:119940503-119940547	NM_194060:19342	Foxo6	INSIDE	1.448	2.395	1510.25	3616.72	3.469	1410.17	4891.28
A_68_P27284036	chr10:80031664-80031708	NM_078477:8355	Klf16	INSIDE	1.448	4.069	2311.53	9405.84	5.892	1831.43	10791.15
A_68_P21375628	chr2:80421369-80421413	NM_016965:-268	Nckap1	PROMOTER	1.448	2.306	83389.97	192305.90	3.338	68067.69	227227.30
A_68_P31208523	chr17:46849258-46849302	NM_145488:869	Pex6	INSIDE	1.448	1.798	581.56	1045.46	2.602	550.63	1432.93
A_68_P21585947	chr2:122174316-122174360	NM_001013829:20316	Shf	DOWNSTREAM	1.448	2.057	1047.19	2154.30	2.979	855.43	2548.27
A_68_P20372367	chr1:78654608-78654652	NM_001136226:-384	Utp14b	PROMOTER	1.448	3.668	1579.09	5791.38	5.311	1255.14	6665.96
A_68_P25507825	chr7:135020108-135020152	NM_146259:207	Zfp668	INSIDE	1.448	3.589	1357.92	4874.13	5.198	1099.79	5716.33
A_68_P25533783	chr7:139508008-139508052	NR_028578:684	Gm10584	INSIDE	1.447	2.447	855.09	2092.47	3.541	701.10	2482.34
A_68_P23339947	chr4:137024556-137024600	NM_008305:-139	Hspg2	PROMOTER	1.447	2.626	2329.40	6116.84	3.800	1884.70	7162.11
A_68_P25111228	chr7:56501603-56501647	NM_175272:66	Nav2	INSIDE	1.447	2.135	576.28	1230.09	3.089	435.18	1344.10
A_68_P25949903	chr8:72635987-72636031	NM_007789:8735	Ncan	INSIDE	1.447	3.122	2376.67	7419.26	4.517	1958.07	8844.04
A_68_P27835020	chr11:58948880-58948924	NM_001171512:975	Obscn	INSIDE	1.447	1.743	2129.04	3710.06	2.522	1679.01	4233.84
A_68_P30250724	chr15:58721209-58721253	NM_175226:447	Rnf139	INSIDE	1.447	2.050	2787.49	5714.56	2.967	2205.36	6543.37
A_68_P31841968	chr18:76400994-76401038	NM_010754:-562	Smad2	PROMOTER	1.447	2.698	713.64	1925.68	3.906	649.33	2536.17
A_68_P31349753	chr17:74397223-74397267	NM_053188:12	Srd5a2	INSIDE	1.447	1.986	2811.36	5582.34	2.873	2314.99	6652.02
A_68_P27833865	chr11:58791958-58792002	NM_053168:363	Trim11	INSIDE	1.447	2.868	2392.15	6859.83	4.148	2095.30	8691.42
A_68_P23429340	chr4:153516608-153516652	NM_021499:150	Wdr8	INSIDE	1.447	1.822	1735.19	3162.07	2.637	1489.55	3928.05
A_68_P22289849	chr3:84063343-84063387	ENSMUST00000147901:-73		PROMOTER	1.447	1.594	959.99	1529.97	2.306	753.69	1737.68
A_68_P22659832	chr3:153607334-153607378	NM_007382:40	Acadm	INSIDE	1.446	1.899	4802.21	9120.06	2.746	3718.40	10209.88
A_68_P31151211	chr17:34088231-34088275	NM_019420:181	B3galt4	INSIDE	1.446	2.006	3132.92	6285.11	2.900	2383.16	6911.87
A_68_P24982502	chr7:17200404-17200448	NM_172739:-84	Grlf1	PROMOTER	1.446	1.896	1027.80	1948.83	2.742	911.75	2500.28
A_68_P25678159	chr8:15011427-15011471	NM_029116:424	Kbtbd11	INSIDE	1.446	2.572	2572.51	6616.93	3.719	2184.53	8123.62
A_68_P28246310	chr12:12948750-12948795	NM_008709:-130	Mycn	PROMOTER	1.446	4.960	400.32	1985.49	7.170	370.26	2654.57
A_68_P21839353	chr2:167906491-167906535	NM_021409:9	Pard6b	INSIDE	1.446	4.552	2533.08	11530.95	6.580	2153.19	14168.50
A_68_P23271971	chr4:124743753-124743797	NM_175246:-162	Snip1	DIVERGENT_PROMOTER	1.446	3.096	1982.96	6139.75	4.479	1676.90	7509.98
A_68_P25362157	chr7:106632148-106632192	NR_002172:-728	Snord15a	PROMOTER	1.446	7.682	550.34	4227.67	11.109	510.90	5675.68
A_68_P20799884	chr1:168309567-168309611	NM_030245:291	Tada1	INSIDE	1.446	1.471	1502.42	2210.58	2.127	1239.05	2635.54
A_68_P27556189	chr11:4087040-4087084	NM_134023:227	Tbc1d10a	INSIDE	1.446	1.590	1408.80	2239.65	2.298	1044.86	2401.22
A_68_P24117669	chr5:136219992-136220036	NM_172541:28	Tmem120a	INSIDE	1.446	2.355	751.09	1768.70	3.406	556.39	1894.83
A_68_P31501880	chr18:12802925-12802969	NM_028341:905	Ttc39c	INSIDE	1.446	2.736	2149.54	5880.10	3.955	1685.22	6664.96
A_68_P31801896	chr18:69358080-69358124	AK080553:328		INSIDE	1.446	1.594	5420.38	8640.82	2.306	3539.97	8162.16
A_68_P26631263	chr9:72951130-72951174	NM_025890:948	2410004A20Rik	INSIDE	1.445	2.753	1723.42	4744.36	3.977	1485.00	5905.53
A_68_P28183249	chr11:120098316-120098360	NM_198423:4078	Bahcc1	INSIDE	1.445	3.233	1865.23	6030.16	4.672	1598.00	7465.76
A_68_P26613990	chr9:69837096-69837140	NM_016787:-189	Bnip2	PROMOTER	1.445	2.128	1610.94	3428.29	3.075	1296.90	3988.08
A_68_P21084948	chr2:27330582-27330626	NM_023336:314	Brd3	INSIDE	1.445	1.974	1939.64	3827.94	2.851	1524.89	4347.46
A_68_P28109101	chr11:107655547-107655591	NM_019431:210	Cacng4	INSIDE	1.445	2.543	2098.32	5336.33	3.675	1621.04	5956.95
A_68_P31097739	chr17:24643626-24643670	NM_027937:17921	Caskin1	INSIDE	1.445	2.261	3569.09	8069.63	3.267	2568.06	8389.01
A_68_P31253493	chr17:56179827-56179871	NM_013733:10	Chaf1a	INSIDE	1.445	1.436	1683.65	2417.12	2.075	1388.40	2880.67
A_68_P28099218	chr11:105898189-105898233	NM_027946:25	Dcaf7	INSIDE	1.445	1.560	1245.12	1942.89	2.255	1031.77	2327.05
A_68_P21978659	chr3:20054868-20054912	NM_013755:105	Gyg	INSIDE	1.445	3.474	1014.97	3526.07	5.021	916.63	4602.41
A_68_P25574932	chr7:146132973-146133017	NM_028708:562	Jakmip3	INSIDE	1.445	2.076	1303.86	2707.15	3.000	1108.06	3324.63
A_68_P30575876	chr16:17815718-17815762	NM_153790:18366	Scarf2	DOWNSTREAM	1.445	1.579	675.06	1065.85	2.282	643.83	1468.97
A_68_P22086571	chr3:41546266-41546310	NM_001081411:148	Sclt1	INSIDE	1.445	2.216	402.28	891.35	3.201	350.35	1121.43

ProbeName	Target position of probe on CpG island microarray	TargetID	GeneSymbol	CpG island Description	Ratio of relative methylation (TiO_2-NP/Vehicle)	Sham group			TiO_2-H group		
						Relative methylation (Cy5/Cy3)	Cy3 signal (Input)	Cy5 signal (MeDIP)	Relative methylation (Cy5/Cy3)	Cy3 signal (Input)	Cy5 signal (MeDIP)
A_68_P21070644	chr2:25079420-25079464	NM_146116:780	Tubb2c	INSIDE	1.445	3.611	2212.18	7987.44	5.218	1676.25	8746.17
A_68_P24974292	chr7:13623438-13623482	NM_145578:-133	Ube2m	PROMOTER	1.445	2.605	1051.12	2738.68	3.765	888.60	3345.28
A_68_P28221330	chr12:8309173-8309217	NM_013527:-434	Gdf7	PROMOTER	1.444	1.733	2430.71	4211.45	2.501	1763.10	4409.85
A_68_P32454253	chrX:68809673-68809717	NM_008253:527	Hmgb3	INSIDE	1.444	1.957	1150.23	2251.28	2.827	601.95	1701.70
A_68_P20619890	chr1:134086976-134087020	NM_001033250:-1014	Lemd1	PROMOTER	1.444	3.065	1704.52	5224.81	4.427	1248.97	5528.78
A_68_P22373271	chr3:100914318-100914362	NM_011197:-251	Ptgfrn	PROMOTER	1.444	1.657	1507.39	2498.00	2.393	1256.15	3006.40
A_68_P24119330	chr5:136559824-136559868	NM_133914:61	Rasa4	INSIDE	1.444	4.408	4686.10	20657.71	6.364	3329.09	21186.54
A_68_P25186098	chr7:73205391-73205435	NM_021336:191	Snrpa1	INSIDE	1.444	2.115	2902.41	6138.04	3.054	2257.62	6894.17
A_68_P24040430	chr5:121161722-121161766	NM_008052:-66	Dtx1	PROMOTER	1.443	2.128	2505.95	5332.54	3.070	1996.93	6129.86
A_68_P31473580	chr18:6490337-6490381	NM_027497:496	Epc1	INSIDE	1.443	1.556	1551.72	2413.95	2.245	1453.93	3264.77
A_68_P28576808	chr12:81744517-81744561	NM_007951:310	Erh	INSIDE	1.443	4.662	1605.88	7487.33	6.729	1315.59	8852.28
A_68_P26065141	chr8:94882239-94882283	NM_018826:566	Irx5	INSIDE	1.443	1.717	2239.54	3846.36	2.478	1825.56	4524.24
A_68_P32508788	chrX:83437640-83437684	NM_007430:549	Nr0b1	INSIDE	1.443	1.851	606.34	1122.20	2.671	315.94	843.85
A_68_P29753344	chr14:80169969-80170013	NM_001042726:1129	Pcdh8	INSIDE	1.443	1.983	980.90	1944.81	2.861	881.14	2520.64
A_68_P21836026	chr2:167363834-167363878	NM_011427:130	Snai1	INSIDE	1.443	2.092	3308.02	6921.51	3.019	2522.35	7614.93
A_68_P31251408	chr17:55621977-55622021	NM_172829:36984	St6gal2	INSIDE	1.443	2.069	709.30	1467.20	2.985	670.32	2001.04
A_68_P31753941	chr18:61008559-61008603	NM_001198984:38	Tcof1	INSIDE	1.443	1.590	814.67	1295.56	2.294	735.15	1686.43
A_68_P23316767	chr4:132833610-132833654	NM_025667:73	Tmem222	INSIDE	1.443	3.044	915.15	2786.12	4.394	784.52	3447.25
A_68_P29613713	chr14:55305483-55305527	NM_001085473:264	Acin1	INSIDE	1.442	1.827	1544.88	2822.00	2.633	1173.53	3090.32
A_68_P23364702	chr4:141102564-141102608	NR_030695:510	B330016D10Rik	INSIDE	1.442	4.796	2115.56	10146.69	6.917	1843.49	12751.85
A_68_P29055347	chr13:55589235-55589279	NM_001177371:181	Dbn1	INSIDE	1.442	2.776	1106.39	3070.84	4.004	931.15	3727.99
A_68_P30373737	chr15:80502053-80502097	NM_145986:-202	Fam83f	PROMOTER	1.442	1.675	1637.22	2741.83	2.416	1702.71	4112.99
A_68_P24449105	chr6:52161725-52161769	NM_010454:-3123	Hoxa6	PROMOTER	1.442	2.003	491.99	985.61	2.889	402.05	1161.53
A_68_P30267538	chr15:61819548-61819592	NM_001177352:2675	Myc	INSIDE	1.442	1.750	2848.24	4984.12	2.523	2256.37	5693.87
A_68_P21069553	chr2:24909996-24910040	NM_001039386:120	Nelf	INSIDE	1.442	7.550	1426.68	10771.43	10.888	1505.80	16395.05
A_68_P27447374	chr10:110602373-110602417	NM_001003717:537	Osbpl8	INSIDE	1.442	4.572	430.88	1970.03	6.594	395.39	2607.36
A_68_P31958010	chr19:11893171-11893215	NM_011502:191	Stx3	INSIDE	1.442	1.540	927.43	1427.99	2.220	880.02	1953.82
A_68_P27468583	chr10:114688211-114688255	NM_025706:317	Tbc1d15	INSIDE	1.442	2.122	1279.35	2714.54	3.061	1029.32	3150.38
A_68_P25836146	chr8:47556498-47556542	NM_007981:125	Acsl1	INSIDE	1.441	1.816	808.68	1468.43	2.617	740.08	1936.95
A_68_P28616635	chr12:88784339-88784383	NM_001102565:429	Alkbh1	INSIDE	1.441	1.623	2599.48	4219.74	2.339	2111.19	4937.21
A_68_P31624567	chr18:36720291-36720335	NM_175375:56	Ankhd1	INSIDE	1.441	1.737	2296.88	3989.94	2.503	1915.71	4794.36
A_68_P30387839	chr15:83180363-83180407	NM_025445:293	Arfgap3	INSIDE	1.441	4.649	671.96	3123.86	6.698	666.26	4462.37
A_68_P25313630	chr7:97592416-97592460	NM_145151:1148	Crebzf	INSIDE	1.441	2.192	1619.69	3550.02	3.158	1301.54	4110.89
A_68_P23236049	chr4:117354422-117354470	NM_023178:384	Dmap1	INSIDE	1.441	2.227	726.54	1618.20	3.210	514.11	1650.34
A_68_P27284764	chr10:80139113-80139157	NM_021462:-4413	Mknk2	PROMOTER	1.441	1.745	511.83	893.20	2.515	494.33	1243.01
A_68_P31621836	chr18:36228394-36228438	NM_001167891:128398	Nrg2	INSIDE	1.441	2.269	1281.16	2907.25	3.271	1086.22	3552.79
A_68_P29228005	chr13:93962476-93962520	NM_133905:-260	Papd4	PROMOTER	1.441	2.111	1789.75	3778.83	3.042	1418.22	4314.68
A_68_P26949006	chr10:12810713-12810757	NM_009538:141	Plagl1	INSIDE	1.441	2.528	588.30	1486.95	3.642	627.94	2286.66
A_68_P26803290	chr9:106331991-106332035	NM_009082:143	Rpl29	INSIDE	1.441	1.653	6921.88	11443.61	2.382	5791.60	13793.26
A_68_P26574137	chr9:62997201-62997245	NM_001163758:-454	Skor1	PROMOTER	1.441	4.119	751.18	3093.92	5.935	672.30	3989.81
A_68_P28781890	chr12:120087139-120087183	NM_177082:2359	Sp8	INSIDE	1.441	1.650	936.66	1545.46	2.377	825.74	1962.99
A_68_P31943259	chr19:8810305-8810349	NM_134139:-2	Wdr74	PROMOTER	1.441	0.437	1483.47	648.57	0.630	1294.98	815.99
A_68_P23319341	chr4:133308432-133308476	NM_001080819:1072	Arid1a	INSIDE	1.440	2.202	1146.43	2524.79	3.172	1025.72	3253.28
A_68_P29941247	chr14:119254329-119254375	NM_001160099:181	Cldn10	INSIDE	1.440	1.864	525.64	980.02	2.684	425.00	1140.85
A_68_P30115251	chr15:31532021-31532065	NM_026546:172	Fam173b	INSIDE	1.440	2.103	1173.93	2468.83	3.028	1082.18	3276.64
A_68_P25729953	chr8:27086780-27086824	NM_029884:414	Hgsnat	INSIDE	1.440	2.990	1651.60	4938.89	4.306	1338.21	5762.28
A_68_P21395111	chr2:84712344-84712388	NM_199223:14483	Rtn4rl2	INSIDE	1.440	1.981	897.15	1777.13	2.853	846.01	2413.72
A_68_P23457021	chr5:5694320-5694364	NM_001103156:-265	Steap2	PROMOTER	1.440	4.252	2226.24	9466.77	6.124	1932.28	11832.98
A_68_P27644759	chr11:23155418-23155462	NM_001035226:-600	Xpo1	PROMOTER	1.440	1.675	1183.87	1982.94	2.412	966.57	2331.03
A_68_P22833088	chr4:34830058-34830102	NM_013889:117	Zfp292	INSIDE	1.440	2.508	1761.58	4417.80	3.611	1647.46	5948.42
A_68_P27243858	chr10:72562474-72562518	ENSMUST00000053544:295		INSIDE	1.440	1.614	867.98	1400.85	2.324	758.66	1763.30

ProbeName	Target position of probe on CpG island microarray	TargetID	GeneSymbol	CpG island Description	Ratio of relative methylation (TiO_2-NP/Vehicle)	Sham group			TiO_2-H group		
						Relative methylation (Cy5/Cy3)	Cy3 signal (Input)	Cy5 signal (MeDIP)	Relative methylation (Cy5/Cy3)	Cy3 signal (Input)	Cy5 signal (MeDIP)
A_68_P25724840	chr8:26127185-26127229	NM_007404:188	Adam9	INSIDE	1.439	2.381	3131.51	7455.70	3.426	2457.23	8418.00
A_68_P22767955	chr4:19969272-19969316	NM_010281:96	Ggh	INSIDE	1.439	2.289	616.67	1411.79	3.294	582.19	1917.86
A_68_P25285302	chr7:91032885-91032929	NM_030705:-55	Mesdc1	DIVERGENT_PROMOTER	1.439	1.504	835.43	1256.80	2.165	719.20	1556.90
A_68_P22422571	chr3:109945742-109945786	NM_001163348:626	Ntng1	INSIDE	1.439	1.492	3545.15	5287.91	2.146	2603.80	5588.93
A_68_P24265237	chr6:17015070-17015114	NM_207176:-56	Tes	PROMOTER	1.439	2.753	677.63	1865.41	3.961	687.12	2721.79
A_68_P27579285	chr11:8564248-8564292	NM_001083587:268	Tns3	INSIDE	1.439	1.503	2113.83	3176.44	2.163	1622.08	3508.07
A_68_P26696139	chr9:85736432-85736476	NM_001164792:-4	Tpbg	PROMOTER	1.439	2.250	684.18	1539.46	3.239	607.52	1967.46
A_68_P25657380	chr8:11758231-11758275	NM_001113518:-77	Arhgef7	PROMOTER	1.438	1.934	2355.29	4554.16	2.780	1938.44	5388.59
A_68_P28731670	chr12:110291859-110291903	NM_001163175:14547	Begain	INSIDE	1.438	3.009	881.12	2650.91	4.326	738.81	3195.76
A_68_P27996044	chr11:87577886-87577930	NM_172449:3866	Bzrap1	INSIDE	1.438	10.339	1173.75	12135.82	14.864	930.53	13830.94
A_68_P30362471	chr15:78633225-78633269	NM_130859:226	Card10	INSIDE	1.438	2.720	1537.93	4183.67	3.911	1301.14	5088.44
A_68_P21768600	chr2:155782266-155782310	NM_001129999:-5	Cep250	PROMOTER	1.438	4.307	2156.21	9286.15	6.193	1805.15	11179.84
A_68_P22875167	chr4:44085230-44085274	NM_001190414:293	Gne	INSIDE	1.438	2.876	1746.94	5024.47	4.137	1460.08	6040.57
A_68_P31244142	chr17:53707297-53707341	NM_020005:1023	Kat2b	INSIDE	1.438	3.857	906.06	3494.38	5.547	732.76	4064.54
A_68_P30397620	chr15:84754050-84754094	NM_016714:215	Nup50	INSIDE	1.438	4.257	1234.08	5253.86	6.121	1122.13	6868.99
A_68_P22729893	chr4:11413533-11413577	NM_001081183:450	1110037F02Rik	INSIDE	1.437	2.024	921.23	1864.51	2.909	803.47	2337.16
A_68_P21635706	chr2:131388108-131388153	NM_013460:-109	Adra1d	PROMOTER	1.437	1.690	2413.40	4079.07	2.429	1981.92	4814.53
A_68_P26229921	chr8:124474582-124474626	NM_001110100:172	Banp	INSIDE	1.437	5.586	4178.75	23342.39	8.029	3019.79	24245.52
A_68_P33007794	chr4_random:128835-128879	NM_001033326:19055	Dhrsx	INSIDE	1.437	6.540	5179.04	33869.01	9.395	3481.04	32704.98
A_68_P24156001	chr5:144076400-144076444	NM_172726:617	E130309D02Rik	INSIDE	1.437	2.264	1046.72	2369.42	3.253	938.73	3053.43
A_68_P26234163	chr8:125075221-125075265	NM_001037298:-13	Fam38a	PROMOTER	1.437	1.552	1816.29	2818.83	2.230	1572.86	3507.22
A_68_P26348586	chr9:21528050-21528094	NM_010700:35	Ldlr	INSIDE	1.437	2.629	1020.50	2682.53	3.778	774.37	2925.79
A_68_P26640623	chr9:74710266-74710310	NM_008262:561	Onecut1	INSIDE	1.437	4.179	2079.83	8692.42	6.006	1664.86	9999.74
A_68_P32139052	chr19:45619644-45619688	NM_020032:15367	Poll	DOWNSTREAM	1.437	1.557	1450.24	2258.63	2.238	1110.43	2484.79
A_68_P22373269	chr3:100913808-100913852	NM_011197:259	Ptgfrn	INSIDE	1.437	4.539	568.12	2578.63	6.521	543.62	3544.99
A_68_P23395558	chr4:148000660-148000704	NM_001003898:423	Tardbp	INSIDE	1.437	1.704	942.11	1605.42	2.448	850.93	2083.30
A_68_P28747159	chr12:113205331-113205376	NM_027360:-165	2010107E04Rik	DIVERGENT_PROMOTER	1.436	4.443	430.49	1912.54	6.380	361.95	2309.12
A_68_P21040812	chr2:18602535-18602579	NM_007552:3912	Bmi1	INSIDE	1.436	2.230	864.18	1926.75	3.203	773.72	2477.95
A_68_P25782096	chr8:36889804-36889848	NM_027998:-213	Cldn23	PROMOTER	1.436	2.860	7034.43	20115.50	4.106	5042.34	20703.83
A_68_P28291022	chr12:25365866-25365910	NM_001004455:772	Cys1	INSIDE	1.436	6.877	15387.49	105817.20	9.873	10241.94	101120.00
A_68_P28739206	chr12:111839872-111839916	NM_030238:290	Dync1h1	INSIDE	1.436	3.659	1091.47	3993.69	5.255	949.14	4987.40
A_68_P29410660	chr14:13177298-13177342	NM_080433:1059	Fezf2	INSIDE	1.436	1.419	1519.40	2156.27	2.038	1195.93	2437.42
A_68_P31159077	chr17:35516814-35516858	NM_001143689:275	H2-Gs10	INSIDE	1.436	2.060	3376.15	6954.00	2.958	2713.51	8025.89
A_68_P26445380	chr9:40609023-40609067	NM_031165:-311	Hspa8	PROMOTER	1.436	3.428	2047.53	7018.47	4.923	1722.88	8481.76
A_68_P29232482	chr13:94827759-94827803	NM_172589:30	Lhfpl2	INSIDE	1.436	1.601	634.81	1016.46	2.300	807.44	1856.79
A_68_P31066197	chr17:15673753-15673797	NM_008799:-9509	Pdcd2	PROMOTER	1.436	1.850	3237.13	5989.84	2.657	2716.52	7216.98
A_68_P31938929	chr19:7343308-7343352	NM_054048:-923	Rcor2	PROMOTER	1.436	1.360	1602.52	2179.14	1.953	1371.04	2678.03
A_68_P20502873	chr1:107252976-107253020	NM_001160368:289	Rnf152	INSIDE	1.436	2.959	1833.21	5423.92	4.248	1574.50	6688.09
A_68_P23281727	chr4:126412759-126412803	NM_198960:733	Tcfap2e	INSIDE	1.436	2.178	5659.61	12327.52	3.128	4685.75	14657.59
A_68_P31092981	chr17:23813793-23813837	NM_001161746:602	Tnfrsf12a	INSIDE	1.436	1.736	3254.07	5648.40	2.492	2768.70	6900.88
A_68_P22889131	chr4:46520297-46520341	NM_029077:28698	Trim14	INSIDE	1.436	2.670	1020.35	2724.65	3.834	923.57	3541.36
A_68_P20936234	chr1:192921983-192922027	NM_030060:-288	Batf3	PROMOTER	1.435	29.784	1166.38	34739.93	42.739	825.04	35261.56
A_68_P26014851	chr8:85913804-85913848	NM_009904:37	Clgn	INSIDE	1.435	1.940	638.89	1239.70	2.785	561.10	1562.71
A_68_P31259849	chr17:57202154-57202198	NM_177638:477	Crb3	INSIDE	1.435	1.695	586.85	994.42	2.432	501.30	1219.36
A_68_P25416987	chr7:118226039-118226083	NM_001040131:484	Eif4g2	INSIDE	1.435	2.014	1141.51	2299.16	2.890	885.24	2558.19
A_68_P31493065	chr18:11054816-11054860	NM_010258:2331	Gata6	INSIDE	1.435	1.635	861.87	1409.53	2.346	825.43	1936.60
A_68_P31708188	chr18:52688501-52688545	NM_010728:840	Lox	INSIDE	1.435	1.560	899.11	1402.22	2.239	709.78	1588.99
A_68_P24658383	chr6:94649752-94649796	NM_008377:365	Lrig1	INSIDE	1.435	2.307	331.57	764.80	3.311	373.13	1235.34
A_68_P24929770	chr6:146591301-146591345	NM_025315:222	Med21	INSIDE	1.435	5.106	823.78	4206.26	7.325	733.79	5374.87
A_68_P23231034	chr4:116480288-116480332	NM_001159581:-28	Mutyh	DIVERGENT_PROMOTER	1.435	2.306	3347.25	7719.85	3.310	2488.78	8238.76
A_68_P29654920	chr14:62950855-62950899	NM_026001:-65	Rnaseh2b	PROMOTER	1.435	1.636	1693.75	2770.46	2.348	1418.53	3330.13

ProbeName	Target position of probe on CpG island microarray	TargetID	GeneSymbol	CpG island Description	Ratio of relative methylation (TiO_2-NP/Vehicle)	Sham group			TiO_2-H group		
						Relative methylation (Cy5/Cy3)	Cy3 signal (Input)	Cy5 signal (MeDIP)	Relative methylation (Cy5/Cy3)	Cy3 signal (Input)	Cy5 signal (MeDIP)
A_68_P27936386	chr11:77029527-77029571	NM_177710:-378	Ssh2	PROMOTER	1.435	4.453	1711.54	7622.33	6.391	1424.23	9102.77
A_68_P30427170	chr15:89672308-89672352	NM_018803:-39	Syt10	PROMOTER	1.435	1.861	449.45	836.50	2.671	474.25	1266.89
A_68_P26845372	chr9:114472029-114472073	NM_001042503:1437	Trim71	INSIDE	1.435	3.231	613.56	1982.19	4.636	571.03	2647.35
A_68_P23349884	chr4:138686603-138686647	NR_033745:328	2310028O11Rik	INSIDE	1.434	3.733	4615.82	17229.37	5.353	3667.75	19633.56
A_68_P28098903	chr11:105850138-105850182	NM_009598:13639	Ace	INSIDE	1.434	7.534	2074.55	15629.93	10.802	1527.36	16498.62
A_68_P30579184	chr16:18348951-18348995	NM_033474:606	Arvcf	INSIDE	1.434	1.813	2856.70	5178.21	2.600	2245.36	5838.14
A_68_P28836396	chr13:14046811-14046855	NM_178640:-108	B3galnt2	PROMOTER	1.434	1.910	1688.04	3223.64	2.739	1365.70	3741.01
A_68_P27172919	chr10:59649414-59649458	NM_016803:32572	Chst3	INSIDE	1.434	2.741	1198.84	3286.30	3.932	959.44	3772.08
A_68_P25542609	chr7:140968445-140968489	NM_133941:20	Dhx32	INSIDE	1.434	2.159	1087.44	2347.25	3.095	979.77	3032.14
A_68_P22722448	chr4:9771474-9771518	NM_013526:-22	Gdf6	PROMOTER	1.434	1.944	2644.16	5140.52	2.789	2155.11	6009.59
A_68_P22129907	chr3:51287845-51287889	NM_016858:-21	Rab33b	PROMOTER	1.434	2.009	1571.71	3158.03	2.881	1413.96	4073.24
A_68_P28774232	chr12:118772473-118772517	NM_175930:17543	Rapgef5	INSIDE	1.434	2.482	682.68	1694.31	3.559	590.93	2102.97
A_68_P27288599	chr10:80748500-80748544	NM_013769:5490	Tjp3	INSIDE	1.434	2.239	797.76	1786.15	3.210	758.90	2436.44
A_68_P30639250	chr16:30008917-30008961	NR_033631:186	4632428C04Rik	INSIDE	1.433	1.966	1133.92	2229.41	2.818	1021.92	2879.83
A_68_P29560848	chr14:41827140-41827184	NM_027464:-98	5730469M10Rik	PROMOTER	1.433	2.133	3828.39	8165.54	3.056	2835.11	8664.27
A_68_P31108899	chr17:26338048-26338092	NM_008113:225	Arhgdig	INSIDE	1.433	3.283	1886.82	6195.22	4.706	1667.91	7848.59
A_68_P24925470	chr6:145811720-145811764	NM_024469:2118	Bhlhe41	INSIDE	1.433	6.170	1965.15	12124.53	8.843	1757.58	15541.76
A_68_P31098015	chr17:24687028-24687072	NM_013630:156	Pkd1	INSIDE	1.433	1.657	615.84	1020.71	2.376	534.61	1269.97
A_68_P29570101	chr14:45949780-45949824	NM_025959:304	Psmc6	INSIDE	1.433	1.411	1408.37	1987.78	2.022	1043.34	2109.70
A_68_P24061314	chr5:124895322-124895366	NM_030241:5406	Setd8	INSIDE	1.433	2.904	696.58	2023.18	4.163	582.53	2425.26
A_68_P27985577	chr11:85650442-85650486	NM_009324:4348	Tbx2	INSIDE	1.433	1.927	2748.21	5296.65	2.763	2035.37	5622.82
A_68_P23339475	chr4:136913438-136913482	NM_009861:192	Cdc42	INSIDE	1.432	6.373	963.58	6140.62	9.125	784.19	7155.96
A_68_P30459322	chr15:95484732-95484776	NM_207533:448	Dbx2	INSIDE	1.432	1.907	1004.97	1916.43	2.731	945.93	2583.68
A_68_P23236052	chr4:117354732-117354776	NM_023178:76	Dmap1	INSIDE	1.432	1.837	2606.68	4788.42	2.630	2143.06	5635.52
A_68_P22881527	chr4:45197858-45197902	NM_001081172:103	Frmpd1	INSIDE	1.432	1.645	593.32	976.03	2.356	603.73	1422.44
A_68_P32022553	chr19:24355018-24355062	NM_008044:36	Fxn	INSIDE	1.432	3.163	980.13	3099.87	4.530	902.11	4086.42
A_68_P31985486	chr19:17431193-17431237	NM_173442:-57	Gcnt1	PROMOTER	1.432	2.980	4805.40	14321.45	4.267	3835.65	16366.47
A_68_P32454254	chrX:68810106-68810150	NM_008253:961	Hmgb3	INSIDE	1.432	2.964	561.61	1664.78	4.246	395.17	1677.91
A_68_P25730818	chr8:27229559-27229603	NM_207659:116	Hook3	INSIDE	1.432	1.751	589.68	1032.40	2.508	525.81	1318.73
A_68_P29469016	chr14:24824250-24824294	NM_010610:-845	Kcnma1	PROMOTER	1.432	2.251	1908.32	4294.72	3.223	1621.56	5226.01
A_68_P27282833	chr10:79844657-79844701	NM_198615:5718	Mex3d	INSIDE	1.432	1.680	460.08	772.90	2.405	345.08	830.04
A_68_P32118288	chr19:42055682-42055726	NM_028152:-78	Mms19	DIVERGENT_PROMOTER	1.432	1.902	1328.02	2526.36	2.724	1108.91	3020.85
A_68_P26015449	chr8:86090778-86090822	NM_025843:144	Ndufb7	INSIDE	1.432	1.721	978.95	1684.31	2.463	824.08	2030.04
A_68_P24360134	chr6:35127844-35127888	NM_027513:251	Nup205	INSIDE	1.432	2.248	1180.52	2653.29	3.218	985.48	3171.57
A_68_P23320011	chr4:133443491-133443535	NM_009097:202	Rps6ka1	INSIDE	1.432	2.188	964.88	2111.38	3.133	907.19	2842.10
A_68_P31254020	chr17:56263726-56263770	NM_013662:9527	Sema6b	INSIDE	1.432	4.319	3412.90	14741.84	6.187	2822.02	17459.94
A_68_P26743637	chr9:95650689-95650733	NM_011643:67	Trpc1	INSIDE	1.432	4.306	1965.66	8463.87	6.165	1585.33	9772.83
A_68_P29958337	chr14:122277966-122278010	NM_026861:161	Ubac2	INSIDE	1.432	2.751	1292.75	3555.77	3.939	1094.81	4312.57
A_68_P27268624	chr10:76978491-76978535	NM_133998:46	1810008A18Rik	INSIDE	1.431	0.469	1440.40	675.72	0.671	1259.75	845.41
A_68_P30482000	chr15:99556370-99556414	NM_183256:344	2310016M24Rik	INSIDE	1.431	1.868	2886.15	5391.15	2.673	2223.93	5944.40
A_68_P25608860	chr7:151924429-151924473	NM_178642:-67	Ano1	PROMOTER	1.431	2.740	1442.97	3953.40	3.920	1301.36	5100.80
A_68_P28082435	chr11:102808047-102808091	NM_011795:-293	C1ql1	PROMOTER	1.431	2.491	1229.97	3064.36	3.565	979.07	3490.33
A_68_P32454734	chrX:68917679-68917723	NM_010340:759	Gpr50	INSIDE	1.431	3.037	1714.79	5208.11	4.346	951.26	4133.82
A_68_P31110190	chr17:26568998-26569042	NM_001081656:17111	Neurl1b	INSIDE	1.431	2.158	1000.89	2160.10	3.089	786.14	2428.58
A_68_P24815137	chr6:124364678-124364722	NM_008995:385	Pex5	INSIDE	1.431	2.394	3352.00	8025.03	3.426	2428.36	8319.20
A_68_P23524108	chr5:20561799-20561843	NM_011203:-205	Ptpn12	PROMOTER	1.431	2.471	4084.29	10094.31	3.535	3490.85	12341.85
A_68_P29855888	chr14:102007522-102007566	NM_001081278:864	Tbc1d4	INSIDE	1.431	1.773	894.75	1586.73	2.537	710.50	1802.46
A_68_P30558456	chr16:13672419-13672463	NM_025653:328	3110001I22Rik	INSIDE	1.430	1.971	580.42	1144.12	2.818	492.14	1386.89
A_68_P24631552	chr6:90259348-90259392	NM_027928:15809	Chst13	INSIDE	1.430	2.267	383.03	868.53	3.243	324.29	1051.80
A_68_P29671620	chr14:65716836-65716880	NM_018788:85	Extl3	INSIDE	1.430	2.093	867.26	1815.24	2.993	790.34	2365.67
A_68_P26905361	chr10:4540405-4540449	NM_025995:-649	Fbxo5	PROMOTER	1.430	1.765	2432.62	4293.20	2.523	1969.64	4970.35

ProbeName	Target position of probe on CpG island microarray	TargetID	GeneSymbol	CpG island Description	Ratio of relative methylation (TiO_2-NP/Vehicle)	Sham group			TiO_2-H group		
						Relative methylation (Cy5/Cy3)	Cy3 signal (Input)	Cy5 signal (MeDIP)	Relative methylation (Cy5/Cy3)	Cy3 signal (Input)	Cy5 signal (MeDIP)
A_68_P29631505	chr14:58694946-58694991	NM_013518:3446	Fgf9	INSIDE	1.430	1.519	1530.88	2325.70	2.173	1248.57	2712.79
A_68_P26406147	chr9:32348923-32348967	NM_008026:9	Fli1	INSIDE	1.430	10.643	1659.07	17657.64	15.216	1332.51	20275.29
A_68_P31215016	chr17:48061356-48061400	NM_001110824:203	Foxp4	INSIDE	1.430	2.065	544.46	1124.39	2.952	431.86	1274.98
A_68_P28307233	chr12:28023729-28023773	NM_009234:3833	Sox11	INSIDE	1.430	1.917	1761.58	3377.42	2.741	1572.18	4309.91
A_68_P20878653	chr1:182872689-182872733	NM_144794:62	Tmem63a	INSIDE	1.430	3.461	1110.82	3844.24	4.949	1171.34	5796.88
A_68_P23296885	chr4:129168578-129168622	NM_001085491:167	1700125D06Rik	INSIDE	1.429	3.440	2205.27	7585.94	4.916	1839.76	9044.09
A_68_P28599343	chr12:85958262-85958306	NM_008992:132	Abcd4	INSIDE	1.429	2.059	496.59	1022.34	2.942	438.31	1289.71
A_68_P29080189	chr13:59658365-59658409	NM_023328:294	Agtpbp1	INSIDE	1.429	2.420	614.39	1486.84	3.458	529.07	1829.46
A_68_P32260333	chrX:11664076-11664120	NM_029510:-6419	Bcor	PROMOTER	1.429	1.463	2105.42	3079.95	2.091	1135.40	2373.82
A_68_P31369865	chr17:78599190-78599234	NM_015800:-375	Crim1	PROMOTER	1.429	3.631	2214.04	8039.99	5.188	1981.30	10278.47
A_68_P27212423	chr10:66998199-66998243	NM_010118:-2396	Egr2	PROMOTER	1.429	1.970	2569.89	5061.85	2.814	2176.70	6125.52
A_68_P27561404	chr11:4998804-4998848	NM_007968:254	Ewsr1	INSIDE	1.429	2.514	1332.85	3351.21	3.594	1126.92	4050.26
A_68_P24038706	chr5:120886174-120886218	NM_008499:4302	Lhx5	INSIDE	1.429	3.656	1390.51	5084.34	5.225	1145.84	5987.14
A_68_P30370814	chr15:80042595-80042639	NM_010795:38466	Mgat3	INSIDE	1.429	3.499	1181.13	4133.12	5.000	930.76	4653.43
A_68_P31207194	chr17:46624614-46624658	NM_001162864:-12	Ttbk1	DIVERGENT_PROMOTER	1.429	4.060	5421.37	22008.46	5.802	4083.42	23694.01
A_68_P26168806	chr8:114060945-114060989	NM_001168623:-1276	Znrf1	PROMOTER	1.429	1.938	1653.27	3204.55	2.771	1338.26	3707.84
A_68_P20370563	chr1:78214303-78214348			Unknown	1.429	1.638	1002.23	1641.76	2.341	768.30	1798.39
A_68_P25465270	chr7:126887848-126887892	ENSMUST00000004569:4431		DOWNSTREAM	1.429	2.309	971.07	2241.91	3.300	817.53	2697.78
A_68_P21977359	chr3:19795207-19795251	NR_027985:358	4632415L05Rik	INSIDE	1.428	1.482	1056.60	1565.64	2.115	866.83	1833.64
A_68_P21745336	chr2:151527951-151527995	NM_001160363:229	5430405G05Rik	INSIDE	1.428	9.637	702.25	6767.77	13.762	645.46	8882.48
A_68_P23542075	chr5:23980487-23980531	NM_139153:22514	Agap3	INSIDE	1.428	4.539	342.01	1552.25	6.483	350.72	2273.86
A_68_P24615404	chr6:87285370-87285414	NM_054041:377	Antxr1	INSIDE	1.428	3.198	3444.45	11013.90	4.568	2856.12	13045.70
A_68_P26022317	chr8:87381112-87381157	NM_025648:250	Farsa	INSIDE	1.428	1.769	496.37	878.07	2.527	424.29	1072.13
A_68_P23582164	chr5:32439192-32439236	NM_008037:370	Fosl2	INSIDE	1.428	2.722	2139.46	5824.04	3.888	2060.63	8011.77
A_68_P24817421	chr6:124811227-124811271	NM_013533:2687	Gpr162	INSIDE	1.428	3.167	1147.31	3633.33	4.524	916.44	4145.62
A_68_P26992095	chr10:21016178-21016222	NM_001145209:416	Hbs1l	INSIDE	1.428	1.591	2034.89	3237.98	2.272	1469.59	3339.49
A_68_P29154201	chr13:76521736-76521780	NM_030174:-650	Mctp1	PROMOTER	1.428	1.812	399.29	723.68	2.588	329.09	851.68
A_68_P30376706	chr15:81020912-81020956	NM_001082536:253	Mkl1	INSIDE	1.428	1.494	2829.65	4228.59	2.135	2432.01	5191.43
A_68_P26319360	chr9:14589324-14589368	NM_018736:196	Mre11a	INSIDE	1.428	4.375	4559.98	19951.51	6.246	3897.17	24342.17
A_68_P32565875	chrX:99045611-99045655	NM_001163610:909	Nhsl2	INSIDE	1.428	2.674	1582.43	4230.92	3.819	1220.26	4660.05
A_68_P21904290	chr2:180234531-180234575	NM_018766:-128	Ntsr1	PROMOTER	1.428	3.278	1431.59	4693.16	4.682	1179.37	5522.04
A_68_P22282897	chr3:82859249-82859293	NM_016784:-189	Plrg1	DIVERGENT_PROMOTER	1.428	1.983	3661.91	7262.64	2.832	2941.09	8329.96
A_68_P21152642	chr2:39046414-39046458	NM_178778:-186	Scai	PROMOTER	1.428	3.775	2173.82	8206.59	5.390	1657.02	8931.67
A_68_P25957635	chr8:74108819-74108863	NM_011977:16015	Slc27a1	INSIDE	1.428	1.924	591.76	1138.79	2.748	566.17	1555.89
A_68_P20218124	chr1:50983912-50983956	NM_019790:-432	Tmeff2	PROMOTER	1.428	1.552	2887.89	4483.16	2.217	2124.27	4710.17
A_68_P32217690	chr19:59244706-59244750	NM_009501:-209	Vax1	PROMOTER	1.428	2.418	3335.67	8065.71	3.453	2981.87	10297.34
A_68_P24594973	chr6:83652821-83652865	NR_002873:8084	Vax2os1	INSIDE	1.428	2.156	1206.72	2602.19	3.079	1009.76	3108.97
A_68_P30214226	chr15:51871837-51871881	NM_175503:206	Aard	INSIDE	1.427	1.916	304.81	584.06	2.733	277.47	758.45
A_68_P28743039	chr12:112510049-112510093	NM_033603:749	Amn	INSIDE	1.427	1.669	1149.82	1918.80	2.381	1011.30	2408.18
A_68_P32576282	chrX:101395577-101395621	NM_001077354:858	C77370	INSIDE	1.427	3.457	1964.90	6793.41	4.933	1171.01	5776.06
A_68_P25806164	chr8:41596758-41596802	NM_011135:-122	Cnot7	DIVERGENT_PROMOTER	1.427	2.515	7808.35	19635.50	3.588	5707.77	20481.14
A_68_P32539668	chrX:91881915-91881959	NM_008179:529	Gspt2	INSIDE	1.427	3.643	222.73	811.43	5.200	158.32	823.29
A_68_P30345339	chr15:75824110-75824154	NM_177922:-66	Mapk15	PROMOTER	1.427	1.919	786.89	1509.80	2.738	701.70	1921.46
A_68_P24981707	chr7:17061268-17061312	NM_008718:839	Npas1	INSIDE	1.427	3.402	567.40	1930.32	4.854	516.92	2509.31
A_68_P26521207	chr9:53345172-53345216	NM_001081152:43	Npat	INSIDE	1.427	2.439	1302.14	3175.88	3.481	978.04	3404.83
A_68_P32745696	chrX:147955251-147955299	NM_177201:60	Phf8	INSIDE	1.427	2.556	706.50	1805.96	3.647	342.37	1248.75
A_68_P31140484	chr17:31702098-31702142	NM_016670:403	Pknox1	INSIDE	1.427	2.201	875.94	1927.71	3.141	748.66	2351.44
A_68_P21132198	chr2:35056008-35056052	NM_026697:610	Rab14	INSIDE	1.427	3.344	1140.50	3814.24	4.773	998.62	4766.42
A_68_P32237966	chrX:6895899-6895943	ENSMUST00000132126:376		INSIDE	1.427	2.569	326.36	838.34	3.665	265.67	973.79
A_68_P29092064	chr13:63341467-63341511	NM_028079:225195	2010111I01Rik	INSIDE	1.426	2.239	3623.94	8114.95	3.193	2653.34	8472.99
A_68_P22862943	chr4:41516655-41516699	NM_001159583:-116	2310028H24Rik	DIVERGENT_PROMOTER	1.426	1.638	902.59	1478.41	2.335	741.87	1732.59

ProbeName	Target position of probe on CpG island microarray	TargetID	GeneSymbol	CpG island Description	Ratio of relative methylation (TiO_2-NP/Vehicle)	Sham group			TiO_2-H group		
						Relative methylation (Cy5/Cy3)	Cy3 signal (Input)	Cy5 signal (MeDIP)	Relative methylation (Cy5/Cy3)	Cy3 signal (Input)	Cy5 signal (MeDIP)
A_68_P27835441	chr11:59015919-59015963	NR_037964:-7	2610507I01Rik	DIVERGENT_PROMOTER	1.426	1.494	5723.64	8553.26	2.131	4391.28	9355.86
A_68_P31154762	chr17:34742611-34742655	NM_001163379:-173	Agpat1	DIVERGENT_PROMOTER	1.426	1.883	1330.03	2504.28	2.685	1020.33	2739.42
A_68_P27902137	chr11:70501182-70501226	NM_001190376:403	Camta2	INSIDE	1.426	1.816	1627.44	2955.45	2.590	1387.32	3593.52
A_68_P30483526	chr15:99869552-99869596	NM_001159361:480	Dip2b	INSIDE	1.426	2.682	882.59	2367.54	3.824	733.53	2805.01
A_68_P23203783	chr4:109656467-109656511	NM_172296:5859	Dmrta2	DOWNSTREAM	1.426	5.320	1612.88	8579.90	7.583	1244.48	9437.45
A_68_P28915538	chr13:30228234-30228278	NM_153546:-102	Mboat1	PROMOTER	1.426	1.618	656.21	1061.57	2.306	544.40	1255.51
A_68_P31257221	chr17:56743113-56743157	NM_001163300:18730	Safb	INSIDE	1.426	1.822	1742.72	3175.71	2.599	1293.65	3362.09
A_68_P26544601	chr9:57788261-57788305	NM_011352:341	Sema7a	INSIDE	1.426	2.228	1064.64	2371.54	3.176	854.68	2714.05
A_68_P28908962	chr13:29043905-29043949	NM_009238:1625	Sox4	INSIDE	1.426	2.876	1566.80	4505.62	4.102	1228.16	5037.66
A_68_P24912677	chr6:143781866-143781912	NM_001113559:376190	Sox5	INSIDE	1.426	1.675	487.02	815.64	2.388	383.16	915.04
A_68_P31253704	chr17:56213984-56214028	NM_024432:406	Ubxn6	INSIDE	1.426	2.904	13803.75	40089.43	4.142	9650.79	39974.27
A_68_P29247842	chr13:97518179-97518223			Unknown	1.426	3.100	3663.27	11355.10	4.420	3179.69	14054.99
A_68_P33005882	A_68_P33005882			Unknown	1.426	3.817	528.30	2016.67	5.443	431.27	2347.38
A_68_P23288223	chr4:127665242-127665286	ENSMUST00000144298:-23		PROMOTER	1.426	2.757	870.54	2400.34	3.933	774.97	3048.08
A_68_P31053612	chr17:12935271-12935317	NR_002853:1118	Airn	INSIDE	1.425	1.664	1280.94	2130.98	2.371	1005.54	2384.52
A_68_P27855080	chr11:62461500-62461544	NM_198861:503	BC046404	INSIDE	1.425	1.607	2660.83	4276.27	2.290	2054.16	4703.79
A_68_P28865538	chr13:20182700-20182744	NM_080288:347	Elmo1	INSIDE	1.425	3.480	2282.78	7943.04	4.958	1633.16	8097.72
A_68_P28814402	chr13:9093309-9093353	NM_172585:180	Larp4b	INSIDE	1.425	4.285	719.83	3084.31	6.105	641.17	3914.29
A_68_P31934662	chr19:6428439-6428483	NM_001205234:9723	Nrxn2	INSIDE	1.425	3.398	357.26	1213.98	4.843	285.18	1381.15
A_68_P25610029	chr7:152101203-152101247	NM_028184:126	Oraov1	INSIDE	1.425	1.363	1473.54	2008.57	1.943	1225.30	2380.48
A_68_P31929908	chr19:5654240-5654284	NM_001164480:9445	Sipa1	INSIDE	1.425	2.172	973.46	2114.71	3.096	854.47	2645.64
A_68_P30544728	chr16:10835113-10835157	NM_001162932:-17	A630055G03Rik	PROMOTER	1.424	4.746	1288.36	6115.03	6.759	1085.06	7334.04
A_68_P21576975	chr2:120556771-120556815	NM_026891:461	Cdan1	INSIDE	1.424	1.974	465.11	918.33	2.811	412.87	1160.65
A_68_P29361890	chr13:118009924-118009968	NM_010330:567	Emb	INSIDE	1.424	1.512	1520.73	2299.62	2.153	1205.35	2595.48
A_68_P32383370	chrX:49967260-49967304	NM_016697:-131	Gpc3	PROMOTER	1.424	3.095	9985.31	30905.65	4.408	4300.38	18956.13
A_68_P26220850	chr8:123061186-123061230	NM_001145896:-100	Gse1	PROMOTER	1.424	2.484	842.06	2092.04	3.537	662.22	2342.54
A_68_P25264894	chr7:87260253-87260297	NM_173011:-38	Idh2	PROMOTER	1.424	3.376	660.39	2229.50	4.806	570.14	2740.19
A_68_P31863637	chr18:80519734-80519778	NM_001190373:41237	Kcng2	INSIDE	1.424	4.185	4794.85	20068.50	5.960	3894.16	23210.21
A_68_P23837519	chr5:81449181-81449225	NM_198702:-1415	Lphn3	PROMOTER	1.424	3.380	834.56	2820.42	4.813	670.50	3227.44
A_68_P20937430	chr1:193141794-193141838	NM_025424:181	Nenf	INSIDE	1.424	3.519	756.69	2663.14	5.011	634.83	3181.09
A_68_P23637083	chr5:42099031-42099075	NM_027295:342	Rab28	INSIDE	1.424	2.210	737.77	1630.38	3.146	643.81	2025.58
A_68_P31836528	chr18:75526822-75526866	NM_001042660:-174	Smad7	PROMOTER	1.424	2.291	514.95	1179.88	3.263	471.35	1538.00
A_68_P27709784	chr11:35793975-35794019	NM_170779:-405	Wwc1	PROMOTER	1.424	2.341	284.11	665.18	3.334	258.24	861.07
A_68_P21290382	chr2:65962425-65962469	NM_015736:404	Galnt3	INSIDE	1.423	2.078	874.59	1817.17	2.956	765.48	2262.47
A_68_P26587426	chr9:65239326-65239370	NM_028974:111	Kbtbd13	INSIDE	1.423	1.711	395.84	677.41	2.435	347.04	845.19
A_68_P21089694	chr2:28061599-28061643	NM_001038612:412	Olfm1	INSIDE	1.423	2.364	1458.26	3447.64	3.365	1185.01	3987.49
A_68_P20053923	chr1:16095671-16095715	NM_133832:-270	Rdh10	DIVERGENT_PROMOTER	1.423	2.090	560.23	1170.67	2.974	551.98	1641.71
A_68_P30140493	chr15:36722578-36722622	NM_011740:1693	Ywhaz	INSIDE	1.423	1.348	2080.18	2803.46	1.918	1581.01	3032.76
A_68_P30245918	chr15:57907631-57907675	NM_001042438:392	Zhx1	INSIDE	1.423	2.738	938.73	2570.11	3.897	833.40	3247.82
A_68_P26074975	chr8:96535903-96535947	NM_011787:616	Amfr	INSIDE	1.422	1.492	1949.22	2908.74	2.123	1624.85	3449.13
A_68_P24046040	chr5:122161910-122161954	NM_009125:315	Atxn2	INSIDE	1.422	2.027	1749.81	3547.00	2.882	1488.38	4290.02
A_68_P32260262	chrX:11655805-11655849	NM_029510:1853	Bcor	INSIDE	1.422	1.371	1911.85	2621.27	1.949	979.23	1908.57
A_68_P25095390	chr7:53088658-53088702	NM_008172:33371	Grin2d	INSIDE	1.422	3.563	1262.40	4498.44	5.067	1114.50	5646.78
A_68_P29613087	chr14:55194747-55194791	NM_010590:1730	Jub	INSIDE	1.422	2.416	2460.45	5944.98	3.436	1982.73	6812.18
A_68_P28095285	chr11:105153865-105153909	NM_008626:-73	Mrc2	PROMOTER	1.422	4.225	3447.09	14562.36	6.007	2430.85	14601.31
A_68_P25092625	chr7:52613255-52613299	NM_029741:9113	Ppfia3	INSIDE	1.422	3.194	663.98	2120.53	4.542	530.13	2407.97
A_68_P25500162	chr7:133520602-133520646	NM_023712:824	Spns1	INSIDE	1.422	2.871	572.14	1642.66	4.083	501.94	2049.58
A_68_P31187133	chr17:43153476-43153520	NM_178589:-5	Tnfrsf21	PROMOTER	1.422	1.782	735.33	1310.66	2.534	663.27	1680.81
A_68_P27365515	chr10:94978265-94978309	NM_080560:491	Ube2n	INSIDE	1.422	3.809	830.13	3161.90	5.418	722.87	3916.47
A_68_P27186307	chr10:61949576-61949620	NM_133672:-45	Vps26a	PROMOTER	1.422	3.902	1404.97	5482.65	5.548	1070.91	5941.57
A_68_P30345267	chr15:75812246-75812290	NM_001163518:447	2410075B13Rik	INSIDE	1.421	1.377	1690.90	2327.70	1.956	1348.63	2637.73

ProbeName	Target position of probe on CpG island microarray	TargetID	GeneSymbol	CpG island Description	Ratio of relative methylation (TiO_2-NP/Vehicle)	Sham group			TiO_2-H group		
						Relative methylation (Cy5/Cy3)	Cy3 signal (Input)	Cy5 signal (MeDIP)	Relative methylation (Cy5/Cy3)	Cy3 signal (Input)	Cy5 signal (MeDIP)
A_68_P26989977	chr10:20672484-20672528	NM_026203:154	Ahi1	INSIDE	1.421	3.099	796.25	2467.77	4.405	848.36	3737.14
A_68_P26841767	chr9:113650601-113650645	NM_001114347:32	Clasp2	INSIDE	1.421	2.491	1132.02	2820.18	3.541	996.51	3528.69
A_68_P23222201	chr4:114574854-114574898	NM_008593:6627	Foxd2	DOWNSTREAM	1.421	3.263	625.49	2040.92	4.638	580.94	2694.51
A_68_P23812523	chr5:76373731-76373775	NM_010612:701	Kdr	INSIDE	1.421	1.552	911.79	1415.15	2.206	757.69	1671.14
A_68_P30378912	chr15:81494223-81494269	NM_145993:-119	L3mbtl2	PROMOTER	1.421	1.964	1468.91	2884.74	2.790	1163.64	3247.14
A_68_P24816267	chr6:124612992-124613036	NM_145130:-107	Lpcat3	PROMOTER	1.421	2.819	6204.39	17491.88	4.007	5172.70	20725.45
A_68_P30382866	chr15:82169438-82169482	NM_008669:-204	Naga	DIVERGENT_PROMOTER	1.421	2.621	903.69	2368.91	3.726	716.73	2670.73
A_68_P27551120	chr11:3189836-3189880	NM_019574:-601	Patz1	PROMOTER	1.421	2.600	566.69	1473.37	3.694	434.89	1606.33
A_68_P30651715	chr16:32277893-32277937	NM_027355:368	Rnf168	INSIDE	1.421	1.480	1228.71	1819.02	2.104	1036.11	2180.33
A_68_P22392367	chr3:104442522-104442566	NM_009196:-46	Slc16a1	PROMOTER	1.421	1.538	3067.84	4719.16	2.186	2499.13	5462.55
A_68_P23607900	chr5:36826413-36826457	NM_001170454:500	Tada2b	INSIDE	1.421	2.162	4646.76	10045.98	3.073	3440.19	10570.87
A_68_P26542618	chr9:57437330-57437374	NM_027895:94	Ulk3	INSIDE	1.421	2.757	987.59	2722.90	3.917	924.14	3620.05
A_68_P22953712	chr4:58956665-58956709	NM_001099323:187	Zkscan16	INSIDE	1.421	2.680	1467.68	3933.53	3.809	1115.69	4249.89
A_68_P29388382	A_68_P29388382			Unknown	1.421	5.787	645.09	3733.38	8.222	598.38	4919.56
A_68_P26872167	chr9:119311485-119311529	NM_007397:-112	Acvr2b	PROMOTER	1.420	1.894	306.96	581.52	2.690	293.65	789.98
A_68_P23990789	chr5:112656382-112656426	NR_003718:1564	Miat	INSIDE	1.420	3.657	3521.20	12877.97	5.195	2718.77	14122.74
A_68_P26144000	chr8:109818386-109818430	NM_018823:1039	Nfat5	INSIDE	1.420	1.406	989.41	1390.67	1.995	906.75	1809.35
A_68_P32071922	chr19:32831644-32831688	NM_008960:-400	Pten	PROMOTER	1.420	3.731	1652.64	6166.00	5.299	1164.64	6171.60
A_68_P26038364	chr8:90484174-90484218	NM_033327:-702	Zfp423	PROMOTER	1.420	2.690	1218.54	3278.46	3.821	1085.88	4149.67
A_68_P22888266	chr4:46358077-46358121	NM_183298:1033	Foxe1	INSIDE	1.419	2.861	1769.83	5064.28	4.061	1543.70	6269.47
A_68_P22885282	chr4:45839676-45839720	NM_018741:1	Igfbpl1	INSIDE	1.419	1.847	961.74	1776.69	2.622	899.33	2357.68
A_68_P25842953	chr8:48761063-48761107	NM_023503:-571	Ing2	PROMOTER	1.419	1.707	607.79	1037.38	2.422	540.57	1309.40
A_68_P26770182	chr9:100446024-100446068	NM_010878:426	Nck1	INSIDE	1.419	1.911	1209.36	2311.66	2.713	970.35	2632.31
A_68_P20282814	chr1:62750008-62750052	NM_001077406:140	Nrp2	INSIDE	1.419	1.496	2768.18	4140.50	2.123	2068.88	4392.13
A_68_P31962487	chr19:12869271-12869315	NR_024093:1321	U05342	INSIDE	1.419	3.008	2234.52	6720.66	4.267	1880.57	8024.34
A_68_P22833091	chr4:34830379-34830423	NM_013889:-203	Zfp292	PROMOTER	1.419	2.359	1941.85	4580.08	3.346	1615.31	5405.37
A_68_P24602878	chr6:85019144-85019188	NM_177077:341	Exoc6b	INSIDE	1.418	2.092	1503.87	3146.46	2.967	1052.23	3121.95
A_68_P24591430	chr6:83052100-83052144	NM_026744:-6979	Mrpl53	PROMOTER	1.418	2.520	513.50	1294.26	3.575	510.65	1825.60
A_68_P26949004	chr10:12810446-12810490	NM_009538:-125	Plagl1	PROMOTER	1.418	2.735	620.49	1697.16	3.880	532.72	2066.77
A_68_P28567253	chr12:80130122-80130166	NM_181073:-5	Plekhh1	PROMOTER	1.418	3.469	750.42	2603.38	4.918	614.61	3022.44
A_68_P23441284	chr4:155265872-155265916	NM_001033490:-23	Pusl1	DIVERGENT_PROMOTER	1.418	2.215	1826.83	4047.07	3.142	1459.89	4586.74
A_68_P30348873	chr15:76350063-76350107	NM_130893:2475	Scrt1	INSIDE	1.418	2.237	2107.41	4714.76	3.172	1649.29	5230.77
A_68_P27807186	chr11:53705037-53705081	NM_011396:147	Slc22a5	INSIDE	1.418	1.943	3064.09	5952.44	2.755	2449.95	6749.51
A_68_P26142730	chr8:109555781-109555825	NM_126165:577	Vps4a	INSIDE	1.418	3.159	742.40	2345.28	4.479	589.76	2641.60
A_68_P22715817	chr4:8619031-8619075	NM_001081417:985	Chd7	INSIDE	1.417	1.514	9548.50	14453.91	2.144	6606.79	14167.87
A_68_P27924600	chr11:74983847-74983891	NM_010430:-111	Hic1	DIVERGENT_PROMOTER	1.417	2.286	1116.06	2550.97	3.238	884.80	2865.09
A_68_P20614486	chr1:133141955-133141999	NM_018750:-221	Rassf5	PROMOTER	1.417	2.281	1232.45	2811.73	3.234	1041.72	3368.61
A_68_P27842503	chr11:60166349-60166393	NM_001039092:37	Tom1l2	INSIDE	1.417	2.063	4182.55	8628.75	2.923	3261.48	9534.34
A_68_P24162463	chr5:145529030-145529074	NM_001081362:-608	Trrap	DIVERGENT_PROMOTER	1.417	3.214	3512.09	11288.62	4.556	2712.99	12359.52
A_68_P25663081	chr8:12672535-12672579	NM_198031:-456	Tubgcp3	PROMOTER	1.417	3.452	2330.06	8043.53	4.891	1945.10	9514.34
A_68_P32751543	chrX:149442021-149442065	NM_001137547:1031	Usp51	INSIDE	1.417	3.561	1928.35	6866.94	5.047	1138.77	5746.86
A_68_P24536898	chr6:71494106-71494150	NM_025783:281	Vps24	INSIDE	1.417	2.320	708.74	1644.28	3.287	567.62	1865.79
A_68_P26074978	chr8:96536308-96536352	NM_011787:210	Amfr	INSIDE	1.416	3.311	1388.52	4596.77	4.687	1274.11	5971.61
A_68_P24062297	chr5:125079741-125079785	NM_011596:115	Atp6v0a2	INSIDE	1.416	1.559	562.67	877.39	2.208	554.78	1224.70
A_68_P29287628	chr13:104711178-104711222	NM_001005868:-606	Erbb2ip	PROMOTER	1.416	1.835	1257.68	2308.12	2.599	1169.33	3039.51
A_68_P25730817	chr8:27229449-27229493	NM_207659:226	Hook3	INSIDE	1.416	2.553	3049.89	7785.12	3.616	2441.25	8826.48
A_68_P32016043	chr19:23210311-23210355	NM_010638:-5383	Klf9	PROMOTER	1.416	4.067	888.34	3613.11	5.759	697.02	4013.92
A_68_P26540423	chr9:56978779-56978823	NM_028636:217	Man2c1	INSIDE	1.416	2.713	653.92	1774.18	3.843	629.24	2417.98
A_68_P25953728	chr8:73267438-73267482	NM_201607:19498	Pde4c	INSIDE	1.416	2.036	858.33	1747.23	2.882	833.15	2401.31
A_68_P32575424	chrX:101176475-101176519	NM_011276:127	Rlim	INSIDE	1.416	2.853	8693.10	24805.08	4.042	6236.87	25207.16
A_68_P21704898	chr2:143837238-143837282	NM_024281:-261	Rrbp1	PROMOTER	1.416	2.615	1426.50	3730.43	3.704	1309.78	4851.46

ProbeName	Target position of probe on CpG island microarray	TargetID	GeneSymbol	CpG island Description	Ratio of relative methylation (TiO_2-NP/Vehicle)	Sham group			TiO_2-H group		
						Relative methylation (Cy5/Cy3)	Cy3 signal (Input)	Cy5 signal (MeDIP)	Relative methylation (Cy5/Cy3)	Cy3 signal (Input)	Cy5 signal (MeDIP)
A_68_P27541473	chr10:127896021-127896065	NM_001114096:-249	Smarcc2	PROMOTER	1.416	1.550	1630.99	2528.10	2.195	1385.29	3040.92
A_68_P27977899	chr11:84327083-84327127	NM_019816:-101	Aatf	PROMOTER	1.415	1.558	523.54	815.46	2.204	444.21	978.92
A_68_P28610312	chr12:87811484-87811528	NM_011934:48913	Esrrb	INSIDE	1.415	3.514	1014.32	3564.09	4.972	806.86	4011.80
A_68_P29075362	chr13:58909694-58909740	NM_001025074:523	Ntrk2	INSIDE	1.415	3.545	1141.26	4045.28	5.014	821.72	4120.05
A_68_P22101761	chr3:45183553-45183597	NM_011043:1255	Pcdh10	INSIDE	1.415	1.576	2007.04	3163.72	2.230	1703.03	3798.26
A_68_P30728925	chr16:45844534-45844578	NM_153412:-65	Phldb2	PROMOTER	1.415	3.345	1302.97	4359.06	4.735	1194.07	5653.92
A_68_P27540451	chr10:127707541-127707585	NM_019963:-69	Stat2	PROMOTER	1.415	1.729	2245.50	3882.21	2.446	1746.72	4272.53
A_68_P28402112	chr12:46175110-46175154	NM_144552:338	Stxbp6	INSIDE	1.415	1.850	3113.93	5759.59	2.617	2470.93	6466.73
A_68_P21920096	chr3:5219661-5219705	NM_030708:1129	Zfhx4	INSIDE	1.415	3.152	1282.92	4043.32	4.461	1384.14	6174.88
A_68_P20319169	chr1:69423807-69423851			Unknown	1.415	2.964	555.40	1646.05	4.193	466.30	1955.39
A_68_P23537869	chr5:23181744-23181788	ENSMUST00000157734:-1567		PROMOTER	1.415	2.503	1679.74	4204.56	3.542	1537.53	5446.32
A_68_P28530097	chr12:73186365-73186409	NM_026038:-86	2810055F11Rik	DIVERGENT_PROMOTER	1.414	1.866	3408.96	6362.14	2.640	2516.52	6642.89
A_68_P22407403	chr3:107398682-107398726	NM_007441:756	Alx3	INSIDE	1.414	2.074	1436.96	2980.63	2.933	1240.18	3637.72
A_68_P30751463	chr16:50432288-50432332	NM_027444:192	Bbx	INSIDE	1.414	1.904	328.99	626.29	2.692	303.84	817.80
A_68_P22151284	chr3:54859150-54859194	NM_007628:-195	Ccna1	PROMOTER	1.414	1.485	1402.96	2083.08	2.099	1405.54	2950.93
A_68_P32287122	chrX:19637760-19637804	NM_021715:1087	Chst7	INSIDE	1.414	2.815	968.09	2725.52	3.980	588.99	2344.32
A_68_P21820018	chr2:164658706-164658751	NM_001038492:356	Ctsa	INSIDE	1.414	2.708	566.82	1534.69	3.830	530.30	2030.87
A_68_P28925097	chr13:31898615-31898660	NM_008592:123	Foxc1	INSIDE	1.414	1.421	1066.47	1515.41	2.009	1209.38	2429.45
A_68_P22954220	chr4:59048156-59048200	NM_025277:151	Gng10	INSIDE	1.414	5.807	989.26	5744.38	8.209	856.33	7029.87
A_68_P30684003	chr16:38088656-38088700	NM_019827:-408	Gsk3b	PROMOTER	1.414	1.435	1193.56	1712.45	2.029	1142.32	2318.16
A_68_P27995150	chr11:87430698-87430742	NM_001045527:55	Hsf5	INSIDE	1.414	3.298	1976.62	6519.18	4.663	1723.73	8036.94
A_68_P25652455	chr8:11009184-11009228	NM_001081212:-776	Irs2	PROMOTER	1.414	2.138	1613.53	3449.83	3.024	1491.62	4510.74
A_68_P23819023	chr5:77380662-77380706	NM_172146:-81	Ppat	PROMOTER	1.414	3.590	3968.51	14247.09	5.075	3214.29	16312.63
A_68_P27921385	chr11:74403747-74403791	NM_001015046:-108	Rap1gap2	PROMOTER	1.414	1.794	920.25	1650.72	2.537	807.88	2049.41
A_68_P20451965	chr1:94807505-94807549	NM_181405:125	Rnpepl1	INSIDE	1.414	3.410	1465.86	4998.88	4.822	1215.11	5859.83
A_68_P26365992	chr9:25060005-25060049	NM_001205367:-13	Septin7	PROMOTER	1.414	2.049	1041.49	2133.69	2.897	923.47	2674.98
A_68_P31850942	chr18:78244032-78244076	NM_001101038:9953	Siglec15	INSIDE	1.414	3.049	393.30	1199.14	4.310	413.77	1783.29
A_68_P23973160	chr5:109058811-109058855	NM_001163531:4	Tmem175	INSIDE	1.414	3.467	380.53	1319.33	4.901	384.66	1885.27
A_68_P25614227	chr8:3496122-3496166	NM_080461:3007	Zfp358	INSIDE	1.414	2.617	496.37	1298.83	3.699	392.65	1452.42
A_68_P20026600	chr1:10222313-10222357	NM_001102430:417	Arfgef1	INSIDE	1.413	1.931	1534.02	2961.89	2.728	1158.33	3160.39
A_68_P29977214	chr15:3976915-3976959	NR_028266:470	BC037032	INSIDE	1.413	2.675	1415.05	3784.72	3.780	1172.61	4432.52
A_68_P25420899	chr7:118923736-118923780	NM_173739:-267	Galntl4	PROMOTER	1.413	3.673	2958.55	10865.71	5.190	2430.84	12615.98
A_68_P26135759	chr8:108282466-108282510	NM_027469:19	Gfod2	INSIDE	1.413	1.523	1541.04	2347.57	2.152	1181.67	2543.51
A_68_P21874393	chr2:174110277-174110321	NM_019690:478	Gnas	INSIDE	1.413	2.154	2046.31	4406.79	3.042	1642.18	4995.92
A_68_P25023656	chr7:29594118-29594162	NM_177301:-1768	Hnrnpl	PROMOTER	1.413	2.687	1331.04	3576.01	3.795	1074.61	4078.44
A_68_P26809623	chr9:107471781-107471825	NM_010489:309	Hyal2	INSIDE	1.413	4.236	5054.64	21410.10	5.986	3419.86	20470.32
A_68_P30478822	chr15:99055021-99055065	NM_010601:-364	Kcnh3	PROMOTER	1.413	3.462	317.84	1100.28	4.891	338.85	1657.16
A_68_P21809534	chr2:162769378-162769422	NM_001081338:200	L3mbtl1	INSIDE	1.413	3.093	968.62	2996.17	4.372	841.69	3679.62
A_68_P24610520	chr6:86475945-86475989	NM_011865:193	Pcbp1	INSIDE	1.413	2.626	1858.75	4881.48	3.710	1609.11	5969.99
A_68_P20442663	chr1:93355837-93355883	NM_011066:45	Per2	INSIDE	1.413	3.657	6446.43	23575.61	5.166	5308.25	27421.77
A_68_P25092684	chr7:52622218-52622262	NM_029741:149	Ppfia3	INSIDE	1.413	1.409	1337.34	1884.40	1.991	1117.01	2223.57
A_68_P23327089	chr4:134731774-134731818	NM_019732:55237	Runx3	INSIDE	1.413	2.139	298.22	637.88	3.022	218.63	660.62
A_68_P23395562	chr4:148001229-148001274	NM_001003898:-146	Tardbp	PROMOTER	1.413	1.494	1928.57	2881.24	2.110	1656.26	3495.28
A_68_P26023340	chr8:87562055-87562099	NM_001122843:1010	Tnpo2	INSIDE	1.413	2.301	2992.19	6884.41	3.251	2164.18	7036.24
A_68_P31118855	chr17:27993453-27993497	NM_001080769:23	Uhrf1bp1	INSIDE	1.413	3.683	777.08	2862.23	5.206	680.20	3541.08
A_68_P24951458	chr7:4945401-4945445	NM_026741:2281	Zfp579	INSIDE	1.413	2.367	3124.98	7396.80	3.345	2462.47	8236.76
A_68_P29021091	chr13:48828074-48828118	BC167198:235103		INSIDE	1.413	2.604	1695.84	4416.64	3.681	1315.86	4843.55
A_68_P25890689	chr8:59819744-59819788	ENSMUST00000132988:8852		DOWNSTREAM	1.413	2.087	997.48	2081.66	2.948	802.38	2365.63
A_68_P32957256	chr17:8476298-8476342	NM_018819:-357	Brp44l	PROMOTER	1.412	2.317	559.09	1295.31	3.271	543.39	1777.50
A_68_P26019658	chr8:86939377-86939421	NM_007578:136	Cacna1a	INSIDE	1.412	3.848	1672.03	6434.41	5.433	1364.80	7414.42
A_68_P30371906	chr15:80203104-80203152	NM_001044308:85461	Cacna1i	INSIDE	1.412	4.922	136.27	670.65	6.947	83.75	581.84

						Sham group			TiO_2-H group		
ProbeName	Target position of probe on CpG island microarray	TargetID	GeneSymbol	CpG island Description	Ratio of relative methylation (TiO_2-NP/Vehicle)	Relative methylation (Cy5/Cy3)	Cy3 signal (Input)	Cy5 signal (MeDIP)	Relative methylation (Cy5/Cy3)	Cy3 signal (Input)	Cy5 signal (MeDIP)
A_68_P22728452	chr4:11117767-11117811	NM_001037134:-712	Ccne2	PROMOTER	1.412	2.320	339.08	786.68	3.277	284.19	931.30
A_68_P28313702	chr12:29236975-29237019	NM_001177964:-3794	Dcdc2c	PROMOTER	1.412	2.750	1290.20	3548.29	3.884	1046.53	4065.16
A_68_P30569150	chr16:16359116-16359160	NM_001025947:-15	Dnm1l	PROMOTER	1.412	10.245	353.34	3619.79	14.461	335.43	4850.62
A_68_P27284176	chr10:80049548-80049592	NM_145421:3516	Fam108a	INSIDE	1.412	2.481	1696.54	4208.31	3.504	1437.16	5035.41
A_68_P23787602	chr5:72048882-72048926	NM_010251:643	Gabra4	INSIDE	1.412	1.663	2656.01	4416.65	2.349	1936.04	4547.33
A_68_P24119633	chr5:136611987-136612031	NM_027891:-64	Lrwd1	DIVERGENT_PROMOTER	1.412	1.868	4287.48	8007.90	2.637	2922.79	7708.54
A_68_P27882408	chr11:67111986-67112033	NM_177369:21384	Myh8	INSIDE	1.412	2.711	235.50	638.38	3.829	202.89	776.82
A_68_P21723786	chr2:147191194-147191239	NM_008780:487	Pax1	INSIDE	1.412	11.427	1168.71	13355.35	16.130	889.81	14352.68
A_68_P20874691	chr1:182186344-182186388	NM_001128605:65	Psen2	INSIDE	1.412	2.020	634.12	1280.71	2.852	529.21	1509.22
A_68_P27902925	chr11:70658259-70658303	NM_019400:16	Rabep1	INSIDE	1.412	1.763	1828.38	3224.09	2.489	1509.22	3757.14
A_68_P28707014	chr12:106270570-106270614	NR_003145:-103	Snhg10	DIVERGENT_PROMOTER	1.412	2.559	751.25	1922.41	3.614	723.34	2614.10
A_68_P32958522	chr17:8627015-8627059	NM_009309:-251	T	PROMOTER	1.412	1.744	1177.05	2052.94	2.463	1001.67	2466.93
A_68_P25952182	chr8:73013396-73013440	NM_182991:-2161	Tmem59l	DIVERGENT_PROMOTER	1.412	2.404	1488.13	3577.32	3.394	1368.06	4642.54
A_68_P22861756	chr4:41296379-41296423	NM_023305:372	Ubap1	INSIDE	1.412	1.899	2409.65	4575.33	2.680	1781.80	4775.54
A_68_P22410425	chr3:107889046-107889090	NM_028779:477	Ampd2	INSIDE	1.411	1.872	3075.40	5756.63	2.641	2414.79	6378.00
A_68_P29695817	chr14:69955362-69955406	NM_026174:177	Entpd4	INSIDE	1.411	1.639	1867.34	3060.75	2.313	1690.78	3910.85
A_68_P30356791	chr15:77786971-77787015	NM_001017983:160	Foxred2	INSIDE	1.411	2.358	2890.26	6815.42	3.328	2449.53	8152.29
A_68_P23757987	chr5:66155002-66155046	NM_001024917:265	N4bp2	INSIDE	1.411	2.078	490.71	1019.55	2.932	461.90	1354.40
A_68_P23057168	chr4:82150339-82150383	NM_001113209:852	Nfib	INSIDE	1.411	2.024	1268.08	2566.46	2.856	1209.56	3454.91
A_68_P25021286	chr7:29124000-29124044	NM_013676:-284	Supt5h	PROMOTER	1.411	2.093	1024.49	2144.66	2.953	951.93	2811.44
A_68_P21101048	chr2:29904402-29904446	NM_001008498:-25	Wdr34	PROMOTER	1.411	2.596	1068.05	2772.33	3.662	950.24	3480.16
A_68_P31152233	chr17:34258866-34258910	NM_010238:-196	Brd2	PROMOTER	1.410	1.630	877.15	1430.03	2.299	840.32	1931.76
A_68_P25083584	chr7:50932175-50932219	NM_145582:4796	Ctu1	INSIDE	1.410	2.203	489.91	1079.14	3.106	410.59	1275.12
A_68_P27281161	chr10:79594591-79594635	NM_001195268:5520	Dos	INSIDE	1.410	2.666	1813.03	4832.69	3.759	1417.47	5328.88
A_68_P30468154	chr15:97077540-97077584	NM_172293:25	Fam113b	INSIDE	1.410	5.074	999.43	5071.38	7.154	834.90	5972.61
A_68_P24817835	chr6:124881155-124881199	NM_001170341:-229	Mlf2	PROMOTER	1.410	2.519	3481.95	8770.99	3.552	3025.57	10748.07
A_68_P25261270	chr7:86610733-86610777	NM_017462:405	Polg	INSIDE	1.410	1.887	2237.30	4221.82	2.661	1842.14	4901.97
A_68_P30576627	chr16:17928257-17928301	NM_153150:34	Slc25a1	INSIDE	1.410	1.496	833.86	1247.16	2.109	719.36	1517.25
A_68_P25235399	chr7:81699616-81699660	NM_001038643:28	Slco3a1	INSIDE	1.410	5.257	915.21	4811.55	7.415	790.12	5858.61
A_68_P30562963	chr16:14705930-14705974	NM_011415:1	Snai2	INSIDE	1.410	1.377	1658.17	2283.48	1.942	1325.17	2573.25
A_68_P30787362	chr16:57606693-57606737	NM_025599:266	2610528E23Rik	INSIDE	1.409	1.377	1366.06	1880.56	1.939	1110.63	2153.52
A_68_P30655032	chr16:32877777-32877821	NM_001159349:-71	Fyttd1	DIVERGENT_PROMOTER	1.409	3.794	690.93	2621.64	5.344	673.85	3601.39
A_68_P32401470	chrX:54756780-54756824	NM_001033360:132	Gpr101	INSIDE	1.409	2.418	865.27	2092.26	3.408	449.92	1533.16
A_68_P27182284	chr10:61246043-61246087	NM_207000:548	H2afy2	INSIDE	1.409	3.237	4096.24	13260.25	4.561	3206.81	14626.86
A_68_P25846169	chr8:49314467-49314511	NM_001145937:445556	Odz3	INSIDE	1.409	3.159	728.27	2300.32	4.451	652.82	2906.04
A_68_P20768946	chr1:162837198-162837242	NM_001024952:679	Rc3h1	INSIDE	1.409	2.773	621.82	1724.47	3.908	536.69	2097.56
A_68_P23294679	chr4:128735519-128735563	NM_029219:26	Rnf19b	INSIDE	1.409	2.908	1340.86	3899.06	4.098	1187.25	4865.85
A_68_P22392370	chr3:104442843-104442887	NM_009196:274	Slc16a1	INSIDE	1.409	3.586	1227.30	4400.60	5.052	1130.60	5711.85
A_68_P27365030	chr10:94878524-94878568	NM_001168656:-428	Socs2	PROMOTER	1.409	2.684	970.86	2605.41	3.781	842.34	3184.83
A_68_P26685862	chr9:83728264-83728308	NM_001110265:-9	Ttk	PROMOTER	1.409	1.703	3577.40	6092.03	2.400	2394.02	5745.88
A_68_P20679676	chr1:145549881-145549925	NM_145991:-88	Cdc73	PROMOTER	1.408	1.476	5325.64	7862.49	2.079	4869.18	10121.53
A_68_P20453266	chr1:94997065-94997109	NM_001110315:1356	Kif1a	INSIDE	1.408	1.643	3795.87	6235.33	2.313	2801.91	6480.75
A_68_P24479790	chr6:57652980-57653024	NM_133737:554	Lancl2	INSIDE	1.408	2.430	794.70	1931.28	3.421	691.36	2364.89
A_68_P22661976	chr3:153991190-153991234	NM_010713:2312	Lhx8	INSIDE	1.408	1.540	3483.43	5364.29	2.168	2801.56	6074.45
A_68_P22793481	chr4:26274038-26274083	NM_172865:-261	Manea	PROMOTER	1.408	2.675	2801.31	7494.27	3.766	2411.79	9082.95
A_68_P27980118	chr11:84694015-84694059	NM_001077636:-249	Pigw	PROMOTER	1.408	1.569	699.70	1097.73	2.209	622.33	1374.67
A_68_P32141866	chr19:46131585-46131629	NM_001081214:578	Pprc1	INSIDE	1.408	1.563	2301.83	3598.49	2.201	1881.74	4142.35
A_68_P32806534	chrX:162917700-162917744	NM_019773:77	Rab9	INSIDE	1.408	2.038	766.79	1562.78	2.870	390.07	1119.42
A_68_P30250720	chr15:58720614-58720658	NM_175226:-147	Rnf139	PROMOTER	1.408	3.567	2289.85	8166.88	5.021	1927.49	9678.51
A_68_P31244849	chr17:53828675-53828719	NM_028232:-56	Sgol1	PROMOTER	1.408	2.141	1821.99	3901.06	3.014	1701.11	5126.64
A_68_P29240371	chr13:96145165-96145209	NM_025630:103	Aggf1	INSIDE	1.407	2.350	738.00	1733.96	3.305	740.57	2447.36

ProbeName	Target position of probe on CpG island microarray	TargetID	GeneSymbol	CpG island Description	Ratio of relative methylation (TiO_2-NP/Vehicle)	Sham group			TiO_2-H group		
						Relative methylation (Cy5/Cy3)	Cy3 signal (Input)	Cy5 signal (MeDIP)	Relative methylation (Cy5/Cy3)	Cy3 signal (Input)	Cy5 signal (MeDIP)
A_68_P25089705	chr7:52109937-52109981	NM_026270:5362	Akt1s1	INSIDE	1.407	2.093	1940.17	4060.15	2.944	1664.79	4901.91
A_68_P32536862	chrX:91225843-91225889	NR_028320:11872	AU015836	DOWNSTREAM	1.407	2.633	433.55	1141.38	3.705	250.43	927.87
A_68_P24055184	chr5:123794688-123794734	NM_029850:254	Bcl7a	INSIDE	1.407	0.431	1429.58	615.62	0.606	1174.64	711.76
A_68_P21569151	chr2:119163754-119163798	NM_019454:12257	Dll4	DOWNSTREAM	1.407	2.068	1210.07	2502.81	2.909	1026.27	2985.64
A_68_P25405199	chr7:116094716-116094760	NM_025344:16810	Eif3f	DOWNSTREAM	1.407	1.942	363.10	704.98	2.731	271.25	740.85
A_68_P20227608	chr1:52557632-52557676	NM_008667:-362	Nab1	PROMOTER	1.407	1.821	1276.26	2324.40	2.562	1135.61	2909.38
A_68_P21842488	chr2:168427130-168427174	NM_001037178:3	Nfatc2	INSIDE	1.407	3.598	4227.71	15210.75	5.063	3218.50	16294.33
A_68_P23894271	chr5:92863563-92863607	NM_183392:641	Nup54	INSIDE	1.407	2.401	2087.01	5011.84	3.380	1927.99	6516.10
A_68_P31156112	chr17:34987286-34987330	NM_001045863:-27	Rdbp	DIVERGENT_PROMOTER	1.407	1.602	936.61	1500.90	2.255	907.16	2045.32
A_68_P31156004	chr17:34973557-34973601	NM_019442:270	Stk19	INSIDE	1.407	2.874	3122.89	8976.41	4.043	2434.15	9842.26
A_68_P26035029	chr8:89996288-89996332	NM_019626:181	Cbln1	INSIDE	1.406	3.564	8407.66	29968.20	5.012	6059.68	30368.11
A_68_P24011284	chr5:116181838-116181882	NM_001080808:-292	Ccdc64	PROMOTER	1.406	1.875	2306.58	4325.59	2.638	1864.46	4917.56
A_68_P31254440	chr17:56323216-56323260	NM_080837:105	D17Wsu104e	INSIDE	1.406	2.193	1001.83	2197.02	3.083	887.19	2735.36
A_68_P27055772	chr10:33927102-33927146	NM_172508:233	Dse	INSIDE	1.406	2.291	1326.27	3038.19	3.220	1109.05	3571.66
A_68_P20637452	chr1:137001893-137001941	NM_001033409:-63	Lgr6	PROMOTER	1.406	6.787	7856.61	53326.08	9.545	5134.92	49011.52
A_68_P31123145	chr17:28712577-28712621	NM_026571:-65	Lhfpl5	PROMOTER	1.406	1.795	762.16	1367.86	2.524	659.41	1664.28
A_68_P27635062	chr11:20991838-20991882	NM_023324:534	Peli1	INSIDE	1.406	2.699	1651.07	4455.42	3.794	1394.60	5290.94
A_68_P29054323	chr13:55423060-55423104	NM_009000:259	Rab24	INSIDE	1.406	2.908	1715.27	4988.57	4.090	1431.38	5853.72
A_68_P23254536	chr4:120689836-120689880	NM_133716:-6	Smap2	PROMOTER	1.406	2.137	1799.58	3844.83	3.003	1896.83	5696.91
A_68_P29964718	chr14:123382401-123382445	NM_028651:61	Tmtc4	INSIDE	1.406	0.451	1631.66	736.13	0.634	1548.36	981.86
A_68_P30592417	chr16:21423184-21423228	NM_001081366:16	Vps8	INSIDE	1.406	1.700	651.68	1107.59	2.390	586.24	1401.27
A_68_P24793727	chr6:119494261-119494305	NM_009525:83	Wnt5b	INSIDE	1.406	1.856	635.81	1180.32	2.610	534.46	1394.95
A_68_P30931895	chr16:85900243-85900287	NM_011782:1106	Adamts5	INSIDE	1.405	1.414	864.81	1223.15	1.988	842.52	1674.73
A_68_P25500905	chr7:133646409-133646453	NM_183020:386	Atxn2l	INSIDE	1.405	3.122	1715.00	5353.63	4.386	1486.82	6521.92
A_68_P31020949	chr17:6079867-6079911	NM_181392:61	Gtf2h5	INSIDE	1.405	1.509	1025.28	1547.57	2.120	822.53	1744.03
A_68_P28431899	chr12:52930265-52930309	NM_144788:237	Hectd1	INSIDE	1.405	3.721	395.03	1469.73	5.229	349.96	1829.86
A_68_P29117879	chr13:69948975-69949019	NM_138596:237	Med10	INSIDE	1.405	2.470	509.97	1259.71	3.470	452.22	1569.13
A_68_P23269531	chr4:124337466-124337523	NM_011141:2606	Pou3f1	INSIDE	1.405	6.180	201.62	1246.01	8.680	203.38	1765.43
A_68_P23238885	chr4:117884835-117884879	NM_011213:79146	Ptprf	INSIDE	1.405	5.342	1806.98	9653.34	7.506	1584.46	11893.57
A_68_P32750676	chrX:149203868-149203912	NM_172441:114	Shroom2	INSIDE	1.405	2.139	894.45	1913.59	3.007	560.54	1685.34
A_68_P24544425	chr6:72908782-72908826	NM_001190327:-62	Tmsb10	PROMOTER	1.405	2.237	1467.27	3282.32	3.144	1175.79	3696.58
A_68_P21620795	chr2:128512977-128513021	NM_008569:133	Anapc1	INSIDE	1.404	1.694	1441.96	2443.33	2.379	1286.45	3060.30
A_68_P32745112	chrX:147779006-147779050	NM_198105:263	Fam120c	INSIDE	1.404	2.058	2046.86	4213.02	2.891	1083.97	3133.24
A_68_P21609427	chr2:126500825-126500869	NM_010249:376	Gabpb1	INSIDE	1.404	5.171	1070.08	5533.26	7.258	919.71	6675.09
A_68_P28577474	chr12:81859527-81859571	NM_001008423:2154	Gm1568	INSIDE	1.404	3.088	946.54	2923.19	4.336	841.54	3648.63
A_68_P23644447	chr5:43625680-43625724	NR_028417:891	Gm7854	INSIDE	1.404	2.190	1122.39	2457.65	3.074	936.76	2879.42
A_68_P32536211	chrX:91077428-91077472	NM_145630:-16	Pdk3	PROMOTER	1.404	1.589	1225.88	1947.97	2.231	635.73	1418.11
A_68_P30575813	chr16:17807076-17807120	NM_153790:9724	Scarf2	INSIDE	1.404	2.276	4243.24	9657.92	3.195	3306.28	10564.39
A_68_P32259784	chrX:11584144-11584188			Unknown	1.404	1.722	6852.99	11799.17	2.417	3456.14	8353.28
A_68_P26885056	chr9:121619308-121619352	NM_026794:-176	Deb1	DIVERGENT_PROMOTER	1.403	1.680	998.84	1677.58	2.356	783.40	1845.53
A_68_P33007792	chr4_random:128621-128665	NM_001033326:18841	Dhrsx	INSIDE	1.403	2.689	4244.61	11413.45	3.771	3341.64	12602.76
A_68_P31936679	chr19:6996326-6996370	NM_007953:-50	Esrra	PROMOTER	1.403	2.076	1004.40	2085.48	2.914	812.87	2368.73
A_68_P31601519	chr18:32322742-32322786	NM_011946:22	Map3k2	INSIDE	1.403	1.491	710.88	1060.03	2.092	704.25	1472.97
A_68_P24757359	chr6:113027455-113027499	NM_028385:-156	Setd5	DIVERGENT_PROMOTER	1.403	3.215	2571.75	8268.07	4.512	2248.85	10147.12
A_68_P31142311	chr17:31992646-31992690	NM_010831:69	Sik1	INSIDE	1.403	1.467	2539.21	3723.82	2.057	2085.33	4290.14
A_68_P29421307	chr14:15536004-15536048	NM_001033270:488	Slc4a7	INSIDE	1.403	3.711	691.17	2564.99	5.208	592.83	3087.32
A_68_P25926490	chr8:67471009-67471053	NM_001163747:-53	Tmem192	PROMOTER	1.403	1.627	811.49	1320.02	2.282	731.54	1669.41
A_68_P24136156	chr5:140020220-140020264	NM_013702:391	Uncx	INSIDE	1.403	2.655	1505.01	3996.43	3.726	1276.00	4753.82
A_68_P24639205	chr6:91423217-91423261	NM_133928:179	Chchd4	INSIDE	1.402	1.712	1123.21	1923.19	2.401	875.72	2102.71
A_68_P26166334	chr8:113581188-113581232	NM_028274:972	Exosc6	INSIDE	1.402	2.006	1168.33	2343.79	2.813	996.51	2802.98
A_68_P31948450	chr19:10176095-10176139	NM_019699:-123	Fads2	PROMOTER	1.402	1.380	1397.41	1929.06	1.936	1083.93	2098.42

ProbeName	Target position of probe on CpG island microarray	TargetID	GeneSymbol	CpG island Description	Ratio of relative methylation (TiO_2-NP/Vehicle)	Sham group			TiO_2-H group		
						Relative methylation (Cy5/Cy3)	Cy3 signal (Input)	Cy5 signal (MeDIP)	Relative methylation (Cy5/Cy3)	Cy3 signal (Input)	Cy5 signal (MeDIP)
A_68_P27591951	chr11:11585898-11585942	NM_001025597:-295	Ikzf1	PROMOTER	1.402	2.070	730.08	1511.22	2.901	599.41	1739.11
A_68_P22139473	chr3:52845232-52845276	NM_175386:-214	Lhfp	PROMOTER	1.402	3.719	2004.22	7453.29	5.213	1562.39	8145.14
A_68_P23316583	chr4:132806890-132806934	NM_016693:10180	Map3k6	INSIDE	1.402	1.507	895.06	1348.55	2.112	732.59	1547.47
A_68_P25478239	chr7:129432560-129432604	NM_008855:-56	Prkcb	PROMOTER	1.402	2.667	505.17	1347.19	3.738	432.90	1618.16
A_68_P20354815	chr1:75543006-75543050	NM_009208:188	Slc4a3	INSIDE	1.402	1.770	518.97	918.77	2.482	430.74	1069.06
A_68_P31469354	chr18:5592137-5592181	NM_011546:270	Zeb1	INSIDE	1.402	2.753	1966.58	5414.65	3.861	1502.43	5801.01
A_68_P28185535	chr11:120459852-120459896	NM_001038230:29	Anapc11	INSIDE	1.401	1.827	749.13	1368.39	2.559	589.68	1509.19
A_68_P25947954	chr8:72315394-72315438	NM_133224:355	Atp13a1	INSIDE	1.401	5.952	863.09	5137.52	8.341	743.89	6204.59
A_68_P31567774	chr18:25911745-25911789	NM_001146292:718	Celf4	INSIDE	1.401	1.931	353.29	682.11	2.706	290.12	784.96
A_68_P30181643	chr15:44451360-44451404	NM_019480:196	Ebag9	INSIDE	1.401	1.552	2230.43	3461.83	2.175	1838.06	3996.93
A_68_P26166328	chr8:113580417-113580461	NM_028274:200	Exosc6	INSIDE	1.401	6.399	499.21	3194.31	8.964	463.31	4152.94
A_68_P32139831	chr19:45734262-45734306	NM_013907:399	Fbxw4	INSIDE	1.401	1.512	587.99	889.29	2.119	532.35	1127.87
A_68_P23577610	chr5:31597885-31597929	NM_022424:344	Fndc4	INSIDE	1.401	2.391	2561.88	6124.91	3.349	2078.32	6959.31
A_68_P25101651	chr7:54101747-54101791	NM_001136069:-683	Ldha	PROMOTER	1.401	2.910	447.71	1302.65	4.075	390.23	1590.14
A_68_P21706641	chr2:144156468-144156512	NM_152947:608	Ovol2	INSIDE	1.401	6.539	4320.91	28254.59	9.164	3330.70	30522.49
A_68_P30140494	chr15:36722668-36722712	NM_011740:1603	Ywhaz	INSIDE	1.401	1.704	2603.52	4437.13	2.388	1951.87	4661.74
A_68_P20349427	chr1:74634546-74634590	NM_029888:34	Zfp142	INSIDE	1.401	1.664	2069.36	3443.62	2.332	1793.48	4182.06
A_68_P31929033	chr19:5490488-5490532	NM_007687:56	Cfl1	INSIDE	1.400	1.869	798.40	1491.89	2.616	769.74	2013.88
A_68_P27533642	chr10:126478457-126478501	NM_001205036:-28	LOC100504608	DIVERGENT_PROMOTER	1.400	3.818	2175.23	8304.52	5.343	2058.67	11000.07
A_68_P27788131	chr11:50172882-50172926	NR_030529:-1523	Mir804	PROMOTER	1.400	2.253	2303.11	5189.26	3.155	1863.17	5879.04
A_68_P27215086	chr10:67442552-67442596	NM_001081346:229	Rtkn2	INSIDE	1.400	2.426	1259.75	3055.84	3.396	1153.03	3916.15
A_68_P22659975	chr3:153636613-153636657	NM_001081263:235	Slc44a5	INSIDE	1.400	1.638	1233.09	2019.54	2.293	1001.03	2295.80
A_68_P24833353	chr6:127511414-127511458			Unknown	1.400	1.740	1231.76	2142.93	2.436	1108.95	2701.05
A_68_P27756941	chr11:44431946-44431990	NM_007897:333	Ebf1	INSIDE	1.399	1.745	394.68	688.61	2.441	362.89	885.87
A_68_P20226307	chr1:52289184-52289228	NM_001081081:870	Gls	INSIDE	1.399	2.484	1200.72	2982.60	3.475	1081.89	3759.12
A_68_P30681056	chr16:37539468-37539512	NM_028812:365	Gtf2e1	INSIDE	1.399	2.322	1872.10	4347.39	3.250	1573.84	5114.56
A_68_P23611441	chr5:37482522-37482566	NM_178394:40449	Jakmip1	INSIDE	1.399	2.144	743.36	1593.77	2.998	643.30	1928.90
A_68_P25263205	chr7:86937995-86938039	NM_008588:460	Mesp1	INSIDE	1.399	3.289	629.69	2070.96	4.600	556.40	2559.24
A_68_P24591487	chr6:83059326-83059370	NM_026744:247	Mrpl53	INSIDE	1.399	1.585	820.52	1300.18	2.217	677.96	1503.31
A_68_P30480281	chr15:99287653-99287697	NM_001001884:505	Nckap5l	INSIDE	1.399	1.686	2209.21	3724.04	2.358	1458.67	3438.90
A_68_P27279098	chr10:79281731-79281775	NM_001161747:25436	Palm	INSIDE	1.399	3.382	602.57	2037.78	4.733	558.73	2644.35
A_68_P31208520	chr17:46848930-46848974	NM_145488:541	Pex6	INSIDE	1.399	2.065	1808.51	3735.42	2.890	1669.19	4823.98
A_68_P27942686	chr11:78142057-78142101	NM_201406:155	Pigs	INSIDE	1.399	2.322	3187.24	7400.05	3.248	2820.21	9160.18
A_68_P20011682	chr1:6204945-6204989	NM_009826:224	Rb1cc1	INSIDE	1.399	6.011	107.23	644.54	8.411	99.08	833.42
A_68_P27926518	chr11:75324220-75324264	NM_001029938:647	Rilp	INSIDE	1.399	4.585	3632.75	16656.98	6.417	2896.51	18586.14
A_68_P23542975	chr5:24107899-24107943	NM_025891:-100	Smarcd3	PROMOTER	1.399	1.953	382.91	747.98	2.732	354.36	968.08
A_68_P20409779	chr1:87915105-87915149	NM_027029:-3390	Spata3	PROMOTER	1.399	2.606	369.43	962.76	3.645	329.67	1201.68
A_68_P29428732	chr14:17198090-17198134	NM_009409:393	Top2b	INSIDE	1.399	3.732	693.33	2587.49	5.223	598.73	3127.03
A_68_P27789375	chr11:50415810-50415854	NM_175643:246	Adamts2	INSIDE	1.398	2.253	2651.57	5973.66	3.151	2404.30	7575.02
A_68_P25065437	chr7:38892310-38892354	NM_007633:177	Ccne1	INSIDE	1.398	1.867	578.86	1080.99	2.611	461.28	1204.19
A_68_P27282061	chr10:79727895-79727939	NM_001122604:181	Dazap1	INSIDE	1.398	3.911	1487.01	5815.28	5.468	1279.79	6997.54
A_68_P23235083	chr4:117223023-117223067	NM_080469:-54	Eri3	PROMOTER	1.398	5.781	754.89	4364.04	8.084	638.76	5163.45
A_68_P29953204	chr14:121435005-121435049	NM_134082:231	Farp1	INSIDE	1.398	2.932	995.92	2920.48	4.099	921.82	3778.72
A_68_P29529251	chr14:35124024-35124068	NM_008133:134	Glud1	INSIDE	1.398	2.579	2250.19	5802.24	3.605	1990.22	7175.04
A_68_P24757357	chr6:113027235-113027279	NR_027010:-18	Gt(ROSA)26Sor	DIVERGENT_PROMOTER	1.398	2.086	588.75	1228.17	2.915	544.79	1588.32
A_68_P24325223	chr6:29162126-29162170	NM_011829:123	Impdh1	INSIDE	1.398	1.871	5279.77	9878.35	2.615	3665.18	9584.02
A_68_P32148149	chr19:47253623-47253667	NM_021360:335	Neurl1a	INSIDE	1.398	2.625	1198.87	3147.37	3.669	959.99	3522.42
A_68_P32565873	chrX:99045311-99045356	NM_001163610:610	Nhsl2	INSIDE	1.398	1.771	1001.28	1772.96	2.475	607.83	1504.35
A_68_P29009460	chr13:46822473-46822517	NM_175749:724	Nup153	INSIDE	1.398	1.677	3751.32	6291.94	2.344	2854.35	6691.14
A_68_P31375202	chr17:79452069-79452113	NM_027455:845	Qpct	INSIDE	1.398	2.331	1879.36	4380.77	3.259	1644.52	5359.97
A_68_P23820630	chr5:77694944-77694988	NM_011263:448	Rest	INSIDE	1.398	1.947	5298.87	10319.06	2.723	4359.51	11869.89

ProbeName	Target position of probe on CpG island microarray	TargetID	GeneSymbol	CpG island Description	Ratio of relative methylation (TiO_2-NP/Vehicle)	Sham group			TiO_2-H group		
						Relative methylation (Cy5/Cy3)	Cy3 signal (Input)	Cy5 signal (MeDIP)	Relative methylation (Cy5/Cy3)	Cy3 signal (Input)	Cy5 signal (MeDIP)
A_68_P21365118	chr2:78709482-78709526	NM_009454:301	Ube2e3	INSIDE	1.398	4.528	1600.81	7247.78	6.330	1381.60	8745.66
A_68_P29448497	chr14:21526842-21526886	NM_027996:91	2310021P13Rik	INSIDE	1.397	1.393	2174.38	3028.32	1.946	1834.85	3570.00
A_68_P20769692	chr1:162965131-162965175	NR_002840:-144	Gas5	DIVERGENT_PROMOTER	1.397	3.524	2582.17	9100.43	4.923	1793.34	8827.97
A_68_P30482737	chr15:99705642-99705686	NM_001113545:223	Lima1	INSIDE	1.397	4.162	1490.10	6202.30	5.814	1170.51	6805.88
A_68_P31236180	chr17:51952405-51952449	NM_001163630:-1047	Satb1	PROMOTER	1.397	2.008	618.50	1242.07	2.805	632.48	1774.17
A_68_P24725198	chr6:106719039-106719083	NM_027296:-105	Trnt1	PROMOTER	1.397	5.081	1506.70	7656.14	7.099	1137.75	8077.29
A_68_P32257886	chrX:11241995-11242039			Unknown	1.397	3.581	665.18	2381.66	5.004	371.14	1857.01
A_68_P28183960	chr11:120209674-120209718	NM_009609:102	Actg1	INSIDE	1.396	1.642	541.23	888.58	2.291	499.59	1144.79
A_68_P21492619	chr2:104966561-104966605	NR_015462:85	AI314831	INSIDE	1.396	2.308	940.63	2170.96	3.222	704.81	2271.21
A_68_P31053607	chr17:12934828-12934873	NR_002853:674	Airn	INSIDE	1.396	2.003	564.26	1130.13	2.796	529.19	1479.46
A_68_P24864229	chr6:134869881-134869925	NM_009875:-516	Cdkn1b	PROMOTER	1.396	1.394	1252.13	1745.42	1.946	1149.29	2237.07
A_68_P22893294	chr4:47221270-47221314	NM_009928:409	Col15a1	INSIDE	1.396	1.957	7873.75	15410.43	2.733	6249.31	17078.71
A_68_P25422971	chr7:119301817-119301861	NM_015814:733	Dkk3	INSIDE	1.396	6.658	4315.43	28730.11	9.294	2768.78	25732.05
A_68_P29863770	chr14:103498866-103498910	NM_015822:-162	Fbxl3	PROMOTER	1.396	2.589	973.82	2521.37	3.613	902.17	3259.82
A_68_P28480021	chr12:62624233-62624277	NM_178714:-1364	Lrfn5	PROMOTER	1.396	1.666	443.87	739.42	2.325	369.37	858.89
A_68_P25091650	chr7:52437190-52437234	NM_053256:848	Pth2	DOWNSTREAM	1.396	1.506	1846.75	2781.72	2.102	1450.07	3048.37
A_68_P27646885	chr11:23565657-23565701	NM_001033654:5	Pus10	INSIDE	1.396	1.369	1802.33	2467.43	1.911	1579.50	3019.08
A_68_P21077274	chr2:26092670-26092714	NM_153559:248	Qsox2	INSIDE	1.396	1.944	2133.27	4147.70	2.715	1864.67	5062.62
A_68_P25092432	chr7:52582657-52582701	NM_175130:6472	Trpm4	INSIDE	1.396	2.555	1622.52	4144.76	3.566	1367.67	4876.75
A_68_P24819389	chr6:125165905-125165949	NM_001080557:328	Vamp1	INSIDE	1.396	1.869	954.47	1783.85	2.609	844.70	2204.17
A_68_P27033518	chr10:28868262-28868306	NM_026138:4483	6330407J23Rik	INSIDE	1.395	1.534	2436.88	3737.10	2.139	1828.86	3912.70
A_68_P23192159	chr4:107356053-107356097	NM_019872:693	Dmrtb1	INSIDE	1.395	1.903	441.89	840.91	2.655	421.38	1118.83
A_68_P27067359	chr10:36693905-36693949	NM_008229:-423	Hdac2	PROMOTER	1.395	2.956	2512.26	7426.68	4.123	1989.08	8200.42
A_68_P31731697	chr18:56867382-56867426	NM_010721:-62	Lmnb1	PROMOTER	1.395	2.458	549.59	1350.75	3.428	488.07	1673.23
A_68_P22038555	chr3:32428477-32428521	NM_024200:95	Mfn1	INSIDE	1.395	3.778	1062.72	4015.10	5.272	1025.16	5404.92
A_68_P32183819	chr19:53750945-53750989	NM_001170847:-829	Rbm20	PROMOTER	1.395	1.383	1942.31	2685.80	1.929	1608.32	3102.19
A_68_P31738737	chr18:58039024-58039069	NM_009194:715	Slc12a2	INSIDE	1.395	1.668	1207.21	2013.50	2.326	1010.92	2351.78
A_68_P32008153	chr19:21852916-21852960	NM_001033759:107	Tmem2	INSIDE	1.395	3.709	1218.89	4520.81	5.173	1023.30	5294.04
A_68_P23874561	A_68_P23874561			Unknown	1.395	7.260	319.56	2320.13	10.131	308.34	3123.78
A_68_P26749715	chr9:96789502-96789546	NM_153420:317	Acpl2	INSIDE	1.394	1.692	12101.53	20476.82	2.358	7410.41	17477.14
A_68_P23315382	chr4:132595283-132595327	NM_146155:27884	Ahdc1	INSIDE	1.394	3.767	896.38	3377.11	5.252	793.29	4166.12
A_68_P21569149	chr2:119163546-119163590	NM_019454:12049	Dll4	DOWNSTREAM	1.394	4.287	1272.36	5454.83	5.977	1115.32	6666.14
A_68_P22081217	chr3:40549685-40549729	NM_011020:172	Hspa4l	INSIDE	1.394	1.992	1461.33	2911.48	2.777	1265.58	3514.48
A_68_P24759766	chr6:113432543-113432587	NM_134159:11116	Il17rc	INSIDE	1.394	4.030	2414.78	9732.27	5.617	1971.48	11073.56
A_68_P31791016	chr18:67448771-67448815	NM_053261:-84	Impa2	PROMOTER	1.394	5.242	21630.25	113388.70	7.309	13337.96	97486.45
A_68_P25954564	chr8:73386897-73386941	NM_032397:-6011	Kcnn1	PROMOTER	1.394	6.923	863.31	5976.70	9.648	731.30	7055.72
A_68_P20080973	chr1:21951507-21951551	NM_001160139:495	Kcnq5	INSIDE	1.394	1.983	1766.27	3502.03	2.764	1556.32	4301.49
A_68_P21203206	chr2:49475493-49475538	NM_008449:682	Kif5c	INSIDE	1.394	2.658	1117.53	2970.46	3.706	947.98	3513.10
A_68_P25954066	chr8:73311865-73311909	NM_199308:4446	Mast3	INSIDE	1.394	1.885	3213.06	6055.79	2.627	2384.39	6264.26
A_68_P24192244	chr5:151476355-151476399	NM_175310:-25	Pds5b	DIVERGENT_PROMOTER	1.394	1.880	2011.69	3782.47	2.620	1713.43	4490.00
A_68_P25114628	chr7:57034222-57034266	NM_133740:517	Prmt3	INSIDE	1.394	2.031	1557.36	3163.39	2.832	1345.01	3809.64
A_68_P26165837	chr8:113494378-113494429	NM_009179:50639	St3gal2	INSIDE	1.394	4.067	281.97	1146.83	5.670	250.48	1420.12
A_68_P25806165	chr8:41596899-41596943	NM_033560:-216	Vps37a	DIVERGENT_PROMOTER	1.394	2.268	3052.89	6923.55	3.162	2440.41	7716.95
A_68_P32414051	chrX:57657552-57657596	NM_001001798:-418	Atp11c	PROMOTER	1.393	3.236	1169.61	3785.34	4.509	898.54	4051.94
A_68_P25091981	chr7:52489690-52489734	NM_007645:4497	Cd37	INSIDE	1.393	3.706	1418.20	5256.43	5.164	1190.30	6146.77
A_68_P25187993	chr7:73532543-73532587	NM_146191:663	Lrrk1	INSIDE	1.393	1.898	3028.57	5747.25	2.644	2787.26	7368.42
A_68_P25016747	chr7:28118579-28118623	NM_001113549:67	Ltbp4	INSIDE	1.393	3.093	2893.09	8948.48	4.309	2424.53	10446.94
A_68_P31866300	chr18:80904546-80904590	NM_001164111:344	Nfatc1	INSIDE	1.393	3.042	622.87	1894.76	4.238	571.47	2421.71
A_68_P29294054	chr13:105844417-105844461	NM_029879:572	Rgs7bp	INSIDE	1.393	4.855	1066.43	5177.25	6.761	999.80	6759.52
A_68_P23012913	chr4:71860924-71860968	NM_011599:1007	Tle1	INSIDE	1.393	1.763	2197.50	3873.44	2.456	1831.01	4496.95
A_68_P20804421	chr1:169238701-169238745	NM_001039483:-78	Tmco1	PROMOTER	1.393	1.455	3612.53	5257.39	2.028	2927.21	5935.77

ProbeName	Target position of probe on CpG island microarray	TargetID	GeneSymbol	CpG island Description	Ratio of relative methylation (TiO_2-NP/Vehicle)	Sham group			TiO_2-H group		
						Relative methylation (Cy5/Cy3)	Cy3 signal (Input)	Cy5 signal (MeDIP)	Relative methylation (Cy5/Cy3)	Cy3 signal (Input)	Cy5 signal (MeDIP)
A_68_P25508612	chr7:135153559-135153603	NM_001079932:5689	Trim72	INSIDE	1.393	3.632	6264.66	22752.62	5.060	4584.63	23199.60
A_68_P30446388	chr15:93166877-93166921	NM_024189:468	Yaf2	INSIDE	1.393	1.789	459.90	822.73	2.492	410.73	1023.67
A_68_P27473557	chr10:115550809-115550853	NM_001161855:143	4933416C03Rik	INSIDE	1.392	1.775	841.21	1492.90	2.471	700.50	1730.61
A_68_P23952640	chr5:104937435-104937479	NM_001166581:86	BC005561	INSIDE	1.392	3.221	917.87	2956.25	4.484	900.73	4038.50
A_68_P31602500	chr18:32537426-32537470	NM_001083334:578	Bin1	INSIDE	1.392	2.016	1367.56	2757.24	2.807	1075.46	3018.73
A_68_P32121152	chr19:42507077-42507121	NM_145123:-825	Crtac1	PROMOTER	1.392	1.346	2500.39	3365.95	1.874	2134.04	3999.26
A_68_P32224194	chr19:60302569-60302613	NM_029648:10	D19Ertd737e	INSIDE	1.392	2.462	712.38	1753.56	3.427	651.94	2234.20
A_68_P21708094	chr2:144425869-144425913	NM_025314:202	Dtd1	INSIDE	1.392	2.707	1902.22	5149.00	3.769	1721.20	6486.54
A_68_P30287849	chr15:65618481-65618525	NM_133766:-100	Efr3a	PROMOTER	1.392	2.069	456.57	944.77	2.880	376.12	1083.26
A_68_P32087899	chr19:36132232-36132276	NM_008315:-404	Htr7	PROMOTER	1.392	3.060	1869.92	5721.76	4.258	1584.05	6744.81
A_68_P26258349	chr8:129107965-129108009	NM_001164598:9350	Irf2bp2	DOWNSTREAM	1.392	4.946	787.26	3893.53	6.883	617.74	4252.15
A_68_P22530174	chr3:130815152-130815196	NM_010703:1786	Lef1	INSIDE	1.392	1.934	1766.84	3416.36	2.691	1447.40	3894.72
A_68_P22289187	chr3:83959390-83959434	NM_029797:296	Mnd1	INSIDE	1.392	3.022	590.88	1785.42	4.205	507.76	2135.18
A_68_P26770183	chr9:100446090-100446134	NM_010878:360	Nck1	INSIDE	1.392	1.694	1160.50	1966.16	2.359	1027.67	2424.48
A_68_P21630523	chr2:130402275-130402319	NM_011025:388	Oxt	INSIDE	1.392	2.016	2820.56	5687.33	2.807	2397.92	6731.12
A_68_P29011801	chr13:47218477-47218521	NM_146042:410	Rnf144b	INSIDE	1.392	1.546	1748.77	2703.86	2.152	1491.46	3209.47
A_68_P23328080	chr4:134908744-134908788	NM_001130477:363	Srrm1	INSIDE	1.392	2.562	1269.99	3254.29	3.567	1131.87	4036.84
A_68_P30784286	chr16:57122130-57122174	NM_138599:326	Tomm70a	INSIDE	1.392	5.873	2134.34	12535.32	8.177	1613.28	13192.50
A_68_P31509672	chr18:14129799-14129843	NM_145492:1422	Zfp521	INSIDE	1.392	1.346	2572.26	3462.43	1.874	2083.95	3905.19
A_68_P28729767	chr12:109996277-109996321			Unknown	1.392	2.215	2800.23	6201.96	3.083	2176.21	6709.01
A_68_P23767822	chr5:67818022-67818066	NM_001164806:994	Bend4	INSIDE	1.391	1.964	2706.33	5314.51	2.732	2302.94	6292.60
A_68_P29787829	chr14:87541024-87541070	NM_019670:-125	Diap3	PROMOTER	1.391	1.480	4821.44	7138.04	2.060	3649.49	7516.66
A_68_P31201910	chr17:45710240-45710284	NM_008302:-52	Hsp90ab1	PROMOTER	1.391	1.931	2513.45	4852.98	2.685	2103.94	5649.86
A_68_P24431605	chr6:49164853-49164897	NM_023670:79	Igf2bp3	INSIDE	1.391	1.783	1060.06	1890.47	2.481	892.70	2215.10
A_68_P20832387	chr1:174255748-174255792	NM_008429:3624	Kcnj9	INSIDE	1.391	3.128	3509.47	10978.33	4.352	2542.31	11064.65
A_68_P31257382	chr17:56766395-56766439	NM_028782:-90	Lonp1	DIVERGENT_PROMOTER	1.391	2.748	5430.44	14925.43	3.824	3946.84	15092.92
A_68_P22190750	chr3:63100022-63100066	NM_008604:251	Mme	INSIDE	1.391	3.594	1374.41	4939.93	4.998	1143.34	5714.37
A_68_P31187137	chr17:43153945-43153989	NM_178589:463	Tnfrsf21	INSIDE	1.391	2.094	2666.64	5583.34	2.912	2260.14	6581.90
A_68_P28818477	chr13:9763394-9763438	NM_001199141:1144	Zmynd11	INSIDE	1.391	2.739	1885.24	5162.99	3.810	1673.44	6376.01
A_68_P32565209	chrX:98893628-98893674	ENSMUST00000073812:-6461		PROMOTER	1.391	3.079	936.61	2883.76	4.284	327.68	1403.82
A_68_P30931273	chr16:85802461-85802505	NM_009621:878	Adamts1	INSIDE	1.390	3.403	1563.18	5318.88	4.729	1329.86	6288.30
A_68_P24593651	chr6:83407070-83407114	NR_028382:15344	B230319C09Rik	DOWNSTREAM	1.390	1.651	1604.03	2648.16	2.295	1366.64	3136.95
A_68_P28191921	chr11:121534336-121534380	NM_178664:107	B3gntl1	INSIDE	1.390	2.023	813.65	1646.24	2.813	683.65	1922.94
A_68_P31166953	chr17:37183108-37183152	NM_019439:220	Gabbr1	INSIDE	1.390	2.246	545.54	1225.43	3.122	433.24	1352.77
A_68_P20293513	chr1:64663621-64663665	NM_025964:100	Mettl21a	INSIDE	1.390	7.059	1748.89	12345.54	9.810	1306.19	12813.78
A_68_P21934677	chr3:8923780-8923824	NM_025434:55	Mrps28	INSIDE	1.390	2.404	1130.38	2717.52	3.342	1012.75	3384.86
A_68_P32082675	chr19:34953035-34953079	NM_023792:-649	Pank1	PROMOTER	1.390	1.862	609.80	1135.72	2.588	620.50	1605.87
A_68_P26760867	chr9:98743140-98743184	NM_001134660:157	Prr23a	INSIDE	1.390	3.725	2610.95	9724.83	5.178	2298.06	11898.87
A_68_P23794550	chr5:73305332-73305376	NM_001113423:-201	Slain2	PROMOTER	1.390	1.421	1679.49	2387.30	1.976	1309.27	2587.76
A_68_P30487544	chr15:100592074-100592119	NM_021530:-81	Slc4a8	PROMOTER	1.390	1.893	878.86	1663.74	2.632	686.91	1807.95
A_68_P25089709	chr7:52110332-52110376	NM_026270:5758	Akt1s1	INSIDE	1.389	1.646	1948.90	3207.99	2.287	1590.76	3637.56
A_68_P30337621	chr15:74350207-74350251	NM_174991:3603	Bai1	INSIDE	1.389	1.830	1937.76	3547.01	2.542	1660.99	4222.91
A_68_P23299543	chr4:129665364-129665408	NM_001199696:3065	Bai2	INSIDE	1.389	4.824	1005.48	4850.09	6.698	818.53	5482.78
A_68_P27289988	chr10:80945144-80945188	NM_176954:288	Celf5	INSIDE	1.389	3.138	7031.28	22066.79	4.360	5090.20	22193.73
A_68_P20997455	chr2:9799133-9799177	NM_008091:1073	Gata3	INSIDE	1.389	2.203	1407.39	3101.06	3.060	1213.21	3711.88
A_68_P27286724	chr10:80448504-80448548	NM_001038655:15584	Gng7	INSIDE	1.389	2.779	5471.48	15203.85	3.859	4309.14	16628.08
A_68_P26346113	chr9:21043510-21043554	NM_001110305:244	Keap1	INSIDE	1.389	2.738	1066.07	2919.34	3.804	977.31	3717.21
A_68_P31462886	chr18:4375807-4375851	NM_026157:239	Mtpap	INSIDE	1.389	1.781	1407.85	2507.38	2.473	1354.18	3349.23
A_68_P23637417	chr5:42154620-42154664	NM_007524:817	Nkx3-2	INSIDE	1.389	1.588	3754.70	5962.47	2.206	2920.74	6441.82
A_68_P31757535	chr18:61559223-61559268	NM_133249:840	Ppargc1b	INSIDE	1.389	2.054	4873.75	10011.88	2.854	3783.56	10798.66
A_68_P21769250	chr2:155890860-155890904	NM_139151:-66	Spag4	PROMOTER	1.389	2.324	596.43	1386.03	3.229	546.75	1765.42

ProbeName	Target position of probe on CpG island microarray	TargetID	GeneSymbol	CpG island Description	Ratio of relative methylation (TiO_2-NP/Vehicle)	Sham group			TiO_2-H group		
						Relative methylation (Cy5/Cy3)	Cy3 signal (Input)	Cy5 signal (MeDIP)	Relative methylation (Cy5/Cy3)	Cy3 signal (Input)	Cy5 signal (MeDIP)
A_68_P27644767	chr11:23156427-23156471	NM_001035226:408	Xpo1	INSIDE	1.389	1.998	524.57	1048.31	2.776	417.92	1160.20
A_68_P28048035	chr11:96858653-96858697			Unknown	1.389	2.884	1597.67	4607.66	4.006	1231.51	4933.87
A_68_P22262794	chr3:79090694-79090738	AK032506:-597		PROMOTER	1.389	1.987	809.05	1607.50	2.761	754.96	2084.19
A_68_P32589659	chrX:105011372-105011416	NM_025921:279	2610002M06Rik	INSIDE	1.388	3.761	4965.52	18676.02	5.219	2295.18	11979.46
A_68_P24134879	chr5:139801058-139801102	NM_172723:338	Adap1	INSIDE	1.388	0.416	3250.82	1352.27	0.577	2894.34	1671.39
A_68_P31516123	chr18:15309505-15309549	NM_134112:429	Kctd1	INSIDE	1.388	1.414	3242.69	4584.00	1.962	2543.33	4989.28
A_68_P28738216	chr12:111684724-111684768	NM_001135001:-642	Ppp2r5c	PROMOTER	1.388	2.129	4451.31	9478.89	2.956	3140.30	9281.26
A_68_P20770876	chr1:163181287-163181331	NM_007453:-11	Prdx6	PROMOTER	1.388	1.612	7474.68	12051.29	2.238	5273.32	11802.44
A_68_P22572447	chr3:138737478-138737522	NM_001040690:663	Rap1gds1	INSIDE	1.388	2.478	2028.06	5026.53	3.440	1758.57	6049.20
A_68_P21557413	chr2:117168443-117168487	NM_011246:149	Rasgrp1	INSIDE	1.388	2.134	448.72	957.44	2.961	404.89	1198.97
A_68_P29715888	chr14:73543452-73543496	NR_033185:883	Rcbtb2	INSIDE	1.388	2.304	1611.48	3712.43	3.198	1358.60	4344.63
A_68_P26823109	chr9:110235990-110236034	NM_001001144:216	Scap	INSIDE	1.388	1.776	1006.73	1787.90	2.465	936.32	2308.19
A_68_P30425916	chr15:89388272-89388316	NM_021423:58007	Shank3	INSIDE	1.388	2.300	963.68	2216.92	3.194	959.09	3063.19
A_68_P30364761	chr15:78993943-78993987	NM_011437:956	Sox10	INSIDE	1.388	1.372	1741.41	2389.96	1.905	1480.70	2820.63
A_68_P23321805	chr4:133805694-133805738	NM_024215:-4210	Zfp593	PROMOTER	1.388	2.078	568.36	1181.10	2.885	373.34	1077.17
A_68_P26350214	chr9:21855080-21855124	NM_010487:1365	Elavl3	INSIDE	1.387	1.879	1127.71	2118.45	2.605	987.56	2572.23
A_68_P31215014	chr17:48061036-48061080	NM_001110824:523	Foxp4	INSIDE	1.387	3.489	1284.81	4483.10	4.841	1017.48	4925.13
A_68_P21874746	chr2:174153286-174153330	NR_003258:-106	Gnas	PROMOTER	1.387	1.563	557.01	870.38	2.168	460.07	997.43
A_68_P27996400	chr11:87630599-87630643	NM_080420:8996	Lpo	INSIDE	1.387	2.331	3454.61	8052.90	3.233	2408.00	7783.99
A_68_P31930919	chr19:5803763-5803807	NR_002847:-1113	Malat1	PROMOTER	1.387	3.632	3709.80	13474.98	5.039	2884.96	14537.26
A_68_P25285292	chr7:91031812-91031856	NM_030705:1017	Mesdc1	INSIDE	1.387	1.854	1987.57	3685.71	2.573	1610.70	4144.00
A_68_P23439968	chr4:155043128-155043172	NM_145124:186	Mib2	INSIDE	1.387	0.439	1003.59	440.94	0.609	1058.62	645.06
A_68_P31542302	chr18:21120354-21120398	NM_026301:17251	Rnf125	INSIDE	1.387	3.232	1806.35	5838.05	4.482	1432.12	6418.86
A_68_P32136240	chr19:45139857-45139901	NM_001178012:17813	Sfxn3	DOWNSTREAM	1.387	2.930	1865.52	5465.30	4.062	1583.58	6432.72
A_68_P27288939	chr10:80795142-80795186	NM_009325:3689	Tbxa2r	INSIDE	1.387	2.434	820.12	1996.51	3.377	677.70	2288.73
A_68_P23255470	chr4:120887662-120887706	ENSMUST00000056635:5		INSIDE	1.387	1.989	1497.95	2979.89	2.760	1185.81	3272.51
A_68_P26617806	chr9:70527432-70527476	NM_007399:647	Adam10	INSIDE	1.386	4.446	897.40	3989.47	6.163	771.76	4756.74
A_68_P27176090	chr10:60149152-60149197	NM_023370:10064	Cdh23	INSIDE	1.386	10.499	320.92	3369.37	14.546	282.00	4102.07
A_68_P25512010	chr7:135831942-135831986	NM_178641:77086	Inpp5f	INSIDE	1.386	3.071	1024.72	3146.72	4.256	926.84	3944.48
A_68_P22934417	chr4:55543327-55543371	NM_010637:1999	Klf4	INSIDE	1.386	2.954	2248.19	6640.92	4.094	1724.91	7061.55
A_68_P31130477	chr17:30026875-30026919	NM_001081160:-2069	Mdga1	PROMOTER	1.386	2.242	770.76	1728.12	3.107	603.50	1875.07
A_68_P27922752	chr11:74651542-74651586	NM_010813:7139	Mnt	INSIDE	1.386	1.680	496.56	834.26	2.329	347.28	808.66
A_68_P29001573	chr13:45484920-45484964	NM_153789:-168	Mylip	PROMOTER	1.386	2.542	1859.71	4727.59	3.523	1466.18	5165.83
A_68_P31112706	chr17:26976161-26976205	NM_008700:2328	Nkx2-5	INSIDE	1.386	1.920	555.70	1067.23	2.663	529.20	1409.09
A_68_P20506812	chr1:108069726-108069770	NM_133821:1303	Phlpp1	INSIDE	1.386	0.442	1093.08	482.98	0.612	988.03	605.05
A_68_P29578876	chr14:47503079-47503123	NM_001037221:461	Samd4	INSIDE	1.386	2.731	1230.87	3361.27	3.786	1130.55	4279.76
A_68_P23081886	chr4:86520283-86520327	NM_139306:-13	Acer2	PROMOTER	1.385	1.376	1471.41	2024.91	1.906	1311.14	2499.13
A_68_P28081265	chr11:102622917-102622961	NM_001110778:186	Adam11	INSIDE	1.385	1.343	4557.53	6122.12	1.860	3493.31	6498.27
A_68_P25954612	chr8:73392998-73393042	NM_026964:4369	Ccdc124	INSIDE	1.385	5.066	149.73	758.54	7.018	132.41	929.27
A_68_P21612764	chr2:127162058-127162102	NM_010090:186	Dusp2	INSIDE	1.385	3.209	1458.87	4681.39	4.445	1289.22	5730.87
A_68_P30480288	chr15:99288482-99288527	NM_001001884:-325	Nckap5l	PROMOTER	1.385	2.314	337.82	781.56	3.205	286.71	918.82
A_68_P26772514	chr9:100935310-100935354	NM_025835:-38	Pccb	PROMOTER	1.385	2.186	736.85	1610.80	3.028	620.75	1879.44
A_68_P28549418	chr12:76696906-76696950	NM_012024:259	Ppp2r5e	INSIDE	1.385	1.423	1358.66	1932.97	1.971	1145.58	2257.54
A_68_P30447921	chr15:93426390-93426434	NM_001033217:-90	Prickle1	PROMOTER	1.385	1.576	625.87	986.42	2.182	603.39	1316.77
A_68_P24092506	chr5:130647910-130647954	NM_001199059:244	Rabgef1	INSIDE	1.385	1.536	704.41	1081.84	2.128	612.31	1302.86
A_68_P26782297	chr9:102737533-102737577	NM_001042607:305	Ryk	INSIDE	1.385	1.916	530.94	1017.41	2.654	555.68	1474.61
A_68_P31386733	chr17:81464391-81464435	NM_028138:13	Thumpd2	INSIDE	1.385	2.672	501.61	1340.18	3.699	445.20	1646.93
A_68_P22594408	chr3:142763801-142763845			Unknown	1.385	2.405	1253.99	3015.34	3.331	1139.39	3794.97
A_68_P27938081	chr11:77329399-77329443	NM_026185:802	Abhd15	INSIDE	1.384	1.651	1075.96	1776.09	2.285	893.15	2041.00
A_68_P31097619	chr17:24625915-24625959	NM_027937:209	Caskin1	INSIDE	1.384	6.079	2677.94	16279.90	8.413	2045.41	17207.47
A_68_P23203782	chr4:109656380-109656424	NM_172296:5773	Dmrta2	DOWNSTREAM	1.384	3.514	1461.45	5135.93	4.864	1229.56	5980.70

ProbeName	Target position of probe on CpG island microarray	TargetID	GeneSymbol	CpG island Description	Ratio of relative methylation (TiO_2-NP/Vehicle)	Sham group			TiO_2-H group		
						Relative methylation (Cy5/Cy3)	Cy3 signal (Input)	Cy5 signal (MeDIP)	Relative methylation (Cy5/Cy3)	Cy3 signal (Input)	Cy5 signal (MeDIP)
A_68_P29626310	chr14:57752221-57752265	NM_008128:-8184	Gjb6	PROMOTER	1.384	2.282	684.04	1561.29	3.159	668.13	2110.85
A_68_P25023673	chr7:29596377-29596421	NM_177301:490	Hnrnpl	INSIDE	1.384	2.347	1864.68	4377.06	3.249	1656.71	5383.10
A_68_P28530098	chr12:73186548-73186592	NM_001205067:-255	Jkamp	DIVERGENT_PROMOTER	1.384	2.022	1860.70	3762.19	2.798	1158.76	3241.72
A_68_P25176079	chr7:71432668-71432712	NM_172742:135	Mtmr10	INSIDE	1.384	2.188	1366.94	2991.38	3.028	1213.24	3673.97
A_68_P25016937	chr7:28149369-28149413	NM_001199235:30135	Spnb4	INSIDE	1.384	4.256	450.84	1918.83	5.890	388.56	2288.49
A_68_P24929724	chr6:146582793-146582837	NM_026281:300	Tm7sf3	INSIDE	1.384	2.488	1029.62	2561.28	3.444	814.79	2806.13
A_68_P20444766	chr1:93698474-93698518	NM_007855:443	Twist2	INSIDE	1.384	2.254	2341.47	5277.33	3.120	1896.89	5918.08
A_68_P31292365	chr17:64199012-64199056			Unknown	1.384	1.783	1971.01	3513.41	2.468	1488.53	3673.51
A_68_P30221125	chr15:53177961-53178005	NM_010162:-244	Ext1	PROMOTER	1.383	1.695	850.39	1441.42	2.345	757.81	1776.96
A_68_P24932468	chr6:147040234-147040278	NM_001081237:660	Klhdc5	INSIDE	1.383	4.799	492.26	2362.45	6.635	486.13	3225.65
A_68_P28246270	chr12:12944193-12944237	NM_008709:4428	Mycn	INSIDE	1.383	1.799	1343.75	2416.97	2.488	1094.02	2721.69
A_68_P31848618	chr18:77803995-77804039	NM_001164504:-141	Rnf165	PROMOTER	1.383	1.500	1536.57	2304.59	2.074	1326.48	2750.58
A_68_P31120665	chr17:28278842-28278886	NM_001004366:-606	Scube3	PROMOTER	1.383	3.106	1273.71	3956.08	4.294	1103.17	4737.50
A_68_P31682245	chr18:47528367-47528411	NM_018744:134	Sema6a	INSIDE	1.383	2.207	659.59	1455.48	3.051	574.22	1751.82
A_68_P25716368	chr8:24522869-24522913	NM_013834:917	Sfrp1	INSIDE	1.383	1.613	2227.30	3593.62	2.231	1935.21	4317.32
A_68_P24039145	chr5:120961107-120961151	NM_001177594:-72	Slc24a6	DIVERGENT_PROMOTER	1.383	1.486	4781.84	7103.62	2.054	3617.40	7431.95
A_68_P23364649	chr4:141094339-141094383	NM_019763:152	Spen	INSIDE	1.383	1.992	2347.37	4676.34	2.756	1958.71	5398.44
A_68_P23238477	chr4:117807306-117807350	NM_009176:167	St3gal3	INSIDE	1.383	2.005	540.61	1083.76	2.773	510.76	1416.34
A_68_P29072110	chr13:58316632-58316676	NM_026842:360	Ubqln1	INSIDE	1.383	2.096	560.88	1175.53	2.898	535.81	1552.68
A_68_P30382917	chr15:82176766-82176812	NM_026914:313	1500032L24Rik	INSIDE	1.382	3.347	912.41	3053.90	4.627	745.99	3451.56
A_68_P26920734	chr10:7500514-7500558	NM_145418:179	BC013529	INSIDE	1.382	1.710	990.54	1693.60	2.363	880.38	2080.26
A_68_P21111660	chr2:31701435-31701479	NM_178887:69	Fibcd1	INSIDE	1.382	2.715	1341.96	3643.49	3.753	1101.84	4135.43
A_68_P22081213	chr3:40549234-40549278	NM_011020:-278	Hspa4l	PROMOTER	1.382	2.443	1011.96	2472.12	3.375	784.50	2647.61
A_68_P28055738	chr11:98195652-98195696	NM_010895:-4715	Neurod2	PROMOTER	1.382	1.669	2668.79	4452.94	2.306	2176.79	5019.22
A_68_P31921055	chr19:4101179-4101223	NM_008851:579	Pitpnm1	INSIDE	1.382	1.719	420.25	722.25	2.375	310.95	738.64
A_68_P26079858	chr8:97381751-97381795	NM_009090:423	Polr2c	INSIDE	1.382	2.436	1507.50	3672.30	3.366	1204.30	4054.04
A_68_P23668870	chr5:48377571-48377615	NM_178804:3199	Slit2	INSIDE	1.382	2.559	2256.35	5774.06	3.537	1713.19	6060.08
A_68_P26504312	chr9:50369341-50369385	NM_025687:11	Tex12	INSIDE	1.382	3.272	1507.58	4933.34	4.523	1344.59	6081.12
A_68_P24115622	chr5:135752511-135752555			Unknown	1.382	2.149	2790.05	5996.38	2.970	2278.78	6767.93
A_68_P31933962	chr19:6320206-6320250	NM_001033342:13772	Cdc42bpg	INSIDE	1.381	3.899	1882.50	7340.23	5.385	1508.45	8123.72
A_68_P32820569	chrX:165758461-165758505	NM_008222:-207	Hccs	PROMOTER	1.381	3.000	691.81	2075.27	4.142	442.18	1831.64
A_68_P25005400	chr7:25318962-25319006	NM_153134:3318	Irgq	INSIDE	1.381	2.764	1793.18	4955.65	3.818	1547.70	5908.62
A_68_P28998903	chr13:44998308-44998352	NM_001205043:171691	Jarid2	INSIDE	1.381	3.750	2610.28	9788.20	5.180	2206.54	11430.73
A_68_P32122315	chr19:42687286-42687330	NM_001164311:-12	Loxl4	PROMOTER	1.381	5.696	2747.26	15647.16	7.863	2171.28	17072.43
A_68_P23985655	chr5:111846403-111846447	NM_001081235:-761	Mn1	PROMOTER	1.381	3.121	952.83	2973.79	4.311	746.99	3220.03
A_68_P23292285	chr4:128332448-128332492	NM_001195130:525	Phc2	INSIDE	1.381	2.352	1220.71	2871.13	3.248	867.57	2817.74
A_68_P21432198	chr2:92885847-92885891	NM_001177536:433	Prdm11	INSIDE	1.381	2.016	2664.89	5372.70	2.784	2130.21	5930.78
A_68_P22883652	chr4:45543353-45543397	NM_001033306:326	Shb	INSIDE	1.381	1.440	1244.64	1792.30	1.989	1018.36	2025.13
A_68_P29936814	chr14:118531918-118531962	NM_029578:47	Tgds	INSIDE	1.381	2.107	848.15	1787.14	2.909	718.51	2090.03
A_68_P20046436	chr1:14496639-14496683			Unknown	1.381	1.790	1532.18	2743.32	2.473	1331.58	3292.41
A_68_P22832218	chr4:34663760-34663804	NM_027041:13673	1700003M02Rik	INSIDE	1.380	2.400	327.86	786.83	3.313	316.86	1049.66
A_68_P23225501	chr4:115410210-115410254	NM_172698:-445	4732418C07Rik	PROMOTER	1.380	1.596	2393.59	3820.41	2.202	1950.35	4295.52
A_68_P23966390	chr5:107760143-107760187	NM_001079873:-48	Brdt	PROMOTER	1.380	1.925	1143.18	2200.75	2.657	1031.75	2741.32
A_68_P30589166	chr16:20716372-20716416	NM_009900:315	Clcn2	INSIDE	1.380	1.526	1428.09	2178.81	2.105	1153.03	2427.48
A_68_P23222742	chr4:114659539-114659583	NM_025647:273	Cmpk1	INSIDE	1.380	5.083	1829.05	9297.78	7.013	1581.23	11088.78
A_68_P31694513	chr18:49915007-49915051	NM_001170960:227	Dtwd2	INSIDE	1.380	4.950	2036.11	10079.53	6.830	1655.93	11309.80
A_68_P22009690	chr3:27052485-27052529	NM_001177625:270	Ect2	INSIDE	1.380	1.652	4969.44	8207.66	2.280	3935.17	8970.84
A_68_P27284175	chr10:80049466-80049510	NM_145421:3598	Fam108a	INSIDE	1.380	4.596	1189.87	5468.94	6.343	1018.45	6459.94
A_68_P22702204	chr4:5571466-5571510	NM_173426:163	Fam110b	INSIDE	1.380	1.694	809.27	1370.98	2.338	736.49	1722.06
A_68_P24429121	chr6:48693073-48693117	NM_008376:4049	Gimap1	INSIDE	1.380	2.233	1776.08	3965.19	3.080	1429.23	4401.83
A_68_P23333240	chr4:135866700-135866744	NM_028871:-168	Hnrnpr	PROMOTER	1.380	2.154	1180.61	2542.84	2.972	1064.75	3164.30

ProbeName	Target position of probe on CpG island microarray	TargetID	GeneSymbol	CpG island Description	Ratio of relative methylation (TiO_2-NP/Vehicle)	Sham group			TiO_2-H group		
						Relative methylation (Cy5/Cy3)	Cy3 signal (Input)	Cy5 signal (MeDIP)	Relative methylation (Cy5/Cy3)	Cy3 signal (Input)	Cy5 signal (MeDIP)
A_68_P28185589	chr11:120469914-120469958	NM_153288:146	Npb	INSIDE	1.380	1.724	626.95	1080.66	2.378	563.76	1340.59
A_68_P23696527	chr5:53133072-53133116	NM_025951:282	Pi4k2b	INSIDE	1.380	3.064	616.45	1889.00	4.230	538.73	2278.62
A_68_P23374912	chr4:142984306-142984350	NM_031377:-15	Pramel1	PROMOTER	1.380	6.083	4431.85	26959.06	8.397	3035.40	25488.68
A_68_P20779531	chr1:164670132-164670176	NM_001081290:533	Prrc2c	INSIDE	1.380	1.464	1019.61	1492.29	2.019	862.65	1741.74
A_68_P30990391	chr16:96212295-96212339	NM_019537:194	Psmg1	INSIDE	1.380	1.645	2589.44	4259.35	2.270	2009.46	4560.84
A_68_P27901831	chr11:70460715-70460759	NM_024211:-241	Slc25a11	DIVERGENT_PROMOTER	1.380	2.844	1614.27	4590.39	3.925	1332.11	5228.40
A_68_P20172918	chr1:40738845-40738889	NM_001033289:310	Slc9a2	INSIDE	1.380	1.589	1993.26	3167.76	2.193	1698.03	3723.11
A_68_P31846337	chr18:77424326-77424370	NM_013666:-237	St8sia5	PROMOTER	1.380	1.438	1106.77	1591.43	1.984	926.23	1837.71
A_68_P22889661	chr4:46627029-46627073	NM_198664:36021	Tbc1d2	INSIDE	1.380	2.524	978.10	2468.45	3.482	838.18	2918.50
A_68_P29506489	chr14:31362538-31362582	NM_009388:226	Tkt	INSIDE	1.380	4.247	1327.69	5639.28	5.860	1239.52	7264.01
A_68_P24155694	chr5:144016617-144016661	NM_017467:15063	Zfp316	INSIDE	1.380	4.227	2787.80	11782.74	5.832	3116.04	18172.71
A_68_P31755200	chr18:61195546-61195590	NM_009880:285	Cdx1	INSIDE	1.379	3.032	739.77	2242.91	4.181	703.01	2939.13
A_68_P25564249	chr7:144390277-144390323	NM_001113414:115828	Ebf3	INSIDE	1.379	8.357	3043.67	25435.00	11.525	2012.21	23191.66
A_68_P24477004	chr6:56782166-56782210	NM_012056:136	Fkbp9	INSIDE	1.379	3.039	1936.77	5886.78	4.190	1684.97	7060.59
A_68_P24129311	chr5:138635844-138635888	NM_177878:325	Mblac1	INSIDE	1.379	1.861	1250.14	2326.95	2.566	1257.67	3227.68
A_68_P30252139	chr15:58912975-58913019	NM_001146180:585	Mtss1	INSIDE	1.379	4.491	2342.42	10520.33	6.192	1909.09	11820.70
A_68_P23281395	chr4:126355065-126355109	NM_011970:200	Psmb2	INSIDE	1.379	1.555	610.53	949.65	2.145	491.93	1055.12
A_68_P30546791	chr16:11203150-11203194	NM_025546:213	Rsl1d1	INSIDE	1.379	2.630	4731.03	12440.96	3.626	4018.95	14572.39
A_68_P26506991	chr9:50816720-50816764	NM_178710:436	Sik2	INSIDE	1.379	1.394	3412.25	4755.79	1.922	2627.82	5049.66
A_68_P30436285	chr15:91403260-91403304	NM_001033633:410	Slc2a13	INSIDE	1.379	6.148	784.46	4822.57	8.478	676.56	5735.66
A_68_P20456741	chr1:95532187-95532231	NM_021537:96	Stk25	INSIDE	1.379	2.702	1331.87	3598.85	3.726	1122.47	4182.07
A_68_P32806692	chrX:162947996-162948040	NM_001007577:392	Tceanc	INSIDE	1.379	2.785	1571.63	4377.73	3.842	810.69	3114.39
A_68_P31385964	chr17:81344186-81344230	NM_026516:237	Tmem178	INSIDE	1.379	1.688	2239.28	3779.05	2.328	1997.79	4650.03
A_68_P31320413	chr17:69190632-69190676	NM_001206661:4179	Tmem200c	INSIDE	1.379	3.280	1315.62	4315.33	4.524	1228.76	5558.54
A_68_P32744218	chrX:147530920-147530964	NM_001164578:144	Tsr2	INSIDE	1.379	1.849	1593.64	2946.93	2.549	884.15	2253.95
A_68_P28029564	chr11:93746411-93746455	NM_001013375:648	Utp18	INSIDE	1.379	3.233	7724.07	24970.39	4.457	6462.51	28801.15
A_68_P29623591	chr14:57190486-57190530	NM_001014996:175	Cenpj	INSIDE	1.378	2.599	1315.91	3419.70	3.581	1026.85	3676.94
A_68_P31620307	chr18:35990504-35990548	NM_133687:1055	Cxxc5	INSIDE	1.378	2.707	1313.88	3556.03	3.729	1077.64	4018.54
A_68_P30678530	chr16:37012233-37012277	NM_001159369:383	Polq	INSIDE	1.378	1.598	710.95	1136.21	2.202	600.92	1323.31
A_68_P24994377	chr7:20204860-20204904	NM_009046:9905	Relb	INSIDE	1.378	2.654	2286.08	6067.75	3.657	1835.48	6713.10
A_68_P27786170	chr11:49838566-49838610	NM_021540:-244	Rnf130	PROMOTER	1.378	1.429	1226.49	1753.24	1.970	1132.76	2231.12
A_68_P31196997	chr17:44873430-44873474	NM_001145920:145	Runx2	INSIDE	1.378	2.438	917.75	2237.63	3.360	915.27	3074.90
A_68_P28332011	chr12:32244102-32244146	NM_011867:710	Slc26a4	INSIDE	1.378	5.869	464.29	2724.78	8.089	473.71	3831.95
A_68_P28087051	chr11:103634875-103634919	NM_009521:-592	Wnt3	PROMOTER	1.378	2.144	2186.97	4688.89	2.955	1748.70	5167.87
A_68_P28703312	chr12:105685478-105685522			Unknown	1.378	4.544	1247.37	5668.27	6.264	1078.33	6754.76
A_68_P31560300	chr18:24625872-24625916	NM_026529:-3477	2700062C07Rik	PROMOTER	1.377	2.091	1837.99	3843.87	2.880	1537.54	4428.68
A_68_P20872226	chr1:181733791-181733835	NM_026375:334	Ahctf1	INSIDE	1.377	1.483	1020.63	1513.38	2.042	953.61	1947.41
A_68_P26641128	chr9:74800966-74801010	NM_001113283:129	BC031353	INSIDE	1.377	1.421	935.86	1330.00	1.957	757.06	1481.86
A_68_P26229677	chr8:124431978-124432022	NM_001033485:268	BC048644	INSIDE	1.377	1.832	3606.16	6605.22	2.522	2702.89	6815.57
A_68_P24993659	chr7:20092410-20092454	NM_177692:1248	Bloc1s3	INSIDE	1.377	1.527	817.62	1248.89	2.103	736.93	1549.64
A_68_P23415125	chr4:151235092-151235136	NM_001081557:763	Camta1	INSIDE	1.377	1.933	1640.21	3170.34	2.661	1284.07	3416.84
A_68_P28175792	chr11:118951266-118951310	NM_007625:-3737	Cbx4	PROMOTER	1.377	1.724	1989.94	3429.86	2.373	1495.37	3548.44
A_68_P30347072	chr15:76077510-76077554	NM_023168:296	Grina	INSIDE	1.377	1.644	4149.13	6822.67	2.264	3082.56	6979.74
A_68_P25770373	chr8:34842329-34842373	NM_001167921:-35	Gtf2e2	PROMOTER	1.377	3.104	596.25	1850.67	4.273	555.40	2373.50
A_68_P23300610	chr4:129815848-129815892	NM_001163027:-75	Hcrtr1	PROMOTER	1.377	3.703	712.34	2637.77	5.099	651.74	3323.44
A_68_P21104807	chr2:30567268-30567312	NR_037272:-9439	Mir3089	PROMOTER	1.377	1.905	1670.97	3182.87	2.623	1314.14	3447.47
A_68_P31110244	chr17:26575843-26575887	NM_001081656:23955	Neurl1b	INSIDE	1.377	2.750	354.52	974.95	3.786	392.75	1486.86
A_68_P31992143	chr19:18706329-18706373	NM_017375:-47	Ostf1	DIVERGENT_PROMOTER	1.377	3.356	665.77	2234.11	4.620	579.61	2677.94
A_68_P27310783	chr10:85334508-85334552	NM_133993:-45	Pwp1	PROMOTER	1.377	4.295	2751.45	11818.80	5.915	2115.68	12513.39
A_68_P21370050	chr2:79475696-79475740	NM_080558:137	Ssfa2	INSIDE	1.377	3.999	1978.05	7909.62	5.507	1706.95	9399.62
A_68_P29647966	chr14:61656769-61656813	NM_001164155:34	Tnfrsf19	INSIDE	1.377	1.838	6260.09	11508.96	2.532	4828.77	12228.67

ProbeName	Target position of probe on CpG island microarray	TargetID	GeneSymbol	CpG island Description	Ratio of relative methylation (TiO_2-NP/Vehicle)	Sham group			TiO_2-H group		
						Relative methylation (Cy5/Cy3)	Cy3 signal (Input)	Cy5 signal (MeDIP)	Relative methylation (Cy5/Cy3)	Cy3 signal (Input)	Cy5 signal (MeDIP)
A_68_P22314483	chr3:88357962-88358006	NM_033526:347	Ubqln4	INSIDE	1.377	1.901	4250.16	8080.01	2.618	3593.31	9407.82
A_68_P24302940	chr6:24615184-24615228	NM_028459:-211	Wasl	PROMOTER	1.377	3.177	5056.14	16063.79	4.373	3996.07	17476.81
A_68_P24164949	chr5:145992823-145992867	NM_028298:217	Zfp655	INSIDE	1.377	2.062	389.23	802.68	2.839	344.22	977.21
A_68_P23434211	chr4:154176443-154176487			Unknown	1.377	1.561	543.93	849.12	2.149	381.65	820.32
A_68_P28430596	chr12:52694381-52694425	NM_001198835:75	Coch	INSIDE	1.376	0.478	1309.17	626.02	0.658	1116.29	734.38
A_68_P29699005	chr14:70475525-70475569	NM_018781:-1705	Egr3	PROMOTER	1.376	1.394	1438.22	2004.90	1.918	1157.31	2219.84
A_68_P26319144	chr9:14556142-14556186	NM_010242:402	Fut4	INSIDE	1.376	3.074	1203.39	3699.80	4.231	1121.20	4744.15
A_68_P26084325	chr8:98157711-98157755	NM_030198:274	Gins3	INSIDE	1.376	1.883	985.87	1856.77	2.592	859.02	2226.91
A_68_P26228892	chr8:124308825-124308869	NM_020605:54388	Jph3	INSIDE	1.376	1.724	995.29	1715.95	2.372	838.23	1988.43
A_68_P25362045	chr7:106617271-106617315	NM_028145:2779	Klhl35	INSIDE	1.376	2.305	526.96	1214.56	3.171	454.24	1440.49
A_68_P23391245	chr4:147278506-147278550	NM_133201:400	Mfn2	INSIDE	1.376	3.309	1814.59	6004.03	4.552	1323.36	6024.14
A_68_P25261415	chr7:86644791-86644835	NR_029818:-5337	Mir9-3	PROMOTER	1.376	3.090	1236.50	3820.81	4.251	1120.30	4762.78
A_68_P23567786	chr5:29804560-29804604	NM_019944:428	Mnx1	INSIDE	1.376	2.546	1206.43	3072.06	3.503	1201.39	4208.33
A_68_P29009458	chr13:46822289-46822335	NM_175749:906	Nup153	INSIDE	1.376	1.969	2573.51	5068.11	2.710	1813.46	4914.81
A_68_P20774854	chr1:163899508-163899552	NM_001039045:212	Pigc	INSIDE	1.376	2.331	1042.59	2430.21	3.208	898.56	2882.98
A_68_P30346606	chr15:76010797-76010841	NM_201394:15322	Plec	INSIDE	1.376	2.047	535.71	1096.39	2.815	444.76	1252.19
A_68_P30566363	chr16:15638071-15638115	NM_011159:134	Prkdc	INSIDE	1.376	1.710	1088.29	1861.23	2.354	930.80	2190.88
A_68_P29256277	chr13:98976271-98976315	NM_012026:-172	Rgnef	PROMOTER	1.376	3.274	1412.57	4624.77	4.504	1243.93	5602.44
A_68_P23576986	chr5:31495832-31495876	NM_153680:178	Snx17	INSIDE	1.376	1.840	2165.65	3985.33	2.533	1787.83	4528.59
A_68_P27579022	chr11:8524378-8524422	NM_001083587:40138	Tns3	INSIDE	1.376	1.548	2177.26	3369.77	2.130	1779.43	3789.85
A_68_P28961200	chr13:38619778-38619822	NM_145367:529	Txndc5	INSIDE	1.376	3.821	5245.05	20040.92	5.259	3912.62	20576.59
A_68_P20833903	chr1:174493113-174493157	NR_027644:3098	Vsig8	INSIDE	1.376	1.793	2249.43	4033.83	2.468	1999.11	4933.52
A_68_P27646190	chr11:23397665-23397709	NM_172391:260	Ahsa2	INSIDE	1.375	1.715	1621.39	2781.47	2.359	1351.90	3189.46
A_68_P29514239	chr14:32644087-32644131	NM_001024604:-507	Ankrd28	PROMOTER	1.375	1.676	1130.69	1895.08	2.304	944.60	2176.30
A_68_P32414052	chrX:57657724-57657768	NM_001001798:-590	Atp11c	PROMOTER	1.375	1.643	538.41	884.38	2.259	296.57	669.91
A_68_P27933360	chr11:76479988-76480032	NM_177182:-5961	Bhlha9	PROMOTER	1.375	3.983	364.29	1450.84	5.478	383.99	2103.54
A_68_P21768605	chr2:155782745-155782804	NM_001129999:481	Cep250	INSIDE	1.375	5.441	118.40	644.25	7.479	128.96	964.52
A_68_P26880500	chr9:120842272-120842316	NM_007614:-223	Ctnnb1	PROMOTER	1.375	2.419	2400.00	5804.42	3.326	2000.36	6653.02
A_68_P22152359	chr3:55046645-55046689	NM_001111053:219	Dclk1	INSIDE	1.375	3.624	8361.76	30303.38	4.984	6503.71	32416.85
A_68_P20137271	chr1:34234808-34234852	NM_133833:166161	Dst	INSIDE	1.375	3.461	716.17	2478.51	4.760	596.28	2838.31
A_68_P28493839	chr12:66176820-66176864	NM_178912:250	Fancm	INSIDE	1.375	2.779	3973.93	11044.06	3.820	3275.45	12511.94
A_68_P25965606	chr8:75876811-75876855	NM_010687:-377	Large	PROMOTER	1.375	2.625	2341.04	6144.85	3.610	1995.36	7202.71
A_68_P30425292	chr15:89288332-89288376	NM_021921:4013	Mapk8ip2	INSIDE	1.375	3.266	229.60	749.97	4.492	227.71	1022.91
A_68_P28269969	chr12:17355438-17355482	NR_037226:-6737	Mir3066	PROMOTER	1.375	2.091	685.61	1433.87	2.876	587.58	1689.64
A_68_P26904675	chr10:4424482-4424526	NM_001161822:364	Rgs17	INSIDE	1.375	1.732	1542.84	2671.65	2.381	1583.48	3770.68
A_68_P31598790	chr18:31769072-31769116	NM_026165:462	Slc25a46	INSIDE	1.375	6.570	998.35	6559.36	9.031	852.54	7699.27
A_68_P32260673	chrX:11717563-11717607	NM_001168321:19897	Bcor	INSIDE	1.374	2.878	826.98	2380.40	3.955	499.68	1976.01
A_68_P23927393	chr5:100468878-100468922	NM_001163035:-112	Enoph1	DIVERGENT_PROMOTER	1.374	1.509	1248.13	1882.91	2.073	955.75	1981.28
A_68_P24143544	chr5:141306207-141306251	NM_010302:157	Gna12	INSIDE	1.374	3.560	907.44	3230.87	4.890	814.68	3984.00
A_68_P30350023	chr15:76528479-76528523	NM_182805:1307	Gpt	INSIDE	1.374	1.770	971.90	1720.43	2.432	862.92	2098.74
A_68_P21794482	chr2:160191240-160191284	NM_010658:1539	Mafb	INSIDE	1.374	1.503	3066.50	4609.26	2.065	2286.40	4721.48
A_68_P32579358	chrX:102316971-102317015	NM_053201:1258	Magee1	INSIDE	1.374	2.382	719.09	1712.51	3.272	498.39	1630.53
A_68_P23006652	chr4:70195652-70195696	NM_172694:288	Megf9	INSIDE	1.374	2.072	963.32	1996.32	2.847	830.41	2363.84
A_68_P21132204	chr2:35056598-35056642	NM_026697:20	Rab14	INSIDE	1.374	1.586	6464.98	10254.22	2.179	5245.70	11430.34
A_68_P25092756	chr7:52632188-52632232	NM_009224:18807	Snrnp70	INSIDE	1.374	3.300	2001.33	6604.55	4.534	1404.80	6368.80
A_68_P32565202	chrX:98892756-98892800	ENSMUST00000073812:-5589		PROMOTER	1.374	6.762	4957.84	33525.38	9.293	2152.12	19999.37
A_68_P22069325	chr3:38355009-38355053	NM_001167883:28708	Ankrd50	INSIDE	1.373	1.625	837.43	1360.69	2.231	684.92	1527.92
A_68_P28050583	chr11:97301455-97301499	NM_021493:-9997	Arhgap23	PROMOTER	1.373	2.380	1152.01	2741.29	3.267	924.38	3019.91
A_68_P22311221	chr3:87801100-87801144	NM_007529:3156	Bcan	INSIDE	1.373	2.311	651.53	1505.78	3.174	611.78	1941.94
A_68_P20350875	chr1:74901471-74901515	NM_009872:-110	Cdk5r2	PROMOTER	1.373	1.914	784.58	1501.78	2.628	687.29	1806.35
A_68_P26526112	chr9:54349310-54349354	NM_172771:101	Dmxl2	INSIDE	1.373	2.680	1768.03	4738.66	3.680	1451.46	5341.14

ProbeName	Target position of probe on CpG island microarray	TargetID	GeneSymbol	CpG island Description	Ratio of relative methylation (TiO_2-NP/Vehicle)	Sham group			TiO_2-H group		
						Relative methylation (Cy5/Cy3)	Cy3 signal (Input)	Cy5 signal (MeDIP)	Relative methylation (Cy5/Cy3)	Cy3 signal (Input)	Cy5 signal (MeDIP)
A_68_P31122729	chr17:28622707-28622751	NM_010220:329	Fkbp5	INSIDE	1.373	1.817	636.24	1155.99	2.495	613.11	1529.42
A_68_P30960667	chr16:91044987-91045031	NM_026110:-384	Gcfc1	PROMOTER	1.373	2.734	1560.00	4264.53	3.753	1215.01	4560.42
A_68_P32539367	chrX:91787344-91787392	NM_019791:45	Maged1	INSIDE	1.373	2.267	4377.50	9923.92	3.112	1911.15	5948.14
A_68_P31425842	chr17:88374222-88374266	NM_010830:-145	Msh6	PROMOTER	1.373	1.615	1220.04	1969.95	2.217	1081.66	2397.57
A_68_P30000047	chr15:8394313-8394357	NM_027707:129	Nipbl	INSIDE	1.373	2.319	673.55	1561.95	3.183	505.75	1609.85
A_68_P31622624	chr18:36356271-36356315	NM_001167891:522	Nrg2	INSIDE	1.373	3.532	1864.27	6583.97	4.848	1729.99	8387.74
A_68_P31934660	chr19:6428244-6428288	NM_001205234:9529	Nrxn2	INSIDE	1.373	4.464	641.73	2864.66	6.130	517.31	3171.36
A_68_P24043140	chr5:121641038-121641082	NM_001109992:346	Ptpn11	INSIDE	1.373	2.484	994.92	2471.65	3.410	895.24	3052.50
A_68_P30477114	chr15:98711909-98711953	NM_026967:-85	Rhebl1	PROMOTER	1.373	1.647	627.51	1033.80	2.262	510.71	1155.47
A_68_P23896973	chr5:93371964-93372008	NM_001077596:44963	Shroom3	INSIDE	1.373	3.531	278.13	982.12	4.847	237.92	1153.27
A_68_P30778635	chr16:55974040-55974084	NM_173026:146	Zbtb11	INSIDE	1.373	2.188	485.07	1061.14	3.003	449.64	1350.30
A_68_P20625950	chr1:135061005-135061049			Unknown	1.373	2.926	2328.63	6813.40	4.016	1832.13	7357.84
A_68_P23573892	chr5:30969470-30969514	NM_007681:218	Cenpa	INSIDE	1.372	2.370	2278.59	5401.33	3.253	1830.57	5955.37
A_68_P31992642	chr19:18787440-18787484	NM_177640:-263	D030056L22Rik	PROMOTER	1.372	2.103	1545.40	3249.62	2.885	1352.69	3903.16
A_68_P26659816	chr9:78329333-78329377	NM_010106:177	Eef1a1	INSIDE	1.372	1.469	695.01	1021.26	2.016	626.18	1262.24
A_68_P23280299	chr4:126146120-126146166	NM_153403:-477	Eif2c1	PROMOTER	1.372	1.714	1628.50	2791.35	2.352	1222.62	2875.57
A_68_P25016727	chr7:28116145-28116189	NM_001113549:2501	Ltbp4	INSIDE	1.372	4.066	1296.28	5270.71	5.579	991.80	5533.22
A_68_P21081858	chr2:26883547-26883591	NM_172659:-50	Slc2a6	PROMOTER	1.372	1.937	521.29	1009.54	2.658	391.90	1041.65
A_68_P20121785	chr1:30930713-30930757	ENSMUST00000046443:-195		PROMOTER	1.372	9.708	671.62	6520.36	13.321	660.88	8803.74
A_68_P27675038	chr11:29447584-29447633	NM_001163521:-341	1700034F02Rik	PROMOTER	1.371	1.827	1378.55	2518.92	2.505	1090.59	2731.52
A_68_P20743645	chr1:158488645-158488689	NM_001136104:-251	Abl2	PROMOTER	1.371	1.999	940.19	1879.19	2.739	830.12	2274.10
A_68_P27868560	chr11:64792596-64792640	NM_023479:82	Elac2	INSIDE	1.371	1.715	2034.10	3487.49	2.351	1612.28	3790.58
A_68_P22892661	chr4:47105201-47105245	NM_172693:398	Galnt12	INSIDE	1.371	2.988	5173.15	15456.04	4.097	3825.02	15672.85
A_68_P31253728	chr17:56219108-56219152	NM_008233:51	Hdgfrp2	INSIDE	1.371	1.367	2289.37	3129.31	1.873	1816.94	3404.02
A_68_P26658189	chr9:77960962-77961006	NM_019987:-60	Ick	PROMOTER	1.371	2.883	1000.73	2884.64	3.952	895.54	3539.20
A_68_P25088776	chr7:51926206-51926250	NM_028021:-5355	Myh14	PROMOTER	1.371	7.363	1447.93	10661.22	10.097	1179.84	11912.56
A_68_P25179906	chr7:72017792-72017836	NM_023239:112	Ndnl2	INSIDE	1.371	2.516	572.71	1440.90	3.449	502.13	1732.03
A_68_P27273029	chr10:77707518-77707562	NM_001081055:-153	Trappc10	PROMOTER	1.371	2.506	572.85	1435.32	3.434	519.86	1785.44
A_68_P27099070	chr10:42894837-42894881	NM_172419:349	9030612E09Rik	INSIDE	1.370	1.604	1165.63	1870.02	2.199	908.73	1997.96
A_68_P24040428	chr5:121161477-121161521	NM_008052:180	Dtx1	INSIDE	1.370	1.414	2522.17	3567.04	1.937	1907.99	3696.06
A_68_P25954902	chr8:73438710-73438754	NM_173013:8860	Mtap1s	INSIDE	1.370	2.956	1750.82	5175.23	4.049	1434.24	5806.80
A_68_P32289246	chrX:20195326-20195370	NM_001167776:464	Rbm10	INSIDE	1.370	2.689	312.43	840.02	3.684	216.81	798.64
A_68_P28599771	chr12:86039461-86039505	NM_001033334:275	Tmem90a	INSIDE	1.370	1.496	1077.57	1611.93	2.049	954.20	1955.36
A_68_P30133050	chr15:35301092-35301136	NM_177151:-186	Vps13b	PROMOTER	1.370	1.559	605.88	944.33	2.136	405.16	865.25
A_68_P24114683	chr5:135565547-135565591			Unknown	1.370	6.231	5435.73	33867.69	8.538	4289.37	36622.50
A_68_P22283292	chr3:82932341-82932385	ENSMUST00000047876:806		INSIDE	1.370	1.540	1362.03	2097.64	2.110	1337.53	2822.76
A_68_P23151254	chr4:100449214-100449258	NM_198037:-47	Cachd1	PROMOTER	1.369	1.924	484.95	932.84	2.632	426.95	1123.94
A_68_P33007797	chr4_random:129202-129246	NM_001033326:19423	Dhrsx	INSIDE	1.369	3.132	1160.60	3634.66	4.289	966.03	4143.07
A_68_P21704238	chr2:143741481-143741525	NM_019771:436	Dstn	INSIDE	1.369	2.307	1017.02	2346.25	3.158	850.15	2685.09
A_68_P27945140	chr11:78565152-78565196	NM_197989:57	Fam58b	INSIDE	1.369	2.862	900.85	2578.09	3.918	754.39	2955.42
A_68_P21562932	chr2:118082494-118082538	NM_027759:186	Fsip1	INSIDE	1.369	1.651	5276.17	8710.22	2.261	4159.80	9404.29
A_68_P28883194	chr13:23830758-23830802	NM_015786:105	Hist1h1c	INSIDE	1.369	2.192	380.66	834.42	3.002	334.35	1003.65
A_68_P28161571	chr11:116704550-116704594	NM_033398:191	Jmjd6	INSIDE	1.369	1.664	2018.24	3357.39	2.278	1617.83	3685.48
A_68_P21122750	chr2:33495287-33495331	NM_010725:723	Lmx1b	INSIDE	1.369	2.211	1750.94	3871.10	3.027	1471.58	4454.33
A_68_P25587306	chr7:148380913-148380957	NM_153777:-107	Lrrc56	PROMOTER	1.369	4.543	360.12	1636.08	6.221	318.47	1981.20
A_68_P32777673	chrX:156426105-156426149	NM_001163085:-238	Map3k15	PROMOTER	1.369	2.374	360.63	856.04	3.249	289.33	939.97
A_68_P20015303	chr1:7078680-7078724	NM_183028:-298	Pcmtd1	PROMOTER	1.369	3.266	2213.15	7228.65	4.471	1704.15	7618.55
A_68_P24344497	chr6:32538021-32538065	NM_175750:150	Plxna4	INSIDE	1.369	2.431	405.17	984.91	3.327	335.28	1115.48
A_68_P28738219	chr12:111685115-111685159	NM_001135001:-252	Ppp2r5c	PROMOTER	1.369	2.398	1728.54	4145.07	3.284	1481.02	4863.54
A_68_P27109370	chr10:44787342-44787386	NM_011156:345	Prep	INSIDE	1.369	2.529	859.35	2173.64	3.462	777.81	2692.76
A_68_P28301681	chr12:27100085-27100129	NM_001081977:15	Rnf144a	INSIDE	1.369	4.189	2490.92	10434.65	5.736	1991.84	11425.37

ProbeName	Target position of probe on CpG island microarray	TargetID	GeneSymbol	CpG island Description	Ratio of relative methylation (TiO_2-NP/Vehicle)	Sham group			TiO_2-H group		
						Relative methylation (Cy5/Cy3)	Cy3 signal (Input)	Cy5 signal (MeDIP)	Relative methylation (Cy5/Cy3)	Cy3 signal (Input)	Cy5 signal (MeDIP)
A_68_P23434595	chr4:154229865-154229909	NM_172878:-422	Ttc34	PROMOTER	1.369	1.886	364.52	687.62	2.581	350.58	905.03
A_68_P21105116	chr2:30627229-30627273			Unknown	1.369	3.274	1242.96	4069.44	4.482	1097.87	4920.44
A_68_P31467175	chr18:5163131-5163175	ENSMUST00000150337:2577		INSIDE	1.369	1.758	2368.93	4165.65	2.408	1900.57	4576.71
A_68_P27971504	chr11:83254254-83254300	NM_025492:3081	1700020L24Rik	INSIDE	1.368	2.805	780.50	2189.34	3.839	560.42	2151.21
A_68_P22892545	chr4:47069865-47069909	NM_001024136:292	Anks6	INSIDE	1.368	1.768	448.56	792.95	2.419	413.33	999.74
A_68_P26169913	chr8:114256102-114256146	NM_001198839:-88	Bcar1	PROMOTER	1.368	1.606	1267.97	2036.39	2.196	1083.80	2380.48
A_68_P21338757	chr2:74520450-74520494	NM_008273:23	Hoxd11	INSIDE	1.368	1.926	1431.74	2757.97	2.636	1127.49	2972.01
A_68_P21137837	chr2:35960605-35960649	NM_001083126:-1046	Lhx6	PROMOTER	1.368	1.750	2410.01	4216.87	2.393	2224.30	5322.58
A_68_P26759567	chr9:98501957-98502001	NM_025485:120	Mrps22	INSIDE	1.368	3.823	1312.87	5019.01	5.230	1138.42	5953.87
A_68_P30670430	chr16:35490785-35490829	NM_028295:153	Pdia5	INSIDE	1.368	2.441	13750.31	33567.02	3.339	10387.86	34680.90
A_68_P30655993	chr16:33056579-33056624	NM_021338:2	Rpl35a	INSIDE	1.368	2.063	1663.09	3431.42	2.822	1331.72	3758.29
A_68_P32396717	chrX:53863261-53863305	NM_172780:271	Slc9a6	INSIDE	1.368	1.889	2597.25	4907.16	2.584	1089.97	2816.44
A_68_P26845378	chr9:114472828-114472872	NM_001042503:637	Trim71	INSIDE	1.368	1.578	472.26	745.14	2.158	428.19	924.04
A_68_P24835542	chr6:127837749-127837793	NM_026743:131	Tspan11	INSIDE	1.368	1.988	2799.60	5564.83	2.719	2337.58	6355.58
A_68_P25005689	chr7:25378891-25378935	NM_001033205:-6252	Zfp575	PROMOTER	1.368	6.844	2705.24	18515.49	9.360	2044.84	19140.21
A_68_P21865360	chr2:172553949-172553993			Unknown	1.368	1.612	1090.82	1757.87	2.204	925.20	2039.16
A_68_P32977628	A_68_P32977628			Unknown	1.368	2.466	2769.34	6828.19	3.373	1619.11	5461.68
A_68_P31292459	chr17:64213804-64213848	ENSMUST00000112829:463		INSIDE	1.368	1.558	2470.65	3849.48	2.132	2091.44	4458.68
A_68_P28706883	chr12:106247987-106248031	NM_172500:11	4831426I19Rik	INSIDE	1.367	1.677	950.15	1593.03	2.292	785.95	1801.11
A_68_P30542888	chr16:10545617-10545661	NM_001204229:207	Clec16a	INSIDE	1.367	1.675	1662.27	2784.25	2.290	1314.90	3011.56
A_68_P25532700	chr7:139346324-139346368	NM_018867:78	Cpxm2	INSIDE	1.367	4.021	2274.54	9147.03	5.496	1804.75	9918.85
A_68_P23349533	chr4:138631065-138631109	NM_021358:-382	Htr6	PROMOTER	1.367	3.012	3354.55	10105.39	4.117	2563.39	10554.44
A_68_P29009466	chr13:46823130-46823174	NM_175749:66	Nup153	INSIDE	1.367	1.694	1586.03	2687.08	2.316	1294.00	2996.38
A_68_P31629950	chr18:37927496-37927540	NM_033595:2285	Pcdhga12	INSIDE	1.367	2.105	393.75	828.98	2.879	371.08	1068.22
A_68_P32240903	chrX:7500233-7500277	NM_013892:3307	Pcsk1n	INSIDE	1.367	3.291	1079.03	3550.77	4.497	639.10	2874.21
A_68_P24117609	chr5:136209766-136209810	NM_008898:44705	Por	INSIDE	1.367	2.931	887.84	2602.55	4.006	739.59	2962.73
A_68_P32462208	chrX:70599329-70599373	NM_001160229:11391	Zfp275	INSIDE	1.367	3.264	322.68	1053.14	4.461	179.68	801.62
A_68_P32145408	chr19:46764549-46764593	NM_025563:175	2010012O05Rik	INSIDE	1.366	4.452	1229.58	5474.42	6.081	1084.00	6591.45
A_68_P27565303	chr11:5762324-5762369	NM_009636:478	Aebp1	INSIDE	1.366	2.865	2978.57	8532.66	3.913	2426.65	9494.28
A_68_P27899584	chr11:70042574-70042618	NM_007528:704	Bcl6b	INSIDE	1.366	2.798	708.68	1983.09	3.823	594.48	2272.99
A_68_P20645802	chr1:138311830-138311874	NM_026500:5	Ddx59	INSIDE	1.366	2.654	593.77	1575.57	3.624	505.83	1833.18
A_68_P27542236	chr10:128027435-128027479	NM_010153:-899	Erbb3	PROMOTER	1.366	2.487	615.00	1529.48	3.397	550.29	1869.55
A_68_P21374844	chr2:80287643-80287687	NM_011356:-111	Frzb	PROMOTER	1.366	2.653	278.41	738.54	3.624	232.57	842.79
A_68_P20122141	chr1:31006006-31006050	NR_002688:527	Gm13363	INSIDE	1.366	3.458	957.64	3311.41	4.722	802.74	3790.64
A_68_P21489524	chr2:104333565-104333609	NM_001145824:1060	Hipk3	INSIDE	1.366	2.527	4985.82	12597.99	3.450	4054.64	13990.46
A_68_P25652450	chr8:11008619-11008663	NM_001081212:-210	Irs2	PROMOTER	1.366	3.243	1037.10	3363.20	4.431	913.30	4046.50
A_68_P30491676	chr15:101242719-101242763	NM_033073:-93	Krt7	PROMOTER	1.366	2.265	2270.88	5144.58	3.096	1817.82	5627.44
A_68_P24656008	chr6:94234014-94234058	NM_001029850:-138	Magi1	PROMOTER	1.366	2.915	3017.70	8797.05	3.983	2358.96	9395.79
A_68_P21071913	chr2:25258906-25258950	NM_008721:326	Npdc1	INSIDE	1.366	4.097	2136.86	8754.80	5.597	1746.94	9778.14
A_68_P26542458	chr9:57408773-57408817	NM_022813:44	Scamp2	INSIDE	1.366	2.322	1662.49	3859.71	3.171	1201.75	3811.15
A_68_P27098031	chr10:42741435-42741479	NM_175407:152880	Sobp	INSIDE	1.366	2.616	436.98	1143.02	3.574	364.82	1303.85
A_68_P27937053	chr11:77161754-77161798	NM_177710:131850	Ssh2	INSIDE	1.366	1.996	388.31	774.94	2.726	401.06	1093.45
A_68_P28087325	chr11:103671166-103671210	NM_009521:35700	Wnt3	INSIDE	1.366	2.438	1160.65	2829.73	3.331	1066.06	3551.03
A_68_P31509674	chr18:14130093-14130137	NM_145492:1128	Zfp521	INSIDE	1.366	2.488	711.15	1769.61	3.399	569.89	1936.82
A_68_P26710067	A_68_P26710067			Unknown	1.366	2.734	9332.13	25516.35	3.736	6010.72	22454.09
A_68_P27892803	chr11:68873789-68873833	NM_028005:534	2310047M10Rik	INSIDE	1.365	1.753	1835.32	3217.20	2.392	1543.35	3691.98
A_68_P30686068	chr16:38452791-38452835	NM_007414:-43	Adprh	DIVERGENT_PROMOTER	1.365	1.811	429.23	777.53	2.473	423.67	1047.63
A_68_P23961351	chr5:106886587-106886631	NM_001005477:577	Barhl2	INSIDE	1.365	5.453	6035.95	32914.14	7.441	4374.52	32552.81
A_68_P29487113	chr14:28242620-28242665	NM_001114879:610	D14Abb1e	INSIDE	1.365	2.278	937.43	2135.62	3.111	760.25	2365.00
A_68_P29699027	chr14:70478576-70478620	NM_018781:1347	Egr3	INSIDE	1.365	2.225	2968.91	6606.06	3.036	1916.90	5820.15
A_68_P22882726	chr4:45421447-45421491	NM_001009949:170	Mcart1	INSIDE	1.365	1.978	1515.41	2998.06	2.701	1358.02	3667.33

ProbeName	Target position of probe on CpG island microarray	TargetID	GeneSymbol	CpG island Description	Ratio of relative methylation (TiO_2-NP/Vehicle)	Sham group			TiO_2-H group		
						Relative methylation (Cy5/Cy3)	Cy3 signal (Input)	Cy5 signal (MeDIP)	Relative methylation (Cy5/Cy3)	Cy3 signal (Input)	Cy5 signal (MeDIP)
A_68_P30884142	chr16:76372860-76372904	NM_173440:412	Nrip1	INSIDE	1.365	1.630	829.32	1351.42	2.224	791.79	1761.14
A_68_P21495967	chr2:105516620-105516664	NM_013627:41	Pax6	INSIDE	1.365	1.404	1409.12	1978.78	1.917	1251.03	2398.75
A_68_P27940746	chr11:77796693-77796737	NM_174852:397	Phf12	INSIDE	1.365	1.576	896.82	1412.96	2.150	747.20	1606.34
A_68_P29482222	chr14:27353534-27353578	NM_032008:-330	Slmap	PROMOTER	1.365	3.503	1597.70	5596.47	4.781	1401.51	6700.50
A_68_P26696151	chr9:85737666-85737710	NM_001164792:1230	Tpbg	INSIDE	1.365	1.601	1639.80	2625.68	2.186	1476.94	3228.44
A_68_P31090924	chr17:23013653-23013697	NM_001110254:-9573	Zfp945	PROMOTER	1.365	3.268	2013.01	6578.76	4.460	1739.67	7759.32
A_68_P29308200	chr13:108680217-108680261	NM_145456:20	Zswim6	INSIDE	1.365	3.452	1029.65	3554.78	4.711	912.24	4297.77
A_68_P22217922	chr3:68674470-68674514	NM_001167996:985	1110032F04Rik	INSIDE	1.364	1.514	3760.24	5692.53	2.065	2765.18	5709.65
A_68_P30339175	chr15:74551980-74552024	NM_198607:339	4930572J05Rik	INSIDE	1.364	4.736	981.45	4647.90	6.460	773.94	5000.06
A_68_P27938078	chr11:77329061-77329105	NM_026185:464	Abhd15	INSIDE	1.364	1.489	948.03	1411.60	2.031	734.04	1491.14
A_68_P28175760	chr11:118947373-118947417	NM_007625:157	Cbx4	INSIDE	1.364	1.991	1552.36	3091.16	2.715	1329.48	3609.96
A_68_P24980083	chr7:16760918-16760963	NM_008627:212	Meis3	INSIDE	1.364	1.724	1918.07	3306.37	2.352	1537.74	3616.61
A_68_P20005799	chr1:4482580-4482624	NM_011441:3892	Sox17	INSIDE	1.364	2.873	1902.00	5463.89	3.918	1576.06	6174.50
A_68_P31201703	chr17:45682394-45682438	NM_001013749:4210	Tmem151b	INSIDE	1.364	2.039	2284.65	4657.56	2.781	1929.60	5367.08
A_68_P25477112	chr7:129211243-129211287	NM_138589:553	Ubfd1	INSIDE	1.364	5.681	694.56	3946.07	7.747	632.13	4896.98
A_68_P20778496	chr1:164501404-164501449	NM_016796:468	Vamp4	INSIDE	1.364	2.376	727.00	1727.60	3.241	610.91	1980.14
A_68_P26158504	chr8:112229332-112229376	NM_001167946:-94	Zfp821	PROMOTER	1.364	1.357	2785.29	3778.49	1.850	2238.75	4141.09
A_68_P27288852	chr10:80783503-80783547	NM_027381:-323	2510012J08Rik	PROMOTER	1.363	1.438	2241.07	3223.18	1.960	1897.65	3720.15
A_68_P32238972	chrX:7150718-7150762	NR_029473:15	4930524L23Rik	INSIDE	1.363	1.425	892.18	1270.99	1.941	467.23	906.99
A_68_P29940455	chr14:119105820-119105864	NM_001033336:-401	Abcc4	PROMOTER	1.363	2.192	444.95	975.45	2.989	386.60	1155.38
A_68_P27543828	chr10:128347300-128347344	NM_007653:401	Cd63	INSIDE	1.363	1.593	2565.47	4086.57	2.172	2080.95	4519.18
A_68_P29454311	chr14:22506206-22506253	NM_001013826:27569	Dupd1	INSIDE	1.363	2.232	524.85	1171.62	3.044	433.34	1318.94
A_68_P21903062	chr2:180068497-180068541	NM_008093:866	Gata5	INSIDE	1.363	3.218	783.22	2520.57	4.386	679.87	2981.68
A_68_P29613086	chr14:55194659-55194703	NM_010590:1818	Jub	INSIDE	1.363	2.489	1979.72	4926.86	3.391	1677.56	5688.85
A_68_P24383013	chr6:39369919-39369963	NM_018810:428	Mkrn1	INSIDE	1.363	2.549	1425.23	3632.38	3.473	1207.82	4195.23
A_68_P21652765	chr2:134612489-134612533	NM_001145830:611	Plcb1	INSIDE	1.363	1.493	4111.13	6137.50	2.034	3304.93	6723.05
A_68_P26818266	chr9:108998036-108998080	NM_172775:109	Plxnb1	INSIDE	1.363	2.414	582.67	1406.34	3.289	549.93	1808.78
A_68_P24537771	chr6:71657856-71657900	NM_178608:204	Reep1	INSIDE	1.363	3.930	1707.76	6711.48	5.355	1465.87	7849.29
A_68_P29871957	chr14:104921836-104921880	NM_026047:25	Rnf219	INSIDE	1.363	2.386	1614.12	3850.86	3.251	1506.69	4898.83
A_68_P23318415	chr4:133155946-133155990	NM_018754:2115	Sfn	DOWNSTREAM	1.363	2.560	2353.04	6023.00	3.488	1731.94	6041.65
A_68_P20412460	chr1:88400352-88400396			Unknown	1.363	1.843	1127.38	2077.21	2.511	924.65	2321.98
A_68_P28914784	chr13:30077124-30077168	NM_010093:786	E2f3	INSIDE	1.362	3.509	1126.80	3954.07	4.779	1102.66	5269.22
A_68_P27281572	chr10:79649620-79649664	NM_007909:7416	Efna2	INSIDE	1.362	2.532	1752.70	4437.39	3.448	1292.57	4456.30
A_68_P23404991	chr4:149610981-149611025	NM_023119:-303	Eno1	PROMOTER	1.362	1.847	622.32	1149.25	2.516	550.40	1384.84
A_68_P24908774	chr6:143115559-143115603	NM_029250:-169	Etnk1	PROMOTER	1.362	1.491	1244.29	1855.55	2.032	1106.83	2248.53
A_68_P23570788	chr5:30441008-30441052	NM_001167879:9295	Fam59b	INSIDE	1.362	1.603	1468.31	2353.99	2.184	1158.64	2530.82
A_68_P23438941	chr4:154865679-154865723	NM_001160016:231	Gnb1	INSIDE	1.362	3.548	2529.70	8975.40	4.831	2211.89	10686.38
A_68_P21717098	chr2:146047771-146047815	NM_016889:60	Insm1	INSIDE	1.362	3.054	1048.62	3202.55	4.161	911.19	3791.03
A_68_P28067976	chr11:100276206-100276250	NM_176830:-95	Leprel4	DIVERGENT_PROMOTER	1.362	2.990	1008.38	3015.14	4.073	901.21	3670.91
A_68_P31106322	chr17:25933806-25933850	NM_133719:162	Metrn	INSIDE	1.362	1.699	689.09	1170.50	2.313	626.86	1449.91
A_68_P20348302	chr1:74438798-74438842	NR_029743:-2063	Mir26b	PROMOTER	1.362	2.584	636.14	1643.58	3.519	801.80	2821.38
A_68_P27901890	chr11:70467341-70467385	NM_011072:790	Pfn1	INSIDE	1.362	3.593	1101.08	3956.38	4.895	1022.58	5005.37
A_68_P20941542	chr1:193916523-193916567	NM_011633:-99	Traf5	PROMOTER	1.362	2.493	3521.84	8780.89	3.395	2869.11	9740.55
A_68_P29117525	chr13:69878696-69878740	NM_001145162:57	Ube2ql1	INSIDE	1.362	2.858	1370.47	3916.86	3.894	1265.64	4928.35
A_68_P30571780	chr16:17070460-17070504	NM_023249:79	Ypel1	INSIDE	1.362	2.294	633.96	1453.98	3.125	532.71	1664.56
A_68_P32564025	chrX:98615354-98615398	NM_001177985:-123	Zmym3	PROMOTER	1.362	1.536	1503.86	2309.65	2.092	872.43	1825.25
A_68_P22291852	chr3:84386166-84386211	NM_001081093:359	Arfip1	INSIDE	1.361	1.874	1862.27	3490.66	2.551	1534.47	3914.95
A_68_P26815969	chr9:108551009-108551053	NM_011790:681	Arih2	INSIDE	1.361	1.913	910.96	1742.30	2.603	809.45	2107.36
A_68_P31845029	chr18:77182994-77183040	NM_001039201:-140	Hdhd2	PROMOTER	1.361	2.124	1149.86	2442.61	2.891	1039.55	3005.11
A_68_P26467130	chr9:44152085-44152129	NM_001110251:-1642	Hmbs	PROMOTER	1.361	2.192	1107.48	2428.13	2.983	1031.07	3075.71
A_68_P27862295	chr11:63735348-63735392	NM_018805:416	Hs3st3b1	INSIDE	1.361	1.969	1565.83	3083.71	2.680	1195.83	3204.44

ProbeName	Target position of probe on CpG island microarray	TargetID	GeneSymbol	CpG island Description	Ratio of relative methylation (TiO_2-NP/Vehicle)	Sham group			TiO_2-H group		
						Relative methylation (Cy5/Cy3)	Cy3 signal (Input)	Cy5 signal (MeDIP)	Relative methylation (Cy5/Cy3)	Cy3 signal (Input)	Cy5 signal (MeDIP)
A_68_P23399811	chr4:148681546-148681590	NM_207682:239	Kif1b	INSIDE	1.361	2.065	441.00	910.65	2.811	399.56	1123.33
A_68_P23164367	chr4:102792385-102792429	NM_001039081:412	Mier1	INSIDE	1.361	1.931	1119.80	2162.68	2.629	899.38	2364.38
A_68_P31425848	chr17:88374947-88374991	NM_010830:579	Msh6	INSIDE	1.361	2.086	1162.99	2425.55	2.839	1098.30	3117.65
A_68_P26772688	chr9:100978469-100978513	NM_001100451:40	Msl2	INSIDE	1.361	1.465	1259.57	1845.10	1.993	1116.02	2224.57
A_68_P27687498	chr11:32121990-32122034	NM_010117:281	Rhbdf1	INSIDE	1.361	1.857	1146.02	2128.65	2.528	942.75	2382.92
A_68_P31003195	chr17:3114834-3114878	NM_134123:-115	Scaf8	PROMOTER	1.361	1.598	476.21	760.78	2.174	473.73	1029.91
A_68_P22411698	chr3:108087081-108087125	NM_019972:53	Sort1	INSIDE	1.361	1.897	3132.01	5941.00	2.581	2374.06	6127.71
A_68_P31958009	chr19:11893089-11893134	NM_011502:272	Stx3	INSIDE	1.361	1.890	2811.58	5314.04	2.573	2393.25	6157.28
A_68_P24946870	chr7:3646016-3646060	NM_024168:-71	Tsen34	PROMOTER	1.361	2.702	3686.60	9962.49	3.677	2903.22	10675.36
A_68_P26685864	chr9:83728415-83728459	NM_001110265:141	Ttk	INSIDE	1.361	1.839	3798.36	6984.55	2.503	3015.76	7548.85
A_68_P26465578	chr9:43893359-43893403	NM_198091:378	Usp2	INSIDE	1.361	1.883	1890.54	3560.74	2.564	1506.49	3862.12
A_68_P20046440	chr1:14497095-14497139			Unknown	1.361	5.292	2590.68	13710.41	7.203	1986.94	14312.50
A_68_P22875972	chr4:44247099-44247143	ENSMUST00000153116:-459		PROMOTER	1.361	1.884	809.90	1525.58	2.563	729.11	1868.62
A_68_P26749718	chr9:96789794-96789838	NM_153420:25	Acpl2	INSIDE	1.360	2.516	1208.35	3040.42	3.423	1060.90	3631.22
A_68_P24639207	chr6:91423387-91423431	NM_133928:9	Chchd4	INSIDE	1.360	1.479	1769.31	2617.20	2.011	1498.62	3014.42
A_68_P25592938	chr7:149280400-149280444	NM_008748:767	Dusp8	INSIDE	1.360	2.194	789.10	1731.38	2.985	574.57	1714.93
A_68_P27560006	chr11:4749005-4749049	NM_010898:504	Nf2	INSIDE	1.360	2.734	1101.42	3011.51	3.719	910.36	3385.51
A_68_P21939653	chr3:9833472-9833516	NM_001195031:185	Pag1	INSIDE	1.360	1.918	767.65	1472.10	2.609	635.03	1656.72
A_68_P24381543	chr6:39067895-39067939	NM_172893:432	Parp12	INSIDE	1.360	2.227	1642.62	3658.04	3.028	1483.86	4493.75
A_68_P28036286	chr11:94902671-94902715	NM_133667:-7	Pdk2	PROMOTER	1.360	2.254	1330.35	2999.21	3.067	1225.86	3759.60
A_68_P32694937	chrX:136145542-136145586	NM_023270:406	Rnf128	INSIDE	1.360	1.800	733.51	1319.95	2.447	365.61	894.79
A_68_P26129567	chr8:107154779-107154823	NM_019662:421	Rrad	INSIDE	1.360	1.446	1182.94	1710.77	1.967	1040.72	2047.28
A_68_P22871354	chr4:43394705-43394749	NM_001037709:-127	Rusc2	PROMOTER	1.360	2.432	951.83	2315.00	3.307	871.20	2881.09
A_68_P31058947	chr17:14416697-14416741	NM_022315:206	Smoc2	INSIDE	1.360	1.903	1483.75	2823.56	2.588	1295.92	3353.82
A_68_P21986766	chr3:21976041-21976085	NM_030732:489	Tbl1xr1	INSIDE	1.360	2.448	1397.89	3421.62	3.328	1312.91	4369.70
A_68_P27365516	chr10:94978402-94978446	NM_080560:629	Ube2n	INSIDE	1.360	2.455	1631.46	4005.41	3.340	1469.29	4906.85
A_68_P20803943	chr1:169156624-169156668	NM_030724:58612	Uck2	INSIDE	1.360	8.497	3753.86	31896.72	11.552	2329.16	26907.04
A_68_P20735710	chr1:156930629-156930673	ENSMUST00000128143:-254		PROMOTER	1.360	1.551	1935.62	3001.40	2.110	1615.07	3407.05
A_68_P31158463	chr17:35373372-35373416	NM_023179:-146	Atp6v1g2	DIVERGENT_PROMOTER	1.359	1.914	2497.39	4780.43	2.600	2011.96	5232.05
A_68_P27796700	chr11:51781220-51781264	NM_029976:110	Cdkn2aipnl	INSIDE	1.359	2.363	1411.32	3335.26	3.211	1298.73	4170.55
A_68_P22412527	chr3:108217228-108217273	NM_001004177:1162	Celsr2	INSIDE	1.359	2.437	327.37	797.66	3.312	271.86	900.52
A_68_P31620310	chr18:35990907-35990952	NM_133687:1458	Cxxc5	INSIDE	1.359	2.002	279.55	559.53	2.721	229.64	624.73
A_68_P24449527	chr6:52210204-52210248	NM_008264:648	Hoxa13	INSIDE	1.359	3.967	1308.24	5189.28	5.389	1142.69	6157.76
A_68_P21006641	chr2:11457093-11457137	NM_001177758:18442	Pfkfb3	INSIDE	1.359	2.507	1694.90	4249.41	3.407	1346.83	4588.87
A_68_P31655273	chr18:42555254-42555298	NM_138945:1026	Pou4f3	INSIDE	1.359	1.688	2210.55	3732.33	2.295	1843.32	4230.40
A_68_P23916643	chr5:98614905-98614949	NM_029947:5039	Prdm8	INSIDE	1.359	1.869	628.31	1174.47	2.540	506.16	1285.51
A_68_P29718153	chr14:73952777-73952821	NM_011506:206	Sucla2	INSIDE	1.359	2.144	427.53	916.50	2.913	397.88	1159.23
A_68_P28974184	chr13:40826354-40826399	NM_001122948:-564	Tcfap2a	PROMOTER	1.359	3.705	5820.36	21562.28	5.033	4575.69	23028.54
A_68_P33005816	A_68_P33005816			Unknown	1.359	2.659	388.66	1033.63	3.615	323.22	1168.46
A_68_P23692834	chr5:52507355-52507399	AK076709:-1175		PROMOTER	1.359	1.615	1889.57	3052.55	2.195	1493.69	3279.23
A_68_P25587958	chr7:148480452-148480496	NM_007878:2570	Drd4	INSIDE	1.358	1.707	1909.73	3259.96	2.318	1638.20	3796.62
A_68_P31769415	chr18:63546129-63546173	NM_001039485:687	Fam38b	INSIDE	1.358	2.290	1864.44	4269.70	3.110	1624.13	5051.65
A_68_P26467611	chr9:44248858-44248902	NM_001033469:71	Foxr1	INSIDE	1.358	2.455	1282.79	3149.56	3.335	957.04	3191.29
A_68_P20058119	chr1:17081907-17081951	NM_020604:6042	Jph1	INSIDE	1.358	2.532	479.42	1213.87	3.439	443.56	1525.24
A_68_P25098809	chr7:53651661-53651705	NM_008421:-155	Kcnc1	PROMOTER	1.358	3.971	520.29	2066.17	5.395	528.12	2848.95
A_68_P31123750	chr17:28826626-28826670	NM_001168508:-1638	Mapk14	PROMOTER	1.358	2.749	1067.98	2935.78	3.733	901.71	3366.23
A_68_P20088276	chr1:23389423-23389467	NM_001081079:570	Ogfrl1	INSIDE	1.358	3.591	630.15	2262.87	4.877	523.96	2555.37
A_68_P29509082	chr14:31814863-31814907	NM_026911:-32	Spcs1	PROMOTER	1.358	7.876	445.18	3506.37	10.697	415.82	4447.88
A_68_P21370052	chr2:79475902-79475946	NM_080558:343	Ssfa2	INSIDE	1.358	1.723	2895.47	4988.92	2.340	2403.07	5623.52
A_68_P24907675	chr6:142912528-142912572	NM_011374:422	St8sia1	INSIDE	1.358	1.772	1785.69	3164.63	2.407	1266.13	3047.71
A_68_P24951676	chr7:4982246-4982290	NM_001033383:10291	Zfp865	INSIDE	1.358	2.423	1233.64	2989.54	3.290	975.09	3207.93

ProbeName	Target position of probe on CpG island microarray	TargetID	GeneSymbol	CpG island Description	Ratio of relative methylation (TiO_2-NP/Vehicle)	Sham group			TiO_2-H group		
						Relative methylation (Cy5/Cy3)	Cy3 signal (Input)	Cy5 signal (MeDIP)	Relative methylation (Cy5/Cy3)	Cy3 signal (Input)	Cy5 signal (MeDIP)
A_68_P27288263	chr10:80702990-80703034	NM_001034895:7105	Zfr2	INSIDE	1.358	3.781	647.33	2447.83	5.134	514.02	2639.05
A_68_P27765615	chr11:45869497-45869541	NM_009616:30	Adam19	INSIDE	1.357	2.475	1814.11	4490.02	3.359	1298.58	4361.56
A_68_P30473398	chr15:97975961-97976005	NM_001170710:151	Asb8	INSIDE	1.357	2.239	2378.64	5324.89	3.038	1972.24	5992.46
A_68_P21897026	chr2:179176969-179177013	NM_009867:-192	Cdh4	PROMOTER	1.357	2.400	941.21	2259.13	3.256	808.82	2633.70
A_68_P25958451	chr8:74233667-74233711	NM_028715:15892	Fcho1	INSIDE	1.357	2.488	2287.70	5691.25	3.376	1781.15	6012.42
A_68_P29228410	chr13:94074181-94074225	NM_011982:-247	Homer1	PROMOTER	1.357	2.763	1506.26	4161.77	3.750	1305.27	4895.03
A_68_P21812073	chr2:163201933-163201977	NM_021566:21755	Jph2	INSIDE	1.357	2.387	517.55	1235.46	3.240	440.74	1427.76
A_68_P31424878	chr17:88145892-88145936	NM_199251:51420	Kcnk12	INSIDE	1.357	1.503	1312.07	1971.76	2.039	1064.52	2170.80
A_68_P22868863	chr4:42929796-42929840	NM_172690:-304	N28178	PROMOTER	1.357	3.146	467.50	1470.79	4.269	417.48	1782.29
A_68_P25050058	chr7:36370084-36370128	NM_145840:495	Rgs9bp	INSIDE	1.357	2.270	661.86	1502.42	3.080	603.50	1858.72
A_68_P30255504	chr15:59479090-59479134	NM_144549:-1096	Trib1	PROMOTER	1.357	1.527	949.03	1449.63	2.073	723.73	1499.94
A_68_P28741417	chr12:112215277-112215321	NM_175207:1933	Ankrd9	INSIDE	1.356	2.526	336.41	849.76	3.425	312.11	1069.03
A_68_P28669124	chr12:99497596-99497640	NM_008079:-71	Galc	DIVERGENT_PROMOTER	1.356	2.292	1424.45	3264.84	3.107	1240.70	3855.38
A_68_P28290299	chr12:25257426-25257470	NM_145890:-8135	Grhl1	PROMOTER	1.356	1.773	1594.35	2827.16	2.404	1278.35	3073.66
A_68_P31255286	chr17:56465347-56465391	NM_172132:-104	Kdm4b	PROMOTER	1.356	3.863	1908.78	7373.43	5.239	1735.55	9092.43
A_68_P22882724	chr4:45421232-45421276	NM_001009949:384	Mcart1	INSIDE	1.356	1.897	1805.70	3425.43	2.572	1310.37	3370.89
A_68_P27795021	chr11:51449266-51449310	NM_025346:110	Rmnd5b	INSIDE	1.356	4.161	516.21	2147.72	5.642	508.89	2871.36
A_68_P20024136	chr1:9788012-9788056	NM_133220:-176	Sgk3	PROMOTER	1.356	4.194	1044.55	4380.35	5.688	908.73	5168.57
A_68_P31381749	chr17:80606236-80606280	NM_001195485:387	Srsf7	INSIDE	1.356	1.494	7234.62	10806.48	2.026	5253.97	10642.61
A_68_P27971718	chr11:83286618-83286662	NM_027427:31	Taf15	INSIDE	1.356	8.044	1308.94	10529.64	10.909	1225.83	13372.08
A_68_P31209392	chr17:47027759-47027803	NM_178385:211	Tbcc	INSIDE	1.356	1.883	3135.97	5904.23	2.553	2378.32	6072.48
A_68_P30484944	chr15:100111219-100111263	NM_183109:-27	Tmprss12	PROMOTER	1.356	2.156	415.05	895.01	2.924	316.24	924.60
A_68_P30328150	chr15:72891418-72891462	NM_029640:-5198	Trappc9	PROMOTER	1.356	1.371	2665.84	3654.67	1.858	2154.79	4004.25
A_68_P32533199	chrX:90533865-90533909	NM_007492:1902	Arx	INSIDE	1.355	2.142	1899.44	4068.56	2.903	1239.21	3597.10
A_68_P31399352	chr17:83913503-83913547	NM_001159529:149	Cox7a2l	INSIDE	1.355	1.556	848.42	1320.11	2.109	742.39	1565.67
A_68_P24423150	chr6:47404743-47404787	NM_012042:442	Cul1	INSIDE	1.355	1.800	723.60	1302.32	2.439	653.62	1593.97
A_68_P23325561	chr4:134479965-134480009	NM_023665:447	D4Wsu53e	INSIDE	1.355	3.398	2142.02	7278.91	4.605	1818.26	8373.31
A_68_P30113005	chr15:31154387-31154431	NM_146057:269	Dap	INSIDE	1.355	1.369	1518.58	2079.26	1.856	1191.38	2211.13
A_68_P24138257	chr5:140384753-140384797	NM_175522:878	Elfn1	INSIDE	1.355	1.525	811.28	1237.10	2.065	768.93	1588.21
A_68_P21338547	chr2:74493903-74493947	NM_007967:3552	Evx2	INSIDE	1.355	3.343	349.06	1166.95	4.530	318.51	1442.81
A_68_P21428052	chr2:92211160-92211204	NM_001166633:11	Gyltl1b	INSIDE	1.355	2.320	1224.27	2840.76	3.144	959.15	3015.32
A_68_P22218631	chr3:68809558-68809602	NR_029529:-4113	Mir15b	PROMOTER	1.355	1.957	1913.91	3745.63	2.652	1423.59	3775.34
A_68_P22342633	chr3:94977194-94977238	NM_011351:8921	Sema6c	INSIDE	1.355	2.157	1867.57	4028.64	2.922	1490.67	4355.66
A_68_P31421397	chr17:87534362-87534421	NM_019654:27373	Socs5	INSIDE	1.355	2.229	258.24	575.61	3.021	242.91	733.71
A_68_P28051188	chr11:97398038-97398082	NM_018873:38380	Srcin1	INSIDE	1.355	2.770	1139.86	3157.30	3.754	924.48	3470.35
A_68_P23223183	chr4:114736145-114736189	NM_011527:4035	Tal1	INSIDE	1.355	3.038	538.70	1636.78	4.117	415.29	1709.74
A_68_P27985546	chr11:85647033-85647077	NM_009324:938	Tbx2	INSIDE	1.355	5.637	4673.73	26343.99	7.638	3623.94	27678.21
A_68_P23297751	chr4:129318057-129318101	NM_001005506:-29	Txlna	PROMOTER	1.355	1.833	5046.79	9250.43	2.483	3828.55	9505.34
A_68_P23358589	chr4:140071100-140071144	NM_001112723:133549	Arhgef10l	INSIDE	1.354	2.587	423.43	1095.63	3.504	399.52	1399.80
A_68_P25274217	chr7:88973759-88973803	NM_146193:537	Btbd1	INSIDE	1.354	2.131	829.29	1767.39	2.887	778.59	2247.43
A_68_P30508958	chr16:4214152-4214196	NM_001025432:-770	Crebbp	PROMOTER	1.354	3.169	968.07	3068.05	4.291	889.88	3818.04
A_68_P23320290	chr4:133524024-133524068	NM_016957:-140	Hmgn2	PROMOTER	1.354	2.333	2847.55	6643.49	3.158	2230.36	7044.42
A_68_P29010691	chr13:47024841-47024885	NM_010617:225	Kif13a	INSIDE	1.354	3.743	2168.68	8117.07	5.068	1869.97	9476.71
A_68_P28206077	chr12:5382789-5382833	NM_001164493:-322	Klhl29	DIVERGENT_PROMOTER	1.354	2.654	871.38	2312.70	3.593	700.41	2516.89
A_68_P24947984	chr7:4101189-4101233	NM_175529:264	Leng9	INSIDE	1.354	1.783	683.29	1218.35	2.414	631.11	1523.52
A_68_P20007250	chr1:4797717-4797761	NM_008866:-235	Lypla1	PROMOTER	1.354	3.212	454.86	1461.19	4.351	455.05	1979.79
A_68_P20849642	chr1:177622972-177623016	NM_010098:-273	Opn3	DIVERGENT_PROMOTER	1.354	3.511	3696.49	12979.44	4.755	2887.33	13729.03
A_68_P31421395	chr17:87534146-87534190	NM_019654:27150	Socs5	INSIDE	1.354	2.103	500.87	1053.44	2.848	331.39	943.73
A_68_P28402111	chr12:46175015-46175059	NM_144552:434	Stxbp6	INSIDE	1.354	2.043	8270.30	16896.14	2.767	6107.35	16899.76
A_68_P30349160	chr15:76406761-76406805	NM_172960:-6	Adck5	PROMOTER	1.353	1.912	1435.20	2744.49	2.587	1309.33	3387.57
A_68_P21901913	chr2:179906128-179906172	NM_019822:-142	Adrm1	PROMOTER	1.353	2.249	536.72	1207.15	3.043	546.14	1661.97

ProbeName	Target position of probe on CpG island microarray	TargetID	GeneSymbol	CpG island Description	Ratio of relative methylation (TiO_2-NP/Vehicle)	Sham group			TiO_2-H group		
						Relative methylation (Cy5/Cy3)	Cy3 signal (Input)	Cy5 signal (MeDIP)	Relative methylation (Cy5/Cy3)	Cy3 signal (Input)	Cy5 signal (MeDIP)
A_68_P29413816	chr14:13655475-13655519	NM_001042617:97	Cadps	INSIDE	1.353	2.294	1232.12	2827.09	3.103	1014.73	3149.20
A_68_P24154206	chr5:143722119-143722163	NM_007984:107	Fscn1	INSIDE	1.353	2.117	1448.25	3066.19	2.864	1282.20	3672.75
A_68_P20799113	chr1:168168950-168168994	NM_175296:-97	Mael	PROMOTER	1.353	2.194	792.66	1738.88	2.967	621.81	1844.92
A_68_P22825640	chr4:33275997-33276041	NM_001034867:694	Pm20d2	INSIDE	1.353	1.769	751.45	1329.68	2.394	836.59	2003.17
A_68_P20636473	chr1:136852289-136852333	NM_001081307:207	Ppp1r12b	INSIDE	1.353	5.648	1800.34	10168.16	7.642	1490.00	11385.87
A_68_P27561411	chr11:4999553-4999597	NM_177370:134	Rhbdd3	INSIDE	1.353	2.588	756.61	1957.93	3.502	639.15	2238.47
A_68_P23317104	chr4:132894918-132894962	NM_199306:290	Wdtc1	INSIDE	1.353	2.205	791.17	1744.65	2.983	755.02	2252.60
A_68_P24951576	chr7:4967342-4967386	NM_025324:255	Zfp524	INSIDE	1.353	3.487	1823.33	6357.48	4.718	1604.79	7571.15
A_68_P21622683	chr2:128864800-128864844	ENSMUST00000145798:16689		INSIDE	1.353	2.007	928.98	1864.50	2.716	839.61	2280.31
A_68_P25357074	chr7:105804627-105804671	NM_172280:431	2210018M11Rik	INSIDE	1.352	3.522	2626.25	9249.25	4.763	2332.64	11110.79
A_68_P28449510	chr12:55963628-55963672	NM_007688:214	Cfl2	INSIDE	1.352	3.331	1397.96	4657.07	4.503	1155.87	5205.38
A_68_P28143432	chr11:113545919-113545963	NM_177777:215	D11Wsu47e	INSIDE	1.352	2.503	3355.22	8398.52	3.385	2468.17	8355.60
A_68_P24501227	chr6:63205942-63205990	NM_008167:-884	Grid2	PROMOTER	1.352	3.292	166.72	548.82	4.452	156.51	696.82
A_68_P31425165	chr17:88198232-88198276	NM_199251:-920	Kcnk12	PROMOTER	1.352	2.405	858.46	2064.29	3.251	787.51	2560.10
A_68_P28516078	chr12:70825634-70825678	NM_145443:205	L2hgdh	INSIDE	1.352	1.922	2372.19	4558.42	2.598	2034.28	5285.87
A_68_P21081081	chr2:26766063-26766107	NM_001033908:78	Med22	INSIDE	1.352	1.373	6011.65	8253.93	1.857	4490.83	8339.26
A_68_P28831284	chr13:12350487-12350531	NM_001081128:-241	Mtr	PROMOTER	1.352	1.899	1347.79	2559.38	2.567	1046.52	2686.24
A_68_P27857775	chr11:62945991-62946035	NM_008885:1001	Pmp22	INSIDE	1.352	3.144	1566.53	4925.80	4.251	1451.94	6172.10
A_68_P21820792	chr2:164793405-164793449	NM_020333:-61	Slc12a5	PROMOTER	1.352	1.914	754.00	1443.04	2.588	676.97	1751.94
A_68_P26238818	chr8:125778428-125778472	NM_020497:356	Zfp276	INSIDE	1.352	1.703	968.26	1649.17	2.302	713.76	1643.32
A_68_P25735806	chr8:28088778-28088822	NM_001101502:993	Zfp703	INSIDE	1.352	1.463	2200.89	3219.55	1.978	1519.14	3004.28
A_68_P22509004	chr3:127201898-127201942	ENSMUST00000104965:-1		PROMOTER	1.352	3.319	1159.32	3847.87	4.486	944.78	4238.11
A_68_P29656176	chr14:63174690-63174734	ENSMUST00000160745:101		INSIDE	1.352	12.068	731.90	8832.82	16.313	732.20	11944.22
A_68_P30568280	chr16:16146642-16146686	NM_146068:262	2310008H04Rik	INSIDE	1.351	3.121	1423.97	4444.92	4.217	1239.77	5228.61
A_68_P22942710	chr4:57003066-57003110	NM_001142965:175	6430704M03Rik	INSIDE	1.351	3.860	405.32	1564.37	5.213	526.59	2744.90
A_68_P29747537	chr14:79124881-79124925	NM_001081336:-6	Dgkh	PROMOTER	1.351	2.255	848.39	1913.52	3.048	683.30	2082.64
A_68_P32541781	chrX:92639710-92639754	NM_175179:447	Fam123b	INSIDE	1.351	2.399	2316.60	5557.38	3.240	1138.92	3690.06
A_68_P28997659	chr13:44826258-44826302	NM_001205043:-359	Jarid2	PROMOTER	1.351	1.460	893.50	1304.83	1.972	875.17	1726.24
A_68_P21421187	chr2:91023173-91023217	NM_001177720:-3716	Madd	PROMOTER	1.351	3.258	1096.95	3574.40	4.403	958.05	4218.22
A_68_P22949803	chr4:58218825-58218869	NM_022814:622	Svep1	INSIDE	1.351	2.522	578.72	1459.31	3.406	486.29	1656.44
A_68_P30566187	chr16:15594249-15594293	NM_001159351:341	Ube2v2	INSIDE	1.351	2.127	4254.61	9048.22	2.872	3286.73	9440.89
A_68_P24091119	chr5:130371741-130371785	NM_183088:170	2410018M08Rik	INSIDE	1.350	4.754	1452.40	6904.13	6.418	1168.93	7501.85
A_68_P21631948	chr2:130665681-130665725	NM_029432:144	4930402H24Rik	INSIDE	1.350	1.883	1526.30	2873.28	2.542	1369.48	3481.37
A_68_P27958472	chr11:80965947-80965991	NM_007384:437	Accn1	INSIDE	1.350	2.931	228.85	670.79	3.958	228.19	903.09
A_68_P23315180	chr4:132568310-132568354	NM_146155:912	Ahdc1	INSIDE	1.350	2.566	577.69	1482.17	3.464	557.57	1931.48
A_68_P24990327	chr7:19479355-19479399	NM_001033314:16377	Ccdc61	INSIDE	1.350	4.732	4065.99	19240.24	6.387	3400.00	21717.34
A_68_P26236516	chr8:125394674-125394718	NM_007662:22423	Cdh15	DOWNSTREAM	1.350	4.613	1240.67	5722.78	6.225	973.31	6058.68
A_68_P27894661	chr11:69182323-69182367	NM_146019:584	Chd3	INSIDE	1.350	1.733	598.91	1037.71	2.340	511.00	1195.65
A_68_P23155324	chr4:101169410-101169454	NM_198412:180	Dnajc6	INSIDE	1.350	1.697	1936.17	3284.88	2.290	1410.10	3228.90
A_68_P23309924	chr4:131604876-131604920	NM_001128606:95	Epb4.1	INSIDE	1.350	4.441	2377.59	10558.25	5.995	2028.13	12159.05
A_68_P26224362	chr8:123609059-123609103	NM_010426:707	Foxf1a	INSIDE	1.350	2.194	1213.76	2662.70	2.961	920.81	2726.96
A_68_P24620344	chr6:88147093-88147137	NM_008090:-1543	Gata2	PROMOTER	1.350	2.203	587.48	1294.48	2.974	532.80	1584.60
A_68_P29613096	chr14:55195734-55195778	NM_010590:742	Jub	INSIDE	1.350	7.990	2719.59	21729.66	10.785	2234.76	24101.80
A_68_P27528046	chr10:125403373-125403417	NM_177152:120	Lrig3	INSIDE	1.350	4.085	1673.97	6838.45	5.516	1338.69	7384.71
A_68_P22209747	chr3:67178066-67178110	NM_001039543:70	Mlf1	INSIDE	1.350	1.503	1142.03	1716.40	2.028	1022.81	2074.71
A_68_P21840226	chr2:168056561-168056605	NM_001160330:461	Mocs3	INSIDE	1.350	4.757	442.98	2107.05	6.422	371.87	2388.16
A_68_P26500015	chr9:49606210-49606254	NM_001081445:942	Ncam1	INSIDE	1.350	2.107	750.15	1580.35	2.844	631.11	1794.89
A_68_P26477175	chr9:45792523-45792567	NM_008775:410	Pafah1b2	INSIDE	1.350	1.712	1861.35	3187.50	2.312	1644.22	3802.17
A_68_P29116256	chr13:69673614-69673658	NM_001169131:-894	Papd7	PROMOTER	1.350	1.569	2724.26	4273.30	2.117	2356.71	4989.79
A_68_P27551126	chr11:3190443-3190488	NM_019574:6	Patz1	INSIDE	1.350	1.532	1497.99	2294.67	2.067	1338.58	2767.13
A_68_P27949793	chr11:79404552-79404596	NM_175543:-139	Rab11fip4	PROMOTER	1.350	2.519	1244.98	3136.67	3.401	1099.13	3737.89

ProbeName	Target position of probe on CpG island microarray	TargetID	GeneSymbol	CpG island Description	Ratio of relative methylation (TiO_2-NP/Vehicle)	Sham group			TiO_2-H group		
						Relative methylation (Cy5/Cy3)	Cy3 signal (Input)	Cy5 signal (MeDIP)	Relative methylation (Cy5/Cy3)	Cy3 signal (Input)	Cy5 signal (MeDIP)
A_68_P27907304	chr11:71565101-71565145	NM_177618:918	Wscd1	INSIDE	1.350	1.817	798.47	1450.58	2.452	704.74	1728.38
A_68_P32111692	chr19:40969658-40969702	NM_028319:486	Zfp518a	INSIDE	1.350	2.005	5300.44	10629.34	2.707	4430.67	11994.26
A_68_P22300151	chr3:85775250-85775294	ENSMUST00000158452:-8518		PROMOTER	1.350	2.485	692.09	1720.00	3.354	680.90	2283.78
A_68_P30382914	chr15:82176511-82176555	NM_026914:57	1500032L24Rik	INSIDE	1.349	4.575	1510.15	6909.63	6.171	1513.47	9339.25
A_68_P24098222	chr5:131916544-131916588	NM_177047:1101647	Auts2	INSIDE	1.349	4.268	378.96	1617.35	5.756	342.54	1971.59
A_68_P30478823	chr15:99055100-99055144	NM_010601:-284	Kcnh3	PROMOTER	1.349	1.660	545.35	905.02	2.239	508.01	1137.66
A_68_P30682957	chr16:37868699-37868743	NM_177093:235	Lrrc58	INSIDE	1.349	2.636	1457.85	3842.19	3.557	1184.30	4211.97
A_68_P24731710	chr6:108090490-108090549	NM_145937:45058	Sumf1	INSIDE	1.349	5.960	233.08	1389.11	8.038	185.59	1491.64
A_68_P29448169	chr14:21480603-21480647	NM_175132:6904	Synpo2l	INSIDE	1.349	1.484	656.87	974.96	2.002	569.66	1140.74
A_68_P23282960	chr4:126645057-126645101	NM_001114399:89	Zmym4	INSIDE	1.349	2.115	2067.77	4373.83	2.854	1783.97	5092.09
A_68_P26663734	chr9:79190352-79190396			Unknown	1.349	2.174	1089.87	2369.86	2.933	884.61	2594.65
A_68_P23593955	chr5:34713045-34713089	ENSMUST00000042234:5		INSIDE	1.349	4.173	510.56	2130.77	5.630	450.09	2534.10
A_68_P31154763	chr17:34742756-34742800	NM_001163379:-27	Agpat1	DIVERGENT_PROMOTER	1.348	1.589	1297.92	2062.07	2.141	1067.47	2285.71
A_68_P24540594	chr6:72185394-72185438	NM_153778:155	Atoh8	INSIDE	1.348	2.927	1388.35	4063.95	3.945	1073.45	4234.55
A_68_P26234162	chr8:125075132-125075176	NM_001037298:75	Fam38a	INSIDE	1.348	2.716	835.72	2269.80	3.661	879.62	3220.14
A_68_P20351021	chr1:74929129-74929173	NM_153111:2832	Fev	INSIDE	1.348	3.448	1564.46	5394.70	4.649	1378.08	6407.18
A_68_P30490428	chr15:101054889-101054933	NM_019518:273	Grasp	INSIDE	1.348	0.394	1524.89	600.94	0.531	1193.36	634.07
A_68_P31489152	chr18:10325147-10325191	NM_001083628:-8	Greb1l	PROMOTER	1.348	4.009	1463.51	5866.73	5.403	1090.28	5890.53
A_68_P26349535	chr9:21722823-21722867	NM_031874:-279	Rab3d	PROMOTER	1.348	1.640	4313.75	7073.43	2.211	2985.88	6600.80
A_68_P31930774	chr19:5771196-5771240	NM_023912:183	Scyl1	INSIDE	1.348	0.402	1293.41	519.44	0.541	1001.35	541.92
A_68_P21623516	chr2:129024287-129024331	NM_001159593:-200	Slc20a1	PROMOTER	1.348	2.524	1562.08	3942.32	3.402	1464.39	4981.84
A_68_P30580561	chr16:18582686-18582730	NM_011532:4354	Tbx1	INSIDE	1.348	1.717	761.91	1308.00	2.315	639.79	1480.99
A_68_P27590826	chr11:11362086-11362130	NM_001185153:314	Zpbp	INSIDE	1.348	6.592	539.80	3558.60	8.889	478.90	4257.03
A_68_P24591142	chr6:83004903-83004947	NM_007517:278	Aup1	INSIDE	1.347	1.420	1180.63	1676.14	1.913	980.14	1874.80
A_68_P27812114	chr11:54601209-54601253	NM_178626:-25	Cdc42se2	PROMOTER	1.347	3.405	812.15	2765.33	4.587	734.19	3367.84
A_68_P31166952	chr17:37183039-37183083	NM_019439:150	Gabbr1	INSIDE	1.347	1.632	1349.72	2202.16	2.198	1103.62	2425.58
A_68_P25301153	chr7:95278950-95278994	NM_001081414:546295	Grm5	INSIDE	1.347	2.153	795.64	1713.16	2.901	698.56	2026.33
A_68_P26348588	chr9:21528254-21528298	NM_010700:239	Ldlr	INSIDE	1.347	4.817	292.19	1407.62	6.490	322.47	2092.75
A_68_P21475550	chr2:102026219-102026263	NM_178886:294	Ldlrad3	INSIDE	1.347	1.932	3546.53	6851.72	2.602	2729.63	7102.60
A_68_P27969031	chr11:82721501-82721545	NM_145431:375	Nle1	INSIDE	1.347	1.508	1683.38	2538.58	2.031	1353.98	2749.68
A_68_P29904272	chr14:112075171-112075215	NM_198865:856	Slitrk5	INSIDE	1.347	2.108	911.50	1921.46	2.839	781.50	2218.40
A_68_P32264678	chrX:12649125-12649169	NM_009481:523	Usp9x	INSIDE	1.347	2.170	1564.01	3393.55	2.922	867.52	2534.63
A_68_P24992394	chr7:19856763-19856807	NM_009499:419	Vasp	INSIDE	1.347	1.565	1471.11	2302.67	2.108	1180.72	2489.50
A_68_P24954288	chr7:6106767-6106811	NM_001013012:785	Zfp787	INSIDE	1.347	1.487	1545.68	2297.86	2.002	1290.12	2582.61
A_68_P24983771	chr7:17470567-17470612	NM_001081655:9924	Dact3	INSIDE	1.346	2.158	696.75	1503.57	2.904	694.21	2016.01
A_68_P23232037	chr4:116692073-116692117	NM_175135:82	Eif2b3	INSIDE	1.346	2.096	1100.19	2306.35	2.822	977.06	2757.37
A_68_P28513502	chr12:70342816-70342860	NM_178253:20	Klhdc1	INSIDE	1.346	2.317	870.11	2016.35	3.120	700.60	2185.64
A_68_P23950022	chr5:104475737-104475781	NM_028794:-271	Nudt9	PROMOTER	1.346	1.393	1131.89	1577.11	1.875	993.45	1862.75
A_68_P28513108	chr12:70260224-70260268	NM_009093:-73	Rps29	DIVERGENT_PROMOTER	1.346	1.580	1551.10	2450.61	2.127	1334.72	2839.08
A_68_P29218721	chr13:91600629-91600673	NM_024186:-51	Ssbp2	PROMOTER	1.346	2.895	1245.08	3604.99	3.896	1126.61	4389.72
A_68_P26805310	chr9:106724832-106724876	NM_001015507:548	Vprbp	INSIDE	1.346	1.700	1624.83	2762.47	2.288	1326.87	3036.44
A_68_P26552554	chr9:59139473-59139517	NM_028121:116	Adpgk	INSIDE	1.345	3.966	546.33	2166.48	5.334	559.45	2984.12
A_68_P27843676	chr11:60352657-60352701	NM_172943:1494	Alkbh5	INSIDE	1.345	1.803	1111.47	2003.81	2.426	936.19	2270.89
A_68_P25587962	chr7:148480819-148480877	NM_007878:2944	Drd4	INSIDE	1.345	2.296	319.06	732.54	3.087	247.41	763.84
A_68_P25827838	chr8:46095952-46095996	NM_001081286:60413	Fat1	INSIDE	1.345	2.345	1020.73	2393.43	3.153	804.20	2535.83
A_68_P31949295	chr19:10315203-10315247	NM_001033481:14	Gm98	INSIDE	1.345	2.226	2762.80	6150.31	2.993	2332.90	6982.74
A_68_P25262701	chr7:86855086-86855130	NM_010626:3964	Kif7	INSIDE	1.345	4.186	342.03	1431.64	5.631	321.35	1809.63
A_68_P31731695	chr18:56867197-56867241	NM_010721:-248	Lmnb1	PROMOTER	1.345	3.064	1742.74	5339.03	4.122	1496.70	6169.25
A_68_P23164324	chr4:102787243-102787287	NM_001039081:-4730	Mier1	PROMOTER	1.345	2.885	1527.16	4405.40	3.881	1239.86	4811.59
A_68_P31100163	chr17:25032650-25032694	NM_023260:608	Mrps34	INSIDE	1.345	1.470	829.83	1219.61	1.977	727.19	1437.34
A_68_P27565429	chr11:5778293-5778337	NM_008894:-55	Pold2	PROMOTER	1.345	1.484	6769.19	10048.55	1.996	4910.58	9801.50

ProbeName	Target position of probe on CpG island microarray	TargetID	GeneSymbol	CpG island Description	Ratio of relative methylation (TiO_2-NP/Vehicle)	Sham group			TiO_2-H group		
						Relative methylation (Cy5/Cy3)	Cy3 signal (Input)	Cy5 signal (MeDIP)	Relative methylation (Cy5/Cy3)	Cy3 signal (Input)	Cy5 signal (MeDIP)
A_68_P29619515	chr14:56341110-56341154	NM_019519:-119	Rabggta	PROMOTER	1.345	1.837	1069.75	1965.49	2.472	872.78	2157.61
A_68_P24428192	chr6:48544047-48544091	NM_001079901:187	Repin1	INSIDE	1.345	1.620	4432.65	7182.98	2.180	3673.76	8008.99
A_68_P30424513	chr15:89145595-89145639	NM_001081030:126	Sbf1	INSIDE	1.345	1.646	977.58	1608.85	2.214	832.48	1842.77
A_68_P29145779	chr13:74487622-74487666	NM_023281:44	Sdha	INSIDE	1.345	1.769	3823.23	6762.77	2.379	3142.66	7474.94
A_68_P32363781	chrX:45638888-45638932	NM_028276:28800	Utp14a	DOWNSTREAM	1.345	4.508	4499.91	20285.34	6.065	2344.00	14216.05
A_68_P28577654	chr12:81892119-81892163	NM_178682:595	4933426M11Rik	INSIDE	1.344	1.873	402.24	753.31	2.517	370.66	932.86
A_68_P30367720	chr15:79489229-79489273	NM_029407:136	4933432B09Rik	INSIDE	1.344	1.571	1644.90	2584.60	2.112	1249.73	2639.32
A_68_P31794495	chr18:68092683-68092727	NM_172631:-206	D18Ertd653e	PROMOTER	1.344	5.098	868.15	4425.93	6.852	728.89	4994.12
A_68_P30346594	chr15:76009406-76009450	NM_201394:16712	Plec	INSIDE	1.344	2.929	1053.43	3084.97	3.935	909.12	3577.10
A_68_P21351539	chr2:76486029-76486073	NM_011871:1	Prkra	INSIDE	1.344	3.292	1449.40	4771.65	4.424	1126.06	4981.41
A_68_P31096042	chr17:24342530-24342574	NM_001163847:-45	Tbc1d24	PROMOTER	1.344	2.127	1128.60	2400.42	2.859	930.85	2661.29
A_68_P32562267	chrX:98254914-98254958	NM_031384:42	Tex11	INSIDE	1.344	2.185	1466.51	3204.23	2.937	759.57	2230.79
A_68_P28614746	chr12:88540796-88540840	NM_001033475:361	Tmed8	INSIDE	1.344	1.730	3353.98	5801.61	2.324	2617.69	6083.77
A_68_P21070953	chr2:25118574-25118618	NM_175286:479	Tprn	INSIDE	1.344	1.619	2392.55	3873.83	2.176	1987.89	4324.89
A_68_P25948524	chr8:72411743-72411787	NM_026818:-473	Cilp2	PROMOTER	1.343	2.962	1181.69	3500.32	3.979	846.69	3368.65
A_68_P23797494	chr5:73797444-73797488	NM_181323:150	Cwh43	INSIDE	1.343	1.827	1940.94	3545.24	2.452	1713.72	4202.67
A_68_P28553890	chr12:77518775-77518819	NM_001163103:211	Gm70	INSIDE	1.343	1.890	847.73	1602.10	2.539	734.25	1864.00
A_68_P26585802	chr9:64949678-64949722	NM_020043:399	Igdcc4	INSIDE	1.343	2.113	2532.28	5349.72	2.838	1862.59	5286.39
A_68_P31220004	chr17:49070978-49071022	NM_027452:-906	Lrfn2	PROMOTER	1.343	2.745	3083.98	8465.89	3.687	2419.79	8921.06
A_68_P20897581	chr1:186635006-186635050	NM_001081361:164	Mosc1	INSIDE	1.343	1.441	1974.58	2846.13	1.936	1701.70	3293.85
A_68_P29591680	chr14:49685967-49686011	NM_144535:-181	Mudeng	PROMOTER	1.343	2.974	782.10	2325.97	3.994	718.01	2868.04
A_68_P22346838	chr3:95799917-95799961	NM_023320:-176	Plekho1	PROMOTER	1.343	3.746	995.77	3729.99	5.032	1010.56	5084.78
A_68_P29177384	chr13:81850152-81850196	NM_001081176:-162	Polr3g	DIVERGENT_PROMOTER	1.343	1.758	1624.81	2856.71	2.361	1401.46	3308.73
A_68_P26701864	chr9:86774762-86774806	NM_013669:423	Snap91	INSIDE	1.343	2.241	2263.56	5071.87	3.009	1966.54	5917.00
A_68_P26770650	chr9:100543637-100543681	NM_009282:-383	Stag1	PROMOTER	1.343	3.029	521.91	1580.85	4.067	522.70	2125.96
A_68_P23892499	chr5:92566763-92566807	NM_019490:-179	Uso1	PROMOTER	1.343	1.352	3090.94	4179.57	1.816	2605.61	4732.47
A_68_P30665799	chr16:34745459-34745503	ENSMUST00000155268:185		INSIDE	1.343	2.697	1401.94	3780.96	3.622	1146.42	4152.87
A_68_P22611613	chr3:145587280-145587324	NM_009740:-39	Bcl10	PROMOTER	1.342	1.846	1465.19	2704.33	2.476	1240.37	3071.52
A_68_P28149797	chr11:114653726-114653770	NM_028055:3458	Btbd17	INSIDE	1.342	1.735	878.36	1524.07	2.329	704.61	1641.20
A_68_P22151285	chr3:54859248-54859292	NM_007628:-293	Ccna1	PROMOTER	1.342	2.456	1542.30	3787.73	3.297	1328.02	4378.25
A_68_P24157327	chr5:144383303-144383347	NM_011182:9	Cyth3	INSIDE	1.342	2.206	732.54	1615.92	2.961	752.32	2227.69
A_68_P32140229	chr19:45817346-45817390	NM_001166361:6	Fgf8	INSIDE	1.342	2.813	896.25	2521.01	3.775	850.84	3212.33
A_68_P28431895	chr12:52929741-52929785	NM_144788:761	Hectd1	INSIDE	1.342	2.458	362.51	891.20	3.299	334.68	1104.11
A_68_P25528897	chr7:138685710-138685754	NM_008257:-744	Hmx3	PROMOTER	1.342	4.723	1970.61	9306.45	6.340	1736.99	11012.01
A_68_P25954891	chr8:73437387-73437431	NM_173013:7536	Mtap1s	INSIDE	1.342	4.074	1656.66	6750.02	5.469	1363.98	7459.09
A_68_P22165653	chr3:57651276-57651320	NM_019410:381	Pfn2	INSIDE	1.342	2.233	966.34	2157.50	2.996	881.45	2640.61
A_68_P32700062	chrX:137133645-137133689	NM_001077364:1395	Tsc22d3	INSIDE	1.342	3.679	648.42	2385.47	4.936	495.78	2447.01
A_68_P25957039	chr8:74005499-74005543	NM_001164679:4446	Ano8	INSIDE	1.341	4.935	612.86	3024.27	6.617	520.56	3444.58
A_68_P32130260	chr19:44144019-44144063	NM_145502:136	Erlin1	INSIDE	1.341	1.536	3085.79	4740.97	2.060	2339.82	4819.63
A_68_P21052788	chr2:20890736-20890780	NR_033225:258	Gm13375	INSIDE	1.341	1.874	3948.02	7398.32	2.513	3064.35	7701.04
A_68_P23344527	chr4:137772352-137772396	NM_001122896:-167	Hp1bp3	PROMOTER	1.341	2.571	1612.56	4146.00	3.449	1364.94	4707.40
A_68_P23333958	chr4:135999081-135999125	NM_008309:19664	Htr1d	INSIDE	1.341	3.546	2662.08	9439.13	4.754	2030.57	9653.41
A_68_P28041933	chr11:95868905-95868949	NM_009951:-1668	Igf2bp1	PROMOTER	1.341	2.490	2490.68	6202.21	3.338	1816.66	6064.22
A_68_P28748394	chr12:113413797-113413841	NM_001097621:29400	Kif26a	INSIDE	1.341	2.524	1151.21	2906.20	3.385	992.17	3358.66
A_68_P25308743	chr7:96781285-96781329	NM_181407:-21	Me3	PROMOTER	1.341	4.545	937.57	4261.60	6.093	730.00	4448.16
A_68_P32778405	chrX:156575888-156575932	NM_008810:358	Pdha1	INSIDE	1.341	1.893	1655.40	3133.51	2.538	761.33	1932.45
A_68_P20335741	chr1:72301294-72301338	NM_023523:29572	Pecr	DOWNSTREAM	1.341	2.547	2164.50	5512.47	3.416	1799.10	6145.68
A_68_P29194041	chr13:85429220-85429264	NM_145452:-151	Rasa1	PROMOTER	1.341	1.531	2338.75	3581.60	2.054	2082.23	4276.98
A_68_P28145843	chr11:113928475-113928519	NM_172800:-1231	Sdk2	PROMOTER	1.341	1.523	863.20	1314.51	2.042	722.08	1474.48
A_68_P31019379	chr17:5841808-5841852	NM_025664:451	Snx9	INSIDE	1.341	2.188	1208.94	2645.75	2.935	1063.24	3120.64
A_68_P28307257	chr12:28026632-28026676	NM_009234:929	Sox11	INSIDE	1.341	2.549	1939.58	4944.88	3.420	1530.73	5234.53

ProbeName	Target position of probe on CpG island microarray	TargetID	GeneSymbol	CpG island Description	Ratio of relative methylation (TiO_2-NP/Vehicle)	Sham group			TiO_2-H group		
						Relative methylation (Cy5/Cy3)	Cy3 signal (Input)	Cy5 signal (MeDIP)	Relative methylation (Cy5/Cy3)	Cy3 signal (Input)	Cy5 signal (MeDIP)
A_68_P21303500	chr2:68309909-68309953	NM_016866:108	Stk39	INSIDE	1.341	0.485	1005.88	488.07	0.651	982.39	639.18
A_68_P24838523	chr6:128305849-128305893	NM_011657:-1	Tulp3	PROMOTER	1.341	1.922	8087.35	15543.80	2.577	5603.71	14438.80
A_68_P30133054	chr15:35301504-35301548	NM_177151:226	Vps13b	INSIDE	1.341	1.790	494.98	886.08	2.400	409.02	981.74
A_68_P31836390	chr18:75503087-75503131			Unknown	1.341	2.940	1694.58	4981.64	3.941	1214.78	4787.82
A_68_P30591270	chr16:21248808-21248852	ENSMUST00000152179:48115		INSIDE	1.341	1.983	537.39	1065.58	2.659	443.74	1179.77
A_68_P33012182	chr9_random:57481-57526	NR_027950:2811	4930526I15Rik	DOWNSTREAM	1.340	1.803	1443.26	2602.19	2.417	1106.91	2675.29
A_68_P25352111	chr7:104886841-104886885	NM_175105:-105	Aqp11	PROMOTER	1.340	1.566	2330.02	3648.13	2.098	1761.88	3695.74
A_68_P20645417	chr1:138242409-138242453	NM_001081360:251	Camsap1l1	INSIDE	1.340	1.793	488.83	876.41	2.402	429.57	1031.69
A_68_P20794602	chr1:167390651-167390695	NM_028759:-78	Dcaf6	DIVERGENT_PROMOTER	1.340	1.682	1369.42	2303.47	2.253	1193.27	2688.65
A_68_P24218728	chr6:6813006-6813052	NM_010057:-305	Dlx6	PROMOTER	1.340	3.124	463.90	1449.06	4.184	343.38	1436.81
A_68_P23590785	chr5:34065022-34065066	NM_001163216:90	Fgfr3	INSIDE	1.340	1.500	1441.38	2161.81	2.010	1196.27	2404.98
A_68_P29257132	chr13:99125852-99125896	NM_008242:1675	Foxd1	INSIDE	1.340	3.574	465.45	1663.66	4.791	406.12	1945.74
A_68_P26803574	chr9:106367984-106368028	NM_001159652:265	Gpr62	INSIDE	1.340	1.427	3088.38	4408.24	1.912	2350.54	4494.43
A_68_P20453280	chr1:94998488-94998532	NM_001110315:-68	Kif1a	PROMOTER	1.340	4.133	1535.83	6347.31	5.537	1108.13	6136.10
A_68_P31442546	chr17:91487752-91487796	NM_020252:4368	Nrxn1	INSIDE	1.340	4.217	690.47	2911.36	5.651	647.49	3658.63
A_68_P26824202	chr9:110434375-110434420	NM_001081340:-703	Setd2	PROMOTER	1.340	1.892	467.14	883.91	2.536	352.86	894.77
A_68_P27176492	chr10:60215582-60215626	NM_023596:-74	Slc29a3	PROMOTER	1.340	2.681	5444.55	14594.76	3.592	3669.82	13180.77
A_68_P27682717	chr11:31269557-31269601	NM_011491:483	Stc2	INSIDE	1.340	1.669	1237.89	2066.49	2.237	1094.87	2449.72
A_68_P32136800	chr19:45228638-45228682	NM_021901:3456	Tlx1	INSIDE	1.340	3.369	748.09	2520.00	4.512	686.92	3099.60
A_68_P32554230	chrX:96412300-96412344			Unknown	1.340	1.351	1922.99	2598.74	1.811	994.30	1800.28
A_68_P30959225	chr16:90831355-90831399	NM_001199210:273	4931408A02Rik	INSIDE	1.339	2.098	570.28	1196.22	2.809	771.21	2166.00
A_68_P23882644	chr5:90795790-90795834	NM_030886:-601	Ankrd17	PROMOTER	1.339	1.716	3282.73	5632.05	2.297	2759.58	6339.29
A_68_P26323900	chr9:15432364-15432408	NM_181816:-9	Ccdc67	PROMOTER	1.339	1.842	1156.73	2130.38	2.466	973.67	2401.32
A_68_P26475231	chr9:45480834-45480878	NM_001081270:242481	Dscaml1	INSIDE	1.339	1.652	1172.34	1936.21	2.211	914.02	2021.00
A_68_P31740998	chr18:58369127-58369171	NM_010181:432	Fbn2	INSIDE	1.339	1.482	3111.41	4611.22	1.985	2320.40	4605.24
A_68_P28082092	chr11:102754466-102754510	NM_010277:4026	Gfap	INSIDE	1.339	1.665	1707.38	2842.56	2.228	1362.45	3036.12
A_68_P24226577	chr6:8460033-8460077	NM_133236:459	Glcci1	INSIDE	1.339	2.909	1410.44	4102.79	3.895	1231.12	4795.38
A_68_P29330809	chr13:112599173-112599217	NM_011945:-3	Map3k1	PROMOTER	1.339	0.427	1060.33	452.65	0.571	1039.45	593.98
A_68_P21839352	chr2:167906373-167906417	NM_021409:-109	Pard6b	PROMOTER	1.339	4.844	374.56	1814.38	6.487	288.36	1870.76
A_68_P23398661	chr4:148473893-148473937	NM_019781:7	Pex14	INSIDE	1.339	1.577	2035.14	3209.18	2.112	1587.54	3353.13
A_68_P32464005	chrX:70905148-70905192	NM_001199351:23	Pnck	INSIDE	1.339	2.115	1153.88	2440.12	2.832	575.03	1628.51
A_68_P28736915	chr12:111426863-111426907	AK044800:1099		INSIDE	1.339	2.545	872.42	2220.13	3.408	737.57	2513.97
A_68_P27281467	chr10:79634488-79634532	NM_027207:-1178	1600002K03Rik	PROMOTER	1.338	3.721	3827.22	14240.89	4.977	3317.73	16513.05
A_68_P30350790	chr15:76648746-76648790	NM_001168288:-168	Arhgap39	PROMOTER	1.338	1.790	1276.86	2286.01	2.395	1173.22	2809.87
A_68_P20331600	chr1:71603721-71603765	NM_026195:13	Atic	INSIDE	1.338	2.362	3141.08	7419.09	3.160	2564.54	8104.76
A_68_P22551964	chr3:135086455-135086499	NM_025902:-79	Cisd2	PROMOTER	1.338	2.027	266.75	540.60	2.711	283.56	768.81
A_68_P28923671	chr13:31650063-31650107	NM_008239:46	Foxq1	INSIDE	1.338	1.997	1114.18	2225.43	2.673	983.38	2628.11
A_68_P24449299	chr6:52184163-52184207	NM_008263:754	Hoxa10	INSIDE	1.338	2.076	455.15	945.07	2.779	425.78	1183.31
A_68_P25092917	chr7:52661860-52661904	NM_010596:553	Kcna7	INSIDE	1.338	2.607	531.73	1386.00	3.487	497.20	1733.82
A_68_P24992159	chr7:19813899-19813943	NM_207525:183	Opa3	INSIDE	1.338	1.911	2633.79	5033.31	2.558	2126.72	5439.79
A_68_P30346607	chr15:76010905-76010949	NM_201394:15214	Plec	INSIDE	1.338	8.869	2737.52	24278.32	11.865	2149.78	25506.15
A_68_P28160474	chr11:116485631-116485675	NM_001167680:-86	Rhbdf2	PROMOTER	1.338	3.174	5177.67	16434.58	4.248	3799.05	16139.05
A_68_P30368252	chr15:79573047-79573091	NM_001205345:-102	Sun2	PROMOTER	1.338	2.064	1709.94	3529.13	2.762	1574.09	4347.12
A_68_P32672209	chrX:131010003-131010047	NM_028958:1005	Taf7l	INSIDE	1.338	1.414	2478.92	3505.23	1.892	1251.69	2368.81
A_68_P31335267	chr17:71948102-71948146	NR_015549:-23	Trmt61b	PROMOTER	1.338	2.084	1345.43	2803.78	2.789	1114.42	3107.82
A_68_P30125733	chr15:33617621-33617665	NM_001085421:-4	Tspyl5	PROMOTER	1.338	2.298	2155.98	4954.41	3.075	1620.37	4982.26
A_68_P28736904	chr12:111425727-111425771	AK044800:-37		DIVERGENT_PROMOTER	1.338	3.048	1148.32	3500.06	4.077	1097.52	4474.22
A_68_P31458282	chr18:3383392-3383436	NM_029402:192	Cul2	INSIDE	1.337	3.211	2117.97	6800.27	4.292	1751.61	7517.97
A_68_P22520451	chr3:129236724-129236768	NM_130450:1443	Elovl6	INSIDE	1.337	4.256	616.20	2622.36	5.692	541.72	3083.33
A_68_P21834174	chr2:167014089-167014133	NM_008420:189	Kcnb1	INSIDE	1.337	1.753	524.44	919.24	2.344	527.04	1235.51
A_68_P27453319	chr10:111708984-111709028	NM_001025581:828	Kcnc2	INSIDE	1.337	2.028	1498.18	3038.83	2.713	1243.51	3373.36

ProbeName	Target position of probe on CpG island microarray	TargetID	GeneSymbol	CpG island Description	Ratio of relative methylation (TiO_2-NP/Vehicle)	Sham group			TiO_2-H group		
						Relative methylation (Cy5/Cy3)	Cy3 signal (Input)	Cy5 signal (MeDIP)	Relative methylation (Cy5/Cy3)	Cy3 signal (Input)	Cy5 signal (MeDIP)
A_68_P20127130	chr1:32229462-32229506	NM_133235:-166	Khdrbs2	PROMOTER	1.337	2.312	2254.58	5212.73	3.091	1987.15	6141.96
A_68_P26133049	chr8:107850154-107850198	NM_177449:0	Lrrc29	INSIDE	1.337	3.266	1227.99	4010.66	4.365	1021.93	4461.19
A_68_P30777937	chr16:55822273-55822317	NM_001159394:-43	Nfkbiz	PROMOTER	1.337	2.175	2129.70	4632.62	2.908	1709.94	4972.36
A_68_P25779457	chr8:36439788-36439832	NM_177741:1016	Ppp1r3b	INSIDE	1.337	2.316	504.42	1168.21	3.097	408.12	1264.11
A_68_P29267242	chr13:100878272-100878316	NM_011353:321	Serf1	INSIDE	1.337	1.446	1531.30	2214.12	1.934	1407.30	2721.17
A_68_P28875421	chr13:22037070-22037115	NM_183014:130	Zfp184	INSIDE	1.337	1.661	896.06	1488.71	2.221	746.96	1658.69
A_68_P21851419	chr2:169956951-169956995	NM_001033299:-252	Zfp217	PROMOTER	1.337	1.763	975.08	1719.13	2.357	860.78	2028.53
A_68_P23296944	chr4:129178242-129178286	NM_001085491:9831	1700125D06Rik	DOWNSTREAM	1.336	3.582	2075.79	7434.50	4.785	1806.94	8646.07
A_68_P28574822	chr12:81361237-81361281	NM_134156:100	Actn1	INSIDE	1.336	3.621	938.72	3399.35	4.840	864.72	4185.02
A_68_P25024597	chr7:29747515-29747559	NM_021895:-237	Actn4	PROMOTER	1.336	1.478	852.53	1259.82	1.975	795.54	1571.06
A_68_P20872222	chr1:181733328-181733372	NM_026375:796	Ahctf1	INSIDE	1.336	2.979	486.39	1448.99	3.980	359.47	1430.54
A_68_P30379106	chr15:81525333-81525377	NM_001164320:2363	Chadl	INSIDE	1.336	4.624	1302.11	6020.74	6.176	1129.61	6976.41
A_68_P31227865	chr17:50432360-50432404	NM_010021:542	Dazl	INSIDE	1.336	2.581	370.51	956.21	3.449	302.26	1042.39
A_68_P31161462	chr17:36016904-36016948	NM_026987:204	Dhx16	INSIDE	1.336	4.865	710.65	3457.34	6.500	533.57	3468.25
A_68_P27937920	chr11:77307416-77307460	NM_001004144:525	Git1	INSIDE	1.336	1.872	3343.68	6260.74	2.501	2574.44	6438.23
A_68_P29141936	chr13:73901069-73901113	NM_011390:-54	Slc12a7	PROMOTER	1.336	5.352	1349.31	7222.09	7.153	1152.16	8241.44
A_68_P27325554	chr10:87922543-87922587	NM_011517:233	Sycp3	INSIDE	1.336	3.252	1820.93	5920.98	4.345	1391.20	6045.37
A_68_P20680088	chr1:145624700-145624744	NM_001159866:315	Uchl5	INSIDE	1.336	1.508	818.20	1233.96	2.016	654.47	1319.14
A_68_P30356301	chr15:77688150-77688194			Unknown	1.336	1.916	1408.52	2698.29	2.560	1253.57	3209.38
A_68_P22747701	chr4:14858053-14858097	ENSMUST00000152024:252		INSIDE	1.336	1.862	1307.55	2434.08	2.487	1130.94	2812.33
A_68_P21092002	chr2:28438501-28438545	NM_148928:277	Gtf3c5	INSIDE	1.335	3.024	1189.90	3598.21	4.036	961.92	3882.22
A_68_P21338754	chr2:74520144-74520188	NM_008273:-283	Hoxd11	PROMOTER	1.335	4.895	964.44	4721.07	6.534	934.03	6103.35
A_68_P21666845	chr2:136941868-136941912	NM_013822:366	Jag1	INSIDE	1.335	1.437	1804.87	2592.90	1.918	1445.41	2772.97
A_68_P23120719	chr4:94719459-94719503	NM_010591:-567	Jun	PROMOTER	1.335	2.009	7357.97	14785.66	2.682	5167.60	13857.99
A_68_P24862701	chr6:134591261-134591305	NM_026371:325	Loh12cr1	INSIDE	1.335	2.315	1231.51	2851.23	3.091	1058.55	3271.87
A_68_P31100078	chr17:25023059-25023105	NM_011956:213	Nubp2	INSIDE	1.335	2.560	1166.37	2985.90	3.419	923.21	3156.12
A_68_P27808204	chr11:53914341-53914385	NM_001136076:-63	P4ha2	PROMOTER	1.335	2.153	739.34	1592.04	2.874	676.91	1945.71
A_68_P22309854	chr3:87572957-87573001	NM_001032413:-103	Pear1	PROMOTER	1.335	2.770	1235.72	3422.55	3.697	1098.60	4061.88
A_68_P25169315	chr7:69608941-69608985	NM_013788:434	Peg12	INSIDE	1.335	2.966	433.72	1286.34	3.961	376.26	1490.24
A_68_P25024908	chr7:29804801-29804845	NM_009109:105348	Ryr1	INSIDE	1.335	1.502	652.69	980.15	2.004	893.28	1790.28
A_68_P25735809	chr8:28089115-28089159	NM_001101502:1329	Zfp703	INSIDE	1.335	2.225	15940.06	35462.73	2.970	13259.88	39380.86
A_68_P20293905	chr1:64737459-64737503	NR_038009:-247	2810408I11Rik	DIVERGENT_PROMOTER	1.334	4.130	700.14	2891.44	5.509	590.88	3254.91
A_68_P21768923	chr2:155833524-155833568	NM_001145351:-3383	6430550D23Rik	PROMOTER	1.334	1.486	1918.07	2850.26	1.982	1568.36	3108.55
A_68_P23352771	chr4:139130470-139130514	NM_001205173:-142	Iffo2	PROMOTER	1.334	1.834	5321.49	9762.17	2.446	3868.28	9463.00
A_68_P25503042	chr7:134072295-134072339	NM_172747:-76	Kctd13	PROMOTER	1.334	2.072	2909.54	6027.35	2.763	2401.59	6636.46
A_68_P28052177	chr11:97560932-97560976	NM_001163307:-256	Pcgf2	PROMOTER	1.334	3.014	323.96	976.35	4.021	264.30	1062.62
A_68_P31257082	chr17:56724139-56724183	NM_001029979:-154	Safb2	DIVERGENT_PROMOTER	1.334	2.397	1326.14	3178.53	3.197	1326.10	4239.77
A_68_P26885415	chr9:121672022-121672066	NM_001166644:2894	Zfp651	INSIDE	1.334	3.001	367.72	1103.34	4.002	323.52	1294.84
A_68_P25264340	chr7:87172009-87172053	NM_175433:917	Zfp710	INSIDE	1.334	2.542	1323.19	3363.81	3.391	1157.96	3926.24
A_68_P32257885	chrX:11241764-11241808			Unknown	1.334	1.843	2993.62	5515.87	2.458	2032.34	4995.40
A_68_P28731504	chr12:110272212-110272256	NM_001163175:34193	Begain	INSIDE	1.333	2.767	351.11	971.62	3.690	331.43	1222.84
A_68_P26880510	chr9:120843613-120843657	NM_001165902:939	Ctnnb1	INSIDE	1.333	2.029	1273.94	2584.83	2.705	1019.34	2757.35
A_68_P26512426	chr9:51771455-51771499	NM_007996:162	Fdx1	INSIDE	1.333	2.638	1623.96	4284.49	3.517	1384.14	4868.40
A_68_P28923686	chr13:31651815-31651859	NM_008239:1798	Foxq1	INSIDE	1.333	2.485	1040.59	2586.20	3.312	840.99	2785.69
A_68_P26035037	chr8:89997425-89997469	NR_033430:736	Gm2694	INSIDE	1.333	3.330	422.27	1406.31	4.441	364.08	1616.82
A_68_P21902331	chr2:179960697-179960741	NM_001081171:-154	Lama5	PROMOTER	1.333	1.805	1144.46	2065.74	2.406	1145.28	2755.15
A_68_P25010203	chr7:26181112-26181156	NM_010719:-128	Lipe	PROMOTER	1.333	2.248	2863.62	6436.32	2.997	2214.24	6635.82
A_68_P20241002	chr1:55294197-55294241	NM_175439:198	Mars2	INSIDE	1.333	2.175	1906.21	4145.37	2.898	1719.83	4984.92
A_68_P31553001	chr18:23198592-23198636	NM_001161483:-1450	Nol4	PROMOTER	1.333	1.905	955.04	1819.39	2.539	751.14	1907.10
A_68_P21114136	chr2:32092306-32092350	NM_145145:126	Pomt1	INSIDE	1.333	1.860	1785.50	3321.77	2.480	1471.03	3648.84
A_68_P24117360	chr5:136164660-136164704	NM_008898:-401	Por	PROMOTER	1.333	1.400	2742.36	3840.04	1.867	2286.08	4267.59

ProbeName	Target position of probe on CpG island microarray	TargetID	GeneSymbol	CpG island Description	Ratio of relative methylation (TiO_2-NP/Vehicle)	Sham group			TiO_2-H group		
						Relative methylation (Cy5/Cy3)	Cy3 signal (Input)	Cy5 signal (MeDIP)	Relative methylation (Cy5/Cy3)	Cy3 signal (Input)	Cy5 signal (MeDIP)
A_68_P30380957	chr15:81846273-81846317	NM_134095:276	Pppde2	INSIDE	1.333	2.057	2296.06	4724.11	2.743	1977.13	5423.42
A_68_P25228919	chr7:80554204-80554248	NM_177740:33821	Rgma	INSIDE	1.333	3.125	1593.04	4978.61	4.164	1257.76	5237.77
A_68_P30575781	chr16:17803149-17803193	NM_153790:5796	Scarf2	INSIDE	1.333	2.172	1337.19	2904.51	2.895	1270.17	3677.27
A_68_P29507582	chr14:31529438-31529482	NM_001166532:660	Sfmbt1	INSIDE	1.333	2.054	2040.89	4192.46	2.737	1920.06	5255.83
A_68_P24120070	chr5:136708234-136708278	NM_018825:12517	Sh2b2	INSIDE	1.333	2.738	276.24	756.47	3.650	197.08	719.35
A_68_P30425529	chr15:89329556-89329600	NM_021423:-709	Shank3	PROMOTER	1.333	3.568	1757.17	6269.47	4.757	1409.19	6703.29
A_68_P23187164	chr4:106585031-106585075	NM_023672:978	Ssbp3	INSIDE	1.333	2.840	979.04	2780.22	3.785	811.06	3069.92
A_68_P30385309	chr15:82729893-82729937	NR_027799:-1023	Tbrg3	PROMOTER	1.333	2.843	332.55	945.29	3.790	351.26	1331.45
A_68_P30572943	chr16:17277156-17277200	NM_177473:786	Tmem191c	INSIDE	1.333	1.574	2471.21	3889.63	2.098	2174.32	4562.63
A_68_P28696867	chr12:104563817-104563863	NM_023049:30371	Asb2	INSIDE	1.332	6.609	655.44	4332.12	8.807	474.02	4174.57
A_68_P26470343	chr9:44728440-44728484	NM_013795:363	Atp5l	INSIDE	1.332	0.502	1215.35	610.51	0.669	1031.13	690.09
A_68_P22523145	chr3:129672979-129673023	NM_025779:124	Ccdc109b	INSIDE	1.332	1.964	752.91	1479.05	2.616	688.48	1801.39
A_68_P31607790	chr18:33623473-33623517	NM_001109989:-93	D0H4S114	PROMOTER	1.332	1.825	540.07	985.73	2.430	490.14	1191.28
A_68_P20422165	chr1:90102210-90102254	NM_008299:91	Dnajb3	INSIDE	1.332	2.473	690.38	1707.28	3.295	585.58	1929.29
A_68_P26658159	chr9:77956440-77956484	NM_023605:1	Fbxo9	INSIDE	1.332	3.803	1019.33	3876.55	5.066	912.99	4625.60
A_68_P20264189	chr1:59539520-59539564	NM_008057:552	Fzd7	INSIDE	1.332	1.795	1363.25	2446.54	2.390	1114.11	2663.22
A_68_P27500269	chr10:120335461-120335505	NM_177092:545	Msrb3	INSIDE	1.332	1.914	1787.68	3421.72	2.550	1542.80	3934.06
A_68_P21722728	chr2:147009701-147009745	NR_030769:-96	Nkx2-2as	PROMOTER	1.332	1.500	810.16	1214.93	1.997	767.80	1533.39
A_68_P31682250	chr18:47528856-47528900	NM_018744:-356	Sema6a	PROMOTER	1.332	1.808	935.61	1691.99	2.409	814.14	1961.36
A_68_P24046794	chr5:122286634-122286678	NM_008507:154	Sh2b3	INSIDE	1.332	2.434	950.55	2313.86	3.242	834.37	2704.65
A_68_P23238480	chr4:117807649-117807693	NM_001161774:-151	St3gal3	PROMOTER	1.332	2.806	2923.17	8200.99	3.736	2211.63	8261.91
A_68_P25576308	chr7:146374357-146374401	NM_021302:15	Stk32c	INSIDE	1.332	2.699	993.13	2680.68	3.596	915.61	3292.55
A_68_P31098879	chr17:24806802-24806846	NM_183149:128	Zfp598	INSIDE	1.332	0.419	1813.18	759.99	0.558	1540.66	860.45
A_68_P26160580	chr8:112603835-112603879	NM_175480:223	Zfp612	INSIDE	1.332	1.437	2261.02	3248.90	1.914	1850.82	3542.32
A_68_P30821643	chr16:64770368-64770412	NM_026273:375	4930453N24Rik	INSIDE	1.331	1.725	1018.18	1756.51	2.296	832.14	1910.38
A_68_P23602808	chr5:36100579-36100623	NM_001177696:72	Ablim2	INSIDE	1.331	1.664	718.64	1195.51	2.214	653.94	1447.58
A_68_P31939551	chr19:7456653-7456699	NM_001033139:840	AI846148	INSIDE	1.331	2.226	646.62	1439.53	2.964	578.22	1713.62
A_68_P24593643	chr6:83406064-83406108	NR_028382:14338	B230319C09Rik	DOWNSTREAM	1.331	1.961	1529.67	2999.72	2.611	1309.39	3418.30
A_68_P32464345	chrX:70961700-70961744	NM_012060:-208	Bcap31	DIVERGENT_PROMOTER	1.331	1.867	2442.66	4561.11	2.485	1185.74	2946.87
A_68_P27360779	chr10:94151657-94151701	NM_029852:144	Ccdc41	INSIDE	1.331	1.374	1423.70	1955.77	1.828	1289.37	2357.44
A_68_P32096195	chr19:37625247-37625291	NM_175353:361	Exoc6	INSIDE	1.331	2.964	518.11	1535.82	3.946	585.86	2311.77
A_68_P26228654	chr8:124277231-124277275	NM_020605:22794	Jph3	INSIDE	1.331	2.378	754.46	1794.34	3.166	785.56	2486.72
A_68_P31515588	chr18:15222716-15222760	NM_001142731:-647	Kctd1	PROMOTER	1.331	3.505	1556.81	5457.17	4.667	1481.41	6913.96
A_68_P28457453	chr12:57636766-57636810	NM_001146198:-695	Nkx2-1	PROMOTER	1.331	2.333	6807.31	15879.70	3.105	5163.41	16032.11
A_68_P20870654	chr1:181447812-181447856	NM_027188:300	Smyd3	INSIDE	1.331	1.548	4397.46	6807.52	2.060	3135.10	6459.42
A_68_P21823565	chr2:165213622-165213666	NM_178411:115	Zfp334	INSIDE	1.331	1.801	1360.15	2449.20	2.397	1128.76	2705.35
A_68_P25504795	chr7:134398033-134398077	NM_177362:10015	Zfp771	INSIDE	1.331	2.801	722.05	2022.40	3.729	710.19	2648.52
A_68_P24183980	chr5:150040675-150040719	AK155259:18562		DOWNSTREAM	1.331	2.303	304.15	700.36	3.066	269.40	825.98
A_68_P27111542	chr10:45205927-45205971	ENSMUST00000099847:-365		PROMOTER	1.331	1.624	2466.59	4006.89	2.162	2163.36	4678.11
A_68_P25027733	chr7:30304368-30304412	ENSMUST00000141713:167		INSIDE	1.331	2.366	489.83	1159.16	3.149	411.67	1296.37
A_68_P21110673	chr2:31544152-31544196	NM_001112703:99	Abl1	INSIDE	1.330	2.593	1393.98	3614.26	3.447	1188.79	4097.93
A_68_P23541452	chr5:23893732-23893776	NM_001002897:4207	Atg9b	INSIDE	1.330	1.663	494.34	822.07	2.212	416.14	920.65
A_68_P27828609	chr11:57822259-57822303	NM_028451:-285	Larp1	PROMOTER	1.330	2.175	1919.17	4173.64	2.893	1632.70	4723.18
A_68_P31938322	chr19:7240010-7240054	NM_134147:108775	Macrod1	INSIDE	1.330	3.244	197.20	639.76	4.316	156.80	676.76
A_68_P29224476	chr13:93300612-93300656	NM_173392:131	Zfyve16	INSIDE	1.330	1.564	1215.33	1900.52	2.079	1033.25	2148.53
A_68_P27921684	chr11:74463331-74463375	NM_001081158:356	1300001I01Rik	INSIDE	1.329	2.131	649.07	1383.17	2.833	529.41	1499.69
A_68_P20861822	chr1:179726539-179726583	NM_007422:80	Adss	INSIDE	1.329	1.576	1110.22	1749.31	2.094	852.39	1785.30
A_68_P26236514	chr8:125394402-125394446	NM_007662:22151	Cdh15	DOWNSTREAM	1.329	2.720	1831.61	4982.14	3.614	1550.16	5602.88
A_68_P26695698	chr9:85642712-85642756	NM_001081282:207	Ibtk	INSIDE	1.329	4.457	487.82	2174.07	5.924	537.96	3186.74
A_68_P28290824	chr12:25336101-25336145	NM_178357:-113	Klf11	PROMOTER	1.329	2.060	906.85	1867.82	2.737	773.95	2118.22
A_68_P24591361	chr6:83037609-83037653	NM_010692:1272	Lbx2	INSIDE	1.329	2.250	737.02	1658.65	2.991	627.50	1877.06

ProbeName	Target position of probe on CpG island microarray	TargetID	GeneSymbol	CpG island Description	Ratio of relative methylation (TiO_2-NP/Vehicle)	Sham group			TiO_2-H group		
						Relative methylation (Cy5/Cy3)	Cy3 signal (Input)	Cy5 signal (MeDIP)	Relative methylation (Cy5/Cy3)	Cy3 signal (Input)	Cy5 signal (MeDIP)
A_68_P31784509	chr18:66162457-66162501	NM_027400:-189	Lman1	PROMOTER	1.329	1.864	2581.80	4812.40	2.477	1946.21	4820.43
A_68_P21747603	chr2:151918965-151919009	NM_001160410:11722	Scrt2	INSIDE	1.329	1.802	874.71	1576.53	2.394	735.01	1759.97
A_68_P26365997	chr9:25060616-25060660	NM_001205367:599	Septin7	INSIDE	1.329	2.708	1613.18	4368.05	3.598	1635.78	5885.79
A_68_P23991345	chr5:112736931-112736975	NM_009419:31226	Tpst2	INSIDE	1.329	2.709	958.79	2597.34	3.600	790.67	2846.33
A_68_P27889184	chr11:68213153-68213197	ENSMUST00000108674:1151		INSIDE	1.329	2.210	6659.31	14716.33	2.936	5271.16	15476.48
A_68_P30425032	chr15:89240650-89240694	NM_001162882:22	1700007E06Rik	INSIDE	1.328	3.837	641.17	2459.90	5.097	593.91	3026.98
A_68_P31304171	chr17:66426811-66426855	NM_001025572:-446	Ankrd12	PROMOTER	1.328	1.740	1184.72	2060.95	2.311	1028.02	2375.55
A_68_P32111327	chr19:40905758-40905802	NM_172839:12	Ccnj	INSIDE	1.328	1.806	337.55	609.64	2.399	338.98	813.34
A_68_P32054108	chr19:29722693-29722737	NM_001081213:196	Ermp1	INSIDE	1.328	1.378	2497.19	3442.12	1.830	2094.02	3833.04
A_68_P24518615	chr6:66846390-66846434	NM_001177558:-472	Gng12	PROMOTER	1.328	3.354	1881.29	6309.00	4.454	1803.62	8032.63
A_68_P20939666	chr1:193542578-193542622	NM_001134829:327	Lpgat1	INSIDE	1.328	2.532	1456.79	3689.26	3.363	1359.30	4571.15
A_68_P26017625	chr8:86535356-86535400	NR_035431:154	Mir1199	DOWNSTREAM	1.328	2.383	289.76	690.47	3.164	247.17	782.08
A_68_P27708863	chr11:35648179-35648223	NM_025936:-170	Rars	PROMOTER	1.328	2.661	1297.55	3453.16	3.534	1109.70	3922.11
A_68_P27277984	chr10:79100620-79100664	NM_001024539:21	Shc2	INSIDE	1.328	2.265	1849.31	4188.43	3.008	1539.84	4632.16
A_68_P27985585	chr11:85651342-85651386	NM_009324:5248	Tbx2	INSIDE	1.328	2.422	537.79	1302.68	3.218	535.62	1723.58
A_68_P23317102	chr4:132894698-132894742	NM_199306:510	Wdtc1	INSIDE	1.328	2.257	1980.31	4469.28	2.997	1700.34	5095.96
A_68_P31054979	chr17:13184902-13184946	NM_001113533:201	Wtap	INSIDE	1.328	1.592	480.14	764.31	2.113	490.52	1036.67
A_68_P31839436	chr18:75979559-75979603	NM_145356:-251	Zbtb7c	PROMOTER	1.328	3.054	1281.61	3913.84	4.056	1122.07	4551.36
A_68_P21863764	chr2:172171330-172171374	NM_025912:276	2010011I20Rik	INSIDE	1.327	2.582	980.11	2530.58	3.425	836.76	2866.20
A_68_P30478152	chr15:98918819-98918863	NM_001024702:-681	C1ql4	PROMOTER	1.327	1.812	1095.73	1985.63	2.406	926.74	2229.38
A_68_P21761656	chr2:154482914-154482958	NM_029362:175	Chmp4b	INSIDE	1.327	2.086	1477.06	3080.69	2.767	1247.02	3450.68
A_68_P21772343	chr2:156439879-156439923	NM_146128:460	Dlgap4	INSIDE	1.327	4.427	527.59	2335.58	5.875	478.94	2813.59
A_68_P23203768	chr4:109654665-109654709	NM_172296:4057	Dmrta2	INSIDE	1.327	2.445	731.29	1788.17	3.245	722.67	2345.00
A_68_P27561405	chr11:4998913-4998957	NM_007968:146	Ewsr1	INSIDE	1.327	1.582	3416.84	5404.07	2.099	2668.71	5601.04
A_68_P21826982	chr2:165818442-165818486	NM_008679:328	Ncoa3	INSIDE	1.327	2.219	749.00	1661.83	2.943	601.78	1771.19
A_68_P29509959	chr14:31952078-31952122	NM_027289:4062	Nt5dc2	INSIDE	1.327	6.149	5256.00	32320.87	8.159	3483.05	28419.71
A_68_P27994102	chr11:87218064-87218108	NM_053269:-89	Rad51c	DIVERGENT_PROMOTER	1.327	2.099	2291.40	4810.14	2.786	1906.29	5310.77
A_68_P28690583	chr12:103521466-103521510	NM_001161365:-362	Rin3	PROMOTER	1.327	3.033	1823.63	5531.38	4.025	1699.66	6841.70
A_68_P26626136	chr9:71959653-71959697	NM_011544:-48	Tcf12	PROMOTER	1.327	2.176	413.81	900.58	2.889	369.21	1066.56
A_68_P24973912	chr7:13556599-13556643	NM_178732:5408	Zfp324	INSIDE	1.327	2.365	596.32	1410.24	3.138	501.39	1573.22
A_68_P22415392	chr3:108743052-108743096	NM_001078646:-400	4921515J06Rik	PROMOTER	1.326	3.943	1193.06	4704.51	5.228	1075.53	5622.93
A_68_P23315375	chr4:132594527-132594571	NM_146155:27128	Ahdc1	INSIDE	1.326	1.780	1395.87	2485.03	2.360	1182.08	2789.86
A_68_P31992643	chr19:18787618-18787662	NM_177640:-85	D030056L22Rik	PROMOTER	1.326	1.684	745.29	1255.19	2.233	604.09	1348.65
A_68_P25371481	chr7:108257732-108257776	NM_001146010:466	Fchsd2	INSIDE	1.326	1.417	1431.97	2028.55	1.879	1190.75	2237.42
A_68_P21393860	chr2:84499188-84499232	NM_001145100:-25	Gm13718	DIVERGENT_PROMOTER	1.326	1.993	461.60	920.05	2.642	366.33	967.97
A_68_P26423570	chr9:35382219-35382263	NM_001167603:8	Gm6762	INSIDE	1.326	2.375	721.29	1713.06	3.149	599.39	1887.46
A_68_P20472800	chr1:99666322-99666367	NM_173760:325	Ppip5k2	INSIDE	1.326	1.502	1205.28	1810.01	1.991	1018.87	2028.53
A_68_P33012164	chr9_random:55526-55570	NM_011154:-516	Ppp2r3d	PROMOTER	1.326	1.883	1207.21	2273.69	2.498	1040.43	2598.63
A_68_P22383500	chr3:102739435-102739479	NM_011516:567	Sycp1	INSIDE	1.326	4.447	800.92	3561.39	5.896	729.86	4303.12
A_68_P20880198	chr1:183142413-183142457	NM_145514:-325	Wdr26	PROMOTER	1.326	3.450	811.74	2800.51	4.576	663.30	3035.36
A_68_P20734230	chr1:156646554-156646598	AK046991:-6703		PROMOTER	1.326	3.352	528.65	1771.80	4.445	611.00	2715.93
A_68_P32744435	chrX:147603926-147603970	ENSMUST00000151778:-294		PROMOTER	1.326	2.441	369.44	901.85	3.236	269.94	873.57
A_68_P26870880	chr9:119071809-119071853	NM_146230:-5619	Acaa1b	PROMOTER	1.325	2.532	209.50	530.41	3.355	199.15	668.13
A_68_P21429508	chr2:92440060-92440104	NM_023850:219	Chst1	INSIDE	1.325	3.221	1274.45	4104.95	4.267	1061.68	4530.38
A_68_P27596686	chr11:12364779-12364823	NM_172496:163	Cobl	INSIDE	1.325	1.630	1174.88	1914.68	2.160	879.30	1899.17
A_68_P23644441	chr5:43624695-43624739	NM_001177379:15	Cpeb2	INSIDE	1.325	1.720	758.50	1304.34	2.279	566.73	1291.42
A_68_P32467042	chrX:71491942-71491986	NM_010227:-91	Flna	DIVERGENT_PROMOTER	1.325	1.736	2304.77	4001.90	2.300	1207.98	2778.69
A_68_P26228891	chr8:124308748-124308792	NM_020605:54312	Jph3	INSIDE	1.325	2.148	1238.21	2660.24	2.846	994.77	2831.00
A_68_P27180702	chr10:60938602-60938646	NM_153542:44	Lrrc20	INSIDE	1.325	2.254	1350.93	3044.50	2.987	1157.71	3458.25
A_68_P24344494	chr6:32537701-32537745	NM_175750:470	Plxna4	INSIDE	1.325	4.779	906.23	4331.14	6.332	817.07	5173.42
A_68_P25261273	chr7:86611105-86611149	NM_017462:33	Polg	INSIDE	1.325	2.466	988.11	2437.11	3.269	783.30	2560.71

ProbeName	Target position of probe on CpG island microarray	TargetID	GeneSymbol	CpG island Description	Ratio of relative methylation (TiO_2-NP/Vehicle)	Sham group			TiO_2-H group		
						Relative methylation (Cy5/Cy3)	Cy3 signal (Input)	Cy5 signal (MeDIP)	Relative methylation (Cy5/Cy3)	Cy3 signal (Input)	Cy5 signal (MeDIP)
A_68_P22373272	chr3:100914442-100914486	NM_011197:-375	Ptgfrn	PROMOTER	1.325	1.846	2116.48	3907.36	2.446	1668.49	4080.89
A_68_P31166410	chr17:37084116-37084160	NM_001099632:4143	Rnf39	INSIDE	1.325	5.307	804.66	4270.32	7.033	666.26	4685.86
A_68_P27502792	chr10:120747561-120747605	NM_029057:663	Tbc1d30	INSIDE	1.325	2.310	2694.13	6222.40	3.061	2143.82	6562.39
A_68_P27273028	chr10:77707431-77707475	NM_001081055:-65	Trappc10	PROMOTER	1.325	3.157	464.09	1465.08	4.183	452.55	1892.91
A_68_P30579667	chr16:18426465-18426509	NM_013711:-23	Txnrd2	PROMOTER	1.325	1.481	783.90	1161.05	1.963	638.69	1253.83
A_68_P20737711	chr1:157264084-157264128	NM_011273:468	Xpr1	INSIDE	1.325	2.450	1346.14	3298.40	3.246	1119.03	3632.03
A_68_P27939246	chr11:77576975-77577019	ENSMUST00000164315:-2108		PROMOTER	1.325	2.885	618.33	1783.74	3.822	584.18	2232.48
A_68_P32362521	chrX:45387855-45387899	NM_013912:153	Apln	INSIDE	1.324	2.085	784.66	1635.84	2.759	531.92	1467.67
A_68_P31013735	chr17:4994646-4994690	NM_001085355:-405	Arid1b	PROMOTER	1.324	4.513	834.47	3766.07	5.977	776.71	4642.31
A_68_P31497270	chr18:11998017-11998061	NM_001146287:256	Cables1	INSIDE	1.324	1.937	792.07	1534.37	2.566	698.72	1792.66
A_68_P31497275	chr18:11998719-11998763	NM_001146287:958	Cables1	INSIDE	1.324	3.779	866.26	3273.55	5.003	818.60	4095.46
A_68_P27856226	chr11:62693049-62693093	NM_026210:79	Fam18b	INSIDE	1.324	1.619	2108.69	3414.57	2.144	1632.20	3499.12
A_68_P28691639	chr12:103707284-103707328	NM_001199004:-37	Golga5	PROMOTER	1.324	6.824	728.83	4973.52	9.036	662.37	5985.37
A_68_P23595697	chr5:35003116-35003160	NM_001080743:111	Grk4	INSIDE	1.324	3.205	811.98	2602.50	4.245	698.69	2965.75
A_68_P26551398	chr9:58883897-58883941	NM_001042752:330	Neo1	INSIDE	1.324	2.352	598.47	1407.40	3.113	584.31	1818.90
A_68_P21099262	chr2:29602819-29602863	NM_001039086:127601	Rapgef1	DOWNSTREAM	1.324	1.773	2158.80	3827.34	2.347	1966.85	4616.65
A_68_P24061815	chr5:124991287-124991331	NM_019770:509	Tmed2	INSIDE	1.324	3.457	830.90	2872.79	4.579	726.16	3324.86
A_68_P27941693	chr11:77978890-77978934	NM_009423:140	Traf4	INSIDE	1.324	2.290	976.67	2236.09	3.031	1005.12	3046.52
A_68_P24974195	chr7:13610408-13610452	NM_011588:930	Trim28	INSIDE	1.324	1.826	4941.50	9023.02	2.417	3820.40	9233.04
A_68_P25805592	chr8:41509363-41509407	NM_178395:216	Zdhhc2	INSIDE	1.324	4.035	2183.74	8811.56	5.341	1808.12	9656.86
A_68_P27282404	chr10:79778048-79778092	NM_011789:13506	Apc2	INSIDE	1.323	2.930	1282.30	3757.48	3.878	1101.62	4272.28
A_68_P28731659	chr12:110290536-110290580	NM_001163175:15869	Begain	INSIDE	1.323	1.482	865.97	1283.25	1.960	693.49	1359.22
A_68_P24172982	chr5:148118030-148118074	NM_007673:773	Cdx2	INSIDE	1.323	1.526	745.96	1137.99	2.019	620.09	1251.70
A_68_P20884270	chr1:183853884-183853931	NM_001083120:96204	Enah	INSIDE	1.323	4.449	283.64	1261.81	5.883	245.23	1442.75
A_68_P30672048	chr16:35770485-35770529	NM_175111:35	Hspbap1	INSIDE	1.323	1.888	504.28	952.34	2.498	500.95	1251.18
A_68_P22301530	chr3:86028536-86028580	NM_001077687:-53	Lrba	PROMOTER	1.323	1.558	2396.45	3734.12	2.062	1823.34	3759.48
A_68_P30477059	chr15:98702362-98702406	NM_001033276:-770	Mll2	PROMOTER	1.323	0.489	1195.94	585.17	0.647	1062.71	687.82
A_68_P22129522	chr3:51220411-51220455	NM_053089:495	Naa15	INSIDE	1.323	3.886	2611.95	10149.18	5.142	2331.12	11986.67
A_68_P29962214	chr14:122933466-122933510	NM_144844:-61	Pcca	PROMOTER	1.323	2.679	806.16	2159.36	3.543	661.97	2345.13
A_68_P27926516	chr11:75323969-75324013	NM_001029938:395	Rilp	INSIDE	1.323	5.409	923.84	4997.02	7.158	875.77	6268.87
A_68_P20502876	chr1:107253429-107253473	NM_001160368:-163	Rnf152	PROMOTER	1.323	1.967	771.60	1518.09	2.603	700.33	1822.83
A_68_P32056100	chr19:30105268-30105312	NM_144873:288	Uhrf2	INSIDE	1.323	1.524	1487.05	2265.92	2.016	1267.08	2555.01
A_68_P27532843	chr10:126338419-126338463	NM_026858:-13	Xrcc6bp1	PROMOTER	1.323	3.405	5351.64	18221.11	4.504	3691.77	16628.77
A_68_P26744829	chr9:95855445-95855489	NM_011916:288	Xrn1	INSIDE	1.323	1.870	757.69	1417.19	2.474	690.29	1707.60
A_68_P25288546	chr7:91558357-91558401	NM_007488:91	Arnt2	INSIDE	1.322	2.099	764.77	1605.49	2.774	727.59	2018.56
A_68_P23909952	chr5:97426279-97426323	NM_080708:-407	Bmp2k	PROMOTER	1.322	2.863	2058.50	5893.04	3.785	1676.29	6345.46
A_68_P21134948	chr2:35516613-35516657	NM_001114124:39134	Dab2ip	INSIDE	1.322	2.312	2135.86	4937.48	3.057	1571.09	4802.63
A_68_P25728861	chr8:26864655-26864699	NM_028102:76	Ddhd2	INSIDE	1.322	3.597	1997.80	7186.27	4.756	1678.32	7982.86
A_68_P23981086	chr5:111082431-111082475	NM_027156:-17	Ddx51	DIVERGENT_PROMOTER	1.322	2.900	612.99	1777.46	3.835	595.78	2284.63
A_68_P23392750	chr4:147533918-147533962	NM_001161851:-236	Fbxo44	PROMOTER	1.322	2.102	1717.08	3610.11	2.779	1242.74	3453.90
A_68_P20306027	chr1:67085323-67085367	NM_001190984:64	Lancl1	INSIDE	1.322	2.292	2434.12	5579.96	3.030	2089.25	6330.04
A_68_P23188354	chr4:106739410-106739454	NM_025500:39	Mrpl37	INSIDE	1.322	2.233	1362.25	3042.43	2.952	1105.58	3263.30
A_68_P25806930	chr8:41720049-41720093	NM_001040699:76	Mtmr7	INSIDE	1.322	1.868	846.82	1581.48	2.469	711.06	1755.95
A_68_P25115908	chr7:57231147-57231191	NM_001037906:449	Nell1	INSIDE	1.322	1.503	757.62	1138.89	1.987	705.90	1402.58
A_68_P20625253	chr1:134942691-134942735	NM_001099276:124	Pik3c2b	INSIDE	1.322	1.948	999.52	1946.72	2.575	823.42	2120.49
A_68_P24774308	chr6:115944222-115944266	NM_026376:779	Plxnd1	INSIDE	1.322	1.750	1046.01	1830.01	2.313	852.82	1972.65
A_68_P23544155	chr5:24348677-24348721	NM_053075:-519	Rheb	PROMOTER	1.322	1.864	2804.58	5226.78	2.464	2232.16	5501.05
A_68_P26429692	chr9:37062811-37062855	NM_001145960:491	Slc37a2	INSIDE	1.322	1.616	5577.69	9012.00	2.136	3945.81	8426.96
A_68_P32287972	chrX:19869101-19869145	NM_177353:-232	Slc9a7	PROMOTER	1.322	2.514	1294.20	3253.30	3.324	852.12	2832.80
A_68_P22317588	chr3:88968981-88969026	NM_001163432:277	Clk2	INSIDE	1.321	1.518	1934.64	2936.72	2.005	1581.67	3170.46
A_68_P29246778	chr13:97313012-97313056	NM_001164222:345	Col4a3bp	INSIDE	1.321	1.625	1179.00	1916.11	2.148	1123.96	2413.79

ProbeName	Target position of probe on CpG island microarray	TargetID	GeneSymbol	CpG island Description	Ratio of relative methylation (TiO_2-NP/Vehicle)	Sham group			TiO_2-H group		
						Relative methylation (Cy5/Cy3)	Cy3 signal (Input)	Cy5 signal (MeDIP)	Relative methylation (Cy5/Cy3)	Cy3 signal (Input)	Cy5 signal (MeDIP)
A_68_P24091776	chr5:130505164-130505208	NM_007761:11	Crcp	INSIDE	1.321	8.956	626.35	5609.50	11.831	547.25	6474.46
A_68_P24058535	chr5:124423625-124423669	NM_145070:10	Hip1r	INSIDE	1.321	2.395	2236.78	5356.70	3.163	1780.95	5632.28
A_68_P25956632	chr8:73905289-73905333	NM_010150:541	Nr2f6	INSIDE	1.321	1.811	433.75	785.73	2.393	366.94	878.09
A_68_P31709378	chr18:52927767-52927811	NM_001199154:-9	Sncaip	PROMOTER	1.321	1.496	4933.51	7378.29	1.976	3616.94	7147.28
A_68_P22363128	chr3:99057574-99057618	NM_009323:-86	Tbx15	PROMOTER	1.321	3.627	5475.86	19861.59	4.790	4531.13	21704.62
A_68_P24543633	chr6:72738096-72738140	NM_001079822:832	Tcf7l1	INSIDE	1.321	3.710	3090.85	11466.74	4.900	2656.84	13017.93
A_68_P26845370	chr9:114471876-114471920	NM_001042503:1589	Trim71	INSIDE	1.321	2.375	839.43	1993.48	3.137	804.38	2523.27
A_68_P27332826	chr10:89208214-89208258	NM_029166:501	Uhrf1bp1l	INSIDE	1.321	4.622	896.95	4146.03	6.104	667.30	4073.18
A_68_P32462207	chrX:70599165-70599209	NM_001160229:11227	Zfp275	INSIDE	1.321	2.975	477.54	1420.49	3.931	285.08	1120.50
A_68_P28011630	chr11:90279544-90279588	AK139414:-6564		PROMOTER	1.321	1.916	1131.88	2168.43	2.532	980.47	2482.21
A_68_P27330521	chr10:88807370-88807414	ENSMUST00000168681:115		INSIDE	1.321	2.051	223.51	458.37	2.710	240.47	651.64
A_68_P22726935	chr4:10801906-10801950	NM_026005:284	2610301B20Rik	INSIDE	1.320	1.516	1758.59	2666.84	2.001	1483.27	2968.67
A_68_P32202573	chr19:56796579-56796623	NM_007419:-261	Adrb1	PROMOTER	1.320	3.560	1510.97	5378.29	4.697	1303.70	6123.66
A_68_P22388171	chr3:103613470-103613514	NM_001163552:53	Ap4b1	INSIDE	1.320	2.902	670.75	1946.43	3.830	684.49	2621.58
A_68_P25657174	chr8:11728216-11728260	NM_001113517:134	Arhgef7	INSIDE	1.320	4.272	2013.22	8601.05	5.640	1690.72	9536.17
A_68_P24925489	chr6:145814071-145814115	NM_024469:-232	Bhlhe41	PROMOTER	1.320	2.428	2408.96	5849.72	3.204	1870.90	5995.06
A_68_P31746198	chr18:59334973-59335017	NM_001081328:1	Chsy3	INSIDE	1.320	0.393	1365.51	536.56	0.519	1369.65	710.52
A_68_P22318693	chr3:89141626-89141670	NM_007910:302	Efna4	INSIDE	1.320	2.758	517.65	1427.61	3.639	477.52	1737.92
A_68_P25637624	chr8:8661448-8661492	NM_010111:-697	Efnb2	PROMOTER	1.320	1.744	681.68	1188.93	2.302	668.01	1538.05
A_68_P22565464	chr3:137527246-137527290	NM_016750:-294	H2afz	PROMOTER	1.320	0.463	2174.78	1007.66	0.612	1794.34	1097.28
A_68_P21338759	chr2:74520621-74520665	NM_008273:193	Hoxd11	INSIDE	1.320	2.060	679.69	1400.29	2.719	543.50	1477.82
A_68_P30514376	chr16:5203769-5203813	NM_013796:315	Nagpa	INSIDE	1.320	2.344	1058.07	2479.86	3.094	1030.70	3189.24
A_68_P26345775	chr9:20970639-20970683	NM_183408:503	Pde4a	INSIDE	1.320	1.653	649.90	1074.00	2.181	628.84	1371.80
A_68_P26700011	chr9:86465251-86465295	NM_001163746:177	Pgm3	INSIDE	1.320	1.631	2316.46	3777.33	2.153	1896.78	4083.99
A_68_P31154947	chr17:34769261-34769305	NM_030890:2652	Prrt1	DOWNSTREAM	1.320	2.189	2412.13	5279.41	2.889	1946.67	5624.40
A_68_P28678694	chr12:101122653-101122697	NM_028354:-50	Tdp1	DIVERGENT_PROMOTER	1.320	3.553	464.84	1651.56	4.689	439.59	2061.40
A_68_P24951679	chr7:4982581-4982625	NM_001033383:10625	Zfp865	INSIDE	1.320	2.406	217.38	523.13	3.175	195.46	620.66
A_68_P20863554	chr1:180299211-180299255			Unknown	1.320	2.271	629.91	1430.31	2.996	579.01	1735.00
A_68_P31661525	chr18:43597987-43598031	ENSMUST00000121805:-68		PROMOTER	1.320	1.411	2721.78	3839.24	1.862	2099.50	3909.95
A_68_P30570068	chr16:16600185-16600230	ENSMUST00000162414:-75		PROMOTER	1.320	3.597	774.75	2786.93	4.748	678.05	3219.19
A_68_P26084574	chr8:98202070-98202114	ENSMUST00000166358:-52		PROMOTER	1.320	8.324	737.08	6135.13	10.985	646.80	7104.85
A_68_P21665423	chr2:136716895-136716939	NM_028201:-37	2210009G21Rik	PROMOTER	1.319	1.987	2290.35	4549.79	2.620	2013.89	5275.58
A_68_P24383687	chr6:39507859-39507903	NM_172477:-47	Dennd2a	PROMOTER	1.319	1.931	2240.18	4324.83	2.546	1784.98	4544.03
A_68_P25520723	chr7:137409825-137409869	NM_010207:476	Fgfr2	INSIDE	1.319	2.219	720.81	1599.73	2.926	714.70	2091.51
A_68_P31347829	chr17:74059599-74059643	NM_027864:171	Galnt14	INSIDE	1.319	5.333	1697.21	9051.21	7.032	1550.96	10906.59
A_68_P24632565	chr6:90412944-90412988	NM_023184:347	Klf15	INSIDE	1.319	2.825	1583.42	4472.47	3.724	1453.82	5414.34
A_68_P26159961	chr8:112485994-112486038	NM_212447:89	Marveld3	INSIDE	1.319	2.326	1310.01	3047.50	3.068	1049.69	3220.71
A_68_P26980661	chr10:19077130-19077174	NM_053008:808	Olig3	INSIDE	1.319	1.602	3972.90	6365.16	2.113	3129.88	6612.40
A_68_P30346610	chr15:76011282-76011326	NM_201394:14836	Plec	INSIDE	1.319	4.220	2274.76	9599.30	5.566	2082.10	11589.26
A_68_P25092682	chr7:52621989-52622033	NM_029741:379	Ppfia3	INSIDE	1.319	3.297	1302.15	4293.37	4.350	1083.03	4711.70
A_68_P20848445	chr1:177422484-177422528	NM_001199003:170	Rgs7	INSIDE	1.319	4.625	1420.26	6569.12	6.099	1880.03	11466.08
A_68_P31256956	chr17:56705945-56705989	NM_001029979:18040	Safb2	INSIDE	1.319	2.169	888.39	1926.95	2.861	748.12	2140.56
A_68_P32021818	chr19:24205720-24205764	NM_001198985:42888	Tjp2	INSIDE	1.319	3.260	1992.82	6497.09	4.300	1836.06	7895.64
A_68_P27886200	chr11:67735519-67735563	NM_173754:115	Usp43	INSIDE	1.319	2.168	1106.12	2397.93	2.859	1010.27	2887.95
A_68_P30499184	chr15:102523520-102523564			Unknown	1.319	2.082	1555.22	3237.48	2.746	1259.57	3458.69
A_68_P25782091	chr8:36889227-36889271	NM_027998:365	Cldn23	INSIDE	1.318	1.908	1666.44	3179.85	2.514	1326.53	3335.34
A_68_P22882282	chr4:45333130-45333174	NM_025513:323	Exosc3	INSIDE	1.318	1.446	2411.88	3486.72	1.906	1944.14	3705.42
A_68_P23577613	chr5:31598167-31598211	NM_022424:62	Fndc4	INSIDE	1.318	1.773	1145.28	2031.03	2.337	1017.31	2376.98
A_68_P23332312	chr4:135700753-135700797	NM_008321:1038	Id3	INSIDE	1.318	2.129	2120.22	4514.37	2.805	1786.75	5012.28
A_68_P24137557	chr5:140251443-140251487	NM_026748:168	Ints1	INSIDE	1.318	1.468	915.06	1342.90	1.934	798.05	1543.68
A_68_P24476402	chr6:56654577-56654621	NM_025520:95	Lsm5	INSIDE	1.318	2.109	1461.25	3081.26	2.780	1251.41	3478.55

ProbeName	Target position of probe on CpG island microarray	TargetID	GeneSymbol	CpG island Description	Ratio of relative methylation (TiO_2-NP/Vehicle)	Sham group			TiO_2-H group		
						Relative methylation (Cy5/Cy3)	Cy3 signal (Input)	Cy5 signal (MeDIP)	Relative methylation (Cy5/Cy3)	Cy3 signal (Input)	Cy5 signal (MeDIP)
A_68_P25210716	chr7:77496064-77496108	NM_009697:9393	Nr2f2	DOWNSTREAM	1.318	1.531	3364.96	5151.09	2.017	2659.38	5363.72
A_68_P24898883	chr6:141198105-141198149	NM_018779:337	Pde3a	INSIDE	1.318	1.967	1181.39	2323.47	2.593	1024.49	2656.20
A_68_P30403196	chr15:85652378-85652422	NM_011105:-237	Pkdrej	PROMOTER	1.318	2.450	1129.50	2767.12	3.228	959.14	3096.33
A_68_P21866928	chr2:172805309-172805353	NM_001083959:-12	Spo11	PROMOTER	1.318	2.220	487.00	1081.11	2.927	445.93	1305.23
A_68_P20147164	chr1:36300875-36300919	NM_198899:251	Uggt1	INSIDE	1.318	1.554	6959.13	10817.93	2.049	5371.50	11004.76
A_68_P26153110	chr8:111316387-111316431	NM_007496:77865	Zfhx3	INSIDE	1.318	1.759	1250.27	2199.65	2.319	1015.92	2356.18
A_68_P24155687	chr5:144015862-144015906	NM_017467:15817	Zfp316	INSIDE	1.318	2.428	1061.06	2576.73	3.200	953.54	3051.26
A_68_P25726105	chr8:26350956-26351000	AK190590:395		INSIDE	1.318	2.020	2706.47	5467.91	2.663	2103.71	5602.89
A_68_P26577166	chr9:63450424-63450468	NM_025857:-15	Aagab	DIVERGENT_PROMOTER	1.317	2.201	789.44	1737.57	2.898	785.77	2277.24
A_68_P32260263	chrX:11655948-11655992	NM_029510:1709	Bcor	INSIDE	1.317	1.450	1662.62	2410.33	1.910	944.99	1804.58
A_68_P26527664	chr9:54613648-54613692	NM_013496:1056	Crabp1	INSIDE	1.317	3.590	700.69	2515.53	4.726	547.75	2588.92
A_68_P26699340	chr9:86360809-86360853	NM_177208:70	Dopey1	INSIDE	1.317	3.081	452.97	1395.49	4.057	466.30	1891.79
A_68_P21517948	chr2:109757926-109757970	NM_172671:145	Lgr4	INSIDE	1.317	2.059	1364.16	2809.25	2.713	1274.38	3457.38
A_68_P32466328	chrX:71330831-71330875	NM_010788:80	Mecp2	INSIDE	1.317	1.608	3044.74	4895.49	2.117	1475.04	3123.20
A_68_P25285286	chr7:91031153-91031197	NM_030705:1677	Mesdc1	INSIDE	1.317	2.271	786.92	1786.81	2.990	595.14	1779.19
A_68_P25780633	chr8:36651446-36651490	NM_001081279:617	Mfhas1	INSIDE	1.317	3.439	1040.64	3578.81	4.530	892.16	4041.59
A_68_P25709495	chr8:23276840-23276884	NM_001162947:-3896	Nek3	PROMOTER	1.317	1.834	1288.04	2362.78	2.415	1231.44	2974.50
A_68_P29753348	chr14:80170386-80170430	NM_001042726:711	Pcdh8	INSIDE	1.317	1.591	863.36	1373.18	2.094	833.06	1744.50
A_68_P28035922	chr11:94852837-94852881	NM_172261:333	Ppp1r9b	INSIDE	1.317	1.664	1090.30	1814.24	2.191	892.40	1955.69
A_68_P24155330	chr5:143942270-143942314	NM_001045482:130	Rbak	INSIDE	1.317	2.620	712.56	1866.56	3.449	625.96	2159.09
A_68_P21817914	chr2:164268773-164268817	NM_011521:-106	Sdc4	PROMOTER	1.317	2.861	1038.43	2970.56	3.767	992.39	3738.59
A_68_P24950759	chr7:4788111-4788155	NM_172737:8166	Shisa7	INSIDE	1.317	2.924	1027.97	3006.28	3.850	868.11	3342.30
A_68_P26477006	chr9:45762946-45762990	NM_172257:364	Sidt2	INSIDE	1.317	1.784	898.87	1603.27	2.348	854.15	2005.80
A_68_P23575087	chr5:31172049-31172093	NM_144525:51	Tmem214	INSIDE	1.317	1.506	7935.28	11952.04	1.984	6042.37	11988.98
A_68_P28575795	chr12:81564283-81564327	NM_133798:223	Exd2	INSIDE	1.316	3.658	5153.17	18848.24	4.815	3636.13	17508.68
A_68_P29346590	chr13:115249262-115249306	NM_008046:-346	Fst	PROMOTER	1.316	1.653	2540.27	4200.22	2.175	2104.93	4578.71
A_68_P29469014	chr14:24824046-24824090	NM_010610:-641	Kcnma1	PROMOTER	1.316	1.484	878.85	1304.58	1.954	776.79	1517.81
A_68_P22934434	chr4:55545364-55545408	NM_010637:-39	Klf4	PROMOTER	1.316	2.394	1702.46	4075.57	3.151	1374.43	4330.43
A_68_P26983898	chr10:19654588-19654632	NM_008580:279	Map3k5	INSIDE	1.316	1.760	1671.56	2942.32	2.317	1513.87	3507.61
A_68_P32134821	chr19:44911447-44911492	NM_011037:79586	Pax2	INSIDE	1.316	1.500	3089.94	4634.51	1.974	2528.66	4991.94
A_68_P22878697	chr4:44723513-44723557	NM_008782:-222	Pax5	PROMOTER	1.316	3.224	1352.76	4361.46	4.242	1245.43	5283.32
A_68_P21834482	chr2:167065917-167065961	NM_008968:99	Ptgis	INSIDE	1.316	1.640	3977.78	6523.45	2.158	2785.35	6011.84
A_68_P27926562	chr11:75328897-75328941	NM_001004157:1876	Scarf1	INSIDE	1.316	2.051	1029.47	2111.34	2.700	897.94	2424.25
A_68_P24024080	chr5:118478322-118478366	NM_021344:512	Tesc	INSIDE	1.316	4.629	945.70	4377.53	6.091	838.74	5108.40
A_68_P31202525	chr17:45822791-45822835	NM_198167:355	Tmem63b	INSIDE	1.316	2.089	1211.65	2530.68	2.750	1011.81	2782.04
A_68_P27836014	chr11:59102302-59102346	NM_009522:1929	Wnt3a	INSIDE	1.316	2.997	382.20	1145.35	3.943	377.15	1487.10
A_68_P31412802	chr17:86199409-86199453	AK142122:208		INSIDE	1.316	1.854	5358.93	9934.05	2.439	3923.47	9570.58
A_68_P23947800	chr5:104084571-104084615	NM_028824:151	1700016H13Rik	INSIDE	1.315	2.463	1484.82	3656.51	3.237	1183.61	3831.77
A_68_P27934861	chr11:76759571-76759615	NM_178645:435	Blmh	INSIDE	1.315	1.589	1623.20	2580.04	2.090	1495.91	3126.22
A_68_P31186620	chr17:43013150-43013194	NM_009847:201	Cd2ap	INSIDE	1.315	1.968	1075.29	2115.69	2.588	976.54	2527.28
A_68_P23309921	chr4:131604501-131604545	NM_001128606:471	Epb4.1	INSIDE	1.315	5.420	2522.16	13670.18	7.125	2069.71	14747.50
A_68_P24046891	chr5:122302921-122302965	NM_175474:3906	Fam109a	INSIDE	1.315	2.427	1047.84	2542.75	3.190	849.76	2711.06
A_68_P29571200	chr14:46149352-46149396	NM_146054:366	Fermt2	INSIDE	1.315	1.812	1239.14	2245.38	2.383	1044.63	2488.84
A_68_P27594244	chr11:11925917-11925961	NM_001177629:2036	Grb10	INSIDE	1.315	4.773	1924.73	9187.03	6.276	1482.37	9303.42
A_68_P20556973	chr1:121318457-121318501	NM_008381:347	Inhbb	INSIDE	1.315	3.089	2105.89	6505.40	4.063	1555.58	6320.74
A_68_P21717097	chr2:146047662-146047707	NM_016889:-48	Insm1	PROMOTER	1.315	1.575	1716.91	2703.90	2.071	1521.54	3150.50
A_68_P26270748	chr8:131209625-131209669	NM_010578:93	Itgb1	INSIDE	1.315	1.669	1173.41	1958.52	2.195	1081.42	2373.62
A_68_P32051829	chr19:29326236-29326280	NM_001048177:-59	Jak2	PROMOTER	1.315	1.953	1573.61	3073.02	2.567	1283.59	3295.20
A_68_P25092919	chr7:52661992-52662036	NM_010596:685	Kcna7	INSIDE	1.315	2.433	798.30	1942.21	3.200	662.46	2119.90
A_68_P30359963	chr15:78267288-78267332	NM_001081367:8253	Kctd17	INSIDE	1.315	2.639	2635.29	6955.47	3.470	1700.55	5901.06
A_68_P20453265	chr1:94996931-94996975	NM_001110315:1490	Kif1a	INSIDE	1.315	3.015	2093.88	6313.38	3.964	1718.09	6810.50

ProbeName	Target position of probe on CpG island microarray	TargetID	GeneSymbol	CpG island Description	Ratio of relative methylation (TiO_2-NP/Vehicle)	Sham group			TiO_2-H group		
						Relative methylation (Cy5/Cy3)	Cy3 signal (Input)	Cy5 signal (MeDIP)	Relative methylation (Cy5/Cy3)	Cy3 signal (Input)	Cy5 signal (MeDIP)
A_68_P30345385	chr15:75829377-75829421	NM_177922:5200	Mapk15	INSIDE	1.315	2.436	4535.10	11047.77	3.204	3380.91	10832.31
A_68_P23592026	chr5:34340043-34340087	NM_001001985:1432	Nat8l	INSIDE	1.315	2.110	377.84	797.40	2.774	325.05	901.76
A_68_P20231212	chr1:53353853-53353897	NM_153556:-34	Pms1	DIVERGENT_PROMOTER	1.315	6.054	2118.56	12825.81	7.960	1553.22	12363.36
A_68_P29094194	chr13:63669536-63669580	NM_008957:-2730	Ptch1	PROMOTER	1.315	1.537	1530.49	2351.68	2.020	1377.86	2783.49
A_68_P26540062	chr9:56920055-56920099	NM_001110350:-4106	Sin3a	PROMOTER	1.315	1.615	959.36	1549.82	2.125	816.42	1734.67
A_68_P22218905	chr3:68848719-68848763	NM_025863:-76	Trim59	PROMOTER	1.315	3.872	903.90	3499.83	5.091	841.47	4284.26
A_68_P30693866	chr16:39984614-39984658	AK082117:-71		PROMOTER	1.315	3.050	1025.21	3126.43	4.011	886.36	3554.84
A_68_P21127431	chr2:34262562-34262606	ENSMUST00000147337:-118		PROMOTER	1.315	2.202	2062.74	4541.97	2.895	1817.90	5263.15
A_68_P27901660	chr11:70429072-70429116	NM_001145537:-301	4930544D05Rik	PROMOTER	1.314	1.840	539.06	992.14	2.418	454.14	1098.33
A_68_P25598323	chr7:150238635-150238679	NM_133655:2	Cd81	INSIDE	1.314	2.764	445.68	1231.72	3.631	409.36	1486.40
A_68_P23339476	chr4:136913526-136913570	NM_009861:104	Cdc42	INSIDE	1.314	1.767	1242.91	2195.86	2.321	1185.61	2751.78
A_68_P21103095	chr2:30271038-30271082	NM_007760:208	Crat	INSIDE	1.314	3.125	1171.17	3660.15	4.106	992.30	4074.11
A_68_P25286994	chr7:91300218-91300262	NM_133722:163	Fam108c	INSIDE	1.314	2.967	933.15	2768.27	3.898	884.59	3447.80
A_68_P26021244	chr8:87225675-87225719	NM_008535:341	Lyl1	INSIDE	1.314	1.708	1373.03	2345.00	2.244	1062.52	2383.86
A_68_P24161575	chr5:145306253-145306297	NM_016789:-481	Nptx2	PROMOTER	1.314	1.650	717.78	1184.51	2.168	601.10	1303.28
A_68_P25377990	chr7:109416158-109416202	NM_009287:-157	Stim1	PROMOTER	1.314	1.883	965.18	1817.67	2.474	815.89	2018.84
A_68_P25182469	chr7:72516013-72516058	NM_001163574:95	Tjp1	INSIDE	1.314	1.683	1133.77	1908.56	2.212	1004.05	2221.14
A_68_P29733542	chr14:76814361-76814405	NM_207652:-1245	Tsc22d1	PROMOTER	1.314	3.076	405.83	1248.14	4.042	371.57	1501.92
A_68_P23610941	chr5:37379997-37380041	NM_011716:203	Wfs1	INSIDE	1.314	2.172	1264.79	2747.47	2.854	1084.02	3093.86
A_68_P31175249	chr17:39985581-39985625	ENSMUST00000062783:170		INSIDE	1.314	3.331	23670.85	78837.49	4.378	17964.65	78645.27
A_68_P31951639	chr19:10680032-10680076	NM_015735:-60	Ddb1	DIVERGENT_PROMOTER	1.313	1.880	1892.66	3557.63	2.468	1408.63	3477.00
A_68_P23202060	chr4:109349261-109349305	NM_007983:51	Faf1	INSIDE	1.313	2.204	899.95	1983.17	2.893	777.80	2249.83
A_68_P28928667	chr13:32430111-32430155	NM_146041:281	Gmds	INSIDE	1.313	4.076	1399.23	5703.60	5.352	1287.51	6890.28
A_68_P31866345	chr18:80909646-80909690	NM_001164111:-4756	Nfatc1	PROMOTER	1.313	5.078	3778.50	19186.36	6.668	3031.32	20211.49
A_68_P26253526	chr8:128258037-128258081	NM_025636:-44	Ntpcr	PROMOTER	1.313	2.307	1712.75	3950.85	3.028	1416.54	4289.92
A_68_P27921383	chr11:74403548-74403592	NM_001015046:90	Rap1gap2	INSIDE	1.313	1.487	2251.45	3348.42	1.953	1938.07	3785.75
A_68_P21099264	chr2:29603006-29603050	NM_001039086:127789	Rapgef1	DOWNSTREAM	1.313	1.473	1343.02	1978.17	1.934	1146.35	2217.20
A_68_P24428223	chr6:48547614-48547658	NM_001079901:3755	Repin1	INSIDE	1.313	3.260	927.15	3022.57	4.281	891.28	3815.31
A_68_P27554682	chr11:3813819-3813863	NM_153142:827	Slc35e4	INSIDE	1.313	2.454	3040.60	7460.32	3.222	2397.68	7725.96
A_68_P32766767	chrX:153929659-153929703	NM_009214:298	Sms	INSIDE	1.313	4.673	667.05	3117.10	6.135	514.12	3154.30
A_68_P23322941	chr4:134024349-134024393	NM_019641:136	Stmn1	INSIDE	1.313	2.772	2406.39	6671.71	3.639	1983.81	7219.85
A_68_P28974160	chr13:40823639-40823683	NM_001122948:2152	Tcfap2a	INSIDE	1.313	1.564	846.12	1323.49	2.054	725.71	1490.63
A_68_P26894032	chr9:123169639-123169683	NM_001002267:247	Tmem158	INSIDE	1.313	2.289	678.84	1554.04	3.007	672.75	2022.72
A_68_P27044453	chr10:31165715-31165759	NM_009413:-9	Tpd52l1	PROMOTER	1.313	1.513	1100.39	1664.77	1.987	926.62	1841.10
A_68_P27086292	chr10:40604319-40604365	NM_031877:1003	Wasf1	INSIDE	1.313	2.172	298.43	648.34	2.852	222.37	634.28
A_68_P30342957	chr15:75446831-75446875	NM_001044718:-262	Zfp41	PROMOTER	1.313	1.573	939.86	1478.67	2.066	768.12	1586.85
A_68_P28435276	chr12:53605530-53605574	AK041016:550		INSIDE	1.313	1.434	4253.89	6099.47	1.883	3479.01	6551.22
A_68_P30425161	chr15:89259781-89259825	NR_004843:556	BC090627	INSIDE	1.312	1.887	932.87	1760.34	2.477	882.45	2185.42
A_68_P30221122	chr15:53177633-53177677	NM_010162:84	Ext1	INSIDE	1.312	5.053	1784.62	9018.40	6.631	1423.70	9441.00
A_68_P30399218	chr15:85036672-85036716	NM_010180:257	Fbln1	INSIDE	1.312	3.036	2885.58	8760.74	3.985	2518.06	10033.89
A_68_P30630403	chr16:28445216-28445260	NM_183064:75	Fgf12	INSIDE	1.312	1.997	736.69	1471.52	2.622	666.78	1748.01
A_68_P29529256	chr14:35124536-35124580	NM_008133:646	Glud1	INSIDE	1.312	2.593	2761.15	7160.42	3.403	2344.05	7976.58
A_68_P27292918	chr10:82092906-82092950	NR_037190:342	Gm9855	INSIDE	1.312	1.873	1576.59	2953.07	2.457	1368.82	3362.63
A_68_P23332339	chr4:135704351-135704395	NM_008321:4636	Id3	DOWNSTREAM	1.312	2.086	3292.78	6869.03	2.737	2434.87	6665.09
A_68_P32451589	chrX:68303308-68303352	NM_001081354:-100	Mamld1	PROMOTER	1.312	2.609	2091.88	5457.59	3.423	1122.91	3843.46
A_68_P28291797	chr12:25516152-25516196	NM_001083341:-289	Mboat2	PROMOTER	1.312	2.255	720.01	1623.57	2.958	643.11	1902.49
A_68_P20625166	chr1:134921738-134921782	NM_008575:165	Mdm4	INSIDE	1.312	1.629	2572.84	4192.26	2.138	2107.13	4505.68
A_68_P25962048	chr8:75072006-75072050	NM_027485:181	Med26	INSIDE	1.312	1.914	984.30	1883.76	2.510	862.74	2165.75
A_68_P21902498	chr2:179992383-179992427	NM_025587:321	Rps21	INSIDE	1.312	0.355	1571.36	558.03	0.466	1223.89	570.12
A_68_P25499767	chr7:133435936-133435980	NM_145587:19826	Sbk1	INSIDE	1.312	2.745	479.90	1317.41	3.602	462.47	1665.81
A_68_P31054977	chr17:13185351-13185395	NM_001113533:-247	Wtap	PROMOTER	1.312	2.763	919.81	2540.98	3.626	814.74	2953.89

ProbeName	Target position of probe on CpG island microarray	TargetID	GeneSymbol	CpG island Description	Ratio of relative methylation (TiO_2-NP/Vehicle)	Sham group			TiO_2-H group		
						Relative methylation (Cy5/Cy3)	Cy3 signal (Input)	Cy5 signal (MeDIP)	Relative methylation (Cy5/Cy3)	Cy3 signal (Input)	Cy5 signal (MeDIP)
A_68_P27927818	chr11:75546834-75546878	NM_009536:468	Ywhae	INSIDE	1.312	1.568	2704.41	4239.29	2.057	2139.87	4401.64
A_68_P24397788	chr6:42300448-42300492	NM_011777:644	Zyx	INSIDE	1.312	1.390	6127.79	8516.22	1.823	4319.77	7876.43
A_68_P29020719	chr13:48764304-48764348	NM_007526:5922	Barx1	DOWNSTREAM	1.311	1.543	1293.74	1996.44	2.023	1056.36	2137.42
A_68_P28109100	chr11:107655405-107655449	NM_019431:352	Cacng4	INSIDE	1.311	1.578	1179.70	1861.41	2.069	816.49	1689.09
A_68_P27990839	chr11:86571533-86571577	NM_001003908:-560	Cltc	PROMOTER	1.311	2.236	964.60	2156.80	2.931	813.45	2384.61
A_68_P20472155	chr1:99558144-99558188	NM_133825:429	D1Ertd622e	INSIDE	1.311	2.736	2311.18	6324.15	3.587	1928.44	6917.33
A_68_P25780174	chr8:36558678-36558722	NM_026067:-113	Eri1	PROMOTER	1.311	4.426	2740.09	12126.52	5.800	2304.83	13367.43
A_68_P26467131	chr9:44152207-44152251	NM_001110251:-1764	Hmbs	PROMOTER	1.311	1.787	1258.18	2248.32	2.342	1130.11	2647.15
A_68_P24634433	chr6:90686814-90686858	NM_001134384:73281	Iqsec1	INSIDE	1.311	1.400	1584.56	2217.82	1.835	1407.07	2581.52
A_68_P24993122	chr7:19989220-19989264	NM_146182:-4037	Klc3	DIVERGENT_PROMOTER	1.311	4.396	754.28	3315.96	5.762	736.59	4244.40
A_68_P26920199	chr10:7401013-7401057	NM_010690:28	Lats1	INSIDE	1.311	3.150	1853.00	5836.10	4.128	1476.45	6094.39
A_68_P32252887	chrX:10294418-10294462	NM_001166635:-50	Mid1ip1	PROMOTER	1.311	3.114	1184.82	3689.99	4.084	805.83	3291.29
A_68_P28941009	chr13:34967839-34967883	NM_013830:498	Prpf4b	INSIDE	1.311	2.099	2031.31	4263.23	2.751	1584.41	4358.19
A_68_P20598625	chr1:129999853-129999897	NM_181750:-8	R3hdm1	DIVERGENT_PROMOTER	1.311	3.304	3560.20	11762.41	4.331	2728.21	11815.83
A_68_P31108132	chr17:26206355-26206399	NM_001162868:-254	Rab11fip3	PROMOTER	1.311	1.420	1282.51	1821.54	1.862	1102.44	2052.41
A_68_P23820629	chr5:77694724-77694768	NM_011263:228	Rest	INSIDE	1.311	1.414	2102.35	2973.67	1.855	1587.82	2945.14
A_68_P29454669	chr14:22569927-22569971	NM_026283:196	Samd8	INSIDE	1.311	2.418	983.65	2378.31	3.169	888.00	2813.80
A_68_P31738735	chr18:58038835-58038879	NM_009194:525	Slc12a2	INSIDE	1.311	3.333	750.33	2500.94	4.368	660.48	2885.31
A_68_P31202526	chr17:45822858-45822902	NM_198167:287	Tmem63b	INSIDE	1.311	1.841	1736.21	3195.60	2.414	1440.02	3475.98
A_68_P27836016	chr11:59102534-59102578	NM_009522:1697	Wnt3a	INSIDE	1.311	2.350	661.87	1555.58	3.081	560.35	1726.20
A_68_P22262797	chr3:79090995-79091039	AK032506:-297		PROMOTER	1.311	2.578	1072.39	2764.16	3.378	876.55	2961.01
A_68_P26813047	chr9:108008622-108008666	NM_007567:84070	Bsn	INSIDE	1.310	3.904	730.57	2852.43	5.115	618.33	3162.45
A_68_P26061569	chr8:94329516-94329560	NM_008393:-4265	Irx3	PROMOTER	1.310	1.559	3461.01	5396.80	2.043	2550.90	5212.10
A_68_P21419651	chr2:90758053-90758097	NM_025576:133	Ptpmt1	INSIDE	1.310	1.531	608.69	932.20	2.007	598.10	1200.20
A_68_P24061764	chr5:124981000-124981044	NM_021430:378	Rilpl1	INSIDE	1.310	2.979	866.48	2581.37	3.903	779.24	3041.07
A_68_P30467109	chr15:96884897-96884941	NM_027052:1469	Slc38a4	INSIDE	1.310	1.925	802.38	1544.79	2.521	700.47	1766.07
A_68_P24389053	chr6:40421556-40421600	NM_028358:165	Ssbp1	INSIDE	1.310	1.995	804.58	1605.29	2.614	633.49	1656.13
A_68_P28740916	chr12:112127614-112127658	NM_001081057:163	Tecpr2	INSIDE	1.310	1.754	2463.40	4320.80	2.297	1891.09	4343.88
A_68_P23575086	chr5:31171940-31171984	NM_144525:-57	Tmem214	PROMOTER	1.310	2.907	2082.81	6054.52	3.809	1773.46	6754.90
A_68_P30351140	chr15:76741911-76741955	NM_001168276:13946	Zfp647	INSIDE	1.310	2.952	809.49	2389.81	3.867	706.45	2731.85
A_68_P24060291	A_68_P24060291			Unknown	1.310	1.809	1709.86	3092.39	2.369	1447.76	3429.46
A_68_P30778641	chr16:55974831-55974875	NR_027965:-122	2310061J03Rik	PROMOTER	1.309	4.204	3177.75	13359.67	5.502	2637.44	14510.06
A_68_P28534551	chr12:74018450-74018494	NM_029444:280	4930447C04Rik	INSIDE	1.309	6.415	2114.90	13567.05	8.395	1681.74	14118.40
A_68_P28603261	chr12:86621337-86621381	NM_025421:27	Acyp1	INSIDE	1.309	1.898	979.89	1859.50	2.485	866.62	2153.45
A_68_P31155039	chr17:34784544-34784588	NM_017406:442	Atf6b	INSIDE	1.309	2.011	1755.10	3529.20	2.632	1452.66	3823.17
A_68_P29050817	chr13:54789017-54789062	NM_001146025:130	Rnf44	INSIDE	1.309	1.521	1711.26	2602.18	1.990	1451.17	2887.46
A_68_P29141935	chr13:73901004-73901048	NM_011390:-118	Slc12a7	PROMOTER	1.309	3.097	3078.56	9533.25	4.055	2465.41	9996.41
A_68_P21836564	chr2:167457070-167457114	NM_023230:413	Ube2v1	INSIDE	1.309	1.998	893.80	1785.54	2.615	710.32	1857.61
A_68_P30474027	chr15:98126645-98126689	NM_173769:-152	Zfp641	PROMOTER	1.309	1.647	1806.18	2975.30	2.157	1612.47	3477.93
A_68_P24603368	chr6:85105219-85105263	ENSMUST00000089584:242		INSIDE	1.309	2.108	542.87	1144.24	2.758	543.40	1498.92
A_68_P21537143	chr2:113688822-113688866	NM_181416:-26	Arhgap11a	PROMOTER	1.308	2.023	1745.08	3529.84	2.646	1416.55	3748.74
A_68_P25506862	chr7:134852430-134852474	NM_009746:-172	Bcl7c	DIVERGENT_PROMOTER	1.308	1.340	4589.62	6148.95	1.752	3607.14	6320.37
A_68_P26350530	chr9:21921461-21921505	NM_170777:-2869	Elof1	PROMOTER	1.308	1.675	1144.83	1917.14	2.191	1033.94	2265.10
A_68_P29241520	chr13:96373925-96373969	NM_010169:14442	F2r	INSIDE	1.308	2.828	360.86	1020.34	3.699	264.49	978.21
A_68_P28156895	chr11:115885168-115885212	NM_008211:628	H3f3b	INSIDE	1.308	2.496	1356.30	3385.90	3.265	1021.21	3334.10
A_68_P31051121	chr17:12511550-12511594	NM_011948:-46	Map3k4	PROMOTER	1.308	1.861	1220.76	2271.33	2.434	1135.96	2765.40
A_68_P27279588	chr10:79361859-79361903	NM_001163276:9803	Med16	INSIDE	1.308	3.352	3661.68	12273.38	4.384	2475.52	10853.27
A_68_P24383012	chr6:39369835-39369879	NM_018810:512	Mkrn1	INSIDE	1.308	2.097	1270.65	2664.88	2.744	1120.41	3074.60
A_68_P22006132	chr3:26230317-26230361	NM_138666:493	Nlgn1	INSIDE	1.308	1.457	1085.32	1581.60	1.906	913.41	1741.38
A_68_P29239503	chr13:95992534-95992578	NM_001170669:449	Pde8b	INSIDE	1.308	4.875	1256.92	6128.05	6.375	1102.46	7028.15
A_68_P21747515	chr2:151907678-151907722	NM_001160410:436	Scrt2	INSIDE	1.308	1.793	351.88	630.97	2.345	387.12	907.73

ProbeName	Target position of probe on CpG island microarray	TargetID	GeneSymbol	CpG island Description	Ratio of relative methylation (TiO_2-NP/Vehicle)	Sham group			TiO_2-H group		
						Relative methylation (Cy5/Cy3)	Cy3 signal (Input)	Cy5 signal (MeDIP)	Relative methylation (Cy5/Cy3)	Cy3 signal (Input)	Cy5 signal (MeDIP)
A_68_P24973913	chr7:13556719-13556763	NM_178732:5528	Zfp324	INSIDE	1.308	1.928	475.96	917.86	2.522	400.57	1010.21
A_68_P24432031	chr6:49269387-49269431	NM_175098:57	Ccdc126	INSIDE	1.307	2.396	1144.52	2742.61	3.132	914.17	2863.03
A_68_P25948479	chr8:72406005-72406049	NM_026818:5265	Cilp2	INSIDE	1.307	2.258	521.32	1177.35	2.952	437.85	1292.49
A_68_P29747539	chr14:79125062-79125106	NM_001081336:-188	Dgkh	PROMOTER	1.307	3.032	1370.59	4155.45	3.964	1149.84	4557.84
A_68_P20566311	chr1:123224390-123224434	NM_133748:-73	Insig2	PROMOTER	1.307	1.804	475.82	858.56	2.358	419.44	989.19
A_68_P21421182	chr2:91022680-91022724	NM_001177720:-3224	Madd	PROMOTER	1.307	3.844	2550.05	9802.18	5.023	2460.76	12359.94
A_68_P27897811	chr11:69715610-69715654	NM_001013414:253	Neurl4	INSIDE	1.307	1.571	1025.78	1611.52	2.053	868.63	1783.29
A_68_P23194284	chr4:107704369-107704413	NM_172874:305	Podn	INSIDE	1.307	3.070	1491.45	4578.12	4.012	1262.85	5066.12
A_68_P20451967	chr1:94807773-94807817	NM_181405:393	Rnpepl1	INSIDE	1.307	1.690	1221.53	2064.62	2.210	1082.61	2392.29
A_68_P21635215	chr2:131318208-131318252	NM_001177833:633	Smox	INSIDE	1.307	1.829	1287.57	2354.76	2.390	1042.16	2490.54
A_68_P25017144	chr7:28189868-28189912	NM_001199236:-8318	Spnb4	PROMOTER	1.307	4.368	2637.82	11522.10	5.709	2009.34	11472.27
A_68_P21866929	chr2:172805440-172805484	NM_001083959:120	Spo11	INSIDE	1.307	2.174	268.48	583.75	2.841	239.13	679.40
A_68_P28161641	chr11:116713805-116713849	NM_011358:582	Srsf2	INSIDE	1.307	2.187	1722.99	3767.62	2.858	1494.57	4271.70
A_68_P31331149	chr17:71202044-71202088	NM_001164075:-992	Tgif1	PROMOTER	1.307	2.750	182.62	502.16	3.593	182.47	655.54
A_68_P24376582	chr6:38249064-38249108	NM_172467:173	Zc3hav1l	INSIDE	1.307	1.599	494.54	790.58	2.089	450.11	940.24
A_68_P29938688	chr14:118836379-118836423			Unknown	1.307	2.306	1426.31	3288.99	3.014	1434.90	4324.77
A_68_P29258006	chr13:99274352-99274396	AK135687:84239		INSIDE	1.307	3.076	834.43	2566.84	4.022	690.55	2777.20
A_68_P25230182	chr7:80763065-80763109	AK158912:183		INSIDE	1.307	1.774	1206.56	2140.97	2.319	1047.81	2429.74
A_68_P27279428	chr10:79337495-79337539	NM_001170935:-137	BC005764	DIVERGENT_PROMOTER	1.306	2.372	2183.07	5178.66	3.098	1624.04	5031.35
A_68_P23250424	chr4:119955496-119955540	NM_194060:4348	Foxo6	INSIDE	1.306	2.779	1829.84	5085.10	3.630	1633.22	5927.82
A_68_P28152362	chr11:115119477-115119521	NM_010350:9059	Grin2c	INSIDE	1.306	3.700	801.28	2964.80	4.834	682.32	3298.37
A_68_P24133865	chr5:139626414-139626458	NM_001081265:260	Heatr2	INSIDE	1.306	2.927	958.50	2805.89	3.824	758.37	2899.90
A_68_P25375722	chr7:108985190-108985234	NM_001122739:1126	Inppl1	INSIDE	1.306	2.147	1896.27	4071.65	2.804	1595.66	4474.06
A_68_P30482202	chr15:99602992-99603036	NM_028015:-68	Lass5	PROMOTER	1.306	1.807	6735.07	12172.06	2.360	5406.62	12758.44
A_68_P26548745	chr9:58430450-58430494	NM_009145:426	Nptn	INSIDE	1.306	1.555	1871.33	2909.89	2.030	1597.79	3244.12
A_68_P31934661	chr19:6428357-6428401	NM_001205234:9641	Nrxn2	INSIDE	1.306	3.818	704.80	2691.21	4.988	557.67	2781.49
A_68_P31943407	chr19:8831518-8831562	NM_016813:-52	Nxf1	PROMOTER	1.306	1.792	398.12	713.49	2.340	313.91	734.54
A_68_P27901903	chr11:70468945-70468989	NM_011072:-814	Pfn1	DIVERGENT_PROMOTER	1.306	2.751	525.83	1446.51	3.591	533.19	1914.91
A_68_P21888792	chr2:177877156-177877200	NM_028806:541	Phactr3	INSIDE	1.306	2.919	920.93	2687.97	3.813	776.29	2960.04
A_68_P21796699	chr2:160557598-160557642	NM_021280:575	Plcg1	INSIDE	1.306	2.503	539.07	1349.13	3.269	414.39	1354.74
A_68_P27080343	chr10:39452167-39452211	NM_011264:223	Rev3l	INSIDE	1.306	2.939	1588.48	4668.40	3.838	1333.46	5118.17
A_68_P20759140	chr1:161161812-161161856	NM_011931:-622	Rfwd2	PROMOTER	1.306	2.737	1207.32	3304.29	3.574	923.39	3300.49
A_68_P31412117	chr17:86087251-86087295	NM_011380:322	Six2	INSIDE	1.306	2.248	430.59	968.15	2.936	459.18	1347.94
A_68_P20940733	chr1:193730478-193730522	NM_009579:-159	Slc30a1	PROMOTER	1.306	1.484	1923.39	2855.27	1.939	1625.46	3152.48
A_68_P31351019	chr17:74738085-74738129	NM_001162870:-220	Spast	PROMOTER	1.306	1.403	2396.18	3361.47	1.832	2038.95	3735.31
A_68_P27513153	chr10:122633430-122633474	NM_027604:527	Usp15	INSIDE	1.306	1.918	1082.46	2076.40	2.505	1018.49	2551.28
A_68_P28931384	chr13:32894661-32894705	NM_030215:784	Wrnip1	INSIDE	1.306	4.902	1428.29	7001.87	6.403	1235.14	7908.42
A_68_P23998433	chr5:113920169-113920213	NM_177292:724	Wscd2	INSIDE	1.306	1.823	1093.18	1992.80	2.380	982.35	2338.42
A_68_P25947916	chr8:72298647-72298691	NM_177899:142	Zfp866	INSIDE	1.306	2.110	1943.12	4099.60	2.755	1700.55	4684.31
A_68_P32537534	chrX:91368825-91368869	NM_001044386:-100	Zfx	PROMOTER	1.306	2.490	1262.96	3144.78	3.253	799.63	2601.35
A_68_P21773774	chr2:156688896-156688940	NM_026124:61	1110008F13Rik	INSIDE	1.305	2.679	1489.12	3989.93	3.496	1365.87	4774.74
A_68_P30382912	chr15:82176300-82176344	NM_026914:-153	1500032L24Rik	PROMOTER	1.305	1.725	423.62	730.66	2.250	406.80	915.36
A_68_P30348811	chr15:76341995-76342039	NM_010046:232	Dgat1	INSIDE	1.305	1.592	3778.90	6017.42	2.078	2896.67	6019.19
A_68_P25328180	chr7:100229175-100229219	NM_021427:808	Fam181b	INSIDE	1.305	1.902	2855.80	5432.99	2.482	2247.40	5578.24
A_68_P30786987	chr16:57549528-57549572	NM_001177871:196	Filip1l	INSIDE	1.305	3.618	528.72	1912.77	4.721	447.30	2111.83
A_68_P24126098	chr5:137952294-137952338	NM_031408:-7791	Gigyf1	DIVERGENT_PROMOTER	1.305	2.386	3572.19	8524.98	3.115	2524.09	7861.33
A_68_P28043432	chr11:96144310-96144354	NM_010461:1114	Hoxb8	INSIDE	1.305	3.033	699.31	2121.00	3.959	652.06	2581.30
A_68_P31208325	chr17:46817475-46817519	NM_001163729:383	Klhdc3	INSIDE	1.305	2.765	2909.67	8043.92	3.607	1830.68	6602.56
A_68_P20633481	chr1:136352243-136352287	NM_153128:133	Klhl12	INSIDE	1.305	2.162	1130.03	2443.36	2.823	1098.53	3100.69
A_68_P25048198	chr7:35988774-35988818	NM_001024707:11568	Lrp3	INSIDE	1.305	1.997	577.52	1153.07	2.605	465.82	1213.33
A_68_P21600379	chr2:124949302-124949346	NM_001162417:72	Myef2	INSIDE	1.305	4.355	681.16	2966.77	5.684	653.17	3712.39

ProbeName	Target position of probe on CpG island microarray	TargetID	GeneSymbol	CpG island Description	Ratio of relative methylation (TiO_2-NP/Vehicle)	Sham group			TiO_2-H group		
						Relative methylation (Cy5/Cy3)	Cy3 signal (Input)	Cy5 signal (MeDIP)	Relative methylation (Cy5/Cy3)	Cy3 signal (Input)	Cy5 signal (MeDIP)
A_68_P30939274	chr16:87354456-87354500	NM_001159331:49	N6amt1	INSIDE	1.305	2.316	635.56	1472.17	3.022	561.50	1696.68
A_68_P30506991	chr16:3884449-3884493	NM_029090:-148	Nat15	PROMOTER	1.305	1.533	1071.15	1641.57	2.000	850.70	1701.57
A_68_P23134316	chr4:97438997-97439041	NM_001122953:-5298	Nfia	PROMOTER	1.305	2.483	2469.27	6131.33	3.240	2088.40	6767.31
A_68_P27165512	chr10:58276626-58276670	NM_172788:542	Sh3rf3	INSIDE	1.305	2.964	964.44	2858.22	3.866	869.92	3363.52
A_68_P23594735	chr5:34856681-34856725	NM_139064:-74	Tnip2	PROMOTER	1.305	1.552	2390.18	3709.44	2.025	2175.02	4403.86
A_68_P29880580	chr14:106505493-106505537	NM_198601:100	Trim52	INSIDE	1.305	2.865	342.53	981.33	3.740	321.89	1203.76
A_68_P20350376	chr1:74818349-74818393	NM_009526:-95	Wnt6	PROMOTER	1.305	1.925	3236.32	6229.09	2.513	2766.33	6950.48
A_68_P29473057	chr14:25598729-25598773			Unknown	1.305	1.632	1487.98	2427.70	2.129	1240.41	2640.40
A_68_P27633575	chr11:20730716-20730761	NM_173752:373	1110067D22Rik	INSIDE	1.304	1.896	842.63	1597.74	2.472	656.57	1623.15
A_68_P23368575	chr4:141794726-141794770	NM_001109685:568	9030409G11Rik	INSIDE	1.304	1.618	3562.45	5765.55	2.111	2853.64	6023.44
A_68_P30811311	chr16:62846647-62846691	NM_026577:198	Arl13b	INSIDE	1.304	1.768	651.89	1152.46	2.305	619.91	1428.60
A_68_P24191337	chr5:151325172-151325216	NM_009765:-3	Brca2	DIVERGENT_PROMOTER	1.304	1.557	865.50	1347.75	2.030	715.03	1451.63
A_68_P30759543	chr16:52032167-52032211	NM_001033238:527	Cblb	INSIDE	1.304	1.647	1773.06	2920.06	2.148	1542.73	3313.43
A_68_P25961304	chr8:74945231-74945275	NM_007944:121	Eps15l1	INSIDE	1.304	2.169	826.17	1792.01	2.829	721.15	2039.93
A_68_P26343222	chr9:20446824-20446868	NM_013911:404	Fbxl12	INSIDE	1.304	1.988	262.22	521.24	2.593	235.93	611.76
A_68_P30631089	chr16:28564952-28564996	NM_010199:188314	Fgf12	INSIDE	1.304	1.619	711.88	1152.60	2.110	726.46	1533.19
A_68_P32704838	chrX:138156435-138156479	NM_010572:3304	Irs4	INSIDE	1.304	1.726	763.07	1317.36	2.252	405.85	913.87
A_68_P20047723	chr1:14745767-14745811	NM_010827:259	Msc	INSIDE	1.304	2.272	1295.95	2944.39	2.963	1215.99	3603.04
A_68_P30082174	chr15:25552690-25552734	NM_019472:408	Myo10	INSIDE	1.304	1.503	1828.18	2747.95	1.960	1526.06	2990.33
A_68_P27302615	chr10:83903859-83903903	NM_001004363:-664	Nuak1	PROMOTER	1.304	2.230	252.78	563.73	2.908	225.44	655.66
A_68_P23916646	chr5:98615304-98615348	NM_029947:5439	Prdm8	INSIDE	1.304	2.608	773.03	2015.71	3.401	663.87	2257.79
A_68_P25585274	chr7:148043495-148043539	NM_053194:221	Ric8	INSIDE	1.304	2.247	7898.19	17748.54	2.931	5357.36	15701.91
A_68_P31253473	chr17:56175813-56175857	NM_013664:165	Sh3gl1	INSIDE	1.304	1.522	1690.98	2574.40	1.985	1484.33	2946.12
A_68_P25412179	chr7:117365148-117365192	NM_009302:-46	Swap70	PROMOTER	1.304	2.077	1858.74	3861.00	2.708	1517.27	4108.26
A_68_P23240689	chr4:118159149-118159193	NM_011587:3284	Tie1	INSIDE	1.304	3.932	1714.91	6742.46	5.127	1394.65	7150.53
A_68_P27359835	chr10:93978114-93978158	NM_172051:535	Tmcc3	INSIDE	1.304	2.635	483.16	1273.23	3.437	593.75	2040.93
A_68_P30401287	chr15:85367949-85367993	NM_001163634:40530	Wnt7b	INSIDE	1.304	1.609	874.13	1406.16	2.098	795.35	1668.37
A_68_P24428351	chr6:48570207-48570251	NM_173429:7050	Zfp775	INSIDE	1.304	2.279	776.68	1769.92	2.971	682.04	2026.12
A_68_P23279474	chr4:125998794-125998839	NM_133883:131	Adprhl2	INSIDE	1.303	1.657	1825.59	3025.00	2.159	1555.04	3357.11
A_68_P24816768	chr6:124695641-124695685	NM_007881:10843	Atn1	INSIDE	1.303	1.954	1543.86	3016.46	2.547	1275.67	3248.71
A_68_P24104853	chr5:133019620-133019664	NM_177047:-1429	Auts2	PROMOTER	1.303	3.198	1277.02	4084.02	4.169	1095.81	4568.02
A_68_P33007787	chr4_random:127945-127989	NM_001033326:18165	Dhrsx	INSIDE	1.303	3.780	8534.81	32260.34	4.923	6191.48	30483.39
A_68_P22787931	chr4:24778185-24778229	NM_001033531:27	Klhl32	INSIDE	1.303	3.093	5015.57	15512.56	4.030	4323.68	17426.10
A_68_P25092990	chr7:52671388-52671432	NM_198190:2346	Ntf5	INSIDE	1.303	1.860	878.37	1634.07	2.424	695.84	1686.60
A_68_P27556014	chr11:4060531-4060575	NM_026175:196	Sf3a1	INSIDE	1.303	1.357	2523.46	3424.69	1.769	2025.03	3582.09
A_68_P20633021	chr1:136258633-136258677	NM_001042485:-78	Tmem183a	PROMOTER	1.303	4.529	599.66	2715.74	5.903	491.50	2901.34
A_68_P25581866	chr7:147222199-147222243	NM_133755:29	Tubgcp2	INSIDE	1.303	1.499	5556.80	8329.12	1.953	4306.14	8411.40
A_68_P21072309	chr2:25316230-25316274	NM_153557:362	BC029214	INSIDE	1.302	1.639	2301.75	3773.20	2.134	1844.86	3937.21
A_68_P26234726	chr8:125154578-125154622	NM_001109873:47475	Cbfa2t3	INSIDE	1.302	3.834	1665.13	6384.10	4.993	1459.81	7289.05
A_68_P27921467	chr11:74424565-74424610	NM_001013784:-8519	E130309D14Rik	PROMOTER	1.302	2.774	1201.41	3332.57	3.611	1111.35	4012.87
A_68_P31322349	chr17:69506595-69506639	NM_013813:467	Epb4.1l3	INSIDE	1.302	2.372	893.64	2119.76	3.089	784.38	2422.93
A_68_P32397421	chrX:53985333-53985377	NM_001077361:221	Fhl1	INSIDE	1.302	1.826	675.77	1233.67	2.378	449.65	1069.16
A_68_P21203199	chr2:49474675-49474719	NM_008449:-137	Kif5c	PROMOTER	1.302	3.019	942.50	2845.14	3.931	728.91	2865.30
A_68_P28515343	chr12:70683664-70683708	NM_001033236:329	Mettl21d	INSIDE	1.302	1.463	1751.96	2563.34	1.905	1419.61	2703.68
A_68_P23577231	chr5:31543485-31543529	NM_147201:216	Nrbp1	INSIDE	1.302	2.442	1253.65	3061.53	3.181	1041.51	3312.58
A_68_P28083321	chr11:102962810-102962854	NM_152813:140	Plcd3	INSIDE	1.302	1.563	959.01	1499.17	2.036	910.88	1854.35
A_68_P22313617	chr3:88213719-88213763	NM_025928:498	Pmf1	INSIDE	1.302	1.683	1017.38	1712.57	2.192	911.68	1998.58
A_68_P25576657	chr7:146434291-146434335	NM_001033206:-68	Pwwp2b	PROMOTER	1.302	3.305	245.94	812.92	4.305	216.44	931.77
A_68_P23896677	chr5:93327277-93327321	NM_001077596:275	Shroom3	INSIDE	1.302	0.417	1538.47	641.67	0.543	1389.54	754.44
A_68_P28050112	chr11:97223519-97223563	NM_138657:-324	Socs7	PROMOTER	1.302	2.092	955.61	1999.16	2.723	865.71	2357.67
A_68_P30572951	chr16:17278027-17278071	NM_177473:1656	Tmem191c	INSIDE	1.302	3.083	959.55	2957.86	4.012	791.47	3175.76

ProbeName	Target position of probe on CpG island microarray	TargetID	GeneSymbol	CpG island Description	Ratio of relative methylation (TiO_2-NP/Vehicle)	Sham group			TiO_2-H group		
						Relative methylation (Cy5/Cy3)	Cy3 signal (Input)	Cy5 signal (MeDIP)	Relative methylation (Cy5/Cy3)	Cy3 signal (Input)	Cy5 signal (MeDIP)
A_68_P31102168	chr17:25325357-25325401	NM_001197024:34	Unkl	INSIDE	1.302	1.874	17884.60	33513.20	2.439	14385.30	35092.38
A_68_P23998981	chr5:114037991-114038035	NM_177292:118546	Wscd2	INSIDE	1.302	1.884	1419.41	2673.71	2.452	1108.04	2716.51
A_68_P28872465	chr13:21454901-21454949	NM_016684:236	Zscan12	INSIDE	1.302	4.229	190.27	804.69	5.505	229.88	1265.40
A_68_P24026284	chr5:118830994-118831046			Unknown	1.302	7.064	3039.22	21468.34	9.200	2114.52	19453.68
A_68_P23756663	chr5:65884143-65884187	NM_133697:-90	1110003E01Rik	PROMOTER	1.301	1.653	1056.90	1747.18	2.151	923.78	1987.37
A_68_P25502308	chr7:133942826-133942870	NM_007438:-80	Aldoa	PROMOTER	1.301	1.675	1348.95	2259.19	2.178	1154.28	2514.55
A_68_P31125954	chr17:29235470-29235514	NM_007669:4776	Cdkn1a	INSIDE	1.301	2.115	423.86	896.58	2.751	346.27	952.67
A_68_P26779715	chr9:102255528-102255572	NM_001168296:1473	Ephb1	INSIDE	1.301	1.468	1362.93	2001.41	1.911	1140.77	2180.07
A_68_P24053334	chr5:123438191-123438235	NM_001005866:330	Kdm2b	INSIDE	1.301	1.681	534.29	898.18	2.188	420.14	919.08
A_68_P21206004	chr2:49921862-49921906	NM_177139:-97	Lypd6	PROMOTER	1.301	1.944	2140.56	4161.88	2.529	1892.45	4785.49
A_68_P27971522	chr11:83256374-83256418	NM_080453:20067	Mmp28	INSIDE	1.301	1.722	1594.45	2746.09	2.241	1420.01	3181.84
A_68_P21107808	chr2:31102041-31102085	NM_019681:620	Ncs1	INSIDE	1.301	2.203	4033.24	8886.65	2.866	2788.72	7991.54
A_68_P29322927	chr13:111184916-111184960	NM_152804:-313	Plk2	PROMOTER	1.301	2.174	1925.22	4185.78	2.828	1678.01	4746.04
A_68_P24116186	chr5:135870022-135870066	NM_148932:372	Pom121	INSIDE	1.301	2.460	1585.33	3899.91	3.201	1422.46	4553.49
A_68_P31633220	chr18:38456665-38456709	NM_001164621:228	Rnf14	INSIDE	1.301	2.208	824.12	1819.88	2.872	707.00	2030.75
A_68_P31682248	chr18:47528673-47528717	NM_018744:-172	Sema6a	PROMOTER	1.301	2.837	1347.16	3822.26	3.692	1134.67	4188.72
A_68_P31207580	chr17:46692523-46692567	NM_020493:567	Srf	INSIDE	1.301	3.279	1018.20	3338.54	4.266	800.24	3414.08
A_68_P28611534	chr12:88019825-88019869	NM_177354:197	Vash1	INSIDE	1.301	2.747	924.25	2539.26	3.575	776.70	2776.44
A_68_P21013703	chr2:12933075-12933119	NM_153155:-605	C1ql3	PROMOTER	1.300	3.616	572.94	2071.92	4.702	490.01	2303.87
A_68_P31156013	chr17:34974519-34974563	NM_001163770:577	Dom3z	INSIDE	1.300	1.778	2885.14	5128.43	2.310	2354.92	5440.15
A_68_P25051003	chr7:36539278-36539322	NM_178704:173	Dpy19l3	INSIDE	1.300	1.902	952.63	1811.46	2.472	758.51	1874.71
A_68_P29241105	chr13:96294931-96294975	NM_007974:243	F2rl1	INSIDE	1.300	1.839	4385.47	8064.38	2.391	3503.02	8375.69
A_68_P31769413	chr18:63545818-63545864	NM_001039485:997	Fam38b	INSIDE	1.300	2.155	1116.41	2406.13	2.802	928.60	2602.19
A_68_P32467043	chrX:71492064-71492108	NM_010227:-213	Flna	DIVERGENT_PROMOTER	1.300	1.615	1616.91	2611.92	2.100	692.07	1453.66
A_68_P20235844	chr1:54251715-54251759	NM_001001883:142	Hecw2	INSIDE	1.300	2.138	1070.30	2288.34	2.778	884.86	2458.49
A_68_P25995580	chr8:82162925-82162969	NM_001081164:-628	Otud4	PROMOTER	1.300	1.462	1863.58	2724.21	1.900	1652.60	3140.65
A_68_P25655776	chr8:11478793-11478843	NM_011227:-319	Rab20	PROMOTER	1.300	6.779	5575.04	37790.58	8.812	3586.03	31600.33
A_68_P25669970	chr8:13676888-13676932	NM_009025:677	Rasa3	INSIDE	1.300	3.010	1860.86	5601.74	3.912	1384.53	5416.69
A_68_P31924878	chr19:4756662-4756706	NM_025717:160	Rbm4b	INSIDE	1.300	3.042	3025.38	9203.08	3.954	2336.29	9236.68
A_68_P21117161	chr2:32597158-32597202	NM_013781:20589	Sh2d3c	INSIDE	1.300	2.954	1767.17	5219.89	3.840	1512.15	5806.58
A_68_P24940169	chr6:148393095-148393139	NM_198967:-242	Tmtc1	PROMOTER	1.300	1.421	1414.33	2010.22	1.847	1231.52	2275.00
A_68_P20886544	chr1:184339098-184339142	NM_173378:-177	Trp53bp2	PROMOTER	1.300	1.665	1389.32	2312.89	2.165	1177.55	2549.02
A_68_P28058995	chr11:98724946-98724990	NM_197940:57	Wipf2	INSIDE	1.300	4.531	809.41	3667.70	5.889	712.49	4195.57
A_68_P25008229	chr7:25861867-25861911	NM_001168506:-335	Zfp574	DIVERGENT_PROMOTER	1.300	1.489	1186.48	1766.74	1.936	937.80	1815.63
A_68_P28741431	chr12:112217094-112217138	NM_175207:115	Ankrd9	INSIDE	1.299	1.699	858.63	1458.56	2.207	827.68	1826.75
A_68_P24925471	chr6:145811847-145811891	NM_024469:1992	Bhlhe41	INSIDE	1.299	1.519	593.93	901.98	1.973	574.00	1132.46
A_68_P29698999	chr14:70474927-70474971	NM_018781:-2303	Egr3	PROMOTER	1.299	1.888	650.22	1227.78	2.453	546.97	1341.85
A_68_P22128838	chr3:51081294-51081338	NM_023502:48593	Elf2	INSIDE	1.299	3.797	7622.44	28944.50	4.934	5912.64	29171.62
A_68_P23145560	chr4:99324186-99324230	NM_010425:1219	Foxd3	INSIDE	1.299	3.025	1622.99	4910.14	3.931	1407.31	5532.08
A_68_P26245549	chr8:126958481-126958525	NM_171824:-666	Pgbd5	PROMOTER	1.299	5.258	1079.47	5675.36	6.828	826.03	5640.24
A_68_P21608665	chr2:126378771-126378815	NM_011978:33	Slc27a2	INSIDE	1.299	1.465	2040.52	2989.91	1.903	1645.65	3132.43
A_68_P22324936	chr3:90193265-90193309	NM_011988:563	Slc27a3	INSIDE	1.299	1.784	1399.12	2496.61	2.319	1273.93	2953.66
A_68_P21754953	chr2:153269988-153270032	NM_001001986:205	8430427H17Rik	INSIDE	1.298	6.644	1832.36	12174.31	8.625	1581.83	13643.01
A_68_P29940447	chr14:119104917-119104961	NM_001033336:503	Abcc4	INSIDE	1.298	0.429	1647.64	707.21	0.557	1343.33	748.52
A_68_P31341454	chr17:72953064-72953108	NM_007439:561	Alk	INSIDE	1.298	1.589	1209.55	1921.98	2.063	1060.01	2186.56
A_68_P31766433	chr18:63081950-63081994	NM_133237:-8	Apcdd1	PROMOTER	1.298	2.567	292.40	750.54	3.331	230.41	767.45
A_68_P22186543	chr3:62143850-62143894	NM_001081295:1174	Arhgef26	INSIDE	1.298	1.668	2148.51	3583.69	2.165	1814.32	3928.50
A_68_P24631553	chr6:90259469-90259513	NM_027928:15689	Chst13	INSIDE	1.298	4.268	498.04	2125.50	5.538	438.54	2428.63
A_68_P28175185	chr11:118860915-118860959	NM_001030291:11435	Enpp7	DOWNSTREAM	1.298	2.472	1553.69	3840.01	3.209	1368.30	4390.89
A_68_P20963944	chr2:3630654-3630698	NM_025626:-53	Fam107b	PROMOTER	1.298	1.903	5388.67	10253.93	2.471	4285.15	10587.07
A_68_P21113218	chr2:31940197-31940241	NM_175511:-993	Fam78a	PROMOTER	1.298	2.621	912.82	2392.38	3.401	769.12	2615.54

ProbeName	Target position of probe on CpG island microarray	TargetID	GeneSymbol	CpG island Description	Ratio of relative methylation (TiO_2-NP/Vehicle)	Sham group			TiO_2-H group		
						Relative methylation (Cy5/Cy3)	Cy3 signal (Input)	Cy5 signal (MeDIP)	Relative methylation (Cy5/Cy3)	Cy3 signal (Input)	Cy5 signal (MeDIP)
A_68_P20405971	chr1:84836410-84836454	NM_025386:17	Fbxo36	INSIDE	1.298	2.008	1333.92	2679.17	2.608	1115.54	2908.78
A_68_P20187368	chr1:43220801-43220845	NM_010212:-16	Fhl2	PROMOTER	1.298	1.716	826.80	1418.44	2.226	680.97	1516.00
A_68_P26891188	chr9:122656242-122656286	NM_001199736:-199	Gm9524	PROMOTER	1.298	3.596	503.58	1810.73	4.666	499.35	2330.23
A_68_P20640026	chr1:137429314-137429358	NM_173437:52596	Nav1	INSIDE	1.298	4.238	2411.73	10220.24	5.501	1980.38	10893.07
A_68_P21527967	chr2:112106072-112106116	NM_133649:-376	Slc12a6	PROMOTER	1.298	1.451	927.01	1345.07	1.884	763.41	1437.92
A_68_P26983380	chr10:19571274-19571318	NM_029529:-31	Slc35d3	PROMOTER	1.298	1.655	1673.61	2769.15	2.148	1423.42	3057.61
A_68_P32582485	chrX:103222578-103222622	NM_001109757:-14	Atp7a	PROMOTER	1.297	2.689	2236.07	6013.78	3.487	1336.28	4659.65
A_68_P24951826	chr7:5008661-5008708	NM_146178:357	Ccdc106	INSIDE	1.297	2.334	1094.43	2554.25	3.028	940.64	2848.39
A_68_P30379091	chr15:81523948-81523992	NM_001164320:3747	Chadl	INSIDE	1.297	6.386	1931.34	12334.19	8.283	1544.16	12790.77
A_68_P22715806	chr4:8617250-8617294	NM_001081417:-795	Chd7	PROMOTER	1.297	2.698	663.02	1788.55	3.499	637.84	2232.01
A_68_P24618503	chr6:87800411-87800455	NM_001109745:668	Cnbp	INSIDE	1.297	2.147	2228.36	4784.93	2.785	1852.11	5157.96
A_68_P28920310	chr13:31065956-31066000	NM_025588:-62	Exoc2	PROMOTER	1.297	1.998	630.43	1259.64	2.592	542.21	1405.48
A_68_P31215002	chr17:48059670-48059714	NM_001110824:1889	Foxp4	INSIDE	1.297	2.173	1470.78	3196.60	2.819	1103.43	3111.00
A_68_P25892513	chr8:60130467-60130511	NM_001167981:1364	Galnt7	INSIDE	1.297	1.735	1025.27	1778.59	2.251	853.93	1921.79
A_68_P24518617	chr6:66846588-66846632	NM_001177558:-274	Gng12	PROMOTER	1.297	2.227	2008.43	4472.91	2.888	1740.11	5025.49
A_68_P28043442	chr11:96145448-96145492	NM_010461:2252	Hoxb8	INSIDE	1.297	2.629	2133.62	5608.86	3.410	1790.42	6104.62
A_68_P23415961	chr4:151383718-151383762	NM_001033352:741	Klhl21	INSIDE	1.297	2.062	866.08	1785.56	2.674	782.25	2091.51
A_68_P20883821	chr1:183772425-183772469	NM_133815:86	Lbr	INSIDE	1.297	1.416	1823.70	2581.48	1.837	1533.40	2816.29
A_68_P24591516	chr6:83065809-83065853	NM_020619:331	Mogs	INSIDE	1.297	1.518	1201.75	1824.53	1.969	1089.85	2146.46
A_68_P27890687	chr11:68505493-68505537	NM_175260:98	Myh10	INSIDE	1.297	1.651	7120.46	11758.59	2.142	5282.58	11312.67
A_68_P32240448	chrX:7419508-7419552	NM_138604:574	Otud5	INSIDE	1.297	1.990	821.81	1635.53	2.582	546.81	1411.90
A_68_P23610095	chr5:37260897-37260941	NM_172994:1110	Ppp2r2c	INSIDE	1.297	3.081	947.02	2918.08	3.996	824.42	3294.65
A_68_P23544149	chr5:24347845-24347889	NM_053075:313	Rheb	INSIDE	1.297	2.678	1112.46	2979.11	3.473	1003.77	3485.93
A_68_P28607918	chr12:87420289-87420333	NM_009368:-319	Tgfb3	DIVERGENT_PROMOTER	1.297	1.625	1813.93	2947.36	2.107	1466.67	3089.80
A_68_P25728011	chr8:26712636-26712680	NM_001001735:-119	Whsc1l1	DIVERGENT_PROMOTER	1.297	2.193	415.37	910.81	2.844	400.11	1137.72
A_68_P31147211	chr17:32966831-32966875	NM_177359:341	Zfp799	INSIDE	1.297	1.526	1016.46	1551.41	1.980	936.86	1855.30
A_68_P26721431	chr9:91250815-91250859	NM_009573:9801	Zic1	DOWNSTREAM	1.297	2.127	558.04	1186.88	2.759	439.03	1211.37
A_68_P30328927	A_68_P30328927			Unknown	1.297	3.543	1419.56	5029.41	4.594	1232.10	5660.00
A_68_P32259779	chrX:11583677-11583722			Unknown	1.297	1.445	836.56	1209.04	1.874	377.74	708.00
A_68_P21840072	chr2:168032731-168032775	NM_009628:-190	Adnp	PROMOTER	1.296	1.524	1047.99	1597.65	1.976	844.77	1669.09
A_68_P25030929	chr7:31099110-31099154	NM_198027:5361	Alkbh6	INSIDE	1.296	2.518	821.79	2069.16	3.264	711.80	2323.13
A_68_P29699202	chr14:70499993-70500037	NM_021328:63	Bin3	INSIDE	1.296	2.412	1652.74	3986.13	3.125	1445.51	4517.72
A_68_P27565933	chr11:5872063-5872107	NM_001174053:93667	Camk2b	INSIDE	1.296	1.572	1338.96	2105.01	2.037	1049.68	2138.32
A_68_P29193442	chr13:85329134-85329178	NM_023243:75	Ccnh	INSIDE	1.296	2.881	820.59	2363.73	3.734	684.11	2554.41
A_68_P26080011	chr8:97400151-97400195	NM_053246:40	Dok4	INSIDE	1.296	2.513	872.35	2191.88	3.257	725.41	2362.32
A_68_P28687870	chr12:103057567-103057611	NM_011812:-323	Fbln5	PROMOTER	1.296	1.498	2819.28	4223.31	1.941	2099.17	4074.35
A_68_P25095551	chr7:53121751-53121795	NM_008172:279	Grin2d	INSIDE	1.296	1.895	489.52	927.67	2.456	452.89	1112.39
A_68_P29628416	chr14:58143558-58143602	NM_145837:-85	Il17d	PROMOTER	1.296	4.077	1297.77	5291.50	5.286	1291.87	6828.41
A_68_P26920439	chr10:7445765-7445809	NM_011835:-11	Katna1	PROMOTER	1.296	1.732	891.38	1543.67	2.244	780.21	1750.68
A_68_P28480022	chr12:62624309-62624353	NM_178714:-1288	Lrfn5	PROMOTER	1.296	2.704	1739.40	4702.88	3.503	1494.33	5234.42
A_68_P25512277	chr7:135883845-135883889	NM_145955:77	Mcmbp	INSIDE	1.296	3.691	1193.78	4406.24	4.784	1081.71	5174.93
A_68_P23749117	chr5:64484174-64484218	NM_025700:8	Pgm1	INSIDE	1.296	1.552	2407.28	3735.03	2.011	1942.84	3906.57
A_68_P23597147	chr5:35291908-35291952	NM_173402:-166	Rgs12	PROMOTER	1.296	1.572	4666.64	7334.58	2.038	3454.23	7038.54
A_68_P22411696	chr3:108086789-108086833	NM_019972:-239	Sort1	PROMOTER	1.296	1.683	2039.03	3431.09	2.180	1716.77	3742.63
A_68_P30680165	chr16:37384543-37384587	NM_001114611:480	Stxbp5l	INSIDE	1.296	1.425	1025.40	1461.51	1.847	864.69	1597.05
A_68_P20803942	chr1:169156556-169156600	NM_030724:58680	Uck2	INSIDE	1.296	7.153	4043.17	28920.81	9.270	2672.35	24771.66
A_68_P27564781	chr11:5640818-5640862	NM_178623:339	Urgcp	INSIDE	1.296	1.533	1403.12	2150.98	1.986	1221.93	2426.95
A_68_P23396198	chr4:148116954-148116998	AK142257:4167		DOWNSTREAM	1.296	1.966	654.23	1286.44	2.548	545.79	1390.71
A_68_P23591272	chr5:34163692-34163736	ENSMUST00000168912:-590		PROMOTER	1.296	3.037	1264.98	3841.96	3.935	1218.12	4793.78
A_68_P31796719	chr18:68459858-68459902	NM_153794:107	4933403F05Rik	INSIDE	1.295	2.068	1237.67	2559.73	2.678	1164.54	3119.03
A_68_P21224222	chr2:53050731-53050775	NM_022989:-365	Arl6ip6	DIVERGENT_PROMOTER	1.295	2.236	707.67	1582.53	2.897	611.90	1772.61

ProbeName	Target position of probe on CpG island microarray	TargetID	GeneSymbol	CpG island Description	Ratio of relative methylation (TiO_2-NP/Vehicle)	Sham group			TiO_2-H group		
						Relative methylation (Cy5/Cy3)	Cy3 signal (Input)	Cy5 signal (MeDIP)	Relative methylation (Cy5/Cy3)	Cy3 signal (Input)	Cy5 signal (MeDIP)
A_68_P27143037	chr10:53317259-53317303	NM_025541:514	Asf1a	INSIDE	1.295	1.670	736.58	1230.13	2.163	660.03	1427.71
A_68_P23609089	chr5:37086829-37086873	NM_001081232:358	D5Ertd579e	INSIDE	1.295	2.159	1653.74	3570.45	2.796	1483.58	4147.77
A_68_P31058444	chr17:14340634-14340678	NM_172826:182	Dact2	INSIDE	1.295	2.031	1656.22	3363.44	2.630	1469.67	3865.24
A_68_P25021026	chr7:29083763-29083810	NM_007866:3018	Dll3	INSIDE	1.295	4.332	459.85	1992.26	5.613	384.31	2156.98
A_68_P28185091	chr11:120392009-120392053	NM_008101:-10	Gcgr	PROMOTER	1.295	4.453	682.96	3041.50	5.768	701.11	4044.11
A_68_P26443399	chr9:40263199-40263243	NM_172768:129	Gramd1b	INSIDE	1.295	3.536	634.59	2243.80	4.579	513.18	2349.66
A_68_P23316586	chr4:132807259-132807303	NM_016693:10548	Map3k6	INSIDE	1.295	2.203	1444.26	3181.78	2.853	1189.96	3394.98
A_68_P32767195	chrX:154036840-154036884	NM_172307:-215	Mbtps2	PROMOTER	1.295	2.882	619.68	1786.06	3.732	436.69	1629.56
A_68_P24171033	chr5:147775400-147775448	NM_029581:-74	Mtif3	PROMOTER	1.295	1.775	735.55	1305.25	2.298	603.64	1387.16
A_68_P26159422	chr8:112392225-112392269	NM_001122594:-256	Phlpp2	PROMOTER	1.295	1.681	833.82	1401.73	2.178	686.14	1494.09
A_68_P20022051	chr1:9290036-9290080	NM_027671:-247	Sntg1	PROMOTER	1.295	2.442	1826.03	4458.62	3.162	1522.51	4813.99
A_68_P31108362	chr17:26250244-26250288	NM_021793:6	Tmem8	INSIDE	1.295	2.581	1564.78	4039.30	3.344	1306.04	4367.59
A_68_P24541202	chr6:72295118-72295162	NM_138592:29	Usp39	INSIDE	1.295	1.490	1146.19	1707.91	1.930	1033.63	1995.15
A_68_P28430598	chr12:52694641-52694685	NM_001198835:335	Coch	INSIDE	1.294	2.078	1764.73	3667.86	2.690	1527.41	4109.17
A_68_P24273894	chr6:18464782-18464826	NM_080285:21	Cttnbp2	INSIDE	1.294	1.778	1181.03	2099.74	2.301	1018.93	2344.69
A_68_P22401913	chr3:106285353-106285397	NM_001093754:26	Dennd2d	INSIDE	1.294	3.081	1640.47	5054.79	3.988	1337.17	5332.95
A_68_P29310173	chr13:109004454-109004498	NM_029001:-121	Elovl7	PROMOTER	1.294	1.521	1764.64	2684.73	1.969	1532.75	3017.55
A_68_P32454246	chrX:68808773-68808817	NM_008253:-373	Hmgb3	PROMOTER	1.294	3.129	1415.47	4428.52	4.047	620.21	2510.02
A_68_P20883818	chr1:183772112-183772156	NM_133815:398	Lbr	INSIDE	1.294	1.415	1047.10	1481.24	1.831	755.53	1383.19
A_68_P31876427	chr18:82645364-82645408	NM_001025245:872	Mbp	INSIDE	1.294	3.051	1072.60	3272.01	3.947	915.05	3611.26
A_68_P25115907	chr7:57231065-57231109	NM_001037906:367	Nell1	INSIDE	1.294	1.685	891.14	1501.82	2.180	793.41	1729.68
A_68_P31112707	chr17:26976313-26976357	NM_008700:2176	Nkx2-5	INSIDE	1.294	1.806	369.56	667.57	2.338	343.85	803.90
A_68_P28072819	chr11:101107662-101107706	NM_019444:37	Ramp2	INSIDE	1.294	2.718	328.94	894.00	3.517	321.48	1130.56
A_68_P28160197	chr11:116442143-116442187	NM_173755:597	Ube2o	INSIDE	1.294	1.724	3669.26	6324.85	2.231	2951.83	6585.43
A_68_P20372370	chr1:78654845-78654889	NM_001136226:-148	Utp14b	PROMOTER	1.294	1.781	1816.14	3234.22	2.304	1502.22	3460.55
A_68_P32246203	chrX:8849653-8849697	NM_023500:-235	Xk	PROMOTER	1.294	1.906	1202.85	2292.65	2.466	709.62	1750.01
A_68_P27913320	chr11:72609515-72609559	NM_001045536:-191	Zzef1	DIVERGENT_PROMOTER	1.294	3.595	2737.74	9842.33	4.653	2173.63	10113.28
A_68_P32321492	chrX:34588621-34588665	NM_173779:-197	Ankrd58	PROMOTER	1.293	1.601	1448.89	2320.04	2.071	629.26	1303.16
A_68_P31351856	chr17:74928018-74928062	NM_007566:406	Birc6	INSIDE	1.293	2.064	1818.05	3752.79	2.669	1579.44	4216.08
A_68_P31746195	chr18:59334616-59334660	NM_001081328:-355	Chsy3	PROMOTER	1.293	6.116	886.14	5419.96	7.909	770.84	6096.84
A_68_P28003125	chr11:88835210-88835254	NM_016706:-16	Coil	PROMOTER	1.293	2.782	1351.04	3759.26	3.599	1133.47	4079.33
A_68_P29570463	chr14:46008598-46008642	NM_019425:-149	Gnpnat1	PROMOTER	1.293	1.955	3987.71	7794.83	2.528	3191.70	8069.56
A_68_P27270903	chr10:77364823-77364867	NM_145152:437	Lrrc3	INSIDE	1.293	1.944	834.13	1621.50	2.514	700.71	1761.68
A_68_P20439698	chr1:92895160-92895204	NM_001111312:-121	Lrrfip1	PROMOTER	1.293	1.583	1984.32	3141.16	2.047	1685.98	3450.53
A_68_P21206010	chr2:49922433-49922477	NM_177139:473	Lypd6	INSIDE	1.293	1.632	1611.87	2631.17	2.111	1345.53	2840.29
A_68_P23229426	chr4:116136323-116136367	NM_001042743:444	Mast2	INSIDE	1.293	2.187	4856.44	10621.51	2.827	3932.85	11119.76
A_68_P30083236	chr15:25711164-25711208	NM_019472:158882	Myo10	INSIDE	1.293	2.101	651.35	1368.72	2.717	574.21	1560.17
A_68_P21853413	chr2:170321616-170321660	NM_001013369:-289	Pfdn4	PROMOTER	1.293	1.661	1576.42	2618.61	2.148	1332.70	2862.35
A_68_P31421199	chr17:87506697-87506741	NM_019654:-300	Socs5	PROMOTER	1.293	1.366	3279.11	4479.12	1.767	2651.67	4684.64
A_68_P28307236	chr12:28024096-28024140	NM_009234:3465	Sox11	INSIDE	1.293	1.883	313.19	589.72	2.435	261.88	637.57
A_68_P20377583	chr1:79758129-79758173	NM_001111279:194	Wdfy1	INSIDE	1.293	0.392	1311.86	514.44	0.507	1043.61	529.16
A_68_P30570067	chr16:16600102-16600146	ENSMUST00000162671:-53		PROMOTER	1.293	2.661	368.27	979.95	3.440	389.81	1341.11
A_68_P25354926	chr7:105457776-105457820	NM_025408:239	Acer3	INSIDE	1.292	2.080	1948.78	4053.49	2.688	1678.46	4512.23
A_68_P24050195	chr5:122872882-122872926	NM_019805:452	Anapc7	INSIDE	1.292	1.992	532.74	1061.01	2.573	509.93	1311.83
A_68_P32533248	chrX:90540027-90540071	NM_007492:8064	Arx	INSIDE	1.292	2.283	3043.46	6947.97	2.950	1738.12	5128.00
A_68_P29338045	chr13:113778996-113779044	NM_001081062:1011	Ccno	INSIDE	1.292	3.069	801.64	2460.34	3.966	758.75	3009.01
A_68_P28575717	chr12:81535921-81535965	NM_177267:1646	Dcaf5	INSIDE	1.292	1.945	761.43	1480.71	2.512	686.74	1725.41
A_68_P32561247	chrX:97972457-97972501	NM_001177780:-131	Dlg3	PROMOTER	1.292	1.830	469.95	860.05	2.365	296.52	701.36
A_68_P26132621	chr8:107793840-107793884	NM_011939:89	Hsf4	INSIDE	1.292	2.204	951.14	2096.24	2.846	787.17	2240.60
A_68_P26348936	chr9:21599583-21599627	NM_145611:3386	Kank2	INSIDE	1.292	2.565	376.16	964.87	3.315	377.67	1251.79
A_68_P27535068	chr10:126725665-126725709	NM_033072:141	Mbd6	INSIDE	1.292	3.226	15005.23	48405.64	4.169	9062.07	37780.41

ProbeName	Target position of probe on CpG island microarray	TargetID	GeneSymbol	CpG island Description	Ratio of relative methylation (TiO_2-NP/Vehicle)	Sham group			TiO_2-H group		
						Relative methylation (Cy5/Cy3)	Cy3 signal (Input)	Cy5 signal (MeDIP)	Relative methylation (Cy5/Cy3)	Cy3 signal (Input)	Cy5 signal (MeDIP)
A_68_P31792975	chr18:67801303-67801347	NM_134138:72	Psmg2	INSIDE	1.292	2.938	3692.97	10849.28	3.797	3209.89	12187.58
A_68_P32567037	chrX:99266374-99266418	NM_183318:250	Rgag4	INSIDE	1.292	2.271	2620.59	5950.26	2.933	1289.14	3780.89
A_68_P23322288	chr4:133898952-133898996	NM_001039677:-10	Slc30a2	PROMOTER	1.292	2.352	2360.47	5551.59	3.038	1847.56	5612.36
A_68_P27841611	chr11:60024072-60024116	NM_011480:10012	Srebf1	INSIDE	1.292	1.462	728.78	1065.73	1.890	589.49	1114.01
A_68_P24287934	chr6:21802232-21802276	NM_173007:261	Tspan12	INSIDE	1.292	1.959	1609.18	3151.72	2.531	1361.68	3446.17
A_68_P23792610	chr5:72971627-72971672	NM_174877:674	Zar1	INSIDE	1.292	1.596	1846.35	2947.34	2.063	1567.68	3233.66
A_68_P31206539	chr17:46520782-46520826	NM_207671:90	Zfp318	INSIDE	1.292	1.937	647.28	1253.67	2.503	594.61	1488.31
A_68_P23962880	chr5:107125598-107125642	NM_026856:229	Zfp644	INSIDE	1.292	1.447	1081.50	1565.06	1.869	941.63	1760.18
A_68_P24951683	chr7:4983086-4983130	NM_001033383:11131	Zfp865	INSIDE	1.292	2.731	914.74	2498.03	3.528	838.17	2957.14
A_68_P32977601	chr12:20821877-20821921	NR_015521:-258	1700030C10Rik	PROMOTER	1.291	1.865	483.32	901.37	2.408	435.24	1047.96
A_68_P23424871	chr4:152806869-152806913	NM_001099299:50049	Ajap1	INSIDE	1.291	2.209	646.85	1428.93	2.853	532.82	1519.98
A_68_P26158883	chr8:112302603-112302647	NM_009677:85	Ap1g1	INSIDE	1.291	1.508	858.11	1294.02	1.947	719.87	1401.74
A_68_P27489850	chr10:118676304-118676348	NM_027994:785	Cand1	INSIDE	1.291	3.668	1251.31	4589.18	4.736	997.46	4723.99
A_68_P21907947	chr2:180763825-180763869	NM_015730:10036	Chrna4	INSIDE	1.291	4.584	4251.08	19486.65	5.916	2753.77	16290.43
A_68_P28156775	chr11:115866293-115866337	NM_001005608:30276	Itgb4	INSIDE	1.291	3.996	1073.42	4289.31	5.161	926.98	4783.77
A_68_P25678290	chr8:15028480-15028524	NM_029116:17478	Kbtbd11	INSIDE	1.291	10.300	1395.55	14373.56	13.299	1184.11	15747.21
A_68_P22201292	chr3:65470413-65470457	NM_001037923:278	Lekr1	INSIDE	1.291	1.788	2086.45	3729.58	2.307	1775.48	4095.78
A_68_P23244387	chr4:118905895-118905939	NM_001042411:-517	Lepre1	PROMOTER	1.291	3.108	1445.26	4491.51	4.011	1243.96	4989.48
A_68_P21006482	chr2:11423738-11423782	NM_001177752:-43	Pfkfb3	PROMOTER	1.291	1.678	388.09	651.03	2.166	300.46	650.91
A_68_P31113240	chr17:27070276-27070320	NM_009343:227	Phf1	INSIDE	1.291	2.976	1739.48	5176.56	3.841	1319.91	5069.64
A_68_P30397290	chr15:84684091-84684135	NM_001081166:2447	Phf21b	INSIDE	1.291	1.396	1057.35	1475.57	1.801	927.49	1670.49
A_68_P23767120	chr5:67698295-67698339	NM_178651:121	Slc30a9	INSIDE	1.291	1.576	868.24	1368.65	2.036	819.72	1668.74
A_68_P21221436	chr2:52601404-52601448	NM_019667:-243	Stam2	PROMOTER	1.291	2.482	1728.93	4290.50	3.205	1486.04	4762.12
A_68_P32241633	chrX:7651543-7651587	NM_011514:322	Suv39h1	INSIDE	1.291	2.502	941.04	2354.96	3.232	573.08	1852.17
A_68_P27504341	chr10:121023507-121023551	NM_019786:322	Tbk1	INSIDE	1.291	1.616	848.44	1370.73	2.086	761.54	1588.48
A_68_P26136330	chr8:108379864-108379908	NM_021513:884	Thap11	INSIDE	1.291	3.037	404.18	1227.39	3.921	424.57	1664.53
A_68_P22749332	chr4:15193464-15193508	NM_181401:520	Tmem64	INSIDE	1.291	4.049	4310.47	17453.31	5.229	3107.10	16246.49
A_68_P24425523	chr6:47997406-47997450	NM_001081382:685	Zfp777	INSIDE	1.291	4.365	1994.40	8706.21	5.635	1644.80	9267.80
A_68_P22820143	chr4:32326406-32326450	ENSMUST00000165661:-4279		PROMOTER	1.291	2.372	3698.56	8771.98	3.061	2955.79	9047.42
A_68_P31112027	chr17:26853342-26853387	NM_029870:770	A930001N09Rik	INSIDE	1.290	4.487	3324.10	14916.56	5.790	2662.85	15417.63
A_68_P31201456	chr17:45643922-45643966	NM_198608:155	Aars2	INSIDE	1.290	1.501	1304.28	1957.55	1.937	1141.63	2211.06
A_68_P29660043	chr14:63859471-63859515	NM_008092:4605	Gata4	INSIDE	1.290	5.842	1535.59	8970.55	7.535	1267.63	9551.91
A_68_P24288409	chr6:21900159-21900203	NM_023626:566	Ing3	INSIDE	1.290	1.750	947.28	1657.65	2.257	655.93	1480.32
A_68_P23154199	chr4:100937872-100937916	NM_146145:-7	Jak1	PROMOTER	1.290	1.360	3369.15	4581.96	1.754	2919.73	5121.17
A_68_P25262926	chr7:86887923-86887967	NM_011068:-33	Pex11a	DIVERGENT_PROMOTER	1.290	1.634	513.12	838.21	2.107	407.28	858.00
A_68_P31302643	chr17:66132754-66132798	NM_001114131:82	Ppp4r1	INSIDE	1.290	2.181	3218.47	7019.49	2.814	3034.14	8537.48
A_68_P20945776	chr1:194660855-194660899	NM_001177794:17065	Sertad4	DOWNSTREAM	1.290	2.106	2255.20	4749.86	2.717	1886.12	5125.42
A_68_P23768850	chr5:67999647-67999692	NM_001033415:548	Shisa3	INSIDE	1.290	2.366	1453.55	3438.65	3.052	1326.83	4049.89
A_68_P30467112	chr15:96885230-96885274	NM_027052:1135	Slc38a4	INSIDE	1.290	1.409	1492.53	2103.70	1.819	1235.31	2246.88
A_68_P20056643	chr1:16655352-16655396	NM_026392:103	Tmem70	INSIDE	1.290	2.740	389.12	1066.34	3.535	415.74	1469.49
A_68_P27635748	chr11:21138636-21138680	NM_139061:-233	Vps54	PROMOTER	1.290	1.623	1586.76	2576.07	2.095	1159.94	2429.62
A_68_P30024033	chr15:12754220-12754264	NM_029998:170	6030458C11Rik	INSIDE	1.289	2.789	479.92	1338.53	3.595	494.68	1778.54
A_68_P27977897	chr11:84326883-84326927	NM_019816:99	Aatf	INSIDE	1.289	2.065	528.48	1091.23	2.661	504.12	1341.36
A_68_P22894883	chr4:47486977-47487022	NM_019998:240	Alg2	INSIDE	1.289	0.444	1285.77	571.38	0.573	1172.33	671.43
A_68_P24816835	chr6:124705909-124705953	NM_007881:575	Atn1	INSIDE	1.289	1.789	6594.29	11798.51	2.307	4687.41	10813.01
A_68_P23637946	chr5:42235648-42235692	NM_001081422:-116	Bod1l	PROMOTER	1.289	2.274	1021.79	2323.87	2.931	906.42	2656.42
A_68_P31792971	chr18:67800851-67800895	NM_001081073:118	Cep76	INSIDE	1.289	2.042	727.58	1485.36	2.632	608.66	1602.26
A_68_P24622150	chr6:88396669-88396713	NM_023060:-157	Eefsec	PROMOTER	1.289	2.045	1396.71	2855.59	2.636	1125.78	2967.78
A_68_P28675869	chr12:100631334-100631378	NM_183186:56928	Foxn3	INSIDE	1.289	1.511	1901.73	2872.71	1.946	1651.92	3215.39
A_68_P28293531	chr12:25783897-25783941	NM_010496:-2961	Id2	PROMOTER	1.289	1.697	431.51	732.39	2.187	412.28	901.78
A_68_P25958333	chr8:74213329-74213373	NM_013564:200	Insl3	INSIDE	1.289	1.549	1098.02	1701.21	1.997	1021.72	2040.68

ProbeName	Target position of probe on CpG island microarray	TargetID	GeneSymbol	CpG island Description	Ratio of relative methylation (TiO_2-NP/Vehicle)	Sham group			TiO_2-H group		
						Relative methylation (Cy5/Cy3)	Cy3 signal (Input)	Cy5 signal (MeDIP)	Relative methylation (Cy5/Cy3)	Cy3 signal (Input)	Cy5 signal (MeDIP)
A_68_P31784507	chr18:66162242-66162286	NM_027400:25	Lman1	INSIDE	1.289	2.079	1219.80	2535.98	2.681	1092.13	2927.77
A_68_P23536416	chr5:22940274-22940318	NM_026984:50	Mll5	INSIDE	1.289	1.599	1101.95	1762.36	2.062	866.53	1786.43
A_68_P27282702	chr10:79819562-79819606	NM_183152:381	Plk5	INSIDE	1.289	3.503	1586.64	5558.53	4.514	1391.65	6282.17
A_68_P29055227	chr13:55573644-55573688	NM_001030296:8039	Prr7	INSIDE	1.289	1.733	780.65	1353.25	2.234	668.25	1492.81
A_68_P21808097	chr2:162486461-162486505	NM_021464:401	Ptprt	INSIDE	1.289	1.636	462.15	756.29	2.109	441.86	931.93
A_68_P23405235	chr4:149655313-149655357	NM_001085492:-690	Rere	PROMOTER	1.289	1.620	542.77	879.49	2.089	483.56	1010.13
A_68_P26684174	chr9:83442213-83442257	NM_172507:290	Sh3bgrl2	INSIDE	1.289	2.398	1482.08	3553.70	3.091	1280.38	3958.13
A_68_P27099065	chr10:42894301-42894345	NM_175407:14	Sobp	INSIDE	1.289	5.791	3868.36	22399.91	7.462	3050.32	22762.69
A_68_P31093840	chr17:23956472-23956516	NM_175229:16341	Srrm2	INSIDE	1.289	2.139	593.78	1270.33	2.757	484.74	1336.42
A_68_P26626133	chr9:71959405-71959449	NM_011544:200	Tcf12	INSIDE	1.289	2.619	913.21	2391.53	3.377	781.52	2639.11
A_68_P27812935	chr11:54769524-54769568	NM_001199275:78	Tnip1	INSIDE	1.289	1.866	1464.37	2732.85	2.406	1183.47	2847.72
A_68_P30570745	chr16:16871029-16871073	NM_011624:67	Top3b	INSIDE	1.289	1.898	1622.30	3079.60	2.446	1432.16	3503.72
A_68_P26593991	chr9:66440276-66440320	NM_144937:489	Usp3	INSIDE	1.289	3.852	611.59	2355.56	4.963	567.75	2817.64
A_68_P27504461	chr10:121043178-121043222	NM_001081056:20172	Xpot	INSIDE	1.289	2.492	2448.39	6101.70	3.213	1885.14	6056.55
A_68_P22834884	chr4:35173017-35173061	NM_001081343:91	3110043O21Rik	INSIDE	1.288	1.629	4198.56	6839.04	2.097	3555.94	7458.02
A_68_P31323856	chr17:69766821-69766865	NR_026848:1056	C030034I22Rik	INSIDE	1.288	0.539	2770.30	1492.87	0.694	2114.29	1467.09
A_68_P29613380	chr14:55250977-55251021	NM_199470:9203	Cdh24	INSIDE	1.288	1.927	487.69	939.59	2.482	393.03	975.56
A_68_P21566996	chr2:118752704-118752748	NM_028117:494	Chst14	INSIDE	1.288	1.697	809.38	1373.30	2.186	689.33	1506.57
A_68_P21100053	chr2:29745687-29745731	NM_001177662:126	Odf2	INSIDE	1.288	1.629	1415.00	2305.63	2.098	1112.08	2333.06
A_68_P23575374	chr5:31210401-31210445	NM_024460:-261	Ost4	DIVERGENT_PROMOTER	1.288	1.409	2672.42	3766.08	1.814	2171.54	3940.14
A_68_P23435280	chr4:154338579-154338623	NM_172990:359	Pank4	INSIDE	1.288	1.476	1246.41	1839.65	1.901	991.41	1885.04
A_68_P24054155	chr5:123591978-123592024	NM_175092:-9362	Rhof	PROMOTER	1.288	4.707	520.70	2450.69	6.062	425.67	2580.47
A_68_P23331867	chr4:135609136-135609180	NM_025919:128	Rpl11	INSIDE	1.288	1.717	884.91	1519.80	2.212	876.07	1938.03
A_68_P25412182	chr7:117365409-117365453	NM_009302:214	Swap70	INSIDE	1.288	1.517	1424.97	2161.70	1.954	1279.91	2500.32
A_68_P32193377	chr19:55390489-55390533	NM_001033573:12	Zdhhc6	INSIDE	1.288	2.787	1228.55	3423.75	3.591	1021.82	3668.88
A_68_P27212542	chr10:67011815-67011859	NM_001005419:-133	Ado	PROMOTER	1.287	2.556	800.10	2044.99	3.289	719.22	2365.29
A_68_P24674233	chr6:97567326-97567370	NM_145148:303	Frmd4b	INSIDE	1.287	1.396	1318.38	1840.78	1.797	1072.41	1926.75
A_68_P27278596	chr10:79198751-79198795	NM_008226:19394	Hcn2	INSIDE	1.287	3.120	1656.68	5169.41	4.014	1286.43	5164.32
A_68_P31149892	chr17:33786930-33786974	NM_001109913:35451	Hnrnpm	INSIDE	1.287	2.029	795.68	1614.10	2.611	714.21	1865.02
A_68_P23927389	chr5:100468389-100468433	NM_016690:-169	Hnrpdl	DIVERGENT_PROMOTER	1.287	2.305	549.11	1265.47	2.966	501.84	1488.28
A_68_P23323460	chr4:134107793-134107837	NM_029100:267	Sepn1	INSIDE	1.287	1.600	1384.81	2215.86	2.060	1151.32	2371.60
A_68_P22149790	chr3:54590088-54590132	NM_019483:30607	Smad9	INSIDE	1.287	3.996	3970.33	15864.16	5.142	2916.86	14999.46
A_68_P29959675	chr14:122506713-122506757	NM_080556:431	Tm9sf2	INSIDE	1.287	1.677	4349.22	7293.71	2.158	3970.40	8567.06
A_68_P25957925	chr8:74152799-74152844	NM_001029873:42835	Unc13a	INSIDE	1.287	2.711	195.03	528.66	3.488	147.91	515.87
A_68_P26884594	chr9:121551770-121551814	NM_011703:-41	Vipr1	DIVERGENT_PROMOTER	1.287	1.605	2744.98	4405.56	2.066	2386.59	4931.14
A_68_P24994117	chr7:20162610-20162654	NM_022409:-3	Zfp296	DIVERGENT_PROMOTER	1.287	1.436	1275.47	1831.12	1.848	932.61	1723.28
A_68_P23270666	chr4:124527512-124527556	NM_001142727:440	1110065P20Rik	INSIDE	1.286	1.856	516.53	958.90	2.387	469.98	1121.94
A_68_P23331196	chr4:135472765-135472809	NM_009924:21468	Cnr2	INSIDE	1.286	3.113	1739.14	5414.11	4.002	1210.68	4845.61
A_68_P27274155	chr10:77888554-77888598	NM_007793:162	Cstb	INSIDE	1.286	2.279	1337.01	3046.89	2.932	1107.48	3246.64
A_68_P31145776	chr17:32629460-32629505	NM_177307:39815	Cyp4f39	INSIDE	1.286	1.667	938.53	1564.61	2.143	826.79	1772.08
A_68_P28924067	chr13:31717371-31717415	NM_010225:-292	Foxf2	PROMOTER	1.286	1.874	3406.06	6383.19	2.410	2661.31	6413.06
A_68_P24864942	chr6:135015624-135015668	NM_181444:-33	Gprc5a	PROMOTER	1.286	2.270	1171.43	2659.29	2.919	1027.49	2999.59
A_68_P21132680	chr2:35138083-35138127	NM_146120:205	Gsn	INSIDE	1.286	2.038	662.85	1351.12	2.621	535.83	1404.57
A_68_P20267137	chr1:60100051-60100095	NM_027407:-141	Ica1l	PROMOTER	1.286	4.945	449.29	2221.94	6.359	437.87	2784.64
A_68_P20007252	chr1:4797954-4797998	NM_008866:3	Lypla1	INSIDE	1.286	2.398	898.17	2153.65	3.085	813.00	2507.85
A_68_P25089969	chr7:52147509-52147554	NM_029365:205	Med25	INSIDE	1.286	2.642	1439.58	3803.16	3.398	1241.53	4219.11
A_68_P31098020	chr17:24687629-24687673	NM_013630:756	Pkd1	INSIDE	1.286	1.763	1160.09	2045.50	2.268	908.85	2061.40
A_68_P31121256	chr17:28369519-28369562	NM_011145:-158	Ppard	PROMOTER	1.286	2.187	2258.23	4939.36	2.812	1803.11	5070.55
A_68_P28112586	chr11:108204519-108204563	NM_011101:662	Prkca	INSIDE	1.286	1.483	698.20	1035.70	1.907	649.45	1238.71
A_68_P29020469	chr13:48720658-48720702	NM_207232:262	Ptpdc1	INSIDE	1.286	1.442	1021.30	1472.37	1.853	920.53	1706.07
A_68_P32790431	chrX:159081429-159081473	NM_178256:83	Reps2	INSIDE	1.286	1.498	1210.21	1812.57	1.926	656.85	1264.84

ProbeName	Target position of probe on CpG island microarray	TargetID	GeneSymbol	CpG island Description	Ratio of relative methylation (TiO_2-NP/Vehicle)	Sham group			TiO_2-H group		
						Relative methylation (Cy5/Cy3)	Cy3 signal (Input)	Cy5 signal (MeDIP)	Relative methylation (Cy5/Cy3)	Cy3 signal (Input)	Cy5 signal (MeDIP)
A_68_P21095252	chr2:28980951-28980995	NM_198033:461	Setx	INSIDE	1.286	2.330	2577.83	6006.83	2.997	2228.89	6679.52
A_68_P23896970	chr5:93371441-93371485	NM_001077596:44439	Shroom3	INSIDE	1.286	2.239	211.30	473.20	2.879	226.36	651.65
A_68_P21584534	chr2:121944006-121944050	NM_145531:94	Spg11	INSIDE	1.286	2.395	517.18	1238.88	3.081	469.57	1446.56
A_68_P25926491	chr8:67471091-67471135	NM_001163747:29	Tmem192	INSIDE	1.286	3.058	3093.79	9459.71	3.933	2762.29	10865.37
A_68_P21578171	chr2:120803024-120803068	NM_175285:249	Tmem62	INSIDE	1.286	2.537	1760.52	4466.90	3.262	1632.42	5325.09
A_68_P21622287	chr2:128793258-128793302	NM_178404:143	Zc3h6	INSIDE	1.286	1.565	1356.36	2122.77	2.013	1157.20	2329.14
A_68_P24919226	chr6:144730238-144730282	ENSMUST00000149451:179		INSIDE	1.286	2.859	569.33	1627.83	3.677	531.69	1955.14
A_68_P29098292	chr13:64414062-64414106	NM_025370:-66	1110018J18Rik	PROMOTER	1.285	1.355	2252.35	3051.57	1.741	2041.83	3554.11
A_68_P24004678	chr5:115017216-115017260	NM_001004180:-1021	BC057022	PROMOTER	1.285	2.606	457.12	1191.42	3.350	396.63	1328.67
A_68_P21569032	chr2:119148703-119148747	NM_019454:-2795	Dll4	PROMOTER	1.285	2.490	1380.47	3437.42	3.201	1138.41	3643.78
A_68_P28703499	chr12:105712671-105712715	NM_010351:-1246	Gsc	PROMOTER	1.285	3.930	554.75	2180.07	5.052	491.97	2485.21
A_68_P27289026	chr10:80809521-80809565	NM_001163165:3629	Hmg20b	INSIDE	1.285	4.034	2130.08	8592.68	5.184	1626.37	8431.46
A_68_P31381046	chr17:80461343-80461387	NM_144802:310	Hnrpll	INSIDE	1.285	2.396	1112.85	2666.55	3.080	1034.85	3187.40
A_68_P29134830	chr13:72769342-72769386	NM_010574:2939	Irx2	INSIDE	1.285	1.524	1010.13	1539.14	1.957	855.34	1674.19
A_68_P25954889	chr8:73437195-73437239	NM_173013:7344	Mtap1s	INSIDE	1.285	2.667	786.17	2096.91	3.428	606.40	2078.92
A_68_P24434513	chr6:49772917-49772962	NM_023456:212	Npy	INSIDE	1.285	2.002	1364.04	2731.28	2.573	1154.82	2970.84
A_68_P30513995	chr16:5132625-5132669	NM_008909:-72	Ppl	PROMOTER	1.285	1.706	2103.56	3589.45	2.192	1768.35	3875.98
A_68_P29612848	chr14:55136330-55136374	NM_013768:-45	Prmt5	PROMOTER	1.285	1.469	1966.21	2887.44	1.887	1657.69	3128.79
A_68_P30152573	chr15:39029866-39029910	NM_053271:11	Rims2	INSIDE	1.285	2.571	456.29	1172.97	3.304	412.49	1362.80
A_68_P30572148	chr16:17132335-17132379	NM_022324:120	Sdf2l1	INSIDE	1.285	2.570	10290.59	26442.68	3.303	8518.47	28132.55
A_68_P23283133	chr4:126698748-126698792	NM_023603:226	Sfpq	INSIDE	1.285	3.389	1636.56	5546.32	4.354	1259.70	5484.83
A_68_P27316752	chr10:86371693-86371737	NM_138673:98973	Stab2	INSIDE	1.285	4.370	8421.16	36796.62	5.617	6135.69	34464.09
A_68_P23814580	chr5:76612959-76613003	NM_011626:76	Tmem165	INSIDE	1.285	1.685	1388.10	2338.44	2.164	1228.09	2657.79
A_68_P21910958	chr2:181231832-181231876	NM_025482:-103	Tpd52l2	PROMOTER	1.285	1.674	1133.21	1897.55	2.152	1003.54	2159.55
A_68_P27992702	chr11:86940963-86941007	NM_197987:406	Trim37	INSIDE	1.285	1.772	2000.17	3544.39	2.278	1698.44	3868.21
A_68_P29220223	chr13:91881212-91881256	NM_028790:109	Acot12	INSIDE	1.284	1.849	419.07	774.72	2.373	421.38	1000.07
A_68_P31315916	chr17:68353564-68353617	NM_172964:-142	Arhgap28	PROMOTER	1.284	2.907	6597.79	19179.08	3.733	4920.44	18368.72
A_68_P20958786	chr1:196802851-196802895	NR_033564:281	C030002c11rik	INSIDE	1.284	1.870	1813.53	3391.95	2.402	1373.06	3297.79
A_68_P31160530	chr17:35837893-35837937	NM_001198833:-379	Ddr1	PROMOTER	1.284	1.753	3487.30	6111.93	2.250	2804.74	6311.79
A_68_P24138655	chr5:140448297-140448346	NM_175522:64425	Elfn1	INSIDE	1.284	6.888	2603.74	17933.33	8.841	1842.32	16287.80
A_68_P28832915	chr13:12658494-12658538	NM_026184:367	Ero1lb	INSIDE	1.284	1.726	2444.08	4218.89	2.217	2013.25	4463.20
A_68_P25025740	chr7:29956305-29956349	NM_182696:918	Ggn	INSIDE	1.284	1.402	2724.49	3818.64	1.800	2078.05	3739.60
A_68_P20226311	chr1:52289826-52289870	NM_001081081:228	Gls	INSIDE	1.284	2.898	629.51	1824.48	3.720	607.14	2258.83
A_68_P32137228	chr19:45310009-45310053	NM_010691:-304	Lbx1	PROMOTER	1.284	1.618	679.06	1098.88	2.078	597.44	1241.49
A_68_P25089971	chr7:52147810-52147854	NM_029365:-96	Med25	DIVERGENT_PROMOTER	1.284	4.218	689.10	2906.36	5.413	637.55	3451.35
A_68_P29060112	chr13:56353073-56353117	NM_010896:430	Neurog1	INSIDE	1.284	1.781	401.02	714.30	2.288	372.65	852.53
A_68_P23796836	chr5:73684343-73684387	NM_001159889:332	Ociad1	INSIDE	1.284	1.697	3145.98	5338.60	2.178	2595.73	5654.75
A_68_P30330338	chr15:73253176-73253220	NM_007982:423	Ptk2	INSIDE	1.284	1.562	2881.52	4501.91	2.006	2153.84	4320.10
A_68_P23341301	chr4:137237373-137237417	NM_001081155:-223	Rap1gap	PROMOTER	1.284	1.813	816.99	1481.18	2.328	650.14	1513.37
A_68_P26324267	chr9:15514415-15514459	NM_172289:224	Slc36a4	INSIDE	1.284	1.755	1059.09	1858.68	2.253	828.99	1867.88
A_68_P29070329	chr13:58009390-58009434	NM_001166464:281	Spock1	INSIDE	1.284	1.595	1483.77	2366.98	2.048	1279.68	2620.80
A_68_P24919225	chr6:144730091-144730135	ENSMUST00000149451:327		INSIDE	1.284	2.637	2798.00	7377.23	3.386	2432.07	8234.41
A_68_P27187160	chr10:62114175-62114219	NM_053183:-250	Ddx50	PROMOTER	1.283	1.466	2415.66	3542.06	1.881	2068.49	3890.16
A_68_P25477105	chr7:129210400-129210444	NM_026140:155	Ears2	INSIDE	1.283	1.671	938.09	1567.18	2.143	859.53	1842.21
A_68_P25503043	chr7:134072430-134072474	NM_172747:60	Kctd13	INSIDE	1.283	1.944	1767.28	3436.40	2.495	1628.44	4062.84
A_68_P23260203	chr4:122674135-122674181	NM_008506:817	Mycl1	INSIDE	1.283	1.556	1779.53	2768.55	1.997	1590.28	3175.45
A_68_P21766702	chr2:155455115-155455159	NM_001085378:18189	Myh7b	INSIDE	1.283	4.662	2897.10	13506.70	5.979	2522.48	15082.95
A_68_P21565318	chr2:118489264-118489308	NM_001033254:-26	Pak6	PROMOTER	1.283	1.421	2123.21	3017.70	1.823	1686.58	3074.60
A_68_P21775763	chr2:157030686-157030730	NM_011249:-438	Rbl1	PROMOTER	1.283	5.703	735.39	4193.93	7.318	689.12	5043.05
A_68_P28550066	chr12:76836469-76836513	NM_030750:226	Sgpp1	INSIDE	1.283	2.237	1332.38	2980.47	2.869	1135.97	3259.63
A_68_P23317228	chr4:132925954-132925998	NM_016981:290	Slc9a1	INSIDE	1.283	2.215	251.74	557.64	2.841	217.01	616.55

ProbeName	Target position of probe on CpG island microarray	TargetID	GeneSymbol	CpG island Description	Ratio of relative methylation (TiO_2-NP/Vehicle)	Sham group			TiO_2-H group		
						Relative methylation (Cy5/Cy3)	Cy3 signal (Input)	Cy5 signal (MeDIP)	Relative methylation (Cy5/Cy3)	Cy3 signal (Input)	Cy5 signal (MeDIP)
A_68_P30980969	chr16:94592388-94592432	NM_009441:65	Ttc3	INSIDE	1.283	3.535	477.73	1688.68	4.536	434.97	1972.96
A_68_P26166795	chr8:113669775-113669819	ENSMUST00000143719:-416		PROMOTER	1.283	1.988	556.56	1106.28	2.549	585.55	1492.73
A_68_P26569950	chr9:62189086-62189130	NM_009672:-41	Anp32a	PROMOTER	1.282	1.417	1170.33	1657.98	1.816	953.72	1732.33
A_68_P31108558	chr17:26275751-26275795	NM_001159598:142	Axin1	INSIDE	1.282	2.387	480.12	1145.83	3.060	484.65	1483.21
A_68_P31926693	chr19:5068431-5068475	NM_054042:375	Cd248	INSIDE	1.282	1.437	1313.35	1887.33	1.842	1138.90	2097.48
A_68_P25654579	chr8:11312543-11312587	NM_009931:262	Col4a1	INSIDE	1.282	1.767	1352.72	2389.83	2.265	1125.80	2549.85
A_68_P22134618	chr3:52073824-52073868	NM_019739:1588	Foxo1	INSIDE	1.282	2.902	460.11	1335.16	3.721	417.80	1554.51
A_68_P29034746	chr13:51942012-51942056	NM_011817:-9	Gadd45g	PROMOTER	1.282	1.683	1520.19	2557.89	2.158	1305.85	2817.78
A_68_P31845028	chr18:77182898-77182942	NM_001039201:-236	Hdhd2	PROMOTER	1.282	2.623	2739.00	7183.68	3.362	2237.94	7524.61
A_68_P25678161	chr8:15011678-15011722	NM_029116:676	Kbtbd11	INSIDE	1.282	1.601	3497.59	5599.17	2.053	2773.68	5694.41
A_68_P30368805	chr15:79659228-79659272	NM_001013360:5513	Npcd	INSIDE	1.282	3.329	1213.59	4040.60	4.268	1060.34	4525.90
A_68_P24974191	chr7:13609857-13609901	NM_011588:378	Trim28	INSIDE	1.282	2.417	434.59	1050.42	3.098	404.21	1252.40
A_68_P26350760	chr9:21992704-21992748	NM_001033813:117	Zfp872	INSIDE	1.282	1.450	5185.29	7521.00	1.860	4458.97	8294.16
A_68_P22900661	chr4:48552753-48552797	NM_001145925:-43	5730528L13Rik	PROMOTER	1.281	2.663	760.51	2025.19	3.410	719.08	2452.02
A_68_P28123544	chr11:110198866-110198910	NM_147219:142	Abca5	INSIDE	1.281	6.630	420.22	2785.88	8.490	371.22	3151.78
A_68_P25601085	chr7:150646964-150647018	NM_001161624:-35	Cdkn1c	PROMOTER	1.281	2.482	817.22	2028.65	3.181	657.97	2092.98
A_68_P20149812	chr1:36748527-36748571	NM_009942:217	Cox5b	INSIDE	1.281	1.763	776.35	1368.61	2.258	718.56	1622.34
A_68_P32982444	chr5:135115258-135115302	NM_033561:-82	Eif4h	PROMOTER	1.281	2.151	1948.99	4192.90	2.757	1917.26	5285.47
A_68_P26550039	chr9:58671843-58671887	NM_001081192:546	Hcn4	INSIDE	1.281	1.486	833.96	1238.94	1.903	723.06	1375.93
A_68_P22450555	chr3:116364918-116364962	NM_008246:655	Hiat1	INSIDE	1.281	2.274	1120.72	2548.42	2.912	1009.70	2940.54
A_68_P24431597	chr6:49163986-49164030	NM_023670:945	Igf2bp3	INSIDE	1.281	2.835	532.69	1510.07	3.632	543.98	1975.72
A_68_P24993775	chr7:20108712-20108756	NM_027116:4655	Nkpd1	INSIDE	1.281	3.360	1843.00	6192.76	4.305	1558.70	6709.62
A_68_P21261721	chr2:60800858-60800902	NM_020296:381	Rbms1	INSIDE	1.281	1.939	677.62	1314.11	2.484	603.57	1499.36
A_68_P26616836	chr9:70350943-70350987	NM_033604:568	Rnf111	INSIDE	1.281	1.438	2034.62	2924.85	1.841	1835.35	3378.55
A_68_P31846338	chr18:77424406-77424450	NM_013666:-157	St8sia5	PROMOTER	1.281	1.503	1026.57	1543.38	1.926	768.49	1480.30
A_68_P25009659	chr7:26100595-26100639	NM_183311:9490	Tmem145	INSIDE	1.281	1.952	1390.26	2713.55	2.500	986.94	2467.19
A_68_P23434596	chr4:154229972-154230016	NM_172878:-314	Ttc34	PROMOTER	1.281	2.702	231.03	624.35	3.462	199.97	692.35
A_68_P28553587	chr12:77469901-77469960	NM_001172104:521	Zbtb25	INSIDE	1.281	2.065	300.12	619.89	2.646	227.00	600.72
A_68_P32977631	A_68_P32977631			Unknown	1.281	9.124	4124.59	37631.81	11.685	2407.80	28135.00
A_68_P20770397	chr1:163100998-163101042	NM_028664:28178	Ankrd45	DOWNSTREAM	1.280	1.629	896.20	1459.67	2.084	790.73	1648.05
A_68_P31793797	chr18:67959692-67959736	NM_027556:-46	Cep192	PROMOTER	1.280	2.076	1096.03	2275.59	2.657	862.87	2293.07
A_68_P25948488	chr8:72407259-72407303	NM_026818:4011	Cilp2	INSIDE	1.280	2.349	1546.96	3634.13	3.007	1315.23	3954.40
A_68_P27996721	chr11:87684998-87685044	NM_007946:4018	Epx	INSIDE	1.280	3.257	1775.56	5782.93	4.170	1261.57	5260.82
A_68_P22317571	chr3:88964184-88964228	NM_008227:-127	Hcn3	DIVERGENT_PROMOTER	1.280	1.471	2372.16	3489.68	1.882	1939.90	3651.76
A_68_P24589808	chr6:82724780-82724828	NM_013820:-356	Hk2	PROMOTER	1.280	1.737	2190.51	3805.46	2.223	1486.69	3305.12
A_68_P21777849	chr2:157385874-157385918	NM_010923:51	Nnat	INSIDE	1.280	1.704	3263.33	5561.08	2.181	2491.35	5434.40
A_68_P24785563	chr6:118146898-118146942	NM_001080780:842	Ret	INSIDE	1.280	2.030	981.12	1991.58	2.598	906.38	2354.71
A_68_P28744650	chr12:112776572-112776616	NR_033168:-2562	Snora28	PROMOTER	1.280	3.566	849.09	3028.13	4.566	712.73	3254.55
A_68_P27985534	chr11:85645565-85645609	NM_009324:-530	Tbx2	PROMOTER	1.280	1.874	1995.43	3738.55	2.399	1641.74	3937.90
A_68_P25182470	chr7:72516116-72516161	NM_001163574:-8	Tjp1	PROMOTER	1.280	2.126	612.28	1301.68	2.722	582.10	1584.61
A_68_P24449885	chr6:52266776-52266820	NR_038163:-1972	5730457N03Rik	PROMOTER	1.279	1.812	2724.97	4937.69	2.317	2335.42	5412.28
A_68_P25274224	chr7:88974543-88974587	NM_146193:-247	Btbd1	PROMOTER	1.279	2.097	1259.03	2639.57	2.682	1127.74	3024.39
A_68_P27284471	chr10:80097175-80097221	NM_001159591:4798	Csnk1g2	INSIDE	1.279	9.978	276.73	2761.19	12.765	229.00	2923.19
A_68_P31677782	chr18:46757349-46757393	NM_010120:13	Eif1a	INSIDE	1.279	1.639	3263.57	5349.50	2.096	2564.13	5374.59
A_68_P30817172	chr16:63857062-63857106	NM_010140:6899	Epha3	INSIDE	1.279	3.489	2355.73	8220.17	4.464	1910.98	8529.90
A_68_P31490816	chr18:10609789-10609833	NM_001081222:540	Esco1	INSIDE	1.279	5.594	1059.49	5927.25	7.158	936.50	6703.49
A_68_P21389688	chr2:83652981-83653025	NM_175514:118	Fam171b	INSIDE	1.279	1.837	780.83	1434.65	2.350	618.57	1453.71
A_68_P24793343	chr6:119429349-119429393	NM_133940:-315	Fbxl14	PROMOTER	1.279	2.408	444.06	1069.15	3.080	406.25	1251.09
A_68_P28419952	chr12:50484269-50484313	NM_008241:297	Foxg1	INSIDE	1.279	1.413	4166.12	5886.56	1.807	3167.10	5724.07
A_68_P28522738	chr12:71926826-71926870	NM_028127:348	Frmd6	INSIDE	1.279	2.393	600.89	1437.64	3.061	610.04	1867.43
A_68_P25307423	chr7:96553263-96553307	NM_008055:409	Fzd4	INSIDE	1.279	1.992	961.71	1916.13	2.549	900.21	2294.38

ProbeName	Target position of probe on CpG island microarray	TargetID	GeneSymbol	CpG island Description	Ratio of relative methylation (TiO_2-NP/Vehicle)	Sham group			TiO_2-H group		
						Relative methylation (Cy5/Cy3)	Cy3 signal (Input)	Cy5 signal (MeDIP)	Relative methylation (Cy5/Cy3)	Cy3 signal (Input)	Cy5 signal (MeDIP)
A_68_P21722284	chr2:146909810-146909854	NM_023504:1249	Nkx2-4	INSIDE	1.279	2.098	482.85	1013.13	2.684	544.87	1462.69
A_68_P29102140	chr13:65277261-65277305	NM_175290:29742	Nlrp4f	DOWNSTREAM	1.279	1.633	1127.59	1840.90	2.089	956.29	1997.35
A_68_P26132671	chr8:107800458-107800502	NM_030152:134	Nol3	INSIDE	1.279	1.735	4572.53	7931.53	2.219	3493.10	7751.45
A_68_P31621829	chr18:36227658-36227702	NM_001167891:129134	Nrg2	INSIDE	1.279	2.408	733.99	1767.10	3.080	682.01	2100.29
A_68_P25435752	chr7:121559588-121559632	NM_011055:843	Pde3b	INSIDE	1.279	2.034	1166.03	2372.10	2.603	982.11	2556.09
A_68_P28567641	chr12:80190421-80190465	NM_029988:215	Pigh	INSIDE	1.279	2.103	1444.07	3037.03	2.690	1247.24	3354.70
A_68_P21819741	chr2:164611806-164611850	NM_133924:308	Snx21	INSIDE	1.279	1.655	1182.12	1956.02	2.116	1064.81	2252.62
A_68_P24118278	chr5:136344278-136344322	NM_021403:50325	Srrm3	INSIDE	1.279	1.738	612.85	1065.06	2.222	577.75	1283.79
A_68_P26242943	chr8:126545298-126545342	NM_133966:-111	Taf5l	DIVERGENT_PROMOTER	1.279	3.384	301.96	1021.92	4.329	350.36	1516.76
A_68_P25731030	chr8:27269296-27269340	NM_199042:678	Thap1	INSIDE	1.279	2.005	828.30	1660.65	2.565	703.16	1803.58
A_68_P20157012	chr1:38053922-38053966	NM_172054:109	Txndc9	INSIDE	1.279	1.835	1136.20	2085.46	2.348	821.10	1928.13
A_68_P32229414	A_68_P32229414			Unknown	1.279	3.806	374.96	1427.10	4.866	313.86	1527.33
A_68_P20286841	chr1:63492318-63492362	NM_001177600:-137	Adam23	PROMOTER	1.278	1.498	981.05	1469.51	1.914	832.07	1592.27
A_68_P32538581	chrX:91612170-91612214	NM_026673:-256	Apoo	PROMOTER	1.278	2.052	1710.91	3511.27	2.623	1026.75	2693.59
A_68_P24760618	chr6:113588447-113588492	NM_001113553:9	Irak2	INSIDE	1.278	3.132	575.96	1803.75	4.004	489.70	1960.68
A_68_P27887969	chr11:68026421-68026465	NM_008744:173886	Ntn1	INSIDE	1.278	2.377	1998.37	4749.87	3.036	1688.80	5128.00
A_68_P29057341	chr13:55937667-55937711	NM_011097:-4902	Pitx1	PROMOTER	1.278	1.527	1218.11	1859.65	1.950	983.27	1917.82
A_68_P23186784	chr4:106512831-106512875	ENSMUST00000151369:7673		DOWNSTREAM	1.278	1.933	747.01	1444.04	2.471	683.53	1688.67
A_68_P27179424	chr10:60708537-60708583	NM_001081127:27626	Adamts14	INSIDE	1.277	1.533	638.06	978.37	1.958	533.27	1044.15
A_68_P22376192	chr3:101407957-101408001	NM_144900:602	Atp1a1	INSIDE	1.277	3.410	530.25	1807.97	4.355	480.96	2094.48
A_68_P27914256	chr11:72774869-72774913	NM_001163336:220	Atp2a3	INSIDE	1.277	1.817	667.00	1212.17	2.321	619.08	1436.67
A_68_P27277812	chr10:79074932-79074976	NM_198614:1809	C2cd4c	INSIDE	1.277	3.920	742.15	2909.48	5.008	576.14	2885.16
A_68_P24990326	chr7:19479287-19479331	NM_001033314:16445	Ccdc61	INSIDE	1.277	2.217	1235.68	2739.02	2.831	1104.20	3126.33
A_68_P23335297	chr4:136250002-136250046	NM_010142:141826	Ephb2	INSIDE	1.277	2.121	2684.95	5694.87	2.709	1659.38	4494.55
A_68_P25420897	chr7:118923563-118923607	NM_173739:-93	Galntl4	PROMOTER	1.277	1.761	1784.11	3141.35	2.249	1590.16	3576.07
A_68_P25661161	chr8:12385043-12385087	NR_027975:-706	Gm5607	PROMOTER	1.277	1.672	686.75	1147.95	2.135	621.96	1327.66
A_68_P21345225	chr2:75497497-75497541	NR_033513:172	Gm6793	INSIDE	1.277	2.445	2267.42	5542.86	3.122	1781.65	5562.96
A_68_P26643352	chr9:75161879-75161923	NM_138719:-148	Gnb5	PROMOTER	1.277	2.194	2021.28	4434.06	2.801	1686.00	4723.04
A_68_P21766453	chr2:155418540-155418584	NM_008180:-120	Gss	PROMOTER	1.277	2.929	578.07	1693.32	3.741	527.26	1972.23
A_68_P31253724	chr17:56218702-56218746	NM_008233:-355	Hdgfrp2	DIVERGENT_PROMOTER	1.277	2.177	8173.74	17795.56	2.781	5076.44	14115.76
A_68_P24448932	chr6:52141182-52141226	NM_008265:498	Hoxa4	INSIDE	1.277	0.494	1588.96	785.36	0.631	1111.80	701.76
A_68_P31930559	chr19:5741845-5741889	NM_008520:963	Ltbp3	INSIDE	1.277	1.351	1925.91	2601.87	1.725	1473.22	2541.36
A_68_P21079215	chr2:26359143-26359187	NM_008714:178	Notch1	INSIDE	1.277	1.925	1536.95	2959.02	2.458	1356.11	3332.74
A_68_P27285560	chr10:80261376-80261420	NM_023900:-27	Plekhj1	DIVERGENT_PROMOTER	1.277	1.521	973.83	1481.23	1.942	773.64	1502.13
A_68_P25392588	chr7:112630344-112630388	NM_028444:345	Prkcdbp	INSIDE	1.277	3.374	1267.87	4278.02	4.308	1103.49	4754.16
A_68_P27915639	chr11:72990519-72990563	NM_001168470:-30	Tmem93	PROMOTER	1.277	2.941	1205.65	3546.23	3.755	942.90	3540.88
A_68_P31155253	chr17:34817071-34817115	NM_031176:9613	Tnxb	INSIDE	1.277	1.444	1128.67	1629.62	1.843	845.95	1559.44
A_68_P28003257	chr11:88861259-88861303	NM_009546:564	Trim25	INSIDE	1.277	1.678	538.09	903.17	2.144	407.52	873.71
A_68_P26822035	chr9:110020257-110020301	NM_133347:-2192	Dhx30	PROMOTER	1.276	1.621	2418.29	3919.46	2.067	2016.41	4168.91
A_68_P28221296	chr12:8305454-8305498	NM_013527:3284	Gdf7	INSIDE	1.276	1.993	1106.61	2204.97	2.542	962.99	2447.49
A_68_P25377912	chr7:109398432-109398476	NM_019566:178	Rhog	INSIDE	1.276	2.636	3077.52	8112.08	3.364	2466.75	8297.91
A_68_P28163661	chr11:117061039-117061083	NM_001113486:86	Septin9	INSIDE	1.276	6.210	1812.09	11252.51	7.923	1642.23	13011.14
A_68_P22749326	chr4:15192772-15192816	NM_181401:-172	Tmem64	PROMOTER	1.276	1.441	953.90	1374.94	1.839	883.54	1625.11
A_68_P21935175	chr3:9004387-9004431	NM_001025263:107	Tpd52	INSIDE	1.276	0.356	1955.86	695.34	0.453	1867.49	846.85
A_68_P21844347	chr2:168780711-168780761	NM_009564:351	Zfp64	INSIDE	1.276	2.304	691.58	1593.36	2.941	591.15	1738.44
A_68_P28740684	chr12:112088879-112088923	NM_001199785:412	Zfp839	INSIDE	1.276	2.023	2566.38	5190.81	2.581	2209.13	5701.65
A_68_P28055487	chr11:98156103-98156147	AK078235:9429		DOWNSTREAM	1.276	4.628	1860.55	8610.39	5.906	1531.71	9046.55
A_68_P27678672	chr11:30168407-30168451	ENSMUST00000149117:-253		PROMOTER	1.276	1.962	1405.65	2757.34	2.502	1156.17	2893.29
A_68_P20091076	chr1:24012273-24012317	NM_026503:185	1110058L19Rik	INSIDE	1.275	1.946	1250.28	2433.50	2.482	1100.40	2731.05
A_68_P24055362	chr5:123823322-123823372	NM_029850:28890	Bcl7a	INSIDE	1.275	1.672	546.43	913.64	2.132	402.49	858.18
A_68_P23280756	chr4:126234616-126234660	NM_175554:415	Clspn	INSIDE	1.275	2.118	687.12	1455.62	2.701	609.16	1645.56

ProbeName	Target position of probe on CpG island microarray	TargetID	GeneSymbol	CpG island Description	Ratio of relative methylation (TiO_2-NP/Vehicle)	Sham group			TiO_2-H group		
						Relative methylation (Cy5/Cy3)	Cy3 signal (Input)	Cy5 signal (MeDIP)	Relative methylation (Cy5/Cy3)	Cy3 signal (Input)	Cy5 signal (MeDIP)
A_68_P31286852	chr17:63231463-63231507	NM_010109:-818	Efna5	PROMOTER	1.275	3.126	2655.06	8298.63	3.985	1978.46	7883.59
A_68_P25151144	chr7:64764458-64764502	NM_176942:899	Gabra5	INSIDE	1.275	5.761	1529.41	8810.58	7.346	1705.22	12526.27
A_68_P30367929	chr15:79521459-79521503	NM_013818:155	Gtpbp1	INSIDE	1.275	2.134	1075.58	2295.73	2.722	858.32	2335.96
A_68_P25088121	chr7:51807262-51807306	NM_009473:2009	Nr1h2	INSIDE	1.275	2.156	1687.94	3638.48	2.747	1592.43	4375.05
A_68_P32119644	chr19:42276497-42276541	NM_018780:224	Sfrp5	INSIDE	1.275	1.789	1631.72	2919.75	2.281	1465.24	3341.72
A_68_P31711733	chr18:53336104-53336148	NM_026386:108	Snx2	INSIDE	1.275	3.207	586.78	1881.92	4.088	568.15	2322.77
A_68_P25591624	chr7:149088169-149088213	NM_023764:121	Tollip	INSIDE	1.275	1.824	1094.03	1995.89	2.327	953.64	2218.76
A_68_P32144304	chr19:46576472-46576516	NM_053100:357	Trim8	INSIDE	1.275	1.774	741.47	1315.62	2.263	685.70	1551.84
A_68_P24951043	chr7:4866972-4867016	NM_170759:176	Zfp628	INSIDE	1.275	2.191	924.82	2025.98	2.794	777.36	2172.04
A_68_P28182346	chr11:119959996-119960041	NM_183137:27	2410002I01Rik	INSIDE	1.274	1.506	2283.20	3438.72	1.919	2093.70	4016.87
A_68_P26372311	chr9:26563473-26563520	NM_029792:4350	B3gat1	INSIDE	1.274	2.541	1171.42	2976.49	3.236	910.98	2947.90
A_68_P25786792	chr8:37677018-37677062	NM_015802:-43	Dlc1	PROMOTER	1.274	1.478	1983.20	2930.27	1.883	1588.70	2991.59
A_68_P23440963	chr4:155222080-155222124	NM_010091:582	Dvl1	INSIDE	1.274	2.082	688.33	1433.32	2.652	610.03	1618.00
A_68_P31149893	chr17:33787074-33787118	NM_001109913:35307	Hnrnpm	INSIDE	1.274	1.836	515.92	947.45	2.339	472.76	1105.65
A_68_P25098815	chr7:53652229-53652273	NM_001112739:384	Kcnc1	INSIDE	1.274	3.041	1970.66	5993.53	3.876	1848.39	7164.51
A_68_P21579145	chr2:120965882-120965926	NM_177846:530	Lcmt2	INSIDE	1.274	2.513	707.60	1778.05	3.201	574.16	1837.65
A_68_P30422819	chr15:88890786-88890830	NM_001002005:653	Panx2	INSIDE	1.274	3.108	1451.93	4512.32	3.959	1241.37	4914.16
A_68_P21817620	chr2:164226294-164226338	NM_009036:-2377	Rbpjl	PROMOTER	1.274	1.849	367.57	679.62	2.355	341.97	805.51
A_68_P23542977	chr5:24108129-24108173	NM_025891:-330	Smarcd3	PROMOTER	1.274	2.707	4042.81	10944.81	3.450	3136.32	10820.35
A_68_P22900940	chr4:48598477-48598521	NM_021436:434	Tmeff1	INSIDE	1.274	2.054	1338.94	2750.62	2.616	1164.37	3046.34
A_68_P25005466	chr7:25332334-25332378	NM_009532:188	Xrcc1	INSIDE	1.274	2.463	793.15	1953.43	3.139	724.62	2274.43
A_68_P22312386	chr3:88004797-88004841	ENSMUST00000167043:274		INSIDE	1.274	2.504	747.37	1871.15	3.191	672.85	2146.76
A_68_P28266975	chr12:16818008-16818052	NM_033270:260	E2f6	INSIDE	1.273	1.452	1602.98	2327.05	1.848	1486.32	2746.33
A_68_P31117097	chr17:27692164-27692208	NM_001025427:-1332	Hmga1	PROMOTER	1.273	5.592	1103.28	6169.78	7.119	944.66	6724.58
A_68_P27887970	chr11:68026508-68026552	NM_008744:173798	Ntn1	INSIDE	1.273	2.431	1447.19	3518.00	3.096	1086.58	3363.77
A_68_P23562013	chr5:28643983-28644027	NM_028234:276	Rbm33	INSIDE	1.273	2.286	779.56	1781.76	2.909	835.37	2430.40
A_68_P21152637	chr2:39045958-39046002	NM_178778:270	Scai	INSIDE	1.273	0.419	1238.25	519.31	0.534	1175.74	627.79
A_68_P21152639	chr2:39046148-39046192	NM_178778:80	Scai	INSIDE	1.273	3.457	742.24	2566.00	4.400	708.97	3119.26
A_68_P25952924	chr8:73125225-73125269	NM_133772:6967	Ssbp4	INSIDE	1.273	0.378	1488.65	562.77	0.481	1291.55	621.35
A_68_P28048905	chr11:97000299-97000343	NM_198100:10706	Tbkbp1	INSIDE	1.273	4.083	981.94	4009.11	5.196	879.99	4572.88
A_68_P21931968	chr3:8327438-8327482			Unknown	1.273	2.348	1046.11	2456.28	2.990	988.62	2956.06
A_68_P25956908	chr8:73985491-73985535	NM_022419:2044	Abhd8	INSIDE	1.272	1.783	504.75	900.04	2.268	422.81	959.03
A_68_P25836149	chr8:47556867-47556911	NM_007981:493	Acsl1	INSIDE	1.272	2.200	593.81	1306.17	2.797	483.29	1351.89
A_68_P29223875	chr13:93196672-93196716	NM_175455:771	Ankrd34b	INSIDE	1.272	1.912	447.72	856.00	2.432	373.23	907.64
A_68_P30927978	chr16:85172880-85172924	NM_001198823:1050	App	INSIDE	1.272	3.762	848.78	3193.13	4.786	858.39	4108.41
A_68_P22019078	chr3:28680105-28680149	NM_177586:-106	Eif5a2	PROMOTER	1.272	2.442	3066.95	7490.76	3.106	2584.70	8027.02
A_68_P29626125	chr14:57723587-57723631	NM_008125:-69	Gjb2	PROMOTER	1.272	2.146	1241.35	2664.05	2.729	1111.81	3034.55
A_68_P23607764	chr5:36807549-36807593	NM_024478:-263	Grpel1	PROMOTER	1.272	1.929	2434.12	4694.94	2.454	1976.42	4849.93
A_68_P20007254	chr1:4798162-4798206	NM_008866:211	Lypla1	INSIDE	1.272	2.741	1174.82	3220.22	3.486	1072.96	3740.63
A_68_P26803474	chr9:106356284-106356328	NM_021567:119	Pcbp4	INSIDE	1.272	2.074	11582.39	24019.00	2.637	9463.05	24957.15
A_68_P29753350	chr14:80170582-80170626	NM_001042726:515	Pcdh8	INSIDE	1.272	1.360	1774.95	2413.95	1.730	1402.06	2425.54
A_68_P25908469	chr8:63703256-63703300	NM_021506:311	Sh3rf1	INSIDE	1.272	1.730	1456.07	2518.56	2.200	1204.66	2650.02
A_68_P24033996	chr5:120121006-120121050	NM_011535:351	Tbx3	INSIDE	1.272	1.906	3551.74	6769.04	2.425	2723.26	6602.77
A_68_P30422255	chr15:88785176-88785220	NM_172818:-350	Ttll8	PROMOTER	1.272	3.622	1178.37	4267.96	4.606	1106.08	5094.90
A_68_P27233861	chr10:70622146-70622190	NM_031397:214	Bicc1	INSIDE	1.271	1.907	1378.71	2629.18	2.423	1169.88	2834.63
A_68_P29024948	chr13:49478604-49478648	NM_001039179:41709	Bicd2	INSIDE	1.271	3.600	1323.63	4765.43	4.576	1283.30	5871.97
A_68_P28602202	chr12:86452173-86452217	NM_030225:412	Dlst	INSIDE	1.271	1.478	1024.73	1514.35	1.878	872.19	1637.60
A_68_P29629404	chr14:58310425-58310469	NM_015771:54514	Lats2	INSIDE	1.271	3.933	1913.49	7525.40	4.999	1497.85	7487.69
A_68_P29725075	chr14:75346986-75347030	NM_001033439:676	Lrch1	INSIDE	1.271	1.478	863.71	1276.21	1.878	742.73	1394.90
A_68_P28094412	chr11:104987932-104987976	NM_172567:216	Mettl2	INSIDE	1.271	1.590	1187.72	1887.89	2.020	992.08	2003.96
A_68_P22355466	chr3:97462027-97462071	NM_182997:-86	Prkab2	PROMOTER	1.271	2.594	4064.98	10546.53	3.297	3055.76	10074.23

ProbeName	Target position of probe on CpG island microarray	TargetID	GeneSymbol	CpG island Description	Ratio of relative methylation (TiO_2-NP/Vehicle)	Sham group			TiO_2-H group		
						Relative methylation (Cy5/Cy3)	Cy3 signal (Input)	Cy5 signal (MeDIP)	Relative methylation (Cy5/Cy3)	Cy3 signal (Input)	Cy5 signal (MeDIP)
A_68_P32760547	chrX:152058267-152058311	NM_001093750:-418	Ptchd1	PROMOTER	1.271	1.505	989.25	1488.49	1.913	642.40	1228.96
A_68_P27561123	chr11:4958083-4958127	NM_145216:-26	Rasl10a	PROMOTER	1.271	1.984	1298.10	2575.37	2.522	1151.64	2904.93
A_68_P31614991	chr18:35000281-35000325	NM_001204914:60	Reep2	INSIDE	1.271	1.453	1795.60	2609.75	1.847	1457.77	2692.58
A_68_P20454240	chr1:95132647-95132691	NM_172463:195	Sned1	INSIDE	1.271	2.447	339.04	829.74	3.110	318.45	990.32
A_68_P27985620	chr11:85655489-85655533	NM_009324:9394	Tbx2	DOWNSTREAM	1.271	2.896	1775.93	5142.72	3.681	1444.85	5319.02
A_68_P21844244	chr2:168760676-168760720	NM_009564:20389	Zfp64	INSIDE	1.271	2.525	723.35	1826.43	3.208	583.75	1872.76
A_68_P31620712	chr18:36047916-36047960			Unknown	1.271	2.389	1053.04	2515.23	3.036	959.34	2912.72
A_68_P22589085	chr3:141832342-141832386	ENSMUST00000098568:-187		PROMOTER	1.271	2.817	1442.63	4064.26	3.581	1242.84	4450.79
A_68_P23197627	chr4:108453252-108453296	ENSMUST00000106658:-166		PROMOTER	1.271	1.677	767.87	1287.99	2.132	659.44	1405.91
A_68_P24173496	chr5:148211773-148211817	NM_010229:271	Flt3	INSIDE	1.270	2.280	4486.84	10227.92	2.894	3602.37	10427.02
A_68_P28189606	chr11:121121098-121121142	NM_001080932:-180	Foxk2	PROMOTER	1.270	2.014	2021.16	4070.78	2.558	1773.00	4534.45
A_68_P27835393	chr11:59005136-59005180	NM_001159410:-3915	Guk1	PROMOTER	1.270	1.522	952.61	1449.87	1.932	713.62	1378.96
A_68_P27794866	chr11:51419856-51419900	NM_001048061:505	Hnrnpab	INSIDE	1.270	1.689	1422.05	2401.77	2.145	1184.57	2541.17
A_68_P28553815	chr12:77504735-77504779	NM_001002012:-406	Hspa2	PROMOTER	1.270	3.540	2968.33	10509.26	4.495	2407.85	10823.02
A_68_P27270906	chr10:77365060-77365104	NM_145152:199	Lrrc3	INSIDE	1.270	4.664	3437.41	16030.98	5.922	2588.99	15331.64
A_68_P27282875	chr10:79849919-79849963	NM_198615:456	Mex3d	INSIDE	1.270	2.059	1297.17	2670.87	2.614	1178.37	3080.78
A_68_P28685553	chr12:102266820-102266864	NR_035421:-6839	Mir1190	PROMOTER	1.270	2.218	751.85	1667.62	2.817	742.38	2091.19
A_68_P22979365	chr4:64785845-64785889	NM_021362:659	Pappa	INSIDE	1.270	1.831	663.79	1215.23	2.326	650.64	1513.29
A_68_P21974614	chr3:19210634-19210678	NM_001122759:666	Pde7a	INSIDE	1.270	2.196	345.63	758.96	2.789	258.80	721.70
A_68_P31157969	chr17:35293395-35293439	NM_001199044:8406	Prrc2a	INSIDE	1.270	4.188	4961.32	20775.58	5.316	3725.98	19808.80
A_68_P23239476	chr4:117963343-117963387	NM_011213:638	Ptprf	INSIDE	1.270	2.880	1084.57	3123.66	3.657	906.10	3313.35
A_68_P26849149	chr9:115219514-115219558	NM_024222:3	Stt3b	INSIDE	1.270	1.386	2025.97	2807.06	1.760	1742.87	3067.12
A_68_P20016444	chr1:7387937-7387981	ENSMUST00000138711:-76		PROMOTER	1.270	1.486	1236.59	1837.32	1.887	1158.30	2186.06
A_68_P28183962	chr11:120210037-120210081	NR_038126:67	0610009L18Rik	INSIDE	1.269	4.261	999.69	4259.56	5.407	821.64	4442.33
A_68_P27277827	chr10:79076635-79076679	NM_198614:107	C2cd4c	INSIDE	1.269	2.229	611.48	1362.80	2.828	548.22	1550.58
A_68_P25272103	chr7:88598756-88598800	NM_007755:784	Cpeb1	INSIDE	1.269	1.513	1220.78	1847.29	1.920	1039.03	1995.01
A_68_P21769577	chr2:155937298-155937342	NM_170588:381	Cpne1	INSIDE	1.269	2.786	2692.57	7501.88	3.535	2306.68	8154.75
A_68_P26519504	chr9:53055815-53055859	NM_029936:381	Ddx10	INSIDE	1.269	3.135	13279.07	41625.28	3.977	8825.63	35102.54
A_68_P23471513	chr5:9161567-9161611	NM_001110327:188	Dmtf1	INSIDE	1.269	3.639	1547.19	5630.00	4.617	1269.72	5862.76
A_68_P26078518	chr8:97125424-97125468	NM_028221:180	Fam192a	INSIDE	1.269	2.561	750.10	1921.00	3.249	704.38	2288.27
A_68_P28185093	chr11:120392227-120392271	NM_008101:208	Gcgr	INSIDE	1.269	1.337	3494.02	4670.89	1.696	2710.09	4596.83
A_68_P20620518	chr1:134195183-134195227	NM_144810:2	Klhdc8a	INSIDE	1.269	3.367	1419.95	4780.62	4.274	1181.68	5050.25
A_68_P22613734	chr3:145947914-145947958	NM_022983:64012	Lpar3	INSIDE	1.269	3.037	1777.67	5398.58	3.852	1454.46	5603.19
A_68_P26983889	chr10:19653542-19653586	NM_008580:-767	Map3k5	PROMOTER	1.269	2.221	637.44	1416.06	2.820	632.97	1785.07
A_68_P27950596	chr11:79524897-79524941	NR_029579:-552	Mir193	PROMOTER	1.269	1.625	1890.18	3071.22	2.062	1514.87	3123.60
A_68_P31920646	chr19:4012495-4012539	NM_133666:209	Ndufv1	INSIDE	1.269	1.636	2741.53	4484.80	2.076	2151.81	4468.15
A_68_P26742494	chr9:95459899-95459943	NM_198414:-315	Paqr9	PROMOTER	1.269	1.829	1103.08	2017.09	2.320	951.41	2207.32
A_68_P22356139	chr3:97555701-97555745	NM_178080:16215	Pde4dip	INSIDE	1.269	2.018	798.15	1611.02	2.561	655.97	1679.97
A_68_P21417955	chr2:90420030-90420074	NM_008982:752	Ptprj	INSIDE	1.269	2.361	639.79	1510.67	2.996	596.15	1786.06
A_68_P27561122	chr11:4958001-4958045	NM_145216:-108	Rasl10a	PROMOTER	1.269	2.261	973.81	2201.50	2.868	916.47	2628.20
A_68_P24428221	chr6:48547445-48547489	NM_001079901:3585	Repin1	INSIDE	1.269	3.192	617.70	1971.72	4.051	520.03	2106.43
A_68_P30345854	chr15:75900190-75900234	NM_134089:-52	Scrib	PROMOTER	1.269	1.957	1230.03	2407.28	2.483	1055.42	2620.94
A_68_P22543381	chr3:133207644-133207688	NM_001040400:-312	Tet2	PROMOTER	1.269	1.723	1710.93	2948.59	2.188	1361.83	2979.10
A_68_P22880595	chr4:45024781-45024825	NM_001163283:482	Zbtb5	INSIDE	1.269	1.704	1217.37	2074.40	2.162	979.76	2118.20
A_68_P24994120	chr7:20162964-20163008	NM_022409:351	Zfp296	INSIDE	1.269	1.584	2185.62	3462.32	2.010	1990.34	4001.45
A_68_P29428130	A_68_P29428130			Unknown	1.269	1.979	352.01	696.65	2.511	296.85	745.48
A_68_P26399344	chr9:31019625-31019669	NM_001102455:-246	Aplp2	PROMOTER	1.268	1.899	2760.89	5242.40	2.408	2143.04	5160.90
A_68_P30281411	chr15:64213769-64213813	NM_010026:691	Asap1	INSIDE	1.268	1.697	805.58	1367.11	2.153	777.04	1672.65
A_68_P29024656	chr13:49437040-49437084	NM_001039179:145	Bicd2	INSIDE	1.268	2.566	703.80	1805.79	3.253	680.14	2212.68
A_68_P30387018	chr15:83002306-83002350	NM_029787:310	Cyb5r3	INSIDE	1.268	0.425	1347.31	572.85	0.539	1026.25	553.23
A_68_P26657027	chr9:77765735-77765781	NM_134255:587	Elovl5	INSIDE	1.268	2.553	1597.62	4078.80	3.238	1347.09	4361.69

ProbeName	Target position of probe on CpG island microarray	TargetID	GeneSymbol	CpG island Description	Ratio of relative methylation (TiO_2-NP/Vehicle)	Sham group			TiO_2-H group		
						Relative methylation (Cy5/Cy3)	Cy3 signal (Input)	Cy5 signal (MeDIP)	Relative methylation (Cy5/Cy3)	Cy3 signal (Input)	Cy5 signal (MeDIP)
A_68_P31936715	chr19:7000722-7000766	NM_008431:8261	Kcnk4	INSIDE	1.268	2.956	1414.46	4181.71	3.750	1184.89	4443.10
A_68_P21101982	chr2:30093777-30093821	NM_177725:510	Lrrc8a	INSIDE	1.268	5.433	963.19	5233.38	6.890	678.57	4675.64
A_68_P23588238	chr5:33678734-33678778	NM_021500:536	Maea	INSIDE	1.268	5.193	3154.45	16380.76	6.583	2359.14	15529.62
A_68_P26192683	chr8:118231489-118231533	NM_001025577:-716	Maf	PROMOTER	1.268	2.220	571.30	1268.01	2.814	440.98	1240.84
A_68_P27282831	chr10:79844492-79844536	NM_198615:5882	Mex3d	INSIDE	1.268	5.592	1593.44	8909.80	7.089	1426.84	10114.79
A_68_P22157981	chr3:55987567-55987611	NM_030595:35	Nbea	INSIDE	1.268	2.882	1223.84	3527.41	3.656	1140.24	4168.63
A_68_P25907378	chr8:63472076-63472120	NM_175089:82	Nek1	INSIDE	1.268	1.575	1075.07	1693.16	1.997	916.59	1830.27
A_68_P24622498	chr6:88450154-88450198	NM_019685:34760	Ruvbl1	DOWNSTREAM	1.268	2.081	3307.01	6881.21	2.639	2688.97	7097.30
A_68_P29578872	chr14:47502657-47502701	NM_001037221:39	Samd4	INSIDE	1.268	2.140	1800.17	3852.57	2.713	1570.51	4260.65
A_68_P20186988	chr1:43155340-43155384	NM_001013025:105	Tgfbrap1	INSIDE	1.268	0.464	1396.70	648.07	0.588	1206.06	709.74
A_68_P20858940	chr1:179252857-179252901			Unknown	1.268	1.746	1430.23	2496.48	2.213	1191.07	2636.16
A_68_P33007024	A_68_P33007024			Unknown	1.268	1.410	1148.79	1619.74	1.788	1035.37	1851.03
A_68_P21761082	chr2:154375340-154375384	NM_029305:723	1700003F12Rik	INSIDE	1.267	1.734	955.07	1656.27	2.197	932.50	2048.66
A_68_P26875484	chr9:119892493-119892537	NM_153287:1262	Csrnp1	INSIDE	1.267	1.523	716.56	1091.23	1.930	627.60	1211.26
A_68_P23342687	chr4:137469337-137469381	NM_199307:51207	Ece1	INSIDE	1.267	2.099	1339.47	2811.71	2.661	1226.65	3263.52
A_68_P21082674	chr2:26997916-26997960	NM_175427:59	Fam163b	INSIDE	1.267	1.972	961.42	1896.33	2.499	847.61	2117.81
A_68_P26612670	chr9:69607179-69607223	NM_022378:1547	Foxb1	INSIDE	1.267	1.394	7936.58	11062.42	1.766	5595.76	9882.10
A_68_P21666842	chr2:136941472-136941517	NM_013822:762	Jag1	INSIDE	1.267	2.336	447.11	1044.55	2.960	367.67	1088.24
A_68_P30148792	chr15:38229751-38229795	NM_013692:690	Klf10	INSIDE	1.267	1.480	1079.76	1598.51	1.875	980.08	1838.08
A_68_P27280137	chr10:79441295-79441339	NM_001003949:5759	ORF61	INSIDE	1.267	3.834	1323.71	5074.57	4.858	905.73	4400.18
A_68_P27265101	chr10:76424431-76424475	NM_021568:240	Pcbp3	INSIDE	1.267	2.148	4139.02	8890.74	2.722	3423.32	9318.77
A_68_P25021471	chr7:29157648-29157692	NM_001083912:11	Plekhg2	INSIDE	1.267	1.721	1857.97	3197.03	2.181	1456.21	3175.64
A_68_P21749123	chr2:152223588-152223632	NM_011438:172	Sox12	INSIDE	1.267	2.628	1477.83	3883.92	3.330	1284.04	4276.02
A_68_P30360706	chr15:78370035-78370079	NM_009218:4719	Sstr3	INSIDE	1.267	2.933	1372.50	4025.76	3.716	1157.76	4301.75
A_68_P22872456	chr4:43575371-43575415	NM_011602:63	Tln1	INSIDE	1.267	2.282	515.18	1175.45	2.892	460.83	1332.58
A_68_P21736746	chr2:149656361-149656405	NM_001085521:-136	Tmem90b	PROMOTER	1.267	1.749	1054.05	1843.43	2.215	949.44	2103.40
A_68_P23142414	chr4:98787172-98787216	NM_026082:412	Dock7	INSIDE	1.266	3.323	691.92	2299.50	4.209	656.21	2761.69
A_68_P23927390	chr5:100468503-100468547	NM_016690:-283	Hnrpdl	DIVERGENT_PROMOTER	1.266	2.734	716.05	1957.66	3.461	636.08	2201.42
A_68_P24449232	chr6:52176211-52176255	NM_010456:1137	Hoxa9	INSIDE	1.266	1.887	3973.77	7499.53	2.389	3242.61	7745.08
A_68_P27736855	chr11:40508399-40508443	NM_134017:-2236	Mat2b	PROMOTER	1.266	1.468	1336.94	1962.56	1.858	1213.79	2255.58
A_68_P27927427	chr11:75469683-75469727	NM_001080775:188	Myo1c	INSIDE	1.266	1.573	3991.67	6280.72	1.993	3457.48	6889.40
A_68_P20826591	chr1:173126357-173126401	NM_025557:21	Pcp4l1	INSIDE	1.266	1.411	1160.84	1637.64	1.786	823.83	1471.40
A_68_P20349748	chr1:74708251-74708295	NM_001014974:-55	Ttll4	PROMOTER	1.266	3.167	1384.94	4386.46	4.009	1261.58	5057.39
A_68_P26082711	chr8:97855540-97855584	NM_024467:288	Zfp319	INSIDE	1.266	5.334	2129.77	11361.27	6.755	1804.34	12188.41
A_68_P23962135	chr5:107003692-107003736	ENSMUST00000131029:20		INSIDE	1.266	1.719	1033.99	1777.88	2.176	1008.44	2194.77
A_68_P28853129	chr13:17786533-17786577	NM_138654:45	5033411D12Rik	INSIDE	1.265	2.260	9494.46	21455.77	2.859	6549.70	18722.36
A_68_P31922426	chr19:4305690-4305734	NM_130863:243	Adrbk1	INSIDE	1.265	1.678	1263.71	2120.16	2.123	1031.50	2189.55
A_68_P32139022	chr19:45616075-45616119	NM_001037758:177873	Btrc	DOWNSTREAM	1.265	2.660	605.24	1610.07	3.365	591.84	1991.53
A_68_P24268748	chr6:17586921-17586965	NM_007604:-155	Capza2	PROMOTER	1.265	2.250	679.09	1528.11	2.847	631.15	1796.93
A_68_P23397277	chr4:148274728-148274772	NM_027195:96250	Casz1	INSIDE	1.265	2.670	383.70	1024.65	3.378	360.02	1216.06
A_68_P23816915	chr5:77017837-77017881	NM_199032:-2880	Cep135	PROMOTER	1.265	2.332	329.41	768.29	2.950	295.18	870.79
A_68_P27677360	chr11:29924702-29924746	NM_146016:1309	Eml6	INSIDE	1.265	1.718	5628.08	9670.61	2.174	4630.52	10066.99
A_68_P24795429	chr6:119797862-119797906	NM_053204:284	Erc1	INSIDE	1.265	2.768	1251.71	3464.60	3.502	970.19	3397.77
A_68_P30395523	chr15:84387987-84388031	NM_177630:245	Ldoc1l	INSIDE	1.265	2.954	1386.49	4096.20	3.736	1180.54	4410.72
A_68_P31074430	chr17:17762134-17762178	NM_172827:-703	Lnpep	PROMOTER	1.265	1.373	4221.16	5796.44	1.737	3037.01	5273.79
A_68_P23296216	chr4:129012377-129012421	NM_009030:216	Rbbp4	INSIDE	1.265	1.605	1135.94	1822.82	2.030	956.85	1942.05
A_68_P31867990	chr18:81169207-81169251	NM_178280:14089	Sall3	INSIDE	1.265	1.995	1760.77	3512.89	2.524	1482.05	3741.43
A_68_P31514145	chr18:14942138-14942182	NM_001100449:407	Taf4b	INSIDE	1.265	3.463	284.18	984.01	4.379	284.97	1247.81
A_68_P26289279	chr9:7764076-7764120	NM_133739:22	Tmem123	INSIDE	1.265	2.132	1412.54	3011.59	2.696	1121.23	3023.12
A_68_P23925842	chr5:100157797-100157841	NR_030692:261	A930011G23Rik	INSIDE	1.264	1.487	1034.17	1537.35	1.879	880.36	1654.13
A_68_P32321495	chrX:34588913-34588957	NM_173779:95	Ankrd58	INSIDE	1.264	2.109	973.77	2053.51	2.666	542.37	1446.06

ProbeName	Target position of probe on CpG island microarray	TargetID	GeneSymbol	CpG island Description	Ratio of relative methylation (TiO_2-NP/Vehicle)	Sham group			TiO_2-H group		
						Relative methylation (Cy5/Cy3)	Cy3 signal (Input)	Cy5 signal (MeDIP)	Relative methylation (Cy5/Cy3)	Cy3 signal (Input)	Cy5 signal (MeDIP)
A_68_P28450023	chr12:56086848-56086892	NM_013815:453	Baz1a	INSIDE	1.264	1.791	538.07	963.65	2.264	489.48	1108.22
A_68_P27894659	chr11:69182111-69182155	NM_146019:796	Chd3	INSIDE	1.264	3.629	2077.29	7538.73	4.588	1704.63	7821.61
A_68_P26790788	chr9:104165092-104165136	NM_001163026:146	Dnajc13	INSIDE	1.264	4.115	2086.32	8584.19	5.201	1759.67	9152.19
A_68_P22019966	chr3:28981333-28981377	NM_001167748:-144	Egfem1	PROMOTER	1.264	2.046	1100.84	2252.74	2.587	967.63	2503.29
A_68_P32152778	chr19:47993679-47993723	NM_001001738:89	Itprip	INSIDE	1.264	2.934	633.02	1857.57	3.710	600.31	2227.22
A_68_P23120705	chr4:94717778-94717822	NM_010591:1113	Jun	INSIDE	1.264	2.591	1013.71	2626.92	3.277	901.41	2953.53
A_68_P29919366	chr14:115439544-115439588	NR_029785:-3326	Mir17	PROMOTER	1.264	3.877	1087.55	4216.78	4.902	939.26	4604.15
A_68_P25326014	chr7:99817883-99817941	NM_029078:510	Pcf11	INSIDE	1.264	2.284	1208.56	2760.68	2.886	956.98	2762.14
A_68_P31757541	chr18:61560019-61560063	NM_133249:45	Ppargc1b	INSIDE	1.264	2.927	236.47	692.04	3.698	227.81	842.44
A_68_P29507005	chr14:31438638-31438682	NM_011103:734	Prkcd	INSIDE	1.264	1.959	5453.51	10681.56	2.475	4311.23	10671.68
A_68_P28469855	chr12:60112957-60113001	NM_009147:26	Sec23a	INSIDE	1.264	2.914	818.11	2383.93	3.684	663.56	2444.38
A_68_P29011270	chr13:47138723-47138767	NM_016785:-158	Tpmt	DIVERGENT_PROMOTER	1.264	2.767	5004.81	13849.25	3.497	4026.38	14078.91
A_68_P21810978	chr2:163029877-163029921	ENSMUST00000128999:-390		PROMOTER	1.264	5.091	1077.20	5484.12	6.437	776.55	4998.35
A_68_P32050812	chr19:29122539-29122583	NM_021299:-168	Ak3	PROMOTER	1.263	1.600	3464.40	5543.36	2.022	2916.88	5896.82
A_68_P28741416	chr12:112215114-112215158	NM_175207:2095	Ankrd9	INSIDE	1.263	2.489	612.82	1525.21	3.143	561.83	1765.86
A_68_P20293018	chr1:64580121-64580165	NM_001037726:765	Creb1	INSIDE	1.263	1.928	1218.33	2348.67	2.434	1055.81	2569.78
A_68_P26317495	chr9:14305304-14305348	NM_023153:264	Cwc15	INSIDE	1.263	1.505	1330.45	2001.97	1.900	1182.32	2246.40
A_68_P27261499	chr10:75807838-75807882	NM_001081419:147	Dip2a	INSIDE	1.263	1.999	2994.62	5987.38	2.526	2628.85	6640.99
A_68_P27594246	chr11:11926108-11926153	NM_001177629:1844	Grb10	INSIDE	1.263	2.147	1896.69	4072.91	2.712	1657.21	4494.92
A_68_P25022017	chr7:29244098-29244142	NM_001141921:6864	Lrfn1	INSIDE	1.263	1.701	829.38	1411.03	2.148	639.56	1374.03
A_68_P31950794	chr19:10532257-10532301	NM_001111140:-341	Lrrc10b	PROMOTER	1.263	1.696	1186.35	2012.17	2.142	876.55	1877.28
A_68_P25187997	chr7:73532946-73532990	NM_146191:259	Lrrk1	INSIDE	1.263	2.602	2788.34	7254.89	3.285	2433.96	7995.91
A_68_P27449323	chr10:110943535-110943582	NM_009344:217	Phlda1	INSIDE	1.263	2.017	1281.53	2584.63	2.548	1117.18	2846.18
A_68_P28103447	chr11:106640770-106640814	NR_027785:37	Polg2	INSIDE	1.263	2.456	1057.25	2596.40	3.102	921.00	2857.29
A_68_P23916645	chr5:98615219-98615263	NM_029947:5353	Prdm8	INSIDE	1.263	1.519	736.27	1118.67	1.919	637.06	1222.39
A_68_P27304471	chr10:84217878-84217922	NM_001024918:-892	Rfx4	PROMOTER	1.263	2.027	838.68	1699.82	2.559	691.37	1769.49
A_68_P26142248	chr8:109459632-109459676	NM_009229:5	Sntb2	INSIDE	1.263	0.525	1169.96	614.08	0.663	1153.47	764.78
A_68_P21864915	chr2:172375828-172375872	NM_001159696:-640	Tcfap2c	PROMOTER	1.263	2.210	2070.51	4576.30	2.792	1806.04	5042.01
A_68_P28078894	chr11:102178324-102178368	NM_001044383:255	Ubtf	INSIDE	1.263	1.610	3048.58	4909.13	2.033	2742.77	5576.66
A_68_P26038357	chr8:90483082-90483126	NM_033327:390	Zfp423	INSIDE	1.263	2.604	584.93	1523.07	3.288	606.53	1994.16
A_68_P25258439	chr7:86135415-86135459			Unknown	1.263	3.364	857.39	2883.87	4.248	692.29	2941.17
A_68_P24850989	chr6:131383710-131383754			Unknown	1.263	1.623	523.16	849.24	2.051	602.17	1234.91
A_68_P24476445	chr6:56664725-56664769	NM_030235:-152	Avl9	PROMOTER	1.262	2.483	2433.00	6041.70	3.135	1876.78	5883.33
A_68_P25496228	chr7:132851532-132851576	NM_001081022:165	D430042O09Rik	INSIDE	1.262	1.711	1108.89	1897.42	2.159	972.86	2100.00
A_68_P28697529	chr12:104664017-104664061	NM_001159502:-48	Ddx24	PROMOTER	1.262	2.138	851.37	1819.85	2.697	805.34	2172.32
A_68_P28610310	chr12:87811236-87811280	NM_011934:48665	Esrrb	INSIDE	1.262	2.462	958.00	2358.63	3.108	774.94	2408.60
A_68_P23333957	chr4:135998981-135999032	NM_008309:19568	Htr1d	INSIDE	1.262	4.376	419.44	1835.45	5.522	339.79	1876.26
A_68_P21147672	chr2:38210172-38210216	NM_010710:3367	Lhx2	INSIDE	1.262	1.957	692.95	1356.06	2.470	602.11	1487.38
A_68_P22552362	chr3:135148859-135148903	NM_027288:306	Manba	INSIDE	1.262	1.413	1505.55	2127.31	1.783	1209.33	2155.79
A_68_P30345376	chr15:75828233-75828277	NM_177922:4056	Mapk15	INSIDE	1.262	1.351	2695.59	3642.36	1.705	2137.07	3644.67
A_68_P29263993	chr13:100286596-100286640	NM_008634:-61	Mtap1b	PROMOTER	1.262	2.033	1083.30	2202.21	2.566	828.90	2126.56
A_68_P28538317	chr12:74685599-74685643	NM_008856:-407	Prkch	PROMOTER	1.262	3.089	473.04	1461.31	3.898	413.37	1611.23
A_68_P26078523	chr8:97125990-97126034	NM_026274:172	Rspry1	INSIDE	1.262	3.000	1418.85	4256.87	3.787	1220.66	4622.56
A_68_P28908966	chr13:29044288-29044332	NM_009238:1241	Sox4	INSIDE	1.262	3.864	1865.85	7210.05	4.877	1550.62	7562.70
A_68_P31149740	chr17:33766012-33766058	NM_026712:-2	Zfp414	PROMOTER	1.262	1.607	934.59	1501.98	2.029	848.57	1721.55
A_68_P32180643	chr19:53216882-53216926	NM_001164100:-341	Add3	PROMOTER	1.261	1.908	2712.37	5174.71	2.406	2168.23	5216.17
A_68_P27217140	chr10:67739840-67739884	NM_023598:1612	Arid5b	INSIDE	1.261	1.572	3850.89	6052.69	1.983	2916.99	5783.27
A_68_P31103785	chr17:25571124-25571168	NM_001163691:-418	Cacna1h	PROMOTER	1.261	2.588	638.28	1652.00	3.263	581.57	1897.41
A_68_P27914667	chr11:72832981-72833025	NM_018883:493	Camkk1	INSIDE	1.261	2.415	2544.85	6146.19	3.044	1941.42	5910.36
A_68_P25537055	chr7:140005461-140005505	NM_212473:-603	Fam53b	PROMOTER	1.261	1.648	622.02	1024.98	2.078	545.76	1134.10
A_68_P27288983	chr10:80800571-80800615	NM_148951:5419	Gipc3	INSIDE	1.261	6.444	1747.06	11257.91	8.125	1635.61	13289.59

ProbeName	Target position of probe on CpG island microarray	TargetID	GeneSymbol	CpG island Description	Ratio of relative methylation (TiO_2-NP/Vehicle)	Sham group			TiO_2-H group		
						Relative methylation (Cy5/Cy3)	Cy3 signal (Input)	Cy5 signal (MeDIP)	Relative methylation (Cy5/Cy3)	Cy3 signal (Input)	Cy5 signal (MeDIP)
A_68_P24182263	chr5:149739992-149740036	NM_153572:209	Katnal1	INSIDE	1.261	2.479	2306.44	5717.72	3.125	1892.74	5915.56
A_68_P22569096	chr3:138152576-138152620	NM_175224:-252	Metap1	PROMOTER	1.261	1.426	2629.55	3750.16	1.799	2263.59	4072.42
A_68_P27277569	chr10:79017872-79017916	NM_027422:-58	Mier2	PROMOTER	1.261	3.251	1824.06	5929.55	4.099	1517.89	6221.82
A_68_P23985066	chr5:111759743-111759787	NM_019640:-18	Pitpnb	PROMOTER	1.261	7.780	3564.89	27736.01	9.814	2821.85	27693.78
A_68_P28107260	chr11:107331896-107331940	NM_145823:116	Pitpnc1	INSIDE	1.261	1.509	2099.88	3168.64	1.902	1617.06	3076.05
A_68_P26747279	chr9:96379047-96379091	NM_011279:-54	Rnf7	PROMOTER	1.261	1.607	1560.64	2508.41	2.027	1285.61	2605.37
A_68_P32752149	chrX:149574498-149574542	NM_001004154:20	Rragb	INSIDE	1.261	1.419	1020.49	1447.74	1.789	467.47	836.21
A_68_P29507583	chr14:31529656-31529700	NM_001166532:878	Sfmbt1	INSIDE	1.261	1.723	762.78	1314.11	2.173	562.94	1223.06
A_68_P23896967	chr5:93371085-93371129	NM_001077596:44083	Shroom3	INSIDE	1.261	2.472	616.95	1525.12	3.116	560.31	1746.12
A_68_P23138232	chr4:98049719-98049763	NM_053157:216	Tm2d1	INSIDE	1.261	1.630	1757.28	2864.79	2.056	1281.48	2635.19
A_68_P28595367	chr12:85262475-85262519	ENSMUST00000085215:298		INSIDE	1.261	2.286	2725.85	6230.43	2.883	2169.66	6254.06
A_68_P28936243	chr13:34129600-34129644	NM_026512:113	Bphl	INSIDE	1.260	1.709	1055.28	1803.53	2.154	920.53	1982.86
A_68_P21013701	chr2:12932877-12932921	NM_153155:-407	C1ql3	PROMOTER	1.260	1.538	741.66	1140.64	1.938	641.86	1243.75
A_68_P30382333	chr15:82074905-82074949	NM_001080158:-160	Cenpm	PROMOTER	1.260	1.400	2750.92	3850.59	1.763	2354.33	4150.79
A_68_P21338895	chr2:74536395-74536439	NM_013555:597	Hoxd9	INSIDE	1.260	4.123	530.12	2185.73	5.196	528.00	2743.38
A_68_P25297068	chr7:94395278-94395322	NM_015760:-4	Nox4	PROMOTER	1.260	2.412	1693.00	4084.35	3.040	1732.65	5267.50
A_68_P31676054	chr18:46440414-46440458	NM_172627:68	Pggt1b	INSIDE	1.260	2.804	3067.69	8600.89	3.533	2521.06	8907.00
A_68_P22261355	chr3:78870478-78870522	NM_001099624:79297	Rapgef2	INSIDE	1.260	2.596	783.77	2034.66	3.271	675.06	2208.21
A_68_P25269481	chr7:88092293-88092339	NM_019951:120	Sec11a	INSIDE	1.260	1.693	937.70	1587.43	2.133	821.00	1751.35
A_68_P25952981	chr8:73133463-73133507	NM_133772:-1271	Ssbp4	DIVERGENT_PROMOTER	1.260	2.255	394.33	889.12	2.842	368.14	1046.24
A_68_P29052709	chr13:55098533-55098577	NM_153131:47762	Unc5a	INSIDE	1.260	4.337	855.43	3709.77	5.466	688.37	3762.59
A_68_P29751627	chr14:79881898-79881942	NM_018765:-845	Wbp4	PROMOTER	1.260	1.991	729.66	1452.92	2.510	807.53	2026.81
A_68_P29308211	chr13:108681537-108681581	NM_145456:-1300	Zswim6	PROMOTER	1.260	2.235	304.86	681.26	2.815	323.56	910.98
A_68_P26616432	chr9:70269348-70269392	NM_007630:-9	Ccnb2	PROMOTER	1.259	1.590	824.91	1311.72	2.003	673.55	1348.96
A_68_P23979393	chr5:110815604-110815648	NM_028596:520	Fbrsl1	INSIDE	1.259	2.320	4562.39	10586.21	2.921	3479.04	10161.39
A_68_P26886029	chr9:121766854-121766898	NM_001112668:-227	Gm9790	PROMOTER	1.259	2.366	1582.81	3745.12	2.979	1311.32	3906.59
A_68_P30252138	chr15:58912850-58912894	NM_001146180:709	Mtss1	INSIDE	1.259	2.192	1103.19	2417.70	2.758	890.65	2456.67
A_68_P28334787	chr12:32745879-32745923	NM_011158:244	Prkar2b	INSIDE	1.259	2.257	1069.46	2413.86	2.843	1028.47	2923.59
A_68_P23198163	chr4:108551696-108551740	NM_023537:44	Rab3b	INSIDE	1.259	1.785	1100.09	1963.71	2.247	927.86	2084.84
A_68_P30182585	chr15:44619330-44619374	NM_001032727:257	Sybu	INSIDE	1.259	1.505	724.39	1090.12	1.895	657.24	1245.38
A_68_P28599772	chr12:86039566-86039610	NM_001033334:169	Tmem90a	INSIDE	1.259	2.630	1669.08	4389.97	3.311	2285.92	7569.48
A_68_P22880599	chr4:45025284-45025328	NM_001163283:-22	Zbtb5	DIVERGENT_PROMOTER	1.259	1.751	453.20	793.46	2.205	395.90	872.84
A_68_P32775150	chrX:155970813-155970857	NM_001033472:235	A830080D01Rik	INSIDE	1.258	3.634	8123.73	29518.31	4.570	3538.68	16172.23
A_68_P23467740	chr5:8367848-8367892	NM_001098225:211	Adam22	INSIDE	1.258	2.932	1894.96	5555.31	3.687	1639.52	6044.47
A_68_P29487115	chr14:28242859-28242903	NM_001114879:848	D14Abb1e	INSIDE	1.258	1.500	807.19	1210.58	1.886	675.25	1273.57
A_68_P22872578	chr4:43591698-43591742	NM_172692:16	Gba2	INSIDE	1.258	1.635	796.77	1302.65	2.056	789.83	1623.99
A_68_P21579146	chr2:120965951-120965995	NM_177846:462	Lcmt2	INSIDE	1.258	1.960	333.19	653.11	2.465	310.18	764.61
A_68_P31491261	chr18:10725960-10726004	NM_144860:360	Mib1	INSIDE	1.258	3.762	332.18	1249.51	4.732	355.47	1682.11
A_68_P23279072	chr4:125933108-125933155	NM_001145970:432	Mtap7d1	INSIDE	1.258	1.636	598.76	979.55	2.058	485.96	1000.11
A_68_P22157982	chr3:55987695-55987739	NM_030595:-93	Nbea	PROMOTER	1.258	2.494	2287.63	5706.15	3.139	1915.11	6011.20
A_68_P30346603	chr15:76010310-76010354	NM_201394:15808	Plec	INSIDE	1.258	2.752	974.82	2683.00	3.463	761.69	2637.56
A_68_P30477986	chr15:98885749-98885793	NM_001163588:166	Prph	INSIDE	1.258	2.841	915.53	2600.58	3.573	788.72	2818.25
A_68_P23230625	chr4:116393777-116393821	NM_146151:239	Tesk2	INSIDE	1.258	1.767	741.54	1310.29	2.223	752.72	1673.26
A_68_P20147880	chr1:36426249-36426293	NM_172652:-244	4632411B12Rik	PROMOTER	1.257	1.900	884.01	1679.62	2.388	794.29	1896.53
A_68_P26073640	chr8:96337458-96337502	NR_026888:-25	4930488L21Rik	PROMOTER	1.257	2.599	716.81	1862.82	3.266	672.10	2195.04
A_68_P24052104	chr5:123213775-123213819	NM_001199676:15623	Camkk2	INSIDE	1.257	8.629	2862.22	24698.88	10.845	1949.78	21144.95
A_68_P32030718	chr19:25684761-25684805	NM_177360:-244	Dmrt3	PROMOTER	1.257	1.993	1456.12	2902.01	2.505	1264.38	3166.83
A_68_P26587298	chr9:65219738-65219782	NM_001111145:10894	Gm514	INSIDE	1.257	2.280	1268.80	2892.48	2.865	991.72	2840.91
A_68_P30963191	chr16:91485340-91485384	NM_010508:-97	Ifnar1	PROMOTER	1.257	0.414	1609.66	666.21	0.520	1385.83	720.98
A_68_P28748395	chr12:113413871-113413915	NM_001097621:29474	Kif26a	INSIDE	1.257	1.780	613.76	1092.79	2.238	538.02	1203.95
A_68_P21794483	chr2:160191360-160191404	NM_010658:1419	Mafb	INSIDE	1.257	1.977	2459.23	4862.52	2.485	1889.67	4694.90

ProbeName	Target position of probe on CpG island microarray	TargetID	GeneSymbol	CpG island Description	Ratio of relative methylation (TiO_2-NP/Vehicle)	Sham group			TiO_2-H group		
						Relative methylation (Cy5/Cy3)	Cy3 signal (Input)	Cy5 signal (MeDIP)	Relative methylation (Cy5/Cy3)	Cy3 signal (Input)	Cy5 signal (MeDIP)
A_68_P24946819	chr7:3637588-3637632	NM_029934:7517	Mboat7	INSIDE	1.257	5.943	2786.89	16563.16	7.473	1934.76	14459.42
A_68_P21820159	chr2:164682781-164682825	NM_011125:406	Pltp	INSIDE	1.257	2.532	811.93	2056.19	3.184	652.01	2076.25
A_68_P23311885	chr4:131901472-131901516	NM_001197082:171	Rcc1	INSIDE	1.257	1.729	1212.95	2097.30	2.174	1098.83	2388.76
A_68_P32672213	chrX:131010426-131010470	NM_028958:581	Taf7l	INSIDE	1.257	1.726	747.30	1290.15	2.170	429.17	931.33
A_68_P28430594	A_68_P28430594			Unknown	1.257	1.403	1638.93	2299.68	1.764	1456.55	2568.90
A_68_P32048665	chr19:28754625-28754669	NM_175459:-79	Glis3	PROMOTER	1.256	2.912	1833.27	5338.16	3.658	1549.37	5666.85
A_68_P25957809	chr8:74135189-74135233	NM_146211:288	Glt25d1	INSIDE	1.256	2.514	1016.92	2556.94	3.159	858.36	2711.51
A_68_P27495356	chr10:119549659-119549703	NM_080446:341	Helb	INSIDE	1.256	1.806	2197.55	3968.05	2.268	1785.92	4050.29
A_68_P29612547	chr14:55083035-55083079	NM_022993:73	Lrp10	INSIDE	1.256	2.007	579.44	1163.20	2.521	550.35	1387.47
A_68_P24754176	chr6:112439420-112439464	NM_001081147:360	Oxtr	INSIDE	1.256	2.240	1047.14	2345.46	2.813	877.56	2468.65
A_68_P20121779	chr1:30929974-30930018	NM_001081080:-9895	Phf3	PROMOTER	1.256	1.671	1353.19	2260.64	2.099	1266.00	2657.44
A_68_P28042484	chr11:95985813-95985857	NM_172799:-9265	Ttll6	PROMOTER	1.256	2.473	1829.70	4525.19	3.107	1517.96	4715.79
A_68_P21739398	chr2:150296010-150296054	NM_001034900:14767	Zfp345	DOWNSTREAM	1.256	4.441	208.20	924.72	5.579	208.77	1164.72
A_68_P23622265	chr5:39075785-39075829	NM_001081144:259	Zfp518b	INSIDE	1.256	2.098	940.70	1973.56	2.636	820.78	2163.48
A_68_P27310644	chr10:85292206-85292250			Unknown	1.256	1.715	413.09	708.65	2.154	340.96	734.47
A_68_P26215030	chr8:122136802-122136846	NM_029428:178	Adad2	INSIDE	1.255	4.440	1500.69	6662.80	5.572	1316.93	7338.24
A_68_P21021381	chr2:14526447-14526491	NM_023116:536	Cacnb2	INSIDE	1.255	2.841	388.59	1103.86	3.566	401.54	1431.70
A_68_P27854485	chr11:62352688-62352732	NM_028448:53	Cenpv	INSIDE	1.255	1.768	431.71	763.10	2.218	370.78	822.39
A_68_P22317585	chr3:88968583-88968627	NM_001163432:-122	Clk2	DIVERGENT_PROMOTER	1.255	1.949	1655.57	3226.14	2.445	1458.68	3567.13
A_68_P23417714	chr4:151657357-151657401	NM_178406:8908	Gpr153	INSIDE	1.255	4.024	896.71	3608.03	5.049	725.94	3665.47
A_68_P27284931	chr10:80165309-80165353	NM_027829:-107	Izumo4	DIVERGENT_PROMOTER	1.255	2.154	3472.99	7480.59	2.703	2761.71	7465.50
A_68_P24112801	chr5:135165012-135165056	NM_010717:-574	Limk1	PROMOTER	1.255	1.398	4835.47	6759.61	1.754	3720.09	6526.85
A_68_P31074428	chr17:17761926-17761970	NM_172827:-495	Lnpep	PROMOTER	1.255	1.640	597.82	980.56	2.058	546.44	1124.66
A_68_P24322961	chr6:28783190-28783234	NM_138682:-1465	Lrrc4	PROMOTER	1.255	3.095	400.64	1239.81	3.884	343.20	1333.09
A_68_P28514078	chr12:70457923-70457967	NM_025441:209	Nemf	INSIDE	1.255	2.336	441.75	1032.10	2.932	386.83	1134.16
A_68_P31935125	chr19:6497430-6497474	NM_001205234:78715	Nrxn2	INSIDE	1.255	2.564	1052.21	2697.86	3.217	881.69	2836.68
A_68_P23308580	chr4:131392321-131392365	NM_001083119:1851	Ptpru	INSIDE	1.255	3.152	2496.09	7867.54	3.955	2080.41	8227.77
A_68_P23298059	chr4:129374001-129374045	NM_199305:60	Tmem39b	INSIDE	1.255	2.662	1338.34	3562.54	3.341	1198.59	4004.59
A_68_P21106256	chr2:30823480-30823525	NM_144884:-64	Tor1a	PROMOTER	1.255	1.826	1543.05	2817.87	2.291	1327.78	3042.61
A_68_P28225920	chr12:9037077-9037121	NM_029321:296	Ttc32	INSIDE	1.255	1.867	652.72	1218.41	2.343	555.85	1302.62
A_68_P26215029	chr8:122136700-122136744	NM_029428:76	Adad2	INSIDE	1.254	2.412	1966.12	4741.77	3.025	1515.18	4583.50
A_68_P26815973	chr9:108551515-108551559	NM_011790:175	Arih2	INSIDE	1.254	3.065	2043.35	6263.27	3.845	1670.50	6423.03
A_68_P24039810	chr5:121063118-121063162	NM_028041:2	Ddx54	INSIDE	1.254	2.009	632.99	1271.96	2.520	567.71	1430.74
A_68_P21039675	chr2:18314331-18314375	NM_001190817:105	Dnajc1	INSIDE	1.254	3.343	1735.14	5801.30	4.191	1635.49	6854.69
A_68_P31961133	chr19:12575663-12575707	NM_172442:802	Dtx4	INSIDE	1.254	2.065	1796.76	3710.69	2.589	1667.77	4318.13
A_68_P24175500	chr5:148537455-148537499	NM_010228:88	Flt1	INSIDE	1.254	1.938	2214.58	4292.48	2.431	1784.27	4337.91
A_68_P31863856	chr18:80560394-80560438	NM_001190373:577	Kcng2	INSIDE	1.254	1.962	862.04	1691.23	2.460	726.69	1787.71
A_68_P27787452	chr11:50044549-50044593	NM_145926:5734	Mgat4b	INSIDE	1.254	1.582	890.40	1408.64	1.983	815.29	1617.05
A_68_P21840225	chr2:168056474-168056518	NM_001160330:375	Mocs3	INSIDE	1.254	1.963	696.25	1366.70	2.461	626.50	1541.96
A_68_P23278957	chr4:125914290-125914334	NM_001145970:19251	Mtap7d1	INSIDE	1.254	2.255	1183.74	2669.29	2.827	1219.20	3447.18
A_68_P22063510	chr3:37318580-37318625	NM_153561:-90	Nudt6	DIVERGENT_PROMOTER	1.254	2.006	6388.36	12815.74	2.515	4755.52	11962.03
A_68_P25953913	chr8:73292527-73292571	NM_008841:8063	Pik3r2	INSIDE	1.254	2.721	1388.85	3779.08	3.413	1039.75	3548.34
A_68_P25737024	chr8:28285216-28285260	NM_001080813:-120	Rab11fip1	PROMOTER	1.254	0.424	1354.69	574.61	0.532	1125.69	598.57
A_68_P31254018	chr17:56263505-56263549	NM_013662:9749	Sema6b	INSIDE	1.254	2.636	2306.70	6081.40	3.306	2033.01	6721.59
A_68_P28613410	chr12:88331231-88331275	NM_001080943:-1626	Zdhhc22	PROMOTER	1.254	1.463	2315.29	3386.43	1.834	1850.91	3395.20
A_68_P31021101	A_68_P31021101			Unknown	1.254	2.723	1778.53	4842.27	3.414	1525.67	5208.72
A_68_P32369652	chrX:47013995-47014044	ENSMUST00000121891:-117		PROMOTER	1.254	3.826	921.42	3525.12	4.797	576.55	2765.75
A_68_P31175229	chr17:39983196-39983240	ENSMUST00000157647:-852		PROMOTER	1.254	2.739	39185.03	107319.50	3.435	30380.84	104357.00
A_68_P25983697	chr8:80041774-80041818	NM_030113:10	Arhgap10	INSIDE	1.253	3.323	12185.98	40496.24	4.165	11147.22	46430.04
A_68_P30373741	chr15:80502565-80502609	NM_145986:310	Fam83f	INSIDE	1.253	1.624	965.76	1568.18	2.034	793.11	1613.53
A_68_P26761440	chr9:98857399-98857443	NM_012020:1395	Foxl2	INSIDE	1.253	1.962	1399.72	2745.84	2.458	1150.67	2828.37

ProbeName	Target position of probe on CpG island microarray	TargetID	GeneSymbol	CpG island Description	Ratio of relative methylation (TiO_2-NP/Vehicle)	Sham group			TiO_2-H group		
						Relative methylation (Cy5/Cy3)	Cy3 signal (Input)	Cy5 signal (MeDIP)	Relative methylation (Cy5/Cy3)	Cy3 signal (Input)	Cy5 signal (MeDIP)
A_68_P25174077	chr7:71036413-71036457	NM_021366:47367	Klf13	INSIDE	1.253	2.627	2645.28	6948.37	3.292	2137.35	7035.35
A_68_P22843574	chr4:36897680-36897724	NM_001165999:1075	Lingo2	INSIDE	1.253	1.871	2352.98	4402.81	2.345	1851.84	4342.34
A_68_P32374346	chrX:48371757-48371801	NM_172413:-583	Rap2c	PROMOTER	1.253	3.212	955.15	3068.30	4.024	599.40	2412.03
A_68_P21071032	chr2:25127662-25127706	NM_023464:254	Ssna1	INSIDE	1.253	1.461	1609.96	2351.59	1.830	1387.39	2539.33
A_68_P25269100	chr7:88020745-88020789	NM_009553:13773	Zscan2	INSIDE	1.253	2.711	817.16	2215.20	3.396	859.95	2920.09
A_68_P22349550	chr3:96279054-96279098	AK136676:11082		DOWNSTREAM	1.253	2.531	2449.39	6200.52	3.173	1818.44	5769.75
A_68_P21320133	chr2:71383777-71383821	NM_010054:1013	Dlx2	INSIDE	1.252	2.504	2332.47	5840.69	3.136	1692.50	5306.90
A_68_P28280007	chr12:21322457-21322501	NM_026347:226	Iah1	INSIDE	1.252	1.925	2547.57	4903.56	2.410	2111.50	5089.48
A_68_P23952342	chr5:104888645-104888689	NM_008861:191	Pkd2	INSIDE	1.252	2.516	1426.52	3589.11	3.150	1279.33	4029.61
A_68_P26609884	chr9:69138237-69138281	NM_013646:636650	Rora	INSIDE	1.252	4.291	1258.44	5400.33	5.372	1049.61	5638.89
A_68_P21721819	chr2:146839148-146839192	NM_011917:375	Xrn2	INSIDE	1.252	1.811	1090.27	1974.33	2.267	938.59	2128.13
A_68_P26721481	chr9:91256506-91256550	NM_009573:4109	Zic1	INSIDE	1.252	1.563	2690.50	4205.14	1.957	2291.20	4482.97
A_68_P26020489	chr8:87082904-87082948	NM_007578:143664	Cacna1a	INSIDE	1.251	2.350	379.98	892.90	2.939	295.37	868.08
A_68_P23396572	chr4:148178339-148178383	NM_027195:-140	Casz1	PROMOTER	1.251	2.175	1480.69	3220.91	2.721	1383.52	3764.80
A_68_P26346393	chr9:21095562-21095606	NM_009878:69	Cdkn2d	INSIDE	1.251	2.226	1410.42	3139.36	2.784	1238.73	3448.90
A_68_P31565719	chr18:25645825-25645869	NM_001146292:266638	Celf4	INSIDE	1.251	1.585	4330.36	6864.65	1.983	3473.65	6888.99
A_68_P25026496	chr7:30093082-30093126	NM_013874:4081	Dpf1	INSIDE	1.251	1.387	2087.54	2895.78	1.735	1802.08	3126.91
A_68_P26804375	chr9:106553157-106553201	NM_001160353:5262	Grm2	INSIDE	1.251	2.376	806.06	1915.49	2.972	647.93	1925.83
A_68_P32507214	chrX:83022249-83022295	NM_008194:-114	Gyk	PROMOTER	1.251	2.527	7443.77	18807.17	3.162	2946.17	9315.16
A_68_P29942854	chr14:119537426-119537470	NM_015820:-38	Hs6st3	PROMOTER	1.251	2.184	1018.57	2224.87	2.734	895.53	2447.96
A_68_P26531859	chr9:55388856-55388900	NM_027397:-77	Isl2	PROMOTER	1.251	1.977	1348.32	2665.63	2.473	1058.16	2616.70
A_68_P29643581	chr14:60870353-60870397	NM_170591:-159	Nupl1	PROMOTER	1.251	1.457	1534.07	2235.00	1.822	1437.44	2619.62
A_68_P27551121	chr11:3189955-3189999	NM_019574:-483	Patz1	PROMOTER	1.251	0.267	2158.38	576.62	0.334	2034.03	679.80
A_68_P22825645	chr4:33276560-33276604	NM_001034867:130	Pm20d2	INSIDE	1.251	1.735	427.62	742.12	2.171	503.58	1093.02
A_68_P22774391	chr4:21616366-21616410	NM_001080771:-3278	Prdm13	PROMOTER	1.251	2.757	2142.24	5906.92	3.449	1784.60	6155.69
A_68_P26044785	chr8:91566664-91566708	NM_021390:1375	Sall1	INSIDE	1.251	3.706	1383.72	5127.86	4.638	1276.13	5918.42
A_68_P28685881	chr12:102321902-102321946	NM_001160214:-12	Smek1	PROMOTER	1.251	1.560	984.82	1536.02	1.951	823.90	1607.71
A_68_P30140501	chr15:36723049-36723093	NM_011740:1223	Ywhaz	INSIDE	1.251	1.936	859.32	1663.60	2.422	757.21	1834.07
A_68_P30421089	chr15:88581968-88582012	NM_181412:-150	Zbed4	PROMOTER	1.251	3.399	901.63	3064.42	4.254	786.58	3345.72
A_68_P24427165	chr6:48388397-48388441	NM_001085415:6671	Zfp467	INSIDE	1.251	1.899	904.07	1717.15	2.375	750.32	1782.38
A_68_P24359960	chr6:35084098-35084143	NM_001164412:-383	Cnot4	PROMOTER	1.250	2.009	374.97	753.48	2.512	264.96	665.48
A_68_P23361674	chr4:140581469-140581513	NM_172122:28086	Crocc	INSIDE	1.250	3.563	1418.80	5054.93	4.452	1161.68	5171.60
A_68_P21428541	chr2:92274385-92274429	NM_009963:-180	Cry2	PROMOTER	1.250	2.007	1118.40	2244.95	2.509	952.02	2389.02
A_68_P28528480	chr12:72932514-72932558	NM_026102:472	Daam1	INSIDE	1.250	1.683	773.54	1302.08	2.103	616.61	1296.98
A_68_P27756940	chr11:44431798-44431842	NM_007897:185	Ebf1	INSIDE	1.250	1.805	632.04	1140.54	2.256	557.00	1256.54
A_68_P25405197	chr7:116094538-116094582	NM_025344:16632	Eif3f	DOWNSTREAM	1.250	1.776	1707.42	3033.02	2.221	1448.00	3215.73
A_68_P23190563	chr4:107107680-107107724	NM_147221:379	Glis1	INSIDE	1.250	2.218	1538.18	3411.78	2.772	1206.59	3344.16
A_68_P21137828	chr2:35959503-35959547	NM_001083126:56	Lhx6	INSIDE	1.250	1.416	1058.01	1498.53	1.770	851.85	1507.96
A_68_P25087543	chr7:51716772-51716816	NM_198250:18938	Lrrc4b	INSIDE	1.250	2.630	1253.41	3295.97	3.286	1017.05	3342.01
A_68_P21375627	chr2:80421060-80421104	NM_016965:40	Nckap1	INSIDE	1.250	1.974	843.92	1665.98	2.467	774.98	1911.64
A_68_P28573810	chr12:81210978-81211022	NM_007564:3000	Zfp36l1	INSIDE	1.250	1.337	2953.82	3948.56	1.671	2208.98	3690.24
A_68_P30534533	chr16:8830219-8830263	NM_001081400:48	1810013L24Rik	INSIDE	1.249	3.202	933.62	2989.05	3.999	846.83	3386.17
A_68_P20425272	chr1:90599100-90599144	NM_177305:-356	Arl4c	PROMOTER	1.249	1.507	2518.27	3794.37	1.882	2142.19	4032.23
A_68_P22408448	chr3:107563100-107563144	NM_001113529:265	Csf1	INSIDE	1.249	1.689	1528.31	2581.97	2.111	1210.48	2554.77
A_68_P24123508	chr5:137357952-137357996	NM_024474:1003	Emid2	INSIDE	1.249	2.263	1647.57	3728.44	2.826	1424.76	4027.07
A_68_P31373922	chr17:79234408-79234452	NM_001081179:291	Heatr5b	INSIDE	1.249	2.023	1507.44	3050.29	2.527	1264.86	3195.69
A_68_P29863085	chr14:103381169-103381213	NM_177715:664	Kctd12	INSIDE	1.249	2.755	709.75	1955.27	3.440	586.77	2018.46
A_68_P29592278	chr14:49792394-49792438	NM_001081430:515	Naa30	INSIDE	1.249	6.468	383.49	2480.53	8.078	375.30	3031.71
A_68_P31633859	chr18:38578724-38578769	NM_022996:118	Ndfip1	INSIDE	1.249	2.818	1181.57	3329.78	3.519	1156.64	4069.83
A_68_P24643139	chr6:92041412-92041456	NM_011630:23	Nr2c2	INSIDE	1.249	2.277	202.79	461.85	2.845	225.16	640.67
A_68_P22085571	chr3:41369106-41369150	NM_001130186:327	Phf17	INSIDE	1.249	2.198	634.87	1395.22	2.745	559.02	1534.59

ProbeName	Target position of probe on CpG island microarray	TargetID	GeneSymbol	CpG island Description	Ratio of relative methylation (TiO_2-NP/Vehicle)	Sham group			TiO_2-H group		
						Relative methylation (Cy5/Cy3)	Cy3 signal (Input)	Cy5 signal (MeDIP)	Relative methylation (Cy5/Cy3)	Cy3 signal (Input)	Cy5 signal (MeDIP)
A_68_P20559397	chr1:121809874-121809922	NM_175106:-153	Tmem177	PROMOTER	1.249	2.032	1437.86	2921.97	2.538	1112.11	2822.68
A_68_P30350974	chr15:76701808-76701852	NM_001007568:35	Zfp251	INSIDE	1.249	2.382	1279.27	3046.72	2.974	1171.82	3485.14
A_68_P32565198	chrX:98892284-98892328	ENSMUST00000073812:-5117		PROMOTER	1.249	4.292	7463.45	32031.83	5.362	2638.28	14146.88
A_68_P32700324	chrX:137199916-137199960	ENSMUST00000112988:328		INSIDE	1.249	3.576	838.25	2997.85	4.468	633.56	2830.49
A_68_P28050436	chr11:97277448-97277492	ENSMUST00000121799:624		INSIDE	1.249	1.790	1266.29	2266.28	2.235	1023.72	2287.88
A_68_P28708611	chr12:106575425-106575469	ENSMUST00000138649:244		INSIDE	1.249	2.424	1565.31	3794.23	3.026	1344.56	4069.29
A_68_P31408618	chr17:85490170-85490214	NM_028576:153	1700106N22Rik	INSIDE	1.248	1.567	624.98	979.26	1.956	588.22	1150.36
A_68_P27473560	chr10:115551150-115551194	NM_001161855:-199	4933416C03Rik	PROMOTER	1.248	2.323	978.87	2274.16	2.898	869.20	2519.38
A_68_P27033507	chr10:28867124-28867168	NM_026138:3345	6330407J23Rik	INSIDE	1.248	1.815	878.14	1594.23	2.266	763.35	1729.89
A_68_P20239572	chr1:54982610-54982654	NM_001081433:599	Ankrd44	INSIDE	1.248	1.767	4378.24	7738.53	2.205	3378.41	7449.77
A_68_P22609989	chr3:145316674-145316720	NM_010516:-3747	Cyr61	PROMOTER	1.248	1.523	622.12	947.61	1.900	519.34	986.94
A_68_P21874518	chr2:174125914-174125958	NM_201617:2577	Gnas	INSIDE	1.248	1.725	449.44	775.29	2.152	375.13	807.45
A_68_P25954565	chr8:73387047-73387091	NM_032397:-6161	Kcnn1	PROMOTER	1.248	1.781	637.74	1136.03	2.223	524.25	1165.42
A_68_P20127131	chr1:32229533-32229577	NM_133235:-96	Khdrbs2	PROMOTER	1.248	2.929	459.35	1345.58	3.655	463.67	1694.54
A_68_P30619231	chr16:26105726-26105770	NM_173379:122	Leprel1	INSIDE	1.248	1.960	2895.74	5676.76	2.446	2358.79	5770.00
A_68_P25279952	chr7:90016468-90016512	NM_175366:648	Mex3b	INSIDE	1.248	1.562	1292.65	2019.13	1.950	1109.58	2163.80
A_68_P26254550	chr8:128421964-128422008	NM_175561:207	Pcnxl2	INSIDE	1.248	1.925	1505.98	2898.63	2.403	1332.47	3201.93
A_68_P27911828	chr11:72303091-72303135	NM_153060:294	Spns2	INSIDE	1.248	2.134	2299.51	4906.38	2.662	1991.47	5302.04
A_68_P33005935	A_68_P33005935			Unknown	1.248	2.549	518.79	1322.28	3.181	515.51	1639.89
A_68_P27283432	chr10:79934830-79934874	ENSMUST00000158603:-4244		PROMOTER	1.248	2.273	1428.07	3245.31	2.835	1292.75	3664.93
A_68_P29335934	chr13:113442566-113442610	NM_001145885:-70	Ddx4	DIVERGENT_PROMOTER	1.247	2.866	962.97	2760.00	3.573	790.58	2824.84
A_68_P27171225	chr10:59342512-59342556	NM_019965:196	Dnajb12	INSIDE	1.247	1.650	849.75	1402.47	2.059	740.09	1523.76
A_68_P27145715	chr10:53795287-53795331	NM_008548:294	Man1a	INSIDE	1.247	2.146	2354.40	5052.54	2.676	2030.45	5432.77
A_68_P24443350	chr6:51220702-51220746	NR_029719:-815	Mir148a	PROMOTER	1.247	2.025	1306.70	2645.72	2.525	1202.64	3036.11
A_68_P26343627	chr9:20532217-20532261	NM_173777:420	Olfm2	INSIDE	1.247	5.191	2293.17	11902.71	6.471	1822.08	11789.95
A_68_P20598628	chr1:130000152-130000196	NM_181750:292	R3hdm1	INSIDE	1.247	2.327	1148.90	2673.45	2.901	966.62	2803.99
A_68_P25504562	chr7:134352166-134352210	NM_144522:-206	Tbc1d10b	DIVERGENT_PROMOTER	1.247	1.536	1015.11	1559.03	1.915	881.88	1688.82
A_68_P27416798	chr10:105011030-105011074	NM_177368:483	Tmtc2	INSIDE	1.247	1.573	1697.16	2668.91	1.960	1221.25	2394.00
A_68_P23141229	chr4:98591079-98591123	NM_146144:600	Usp1	INSIDE	1.247	1.659	1472.95	2443.54	2.068	1301.96	2692.76
A_68_P23196091	chr4:108132080-108132124	NM_175472:72	Zcchc11	INSIDE	1.247	2.526	1748.34	4416.82	3.149	1504.96	4739.78
A_68_P24783630	chr6:117791108-117791152	NM_177684:-129	Zfp637	PROMOTER	1.247	1.687	1611.83	2718.42	2.103	1410.60	2965.94
A_68_P26888895	chr9:122260872-122260916	NM_026179:161	Abhd5	INSIDE	1.246	3.749	970.64	3638.97	4.671	871.24	4069.51
A_68_P31259474	chr17:57136971-57137015	NM_025538:231	Alkbh7	INSIDE	1.246	1.713	2572.73	4408.24	2.136	2124.39	4536.82
A_68_P27897123	chr11:69609071-69609115	NM_009601:347	Chrnb1	INSIDE	1.246	1.952	1126.95	2200.28	2.432	899.88	2188.72
A_68_P25804898	chr8:41393101-41393145	NM_030110:-282	Efha2	PROMOTER	1.246	2.471	304.12	751.41	3.079	260.66	802.66
A_68_P23416685	chr4:151500131-151500175	NM_019585:2882	Espn	INSIDE	1.246	1.569	1559.72	2447.18	1.955	1150.05	2248.46
A_68_P28463194	chr12:58643991-58644035	NM_008259:3096	Foxa1	INSIDE	1.246	1.651	1395.56	2304.44	2.057	1202.32	2473.16
A_68_P23300608	chr4:129815629-129815674	NM_001163027:144	Hcrtr1	INSIDE	1.246	3.318	971.37	3223.16	4.134	849.45	3511.44
A_68_P29604766	chr14:52724118-52724162	NM_001170981:-437	Hnrnpc	DIVERGENT_PROMOTER	1.246	1.788	1184.33	2117.22	2.227	871.76	1941.82
A_68_P24051357	chr5:123064217-123064261	NM_009879:289	Ift81	INSIDE	1.246	1.726	1134.07	1956.96	2.151	967.09	2079.93
A_68_P26374351	chr9:26962634-26962678	NM_023277:350	Jam3	INSIDE	1.246	3.139	541.16	1698.71	3.912	521.35	2039.76
A_68_P28105679	chr11:107050342-107050386	NM_001161329:331	Nol11	INSIDE	1.246	2.317	3249.95	7530.63	2.888	2457.12	7095.70
A_68_P22878647	chr4:44717142-44717186	NM_008782:6148	Pax5	INSIDE	1.246	1.638	1243.00	2036.06	2.041	1224.49	2499.58
A_68_P31113239	chr17:27070188-27070232	NM_009343:139	Phf1	INSIDE	1.246	1.438	2313.67	3327.70	1.793	1950.38	3496.13
A_68_P30423673	chr15:89004061-89004105	NM_001159521:198	Plxnb2	INSIDE	1.246	3.391	1934.89	6561.95	4.225	1570.38	6635.07
A_68_P24694202	chr6:100784182-100784226	NM_182939:573	Ppp4r2	INSIDE	1.246	1.430	2138.05	3058.07	1.782	1461.44	2604.03
A_68_P26136629	chr8:108424268-108424312	NM_173432:-83	Pskh1	PROMOTER	1.246	2.322	720.89	1673.83	2.893	633.05	1831.72
A_68_P27836018	chr11:59102804-59102848	NM_009522:1427	Wnt3a	INSIDE	1.246	2.282	2968.11	6773.89	2.844	2429.64	6909.10
A_68_P28037459	chr11:95084648-95084692	ENSMUST00000150818:2276		INSIDE	1.246	5.041	2103.01	10600.95	6.282	1573.34	9884.37
A_68_P22503483	chr3:126300156-126300200	NM_001025438:288	Camk2d	INSIDE	1.245	1.465	916.26	1342.64	1.825	793.31	1447.43
A_68_P27234933	chr10:70807476-70807520	NM_134007:99	Cisd1	INSIDE	1.245	2.459	1162.36	2858.70	3.062	1080.51	3308.04

ProbeName	Target position of probe on CpG island microarray	TargetID	GeneSymbol	CpG island Description	Ratio of relative methylation (TiO_2-NP/Vehicle)	Sham group			TiO_2-H group		
						Relative methylation (Cy5/Cy3)	Cy3 signal (Input)	Cy5 signal (MeDIP)	Relative methylation (Cy5/Cy3)	Cy3 signal (Input)	Cy5 signal (MeDIP)
A_68_P22614660	chr3:146113432-146113476	NM_028836:-2	Ctbs	PROMOTER	1.245	1.902	2950.13	5611.33	2.368	2334.24	5526.49
A_68_P25394427	chr7:112936384-112936433	NM_001162943:-344	Dchs1	PROMOTER	1.245	3.206	1009.19	3234.97	3.991	648.44	2587.87
A_68_P23283633	chr4:126803154-126803198	NM_001099319:-113	Gm12942	PROMOTER	1.245	1.513	956.11	1446.22	1.884	785.53	1479.87
A_68_P28577469	chr12:81858735-81858779	NM_001008423:2946	Gm1568	INSIDE	1.245	3.214	1389.69	4466.02	4.000	1106.08	4424.17
A_68_P27163122	chr10:57786302-57786346	NM_026148:111	Lims1	INSIDE	1.245	3.692	656.53	2423.74	4.598	610.74	2808.12
A_68_P25189684	chr7:73834910-73834954	NM_001191001:158	Lins	INSIDE	1.245	1.707	1319.84	2252.58	2.124	1110.91	2359.62
A_68_P25031209	chr7:31145387-31145431	NM_175478:2383	Lrfn3	INSIDE	1.245	2.575	466.90	1202.28	3.205	454.22	1455.67
A_68_P27284768	chr10:80139566-80139610	NM_021462:-4867	Mknk2	PROMOTER	1.245	1.506	1548.45	2331.61	1.874	1166.29	2185.59
A_68_P29585963	chr14:48740738-48740782	NM_033602:217	Peli2	INSIDE	1.245	1.701	1478.59	2515.67	2.118	1338.33	2834.88
A_68_P25026383	chr7:30074297-30074341	NM_026731:-20	Ppp1r14a	DIVERGENT_PROMOTER	1.245	2.661	1096.87	2918.96	3.313	907.54	3006.67
A_68_P26349533	chr9:21722608-21722652	NM_031874:-65	Rab3d	PROMOTER	1.245	1.353	3484.91	4713.97	1.685	3009.85	5070.84
A_68_P30575874	chr16:17815530-17815575	NM_153790:18178	Scarf2	DOWNSTREAM	1.245	4.568	1222.80	5585.25	5.685	1053.27	5987.68
A_68_P29421305	chr14:15535746-15535790	NM_001033270:230	Slc4a7	INSIDE	1.245	2.894	1173.65	3397.03	3.603	974.75	3512.40
A_68_P21749133	chr2:152224628-152224672	NM_011438:-868	Sox12	PROMOTER	1.245	1.800	485.24	873.51	2.241	443.37	993.47
A_68_P32672216	chrX:131010833-131010877	NM_028958:175	Taf7l	INSIDE	1.245	1.871	582.99	1090.56	2.328	337.70	786.28
A_68_P28536526	chr12:74387399-74387443	NM_029580:278	Trmt5	INSIDE	1.245	0.452	1178.56	532.24	0.562	1006.04	565.85
A_68_P29950628	A_68_P29950628			Unknown	1.245	2.174	1561.10	3393.72	2.706	1412.12	3820.79
A_68_P28725239	chr12:109244424-109244468	NM_001079883:-2822	Bcl11b	PROMOTER	1.244	1.391	1623.33	2257.30	1.730	1382.98	2392.33
A_68_P20456920	chr1:95583181-95583225	NM_016778:932	Bok	INSIDE	1.244	2.225	647.81	1441.29	2.768	599.15	1658.48
A_68_P31620309	chr18:35990795-35990839	NM_133687:1345	Cxxc5	INSIDE	1.244	1.769	1890.23	3343.00	2.201	1524.20	3354.40
A_68_P27281217	chr10:79602092-79602136	NR_036582:-2	Dos	DIVERGENT_PROMOTER	1.244	1.549	1805.92	2797.64	1.927	1438.56	2772.35
A_68_P26132769	chr8:107814164-107814208	NM_177788:5812	Exoc3l	INSIDE	1.244	1.571	1164.82	1829.53	1.954	1012.60	1978.53
A_68_P28080758	chr11:102526866-102526910	NM_001127576:-13	Gm1564	PROMOTER	1.244	2.990	2089.75	6248.75	3.719	1642.64	6108.78
A_68_P27812448	chr11:54680388-54680432	NM_008248:471	Hint1	INSIDE	1.244	1.744	1484.50	2588.72	2.170	1308.37	2838.89
A_68_P20739612	chr1:157589907-157589951	NM_010712:-771	Lhx4	PROMOTER	1.244	2.251	1070.22	2408.98	2.801	998.26	2795.74
A_68_P29725076	chr14:75347080-75347124	NM_001033439:582	Lrch1	INSIDE	1.244	5.256	911.20	4789.64	6.541	752.47	4922.20
A_68_P32266420	chrX:13063059-13063103	NM_173415:18283	Nyx	INSIDE	1.244	1.668	1069.58	1784.24	2.076	619.59	1286.00
A_68_P31655270	chr18:42554978-42555022	NM_138945:750	Pou4f3	INSIDE	1.244	0.483	1446.10	698.58	0.601	1248.73	750.64
A_68_P31735662	chr18:57522846-57522891	NM_028447:8482	Prrc1	INSIDE	1.244	3.999	2807.67	11228.79	4.976	1990.29	9904.65
A_68_P29518735	chr14:33411263-33411307	NM_001001796:-1828	Prrxl1	PROMOTER	1.244	2.059	2013.62	4146.23	2.562	1813.30	4645.67
A_68_P30375546	chr15:80808340-80808384	NM_134091:168	Sgsm3	INSIDE	1.244	2.090	1155.13	2414.38	2.599	1148.50	2985.52
A_68_P24009033	chr5:115777444-115777488	NM_025573:281	Srsf9	INSIDE	1.244	0.487	1268.23	617.57	0.606	974.10	589.95
A_68_P20880193	chr1:183141557-183141601	NM_145514:531	Wdr26	INSIDE	1.244	3.488	599.71	2091.94	4.339	550.61	2389.35
A_68_P30571778	chr16:17070255-17070299	NM_023249:-127	Ypel1	PROMOTER	1.244	2.528	5306.44	13416.66	3.145	3892.40	12240.73
A_68_P24098211	chr5:131915292-131915336	NM_177047:1102899	Auts2	INSIDE	1.243	2.259	711.67	1607.87	2.809	697.07	1957.96
A_68_P31092908	chr17:23803677-23803721	NM_153784:6209	Ccdc64b	INSIDE	1.243	2.159	309.19	667.51	2.683	266.22	714.13
A_68_P29957984	chr14:122197004-122197048	NM_134074:-70	Dock9	PROMOTER	1.243	2.182	628.20	1370.81	2.712	531.98	1442.63
A_68_P23363269	chr4:140864959-140865003	NM_010139:7826	Epha2	INSIDE	1.243	4.222	3321.64	14025.22	5.249	2268.17	11906.38
A_68_P21315048	chr2:70499769-70499813	NM_027352:225	Gorasp2	INSIDE	1.243	5.489	1053.46	5782.87	6.822	936.99	6391.96
A_68_P27210043	chr10:66590012-66590056	NM_207221:29	Jmjd1c	INSIDE	1.243	1.880	720.26	1353.78	2.337	648.50	1515.27
A_68_P28744817	chr12:112812887-112812931	NM_021516:188	Mark3	INSIDE	1.243	2.508	904.56	2268.92	3.119	748.34	2333.79
A_68_P27281296	chr10:79612763-79612807	NM_021565:1750	Midn	INSIDE	1.243	3.371	2199.07	7414.03	4.191	1821.78	7635.18
A_68_P25025268	chr7:29871232-29871276	NR_035489:-2541	Mir1963	PROMOTER	1.243	2.310	386.85	893.59	2.871	287.15	824.27
A_68_P28055691	chr11:98188926-98188970	NM_010895:2011	Neurod2	INSIDE	1.243	1.630	673.71	1098.28	2.027	617.22	1250.96
A_68_P22509731	chr3:127336864-127336908	NM_009718:824	Neurog2	INSIDE	1.243	1.829	1273.66	2329.76	2.274	1064.78	2421.49
A_68_P23792520	chr5:72950217-72950261	NM_133921:646	Nfxl1	INSIDE	1.243	1.775	1114.16	1977.16	2.206	1059.70	2337.52
A_68_P22946060	chr4:57580871-57580915	NM_172868:-227	Palm2	PROMOTER	1.243	1.953	2016.46	3937.84	2.428	1923.99	4672.15
A_68_P22031102	chr3:30868233-30868277	NM_001165954:83	Phc3	INSIDE	1.243	1.598	2356.87	3767.43	1.987	1957.54	3889.76
A_68_P21351534	chr2:76485547-76485591	NM_011871:483	Prkra	INSIDE	1.243	1.991	753.34	1500.29	2.476	620.54	1536.70
A_68_P26826272	chr9:110760480-110760524	NM_146227:32	Prss50	INSIDE	1.243	1.802	1509.79	2720.03	2.239	1273.09	2850.40
A_68_P30213989	chr15:51823378-51823422	NM_009009:-94	Rad21	PROMOTER	1.243	2.424	512.24	1241.62	3.012	568.55	1712.35

ProbeName	Target position of probe on CpG island microarray	TargetID	GeneSymbol	CpG island Description	Ratio of relative methylation (TiO_2-NP/Vehicle)	Sham group			TiO_2-H group		
						Relative methylation (Cy5/Cy3)	Cy3 signal (Input)	Cy5 signal (MeDIP)	Relative methylation (Cy5/Cy3)	Cy3 signal (Input)	Cy5 signal (MeDIP)
A_68_P27695163	chr11:33413461-33413505	NM_023146:264	Ranbp17	INSIDE	1.243	1.689	724.83	1224.46	2.100	720.91	1514.26
A_68_P26240713	chr8:126177658-126177702	NM_133955:-148	Rhou	PROMOTER	1.243	1.957	2592.72	5073.24	2.432	2129.31	5178.14
A_68_P30489267	chr15:100876705-100876749	NM_011323:110025	Scn8a	DOWNSTREAM	1.243	1.454	952.86	1385.49	1.808	754.50	1363.75
A_68_P22829976	chr4:34136877-34136921	NM_026293:144	Spaca1	INSIDE	1.243	2.106	273.16	575.34	2.617	269.97	706.62
A_68_P23325212	chr4:134408532-134408576	NM_025382:706	Tmem57	INSIDE	1.243	1.885	1913.71	3606.48	2.343	1637.27	3836.66
A_68_P29478600	chr14:26427576-26427620	NM_183208:148928	Zmiz1	INSIDE	1.243	1.722	442.38	761.73	2.140	380.10	813.50
A_68_P20625946	chr1:135060523-135060567			Unknown	1.243	4.473	841.22	3762.62	5.561	676.78	3763.47
A_68_P25591880	chr7:149136294-149136338	NM_001009929:326	Brsk2	INSIDE	1.242	5.492	589.54	3237.99	6.822	507.97	3465.37
A_68_P24980672	chr7:16871856-16871900	NM_172297:-1870	Ccdc9	PROMOTER	1.242	2.206	5364.89	11837.58	2.740	3756.48	10293.89
A_68_P24052807	chr5:123349777-123349821	NM_013910:561	Kdm2b	INSIDE	1.242	3.035	237.11	719.64	3.768	237.92	896.55
A_68_P22601418	chr3:143865112-143865156	NM_001161770:162	Lmo4	INSIDE	1.242	1.792	3344.93	5992.95	2.226	2452.77	5459.35
A_68_P27931782	chr11:76212095-76212139	NM_008750:527	Nxn	INSIDE	1.242	1.396	2403.92	3356.63	1.734	2185.04	3789.94
A_68_P31958230	chr19:11943487-11943531	NM_001011775:2196	Olfr1419	DOWNSTREAM	1.242	2.053	903.37	1854.28	2.550	799.94	2039.68
A_68_P26587151	chr9:65194360-65194404	NM_016688:508	Pdcd7	INSIDE	1.242	4.360	439.00	1914.24	5.417	406.00	2199.31
A_68_P24206536	chr6:4697665-4697715	NM_001040611:385	Peg10	INSIDE	1.242	1.805	383.28	691.98	2.242	450.16	1009.31
A_68_P24949867	chr7:4610509-4610553	NM_172894:22	Ppp6r1	INSIDE	1.242	1.439	2039.58	2935.55	1.788	1631.68	2917.31
A_68_P29605620	chr14:52877501-52877545	NM_172601:21548	Rab2b	DOWNSTREAM	1.242	2.516	539.52	1357.42	3.125	467.97	1462.18
A_68_P27054670	chr10:33739429-33739473	NM_025614:-28	Rwdd1	PROMOTER	1.242	1.898	692.21	1314.01	2.358	694.41	1637.11
A_68_P30463773	chr15:96291502-96291546	NM_028148:-250	Scaf11	PROMOTER	1.242	1.863	1099.28	2047.92	2.314	971.70	2248.83
A_68_P32144298	chr19:46575400-46575444	NM_053100:-715	Trim8	PROMOTER	1.242	3.957	1474.95	5836.50	4.916	1317.92	6478.51
A_68_P30665016	chr16:34573818-34573862	ENSMUST00000114961:-222		PROMOTER	1.242	1.649	616.46	1016.40	2.048	500.75	1025.41
A_68_P32237967	chrX:6895968-6896012	ENSMUST00000132126:306		INSIDE	1.242	2.576	431.86	1112.67	3.199	303.68	971.44
A_68_P24686642	chr6:99471242-99471286	ENSMUST00000155466:145527		INSIDE	1.242	2.325	566.03	1315.96	2.888	436.63	1260.97
A_68_P28612730	chr12:88224807-88224851	NM_145836:936	6430527G18Rik	INSIDE	1.241	1.521	1897.55	2886.17	1.887	1505.53	2841.36
A_68_P27282652	chr10:79811999-79812043	NM_001113548:-829	Adamtsl5	DIVERGENT_PROMOTER	1.241	2.558	1695.30	4335.84	3.175	1465.16	4651.50
A_68_P30281416	chr15:64214267-64214311	NM_010026:193	Asap1	INSIDE	1.241	2.017	1051.04	2120.28	2.503	1004.74	2515.13
A_68_P25503172	chr7:134093232-134093276	NM_001039645:-159	Asphd1	DIVERGENT_PROMOTER	1.241	1.574	3098.78	4876.18	1.953	2367.27	4623.35
A_68_P25371475	chr7:108256912-108256956	NM_001146010:-354	Fchsd2	PROMOTER	1.241	2.611	1581.60	4129.11	3.239	1347.12	4362.84
A_68_P24501238	chr6:63207151-63207195	NM_008167:322	Grid2	INSIDE	1.241	1.559	899.11	1401.34	1.934	817.59	1580.99
A_68_P20897050	chr1:186542380-186542424	NM_008250:13970	Hlx	DOWNSTREAM	1.241	2.146	1253.58	2690.62	2.664	1034.80	2757.03
A_68_P20864309	chr1:180460129-180460173	NM_001161665:895	Kif26b	INSIDE	1.241	1.727	437.24	755.19	2.143	415.39	890.07
A_68_P21615602	chr2:127614486-127614530	NM_016902:82	Nphp1	INSIDE	1.241	1.532	1048.58	1606.01	1.901	976.74	1856.37
A_68_P22325404	chr3:90269294-90269338	NM_008727:472	Npr1	INSIDE	1.241	2.781	1212.93	3373.39	3.453	1057.74	3652.19
A_68_P28397148	chr12:45370156-45370200	NM_026164:38	Pnpla8	INSIDE	1.241	1.617	1845.78	2984.75	2.007	1449.14	2908.38
A_68_P24152257	chr5:143026729-143026773	NM_178702:281	Radil	INSIDE	1.241	2.493	1926.80	4803.14	3.093	1706.90	5279.37
A_68_P23618265	chr5:38611458-38611503	NM_029162:187	Zbtb49	INSIDE	1.241	1.465	1245.44	1824.82	1.818	1165.60	2119.63
A_68_P23588821	chr5:33773068-33773112	ENSMUST00000030994:754		DOWNSTREAM	1.241	1.561	1828.78	2855.12	1.938	1536.19	2977.50
A_68_P27339936	chr10:90545573-90545617	NM_001042558:-106	Apaf1	DIVERGENT_PROMOTER	1.240	2.561	670.67	1717.48	3.176	634.13	2013.89
A_68_P32233091	chrX:5660358-5660402	NM_001033211:158	AU022751	INSIDE	1.240	2.810	373.95	1050.64	3.484	219.06	763.29
A_68_P21324623	chr2:72124103-72124147	NM_178084:367	B230120H23Rik	INSIDE	1.240	1.622	433.08	702.37	2.011	419.95	844.59
A_68_P28183231	chr11:120096349-120096393	NM_198423:2110	Bahcc1	INSIDE	1.240	1.576	463.59	730.80	1.954	443.92	867.52
A_68_P23260770	chr4:122782660-122782704	NM_007559:275	Bmp8b	INSIDE	1.240	1.667	1952.32	3254.69	2.067	1560.75	3225.52
A_68_P29289426	chr13:105018966-105019010	NM_181061:-46	Cenpk	DIVERGENT_PROMOTER	1.240	2.914	265.54	773.76	3.613	266.41	962.60
A_68_P23609093	chr5:37087355-37087399	NM_001081232:-168	D5Ertd579e	PROMOTER	1.240	4.486	2650.67	11889.93	5.561	2185.60	12154.40
A_68_P28525540	chr12:72419394-72419438	NM_001190466:8546	Dact1	INSIDE	1.240	2.243	909.41	2040.06	2.781	713.15	1983.48
A_68_P28705232	chr12:105990506-105990550	NM_148948:-366	Dicer1	PROMOTER	1.240	1.712	1413.42	2420.17	2.123	1292.42	2744.31
A_68_P31694858	chr18:49992481-49992525	NM_001081371:-164	Dmxl1	PROMOTER	1.240	4.242	273.20	1158.83	5.261	260.32	1369.40
A_68_P21052793	chr2:20891228-20891272	NR_033225:750	Gm13375	INSIDE	1.240	1.514	1545.28	2339.43	1.878	1297.01	2435.35
A_68_P26220843	chr8:123060419-123060463	NM_001145896:-868	Gse1	PROMOTER	1.240	2.353	2561.25	6026.72	2.918	2050.43	5983.03
A_68_P28452663	chr12:56701421-56701465	NM_020287:1539	Insm2	INSIDE	1.240	1.746	2136.04	3729.95	2.166	1950.16	4223.07
A_68_P20925497	chr1:191167558-191167602	NM_001159850:-348	Kcnk2	PROMOTER	1.240	1.742	1493.38	2602.05	2.160	1321.58	2854.22

ProbeName	Target position of probe on CpG island microarray	TargetID	GeneSymbol	CpG island Description	Ratio of relative methylation (TiO_2-NP/Vehicle)	Sham group			TiO_2-H group		
						Relative methylation (Cy5/Cy3)	Cy3 signal (Input)	Cy5 signal (MeDIP)	Relative methylation (Cy5/Cy3)	Cy3 signal (Input)	Cy5 signal (MeDIP)
A_68_P26537382	chr9:56483766-56483810	NM_181074:49272	Lingo1	INSIDE	1.240	3.194	3619.85	11560.74	3.960	3342.47	13236.53
A_68_P24796449	chr6:119987179-119987223	NR_030487:-2871	Mir706	PROMOTER	1.240	2.561	1001.63	2564.95	3.175	901.57	2862.28
A_68_P27222783	chr10:68732909-68732953	NM_001081347:57526	Rhobtb1	INSIDE	1.240	1.956	1458.67	2853.35	2.426	1110.64	2694.65
A_68_P31383398	chr17:80879916-80879960	NM_009231:-145	Sos1	PROMOTER	1.240	1.703	1343.08	2287.37	2.111	1149.16	2425.95
A_68_P27915637	chr11:72990334-72990378	NM_001168470:154	Tmem93	INSIDE	1.240	2.253	981.87	2211.77	2.793	842.99	2354.21
A_68_P20023923	chr1:9738247-9738291	NM_173443:195	Vcpip1	INSIDE	1.240	0.494	1117.23	551.58	0.612	914.46	559.92
A_68_P27287685	chr10:80606918-80606962	NM_010731:7925	Zbtb7a	INSIDE	1.240	2.668	269.30	718.38	3.308	250.24	827.84
A_68_P26199435	chr8:119387454-119387498			Unknown	1.240	1.721	748.78	1288.47	2.133	648.06	1382.53
A_68_P23542162	chr5:23991041-23991085	NM_139153:33068	Agap3	INSIDE	1.239	1.481	1333.93	1975.65	1.836	1228.96	2255.86
A_68_P28025136	chr11:92960730-92960774	NM_028296:149	Car10	INSIDE	1.239	1.670	1646.14	2749.64	2.069	1290.25	2669.70
A_68_P22340619	chr3:94583028-94583072	NM_001037711:7387	Cgn	INSIDE	1.239	3.622	428.70	1552.64	4.489	360.99	1620.52
A_68_P28150128	chr11:114713294-114713338	NM_147217:-27	Gprc5c	PROMOTER	1.239	1.601	3772.47	6039.25	1.984	2980.61	5912.21
A_68_P29059426	chr13:56236410-56236454	NM_001159513:479	H2afy	INSIDE	1.239	1.965	697.29	1370.08	2.434	566.58	1378.97
A_68_P26142251	chr8:109460182-109460226	NM_009229:555	Sntb2	INSIDE	1.239	1.946	3526.37	6863.93	2.411	2992.61	7214.83
A_68_P24951724	chr7:4989811-4989856	NM_001039532:215	Zfp784	INSIDE	1.239	1.886	8734.42	16475.17	2.336	6341.85	14817.50
A_68_P29056514	chr13:55794315-55794359	NM_181278:-148	B230219D22Rik	PROMOTER	1.238	2.236	890.15	1990.52	2.767	799.64	2212.84
A_68_P27258520	chr10:75226966-75227010	NM_172549:114	Cabin1	INSIDE	1.238	0.516	1214.82	627.29	0.639	973.45	622.34
A_68_P31834477	chr18:75178883-75178927	NM_027727:479	Dym	INSIDE	1.238	4.146	1322.65	5483.98	5.131	1121.13	5752.82
A_68_P29928905	chr14:117325692-117325736	NM_001079844:1178	Gpc6	INSIDE	1.238	2.202	269.73	593.81	2.726	255.58	696.83
A_68_P20877961	chr1:182743984-182744028	NM_008210:-272	H3f3a	PROMOTER	1.238	2.192	1350.01	2958.78	2.714	1176.66	3193.29
A_68_P29097470	chr13:64263694-64263738	NM_019986:543	Habp4	INSIDE	1.238	0.435	1755.82	764.58	0.539	1625.97	876.25
A_68_P23540977	chr5:23830831-23830875	NM_013569:26570	Kcnh2	INSIDE	1.238	1.856	726.25	1347.81	2.298	508.36	1168.28
A_68_P20620520	chr1:134195429-134195473	NM_144810:248	Klhdc8a	INSIDE	1.238	1.485	630.48	936.52	1.839	596.56	1096.87
A_68_P31600354	chr18:32098983-32099027	NM_144862:7844	Lims2	INSIDE	1.238	3.081	745.99	2298.21	3.813	623.46	2377.42
A_68_P30004455	chr15:9070632-9070676	NM_177178:329	Lmbrd2	INSIDE	1.238	1.985	935.03	1855.86	2.457	836.88	2056.07
A_68_P23134366	chr4:97445300-97445344	NM_001122953:1006	Nfia	INSIDE	1.238	2.670	535.17	1429.03	3.307	462.23	1528.55
A_68_P21565421	chr2:118502775-118502819	NM_001145854:-185	Pak6	PROMOTER	1.238	1.450	3479.05	5043.21	1.795	2846.84	5110.26
A_68_P31023590	chr17:7182740-7182784	NM_026611:-446	Rnaset2b	PROMOTER	1.238	7.390	18851.93	139315.30	9.147	12639.12	115612.90
A_68_P30671369	chr16:35651290-35651334	NM_013661:109865	Sema5b	INSIDE	1.238	2.393	399.51	956.14	2.963	374.12	1108.49
A_68_P32149065	chr19:47388402-47388446	NM_001164717:150477	Sh3pxd2a	INSIDE	1.238	2.786	576.32	1605.88	3.451	449.39	1550.75
A_68_P27851930	chr11:61890326-61890370	NM_001029936:-250	Specc1	PROMOTER	1.238	1.892	6923.25	13101.79	2.342	5444.28	12750.40
A_68_P32136814	chr19:45230529-45230573	NM_021901:5346	Tlx1	INSIDE	1.238	0.447	1308.50	585.31	0.554	1010.63	559.58
A_68_P26292116	chr9:8544357-8544401	NM_013838:237	Trpc6	INSIDE	1.238	2.452	394.17	966.68	3.036	400.80	1216.79
A_68_P25715449	chr8:24318844-24318888	NM_018743:59	Agpat6	INSIDE	1.237	1.793	699.64	1254.42	2.218	634.63	1407.82
A_68_P29675826	chr14:66456589-66456633	NM_145944:472	Ccdc25	INSIDE	1.237	1.602	921.96	1477.40	1.983	914.05	1812.27
A_68_P28177060	chr11:119161422-119161466	NM_138669:-87	Eif4a3	PROMOTER	1.237	1.870	1394.98	2609.17	2.313	1155.28	2672.57
A_68_P23973616	chr5:109124200-109124244	NM_001164259:975	Fgfrl1	INSIDE	1.237	1.930	4703.58	9079.96	2.388	3704.93	8846.37
A_68_P21338950	chr2:74542960-74543004	NM_008276:-563	Hoxd8	PROMOTER	1.237	3.289	792.64	2606.66	4.067	771.79	3139.04
A_68_P21103437	chr2:30328220-30328264	NM_030244:1477	Ier5l	INSIDE	1.237	1.783	969.41	1728.04	2.206	873.63	1926.91
A_68_P26348964	chr9:21603129-21603173	NM_145611:-160	Kank2	PROMOTER	1.237	2.092	1105.14	2311.91	2.588	917.52	2374.59
A_68_P25022020	chr7:29244408-29244453	NM_001141921:7174	Lrfn1	INSIDE	1.237	2.756	999.80	2755.87	3.410	851.80	2904.46
A_68_P29226007	chr13:93614754-93614798	NM_001162945:35	Mtx3	INSIDE	1.237	5.397	1440.29	7772.75	6.675	1232.82	8229.37
A_68_P30458581	chr15:95359007-95359051	NM_016743:109	Nell2	INSIDE	1.237	0.414	1492.61	618.14	0.512	1266.69	648.80
A_68_P25370976	chr7:108160468-108160512	NM_008773:15	P2ry2	INSIDE	1.237	4.261	1849.73	7881.62	5.271	1535.93	8095.53
A_68_P30423565	chr15:88991328-88991372	NM_001159521:12930	Plxnb2	INSIDE	1.237	2.121	368.90	782.47	2.624	282.04	739.96
A_68_P20740069	chr1:157660136-157660180	NM_001024945:-129	Qsox1	PROMOTER	1.237	1.817	479.57	871.24	2.247	523.68	1176.62
A_68_P28956973	chr13:37949256-37949300	NM_001039188:30017	Rreb1	INSIDE	1.237	1.816	446.55	811.04	2.248	426.08	957.66
A_68_P28830403	chr13:12198675-12198719	NM_023868:516	Ryr2	INSIDE	1.237	2.275	854.64	1944.66	2.815	839.04	2362.14
A_68_P20945911	chr1:194681059-194681103	NM_001177794:-3139	Sertad4	PROMOTER	1.237	2.947	1351.92	3984.15	3.645	1205.19	4392.81
A_68_P26710064	chr9:88333383-88333427	NM_172926:385	Snx14	INSIDE	1.237	2.074	1749.58	3628.84	2.566	1420.74	3645.36
A_68_P26626138	chr9:71959964-71960008	NM_011544:-360	Tcf12	PROMOTER	1.237	2.531	2042.50	5168.92	3.132	1681.02	5264.45

ProbeName	Target position of probe on CpG island microarray	TargetID	GeneSymbol	CpG island Description	Ratio of relative methylation (TiO_2-NP/Vehicle)	Sham group			TiO_2-H group		
						Relative methylation (Cy5/Cy3)	Cy3 signal (Input)	Cy5 signal (MeDIP)	Relative methylation (Cy5/Cy3)	Cy3 signal (Input)	Cy5 signal (MeDIP)
A_68_P26544600	A_68_P26544600			Unknown	1.237	3.490	1101.16	3843.33	4.316	987.12	4260.49
A_68_P31215274	chr17:48112426-48112470			Unknown	1.237	1.731	789.53	1366.32	2.141	690.46	1478.42
A_68_P32670206	chrX:130593674-130593718	NM_133196:-191	Cstf2	PROMOTER	1.236	2.152	871.71	1875.76	2.660	632.77	1683.00
A_68_P24449035	chr6:52153690-52153734	NM_010453:874	Hoxa5	INSIDE	1.236	1.981	1294.73	2564.25	2.449	1072.42	2625.86
A_68_P28043567	chr11:96160575-96160619	NM_008269:112	Hoxb6	INSIDE	1.236	1.992	1521.36	3030.19	2.463	1248.37	3074.14
A_68_P26640632	chr9:74711201-74711245	NM_008262:1495	Onecut1	INSIDE	1.236	2.985	3357.89	10022.92	3.690	2778.40	10253.04
A_68_P20241610	chr1:55463210-55463254	NM_001114663:443	Plcl1	INSIDE	1.236	1.632	915.80	1494.27	2.016	850.74	1715.41
A_68_P31600225	chr18:32070729-32070773	NM_026006:807	Sft2d3	INSIDE	1.236	1.597	914.21	1460.16	1.973	872.48	1721.73
A_68_P27966237	chr11:82248208-82248252	NM_023438:45829	Tmem132e	INSIDE	1.236	3.714	1503.42	5583.86	4.591	1268.33	5822.68
A_68_P28261138	chr12:15822842-15822886	NM_144551:727	Trib2	INSIDE	1.236	3.276	625.97	2050.90	4.049	686.49	2779.48
A_68_P27910151	chr11:72020571-72020617	NM_026559:-461	Txndc17	DIVERGENT_PROMOTER	1.236	1.705	607.46	1035.89	2.108	526.85	1110.75
A_68_P26948225	chr10:12642644-12642688	NR_038027:259	B230208H11Rik	INSIDE	1.235	1.796	495.93	890.72	2.219	441.49	979.68
A_68_P28153095	chr11:115228977-115229021	NM_175454:35	C630004H02Rik	INSIDE	1.235	3.505	877.03	3073.74	4.329	1029.77	4457.52
A_68_P28170165	chr11:118110109-118110153	NM_001112699:-224	Cyth1	PROMOTER	1.235	2.764	1221.01	3375.34	3.413	997.16	3403.27
A_68_P30590962	chr16:21204783-21204827	NM_010143:-63	Ephb3	PROMOTER	1.235	3.268	710.78	2322.96	4.035	565.11	2280.23
A_68_P29996508	chr15:7763780-7763824	NM_010275:2792	Gdnf	INSIDE	1.235	1.708	684.74	1169.37	2.108	599.98	1265.04
A_68_P32767190	chrX:154036239-154036283	NM_172307:387	Mbtps2	INSIDE	1.235	1.745	631.98	1102.73	2.155	329.06	709.18
A_68_P28052184	chr11:97561645-97561689	NM_001163308:-704	Pcgf2	PROMOTER	1.235	1.718	1571.01	2698.74	2.122	1284.56	2726.03
A_68_P28055914	chr11:98219976-98220020	NM_021547:301	Stard3	INSIDE	1.235	1.966	797.08	1566.71	2.428	635.47	1542.91
A_68_P23317937	chr4:133054043-133054087	NM_001081156:401	Trnp1	INSIDE	1.235	3.366	947.42	3188.70	4.157	842.58	3502.95
A_68_P24874652	chr6:136776509-136776553	NM_021714:207	Wbp11	INSIDE	1.235	1.527	3699.15	5646.81	1.884	2809.48	5294.46
A_68_P25600929	chr7:150624243-150624287			Unknown	1.235	3.584	722.05	2587.69	4.426	654.92	2898.72
A_68_P21565885	chr2:118571382-118571426	NR_030716:-93	5430417L22Rik	PROMOTER	1.234	1.916	1461.77	2800.90	2.364	1291.48	3053.51
A_68_P27033510	chr10:28867430-28867474	NM_026138:3651	6330407J23Rik	INSIDE	1.234	1.641	1994.17	3273.28	2.025	1639.25	3319.61
A_68_P24469556	chr6:55402442-55402486	NM_001025372:491	Adcyap1r1	INSIDE	1.234	1.418	837.79	1187.83	1.749	722.95	1264.50
A_68_P29342444	chr13:114585071-114585115	NM_172595:377	Arl15	INSIDE	1.234	1.500	3228.18	4840.67	1.850	2735.40	5061.43
A_68_P21324621	chr2:72123755-72123799	NM_178084:19	B230120H23Rik	INSIDE	1.234	1.529	797.45	1219.18	1.886	690.20	1301.67
A_68_P27100735	chr10:43231159-43231203	NM_199028:32235	Bend3	INSIDE	1.234	2.682	750.18	2012.34	3.311	643.42	2130.22
A_68_P21040743	chr2:18593952-18593996	NM_147778:-114	Commd3	PROMOTER	1.234	1.603	2488.35	3988.75	1.978	2335.94	4621.12
A_68_P27286404	chr10:80392789-80392833	NM_008655:-25	Gadd45b	PROMOTER	1.234	1.669	2540.30	4238.98	2.059	2173.09	4473.86
A_68_P27794864	chr11:51419555-51419599	NM_001048061:807	Hnrnpab	INSIDE	1.234	1.411	1693.90	2390.86	1.741	1472.33	2563.46
A_68_P20241001	chr1:55294058-55294102	NM_175439:60	Mars2	INSIDE	1.234	1.581	695.13	1099.14	1.951	638.40	1245.25
A_68_P21240022	chr2:56969332-56969376	NM_013613:-1905	Nr4a2	PROMOTER	1.234	2.574	1506.44	3877.61	3.175	1177.32	3738.28
A_68_P20937889	chr1:193221406-193221450	NM_144880:-508	Ppp2r5a	PROMOTER	1.234	2.008	1221.32	2452.48	2.478	1000.57	2479.18
A_68_P20793343	chr1:167124694-167124739	NM_145512:-152	Sft2d2	PROMOTER	1.234	2.186	836.65	1829.18	2.699	732.23	1975.97
A_68_P21820991	chr2:164823054-164823098	NM_020333:29589	Slc12a5	INSIDE	1.234	1.746	1633.82	2852.86	2.156	1806.18	3893.38
A_68_P32196168	chr19:55816042-55816086	NM_001142918:-235	Tcf7l2	PROMOTER	1.234	1.752	1637.11	2867.52	2.161	1401.68	3029.05
A_68_P29733543	chr14:76814437-76814481	NM_207652:-1169	Tsc22d1	PROMOTER	1.234	1.886	1653.09	3117.66	2.327	1401.61	3261.57
A_68_P31982218	chr19:16855459-16855503	NM_173028:-63	Vps13a	PROMOTER	1.234	3.153	470.62	1483.96	3.892	432.33	1682.66
A_68_P24954137	chr7:6083887-6083931	NM_001013012:23665	Zfp787	INSIDE	1.234	3.739	3994.65	14935.73	4.614	3130.49	14442.68
A_68_P20162421	chr1:38892803-38892847	ENSMUST00000147695:111		INSIDE	1.234	2.999	484.34	1452.42	3.700	522.16	1931.96
A_68_P23268972	chr4:124252405-124252449	ENSMUST00000164308:5994		INSIDE	1.234	1.647	863.46	1421.78	2.033	734.34	1492.62
A_68_P25713873	chr8:24085890-24085934	NM_001110783:559	Ank1	INSIDE	1.233	1.576	1016.08	1601.02	1.943	838.45	1629.00
A_68_P21080072	chr2:26484180-26484224	NM_019833:226	Fam69b	INSIDE	1.233	3.820	381.45	1457.15	4.709	331.17	1559.50
A_68_P28043916	chr11:96202147-96202192	NM_010458:-2913	Hoxb3	PROMOTER	1.233	1.393	1248.94	1739.30	1.717	1151.69	1976.87
A_68_P22080609	chr3:40439835-40439879	NM_175515:168	Intu	INSIDE	1.233	2.460	373.11	917.90	3.034	356.07	1080.28
A_68_P31100484	chr17:25073844-25073888	NM_001163447:56	Mapk8ip3	INSIDE	1.233	1.565	808.90	1266.33	1.931	789.98	1525.30
A_68_P22357697	chr3:97817451-97817495	NM_010928:12	Notch2	INSIDE	1.233	2.887	1140.75	3293.65	3.560	1010.67	3598.29
A_68_P25601377	chr7:150688210-150688254	NM_009434:197	Phlda2	INSIDE	1.233	1.626	608.67	989.95	2.006	542.58	1088.51
A_68_P31599575	chr18:31948663-31948707	NM_027002:-128	Polr2d	PROMOTER	1.233	2.408	4034.10	9714.80	2.969	3275.83	9724.90
A_68_P30477989	chr15:98886157-98886201	NM_001163588:574	Prph	INSIDE	1.233	1.503	1733.54	2605.56	1.853	1555.19	2881.91

ProbeName	Target position of probe on CpG island microarray	TargetID	GeneSymbol	CpG island Description	Ratio of relative methylation (TiO_2-NP/Vehicle)	Sham group			TiO_2-H group		
						Relative methylation (Cy5/Cy3)	Cy3 signal (Input)	Cy5 signal (MeDIP)	Relative methylation (Cy5/Cy3)	Cy3 signal (Input)	Cy5 signal (MeDIP)
A_68_P28042231	chr11:95926737-95926781	NM_172300:-80	Ube2z	PROMOTER	1.233	2.073	281.69	584.05	2.556	392.09	1002.33
A_68_P27947946	chr11:79068163-79068207	NM_019653:13	Wsb1	INSIDE	1.233	2.154	2856.62	6154.39	2.657	2088.05	5547.64
A_68_P21067995	chr2:24618544-24618589	NM_001042528:1	Cacna1b	INSIDE	1.232	1.428	1148.66	1640.72	1.759	1047.72	1843.10
A_68_P32111328	chr19:40905903-40905947	NM_172839:156	Ccnj	INSIDE	1.232	2.153	771.12	1660.53	2.652	689.25	1828.14
A_68_P23448647	chr5:4104421-4104465	NM_020010:255	Cyp51	INSIDE	1.232	2.400	1632.82	3918.57	2.957	1271.28	3759.14
A_68_P22288625	chr3:83843924-83843968	NM_172681:137	D930015E06Rik	INSIDE	1.232	1.536	1161.00	1783.82	1.893	946.27	1791.52
A_68_P23081271	chr4:86394332-86394376	NM_001081014:-104	Dennd4c	PROMOTER	1.232	1.610	1405.26	2262.23	1.983	1205.91	2391.42
A_68_P23142413	chr4:98786997-98787041	NM_026082:588	Dock7	INSIDE	1.232	2.294	716.58	1644.00	2.826	638.27	1804.07
A_68_P21113255	chr2:31944229-31944273	NM_175511:-5025	Fam78a	DIVERGENT_PROMOTER	1.232	1.563	852.24	1331.80	1.925	804.68	1548.84
A_68_P26458699	chr9:42752539-42752583	NM_175481:-106	Grik4	PROMOTER	1.232	2.405	357.87	860.78	2.963	360.24	1067.29
A_68_P24868562	chr6:135682934-135682978	NM_008171:440573	Grin2b	INSIDE	1.232	1.918	500.98	960.81	2.363	403.88	954.55
A_68_P32152395	chr19:47939790-47939835	NM_030051:-222	Gsto2	PROMOTER	1.232	3.284	741.51	2434.96	4.047	623.46	2523.27
A_68_P32095551	chr19:37509175-37509219	NM_008245:-134	Hhex	PROMOTER	1.232	1.399	1105.66	1546.39	1.723	1015.80	1750.62
A_68_P30477336	chr15:98748107-98748151	NM_029098:401	Lmbr1l	INSIDE	1.232	2.239	1327.10	2971.37	2.758	1218.06	3359.49
A_68_P23896966	chr5:93370979-93371023	NM_001077596:43977	Shroom3	INSIDE	1.232	3.027	621.66	1881.46	3.729	603.54	2250.80
A_68_P26031095	chr8:89269939-89269983	NM_009172:-55	Siah1a	PROMOTER	1.232	2.392	492.98	1179.08	2.946	436.18	1284.95
A_68_P27365031	chr10:94878605-94878649	NM_001168656:-508	Socs2	PROMOTER	1.232	1.955	1850.53	3617.23	2.409	1495.91	3602.94
A_68_P28745892	A_68_P28745892			Unknown	1.232	4.201	312.61	1313.32	5.178	301.70	1562.09
A_68_P21594122	chr2:123915605-123915649	ENSMUST00000137172:-78		PROMOTER	1.232	1.652	1954.46	3229.10	2.036	1701.34	3463.88
A_68_P31619430	chr18:35813598-35813642	NM_027222:-4599	2010001M09Rik	PROMOTER	1.231	2.219	312.19	692.64	2.732	275.04	751.27
A_68_P24430829	chr6:49023914-49023958	NM_029353:143	2410003K15Rik	INSIDE	1.231	1.648	426.66	703.25	2.029	398.52	808.62
A_68_P25952414	chr8:73046706-73046750	NM_029366:351	2810422J05Rik	INSIDE	1.231	1.634	3089.90	5050.44	2.013	2292.98	4615.03
A_68_P27282604	chr10:79805724-79805768	NM_025629:5411	Adamtsl5	INSIDE	1.231	1.712	736.33	1260.42	2.107	684.01	1441.16
A_68_P26741570	chr9:95307111-95307155	NM_018763:557	Chst2	INSIDE	1.231	1.791	453.81	812.60	2.204	372.06	820.11
A_68_P27475788	chr10:115911221-115911265	NM_021452:-663	Kcnmb4	PROMOTER	1.231	3.008	1683.22	5062.79	3.704	1542.11	5711.26
A_68_P25022022	chr7:29244663-29244707	NM_001141921:7428	Lrfn1	INSIDE	1.231	2.420	534.11	1292.35	2.979	495.36	1475.77
A_68_P21822913	chr2:165113069-165113113	NM_144893:237	Slc35c2	INSIDE	1.231	0.446	1241.72	553.31	0.549	946.93	519.56
A_68_P24820215	chr6:125311848-125311892	NM_011609:12130	Tnfrsf1a	INSIDE	1.231	2.922	753.59	2201.62	3.597	607.65	2185.54
A_68_P24951680	chr7:4982714-4982758	NM_001033383:10759	Zfp865	INSIDE	1.231	2.091	297.20	621.40	2.573	274.77	706.99
A_68_P21797522	chr2:160698309-160698353	NM_177263:396	Zhx3	INSIDE	1.231	1.339	3465.47	4639.79	1.648	2675.65	4409.78
A_68_P21833422	chr2:166888209-166888253	NM_001081005:-203	1500012F01Rik	PROMOTER	1.230	2.330	544.13	1267.81	2.866	487.60	1397.67
A_68_P30468157	chr15:97078001-97078045	NM_001164563:-304	Amigo2	PROMOTER	1.230	1.493	1005.64	1501.01	1.835	1008.90	1851.63
A_68_P30502976	chr15:103070313-103070357	NM_001076789:-87	Cbx5	DIVERGENT_PROMOTER	1.230	0.450	1455.01	655.15	0.554	1164.57	645.05
A_68_P31157311	chr17:35195952-35195996	NM_001190449:-5	Ddah2	PROMOTER	1.230	1.507	3276.84	4938.01	1.854	2586.63	4794.62
A_68_P29361885	chr13:118009364-118009408	NM_010330:7	Emb	INSIDE	1.230	2.294	840.00	1927.26	2.822	735.72	2076.20
A_68_P28171532	chr11:118338109-118338153	NM_172573:-143	Engase	PROMOTER	1.230	1.412	1846.85	2607.24	1.736	1476.00	2562.76
A_68_P22071607	chr3:38786088-38786133	NM_183221:249	Fat4	INSIDE	1.230	2.228	694.26	1546.83	2.740	525.85	1440.62
A_68_P25007848	chr7:25795874-25795918	NM_008168:61492	Grik5	INSIDE	1.230	2.823	3495.69	9868.19	3.473	2584.16	8974.15
A_68_P25196753	chr7:75097151-75097195	NM_010513:30	Igf1r	INSIDE	1.230	4.008	1503.59	6027.14	4.931	1262.54	6225.99
A_68_P29952578	chr14:121310982-121311026	NM_023579:589	Ipo5	INSIDE	1.230	1.677	963.91	1616.15	2.062	792.05	1633.04
A_68_P28271091	chr12:17551967-17552011	NM_013614:310	Odc1	INSIDE	1.230	2.162	2582.31	5583.53	2.660	2122.93	5647.21
A_68_P31066146	chr17:15663980-15664024	NM_008799:263	Pdcd2	INSIDE	1.230	2.695	857.19	2310.36	3.316	750.54	2488.78
A_68_P30578589	chr16:18248400-18248444	NM_011239:365	Ranbp1	INSIDE	1.230	1.705	2260.66	3854.55	2.097	1923.63	4034.56
A_68_P21706316	chr2:144096174-144096218	NR_030762:-4258	Snord17	PROMOTER	1.230	2.303	904.99	2083.99	2.832	801.34	2269.41
A_68_P26455637	chr9:42280374-42280418	NM_173038:-87	Tbcel	PROMOTER	1.230	1.651	824.04	1360.52	2.031	733.44	1489.33
A_68_P27900651	chr11:70245942-70245986	NM_145429:-190	Arrb2	PROMOTER	1.229	1.546	1553.06	2400.75	1.900	1488.33	2827.45
A_68_P21285104	chr2:65076845-65076889	NM_027225:-183	Cobll1	PROMOTER	1.229	0.412	1598.81	658.86	0.506	1269.58	642.91
A_68_P27183508	chr10:61441806-61441850	NM_007731:28	Col13a1	INSIDE	1.229	1.704	2705.65	4610.97	2.094	2021.23	4232.27
A_68_P24620288	chr6:88140437-88140481	NM_008090:-8199	Gata2	PROMOTER	1.229	3.065	3155.55	9672.95	3.768	2633.69	9923.04
A_68_P31624332	chr18:36675376-36675420	NM_010415:61	Hbegf	INSIDE	1.229	2.145	1199.99	2573.73	2.637	1439.23	3794.92
A_68_P20228742	chr1:52784064-52784108	NM_133829:76	Mfsd6	INSIDE	1.229	1.361	2780.57	3783.89	1.673	2266.05	3790.85

ProbeName	Target position of probe on CpG island microarray	TargetID	GeneSymbol	CpG island Description	Ratio of relative methylation (TiO_2-NP/Vehicle)	Sham group			TiO_2-H group		
						Relative methylation (Cy5/Cy3)	Cy3 signal (Input)	Cy5 signal (MeDIP)	Relative methylation (Cy5/Cy3)	Cy3 signal (Input)	Cy5 signal (MeDIP)
A_68_P30368847	chr15:79664630-79664674	NM_001013360:111	Npcd	INSIDE	1.229	1.521	3801.75	5782.91	1.869	3069.43	5738.13
A_68_P30961945	chr16:91270311-91270355	NM_016968:319	Olig1	INSIDE	1.229	1.423	866.91	1233.81	1.749	745.77	1304.37
A_68_P25730820	chr8:27229765-27229809	NM_029965:-65	Rnf170	DIVERGENT_PROMOTER	1.229	1.842	464.17	854.77	2.263	362.99	821.45
A_68_P24630040	chr6:89785772-89785816	NM_053221:9737	Vmn1r42	DOWNSTREAM	1.229	2.967	1374.81	4078.92	3.646	1256.51	4581.76
A_68_P27568222	chr11:6289791-6289835	NM_001005867:-5315	Zmiz2	PROMOTER	1.229	2.068	685.07	1416.68	2.541	569.82	1447.76
A_68_P20718903	chr1:153936776-153936820	NM_197990:652	1700025G04Rik	INSIDE	1.228	2.036	4522.29	9208.00	2.501	3098.69	7750.10
A_68_P22905957	chr4:49610300-49610344	NM_025944:420	2810432L12Rik	INSIDE	1.228	1.681	1179.40	1982.04	2.063	985.94	2033.96
A_68_P22151288	chr3:54859609-54859653	NM_007628:-653	Ccna1	PROMOTER	1.228	2.400	1122.33	2693.33	2.947	1034.44	3048.09
A_68_P20910365	chr1:188791961-188792005	NM_033077:688	D1Pas1	INSIDE	1.228	3.475	1136.98	3950.91	4.268	881.33	3761.77
A_68_P24133866	chr5:139626612-139626656	NM_001081265:458	Heatr2	INSIDE	1.228	2.083	766.79	1596.99	2.557	608.20	1555.28
A_68_P25031211	chr7:31145626-31145670	NM_175478:2143	Lrfn3	INSIDE	1.228	2.437	1319.81	3216.65	2.993	1079.76	3231.79
A_68_P30343816	chr15:75578159-75578203	NM_194350:172	Mafa	INSIDE	1.228	2.750	2383.80	6555.74	3.378	2098.13	7087.88
A_68_P28671530	chr12:99927621-99927665	NM_011877:45213	Ptpn21	INSIDE	1.228	3.152	2204.42	6948.39	3.870	1444.78	5591.59
A_68_P22826130	chr4:33397227-33397271	NM_011884:-37	Rngtt	PROMOTER	1.228	3.959	1538.77	6091.71	4.862	1236.26	6010.36
A_68_P22286846	chr3:83570454-83570498	NM_009144:234	Sfrp2	INSIDE	1.228	1.617	940.06	1520.06	1.986	795.89	1580.52
A_68_P28781896	chr12:120087840-120087884	NM_177082:3061	Sp8	INSIDE	1.228	3.215	1362.52	4380.64	3.949	1219.70	4816.19
A_68_P27787326	chr11:50024305-50024349	NM_011018:-34	Sqstm1	PROMOTER	1.228	2.761	550.75	1520.66	3.390	434.71	1473.52
A_68_P21864909	chr2:172375260-172375304	NM_001159696:-1208	Tcfap2c	PROMOTER	1.228	2.180	691.57	1507.33	2.676	597.23	1597.91
A_68_P21610504	chr2:126701750-126701794	NM_001164325:225	Trpm7	INSIDE	1.228	2.123	1630.06	3460.12	2.607	1496.67	3902.35
A_68_P29604340	chr14:52639614-52639658	NM_027248:772	Zfp219	INSIDE	1.228	1.544	499.19	770.83	1.897	451.01	855.39
A_68_P25098516	chr7:53604272-53604316	AK015435:95		INSIDE	1.228	2.251	1736.49	3908.75	2.764	1378.33	3809.61
A_68_P30462687	chr15:96117084-96117128	NM_175251:-846	Arid2	PROMOTER	1.227	1.901	933.88	1775.24	2.333	872.69	2035.94
A_68_P32071736	chr19:32786770-32786814	NM_026487:-4	Atad1	PROMOTER	1.227	0.522	1477.87	770.94	0.640	1242.71	795.14
A_68_P25503356	chr7:134119822-134119866	NM_026638:-18	Cdipt	PROMOTER	1.227	1.884	912.21	1718.37	2.312	756.91	1749.82
A_68_P22954049	chr4:59016297-59016341	NM_001033165:254	Dnajc25	INSIDE	1.227	2.696	701.18	1890.34	3.308	642.03	2123.55
A_68_P27003034	chr10:23070344-23070388	NM_010167:-657	Eya4	PROMOTER	1.227	1.943	1594.66	3098.56	2.384	1430.38	3410.50
A_68_P22369563	chr3:100293548-100293593	NM_001142952:-455	Fam46c	PROMOTER	1.227	1.472	667.29	981.98	1.806	519.22	937.91
A_68_P25504682	chr7:134376720-134376764	NR_030674:-98	Gm4532	DIVERGENT_PROMOTER	1.227	2.153	993.33	2138.67	2.642	824.46	2178.34
A_68_P29982185	chr15:5093533-5093577	NM_001013367:-306	Prkaa1	PROMOTER	1.227	2.084	601.67	1254.00	2.558	518.63	1326.80
A_68_P30463770	chr15:96290889-96290933	NM_028148:364	Scaf11	INSIDE	1.227	2.041	1279.57	2611.67	2.503	1313.24	3287.52
A_68_P30348909	chr15:76354254-76354298	NM_130893:-1717	Scrt1	PROMOTER	1.227	2.435	854.93	2081.43	2.987	702.24	2097.31
A_68_P23403484	chr4:149329133-149329178	NM_029035:-40	Spsb1	PROMOTER	1.227	1.543	1454.34	2244.66	1.894	1259.78	2386.46
A_68_P22323362	chr3:89884192-89884236	NM_022314:7599	Tpm3	INSIDE	1.227	2.084	4837.23	10081.20	2.556	3971.26	10152.08
A_68_P31301680	chr17:65962034-65962078	NM_013933:839	Vapa	INSIDE	1.227	1.753	516.65	905.61	2.151	511.07	1099.12
A_68_P24049903	chr5:122804592-122804636	NM_019780:193	Vps29	INSIDE	1.227	1.577	1546.24	2438.78	1.936	1363.23	2638.68
A_68_P23195376	chr4:107973405-107973449	NM_001033634:269	Zyg11b	INSIDE	1.227	1.496	4893.35	7321.19	1.835	3773.54	6924.85
A_68_P22322663	chr3:89764566-89764610	NM_001081182:2842	Atp8b2	INSIDE	1.226	2.640	457.10	1206.63	3.236	449.16	1453.58
A_68_P27903678	chr11:70796278-70796322	NM_007573:228	C1qbp	INSIDE	1.226	2.295	1922.10	4410.66	2.814	1771.75	4986.10
A_68_P20166302	chr1:39593044-39593088	NM_028043:420	D1Bwg0212e	INSIDE	1.226	2.595	718.46	1864.56	3.183	706.12	2247.34
A_68_P23365990	chr4:141346639-141346683	NM_172338:-101	Dnajc16	DIVERGENT_PROMOTER	1.226	1.835	1744.87	3201.47	2.250	1531.30	3444.89
A_68_P23613128	chr5:37729744-37729788	NM_145920:50	Evc2	INSIDE	1.226	2.168	750.76	1627.35	2.657	585.43	1555.35
A_68_P23927135	chr5:100407424-100407468	NM_001077265:511	Hnrnpd	INSIDE	1.226	0.432	1994.52	862.44	0.530	1578.39	836.56
A_68_P30342689	chr15:75396844-75396888	NM_001135689:212	Ly6h	INSIDE	1.226	2.454	1590.39	3903.43	3.008	1369.01	4118.41
A_68_P27500270	chr10:120335605-120335649	NM_177092:401	Msrb3	INSIDE	1.226	2.705	1388.11	3755.11	3.316	1316.25	4365.29
A_68_P28514079	chr12:70458006-70458050	NM_025441:125	Nemf	INSIDE	1.226	2.550	1071.88	2733.09	3.127	986.98	3086.25
A_68_P31867986	chr18:81168668-81168712	NM_178280:14627	Sall3	INSIDE	1.226	2.009	721.32	1449.13	2.462	616.64	1518.47
A_68_P32672211	chrX:131010176-131010220	NM_028958:831	Taf7l	INSIDE	1.226	2.102	3097.66	6512.06	2.577	1611.16	4151.38
A_68_P27507137	chr10:121534278-121534322	NM_153059:-142	Tmem5	PROMOTER	1.226	2.309	2895.69	6687.54	2.832	2106.24	5964.45
A_68_P27287684	chr10:80606810-80606854	NM_010731:7817	Zbtb7a	INSIDE	1.226	1.856	369.15	684.96	2.275	346.56	788.41
A_68_P28050434	chr11:97277178-97277222	ENSMUST00000121799:354		INSIDE	1.226	1.575	872.23	1374.09	1.932	737.13	1424.27
A_68_P21138537	chr2:36066471-36066515	ENSMUST00000128242:201		INSIDE	1.226	1.921	686.54	1319.17	2.356	618.62	1457.47

ProbeName	Target position of probe on CpG island microarray	TargetID	GeneSymbol	CpG island Description	Ratio of relative methylation (TiO_2-NP/Vehicle)	Sham group			TiO_2-H group		
						Relative methylation (Cy5/Cy3)	Cy3 signal (Input)	Cy5 signal (MeDIP)	Relative methylation (Cy5/Cy3)	Cy3 signal (Input)	Cy5 signal (MeDIP)
A_68_P28183237	chr11:120096983-120097028	NM_198423:2745	Bahcc1	INSIDE	1.225	2.444	219.70	536.84	2.992	196.95	589.36
A_68_P28160638	chr11:116515525-116515569	NM_030206:81	Cygb	INSIDE	1.225	1.769	2344.97	4147.39	2.167	1835.19	3976.36
A_68_P27992419	chr11:86887561-86887605	NM_025638:57	Gdpd1	INSIDE	1.225	2.144	824.01	1766.68	2.627	735.40	1932.04
A_68_P31929500	chr19:5567652-5567696	NM_001033448:-399	Gm962	DIVERGENT_PROMOTER	1.225	1.689	1417.64	2394.41	2.069	1199.60	2482.25
A_68_P30540355	chr16:9994229-9994273	NM_008170:-1624	Grin2a	PROMOTER	1.225	1.693	1423.74	2410.40	2.074	1225.16	2540.53
A_68_P29247522	chr13:97440489-97440533	NM_008255:381	Hmgcr	INSIDE	1.225	1.577	1808.36	2852.06	1.932	1518.68	2933.52
A_68_P28997661	chr13:44826453-44826497	NM_001205043:-165	Jarid2	PROMOTER	1.225	1.839	1510.37	2777.67	2.252	1365.55	3075.17
A_68_P32218824	chr19:59420634-59420678	NM_001033222:-386	Pdzd8	PROMOTER	1.225	1.969	962.38	1895.01	2.412	839.52	2025.30
A_68_P29479231	chr14:26513717-26513761	NM_134084:83	Ppif	INSIDE	1.225	1.353	3010.47	4073.36	1.658	2550.60	4228.16
A_68_P24092504	chr5:130647587-130647631	NM_001199059:-80	Rabgef1	PROMOTER	1.225	2.954	1188.45	3510.89	3.618	968.47	3504.16
A_68_P21920095	chr3:5219581-5219625	NM_030708:1049	Zfhx4	INSIDE	1.225	1.664	914.63	1521.85	2.039	800.95	1632.75
A_68_P20872225	chr1:181733706-181733750	NM_026375:418	Ahctf1	INSIDE	1.224	1.839	948.71	1744.62	2.251	850.17	1914.11
A_68_P28576088	chr12:81620372-81620416	NM_001081421:418	Galntl1	INSIDE	1.224	2.315	1570.96	3637.15	2.835	1387.99	3934.54
A_68_P20432981	chr1:91825501-91825545	NM_010262:2229	Gbx2	INSIDE	1.224	1.784	1245.97	2222.38	2.183	1048.73	2289.06
A_68_P30659188	chr16:33684818-33684862	NM_175256:289	Heg1	INSIDE	1.224	1.761	634.91	1117.88	2.156	557.05	1200.85
A_68_P31834167	chr18:75120830-75120874	NM_010720:65	Lipg	INSIDE	1.224	1.785	2507.39	4475.37	2.185	2116.70	4625.96
A_68_P31099061	chr17:24833259-24833303	NM_027988:102	Noxo1	INSIDE	1.224	1.704	3566.40	6076.17	2.085	2770.54	5776.03
A_68_P20354546	chr1:75502170-75502214	NM_178884:835	Obsl1	INSIDE	1.224	1.496	1182.11	1767.89	1.830	1036.25	1896.50
A_68_P29618379	chr14:56159407-56159453	NM_028994:328	Pck2	INSIDE	1.224	2.157	292.61	631.02	2.640	313.77	828.25
A_68_P21250387	chr2:58999594-58999638	NM_026361:710	Pkp4	INSIDE	1.224	3.545	527.38	1869.54	4.339	529.44	2297.29
A_68_P28746794	chr12:113146363-113146407	NM_011625:-118	Ppp1r13b	PROMOTER	1.224	2.010	433.82	871.86	2.460	428.59	1054.41
A_68_P31106620	chr17:25974088-25974132	NM_144816:-38	Rhbdl1	PROMOTER	1.224	2.405	2457.35	5909.73	2.944	1910.25	5623.49
A_68_P23699213	chr5:53604822-53604866	NM_172710:-153	Sel1l3	PROMOTER	1.224	1.517	2365.84	3590.14	1.858	2017.93	3748.93
A_68_P23440102	chr4:155079253-155079297	NM_026899:351	Ssu72	INSIDE	1.224	4.098	850.86	3486.75	5.017	726.50	3644.95
A_68_P20998558	chr2:9969914-9969958	NM_027748:300	Taf3	INSIDE	1.224	1.790	1109.09	1985.04	2.191	884.61	1938.57
A_68_P28176626	chr11:119089661-119089705	NM_172443:131	Tbc1d16	INSIDE	1.224	2.327	1982.20	4612.60	2.849	1677.17	4778.38
A_68_P31213969	chr17:47875277-47875321	NM_001161723:419	Tcfeb	INSIDE	1.224	2.946	345.45	1017.55	3.607	288.05	1038.93
A_68_P31386730	chr17:81464149-81464193	NM_028138:255	Thumpd2	INSIDE	1.224	1.716	1027.58	1763.14	2.100	991.87	2082.99
A_68_P30963823	chr16:91597831-91597875	NM_030018:73	Tmem50b	INSIDE	1.224	1.669	1208.84	2017.90	2.043	1008.54	2060.89
A_68_P26644964	chr9:75459172-75459216	NM_016711:-62	Tmod2	PROMOTER	1.224	2.097	2242.67	4703.79	2.567	1736.67	4458.86
A_68_P31542075	chr18:21054261-21054305	NM_177038:297	Trappc8	INSIDE	1.224	1.757	687.06	1207.20	2.151	636.80	1369.72
A_68_P20551186	chr1:120207831-120207875	NM_011650:-143	Tsn	PROMOTER	1.224	1.743	552.94	963.58	2.132	480.37	1024.28
A_68_P22630557	chr3:148650752-148650796	ENSMUST00000098518:1506		INSIDE	1.224	1.768	9995.66	17669.51	2.163	6813.05	14739.70
A_68_P30671880	chr16:35735579-35735623	NM_153550:33842	Dirc2	INSIDE	1.223	2.148	1465.32	3147.91	2.626	1190.41	3126.37
A_68_P30981934	chr16:94748127-94748171	NM_007834:88	Dscr3	INSIDE	1.223	1.877	1063.38	1995.83	2.295	956.24	2194.75
A_68_P21585581	chr2:122124764-122124808	NM_025777:151	Duoxa2	INSIDE	1.223	14.281	862.74	12320.62	17.466	674.94	11788.86
A_68_P24142816	chr5:141178207-141178251	NM_028833:104	Iqce	INSIDE	1.223	2.460	793.10	1950.80	3.009	623.60	1876.61
A_68_P24112797	chr5:135164410-135164454	NM_010717:28	Limk1	INSIDE	1.223	3.946	1189.66	4694.60	4.826	1031.39	4977.17
A_68_P23588234	chr5:33678349-33678393	NM_021500:150	Maea	INSIDE	1.223	2.187	3783.12	8272.60	2.674	3137.32	8388.58
A_68_P29116722	chr13:69750305-69750349	NM_175283:15	Srd5a1	INSIDE	1.223	1.700	2550.30	4335.32	2.079	1885.09	3918.71
A_68_P21579355	chr2:120997099-120997143	NM_153387:179	Tubgcp4	INSIDE	1.223	1.905	1073.76	2045.52	2.330	891.10	2076.03
A_68_P32326600	chrX:35542695-35542742	NM_001079513:-251	Zbtb33	PROMOTER	1.223	2.961	616.99	1826.62	3.621	430.10	1557.23
A_68_P28741418	chr12:112215432-112215476	NM_175207:1777	Ankrd9	INSIDE	1.222	2.621	1325.34	3473.50	3.204	1058.32	3390.41
A_68_P21425706	chr2:91805641-91805685	NM_138306:-1942	Dgkz	PROMOTER	1.222	1.905	683.84	1302.55	2.327	598.46	1392.86
A_68_P32743940	chrX:147482252-147482296	NM_008001:499	Fgd1	INSIDE	1.222	2.341	449.34	1051.74	2.861	291.76	834.66
A_68_P20455651	chr1:95342033-95342077	NM_133808:33331	Hdlbp	INSIDE	1.222	1.584	1424.42	2256.09	1.936	1236.06	2393.27
A_68_P29717288	chr14:73784719-73784763	NM_008410:338	Itm2b	INSIDE	1.222	4.968	7211.91	35827.01	6.068	5508.43	33427.36
A_68_P20510207	chr1:108656347-108656391	NM_027534:-49	Kdsr	PROMOTER	1.222	1.514	1734.11	2625.89	1.851	1781.69	3297.17
A_68_P22171315	chr3:58809811-58809855	NM_177855:-1067	Med12l	PROMOTER	1.222	2.528	570.34	1442.07	3.091	511.31	1580.24
A_68_P31208519	chr17:46848833-46848877	NM_145488:443	Pex6	INSIDE	1.222	3.867	962.67	3722.62	4.726	920.14	4348.54
A_68_P32142309	chr19:46222327-46222371	NM_008852:467	Pitx3	INSIDE	1.222	2.179	2169.91	4727.81	2.662	1695.21	4512.46

ProbeName	Target position of probe on CpG island microarray	TargetID	GeneSymbol	CpG island Description	Ratio of relative methylation (TiO_2-NP/Vehicle)	Sham group			TiO_2-H group		
						Relative methylation (Cy5/Cy3)	Cy3 signal (Input)	Cy5 signal (MeDIP)	Relative methylation (Cy5/Cy3)	Cy3 signal (Input)	Cy5 signal (MeDIP)
A_68_P25091653	chr7:52437711-52437755	NM_053256:1368	Pth2	DOWNSTREAM	1.222	3.688	630.07	2323.72	4.506	511.43	2304.57
A_68_P24061235	chr5:124875362-124875406	NM_001081203:539	Sbno1	INSIDE	1.222	4.184	1675.42	7009.98	5.115	1312.24	6711.56
A_68_P27926563	chr11:75328969-75329013	NM_001004157:1948	Scarf1	INSIDE	1.222	2.748	594.63	1634.26	3.359	535.87	1799.89
A_68_P22169673	chr3:58495733-58495777	NM_009174:556	Siah2	INSIDE	1.222	2.583	943.81	2438.21	3.156	914.83	2887.66
A_68_P28781791	chr12:120075046-120075090	NM_177082:-9733	Sp8	PROMOTER	1.222	1.748	2047.32	3578.11	2.136	1760.30	3760.04
A_68_P31479221	chr18:7868637-7868681	NR_027465:-197	Wac	PROMOTER	1.222	2.412	621.15	1498.38	2.947	593.00	1747.85
A_68_P22408056	chr3:107499381-107499425	NM_145542:64	Ahcyl1	INSIDE	1.221	3.804	604.76	2300.36	4.643	582.18	2702.99
A_68_P21068910	chr2:24790687-24790731	NM_001162485:93	Arrdc1	INSIDE	1.221	1.760	1978.72	3481.87	2.148	1658.76	3563.63
A_68_P21566800	chr2:118728429-118728473	NM_001045523:1100	Bahd1	INSIDE	1.221	1.529	1105.76	1691.24	1.867	1087.07	2030.10
A_68_P25661488	chr8:12430745-12430789	NR_027975:44996	Gm5607	INSIDE	1.221	1.440	3591.20	5170.10	1.758	2564.52	4508.14
A_68_P21283784	chr2:64860481-64860525	NM_016719:321	Grb14	INSIDE	1.221	1.616	2437.16	3939.07	1.974	2045.12	4037.12
A_68_P32215806	chr19:58935621-58935665	NM_175199:-168	Hspa12a	PROMOTER	1.221	1.792	1191.09	2134.23	2.188	1002.16	2192.71
A_68_P24818876	chr6:125081706-125081750	NM_138747:-172	Nop2	PROMOTER	1.221	1.805	1792.87	3236.23	2.204	1450.08	3195.48
A_68_P26011767	chr8:85388102-85388146	NM_177378:870	Rnf150	INSIDE	1.221	1.556	700.95	1090.48	1.900	616.87	1172.03
A_68_P28167721	chr11:117734583-117734627	NM_027919:236	Tha1	INSIDE	1.221	1.577	609.44	961.04	1.926	572.04	1101.77
A_68_P29260367	chr13:99696244-99696288	NM_178716:73	Tnpo1	INSIDE	1.221	2.206	1171.67	2585.16	2.694	958.39	2582.00
A_68_P21836563	chr2:167456985-167457029	NM_023230:499	Ube2v1	INSIDE	1.221	2.095	1821.01	3814.31	2.557	1477.22	3776.89
A_68_P31625689	chr18:36920123-36920167	NM_026464:252	Wdr55	INSIDE	1.221	1.417	2263.84	3206.83	1.730	1740.24	3010.16
A_68_P31408615	chr17:85489810-85489854	NM_028576:-207	1700106N22Rik	DIVERGENT_PROMOTER	1.220	1.724	2932.18	5055.40	2.103	2255.24	4741.77
A_68_P31331955	chr17:71351299-71351343	NM_026064:553	2900073G15Rik	INSIDE	1.220	2.185	2941.98	6429.19	2.667	2397.31	6393.55
A_68_P32957258	chr17:8476452-8476496	NM_018819:-203	Brp44l	PROMOTER	1.220	1.425	1595.27	2273.87	1.739	1415.48	2461.45
A_68_P24798986	chr6:120481161-120481205	NM_144815:135	Cecr5	INSIDE	1.220	1.599	1030.33	1647.33	1.950	902.76	1760.81
A_68_P21477095	chr2:102291921-102291965	NM_010218:7	Fjx1	INSIDE	1.220	1.914	597.28	1143.08	2.334	524.22	1223.61
A_68_P25087550	chr7:51717698-51717742	NM_198250:19864	Lrrc4b	INSIDE	1.220	1.787	967.20	1728.71	2.181	847.95	1849.50
A_68_P23334088	chr4:136025956-136026001	NM_024452:303	Luzp1	INSIDE	1.220	3.249	216.59	703.69	3.964	185.84	736.66
A_68_P26041378	chr8:91045300-91045344	NM_001163660:-6209	Nkd1	PROMOTER	1.220	3.426	457.24	1566.51	4.181	391.00	1634.88
A_68_P30542114	chr16:10411911-10411955	NM_011955:-98	Nubp1	PROMOTER	1.220	2.917	1464.43	4272.17	3.559	1146.64	4081.18
A_68_P32240644	chrX:7455750-7455794	NM_138606:341	Pim2	INSIDE	1.220	1.739	2037.48	3542.39	2.122	956.24	2028.70
A_68_P31253472	chr17:56175685-56175729	NM_013664:293	Sh3gl1	INSIDE	1.220	2.499	680.59	1700.67	3.048	629.49	1918.47
A_68_P26644962	chr9:75459020-75459064	NM_016711:90	Tmod2	INSIDE	1.220	2.056	1367.80	2811.58	2.509	1144.93	2872.09
A_68_P23111973	chr4:93001689-93001733	NM_026954:492	Tusc1	INSIDE	1.220	3.702	429.98	1591.87	4.517	408.78	1846.61
A_68_P27181846	chr10:61159115-61159159	NM_027912:875	Tysnd1	INSIDE	1.220	2.673	839.86	2244.68	3.262	782.79	2553.38
A_68_P20827837	chr1:173341785-173341830	NM_009480:-4	Usf1	PROMOTER	1.220	1.481	1011.33	1497.82	1.807	846.49	1529.96
A_68_P30378286	chr15:81353764-81353808			Unknown	1.220	2.355	872.83	2055.76	2.875	813.49	2338.46
A_68_P20874334	chr1:182126153-182126197	NM_023341:-23	Adck3	PROMOTER	1.219	2.146	957.16	2053.87	2.616	802.24	2098.32
A_68_P25370481	chr7:108079952-108079996	NM_001081116:701	Arhgef17	INSIDE	1.219	3.161	666.69	2107.10	3.852	562.87	2168.40
A_68_P32260375	chrX:11668929-11668973	NM_001168321:68531	Bcor	INSIDE	1.219	1.666	453.85	756.20	2.031	284.92	578.67
A_68_P21565620	chr2:118529638-118529682	NM_001081971:39	Gm1337	INSIDE	1.219	3.180	825.42	2625.01	3.876	759.57	2944.15
A_68_P28041811	chr11:95852863-95852907	NM_009951:14374	Igf2bp1	INSIDE	1.219	1.554	1463.15	2274.31	1.895	1125.11	2132.18
A_68_P32082882	chr19:34996734-34996778	NM_183046:-91	Kif20b	PROMOTER	1.219	1.637	663.28	1085.95	1.996	570.64	1139.14
A_68_P27624062	chr11:18927325-18927369	NM_001193271:-8374	Meis1	PROMOTER	1.219	2.226	514.50	1145.18	2.714	467.18	1268.00
A_68_P23278955	chr4:125914068-125914112	NM_001145970:19473	Mtap7d1	INSIDE	1.219	2.742	1776.84	4872.62	3.343	1522.05	5087.55
A_68_P23236917	chr4:117540757-117540801	NM_008135:32916	Slc6a9	INSIDE	1.219	2.076	2217.44	4602.91	2.530	1955.98	4949.45
A_68_P27966235	chr11:82248010-82248054	NM_023438:45631	Tmem132e	INSIDE	1.219	1.387	1092.69	1515.42	1.690	798.51	1349.42
A_68_P31620889	chr18:36079889-36079933	AK021191:7125		DOWNSTREAM	1.219	2.143	940.11	2014.98	2.613	879.30	2297.45
A_68_P27971499	chr11:83253762-83253806	NM_025492:2589	1700020L24Rik	INSIDE	1.218	2.503	284.99	713.23	3.049	277.22	845.18
A_68_P23545744	chr5:24606762-24606806	NR_027851:-8	2900005J15Rik	DIVERGENT_PROMOTER	1.218	1.546	1476.11	2281.50	1.882	1334.20	2511.10
A_68_P27257131	chr10:75022752-75022796	NM_198860:301	AI646023	INSIDE	1.218	1.899	1136.89	2158.93	2.312	950.89	2198.63
A_68_P26234305	chr8:125100749-125100793	NM_009698:37	Aprt	INSIDE	1.218	3.940	10683.97	42096.27	4.798	6517.13	31271.70
A_68_P28153160	chr11:115243190-115243234	NM_001080929:-17	Cdr2l	PROMOTER	1.218	1.777	1294.07	2299.80	2.165	1141.67	2471.44
A_68_P29618487	chr14:56179252-56179296	NM_001199009:-485	Dcaf11	PROMOTER	1.218	1.689	1309.25	2211.35	2.057	867.90	1784.85

ProbeName	Target position of probe on CpG island microarray	TargetID	GeneSymbol	CpG island Description	Ratio of relative methylation (TiO_2-NP/Vehicle)	Sham group			TiO_2-H group		
						Relative methylation (Cy5/Cy3)	Cy3 signal (Input)	Cy5 signal (MeDIP)	Relative methylation (Cy5/Cy3)	Cy3 signal (Input)	Cy5 signal (MeDIP)
A_68_P27572316	chr11:7113730-7113774	NM_008343:174	Igfbp3	INSIDE	1.218	1.530	1434.05	2193.49	1.863	1104.16	2057.00
A_68_P22934420	chr4:55543726-55543770	NM_010637:1599	Klf4	INSIDE	1.218	1.693	1092.36	1849.56	2.062	990.88	2043.25
A_68_P26537258	chr9:56467405-56467449	NM_181074:65634	Lingo1	INSIDE	1.218	5.982	3327.82	19905.82	7.288	1983.17	14453.23
A_68_P27542076	chr10:128003183-128003227	NM_011119:-214	Pa2g4	PROMOTER	1.218	2.281	653.65	1491.04	2.778	574.51	1596.07
A_68_P22081520	chr3:40603976-40604020	NM_011495:126	Plk4	INSIDE	1.218	3.028	394.07	1193.38	3.690	370.14	1365.71
A_68_P32227660	chr19:60950291-60950335	NM_007452:129	Prdx3	INSIDE	1.218	1.888	3592.01	6780.73	2.300	2633.46	6057.23
A_68_P31868119	chr18:81184645-81184689	NM_178280:-1349	Sall3	PROMOTER	1.218	1.827	659.62	1205.02	2.225	516.93	1150.13
A_68_P26084861	chr8:98239992-98240036	NM_001035123:202	Setd6	INSIDE	1.218	2.990	3190.57	9539.16	3.641	2531.08	9214.92
A_68_P25957631	chr8:74108344-74108388	NM_011977:15541	Slc27a1	INSIDE	1.218	1.556	575.66	895.51	1.894	408.88	774.58
A_68_P32147463	chr19:47142891-47142935	NM_177342:675	Taf5	INSIDE	1.218	1.585	565.84	897.04	1.931	511.88	988.40
A_68_P32557245	chrX:97016277-97016321	NM_001081283:-109	Tmem28	PROMOTER	1.218	2.729	424.89	1159.72	3.325	264.69	880.23
A_68_P24777345	chr6:116578587-116578631	NM_026057:387	Zfp422	INSIDE	1.218	1.363	1560.09	2127.07	1.661	1178.73	1958.03
A_68_P23611946	chr5:37554326-37554370	AK036147:261		INSIDE	1.218	3.156	715.66	2258.85	3.844	653.45	2511.74
A_68_P22312389	chr3:88005037-88005081	ENSMUST00000167043:514		INSIDE	1.218	3.674	920.17	3380.42	4.476	866.24	3876.91
A_68_P29249239	chr13:97841732-97841776	NM_001100458:157	Fam169a	INSIDE	1.217	1.529	544.84	832.80	1.860	511.23	950.80
A_68_P26809635	chr9:107473258-107473302	NM_010489:1787	Hyal2	INSIDE	1.217	2.344	959.28	2248.38	2.852	703.05	2005.23
A_68_P24804559	chr6:121423187-121423231	NM_001033354:488	Iqsec3	INSIDE	1.217	2.100	466.46	979.71	2.557	493.04	1260.74
A_68_P31863853	chr18:80560016-80560060	NM_001190373:955	Kcng2	INSIDE	1.217	1.620	1215.45	1968.48	1.970	1088.15	2144.11
A_68_P26144747	chr8:109949061-109949105	NM_026277:-144	Nob1	PROMOTER	1.217	2.368	1208.29	2860.91	2.882	1033.59	2978.72
A_68_P27357768	chr10:93610732-93610776	NM_011629:79	Nr2c1	INSIDE	1.217	1.752	1829.57	3205.03	2.132	1420.83	3029.79
A_68_P27924779	chr11:75008788-75008832	NM_177708:1316	Rtn4rl1	INSIDE	1.217	1.578	570.41	900.06	1.920	488.48	937.69
A_68_P25024912	chr7:29805278-29805322	NM_009109:104870	Ryr1	INSIDE	1.217	2.133	767.95	1638.34	2.596	699.79	1816.35
A_68_P27552578	chr11:3435772-3435816	NM_001159284:3501	Smtn	INSIDE	1.217	2.168	853.02	1849.67	2.640	794.44	2097.06
A_68_P26696148	chr9:85737347-85737391	NM_001164792:910	Tpbg	INSIDE	1.217	2.215	911.09	2017.91	2.694	839.94	2263.20
A_68_P23763266	chr5:67067724-67067768	NM_011670:387	Uchl1	INSIDE	1.217	4.045	1721.59	6962.99	4.921	1280.87	6303.08
A_68_P27563477	chr11:5344468-5344513	NM_001080924:360	Znrf3	INSIDE	1.217	1.546	1341.80	2074.88	1.881	1209.54	2275.51
A_68_P25097408	chr7:53435337-53435381	NM_011510:45	Abcc8	INSIDE	1.216	1.856	2952.84	5480.32	2.257	2330.10	5258.56
A_68_P26665701	chr9:79566357-79566401	NM_007730:107	Col12a1	INSIDE	1.216	1.596	2430.74	3880.54	1.942	1980.78	3846.77
A_68_P30579148	chr16:18343878-18343922	NM_138583:125	D16H22S680E	INSIDE	1.216	1.339	2497.85	3343.59	1.628	2201.48	3583.74
A_68_P31227863	chr17:50432140-50432184	NM_010021:762	Dazl	INSIDE	1.216	1.362	4813.80	6558.69	1.657	3976.66	6588.76
A_68_P32119984	chr19:42330495-42330539	NM_027694:288	Golga7b	INSIDE	1.216	0.494	1290.49	638.10	0.601	1069.39	642.92
A_68_P30021000	chr15:12251385-12251429	NM_025673:156	Golph3	INSIDE	1.216	1.905	727.77	1386.64	2.316	729.80	1690.18
A_68_P29363819	chr13:118391652-118391696	NM_010408:548	Hcn1	INSIDE	1.216	1.927	1040.13	2004.48	2.343	997.56	2337.07
A_68_P24137556	chr5:140251303-140251347	NM_026748:308	Ints1	INSIDE	1.216	0.473	2140.77	1012.17	0.575	1721.04	989.59
A_68_P27922696	chr11:74645200-74645244	NM_010813:797	Mnt	INSIDE	1.216	2.877	680.70	1958.34	3.499	740.74	2591.54
A_68_P23523153	chr5:20387713-20387757	NM_172992:208	Phtf2	INSIDE	1.216	1.648	1303.91	2149.11	2.004	1272.49	2549.94
A_68_P32118987	chr19:42164891-42164935	NM_145501:-12	Pi4k2a	DIVERGENT_PROMOTER	1.216	1.502	1258.15	1889.56	1.827	1113.07	2033.48
A_68_P29648701	chr14:61792097-61792141	NM_172809:34825	Sacs	INSIDE	1.216	1.769	1343.37	2376.27	2.151	1227.85	2641.07
A_68_P25090603	chr7:52263124-52263168	NM_001008422:8473	Scaf1	INSIDE	1.216	2.144	741.55	1589.61	2.608	576.21	1502.49
A_68_P26887823	chr9:122075943-122075987	NM_001164572:49581	Snrk	INSIDE	1.216	1.852	1244.00	2304.39	2.252	962.59	2167.89
A_68_P30019344	chr15:11925168-11925212	NM_011294:572	Sub1	INSIDE	1.216	1.948	1157.79	2255.22	2.369	959.97	2273.81
A_68_P31209393	chr17:47027920-47027964	NM_178385:373	Tbcc	INSIDE	1.216	2.067	705.06	1457.22	2.514	584.66	1469.91
A_68_P24459355	chr6:53770847-53770891	NM_025817:-49	Tril	PROMOTER	1.216	1.561	2820.71	4403.22	1.898	2033.29	3858.81
A_68_P22142065	chr3:53282814-53282858	NM_173382:15098	2810046L04Rik	INSIDE	1.215	4.242	6919.45	29351.52	5.153	5009.68	25813.97
A_68_P29049838	chr13:54605642-54605686	NM_176987:499	4732471D19Rik	INSIDE	1.215	2.243	677.44	1519.76	2.726	635.08	1731.27
A_68_P23154903	chr4:101092599-101092643	NM_009647:313	Ak4	INSIDE	1.215	2.763	3503.47	9678.66	3.358	3174.14	10657.39
A_68_P30357052	chr15:77825727-77825771	NM_007583:123962	Cacng2	INSIDE	1.215	1.776	826.86	1468.79	2.159	594.32	1283.06
A_68_P24040307	chr5:121144585-121144629	NM_008052:17072	Dtx1	INSIDE	1.215	1.943	542.80	1054.74	2.360	448.67	1058.88
A_68_P31928722	chr19:5448027-5448071	NM_010235:351	Fosl1	INSIDE	1.215	1.594	1667.83	2659.35	1.937	1372.21	2658.55
A_68_P24226580	chr6:8460315-8460359	NM_133236:741	Glcci1	INSIDE	1.215	2.075	1530.55	3175.74	2.522	1327.77	3348.11
A_68_P26467075	chr9:44143629-44143673	NM_010436:853	H2afx	INSIDE	1.215	2.114	1285.43	2717.19	2.568	1028.36	2640.43

ProbeName	Target position of probe on CpG island microarray	TargetID	GeneSymbol	CpG island Description	Ratio of relative methylation (TiO_2-NP/Vehicle)	Sham group			TiO_2-H group		
						Relative methylation (Cy5/Cy3)	Cy3 signal (Input)	Cy5 signal (MeDIP)	Relative methylation (Cy5/Cy3)	Cy3 signal (Input)	Cy5 signal (MeDIP)
A_68_P22603829	chr3:144232802-144232849	NM_011828:355	Hs2st1	INSIDE	1.215	1.906	1245.14	2373.51	2.316	1107.93	2565.60
A_68_P32465997	chrX:71268691-71268735	NM_001177975:545	Irak1	INSIDE	1.215	1.954	516.82	1009.72	2.374	346.10	821.78
A_68_P28099128	chr11:105880140-105880184	NM_001037712:10646	Kcnh6	INSIDE	1.215	1.598	808.37	1291.42	1.942	662.97	1287.23
A_68_P31259699	chr17:57171306-57171350	NM_010613:-398	Khsrp	PROMOTER	1.215	1.414	3773.68	5335.16	1.718	2951.95	5072.48
A_68_P27558539	chr11:4494769-4494813	NM_028860:28	Mtmr3	INSIDE	1.215	2.817	925.25	2606.01	3.423	893.53	3058.31
A_68_P29654922	chr14:62951071-62951115	NM_026001:151	Rnaseh2b	INSIDE	1.215	1.929	1085.90	2094.21	2.342	927.31	2172.07
A_68_P21311406	chr2:69699566-69699610	NM_001110145:-30	Ssb	PROMOTER	1.215	1.657	1330.55	2204.31	2.014	1081.60	2177.89
A_68_P20804307	chr1:169213914-169213958	NM_030724:1322	Uck2	INSIDE	1.215	1.487	2282.46	3393.54	1.806	2003.04	3617.56
A_68_P25728007	chr8:26712095-26712139	NM_001001735:-661	Whsc1l1	DIVERGENT_PROMOTER	1.215	2.175	764.67	1662.89	2.643	689.20	1821.55
A_68_P32369655	chrX:47014344-47014388	ENSMUST00000121891:230		INSIDE	1.215	1.793	411.42	737.83	2.179	257.78	561.62
A_68_P29147535	chr13:74946041-74946085	NM_009817:-693	Cast	PROMOTER	1.214	2.119	2732.05	5789.63	2.572	2368.41	6090.97
A_68_P31186625	chr17:43013785-43013829	NM_009847:-433	Cd2ap	PROMOTER	1.214	1.709	1088.08	1859.39	2.074	913.37	1894.27
A_68_P26844378	chr9:114293915-114293959	NM_019922:5894	Crtap	INSIDE	1.214	2.702	590.24	1594.85	3.280	494.84	1623.18
A_68_P29628539	chr14:58161485-58161529	NM_145837:17841	Il17d	INSIDE	1.214	2.918	1027.70	2998.40	3.542	990.17	3506.85
A_68_P20168281	chr1:39958327-39958371	NM_008696:591	Map4k4	INSIDE	1.214	1.487	1182.43	1758.56	1.805	992.43	1791.73
A_68_P29330292	chr13:112515172-112515216	NM_172593:38809	Mier3	DOWNSTREAM	1.214	1.384	1262.53	1747.10	1.680	969.96	1629.70
A_68_P25809390	chr8:42218830-42218874	NM_001005863:228	Mtus1	INSIDE	1.214	1.551	2016.19	3126.23	1.883	1817.19	3421.10
A_68_P23322026	chr4:133842693-133842737	NM_001163794:454	Pdik1l	INSIDE	1.214	1.965	938.36	1844.12	2.385	967.74	2308.52
A_68_P30861550	chr16:72663819-72663863	NM_019413:447	Robo1	INSIDE	1.214	1.608	1630.32	2621.63	1.952	1304.27	2545.63
A_68_P21095249	chr2:28980679-28980724	NM_198033:190	Setx	INSIDE	1.214	1.550	557.70	864.59	1.882	546.02	1027.61
A_68_P24083090	chr5:128938897-128938941	NM_172885:19	Tmem132d	INSIDE	1.214	2.527	910.69	2301.06	3.069	914.61	2806.50
A_68_P31292453	chr17:64213171-64213215	ENSMUST00000050753:-52		DIVERGENT_PROMOTER	1.214	2.671	1169.59	3123.83	3.241	1007.04	3264.13
A_68_P21741661	chr2:150730134-150730178	NM_024465:311	Abhd12	INSIDE	1.213	2.015	1271.67	2562.23	2.444	1093.83	2672.89
A_68_P21438435	chr2:93851039-93851083	NM_026944:-173	Alkbh3	PROMOTER	1.213	2.163	764.64	1653.54	2.624	605.84	1589.68
A_68_P30925872	chr16:84835744-84835788	NM_016755:53	Atp5j	INSIDE	1.213	1.839	525.84	966.88	2.231	557.29	1243.15
A_68_P21419476	chr2:90729680-90729724	NM_026161:3760	C1qtnf4	INSIDE	1.213	0.463	1193.16	551.85	0.561	1003.74	563.11
A_68_P26426473	chr9:36533774-36533818	NM_007691:447	Chek1	INSIDE	1.213	1.364	1897.82	2589.22	1.655	1527.98	2529.17
A_68_P29060387	chr13:56397406-56397450	NM_019568:484	Cxcl14	INSIDE	1.213	1.686	1021.51	1722.57	2.045	971.78	1987.59
A_68_P21374004	chr2:80155781-80155825	NM_024181:180	Dnajc10	INSIDE	1.213	1.756	2330.78	4092.81	2.130	2039.39	4342.95
A_68_P31097233	chr17:24563466-24563510	NM_010023:-139	Eci1	PROMOTER	1.213	2.568	898.95	2308.29	3.115	793.32	2471.08
A_68_P24908778	chr6:143116037-143116081	NM_029250:309	Etnk1	INSIDE	1.213	2.765	730.28	2018.90	3.352	614.99	2061.52
A_68_P27930555	chr11:76015561-76015605	NM_027773:25	Fam57a	INSIDE	1.213	1.859	3970.10	7381.69	2.256	3291.20	7424.16
A_68_P31106390	chr17:25946464-25946508	NM_001164225:457	Fbxl16	INSIDE	1.213	1.667	3314.97	5525.25	2.021	2685.30	5427.25
A_68_P29257124	chr13:99124764-99124808	NM_008242:587	Foxd1	INSIDE	1.213	1.803	2041.75	3680.27	2.186	1641.97	3588.96
A_68_P24112561	chr5:134789297-134789341	NM_001080746:1298	Gtf2i	INSIDE	1.213	0.550	1194.65	657.02	0.667	1019.57	680.40
A_68_P26020983	chr8:87186771-87186818	NM_010499:-43	Ier2	PROMOTER	1.213	1.382	1896.58	2621.84	1.677	1520.49	2549.89
A_68_P27284096	chr10:80039600-80039644	NM_078477:419	Klf16	INSIDE	1.213	2.167	1393.30	3019.67	2.629	1153.65	3033.17
A_68_P23657851	chr5:46061284-46061329	NM_019438:143	Ncapg	INSIDE	1.213	1.742	566.02	985.83	2.113	476.62	1007.31
A_68_P33012160	chr9_random:55072-55116	NM_011154:-62	Ppp2r3d	PROMOTER	1.213	3.675	3654.69	13431.85	4.460	2786.60	12427.65
A_68_P30478003	chr15:98887633-98887677	NM_001163588:2050	Prph	INSIDE	1.213	1.957	730.48	1429.75	2.374	537.81	1276.62
A_68_P24061317	chr5:124895849-124895893	NM_030241:5932	Setd8	INSIDE	1.213	1.415	1452.31	2055.36	1.717	1198.19	2057.02
A_68_P27466129	chr10:114238135-114238179	NM_146241:270	Trhde	INSIDE	1.213	1.844	807.67	1489.00	2.237	817.83	1829.36
A_68_P27969172	chr11:82744813-82744857	NM_178680:20080	Unc45b	INSIDE	1.213	2.008	3402.11	6832.57	2.436	2986.49	7276.05
A_68_P28677276	chr12:100866117-100866161	ENSMUST00000137842:64088		DOWNSTREAM	1.213	2.071	457.34	947.17	2.512	418.69	1051.83
A_68_P20876218	chr1:182413556-182413600	NM_001081227:57	6330403A02Rik	INSIDE	1.212	2.568	721.17	1852.23	3.112	593.47	1846.75
A_68_P26595206	chr9:66643069-66643113	NM_177583:140	Aph1b	INSIDE	1.212	1.438	1281.51	1842.73	1.743	1122.10	1955.36
A_68_P28050945	chr11:97362080-97362124	NM_021493:50629	Arhgap23	INSIDE	1.212	2.861	701.92	2008.51	3.467	634.80	2201.14
A_68_P28724521	chr12:109154706-109154750	NM_001079883:86896	Bcl11b	INSIDE	1.212	2.803	415.06	1163.41	3.398	400.50	1361.00
A_68_P31161463	chr17:36016977-36017021	NM_026987:276	Dhx16	INSIDE	1.212	2.333	789.17	1840.98	2.828	812.59	2298.12
A_68_P23342692	chr4:137469822-137469866	NM_199307:51693	Ece1	INSIDE	1.212	1.580	696.00	1099.95	1.915	588.29	1126.84
A_68_P27181452	chr10:61088878-61088922	NM_001177511:30669	Npffr1	INSIDE	1.212	1.575	804.23	1266.92	1.909	662.94	1265.87

ProbeName	Target position of probe on CpG island microarray	TargetID	GeneSymbol	CpG island Description	Ratio of relative methylation (TiO_2-NP/Vehicle)	Sham group			TiO_2-H group		
						Relative methylation (Cy5/Cy3)	Cy3 signal (Input)	Cy5 signal (MeDIP)	Relative methylation (Cy5/Cy3)	Cy3 signal (Input)	Cy5 signal (MeDIP)
A_68_P29589458	chr14:49293418-49293462	NR_029384:4478	Otx2os1	INSIDE	1.212	1.884	611.37	1151.77	2.283	544.58	1243.36
A_68_P32113657	chr19:41281416-41281460	NM_011904:-174	Tll2	PROMOTER	1.212	1.720	1625.52	2795.99	2.085	1519.56	3167.62
A_68_P28593635	chr12:84937847-84937891	NM_183154:229	Zfyve1	INSIDE	1.212	1.458	1015.85	1480.99	1.768	874.06	1544.95
A_68_P31103786	chr17:25571232-25571276	NM_001163691:-526	Cacna1h	PROMOTER	1.211	2.155	1772.87	3820.35	2.610	1475.59	3851.48
A_68_P26822034	chr9:110020069-110020113	NM_133347:-2004	Dhx30	PROMOTER	1.211	1.750	677.52	1185.81	2.119	545.30	1155.61
A_68_P31099856	chr17:24987535-24987579	NM_001159626:-55	Hagh	PROMOTER	1.211	2.844	1136.89	3233.30	3.444	948.52	3266.92
A_68_P25264890	chr7:87259796-87259840	NM_173011:418	Idh2	INSIDE	1.211	2.221	7254.05	16113.71	2.690	5105.30	13733.74
A_68_P20285216	chr1:63160901-63160945	NM_001081436:-81	Ino80d	PROMOTER	1.211	2.238	1355.93	3034.20	2.709	1215.97	3294.23
A_68_P24053338	chr5:123438550-123438594	NM_001005866:-30	Kdm2b	PROMOTER	1.211	1.770	15591.49	27603.73	2.145	10896.96	23371.47
A_68_P20641807	chr1:137715358-137715402	NM_133664:206	Lad1	INSIDE	1.211	1.868	456.66	853.19	2.263	426.36	965.01
A_68_P26134881	chr8:108138096-108138140	NR_035492:-1247	Mir1966	PROMOTER	1.211	2.300	1524.62	3507.30	2.786	1216.70	3389.50
A_68_P21006475	chr2:11423041-11423085	NM_001177752:655	Pfkfb3	INSIDE	1.211	2.120	2292.87	4860.41	2.567	1992.15	5114.79
A_68_P27287755	chr10:80616237-80616281	NM_021501:14207	Pias4	DOWNSTREAM	1.211	3.414	959.62	3276.19	4.133	777.76	3214.36
A_68_P27469298	chr10:114821572-114821616	NM_025780:-103	Thap2	DIVERGENT_PROMOTER	1.211	1.721	4851.98	8349.37	2.084	4025.04	8389.94
A_68_P26217893	A_68_P26217893			Unknown	1.211	2.282	1996.67	4556.17	2.763	1930.64	5334.78
A_68_P28031039	chr11:94010311-94010355	ENSMUST00000155965:15517		INSIDE	1.211	1.452	1984.94	2881.26	1.757	1590.97	2795.89
A_68_P21565892	chr2:118572266-118572310	NR_030716:791	5430417L22Rik	INSIDE	1.210	0.404	2818.48	1139.86	0.489	1636.87	801.03
A_68_P30434244	chr15:91030048-91030092	NM_011994:-7832	Abcd2	PROMOTER	1.210	2.404	1423.99	3423.11	2.908	1133.33	3295.15
A_68_P20241456	chr1:55420453-55420497	NM_029267:-161	Boll	PROMOTER	1.210	1.560	515.69	804.43	1.887	491.67	927.74
A_68_P24980698	chr7:16881008-16881052	NM_001136471:-8886	Ccdc9	PROMOTER	1.210	2.242	1518.35	3403.93	2.713	1309.25	3552.51
A_68_P22889575	chr4:46614883-46614927	NM_001164804:-103	Coro2a	PROMOTER	1.210	3.004	1884.05	5659.50	3.636	1331.83	4841.95
A_68_P25842219	chr8:48618727-48618771	NM_177240:76	D030016E14Rik	INSIDE	1.210	2.187	1753.26	3834.54	2.647	1577.35	4175.02
A_68_P30364005	chr15:78873228-78873272	NM_015738:936	Galr3	INSIDE	1.210	2.816	647.24	1822.33	3.408	585.40	1995.13
A_68_P27087303	chr10:40791899-40791943	NM_199058:-530	Gpr6	PROMOTER	1.210	1.633	992.17	1620.21	1.976	872.03	1722.94
A_68_P24156175	chr5:144119353-144119397	NM_133355:-6212	Grid2ip	PROMOTER	1.210	5.490	5258.33	28868.55	6.646	5041.27	33502.05
A_68_P29138629	chr13:73397900-73397944	NM_018885:-22	Irx4	PROMOTER	1.210	2.940	1412.02	4151.56	3.556	1281.35	4557.11
A_68_P21902329	chr2:179960496-179960540	NM_001081171:46	Lama5	INSIDE	1.210	2.391	1073.73	2567.44	2.893	912.09	2639.11
A_68_P28154416	chr11:115473786-115473830	NM_027162:9	Mif4gd	INSIDE	1.210	2.950	1262.79	3725.77	3.569	1068.60	3814.23
A_68_P31227302	chr17:50329542-50329586	NM_181397:258	Rftn1	INSIDE	1.210	3.297	1539.25	5075.30	3.991	1407.06	5615.56
A_68_P22317135	chr3:88894988-88895032	NM_001083808:-1057	Rusc1	PROMOTER	1.210	1.574	2138.77	3365.96	1.905	1700.57	3238.87
A_68_P25090607	chr7:52263642-52263686	NM_001008422:7955	Scaf1	INSIDE	1.210	1.532	774.04	1185.85	1.853	605.87	1122.84
A_68_P21707841	chr2:144382255-144382299	NM_019787:264	Sec23b	INSIDE	1.210	3.903	1293.78	5049.14	4.724	963.28	4550.43
A_68_P30651947	chr16:32333125-32333169	NM_177633:809	Ubxn7	INSIDE	1.210	3.221	437.20	1408.30	3.897	433.52	1689.37
A_68_P32039606	chr19:27293512-27293556	NM_001161420:2025	Vldlr	INSIDE	1.210	1.516	1207.62	1830.62	1.834	894.67	1640.91
A_68_P23938661	chr5:102498072-102498116	NM_172882:846	Wdfy3	INSIDE	1.210	1.857	557.00	1034.33	2.246	478.19	1074.16
A_68_P25844871	chr8:49075873-49075917	NM_133791:11	Wwc2	INSIDE	1.210	1.721	828.75	1425.91	2.081	699.20	1455.14
A_68_P33005807	A_68_P33005807			Unknown	1.210	3.543	902.80	3198.78	4.287	789.06	3382.59
A_68_P31019377	A_68_P31019377			Unknown	1.210	2.532	255.63	647.25	3.064	242.82	743.88
A_68_P29848865	chr14:100684720-100684764	AK021307:-1136		PROMOTER	1.210	1.771	1294.44	2292.44	2.143	1027.10	2200.77
A_68_P28275058	chr12:19181894-19181939	BC012645:412612		INSIDE	1.210	2.183	28311.06	61795.69	2.642	22174.70	58588.32
A_68_P23435929	chr4:154417040-154417084	ENSMUST00000135665:201		INSIDE	1.210	1.651	723.71	1194.90	1.998	663.70	1326.21
A_68_P27473559	chr10:115551010-115551054	NM_001161855:-59	4933416C03Rik	PROMOTER	1.209	2.550	1628.90	4153.49	3.084	1336.31	4121.08
A_68_P27804987	chr11:53292850-53292894	NM_183173:825	Ankrd43	INSIDE	1.209	5.455	270.63	1476.23	6.595	264.40	1743.64
A_68_P27083519	chr10:40068940-40068984	NM_001168304:-151	Cdk19	PROMOTER	1.209	1.484	1031.52	1530.91	1.794	818.88	1468.75
A_68_P29060392	chr13:56397929-56397973	NM_019568:-38	Cxcl14	PROMOTER	1.209	3.247	15148.05	49182.02	3.926	10128.64	39765.74
A_68_P23541891	chr5:23951016-23951060	NM_023229:15	Fastk	INSIDE	1.209	2.456	822.35	2019.51	2.970	758.13	2251.79
A_68_P25265269	chr7:87343641-87343685	NM_013659:11936	Sema4b	INSIDE	1.209	2.280	2248.75	5126.41	2.755	1818.43	5010.02
A_68_P29512056	chr14:32249107-32249151	NM_011894:91	Sh3bp5	INSIDE	1.209	1.919	380.32	729.82	2.321	318.48	739.05
A_68_P32775569	chrX:156065756-156065800	NM_001135727:439	Sh3kbp1	INSIDE	1.209	1.410	1561.88	2202.85	1.705	702.29	1197.31
A_68_P27138493	chr10:52410426-52410470	NM_178675:142	Slc35f1	INSIDE	1.209	3.200	2916.10	9331.08	3.869	2239.55	8664.61
A_68_P23801182	chr5:74489320-74489364	NR_031758:-212	Snora26	PROMOTER	1.209	1.452	2144.70	3113.99	1.755	1872.65	3285.93

ProbeName	Target position of probe on CpG island microarray	TargetID	GeneSymbol	CpG island Description	Ratio of relative methylation (TiO_2-NP/Vehicle)	Sham group			TiO_2-H group		
						Relative methylation (Cy5/Cy3)	Cy3 signal (Input)	Cy5 signal (MeDIP)	Relative methylation (Cy5/Cy3)	Cy3 signal (Input)	Cy5 signal (MeDIP)
A_68_P21900382	chr2:179666703-179666747	NM_001081092:44627	Taf4a	INSIDE	1.209	3.692	580.06	2141.42	4.464	546.36	2438.88
A_68_P29919369	A_68_P29919369			Unknown	1.209	2.929	205.40	601.66	3.542	138.16	489.39
A_68_P24513207	chr6:65622605-65622649	NM_172399:1022	A930038C07Rik	INSIDE	1.208	1.483	2336.85	3466.32	1.792	1795.97	3219.23
A_68_P23897550	chr5:93473495-93473539	NM_175270:532	Ankrd56	INSIDE	1.208	1.720	1966.63	3381.90	2.077	1634.43	3394.44
A_68_P24172968	chr5:148116746-148116790	NM_007673:2057	Cdx2	INSIDE	1.208	1.434	1086.45	1558.01	1.733	932.64	1616.02
A_68_P31694860	chr18:49992722-49992766	NM_001081371:78	Dmxl1	INSIDE	1.208	1.585	483.46	766.18	1.914	673.09	1288.30
A_68_P24334780	chr6:30908766-30908810	NM_001135093:202	Klf14	INSIDE	1.208	4.077	338.89	1381.55	4.924	339.69	1672.63
A_68_P24443356	chr6:51221246-51221290	NR_029719:-1359	Mir148a	PROMOTER	1.208	2.358	470.13	1108.49	2.848	435.21	1239.41
A_68_P31157234	chr17:35183191-35183235	NM_013600:339	Msh5	INSIDE	1.208	2.753	972.47	2676.94	3.327	834.02	2774.41
A_68_P30363152	chr15:78744712-78744756	NM_020271:386	Pdxp	INSIDE	1.208	4.206	844.10	3550.39	5.081	772.17	3923.59
A_68_P29055223	chr13:55573203-55573247	NM_001030296:7597	Prr7	INSIDE	1.208	1.695	3482.14	5903.44	2.048	3195.29	6544.11
A_68_P31227303	chr17:50329691-50329735	NM_181397:110	Rftn1	INSIDE	1.208	1.540	1156.27	1780.47	1.861	1041.43	1937.95
A_68_P26823386	chr9:110283633-110283677	NM_001001144:47858	Scap	INSIDE	1.208	2.701	3742.13	10105.67	3.263	2934.74	9574.77
A_68_P30425918	chr15:89388530-89388574	NM_021423:58265	Shank3	INSIDE	1.208	1.421	945.84	1344.26	1.716	779.53	1337.99
A_68_P26822092	chr9:110034844-110034888	NM_009211:339	Smarcc1	INSIDE	1.208	1.539	1277.09	1964.84	1.858	1241.54	2307.05
A_68_P29937468	chr14:118633821-118633865	NM_177753:2410	Sox21	INSIDE	1.208	1.399	4476.06	6261.92	1.689	3426.76	5789.21
A_68_P22825734	chr4:33296280-33296324	NM_177774:337	Srsf12	INSIDE	1.208	3.249	1547.76	5029.04	3.925	1319.07	5177.96
A_68_P27187528	chr10:62188471-62188515	NM_001033260:355	Stox1	INSIDE	1.208	3.141	1315.43	4132.20	3.795	1190.73	4519.24
A_68_P31260743	chr17:57390196-57390242	NM_134125:1345	Trip10	INSIDE	1.208	1.805	318.17	574.32	2.180	310.56	676.97
A_68_P30453204	chr15:94420006-94420050	NM_008971:227	Twf1	INSIDE	1.208	2.659	2003.30	5327.44	3.214	1751.51	5628.69
A_68_P25411548	chr7:117205451-117205495	NM_009281:257	Zfp143	INSIDE	1.208	1.727	440.26	760.22	2.087	352.21	734.98
A_68_P20184572	chr1:42741537-42741581	NR_027826:10112	2610017I09Rik	INSIDE	1.207	1.596	4694.31	7490.19	1.926	3365.88	6482.10
A_68_P28432829	chr12:53107422-53107466	NM_029545:44	6530401N04Rik	INSIDE	1.207	1.922	2211.06	4250.74	2.320	1858.65	4311.98
A_68_P26617800	chr9:70526556-70526600	NM_007399:-229	Adam10	PROMOTER	1.207	1.603	884.87	1418.57	1.935	833.21	1612.13
A_68_P27282385	chr10:79775990-79776034	NM_011789:11448	Apc2	INSIDE	1.207	3.154	1399.50	4414.39	3.808	1096.28	4174.19
A_68_P20456915	chr1:95582543-95582587	NM_016778:294	Bok	INSIDE	1.207	0.410	1341.70	549.63	0.494	1081.27	534.44
A_68_P24000794	chr5:114358647-114358691	NM_011779:47	Coro1c	INSIDE	1.207	2.767	1982.64	5485.08	3.339	1802.46	6017.90
A_68_P20383169	chr1:80754785-80754829	NM_175291:322	Dock10	INSIDE	1.207	0.430	1340.73	575.89	0.519	1067.11	553.45
A_68_P21113209	chr2:31939133-31939177	NM_175511:71	Fam78a	INSIDE	1.207	1.740	1448.64	2520.25	2.101	1131.20	2376.28
A_68_P27286395	chr10:80391635-80391679	NM_008655:-1179	Gadd45b	PROMOTER	1.207	0.490	1172.23	574.50	0.592	1005.25	594.86
A_68_P28989506	chr13:43399105-43399149	NM_001033399:415	Gfod1	INSIDE	1.207	2.520	390.30	983.69	3.043	356.18	1083.82
A_68_P27279738	chr10:79384372-79384416	NM_053244:4679	Kiss1r	INSIDE	1.207	1.419	1165.95	1655.02	1.714	909.30	1558.27
A_68_P31475770	chr18:7004914-7004958	NM_177595:-159	Mkx	PROMOTER	1.207	1.835	1192.33	2188.08	2.216	1074.98	2381.95
A_68_P24990783	chr7:19576629-19576673	NM_145579:57	Mypop	INSIDE	1.207	1.422	1419.62	2018.55	1.717	1119.76	1922.35
A_68_P24428193	chr6:48544163-48544207	NM_001079901:303	Repin1	INSIDE	1.207	2.575	964.64	2484.34	3.107	904.20	2809.72
A_68_P26129565	chr8:107154553-107154597	NM_019662:647	Rrad	INSIDE	1.207	1.390	1404.33	1952.16	1.678	1122.45	1883.78
A_68_P28187710	chr11:120810691-120810735	NM_001038653:332	Slc16a3	INSIDE	1.207	1.707	1225.51	2092.38	2.061	974.29	2008.14
A_68_P28550363	chr12:76919145-76919189	NM_001005510:-138	Syne2	PROMOTER	1.207	1.708	609.14	1040.52	2.061	561.50	1157.31
A_68_P24061812	chr5:124990693-124990737	NM_019770:-85	Tmed2	DIVERGENT_PROMOTER	1.207	2.616	542.07	1417.98	3.158	522.03	1648.36
A_68_P24951465	chr7:4946322-4946366	NM_026741:1359	Zfp579	INSIDE	1.207	4.924	2513.06	12375.01	5.946	1889.30	11233.07
A_68_P25947784	chr8:72240592-72240636	NM_181274:-229	Zfp869	PROMOTER	1.207	1.837	1966.78	3612.26	2.217	1810.30	4012.74
A_68_P24050007	chr5:122822159-122822211	NM_026883:-212	1500011H22Rik	DIVERGENT_PROMOTER	1.206	2.053	780.57	1602.90	2.476	681.98	1688.26
A_68_P30478228	chr15:98928659-98928703	NM_176835:-1234	Dnajc22	PROMOTER	1.206	2.299	897.95	2064.41	2.773	824.16	2285.22
A_68_P23796632	chr5:73647376-73647420	NM_028194:459	Fryl	INSIDE	1.206	1.984	841.94	1670.48	2.392	839.41	2008.04
A_68_P27271932	chr10:77532109-77532153	NM_015790:18	Icosl	INSIDE	1.206	1.418	1348.92	1912.53	1.709	1009.89	1726.26
A_68_P32028431	chr19:25312079-25312123	NM_181404:409	Kank1	INSIDE	1.206	1.823	499.82	910.94	2.198	409.07	899.05
A_68_P24333873	chr6:30688134-30688178	NR_029900:-3142	Mir335	PROMOTER	1.206	1.587	712.42	1130.79	1.914	629.86	1205.73
A_68_P30681786	chr16:37654577-37654621	NM_026610:-144	Ndufb4	PROMOTER	1.206	1.454	1249.36	1816.82	1.754	1077.66	1889.79
A_68_P26137546	chr8:108583679-108583723	NM_010901:198	Nfatc3	INSIDE	1.206	1.683	375.65	632.26	2.030	367.52	746.12
A_68_P29350211	chr13:115880342-115880386	NM_134058:2	Pelo	INSIDE	1.206	2.189	1298.94	2842.80	2.639	1143.00	3015.91
A_68_P24046797	chr5:122286984-122287028	NM_008507:-196	Sh2b3	PROMOTER	1.206	2.268	937.38	2125.58	2.734	884.39	2417.72

ProbeName	Target position of probe on CpG island microarray	TargetID	GeneSymbol	CpG island Description	Ratio of relative methylation (TiO_2-NP/Vehicle)	Sham group			TiO_2-H group		
						Relative methylation (Cy5/Cy3)	Cy3 signal (Input)	Cy5 signal (MeDIP)	Relative methylation (Cy5/Cy3)	Cy3 signal (Input)	Cy5 signal (MeDIP)
A_68_P23991346	chr5:112737009-112737053	NM_009419:31304	Tpst2	INSIDE	1.206	1.844	2178.30	4017.56	2.224	1731.64	3850.95
A_68_P30176278	chr15:43309169-43309213	NM_025736:416	Ttc35	INSIDE	1.206	11.506	2959.95	34057.52	13.876	2316.18	32140.01
A_68_P21047273	chr2:19831293-19831337	ENSMUST00000114610:-92		PROMOTER	1.206	4.426	4936.41	21846.20	5.335	3571.91	19057.88
A_68_P23364695	chr4:141101558-141101602	NR_030695:-496	B330016D10Rik	DIVERGENT_PROMOTER	1.205	1.858	1020.09	1895.39	2.239	931.31	2084.81
A_68_P24120336	chr5:136750661-136750705	NM_198602:292593	Cux1	INSIDE	1.205	2.752	433.57	1193.27	3.317	439.37	1457.35
A_68_P20660113	chr1:140860423-140860467	NM_181347:-3853	Dennd1b	PROMOTER	1.205	3.266	647.57	2114.75	3.934	629.41	2476.36
A_68_P21319994	chr2:71367531-71367575	NM_010053:51	Dlx1	INSIDE	1.205	1.660	7033.80	11673.78	2.000	6150.28	12300.98
A_68_P28577471	chr12:81858975-81859019	NM_001008423:2706	Gm1568	INSIDE	1.205	2.435	413.84	1007.83	2.935	368.66	1082.17
A_68_P22565467	chr3:137527631-137527675	NM_016750:90	H2afz	INSIDE	1.205	2.434	971.95	2365.24	2.932	805.68	2362.05
A_68_P27495310	chr10:119542700-119542745	NM_080446:7299	Helb	INSIDE	1.205	2.602	278.34	724.37	3.137	280.18	878.96
A_68_P21908761	chr2:180869735-180869779	NM_001003824:174	Kcnq2	INSIDE	1.205	1.570	496.64	779.92	1.893	465.00	880.09
A_68_P24333876	chr6:30688404-30688448	NR_029900:-2872	Mir335	PROMOTER	1.205	3.232	1916.77	6194.18	3.895	1405.29	5473.62
A_68_P25588916	chr7:148641001-148641045	NM_001163689:-64	Pnpla2	PROMOTER	1.205	1.681	1026.23	1725.53	2.026	791.87	1604.40
A_68_P23921376	chr5:99466286-99466330	NM_008926:-210	Prkg2	PROMOTER	1.205	1.426	1398.71	1994.05	1.718	1254.18	2154.94
A_68_P31868010	chr18:81171392-81171436	NM_178280:11903	Sall3	INSIDE	1.205	3.097	1061.96	3289.01	3.733	1010.19	3771.19
A_68_P25805590	chr8:41509132-41509176	NM_178395:-14	Zdhhc2	PROMOTER	1.205	4.513	663.48	2994.06	5.436	694.49	3775.25
A_68_P24973911	chr7:13556484-13556528	NM_178732:5294	Zfp324	INSIDE	1.205	5.259	2625.33	13807.29	6.336	2500.36	15842.21
A_68_P31939719	chr19:7496867-7496911	NM_175381:4774	2700081O15Rik	INSIDE	1.204	3.596	2149.64	7729.16	4.330	1843.47	7981.71
A_68_P26158882	chr8:112302510-112302554	NM_009677:-7	Ap1g1	PROMOTER	1.204	2.368	1138.42	2695.58	2.851	981.29	2797.89
A_68_P23979252	chr5:110792613-110792657	NM_028596:23512	Fbrsl1	INSIDE	1.204	1.543	1002.79	1547.66	1.859	859.18	1596.97
A_68_P23248723	chr4:119705546-119705590	NM_010657:218286	Hivep3	INSIDE	1.204	2.857	1045.25	2986.40	3.440	953.42	3279.53
A_68_P30511776	chr16:4726510-4726554	NM_001136066:172	Hmox2	INSIDE	1.204	1.684	875.43	1474.02	2.027	709.15	1437.15
A_68_P30956553	chr16:90386404-90386448	NM_015755:-215	Hunk	PROMOTER	1.204	1.462	1047.74	1531.37	1.759	927.71	1632.30
A_68_P27109368	chr10:44786970-44787014	NM_011156:-27	Prep	PROMOTER	1.204	1.724	1058.95	1826.06	2.076	912.49	1893.90
A_68_P32583451	chrX:103414842-103414886	NM_001001176:317	Taf9b	INSIDE	1.204	1.674	577.58	966.85	2.016	326.37	657.81
A_68_P31936612	chr19:6984205-6984249	NM_001166370:39	Trmt112	INSIDE	1.204	1.955	1393.20	2723.52	2.353	1313.17	3090.40
A_68_P26885417	chr9:121672235-121672279	NM_001166644:3106	Zfp651	INSIDE	1.204	1.875	480.33	900.61	2.258	441.82	997.64
A_68_P25579476	chr7:146846040-146846084	ENSMUST00000118750:-8		PROMOTER	1.204	2.417	2631.11	6360.44	2.909	2065.82	6010.26
A_68_P21079933	chr2:26459306-26459350	NM_026212:402	Agpat2	INSIDE	1.203	1.892	656.03	1241.32	2.277	605.01	1377.37
A_68_P20975262	chr2:5635173-5635217	NM_177343:516	Camk1d	INSIDE	1.203	5.198	1437.93	7474.26	6.253	1158.72	7245.33
A_68_P28745355	chr12:112910496-112910540	NM_021273:31	Ckb	INSIDE	1.203	2.306	1940.40	4474.67	2.773	1519.96	4215.19
A_68_P20258855	chr1:58480898-58480942	NM_001042634:12	Clk1	INSIDE	1.203	1.725	1869.07	3224.53	2.076	1666.37	3458.95
A_68_P25268092	chr7:87833284-87833328	NM_173863:457	Crtc3	INSIDE	1.203	2.888	1369.27	3954.51	3.475	1189.59	4133.44
A_68_P23415470	chr4:151308129-151308173	NM_172704:322	Dnajc11	INSIDE	1.203	2.195	442.74	971.92	2.641	370.30	978.07
A_68_P25596358	chr7:149845220-149845264	NR_002855:-355	Igf2as	DIVERGENT_PROMOTER	1.203	2.933	1135.39	3330.63	3.528	965.24	3405.49
A_68_P25952869	chr8:73118953-73118997	NM_023627:595	Isyna1	INSIDE	1.203	2.305	1975.31	4554.06	2.773	1604.48	4450.01
A_68_P23588237	chr5:33678644-33678688	NM_021500:446	Maea	INSIDE	1.203	4.465	1242.80	5549.32	5.371	1051.07	5645.77
A_68_P23978674	chr5:110698757-110698801	NM_001163538:140	Pgam5	INSIDE	1.203	2.077	828.18	1720.26	2.500	694.43	1735.81
A_68_P31254024	chr17:56264131-56264175	NM_013662:9123	Sema6b	INSIDE	1.203	2.478	2592.56	6425.25	2.981	1974.26	5884.48
A_68_P27095211	chr10:42222280-42222324	NM_017472:443	Snx3	INSIDE	1.203	2.859	1344.03	3842.41	3.439	1300.70	4472.92
A_68_P26163688	chr8:113142314-113142358	NM_146216:-201	Vac14	PROMOTER	1.203	2.251	1513.09	3406.72	2.709	1273.76	3451.24
A_68_P25499302	chr7:133343826-133343870	NM_028816:74	Xpo6	INSIDE	1.203	1.465	777.14	1138.55	1.763	858.23	1513.19
A_68_P31144038	A_68_P31144038			Unknown	1.203	2.582	568.68	1468.28	3.105	478.04	1484.52
A_68_P31159052	chr17:35496088-35496132	M19687:92295		INSIDE	1.203	3.619	3325.24	12035.17	4.353	2816.53	12261.71
A_68_P28573972	chr12:81233698-81233742	NR_033514:111	2310015A10Rik	INSIDE	1.202	2.767	587.25	1624.69	3.327	515.19	1713.80
A_68_P28181737	chr11:119873242-119873286	NM_001198787:29831	Aatk	INSIDE	1.202	2.499	988.52	2469.91	3.003	803.88	2414.16
A_68_P22411290	chr3:108013838-108013882	NM_175183:-8	Atxn7l2	PROMOTER	1.202	2.468	1955.92	4827.76	2.966	1551.88	4602.91
A_68_P27275481	chr10:78215248-78215292	NM_027630:542	Ccdc105	INSIDE	1.202	2.253	489.86	1103.46	2.708	424.64	1149.89
A_68_P26674982	chr9:81526714-81526758	NM_010482:-577	Htr1b	PROMOTER	1.202	1.528	944.96	1443.82	1.837	822.85	1511.50
A_68_P28161572	chr11:116704633-116704677	NM_033398:109	Jmjd6	INSIDE	1.202	2.083	2133.37	4443.18	2.503	1716.89	4298.09
A_68_P30114903	chr15:31460728-31460772	NM_172606:42	Marchf6	INSIDE	1.202	1.560	2000.26	3121.15	1.876	1724.98	3236.31

ProbeName	Target position of probe on CpG island microarray	TargetID	GeneSymbol	CpG island Description	Ratio of relative methylation (TiO_2-NP/Vehicle)	Sham group			TiO_2-H group		
						Relative methylation (Cy5/Cy3)	Cy3 signal (Input)	Cy5 signal (MeDIP)	Relative methylation (Cy5/Cy3)	Cy3 signal (Input)	Cy5 signal (MeDIP)
A_68_P27279615	chr10:79365943-79365988	NM_001163276:5718	Med16	INSIDE	1.202	2.420	2154.97	5214.57	2.908	1617.33	4703.97
A_68_P25957228	chr8:74035689-74035737	NM_032398:-44	Plvap	PROMOTER	1.202	2.105	429.88	905.07	2.531	413.76	1047.38
A_68_P24538898	chr6:71858964-71859010	NM_009088:-60	Polr1a	DIVERGENT_PROMOTER	1.202	1.929	539.01	1039.80	2.319	435.79	1010.74
A_68_P27287000	chr10:80494188-80494232	NM_134135:2447	Slc39a3	INSIDE	1.202	4.007	1550.65	6213.92	4.818	1290.24	6216.36
A_68_P23185610	chr4:106311623-106311667	NM_177667:16591	Ttc22	INSIDE	1.202	2.320	1270.77	2948.47	2.788	1024.68	2856.75
A_68_P27255926	chr10:74779688-74779732	NM_009630:23	Adora2a	INSIDE	1.201	1.376	3824.63	5261.75	1.653	2937.97	4855.30
A_68_P26888669	chr9:122203192-122203236	NM_133979:278	Ano10	INSIDE	1.201	1.714	2282.07	3911.22	2.058	1951.61	4016.58
A_68_P27869724	chr11:64976171-64976215	NM_001099288:271	Arhgap44	INSIDE	1.201	1.935	1509.74	2921.23	2.324	1336.19	3105.08
A_68_P26544129	chr9:57684523-57684567	NM_019689:-2503	Arid3b	PROMOTER	1.201	1.934	472.62	913.96	2.322	389.11	903.36
A_68_P28732762	chr12:110698103-110698147	NM_001190703:6460	Dlk1	INSIDE	1.201	1.565	508.18	795.43	1.879	438.73	824.48
A_68_P28842025	chr13:15555334-15555378	NM_008130:-199	Gli3	PROMOTER	1.201	2.068	1512.51	3128.27	2.483	1353.34	3360.74
A_68_P31978250	chr19:16207196-16207240	NM_008139:-102	Gnaq	PROMOTER	1.201	2.136	1515.67	3237.78	2.566	1259.86	3233.00
A_68_P28156587	chr11:115836535-115836579	NM_001005608:518	Itgb4	INSIDE	1.201	1.793	951.18	1705.87	2.154	799.94	1722.72
A_68_P24052806	chr5:123349603-123349647	NM_013910:735	Kdm2b	INSIDE	1.201	1.905	668.87	1274.50	2.289	594.00	1359.42
A_68_P31614616	chr18:34937211-34937255	NM_001081256:571	Kdm3b	INSIDE	1.201	3.930	2091.99	8222.40	4.721	1702.98	8039.95
A_68_P30345383	chr15:75829147-75829191	NM_177922:4970	Mapk15	INSIDE	1.201	2.222	3250.31	7222.26	2.669	2395.77	6395.16
A_68_P30512463	chr16:4886291-4886335	NM_029657:61	Mgrn1	INSIDE	1.201	0.492	1704.49	839.26	0.591	1353.17	800.34
A_68_P23391037	chr4:147242647-147242691	NM_001025365:160	Miip	INSIDE	1.201	1.505	804.89	1211.57	1.808	594.37	1074.79
A_68_P32383984	chrX:50097577-50097621	NR_029657:-1854	Mir106a	PROMOTER	1.201	1.595	826.61	1318.74	1.916	481.03	921.51
A_68_P23893298	chr5:92706005-92706049	NM_001163687:1181	Naaa	INSIDE	1.201	3.103	919.71	2854.17	3.726	788.33	2937.06
A_68_P29694800	chr14:69793093-69793137	NM_010920:3256	Nkx2-6	INSIDE	1.201	1.401	1432.03	2005.64	1.682	1249.73	2101.65
A_68_P21239896	chr2:56954902-56954946	NM_013613:12525	Nr4a2	DOWNSTREAM	1.201	2.020	672.20	1357.99	2.427	561.07	1361.53
A_68_P23950024	chr5:104475955-104475999	NM_028794:-53	Nudt9	PROMOTER	1.201	2.443	749.67	1831.59	2.934	647.10	1898.32
A_68_P27542078	chr10:128003336-128003380	NM_011119:-368	Pa2g4	PROMOTER	1.201	1.971	4498.71	8868.12	2.367	3408.54	8067.44
A_68_P25587733	chr7:148445537-148445581	NM_001081118:30872	Phrf1	INSIDE	1.201	4.271	960.13	4101.12	5.129	862.30	4422.98
A_68_P21044973	chr2:19367231-19367275	NM_018809:-37	Ptf1a	PROMOTER	1.201	1.546	753.98	1165.95	1.857	632.19	1174.06
A_68_P22378033	chr3:101728452-101728496	NM_001039371:-98	Slc22a15	PROMOTER	1.201	1.422	1112.23	1581.70	1.708	910.47	1555.27
A_68_P30137263	chr15:36140403-36140447	NM_012031:31140	Spag1	INSIDE	1.201	4.975	1552.37	7722.78	5.976	1363.33	8147.36
A_68_P28740618	chr12:112078936-112078980	NM_011973:191	Stk30	INSIDE	1.201	1.460	1162.40	1697.03	1.753	998.21	1749.57
A_68_P21431327	chr2:92755255-92755299	NM_030725:19	Syt13	INSIDE	1.201	2.702	815.33	2203.34	3.246	732.49	2377.70
A_68_P26447523	chr9:40965396-40965440	NM_176860:159	Ubash3b	INSIDE	1.201	1.514	1986.08	3007.57	1.819	1579.76	2873.94
A_68_P28765898	chr12:117316198-117316242	NM_009511:25	Vipr2	INSIDE	1.201	2.137	1336.38	2855.37	2.565	1051.81	2698.41
A_68_P25947915	chr8:72298581-72298625	NM_177899:208	Zfp866	INSIDE	1.201	1.730	1494.48	2584.81	2.076	1491.94	3097.87
A_68_P25106560	chr7:55392877-55392921			Unknown	1.201	1.374	1979.16	2718.52	1.650	1660.38	2740.15
A_68_P27977898	chr11:84326983-84327029	NM_019816:-3	Aatf	PROMOTER	1.200	1.495	986.76	1475.63	1.795	842.45	1511.81
A_68_P21007911	chr2:11699878-11699922	NM_177268:521	Ankrd16	INSIDE	1.200	1.428	2346.46	3351.49	1.714	1802.10	3089.34
A_68_P23887087	chr5:91568591-91568635	NM_009704:-28	Areg	PROMOTER	1.200	1.490	1128.74	1681.98	1.789	1004.59	1796.85
A_68_P31013734	chr17:4994384-4994428	NM_001085355:-667	Arid1b	PROMOTER	1.200	1.402	1052.67	1475.81	1.682	1016.82	1710.06
A_68_P26078866	chr8:97190855-97190900	NM_024269:-128	Arl2bp	PROMOTER	1.200	1.657	1141.95	1892.25	1.989	936.67	1862.85
A_68_P28685559	chr12:102267503-102267547	NM_026681:-331	Ccdc88c	PROMOTER	1.200	1.599	676.07	1081.01	1.919	550.69	1057.01
A_68_P27921612	chr11:74452062-74452106	NM_001013784:18978	E130309D14Rik	INSIDE	1.200	2.223	724.51	1610.30	2.667	632.73	1687.51
A_68_P31121752	chr17:28450748-28450792	NM_001163819:296	Fance	INSIDE	1.200	1.754	642.77	1127.42	2.105	622.22	1309.88
A_68_P27288984	chr10:80800721-80800765	NM_148951:5269	Gipc3	INSIDE	1.200	2.025	1090.07	2207.29	2.429	963.35	2340.28
A_68_P30777939	chr16:55822496-55822540	NM_001159394:-267	Nfkbiz	PROMOTER	1.200	1.556	764.68	1189.63	1.866	685.66	1279.72
A_68_P30424834	chr15:89204309-89204353	NM_001111288:-81	Sco2	PROMOTER	1.200	2.430	3102.64	7540.79	2.915	2493.70	7270.00
A_68_P20354816	chr1:75543154-75543198	NM_009208:336	Slc4a3	INSIDE	1.200	1.830	869.00	1590.61	2.196	677.53	1487.88
A_68_P27542561	chr10:128106235-128106279	NM_173733:4718	Suox	DOWNSTREAM	1.200	1.712	1991.25	3408.27	2.055	1665.84	3422.59
A_68_P23594736	chr5:34856754-34856798	NM_139064:-148	Tnip2	PROMOTER	1.200	1.639	992.62	1626.68	1.966	873.30	1716.85
A_68_P21774854	chr2:156865842-156865886	NM_001164663:39137	9830001H06Rik	INSIDE	1.199	2.038	437.35	891.42	2.443	395.35	965.81
A_68_P30747802	chr16:49855795-49855839	NM_010581:50	Cd47	INSIDE	1.199	2.040	634.47	1294.02	2.445	551.19	1347.74
A_68_P27303131	chr10:83997120-83997164	NM_175451:-509	Ckap4	PROMOTER	1.199	1.871	836.19	1564.72	2.243	735.25	1649.43

ProbeName	Target position of probe on CpG island microarray	TargetID	GeneSymbol	CpG island Description	Ratio of relative methylation (TiO_2-NP/Vehicle)	Sham group			TiO_2-H group		
						Relative methylation (Cy5/Cy3)	Cy3 signal (Input)	Cy5 signal (MeDIP)	Relative methylation (Cy5/Cy3)	Cy3 signal (Input)	Cy5 signal (MeDIP)
A_68_P32126462	chr19:43515774-43515818	NM_031396:871	Cnnm1	INSIDE	1.199	2.659	1402.10	3728.82	3.189	1245.96	3973.03
A_68_P31715735	chr18:54022118-54022162	NM_152809:374	Csnk1g3	INSIDE	1.199	2.101	1200.08	2521.09	2.519	994.27	2504.13
A_68_P24593883	chr6:83447756-83447800	NM_001162521:9185	Dguok	INSIDE	1.199	1.520	847.49	1288.42	1.823	674.59	1229.91
A_68_P24156003	chr5:144076609-144076653	NM_172726:409	E130309D02Rik	INSIDE	1.199	1.446	1100.55	1591.31	1.733	993.09	1721.15
A_68_P26804376	chr9:106553310-106553354	NM_001160353:5108	Grm2	INSIDE	1.199	1.780	492.34	876.19	2.135	373.69	797.69
A_68_P26430295	chr9:37192346-37192390	NM_175189:17178	Hepacam	INSIDE	1.199	1.859	1049.45	1951.42	2.230	889.29	1983.10
A_68_P29942857	chr14:119537874-119537918	NM_015820:410	Hs6st3	INSIDE	1.199	1.657	555.66	920.94	1.988	508.63	1011.00
A_68_P26772687	chr9:100978284-100978328	NM_001100451:-144	Msl2	PROMOTER	1.199	6.874	1267.60	8713.53	8.242	749.65	6178.30
A_68_P29075365	chr13:58909956-58910000	NM_001025074:785	Ntrk2	INSIDE	1.199	2.290	1856.49	4250.45	2.744	1528.82	4195.30
A_68_P32114803	chr19:41459691-41459735	NM_031376:-152	Pik3ap1	PROMOTER	1.199	2.753	2209.03	6082.22	3.302	1929.94	6372.56
A_68_P31925249	chr19:4811087-4811131	NM_019869:526	Rbm14	INSIDE	1.199	3.061	2761.21	8452.75	3.671	2371.79	8707.75
A_68_P24153528	chr5:143576639-143576683	NM_001122730:2406	Tnrc18	INSIDE	1.199	1.632	671.58	1095.86	1.956	607.97	1189.09
A_68_P33004939	A_68_P33004939			Unknown	1.199	2.169	215.84	468.23	2.600	309.92	805.91
A_68_P22348135	chr3:96001508-96001552	NM_026975:-21	Bola1	PROMOTER	1.198	1.846	874.79	1614.54	2.211	705.40	1559.71
A_68_P24268751	chr6:17587317-17587361	NM_007604:241	Capza2	INSIDE	1.198	1.965	973.94	1914.05	2.354	819.49	1928.91
A_68_P23397601	chr4:148312774-148312818	NM_027195:134296	Casz1	INSIDE	1.198	2.111	2234.17	4717.09	2.530	1612.59	4079.45
A_68_P26761428	chr9:98856199-98856243	NM_012020:195	Foxl2	INSIDE	1.198	3.325	1323.32	4400.14	3.985	1143.24	4555.85
A_68_P21375629	chr2:80421545-80421589	NM_016965:-444	Nckap1	PROMOTER	1.198	2.202	1385.35	3051.00	2.637	1195.45	3152.93
A_68_P21100049	chr2:29745376-29745420	NM_001177662:-184	Odf2	PROMOTER	1.198	0.535	1223.75	654.86	0.641	977.58	626.78
A_68_P20506809	chr1:108069287-108069331	NM_133821:863	Phlpp1	INSIDE	1.198	0.362	2336.90	846.47	0.434	1997.62	867.09
A_68_P21928446	chr3:7366713-7366757	NM_008862:131	Pkia	INSIDE	1.198	1.586	1508.69	2392.07	1.899	1393.98	2646.76
A_68_P21149538	chr2:38499224-38499268	NM_011187:180	Psmb7	INSIDE	1.198	1.479	3601.32	5327.07	1.772	3034.55	5377.73
A_68_P26044790	chr8:91567290-91567334	NM_021390:749	Sall1	INSIDE	1.198	1.794	2770.37	4969.06	2.149	2217.58	4766.64
A_68_P24640499	chr6:91634367-91634411	NM_009320:328	Slc6a6	INSIDE	1.198	1.647	1530.11	2519.63	1.973	1271.29	2507.95
A_68_P26228070	chr8:124175318-124175362	NM_080855:493	Zcchc14	INSIDE	1.198	2.972	1405.77	4177.65	3.560	1250.98	4453.92
A_68_P24424843	chr6:47855408-47855452	NM_146175:27877	Zfp282	INSIDE	1.198	2.585	2972.27	7683.04	3.098	2282.90	7071.57
A_68_P24155214	chr5:143919270-143919314	ENSMUST00000157346:-2486		PROMOTER	1.198	2.470	724.17	1788.53	2.960	700.45	2073.15
A_68_P22953595	chr4:58924697-58924741	NM_172381:879	AI314180	INSIDE	1.197	1.747	2148.60	3754.12	2.092	1726.90	3611.82
A_68_P25524391	chr7:137910411-137910455	NM_001081038:-7150	Btbd16	PROMOTER	1.197	2.439	1590.49	3878.90	2.919	1337.47	3903.97
A_68_P22822456	chr4:32702176-32702220	NM_001122978:-249	Casp8ap2	PROMOTER	1.197	1.979	2193.11	4340.85	2.370	1706.66	4044.52
A_68_P31715220	chr18:53904386-53904430	NM_178686:-207	Cep120	PROMOTER	1.197	0.511	1571.24	802.47	0.611	1284.34	785.03
A_68_P33008827	chr4_random:110161-110205	NM_001033326:381	Dhrsx	INSIDE	1.197	2.014	2401.18	4836.73	2.412	2218.64	5350.53
A_68_P28577477	chr12:81859800-81859844	NM_001008423:1880	Gm1568	INSIDE	1.197	3.137	857.75	2690.73	3.756	782.02	2937.65
A_68_P32245741	chrX:8777677-8777721	NM_173414:600	Lancl3	INSIDE	1.197	2.141	1388.26	2972.43	2.564	798.82	2048.01
A_68_P23156221	chr4:101320393-101320437	NM_175036:27	Leprot	INSIDE	1.197	1.563	1363.66	2131.28	1.871	1274.77	2384.91
A_68_P28565714	chr12:79850102-79850146	NM_019579:191	Mpp5	INSIDE	1.197	1.828	1176.34	2150.33	2.188	1086.67	2377.44
A_68_P21150017	chr2:38567253-38567297	NM_139051:2788	Nr5a1	INSIDE	1.197	2.582	1941.44	5012.63	3.091	1513.12	4677.30
A_68_P31442544	chr17:91487486-91487530	NM_020252:4634	Nrxn1	INSIDE	1.197	3.043	312.05	949.49	3.641	342.10	1245.62
A_68_P27542073	chr10:128002710-128002754	NM_011119:258	Pa2g4	INSIDE	1.197	1.424	7265.17	10347.92	1.705	5235.63	8928.44
A_68_P29246773	chr13:97312251-97312295	NM_012048:168	Polk	INSIDE	1.197	2.587	1124.61	2909.53	3.098	983.35	3046.21
A_68_P31157970	chr17:35293556-35293603	NM_001199044:8243	Prrc2a	INSIDE	1.197	2.537	350.79	889.93	3.037	289.97	880.53
A_68_P30489328	chr15:100884313-100884357	NM_011323:117633	Scn8a	DOWNSTREAM	1.197	2.815	1023.14	2880.41	3.371	918.95	3097.88
A_68_P26574111	chr9:62993729-62993773	NM_172446:1037	Skor1	INSIDE	1.197	1.821	586.46	1067.98	2.180	467.76	1019.77
A_68_P28889208	chr13:24923670-24923714	NM_019551:165	Tdp2	INSIDE	1.197	1.784	4814.88	8590.57	2.135	4055.02	8658.86
A_68_P30363491	chr15:78798091-78798135	NM_001039155:19959	Triobp	INSIDE	1.197	3.848	3215.14	12370.25	4.607	2187.53	10078.29
A_68_P27895906	chr11:69395043-69395087	NM_001127233:1204	Trp53	INSIDE	1.197	3.025	950.02	2874.19	3.622	838.58	3037.04
A_68_P24630036	chr6:89785323-89785367	NM_053221:10187	Vmn1r42	DOWNSTREAM	1.197	3.380	1101.90	3724.54	4.047	882.11	3570.12
A_68_P24951463	chr7:4946096-4946140	NM_026741:1585	Zfp579	INSIDE	1.197	2.466	699.81	1725.54	2.951	533.42	1574.09
A_68_P28737719	chr12:111558035-111558079	AK200124:417495		INSIDE	1.197	3.062	1165.11	3567.74	3.665	932.54	3417.82
A_68_P21133893	chr2:35347074-35347118	ENSMUST00000121664:-57		PROMOTER	1.197	3.254	622.32	2025.07	3.896	566.77	2208.36
A_68_P24460750	chr6:53989374-53989418	NM_001163640:-529	Chn2	PROMOTER	1.196	2.194	803.20	1762.22	2.624	792.44	2079.40

ProbeName	Target position of probe on CpG island microarray	TargetID	GeneSymbol	CpG island Description	Ratio of relative methylation (TiO_2-NP/Vehicle)	Sham group			TiO_2-H group		
						Relative methylation (Cy5/Cy3)	Cy3 signal (Input)	Cy5 signal (MeDIP)	Relative methylation (Cy5/Cy3)	Cy3 signal (Input)	Cy5 signal (MeDIP)
A_68_P29841645	chr14:99499049-99499093	NM_028315:-81	Dis3	PROMOTER	1.196	2.390	679.87	1624.88	2.859	520.43	1488.11
A_68_P32030095	chr19:25581366-25581410	NM_015826:1193	Dmrt1	INSIDE	1.196	3.020	447.64	1351.77	3.611	400.99	1447.95
A_68_P25537310	chr7:140050852-140050896	NM_198017:-33	Fam175b	DIVERGENT_PROMOTER	1.196	2.728	877.71	2394.66	3.264	839.35	2739.59
A_68_P23246064	chr4:119212892-119212936	NM_172699:622	Foxj3	INSIDE	1.196	1.754	832.79	1460.53	2.098	764.35	1603.68
A_68_P28529995	chr12:73171043-73171090	NM_181752:912	Gpr135	INSIDE	1.196	3.578	898.42	3214.43	4.281	786.93	3368.51
A_68_P31125155	chr17:29089634-29089678	NM_025888:-504	Kctd20	PROMOTER	1.196	1.802	1349.17	2431.22	2.155	1174.92	2532.27
A_68_P22674476	chr3:156223832-156223876	NM_001039094:-903	Negr1	PROMOTER	1.196	1.802	6783.26	12224.22	2.155	5207.52	11222.95
A_68_P22774296	chr4:21605518-21605562	NM_001080771:7570	Prdm13	INSIDE	1.196	1.873	556.18	1041.73	2.241	491.28	1100.81
A_68_P31713995	chr18:53712057-53712101	NM_001033281:87879	Prdm6	INSIDE	1.196	1.476	662.09	977.41	1.766	603.83	1066.11
A_68_P23164321	chr4:102786815-102786859	NM_146254:68	Wdr78	INSIDE	1.196	1.367	1752.93	2395.52	1.635	1521.03	2486.26
A_68_P22217918	chr3:68673912-68673956	NM_001167996:427	1110032F04Rik	INSIDE	1.195	6.444	642.03	4137.02	7.697	847.75	6525.18
A_68_P32579167	chrX:102274990-102275034	NM_026312:-101	2610029G23Rik	DIVERGENT_PROMOTER	1.195	3.215	226.15	727.06	3.843	194.91	749.06
A_68_P24091172	chr5:130384666-130384710	NM_026916:279	4930579G22Rik	INSIDE	1.195	1.904	685.49	1305.42	2.275	571.90	1301.08
A_68_P28752162	chr12:113974838-113974882	NM_001024602:14476	AW555464	INSIDE	1.195	3.181	4019.01	12786.48	3.801	3145.97	11959.15
A_68_P24735108	chr6:108610209-108610253	NM_011498:-392	Bhlhe40	PROMOTER	1.195	2.015	812.46	1637.30	2.409	752.79	1813.19
A_68_P20827298	chr1:173259152-173259196	NM_001128609:-101	Dedd	PROMOTER	1.195	1.451	1647.59	2390.77	1.734	1221.32	2117.55
A_68_P32219569	chr19:59533670-59533714	NM_010132:513	Emx2	INSIDE	1.195	2.136	668.95	1428.72	2.553	525.69	1342.02
A_68_P23363268	chr4:140864869-140864913	NM_010139:7736	Epha2	INSIDE	1.195	2.445	1188.37	2905.43	2.923	1078.11	3151.13
A_68_P26134297	chr8:108042949-108042993	NM_008289:325	Hsd11b2	INSIDE	1.195	3.040	568.80	1728.95	3.631	533.68	1937.83
A_68_P27804079	chr11:53113636-53113680	NM_008300:323	Hspa4	INSIDE	1.195	1.552	1285.21	1994.36	1.855	1131.28	2098.55
A_68_P26500007	chr9:49605368-49605412	NM_001081445:1784	Ncam1	INSIDE	1.195	3.028	1201.60	3638.90	3.618	954.29	3452.15
A_68_P29237468	chr13:95638619-95638663	NM_011021:-6941	Otp	PROMOTER	1.195	2.010	508.94	1022.87	2.402	443.75	1065.93
A_68_P29619051	chr14:56262543-56262587	NM_028780:79	Tm9sf1	INSIDE	1.195	2.774	351.60	975.50	3.316	288.82	957.78
A_68_P21084626	chr2:27282675-27282719	NM_009500:-351	Vav2	PROMOTER	1.195	3.594	984.63	3538.43	4.296	828.94	3560.77
A_68_P26511312	A_68_P26511312			Unknown	1.195	3.374	516.49	1742.85	4.032	434.75	1752.71
A_68_P20872224	chr1:181733633-181733677	NM_026375:492	Ahctf1	INSIDE	1.194	2.197	998.52	2193.43	2.622	861.46	2258.66
A_68_P21144181	chr2:37649266-37649310	NM_001163566:17520	Crb2	INSIDE	1.194	3.612	2212.31	7990.61	4.313	1562.42	6738.56
A_68_P29241525	chr13:96374455-96374499	NM_010169:13912	F2r	INSIDE	1.194	2.824	420.10	1186.56	3.373	383.43	1293.37
A_68_P28071798	chr11:100941289-100941333	NM_010475:1586	Hsd17b1	INSIDE	1.194	1.542	2070.49	3193.21	1.841	1700.89	3132.07
A_68_P27894856	chr11:69213335-69213379	NM_001017426:13821	Kdm6b	INSIDE	1.194	2.139	1525.97	3263.77	2.555	1189.26	3038.14
A_68_P20659550	chr1:140738110-140738154	NM_001025565:889	Lhx9	INSIDE	1.194	1.416	2120.04	3002.21	1.690	1611.22	2723.76
A_68_P25587562	chr7:148414623-148414668	NM_001081118:-41	Phrf1	DIVERGENT_PROMOTER	1.194	2.212	755.34	1670.63	2.640	589.72	1556.85
A_68_P28164255	chr11:117151945-117151989	NM_001113487:24407	Septin9	INSIDE	1.194	2.321	353.04	819.56	2.771	327.48	907.53
A_68_P21836029	chr2:167364249-167364293	NM_011427:544	Snai1	INSIDE	1.194	1.894	2099.09	3976.36	2.262	1770.36	4005.22
A_68_P25983693	chr8:80041310-80041354	NR_028125:378	0610038B21Rik	INSIDE	1.193	2.324	912.20	2119.71	2.771	812.28	2250.88
A_68_P29419197	chr14:14844919-14844966	NM_139227:-62	Atxn7	PROMOTER	1.193	2.249	1293.71	2909.69	2.684	1082.05	2903.93
A_68_P21094146	chr2:28771793-28771837	NM_019446:127	Barhl1	INSIDE	1.193	1.706	1434.26	2447.18	2.036	1360.93	2770.20
A_68_P21969034	chr3:17954954-17954998	NM_021560:652	Bhlhe22	INSIDE	1.193	1.926	666.69	1284.08	2.298	597.03	1372.19
A_68_P26880501	chr9:120842440-120842484	NM_007614:-55	Ctnnb1	PROMOTER	1.193	1.575	743.11	1170.28	1.878	744.78	1398.97
A_68_P27311664	chr10:85484474-85484519	NM_153195:-177	Fbxo7	DIVERGENT_PROMOTER	1.193	1.731	2136.20	3696.93	2.065	1924.36	3974.43
A_68_P28675866	chr12:100630793-100630837	NM_183186:57470	Foxn3	INSIDE	1.193	0.534	1414.50	755.58	0.637	1164.91	742.37
A_68_P26248332	chr8:127421933-127421977	NM_001111141:169	Gm505	INSIDE	1.193	2.011	358.67	721.22	2.400	303.45	728.20
A_68_P25958332	chr8:74213234-74213278	NM_013564:106	Insl3	INSIDE	1.193	2.543	406.36	1033.31	3.033	388.94	1179.47
A_68_P30575359	chr16:17722895-17722939	NM_001040683:107	Med15	INSIDE	1.193	1.761	1883.39	3316.02	2.100	1585.20	3329.50
A_68_P22317891	chr3:89018021-89018065	NM_001161824:215	Mtx1	INSIDE	1.193	3.137	1233.42	3869.11	3.741	972.58	3638.84
A_68_P27560497	chr11:4847206-4847250	NM_010904:839	Nefh	INSIDE	1.193	2.497	338.55	845.38	2.979	365.52	1088.94
A_68_P23134368	chr4:97445476-97445520	NM_001122953:1182	Nfia	INSIDE	1.193	2.120	2538.36	5380.76	2.530	2274.70	5754.40
A_68_P27980110	chr11:84693182-84693228	NM_027388:493	Pigw	INSIDE	1.193	3.518	381.69	1342.88	4.197	397.58	1668.78
A_68_P31201639	chr17:45672060-45672104	NM_013688:11700	Tcte1	INSIDE	1.193	2.517	1119.73	2818.45	3.003	934.86	2807.65
A_68_P29560593	chr14:41780203-41780247	NM_145928:-128	Tspan14	PROMOTER	1.193	1.339	2742.41	3670.95	1.597	2420.12	3865.19
A_68_P32320541	chrX:34414442-34414486	NM_019668:104	Ube2a	INSIDE	1.193	2.080	850.63	1769.42	2.482	613.74	1523.27

						Sham group			TiO_2-H group		
ProbeName	Target position of probe on CpG island microarray	TargetID	GeneSymbol	CpG island Description	Ratio of relative methylation (TiO_2-NP/Vehicle)	Relative methylation (Cy5/Cy3)	Cy3 signal (Input)	Cy5 signal (MeDIP)	Relative methylation (Cy5/Cy3)	Cy3 signal (Input)	Cy5 signal (MeDIP)
A_68_P30133052	chr15:35301302-35301346	NM_177151:24	Vps13b	INSIDE	1.193	2.685	540.21	1450.48	3.202	524.15	1678.44
A_68_P28706884	chr12:106248180-106248224	NM_172500:-183	4831426I19Rik	PROMOTER	1.192	1.893	1837.17	3477.10	2.256	1579.06	3561.97
A_68_P26920198	chr10:7400931-7400975	NR_033219:4	BC020402	INSIDE	1.192	2.316	925.05	2142.05	2.760	836.31	2307.98
A_68_P22412543	chr3:108218968-108219012	NM_001004177:-578	Celsr2	PROMOTER	1.192	3.067	731.38	2243.17	3.655	618.00	2259.06
A_68_P26816896	chr9:108735008-108735052	NM_080437:6380	Celsr3	INSIDE	1.192	0.489	1330.34	649.95	0.583	956.08	556.91
A_68_P27941268	chr11:77893553-77893597	NM_022313:311	Eral1	INSIDE	1.192	1.418	2251.05	3192.11	1.691	1829.04	3092.02
A_68_P26458361	chr9:42705530-42705574	NM_175481:46902	Grik4	INSIDE	1.192	4.344	2887.47	12541.93	5.176	2200.02	11387.69
A_68_P23197991	chr4:108520598-108520642	NM_029571:159	Kti12	INSIDE	1.192	0.580	1232.03	714.76	0.692	1317.47	911.39
A_68_P28246268	chr12:12944044-12944088	NM_008709:4576	Mycn	INSIDE	1.192	3.402	588.88	2003.09	4.055	585.54	2374.45
A_68_P30961948	chr16:91270536-91270580	NM_016968:545	Olig1	INSIDE	1.192	2.525	1532.09	3867.92	3.010	1309.77	3942.72
A_68_P21626037	chr2:129514047-129514091	NM_018863:1536	Pdyn	INSIDE	1.192	5.092	2907.65	14805.35	6.069	2024.43	12285.94
A_68_P22825644	chr4:33276387-33276431	NM_001034867:304	Pm20d2	INSIDE	1.192	2.991	766.50	2292.29	3.566	706.84	2520.66
A_68_P22825900	chr4:33334938-33334982	NM_001033225:802	Pnrc1	INSIDE	1.192	1.788	1579.02	2822.94	2.131	1408.37	3001.07
A_68_P21604779	chr2:125608569-125608613	NM_177608:16	Secisbp2l	INSIDE	1.192	1.335	3704.89	4946.48	1.591	3098.16	4930.71
A_68_P25504896	chr7:134417212-134417256	NM_009266:339	Sephs2	INSIDE	1.192	3.351	485.04	1625.54	3.995	417.65	1668.70
A_68_P31093838	chr17:23956287-23956338	NM_175229:16159	Srrm2	INSIDE	1.192	2.276	367.58	836.48	2.713	295.98	802.86
A_68_P27466138	chr10:114239189-114239233	NM_146241:-784	Trhde	PROMOTER	1.192	2.823	1122.41	3169.11	3.365	972.02	3270.88
A_68_P25951333	chr8:72877019-72877063	NM_001122829:132	Upf1	INSIDE	1.192	3.104	613.15	1903.27	3.699	563.05	2082.45
A_68_P20319167	chr1:69423580-69423624			Unknown	1.192	1.903	657.24	1251.01	2.269	646.37	1466.36
A_68_P21533269	chr2:113057403-113057447	ENSMUST00000128192:-171		PROMOTER	1.192	2.249	1327.52	2985.22	2.681	1192.00	3195.92
A_68_P30927981	chr16:85173236-85173280	NM_001198823:694	App	INSIDE	1.191	2.061	1193.89	2460.99	2.455	1120.86	2751.57
A_68_P28074534	chr11:101413318-101413362	NM_009764:-71	Brca1	DIVERGENT_PROMOTER	1.191	1.978	790.37	1563.53	2.356	631.98	1489.22
A_68_P26423439	chr9:35366246-35366290	NM_013932:-213	Ddx25	DIVERGENT_PROMOTER	1.191	0.397	1534.71	609.30	0.473	1395.67	659.85
A_68_P24991559	chr7:19703647-19703691	NM_175530:-1539	Fbxo46	PROMOTER	1.191	2.506	1033.56	2590.50	2.985	781.12	2331.61
A_68_P20187366	chr1:43220621-43220665	NM_010212:164	Fhl2	INSIDE	1.191	1.713	659.20	1129.27	2.040	561.46	1145.29
A_68_P24135227	chr5:139856761-139856805	NM_001038703:-9075	Gpr146	PROMOTER	1.191	1.806	1308.77	2363.14	2.151	1151.99	2477.47
A_68_P26039231	chr8:90662304-90662348	NM_172757:543	Heatr3	INSIDE	1.191	1.426	1290.16	1839.73	1.699	1073.19	1822.86
A_68_P23331512	chr4:135528527-135528571	NM_011942:-39	Lypla2	PROMOTER	1.191	1.682	872.93	1468.63	2.004	736.21	1475.27
A_68_P27278629	chr10:79202122-79202166	NM_172551:7182	Polrmt	INSIDE	1.191	2.210	1590.85	3515.45	2.632	1177.78	3099.80
A_68_P31863198	chr18:80453249-80453293	NM_001164423:226	Pqlc1	INSIDE	1.191	1.555	491.19	763.82	1.853	392.29	726.80
A_68_P28523608	chr12:72076024-72076068	NM_011184:437	Psma3	INSIDE	1.191	2.132	680.54	1451.09	2.540	587.20	1491.59
A_68_P30401591	chr15:85408071-85408115	NM_001163634:408	Wnt7b	INSIDE	1.191	1.743	605.16	1054.96	2.077	590.37	1225.99
A_68_P24054285	chr5:123610907-123610951	ENSMUST00000162839:-2462		PROMOTER	1.191	2.607	1978.07	5156.25	3.106	1583.43	4917.43
A_68_P32145406	chr19:46764395-46764439	NM_025563:21	2010012O05Rik	INSIDE	1.190	1.643	671.21	1102.64	1.954	621.95	1215.38
A_68_P25394423	chr7:112935862-112935906	NM_001162943:180	Dchs1	INSIDE	1.190	1.440	1037.34	1494.04	1.713	954.00	1634.50
A_68_P26286958	chr9:7184623-7184667	NM_029775:79	Dcun1d5	INSIDE	1.190	4.112	566.12	2328.00	4.893	516.28	2526.06
A_68_P20294256	chr1:64783172-64783216	NM_001042659:1130	Fzd5	INSIDE	1.190	1.474	929.98	1371.07	1.754	749.64	1315.21
A_68_P20644881	chr1:138157260-138157304	NM_001101516:168	Gpr25	INSIDE	1.190	2.591	1119.21	2899.74	3.083	1048.01	3231.41
A_68_P27995148	chr11:87430476-87430520	NM_001045527:-167	Hsf5	PROMOTER	1.190	2.895	616.40	1784.54	3.446	559.56	1928.25
A_68_P25086723	chr7:51568193-51568237	NM_001034115:2581	Shank1	INSIDE	1.190	2.230	648.03	1444.80	2.653	497.98	1321.24
A_68_P32427541	chrX:61528987-61529031	NM_178740:1163	Slitrk4	INSIDE	1.190	2.126	2222.11	4724.22	2.529	1123.55	2841.52
A_68_P28155493	chr11:115676194-115676238	NM_029557:164	Tsen54	INSIDE	1.190	1.749	1975.10	3455.21	2.081	1574.95	3277.99
A_68_P23185485	chr4:106295363-106295407	NM_177667:331	Ttc22	INSIDE	1.190	3.357	249.85	838.66	3.995	242.24	967.81
A_68_P24425394	chr6:47974652-47974696	NM_001081382:23439	Zfp777	INSIDE	1.190	1.804	1045.36	1886.35	2.148	969.37	2082.10
A_68_P21105114	chr2:30626927-30626971			Unknown	1.190	2.951	569.73	1681.23	3.512	533.67	1873.98
A_68_P20046438	chr1:14496853-14496897			Unknown	1.190	2.234	898.78	2008.12	2.658	826.49	2196.89
A_68_P24856098	chr6:133055497-133055541	NM_001163445:262	2700089E24Rik	INSIDE	1.189	2.511	1150.75	2889.25	2.986	998.02	2980.26
A_68_P22953596	chr4:58924953-58924997	NM_172381:623	AI314180	INSIDE	1.189	3.328	283.12	942.33	3.958	280.63	1110.79
A_68_P24059412	chr5:124571591-124571635	NM_011256:94815	Pitpnm2	INSIDE	1.189	3.406	921.50	3138.28	4.049	794.11	3215.65
A_68_P28164070	chr11:117127735-117127779	NM_001113487:197	Septin9	INSIDE	1.189	1.800	1115.46	2008.19	2.140	1035.90	2216.58
A_68_P28751511	chr12:113882861-113882905	NM_001161737:-156	Siva1	PROMOTER	1.189	1.556	4921.13	7657.49	1.849	3920.30	7250.40

ProbeName	Target position of probe on CpG island microarray	TargetID	GeneSymbol	CpG island Description	Ratio of relative methylation (TiO_2-NP/Vehicle)	Sham group			TiO_2-H group		
						Relative methylation (Cy5/Cy3)	Cy3 signal (Input)	Cy5 signal (MeDIP)	Relative methylation (Cy5/Cy3)	Cy3 signal (Input)	Cy5 signal (MeDIP)
A_68_P24155692	chr5:144016313-144016357	NM_017467:15367	Zfp316	INSIDE	1.189	2.120	724.33	1535.30	2.519	615.54	1550.65
A_68_P24837689	chr6:128150353-128150397	ENSMUST00000071101:267		INSIDE	1.189	0.443	1350.21	598.63	0.527	1159.05	610.93
A_68_P27508735	chr10:121885914-121885958	NM_016847:382	Avpr1a	INSIDE	1.188	1.766	1349.40	2383.62	2.099	1122.68	2356.89
A_68_P20561474	chr1:122237586-122237630	NM_207233:450	C1ql2	INSIDE	1.188	1.979	1061.38	2100.76	2.351	921.90	2167.00
A_68_P25948475	chr8:72405501-72405545	NM_026818:5769	Cilp2	INSIDE	1.188	2.696	442.98	1194.29	3.202	421.83	1350.75
A_68_P21877294	chr2:174586233-174586277	NM_007903:-19	Edn3	PROMOTER	1.188	7.053	834.30	5884.22	8.380	743.66	6231.99
A_68_P21339409	chr2:74600922-74600966	NM_010467:-92	Hoxd1	PROMOTER	1.188	2.063	905.17	1867.31	2.450	780.69	1912.95
A_68_P21809984	chr2:162843268-162843312	NM_172150:83	Ift52	INSIDE	1.188	2.270	792.72	1799.20	2.695	724.18	1951.98
A_68_P24759668	chr6:113419933-113419977	NM_001034029:11066	Il17re	INSIDE	1.188	2.014	1210.10	2436.55	2.391	1148.67	2746.92
A_68_P24053123	chr5:123401996-123402040	NM_001005866:36524	Kdm2b	INSIDE	1.188	1.526	4698.48	7171.64	1.814	3737.51	6779.76
A_68_P20726276	chr1:155179693-155179737	NM_010683:202	Lamc1	INSIDE	1.188	3.524	1316.97	4641.04	4.186	1311.92	5491.91
A_68_P25266278	chr7:87508116-87508160	NM_172903:8123	Man2a2	INSIDE	1.188	2.501	995.22	2488.95	2.972	678.20	2015.69
A_68_P28052461	chr11:97605654-97605698	NM_054051:342	Pip4k2b	INSIDE	1.188	1.696	5214.13	8844.54	2.016	3995.07	8053.93
A_68_P24007303	chr5:115460994-115461038	NM_029012:-516	Sppl3	PROMOTER	1.188	2.151	1644.55	3538.22	2.556	1442.32	3686.58
A_68_P28755239	chr12:114425974-114426018	NM_153776:1883	Tmem121	INSIDE	1.188	1.731	675.00	1168.54	2.056	594.31	1221.78
A_68_P23984847	chr5:111706473-111706517	NM_024477:397673	Ttc28	INSIDE	1.188	2.347	483.10	1133.91	2.789	440.81	1229.29
A_68_P22878937	chr4:44769646-44769690	NM_138590:238	Zcchc7	INSIDE	1.188	1.381	1864.27	2575.32	1.641	1623.92	2664.81
A_68_P31623248	chr18:36479879-36479923	AK015580:1246		DOWNSTREAM	1.188	1.538	1715.45	2638.67	1.827	1513.76	2765.96
A_68_P31939720	chr19:7496955-7496999	NM_175381:4862	2700081O15Rik	INSIDE	1.187	1.668	5836.06	9734.12	1.981	3783.49	7493.24
A_68_P26351648	chr9:22193305-22193349	NM_028390:324	Anln	INSIDE	1.187	2.217	622.15	1379.04	2.631	581.35	1529.81
A_68_P21078053	chr2:26208046-26208090	NM_001037747:6999	Card9	INSIDE	1.187	2.198	861.78	1894.55	2.609	694.89	1812.96
A_68_P27278709	chr10:79217725-79217769	NM_023304:-117	Fgf22	DIVERGENT_PROMOTER	1.187	1.474	6133.92	9039.58	1.750	4083.47	7146.00
A_68_P31214873	chr17:48041518-48041563	NM_001110824:20041	Foxp4	INSIDE	1.187	5.301	3964.68	21018.71	6.292	2851.71	17943.38
A_68_P22864174	chr4:41702682-41702730	NM_016658:581	Galt	INSIDE	1.187	1.593	1093.96	1742.69	1.890	730.83	1381.41
A_68_P20769691	chr1:162965037-162965081	NR_002840:-238	Gas5	DIVERGENT_PROMOTER	1.187	3.134	1806.28	5660.63	3.721	1652.58	6149.38
A_68_P32240896	chrX:7499453-7499497	NM_013892:2527	Pcsk1n	INSIDE	1.187	1.443	2129.59	3072.44	1.713	1105.82	1893.79
A_68_P24992917	chr7:19955431-19955475	NM_001010836:8888	Ppp1r13l	INSIDE	1.187	1.593	1452.51	2313.95	1.891	1275.53	2412.50
A_68_P23545737	chr5:24606038-24606082	NM_145401:400	Prkag2	INSIDE	1.187	3.249	392.97	1276.68	3.857	367.32	1416.68
A_68_P26983364	chr10:19569406-19569450	NM_029529:1837	Slc35d3	INSIDE	1.187	1.625	1304.20	2118.67	1.928	1016.82	1960.91
A_68_P31163430	chr17:36408687-36408731	NM_178281:-330	Trim39	PROMOTER	1.187	1.483	962.34	1427.45	1.761	804.56	1416.46
A_68_P22129169	chr3:51144329-51144373	ENSMUST00000091144:138		INSIDE	1.187	2.044	1327.56	2713.40	2.426	1121.58	2720.45
A_68_P20286429	chr1:63411957-63412001	ENSMUST00000120413:-39		PROMOTER	1.187	2.465	915.34	2256.42	2.925	833.13	2437.20
A_68_P23467743	chr5:8368197-8368241	NM_001098225:-137	Adam22	PROMOTER	1.186	2.060	1137.28	2343.04	2.444	1056.57	2581.90
A_68_P26234239	chr8:125091584-125091628	NM_026014:-308	Cdt1	PROMOTER	1.186	1.887	1759.05	3319.18	2.238	1454.65	3254.82
A_68_P32769557	chrX:154481653-154481697	NM_177751:-632	Cnksr2	PROMOTER	1.186	2.112	1061.37	2242.13	2.505	635.68	1592.39
A_68_P24388781	chr6:40385401-40385445	NM_175528:710	E330009J07Rik	INSIDE	1.186	2.071	3956.35	8192.25	2.455	3097.27	7604.23
A_68_P21680290	chr2:139503712-139503756	NM_001126490:-179	Ism1	PROMOTER	1.186	1.578	802.04	1265.39	1.871	681.97	1275.83
A_68_P21206006	chr2:49922023-49922067	NM_177139:63	Lypd6	INSIDE	1.186	2.356	1095.68	2581.11	2.793	980.41	2738.11
A_68_P29919368	chr14:115439764-115439808	NR_029785:-3106	Mir17	PROMOTER	1.186	2.291	831.94	1905.57	2.718	868.55	2360.38
A_68_P25981542	chr8:79709972-79710016	NM_001083906:283588	Nr3c2	INSIDE	1.186	1.934	415.77	804.02	2.294	319.17	732.29
A_68_P22356140	chr3:97555778-97555823	NM_178080:16137	Pde4dip	INSIDE	1.186	2.617	519.58	1359.96	3.104	402.47	1249.13
A_68_P26744223	chr9:95745481-95745525	NM_001033210:196	Pls1	INSIDE	1.186	2.495	2416.27	6029.45	2.960	2154.27	6375.87
A_68_P21843419	chr2:168591449-168591493	NM_175303:1231	Sall4	INSIDE	1.186	1.774	1288.52	2285.22	2.104	1112.90	2340.99
A_68_P20945912	chr1:194681174-194681218	NM_001177794:-3255	Sertad4	PROMOTER	1.186	3.961	384.23	1521.92	4.697	425.63	1999.37
A_68_P27947942	chr11:79067536-79067580	NM_019653:639	Wsb1	INSIDE	1.186	2.064	1868.53	3856.69	2.448	1572.98	3850.16
A_68_P21721815	chr2:146838797-146838841	NM_011917:23	Xrn2	INSIDE	1.186	1.589	1574.55	2502.68	1.884	1402.54	2642.84
A_68_P30402203	chr15:85502100-85502144	AK038224:-5049		PROMOTER	1.186	1.807	304.07	549.58	2.144	261.43	560.43
A_68_P22941605	chr4:56814945-56814989	NM_001081420:-234	BC026590	PROMOTER	1.185	1.675	1300.10	2177.03	1.985	1042.38	2069.03
A_68_P23607906	chr5:36827371-36827415	NM_025725:156	Ccdc96	INSIDE	1.185	1.484	749.61	1112.23	1.758	597.66	1050.89
A_68_P31921839	chr19:4214705-4214749	NM_019952:335	Clcf1	INSIDE	1.185	2.233	1026.28	2291.78	2.647	902.86	2389.76
A_68_P21752059	chr2:152769043-152769087	NM_001159376:8254	Dusp15	INSIDE	1.185	2.077	342.13	710.58	2.461	358.37	881.85

ProbeName	Target position of probe on CpG island microarray	TargetID	GeneSymbol	CpG island Description	Ratio of relative methylation (TiO_2-NP/Vehicle)	Sham group			TiO_2-H group		
						Relative methylation (Cy5/Cy3)	Cy3 signal (Input)	Cy5 signal (MeDIP)	Relative methylation (Cy5/Cy3)	Cy3 signal (Input)	Cy5 signal (MeDIP)
A_68_P22957376	chr4:59638970-59639014	NM_153158:-100	E130308A19Rik	PROMOTER	1.185	2.119	1021.97	2165.07	2.511	939.08	2357.65
A_68_P27640630	chr11:22186736-22186780	NM_153078:-917	Ehbp1	PROMOTER	1.185	2.496	1389.99	3469.37	2.958	1241.61	3672.92
A_68_P27180609	chr10:60915255-60915299	NM_010124:141	Eif4ebp2	INSIDE	1.185	1.630	1554.18	2534.08	1.932	1249.09	2413.82
A_68_P31927969	chr19:5307468-5307512	NM_001024717:9160	Gal3st3	INSIDE	1.185	3.004	403.44	1212.03	3.559	387.39	1378.67
A_68_P29072859	chr13:58460443-58460487	NM_175214:-4241	Kif27	PROMOTER	1.185	1.384	2947.81	4079.93	1.641	2500.69	4102.47
A_68_P31930563	chr19:5742280-5742324	NM_008520:1399	Ltbp3	INSIDE	1.185	2.833	929.09	2632.34	3.358	853.78	2866.75
A_68_P30340463	chr15:74785980-74786024	NM_001164040:467	Ly6e	INSIDE	1.185	2.730	21731.91	59321.27	3.234	14707.19	47568.69
A_68_P32266424	chrX:13063560-13063604	NM_173415:18785	Nyx	INSIDE	1.185	4.530	363.54	1646.94	5.366	262.53	1408.85
A_68_P20782634	chr1:165242803-165242847	NM_011127:957	Prrx1	INSIDE	1.185	2.155	542.24	1168.54	2.553	451.77	1153.34
A_68_P25588867	chr7:148633309-148633353	NM_026020:-218	Rplp2	DIVERGENT_PROMOTER	1.185	1.692	1470.19	2486.98	2.004	1106.43	2217.30
A_68_P26887821	chr9:122075732-122075776	NM_001164572:49371	Snrk	INSIDE	1.185	1.881	1028.17	1934.07	2.228	775.75	1728.57
A_68_P31421396	chr17:87534273-87534317	NM_019654:27276	Socs5	INSIDE	1.185	3.050	2188.27	6674.15	3.615	1812.63	6553.10
A_68_P21864919	chr2:172376460-172376504	NM_001159696:-8	Tcfap2c	PROMOTER	1.185	1.716	507.49	870.94	2.033	376.61	765.74
A_68_P24819777	chr6:125236044-125236088	NM_009446:-6	Tuba3a	PROMOTER	1.185	1.612	739.24	1191.95	1.911	570.68	1090.31
A_68_P20977354	chr2:6022037-6022081	NM_001159657:29173	5430407P10Rik	INSIDE	1.184	2.323	409.44	950.97	2.749	347.66	955.67
A_68_P24010911	chr5:116112644-116112688	NM_001080808:68902	Ccdc64	INSIDE	1.184	3.239	3818.08	12366.38	3.833	3197.66	12257.70
A_68_P27934411	chr11:76660318-76660362	NM_007754:170	Cpd	INSIDE	1.184	0.507	1023.03	519.18	0.601	907.53	545.40
A_68_P25949731	chr8:72608268-72608312	NM_177900:863	Hapln4	INSIDE	1.184	1.438	804.93	1157.12	1.703	651.75	1109.70
A_68_P27315613	chr10:86168167-86168211	NM_011631:1	Hsp90b1	INSIDE	1.184	1.564	4080.66	6382.68	1.853	3268.24	6054.53
A_68_P21420371	chr2:90894443-90894487	NM_008948:292	Psmc3	INSIDE	1.184	3.449	2662.54	9182.12	4.083	2021.47	8254.26
A_68_P32019129	chr19:23762386-23762431	NM_028208:519	Ptar1	INSIDE	1.184	2.079	290.24	603.33	2.462	254.60	626.81
A_68_P25025138	chr7:29849498-29849542	NM_009109:60650	Ryr1	INSIDE	1.184	2.703	1634.30	4417.20	3.199	1327.01	4245.69
A_68_P26750366	chr9:96896367-96896411	NM_145134:22386	Spsb4	INSIDE	1.184	2.871	457.80	1314.36	3.400	410.59	1395.93
A_68_P28678695	chr12:101122821-101122865	NM_028354:118	Tdp1	INSIDE	1.184	3.712	433.79	1610.22	4.397	383.01	1683.92
A_68_P31092442	chr17:23713394-23713438	NM_011747:23038	Zfp13	INSIDE	1.184	1.502	1815.08	2726.65	1.779	1364.63	2427.39
A_68_P32674749	chrX:131543372-131543416	NM_175446:354	Zmat1	INSIDE	1.184	2.635	546.21	1438.99	3.118	325.94	1016.40
A_68_P28752686	chr12:114047096-114047140	NM_145450:-67	BC022687	PROMOTER	1.183	1.855	823.29	1526.84	2.193	617.78	1354.87
A_68_P28313704	chr12:29237167-29237211	NM_001177964:-3986	Dcdc2c	PROMOTER	1.183	2.331	826.07	1925.55	2.759	692.29	1909.80
A_68_P21819504	chr2:164571506-164571550	NM_133763:14	Dnttip1	INSIDE	1.183	2.527	1027.54	2596.88	2.991	918.72	2747.56
A_68_P29703988	chr14:71289674-71289718	NM_008115:-240	Gfra2	PROMOTER	1.183	1.804	546.38	985.88	2.134	428.67	914.97
A_68_P24133864	chr5:139626346-139626390	NM_001081265:192	Heatr2	INSIDE	1.183	2.951	1728.63	5102.00	3.490	1598.98	5580.66
A_68_P31244136	chr17:53706802-53706846	NM_020005:529	Kat2b	INSIDE	1.183	2.247	692.47	1556.13	2.657	577.68	1535.14
A_68_P30795699	chr16:59471732-59471776	NM_025910:154	Mina	INSIDE	1.183	1.667	1395.38	2326.12	1.972	1287.78	2538.87
A_68_P27708454	chr11:35583064-35583108	NM_145962:90	Pank3	INSIDE	1.183	2.247	5735.70	12885.78	2.658	4833.75	12850.05
A_68_P21612440	chr2:127096105-127096149	NM_139308:162	Stard7	INSIDE	1.183	4.366	1182.72	5163.27	5.165	911.91	4710.40
A_68_P28074032	chr11:101327214-101327258	NM_012037:277	Vat1	INSIDE	1.183	1.883	287.23	540.81	2.227	251.38	559.85
A_68_P20859688	chr1:179372159-179372203	NM_001012330:-2611	Zfp238	PROMOTER	1.183	0.499	1499.90	748.11	0.590	1220.01	719.64
A_68_P21104695	chr2:30549015-30549059	ENSMUST00000146702:63862		DOWNSTREAM	1.183	2.707	639.24	1730.46	3.203	529.32	1695.54
A_68_P24864121	chr6:134848481-134848525	NR_029441:524	2810454H06Rik	INSIDE	1.182	1.563	488.17	763.02	1.848	498.94	922.13
A_68_P31331958	chr17:71351874-71351918	NM_026064:-23	2900073G15Rik	PROMOTER	1.182	1.855	858.64	1592.84	2.193	746.12	1636.45
A_68_P26019649	chr8:86938288-86938332	NM_007578:-952	Cacna1a	PROMOTER	1.182	2.444	815.37	1992.98	2.888	781.70	2257.55
A_68_P27764452	chr11:45665495-45665539	NM_001045520:51	Clint1	INSIDE	1.182	1.525	772.74	1178.77	1.804	801.22	1445.12
A_68_P23192160	chr4:107356153-107356197	NM_019872:593	Dmrtb1	INSIDE	1.182	3.323	619.94	2059.90	3.929	545.95	2144.78
A_68_P20825164	chr1:172815355-172815399	NM_023173:295	Dusp12	INSIDE	1.182	1.396	1760.97	2457.70	1.649	1559.16	2571.46
A_68_P27893912	chr11:69049971-69050015	NM_008192:532	Gucy2e	INSIDE	1.182	2.340	772.44	1807.42	2.767	710.63	1966.12
A_68_P25274810	chr7:89078740-89078784	NM_013886:583	Hdgfrp3	INSIDE	1.182	2.374	1065.61	2529.93	2.806	985.69	2765.69
A_68_P31558687	chr18:24280026-24280070	NM_172625:272	Ino80c	INSIDE	1.182	2.458	2861.81	7033.48	2.905	2881.99	8371.38
A_68_P27828613	chr11:57822962-57823006	NM_028451:419	Larp1	INSIDE	1.182	5.529	528.11	2919.82	6.538	413.35	2702.39
A_68_P24159776	chr5:144934512-144934556	NM_001081109:73230	Lmtk2	INSIDE	1.182	2.453	632.54	1551.69	2.900	510.60	1480.78
A_68_P21240085	chr2:56976720-56976764	NM_001139509:-328	Nr4a2	PROMOTER	1.182	2.356	1070.53	2521.96	2.784	963.40	2682.54
A_68_P32147398	chr19:47125047-47125091	NM_027654:267	Pcgf6	INSIDE	1.182	1.655	499.17	825.88	1.956	465.54	910.51

ProbeName	Target position of probe on CpG island microarray	TargetID	GeneSymbol	CpG island Description	Ratio of relative methylation (TiO_2-NP/Vehicle)	Sham group			TiO_2-H group		
						Relative methylation (Cy5/Cy3)	Cy3 signal (Input)	Cy5 signal (MeDIP)	Relative methylation (Cy5/Cy3)	Cy3 signal (Input)	Cy5 signal (MeDIP)
A_68_P22518661	chr3:128907456-128907500	NM_001042502:-9376	Pitx2	PROMOTER	1.182	2.649	2229.61	5905.84	3.131	1841.62	5765.92
A_68_P31802711	chr18:69505644-69505688	NM_001083967:292	Tcf4	INSIDE	1.182	1.646	747.31	1230.06	1.946	626.15	1218.63
A_68_P22510646	chr3:127492950-127492994	NM_145133:142	Tifa	INSIDE	1.182	1.546	1220.98	1887.04	1.828	1104.80	2019.08
A_68_P25411545	chr7:117205191-117205235	NM_009281:-3	Zfp143	PROMOTER	1.182	3.087	10187.97	31449.07	3.649	7588.74	27692.57
A_68_P24800508	chr6:120786336-120786380	NM_153516:111	Bcl2l13	INSIDE	1.181	3.461	2660.20	9207.73	4.089	1988.63	8131.17
A_68_P28944866	chr13:35832734-35832778	NM_001123386:-514	Cdyl	PROMOTER	1.181	2.282	1083.90	2473.82	2.696	879.59	2371.61
A_68_P25313627	chr7:97591983-97592027	NM_145151:714	Crebzf	INSIDE	1.181	2.402	1157.23	2779.37	2.835	1094.65	3103.85
A_68_P24951534	chr7:4960187-4960231	NM_001110330:4636	Fiz1	INSIDE	1.181	2.640	762.45	2012.68	3.116	749.75	2336.50
A_68_P31075179	chr17:17987266-17987310	NM_008215:4864	Has1	INSIDE	1.181	2.604	1723.66	4488.49	3.075	1390.40	4275.85
A_68_P23192848	chr4:107475278-107475322	NM_001080926:437	Lrp8	INSIDE	1.181	1.593	933.55	1487.56	1.883	822.84	1549.00
A_68_P32181745	chr19:53388277-53388321	NM_001008542:3303	Mxi1	INSIDE	1.181	1.535	823.75	1264.39	1.813	757.53	1373.06
A_68_P22875663	chr4:44181273-44181317	NM_001038993:-139	Rnf38	PROMOTER	1.181	2.728	478.17	1304.55	3.221	446.94	1439.71
A_68_P27923093	chr11:74710538-74710582	NM_197943:22	Sgsm2	INSIDE	1.181	1.988	1772.45	3524.28	2.348	1486.53	3490.62
A_68_P28071034	chr11:100800458-100800502	NM_011486:345	Stat3	INSIDE	1.181	1.405	1214.34	1706.46	1.659	1050.90	1743.59
A_68_P23966229	chr5:107717967-107718011	NM_011578:626	Tgfbr3	INSIDE	1.181	2.620	1501.38	3933.04	3.093	1306.12	4040.12
A_68_P21739582	chr2:150351755-150351799	ENSMUST00000119514:4948		DOWNSTREAM	1.181	1.374	1832.06	2517.64	1.623	1698.13	2755.29
A_68_P27899783	chr11:70068622-70068666	NM_007440:199	Alox12	INSIDE	1.180	1.346	2549.15	3430.63	1.588	1853.75	2943.08
A_68_P30990992	chr16:96303585-96303629	NM_145125:429	Brwd1	INSIDE	1.180	2.371	2169.95	5145.85	2.797	2021.10	5653.66
A_68_P32316566	chrX:33565537-33565581	NM_001009947:136732	Dock11	INSIDE	1.180	3.352	858.77	2878.78	3.956	606.25	2398.36
A_68_P28051738	chr11:97490235-97490279	NM_175332:774	E130012A19Rik	INSIDE	1.180	1.988	1857.30	3691.41	2.345	1690.47	3964.67
A_68_P30478816	chr15:99054388-99054432	NM_010601:-996	Kcnh3	PROMOTER	1.180	1.554	2388.94	3713.30	1.833	1842.63	3378.35
A_68_P32396635	chrX:53850948-53850992	NM_146234:126	Mmgt1	INSIDE	1.180	1.481	866.96	1284.08	1.748	415.55	726.20
A_68_P24992504	chr7:19878717-19878763	NM_001025364:2293	Rtn2	INSIDE	1.180	3.278	1027.12	3366.73	3.868	788.62	3050.45
A_68_P27340134	chr10:90586370-90586414	NM_133668:316	Slc25a3	INSIDE	1.180	0.543	1031.27	559.70	0.640	899.50	575.84
A_68_P28052666	chr11:97643069-97643113	NR_028078:-10	Snora21	PROMOTER	1.180	3.549	623.28	2212.08	4.189	494.91	2072.95
A_68_P20353686	chr1:75384915-75384959	NM_001085370:2752	Speg	INSIDE	1.180	1.553	1206.68	1874.30	1.833	1135.92	2082.23
A_68_P31335315	chr17:71965807-71965851	NM_175639:274	Wdr43	INSIDE	1.180	2.560	1507.13	3858.32	3.022	1328.52	4014.40
A_68_P23195378	chr4:107973651-107973695	NM_001033634:23	Zyg11b	INSIDE	1.180	1.535	3893.30	5977.24	1.811	2816.66	5101.46
A_68_P33005897	A_68_P33005897			Unknown	1.180	2.981	617.20	1839.84	3.518	536.57	1887.64
A_68_P31620715	chr18:36048154-36048198			Unknown	1.180	2.156	932.07	2009.85	2.545	857.65	2182.47
A_68_P21839558	chr2:167942901-167942945			Unknown	1.180	6.706	1506.48	10102.30	7.911	1155.79	9143.71
A_68_P32373281	chrX:48194954-48194998	NM_133729:364	2610018G03Rik	INSIDE	1.179	1.657	911.24	1509.50	1.953	537.77	1050.16
A_68_P29523397	chr14:34133413-34133457	NM_153800:103426	Arhgap22	INSIDE	1.179	1.504	1672.24	2514.42	1.773	1478.82	2621.22
A_68_P20753946	chr1:160292678-160292722	NM_001205204:266	Astn1	INSIDE	1.179	4.381	602.88	2640.95	5.163	524.62	2708.50
A_68_P21814762	chr2:163679681-163679725	NM_001030292:217	Kcnk15	INSIDE	1.179	2.991	712.02	2129.53	3.528	623.69	2200.14
A_68_P22201243	chr3:65463433-65463477	NM_001166659:-6695	Lekr1	PROMOTER	1.179	3.237	796.34	2577.44	3.815	737.08	2811.72
A_68_P31923503	chr19:4509996-4510046	NM_001162946:-451	Pcx	PROMOTER	1.179	1.900	1097.27	2085.11	2.241	755.88	1694.03
A_68_P30650135	chr16:31932946-31932990	NM_172822:-968	Pigz	PROMOTER	1.179	2.748	258.77	711.14	3.240	282.25	914.61
A_68_P21110366	chr2:31493479-31493523	NM_001123362:-2056	Prdm12	PROMOTER	1.179	1.786	1397.58	2496.15	2.105	1209.03	2545.39
A_68_P29605617	chr14:52877185-52877229	NM_172601:21864	Rab2b	DOWNSTREAM	1.179	1.397	2622.90	3663.30	1.647	2105.01	3466.29
A_68_P20011684	chr1:6205145-6205189	NM_009826:424	Rb1cc1	INSIDE	1.179	1.886	402.52	759.02	2.224	360.74	802.11
A_68_P28957533	chr13:38023055-38023099	NM_001177869:42337	Rreb1	INSIDE	1.179	2.465	1137.56	2804.27	2.907	957.77	2784.12
A_68_P21820793	chr2:164793533-164793577	NM_020333:67	Slc12a5	INSIDE	1.179	1.665	3206.47	5337.49	1.963	2511.42	4930.66
A_68_P27848100	chr11:61191430-61191474	NM_026183:125	Slc47a1	INSIDE	1.179	2.168	675.67	1464.89	2.556	640.54	1637.46
A_68_P29955473	chr14:121778112-121778156	NM_145465:318	Stk24	INSIDE	1.179	1.922	942.99	1812.06	2.265	894.12	2024.94
A_68_P32562270	chrX:98255164-98255208	NM_031384:-208	Tex11	PROMOTER	1.179	2.236	1378.80	3082.92	2.637	576.46	1520.01
A_68_P23991123	chr5:112705779-112705823	NM_009419:74	Tpst2	INSIDE	1.179	2.513	759.96	1909.76	2.963	687.45	2037.12
A_68_P25663078	chr8:12672178-12672222	NM_198031:-100	Tubgcp3	PROMOTER	1.179	1.896	290.89	551.43	2.236	246.83	551.84
A_68_P32237968	chrX:6896043-6896087	ENSMUST00000132126:232		INSIDE	1.179	1.936	528.59	1023.57	2.283	379.29	865.96
A_68_P30567433	chr16:15887647-15887691	NM_007679:290	Cebpd	INSIDE	1.178	0.523	1530.76	800.83	0.617	1133.01	698.52
A_68_P25827831	chr8:46095191-46095238	NM_001081286:59653	Fat1	INSIDE	1.178	2.745	210.19	577.07	3.233	197.04	637.04

ProbeName	Target position of probe on CpG island microarray	TargetID	GeneSymbol	CpG island Description	Ratio of relative methylation (TiO_2-NP/Vehicle)	Sham group			TiO_2-H group		
						Relative methylation (Cy5/Cy3)	Cy3 signal (Input)	Cy5 signal (MeDIP)	Relative methylation (Cy5/Cy3)	Cy3 signal (Input)	Cy5 signal (MeDIP)
A_68_P26572465	chr9:62659122-62659166	NM_010193:311	Fem1b	INSIDE	1.178	1.743	2653.03	4623.82	2.054	2281.84	4686.31
A_68_P32745698	chrX:147955401-147955445	NM_177201:208	Phf8	INSIDE	1.178	2.814	562.00	1581.30	3.315	362.33	1201.23
A_68_P21431994	chr2:92855345-92855389	NM_001177536:30935	Prdm11	INSIDE	1.178	1.386	1167.56	1618.76	1.633	1102.41	1800.08
A_68_P28096908	chr11:105451551-105451595	NM_181071:273	Tanc2	INSIDE	1.178	2.084	3086.66	6433.49	2.455	2486.33	6103.63
A_68_P27798805	chr11:52175093-52175137	NM_011694:498	Vdac1	INSIDE	1.178	0.536	1313.28	703.50	0.631	1043.15	658.33
A_68_P31599638	chr18:31963974-31964018	NM_001170966:286	Wdr33	INSIDE	1.178	1.648	866.41	1427.64	1.942	817.58	1587.44
A_68_P28577649	chr12:81891588-81891632	NM_178682:65	4933426M11Rik	INSIDE	1.177	1.454	1063.69	1546.33	1.712	989.54	1693.60
A_68_P24160788	chr5:145119007-145119051	NM_025833:-47	Baiap2l1	PROMOTER	1.177	2.903	2923.69	8488.36	3.417	2530.79	8647.03
A_68_P27170203	chr10:59165375-59165419	NM_144822:14	Cbara1	INSIDE	1.177	1.810	2896.74	5244.33	2.131	2134.20	4548.60
A_68_P21853424	chr2:170322747-170322791	NM_009996:-130	Cyp24a1	PROMOTER	1.177	1.871	4193.27	7846.27	2.202	3098.94	6822.80
A_68_P20831242	chr1:174078262-174078306	NM_153555:139	Dcaf8	INSIDE	1.177	2.111	2711.99	5725.63	2.485	2520.61	6262.84
A_68_P31106387	chr17:25946215-25946259	NM_001164225:207	Fbxl16	INSIDE	1.177	3.750	590.90	2215.86	4.414	538.78	2378.10
A_68_P31936769	chr19:7008810-7008854	NM_008431:173	Kcnk4	INSIDE	1.177	1.592	2284.28	3637.05	1.874	1926.29	3610.47
A_68_P21077008	chr2:26058081-26058125	NM_001039653:3974	Lhx3	INSIDE	1.177	1.999	1203.82	2406.55	2.352	1068.07	2512.45
A_68_P27551861	chr11:3309133-3309177	NM_010718:84	Limk2	INSIDE	1.177	1.784	2180.04	3888.87	2.100	1716.91	3606.21
A_68_P24027257	chr5:119011484-119011528	NM_172424:779	Med13l	INSIDE	1.177	1.739	1121.26	1949.53	2.047	866.97	1774.75
A_68_P20240461	chr1:55187949-55187993	NM_025283:-118	Mobkl3	PROMOTER	1.177	1.892	1017.01	1924.51	2.227	902.22	2009.10
A_68_P23430797	chr4:153715735-153715779	NM_001177995:295226	Prdm16	INSIDE	1.177	4.816	4127.11	19877.44	5.671	3108.68	17628.89
A_68_P25089827	chr7:52125023-52125067	NM_133949:114	Ptov1	INSIDE	1.177	2.339	1331.25	3114.42	2.753	1224.34	3370.60
A_68_P24464123	chr6:54516358-54516402	NM_027268:-4	Scrn1	PROMOTER	1.177	1.939	1267.41	2457.38	2.282	1200.89	2739.99
A_68_P26983373	chr10:19570476-19570520	NM_029529:767	Slc35d3	INSIDE	1.177	5.374	1943.91	10446.65	6.327	1830.25	11580.90
A_68_P30479870	chr15:99223391-99223435	NM_001171035:-237	Tmbim6	PROMOTER	1.177	2.258	2572.45	5808.41	2.657	1754.23	4660.94
A_68_P27286198	chr10:80360560-80360604	NM_001081688:18022	Tmprss9	INSIDE	1.177	2.091	1863.20	3896.65	2.462	1467.24	3612.94
A_68_P27636577	chr11:21270655-21270699	NM_139297:206	Ugp2	INSIDE	1.177	18.291	4294.76	78553.37	21.521	3151.00	67811.32
A_68_P30351142	chr15:76742082-76742126	NM_001168276:13774	Zfp647	INSIDE	1.177	2.112	683.51	1443.70	2.487	546.51	1359.20
A_68_P28724518	chr12:109154385-109154429	NM_001079883:87218	Bcl11b	INSIDE	1.176	2.405	3047.11	7327.36	2.828	2396.16	6775.97
A_68_P21821742	chr2:164941702-164941746	NM_174988:118513	Cdh22	INSIDE	1.176	2.712	2489.47	6752.49	3.189	1734.16	5529.61
A_68_P24818636	chr6:125046050-125046094	NM_145979:-108	Chd4	PROMOTER	1.176	2.328	6303.65	14674.87	2.737	4637.39	12692.72
A_68_P26519505	chr9:53055899-53055943	NM_029936:297	Ddx10	INSIDE	1.176	3.135	8812.13	27625.80	3.687	6282.21	23162.20
A_68_P28925109	chr13:31900260-31900304	NM_008592:1768	Foxc1	INSIDE	1.176	0.490	2394.15	1172.73	0.576	2210.88	1273.86
A_68_P24052688	chr5:123330338-123330382	NM_013910:19999	Kdm2b	INSIDE	1.176	2.162	2575.04	5567.15	2.543	2090.33	5315.33
A_68_P28513753	chr12:70397631-70397675	NM_027117:-15	Klhdc2	PROMOTER	1.176	1.827	1015.23	1854.31	2.147	926.60	1989.82
A_68_P22210771	chr3:67386632-67386676	NM_025813:-35	Mfsd1	PROMOTER	1.176	3.689	840.82	3102.06	4.337	618.77	2683.79
A_68_P21904884	chr2:180324017-180324061	NM_031373:-73	Ogfr	PROMOTER	1.176	1.986	1002.71	1991.18	2.336	858.38	2005.34
A_68_P24053485	chr5:123465296-123465340	NM_175423:236	Orai1	INSIDE	1.176	3.759	660.33	2482.04	4.421	653.26	2887.93
A_68_P32326382	chrX:35474320-35474364	NM_001185002:326	Rhox13	INSIDE	1.176	2.115	738.67	1562.32	2.487	388.24	965.44
A_68_P22324939	chr3:90193494-90193538	NM_011988:333	Slc27a3	INSIDE	1.176	2.058	965.88	1988.26	2.421	937.30	2269.29
A_68_P31056399	chr17:13898251-13898295	NM_022311:129	Tcte2	INSIDE	1.176	2.522	2553.85	6439.65	2.967	2185.59	6483.62
A_68_P25032389	chr7:31373349-31373393	NM_029274:375	Wbp7	INSIDE	1.176	1.721	5578.90	9599.10	2.024	4074.99	8248.28
A_68_P28593636	chr12:84937949-84937993	NM_183154:127	Zfyve1	INSIDE	1.176	1.501	3434.07	5155.41	1.766	3008.02	5312.60
A_68_P21071713	chr2:25228424-25228468	NM_001081085:606	2010317E24Rik	INSIDE	1.175	3.315	480.47	1592.85	3.895	435.00	1694.37
A_68_P23391467	chr4:147315560-147315604	NM_029841:579	2510039O18Rik	INSIDE	1.175	2.276	1203.32	2738.18	2.674	1099.53	2940.23
A_68_P21900711	chr2:179711603-179711647	NR_033782:67	4921531C22Rik	INSIDE	1.175	1.834	3091.93	5670.25	2.154	2597.42	5596.03
A_68_P21346286	chr2:75670175-75670220	NM_172666:-36	Agps	PROMOTER	1.175	4.022	1651.64	6643.53	4.726	1444.21	6825.85
A_68_P24816767	chr6:124695514-124695558	NM_007881:10969	Atn1	INSIDE	1.175	2.735	1605.21	4389.64	3.214	1331.81	4280.50
A_68_P31794502	chr18:68093477-68093521	NM_172631:588	D18Ertd653e	INSIDE	1.175	1.654	2675.89	4424.72	1.942	2205.60	4283.81
A_68_P24123516	chr5:137358920-137358964	NM_024474:35	Emid2	INSIDE	1.175	1.492	1062.72	1585.22	1.753	973.25	1706.07
A_68_P31544268	chr18:21458551-21458595	NM_001033445:68	Fam59a	INSIDE	1.175	1.876	993.04	1862.59	2.204	813.80	1793.40
A_68_P26761427	chr9:98856071-98856115	NM_012020:67	Foxl2	INSIDE	1.175	2.878	1162.33	3345.53	3.382	974.36	3294.88
A_68_P22248625	chr3:75760605-75760649	NM_175193:127	Golim4	INSIDE	1.175	1.576	1553.47	2448.55	1.851	1182.10	2188.51
A_68_P31425150	chr17:88196845-88196889	NM_199251:468	Kcnk12	INSIDE	1.175	2.834	594.01	1683.27	3.329	584.56	1945.98

ProbeName	Target position of probe on CpG island microarray	TargetID	GeneSymbol	CpG island Description	Ratio of relative methylation (TiO_2-NP/Vehicle)	Sham group			TiO_2-H group		
						Relative methylation (Cy5/Cy3)	Cy3 signal (Input)	Cy5 signal (MeDIP)	Relative methylation (Cy5/Cy3)	Cy3 signal (Input)	Cy5 signal (MeDIP)
A_68_P26811597	chr9:107790348-107790392	NM_028369:-89	Mon1a	PROMOTER	1.175	1.496	674.87	1009.67	1.758	556.77	979.04
A_68_P21351542	chr2:76486311-76486355	NM_011871:-281	Prkra	DIVERGENT_PROMOTER	1.175	2.351	3629.28	8530.82	2.762	2854.07	7882.19
A_68_P22405721	chr3:107137047-107137091	NM_001045807:-861	Rbm15	PROMOTER	1.175	1.853	658.70	1220.27	2.177	654.33	1424.52
A_68_P21078764	chr2:26300667-26300711	NM_153125:48	Sec16a	INSIDE	1.175	3.699	2327.97	8611.71	4.347	1921.31	8352.09
A_68_P32320134	chrX:34335518-34335562	NM_007451:-106	Slc25a5	PROMOTER	1.175	1.383	1384.81	1915.45	1.625	857.68	1393.69
A_68_P31058955	chr17:14417592-14417636	NM_022315:1102	Smoc2	INSIDE	1.175	1.933	1349.92	2609.07	2.271	1247.15	2832.86
A_68_P24663948	chr6:95668305-95668349	NM_011507:505	Suclg2	INSIDE	1.175	1.811	758.30	1372.99	2.128	625.67	1331.39
A_68_P30595261	chr16:21947632-21947676	NM_025693:-29	Tmem41a	PROMOTER	1.175	2.319	1071.51	2484.48	2.724	928.62	2529.91
A_68_P30023613	chr15:12669624-12669668	ENSMUST00000096893:33		INSIDE	1.175	4.322	408.86	1767.12	5.079	375.46	1907.07
A_68_P26225640	chr8:123797224-123797270	ENSMUST00000127664:1362506		INSIDE	1.175	3.168	620.19	1964.88	3.724	516.68	1924.10
A_68_P22082007	chr3:40698419-40698463	NM_026622:242	3110057O12Rik	INSIDE	1.174	1.574	597.20	939.78	1.847	535.28	988.92
A_68_P25354430	chr7:105342884-105342928	NM_001081167:5079	B3gnt6	INSIDE	1.174	2.240	405.30	907.91	2.629	372.63	979.71
A_68_P20329006	chr1:71149346-71149390	NM_007525:178	Bard1	INSIDE	1.174	1.798	999.83	1797.81	2.111	995.65	2102.21
A_68_P26236468	chr8:125388849-125388893	NM_007662:16597	Cdh15	INSIDE	1.174	1.539	844.80	1299.78	1.806	749.67	1354.25
A_68_P25009389	chr7:26063544-26063588	NM_001110131:-3631	Cic	PROMOTER	1.174	2.985	1331.06	3972.77	3.505	1095.19	3839.02
A_68_P29455231	chr14:22667975-22668019	NM_026965:136	Comtd1	INSIDE	1.174	2.696	15959.49	43034.38	3.165	13321.09	42167.36
A_68_P23203754	chr4:109653015-109653059	NM_172296:2407	Dmrta2	INSIDE	1.174	1.562	2790.54	4358.82	1.834	2191.03	4019.43
A_68_P31343015	chr17:73268700-73268744	NM_029999:1078	Lbh	INSIDE	1.174	1.550	1411.15	2187.14	1.820	1173.68	2135.68
A_68_P31601518	chr18:32322635-32322679	NM_011946:-86	Map3k2	PROMOTER	1.174	2.837	510.27	1447.68	3.330	529.79	1764.37
A_68_P22898222	chr4:48064747-48064791	NM_015743:649	Nr4a3	INSIDE	1.174	2.191	2419.99	5301.69	2.571	1900.45	4886.03
A_68_P22309969	chr3:87587605-87587650	NM_001033124:11457	Ntrk1	INSIDE	1.174	2.652	400.59	1062.18	3.114	355.54	1107.04
A_68_P29471619	chr14:25306273-25306317	NM_001081247:-26	Polr3a	DIVERGENT_PROMOTER	1.174	1.551	624.42	968.48	1.820	553.74	1007.87
A_68_P26044775	chr8:91565596-91565640	NM_021390:2443	Sall1	INSIDE	1.174	1.454	2192.95	3188.24	1.706	1881.28	3210.37
A_68_P22338877	chr3:94217455-94217499	NM_028307:237	Tdrkh	INSIDE	1.174	1.939	5021.73	9735.26	2.275	3385.58	7702.92
A_68_P24776845	chr6:116411172-116411216	NM_009662:2	Alox5	INSIDE	1.173	2.585	1718.19	4441.70	3.033	1492.39	4526.74
A_68_P25756352	chr8:32261404-32261448	NM_144927:639	BC019943	INSIDE	1.173	2.060	437.02	900.07	2.416	377.37	911.55
A_68_P26346396	chr9:21095892-21095936	NM_009878:-261	Cdkn2d	PROMOTER	1.173	2.361	291.74	688.84	2.769	256.26	709.62
A_68_P24817997	chr6:124915301-124915345	NM_012003:93	Cops7a	INSIDE	1.173	1.540	3010.61	4637.26	1.806	2495.11	4506.97
A_68_P30082169	chr15:25552086-25552130	NM_019472:-196	Myo10	PROMOTER	1.173	3.038	16835.81	51145.46	3.564	9923.02	35369.00
A_68_P30094309	chr15:27677579-27677623	NM_001081302:278003	Trio	INSIDE	1.173	3.190	1948.03	6215.01	3.742	1440.71	5391.71
A_68_P23692818	chr5:52505349-52505393	AK076709:-3181		PROMOTER	1.173	1.986	1069.74	2124.12	2.330	1094.81	2550.44
A_68_P23819409	chr5:77445107-77445151	NM_027970:81	1700023E05Rik	INSIDE	1.172	2.182	1709.35	3729.44	2.558	1432.96	3665.36
A_68_P27996046	chr11:87578150-87578194	NM_172449:4130	Bzrap1	INSIDE	1.172	1.607	903.83	1452.83	1.884	843.58	1589.08
A_68_P27315614	chr10:86168270-86168314	NM_011631:-103	Hsp90b1	DIVERGENT_PROMOTER	1.172	1.377	4402.15	6062.93	1.614	3113.80	5025.96
A_68_P28099477	chr11:105946015-105946059	NM_011947:-179	Map3k3	PROMOTER	1.172	2.104	800.72	1685.03	2.467	712.88	1758.86
A_68_P25957202	chr8:74031641-74031685	NM_032398:4006	Plvap	INSIDE	1.172	1.827	338.17	617.81	2.140	306.47	656.00
A_68_P30479249	chr15:99125815-99125859	NM_018786:-3	Prpf40b	DIVERGENT_PROMOTER	1.172	2.164	4875.43	10549.88	2.535	3676.80	9321.97
A_68_P20410046	chr1:87961433-87961477	NM_027357:261	Psmd1	INSIDE	1.172	1.858	1165.07	2164.95	2.179	1015.82	2213.19
A_68_P29507209	chr14:31467715-31467759	NM_177815:176	Rft1	INSIDE	1.172	2.007	760.90	1527.20	2.352	731.05	1719.45
A_68_P22871711	chr4:43454970-43455014	NM_011571:-156	Tesk1	PROMOTER	1.172	1.521	1802.41	2741.86	1.782	1492.29	2659.59
A_68_P30657576	chr16:33380868-33380912	NM_011749:30	Zfp148	INSIDE	1.172	1.592	553.78	881.68	1.866	507.34	946.92
A_68_P31156110	A_68_P31156110			Unknown	1.172	2.907	307.77	894.64	3.406	290.75	990.41
A_68_P24043268	chr5:121670665-121670709	ENSMUST00000119892:459		INSIDE	1.172	2.367	886.12	2097.88	2.775	735.38	2040.82
A_68_P24798987	chr6:120481227-120481271	NM_144815:69	Cecr5	INSIDE	1.171	2.505	1351.25	3384.32	2.933	1147.76	3366.51
A_68_P27281433	chr10:79630600-79630644	NM_007705:37	Cirbp	INSIDE	1.171	2.359	1774.83	4186.77	2.763	1355.97	3746.92
A_68_P26841770	chr9:113650921-113650965	NM_001114347:352	Clasp2	INSIDE	1.171	1.780	575.59	1024.84	2.085	553.87	1154.56
A_68_P28185233	chr11:120410651-120410695	NM_001033231:369	Fam195b	INSIDE	1.171	5.275	2908.91	15344.37	6.175	2105.91	13004.68
A_68_P28054695	chr11:98011746-98011790	NM_028149:-838	Fbxl20	PROMOTER	1.171	2.520	907.52	2286.60	2.950	885.19	2611.29
A_68_P21339003	chr2:74549994-74550038	NM_010468:-33	Hoxd3	PROMOTER	1.171	4.305	423.13	1821.49	5.043	413.21	2083.60
A_68_P27470617	chr10:115024975-115025019	NM_010195:-160	Lgr5	PROMOTER	1.171	2.026	1166.86	2364.64	2.374	965.12	2291.21
A_68_P26580973	chr9:64101378-64101422	NM_008927:12	Map2k1	INSIDE	1.171	2.516	653.51	1644.18	2.947	653.18	1924.87

ProbeName	Target position of probe on CpG island microarray	TargetID	GeneSymbol	CpG island Description	Ratio of relative methylation (TiO_2-NP/Vehicle)	Sham group			TiO_2-H group		
						Relative methylation (Cy5/Cy3)	Cy3 signal (Input)	Cy5 signal (MeDIP)	Relative methylation (Cy5/Cy3)	Cy3 signal (Input)	Cy5 signal (MeDIP)
A_68_P31158451	chr17:35371961-35372005	NM_010909:778	Nfkbil1	INSIDE	1.171	6.490	6156.92	39955.50	7.596	4445.24	33768.05
A_68_P30343472	chr15:75534891-75534935	NM_001163465:195	Rhpn1	INSIDE	1.171	1.559	1227.30	1913.77	1.826	991.26	1810.51
A_68_P27284271	chr10:80069708-80069752	NM_019575:4104	Scamp4	INSIDE	1.171	2.566	1122.20	2879.64	3.004	872.37	2620.38
A_68_P25092755	chr7:52631986-52632030	NM_009224:19009	Snrnp70	INSIDE	1.171	5.045	1420.34	7165.66	5.906	1160.11	6851.10
A_68_P24974188	chr7:13609447-13609491	NM_011588:-32	Trim28	PROMOTER	1.171	10.235	957.80	9803.35	11.981	634.32	7599.91
A_68_P21781777	chr2:158055194-158055238	NM_001131021:-258	D630003M21Rik	PROMOTER	1.170	1.945	911.27	1772.15	2.276	924.04	2102.71
A_68_P31065253	chr17:15511986-15512030	NM_007865:779	Dll1	INSIDE	1.170	2.302	597.27	1375.18	2.695	534.13	1439.26
A_68_P29247524	chr13:97440758-97440802	NM_008255:111	Hmgcr	INSIDE	1.170	1.987	621.79	1235.56	2.324	585.32	1360.45
A_68_P21309976	chr2:69423831-69423875	NM_001081088:272	Lrp2	INSIDE	1.170	1.522	547.70	833.67	1.781	478.90	852.87
A_68_P32385500	chrX:50410153-50410197	NR_029756:-2648	Mir322	PROMOTER	1.170	4.313	8848.37	38161.07	5.048	4339.45	21905.66
A_68_P22209749	chr3:67178298-67178342	NM_001039543:302	Mlf1	INSIDE	1.170	3.526	1301.46	4589.44	4.126	912.46	3765.06
A_68_P27708457	chr11:35583330-35583374	NM_145962:356	Pank3	INSIDE	1.170	2.292	488.97	1120.79	2.682	449.18	1204.82
A_68_P25908468	chr8:63703117-63703161	NM_021506:171	Sh3rf1	INSIDE	1.170	2.328	1964.55	4573.12	2.723	1756.40	4783.53
A_68_P30366259	chr15:79233437-79233481	NM_172608:275	Tmem184b	INSIDE	1.170	1.995	1749.87	3491.67	2.335	1513.75	3534.59
A_68_P22856569	chr4:40215391-40215435	NM_134097:1462	Topors	INSIDE	1.170	2.390	438.18	1047.15	2.796	526.87	1472.93
A_68_P22372024	chr3:100726413-100726457	NM_001165953:19	Trim45	INSIDE	1.170	1.685	1086.90	1831.42	1.971	855.83	1687.00
A_68_P20820922	chr1:172105070-172105114	NM_133806:-15	Uap1	PROMOTER	1.170	2.249	1284.18	2888.59	2.632	1193.52	3141.16
A_68_P27287713	chr10:80611280-80611324	NM_010731:12287	Zbtb7a	INSIDE	1.170	3.948	3896.85	15386.58	4.619	2916.87	13473.78
A_68_P24951459	chr7:4945480-4945524	NM_026741:2201	Zfp579	INSIDE	1.170	2.055	1189.59	2445.08	2.404	912.48	2193.44
A_68_P27282448	chr10:79783216-79783260	NM_025521:55	2310011J03Rik	INSIDE	1.169	1.578	1045.47	1649.40	1.845	762.14	1405.83
A_68_P28724526	chr12:109155259-109155303	NM_001079883:86344	Bcl11b	INSIDE	1.169	2.975	2007.04	5971.63	3.477	1626.65	5655.68
A_68_P24735103	chr6:108609619-108609663	NM_011498:-982	Bhlhe40	PROMOTER	1.169	1.528	1307.76	1998.29	1.786	1253.00	2237.58
A_68_P22019968	chr3:28981553-28981597	NM_001167748:76	Egfem1	INSIDE	1.169	2.778	604.99	1680.69	3.248	488.45	1586.50
A_68_P22843581	chr4:36898671-36898715	NM_001165999:85	Lingo2	INSIDE	1.169	1.717	4110.15	7055.25	2.007	3340.56	6704.30
A_68_P24117100	chr5:136108637-136108681	NM_146002:135	Rhbdd2	INSIDE	1.169	1.574	1408.35	2216.47	1.841	1233.96	2271.16
A_68_P26745887	chr9:96096585-96096629	NM_001184706:-87	Tfdp2	PROMOTER	1.169	2.006	1202.81	2412.23	2.344	1018.95	2388.52
A_68_P29225757	chr13:93564876-93564920	NM_011582:-125	Thbs4	PROMOTER	1.169	1.814	2878.95	5221.48	2.120	2240.45	4750.73
A_68_P29052393	chr13:55057473-55057517	NM_153131:6702	Unc5a	INSIDE	1.169	1.916	2710.68	5193.47	2.240	2155.21	4827.54
A_68_P27281235	chr10:79605180-79605224	NM_025313:143	Atp5d	INSIDE	1.168	1.399	2343.57	3277.90	1.634	1722.01	2813.61
A_68_P26528581	chr9:54798811-54798855	NM_145229:-71	AY074887	PROMOTER	1.168	3.821	432.71	1653.22	4.464	375.19	1674.78
A_68_P21906145	chr2:180511577-180511621	NM_080641:7	Bhlhe23	INSIDE	1.168	3.176	1016.80	3228.86	3.709	938.50	3480.51
A_68_P31103375	chr17:25522729-25522773	NM_001163691:47978	Cacna1h	INSIDE	1.168	7.111	2787.64	19822.74	8.305	2027.47	16837.58
A_68_P31106875	chr17:26005655-26005699	NM_026633:7	Fam195a	INSIDE	1.168	1.552	1377.19	2137.56	1.813	1187.50	2153.50
A_68_P20819671	chr1:171899591-171899635	NM_010476:-276	Hsd17b7	PROMOTER	1.168	1.597	633.28	1011.18	1.866	492.61	919.04
A_68_P27806466	chr11:53583987-53584031	NM_001159396:34	Irf1	INSIDE	1.168	3.227	649.35	2095.55	3.768	517.44	1949.59
A_68_P28036508	chr11:94937511-94937555	NM_013565:496	Itga3	INSIDE	1.168	2.307	406.22	936.96	2.695	311.03	838.11
A_68_P25362046	chr7:106617483-106617527	NM_028145:2991	Klhl35	INSIDE	1.168	6.002	474.79	2849.66	7.011	377.26	2645.16
A_68_P21826984	chr2:165818646-165818690	NM_008679:532	Ncoa3	INSIDE	1.168	6.635	13235.85	87819.52	7.746	8271.53	64074.80
A_68_P24119777	chr5:136646370-136646414	NM_178751:134	Orai2	INSIDE	1.168	2.450	2503.77	6134.38	2.861	2280.77	6526.27
A_68_P31626159	chr18:37091821-37091865	NM_198117:-7016	Pcdha2	PROMOTER	1.168	2.249	717.03	1612.58	2.627	574.12	1508.33
A_68_P31207759	chr17:46728766-46728810	NM_175168:37665	Ptk7	INSIDE	1.168	6.175	701.98	4334.54	7.211	650.35	4689.63
A_68_P20871442	chr1:181598207-181598251	NM_178653:-133	Sccpdh	PROMOTER	1.168	3.031	1098.04	3327.82	3.540	980.73	3472.20
A_68_P28726325	chr12:109417342-109417386	NM_028262:130	Setd3	INSIDE	1.168	4.935	671.05	3311.79	5.765	904.36	5213.30
A_68_P23437238	chr4:154596264-154596308	NM_011385:358	Ski	INSIDE	1.168	0.435	1575.44	684.98	0.508	1205.52	612.25
A_68_P27286997	chr10:80493927-80493971	NM_134135:2709	Slc39a3	INSIDE	1.168	2.052	1488.23	3054.04	2.396	1327.30	3180.33
A_68_P23694282	chr5:52759804-52759848	NM_011435:4784	Sod3	INSIDE	1.168	1.736	506.10	878.76	2.028	389.37	789.52
A_68_P25183831	chr7:72789891-72789935	NM_172310:129	Tarsl2	INSIDE	1.168	1.987	379.50	753.98	2.320	347.16	805.30
A_68_P25049869	chr7:36322564-36322608	NM_028034:177	Tdrd12	INSIDE	1.168	2.954	1133.07	3346.67	3.451	966.03	3333.35
A_68_P21394207	chr2:84555063-84555107	NM_144887:237	Zdhhc5	INSIDE	1.168	1.569	2698.58	4235.31	1.834	2214.63	4061.16
A_68_P26154270	chr8:111471142-111471186	NM_007496:232621	Zfhx3	INSIDE	1.168	2.752	1731.08	4763.11	3.214	1537.66	4941.51
A_68_P21844345	chr2:168780474-168780518	NM_009564:591	Zfp64	INSIDE	1.168	1.868	1000.90	1869.68	2.181	927.33	2022.89

ProbeName	Target position of probe on CpG island microarray	TargetID	GeneSymbol	CpG island Description	Ratio of relative methylation (TiO_2-NP/Vehicle)	Sham group			TiO_2-H group		
						Relative methylation (Cy5/Cy3)	Cy3 signal (Input)	Cy5 signal (MeDIP)	Relative methylation (Cy5/Cy3)	Cy3 signal (Input)	Cy5 signal (MeDIP)
A_68_P24428352	chr6:48570342-48570386	NM_173429:7186	Zfp775	INSIDE	1.168	2.564	241.84	619.97	2.995	209.69	628.03
A_68_P26240063	A_68_P26240063			Unknown	1.168	2.955	629.58	1860.64	3.451	522.78	1804.31
A_68_P29625000	chr14:57484103-57484147	AK209334:-600		PROMOTER	1.168	1.832	1452.19	2660.45	2.140	1092.17	2337.65
A_68_P29090576	chr13:63066071-63066115	ENSMUST00000170896:-160		PROMOTER	1.168	5.055	1004.60	5078.19	5.903	870.98	5141.53
A_68_P22311243	chr3:87803866-87803910	NM_007529:390	Bcan	INSIDE	1.167	1.480	1001.50	1482.50	1.728	839.77	1451.22
A_68_P20561482	chr1:122238470-122238514	NM_207233:1334	C1ql2	INSIDE	1.167	2.067	2220.70	4589.35	2.412	1710.42	4125.26
A_68_P32670209	chrX:130594062-130594106	NM_133196:197	Cstf2	INSIDE	1.167	2.180	771.48	1681.60	2.545	479.05	1218.98
A_68_P28069094	chr11:100480931-100480975	NM_019795:530	Dnajc7	INSIDE	1.167	1.629	2233.29	3637.65	1.901	1724.85	3278.49
A_68_P26885391	chr9:121669059-121669103	NR_033503:-19	E530011L22Rik	DIVERGENT_PROMOTER	1.167	1.499	684.62	1026.23	1.749	664.94	1162.84
A_68_P21071367	chr2:25174641-25174685	NM_001177656:21	Grin1	INSIDE	1.167	0.389	1355.28	527.85	0.454	1194.69	542.84
A_68_P28152361	chr11:115119374-115119418	NM_010350:9161	Grin2c	INSIDE	1.167	2.191	1420.38	3111.90	2.557	1285.03	3285.26
A_68_P30339717	chr15:74630092-74630141	NM_029627:282	Ly6k	INSIDE	1.167	4.372	698.14	3052.55	5.103	564.33	2879.52
A_68_P24613352	chr6:86959758-86959802	NM_001170591:-49	Nfu1	PROMOTER	1.167	4.595	2845.89	13077.41	5.363	2377.80	12751.89
A_68_P28058395	chr11:98629793-98629837	NM_145434:6742	Nr1d1	INSIDE	1.167	3.396	1062.87	3609.55	3.962	842.93	3340.04
A_68_P28802627	chr13:6547373-6547417	NM_145131:-8	Pitrm1	PROMOTER	1.167	1.505	2063.02	3105.60	1.757	1640.16	2882.03
A_68_P26079046	chr8:97219612-97219656	NM_026385:508	Pllp	INSIDE	1.167	2.656	870.94	2313.17	3.099	776.48	2406.25
A_68_P25089777	chr7:52119118-52119162	NM_133949:6018	Ptov1	INSIDE	1.167	1.956	2459.03	4808.72	2.282	2127.62	4854.25
A_68_P28159202	chr11:116274533-116274577	NM_027258:-208	Rnf157	PROMOTER	1.167	1.474	1007.94	1485.30	1.720	877.84	1510.09
A_68_P31738728	chr18:58037843-58037887	NM_009194:-467	Slc12a2	PROMOTER	1.167	2.167	1231.22	2668.08	2.528	1280.23	3236.80
A_68_P24771682	chr6:115494959-115495004	NM_199033:260	Tsen2	INSIDE	1.167	2.768	405.89	1123.44	3.231	315.88	1020.68
A_68_P23334946	chr4:136194010-136194054	ENSMUST00000146123:15526		DOWNSTREAM	1.167	1.990	610.95	1215.51	2.322	582.43	1352.40
A_68_P22169031	chr3:58381260-58381314	NM_001040396:707	2810407C02Rik	INSIDE	1.166	1.598	622.06	993.85	1.863	477.32	889.12
A_68_P28182974	chr11:120050877-120050921	NR_030682:272	2810410L24Rik	INSIDE	1.166	7.043	915.25	6446.36	8.209	767.59	6301.25
A_68_P31925522	chr19:4867768-4867812	NM_013456:10094	Actn3	INSIDE	1.166	2.112	1914.48	4044.22	2.462	1467.45	3613.51
A_68_P24994775	chr7:20282050-20282094	NM_009696:2443	Apoe	INSIDE	1.166	2.448	987.41	2416.97	2.854	862.79	2461.98
A_68_P21754299	chr2:153171488-153171547	NM_001039939:-357	Asxl1	PROMOTER	1.166	3.197	254.38	813.28	3.726	235.13	876.21
A_68_P20740948	chr1:157820278-157820322	NM_001039184:85	Cep350	INSIDE	1.166	2.097	890.49	1866.95	2.444	902.34	2205.19
A_68_P25368311	chr7:107685728-107685772	NM_183270:137	Chchd8	INSIDE	1.166	2.320	1049.99	2435.87	2.704	1152.50	3116.64
A_68_P20166303	chr1:39593180-39593224	NM_028043:556	D1Bwg0212e	INSIDE	1.166	3.176	494.81	1571.64	3.703	461.40	1708.52
A_68_P20600406	chr1:130314008-130314052	NM_177445:-37	Dars	PROMOTER	1.166	2.678	1238.05	3315.33	3.122	990.98	3093.92
A_68_P33015610	chr1_random:49416-49460	NM_026866:135900	Disp1	INSIDE	1.166	2.435	569.47	1386.91	2.841	520.89	1479.79
A_68_P22872021	chr4:43512609-43512653	NM_001013377:-98	E130306D19Rik	DIVERGENT_PROMOTER	1.166	2.778	918.02	2550.20	3.240	772.64	2503.26
A_68_P27921506	chr11:74432896-74432940	NM_001013784:-188	E130309D14Rik	PROMOTER	1.166	1.844	1715.91	3164.87	2.150	1387.26	2982.81
A_68_P23278481	chr4:125826881-125826925	NM_172145:1656	Fam176b	INSIDE	1.166	1.424	2365.06	3367.83	1.660	1762.93	2926.81
A_68_P24544189	chr6:72848961-72849005	NM_019715:991	Kcmf1	INSIDE	1.166	1.930	716.29	1382.63	2.251	665.98	1498.86
A_68_P22344954	chr3:95463365-95463409	NM_008562:744	Mcl1	INSIDE	1.166	3.785	665.65	2519.18	4.411	643.65	2839.10
A_68_P31866306	chr18:80905336-80905380	NM_001164111:-446	Nfatc1	PROMOTER	1.166	2.099	1000.58	2100.11	2.448	951.49	2329.55
A_68_P29620064	chr14:56444394-56444438	NM_001168346:785	Nfatc4	INSIDE	1.166	1.624	956.60	1553.90	1.893	843.96	1597.94
A_68_P31442543	chr17:91487324-91487368	NM_020252:4796	Nrxn1	INSIDE	1.166	2.653	902.84	2395.25	3.093	789.77	2442.60
A_68_P27280128	chr10:79440201-79440245	NM_001003949:6853	ORF61	INSIDE	1.166	2.892	514.13	1486.75	3.371	469.56	1582.83
A_68_P23523156	chr5:20387967-20388011	NM_172992:-46	Phtf2	DIVERGENT_PROMOTER	1.166	2.800	509.68	1427.33	3.265	465.37	1519.46
A_68_P31375073	chr17:79420257-79420301	NM_001171004:-122	Prkd3	PROMOTER	1.166	1.924	901.90	1735.33	2.243	985.30	2210.25
A_68_P23308456	chr4:131375306-131375350	NM_001083119:18865	Ptpru	INSIDE	1.166	2.395	1070.24	2563.65	2.792	849.34	2371.43
A_68_P23301754	chr4:130219409-130219453	NM_001159603:157	Pum1	INSIDE	1.166	1.593	2943.81	4688.97	1.857	2433.48	4518.09
A_68_P27971356	chr11:83232242-83232286	NM_001013386:8691	Rasl10b	INSIDE	1.166	1.888	560.46	1058.16	2.202	505.04	1112.08
A_68_P23269838	chr4:124386319-124386363	NM_029157:-5764	Sf3a3	PROMOTER	1.166	2.270	1051.12	2385.68	2.645	923.29	2442.51
A_68_P28469868	chr12:60114568-60114612	NM_025656:211	Sip1	INSIDE	1.166	3.562	947.64	3375.86	4.154	750.96	3119.53
A_68_P23437240	chr4:154596449-154596493	NM_011385:174	Ski	INSIDE	1.166	3.145	1421.01	4469.39	3.668	1317.50	4832.49
A_68_P31190360	chr17:43766712-43766757	NM_001161366:514	Tdrd6	INSIDE	1.166	3.015	1388.38	4186.14	3.517	1101.66	3874.57
A_68_P29733539	chr14:76814024-76814068	NM_207652:-1581	Tsc22d1	PROMOTER	1.166	1.597	1582.73	2527.26	1.862	1241.80	2312.65
A_68_P21365113	chr2:78708721-78708765	NM_009454:-461	Ube2e3	PROMOTER	1.166	1.490	728.34	1085.41	1.737	674.61	1171.98

ProbeName	Target position of probe on CpG island microarray	TargetID	GeneSymbol	CpG island Description	Ratio of relative methylation (TiO_2-NP/Vehicle)	Sham group			TiO_2-H group		
						Relative methylation (Cy5/Cy3)	Cy3 signal (Input)	Cy5 signal (MeDIP)	Relative methylation (Cy5/Cy3)	Cy3 signal (Input)	Cy5 signal (MeDIP)
A_68_P27804989	chr11:53293020-53293064	NM_183173:655	Ankrd43	INSIDE	1.165	1.588	776.88	1233.52	1.849	718.72	1329.25
A_68_P26813124	chr9:108017763-108017807	NM_007567:74930	Bsn	INSIDE	1.165	2.238	593.78	1328.85	2.606	504.27	1314.23
A_68_P29265995	chr13:100670678-100670722	NM_001081493:-62	Cartpt	PROMOTER	1.165	1.491	1525.60	2274.37	1.736	1340.60	2327.63
A_68_P20148459	chr1:36529023-36529067	NM_033570:603	Cnnm4	INSIDE	1.165	1.785	745.44	1330.67	2.080	617.23	1283.54
A_68_P25394242	chr7:112911135-112911179	NM_001162943:24908	Dchs1	INSIDE	1.165	2.578	2574.72	6638.59	3.005	1994.20	5992.69
A_68_P26656976	chr9:77758360-77758404	NM_134255:-6789	Elovl5	PROMOTER	1.165	2.843	775.36	2204.73	3.313	719.40	2383.17
A_68_P26919702	chr10:7310204-7310248	NM_172784:629	Lrp11	INSIDE	1.165	2.684	499.30	1340.24	3.126	444.91	1390.92
A_68_P28744821	chr12:112813339-112813383	NM_021516:640	Mark3	INSIDE	1.165	0.486	1189.00	578.41	0.567	1016.12	575.83
A_68_P31827985	chr18:73975089-73975133	NM_145494:-64	Me2	PROMOTER	1.165	6.271	892.85	5598.90	7.304	890.11	6500.97
A_68_P28071734	chr11:100931359-100931403	NM_013792:-27	Naglu	PROMOTER	1.165	2.415	1017.05	2456.08	2.813	926.57	2606.71
A_68_P25953245	chr8:73183706-73183750	NM_023217:-148	Pgpep1	PROMOTER	1.165	1.716	7569.75	12989.11	1.998	5233.01	10458.06
A_68_P25265268	chr7:87343468-87343512	NM_013659:11764	Sema4b	INSIDE	1.165	4.737	898.99	4258.33	5.516	879.43	4851.20
A_68_P22963535	chr4:61947389-61947433	NM_025286:-68	Slc31a2	PROMOTER	1.165	2.140	906.91	1940.79	2.494	893.96	2229.65
A_68_P21556057	chr2:116947191-116947235	NM_033524:27	Spred1	INSIDE	1.165	3.570	303.39	1083.24	4.158	285.58	1187.43
A_68_P32775152	chrX:155971013-155971057	NM_001033472:435	A830080D01Rik	INSIDE	1.164	2.538	1217.05	3088.99	2.953	922.06	2722.95
A_68_P27285629	chr10:80269928-80269972	NM_007445:1958	Amh	INSIDE	1.164	1.844	717.78	1323.69	2.146	631.50	1355.50
A_68_P25372490	chr7:108450530-108450574	NM_001111111:50	Atg16l2	INSIDE	1.164	1.840	694.52	1277.58	2.141	577.71	1236.66
A_68_P22871971	chr4:43506143-43506188	NM_001037913:-71	Ccdc107	DIVERGENT_PROMOTER	1.164	3.292	448.35	1475.76	3.832	370.94	1421.41
A_68_P27303130	chr10:83997039-83997083	NM_175451:-427	Ckap4	PROMOTER	1.164	1.563	983.03	1536.56	1.820	841.91	1532.39
A_68_P29335933	chr13:113442449-113442493	NM_001145885:48	Ddx4	INSIDE	1.164	3.803	895.02	3403.52	4.425	799.64	3538.47
A_68_P25776099	chr8:35872603-35872647	NM_176933:1961	Dusp4	INSIDE	1.164	2.837	620.70	1760.80	3.303	563.63	1861.79
A_68_P26983899	chr10:19654713-19654757	NM_008580:403	Map3k5	INSIDE	1.164	2.398	3140.19	7531.51	2.792	2610.10	7287.64
A_68_P25025267	chr7:29871114-29871158	NR_035489:-2423	Mir1963	PROMOTER	1.164	3.748	884.50	3315.12	4.364	694.22	3029.26
A_68_P28790400	chr13:3892775-3892819	NM_001047159:28	Net1	INSIDE	1.164	1.641	540.23	886.63	1.911	477.41	912.36
A_68_P25846475	chr8:49362088-49362132	NM_001145937:397934	Odz3	INSIDE	1.164	2.003	587.65	1176.83	2.332	528.97	1233.48
A_68_P26018119	chr8:86618743-86618787	NM_009055:28030	Rfx1	INSIDE	1.164	2.584	763.92	1973.77	3.008	717.04	2156.87
A_68_P30425657	chr15:89354548-89354592	NM_021423:24283	Shank3	INSIDE	1.164	2.008	560.78	1126.20	2.337	621.33	1451.99
A_68_P28332009	chr12:32243915-32243959	NM_011867:898	Slc26a4	INSIDE	1.164	2.034	667.24	1356.89	2.366	587.76	1390.74
A_68_P28047897	chr11:96838592-96838636	NM_001080964:388	Sp2	INSIDE	1.164	4.326	886.05	3833.34	5.036	857.14	4316.19
A_68_P20886547	chr1:184339477-184339521	NM_173378:201	Trp53bp2	INSIDE	1.164	1.427	1103.43	1574.76	1.662	1022.68	1699.44
A_68_P24874649	chr6:136776165-136776209	NM_021714:551	Wbp11	INSIDE	1.164	1.634	1191.71	1947.50	1.902	999.15	1900.43
A_68_P20412363	chr1:88383877-88383921			Unknown	1.164	2.015	1185.95	2389.44	2.345	1015.60	2381.90
A_68_P32101745	chr19:38555886-38555930	ENSMUST00000158315:-8216		PROMOTER	1.164	1.605	1073.85	1723.43	1.869	895.69	1673.74
A_68_P24045560	chr5:122068966-122069010	NM_178799:-41	Acad12	PROMOTER	1.163	1.860	372.25	692.22	2.163	353.93	765.45
A_68_P21021376	chr2:14525863-14525907	NM_023116:-48	Cacnb2	PROMOTER	1.163	1.602	1155.81	1851.38	1.863	1025.78	1911.28
A_68_P32315763	chrX:33428723-33428767	NM_001009947:-82	Dock11	PROMOTER	1.163	2.910	459.91	1338.39	3.385	251.06	849.78
A_68_P26896523	chr9:123626629-123626673	NM_033322:25	Lztfl1	INSIDE	1.163	1.494	1426.66	2131.75	1.738	1060.48	1843.48
A_68_P24132966	chr5:139470282-139470326	NM_008808:603	Pdgfa	INSIDE	1.163	1.573	5580.03	8775.96	1.829	4162.00	7610.81
A_68_P28159703	chr11:116360161-116360205	NM_026364:-8522	Prpsap1	PROMOTER	1.163	1.550	1351.56	2094.93	1.802	1066.40	1922.01
A_68_P27045328	chr10:31328333-31328377	NM_001146349:1177	Rnf217	INSIDE	1.163	2.238	510.93	1143.60	2.604	475.56	1238.40
A_68_P25408486	chr7:116662855-116662899	NR_028079:-769	Snora3	PROMOTER	1.163	1.854	1941.16	3599.16	2.156	1582.30	3412.06
A_68_P21265074	chr2:61431520-61431564	NM_001164071:389	Tank	INSIDE	1.163	1.521	851.53	1295.35	1.768	729.75	1290.55
A_68_P25056438	chr7:37480533-37480577	NM_172298:-2582	Tshz3	PROMOTER	1.163	1.583	4047.07	6407.55	1.841	3162.14	5820.64
A_68_P29072113	chr13:58317002-58317046	NM_026842:-10	Ubqln1	PROMOTER	1.163	2.057	600.52	1235.01	2.391	547.77	1309.98
A_68_P28152749	chr11:115179633-115179677	NM_176847:3578	Ush1g	INSIDE	1.163	1.541	3067.01	4726.19	1.792	2464.29	4416.65
A_68_P21583235	chr2:121692691-121692735	NM_001205371:-728	Casc4	PROMOTER	1.162	2.770	373.35	1034.29	3.219	360.94	1162.01
A_68_P25672257	chr8:14096396-14096440	NM_001145965:544	Dlgap2	INSIDE	1.162	3.953	809.97	3202.12	4.595	729.25	3350.76
A_68_P28958812	chr13:38243505-38243549	NM_023842:364	Dsp	INSIDE	1.162	2.133	585.73	1249.13	2.478	505.13	1251.96
A_68_P20192817	chr1:44204965-44205009	NM_011729:398	Ercc5	INSIDE	1.162	2.004	1968.31	3943.75	2.328	1667.14	3880.40
A_68_P21582987	chr2:121632660-121632704	NM_172673:111	Frmd5	INSIDE	1.162	1.775	4081.34	7245.41	2.063	3367.87	6947.58
A_68_P28107590	chr11:107409081-107409125	NM_198298:-171	Helz	PROMOTER	1.162	1.765	2408.20	4251.53	2.051	2018.51	4139.88

ProbeName	Target position of probe on CpG island microarray	TargetID	GeneSymbol	CpG island Description	Ratio of relative methylation (TiO_2-NP/Vehicle)	Sham group			TiO_2-H group		
						Relative methylation (Cy5/Cy3)	Cy3 signal (Input)	Cy5 signal (MeDIP)	Relative methylation (Cy5/Cy3)	Cy3 signal (Input)	Cy5 signal (MeDIP)
A_68_P20633482	chr1:136352339-136352383	NM_153128:229	Klhl12	INSIDE	1.162	1.746	576.39	1006.33	2.029	540.89	1097.33
A_68_P27284766	chr10:80139373-80139417	NM_021462:-4673	Mknk2	PROMOTER	1.162	0.489	1718.43	839.46	0.568	1357.70	770.68
A_68_P31478454	chr18:7626883-7626927	NM_001081287:-43	Mpp7	PROMOTER	1.162	1.478	1676.34	2477.40	1.717	1396.87	2398.57
A_68_P30083235	chr15:25711092-25711136	NM_019472:158810	Myo10	INSIDE	1.162	2.489	663.44	1651.06	2.892	565.16	1634.44
A_68_P24084799	chr5:129242041-129242085	NM_021311:-58	Piwil1	PROMOTER	1.162	2.744	1410.40	3870.44	3.188	1125.43	3587.53
A_68_P30513996	chr16:5132766-5132810	NM_008909:-214	Ppl	PROMOTER	1.162	1.562	909.88	1421.00	1.815	807.16	1464.79
A_68_P23538313	chr5:23289297-23289341	NM_178403:161	Pus7	INSIDE	1.162	1.354	1984.53	2687.86	1.574	1618.98	2548.35
A_68_P23993051	chr5:113005885-113005929	NM_019982:299	Sez6l	INSIDE	1.162	1.662	832.98	1384.55	1.932	762.18	1472.36
A_68_P23576449	chr5:31395933-31395977	NM_011773:-54	Slc30a3	PROMOTER	1.162	1.333	4714.24	6282.60	1.549	3547.57	5495.42
A_68_P27287686	chr10:80607070-80607114	NM_010731:8077	Zbtb7a	INSIDE	1.162	2.345	1634.05	3831.22	2.726	1251.88	3412.07
A_68_P25261702	chr7:86680581-86680625	ENSMUST00000141822:35600		DOWNSTREAM	1.162	2.394	1222.47	2926.70	2.783	1069.30	2975.58
A_68_P32538580	chrX:91612075-91612119	NM_026673:-352	Apoo	PROMOTER	1.161	1.776	931.76	1654.73	2.063	493.39	1017.63
A_68_P23350169	chr4:138748940-138748984	NM_001037761:69	Capzb	INSIDE	1.161	0.498	2606.24	1296.94	0.578	2247.46	1298.31
A_68_P26127991	chr8:106864790-106864834	NM_024217:319	Cmtm3	INSIDE	1.161	2.268	1053.71	2389.46	2.633	885.32	2331.31
A_68_P29578364	chr14:47407974-47408018	NM_009919:36	Cnih	INSIDE	1.161	0.594	2979.49	1769.89	0.690	2436.84	1681.31
A_68_P24158342	chr5:144578471-144578515	NM_013557:-168	Eif2ak1	PROMOTER	1.161	1.445	4526.62	6541.93	1.678	3554.32	5963.62
A_68_P26612669	chr9:69607113-69607157	NM_022378:1613	Foxb1	INSIDE	1.161	1.745	5516.67	9628.09	2.026	4452.61	9019.66
A_68_P26224602	chr8:123640403-123640447	NM_013519:354	Foxc2	INSIDE	1.161	1.785	647.47	1155.93	2.073	590.03	1223.19
A_68_P32823843	chrX:166435357-166435401	NM_183151:117825	Mid1	DOWNSTREAM	1.161	1.952	20431.29	39877.87	2.267	17302.79	39218.31
A_68_P21742433	chr2:150864640-150864699	NM_026086:446	Nanp	INSIDE	1.161	1.808	605.21	1094.50	2.100	547.92	1150.48
A_68_P32266421	chrX:13063192-13063236	NM_173415:18417	Nyx	INSIDE	1.161	1.926	755.79	1455.54	2.236	365.90	818.02
A_68_P21775756	chr2:157029918-157029962	NM_011249:330	Rbl1	INSIDE	1.161	1.688	1073.55	1812.10	1.960	892.77	1750.20
A_68_P23405236	chr4:149655404-149655448	NM_001085492:-598	Rere	PROMOTER	1.161	3.151	649.22	2045.76	3.659	603.89	2209.74
A_68_P25050061	chr7:36370407-36370451	NM_145840:173	Rgs9bp	INSIDE	1.161	1.472	671.23	988.26	1.710	564.62	965.42
A_68_P30465077	chr15:96529839-96529883	NM_175121:269	Slc38a2	INSIDE	1.161	1.661	1754.43	2914.12	1.929	1551.69	2992.48
A_68_P20005795	chr1:4482172-4482216	NM_011441:4300	Sox17	INSIDE	1.161	1.784	876.78	1564.43	2.072	771.57	1598.41
A_68_P31752325	chr18:60755946-60755990	NM_177340:27991	Synpo	INSIDE	1.161	1.988	453.13	900.73	2.308	358.69	827.98
A_68_P23416140	chr4:151406689-151406733	NM_031867:5889	Tas1r1	INSIDE	1.161	2.454	1721.04	4222.81	2.848	1236.85	3523.10
A_68_P29048726	chr13:54398661-54398705	AK086128:102013		DOWNSTREAM	1.161	2.343	1399.89	3279.85	2.719	1321.84	3594.11
A_68_P23425242	chr4:152856847-152856891	NM_001099299:71	Ajap1	INSIDE	1.160	1.544	554.00	855.46	1.791	480.57	860.66
A_68_P23210414	chr4:111087603-111087647	NM_026279:14	Bend5	INSIDE	1.160	2.318	2336.90	5416.06	2.688	1735.98	4665.75
A_68_P24093838	chr5:130925235-130925279	NM_021371:595	Caln1	INSIDE	1.160	1.977	954.06	1886.18	2.294	859.31	1971.33
A_68_P30459324	chr15:95485068-95485112	NM_207533:112	Dbx2	INSIDE	1.160	1.935	1460.66	2826.98	2.245	1208.85	2714.17
A_68_P32651795	chrX:126284318-126284362	NM_172493:63	Diap2	INSIDE	1.160	1.606	1484.43	2384.13	1.864	720.63	1343.02
A_68_P29250235	chr13:98010886-98010930	NM_007930:-151	Enc1	PROMOTER	1.160	2.613	1346.21	3517.81	3.032	1206.68	3659.00
A_68_P20441864	chr1:93242302-93242346	NM_001033292:23673	Espnl	INSIDE	1.160	1.865	373.50	696.57	2.163	298.95	646.52
A_68_P26617706	chr9:70505115-70505159	NM_172772:-155	Fam63b	PROMOTER	1.160	4.001	1407.09	5629.81	4.640	1348.15	6256.02
A_68_P31936818	chr19:7015888-7015932	NM_001177360:-227	Gpr137	PROMOTER	1.160	0.476	1920.49	914.03	0.552	1627.59	898.76
A_68_P21680297	chr2:139504517-139504561	NM_001126490:625	Ism1	INSIDE	1.160	2.306	530.37	1223.07	2.675	493.09	1318.92
A_68_P21074761	chr2:25733684-25733728	NM_175462:326	Kcnt1	INSIDE	1.160	1.958	764.40	1496.40	2.270	584.33	1326.47
A_68_P27767869	chr11:46250544-46250588	NM_001104556:104	Med7	INSIDE	1.160	1.894	1120.01	2121.24	2.198	862.62	1895.61
A_68_P27922353	chr11:74584217-74584261	NM_026197:-126	Mettl16	PROMOTER	1.160	1.855	2512.12	4659.88	2.153	1995.23	4294.96
A_68_P32774619	chrX:155852596-155852640	NM_001081124:109	Mtap7d2	INSIDE	1.160	2.861	1001.79	2866.21	3.319	714.12	2369.83
A_68_P24992918	chr7:19955528-19955572	NM_001010836:8986	Ppp1r13l	INSIDE	1.160	1.914	985.22	1885.39	2.220	877.85	1948.64
A_68_P28058848	chr11:98698706-98698750	NM_001080925:630	Rapgefl1	INSIDE	1.160	1.437	1326.86	1906.16	1.666	1019.99	1699.69
A_68_P28037688	chr11:95120636-95120680	NM_053093:-2184	Tac4	PROMOTER	1.160	1.579	845.68	1335.31	1.831	730.18	1337.12
A_68_P29095533	chr13:63916826-63916870	NM_023507:207	0610007P08Rik	INSIDE	1.159	2.493	997.04	2485.63	2.888	929.73	2685.42
A_68_P30404613	chr15:85862950-85862994	NM_009886:1235	Celsr1	INSIDE	1.159	3.177	1485.04	4717.54	3.680	1245.53	4584.13
A_68_P22649277	chr3:151872936-151872980	NM_027287:305	Dnajb4	INSIDE	1.159	2.736	1124.88	3077.72	3.171	1025.47	3252.22
A_68_P26137129	chr8:108503182-108503226	NM_027960:115	Dpep3	INSIDE	1.159	6.067	6485.20	39344.93	7.029	4215.29	29628.86
A_68_P25592947	chr7:149281374-149281418	NM_008748:-207	Dusp8	PROMOTER	1.159	1.443	3302.17	4763.96	1.672	2564.44	4286.69

ProbeName	Target position of probe on CpG island microarray	TargetID	GeneSymbol	CpG island Description	Ratio of relative methylation (TiO_2-NP/Vehicle)	Sham group			TiO_2-H group		
						Relative methylation (Cy5/Cy3)	Cy3 signal (Input)	Cy5 signal (MeDIP)	Relative methylation (Cy5/Cy3)	Cy3 signal (Input)	Cy5 signal (MeDIP)
A_68_P24131504	chr5:139231132-139231176	NM_030565:120	Fam20c	INSIDE	1.159	1.484	1863.85	2765.61	1.719	1547.42	2660.61
A_68_P28940417	chr13:34839281-34839325	NM_138746:7792	Fam50b	INSIDE	1.159	2.694	1092.47	2942.64	3.122	872.89	2725.40
A_68_P28558330	chr12:78339604-78339648	NM_016893:-381	Fut8	PROMOTER	1.159	1.710	622.26	1064.14	1.982	558.95	1107.99
A_68_P23417713	chr4:151657232-151657276	NM_178406:8784	Gpr153	INSIDE	1.159	1.729	995.04	1720.92	2.005	855.70	1715.49
A_68_P26991381	chr10:20879458-20879502	NM_001198914:1310	Myb	INSIDE	1.159	2.304	1496.69	3448.80	2.672	2098.36	5605.77
A_68_P25088768	chr7:51924794-51924838	NM_028021:-3943	Myh14	PROMOTER	1.159	3.492	1828.66	6386.37	4.048	1540.49	6236.61
A_68_P32147200	chr19:47089737-47089781	NM_001164363:-79	Nt5c2	PROMOTER	1.159	4.456	652.74	2908.64	5.164	608.74	3143.35
A_68_P24464318	chr6:54545243-54545287	NM_001001335:-7876	Plekha8	PROMOTER	1.159	3.022	388.51	1174.15	3.503	345.22	1209.42
A_68_P27646888	chr11:23565964-23566008	NM_028304:11	Pus10	INSIDE	1.159	1.528	906.17	1384.92	1.771	795.22	1408.44
A_68_P23538314	chr5:23289429-23289473	NM_178403:29	Pus7	INSIDE	1.159	0.492	2412.81	1186.80	0.570	1999.98	1139.89
A_68_P31096075	chr17:24345873-24345917	NM_001163847:-3387	Tbc1d24	PROMOTER	1.159	2.273	1696.78	3856.21	2.633	1542.43	4061.20
A_68_P23416613	chr4:151490095-151490139	NM_033042:-335	Tnfrsf25	PROMOTER	1.159	2.088	1440.34	3006.97	2.420	1165.13	2819.27
A_68_P24153998	chr5:143668502-143668546	NM_007393:-121	Actb	PROMOTER	1.158	1.736	1188.70	2063.87	2.011	986.52	1983.81
A_68_P27899598	chr11:70044019-70044063	NM_007528:-740	Bcl6b	DIVERGENT_PROMOTER	1.158	2.917	691.45	2016.72	3.378	604.63	2042.55
A_68_P30567430	chr16:15887377-15887421	NM_007679:20	Cebpd	INSIDE	1.158	1.751	3930.87	6882.25	2.028	3060.61	6207.85
A_68_P30981937	chr16:94748428-94748472	NM_007834:-214	Dscr3	PROMOTER	1.158	1.942	755.82	1467.54	2.248	708.23	1591.80
A_68_P25565283	chr7:144514346-144514390	NM_001113414:-8240	Ebf3	PROMOTER	1.158	1.540	850.97	1310.83	1.784	787.12	1404.50
A_68_P30345479	chr15:75839800-75839844	NM_001168253:106	Fam83h	INSIDE	1.158	4.882	1616.96	7894.66	5.655	1461.34	8264.06
A_68_P31099059	chr17:24832929-24832973	NM_023040:151	Gfer	INSIDE	1.158	2.092	392.30	820.80	2.423	319.25	773.47
A_68_P29138631	chr13:73398065-73398109	NM_018885:142	Irx4	INSIDE	1.158	2.495	449.44	1121.21	2.889	488.88	1412.60
A_68_P31936764	chr19:7008234-7008278	NM_008431:749	Kcnk4	INSIDE	1.158	2.114	2046.22	4325.38	2.449	1656.81	4057.10
A_68_P27897675	chr11:69694588-69694632	NM_153143:-123	Kctd11	PROMOTER	1.158	1.503	1277.15	1919.13	1.740	1213.74	2112.29
A_68_P32281788	chrX:17740669-17740713	NM_009483:898	Kdm6a	INSIDE	1.158	0.413	1681.79	694.05	0.478	773.20	369.41
A_68_P26017626	chr8:86535448-86535492	NR_035431:62	Mir1199	INSIDE	1.158	3.710	1220.15	4526.23	4.295	1066.78	4581.71
A_68_P26610634	chr9:69245687-69245731	NM_145618:-96	Narg2	PROMOTER	1.158	3.492	373.65	1304.78	4.044	363.11	1468.53
A_68_P31938622	chr19:7280556-7280600	NM_134150:196	Otub1	INSIDE	1.158	1.461	1112.23	1625.01	1.692	1141.24	1931.34
A_68_P22774300	chr4:21606032-21606076	NM_001080771:7056	Prdm13	INSIDE	1.158	3.298	558.32	1841.30	3.818	482.94	1844.03
A_68_P21086001	chr2:27532393-27532437	NM_011305:-306	Rxra	PROMOTER	1.158	0.528	1452.89	766.40	0.611	1215.90	742.78
A_68_P32126392	chr19:43502033-43502077			Unknown	1.158	3.022	746.70	2256.29	3.500	628.86	2200.90
A_68_P32473671	chrX:73211910-73211954	ENSMUST00000121090:-7625		PROMOTER	1.158	2.230	1777.83	3965.40	2.582	726.23	1875.48
A_68_P23195470	chr4:108000920-108000964	NM_027250:186	2010305A19Rik	INSIDE	1.157	1.465	5344.99	7832.16	1.696	3928.29	6662.37
A_68_P30475044	chr15:98364664-98364708	NM_133714:-34	2310037I24Rik	PROMOTER	1.157	1.404	1906.96	2678.06	1.625	1780.35	2893.71
A_68_P31103782	chr17:25570573-25570617	NM_001163691:134	Cacna1h	INSIDE	1.157	2.787	623.68	1737.90	3.225	543.74	1753.39
A_68_P21021384	chr2:14526701-14526745	NM_023116:790	Cacnb2	INSIDE	1.157	1.420	2744.64	3898.10	1.644	2168.95	3564.77
A_68_P30978138	chr16:93883923-93883967	NM_028083:-201	Chaf1b	PROMOTER	1.157	2.705	2183.38	5906.71	3.131	1634.23	5116.40
A_68_P22729161	chr4:11249125-11249169	NM_001081201:132	Dpy19l4	INSIDE	1.157	1.628	735.55	1197.84	1.884	634.65	1195.85
A_68_P31252967	chr17:56095790-56095834	NM_015766:3767	Ebi3	INSIDE	1.157	2.913	618.38	1801.32	3.369	543.91	1832.44
A_68_P25328176	chr7:100228697-100228741	NM_021427:330	Fam181b	INSIDE	1.157	2.624	2630.72	6902.01	3.035	2185.13	6631.03
A_68_P23546246	chr5:24728813-24728857	NM_144908:124	Galnt11	INSIDE	1.157	2.125	443.46	942.42	2.459	446.08	1096.73
A_68_P28091896	chr11:104469667-104469711	NM_016780:375	Itgb3	INSIDE	1.157	2.102	2504.57	5263.47	2.432	2020.52	4913.52
A_68_P22686351	chr3:158224713-158224757	NM_001081358:451	Lrrc7	INSIDE	1.157	0.399	1406.49	561.65	0.462	1064.24	491.81
A_68_P25169129	chr7:69564808-69564852	NM_011746:195	Mkrn3	INSIDE	1.157	2.341	1013.95	2373.88	2.710	832.69	2256.34
A_68_P32266423	chrX:13063488-13063532	NM_173415:18713	Nyx	INSIDE	1.157	4.248	599.53	2546.75	4.914	483.60	2376.30
A_68_P24832848	chr6:127403924-127403968	NM_181402:206	Parp11	INSIDE	1.157	1.601	1326.84	2124.07	1.851	1212.37	2244.69
A_68_P22101766	chr3:45184223-45184267	NM_011043:1925	Pcdh10	INSIDE	1.157	1.610	981.14	1579.28	1.862	738.83	1375.94
A_68_P24479719	chr6:57641697-57641742	NM_025574:353	Pigy	INSIDE	1.157	2.388	1735.35	4144.24	2.763	1623.52	4485.24
A_68_P30377510	chr15:81191319-81191363	NM_011399:-145	Slc25a17	PROMOTER	1.157	0.517	1028.75	532.34	0.599	806.35	482.61
A_68_P25016939	chr7:28149566-28149610	NM_001199235:29937	Spnb4	INSIDE	1.157	2.209	701.16	1548.84	2.556	605.31	1546.98
A_68_P31175211	A_68_P31175211			Unknown	1.157	6.601	10784.75	71187.93	7.637	7274.03	55553.92
A_68_P31498901	chr18:12279785-12279829	NM_178801:156	6030446N20Rik	INSIDE	1.156	2.003	2839.72	5686.95	2.315	2295.22	5313.65
A_68_P22682766	chr3:157610204-157610248	NM_001013806:-203	Ankrd13c	PROMOTER	1.156	2.091	542.69	1134.73	2.416	546.11	1319.62

ProbeName	Target position of probe on CpG island microarray	TargetID	GeneSymbol	CpG island Description	Ratio of relative methylation (TiO_2-NP/Vehicle)	Sham group			TiO_2-H group		
						Relative methylation (Cy5/Cy3)	Cy3 signal (Input)	Cy5 signal (MeDIP)	Relative methylation (Cy5/Cy3)	Cy3 signal (Input)	Cy5 signal (MeDIP)
A_68_P30533733	chr16:8672342-8672386	NM_025821:-118	Carhsp1	PROMOTER	1.156	2.730	1314.80	3588.98	3.155	1185.90	3741.36
A_68_P29976942	chr15:3929549-3929593	NM_134099:3	Fbxo4	INSIDE	1.156	3.256	1137.38	3703.72	3.764	950.74	3578.57
A_68_P32104291	chr19:39005160-39005205	NM_008234:-297	Hells	PROMOTER	1.156	1.515	759.51	1150.45	1.751	671.56	1175.95
A_68_P21339338	chr2:74590901-74590945	NM_010468:40873	Hoxd3	DOWNSTREAM	1.156	2.826	221.94	627.30	3.268	213.91	699.14
A_68_P27971658	chr11:83276246-83276290	NM_080453:195	Mmp28	INSIDE	1.156	2.247	567.02	1274.19	2.597	502.23	1304.47
A_68_P20237359	chr1:54614549-54614593	NM_001163314:-44	Pgap1	PROMOTER	1.156	1.681	1484.85	2495.30	1.942	1145.74	2225.53
A_68_P23794549	chr5:73305228-73305272	NM_001113423:-305	Slain2	PROMOTER	1.156	1.579	1282.97	2025.55	1.825	1051.06	1918.03
A_68_P23278183	chr4:125781499-125781543	NM_001145827:320	Stk40	INSIDE	1.156	0.471	1316.57	619.55	0.544	1027.19	558.54
A_68_P22039928	chr3:32716452-32716496	NM_001013024:-73	Usp13	PROMOTER	1.156	1.465	870.46	1274.93	1.693	669.07	1132.52
A_68_P26257412	chr8:128946745-128946789	NM_174987:366	1810063B05Rik	INSIDE	1.155	2.405	681.37	1638.65	2.777	568.60	1578.98
A_68_P28534553	chr12:74018698-74018742	NM_029444:32	4930447C04Rik	INSIDE	1.155	3.989	7108.18	28356.46	4.609	5976.23	27542.38
A_68_P23542800	chr5:24082936-24082980	NM_001190443:327	Abcf2	INSIDE	1.155	0.589	1560.59	919.10	0.680	1203.93	818.89
A_68_P28745653	chr12:112951441-112951485	NM_027404:5	Bag5	INSIDE	1.155	1.726	982.62	1696.40	1.994	868.71	1731.79
A_68_P28731506	chr12:110272427-110272471	NM_001163175:33979	Begain	INSIDE	1.155	2.366	1683.56	3983.20	2.733	1420.80	3883.43
A_68_P29056138	chr13:55724621-55724665	NM_007596:277	Caml	INSIDE	1.155	2.478	1255.29	3110.73	2.862	1096.60	3138.88
A_68_P21958960	chr3:14886530-14886574	NM_009801:127	Car2	INSIDE	1.155	1.353	2604.46	3524.53	1.563	2263.90	3537.82
A_68_P24818640	chr6:125046428-125046472	NM_145979:270	Chd4	INSIDE	1.155	1.801	701.15	1263.00	2.080	738.31	1535.56
A_68_P26885385	chr9:121668453-121668497	NR_033503:587	E530011L22Rik	INSIDE	1.155	1.646	856.34	1409.34	1.900	669.58	1272.38
A_68_P32397419	chrX:53984962-53985006	NM_001077361:-149	Fhl1	PROMOTER	1.155	3.065	1977.99	6062.99	3.541	1065.91	3773.98
A_68_P28099479	chr11:105946314-105946358	NM_011947:121	Map3k3	INSIDE	1.155	1.514	1454.11	2200.96	1.749	1247.97	2182.65
A_68_P21630447	chr2:130390003-130390047	NM_207207:532	Mrps26	INSIDE	1.155	3.831	1830.39	7011.35	4.426	1511.55	6690.00
A_68_P32066442	chr19:31838948-31838992	NM_001013833:553	Prkg1	INSIDE	1.155	2.084	5310.62	11069.05	2.408	3956.50	9525.71
A_68_P31256043	chr17:56567276-56567320	NM_011218:48605	Ptprs	INSIDE	1.155	3.724	3535.54	13167.54	4.301	2675.37	11505.97
A_68_P27165504	chr10:58275815-58275859	NM_172788:-270	Sh3rf3	PROMOTER	1.155	0.536	969.40	519.69	0.619	859.35	532.21
A_68_P25409835	chr7:116895803-116895847	NM_020050:-47	Tmem9b	PROMOTER	1.155	1.829	513.43	938.86	2.111	423.64	894.50
A_68_P21936415	chr3:9251283-9251327	NM_177660:738	Zbtb10	INSIDE	1.155	3.331	869.86	2897.25	3.848	782.06	3009.73
A_68_P31841471	chr18:76305627-76305671	NM_145356:325817	Zbtb7c	INSIDE	1.155	2.249	566.33	1273.67	2.598	495.76	1288.20
A_68_P23416422	chr4:151460754-151460798	ENSMUST00000118648:-190		PROMOTER	1.155	2.205	765.62	1688.31	2.548	701.03	1786.12
A_68_P32410982	chrX:56820979-56821023	ENSMUST00000145767:-2		PROMOTER	1.155	1.591	1587.32	2525.98	1.839	819.86	1507.50
A_68_P31791928	chr18:67609055-67609099	NM_027130:-286	Afg3l2	PROMOTER	1.154	5.143	12828.58	65972.89	5.933	8047.74	47750.72
A_68_P23763013	chr5:67009680-67009724	NM_009686:208	Apbb2	INSIDE	1.154	2.668	730.51	1948.79	3.079	625.01	1924.45
A_68_P23237353	chr4:117599338-117599382	NM_009711:3008	Artn	INSIDE	1.154	2.737	650.07	1779.35	3.159	563.57	1780.24
A_68_P32397416	chrX:53984482-53984526	NM_010211:-459	Fhl1	PROMOTER	1.154	2.692	598.90	1612.02	3.106	371.04	1152.36
A_68_P25189681	chr7:73834635-73834679	NM_001191001:-118	Lins	DIVERGENT_PROMOTER	1.154	2.834	1821.77	5162.80	3.271	1294.19	4233.66
A_68_P24444194	chr6:51382876-51382920	NM_010903:230	Nfe2l3	INSIDE	1.154	1.555	2333.32	3627.26	1.794	1802.04	3233.76
A_68_P30346600	chr15:76010013-76010057	NM_201394:16106	Plec	INSIDE	1.154	3.116	695.31	2166.92	3.597	533.84	1920.38
A_68_P31152721	chr17:34341412-34341456	NM_011530:11	Tap2	INSIDE	1.154	1.589	558.74	887.67	1.833	495.39	907.98
A_68_P21084955	chr2:27331406-27331450	NM_001113573:-235	Brd3	PROMOTER	1.153	2.112	683.68	1444.11	2.435	654.59	1593.77
A_68_P26317082	chr9:14185179-14185223	NM_028013:486	Endod1	INSIDE	1.153	1.726	1018.61	1757.62	1.989	889.30	1769.04
A_68_P24154218	chr5:143723570-143723614	NM_007984:1559	Fscn1	INSIDE	1.153	1.589	527.00	837.53	1.832	385.81	706.67
A_68_P27280645	chr10:79516852-79516896	NM_001037741:93	Gpx4	INSIDE	1.153	6.485	420.35	2725.92	7.474	416.77	3115.07
A_68_P25770032	chr8:34763961-34764005	NM_010344:273	Gsr	INSIDE	1.153	4.055	2576.34	10447.66	4.676	2207.67	10323.87
A_68_P32820568	chrX:165758296-165758340	NM_008222:-43	Hccs	PROMOTER	1.153	2.079	470.76	978.51	2.397	273.15	654.67
A_68_P31876422	chr18:82644757-82644801	NM_001025245:264	Mbp	INSIDE	1.153	2.947	1321.25	3894.28	3.398	1102.20	3744.89
A_68_P20354530	chr1:75500243-75500287	NM_178884:2763	Obsl1	INSIDE	1.153	2.068	1461.22	3022.31	2.384	1186.06	2827.44
A_68_P30346578	chr15:76007226-76007270	NM_201394:18892	Plec	INSIDE	1.153	2.602	1583.85	4121.26	3.001	1283.60	3852.48
A_68_P25586915	chr7:148317107-148317151	NM_013782:-56	Ptdss2	PROMOTER	1.153	1.639	1023.62	1678.04	1.891	823.34	1556.71
A_68_P27190661	chr10:62801691-62801735	NM_001159590:31	Sirt1	INSIDE	1.153	1.703	1161.69	1978.28	1.963	884.11	1735.58
A_68_P30580600	chr16:18586533-18586577	NM_011532:508	Tbx1	INSIDE	1.153	1.395	1737.24	2423.83	1.608	1351.56	2173.74
A_68_P20228140	chr1:52687403-52687448	NM_001142647:-123	Tmem194b	PROMOTER	1.153	1.901	1178.02	2239.49	2.192	1077.49	2362.21
A_68_P23440602	chr4:155158465-155158509	NM_001033394:1497	Tmem88b	INSIDE	1.153	1.632	988.17	1612.51	1.881	909.92	1711.80

ProbeName	Target position of probe on CpG island microarray	TargetID	GeneSymbol	CpG island Description	Ratio of relative methylation (TiO_2-NP/Vehicle)	Sham group			TiO_2-H group		
						Relative methylation (Cy5/Cy3)	Cy3 signal (Input)	Cy5 signal (MeDIP)	Relative methylation (Cy5/Cy3)	Cy3 signal (Input)	Cy5 signal (MeDIP)
A_68_P23984844	chr5:111706164-111706208	NM_024477:397365	Ttc28	INSIDE	1.153	2.010	1297.30	2606.94	2.317	987.23	2287.64
A_68_P23409800	chr4:150431562-150431606	NM_009498:478	Vamp3	INSIDE	1.153	1.425	1745.14	2486.69	1.643	1553.07	2551.70
A_68_P27863350	chr11:63892884-63892928	NM_178379:68	Cox10	INSIDE	1.152	0.551	1745.95	962.02	0.635	1551.37	985.07
A_68_P30106581	chr15:30102269-30102313	NM_008729:-57	Ctnnd2	PROMOTER	1.152	1.929	560.44	1080.84	2.221	541.42	1202.42
A_68_P31830235	chr18:74376174-74376218	NM_028868:331	Cxxc1	INSIDE	1.152	0.571	1521.91	868.85	0.658	1252.81	824.05
A_68_P28075716	chr11:101646089-101646133	NM_008815:514	Etv4	INSIDE	1.152	1.778	1326.32	2358.77	2.049	1114.10	2282.99
A_68_P31129059	chr17:29797809-29797853	NM_028791:285	Ftsjd2	INSIDE	1.152	8.814	1656.89	14604.27	10.155	1605.52	16303.57
A_68_P28080759	chr11:102527010-102527054	NM_001127576:131	Gm1564	INSIDE	1.152	2.624	1077.03	2825.96	3.023	1089.15	3292.33
A_68_P31114981	chr17:27340635-27340679	NM_146075:727	Lemd2	INSIDE	1.152	1.503	615.33	924.93	1.732	482.80	836.27
A_68_P21147646	chr2:38207402-38207446	NM_010710:597	Lhx2	INSIDE	1.152	1.841	1141.04	2100.71	2.121	958.31	2032.12
A_68_P27787407	chr11:50038771-50038815	NM_145926:-44	Mgat4b	PROMOTER	1.152	3.262	814.85	2658.43	3.757	726.36	2728.77
A_68_P22747170	chr4:14753655-14753699	NM_152812:58	Otud6b	INSIDE	1.152	1.698	514.41	873.62	1.957	368.39	720.91
A_68_P22825737	chr4:33296673-33296717	NM_177774:729	Srsf12	INSIDE	1.152	1.580	1188.05	1876.62	1.820	924.49	1682.97
A_68_P31206911	chr17:46582449-46582493	NM_001162864:42154	Ttbk1	INSIDE	1.152	2.193	1789.25	3924.04	2.526	1399.77	3536.41
A_68_P21839206	chr2:167875978-167876022			Unknown	1.152	1.581	1258.23	1989.21	1.821	1086.28	1978.20
A_68_P26215510	chr8:122223902-122223946	NM_026922:16	Atp2c2	INSIDE	1.151	2.071	535.12	1108.12	2.383	438.83	1045.53
A_68_P27534326	chr10:126603018-126603062	NM_027739:154	B4galnt1	INSIDE	1.151	3.016	1466.51	4423.32	3.473	1157.42	4019.34
A_68_P28198046	chr12:3982083-3982127	NM_001082483:56811	Efr3b	INSIDE	1.151	3.068	3248.42	9965.24	3.532	2458.78	8684.08
A_68_P21874508	chr2:174124774-174124818	NM_201617:1437	Gnas	INSIDE	1.151	3.335	2389.18	7967.92	3.838	1975.22	7580.59
A_68_P28529997	chr12:73171347-73171391	NM_181752:610	Gpr135	INSIDE	1.151	1.732	1530.24	2650.15	1.994	1318.66	2629.36
A_68_P23812527	chr5:76374231-76374275	NM_010612:201	Kdr	INSIDE	1.151	1.548	795.11	1231.04	1.782	694.36	1237.52
A_68_P30127461	chr15:34168406-34168450	NM_033521:648	Laptm4b	INSIDE	1.151	2.387	1148.37	2741.12	2.747	1087.40	2987.28
A_68_P23264337	chr4:123364300-123364344	NM_001199136:-2719	Macf1	PROMOTER	1.151	2.167	353.42	765.78	2.494	283.89	708.15
A_68_P27287761	chr10:80616838-80616882	NM_021501:13605	Pias4	INSIDE	1.151	3.003	2071.67	6220.81	3.458	1748.95	6047.29
A_68_P27385038	chr10:98569722-98569766	NM_027740:-60	Poc1b	PROMOTER	1.151	1.788	601.13	1075.03	2.059	609.33	1254.53
A_68_P23440604	chr4:155158617-155158661	NM_001033394:1345	Tmem88b	INSIDE	1.151	1.362	2051.50	2794.00	1.568	1740.74	2729.08
A_68_P25842775	chr8:48732135-48732179			Unknown	1.151	2.042	369.10	753.62	2.350	367.87	864.34
A_68_P22150498	chr3:54719703-54719747	NM_177854:85	6030405A18Rik	INSIDE	1.150	1.446	1113.81	1610.68	1.663	916.62	1524.09
A_68_P20241449	chr1:55419481-55419525	NM_001113367:49	Boll	INSIDE	1.150	1.747	682.79	1193.15	2.010	583.53	1172.82
A_68_P24046889	chr5:122302699-122302743	NM_175474:3684	Fam109a	INSIDE	1.150	2.068	362.88	750.40	2.379	270.56	643.62
A_68_P27088635	chr10:41023104-41023148	NM_133999:-79	Fig4	PROMOTER	1.150	1.517	1590.68	2413.02	1.744	1381.33	2408.82
A_68_P23968414	chr5:108150872-108150916	NM_010278:2469	Gfi1	INSIDE	1.150	2.940	832.39	2447.48	3.382	726.42	2456.58
A_68_P26891187	chr9:122656143-122656187	NM_001199736:-299	Gm9524	PROMOTER	1.150	2.222	872.48	1938.92	2.555	708.56	1810.08
A_68_P26345743	chr9:20966106-20966150	NM_183408:-4029	Pde4a	PROMOTER	1.150	2.230	951.45	2121.40	2.565	804.78	2064.01
A_68_P30369712	chr15:79843045-79843089	NM_011057:2172	Pdgfb	INSIDE	1.150	2.543	1051.94	2674.89	2.924	878.09	2567.18
A_68_P30346548	chr15:76003699-76003743	NM_201394:22420	Plec	INSIDE	1.150	2.013	1564.84	3150.25	2.315	1141.32	2642.48
A_68_P24085130	chr5:129287884-129287928	NM_001081388:171331	Rimbp2	INSIDE	1.150	1.834	1114.28	2043.87	2.110	882.95	1863.05
A_68_P26845374	chr9:114472361-114472405	NM_001042503:1105	Trim71	INSIDE	1.150	1.412	1198.14	1691.77	1.624	945.59	1536.07
A_68_P23297753	chr4:129318253-129318297	NM_001005506:-225	Txlna	PROMOTER	1.150	0.526	2103.56	1106.73	0.605	1736.42	1050.79
A_68_P24540295	chr6:72139369-72139413			Unknown	1.150	2.265	2184.93	4948.60	2.605	1735.39	4520.00
A_68_P28183973	chr11:120211378-120211422	NR_038126:1409	0610009L18Rik	INSIDE	1.149	1.619	434.50	703.53	1.861	401.51	747.19
A_68_P25505914	chr7:134656178-134656222	NR_024331:183	1700008J07Rik	INSIDE	1.149	1.740	2414.76	4202.83	1.999	1954.02	3906.12
A_68_P21706322	chr2:144096823-144096872	NM_028984:77	8430406I07Rik	INSIDE	1.149	1.735	745.56	1293.37	1.993	638.65	1273.05
A_68_P28075091	chr11:101527015-101527059	NM_025404:182	Arl4d	INSIDE	1.149	1.538	1562.43	2402.64	1.767	1382.73	2443.16
A_68_P21907945	chr2:180763668-180763712	NM_015730:10192	Chrna4	INSIDE	1.149	4.048	1725.01	6982.32	4.649	1162.89	5406.42
A_68_P23200238	chr4:108953316-108953360	NM_007943:459	Eps15	INSIDE	1.149	2.136	675.25	1442.32	2.454	644.64	1581.66
A_68_P30988899	chr16:95923500-95923544	NM_011809:-491	Ets2	PROMOTER	1.149	2.001	716.67	1434.05	2.298	614.74	1412.93
A_68_P20553862	chr1:120733925-120733969	NM_001081125:216250	Gli2	INSIDE	1.149	3.651	1064.38	3885.94	4.196	797.16	3344.76
A_68_P27278161	chr10:79132524-79132568	NM_148934:392	Gm16517	INSIDE	1.149	1.454	1137.15	1653.78	1.671	961.73	1607.37
A_68_P31829405	chr18:74223815-74223859	NM_172632:767	Mapk4	INSIDE	1.149	1.704	1577.83	2689.19	1.959	1325.20	2596.28
A_68_P28058593	chr11:98657535-98657579	NM_028722:474	Msl1	INSIDE	1.149	0.547	1212.22	662.73	0.628	1001.63	629.01

ProbeName	Target position of probe on CpG island microarray	TargetID	GeneSymbol	CpG island Description	Ratio of relative methylation (TiO_2-NP/Vehicle)	Sham group			TiO_2-H group		
						Relative methylation (Cy5/Cy3)	Cy3 signal (Input)	Cy5 signal (MeDIP)	Relative methylation (Cy5/Cy3)	Cy3 signal (Input)	Cy5 signal (MeDIP)
A_68_P22278907	chr3:82162344-82162388	NM_001081230:373	Mtap9	INSIDE	1.149	1.687	2298.93	3877.82	1.939	1837.89	3562.99
A_68_P24758829	chr6:113276600-113276644	NM_010957:-347	Ogg1	PROMOTER	1.149	0.467	1435.35	669.83	0.536	1065.39	571.35
A_68_P25354129	chr7:105293827-105293871	NM_011010:109	Omp	INSIDE	1.149	2.062	480.69	991.22	2.370	372.61	882.90
A_68_P30346547	chr15:76003595-76003639	NM_201394:22524	Plec	INSIDE	1.149	2.174	969.23	2107.08	2.498	679.40	1696.82
A_68_P25406005	chr7:116226702-116226746	NM_001038624:114	Ric3	INSIDE	1.149	1.974	926.07	1827.95	2.267	797.64	1808.44
A_68_P23467999	chr5:8423016-8423060	NM_178766:189	Slc25a40	INSIDE	1.149	1.835	449.82	825.36	2.108	413.55	871.65
A_68_P20172913	chr1:40738293-40738337	NM_001033289:-242	Slc9a2	PROMOTER	1.149	2.329	495.35	1153.59	2.677	517.14	1384.28
A_68_P21330558	chr2:73115586-73115630	NM_001005343:5626	Sp9	DOWNSTREAM	1.149	2.730	397.69	1085.74	3.136	382.48	1199.60
A_68_P24760943	chr6:113647199-113647243	NM_001033463:-272	Tatdn2	PROMOTER	1.149	1.568	642.60	1007.29	1.801	571.71	1029.74
A_68_P20405960	chr1:84835061-84835105	NM_133975:797	Trip12	INSIDE	1.149	1.573	1164.17	1830.72	1.807	964.18	1741.94
A_68_P24066624	chr5:125869903-125869947	NM_019639:463	Ubc	INSIDE	1.149	1.614	2006.21	3237.36	1.854	1637.33	3035.47
A_68_P28992608	chr13:43960038-43960082	ENSMUST00000158482:-8110		PROMOTER	1.149	1.561	596.13	930.69	1.794	545.88	979.56
A_68_P32373279	chrX:48194718-48194762	NM_133729:128	2610018G03Rik	INSIDE	1.148	2.034	1137.10	2312.60	2.336	595.41	1390.72
A_68_P26158735	chr8:112261518-112261562	NM_001080930:99	Atxn1l	INSIDE	1.148	0.501	3757.68	1882.24	0.575	3104.61	1785.73
A_68_P26920733	chr10:7500333-7500377	NM_145418:361	BC013529	INSIDE	1.148	0.424	2350.01	996.08	0.487	1973.35	960.28
A_68_P27796930	chr11:51817853-51817897	NM_001166655:152	Cdkl3	INSIDE	1.148	1.460	1417.02	2069.52	1.677	1171.54	1965.02
A_68_P26585433	chr9:64880240-64880284	NM_028906:-2	Dpp8	PROMOTER	1.148	2.027	697.06	1413.25	2.328	589.23	1371.96
A_68_P30540365	chr16:9995470-9995514	NM_008170:-2866	Grin2a	PROMOTER	1.148	1.424	4960.74	7064.25	1.635	3680.85	6018.02
A_68_P26954153	chr10:13685444-13685488	NM_010437:-718	Hivep2	PROMOTER	1.148	1.784	1339.21	2388.49	2.047	1110.57	2273.65
A_68_P21890549	chr2:178142357-178142402	NM_177191:-16	Sycp2	PROMOTER	1.148	4.637	255.79	1186.08	5.325	222.49	1184.80
A_68_P31385965	chr17:81344280-81344324	NM_026516:331	Tmem178	INSIDE	1.148	2.017	796.65	1606.93	2.316	677.17	1568.23
A_68_P32467741	chrX:71613538-71613585	NM_145405:305	Ubl4	INSIDE	1.148	1.561	1085.93	1694.69	1.792	587.05	1052.18
A_68_P22715608	chr4:8574757-8574801	AK084737:-179		PROMOTER	1.148	1.951	3467.41	6763.96	2.240	3093.96	6931.47
A_68_P26055738	chr8:93432239-93432283	NM_177224:79527	Chd9	INSIDE	1.147	1.525	696.37	1062.00	1.748	661.38	1156.43
A_68_P32219657	chr19:59543612-59543656	NM_010132:10455	Emx2	DOWNSTREAM	1.147	2.613	671.78	1755.50	2.998	603.73	1809.84
A_68_P26234160	chr8:125074869-125074913	NM_001037298:339	Fam38a	INSIDE	1.147	2.912	744.90	2168.89	3.339	648.66	2166.20
A_68_P27278594	chr10:79198492-79198536	NM_008226:19136	Hcn2	INSIDE	1.147	2.836	798.73	2265.34	3.254	714.36	2324.19
A_68_P25656208	chr8:11555566-11555610	NM_011919:-477	Ing1	DIVERGENT_PROMOTER	1.147	1.485	1406.37	2088.15	1.703	968.52	1649.07
A_68_P30660050	chr16:33829808-33829852	NM_001145884:80	Itgb5	INSIDE	1.147	0.524	1221.84	640.16	0.601	1071.45	643.74
A_68_P20725327	chr1:155033029-155033073	NM_008485:527	Lamc2	INSIDE	1.147	1.443	2155.58	3109.55	1.655	1888.68	3126.03
A_68_P24817390	chr6:124807543-124807587	NM_013534:141	Leprel2	INSIDE	1.147	2.965	603.53	1789.17	3.401	555.26	1888.39
A_68_P30086030	chr15:26239396-26239440	NM_177597:592	Marchf11	INSIDE	1.147	1.701	1533.20	2607.97	1.951	1305.56	2547.75
A_68_P26824824	chr9:110532605-110532649	NM_183276:24039	Nbeal2	INSIDE	1.147	2.999	4559.26	13673.66	3.441	3199.65	11009.77
A_68_P25507199	chr7:134913679-134913723	NM_198424:372	Orai3	INSIDE	1.147	1.420	1173.39	1666.07	1.629	908.09	1478.88
A_68_P30369639	chr15:79832333-79832377	NM_011057:12884	Pdgfb	INSIDE	1.147	2.403	314.82	756.55	2.757	274.89	757.78
A_68_P22727584	chr4:10935394-10935438	NM_175175:-650	Plekhf2	PROMOTER	1.147	4.620	3714.69	17162.09	5.301	2901.09	15379.18
A_68_P27997510	chr11:87861570-87861615	NM_001078167:420	Srsf1	INSIDE	1.147	1.352	4076.03	5511.77	1.551	2952.90	4579.51
A_68_P24539866	chr6:72047427-72047471	NM_001035228:-158	St3gal5	PROMOTER	1.147	1.742	1402.30	2443.15	1.999	1239.77	2478.14
A_68_P24951681	chr7:4982839-4982883	NM_001033383:10883	Zfp865	INSIDE	1.147	3.014	1643.98	4955.44	3.459	1375.69	4758.27
A_68_P32579462	chrX:102347389-102347433	AK136781:-2300		PROMOTER	1.147	2.156	1328.61	2864.61	2.473	723.39	1788.92
A_68_P31122888	chr17:28655312-28655356	ENSMUST00000114792:-865		DIVERGENT_PROMOTER	1.147	2.051	1694.27	3475.54	2.353	1360.97	3201.97
A_68_P27371447	chr10:96079236-96079280	NM_007569:-376	Btg1	PROMOTER	1.146	1.747	651.75	1138.68	2.002	568.25	1137.36
A_68_P29413813	chr14:13654802-13654846	NM_001042617:769	Cadps	INSIDE	1.146	2.063	495.72	1022.87	2.365	395.06	934.44
A_68_P23881742	chr5:90652641-90652685	NM_001033310:359	Cox18	INSIDE	1.146	2.644	1093.43	2891.44	3.030	953.31	2888.81
A_68_P30597632	chr16:22439432-22439476	NM_023794:189	Etv5	INSIDE	1.146	1.540	1122.92	1729.36	1.765	1004.04	1772.61
A_68_P23417715	chr4:151657468-151657512	NM_178406:9020	Gpr153	INSIDE	1.146	3.479	875.71	3046.49	3.985	765.58	3051.19
A_68_P31927058	chr19:5118339-5118383	NM_008451:48	Klc2	INSIDE	1.146	2.108	920.18	1939.91	2.415	874.32	2111.86
A_68_P31304303	chr17:66450941-66450985	NM_028388:-131	Ndufv2	DIVERGENT_PROMOTER	1.146	2.833	293.43	831.27	3.246	309.33	1004.13
A_68_P29516148	chr14:33015159-33015203	NM_011960:24	Parg	INSIDE	1.146	1.848	1409.37	2604.50	2.117	1204.44	2550.01
A_68_P28159662	chr11:116351581-116351625	NM_026364:58	Prpsap1	INSIDE	1.146	0.562	1876.10	1054.65	0.644	1526.63	983.73
A_68_P31256044	chr17:56567422-56567466	NM_011218:48459	Ptprs	INSIDE	1.146	2.105	728.87	1534.11	2.411	633.36	1527.06

ProbeName	Target position of probe on CpG island microarray	TargetID	GeneSymbol	CpG island Description	Ratio of relative methylation (TiO_2-NP/Vehicle)	Sham group			TiO_2-H group		
						Relative methylation (Cy5/Cy3)	Cy3 signal (Input)	Cy5 signal (MeDIP)	Relative methylation (Cy5/Cy3)	Cy3 signal (Input)	Cy5 signal (MeDIP)
A_68_P27278801	chr10:79229322-79229366	NM_144528:322	Rnf126	INSIDE	1.146	1.758	1376.94	2420.31	2.014	1173.18	2362.58
A_68_P28267488	chr12:16902116-16902160	NM_009072:355	Rock2	INSIDE	1.146	1.705	1265.22	2157.44	1.954	1185.50	2316.01
A_68_P31857883	chr18:79306004-79306048	NM_053099:104	Setbp1	INSIDE	1.146	2.809	318.05	893.29	3.219	277.81	894.35
A_68_P31335036	chr17:71901525-71901569	NM_001142631:146	Spdya	INSIDE	1.146	1.884	1423.82	2682.70	2.159	1190.81	2571.49
A_68_P26495378	chr9:48863573-48863617	NM_012039:-91	Zw10	PROMOTER	1.146	0.584	1322.24	772.49	0.669	1055.85	706.74
A_68_P31157494	chr17:35226276-35226320	NM_178592:63	Abhd16a	INSIDE	1.145	2.161	1166.14	2519.81	2.473	1036.03	2562.23
A_68_P26595208	chr9:66643280-66643324	NM_177583:-72	Aph1b	PROMOTER	1.145	1.349	2023.61	2730.84	1.545	1609.91	2486.73
A_68_P20910361	chr1:188791550-188791594	NM_033077:278	D1Pas1	INSIDE	1.145	2.999	898.09	2693.55	3.434	737.92	2534.04
A_68_P27995151	chr11:87430824-87430868	NM_001045527:181	Hsf5	INSIDE	1.145	2.852	1092.69	3116.31	3.265	914.23	2984.58
A_68_P25176744	chr7:71536910-71536954	NM_026483:190	Mphosph10	INSIDE	1.145	1.415	1901.07	2690.74	1.621	1565.50	2536.98
A_68_P21766706	chr2:155455507-155455551	NM_001085378:18581	Myh7b	INSIDE	1.145	1.445	2078.49	3002.81	1.654	1648.52	2727.10
A_68_P21826985	chr2:165818808-165818852	NM_008679:694	Ncoa3	INSIDE	1.145	9.319	16574.18	154453.60	10.673	9712.30	103662.90
A_68_P26143993	chr8:109817338-109817382	NM_018823:-9	Nfat5	PROMOTER	1.145	2.190	1725.82	3780.06	2.509	1593.99	3998.84
A_68_P27638841	chr11:21893689-21893733	NM_011023:7944	Otx1	DOWNSTREAM	1.145	1.951	665.39	1298.11	2.234	529.96	1184.10
A_68_P30422883	chr15:88898977-88899021	NM_001002005:8843	Panx2	INSIDE	1.145	1.689	1262.48	2131.96	1.933	965.63	1866.86
A_68_P24424497	chr6:47780471-47780515	NM_177882:12	Zfp786	INSIDE	1.145	2.292	1671.68	3831.11	2.624	1432.27	3757.89
A_68_P22202961	chr3:65778787-65778831	AK133062:4973		DOWNSTREAM	1.145	1.377	1272.11	1751.64	1.577	1114.13	1757.07
A_68_P26686357	chr9:83818668-83818712	ENSMUST00000034801:-66		PROMOTER	1.145	1.667	2198.69	3665.55	1.909	1807.73	3450.68
A_68_P23349887	chr4:138686949-138686993	NR_033745:-18	2310028O11Rik	PROMOTER	1.144	1.610	668.37	1076.29	1.842	629.81	1160.18
A_68_P23470075	chr5:8893888-8893932	NM_008830:190	Abcb4	INSIDE	1.144	1.832	1594.06	2919.94	2.096	1304.42	2733.61
A_68_P24098220	chr5:131916295-131916339	NM_177047:1101897	Auts2	INSIDE	1.144	1.938	599.95	1162.40	2.217	724.89	1606.87
A_68_P30379107	chr15:81525500-81525544	NM_001164320:2195	Chadl	INSIDE	1.144	1.708	2331.94	3983.76	1.954	1946.06	3803.48
A_68_P26756439	chr9:97933339-97933383	NM_022319:226	Clstn2	INSIDE	1.144	2.092	2083.53	4358.32	2.393	1768.44	4231.25
A_68_P22874796	chr4:44025897-44025941	NM_001080384:404	Clta	INSIDE	1.144	0.539	2883.94	1554.15	0.616	2250.60	1387.39
A_68_P28732761	chr12:110697987-110698031	NM_001190703:6344	Dlk1	INSIDE	1.144	1.561	1106.10	1726.58	1.786	1061.40	1895.27
A_68_P22483665	chr3:122322229-122322273	NM_001114665:335	Fnbp1l	INSIDE	1.144	3.310	856.91	2836.36	3.787	766.56	2903.14
A_68_P22448210	chr3:115955054-115955098	NM_022427:1326	Gpr88	INSIDE	1.144	1.863	1132.96	2110.22	2.131	969.20	2065.07
A_68_P20897164	chr1:186556642-186556686	NM_008250:-292	Hlx	PROMOTER	1.144	1.773	462.95	820.70	2.027	460.42	933.37
A_68_P28068270	chr11:100333380-100333424	NM_172565:694	Klhl11	INSIDE	1.144	1.816	2440.30	4431.08	2.077	1895.71	3937.90
A_68_P21548580	chr2:115686166-115686210	NM_001159569:204169	Meis2	DOWNSTREAM	1.144	0.554	1024.15	567.65	0.634	825.09	523.27
A_68_P20293515	chr1:64663797-64663841	NM_025964:-76	Mettl21a	PROMOTER	1.144	1.729	429.27	742.27	1.979	394.61	780.78
A_68_P21339747	chr2:74663863-74663907	NM_016804:16	Mtx2	INSIDE	1.144	2.190	327.21	716.61	2.505	306.73	768.40
A_68_P28523604	chr12:72075575-72075619	NM_011184:-13	Psma3	PROMOTER	1.144	2.010	631.63	1269.65	2.299	596.95	1372.34
A_68_P21261141	chr2:60719359-60719403	NM_001141931:115	Rbms1	INSIDE	1.144	2.191	282.47	618.89	2.505	256.38	642.33
A_68_P22336391	chr3:93247585-93247629	NM_001163098:1355	Tchh	INSIDE	1.144	2.137	1454.63	3109.17	2.446	1219.93	2983.87
A_68_P21070954	chr2:25118703-25118747	NM_175286:607	Tprn	INSIDE	1.144	1.533	1113.00	1705.69	1.753	983.21	1723.33
A_68_P27473556	chr10:115550730-115550774	NM_001161855:221	4933416C03Rik	INSIDE	1.143	2.052	496.92	1019.64	2.344	432.89	1014.90
A_68_P28609993	chr12:87762632-87762676	NM_011934:61	Esrrb	INSIDE	1.143	1.759	916.15	1611.57	2.010	795.42	1598.60
A_68_P22888253	chr4:46356368-46356412	NM_183298:-675	Foxe1	PROMOTER	1.143	1.645	770.29	1267.12	1.881	699.15	1314.76
A_68_P20556971	chr1:121318266-121318310	NM_008381:537	Inhbb	INSIDE	1.143	1.555	965.48	1501.06	1.777	918.01	1630.88
A_68_P26258418	chr8:129116571-129116616	NM_001164598:743	Irf2bp2	INSIDE	1.143	2.685	391.89	1052.05	3.069	353.53	1084.99
A_68_P31936712	chr19:7000456-7000500	NM_008431:8527	Kcnk4	INSIDE	1.143	1.595	1158.65	1847.83	1.822	1029.44	1876.10
A_68_P24045149	chr5:121995337-121995381	NM_010765:543	Mapkapk5	INSIDE	1.143	2.283	490.57	1119.86	2.608	472.92	1233.48
A_68_P20935797	chr1:192849641-192849685	NM_001081259:407	Mfsd7b	INSIDE	1.143	1.410	1694.90	2389.78	1.612	1293.74	2085.69
A_68_P22944674	chr4:57313119-57313163	NM_011207:1569	Ptpn3	INSIDE	1.143	1.537	1616.30	2484.45	1.756	1493.44	2622.79
A_68_P26748152	chr9:96531410-96531454	NM_053268:490	Rasa2	INSIDE	1.143	1.840	1099.73	2022.97	2.103	944.73	1986.30
A_68_P28301682	chr12:27100225-27100269	NM_001081977:-125	Rnf144a	PROMOTER	1.143	1.674	1893.27	3169.01	1.913	1653.53	3163.47
A_68_P27284240	chr10:80065867-80065911	NM_019575:262	Scamp4	INSIDE	1.143	0.461	1258.91	580.86	0.527	848.67	447.41
A_68_P22031739	chr3:30993591-30993635	NM_001039090:-370	Skil	PROMOTER	1.143	2.519	873.02	2198.71	2.878	780.35	2245.49
A_68_P32036072	chr19:26679451-26679495	NM_011416:-177	Smarca2	PROMOTER	1.143	1.648	1021.62	1683.35	1.883	841.48	1584.64
A_68_P31962490	chr19:12869772-12869816	NR_024093:819	U05342	INSIDE	1.143	2.436	901.28	2195.20	2.784	866.72	2413.08

ProbeName	Target position of probe on CpG island microarray	TargetID	GeneSymbol	CpG island Description	Ratio of relative methylation (TiO_2-NP/Vehicle)	Sham group			TiO_2-H group		
						Relative methylation (Cy5/Cy3)	Cy3 signal (Input)	Cy5 signal (MeDIP)	Relative methylation (Cy5/Cy3)	Cy3 signal (Input)	Cy5 signal (MeDIP)
A_68_P21779874	chr2:157740919-157740963	NM_198627:552	Vstm2l	INSIDE	1.143	3.186	3156.91	10059.25	3.643	2160.54	7870.49
A_68_P21076583	chr2:25994281-25994325	ENSMUST00000163836:20		INSIDE	1.143	2.486	5233.91	13009.34	2.842	4058.83	11535.03
A_68_P27281469	chr10:79634617-79634661	NM_027207:-1050	1600002K03Rik	PROMOTER	1.142	3.140	1389.69	4363.61	3.586	1101.78	3950.74
A_68_P20416762	chr1:89161364-89161408	NM_028889:448	Efhd1	INSIDE	1.142	1.890	1935.79	3658.56	2.158	1768.27	3816.05
A_68_P24138290	chr5:140388742-140388786	NM_175522:4868	Elfn1	INSIDE	1.142	1.382	1371.99	1896.57	1.578	1171.16	1848.62
A_68_P24138664	chr5:140449200-140449245	NM_175522:65326	Elfn1	INSIDE	1.142	2.327	1456.27	3388.96	2.657	1164.39	3093.79
A_68_P23392749	chr4:147533784-147533828	NM_001161851:-102	Fbxo44	PROMOTER	1.142	1.388	1996.76	2771.44	1.585	1604.84	2543.39
A_68_P24325253	chr6:29166069-29166113	NM_011829:-3819	Impdh1	PROMOTER	1.142	2.299	291.55	670.30	2.625	305.52	802.08
A_68_P32141807	chr19:46119714-46119758	NM_001113408:-35	Ldb1	PROMOTER	1.142	1.702	613.53	1044.20	1.944	582.83	1132.92
A_68_P27782065	chr11:49057416-49057460	NM_001110148:-254	Mgat1	PROMOTER	1.142	1.465	1555.95	2279.67	1.673	1283.27	2146.85
A_68_P23392001	chr4:147413122-147413166	NM_010840:-41	Mthfr	DIVERGENT_PROMOTER	1.142	1.696	1563.37	2652.04	1.938	1223.33	2370.34
A_68_P32231475	chrX:5045956-5046000	NM_010850:405	Mycs	INSIDE	1.142	2.263	636.59	1440.54	2.584	348.50	900.51
A_68_P22649654	chr3:151928897-151928941	NM_199465:366	Nexn	INSIDE	1.142	1.382	1293.67	1787.47	1.578	1242.78	1961.73
A_68_P26742969	chr9:95537953-95537997	NM_029620:-72	Pcolce2	PROMOTER	1.142	1.517	554.55	841.36	1.733	513.29	889.64
A_68_P22446820	chr3:115710167-115710211	NM_023214:136	Slc30a7	INSIDE	1.142	1.587	1582.11	2511.14	1.813	1320.18	2393.82
A_68_P23308817	chr4:131429521-131429565	NM_020587:-11	Srsf4	PROMOTER	1.142	3.553	499.71	1775.50	4.058	484.82	1967.52
A_68_P25584910	chr7:147973596-147973640	NM_001143765:135	Syce1	INSIDE	1.142	3.011	2858.30	8606.63	3.438	2540.67	8734.54
A_68_P28687318	chr12:102956856-102956900	NM_001082976:-166	Tc2n	PROMOTER	1.142	2.347	4922.48	11551.08	2.679	3757.29	10065.84
A_68_P22856580	chr4:40216716-40216760	NM_134097:136	Topors	INSIDE	1.142	0.529	1290.69	682.21	0.604	1070.61	646.32
A_68_P27033509	chr10:28867346-28867390	NM_026138:3567	6330407J23Rik	INSIDE	1.141	1.889	1801.19	3401.94	2.154	1654.05	3563.34
A_68_P20026603	chr1:10222944-10222988	NM_001102430:-215	Arfgef1	PROMOTER	1.141	1.817	421.57	766.16	2.073	410.39	850.71
A_68_P32268349	chrX:13423877-13423921	NM_009806:-231	Cask	PROMOTER	1.141	2.936	1476.93	4335.55	3.349	937.02	3138.00
A_68_P20619647	chr1:134035982-134036026	NM_008795:258	Cdk18	INSIDE	1.141	2.748	897.73	2467.03	3.137	833.16	2613.40
A_68_P26238529	chr8:125736395-125736439	NM_145606:272	Chmp1a	INSIDE	1.141	1.789	1769.26	3164.82	2.041	1278.91	2610.54
A_68_P23192163	chr4:107356485-107356529	NM_019872:261	Dmrtb1	INSIDE	1.141	3.490	350.53	1223.28	3.983	299.05	1191.00
A_68_P28378464	chr12:41172641-41172685	NM_172803:23	Dock4	INSIDE	1.141	2.323	320.99	745.72	2.651	257.71	683.13
A_68_P24449831	chr6:52260423-52260467	NM_007966:-3047	Evx1	PROMOTER	1.141	4.313	3128.82	13495.28	4.920	2577.10	12680.36
A_68_P27995152	chr11:87431026-87431070	NM_001045527:383	Hsf5	INSIDE	1.141	2.267	1056.51	2395.17	2.587	918.12	2374.93
A_68_P20335537	chr1:72258509-72258553	NM_001005423:351	Mreg	INSIDE	1.141	1.377	1522.96	2097.51	1.572	1252.36	1968.69
A_68_P25581955	chr7:147234797-147234841	NM_010836:169	Msx3	INSIDE	1.141	0.549	1314.54	721.46	0.626	1005.80	629.87
A_68_P23393398	chr4:147645613-147645662	NM_001083342:16437	Ptchd2	INSIDE	1.141	2.603	450.79	1173.19	2.970	402.37	1195.09
A_68_P26816015	chr9:108564655-108564699	NM_020520:248	Slc25a20	INSIDE	1.141	3.193	1361.74	4348.06	3.644	1274.91	4646.06
A_68_P25954770	chr8:73416471-73416515	NM_053248:164	Slc5a5	INSIDE	1.141	1.687	1035.00	1745.83	1.925	923.23	1777.23
A_68_P22367896	chr3:99966241-99966285	NM_175552:64	Wdr3	INSIDE	1.141	0.401	1678.24	672.19	0.457	1461.15	667.98
A_68_P23154332	chr4:100965654-100965698	ENSMUST00000131864:28674		INSIDE	1.141	16.638	2625.93	43691.16	18.986	1836.30	34864.06
A_68_P24127046	chr5:138125901-138125945	NM_145566:-1	Agfg2	PROMOTER	1.140	2.657	14382.74	38221.34	3.029	10803.47	32723.97
A_68_P31549437	chr18:22502988-22503032	NM_001167777:-579	Asxl3	PROMOTER	1.140	1.640	1240.83	2034.48	1.869	981.96	1834.84
A_68_P26845718	chr9:114549214-114549258	NM_153585:82	Cnot10	INSIDE	1.140	0.515	1234.90	635.71	0.587	937.77	550.11
A_68_P21144122	chr2:37642341-37642385	NM_001163566:10594	Crb2	INSIDE	1.140	1.372	1424.99	1955.46	1.564	1157.99	1811.65
A_68_P26581359	chr9:64188861-64188905	NM_001177784:-302	Dis3l	PROMOTER	1.140	1.862	509.42	948.60	2.124	481.01	1021.52
A_68_P31398414	chr17:83750191-83750235	NM_001114361:-58	Eml4	PROMOTER	1.140	2.021	520.98	1052.64	2.303	540.18	1244.08
A_68_P29130720	chr13:72100566-72100611	NM_010573:583	Irx1	INSIDE	1.140	1.807	1014.61	1833.12	2.060	1215.21	2503.18
A_68_P24053344	chr5:123439364-123439408	NM_001003953:-285	Kdm2b	PROMOTER	1.140	2.657	8288.90	22026.62	3.030	5752.37	17428.50
A_68_P26082829	chr8:97876209-97876253	NM_008609:-6	Mmp15	PROMOTER	1.140	2.947	1460.00	4302.18	3.359	1203.22	4041.79
A_68_P25326013	chr7:99817798-99817842	NM_029078:602	Pcf11	INSIDE	1.140	1.806	973.23	1757.77	2.059	777.02	1600.24
A_68_P24627788	chr6:89311893-89311937	NM_008881:693	Plxna1	INSIDE	1.140	1.583	1143.52	1810.26	1.805	1090.70	1968.78
A_68_P25508221	chr7:135079319-135079363	NM_001081374:10899	Prss36	INSIDE	1.140	2.897	3483.02	10091.90	3.303	2870.21	9479.93
A_68_P31256054	chr17:56568541-56568585	NM_011218:47341	Ptprs	INSIDE	1.140	3.934	2134.59	8396.48	4.484	1636.49	7337.60
A_68_P23922674	chr5:99681727-99681771	NM_145839:198	Rasgef1b	INSIDE	1.140	1.858	1720.84	3198.16	2.118	1481.41	3138.20
A_68_P23327086	chr4:134731348-134731392	NM_019732:54811	Runx3	INSIDE	1.140	1.618	2503.94	4051.62	1.845	2053.89	3790.26
A_68_P21747605	chr2:151919256-151919300	NM_001160410:12014	Scrt2	INSIDE	1.140	1.697	1345.80	2283.95	1.935	1177.80	2279.37

ProbeName	Target position of probe on CpG island microarray	TargetID	GeneSymbol	CpG island Description	Ratio of relative methylation (TiO_2-NP/Vehicle)	Sham group			TiO_2-H group		
						Relative methylation (Cy5/Cy3)	Cy3 signal (Input)	Cy5 signal (MeDIP)	Relative methylation (Cy5/Cy3)	Cy3 signal (Input)	Cy5 signal (MeDIP)
A_68_P20151303	chr1:36996367-36996411	NM_018872:-16	Tmem131	PROMOTER	1.140	2.394	2960.76	7088.56	2.729	2089.12	5700.62
A_68_P31002108	chr16:98183316-98183360	NM_001081684:448	Zfp295	INSIDE	1.140	3.915	2251.56	8814.55	4.463	1684.45	7518.43
A_68_P28053992	chr11:97884169-97884213	NM_145121:-249	Cacnb1	DIVERGENT_PROMOTER	1.139	1.455	4643.19	6755.89	1.658	3410.85	5654.04
A_68_P32383366	chrX:49966645-49966690	NM_016697:484	Gpc3	INSIDE	1.139	1.660	1330.07	2207.32	1.890	806.06	1523.15
A_68_P26550038	chr9:58671669-58671713	NM_001081192:372	Hcn4	INSIDE	1.139	0.507	1451.40	736.01	0.578	1245.16	719.43
A_68_P21321484	chr2:71625261-71625305	NM_008397:143	Itga6	INSIDE	1.139	2.643	523.77	1384.25	3.011	438.63	1320.81
A_68_P32136345	chr19:45154436-45154480	NM_178929:3830	Kazald1	DOWNSTREAM	1.139	1.859	3426.28	6369.98	2.117	2584.79	5472.58
A_68_P20348303	chr1:74438925-74438969	NR_029743:-1937	Mir26b	PROMOTER	1.139	0.527	1036.31	545.76	0.600	838.60	502.94
A_68_P24051802	chr5:123157935-123157979	NM_011026:391	P2rx4	INSIDE	1.139	2.251	1115.71	2511.71	2.563	987.41	2531.14
A_68_P24046733	chr5:122278943-122278987	NM_008507:7846	Sh2b3	INSIDE	1.139	3.173	1425.63	4522.86	3.613	1344.28	4857.28
A_68_P23608246	chr5:36873249-36873293	NM_001113364:2	Tbc1d14	INSIDE	1.139	1.530	938.48	1436.30	1.743	846.96	1476.35
A_68_P26891892	chr9:122795954-122795998	NM_001177505:-1612	Zfp167	PROMOTER	1.139	2.196	1661.44	3648.76	2.501	1448.05	3622.28
A_68_P32672910	chrX:131160541-131160585	ENSMUST00000137737:-279		PROMOTER	1.139	2.091	913.69	1910.42	2.382	512.07	1219.71
A_68_P28743345	chr12:112556826-112556880	NM_183016:59077	Cdc42bpb	INSIDE	1.138	1.685	641.65	1081.46	1.918	571.57	1096.48
A_68_P23200235	chr4:108952879-108952923	NM_007943:21	Eps15	INSIDE	1.138	1.769	2860.81	5060.18	2.013	2362.74	4755.71
A_68_P27141570	chr10:53018974-53019019	NM_001204983:80661	Gm9766	INSIDE	1.138	1.796	484.83	870.64	2.044	421.73	861.87
A_68_P28043344	chr11:96133745-96133789	NM_008270:1123	Hoxb9	INSIDE	1.138	2.292	551.78	1264.92	2.609	512.96	1338.40
A_68_P27902199	chr11:70513617-70513661	NM_213729:19	Inca1	INSIDE	1.138	1.931	1131.10	2183.78	2.197	1057.60	2324.01
A_68_P30682958	chr16:37868795-37868839	NM_177093:331	Lrrc58	INSIDE	1.138	2.125	405.07	860.73	2.418	408.08	986.86
A_68_P27011027	chr10:24589834-24589878	NM_001166416:65	Med23	INSIDE	1.138	1.418	1286.73	1825.18	1.614	1096.31	1769.01
A_68_P28269967	chr12:17355126-17355170	NM_001008421:-150	Nol10	PROMOTER	1.138	1.505	2601.68	3914.40	1.713	2127.26	3643.00
A_68_P23409032	chr4:150283998-150284042	NM_020569:10	Park7	INSIDE	1.138	1.564	1909.68	2987.47	1.780	1617.75	2879.64
A_68_P31632591	chr18:38357539-38357583	NM_029357:11856	Pcdh1	INSIDE	1.138	3.034	1272.71	3861.54	3.452	901.81	3113.24
A_68_P30423564	chr15:88991163-88991207	NM_001159521:13096	Plxnb2	INSIDE	1.138	3.484	884.01	3079.79	3.966	776.42	3078.92
A_68_P25771677	chr8:35040417-35040461	NM_001042674:-125	Rbpms	PROMOTER	1.138	1.564	624.97	977.15	1.780	778.65	1385.70
A_68_P25025186	chr7:29856672-29856716	NM_009109:53476	Ryr1	INSIDE	1.138	1.893	711.20	1346.42	2.154	526.30	1133.49
A_68_P21843418	chr2:168591344-168591388	NM_175303:1335	Sall4	INSIDE	1.138	3.052	1325.71	4045.75	3.472	1107.27	3843.90
A_68_P31709384	chr18:52928549-52928593	NM_001199154:773	Sncaip	INSIDE	1.138	1.390	2108.75	2931.07	1.581	1716.34	2713.76
A_68_P24432386	chr6:49345471-49345515	NM_029916:-110	Stk31	PROMOTER	1.138	2.814	977.94	2752.31	3.204	750.59	2404.82
A_68_P21748556	chr2:152119601-152119645	NM_024196:15	Tbc1d20	INSIDE	1.138	2.451	430.45	1054.87	2.789	382.58	1067.10
A_68_P27641463	chr11:22412449-22412493	NM_153596:185	Tmem17	INSIDE	1.138	3.343	1232.01	4118.01	3.802	1073.40	4081.58
A_68_P23185612	chr4:106311841-106311885	NM_177667:16809	Ttc22	INSIDE	1.138	2.496	1130.44	2821.23	2.841	981.37	2788.35
A_68_P20827839	chr1:173341981-173342025	NM_009480:191	Usf1	INSIDE	1.138	1.812	1426.53	2585.04	2.063	1148.99	2370.41
A_68_P30401334	chr15:85374054-85374098	NM_001163634:34424	Wnt7b	INSIDE	1.138	2.942	1235.10	3634.16	3.348	1139.66	3816.09
A_68_P33013883	chrUn_random:3232956-3233000			Unknown	1.138	2.077	846.94	1758.82	2.363	678.87	1603.83
A_68_P27340461	chr10:90644609-90644653	ENSMUST00000099357:-9871		PROMOTER	1.138	0.507	1087.58	550.98	0.576	969.83	558.94
A_68_P31939680	chr19:7491931-7491975	NM_175381:-162	2700081O15Rik	PROMOTER	1.137	1.690	1418.97	2398.14	1.922	1138.96	2189.30
A_68_P22398700	chr3:105507744-105507788	NM_001163356:-609	6530418L21Rik	PROMOTER	1.137	2.276	691.12	1573.21	2.587	624.36	1615.33
A_68_P27339935	chr10:90545393-90545437	NM_001042558:74	Apaf1	INSIDE	1.137	0.475	1087.17	516.72	0.540	1031.02	556.96
A_68_P22058858	chr3:36470838-36470882	NM_009828:58	Ccna2	INSIDE	1.137	2.578	1330.56	3430.10	2.930	1130.50	3312.56
A_68_P20139530	chr1:34636521-34636565	NM_213727:5	Fam123c	INSIDE	1.137	1.978	1105.59	2186.87	2.248	984.97	2214.32
A_68_P22450556	chr3:116365109-116365153	NM_008246:465	Hiat1	INSIDE	1.137	2.950	661.58	1951.71	3.354	600.60	2014.56
A_68_P21309973	chr2:69423496-69423540	NM_001081088:606	Lrp2	INSIDE	1.137	1.706	653.63	1115.20	1.940	655.63	1271.97
A_68_P21044978	chr2:19367856-19367900	NM_018809:589	Ptf1a	INSIDE	1.137	1.407	1024.31	1440.82	1.600	846.03	1353.62
A_68_P29622735	chr14:57021569-57021613	NM_001033043:57	Rnf17	INSIDE	1.137	3.045	2228.51	6786.90	3.464	1777.25	6156.76
A_68_P26750481	chr9:96919029-96919073	NM_145134:-276	Spsb4	PROMOTER	1.137	1.712	833.14	1426.16	1.946	713.38	1388.25
A_68_P28166384	chr11:117515968-117516012	NM_198022:388	Tnrc6c	INSIDE	1.137	1.546	1191.84	1842.42	1.757	1029.05	1808.44
A_68_P24638741	chr6:91344683-91344727	NM_009527:16659	Wnt7a	INSIDE	1.137	2.113	258.94	547.12	2.403	210.85	506.75
A_68_P29373467	chr14:4829490-4829534	ENSMUST00000165619:-301		PROMOTER	1.137	2.836	3584.78	10166.21	3.224	3373.19	10874.69
A_68_P31746201	chr18:59335430-59335474	NM_001081328:459	Chsy3	INSIDE	1.136	0.479	1428.60	683.91	0.544	1166.90	634.86
A_68_P25948480	chr8:72406164-72406208	NM_026818:5105	Cilp2	INSIDE	1.136	2.662	565.04	1504.40	3.024	564.02	1705.81

ProbeName	Target position of probe on CpG island microarray	TargetID	GeneSymbol	CpG island Description	Ratio of relative methylation (TiO_2-NP/Vehicle)	Sham group			TiO_2-H group		
						Relative methylation (Cy5/Cy3)	Cy3 signal (Input)	Cy5 signal (MeDIP)	Relative methylation (Cy5/Cy3)	Cy3 signal (Input)	Cy5 signal (MeDIP)
A_68_P29334873	chr13:113254637-113254681	NM_010560:381	Il6st	INSIDE	1.136	1.546	2638.65	4079.29	1.756	2019.87	3547.22
A_68_P24804565	chr6:121423791-121423835	NM_001033354:-116	Iqsec3	PROMOTER	1.136	0.548	1171.87	641.70	0.622	977.95	608.46
A_68_P21239895	chr2:56954793-56954837	NM_013613:12635	Nr4a2	DOWNSTREAM	1.136	0.561	1099.56	616.62	0.637	1074.70	684.82
A_68_P22778509	chr4:22415123-22415167	NM_008899:134	Pou3f2	INSIDE	1.136	1.829	762.99	1395.40	2.077	650.63	1351.26
A_68_P30390277	chr15:83555041-83555085	NM_022723:389	Scube1	INSIDE	1.136	2.018	1445.64	2917.40	2.293	1238.70	2840.08
A_68_P21116239	chr2:32442606-32442650	NM_011373:-381	St6galnac4	PROMOTER	1.136	1.902	470.62	895.15	2.161	458.76	991.20
A_68_P24880088	chr6:137683751-137683795	NM_011499:170	Strap	INSIDE	1.136	2.621	467.53	1225.55	2.977	490.12	1459.09
A_68_P31254298	chr17:56301931-56301975	NM_025566:39	Tnfaip8l1	INSIDE	1.136	1.441	1132.24	1631.07	1.637	894.21	1463.53
A_68_P28787946	chr13:3358712-3358756			Unknown	1.136	2.407	840.16	2022.29	2.734	774.05	2116.13
A_68_P22039461	chr3:32608077-32608121	NM_019673:631	Actl6a	INSIDE	1.135	1.884	860.68	1621.38	2.139	815.82	1744.81
A_68_P24994776	chr7:20282146-20282190	NM_009696:2347	Apoe	INSIDE	1.135	2.429	696.47	1691.65	2.756	624.91	1722.34
A_68_P23262160	chr4:123020018-123020062	NM_007558:6	Bmp8a	INSIDE	1.135	0.545	1865.12	1016.77	0.619	1467.31	907.87
A_68_P26813082	chr9:108012831-108012875	NM_007567:79862	Bsn	INSIDE	1.135	2.328	1586.69	3694.61	2.642	1263.38	3337.69
A_68_P21580764	chr2:121239313-121239357	NM_153075:194	Catsper2	INSIDE	1.135	1.616	4136.12	6685.98	1.835	3328.44	6108.71
A_68_P25601118	chr7:150651001-150651045	NM_001161624:-4067	Cdkn1c	DIVERGENT_PROMOTER	1.135	3.697	454.16	1679.14	4.198	443.60	1862.18
A_68_P27476526	chr10:116018789-116018833	NM_001037846:-243	Cnot2	PROMOTER	1.135	3.158	1799.87	5683.88	3.585	1572.05	5636.01
A_68_P21706813	chr2:144194864-144194908	NM_181417:116	Csrp2bp	INSIDE	1.135	1.860	547.64	1018.78	2.111	480.48	1014.22
A_68_P29737940	chr14:77556610-77556654	NM_172813:10	Enox1	INSIDE	1.135	1.449	758.66	1099.67	1.646	686.66	1130.12
A_68_P28928669	chr13:32430434-32430478	NM_146041:-43	Gmds	PROMOTER	1.135	0.534	1486.36	793.48	0.606	1327.02	804.05
A_68_P21933421	chr3:8667100-8667144	NM_010423:-84	Hey1	PROMOTER	1.135	2.545	573.08	1458.68	2.888	516.10	1490.73
A_68_P25528245	chr7:138553702-138553746	NM_175115:268	Ikzf5	INSIDE	1.135	0.399	1405.83	560.46	0.453	1239.71	561.09
A_68_P27484949	chr10:117578950-117578994	NM_001162904:130	Mdm1	INSIDE	1.135	1.405	1467.01	2061.57	1.595	1226.56	1956.14
A_68_P24894481	chr6:140372708-140372752	NM_144920:111	Plekha5	INSIDE	1.135	1.474	1618.55	2385.49	1.673	1491.83	2496.12
A_68_P31953479	chr19:10969598-10969642	NM_134129:-161	Prpf19	PROMOTER	1.135	1.778	396.41	704.95	2.019	365.25	737.54
A_68_P24125229	chr5:137774819-137774863	NM_031406:-30	Slc12a9	PROMOTER	1.135	1.499	6017.14	9021.20	1.701	3977.16	6766.42
A_68_P32319890	chrX:34283973-34284017	NM_001085497:341	Slc25a43	INSIDE	1.135	1.505	1027.93	1547.46	1.709	543.60	929.16
A_68_P30360708	chr15:78370197-78370241	NM_009218:4557	Sstr3	INSIDE	1.135	1.654	888.91	1470.41	1.878	768.68	1443.43
A_68_P26470629	chr9:44773701-44773745	NM_145400:-39	Ube4a	PROMOTER	1.135	1.939	1914.96	3712.77	2.200	1453.61	3198.39
A_68_P20505871	chr1:107887105-107887149	NM_001122675:144	Zcchc2	INSIDE	1.135	2.550	1079.51	2753.24	2.894	823.13	2382.24
A_68_P32975879	A_68_P32975879			Unknown	1.135	1.732	929.03	1608.98	1.966	944.61	1857.17
A_68_P32983000	A_68_P32983000			Unknown	1.135	2.262	775.27	1753.61	2.568	794.92	2041.58
A_68_P26080209	chr8:97437362-97437406	NM_001033533:4614	Ccdc102a	INSIDE	1.134	1.537	990.63	1522.27	1.743	796.04	1387.28
A_68_P29613438	chr14:55260166-55260210	NM_199470:13	Cdh24	INSIDE	1.134	1.897	737.77	1399.69	2.151	683.00	1469.00
A_68_P24798785	chr6:120443882-120443926	NM_033567:-79	Cecr6	PROMOTER	1.134	1.745	478.57	835.24	1.978	448.44	887.20
A_68_P26505264	chr9:50536185-50536229	NM_178118:-117	Dixdc1	PROMOTER	1.134	1.567	701.53	1099.40	1.777	604.16	1073.40
A_68_P26523445	chr9:53822873-53822917	NM_177769:214	Elmod1	INSIDE	1.134	0.553	1270.53	702.32	0.627	1094.15	685.93
A_68_P28675862	chr12:100630292-100630340	NM_183186:57968	Foxn3	INSIDE	1.134	1.880	295.02	554.51	2.131	296.99	632.87
A_68_P24658382	chr6:94649670-94649714	NM_008377:447	Lrig1	INSIDE	1.134	1.895	379.50	719.02	2.149	322.08	692.01
A_68_P24009588	chr5:115890672-115890717	NM_008629:11001	Msi1	INSIDE	1.134	4.011	469.76	1884.44	4.551	374.48	1704.11
A_68_P31629193	chr18:37829497-37829541	NM_033586:-4470	Pcdhga3	PROMOTER	1.134	2.242	516.06	1156.85	2.543	426.68	1085.05
A_68_P32536213	chrX:91077623-91077667	NM_145630:-210	Pdk3	PROMOTER	1.134	1.632	1149.18	1875.44	1.850	558.78	1033.82
A_68_P24059429	chr5:124573689-124573733	NM_011256:92717	Pitpnm2	INSIDE	1.134	3.249	669.87	2176.50	3.685	567.36	2090.99
A_68_P30729957	chr16:46010656-46010700	NM_001134480:-152	Plexd2	PROMOTER	1.134	1.632	1086.91	1773.71	1.850	987.21	1826.10
A_68_P28740620	chr12:112079116-112079160	NM_011973:11	Stk30	INSIDE	1.134	2.221	355.68	789.89	2.518	323.07	813.64
A_68_P22955049	chr4:59202246-59202290	NM_011673:-153	Ugcg	PROMOTER	1.134	2.748	474.71	1304.46	3.115	474.73	1478.80
A_68_P24427163	chr6:48388218-48388262	NM_001085415:6849	Zfp467	INSIDE	1.134	1.788	1596.39	2854.54	2.027	1261.90	2558.29
A_68_P21934023	chr3:8767607-8767651			Unknown	1.134	2.092	3179.25	6649.98	2.372	2285.15	5419.68
A_68_P32410984	chrX:56821241-56821285	ENSMUST00000119833:-14		PROMOTER	1.134	1.938	432.75	838.84	2.198	268.01	589.07
A_68_P23391463	chr4:147315197-147315241	NM_029841:215	2510039O18Rik	INSIDE	1.133	1.956	904.26	1768.69	2.216	743.95	1648.92
A_68_P21776100	chr2:157105079-157105123	NM_001039557:185	4922505G16Rik	INSIDE	1.133	1.601	932.72	1493.56	1.814	809.44	1468.62
A_68_P23447647	chr5:3928400-3928444	NM_194462:237	Akap9	INSIDE	1.133	1.419	1319.18	1871.49	1.607	1160.21	1865.01

ProbeName	Target position of probe on CpG island microarray	TargetID	GeneSymbol	CpG island Description	Ratio of relative methylation (TiO_2-NP/Vehicle)	Sham group			TiO_2-H group		
						Relative methylation (Cy5/Cy3)	Cy3 signal (Input)	Cy5 signal (MeDIP)	Relative methylation (Cy5/Cy3)	Cy3 signal (Input)	Cy5 signal (MeDIP)
A_68_P21673348	chr2:138082206-138082250	NM_001025431:-91	Btbd3	PROMOTER	1.133	3.215	1850.87	5950.56	3.642	1537.36	5599.36
A_68_P25047616	chr7:35905038-35905082	NM_007678:749	Cebpa	INSIDE	1.133	0.539	4210.42	2271.41	0.611	3692.96	2256.50
A_68_P20148716	chr1:36568774-36568818	NM_001039551:76	Cnnm3	INSIDE	1.133	3.034	2912.87	8838.60	3.437	2339.06	8038.66
A_68_P25083573	chr7:50930678-50930722	NM_145582:3300	Ctu1	INSIDE	1.133	2.076	826.67	1716.12	2.352	763.67	1795.91
A_68_P31620540	chr18:36020339-36020383	NM_133687:30889	Cxxc5	INSIDE	1.133	3.049	1031.39	3144.98	3.455	827.36	2858.70
A_68_P25496227	chr7:132851431-132851475	NM_001081022:63	D430042O09Rik	INSIDE	1.133	2.950	470.41	1387.85	3.341	428.30	1431.11
A_68_P29626122	chr14:57723066-57723110	NM_008125:451	Gjb2	INSIDE	1.133	0.447	1816.05	812.43	0.507	1475.09	747.34
A_68_P21817704	chr2:164239982-164240026	NM_013592:-9890	Matn4	PROMOTER	1.133	2.320	2606.22	6046.12	2.629	1994.86	5244.03
A_68_P28246306	chr12:12948314-12948358	NM_008709:306	Mycn	INSIDE	1.133	1.839	1193.87	2195.91	2.084	1001.15	2086.53
A_68_P22355468	chr3:97462189-97462238	NM_182997:79	Prkab2	INSIDE	1.133	1.784	1722.40	3073.42	2.021	1329.38	2686.69
A_68_P24325039	chr6:29129114-29129158	NM_001101443:448	Prrt4	INSIDE	1.133	2.009	351.20	705.47	2.276	402.57	916.34
A_68_P22383501	chr3:102739596-102739640	NM_011516:405	Sycp1	INSIDE	1.133	2.771	1127.57	3124.35	3.139	952.37	2989.38
A_68_P28936502	chr13:34169897-34169941	NM_009450:-41	Tubb2a	PROMOTER	1.133	1.555	1520.94	2364.84	1.762	1313.99	2314.93
A_68_P20286081	chr1:63320021-63320065	NM_028673:200	Zdbf2	INSIDE	1.133	1.418	1173.03	1663.37	1.607	1090.62	1752.33
A_68_P28603808	A_68_P28603808			Unknown	1.133	2.003	919.76	1842.41	2.269	826.92	1876.64
A_68_P26467841	chr9:44291334-44291378	ENSMUST00000136322:4496		DOWNSTREAM	1.133	2.280	1789.97	4082.00	2.584	1453.44	3755.63
A_68_P23315558	chr4:132617847-132617891	NM_146155:50448	Ahdc1	INSIDE	1.132	2.539	2589.28	6574.24	2.874	2032.85	5841.54
A_68_P31613237	chr18:34666453-34666497	NM_146084:3	Fam13b	INSIDE	1.132	1.462	2874.26	4203.33	1.655	2339.14	3870.69
A_68_P23969766	chr5:108415737-108415781	NM_026062:338	Fam69a	INSIDE	1.132	1.456	2521.27	3670.94	1.648	2070.27	3412.25
A_68_P27289254	chr10:80841374-80841418	NM_019757:-281	Fzr1	DIVERGENT_PROMOTER	1.132	1.610	9507.21	15305.59	1.822	6175.11	11253.25
A_68_P22785943	chr4:24423379-24423423	NM_199467:-208	Mms22l	PROMOTER	1.132	2.328	1320.57	3073.64	2.635	1149.53	3028.70
A_68_P23348674	chr4:138469719-138469763	NM_028453:122	Otud3	INSIDE	1.132	4.680	283.97	1328.95	5.299	266.72	1413.40
A_68_P25435746	chr7:121558834-121558878	NM_011055:89	Pde3b	INSIDE	1.132	1.823	732.04	1334.45	2.063	693.75	1431.19
A_68_P22312796	chr3:88058579-88058623	NM_021375:6	Rhbg	INSIDE	1.132	0.517	1728.80	893.81	0.585	1309.53	766.10
A_68_P28517710	chr12:71088293-71088337	NM_022028:-325	Sav1	PROMOTER	1.132	2.472	1362.43	3367.50	2.797	1247.11	3487.91
A_68_P30370350	chr15:79963500-79963544	NM_025609:-61	Tab1	PROMOTER	1.132	2.273	1546.40	3514.63	2.573	1215.44	3127.29
A_68_P20552782	chr1:120524517-120524561	NM_023755:17	Tcfcp2l1	INSIDE	1.132	2.165	479.23	1037.66	2.452	440.91	1081.05
A_68_P27355937	chr10:93297003-93297047	NM_183199:2725	Usp44	INSIDE	1.132	1.866	1594.73	2975.74	2.112	1420.31	2999.49
A_68_P26350324	chr9:21875178-21875222	NM_177318:589	Zfp653	INSIDE	1.132	2.191	1484.34	3252.24	2.480	1254.36	3110.43
A_68_P27243854	chr10:72562038-72562082	ENSMUST00000123398:-29		PROMOTER	1.132	1.546	813.55	1257.52	1.750	691.43	1209.73
A_68_P30980681	chr16:94535120-94535164	ENSMUST00000163193:28		INSIDE	1.132	2.204	421.03	927.89	2.495	443.79	1107.48
A_68_P25956922	chr8:73987506-73987550	NM_022419:28	Abhd8	INSIDE	1.131	2.020	1162.52	2348.78	2.286	989.73	2262.10
A_68_P31792809	chr18:67770642-67770686	NM_001081073:30326	Cep76	DOWNSTREAM	1.131	2.718	912.10	2478.85	3.075	868.89	2671.83
A_68_P21144177	chr2:37648771-37648815	NM_001163566:17024	Crb2	INSIDE	1.131	1.884	942.70	1775.59	2.130	797.93	1699.64
A_68_P22478235	chr3:121426699-121426743	NM_010171:266	F3	INSIDE	1.131	3.407	867.23	2954.66	3.854	775.43	2988.73
A_68_P31769419	chr18:63546533-63546577	NM_001039485:283	Fam38b	INSIDE	1.131	1.997	581.71	1161.72	2.258	567.09	1280.64
A_68_P24084077	chr5:129107075-129107119	NM_175284:116	Fzd10	INSIDE	1.131	1.604	2129.45	3415.30	1.815	1811.96	3288.13
A_68_P25101369	chr7:54051464-54051508	NM_008186:23	Gtf2h1	INSIDE	1.131	1.661	936.05	1554.75	1.879	896.20	1683.89
A_68_P20735838	chr1:156946384-156946428	NM_010500:360	Ier5	INSIDE	1.131	2.201	1244.46	2738.95	2.490	1188.58	2959.83
A_68_P24990860	chr7:19590227-19590271	NM_178757:835	Irf2bp1	INSIDE	1.131	1.763	1837.61	3238.94	1.993	1762.95	3514.40
A_68_P25048200	chr7:35989129-35989173	NM_001024707:11214	Lrp3	INSIDE	1.131	2.902	1181.12	3427.53	3.281	1136.90	3730.09
A_68_P31950785	chr19:10531094-10531138	NM_001111140:821	Lrrc10b	INSIDE	1.131	2.153	1053.08	2267.75	2.435	839.23	2043.34
A_68_P31123769	chr17:28828900-28828944	NM_001168513:-590	Mapk14	PROMOTER	1.131	0.473	1171.18	553.93	0.535	945.80	505.85
A_68_P23912413	chr5:97821466-97821510	NM_001033191:-139	Naa11	PROMOTER	1.131	3.127	490.68	1534.16	3.537	462.38	1635.47
A_68_P27938850	chr11:77500718-77500762	NM_001024205:1100	Nufip2	INSIDE	1.131	1.901	799.40	1519.34	2.149	761.15	1635.71
A_68_P20506805	chr1:108068539-108068583	NM_133821:115	Phlpp1	INSIDE	1.131	1.759	960.77	1689.57	1.989	957.95	1905.39
A_68_P29614457	chr14:55433063-55433107	ENSMUST00000095853:-2239		PROMOTER	1.131	3.954	388.67	1536.94	4.472	346.70	1550.30
A_68_P23964211	chr5:107393205-107393249	NM_009863:-114	Cdc7	PROMOTER	1.130	0.493	1524.97	751.29	0.557	1224.76	681.98
A_68_P30978325	chr16:93919893-93919937	NM_001165925:9898	Cldn14	INSIDE	1.130	2.372	949.97	2253.51	2.680	775.30	2077.44
A_68_P23335797	chr4:136327232-136327276	NM_010142:64596	Ephb2	INSIDE	1.130	2.063	874.58	1804.27	2.330	651.95	1519.16
A_68_P20802634	chr1:168931803-168931847	NM_001160261:277	Fam78b	INSIDE	1.130	0.316	1915.80	605.47	0.357	1718.61	613.93

ProbeName	Target position of probe on CpG island microarray	TargetID	GeneSymbol	CpG island Description	Ratio of relative methylation (TiO_2-NP/Vehicle)	Sham group			TiO_2-H group		
						Relative methylation (Cy5/Cy3)	Cy3 signal (Input)	Cy5 signal (MeDIP)	Relative methylation (Cy5/Cy3)	Cy3 signal (Input)	Cy5 signal (MeDIP)
A_68_P28252842	chr12:14156956-14157000	NM_029007:1866	Fam84a	INSIDE	1.130	2.618	555.34	1453.73	2.959	554.02	1639.09
A_68_P24023710	chr5:118426893-118426937	NM_145564:136	Fbxo21	INSIDE	1.130	3.068	500.83	1536.34	3.465	508.89	1763.37
A_68_P20659571	chr1:140740389-140740433	NM_001025565:-1389	Lhx9	PROMOTER	1.130	1.831	1299.75	2379.48	2.069	1055.41	2183.13
A_68_P30397301	chr15:84685820-84685864	NM_001081166:717	Phf21b	INSIDE	1.130	3.178	1138.98	3619.84	3.592	842.94	3028.01
A_68_P25988741	chr8:80959478-80959522	NM_138944:1051	Pou4f2	INSIDE	1.130	3.656	4305.55	15740.83	4.132	3111.38	12857.22
A_68_P28768141	chr12:117724105-117724149	NM_011215:-66	Ptprn2	PROMOTER	1.130	1.583	1074.15	1700.39	1.789	1006.04	1800.06
A_68_P24995080	chr7:20334701-20334745	NM_008990:200	Pvrl2	INSIDE	1.130	1.346	2087.53	2810.35	1.522	1636.76	2490.75
A_68_P30602965	chr16:23431568-23431612	NM_001004151:2385	Rtp1	INSIDE	1.130	2.379	1365.49	3247.93	2.689	1055.37	2837.82
A_68_P32464062	chrX:70918709-70918753	NM_001142809:259	Slc6a8	INSIDE	1.130	2.245	1005.50	2257.49	2.538	600.47	1523.78
A_68_P24946871	chr7:3646125-3646169	NM_024168:37	Tsen34	INSIDE	1.130	1.826	930.72	1699.85	2.064	796.80	1644.50
A_68_P29052703	chr13:55097861-55097905	NM_153131:47090	Unc5a	INSIDE	1.130	1.820	850.05	1547.27	2.057	709.55	1459.72
A_68_P27287691	chr10:80607646-80607690	NM_010731:8653	Zbtb7a	INSIDE	1.130	2.238	891.42	1995.35	2.530	738.06	1866.95
A_68_P23246906	chr4:119406428-119406472	ENSMUST00000123698:5		INSIDE	1.130	1.603	645.40	1034.34	1.811	645.94	1169.68
A_68_P20140407	chr1:34807751-34807795	ENSMUST00000159021:47		INSIDE	1.130	1.687	1158.01	1953.28	1.907	1050.77	2003.67
A_68_P22831248	chr4:34498476-34498520	NM_001007589:635	Akirin2	INSIDE	1.129	1.711	696.00	1190.84	1.932	724.90	1400.82
A_68_P28118513	chr11:109287169-109287213	NM_025275:-69	Amz2	PROMOTER	1.129	3.757	13200.16	49595.05	4.243	9103.96	38625.81
A_68_P30379101	chr15:81524789-81524833	NM_001164320:2907	Chadl	INSIDE	1.129	2.269	506.69	1149.46	2.561	510.76	1308.12
A_68_P23048394	chr4:80557447-80557491	NM_026821:879	D4Bwg0951e	INSIDE	1.129	1.531	1289.37	1974.09	1.729	1187.68	2053.84
A_68_P32571131	chrX:100129101-100129145	NR_026596:440	Gm5126	INSIDE	1.129	1.972	1117.42	2203.20	2.226	614.16	1367.03
A_68_P29616399	chr14:55732726-55732770	NM_177049:3024	Jph4	INSIDE	1.129	2.261	824.86	1865.14	2.552	722.05	1842.75
A_68_P23540978	chr5:23830927-23830971	NM_013569:26474	Kcnh2	INSIDE	1.129	2.988	2107.06	6296.46	3.375	1674.04	5649.80
A_68_P27812422	chr11:54673996-54674040	NM_029327:75	Lyrm7	INSIDE	1.129	1.429	3304.07	4720.10	1.613	2508.60	4045.64
A_68_P23760677	chr5:66651381-66651425	NM_027602:39	Nsun7	INSIDE	1.129	2.403	1435.65	3449.80	2.713	1227.49	3329.77
A_68_P25046335	chr7:35697613-35697657	NM_008820:209	Pepd	INSIDE	1.129	1.376	1225.76	1686.86	1.554	1149.70	1786.12
A_68_P30421717	chr15:88692777-88692821	NM_145478:175	Pim3	INSIDE	1.129	1.394	1000.10	1393.68	1.574	916.11	1441.65
A_68_P31255958	chr17:56556525-56556570	NM_011218:59356	Ptprs	INSIDE	1.129	4.864	4355.83	21185.90	5.492	2882.79	15830.97
A_68_P26750957	chr9:97011068-97011112	NM_138756:370	Slc25a36	INSIDE	1.129	2.684	511.45	1372.60	3.031	488.22	1479.94
A_68_P31320416	chr17:69191045-69191089	NM_001206661:4591	Tmem200c	INSIDE	1.129	2.166	601.76	1303.63	2.446	534.97	1308.47
A_68_P21578172	chr2:120803100-120803144	NM_175285:325	Tmem62	INSIDE	1.129	1.454	1105.95	1608.00	1.642	921.30	1512.60
A_68_P27033726	chr10:28916932-28916976	NM_026138:53153	6330407J23Rik	INSIDE	1.128	1.859	748.58	1391.81	2.098	668.17	1401.64
A_68_P27261497	chr10:75807668-75807712	NM_001081419:317	Dip2a	INSIDE	1.128	3.131	272.03	851.86	3.531	272.92	963.72
A_68_P24114557	chr5:135540470-135540514	NM_025362:417	Dnajc30	INSIDE	1.128	2.133	1776.01	3788.39	2.406	1434.69	3452.13
A_68_P26426831	chr9:36604670-36604714	NM_007915:-39	Ei24	PROMOTER	1.128	1.592	1012.32	1611.88	1.796	922.58	1657.33
A_68_P27325079	chr10:87842398-87842442	NM_001004164:264	Gnptab	INSIDE	1.128	1.342	2931.35	3934.67	1.514	2436.78	3689.79
A_68_P30349001	chr15:76369402-76369446	NM_029643:52	Gpr172b	INSIDE	1.128	1.357	6927.26	9402.20	1.531	4481.62	6861.63
A_68_P29531951	chr14:35632882-35632926	NM_008166:-417	Grid1	PROMOTER	1.128	1.842	492.01	906.46	2.077	427.17	887.38
A_68_P30659189	chr16:33684918-33684962	NM_175256:389	Heg1	INSIDE	1.128	2.272	528.67	1201.19	2.562	558.98	1432.02
A_68_P25952861	chr8:73118130-73118174	NM_023627:-227	Isyna1	PROMOTER	1.128	2.026	3018.72	6117.16	2.286	2862.07	6543.52
A_68_P26164569	chr8:113262564-113262608	NM_198625:17203	Mtss1l	INSIDE	1.128	2.465	381.11	939.34	2.780	287.14	798.36
A_68_P31334901	chr17:71876213-71876257	NM_023294:-37	Ndc80	PROMOTER	1.128	2.978	1733.43	5161.62	3.359	1504.81	5054.96
A_68_P27095769	chr10:42311289-42311333	NM_152229:-7916	Nr2e1	PROMOTER	1.128	2.252	814.51	1833.99	2.540	721.19	1832.09
A_68_P30346946	chr15:76059550-76059594	NM_201385:355	Plec	INSIDE	1.128	1.776	652.58	1158.87	2.004	605.70	1213.76
A_68_P29507012	chr14:31439384-31439428	NM_011103:-12	Prkcd	PROMOTER	1.128	1.764	1693.98	2988.38	1.991	1341.56	2670.65
A_68_P27680407	chr11:30671725-30671769	NM_134013:-28	Psme4	PROMOTER	1.128	3.983	557.55	2220.92	4.491	488.03	2191.96
A_68_P30964118	chr16:91647937-91647981	NM_019973:-110	Son	DIVERGENT_PROMOTER	1.128	1.459	719.73	1049.93	1.646	583.50	960.53
A_68_P24136155	chr5:140020104-140020148	NM_013702:275	Uncx	INSIDE	1.128	2.398	418.23	1002.95	2.705	376.69	1019.00
A_68_P22682765	chr3:157610022-157610066	NM_001013806:-385	Ankrd13c	PROMOTER	1.127	2.003	281.45	563.66	2.258	262.83	593.44
A_68_P27383822	chr10:98377388-98377432	NM_026482:-375	Atp2b1	PROMOTER	1.127	1.958	1082.85	2120.25	2.206	1071.78	2364.26
A_68_P23262157	chr4:123019781-123019825	NM_007558:244	Bmp8a	INSIDE	1.127	1.526	2048.80	3126.73	1.721	1635.69	2814.47
A_68_P25601079	chr7:150645980-150646024	NM_001161624:953	Cdkn1c	INSIDE	1.127	2.138	1118.53	2391.11	2.410	926.98	2233.96
A_68_P25416676	chr7:118172200-118172244	NM_009431:-242	Ctr9	PROMOTER	1.127	2.911	2509.27	7304.92	3.281	1987.88	6521.38

ProbeName	Target position of probe on CpG island microarray	TargetID	GeneSymbol	CpG island Description	Ratio of relative methylation (TiO_2-NP/Vehicle)	Sham group			TiO_2-H group		
						Relative methylation (Cy5/Cy3)	Cy3 signal (Input)	Cy5 signal (MeDIP)	Relative methylation (Cy5/Cy3)	Cy3 signal (Input)	Cy5 signal (MeDIP)
A_68_P26702587	chr9:86916837-86916881	NM_024195:-5	Cyb5r4	PROMOTER	1.127	1.823	754.56	1375.67	2.056	720.12	1480.25
A_68_P32121690	chr19:42593224-42593268	NM_177464:-48	D19Ertd386e	PROMOTER	1.127	1.817	1137.00	2065.65	2.048	1086.35	2224.81
A_68_P25804901	chr8:41393403-41393447	NM_030110:20	Efha2	INSIDE	1.127	2.683	495.32	1329.13	3.025	443.06	1340.16
A_68_P21978661	chr3:20055140-20055184	NM_013755:-167	Gyg	PROMOTER	1.127	1.955	1641.92	3209.14	2.202	1347.68	2967.28
A_68_P24874508	chr6:136756818-136756862	NM_177688:72	H2afj	INSIDE	1.127	1.433	1278.02	1831.51	1.615	1066.15	1721.33
A_68_P32317010	chrX:33652351-33652395	NM_133990:239	Il13ra1	INSIDE	1.127	1.899	4506.61	8558.69	2.140	2304.04	4930.72
A_68_P29629465	chr14:58318680-58318724	NM_015771:46258	Lats2	INSIDE	1.127	2.930	504.95	1479.46	3.301	495.60	1636.15
A_68_P22530171	chr3:130814691-130814735	NM_010703:1324	Lef1	INSIDE	1.127	1.429	2297.82	3284.60	1.610	1885.12	3035.82
A_68_P24947963	chr7:4098615-4098659	NM_172736:9979	Leng8	INSIDE	1.127	1.830	439.99	805.26	2.063	385.96	796.09
A_68_P25962049	chr8:75072127-75072171	NM_027485:61	Med26	INSIDE	1.127	1.502	3907.00	5867.53	1.693	3200.25	5416.89
A_68_P21117012	chr2:32568363-32568407	NR_037217:24	Mir2861	INSIDE	1.127	3.167	835.73	2647.07	3.571	799.84	2856.20
A_68_P30489319	chr15:100883076-100883120	NM_011323:116397	Scn8a	DOWNSTREAM	1.127	2.092	1309.64	2740.06	2.358	1253.14	2955.06
A_68_P23330839	chr4:135411815-135411861	NM_001080387:-168	Srsf10	PROMOTER	1.127	1.682	3204.38	5389.99	1.896	2995.58	5681.04
A_68_P23012916	chr4:71861284-71861328	NM_011599:647	Tle1	INSIDE	1.127	2.671	1196.33	3194.96	3.009	952.46	2866.07
A_68_P25507829	chr7:135020578-135020622	NM_146259:-263	Zfp668	DIVERGENT_PROMOTER	1.127	2.046	823.32	1684.78	2.307	742.75	1713.48
A_68_P28724524	chr12:109155065-109155109	NM_001079883:86538	Bcl11b	INSIDE	1.126	2.863	1903.33	5449.60	3.223	1611.25	5192.94
A_68_P27170202	chr10:59165196-59165240	NM_144822:-164	Cbara1	PROMOTER	1.126	2.006	4977.36	9985.92	2.258	3719.38	8399.22
A_68_P30398340	chr15:84867516-84867560	NM_177067:47	Fam118a	INSIDE	1.126	2.443	1611.58	3937.51	2.752	1407.99	3874.08
A_68_P21903071	chr2:180069548-180069594	NM_008093:-186	Gata5	PROMOTER	1.126	1.623	1135.94	1843.92	1.827	976.85	1785.10
A_68_P26803568	chr9:106367195-106367239	NM_001159652:1055	Gpr62	INSIDE	1.126	1.666	386.43	643.71	1.875	384.51	720.93
A_68_P23609898	chr5:37221813-37221857	NM_008550:54	Man2b2	INSIDE	1.126	2.762	327.00	903.21	3.110	277.74	863.68
A_68_P23165465	chr4:102986727-102986771	NM_025909:298	Oma1	INSIDE	1.126	5.112	1188.71	6077.25	5.755	1120.92	6450.42
A_68_P27898397	chr11:69810791-69810835	NM_030064:1540	Phf23	INSIDE	1.126	2.066	7259.79	15002.29	2.327	5141.65	11964.06
A_68_P26463563	chr9:43552020-43552064	NM_021424:-616	Pvrl1	PROMOTER	1.126	2.202	1633.90	3597.46	2.479	1477.24	3661.51
A_68_P29043022	chr13:53381392-53381436	NM_013846:64	Ror2	INSIDE	1.126	3.425	617.86	2116.16	3.856	536.08	2067.12
A_68_P27138496	chr10:52410751-52410795	NM_178675:466	Slc35f1	INSIDE	1.126	1.990	1572.59	3130.09	2.241	1336.97	2995.73
A_68_P31092361	chr17:23694750-23694794	NM_001033496:6421	Zfp213	INSIDE	1.126	1.829	798.17	1460.02	2.059	675.76	1391.72
A_68_P26721513	chr9:91260697-91260741	NM_009573:-81	Zic1	DIVERGENT_PROMOTER	1.126	1.830	844.72	1545.93	2.060	792.22	1631.95
A_68_P26397765	chr9:30750581-30750625	NM_013906:455	Adamts8	INSIDE	1.125	2.011	278.48	559.91	2.263	326.96	739.84
A_68_P20415724	chr1:88995295-88995339	NM_001081082:2865	Alpi	INSIDE	1.125	2.535	1740.25	4411.02	2.851	1512.82	4312.72
A_68_P31126995	chr17:29405863-29405907	NM_030561:152	BC004004	INSIDE	1.125	4.399	837.45	3684.17	4.947	928.52	4593.58
A_68_P30577277	chr16:18066405-18066449	NM_010047:13474	Dgcr6	INSIDE	1.125	2.111	622.10	1313.52	2.375	589.46	1399.75
A_68_P26467018	chr9:44134953-44134997	NM_007875:47	Dpagt1	INSIDE	1.125	1.704	1665.61	2838.60	1.917	1318.30	2527.30
A_68_P22143243	chr3:53461165-53461209	NM_172862:91	Frem2	INSIDE	1.125	0.561	1229.20	689.04	0.631	1013.81	639.35
A_68_P29919651	chr14:115491767-115491811	NM_175500:352	Gpc5	INSIDE	1.125	1.438	1690.74	2430.92	1.618	1455.25	2354.06
A_68_P29363818	chr13:118391526-118391570	NM_010408:422	Hcn1	INSIDE	1.125	1.481	1062.38	1572.93	1.666	931.13	1551.41
A_68_P28041908	chr11:95866113-95866157	NM_009951:1124	Igf2bp1	INSIDE	1.125	1.694	717.37	1215.34	1.906	660.05	1258.22
A_68_P26348939	chr9:21599872-21599916	NM_145611:3096	Kank2	INSIDE	1.125	3.439	382.15	1314.14	3.868	340.80	1318.37
A_68_P29330802	chr13:112598180-112598224	NM_011945:989	Map3k1	INSIDE	1.125	1.958	1148.76	2249.64	2.204	1021.70	2251.52
A_68_P22171318	chr3:58810124-58810168	NM_177855:-753	Med12l	PROMOTER	1.125	4.283	1021.22	4374.12	4.820	881.59	4249.61
A_68_P29020016	chr13:48641931-48641975	NR_029725:-8311	Mirlet7a-1	PROMOTER	1.125	0.466	1396.43	651.43	0.525	1184.86	621.63
A_68_P31478451	chr18:7626517-7626561	NM_001081287:323	Mpp7	INSIDE	1.125	1.504	2215.94	3333.11	1.693	1868.73	3163.29
A_68_P30506992	chr16:3884518-3884562	NM_029090:-78	Nat15	PROMOTER	1.125	2.018	1018.83	2056.29	2.271	959.87	2180.16
A_68_P21096075	chr2:29107688-29107733	NM_133500:-4150	Ntng2	PROMOTER	1.125	1.354	3110.70	4212.44	1.523	2243.73	3417.78
A_68_P30392999	chr15:83998465-83998509	NM_054088:241	Pnpla3	INSIDE	1.125	1.500	1090.90	1636.51	1.688	843.21	1423.21
A_68_P24010226	chr5:116009731-116009775	NM_007475:277	Rplp0	INSIDE	1.125	1.643	987.19	1622.35	1.849	821.63	1518.89
A_68_P30345849	chr15:75899507-75899551	NM_134089:632	Scrib	INSIDE	1.125	1.881	480.74	904.32	2.117	420.75	890.66
A_68_P30572143	chr16:17131714-17131758	NM_022324:740	Sdf2l1	INSIDE	1.125	2.034	1252.01	2546.19	2.288	964.51	2207.23
A_68_P31253057	chr17:56110074-56110118	NM_001159523:192	Shd	INSIDE	1.125	1.748	842.82	1472.92	1.967	711.24	1398.88
A_68_P22049386	chr3:34548677-34548721	NM_011443:-228	Sox2	PROMOTER	1.125	1.999	2345.89	4690.46	2.249	1956.16	4399.36
A_68_P28137857	chr11:112646792-112646839	NM_011448:3292	Sox9	INSIDE	1.125	1.925	389.88	750.50	2.165	311.62	674.72

ProbeName	Target position of probe on CpG island microarray	TargetID	GeneSymbol	CpG island Description	Ratio of relative methylation (TiO_2-NP/Vehicle)	Sham group			TiO_2-H group		
						Relative methylation (Cy5/Cy3)	Cy3 signal (Input)	Cy5 signal (MeDIP)	Relative methylation (Cy5/Cy3)	Cy3 signal (Input)	Cy5 signal (MeDIP)
A_68_P21328888	chr2:72818835-72818879	NM_001018042:-353	Sp3	PROMOTER	1.125	1.616	663.56	1072.08	1.818	670.89	1219.78
A_68_P27316753	chr10:86371829-86371873	NM_138673:98837	Stab2	INSIDE	1.125	3.402	3564.78	12127.28	3.827	2463.71	9429.19
A_68_P29619289	chr14:56300469-56300513	NM_145705:164	Tinf2	INSIDE	1.125	0.541	966.05	522.83	0.609	872.28	531.09
A_68_P23253640	chr4:120527812-120527856	ENSMUST00000106283:-6547		PROMOTER	1.125	0.498	1439.94	716.85	0.560	1221.75	684.51
A_68_P30753194	chr16:50732861-50732905	NR_028300:4	5330426P16Rik	INSIDE	1.124	1.619	1755.53	2842.94	1.821	1473.33	2682.76
A_68_P21477087	chr2:102290962-102291006	NM_010218:965	Fjx1	INSIDE	1.124	1.437	1031.17	1481.36	1.615	916.58	1480.38
A_68_P23404325	chr4:149475753-149475801	NM_177366:14165	Gpr157	INSIDE	1.124	2.965	880.82	2611.72	3.334	642.22	2140.87
A_68_P31936713	chr19:7000522-7000566	NM_008431:8461	Kcnk4	INSIDE	1.124	1.523	707.45	1077.34	1.712	615.21	1053.30
A_68_P22629212	chr3:148482607-148482651	NM_001081298:134971	Lphn2	INSIDE	1.124	2.333	916.79	2139.02	2.622	775.73	2034.33
A_68_P29360869	chr13:117813423-117813467	NM_001081009:879	Parp8	INSIDE	1.124	2.116	964.43	2041.21	2.380	921.32	2192.53
A_68_P21250383	chr2:58999148-58999192	NM_026361:264	Pkp4	INSIDE	1.124	2.186	862.41	1885.42	2.458	739.05	1816.46
A_68_P23236665	chr4:117508655-117508699	NM_008135:814	Slc6a9	INSIDE	1.124	1.965	2599.01	5107.11	2.208	1728.01	3815.76
A_68_P20002770	chr1:3661760-3661804	NM_001011874:-203	Xkr4	PROMOTER	1.124	2.102	577.43	1214.00	2.363	544.73	1286.96
A_68_P20286079	chr1:63319663-63319707	NM_028673:-158	Zdbf2	PROMOTER	1.124	0.562	1637.97	921.24	0.632	1465.82	926.32
A_68_P24155695	chr5:144016767-144016811	NM_017467:14913	Zfp316	INSIDE	1.124	2.209	2534.23	5598.63	2.483	2186.21	5428.60
A_68_P21844246	chr2:168760814-168760858	NM_009564:20251	Zfp64	INSIDE	1.124	3.814	2479.37	9455.26	4.287	2093.05	8972.35
A_68_P22360098	chr3:98232509-98232553	NM_172863:46127	Zfp697	INSIDE	1.124	2.797	707.86	1980.00	3.144	553.85	1741.06
A_68_P27835192	chr11:58977171-58977215	NM_173785:52	A230051G13Rik	INSIDE	1.123	1.786	433.89	775.06	2.005	412.37	826.87
A_68_P31016357	chr17:5342164-5342208	NM_001085355:347113	Arid1b	INSIDE	1.123	2.644	663.18	1753.23	2.969	479.02	1422.15
A_68_P23244382	chr8:118905317-118905361	NM_001012400:-9	AU022252	DIVERGENT_PROMOTER	1.123	1.442	1404.68	2026.13	1.620	1063.93	1723.14
A_68_P25952217	chr8:73018186-73018230	NM_018827:1154	Crlf1	INSIDE	1.123	1.798	5505.74	9900.80	2.020	4030.02	8140.75
A_68_P25416832	chr7:118198930-118198974	NM_009431:26488	Ctr9	INSIDE	1.123	2.798	260.89	730.04	3.142	239.10	751.26
A_68_P31329272	chr17:70865492-70865536	NM_001128180:-5935	Dlgap1	PROMOTER	1.123	2.935	1908.73	5602.06	3.295	1435.61	4730.44
A_68_P30087404	chr15:26473244-26473288	NM_176959:352053	Fbxl7	INSIDE	1.123	1.753	538.92	944.86	1.969	483.75	952.54
A_68_P30367928	chr15:79521367-79521411	NM_013818:63	Gtpbp1	INSIDE	1.123	3.069	635.17	1949.07	3.446	634.33	2185.78
A_68_P31075109	chr17:17975189-17975234	NM_001162909:7273	Ncrna00085	INSIDE	1.123	3.469	605.57	2100.56	3.894	558.81	2176.19
A_68_P30558912	chr16:13780955-13780999	NM_001039521:185	Rrn3	INSIDE	1.123	2.433	258.03	627.69	2.732	280.10	765.35
A_68_P31857887	chr18:79306987-79307031	NM_053099:-878	Setbp1	PROMOTER	1.123	0.497	1281.86	636.88	0.558	1098.54	612.95
A_68_P21911426	chr2:181316540-181316584	NM_026765:116	Uckl1	INSIDE	1.123	2.656	1267.55	3367.00	2.983	1110.50	3312.11
A_68_P32129546	chr19:44014604-44014648	ENSMUST00000122692:7821		DOWNSTREAM	1.123	1.731	2898.59	5017.33	1.943	2287.84	4445.72
A_68_P21040892	chr2:18611762-18611806	NM_001001334:-8864	BC061194	PROMOTER	1.122	0.519	1628.27	844.76	0.582	1449.80	843.79
A_68_P27278227	chr10:79144709-79144753	NM_177613:-209	Cdc34	PROMOTER	1.122	0.465	1950.56	906.64	0.522	1612.26	840.94
A_68_P31125955	chr17:29235539-29235583	NM_007669:4844	Cdkn1a	INSIDE	1.122	2.972	502.58	1493.77	3.336	409.77	1366.84
A_68_P28075715	chr11:101645928-101645972	NM_008815:674	Etv4	INSIDE	1.122	2.141	970.35	2077.23	2.402	990.51	2379.56
A_68_P31876064	chr18:82575063-82575107	NM_008082:1085	Galr1	INSIDE	1.122	1.995	1520.40	3032.62	2.238	1261.61	2824.04
A_68_P30347942	chr15:76211135-76211179	NM_001162489:-9304	Heatr7a	PROMOTER	1.122	1.474	1749.03	2578.93	1.655	1362.20	2254.57
A_68_P28044023	chr11:96214672-96214716	NM_134032:1749	Hoxb2	INSIDE	1.122	1.727	938.09	1620.24	1.938	789.67	1529.99
A_68_P26674972	chr9:81525625-81525671	NM_010482:511	Htr1b	INSIDE	1.122	1.738	911.08	1583.83	1.951	819.14	1597.96
A_68_P24479788	chr6:57652816-57652860	NM_133737:390	Lancl2	INSIDE	1.122	1.545	2724.07	4208.06	1.734	2402.54	4165.97
A_68_P24652024	chr6:93628341-93628385	NM_001083320:235322	Magi1	INSIDE	1.122	3.051	846.76	2583.12	3.423	681.07	2331.16
A_68_P31779043	chr18:65243488-65243532	NM_031881:60330	Nedd4l	INSIDE	1.122	2.066	1240.90	2563.21	2.317	1049.16	2430.79
A_68_P27709960	chr11:35821756-35821800	NM_011856:935967	Odz2	INSIDE	1.122	2.553	741.30	1892.76	2.865	568.48	1628.59
A_68_P28168391	chr11:117848501-117848545	NM_133757:352	Pgs1	INSIDE	1.122	1.519	1115.17	1694.49	1.706	956.84	1632.00
A_68_P29471618	chr14:25306180-25306224	NM_001081247:66	Polr3a	INSIDE	1.122	1.601	518.23	829.50	1.795	439.62	789.19
A_68_P31407761	chr17:85357878-85357922	NM_001159496:560	Ppm1b	INSIDE	1.122	4.425	755.98	3345.11	4.965	671.37	3333.25
A_68_P21095251	chr2:28980877-28980921	NM_198033:387	Setx	INSIDE	1.122	1.525	642.35	979.81	1.711	537.64	919.91
A_68_P29664628	chr14:64563075-64563120	NM_011446:555	Sox7	INSIDE	1.122	2.820	273.05	769.89	3.164	232.25	734.88
A_68_P30580644	chr16:18592043-18592087	NM_011532:-5002	Tbx1	PROMOTER	1.122	0.571	1145.30	653.68	0.640	948.06	606.86
A_68_P25102684	chr7:54305881-54305925	NM_026436:-107	Tmem86a	PROMOTER	1.122	2.063	564.73	1164.84	2.315	582.82	1349.42
A_68_P24425525	chr6:47997586-47997630	NM_001081382:505	Zfp777	INSIDE	1.122	1.448	766.77	1110.37	1.625	685.61	1114.02
A_68_P25600984	chr7:150631945-150631989			Unknown	1.122	1.398	1065.69	1490.18	1.569	1054.23	1654.22

ProbeName	Target position of probe on CpG island microarray	TargetID	GeneSymbol	CpG island Description	Ratio of relative methylation (TiO_2-NP/Vehicle)	Sham group			TiO_2-H group		
						Relative methylation (Cy5/Cy3)	Cy3 signal (Input)	Cy5 signal (MeDIP)	Relative methylation (Cy5/Cy3)	Cy3 signal (Input)	Cy5 signal (MeDIP)
A_68_P24093843	chr5:130925785-130925831	NM_021371:1147	Caln1	INSIDE	1.121	3.360	181.75	610.69	3.767	153.56	578.45
A_68_P28331591	chr12:32183927-32183971	NM_134048:457	Cbll1	INSIDE	1.121	1.762	3052.27	5376.65	1.974	2421.00	4780.18
A_68_P23201930	chr4:109330255-109330299	NM_007671:7701	Cdkn2c	DOWNSTREAM	1.121	1.866	1386.97	2588.58	2.091	1206.84	2523.92
A_68_P24994187	chr7:20172257-20172301	NM_016680:17539	Clasrp	INSIDE	1.121	2.314	336.27	778.07	2.594	293.16	760.49
A_68_P26221731	chr8:123191547-123191591	NM_010926:444	Cox4nb	INSIDE	1.121	1.526	601.10	917.29	1.711	524.80	897.81
A_68_P23190560	chr4:107107097-107107141	NM_147221:-205	Glis1	PROMOTER	1.121	2.399	433.66	1040.29	2.688	422.84	1136.72
A_68_P30580075	chr16:18498740-18498784	NM_001081682:-98	Gnb1l	PROMOTER	1.121	4.066	4129.99	16792.92	4.560	3299.08	15042.29
A_68_P27278461	chr10:79180701-79180746	NM_008226:1345	Hcn2	INSIDE	1.121	3.153	2683.44	8461.71	3.534	1931.89	6826.56
A_68_P32094346	chr19:37282376-37282420	NM_001164337:364	Marchf5	INSIDE	1.121	2.081	1008.82	2099.36	2.333	912.38	2128.66
A_68_P30422455	chr15:88813114-88813158	NM_031260:-287	Mov10l1	DIVERGENT_PROMOTER	1.121	2.128	2273.16	4837.85	2.385	1706.98	4070.87
A_68_P20961511	chr2:3201548-3201592	NM_008708:11	Nmt2	INSIDE	1.121	2.092	330.52	691.59	2.346	364.06	854.21
A_68_P20797853	chr1:167932451-167932495	NM_011137:293	Pou2f1	INSIDE	1.121	1.428	1479.75	2112.56	1.601	1246.58	1995.60
A_68_P31929630	chr19:5602393-5602437	NM_026616:542	Rnaseh2c	INSIDE	1.121	1.413	4440.56	6275.22	1.585	3018.56	4783.04
A_68_P23896968	chr5:93371161-93371205	NM_001077596:44159	Shroom3	INSIDE	1.121	2.532	920.68	2330.91	2.838	816.76	2317.61
A_68_P20870658	chr1:181448224-181448272	NM_027188:-114	Smyd3	PROMOTER	1.121	4.340	590.33	2562.23	4.865	547.57	2663.98
A_68_P32246204	chrX:8849773-8849817	NM_023500:-115	Xk	PROMOTER	1.121	3.704	336.44	1246.18	4.152	218.63	907.79
A_68_P21565889	chr2:118571987-118572031	NR_030716:511	5430417L22Rik	INSIDE	1.120	1.486	610.76	907.70	1.664	471.16	784.02
A_68_P26744297	chr9:95758279-95758323	NM_019864:285	Atr	INSIDE	1.120	1.587	748.90	1188.77	1.778	597.84	1063.13
A_68_P28752184	chr12:113977018-113977062	NM_001024602:16656	AW555464	INSIDE	1.120	2.674	1336.86	3574.81	2.994	1027.27	3075.56
A_68_P31382676	chr17:80772525-80772569	NR_028385:336	Gm10190	INSIDE	1.120	2.192	553.15	1212.38	2.454	513.84	1260.95
A_68_P22954219	chr4:59048073-59048117	NM_025277:67	Gng10	INSIDE	1.120	4.538	1394.87	6330.21	5.085	1370.41	6968.52
A_68_P25958331	chr8:74213094-74213138	NM_013564:-34	Insl3	PROMOTER	1.120	1.626	823.07	1338.24	1.821	701.25	1276.68
A_68_P22481651	chr3:121996059-121996103	NR_030439:541	Mir760	DOWNSTREAM	1.120	2.126	448.39	953.45	2.381	459.44	1093.81
A_68_P21742435	chr2:150864820-150864864	NM_026086:273	Nanp	INSIDE	1.120	1.852	881.80	1633.27	2.074	781.06	1620.22
A_68_P21907359	chr2:180689094-180689138	NM_001141933:288	Nkain4	INSIDE	1.120	2.360	1131.09	2669.47	2.644	917.62	2426.02
A_68_P27889317	chr11:68245685-68245729	NM_177320:80	Pik3r5	INSIDE	1.120	1.729	506.43	875.69	1.936	471.89	913.70
A_68_P24059411	chr5:124571440-124571484	NM_011256:94965	Pitpnm2	INSIDE	1.120	2.346	567.72	1331.95	2.627	546.52	1435.45
A_68_P23365303	chr4:141219467-141219511	NM_001033150:542	Plekhm2	INSIDE	1.120	1.622	1466.94	2378.87	1.816	1239.60	2251.09
A_68_P32599601	chrX:108577069-108577113	NM_025949:74464	Rps6ka6	INSIDE	1.120	1.643	1972.86	3241.21	1.841	1166.85	2147.90
A_68_P25026631	chr7:30117136-30117180	NM_001081028:173321	Sipa1l3	INSIDE	1.120	3.059	2587.23	7913.82	3.427	1948.58	6678.30
A_68_P27214753	chr10:67374987-67375031	NM_178679:402	Zfp365	INSIDE	1.120	1.797	601.61	1081.00	2.012	528.76	1063.92
A_68_P26202777	chr8:119960582-119960626	NM_028941:87535	4933407C03Rik	INSIDE	1.119	2.332	584.79	1363.68	2.610	480.30	1253.47
A_68_P20977505	chr2:6051162-6051206	NM_001159657:47	5430407P10Rik	INSIDE	1.119	2.389	8410.86	20096.31	2.674	7513.35	20090.52
A_68_P20770260	chr1:163072965-163073010	NM_028664:145	Ankrd45	INSIDE	1.119	0.529	1247.57	660.19	0.592	974.04	576.73
A_68_P23915348	chr5:98364470-98364514	NM_133738:95489	Antxr2	INSIDE	1.119	2.120	1242.38	2633.50	2.372	1133.00	2687.86
A_68_P30462677	chr15:96115709-96115753	NM_175251:-2222	Arid2	PROMOTER	1.119	1.739	718.22	1249.29	1.946	719.96	1400.90
A_68_P20958788	chr1:196803083-196803130	NR_033564:47	C030002c11rik	INSIDE	1.119	2.151	717.23	1542.80	2.408	601.27	1447.62
A_68_P24420943	chr6:46994568-46994616	NM_001004357:1984533	Cntnap2	INSIDE	1.119	3.359	2278.37	7652.07	3.758	1893.91	7116.73
A_68_P26802913	chr9:106271467-106271511	NM_153459:526	Dusp7	INSIDE	1.119	0.573	1527.48	874.68	0.641	1155.71	740.71
A_68_P26200115	chr8:119516976-119517022	NM_026572:351	Gcsh	INSIDE	1.119	1.450	1471.21	2133.09	1.622	1306.36	2119.18
A_68_P26567943	chr9:61794500-61794544	NM_024245:84	Kif23	INSIDE	1.119	1.472	1282.91	1888.45	1.647	1028.16	1693.63
A_68_P29725081	chr14:75347579-75347623	NM_001033439:84	Lrch1	INSIDE	1.119	1.622	3333.98	5409.35	1.815	2695.17	4892.12
A_68_P21309974	chr2:69423570-69423614	NM_001081088:532	Lrp2	INSIDE	1.119	1.903	1382.58	2631.31	2.131	1206.67	2570.83
A_68_P21742300	chr2:150835045-150835089	NM_207204:68	Ninl	INSIDE	1.119	2.545	799.09	2033.39	2.847	712.29	2027.63
A_68_P23295438	chr4:128866763-128866807	NM_029036:-58	S100pbp	DIVERGENT_PROMOTER	1.119	1.405	2416.59	3394.19	1.571	2105.69	3308.04
A_68_P25586715	chr7:148286395-148286439	NM_023059:29	Sigirr	INSIDE	1.119	1.568	807.70	1266.29	1.754	725.84	1273.46
A_68_P25102014	chr7:54175443-54175487	NM_021884:-164	Tsg101	PROMOTER	1.119	1.970	1145.27	2256.14	2.205	988.56	2179.73
A_68_P30149950	chr15:38448838-38448882	NM_001102458:161	Azin1	INSIDE	1.118	0.528	991.93	523.53	0.590	861.13	508.13
A_68_P28752688	chr12:114047423-114047467	NM_145450:259	BC022687	INSIDE	1.118	1.656	1170.68	1939.07	1.851	933.36	1728.11
A_68_P21485631	chr2:103637120-103637164	NM_001111289:93	Caprin1	INSIDE	1.118	0.506	2067.90	1047.34	0.566	1607.56	910.60
A_68_P27934410	chr11:76660169-76660213	NM_007754:320	Cpd	INSIDE	1.118	2.471	825.48	2039.58	2.762	706.44	1951.44

ProbeName	Target position of probe on CpG island microarray	TargetID	GeneSymbol	CpG island Description	Ratio of relative methylation (TiO_2-NP/Vehicle)	Sham group			TiO_2-H group		
						Relative methylation (Cy5/Cy3)	Cy3 signal (Input)	Cy5 signal (MeDIP)	Relative methylation (Cy5/Cy3)	Cy3 signal (Input)	Cy5 signal (MeDIP)
A_68_P28051739	chr11:97490327-97490371	NM_175332:682	E130012A19Rik	INSIDE	1.118	2.757	390.68	1077.25	3.082	352.10	1084.99
A_68_P27278197	chr10:79138491-79138535	NM_148934:6358	Gm16517	INSIDE	1.118	3.378	1917.71	6478.74	3.776	1572.14	5935.78
A_68_P26682077	chr9:83039520-83039564	NM_026122:672	Hmgn3	INSIDE	1.118	1.602	1610.26	2579.30	1.790	1387.80	2484.26
A_68_P27865437	chr11:64248821-64248865	NM_178870:9	Hs3st3a1	INSIDE	1.118	1.960	1164.46	2282.50	2.191	988.93	2166.46
A_68_P22698900	chr4:4720461-4720505	NM_177730:-29	Impad1	PROMOTER	1.118	2.283	305.10	696.60	2.553	307.91	786.01
A_68_P21834186	chr2:167015822-167015866	NM_008420:-1545	Kcnb1	PROMOTER	1.118	1.765	846.31	1493.40	1.973	779.28	1537.66
A_68_P25034375	chr7:31758234-31758278	NM_001164184:232	Lsr	INSIDE	1.118	3.748	1408.83	5279.87	4.188	1133.35	4746.87
A_68_P29154200	chr13:76521644-76521688	NM_030174:-742	Mctp1	PROMOTER	1.118	1.674	586.81	982.13	1.871	551.60	1032.07
A_68_P29269010	chr13:101322324-101322368	NM_008756:107	Ocln	INSIDE	1.118	2.393	1313.83	3144.51	2.675	1164.18	3114.39
A_68_P28974190	chr13:40827038-40827082	NM_001122948:-1248	Tcfap2a	PROMOTER	1.118	1.546	2189.51	3385.25	1.729	1854.80	3206.15
A_68_P33007019	A_68_P33007019			Unknown	1.118	1.718	7636.02	13119.22	1.921	6500.10	12484.72
A_68_P32977626	A_68_P32977626			Unknown	1.118	2.148	250.87	538.96	2.402	196.25	471.41
A_68_P29056007	chr13:55701465-55701509	NM_146045:15	B4galt7	INSIDE	1.117	1.664	2212.00	3681.16	1.859	1699.60	3159.23
A_68_P30836894	chr16:67620955-67620999	NM_001145977:177	Cadm2	INSIDE	1.117	2.724	3542.30	9649.26	3.044	2838.72	8641.29
A_68_P20432996	chr1:91827206-91827250	NM_010262:523	Gbx2	INSIDE	1.117	1.937	4102.88	7948.25	2.163	3588.59	7763.11
A_68_P25842950	chr8:48760715-48760759	NM_023503:-223	Ing2	PROMOTER	1.117	1.899	1125.08	2136.16	2.121	1007.55	2136.62
A_68_P26228656	chr8:124277409-124277453	NM_020605:22972	Jph3	INSIDE	1.117	3.656	1070.93	3915.12	4.084	930.01	3797.91
A_68_P27285769	chr10:80298722-80298766	NM_001013758:8040	Lingo3	INSIDE	1.117	2.229	892.09	1988.62	2.489	823.80	2050.59
A_68_P24065425	chr5:125658527-125658571	NM_011424:1036	Ncor2	INSIDE	1.117	1.615	1164.70	1880.86	1.805	993.57	1793.04
A_68_P27289568	chr10:80882945-80882989	NM_008688:6952	Nfic	INSIDE	1.117	2.828	1756.96	4969.52	3.159	1329.80	4200.48
A_68_P32089884	chr19:36484605-36484649	NM_029508:31070	Pcgf5	INSIDE	1.117	3.238	263.75	853.95	3.617	289.07	1045.50
A_68_P29341546	chr13:114408820-114408864	NM_130796:-70	Snx18	PROMOTER	1.117	2.215	373.58	827.36	2.475	355.67	880.15
A_68_P28051298	chr11:97413135-97413179	NM_018873:23284	Srcin1	INSIDE	1.117	2.247	1005.72	2260.14	2.510	860.66	2160.05
A_68_P24593027	chr6:83318037-83318081	NM_183138:73614	Tet3	INSIDE	1.117	1.521	737.64	1121.77	1.699	697.04	1183.97
A_68_P29998443	chr15:8119317-8119361	NM_001162906:233	2410089E03Rik	INSIDE	1.116	1.501	1002.48	1505.21	1.675	847.70	1419.87
A_68_P26601253	chr9:67679488-67679532	NM_001163143:627	C2cd4a	INSIDE	1.116	0.579	2142.64	1240.46	0.646	1646.71	1064.16
A_68_P31103373	chr17:25522563-25522607	NM_001163691:48144	Cacna1h	INSIDE	1.116	2.200	1594.60	3508.64	2.456	1271.47	3122.18
A_68_P27101045	chr10:43298595-43298639	NM_009846:-358	Cd24a	PROMOTER	1.116	1.659	1624.72	2695.90	1.851	1304.70	2414.99
A_68_P25582339	chr7:147302298-147302342	NM_053119:2	Echs1	INSIDE	1.116	1.380	2731.68	3768.61	1.539	2205.72	3394.84
A_68_P28198383	chr12:4038752-4038796	NM_001082483:141	Efr3b	INSIDE	1.116	1.659	1178.53	1955.39	1.852	906.67	1679.13
A_68_P28577470	chr12:81858836-81858880	NM_001008423:2844	Gm1568	INSIDE	1.116	3.116	425.19	1325.03	3.477	397.47	1382.16
A_68_P24326993	chr6:29457921-29457965	NM_001029985:10	Kcp	INSIDE	1.116	1.460	1608.78	2348.02	1.630	1361.76	2219.01
A_68_P30349814	chr15:76497752-76497796	NM_010630:6704	Kifc2	INSIDE	1.116	2.587	1147.70	2968.96	2.888	958.87	2768.77
A_68_P22218636	chr3:68810008-68810052	NR_029529:-3663	Mir15b	PROMOTER	1.116	2.535	1158.84	2937.45	2.829	999.36	2827.23
A_68_P30132612	chr15:35225823-35225867	NM_054049:-22	Osr2	PROMOTER	1.116	2.510	1294.26	3248.09	2.800	1302.66	3647.52
A_68_P30346580	chr15:76007443-76007487	NM_201394:18676	Plec	INSIDE	1.116	2.179	505.18	1100.96	2.433	424.99	1033.88
A_68_P26022782	chr8:87469669-87469713	NM_144929:-1199	Rtbdn	DIVERGENT_PROMOTER	1.116	2.277	731.92	1666.68	2.541	676.60	1719.38
A_68_P31677778	chr18:46756824-46756868	NM_025698:343	Tmed7	INSIDE	1.116	2.791	628.93	1755.62	3.116	540.29	1683.46
A_68_P28166387	chr11:117516412-117516456	NM_198022:832	Tnrc6c	INSIDE	1.116	5.767	469.60	2708.04	6.435	501.11	3224.86
A_68_P25008268	chr7:25866633-25866677	NM_175477:4390	Zfp574	INSIDE	1.116	1.888	1529.88	2887.88	2.107	1202.26	2532.57
A_68_P20049945	chr1:15277016-15277060			Unknown	1.116	2.104	4269.61	8982.19	2.349	3589.69	8431.53
A_68_P28751869	chr12:113931759-113931803	AK028601:1		INSIDE	1.116	1.522	1468.97	2235.45	1.699	1275.23	2166.20
A_68_P23437422	chr4:154624331-154624375	NM_001190445:278	2610002J02Rik	INSIDE	1.115	1.638	5100.60	8357.15	1.828	3421.28	6253.01
A_68_P26548139	chr9:58335761-58335806	NM_176921:-626	6030419C18Rik	PROMOTER	1.115	1.504	2498.16	3758.16	1.677	2009.98	3371.06
A_68_P32151689	chr19:47806170-47806214	NM_026377:-53	6330577E15Rik	PROMOTER	1.115	1.534	915.79	1404.87	1.711	802.12	1372.21
A_68_P20825059	chr1:172797927-172797976	NM_001081304:-49	Atf6	PROMOTER	1.115	1.508	2595.38	3913.20	1.682	1975.76	3322.78
A_68_P29977215	chr15:3977091-3977135	NR_028266:294	BC037032	INSIDE	1.115	1.651	487.07	804.29	1.841	442.14	813.92
A_68_P28056294	chr11:98274418-98274462	NM_001003817:643	Erbb2	INSIDE	1.115	1.754	1283.51	2251.25	1.955	1241.19	2426.34
A_68_P26132791	chr8:107817394-107817438	NM_177788:2582	Exoc3l	INSIDE	1.115	2.419	1005.77	2432.82	2.696	896.78	2417.66
A_68_P23917032	chr5:98683315-98683359	NM_010203:134	Fgf5	INSIDE	1.115	1.879	1700.68	3195.80	2.095	1375.73	2881.99
A_68_P27946742	chr11:78858560-78858604	NM_013571:101274	Ksr1	INSIDE	1.115	3.805	2731.57	10394.15	4.241	1933.37	8200.24

ProbeName	Target position of probe on CpG island microarray	TargetID	GeneSymbol	CpG island Description	Ratio of relative methylation (TiO_2-NP/Vehicle)	Sham group			TiO_2-H group		
						Relative methylation (Cy5/Cy3)	Cy3 signal (Input)	Cy5 signal (MeDIP)	Relative methylation (Cy5/Cy3)	Cy3 signal (Input)	Cy5 signal (MeDIP)
A_68_P26643864	chr9:75257719-75257763	NM_027418:-574	Mapk6	PROMOTER	1.115	1.868	2591.67	4841.21	2.083	2165.48	4510.34
A_68_P22558740	chr3:136333384-136333428	NM_008913:-327	Ppp3ca	PROMOTER	1.115	1.937	1599.62	3097.88	2.159	1304.06	2815.72
A_68_P24063269	A_68_P24063269			Unknown	1.115	4.671	149.10	696.49	5.208	139.94	728.81
A_68_P27929276	chr11:75780169-75780213			Unknown	1.115	1.595	1215.60	1938.81	1.779	1111.80	1977.86
A_68_P23265703	chr4:123667997-123668041			Unknown	1.115	3.126	453.85	1418.94	3.486	376.38	1312.22
A_68_P26940976	chr10:11211956-11212000			Unknown	1.115	3.364	7792.34	26213.94	3.750	5605.59	21021.23
A_68_P31422207	chr17:87682043-87682087	NR_015506:90	4833418N02Rik	INSIDE	1.114	1.689	2165.24	3657.71	1.883	2014.50	3792.68
A_68_P24045429	chr5:122043432-122043476	NM_009656:379	Aldh2	INSIDE	1.114	1.963	787.37	1545.57	2.187	717.29	1568.84
A_68_P27854482	chr11:62352254-62352298	NM_028448:487	Cenpv	INSIDE	1.114	1.346	5253.97	7074.21	1.499	4201.58	6299.37
A_68_P22318564	chr3:89125165-89125211	NM_010108:1613	Efna3	INSIDE	1.114	2.233	276.32	616.94	2.487	261.03	649.22
A_68_P23854795	chr5:84846156-84846200	NM_007937:229	Epha5	INSIDE	1.114	2.305	1034.51	2384.41	2.568	884.68	2271.42
A_68_P25369178	chr7:107855525-107855569	NM_178764:331	Fam168a	INSIDE	1.114	3.651	10253.38	37438.04	4.068	7168.98	29160.87
A_68_P21477091	chr2:102291430-102291474	NM_010218:497	Fjx1	INSIDE	1.114	4.246	486.82	2067.13	4.729	495.88	2345.01
A_68_P24613523	chr6:86992531-86992575	NM_013528:-287	Gfpt1	PROMOTER	1.114	1.609	2066.64	3326.16	1.793	1834.27	3288.07
A_68_P25730815	chr8:27229276-27229320	NM_207659:398	Hook3	INSIDE	1.114	2.140	1155.47	2472.37	2.384	1065.43	2540.00
A_68_P28918887	chr13:30842448-30842492	NM_013674:1344	Irf4	INSIDE	1.114	1.813	1241.18	2250.41	2.021	1048.66	2118.97
A_68_P23753093	chr5:65213285-65213329	NM_008453:18545	Klf3	INSIDE	1.114	2.832	878.61	2488.43	3.154	748.01	2359.37
A_68_P29629463	chr14:58318497-58318541	NM_015771:46442	Lats2	INSIDE	1.114	4.979	903.70	4499.29	5.545	858.88	4762.66
A_68_P29233343	chr13:94943998-94944042	NM_172589:116270	Lhfpl2	INSIDE	1.114	0.567	1271.89	721.79	0.632	938.13	592.89
A_68_P31827984	chr18:73974953-73974997	NM_145494:72	Me2	INSIDE	1.114	3.584	349.24	1251.76	3.994	323.03	1290.08
A_68_P22615133	chr3:146184298-146184342	NM_027371:67	Rpf1	INSIDE	1.114	1.483	725.59	1075.86	1.652	667.49	1102.41
A_68_P20616239	chr1:133423080-133423124	NM_001081011:836	Srgap2	INSIDE	1.114	2.210	999.85	2209.17	2.461	888.08	2185.28
A_68_P27641464	chr11:22412537-22412581	NM_153596:273	Tmem17	INSIDE	1.114	2.681	566.97	1520.28	2.988	581.37	1737.13
A_68_P27287710	chr10:80610694-80610738	NM_010731:11701	Zbtb7a	INSIDE	1.114	4.580	942.47	4316.84	5.104	733.20	3742.34
A_68_P30423043	chr15:88919652-88919696	NM_027905:138	1300018J18Rik	INSIDE	1.113	3.607	327.03	1179.61	4.016	302.25	1213.94
A_68_P31939676	chr19:7491566-7491610	NM_175381:-526	2700081O15Rik	PROMOTER	1.113	1.862	501.91	934.69	2.073	520.79	1079.53
A_68_P33007147	chr9_random:49997-50041	NR_015516:-3390	4930526I15Rik	PROMOTER	1.113	2.617	1448.49	3790.79	2.912	1076.05	3133.92
A_68_P24946543	chr7:3597351-3597395	NM_146176:502	Cnot3	INSIDE	1.113	2.337	873.23	2040.34	2.601	708.26	1842.52
A_68_P33015614	chr1_random:49829-49873	NM_026866:135488	Disp1	INSIDE	1.113	2.077	392.09	814.30	2.311	386.05	892.35
A_68_P22777889	chr4:22284762-22284806	NM_172988:73	Fbxl4	INSIDE	1.113	3.532	1100.87	3887.93	3.930	966.40	3798.19
A_68_P26886027	chr9:121766646-121766690	NM_001112668:-19	Gm9790	PROMOTER	1.113	1.494	1377.26	2058.08	1.663	1255.35	2087.66
A_68_P22592457	chr3:142428332-142428376	NM_145546:144	Gtf2b	INSIDE	1.113	1.688	970.57	1638.19	1.878	920.68	1728.99
A_68_P25958255	chr8:74203600-74203644	NM_001190830:3341	Jak3	INSIDE	1.113	2.852	1118.31	3189.12	3.175	975.45	3096.71
A_68_P25022783	chr7:29383081-29383125	NM_027470:101	Pak4	INSIDE	1.113	1.474	2496.37	3680.31	1.641	1917.92	3146.56
A_68_P23891543	chr5:92392231-92392285	NM_026557:-164	Rchy1	PROMOTER	1.113	1.683	979.89	1648.88	1.873	723.63	1355.19
A_68_P31119183	chr17:28044657-28044701	NM_026836:-9	Taf11	DIVERGENT_PROMOTER	1.113	1.776	1270.30	2256.34	1.978	1129.63	2233.95
A_68_P29033261	chr13:51690524-51690568			Unknown	1.113	3.699	6892.75	25497.87	4.117	5071.89	20882.56
A_68_P29339701	chr13:114083707-114083751			Unknown	1.113	1.442	1363.39	1965.56	1.605	1129.48	1813.02
A_68_P29583268	chr14:48269057-48269101	BC075697:10		INSIDE	1.113	1.509	989.59	1493.78	1.681	941.39	1582.04
A_68_P27503018	chr10:120788390-120788434	ENSMUST00000078515:8085		DOWNSTREAM	1.113	2.683	371.36	996.39	2.986	351.03	1048.27
A_68_P23915350	chr5:98364657-98364701	NM_133738:95303	Antxr2	INSIDE	1.112	2.120	953.19	2021.14	2.359	921.02	2172.29
A_68_P24593642	chr6:83405946-83405990	NR_028382:14220	B230319C09Rik	DOWNSTREAM	1.112	1.877	751.11	1409.48	2.087	670.10	1398.28
A_68_P30404229	chr15:85809217-85809261	NM_009886:54969	Celsr1	INSIDE	1.112	3.007	1997.02	6005.57	3.346	1606.39	5374.18
A_68_P32982273	chr5:134998822-134998866	NM_001039162:29460	Clip2	INSIDE	1.112	1.982	2247.68	4454.57	2.203	1690.13	3724.02
A_68_P28056291	chr11:98274041-98274085	NM_001003817:265	Erbb2	INSIDE	1.112	2.516	612.86	1541.83	2.796	528.87	1478.90
A_68_P30468548	chr15:97154022-97154066	NM_172293:76507	Fam113b	INSIDE	1.112	1.571	831.11	1305.82	1.746	653.46	1141.25
A_68_P20091644	chr1:24107112-24107156	NM_026604:46	Fam135a	INSIDE	1.112	2.543	815.70	2074.07	2.827	776.47	2195.41
A_68_P23222369	chr4:114598689-114598733	NM_015758:-92	Foxe3	PROMOTER	1.112	2.392	2267.12	5423.63	2.659	1717.67	4568.11
A_68_P29130723	chr13:72100965-72101009	NM_010573:185	Irx1	INSIDE	1.112	0.481	2187.76	1052.03	0.535	1882.94	1006.85
A_68_P31343017	chr17:73268907-73268952	NM_029999:1285	Lbh	INSIDE	1.112	1.699	431.13	732.52	1.890	421.40	796.33
A_68_P25535616	chr7:139802586-139802630	NM_029609:283	Lhpp	INSIDE	1.112	2.313	1321.17	3055.92	2.572	1145.32	2945.89

ProbeName	Target position of probe on CpG island microarray	TargetID	GeneSymbol	CpG island Description	Ratio of relative methylation (TiO_2-NP/Vehicle)	Sham group			TiO_2-H group		
						Relative methylation (Cy5/Cy3)	Cy3 signal (Input)	Cy5 signal (MeDIP)	Relative methylation (Cy5/Cy3)	Cy3 signal (Input)	Cy5 signal (MeDIP)
A_68_P31256024	chr17:56564825-56564869	NM_011218:51057	Ptprs	INSIDE	1.112	1.606	936.97	1504.88	1.786	871.52	1556.46
A_68_P20785985	chr1:165859996-165860040	NM_028776:-176	Scyl3	PROMOTER	1.112	1.426	1040.08	1483.08	1.585	915.40	1450.89
A_68_P20826332	chr1:173080599-173080643	NM_025321:114	Sdhc	INSIDE	1.112	1.778	1525.95	2713.81	1.978	1417.45	2803.12
A_68_P31320372	chr17:69186496-69186540	NM_001206661:43	Tmem200c	INSIDE	1.112	2.543	813.95	2069.61	2.828	756.39	2139.13
A_68_P24838525	chr6:128306097-128306141	NM_011657:-249	Tulp3	PROMOTER	1.112	2.846	2515.29	7157.50	3.163	2156.74	6821.81
A_68_P24091287	chr5:130417556-130417600	NM_001001327:-404	Vkorc1l1	PROMOTER	1.112	2.185	13082.56	28590.68	2.430	8531.87	20730.25
A_68_P25264703	chr7:87226955-87226999	NM_175433:55863	Zfp710	INSIDE	1.112	2.269	1689.27	3833.05	2.523	1262.18	3184.37
A_68_P30639624	A_68_P30639624			Unknown	1.112	2.441	701.47	1712.38	2.715	571.63	1551.86
A_68_P27678668	chr11:30167911-30167955	ENSMUST00000006629:237		INSIDE	1.112	1.894	1065.55	2018.57	2.107	1017.12	2143.22
A_68_P21224227	chr2:53051236-53051280	NM_022989:141	Arl6ip6	INSIDE	1.111	1.879	1413.11	2655.81	2.088	1235.80	2580.52
A_68_P21040931	chr2:18616371-18616415	NM_001001334:-4256	BC061194	PROMOTER	1.111	0.540	1195.96	645.92	0.600	956.04	573.79
A_68_P24820861	chr6:125444215-125444259	NM_007657:537	Cd9	INSIDE	1.111	1.639	1070.63	1754.88	1.821	845.55	1539.34
A_68_P27263124	chr10:76077361-76077405	NM_146007:8767	Col6a2	INSIDE	1.111	2.028	1160.83	2353.72	2.253	925.57	2084.92
A_68_P22310619	chr3:87710732-87710776	NM_008231:512	Hdgf	INSIDE	1.111	1.483	1273.84	1889.26	1.648	1130.10	1862.26
A_68_P20945385	chr1:194596745-194596789	NM_144881:647	Hhat	INSIDE	1.111	1.714	2974.03	5097.13	1.905	2328.39	4434.95
A_68_P21833676	chr2:166930579-166930623	NM_008420:83699	Kcnb1	INSIDE	1.111	2.670	1288.49	3440.02	2.966	937.32	2780.49
A_68_P25088268	chr7:51844686-51844730	NM_008422:-1547	Kcnc3	PROMOTER	1.111	1.651	3342.49	5518.85	1.835	2543.23	4666.63
A_68_P29011273	chr13:47139014-47139058	NM_172262:129	Kdm1b	INSIDE	1.111	1.637	715.65	1171.28	1.818	697.83	1268.99
A_68_P22171326	chr3:58811151-58811195	NM_177855:273	Med12l	INSIDE	1.111	1.508	1081.75	1630.89	1.675	971.78	1627.37
A_68_P24027248	chr5:119010105-119010149	NM_172424:-601	Med13l	PROMOTER	1.111	4.378	748.09	3275.00	4.862	1173.50	5705.17
A_68_P20759145	chr1:161162430-161162474	NM_011931:-4	Rfwd2	PROMOTER	1.111	2.955	1143.70	3379.93	3.284	963.50	3164.05
A_68_P29047303	chr13:54167121-54167165	NM_027324:-71	Sfxn1	PROMOTER	1.111	3.541	1370.09	4851.75	3.934	1193.53	4694.97
A_68_P25034246	chr7:31740698-31740742	NM_011680:1102	Usf2	INSIDE	1.111	2.193	1123.73	2463.85	2.436	962.08	2343.40
A_68_P31257914	chr17:56856378-56856423	NM_178926:-56	Vmac	PROMOTER	1.111	4.449	2235.16	9945.32	4.945	1614.99	7986.65
A_68_P25089145	chr7:52004212-52004256	NM_133945:236	Vrk3	INSIDE	1.111	1.937	493.34	955.39	2.152	458.75	987.13
A_68_P23338967	chr4:136832827-136832871	NM_009523:-701	Wnt4	PROMOTER	1.111	1.700	2364.74	4021.18	1.890	2049.24	3872.91
A_68_P24319362	chr6:28210996-28211040	NM_001081678:583	Zfp800	INSIDE	1.111	1.471	1695.37	2494.57	1.634	1408.26	2301.25
A_68_P27258519	chr10:75226844-75226888	NM_172549:236	Cabin1	INSIDE	1.110	1.792	1206.26	2162.03	1.990	1061.42	2111.82
A_68_P31378406	chr17:80014550-80014594	NM_201361:333	Fam82a1	INSIDE	1.110	2.626	879.04	2308.04	2.914	800.64	2333.02
A_68_P25419719	chr7:118742491-118742535	NM_173739:180979	Galntl4	INSIDE	1.110	2.155	766.62	1652.06	2.392	643.39	1539.13
A_68_P22531296	chr3:130974638-130974682	NM_008212:295	Hadh	INSIDE	1.110	2.299	1623.78	3733.83	2.553	1346.68	3438.03
A_68_P29101874	chr13:65214224-65214268	NM_001083901:44	Hiatl1	INSIDE	1.110	1.621	2617.70	4243.51	1.799	2256.35	4058.67
A_68_P27704165	chr11:34935121-34935165	NM_011412:185	Slit3	INSIDE	1.110	0.571	1362.09	777.29	0.633	1173.57	743.22
A_68_P20510432	chr1:108693123-108693167	NM_009190:158	Vps4b	INSIDE	1.110	1.691	5543.94	9374.80	1.877	4039.01	7580.02
A_68_P27532857	chr10:126342966-126343010	NM_026858:-4561	Xrcc6bp1	PROMOTER	1.110	1.431	857.69	1227.16	1.588	825.60	1311.03
A_68_P28751778	chr12:113917322-113917366	NM_001100460:294	Zbtb42	INSIDE	1.110	1.609	472.21	759.56	1.785	461.88	824.44
A_68_P26152541	chr8:111238563-111238607	NM_007496:41	Zfhx3	INSIDE	1.110	2.268	1060.03	2404.62	2.518	883.51	2225.09
A_68_P21068971	chr2:24805706-24805750	NM_026021:407	Zmynd19	INSIDE	1.110	1.706	1159.62	1978.09	1.893	1062.88	2011.82
A_68_P20250390	chr1:57028403-57028447	NR_024325:112	9130024F11Rik	INSIDE	1.109	1.990	1624.83	3233.13	2.207	1255.14	2769.81
A_68_P21908931	chr2:180891580-180891624	NM_007906:118	Eef1a2	INSIDE	1.109	2.085	1456.02	3035.20	2.312	1094.41	2529.79
A_68_P27835271	chr11:58990590-58990634	NM_175452:5716	Gjc2	INSIDE	1.109	1.458	5359.93	7816.85	1.617	4170.42	6745.25
A_68_P20644876	chr1:138156784-138156828	NM_001101516:644	Gpr25	INSIDE	1.109	1.402	1287.57	1804.58	1.555	1133.29	1761.95
A_68_P28150134	chr11:114713892-114713936	NM_147217:571	Gprc5c	INSIDE	1.109	2.561	1014.17	2597.21	2.840	972.71	2762.72
A_68_P24449037	chr6:52153900-52153944	NM_010453:664	Hoxa5	INSIDE	1.109	1.494	943.76	1409.66	1.657	860.36	1425.53
A_68_P27804081	chr11:53113814-53113858	NM_008300:145	Hspa4	INSIDE	1.109	1.404	1064.35	1494.83	1.558	973.51	1516.26
A_68_P25259447	chr7:86293791-86293835	NM_001045489:134	Mfge8	INSIDE	1.109	3.213	1304.45	4190.87	3.563	1168.73	4163.92
A_68_P28161653	chr11:116715108-116715152	NM_178620:-198	Mfsd11	DIVERGENT_PROMOTER	1.109	1.625	670.74	1089.79	1.802	629.73	1134.94
A_68_P21070358	chr2:25036517-25036561	NM_025980:261	Nrarp	INSIDE	1.109	2.701	453.29	1224.50	2.995	517.98	1551.53
A_68_P31029165	chr17:8993570-8993614	NM_011866:-1017	Pde10a	PROMOTER	1.109	2.698	1862.75	5025.80	2.992	1458.19	4363.34
A_68_P24956666	chr7:6682979-6683023	NM_008817:130	Peg3	INSIDE	1.109	3.275	1574.87	5158.22	3.633	1437.93	5223.32
A_68_P25614471	chr8:3537772-3537816	NM_001122818:22411	Pnpla6	INSIDE	1.109	2.222	1529.41	3397.94	2.464	1163.71	2867.45

ProbeName	Target position of probe on CpG island microarray	TargetID	GeneSymbol	CpG island Description	Ratio of relative methylation (TiO_2-NP/Vehicle)	Sham group			TiO_2-H group		
						Relative methylation (Cy5/Cy3)	Cy3 signal (Input)	Cy5 signal (MeDIP)	Relative methylation (Cy5/Cy3)	Cy3 signal (Input)	Cy5 signal (MeDIP)
A_68_P30426168	chr15:89422228-89422272	NM_026817:104	Rabl2	INSIDE	1.109	2.122	2315.54	4913.31	2.353	1862.89	4382.98
A_68_P21395216	chr2:84726819-84726863	NM_199223:9	Rtn4rl2	INSIDE	1.109	2.606	752.21	1959.94	2.889	728.75	2105.30
A_68_P31254017	chr17:56263391-56263435	NM_013662:9863	Sema6b	INSIDE	1.109	2.153	518.00	1115.45	2.388	524.22	1252.00
A_68_P22081036	chr3:40513212-40513256	NM_178386:442	Slc25a31	INSIDE	1.109	1.885	1023.93	1929.70	2.090	829.68	1734.17
A_68_P26133220	chr8:107872806-107872850	NM_001081332:671	Slc9a5	INSIDE	1.109	1.627	1288.32	2096.66	1.805	1116.65	2015.82
A_68_P27704164	chr11:34934976-34935020	NM_011412:41	Slit3	INSIDE	1.109	1.592	762.74	1214.01	1.766	751.57	1327.02
A_68_P28862224	chr13:19487507-19487551	NM_024270:93	Stard3nl	INSIDE	1.109	1.643	627.75	1031.42	1.822	530.04	965.89
A_68_P32008254	chr19:21867188-21867232	NM_001033759:14379	Tmem2	INSIDE	1.109	2.746	1146.65	3148.31	3.046	941.66	2868.34
A_68_P25772480	chr8:35217817-35217861	NM_026432:222	Tmem66	INSIDE	1.109	2.320	1364.74	3166.46	2.572	1172.19	3014.98
A_68_P21568845	chr2:119119812-119119856	NM_172269:5357	Vps18	INSIDE	1.109	2.941	487.14	1432.60	3.260	466.95	1522.27
A_68_P26494151	chr9:48644017-48644061	NM_001033324:12	Zbtb16	INSIDE	1.109	1.612	2057.02	3315.34	1.788	1692.83	3026.31
A_68_P33005900	A_68_P33005900			Unknown	1.109	1.915	696.22	1333.10	2.123	570.40	1211.18
A_68_P23692813	chr5:52504803-52504847	AK076709:-3727		PROMOTER	1.109	1.371	6727.97	9221.92	1.520	5127.52	7791.56
A_68_P31323975	chr17:69788589-69788633	NM_001145192:-55	A330050F15Rik	PROMOTER	1.108	1.828	865.81	1582.90	2.025	805.30	1630.86
A_68_P28694107	chr12:104116017-104116061	NM_172806:578	Btbd7	INSIDE	1.108	1.799	394.99	710.61	1.994	387.44	772.43
A_68_P24123515	chr5:137358798-137358842	NM_024474:157	Emid2	INSIDE	1.108	2.190	1671.07	3659.78	2.426	1545.38	3749.28
A_68_P30345427	chr15:75834015-75834059	NM_001168253:5892	Fam83h	INSIDE	1.108	1.663	484.95	806.25	1.843	533.86	983.69
A_68_P25375734	chr7:108986819-108986865	NM_010567:-102	Inppl1	PROMOTER	1.108	1.635	1188.56	1943.67	1.813	982.06	1780.17
A_68_P31937503	chr19:7131378-7131422	NM_134147:143	Macrod1	INSIDE	1.108	1.892	580.98	1099.28	2.096	535.47	1122.49
A_68_P21842270	chr2:168396257-168396301	NM_001136073:19413	Nfatc2	INSIDE	1.108	2.997	1744.82	5229.33	3.321	1309.91	4350.47
A_68_P32140286	chr19:45824101-45824145	NM_008723:-69	Npm3	PROMOTER	1.108	2.303	1914.06	4407.19	2.551	1729.97	4412.58
A_68_P31140482	chr17:31701718-31701762	NM_016670:23	Pknox1	INSIDE	1.108	2.986	1230.45	3674.08	3.308	1067.44	3530.61
A_68_P22149940	chr3:54611394-54611438	NM_133231:297	Rfxap	INSIDE	1.108	4.069	1021.37	4156.08	4.511	888.33	4006.88
A_68_P31940082	chr19:7557921-7557965	NM_001003933:-163	Rtn3	PROMOTER	1.108	1.473	2339.36	3446.39	1.632	1985.18	3240.41
A_68_P21115505	chr2:32306645-32306689	NM_001164357:324	Slc25a25	INSIDE	1.108	2.834	648.75	1838.38	3.140	581.61	1826.00
A_68_P28170836	chr11:118216495-118216539	NM_011594:209	Timp2	INSIDE	1.108	2.021	1945.18	3931.72	2.240	1600.28	3584.67
A_68_P31156941	chr17:35137890-35137934	NM_011690:61	Vars	INSIDE	1.108	2.185	750.17	1639.09	2.422	665.46	1611.67
A_68_P32246512	chrX:8905191-8905235	ENSMUST00000119858:22		INSIDE	1.108	2.126	874.93	1860.11	2.357	580.97	1369.12
A_68_P21755514	chr2:153356022-153356066	NM_001134300:-337	8430427H17Rik	PROMOTER	1.107	1.564	959.22	1500.40	1.732	866.99	1501.73
A_68_P28853784	chr13:17896191-17896235	NM_001081058:719	Cdk13	INSIDE	1.107	2.998	479.27	1436.69	3.319	441.55	1465.56
A_68_P26132792	chr8:107817484-107817528	NM_177788:2492	Exoc3l	INSIDE	1.107	1.911	1111.55	2124.45	2.116	938.78	1986.60
A_68_P30720007	chr16:44173365-44173409	NM_001029889:-123	Gm608	PROMOTER	1.107	1.694	742.62	1257.86	1.874	696.52	1305.50
A_68_P25025266	chr7:29871013-29871057	NR_035489:-2321	Mir1963	PROMOTER	1.107	2.833	743.46	2106.55	3.137	586.02	1838.43
A_68_P32565872	chrX:99045232-99045276	NM_001163610:531	Nhsl2	INSIDE	1.107	2.059	1487.49	3062.05	2.278	906.03	2064.14
A_68_P29050855	chr13:54795009-54795053	NM_001146027:-5469	Rnf44	PROMOTER	1.107	1.698	982.67	1668.84	1.879	968.95	1821.05
A_68_P32319888	chrX:34283671-34283715	NM_001085497:39	Slc25a43	INSIDE	1.107	2.735	2843.65	7776.65	3.027	1960.98	5935.99
A_68_P29421304	chr14:15535576-15535620	NM_001033270:60	Slc4a7	INSIDE	1.107	1.777	711.88	1265.01	1.966	590.96	1162.03
A_68_P27850829	chr11:61668825-61668869	NM_013881:-252	Ulk2	PROMOTER	1.107	0.562	1006.84	565.56	0.622	860.57	535.23
A_68_P30364132	chr15:78893377-78893421	NM_144849:-109	Ankrd54	PROMOTER	1.106	0.513	1153.75	591.46	0.567	1071.11	607.43
A_68_P24925473	chr6:145812067-145812111	NM_024469:1772	Bhlhe41	INSIDE	1.106	1.479	662.10	979.16	1.636	647.99	1060.21
A_68_P29487613	chr14:28321574-28321618	NM_177111:50	Ccdc66	INSIDE	1.106	1.514	2246.13	3401.48	1.674	2001.96	3351.59
A_68_P26240288	chr8:126039003-126039047	NM_001170976:331	Dbndd1	INSIDE	1.106	2.034	1752.77	3565.88	2.249	1507.31	3390.23
A_68_P27285230	chr10:80218002-80218046	NM_199322:74	Dot1l	INSIDE	1.106	3.121	897.86	2801.90	3.453	920.91	3179.56
A_68_P25505763	chr7:134622752-134622796	NM_010183:-5960	Fbrs	PROMOTER	1.106	1.964	398.67	782.89	2.172	328.57	713.71
A_68_P21768525	chr2:155768031-155768075	NM_008109:3048	Gdf5	INSIDE	1.106	1.522	934.61	1422.75	1.684	834.90	1405.67
A_68_P26805690	chr9:106788802-106788846	NM_175402:507	Rbm15b	INSIDE	1.106	0.424	1238.52	525.38	0.469	916.20	429.83
A_68_P27282554	chr10:79798280-79798324	NM_001204931:5413	Reep6	INSIDE	1.106	1.879	771.59	1449.88	2.079	716.98	1490.65
A_68_P27841610	chr11:60023907-60023951	NM_011480:10178	Srebf1	INSIDE	1.106	0.621	1827.24	1134.82	0.687	1503.79	1032.59
A_68_P24769156	chr6:115084797-115084841	NM_013681:-101	Syn2	PROMOTER	1.106	2.022	651.30	1317.15	2.238	613.98	1373.89
A_68_P25009105	chr7:26010680-26010724	NM_175436:4233	Zfp526	INSIDE	1.106	2.461	2457.13	6046.14	2.722	1969.01	5358.73
A_68_P26465230	chr9:43829019-43829063			Unknown	1.106	5.934	5061.55	30033.83	6.563	3710.44	24350.97

ProbeName	Target position of probe on CpG island microarray	TargetID	GeneSymbol	CpG island Description	Ratio of relative methylation (TiO_2-NP/Vehicle)	Sham group			TiO_2-H group		
						Relative methylation (Cy5/Cy3)	Cy3 signal (Input)	Cy5 signal (MeDIP)	Relative methylation (Cy5/Cy3)	Cy3 signal (Input)	Cy5 signal (MeDIP)
A_68_P25008855	chr7:25964863-25964907	X53654:547486		INSIDE	1.106	2.800	1755.03	4913.95	3.097	1511.21	4680.03
A_68_P21096084	chr2:29108807-29108851	NR_029460:-315	6530402F18Rik	PROMOTER	1.105	1.540	995.75	1533.33	1.702	875.16	1489.28
A_68_P24864545	chr6:134933738-134933782	NM_001109914:1742	Apold1	INSIDE	1.105	2.109	872.81	1840.91	2.331	797.24	1858.36
A_68_P22063090	chr3:37247843-37247887	NM_008006:290	Fgf2	INSIDE	1.105	0.582	1124.57	654.65	0.643	966.51	621.79
A_68_P20842598	chr1:176433545-176433589	NM_019445:1611	Fmn2	INSIDE	1.105	1.845	466.98	861.72	2.038	446.33	909.67
A_68_P23787823	chr5:72092225-72092269	NM_008069:992	Gabrb1	INSIDE	1.105	1.907	1544.31	2944.82	2.108	1314.96	2771.47
A_68_P21874504	chr2:174124338-174124382	NM_201617:1001	Gnas	INSIDE	1.105	1.951	1454.41	2837.99	2.157	1281.48	2763.87
A_68_P26443402	chr9:40263578-40263622	NM_172768:-251	Gramd1b	PROMOTER	1.105	1.678	961.62	1613.64	1.854	810.99	1503.22
A_68_P29228407	chr13:94073846-94073890	NM_011982:-581	Homer1	PROMOTER	1.105	0.486	1975.44	959.12	0.537	1577.57	846.52
A_68_P29284632	chr13:104123896-104123940	NM_175171:654	Mast4	INSIDE	1.105	0.548	1148.42	629.89	0.606	952.70	577.30
A_68_P27782070	chr11:49057978-49058022	NM_001110149:-2978	Mgat1	PROMOTER	1.105	1.640	1394.95	2287.59	1.812	1127.48	2043.39
A_68_P24538896	chr6:71858733-71858777	NM_027275:2	Ptcd3	INSIDE	1.105	1.680	550.76	925.12	1.855	423.21	785.21
A_68_P30012299	chr15:10644337-10644381	NM_030690:-1063	Rai14	PROMOTER	1.105	3.691	417.90	1542.63	4.078	397.96	1623.02
A_68_P23359680	chr4:140258068-140258112	NM_173867:703	Rcc2	INSIDE	1.105	1.865	1277.22	2382.41	2.061	1180.17	2432.10
A_68_P31251409	chr17:55622134-55622178	NM_172829:37142	St6gal2	INSIDE	1.105	1.806	706.85	1276.39	1.995	636.46	1269.98
A_68_P26217895	chr8:122625358-122625402	NM_133967:-8	Zdhhc7	PROMOTER	1.105	5.049	1780.50	8989.42	5.579	1358.84	7581.56
A_68_P21076584	chr2:25994375-25994419	ENSMUST00000163836:-74		PROMOTER	1.105	3.597	3267.88	11755.05	3.974	2185.98	8686.31
A_68_P26826442	chr9:110782965-110783009	NM_001146060:161	Als2cl	INSIDE	1.104	1.915	766.60	1468.20	2.113	666.98	1409.64
A_68_P24444443	chr6:51421130-51421174	NM_007624:538	Cbx3	INSIDE	1.104	3.429	744.19	2551.92	3.784	652.98	2471.20
A_68_P27294770	chr10:82448262-82448306	NM_021439:43	Chst11	INSIDE	1.104	1.350	1816.22	2452.11	1.491	1474.67	2198.48
A_68_P26864772	chr9:118059149-118059193	NM_026442:144	Cmc1	INSIDE	1.104	1.605	826.23	1326.09	1.772	748.09	1325.65
A_68_P31378939	chr17:80115332-80115376	NM_009994:-973	Cyp1b1	PROMOTER	1.104	2.163	414.69	897.11	2.388	331.30	791.21
A_68_P32054106	chr19:29722522-29722566	NM_001081213:366	Ermp1	INSIDE	1.104	1.684	908.11	1528.86	1.858	782.43	1453.68
A_68_P29671483	chr14:65695129-65695173	NM_018788:21793	Extl3	INSIDE	1.104	1.641	779.48	1279.35	1.813	745.01	1350.49
A_68_P30925475	chr16:84774232-84774276	NM_023844:-113	Jam2	PROMOTER	1.104	2.066	9365.24	19347.40	2.280	6895.73	15721.68
A_68_P31863476	chr18:80492765-80492809	NM_001190373:68207	Kcng2	INSIDE	1.104	1.948	911.09	1775.10	2.150	774.58	1665.38
A_68_P20799112	chr1:168168830-168168874	NM_175296:23	Mael	INSIDE	1.104	2.957	858.62	2538.82	3.265	785.52	2564.93
A_68_P23278937	chr4:125911565-125911609	NM_001145970:21977	Mtap7d1	INSIDE	1.104	2.305	1298.98	2994.22	2.544	973.72	2476.84
A_68_P31110189	chr17:26568886-26568930	NM_001081656:16999	Neurl1b	INSIDE	1.104	2.048	2086.40	4272.27	2.261	1670.55	3777.10
A_68_P21070359	chr2:25036616-25036660	NM_025980:361	Nrarp	INSIDE	1.104	2.327	345.04	802.90	2.570	281.12	722.45
A_68_P32114045	chr19:41338252-41338296	NM_133352:220	Tm9sf3	INSIDE	1.104	2.466	1610.59	3971.51	2.723	1259.07	3428.79
A_68_P30572940	chr16:17276845-17276889	NM_177473:474	Tmem191c	INSIDE	1.104	1.855	1979.74	3671.82	2.048	1850.86	3791.39
A_68_P24786876	chr6:118405064-118405108	NM_028335:438	Zfp248	INSIDE	1.104	1.709	3107.39	5310.41	1.886	2581.80	4868.97
A_68_P25008234	chr7:25862568-25862612	NM_175477:326	Zfp574	INSIDE	1.104	1.873	12504.21	23422.91	2.068	8698.07	17990.66
A_68_P32462526	chrX:70667546-70667590	NM_009566:11127	Zfp92	INSIDE	1.104	1.759	1280.97	2252.95	1.941	667.88	1296.26
A_68_P24058182	chr5:124357285-124357329	NM_026603:23	Denr	INSIDE	1.103	2.189	1582.48	3463.38	2.415	1266.48	3058.66
A_68_P20850632	chr1:177810893-177810937	NM_012012:6	Exo1	INSIDE	1.103	1.842	1017.41	1874.43	2.033	796.67	1619.51
A_68_P28187038	chr11:120686207-120686251	NM_007988:-367	Fasn	PROMOTER	1.103	0.548	1132.34	620.47	0.605	938.16	567.18
A_68_P24172328	chr5:148001784-148001828	NM_008178:1535	Gsx1	INSIDE	1.103	1.456	2049.90	2985.10	1.607	1790.45	2876.73
A_68_P23340287	chr4:137074439-137074483	NM_008305:49743	Hspg2	INSIDE	1.103	4.064	2019.25	8206.19	4.481	1749.57	7840.43
A_68_P21840417	chr2:168094618-168094662	NM_001081134:191	Kcng1	INSIDE	1.103	2.324	1081.48	2513.03	2.562	913.04	2339.52
A_68_P25349938	chr7:104480832-104480876	NM_001039039:22	Kctd21	INSIDE	1.103	3.201	1944.26	6224.40	3.531	1557.09	5498.11
A_68_P23756015	chr5:65770784-65770828	NM_031180:31157	Klb	INSIDE	1.103	1.735	519.81	901.72	1.913	413.66	791.47
A_68_P27284038	chr10:80031829-80031873	NM_078477:8191	Klf16	INSIDE	1.103	3.309	1243.59	4115.42	3.649	1061.80	3874.02
A_68_P31669144	chr18:44971731-44971775	NM_001085373:84	Mcc	INSIDE	1.103	2.227	1592.36	3546.67	2.456	1375.47	3378.05
A_68_P22422565	chr3:109945185-109945229	NM_001163348:1184	Ntng1	INSIDE	1.103	3.408	1935.24	6595.25	3.759	1599.62	6012.72
A_68_P31626662	chr18:37166261-37166305	NM_009960:-4229	Pcdha11	PROMOTER	1.103	1.866	459.18	856.91	2.058	466.83	960.53
A_68_P21587820	chr2:122564356-122564400	NM_019788:138	Pldn	INSIDE	1.103	2.086	722.62	1507.71	2.302	687.50	1582.35
A_68_P20770872	chr1:163180941-163180985	NM_007453:335	Prdx6	INSIDE	1.103	2.159	804.81	1737.89	2.381	686.81	1635.46
A_68_P22933503	chr4:55362594-55362638	NM_009011:-297	Rad23b	PROMOTER	1.103	2.309	1582.80	3655.15	2.547	1407.10	3583.73
A_68_P26533944	chr9:55889943-55889987	NM_011992:313	Rcn2	INSIDE	1.103	1.907	494.90	943.62	2.104	460.62	969.11

ProbeName	Target position of probe on CpG island microarray	TargetID	GeneSymbol	CpG island Description	Ratio of relative methylation (TiO_2-NP/Vehicle)	Sham group			TiO_2-H group		
						Relative methylation (Cy5/Cy3)	Cy3 signal (Input)	Cy5 signal (MeDIP)	Relative methylation (Cy5/Cy3)	Cy3 signal (Input)	Cy5 signal (MeDIP)
A_68_P32957490	chr17:8138877-8138921	NM_025789:420	Rsph3a	INSIDE	1.103	2.242	852.02	1910.14	2.472	726.98	1797.16
A_68_P28535257	chr12:74145983-74146027	NM_009189:1695	Six1	INSIDE	1.103	2.991	754.03	2255.40	3.300	687.27	2267.71
A_68_P23322289	chr4:133899035-133899079	NM_001039677:72	Slc30a2	INSIDE	1.103	1.516	2312.37	3505.26	1.672	1969.05	3292.32
A_68_P28554953	chr12:77707492-77707536	NM_013675:104020	Spnb1	INSIDE	1.103	2.183	249.54	544.80	2.408	247.06	594.86
A_68_P28054165	chr11:97913498-97913542	NM_146028:1256	Stac2	INSIDE	1.103	2.089	1059.53	2213.23	2.304	949.59	2188.28
A_68_P32264675	chrX:12648851-12648895	NM_009481:249	Usp9x	INSIDE	1.103	1.647	658.30	1083.97	1.817	383.54	696.87
A_68_P30402004	chr15:85476715-85476759	AK037366:-9359		PROMOTER	1.103	1.928	1119.56	2158.66	2.126	969.47	2061.56
A_68_P22502227	chr3:126066719-126066763	NM_173451:-29	Arsj	PROMOTER	1.102	1.933	303.83	587.19	2.130	249.28	531.08
A_68_P29335931	chr13:113442236-113442280	NM_001145885:260	Ddx4	INSIDE	1.102	3.403	910.20	3097.74	3.751	786.67	2950.92
A_68_P23336931	chr4:136501821-136501865	NM_007939:10889	Epha8	INSIDE	1.102	2.063	1072.71	2213.36	2.274	887.48	2018.32
A_68_P29034745	chr13:51941936-51941980	NM_011817:-85	Gadd45g	PROMOTER	1.102	1.681	652.55	1096.73	1.852	576.13	1066.84
A_68_P28431901	chr12:52930414-52930458	NM_144788:87	Hectd1	INSIDE	1.102	1.880	886.21	1665.80	2.072	709.03	1468.91
A_68_P31927056	chr19:5118110-5118154	NM_008451:276	Klc2	INSIDE	1.102	0.614	2991.61	1836.84	0.677	2461.05	1665.73
A_68_P23547785	chr5:25003901-25003945	NM_001081383:679	Mll3	INSIDE	1.102	1.838	799.20	1468.63	2.025	717.99	1454.21
A_68_P31442548	chr17:91487890-91487934	NM_020252:4230	Nrxn1	INSIDE	1.102	1.986	552.40	1096.84	2.188	520.29	1138.44
A_68_P22031104	chr3:30868422-30868466	NM_001165954:-107	Phc3	PROMOTER	1.102	1.461	1841.08	2689.15	1.610	1606.19	2586.35
A_68_P24772420	chr6:115626105-115626149	NM_029780:527	Raf1	INSIDE	1.102	7.291	363.41	2649.50	8.034	348.64	2801.01
A_68_P23364274	chr4:141028926-141028970	NM_019763:65564	Spen	INSIDE	1.102	2.785	869.68	2421.76	3.069	680.25	2087.65
A_68_P26770338	chr9:100471400-100471444	NM_001101483:82	Tmem22	INSIDE	1.102	1.946	712.95	1387.47	2.144	671.46	1439.48
A_68_P25957477	chr8:74082522-74082566	NM_001100462:226	Tmem221	INSIDE	1.102	1.820	461.17	839.50	2.006	469.93	942.64
A_68_P25670555	chr8:13785828-13785872	NM_025924:236	Upf3a	INSIDE	1.102	2.531	970.82	2457.33	2.789	760.60	2121.07
A_68_P28611612	chr12:88030123-88030167	NM_177354:10495	Vash1	INSIDE	1.102	1.790	383.02	685.50	1.972	296.87	585.30
A_68_P21780089	chr2:157770092-157770136	NM_198627:29726	Vstm2l	INSIDE	1.102	1.909	897.85	1713.98	2.103	776.45	1632.71
A_68_P32959961	A_68_P32959961			Unknown	1.102	3.353	574.19	1925.52	3.696	463.32	1712.28
A_68_P24980670	chr7:16871649-16871693	NM_172297:-1662	Ccdc9	PROMOTER	1.101	6.756	15064.41	101777.60	7.440	9753.59	72563.67
A_68_P21425707	chr2:91805748-91805792	NM_138306:-2050	Dgkz	PROMOTER	1.101	1.803	482.99	870.86	1.986	437.06	867.93
A_68_P28158451	chr11:116144755-116144799	NM_010254:2524	Galr2	INSIDE	1.101	1.881	693.52	1304.57	2.072	601.76	1246.70
A_68_P30650845	chr16:32079162-32079206	NM_177326:175	Pak2	INSIDE	1.101	1.803	410.07	739.45	1.985	315.90	627.13
A_68_P29360872	chr13:117813736-117813780	NM_001081009:565	Parp8	INSIDE	1.101	1.705	973.67	1660.05	1.877	875.74	1643.90
A_68_P23152968	chr4:100741662-100741706	NM_183024:42	Raver2	INSIDE	1.101	2.029	550.37	1116.75	2.235	498.47	1114.08
A_68_P30960424	chr16:91011840-91011884	NM_001164483:-309	Synj1	PROMOTER	1.101	1.603	906.67	1453.61	1.766	729.98	1288.90
A_68_P27850820	chr11:61667944-61667988	NM_013881:628	Ulk2	INSIDE	1.101	2.688	914.17	2457.72	2.961	889.66	2634.44
A_68_P31323666	chr17:69732894-69732938	NM_009547:-401	Zfp161	PROMOTER	1.101	2.115	650.27	1375.15	2.328	598.19	1392.54
A_68_P30501994	chr15:102916894-102916938	AF216290:299350		INSIDE	1.101	1.419	1289.12	1829.43	1.563	1012.20	1581.61
A_68_P26080208	chr8:97437258-97437302	NM_001033533:4718	Ccdc102a	INSIDE	1.100	1.828	1651.99	3020.05	2.011	1302.23	2618.49
A_68_P23609088	chr5:37086702-37086746	NM_001081232:484	D5Ertd579e	INSIDE	1.100	2.415	917.45	2215.42	2.656	1094.76	2908.15
A_68_P28003591	chr11:88921871-88921915	NM_019505:170	Dgke	INSIDE	1.100	2.485	910.49	2262.26	2.732	793.91	2169.23
A_68_P23203770	chr4:109654911-109654955	NM_172296:4303	Dmrta2	INSIDE	1.100	1.666	1054.18	1755.89	1.832	918.82	1683.71
A_68_P21601164	chr2:125073520-125073564	NM_023595:292	Dut	INSIDE	1.100	0.462	1115.63	515.10	0.508	843.03	428.12
A_68_P23577614	chr5:31598329-31598373	NM_022424:-100	Fndc4	DIVERGENT_PROMOTER	1.100	1.478	2212.63	3271.36	1.626	2122.88	3452.10
A_68_P25098810	chr7:53651744-53651788	NM_008421:-71	Kcnc1	PROMOTER	1.100	1.496	739.26	1105.94	1.646	673.61	1108.57
A_68_P31123766	chr17:28828497-28828541	NM_001168513:-994	Mapk14	PROMOTER	1.100	2.326	351.56	817.65	2.558	328.17	839.55
A_68_P20648918	chr1:138845713-138845757	NM_001159769:4473	Nr5a2	INSIDE	1.100	3.578	750.05	2683.52	3.936	640.73	2521.92
A_68_P25089776	chr7:52119038-52119082	NM_133949:6098	Ptov1	INSIDE	1.100	2.535	3159.37	8008.01	2.788	2292.23	6390.84
A_68_P26823557	chr9:110310713-110310757	NM_001081043:-20	Ptpn23	PROMOTER	1.100	1.495	2176.48	3253.83	1.644	1839.93	3024.47
A_68_P21217116	chr2:51928627-51928671	NM_175238:292	Rif1	INSIDE	1.100	1.938	608.48	1179.52	2.132	568.79	1212.67
A_68_P21116406	chr2:32470512-32470556	NM_001025310:8054	St6galnac6	INSIDE	1.100	2.330	316.04	736.39	2.563	277.08	710.03
A_68_P31152725	chr17:34341728-34341772	NM_011530:327	Tap2	INSIDE	1.100	1.508	3651.15	5504.36	1.659	2731.87	4532.18
A_68_P20303907	chr1:66724032-66724076	NM_175510:209034	Unc80	INSIDE	1.100	1.633	876.45	1431.61	1.797	772.37	1388.03
A_68_P31092362	chr17:23694847-23694891	NM_001033496:6325	Zfp213	INSIDE	1.100	2.518	705.98	1778.01	2.770	576.10	1595.99
A_68_P24818156	chr6:124959237-124959281	NM_175557:-564	Zfp384	PROMOTER	1.100	2.394	1042.11	2495.18	2.634	951.09	2505.19

ProbeName	Target position of probe on CpG island microarray	TargetID	GeneSymbol	CpG island Description	Ratio of relative methylation (TiO_2-NP/Vehicle)	Sham group			TiO_2-H group		
						Relative methylation (Cy5/Cy3)	Cy3 signal (Input)	Cy5 signal (MeDIP)	Relative methylation (Cy5/Cy3)	Cy3 signal (Input)	Cy5 signal (MeDIP)
A_68_P31940131	chr19:7569296-7569340	NM_146091:391	Atl3	INSIDE	1.099	3.962	334.35	1324.64	4.353	306.89	1335.74
A_68_P25354429	chr7:105342798-105342842	NM_001081167:5165	B3gnt6	INSIDE	1.099	3.040	598.15	1818.66	3.343	619.13	2069.54
A_68_P28001993	chr11:88580214-88580258	NR_038058:280	C030037D09Rik	INSIDE	1.099	1.666	2176.43	3626.51	1.831	1482.29	2713.92
A_68_P23196907	chr4:108292428-108292472	NM_177045:-110	Cc2d1b	PROMOTER	1.099	1.563	2440.59	3813.62	1.717	2187.95	3756.16
A_68_P30368454	chr15:79604585-79604629	NM_017470:291	Dnalc4	INSIDE	1.099	1.521	3573.91	5436.54	1.671	2808.06	4692.63
A_68_P23927392	chr5:100468747-100468791	NM_001163035:-244	Enoph1	DIVERGENT_PROMOTER	1.099	0.360	2153.59	775.41	0.396	1821.03	720.89
A_68_P24795427	chr6:119797721-119797765	NM_053204:426	Erc1	INSIDE	1.099	1.919	2615.82	5019.27	2.109	2223.30	4689.08
A_68_P29671484	chr14:65695263-65695307	NM_018788:21659	Extl3	INSIDE	1.099	2.747	2218.76	6093.92	3.019	1738.86	5249.36
A_68_P22384167	chr3:102862328-102862372	NM_010937:143	Nras	INSIDE	1.099	1.740	700.71	1219.30	1.912	520.51	995.42
A_68_P24053562	chr5:123479427-123479471	NM_175423:14366	Orai1	INSIDE	1.099	2.923	1394.74	4077.28	3.213	1101.22	3538.22
A_68_P30380707	chr15:81790992-81791036	NM_013872:283	Pmm1	INSIDE	1.099	1.900	2072.56	3936.86	2.088	1585.63	3310.85
A_68_P28854348	chr13:18035877-18035921	NM_019491:153	Rala	INSIDE	1.099	2.752	261.74	720.37	3.026	245.21	741.94
A_68_P29507577	chr14:31528572-31528616	NM_001166532:-206	Sfmbt1	PROMOTER	1.099	2.466	795.22	1960.94	2.709	752.75	2039.13
A_68_P20370951	chr1:78306769-78306813	NM_001004173:-130	Sgpp2	PROMOTER	1.099	2.099	2689.27	5645.51	2.308	2342.46	5405.30
A_68_P31201839	chr17:45701335-45701379	NM_028662:256	Slc35b2	INSIDE	1.099	2.148	1556.77	3343.46	2.360	1346.47	3177.94
A_68_P25102242	chr7:54213694-54213738	NM_001040695:172	Uevld	INSIDE	1.099	1.382	3548.99	4905.88	1.519	2749.59	4175.48
A_68_P27340460	chr10:90644534-90644578	ENSMUST00000099357:-9797		PROMOTER	1.099	0.528	1456.29	768.86	0.580	1220.19	707.86
A_68_P25083583	chr7:50932042-50932086	NM_145582:4664	Ctu1	INSIDE	1.098	3.050	1089.78	3324.31	3.348	875.97	2932.86
A_68_P24122083	chr5:137041608-137041652	NM_198602:1645	Cux1	INSIDE	1.098	2.278	1845.87	4204.99	2.500	1407.34	3518.95
A_68_P22194043	chr3:63709285-63709329	NM_177856:24001	E130311K13Rik	DOWNSTREAM	1.098	2.201	745.28	1640.29	2.416	676.66	1635.08
A_68_P21393859	chr2:84499121-84499165	NM_001145100:-93	Gm13718	DIVERGENT_PROMOTER	1.098	2.504	1253.81	3139.02	2.749	1100.49	3024.84
A_68_P31055442	chr17:13108620-13108664	NM_026310:315	Mrpl18	INSIDE	1.098	1.658	793.46	1315.49	1.821	681.97	1241.58
A_68_P25377736	chr7:109371755-109371799	NM_145583:189	Pgap2	INSIDE	1.098	2.106	2797.72	5893.33	2.313	2369.32	5479.69
A_68_P25586914	chr7:148317021-148317066	NM_013782:-141	Ptdss2	PROMOTER	1.098	1.819	1844.35	3355.55	1.998	1635.86	3269.24
A_68_P23308458	chr4:131375508-131375552	NM_001083119:18663	Ptpru	INSIDE	1.098	2.372	760.89	1805.20	2.604	660.89	1720.93
A_68_P21077277	chr2:26092977-26093024	NM_153559:-60	Qsox2	PROMOTER	1.098	1.708	10144.92	17331.42	1.876	7141.09	13395.64
A_68_P24622497	chr6:88450007-88450051	NM_019685:34612	Ruvbl1	DOWNSTREAM	1.098	1.850	654.79	1211.43	2.031	730.77	1484.45
A_68_P30575812	chr16:17806955-17806999	NM_153790:9602	Scarf2	INSIDE	1.098	2.817	590.69	1663.81	3.093	535.63	1656.69
A_68_P28376736	chr12:40860722-40860766	NM_001146196:71	Scin	INSIDE	1.098	0.421	2229.60	938.53	0.462	1878.47	868.56
A_68_P22081032	chr3:40512823-40512867	NM_178386:52	Slc25a31	INSIDE	1.098	1.842	508.57	936.79	2.022	413.76	836.71
A_68_P21749134	chr2:152224754-152224798	NM_011438:-994	Sox12	PROMOTER	1.098	3.214	1100.66	3537.74	3.530	986.23	3481.83
A_68_P25016990	chr7:28157258-28157302	NM_001199235:22245	Spnb4	INSIDE	1.098	1.989	877.29	1745.03	2.185	763.84	1668.85
A_68_P22774760	chr4:21703216-21703260	NM_152825:-178	Usp45	DIVERGENT_PROMOTER	1.098	0.492	2362.77	1161.73	0.540	1998.06	1079.15
A_68_P23195052	chr4:107890429-107890473	NM_001167936:77	Zyg11a	INSIDE	1.098	1.467	1114.49	1634.49	1.611	1005.88	1620.10
A_68_P29072930	chr13:58486645-58486689	NM_027335:-80	2210016F16Rik	PROMOTER	1.097	2.187	459.36	1004.70	2.398	382.48	917.31
A_68_P25657382	chr8:11758359-11758403	NM_001113518:51	Arhgef7	INSIDE	1.097	0.424	1608.98	682.35	0.465	1259.32	586.04
A_68_P28853785	chr13:17896465-17896509	NM_001081058:445	Cdk13	INSIDE	1.097	2.067	838.02	1732.39	2.267	825.78	1872.27
A_68_P21907946	chr2:180763743-180763787	NM_015730:10118	Chrna4	INSIDE	1.097	4.949	2893.12	14319.29	5.430	1954.99	10615.68
A_68_P25565163	chr7:144500856-144500900	NM_001113414:5250	Ebf3	INSIDE	1.097	1.600	496.65	794.46	1.754	523.84	918.83
A_68_P30682953	chr16:37868211-37868255	NM_177093:-253	Lrrc58	PROMOTER	1.097	1.571	509.51	800.26	1.723	491.48	846.94
A_68_P29446633	chr14:21213077-21213121	NM_025440:-321	Mrps16	PROMOTER	1.097	1.644	1409.37	2316.45	1.803	1079.66	1947.07
A_68_P20727255	chr1:155396749-155396793	NM_028749:74	Npl	INSIDE	1.097	2.016	1154.50	2327.54	2.211	972.67	2150.41
A_68_P32089882	chr19:36484156-36484200	NM_029508:30622	Pcgf5	INSIDE	1.097	2.651	3345.17	8868.63	2.907	2595.90	7547.26
A_68_P29585964	chr14:48740853-48740897	NM_033602:331	Peli2	INSIDE	1.097	1.487	4036.71	6001.97	1.631	3043.00	4963.55
A_68_P26977747	chr10:18564707-18564751	NM_022032:-148	Perp	PROMOTER	1.097	2.292	1862.10	4267.04	2.513	1552.44	3901.69
A_68_P24983338	chr7:17396479-17396523	NM_001039878:-4737	Strn4	PROMOTER	1.097	1.683	1000.44	1683.82	1.847	929.11	1715.74
A_68_P26037060	chr8:90304857-90304901	NM_033327:178616	Zfp423	INSIDE	1.097	1.717	665.72	1143.01	1.883	532.01	1002.04
A_68_P26974631	chr10:18038563-18038607			Unknown	1.097	2.785	371.63	1034.90	3.054	372.92	1139.01
A_68_P27630019	chr11:20012621-20012665	NM_146243:312	Actr2	INSIDE	1.096	1.651	700.88	1156.98	1.810	707.93	1281.04
A_68_P27649727	chr11:24064666-24064712	NM_001159290:83994	Bcl11a	INSIDE	1.096	2.574	695.18	1789.56	2.822	572.83	1616.49
A_68_P28724520	chr12:109154600-109154644	NM_001079883:87002	Bcl11b	INSIDE	1.096	2.780	925.70	2573.37	3.048	766.16	2335.01

ProbeName	Target position of probe on CpG island microarray	TargetID	GeneSymbol	CpG island Description	Ratio of relative methylation (TiO_2-NP/Vehicle)	Sham group			TiO_2-H group		
						Relative methylation (Cy5/Cy3)	Cy3 signal (Input)	Cy5 signal (MeDIP)	Relative methylation (Cy5/Cy3)	Cy3 signal (Input)	Cy5 signal (MeDIP)
A_68_P28175451	chr11:118902630-118902674	NM_013926:-425	Cbx8	PROMOTER	1.096	1.609	1004.31	1615.95	1.763	885.39	1561.05
A_68_P22058857	chr3:36470723-36470767	NM_009828:174	Ccna2	INSIDE	1.096	2.081	370.59	771.15	2.280	364.34	830.64
A_68_P27543829	chr10:128347422-128347466	NM_007653:523	Cd63	INSIDE	1.096	1.574	2072.01	3262.23	1.726	1611.29	2780.36
A_68_P26078038	chr8:97056657-97056701	NM_153507:-249	Cpne2	PROMOTER	1.096	2.363	813.79	1922.83	2.589	798.90	2068.14
A_68_P28540763	chr12:75050763-75050807	NM_010431:41931	Hif1a	DOWNSTREAM	1.096	1.718	901.34	1548.12	1.882	797.19	1500.56
A_68_P23295050	chr4:128798908-128798952	NM_010471:-395	Hpca	PROMOTER	1.096	5.900	2693.09	15889.35	6.465	2172.96	14048.83
A_68_P28445676	chr12:55169720-55169764	NM_013780:820079	Npas3	INSIDE	1.096	1.652	1977.23	3265.80	1.811	1565.36	2834.71
A_68_P32508786	chrX:83437353-83437397	NM_007430:261	Nr0b1	INSIDE	1.096	1.836	787.54	1446.03	2.012	488.35	982.73
A_68_P27980113	chr11:84693476-84693520	NM_027388:199	Pigw	INSIDE	1.096	2.400	890.02	2135.74	2.630	823.70	2166.42
A_68_P25972618	chr8:77737845-77737889	NM_029182:24	Rasd2	INSIDE	1.096	3.637	1104.94	4018.87	3.986	966.16	3851.25
A_68_P30348905	chr15:76353796-76353840	NM_130893:-1259	Scrt1	PROMOTER	1.096	0.649	2464.40	1599.58	0.711	1953.71	1389.45
A_68_P22342629	chr3:94976833-94976877	NM_011351:8559	Sema6c	INSIDE	1.096	2.654	387.34	1027.96	2.909	383.05	1114.28
A_68_P25962758	chr8:75274038-75274082	NM_009188:26874	Sin3b	INSIDE	1.096	2.999	195.17	585.41	3.287	182.85	601.09
A_68_P27911315	chr11:72224926-72224970	NM_177776:267	Smtnl2	INSIDE	1.096	1.502	625.39	939.38	1.646	533.55	878.30
A_68_P22383503	chr3:102739831-102739875	NM_011516:171	Sycp1	INSIDE	1.096	1.980	389.50	771.28	2.171	393.12	853.45
A_68_P32241861	chrX:7706354-7706398	NR_029428:3597	Wdr13	INSIDE	1.096	2.726	708.04	1930.07	2.987	452.32	1351.18
A_68_P25735820	chr8:28090231-28090275	NM_001101502:2445	Zfp703	INSIDE	1.096	2.315	1698.88	3932.82	2.536	1461.03	3705.63
A_68_P22609446	chr3:145239414-145239458	NM_001081094:265	Znhit6	INSIDE	1.096	1.863	3505.74	6532.24	2.042	2613.34	5337.02
A_68_P28751871	chr12:113931919-113931963	AK028601:-159		PROMOTER	1.096	1.846	608.14	1122.38	2.023	560.09	1132.81
A_68_P26766898	chr9:99847864-99847908	ENSMUST00000104307:3759		DOWNSTREAM	1.096	2.399	459.61	1102.62	2.630	369.52	971.74
A_68_P27971501	chr11:83253964-83254008	NM_025492:2791	1700020L24Rik	INSIDE	1.095	1.590	771.86	1227.18	1.741	713.71	1242.38
A_68_P31259472	chr17:57136735-57136779	NM_025538:-5	Alkbh7	PROMOTER	1.095	2.824	2326.47	6569.20	3.093	1874.19	5796.53
A_68_P22503477	chr3:126299555-126299599	NM_001025438:-314	Camk2d	PROMOTER	1.095	0.571	1547.58	883.86	0.626	1330.01	831.96
A_68_P20716449	chr1:153602522-153602566	NM_001039644:41	Edem3	INSIDE	1.095	1.760	1489.59	2621.41	1.927	1380.28	2660.08
A_68_P28059762	chr11:98843848-98843892	NM_178596:460	Gjd3	INSIDE	1.095	3.871	483.38	1871.34	4.237	451.17	1911.80
A_68_P22141943	chr3:53267216-53267260	NM_172501:-58	Nhlrc3	DIVERGENT_PROMOTER	1.095	1.523	1173.78	1787.53	1.668	1090.97	1819.98
A_68_P20474869	chr1:99992118-99992162	NM_013626:69	Pam	INSIDE	1.095	2.214	936.07	2072.47	2.424	900.91	2183.96
A_68_P30346596	chr15:76009591-76009635	NM_201394:16528	Plec	INSIDE	1.095	3.534	655.50	2316.62	3.871	479.63	1856.44
A_68_P24171758	chr5:147889132-147889176	NM_181730:11	Polr1d	INSIDE	1.095	1.643	771.88	1268.14	1.800	718.92	1293.89
A_68_P29989756	chr15:6659356-6659404	NM_030168:1000	Rictor	INSIDE	1.095	2.369	506.49	1200.04	2.594	410.35	1064.66
A_68_P29676506	chr14:66572378-66572422	NM_172604:181	Scara3	INSIDE	1.095	1.636	551.54	902.35	1.791	478.77	857.42
A_68_P29244711	chr13:96901122-96901166	NM_029210:1388	Sv2c	INSIDE	1.095	1.659	2464.91	4089.63	1.817	1941.61	3526.98
A_68_P21890547	chr2:178142154-178142198	NM_177191:187	Sycp2	INSIDE	1.095	2.740	1751.06	4798.67	3.000	1329.81	3988.97
A_68_P25195710	chr7:74904616-74904660	NM_183312:-10	Synm	PROMOTER	1.095	1.485	948.71	1408.90	1.626	811.41	1319.28
A_68_P21817998	chr2:164286400-164286444	NM_025575:-48	Sys1	PROMOTER	1.095	0.439	1184.32	520.46	0.481	1029.52	495.43
A_68_P29880583	chr14:106505860-106505904	NM_198601:468	Trim52	INSIDE	1.095	2.842	209.31	594.90	3.112	213.04	662.96
A_68_P23865420	chr5:87233089-87233133	NM_177680:-404	Ythdc1	PROMOTER	1.095	1.800	1699.39	3058.05	1.971	1322.24	2605.80
A_68_P28396998	A_68_P28396998			Unknown	1.095	2.358	307.01	723.80	2.582	258.93	668.60
A_68_P23443314	chr5:3100163-3100207	ENSMUST00000161709:9416		DOWNSTREAM	1.095	2.099	627.81	1317.50	2.298	602.79	1385.26
A_68_P27083333	chr10:40022153-40022197	NM_009665:-180	Amd1	PROMOTER	1.094	0.552	2098.05	1158.94	0.604	1648.03	996.14
A_68_P24797812	chr6:120244301-120244345	NM_198884:255	B4galnt3	INSIDE	1.094	1.764	1475.74	2603.83	1.931	1294.51	2499.84
A_68_P32145750	chr19:46835351-46835395	NM_001102471:-726	Cnnm2	PROMOTER	1.094	2.382	953.34	2270.87	2.606	904.07	2356.42
A_68_P26584125	chr9:64659762-64659806	NM_001162917:967	Dennd4a	INSIDE	1.094	1.796	801.75	1439.91	1.965	720.56	1416.04
A_68_P32561248	chrX:97972645-97972689	NM_001177780:57	Dlg3	INSIDE	1.094	2.285	678.85	1551.46	2.500	326.80	817.04
A_68_P21221948	chr2:52717600-52717648	NM_172409:723	Fmnl2	INSIDE	1.094	1.605	686.17	1101.62	1.757	570.74	1002.80
A_68_P26512824	chr9:51856849-51856893	NR_023357:-60	Gm6981	PROMOTER	1.094	1.575	466.03	734.16	1.723	475.43	819.33
A_68_P31828825	chr18:74129885-74129929	NM_172632:94697	Mapk4	INSIDE	1.094	1.973	848.06	1673.02	2.158	728.11	1570.94
A_68_P26082830	chr8:97876281-97876325	NM_008609:66	Mmp15	INSIDE	1.094	1.684	465.56	784.15	1.843	396.54	730.78
A_68_P26789126	chr9:103905204-103905248	NM_172460:353	Nphp3	INSIDE	1.094	1.460	989.50	1444.25	1.597	820.31	1310.40
A_68_P31793419	chr18:67883751-67883795	NM_001127177:503	Ptpn2	INSIDE	1.094	0.472	1280.61	605.05	0.517	1073.88	555.17
A_68_P27359833	chr10:93977922-93977966	NM_172051:343	Tmcc3	INSIDE	1.094	1.744	755.86	1318.46	1.908	613.35	1170.34

ProbeName	Target position of probe on CpG island microarray	TargetID	GeneSymbol	CpG island Description	Ratio of relative methylation (TiO_2-NP/Vehicle)	Sham group			TiO_2-H group		
						Relative methylation (Cy5/Cy3)	Cy3 signal (Input)	Cy5 signal (MeDIP)	Relative methylation (Cy5/Cy3)	Cy3 signal (Input)	Cy5 signal (MeDIP)
A_68_P24153530	chr5:143576863-143576907	NM_001122730:2182	Tnrc18	INSIDE	1.094	0.454	1851.58	840.83	0.497	1583.04	786.50
A_68_P22889130	chr4:46520205-46520249	NM_029077:28790	Trim14	INSIDE	1.094	1.548	585.74	906.90	1.693	529.81	897.02
A_68_P28693527	chr12:103996226-103996270	NM_025666:64	Ubr7	INSIDE	1.094	2.063	1864.81	3847.91	2.258	1501.32	3389.55
A_68_P20804308	chr1:169214081-169214125	NM_030724:1156	Uck2	INSIDE	1.094	2.333	233.12	543.97	2.553	191.55	488.96
A_68_P27541965	chr10:127984764-127984808	NM_134003:14	Zc3h10	INSIDE	1.094	1.926	2235.07	4304.13	2.106	1881.60	3962.33
A_68_P27613618	chr11:16851175-16851219	NR_015469:-100	2810442I21Rik	DIVERGENT_PROMOTER	1.093	1.475	1387.50	2047.09	1.612	1126.38	1816.10
A_68_P23541453	chr5:23893834-23893878	NM_001002897:4105	Atg9b	INSIDE	1.093	2.059	1197.92	2466.67	2.252	991.76	2233.05
A_68_P30365376	chr15:79092452-79092496	NM_177580:23465	Baiap2l2	INSIDE	1.093	1.453	872.48	1267.51	1.589	688.86	1094.32
A_68_P21013690	chr2:12931726-12931771	NM_153155:743	C1ql3	INSIDE	1.093	0.569	2269.11	1292.14	0.623	1999.12	1244.55
A_68_P20408506	chr1:87690361-87690405	NM_133781:361	Cab39	INSIDE	1.093	1.478	2113.71	3124.57	1.615	1995.21	3223.17
A_68_P29098741	chr13:64533952-64533996	NM_053180:114	Cdk20	INSIDE	1.093	1.674	809.96	1355.95	1.830	691.26	1265.07
A_68_P21907944	chr2:180763528-180763574	NM_015730:10332	Chrna4	INSIDE	1.093	3.030	1712.53	5188.97	3.312	1144.44	3790.51
A_68_P23417710	chr4:151656882-151656926	NM_178406:8434	Gpr153	INSIDE	1.093	2.321	1424.22	3305.77	2.536	1080.51	2740.29
A_68_P23415954	chr4:151382992-151383036	NM_001033352:15	Klhl21	INSIDE	1.093	1.545	1367.30	2112.56	1.689	1179.60	1991.84
A_68_P25193398	chr7:74517805-74517849	NM_001033713:-82	Mef2a	PROMOTER	1.093	1.375	1259.58	1732.04	1.502	1051.74	1580.16
A_68_P30506994	chr16:3884902-3884946	NM_029090:306	Nat15	INSIDE	1.093	1.522	1897.45	2888.34	1.664	1407.78	2342.56
A_68_P21006642	chr2:11457159-11457203	NM_001177758:18376	Pfkfb3	INSIDE	1.093	0.580	1354.46	785.05	0.633	1056.28	669.06
A_68_P21432203	chr2:92886316-92886360	NM_001177536:-37	Prdm11	PROMOTER	1.093	2.537	1023.81	2597.57	2.773	832.37	2308.57
A_68_P21866930	chr2:172805522-172805566	NM_001083959:202	Spo11	INSIDE	1.093	3.377	485.14	1638.48	3.690	497.32	1835.29
A_68_P32022083	chr19:24248437-24248481	NM_001198985:172	Tjp2	INSIDE	1.093	2.134	1806.97	3856.71	2.333	1673.90	3904.92
A_68_P28553607	chr12:77472331-77472375	NM_178744:1100	Zbtb1	INSIDE	1.093	1.590	2262.82	3597.87	1.738	1619.54	2814.80
A_68_P25505251	chr7:134487065-134487109	NM_146202:1742	Zfp768	INSIDE	1.093	2.159	1738.56	3753.23	2.359	1355.70	3198.30
A_68_P26559434	chr9:60369978-60370022	ENSMUST00000140824:-147		PROMOTER	1.093	2.735	343.89	940.40	2.989	326.64	976.22
A_68_P21483986	chr2:103406434-103406478	NM_178890:-10	Abtb2	PROMOTER	1.092	1.590	2469.94	3927.43	1.736	1790.45	3108.70
A_68_P23342103	chr4:137352189-137352233	NM_007431:82	Alpl	INSIDE	1.092	2.207	885.02	1953.63	2.410	865.49	2086.12
A_68_P32384237	chrX:50144177-50144221	NM_001034059:-178	Ccdc160	PROMOTER	1.092	1.926	680.66	1310.80	2.104	395.01	830.95
A_68_P26543679	chr9:57613020-57613064	NM_007713:625	Clk3	INSIDE	1.092	2.246	713.12	1601.84	2.453	624.13	1531.21
A_68_P26249217	chr8:127577984-127578028	NM_174853:-88	Disc1	PROMOTER	1.092	1.405	2176.29	3057.95	1.535	1676.53	2572.87
A_68_P27094078	chr10:41995833-41995877	NM_019740:694	Foxo3	INSIDE	1.092	1.946	1132.38	2203.31	2.125	996.06	2116.45
A_68_P24449405	chr6:52196592-52196638	NM_010450:-848	Hoxa11	PROMOTER	1.092	2.078	254.53	528.92	2.269	206.42	468.39
A_68_P24449194	chr6:52171860-52171904	NM_010455:-3310	Hoxa7	PROMOTER	1.092	2.981	816.63	2434.60	3.255	710.25	2311.83
A_68_P27160596	chr10:57206710-57206754	NM_008297:542	Hsf2	INSIDE	1.092	1.967	679.85	1336.92	2.146	676.80	1452.75
A_68_P25259449	chr7:86294014-86294058	NM_001045489:-90	Mfge8	PROMOTER	1.092	1.683	965.64	1625.51	1.838	854.34	1569.95
A_68_P26129004	chr8:107058523-107058567	NM_144931:-7	Nae1	DIVERGENT_PROMOTER	1.092	1.827	635.44	1161.02	1.996	610.90	1219.32
A_68_P32240895	chrX:7499312-7499356	NM_013892:2387	Pcsk1n	INSIDE	1.092	1.881	702.32	1321.14	2.054	388.47	797.86
A_68_P28197874	chr12:3960028-3960072	NM_008895:5100	Pomc	INSIDE	1.092	1.566	1562.96	2447.85	1.710	1350.78	2310.05
A_68_P28112588	chr11:108204767-108204813	NM_011101:412	Prkca	INSIDE	1.092	1.524	701.76	1069.68	1.664	622.72	1036.41
A_68_P21005390	chr2:11212009-11212053	NM_008859:118022	Prkcq	INSIDE	1.092	2.726	459.91	1253.81	2.977	423.30	1260.32
A_68_P25427818	chr7:120097419-120097463	NM_175279:-35	Rassf10	PROMOTER	1.092	1.871	1730.24	3236.87	2.043	1421.81	2904.66
A_68_P31985666	chr19:17468871-17468915	NM_019437:360	Rfk	INSIDE	1.092	2.565	529.91	1359.11	2.801	556.06	1557.61
A_68_P22336986	chr3:93359142-93359186	NM_009112:126	S100a10	INSIDE	1.092	1.758	1614.85	2839.13	1.920	1482.65	2846.78
A_68_P20585385	chr1:127457901-127457945	NM_028787:330	Slc35f5	INSIDE	1.092	1.638	1881.03	3081.93	1.789	1488.57	2662.31
A_68_P21875635	chr2:174298480-174298524	NM_025531:-60	Slmo2	PROMOTER	1.092	1.905	405.20	771.86	2.081	405.11	842.84
A_68_P26142249	chr8:109459792-109459836	NM_009229:165	Sntb2	INSIDE	1.092	2.236	1254.93	2806.20	2.441	1215.30	2966.75
A_68_P27332824	chr10:89208075-89208119	NM_029166:361	Uhrf1bp1l	INSIDE	1.092	2.596	597.77	1551.87	2.834	563.65	1597.36
A_68_P31208848	chr17:46910008-46910052	NM_025966:447	2310039H08Rik	INSIDE	1.091	1.438	944.27	1358.31	1.569	932.43	1463.08
A_68_P27649725	chr11:24064408-24064452	NM_001159290:83736	Bcl11a	INSIDE	1.091	2.063	1958.95	4041.01	2.251	1478.96	3329.41
A_68_P29511373	chr14:32150285-32150329	NM_009796:397	Capn7	INSIDE	1.091	3.179	1243.34	3952.01	3.468	1100.41	3816.28
A_68_P29338036	chr13:113777997-113778041	NM_001081062:9	Ccno	INSIDE	1.091	0.430	1682.61	722.85	0.469	1393.68	653.36
A_68_P32229422	chr19:61304541-61304585	NM_009970:-241	Csf2ra	PROMOTER	1.091	2.262	925.24	2092.53	2.468	849.59	2096.72
A_68_P27446113	chr10:110357232-110357276	NM_007792:23	Csrp2	INSIDE	1.091	0.558	2555.40	1425.10	0.608	1897.56	1154.62

ProbeName	Target position of probe on CpG island microarray	TargetID	GeneSymbol	CpG island Description	Ratio of relative methylation (TiO_2-NP/Vehicle)	Sham group			TiO_2-H group		
						Relative methylation (Cy5/Cy3)	Cy3 signal (Input)	Cy5 signal (MeDIP)	Relative methylation (Cy5/Cy3)	Cy3 signal (Input)	Cy5 signal (MeDIP)
A_68_P25462320	chr7:126327951-126327995	NM_001024138:-84	Gpr139	PROMOTER	1.091	1.512	939.92	1421.21	1.650	736.08	1214.59
A_68_P23573588	chr5:30924821-30924865	NM_010608:34300	Kcnk3	INSIDE	1.091	1.839	544.11	1000.38	2.006	558.17	1119.66
A_68_P25349937	chr7:104480730-104480774	NM_001039039:-80	Kctd21	DIVERGENT_PROMOTER	1.091	1.668	877.16	1462.83	1.819	815.43	1483.00
A_68_P25784752	chr8:37311896-37311940	NM_001081150:652	Lonrf1	INSIDE	1.091	1.739	541.73	942.17	1.898	526.66	999.42
A_68_P20023663	chr1:9690009-9690053	NM_008651:260	Mybl1	INSIDE	1.091	0.519	3635.95	1887.26	0.566	2821.43	1597.30
A_68_P24117615	chr5:136210361-136210405	NM_008898:45299	Por	INSIDE	1.091	1.734	584.65	1014.05	1.892	524.31	992.21
A_68_P26594001	chr9:66441281-66441325	NM_144937:-515	Usp3	PROMOTER	1.091	0.576	2182.33	1257.82	0.629	1688.66	1062.32
A_68_P23059230	chr4:82505564-82505608	NM_026647:-21	Zdhhc21	PROMOTER	1.091	1.889	970.77	1833.93	2.061	880.93	1815.98
A_68_P29224480	chr13:93301031-93301075	NM_173392:-287	Zfyve16	PROMOTER	1.091	0.464	1398.64	648.88	0.506	1184.82	599.58
A_68_P24908454	chr6:143048465-143048509	NM_001109688:141	5730419I09Rik	INSIDE	1.090	1.465	894.46	1309.98	1.597	742.86	1186.33
A_68_P32350054	chrX:41720980-41721024	NM_175539:512	Dcaf12l2	INSIDE	1.090	1.457	1795.18	2615.06	1.588	941.44	1495.00
A_68_P26227567	chr8:124102726-124102770	NM_133765:-42	Fbxo31	PROMOTER	1.090	2.066	2748.15	5677.93	2.252	2044.88	4604.11
A_68_P23577609	chr5:31597809-31597853	NM_022424:420	Fndc4	INSIDE	1.090	1.739	9346.16	16252.36	1.896	7226.94	13702.41
A_68_P28577468	chr12:81858602-81858646	NM_001008423:3078	Gm1568	INSIDE	1.090	1.986	1107.37	2199.70	2.164	880.52	1905.66
A_68_P28186730	chr11:120645846-120645891	NM_145370:40	Gps1	INSIDE	1.090	2.505	242.19	606.71	2.730	205.36	560.59
A_68_P27924566	chr11:74979860-74979904	NM_001098203:1772	Hic1	INSIDE	1.090	0.538	1490.23	801.41	0.586	1093.12	641.03
A_68_P23333246	chr4:135867340-135867384	NM_028871:472	Hnrnpr	INSIDE	1.090	1.697	965.19	1638.13	1.850	792.11	1465.12
A_68_P27353693	chr10:92916270-92916314	NM_008517:152	Lta4h	INSIDE	1.090	0.582	1397.76	813.35	0.634	1224.43	776.44
A_68_P32148146	chr19:47253257-47253301	NM_021360:-31	Neurl1a	PROMOTER	1.090	1.559	707.46	1102.93	1.699	612.61	1040.68
A_68_P31935410	chr19:6531540-6531584	NM_001205234:112825	Nrxn2	INSIDE	1.090	0.543	1937.03	1051.72	0.592	1523.40	901.80
A_68_P25427821	chr7:120097865-120097909	NM_175279:411	Rassf10	INSIDE	1.090	1.749	1317.82	2304.86	1.907	1209.87	2307.04
A_68_P22741779	chr4:13670817-13670861	NM_001111027:-7605	Runx1t1	PROMOTER	1.090	1.428	1342.89	1918.06	1.557	1071.37	1668.37
A_68_P25586717	chr7:148286592-148286636	NM_023059:-169	Sigirr	PROMOTER	1.090	1.757	835.30	1467.72	1.916	735.86	1409.86
A_68_P26766608	chr9:99770906-99770951	NM_011440:5661	Sox14	DOWNSTREAM	1.090	1.814	1250.11	2267.81	1.978	960.91	1900.56
A_68_P21809439	chr2:162757267-162757311	NM_026499:45	Srsf6	INSIDE	1.090	3.456	1093.28	3778.55	3.767	948.14	3571.23
A_68_P25947574	chr8:72149082-72149126	NM_001045553:-30	Zfp868	PROMOTER	1.090	0.489	1490.68	728.48	0.532	1439.65	766.58
A_68_P30413820	chr15:87375020-87375064	ENSMUST00000169110:139		INSIDE	1.090	1.546	718.08	1109.84	1.684	666.87	1123.01
A_68_P24830751	chr6:127059602-127059646	NM_177003:-54	9630033F20Rik	PROMOTER	1.089	0.585	1703.63	996.57	0.637	1313.56	836.50
A_68_P27282593	chr10:79804404-79804448	NM_001113548:6765	Adamtsl5	INSIDE	1.089	1.727	2643.31	4565.54	1.882	2176.75	4095.73
A_68_P26291034	chr9:8134431-8134475	NM_001045524:-158	AK129341	PROMOTER	1.089	2.008	727.17	1460.48	2.188	635.94	1391.47
A_68_P24758539	chr6:113233198-113233242	NM_170673:920	Cpne9	INSIDE	1.089	1.342	2195.07	2945.46	1.461	1824.69	2666.41
A_68_P30896820	chr16:78576898-78576942	NM_025967:-7	D16Ertd472e	PROMOTER	1.089	1.466	763.07	1118.89	1.596	636.63	1016.24
A_68_P25410983	chr7:117103395-117103439	NM_021494:520	Dennd5a	INSIDE	1.089	2.208	1236.91	2731.21	2.404	1097.45	2638.69
A_68_P28672826	chr12:100139771-100139815	NM_001081191:-98	Eml5	PROMOTER	1.089	2.423	1085.33	2629.26	2.637	973.42	2567.26
A_68_P29644194	chr14:60997825-60997869	NM_001164705:724	Fam123a	INSIDE	1.089	1.764	919.47	1621.70	1.921	815.23	1566.47
A_68_P32678434	chrX:132373604-132373648	NM_001163015:54	Gprasp2	INSIDE	1.089	2.036	996.22	2028.35	2.217	634.40	1406.66
A_68_P21080306	chr2:26516993-26517037	NM_010695:9788	Lcn4	DOWNSTREAM	1.089	2.024	541.27	1095.45	2.203	559.93	1233.66
A_68_P20337470	chr1:72583581-72583625	NM_001045533:-464	Marchf4	PROMOTER	1.089	4.420	1932.71	8541.63	4.815	1637.09	7882.05
A_68_P27788133	chr11:50173073-50173117	NR_030529:-1713	Mir804	PROMOTER	1.089	1.605	2614.47	4196.32	1.748	2064.20	3608.20
A_68_P27560009	chr11:4749259-4749303	NM_010898:250	Nf2	INSIDE	1.089	2.234	1172.00	2618.64	2.434	1032.93	2513.90
A_68_P27922135	chr11:74536870-74536914	NR_037610:468	Pafah1b1	INSIDE	1.089	1.440	2095.50	3017.17	1.568	1805.45	2830.26
A_68_P28594554	chr12:85113181-85113234	NM_001205343:8624	Papln	INSIDE	1.089	2.860	390.19	1115.97	3.115	341.98	1065.20
A_68_P26542154	chr9:57352236-57352280	NM_133982:350	Rpp25	INSIDE	1.089	0.584	2362.39	1379.66	0.636	1965.79	1250.12
A_68_P28036073	chr11:94871704-94871750	NM_146025:534	Samd14	INSIDE	1.089	2.107	5933.03	12498.82	2.295	3951.23	9067.87
A_68_P21075769	chr2:25876845-25876892	NM_133835:412	Ubac1	INSIDE	1.089	1.482	1130.86	1675.83	1.614	883.53	1425.81
A_68_P23254147	chr4:120624114-120624158	NM_001005788:170	Zfp69	INSIDE	1.089	2.275	2074.37	4718.46	2.476	1737.50	4302.89
A_68_P20286846	chr1:63492907-63492951	NM_001177600:451	Adam23	INSIDE	1.088	1.815	1485.81	2697.48	1.975	1305.15	2578.00
A_68_P23542163	chr5:23991120-23991164	NM_139153:33148	Agap3	INSIDE	1.088	1.597	1168.11	1864.92	1.737	1061.88	1843.97
A_68_P27103326	chr10:43724148-43724192	NM_172393:482	Aim1	INSIDE	1.088	2.573	1166.37	3000.62	2.798	972.81	2721.78
A_68_P25946258	chr8:71612629-71612673	NM_007509:16	Atp6v1b2	INSIDE	1.088	2.878	669.21	1926.00	3.130	630.15	1972.48
A_68_P20900640	chr1:187165153-187165197	NM_011794:9137	Bpnt1	INSIDE	1.088	2.192	702.20	1539.01	2.385	563.89	1345.10

ProbeName	Target position of probe on CpG island microarray	TargetID	GeneSymbol	CpG island Description	Ratio of relative methylation (TiO_2-NP/Vehicle)	Sham group			TiO_2-H group		
						Relative methylation (Cy5/Cy3)	Cy3 signal (Input)	Cy5 signal (MeDIP)	Relative methylation (Cy5/Cy3)	Cy3 signal (Input)	Cy5 signal (MeDIP)
A_68_P24008368	chr5:115643546-115643590	NM_013879:-7438	Cabp1	PROMOTER	1.088	0.494	1592.32	785.95	0.537	1390.94	746.97
A_68_P29651395	chr14:62301504-62301548	NR_028264:-316	Dleu2	PROMOTER	1.088	1.774	939.19	1666.15	1.931	839.18	1620.32
A_68_P20317719	chr1:69153647-69153691	NM_010154:965	Erbb4	INSIDE	1.088	2.366	689.52	1631.39	2.575	585.82	1508.69
A_68_P23582140	chr5:32435627-32435671	NM_008037:-3196	Fosl2	PROMOTER	1.088	1.493	642.03	958.50	1.624	519.60	844.07
A_68_P24990912	chr7:19597699-19597743	NM_008260:11168	Foxa3	DOWNSTREAM	1.088	3.928	207.60	815.53	4.273	221.23	945.35
A_68_P29531946	chr14:35632404-35632448	NM_008166:-895	Grid1	PROMOTER	1.088	1.695	625.50	1059.92	1.843	535.61	987.18
A_68_P25021327	chr7:29136106-29136150	NR_028129:-430	LOC100302567	PROMOTER	1.088	2.380	5000.00	11900.01	2.590	3683.82	9542.52
A_68_P28056218	chr11:98261638-98261682	NM_001033537:144	Pgap3	INSIDE	1.088	1.799	1283.50	2309.61	1.958	1110.18	2173.68
A_68_P24170388	chr5:147656591-147656635	NM_026864:-34	Rasl11a	PROMOTER	1.088	2.313	438.53	1014.12	2.517	536.51	1350.29
A_68_P25499763	chr7:133435519-133435563	NM_145587:19408	Sbk1	INSIDE	1.088	2.004	1195.03	2395.20	2.180	992.60	2164.17
A_68_P32672217	chrX:131010986-131011030	NM_028958:21	Taf7l	INSIDE	1.088	1.990	1340.55	2667.57	2.165	535.73	1159.63
A_68_P26752041	chr9:97266267-97266311	NM_030219:4089	Trim42	INSIDE	1.088	2.208	574.16	1267.98	2.403	518.00	1244.76
A_68_P31889610	chr18:84759292-84759336	NM_001033341:-418	Zfp407	PROMOTER	1.088	2.542	1038.85	2640.85	2.765	861.68	2382.47
A_68_P25735815	chr8:28089687-28089731	NM_001101502:1901	Zfp703	INSIDE	1.088	0.611	3527.33	2156.10	0.665	2599.19	1728.05
A_68_P28677373	chr12:100883228-100883278	ENSMUST00000158736:-70		PROMOTER	1.088	2.570	228.82	588.11	2.797	197.12	551.38
A_68_P32443537	chrX:66613822-66613866	NM_008032:339	Aff2	INSIDE	1.087	1.829	895.87	1638.81	1.989	441.52	878.02
A_68_P30462686	chr15:96116941-96116985	NM_175251:-990	Arid2	PROMOTER	1.087	3.061	370.46	1134.12	3.329	336.06	1118.76
A_68_P23069979	chr4:84320697-84320741	NM_172870:272	Bnc2	INSIDE	1.087	1.825	748.75	1366.52	1.984	669.67	1328.41
A_68_P20910362	chr1:188791704-188791749	NM_033077:432	D1Pas1	INSIDE	1.087	1.845	735.33	1356.69	2.005	591.72	1186.32
A_68_P30630419	chr16:28446947-28446991	NM_183064:-1655	Fgf12	PROMOTER	1.087	2.102	459.89	966.85	2.286	384.98	879.93
A_68_P32563072	chrX:98449525-98449570	NM_018789:-319	Foxo4	PROMOTER	1.087	3.550	1639.04	5817.97	3.859	824.20	3180.59
A_68_P25348906	chr7:104230072-104230116	NM_001162477:-166	Gab2	PROMOTER	1.087	6.600	8024.21	52957.00	7.176	6424.64	46106.17
A_68_P21932362	chr3:8462856-8462900	NR_033512:220	Gm6194	INSIDE	1.087	1.934	1388.70	2686.41	2.103	980.84	2063.11
A_68_P30473864	chr15:98087423-98087467	NM_027304:294	H1fnt	INSIDE	1.087	2.348	699.76	1643.17	2.551	634.24	1618.21
A_68_P29334866	chr13:113253950-113253994	NM_010560:-305	Il6st	PROMOTER	1.087	1.445	763.05	1102.54	1.571	729.30	1145.57
A_68_P20229132	chr1:52874292-52874338	NM_008384:218	Inpp1	INSIDE	1.087	1.627	1632.95	2656.39	1.768	1277.61	2259.43
A_68_P28291801	chr12:25516771-25516815	NM_001083341:329	Mboat2	INSIDE	1.087	2.263	634.38	1435.69	2.461	555.62	1367.22
A_68_P25263202	chr7:86937639-86937683	NM_008588:816	Mesp1	INSIDE	1.087	1.465	838.96	1228.99	1.592	743.62	1184.00
A_68_P29751162	chr14:79790477-79790521	NM_025832:-23	Naa16	DIVERGENT_PROMOTER	1.087	2.084	720.03	1500.88	2.265	677.17	1534.05
A_68_P31207758	chr17:46728606-46728650	NM_175168:37825	Ptk7	INSIDE	1.087	2.213	950.09	2102.09	2.405	812.40	1954.23
A_68_P21106144	chr2:30808670-30808714	NM_133673:172	Tor1b	INSIDE	1.087	0.599	1166.94	699.08	0.651	916.87	597.27
A_68_P31372685	chr17:79024354-79024402	NM_001197028:116976	Vit	INSIDE	1.087	1.873	531.47	995.41	2.036	423.84	863.13
A_68_P32005487	chr19:21346304-21346348	NM_009551:-441	Zfand5	PROMOTER	1.087	1.405	1110.67	1560.54	1.527	935.22	1428.32
A_68_P31002112	chr16:98183669-98183713	NM_001081684:96	Zfp295	INSIDE	1.087	0.575	1056.91	607.77	0.625	866.23	541.67
A_68_P23145844	chr4:99382494-99382538	NM_001081264:196	Alg6	INSIDE	1.086	1.446	1249.49	1807.04	1.570	1044.81	1640.30
A_68_P32592613	chrX:106029430-106029474	NM_001081477:242	Brwd3	INSIDE	1.086	1.470	2860.43	4205.73	1.596	1298.59	2073.00
A_68_P22412539	chr3:108218569-108218613	NM_001004177:-178	Celsr2	PROMOTER	1.086	1.660	1216.24	2018.81	1.803	1164.73	2099.47
A_68_P27865444	chr11:64249527-64249571	NR_028445:628	F930015N05Rik	INSIDE	1.086	2.764	847.34	2342.45	3.002	908.25	2726.73
A_68_P25371472	chr7:108256614-108256658	NM_001146010:-652	Fchsd2	PROMOTER	1.086	1.650	1217.58	2008.60	1.791	1062.57	1903.24
A_68_P25770034	chr8:34764345-34764389	NM_010344:657	Gsr	INSIDE	1.086	1.433	1458.70	2090.94	1.557	1147.76	1786.55
A_68_P20193922	chr1:44608989-44609033	NM_028450:495	Gulp1	INSIDE	1.086	1.372	1587.56	2177.72	1.490	1363.86	2032.28
A_68_P22397119	chr3:105261830-105261874	NM_019931:119	Kcnd3	INSIDE	1.086	2.245	244.99	549.95	2.437	294.15	716.85
A_68_P25713199	chr8:23970569-23970613	NM_001081149:580	Myst3	INSIDE	1.086	1.479	1748.30	2585.19	1.606	1548.07	2486.77
A_68_P26022002	chr8:87323170-87323214	NM_001081982:1047	Nfix	INSIDE	1.086	1.628	834.82	1358.74	1.768	700.56	1238.27
A_68_P22878645	chr4:44716965-44717009	NM_008782:6326	Pax5	INSIDE	1.086	1.393	1322.38	1841.84	1.513	1132.00	1712.39
A_68_P31632592	chr18:38357632-38357676	NM_029357:11762	Pcdh1	INSIDE	1.086	2.484	920.03	2285.68	2.699	758.20	2046.08
A_68_P31252244	chr17:55852024-55852068	NM_028370:-120	Pot1b	PROMOTER	1.086	2.296	685.00	1572.78	2.493	624.59	1557.07
A_68_P26801800	chr9:106101470-106101514	NM_198931:-222	Ppm1m	PROMOTER	1.086	1.613	565.30	911.76	1.751	504.35	883.04
A_68_P28991175	chr13:43711506-43711550	NM_183204:363	Rnf182	INSIDE	1.086	2.249	764.36	1719.41	2.443	661.77	1616.44
A_68_P26133225	chr8:107873334-107873378	NM_001081332:1199	Slc9a5	INSIDE	1.086	0.610	1579.39	962.75	0.662	1449.88	960.12
A_68_P28159868	chr11:116395378-116395422	NM_011451:-653	Sphk1	PROMOTER	1.086	1.603	1490.45	2389.48	1.741	1146.65	1996.62

ProbeName	Target position of probe on CpG island microarray	TargetID	GeneSymbol	CpG island Description	Ratio of relative methylation (TiO_2-NP/Vehicle)	Sham group			TiO_2-H group		
						Relative methylation (Cy5/Cy3)	Cy3 signal (Input)	Cy5 signal (MeDIP)	Relative methylation (Cy5/Cy3)	Cy3 signal (Input)	Cy5 signal (MeDIP)
A_68_P31752326	chr18:60756117-60756161	NM_177340:27821	Synpo	INSIDE	1.086	2.221	1256.85	2791.85	2.411	1149.92	2772.93
A_68_P27356011	chr10:93308901-93308948	NM_183199:14625	Usp44	INSIDE	1.086	3.954	748.41	2959.48	4.293	558.82	2399.15
A_68_P21394210	chr2:84555540-84555584	NM_144887:-241	Zdhhc5	PROMOTER	1.086	2.374	1388.58	3297.07	2.578	1267.29	3267.24
A_68_P24783631	chr6:117791197-117791241	NM_177684:-41	Zfp637	PROMOTER	1.086	1.775	363.93	645.98	1.928	355.12	684.55
A_68_P27543827	chr10:128347191-128347235	NM_007653:291	Cd63	INSIDE	1.085	2.181	1226.97	2676.50	2.366	1107.11	2619.12
A_68_P26248668	chr8:127473510-127473554	NM_053207:-378	Egln1	PROMOTER	1.085	3.244	545.00	1767.81	3.519	514.37	1810.32
A_68_P29054917	chr13:55521884-55521928	NM_021489:6257	F12	INSIDE	1.085	4.411	3153.12	13907.92	4.784	2278.57	10899.88
A_68_P27278518	chr10:79187320-79187368	NM_008226:7966	Hcn2	INSIDE	1.085	3.426	343.67	1177.56	3.718	252.43	938.61
A_68_P26537259	chr9:56467537-56467581	NM_181074:65502	Lingo1	INSIDE	1.085	3.955	2587.53	10233.74	4.291	1897.13	8140.56
A_68_P21842273	chr2:168396596-168396647	NM_001136073:19070	Nfatc2	INSIDE	1.085	5.580	730.27	4075.04	6.053	557.44	3374.42
A_68_P31926006	chr19:4942839-4942883	NM_172835:232	Peli3	INSIDE	1.085	2.397	10563.21	25319.83	2.600	7828.38	20352.98
A_68_P27287760	chr10:80616735-80616779	NM_021501:13709	Pias4	INSIDE	1.085	2.793	1564.14	4368.36	3.029	1257.72	3809.65
A_68_P21351536	chr2:76485728-76485772	NM_011871:301	Prkra	INSIDE	1.085	1.350	1911.62	2581.54	1.465	1645.67	2410.74
A_68_P21608666	chr2:126378873-126378917	NM_011978:135	Slc27a2	INSIDE	1.085	2.275	463.89	1055.40	2.468	580.43	1432.62
A_68_P20901345	chr1:187279321-187279365	NM_001033286:616	Slc30a10	INSIDE	1.085	1.453	2158.64	3137.54	1.576	1919.78	3026.20
A_68_P30486835	chr15:100467504-100467548	NM_001033872:-230	Smagp	PROMOTER	1.085	1.373	2862.18	3929.02	1.490	2427.87	3617.65
A_68_P32557249	chrX:97016733-97016777	NM_001081283:347	Tmem28	INSIDE	1.085	2.341	759.64	1778.27	2.539	493.97	1254.15
A_68_P26253146	chr8:128193938-128193982	NM_028908:243	4933403G14Rik	INSIDE	1.084	2.367	406.41	961.89	2.566	386.04	990.76
A_68_P27320008	chr10:86956714-86956759	NM_008553:-331	Ascl1	PROMOTER	1.084	2.454	354.66	870.18	2.661	320.68	853.27
A_68_P26372312	chr9:26563608-26563652	NM_029792:4484	B3gat1	INSIDE	1.084	1.764	438.16	773.11	1.913	321.66	615.24
A_68_P24372703	chr6:37391889-37391933	NM_178661:238	Creb3l2	INSIDE	1.084	2.131	1571.80	3349.57	2.311	1302.35	3009.34
A_68_P23244385	chr4:118905637-118905681	NM_001042411:-775	Lepre1	PROMOTER	1.084	0.546	1114.00	607.96	0.591	1081.07	639.37
A_68_P32579350	chrX:102316061-102316105	NM_053201:348	Magee1	INSIDE	1.084	2.179	794.27	1730.67	2.362	509.38	1203.08
A_68_P25809394	chr8:42219374-42219418	NM_001005863:-316	Mtus1	PROMOTER	1.084	0.486	1338.27	650.56	0.527	1201.07	632.62
A_68_P22313146	chr3:88137030-88137074	NM_024246:1303	Tmem79	INSIDE	1.084	2.012	1491.70	3001.41	2.182	1211.37	2642.72
A_68_P22313144	chr3:88136817-88136861	NM_024246:1517	Tmem79	INSIDE	1.084	2.398	1419.15	3403.77	2.600	899.90	2339.69
A_68_P20352743	chr1:75216251-75216295	NM_009447:-444	Tuba4a	PROMOTER	1.084	1.351	2017.00	2723.97	1.464	1510.56	2211.38
A_68_P28220739	chr12:8214991-8215035	NM_001167767:80	1110057K04Rik	INSIDE	1.083	1.464	3392.89	4967.85	1.585	2514.03	3984.88
A_68_P20086662	chr1:23109142-23109186	NM_025746:-72	4933415F23Rik	PROMOTER	1.083	1.897	442.90	839.98	2.054	489.22	1004.88
A_68_P28889024	chr13:24894047-24894092	NM_024473:544	BC005537	INSIDE	1.083	2.116	1408.04	2979.88	2.292	1418.33	3250.86
A_68_P31485628	chr18:9449839-9449883	NM_026484:288	Ccny	INSIDE	1.083	1.420	1567.32	2225.76	1.538	1500.15	2306.85
A_68_P25637611	chr8:8660029-8660073	NM_010111:723	Efnb2	INSIDE	1.083	1.391	1911.18	2659.29	1.507	1558.59	2348.95
A_68_P23969768	chr5:108415971-108416015	NM_026062:104	Fam69a	INSIDE	1.083	1.615	3737.05	6035.04	1.749	2970.71	5195.43
A_68_P26572467	chr9:62659271-62659315	NM_010193:163	Fem1b	INSIDE	1.083	1.740	842.93	1466.63	1.884	779.02	1467.71
A_68_P25005396	chr7:25318615-25318659	NM_153134:2970	Irgq	INSIDE	1.083	2.746	2862.77	7861.91	2.975	2176.56	6474.47
A_68_P21680294	chr2:139504121-139504165	NM_001126490:229	Ism1	INSIDE	1.083	2.270	583.28	1323.92	2.457	592.64	1456.33
A_68_P32040191	chr19:27397873-27397917	NM_183179:786	Kcnv2	INSIDE	1.083	2.107	802.13	1689.80	2.281	676.91	1543.98
A_68_P29509958	chr14:31951947-31951991	NM_027289:3930	Nt5dc2	INSIDE	1.083	3.944	1570.26	6193.19	4.270	1095.93	4679.34
A_68_P27922136	chr11:74536967-74537011	NR_037610:372	Pafah1b1	INSIDE	1.083	0.512	1119.25	572.50	0.554	965.78	535.17
A_68_P32668460	chrX:130222185-130222229	NM_001105245:1326	Pcdh19	INSIDE	1.083	2.529	871.95	2205.54	2.740	550.74	1508.80
A_68_P27287762	chr10:80616963-80617007	NM_021501:13481	Pias4	INSIDE	1.083	2.001	826.10	1652.81	2.167	690.08	1495.39
A_68_P23435436	chr4:154357832-154357876	NM_001113360:27239	Plch2	INSIDE	1.083	1.624	1346.07	2185.98	1.759	1013.35	1782.85
A_68_P25025803	chr7:29965431-29965475	NM_026545:240	Psmd8	INSIDE	1.083	1.646	842.12	1386.40	1.783	665.51	1186.81
A_68_P27286999	chr10:80494095-80494139	NM_134135:2541	Slc39a3	INSIDE	1.083	2.901	805.63	2337.38	3.141	671.47	2109.37
A_68_P31093843	chr17:23956823-23956878	NM_175229:16697	Srrm2	INSIDE	1.083	3.064	209.21	640.93	3.319	205.01	680.48
A_68_P27504467	chr10:121043954-121043999	NM_001081056:19396	Xpot	INSIDE	1.083	3.310	2133.85	7062.86	3.586	1523.13	5462.33
A_68_P23588853	chr5:33777078-33777122	ENSMUST00000030994:-3256		PROMOTER	1.083	3.685	1717.52	6329.01	3.992	1460.63	5830.60
A_68_P26218687	chr8:122754031-122754075	ENSMUST00000127664:319312		INSIDE	1.083	1.449	1034.17	1498.69	1.570	934.02	1466.36
A_68_P24766863	chr6:114688424-114688468	NM_028835:95304	Atg7	INSIDE	1.082	2.256	544.41	1228.36	2.441	594.42	1450.90
A_68_P24908776	chr6:143115770-143115814	NM_029250:43	Etnk1	INSIDE	1.082	0.615	1151.54	708.24	0.665	1122.82	746.97
A_68_P25307415	chr7:96552181-96552225	NM_008055:-673	Fzd4	PROMOTER	1.082	1.848	1413.66	2611.81	2.000	1161.64	2322.89

ProbeName	Target position of probe on CpG island microarray	TargetID	GeneSymbol	CpG island Description	Ratio of relative methylation (TiO_2-NP/Vehicle)	Sham group			TiO_2-H group		
						Relative methylation (Cy5/Cy3)	Cy3 signal (Input)	Cy5 signal (MeDIP)	Relative methylation (Cy5/Cy3)	Cy3 signal (Input)	Cy5 signal (MeDIP)
A_68_P24126245	chr5:137972339-137972383	NM_010312:2097	Gnb2	INSIDE	1.082	1.768	1605.49	2839.14	1.913	1433.29	2741.26
A_68_P28099139	chr11:105881776-105881820	NM_001037712:12282	Kcnh6	INSIDE	1.082	1.660	2806.09	4657.08	1.795	2058.56	3695.78
A_68_P27365816	chr10:95026259-95026303	NR_037212:-4325	Mir3058	PROMOTER	1.082	1.476	1941.81	2866.07	1.597	1704.17	2721.57
A_68_P23792521	chr5:72950400-72950444	NM_133921:462	Nfxl1	INSIDE	1.082	2.029	395.58	802.48	2.195	382.97	840.77
A_68_P31397730	chr17:83614079-83614123	NM_134117:-522	Pkdcc	PROMOTER	1.082	0.519	3450.30	1790.20	0.562	2733.35	1535.13
A_68_P30330340	chr15:73253433-73253477	NM_007982:167	Ptk2	INSIDE	1.082	1.694	1461.10	2475.21	1.833	1438.43	2636.18
A_68_P31631090	chr18:38115096-38115140	NM_153793:-94	Rell2	DIVERGENT_PROMOTER	1.082	1.446	810.48	1171.94	1.564	644.06	1007.62
A_68_P32036078	chr19:26680160-26680204	NM_011416:533	Smarca2	INSIDE	1.082	5.509	565.99	3117.79	5.958	567.26	3379.61
A_68_P27282400	chr10:79777704-79777748	NM_011789:13162	Apc2	INSIDE	1.081	4.071	1006.26	4096.37	4.403	886.28	3901.88
A_68_P29501750	chr14:30535873-30535917	NM_009785:-844	Cacna2d3	PROMOTER	1.081	1.626	1086.12	1766.39	1.758	959.50	1686.37
A_68_P24818635	chr6:125045930-125045974	NM_145979:-228	Chd4	PROMOTER	1.081	1.450	2836.15	4112.99	1.568	2092.42	3280.68
A_68_P24850799	chr6:131338430-131338474	NM_011733:16	Csda	INSIDE	1.081	2.069	1929.61	3991.90	2.236	1746.32	3904.71
A_68_P27187153	chr10:62113528-62113572	NM_053183:396	Ddx50	INSIDE	1.081	2.048	1143.95	2342.41	2.214	922.86	2043.25
A_68_P27835392	chr11:59005058-59005102	NM_001159410:-3837	Guk1	PROMOTER	1.081	0.434	1538.20	667.38	0.469	1318.39	618.25
A_68_P27482567	chr10:117147762-117147806	NM_010786:-12	Mdm2	PROMOTER	1.081	2.078	1021.72	2123.29	2.246	939.52	2110.48
A_68_P21967992	chr3:17694822-17694866	NR_029814:-817	Mir124a-2	PROMOTER	1.081	1.532	1068.35	1636.40	1.655	878.26	1453.74
A_68_P23657845	chr5:46060700-46060744	NM_019438:-441	Ncapg	PROMOTER	1.081	0.395	1322.04	522.10	0.427	1034.57	441.49
A_68_P21826979	chr2:165818066-165818110	NM_008679:-48	Ncoa3	PROMOTER	1.081	3.898	1479.50	5766.38	4.215	1277.66	5385.51
A_68_P29510249	chr14:31989749-31989793	NM_022656:30242	Nisch	INSIDE	1.081	2.747	3074.59	8445.35	2.970	2229.52	6620.63
A_68_P32114751	chr19:41450414-41450458	NM_031376:9124	Pik3ap1	INSIDE	1.081	2.173	470.08	1021.30	2.350	468.06	1099.76
A_68_P24979913	chr7:16730734-16730778	NM_148946:15108	Slc8a2	INSIDE	1.081	3.422	4275.22	14628.45	3.698	3396.45	12558.68
A_68_P23364645	chr4:141093735-141093779	NM_019763:756	Spen	INSIDE	1.081	1.702	425.82	724.88	1.841	412.57	759.45
A_68_P31093844	chr17:23956989-23957033	NM_175229:16857	Srrm2	INSIDE	1.081	2.495	1154.37	2880.62	2.698	879.58	2372.82
A_68_P25195702	chr7:74903601-74903645	NM_183312:1006	Synm	INSIDE	1.081	1.720	402.89	693.15	1.860	343.64	639.16
A_68_P25957926	chr8:74152893-74152939	NM_001029873:42740	Unc13a	INSIDE	1.081	2.049	290.24	594.71	2.216	257.69	570.92
A_68_P29604236	chr14:52626490-52626534	NM_027248:13896	Zfp219	INSIDE	1.081	3.110	922.81	2870.24	3.361	889.59	2989.75
A_68_P30708314	chr16:42190481-42190525	ENSMUST00000099761:657353		DOWNSTREAM	1.081	1.478	761.15	1125.35	1.598	672.20	1074.34
A_68_P24944594	chr6:149302348-149302392	ENSMUST00000121073:-22		DIVERGENT_PROMOTER	1.081	2.873	1197.78	3441.02	3.105	1060.99	3294.70
A_68_P30575977	chr16:17834072-17834116	NR_027959:883	B830017H08Rik	INSIDE	1.080	2.123	423.75	899.43	2.293	375.44	860.84
A_68_P23201926	chr4:109329790-109329834	NM_007671:8165	Cdkn2c	DOWNSTREAM	1.080	1.387	1720.82	2386.25	1.498	1604.44	2403.75
A_68_P28158071	chr11:116083426-116083470	NM_025276:15957	Evpl	INSIDE	1.080	1.744	649.04	1131.68	1.883	565.73	1065.47
A_68_P29631462	chr14:58689892-58689936	NM_013518:-1608	Fgf9	PROMOTER	1.080	3.630	1037.56	3765.94	3.921	926.49	3632.53
A_68_P25348904	chr7:104229911-104229955	NM_001162477:-328	Gab2	PROMOTER	1.080	2.251	784.80	1766.58	2.430	662.88	1610.91
A_68_P25885333	chr8:58773316-58773360	NM_008278:-10	Hpgd	PROMOTER	1.080	2.028	1609.36	3264.26	2.191	1324.83	2902.42
A_68_P31113973	chr17:27194296-27194340	NM_080553:70	Itpr3	INSIDE	1.080	1.466	1219.41	1787.40	1.583	1010.75	1599.96
A_68_P31304302	chr17:66450798-66450842	NM_028388:11	Ndufv2	INSIDE	1.080	2.986	664.31	1983.83	3.226	630.97	2035.74
A_68_P22101773	chr3:45184916-45184960	NM_011043:2619	Pcdh10	INSIDE	1.080	1.638	1512.32	2477.22	1.769	1373.93	2430.73
A_68_P22593188	chr3:142544925-142544969	NM_178654:22	Pkn2	INSIDE	1.080	2.339	460.27	1076.56	2.525	454.22	1146.90
A_68_P32479509	chrX:75041437-75041481	NM_016979:-159	Prkx	PROMOTER	1.080	2.647	639.09	1691.40	2.857	431.93	1234.13
A_68_P29262747	chr13:100114515-100114559	NM_026873:97	Ptcd2	INSIDE	1.080	1.599	667.57	1067.12	1.727	623.50	1076.51
A_68_P22378031	chr3:101728081-101728125	NM_001039371:274	Slc22a15	INSIDE	1.080	2.650	958.43	2539.64	2.863	722.35	2068.17
A_68_P21901051	chr2:179777462-179777506	NM_178750:297	Ss18l1	INSIDE	1.080	1.566	2947.62	4615.63	1.691	2650.81	4483.44
A_68_P31826836	chr18:73782887-73782931	ENSMUST00000103753:-1673		PROMOTER	1.080	1.586	581.03	921.63	1.713	522.88	895.68
A_68_P31422206	chr17:87681968-87682012	NR_015506:164	4833418N02Rik	INSIDE	1.079	1.609	915.83	1473.86	1.737	770.39	1338.11
A_68_P21774855	chr2:156866013-156866057	NM_001164663:38967	9830001H06Rik	INSIDE	1.079	3.132	487.81	1527.71	3.380	415.22	1403.27
A_68_P31599408	chr18:31920327-31920372	NM_153515:816	Ammecr1l	INSIDE	1.079	1.533	1633.84	2504.64	1.654	1132.02	1872.67
A_68_P26459688	chr9:42913910-42913954	NM_027144:-131	Arhgef12	PROMOTER	1.079	2.318	406.31	941.75	2.502	531.04	1328.68
A_68_P31564332	chr18:25327565-25327609	NM_001033532:66	AW554918	INSIDE	1.079	1.955	595.66	1164.41	2.109	551.44	1163.19
A_68_P25948481	chr8:72406321-72406365	NM_026818:4949	Cilp2	INSIDE	1.079	2.303	966.50	2225.58	2.484	875.81	2175.71
A_68_P24122107	chr5:137044683-137044727	NM_009986:-1403	Cux1	PROMOTER	1.079	1.641	442.58	726.35	1.771	436.97	773.82
A_68_P30010734	chr15:10399494-10399539	NM_030046:755	Dnajc21	INSIDE	1.079	2.893	470.80	1361.79	3.121	503.18	1570.42

ProbeName	Target position of probe on CpG island microarray	TargetID	GeneSymbol	CpG island Description	Ratio of relative methylation (TiO_2-NP/Vehicle)	Sham group			TiO_2-H group		
						Relative methylation (Cy5/Cy3)	Cy3 signal (Input)	Cy5 signal (MeDIP)	Relative methylation (Cy5/Cy3)	Cy3 signal (Input)	Cy5 signal (MeDIP)
A_68_P25565208	chr7:144505952-144505996	NM_001113414:154	Ebf3	INSIDE	1.079	2.957	598.24	1769.23	3.192	563.20	1797.99
A_68_P20562756	chr1:122499449-122499493	NM_010133:407	En1	INSIDE	1.079	1.480	1523.21	2254.00	1.597	1317.12	2103.82
A_68_P31214871	chr17:48041296-48041340	NM_001110824:20263	Foxp4	INSIDE	1.079	1.418	1234.48	1750.63	1.531	971.42	1486.99
A_68_P30580077	chr16:18498909-18498953	NM_023120:-33	Gnb1l	PROMOTER	1.079	1.525	921.52	1405.22	1.645	843.32	1387.48
A_68_P30482739	chr15:99705810-99705854	NM_001113545:55	Lima1	INSIDE	1.079	1.401	2145.87	3006.05	1.511	1822.19	2753.12
A_68_P29425004	chr14:16271153-16271197	NM_146052:327	Lrrc3b	INSIDE	1.079	1.974	1095.17	2161.49	2.129	925.23	1970.21
A_68_P27287759	chr10:80616606-80616650	NM_021501:13837	Pias4	DOWNSTREAM	1.079	2.959	692.09	2048.17	3.193	636.26	2031.37
A_68_P30390280	chr15:83555410-83555454	NM_022723:19	Scube1	INSIDE	1.079	2.419	753.21	1821.69	2.608	764.83	1995.01
A_68_P26591325	chr9:65972739-65972783	NM_019727:-67	Snx1	DIVERGENT_PROMOTER	1.079	1.924	2572.37	4949.01	2.076	2215.56	4599.79
A_68_P20322000	chr1:69873617-69873661	NM_029160:77	Spag16	INSIDE	1.079	0.538	1306.32	703.40	0.581	1074.64	624.13
A_68_P24593030	chr6:83318306-83318350	NM_183138:73344	Tet3	INSIDE	1.079	2.358	790.16	1863.46	2.545	541.70	1378.72
A_68_P27848242	chr11:61221514-61221558	ENSMUST00000121719:8781		DOWNSTREAM	1.079	1.971	588.37	1159.59	2.126	543.62	1155.95
A_68_P26347944	chr9:21397144-21397188	NM_178619:1	1810026J23Rik	INSIDE	1.078	1.400	1296.34	1814.42	1.509	1053.54	1589.38
A_68_P26242727	chr8:126506886-126506930	NM_019552:114	Abcb10	INSIDE	1.078	1.899	585.73	1112.47	2.047	555.28	1136.82
A_68_P25597930	chr7:150154345-150154389	NM_008554:803	Ascl2	INSIDE	1.078	1.679	552.15	927.15	1.809	510.03	922.81
A_68_P29369780	chr13:119503164-119503208	NM_008002:-319	Fgf10	PROMOTER	1.078	1.516	1114.00	1688.30	1.634	928.25	1516.71
A_68_P29071667	chr13:58229346-58229390	NM_029872:549	Hnrnpa0	INSIDE	1.078	1.940	1563.14	3032.76	2.092	1369.68	2865.29
A_68_P30433466	chr15:90879641-90879685	NM_001109040:717	Kif21a	INSIDE	1.078	2.587	1505.35	3893.82	2.788	1061.34	2958.71
A_68_P24385097	chr6:39761053-39761097	NM_001010930:-139	Mrps33	PROMOTER	1.078	1.674	1580.80	2646.78	1.804	1307.30	2358.52
A_68_P20418213	chr1:89406454-89406498	NM_019867:461	Ngef	INSIDE	1.078	0.554	1066.15	590.96	0.598	894.26	534.44
A_68_P27483042	chr10:117229727-117229771	NM_134010:13	Nup107	INSIDE	1.078	1.711	723.11	1237.56	1.846	720.16	1329.08
A_68_P28946211	chr13:36060705-36060749	NM_029628:952	Ppp1r3g	INSIDE	1.078	2.598	870.26	2261.21	2.800	730.42	2045.41
A_68_P25427828	chr7:120098728-120098772	NM_175279:1275	Rassf10	INSIDE	1.078	1.446	734.25	1062.04	1.560	649.71	1013.32
A_68_P26872698	chr9:119394966-119395010	NM_021544:93146	Scn5a	INSIDE	1.078	1.925	817.81	1573.94	2.075	645.15	1338.74
A_68_P27630973	chr11:20232357-20232401	NM_018861:338	Slc1a4	INSIDE	1.078	0.540	1228.82	663.41	0.582	1068.08	621.84
A_68_P28357645	chr12:37043657-37043701	NM_025312:2923	Sostdc1	INSIDE	1.078	1.680	667.21	1121.04	1.811	597.50	1082.35
A_68_P31802712	chr18:69505723-69505767	NM_001083967:370	Tcf4	INSIDE	1.078	0.628	1732.31	1088.73	0.678	1465.99	993.56
A_68_P31201713	chr17:45683888-45683932	NM_001013749:2716	Tmem151b	INSIDE	1.078	1.767	562.80	994.66	1.905	442.37	842.69
A_68_P28159935	chr11:116403194-116403238	NM_173755:39545	Ube2o	INSIDE	1.078	2.007	1088.81	2185.09	2.164	951.58	2058.87
A_68_P25349897	chr7:104474502-104474546	NM_001177412:-50	Usp35	DIVERGENT_PROMOTER	1.078	2.459	1831.48	4503.34	2.651	1575.47	4175.82
A_68_P22964623	chr4:62131950-62131994	NM_023597:-66	Wdr31	DIVERGENT_PROMOTER	1.078	2.056	574.20	1180.53	2.217	512.48	1135.98
A_68_P29703312	chr14:71166447-71166491	NM_023045:-33	Xpo7	DIVERGENT_PROMOTER	1.078	2.789	409.11	1140.91	3.006	359.01	1079.07
A_68_P31092440	chr17:23713162-23713206	NM_011747:23270	Zfp13	INSIDE	1.078	2.504	1189.02	2977.49	2.701	965.76	2608.10
A_68_P28336342	chr12:33064298-33064342	NM_001162903:667	2010109K11Rik	INSIDE	1.077	2.382	546.52	1302.02	2.566	493.92	1267.26
A_68_P22142066	chr3:53282945-53282989	NM_173382:15228	2810046L04Rik	INSIDE	1.077	2.661	2085.95	5549.77	2.865	1645.65	4715.00
A_68_P24008812	chr5:115729714-115729758	NM_026504:26	Coq5	INSIDE	1.077	1.747	671.62	1173.45	1.882	655.67	1233.76
A_68_P28446510	chr12:55304616-55304660	NM_028133:223	Egln3	INSIDE	1.077	2.054	522.12	1072.55	2.212	508.72	1125.28
A_68_P21874503	chr2:174124272-174124316	NM_201617:935	Gnas	INSIDE	1.077	1.529	684.87	1046.89	1.646	615.93	1013.59
A_68_P25090529	chr7:52253176-52253220	NM_016849:169	Irf3	INSIDE	1.077	3.535	1325.06	4684.13	3.806	1166.81	4441.09
A_68_P21840390	chr2:168088577-168088621	NM_001081134:6233	Kcng1	INSIDE	1.077	3.037	4213.25	12796.91	3.271	3132.88	10248.27
A_68_P26321969	chr9:15083907-15083951	NM_144933:383	Med17	INSIDE	1.077	2.562	373.86	957.76	2.758	307.90	849.21
A_68_P32266426	chrX:13063861-13063905	NM_173415:19085	Nyx	INSIDE	1.077	2.471	535.52	1323.03	2.660	274.70	730.75
A_68_P20614487	chr1:133142076-133142120	NM_018750:-343	Rassf5	PROMOTER	1.077	0.578	1301.26	751.59	0.622	1038.32	646.09
A_68_P25414288	chr7:117758585-117758629	NM_177324:-172	Sbf2	PROMOTER	1.077	2.206	783.19	1727.71	2.377	731.47	1738.51
A_68_P21266438	chr2:61649821-61649865	NM_009322:7333	Tbr1	INSIDE	1.077	2.900	603.50	1750.02	3.123	553.44	1728.40
A_68_P28538087	chr12:74646709-74646753	NM_178715:652	Tmem30b	INSIDE	1.077	2.743	759.87	2084.56	2.955	637.27	1883.10
A_68_P26154295	chr8:111474184-111474229	NM_007496:235663	Zfhx3	INSIDE	1.077	3.115	1123.79	3500.89	3.356	883.92	2966.63
A_68_P31092622	chr17:23747282-23747326	NM_001033425:9482	Zscan10	INSIDE	1.077	1.982	747.96	1482.47	2.135	606.33	1294.51
A_68_P32554231	chrX:96412395-96412439			Unknown	1.077	2.034	2212.71	4501.10	2.192	1277.74	2800.61
A_68_P29102523	chr13:65361108-65361152	ENSMUST00000099425:284		INSIDE	1.077	1.912	464.78	888.47	2.058	410.84	845.70
A_68_P23801180	chr5:74488973-74489017	NR_015531:-113	2700023E23Rik	PROMOTER	1.076	2.452	1241.04	3042.83	2.639	1069.01	2821.04

ProbeName	Target position of probe on CpG island microarray	TargetID	GeneSymbol	CpG island Description	Ratio of relative methylation (TiO_2-NP/Vehicle)	Sham group			TiO_2-H group		
						Relative methylation (Cy5/Cy3)	Cy3 signal (Input)	Cy5 signal (MeDIP)	Relative methylation (Cy5/Cy3)	Cy3 signal (Input)	Cy5 signal (MeDIP)
A_68_P31499176	chr18:12327130-12327174	NM_029623:-86	3110002H16Rik	PROMOTER	1.076	1.704	536.63	914.34	1.834	497.05	911.51
A_68_P32143650	chr19:46469545-46469589	NM_016860:659	Actr1a	INSIDE	1.076	4.088	3386.97	13844.71	4.397	2397.83	10544.04
A_68_P30645474	chr16:31081440-31081484	NM_198626:56	AI480653	INSIDE	1.076	3.419	762.59	2607.41	3.680	634.02	2333.29
A_68_P24735709	chr6:108733062-108733106	NM_026011:32	Arl8b	INSIDE	1.076	0.643	1563.37	1004.57	0.692	1296.01	896.31
A_68_P24800930	chr6:120866663-120866707	NM_007544:154	Bid	INSIDE	1.076	1.426	2162.82	3085.01	1.535	1746.23	2681.08
A_68_P24631554	chr6:90259619-90259663	NM_027928:15539	Chst13	INSIDE	1.076	2.274	555.12	1262.56	2.447	549.58	1344.71
A_68_P25371478	chr7:108257374-108257418	NM_001146010:108	Fchsd2	INSIDE	1.076	1.650	2019.03	3330.68	1.775	1526.09	2708.13
A_68_P30631086	chr16:28564499-28564543	NM_010199:188768	Fgf12	INSIDE	1.076	2.233	764.97	1708.10	2.402	672.14	1614.74
A_68_P28293548	chr12:25785727-25785771	NM_010496:-4791	Id2	PROMOTER	1.076	2.409	2706.49	6519.67	2.592	2173.80	5634.35
A_68_P25095112	chr7:53050502-53050546	NM_001005511:11208	Lmtk3	INSIDE	1.076	2.825	935.66	2643.11	3.039	808.11	2455.56
A_68_P23428418	chr4:153386065-153386109	NM_201226:175	Lrrc47	INSIDE	1.076	4.769	644.55	3073.55	5.130	530.64	2722.12
A_68_P25424701	chr7:119571229-119571273	NM_020606:31	Parva	INSIDE	1.076	1.728	716.98	1238.97	1.860	583.11	1084.57
A_68_P27230712	chr10:70062823-70062867	NM_178621:-805	Phyhipl	PROMOTER	1.076	1.509	1085.49	1637.65	1.624	951.35	1544.51
A_68_P27901222	chr11:70353684-70353728	NM_008876:41	Pld2	INSIDE	1.076	0.586	1130.51	662.51	0.630	929.07	585.65
A_68_P31107656	chr17:26122124-26122168	NM_153140:36659	Rab11fip3	DOWNSTREAM	1.076	1.519	1016.75	1544.33	1.635	871.14	1424.14
A_68_P26579233	chr9:63801395-63801439	NM_008542:68450	Smad6	INSIDE	1.076	1.661	828.55	1376.32	1.787	785.52	1403.60
A_68_P27992700	chr11:86940726-86940770	NM_197987:170	Trim37	INSIDE	1.076	6.146	231.32	1421.65	6.615	258.04	1707.03
A_68_P24428347	chr6:48569751-48569795	NM_173429:6594	Zfp775	INSIDE	1.076	1.977	783.47	1549.15	2.128	604.91	1287.51
A_68_P20038596	A_68_P20038596			Unknown	1.076	1.638	817.49	1339.39	1.762	747.56	1317.46
A_68_P24059259	chr5:124540142-124540186	NM_019875:5643	Abcb9	INSIDE	1.075	2.290	1497.55	3429.39	2.462	1143.95	2816.15
A_68_P22321216	chr3:89519299-89519343	NM_001038587:377	Adar	INSIDE	1.075	2.404	904.82	2175.30	2.585	816.35	2110.58
A_68_P28751741	chr12:113912366-113912410	NM_001165894:99	Akt1	INSIDE	1.075	1.850	565.86	1046.91	1.989	554.20	1102.27
A_68_P30542889	chr16:10545693-10545737	NM_001204229:283	Clec16a	INSIDE	1.075	0.565	1526.95	862.85	0.607	1143.88	694.64
A_68_P23279249	chr4:125963943-125963987	NM_199473:-73	Col8a2	PROMOTER	1.075	3.385	2655.88	8990.38	3.638	2397.93	8724.28
A_68_P26543014	chr9:57493074-57493118	NM_007783:-109	Csk	PROMOTER	1.075	0.482	1192.25	575.02	0.518	985.15	510.72
A_68_P31161105	chr17:35960212-35960256	NM_008027:-67	Flot1	PROMOTER	1.075	1.463	899.60	1315.98	1.573	860.01	1353.00
A_68_P29257128	chr13:99125244-99125288	NM_008242:1067	Foxd1	INSIDE	1.075	1.950	1163.48	2269.12	2.096	1029.83	2159.02
A_68_P25951930	chr8:72975613-72975657	NM_001122830:25208	Klhl26	INSIDE	1.075	1.620	904.54	1465.51	1.742	855.74	1490.99
A_68_P24378360	chr6:38586324-38586368	NM_001033171:893	Klrg2	INSIDE	1.075	0.557	1057.67	589.12	0.599	965.04	577.67
A_68_P28582490	chr12:82833202-82833246	NM_001174107:48933	Map3k9	INSIDE	1.075	2.320	342.41	794.50	2.493	331.86	827.41
A_68_P27854128	chr11:62271118-62271162	NM_011308:-306	Ncor1	DIVERGENT_PROMOTER	1.075	2.116	518.80	1097.80	2.275	474.78	1080.12
A_68_P24477532	chr6:56873994-56874038	NM_026004:-90	Nt5c3	PROMOTER	1.075	2.384	315.92	753.10	2.563	321.98	825.27
A_68_P30346595	chr15:76009519-76009563	NM_201394:16600	Plec	INSIDE	1.075	3.383	964.85	3263.94	3.637	848.06	3084.08
A_68_P30651713	chr16:32277691-32277735	NM_027355:166	Rnf168	INSIDE	1.075	1.398	1089.18	1522.73	1.503	1021.13	1534.57
A_68_P23491409	chr5:14025282-14025326	NM_011348:29	Sema3e	INSIDE	1.075	1.411	2172.41	3065.14	1.517	1906.94	2892.16
A_68_P25584911	chr7:147973663-147973707	NM_001143765:69	Syce1	INSIDE	1.075	2.629	765.88	2013.22	2.826	675.00	1907.22
A_68_P24374363	chr6:37821300-37821344	NM_145076:512	Trim24	INSIDE	1.075	2.037	2161.44	4403.23	2.191	1896.05	4153.95
A_68_P30363494	chr15:78798409-78798453	NM_001039155:20277	Triobp	INSIDE	1.075	2.911	4576.66	13321.16	3.128	3181.12	9950.05
A_68_P31323665	chr17:69732730-69732774	NM_009547:-565	Zfp161	PROMOTER	1.075	0.626	3053.22	1909.84	0.672	2725.38	1832.44
A_68_P27033722	chr10:28916520-28916567	NM_026138:52742	6330407J23Rik	INSIDE	1.074	1.710	1249.87	2137.50	1.837	1023.13	1879.42
A_68_P25614033	chr8:3452130-3452174	NM_133962:59145	Arhgef18	INSIDE	1.074	1.960	997.98	1956.00	2.106	795.20	1674.63
A_68_P31038473	chr17:10512600-10512644	NR_037588:-161	B930003M22Rik	DIVERGENT_PROMOTER	1.074	1.612	470.29	758.22	1.732	458.84	794.52
A_68_P28724519	chr12:109154477-109154521	NM_001079883:87126	Bcl11b	INSIDE	1.074	2.790	2650.28	7393.52	2.996	2141.19	6414.49
A_68_P23397278	chr4:148274906-148274950	NM_027195:96428	Casz1	INSIDE	1.074	3.590	4243.14	15231.23	3.855	3263.12	12578.86
A_68_P25637616	chr8:8660606-8660650	NM_010111:145	Efnb2	INSIDE	1.074	1.495	1235.25	1846.59	1.606	1131.29	1816.36
A_68_P23227298	chr4:115690557-115690601	NM_010173:-71	Faah	PROMOTER	1.074	2.041	563.15	1149.64	2.192	547.15	1199.43
A_68_P28577489	chr12:81861416-81861460	NM_001008423:264	Gm1568	INSIDE	1.074	2.530	1439.51	3642.27	2.717	1197.06	3252.59
A_68_P24637119	chr6:91106721-91106765	NM_144919:-66	Hdac11	PROMOTER	1.074	2.520	1255.28	3162.76	2.707	1083.44	2933.12
A_68_P24483218	chr6:58781700-58781744	NM_028705:-1971	Herc3	PROMOTER	1.074	2.150	795.93	1711.36	2.310	686.71	1586.40
A_68_P26400214	chr9:31193743-31193787	NM_172766:-12	Nfrkb	PROMOTER	1.074	2.276	1066.76	2427.81	2.445	924.86	2261.53
A_68_P23435438	chr4:154358119-154358163	NM_001113360:26953	Plch2	INSIDE	1.074	2.426	638.97	1550.26	2.605	558.84	1455.73

ProbeName	Target position of probe on CpG island microarray	TargetID	GeneSymbol	CpG island Description	Ratio of relative methylation (TiO_2-NP/Vehicle)	Sham group			TiO_2-H group		
						Relative methylation (Cy5/Cy3)	Cy3 signal (Input)	Cy5 signal (MeDIP)	Relative methylation (Cy5/Cy3)	Cy3 signal (Input)	Cy5 signal (MeDIP)
A_68_P25499768	chr7:133436020-133436064	NM_145587:19910	Sbk1	INSIDE	1.074	2.866	705.12	2020.95	3.079	598.49	1842.91
A_68_P25090605	chr7:52263349-52263393	NM_001008422:8249	Scaf1	INSIDE	1.074	2.232	558.85	1247.24	2.396	494.64	1185.27
A_68_P31202055	chr17:45732191-45732235	NM_001199113:-1623	Slc29a1	PROMOTER	1.074	1.837	1381.82	2537.95	1.973	1142.71	2254.56
A_68_P29286847	chr13:104563945-104563989	NM_172592:-9304	Srek1	PROMOTER	1.074	1.489	2351.32	3500.96	1.599	1665.93	2663.25
A_68_P31175226	chr17:39982856-39982900	ENSMUST00000157647:-1192		PROMOTER	1.074	2.067	51240.87	105902.50	2.220	40303.34	89483.25
A_68_P23438433	chr4:154783299-154783343	NM_001166029:22	2010015L04Rik	INSIDE	1.073	0.571	1323.91	755.64	0.613	1052.35	644.56
A_68_P30728224	chr16:45742850-45742894	NM_172511:147	Abhd10	INSIDE	1.073	1.996	1583.16	3160.57	2.142	1394.50	2986.70
A_68_P28071366	chr11:100870700-100870746	NM_016920:-46	Atp6v0a1	PROMOTER	1.073	6.364	1580.45	10058.17	6.830	1232.12	8415.00
A_68_P32289635	chrX:20265624-20265668	NM_011049:28	Cdk16	INSIDE	1.073	2.414	723.96	1747.80	2.590	412.28	1067.70
A_68_P30486146	chr15:100325931-100325975	NM_153407:-282	Csrnp2	PROMOTER	1.073	0.584	1409.79	822.86	0.626	1104.17	691.70
A_68_P29241522	chr13:96374151-96374199	NM_010169:14214	F2r	INSIDE	1.073	3.436	1403.80	4823.40	3.685	1042.39	3841.51
A_68_P32028427	chr19:25311713-25311757	NM_181404:43	Kank1	INSIDE	1.073	1.592	1172.85	1867.71	1.708	1084.53	1852.50
A_68_P24776189	chr6:116288297-116288341	NM_027920:178	Marchf8	INSIDE	1.073	1.683	1662.62	2798.91	1.807	1480.71	2675.06
A_68_P24333874	chr6:30688204-30688248	NR_029900:-3072	Mir335	PROMOTER	1.073	2.065	405.20	836.80	2.217	366.46	812.31
A_68_P31127485	chr17:29484900-29484944	NM_019880:-73	Mtch1	PROMOTER	1.073	1.846	1536.11	2836.39	1.982	1213.50	2405.24
A_68_P25846164	chr8:49313858-49313902	NM_001145937:446164	Odz3	INSIDE	1.073	1.720	1092.17	1878.99	1.846	823.93	1521.27
A_68_P26573399	chr9:62828500-62828544	NM_019663:164	Pias1	INSIDE	1.073	1.763	2345.69	4134.86	1.892	2086.12	3946.98
A_68_P22522616	chr3:129582033-129582077	NM_183423:-2047	Pla2g12a	PROMOTER	1.073	7.093	13889.41	98523.11	7.608	8851.94	67343.55
A_68_P28186391	chr11:120582930-120582974	NM_133223:171	Rac3	INSIDE	1.073	2.156	1843.94	3976.11	2.313	1501.64	3473.02
A_68_P32790793	chrX:159198362-159198406	NM_009031:81	Rbbp7	INSIDE	1.073	2.663	296.30	789.04	2.856	199.67	570.28
A_68_P29871953	chr14:104921479-104921523	NM_026047:383	Rnf219	INSIDE	1.073	1.629	465.37	758.21	1.748	428.73	749.31
A_68_P21095250	chr2:28980805-28980849	NM_198033:315	Setx	INSIDE	1.073	2.593	1350.45	3501.48	2.782	1105.95	3077.07
A_68_P20038599	chr1:12982807-12982851	NM_172841:-1612	Slco5a1	PROMOTER	1.073	3.350	6202.21	20776.82	3.594	4614.42	16586.42
A_68_P32464772	chrX:71032781-71032825	NM_009279:321	Ssr4	INSIDE	1.073	1.494	1004.81	1501.53	1.603	588.72	943.93
A_68_P32341134	chrX:39503420-39503464	NM_021465:-435	Stag2	PROMOTER	1.073	2.332	627.42	1463.19	2.502	425.17	1063.94
A_68_P24636064	chr6:90937651-90937695			Unknown	1.073	1.900	1116.61	2121.45	2.038	973.01	1982.95
A_68_P21836345	chr2:167422781-167422825	ENSMUST00000127006:43989		INSIDE	1.073	3.956	1989.37	7869.04	4.246	1491.25	6331.42
A_68_P26807592	chr9:107199260-107199304	NM_009895:263	Cish	INSIDE	1.072	1.520	3516.66	5345.27	1.629	2706.95	4410.47
A_68_P30495773	chr15:101904768-101904812	NM_145625:587	Eif4b	INSIDE	1.072	0.291	1925.63	560.02	0.312	1710.09	533.01
A_68_P24112562	chr5:134789379-134789423	NM_001080746:1216	Gtf2i	INSIDE	1.072	2.096	790.98	1658.23	2.248	707.85	1591.16
A_68_P24634435	chr6:90687050-90687094	NM_001134384:73045	Iqsec1	INSIDE	1.072	2.378	505.07	1200.99	2.549	519.28	1323.41
A_68_P32016045	chr19:23210547-23210591	NM_010638:-5147	Klf9	PROMOTER	1.072	2.519	610.54	1538.06	2.700	608.50	1643.02
A_68_P27971661	chr11:83276581-83276625	NM_080453:-139	Mmp28	PROMOTER	1.072	1.355	1879.87	2547.96	1.454	1684.99	2449.36
A_68_P29368026	chr13:119175523-119175567	NM_021556:515	Mrps30	INSIDE	1.072	2.987	863.92	2580.19	3.202	800.26	2562.16
A_68_P32266425	chrX:13063695-13063739	NM_173415:18919	Nyx	INSIDE	1.072	2.416	982.29	2372.88	2.589	562.57	1456.42
A_68_P29056696	chr13:55828862-55828906	NM_028281:156	Pcbd2	INSIDE	1.072	2.246	597.22	1341.65	2.408	601.40	1448.34
A_68_P31106582	chr17:25969835-25969879	NM_019719:450	Stub1	INSIDE	1.072	2.136	1107.61	2366.10	2.289	952.01	2179.23
A_68_P26037062	chr8:90305072-90305116	NM_033327:178400	Zfp423	INSIDE	1.072	2.129	646.97	1377.45	2.282	570.51	1302.04
A_68_P27534005	chr10:126528987-126529031	NM_001033263:13046	Agap2	INSIDE	1.071	1.944	12855.71	24996.72	2.083	9244.10	19256.38
A_68_P27298385	chr10:83111453-83111497	NM_145220:-65	Appl2	PROMOTER	1.071	1.942	435.80	846.21	2.079	326.23	678.12
A_68_P28450019	chr12:56086318-56086362	NM_013815:983	Baz1a	INSIDE	1.071	1.656	544.69	901.83	1.774	512.86	909.78
A_68_P30480361	chr15:99300972-99301016	NM_029236:4167	Bcdin3d	INSIDE	1.071	2.525	648.39	1637.22	2.704	575.58	1556.42
A_68_P27674147	chr11:29273422-29273466	NM_176841:-330	Ccdc88a	PROMOTER	1.071	2.303	923.17	2126.28	2.466	864.44	2131.50
A_68_P28853776	chr13:17895335-17895379	NM_001081058:1575	Cdk13	INSIDE	1.071	2.216	564.66	1251.35	2.374	463.42	1100.04
A_68_P31227869	chr17:50432800-50432844	NM_010021:102	Dazl	INSIDE	1.071	2.893	591.71	1711.96	3.098	486.05	1505.99
A_68_P30483524	chr15:99869292-99869336	NM_001159361:220	Dip2b	INSIDE	1.071	3.295	1462.67	4819.15	3.528	1311.32	4626.49
A_68_P23317858	chr4:133042766-133042810	NM_175307:6741	Fam46b	INSIDE	1.071	1.791	473.57	848.26	1.919	427.24	819.90
A_68_P26550035	chr9:58671367-58671411	NM_001081192:70	Hcn4	INSIDE	1.071	1.689	1976.89	3338.64	1.809	1485.12	2686.89
A_68_P25005393	chr7:25318304-25318348	NM_153134:2660	Irgq	INSIDE	1.071	1.927	555.56	1070.73	2.063	482.73	996.05
A_68_P29725072	chr14:75346702-75346746	NM_001033439:960	Lrch1	INSIDE	1.071	1.657	818.49	1356.26	1.774	803.53	1425.41
A_68_P21421185	chr2:91022943-91022987	NM_001177720:-3486	Madd	PROMOTER	1.071	3.002	1125.65	3379.66	3.216	975.03	3135.34

ProbeName	Target position of probe on CpG island microarray	TargetID	GeneSymbol	CpG island Description	Ratio of relative methylation (TiO_2-NP/Vehicle)	Sham group			TiO_2-H group		
						Relative methylation (Cy5/Cy3)	Cy3 signal (Input)	Cy5 signal (MeDIP)	Relative methylation (Cy5/Cy3)	Cy3 signal (Input)	Cy5 signal (MeDIP)
A_68_P25956626	chr8:73904570-73904614	NM_010150:1259	Nr2f6	INSIDE	1.071	1.990	658.39	1310.35	2.131	581.25	1238.39
A_68_P21095669	chr2:29050154-29050198	NM_133500:53384	Ntng2	DOWNSTREAM	1.071	1.693	1465.30	2481.14	1.814	1219.03	2211.03
A_68_P26504100	chr9:50336652-50336696	NM_011220:72	Pts	INSIDE	1.071	2.247	513.32	1153.34	2.406	472.49	1136.98
A_68_P26165185	chr8:113370432-113370477	NR_028559:-7956	Snord111	PROMOTER	1.071	0.562	1168.45	656.78	0.602	1106.26	665.91
A_68_P30253390	chr15:59146277-59146321	NM_009270:-348	Sqle	PROMOTER	1.071	1.662	851.93	1415.64	1.780	723.87	1288.32
A_68_P31612301	chr18:34490799-34490843	NM_025527:22	Srp19	INSIDE	1.071	2.056	1161.83	2389.23	2.201	975.79	2148.17
A_68_P30250405	chr15:58655411-58655455	NM_175212:-450	Tmem65	PROMOTER	1.071	1.806	830.32	1499.75	1.935	683.58	1322.54
A_68_P24428346	chr6:48569573-48569617	NM_173429:6416	Zfp775	INSIDE	1.071	1.804	411.54	742.26	1.931	314.33	607.02
A_68_P32462527	chrX:70667702-70667746	NM_009566:11283	Zfp92	INSIDE	1.071	1.685	1026.63	1729.37	1.804	494.28	891.53
A_68_P32783799	chrX:157736143-157736189	ENSMUST00000141946:10779		INSIDE	1.071	3.167	1363.28	4317.58	3.391	568.23	1926.81
A_68_P32319554	chrX:34185327-34185371	NM_001081956:61	Akap17b	INSIDE	1.070	1.844	825.37	1521.95	1.973	477.44	941.88
A_68_P24046043	chr5:122162421-122162465	NM_009125:825	Atxn2	INSIDE	1.070	2.130	1666.70	3549.36	2.279	1404.11	3200.24
A_68_P24946541	chr7:3597064-3597108	NM_146176:216	Cnot3	INSIDE	1.070	1.908	461.46	880.33	2.041	361.20	737.32
A_68_P23417815	chr4:151671322-151671366	NM_133788:22	Icmt	INSIDE	1.070	2.202	809.29	1782.35	2.357	727.75	1715.59
A_68_P21908760	chr2:180869636-180869680	NM_001003824:272	Kcnq2	INSIDE	1.070	2.253	545.18	1228.40	2.412	503.17	1213.43
A_68_P25954900	chr8:73438460-73438504	NM_173013:8610	Mtap1s	INSIDE	1.070	2.439	1248.31	3044.72	2.609	1034.02	2697.92
A_68_P26133402	chr8:107898609-107898653	NM_001081333:-650	Plekhg4	PROMOTER	1.070	1.966	2703.44	5315.59	2.104	2276.92	4791.54
A_68_P26453529	chr9:41932012-41932056	NM_011436:338	Sorl1	INSIDE	1.070	2.168	652.09	1413.91	2.319	624.90	1449.43
A_68_P25508613	chr7:135153669-135153713	NM_001079932:5799	Trim72	INSIDE	1.070	2.699	4632.02	12500.85	2.887	3522.92	10169.13
A_68_P24124221	chr5:137507075-137507119	NM_001039385:932	Vgf	INSIDE	1.070	1.416	899.29	1273.07	1.514	896.06	1356.98
A_68_P31925604	chr19:4878831-4878875	NM_001168517:163	Zdhhc24	INSIDE	1.070	1.825	2497.29	4557.02	1.952	2035.55	3973.72
A_68_P26077485	chr8:96958377-96958421	NR_015514:605	9330175E14Rik	INSIDE	1.069	2.252	3191.46	7186.08	2.407	2541.57	6118.28
A_68_P29523396	chr14:34133331-34133375	NM_153800:103344	Arhgap22	INSIDE	1.069	0.613	1444.11	885.39	0.655	1243.71	814.90
A_68_P31369867	chr17:78599714-78599759	NM_015800:149	Crim1	INSIDE	1.069	1.578	1214.56	1916.59	1.688	1156.97	1952.47
A_68_P29335935	chr13:113442657-113442701	NM_001145885:-160	Ddx4	DIVERGENT_PROMOTER	1.069	2.259	452.55	1022.28	2.415	435.72	1052.33
A_68_P25547706	chr7:141862876-141862920	NM_001033420:529	Dock1	INSIDE	1.069	1.753	929.22	1629.38	1.874	864.22	1619.67
A_68_P27997243	chr11:87800391-87800435	NM_001168472:-325	Dynll2	PROMOTER	1.069	0.479	2199.34	1054.57	0.513	1883.23	965.68
A_68_P26986428	chr10:20067570-20067614	NM_027930:-32	Fam54a	PROMOTER	1.069	1.670	836.41	1396.86	1.785	790.88	1411.88
A_68_P26761435	chr9:98856857-98856901	NM_012020:853	Foxl2	INSIDE	1.069	0.518	1493.26	773.99	0.554	1279.99	709.43
A_68_P28748130	chr12:113382569-113382613	NM_001097621:-1828	Kif26a	PROMOTER	1.069	2.404	349.89	841.26	2.571	297.08	763.77
A_68_P23809993	chr5:75971506-75971550	NM_021099:513	Kit	INSIDE	1.069	1.780	420.38	748.18	1.903	412.88	785.80
A_68_P27262524	chr10:75994246-75994290	NM_146006:-103	Lss	PROMOTER	1.069	1.923	491.53	945.06	2.056	441.10	906.99
A_68_P28178600	chr11:119411075-119411119	NM_008730:-1962	Nptx1	PROMOTER	1.069	1.757	638.48	1122.01	1.878	636.03	1194.77
A_68_P27280129	chr10:79440293-79440337	NM_001003949:6761	ORF61	INSIDE	1.069	3.303	2255.16	7447.80	3.530	1815.09	6407.46
A_68_P31846729	chr18:77493099-77493143	NM_013666:68535	St8sia5	INSIDE	1.069	2.766	1461.26	4042.37	2.958	1155.86	3419.28
A_68_P26023336	chr8:87561629-87561686	NM_001122843:591	Tnpo2	INSIDE	1.069	3.364	268.81	904.32	3.596	224.13	805.87
A_68_P27234635	chr10:70747809-70747853	NM_145420:180	Ube2d1	INSIDE	1.069	0.596	1429.21	852.29	0.637	1296.95	826.80
A_68_P22417418	chr3:109143649-109143693	NM_020505:70	Vav3	INSIDE	1.069	2.461	389.83	959.42	2.631	408.05	1073.51
A_68_P23416419	chr4:151460529-151460574	ENSMUST00000118648:-415		PROMOTER	1.069	3.478	984.81	3424.77	3.718	797.52	2965.00
A_68_P24686643	chr6:99471350-99471394	ENSMUST00000155466:145419		INSIDE	1.069	0.613	2466.91	1512.10	0.655	2035.70	1333.82
A_68_P24006302	chr5:115263981-115264025	NR_027817:-17	1500011B03Rik	PROMOTER	1.068	1.838	1942.54	3571.14	1.964	1463.30	2873.76
A_68_P27812108	chr11:54600567-54600611	NM_178626:617	Cdc42se2	INSIDE	1.068	0.594	1334.22	792.35	0.634	1091.79	692.69
A_68_P24138647	chr5:140447479-140447523	NM_175522:63604	Elfn1	INSIDE	1.068	2.193	901.68	1977.41	2.343	824.47	1931.59
A_68_P30361918	chr15:78549192-78549236	NM_183141:-671	Elfn2	PROMOTER	1.068	1.752	2668.57	4674.10	1.871	1948.84	3646.39
A_68_P29287630	chr13:104711739-104711783	NM_001005868:-1166	Erbb2ip	PROMOTER	1.068	5.682	3000.78	17049.53	6.068	2284.84	13864.31
A_68_P23969235	chr5:108298266-108298310	NM_007964:5838	Evi5	INSIDE	1.068	1.708	1535.23	2622.70	1.824	1358.86	2478.50
A_68_P24620355	chr6:88148400-88148444	NM_008090:-235	Gata2	PROMOTER	1.068	2.105	1239.12	2608.22	2.248	1173.52	2637.73
A_68_P29097469	chr13:64263530-64263574	NM_019986:379	Habp4	INSIDE	1.068	2.541	414.14	1052.23	2.714	445.10	1207.80
A_68_P27812443	chr11:54679796-54679846	NM_008248:-119	Hint1	DIVERGENT_PROMOTER	1.068	2.105	261.60	550.65	2.249	216.90	487.70
A_68_P28043747	chr11:96181632-96181676	NM_010459:2074	Hoxb4	INSIDE	1.068	1.769	556.46	984.65	1.890	535.78	1012.81
A_68_P21137728	chr2:35946930-35946974	NM_001083126:12628	Lhx6	INSIDE	1.068	1.733	654.95	1135.16	1.852	542.36	1004.23

ProbeName	Target position of probe on CpG island microarray	TargetID	GeneSymbol	CpG island Description	Ratio of relative methylation (TiO_2-NP/Vehicle)	Sham group			TiO_2-H group		
						Relative methylation (Cy5/Cy3)	Cy3 signal (Input)	Cy5 signal (MeDIP)	Relative methylation (Cy5/Cy3)	Cy3 signal (Input)	Cy5 signal (MeDIP)
A_68_P23189553	chr4:106926047-106926091	NM_029985:70	Lrrc42	INSIDE	1.068	1.518	1928.94	2927.35	1.621	1541.12	2497.59
A_68_P31503253	chr18:13020852-13020896	NM_207530:79355	Osbpl1a	INSIDE	1.068	2.496	918.50	2292.39	2.665	824.15	2196.29
A_68_P21818280	chr2:164332796-164332840	NM_133779:9794	Pigt	INSIDE	1.068	3.026	1989.20	6018.73	3.232	1197.74	3870.93
A_68_P29681987	chr14:67530058-67530102	NM_175498:36	Pnma2	INSIDE	1.068	0.635	2489.00	1581.34	0.679	2020.23	1370.76
A_68_P25574426	chr7:146038756-146038800	NM_026391:710	Ppp2r2d	INSIDE	1.068	2.181	1217.08	2654.37	2.328	966.88	2251.22
A_68_P27807840	chr11:53841165-53841209	NM_019687:406	Slc22a4	INSIDE	1.068	1.794	1193.11	2140.03	1.916	1017.07	1948.55
A_68_P24983341	chr7:17396988-17397032	NM_001039878:-4227	Strn4	PROMOTER	1.068	1.901	2335.08	4438.41	2.029	1975.72	4009.50
A_68_P22383505	chr3:102740035-102740079	NM_011516:-33	Sycp1	PROMOTER	1.068	1.583	1047.06	1657.13	1.690	899.22	1519.52
A_68_P30574322	chr16:17530755-17530799	NM_026909:369	Thap7	INSIDE	1.068	0.578	2458.96	1420.16	0.617	1782.36	1099.53
A_68_P30991291	chr16:96366989-96367033	NM_207301:-15	Wrb	PROMOTER	1.068	1.414	1096.66	1551.14	1.511	874.97	1322.03
A_68_P31342569	chr17:73186874-73186918	NM_027166:853	Ypel5	INSIDE	1.068	1.492	1088.88	1624.08	1.592	943.62	1502.60
A_68_P28950039	chr13:36670467-36670511	AK135154:190947		INSIDE	1.068	1.815	599.71	1088.65	1.939	579.69	1124.26
A_68_P24619475	chr6:87994860-87994904	ENSMUST00000113598:129		INSIDE	1.068	1.506	781.69	1177.05	1.608	776.84	1249.39
A_68_P28182344	chr11:119959816-119959860	NM_183137:207	2410002I01Rik	INSIDE	1.067	0.635	2000.66	1269.49	0.677	1506.96	1020.30
A_68_P20354314	chr1:75472567-75472611	NM_001001565:3458	Chpf	INSIDE	1.067	2.115	855.37	1809.19	2.256	586.08	1322.21
A_68_P32567803	chrX:99446637-99446681	NM_007709:450	Cited1	INSIDE	1.067	1.578	1156.58	1824.62	1.683	574.61	966.80
A_68_P27897959	chr11:69734325-69734369	NM_001166590:-214	Eif5a	PROMOTER	1.067	0.532	1355.27	721.46	0.568	1116.84	634.31
A_68_P21202298	chr2:49306701-49306745	NM_172663:-283	Epc2	PROMOTER	1.067	2.469	688.54	1699.97	2.635	602.74	1588.28
A_68_P27594240	chr11:11925551-11925597	NM_001177629:2400	Grb10	INSIDE	1.067	1.888	872.01	1645.98	2.014	699.64	1409.01
A_68_P24798117	chr6:120314589-120314634	NM_145997:495	Kdm5a	INSIDE	1.067	2.106	450.76	949.53	2.248	385.06	865.43
A_68_P23658795	chr5:46248998-46249042	NM_178142:-241	Lcorl	PROMOTER	1.067	1.616	1180.70	1908.36	1.724	993.44	1712.92
A_68_P26980659	chr10:19076945-19076989	NM_053008:622	Olig3	INSIDE	1.067	1.491	3743.93	5581.89	1.591	2784.14	4430.55
A_68_P26815241	chr9:108420024-108420068	NM_001114119:629	Qrich1	INSIDE	1.067	1.618	489.89	792.64	1.727	464.16	801.48
A_68_P32599600	chrX:108576921-108576965	NM_025949:74612	Rps6ka6	INSIDE	1.067	2.037	1127.39	2295.95	2.172	737.79	1602.60
A_68_P23338973	chr4:136833633-136833677	NM_009523:105	Wnt4	INSIDE	1.067	2.461	590.60	1453.17	2.626	506.47	1330.03
A_68_P26023270	chr8:87551092-87551137	NM_026760:383	2310036O22Rik	INSIDE	1.066	1.773	1876.67	3326.65	1.890	1547.29	2924.30
A_68_P28167621	chr11:117722126-117722170	NM_001085535:83	Cldn27	INSIDE	1.066	2.088	290.76	607.19	2.226	278.10	619.04
A_68_P24906446	chr6:142705458-142705502	NM_009908:275	Cmas	INSIDE	1.066	0.522	1282.60	669.67	0.557	1036.94	577.15
A_68_P27263162	chr10:76086244-76086288	NM_146007:-117	Col6a2	PROMOTER	1.066	1.768	1103.03	1950.02	1.884	880.30	1658.62
A_68_P27272484	chr10:77632233-77632277	NM_138601:259	D10Jhu81e	INSIDE	1.066	0.591	1279.44	756.61	0.630	1051.98	662.99
A_68_P29446598	chr14:21208189-21208233	NM_134081:-78	Dnajc9	PROMOTER	1.066	2.422	1119.97	2712.50	2.582	978.53	2526.78
A_68_P25827947	chr8:46108650-46108696	NM_001081286:73111	Fat1	INSIDE	1.066	2.500	368.52	921.47	2.665	345.10	919.73
A_68_P32139834	chr19:45734660-45734704	NM_013907:1	Fbxw4	INSIDE	1.066	1.489	2500.73	3723.63	1.587	1882.33	2987.01
A_68_P20510204	chr1:108656008-108656052	NM_027534:289	Kdsr	INSIDE	1.066	1.579	1322.12	2087.69	1.683	1187.20	1997.81
A_68_P25016729	chr7:28116317-28116361	NM_001113549:2329	Ltbp4	INSIDE	1.066	1.541	854.92	1317.85	1.642	708.89	1164.33
A_68_P25953978	chr8:73300295-73300339	NM_008841:295	Pik3r2	INSIDE	1.066	1.444	1291.32	1864.47	1.539	1033.67	1590.69
A_68_P24117614	chr5:136210246-136210290	NM_008898:45185	Por	INSIDE	1.066	2.178	1217.63	2651.88	2.321	1026.64	2382.86
A_68_P21091624	chr2:28389115-28389159	NM_001145835:153	Ralgds	INSIDE	1.066	1.744	712.69	1243.09	1.859	642.17	1193.73
A_68_P25094160	chr7:52888072-52888116	NM_028544:5188	Rasip1	INSIDE	1.066	2.168	737.80	1599.44	2.311	691.74	1598.91
A_68_P31256957	chr17:56706054-56706098	NM_001029979:17930	Safb2	INSIDE	1.066	2.415	366.58	885.23	2.574	357.62	920.35
A_68_P27167593	chr10:58684415-58684459	NM_001024910:159	Septin10	INSIDE	1.066	2.713	435.09	1180.55	2.893	430.32	1244.77
A_68_P23590297	chr5:33995249-33995293	NM_009193:-315	Slbp	PROMOTER	1.066	0.513	1039.37	533.38	0.547	929.15	508.24
A_68_P23353414	chr4:139225201-139225245	NM_031873:15770	Tas1r2	INSIDE	1.066	1.735	630.17	1093.56	1.851	531.26	983.19
A_68_P23998980	chr5:114037914-114037958	NM_177292:118470	Wscd2	INSIDE	1.066	2.233	1300.40	2903.15	2.380	998.19	2375.29
A_68_P31669245	chr18:44988148-44988192	NM_001163013:-148	Ythdc2	PROMOTER	1.066	1.960	568.50	1114.09	2.090	490.84	1025.65
A_68_P22630568	chr3:148652007-148652051	ENSMUST00000098518:252		INSIDE	1.066	1.830	765.09	1400.31	1.951	741.71	1447.07
A_68_P23978457	chr5:110660379-110660423	NM_027922:341	Ankle2	INSIDE	1.065	1.420	1244.39	1767.16	1.512	1051.58	1589.95
A_68_P31926489	chr19:5039460-5039504	NM_175383:657	B3gnt1	INSIDE	1.065	1.696	2170.05	3679.70	1.806	1716.78	3101.32
A_68_P25093322	chr7:52721892-52721936	NM_007527:354	Bax	INSIDE	1.065	1.464	1172.94	1717.28	1.559	999.87	1558.81
A_68_P20137272	chr1:34234902-34234946	NM_133833:166255	Dst	INSIDE	1.065	3.338	574.40	1917.21	3.555	529.93	1884.15
A_68_P29712956	chr14:72931825-72931869	NM_207636:177964	Fndc3a	DOWNSTREAM	1.065	1.912	1643.13	3140.92	2.036	1410.63	2871.85

ProbeName	Target position of probe on CpG island microarray	TargetID	GeneSymbol	CpG island Description	Ratio of relative methylation (TiO_2-NP/Vehicle)	Sham group			TiO_2-H group		
						Relative methylation (Cy5/Cy3)	Cy3 signal (Input)	Cy5 signal (MeDIP)	Relative methylation (Cy5/Cy3)	Cy3 signal (Input)	Cy5 signal (MeDIP)
A_68_P23250423	chr4:119955345-119955389	NM_194060:4500	Foxo6	INSIDE	1.065	1.435	2217.29	3182.43	1.529	1976.76	3021.94
A_68_P28558329	chr12:78339501-78339545	NM_016893:-485	Fut8	PROMOTER	1.065	1.640	679.96	1115.02	1.746	554.76	968.50
A_68_P28577467	chr12:81858507-81858551	NM_001008423:3174	Gm1568	INSIDE	1.065	2.221	793.23	1761.72	2.365	672.98	1591.65
A_68_P26891191	chr9:122656494-122656538	NM_001199736:53	Gm9524	INSIDE	1.065	2.823	1069.85	3019.78	3.007	871.87	2621.91
A_68_P21874515	chr2:174125570-174125614	NM_201617:2233	Gnas	INSIDE	1.065	1.514	633.41	959.05	1.613	589.31	950.39
A_68_P25011764	chr7:26539484-26539528	NM_178089:214	Hnrnpul1	INSIDE	1.065	0.533	1101.26	587.23	0.568	928.86	527.38
A_68_P26026700	chr8:88364563-88364607	NM_028007:264	Itfg1	INSIDE	1.065	1.915	835.48	1599.64	2.038	745.02	1518.50
A_68_P29616394	chr14:55732300-55732344	NM_177049:3450	Jph4	INSIDE	1.065	2.290	349.86	801.22	2.438	357.50	871.64
A_68_P24816534	chr6:124662607-124662651	NM_007531:322	Phb2	INSIDE	1.065	0.507	2195.04	1112.58	0.540	1679.32	906.21
A_68_P27034633	chr10:29081479-29081523	NM_001110196:748	Rnf146	INSIDE	1.065	0.588	1387.27	816.22	0.627	1299.81	814.71
A_68_P29675225	chr14:66349795-66349839	NM_001168318:64577	Scara5	INSIDE	1.065	1.946	850.29	1654.80	2.073	748.60	1551.83
A_68_P22523675	chr3:129763103-129763147	NM_207209:701	Sec24b	INSIDE	1.065	2.126	1415.44	3008.78	2.264	1355.15	3067.49
A_68_P28730170	chr12:110065141-110065185	NM_181328:8924	Slc25a29	INSIDE	1.065	2.184	557.17	1216.63	2.324	500.81	1164.10
A_68_P31099197	chr17:24856900-24856944	NR_027900:-12	Snhg9	DIVERGENT_PROMOTER	1.065	0.622	2445.04	1519.80	0.662	1921.63	1272.48
A_68_P21813370	chr2:163428210-163428254	NM_029512:183	Ttpal	INSIDE	1.065	1.433	2253.30	3229.02	1.525	1857.85	2834.10
A_68_P27446662	chr10:110447016-110447060	NM_172554:84	Zdhhc17	INSIDE	1.065	1.596	821.53	1310.95	1.699	822.01	1396.54
A_68_P26736547	chr9:94438241-94438285	NM_001033145:238	1190002N15Rik	INSIDE	1.064	1.403	1025.81	1438.88	1.492	993.80	1482.93
A_68_P21084956	chr2:27331496-27331540	NM_001113573:-325	Brd3	PROMOTER	1.064	1.825	398.45	727.09	1.942	348.84	677.42
A_68_P25520724	chr7:137409910-137409954	NM_010207:390	Fgfr2	INSIDE	1.064	2.159	742.01	1601.66	2.296	666.96	1531.48
A_68_P25174403	chr7:71083373-71083417	NM_021366:407	Klf13	INSIDE	1.064	0.462	1384.41	639.74	0.492	1243.27	611.15
A_68_P27560004	chr11:4748803-4748847	NM_010898:706	Nf2	INSIDE	1.064	0.586	1353.85	793.15	0.623	1150.75	717.31
A_68_P28457435	chr12:57634322-57634366	NM_001146198:1749	Nkx2-1	INSIDE	1.064	1.621	531.31	861.22	1.724	569.79	982.34
A_68_P27287757	chr10:80616441-80616485	NM_021501:14003	Pias4	DOWNSTREAM	1.064	3.121	6991.11	21817.94	3.320	4931.95	16373.16
A_68_P21568534	chr2:119063096-119063140	NM_016907:23	Spint1	INSIDE	1.064	2.479	675.39	1674.53	2.639	588.94	1554.16
A_68_P26626134	chr9:71959499-71959543	NM_011544:106	Tcf12	INSIDE	1.064	3.050	214.12	652.99	3.245	195.47	634.31
A_68_P25393403	chr7:112781845-112781889	NM_018880:147	Trim3	INSIDE	1.064	1.435	13126.83	18842.37	1.528	9134.82	13955.10
A_68_P30094472	chr15:27703695-27703739	NM_001081302:251887	Trio	INSIDE	1.064	2.354	1311.68	3088.05	2.505	1110.35	2781.30
A_68_P20737715	chr1:157264567-157264611	NM_011273:-14	Xpr1	PROMOTER	1.064	1.456	1651.21	2404.07	1.549	1360.31	2107.17
A_68_P26885416	chr9:121672100-121672144	NM_001166644:2972	Zfp651	INSIDE	1.064	2.065	496.15	1024.62	2.197	480.38	1055.22
A_68_P27977895	chr11:84326684-84326729	NM_019816:297	Aatf	INSIDE	1.063	0.543	1178.74	640.54	0.578	1054.55	609.34
A_68_P20633262	chr1:136311950-136311994	NM_028320:-71	Adipor1	PROMOTER	1.063	0.393	1840.01	723.62	0.418	1581.37	660.79
A_68_P28153228	chr11:115255096-115255140	NM_001080929:11889	Cdr2l	INSIDE	1.063	1.533	647.86	992.88	1.629	574.04	935.20
A_68_P21633705	chr2:131005466-131005510	NM_007682:260	Cenpb	INSIDE	1.063	2.135	1286.26	2746.41	2.270	1141.88	2591.50
A_68_P27303129	chr10:83996944-83996988	NM_175451:-333	Ckap4	PROMOTER	1.063	1.501	1001.56	1503.09	1.595	874.68	1395.05
A_68_P32364770	chrX:45807712-45807756	NM_019680:8575	Elf4	INSIDE	1.063	1.586	2361.48	3744.24	1.685	1202.67	2027.07
A_68_P32743829	chrX:147451959-147452003	NM_198110:-115	Gnl3l	PROMOTER	1.063	1.734	1767.66	3065.15	1.844	921.10	1698.05
A_68_P32152399	chr19:47940223-47940267	NM_026619:-39	Gsto2	PROMOTER	1.063	2.550	815.09	2078.66	2.711	740.63	2007.90
A_68_P24623143	chr6:88577556-88577600	NM_001142724:-139	Kbtbd12	PROMOTER	1.063	1.804	2698.05	4866.07	1.917	2325.06	4456.69
A_68_P22406473	chr3:107262834-107262878	NM_145922:-1040	Kcnc4	PROMOTER	1.063	3.764	355.56	1338.39	4.003	321.77	1288.04
A_68_P20939667	chr1:193542758-193542803	NM_001134829:507	Lpgat1	INSIDE	1.063	2.502	803.87	2011.04	2.660	639.01	1700.02
A_68_P31442545	chr17:91487617-91487661	NM_020252:4504	Nrxn1	INSIDE	1.063	2.714	1118.21	3034.47	2.883	968.72	2793.29
A_68_P27648122	chr11:23794713-23794757	NM_172555:536	Papolg	INSIDE	1.063	1.539	3514.20	5409.15	1.636	2725.59	4458.97
A_68_P22774297	chr4:21605606-21605652	NM_001080771:7482	Prdm13	INSIDE	1.063	0.526	991.35	521.58	0.559	823.33	460.63
A_68_P25100826	chr7:53965778-53965822	NM_030233:221	Saal1	INSIDE	1.063	1.377	3165.68	4358.13	1.463	2524.93	3693.33
A_68_P20457300	chr1:95651043-95651087	NM_025920:351	Thap4	INSIDE	1.063	1.678	1225.61	2056.95	1.784	1090.13	1944.65
A_68_P22360091	chr3:98231564-98231608	NM_172863:45183	Zfp697	INSIDE	1.063	2.487	9225.38	22946.94	2.643	6303.91	16664.00
A_68_P27268023	chr10:76880611-76880655	NM_001024837:386	Adarb1	INSIDE	1.062	2.130	274.09	583.67	2.261	257.87	583.08
A_68_P28776185	chr12:119082606-119082650	NM_146040:295	Cdca7l	INSIDE	1.062	2.321	618.93	1436.62	2.465	588.78	1451.09
A_68_P30431386	chr15:90509992-90510036	NM_025815:-195	Cpne8	PROMOTER	1.062	1.646	1667.61	2744.88	1.747	1316.08	2299.69
A_68_P23362416	chr4:140703968-140704012	NM_172518:154	Fbxo42	INSIDE	1.062	2.346	1727.15	4051.36	2.492	1524.92	3800.28
A_68_P21811851	chr2:163164903-163164947	NM_021566:58785	Jph2	INSIDE	1.062	4.607	12198.30	56200.61	4.892	8024.74	39258.66

ProbeName	Target position of probe on CpG island microarray	TargetID	GeneSymbol	CpG island Description	Ratio of relative methylation (TiO_2-NP/Vehicle)	Sham group			TiO_2-H group		
						Relative methylation (Cy5/Cy3)	Cy3 signal (Input)	Cy5 signal (MeDIP)	Relative methylation (Cy5/Cy3)	Cy3 signal (Input)	Cy5 signal (MeDIP)
A_68_P31125154	chr17:29089463-29089507	NM_025888:-676	Kctd20	PROMOTER	1.062	2.042	1483.60	3029.40	2.169	1363.23	2956.52
A_68_P22006133	chr3:26230419-26230463	NM_138666:391	Nlgn1	INSIDE	1.062	1.470	2019.90	2969.89	1.561	1737.47	2712.86
A_68_P21628850	chr2:130099716-130099773	NM_024193:-403	Nop56	PROMOTER	1.062	2.046	1007.80	2062.32	2.174	799.96	1739.26
A_68_P30397307	chr15:84686477-84686521	NM_001081166:61	Phf21b	INSIDE	1.062	2.626	366.48	962.46	2.790	366.20	1021.60
A_68_P31934550	chr19:6413237-6413281	NM_011242:12676	Rasgrp2	INSIDE	1.062	2.376	4064.02	9656.89	2.523	3087.76	7789.77
A_68_P21867216	chr2:172847313-172847357	NM_019547:-68	Rbm38	PROMOTER	1.062	1.909	1387.85	2650.05	2.027	1160.68	2352.58
A_68_P27286998	chr10:80494022-80494066	NM_134135:2613	Slc39a3	INSIDE	1.062	2.534	1329.83	3370.15	2.691	1130.74	3042.74
A_68_P26750365	chr9:96896276-96896320	NM_145134:22476	Spsb4	INSIDE	1.062	2.697	275.25	742.40	2.865	327.43	938.18
A_68_P20736178	chr1:157006310-157006354	NM_021433:500	Stx6	INSIDE	1.062	1.602	1110.11	1778.35	1.702	952.30	1620.80
A_68_P30015972	chr15:11313703-11313747	NM_033074:15689	Tars	INSIDE	1.062	1.777	1080.26	1919.16	1.887	851.29	1606.72
A_68_P21658149	chr2:135485033-135485077	ENSMUST00000139480:-235		PROMOTER	1.062	3.161	598.57	1891.91	3.358	559.15	1877.62
A_68_P28939817	chr13:34744234-34744278	NM_025831:294	1300014I06Rik	INSIDE	1.061	2.666	808.78	2156.33	2.829	733.80	2075.72
A_68_P24006039	chr5:115224465-115224516	NR_027872:502	4930515G01Rik	INSIDE	1.061	2.045	565.94	1157.19	2.170	532.57	1155.79
A_68_P20395057	chr1:82835935-82835979	NM_010472:-101	Agfg1	PROMOTER	1.061	1.362	1745.30	2376.30	1.445	1464.82	2116.42
A_68_P28175391	chr11:118895720-118895764	NM_007623:11400	Cbx2	DOWNSTREAM	1.061	0.593	1506.85	893.24	0.629	1058.78	666.16
A_68_P22412513	chr3:108215712-108215756	NM_001004177:2678	Celsr2	INSIDE	1.061	2.553	789.99	2016.61	2.709	618.72	1676.15
A_68_P25603237	chr7:151015959-151016003	NM_007856:6909	Dhcr7	INSIDE	1.061	1.650	1159.42	1912.59	1.750	1010.83	1768.51
A_68_P22858439	chr4:40670130-40670174	NM_001164672:190	Dnaja1	INSIDE	1.061	1.820	950.46	1729.82	1.931	905.48	1748.68
A_68_P29287625	chr13:104710556-104710600	NM_001005868:16	Erbb2ip	INSIDE	1.061	1.864	599.15	1116.96	1.978	499.04	986.94
A_68_P21909701	chr2:181022187-181022231	NM_198169:463	Gmeb2	INSIDE	1.061	1.359	2571.75	3495.74	1.442	1885.13	2718.02
A_68_P30142979	chr15:37162921-37162965	NM_026496:152	Grhl2	INSIDE	1.061	1.924	2064.37	3972.78	2.043	1671.31	3413.99
A_68_P29469007	chr14:24822857-24822901	NM_010610:549	Kcnma1	INSIDE	1.061	1.588	7468.40	11856.67	1.685	5299.67	8928.22
A_68_P25187996	chr7:73532827-73532871	NM_146191:379	Lrrk1	INSIDE	1.061	2.385	930.46	2218.78	2.531	863.50	2185.34
A_68_P27845570	chr11:60745579-60745623	NM_008928:42	Map2k3	INSIDE	1.061	0.589	1373.01	808.14	0.624	1114.73	696.08
A_68_P30083232	chr15:25710801-25710845	NM_019472:158518	Myo10	INSIDE	1.061	3.772	279.12	1052.73	4.000	252.46	1009.88
A_68_P20184670	chr1:42754004-42754048	NM_008900:36	Pou3f3	INSIDE	1.061	1.445	1123.59	1623.28	1.532	1003.27	1537.18
A_68_P26805183	chr9:106691283-106691327	NM_030730:240	Rad54l2	INSIDE	1.061	1.653	570.21	942.66	1.754	543.20	952.57
A_68_P24992476	chr7:19872340-19872384	NM_001025364:-4085	Rtn2	PROMOTER	1.061	1.809	631.69	1142.92	1.920	563.16	1081.39
A_68_P20870656	chr1:181448020-181448064	NM_027188:92	Smyd3	INSIDE	1.061	1.370	3062.67	4196.61	1.454	2340.54	3404.07
A_68_P20005797	chr1:4482373-4482417	NM_011441:4100	Sox17	INSIDE	1.061	1.589	1431.99	2275.11	1.685	1269.05	2138.80
A_68_P31953435	chr19:10956222-10956266	NM_134142:-11	Tmem109	PROMOTER	1.061	1.493	664.98	992.99	1.585	558.09	884.31
A_68_P32709177	chrX:139116370-139116414	NM_001199360:124	Tmem164	INSIDE	1.061	2.766	3568.80	9870.08	2.933	1703.12	4996.04
A_68_P25056444	chr7:37481116-37481160	NM_172298:-1998	Tshz3	PROMOTER	1.061	1.823	774.46	1411.53	1.933	782.37	1512.34
A_68_P21779875	chr2:157741034-157741081	NM_198627:669	Vstm2l	INSIDE	1.061	2.846	1593.58	4535.74	3.020	1121.35	3386.19
A_68_P31479223	chr18:7868850-7868894	NM_001146298:-322	Wac	PROMOTER	1.061	1.841	1009.63	1858.37	1.953	961.15	1876.87
A_68_P25272791	chr7:88716304-88716348	NM_001004185:149	Whamm	INSIDE	1.061	2.277	502.90	1145.19	2.416	441.36	1066.21
A_68_P22970352	chr4:63077484-63077528	NM_001008797:16850	Whrn	INSIDE	1.061	3.007	1577.22	4743.33	3.191	1120.71	3576.58
A_68_P28751779	chr12:113917392-113917436	NM_001100460:364	Zbtb42	INSIDE	1.061	2.635	1111.42	2928.60	2.795	968.56	2707.36
A_68_P29656177	chr14:63174859-63174903	ENSMUST00000160745:269		INSIDE	1.061	1.420	3267.38	4640.35	1.507	2526.30	3807.42
A_68_P21833421	chr2:166888085-166888129	NM_001081005:-327	1500012F01Rik	PROMOTER	1.060	1.588	1268.52	2014.59	1.683	1043.61	1756.61
A_68_P21528938	chr2:112295037-112295081	NM_133749:-123	2900064A13Rik	PROMOTER	1.060	1.852	740.97	1372.23	1.963	722.01	1417.44
A_68_P30743457	chr16:48994281-48994325	NM_172616:2	C330027C09Rik	INSIDE	1.060	2.351	491.36	1155.44	2.492	454.13	1131.90
A_68_P31208114	chr17:46787782-46787826	NM_025611:518	Cul7	INSIDE	1.060	1.970	4081.36	8039.80	2.089	2929.86	6120.46
A_68_P28525542	chr12:72419588-72419632	NM_001190466:8740	Dact1	INSIDE	1.060	3.038	767.91	2332.87	3.221	664.44	2140.12
A_68_P27924584	chr11:74982007-74982051	NM_001098203:-374	Hic1	PROMOTER	1.060	1.368	1812.80	2479.49	1.451	1430.02	2074.25
A_68_P23333242	chr4:135866928-135866972	NM_028871:60	Hnrnpr	INSIDE	1.060	1.841	577.01	1062.14	1.952	501.30	978.34
A_68_P23949339	chr5:104339900-104339944	NM_178741:326	Klhl8	INSIDE	1.060	1.633	1033.11	1686.87	1.730	907.33	1570.10
A_68_P29725073	chr14:75346827-75346871	NM_001033439:836	Lrch1	INSIDE	1.060	0.569	1359.68	773.58	0.603	1192.40	719.43
A_68_P23334046	chr4:136018440-136018484	NM_024452:-7213	Luzp1	PROMOTER	1.060	2.968	516.83	1533.93	3.145	457.54	1439.03
A_68_P28744816	chr12:112812796-112812840	NM_021516:98	Mark3	INSIDE	1.060	1.445	2876.14	4157.17	1.532	2477.93	3796.47
A_68_P27154487	chr10:55835680-55835724	NM_001163833:8980	Msl3l2	INSIDE	1.060	0.517	1013.81	524.34	0.548	885.39	485.56

ProbeName	Target position of probe on CpG island microarray	TargetID	GeneSymbol	CpG island Description	Ratio of relative methylation (TiO_2-NP/Vehicle)	Sham group			TiO_2-H group		
						Relative methylation (Cy5/Cy3)	Cy3 signal (Input)	Cy5 signal (MeDIP)	Relative methylation (Cy5/Cy3)	Cy3 signal (Input)	Cy5 signal (MeDIP)
A_68_P32395552	chrX:53627636-53627680	NR_028086:-104	Ncrna00086	PROMOTER	1.060	1.889	3488.88	6590.57	2.003	1753.31	3512.07
A_68_P23310229	chr4:131669398-131669442	NM_013622:30981	Oprd1	INSIDE	1.060	1.686	860.07	1450.16	1.787	757.83	1354.48
A_68_P24956667	chr7:6683071-6683115	NM_008817:38	Peg3	INSIDE	1.060	1.978	1601.52	3167.82	2.096	1446.95	3032.79
A_68_P31207976	chr17:46766512-46766556	NM_175168:-81	Ptk7	PROMOTER	1.060	3.291	776.86	2556.42	3.487	550.58	1920.14
A_68_P27478506	chr10:116387438-116387482	NM_001003950:-24	Rab3ip	DIVERGENT_PROMOTER	1.060	2.100	451.75	948.55	2.225	407.29	906.15
A_68_P21875633	chr2:174298255-174298299	NM_025531:166	Slmo2	INSIDE	1.060	1.722	905.44	1558.74	1.825	723.15	1319.59
A_68_P25507646	chr7:134985207-134985251	NM_009294:-93	Stx4a	PROMOTER	1.060	2.442	814.24	1988.54	2.588	724.96	1876.17
A_68_P23429326	chr4:153514732-153514776	NM_011642:-437	Trp73	DIVERGENT_PROMOTER	1.060	0.492	1158.68	570.25	0.522	761.71	397.32
A_68_P30476460	chr15:98620564-98620608	NM_021279:299	Wnt1	INSIDE	1.060	1.439	1148.78	1652.99	1.525	955.89	1457.45
A_68_P25008265	chr7:25866336-25866380	NM_175477:4094	Zfp574	INSIDE	1.060	3.211	2802.17	8997.55	3.403	2116.24	7200.92
A_68_P30593947	chr16:21694402-21694446	NM_001001881:314	2510009E07Rik	INSIDE	1.059	1.999	885.99	1770.92	2.117	742.17	1571.17
A_68_P31053603	chr17:12934351-12934395	NR_002853:196	Airn	INSIDE	1.059	1.529	692.12	1058.04	1.618	532.10	861.14
A_68_P27540826	chr10:127774827-127774871	NM_026444:-39	Cs	PROMOTER	1.059	1.523	1535.64	2339.05	1.614	1391.23	2244.95
A_68_P29098528	chr13:64471285-64471329	NM_009984:308	Ctsl	INSIDE	1.059	0.500	1138.75	568.84	0.529	1060.20	561.09
A_68_P25587954	chr7:148479800-148479844	NM_007878:1918	Drd4	INSIDE	1.059	1.829	1443.30	2639.20	1.937	1062.54	2058.48
A_68_P22019967	chr3:28981485-28981529	NM_001167748:8	Egfem1	INSIDE	1.059	2.634	290.88	766.04	2.790	316.41	882.72
A_68_P20741944	chr1:158134876-158134920	NM_177838:258	Fam163a	INSIDE	1.059	1.727	968.26	1671.80	1.829	885.33	1619.25
A_68_P25827954	chr8:46109471-46109515	NM_001081286:73931	Fat1	INSIDE	1.059	2.821	481.61	1358.76	2.987	389.89	1164.46
A_68_P26614159	chr9:69860258-69860302	NM_001039519:-76	Gtf2a2	PROMOTER	1.059	1.626	2138.39	3476.20	1.721	1760.89	3031.25
A_68_P29952571	chr14:121309972-121310016	NM_023579:-421	Ipo5	PROMOTER	1.059	2.136	1579.28	3373.73	2.263	1389.96	3145.78
A_68_P26022956	chr8:87501679-87501723	NM_008416:947	Junb	INSIDE	1.059	1.397	1255.84	1753.94	1.479	1081.04	1598.85
A_68_P26159963	chr8:112486245-112486304	NM_212447:-169	Marveld3	PROMOTER	1.059	3.847	195.21	751.04	4.074	144.33	588.01
A_68_P22569091	chr3:138151998-138152042	NM_175224:326	Metap1	INSIDE	1.059	1.496	1236.11	1849.05	1.584	1295.42	2051.42
A_68_P24140928	chr5:140807887-140807931	NM_008637:33	Nudt1	INSIDE	1.059	1.682	3017.50	5075.70	1.782	2160.48	3850.28
A_68_P26261059	chr8:129588231-129588275	NM_001122850:297	Pard3	INSIDE	1.059	2.957	1688.89	4993.65	3.131	1505.81	4715.29
A_68_P31629192	chr18:37829317-37829361	NM_033586:-4650	Pcdhga3	PROMOTER	1.059	1.846	603.99	1114.93	1.956	533.65	1043.59
A_68_P26240712	chr8:126177554-126177598	NM_133955:-252	Rhou	PROMOTER	1.059	1.534	2986.59	4580.97	1.624	2510.70	4077.90
A_68_P26996376	chr10:21713866-21713910	NM_011361:-583	Sgk1	PROMOTER	1.059	1.983	1151.96	2283.92	2.100	1056.35	2218.78
A_68_P32148715	chr19:47341968-47342012	NM_001164717:196911	Sh3pxd2a	INSIDE	1.059	1.547	774.55	1198.18	1.639	701.42	1149.35
A_68_P31056398	chr17:13898109-13898153	NM_022311:271	Tcte2	INSIDE	1.059	1.539	1718.22	2644.64	1.629	1469.46	2394.26
A_68_P20742450	chr1:158233703-158233747	NM_001134741:-245	Tdrd5	DIVERGENT_PROMOTER	1.059	2.025	1011.27	2047.58	2.143	886.64	1900.28
A_68_P27709779	chr11:35793475-35793519	NM_170779:95	Wwc1	INSIDE	1.059	0.536	1425.82	764.12	0.567	1318.86	748.40
A_68_P31092364	chr17:23695056-23695100	NM_001033496:6115	Zfp213	INSIDE	1.059	1.918	698.09	1339.16	2.031	565.09	1147.84
A_68_P22411476	chr3:108042892-108042936	ENSMUST00000119163:-85		PROMOTER	1.059	2.014	896.15	1804.52	2.133	703.63	1500.63
A_68_P22169028	chr3:58380988-58381032	NM_001040396:431	2810407C02Rik	INSIDE	1.058	4.492	626.81	2815.45	4.751	546.52	2596.71
A_68_P22888785	chr4:46463681-46463725	NM_130889:-286	Anp32b	PROMOTER	1.058	1.571	1536.02	2413.20	1.663	1314.47	2185.39
A_68_P28091583	chr11:104411776-104411820	NM_145436:136	Cdc27	INSIDE	1.058	1.646	659.82	1086.32	1.742	586.91	1022.41
A_68_P23342965	chr4:137513918-137513962	NM_199307:95789	Ece1	INSIDE	1.058	2.303	255.87	589.17	2.437	195.40	476.10
A_68_P31493063	chr18:11054641-11054685	NM_010258:2155	Gata6	INSIDE	1.058	2.204	806.82	1778.10	2.332	668.66	1559.61
A_68_P25951136	chr8:72854054-72854098	NM_008107:413	Gdf1	INSIDE	1.058	1.735	818.33	1419.97	1.835	633.07	1161.78
A_68_P23274586	chr4:125167342-125167386	NM_001081097:-710	Grik3	PROMOTER	1.058	1.939	942.42	1827.71	2.051	774.82	1589.31
A_68_P25512006	chr7:135831552-135831596	NM_178641:76696	Inpp5f	INSIDE	1.058	1.995	1990.64	3971.85	2.111	1415.63	2988.14
A_68_P21571260	chr2:119570038-119570082	NM_146125:1988	Itpka	INSIDE	1.058	1.756	1020.41	1791.98	1.857	737.20	1369.33
A_68_P31847801	chr18:77680289-77680333	NM_172834:159614	Loxhd1	INSIDE	1.058	2.145	751.68	1612.64	2.271	589.52	1338.68
A_68_P26133403	chr8:107898777-107898821	NM_001081333:-482	Plekhg4	PROMOTER	1.058	2.482	697.04	1729.99	2.626	547.69	1438.39
A_68_P20779537	chr1:164670744-164670788	NM_001081290:-79	Prrc2c	PROMOTER	1.058	2.054	463.32	951.72	2.173	524.43	1139.81
A_68_P24085128	chr5:129287634-129287678	NM_001081388:171581	Rimbp2	INSIDE	1.058	3.773	684.36	2581.84	3.992	544.94	2175.21
A_68_P28958207	chr13:38129003-38129047	NM_024242:167	Riok1	INSIDE	1.058	1.391	10890.04	15142.99	1.471	7834.30	11522.48
A_68_P29517812	chr14:33278202-33278246	NM_021712:-188	Slc18a3	PROMOTER	1.058	2.412	641.13	1546.44	2.551	537.59	1371.62
A_68_P24981575	chr7:17038564-17038608	NM_026938:459	Tmem160	INSIDE	1.058	1.627	2953.81	4807.15	1.722	2303.19	3966.17
A_68_P25506497	chr7:134754654-134754698	NM_177226:3271	Zfp629	INSIDE	1.058	2.535	994.16	2520.31	2.683	840.85	2255.58

ProbeName	Target position of probe on CpG island microarray	TargetID	GeneSymbol	CpG island Description	Ratio of relative methylation (TiO_2-NP/Vehicle)	Sham group			TiO_2-H group		
						Relative methylation (Cy5/Cy3)	Cy3 signal (Input)	Cy5 signal (MeDIP)	Relative methylation (Cy5/Cy3)	Cy3 signal (Input)	Cy5 signal (MeDIP)
A_68_P27581769	chr11:9050712-9050764	ENSMUST00000121861:-138		PROMOTER	1.058	3.527	343.26	1210.85	3.731	231.03	862.01
A_68_P27932459	chr11:76308651-76308695	NM_198018:14249	Abr	INSIDE	1.057	0.652	2263.69	1474.90	0.689	1685.87	1161.15
A_68_P20430407	chr1:91477732-91477776	NM_001037136:126369	Agap1	INSIDE	1.057	2.516	228.92	575.93	2.660	225.27	599.13
A_68_P27089722	chr10:41239137-41239181	NM_016898:-147	Cd164	PROMOTER	1.057	0.442	1605.16	708.75	0.467	1296.73	605.32
A_68_P23327246	chr4:134762197-134762241	NM_013885:66457	Clic4	DOWNSTREAM	1.057	0.528	1258.89	664.21	0.558	817.20	455.72
A_68_P26754210	chr9:97601436-97601480	NM_022319:332128	Clstn2	INSIDE	1.057	2.034	3961.10	8057.47	2.150	3618.28	7780.61
A_68_P32007551	chr19:21727608-21727652	NM_146096:-168	Fam108b	DIVERGENT_PROMOTER	1.057	2.358	1529.81	3607.88	2.492	1308.71	3260.86
A_68_P20832290	chr1:174242716-174242760	NM_080419:201	Igsf8	INSIDE	1.057	1.361	2664.04	3624.75	1.438	2007.34	2886.58
A_68_P25953856	chr8:73285042-73285086	NM_183170:-244	Mpv17l2	PROMOTER	1.057	1.367	2760.76	3774.32	1.445	2194.05	3171.31
A_68_P31108289	chr17:26232390-26232434	NM_019731:3	Nme4	INSIDE	1.057	3.019	629.76	1901.12	3.190	655.49	2090.71
A_68_P20823479	chr1:172519702-172519746	NM_001109985:256	Nos1ap	INSIDE	1.057	1.571	561.61	882.04	1.659	578.11	959.34
A_68_P27553494	chr11:3605439-3605483	NM_152818:158446	Osbp2	INSIDE	1.057	1.867	1416.13	2643.41	1.974	1031.88	2036.59
A_68_P22741774	chr4:13670169-13670215	NM_001111026:-256	Runx1t1	PROMOTER	1.057	1.369	1717.95	2352.57	1.448	1428.54	2068.48
A_68_P23999962	chr5:114221210-114221254	NM_016926:426	Sart3	INSIDE	1.057	1.502	930.30	1397.20	1.588	821.62	1304.54
A_68_P27807841	chr11:53841272-53841316	NM_019687:298	Slc22a4	INSIDE	1.057	1.500	1050.08	1575.54	1.587	882.39	1400.04
A_68_P28730243	chr12:110074111-110074155	NM_181328:-46	Slc25a29	DIVERGENT_PROMOTER	1.057	1.662	478.86	795.81	1.756	448.89	788.30
A_68_P20723681	chr1:154749520-154749564	NM_001005507:234	Smg7	INSIDE	1.057	1.688	2507.49	4232.90	1.785	2284.79	4077.93
A_68_P26447521	chr9:40965188-40965232	NM_176860:367	Ubash3b	INSIDE	1.057	0.477	1290.92	615.80	0.504	1015.36	511.84
A_68_P30351141	chr15:76741990-76742034	NM_001168276:13866	Zfp647	INSIDE	1.057	2.842	320.76	911.60	3.003	313.80	942.27
A_68_P28523846	chr12:72117418-72117462	NM_001081195:487	Arid4a	INSIDE	1.056	1.416	2031.79	2876.65	1.494	1627.43	2432.05
A_68_P28453876	chr12:56937237-56937281	NM_001037756:-94	Brms1l	PROMOTER	1.056	0.462	1942.78	897.35	0.488	1488.85	726.42
A_68_P20887293	chr1:184447465-184447509	NM_009794:128	Capn2	INSIDE	1.056	0.590	1254.56	740.30	0.623	981.52	611.84
A_68_P24326228	chr6:29352876-29352920	NM_001201378:3973	Ccdc136	INSIDE	1.056	2.108	529.94	1117.19	2.227	529.06	1178.27
A_68_P25601117	chr7:150650897-150650941	NM_001161624:-3963	Cdkn1c	DIVERGENT_PROMOTER	1.056	2.187	539.69	1180.27	2.309	488.56	1128.06
A_68_P26504859	chr9:50467279-50467323	NM_145614:585	Dlat	INSIDE	1.056	0.387	1993.49	771.49	0.409	1668.74	681.68
A_68_P30175461	chr15:43113914-43113958	NM_008388:346	Eif3e	INSIDE	1.056	1.371	7102.04	9737.71	1.448	5870.64	8499.43
A_68_P27849837	chr11:61498300-61498344	NM_178618:411	Fam83g	INSIDE	1.056	1.530	2112.87	3232.09	1.615	1749.23	2825.58
A_68_P23979337	chr5:110807040-110807084	NM_028596:9084	Fbrsl1	INSIDE	1.056	2.950	3275.52	9661.68	3.116	2391.06	7451.09
A_68_P20457531	chr1:95700822-95700866	NM_025454:303	Ing5	INSIDE	1.056	2.268	1986.89	4506.64	2.394	1654.74	3961.71
A_68_P27927253	chr11:75444214-75444258	NM_008916:-285	Inpp5k	PROMOTER	1.056	1.434	1820.73	2610.98	1.515	1628.18	2466.08
A_68_P24009511	chr5:115880792-115880836	NM_008629:1121	Msi1	INSIDE	1.056	2.141	2233.33	4781.69	2.260	1746.57	3947.79
A_68_P30083239	chr15:25711450-25711494	NM_019472:159168	Myo10	INSIDE	1.056	2.264	375.50	850.17	2.390	318.95	762.46
A_68_P23134167	chr4:97418430-97418474	NM_001122952:169819	Nfia	INSIDE	1.056	1.607	573.29	921.14	1.696	592.97	1005.80
A_68_P27922137	chr11:74537095-74537139	NR_037610:244	Pafah1b1	INSIDE	1.056	0.573	1423.04	815.03	0.605	1159.20	700.99
A_68_P22696143	chr4:4065948-4065992	NM_001002927:-378	Penk	PROMOTER	1.056	2.076	603.07	1252.11	2.192	548.77	1202.84
A_68_P30558910	chr16:13780799-13780843	NM_001039521:29	Rrn3	INSIDE	1.056	2.008	525.43	1055.06	2.120	481.71	1021.39
A_68_P28635987	chr12:92885819-92885865	NM_175367:139034	Ston2	INSIDE	1.056	1.874	351.83	659.28	1.978	282.14	558.15
A_68_P27340406	chr10:90633535-90633579	NM_001080129:771	Tmpo	INSIDE	1.056	0.566	1391.68	788.31	0.598	1222.33	731.42
A_68_P23394412	chr4:147818678-147818722	NM_027873:160	Ubiad1	INSIDE	1.056	1.902	1383.96	2632.85	2.010	1072.93	2156.11
A_68_P31554072	chr18:23469485-23469529	X95227:781		INSIDE	1.056	2.309	2242.99	5179.88	2.438	1865.05	4547.67
A_68_P25906688	chr8:63369041-63369085	NM_028299:-185	2700029M09Rik	PROMOTER	1.055	1.602	1276.12	2044.51	1.690	1232.98	2084.30
A_68_P28269862	chr12:17331276-17331320	NM_001159632:238	Atp6v1c2	INSIDE	1.055	1.599	1138.87	1821.02	1.687	927.34	1564.40
A_68_P32268348	chrX:13423751-13423795	NM_009806:-105	Cask	PROMOTER	1.055	2.579	462.92	1193.84	2.721	297.99	810.83
A_68_P31125934	chr17:29232562-29232606	NM_007669:1868	Cdkn1a	INSIDE	1.055	1.586	1440.75	2285.51	1.673	1257.85	2104.35
A_68_P21836888	chr2:167514985-167515029	NM_009883:592	Cebpb	INSIDE	1.055	3.231	264.49	854.57	3.408	241.91	824.38
A_68_P21746889	chr2:151796098-151796142	NM_028666:3590	Fam110a	INSIDE	1.055	3.222	3512.24	11317.41	3.399	2684.79	9124.54
A_68_P31740999	chr18:58369270-58369314	NM_010181:288	Fbn2	INSIDE	1.055	0.649	1970.59	1278.49	0.685	1453.34	995.20
A_68_P32143277	chr19:46403804-46403848	NM_133694:1153	Fbxl15	INSIDE	1.055	2.039	1473.79	3004.47	2.151	1315.25	2829.17
A_68_P30630415	chr16:28446508-28446552	NM_183064:-1217	Fgf12	PROMOTER	1.055	1.827	1937.39	3539.41	1.927	1790.36	3449.45
A_68_P32401468	chrX:54756573-54756617	NM_001033360:340	Gpr101	INSIDE	1.055	2.029	1236.25	2507.86	2.140	643.32	1376.75
A_68_P31117112	chr17:27693865-27693909	NM_001166476:289	Hmga1-rs1	INSIDE	1.055	15.964	895.81	14301.03	16.843	740.24	12468.17

ProbeName	Target position of probe on CpG island microarray	TargetID	GeneSymbol	CpG island Description	Ratio of relative methylation (TiO_2-NP/Vehicle)	Sham group			TiO_2-H group		
						Relative methylation (Cy5/Cy3)	Cy3 signal (Input)	Cy5 signal (MeDIP)	Relative methylation (Cy5/Cy3)	Cy3 signal (Input)	Cy5 signal (MeDIP)
A_68_P24990865	chr7:19590861-19590905	NM_178757:1469	Irf2bp1	INSIDE	1.055	2.267	1171.62	2656.17	2.392	1123.33	2686.48
A_68_P30660049	chr16:33829724-33829768	NM_001145884:-4	Itgb5	PROMOTER	1.055	1.430	886.09	1266.92	1.508	801.75	1208.98
A_68_P20451521	chr1:94745114-94745158	NR_029559:-1818	Mir149	PROMOTER	1.055	2.266	13460.81	30504.50	2.391	9543.91	22820.53
A_68_P24009623	chr5:115897106-115897158	NM_008629:17439	Msi1	INSIDE	1.055	2.116	312.79	661.78	2.232	260.78	581.96
A_68_P31110247	chr17:26576151-26576195	NM_001081656:24263	Neurl1b	INSIDE	1.055	2.392	794.24	1899.89	2.523	771.29	1945.87
A_68_P27638931	chr11:21903874-21903918	NM_011023:-2242	Otx1	PROMOTER	1.055	1.400	1731.64	2424.22	1.477	1488.25	2197.65
A_68_P22567229	chr3:137806950-137806994	NM_175389:471	Rg9mtd2	INSIDE	1.055	2.151	521.04	1120.51	2.270	467.62	1061.40
A_68_P27290119	chr10:80961961-80962005	NM_010102:900	S1pr4	INSIDE	1.055	1.517	951.89	1444.04	1.600	822.44	1315.83
A_68_P26022400	chr8:87396607-87396651	NM_001168244:472	Syce2	INSIDE	1.055	2.054	743.06	1526.57	2.167	689.01	1493.41
A_68_P31020006	chr17:5941362-5941406	NM_001113352:105	Synj2	INSIDE	1.055	2.227	728.88	1623.51	2.351	685.94	1612.60
A_68_P21571904	chr2:119698870-119698914	ENSMUST00000120952:5247		DOWNSTREAM	1.055	4.291	779.40	3344.27	4.526	596.63	2700.21
A_68_P27680364	chr11:30655100-30655144	ENSMUST00000121590:-161		PROMOTER	1.055	3.041	761.54	2315.47	3.209	711.79	2284.05
A_68_P26202775	chr8:119960432-119960476	NM_028941:87385	4933407C03Rik	INSIDE	1.054	3.269	2328.27	7610.33	3.445	1725.53	5943.98
A_68_P23069985	chr4:84321596-84321640	NM_172870:-628	Bnc2	PROMOTER	1.054	1.518	1443.33	2191.06	1.600	1132.77	1812.62
A_68_P26020781	chr8:87141111-87141156	NM_007578:201871	Cacna1a	INSIDE	1.054	1.955	1845.60	3608.54	2.061	1385.11	2854.61
A_68_P24063260	chr5:125342106-125342150	NM_144819:463	Ccdc92	INSIDE	1.054	1.558	1062.96	1655.96	1.642	875.82	1438.27
A_68_P26875427	chr9:119883344-119883388	NM_153287:10410	Csrnp1	INSIDE	1.054	4.579	401.52	1838.77	4.825	340.88	1644.75
A_68_P22569292	chr3:138190910-138190954	NM_007917:1778	Eif4e	INSIDE	1.054	1.403	1694.19	2377.79	1.479	1461.31	2161.80
A_68_P24084081	chr5:129107610-129107654	NM_175284:652	Fzd10	INSIDE	1.054	1.559	2088.20	3254.69	1.643	1626.70	2673.19
A_68_P22565471	chr3:137528339-137528383	NM_016750:798	H2afz	INSIDE	1.054	1.339	3314.99	4440.02	1.411	2522.63	3559.91
A_68_P25186205	chr7:73224603-73224647	NM_024439:90	H47	INSIDE	1.054	1.539	954.93	1469.86	1.622	799.43	1296.53
A_68_P20600072	chr1:130256315-130256359	NM_008567:-103	Mcm6	PROMOTER	1.054	0.409	1603.33	656.47	0.432	1304.49	563.18
A_68_P21150010	chr2:38566321-38566365	NM_139051:3720	Nr5a1	INSIDE	1.054	0.476	1375.42	654.74	0.502	1174.48	589.04
A_68_P27896908	chr11:69571787-69571831	NM_009089:-83	Polr2a	PROMOTER	1.054	1.652	780.39	1288.81	1.741	709.33	1234.91
A_68_P23814584	chr5:76613501-76613545	NM_011626:618	Tmem165	INSIDE	1.054	5.120	893.40	4574.28	5.397	871.61	4704.38
A_68_P24630039	chr6:89785616-89785660	NM_053221:9893	Vmn1r42	DOWNSTREAM	1.054	2.230	1167.94	2603.99	2.351	1040.78	2446.92
A_68_P30509169	chr16:4268211-4268255			Unknown	1.054	2.676	320.66	858.12	2.821	314.92	888.35
A_68_P21041599	chr2:18722024-18722068	AK018938:-386		PROMOTER	1.054	2.464	1024.88	2525.63	2.597	863.48	2242.86
A_68_P26253145	chr8:128193794-128193838	NM_028908:99	4933403G14Rik	INSIDE	1.053	2.251	1672.01	3764.39	2.370	1482.81	3513.91
A_68_P26617807	chr9:70527537-70527581	NM_007399:751	Adam10	INSIDE	1.053	1.490	1177.32	1753.86	1.568	890.70	1396.93
A_68_P27566543	chr11:5965060-5965104	NM_001174053:669	Camk2b	INSIDE	1.053	2.056	1793.20	3686.79	2.165	1521.95	3295.16
A_68_P21836882	chr2:167514407-167514451	NM_009883:14	Cebpb	INSIDE	1.053	1.895	1684.07	3191.26	1.995	1550.56	3093.73
A_68_P20442010	chr1:93263130-93263174	NM_173395:146	Fam132b	INSIDE	1.053	0.591	1211.65	715.90	0.622	950.85	591.53
A_68_P20025037	chr1:9957276-9957320	NM_145692:41903	Lrrc67	DOWNSTREAM	1.053	1.443	1283.38	1851.83	1.519	1099.49	1669.96
A_68_P22705748	chr4:6380821-6380865	NM_010945:576	Nsmaf	INSIDE	1.053	1.963	953.82	1872.73	2.067	863.77	1785.63
A_68_P30346556	chr15:76004532-76004576	NM_201394:21586	Plec	INSIDE	1.053	2.876	325.47	936.00	3.028	257.29	779.00
A_68_P29950625	chr14:120877439-120877483	NM_029519:-222	Rap2a	PROMOTER	1.053	1.376	1520.92	2092.20	1.448	1218.89	1764.88
A_68_P25265265	chr7:87343187-87343231	NM_013659:11482	Sema4b	INSIDE	1.053	1.391	1203.42	1674.53	1.465	939.80	1377.09
A_68_P21999683	chr3:25043045-25043089	AK054291:117		INSIDE	1.053	2.391	1303.05	3115.62	2.517	1121.31	2822.76
A_68_P27212538	chr10:67011354-67011398	NM_001005419:327	Ado	INSIDE	1.052	2.588	613.18	1587.05	2.722	568.66	1547.97
A_68_P24797811	chr6:120244232-120244276	NM_198884:323	B4galnt3	INSIDE	1.052	3.257	1237.02	4029.08	3.428	1061.68	3639.20
A_68_P27183503	chr10:61441267-61441311	NM_007731:568	Col13a1	INSIDE	1.052	1.653	1199.32	1982.09	1.738	1046.49	1818.82
A_68_P25587956	chr7:148480038-148480082	NM_007878:2156	Drd4	INSIDE	1.052	1.413	1485.78	2099.20	1.486	1150.20	1709.28
A_68_P27561403	chr11:4998729-4998773	NM_007968:330	Ewsr1	INSIDE	1.052	0.612	1267.66	776.17	0.644	1084.66	698.96
A_68_P24290982	chr6:22306343-22306387	NM_138587:-283	Fam3c	PROMOTER	1.052	0.643	1268.33	815.93	0.677	1112.26	752.57
A_68_P26248333	chr8:127422026-127422070	NM_001111141:263	Gm505	INSIDE	1.052	1.953	2039.54	3984.00	2.055	1734.51	3563.75
A_68_P20391719	chr1:82287804-82287848	NM_010570:188	Irs1	INSIDE	1.052	1.899	821.78	1560.92	1.999	696.01	1391.34
A_68_P25034849	chr7:31852601-31852645	NM_144556:7669	Lgi4	INSIDE	1.052	2.127	1036.33	2204.40	2.238	956.97	2141.61
A_68_P28155818	chr11:115719846-115719890	NM_145438:34497	Llgl2	DOWNSTREAM	1.052	1.669	1419.85	2369.94	1.757	1131.73	1987.96
A_68_P31112804	chr17:26988009-26988053	NM_008700:-9520	Nkx2-5	PROMOTER	1.052	0.586	1144.98	671.45	0.617	835.57	515.69
A_68_P28068129	chr11:100302265-100302309	NM_026561:117	Nt5c3l	INSIDE	1.052	0.487	1655.61	805.64	0.512	1315.62	673.52

ProbeName	Target position of probe on CpG island microarray	TargetID	GeneSymbol	CpG island Description	Ratio of relative methylation (TiO_2-NP/Vehicle)	Sham group			TiO_2-H group		
						Relative methylation (Cy5/Cy3)	Cy3 signal (Input)	Cy5 signal (MeDIP)	Relative methylation (Cy5/Cy3)	Cy3 signal (Input)	Cy5 signal (MeDIP)
A_68_P21095897	chr2:29083296-29083340	NM_133500:20242	Ntng2	INSIDE	1.052	1.704	513.89	875.65	1.793	421.70	755.91
A_68_P31757527	chr18:61558226-61558270	NM_133249:1837	Ppargc1b	INSIDE	1.052	1.715	1483.80	2544.46	1.803	1200.70	2165.31
A_68_P25502256	chr7:133936075-133936119	NM_019674:-111	Ppp4c	PROMOTER	1.052	1.828	872.59	1594.69	1.923	761.80	1465.02
A_68_P31375071	chr17:79419979-79420023	NM_001171004:156	Prkd3	INSIDE	1.052	1.482	1005.33	1490.25	1.559	937.40	1461.61
A_68_P29716897	chr14:73725909-73725953	NM_009029:-332	Rb1	PROMOTER	1.052	2.514	1631.85	4102.83	2.645	1447.63	3828.78
A_68_P21750310	chr2:152460214-152460258	NM_009047:7493	Rem1	INSIDE	1.052	1.755	525.16	921.46	1.845	396.97	732.43
A_68_P27284267	chr10:80069318-80069362	NM_019575:3714	Scamp4	INSIDE	1.052	2.011	822.67	1654.62	2.115	638.23	1349.88
A_68_P20946921	chr1:194861981-194862025	NM_181546:-110	Syt14	PROMOTER	1.052	1.607	2183.09	3508.01	1.691	1720.17	2908.36
A_68_P32340726	chrX:39421609-39421653	NM_009688:618	Xiap	INSIDE	1.052	1.914	570.87	1092.78	2.014	324.52	653.44
A_68_P20141345	chr1:34986894-34986938			Unknown	1.052	2.407	845.36	2034.56	2.531	767.32	1942.04
A_68_P32975994	chr12:18394285-18394329	ENSMUST00000080502:-254		PROMOTER	1.052	1.915	940.51	1800.94	2.015	983.34	1981.19
A_68_P21587204	chr2:122456531-122456575	NM_001004174:-7070	AA467197	PROMOTER	1.051	1.751	540.62	946.38	1.839	454.27	835.39
A_68_P21317589	chr2:70956028-70956072	NM_028593:-60	Cybrd1	PROMOTER	1.051	1.795	2991.99	5371.34	1.887	2390.59	4510.55
A_68_P32553702	chrX:96332024-96332068	NM_010110:578	Efnb1	INSIDE	1.051	2.353	702.59	1653.05	2.473	475.70	1176.47
A_68_P22045622	chr3:33919516-33919560	NM_001113188:538	Fxr1	INSIDE	1.051	1.621	2612.45	4233.95	1.703	2544.75	4334.78
A_68_P23600191	chr5:35731800-35731844	NM_010445:57	Hmx1	INSIDE	1.051	2.439	829.20	2022.42	2.564	790.07	2026.07
A_68_P27902205	chr11:70514397-70514441	NM_153103:369	Kif1c	INSIDE	1.051	0.629	1261.57	794.05	0.662	1082.59	716.25
A_68_P28049242	chr11:97048472-97048516	NM_008379:712	Kpnb1	INSIDE	1.051	1.380	1851.96	2556.02	1.450	1415.02	2051.84
A_68_P29629466	chr14:58318760-58318804	NM_015771:46178	Lats2	INSIDE	1.051	2.590	486.01	1258.84	2.723	490.72	1336.23
A_68_P25094514	chr7:52949352-52949396	NM_001033356:9591	Ntn5	INSIDE	1.051	3.158	680.85	2150.12	3.318	570.34	1892.21
A_68_P26609887	chr9:69138471-69138515	NM_013646:636884	Rora	INSIDE	1.051	2.963	682.70	2023.12	3.113	648.29	2018.35
A_68_P23768851	chr5:67999755-67999799	NM_001033415:655	Shisa3	INSIDE	1.051	0.329	1560.48	514.04	0.346	1366.36	473.21
A_68_P27765282	chr11:45793719-45793763	NM_173384:-71	Sox30	PROMOTER	1.051	1.575	1214.37	1912.48	1.654	973.82	1611.11
A_68_P21833061	chr2:166821891-166821935	NM_001109905:-134	Stau1	PROMOTER	1.051	0.519	1805.84	937.10	0.545	1488.97	812.04
A_68_P29052711	chr13:55098855-55098899	NM_153131:48084	Unc5a	INSIDE	1.051	2.447	2647.86	6479.58	2.572	2006.29	5160.56
A_68_P27564655	chr11:5616910-5616954	NM_178623:24247	Urgcp	INSIDE	1.051	2.198	475.93	1046.27	2.310	430.77	994.96
A_68_P27926119	chr11:75258911-75258955	NM_138950:9287	Wdr81	INSIDE	1.051	2.013	1915.73	3855.87	2.115	1500.98	3174.13
A_68_P22148835	chr3:54437544-54437588	ENSMUST00000172420:2206		INSIDE	1.051	2.079	592.78	1232.16	2.184	496.45	1084.27
A_68_P32070028	chr19:32463753-32463797	NR_033198:69	2700046G09Rik	INSIDE	1.050	0.424	1428.36	605.23	0.445	1170.14	520.77
A_68_P26202778	chr8:119960709-119960753	NM_028941:87661	4933407C03Rik	INSIDE	1.050	1.915	383.68	734.86	2.011	334.99	673.69
A_68_P30512949	chr16:4964346-4964390	NM_028301:-38	Anks3	PROMOTER	1.050	2.010	2053.66	4127.84	2.111	1559.87	3292.99
A_68_P25957040	chr8:74005621-74005665	NM_001164679:4324	Ano8	INSIDE	1.050	3.611	1476.63	5332.22	3.793	1238.30	4696.38
A_68_P21013692	chr2:12931915-12931959	NM_153155:555	C1ql3	INSIDE	1.050	1.576	1567.65	2470.71	1.654	1278.29	2114.85
A_68_P26235087	chr8:125202646-125202690	NM_001109873:-593	Cbfa2t3	PROMOTER	1.050	1.683	682.28	1148.03	1.767	573.00	1012.51
A_68_P28727030	chr12:109512594-109512638	NM_001159910:1011	Ccdc85c	INSIDE	1.050	0.453	1493.68	676.58	0.476	1113.60	529.76
A_68_P30540341	chr16:9992483-9992527	NM_008170:122	Grin2a	INSIDE	1.050	1.990	1252.76	2493.26	2.090	1058.42	2212.56
A_68_P20587179	chr1:127808497-127808541	NM_145100:273	Lypd1	INSIDE	1.050	2.114	790.24	1670.53	2.219	786.51	1745.39
A_68_P27282701	chr10:79819469-79819513	NM_183152:287	Plk5	INSIDE	1.050	1.401	1074.47	1505.53	1.471	923.17	1357.77
A_68_P24117611	chr5:136209975-136210019	NM_008898:44913	Por	INSIDE	1.050	2.162	762.60	1648.69	2.269	697.84	1583.39
A_68_P23012915	chr4:71861101-71861145	NM_011599:831	Tle1	INSIDE	1.050	1.865	1133.65	2114.25	1.958	1008.58	1975.09
A_68_P32143438	chr19:46431466-46431510	NM_029186:119	Tmem180	INSIDE	1.050	1.925	722.99	1391.54	2.021	591.88	1196.06
A_68_P32144311	chr19:46577241-46577285	NM_053100:1125	Trim8	INSIDE	1.050	1.599	754.38	1206.13	1.678	670.76	1125.53
A_68_P30351138	chr15:76741648-76741692	NM_001168276:14208	Zfp647	INSIDE	1.050	1.826	512.82	936.61	1.917	428.50	821.38
A_68_P27288888	chr10:80788031-80788075	NM_027381:4205	2510012J08Rik	INSIDE	1.049	2.327	867.89	2019.48	2.441	819.38	2000.17
A_68_P22128531	chr3:51028825-51028869	NM_009834:478	Ccrn4l	INSIDE	1.049	2.349	1254.26	2945.68	2.463	1059.50	2610.00
A_68_P28992095	chr13:43880702-43880746	NM_009856:249	Cd83	INSIDE	1.049	1.769	1338.22	2367.69	1.855	1251.62	2322.16
A_68_P20741943	chr1:158134761-158134805	NM_177838:374	Fam163a	INSIDE	1.049	0.681	2702.78	1839.89	0.714	2009.85	1435.20
A_68_P29571201	chr14:46149666-46149710	NM_146054:52	Fermt2	INSIDE	1.049	1.977	456.96	903.43	2.074	472.78	980.78
A_68_P27812595	chr11:54716247-54716291	NM_001083929:-86	Gpx3	PROMOTER	1.049	1.768	979.29	1731.09	1.854	844.59	1566.24
A_68_P31255288	chr17:56465651-56465695	NM_172132:200	Kdm4b	INSIDE	1.049	3.446	1077.34	3712.56	3.616	972.87	3517.87
A_68_P21111931	chr2:31742789-31742833	NM_011836:10	Lamc3	INSIDE	1.049	4.163	2263.15	9421.92	4.369	1664.87	7274.08

ProbeName	Target position of probe on CpG island microarray	TargetID	GeneSymbol	CpG island Description	Ratio of relative methylation (TiO_2-NP/Vehicle)	Sham group			TiO_2-H group		
						Relative methylation (Cy5/Cy3)	Cy3 signal (Input)	Cy5 signal (MeDIP)	Relative methylation (Cy5/Cy3)	Cy3 signal (Input)	Cy5 signal (MeDIP)
A_68_P23839618	chr5:81758787-81758831	NM_198702:308191	Lphn3	INSIDE	1.049	1.859	1201.07	2233.14	1.950	1092.70	2130.77
A_68_P23324341	chr4:134259355-134259399	NM_207237:829	Man1c1	INSIDE	1.049	2.118	1675.38	3548.46	2.222	1487.47	3305.27
A_68_P27971660	chr11:83276484-83276528	NM_080453:-43	Mmp28	PROMOTER	1.049	1.399	1238.65	1732.52	1.468	1036.90	1521.65
A_68_P23617869	chr5:38550496-38550540	NM_010942:188	Nsg1	INSIDE	1.049	0.548	1884.92	1033.36	0.575	1339.06	770.13
A_68_P25089828	chr7:52125095-52125139	NM_133949:42	Ptov1	INSIDE	1.049	1.362	1562.37	2128.62	1.430	1255.48	1795.11
A_68_P28774234	chr12:118772641-118772685	NM_175930:17711	Rapgef5	INSIDE	1.049	2.364	335.52	793.07	2.481	270.92	672.04
A_68_P25349895	chr7:104474236-104474280	NM_001177412:216	Usp35	INSIDE	1.049	2.001	604.08	1208.82	2.099	669.02	1404.04
A_68_P30401628	chr15:85412235-85412279	NM_009528:-5	Wnt7b	PROMOTER	1.049	3.132	351.23	1100.02	3.286	387.70	1274.05
A_68_P23321770	chr4:133801322-133801366	NM_024215:162	Zfp593	INSIDE	1.049	1.984	1228.98	2437.74	2.080	1050.59	2185.13
A_68_P26465225	chr9:43828528-43828572			Unknown	1.049	4.163	480.45	2000.05	4.367	447.19	1952.97
A_68_P28089255	chr11:103995098-103995142	NM_007762:925	Crhr1	INSIDE	1.048	2.180	1742.31	3797.37	2.284	1525.84	3485.54
A_68_P30367146	chr15:79376680-79376724	NM_199079:287	Ddx17	INSIDE	1.048	1.518	1520.01	2306.90	1.591	1297.53	2064.19
A_68_P32463901	chrX:70885738-70885782	NM_029352:981	Dusp9	INSIDE	1.048	2.255	1021.84	2304.06	2.364	782.32	1849.10
A_68_P25565214	chr7:144506892-144506936	NM_001113414:-786	Ebf3	PROMOTER	1.048	1.828	481.15	879.64	1.917	463.32	888.02
A_68_P23200234	chr4:108952731-108952775	NM_007943:-127	Eps15	PROMOTER	1.048	1.629	2198.26	3581.63	1.707	1811.87	3093.69
A_68_P25828113	chr8:46129756-46129800	NM_001081286:94217	Fat1	INSIDE	1.048	3.776	2422.66	9146.86	3.955	1772.67	7010.95
A_68_P26141795	chr8:109394997-109395041	NM_008217:877	Has3	INSIDE	1.048	2.766	1222.06	3379.71	2.899	903.80	2619.99
A_68_P20156459	chr1:37947346-37947390	NM_027098:-29	Mrpl30	DIVERGENT_PROMOTER	1.048	0.644	2410.00	1552.09	0.675	1960.83	1323.03
A_68_P24993781	chr7:20109373-20109417	NM_027116:5315	Nkpd1	INSIDE	1.048	2.383	1383.75	3297.38	2.496	1230.05	3070.42
A_68_P24754175	chr6:112439230-112439274	NM_001081147:550	Oxtr	INSIDE	1.048	1.991	636.99	1268.46	2.087	605.87	1264.22
A_68_P24618287	chr6:87761915-87761959	NM_001039394:-163	Rab43	PROMOTER	1.048	3.263	6897.71	22505.88	3.419	4611.99	15767.68
A_68_P31925086	chr19:4787882-4787926	NM_009032:5973	Rbm4	INSIDE	1.048	3.117	2028.35	6322.46	3.267	1572.73	5137.99
A_68_P27641466	chr11:22412783-22412827	NM_153596:519	Tmem17	INSIDE	1.048	0.584	1407.57	821.64	0.612	1229.51	752.42
A_68_P26644961	chr9:75458869-75458913	NM_016711:242	Tmod2	INSIDE	1.048	2.402	631.49	1517.09	2.519	584.43	1471.94
A_68_P29097418	chr13:64254293-64254337	NM_175494:193	Zfp367	INSIDE	1.048	1.750	908.79	1590.78	1.835	743.70	1364.40
A_68_P24862403	A_68_P24862403			Unknown	1.048	20.204	320.02	6465.77	21.166	244.54	5176.12
A_68_P26584120	chr9:64659199-64659243	NM_001162917:403	Dennd4a	INSIDE	1.047	0.596	1289.86	768.18	0.624	1185.43	739.14
A_68_P23981795	chr5:111199426-111199470	NM_029337:288	Ep400	INSIDE	1.047	4.730	11570.70	54734.01	4.952	8287.44	41043.16
A_68_P23270839	chr4:124558845-124558889	NM_177671:-162	Epha10	PROMOTER	1.047	0.668	1790.53	1196.25	0.699	1538.33	1075.72
A_68_P28492940	chr12:66018887-66018931	NM_001025605:11	Gm527	INSIDE	1.047	2.490	1105.72	2753.14	2.607	999.15	2604.59
A_68_P24904451	chr6:142363030-142363089	NM_145572:58570	Gys2	DOWNSTREAM	1.047	2.695	1234.14	3325.79	2.822	795.13	2243.52
A_68_P31208331	chr17:46818042-46818086	NM_010787:-21	Mea1	DIVERGENT_PROMOTER	1.047	2.264	4024.30	9112.49	2.370	2451.76	5811.57
A_68_P28057821	chr11:98544135-98544179	NM_009439:289	Psmd3	INSIDE	1.047	1.824	2282.55	4163.46	1.909	1794.48	3426.56
A_68_P23018374	chr4:73436830-73436874	NM_001017427:-346	Rasef	PROMOTER	1.047	2.450	436.09	1068.61	2.565	439.75	1128.06
A_68_P21000493	chr2:10294275-10294319	NM_001198809:234	Sfmbt2	INSIDE	1.047	1.768	1264.93	2236.73	1.852	1200.31	2222.74
A_68_P32005495	chr19:21347202-21347246	NM_009551:457	Zfand5	INSIDE	1.047	1.709	350.84	599.41	1.789	366.47	655.58
A_68_P24951677	chr7:4982359-4982403	NM_001033383:10403	Zfp865	INSIDE	1.047	2.175	704.58	1532.78	2.278	649.15	1478.81
A_68_P26948855	chr10:12780660-12780705	FJ425893:373		INSIDE	1.047	1.874	860.53	1613.05	1.962	740.60	1453.24
A_68_P23327635	chr4:134828183-134828227	NM_013885:471	Clic4	INSIDE	1.046	2.012	680.57	1369.61	2.105	665.49	1401.01
A_68_P27289088	chr10:80820220-80820264	NM_028657:-72	F630110N24Rik	DIVERGENT_PROMOTER	1.046	1.901	475.19	903.48	1.988	471.41	937.16
A_68_P31159262	chr17:35607004-35607048	NM_010391:-7	H2-Q10	PROMOTER	1.046	2.079	1028.06	2137.54	2.175	924.66	2010.83
A_68_P20799111	chr1:168168733-168168779	NM_175296:119	Mael	INSIDE	1.046	2.369	332.64	788.10	2.478	283.30	702.14
A_68_P30346618	chr15:76012196-76012240	NM_201394:13922	Plec	INSIDE	1.046	1.801	369.10	664.84	1.883	368.36	693.77
A_68_P22825897	chr4:33334510-33334554	NM_001033225:1230	Pnrc1	INSIDE	1.046	0.642	1333.43	856.62	0.672	1142.52	767.72
A_68_P26167600	chr8:113824005-113824049	NM_146218:96	Rfwd3	INSIDE	1.046	0.555	3761.29	2086.67	0.580	3013.35	1747.97
A_68_P32562269	chrX:98255087-98255141	NM_031384:-136	Tex11	PROMOTER	1.046	2.096	1224.91	2567.23	2.193	466.72	1023.46
A_68_P24428350	chr6:48570111-48570155	NM_173429:6954	Zfp775	INSIDE	1.046	2.360	1162.00	2742.71	2.468	971.23	2397.32
A_68_P28183976	chr11:120211624-120211668	NR_038126:1655	0610009L18Rik	INSIDE	1.045	2.195	665.37	1460.41	2.295	601.76	1380.79
A_68_P21068909	chr2:24790606-24790650	NM_001162485:173	Arrdc1	INSIDE	1.045	0.467	1546.09	722.38	0.488	1310.47	639.65
A_68_P29613381	chr14:55251101-55251145	NM_199470:9079	Cdh24	INSIDE	1.045	2.153	1071.20	2306.36	2.249	952.55	2142.57
A_68_P27954535	chr11:80290099-80290143	NM_009871:-427	Cdk5r1	PROMOTER	1.045	1.772	957.36	1696.41	1.852	825.51	1529.14

ProbeName	Target position of probe on CpG island microarray	TargetID	GeneSymbol	CpG island Description	Ratio of relative methylation (TiO_2-NP/Vehicle)	Sham group			TiO_2-H group		
						Relative methylation (Cy5/Cy3)	Cy3 signal (Input)	Cy5 signal (MeDIP)	Relative methylation (Cy5/Cy3)	Cy3 signal (Input)	Cy5 signal (MeDIP)
A_68_P26504860	chr9:50467402-50467446	NM_145614:461	Dlat	INSIDE	1.045	0.604	1224.75	740.09	0.632	1142.35	721.57
A_68_P30512416	chr16:4879609-4879653	NM_145359:221	Fam100a	INSIDE	1.045	0.613	1038.60	636.17	0.640	901.87	577.51
A_68_P24112572	chr5:134790667-134790711	NM_001080746:-72	Gtf2i	PROMOTER	1.045	2.524	1564.41	3949.03	2.638	1374.38	3625.59
A_68_P26021243	chr8:87225571-87225617	NM_008535:239	Lyl1	INSIDE	1.045	1.688	1632.11	2755.34	1.764	1303.98	2300.28
A_68_P31556967	chr18:23962508-23962552	NM_153058:60	Mapre2	INSIDE	1.045	1.384	2864.47	3964.89	1.447	2179.63	3153.12
A_68_P27855154	chr11:62478652-62478704	NM_175002:16513	Mmgt2	INSIDE	1.045	1.836	365.93	671.76	1.919	332.43	637.78
A_68_P29314989	chr13:109906595-109906639	NM_011056:462246	Pde4d	INSIDE	1.045	0.634	1460.52	925.84	0.662	1194.99	791.55
A_68_P26350663	chr9:21961255-21961299	NM_001082532:-13	Pigyl	PROMOTER	1.045	3.047	1304.98	3976.91	3.184	1021.61	3252.37
A_68_P21749132	chr2:152224537-152224581	NM_011438:-776	Sox12	PROMOTER	1.045	2.310	611.12	1411.45	2.413	577.78	1393.90
A_68_P25030459	chr7:31016969-31017013	NM_025548:58	Tbcb	INSIDE	1.045	0.482	1388.93	669.17	0.504	1198.39	603.56
A_68_P27644762	chr11:23155768-23155812	NM_001035226:-250	Xpo1	PROMOTER	1.045	0.679	2353.67	1597.27	0.709	1775.56	1258.75
A_68_P28751777	chr12:113917151-113917195	NM_001100460:122	Zbtb42	INSIDE	1.045	2.009	869.55	1746.68	2.099	848.73	1781.51
A_68_P24596107	chr6:83864866-83864910	NM_001166371:542	Zfml	INSIDE	1.045	1.530	722.16	1104.90	1.598	583.64	932.73
A_68_P26038359	chr8:90483614-90483658	NM_033327:-142	Zfp423	PROMOTER	1.045	1.373	1466.81	2014.03	1.435	1255.04	1801.28
A_68_P26235083	A_68_P26235083			Unknown	1.045	2.431	553.02	1344.46	2.541	473.46	1202.92
A_68_P23334942	chr4:136193562-136193606	ENSMUST00000146123:15078		INSIDE	1.045	0.585	1122.30	656.00	0.611	1046.54	639.31
A_68_P21969036	chr3:17955303-17955347	NM_021560:1000	Bhlhe22	INSIDE	1.044	1.685	837.92	1412.20	1.760	807.05	1420.08
A_68_P26135495	chr8:108228498-108228542	NM_198299:3548	E130303B06Rik	INSIDE	1.044	3.241	897.85	2910.09	3.385	658.64	2229.34
A_68_P24993062	chr7:19979665-19979709	NM_007949:12299	Ercc2	INSIDE	1.044	2.893	276.61	800.13	3.019	227.67	687.30
A_68_P23416734	chr4:151506423-151506467	NM_019585:-3410	Espn	PROMOTER	1.044	1.554	1907.23	2964.13	1.622	1427.44	2315.43
A_68_P24046863	chr5:122299245-122299289	NM_175474:230	Fam109a	INSIDE	1.044	1.865	1301.20	2427.36	1.948	1093.39	2129.73
A_68_P25531564	chr7:139158932-139158976	NM_173410:812	Gpr26	INSIDE	1.044	0.575	1305.07	749.95	0.600	1046.30	627.96
A_68_P28605005	chr12:86939999-86940043	NM_001205052:-34	Jdp2	PROMOTER	1.044	1.828	1103.33	2017.21	1.909	1020.13	1947.39
A_68_P26022553	chr8:87426545-87426589	NM_010635:740	Klf1	INSIDE	1.044	1.392	1297.55	1806.29	1.454	1080.75	1571.32
A_68_P30148787	chr15:38229176-38229220	NM_013692:1264	Klf10	INSIDE	1.044	2.644	386.74	1022.47	2.761	400.73	1106.49
A_68_P25951938	chr8:72976513-72976557	NM_001122830:24308	Klhl26	INSIDE	1.044	2.053	551.03	1131.07	2.142	496.89	1064.42
A_68_P29269304	chr13:101386895-101386939	NM_178410:-17	Marveld2	PROMOTER	1.044	1.795	1108.65	1990.45	1.874	1096.50	2054.39
A_68_P30083234	chr15:25710990-25711034	NM_019472:158708	Myo10	INSIDE	1.044	3.394	281.59	955.84	3.545	230.09	815.76
A_68_P30480087	chr15:99256378-99256422	NM_001001884:31779	Nckap5l	INSIDE	1.044	2.160	708.07	1529.56	2.255	586.30	1322.29
A_68_P23544555	chr5:24414575-24414619	NM_001170555:-269	Prkag2	PROMOTER	1.044	2.782	288.31	802.23	2.906	255.47	742.26
A_68_P25094157	chr7:52887692-52887738	NM_028544:4808	Rasip1	INSIDE	1.044	1.716	1975.86	3390.32	1.792	1625.60	2913.17
A_68_P27833618	chr11:58752100-58752144	NM_022423:286	Rnf187	INSIDE	1.044	1.443	944.71	1363.65	1.507	868.54	1309.22
A_68_P27798413	chr11:52096958-52097002	NM_009331:-1228	Tcf7	PROMOTER	1.044	0.571	928.18	529.67	0.596	864.90	515.51
A_68_P26845368	chr9:114471438-114471482	NM_001042503:2027	Trim71	INSIDE	1.044	2.685	604.08	1621.88	2.804	480.27	1346.57
A_68_P22970877	chr4:63156474-63156518	NM_001008791:489	Whrn	INSIDE	1.044	1.760	3493.85	6149.62	1.837	2795.05	5134.03
A_68_P20859687	chr1:179372004-179372048	NM_001012330:-2765	Zfp238	PROMOTER	1.044	0.546	1085.20	592.60	0.570	902.20	514.48
A_68_P32634536	chrX:120217471-120217515	NM_028426:325	3110007F17Rik	INSIDE	1.043	1.383	4445.64	6147.00	1.442	2163.02	3119.10
A_68_P24800466	chr6:120772672-120772716	NM_007510:9	Atp6v1e1	INSIDE	1.043	1.400	1144.93	1602.54	1.460	1022.63	1493.49
A_68_P24863574	chr6:134742286-134742330	NM_001048054:338	Dusp16	INSIDE	1.043	1.876	587.19	1101.66	1.958	575.64	1126.84
A_68_P25564250	chr7:144390350-144390394	NM_001113414:115756	Ebf3	INSIDE	1.043	4.902	2181.37	10692.79	5.111	1666.03	8514.54
A_68_P22569283	chr3:138189837-138189881	NM_007917:704	Eif4e	INSIDE	1.043	2.081	415.97	865.56	2.170	383.89	832.98
A_68_P24466826	chr6:54988021-54988065	NM_180678:48	Gars	INSIDE	1.043	2.327	1113.92	2592.41	2.426	1037.95	2518.29
A_68_P24448669	chr6:52108855-52108899	NM_010449:-560	Hoxa1	PROMOTER	1.043	0.342	1799.12	614.87	0.357	1544.28	550.61
A_68_P23756016	chr5:65770881-65770930	NM_031180:31256	Klb	INSIDE	1.043	2.516	232.44	584.88	2.624	208.75	547.83
A_68_P26537261	chr9:56467792-56467836	NM_181074:65246	Lingo1	INSIDE	1.043	1.712	2456.36	4205.16	1.786	1592.78	2844.00
A_68_P27542074	chr10:128002848-128002892	NM_011119:120	Pa2g4	INSIDE	1.043	0.572	1497.55	856.73	0.597	1231.77	735.32
A_68_P31629586	chr18:37881660-37881706	NM_033591:-3677	Pcdhga8	PROMOTER	1.043	1.839	491.62	903.97	1.917	419.24	803.83
A_68_P26293554	chr9:8901675-8901719	NM_008829:1864	Pgr	INSIDE	1.043	2.294	779.59	1788.72	2.392	749.73	1793.51
A_68_P25972608	chr8:77736812-77736856	NM_029182:-1008	Rasd2	PROMOTER	1.043	0.641	3512.42	2251.36	0.668	2725.01	1821.52
A_68_P33007022	A_68_P33007022			Unknown	1.043	2.062	31059.58	64030.31	2.150	25863.33	55609.81
A_68_P24127513	chr5:138220985-138221029	NM_027242:861	2010007H12Rik	INSIDE	1.042	1.733	1168.45	2025.09	1.807	928.40	1677.37

ProbeName	Target position of probe on CpG island microarray	TargetID	GeneSymbol	CpG island Description	Ratio of relative methylation (TiO_2-NP/Vehicle)	Sham group			TiO_2-H group		
						Relative methylation (Cy5/Cy3)	Cy3 signal (Input)	Cy5 signal (MeDIP)	Relative methylation (Cy5/Cy3)	Cy3 signal (Input)	Cy5 signal (MeDIP)
A_68_P30778637	chr16:55974341-55974385	NR_027965:368	2310061J03Rik	INSIDE	1.042	1.663	1118.02	1859.62	1.733	996.69	1727.51
A_68_P22142064	chr3:53282739-53282783	NM_173382:15022	2810046L04Rik	INSIDE	1.042	3.519	5006.99	17618.96	3.666	3465.81	12705.92
A_68_P32233088	chrX:5660010-5660054	NM_001033211:506	AU022751	INSIDE	1.042	1.522	3159.39	4809.60	1.586	1454.19	2305.82
A_68_P21067991	chr2:24618118-24618162	NM_001042528:427	Cacna1b	INSIDE	1.042	1.615	2750.23	4441.73	1.683	2350.54	3955.88
A_68_P31921836	chr19:4214356-4214400	NM_019952:-13	Clcf1	PROMOTER	1.042	1.836	1029.34	1890.17	1.913	958.78	1834.13
A_68_P33015620	chr1_random:50449-50497	NM_026866:134866	Disp1	INSIDE	1.042	2.771	442.48	1226.17	2.889	347.14	1002.75
A_68_P23392782	chr4:147538404-147538448	NM_176848:3650	Fbxo2	INSIDE	1.042	1.866	871.05	1625.16	1.944	765.16	1487.79
A_68_P22448209	chr3:115954867-115954911	NM_022427:1514	Gpr88	INSIDE	1.042	2.531	539.14	1364.84	2.639	501.70	1323.88
A_68_P22210129	chr3:67267888-67267932	NM_016753:-81	Lxn	PROMOTER	1.042	1.515	927.53	1404.92	1.578	734.14	1158.50
A_68_P30000056	chr15:8395496-8395540	NM_027707:-1055	Nipbl	PROMOTER	1.042	1.520	857.10	1303.04	1.584	834.71	1321.84
A_68_P25092986	chr7:52670982-52671026	NM_198190:1940	Ntf5	INSIDE	1.042	2.173	407.44	885.24	2.265	367.05	831.33
A_68_P25499770	chr7:133436200-133436244	NM_145587:20090	Sbk1	INSIDE	1.042	2.112	1006.85	2126.92	2.201	796.38	1753.09
A_68_P26142247	chr8:109459545-109459589	NM_009229:-83	Sntb2	PROMOTER	1.042	2.493	736.32	1835.98	2.599	644.29	1674.54
A_68_P24163027	chr5:145612055-145612099	NM_001081362:82416	Trrap	INSIDE	1.042	1.919	830.14	1592.73	1.998	577.01	1153.12
A_68_P29097416	chr13:64254085-64254129	NM_175494:401	Zfp367	INSIDE	1.042	3.502	925.04	3239.57	3.648	847.03	3089.96
A_68_P23299680	chr4:129684126-129684170	NM_001199696:21827	Bai2	INSIDE	1.041	2.939	565.69	1662.69	3.059	499.91	1529.35
A_68_P21646221	chr2:133378682-133378726	NM_007553:-230	Bmp2	PROMOTER	1.041	2.647	1696.40	4490.61	2.755	1388.78	3825.80
A_68_P23351275	chr4:138908526-138908570	NM_001039200:41	C230096C10Rik	INSIDE	1.041	1.718	2977.06	5115.51	1.789	2277.70	4075.38
A_68_P24631550	chr6:90259089-90259133	NM_027928:16069	Chst13	INSIDE	1.041	1.923	930.71	1790.20	2.002	772.41	1546.42
A_68_P22305775	chr3:86723959-86724003	NM_001195496:826	Dclk2	INSIDE	1.041	1.575	1090.96	1718.79	1.640	975.17	1599.18
A_68_P23280649	chr4:126210358-126210402	NM_153177:322	Eif2c4	INSIDE	1.041	2.923	1288.88	3767.39	3.042	1349.21	4104.17
A_68_P28563526	chr12:79327709-79327753	NM_145965:89	Gphn	INSIDE	1.041	1.667	692.64	1154.90	1.736	666.40	1156.92
A_68_P21834173	chr2:167013977-167014021	NM_008420:301	Kcnb1	INSIDE	1.041	2.569	1455.04	3738.70	2.676	1336.33	3575.85
A_68_P25951931	chr8:72975757-72975801	NM_001122830:25064	Klhl26	INSIDE	1.041	2.492	367.21	915.26	2.596	336.42	873.19
A_68_P31130476	chr17:30026783-30026828	NM_001081160:-1978	Mdga1	PROMOTER	1.041	1.819	668.52	1215.82	1.893	543.92	1029.49
A_68_P26017636	chr8:86536527-86536571	NR_035431:-1016	Mir1199	DIVERGENT_PROMOTER	1.041	0.572	1480.52	846.54	0.595	1269.41	755.29
A_68_P26164570	chr8:113262638-113262682	NM_198625:17277	Mtss1l	INSIDE	1.041	1.891	354.50	670.49	1.968	342.76	674.59
A_68_P20474868	chr1:99991929-99991973	NM_013626:259	Pam	INSIDE	1.041	0.520	2382.96	1240.10	0.542	2136.84	1157.41
A_68_P26504618	chr9:50425465-50425510	NM_028300:62	Pih1d2	INSIDE	1.041	1.732	855.79	1482.45	1.804	815.32	1470.88
A_68_P26823384	chr9:110283423-110283467	NM_001001144:47648	Scap	INSIDE	1.041	2.276	508.24	1156.95	2.369	440.12	1042.78
A_68_P22218623	chr3:68808767-68808811	NM_133786:-105	Smc4	DIVERGENT_PROMOTER	1.041	1.704	932.28	1588.71	1.774	863.15	1531.25
A_68_P22336393	chr3:93247754-93247798	NM_001163098:1525	Tchh	INSIDE	1.041	1.830	872.91	1597.72	1.905	701.58	1336.32
A_68_P25011333	chr7:26472908-26472952	NM_011577:910	Tgfb1	INSIDE	1.041	1.606	653.23	1049.15	1.671	561.79	938.88
A_68_P29880578	chr14:106505252-106505296	NM_198601:-140	Trim52	PROMOTER	1.041	2.026	319.67	647.56	2.108	265.76	560.32
A_68_P24124211	chr5:137505950-137505994	NM_001039385:-192	Vgf	PROMOTER	1.041	1.723	843.90	1454.12	1.794	762.20	1367.58
A_68_P31092444	chr17:23713564-23713608	NM_011747:22868	Zfp13	INSIDE	1.041	2.787	934.23	2603.98	2.903	790.19	2293.70
A_68_P24065807	chr5:125726086-125726130			Unknown	1.041	0.418	1395.60	583.26	0.435	1041.88	453.23
A_68_P31149281	chr17:33661172-33661216	NM_172619:54	Adamts10	INSIDE	1.040	0.619	1420.75	880.04	0.644	1104.84	711.93
A_68_P26023161	chr8:87535204-87535248	NM_001130193:3004	Best2	INSIDE	1.040	1.483	1355.93	2011.05	1.542	1274.61	1965.91
A_68_P32268345	chrX:13423285-13423329	NM_009806:361	Cask	INSIDE	1.040	2.319	513.28	1190.17	2.412	330.41	796.96
A_68_P23336932	chr4:136501941-136501985	NM_007939:10769	Epha8	INSIDE	1.040	2.435	1675.12	4078.45	2.532	1277.19	3233.46
A_68_P30720006	chr16:44173216-44173260	NM_001029889:-271	Gm608	PROMOTER	1.040	1.359	2434.22	3307.92	1.414	1899.95	2685.64
A_68_P28748382	chr12:113412288-113412332	NM_001097621:27892	Kif26a	INSIDE	1.040	2.659	685.56	1822.57	2.766	590.06	1632.09
A_68_P24378364	chr6:38586770-38586814	NM_001033171:447	Klrg2	INSIDE	1.040	2.189	1205.08	2637.46	2.276	1086.49	2472.73
A_68_P22210124	chr3:67267480-67267524	NM_016753:327	Lxn	INSIDE	1.040	0.625	1803.38	1127.06	0.650	1374.83	893.78
A_68_P27279099	chr10:79281830-79281874	NM_001161747:25536	Palm	INSIDE	1.040	2.752	274.85	756.34	2.862	195.87	560.53
A_68_P30346634	chr15:76014247-76014291	NM_201394:11872	Plec	INSIDE	1.040	2.761	1003.49	2770.64	2.873	761.57	2187.78
A_68_P21417953	chr2:90419832-90419876	NM_008982:950	Ptprj	INSIDE	1.040	1.979	1396.89	2764.15	2.057	1241.18	2553.45
A_68_P29675226	chr14:66349915-66349959	NM_001168318:64697	Scara5	INSIDE	1.040	1.882	563.51	1060.26	1.956	485.53	949.92
A_68_P32168795	chr19:50753385-50753429	NM_021377:-304	Sorcs1	PROMOTER	1.040	1.427	1397.99	1994.69	1.484	1288.34	1912.38
A_68_P31513732	chr18:14840635-14840679	NM_001161369:767	Ss18	INSIDE	1.040	1.438	1617.10	2325.69	1.496	1518.86	2272.81

ProbeName	Target position of probe on CpG island microarray	TargetID	GeneSymbol	CpG island Description	Ratio of relative methylation (TiO_2-NP/Vehicle)	Sham group			TiO_2-H group		
						Relative methylation (Cy5/Cy3)	Cy3 signal (Input)	Cy5 signal (MeDIP)	Relative methylation (Cy5/Cy3)	Cy3 signal (Input)	Cy5 signal (MeDIP)
A_68_P21612437	chr2:127095568-127095612	NM_139308:-374	Stard7	PROMOTER	1.040	1.435	1214.92	1742.80	1.492	1099.80	1640.49
A_68_P25614230	chr8:3496425-3496469	NM_080461:3309	Zfp358	INSIDE	1.040	2.652	2418.50	6414.67	2.757	1936.61	5340.06
A_68_P22630570	chr3:148652214-148652258	ENSMUST00000098518:44		INSIDE	1.040	1.400	2742.44	3840.44	1.456	2214.13	3223.95
A_68_P20936908	chr1:193048383-193048427	ENSMUST00000139056:52		INSIDE	1.040	1.412	996.51	1406.78	1.469	864.50	1269.72
A_68_P20456909	chr1:95582007-95582051	NM_016778:-242	Bok	PROMOTER	1.039	1.853	1276.27	2365.49	1.926	1214.63	2339.16
A_68_P23818508	chr5:77287446-77287490	NM_001163793:17845	C530008M17Rik	INSIDE	1.039	1.758	869.28	1528.10	1.826	659.57	1204.48
A_68_P23440734	chr4:155186580-155186624	NM_207678:5	Ccnl2	INSIDE	1.039	0.435	2062.61	896.78	0.452	1736.83	784.33
A_68_P26843397	chr9:113935916-113935960	NM_178624:-69	Fbxl2	PROMOTER	1.039	0.578	1245.84	720.25	0.601	1178.26	707.58
A_68_P20553868	chr1:120734527-120734571	NM_001081125:215648	Gli2	INSIDE	1.039	2.093	1221.98	2557.06	2.173	847.19	1841.31
A_68_P23438947	chr4:154866365-154866409	NM_001160016:917	Gnb1	INSIDE	1.039	0.439	2353.45	1032.60	0.456	1960.08	893.17
A_68_P26867068	chr9:118468753-118468797	NM_018748:53339	Golga4	INSIDE	1.039	2.785	1156.63	3221.46	2.893	928.84	2687.11
A_68_P25525510	chr7:138079636-138079680	NM_019564:-58	Htra1	PROMOTER	1.039	1.463	1067.31	1561.54	1.520	886.73	1348.05
A_68_P32029536	chr19:25484468-25484512	NM_181404:172799	Kank1	INSIDE	1.039	2.607	1038.04	2706.33	2.709	826.18	2238.26
A_68_P25018409	chr7:28443259-28443303	NM_001081292:16337	Map3k10	INSIDE	1.039	1.733	2167.04	3754.61	1.800	1817.53	3271.69
A_68_P25954142	chr8:73321226-73321270	NM_199308:-4916	Mast3	PROMOTER	1.039	2.706	4368.49	11819.08	2.811	2760.84	7760.00
A_68_P22039667	chr3:32635840-32635884	NM_025316:-122	Ndufb5	DIVERGENT_PROMOTER	1.039	1.470	1598.16	2348.95	1.527	1266.94	1934.85
A_68_P27261658	chr10:75837803-75837847	NM_008787:67833	Pcnt	INSIDE	1.039	5.183	127.46	660.66	5.388	126.38	680.94
A_68_P27362365	chr10:94407441-94407485	NM_018797:-250	Plxnc1	PROMOTER	1.039	1.774	567.67	1007.33	1.844	450.72	831.11
A_68_P20598630	chr1:130000293-130000337	NM_181750:432	R3hdm1	INSIDE	1.039	3.943	390.35	1538.97	4.098	433.83	1777.67
A_68_P21747682	chr2:151931567-151931611	NM_029688:123	Srxn1	INSIDE	1.039	0.441	2156.03	950.82	0.458	1629.59	746.88
A_68_P29734164	chr14:76904319-76904363	NM_009366:24	Tsc22d1	INSIDE	1.039	1.787	470.76	841.10	1.857	446.09	828.18
A_68_P23185482	chr4:106295106-106295150	NM_177667:75	Ttc22	INSIDE	1.039	3.272	1376.86	4504.94	3.399	1162.89	3953.08
A_68_P28160202	chr11:116442703-116442747	NM_173755:37	Ube2o	INSIDE	1.039	2.036	1996.47	4063.88	2.114	1789.66	3784.15
A_68_P30571779	chr16:17070377-17070421	NM_023249:-5	Ypel1	PROMOTER	1.039	3.962	934.43	3702.52	4.115	866.05	3564.00
A_68_P28939815	chr13:34744074-34744118	NM_025831:454	1300014I06Rik	INSIDE	1.038	0.626	1166.07	730.49	0.650	995.92	647.60
A_68_P28420020	chr12:50492378-50492422	NR_026733:1762	3110039M20Rik	INSIDE	1.038	1.364	1668.80	2275.82	1.415	1462.19	2068.92
A_68_P28198915	chr12:4133424-4133468	NM_138305:-706	Adcy3	PROMOTER	1.038	1.655	1073.26	1776.42	1.719	945.52	1625.09
A_68_P25415075	chr7:117912319-117912363	NM_009667:-3777	Ampd3	PROMOTER	1.038	3.083	473.86	1460.75	3.201	423.67	1356.15
A_68_P25537314	chr7:140051211-140051255	NM_198017:325	Fam175b	INSIDE	1.038	1.511	726.58	1097.76	1.569	590.46	926.24
A_68_P27284855	chr10:80154115-80154159	NM_172457:10429	Mobkl2a	INSIDE	1.038	1.781	498.65	888.18	1.848	439.66	812.52
A_68_P20377655	chr1:79772877-79772921	NM_001081210:306	Mrpl44	INSIDE	1.038	2.123	914.60	1941.32	2.204	839.76	1850.73
A_68_P23260198	chr4:122673656-122673700	NM_008506:337	Mycl1	INSIDE	1.038	2.448	1007.83	2466.73	2.540	904.36	2296.66
A_68_P28178596	chr11:119410661-119410705	NM_008730:-1548	Nptx1	PROMOTER	1.038	1.426	839.25	1196.76	1.481	745.33	1103.61
A_68_P26742500	chr9:95460884-95460928	NM_198414:671	Paqr9	INSIDE	1.038	2.436	1252.77	3052.29	2.529	1258.48	3182.23
A_68_P27278598	chr10:79198902-79198960	NM_172551:10396	Polrmt	INSIDE	1.038	3.322	984.85	3272.10	3.450	777.88	2683.31
A_68_P23649335	chr5:44492359-44492403	NM_001163584:-6373	Prom1	PROMOTER	1.038	1.951	3737.68	7292.88	2.025	2978.63	6031.96
A_68_P27287274	chr10:80542595-80542639	NM_022653:9789	Thop1	INSIDE	1.038	4.436	1488.23	6602.37	4.604	1068.13	4917.69
A_68_P23340750	chr4:137150373-137150417	NM_130879:291	Usp48	INSIDE	1.038	0.464	2020.14	938.28	0.482	1501.88	724.18
A_68_P31120933	chr17:28314032-28314076	NM_172617:-308	Zfp523	PROMOTER	1.038	2.786	696.21	1939.46	2.893	639.86	1850.80
A_68_P25769255	chr8:34627820-34627864	ENSMUST00000124496:47		INSIDE	1.038	3.548	786.16	2788.97	3.682	751.30	2766.48
A_68_P32097048	chr19:37767375-37767419	NM_001105201:7227	Cyp26c1	INSIDE	1.037	0.617	1787.14	1103.34	0.640	1365.30	874.17
A_68_P28558336	chr12:78340320-78340364	NM_016893:335	Fut8	INSIDE	1.037	2.225	639.18	1422.50	2.308	614.72	1418.76
A_68_P29612959	chr14:55172748-55172792	NM_145462:428	Haus4	INSIDE	1.037	1.658	982.69	1629.49	1.719	936.91	1610.96
A_68_P28067971	chr11:100275626-100275670	NM_176830:485	Leprel4	INSIDE	1.037	1.591	1039.17	1653.26	1.650	1005.57	1659.17
A_68_P23837517	chr5:81448669-81448713	NM_198702:-1927	Lphn3	PROMOTER	1.037	2.101	632.54	1328.75	2.179	663.81	1446.45
A_68_P30447287	chr15:93333954-93333998	NM_001033217:92346	Prickle1	INSIDE	1.037	3.695	766.22	2830.95	3.832	677.95	2597.98
A_68_P24983629	chr7:17441116-17441160	NM_178900:12725	Prkd2	INSIDE	1.037	3.555	992.75	3529.03	3.685	790.63	2913.61
A_68_P26349530	chr9:21722254-21722298	NM_031874:289	Rab3d	INSIDE	1.037	1.541	639.79	985.72	1.598	696.64	1113.24
A_68_P26816016	chr9:108564748-108564792	NM_020520:342	Slc25a20	INSIDE	1.037	3.447	447.33	1541.75	3.574	449.75	1607.19
A_68_P26042147	chr8:91151173-91151217	NM_027840:8833	Snx20	INSIDE	1.037	2.059	367.65	756.90	2.135	339.76	725.27
A_68_P21266382	chr2:61643297-61643341	NM_009322:809	Tbr1	INSIDE	1.037	0.446	1421.80	634.29	0.463	1192.75	551.66

ProbeName	Target position of probe on CpG island microarray	TargetID	GeneSymbol	CpG island Description	Ratio of relative methylation (TiO_2-NP/Vehicle)	Sham group			TiO_2-H group		
						Relative methylation (Cy5/Cy3)	Cy3 signal (Input)	Cy5 signal (MeDIP)	Relative methylation (Cy5/Cy3)	Cy3 signal (Input)	Cy5 signal (MeDIP)
A_68_P25701520	chr8:18950897-18950941	NM_001113350:55	Xkr5	INSIDE	1.037	1.822	1512.55	2756.17	1.889	1278.15	2414.99
A_68_P24951573	chr7:4966992-4967036	NM_025324:-95	Zfp524	DIVERGENT_PROMOTER	1.037	1.743	1867.69	3256.12	1.809	1541.37	2787.69
A_68_P27590827	chr11:11362280-11362324	NM_001185153:120	Zpbp	INSIDE	1.037	1.561	685.69	1070.22	1.618	651.09	1053.62
A_68_P31116860	chr17:27650768-27650812	ENSMUST00000088073:-504		DIVERGENT_PROMOTER	1.037	1.512	879.05	1329.36	1.568	733.42	1149.87
A_68_P31791923	chr18:67608414-67608458	NM_027130:354	Afg3l2	INSIDE	1.036	1.438	1375.24	1977.83	1.490	1222.52	1821.31
A_68_P32710890	chrX:139401516-139401560	NM_019496:-267	Ammecr1	PROMOTER	1.036	4.018	347.15	1394.69	4.162	215.96	898.81
A_68_P27278226	chr10:79144635-79144681	NM_177613:-281	Cdc34	PROMOTER	1.036	1.428	1182.18	1688.34	1.479	1022.12	1511.95
A_68_P23448649	chr5:4104754-4104798	NM_020010:-79	Cyp51	PROMOTER	1.036	0.596	1307.18	778.61	0.617	1031.83	636.76
A_68_P31258212	chr17:56901669-56901713	NM_144858:-2483	Dus3l	DIVERGENT_PROMOTER	1.036	1.343	2116.79	2841.86	1.391	1599.41	2224.80
A_68_P28187033	chr11:120685676-120685720	NM_007988:163	Fasn	INSIDE	1.036	1.650	2813.18	4640.61	1.710	2482.17	4243.68
A_68_P24115931	chr5:135825936-135825980	NM_033571:-97	Fkbp6	DIVERGENT_PROMOTER	1.036	2.488	621.20	1545.26	2.578	519.02	1338.02
A_68_P21874405	chr2:174111730-174111779	NM_019690:1934	Gnas	INSIDE	1.036	1.839	537.74	988.94	1.905	404.64	770.93
A_68_P28997685	chr13:44829480-44829524	NM_001205043:2863	Jarid2	INSIDE	1.036	0.548	1286.57	705.20	0.568	1014.16	576.16
A_68_P26643862	chr9:75257222-75257266	NM_027418:-78	Mapk6	PROMOTER	1.036	2.289	799.88	1830.75	2.371	724.01	1716.95
A_68_P23345718	chr4:137990544-137990588	NM_026689:-20	Mul1	PROMOTER	1.036	1.630	2063.11	3362.57	1.689	1589.54	2684.37
A_68_P31866222	chr18:80894933-80894977	NM_001164111:9958	Nfatc1	INSIDE	1.036	2.134	555.22	1185.09	2.212	487.50	1078.39
A_68_P30346558	chr15:76004732-76004776	NM_201394:21386	Plec	INSIDE	1.036	3.094	237.53	734.95	3.206	181.82	582.85
A_68_P26240715	chr8:126178067-126178111	NM_133955:260	Rhou	INSIDE	1.036	0.536	1978.12	1059.92	0.555	1563.45	868.16
A_68_P23234754	chr4:117166920-117166964	NM_025739:2578	Rnf220	INSIDE	1.036	1.395	1066.48	1488.12	1.446	905.07	1308.34
A_68_P29041945	chr13:53206732-53206776	NM_013846:174724	Ror2	INSIDE	1.036	2.572	1137.57	2926.33	2.665	813.07	2166.50
A_68_P20634554	chr1:136543381-136543425	NM_009307:145	Syt2	INSIDE	1.036	1.661	576.40	957.34	1.721	519.74	894.59
A_68_P25615129	chr8:3679115-3679159	NM_025701:2660	Trappc5	INSIDE	1.036	3.334	647.76	2159.57	3.454	608.65	2102.57
A_68_P32753664	chrX:149932965-149933009	NM_018798:212	Ubqln2	INSIDE	1.036	2.845	611.01	1738.17	2.948	373.69	1101.56
A_68_P27645074	chr11:23207052-23207096	NM_001190401:180	Usp34	INSIDE	1.036	1.397	2055.29	2871.92	1.448	1681.68	2435.10
A_68_P20350471	chr1:74830842-74830886	NM_009526:12399	Wnt6	INSIDE	1.036	2.541	844.23	2144.90	2.633	769.42	2026.00
A_68_P32403976	chrX:55291949-55291993	NM_009575:8166	Zic3	DOWNSTREAM	1.036	1.984	1757.44	3486.15	2.055	950.45	1952.99
A_68_P31022096	chr17:6257118-6257162	ENSMUST00000121576:3238		DOWNSTREAM	1.036	0.620	2682.79	1664.52	0.643	2215.00	1424.37
A_68_P28050435	chr11:97277300-97277344	ENSMUST00000121799:476		INSIDE	1.036	1.627	1294.40	2106.35	1.685	1108.73	1868.33
A_68_P27813411	chr11:54846956-54847000	NM_001110211:-5	Anxa6	PROMOTER	1.035	0.698	2470.01	1722.97	0.722	1877.85	1355.44
A_68_P24761384	chr6:113709003-113709047	NM_001036684:132346	Atp2b2	INSIDE	1.035	2.056	579.35	1190.93	2.127	442.24	940.81
A_68_P28752037	chr12:113960819-113960863	NM_001024602:456	AW555464	INSIDE	1.035	1.627	566.01	921.04	1.684	538.58	906.72
A_68_P26844380	chr9:114294100-114294144	NM_019922:5708	Crtap	INSIDE	1.035	2.128	491.74	1046.51	2.203	392.87	865.70
A_68_P22288618	chr3:83843229-83843273	NM_172681:833	D930015E06Rik	INSIDE	1.035	0.591	1843.04	1088.83	0.611	1386.08	847.12
A_68_P28525539	chr12:72419195-72419245	NM_001190466:8350	Dact1	INSIDE	1.035	3.546	626.62	2222.31	3.671	501.31	1840.30
A_68_P26674979	chr9:81526399-81526443	NM_010482:-261	Htr1b	PROMOTER	1.035	1.798	869.89	1563.66	1.860	717.48	1334.53
A_68_P27282840	chr10:79845290-79845334	NM_198615:5084	Mex3d	INSIDE	1.035	2.294	1765.78	4050.84	2.374	1251.80	2971.21
A_68_P23585506	chr5:33128194-33128238	NM_177298:59	Pisd	INSIDE	1.035	1.408	4637.86	6529.07	1.456	3578.25	5211.23
A_68_P26804663	chr9:106587992-106588036	NM_011573:-16	Tex264	PROMOTER	1.035	2.154	942.34	2030.14	2.230	738.81	1647.26
A_68_P27564657	chr11:5617071-5617115	NM_178623:24087	Urgcp	INSIDE	1.035	3.082	624.35	1924.52	3.190	544.05	1735.43
A_68_P28611535	chr12:88019970-88020014	NM_177354:343	Vash1	INSIDE	1.035	1.527	597.85	913.12	1.581	572.44	905.30
A_68_P32455383	chrX:69062125-69062169	NM_001081356:29	Vma21	INSIDE	1.035	1.730	832.67	1440.21	1.790	468.95	839.62
A_68_P21779867	chr2:157739997-157740041	NM_198627:-370	Vstm2l	PROMOTER	1.035	1.567	7678.84	12031.63	1.622	5888.38	9553.04
A_68_P24097667	chr5:131782979-131783023	NM_145218:392	Wbscr17	INSIDE	1.035	1.617	584.26	944.73	1.674	430.13	719.94
A_68_P23396095	chr4:148098538-148098582	ENSMUST00000139895:10749		DOWNSTREAM	1.035	1.650	1767.98	2917.94	1.708	1445.35	2469.34
A_68_P26430868	chr9:37296656-37296700	NM_146222:-227	BC024479	PROMOTER	1.034	2.113	814.37	1720.44	2.185	716.10	1565.02
A_68_P32680074	chrX:132749644-132749688	NM_009052:386	Bex1	INSIDE	1.034	1.702	1050.85	1788.73	1.760	644.26	1133.76
A_68_P31111039	chr17:26698374-26698418	NM_026170:-60	Ergic1	PROMOTER	1.034	0.698	2460.87	1716.89	0.721	1979.24	1427.78
A_68_P24046890	chr5:122302790-122302834	NM_175474:3776	Fam109a	INSIDE	1.034	2.385	1289.17	3074.46	2.467	1194.99	2948.05
A_68_P28266915	chr12:16807626-16807670	NM_015764:44	Greb1	INSIDE	1.034	1.747	1038.38	1814.19	1.806	1010.22	1824.18
A_68_P25285298	chr7:91032485-91032529	NM_030705:345	Mesdc1	INSIDE	1.034	1.865	338.08	630.54	1.928	308.74	595.31
A_68_P21840229	chr2:168056835-168056879	NM_001160330:735	Mocs3	INSIDE	1.034	3.492	763.98	2667.45	3.609	780.08	2815.64

ProbeName	Target position of probe on CpG island microarray	TargetID	GeneSymbol	CpG island Description	Ratio of relative methylation (TiO_2-NP/Vehicle)	Sham group			TiO_2-H group		
						Relative methylation (Cy5/Cy3)	Cy3 signal (Input)	Cy5 signal (MeDIP)	Relative methylation (Cy5/Cy3)	Cy3 signal (Input)	Cy5 signal (MeDIP)
A_68_P20936019	chr1:192887145-192887189	NM_198654:267	Nsl1	INSIDE	1.034	1.556	689.60	1072.86	1.608	623.14	1002.03
A_68_P28941007	chr13:34967553-34967597	NM_013830:212	Prpf4b	INSIDE	1.034	0.472	1266.44	597.40	0.488	1056.25	515.21
A_68_P25499769	chr7:133436111-133436155	NM_145587:20000	Sbk1	INSIDE	1.034	2.798	709.88	1986.10	2.892	637.95	1845.02
A_68_P26823380	chr9:110283014-110283058	NM_001001144:47240	Scap	INSIDE	1.034	2.914	1065.15	3104.32	3.013	827.75	2493.91
A_68_P24764863	chr6:114232692-114232736	NM_178703:86	Slc6a1	INSIDE	1.034	1.392	3900.18	5428.39	1.440	2471.85	3558.28
A_68_P28048175	chr11:96880255-96880299	NM_031183:5394	Sp6	INSIDE	1.034	2.620	533.59	1398.23	2.711	486.20	1317.99
A_68_P25283749	chr7:90780539-90780583	NM_023377:34	Stard5	INSIDE	1.034	0.561	1071.33	601.53	0.581	928.65	539.09
A_68_P22949805	chr4:58219063-58219107	NM_022814:384	Svep1	INSIDE	1.034	4.156	1248.61	5189.78	4.299	1134.26	4876.02
A_68_P25409834	chr7:116895598-116895642	NM_020050:157	Tmem9b	INSIDE	1.034	2.971	944.98	2807.67	3.073	830.74	2552.76
A_68_P23400514	chr4:148800490-148800534	NM_022022:228	Ube4b	INSIDE	1.034	1.679	4742.69	7961.41	1.735	4031.17	6995.60
A_68_P29650713	chr14:62175501-62175545	NM_025697:201	6330409N04Rik	INSIDE	1.033	3.506	910.36	3191.78	3.623	774.37	2805.39
A_68_P25960290	chr8:74764202-74764246	NM_007456:194	Ap1m1	INSIDE	1.033	1.751	901.83	1578.83	1.809	790.96	1430.45
A_68_P27180607	chr10:60915001-60915045	NM_010124:395	Eif4ebp2	INSIDE	1.033	1.604	895.74	1437.11	1.657	817.39	1354.26
A_68_P25979792	chr8:79423193-79423237	NR_028579:858	Gm10649	INSIDE	1.033	1.714	524.68	899.44	1.772	434.27	769.35
A_68_P31116317	chr17:27571773-27571817	NM_001013385:68455	Grm4	INSIDE	1.033	2.342	1021.64	2392.55	2.419	829.42	2006.29
A_68_P24637120	chr6:91106803-91106847	NM_144919:16	Hdac11	INSIDE	1.033	1.558	1397.49	2177.90	1.610	1268.38	2041.58
A_68_P30991214	chr16:96349614-96349659	NM_008251:-304	Hmgn1	PROMOTER	1.033	0.585	1727.85	1009.99	0.604	1372.90	828.79
A_68_P24132969	chr5:139470681-139470725	NM_008808:205	Pdgfa	INSIDE	1.033	4.776	2010.46	9602.70	4.932	1705.12	8409.32
A_68_P30423621	chr15:88997899-88997943	NM_001159521:6360	Plxnb2	INSIDE	1.033	1.589	1142.49	1815.02	1.642	1039.96	1707.18
A_68_P31257077	chr17:56723655-56723699	NM_001029979:330	Safb2	INSIDE	1.033	0.625	2293.84	1434.48	0.646	1865.71	1204.66
A_68_P24153011	chr5:143194631-143194675	NM_146257:16598	Slc29a4	INSIDE	1.033	3.144	6262.46	19691.47	3.248	4624.55	15019.87
A_68_P25425958	chr7:119823278-119823322	NM_001166584:467	Tead1	INSIDE	1.033	0.500	1122.70	560.84	0.516	1034.62	533.95
A_68_P24761358	chr6:113705242-113705289	NM_001036684:136105	Atp2b2	INSIDE	1.032	2.418	240.07	580.50	2.496	224.38	560.05
A_68_P29977063	chr15:3945932-3945978	NM_145930:-202	AW549877	PROMOTER	1.032	1.823	1176.72	2145.28	1.881	862.16	1622.06
A_68_P24791288	chr6:119058455-119058499	NM_001159533:-269	Cacna1c	PROMOTER	1.032	2.194	601.87	1320.49	2.264	535.79	1213.15
A_68_P25601084	chr7:150646850-150646894	NM_001161624:83	Cdkn1c	INSIDE	1.032	1.796	1092.50	1961.75	1.853	1020.12	1890.63
A_68_P24597420	chr6:84085413-84085457	NM_021469:116052	Dysf	INSIDE	1.032	2.164	995.28	2154.09	2.233	803.20	1793.80
A_68_P28940414	chr13:34838852-34838896	NM_138746:7364	Fam50b	INSIDE	1.032	2.562	747.74	1915.36	2.643	659.01	1741.99
A_68_P22291214	chr3:84284017-84284061	NM_001205355:-625	Fhdc1	PROMOTER	1.032	0.660	2215.56	1461.68	0.681	1695.82	1154.59
A_68_P26587423	chr9:65239016-65239060	NM_028974:421	Kbtbd13	INSIDE	1.032	1.553	733.37	1139.15	1.603	665.63	1067.01
A_68_P25954428	chr8:73366677-73366723	NM_032397:14207	Kcnn1	INSIDE	1.032	2.860	277.02	792.18	2.952	224.19	661.88
A_68_P31777748	chr18:65047791-65047835	NM_001114386:403	Nedd4l	INSIDE	1.032	2.290	1227.91	2811.75	2.364	1109.68	2623.49
A_68_P27280127	chr10:79440093-79440137	NM_001003949:6961	ORF61	INSIDE	1.032	2.253	781.01	1759.84	2.326	697.31	1622.13
A_68_P27993896	chr11:87171950-87171994	NM_177167:524	Ppm1e	INSIDE	1.032	0.546	1676.43	915.18	0.563	1398.89	787.82
A_68_P31938928	chr19:7343225-7343269	NM_054048:-1007	Rcor2	PROMOTER	1.032	1.538	1092.18	1679.46	1.588	1006.77	1598.35
A_68_P28755238	chr12:114425814-114425858	NM_153776:1723	Tmem121	INSIDE	1.032	0.606	1071.35	648.71	0.625	848.34	529.96
A_68_P31092443	chr17:23713479-23713523	NM_011747:22954	Zfp13	INSIDE	1.032	1.768	1244.52	2200.40	1.825	980.74	1790.06
A_68_P25005236	chr7:25292275-25292319	NM_146183:191	Zfp428	INSIDE	1.032	0.421	1788.70	752.68	0.434	1410.15	612.15
A_68_P28175550	chr11:118914821-118914865			Unknown	1.032	0.416	2274.58	947.24	0.430	1714.19	736.82
A_68_P31929125	chr19:5503714-5503758	NM_023781:51	1700020D05Rik	INSIDE	1.031	1.782	751.37	1338.60	1.838	584.78	1074.55
A_68_P25656693	chr8:11636046-11636090	NM_133971:-314	Ankrd10	PROMOTER	1.031	1.475	4424.38	6526.53	1.520	3366.26	5117.39
A_68_P25614032	chr8:3451969-3452013	NM_133962:58983	Arhgef18	INSIDE	1.031	2.770	694.50	1923.90	2.857	611.76	1747.75
A_68_P30026214	chr15:13103007-13103051	NM_007666:366	Cdh6	INSIDE	1.031	3.209	3263.97	10475.47	3.308	2614.19	8647.59
A_68_P29578607	chr14:47452213-47452258	NM_026832:317	Cgrrf1	INSIDE	1.031	2.074	2204.15	4571.36	2.139	1837.85	3930.78
A_68_P23327636	chr4:134828309-134828353	NM_013885:345	Clic4	INSIDE	1.031	0.466	1285.36	599.31	0.481	1328.66	638.97
A_68_P29487107	chr14:28241923-28241967	NM_001114879:-88	D14Abb1e	PROMOTER	1.031	1.647	607.28	1000.34	1.699	551.34	936.48
A_68_P30414446	chr15:87454576-87454620	NM_134096:-1061	Fam19a5	PROMOTER	1.031	1.865	1069.29	1994.25	1.923	974.99	1874.71
A_68_P23438375	chr4:154771703-154771749	NM_008072:452	Gabrd	INSIDE	1.031	1.985	466.35	925.85	2.047	386.00	790.26
A_68_P28041930	chr11:95868489-95868534	NM_009951:-1253	Igf2bp1	PROMOTER	1.031	2.012	490.71	987.56	2.074	458.50	950.99
A_68_P28605006	chr12:86940162-86940206	NM_030887:-181	Jdp2	PROMOTER	1.031	1.751	1925.30	3371.83	1.805	1656.70	2990.08
A_68_P31779041	chr18:65243342-65243386	NM_031881:60184	Nedd4l	INSIDE	1.031	2.770	1161.82	3217.72	2.856	880.80	2515.17

ProbeName	Target position of probe on CpG island microarray	TargetID	GeneSymbol	CpG island Description	Ratio of relative methylation (TiO_2-NP/Vehicle)	Sham group			TiO_2-H group		
						Relative methylation (Cy5/Cy3)	Cy3 signal (Input)	Cy5 signal (MeDIP)	Relative methylation (Cy5/Cy3)	Cy3 signal (Input)	Cy5 signal (MeDIP)
A_68_P26551405	chr9:58884860-58884904	NM_001042752:-634	Neo1	PROMOTER	1.031	1.443	882.00	1273.04	1.489	889.29	1323.91
A_68_P25088122	chr7:51807387-51807431	NM_009473:1885	Nr1h2	INSIDE	1.031	1.923	1669.20	3209.82	1.982	1448.21	2870.47
A_68_P31626610	chr18:37159323-37159367	NM_009961:-5629	Pcdha10	PROMOTER	1.031	1.794	857.81	1539.11	1.849	649.94	1202.03
A_68_P27230709	chr10:70062556-70062600	NM_178621:-539	Phyhipl	PROMOTER	1.031	2.478	392.58	972.95	2.555	363.29	928.34
A_68_P28085165	chr11:103273873-103273917	NM_183034:84	Plekhm1	INSIDE	1.031	0.540	1694.59	914.47	0.556	1399.44	778.57
A_68_P21870537	chr2:173411492-173411536	NR_027957:73526	Ppp4r1l-ps	INSIDE	1.031	1.561	4188.44	6538.08	1.609	3094.03	4979.29
A_68_P25457393	chr7:125387260-125387304	NM_001031814:-131	Smg1	DIVERGENT_PROMOTER	1.031	1.808	2888.02	5220.40	1.864	1957.15	3648.09
A_68_P22049387	chr3:34548813-34548857	NM_011443:-92	Sox2	PROMOTER	1.031	2.841	1505.07	4275.21	2.929	1248.18	3655.46
A_68_P25261546	chr7:86660511-86660555	ENSMUST00000150985:415		INSIDE	1.031	1.979	1308.76	2590.42	2.041	1185.14	2418.82
A_68_P24686651	chr6:99472059-99472103	ENSMUST00000155466:144711		INSIDE	1.031	1.535	1974.88	3031.03	1.582	1741.99	2755.71
A_68_P30413823	chr15:87375395-87375439	ENSMUST00000169110:513		INSIDE	1.031	2.093	961.66	2012.79	2.157	899.73	1940.63
A_68_P32290254	chrX:20425761-20425805	NM_001159645:114	Araf	INSIDE	1.030	2.332	2458.89	5733.16	2.401	1085.01	2604.74
A_68_P26132519	chr8:107778394-107778438	NM_178879:635	B3gnt9-ps	INSIDE	1.030	1.638	1660.69	2720.64	1.688	1382.40	2333.64
A_68_P28731502	chr12:110271962-110272006	NM_001163175:34443	Begain	INSIDE	1.030	2.963	1096.64	3249.30	3.052	976.94	2981.76
A_68_P20969749	chr2:4639031-4639075	NM_001190400:176	Bend7	INSIDE	1.030	2.808	866.13	2432.12	2.894	794.12	2297.88
A_68_P24397197	chr6:42214753-42214798	NM_007610:-262	Casp2	PROMOTER	1.030	2.111	410.37	866.29	2.173	344.71	749.19
A_68_P30382334	chr15:82074972-82075016	NM_001080158:-228	Cenpm	PROMOTER	1.030	1.446	876.38	1267.41	1.490	759.77	1131.79
A_68_P27212449	chr10:67001247-67001291	NM_010118:652	Egr2	INSIDE	1.030	1.820	2986.82	5436.32	1.875	2064.55	3870.68
A_68_P20091641	chr1:24106778-24106822	NM_026604:380	Fam135a	INSIDE	1.030	2.049	898.59	1841.41	2.111	786.19	1659.41
A_68_P27813684	chr11:54911411-54911457	NM_010299:-52	Gm2a	PROMOTER	1.030	1.568	1881.73	2950.07	1.615	1496.02	2416.33
A_68_P27572312	chr11:7113377-7113421	NM_008343:528	Igfbp3	INSIDE	1.030	1.451	1149.90	1668.58	1.494	1051.19	1570.37
A_68_P30366726	chr15:79315953-79315997	NM_008427:19697	Kcnj4	INSIDE	1.030	3.981	698.63	2781.50	4.102	558.33	2290.29
A_68_P31491264	chr18:10726495-10726539	NM_144860:894	Mib1	INSIDE	1.030	1.556	1000.29	1556.00	1.602	881.42	1412.41
A_68_P24627746	chr6:89306761-89306805	NM_008881:5825	Plxna1	INSIDE	1.030	3.123	1312.80	4100.27	3.218	1051.69	3384.75
A_68_P28599774	chr12:86039811-86039855	NM_001033334:-75	Tmem90a	PROMOTER	1.030	2.358	2319.90	5470.56	2.430	1980.16	4810.80
A_68_P26721504	chr9:91259471-91259515	NM_009573:1145	Zic1	INSIDE	1.030	1.441	996.30	1435.20	1.484	894.71	1327.57
A_68_P30501997	chr15:102917300-102917348	AF216290:299758		INSIDE	1.030	1.945	657.00	1277.56	2.004	611.37	1224.95
A_68_P28153581	chr11:115309405-115309449	NM_001033775:7568	4933422H20Rik	INSIDE	1.029	5.051	1178.85	5954.61	5.198	1109.58	5767.92
A_68_P26756438	chr9:97933240-97933284	NM_022319:324	Clstn2	INSIDE	1.029	2.744	1649.89	4526.86	2.822	1299.56	3667.51
A_68_P29644189	chr14:60997251-60997301	NM_001164705:154	Fam123a	INSIDE	1.029	1.827	441.69	806.97	1.881	406.27	764.14
A_68_P25671124	chr8:13907951-13907995	NM_025785:167	Fbxo25	INSIDE	1.029	1.811	1804.36	3267.53	1.864	1521.83	2835.98
A_68_P28707017	chr12:106270861-106270905	NM_028419:-16	Glrx5	DIVERGENT_PROMOTER	1.029	1.782	845.17	1505.92	1.833	596.79	1094.13
A_68_P24932466	chr6:147039958-147040002	NM_001081237:384	Klhdc5	INSIDE	1.029	1.895	1143.85	2168.01	1.950	1026.11	2000.51
A_68_P24333872	chr6:30688012-30688056	NM_008590:-28	Mest	PROMOTER	1.029	2.211	2738.46	6053.36	2.274	2072.98	4714.91
A_68_P30369637	chr15:79832097-79832141	NM_011057:13120	Pdgfb	INSIDE	1.029	2.780	1506.62	4189.03	2.860	1458.62	4171.71
A_68_P32065918	chr19:31738447-31738491	NM_011160:392	Prkg1	INSIDE	1.029	0.667	1407.21	938.85	0.687	1170.65	803.78
A_68_P20930617	chr1:191994889-191994933	NM_008937:-351	Prox1	PROMOTER	1.029	1.873	704.80	1320.18	1.927	573.73	1105.50
A_68_P31067739	chr17:15963621-15963665	NM_178615:-92	Rgmb	PROMOTER	1.029	1.495	2763.43	4131.25	1.538	2182.18	3356.38
A_68_P30955585	chr16:90220935-90220979	NM_011434:-30	Sod1	PROMOTER	1.029	1.353	4048.46	5476.61	1.392	2993.45	4168.26
A_68_P30721026	chr16:44347402-44347446	NM_144550:-89	Spice1	PROMOTER	1.029	1.435	2417.05	3467.50	1.477	1935.51	2858.10
A_68_P23416137	chr4:151406428-151406472	NM_031867:6149	Tas1r1	INSIDE	1.029	2.737	646.19	1768.58	2.817	533.31	1502.54
A_68_P28755248	chr12:114426981-114427025	NM_153776:2889	Tmem121	INSIDE	1.029	2.173	1000.10	2173.21	2.236	691.94	1546.86
A_68_P23984845	chr5:111706231-111706275	NM_024477:397431	Ttc28	INSIDE	1.029	2.617	807.63	2113.17	2.693	666.53	1794.99
A_68_P24424708	chr6:47827424-47827468	NM_146175:-107	Zfp282	PROMOTER	1.029	2.437	522.35	1272.86	2.507	523.32	1311.98
A_68_P22630578	chr3:148653042-148653086	ENSMUST00000098518:-784		PROMOTER	1.029	0.613	1707.96	1046.38	0.630	1327.81	836.92
A_68_P31292456	chr17:64213464-64213508	ENSMUST00000112829:123		INSIDE	1.029	1.843	910.38	1678.06	1.896	954.33	1809.76
A_68_P23361671	chr4:140581070-140581114	NM_172122:28484	Crocc	INSIDE	1.028	2.620	842.33	2207.10	2.694	726.82	1957.86
A_68_P31827569	chr18:73913791-73913835	NM_053255:321	Elac1	INSIDE	1.028	1.683	749.08	1260.90	1.731	697.67	1207.77
A_68_P29050429	chr13:54723129-54723173	NM_178397:6	Faf2	INSIDE	1.028	2.981	599.81	1787.77	3.065	558.94	1713.31
A_68_P25727490	chr8:26629802-26629846	NM_001079908:581	Fgfr1	INSIDE	1.028	2.094	893.57	1870.77	2.152	775.80	1669.74
A_68_P26348935	chr9:21599484-21599528	NM_145611:3484	Kank2	INSIDE	1.028	2.743	331.55	909.31	2.821	275.87	778.14

ProbeName	Target position of probe on CpG island microarray	TargetID	GeneSymbol	CpG island Description	Ratio of relative methylation (TiO_2-NP/Vehicle)	Sham group			TiO_2-H group		
						Relative methylation (Cy5/Cy3)	Cy3 signal (Input)	Cy5 signal (MeDIP)	Relative methylation (Cy5/Cy3)	Cy3 signal (Input)	Cy5 signal (MeDIP)
A_68_P25926103	chr8:67373325-67373369	NM_178633:370	Klhl2	INSIDE	1.028	0.540	1851.67	999.81	0.555	1586.97	881.25
A_68_P27289569	chr10:80883025-80883071	NM_008688:6870	Nfic	INSIDE	1.028	1.833	3172.79	5816.68	1.884	2464.77	4644.44
A_68_P26345851	chr9:20988585-20988629	NM_183408:18449	Pde4a	INSIDE	1.028	1.967	2097.02	4125.40	2.022	1792.47	3623.98
A_68_P24117821	chr5:136253656-136253701	NM_029659:577	Styxl1	INSIDE	1.028	2.078	537.56	1117.04	2.136	435.59	930.37
A_68_P31542078	chr18:21054518-21054562	NM_177038:39	Trappc8	INSIDE	1.028	1.560	747.21	1166.00	1.605	800.51	1284.73
A_68_P23246907	chr4:119406501-119406545	ENSMUST00000123698:77		INSIDE	1.028	2.880	473.12	1362.40	2.959	494.46	1463.14
A_68_P28078743	chr11:102157246-102157290	NM_001098836:675	Atxn7l3	INSIDE	1.027	0.526	1465.70	771.14	0.540	1137.92	614.75
A_68_P25784088	chr8:37210172-37210216	NM_172911:52313	D8Ertd82e	INSIDE	1.027	1.990	424.02	843.70	2.043	376.04	768.22
A_68_P25301154	chr7:95279055-95279099	NM_001081414:546399	Grm5	INSIDE	1.027	1.437	1700.77	2444.06	1.476	1368.65	2019.52
A_68_P29510250	chr14:31989817-31989861	NM_022656:30174	Nisch	INSIDE	1.027	2.494	2451.47	6113.60	2.561	1754.26	4493.47
A_68_P30636603	chr16:29579592-29579636	NM_001199177:195	Opa1	INSIDE	1.027	1.518	1031.24	1565.35	1.558	833.61	1299.02
A_68_P30346604	chr15:76010482-76010526	NM_201394:15636	Plec	INSIDE	1.027	4.748	1550.08	7360.37	4.877	1184.22	5774.88
A_68_P26815238	chr9:108419578-108419622	NM_001114119:183	Qrich1	INSIDE	1.027	1.938	7807.01	15130.27	1.990	6392.04	12718.33
A_68_P27277981	chr10:79100167-79100211	NM_001024539:475	Shc2	INSIDE	1.027	0.527	1308.34	689.71	0.541	1099.81	595.21
A_68_P27287001	chr10:80494270-80494314	NM_134135:2365	Slc39a3	INSIDE	1.027	2.104	748.31	1574.15	2.160	627.41	1354.92
A_68_P21303492	chr2:68308957-68309001	NM_016866:1060	Stk39	INSIDE	1.027	1.759	969.76	1705.49	1.805	902.91	1630.02
A_68_P25747456	chr8:30329801-30329845	NM_153135:286	Unc5d	INSIDE	1.027	0.640	1231.07	787.39	0.657	995.09	653.57
A_68_P27913323	chr11:72609942-72609986	NM_001045536:237	Zzef1	INSIDE	1.027	1.574	719.01	1132.08	1.616	650.65	1051.67
A_68_P24319038	chr6:28165391-28165435	ENSMUST00000143099:-142		PROMOTER	1.027	2.278	470.05	1070.56	2.339	462.41	1081.67
A_68_P32233086	chrX:5659825-5659869	NM_001033211:692	AU022751	INSIDE	1.026	2.302	794.90	1830.10	2.362	377.97	892.88
A_68_P26478981	chr9:46091218-46091262	NM_146000:146	Bud13	INSIDE	1.026	1.535	2546.08	3907.83	1.575	2083.47	3282.14
A_68_P32289636	chrX:20265804-20265848	NM_011049:208	Cdk16	INSIDE	1.026	2.864	421.62	1207.52	2.940	302.01	887.85
A_68_P30478218	chr15:98927458-98927502	NM_176835:-2434	Dnajc22	DIVERGENT_PROMOTER	1.026	1.578	1156.73	1825.76	1.620	955.25	1547.19
A_68_P27352992	chr10:92773316-92773360	NM_013508:566	Elk3	INSIDE	1.026	2.203	3554.40	7828.68	2.260	2902.43	6558.71
A_68_P23314728	chr4:132478506-132478550	NM_001163792:-62	Fam76a	PROMOTER	1.026	0.523	1280.54	670.25	0.537	1036.71	556.84
A_68_P27190083	chr10:62707080-62707124	NM_026101:558	Herc4	INSIDE	1.026	1.765	677.61	1195.73	1.810	589.06	1066.27
A_68_P25951932	chr8:72975857-72975901	NM_001122830:24964	Klhl26	INSIDE	1.026	2.361	764.72	1805.58	2.422	631.47	1529.65
A_68_P31211857	chr17:47506227-47506271	NM_183086:103	Mrps10	INSIDE	1.026	2.186	1619.25	3539.48	2.244	1374.76	3084.54
A_68_P30346581	chr15:76007523-76007567	NM_201394:18596	Plec	INSIDE	1.026	2.287	801.30	1832.20	2.347	674.74	1583.46
A_68_P25471035	chr7:128061434-128061478	NM_001164096:199	Polr3e	INSIDE	1.026	1.602	527.68	845.45	1.644	576.54	948.10
A_68_P21820739	chr2:164786459-164786503	NM_020333:-7007	Slc12a5	PROMOTER	1.026	2.368	619.14	1465.91	2.430	599.66	1457.04
A_68_P26876104	chr9:120020031-120020078	NM_144793:538	Slc25a38	INSIDE	1.026	2.314	1098.90	2542.73	2.373	861.23	2043.90
A_68_P27942761	chr11:78156911-78156955	NM_011676:-91	Unc119	PROMOTER	1.026	2.187	402.10	879.19	2.243	358.93	805.03
A_68_P31879481	chr18:83126670-83126714	NM_001177464:42669	Zfp516	INSIDE	1.026	2.442	969.19	2366.47	2.504	806.05	2018.55
A_68_P26749717	chr9:96789706-96789750	NM_153420:113	Acpl2	INSIDE	1.025	0.604	3115.70	1882.41	0.619	2338.69	1447.60
A_68_P32769556	chrX:154481512-154481556	NM_177751:-492	Cnksr2	PROMOTER	1.025	3.574	639.33	2284.70	3.664	381.93	1399.59
A_68_P20870811	chr1:181476719-181476763	NM_146105:81	Cnst	INSIDE	1.025	2.397	742.11	1778.92	2.456	670.12	1646.15
A_68_P29257121	chr13:99124384-99124428	NM_008242:207	Foxd1	INSIDE	1.025	1.950	1538.04	2999.29	1.999	1137.47	2273.92
A_68_P24451816	chr6:52590190-52590234	NM_145567:82	Hibadh	INSIDE	1.025	0.427	1427.75	609.72	0.438	1318.86	577.21
A_68_P21717108	chr2:146049089-146049133	NM_016889:1378	Insm1	INSIDE	1.025	0.527	2024.34	1066.68	0.540	1592.62	860.26
A_68_P32281774	chrX:17738988-17739032	NM_009483:-782	Kdm6a	PROMOTER	1.025	0.463	1210.69	560.30	0.474	635.66	301.50
A_68_P21080307	chr2:26517162-26517206	NM_010695:9618	Lcn4	DOWNSTREAM	1.025	2.560	1240.57	3175.65	2.623	1124.59	2949.77
A_68_P24045146	chr5:121995041-121995098	NM_010765:832	Mapkapk5	INSIDE	1.025	2.178	464.60	1012.04	2.233	450.66	1006.30
A_68_P26313303	chr9:13553960-13554004	NM_023858:358	Mtmr2	INSIDE	1.025	0.532	1192.30	634.45	0.546	986.86	538.38
A_68_P21842271	chr2:168396389-168396433	NM_001136073:19281	Nfatc2	INSIDE	1.025	3.087	2554.74	7885.31	3.164	2163.59	6845.88
A_68_P26400213	chr9:31193594-31193638	NM_172766:-160	Nfrkb	PROMOTER	1.025	0.444	1532.71	681.01	0.455	1276.68	581.26
A_68_P28185532	chr11:120459254-120459298	NM_011568:403	Thoc4	INSIDE	1.025	1.576	822.59	1296.05	1.614	755.28	1219.32
A_68_P30484946	chr15:100111437-100111481	NM_183109:191	Tmprss12	INSIDE	1.025	2.275	1109.55	2523.83	2.332	902.42	2104.41
A_68_P28013351	chr11:90548942-90548986	NM_028011:-49	Tom1l1	PROMOTER	1.025	1.749	1225.06	2142.89	1.794	1077.29	1932.23
A_68_P21476822	chr2:102240840-102240884	NM_020267:195	Trim44	INSIDE	1.025	2.329	538.38	1253.89	2.386	533.37	1272.82
A_68_P26699327	chr9:86358534-86358578	NM_027394:-33	Ube2cbp	DIVERGENT_PROMOTER	1.025	1.970	1186.50	2337.17	2.020	1008.33	2036.52

ProbeName	Target position of probe on CpG island microarray	TargetID	GeneSymbol	CpG island Description	Ratio of relative methylation (TiO_2-NP/Vehicle)	Sham group			TiO_2-H group		
						Relative methylation (Cy5/Cy3)	Cy3 signal (Input)	Cy5 signal (MeDIP)	Relative methylation (Cy5/Cy3)	Cy3 signal (Input)	Cy5 signal (MeDIP)
A_68_P30568844	chr16:16303089-16303133	NM_198246:53	Yars2	INSIDE	1.025	2.030	597.55	1213.15	2.081	551.38	1147.44
A_68_P26335718	chr9:17902323-17902375			Unknown	1.025	2.554	670.70	1712.81	2.617	610.72	1598.02
A_68_P25032016	chr7:31319911-31319955	NM_178252:96	Arhgap33	INSIDE	1.024	0.503	4569.67	2299.00	0.515	3116.57	1605.22
A_68_P24758532	chr6:113232291-113232335	NM_170673:12	Cpne9	INSIDE	1.024	3.063	1063.33	3257.38	3.138	931.86	2924.51
A_68_P25952211	chr8:73017252-73017296	NM_018827:220	Crlf1	INSIDE	1.024	1.760	958.09	1686.29	1.802	809.04	1457.87
A_68_P30964443	chr16:91688927-91688971	NM_021720:25	Donson	INSIDE	1.024	1.674	976.54	1634.92	1.714	838.75	1437.66
A_68_P24295389	chr6:23198493-23198537	NM_028462:-250	Fezf1	PROMOTER	1.024	1.744	1782.75	3108.58	1.786	1553.11	2774.09
A_68_P23190570	chr4:107108505-107108549	NM_147221:1203	Glis1	INSIDE	1.024	1.676	717.73	1202.66	1.715	719.66	1234.45
A_68_P27289052	chr10:80813045-80813089	NM_001163165:105	Hmg20b	INSIDE	1.024	0.441	1373.11	606.19	0.452	1150.76	520.41
A_68_P27288649	chr10:80755618-80755662	NM_001146687:-76	Pip5k1c	DIVERGENT_PROMOTER	1.024	3.049	918.73	2801.60	3.123	855.35	2671.26
A_68_P21784244	chr2:158493590-158493634	NM_001159662:742	Ppp1r16b	INSIDE	1.024	1.652	786.31	1299.15	1.692	677.23	1146.21
A_68_P32066440	chr19:31838752-31838796	NM_001013833:749	Prkg1	INSIDE	1.024	1.417	1912.14	2709.03	1.450	1645.24	2385.95
A_68_P27950228	chr11:79474495-79474539	NM_175543:69803	Rab11fip4	INSIDE	1.024	2.273	633.82	1440.51	2.328	563.80	1312.67
A_68_P28325481	chr12:31058300-31058344	NM_172951:-82	Sntg2	PROMOTER	1.024	1.787	11660.29	20842.30	1.830	8641.24	15812.10
A_68_P24444652	chr6:51473918-51473962	NM_001127349:39	Snx10	INSIDE	1.024	1.786	1187.00	2120.55	1.829	997.31	1823.87
A_68_P20946919	chr1:194861703-194861747	NM_181546:168	Syt14	INSIDE	1.024	1.506	2431.84	3661.40	1.541	2025.70	3121.60
A_68_P32147459	chr19:47142283-47142327	NM_177342:67	Taf5	INSIDE	1.024	2.082	628.45	1308.67	2.133	529.81	1130.04
A_68_P32114047	chr19:41338628-41338672	NM_133352:-156	Tm9sf3	PROMOTER	1.024	1.671	514.03	858.99	1.712	485.70	831.46
A_68_P23409802	chr4:150431872-150431916	NM_009498:168	Vamp3	INSIDE	1.024	3.257	538.28	1753.27	3.334	532.25	1774.42
A_68_P30446389	chr15:93167185-93167229	NM_024189:160	Yaf2	INSIDE	1.024	2.908	664.21	1931.51	2.979	706.23	2103.81
A_68_P23591264	chr5:34162817-34162861	ENSMUST00000075812:-535		PROMOTER	1.024	2.351	611.03	1436.53	2.407	634.60	1527.66
A_68_P24197924	chr6:3151637-3151681	ENSMUST00000158475:442		DOWNSTREAM	1.024	3.131	1179.43	3692.58	3.206	902.62	2893.64
A_68_P32362519	chrX:45387638-45387682	NM_013912:369	Apln	INSIDE	1.023	2.283	2640.04	6026.68	2.336	1375.86	3214.14
A_68_P23295632	chr4:128901619-128901663	NM_001033189:24047	C77080	INSIDE	1.023	1.562	1033.23	1613.46	1.598	826.12	1320.32
A_68_P27172912	chr10:59648749-59648793	NM_016803:33238	Chst3	INSIDE	1.023	2.004	2010.02	4028.20	2.050	1746.71	3580.00
A_68_P27995153	chr11:87431204-87431248	NM_001045527:561	Hsf5	INSIDE	1.023	2.076	1308.97	2716.89	2.124	921.70	1957.46
A_68_P31156853	chr17:35115138-35115182	NM_013558:5513	Hspa1l	INSIDE	1.023	2.745	593.03	1628.04	2.809	522.76	1468.56
A_68_P30350209	chr15:76552567-76552611	NM_198119:15	Lrrc24	INSIDE	1.023	0.520	1141.47	593.08	0.532	895.77	476.12
A_68_P30339720	chr15:74630372-74630416	NM_029627:4	Ly6k	INSIDE	1.023	3.400	904.03	3073.26	3.479	750.71	2612.02
A_68_P24135186	chr5:139848163-139848207	NR_029768:-2485	Mir339	DIVERGENT_PROMOTER	1.023	0.628	1396.32	876.46	0.642	1192.55	765.78
A_68_P29263992	chr13:100286417-100286461	NM_008634:119	Mtap1b	INSIDE	1.023	2.087	1145.37	2390.55	2.135	985.53	2103.78
A_68_P32218818	chr19:59419895-59419939	NM_001033222:354	Pdzd8	INSIDE	1.023	2.667	901.00	2402.85	2.729	836.30	2281.91
A_68_P21765151	chr2:155183097-155183141	NM_001004721:42	Pigu	INSIDE	1.023	2.182	1897.17	4139.78	2.232	1561.28	3484.34
A_68_P28314232	chr12:29320359-29320403	NM_011300:438	Rps7	INSIDE	1.023	1.618	766.41	1239.93	1.656	698.92	1157.19
A_68_P26346144	chr9:21048991-21049035	NM_053190:3875	S1pr5	INSIDE	1.023	0.377	2938.41	1107.13	0.385	2326.68	896.81
A_68_P22523678	chr3:129763488-129763532	NM_207209:315	Sec24b	INSIDE	1.023	3.911	228.74	894.54	4.000	298.89	1195.53
A_68_P23364646	chr4:141093876-141093920	NM_019763:614	Spen	INSIDE	1.023	2.693	357.16	961.65	2.754	375.58	1034.38
A_68_P30230210	chr15:54903527-54903571	NM_001081288:159	Taf2	INSIDE	1.023	1.421	1168.33	1660.71	1.454	907.72	1320.09
A_68_P24874567	chr6:136765429-136765473	NM_021714:11287	Wbp11	INSIDE	1.023	1.744	1602.22	2794.05	1.785	1361.23	2429.36
A_68_P20305039	chr1:66909588-66909632	NM_007381:231	Acadl	INSIDE	1.022	2.083	775.59	1615.21	2.128	729.79	1553.21
A_68_P30338800	chr15:74502963-74503007	NM_018790:16	Arc	INSIDE	1.022	0.564	2107.27	1189.10	0.577	1601.99	924.05
A_68_P24011285	chr5:116182026-116182070	NM_001080808:-480	Ccdc64	PROMOTER	1.022	1.838	744.45	1368.58	1.878	694.82	1305.21
A_68_P20829087	chr1:173609897-173609941	NM_007649:-2267	Cd48	PROMOTER	1.022	2.030	951.66	1931.77	2.076	754.70	1566.40
A_68_P28672821	chr12:100139294-100139338	NM_001081191:378	Eml5	INSIDE	1.022	1.885	1262.34	2379.11	1.926	1135.88	2187.85
A_68_P27534775	chr10:126676335-126676379	NM_001039000:24063	Kif5a	INSIDE	1.022	1.997	980.39	1957.75	2.041	769.83	1570.90
A_68_P32148598	chr19:47327938-47327982	NM_001163480:24628	Neurl1a	INSIDE	1.022	3.550	330.76	1174.16	3.626	273.57	992.00
A_68_P22878716	chr4:44725913-44725957	NM_008782:-2622	Pax5	PROMOTER	1.022	0.413	1857.96	766.86	0.422	1444.18	609.07
A_68_P26815232	chr9:108418960-108419004	NM_001114119:-435	Qrich1	PROMOTER	1.022	2.148	2467.90	5301.53	2.195	1856.56	4074.34
A_68_P25669975	chr8:13677504-13677548	NM_009025:61	Rasa3	INSIDE	1.022	0.549	1636.73	898.26	0.561	1411.62	791.60
A_68_P25989168	chr8:81033011-81033055	NM_029736:-201	Slc10a7	DIVERGENT_PROMOTER	1.022	0.617	1545.93	954.29	0.631	1180.52	744.77
A_68_P27682721	chr11:31270001-31270045	NM_011491:39	Stc2	INSIDE	1.022	1.666	1754.05	2922.98	1.704	1305.15	2223.57

ProbeName	Target position of probe on CpG island microarray	TargetID	GeneSymbol	CpG island Description	Ratio of relative methylation (TiO_2-NP/Vehicle)	Sham group			TiO_2-H group		
						Relative methylation (Cy5/Cy3)	Cy3 signal (Input)	Cy5 signal (MeDIP)	Relative methylation (Cy5/Cy3)	Cy3 signal (Input)	Cy5 signal (MeDIP)
A_68_P26710363	chr9:88377377-88377421	NM_019796:-163	Syncrip	PROMOTER	1.022	1.617	1014.18	1640.33	1.653	849.59	1404.27
A_68_P31023472	chr17:7165401-7165445	NM_147155:83	Tagap1	INSIDE	1.022	0.631	1034.27	652.39	0.645	938.77	605.23
A_68_P22894263	chr4:47366193-47366237	NM_009370:38	Tgfbr1	INSIDE	1.022	0.436	1812.93	790.28	0.445	1688.42	752.15
A_68_P20620973	chr1:134257439-134257483	NM_178874:30398	Tmcc2	INSIDE	1.022	5.315	2714.33	14425.84	5.429	1737.92	9435.27
A_68_P25615130	chr8:3679220-3679265	NM_025701:2766	Trappc5	INSIDE	1.022	4.629	1951.75	9033.75	4.730	1661.71	7859.64
A_68_P27850825	chr11:61668376-61668420	NM_013881:196	Ulk2	INSIDE	1.022	2.009	466.65	937.38	2.052	487.87	1001.29
A_68_P25505249	chr7:134486849-134486893	NM_146202:1958	Zfp768	INSIDE	1.022	1.800	1009.04	1816.27	1.839	713.76	1312.88
A_68_P31219172	chr17:48925673-48925717			Unknown	1.022	3.770	1278.87	4820.77	3.853	939.74	3621.19
A_68_P25354924	chr7:105457494-105457538	NM_025408:521	Acer3	INSIDE	1.021	1.431	1908.32	2730.38	1.461	1415.53	2068.19
A_68_P31152214	chr17:34256524-34256568	NR_037970:299	Brd2	INSIDE	1.021	1.500	1232.66	1849.54	1.532	994.53	1523.14
A_68_P31676223	chr18:46471587-46471631	NM_001160399:-26	Ccdc112	PROMOTER	1.021	2.183	2824.92	6165.53	2.229	2178.65	4855.79
A_68_P25578900	chr7:146768561-146768605	NR_027857:114	Nkx6-2	INSIDE	1.021	2.125	841.90	1789.21	2.169	681.78	1478.80
A_68_P29237596	chr13:95653350-95653394	NM_011021:7791	Otp	INSIDE	1.021	2.595	1338.12	3471.96	2.649	1169.82	3098.28
A_68_P30422817	chr15:88890519-88890563	NM_001002005:385	Panx2	INSIDE	1.021	2.000	586.15	1172.43	2.041	638.26	1302.89
A_68_P29518733	chr14:33411046-33411090	NM_001001796:-2044	Prrxl1	PROMOTER	1.021	1.564	1602.67	2505.86	1.597	1446.97	2310.76
A_68_P24618288	chr6:87762030-87762074	NM_001039394:-279	Rab43	PROMOTER	1.021	2.506	16931.45	42428.15	2.559	10823.86	27701.08
A_68_P25370036	chr7:108011436-108011480	NM_177073:469	Relt	INSIDE	1.021	0.423	1289.14	545.59	0.432	1047.76	452.94
A_68_P24155044	chr5:143874288-143874332	NM_080561:389	Rnf216	INSIDE	1.021	2.000	707.15	1414.39	2.043	652.90	1333.75
A_68_P27340133	chr10:90586197-90586241	NM_133668:490	Slc25a3	INSIDE	1.021	1.459	820.30	1196.64	1.489	757.39	1127.91
A_68_P23570613	chr5:30400253-30400312	NM_021288:-117	Tyms	PROMOTER	1.021	4.723	264.46	1249.07	4.821	294.08	1417.62
A_68_P28178429	chr11:119385860-119385904			Unknown	1.021	2.305	928.70	2140.93	2.353	928.24	2184.23
A_68_P29509887	chr14:31944357-31944401	NR_024069:-2262	2010107H07Rik	DIVERGENT_PROMOTER	1.020	0.477	1480.76	706.82	0.487	1415.77	689.47
A_68_P20977349	chr2:6021517-6021561	NM_001159657:29693	5430407P10Rik	INSIDE	1.020	2.215	739.59	1638.06	2.258	685.79	1548.66
A_68_P26362426	chr9:24307746-24307790	NM_172920:-184	Dpy19l1	PROMOTER	1.020	3.375	194.09	655.01	3.441	187.45	645.09
A_68_P28728198	chr12:109701817-109701861	NM_001043335:40813	Eml1	INSIDE	1.020	2.002	616.84	1234.94	2.042	469.66	958.84
A_68_P23235082	chr4:117222946-117222990	NM_080469:-130	Eri3	PROMOTER	1.020	2.103	1053.77	2216.36	2.145	981.45	2104.96
A_68_P27503063	chr10:120802153-120802197	NM_029364:29	Gns	INSIDE	1.020	0.450	2215.37	997.29	0.459	1951.91	896.60
A_68_P31730289	chr18:56591860-56591904	NM_026240:97	Gramd3	INSIDE	1.020	2.011	1369.11	2753.57	2.051	1277.02	2619.72
A_68_P27568544	chr11:6344567-6344611	NM_029938:-142	H2afv	PROMOTER	1.020	0.607	1227.61	745.36	0.619	945.58	585.77
A_68_P28103517	chr11:106649497-106649541	NR_037224:-5445	Mir3064	PROMOTER	1.020	0.545	1238.11	674.83	0.556	953.12	529.81
A_68_P30456842	chr15:95059493-95059537	NM_016743:299623	Nell2	INSIDE	1.020	2.582	638.71	1649.12	2.633	579.90	1526.61
A_68_P26345840	chr9:20986196-20986240	NM_183408:16061	Pde4a	INSIDE	1.020	0.475	1150.47	546.32	0.484	948.82	459.62
A_68_P26138375	chr8:108734930-108734974	NM_145404:-1	Prmt7	DIVERGENT_PROMOTER	1.020	1.695	2048.09	3470.53	1.728	1800.77	3112.18
A_68_P31121838	chr17:28466106-28466150	NM_011287:713	Rpl10a	INSIDE	1.020	2.448	890.67	2180.77	2.496	829.73	2071.29
A_68_P31052340	chr17:12700162-12700206	NM_011395:386	Slc22a3	INSIDE	1.020	0.594	1901.85	1129.57	0.606	1524.43	923.15
A_68_P22477034	chr3:121235139-121235183	NM_145394:102	Slc44a3	INSIDE	1.020	1.802	889.15	1602.51	1.839	788.96	1451.00
A_68_P22314617	chr3:88383670-88383714	NM_025448:100	Ssr2	INSIDE	1.020	1.438	1292.82	1859.69	1.467	1187.67	1742.19
A_68_P31846341	chr18:77424704-77424748	NM_013666:141	St8sia5	INSIDE	1.020	1.891	1384.89	2619.23	1.930	1245.02	2402.77
A_68_P25102013	chr7:54175369-54175413	NM_021884:-90	Tsg101	PROMOTER	1.020	1.822	757.12	1379.50	1.858	780.55	1450.46
A_68_P28344355	chr12:34642855-34642899	NM_011658:341	Twist1	INSIDE	1.020	2.068	623.88	1290.22	2.108	634.56	1337.93
A_68_P23576666	chr5:31440580-31440624	NM_021290:666	Ucn	INSIDE	1.020	1.614	535.03	863.51	1.646	540.28	889.50
A_68_P31926824	chr19:5088863-5088907	NM_026553:347	Yif1a	INSIDE	1.020	1.689	1402.00	2367.45	1.723	1228.88	2117.32
A_68_P24057005	chr5:124134134-124134178	NM_019765:144	Clip1	INSIDE	1.019	1.432	958.82	1373.43	1.460	789.24	1152.39
A_68_P25564251	chr7:144390467-144390511	NM_001113414:115640	Ebf3	INSIDE	1.019	3.218	2651.42	8531.69	3.278	1957.18	6416.54
A_68_P23270865	chr4:124562969-124563014	NM_177671:3963	Epha10	INSIDE	1.019	2.145	554.25	1188.79	2.186	504.52	1103.07
A_68_P26134296	chr8:108042857-108042901	NM_008289:233	Hsd11b2	INSIDE	1.019	2.457	648.37	1593.00	2.503	574.47	1437.86
A_68_P22397063	chr3:105255456-105255500	NM_019931:-6255	Kcnd3	PROMOTER	1.019	0.387	1741.27	674.03	0.394	1388.21	547.61
A_68_P25991119	chr8:81344708-81344752	NM_001191004:321	Lsm6	INSIDE	1.019	1.745	1208.81	2108.85	1.777	1009.64	1794.12
A_68_P27787504	chr11:50050408-50050452	NM_008521:1543	Ltc4s	INSIDE	1.019	1.655	1603.64	2653.39	1.686	1361.70	2295.41
A_68_P31934086	chr19:6341582-6341626	NM_009006:355	Map4k2	INSIDE	1.019	1.587	936.29	1486.19	1.617	812.32	1313.93
A_68_P27796190	chr11:51671242-51671287	NM_199299:-281	Phf15	PROMOTER	1.019	3.109	446.27	1387.51	3.168	359.05	1137.33

ProbeName	Target position of probe on CpG island microarray	TargetID	GeneSymbol	CpG island Description	Ratio of relative methylation (TiO_2-NP/Vehicle)	Sham group			TiO_2-H group		
						Relative methylation (Cy5/Cy3)	Cy3 signal (Input)	Cy5 signal (MeDIP)	Relative methylation (Cy5/Cy3)	Cy3 signal (Input)	Cy5 signal (MeDIP)
A_68_P25988753	chr8:80961280-80961324	NM_138944:-751	Pou4f2	PROMOTER	1.019	4.509	2900.66	13080.03	4.593	2072.27	9518.50
A_68_P21834481	chr2:167065786-167065830	NM_008968:229	Ptgis	INSIDE	1.019	0.384	1726.09	662.74	0.391	1416.61	554.37
A_68_P29050814	chr13:54788639-54788683	NM_001146025:509	Rnf44	INSIDE	1.019	2.736	1084.01	2965.45	2.789	783.01	2183.69
A_68_P31256955	chr17:56705842-56705886	NM_001029979:18142	Safb2	INSIDE	1.019	1.925	1834.39	3531.08	1.961	1461.14	2865.45
A_68_P27280661	chr10:79520418-79520462	NM_183426:45007	Sbno2	INSIDE	1.019	1.509	1079.23	1628.19	1.537	847.31	1302.73
A_68_P32136769	chr19:45224972-45225016	NM_021901:-210	Tlx1	PROMOTER	1.019	0.630	1171.63	738.08	0.642	1055.56	677.73
A_68_P25272788	chr7:88716073-88716117	NM_001004185:-83	Whamm	DIVERGENT_PROMOTER	1.019	3.711	761.27	2825.20	3.780	706.19	2669.66
A_68_P28104776	chr11:106889479-106889523	NM_001163473:-36	1810010H24Rik	PROMOTER	1.018	2.485	350.78	871.55	2.529	343.57	868.95
A_68_P28050940	chr11:97361543-97361587	NM_021493:50091	Arhgap23	INSIDE	1.018	2.543	1212.72	3084.07	2.588	1056.17	2733.61
A_68_P31616439	chr18:35278605-35278649	NM_009818:61	Ctnna1	INSIDE	1.018	0.554	1713.40	948.65	0.563	1351.12	761.35
A_68_P25083585	chr7:50932262-50932306	NM_145582:4884	Ctu1	INSIDE	1.018	2.581	787.80	2033.08	2.627	739.20	1941.94
A_68_P31933640	chr19:6277337-6277381	NM_010119:463	Ehd1	INSIDE	1.018	0.512	1828.20	935.72	0.521	1528.88	796.26
A_68_P22737304	chr4:12834151-12834195	NM_001171801:189	Gm11818	INSIDE	1.018	2.482	519.50	1289.49	2.527	498.95	1260.72
A_68_P32571133	chrX:100129283-100129327	NR_026596:258	Gm5126	INSIDE	1.018	1.708	811.88	1386.57	1.739	476.40	828.52
A_68_P23355140	chr4:139521655-139521700	NM_173427:2264	Klhdc7a	INSIDE	1.018	3.061	1402.90	4294.47	3.115	1090.17	3396.27
A_68_P25087546	chr7:51717127-51717171	NM_198250:19292	Lrrc4b	INSIDE	1.018	1.630	1109.24	1808.35	1.659	978.40	1623.26
A_68_P21842275	chr2:168396762-168396807	NM_001136073:18907	Nfatc2	INSIDE	1.018	2.770	762.29	2111.55	2.819	649.90	1832.23
A_68_P22309971	chr3:87587854-87587898	NM_001033124:11208	Ntrk1	INSIDE	1.018	2.081	936.35	1948.86	2.118	789.74	1672.89
A_68_P24636899	chr6:91066454-91066498	NM_018815:344	Nup210	INSIDE	1.018	1.547	926.76	1433.54	1.575	746.26	1175.41
A_68_P31229018	chr17:50648295-50648339	NM_013880:-555	Plcl2	PROMOTER	1.018	2.163	537.99	1163.90	2.203	566.54	1248.21
A_68_P24117608	chr5:136209662-136209706	NM_008898:44601	Por	INSIDE	1.018	3.740	3233.32	12093.06	3.808	2181.72	8308.72
A_68_P23298637	chr4:129497229-129497273	NM_008974:-472	Ptp4a2	PROMOTER	1.018	2.011	1478.98	2974.61	2.048	1235.54	2530.55
A_68_P27926514	chr11:75323805-75323849	NM_001029938:231	Rilp	INSIDE	1.018	3.142	647.09	2032.91	3.197	585.68	1872.45
A_68_P23339118	chr4:136852527-136852571	NM_009523:18999	Wnt4	INSIDE	1.018	1.855	472.09	875.67	1.888	419.03	790.93
A_68_P29235876	chr13:95365929-95365973			Unknown	1.018	2.202	920.47	2026.61	2.242	830.88	1862.54
A_68_P30711910	chr16:42875216-42875260	ENSMUST00000142270:462		INSIDE	1.018	1.734	1375.71	2386.15	1.765	1192.57	2105.32
A_68_P25352335	chr7:104937048-104937092	ENSMUST00000172423:20		INSIDE	1.018	0.657	1925.05	1264.30	0.669	1705.34	1140.52
A_68_P25608516	chr7:151864802-151864846	NM_178642:59559	Ano1	INSIDE	1.017	2.007	322.54	647.47	2.042	299.49	611.62
A_68_P25429184	chr7:120351143-120351187	NM_007489:186	Arntl	INSIDE	1.017	1.853	377.53	699.52	1.884	392.14	738.73
A_68_P24172963	chr5:148116041-148116085	NM_007673:2763	Cdx2	INSIDE	1.017	1.417	1109.13	1571.40	1.442	930.91	1341.94
A_68_P24056166	chr5:123974219-123974263	NM_023232:-67	Diablo	PROMOTER	1.017	0.339	1819.62	616.63	0.345	1453.42	501.13
A_68_P24138646	chr5:140447394-140447438	NM_175522:63520	Elfn1	INSIDE	1.017	2.221	2382.98	5291.46	2.259	1880.37	4247.98
A_68_P31161106	chr17:35960328-35960372	NM_008027:49	Flot1	INSIDE	1.017	1.451	1122.42	1629.13	1.476	989.82	1460.69
A_68_P29228414	chr13:94074555-94074599	NM_152134:-3412	Homer1	PROMOTER	1.017	1.551	1198.09	1858.08	1.578	1033.15	1629.84
A_68_P28290825	chr12:25336262-25336306	NM_178357:49	Klf11	INSIDE	1.017	2.733	830.83	2270.37	2.778	740.79	2057.97
A_68_P21818285	chr2:164333404-164333448	NM_133779:10402	Pigt	INSIDE	1.017	2.149	956.00	2054.27	2.185	851.94	1861.28
A_68_P24120143	chr5:136720679-136720723	NM_018825:73	Sh2b2	INSIDE	1.017	2.003	1292.96	2589.23	2.036	1186.73	2416.36
A_68_P31850941	chr18:78243939-78243983	NM_001101038:10047	Siglec15	INSIDE	1.017	1.549	657.87	1018.73	1.575	623.08	981.63
A_68_P23364273	chr4:141028839-141028883	NM_019763:65652	Spen	INSIDE	1.017	2.210	2740.52	6057.52	2.247	1916.89	4307.02
A_68_P25195708	chr7:74904333-74904377	NM_183312:274	Synm	INSIDE	1.017	1.519	1274.06	1935.50	1.546	1125.34	1739.29
A_68_P20141348	chr1:34987329-34987373			Unknown	1.017	1.967	1236.31	2431.97	2.001	1052.53	2106.05
A_68_P26128335	chr8:106919047-106919091	NM_153582:528	Cmtm4	INSIDE	1.016	1.850	894.07	1654.47	1.880	844.66	1587.76
A_68_P28525538	chr12:72419084-72419128	NM_001190466:8236	Dact1	INSIDE	1.016	1.753	794.77	1393.56	1.782	639.90	1140.24
A_68_P31286859	chr17:63232405-63232449	NM_010109:-1760	Efna5	PROMOTER	1.016	1.874	331.66	621.60	1.903	330.00	628.16
A_68_P25369179	chr7:107855638-107855683	NM_178764:445	Fam168a	INSIDE	1.016	8.960	2670.60	23928.05	9.103	1674.27	15240.13
A_68_P25048201	chr7:35989266-35989310	NM_001024707:11076	Lrp3	INSIDE	1.016	2.233	829.96	1853.41	2.268	689.75	1564.32
A_68_P26575781	chr9:63225502-63225546	NM_011840:135	Map2k5	INSIDE	1.016	1.555	984.16	1530.81	1.580	901.52	1424.54
A_68_P25354128	chr7:105293642-105293686	NM_011010:293	Omp	INSIDE	1.016	2.863	1432.66	4101.71	2.909	1168.14	3397.84
A_68_P29041946	chr13:53206812-53206856	NM_013846:174644	Ror2	INSIDE	1.016	2.057	2601.12	5351.79	2.091	1847.13	3862.63
A_68_P31254141	chr17:56279185-56279230	NM_013662:-5932	Sema6b	PROMOTER	1.016	3.127	259.03	810.09	3.178	280.49	891.52
A_68_P31850954	chr18:78245651-78245695	NM_001101038:8335	Siglec15	INSIDE	1.016	1.909	1558.53	2975.74	1.940	1387.78	2692.01

ProbeName	Target position of probe on CpG island microarray	TargetID	GeneSymbol	CpG island Description	Ratio of relative methylation (TiO_2-NP/Vehicle)	Sham group			TiO_2-H group		
						Relative methylation (Cy5/Cy3)	Cy3 signal (Input)	Cy5 signal (MeDIP)	Relative methylation (Cy5/Cy3)	Cy3 signal (Input)	Cy5 signal (MeDIP)
A_68_P23339119	chr4:136852669-136852713	NM_009523:19141	Wnt4	INSIDE	1.016	3.092	498.27	1540.41	3.140	516.62	1622.32
A_68_P21839557	chr2:167942759-167942803			Unknown	1.016	3.053	581.51	1775.15	3.101	529.29	1641.09
A_68_P21037845	chr2:17953722-17953766	NR_028376:5624	A930004D18Rik	INSIDE	1.015	2.053	566.94	1164.05	2.083	451.20	939.92
A_68_P21441217	chr2:94278208-94278252	NM_007466:74	Api5	INSIDE	1.015	0.568	1743.55	989.72	0.576	1415.17	815.23
A_68_P27383821	chr10:98377167-98377211	NM_026482:-597	Atp2b1	PROMOTER	1.015	1.770	607.96	1076.19	1.797	605.89	1088.74
A_68_P26807071	chr9:107096201-107096245	NM_153413:38018	Dock3	INSIDE	1.015	1.745	792.40	1382.94	1.771	736.95	1305.00
A_68_P20146386	chr1:36160613-36160657	NM_015818:35390	Hs6st1	INSIDE	1.015	2.158	869.63	1876.25	2.190	750.58	1644.13
A_68_P28748144	chr12:113384449-113384493	NM_001097621:52	Kif26a	INSIDE	1.015	2.190	831.65	1821.24	2.222	717.14	1593.27
A_68_P23753092	chr5:65213185-65213229	NM_008453:18445	Klf3	INSIDE	1.015	2.258	573.82	1295.52	2.292	440.10	1008.89
A_68_P26017699	chr8:86550056-86550100	NM_028877:4706	Palm3	INSIDE	1.015	1.803	1404.78	2532.57	1.830	1228.64	2248.92
A_68_P25094417	chr7:52934632-52934676	NM_019934:274	Sec1	INSIDE	1.015	2.394	867.21	2075.92	2.429	757.04	1839.20
A_68_P27927812	chr11:75546197-75546241	NM_009536:-170	Ywhae	PROMOTER	1.015	1.773	1414.14	2507.42	1.800	1256.66	2261.99
A_68_P21067989	chr2:24617842-24617886	NM_001042528:703	Cacna1b	INSIDE	1.014	1.377	4255.00	5859.70	1.397	3168.92	4426.03
A_68_P23316406	chr4:132776655-132776700	NM_027152:-46	Cd164l2	DIVERGENT_PROMOTER	1.014	3.110	397.54	1236.39	3.154	313.71	989.46
A_68_P26844287	chr9:114277292-114277336	NM_019922:22516	Crtap	DOWNSTREAM	1.014	1.733	1719.52	2979.48	1.757	1524.39	2678.85
A_68_P32982442	chr5:135115011-135115055	NM_033561:166	Eif4h	INSIDE	1.014	0.546	1207.47	659.26	0.554	916.99	507.75
A_68_P28940413	chr13:34838757-34838801	NM_138746:7268	Fam50b	INSIDE	1.014	2.543	368.97	938.27	2.579	336.41	867.71
A_68_P25666781	chr8:13200390-13200434	NM_025768:212	Grtp1	INSIDE	1.014	1.465	1125.60	1649.24	1.485	1047.05	1555.01
A_68_P29616396	chr14:55732459-55732503	NM_177049:3292	Jph4	INSIDE	1.014	2.253	1169.56	2635.17	2.285	1041.20	2379.30
A_68_P27947419	chr11:78959894-78959938	NM_013571:-60	Ksr1	PROMOTER	1.014	1.502	3094.52	4648.20	1.523	2556.05	3892.26
A_68_P25368691	chr7:107757091-107757135	NR_003559:-602	Mrpl48	PROMOTER	1.014	0.603	1410.81	851.24	0.612	1116.13	683.04
A_68_P22342235	chr3:94910203-94910247	NM_008847:556	Pip5k1a	INSIDE	1.014	0.569	1403.59	798.30	0.576	1035.68	597.02
A_68_P24054126	chr5:123588945-123588989	NM_175092:-6328	Rhof	PROMOTER	1.014	1.924	660.05	1269.78	1.952	657.46	1283.12
A_68_P26476666	chr9:45714950-45714994	NM_178709:-12	Rnf214	PROMOTER	1.014	1.892	728.81	1378.68	1.918	644.17	1235.64
A_68_P31093842	chr17:23956720-23956764	NM_175229:16589	Srrm2	INSIDE	1.014	3.328	834.28	2776.45	3.373	591.45	1994.92
A_68_P30363681	chr15:78823328-78823373	NM_001024716:9865	Triobp	INSIDE	1.014	2.259	1654.09	3736.50	2.290	1179.71	2701.38
A_68_P21365117	chr2:78709405-78709449	NM_009454:223	Ube2e3	INSIDE	1.014	2.795	780.54	2181.78	2.834	746.57	2115.65
A_68_P26217007	chr8:122480845-122480889	NM_009462:46115	Usp10	INSIDE	1.014	3.210	1206.90	3874.09	3.254	1113.49	3623.31
A_68_P30633251	chr16:28929310-28929354	NM_177718:452	1600021P15Rik	INSIDE	1.013	1.395	1324.84	1847.92	1.413	1015.53	1434.97
A_68_P28183252	chr11:120098764-120098808	NM_198423:4526	Bahcc1	INSIDE	1.013	1.649	671.53	1107.56	1.672	654.90	1094.68
A_68_P28724522	chr12:109154828-109154872	NM_001079883:86774	Bcl11b	INSIDE	1.013	1.999	461.41	922.13	2.024	421.72	853.50
A_68_P24318693	chr6:28084879-28084923	NM_008174:-531	Grm8	PROMOTER	1.013	0.574	1194.61	685.64	0.581	921.40	535.66
A_68_P23435246	chr4:154334733-154334777	NM_010419:-277	Hes5	PROMOTER	1.013	2.014	989.10	1992.23	2.041	922.83	1883.36
A_68_P22344946	chr3:95462398-95462442	NM_008562:-222	Mcl1	PROMOTER	1.013	1.635	484.53	792.15	1.656	511.46	846.92
A_68_P31621832	chr18:36227944-36227988	NM_001167891:128848	Nrg2	INSIDE	1.013	2.233	1152.09	2572.24	2.261	1044.89	2362.36
A_68_P21782730	chr2:158236015-158236059	NM_177658:448	Ralgapb	INSIDE	1.013	1.807	537.36	970.97	1.831	504.21	923.07
A_68_P23896972	chr5:93371791-93371835	NM_001077596:44789	Shroom3	INSIDE	1.013	3.740	570.92	2135.49	3.790	622.83	2360.71
A_68_P21973812	chr3:19062717-19062761	NM_028840:327	Armc1	INSIDE	1.012	1.519	1032.29	1568.44	1.538	849.62	1306.98
A_68_P32233090	chrX:5660210-5660254	NM_001033211:306	AU022751	INSIDE	1.012	2.497	646.75	1615.14	2.527	366.40	925.79
A_68_P26349690	chr9:21752178-21752222	NM_144935:9483	BC018242	INSIDE	1.012	2.016	1399.69	2821.67	2.040	1070.25	2182.84
A_68_P20499743	chr1:106665396-106665440	NM_011800:23	Cdh20	INSIDE	1.012	0.615	1911.54	1175.65	0.622	1635.89	1017.94
A_68_P23976362	chr5:109987925-109987969	NM_001164735:66	Crlf2	INSIDE	1.012	0.465	1240.84	576.63	0.470	1004.97	472.64
A_68_P31614615	chr18:34937144-34937188	NM_001081256:505	Kdm3b	INSIDE	1.012	1.790	473.38	847.35	1.812	473.69	858.42
A_68_P25950174	chr8:72682451-72682495	NM_001045484:5796	Mef2b	INSIDE	1.012	1.931	2110.83	4075.30	1.954	1855.97	3626.75
A_68_P23278933	chr4:125911136-125911183	NM_001145970:22404	Mtap7d1	INSIDE	1.012	2.787	630.04	1755.81	2.821	546.32	1541.15
A_68_P24950724	chr7:4781725-4781769	NM_172737:14552	Shisa7	INSIDE	1.012	2.196	3129.99	6872.76	2.222	2439.66	5421.08
A_68_P30486832	chr15:100467219-100467263	NM_174992:46	Smagp	INSIDE	1.012	3.127	692.71	2166.03	3.164	701.87	2220.55
A_68_P31923332	chr19:4477049-4477093	NM_134164:73	Syt12	INSIDE	1.012	1.926	639.69	1231.78	1.948	604.18	1177.16
A_68_P25009655	chr7:26100173-26100217	NM_183311:9068	Tmem145	INSIDE	1.012	2.226	6421.23	14294.07	2.253	4633.53	10440.78
A_68_P21836349	chr2:167423229-167423273	NM_023230:34255	Ube2v1	DOWNSTREAM	1.012	1.669	2968.08	4953.87	1.689	2335.93	3945.19
A_68_P26342783	A_68_P26342783			Unknown	1.012	1.466	1444.83	2118.13	1.484	1277.74	1895.80

ProbeName	Target position of probe on CpG island microarray	TargetID	GeneSymbol	CpG island Description	Ratio of relative methylation (TiO_2-NP/Vehicle)	Sham group			TiO_2-H group		
						Relative methylation (Cy5/Cy3)	Cy3 signal (Input)	Cy5 signal (MeDIP)	Relative methylation (Cy5/Cy3)	Cy3 signal (Input)	Cy5 signal (MeDIP)
A_68_P26580034	chr9:63932732-63932776	AK011166:4187		INSIDE	1.012	1.539	1307.95	2012.57	1.557	1070.35	1666.64
A_68_P24067081	chr5:125956133-125956177	NM_030210:-88	Aacs	PROMOTER	1.011	1.365	4018.89	5484.01	1.380	2988.95	4124.90
A_68_P23005030	chr4:69910440-69910484	NM_145990:160939	Cdk5rap2	INSIDE	1.011	2.938	699.38	2054.96	2.971	679.00	2017.47
A_68_P24568082	chr6:77929708-77929753	NM_001109764:-69	Ctnna2	PROMOTER	1.011	1.909	690.35	1317.99	1.930	598.55	1155.48
A_68_P21612765	chr2:127162244-127162288	NM_010090:372	Dusp2	INSIDE	1.011	1.642	535.98	880.06	1.659	490.61	814.12
A_68_P27093482	chr10:41917067-41917111	NM_019740:79460	Foxo3	INSIDE	1.011	2.142	1238.06	2652.45	2.166	934.39	2024.22
A_68_P26745534	chr9:96020049-96020093	NM_177352:223	Gk5	INSIDE	1.011	0.543	1819.24	987.26	0.548	1628.24	892.95
A_68_P23226010	chr4:115500724-115500768	NM_175308:50	Mobkl2c	INSIDE	1.011	0.535	1002.96	536.94	0.541	801.46	433.77
A_68_P20347699	chr1:74331738-74331785	NM_025580:154	Pnkd	INSIDE	1.011	1.691	758.95	1283.02	1.709	559.87	956.66
A_68_P24951460	chr7:4945585-4945629	NM_026741:2097	Zfp579	INSIDE	1.011	2.230	1910.44	4259.86	2.254	1348.44	3038.83
A_68_P26017563	chr8:86525640-86525684	NM_183097:58	1700067K01Rik	INSIDE	1.010	2.204	752.06	1657.50	2.227	753.10	1677.15
A_68_P32362518	chrX:45387551-45387595	NM_013912:457	Apln	INSIDE	1.010	2.013	1801.68	3626.97	2.033	884.07	1796.93
A_68_P23440845	chr4:155205317-155205361	NM_025338:-416	Aurkaip1	PROMOTER	1.010	0.508	1126.88	572.90	0.514	957.57	491.81
A_68_P29530306	chr14:35315823-35315867	NM_009758:-112	Bmpr1a	PROMOTER	1.010	3.763	505.08	1900.53	3.799	552.53	2098.93
A_68_P29696921	chr14:70132430-70132474	NM_134078:-75	Chmp7	PROMOTER	1.010	1.422	2147.44	3053.09	1.436	1764.10	2533.06
A_68_P21144178	chr2:37648888-37648932	NM_001163566:17142	Crb2	INSIDE	1.010	2.973	1359.61	4041.92	3.003	1095.21	3289.18
A_68_P31630972	chr18:38094826-38094871	NM_007858:217	Diap1	INSIDE	1.010	3.595	557.32	2003.72	3.632	562.94	2044.53
A_68_P23284224	chr4:126913454-126913498	NM_198618:66962	Dlgap3	INSIDE	1.010	1.495	1391.98	2080.57	1.510	1181.16	1783.87
A_68_P32027007	chr19:25073867-25073911	NM_028785:-130	Dock8	PROMOTER	1.010	4.875	260.05	1267.68	4.923	279.03	1373.52
A_68_P25286998	chr7:91300735-91300779	NM_133722:-353	Fam108c	PROMOTER	1.010	1.437	1560.32	2242.21	1.452	1184.32	1719.21
A_68_P24046893	chr5:122303149-122303193	NM_175474:4134	Fam109a	INSIDE	1.010	2.419	593.54	1435.60	2.443	537.66	1313.34
A_68_P27497672	chr10:119912710-119912754	NM_010441:1259	Hmga2	INSIDE	1.010	1.607	890.13	1430.01	1.623	900.92	1462.32
A_68_P27788208	chr11:50191636-50191680	NM_021510:438	Hnrnph1	INSIDE	1.010	0.473	1172.77	555.28	0.478	972.22	464.97
A_68_P28293530	chr12:25783788-25783832	NM_010496:-2853	Id2	PROMOTER	1.010	2.008	269.06	540.19	2.028	285.86	579.62
A_68_P31614612	chr18:34936814-34936858	NM_001081256:175	Kdm3b	INSIDE	1.010	0.550	1664.94	916.45	0.556	1670.41	928.88
A_68_P27285759	chr10:80297631-80297675	NM_001013758:9132	Lingo3	INSIDE	1.010	1.710	684.92	1171.13	1.728	563.57	973.59
A_68_P29661996	chr14:64162753-64162797	NM_177594:16	Mtmr9	INSIDE	1.010	1.836	2333.69	4285.60	1.855	1564.53	2902.65
A_68_P24983630	chr7:17441193-17441237	NM_178900:12801	Prkd2	INSIDE	1.010	2.522	1875.45	4729.09	2.547	1467.04	3736.58
A_68_P24459349	chr6:53770114-53770158	NM_025817:683	Tril	INSIDE	1.010	1.998	2589.86	5174.29	2.018	2293.26	4628.66
A_68_P32144292	chr19:46574510-46574557	NM_053100:-1604	Trim8	PROMOTER	1.010	0.525	1206.72	633.26	0.530	1104.49	585.53
A_68_P27234637	chr10:70747983-70748027	NM_145420:6	Ube2d1	INSIDE	1.010	1.772	519.76	921.14	1.791	465.17	832.92
A_68_P27564654	chr11:5616764-5616808	NM_178623:24393	Urgcp	INSIDE	1.010	2.461	312.41	768.82	2.486	349.49	868.85
A_68_P29601197	chr14:51978178-51978222	ENSMUST00000162079:3332		DOWNSTREAM	1.010	1.722	1202.64	2070.65	1.738	1030.84	1791.81
A_68_P31103776	chr17:25569955-25569999	NM_001163691:752	Cacna1h	INSIDE	1.009	1.427	1370.68	1955.87	1.440	1124.35	1618.75
A_68_P30330813	chr15:73342857-73342901	NM_001081066:-111	Dennd3	PROMOTER	1.009	1.664	2966.96	4937.93	1.679	2315.90	3888.75
A_68_P31932438	chr19:6058043-6058087	NM_001190436:98	Fau	INSIDE	1.009	1.496	3182.57	4760.79	1.509	2526.55	3812.69
A_68_P23193164	chr4:107518873-107518917	NM_001080926:44031	Lrp8	INSIDE	1.009	1.953	1565.41	3057.58	1.971	1287.31	2537.44
A_68_P25954896	chr8:73437919-73437963	NM_173013:8068	Mtap1s	INSIDE	1.009	1.754	700.91	1229.73	1.771	486.55	861.71
A_68_P29162262	chr13:78334257-78334301	NM_010151:3965	Nr2f1	INSIDE	1.009	0.548	996.14	545.77	0.553	902.03	498.63
A_68_P30422879	chr15:88898490-88898534	NM_001002005:8357	Panx2	INSIDE	1.009	1.660	824.96	1369.59	1.675	671.27	1124.22
A_68_P29059921	chr13:56322911-56322955	ENSMUST00000127754:22369		DOWNSTREAM	1.009	1.948	1010.11	1967.44	1.966	849.57	1670.45
A_68_P21615625	chr2:127618048-127618092	NR_015476:154	1500011K16Rik	INSIDE	1.008	1.715	1826.80	3132.61	1.729	1407.15	2433.34
A_68_P33007150	chr9_random:50340-50384	NR_015516:-3046	4930526I15Rik	PROMOTER	1.008	3.159	4502.30	14222.42	3.184	2977.33	9479.68
A_68_P24138255	chr5:140384458-140384502	NM_175522:584	Elfn1	INSIDE	1.008	2.956	360.77	1066.50	2.981	288.15	859.02
A_68_P27284174	chr10:80049350-80049394	NM_145421:3714	Fam108a	INSIDE	1.008	2.250	444.84	1001.10	2.270	412.50	936.18
A_68_P28603297	chr12:86629542-86629586	NM_172414:24	Fam164c	INSIDE	1.008	1.465	1028.09	1505.74	1.477	837.25	1236.28
A_68_P23253012	chr4:120419677-120419721	NM_001081142:83	Kcnq4	INSIDE	1.008	2.915	864.16	2518.82	2.937	827.59	2430.77
A_68_P25368689	chr7:107756875-107756919	NR_003559:-386	Mrpl48	PROMOTER	1.008	1.444	2725.65	3935.18	1.456	1948.57	2836.21
A_68_P21821257	chr2:164860379-164860423	NM_144892:-121	Ncoa5	PROMOTER	1.008	0.651	1672.78	1088.72	0.656	1413.82	927.64
A_68_P29448699	chr14:21553663-21553707	NM_010811:100	Ndst2	INSIDE	1.008	1.733	609.99	1057.13	1.747	594.94	1039.31
A_68_P31633051	chr18:38428772-38428818	NM_017378:15261	Pcdh12	INSIDE	1.008	2.192	395.43	866.62	2.208	324.50	716.63

ProbeName	Target position of probe on CpG island microarray	TargetID	GeneSymbol	CpG island Description	Ratio of relative methylation (TiO_2-NP/Vehicle)	Sham group			TiO_2-H group		
						Relative methylation (Cy5/Cy3)	Cy3 signal (Input)	Cy5 signal (MeDIP)	Relative methylation (Cy5/Cy3)	Cy3 signal (Input)	Cy5 signal (MeDIP)
A_68_P29637348	chr14:59820302-59820347	NM_027764:260	Rcbtb1	INSIDE	1.008	1.812	415.53	752.95	1.827	419.01	765.41
A_68_P29041999	chr13:53214193-53214237	NM_013846:167264	Ror2	INSIDE	1.008	2.409	2226.22	5362.56	2.429	1769.41	4297.48
A_68_P23236667	chr4:117508851-117508895	NM_008135:1010	Slc6a9	INSIDE	1.008	1.790	2295.98	4109.55	1.805	1805.86	3259.59
A_68_P32672206	chrX:131009632-131009676	NM_028958:1375	Taf7l	INSIDE	1.008	1.980	655.92	1298.44	1.995	289.32	577.28
A_68_P31155254	chr17:34817160-34817204	NM_031176:9703	Tnxb	INSIDE	1.008	2.338	1740.76	4069.74	2.356	1394.26	3285.44
A_68_P30588066	chr16:20535808-20535852	NM_009679:254	Ap2m1	INSIDE	1.007	1.478	1137.84	1681.89	1.488	1032.94	1537.22
A_68_P31118685	chr17:27957394-27957438	NM_001033279:71	D17Wsu92e	INSIDE	1.007	1.898	964.71	1831.38	1.912	926.36	1771.00
A_68_P27093484	chr10:41917285-41917329	NM_019740:79242	Foxo3	INSIDE	1.007	2.237	990.21	2215.21	2.252	784.37	1766.72
A_68_P31936820	chr19:7016078-7016122	NM_001177362:-103	Gpr137	DIVERGENT_PROMOTER	1.007	1.876	486.50	912.75	1.890	440.05	831.78
A_68_P23417748	chr4:151661232-151661276	NM_008237:4517	Hes3	INSIDE	1.007	1.432	1667.53	2387.37	1.441	1274.33	1836.72
A_68_P27279590	chr10:79362078-79362122	NM_001163276:9583	Med16	INSIDE	1.007	1.578	761.35	1201.04	1.588	527.52	837.75
A_68_P22038556	chr3:32428603-32428647	NM_024200:221	Mfn1	INSIDE	1.007	0.557	1335.70	744.06	0.561	1182.42	663.26
A_68_P23615665	chr5:38208583-38208627	NM_010835:7220	Msx1	DOWNSTREAM	1.007	1.433	2007.23	2877.15	1.443	1648.83	2380.03
A_68_P27909148	chr11:71864535-71864579	NM_001024927:84835	Pitpnm3	INSIDE	1.007	1.668	4574.97	7632.05	1.680	4016.57	6749.75
A_68_P31023591	chr17:7182888-7182932	NM_026611:-298	Rnaset2b	PROMOTER	1.007	4.818	4524.14	21797.33	4.850	3298.44	15998.23
A_68_P31351304	chr17:74794846-74794890	NM_144798:-103	Slc30a6	PROMOTER	1.007	0.606	1054.37	638.52	0.610	871.46	531.30
A_68_P29699983	chr14:70605493-70605537	NM_011366:1930	Sorbs3	INSIDE	1.007	0.670	1342.98	899.67	0.675	1134.47	765.68
A_68_P25009106	chr7:26010791-26010835	NM_175436:4343	Zfp526	INSIDE	1.007	2.151	1569.87	3377.11	2.167	1196.81	2593.15
A_68_P25504741	chr7:134388254-134388298	NM_177362:237	Zfp771	INSIDE	1.007	0.505	1146.43	579.16	0.509	1017.06	517.56
A_68_P30665796	chr16:34745100-34745144	ENSMUST00000155268:-173		PROMOTER	1.007	2.874	274.14	787.92	2.894	278.81	806.94
A_68_P23155321	chr4:101169179-101169223	NM_198412:-52	Dnajc6	PROMOTER	1.006	2.036	954.91	1944.31	2.049	846.14	1733.69
A_68_P27550688	chr11:3102520-3102564	NM_023743:-43	Eif4enif1	PROMOTER	1.006	1.707	616.43	1052.56	1.719	621.81	1068.60
A_68_P31490817	chr18:10609887-10609931	NM_001081222:442	Esco1	INSIDE	1.006	1.449	909.60	1318.25	1.459	858.72	1252.47
A_68_P21278821	chr2:63936348-63936392	NM_021716:-301	Fign	PROMOTER	1.006	1.576	1081.45	1704.32	1.585	1018.56	1614.78
A_68_P23451693	chr5:4758175-4758219	NM_021457:20	Fzd1	INSIDE	1.006	1.814	1211.79	2198.78	1.825	993.84	1814.15
A_68_P30143280	chr15:37209165-37209209	NM_026496:46396	Grhl2	INSIDE	1.006	2.350	234.00	549.91	2.365	201.84	477.27
A_68_P23340289	chr4:137074647-137074691	NM_008305:49951	Hspg2	INSIDE	1.006	3.265	739.05	2413.32	3.284	579.80	1903.98
A_68_P29620045	chr14:56442427-56442471	NM_001168346:-1183	Nfatc4	PROMOTER	1.006	2.332	454.75	1060.33	2.347	383.31	899.49
A_68_P25370888	chr7:108147418-108147462	NM_008773:13065	P2ry2	INSIDE	1.006	8.220	2586.95	21264.25	8.265	1766.71	14602.70
A_68_P27215088	chr10:67442788-67442832	NM_001081346:465	Rtkn2	INSIDE	1.006	0.540	1011.31	546.00	0.543	835.68	453.98
A_68_P26137064	chr8:108489850-108489894	NM_009195:67	Slc12a4	INSIDE	1.006	0.456	2060.77	939.01	0.458	1391.72	637.74
A_68_P26814194	chr9:108208208-108208252	NM_133986:52	Tcta	INSIDE	1.006	1.690	894.57	1511.87	1.700	735.98	1251.44
A_68_P31190361	chr17:43766788-43766832	NM_001161366:438	Tdrd6	INSIDE	1.006	3.590	951.40	3415.20	3.611	912.93	3296.31
A_68_P26596757	chr9:66897011-66897055	NM_001164249:-12	Tpm1	PROMOTER	1.006	1.442	3824.26	5514.37	1.451	2927.24	4247.17
A_68_P22037675	chr3:32264764-32264808	NM_009517:-199	Zmat3	PROMOTER	1.006	1.633	996.45	1627.40	1.643	880.81	1446.98
A_68_P20722811	chr1:154613676-154613720	NM_026369:27	Arpc5	INSIDE	1.005	1.558	713.77	1112.18	1.566	616.26	964.87
A_68_P29020671	chr13:48758693-48758737	NM_007526:310	Barx1	INSIDE	1.005	1.639	999.27	1637.75	1.647	923.83	1521.97
A_68_P23295624	chr4:128900663-128900707	NM_001033189:25003	C77080	INSIDE	1.005	1.626	781.47	1270.36	1.634	673.90	1100.85
A_68_P21073094	chr2:25433567-25433611	NM_001005424:2031	Gm996	INSIDE	1.005	1.888	904.89	1708.26	1.898	938.00	1780.18
A_68_P20146387	chr1:36160737-36160781	NM_015818:35514	Hs6st1	INSIDE	1.005	2.040	583.09	1189.37	2.051	506.11	1037.92
A_68_P31096079	chr17:24346336-24346380	NM_010947:-26	Ntn3	PROMOTER	1.005	1.464	1130.61	1655.35	1.472	1167.37	1718.29
A_68_P23543338	chr5:24191818-24191862	NM_016736:208	Nub1	INSIDE	1.005	1.918	911.88	1749.16	1.928	782.21	1508.27
A_68_P20971762	chr2:4985136-4985180	NM_181848:-174	Optn	PROMOTER	1.005	2.105	5285.74	11125.04	2.114	4344.30	9185.21
A_68_P22590676	chr3:142058482-142058526	NM_001190857:-3690	Pdlim5	PROMOTER	1.005	0.719	2330.56	1674.66	0.722	1825.16	1317.80
A_68_P26630945	chr9:72887670-72887714	NM_018889:-186	Pigb	DIVERGENT_PROMOTER	1.005	3.054	642.40	1961.74	3.068	708.79	2174.67
A_68_P28314292	chr12:29334596-29334640	NM_011275:152	Rnaseh1	INSIDE	1.005	0.588	1334.25	785.20	0.591	1063.12	628.47
A_68_P23668846	chr5:48374552-48374596	NM_178804:181	Slit2	INSIDE	1.005	0.554	924.62	512.65	0.557	731.04	407.43
A_68_P25670557	chr8:13786121-13786165	NM_025924:528	Upf3a	INSIDE	1.005	0.547	1048.31	573.12	0.549	827.64	454.76
A_68_P25614226	chr8:3495954-3495998	NM_080461:2839	Zfp358	INSIDE	1.005	3.586	411.15	1474.50	3.606	414.49	1494.65
A_68_P25714452	chr8:24168992-24169036	NM_031158:268	Ank1	INSIDE	1.004	1.617	1589.37	2570.48	1.624	1204.46	1956.55
A_68_P21067471	chr2:24534614-24534658	NM_001042528:83931	Cacna1b	INSIDE	1.004	2.402	766.26	1840.88	2.412	629.44	1518.37

ProbeName	Target position of probe on CpG island microarray	TargetID	GeneSymbol	CpG island Description	Ratio of relative methylation (TiO_2-NP/Vehicle)	Sham group			TiO_2-H group		
						Relative methylation (Cy5/Cy3)	Cy3 signal (Input)	Cy5 signal (MeDIP)	Relative methylation (Cy5/Cy3)	Cy3 signal (Input)	Cy5 signal (MeDIP)
A_68_P25392337	chr7:112583863-112583907	NM_007627:9551	Cckbr	INSIDE	1.004	3.326	1134.41	3772.78	3.338	903.46	3015.59
A_68_P31755198	chr18:61195223-61195267	NM_009880:609	Cdx1	INSIDE	1.004	1.758	498.10	875.64	1.764	444.91	785.00
A_68_P27286398	chr10:80392047-80392091	NM_008655:-767	Gadd45b	PROMOTER	1.004	0.557	1109.90	618.49	0.560	881.38	493.28
A_68_P22344501	chr3:95363489-95363533	NM_026489:-88	Hormad1	PROMOTER	1.004	1.968	538.50	1059.68	1.976	506.20	1000.35
A_68_P30672049	chr16:35770608-35770652	NM_175111:159	Hspbap1	INSIDE	1.004	1.495	1440.81	2154.42	1.501	1329.84	1995.72
A_68_P26162181	chr8:112889462-112889508	NM_172916:98608	Hydin	INSIDE	1.004	2.920	1290.45	3767.60	2.931	965.91	2831.17
A_68_P26586059	chr9:64989179-64989223	NM_008988:205	Igdcc3	INSIDE	1.004	2.150	1190.39	2559.14	2.158	1077.88	2326.10
A_68_P23229577	chr4:116180041-116180085	NM_008389:-91	Ipp	PROMOTER	1.004	0.605	1228.39	742.84	0.607	1041.42	632.32
A_68_P22201289	chr3:65469993-65470037	NM_001166659:-135	Lekr1	PROMOTER	1.004	3.210	3808.32	12225.65	3.225	2718.05	8764.56
A_68_P23297038	chr4:129191512-129191557	NM_010807:710	Marcksl1	INSIDE	1.004	1.516	2234.44	3387.35	1.522	1760.81	2679.96
A_68_P25210675	chr7:77491224-77491268	NM_009697:14233	Nr2f2	DOWNSTREAM	1.004	1.966	1162.56	2285.15	1.973	932.26	1838.90
A_68_P24984114	chr7:17532348-17532392	NM_001099636:2340	Pnmal2	INSIDE	1.004	0.346	1548.55	536.49	0.348	1202.49	418.06
A_68_P24117613	chr5:136210132-136210176	NM_008898:45071	Por	INSIDE	1.004	2.407	583.53	1404.36	2.416	544.81	1316.22
A_68_P20855860	chr1:178744266-178744310	NM_029756:-654	Sdccag8	DIVERGENT_PROMOTER	1.004	1.776	2194.73	3897.06	1.783	1890.77	3371.18
A_68_P24009124	chr5:115791228-115791272	NM_026933:-5	Triap1	DIVERGENT_PROMOTER	1.004	0.522	1318.21	688.47	0.524	1140.89	598.14
A_68_P22766407	chr4:19635843-19635887	NM_177327:276	Wwp1	INSIDE	1.004	0.469	1326.52	622.78	0.471	1278.87	602.88
A_68_P23577042	chr5:31502796-31502840	NM_001177901:1597	Zfp513	INSIDE	1.004	3.891	4411.39	17165.75	3.905	3484.29	13606.99
A_68_P29339700	chr13:114083617-114083661			Unknown	1.004	1.401	1770.64	2479.85	1.406	1500.91	2110.57
A_68_P25065735	chr7:38968873-38968917	NM_028166:89	1600014C10Rik	INSIDE	1.003	0.637	1185.99	754.94	0.639	909.21	580.67
A_68_P27033723	chr10:28916701-28916745	NM_026138:52921	6330407J23Rik	INSIDE	1.003	1.906	1741.22	3318.11	1.911	1379.64	2636.34
A_68_P22322689	chr3:89767337-89767382	NM_001081182:71	Atp8b2	INSIDE	1.003	1.651	2632.80	4347.19	1.657	2094.98	3471.03
A_68_P24157328	chr5:144383397-144383441	NM_011182:103	Cyth3	INSIDE	1.003	1.920	768.05	1474.66	1.926	649.74	1251.70
A_68_P23103729	chr4:91037950-91037994	NM_010486:774	Elavl2	INSIDE	1.003	0.666	2011.99	1339.58	0.668	1748.82	1168.08
A_68_P25954892	chr8:73437474-73437518	NM_173013:7624	Mtap1s	INSIDE	1.003	3.008	679.04	2042.53	3.018	517.91	1563.17
A_68_P30346611	chr15:76011370-76011414	NM_201394:14748	Plec	INSIDE	1.003	3.411	558.28	1904.44	3.420	481.25	1646.09
A_68_P28035919	chr11:94852484-94852528	NM_172261:-19	Ppp1r9b	PROMOTER	1.003	4.171	547.41	2283.01	4.185	542.52	2270.32
A_68_P27674870	chr11:29415061-29415105	NM_001163454:-49	Prorsd1	PROMOTER	1.003	0.668	1582.33	1056.42	0.670	1375.76	921.61
A_68_P26471637	chr9:44946814-44946858	NM_001013390:-288	Scn4b	DIVERGENT_PROMOTER	1.003	0.507	1123.27	569.87	0.509	1003.86	510.81
A_68_P20616238	chr1:133422989-133423033	NM_001081011:928	Srgap2	INSIDE	1.003	0.711	4267.27	3034.24	0.713	3794.25	2706.89
A_68_P24755317	chr6:112677286-112677330	NM_080448:219952	Srgap3	INSIDE	1.003	3.678	1760.66	6475.38	3.687	1153.35	4252.87
A_68_P32008255	chr19:21867281-21867325	NM_001033759:14471	Tmem2	INSIDE	1.003	3.014	1349.89	4068.02	3.024	1140.00	3447.19
A_68_P28280591	chr12:21423034-21423078	NM_011739:241	Ywhaq	INSIDE	1.003	0.599	1853.43	1110.18	0.601	1525.07	915.92
A_68_P29072927	chr13:58486285-58486329	NM_027335:280	2210016F16Rik	INSIDE	1.002	1.425	1227.37	1748.57	1.427	1112.04	1586.72
A_68_P21311081	chr2:69627424-69627468	NM_001077684:97	4930578N16Rik	INSIDE	1.002	1.952	637.55	1244.40	1.956	569.84	1114.80
A_68_P27234934	chr10:70807560-70807604	NM_134007:15	Cisd1	INSIDE	1.002	2.009	1802.07	3620.55	2.013	1646.42	3314.53
A_68_P23999130	chr5:114064140-114064194	NM_008153:36233	Cmklr1	INSIDE	1.002	7.755	1360.90	10553.40	7.770	1067.04	8290.79
A_68_P31458150	chr18:3337100-3337144	NM_001110850:465	Crem	INSIDE	1.002	1.481	868.72	1286.63	1.484	802.48	1191.11
A_68_P20613816	chr1:133034598-133034642	NM_145508:191	Dyrk3	INSIDE	1.002	1.349	2436.63	3286.10	1.351	2270.32	3067.97
A_68_P24388787	chr6:40386026-40386070	NM_175528:84	E330009J07Rik	INSIDE	1.002	2.277	486.02	1106.42	2.281	506.50	1155.30
A_68_P23445681	chr5:3543833-3543877	NM_001042501:22	Fam133b	INSIDE	1.002	0.641	2179.93	1396.43	0.642	1670.71	1071.98
A_68_P21565622	chr2:118529841-118529885	NM_001081971:-163	Gm1337	PROMOTER	1.002	2.729	419.05	1143.54	2.733	349.67	955.76
A_68_P24828004	chr6:126593418-126593462	NM_010595:2379	Kcna1	INSIDE	1.002	1.765	859.28	1516.22	1.768	709.71	1255.05
A_68_P29106491	chr13:67034356-67034400	NM_025547:-370	Mterfd1	PROMOTER	1.002	3.182	413.13	1314.68	3.187	459.25	1463.80
A_68_P26350702	chr9:21969587-21969631	NM_001082532:8319	Pigyl	DOWNSTREAM	1.002	2.022	1436.01	2902.98	2.026	1195.41	2421.70
A_68_P21349792	chr2:76208467-76208511	NM_153405:448	Rbm45	INSIDE	1.002	1.506	2977.07	4484.37	1.509	2362.36	3564.91
A_68_P29637346	chr14:59820048-59820093	NM_027764:6	Rcbtb1	INSIDE	1.002	1.954	323.30	631.79	1.958	313.85	614.51
A_68_P30363606	chr15:78813601-78813645	NM_001024716:137	Triobp	INSIDE	1.002	2.081	942.81	1961.67	2.085	878.42	1831.90
A_68_P20820924	chr1:172105271-172105315	NM_133806:-215	Uap1	PROMOTER	1.002	0.575	1316.76	756.52	0.575	1083.12	623.33
A_68_P21149699	chr2:38527835-38527879	ENSMUST00000152645:-200		PROMOTER	1.002	2.125	837.78	1780.58	2.130	683.64	1456.02
A_68_P32683271	chrX:133462182-133462226	NM_027067:341	1700014N06Rik	INSIDE	1.001	4.503	1528.00	6879.82	4.505	686.58	3092.99
A_68_P24760449	chr6:113554951-113554995	NM_133937:207	6720456B07Rik	INSIDE	1.001	0.578	1938.36	1120.30	0.579	1620.54	937.64

ProbeName	Target position of probe on CpG island microarray	TargetID	GeneSymbol	CpG island Description	Ratio of relative methylation (TiO_2-NP/Vehicle)	Sham group			TiO_2-H group		
						Relative methylation (Cy5/Cy3)	Cy3 signal (Input)	Cy5 signal (MeDIP)	Relative methylation (Cy5/Cy3)	Cy3 signal (Input)	Cy5 signal (MeDIP)
A_68_P25708739	chr8:23123882-23123926	NM_007511:46642	Atp7b	INSIDE	1.001	1.710	977.14	1670.45	1.712	846.17	1448.32
A_68_P31933858	chr19:6306849-6306893	NM_001033342:414	Cdc42bpg	INSIDE	1.001	1.793	1243.97	2230.48	1.795	1069.75	1920.01
A_68_P22355190	chr3:97413997-97414041	NM_026539:95	Chd1l	INSIDE	1.001	2.590	611.24	1582.88	2.592	610.65	1582.68
A_68_P27281410	chr10:79627471-79627515	NM_007705:-3093	Cirbp	PROMOTER	1.001	1.936	1128.46	2184.35	1.938	903.92	1751.96
A_68_P30507167	chr16:3908948-3908992	NM_029738:-38	Cluap1	DIVERGENT_PROMOTER	1.001	0.583	1104.12	644.07	0.584	908.10	530.47
A_68_P27952486	chr11:79894373-79894417	NM_018776:63	Crlf3	INSIDE	1.001	1.979	1368.96	2708.73	1.980	1208.10	2392.53
A_68_P22954048	chr4:59016217-59016261	NM_001033165:174	Dnajc25	INSIDE	1.001	1.826	408.85	746.45	1.828	358.06	654.36
A_68_P25564248	chr7:144390170-144390214	NM_001113414:115936	Ebf3	INSIDE	1.001	4.113	3266.90	13437.90	4.119	2477.94	10206.66
A_68_P26617704	chr9:70504900-70504944	NM_172772:59	Fam63b	INSIDE	1.001	2.570	697.55	1792.65	2.573	769.23	1979.39
A_68_P29928896	chr14:117324667-117324711	NM_001079844:152	Gpc6	INSIDE	1.001	1.768	1998.31	3532.86	1.769	1657.61	2932.72
A_68_P27568543	chr11:6344478-6344522	NM_029938:-54	H2afv	PROMOTER	1.001	0.412	1695.25	698.80	0.413	1425.40	588.36
A_68_P29942852	chr14:119537211-119537255	NM_015820:-254	Hs6st3	PROMOTER	1.001	2.083	685.49	1428.16	2.084	615.86	1283.75
A_68_P32746465	chrX:148238176-148238221	NM_021523:374	Huwe1	INSIDE	1.001	2.184	3499.70	7644.24	2.186	1699.28	3714.16
A_68_P20739568	chr1:157584694-157584738	NM_010712:4441	Lhx4	INSIDE	1.001	0.634	1644.18	1041.84	0.634	1262.79	801.23
A_68_P26537376	chr9:56482845-56482889	NM_181074:50194	Lingo1	INSIDE	1.001	0.537	1729.64	929.27	0.538	1418.17	762.47
A_68_P20348301	chr1:74438711-74438755	NR_029743:-2151	Mir26b	PROMOTER	1.001	5.814	793.29	4612.24	5.821	763.86	4446.28
A_68_P24191791	chr5:151397312-151397356	NM_133898:-234	N4bp2l1	PROMOTER	1.001	1.664	588.10	978.45	1.666	568.62	947.41
A_68_P31866221	chr18:80894853-80894897	NM_001164111:10038	Nfatc1	INSIDE	1.001	1.650	1020.30	1683.16	1.652	770.54	1272.96
A_68_P29054897	chr13:55516522-55516566	NM_029303:49	Pfn3	INSIDE	1.001	0.520	1325.73	689.16	0.520	1006.69	523.58
A_68_P27568687	chr11:6375581-6375625	NM_011221:318	Purb	INSIDE	1.001	1.893	476.34	901.68	1.895	503.04	953.43
A_68_P21782731	chr2:158236154-158236198	NM_177658:588	Ralgapb	INSIDE	1.001	2.788	2187.42	6099.31	2.790	1832.50	5112.94
A_68_P31138857	chr17:31433598-31433642	NM_153062:1193	Slc37a1	INSIDE	1.001	1.916	1401.63	2684.97	1.918	1134.51	2176.18
A_68_P22543384	chr3:133208009-133208053	NM_001040400:-676	Tet2	PROMOTER	1.001	2.501	932.77	2333.05	2.505	819.82	2053.37
A_68_P22820140	chr4:32326028-32326072	ENSMUST00000149201:-1		PROMOTER	1.001	1.440	1105.42	1592.01	1.441	1016.43	1464.60
A_68_P20562758	chr1:122499691-122499735	NM_010133:649	En1	INSIDE	1.000	2.775	1810.25	5024.30	2.774	1534.25	4256.35
A_68_P26804353	chr9:106550228-106550272	NM_001160353:8190	Grm2	INSIDE	1.000	1.849	396.78	733.67	1.849	337.51	624.06
A_68_P31053794	chr17:12962250-12962294	NM_010515:300	Igf2r	INSIDE	1.000	2.114	812.96	1718.60	2.115	748.12	1581.99
A_68_P27340038	chr10:90564802-90564859	NM_027078:19047	Ikbip	INSIDE	1.000	4.183	770.88	3224.39	4.185	644.40	2696.50
A_68_P29334869	chr13:113254155-113254199	NM_010560:-101	Il6st	PROMOTER	1.000	2.473	720.39	1781.77	2.473	643.17	1590.61
A_68_P32147196	chr19:47089352-47089396	NM_146100:187	Ina	INSIDE	1.000	1.629	591.63	964.03	1.630	530.30	864.16
A_68_P25111232	chr7:56502025-56502069	NM_175272:488	Nav2	INSIDE	1.000	1.904	1561.73	2973.72	1.905	1228.89	2340.61
A_68_P30165311	chr15:41279202-41279246	NM_001130166:197	Oxr1	INSIDE	1.000	1.483	1196.71	1774.96	1.483	1051.71	1559.45
A_68_P20641451	chr1:137662928-137662972	NM_013750:289	Phlda3	INSIDE	1.000	1.648	653.33	1076.81	1.648	647.38	1066.93
A_68_P24984543	chr7:17612915-17612959	NM_011155:327	Ppp5c	INSIDE	1.000	2.526	813.45	2055.03	2.526	786.34	1986.38
A_68_P25090467	chr7:52241632-52241676	NM_019830:48	Prmt1	INSIDE	1.000	0.563	2673.91	1505.32	0.563	2064.17	1161.69
A_68_P31255961	chr17:56556971-56557015	NM_011218:58911	Ptprs	INSIDE	1.000	2.726	2495.12	6800.53	2.726	1799.25	4905.24
A_68_P26822090	chr9:110034578-110034622	NM_009211:73	Smarcc1	INSIDE	1.000	0.587	1716.41	1007.15	0.587	1489.01	873.48
A_68_P26752043	chr9:97266499-97266543	NM_030219:3857	Trim42	INSIDE	1.000	3.580	2124.53	7605.45	3.580	1367.72	4896.73
A_68_P21911428	chr2:181316733-181316777	NM_026765:-76	Uckl1	PROMOTER	1.000	0.550	2365.67	1300.63	0.550	1911.27	1051.32
A_68_P24638867	chr6:91361318-91361362	NM_009527:23	Wnt7a	INSIDE	1.000	0.534	1200.04	640.63	0.534	943.05	503.65
A_68_P21622216	chr2:128769433-128769477	NM_020594:302	Zc3h8	INSIDE	1.000	0.661	3469.05	2291.63	0.661	2788.06	1841.73
A_68_P31159051	chr17:35495987-35496031	M19687:92193		INSIDE	1.000	1.623	852.55	1383.39	1.622	692.14	1122.72
A_68_P31632850	chr18:38398968-38399012	NR_015475:10862	1700086O06Rik	INSIDE	0.999	2.657	1930.10	5128.40	2.653	1700.15	4510.89
A_68_P22888515	chr4:46402231-46402275	NM_029086:43	5830415F09Rik	INSIDE	0.999	0.586	3453.67	2025.56	0.586	2807.81	1645.52
A_68_P25509658	chr7:135381901-135381945	NM_177001:-377	9130023H24Rik	PROMOTER	0.999	1.842	1505.89	2773.86	1.840	1308.44	2406.96
A_68_P26397637	chr9:30728994-30729041	NM_001024139:1020	Adamts15	INSIDE	0.999	2.179	523.50	1140.66	2.176	427.05	929.30
A_68_P25589622	chr7:148748160-148748204	NM_007459:104	Ap2a2	INSIDE	0.999	2.413	690.05	1665.06	2.411	645.41	1556.04
A_68_P28517159	chr12:70993954-70993998	NM_178628:-115	Atl1	PROMOTER	0.999	2.421	257.97	624.52	2.419	228.35	552.45
A_68_P28183246	chr11:120098017-120098061	NM_198423:3778	Bahcc1	INSIDE	0.999	0.671	2111.42	1416.75	0.670	1828.52	1225.29
A_68_P21751444	chr2:152656729-152656773	NM_009743:668	Bcl2l1	INSIDE	0.999	0.483	1434.21	692.88	0.483	1306.97	630.63
A_68_P25951052	chr8:72839724-72839768	NM_001163282:73	Gdf1	INSIDE	0.999	0.549	1349.95	741.73	0.549	1069.18	586.87

ProbeName	Target position of probe on CpG island microarray	TargetID	GeneSymbol	CpG island Description	Ratio of relative methylation (TiO_2-NP/Vehicle)	Sham group			TiO_2-H group		
						Relative methylation (Cy5/Cy3)	Cy3 signal (Input)	Cy5 signal (MeDIP)	Relative methylation (Cy5/Cy3)	Cy3 signal (Input)	Cy5 signal (MeDIP)
A_68_P27278193	chr10:79138046-79138092	NM_148934:5914	Gm16517	INSIDE	0.999	2.462	415.58	1023.07	2.459	315.78	776.61
A_68_P27279643	chr10:79371605-79371649	NM_001163276:57	Med16	INSIDE	0.999	3.411	4547.19	15510.39	3.409	4065.17	13858.61
A_68_P23260200	chr4:122673850-122673894	NM_008506:531	Mycl1	INSIDE	0.999	2.358	2423.27	5714.37	2.357	2125.77	5009.77
A_68_P30497261	chr15:102156542-102156586	NM_027044:18	Pfdn5	INSIDE	0.999	0.647	2071.67	1340.46	0.646	1745.19	1127.84
A_68_P26750484	chr9:96919484-96919528	NM_145134:-732	Spsb4	PROMOTER	0.999	2.141	1284.14	2749.85	2.139	1093.79	2340.05
A_68_P27845269	chr11:60692535-60692579	NM_001168507:-16	Tmem11	PROMOTER	0.999	0.550	1323.77	727.90	0.549	969.23	532.31
A_68_P23240958	chr4:118214708-118214752	NM_172383:1601	Tmem125	INSIDE	0.999	2.109	1245.79	2627.70	2.107	992.87	2092.00
A_68_P24067083	chr5:125956377-125956421	NM_030210:156	Aacs	INSIDE	0.998	0.603	1336.15	805.51	0.602	1025.60	616.95
A_68_P30762452	chr16:52452809-52452853	NM_009655:280	Alcam	INSIDE	0.998	0.339	1713.94	580.70	0.338	1668.29	564.12
A_68_P23576264	chr5:31371183-31371228	NM_023525:14022	Cad	INSIDE	0.998	3.213	598.79	1923.96	3.207	454.70	1458.27
A_68_P27924577	chr11:74981143-74981187	NM_001098203:490	Hic1	INSIDE	0.998	2.270	565.78	1284.39	2.267	434.21	984.21
A_68_P23333956	chr4:135998848-135998894	NM_008309:19432	Htr1d	INSIDE	0.998	2.131	351.60	749.42	2.127	252.44	536.90
A_68_P31829406	chr18:74223918-74223962	NM_172632:663	Mapk4	INSIDE	0.998	1.442	2017.22	2908.79	1.439	1555.75	2239.24
A_68_P30368654	chr15:79634541-79634585	NM_030689:577	Nptxr	INSIDE	0.998	1.960	489.40	959.03	1.956	468.66	916.49
A_68_P29116252	chr13:69672648-69672692	NM_001169131:72	Papd7	INSIDE	0.998	3.351	553.07	1853.12	3.345	451.07	1509.02
A_68_P23576466	chr5:31397923-31397967	NM_011773:-2044	Slc30a3	PROMOTER	0.998	1.857	619.34	1150.15	1.854	570.84	1058.10
A_68_P29238030	chr13:95746505-95746549	NM_172590:224	Wdr41	INSIDE	0.998	1.934	361.03	698.29	1.929	334.92	646.20
A_68_P29614458	chr14:55433137-55433182	ENSMUST00000095853:-2164		PROMOTER	0.998	2.005	1026.89	2058.78	2.001	872.72	1745.93
A_68_P24973686	chr7:13514022-13514066	NM_029809:1280	2310014L17Rik	INSIDE	0.997	1.983	2957.50	5863.54	1.978	2257.46	4464.18
A_68_P28098902	chr11:105849980-105850024	NM_009598:13481	Ace	INSIDE	0.997	3.371	2790.42	9406.29	3.361	1956.84	6576.26
A_68_P28751739	chr12:113912106-113912150	NM_001165894:359	Akt1	INSIDE	0.997	1.867	988.87	1845.84	1.862	865.73	1611.66
A_68_P31500013	chr18:12464044-12464088	NM_001190371:163	Ankrd29	INSIDE	0.997	0.530	1194.05	633.15	0.529	906.64	479.31
A_68_P20722814	chr1:154614048-154614092	NM_026369:399	Arpc5	INSIDE	0.997	0.508	1445.23	733.62	0.506	1186.65	600.79
A_68_P23332635	chr4:135762482-135762526	NM_001008232:225	Asap3	INSIDE	0.997	1.446	2295.55	3318.93	1.441	1657.39	2388.62
A_68_P20294242	chr1:64781496-64781540	NM_001042659:2806	Fzd5	INSIDE	0.997	2.794	629.47	1758.50	2.784	552.56	1538.30
A_68_P28553888	chr12:77518476-77518520	NM_001163103:-87	Gm70	PROMOTER	0.997	1.733	679.79	1178.21	1.727	697.08	1204.08
A_68_P31259627	chr17:57162083-57162127	NM_010613:8826	Khsrp	INSIDE	0.997	2.439	1125.22	2744.81	2.431	860.59	2092.42
A_68_P30365873	chr15:79178383-79178427	NM_010755:297	Maff	INSIDE	0.997	1.377	3938.23	5423.92	1.374	2859.41	3928.10
A_68_P30370811	chr15:80042269-80042313	NM_010795:38140	Mgat3	INSIDE	0.997	2.631	865.28	2276.38	2.622	728.36	1909.78
A_68_P29116249	chr13:69672357-69672401	NM_001169131:364	Papd7	INSIDE	0.997	2.373	430.14	1020.63	2.366	473.01	1119.05
A_68_P24980577	chr7:16858327-16858371	NM_001136270:693	Prr24	INSIDE	0.997	1.584	735.36	1165.10	1.580	698.37	1103.34
A_68_P32071925	chr19:32832040-32832084	NM_008960:-4	Pten	PROMOTER	0.997	0.393	2158.87	847.85	0.392	1626.33	636.79
A_68_P26872699	chr9:119395083-119395128	NM_021544:93029	Scn5a	INSIDE	0.997	6.691	1675.87	11213.76	6.669	1240.89	8275.00
A_68_P28054174	chr11:97914443-97914487	NM_146028:312	Stac2	INSIDE	0.997	1.516	1351.03	2048.05	1.511	1116.39	1687.37
A_68_P21910189	chr2:181100664-181100708	NM_001048148:559	Zgpat	INSIDE	0.997	1.533	1047.54	1605.55	1.528	912.86	1394.56
A_68_P21967925	chr3:17686263-17686307	NR_033490:-3636	2610100L16Rik	PROMOTER	0.996	1.832	700.79	1283.61	1.824	671.51	1224.61
A_68_P26809314	chr9:107429770-107429814	NM_001174047:127582	Cacna2d2	INSIDE	0.996	2.280	4757.54	10847.27	2.270	3392.77	7701.93
A_68_P23193626	chr4:107596160-107596204	NM_009949:12	Cpt2	INSIDE	0.996	0.568	1778.95	1010.03	0.566	1435.54	812.00
A_68_P27386041	chr10:98726685-98726729	NM_026268:842	Dusp6	INSIDE	0.996	1.374	1779.75	2445.20	1.369	1581.44	2164.98
A_68_P27479722	chr10:116585133-116585177	NM_177798:376	Frs2	INSIDE	0.996	1.876	4085.68	7664.93	1.868	3157.32	5897.78
A_68_P27790976	chr11:50673440-50673484	NM_173372:9276	Grm6	INSIDE	0.996	3.290	1696.71	5582.56	3.277	1165.85	3820.51
A_68_P28727296	chr12:109556884-109556928	NM_001044380:12427	Hhipl1	INSIDE	0.996	1.574	2623.55	4129.92	1.567	2017.20	3161.77
A_68_P21261715	chr2:60800332-60800376	NM_020296:907	Rbms1	INSIDE	0.996	0.371	3510.84	1303.82	0.370	1038.29	384.24
A_68_P21297863	chr2:67403728-67403772			Unknown	0.996	2.896	374.84	1085.70	2.886	345.96	998.55
A_68_P32565208	chrX:98893525-98893573	ENSMUST00000073812:-6359		PROMOTER	0.996	4.092	858.36	3512.21	4.077	365.29	1489.31
A_68_P23562832	chr5:28785049-28785093	NR_015562:-8453	9530036O11Rik	PROMOTER	0.995	1.861	900.53	1675.67	1.852	815.55	1510.14
A_68_P23315555	chr4:132617504-132617548	NM_146155:50106	Ahdc1	INSIDE	0.995	2.198	2866.16	6300.82	2.187	2036.49	4454.39
A_68_P31304169	chr17:66426588-66426632	NM_001025572:-224	Ankrd12	PROMOTER	0.995	1.367	2946.48	4027.20	1.360	2267.77	3083.51
A_68_P31015191	chr17:5189996-5190040	NM_001085355:194945	Arid1b	INSIDE	0.995	1.921	867.83	1667.48	1.911	812.52	1552.65
A_68_P26505535	chr9:50576596-50576640	NM_198675:276	Fdxacb1	INSIDE	0.995	1.526	1064.15	1623.66	1.518	972.54	1475.91
A_68_P30673113	chr16:35983288-35983332	NM_008465:-138	Kpna1	PROMOTER	0.995	2.069	641.80	1328.17	2.060	610.60	1257.66

ProbeName	Target position of probe on CpG island microarray	TargetID	GeneSymbol	CpG island Description	Ratio of relative methylation (TiO_2-NP/Vehicle)	Sham group			TiO_2-H group		
						Relative methylation (Cy5/Cy3)	Cy3 signal (Input)	Cy5 signal (MeDIP)	Relative methylation (Cy5/Cy3)	Cy3 signal (Input)	Cy5 signal (MeDIP)
A_68_P27528047	chr10:125403454-125403498	NM_177152:202	Lrig3	INSIDE	0.995	1.897	1111.46	2108.73	1.888	1074.76	2028.77
A_68_P22569094	chr3:138152230-138152274	NM_175224:94	Metap1	INSIDE	0.995	0.558	1421.82	793.85	0.556	1294.07	719.08
A_68_P23230187	chr4:116299629-116299673	NM_001081475:452	Nasp	INSIDE	0.995	2.444	818.05	1998.97	2.431	775.57	1885.28
A_68_P21322071	chr2:71711579-71711623	NM_172665:272	Pdk1	INSIDE	0.995	1.869	408.41	763.22	1.860	451.21	839.30
A_68_P29701214	chr14:70828882-70828926	NM_021308:-3	Piwil2	PROMOTER	0.995	2.654	1207.94	3206.23	2.641	914.92	2416.33
A_68_P20412582	chr1:88421547-88421591	NM_008972:-1742	Ptma	PROMOTER	0.995	0.620	1245.87	771.94	0.616	971.73	598.81
A_68_P25957632	chr8:74108446-74108490	NM_011977:15643	Slc27a1	INSIDE	0.995	1.632	1076.80	1757.14	1.623	876.30	1422.62
A_68_P24983334	chr7:17396097-17396141	NM_001039878:-5119	Strn4	PROMOTER	0.995	1.522	1647.32	2507.44	1.515	1366.68	2070.67
A_68_P24757154	chr6:112996444-112996488	NM_008188:146	Thumpd3	INSIDE	0.995	1.737	4296.78	7462.07	1.729	3293.29	5693.23
A_68_P27017611	chr10:25798724-25798768	NM_029881:112	Tmem200a	INSIDE	0.995	2.407	411.15	989.66	2.395	378.57	906.74
A_68_P28272753	chr12:17888729-17888773	ENSMUST00000169996:-252		PROMOTER	0.995	3.529	636.40	2245.90	3.512	605.21	2125.64
A_68_P23319352	chr4:133309797-133309841	NM_001080819:-292	Arid1a	PROMOTER	0.994	2.271	259.06	588.20	2.257	268.11	605.25
A_68_P26020866	chr8:87163478-87163522	NM_007578:224238	Cacna1a	INSIDE	0.994	3.412	181.89	620.63	3.391	273.43	927.22
A_68_P25392336	chr7:112583704-112583750	NM_007627:9393	Cckbr	INSIDE	0.994	1.993	772.15	1538.80	1.982	691.06	1369.49
A_68_P21080069	chr2:26483908-26483952	NM_019833:-46	Fam69b	PROMOTER	0.994	2.648	1676.41	4438.84	2.632	1413.20	3719.47
A_68_P23980412	chr5:110973428-110973472	NM_198306:87	Galnt9	INSIDE	0.994	2.402	1560.80	3749.46	2.389	1323.60	3162.07
A_68_P22448207	chr3:115954695-115954739	NM_022427:1686	Gpr88	INSIDE	0.994	3.688	1626.77	5999.23	3.666	1173.73	4302.61
A_68_P30473862	chr15:98087209-98087253	NM_027304:508	H1fnt	INSIDE	0.994	2.372	828.18	1964.16	2.357	742.30	1749.38
A_68_P20146151	chr1:36125666-36125710	NM_015818:444	Hs6st1	INSIDE	0.994	1.782	1059.26	1887.91	1.771	864.25	1530.45
A_68_P20058117	chr1:17081719-17081763	NM_020604:6230	Jph1	INSIDE	0.994	2.415	432.87	1045.23	2.400	437.87	1050.67
A_68_P30365872	chr15:79178280-79178324	NM_010755:195	Maff	INSIDE	0.994	1.455	860.84	1252.58	1.447	853.34	1234.36
A_68_P27279642	chr10:79371508-79371552	NM_001163276:153	Med16	INSIDE	0.994	1.480	5316.32	7868.73	1.472	4037.00	5940.95
A_68_P31918144	chr19:3575377-3575421	NM_001164159:351	Ppp6r3	INSIDE	0.994	2.928	226.67	663.80	2.910	224.20	652.39
A_68_P30577449	chr16:18088865-18088909	NM_011172:397	Prodh	INSIDE	0.994	0.663	3212.32	2130.88	0.660	2666.40	1758.78
A_68_P26145652	chr8:110111876-110111920	NM_010817:484	Psmd7	INSIDE	0.994	1.754	1356.42	2379.29	1.744	984.47	1716.67
A_68_P29094190	chr13:63669109-63669153	NM_008957:-2302	Ptch1	PROMOTER	0.994	0.643	1264.45	812.72	0.639	1079.27	689.55
A_68_P20826099	chr1:173032693-173032737			Unknown	0.994	1.419	4354.08	6179.40	1.410	3366.74	4747.43
A_68_P23236238	chr4:117402055-117402099	ENSMUST00000119463:-172		PROMOTER	0.994	3.803	377.85	1436.81	3.780	332.07	1255.28
A_68_P23358590	chr4:140071282-140071326	NM_001112723:133367	Arhgef10l	INSIDE	0.993	2.016	607.45	1224.71	2.003	460.13	921.44
A_68_P23069061	chr4:84191429-84191473	NM_172870:129540	Bnc2	INSIDE	0.993	2.050	730.17	1496.77	2.035	684.91	1393.86
A_68_P20162792	chr1:38954736-38954780	NM_145142:247	Chst10	INSIDE	0.993	1.434	1466.35	2103.29	1.425	1325.92	1889.34
A_68_P21566331	chr2:118640254-118640305	NM_026412:541	D2Ertd750e	INSIDE	0.993	3.780	189.64	716.82	3.754	202.56	760.45
A_68_P20884849	chr1:183950484-183950528	NM_001083120:-395	Enah	PROMOTER	0.993	2.101	3415.11	7174.11	2.087	2834.83	5915.61
A_68_P25095110	chr7:53050321-53050365	NM_001005511:11026	Lmtk3	INSIDE	0.993	1.941	685.01	1329.93	1.928	599.14	1155.30
A_68_P26164574	chr8:113263014-113263058	NM_198625:17653	Mtss1l	INSIDE	0.993	2.608	774.49	2019.64	2.591	637.24	1650.87
A_68_P32065927	chr19:31739233-31739277	NM_011160:-394	Prkg1	PROMOTER	0.993	2.083	7892.51	16437.63	2.067	5044.98	10428.48
A_68_P27949797	chr11:79405082-79405126	NM_175543:391	Rab11fip4	INSIDE	0.993	3.847	243.01	934.92	3.821	240.95	920.65
A_68_P27804897	chr11:53277337-53277381	NM_027917:6652	Shroom1	INSIDE	0.993	3.196	478.62	1529.53	3.173	432.98	1373.82
A_68_P30425030	chr15:89240388-89240432	NM_001162882:284	1700007E06Rik	INSIDE	0.992	4.670	1754.18	8192.88	4.632	1273.24	5897.68
A_68_P28990875	chr13:43655667-43655711	NM_001081059:-128	Ccdc90a	PROMOTER	0.992	1.488	2361.83	3513.87	1.476	1911.69	2822.43
A_68_P26055365	chr8:93352513-93352557	NM_177224:-199	Chd9	PROMOTER	0.992	2.663	568.34	1513.73	2.642	535.13	1413.73
A_68_P32982272	chr5:134998625-134998669	NM_001039162:29658	Clip2	INSIDE	0.992	2.331	2254.36	5255.17	2.312	1835.41	4244.26
A_68_P27284503	chr10:80101463-80101507	NM_001159591:9084	Csnk1g2	INSIDE	0.992	2.078	1387.24	2882.12	2.061	995.37	2051.67
A_68_P22019081	chr3:28680448-28680492	NM_177586:238	Eif5a2	INSIDE	0.992	0.645	1422.03	917.80	0.640	1312.04	839.68
A_68_P29237510	chr13:95643777-95643821	NM_011021:-1783	Otp	PROMOTER	0.992	1.508	1105.73	1667.74	1.497	944.99	1414.31
A_68_P29437783	chr14:19103060-19103104	NM_025586:418	Rpl15	INSIDE	0.992	0.499	1339.28	668.87	0.495	1211.93	600.36
A_68_P21293424	chr2:66472811-66472855	NM_018852:177	Scn9a	INSIDE	0.992	0.633	1601.71	1014.38	0.628	1282.15	805.12
A_68_P25100396	chr7:53895073-53895117	NM_013789:83	Sergef	INSIDE	0.992	1.898	771.68	1464.98	1.882	671.26	1263.58
A_68_P31958012	chr19:11893471-11893515	NM_011502:-109	Stx3	PROMOTER	0.992	2.210	922.69	2038.75	2.191	894.54	1959.78
A_68_P32021816	chr19:24205473-24205517	NM_001198985:43136	Tjp2	INSIDE	0.992	3.244	1594.51	5172.06	3.218	1164.87	3748.69
A_68_P28747158	chr12:113205217-113205261	NM_027360:-50	2010107E04Rik	DIVERGENT_PROMOTER	0.991	1.582	1091.43	1727.06	1.569	962.54	1510.08

ProbeName	Target position of probe on CpG island microarray	TargetID	GeneSymbol	CpG island Description	Ratio of relative methylation (TiO_2-NP/Vehicle)	Sham group			TiO_2-H group		
						Relative methylation (Cy5/Cy3)	Cy3 signal (Input)	Cy5 signal (MeDIP)	Relative methylation (Cy5/Cy3)	Cy3 signal (Input)	Cy5 signal (MeDIP)
A_68_P30362447	chr15:78629670-78629714	NM_130859:3780	Card10	INSIDE	0.991	2.938	2744.23	8062.68	2.913	2408.44	7015.37
A_68_P27172914	chr10:59648890-59648934	NM_016803:33096	Chst3	INSIDE	0.991	2.824	436.94	1233.82	2.799	379.51	1062.24
A_68_P26841766	chr9:113650462-113650506	NM_001114347:-106	Clasp2	PROMOTER	0.991	1.484	956.75	1419.92	1.471	794.79	1168.91
A_68_P32145200	chr19:46728723-46728767	NM_001177813:30854	D19Wsu162e	INSIDE	0.991	2.603	856.94	2230.90	2.579	754.84	1947.05
A_68_P30590964	chr16:21204925-21204969	NM_010143:79	Ephb3	INSIDE	0.991	0.692	1919.35	1329.08	0.686	1639.78	1125.32
A_68_P24951532	chr7:4959919-4959963	NM_001110330:4904	Fiz1	INSIDE	0.991	1.724	2321.68	4002.67	1.708	2045.57	3494.79
A_68_P30083238	chr15:25711367-25711411	NM_019472:159084	Myo10	INSIDE	0.991	1.799	980.50	1763.81	1.783	779.10	1389.16
A_68_P25353646	chr7:105227755-105227799	NM_008663:40227	Myo7a	INSIDE	0.991	1.580	894.89	1413.47	1.565	796.39	1246.73
A_68_P23134314	chr4:97438807-97438855	NM_001122953:-5486	Nfia	PROMOTER	0.991	1.589	561.28	892.12	1.575	521.62	821.32
A_68_P21580770	chr2:121240019-121240063	NM_007952:403	Pdia3	INSIDE	0.991	0.715	1728.78	1235.54	0.709	1475.76	1045.67
A_68_P25090608	chr7:52263818-52263862	NM_001008422:7779	Scaf1	INSIDE	0.991	1.607	1196.28	1922.74	1.592	956.34	1522.78
A_68_P22342628	chr3:94976704-94976748	NM_011351:8431	Sema6c	INSIDE	0.991	1.756	720.73	1265.74	1.741	642.58	1118.69
A_68_P28755250	chr12:114427183-114427227	NM_153776:3091	Tmem121	INSIDE	0.991	3.686	1213.87	4474.68	3.655	1068.91	3906.50
A_68_P30566189	chr16:15594510-15594554	NM_001159351:79	Ube2v2	INSIDE	0.991	1.415	2170.14	3071.20	1.403	1695.05	2378.10
A_68_P24118487	chr5:136386793-136386840	NM_018871:23695	Ywhag	INSIDE	0.991	2.762	310.23	856.83	2.736	245.32	671.20
A_68_P32603679	chrX:109714349-109714393	NM_177747:236	Zfp711	INSIDE	0.991	2.455	464.94	1141.53	2.434	289.87	705.61
A_68_P26232729	chr8:124860750-124860794	NM_009569:54732	Zfpm1	INSIDE	0.991	1.687	666.89	1125.17	1.672	568.14	949.98
A_68_P26276224	chr9:4260268-4260312			Unknown	0.991	1.504	1229.05	1848.69	1.490	927.93	1382.64
A_68_P21774735	chr2:156846821-156846865	NM_001164663:58159	9830001H06Rik	INSIDE	0.990	1.965	425.28	835.52	1.946	418.09	813.42
A_68_P27383819	chr10:98376903-98376947	NM_026482:-861	Atp2b1	PROMOTER	0.990	1.436	1235.63	1774.96	1.422	1076.44	1530.53
A_68_P25586497	chr7:148254408-148254452	NM_177897:7258	B4galnt4	INSIDE	0.990	2.607	495.52	1291.70	2.580	446.78	1152.81
A_68_P28175482	chr11:118906328-118906372	NM_013926:-4123	Cbx8	PROMOTER	0.990	1.958	739.25	1447.66	1.939	674.22	1307.55
A_68_P23418119	chr4:151713113-151713157	NM_001081376:375	Chd5	INSIDE	0.990	2.392	2509.82	6002.41	2.367	2237.51	5297.09
A_68_P20553863	chr1:120734057-120734104	NM_001081125:216116	Gli2	INSIDE	0.990	2.316	1210.84	2804.00	2.293	959.38	2199.99
A_68_P23977824	chr5:110537149-110537193	NM_145147:46	Gtpbp6	INSIDE	0.990	1.441	3217.96	4637.99	1.427	2416.16	3448.39
A_68_P26346744	chr9:21172242-21172286	NM_001042708:-182	Ilf3	PROMOTER	0.990	0.575	1430.63	822.38	0.569	1082.06	615.89
A_68_P21117014	chr2:32568718-32568762	NR_037217:-332	Mir2861	DIVERGENT_PROMOTER	0.990	1.400	1642.46	2298.77	1.385	1568.19	2172.01
A_68_P24810881	chr6:122824922-122824967	NM_026267:370	Necap1	INSIDE	0.990	0.631	3086.28	1948.66	0.625	2397.67	1499.10
A_68_P30368806	chr15:79659338-79659382	NM_001013360:5403	Npcd	INSIDE	0.990	2.081	1115.25	2321.14	2.060	916.46	1887.95
A_68_P29697194	chr14:70196895-70196939	NM_153514:8436	Rhobtb2	INSIDE	0.990	2.714	2553.13	6929.77	2.687	1854.22	4981.90
A_68_P30602963	chr16:23431338-23431382	NM_001004151:2155	Rtp1	INSIDE	0.990	2.822	258.41	729.15	2.793	243.89	681.29
A_68_P27877796	chr11:66340159-66340203	NM_001034874:-552	Shisa6	PROMOTER	0.990	2.622	1594.17	4180.25	2.597	1209.43	3140.43
A_68_P28100604	chr11:106133705-106133749	NM_031878:-4780	Smarcd2	PROMOTER	0.990	1.482	2228.00	3301.56	1.468	1870.66	2745.54
A_68_P26453530	chr9:41932147-41932191	NM_011436:204	Sorl1	INSIDE	0.990	1.637	761.75	1246.69	1.620	667.80	1082.07
A_68_P29493964	chr14:29331522-29331566	NM_009524:12886	Wnt5a	INSIDE	0.990	1.819	1162.20	2113.59	1.800	998.13	1796.91
A_68_P26020864	A_68_P26020864			Unknown	0.990	1.619	578.31	936.50	1.604	527.62	846.20
A_68_P29309086	chr13:108834463-108834507	NM_001048250:-183	2810008M24Rik	PROMOTER	0.989	1.482	2401.68	3558.36	1.465	2094.99	3070.03
A_68_P22415393	chr3:108743147-108743191	NM_001078646:-306	4921515J06Rik	PROMOTER	0.989	3.883	830.22	3223.45	3.839	712.46	2735.20
A_68_P30339173	chr15:74551790-74551834	NM_198607:149	4930572J05Rik	INSIDE	0.989	1.603	1174.18	1882.01	1.585	999.82	1584.42
A_68_P23891971	chr5:92471843-92471887	NM_177270:168	Cdkl2	INSIDE	0.989	1.827	362.26	661.98	1.807	349.71	631.87
A_68_P24141815	chr5:141000477-141000521	NM_021528:18936	Chst12	INSIDE	0.989	2.683	816.95	2191.62	2.654	781.86	2074.84
A_68_P26572466	chr9:62659190-62659234	NM_010193:243	Fem1b	INSIDE	0.989	2.004	1302.10	2609.44	1.981	1173.92	2325.73
A_68_P28080241	chr11:102417779-102417823	NM_001159492:-328	Gpatch8	PROMOTER	0.989	1.658	840.79	1394.44	1.640	747.80	1226.57
A_68_P31114358	chr17:27252888-27252932	NM_080553:58662	Itpr3	INSIDE	0.989	2.351	1104.54	2596.50	2.326	798.30	1856.78
A_68_P26587427	chr9:65239407-65239451	NM_028974:31	Kbtbd13	INSIDE	0.989	1.909	3214.16	6135.23	1.888	2567.53	4848.61
A_68_P21475551	chr2:102026439-102026483	NM_178886:74	Ldlrad3	INSIDE	0.989	2.798	3455.55	9667.91	2.767	3205.43	8867.99
A_68_P26507495	chr9:50911542-50911586	NR_029654:-349	Mir34c	PROMOTER	0.989	1.836	752.30	1381.07	1.816	742.77	1349.08
A_68_P21840231	chr2:168057062-168057106	NM_001160330:963	Mocs3	INSIDE	0.989	3.064	873.74	2677.58	3.030	775.97	2351.26
A_68_P31779044	chr18:65243606-65243650	NM_031881:60448	Nedd4l	INSIDE	0.989	2.579	570.33	1470.94	2.551	530.92	1354.30
A_68_P28185956	chr11:120522369-120522413	NM_175263:-239	Notum	PROMOTER	0.989	0.606	1165.65	705.92	0.599	984.16	589.61
A_68_P20768472	chr1:162722548-162722592	NM_013862:499	Rabgap1l	INSIDE	0.989	0.603	1760.02	1061.95	0.597	1518.06	905.93

ProbeName	Target position of probe on CpG island microarray	TargetID	GeneSymbol	CpG island Description	Ratio of relative methylation (TiO_2-NP/Vehicle)	Sham group			TiO_2-H group		
						Relative methylation (Cy5/Cy3)	Cy3 signal (Input)	Cy5 signal (MeDIP)	Relative methylation (Cy5/Cy3)	Cy3 signal (Input)	Cy5 signal (MeDIP)
A_68_P23701541	chr5:53980854-53980898	NM_009035:-577	Rbpj	PROMOTER	0.989	2.061	830.34	1711.25	2.039	901.43	1837.68
A_68_P27807187	chr11:53705109-53705153	NM_011396:75	Slc22a5	INSIDE	0.989	0.658	1648.46	1085.07	0.651	1324.12	862.34
A_68_P31201708	chr17:45682853-45682897	NM_001013749:3752	Tmem151b	INSIDE	0.989	1.734	1153.69	2000.09	1.714	1012.03	1734.82
A_68_P31213639	chr17:47826066-47826110	NM_001164729:-753	Tomm6	DIVERGENT_PROMOTER	0.989	1.579	5728.67	9043.05	1.561	3958.98	6180.54
A_68_P24428426	chr6:48579284-48579328	NR_027236:1141	AI854703	INSIDE	0.988	1.547	1267.20	1960.61	1.529	1086.86	1662.15
A_68_P32319552	chrX:34185014-34185058	NM_001081956:373	Akap17b	INSIDE	0.988	2.118	578.43	1225.19	2.093	337.18	705.86
A_68_P25471454	chr7:128125669-128125713	NM_007672:136	Cdr2	INSIDE	0.988	1.885	713.74	1345.06	1.861	641.60	1194.29
A_68_P25956954	chr8:73993612-73993656	NM_025600:542	Dda1	INSIDE	0.988	2.090	419.93	877.79	2.065	348.89	720.56
A_68_P29241526	chr13:96374600-96374644	NM_010169:13766	F2r	INSIDE	0.988	2.375	420.52	998.80	2.346	341.74	801.61
A_68_P21874509	chr2:174124950-174124994	NM_201617:1613	Gnas	INSIDE	0.988	1.802	1248.85	2250.76	1.780	1032.70	1838.30
A_68_P31116850	chr17:27649440-27649484	NM_001013385:-9213	Grm4	PROMOTER	0.988	0.464	1307.14	606.05	0.458	1107.42	507.50
A_68_P23415960	chr4:151383595-151383639	NM_001033352:617	Klhl21	INSIDE	0.988	2.642	365.93	966.95	2.610	381.00	994.36
A_68_P22629215	chr3:148482907-148482960	NM_001081298:134666	Lphn2	INSIDE	0.988	2.062	396.38	817.25	2.036	345.95	704.50
A_68_P30350167	chr15:76546156-76546200	NM_198119:6425	Lrrc24	INSIDE	0.988	0.557	993.26	553.73	0.551	692.99	381.75
A_68_P22312500	chr3:88018418-88018462	NR_037276:-652	Mir3093	PROMOTER	0.988	0.606	1184.88	718.54	0.599	1037.70	621.45
A_68_P24466502	chr6:54922369-54922413	NM_001171007:-735	Nod1	PROMOTER	0.988	0.532	1305.33	694.96	0.526	1238.16	651.16
A_68_P31375069	chr17:79419723-79419767	NM_001171004:412	Prkd3	INSIDE	0.988	2.266	1129.26	2559.25	2.238	1105.71	2474.92
A_68_P26629967	chr9:72704625-72704669	NM_175485:49566	Prtg	INSIDE	0.988	2.087	1229.92	2566.27	2.062	1035.26	2134.25
A_68_P26892620	chr9:122930391-122930435	NM_001164823:-31	Tmem42	PROMOTER	0.988	1.660	1637.69	2718.51	1.640	1357.42	2226.35
A_68_P24972906	chr7:13366070-13366114	NM_001017955:-5085	Zscan18	PROMOTER	0.988	1.737	885.58	1537.96	1.715	816.81	1400.82
A_68_P31219173	chr17:48925797-48925845			Unknown	0.988	2.862	1780.84	5096.60	2.828	1310.40	3705.23
A_68_P20121784	chr1:30930609-30930653	ENSMUST00000046443:-91		PROMOTER	0.988	2.362	671.98	1587.49	2.335	631.87	1475.50
A_68_P20162424	chr1:38893271-38893315	ENSMUST00000147695:-357		PROMOTER	0.988	1.663	900.96	1498.38	1.644	918.20	1509.18
A_68_P25545210	chr7:141416870-141416914	NM_007400:-112	Adam12	PROMOTER	0.987	0.527	3050.15	1608.49	0.520	2311.17	1202.90
A_68_P27100727	chr10:43230406-43230450	NM_199028:31483	Bend3	INSIDE	0.987	1.976	388.54	767.63	1.949	336.31	655.53
A_68_P23904971	chr5:96590602-96590646	NM_178854:150	Cnot6l	INSIDE	0.987	2.438	17179.15	41888.87	2.407	12504.52	30097.85
A_68_P27594243	chr11:11925830-11925874	NM_001177629:2122	Grb10	INSIDE	0.987	1.542	1367.93	2109.57	1.522	1134.56	1726.73
A_68_P27340039	chr10:90564881-90564925	NM_027078:19119	Ikbip	INSIDE	0.987	4.260	1141.69	4863.62	4.203	888.96	3736.56
A_68_P27235135	chr10:70844350-70844394	NM_027184:33832	Ipmk	INSIDE	0.987	1.742	677.75	1180.30	1.719	606.33	1041.97
A_68_P22314304	chr3:88335424-88335468	NM_001029890:-870	Mex3a	PROMOTER	0.987	1.540	2407.80	3708.04	1.520	1959.91	2979.61
A_68_P27282834	chr10:79844741-79844785	NM_198615:5634	Mex3d	INSIDE	0.987	2.711	1230.65	3336.19	2.676	1166.58	3122.22
A_68_P26381826	chr9:28210925-28210969	NM_177906:612093	Opcml	INSIDE	0.987	1.813	1215.38	2203.13	1.790	1027.37	1838.58
A_68_P30012298	chr15:10644202-10644246	NM_030690:-929	Rai14	PROMOTER	0.987	0.565	1508.75	851.97	0.557	1348.68	751.79
A_68_P31256954	chr17:56705768-56705812	NM_001029979:18216	Safb2	INSIDE	0.987	2.550	2205.86	5625.21	2.517	1821.96	4585.77
A_68_P27325551	chr10:87922155-87922200	NM_011517:-154	Sycp3	PROMOTER	0.987	2.836	1525.67	4326.28	2.798	1185.52	3317.35
A_68_P29039174	chr13:52733712-52733756	NM_011518:41484	Sykb	INSIDE	0.987	1.718	1739.03	2988.28	1.696	1396.85	2369.41
A_68_P30140502	chr15:36723196-36723240	NM_011740:1075	Ywhaz	INSIDE	0.987	0.545	1024.90	558.38	0.538	905.44	486.70
A_68_P25637837	chr8:8690176-8690220	NM_176849:339	Arglu1	INSIDE	0.986	2.475	501.48	1241.14	2.440	479.12	1169.03
A_68_P25961590	chr8:74998787-74998831	NM_138585:324	Cherp	INSIDE	0.986	0.477	1545.33	737.31	0.470	1282.48	603.28
A_68_P24122082	chr5:137041507-137041551	NM_198602:1747	Cux1	INSIDE	0.986	0.558	1320.70	736.48	0.550	1047.86	576.37
A_68_P31537508	chr18:20217493-20217537	NM_013505:492	Dsc2	INSIDE	0.986	1.542	3097.57	4775.46	1.520	2366.96	3597.67
A_68_P32152396	chr19:47939880-47939924	NM_030051:-132	Gsto2	PROMOTER	0.986	0.455	1277.20	580.60	0.448	948.67	425.33
A_68_P25004860	chr7:25217492-25217536	NM_199013:13187	Irgc1	INSIDE	0.986	3.089	315.26	973.74	3.046	319.86	974.28
A_68_P25096864	chr7:53356069-53356113	NM_010602:-483	Kcnj11	PROMOTER	0.986	1.440	1508.67	2171.80	1.419	1155.14	1639.24
A_68_P31847802	chr18:77680387-77680431	NM_172834:159712	Loxhd1	INSIDE	0.986	3.351	903.31	3027.01	3.304	787.80	2602.59
A_68_P25370886	chr7:108147189-108147233	NM_008773:13295	P2ry2	INSIDE	0.986	3.180	1765.03	5612.30	3.136	1453.99	4560.23
A_68_P28323159	chr12:30687550-30687594	NM_181395:64672	Pxdn	INSIDE	0.986	2.115	1291.19	2730.50	2.085	957.93	1997.51
A_68_P32135694	chr19:45063945-45063989	NM_011976:133	Sema4g	INSIDE	0.986	2.952	1421.68	4196.36	2.909	1054.56	3067.81
A_68_P21133066	chr2:35192576-35192620	NM_013515:-69	Stom	PROMOTER	0.986	3.443	1961.74	6753.71	3.393	1605.99	5449.21
A_68_P26785091	chr9:103207385-103207429	NM_176979:-250	Topbp1	PROMOTER	0.986	1.447	1636.94	2369.01	1.428	1412.41	2016.41
A_68_P20350605	chr1:74847285-74847329	NM_009518:8714	Wnt10a	INSIDE	0.986	1.688	608.84	1027.91	1.665	595.63	991.60

ProbeName	Target position of probe on CpG island microarray	TargetID	GeneSymbol	CpG island Description	Ratio of relative methylation (TiO_2-NP/Vehicle)	Sham group			TiO_2-H group		
						Relative methylation (Cy5/Cy3)	Cy3 signal (Input)	Cy5 signal (MeDIP)	Relative methylation (Cy5/Cy3)	Cy3 signal (Input)	Cy5 signal (MeDIP)
A_68_P27541020	chr10:127814573-127814617	NM_172790:415	Ankrd52	INSIDE	0.985	0.631	1965.33	1239.93	0.622	1591.53	989.50
A_68_P24114167	chr5:135462404-135462448	NM_009902:343	Cldn3	INSIDE	0.985	2.183	1046.48	2284.14	2.150	760.25	1634.22
A_68_P26224695	chr8:123651891-123651935	NM_008024:328	Foxl1	INSIDE	0.985	1.820	560.55	1019.98	1.792	564.55	1011.86
A_68_P21901458	chr2:179839037-179839081	NM_133849:-131	Hrh3	PROMOTER	0.985	0.618	7051.52	4360.75	0.609	5215.24	3177.94
A_68_P21309975	chr2:69423669-69423713	NM_001081088:434	Lrp2	INSIDE	0.985	0.495	1031.62	510.89	0.488	895.81	437.07
A_68_P28523609	chr12:72076112-72076156	NM_011184:525	Psma3	INSIDE	0.985	2.430	1255.42	3050.49	2.394	1012.48	2424.02
A_68_P25035179	chr7:31911519-31911563	NM_011322:424	Scn1b	INSIDE	0.985	0.534	2896.12	1547.08	0.526	2303.42	1212.61
A_68_P30465080	chr15:96530202-96530246	NM_175121:-95	Slc38a2	PROMOTER	0.985	1.470	1227.30	1804.41	1.449	1141.11	1653.36
A_68_P31096054	chr17:24343797-24343841	NM_001163847:-1311	Tbc1d24	PROMOTER	0.985	1.682	848.25	1426.70	1.657	666.97	1104.98
A_68_P26398235	chr9:30838331-30838375	NM_001115130:124	Zbtb44	INSIDE	0.985	2.720	648.26	1763.40	2.680	666.22	1785.42
A_68_P32783800	chrX:157736299-157736343	ENSMUST00000141946:10933		INSIDE	0.985	1.914	995.11	1904.90	1.885	437.04	823.86
A_68_P27212535	chr10:67011075-67011119	NM_001005419:607	Ado	INSIDE	0.984	1.617	666.68	1077.75	1.591	637.98	1014.87
A_68_P30371309	chr15:80118848-80118907	NM_001044308:1210	Cacna1i	INSIDE	0.984	5.900	779.90	4601.09	5.802	448.47	2602.24
A_68_P26129047	chr8:107064724-107064768	NM_053070:40	Car7	INSIDE	0.984	1.474	916.78	1351.33	1.450	845.35	1225.81
A_68_P20350878	chr1:74901948-74901992	NM_009872:368	Cdk5r2	INSIDE	0.984	0.588	1237.48	727.57	0.578	1135.59	656.87
A_68_P21177697	chr2:44412424-44412468	NM_172662:304696	Gtdc1	DOWNSTREAM	0.984	1.679	1570.50	2637.29	1.652	1391.55	2299.44
A_68_P23912410	chr5:97820982-97821026	NM_001033191:345	Naa11	INSIDE	0.984	2.256	490.34	1106.15	2.221	444.91	988.06
A_68_P21350021	chr2:76245111-76245155	NM_145525:538	Osbpl6	INSIDE	0.984	1.465	1364.17	1998.50	1.442	1121.09	1616.89
A_68_P27924665	chr11:74992221-74992265	NM_027136:68	Ovca2	INSIDE	0.984	1.688	562.63	949.91	1.661	630.56	1047.52
A_68_P24117610	chr5:136209906-136209950	NM_008898:44845	Por	INSIDE	0.984	2.490	1005.36	2503.21	2.450	783.90	1920.31
A_68_P24770500	chr6:115312177-115312222	NM_001127330:961	Pparg	INSIDE	0.984	2.309	1915.39	4422.92	2.273	1607.28	3653.70
A_68_P27675740	chr11:29593694-29593738	NM_194051:-57	Rtn4	PROMOTER	0.984	1.512	1435.95	2171.48	1.488	1135.39	1689.32
A_68_P31253476	chr17:56176213-56176257	NM_013664:-235	Sh3gl1	DIVERGENT_PROMOTER	0.984	0.587	1319.85	774.32	0.577	1060.50	612.42
A_68_P20156313	chr1:37921860-37921904	NM_207228:42	Tsga10	INSIDE	0.984	1.487	1303.87	1938.34	1.463	1176.17	1720.15
A_68_P25735811	chr8:28089322-28089366	NM_001101502:1537	Zfp703	INSIDE	0.984	2.211	722.22	1596.76	2.177	719.20	1565.39
A_68_P29477491	chr14:26279234-26279278	NM_183208:586	Zmiz1	INSIDE	0.984	0.604	984.40	594.36	0.594	968.91	575.69
A_68_P20644617	chr1:138112450-138112494	NM_028872:18369	5730559C18Rik	INSIDE	0.983	2.441	1365.18	3332.74	2.399	1098.98	2636.37
A_68_P25024595	chr7:29747291-29747335	NM_021895:-13	Actn4	PROMOTER	0.983	2.055	665.06	1366.71	2.021	658.59	1330.70
A_68_P21007915	chr2:11700413-11700457	NM_177268:1055	Ankrd16	INSIDE	0.983	0.592	2052.73	1215.02	0.582	1741.63	1013.63
A_68_P26126978	chr8:106666653-106666697	NM_009868:41150	Cdh5	INSIDE	0.983	3.899	1326.26	5170.47	3.831	1011.90	3876.40
A_68_P21768603	chr2:155782523-155782567	NM_001129999:251	Cep250	INSIDE	0.983	1.502	1093.14	1641.73	1.476	1008.19	1487.72
A_68_P23815279	chr5:76733734-76733778	NM_007715:-183	Clock	PROMOTER	0.983	2.763	1443.94	3990.20	2.717	1235.94	3358.32
A_68_P33007788	chr4_random:128082-128126	NM_001033326:18303	Dhrsx	INSIDE	0.983	3.352	7826.58	26236.45	3.294	5918.35	19497.41
A_68_P25547702	chr7:141862476-141862520	NM_001033420:129	Dock1	INSIDE	0.983	2.493	457.48	1140.66	2.451	472.49	1158.29
A_68_P32127591	chr19:43689143-43689187	NM_008699:2350	Nkx2-3	INSIDE	0.983	1.958	1635.82	3203.36	1.925	1398.23	2691.74
A_68_P24161581	chr5:145306987-145307031	NM_016789:253	Nptx2	INSIDE	0.983	2.154	644.33	1388.03	2.118	590.96	1251.53
A_68_P31256442	chr17:56615968-56616012	NM_011218:-87	Ptprs	PROMOTER	0.983	1.977	580.47	1147.41	1.944	511.02	993.22
A_68_P21098345	chr2:29475651-29475695	NM_001039086:433	Rapgef1	INSIDE	0.983	1.737	1341.38	2329.43	1.706	1157.13	1974.37
A_68_P23364322	chr4:141034341-141034385	NM_019763:60150	Spen	INSIDE	0.983	1.865	847.60	1580.36	1.834	654.27	1199.61
A_68_P23330842	chr4:135412186-135412230	NM_001080387:202	Srsf10	INSIDE	0.983	0.521	1242.43	647.70	0.512	1088.35	557.75
A_68_P20016448	chr1:7388275-7388319	ENSMUST00000138711:-414		PROMOTER	0.983	1.639	587.94	963.72	1.611	509.66	820.91
A_68_P26291033	chr9:8134257-8134301	NM_001045524:16	AK129341	INSIDE	0.982	1.967	652.48	1283.63	1.932	584.75	1129.52
A_68_P32316569	chrX:33565828-33565872	NM_001009947:137024	Dock11	INSIDE	0.982	2.289	707.75	1620.24	2.248	378.57	851.21
A_68_P20352250	chr1:75139665-75139709	NM_170755:327	Fam134a	INSIDE	0.982	1.521	1512.86	2300.82	1.493	1259.14	1879.97
A_68_P30514665	chr16:5255833-5255877	NM_027446:195	Fam86	INSIDE	0.982	1.983	1225.66	2430.13	1.946	1036.48	2017.19
A_68_P25371479	chr7:108257513-108257557	NM_001146010:246	Fchsd2	INSIDE	0.982	1.998	6387.90	12762.35	1.961	4591.22	9004.18
A_68_P27543732	chr10:128328704-128328748	NM_010272:48	Gdf11	INSIDE	0.982	2.630	937.32	2465.40	2.584	847.16	2189.11
A_68_P22843572	chr4:36897429-36897473	NM_001165999:1327	Lingo2	INSIDE	0.982	0.645	1435.10	925.78	0.634	1199.28	759.94
A_68_P31930680	chr19:5757972-5758016	NM_008520:17091	Ltbp3	INSIDE	0.982	1.847	483.48	893.10	1.813	444.87	806.68
A_68_P28946344	chr13:36083419-36083472	NM_201358:125781	Lyrm4	INSIDE	0.982	5.879	1093.48	6428.24	5.775	840.65	4854.98
A_68_P27971539	chr11:83258360-83258404	NM_080453:18081	Mmp28	INSIDE	0.982	1.664	670.72	1115.84	1.633	551.82	901.11

ProbeName	Target position of probe on CpG island microarray	TargetID	GeneSymbol	CpG island Description	Ratio of relative methylation (TiO_2-NP/Vehicle)	Sham group			TiO_2-H group		
						Relative methylation (Cy5/Cy3)	Cy3 signal (Input)	Cy5 signal (MeDIP)	Relative methylation (Cy5/Cy3)	Cy3 signal (Input)	Cy5 signal (MeDIP)
A_68_P24161578	chr5:145306716-145306760	NM_016789:-17	Nptx2	PROMOTER	0.982	2.101	570.06	1197.44	2.062	580.45	1196.88
A_68_P31442547	chr17:91487821-91487865	NM_020252:4300	Nrxn1	INSIDE	0.982	3.202	406.13	1300.31	3.143	359.60	1130.32
A_68_P28103446	chr11:106640661-106640705	NR_027785:147	Polg2	INSIDE	0.982	1.592	592.32	942.93	1.564	572.64	895.33
A_68_P29073065	chr13:58504155-58504199	NM_001168248:219	Rmi1	INSIDE	0.982	0.545	1789.99	976.38	0.535	1472.97	788.63
A_68_P26873497	chr9:119518976-119519024	NM_001205321:109150	Scn10a	INSIDE	0.982	2.249	395.25	888.94	2.208	352.59	778.39
A_68_P26365991	chr9:25059906-25059950	NM_001205367:-111	Septin7	PROMOTER	0.982	0.432	1574.16	680.02	0.424	1299.28	551.07
A_68_P21116240	chr2:32442707-32442751	NM_011373:-281	St6galnac4	PROMOTER	0.982	1.714	3043.54	5215.76	1.683	2300.64	3871.68
A_68_P23309116	chr4:131477634-131477678	NM_001201367:-29	Tmem200b	PROMOTER	0.982	3.207	560.76	1798.08	3.147	490.19	1542.84
A_68_P25452560	chr7:124524755-124524799	NM_175645:284	Xylt1	INSIDE	0.982	5.654	1157.92	6546.38	5.552	859.09	4769.48
A_68_P31932464	chr19:6061914-6061958	NM_013859:730	Znhit2-ps	INSIDE	0.982	0.578	1225.76	708.18	0.567	1047.98	594.59
A_68_P27282330	chr10:79767570-79767614	NM_011789:3028	Apc2	INSIDE	0.981	2.687	2411.80	6480.44	2.637	2069.36	5456.03
A_68_P27298383	chr10:83111269-83111313	NM_145220:119	Appl2	INSIDE	0.981	1.653	611.68	1011.10	1.621	543.45	881.12
A_68_P20456918	chr1:95582954-95582998	NM_016778:706	Bok	INSIDE	0.981	1.672	797.07	1332.57	1.639	709.43	1163.04
A_68_P23498122	chr5:15441040-15441084	NM_001110843:554	Cacna2d1	INSIDE	0.981	3.337	461.34	1539.61	3.275	428.80	1404.37
A_68_P27543294	chr10:128242682-128242726	NM_028873:-27	Dnajc14	DIVERGENT_PROMOTER	0.981	2.385	701.76	1673.61	2.340	607.95	1422.84
A_68_P20351022	chr1:74929216-74929260	NM_153111:2744	Fev	INSIDE	0.981	2.452	1021.02	2503.69	2.406	878.87	2114.20
A_68_P26319136	chr9:14555274-14555318	NM_010242:1270	Fut4	INSIDE	0.981	1.700	938.39	1594.98	1.668	793.74	1323.68
A_68_P25842942	chr8:48759661-48759705	NM_023503:831	Ing2	INSIDE	0.981	1.818	1503.05	2733.00	1.784	1379.85	2461.33
A_68_P27843264	chr11:60290404-60290448	NM_001103171:7586	Myo15	INSIDE	0.981	2.251	944.66	2126.86	2.208	790.14	1744.35
A_68_P29700850	chr14:70751126-70751170	NM_001135152:-2908	Slc39a14	PROMOTER	0.981	1.396	3974.76	5547.45	1.369	3527.42	4830.41
A_68_P21831890	chr2:166612210-166612254	NM_023815:-7034	Trp53rk	PROMOTER	0.981	1.675	591.75	991.20	1.643	505.29	830.18
A_68_P22349909	chr3:96361476-96361535	NM_001009935:-374	Txnip	PROMOTER	0.981	2.409	240.78	579.98	2.362	193.65	457.45
A_68_P25499303	chr7:133343931-133343975	NM_028816:-30	Xpo6	PROMOTER	0.981	2.118	1438.07	3045.89	2.078	1286.10	2672.31
A_68_P28608874	chr12:87585109-87585153	NM_027405:2303	1700020O03Rik	INSIDE	0.980	2.404	1351.32	3248.52	2.355	1093.47	2575.42
A_68_P31849429	chr18:77952019-77952063	NM_178670:709	8030462N17Rik	INSIDE	0.980	1.975	1675.16	3307.91	1.935	1583.02	3062.53
A_68_P24469555	chr6:55402357-55402401	NM_001025372:405	Adcyap1r1	INSIDE	0.980	0.596	1623.90	968.08	0.584	1361.30	795.29
A_68_P32385501	chrX:50410354-50410398	NR_038184:-9	C430049B03Rik	PROMOTER	0.980	2.333	2763.72	6446.59	2.286	1385.34	3166.64
A_68_P22822458	chr4:32702338-32702382	NM_001122978:-87	Casp8ap2	PROMOTER	0.980	1.454	1112.22	1617.57	1.425	940.84	1340.41
A_68_P24122109	chr5:137044884-137044928	NM_009986:-1605	Cux1	PROMOTER	0.980	1.796	1029.52	1848.75	1.760	1002.70	1765.00
A_68_P23325563	chr4:134480163-134480207	NM_023665:645	D4Wsu53e	INSIDE	0.980	2.435	330.16	803.87	2.387	339.16	809.61
A_68_P23192161	chr4:107356258-107356302	NM_019872:487	Dmrtb1	INSIDE	0.980	2.526	702.73	1774.96	2.474	651.76	1612.74
A_68_P26319147	chr9:14556469-14556513	NM_010242:76	Fut4	INSIDE	0.980	2.217	666.39	1477.29	2.173	654.42	1421.96
A_68_P24537468	chr6:71583029-71583078	NM_001038695:-154	Kdm3a	PROMOTER	0.980	3.072	2362.22	7256.83	3.009	1491.92	4489.71
A_68_P25034847	chr7:31852339-31852383	NM_144556:7407	Lgi4	INSIDE	0.980	1.931	685.05	1322.98	1.894	571.57	1082.27
A_68_P23193224	chr4:107526867-107526911	NM_001080926:52025	Lrp8	INSIDE	0.980	1.745	678.85	1184.64	1.710	524.48	896.80
A_68_P22222512	chr3:69526351-69526395	NM_133787:396	Nmd3	INSIDE	0.980	0.562	1321.36	742.32	0.551	1101.43	606.69
A_68_P30346616	chr15:76011981-76012026	NM_201394:14137	Plec	INSIDE	0.980	3.494	444.43	1552.78	3.423	351.97	1204.88
A_68_P23984174	chr5:111611967-111612011	NM_024477:303167	Ttc28	INSIDE	0.980	1.823	494.07	900.72	1.786	446.67	797.79
A_68_P27287687	chr10:80607189-80607233	NM_010731:8195	Zbtb7a	INSIDE	0.980	2.194	1251.84	2747.00	2.150	909.51	1955.09
A_68_P20319175	chr1:69424592-69424636			Unknown	0.980	1.944	1354.40	2632.93	1.906	1157.69	2206.49
A_68_P23368579	chr4:141795272-141795316	NM_001109685:22	9030409G11Rik	INSIDE	0.979	0.536	1130.87	606.50	0.525	963.86	506.31
A_68_P26349675	chr9:21748731-21748775	NM_144935:6035	BC018242	INSIDE	0.979	2.028	2302.75	4669.95	1.986	1619.03	3214.96
A_68_P32097107	chr19:37773571-37773615	NM_007811:1295	Cyp26a1	INSIDE	0.979	0.471	1173.84	553.24	0.462	971.49	448.39
A_68_P32227249	chr19:60866612-60866656	NM_010123:-38	Eif3a	PROMOTER	0.979	1.523	952.50	1450.73	1.491	853.67	1272.68
A_68_P28865543	chr13:20183326-20183370	NM_080288:973	Elmo1	INSIDE	0.979	1.481	1756.61	2601.79	1.451	1422.94	2064.20
A_68_P25780170	chr8:36558186-36558230	NM_026067:379	Eri1	INSIDE	0.979	0.426	1745.28	743.68	0.417	1319.80	550.56
A_68_P25087552	chr7:51718008-51718052	NM_198250:20174	Lrrc4b	INSIDE	0.979	1.656	1325.45	2194.73	1.620	1247.49	2021.43
A_68_P25601671	chr7:150734944-150734988	NM_008672:29	Nap1l4	INSIDE	0.979	0.592	2591.99	1534.04	0.579	2194.30	1271.39
A_68_P30473222	chr15:97951292-97951336	NM_021514:8824	Pfkm	INSIDE	0.979	1.863	545.73	1016.79	1.823	461.51	841.56
A_68_P26203591	chr8:120114052-120114099	NM_172285:91885	Plcg2	INSIDE	0.979	3.164	265.78	841.05	3.097	227.03	703.15
A_68_P31154936	chr17:34768097-34768141	NM_030890:1488	Prrt1	INSIDE	0.979	2.065	2168.30	4478.42	2.021	1633.94	3302.25

ProbeName	Target position of probe on CpG island microarray	TargetID	GeneSymbol	CpG island Description	Ratio of relative methylation (TiO_2-NP/Vehicle)	Sham group			TiO_2-H group		
						Relative methylation (Cy5/Cy3)	Cy3 signal (Input)	Cy5 signal (MeDIP)	Relative methylation (Cy5/Cy3)	Cy3 signal (Input)	Cy5 signal (MeDIP)
A_68_P30348895	chr15:76352647-76352691	NM_130893:-109	Scrt1	PROMOTER	0.979	3.249	1080.22	3510.05	3.180	978.98	3112.86
A_68_P21584533	chr2:121943910-121943954	NM_145531:190	Spg11	INSIDE	0.979	2.006	585.34	1173.93	1.963	566.39	1112.05
A_68_P23996936	chr5:113649993-113650037	NM_001033428:-5914	Tmem211	PROMOTER	0.979	0.612	2611.36	1597.81	0.599	2278.23	1364.43
A_68_P29051403	chr13:54890672-54890716	NM_028841:-71	Tspan17	PROMOTER	0.979	1.507	4407.64	6640.25	1.475	3326.99	4906.93
A_68_P23756860	chr5:65929084-65929128	NM_016786:607	Ube2k	INSIDE	0.979	0.642	2315.85	1486.66	0.629	1880.74	1182.58
A_68_P30572505	chr16:17201070-17201114	NM_009456:493	Ube2l3	INSIDE	0.979	2.566	2010.01	5156.91	2.513	1795.94	4512.98
A_68_P24007964	chr5:115577031-115577075	NM_175352:7932	Unc119b	INSIDE	0.979	2.099	973.47	2043.29	2.054	724.83	1488.77
A_68_P26891838	chr9:122775033-122775077	NM_173364:70	Zfp445	INSIDE	0.979	0.664	1036.49	687.72	0.649	953.74	619.36
A_68_P25505248	chr7:134486671-134486730	NM_146202:2128	Zfp768	INSIDE	0.979	6.394	365.83	2339.20	6.262	286.34	1792.97
A_68_P20637767	chr1:137043374-137043418	NM_026823:-14	Arl8a	PROMOTER	0.978	1.852	842.77	1561.15	1.812	798.09	1446.17
A_68_P21866714	chr2:172766163-172766207	NM_007557:-390	Bmp7	PROMOTER	0.978	1.710	2912.63	4981.20	1.672	2002.53	3348.33
A_68_P28153230	chr11:115255314-115255358	NM_001080929:12107	Cdr2l	INSIDE	0.978	2.087	1160.03	2420.95	2.041	962.20	1963.40
A_68_P28685876	chr12:102321244-102321288	NR_038047:606	D130020L05Rik	INSIDE	0.978	1.571	845.94	1329.10	1.536	916.73	1408.13
A_68_P32206764	chr19:57435985-57436029	NM_145505:508	Fam160b1	INSIDE	0.978	1.877	766.09	1437.58	1.835	641.15	1176.82
A_68_P31927963	chr19:5306888-5306932	NM_001024717:8580	Gal3st3	INSIDE	0.978	1.720	1482.12	2548.76	1.683	1307.41	2199.86
A_68_P23404326	chr4:149475854-149475898	NM_177366:14265	Gpr157	INSIDE	0.978	2.830	193.87	548.74	2.768	190.58	527.50
A_68_P28703478	chr12:105710469-105710513	NM_010351:956	Gsc	INSIDE	0.978	0.389	2081.81	810.77	0.381	1827.05	695.76
A_68_P31304340	chr17:66460894-66460938	NM_001037757:-68	ORF19	PROMOTER	0.978	0.395	1369.36	541.08	0.386	1096.00	423.59
A_68_P23322025	chr4:133842588-133842632	NM_001163794:558	Pdik1l	INSIDE	0.978	0.636	1898.42	1207.09	0.622	1390.50	864.79
A_68_P24777648	chr6:116623614-116623658	NM_178045:218	Rassf4	INSIDE	0.978	1.595	1647.73	2628.07	1.560	1354.69	2112.89
A_68_P30956056	chr16:90283863-90283907	NM_178923:786	Scaf4	INSIDE	0.978	1.505	968.35	1457.62	1.473	817.58	1204.02
A_68_P26887499	chr9:122026546-122026590	NM_001164572:185	Snrk	INSIDE	0.978	2.202	834.93	1838.70	2.155	752.48	1621.30
A_68_P22059401	chr3:36570355-36570399	NM_019510:18713	Trpc3	INSIDE	0.978	2.619	791.95	2074.36	2.561	664.29	1701.35
A_68_P31016358	chr17:5342260-5342304	NM_001085355:347209	Arid1b	INSIDE	0.977	3.958	1777.33	7034.09	3.867	1236.33	4780.80
A_68_P23440035	chr4:155068628-155068672	NM_178699:200	B930041F14Rik	INSIDE	0.977	2.625	577.01	1514.58	2.565	549.08	1408.63
A_68_P22412463	chr3:108209838-108209882	NM_001004177:8552	Celsr2	INSIDE	0.977	3.198	2283.82	7304.78	3.125	1809.95	5656.47
A_68_P21425367	chr2:91762276-91762320	NM_007699:-47	Chrm4	PROMOTER	0.977	0.585	1082.72	633.06	0.571	830.94	474.49
A_68_P25948482	chr8:72406463-72406507	NM_026818:4807	Cilp2	INSIDE	0.977	1.583	988.34	1564.68	1.547	916.61	1417.58
A_68_P29140850	chr13:73741332-73741376	NM_146047:-95	Clptm1l	PROMOTER	0.977	3.296	1111.79	3664.29	3.221	902.09	2905.54
A_68_P31150930	chr17:34048600-34048644	NM_001199733:2077	Daxx	INSIDE	0.977	2.635	446.63	1176.68	2.575	432.74	1114.31
A_68_P26581361	chr9:64189105-64189149	NM_001001295:-62	Dis3l	PROMOTER	0.977	0.556	1025.28	570.46	0.543	945.90	514.00
A_68_P21877293	chr2:174586128-174586172	NM_007903:-123	Edn3	PROMOTER	0.977	2.024	1003.70	2031.23	1.978	878.99	1738.72
A_68_P23363267	chr4:140864773-140864817	NM_010139:7640	Epha2	INSIDE	0.977	2.644	875.68	2315.66	2.584	763.89	1974.11
A_68_P29083939	chr13:60277184-60277228	NM_008086:1690	Gas1	INSIDE	0.977	0.670	2245.18	1505.13	0.655	1665.53	1090.72
A_68_P20679836	chr1:145586729-145586773	NM_001038594:-826	Glrx2	PROMOTER	0.977	0.615	1449.85	891.55	0.601	1225.25	735.82
A_68_P21338760	chr2:74520854-74520898	NM_008273:427	Hoxd11	INSIDE	0.977	2.046	572.41	1171.33	1.998	554.02	1107.19
A_68_P26674975	chr9:81525929-81525973	NM_010482:209	Htr1b	INSIDE	0.977	0.622	1087.38	676.59	0.608	832.45	506.22
A_68_P26423542	chr9:35378046-35378090	NM_029762:-414	Hyls1	PROMOTER	0.977	1.684	2019.99	3401.85	1.645	1561.96	2570.02
A_68_P25031178	chr7:31141047-31141091	NM_175478:6723	Lrfn3	INSIDE	0.977	2.315	750.13	1736.85	2.261	577.61	1306.04
A_68_P28095691	chr11:105224580-105224624	NM_172568:93447	Marchf10	INSIDE	0.977	3.035	583.50	1770.92	2.964	478.99	1419.92
A_68_P24591434	chr6:83052524-83052568	NM_026744:-6555	Mrpl53	PROMOTER	0.977	0.487	1127.49	548.90	0.476	917.90	436.65
A_68_P24974396	chr7:13639929-13639973	NM_145819:163	Mzf1	INSIDE	0.977	0.684	1426.34	975.04	0.668	1160.36	775.00
A_68_P21820162	chr2:164683144-164683188	NM_011125:42	Pltp	INSIDE	0.977	0.491	1594.58	783.21	0.480	1299.47	623.55
A_68_P27267071	chr10:76728631-76728675	NM_030262:6608	Pofut2	INSIDE	0.977	2.304	738.07	1700.60	2.251	591.94	1332.31
A_68_P32957128	chr17:8534244-8534288	NM_178774:663	Prr18	INSIDE	0.977	1.822	1223.40	2229.03	1.781	1129.82	2012.17
A_68_P26825466	chr9:110629375-110629419	NM_001083936:15718	Pth1r	INSIDE	0.977	3.170	3255.82	10320.97	3.097	2223.98	6888.59
A_68_P21843310	chr2:168575642-168575686	NM_175303:17037	Sall4	INSIDE	0.977	1.713	525.29	899.66	1.674	475.38	795.59
A_68_P21116407	chr2:32470667-32470711	NM_001025310:8208	St6galnac6	INSIDE	0.977	3.117	1219.51	3800.73	3.045	1024.38	3118.84
A_68_P27507136	chr10:121534175-121534219	NM_153059:-38	Tmem5	PROMOTER	0.977	1.616	1674.61	2706.35	1.580	1416.59	2237.63
A_68_P22059402	chr3:36570442-36570486	NM_019510:18625	Trpc3	INSIDE	0.977	2.023	1040.82	2105.14	1.976	850.40	1680.54
A_68_P30379485	chr15:81602412-81602456	NM_001081016:27157	Zc3h7b	INSIDE	0.977	2.220	1012.45	2247.43	2.168	780.10	1691.56

ProbeName	Target position of probe on CpG island microarray	TargetID	GeneSymbol	CpG island Description	Ratio of relative methylation (TiO_2-NP/Vehicle)	Sham group			TiO_2-H group		
						Relative methylation (Cy5/Cy3)	Cy3 signal (Input)	Cy5 signal (MeDIP)	Relative methylation (Cy5/Cy3)	Cy3 signal (Input)	Cy5 signal (MeDIP)
A_68_P27781839	chr11:49017033-49017077	NM_001024846:53	Zfp62	INSIDE	0.977	2.007	2219.29	4454.45	1.961	1945.58	3814.57
A_68_P29059922	chr13:56322977-56323021	ENSMUST00000127754:22303		DOWNSTREAM	0.977	2.973	957.88	2847.64	2.903	849.62	2466.71
A_68_P23576262	chr5:31370994-31371038	NM_023525:13833	Cad	INSIDE	0.976	1.979	1458.58	2886.34	1.932	1161.40	2243.57
A_68_P21114928	chr2:32218406-32218450	NM_028412:-325	Ciz1	DIVERGENT_PROMOTER	0.976	1.869	506.90	947.20	1.824	508.52	927.35
A_68_P27278592	chr10:79198242-79198286	NM_008226:18886	Hcn2	INSIDE	0.976	1.766	1097.74	1938.15	1.724	1007.25	1736.10
A_68_P32147194	chr19:47089078-47089122	NM_146100:-87	Ina	PROMOTER	0.976	0.523	3667.21	1916.31	0.510	3001.83	1531.16
A_68_P21147645	chr2:38207336-38207380	NM_010710:531	Lhx2	INSIDE	0.976	0.632	864.99	546.87	0.617	799.66	493.45
A_68_P32135962	chr19:45100671-45100715	NM_001130526:8068	Lzts2	INSIDE	0.976	3.964	1514.94	6005.14	3.870	1213.70	4696.96
A_68_P30370604	chr15:80003678-80003722	NM_010795:-450	Mgat3	PROMOTER	0.976	2.172	11821.68	25672.58	2.119	8110.50	17184.88
A_68_P28174637	chr11:118770429-118770473	NM_001024931:436	Rbfox3	INSIDE	0.976	0.661	4278.64	2827.43	0.645	3798.84	2451.21
A_68_P25090604	chr7:52263215-52263259	NM_001008422:8383	Scaf1	INSIDE	0.976	2.000	1363.86	2727.31	1.952	1035.03	2020.16
A_68_P28469857	chr12:60113204-60113248	NM_009147:-222	Sec23a	DIVERGENT_PROMOTER	0.976	1.644	6327.96	10405.71	1.605	4641.74	7448.01
A_68_P25049870	chr7:36322643-36322687	NM_028034:99	Tdrd12	INSIDE	0.976	3.088	6114.63	18884.77	3.015	4637.15	13983.05
A_68_P30849198	A_68_P30849198			Unknown	0.976	3.231	305.26	986.23	3.154	328.75	1036.88
A_68_P23226517	chr4:115570669-115570713	ENSMUST00000165938:7420		INSIDE	0.976	1.480	2350.66	3479.43	1.445	1926.69	2783.98
A_68_P27686041	chr11:31865736-31865780	NM_026262:126	4930524B15Rik	INSIDE	0.975	3.018	695.12	2098.16	2.944	570.19	1678.47
A_68_P30338797	chr15:74502578-74502622	NM_018790:400	Arc	INSIDE	0.975	1.540	1185.35	1825.67	1.502	1091.35	1639.54
A_68_P31067114	chr17:15867919-15867967	NM_007690:26012	Chd1	INSIDE	0.975	2.854	324.90	927.39	2.784	305.46	850.25
A_68_P29671492	chr14:65696284-65696328	NM_018788:20637	Extl3	INSIDE	0.975	2.313	280.87	649.69	2.256	233.85	527.46
A_68_P27185079	chr10:61732442-61732486	NM_001146100:70705	Hk1	INSIDE	0.975	4.017	4116.51	16537.39	3.916	2906.27	11380.05
A_68_P23637418	chr5:42154713-42154757	NM_007524:725	Nkx3-2	INSIDE	0.975	0.641	1306.67	837.10	0.624	1040.15	649.42
A_68_P30421712	chr15:88692211-88692255	NM_145478:-391	Pim3	PROMOTER	0.975	0.602	1174.67	707.32	0.587	937.72	550.57
A_68_P30346543	chr15:76003047-76003091	NM_201394:23072	Plec	INSIDE	0.975	1.828	787.27	1439.32	1.782	693.63	1235.86
A_68_P30447267	chr15:93331510-93331555	NM_001033217:94790	Prickle1	INSIDE	0.975	2.282	442.94	1010.69	2.224	375.40	835.01
A_68_P21747514	chr2:151907523-151907567	NM_001160410:280	Scrt2	INSIDE	0.975	0.649	1094.32	710.30	0.633	926.81	586.47
A_68_P26239400	chr8:125877888-125877932	NM_172287:21298	Spire2	INSIDE	0.975	3.363	569.51	1915.37	3.278	381.42	1250.24
A_68_P25425957	chr7:119823141-119823185	NM_001166584:329	Tead1	INSIDE	0.975	2.037	684.18	1393.77	1.986	652.73	1296.55
A_68_P25026078	chr7:30023443-30023487	NM_001110201:123	Yif1b	INSIDE	0.975	0.662	2872.41	1900.29	0.645	2226.40	1436.72
A_68_P24118492	chr5:136387366-136387410	NM_018871:23123	Ywhag	INSIDE	0.975	2.877	598.14	1720.58	2.805	533.54	1496.64
A_68_P24951462	chr7:4945928-4945972	NM_026741:1753	Zfp579	INSIDE	0.975	1.980	447.72	886.71	1.932	421.27	813.87
A_68_P30602964	A_68_P30602964			Unknown	0.975	3.257	472.59	1539.02	3.175	456.35	1448.71
A_68_P28094596	chr11:105026660-105026704	ENSMUST00000118770:1252		INSIDE	0.975	2.859	451.76	1291.45	2.787	369.47	1029.71
A_68_P23340288	chr4:137074524-137074568	NM_008305:49829	Hspg2	INSIDE	0.974	2.648	2867.07	7590.62	2.579	2334.33	6021.37
A_68_P25044863	chr7:35439623-35439667	NM_146188:-1784	Kctd15	PROMOTER	0.974	0.607	867.27	526.51	0.591	726.25	429.41
A_68_P29177385	chr13:81850332-81850377	NM_028372:-61	Mblac2	DIVERGENT_PROMOTER	0.974	0.339	2536.50	860.53	0.331	1973.15	652.29
A_68_P25954890	chr8:73437278-73437322	NM_173013:7428	Mtap1s	INSIDE	0.974	2.538	1489.36	3779.47	2.471	1139.43	2815.52
A_68_P29482962	chr14:27489116-27489160	NM_178668:194	Pde12	INSIDE	0.974	0.620	1054.13	653.29	0.603	913.26	551.02
A_68_P24984542	chr7:17612840-17612884	NM_011155:401	Ppp5c	INSIDE	0.974	2.416	1936.40	4678.89	2.353	1557.84	3665.03
A_68_P31311925	chr17:67704118-67704162	NM_008984:-341	Ptprm	PROMOTER	0.974	0.640	1013.74	648.50	0.623	863.34	537.95
A_68_P30365304	chr15:79082520-79082564	NM_020516:2636	Slc16a8	INSIDE	0.974	1.490	1849.42	2754.95	1.451	1669.98	2422.87
A_68_P32143399	chr19:46423459-46423503	NM_029186:-7889	Tmem180	PROMOTER	0.974	1.987	383.91	762.94	1.936	368.41	713.20
A_68_P22973922	chr4:63678515-63678559	NM_011607:29513	Tnc	INSIDE	0.974	2.302	1958.86	4509.35	2.242	1621.52	3636.18
A_68_P23204068	chr4:109723690-109723734			Unknown	0.974	2.594	696.53	1806.75	2.526	702.31	1774.22
A_68_P31623411	chr18:36508072-36508116	NM_001081365:-183	0610010O12Rik	PROMOTER	0.973	1.908	2562.23	4888.73	1.857	1780.99	3307.21
A_68_P31619440	chr18:35814836-35814880	NM_027222:-5837	2010001M09Rik	PROMOTER	0.973	0.503	1312.48	660.13	0.489	1101.28	538.76
A_68_P21037978	chr2:17969077-17969121	NM_028317:978	2810030E01Rik	INSIDE	0.973	3.426	2318.78	7943.93	3.335	1828.23	6097.29
A_68_P26521622	chr9:53418206-53418250	NM_144784:227	Acat1	INSIDE	0.973	1.614	810.98	1308.68	1.571	834.70	1311.19
A_68_P32020401	chr19:23968179-23968223	NM_177034:134835	Apba1	INSIDE	0.973	2.201	1184.79	2607.64	2.142	992.30	2125.58
A_68_P22186537	chr3:62143246-62143290	NM_001081295:570	Arhgef26	INSIDE	0.973	0.638	963.31	614.91	0.621	959.28	595.76
A_68_P31977527	chr19:16059339-16059383	NM_198019:119	Cep78	INSIDE	0.973	0.683	1481.56	1012.63	0.665	1291.48	858.95
A_68_P30511521	chr16:4679480-4679524	NM_030205:218	Coro7	INSIDE	0.973	1.445	2711.21	3917.38	1.405	2028.75	2851.38

ProbeName	Target position of probe on CpG island microarray	TargetID	GeneSymbol	CpG island Description	Ratio of relative methylation (TiO_2-NP/Vehicle)	Sham group			TiO_2-H group		
						Relative methylation (Cy5/Cy3)	Cy3 signal (Input)	Cy5 signal (MeDIP)	Relative methylation (Cy5/Cy3)	Cy3 signal (Input)	Cy5 signal (MeDIP)
A_68_P30576507	chr16:17911333-17911377	NM_001081633:87	Dgcr14	INSIDE	0.973	2.101	834.08	1752.47	2.045	708.67	1448.95
A_68_P27490918	chr10:118890399-118890443	NM_133442:-949	Grip1	PROMOTER	0.973	0.502	1232.00	618.41	0.488	1005.16	490.87
A_68_P25945210	chr8:71405256-71405300	NM_008509:825	Lpl	INSIDE	0.973	0.518	2080.67	1077.04	0.503	1764.77	888.48
A_68_P26506085	chr9:50665071-50665115	NM_028614:53	Ppp2r1b	INSIDE	0.973	1.439	2614.80	3762.33	1.400	2172.63	3042.22
A_68_P21838401	chr2:167757841-167757885	NM_011201:36	Ptpn1	INSIDE	0.973	0.517	1051.70	544.18	0.503	1023.82	515.47
A_68_P24428216	chr6:48546831-48546875	NM_001079901:2971	Repin1	INSIDE	0.973	2.168	619.59	1343.25	2.108	512.76	1081.08
A_68_P27222447	chr10:68675289-68675333	NM_001081347:-94	Rhobtb1	PROMOTER	0.973	2.827	932.84	2636.79	2.751	756.59	2081.29
A_68_P30118686	chr15:32174840-32174884	NM_009154:295	Sema5a	INSIDE	0.973	0.599	910.67	545.19	0.583	707.60	412.35
A_68_P27552579	chr11:3435873-3435917	NM_001159284:3401	Smtn	INSIDE	0.973	1.752	588.70	1031.64	1.705	579.55	988.06
A_68_P20345417	chr1:73953929-73953973	NM_027884:217071	Tns1	DOWNSTREAM	0.973	2.227	855.46	1905.15	2.168	732.78	1588.49
A_68_P25506523	chr7:134758122-134758166	NM_177226:-197	Zfp629	PROMOTER	0.973	0.526	1502.29	790.72	0.512	1249.45	640.12
A_68_P30560721	chr16:14159223-14159267	NM_001081154:123	4921513D23Rik	INSIDE	0.972	1.435	2759.99	3961.06	1.395	1903.99	2655.38
A_68_P23602806	chr5:36100352-36100396	NM_001177696:-154	Ablim2	PROMOTER	0.972	1.753	649.81	1138.80	1.704	549.81	936.86
A_68_P28688688	chr12:103196108-103196152	NM_029705:323	Atxn3	INSIDE	0.972	1.468	1945.95	2856.10	1.427	1782.70	2544.49
A_68_P33015625	chr1_random:51092-51145	NM_026866:134220	Disp1	INSIDE	0.972	4.052	232.86	943.57	3.940	203.36	801.25
A_68_P27849839	chr11:61498513-61498557	NM_178618:623	Fam83g	INSIDE	0.972	1.531	2052.44	3143.21	1.489	1596.08	2376.43
A_68_P21874750	chr2:174153901-174153945	NM_001077510:-1667	Gnas	PROMOTER	0.972	1.869	1350.12	2523.31	1.816	1243.89	2259.27
A_68_P23753095	chr5:65213443-65213487	NM_008453:18703	Klf3	INSIDE	0.972	2.420	4090.97	9899.64	2.352	3096.05	7281.08
A_68_P27180706	chr10:60938993-60939037	NM_153542:434	Lrrc20	INSIDE	0.972	1.527	1138.09	1738.17	1.484	1050.16	1558.88
A_68_P26551402	chr9:58884501-58884545	NM_001042752:-274	Neo1	PROMOTER	0.972	3.181	2515.51	8000.97	3.092	2099.48	6492.63
A_68_P21823566	chr2:165213753-165213797	NM_178411:-15	Zfp334	PROMOTER	0.972	0.615	1111.51	683.48	0.598	920.96	550.71
A_68_P24951457	chr7:4945305-4945349	NM_026741:2377	Zfp579	INSIDE	0.972	1.821	1574.76	2867.78	1.770	1306.81	2312.55
A_68_P23320094	chr4:133466780-133466824	AK015234:96		INSIDE	0.972	1.863	826.65	1539.80	1.811	730.81	1323.62
A_68_P26215962	chr8:122292157-122292201	NM_028883:10138	4632415K11Rik	INSIDE	0.971	2.449	626.16	1533.72	2.378	518.40	1232.88
A_68_P27473558	chr10:115550897-115550941	NM_001161855:55	4933416C03Rik	INSIDE	0.971	1.953	1190.25	2324.22	1.897	1072.90	2035.22
A_68_P28171533	chr11:118338257-118338301	NM_172573:5	Engase	INSIDE	0.971	0.462	1195.86	552.07	0.448	1010.57	452.84
A_68_P24024962	chr5:118605340-118605384	NM_172721:105	Fbxw8	INSIDE	0.971	1.612	660.95	1065.53	1.566	626.27	980.71
A_68_P29660046	chr14:63859832-63859876	NM_008092:4243	Gata4	INSIDE	0.971	2.882	834.39	2404.36	2.798	736.16	2059.75
A_68_P21874752	chr2:174154225-174154269	NM_001077510:-1343	Gnas	PROMOTER	0.971	2.941	634.88	1867.23	2.855	572.98	1635.85
A_68_P28266914	chr12:16807531-16807575	NM_015764:140	Greb1	INSIDE	0.971	2.246	630.05	1414.97	2.181	603.58	1316.49
A_68_P21840418	chr2:168094705-168094749	NM_001081134:105	Kcng1	INSIDE	0.971	3.398	1086.03	3689.96	3.301	884.92	2920.90
A_68_P22530124	chr3:130809206-130809257	NM_010703:-4157	Lef1	PROMOTER	0.971	1.913	454.89	870.06	1.858	426.51	792.38
A_68_P32135963	chr19:45100793-45100839	NM_001130526:8192	Lzts2	INSIDE	0.971	1.825	849.89	1551.07	1.772	641.09	1135.76
A_68_P29437674	chr14:19071169-19071213	NM_011584:430	Nr1d2	INSIDE	0.971	0.578	1302.30	753.13	0.562	1184.57	665.39
A_68_P26825588	chr9:110645482-110645526	NM_001083936:-390	Pth1r	PROMOTER	0.971	0.599	3626.38	2172.34	0.582	2620.71	1525.09
A_68_P20878434	chr1:182834646-182834690	NM_133705:264	Pycr2	INSIDE	0.971	3.018	557.10	1681.11	2.930	528.81	1549.31
A_68_P32099687	chr19:38199117-38199161	NM_011255:163	Rbp4	INSIDE	0.971	0.608	907.04	551.75	0.590	788.52	465.61
A_68_P27795628	chr11:51577154-51577198	NM_025535:12	Sar1b	INSIDE	0.971	1.390	3060.05	4254.21	1.350	2587.65	3494.02
A_68_P30575738	chr16:17797523-17797567	NM_153790:170	Scarf2	INSIDE	0.971	2.068	6359.23	13153.07	2.009	4977.00	9996.54
A_68_P22286845	chr3:83570356-83570400	NM_009144:136	Sfrp2	INSIDE	0.971	1.885	816.44	1538.69	1.830	721.69	1320.64
A_68_P28116268	chr11:108873014-108873058	ENSMUST00000119655:4072		DOWNSTREAM	0.971	1.853	1103.12	2044.13	1.799	840.74	1512.54
A_68_P25087019	chr7:51615634-51615678	NM_027049:14744	1700008O03Rik	INSIDE	0.970	0.615	983.82	605.52	0.597	851.36	508.18
A_68_P28525479	chr12:72411396-72411440	NM_001190466:548	Dact1	INSIDE	0.970	1.623	5022.66	8149.95	1.573	3551.70	5587.56
A_68_P28157767	chr11:116029485-116029529	NM_172571:-14	Fbf1	PROMOTER	0.970	5.003	341.38	1707.97	4.851	315.61	1531.16
A_68_P29101872	chr13:65213921-65213965	NM_001083901:348	Hiatl1	INSIDE	0.970	2.047	374.73	766.90	1.984	340.45	675.61
A_68_P27950544	chr11:79517837-79517881	NR_029579:-7612	Mir193	PROMOTER	0.970	1.806	857.19	1547.81	1.752	653.94	1145.67
A_68_P24055453	chr5:123845311-123845355	NM_177582:505	Mlxip	INSIDE	0.970	0.648	1420.28	920.71	0.629	1159.24	729.07
A_68_P31632606	chr18:38358924-38358968	NM_029357:10470	Pcdh1	INSIDE	0.970	2.628	1795.93	4719.59	2.549	1291.78	3292.12
A_68_P32745693	chrX:147954890-147954934	NM_177201:-302	Phf8	PROMOTER	0.970	2.347	3293.57	7730.06	2.277	1522.83	3467.79
A_68_P25972617	chr8:77737759-77737803	NM_029182:-62	Rasd2	PROMOTER	0.970	2.110	1152.07	2430.50	2.047	1049.70	2149.08
A_68_P27275565	chr10:78243326-78243370	NM_009200:108	Slc1a6	INSIDE	0.970	1.966	597.28	1174.40	1.908	617.78	1178.55

ProbeName	Target position of probe on CpG island microarray	TargetID	GeneSymbol	CpG island Description	Ratio of relative methylation (TiO_2-NP/Vehicle)	Sham group			TiO_2-H group		
						Relative methylation (Cy5/Cy3)	Cy3 signal (Input)	Cy5 signal (MeDIP)	Relative methylation (Cy5/Cy3)	Cy3 signal (Input)	Cy5 signal (MeDIP)
A_68_P24092031	chr5:130555095-130555139	NM_001130476:-239	Tpst1	PROMOTER	0.970	3.754	1010.19	3791.84	3.639	843.27	3068.80
A_68_P29438117	chr14:19159490-19159534	NM_009455:4846	Ube2e1	INSIDE	0.970	1.437	1631.20	2343.89	1.393	1450.95	2021.53
A_68_P20191088	chr1:43884115-43884159	NM_026430:417	Uxs1	INSIDE	0.970	1.583	1301.83	2060.39	1.535	1120.06	1719.28
A_68_P20350533	chr1:74838685-74838729	NM_009518:114	Wnt10a	INSIDE	0.970	0.740	2570.24	1900.83	0.717	2048.58	1469.73
A_68_P31939674	chr19:7491304-7491348	NM_175381:-788	2700081O15Rik	PROMOTER	0.969	1.520	965.20	1466.89	1.473	876.30	1291.04
A_68_P22150381	chr3:54703304-54703351	NM_177854:16482	6030405A18Rik	INSIDE	0.969	3.070	393.95	1209.27	2.975	324.90	966.51
A_68_P23417009	chr4:151552981-151553025	NM_001146057:-7240	Acot7	PROMOTER	0.969	1.607	1097.55	1763.26	1.556	935.98	1456.63
A_68_P27809325	chr11:54117457-54117501	NM_001033597:-227	Acsl6	PROMOTER	0.969	1.779	3088.94	5494.13	1.723	2481.90	4276.18
A_68_P21105432	chr2:30679697-30679741	NM_133346:4102	Asb6	INSIDE	0.969	2.592	539.86	1399.11	2.511	410.86	1031.65
A_68_P32536864	chrX:91226158-91226202	NR_028320:12186	AU015836	DOWNSTREAM	0.969	2.802	483.67	1355.22	2.714	255.56	693.61
A_68_P27649723	chr11:24064200-24064244	NM_001159290:83528	Bcl11a	INSIDE	0.969	2.943	1208.68	3556.93	2.851	1018.30	2903.27
A_68_P28452660	chr12:56701063-56701107	NM_020287:1181	Insm2	INSIDE	0.969	0.396	1845.14	731.48	0.384	1511.96	580.89
A_68_P32029537	chr19:25484593-25484638	NM_181404:172924	Kank1	INSIDE	0.969	3.483	2154.07	7501.80	3.374	1536.95	5185.68
A_68_P27267070	chr10:76728491-76728535	NM_030262:6468	Pofut2	INSIDE	0.969	2.232	1263.16	2819.82	2.163	1016.41	2198.40
A_68_P24428218	chr6:48547068-48547112	NM_001079901:3209	Repin1	INSIDE	0.969	2.236	1659.60	3710.07	2.165	1377.35	2982.11
A_68_P25228918	chr7:80554115-80554159	NM_177740:33731	Rgma	INSIDE	0.969	2.092	1099.96	2301.04	2.027	810.80	1643.35
A_68_P23339116	chr4:136852284-136852328	NM_009523:18757	Wnt4	INSIDE	0.969	4.338	1849.93	8025.39	4.202	1426.82	5995.50
A_68_P27287689	chr10:80607406-80607450	NM_010731:8413	Zbtb7a	INSIDE	0.969	1.930	911.26	1758.90	1.871	733.71	1372.55
A_68_P27686040	chr11:31865634-31865678	NM_026262:24	4930524B15Rik	INSIDE	0.968	2.096	2028.28	4251.89	2.029	1642.47	3332.84
A_68_P28612729	chr12:88224702-88224747	NM_145836:1040	6430527G18Rik	INSIDE	0.968	0.417	1236.63	515.22	0.403	1032.77	416.67
A_68_P28724527	chr12:109155388-109155432	NM_001079883:86214	Bcl11b	INSIDE	0.968	2.041	561.66	1146.15	1.976	454.74	898.70
A_68_P23818501	chr5:77286574-77286618	NM_001163793:16973	C530008M17Rik	INSIDE	0.968	3.192	3341.82	10666.19	3.091	2226.22	6881.31
A_68_P26844413	chr9:114299730-114299774	NM_019922:78	Crtap	INSIDE	0.968	1.794	1238.57	2222.39	1.737	1120.55	1946.50
A_68_P31227870	chr17:50432887-50432931	NM_010021:16	Dazl	INSIDE	0.968	2.598	1094.53	2843.43	2.515	1119.65	2816.38
A_68_P30642684	chr16:30601415-30601459	NM_177632:1628	Fam43a	INSIDE	0.968	1.593	696.07	1109.15	1.542	591.69	912.32
A_68_P32026433	chr19:24974465-24974509	NM_008022:1313	Foxd4	INSIDE	0.968	0.614	2497.57	1532.41	0.594	2040.07	1212.09
A_68_P24674234	chr6:97567426-97567470	NM_145148:203	Frmd4b	INSIDE	0.968	1.614	1134.36	1830.40	1.562	960.93	1501.12
A_68_P23417689	chr4:151654150-151654194	NM_178406:5702	Gpr153	INSIDE	0.968	2.088	484.12	1011.08	2.021	413.55	835.63
A_68_P31313936	chr17:68046719-68046763	NM_008480:136	Lama1	INSIDE	0.968	2.073	687.64	1425.35	2.007	726.58	1458.48
A_68_P25031177	chr7:31140943-31140987	NM_175478:6827	Lrfn3	INSIDE	0.968	2.651	681.89	1807.68	2.566	563.90	1446.71
A_68_P23326916	chr4:134708430-134708475	NM_019732:31893	Runx3	INSIDE	0.968	3.717	197.36	733.68	3.599	176.25	634.34
A_68_P21018171	chr2:13715028-13715072	NM_145838:97	St8sia6	INSIDE	0.968	0.641	2543.71	1630.95	0.620	2023.22	1255.36
A_68_P25959800	chr8:74659745-74659789	NM_001001491:576	Tpm4	INSIDE	0.968	0.745	4219.91	3142.70	0.721	3224.85	2324.62
A_68_P30363605	chr15:78813442-78813487	NM_001024716:-21	Triobp	PROMOTER	0.968	2.827	243.95	689.73	2.737	220.11	602.41
A_68_P27365512	chr10:94977939-94977984	NM_080560:166	Ube2n	INSIDE	0.968	2.585	249.26	644.23	2.502	214.67	537.14
A_68_P24168282	chr5:147197029-147197073	NM_145155:469	Wasf3	INSIDE	0.968	0.480	2103.45	1008.98	0.464	1741.66	808.88
A_68_P25008271	chr7:25866888-25866932	NM_175477:4646	Zfp574	INSIDE	0.968	1.565	762.61	1193.64	1.516	665.04	1008.12
A_68_P32565627	chrX:98989595-98989639	ENSMUST00000151231:-378		DIVERGENT_PROMOTER	0.968	4.679	438.11	2050.04	4.528	224.84	1017.97
A_68_P27899786	chr11:70068857-70068903	NM_007440:-37	Alox12	PROMOTER	0.967	3.348	1152.40	3858.56	3.238	1063.01	3441.50
A_68_P27503065	chr10:120802501-120802545	NM_029364:377	Gns	INSIDE	0.967	1.927	842.41	1623.03	1.863	818.41	1524.87
A_68_P27278589	chr10:79197977-79198021	NM_008226:18620	Hcn2	INSIDE	0.967	1.636	723.89	1184.53	1.583	647.23	1024.38
A_68_P25599895	chr7:150481208-150481262	NR_001461:1218	Kcnq1ot1	INSIDE	0.967	2.362	3496.10	8256.61	2.284	2538.78	5797.90
A_68_P21816616	chr2:163996879-163996923	NM_008435:-51	Kcns1	PROMOTER	0.967	1.663	1066.31	1773.00	1.607	941.67	1513.49
A_68_P25951933	chr8:72975932-72975976	NM_001122830:24888	Klhl26	INSIDE	0.967	2.973	391.33	1163.61	2.876	331.47	953.16
A_68_P26216538	chr8:122394181-122394225	NM_146219:7998	Klhl36	INSIDE	0.967	2.742	396.15	1086.42	2.652	320.98	851.10
A_68_P27950594	chr11:79524651-79524695	NR_029579:-798	Mir193	PROMOTER	0.967	0.620	2065.30	1280.80	0.600	1682.46	1008.83
A_68_P28055768	chr11:98200119-98200163	NM_010895:-9181	Neurod2	DIVERGENT_PROMOTER	0.967	0.422	1482.50	625.98	0.408	1382.24	564.18
A_68_P29056694	chr13:55828552-55828596	NM_028281:-154	Pcbd2	PROMOTER	0.967	3.181	542.75	1726.57	3.078	548.22	1687.25
A_68_P24994416	chr7:20214165-20214209	NM_009046:601	Relb	INSIDE	0.967	2.034	1502.25	3055.02	1.967	1131.46	2226.11
A_68_P24950762	chr7:4788423-4788469	NM_172737:7852	Shisa7	INSIDE	0.967	3.937	2138.58	8419.16	3.806	1674.67	6373.12
A_68_P26849143	chr9:115218715-115218759	NM_024222:803	Stt3b	INSIDE	0.967	1.821	859.50	1565.13	1.761	790.59	1392.07

ProbeName	Target position of probe on CpG island microarray	TargetID	GeneSymbol	CpG island Description	Ratio of relative methylation (TiO_2-NP/Vehicle)	Sham group			TiO_2-H group		
						Relative methylation (Cy5/Cy3)	Cy3 signal (Input)	Cy5 signal (MeDIP)	Relative methylation (Cy5/Cy3)	Cy3 signal (Input)	Cy5 signal (MeDIP)
A_68_P23331726	chr4:135577649-135577693	NM_013736:-106	Tceb3	PROMOTER	0.967	1.640	2011.60	3299.36	1.587	1605.53	2547.20
A_68_P26323063	chr9:15297918-15297962	AK146267:65		INSIDE	0.967	2.930	319.57	936.38	2.833	315.23	893.07
A_68_P21326874	chr2:72472122-72472166	ENSMUST00000120282:152		INSIDE	0.967	2.729	563.77	1538.69	2.638	482.34	1272.48
A_68_P30665798	chr16:34745356-34745400	ENSMUST00000155268:83		INSIDE	0.967	2.005	1256.21	2518.85	1.938	1092.85	2118.02
A_68_P21774856	chr2:156866097-156866141	NM_001164663:38883	9830001H06Rik	INSIDE	0.966	2.841	512.26	1455.22	2.745	425.38	1167.58
A_68_P23320879	chr4:133628861-133628905	NM_001162970:4516	Aim1l	INSIDE	0.966	1.869	858.03	1603.98	1.806	718.83	1297.94
A_68_P32533284	chrX:90544387-90544431	NM_007492:12424	Arx	DOWNSTREAM	0.966	1.941	861.31	1671.88	1.874	610.59	1144.38
A_68_P27173579	chr10:59766022-59766066	NM_023370:393194	Cdh23	INSIDE	0.966	2.433	773.97	1883.27	2.351	600.60	1412.11
A_68_P25020922	chr7:29062595-29062639	NM_001177427:-108	Eid2b	PROMOTER	0.966	1.803	848.63	1530.30	1.742	688.20	1198.81
A_68_P24003588	chr5:114830147-114830191	NM_001159941:346	Kctd10	INSIDE	0.966	2.081	10672.79	22213.64	2.011	6353.84	12779.78
A_68_P31922935	chr19:4397029-4397073	NM_001001984:27	Kdm2a	INSIDE	0.966	1.675	8529.86	14284.03	1.618	5892.29	9532.24
A_68_P31601517	chr18:32322542-32322586	NM_011946:-178	Map3k2	PROMOTER	0.966	1.986	334.24	663.95	1.918	394.56	756.93
A_68_P24990477	chr7:19511769-19511813	NM_001029877:554	Nova2	INSIDE	0.966	1.589	2661.36	4228.37	1.535	1824.74	2800.68
A_68_P27181450	chr10:61088648-61088692	NM_001177511:30439	Npffr1	INSIDE	0.966	0.577	1670.78	964.19	0.557	1354.64	755.13
A_68_P25953724	chr8:73267060-73267104	NM_201607:19120	Pde4c	INSIDE	0.966	2.033	643.26	1307.89	1.965	544.13	1069.02
A_68_P22522615	chr3:129581952-129581996	NM_183423:-2127	Pla2g12a	PROMOTER	0.966	7.610	7942.28	60438.52	7.351	4845.14	35618.85
A_68_P23430799	chr4:153715942-153715986	NM_001177995:295018	Prdm16	INSIDE	0.966	5.873	4321.20	25379.65	5.673	3110.20	17644.01
A_68_P31256001	chr17:56562089-56562133	NM_011218:53793	Ptprs	INSIDE	0.966	1.883	390.47	735.39	1.819	354.17	644.21
A_68_P22523680	chr3:129763666-129763710	NM_207209:137	Sec24b	INSIDE	0.966	0.599	2451.45	1468.27	0.578	2098.65	1213.61
A_68_P32203282	chr19:56901047-56901091	NM_001002241:-4133	Tdrd1	PROMOTER	0.966	2.627	1661.33	4363.70	2.538	1401.68	3557.37
A_68_P29049666	chr13:54569805-54569849	NM_028597:375	Thoc3	INSIDE	0.966	3.584	11387.77	40816.57	3.462	7150.73	24752.26
A_68_P32700063	chrX:137133904-137133948	NM_001077364:1135	Tsc22d3	INSIDE	0.966	3.058	458.56	1402.22	2.954	315.96	933.49
A_68_P21931967	chr3:8327343-8327387			Unknown	0.966	3.401	1379.32	4691.57	3.287	1266.35	4162.24
A_68_P24944609	chr6:149310012-149310056	ENSMUST00000120260:-173		DIVERGENT_PROMOTER	0.966	2.507	614.97	1541.84	2.423	559.63	1355.90
A_68_P28184669	chr11:120319314-120319358	NM_025560:41	1810049H13Rik	INSIDE	0.965	1.683	696.59	1172.54	1.625	646.08	1049.91
A_68_P20412921	chr1:88483574-88483618	NM_172974:-78	Cops7b	DIVERGENT_PROMOTER	0.965	1.634	769.01	1256.72	1.578	612.63	966.60
A_68_P32467036	chrX:71491383-71491427	NM_010227:469	Flna	INSIDE	0.965	1.705	3377.47	5757.93	1.645	1360.61	2238.20
A_68_P22661983	chr3:153991849-153991908	NM_010713:1646	Lhx8	INSIDE	0.965	2.132	1134.10	2418.22	2.057	981.86	2019.46
A_68_P23955549	chr5:105910183-105910227	NM_001033550:65411	Lrrc8b	INSIDE	0.965	2.192	617.55	1353.45	2.116	554.46	1173.09
A_68_P24946818	chr7:3637479-3637523	NM_029934:7627	Mboat7	INSIDE	0.965	3.062	1927.97	5902.77	2.955	1574.12	4651.46
A_68_P27843267	chr11:60290696-60290740	NM_001103171:7878	Myo15	INSIDE	0.965	1.793	1689.47	3029.45	1.731	1177.08	2036.99
A_68_P29237614	chr13:95655500-95655544	NM_011021:9941	Otp	DOWNSTREAM	0.965	2.514	220.86	555.15	2.426	219.41	532.18
A_68_P26492040	chr9:48288627-48288671	NM_024233:68	Rexo2	INSIDE	0.965	0.662	3589.28	2375.05	0.639	3021.85	1929.84
A_68_P32415805	chrX:58146252-58146296	NM_009237:331	Sox3	INSIDE	0.965	2.384	899.27	2143.43	2.300	498.80	1147.02
A_68_P23416139	chr4:151406623-151406667	NM_031867:5955	Tas1r1	INSIDE	0.965	2.616	2348.04	6143.61	2.526	1773.68	4479.83
A_68_P27943938	chr11:78349603-78349647	NM_001159392:138	Tnfaip1	INSIDE	0.965	0.517	1417.60	733.32	0.499	1037.53	517.97
A_68_P24002087	chr5:114580701-114580745	NM_011677:-442	Ung	PROMOTER	0.965	0.327	1848.49	603.84	0.315	1538.96	485.30
A_68_P30511262	chr16:4640285-4640329	NM_139307:362	Vasn	INSIDE	0.965	0.597	1099.81	656.89	0.576	985.82	568.05
A_68_P29390921	chr14:9498720-9498764	NM_028934:62	4930452B06Rik	INSIDE	0.964	0.733	2147.08	1574.57	0.707	1850.66	1308.08
A_68_P23293013	chr4:128444472-128444516	NM_001009819:7993	A3galt2	INSIDE	0.964	1.601	921.37	1475.32	1.543	656.77	1013.69
A_68_P22482405	chr3:122123152-122123204	NM_013867:481	Bcar3	INSIDE	0.964	3.352	284.01	951.99	3.231	218.22	705.12
A_68_P23574101	chr5:31013677-31013721	NM_023047:-569	Dpysl5	PROMOTER	0.964	2.119	640.71	1357.36	2.042	550.25	1123.69
A_68_P27143973	chr10:53471309-53471353	NM_001081428:-615	Fam184a	PROMOTER	0.964	0.752	6136.58	4614.28	0.725	4835.20	3505.87
A_68_P28940416	chr13:34839156-34839200	NM_138746:7668	Fam50b	INSIDE	0.964	2.220	1030.54	2287.50	2.140	843.92	1805.91
A_68_P26658191	chr9:77961162-77961206	NM_019987:140	Ick	INSIDE	0.964	0.562	2159.68	1214.59	0.542	1848.77	1002.06
A_68_P33012165	chr9_random:55589-55633	NM_011154:-578	Ppp2r3d	PROMOTER	0.964	0.513	1365.48	700.53	0.495	1157.80	572.79
A_68_P20412584	chr1:88421724-88421768	NM_008972:-1564	Ptma	PROMOTER	0.964	0.590	1192.90	703.68	0.569	943.08	536.36
A_68_P23308588	chr4:131393415-131393459	NM_001083119:757	Ptpru	INSIDE	0.964	4.245	16927.72	71856.67	4.093	10362.62	42409.37
A_68_P25025681	chr7:29946628-29946672	NM_182927:7016	Spred3	INSIDE	0.964	2.362	2182.95	5156.37	2.277	1803.38	4105.54
A_68_P23395335	chr4:147966100-147966144	NM_009272:501	Srm	INSIDE	0.964	1.600	1672.56	2676.41	1.543	1587.41	2449.95
A_68_P23608584	chr5:36924656-36924700	NM_133910:368	Tbc1d14	INSIDE	0.964	1.411	11401.18	16081.94	1.359	7749.20	10534.57

ProbeName	Target position of probe on CpG island microarray	TargetID	GeneSymbol	CpG island Description	Ratio of relative methylation (TiO_2-NP/Vehicle)	Sham group			TiO_2-H group		
						Relative methylation (Cy5/Cy3)	Cy3 signal (Input)	Cy5 signal (MeDIP)	Relative methylation (Cy5/Cy3)	Cy3 signal (Input)	Cy5 signal (MeDIP)
A_68_P31092363	chr17:23694927-23694971	NM_001033496:6245	Zfp213	INSIDE	0.964	2.598	1121.66	2913.77	2.505	900.96	2256.49
A_68_P21914376	chr3:3309284-3309328			Unknown	0.964	3.811	1027.34	3915.69	3.676	868.98	3194.30
A_68_P26403086	chr9:31839692-31839736			Unknown	0.964	0.617	1749.03	1079.72	0.595	1490.88	887.49
A_68_P26017564	chr8:86525774-86525820	NM_183097:192	1700067K01Rik	INSIDE	0.963	1.930	332.98	642.60	1.859	320.28	595.36
A_68_P22314994	chr3:88448009-88448053	NM_001198912:22715	Arhgef2	INSIDE	0.963	2.591	883.10	2288.09	2.494	829.48	2069.13
A_68_P28788966	chr13:3610442-3610486	NM_134063:-110	BC016423	PROMOTER	0.963	2.094	1375.50	2880.73	2.017	1151.33	2322.70
A_68_P24055363	chr5:123823490-123823534	NM_029850:29056	Bcl7a	INSIDE	0.963	2.687	2360.30	6341.13	2.586	1809.47	4679.46
A_68_P26055369	chr8:93352945-93352989	NM_177224:233	Chd9	INSIDE	0.963	1.857	723.30	1342.95	1.788	650.44	1163.16
A_68_P26349278	chr9:21656710-21656754	NM_177030:347	Dock6	INSIDE	0.963	2.223	462.16	1027.19	2.140	412.57	883.05
A_68_P32750744	chrX:149216782-149216826	NM_010951:341	Gpr143	INSIDE	0.963	1.507	1631.26	2457.98	1.452	937.50	1360.83
A_68_P29589396	chr14:49286130-49286176	NR_029384:-2810	Otx2os1	DIVERGENT_PROMOTER	0.963	3.952	186.22	735.89	3.805	161.62	615.03
A_68_P24898887	chr6:141198621-141198665	NM_018779:853	Pde3a	INSIDE	0.963	1.821	1527.37	2781.61	1.753	1246.35	2185.34
A_68_P31127303	chr17:29459319-29459363	NM_023734:3514	Pi16	INSIDE	0.963	3.564	6569.63	23416.27	3.433	4080.06	14007.31
A_68_P29701213	chr14:70828758-70828802	NM_021308:121	Piwil2	INSIDE	0.963	1.752	1106.00	1937.24	1.687	844.62	1424.66
A_68_P24424178	chr6:47601643-47601687	NR_002841:3429	Rn4.5s	DOWNSTREAM	0.963	1.971	5917.35	11664.09	1.899	5060.23	9607.70
A_68_P30489330	chr15:100884551-100884595	NM_011323:117871	Scn8a	DOWNSTREAM	0.963	1.855	882.90	1637.64	1.786	764.43	1365.66
A_68_P27911312	chr11:72224674-72224718	NM_177776:519	Smtnl2	INSIDE	0.963	1.476	5700.32	8414.10	1.421	4051.52	5758.33
A_68_P22386748	chr3:103380204-103380248	NM_018800:1023	Syt6	INSIDE	0.963	2.268	1445.24	3277.60	2.184	1339.00	2924.00
A_68_P30370355	chr15:79963981-79964036	NM_025609:425	Tab1	INSIDE	0.963	1.930	1201.95	2319.81	1.859	863.31	1604.63
A_68_P32203280	chr19:56900735-56900779	NM_001002240:-8	Tdrd1	PROMOTER	0.963	2.616	829.11	2169.16	2.520	640.53	1614.19
A_68_P26082679	chr8:97852246-97852290	NM_024467:3582	Zfp319	INSIDE	0.963	1.545	1141.63	1763.91	1.488	943.23	1403.44
A_68_P33007655	A_68_P33007655			Unknown	0.963	1.687	3501.59	5908.89	1.625	3706.54	6023.41
A_68_P29018252	chr13:48323302-48323346	ENSMUST00000143259:34668		INSIDE	0.963	0.456	1506.78	686.38	0.439	1250.98	548.78
A_68_P27100725	chr10:43230190-43230234	NM_199028:31267	Bend3	INSIDE	0.962	1.880	643.01	1208.91	1.810	472.27	854.59
A_68_P20528694	chr1:113761142-113761186	NM_001081316:331	Dsel	INSIDE	0.962	2.025	607.10	1229.49	1.949	594.41	1158.61
A_68_P24620357	chr6:88148628-88148672	NM_008090:-7	Gata2	PROMOTER	0.962	1.839	1623.52	2985.55	1.768	1387.12	2452.93
A_68_P21874751	chr2:174154053-174154097	NM_001077510:-1515	Gnas	PROMOTER	0.962	2.056	634.46	1304.31	1.978	668.48	1322.40
A_68_P24159777	chr5:144934590-144934634	NM_001081109:73308	Lmtk2	INSIDE	0.962	2.531	1229.86	3112.97	2.436	1028.45	2505.18
A_68_P21102108	chr2:30111813-30111859	NM_177725:18548	Lrrc8a	INSIDE	0.962	2.719	527.34	1433.95	2.617	456.35	1194.17
A_68_P30961946	chr16:91270379-91270423	NM_016968:387	Olig1	INSIDE	0.962	2.180	3404.57	7422.90	2.098	2602.93	5462.02
A_68_P20474071	chr1:99873584-99873628	NM_013626:118603	Pam	INSIDE	0.962	1.860	588.27	1094.41	1.790	514.69	921.12
A_68_P32114752	chr19:41450572-41450616	NM_031376:8966	Pik3ap1	INSIDE	0.962	1.895	907.28	1719.06	1.823	776.69	1415.58
A_68_P23271481	chr4:124664358-124664402	NM_138683:707	Rspo1	INSIDE	0.962	0.580	1089.79	632.44	0.558	934.84	522.03
A_68_P31123428	chr17:28758772-28758816	NM_016795:605	Srpk1	INSIDE	0.962	0.704	2057.92	1448.38	0.677	1632.02	1104.51
A_68_P25971567	chr8:77558116-77558160	NM_001136259:554	Tom1	INSIDE	0.962	0.608	1510.12	918.48	0.585	1209.47	707.74
A_68_P21875504	chr2:174282882-174282926	NM_001080971:6709	Tubb1	INSIDE	0.962	3.783	1344.89	5087.13	3.640	1020.41	3714.05
A_68_P31301685	chr17:65962800-65962844	NM_013933:73	Vapa	INSIDE	0.962	0.710	1375.96	977.26	0.683	1181.22	807.34
A_68_P28599174	chr12:85930850-85930894	NM_007701:20071	Vsx2	INSIDE	0.962	1.642	528.35	867.58	1.579	757.69	1196.38
A_68_P24424841	chr6:47855192-47855236	NM_146175:27661	Zfp282	INSIDE	0.962	1.687	1379.08	2326.54	1.623	1051.04	1706.17
A_68_P31219171	chr17:48925588-48925633			Unknown	0.962	1.856	595.33	1105.13	1.785	492.10	878.55
A_68_P24761357	chr6:113705166-113705210	NM_001036684:136182	Atp2b2	INSIDE	0.961	2.519	525.97	1324.93	2.420	444.96	1076.97
A_68_P25272114	chr7:88600003-88600048	NM_007755:-463	Cpeb1	PROMOTER	0.961	2.508	992.10	2488.33	2.411	823.50	1985.70
A_68_P21082466	chr2:26968285-26968329	NM_175427:29691	Fam163b	INSIDE	0.961	3.705	5130.49	19006.68	3.560	3391.22	12072.19
A_68_P21621960	chr2:128718146-128718190	NM_024237:28501	Fbln7	INSIDE	0.961	1.719	1152.19	1981.03	1.653	1082.09	1788.55
A_68_P21122756	chr2:33495852-33495899	NM_010725:156	Lmx1b	INSIDE	0.961	2.376	896.77	2130.85	2.284	673.54	1538.17
A_68_P25021325	chr7:29135852-29135896	NR_028129:-684	LOC100302567	PROMOTER	0.961	1.620	1713.15	2775.30	1.556	1245.43	1938.27
A_68_P28554434	chr12:77634454-77634498	NM_153804:-70	Plekhg3	PROMOTER	0.961	0.643	1525.63	980.27	0.617	1359.06	839.04
A_68_P30447392	chr15:93349977-93350021	NM_001033217:76324	Prickle1	INSIDE	0.961	3.097	1119.09	3465.84	2.975	1056.04	3141.88
A_68_P21227529	chr2:53937298-53937342	NM_023396:643	Rprm	INSIDE	0.961	1.937	1088.69	2108.54	1.862	991.20	1845.20
A_68_P28291143	chr12:25392901-25392945	NM_009104:-196	Rrm2	PROMOTER	0.961	0.648	4211.50	2730.99	0.623	3364.06	2096.64
A_68_P28469854	chr12:60112791-60112835	NM_009147:192	Sec23a	INSIDE	0.961	0.346	1613.18	558.72	0.333	1264.54	420.80

ProbeName	Target position of probe on CpG island microarray	TargetID	GeneSymbol	CpG island Description	Ratio of relative methylation (TiO_2-NP/Vehicle)	Sham group			TiO_2-H group		
						Relative methylation (Cy5/Cy3)	Cy3 signal (Input)	Cy5 signal (MeDIP)	Relative methylation (Cy5/Cy3)	Cy3 signal (Input)	Cy5 signal (MeDIP)
A_68_P26752042	chr9:97266348-97266392	NM_030219:4007	Trim42	INSIDE	0.961	2.286	1445.52	3304.90	2.197	1218.61	2677.25
A_68_P22485525	chr3:122687267-122687311	NM_133857:77	Usp53	INSIDE	0.961	0.525	2546.71	1336.85	0.504	1892.18	954.49
A_68_P26494144	A_68_P26494144			Unknown	0.961	2.117	1431.31	3029.98	2.034	1248.77	2540.55
A_68_P28175548	chr11:118914534-118914580			Unknown	0.961	1.700	648.78	1103.06	1.633	538.68	879.74
A_68_P23523567	chr5:20457129-20457173	NR_030699:344	A630072M18Rik	INSIDE	0.960	2.090	944.95	1974.83	2.007	815.25	1636.07
A_68_P24617814	chr6:87681107-87681152	NM_001159697:267	Ccdc48	INSIDE	0.960	1.855	826.58	1533.44	1.782	719.54	1281.91
A_68_P27172913	chr10:59648822-59648866	NM_016803:33164	Chst3	INSIDE	0.960	2.228	451.05	1004.98	2.139	347.59	743.61
A_68_P26221740	chr8:123192627-123192671	NM_009941:459	Cox4i1	INSIDE	0.960	0.620	1248.33	774.28	0.596	962.51	573.36
A_68_P24193750	chr5:151755531-151755575	NM_013823:371	Kl	INSIDE	0.960	0.401	1398.75	560.46	0.385	1214.61	467.10
A_68_P27270908	chr10:77365226-77365270	NM_145152:33	Lrrc3	INSIDE	0.960	1.586	5274.44	8363.88	1.522	3710.46	5647.16
A_68_P21127263	chr2:34226980-34227024	NM_016768:554	Pbx3	INSIDE	0.960	0.667	1242.41	828.21	0.640	1096.85	701.82
A_68_P31207974	chr17:46766268-46766312	NM_175168:163	Ptk7	INSIDE	0.960	0.658	1815.69	1195.39	0.632	1476.68	932.97
A_68_P20370953	chr1:78306980-78307024	NM_001004173:82	Sgpp2	INSIDE	0.960	0.643	1754.94	1128.30	0.617	1602.42	989.17
A_68_P28176530	chr11:119072055-119072099	NM_172443:17737	Tbc1d16	INSIDE	0.960	18.837	1910.68	35990.56	18.076	1455.53	26310.09
A_68_P27965916	chr11:82200818-82200862	NM_023438:-1561	Tmem132e	PROMOTER	0.960	0.512	1179.51	604.47	0.492	1055.62	519.23
A_68_P26247306	chr8:127245477-127245521	NM_145607:377	Ttc13	INSIDE	0.960	0.634	1928.51	1221.94	0.608	1659.64	1009.76
A_68_P23424872	chr4:152806992-152807036	NM_001099299:49925	Ajap1	INSIDE	0.959	2.196	1927.70	4232.39	2.106	1556.44	3277.19
A_68_P27298384	chr10:83111351-83111395	NM_145220:37	Appl2	INSIDE	0.959	0.551	1183.75	652.63	0.529	955.54	505.35
A_68_P29338035	chr13:113777889-113777933	NM_001081062:-99	Ccno	PROMOTER	0.959	1.858	648.69	1205.01	1.781	623.25	1109.76
A_68_P33015621	chr1_random:50610-50662	NM_026866:134702	Disp1	INSIDE	0.959	2.720	779.42	2120.03	2.607	615.67	1605.19
A_68_P25006992	chr7:25654882-25654927	NM_027732:-171	Dmrtc2	DIVERGENT_PROMOTER	0.959	2.247	829.70	1864.04	2.155	625.27	1347.61
A_68_P23335794	chr4:136326887-136326931	NM_010142:64942	Ephb2	INSIDE	0.959	3.026	2754.47	8334.68	2.901	2143.54	6218.37
A_68_P22293166	chr3:84619656-84619700	NM_001177773:180	Fbxw7	INSIDE	0.959	0.621	2197.02	1364.24	0.595	1721.42	1025.02
A_68_P24112566	chr5:134790011-134790055	NM_001080746:584	Gtf2i	INSIDE	0.959	1.604	864.13	1385.83	1.539	782.29	1203.64
A_68_P31113975	chr17:27194456-27194500	NM_080553:230	Itpr3	INSIDE	0.959	2.019	400.59	808.86	1.937	400.96	776.62
A_68_P28547082	chr12:76277823-76277867	NM_172805:475	Kcnh5	INSIDE	0.959	4.953	805.27	3988.38	4.751	744.42	3536.76
A_68_P26346082	chr9:21038221-21038265	NM_001110305:5534	Keap1	INSIDE	0.959	2.452	324.58	796.01	2.353	286.58	674.21
A_68_P27988569	chr11:86171411-86171455	NM_001080931:-405	Med13	PROMOTER	0.959	2.829	566.59	1602.62	2.712	538.16	1459.64
A_68_P30422884	chr15:88899097-88899141	NM_001002005:8963	Panx2	INSIDE	0.959	3.241	2929.63	9493.74	3.108	2032.62	6318.18
A_68_P31868009	chr18:81171208-81171252	NM_178280:12087	Sall3	INSIDE	0.959	2.177	435.61	948.22	2.089	400.11	835.66
A_68_P27942518	chr11:78114962-78115006	NM_017407:-108	Spag5	PROMOTER	0.959	0.500	1638.13	818.31	0.479	1327.25	635.91
A_68_P32114044	chr19:41338030-41338074	NM_133352:442	Tm9sf3	INSIDE	0.959	1.935	713.01	1379.85	1.856	642.13	1191.85
A_68_P25062054	chr7:38352951-38352995	NM_172385:201799	Zfp536	INSIDE	0.959	2.206	1504.60	3319.42	2.117	1255.26	2656.78
A_68_P33007021	A_68_P33007021			Unknown	0.959	1.848	19506.44	36045.89	1.772	15585.65	27618.44
A_68_P26323069	chr9:15298668-15298712	AK146267:815		INSIDE	0.959	1.413	2726.28	3853.27	1.356	1913.02	2593.92
A_68_P32288516	chrX:20002305-20002349	ENSMUST00000115384:-487		PROMOTER	0.959	1.727	690.49	1192.30	1.656	481.95	797.99
A_68_P22455157	chr3:117278382-117278426	NM_029425:26	4833424O15Rik	INSIDE	0.958	0.575	1998.63	1148.34	0.551	1726.16	950.40
A_68_P32795516	chrX:160347102-160347146	NM_026887:33	Ap1s2	INSIDE	0.958	1.975	1139.31	2249.72	1.893	776.14	1468.95
A_68_P22338536	chr3:94167827-94167871	NM_001136117:1483	C2cd4d	INSIDE	0.958	1.814	834.22	1513.20	1.739	827.50	1438.61
A_68_P21070566	chr2:25066778-25066822	NM_021393:209	Cobra1	INSIDE	0.958	1.451	2238.87	3248.01	1.390	1853.17	2576.21
A_68_P28575259	chr12:81440889-81440933	NM_177267:96678	Dcaf5	INSIDE	0.958	2.806	1783.02	5003.84	2.690	1431.13	3849.20
A_68_P28158193	chr11:116099596-116099640	NM_025276:-213	Evpl	PROMOTER	0.958	2.085	877.97	1830.20	1.997	809.10	1615.42
A_68_P20294244	chr1:64781733-64781777	NM_001042659:2570	Fzd5	INSIDE	0.958	4.143	704.93	2920.67	3.969	559.53	2220.89
A_68_P21750610	chr2:152512595-152512639	NM_025543:-267	Mcts2	PROMOTER	0.958	2.084	1033.76	2154.25	1.997	871.73	1741.04
A_68_P27843266	chr11:60290609-60290656	NM_001103171:7792	Myo15	INSIDE	0.958	2.232	981.08	2189.87	2.137	738.41	1578.32
A_68_P28185382	chr11:120434920-120434964	NM_011032:-692	P4hb	PROMOTER	0.958	0.560	954.84	534.66	0.536	717.26	384.58
A_68_P24117612	chr5:136210056-136210100	NM_008898:44995	Por	INSIDE	0.958	2.450	458.74	1123.78	2.346	371.55	871.64
A_68_P25728673	chr8:26830531-26830575	NM_028000:33	Ppapdc1b	INSIDE	0.958	3.163	1901.11	6013.56	3.031	1711.61	5187.95
A_68_P21817577	chr2:164219124-164219168	NM_009036:-9547	Rbpjl	PROMOTER	0.958	1.762	479.37	844.54	1.688	438.85	740.97
A_68_P21750312	chr2:152460407-152460451	NM_009047:7685	Rem1	INSIDE	0.958	2.295	837.19	1921.38	2.199	827.94	1820.40
A_68_P26539387	chr9:56775942-56775986	NM_175483:214	Snx33	INSIDE	0.958	0.526	1225.75	644.99	0.504	1010.84	509.51

ProbeName	Target position of probe on CpG island microarray	TargetID	GeneSymbol	CpG island Description	Ratio of relative methylation (TiO_2-NP/Vehicle)	Sham group			TiO_2-H group		
						Relative methylation (Cy5/Cy3)	Cy3 signal (Input)	Cy5 signal (MeDIP)	Relative methylation (Cy5/Cy3)	Cy3 signal (Input)	Cy5 signal (MeDIP)
A_68_P30498346	chr15:102348908-102348952	NM_009319:254	Tarbp2	INSIDE	0.958	1.938	3148.64	6103.39	1.856	2604.36	4834.94
A_68_P23317934	chr4:133053638-133053682	NM_001081156:805	Trnp1	INSIDE	0.958	1.567	1644.13	2576.55	1.501	1268.42	1903.77
A_68_P29477492	chr14:26279368-26279412	NM_183208:720	Zmiz1	INSIDE	0.958	0.534	1044.11	557.57	0.512	755.70	386.78
A_68_P25357072	chr7:105804387-105804431	NM_172280:671	2210018M11Rik	INSIDE	0.957	0.434	1612.29	699.63	0.415	1309.20	543.56
A_68_P25730019	chr8:27096869-27096915	NM_029037:7701	4930444A02Rik	INSIDE	0.957	3.339	2049.67	6843.96	3.196	1564.71	5001.28
A_68_P24127312	chr5:138182456-138182500	NM_175521:-1252	6430598A04Rik	DIVERGENT_PROMOTER	0.957	2.453	6825.66	16740.28	2.347	4518.49	10605.07
A_68_P28696863	chr12:104563389-104563437	NM_023049:30799	Asb2	INSIDE	0.957	2.091	465.93	974.27	2.000	430.02	860.18
A_68_P25047622	chr7:35905690-35905734	NM_007678:1401	Cebpa	INSIDE	0.957	0.662	1425.63	943.10	0.633	1083.91	685.89
A_68_P20884269	chr1:183853674-183853718	NM_001083120:96415	Enah	INSIDE	0.957	1.579	1538.17	2428.25	1.511	1161.41	1754.80
A_68_P20941838	chr1:193960926-193960970	NR_033536:169	Gm10516	INSIDE	0.957	0.596	1299.74	774.12	0.570	1083.37	617.57
A_68_P32119985	chr19:42330579-42330623	NM_027694:372	Golga7b	INSIDE	0.957	1.869	2309.86	4317.51	1.790	1858.20	3325.41
A_68_P29616393	chr14:55732216-55732260	NM_177049:3534	Jph4	INSIDE	0.957	2.803	686.73	1925.01	2.683	683.02	1832.50
A_68_P20864297	chr1:180458955-180458999	NM_001161665:-279	Kif26b	PROMOTER	0.957	1.631	665.77	1086.07	1.561	596.48	931.03
A_68_P21071165	chr2:25146200-25146244	NM_146117:792	Lrrc26	INSIDE	0.957	0.630	1622.06	1022.20	0.603	1199.75	723.67
A_68_P22006136	chr3:26230775-26230819	NM_138666:35	Nlgn1	INSIDE	0.957	0.672	2414.71	1621.73	0.642	1915.82	1230.87
A_68_P30139533	chr15:36539361-36539405	NM_008774:-654	Pabpc1	PROMOTER	0.957	2.235	744.22	1663.29	2.139	709.18	1517.28
A_68_P28957530	chr13:38022811-38022855	NM_001177869:42093	Rreb1	INSIDE	0.957	1.650	2230.16	3680.80	1.580	1744.40	2756.46
A_68_P30953522	chr16:89885052-89885096	NM_009384:75998	Tiam1	INSIDE	0.957	2.368	1041.42	2466.41	2.266	930.20	2107.46
A_68_P24974196	chr7:13610514-13610558	NM_011588:1036	Trim28	INSIDE	0.957	1.517	1684.95	2555.76	1.452	1209.98	1757.00
A_68_P31119041	chr17:28024335-28024379	NM_001080769:30905	Uhrf1bp1	INSIDE	0.957	3.042	909.15	2765.72	2.913	785.87	2289.00
A_68_P32241860	chrX:7706225-7706269	NR_029428:3727	Wdr13	INSIDE	0.957	3.661	1399.37	5123.30	3.502	630.03	2206.64
A_68_P25029972	chr7:30906920-30906964	NM_001081007:-24	Zfp382	PROMOTER	0.957	1.598	936.59	1496.75	1.529	802.25	1226.46
A_68_P25506496	chr7:134754584-134754628	NM_177226:3341	Zfp629	INSIDE	0.957	2.684	517.66	1389.16	2.568	466.53	1198.11
A_68_P30959217	chr16:90830454-90830498	NM_001199210:-627	4931408A02Rik	PROMOTER	0.956	1.735	2441.84	4235.63	1.659	1940.22	3218.64
A_68_P22831245	chr4:34498019-34498063	NM_001007589:177	Akirin2	INSIDE	0.956	2.089	599.92	1253.27	1.997	582.67	1163.50
A_68_P26813123	chr9:108017642-108017686	NM_007567:75050	Bsn	INSIDE	0.956	3.257	2356.65	7676.63	3.114	1652.23	5145.64
A_68_P27565934	chr11:5872237-5872281	NM_001174053:93493	Camk2b	INSIDE	0.956	1.725	1149.19	1982.17	1.649	892.23	1471.31
A_68_P25186368	chr7:73253968-73254012	NM_001081163:-410	Chsy1	PROMOTER	0.956	1.555	1168.42	1816.54	1.487	970.19	1442.53
A_68_P31951462	chr19:10651912-10651957	NM_201351:-278	Cybasc3	DIVERGENT_PROMOTER	0.956	0.607	969.01	588.52	0.580	831.83	482.73
A_68_P23366443	chr4:141430949-141430993	NM_025994:-135	Efhd2	PROMOTER	0.956	2.508	640.76	1606.84	2.397	569.55	1365.24
A_68_P21229088	chr2:54288519-54288563	NM_173030:-257	Galnt13	PROMOTER	0.956	0.722	1814.71	1311.03	0.691	1669.13	1153.38
A_68_P27290411	chr10:81007514-81007558	NM_010301:255	Gna11	INSIDE	0.956	2.305	2034.66	4690.58	2.204	1745.69	3846.83
A_68_P22344500	chr3:95363378-95363422	NM_026489:-198	Hormad1	PROMOTER	0.956	1.949	602.46	1174.31	1.864	522.17	973.35
A_68_P32537944	chrX:91480391-91480435	NM_001039059:144	Klhl15	INSIDE	0.956	1.934	1279.09	2473.33	1.848	728.56	1346.52
A_68_P22659609	chr3:153568957-153569001	NM_031870:124	Msh4	INSIDE	0.956	2.029	1639.52	3326.21	1.939	1324.55	2568.78
A_68_P32319272	chrX:34138495-34138539	NM_016783:297	Pgrmc1	INSIDE	0.956	2.274	2937.00	6678.02	2.173	1297.31	2819.63
A_68_P21843325	chr2:168577925-168577969	NM_175303:14755	Sall4	INSIDE	0.956	2.520	1293.03	3258.06	2.409	1032.64	2487.51
A_68_P22207890	chr3:66785065-66785109	NM_013665:607	Shox2	INSIDE	0.956	7.540	600.45	4527.71	7.207	538.83	3883.61
A_68_P20723684	chr1:154750088-154750132	NM_001005507:-334	Smg7	PROMOTER	0.956	3.453	995.18	3436.09	3.302	963.71	3181.71
A_68_P28607920	chr12:87420588-87420632	NM_009368:-619	Tgfb3	DIVERGENT_PROMOTER	0.956	0.485	1808.96	877.50	0.464	1498.61	695.11
A_68_P23463653	chr5:7344782-7344826	NM_001163223:-426	Zfp804b	PROMOTER	0.956	1.985	832.43	1651.99	1.898	889.98	1689.06
A_68_P26304291	chr9:11277863-11277907			Unknown	0.956	2.403	510.57	1227.14	2.298	451.74	1038.07
A_68_P28178206	chr11:119352324-119352368	NM_001164636:-314	A730011L01Rik	PROMOTER	0.955	0.510	1544.29	787.09	0.487	1180.90	574.55
A_68_P27533697	chr10:126486074-126486118	NM_010009:795	Cyp27b1	INSIDE	0.955	0.621	1069.91	664.84	0.594	917.02	544.42
A_68_P24156008	chr5:144077206-144077250	NM_172726:-189	E130309D02Rik	PROMOTER	0.955	0.616	1817.82	1119.85	0.589	1371.11	807.03
A_68_P28748275	chr12:113399969-113400013	NM_001097621:15572	Kif26a	INSIDE	0.955	3.309	2655.44	8786.70	3.160	2009.42	6350.13
A_68_P21137730	chr2:35947180-35947224	NM_001083126:12378	Lhx6	INSIDE	0.955	1.893	979.93	1854.85	1.808	824.89	1491.21
A_68_P26709438	chr9:88221992-88222036	NM_011851:-432	Nt5e	PROMOTER	0.955	1.607	1083.63	1741.06	1.535	929.26	1426.31
A_68_P26242532	chr8:126472872-126472916	NM_172288:271	Nup133	INSIDE	0.955	1.490	1427.66	2126.63	1.422	1213.57	1725.67
A_68_P24381545	chr6:39068163-39068207	NM_172893:164	Parp12	INSIDE	0.955	3.098	338.47	1048.60	2.957	338.51	1001.13
A_68_P31097497	chr17:24607518-24607562	NM_025954:123	Pgp	INSIDE	0.955	0.536	1698.35	910.79	0.512	1297.88	664.88

ProbeName	Target position of probe on CpG island microarray	TargetID	GeneSymbol	CpG island Description	Ratio of relative methylation (TiO_2-NP/Vehicle)	Sham group			TiO_2-H group		
						Relative methylation (Cy5/Cy3)	Cy3 signal (Input)	Cy5 signal (MeDIP)	Relative methylation (Cy5/Cy3)	Cy3 signal (Input)	Cy5 signal (MeDIP)
A_68_P25505712	chr7:134615346-134615390	NM_145589:241	Prr14	INSIDE	0.955	0.447	1552.59	693.42	0.427	1363.49	581.63
A_68_P28863269	chr13:19715200-19715245	NM_016687:179	Sfrp4	INSIDE	0.955	2.584	369.66	955.19	2.467	289.85	715.04
A_68_P23158194	chr4:101760223-101760267	ENSMUST00000106911:97		INSIDE	0.955	0.667	1763.67	1177.15	0.637	1724.37	1098.96
A_68_P25090215	chr7:52185401-52185445	NM_001077264:-562	Ap2a1	PROMOTER	0.954	0.460	1559.85	717.83	0.439	1358.75	596.73
A_68_P29523393	chr14:34133081-34133125	NM_153800:103094	Arhgap22	INSIDE	0.954	0.534	1814.67	968.68	0.509	1356.39	690.72
A_68_P32233092	chrX:5660427-5660471	NM_001033211:90	AU022751	INSIDE	0.954	2.287	618.24	1413.85	2.182	293.52	640.38
A_68_P27892695	chr11:68853469-68853515	NM_011496:-5652	Aurkb	PROMOTER	0.954	1.516	1056.03	1600.72	1.447	832.58	1204.37
A_68_P28047197	chr11:96723274-96723318	NM_019877:12107	Copz2	DOWNSTREAM	0.954	1.615	2150.86	3473.84	1.541	1550.99	2389.77
A_68_P24383682	chr6:39507280-39507324	NM_172477:531	Dennd2a	INSIDE	0.954	0.501	2609.72	1308.07	0.478	2051.52	981.46
A_68_P31927972	chr19:5307824-5307868	NM_001024717:9516	Gal3st3	INSIDE	0.954	1.799	1078.67	1940.44	1.717	883.85	1517.30
A_68_P26270752	chr8:131210063-131210107	NM_010578:531	Itgb1	INSIDE	0.954	0.456	2072.96	944.76	0.435	1898.93	825.54
A_68_P25031210	chr7:31145533-31145577	NM_175478:2237	Lrfn3	INSIDE	0.954	2.155	555.19	1196.53	2.056	473.92	974.43
A_68_P30477058	chr15:98702295-98702339	NM_001033276:-702	Mll2	PROMOTER	0.954	1.816	978.69	1777.24	1.733	830.55	1439.58
A_68_P20075274	chr1:20925596-20925640	NM_028829:44916	Paqr8	INSIDE	0.954	2.264	1098.57	2487.55	2.160	799.23	1726.58
A_68_P28583198	chr12:82961466-82961510	NM_018814:472	Pcnx	INSIDE	0.954	0.677	1769.84	1197.56	0.646	1493.74	964.70
A_68_P21871014	chr2:173485531-173485575	NM_024436:207	Rab22a	INSIDE	0.954	1.968	3089.01	6079.67	1.878	2650.30	4976.20
A_68_P21142185	chr2:37307958-37308002	NM_001033960:207	Rabgap1	INSIDE	0.954	0.531	1686.60	896.29	0.507	1461.38	740.76
A_68_P23415822	chr4:151362674-151362718	NM_001145929:399	Thap3	INSIDE	0.954	0.720	1816.31	1308.55	0.687	1408.32	967.60
A_68_P24431877	chr6:49214057-49214101	NM_198102:-27	Tra2a	PROMOTER	0.954	0.660	1099.37	725.21	0.629	994.06	625.68
A_68_P30094307	chr15:27677421-27677465	NM_001081302:278161	Trio	INSIDE	0.954	3.859	3487.62	13459.28	3.680	2485.40	9146.76
A_68_P28693529	chr12:103996499-103996543	NM_025666:336	Ubr7	INSIDE	0.954	0.483	1510.97	730.41	0.461	1248.64	575.72
A_68_P28202958	chr12:4913875-4913919	NM_145441:430	Ubxn2a	INSIDE	0.954	2.060	1176.76	2424.71	1.965	1060.07	2083.33
A_68_P24123997	chr5:137463625-137463669	NM_027318:106	Znhit1	INSIDE	0.954	0.685	1397.38	957.66	0.654	1122.40	733.96
A_68_P24159892	chr5:144952166-144952210	NM_010800:1034	Bhlha15	INSIDE	0.953	1.540	1328.00	2045.23	1.468	953.73	1399.66
A_68_P21483578	chr2:103325096-103325140	NM_009804:192	Cat	INSIDE	0.953	0.627	1379.71	864.72	0.597	1074.01	641.70
A_68_P31919589	chr19:3852192-3852236	NM_001025566:442	Chka	INSIDE	0.953	0.554	1239.57	687.30	0.528	1117.50	590.59
A_68_P31227866	chr17:50432495-50432539	NM_010021:408	Dazl	INSIDE	0.953	1.651	656.14	1083.06	1.573	638.53	1004.26
A_68_P28221326	chr12:8308667-8308711	NM_013527:72	Gdf7	INSIDE	0.953	2.106	446.54	940.46	2.007	388.19	779.04
A_68_P25512009	chr7:135831848-135831892	NM_178641:76992	Inpp5f	INSIDE	0.953	1.530	1504.42	2302.15	1.459	1268.63	1850.90
A_68_P25004857	chr7:25217107-25217151	NM_199013:13573	Irgc1	INSIDE	0.953	2.736	980.10	2681.66	2.607	728.21	1898.59
A_68_P25088775	chr7:51926026-51926070	NM_028021:-5175	Myh14	PROMOTER	0.953	0.676	1210.01	818.09	0.644	1067.81	687.88
A_68_P20412597	chr1:88423071-88423115	NM_008972:-218	Ptma	PROMOTER	0.953	0.442	1205.24	533.08	0.421	928.24	391.11
A_68_P29751623	chr14:79881191-79881235	NM_018765:-137	Wbp4	PROMOTER	0.953	2.138	413.32	883.59	2.037	297.03	604.94
A_68_P24951675	chr7:4982142-4982186	NM_001033383:10187	Zfp865	INSIDE	0.953	2.251	2039.47	4591.47	2.145	1611.87	3457.32
A_68_P26663736	chr9:79190551-79190595			Unknown	0.953	2.576	592.54	1526.10	2.455	570.51	1400.47
A_68_P24159893	chr5:144952289-144952338	NM_010800:1159	Bhlha15	INSIDE	0.952	2.218	774.51	1717.66	2.111	642.24	1356.00
A_68_P31977525	chr19:16059111-16059155	NM_198019:347	Cep78	INSIDE	0.952	0.619	1873.65	1160.34	0.590	1556.63	918.11
A_68_P21911108	chr2:181255242-181255287	NM_016775:55	Dnajc5	INSIDE	0.952	2.089	1414.50	2954.99	1.989	1199.12	2385.43
A_68_P32092667	chr19:36993325-36993369	NM_028263:743	Fgfbp3	INSIDE	0.952	2.674	602.46	1610.76	2.546	538.83	1371.93
A_68_P23283635	chr4:126803524-126803568	NM_001099319:257	Gm12942	INSIDE	0.952	2.539	521.49	1323.85	2.418	465.84	1126.25
A_68_P27235136	chr10:70844509-70844565	NM_027184:33996	Ipmk	INSIDE	0.952	1.625	774.29	1258.02	1.547	586.96	907.95
A_68_P21583617	chr2:121781672-121781716	NM_008545:134	Mageb3	INSIDE	0.952	2.052	920.31	1888.65	1.953	861.95	1683.21
A_68_P23278934	chr4:125911232-125911279	NM_001145970:22308	Mtap7d1	INSIDE	0.952	4.289	283.33	1215.24	4.082	230.80	942.10
A_68_P24171760	chr5:147889385-147889429	NM_181730:263	Polr1d	INSIDE	0.952	1.454	2501.83	3638.73	1.384	2189.87	3031.72
A_68_P25481817	chr7:130113128-130113172	NM_175023:-927	Rbbp6	PROMOTER	0.952	0.619	2123.82	1313.92	0.589	1730.51	1019.23
A_68_P26248944	chr8:127536746-127536790	NM_016909:-128	Tsnax	PROMOTER	0.952	0.690	1878.23	1295.18	0.657	1486.63	976.34
A_68_P27932455	chr11:76308282-76308326	NM_198018:14617	Abr	INSIDE	0.951	1.907	1602.66	3056.94	1.814	1368.07	2482.01
A_68_P27804993	chr11:53293588-53293632	NM_183173:87	Ankrd43	INSIDE	0.951	0.662	1292.64	856.03	0.630	1097.01	690.96
A_68_P26169910	chr8:114255749-114255793	NM_001198839:266	Bcar1	INSIDE	0.951	2.050	805.29	1650.51	1.948	787.49	1534.13
A_68_P26336206	chr9:18096974-18097018	NM_025844:286	Chordc1	INSIDE	0.951	1.479	2032.93	3006.03	1.406	1760.63	2475.95
A_68_P23284714	chr4:127003444-127003488	NM_008126:2903	Gjb3	INSIDE	0.951	1.869	1017.67	1902.08	1.777	853.97	1517.74

ProbeName	Target position of probe on CpG island microarray	TargetID	GeneSymbol	CpG island Description	Ratio of relative methylation (TiO_2-NP/Vehicle)	Sham group			TiO_2-H group		
						Relative methylation (Cy5/Cy3)	Cy3 signal (Input)	Cy5 signal (MeDIP)	Relative methylation (Cy5/Cy3)	Cy3 signal (Input)	Cy5 signal (MeDIP)
A_68_P27339938	chr10:90545771-90545815	NM_026166:9	Ikbip	INSIDE	0.951	2.719	1586.47	4314.39	2.587	1367.46	3537.94
A_68_P26700781	chr9:86589673-86589717	NM_001198933:-8488	Me1	PROMOTER	0.951	1.841	799.09	1470.86	1.750	812.26	1421.83
A_68_P25285295	chr7:91032191-91032235	NM_030705:639	Mesdc1	INSIDE	0.951	2.221	496.80	1103.61	2.112	431.29	910.98
A_68_P29612018	chr14:54980756-54980800	NM_026936:254	Oxa1l	INSIDE	0.951	1.774	864.11	1532.91	1.687	718.72	1212.76
A_68_P23401742	chr4:149029621-149029665	NM_001164052:7783	Pik3cd	INSIDE	0.951	2.400	2923.05	7015.79	2.283	2391.86	5460.81
A_68_P24025453	chr5:118687359-118687403	NM_172998:7490	Rnft2	INSIDE	0.951	2.926	589.17	1724.16	2.784	607.63	1691.77
A_68_P31257220	chr17:56743025-56743069	NM_001163300:18642	Safb	INSIDE	0.951	1.909	1289.90	2462.09	1.815	1019.44	1850.18
A_68_P21099539	chr2:29658324-29658368	NM_011989:147	Slc27a4	INSIDE	0.951	0.619	2874.42	1779.42	0.589	2197.48	1293.98
A_68_P27287199	chr10:80532582-80532627	NM_022653:-223	Thop1	DIVERGENT_PROMOTER	0.951	2.961	231.60	685.65	2.816	225.48	634.86
A_68_P23984450	chr5:111652272-111652316	NM_024477:343473	Ttc28	INSIDE	0.951	1.731	647.17	1120.45	1.646	552.58	909.65
A_68_P25805587	chr8:41508783-41508827	NM_178395:-364	Zdhhc2	PROMOTER	0.951	0.625	1129.17	706.27	0.595	942.09	560.54
A_68_P29614459	chr14:55433246-55433296	ENSMUST00000095853:-2053		PROMOTER	0.951	3.890	420.99	1637.70	3.698	486.05	1797.33
A_68_P28680132	chr12:101369299-101369345	NM_183155:28541	BC002230	INSIDE	0.950	2.143	523.21	1121.15	2.036	411.71	838.16
A_68_P24120339	chr5:136751005-136751049	NM_198602:292249	Cux1	INSIDE	0.950	1.844	1299.39	2395.50	1.752	1079.89	1891.46
A_68_P27188599	chr10:62409837-62409881	NM_177372:82	Dna2	INSIDE	0.950	1.689	1171.92	1979.90	1.605	1063.89	1707.23
A_68_P28947123	chr13:36209265-36209309	NM_001039189:7	Fars2	INSIDE	0.950	1.652	618.92	1022.24	1.570	650.19	1020.61
A_68_P22209918	chr3:67234256-67234300	NM_138591:242	Gfm1	INSIDE	0.950	1.607	1082.84	1740.46	1.527	932.35	1423.71
A_68_P24143542	chr5:141305948-141305992	NM_010302:415	Gna12	INSIDE	0.950	0.560	1264.38	707.97	0.532	1040.87	553.58
A_68_P26085826	chr8:98412091-98412135	NM_010325:153	Got2	INSIDE	0.950	0.630	953.08	600.12	0.598	794.94	475.58
A_68_P29616397	chr14:55732548-55732592	NM_177049:3202	Jph4	INSIDE	0.950	2.187	675.81	1478.30	2.078	644.51	1339.45
A_68_P25954895	chr8:73437847-73437891	NM_173013:7996	Mtap1s	INSIDE	0.950	2.055	878.16	1805.04	1.953	758.42	1481.49
A_68_P29116251	chr13:69672583-69672627	NM_001169131:138	Papd7	INSIDE	0.950	4.069	298.07	1212.90	3.865	319.70	1235.70
A_68_P22614374	chr3:146068079-146068128	NM_138744:453	Ssx2ip	INSIDE	0.950	1.775	2065.99	3667.38	1.687	1556.20	2624.72
A_68_P26948303	chr10:12661129-12661173	NM_001163590:22915	Stx11	INSIDE	0.950	1.690	839.17	1418.24	1.605	735.18	1179.75
A_68_P28755249	chr12:114427080-114427124	NM_153776:2989	Tmem121	INSIDE	0.950	2.139	520.04	1112.54	2.032	460.61	936.02
A_68_P23316766	chr4:132833535-132833579	NM_025667:149	Tmem222	INSIDE	0.950	1.648	1050.47	1731.49	1.565	910.33	1424.80
A_68_P21610507	chr2:126702013-126702057	NM_001164325:-37	Trpm7	PROMOTER	0.950	0.682	1252.94	854.32	0.647	1117.65	723.63
A_68_P32978434	A_68_P32978434			Unknown	0.950	2.538	1628.55	4133.13	2.412	1362.62	3286.50
A_68_P26742255	chr9:95412044-95412088	NM_001114977:349	2610101N10Rik	INSIDE	0.949	0.503	1420.62	714.88	0.477	1209.80	577.56
A_68_P22314995	chr3:88448116-88448160	NM_001198912:22823	Arhgef2	INSIDE	0.949	2.164	488.16	1056.49	2.054	483.02	991.95
A_68_P28051740	chr11:97490459-97490503	NM_175332:550	E130012A19Rik	INSIDE	0.949	2.035	1804.19	3671.74	1.932	1676.51	3238.19
A_68_P21874502	chr2:174124098-174124142	NM_201617:761	Gnas	INSIDE	0.949	1.663	2169.11	3608.23	1.578	1782.20	2813.05
A_68_P32748068	chrX:148579482-148579526	NM_001114664:694	Iqsec2	INSIDE	0.949	1.801	1310.24	2359.55	1.709	738.33	1261.54
A_68_P31150605	chr17:33954480-33954524	NM_030697:7035	Kank3	INSIDE	0.949	2.462	404.17	995.07	2.337	345.74	807.85
A_68_P23752950	chr5:65195357-65195401	NM_008453:617	Klf3	INSIDE	0.949	0.565	948.37	536.21	0.537	780.04	418.58
A_68_P25048197	chr7:35988690-35988734	NM_001024707:11652	Lrp3	INSIDE	0.949	1.793	1306.48	2342.27	1.702	978.70	1665.35
A_68_P31828826	chr18:74130007-74130051	NM_172632:94575	Mapk4	INSIDE	0.949	2.051	603.40	1237.87	1.946	501.29	975.45
A_68_P24090499	chr5:130221338-130221382	NM_025450:-42	Mrps17	DIVERGENT_PROMOTER	0.949	2.952	684.66	2021.29	2.801	646.85	1811.85
A_68_P25360747	chr7:106416712-106416756	NM_001043355:-121	Mtap6	PROMOTER	0.949	1.784	654.16	1166.75	1.693	577.95	978.30
A_68_P25091030	chr7:52331941-52331985	NM_001163684:14164	Nosip	INSIDE	0.949	2.088	934.96	1951.95	1.982	747.48	1481.39
A_68_P23198685	chr4:108673245-108673289	NM_146150:-143	Nrd1	PROMOTER	0.949	1.625	1514.57	2461.00	1.543	1281.69	1977.19
A_68_P27709961	chr11:35821845-35821890	NM_011856:935878	Odz2	INSIDE	0.949	1.790	925.66	1656.62	1.698	763.42	1296.14
A_68_P28224326	chr12:8779101-8779145	NM_011519:921	Sdc1	INSIDE	0.949	0.592	1117.41	661.81	0.562	940.49	528.65
A_68_P26316375	chr9:14081197-14081241	NM_030261:474	Sesn3	INSIDE	0.949	1.473	3222.18	4745.89	1.398	2368.00	3310.02
A_68_P26540064	chr9:56920273-56920317	NM_001110350:-3888	Sin3a	PROMOTER	0.949	1.901	721.72	1371.67	1.803	668.74	1205.53
A_68_P27468584	chr10:114688310-114688354	NM_025706:217	Tbc1d15	INSIDE	0.949	0.726	1912.87	1388.53	0.689	1706.58	1175.99
A_68_P32288520	chrX:20002860-20002904	ENSMUST00000136093:-31		PROMOTER	0.949	2.872	1179.57	3388.24	2.725	868.15	2365.54
A_68_P31939730	chr19:7498138-7498182	NM_175381:6046	2700081O15Rik	INSIDE	0.948	0.539	969.76	522.24	0.510	837.73	427.62
A_68_P29450644	chr14:21895153-21895197	NM_134079:23320	Adk	INSIDE	0.948	1.986	861.34	1710.25	1.882	737.54	1387.95
A_68_P26822031	chr9:110019702-110019746	NM_133347:-1638	Dhx30	PROMOTER	0.948	1.768	1211.54	2142.19	1.676	1108.21	1857.76
A_68_P23553583	chr5:27144126-27144170	NM_010075:252	Dpp6	INSIDE	0.948	0.484	1113.86	538.90	0.459	945.83	433.82

ProbeName	Target position of probe on CpG island microarray	TargetID	GeneSymbol	CpG island Description	Ratio of relative methylation (TiO_2-NP/Vehicle)	Sham group			TiO_2-H group		
						Relative methylation (Cy5/Cy3)	Cy3 signal (Input)	Cy5 signal (MeDIP)	Relative methylation (Cy5/Cy3)	Cy3 signal (Input)	Cy5 signal (MeDIP)
A_68_P32408596	chrX:56387279-56387323	NM_010200:313	Fgf13	INSIDE	0.948	2.347	2716.44	6374.24	2.225	1557.16	3464.98
A_68_P25087544	chr7:51716906-51716950	NM_198250:19072	Lrrc4b	INSIDE	0.948	2.165	1247.86	2701.19	2.053	985.60	2023.04
A_68_P23536419	chr5:22940563-22940607	NM_026984:338	Mll5	INSIDE	0.948	0.610	1181.44	720.77	0.579	936.20	541.73
A_68_P27278628	chr10:79202047-79202091	NM_172551:7258	Polrmt	INSIDE	0.948	2.075	1313.39	2725.53	1.968	1085.54	2136.67
A_68_P31862907	chr18:80397180-80397224	NM_199197:156	Rbfa	INSIDE	0.948	1.621	828.14	1342.67	1.537	713.65	1096.65
A_68_P31196988	chr17:44872166-44872210	NM_001145920:1409	Runx2	INSIDE	0.948	0.593	1390.44	824.05	0.562	1029.09	578.09
A_68_P27228616	chr10:69707646-69707690	NM_025807:-355	Slc16a9	PROMOTER	0.948	0.436	1218.78	531.92	0.414	1025.53	424.23
A_68_P25075290	chr7:48158061-48158105	NM_021387:3435	Vstm2b	INSIDE	0.948	1.871	1151.28	2154.08	1.775	958.72	1701.34
A_68_P28729930	chr12:110032477-110032521	NM_009537:978	Yy1	INSIDE	0.948	1.817	1001.90	1820.51	1.723	872.94	1504.05
A_68_P24631963	chr6:90319610-90319654	NM_030260:145	Zxdc	INSIDE	0.948	1.656	650.36	1077.09	1.570	636.97	1000.00
A_68_P25730016	chr8:27096591-27096635	NM_029037:7981	4930444A02Rik	INSIDE	0.947	2.557	802.93	2053.10	2.420	671.46	1625.27
A_68_P30357051	chr15:77825627-77825674	NM_007583:124060	Cacng2	INSIDE	0.947	3.122	698.41	2180.23	2.958	746.80	2208.75
A_68_P24798777	chr6:120442544-120442588	NM_033567:1259	Cecr6	INSIDE	0.947	0.623	1083.29	674.47	0.589	945.78	557.54
A_68_P21221950	chr2:52717844-52717888	NM_172409:965	Fmnl2	INSIDE	0.947	0.511	1484.84	758.85	0.484	1205.24	583.53
A_68_P31978255	chr19:16207934-16207978	NM_008139:636	Gnaq	INSIDE	0.947	2.497	3619.36	9037.74	2.366	3063.27	7246.82
A_68_P26220842	chr8:123060303-123060347	NM_001145896:-984	Gse1	PROMOTER	0.947	0.653	2028.37	1324.77	0.619	1745.02	1079.87
A_68_P32378027	chrX:49034445-49034489	NM_015819:-812	Hs6st2	PROMOTER	0.947	1.945	846.63	1646.45	1.841	582.14	1071.84
A_68_P23252661	chr4:120370848-120370892	NM_001081142:48911	Kcnq4	INSIDE	0.947	3.449	3645.73	12574.45	3.267	2683.92	8768.79
A_68_P32697224	chrX:136596652-136596696	NM_001081668:433	Nup62cl	INSIDE	0.947	2.098	2824.97	5927.37	1.987	1577.19	3134.01
A_68_P24697527	chr6:101327639-101327683	NM_018884:231	Pdzrn3	INSIDE	0.947	1.637	703.14	1151.28	1.551	652.32	1011.68
A_68_P28100896	chr11:106181914-106181958	NM_133199:28768	Scn4a	INSIDE	0.947	2.129	476.92	1015.42	2.016	381.97	770.16
A_68_P23893507	chr5:92739062-92739106	NM_172713:-34	Sdad1	PROMOTER	0.947	0.613	1081.42	662.41	0.580	898.38	520.93
A_68_P22031740	chr3:30993764-30993808	NM_001039090:-196	Skil	PROMOTER	0.947	2.467	473.40	1167.79	2.336	458.60	1071.50
A_68_P30015973	chr15:11313872-11313916	NM_033074:15519	Tars	INSIDE	0.947	1.980	419.00	829.46	1.874	312.13	585.04
A_68_P32226231	chr19:60657044-60657088	NM_001172096:-140	2700078E11Rik	PROMOTER	0.946	0.663	1307.06	866.11	0.627	1010.77	633.57
A_68_P22142067	chr3:53283022-53283066	NM_173382:15306	2810046L04Rik	INSIDE	0.946	2.409	818.74	1972.34	2.278	753.75	1717.07
A_68_P31096787	chr17:24488644-24488688	NM_001039581:-324	Abca3	DIVERGENT_PROMOTER	0.946	0.612	908.95	556.50	0.579	715.47	414.61
A_68_P24980794	chr7:16899349-16899393	NM_133234:4439	Bbc3	INSIDE	0.946	0.403	1364.78	550.52	0.382	1106.48	422.27
A_68_P27933408	chr11:76486478-76486522	NM_177182:529	Bhlha9	INSIDE	0.946	0.455	1610.89	733.29	0.431	1333.24	574.16
A_68_P26022160	chr8:87356049-87356093	NM_201227:-9348	Dand5	PROMOTER	0.946	0.503	1731.61	871.30	0.476	1431.56	681.49
A_68_P28036777	chr11:94982683-94982727	NM_010055:1274	Dlx3	INSIDE	0.946	0.596	1243.10	740.84	0.564	1090.73	614.81
A_68_P27093483	chr10:41917159-41917203	NM_019740:79368	Foxo3	INSIDE	0.946	1.894	1674.37	3170.51	1.792	1248.38	2236.58
A_68_P31515587	chr18:15222623-15222667	NM_001142731:-553	Kctd1	PROMOTER	0.946	2.206	1302.36	2873.42	2.087	1080.43	2254.95
A_68_P24921913	chr6:145198164-145198208	NM_021284:565	Kras	INSIDE	0.946	0.713	1827.39	1302.38	0.674	1510.40	1018.27
A_68_P25025265	chr7:29870930-29870974	NR_035489:-2239	Mir1963	PROMOTER	0.946	2.665	705.36	1879.64	2.521	621.96	1567.73
A_68_P28961841	chr13:38726802-38726846	NM_139063:154	Muted	INSIDE	0.946	0.497	2137.73	1061.59	0.470	1669.08	784.06
A_68_P31110246	chr17:26576080-26576124	NM_001081656:24193	Neurl1b	INSIDE	0.946	1.773	745.70	1322.39	1.677	688.70	1154.89
A_68_P27261657	chr10:75837670-75837714	NM_008787:67965	Pcnt	INSIDE	0.946	3.115	792.24	2468.18	2.948	773.42	2280.40
A_68_P25169318	chr7:69609337-69609381	NM_013788:38	Peg12	INSIDE	0.946	3.281	735.56	2413.63	3.106	744.26	2311.47
A_68_P21005392	chr2:11212245-11212289	NM_008859:118258	Prkcq	INSIDE	0.946	2.137	735.89	1572.82	2.023	631.92	1278.11
A_68_P28159661	chr11:116351482-116351526	NM_026364:156	Prpsap1	INSIDE	0.946	3.643	7548.92	27497.03	3.446	5932.00	20439.50
A_68_P26540069	chr9:56920831-56920875	NM_001110350:-3330	Sin3a	PROMOTER	0.946	1.459	2615.39	3815.11	1.380	2020.45	2788.38
A_68_P21682258	chr2:139892550-139892594	NM_001159640:-51	Tasp1	PROMOTER	0.946	0.610	1467.19	894.95	0.577	1226.02	707.29
A_68_P21747902	chr2:151968924-151968968	NM_009328:-398	Tcf15	PROMOTER	0.946	0.621	1002.71	622.91	0.587	803.39	471.93
A_68_P22860681	chr4:41082365-41082409	NM_026275:-667	Ube2r2	PROMOTER	0.946	2.082	2362.25	4917.10	1.970	2012.58	3964.55
A_68_P32753662	chrX:149932604-149932648	NM_018798:-148	Ubqln2	PROMOTER	0.946	2.655	486.17	1291.01	2.511	283.40	711.64
A_68_P31087860	chr17:22058016-22058060	NM_001199048:41393	Zfp942	DOWNSTREAM	0.946	2.173	1132.14	2460.65	2.055	1150.69	2364.87
A_68_P24449794	chr6:52256292-52256336	NR_038163:8512	5730457N03Rik	DOWNSTREAM	0.945	0.588	1786.96	1051.20	0.556	1363.13	758.06
A_68_P25430230	chr7:120513248-120513292	NM_133700:-417	Btbd10	PROMOTER	0.945	2.884	1010.02	2912.97	2.725	953.11	2597.00
A_68_P26581355	chr9:64188489-64188533	NM_172519:-246	Dis3l	PROMOTER	0.945	0.524	1460.70	765.96	0.496	1186.66	588.05
A_68_P30588851	chr16:20672864-20672908	NM_001005331:65	Eif4g1	INSIDE	0.945	0.596	2425.81	1445.29	0.563	1983.47	1116.34

ProbeName	Target position of probe on CpG island microarray	TargetID	GeneSymbol	CpG island Description	Ratio of relative methylation (TiO_2-NP/Vehicle)	Sham group			TiO_2-H group		
						Relative methylation (Cy5/Cy3)	Cy3 signal (Input)	Cy5 signal (MeDIP)	Relative methylation (Cy5/Cy3)	Cy3 signal (Input)	Cy5 signal (MeDIP)
A_68_P26224694	chr8:123651817-123651861	NM_008024:254	Foxl1	INSIDE	0.945	0.587	1549.82	910.50	0.555	1383.76	768.08
A_68_P20679834	chr1:145586536-145586580	NM_001038592:-139	Glrx2	PROMOTER	0.945	2.550	1450.77	3699.63	2.410	1244.38	2999.39
A_68_P24131923	chr5:139297423-139297474	NM_001195128:1415	Gm5294	INSIDE	0.945	0.615	3755.96	2308.38	0.581	2559.10	1486.48
A_68_P24176695	chr5:148768968-148769012	NM_029920:95	Mtus2	INSIDE	0.945	0.506	1537.91	777.52	0.478	1269.36	606.38
A_68_P21078907	chr2:26321004-26321048	NM_008714:38316	Notch1	INSIDE	0.945	2.106	1488.18	3134.76	1.991	1104.51	2199.57
A_68_P27833619	chr11:58752222-58752266	NM_022423:164	Rnf187	INSIDE	0.945	2.634	464.91	1224.41	2.490	437.20	1088.45
A_68_P22049380	chr3:34548131-34548175	NM_011443:-774	Sox2	PROMOTER	0.945	1.760	839.32	1476.83	1.663	656.92	1092.73
A_68_P27985859	chr11:85699856-85699900	NM_011536:-3686	Tbx4	PROMOTER	0.945	1.881	2279.64	4287.94	1.777	1920.82	3413.98
A_68_P21612304	chr2:127073616-127073660	NM_175145:-72	Tmem127	DIVERGENT_PROMOTER	0.945	24.852	5414.66	134564.50	23.497	3681.31	86499.55
A_68_P31155255	chr17:34817289-34817333	NM_031176:9831	Tnxb	INSIDE	0.945	1.721	1001.58	1723.96	1.627	759.94	1236.25
A_68_P22168172	chr3:58220477-58220521	NM_001081229:888	Tsc22d2	INSIDE	0.945	1.751	807.72	1414.13	1.655	739.43	1223.44
A_68_P29081872	chr13:59924315-59924359	NM_153538:172	Zcchc6	INSIDE	0.945	1.601	1031.74	1651.67	1.513	928.02	1403.84
A_68_P26233343	chr8:124956522-124956566	NM_007806:296	Cyba	INSIDE	0.944	1.813	777.61	1409.71	1.711	731.03	1250.78
A_68_P26659375	chr9:78243556-78243600	NM_001191044:-5	Ddx43	PROMOTER	0.944	3.376	724.26	2445.23	3.186	759.30	2419.45
A_68_P24795426	chr6:119797651-119797695	NM_053204:496	Erc1	INSIDE	0.944	1.456	6328.99	9215.64	1.375	4864.59	6689.62
A_68_P24990861	chr7:19590354-19590398	NM_178757:963	Irf2bp1	INSIDE	0.944	2.185	636.71	1391.04	2.062	461.79	952.33
A_68_P28185783	chr11:120494397-120494441	NM_010756:443	Mafg	INSIDE	0.944	1.615	805.42	1300.70	1.524	836.72	1275.08
A_68_P28875768	chr13:22097992-22098036	NM_019429:3596	Prss16	INSIDE	0.944	1.533	1141.69	1749.83	1.447	996.14	1441.49
A_68_P21115159	chr2:32252005-32252049	NM_133783:617	Ptges2	INSIDE	0.944	2.056	756.72	1555.81	1.942	694.65	1348.76
A_68_P30496912	chr15:102087767-102087811	NM_011244:126	Rarg	INSIDE	0.944	1.689	1292.35	2182.69	1.594	1127.42	1796.95
A_68_P25499765	chr7:133435758-133435803	NM_145587:19648	Sbk1	INSIDE	0.944	2.339	468.23	1095.39	2.208	434.13	958.77
A_68_P29699984	chr14:70605667-70605711	NM_011366:1756	Sorbs3	INSIDE	0.944	1.707	797.44	1361.28	1.611	627.37	1010.57
A_68_P30250398	chr15:58654714-58654758	NM_175212:246	Tmem65	INSIDE	0.944	2.123	577.90	1227.09	2.005	528.23	1059.01
A_68_P24956428	chr7:6649272-6649316	NM_011769:-151	Zim1	PROMOTER	0.944	1.981	468.49	927.94	1.870	433.58	810.59
A_68_P23093077	chr4:88583855-88583899			Unknown	0.944	1.678	1118.47	1876.96	1.584	903.53	1431.23
A_68_P23747547	chr5:64203832-64203876	NM_029554:121	0610040J01Rik	INSIDE	0.943	2.505	463.36	1160.70	2.362	410.75	970.19
A_68_P22832217	chr4:34663673-34663717	NM_027041:13761	1700003M02Rik	INSIDE	0.943	2.878	323.36	930.79	2.715	270.54	734.41
A_68_P31124400	chr17:28943442-28943486	NM_001081315:5394	Brpf3	INSIDE	0.943	1.609	1023.69	1647.24	1.518	758.95	1151.74
A_68_P24610262	chr6:86422458-86422502	NR_027370:33841	C87436	DOWNSTREAM	0.943	1.442	3397.02	4898.03	1.360	2426.23	3299.22
A_68_P27172918	chr10:59649296-59649341	NM_016803:32690	Chst3	INSIDE	0.943	1.922	663.70	1275.84	1.813	487.75	884.24
A_68_P26017804	chr8:86566375-86566419	NM_016671:78	Il27ra	INSIDE	0.943	0.695	1628.08	1131.01	0.655	1304.68	854.36
A_68_P33007165	chr8:121970673-121970717	NM_054095:76	Necab2	INSIDE	0.943	0.679	1627.56	1104.46	0.640	1443.05	923.56
A_68_P21098346	chr2:29475741-29475785	NM_001039086:523	Rapgef1	INSIDE	0.943	0.431	1349.14	581.07	0.406	1137.69	462.01
A_68_P26823381	chr9:110283194-110283238	NM_001001144:47420	Scap	INSIDE	0.943	2.046	2323.51	4752.96	1.929	1818.29	3507.89
A_68_P22323187	chr3:89856233-89856277	NM_028475:143	Ubap2l	INSIDE	0.943	1.791	883.68	1583.05	1.689	550.99	930.46
A_68_P32462209	chrX:70599489-70599533	NM_001160229:11551	Zfp275	INSIDE	0.943	2.784	440.74	1227.21	2.625	246.32	646.50
A_68_P24955007	chr7:6282885-6282929	NM_001033249:-870	Zfp583	PROMOTER	0.943	0.413	1286.23	531.11	0.389	1166.04	453.82
A_68_P23593510	chr5:34631368-34631412	NM_001015039:-417	Zfyve28	PROMOTER	0.943	0.487	1090.78	531.29	0.459	924.19	424.44
A_68_P28175593	chr11:118919618-118919662			Unknown	0.943	0.513	1413.07	724.85	0.484	1174.46	568.05
A_68_P29998441	chr15:8118989-8119033	NM_001162906:-95	2410089E03Rik	PROMOTER	0.942	3.178	846.38	2689.70	2.995	756.40	2265.47
A_68_P27262974	chr10:76059438-76059482	NM_146007:26689	Col6a2	INSIDE	0.942	2.102	2516.49	5290.32	1.980	1808.44	3580.44
A_68_P31227872	chr17:50433053-50433097	NM_010021:-150	Dazl	PROMOTER	0.942	1.816	1051.38	1909.44	1.710	818.52	1399.97
A_68_P27612237	chr11:16652694-16652738	NM_007912:511	Egfr	INSIDE	0.942	0.501	1344.60	673.73	0.472	1170.83	552.86
A_68_P20644879	chr1:138157097-138157141	NM_001101516:332	Gpr25	INSIDE	0.942	2.683	228.53	613.12	2.528	235.83	596.15
A_68_P23753094	chr5:65213362-65213406	NM_008453:18623	Klf3	INSIDE	0.942	2.863	2103.00	6021.37	2.697	1693.60	4567.94
A_68_P31130219	chr17:29989367-29989411	NM_001081160:35439	Mdga1	INSIDE	0.942	2.105	2321.67	4886.79	1.982	1727.19	3422.96
A_68_P22373051	chr3:100881021-100881065	NM_011197:33047	Ptgfrn	INSIDE	0.942	2.037	1378.53	2807.74	1.919	1035.87	1988.00
A_68_P23457020	chr5:5694251-5694295	NM_001103156:-195	Steap2	PROMOTER	0.942	0.688	3441.84	2368.77	0.648	2758.68	1788.69
A_68_P32700064	chrX:137133979-137134023	NM_001077364:1061	Tsc22d3	INSIDE	0.942	1.946	760.45	1480.05	1.833	494.69	906.81
A_68_P30716739	chr16:43618530-43618574	NM_019778:371156	Zbtb20	INSIDE	0.942	2.334	388.90	907.50	2.199	345.38	759.57
A_68_P25264699	chr7:87226574-87226619	NM_175433:55483	Zfp710	INSIDE	0.942	4.519	1354.71	6122.23	4.259	939.94	4003.00

ProbeName	Target position of probe on CpG island microarray	TargetID	GeneSymbol	CpG island Description	Ratio of relative methylation (TiO_2-NP/Vehicle)	Sham group			TiO_2-H group		
						Relative methylation (Cy5/Cy3)	Cy3 signal (Input)	Cy5 signal (MeDIP)	Relative methylation (Cy5/Cy3)	Cy3 signal (Input)	Cy5 signal (MeDIP)
A_68_P32975877	A_68_P32975877			Unknown	0.942	1.893	1860.06	3521.33	1.784	1384.30	2469.45
A_68_P28741419	chr12:112215560-112215604	NM_175207:1649	Ankrd9	INSIDE	0.941	1.626	793.35	1289.94	1.531	611.01	935.23
A_68_P27143036	chr10:53317089-53317133	NM_025541:344	Asf1a	INSIDE	0.941	1.458	2012.07	2934.53	1.372	1630.53	2237.28
A_68_P28525477	chr12:72411137-72411181	NM_001190466:288	Dact1	INSIDE	0.941	2.192	619.26	1357.53	2.063	506.29	1044.40
A_68_P32006563	chr19:21547067-21547111	NM_010266:63	Gda	INSIDE	0.941	1.768	627.23	1108.87	1.664	557.66	928.16
A_68_P24636903	chr6:91066915-91066959	NM_018815:-116	Nup210	PROMOTER	0.941	0.454	1336.05	606.29	0.427	1065.66	454.90
A_68_P30587014	chr16:20302209-20302253	NM_001005767:205	Parl	INSIDE	0.941	0.437	1815.83	792.98	0.411	1510.87	621.17
A_68_P24759852	chr6:113445089-113445133	NM_172487:6702	Prrt3	INSIDE	0.941	1.771	1120.24	1984.17	1.667	949.71	1582.89
A_68_P20427304	chr1:90967161-90967205	NM_133816:146	Sh3bp4	INSIDE	0.941	0.657	988.01	648.98	0.618	945.76	584.65
A_68_P24111847	chr5:134652378-134652422	NM_033572:237	Wbscr16	INSIDE	0.941	0.528	1901.87	1003.70	0.497	1656.34	822.63
A_68_P28553591	chr12:77470382-77470426	NM_001172104:47	Zbtb25	INSIDE	0.941	2.515	892.11	2243.53	2.368	891.52	2110.76
A_68_P29224475	chr13:93300495-93300539	NM_173392:249	Zfyve16	INSIDE	0.941	0.704	2025.36	1426.24	0.662	1802.78	1194.08
A_68_P33005898	A_68_P33005898			Unknown	0.941	3.460	240.81	833.25	3.256	260.17	847.15
A_68_P27883391	chr11:67269235-67269279	ENSMUST00000136796:31		INSIDE	0.941	0.385	1467.72	564.62	0.362	1146.14	414.74
A_68_P25594226	chr7:149558251-149558295	NM_177265:-108	6330512M04Rik	PROMOTER	0.940	3.184	425.70	1355.52	2.993	390.45	1168.47
A_68_P28050586	chr11:97301883-97301927	NM_021493:-9569	Arhgap23	PROMOTER	0.940	1.598	1122.42	1794.16	1.502	997.97	1499.32
A_68_P24098743	chr5:132014457-132014501	NM_177047:1003735	Auts2	INSIDE	0.940	0.583	1364.72	795.42	0.548	1102.83	604.53
A_68_P27254388	chr10:74523771-74523815	NM_001081412:152	Bcr	INSIDE	0.940	3.177	694.76	2207.09	2.987	739.58	2209.46
A_68_P27100736	chr10:43231246-43231290	NM_199028:32323	Bend3	INSIDE	0.940	3.083	694.43	2140.99	2.897	666.21	1930.18
A_68_P28743718	chr12:112615438-112615482	NM_183016:469	Cdc42bpb	INSIDE	0.940	1.894	626.13	1185.97	1.781	532.96	949.31
A_68_P26224343	chr8:123606757-123606801	NM_010426:-1595	Foxf1a	PROMOTER	0.940	0.497	2217.09	1100.96	0.467	1745.45	814.80
A_68_P25151569	chr7:64846153-64846197	NM_008071:-368	Gabrb3	PROMOTER	0.940	0.518	2187.00	1132.17	0.486	1706.30	830.00
A_68_P25891572	chr8:59990950-59990994	NM_008252:333	Hmgb2	INSIDE	0.940	2.112	1066.80	2253.47	1.985	881.81	1750.02
A_68_P23985682	chr5:111849354-111849398	NM_001081235:2191	Mn1	INSIDE	0.940	2.106	673.48	1418.48	1.979	605.51	1198.55
A_68_P23429470	chr4:153534460-153534504	NM_026388:311	Tprgl	INSIDE	0.940	2.046	9904.95	20266.06	1.922	7726.65	14853.14
A_68_P30665800	chr16:34745530-34745574	ENSMUST00000155268:257		INSIDE	0.940	2.629	260.28	684.36	2.472	242.63	599.77
A_68_P24172798	chr5:148086398-148086442	NR_038157:-3323	2210019I11Rik	PROMOTER	0.939	0.494	1607.45	794.38	0.464	1341.10	622.45
A_68_P27288887	chr10:80787956-80788000	NM_027381:4131	2510012J08Rik	INSIDE	0.939	1.794	766.38	1374.67	1.684	580.83	977.83
A_68_P21646214	chr2:133377926-133377970	NM_007553:-986	Bmp2	PROMOTER	0.939	3.550	300.44	1066.54	3.335	307.00	1023.73
A_68_P30404602	chr15:85861703-85861747	NM_009886:2483	Celsr1	INSIDE	0.939	1.756	734.87	1290.40	1.648	577.03	951.18
A_68_P26526448	chr9:54407958-54408002	NM_019686:-94	Cib2	PROMOTER	0.939	2.155	860.64	1854.25	2.023	754.91	1527.00
A_68_P22317611	chr3:88972711-88972761	NM_001163432:4010	Clk2	INSIDE	0.939	2.947	1251.65	3688.96	2.767	898.68	2486.30
A_68_P28576092	chr12:81620747-81620791	NM_001081421:792	Galntl1	INSIDE	0.939	0.571	1472.51	840.98	0.536	1193.43	639.99
A_68_P26035032	chr8:89996819-89996863	NR_033430:130	Gm2694	INSIDE	0.939	0.691	1843.43	1273.49	0.649	1475.11	957.12
A_68_P25394035	chr7:112885416-112885460	NM_010562:200	Ilk	INSIDE	0.939	0.617	1056.24	652.07	0.580	746.90	433.14
A_68_P22129521	chr3:51220268-51220312	NM_053089:353	Naa15	INSIDE	0.939	0.554	1466.45	812.38	0.520	1114.27	579.70
A_68_P32147396	chr19:47124826-47124870	NM_027654:487	Pcgf6	INSIDE	0.939	0.644	1518.78	977.47	0.604	1259.92	761.24
A_68_P31121662	chr17:28434204-28434248	NM_011145:64528	Ppard	INSIDE	0.939	2.365	562.99	1331.48	2.220	509.14	1130.42
A_68_P32183828	chr19:53752058-53752102	NM_001170847:285	Rbm20	INSIDE	0.939	2.127	508.92	1082.35	1.997	425.19	849.10
A_68_P25588800	chr7:148623401-148623445	NM_001177576:-2929	Slc25a22	PROMOTER	0.939	0.650	1965.89	1277.03	0.610	1442.70	879.97
A_68_P24320421	chr6:28430461-28430505	NM_019776:135	Snd1	INSIDE	0.939	1.776	1401.21	2488.93	1.667	1109.64	1850.05
A_68_P29478589	chr14:26426247-26426291	NM_183208:147598	Zmiz1	INSIDE	0.939	0.609	1480.53	900.95	0.571	1087.21	621.27
A_68_P27360776	chr10:94151341-94151385	NR_015524:-4	4932415G12Rik	DIVERGENT_PROMOTER	0.938	6.981	1292.11	9020.13	6.550	1011.53	6625.23
A_68_P29049158	chr13:54472997-54473041	NM_009946:306	Cplx2	INSIDE	0.938	0.694	1999.40	1387.98	0.651	1800.75	1172.88
A_68_P29611636	chr14:54873516-54873560	NM_001113358:66	Dad1	INSIDE	0.938	0.385	1904.44	733.75	0.361	1358.99	491.17
A_68_P23365989	chr4:141346549-141346593	NM_172338:-11	Dnajc16	DIVERGENT_PROMOTER	0.938	2.433	490.62	1193.88	2.283	512.72	1170.76
A_68_P28329060	chr12:31758731-31758776	NM_027828:-79	Fam110c	PROMOTER	0.938	0.704	1708.18	1202.26	0.660	1320.20	871.64
A_68_P23320288	chr4:133523806-133523851	NM_016957:78	Hmgn2	INSIDE	0.938	2.021	591.76	1196.07	1.896	465.46	882.40
A_68_P28204868	chr12:5216846-5216890	NM_001164493:165620	Klhl29	INSIDE	0.938	2.283	1485.62	3391.05	2.141	1312.03	2809.20
A_68_P25031175	chr7:31140774-31140818	NM_175478:6995	Lrfn3	INSIDE	0.938	2.359	656.86	1549.79	2.213	559.69	1238.72
A_68_P21776626	chr2:157193205-157193256	NM_026968:-99	Manbal	PROMOTER	0.938	2.343	438.75	1027.93	2.198	324.14	712.58

ProbeName	Target position of probe on CpG island microarray	TargetID	GeneSymbol	CpG island Description	Ratio of relative methylation (TiO_2-NP/Vehicle)	Sham group			TiO_2-H group		
						Relative methylation (Cy5/Cy3)	Cy3 signal (Input)	Cy5 signal (MeDIP)	Relative methylation (Cy5/Cy3)	Cy3 signal (Input)	Cy5 signal (MeDIP)
A_68_P30425291	chr15:89288179-89288223	NM_021921:3859	Mapk8ip2	INSIDE	0.938	2.722	1156.29	3147.71	2.554	1011.50	2582.99
A_68_P27787485	chr11:50048388-50048432	NM_145926:9574	Mgat4b	INSIDE	0.938	2.287	658.22	1505.65	2.145	588.42	1262.21
A_68_P23439845	chr4:155027518-155027562	NM_011985:-47	Mmp23	PROMOTER	0.938	0.658	1282.06	843.71	0.617	1014.34	625.97
A_68_P21818281	chr2:164332907-164332953	NM_133779:9906	Pigt	INSIDE	0.938	3.599	2072.70	7458.85	3.374	1373.13	4633.39
A_68_P30423624	chr15:88998188-88998232	NM_001159521:6070	Plxnb2	INSIDE	0.938	2.078	394.84	820.55	1.949	398.88	777.55
A_68_P21261714	chr2:60800138-60800182	NM_020296:1101	Rbms1	INSIDE	0.938	0.649	1591.36	1032.82	0.609	1264.91	769.77
A_68_P31067775	chr17:15968105-15968150	NM_178615:-4577	Rgmb	PROMOTER	0.938	1.714	1439.57	2467.33	1.608	1291.47	2076.14
A_68_P20165363	chr1:39424634-39424678	NM_053257:-39	Rpl31	PROMOTER	0.938	0.592	3993.95	2363.20	0.555	3084.49	1711.13
A_68_P29614999	chr14:55531847-55531891	NM_021551:101	Slc22a17	INSIDE	0.938	0.679	1894.38	1287.19	0.637	1647.96	1050.22
A_68_P30964218	chr16:91660311-91660368	NM_019973:12271	Son	INSIDE	0.938	3.343	421.83	1410.38	3.137	362.66	1137.51
A_68_P26363428	chr9:24578465-24578509	NM_194263:261	Tbx20	INSIDE	0.938	1.490	1627.26	2424.94	1.397	1295.62	1810.08
A_68_P23240688	chr4:118159059-118159103	NM_011587:3374	Tie1	INSIDE	0.938	2.368	1221.78	2893.54	2.222	997.05	2215.06
A_68_P27056140	chr10:34001966-34002010	NM_009433:-7	Tspyl1	PROMOTER	0.938	1.563	1500.06	2344.63	1.466	1331.59	1951.57
A_68_P27186305	chr10:61949424-61949468	NM_001113355:-100	Vps26a	PROMOTER	0.938	0.550	1760.19	968.97	0.516	1636.84	845.10
A_68_P24424641	chr6:47816485-47816534	NM_027477:30850	Zfp398	INSIDE	0.938	2.112	455.96	962.78	1.981	379.81	752.54
A_68_P24424642	chr6:47816553-47816601	NM_027477:30917	Zfp398	INSIDE	0.938	2.422	373.19	903.88	2.273	305.03	693.27
A_68_P30796632	chr16:59600772-59600816			Unknown	0.938	3.041	295.36	898.13	2.852	286.72	817.81
A_68_P24541219	chr6:72297667-72297711	NM_026696:378	0610030E20Rik	INSIDE	0.937	0.668	4380.83	2925.49	0.626	3699.06	2314.44
A_68_P30941181	chr16:87699207-87699251	NM_007520:30	Bach1	INSIDE	0.937	0.725	4884.89	3542.67	0.680	4048.14	2751.72
A_68_P27489857	chr10:118677199-118677243	NM_027994:-109	Cand1	PROMOTER	0.937	0.577	1470.72	847.95	0.540	1333.66	720.51
A_68_P31125929	chr17:29231859-29231903	NM_007669:1164	Cdkn1a	INSIDE	0.937	0.527	1029.13	542.00	0.493	822.09	405.52
A_68_P27263123	chr10:76077247-76077291	NM_146007:8881	Col6a2	INSIDE	0.937	2.296	1694.67	3891.28	2.152	1389.29	2990.21
A_68_P26715672	chr9:89949316-89949360	NM_007801:234	Ctsh	INSIDE	0.937	0.723	2180.24	1576.89	0.678	1842.92	1248.80
A_68_P27271712	chr10:77497354-77497398	NM_001081695:-7655	Dnmt3l	PROMOTER	0.937	3.002	1086.70	3261.85	2.812	968.73	2724.14
A_68_P25641011	chr8:9207733-9207777	NM_173446:563269	Fam155a	INSIDE	0.937	3.319	310.90	1031.91	3.109	278.82	866.98
A_68_P32241740	chrX:7674050-7674094	NR_003632:-140	Gm6787	DIVERGENT_PROMOTER	0.937	1.980	1381.70	2735.60	1.856	733.67	1361.36
A_68_P29356808	chr13:117099881-117099929	NM_021459:-8	Isl1	PROMOTER	0.937	0.512	1546.76	791.45	0.479	1258.06	603.15
A_68_P29054505	chr13:55452830-55452879	NM_025828:11290	Lman2	INSIDE	0.937	2.208	562.31	1241.77	2.070	513.04	1062.09
A_68_P24990780	chr7:19576294-19576338	NM_145579:-277	Mypop	PROMOTER	0.937	2.314	374.98	867.62	2.169	355.93	772.02
A_68_P26824898	chr9:110540931-110540975	NM_183276:15713	Nbeal2	INSIDE	0.937	1.873	693.29	1298.55	1.755	637.89	1119.29
A_68_P31629941	chr18:37926527-37926571	NM_033595:1315	Pcdhga12	INSIDE	0.937	1.884	771.43	1453.18	1.765	703.20	1241.41
A_68_P31236181	chr17:51952553-51952597	NM_001163630:-1195	Satb1	PROMOTER	0.937	2.753	402.23	1107.43	2.581	394.86	1019.18
A_68_P30979599	chr16:94347306-94347350	NM_011377:261824	Sim2	INSIDE	0.937	3.122	497.54	1553.49	2.927	515.43	1508.75
A_68_P22324940	chr3:90193568-90193612	NM_011988:259	Slc27a3	INSIDE	0.937	2.306	459.79	1060.23	2.160	506.46	1094.18
A_68_P28550370	chr12:76919981-76920025	NM_001005510:698	Syne2	INSIDE	0.937	0.376	2522.86	949.35	0.353	2087.13	736.02
A_68_P31926782	chr19:5082847-5082891	NM_001001885:2609	Tmem151a	INSIDE	0.937	3.548	882.26	3130.47	3.326	808.60	2689.12
A_68_P25393299	chr7:112766364-112766408	NM_018880:15627	Trim3	INSIDE	0.937	2.642	851.71	2250.06	2.476	718.54	1778.87
A_68_P32791134	chrX:159266854-159266898	NM_178935:455	Txlng	INSIDE	0.937	2.277	6885.64	15678.71	2.133	3336.07	7114.19
A_68_P24630035	chr6:89785240-89785284	NM_053221:10269	Vmn1r42	DOWNSTREAM	0.937	3.436	600.88	2064.41	3.220	554.95	1786.73
A_68_P23244792	chr4:118967244-118967289	NM_011732:-148	Ybx1	PROMOTER	0.937	1.855	3083.95	5719.25	1.737	1938.79	3368.39
A_68_P24118629	chr5:136410508-136410552	NM_018871:-19	Ywhag	PROMOTER	0.937	2.420	1234.43	2987.44	2.267	985.74	2234.22
A_68_P25030073	chr7:30928817-30928863	NM_001081007:21874	Zfp382	DOWNSTREAM	0.937	3.328	614.01	2043.25	3.119	441.65	1377.62
A_68_P24142834	chr5:141180937-141180981	NM_172724:-18	Baat1	DIVERGENT_PROMOTER	0.936	1.705	2460.35	4194.40	1.596	1895.74	3025.52
A_68_P31158872	chr17:35457951-35457995	NM_010390:470	H2-Q1	INSIDE	0.936	0.566	1044.41	590.81	0.529	918.60	486.28
A_68_P27285763	chr10:80298067-80298111	NM_001013758:8696	Lingo3	INSIDE	0.936	2.028	638.15	1294.23	1.898	564.92	1072.28
A_68_P25031174	chr7:31140698-31140742	NM_175478:7071	Lrfn3	INSIDE	0.936	2.191	464.69	1017.98	2.050	428.31	878.23
A_68_P24008162	chr5:115607960-115608004	NM_175403:203	Mlec	INSIDE	0.936	2.904	7081.42	20564.08	2.717	5209.97	14154.52
A_68_P27854122	chr11:62270378-62270422	NM_011308:434	Ncor1	INSIDE	0.936	0.686	2475.11	1697.00	0.642	1891.41	1213.61
A_68_P23253519	chr4:120498120-120498164	NM_008692:178	Nfyc	INSIDE	0.936	0.647	963.60	623.07	0.605	831.37	502.91
A_68_P28683723	chr12:101962977-101963021	NM_153587:240	Rps6ka5	INSIDE	0.936	0.630	1066.04	671.42	0.590	909.81	536.44
A_68_P26783276	chr9:102911099-102911143	NM_033314:302	Slco2a1	INSIDE	0.936	0.589	1020.16	601.37	0.552	903.70	498.77

ProbeName	Target position of probe on CpG island microarray	TargetID	GeneSymbol	CpG island Description	Ratio of relative methylation (TiO_2-NP/Vehicle)	Sham group			TiO_2-H group		
						Relative methylation (Cy5/Cy3)	Cy3 signal (Input)	Cy5 signal (MeDIP)	Relative methylation (Cy5/Cy3)	Cy3 signal (Input)	Cy5 signal (MeDIP)
A_68_P27098030	chr10:42741219-42741263	NM_175407:153096	Sobp	INSIDE	0.936	5.340	375.61	2005.75	4.998	362.83	1813.31
A_68_P28614749	chr12:88541116-88541160	NM_001033475:41	Tmed8	INSIDE	0.936	2.255	472.30	1065.01	2.110	453.31	956.47
A_68_P26894029	chr9:123169283-123169327	NM_001002267:603	Tmem158	INSIDE	0.936	2.848	821.54	2339.84	2.665	868.02	2313.21
A_68_P29260159	chr13:99660900-99660944	NM_001048267:75	Tnpo1	INSIDE	0.936	1.803	1558.28	2810.13	1.688	1428.62	2410.99
A_68_P27779788	chr11:48567252-48567296	ENSMUST00000120566:-58		PROMOTER	0.936	2.510	1291.20	3241.30	2.350	1089.83	2560.82
A_68_P24648069	chr6:92893420-92893464	NR_015530:2867	9530026P05Rik	INSIDE	0.935	2.223	648.74	1442.12	2.078	649.95	1350.92
A_68_P28731507	chr12:110272513-110272557	NM_001163175:33893	Begain	INSIDE	0.935	1.815	1673.06	3036.33	1.698	1227.85	2084.55
A_68_P28175417	chr11:118898832-118898876	NM_013926:3373	Cbx8	DOWNSTREAM	0.935	1.706	1775.60	3028.48	1.595	1676.58	2673.53
A_68_P23343156	chr4:137550305-137550349	NM_172703:942	Eif4g3	INSIDE	0.935	1.938	527.20	1021.48	1.812	402.54	729.51
A_68_P23335793	chr4:136326759-136326803	NM_010142:65070	Ephb2	INSIDE	0.935	2.346	1211.20	2841.44	2.193	1012.31	2219.81
A_68_P22324052	chr3:90035906-90035950	NM_206924:410	Jtb	INSIDE	0.935	1.731	1141.67	1975.86	1.618	940.23	1521.04
A_68_P21842277	chr2:168397045-168397089	NM_001136073:18625	Nfatc2	INSIDE	0.935	1.905	620.83	1182.57	1.781	499.77	890.31
A_68_P24209922	chr6:5248441-5248485	NM_183308:-89	Pon2	PROMOTER	0.935	0.470	3231.83	1519.48	0.439	2566.20	1127.64
A_68_P23974099	chr5:109203826-109203870	NM_026109:20534	Tmed11	DOWNSTREAM	0.935	2.850	1603.10	4568.82	2.666	1266.35	3375.87
A_68_P26154297	chr8:111474389-111474435	NM_007496:235869	Zfhx3	INSIDE	0.935	1.628	1147.23	1867.33	1.522	809.30	1232.06
A_68_P24428349	chr6:48570036-48570080	NM_173429:6880	Zfp775	INSIDE	0.935	1.984	1028.78	2041.22	1.855	703.44	1304.96
A_68_P31461137	chr18:4052694-4052738			Unknown	0.935	2.136	793.43	1694.72	1.998	711.94	1422.47
A_68_P23999131	chr5:114064213-114064257	NM_008153:36165	Cmklr1	INSIDE	0.934	5.924	1827.40	10825.68	5.532	1166.26	6451.88
A_68_P31927966	chr19:5307193-5307237	NM_001024717:8884	Gal3st3	INSIDE	0.934	1.984	1127.02	2235.73	1.852	899.33	1665.99
A_68_P26804349	chr9:106549876-106549920	NM_001160353:8542	Grm2	INSIDE	0.934	3.614	2231.27	8064.18	3.377	1690.72	5709.25
A_68_P26346083	chr9:21038393-21038437	NM_001110305:5362	Keap1	INSIDE	0.934	2.544	1105.00	2811.50	2.376	919.04	2183.83
A_68_P25595364	chr7:149718822-149718866	NM_011288:-177	Mrpl23	PROMOTER	0.934	1.724	6237.18	10749.81	1.609	4530.69	7289.82
A_68_P30346546	chr15:76003525-76003570	NM_201394:22593	Plec	INSIDE	0.934	2.739	704.59	1930.14	2.560	676.47	1731.43
A_68_P32467532	chrX:71574336-71574380	NM_008883:-46	Plxna3	PROMOTER	0.934	1.948	691.33	1346.47	1.819	358.24	651.48
A_68_P25090600	chr7:52262845-52262889	NM_001008422:8753	Scaf1	INSIDE	0.934	2.426	1818.93	4413.07	2.267	1230.69	2790.08
A_68_P20354814	chr1:75542871-75542915	NM_009208:52	Slc4a3	INSIDE	0.934	4.528	2101.91	9518.27	4.228	1820.53	7696.53
A_68_P27355934	chr10:93296766-93296810	NM_183199:2489	Usp44	INSIDE	0.934	0.552	1809.82	999.40	0.516	1590.17	820.14
A_68_P27287690	chr10:80607523-80607567	NM_010731:8529	Zbtb7a	INSIDE	0.934	2.216	1502.36	3329.37	2.070	1182.19	2447.57
A_68_P24155560	chr5:143997116-143997160	NM_177681:252	Zfp12	INSIDE	0.934	0.686	1519.94	1042.31	0.641	1238.68	793.61
A_68_P25063505	chr7:38555583-38555627	NM_172385:-833	Zfp536	PROMOTER	0.934	0.725	3633.79	2632.87	0.676	3039.66	2056.33
A_68_P28202317	chr12:4775790-4775834	NM_175431:261	A830093I24Rik	INSIDE	0.933	1.687	661.19	1115.10	1.574	651.77	1025.80
A_68_P23145841	chr4:99382126-99382170	NM_001081264:-172	Alg6	PROMOTER	0.933	0.698	3490.44	2436.86	0.651	2894.75	1884.59
A_68_P30647300	chr16:31430146-31430190	NM_001122683:1330	Bdh1	INSIDE	0.933	0.572	1685.85	963.80	0.533	1290.62	688.28
A_68_P31096346	chr17:24388277-24388321	NM_007634:56	Ccnf	INSIDE	0.933	2.667	523.87	1397.08	2.487	511.19	1271.30
A_68_P25272110	chr7:88599409-88599453	NM_007755:132	Cpeb1	INSIDE	0.933	0.707	1233.45	872.65	0.660	1098.16	725.09
A_68_P26165985	chr8:113521608-113521652	NM_007916:93	Ddx19a	INSIDE	0.933	0.613	1696.24	1039.05	0.572	1386.22	792.50
A_68_P26458701	chr9:42752890-42752934	NM_175481:-458	Grik4	PROMOTER	0.933	1.654	1478.80	2446.64	1.543	1277.31	1970.99
A_68_P28751166	chr12:113838256-113838300	NM_198411:11284	Inf2	INSIDE	0.933	2.107	1940.99	4090.03	1.966	1420.36	2791.97
A_68_P28998905	chr13:44998485-44998530	NM_001205043:171868	Jarid2	INSIDE	0.933	1.880	625.81	1176.60	1.754	509.95	894.22
A_68_P25004550	chr7:25162275-25162319	NM_001163510:7015	Kcnn4	INSIDE	0.933	1.625	1515.69	2463.23	1.517	1195.10	1812.96
A_68_P30422457	chr15:88813275-88813319	NM_031260:-127	Mov10l1	DIVERGENT_PROMOTER	0.933	2.528	492.30	1244.42	2.358	425.08	1002.55
A_68_P31029176	chr17:8995030-8995074	NM_011866:443	Pde10a	INSIDE	0.933	1.958	2235.98	4378.14	1.827	1809.79	3306.25
A_68_P21911589	chr2:181335871-181335915	NM_133701:-131	Prpf6	DIVERGENT_PROMOTER	0.933	0.415	1426.10	591.41	0.387	1053.17	407.30
A_68_P24090824	chr5:130292894-130292938	NM_133900:213	Psph	INSIDE	0.933	0.445	1349.08	599.96	0.415	1179.34	489.59
A_68_P26022784	chr8:87469901-87469945	NM_144929:-967	Rtbdn	DIVERGENT_PROMOTER	0.933	0.673	1056.43	711.02	0.628	927.78	582.88
A_68_P27550650	chr11:3093527-3093573	NM_030207:-84	Sfi1	DIVERGENT_PROMOTER	0.933	1.890	1565.66	2958.66	1.764	1376.07	2427.19
A_68_P23278806	chr4:125879586-125879630	NM_146153:346	Thrap3	INSIDE	0.933	0.483	2144.94	1036.48	0.451	1968.26	887.61
A_68_P31771010	chr18:63852103-63852147	NM_016792:-111	Txnl1	PROMOTER	0.933	0.627	1325.06	830.80	0.585	1097.23	642.18
A_68_P21977355	chr3:19794775-19794819	NR_027985:-74	4632415L05Rik	PROMOTER	0.932	0.579	1746.18	1011.07	0.539	1473.29	794.74
A_68_P28182256	chr11:119948049-119948093	NM_009734:71	Azi1	INSIDE	0.932	2.022	479.20	968.95	1.884	462.83	871.98
A_68_P26156363	chr8:111864572-111864616	NR_002928:214	Gm1943	INSIDE	0.932	1.848	692.34	1279.78	1.723	525.75	905.69

ProbeName	Target position of probe on CpG island microarray	TargetID	GeneSymbol	CpG island Description	Ratio of relative methylation (TiO_2-NP/Vehicle)	Sham group			TiO_2-H group		
						Relative methylation (Cy5/Cy3)	Cy3 signal (Input)	Cy5 signal (MeDIP)	Relative methylation (Cy5/Cy3)	Cy3 signal (Input)	Cy5 signal (MeDIP)
A_68_P26804350	chr9:106549989-106550033	NM_001160353:8430	Grm2	INSIDE	0.932	4.656	2018.50	9397.18	4.339	1393.34	6045.26
A_68_P29628537	chr14:58161247-58161291	NM_145837:17603	Il17d	INSIDE	0.932	1.992	854.92	1702.76	1.857	778.25	1445.41
A_68_P32319267	chrX:34137999-34138043	NM_016783:-199	Pgrmc1	PROMOTER	0.932	1.816	949.99	1725.34	1.693	489.37	828.28
A_68_P22825907	chr4:33335613-33335657	NM_001033225:128	Pnrc1	INSIDE	0.932	0.537	1116.26	599.04	0.500	856.03	428.22
A_68_P23373725	chr4:142752302-142752346	NM_001081355:50288	Prdm2	INSIDE	0.932	0.401	1598.97	640.41	0.373	1364.42	509.43
A_68_P31251406	chr17:55621810-55621854	NM_172829:36818	St6gal2	INSIDE	0.932	3.147	180.23	567.20	2.932	193.92	568.55
A_68_P29056640	chr13:55819313-55819357	NM_175150:3324	Txndc15	INSIDE	0.932	2.212	703.56	1556.21	2.062	581.00	1198.22
A_68_P29023550	chr13:49243481-49243525	NM_029361:-119	Wnk2	PROMOTER	0.932	1.551	985.13	1528.36	1.446	875.99	1266.83
A_68_P21105204	chr2:30640432-30640476	ENSMUST00000139968:18701		DOWNSTREAM	0.932	1.983	782.44	1551.78	1.848	749.69	1385.48
A_68_P26559432	chr9:60369799-60369843	ENSMUST00000140824:33		INSIDE	0.932	3.850	666.42	2566.04	3.589	542.76	1947.87
A_68_P24059291	chr5:124545373-124545417	NM_019875:413	Abcb9	INSIDE	0.931	0.612	1460.57	893.62	0.570	1181.60	673.24
A_68_P24925494	chr6:145814701-145814745	NM_024469:-862	Bhlhe41	PROMOTER	0.931	0.659	2763.85	1820.61	0.614	2089.35	1282.03
A_68_P23397715	chr4:148326702-148326746	NM_001159344:148224	Casz1	INSIDE	0.931	0.597	1184.56	707.77	0.556	1037.62	576.89
A_68_P31157314	chr17:35196243-35196287	NM_001190449:285	Ddah2	INSIDE	0.931	1.471	3333.08	4902.54	1.369	2560.89	3505.95
A_68_P25678286	chr8:15027955-15027999	NM_029116:16952	Kbtbd11	INSIDE	0.931	1.887	642.03	1211.62	1.757	630.98	1108.63
A_68_P29629403	chr14:58310348-58310392	NM_015771:54590	Lats2	INSIDE	0.931	2.836	1282.63	3637.22	2.641	985.18	2601.76
A_68_P25034848	chr7:31852435-31852479	NM_144556:7503	Lgi4	INSIDE	0.931	3.337	754.59	2518.40	3.107	693.17	2153.84
A_68_P23912412	chr5:97821337-97821381	NM_001033191:-9	Naa11	PROMOTER	0.931	3.327	1216.16	4045.88	3.096	1061.21	3285.11
A_68_P22859777	chr4:40918239-40918283	NM_023739:285	Nfx1	INSIDE	0.931	1.798	497.02	893.71	1.674	475.58	796.10
A_68_P31862859	chr18:80389409-80389453	NM_199197:7928	Rbfa	INSIDE	0.931	2.547	525.61	1338.69	2.372	416.05	986.71
A_68_P24085131	chr5:129287972-129288016	NM_001081388:171243	Rimbp2	INSIDE	0.931	2.217	1299.57	2881.31	2.065	1058.34	2185.31
A_68_P29244710	chr13:96901056-96901100	NM_029210:1454	Sv2c	INSIDE	0.931	0.654	3816.22	2494.89	0.608	3071.71	1868.66
A_68_P30370136	chr15:79922200-79922244	NM_009303:459	Syngr1	INSIDE	0.931	0.521	1226.39	639.08	0.485	1012.89	491.51
A_68_P24118489	chr5:136387042-136387086	NM_018871:23447	Ywhag	INSIDE	0.931	1.985	1221.53	2424.51	1.849	816.05	1508.54
A_68_P23590587	chr5:34038039-34038083	AK172441:87326		INSIDE	0.931	1.916	856.24	1640.37	1.783	841.39	1500.23
A_68_P24513209	chr6:65622803-65622847	NM_172399:1220	A930038C07Rik	INSIDE	0.930	1.907	1171.40	2234.16	1.773	1043.11	1849.37
A_68_P29050152	chr13:54676736-54676780	NM_019968:385	Arl10	INSIDE	0.930	0.707	1562.67	1104.27	0.657	1288.45	847.01
A_68_P22716231	chr4:8679020-8679064	NM_001081417:60975	Chd7	INSIDE	0.930	2.694	1432.17	3858.30	2.506	1180.21	2958.14
A_68_P33007790	chr4_random:128337-128381	NM_001033326:18557	Dhrsx	INSIDE	0.930	3.315	8590.18	28473.79	3.083	6402.68	19739.27
A_68_P20416040	chr1:89052463-89052507	NM_021306:-882	Ecel1	PROMOTER	0.930	1.703	2901.99	4942.58	1.583	2632.76	4168.89
A_68_P27497201	chr10:119851658-119851707	NM_010441:62309	Hmga2	INSIDE	0.930	4.452	413.71	1841.97	4.142	321.53	1331.82
A_68_P23527252	chr5:21181527-21181571	NM_178728:25615	Napepld	INSIDE	0.930	2.249	1264.18	2843.54	2.093	928.53	1943.21
A_68_P28433223	chr12:53198848-53198892	NM_029760:138	Nubpl	INSIDE	0.930	2.245	1770.08	3973.89	2.087	1560.57	3257.11
A_68_P25021461	chr7:29156631-29156675	NM_001083912:1029	Plekhg2	INSIDE	0.930	0.351	1825.35	640.18	0.326	1453.40	474.25
A_68_P23374048	chr4:142802256-142802300	NM_001081355:334	Prdm2	INSIDE	0.930	0.538	1431.28	770.08	0.501	1201.33	601.28
A_68_P23232473	chr4:116782119-116782163	NM_008958:13180	Ptch2	INSIDE	0.930	2.186	926.65	2025.30	2.033	800.17	1626.93
A_68_P25090596	chr7:52262461-52262505	NM_001008422:9137	Scaf1	INSIDE	0.930	2.752	1095.77	3015.02	2.559	862.57	2207.06
A_68_P27259388	chr10:75383953-75383997	NM_001161853:385	Smarcb1	INSIDE	0.930	1.947	669.46	1303.24	1.811	679.70	1230.63
A_68_P31357836	A_68_P31357836			Unknown	0.930	0.736	4229.64	3111.36	0.684	3439.40	2353.71
A_68_P27534320	chr10:126602342-126602386	NM_027739:-522	B4galnt1	PROMOTER	0.929	0.363	1781.55	646.66	0.337	1241.86	418.56
A_68_P28743216	chr12:112537725-112537769	NM_183016:78183	Cdc42bpb	INSIDE	0.929	2.500	824.77	2061.80	2.322	701.96	1630.17
A_68_P26699344	chr9:86361211-86361255	NM_177208:472	Dopey1	INSIDE	0.929	1.839	831.39	1528.75	1.709	764.75	1306.93
A_68_P28069617	chr11:100570105-100570149	NM_001038010:3655	Kat2a	INSIDE	0.929	3.539	1048.39	3710.05	3.288	958.51	3151.62
A_68_P27281281	chr10:79610845-79610889	NM_021565:-168	Midn	PROMOTER	0.929	0.590	1110.44	654.95	0.548	830.43	454.82
A_68_P26877097	chr9:120213366-120213410	NM_144557:198	Myrip	INSIDE	0.929	0.546	942.90	514.81	0.507	826.47	419.10
A_68_P27931785	chr11:76212519-76212563	NM_008750:103	Nxn	INSIDE	0.929	1.568	1469.67	2303.85	1.457	1224.93	1784.29
A_68_P24324978	chr6:29121084-29121128	NM_001101443:8478	Prrt4	INSIDE	0.929	2.009	875.60	1758.77	1.866	818.63	1527.59
A_68_P31924141	chr19:4625447-4625491	NM_023131:149	Rce1	INSIDE	0.929	0.536	1535.67	823.65	0.498	1258.35	627.22
A_68_P22342626	chr3:94976422-94976466	NM_011351:8149	Sema6c	INSIDE	0.929	1.749	1088.72	1904.39	1.625	863.85	1403.55
A_68_P20618599	chr1:133859814-133859858	NM_145977:-7349	Slc45a3	PROMOTER	0.929	1.697	964.00	1636.14	1.576	936.57	1476.01
A_68_P30546007	chr16:11066704-11066748	NM_009223:336	Snn	INSIDE	0.929	0.652	1869.64	1218.92	0.606	1336.51	809.76

ProbeName	Target position of probe on CpG island microarray	TargetID	GeneSymbol	CpG island Description	Ratio of relative methylation (TiO_2-NP/Vehicle)	Sham group			TiO_2-H group		
						Relative methylation (Cy5/Cy3)	Cy3 signal (Input)	Cy5 signal (MeDIP)	Relative methylation (Cy5/Cy3)	Cy3 signal (Input)	Cy5 signal (MeDIP)
A_68_P30351139	chr15:76741794-76741838	NM_001168276:14062	Zfp647	INSIDE	0.929	2.321	725.82	1684.29	2.156	593.98	1280.38
A_68_P31256677	chr17:56651893-56651937	NM_011483:-8	Znrf4	PROMOTER	0.929	2.174	382.88	832.54	2.019	365.16	737.28
A_68_P23498118	chr5:15440585-15440629	NM_001110843:98	Cacna2d1	INSIDE	0.928	2.072	602.76	1249.19	1.923	588.45	1131.86
A_68_P27489859	chr10:118677390-118677434	NM_027994:-301	Cand1	PROMOTER	0.928	2.069	740.55	1531.99	1.920	574.47	1103.04
A_68_P22318561	chr3:89124750-89124794	NM_010108:2029	Efna3	INSIDE	0.928	0.668	1025.98	685.77	0.620	851.48	527.99
A_68_P28924077	chr13:31718533-31718577	NM_010225:870	Foxf2	INSIDE	0.928	0.496	1489.10	738.07	0.460	1143.83	526.10
A_68_P32539665	chrX:91881517-91881561	NM_008179:131	Gspt2	INSIDE	0.928	1.689	1639.76	2769.73	1.567	971.22	1522.34
A_68_P21339292	chr2:74584954-74584998	NM_010468:34927	Hoxd3	INSIDE	0.928	0.624	3452.72	2153.79	0.579	2887.64	1671.32
A_68_P28060086	chr11:98903124-98903168	NM_010517:573	Igfbp4	INSIDE	0.928	0.563	993.45	559.50	0.523	775.11	405.05
A_68_P22540996	chr3:132754802-132754846	NM_027927:-92	Ints12	DIVERGENT_PROMOTER	0.928	0.517	1457.16	752.89	0.480	1148.49	550.95
A_68_P30619233	chr16:26105938-26105982	NM_173379:-90	Leprel1	PROMOTER	0.928	1.709	1075.12	1837.55	1.586	946.38	1500.80
A_68_P32024331	chr19:24630850-24630894	NM_008846:-555	Pip5k1b	PROMOTER	0.928	0.456	1485.31	677.37	0.423	1114.58	471.79
A_68_P21395217	chr2:84726953-84726997	NM_199223:-125	Rtn4rl2	PROMOTER	0.928	2.484	408.60	1014.77	2.305	449.17	1035.33
A_68_P27926564	chr11:75329061-75329105	NM_001004157:2040	Scarf1	INSIDE	0.928	2.616	981.75	2567.92	2.426	735.34	1784.28
A_68_P25661263	chr8:12397648-12397692	NM_009233:2152	Sox1	INSIDE	0.928	0.677	1109.99	751.95	0.628	947.23	595.23
A_68_P30296513	chr15:67008341-67008385	NM_009177:82	St3gal1	INSIDE	0.928	0.665	1619.75	1076.98	0.617	1409.80	869.46
A_68_P26154294	chr8:111474065-111474109	NM_007496:235543	Zfhx3	INSIDE	0.928	2.331	788.56	1838.29	2.164	617.30	1335.93
A_68_P20135161	chr1:33871021-33871065	NM_133817:230	Zfp451	INSIDE	0.928	0.714	2008.21	1433.43	0.662	1487.99	985.37
A_68_P25956909	chr8:73985635-73985679	NM_022419:1900	Abhd8	INSIDE	0.927	2.496	2338.73	5837.75	2.315	1796.08	4157.61
A_68_P29613379	chr14:55250870-55250914	NM_199470:9309	Cdh24	INSIDE	0.927	1.651	1360.45	2245.70	1.531	1158.87	1773.77
A_68_P29241611	chr13:96388009-96388053	NM_010169:358	F2r	INSIDE	0.927	0.543	1303.58	708.17	0.504	1181.02	595.02
A_68_P27897169	chr11:69615077-69615121	NM_010198:29	Fgf11	INSIDE	0.927	0.651	1935.64	1260.82	0.604	1357.33	819.78
A_68_P21091204	chr2:28302022-28302066	NM_001005420:-1416	Gm347	PROMOTER	0.927	0.715	1716.94	1227.65	0.663	1625.41	1076.85
A_68_P25004864	chr7:25218003-25218050	NM_199013:12675	Irgc1	INSIDE	0.927	2.427	637.91	1548.09	2.249	502.25	1129.74
A_68_P26644019	chr9:75289708-75289753	NM_001039522:400	Leo1	INSIDE	0.927	2.108	671.27	1414.83	1.954	588.84	1150.66
A_68_P22878699	chr4:44723813-44723857	NM_008782:-522	Pax5	PROMOTER	0.927	0.628	1207.35	757.62	0.582	984.37	572.80
A_68_P25377918	chr7:109399091-109399135	NM_019566:-480	Rhog	PROMOTER	0.927	1.740	1309.38	2278.08	1.612	989.03	1594.61
A_68_P21843353	chr2:168581710-168581754	NM_175303:10969	Sall4	INSIDE	0.927	2.251	288.60	649.77	2.086	270.97	565.35
A_68_P30348872	chr15:76349910-76349954	NM_130893:2627	Scrt1	INSIDE	0.927	2.304	1037.55	2390.39	2.136	935.21	1997.37
A_68_P26885414	chr9:121671950-121671994	NM_001166644:2822	Zfp651	INSIDE	0.927	2.660	602.31	1602.43	2.467	441.37	1088.84
A_68_P29308209	chr13:108681343-108681387	NM_145456:-1106	Zswim6	PROMOTER	0.927	1.590	963.81	1532.68	1.475	822.39	1212.89
A_68_P32473670	chrX:73211772-73211816	ENSMUST00000121090:-7487		PROMOTER	0.927	1.530	2064.85	3159.89	1.419	1011.59	1434.99
A_68_P21901912	chr2:179906060-179906104	NM_019822:-210	Adrm1	PROMOTER	0.926	3.610	757.83	2735.99	3.341	669.25	2236.27
A_68_P27899784	chr11:70068702-70068746	NM_007440:119	Alox12	INSIDE	0.926	0.627	1208.42	758.19	0.581	1002.89	582.54
A_68_P29056517	chr13:55794620-55794664	NM_181278:158	B230219D22Rik	INSIDE	0.926	1.805	391.42	706.59	1.671	477.72	798.38
A_68_P26221735	chr8:123191978-123192022	NM_010926:12	Cox4nb	INSIDE	0.926	0.674	1815.53	1223.91	0.625	1463.23	913.79
A_68_P24602880	chr6:85019421-85019465	NM_177077:65	Exoc6b	INSIDE	0.926	1.706	1531.81	2613.24	1.579	1418.22	2239.40
A_68_P25835331	chr8:47377554-47377598	NM_173789:2449	Helt	INSIDE	0.926	2.060	1321.32	2721.37	1.907	1003.71	1914.36
A_68_P26258434	chr8:129118421-129118465	NM_001164598:-1106	Irf2bp2	PROMOTER	0.926	2.005	609.11	1221.10	1.857	550.31	1021.94
A_68_P29054504	chr13:55452758-55452802	NM_025828:11364	Lman2	INSIDE	0.926	2.259	732.69	1655.06	2.091	598.16	1250.98
A_68_P27787411	chr11:50039460-50039504	NM_145926:646	Mgat4b	INSIDE	0.926	5.290	410.60	2172.16	4.897	343.75	1683.29
A_68_P29044154	chr13:53571404-53571448	NM_013601:-3277	Msx2	PROMOTER	0.926	1.539	3706.05	5702.14	1.425	3328.32	4743.76
A_68_P25954893	chr8:73437576-73437620	NM_173013:7726	Mtap1s	INSIDE	0.926	3.681	528.86	1946.62	3.407	419.85	1430.30
A_68_P26991388	chr10:20880481-20880525	NM_001198914:288	Myb	INSIDE	0.926	1.571	985.08	1548.04	1.455	930.29	1353.33
A_68_P20189690	chr1:43610952-43610996	NM_010879:108379	Nck2	INSIDE	0.926	1.909	809.49	1545.16	1.767	636.36	1124.56
A_68_P28621835	chr12:90498748-90498792	NM_172544:306937	Nrxn3	INSIDE	0.926	2.186	1067.40	2333.15	2.023	884.84	1790.11
A_68_P22309972	chr3:87587929-87587973	NM_001033124:11134	Ntrk1	INSIDE	0.926	3.167	1827.72	5788.41	2.932	1500.15	4398.10
A_68_P30346561	chr15:76005231-76005275	NM_201394:20888	Plec	INSIDE	0.926	2.334	1017.33	2373.96	2.161	788.14	1702.87
A_68_P24324924	chr6:29114488-29114532	NM_133925:214	Rbm28	INSIDE	0.926	0.629	3325.73	2091.72	0.582	2489.29	1449.67
A_68_P24428219	chr6:48547191-48547235	NM_001079901:3331	Repin1	INSIDE	0.926	2.259	1449.45	3274.75	2.092	1083.81	2267.57
A_68_P32694935	chrX:136145294-136145338	NM_023270:158	Rnf128	INSIDE	0.926	3.100	899.92	2789.55	2.871	675.38	1938.79

						Sham group			TiO_2-H group		
ProbeName	Target position of probe on CpG island microarray	TargetID	GeneSymbol	CpG island Description	Ratio of relative methylation (TiO_2-NP/Vehicle)	Relative methylation (Cy5/Cy3)	Cy3 signal (Input)	Cy5 signal (MeDIP)	Relative methylation (Cy5/Cy3)	Cy3 signal (Input)	Cy5 signal (MeDIP)
A_68_P24604452	chr6:85283101-85283145	NM_178639:294	Sfxn5	INSIDE	0.926	0.643	875.11	562.71	0.595	756.02	450.19
A_68_P28151703	chr11:115025252-115025296	NM_012030:620	Slc9a3r1	INSIDE	0.926	0.600	1635.43	981.75	0.556	1136.03	631.70
A_68_P25183829	chr7:72789715-72789759	NM_172310:-47	Tarsl2	PROMOTER	0.926	2.044	13789.93	28181.66	1.892	9572.49	18111.79
A_68_P21105306	chr2:30656954-30656998	NM_198000:251	1700001O22Rik	INSIDE	0.925	2.505	2026.51	5076.77	2.316	1721.46	3987.51
A_68_P27033724	chr10:28916766-28916810	NM_026138:52987	6330407J23Rik	INSIDE	0.925	2.092	1805.80	3777.61	1.936	1478.82	2862.56
A_68_P30371903	chr15:80202683-80202727	NM_001044308:85037	Cacna1i	INSIDE	0.925	2.667	998.46	2662.87	2.468	786.47	1941.09
A_68_P20910363	chr1:188791777-188791821	NM_033077:504	D1Pas1	INSIDE	0.925	1.733	689.41	1194.66	1.604	577.34	925.83
A_68_P20149760	chr1:36739833-36739877	NM_001126046:174	Fam178b	INSIDE	0.925	2.814	1267.41	3566.45	2.602	1126.79	2932.09
A_68_P31927961	chr19:5306691-5306735	NM_001024717:8382	Gal3st3	INSIDE	0.925	2.240	1301.78	2915.80	2.073	1039.13	2153.91
A_68_P21874743	chr2:174152892-174152936	NR_003258:-500	Gnas	PROMOTER	0.925	3.703	4265.92	15798.12	3.427	2818.28	9657.38
A_68_P21339005	chr2:74550245-74550289	NM_010468:217	Hoxd3	INSIDE	0.925	0.601	2203.74	1323.68	0.556	1732.19	962.30
A_68_P27865441	chr11:64249280-64249324	NM_178870:469	Hs3st3a1	INSIDE	0.925	0.549	1280.62	703.58	0.508	985.05	500.45
A_68_P26258420	chr8:129116753-129116797	NM_001164598:562	Irf2bp2	INSIDE	0.925	1.589	1252.23	1989.99	1.471	1071.28	1575.59
A_68_P24428220	chr6:48547266-48547310	NM_001079901:3407	Repin1	INSIDE	0.925	2.499	661.65	1653.19	2.310	576.30	1331.30
A_68_P21081267	chr2:26788591-26788635	NM_011512:419	Surf4	INSIDE	0.925	0.675	1431.85	965.83	0.624	1350.10	842.51
A_68_P31950560	chr19:10500789-10500833	NM_018801:37231	Syt7	INSIDE	0.925	1.812	1635.94	2963.67	1.675	1198.81	2008.51
A_68_P26883519	chr9:121363673-121363717	NM_175114:87619	Trak1	INSIDE	0.925	2.819	989.40	2788.91	2.608	705.00	1838.77
A_68_P27466130	chr10:114238213-114238257	NM_146241:192	Trhde	INSIDE	0.925	0.675	1117.27	754.17	0.624	895.70	559.19
A_68_P26513689	chr9:51976512-51976556	NM_001162921:-318	Zc3h12c	PROMOTER	0.925	2.048	839.46	1719.39	1.895	710.91	1347.18
A_68_P26238819	chr8:125778613-125778657	NM_020497:540	Zfp276	INSIDE	0.925	0.601	1523.49	915.89	0.556	1187.33	660.04
A_68_P23253637	chr4:120527431-120527475	ENSMUST00000106283:-6929		PROMOTER	0.925	1.768	1223.90	2163.39	1.635	1053.28	1722.40
A_68_P32151687	chr19:47805901-47805945	NM_026377:-323	6330577E15Rik	PROMOTER	0.924	0.610	1914.37	1167.07	0.563	1715.17	966.13
A_68_P28053987	chr11:97883567-97883611	NM_001159320:-3902	Cacnb1	PROMOTER	0.924	0.549	1904.28	1044.56	0.507	1410.05	714.97
A_68_P27563899	chr11:5441997-5442041	NM_134033:202	Ccdc117	INSIDE	0.924	1.617	915.50	1480.74	1.495	799.03	1194.48
A_68_P26844379	chr9:114294023-114294067	NM_019922:5786	Crtap	INSIDE	0.924	1.663	982.86	1634.68	1.537	846.08	1300.38
A_68_P28170155	chr11:118108984-118109028	NM_001112699:900	Cyth1	INSIDE	0.924	0.568	1606.36	911.99	0.525	1191.10	624.81
A_68_P31794503	chr18:68093621-68093666	NM_172631:733	D18Ertd653e	INSIDE	0.924	1.920	1540.79	2958.23	1.774	1194.60	2119.18
A_68_P31058441	chr17:14340236-14340280	NM_172826:580	Dact2	INSIDE	0.924	0.624	1288.87	803.71	0.576	1079.74	622.11
A_68_P22019077	chr3:28680006-28680050	NM_177586:-204	Eif5a2	PROMOTER	0.924	0.492	1295.33	637.57	0.455	1061.91	482.74
A_68_P28940412	chr13:34838589-34838633	NM_138746:7100	Fam50b	INSIDE	0.924	2.203	651.73	1435.87	2.037	544.01	1107.91
A_68_P27093485	chr10:41917381-41917425	NM_019740:79146	Foxo3	INSIDE	0.924	1.936	2076.54	4020.95	1.790	1616.27	2893.07
A_68_P20969135	chr2:4525120-4525164	NM_001177844:44343	Frmd4a	INSIDE	0.924	2.158	1092.22	2356.97	1.993	983.41	1960.07
A_68_P22204191	chr3:66024790-66024834	NM_008987:1004	Ptx3	INSIDE	0.924	2.248	411.46	924.91	2.077	392.56	815.37
A_68_P21116405	chr2:32470425-32470469	NM_001025310:7966	St6galnac6	INSIDE	0.924	2.819	1322.82	3729.64	2.605	1030.62	2684.68
A_68_P23337641	chr4:136604483-136604527	NM_198248:106	Zbtb40	INSIDE	0.924	2.449	759.46	1860.03	2.262	671.94	1519.87
A_68_P21775102	chr2:156905117-156905161	NM_001164663:-137	9830001H06Rik	DIVERGENT_PROMOTER	0.923	1.567	1177.07	1844.63	1.446	1140.98	1649.65
A_68_P26466976	chr9:44127791-44127835	NM_027909:553	C2cd2l	INSIDE	0.923	0.421	1601.40	673.48	0.388	1416.07	549.95
A_68_P26140463	chr8:109127687-109127731	NM_009864:441	Cdh1	INSIDE	0.923	2.015	777.32	1566.55	1.860	679.54	1263.96
A_68_P21144179	chr2:37648980-37649024	NM_001163566:17234	Crb2	INSIDE	0.923	1.520	1714.39	2605.17	1.402	1444.54	2025.88
A_68_P26885587	chr9:121697963-121698007	NR_027967:2254	Hhatl	INSIDE	0.923	2.123	346.73	736.06	1.960	310.70	608.94
A_68_P21840386	chr2:168088202-168088246	NM_001081134:6607	Kcng1	INSIDE	0.923	1.912	410.37	784.49	1.764	397.74	701.74
A_68_P23297927	chr4:129349792-129349836	NM_008468:197	Kpna6	INSIDE	0.923	0.456	2193.16	999.59	0.421	1728.92	727.21
A_68_P23264336	chr4:123364187-123364231	NM_001199136:-2605	Macf1	PROMOTER	0.923	0.647	880.14	569.05	0.597	781.60	466.56
A_68_P23542978	chr5:24108266-24108310	NM_025891:-468	Smarcd3	PROMOTER	0.923	2.188	676.38	1480.03	2.020	578.55	1168.40
A_68_P20005806	chr1:4483262-4483306	NM_011441:3210	Sox17	INSIDE	0.923	0.554	1089.43	604.00	0.512	873.68	447.25
A_68_P27502796	chr10:120748000-120748044	NM_029057:223	Tbc1d30	INSIDE	0.923	0.576	918.78	528.83	0.531	838.23	445.13
A_68_P26564384	chr9:61220668-61220712	NM_001083927:518	Tle3	INSIDE	0.923	0.685	2351.62	1611.64	0.633	1823.98	1154.16
A_68_P26373639	chr9:26837630-26837674	NM_178027:27	Vps26b	INSIDE	0.923	0.693	1163.43	806.21	0.640	1047.73	670.29
A_68_P24994121	chr7:20163073-20163117	NM_022409:459	Zfp296	INSIDE	0.923	0.624	1155.04	720.23	0.576	953.90	549.13
A_68_P29477473	chr14:26277283-26277327	NM_183208:-1366	Zmiz1	PROMOTER	0.923	0.536	1061.53	569.14	0.495	909.18	450.08
A_68_P23990398	chr5:112583018-112583062	AK006010:-2216		PROMOTER	0.923	0.662	1508.11	998.75	0.611	1096.29	670.34

ProbeName	Target position of probe on CpG island microarray	TargetID	GeneSymbol	CpG island Description	Ratio of relative methylation (TiO_2-NP/Vehicle)	Sham group			TiO_2-H group		
						Relative methylation (Cy5/Cy3)	Cy3 signal (Input)	Cy5 signal (MeDIP)	Relative methylation (Cy5/Cy3)	Cy3 signal (Input)	Cy5 signal (MeDIP)
A_68_P24127507	chr5:138220239-138220283	NM_027242:115	2010007H12Rik	INSIDE	0.922	0.600	1014.62	608.72	0.553	967.60	535.52
A_68_P28083320	chr11:102962675-102962719	NM_152813:276	Plcd3	INSIDE	0.922	0.649	1279.19	830.60	0.598	1018.30	609.38
A_68_P29055171	chr13:55565839-55565883	NM_001030296:233	Prr7	INSIDE	0.922	0.514	1205.22	619.86	0.474	1011.87	479.77
A_68_P31868008	chr18:81171137-81171181	NM_178280:12159	Sall3	INSIDE	0.922	2.704	724.41	1958.57	2.492	821.88	2047.98
A_68_P27631988	chr11:20443335-20443379	NM_021372:101	Sertad2	INSIDE	0.922	1.936	4953.85	9591.84	1.785	3999.43	7138.26
A_68_P21901054	chr2:179777765-179777809	NM_178750:599	Ss18l1	INSIDE	0.922	2.119	1100.80	2333.01	1.954	931.45	1819.68
A_68_P28037690	chr11:95120921-95120965	NM_053093:-1900	Tac4	PROMOTER	0.922	2.320	653.20	1515.62	2.140	608.61	1302.34
A_68_P24427168	chr6:48388705-48388749	NM_001085415:6363	Zfp467	INSIDE	0.922	1.820	2146.73	3907.02	1.678	1664.83	2793.47
A_68_P23470208	chr5:8912735-8912783	NM_008830:19038	Abcb4	INSIDE	0.921	3.070	683.03	2096.73	2.827	568.93	1608.38
A_68_P20395058	chr1:82836029-82836073	NM_010472:-7	Agfg1	PROMOTER	0.921	2.685	501.22	1345.70	2.472	466.34	1152.64
A_68_P28859760	chr13:19040273-19040317	NM_175007:55	Amph	INSIDE	0.921	0.646	900.90	581.85	0.595	743.02	442.05
A_68_P31932937	chr19:6141281-6141325	NM_019722:-165	Arl2	PROMOTER	0.921	3.505	455.29	1595.64	3.229	409.41	1321.83
A_68_P28731505	chr12:110272292-110272336	NM_001163175:34113	Begain	INSIDE	0.921	2.646	811.72	2147.78	2.438	730.43	1780.89
A_68_P31103374	chr17:25522637-25522681	NM_001163691:48070	Cacna1h	INSIDE	0.921	2.261	1944.76	4396.13	2.081	1563.35	3253.84
A_68_P24114171	chr5:135462844-135462888	NM_009902:783	Cldn3	INSIDE	0.921	1.729	2960.72	5119.20	1.593	2373.01	3780.02
A_68_P22398586	chr3:105490205-105490249	NM_017397:263	Ddx20	INSIDE	0.921	2.017	392.44	791.43	1.857	423.60	786.51
A_68_P25957754	chr8:74126853-74126897	NM_001166213:5330	Fam129c	INSIDE	0.921	2.138	12168.65	26011.80	1.969	8217.01	16177.09
A_68_P31123770	chr17:28829032-28829076	NM_001168513:-458	Mapk14	PROMOTER	0.921	0.641	1683.02	1078.68	0.590	1428.15	843.26
A_68_P25025269	chr7:29871316-29871362	NR_035489:-2625	Mir1963	PROMOTER	0.921	3.396	613.42	2083.33	3.129	507.96	1589.35
A_68_P21722744	chr2:147011624-147011668	NM_001077632:492	Nkx2-2	INSIDE	0.921	1.968	727.42	1431.29	1.813	595.33	1079.31
A_68_P32218816	chr19:59419677-59419721	NM_001033222:572	Pdzd8	INSIDE	0.921	0.523	1170.46	611.96	0.481	929.73	447.62
A_68_P23593814	chr5:34679655-34679699	NM_011278:638	Rnf4	INSIDE	0.921	0.482	1158.62	559.03	0.444	1094.18	485.99
A_68_P24320418	chr6:28429971-28430015	NM_019776:-355	Snd1	PROMOTER	0.921	0.612	904.85	553.79	0.564	814.37	458.93
A_68_P32562268	chrX:98255023-98255069	NM_031384:-68	Tex11	PROMOTER	0.921	2.023	1132.45	2290.77	1.862	442.25	823.68
A_68_P27845262	chr11:60691840-60691884	NM_001168507:678	Tmem11	INSIDE	0.921	0.622	3974.17	2471.33	0.573	3224.08	1846.28
A_68_P22889233	chr4:46548820-46548864	NM_029077:174	Trim14	INSIDE	0.921	1.728	1082.48	1870.15	1.591	913.12	1452.64
A_68_P24836602	chr6:127984334-127984378	NM_175414:109240	Tspan9	INSIDE	0.921	1.910	1058.84	2022.13	1.759	943.96	1660.64
A_68_P29256602	chr13:99032998-99033042	NM_178918:-73	Utp15	DIVERGENT_PROMOTER	0.921	1.797	514.75	925.17	1.655	497.54	823.52
A_68_P32977629	A_68_P32977629			Unknown	0.921	2.962	2336.85	6921.36	2.728	1541.95	4207.14
A_68_P28092301	chr11:104547023-104547067	ENSMUST00000148007:24		INSIDE	0.921	1.836	938.30	1723.10	1.692	867.86	1468.51
A_68_P28574820	chr12:81360887-81360931	NM_134156:450	Actn1	INSIDE	0.920	1.792	1836.91	3291.99	1.648	1515.90	2498.01
A_68_P24325868	chr6:29297894-29297938	NM_007594:-202	Calu	PROMOTER	0.920	0.629	1414.62	889.23	0.578	1340.39	774.96
A_68_P30778319	chr16:55905763-55905807	NM_028815:29177	Cep97	INSIDE	0.920	2.708	864.84	2341.89	2.492	772.19	1924.47
A_68_P20148455	chr1:36528583-36528627	NM_033570:163	Cnnm4	INSIDE	0.920	1.936	859.43	1663.84	1.781	774.69	1379.84
A_68_P25005643	chr7:25370538-25370582	NM_023154:-2001	Ethe1	PROMOTER	0.920	2.028	1563.68	3170.59	1.865	1194.52	2227.83
A_68_P23145550	chr4:99323204-99323248	NM_010425:237	Foxd3	INSIDE	0.920	0.511	1677.83	857.45	0.470	1431.85	673.51
A_68_P24133860	chr5:139625951-139625996	NM_001081265:-203	Heatr2	PROMOTER	0.920	3.659	8339.11	30511.15	3.366	4849.44	16322.30
A_68_P26228653	chr8:124277085-124277129	NM_020605:22648	Jph3	INSIDE	0.920	3.301	272.80	900.46	3.037	203.59	618.29
A_68_P31113143	chr17:27053838-27053882	NM_053173:-175	Kifc5b	PROMOTER	0.920	0.578	1304.57	753.65	0.531	985.08	523.54
A_68_P24324309	chr6:29010153-29010197	NM_008493:-46	Lep	PROMOTER	0.920	2.123	2161.27	4589.25	1.954	1707.95	3337.86
A_68_P28565711	chr12:79849726-79849770	NM_019579:-185	Mpp5	PROMOTER	0.920	1.769	700.74	1239.95	1.629	663.69	1080.89
A_68_P31256052	chr17:56568252-56568296	NM_011218:47629	Ptprs	INSIDE	0.920	3.075	561.43	1726.20	2.829	522.54	1478.44
A_68_P31947894	chr19:10092837-10092881	NM_144538:141	Rab3il1	INSIDE	0.920	0.690	3336.68	2303.44	0.635	2711.30	1721.75
A_68_P32070014	chr19:32462198-32462242	NM_144792:724	Sgms1	INSIDE	0.920	0.712	2112.33	1503.37	0.655	1594.68	1044.16
A_68_P24979911	chr7:16730509-16730553	NM_148946:14882	Slc8a2	INSIDE	0.920	1.764	1388.49	2449.16	1.623	1108.67	1799.57
A_68_P23650101	chr5:44617682-44617726	NM_173764:141	Tapt1	INSIDE	0.920	1.921	750.67	1442.21	1.767	714.94	1263.53
A_68_P26940975	chr10:11211781-11211825			Unknown	0.920	3.073	1680.13	5162.79	2.828	1400.89	3961.28
A_68_P30671438	A_68_P30671438			Unknown	0.920	1.812	1071.22	1941.35	1.667	961.31	1602.97
A_68_P25730018	chr8:27096782-27096826	NM_029037:7789	4930444A02Rik	INSIDE	0.919	2.917	1340.66	3911.08	2.682	1135.86	3046.79
A_68_P20086661	chr1:23108998-23109042	NM_025746:72	4933415F23Rik	INSIDE	0.919	2.250	473.59	1065.65	2.068	431.60	892.56
A_68_P25677778	chr8:14962588-14962634	NM_001037736:50894	Arhgef10	INSIDE	0.919	2.919	990.48	2891.20	2.682	718.70	1927.50

ProbeName	Target position of probe on CpG island microarray	TargetID	GeneSymbol	CpG island Description	Ratio of relative methylation (TiO_2-NP/Vehicle)	Sham group			TiO_2-H group		
						Relative methylation (Cy5/Cy3)	Cy3 signal (Input)	Cy5 signal (MeDIP)	Relative methylation (Cy5/Cy3)	Cy3 signal (Input)	Cy5 signal (MeDIP)
A_68_P25957004	chr8:74000092-74000136	NM_025600:7022	Dda1	INSIDE	0.919	1.657	3085.78	5112.48	1.523	2569.43	3912.55
A_68_P30361554	chr15:78502409-78502453	NM_183141:46113	Elfn2	INSIDE	0.919	1.906	780.06	1486.59	1.752	708.23	1240.82
A_68_P26885589	chr9:121698137-121698187	NR_027967:2076	Hhatl	INSIDE	0.919	3.102	802.68	2489.87	2.849	625.77	1783.02
A_68_P31828823	chr18:74129671-74129715	NM_172632:94911	Mapk4	INSIDE	0.919	1.826	692.70	1265.01	1.678	590.55	990.84
A_68_P28621837	chr12:90498937-90498981	NM_172544:307125	Nrxn3	INSIDE	0.919	2.469	1976.42	4878.99	2.269	1430.31	3245.83
A_68_P22309970	chr3:87587747-87587797	NM_001033124:11312	Ntrk1	INSIDE	0.919	2.231	527.81	1177.69	2.051	428.47	878.99
A_68_P22165658	chr3:57651977-57652021	NM_019410:-319	Pfn2	PROMOTER	0.919	0.648	2123.25	1375.69	0.595	1737.79	1034.59
A_68_P23361257	chr4:140517377-140517421	NM_023374:213	Sdhb	INSIDE	0.919	1.651	757.26	1250.61	1.518	665.89	1011.16
A_68_P21054133	chr2:21127231-21127275	NM_001001297:-98	Thnsl1	DIVERGENT_PROMOTER	0.919	0.730	2338.84	1706.39	0.671	1924.01	1290.21
A_68_P32683274	chrX:133462451-133462502	NM_027067:613	1700014N06Rik	INSIDE	0.918	2.891	585.23	1692.17	2.655	289.14	767.62
A_68_P30484701	chr15:100058334-100058378	NM_007497:67	Atf1	INSIDE	0.918	0.649	794.37	515.70	0.596	714.51	425.61
A_68_P30337697	chr15:74359726-74359770	NM_174991:13123	Bai1	INSIDE	0.918	2.542	3558.01	9045.16	2.333	2257.02	5265.51
A_68_P27228309	chr10:69651754-69651798	NM_001111121:91908	Ccdc6	INSIDE	0.918	1.657	1587.61	2631.07	1.521	1246.15	1895.54
A_68_P24376401	chr6:38203728-38203772	NM_194061:259	D630045J12Rik	INSIDE	0.918	1.605	3985.68	6398.21	1.473	3028.58	4461.45
A_68_P24046892	chr5:122303021-122303065	NM_175474:4006	Fam109a	INSIDE	0.918	2.456	1651.48	4056.63	2.255	1259.89	2840.97
A_68_P26065154	chr8:94883835-94883879	NM_018826:2162	Irx5	INSIDE	0.918	0.643	1283.13	824.75	0.590	1241.43	732.50
A_68_P21842274	chr2:168396695-168396739	NM_001136073:18975	Nfatc2	INSIDE	0.918	3.193	485.87	1551.40	2.930	526.18	1541.56
A_68_P24643141	chr6:92041701-92041746	NM_011630:312	Nr2c2	INSIDE	0.918	2.773	368.84	1022.84	2.545	354.06	901.20
A_68_P28159702	chr11:116360052-116360096	NM_026364:-8414	Prpsap1	PROMOTER	0.918	1.562	1892.27	2956.49	1.434	1514.92	2172.83
A_68_P21635428	chr2:131346491-131346535	NM_001177833:28915	Smox	INSIDE	0.918	1.877	1749.46	3284.60	1.723	1417.18	2442.32
A_68_P24912679	chr6:143782088-143782132	NM_001113559:375968	Sox5	INSIDE	0.918	2.312	1433.26	3314.02	2.122	1134.07	2407.00
A_68_P21431326	chr2:92755177-92755221	NM_030725:-59	Syt13	PROMOTER	0.918	0.484	1817.99	880.42	0.445	1664.21	739.91
A_68_P32147506	chr19:47149599-47149649	NM_177342:7387	Taf5	INSIDE	0.918	2.631	599.92	1578.10	2.414	528.51	1275.59
A_68_P28524377	chr12:72212495-72212539	NM_029227:102	Tomm20l	INSIDE	0.918	1.551	1591.20	2468.23	1.424	1402.54	1997.90
A_68_P24951591	chr7:4969560-4969604	NM_025324:2473	Zfp524	INSIDE	0.918	2.127	1471.39	3129.84	1.953	1061.33	2072.31
A_68_P25027730	chr7:30304085-30304129	ENSMUST00000141713:-117		PROMOTER	0.918	2.141	593.13	1270.08	1.965	539.71	1060.76
A_68_P31632916	chr18:38409853-38409897	NR_015475:-22	1700086O06Rik	DIVERGENT_PROMOTER	0.917	0.416	1355.48	563.97	0.381	1069.20	407.73
A_68_P21566910	chr2:118741704-118741748	NM_001045523:14376	Bahd1	INSIDE	0.917	1.807	970.43	1753.47	1.658	736.05	1220.16
A_68_P21821741	chr2:164941626-164941673	NM_174988:118588	Cdh22	INSIDE	0.917	2.649	398.23	1054.83	2.428	331.88	805.89
A_68_P21605248	chr2:125684747-125684791	NM_009939:-14	Cops2	DIVERGENT_PROMOTER	0.917	2.064	4036.12	8328.99	1.892	3090.50	5847.58
A_68_P23284203	chr4:126911090-126911134	NM_198618:64598	Dlgap3	INSIDE	0.917	3.055	949.79	2902.01	2.802	867.32	2429.83
A_68_P30981935	chr16:94748214-94748258	NM_007834:0	Dscr3	INSIDE	0.917	1.851	1196.58	2215.01	1.698	912.55	1549.24
A_68_P26585941	chr9:64971903-64971947	NM_020043:22623	Igdcc4	INSIDE	0.917	1.778	654.85	1164.43	1.631	592.66	966.37
A_68_P26546334	chr9:58046265-58046309	NM_001161535:3236	Islr2	INSIDE	0.917	0.582	2192.49	1276.24	0.534	1704.50	909.84
A_68_P23303541	chr4:130506143-130506190	NM_010769:5867	Matn1	INSIDE	0.917	4.825	771.32	3721.94	4.425	633.88	2805.09
A_68_P23088454	chr4:87679337-87679381	NM_027326:-47	Mllt3	PROMOTER	0.917	0.673	1179.48	793.40	0.617	1042.83	643.36
A_68_P29619205	chr14:56290728-56290772	NM_008683:-7	Nedd8	DIVERGENT_PROMOTER	0.917	0.531	1414.85	751.63	0.487	1021.37	497.47
A_68_P28514077	chr12:70457809-70457853	NM_025441:323	Nemf	INSIDE	0.917	0.674	1301.03	877.22	0.618	1102.99	682.05
A_68_P25090602	chr7:52263044-52263088	NM_001008422:8553	Scaf1	INSIDE	0.917	2.656	1165.01	3094.49	2.435	904.05	2201.41
A_68_P26540061	chr9:56919951-56919995	NM_001110350:-4210	Sin3a	PROMOTER	0.917	2.642	936.69	2475.05	2.422	713.46	1728.21
A_68_P21986758	chr3:21974743-21974787	NM_030732:-809	Tbl1xr1	PROMOTER	0.917	2.090	871.44	1821.59	1.916	889.85	1705.01
A_68_P22168164	chr3:58219493-58219537	NM_001081229:-96	Tsc22d2	PROMOTER	0.917	0.522	1104.35	576.37	0.479	1013.28	484.90
A_68_P32203591	chr19:56949104-56949148	NM_172840:221	Vwa2	INSIDE	0.917	0.442	1255.21	554.21	0.405	1057.06	427.97
A_68_P25021517	chr7:29163975-29164019	NM_011756:251	Zfp36	INSIDE	0.917	0.578	1544.11	893.06	0.530	1323.56	701.65
A_68_P27288262	chr10:80702920-80702964	NM_001034895:7035	Zfr2	INSIDE	0.917	1.944	698.85	1358.24	1.783	681.95	1215.59
A_68_P24631966	chr6:90319994-90320038	NM_030260:529	Zxdc	INSIDE	0.917	0.578	1009.86	583.85	0.530	867.87	459.88
A_68_P22185882	chr3:61963377-61963421			Unknown	0.917	3.084	657.13	2026.33	2.829	540.90	1530.11
A_68_P25190566	chr7:73985093-73985137	NM_001033877:494	Adamts17	INSIDE	0.916	2.066	1073.93	2218.32	1.892	931.62	1762.53
A_68_P28741429	chr12:112216865-112216909	NM_175207:345	Ankrd9	INSIDE	0.916	1.627	678.59	1104.03	1.491	698.10	1040.77
A_68_P29484378	chr14:27783198-27783242	NM_145221:517	Appl1	INSIDE	0.916	0.628	3298.86	2071.03	0.575	2583.83	1485.80
A_68_P27100734	chr10:43231084-43231128	NM_199028:32161	Bend3	INSIDE	0.916	1.797	615.40	1106.09	1.646	512.34	843.26

ProbeName	Target position of probe on CpG island microarray	TargetID	GeneSymbol	CpG island Description	Ratio of relative methylation (TiO_2-NP/Vehicle)	Sham group			TiO_2-H group		
						Relative methylation (Cy5/Cy3)	Cy3 signal (Input)	Cy5 signal (MeDIP)	Relative methylation (Cy5/Cy3)	Cy3 signal (Input)	Cy5 signal (MeDIP)
A_68_P25430225	chr7:120512643-120512687	NM_133700:189	Btbd10	INSIDE	0.916	0.561	1769.13	992.57	0.514	1503.52	772.39
A_68_P28171198	chr11:118280168-118280212	NM_001025617:176	Cant1	INSIDE	0.916	0.647	2755.70	1783.83	0.593	2199.21	1304.61
A_68_P24820867	chr6:125444742-125444786	NM_007657:9	Cd9	INSIDE	0.916	2.147	2772.20	5951.48	1.967	2263.15	4451.33
A_68_P27266462	chr10:76628409-76628453	NM_009929:845	Col18a1	INSIDE	0.916	0.616	1428.79	880.26	0.565	1250.35	705.97
A_68_P29698998	chr14:70474791-70474835	NM_018781:-2439	Egr3	PROMOTER	0.916	0.521	1351.15	703.35	0.477	1329.65	634.18
A_68_P20442328	chr1:93309360-93309404	NM_019479:417	Hes6	INSIDE	0.916	0.688	1596.55	1098.15	0.630	1328.47	836.90
A_68_P28293526	chr12:25783349-25783393	NM_010496:-2413	Id2	PROMOTER	0.916	1.739	3890.73	6767.63	1.593	3011.29	4796.95
A_68_P20556966	chr1:121317400-121317444	NM_008381:1403	Inhbb	INSIDE	0.916	0.693	1725.04	1195.27	0.634	1378.94	874.78
A_68_P21812069	chr2:163201531-163201575	NM_021566:22157	Jph2	INSIDE	0.916	2.714	715.06	1940.86	2.485	678.76	1687.06
A_68_P25960691	chr8:74843873-74843917	NM_008452:934	Klf2	INSIDE	0.916	1.986	675.18	1340.86	1.819	596.70	1085.18
A_68_P27880946	chr11:66908183-66908227	NM_001099635:16403	Myh3	INSIDE	0.916	2.908	275.28	800.52	2.665	275.51	734.13
A_68_P32552416	chrX:96086453-96086497	NM_052976:-150	Ophn1	PROMOTER	0.916	2.159	790.82	1707.24	1.978	565.28	1118.07
A_68_P27553983	chr11:3674675-3674719	NM_152818:89210	Osbp2	INSIDE	0.916	2.192	1166.26	2556.72	2.008	993.25	1994.00
A_68_P23234765	chr4:117168524-117168568	NM_025739:974	Rnf220	INSIDE	0.916	0.675	1152.63	778.22	0.618	969.97	599.77
A_68_P25016961	chr7:28152919-28152968	NM_001199235:26582	Spnb4	INSIDE	0.916	4.566	1382.05	6310.84	4.181	998.02	4172.60
A_68_P20191095	chr1:43884838-43884882	NM_026430:-307	Uxs1	PROMOTER	0.916	0.521	1162.04	605.62	0.477	941.87	449.59
A_68_P25805594	chr8:41509577-41509621	NM_178395:430	Zdhhc2	INSIDE	0.916	0.473	1937.26	915.80	0.433	1596.11	690.88
A_68_P25004348	chr7:25118652-25118696	NM_001163810:310	1700008P20Rik	INSIDE	0.915	1.962	772.49	1515.53	1.794	615.01	1103.54
A_68_P25260060	chr7:86418215-86418259	NM_018811:85	Abhd2	INSIDE	0.915	0.580	878.58	509.34	0.530	794.98	421.51
A_68_P32233080	chrX:5659260-5659304	NM_001033211:1256	AU022751	INSIDE	0.915	2.228	799.75	1782.09	2.038	347.59	708.37
A_68_P27183499	chr10:61440751-61440795	NM_007731:1084	Col13a1	INSIDE	0.915	0.683	5777.62	3943.80	0.625	4263.72	2663.04
A_68_P25755967	chr8:32200134-32200178	NM_025869:23	Dusp26	INSIDE	0.915	3.055	317.69	970.63	2.797	319.89	894.73
A_68_P27640628	chr11:22186580-22186624	NM_153078:-761	Ehbp1	PROMOTER	0.915	0.588	1787.92	1051.22	0.538	1426.37	767.05
A_68_P29702035	chr14:70954292-70954336	NM_021877:452	Hr	INSIDE	0.915	0.592	1273.02	753.64	0.542	941.22	510.03
A_68_P28149754	chr11:114647920-114647964	NM_001102615:21240	Kif19a	INSIDE	0.915	2.153	384.92	828.78	1.970	379.74	748.13
A_68_P26919699	chr10:7309827-7309871	NM_172784:251	Lrp11	INSIDE	0.915	2.778	188.46	523.47	2.541	237.78	604.10
A_68_P27356288	chr10:93352563-93352607	NM_019648:1363	Metap2	INSIDE	0.915	0.516	1843.03	951.06	0.472	1732.10	818.08
A_68_P27857774	chr11:62945885-62945929	NM_008885:895	Pmp22	INSIDE	0.915	0.587	1384.16	811.85	0.537	1023.54	549.40
A_68_P25025139	chr7:29849652-29849696	NM_009109:60496	Ryr1	INSIDE	0.915	2.309	420.88	971.83	2.112	385.55	814.32
A_68_P26872706	chr9:119395898-119395942	NM_021544:92214	Scn5a	INSIDE	0.915	2.434	538.01	1309.61	2.227	409.24	911.22
A_68_P25588801	chr7:148623566-148623613	NM_001177576:-3096	Slc25a22	PROMOTER	0.915	2.258	927.83	2094.59	2.065	791.15	1633.90
A_68_P24593031	chr6:83318401-83318445	NM_183138:73250	Tet3	INSIDE	0.915	2.163	2635.46	5701.62	1.979	1852.19	3666.35
A_68_P27537442	chr10:127114141-127114185	NM_173732:42	Tmem194	INSIDE	0.915	0.405	5104.16	2065.48	0.370	3901.48	1445.03
A_68_P20354370	chr1:75479524-75479568	NM_177056:3440	Tmem198	INSIDE	0.915	2.226	1183.36	2633.61	2.036	891.58	1815.56
A_68_P26743635	chr9:95650332-95650376	NM_011643:423	Trpc1	INSIDE	0.915	0.635	1640.21	1041.39	0.581	1449.49	842.05
A_68_P22038455	chr3:32409802-32409846	NM_144519:311	Zfp639	INSIDE	0.915	2.020	348.30	703.67	1.848	356.15	658.24
A_68_P33007654	A_68_P33007654			Unknown	0.915	2.377	1514.86	3600.31	2.175	1622.01	3527.90
A_68_P28194146	chr12:3235815-3235859	NR_015551:46	1700012B15Rik	INSIDE	0.914	0.691	1237.93	855.77	0.632	1004.35	634.45
A_68_P22376196	chr3:101408366-101408410	NM_144900:192	Atp1a1	INSIDE	0.914	2.095	624.80	1308.72	1.915	553.33	1059.59
A_68_P28731503	chr12:110272067-110272111	NM_001163175:34339	Begain	INSIDE	0.914	3.240	566.15	1834.45	2.963	529.39	1568.50
A_68_P25585266	chr7:148042623-148042667	NM_018742:-362	Bet1l	DIVERGENT_PROMOTER	0.914	0.608	3728.44	2266.10	0.556	2848.61	1582.93
A_68_P26475229	chr9:45480644-45480688	NM_001081270:242291	Dscaml1	INSIDE	0.914	1.947	485.53	945.16	1.778	388.13	690.22
A_68_P31961135	chr19:12575854-12575898	NM_172442:610	Dtx4	INSIDE	0.914	2.018	1040.25	2099.57	1.844	920.23	1697.29
A_68_P29625742	chr14:57655527-57655571	NM_016975:21234	Gja3	INSIDE	0.914	3.116	2408.74	7505.58	2.847	1902.11	5415.66
A_68_P27594323	chr11:11936669-11936713	NM_001177629:-8716	Grb10	PROMOTER	0.914	1.988	1197.26	2379.78	1.817	1118.25	2031.78
A_68_P23080768	chr4:86257578-86257622	NM_173400:326	Haus6	INSIDE	0.914	0.692	1172.91	812.12	0.633	1036.91	656.16
A_68_P27828619	chr11:57823508-57823552	NM_028451:965	Larp1	INSIDE	0.914	2.673	2151.18	5750.12	2.443	1850.51	4520.88
A_68_P23229425	chr4:116136181-116136225	NM_001042743:586	Mast2	INSIDE	0.914	3.392	19747.02	66976.33	3.100	12848.53	39828.77
A_68_P27995000	chr11:87409082-87409126	NM_133215:3386	Mtmr4	INSIDE	0.914	1.967	515.81	1014.43	1.798	453.49	815.55
A_68_P21909034	chr2:180922268-180922312	NM_025598:243	Ppdpf	INSIDE	0.914	0.597	1292.55	771.74	0.546	1078.22	588.64
A_68_P27841039	chr11:59953284-59953328	NM_001037764:-277	Rai1	PROMOTER	0.914	0.413	1677.63	693.21	0.378	1332.67	503.35

ProbeName	Target position of probe on CpG island microarray	TargetID	GeneSymbol	CpG island Description	Ratio of relative methylation (TiO_2-NP/Vehicle)	Sham group			TiO_2-H group		
						Relative methylation (Cy5/Cy3)	Cy3 signal (Input)	Cy5 signal (MeDIP)	Relative methylation (Cy5/Cy3)	Cy3 signal (Input)	Cy5 signal (MeDIP)
A_68_P27943625	chr11:78301348-78301392	NM_001168521:9886	Sarm1	INSIDE	0.914	2.161	684.88	1480.18	1.975	660.90	1305.19
A_68_P32116949	chr19:41817858-41817902	NM_015748:466	Slit1	INSIDE	0.914	0.633	1233.86	781.56	0.579	1014.56	587.63
A_68_P21635427	chr2:131346365-131346409	NM_001177833:28789	Smox	INSIDE	0.914	1.979	427.09	845.07	1.809	401.49	726.30
A_68_P22829978	chr4:34137124-34137168	NM_026293:-104	Spaca1	PROMOTER	0.914	2.080	1572.08	3270.31	1.902	1197.05	2276.96
A_68_P21370051	chr2:79475802-79475846	NM_080558:243	Ssfa2	INSIDE	0.914	0.666	924.67	615.91	0.609	797.06	485.14
A_68_P26948442	chr10:12684445-12684489	NM_001163590:-401	Stx11	PROMOTER	0.914	0.344	1753.27	602.35	0.314	1441.73	452.60
A_68_P23240955	chr4:118214466-118214510	NM_172383:1843	Tmem125	INSIDE	0.914	2.009	918.78	1845.82	1.835	837.11	1536.48
A_68_P24092032	chr5:130555232-130555276	NM_001130476:-101	Tpst1	PROMOTER	0.914	1.707	2098.62	3581.88	1.560	1867.56	2912.99
A_68_P31862936	chr18:80403608-80403652	NM_001042408:25	Txnl4a	INSIDE	0.914	0.452	1296.66	586.40	0.414	1071.83	443.25
A_68_P26217006	chr8:122480748-122480792	NM_009462:46019	Usp10	INSIDE	0.914	4.372	543.87	2377.79	3.997	496.70	1985.08
A_68_P24425593	chr6:48014031-48014075	NM_001163475:22540	Zfp746	INSIDE	0.914	1.687	1279.20	2157.94	1.542	867.90	1338.47
A_68_P25992688	chr8:81636396-81636453	NM_178267:84089	Zfp827	INSIDE	0.914	2.145	621.65	1333.70	1.960	565.72	1108.89
A_68_P23971471	chr5:108796094-108796138	ENSMUST00000100944:-2760		PROMOTER	0.914	0.503	1333.30	670.04	0.460	1135.89	521.95
A_68_P27670840	chr11:28483846-28483890	NM_001166661:428	Ccdc85a	INSIDE	0.913	1.875	1347.24	2526.48	1.711	1118.10	1913.35
A_68_P23936790	chr5:102193955-102193999	NM_173370:-172	Cds1	PROMOTER	0.913	2.051	460.31	943.91	1.873	389.65	729.83
A_68_P20938884	chr1:193399786-193399830	NM_178632:196	Ints7	INSIDE	0.913	0.605	905.88	548.13	0.552	698.91	386.01
A_68_P24517582	chr6:66485182-66485226	NM_019499:-257	Mad2l1	PROMOTER	0.913	1.605	1635.32	2625.02	1.465	1352.44	1981.72
A_68_P29618280	chr14:56139420-56139464	NM_008736:3586	Nrl	INSIDE	0.913	2.239	658.22	1473.69	2.044	644.40	1317.06
A_68_P31632605	chr18:38358832-38358876	NM_029357:10562	Pcdh1	INSIDE	0.913	2.962	3588.90	10630.72	2.704	2750.09	7435.50
A_68_P31924876	chr19:4756468-4756512	NM_025717:-34	Rbm4b	PROMOTER	0.913	3.544	477.60	1692.55	3.235	498.05	1610.97
A_68_P23523568	chr5:20457296-20457340	NM_001080977:322	Rsbn1l	INSIDE	0.913	0.553	1654.69	915.68	0.505	1401.61	708.14
A_68_P27804720	chr11:53244141-53244185	NM_025352:171	Uqcrq	INSIDE	0.913	0.554	1948.20	1078.99	0.506	1537.84	777.74
A_68_P23244789	chr4:118966777-118966821	NM_011732:320	Ybx1	INSIDE	0.913	5.471	1792.55	9806.91	4.995	1327.80	6632.44
A_68_P26154271	chr8:111471277-111471321	NM_007496:232755	Zfhx3	INSIDE	0.913	2.178	895.93	1951.03	1.989	812.63	1616.01
A_68_P25009109	chr7:26011119-26011163	NM_175436:4671	Zfp526	INSIDE	0.913	4.032	1513.46	6101.83	3.683	1116.70	4112.43
A_68_P25992137	chr8:81554288-81554333	NM_178267:1975	Zfp827	INSIDE	0.913	0.533	1115.14	593.96	0.486	976.80	475.19
A_68_P26345262	chr9:20871580-20871624	NM_001103168:-65	Zglp1	PROMOTER	0.913	2.273	1245.74	2831.65	2.075	1027.99	2133.35
A_68_P25022915	chr7:29415604-29415648	NM_175319:408	C330005M16Rik	INSIDE	0.912	0.651	1298.54	845.58	0.594	1163.18	690.56
A_68_P26699343	chr9:86361138-86361182	NM_177208:400	Dopey1	INSIDE	0.912	2.230	398.53	888.65	2.033	465.42	946.08
A_68_P29142607	chr13:73984759-73984803	NM_028186:299	Nkd2	INSIDE	0.912	0.701	2323.13	1627.44	0.639	1701.10	1087.35
A_68_P20088278	chr1:23389719-23389763	NM_001081079:274	Ogfrl1	INSIDE	0.912	2.155	479.67	1033.64	1.965	435.94	856.55
A_68_P23415894	chr4:151370837-151370881	NM_172705:-570	Phf13	PROMOTER	0.912	0.577	1258.98	726.71	0.527	968.80	510.13
A_68_P24428217	chr6:48546955-48546999	NM_001079901:3095	Repin1	INSIDE	0.912	2.121	1402.80	2975.10	1.934	1375.97	2660.65
A_68_P28530875	chr12:73337837-73337881	NM_001007596:-147	Rtn1	PROMOTER	0.912	0.539	1114.54	601.20	0.492	836.05	411.21
A_68_P32182248	chr19:53464624-53464668	NM_172429:417	Smndc1	INSIDE	0.912	0.613	1466.11	899.21	0.560	1378.24	771.32
A_68_P31834367	chr18:75160274-75160318	NR_028552:-425	Snord58b	PROMOTER	0.912	0.653	1017.14	664.69	0.596	894.02	532.84
A_68_P26398840	chr9:30939131-30939175	NM_011176:232	St14	INSIDE	0.912	0.608	1464.50	889.92	0.554	1174.70	651.04
A_68_P21266476	chr2:61654143-61654187	NM_009322:11655	Tbr1	DOWNSTREAM	0.912	2.241	595.49	1334.19	2.044	707.44	1445.73
A_68_P32393596	chrX:52896212-52896256	ENSMUST00000163353:91502		INSIDE	0.912	2.117	2437.75	5160.65	1.931	881.98	1703.23
A_68_P22510980	chr3:127540779-127540823	NM_145964:-390	Ap1ar	PROMOTER	0.911	3.221	314.24	1012.20	2.933	333.22	977.43
A_68_P26781225	chr9:102528131-102528175	NM_001081122:302	Cep63	INSIDE	0.911	3.181	2230.97	7096.27	2.897	1829.44	5299.82
A_68_P27261496	chr10:75807584-75807628	NM_001081419:401	Dip2a	INSIDE	0.911	1.795	1383.18	2482.49	1.636	1131.51	1850.90
A_68_P28054686	chr11:98010707-98010751	NM_028149:202	Fbxl20	INSIDE	0.911	1.890	2035.27	3846.59	1.723	1624.25	2797.78
A_68_P25004863	chr7:25217918-25217962	NM_199013:12761	Irgc1	INSIDE	0.911	2.087	327.08	682.46	1.901	307.40	584.23
A_68_P29863084	chr14:103380991-103381035	NM_177715:842	Kctd12	INSIDE	0.911	1.894	528.55	1001.18	1.725	511.94	883.02
A_68_P31827982	chr18:73974763-73974807	NM_145494:262	Me2	INSIDE	0.911	0.498	1563.41	777.81	0.453	1366.81	619.14
A_68_P21840223	chr2:168056306-168056350	NM_001160330:207	Mocs3	INSIDE	0.911	2.755	525.13	1446.59	2.509	439.79	1103.43
A_68_P26821058	chr9:109833931-109833975	NM_001205331:-325	Mtap4	PROMOTER	0.911	2.030	1203.28	2442.62	1.849	1202.70	2224.21
A_68_P22741777	chr4:13670508-13670552	NM_001111027:-7913	Runx1t1	PROMOTER	0.911	2.605	1098.61	2861.99	2.373	870.09	2064.83
A_68_P24215193	chr6:6166905-6166949	NM_001177572:247	Slc25a13	INSIDE	0.911	0.546	1122.71	613.06	0.498	919.27	457.45
A_68_P26978752	chr10:18735310-18735354	NM_009397:-116	Tnfaip3	PROMOTER	0.911	1.689	879.64	1485.94	1.539	860.43	1323.78

ProbeName	Target position of probe on CpG island microarray	TargetID	GeneSymbol	CpG island Description	Ratio of relative methylation (TiO_2-NP/Vehicle)	Sham group			TiO_2-H group		
						Relative methylation (Cy5/Cy3)	Cy3 signal (Input)	Cy5 signal (MeDIP)	Relative methylation (Cy5/Cy3)	Cy3 signal (Input)	Cy5 signal (MeDIP)
A_68_P31156690	chr17:35089452-35089496	NR_015536:-58	1110038B12Rik	PROMOTER	0.910	0.466	1142.58	532.88	0.425	1012.88	430.05
A_68_P21537142	chr2:113688722-113688766	NM_181416:74	Arhgap11a	INSIDE	0.910	0.679	2069.45	1405.16	0.618	1635.40	1010.55
A_68_P22338537	chr3:94167894-94167938	NM_001136117:1551	C2cd4d	INSIDE	0.910	1.686	821.80	1385.84	1.534	695.46	1067.17
A_68_P27275480	chr10:78215153-78215197	NM_027630:638	Ccdc105	INSIDE	0.910	0.525	1019.24	534.96	0.478	818.26	390.88
A_68_P30331033	chr15:73377952-73378002	NM_001081066:34987	Dennd3	INSIDE	0.910	1.866	798.39	1490.00	1.698	668.94	1135.73
A_68_P25507079	chr7:134895761-134895805	NM_172748:5494	Fbxl19	INSIDE	0.910	1.923	1984.65	3817.08	1.751	1680.12	2941.83
A_68_P28080761	chr11:102527193-102527237	NM_001127576:313	Gm1564	INSIDE	0.910	2.213	643.54	1424.34	2.013	620.00	1248.19
A_68_P23321826	chr4:133808110-133808154	NM_001099296:1889	Grrp1	INSIDE	0.910	0.591	929.60	549.80	0.538	732.35	394.12
A_68_P28748276	chr12:113400089-113400133	NM_001097621:15692	Kif26a	INSIDE	0.910	3.526	3820.00	13468.84	3.207	2800.83	8981.82
A_68_P24563506	chr6:77193106-77193150	NM_028880:418	Lrrtm1	INSIDE	0.910	0.392	1593.82	624.59	0.357	1187.55	423.71
A_68_P27279614	chr10:79365826-79365870	NM_001163276:5835	Med16	INSIDE	0.910	2.087	2603.62	5433.87	1.900	1788.88	3399.11
A_68_P22785945	chr4:24423615-24423659	NM_199467:28	Mms22l	INSIDE	0.910	1.637	981.98	1607.63	1.489	825.95	1230.23
A_68_P28189462	chr11:121098436-121098480	NM_026272:-91	Narf	DIVERGENT_PROMOTER	0.910	1.511	2766.99	4180.81	1.376	2032.38	2795.90
A_68_P27262051	chr10:75905675-75905719	NM_008787:-39	Pcnt	DIVERGENT_PROMOTER	0.910	0.519	1174.90	609.59	0.472	841.86	397.48
A_68_P21630861	chr2:130460763-130460807	NM_197945:7755	Prosapip1	INSIDE	0.910	1.758	3024.45	5317.67	1.599	2429.11	3885.29
A_68_P26585375	chr9:64868889-64868933	NM_021345:614	Ptplad1	INSIDE	0.910	0.530	1701.69	902.59	0.482	1509.48	728.27
A_68_P32050980	chr19:29175604-29175648	NM_021525:-238	Rcl1	PROMOTER	0.910	0.603	1026.50	619.10	0.549	902.23	495.32
A_68_P24979891	chr7:16726357-16726401	NM_148946:10730	Slc8a2	INSIDE	0.910	1.947	710.34	1383.30	1.772	565.83	1002.69
A_68_P27673285	chr11:29073225-29073269	NM_134034:340	Smek2	INSIDE	0.910	0.600	940.64	564.26	0.546	688.87	376.24
A_68_P23814579	chr5:76612836-76612880	NM_011626:-46	Tmem165	PROMOTER	0.910	1.780	983.59	1750.47	1.620	848.78	1374.83
A_68_P31161191	chr17:35974038-35974082	NM_011655:1186	Tubb5	INSIDE	0.910	2.097	798.25	1673.90	1.909	767.73	1465.82
A_68_P20827835	chr1:173341606-173341650	NM_009480:-183	Usf1	PROMOTER	0.910	2.674	926.55	2477.22	2.434	905.46	2204.16
A_68_P24992397	chr7:19857058-19857102	NM_009499:123	Vasp	INSIDE	0.910	0.442	1371.75	606.21	0.402	1152.11	463.28
A_68_P24864287	chr6:134876970-134877014	NR_037955:-2117	1190002F15Rik	PROMOTER	0.909	0.666	2170.95	1445.29	0.605	1924.43	1164.33
A_68_P30927369	chr16:85080522-85080569	NM_001198823:93407	App	INSIDE	0.909	6.198	8581.05	53183.60	5.631	5463.68	30767.67
A_68_P21843263	chr2:168567207-168567251	NM_015731:72	Atp9a	INSIDE	0.909	2.755	438.76	1208.83	2.505	434.09	1087.42
A_68_P28752187	chr12:113977286-113977330	NM_001024602:16924	AW555464	INSIDE	0.909	2.120	2107.47	4467.78	1.928	1826.84	3521.44
A_68_P22775355	chr4:21807011-21807055	NM_172687:211	Coq3	INSIDE	0.909	1.753	1012.28	1774.71	1.594	884.00	1409.17
A_68_P25542608	chr7:140968333-140968377	NM_133941:132	Dhx32	INSIDE	0.909	1.950	556.22	1084.43	1.773	565.66	1002.75
A_68_P25020568	chr7:28964442-28964486	NM_001037957:-37	Dyrk1b	PROMOTER	0.909	1.761	985.77	1735.57	1.600	798.92	1278.50
A_68_P26161391	chr8:112741858-112741902	NM_146215:21	Ftsjd1	INSIDE	0.909	1.633	1996.24	3260.66	1.484	1509.42	2240.03
A_68_P29625743	chr14:57655656-57655700	NM_016975:21104	Gja3	INSIDE	0.909	3.413	1377.89	4702.19	3.101	1178.94	3655.34
A_68_P26972773	chr10:17667513-17667557	NM_001033432:339	Heca	INSIDE	0.909	1.907	736.48	1404.12	1.732	745.59	1291.66
A_68_P31114337	chr17:27250538-27250582	NM_080553:56312	Itpr3	INSIDE	0.909	1.969	1001.53	1972.40	1.790	941.90	1685.78
A_68_P22530128	chr3:130809594-130809638	NM_010703:-3772	Lef1	PROMOTER	0.909	1.676	908.56	1522.81	1.523	779.62	1187.66
A_68_P21817705	chr2:164240088-164240132	NM_013592:-9996	Matn4	PROMOTER	0.909	2.218	2413.48	5353.73	2.017	1752.76	3535.70
A_68_P32466325	chrX:71330416-71330460	NM_010788:494	Mecp2	INSIDE	0.909	2.086	2623.90	5474.29	1.896	1715.46	3252.78
A_68_P27944883	chr11:78510477-78510521	NM_008702:429	Nlk	INSIDE	0.909	1.523	2650.19	4035.83	1.384	2052.07	2839.85
A_68_P30558434	chr16:13668204-13668248	NM_028761:37	Parn	INSIDE	0.909	0.668	1822.33	1217.49	0.607	1324.03	803.90
A_68_P24983897	chr7:17492695-17492739	NM_008967:878	Ptgir	INSIDE	0.909	2.573	547.58	1408.99	2.338	467.01	1092.00
A_68_P28322713	chr12:30623311-30623355	NM_181395:432	Pxdn	INSIDE	0.909	2.156	1897.49	4091.68	1.961	1481.79	2905.50
A_68_P22347939	chr3:95976249-95976293	NM_153053:-202	Sf3b4	PROMOTER	0.909	0.535	1186.04	634.86	0.487	872.25	424.46
A_68_P25962756	chr8:75273840-75273884	NM_009188:26676	Sin3b	INSIDE	0.909	3.118	851.47	2655.29	2.835	739.59	2097.00
A_68_P27833774	chr11:58775271-58775315	NM_031172:-1990	Trim17	PROMOTER	0.909	1.774	1945.03	3450.16	1.613	1429.05	2304.92
A_68_P23984176	chr5:111612120-111612164	NM_024477:303321	Ttc28	INSIDE	0.909	2.073	899.00	1863.63	1.884	771.92	1454.45
A_68_P30668283	chr16:35156994-35157038	NM_001012765:1295	Adcy5	INSIDE	0.908	1.952	933.76	1822.48	1.772	761.45	1349.46
A_68_P28115698	chr11:108783986-108784030	NM_015732:2346	Axin2	INSIDE	0.908	0.551	1565.96	863.45	0.501	1274.13	638.17
A_68_P26170009	chr8:114267322-114267366	NM_009954:405	Bcar1	INSIDE	0.908	0.713	2571.24	1832.86	0.647	2060.27	1333.80
A_68_P28778249	chr12:119437163-119437207	NM_010060:332	Dnahc11	INSIDE	0.908	0.645	1768.75	1140.10	0.585	1504.64	880.82
A_68_P24591568	chr6:83072012-83072056	NM_023547:2989	Ino80b	INSIDE	0.908	0.613	2332.76	1430.36	0.557	1833.55	1020.97
A_68_P21019040	chr2:13976882-13976926	NM_001012396:758	Ptpla	INSIDE	0.908	0.725	2669.08	1935.24	0.658	2280.55	1501.13

ProbeName	Target position of probe on CpG island microarray	TargetID	GeneSymbol	CpG island Description	Ratio of relative methylation (TiO_2-NP/Vehicle)	Sham group			TiO_2-H group		
						Relative methylation (Cy5/Cy3)	Cy3 signal (Input)	Cy5 signal (MeDIP)	Relative methylation (Cy5/Cy3)	Cy3 signal (Input)	Cy5 signal (MeDIP)
A_68_P25090599	chr7:52262741-52262785	NM_001008422:8857	Scaf1	INSIDE	0.908	2.559	2295.19	5874.15	2.324	1620.01	3765.56
A_68_P28184908	chr11:120353660-120353704	NM_013770:532	Slc25a10	INSIDE	0.908	0.616	1668.99	1027.60	0.559	1251.74	699.69
A_68_P23541736	chr5:23931307-23931351	NM_009207:279	Slc4a2	INSIDE	0.908	0.395	1683.88	665.86	0.359	1530.19	549.44
A_68_P23364328	chr4:141035058-141035110	NM_019763:59428	Spen	INSIDE	0.908	2.938	388.35	1140.81	2.667	331.92	885.26
A_68_P28034316	chr11:94538398-94538442	NM_145828:387	Xylt2	INSIDE	0.908	2.566	1878.56	4820.87	2.330	1638.79	3818.57
A_68_P26037061	chr8:90304956-90305000	NM_033327:178516	Zfp423	INSIDE	0.908	2.265	742.47	1681.34	2.057	693.16	1426.02
A_68_P21812331	chr2:163245232-163245276	NM_025699:-48	3230401D17Rik	PROMOTER	0.907	0.663	1874.69	1243.38	0.602	1543.60	928.67
A_68_P31939556	chr19:7457214-7457258	NM_001033139:280	AI846148	INSIDE	0.907	0.396	1586.15	628.36	0.359	1246.60	447.79
A_68_P24248437	chr6:13627972-13628016	NM_175312:-28	B630005N14Rik	PROMOTER	0.907	0.692	2585.36	1789.63	0.628	1903.83	1195.83
A_68_P23391997	chr4:147412754-147412798	NM_011929:100	Clcn6	INSIDE	0.907	2.323	8775.74	20383.07	2.107	6035.76	12718.59
A_68_P25951438	chr8:72898648-72898692	NM_016685:1224	Comp	INSIDE	0.907	2.032	1190.57	2419.66	1.843	1090.65	2010.15
A_68_P24138652	chr5:140448047-140448091	NM_175522:64172	Elfn1	INSIDE	0.907	1.636	1748.14	2860.58	1.484	1296.65	1923.62
A_68_P25181442	chr7:72300864-72300908	NM_183087:528	Fam189a1	INSIDE	0.907	1.704	1346.92	2294.50	1.545	1256.31	1940.80
A_68_P22325240	chr3:90237336-90237380	NM_145540:200	Ints3	INSIDE	0.907	0.731	3096.03	2264.53	0.663	2335.88	1549.27
A_68_P30346608	chr15:76011006-76011051	NM_201394:15112	Plec	INSIDE	0.907	3.573	300.62	1074.11	3.240	271.39	879.35
A_68_P21870545	chr2:173412385-173412429	NR_027957:72634	Ppp4r1l-ps	INSIDE	0.907	2.570	582.18	1495.94	2.330	563.48	1312.99
A_68_P31209386	chr17:47026859-47026903	NM_178385:-689	Tbcc	PROMOTER	0.907	0.685	2550.38	1747.93	0.622	2038.89	1267.68
A_68_P32557247	chrX:97016551-97016595	NM_001081283:165	Tmem28	INSIDE	0.907	1.859	752.17	1398.16	1.685	448.35	755.63
A_68_P32707836	chrX:138824951-138824995	NM_001033600:106	Acsl4	INSIDE	0.906	2.755	794.68	2188.96	2.495	395.71	987.47
A_68_P21105431	chr2:30679624-30679668	NM_133346:4174	Asb6	INSIDE	0.906	2.312	353.79	818.00	2.095	303.06	634.88
A_68_P21566912	chr2:118741928-118741972	NM_001045523:14600	Bahd1	INSIDE	0.906	2.900	1093.85	3172.31	2.627	900.69	2366.29
A_68_P27258522	chr10:75227236-75227280	NM_172549:-156	Cabin1	PROMOTER	0.906	2.163	7305.06	15800.65	1.959	6322.41	12388.58
A_68_P30822066	chr16:64852004-64852048	NM_178647:116	Cggbp1	INSIDE	0.906	1.861	502.75	935.83	1.686	503.16	848.20
A_68_P29086260	chr13:60703649-60703693	NM_134062:99	Dapk1	INSIDE	0.906	0.689	1900.58	1310.34	0.624	1481.97	925.31
A_68_P28737545	chr12:111517127-111517171	NM_172119:-291	Dio3	DIVERGENT_PROMOTER	0.906	0.584	2005.42	1170.28	0.529	1681.27	888.68
A_68_P24046861	chr5:122298939-122298983	NM_175474:-76	Fam109a	PROMOTER	0.906	1.564	2577.92	4032.65	1.418	2161.26	3064.60
A_68_P23317857	chr4:133042635-133042679	NM_175307:6609	Fam46b	INSIDE	0.906	2.028	886.69	1798.38	1.838	688.39	1265.27
A_68_P29976940	chr15:3929308-3929352	NM_134099:243	Fbxo4	INSIDE	0.906	1.989	508.18	1010.70	1.802	516.88	931.16
A_68_P23582143	chr5:32435937-32435982	NM_008037:-2885	Fosl2	PROMOTER	0.906	1.991	4053.18	8070.65	1.803	2564.52	4623.98
A_68_P23451688	chr5:4757569-4757613	NM_021457:626	Fzd1	INSIDE	0.906	0.703	1243.83	873.91	0.637	1152.83	734.12
A_68_P20156379	chr1:37932274-37932318	NM_001037918:3246	Lipt1	INSIDE	0.906	3.342	1954.19	6530.44	3.028	1374.07	4160.95
A_68_P31217292	chr17:48548926-48548970	NM_001110832:197	Nfya	INSIDE	0.906	0.632	3533.57	2231.54	0.572	2749.24	1573.47
A_68_P27290118	chr10:80961853-80961897	NM_010102:1008	S1pr4	INSIDE	0.906	2.200	1741.43	3831.92	1.993	1381.49	2753.88
A_68_P26885412	chr9:121671713-121671757	NM_001166644:2584	Zfp651	INSIDE	0.906	1.948	1133.07	2206.85	1.766	827.12	1460.32
A_68_P28092303	chr11:104547246-104547290	ENSMUST00000148007:248		INSIDE	0.906	2.010	534.15	1073.54	1.820	485.49	883.83
A_68_P27228311	chr10:69652022-69652071	NM_001111121:92178	Ccdc6	INSIDE	0.905	2.857	1630.69	4659.48	2.585	1336.94	3456.22
A_68_P23203727	chr4:109650158-109650202	NM_172296:-449	Dmrta2	PROMOTER	0.905	0.388	2052.51	796.11	0.351	1531.56	537.35
A_68_P24133858	chr5:139625772-139625819	NM_001081265:-381	Heatr2	PROMOTER	0.905	10.675	1522.33	16250.91	9.660	739.67	7145.52
A_68_P27804076	chr11:53113307-53113351	NM_008300:653	Hspa4	INSIDE	0.905	0.474	1240.25	587.97	0.429	1155.47	495.84
A_68_P21148580	chr2:38367238-38367282	NM_001159631:-135	Nek6	PROMOTER	0.905	2.049	513.90	1052.73	1.854	478.03	886.06
A_68_P24755177	chr6:112646572-112646616	NM_001167730:70	Rad18	INSIDE	0.905	0.676	3400.51	2297.13	0.612	2775.26	1697.42
A_68_P27045329	chr10:31328483-31328527	NM_001146349:1027	Rnf217	INSIDE	0.905	0.699	1813.12	1266.87	0.632	1643.81	1039.44
A_68_P26504535	chr9:50411810-50411854	NM_025848:122	Sdhd	INSIDE	0.905	0.618	1345.33	831.18	0.559	1101.25	615.85
A_68_P20970770	chr2:4802809-4802853	NM_175400:221	Sephs1	INSIDE	0.905	2.107	410.64	865.22	1.907	444.75	848.33
A_68_P27392327	chr10:99950250-99950294	NM_001110013:-291	Tmtc3	DIVERGENT_PROMOTER	0.905	0.714	1948.72	1392.34	0.646	1649.39	1066.13
A_68_P25589082	chr7:148661081-148661125	NM_053082:-36	Tspan4	DIVERGENT_PROMOTER	0.905	0.735	2592.32	1904.80	0.665	1966.21	1306.94
A_68_P29998080	chr15:8049104-8049148	NM_001081402:83	Wdr70	INSIDE	0.905	0.636	3185.56	2025.95	0.575	2715.84	1562.79
A_68_P29339698	chr13:114083264-114083308			Unknown	0.905	1.663	1699.17	2826.00	1.504	1343.05	2020.54
A_68_P27283430	chr10:79934572-79934616	ENSMUST00000158603:-4502		PROMOTER	0.905	1.944	3361.98	6536.15	1.760	2444.37	4303.02
A_68_P30480392	chr15:99304984-99305028	NM_029236:155	Bcdin3d	INSIDE	0.904	1.722	883.64	1521.24	1.556	645.36	1004.00
A_68_P22394779	chr3:104855704-104855748	NM_001163332:145	Cttnbp2nl	INSIDE	0.904	0.370	1552.02	574.19	0.334	1356.59	453.68

ProbeName	Target position of probe on CpG island microarray	TargetID	GeneSymbol	CpG island Description	Ratio of relative methylation (TiO_2-NP/Vehicle)	Sham group			TiO_2-H group		
						Relative methylation (Cy5/Cy3)	Cy3 signal (Input)	Cy5 signal (MeDIP)	Relative methylation (Cy5/Cy3)	Cy3 signal (Input)	Cy5 signal (MeDIP)
A_68_P28248077	chr12:13255581-13255625	NM_134040:378	Ddx1	INSIDE	0.904	0.703	1863.96	1309.78	0.635	1289.02	818.79
A_68_P30591036	chr16:21214742-21214786	NM_010143:9897	Ephb3	INSIDE	0.904	2.117	544.65	1153.15	1.915	489.24	936.86
A_68_P26525031	chr9:54134407-54134451	NM_177350:136	Gldn	INSIDE	0.904	1.911	491.41	938.89	1.727	483.51	835.00
A_68_P28048330	chr11:96903095-96903139	NM_026154:217	Mrpl10	INSIDE	0.904	0.607	1074.06	652.45	0.549	860.68	472.57
A_68_P31553011	chr18:23199794-23199838	NM_001161483:-2652	Nol4	PROMOTER	0.904	1.994	1599.86	3190.22	1.803	1407.92	2538.13
A_68_P31438303	chr17:90854426-90854470	NM_020252:637694	Nrxn1	INSIDE	0.904	0.424	1332.23	564.50	0.383	1057.41	405.00
A_68_P29479232	chr14:26513832-26513876	NM_134084:199	Ppif	INSIDE	0.904	0.637	1020.39	650.41	0.576	869.78	500.94
A_68_P32020395	chr19:23967551-23967595	NM_177034:134207	Apba1	INSIDE	0.903	2.464	943.55	2325.28	2.226	1008.25	2244.67
A_68_P26459693	chr9:42914668-42914712	NM_027144:-889	Arhgef12	PROMOTER	0.903	0.557	1284.33	715.07	0.503	1026.73	516.44
A_68_P23541451	chr5:23893582-23893626	NM_001002897:4357	Atg9b	INSIDE	0.903	0.607	1083.47	657.93	0.548	891.06	488.67
A_68_P30371304	chr15:80118357-80118401	NM_001044308:711	Cacna1i	INSIDE	0.903	0.574	1227.16	704.92	0.518	974.57	505.26
A_68_P31715219	chr18:53904261-53904305	NM_178686:-81	Cep120	PROMOTER	0.903	0.715	2087.69	1493.30	0.646	1624.30	1049.36
A_68_P27186923	chr10:62065160-62065204	NM_019553:-136	Ddx21	PROMOTER	0.903	0.433	1536.72	665.65	0.391	1159.76	453.82
A_68_P25495195	chr7:132695872-132695916	NM_001008700:99	Il4ra	INSIDE	0.903	1.675	2603.14	4359.57	1.513	2092.22	3164.90
A_68_P29616401	chr14:55732942-55732986	NM_177049:2808	Jph4	INSIDE	0.903	1.665	910.41	1516.24	1.504	819.24	1232.47
A_68_P27425028	chr10:106709567-106709611	NM_001033223:702	Lin7a	INSIDE	0.903	2.113	494.63	1045.37	1.908	464.65	886.69
A_68_P25574331	chr7:146022127-146022171	NM_001045483:15802	Mapk1ip1	DOWNSTREAM	0.903	1.898	2004.32	3804.27	1.713	1629.68	2792.29
A_68_P26349890	chr9:21792524-21792568	NM_023622:1351	Rgl3	INSIDE	0.903	0.568	1475.03	838.36	0.513	1197.76	614.81
A_68_P22393170	chr3:104591895-104591939	NM_007484:-35	Rhoc	PROMOTER	0.903	1.881	596.53	1122.31	1.699	537.18	912.78
A_68_P26430601	chr9:37237135-37237179	NM_001164767:3604	Robo3	INSIDE	0.903	2.151	1497.53	3220.60	1.943	1137.22	2209.24
A_68_P21101134	chr2:29922658-29922702	NM_001204875:687	Set	INSIDE	0.903	0.608	1618.39	983.76	0.549	1283.71	704.91
A_68_P27549890	chr10:129881898-129881942	NM_001103365:11030	Vmn2r86	DOWNSTREAM	0.903	3.186	366.71	1168.41	2.876	357.92	1029.36
A_68_P21113503	chr2:31993634-31993679			Unknown	0.903	2.446	257.61	630.19	2.208	237.53	524.56
A_68_P22048260	chr3:34353370-34353414			Unknown	0.903	1.891	1781.39	3368.16	1.707	1383.72	2362.60
A_68_P33005942	A_68_P33005942			Unknown	0.903	4.743	113.68	539.15	4.283	107.72	461.42
A_68_P24784500	chr6:117961807-117961851	ENSMUST00000032230:437		INSIDE	0.903	0.668	1435.10	958.74	0.603	1309.60	789.95
A_68_P31305846	chr17:66728820-66728864	NM_001114098:70248	1110012J17Rik	INSIDE	0.902	2.008	1910.28	3836.62	1.811	1495.39	2708.51
A_68_P26248108	chr8:127386840-127386884	NM_025615:67	2810004N23Rik	INSIDE	0.902	0.727	2122.54	1542.62	0.655	1554.36	1018.71
A_68_P22407413	chr3:107399619-107399663	NM_007441:1692	Alx3	INSIDE	0.902	0.538	1989.04	1069.92	0.485	1645.56	798.83
A_68_P27535079	chr10:126727356-126727401	NM_007837:-470	Ddit3	DIVERGENT_PROMOTER	0.902	2.347	2221.75	5214.23	2.117	1498.84	3173.13
A_68_P23981088	chr5:111082625-111082669	NM_027156:177	Ddx51	INSIDE	0.902	0.552	1720.43	949.02	0.498	1508.03	750.33
A_68_P22582960	chr3:140874546-140874590	NM_008811:751	Pdha2	INSIDE	0.902	2.385	1033.07	2463.77	2.150	879.31	1890.70
A_68_P26238678	chr8:125759558-125759602	NM_030176:529	Spata2L	INSIDE	0.902	0.646	1285.20	830.44	0.583	1030.59	600.50
A_68_P25094660	chr7:52968835-52968879	NM_020011:1721	Sphk2	INSIDE	0.902	3.189	1211.78	3863.88	2.877	940.44	2705.92
A_68_P30792109	chr16:58523405-58523449	NM_018784:-1	St3gal6	PROMOTER	0.902	0.701	1841.04	1290.41	0.632	1504.53	951.14
A_68_P31187138	chr17:43154011-43154055	NM_178589:529	Tnfrsf21	INSIDE	0.902	0.647	902.73	584.02	0.584	824.56	481.17
A_68_P24153574	chr5:143581988-143582032	NM_001122730:-2944	Tnrc18	PROMOTER	0.902	1.942	2678.39	5201.19	1.751	2110.42	3695.86
A_68_P20804316	chr1:169215214-169215258	NM_030724:22	Uck2	INSIDE	0.902	0.533	1248.47	665.67	0.481	1113.48	535.48
A_68_P24974110	chr7:13593210-13593254	NM_001024699:1917	Zbtb45	INSIDE	0.902	2.045	2511.70	5136.71	1.844	2214.64	4084.48
A_68_P25379198	chr7:109661711-109661755	ENSMUST00000097222:7806		DOWNSTREAM	0.902	2.439	1320.62	3221.37	2.199	1165.99	2564.30
A_68_P23637947	chr5:42235776-42235820	NM_001081422:-244	Bod1l	PROMOTER	0.901	2.309	388.61	897.37	2.080	395.00	821.58
A_68_P25582099	chr7:147256517-147256561	NM_001190386:11662	Caly	INSIDE	0.901	2.346	2170.02	5090.76	2.114	1610.82	3405.00
A_68_P30589272	chr16:20733318-20733362	NM_009893:141	Chrd	INSIDE	0.901	2.224	921.15	2048.58	2.003	723.35	1448.94
A_68_P24000793	chr5:114358500-114358544	NM_011779:193	Coro1c	INSIDE	0.901	3.678	493.88	1816.37	3.314	528.25	1750.75
A_68_P20456055	chr1:95408562-95408606	NM_145519:-96	Farp2	PROMOTER	0.901	0.592	1047.66	619.92	0.533	927.51	494.68
A_68_P23591963	chr5:34325816-34325860	NM_001033458:-284	Gm1673	PROMOTER	0.901	0.680	1493.76	1015.53	0.612	1125.13	688.85
A_68_P30490485	chr15:101062094-101062138	NM_019518:7479	Grasp	INSIDE	0.901	2.236	1736.64	3882.73	2.015	1316.52	2652.36
A_68_P26622299	chr9:71333296-71333340	NM_001164793:-553	Grinl1a	PROMOTER	0.901	2.436	960.07	2338.90	2.194	927.00	2034.27
A_68_P30547090	chr16:11254627-11254671	NR_035466:-110	Mir1945	PROMOTER	0.901	0.648	1850.35	1199.02	0.584	1587.98	927.44
A_68_P26884592	chr9:121551542-121551586	NM_011703:-269	Vipr1	DIVERGENT_PROMOTER	0.901	0.544	1221.70	664.47	0.490	1002.70	491.16
A_68_P31206541	chr17:46521167-46521211	NM_207671:474	Zfp318	INSIDE	0.901	1.709	772.27	1319.66	1.540	796.07	1226.29

ProbeName	Target position of probe on CpG island microarray	TargetID	GeneSymbol	CpG island Description	Ratio of relative methylation (TiO_2-NP/Vehicle)	Sham group			TiO_2-H group		
						Relative methylation (Cy5/Cy3)	Cy3 signal (Input)	Cy5 signal (MeDIP)	Relative methylation (Cy5/Cy3)	Cy3 signal (Input)	Cy5 signal (MeDIP)
A_68_P25614225	chr8:3495864-3495908	NM_080461:2749	Zfp358	INSIDE	0.901	4.545	642.47	2919.93	4.093	572.13	2341.82
A_68_P22283295	chr3:82932608-82932652	ENSMUST00000047876:538		INSIDE	0.901	1.714	736.83	1263.15	1.544	630.15	973.10
A_68_P27288864	chr10:80785360-80785404	NM_027381:1535	2510012J08Rik	INSIDE	0.900	2.937	293.82	863.01	2.644	274.55	725.94
A_68_P31731123	chr18:56732742-56732786	NM_001127338:-171	Aldh7a1	PROMOTER	0.900	0.493	1137.97	560.60	0.443	887.95	393.67
A_68_P28183243	chr11:120097692-120097736	NM_198423:3454	Bahcc1	INSIDE	0.900	0.639	2039.68	1303.20	0.575	1578.37	907.40
A_68_P26486126	chr9:47339043-47339087	NM_001025600:630	Cadm1	INSIDE	0.900	0.563	2852.15	1605.16	0.507	2135.41	1081.73
A_68_P23202005	chr4:109338819-109338863	NM_007671:-863	Cdkn2c	PROMOTER	0.900	0.545	1437.43	783.03	0.490	1076.21	527.60
A_68_P21144120	chr2:37642065-37642109	NM_001163566:10318	Crb2	INSIDE	0.900	2.021	477.45	965.13	1.819	458.74	834.29
A_68_P25098704	chr7:53632208-53632252	NM_010866:387	Myod1	INSIDE	0.900	0.679	1345.36	913.65	0.611	1097.88	670.94
A_68_P23134165	chr4:97418263-97418307	NM_001122952:169651	Nfia	INSIDE	0.900	0.661	1100.36	727.53	0.595	912.53	542.95
A_68_P21585951	chr2:122174819-122174863	NM_001013829:19814	Shf	INSIDE	0.900	2.019	691.97	1396.89	1.816	601.03	1091.52
A_68_P26983374	chr10:19570550-19570594	NM_029529:693	Slc35d3	INSIDE	0.900	1.575	1408.52	2217.98	1.418	1236.93	1753.54
A_68_P24320419	chr6:28430125-28430169	NM_019776:-201	Snd1	PROMOTER	0.900	2.586	1223.49	3163.65	2.327	1010.25	2350.64
A_68_P32109308	chr19:40587948-40587992	NM_178362:256	Sorbs1	INSIDE	0.900	0.645	1287.29	829.66	0.580	1027.77	596.23
A_68_P20914290	chr1:189431183-189431227	ENSMUST00000127489:-1465		PROMOTER	0.900	0.699	2367.05	1653.64	0.629	1842.90	1158.96
A_68_P21179669	chr2:44783090-44783134	ENSMUST00000129240:-328		PROMOTER	0.900	1.895	865.95	1641.34	1.706	885.74	1511.24
A_68_P24050675	chr5:122951393-122951437	NM_009722:820	Atp2a2	INSIDE	0.899	0.618	948.84	586.12	0.555	880.06	488.51
A_68_P32260365	chrX:11667413-11667459	NM_029510:-9757	Bcor	PROMOTER	0.899	0.565	1720.60	972.22	0.508	787.97	400.48
A_68_P31227868	chr17:50432726-50432770	NM_010021:176	Dazl	INSIDE	0.899	2.452	462.45	1134.09	2.204	444.40	979.31
A_68_P20553864	chr1:120734165-120734209	NM_001081125:216010	Gli2	INSIDE	0.899	2.520	727.31	1832.81	2.266	716.99	1625.05
A_68_P25362936	chr7:106774598-106774642	NM_001195529:993	Gm4980	INSIDE	0.899	1.972	446.92	881.38	1.773	446.42	791.68
A_68_P25031176	chr7:31140849-31140896	NM_175478:6919	Lrfn3	INSIDE	0.899	3.149	478.42	1506.49	2.832	405.84	1149.19
A_68_P31050850	chr17:12464627-12464673	NM_011948:46876	Map3k4	INSIDE	0.899	1.679	965.89	1621.77	1.509	681.93	1029.26
A_68_P25223253	chr7:79451535-79451579	NM_001024703:-75	Mctp2	PROMOTER	0.899	2.467	417.02	1028.97	2.217	456.80	1012.71
A_68_P29226006	chr13:93614678-93614722	NM_001162945:-41	Mtx3	PROMOTER	0.899	2.326	476.87	1109.03	2.091	418.70	875.50
A_68_P21843327	chr2:168578173-168578217	NM_175303:14507	Sall4	INSIDE	0.899	1.989	526.47	1047.37	1.788	448.48	801.69
A_68_P26983368	chr10:19569800-19569844	NM_029529:1443	Slc35d3	INSIDE	0.899	0.555	1517.26	841.51	0.498	1246.99	621.42
A_68_P26239285	chr8:125856857-125856901	NM_172287:266	Spire2	INSIDE	0.899	0.401	2039.35	817.12	0.360	1666.75	600.40
A_68_P30360707	chr15:78370109-78370155	NM_009218:4643	Sstr3	INSIDE	0.899	1.974	1226.45	2421.06	1.774	975.72	1731.41
A_68_P27290697	chr10:81050580-81050624	NM_019725:12326	Tle2	INSIDE	0.899	1.789	979.04	1751.51	1.607	858.15	1379.43
A_68_P24974294	chr7:13623604-13623648	NM_001168469:-7	Ube2m	PROMOTER	0.899	1.673	1311.96	2195.52	1.505	1046.81	1575.00
A_68_P21797527	chr2:160698949-160698993	NM_177263:-244	Zhx3	DIVERGENT_PROMOTER	0.899	0.696	1569.59	1093.10	0.626	1493.34	934.84
A_68_P30353821	chr15:77200742-77200786	BC100417:676		INSIDE	0.899	2.384	882.16	2102.68	2.143	739.78	1585.34
A_68_P31678552	chr18:46901420-46901464	NM_009681:-128	Ap3s1	DIVERGENT_PROMOTER	0.898	0.598	1436.08	858.15	0.537	1146.25	615.14
A_68_P27788007	chr11:50139083-50139127	NM_001110499:71	Canx	INSIDE	0.898	0.435	2047.06	890.59	0.391	1681.03	656.54
A_68_P27921607	chr11:74451400-74451447	NM_001013784:18317	E130309D14Rik	INSIDE	0.898	0.672	1060.70	713.04	0.603	922.67	556.72
A_68_P32461571	chrX:70473910-70473954	NM_007978:288	F8a	INSIDE	0.898	2.270	536.08	1216.71	2.038	401.25	817.74
A_68_P25827950	chr8:46108981-46109030	NM_001081286:73444	Fat1	INSIDE	0.898	3.184	318.98	1015.61	2.858	310.74	888.21
A_68_P25954495	chr8:73376456-73376500	NM_032397:4429	Kcnn1	INSIDE	0.898	3.380	671.05	2268.10	3.037	596.84	1812.33
A_68_P24378363	chr6:38586630-38586674	NM_001033171:587	Klrg2	INSIDE	0.898	0.651	1272.65	828.50	0.585	1154.95	675.12
A_68_P32016088	chr19:23216510-23216554	NR_035423:-7388	Mir1192	PROMOTER	0.898	0.665	1387.25	922.58	0.597	1122.97	670.34
A_68_P32697218	chrX:136595877-136595921	NM_001081668:1209	Nup62cl	INSIDE	0.898	2.127	554.82	1180.11	1.910	396.66	757.56
A_68_P30346601	chr15:76010142-76010186	NM_201394:15976	Plec	INSIDE	0.898	2.830	425.34	1203.82	2.541	338.94	861.08
A_68_P24008563	chr5:115685673-115685717	NM_026398:-165	Pop5	PROMOTER	0.898	1.617	1220.32	1972.67	1.452	1061.02	1540.15
A_68_P28186726	chr11:120645198-120645242	NM_009053:298	Rfng	INSIDE	0.898	0.553	1266.87	700.90	0.497	1152.82	573.05
A_68_P22875662	chr4:44181111-44181156	NM_175201:-723	Rnf38	PROMOTER	0.898	2.601	622.41	1618.74	2.335	610.15	1424.68
A_68_P25957633	chr8:74108592-74108640	NM_011977:15791	Slc27a1	INSIDE	0.898	2.098	924.15	1938.48	1.883	694.71	1308.17
A_68_P27629545	chr11:19920766-19920810	NM_033523:96344	Spred2	INSIDE	0.898	2.268	647.00	1467.11	2.036	553.21	1126.51
A_68_P25025683	chr7:29946879-29946923	NM_182927:6766	Spred3	INSIDE	0.898	1.910	1121.38	2141.97	1.716	872.72	1497.47
A_68_P20444768	chr1:93698701-93698745	NM_007855:669	Twist2	INSIDE	0.898	0.723	1562.40	1129.79	0.650	1247.70	810.64
A_68_P23364203	chr4:141020484-141020528	NM_009541:19919	Zbtb17	INSIDE	0.898	1.955	638.54	1248.27	1.755	599.83	1052.72

ProbeName	Target position of probe on CpG island microarray	TargetID	GeneSymbol	CpG island Description	Ratio of relative methylation (TiO_2-NP/Vehicle)	Sham group			TiO_2-H group		
						Relative methylation (Cy5/Cy3)	Cy3 signal (Input)	Cy5 signal (MeDIP)	Relative methylation (Cy5/Cy3)	Cy3 signal (Input)	Cy5 signal (MeDIP)
A_68_P21937419	chr3:9452272-9452316	NM_133218:157791	Zfp704	INSIDE	0.898	2.019	399.00	805.54	1.813	393.61	713.51
A_68_P24385305	chr6:39822036-39822080			Unknown	0.898	0.620	1908.35	1183.69	0.557	1446.29	805.78
A_68_P29002651	chr13:45663653-45663697	NM_009124:396671	Atxn1	INSIDE	0.897	3.826	2945.82	11270.84	3.432	2389.25	8200.46
A_68_P31103376	chr17:25522874-25522918	NM_001163691:47832	Cacna1h	INSIDE	0.897	2.191	423.71	928.39	1.966	354.80	697.60
A_68_P30369297	chr15:79762789-79762833	NM_144811:266	Cbx7	INSIDE	0.897	1.947	2026.83	3945.98	1.747	1624.22	2837.25
A_68_P31792810	chr18:67770708-67770752	NM_001081073:30260	Cep76	DOWNSTREAM	0.897	0.570	924.59	527.08	0.511	882.98	451.34
A_68_P22291209	chr3:84283512-84283556	NM_001205355:-121	Fhdc1	PROMOTER	0.897	0.367	2113.36	776.24	0.329	1625.63	535.36
A_68_P27800934	chr11:52578718-52578762	NM_177059:533	Fstl4	INSIDE	0.897	0.684	3671.47	2510.26	0.613	2769.31	1698.85
A_68_P26156364	chr8:111864649-111864693	NR_002928:138	Gm1943	INSIDE	0.897	3.628	1239.58	4497.27	3.255	893.95	2910.05
A_68_P24428224	chr6:48547697-48547741	NM_001079901:3837	Repin1	INSIDE	0.897	2.619	338.79	887.28	2.350	315.97	742.40
A_68_P22824534	chr4:33070413-33070457	NM_027491:462	Rragd	INSIDE	0.897	2.132	656.19	1399.32	1.913	696.22	1331.73
A_68_P27807573	chr11:53794024-53794068	NM_019723:-517	Slc22a21	PROMOTER	0.897	1.627	979.41	1593.89	1.459	837.46	1222.22
A_68_P24127578	chr5:138229494-138229544	NM_001005426:489	Zcwpw1	INSIDE	0.897	6.519	1167.22	7609.32	5.846	817.42	4778.26
A_68_P32536916	chrX:91234250-91234302	ENSMUST00000118140:-118		PROMOTER	0.897	1.915	843.43	1615.10	1.717	406.50	697.91
A_68_P25833740	chr8:47085210-47085254	NM_001039562:-28	Ankrd37	PROMOTER	0.896	0.573	901.59	516.87	0.514	718.51	369.15
A_68_P30927365	chr16:85080178-85080222	NM_001198823:93752	App	INSIDE	0.896	2.908	843.05	2451.72	2.605	703.09	1831.89
A_68_P32782610	chrX:157432513-157432557	NM_001024624:79	Cdkl5	INSIDE	0.896	1.581	3556.58	5624.24	1.416	1697.33	2404.24
A_68_P31075177	chr17:17987055-17987099	NM_008215:5076	Has1	INSIDE	0.896	1.671	2408.65	4025.72	1.498	2203.82	3300.89
A_68_P23080770	chr4:86257737-86257781	NM_173400:168	Haus6	INSIDE	0.896	0.405	1715.87	695.31	0.363	1289.28	467.85
A_68_P32029538	chr19:25484679-25484723	NM_181404:173009	Kank1	INSIDE	0.896	4.044	2173.99	8790.99	3.624	1640.19	5943.97
A_68_P32140494	chr19:45857174-45857218	NM_023799:585	Mgea5	INSIDE	0.896	1.552	1486.20	2306.76	1.390	1265.56	1759.58
A_68_P20451520	chr1:94744954-94744998	NR_029559:-1978	Mir149	PROMOTER	0.896	2.155	8283.84	17848.09	1.930	5640.09	10883.24
A_68_P25949902	chr8:72635905-72635949	NM_007789:8817	Ncan	INSIDE	0.896	1.825	1815.98	3315.00	1.636	1587.47	2596.39
A_68_P30456843	chr15:95059574-95059618	NM_016743:299541	Nell2	INSIDE	0.896	2.057	763.90	1571.61	1.843	702.66	1295.33
A_68_P22695187	chr4:3865199-3865243	NM_019969:332	Plag1	INSIDE	0.896	1.656	3559.54	5893.17	1.483	2568.88	3809.52
A_68_P22204193	chr3:66025009-66025053	NM_008987:1222	Ptx3	INSIDE	0.896	2.451	1646.55	4035.73	2.197	1262.02	2772.17
A_68_P24992474	chr7:19872133-19872177	NM_001025364:-4293	Rtn2	PROMOTER	0.896	2.827	968.96	2739.17	2.533	874.12	2214.03
A_68_P28307255	chr12:28026369-28026413	NM_009234:1193	Sox11	INSIDE	0.896	1.919	1302.54	2499.73	1.719	1124.74	1933.78
A_68_P31381746	chr17:80605842-80605886	NM_001195485:781	Srsf7	INSIDE	0.896	0.611	1187.90	726.08	0.548	1110.29	607.92
A_68_P23353413	chr4:139225072-139225116	NM_031873:15642	Tas1r2	INSIDE	0.896	3.467	797.66	2765.31	3.105	678.03	2105.13
A_68_P27992698	chr11:86940439-86940483	NM_197987:-118	Trim37	PROMOTER	0.896	0.486	1740.04	846.25	0.436	1509.53	657.48
A_68_P21622817	chr2:128891746-128891790	NM_027192:86	Ttl	INSIDE	0.896	0.642	1383.93	888.00	0.575	1104.45	634.85
A_68_P21137234	chr2:35866956-35867000			Unknown	0.896	0.479	1347.83	645.43	0.429	1116.62	479.17
A_68_P20641035	chr1:137584675-137584719	ENSMUST00000112103:-14		PROMOTER	0.896	2.874	408.73	1174.71	2.575	359.50	925.58
A_68_P31175219	chr17:39982102-39982146	ENSMUST00000157647:-1946		PROMOTER	0.896	3.289	218348.60	718222.00	2.948	151257.30	445937.40
A_68_P31939675	chr19:7491423-7491467	NM_175381:-670	2700081O15Rik	PROMOTER	0.895	0.652	789.95	515.00	0.583	720.18	420.01
A_68_P31150730	chr17:33980032-33980076	NM_019421:19	Cd320	INSIDE	0.895	2.172	1440.50	3129.12	1.944	1156.43	2247.57
A_68_P24122090	chr5:137042535-137042579	NM_198602:719	Cux1	INSIDE	0.895	0.515	1632.00	839.84	0.461	1315.10	605.95
A_68_P21840413	chr2:168094194-168094238	NM_001081134:615	Kcng1	INSIDE	0.895	2.129	1437.87	3061.37	1.905	990.42	1886.70
A_68_P29330398	chr13:112545643-112545687	NM_011945:53527	Map3k1	INSIDE	0.895	2.390	1140.16	2724.47	2.139	810.08	1732.84
A_68_P25279961	chr7:90017553-90017603	NM_175366:1736	Mex3b	INSIDE	0.895	0.500	1161.42	580.62	0.448	956.32	428.03
A_68_P24591425	chr6:83051529-83051573	NM_026744:-7551	Mrpl53	PROMOTER	0.895	0.601	1376.95	827.31	0.538	1073.82	577.68
A_68_P20413388	chr1:88567300-88567344	NM_010933:-174	Nppc	PROMOTER	0.895	1.763	1139.18	2008.72	1.579	961.93	1518.56
A_68_P31256421	chr17:56613568-56613612	NM_011218:2313	Ptprs	INSIDE	0.895	0.589	1960.16	1154.00	0.527	1435.34	756.32
A_68_P23359682	chr4:140258313-140258357	NM_173867:947	Rcc2	INSIDE	0.895	0.635	1178.32	748.79	0.569	1066.09	606.26
A_68_P30605570	chr16:23927739-23927783	NM_001008230:3120	Rtp2	INSIDE	0.895	2.054	418.35	859.20	1.838	359.45	660.59
A_68_P21843350	chr2:168581447-168581491	NM_175303:11233	Sall4	INSIDE	0.895	2.566	1164.25	2987.90	2.297	943.35	2166.60
A_68_P29675223	chr14:66349464-66349508	NM_001168318:64247	Scara5	INSIDE	0.895	2.822	397.03	1120.57	2.527	312.90	790.63
A_68_P27814243	chr11:55017874-55017918	NM_153139:55	Slc36a1	INSIDE	0.895	1.717	1001.84	1720.05	1.537	900.53	1384.20
A_68_P32116952	chr19:41818141-41818185	NM_015748:184	Slit1	INSIDE	0.895	1.783	783.34	1396.69	1.596	638.32	1018.56
A_68_P31201715	chr17:45684089-45684133	NM_001013749:2516	Tmem151b	INSIDE	0.895	3.892	1793.01	6978.43	3.482	1251.09	4356.57

ProbeName	Target position of probe on CpG island microarray	TargetID	GeneSymbol	CpG island Description	Ratio of relative methylation (TiO_2-NP/Vehicle)	Sham group			TiO_2-H group		
						Relative methylation (Cy5/Cy3)	Cy3 signal (Input)	Cy5 signal (MeDIP)	Relative methylation (Cy5/Cy3)	Cy3 signal (Input)	Cy5 signal (MeDIP)
A_68_P22059534	chr3:36588360-36588404	NM_019510:707	Trpc3	INSIDE	0.895	0.586	934.55	547.83	0.525	732.05	384.19
A_68_P23185611	chr4:106311721-106311765	NM_177667:16689	Ttc22	INSIDE	0.895	3.008	642.71	1932.98	2.691	546.04	1469.33
A_68_P26082676	chr8:97851882-97851927	NM_024467:3946	Zfp319	INSIDE	0.895	2.044	367.33	750.93	1.831	361.52	661.78
A_68_P25008270	chr7:25866801-25866845	NM_175477:4558	Zfp574	INSIDE	0.895	1.741	883.35	1537.77	1.558	738.85	1151.44
A_68_P24768311	chr6:114919698-114919742	ENSMUST00000164670:-499		PROMOTER	0.895	0.629	923.01	580.32	0.563	727.46	409.57
A_68_P23278549	chr4:125840190-125840234	NM_025856:-303	1700029G01Rik	PROMOTER	0.894	1.674	4006.90	6707.24	1.496	3264.99	4884.42
A_68_P23327317	chr4:134774491-134774538	NM_013885:54161	Clic4	INSIDE	0.894	3.881	1064.28	4130.46	3.471	779.07	2703.87
A_68_P21905702	chr2:180445146-180445190	NM_177852:-464	Dido1	DIVERGENT_PROMOTER	0.894	2.009	1084.89	2179.82	1.795	1063.16	1908.88
A_68_P29047198	chr13:54151095-54151139	NM_010076:-89	Drd1a	PROMOTER	0.894	0.651	1388.26	903.94	0.582	1169.17	680.59
A_68_P26658153	chr9:77955869-77955913	NM_023605:573	Fbxo9	INSIDE	0.894	0.691	2457.81	1697.89	0.617	1843.60	1138.00
A_68_P23804519	chr5:75016038-75016082	NM_001159578:57837	Lnx1	INSIDE	0.894	1.830	652.45	1194.16	1.637	495.91	811.86
A_68_P27782124	chr11:49064057-49064101	NM_001110150:25	Mgat1	INSIDE	0.894	0.674	1668.03	1123.92	0.602	1420.33	855.57
A_68_P21722730	chr2:147009969-147010013	NR_030769:172	Nkx2-2as	INSIDE	0.894	0.645	1106.53	713.17	0.576	884.23	509.32
A_68_P26017400	chr8:86496806-86496851	NM_008854:-48	Prkaca	PROMOTER	0.894	0.603	2236.05	1347.68	0.539	1833.58	987.45
A_68_P29715884	chr14:73542895-73542939	NR_033185:325	Rcbtb2	INSIDE	0.894	1.878	520.24	977.24	1.680	437.04	734.18
A_68_P28081368	chr11:102635887-102635931	NM_001110778:13156	Adam11	INSIDE	0.893	0.551	1013.64	558.08	0.492	808.55	397.64
A_68_P21781773	chr2:158054637-158054681	NM_001131021:300	D630003M21Rik	INSIDE	0.893	2.303	368.89	849.66	2.057	315.97	649.85
A_68_P26345299	chr9:20878051-20878095	NM_001039824:-114	Fdx1l	PROMOTER	0.893	0.671	1223.88	821.37	0.599	1042.72	624.58
A_68_P30363893	chr15:78858397-78858441	NM_008197:-223	H1f0	PROMOTER	0.893	1.973	784.65	1548.01	1.762	719.15	1267.09
A_68_P25466057	chr7:127039834-127039878	NM_029610:51	Lyrm1	INSIDE	0.893	0.530	2019.19	1070.06	0.473	1766.05	835.71
A_68_P30232956	chr15:55389148-55389192	NM_001168250:208	Mtbp	INSIDE	0.893	0.587	1426.30	836.89	0.524	1210.81	634.41
A_68_P31958229	chr19:11943317-11943361	NM_001011775:2366	Olfr1419	DOWNSTREAM	0.893	2.282	713.00	1627.28	2.038	571.50	1164.93
A_68_P31632687	chr18:38369690-38369734	NM_029357:-296	Pcdh1	PROMOTER	0.893	0.561	1628.03	912.99	0.501	1460.88	731.77
A_68_P24269923	chr6:17790613-17790657	NM_001083315:91419	St7	INSIDE	0.893	2.905	481.50	1398.61	2.594	490.75	1272.89
A_68_P27288943	chr10:80795596-80795640	NM_009325:4143	Tbxa2r	INSIDE	0.893	2.235	2492.34	5570.41	1.996	1690.73	3374.13
A_68_P20346777	chr1:74145308-74145352	NM_027884:25691	Tns1	INSIDE	0.893	1.733	1088.13	1885.75	1.548	960.92	1487.36
A_68_P30094310	chr15:27677668-27677712	NM_001081302:277913	Trio	INSIDE	0.893	2.331	1235.93	2881.51	2.083	969.16	2018.62
A_68_P21875502	chr2:174282709-174282753	NM_001080971:6535	Tubb1	INSIDE	0.893	2.013	1148.52	2312.36	1.798	876.83	1576.79
A_68_P29052330	chr13:55050305-55050349	NM_153131:-466	Unc5a	PROMOTER	0.893	2.652	390.21	1034.86	2.368	388.66	920.17
A_68_P26422483	chr9:35229495-35229539	ENSMUST00000154652:-1656		PROMOTER	0.893	0.616	930.36	573.49	0.550	854.23	470.02
A_68_P27933405	chr11:76486147-76486191	NM_177182:197	Bhlha9	INSIDE	0.892	1.588	1183.80	1879.78	1.416	1013.72	1435.16
A_68_P26132607	chr8:107792187-107792231	NM_015821:3661	Fbxl8	INSIDE	0.892	0.590	1532.96	904.80	0.527	1271.91	669.90
A_68_P23417739	chr4:151660365-151660409	NM_008237:5385	Hes3	INSIDE	0.892	2.008	4636.67	9310.22	1.792	3243.52	5812.43
A_68_P28748413	chr12:113416336-113416380	NM_001097621:31940	Kif26a	INSIDE	0.892	2.558	1489.18	3809.76	2.282	1364.44	3113.55
A_68_P29681980	chr14:67529185-67529229	NM_175498:-838	Pnma2	PROMOTER	0.892	0.705	1313.08	926.03	0.629	1081.10	680.19
A_68_P23326918	chr4:134708841-134708885	NM_019732:32303	Runx3	INSIDE	0.892	0.590	2478.02	1462.88	0.527	2063.71	1087.31
A_68_P24354189	chr6:34126897-34126941	NM_021435:136	Slc35b4	INSIDE	0.892	0.665	1656.19	1102.03	0.594	1227.73	728.85
A_68_P26504313	chr9:50369450-50369494	NM_025687:-99	Tex12	DIVERGENT_PROMOTER	0.892	2.725	463.21	1262.40	2.430	446.03	1083.89
A_68_P21765248	chr2:155207645-155207689	NM_178111:75	Trp53inp2	INSIDE	0.892	2.304	401.92	926.02	2.055	410.90	844.41
A_68_P27756066	chr11:44283825-44283869	NM_024475:204	Ublcp1	INSIDE	0.892	0.520	1475.52	767.30	0.464	1167.52	541.77
A_68_P27756065	chr11:44283688-44283732	NM_024475:340	Ublcp1	INSIDE	0.892	0.641	1138.10	729.61	0.572	978.87	560.05
A_68_P31841470	chr18:76305467-76305511	NM_145356:325657	Zbtb7c	INSIDE	0.892	1.724	2962.34	5106.33	1.538	2348.04	3610.50
A_68_P27282338	chr10:79768997-79769041	NM_011789:4454	Apc2	INSIDE	0.891	1.718	939.99	1615.24	1.531	807.03	1235.49
A_68_P24761303	chr6:113698666-113698710	NM_001036684:142682	Atp2b2	INSIDE	0.891	2.564	3246.56	8323.77	2.285	2163.76	4943.27
A_68_P29002652	chr13:45663747-45663791	NM_009124:396577	Atxn1	INSIDE	0.891	3.810	2675.42	10194.42	3.395	1946.25	6607.47
A_68_P30404228	chr15:85809105-85809153	NM_009886:55079	Celsr1	INSIDE	0.891	3.412	780.39	2662.54	3.039	619.98	1884.14
A_68_P21706949	chr2:144219429-144219473	NM_181417:24680	Csrp2bp	INSIDE	0.891	3.298	2238.78	7383.22	2.939	1705.62	5012.16
A_68_P23284104	chr4:126892104-126892148	NM_198618:45612	Dlgap3	INSIDE	0.891	1.552	1494.79	2319.61	1.383	1290.25	1784.79
A_68_P20941836	chr1:193960762-193960806	NR_033536:5	Gm10516	INSIDE	0.891	2.676	2122.15	5679.21	2.383	1429.62	3407.41
A_68_P28067856	chr11:100259165-100259209	NM_010593:-82	Jup	PROMOTER	0.891	0.696	1379.78	960.29	0.620	1164.11	721.74
A_68_P25285284	chr7:91030916-91030960	NM_030705:1913	Mesdc1	INSIDE	0.891	2.069	982.31	2032.07	1.844	883.45	1628.94

ProbeName	Target position of probe on CpG island microarray	TargetID	GeneSymbol	CpG island Description	Ratio of relative methylation (TiO_2-NP/Vehicle)	Sham group			TiO_2-H group		
						Relative methylation (Cy5/Cy3)	Cy3 signal (Input)	Cy5 signal (MeDIP)	Relative methylation (Cy5/Cy3)	Cy3 signal (Input)	Cy5 signal (MeDIP)
A_68_P31897793	chr18:86564837-86564881	NM_144946:515	Neto1	INSIDE	0.891	0.590	2052.63	1211.32	0.526	1606.27	844.86
A_68_P27614517	chr11:17059599-17059643	NM_024459:320	Ppp3r1	INSIDE	0.891	3.572	2969.67	10608.45	3.184	2643.72	8417.17
A_68_P23294937	chr4:128782610-128782654	NM_025452:-141	Tmem54	PROMOTER	0.891	1.716	4090.85	7021.22	1.529	3080.52	4709.00
A_68_P30401942	chr15:85465263-85465307			Unknown	0.891	0.643	1105.37	710.49	0.573	982.58	562.67
A_68_P30380760	chr15:81810911-81810955	NM_025503:-37	1700029P11Rik	PROMOTER	0.890	4.691	4260.78	19987.46	4.175	2898.60	12100.68
A_68_P24735712	chr6:108733427-108733471	NM_026011:396	Arl8b	INSIDE	0.890	1.975	2932.06	5790.35	1.757	2230.39	3919.63
A_68_P25591879	chr7:149136030-149136074	NM_001009929:62	Brsk2	INSIDE	0.890	0.464	1594.94	740.83	0.414	1472.64	609.10
A_68_P31113353	chr17:27083739-27083783	NM_026948:-7337	Cuta	PROMOTER	0.890	0.583	1954.15	1140.09	0.519	1695.82	880.82
A_68_P32351250	chrX:42142934-42142979	NM_001190718:382	Dcaf12l1	INSIDE	0.890	2.434	1406.27	3422.22	2.166	740.86	1605.04
A_68_P20351507	chr1:74997610-74997654	NM_010544:593	Ihh	INSIDE	0.890	0.665	1540.56	1024.81	0.592	1185.68	701.65
A_68_P30456841	chr15:95059370-95059414	NM_016743:299745	Nell2	INSIDE	0.890	2.339	404.52	946.26	2.083	371.13	773.09
A_68_P24020331	chr5:117838565-117838609	NM_028128:446	Rfc5	INSIDE	0.890	1.669	876.74	1463.34	1.485	767.04	1139.27
A_68_P27924771	chr11:75007879-75007923	NM_177708:406	Rtn4rl1	INSIDE	0.890	0.352	2486.62	874.84	0.313	2041.80	639.69
A_68_P26828802	chr9:111213792-111213836	NM_001164659:-428	Trank1	PROMOTER	0.890	1.666	1935.66	3225.03	1.483	1523.41	2259.64
A_68_P26153108	chr8:111316106-111316150	NM_007496:77585	Zfhx3	INSIDE	0.890	1.954	1169.80	2285.54	1.739	897.51	1560.72
A_68_P32288521	chrX:20002973-20003017	ENSMUST00000136093:81		INSIDE	0.890	2.401	1636.55	3928.69	2.137	885.10	1891.18
A_68_P22789805	chr4:25208818-25208862	NM_026194:128	1810074P20Rik	INSIDE	0.889	0.493	1201.30	592.31	0.439	1106.56	485.27
A_68_P28574819	chr12:81360793-81360837	NM_134156:544	Actn1	INSIDE	0.889	2.440	802.41	1957.68	2.169	843.03	1828.88
A_68_P28853793	chr13:17897267-17897311	NM_001081058:-357	Cdk13	PROMOTER	0.889	0.605	1140.77	689.66	0.537	969.01	520.64
A_68_P20613763	chr1:133026416-133026460	NM_145508:8373	Dyrk3	INSIDE	0.889	3.548	1432.28	5081.17	3.153	1217.69	3839.20
A_68_P26156365	chr8:111864724-111864768	NR_002928:62	Gm1943	INSIDE	0.889	3.269	1637.77	5353.48	2.905	1259.29	3657.66
A_68_P30404769	chr15:85889475-85889519	NM_001205353:340	Gramd4	INSIDE	0.889	3.116	2346.73	7313.48	2.771	1889.15	5235.64
A_68_P30366725	chr15:79315828-79315872	NM_008427:19821	Kcnj4	INSIDE	0.889	2.299	344.63	792.26	2.044	276.13	564.42
A_68_P23439339	chr4:154938015-154938059	NM_001159637:113	Nadk	INSIDE	0.889	1.566	1469.01	2300.80	1.393	1343.84	1871.65
A_68_P23435281	chr4:154338676-154338720	NM_172990:457	Pank4	INSIDE	0.889	0.589	1099.66	648.16	0.524	846.94	444.04
A_68_P24126059	chr5:137943930-137943974	NM_028753:-295	Pop7	PROMOTER	0.889	0.698	1459.67	1019.42	0.621	1105.83	686.23
A_68_P20172917	chr1:40738757-40738801	NM_001033289:222	Slc9a2	INSIDE	0.889	2.232	17246.89	38489.60	1.983	13377.93	26533.75
A_68_P21568535	chr2:119063252-119063296	NM_016907:179	Spint1	INSIDE	0.889	0.632	837.28	529.12	0.562	642.68	361.04
A_68_P26529575	chr9:54997611-54997655	NM_180600:457	Ube2q2	INSIDE	0.889	2.040	504.80	1029.68	1.813	467.03	846.80
A_68_P30401621	chr15:85411484-85411528	NM_001163633:-347	Wnt7b	PROMOTER	0.889	1.822	549.87	1001.87	1.619	632.13	1023.39
A_68_P26549614	chr9:58589155-58589200	NM_028598:189	2410076I21Rik	INSIDE	0.888	2.457	728.87	1791.15	2.181	717.38	1564.61
A_68_P31630976	chr18:38095246-38095290	NM_007858:-203	Diap1	PROMOTER	0.888	0.580	1141.50	662.41	0.515	988.59	509.44
A_68_P28036751	chr11:94979621-94979665	NM_010055:-1788	Dlx3	PROMOTER	0.888	2.004	725.68	1454.37	1.779	642.02	1142.29
A_68_P31156414	chr17:35042317-35042361	NM_145830:6420	Ehmt2	INSIDE	0.888	4.497	1428.50	6424.10	3.994	1083.27	4326.21
A_68_P26350220	chr9:21855814-21855858	NM_010487:631	Elavl3	INSIDE	0.888	0.451	1257.45	567.25	0.400	1010.05	404.47
A_68_P25958570	chr8:74249375-74249419	NM_028715:184	Fcho1	INSIDE	0.888	0.614	3441.64	2111.50	0.545	2579.42	1404.99
A_68_P30547079	chr16:11253448-11253492	NM_001130008:948	Gspt1	INSIDE	0.888	2.249	1059.18	2382.58	1.998	942.43	1883.18
A_68_P30347940	chr15:76210921-76210965	NM_001162489:-9518	Heatr7a	PROMOTER	0.888	0.604	1978.08	1194.86	0.536	1697.95	910.92
A_68_P28431905	chr12:52930950-52930994	NM_144788:-449	Hectd1	PROMOTER	0.888	2.789	638.70	1781.57	2.477	583.04	1444.26
A_68_P31259626	chr17:57161992-57162036	NM_010613:8916	Khsrp	INSIDE	0.888	1.776	843.68	1498.78	1.578	715.60	1129.16
A_68_P30347566	chr15:76158037-76158081	NR_028283:-95	LOC100302626	PROMOTER	0.888	0.623	2017.93	1256.67	0.553	1538.90	850.92
A_68_P32143075	chr19:46380056-46380100	NM_019408:-106	Nfkb2	PROMOTER	0.888	0.545	1261.39	687.85	0.484	1086.06	525.93
A_68_P30371156	chr15:80094548-80094592	NM_175109:166	Rps19bp1	INSIDE	0.888	0.494	2262.20	1117.65	0.439	1985.43	870.72
A_68_P23794551	chr5:73305609-73305653	NM_001113423:75	Slain2	INSIDE	0.888	0.495	1486.99	735.78	0.439	1346.56	591.65
A_68_P24118205	chr5:136333004-136333048	NM_021403:39051	Srrm3	INSIDE	0.888	2.079	4327.95	8996.79	1.845	3515.61	6487.98
A_68_P27325553	chr10:87922455-87922499	NM_011517:145	Sycp3	INSIDE	0.888	3.006	771.95	2320.57	2.670	653.58	1744.77
A_68_P26736546	chr9:94438103-94438147	NM_001033145:376	1190002N15Rik	INSIDE	0.887	1.945	419.62	816.24	1.726	405.19	699.31
A_68_P25591876	chr7:149135444-149135488	NM_029426:-189	Brsk2	PROMOTER	0.887	0.716	2210.20	1583.34	0.636	1747.49	1110.87
A_68_P24990419	chr7:19495688-19495732	NM_001033314:43	Ccdc61	INSIDE	0.887	0.481	1236.84	594.90	0.427	1035.96	442.11
A_68_P22716232	chr4:8679096-8679140	NM_001081417:61051	Chd7	INSIDE	0.887	1.567	2761.49	4327.97	1.390	2036.95	2832.29
A_68_P33015613	chr1_random:49705-49759	NM_026866:135606	Disp1	INSIDE	0.887	2.701	799.45	2159.29	2.396	656.93	1574.22

ProbeName	Target position of probe on CpG island microarray	TargetID	GeneSymbol	CpG island Description	Ratio of relative methylation (TiO_2-NP/Vehicle)	Sham group			TiO_2-H group		
						Relative methylation (Cy5/Cy3)	Cy3 signal (Input)	Cy5 signal (MeDIP)	Relative methylation (Cy5/Cy3)	Cy3 signal (Input)	Cy5 signal (MeDIP)
A_68_P28988894	chr13:43295868-43295912	NM_001033399:103651	Gfod1	INSIDE	0.887	1.982	525.10	1040.66	1.758	510.51	897.42
A_68_P27470614	chr10:115024633-115024677	NM_010195:182	Lgr5	INSIDE	0.887	0.578	1660.17	960.29	0.513	1335.74	685.04
A_68_P24950637	chr7:4764223-4764267	NM_133777:-302	Ube2s	PROMOTER	0.887	0.650	3030.13	1970.06	0.576	2277.73	1313.08
A_68_P25261545	chr7:86660396-86660440	ENSMUST00000150985:301		INSIDE	0.887	1.610	1105.60	1780.46	1.428	1076.53	1537.40
A_68_P27937764	chr11:77285096-77285140	NM_172945:18062	Ankrd13b	INSIDE	0.886	2.252	1376.19	3098.78	1.996	988.62	1972.84
A_68_P23299544	chr4:129665518-129665562	NM_001199696:3219	Bai2	INSIDE	0.886	0.625	2777.98	1735.37	0.554	2143.22	1186.57
A_68_P21067987	chr2:24617651-24617695	NM_001042528:895	Cacna1b	INSIDE	0.886	0.644	1720.71	1107.91	0.571	1421.25	811.02
A_68_P28155404	chr11:115665077-115665121	NM_080643:9808	Caskin2	INSIDE	0.886	2.163	2130.26	4607.16	1.917	1532.01	2936.73
A_68_P29625864	chr14:57676795-57676839	NM_016975:-34	Gja3	PROMOTER	0.886	1.641	940.77	1543.68	1.454	891.00	1295.38
A_68_P20058116	chr1:17081618-17081662	NM_020604:6330	Jph1	INSIDE	0.886	3.210	702.48	2254.85	2.843	720.91	2049.42
A_68_P21698986	chr2:142727237-142727281	NM_001081133:-58	Kif16b	PROMOTER	0.886	1.644	1231.82	2025.60	1.458	1125.94	1641.14
A_68_P25016757	chr7:28119959-28120009	NM_001113549:-1317	Ltbp4	PROMOTER	0.886	2.199	293.92	646.48	1.950	287.26	560.09
A_68_P28445669	chr12:55168877-55168921	NM_013780:819235	Npas3	INSIDE	0.886	0.696	1409.32	981.05	0.617	1159.64	715.51
A_68_P30346612	chr15:76011489-76011533	NM_201394:14630	Plec	INSIDE	0.886	2.690	867.67	2333.74	2.382	770.66	1835.71
A_68_P23194206	chr4:107694030-107694074	NM_172874:10643	Podn	INSIDE	0.886	3.121	1472.77	4597.03	2.767	1025.11	2836.50
A_68_P23982395	chr5:111308888-111308932	NM_024477:89	Ttc28	INSIDE	0.886	4.985	179.99	897.17	4.414	203.68	899.08
A_68_P25614229	chr8:3496319-3496363	NM_080461:3203	Zfp358	INSIDE	0.886	2.017	1133.62	2286.22	1.786	965.21	1723.89
A_68_P27830383	chr11:58136875-58136919	NM_178761:-48	Zfp672	DIVERGENT_PROMOTER	0.886	0.404	1305.86	527.96	0.358	1079.79	386.65
A_68_P30402119	chr15:85491531-85491575	AK083890:-4159		PROMOTER	0.886	2.426	472.99	1147.30	2.149	375.78	807.66
A_68_P31374374	chr17:79315892-79315936	NM_001177402:76	1110001A16Rik	INSIDE	0.885	1.757	2935.54	5157.45	1.556	2191.49	3409.07
A_68_P26760984	chr9:98765311-98765355	NM_028033:147	2410012M07Rik	INSIDE	0.885	2.311	9977.79	23062.86	2.047	7501.02	15352.54
A_68_P32053390	chr19:29597209-29597253	NM_001081319:459	C030046E11Rik	INSIDE	0.885	0.649	2249.76	1459.61	0.574	1757.46	1009.35
A_68_P27750784	chr11:43304478-43304526	NM_026979:16695	C1qtnf2	INSIDE	0.885	2.216	685.37	1518.99	1.961	547.19	1072.89
A_68_P32982271	chr5:134998519-134998564	NM_001039162:29763	Clip2	INSIDE	0.885	2.051	545.95	1119.78	1.816	392.01	711.91
A_68_P25090601	chr7:52262939-52262983	NM_001008422:8659	Scaf1	INSIDE	0.885	2.254	2312.44	5212.22	1.994	1685.86	3361.29
A_68_P28730244	chr12:110074293-110074337	NM_181328:-228	Slc25a29	DIVERGENT_PROMOTER	0.885	0.477	1790.65	854.77	0.423	1281.74	541.57
A_68_P25807722	chr8:41947794-41947838	NM_001044740:70	Slc7a2	INSIDE	0.885	2.787	5287.51	14737.81	2.466	4103.96	10120.58
A_68_P24604965	chr6:85382281-85382325	NM_144918:333	Smyd5	INSIDE	0.885	1.685	1943.30	3274.36	1.492	1482.54	2211.25
A_68_P25183828	chr7:72789560-72789604	NM_172310:-201	Tarsl2	PROMOTER	0.885	2.050	11629.38	23838.43	1.814	8013.07	14539.39
A_68_P31092360	chr17:23694657-23694701	NM_001033496:6515	Zfp213	INSIDE	0.885	1.838	1238.42	2275.95	1.627	962.24	1565.67
A_68_P28048036	chr11:96858742-96858786			Unknown	0.885	3.501	1930.90	6760.89	3.099	1182.97	3666.53
A_68_P20738463	chr1:157405630-157405674	NM_026683:403	Acbd6	INSIDE	0.884	0.580	1438.63	833.74	0.512	1286.86	659.21
A_68_P31383665	chr17:80942894-80942947	NM_001033443:20254	Cdkl4	INSIDE	0.884	1.995	466.18	930.16	1.764	403.77	712.27
A_68_P23332455	chr4:135728739-135728783	NM_177733:452	E2f2	INSIDE	0.884	1.985	826.98	1641.55	1.754	909.17	1594.89
A_68_P29604237	chr14:52626582-52626626	NM_001033805:-9935	G630016D24Rik	PROMOTER	0.884	2.243	517.83	1161.49	1.983	448.36	889.14
A_68_P24518613	chr6:66846160-66846204	NM_001177556:-208	Gng12	PROMOTER	0.884	1.634	2320.89	3791.60	1.444	1765.80	2550.04
A_68_P29059423	chr13:56236168-56236212	NM_001159513:721	H2afy	INSIDE	0.884	2.219	656.16	1455.91	1.961	616.69	1209.22
A_68_P25004862	chr7:25217754-25217798	NM_199013:12925	Irgc1	INSIDE	0.884	2.503	2781.22	6960.94	2.213	2083.35	4609.51
A_68_P27284037	chr10:80031748-80031792	NM_078477:8271	Klf16	INSIDE	0.884	2.100	1506.71	3164.06	1.857	1194.85	2219.18
A_68_P29842701	chr14:99698407-99698451	NM_009769:519	Klf5	INSIDE	0.884	2.429	574.82	1396.19	2.148	590.05	1267.57
A_68_P24979591	chr7:16684155-16684199	NM_025898:185	Napa	INSIDE	0.884	1.766	825.10	1457.00	1.561	741.22	1156.81
A_68_P29654921	chr14:62950975-62951019	NM_026001:55	Rnaseh2b	INSIDE	0.884	0.689	1285.98	885.54	0.609	1078.20	656.31
A_68_P20871446	chr1:181598547-181598591	NM_178653:207	Sccpdh	INSIDE	0.884	2.382	847.75	2019.24	2.105	736.66	1550.54
A_68_P23187163	chr4:106584892-106584936	NM_023672:840	Ssbp3	INSIDE	0.884	2.156	551.29	1188.82	1.906	505.26	962.94
A_68_P30385304	chr15:82729412-82729456	NR_027799:-543	Tbrg3	PROMOTER	0.884	0.586	3052.25	1789.00	0.518	2394.48	1240.03
A_68_P31215267	chr17:48111395-48111439			Unknown	0.884	2.859	671.03	1918.26	2.528	594.96	1503.78
A_68_P20530739	chr1:114313105-114313149			Unknown	0.884	0.630	2691.13	1694.34	0.557	2143.10	1192.92
A_68_P22407294	chr3:107385858-107385902			Unknown	0.884	0.645	2990.34	1927.42	0.570	2150.88	1226.01
A_68_P20977503	chr2:6050985-6051029	NM_001159657:225	5430407P10Rik	INSIDE	0.883	0.453	2161.70	979.51	0.400	1714.00	685.98
A_68_P28752032	chr12:113960100-113960144	NM_001024602:-262	AW555464	PROMOTER	0.883	0.612	1264.74	773.71	0.540	1012.69	547.17
A_68_P27891998	chr11:68715228-68715272	NR_003373:160	Gm15772	INSIDE	0.883	1.731	988.57	1711.66	1.529	904.83	1383.55

ProbeName	Target position of probe on CpG island microarray	TargetID	GeneSymbol	CpG island Description	Ratio of relative methylation (TiO_2-NP/Vehicle)	Sham group			TiO_2-H group		
						Relative methylation (Cy5/Cy3)	Cy3 signal (Input)	Cy5 signal (MeDIP)	Relative methylation (Cy5/Cy3)	Cy3 signal (Input)	Cy5 signal (MeDIP)
A_68_P31863473	chr18:80492422-80492466	NM_001190373:68549	Kcng2	INSIDE	0.883	2.139	537.51	1149.63	1.888	492.94	930.62
A_68_P28748273	chr12:113399710-113399754	NM_001097621:15314	Kif26a	INSIDE	0.883	2.716	962.74	2615.04	2.399	697.58	1673.63
A_68_P24171440	chr5:147840732-147840776	NM_080795:47394	Lnx2	INSIDE	0.883	2.496	2153.82	5375.62	2.203	1763.11	3884.53
A_68_P25022019	chr7:29244304-29244350	NM_001141921:7070	Lrfn1	INSIDE	0.883	1.921	859.40	1651.15	1.696	742.61	1259.68
A_68_P25343508	chr7:103358546-103358591	NM_011858:-578	Odz4	PROMOTER	0.883	2.973	4745.77	14108.42	2.626	3949.80	10372.35
A_68_P28085167	chr11:103274059-103274103	NM_183034:-102	Plekhm1	PROMOTER	0.883	2.758	598.75	1651.22	2.435	607.68	1479.59
A_68_P24949868	chr7:4610619-4610663	NM_172894:-88	Ppp6r1	PROMOTER	0.883	0.488	1643.60	801.32	0.430	1299.87	559.59
A_68_P22412277	chr3:108186677-108186721	NM_001190161:-23	Psrc1	PROMOTER	0.883	1.916	1007.11	1929.68	1.692	851.01	1439.80
A_68_P29106487	chr13:67033811-67033855	NM_008959:67	Ptdss1	INSIDE	0.883	0.563	1801.66	1014.59	0.497	1475.39	733.34
A_68_P27687432	chr11:32113340-32113384	NM_010117:8931	Rhbdf1	INSIDE	0.883	2.498	1619.09	4043.93	2.206	1199.30	2645.07
A_68_P26522398	chr9:53619438-53619482	NM_028060:119	Slc35f2	INSIDE	0.883	0.467	1630.81	761.64	0.412	1242.05	512.29
A_68_P23364321	chr4:141034275-141034319	NM_019763:60216	Spen	INSIDE	0.883	2.405	879.25	2114.58	2.123	734.20	1558.94
A_68_P29455872	chr14:22804750-22804794	NM_145459:4051	Zfp503	INSIDE	0.883	0.494	1055.14	521.10	0.436	868.70	378.86
A_68_P28724516	chr12:109154117-109154161	NM_001079883:87486	Bcl11b	INSIDE	0.882	2.757	551.62	1520.91	2.432	492.18	1196.84
A_68_P29941712	chr14:119322120-119322164	NM_025943:247	Dzip1	INSIDE	0.882	0.652	2030.30	1324.24	0.576	1648.09	948.50
A_68_P24620282	chr6:88139868-88139912	NM_008090:-8767	Gata2	PROMOTER	0.882	0.601	1261.08	758.45	0.530	1063.46	563.81
A_68_P28516080	chr12:70825874-70825918	NM_145443:-35	L2hgdh	DIVERGENT_PROMOTER	0.882	0.558	1239.16	691.65	0.493	1025.60	505.12
A_68_P27854124	chr11:62270784-62270828	NM_011308:28	Ncor1	INSIDE	0.882	1.715	2120.57	3636.39	1.512	1814.20	2743.19
A_68_P26136321	chr8:108378956-108379000	NM_021513:-24	Thap11	DIVERGENT_PROMOTER	0.882	0.608	1669.23	1014.36	0.536	1534.73	822.50
A_68_P24427164	chr6:48388295-48388339	NM_001085415:6773	Zfp467	INSIDE	0.882	1.709	1225.02	2093.33	1.507	995.08	1499.35
A_68_P25505621	chr7:134587797-134587841	NM_175163:4854	Zfp689	INSIDE	0.882	2.613	1207.11	3154.03	2.305	1017.64	2345.25
A_68_P22086836	chr3:41643672-41643716			Unknown	0.882	3.462	1857.26	6429.11	3.054	1641.30	5011.80
A_68_P23383664	chr4:145167106-145167158	ENSMUST00000117838:63		INSIDE	0.882	3.054	582.83	1779.91	2.694	430.09	1158.66
A_68_P27288891	chr10:80788278-80788322	NM_027381:4453	2510012J08Rik	INSIDE	0.881	2.577	5444.06	14031.83	2.271	4378.63	9941.86
A_68_P23542802	chr5:24083177-24083221	NM_001190443:87	Abcf2	INSIDE	0.881	0.564	1243.51	701.42	0.497	942.44	468.12
A_68_P24298740	chr6:23788658-23788702	NM_153163:620	Cadps2	INSIDE	0.881	0.559	1377.29	769.96	0.493	1170.14	576.51
A_68_P20354307	chr1:75471829-75471873	NM_001001565:4196	Chpf	INSIDE	0.881	2.373	427.64	1014.80	2.091	433.24	905.96
A_68_P21134369	chr2:35438463-35438507	NM_001114125:24507	Dab2ip	INSIDE	0.881	2.114	729.69	1542.34	1.862	671.43	1249.92
A_68_P25021008	chr7:29079932-29079976	NM_007866:6850	Dll3	INSIDE	0.881	0.673	1733.19	1165.99	0.593	1420.20	841.87
A_68_P26761431	chr9:98856538-98856582	NM_012020:535	Foxl2	INSIDE	0.881	0.643	928.63	596.97	0.566	839.59	475.53
A_68_P28087337	chr11:103672587-103672632	NM_009521:37121	Wnt3	INSIDE	0.881	1.767	842.47	1488.64	1.556	640.74	997.27
A_68_P26913056	chr10:5959839-5959883	NM_001033466:1428	Zbtb2	INSIDE	0.881	1.987	718.02	1426.92	1.750	687.92	1203.76
A_68_P29285955	chr13:104383068-104383112	BC099526:114623		INSIDE	0.881	2.201	742.43	1634.06	1.939	619.61	1201.68
A_68_P30665009	chr16:34572678-34572722	ENSMUST00000114961:918		INSIDE	0.881	0.508	1110.04	564.23	0.448	896.30	401.28
A_68_P26221248	chr8:123112748-123112792	NM_178856:205	Gins2	INSIDE	0.880	0.420	1921.46	807.71	0.370	1507.14	557.72
A_68_P27286894	chr10:80477702-80477746	NM_010319:-54	Gng7	PROMOTER	0.880	0.648	1241.59	803.96	0.570	894.68	509.65
A_68_P26847145	chr9:114842599-114842643	NM_175380:485	Gpd1l	INSIDE	0.880	0.562	987.56	554.65	0.494	807.18	398.85
A_68_P21750931	chr2:152565335-152565379	NM_010495:3347	Id1	DOWNSTREAM	0.880	0.597	1356.17	809.72	0.526	1255.72	659.90
A_68_P30343807	chr15:75577030-75577074	NM_194350:1300	Mafa	DOWNSTREAM	0.880	2.352	708.34	1666.16	2.069	642.84	1329.94
A_68_P30086028	chr15:26239162-26239206	NM_177597:358	Marchf11	INSIDE	0.880	1.952	531.23	1036.89	1.718	459.45	789.12
A_68_P22509668	chr3:127328592-127328639	NM_009718:-7447	Neurog2	PROMOTER	0.880	1.605	1673.53	2685.52	1.412	1283.43	1811.95
A_68_P24643138	chr6:92041251-92041295	NM_011630:-139	Nr2c2	PROMOTER	0.880	2.086	473.24	987.24	1.835	516.44	947.91
A_68_P26747582	chr9:96437440-96437484	NM_053268:94460	Rasa2	DOWNSTREAM	0.880	2.073	1342.99	2784.05	1.825	1210.32	2208.29
A_68_P22063905	chr3:37380234-37380278	NM_001163511:61055	Spata5	INSIDE	0.880	2.127	517.41	1100.28	1.871	461.17	862.66
A_68_P24019921	chr5:117769319-117769363	NM_001033311:66	Vsig10	INSIDE	0.880	0.630	1377.61	867.31	0.554	1042.02	577.15
A_68_P24793131	chr6:119390505-119390549	NM_009525:103839	Wnt5b	INSIDE	0.880	1.948	968.91	1887.87	1.715	832.27	1426.99
A_68_P31620633	chr18:36033456-36033500			Unknown	0.880	0.555	1099.80	610.31	0.488	909.51	443.96
A_68_P23962133	chr5:107003460-107003504	ENSMUST00000131029:252		INSIDE	0.880	2.076	767.97	1594.15	1.826	704.15	1285.79
A_68_P28608876	chr12:87585301-87585347	NM_027405:2497	1700020O03Rik	INSIDE	0.879	1.996	563.91	1125.56	1.755	455.50	799.53
A_68_P23415137	chr4:151236473-151236517	NM_001081557:-617	Camta1	PROMOTER	0.879	0.534	1227.32	655.38	0.469	987.73	463.39
A_68_P33015624	chr1_random:51004-51048	NM_026866:134312	Disp1	INSIDE	0.879	2.084	598.72	1247.50	1.832	487.48	892.85

ProbeName	Target position of probe on CpG island microarray	TargetID	GeneSymbol	CpG island Description	Ratio of relative methylation (TiO_2-NP/Vehicle)	Sham group			TiO_2-H group		
						Relative methylation (Cy5/Cy3)	Cy3 signal (Input)	Cy5 signal (MeDIP)	Relative methylation (Cy5/Cy3)	Cy3 signal (Input)	Cy5 signal (MeDIP)
A_68_P21007902	chr2:11698864-11698908	NM_015792:268	Fbxo18	INSIDE	0.879	0.468	1190.76	557.58	0.412	918.09	378.05
A_68_P24025035	chr5:118619751-118619795	NM_007545:0	Hrk	INSIDE	0.879	2.156	2006.56	4326.29	1.895	1761.89	3338.29
A_68_P26270746	chr8:131209434-131209478	NM_010578:-97	Itgb1	PROMOTER	0.879	0.497	2107.81	1048.05	0.437	1682.40	735.33
A_68_P31930917	chr19:5803602-5803646	NR_002847:-953	Malat1	PROMOTER	0.879	0.512	2164.12	1107.04	0.449	1779.38	799.77
A_68_P24065430	chr5:125659058-125659102	NM_011424:504	Ncor2	INSIDE	0.879	0.637	892.22	568.14	0.560	784.37	438.91
A_68_P23184551	chr4:106136489-106136533	NM_153565:420	Pcsk9	INSIDE	0.879	0.527	1329.89	701.39	0.464	1209.62	560.90
A_68_P26843527	chr9:113966461-113966505	NM_001101510:11	Susd5	INSIDE	0.879	1.680	1375.33	2310.10	1.476	1348.70	1991.20
A_68_P31101670	chr17:25252921-25252965	NM_001163661:-30	Telo2	DIVERGENT_PROMOTER	0.879	2.479	1251.80	3103.49	2.178	937.42	2041.89
A_68_P28031427	chr11:94073436-94073480	NM_009427:691	Tob1	INSIDE	0.879	1.841	522.92	962.51	1.618	493.48	798.44
A_68_P25092433	chr7:52582778-52582822	NM_175130:6350	Trpm4	INSIDE	0.879	3.047	1093.75	3332.89	2.680	958.23	2567.65
A_68_P31403188	chr17:84585249-84585293	NM_001001806:2017	Zfp36l2	INSIDE	0.879	0.746	3473.86	2590.14	0.656	2997.06	1964.78
A_68_P22341860	chr3:94819980-94820024	NM_030074:-842	Zfp687	PROMOTER	0.879	0.596	946.71	564.18	0.524	835.88	437.77
A_68_P29502989	chr14:30791625-30791669	NM_027493:124	Actr8	INSIDE	0.878	2.037	1741.90	3547.43	1.788	1385.59	2477.26
A_68_P27226082	chr10:69284986-69285030	NM_146005:288553	Ank3	INSIDE	0.878	2.411	636.18	1533.98	2.118	503.14	1065.59
A_68_P32262595	chrX:12165037-12165081	NM_027439:174	Atp6ap2	INSIDE	0.878	2.231	2195.93	4898.77	1.959	919.05	1800.24
A_68_P28221298	chr12:8305600-8305644	NM_013527:3138	Gdf7	INSIDE	0.878	2.409	1459.90	3517.04	2.116	1270.55	2688.73
A_68_P32016033	chr19:23209323-23209367	NM_010638:-6371	Klf9	PROMOTER	0.878	1.812	5407.44	9800.32	1.591	3861.51	6143.24
A_68_P21037798	chr2:17948361-17948405	NR_028378:-493	LOC100034739	PROMOTER	0.878	0.475	1486.45	705.99	0.417	1338.60	558.44
A_68_P31127481	chr17:29484296-29484340	NM_019880:531	Mtch1	INSIDE	0.878	2.942	9126.89	26850.41	2.584	5692.52	14707.71
A_68_P24483715	chr6:58856617-58856661	NM_021432:482	Nap1l5	INSIDE	0.878	1.600	1411.86	2258.41	1.405	1079.94	1516.88
A_68_P31421200	chr17:87506806-87506850	NM_019654:-190	Socs5	PROMOTER	0.878	1.941	1005.91	1952.40	1.705	827.43	1410.48
A_68_P22385232	chr3:103083556-103083600	NM_001079830:363	Trim33	INSIDE	0.878	2.213	475.63	1052.78	1.943	457.62	889.07
A_68_P22382655	chr3:102538658-102538702	NM_027533:-12	Tspan2	PROMOTER	0.878	1.666	1465.85	2442.37	1.463	1047.61	1532.58
A_68_P26036156	chr8:90184417-90184461	NM_033327:299056	Zfp423	DOWNSTREAM	0.878	0.672	1609.80	1082.00	0.590	1253.79	739.63
A_68_P23424870	chr4:152806765-152806809	NM_001099299:50153	Ajap1	INSIDE	0.877	2.823	844.85	2385.25	2.475	778.38	1926.29
A_68_P23767825	chr5:67818729-67818773	NM_001164806:288	Bend4	INSIDE	0.877	1.872	890.42	1667.27	1.642	799.12	1312.05
A_68_P27673731	chr11:29147082-29147126	NM_025740:168	Ccdc104	INSIDE	0.877	0.607	2592.11	1573.95	0.533	2048.30	1091.15
A_68_P21822532	chr2:165058963-165059007	NM_174988:1253	Cdh22	INSIDE	0.877	0.600	2771.82	1663.64	0.526	2139.77	1126.16
A_68_P23270849	chr4:124559951-124559995	NM_177671:944	Epha10	INSIDE	0.877	0.650	1196.97	778.12	0.570	1084.26	618.41
A_68_P26156366	chr8:111864799-111864851	NR_002928:-16	Gm1943	PROMOTER	0.877	2.203	530.44	1168.49	1.932	457.17	883.10
A_68_P26804348	chr9:106549759-106549803	NM_001160353:8660	Grm2	INSIDE	0.877	2.262	1147.04	2594.88	1.983	949.81	1883.67
A_68_P28043433	chr11:96144433-96144477	NM_010461:1236	Hoxb8	INSIDE	0.877	0.594	1738.00	1031.92	0.521	1465.23	763.06
A_68_P22957168	chr4:59594155-59594199	NM_024255:-258	Hsdl2	PROMOTER	0.877	2.022	2614.58	5286.23	1.773	2108.68	3737.94
A_68_P23611442	chr5:37482587-37482631	NM_178394:40513	Jakmip1	INSIDE	0.877	2.693	235.16	633.34	2.361	203.14	479.69
A_68_P25406536	chr7:116313170-116313214	NM_057173:630	Lmo1	INSIDE	0.877	0.608	969.60	589.95	0.533	764.70	407.94
A_68_P27535213	chr10:126748669-126748713	NM_001003913:152	Mars	INSIDE	0.877	0.679	1274.04	864.85	0.596	1121.64	668.03
A_68_P20506811	chr1:108069599-108069643	NM_133821:1175	Phlpp1	INSIDE	0.877	0.503	1391.74	700.18	0.441	1156.96	510.72
A_68_P30346972	chr15:76062477-76062521	NM_001163542:-690	Plec	PROMOTER	0.877	0.506	1128.23	570.93	0.444	875.00	388.38
A_68_P30969342	chr16:92466123-92466167	NM_001081549:247	Rcan1	INSIDE	0.877	0.664	1600.56	1063.38	0.583	1306.95	761.58
A_68_P28535267	chr12:74146949-74146993	NM_009189:729	Six1	INSIDE	0.877	0.672	984.82	661.31	0.589	732.01	431.03
A_68_P25957634	chr8:74108737-74108781	NM_011977:15933	Slc27a1	INSIDE	0.877	2.589	774.69	2006.03	2.272	641.74	1458.11
A_68_P27911318	chr11:72225271-72225315	NM_177776:-77	Smtnl2	PROMOTER	0.877	0.443	2457.74	1088.17	0.388	2042.36	792.63
A_68_P22314616	chr3:88383591-88383635	NM_025448:20	Ssr2	INSIDE	0.877	0.467	1128.02	527.09	0.410	939.00	384.58
A_68_P21815997	chr2:163899961-163900005	NM_021420:69	Stk4	INSIDE	0.877	0.627	927.36	581.07	0.550	816.48	448.66
A_68_P21747903	chr2:151969066-151969110	NM_009328:-256	Tcf15	PROMOTER	0.877	0.522	1197.47	624.63	0.457	988.92	452.27
A_68_P22894265	chr4:47366582-47366626	NM_009370:428	Tgfbr1	INSIDE	0.877	1.831	1099.21	2012.41	1.606	1002.71	1610.29
A_68_P29023542	chr13:49242589-49242633	NM_029361:773	Wnk2	INSIDE	0.877	1.866	949.60	1771.74	1.637	868.31	1421.13
A_68_P27563766	chr11:5420720-5420764	NM_013842:-227	Xbp1	PROMOTER	0.877	0.475	1171.89	556.12	0.416	995.63	414.25
A_68_P25842776	chr8:48732206-48732250			Unknown	0.877	2.461	489.53	1204.91	2.157	461.34	995.28
A_68_P28183803	chr11:120166774-120166818			Unknown	0.877	0.609	2320.91	1414.24	0.534	1954.13	1043.99
A_68_P27288892	chr10:80788388-80788440	NM_027381:4567	2510012J08Rik	INSIDE	0.876	3.218	1342.72	4321.14	2.820	1074.69	3030.46

ProbeName	Target position of probe on CpG island microarray	TargetID	GeneSymbol	CpG island Description	Ratio of relative methylation (TiO_2-NP/Vehicle)	Sham group			TiO_2-H group		
						Relative methylation (Cy5/Cy3)	Cy3 signal (Input)	Cy5 signal (MeDIP)	Relative methylation (Cy5/Cy3)	Cy3 signal (Input)	Cy5 signal (MeDIP)
A_68_P26553627	chr9:59333545-59333589	NM_019927:615	Arih1	INSIDE	0.876	0.420	1804.53	757.95	0.368	1462.24	538.00
A_68_P25617307	chr8:4325222-4325266	NR_033527:35	Ccl25	INSIDE	0.876	0.498	1128.92	562.59	0.437	946.00	413.20
A_68_P24120337	chr5:136750787-136750831	NM_198602:292467	Cux1	INSIDE	0.876	2.919	259.86	758.47	2.556	282.14	721.23
A_68_P31150931	chr17:34048707-34048751	NM_001199733:2183	Daxx	INSIDE	0.876	2.611	597.45	1559.83	2.286	573.32	1310.69
A_68_P21569026	chr2:119148000-119148050	NM_019454:-3495	Dll4	PROMOTER	0.876	0.654	977.03	638.96	0.573	750.88	430.09
A_68_P28429305	chr12:52449483-52449527	NM_001015099:288	G2e3	INSIDE	0.876	2.516	312.43	786.10	2.205	307.14	677.14
A_68_P24451818	chr6:52590402-52590446	NM_145567:-130	Hibadh	PROMOTER	0.876	3.545	1061.80	3764.10	3.105	841.52	2613.18
A_68_P27340040	chr10:90564963-90565014	NM_027078:19205	Ikbip	INSIDE	0.876	3.832	516.65	1979.63	3.357	422.66	1418.85
A_68_P23318790	chr4:133228292-133228336	NM_001145955:248	Pigv	INSIDE	0.876	0.628	1537.22	965.66	0.550	1378.04	758.04
A_68_P29701215	chr14:70829074-70829118	NM_021308:-195	Piwil2	PROMOTER	0.876	1.668	3250.31	5422.05	1.462	2693.08	3936.18
A_68_P22405767	chr3:107145905-107145949	NM_001045807:-9719	Rbm15	PROMOTER	0.876	3.020	324.18	979.09	2.645	317.58	839.86
A_68_P30575793	chr16:17804490-17804534	NM_153790:7138	Scarf2	INSIDE	0.876	0.597	1746.76	1042.25	0.523	1495.73	781.76
A_68_P28038654	chr11:95275393-95275437	NM_025287:18	Spop	INSIDE	0.876	1.642	2808.11	4609.75	1.437	2234.70	3211.87
A_68_P27340414	chr10:90634556-90634600	NM_001080129:-251	Tmpo	PROMOTER	0.876	0.733	2305.27	1689.13	0.642	1912.46	1228.07
A_68_P29052710	chr13:55098681-55098725	NM_153131:47910	Unc5a	INSIDE	0.876	2.258	1458.83	3293.84	1.978	1203.00	2378.94
A_68_P27285197	chr10:80204884-80204928	NM_007460:50	Ap3d1	INSIDE	0.875	0.663	959.79	636.45	0.580	776.06	450.31
A_68_P26356129	chr9:23027849-23027893	NM_028472:351	Bmper	INSIDE	0.875	0.657	1299.35	853.82	0.575	1130.27	649.66
A_68_P33007789	chr4_random:128217-128261	NM_001033326:18437	Dhrsx	INSIDE	0.875	1.882	2204.84	4149.44	1.647	1811.37	2982.56
A_68_P25108777	chr7:56136031-56136075	NM_001013368:359	E2f8	INSIDE	0.875	0.607	1125.83	683.74	0.531	1032.82	548.73
A_68_P25005646	chr7:25370925-25370969	NM_023154:-1615	Ethe1	PROMOTER	0.875	2.250	5331.10	11992.35	1.969	3886.69	7654.13
A_68_P25827402	chr8:46036102-46036161	NM_001081286:570	Fat1	INSIDE	0.875	2.330	1150.14	2680.06	2.040	820.64	1674.08
A_68_P31927962	chr19:5306803-5306849	NM_001024717:8496	Gal3st3	INSIDE	0.875	2.837	379.24	1075.97	2.481	338.69	840.40
A_68_P31927965	chr19:5307061-5307105	NM_001024717:8752	Gal3st3	INSIDE	0.875	2.051	1187.86	2436.13	1.795	1079.42	1938.05
A_68_P30347069	chr15:76077165-76077209	NM_023168:-50	Grina	DIVERGENT_PROMOTER	0.875	0.737	4098.15	3022.21	0.646	3129.19	2020.11
A_68_P27995154	chr11:87431401-87431445	NM_001045527:757	Hsf5	INSIDE	0.875	2.391	1279.29	3059.40	2.091	1056.67	2209.95
A_68_P22885280	chr4:45839410-45839454	NM_018741:267	Igfbpl1	INSIDE	0.875	1.661	1465.95	2435.45	1.454	1263.17	1836.21
A_68_P20725329	chr1:155033264-155033308	NM_008485:291	Lamc2	INSIDE	0.875	0.624	971.45	606.51	0.546	824.56	450.51
A_68_P27911829	chr11:72303431-72303475	NM_153060:-46	Spns2	PROMOTER	0.875	2.874	284.38	817.41	2.516	265.35	667.65
A_68_P26234571	chr8:125135662-125135707	NM_021502:159	Trappc2l	INSIDE	0.875	0.679	1759.88	1194.45	0.594	1268.27	752.95
A_68_P24874653	chr6:136776590-136776634	NM_021714:125	Wbp11	INSIDE	0.875	0.519	2131.40	1106.10	0.454	1793.56	814.49
A_68_P30558815	chr16:13754812-13754856	AK044312:176		INSIDE	0.875	0.605	1619.73	979.75	0.529	1199.97	635.29
A_68_P21705754	chr2:143991432-143991476	ENSMUST00000099292:23572		DOWNSTREAM	0.875	0.586	1016.88	595.40	0.512	857.74	439.39
A_68_P31762240	chr18:62339500-62339544	NM_007420:91	Adrb2	INSIDE	0.874	2.929	2026.26	5934.03	2.560	1798.58	4605.07
A_68_P30404230	chr15:85809331-85809375	NM_009886:54855	Celsr1	INSIDE	0.874	2.855	1537.19	4387.99	2.496	1131.76	2825.03
A_68_P26127987	chr8:106864391-106864435	NM_024217:-81	Cmtm3	PROMOTER	0.874	4.979	12296.25	61227.81	4.354	7343.52	31972.60
A_68_P23246056	chr4:119212030-119212074	NM_172699:-240	Foxj3	DIVERGENT_PROMOTER	0.874	0.603	1433.67	863.88	0.527	1228.07	647.03
A_68_P25971957	chr8:77617610-77617654	NM_010442:116	Hmox1	INSIDE	0.874	0.641	893.64	572.73	0.560	807.91	452.65
A_68_P30650136	chr16:31933025-31933069	NM_172822:-890	Pigz	PROMOTER	0.874	0.355	1614.70	573.00	0.310	1301.58	403.71
A_68_P30489220	chr15:100870918-100870964	NM_011323:104239	Scn8a	INSIDE	0.874	6.479	1711.41	11087.85	5.661	1228.12	6952.74
A_68_P25021510	chr7:29163296-29163340	NM_011756:929	Zfp36	INSIDE	0.874	3.055	2225.08	6796.90	2.670	1755.38	4686.07
A_68_P24319372	chr6:28212314-28212358	NM_001081678:-735	Zfp800	PROMOTER	0.874	1.957	1196.51	2341.41	1.711	1061.10	1815.23
A_68_P25947616	chr8:72178703-72178747	NM_001177527:270	Zfp964	INSIDE	0.874	1.646	1082.53	1781.71	1.438	1051.95	1513.00
A_68_P31092624	chr17:23747448-23747492	NM_001033425:9648	Zscan10	INSIDE	0.874	1.828	2065.27	3775.57	1.599	1601.32	2559.97
A_68_P31157813	chr17:35272266-35272310	NM_057171:102	Bag6	INSIDE	0.873	0.617	1039.82	641.14	0.538	714.78	384.63
A_68_P32287114	chrX:19637038-19637082	NM_021715:365	Chst7	INSIDE	0.873	1.949	1259.55	2454.89	1.701	643.37	1094.32
A_68_P20825163	chr1:172815285-172815329	NM_023173:365	Dusp12	INSIDE	0.873	0.698	1268.96	885.60	0.609	1048.39	638.71
A_68_P31114356	chr17:27252698-27252742	NM_080553:58472	Itpr3	INSIDE	0.873	2.966	744.82	2209.27	2.588	511.35	1323.55
A_68_P28997653	chr13:44825548-44825592	NM_001205044:-572	Jarid2	PROMOTER	0.873	2.320	394.62	915.52	2.025	388.99	787.65
A_68_P22934428	chr4:55544566-55544610	NM_010637:759	Klf4	INSIDE	0.873	0.659	899.77	592.68	0.575	783.94	450.72
A_68_P22009790	chr3:27081996-27082040	NM_178772:93	Nceh1	INSIDE	0.873	0.389	1543.25	600.22	0.340	1256.59	426.71
A_68_P23419144	chr4:151852440-151852484	NM_153424:212	Nphp4	INSIDE	0.873	0.550	2401.43	1319.96	0.480	1842.79	884.19

ProbeName	Target position of probe on CpG island microarray	TargetID	GeneSymbol	CpG island Description	Ratio of relative methylation (TiO_2-NP/Vehicle)	Sham group			TiO_2-H group		
						Relative methylation (Cy5/Cy3)	Cy3 signal (Input)	Cy5 signal (MeDIP)	Relative methylation (Cy5/Cy3)	Cy3 signal (Input)	Cy5 signal (MeDIP)
A_68_P27921013	chr11:74336837-74336884	NM_001015046:66800	Rap1gap2	INSIDE	0.873	0.728	2592.51	1888.10	0.635	2117.39	1345.49
A_68_P23294684	chr4:128736191-128736235	NM_029219:698	Rnf19b	INSIDE	0.873	0.610	1738.68	1060.61	0.533	1508.23	803.54
A_68_P30953525	chr16:89885381-89885425	NM_009384:75670	Tiam1	INSIDE	0.873	2.170	379.66	823.93	1.894	358.22	678.40
A_68_P23991347	chr5:112737115-112737159	NM_009419:31410	Tpst2	INSIDE	0.873	2.471	1425.99	3522.92	2.156	1061.89	2289.65
A_68_P24084073	chr5:129106395-129106439	NR_015517:146	5930412G12Rik	INSIDE	0.872	0.640	846.04	541.27	0.558	667.89	372.70
A_68_P24925496	chr6:145814867-145814912	NM_024469:-1029	Bhlhe41	PROMOTER	0.872	0.637	1531.95	975.27	0.555	1233.23	685.00
A_68_P27839286	chr11:59653097-59653142	NM_011991:150	Cops3	INSIDE	0.872	0.543	1527.69	830.07	0.474	1156.18	547.89
A_68_P30361555	chr15:78502500-78502544	NM_183141:46021	Elfn2	INSIDE	0.872	2.885	447.59	1291.39	2.515	372.33	936.37
A_68_P31116775	chr17:27639850-27639894	NM_001013385:377	Grm4	INSIDE	0.872	1.946	1504.02	2926.97	1.697	1096.70	1861.37
A_68_P29619757	chr14:56381299-56381343	NM_020490:1056	Ltb4r2	INSIDE	0.872	0.677	1255.29	849.54	0.590	1015.33	599.05
A_68_P32181858	chr19:53404585-53404629	NM_001008543:-329	Mxi1	PROMOTER	0.872	2.460	4597.46	11311.27	2.145	3146.55	6750.54
A_68_P32143068	chr19:46379305-46379349	NM_019408:-858	Nfkb2	PROMOTER	0.872	0.497	1151.85	572.46	0.433	953.31	413.09
A_68_P20454943	chr1:95239662-95239706	NM_080850:-319	Pask	DIVERGENT_PROMOTER	0.872	0.699	2110.67	1475.09	0.609	1597.99	973.57
A_68_P27796193	chr11:51671506-51671550	NM_199299:-545	Phf15	PROMOTER	0.872	1.654	4153.28	6869.35	1.443	3366.99	4858.72
A_68_P30447284	chr15:93333655-93333701	NM_001033217:92644	Prickle1	INSIDE	0.872	2.194	1071.72	2351.23	1.914	890.92	1704.81
A_68_P24155045	chr5:143874478-143874522	NM_080561:199	Rnf216	INSIDE	0.872	1.614	1402.68	2264.03	1.408	1365.53	1923.04
A_68_P25086971	chr7:51608782-51608826	NM_001034115:43171	Shank1	INSIDE	0.872	1.675	1856.04	3109.02	1.461	1463.80	2138.35
A_68_P31349750	chr17:74396973-74397017	NM_053188:262	Srd5a2	INSIDE	0.872	0.443	1808.29	800.22	0.386	1424.46	549.98
A_68_P22889660	chr4:46626863-46626907	NM_198664:36187	Tbc1d2	INSIDE	0.872	3.262	795.20	2593.92	2.845	690.76	1965.00
A_68_P27288944	chr10:80795734-80795778	NM_009325:4281	Tbxa2r	INSIDE	0.872	2.451	3048.99	7472.80	2.137	2148.70	4591.81
A_68_P20066645	chr1:19204117-19204161	NM_001025305:2004	Tcfap2b	INSIDE	0.872	2.066	3565.75	7367.77	1.801	2815.05	5069.63
A_68_P20620974	chr1:134257507-134257551	NM_178874:30330	Tmcc2	INSIDE	0.872	5.327	1974.48	10518.96	4.646	1297.05	6026.06
A_68_P29455105	chr14:22650386-22650430	NM_011695:-374	Vdac2	PROMOTER	0.872	2.870	2625.64	7534.59	2.504	2167.00	5425.31
A_68_P25003662	chr7:24919226-24919270	NM_019941:67	Zfp235	INSIDE	0.872	0.651	1428.14	930.36	0.568	1281.49	727.82
A_68_P27268625	chr10:76978607-76978651	NM_133998:-70	1810008A18Rik	PROMOTER	0.871	0.632	870.27	550.25	0.551	736.48	405.68
A_68_P24153993	chr5:143667831-143667875	NM_007393:551	Actb	INSIDE	0.871	0.429	3209.67	1378.50	0.374	2907.59	1087.34
A_68_P21866711	chr2:172765860-172765904	NM_007557:-88	Bmp7	PROMOTER	0.871	1.565	2928.19	4583.07	1.364	2096.07	2858.44
A_68_P26445781	chr9:40686287-40686331	NM_178245:4099	Bsx	DOWNSTREAM	0.871	0.413	1713.08	706.96	0.360	1469.31	528.30
A_68_P31933967	chr19:6320701-6320745	NM_001033342:14266	Cdc42bpg	INSIDE	0.871	1.835	720.74	1322.88	1.598	573.85	917.16
A_68_P23363270	chr4:140865058-140865102	NM_010139:7926	Epha2	INSIDE	0.871	3.984	1655.48	6595.09	3.471	1201.84	4171.69
A_68_P29287623	chr13:104710322-104710366	NM_001005868:250	Erbb2ip	INSIDE	0.871	0.677	1248.07	845.51	0.590	1128.33	665.73
A_68_P20897124	chr1:186551680-186551724	NM_008250:4670	Hlx	INSIDE	0.871	0.606	1000.50	606.02	0.527	837.16	441.43
A_68_P29138729	chr13:73409301-73409345	NM_018885:11378	Irx4	DOWNSTREAM	0.871	0.585	1699.06	994.40	0.510	1628.36	829.90
A_68_P20025041	chr1:9957764-9957808	NM_145692:41415	Lrrc67	DOWNSTREAM	0.871	0.609	2970.43	1809.75	0.531	2503.54	1328.63
A_68_P30370810	chr15:80042202-80042246	NM_010795:38074	Mgat3	INSIDE	0.871	2.058	719.66	1480.88	1.791	530.71	950.66
A_68_P24053561	chr5:123479352-123479396	NM_175423:14292	Orai1	INSIDE	0.871	1.968	581.61	1144.85	1.715	445.50	764.19
A_68_P24176179	chr5:148674196-148674240	NM_025624:2015	Pomp	INSIDE	0.871	2.050	1275.08	2613.68	1.786	907.28	1620.27
A_68_P24991726	chr7:19735138-19735182	NM_026943:-26	Snrpd2	DIVERGENT_PROMOTER	0.871	0.725	3973.23	2880.75	0.632	2969.44	1876.17
A_68_P29030714	chr13:51196855-51196899	NM_146043:610	Spin1	INSIDE	0.871	0.525	1982.85	1041.34	0.457	1672.65	765.19
A_68_P27540232	chr10:127669379-127669423	NM_011589:-5721	Timeless	PROMOTER	0.871	3.062	436.90	1337.93	2.667	404.36	1078.51
A_68_P22200383	chr3:65331975-65332019	NM_178892:-372	Tiparp	PROMOTER	0.871	0.627	923.82	578.98	0.546	882.28	481.56
A_68_P24076592	chr5:127723010-127723054	NM_175432:837	Tmem132c	INSIDE	0.871	0.387	1582.26	611.72	0.337	1427.04	480.36
A_68_P23610768	chr5:37358352-37358396	NM_011716:21847	Wfs1	INSIDE	0.871	1.656	1316.95	2180.30	1.442	949.83	1370.11
A_68_P25008233	chr7:25862458-25862502	NM_175477:216	Zfp574	INSIDE	0.871	0.392	1481.03	580.40	0.341	1130.08	385.78
A_68_P25505624	chr7:134588136-134588180	NM_175163:4514	Zfp689	INSIDE	0.871	2.485	1382.72	3435.98	2.165	1050.76	2274.70
A_68_P32554229	chrX:96412142-96412186			Unknown	0.871	2.003	774.08	1550.49	1.744	446.97	779.67
A_68_P30575402	chr16:17734359-17734403			Unknown	0.871	2.173	417.98	908.32	1.893	407.78	771.86
A_68_P24015106	chr5:116894713-116894757	NR_015595:6004	2410137F16Rik	INSIDE	0.870	1.855	751.77	1394.82	1.615	588.51	950.44
A_68_P20872229	chr1:181734124-181734173	NM_026375:-2	Ahctf1	PROMOTER	0.870	0.632	969.51	612.75	0.550	722.46	397.46
A_68_P27383825	chr10:98377784-98377828	NM_026482:21	Atp2b1	INSIDE	0.870	2.508	706.86	1772.64	2.183	598.46	1306.38
A_68_P25374661	chr7:108812468-108812512	NM_009191:209	Clpb	INSIDE	0.870	0.567	1128.62	639.93	0.493	963.78	475.32

ProbeName	Target position of probe on CpG island microarray	TargetID	GeneSymbol	CpG island Description	Ratio of relative methylation (TiO_2-NP/Vehicle)	Sham group			TiO_2-H group		
						Relative methylation (Cy5/Cy3)	Cy3 signal (Input)	Cy5 signal (MeDIP)	Relative methylation (Cy5/Cy3)	Cy3 signal (Input)	Cy5 signal (MeDIP)
A_68_P27479308	chr10:116511163-116511209	NM_177798:74344	Frs2	INSIDE	0.870	1.872	641.53	1201.21	1.629	554.31	902.71
A_68_P25724985	chr8:26149132-26149176	NM_001081187:280	Htra4	INSIDE	0.870	2.906	777.34	2258.56	2.528	736.51	1862.15
A_68_P27987931	chr11:86070791-86070835	NM_027421:258	Ints2	INSIDE	0.870	0.667	1556.45	1037.85	0.580	1345.03	780.48
A_68_P30366728	chr15:79316188-79316232	NM_008427:19461	Kcnj4	INSIDE	0.870	2.719	1525.52	4148.39	2.366	1088.64	2575.47
A_68_P29865207	chr14:103745609-103745653	NM_207215:387	Mycbp2	INSIDE	0.870	0.620	2462.07	1527.23	0.540	2071.53	1117.82
A_68_P27897405	chr11:69647501-69647545	NM_198862:829	Nlgn2	INSIDE	0.870	1.934	703.79	1361.32	1.682	582.86	980.53
A_68_P22325408	chr3:90269752-90269796	NM_008727:14	Npr1	INSIDE	0.870	0.602	1107.03	666.74	0.524	1072.52	562.03
A_68_P27614818	chr11:17111314-17111358	NM_025443:256	Pno1	INSIDE	0.870	0.709	2298.36	1628.53	0.616	1845.26	1136.89
A_68_P24795626	chr6:119852843-119852887	NM_001166383:149	Rad52	INSIDE	0.870	0.686	1194.88	820.04	0.597	1143.92	683.17
A_68_P25091259	chr7:52379751-52379795	NM_013725:-13	Rps11	PROMOTER	0.870	0.575	1341.90	771.36	0.500	984.21	491.94
A_68_P22488493	chr3:123211082-123211126	NR_028573:-120	Snora24	PROMOTER	0.870	0.626	1027.43	642.68	0.545	877.38	477.74
A_68_P31155256	chr17:34817442-34817486	NM_031176:9985	Tnxb	INSIDE	0.870	1.717	900.21	1545.81	1.494	764.78	1142.74
A_68_P25614228	chr8:3496226-3496270	NM_080461:3111	Zfp358	INSIDE	0.870	1.935	571.83	1106.36	1.683	458.61	772.03
A_68_P32975995	chr12:18394467-18394511	ENSMUST00000080502:-72		PROMOTER	0.870	2.392	608.84	1456.18	2.081	630.39	1312.15
A_68_P28854600	chr13:18084983-18085027	NM_025904:181	1600012F09Rik	INSIDE	0.869	0.701	1274.65	894.08	0.609	1057.37	644.15
A_68_P27282332	chr10:79767774-79767818	NM_011789:3232	Apc2	INSIDE	0.869	2.388	3656.87	8733.89	2.076	2699.25	5604.91
A_68_P28046785	chr11:96650631-96650675	NM_007622:203	Cbx1	INSIDE	0.869	0.494	1413.61	698.11	0.429	1106.79	474.91
A_68_P21708093	chr2:144425785-144425829	NM_025314:118	Dtd1	INSIDE	0.869	0.681	1069.50	727.94	0.591	925.92	547.53
A_68_P31424357	chr17:88039524-88039571	NM_008532:4229	Epcam	INSIDE	0.869	2.625	372.84	978.86	2.281	387.07	883.04
A_68_P26421276	chr9:35018562-35018606	NM_172291:-74	Foxred1	DIVERGENT_PROMOTER	0.869	0.437	1548.43	676.67	0.380	1250.58	475.02
A_68_P21753005	chr2:152934198-152934242	NM_001172117:17	Hck	INSIDE	0.869	0.599	914.05	547.48	0.520	830.11	432.03
A_68_P21722291	chr2:146910728-146910772	NM_023504:331	Nkx2-4	INSIDE	0.869	2.664	751.97	2003.09	2.314	707.09	1635.90
A_68_P30542115	chr16:10411996-10412040	NM_011955:-12	Nubp1	PROMOTER	0.869	0.668	944.17	631.01	0.581	860.32	499.49
A_68_P21089695	chr2:28061711-28061755	NM_001038612:524	Olfm1	INSIDE	0.869	1.627	1541.35	2507.71	1.415	1384.94	1959.01
A_68_P21870540	chr2:173411809-173411854	NR_027957:73209	Ppp4r1l-ps	INSIDE	0.869	2.777	309.12	858.50	2.414	214.41	517.69
A_68_P31735661	chr18:57522721-57522765	NM_028447:8356	Prrc1	INSIDE	0.869	2.775	1221.69	3390.28	2.413	805.85	1944.21
A_68_P27898299	chr11:69795072-69795116	NM_018740:-357	Rai12	PROMOTER	0.869	0.483	1406.71	679.02	0.420	1215.32	509.86
A_68_P24939590	chr6:148303285-148303329	NR_003634:129	Rps4y2	INSIDE	0.869	1.918	1410.21	2704.71	1.666	1243.03	2070.60
A_68_P26569718	chr9:62129773-62129817	NM_025721:192	Spesp1	INSIDE	0.869	2.798	575.89	1611.10	2.430	502.60	1221.21
A_68_P30506930	chr16:3872290-3872334	NM_001033159:62	Zfp597	INSIDE	0.869	2.310	5535.65	12789.94	2.008	4065.17	8162.34
A_68_P26640510	chr9:74697051-74697095	ENSMUST00000160842:271		INSIDE	0.869	1.670	1508.71	2518.81	1.451	1391.82	2019.66
A_68_P20146384	chr1:36160417-36160461	NM_015818:35194	Hs6st1	INSIDE	0.868	1.963	978.06	1920.18	1.704	850.90	1449.85
A_68_P21812072	chr2:163201808-163201852	NM_021566:21879	Jph2	INSIDE	0.868	2.466	744.13	1835.07	2.140	751.71	1608.81
A_68_P21078908	chr2:26321156-26321200	NM_008714:38164	Notch1	INSIDE	0.868	1.835	1854.93	3403.59	1.592	1521.03	2421.85
A_68_P27209838	chr10:66558947-66558991	NM_001204915:768	Reep3	INSIDE	0.868	0.655	1130.56	740.18	0.569	906.29	515.27
A_68_P24085132	chr5:129288040-129288085	NM_001081388:171175	Rimbp2	INSIDE	0.868	2.478	919.33	2278.16	2.150	759.17	1632.17
A_68_P30642390	chr16:30550650-30550694	NM_172614:-8	Tmem44	PROMOTER	0.868	5.105	435.99	2225.66	4.433	505.33	2240.22
A_68_P25029338	chr7:30768885-30768929	NM_001033355:-67	Zfp568	PROMOTER	0.868	1.929	615.79	1187.88	1.674	555.88	930.78
A_68_P22061573	chr3:36962572-36962616	NM_009350:17	Adad1	INSIDE	0.867	2.272	469.16	1066.08	1.971	460.17	906.95
A_68_P27334199	chr10:89512717-89512765	NM_001128086:176487	Anks1b	INSIDE	0.867	2.633	2012.36	5298.50	2.284	1533.30	3501.44
A_68_P26569974	chr9:62191922-62191966	NM_009672:2795	Anp32a	INSIDE	0.867	1.922	1060.27	2037.41	1.666	813.61	1355.70
A_68_P25601077	chr7:150645470-150645514	NM_001161624:1463	Cdkn1c	INSIDE	0.867	2.175	346.17	752.85	1.886	327.91	618.38
A_68_P24631555	chr6:90259775-90259819	NM_027928:15383	Chst13	INSIDE	0.867	2.598	454.40	1180.42	2.252	499.07	1123.66
A_68_P26141977	chr8:109417130-109417174	NM_145412:341	Chtf8	INSIDE	0.867	1.566	4050.16	6344.42	1.357	3176.69	4312.33
A_68_P26813992	chr9:108165850-108165894	NM_010017:195	Dag1	INSIDE	0.867	4.360	4840.93	21107.51	3.782	3687.16	13944.75
A_68_P28431897	chr12:52930027-52930071	NM_144788:475	Hectd1	INSIDE	0.867	0.586	2107.41	1235.91	0.509	1887.39	959.89
A_68_P23088446	chr4:87678217-87678261	NM_027326:1073	Mllt3	INSIDE	0.867	1.911	9013.93	17224.68	1.656	5695.10	9432.97
A_68_P28621836	chr12:90498827-90498871	NM_172544:307015	Nrxn3	INSIDE	0.867	2.348	1035.06	2430.21	2.035	841.98	1713.61
A_68_P30397622	chr15:84754271-84754315	NM_016714:435	Nup50	INSIDE	0.867	0.701	1818.50	1275.42	0.608	1451.79	883.20
A_68_P27553886	chr11:3661741-3661785	NM_152818:102144	Osbp2	INSIDE	0.867	0.611	973.22	594.88	0.530	773.97	410.38
A_68_P31951137	chr19:10599680-10599724	NM_025333:-3	Sdhaf2	DIVERGENT_PROMOTER	0.867	0.595	2108.23	1255.39	0.516	1676.01	865.31

ProbeName	Target position of probe on CpG island microarray	TargetID	GeneSymbol	CpG island Description	Ratio of relative methylation (TiO_2-NP/Vehicle)	Sham group			TiO_2-H group		
						Relative methylation (Cy5/Cy3)	Cy3 signal (Input)	Cy5 signal (MeDIP)	Relative methylation (Cy5/Cy3)	Cy3 signal (Input)	Cy5 signal (MeDIP)
A_68_P31197209	chr17:44913929-44913973	NM_178652:-169	Supt3h	PROMOTER	0.867	0.694	1209.12	838.88	0.602	1110.31	667.90
A_68_P25264700	chr7:87226640-87226684	NM_175433:55549	Zfp710	INSIDE	0.867	5.599	2146.15	12015.37	4.854	1439.13	6985.40
A_68_P21041402	chr2:18688499-18688543	ENSMUST00000119424:7910		DOWNSTREAM	0.867	0.662	1346.87	891.74	0.574	911.08	523.20
A_68_P30280956	chr15:64144021-64144065	NM_010026:70439	Asap1	INSIDE	0.866	0.518	1540.98	798.96	0.449	1183.54	531.62
A_68_P31155037	chr17:34784214-34784258	NM_017406:112	Atf6b	INSIDE	0.866	0.636	1437.71	914.68	0.551	1213.68	668.46
A_68_P27566548	chr11:5966021-5966065	NM_001174053:-291	Camk2b	PROMOTER	0.866	0.565	1963.07	1108.52	0.489	1621.10	792.99
A_68_P24011288	chr5:116182625-116182669	NM_001080808:-1078	Ccdc64	PROMOTER	0.866	0.458	2056.27	941.30	0.396	1749.99	693.52
A_68_P32467106	chrX:71500485-71500529	NM_007927:329	Emd	INSIDE	0.866	2.166	688.32	1491.24	1.875	524.65	983.86
A_68_P23323334	chr4:134090623-134090667	NM_029759:-1977	Fam54b	PROMOTER	0.866	4.102	229.87	942.85	3.552	284.84	1011.86
A_68_P23792662	chr5:72978528-72978572	NM_001024147:239	Gm5868	INSIDE	0.866	1.574	4410.48	6944.21	1.363	3337.78	4550.13
A_68_P32040189	chr19:27397675-27397720	NM_183179:589	Kcnv2	INSIDE	0.866	2.631	288.53	759.26	2.279	222.11	506.17
A_68_P27897481	chr11:69659900-69659944	NM_023564:-196	Plscr3	PROMOTER	0.866	0.429	2485.48	1066.58	0.372	1987.56	738.94
A_68_P28671531	chr12:99927688-99927737	NM_011877:45143	Ptpn21	INSIDE	0.866	2.361	1448.94	3420.54	2.044	1165.78	2382.44
A_68_P31862860	chr18:80389507-80389558	NM_199197:7826	Rbfa	INSIDE	0.866	3.521	778.32	2740.13	3.050	657.44	2005.38
A_68_P29434939	chr14:18492981-18493025	NM_001113417:-471	Thrb	PROMOTER	0.866	2.355	1553.79	3658.48	2.039	1308.39	2667.18
A_68_P31403195	chr17:84586228-84586272	NM_001001806:1037	Zfp36l2	INSIDE	0.866	2.433	509.64	1239.84	2.107	482.28	1016.21
A_68_P32126393	chr19:43502105-43502149			Unknown	0.866	3.073	586.60	1802.37	2.662	528.52	1406.92
A_68_P31305847	chr17:66728898-66728942	NM_001114098:70170	1110012J17Rik	INSIDE	0.865	2.267	1452.35	3291.76	1.960	1052.98	2064.32
A_68_P23315717	chr4:132641057-132641101	NM_146155:73658	Ahdc1	DOWNSTREAM	0.865	0.611	1018.31	621.88	0.528	885.12	467.45
A_68_P24460753	chr6:53989704-53989748	NM_001163640:-199	Chn2	PROMOTER	0.865	0.558	1024.92	572.28	0.483	865.45	417.93
A_68_P28038132	chr11:95198164-95198208	NM_172543:-145	Fam117a	PROMOTER	0.865	0.656	891.88	585.28	0.567	769.31	436.55
A_68_P26227387	chr8:124078337-124078381	NM_133765:24348	Fbxo31	INSIDE	0.865	2.490	548.54	1366.02	2.153	472.00	1016.38
A_68_P24863967	chr6:134819942-134819986	NM_001167697:17913	Gpr19	INSIDE	0.865	3.536	676.30	2391.39	3.059	536.40	1641.09
A_68_P30363243	chr15:78758427-78758471	NM_008495:1294	Lgals1	INSIDE	0.865	0.692	1760.21	1218.25	0.599	1375.37	823.36
A_68_P25266277	chr7:87508028-87508072	NM_172903:8211	Man2a2	INSIDE	0.865	1.912	1932.47	3694.81	1.654	1330.30	2200.65
A_68_P20168276	chr1:39957440-39957484	NM_008696:-295	Map4k4	PROMOTER	0.865	2.016	752.92	1518.24	1.745	589.78	1029.15
A_68_P21731906	chr2:148558116-148558160	NM_019632:18	Napb	INSIDE	0.865	0.573	972.05	556.92	0.496	804.00	398.66
A_68_P29998112	chr15:8059193-8059237	NM_133227:-98	Nup155	PROMOTER	0.865	1.706	3614.97	6165.40	1.475	2486.84	3666.92
A_68_P27137646	chr10:52137376-52137420	NM_030250:46	Nus1	INSIDE	0.865	2.089	13326.36	27839.76	1.807	9593.03	17333.46
A_68_P27080349	chr10:39452815-39452859	NM_011264:871	Rev3l	INSIDE	0.865	0.688	2277.62	1566.19	0.594	1867.17	1110.00
A_68_P27034632	chr10:29081402-29081446	NM_001110196:824	Rnf146	INSIDE	0.865	0.631	993.92	626.90	0.545	766.87	418.15
A_68_P20233541	chr1:53841640-53841684	NM_133810:397	Stk17b	INSIDE	0.865	0.730	2383.44	1739.64	0.632	1961.17	1238.69
A_68_P23974101	chr5:109204043-109204087	NM_026109:20318	Tmed11	DOWNSTREAM	0.865	1.652	1295.03	2139.48	1.429	1013.24	1448.21
A_68_P27285909	chr10:80318182-80318226	NM_175195:185	3110056O03Rik	INSIDE	0.864	0.673	1710.84	1151.81	0.582	1581.99	919.98
A_68_P23320878	chr4:133628753-133628797	NM_001162970:4408	Aim1l	INSIDE	0.864	2.216	583.81	1293.70	1.915	433.60	830.45
A_68_P22384844	chr3:102975721-102975767	NM_026602:111	Bcas2	INSIDE	0.864	2.678	1801.70	4825.71	2.314	1251.85	2897.17
A_68_P21646235	chr2:133380368-133380412	NM_007553:1456	Bmp2	INSIDE	0.864	0.583	977.78	570.09	0.504	846.56	426.65
A_68_P28752828	chr12:114067098-114067142	NM_028023:480	Cdca4	INSIDE	0.864	0.482	1426.83	687.84	0.416	1138.69	474.11
A_68_P27091225	chr10:41529755-41529799	NM_026643:-340	Cep57l1	DIVERGENT_PROMOTER	0.864	0.600	1175.86	705.05	0.518	947.80	491.12
A_68_P28727387	chr12:109572709-109572753	NM_010010:140	Cyp46a1	INSIDE	0.864	0.563	1057.15	595.44	0.487	838.21	407.83
A_68_P27288015	chr10:80655147-80655191	NM_007828:8506	Dapk3	INSIDE	0.864	2.019	530.06	1070.21	1.744	459.21	800.82
A_68_P27010233	chr10:24431686-24431730	NM_008813:200	Enpp1	INSIDE	0.864	0.582	898.67	523.34	0.503	737.01	370.83
[truncated: 2,139,093 more chars]
