# Supplementary material for: Effects of Prenatal Exposure to Titanium Dioxide Nanoparticles on DNA Methylation and Gene Expression Profile in the Mouse Brain
Source: Front Toxicol. 2021 Oct 8;3:705910. doi: 10.3389/ftox.2021.705910 (PMC8915839; doi:10.3389/ftox.2021.705910)
Supplement: Supplementary file 9 [file Table12.pdf]

Supplementary Table 12.

GO terms enriched in the genes that showed differential expression accompanied by altered DNA methylation commonly in male (M) and female (F) offspring in the TiO_2-H group.

ID	GO term	Sex	Enrichment factor	p-value
GO:0003779	actin binding	M	3.28	0.019
		F	2.73	0.037
GO:0008285	negative regulation of cell population proliferation	M	3.07	0.024
		F	3.58	0.004
GO:0007275	multicellular organism development	M	2.63	0.002
		F	3.48	< 0.001
GO:0003700	DNA-binding transcription factor activity	M	2.63	0.018
		F	5.01	< 0.001
GO:0043565	sequence-specific DNA binding	M	2.50	0.033
		F	4.17	< 0.001
GO:0030054	cell junction	M	2.42	0.012
		F	2.24	0.014
GO:0006355	regulation of transcription, DNA-templated	M	2.34	0.011
		F	3.31	< 0.001
