# Supplementary material for: Effects of Prenatal Exposure to Titanium Dioxide Nanoparticles on DNA Methylation and Gene Expression Profile in the Mouse Brain
Source: Front Toxicol. 2021 Oct 8;3:705910. doi: 10.3389/ftox.2021.705910 (PMC8915839; doi:10.3389/ftox.2021.705910)
Supplement: Supplementary file 10 [file Table7.pdf]

Supplementary Table 7. The genes of which DNA methylation were decreased in the brain of female offspring prenatally exposed to TiO_2-NP.

ProbeName	Target position of probe on CpG island microarray	TargetID	GeneSymbol	CpG island Description	Ratio of relative methylation (TiO2-NP/Vehicle)	Sham group			TiO_2-H group		
						Relative methylation (Cy-5/Cy-3)	Cy-3 signal (Input)	Cy-5 signal (MeDIP)	Relative methylation (Cy-5/Cy-3)	Cy-3 signal (Input)	Cy-5 signal (MeDIP)
A_68_P31102483	chr17:25377069-25377113	NM_001163718:-24	0610007P22Rik	DIVERGENT_PROMOTER	0.275	0.365	1564.68	570.88	0.100	1276.81	127.95
A_68_P27795275	chr11:51502704-51502761	NM_025319:-596	0610009B22Rik	PROMOTER	0.255	0.349	1652.35	576.76	0.089	1336.07	119.14
A_68_P28202484	chr12:4824078-4824122	NM_025323:-313	0610009D07Rik	PROMOTER	0.406	0.608	1944.10	1181.96	0.247	1603.98	395.49
A_68_P28183969	chr11:120210906-120210950	NR_038126:937	0610009L18Rik	INSIDE	0.221	0.653	1539.12	1004.95	0.145	1088.27	157.27
A_68_P27646750	chr11:23533697-23533742	NM_027860:-88	0610010F05Rik	PROMOTER	0.439	0.620	1612.47	1000.42	0.272	1363.85	371.50
A_68_P27899662	chr11:70051263-70051307	NM_001177601:132	0610010K14Rik	INSIDE	0.238	0.356	1752.37	623.05	0.085	1486.63	125.86
A_68_P31623418	chr18:36508869-36508913	NM_001081365:613	0610010O12Rik	INSIDE	0.420	0.560	1256.22	703.63	0.235	1148.09	270.04
A_68_P24541217	chr6:72297432-72297476	NM_026696:144	0610030E20Rik	INSIDE	0.473	0.544	1173.35	638.12	0.257	961.45	247.19
A_68_P24541218	chr6:72297585-72297629	NM_026696:296	0610030E20Rik	INSIDE	0.506	0.640	1200.57	768.92	0.324	976.07	316.47
A_68_P23747544	chr5:64203539-64203583	NM_029554:-173	0610040J01Rik	PROMOTER	0.407	0.325	2198.01	714.47	0.132	1814.50	239.97
A_68_P24377801	chr6:38484955-38484999	NM_025363:116	1110001J03Rik	INSIDE	0.560	0.622	1455.55	905.08	0.348	1209.77	421.18
A_68_P28448909	chr12:55757202-55757246	NM_134054:335	1110002B05Rik	INSIDE	0.588	0.474	2137.50	1012.68	0.279	1647.80	459.13
A_68_P28448910	chr12:55757351-55757395	NM_134054:187	1110002B05Rik	INSIDE	0.404	0.509	1885.11	959.65	0.206	1424.62	293.29
A_68_P23756658	chr5:65883543-65883594	NM_133697:506	1110003E01Rik	INSIDE	0.082	1.376	1428.44	1965.72	0.113	1055.44	119.46
A_68_P23756666	chr5:65884517-65884566	NM_133697:-467	1110003E01Rik	PROMOTER	0.062	1.900	2135.45	4057.75	0.119	1475.15	175.18
A_68_P30959868	chr16:90934441-90934485	NM_026502:632	1110004E09Rik	INSIDE	0.102	1.625	2187.27	3554.32	0.166	1532.00	254.74
A_68_P30959870	chr16:90934584-90934628	NM_026502:488	1110004E09Rik	INSIDE	0.197	1.493	5615.36	8385.98	0.294	3441.60	1013.47
A_68_P30959877	chr16:90935364-90935408	NM_026502:-292	1110004E09Rik	PROMOTER	0.549	0.655	975.44	638.67	0.359	806.84	289.87
A_68_P24010682	chr5:116081413-116081457	NR_027810:391	1110006O24Rik	INSIDE	0.465	0.585	1981.41	1158.76	0.272	1367.20	372.10
A_68_P24010683	chr5:116081567-116081611	NR_027810:237	1110006O24Rik	INSIDE	0.543	0.480	3967.39	1902.82	0.261	3024.88	788.20
A_68_P24039804	chr5:121062475-121062523	NM_029096:100	1110008J03Rik	INSIDE	0.262	0.569	1261.98	717.52	0.149	882.62	131.63
A_68_P24039806	chr5:121062707-121062753	NM_029096:-132	1110008J03Rik	DIVERGENT_PROMOTER	0.173	0.605	1671.34	1011.87	0.104	1153.56	120.49
A_68_P24039807	chr5:121062809-121062858	NM_029096:-235	1110008J03Rik	DIVERGENT_PROMOTER	0.085	0.741	2716.71	2012.92	0.063	1912.35	120.62
A_68_P21115063	chr2:32237069-32237113	NM_198001:345	1110008P14Rik	INSIDE	0.487	0.635	813.33	516.47	0.309	625.13	193.40
A_68_P21115067	chr2:32237497-32237541	NM_198001:-83	1110008P14Rik	PROMOTER	0.474	0.452	3164.68	1431.07	0.215	2248.98	482.49
A_68_P31305849	chr17:66729099-66729143	NM_001114098:69970	1110012J17Rik	INSIDE	0.664	2.326	1921.71	4469.38	1.544	1357.88	2095.95
A_68_P22862527	chr4:41454006-41454050	NM_028624:769	1110017D15Rik	INSIDE	0.346	0.574	1866.88	1070.67	0.199	1492.32	296.37
A_68_P28601430	chr12:86311556-86311600	NM_178065:258	1110018G07Rik	INSIDE	0.141	0.694	2777.69	1926.56	0.098	2013.51	197.55
A_68_P30003953	chr15:9000818-9000866	NM_001040395:-166	1110020G09Rik	PROMOTER	0.283	0.592	1047.71	620.19	0.167	721.25	120.70
A_68_P30003954	chr15:9000908-9000952	NM_001040395:-78	1110020G09Rik	PROMOTER	0.298	0.579	2192.77	1270.55	0.173	1518.52	262.25
A_68_P31129446	chr17:29853826-29853870	NM_001163741:114	1110021J02Rik	INSIDE	0.241	1.895	2742.01	5195.22	0.457	1743.70	797.18
A_68_P31129448	chr17:29854041-29854085	NM_001163741:-100	1110021J02Rik	PROMOTER	0.167	1.646	4708.75	7749.57	0.275	3153.45	868.55
A_68_P20304762	chr1:66863250-66863306	NM_001122738:891	1110028C15Rik	INSIDE	0.253	0.432	1501.38	648.86	0.109	1084.27	118.50
A_68_P20304765	chr1:66863691-66863735	NM_001122738:457	1110028C15Rik	INSIDE	0.631	0.632	979.39	619.21	0.399	800.69	319.66
A_68_P28189252	chr11:121066090-121066134	NM_025402:-150	1110031I02Rik	DIVERGENT_PROMOTER	0.448	0.729	3435.64	2503.65	0.326	2421.51	789.79
A_68_P28189253	chr11:121066168-121066212	NM_025402:-228	1110031I02Rik	DIVERGENT_PROMOTER	0.647	0.674	2836.41	1911.52	0.436	2309.43	1007.33
A_68_P22217911	chr3:68673203-68673247	NM_001167996:-283	1110032F04Rik	PROMOTER	0.603	0.508	1114.32	566.44	0.306	941.56	288.50
A_68_P20252951	chr1:57463816-57463860	NM_001037742:-320	1110034B05Rik	PROMOTER	0.284	0.480	1330.48	639.27	0.137	1073.59	146.60
A_68_P27256916	chr10:74980191-74980235	NM_175133:-145	1110038D17Rik	DIVERGENT_PROMOTER	0.610	0.716	1785.62	1279.09	0.437	1457.66	637.16
A_68_P30351330	chr15:76779157-76779201	NM_054099:205	1110038F14Rik	INSIDE	0.618	0.466	1212.67	565.59	0.288	1047.35	301.82
A_68_P30351332	chr15:76779347-76779391	NM_054099:395	1110038F14Rik	INSIDE	0.575	0.453	1546.39	700.56	0.261	1351.00	352.07
A_68_P23331579	chr4:135543347-135543391	NM_025411:-209	1110049F12Rik	PROMOTER	0.559	0.640	1505.82	963.38	0.358	1300.10	465.03

ProbeName	Target position of probe on CpG island microarray	TargetID	GeneSymbol	CpG island Description	Ratio of relative methylation (TiO2-NP/Vehicle)	Sham group			TiO_2-H group		
						Relative methylation (Cy-5/Cy-3)	Cy-3 signal (Input)	Cy-5 signal (MeDIP)	Relative methylation (Cy-5/Cy-3)	Cy-3 signal (Input)	Cy-5 signal (MeDIP)
A_68_P22958317	chr4:59796668-59796712	NM_001013577:37	1110054O05Rik	INSIDE	0.275	0.528	5380.06	2838.36	0.145	4209.44	611.65
A_68_P32007549	chr19:21727415-21727459	NM_025423:-155	1110059E24Rik	DIVERGENT_PROMOTER	0.387	0.465	1153.75	536.60	0.180	781.26	140.74
A_68_P26892246	chr9:122859846-122859890	NM_025419:250	1110059G10Rik	INSIDE	0.339	0.569	2083.75	1185.89	0.193	1729.49	333.35
A_68_P26892247	chr9:122859927-122859971	NM_025419:170	1110059G10Rik	INSIDE	0.614	0.597	1193.62	712.33	0.366	952.22	348.99
A_68_P26892248	chr9:122860010-122860061	NM_025419:83	1110059G10Rik	INSIDE	0.129	0.610	2098.94	1280.17	0.078	1497.80	117.56
A_68_P27633580	chr11:20731422-20731466	NM_173752:-333	1110067D22Rik	PROMOTER	0.286	0.403	2112.75	851.46	0.115	1634.76	188.73
A_68_P24864290	chr6:134877311-134877355	NR_037955:-1777	1190002F15Rik	PROMOTER	0.462	0.538	992.32	533.45	0.248	857.11	212.84
A_68_P24864301	chr6:134878457-134878501	NR_037955:-631	1190002F15Rik	PROMOTER	0.315	0.694	1887.29	1309.59	0.219	1288.40	281.77
A_68_P29750655	chr14:79700758-79700803	NM_025427:662	1190002H23Rik	INSIDE	0.424	0.631	2121.04	1338.95	0.267	1624.80	434.42
A_68_P29750659	chr14:79701095-79701139	NM_025427:326	1190002H23Rik	INSIDE	0.404	0.574	1404.21	805.56	0.232	1064.58	246.87
A_68_P20714686	chr1:153275868-153275912	NM_026876:113	1190005F20Rik	INSIDE	0.454	0.485	2219.62	1075.83	0.220	1708.71	376.26
A_68_P27292892	chr10:82085954-82085998	NM_001135567:-3	1190007I07Rik	DIVERGENT_PROMOTER	0.568	0.611	1061.20	648.32	0.347	864.90	300.19
A_68_P28743981	chr12:112661620-112661664	NM_028807:6002	1200009I06Rik	INSIDE	0.292	0.533	994.97	530.58	0.156	775.75	120.64
A_68_P28744028	chr12:112667836-112667880	NM_028807:12218	1200009I06Rik	INSIDE	0.526	0.649	1883.15	1222.35	0.341	1429.66	488.04
A_68_P27992494	chr11:86900082-86900126	NM_024262:175	1200011M11Rik	INSIDE	0.557	0.685	3633.35	2489.28	0.381	2646.03	1009.26
A_68_P27992495	chr11:86900155-86900199	NM_024262:103	1200011M11Rik	INSIDE	0.213	0.674	1512.21	1018.51	0.143	1136.18	162.92
A_68_P27921678	chr11:74462698-74462742	NM_001081158:-276	1300001I01Rik	PROMOTER	0.226	0.460	1396.56	643.09	0.104	1256.78	130.76
A_68_P22885561	chr4:45896280-45896324	NM_028788:34484	1300002K09Rik	INSIDE	0.518	3.420	921.96	3153.51	1.773	691.69	1226.57
A_68_P27298723	chr10:83185538-83185582	NM_001145198:-49	1500009L16Rik	PROMOTER	0.264	0.633	3031.30	1917.79	0.167	2286.84	382.04
A_68_P31759410	chr18:61886034-61886078	NR_029432:13	1500015A07Rik	INSIDE	0.187	0.441	1836.90	809.51	0.083	1401.31	115.76
A_68_P27977999	chr11:84339401-84339452	NR_038057:265	1500016L03Rik	INSIDE	0.148	0.520	2271.78	1180.29	0.077	1698.36	130.30
A_68_P27978000	chr11:84339607-84339651	NR_038057:467	1500016L03Rik	INSIDE	0.576	0.596	1987.31	1184.09	0.343	1411.79	484.72
A_68_P30382913	chr15:82176419-82176463	NM_026914:-35	1500032L24Rik	PROMOTER	0.462	0.645	963.38	621.00	0.298	704.86	209.96
A_68_P30382915	chr15:82176602-82176646	NM_026914:149	1500032L24Rik	INSIDE	0.361	0.585	1133.20	662.59	0.211	926.82	195.82
A_68_P30633252	chr16:28929399-28929443	NM_177718:364	1600021P15Rik	INSIDE	0.231	1.334	3525.37	4702.95	0.309	2392.68	738.53
A_68_P30633253	chr16:28929529-28929575	NM_177718:232	1600021P15Rik	INSIDE	0.040	3.548	1827.40	6483.44	0.143	1069.02	152.71
A_68_P21904833	chr2:180317770-180317815	NM_028479:1784	1600027N09Rik	INSIDE	0.285	0.714	1518.90	1083.89	0.203	1141.79	231.82
A_68_P21105254	chr2:30650269-30650313	NM_198000:6937	1700001O22Rik	DOWNSTREAM	0.221	0.462	1621.13	748.23	0.102	1197.51	122.03
A_68_P21105302	chr2:30656577-30656621	NM_198000:629	1700001O22Rik	INSIDE	0.033	3.922	16457.71	64553.78	0.130	10033.62	1305.90
A_68_P25505909	chr7:134655506-134655551	NR_024331:855	1700008J07Rik	INSIDE	0.096	0.686	2461.44	1688.69	0.066	1760.20	115.85
A_68_P25505911	chr7:134655737-134655781	NR_024331:625	1700008J07Rik	INSIDE	0.248	0.511	1287.65	658.42	0.127	1048.62	133.01
A_68_P25505917	chr7:134656518-134656562	NR_024331:-157	1700008J07Rik	PROMOTER	0.358	0.593	1447.69	857.94	0.212	1201.80	254.90
A_68_P25087020	chr7:51615740-51615784	NM_027049:14638	1700008O03Rik	INSIDE	0.377	0.531	1102.21	585.73	0.200	868.73	174.08
A_68_P25417176	chr7:118266442-118266486	NR_015573:-275	1700012D14Rik	PROMOTER	0.661	0.473	1157.80	547.26	0.312	1034.55	323.09
A_68_P26541144	chr9:57109788-57109832	NM_028820:596	1700017B05Rik	INSIDE	0.424	0.699	1446.44	1010.98	0.297	1101.96	326.77
A_68_P26541149	chr9:57110346-57110390	NM_028820:38	1700017B05Rik	INSIDE	0.599	0.630	1570.04	989.76	0.378	1379.00	520.84
A_68_P25614137	chr8:3476402-3476449	NM_029598:5564	1700019B03Rik	INSIDE	0.276	2.077	1712.91	3558.55	0.574	1103.08	633.60
A_68_P28607941	chr12:87423506-87423550	NM_001199843:18	1700019E19Rik	INSIDE	0.213	0.647	1038.30	672.16	0.138	855.70	118.19
A_68_P28607942	chr12:87423668-87423712	NM_001199843:180	1700019E19Rik	INSIDE	0.470	0.595	2058.51	1224.67	0.280	1637.92	458.01
A_68_P20412114	chr1:88330989-88331033	NM_027966:8107	1700019O17Rik	DOWNSTREAM	0.641	0.597	2890.85	1724.47	0.382	2337.87	893.83
A_68_P28608855	chr12:87582934-87582978	NM_027405:129	1700020O03Rik	INSIDE	0.352	0.698	4032.55	2815.89	0.246	3252.59	799.85
A_68_P27100908	chr10:43260523-43260567	NM_026411:256	1700021F05Rik	INSIDE	0.656	0.624	1409.83	879.04	0.409	1221.30	499.63
A_68_P21092715	chr2:28555096-28555140	NM_027283:53	1700026L06Rik	INSIDE	0.491	0.650	1138.32	739.99	0.319	940.14	300.02
A_68_P21092717	chr2:28555343-28555389	NM_027283:-195	1700026L06Rik	DIVERGENT_PROMOTER	0.178	0.655	1843.17	1207.22	0.116	1391.48	162.00

ProbeName	Target position of probe on CpG island microarray	TargetID	GeneSymbol	CpG island Description	Ratio of relative methylation (TiO2-NP/Vehicle)	Sham group			TiO_2-H group		
						Relative methylation (Cy-5/Cy-3)	Cy-3 signal (Input)	Cy-5 signal (MeDIP)	Relative methylation (Cy-5/Cy-3)	Cy-3 signal (Input)	Cy-5 signal (MeDIP)
A_68_P20192515	chr1:44159202-44159246	NM_029368:9	1700029F09Rik	INSIDE	0.468	0.483	1667.14	805.58	0.226	1423.63	321.98
A_68_P23278552	chr4:125840552-125840597	NM_025856:-665	1700029G01Rik	PROMOTER	0.133	0.599	1894.74	1134.87	0.080	1477.44	117.46
A_68_P26200041	chr8:119502652-119502696	NM_027103:169	1700030J22Rik	INSIDE	0.301	0.583	1336.08	779.09	0.175	1050.21	184.17
A_68_P25961390	chr8:74967839-74967883	NM_028170:82	1700030K09Rik	INSIDE	0.184	1.398	2528.19	3534.89	0.257	1772.52	456.11
A_68_P27675036	chr11:29447352-29447406	NM_001163521:-571	1700034F02Rik	PROMOTER	0.168	0.691	1265.71	874.46	0.116	1063.16	123.63
A_68_P31891632	chr18:85121550-85121594	NM_025969:-656	1700034H14Rik	PROMOTER	0.552	0.478	2856.82	1365.88	0.264	2229.58	588.18
A_68_P24605099	chr6:85401740-85401787	NR_028081:201	1700040I03Rik	INSIDE	0.281	0.630	892.75	562.20	0.177	656.57	116.23
A_68_P24605102	chr6:85402096-85402141	NR_028081:-154	1700040I03Rik	PROMOTER	0.105	0.516	2808.24	1449.38	0.054	2203.86	119.42
A_68_P28450732	chr12:56225370-56225414	NM_001100116:-122	1700047I17Rik2	PROMOTER	0.481	0.695	1744.69	1213.31	0.334	1633.27	545.84
A_68_P28450735	chr12:56225735-56225781	NM_001100116:244	1700047I17Rik2	INSIDE	0.242	0.608	994.36	604.88	0.147	796.08	117.20
A_68_P20893085	chr1:185857220-185857264	NM_028516:635	1700056E22Rik	INSIDE	0.632	0.641	1625.01	1041.74	0.405	1221.15	494.90
A_68_P20893091	chr1:185857997-185858041	NM_028516:-141	1700056E22Rik	DIVERGENT_PROMOTER	0.437	0.657	861.33	565.99	0.287	713.63	205.10
A_68_P31215873	chr17:48232887-48232931	NM_026625:6327	1700067P10Rik	DOWNSTREAM	0.379	0.730	1820.06	1328.36	0.277	1416.41	391.83
A_68_P31215882	chr17:48233958-48234004	NM_026625:7399	1700067P10Rik	DOWNSTREAM	0.288	0.584	897.20	524.07	0.168	720.02	121.27
A_68_P28091093	chr11:104304028-104304074	NM_001081045:-445	1700081L11Rik	PROMOTER	0.217	0.441	1552.06	684.41	0.096	1209.75	115.88
A_68_P21101976	chr2:30093104-30093148	NR_028299:25	1700084E18Rik	INSIDE	0.233	0.511	1287.13	657.70	0.119	1021.28	121.43
A_68_P31632918	chr18:38410055-38410099	NR_027904:143	1700086O06Rik	INSIDE	0.664	0.533	1071.74	570.96	0.354	893.60	316.12
A_68_P23547918	chr5:25035180-25035225	NR_027388:-633	1700096K18Rik	PROMOTER	0.516	0.509	1014.77	516.90	0.263	909.77	239.35
A_68_P23547920	chr5:25035394-25035438	NR_027388:-419	1700096K18Rik	PROMOTER	0.433	0.574	3576.99	2052.69	0.249	2768.49	688.25
A_68_P23547921	chr5:25035545-25035589	NR_027388:-269	1700096K18Rik	PROMOTER	0.298	0.568	3000.72	1704.54	0.169	2111.86	357.66
A_68_P27317703	chr10:86521047-86521091	NM_029685:278	1700113H08Rik	INSIDE	0.624	0.575	1156.61	665.29	0.359	870.51	312.44
A_68_P29613709	chr14:55305052-55305096	NM_021437:67	1700123O20Rik	INSIDE	0.209	0.597	1182.48	705.82	0.125	972.83	121.36
A_68_P23296884	chr4:129168378-129168422	NM_001085491:-33	1700125D06Rik	DIVERGENT_PROMOTER	0.201	0.683	1197.36	817.50	0.137	877.02	120.53
A_68_P23296886	chr4:129168678-129168722	NM_001085491:267	1700125D06Rik	INSIDE	0.230	0.414	1808.23	748.83	0.095	1441.27	137.45
A_68_P23296887	chr4:129168766-129168810	NM_001085491:355	1700125D06Rik	INSIDE	0.345	0.541	2189.77	1184.01	0.187	1498.37	279.57
A_68_P27268621	chr10:76978260-76978304	NM_133998:276	1810008A18Rik	INSIDE	0.450	0.667	4574.43	3053.04	0.301	3239.55	973.60
A_68_P27268626	chr10:76978772-76978818	NM_133998:-236	1810008A18Rik	PROMOTER	0.088	0.724	2614.28	1893.48	0.064	1833.69	117.28
A_68_P27945543	chr11:78640074-78640118	NM_001076681:0	1810012P15Rik	INSIDE	0.424	0.513	1366.42	701.09	0.218	1211.86	263.79
A_68_P27945544	chr11:78640263-78640307	NM_001076681:188	1810012P15Rik	INSIDE	0.270	0.607	862.96	523.41	0.164	763.48	124.95
A_68_P23699553	chr5:53658148-53658198	NM_001145433:-172	1810013D10Rik	PROMOTER	0.219	0.673	1273.30	857.06	0.147	1110.20	163.35
A_68_P30534541	chr16:8831297-8831343	NM_001081400:1128	1810013L24Rik	INSIDE	0.460	0.671	952.04	638.54	0.309	786.53	242.84
A_68_P21957614	chr3:14611075-14611119	NM_001099674:160	1810022K09Rik	INSIDE	0.140	1.474	1437.04	2118.18	0.207	1109.71	229.17
A_68_P21957615	chr3:14611165-14611209	NM_001099674:70	1810022K09Rik	INSIDE	0.169	1.627	10250.23	16677.58	0.275	6020.26	1657.59
A_68_P21957616	chr3:14611261-14611305	NM_001099674:-26	1810022K09Rik	PROMOTER	0.055	1.711	8239.94	14100.84	0.094	4815.05	454.13
A_68_P25933301	chr8:69010356-69010400	NM_025465:28	1810029B16Rik	INSIDE	0.233	0.708	3416.36	2418.37	0.165	2503.84	413.15
A_68_P25933302	chr8:69010455-69010506	NM_025465:-74	1810029B16Rik	PROMOTER	0.242	0.675	1746.74	1178.53	0.164	1330.58	217.58
A_68_P20352243	chr1:75138899-75138957	NM_026977:14	1810031K17Rik	INSIDE	0.264	0.622	1005.25	625.04	0.164	715.96	117.57
A_68_P30394507	chr15:84237076-84237131	NM_001163145:40424	1810041L15Rik	INSIDE	0.649	5.609	2320.61	13015.88	3.642	1346.29	4902.97
A_68_P30394508	chr15:84237151-84237195	NM_001163145:40355	1810041L15Rik	INSIDE	0.495	3.611	5381.34	19431.95	1.786	3483.19	6219.70
A_68_P28182349	chr11:119960272-119960316	NM_001110242:47	1810043H04Rik	INSIDE	0.308	0.394	1425.75	561.74	0.121	1015.23	123.11
A_68_P28182350	chr11:119960353-119960397	NM_001110242:127	1810043H04Rik	INSIDE	0.641	0.643	2765.61	1779.32	0.412	2015.50	830.58
A_68_P28182351	chr11:119960438-119960482	NM_001110242:213	1810043H04Rik	INSIDE	0.420	0.433	1670.26	723.07	0.182	1218.38	221.64
A_68_P28182352	chr11:119960513-119960557	NM_001110242:287	1810043H04Rik	INSIDE	0.424	0.680	1235.58	839.70	0.288	927.01	267.38
A_68_P28056476	chr11:98300031-98300075	NM_025559:250	1810046J19Rik	INSIDE	0.314	0.612	1741.14	1065.91	0.192	1258.27	241.81

ProbeName	Target position of probe on CpG island microarray	TargetID	GeneSymbol	CpG island Description	Ratio of relative methylation (TiO2-NP/Vehicle)	Sham group			TiO_2-H group		
						Relative methylation (Cy-5/Cy-3)	Cy-3 signal (Input)	Cy-5 signal (MeDIP)	Relative methylation (Cy-5/Cy-3)	Cy-3 signal (Input)	Cy-5 signal (MeDIP)
A_68_P28532685	chr12:73637141-73637185	NM_026327:-181	1810048J11Rik	PROMOTER	0.316	0.558	1404.89	784.23	0.176	1118.08	196.99
A_68_P31918868	chr19:3708036-3708080	NM_028077:-274	1810055G02Rik	PROMOTER	0.337	0.436	1462.67	638.10	0.147	1340.36	196.90
A_68_P31918872	chr19:3708434-3708478	NM_028077:124	1810055G02Rik	INSIDE	0.420	0.582	1229.51	715.50	0.245	1080.36	264.31
A_68_P31918873	chr19:3708527-3708572	NM_028077:217	1810055G02Rik	INSIDE	0.216	0.664	9223.46	6124.10	0.144	6444.52	925.99
A_68_P29445005	chr14:20902070-20902114	NM_026209:302	1810063B07Rik	INSIDE	0.254	0.388	1402.98	544.17	0.099	1187.66	117.17
A_68_P31619432	chr18:35813900-35813944	NM_027222:-4901	2010001M09Rik	PROMOTER	0.301	0.538	1419.87	764.60	0.162	1072.14	173.69
A_68_P24127511	chr5:138220691-138220735	NM_027242:567	2010007H12Rik	INSIDE	0.288	0.582	1030.64	599.67	0.168	736.02	123.52
A_68_P21610826	chr2:126758946-126758990	NM_023220:3	2010106G01Rik	INSIDE	0.244	0.617	1130.54	697.54	0.151	890.64	134.16
A_68_P28747155	chr12:113204858-113204902	NM_027360:308	2010107E04Rik	INSIDE	0.432	0.537	1594.75	856.04	0.232	1321.24	306.72
A_68_P28747156	chr12:113205010-113205054	NM_027360:156	2010107E04Rik	INSIDE	0.604	0.507	1478.02	750.02	0.306	1230.96	376.99
A_68_P29509868	chr14:31941877-31941922	NR_024069:217	2010107H07Rik	INSIDE	0.130	0.704	2899.08	2041.86	0.091	1914.49	174.97
A_68_P29509884	chr14:31943948-31943992	NR_024069:-1854	2010107H07Rik	DIVERGENT_PROMOTER	0.241	0.517	1129.05	583.15	0.124	982.77	122.29
A_68_P29509891	chr14:31944757-31944801	NR_024069:-2662	2010107H07Rik	DIVERGENT_PROMOTER	0.601	5.677	1068.55	6065.94	3.414	867.90	2962.98
A_68_P28336337	chr12:33063533-33063581	NM_001162903:-97	2010109K11Rik	PROMOTER	0.334	1.633	1193.02	1947.75	0.545	864.99	471.39
A_68_P23195467	chr4:108000626-108000670	NM_027250:-108	2010305A19Rik	PROMOTER	0.279	0.516	1602.85	827.23	0.144	1309.24	188.61
A_68_P23195471	chr4:108000991-108001035	NM_027250:256	2010305A19Rik	INSIDE	0.457	0.734	5938.42	4356.88	0.336	4483.10	1504.40
A_68_P21071706	chr2:25227652-25227698	NM_001081085:-166	2010317E24Rik	DIVERGENT_PROMOTER	0.263	0.408	1396.16	569.53	0.107	1148.30	123.05
A_68_P26584997	chr9:64808465-64808509	NM_001077631:-152	2010321M09Rik	DIVERGENT_PROMOTER	0.425	0.646	1408.86	910.69	0.275	1177.75	323.77
A_68_P26584998	chr9:64808639-64808683	NM_175153:-83	2010321M09Rik	PROMOTER	0.361	0.740	2850.46	2110.58	0.268	2164.57	579.04
A_68_P26584999	chr9:64808747-64808792	NM_175153:26	2010321M09Rik	INSIDE	0.108	1.509	6771.50	10220.88	0.163	4637.15	755.05
A_68_P21665424	chr2:136717006-136717053	NM_028201:76	2210009G21Rik	INSIDE	0.216	0.475	1486.99	705.78	0.103	1146.17	117.69
A_68_P23189064	chr4:106850847-106850891	NM_025617:103	2210012G02Rik	INSIDE	0.213	0.526	2225.36	1170.43	0.112	1595.14	178.77
A_68_P24172796	chr5:148086210-148086254	NR_038157:-3135	2210019I11Rik	PROMOTER	0.625	0.522	1982.01	1035.05	0.326	1761.65	575.12
A_68_P30403116	chr15:85641498-85641553	NM_001164625:602	2210021J22Rik	INSIDE	0.226	0.701	2134.13	1496.30	0.158	1606.59	254.60
A_68_P30403119	chr15:85641848-85641892	NM_001164625:257	2210021J22Rik	INSIDE	0.538	0.540	1398.88	754.73	0.290	1111.34	322.74
A_68_P30403120	chr15:85641984-85642028	NM_001164625:121	2210021J22Rik	INSIDE	0.157	0.639	2838.89	1814.25	0.101	2116.42	212.73
A_68_P30403121	chr15:85642175-85642219	NM_001164625:-69	2210021J22Rik	PROMOTER	0.312	0.575	3923.86	2255.17	0.179	2919.46	523.95
A_68_P29157707	chr13:77274878-77274925	NM_001081353:105	2210408I21Rik	INSIDE	0.222	0.579	1285.00	743.45	0.129	909.28	116.89
A_68_P29157708	chr13:77274951-77274995	NM_001081353:176	2210408I21Rik	INSIDE	0.329	0.475	1619.18	769.42	0.156	1366.62	213.73
A_68_P21070791	chr2:25096537-25096581	NM_026415:-1141	2310002J15Rik	DIVERGENT_PROMOTER	0.327	0.621	1681.19	1044.39	0.203	1276.40	259.49
A_68_P21682771	chr2:139996347-139996391	NM_027093:-13	2310003L22Rik	DIVERGENT_PROMOTER	0.255	0.594	926.68	550.90	0.152	801.64	121.71
A_68_P27282446	chr10:79783036-79783080	NM_025521:235	2310011J03Rik	INSIDE	0.176	0.489	1952.13	955.33	0.086	1409.78	121.28
A_68_P27282447	chr10:79783116-79783160	NM_025521:155	2310011J03Rik	INSIDE	0.433	0.599	5286.50	3169.06	0.260	3798.53	986.03
A_68_P31161374	chr17:36002854-36002898	NM_001146710:-196	2310014H01Rik	PROMOTER	0.519	0.538	1848.47	994.37	0.279	1230.22	343.55
A_68_P24973681	chr7:13513425-13513470	NM_029809:683	2310014L17Rik	INSIDE	0.446	0.693	1574.98	1091.79	0.309	1150.74	355.56
A_68_P24973684	chr7:13513821-13513865	NM_029809:1078	2310014L17Rik	INSIDE	0.385	1.494	1749.65	2614.53	0.576	1454.10	837.63
A_68_P24973685	chr7:13513884-13513935	NM_029809:1145	2310014L17Rik	INSIDE	0.332	1.825	1243.46	2269.35	0.606	918.27	556.08
A_68_P28573970	chr12:81233412-81233456	NR_033514:397	2310015A10Rik	INSIDE	0.461	0.637	1584.81	1008.89	0.293	1205.71	353.78
A_68_P24325497	chr6:29222598-29222642	NM_023516:-5	2310016C08Rik	PROMOTER	0.118	0.676	2101.49	1420.29	0.080	1546.45	123.23
A_68_P24325499	chr6:29222771-29222816	NM_023516:168	2310016C08Rik	INSIDE	0.325	0.492	1529.09	752.07	0.160	1159.55	185.56
A_68_P24325500	chr6:29222845-29222889	NM_023516:241	2310016C08Rik	INSIDE	0.266	0.519	1763.29	915.53	0.138	1391.52	191.86
A_68_P30481998	chr15:99556153-99556197	NM_183256:126	2310016M24Rik	INSIDE	0.485	0.664	2354.99	1564.25	0.322	1729.37	556.70
A_68_P30481999	chr15:99556228-99556272	NM_183256:202	2310016M24Rik	INSIDE	0.439	0.660	1722.97	1137.77	0.290	1309.47	379.55
A_68_P29448496	chr14:21526704-21526748	NM_027996:-47	2310021P13Rik	PROMOTER	0.432	0.493	1547.75	762.95	0.213	1138.01	242.17

ProbeName	Target position of probe on CpG island microarray	TargetID	GeneSymbol	CpG island Description	Ratio of relative methylation (TiO2-NP/Vehicle)	Sham group			TiO_2-H group		
						Relative methylation (Cy-5/Cy-3)	Cy-3 signal (Input)	Cy-5 signal (MeDIP)	Relative methylation (Cy-5/Cy-3)	Cy-3 signal (Input)	Cy-5 signal (MeDIP)
A_68_P26246901	chr8:127187263-127187307	NM_175149:-15	2310022B05Rik	PROMOTER	0.372	0.509	2820.86	1436.60	0.190	2109.31	399.83
A_68_P23349878	chr4:138686042-138686091	NR_033745:886	2310028O11Rik	INSIDE	0.207	0.647	2238.73	1447.58	0.134	1701.77	227.33
A_68_P23349880	chr4:138686224-138686268	NR_033745:706	2310028O11Rik	INSIDE	0.300	0.640	828.17	529.84	0.192	621.75	119.45
A_68_P23349886	chr4:138686821-138686865	NR_033745:110	2310028O11Rik	INSIDE	0.280	0.548	2023.91	1109.13	0.153	1605.35	246.22
A_68_P22727922	chr4:11003062-11003106	NM_001085493:267	2310030N02Rik	INSIDE	0.458	0.608	1158.70	704.54	0.279	1073.45	299.16
A_68_P22727924	chr4:11003258-11003303	NM_001085493:71	2310030N02Rik	INSIDE	0.639	0.508	1185.00	602.42	0.325	1010.87	328.61
A_68_P22727925	chr4:11003436-11003481	NM_001085493:-107	2310030N02Rik	PROMOTER	0.124	0.484	3046.12	1474.87	0.060	2014.35	120.50
A_68_P27835480	chr11:59021879-59021923	NM_024210:38	2310033P09Rik	INSIDE	0.396	0.576	2695.23	1552.55	0.228	1958.86	446.66
A_68_P20504209	chr1:107560642-107560686	NM_029349:227	2310035C23Rik	INSIDE	0.470	0.554	1750.35	969.90	0.260	1233.58	320.97
A_68_P20504214	chr1:107561226-107561270	NM_029349:811	2310035C23Rik	INSIDE	0.140	0.684	2519.03	1723.27	0.096	1977.98	189.90
A_68_P28613111	chr12:88288337-88288381	NM_173735:66	2310044G17Rik	INSIDE	0.331	0.515	2181.83	1123.70	0.170	1614.00	274.96
A_68_P28613115	chr12:88288746-88288790	NM_173735:476	2310044G17Rik	INSIDE	0.225	1.409	2822.27	3976.08	0.317	1969.41	624.83
A_68_P26349728	chr9:21760084-21760135	NM_025870:-87	2310047B19Rik	PROMOTER	0.383	0.671	975.05	654.41	0.257	709.65	182.60
A_68_P27892798	chr11:68873155-68873199	NM_028005:-100	2310047M10Rik	PROMOTER	0.253	0.442	1362.85	602.21	0.112	1039.25	116.22
A_68_P27892804	chr11:68873951-68873999	NM_028005:698	2310047M10Rik	INSIDE	0.224	0.393	1791.71	703.54	0.088	1346.38	118.62
A_68_P27288030	chr10:80657437-80657487	NR_015477:27	2310050B05Rik	INSIDE	0.258	0.577	951.66	548.79	0.149	792.99	117.91
A_68_P25527991	chr7:138506051-138506095	NM_026655:140	2310057M21Rik	INSIDE	0.451	0.559	1087.60	607.53	0.252	826.45	208.07
A_68_P26199699	chr8:119445245-119445289	NM_026844:70	2310061C15Rik	INSIDE	0.593	0.631	1993.24	1257.71	0.374	1502.66	561.86
A_68_P28155137	chr11:115626646-115626697	NM_028014:-75	2310067B10Rik	PROMOTER	0.212	0.619	3676.80	2274.87	0.131	2447.94	321.31
A_68_P28155138	chr11:115626737-115626781	NM_028014:12	2310067B10Rik	INSIDE	0.222	0.657	1110.69	729.25	0.146	842.23	122.66
A_68_P30483171	chr15:99804219-99804265	NR_028124:-524	2310068J16Rik	PROMOTER	0.353	0.557	1147.31	638.54	0.196	923.45	181.41
A_68_P23601494	chr5:35917751-35917795	NM_030208:-53	2310079F23Rik	DIVERGENT_PROMOTER	0.613	0.579	1261.35	730.68	0.355	978.75	347.82
A_68_P23601495	chr5:35917857-35917901	NM_030208:-159	2310079F23Rik	DIVERGENT_PROMOTER	0.264	0.467	1151.30	537.11	0.123	955.93	117.62
A_68_P25376019	chr7:109054202-109054254	NM_025605:-122	2400001E08Rik	PROMOTER	0.110	0.718	2195.53	1577.26	0.079	1625.39	128.69
A_68_P25376020	chr7:109054295-109054339	NM_025605:-34	2400001E08Rik	PROMOTER	0.373	0.598	1246.58	744.90	0.223	882.36	196.69
A_68_P25376021	chr7:109054423-109054467	NM_025605:94	2400001E08Rik	INSIDE	0.448	0.552	1499.79	827.77	0.247	1242.59	307.04
A_68_P26158468	chr8:112217560-112217604	NM_028018:-388	2400003C14Rik	PROMOTER	0.452	0.537	1559.31	838.02	0.243	1307.15	317.74
A_68_P21864322	chr2:172265854-172265898	NM_025542:-201	2410001C21Rik	PROMOTER	0.376	0.593	1408.80	834.79	0.223	1038.56	231.34
A_68_P21864323	chr2:172265966-172266010	NM_025542:-89	2410001C21Rik	PROMOTER	0.281	0.572	1502.08	859.66	0.161	1181.28	190.27
A_68_P24430831	chr6:49024182-49024226	NM_029353:411	2410003K15Rik	INSIDE	0.337	0.620	1526.36	946.80	0.209	1324.44	277.11
A_68_P24430832	chr6:49024262-49024309	NM_029353:492	2410003K15Rik	INSIDE	0.409	0.678	1040.49	705.25	0.277	822.87	228.27
A_68_P31609276	chr18:33954463-33954507	NR_038151:-61	2410004N09Rik	PROMOTER	0.560	0.719	2316.79	1666.26	0.403	1838.05	739.95
A_68_P31609279	chr18:33954809-33954858	NR_038151:288	2410004N09Rik	INSIDE	0.288	0.536	1097.38	588.01	0.154	778.08	120.08
A_68_P31257262	chr17:56749026-56749070	NM_153152:146	2410015M20Rik	INSIDE	0.353	0.592	4505.74	2666.45	0.209	3180.53	664.58
A_68_P31257263	chr17:56749175-56749221	NM_153152:-4	2410015M20Rik	DIVERGENT_PROMOTER	0.311	0.650	880.87	572.13	0.202	647.21	130.60
A_68_P28595538	chr12:85292535-85292579	NM_023633:999	2410016O06Rik	INSIDE	0.235	0.678	1223.41	829.37	0.159	965.04	153.78
A_68_P31162735	chr17:36282108-36282152	NR_033517:168	2410017I17Rik	INSIDE	0.210	0.339	2071.48	702.04	0.071	1698.86	121.17
A_68_P24091117	chr5:130371504-130371553	NM_183088:-64	2410018M08Rik	DIVERGENT_PROMOTER	0.277	0.508	1104.46	561.61	0.141	859.59	121.15
A_68_P24464804	chr6:54631784-54631828	NM_026629:41	2410066E13Rik	INSIDE	0.542	0.375	1375.53	515.97	0.203	1127.07	229.13
A_68_P24464806	chr6:54631922-54631966	NM_026629:179	2410066E13Rik	INSIDE	0.435	0.525	1947.35	1021.62	0.228	1774.77	404.87
A_68_P24464807	chr6:54632035-54632079	NM_026629:291	2410066E13Rik	INSIDE	0.632	0.590	1040.73	613.55	0.373	893.50	333.04
A_68_P29998440	chr15:8118886-8118930	NM_001162906:-197	2410089E03Rik	PROMOTER	0.351	0.576	1234.81	711.34	0.202	1166.72	235.67
A_68_P31992375	chr19:18745959-18746006	NM_026120:713	2410127L17Rik	INSIDE	0.163	0.490	1897.14	929.53	0.080	1429.31	113.89
A_68_P23297491	chr4:129278370-129278414	NM_029748:442	2510006D16Rik	INSIDE	0.564	0.554	1089.87	604.22	0.313	906.89	283.60

ProbeName	Target position of probe on CpG island microarray	TargetID	GeneSymbol	CpG island Description	Ratio of relative methylation (TiO2-NP/Vehicle)	Sham group			TiO_2-H group		
						Relative methylation (Cy-5/Cy-3)	Cy-3 signal (Input)	Cy-5 signal (MeDIP)	Relative methylation (Cy-5/Cy-3)	Cy-3 signal (Input)	Cy-5 signal (MeDIP)
A_68_P27288850	chr10:80783292-80783342	NM_027381:-531	2510012J08Rik	PROMOTER	0.122	1.612	9780.00	15761.68	0.196	5849.40	1146.91
A_68_P23391468	chr4:147315734-147315778	NM_029841:753	2510039O18Rik	INSIDE	0.607	0.686	1454.28	997.18	0.416	1186.43	493.83
A_68_P26822981	chr9:110207579-110207626	NM_001081381:-93	2610002I17Rik	PROMOTER	0.267	0.433	1295.83	560.67	0.116	1015.06	117.37
A_68_P26822982	chr9:110207662-110207706	NM_001081381:-11	2610002I17Rik	PROMOTER	0.519	0.491	1155.40	567.39	0.255	918.98	234.03
A_68_P26822985	chr9:110207937-110207981	NM_001081381:263	2610002I17Rik	INSIDE	0.295	0.546	2450.33	1336.68	0.161	1753.18	282.25
A_68_P23437421	chr4:154624224-154624268	NM_001190445:172	2610002J02Rik	INSIDE	0.374	0.492	3337.24	1641.54	0.184	2477.98	456.36
A_68_P32589660	chrX:105011451-105011503	NM_025921:197	2610002M06Rik	INSIDE	0.070	2.499	2043.26	5105.76	0.175	685.55	119.65
A_68_P20184658	chr1:42752112-42752171	NR_027826:-471	2610017I09Rik	DIVERGENT_PROMOTER	0.097	2.128	723.64	1539.94	0.207	577.58	119.60
A_68_P25671550	chr8:13975017-13975061	NM_173744:-261	2610019F03Rik	PROMOTER	0.443	0.550	1658.24	911.69	0.244	1268.81	309.06
A_68_P28140558	chr11:113062660-113062712	NR_015556:466	2610035D17Rik	INSIDE	0.223	0.458	1392.18	637.90	0.102	1134.67	115.94
A_68_P28140562	chr11:113063090-113063135	NR_015556:40	2610035D17Rik	INSIDE	0.299	1.773	1746.44	3096.57	0.529	1170.83	619.76
A_68_P30958535	chr16:90727405-90727449	NM_025642:190	2610039C10Rik	INSIDE	0.237	0.541	1226.40	663.48	0.128	955.47	122.43
A_68_P30958537	chr16:90727677-90727721	NM_025642:-82	2610039C10Rik	PROMOTER	0.443	0.419	2239.70	938.37	0.186	1728.55	320.74
A_68_P21967930	chr3:17686893-17686937	NR_033490:-3006	2610100L16Rik	PROMOTER	0.603	0.621	1124.67	697.86	0.374	890.64	332.99
A_68_P29699589	chr14:70552945-70552991	NM_146055:630	2610301G19Rik	INSIDE	0.423	5.739	4124.45	23669.03	2.427	2441.32	5924.78
A_68_P29699593	chr14:70553524-70553568	NM_146055:52	2610301G19Rik	INSIDE	0.226	0.660	2993.10	1974.52	0.149	2377.12	354.63
A_68_P29699594	chr14:70553626-70553670	NM_146055:-50	2610301G19Rik	PROMOTER	0.391	0.531	1427.31	757.25	0.207	1115.67	231.50
A_68_P27942254	chr11:78075324-78075368	NM_001002004:91	2610507B11Rik	INSIDE	0.517	0.572	1212.73	693.61	0.296	959.54	283.96
A_68_P27942255	chr11:78075391-78075435	NM_001002004:157	2610507B11Rik	INSIDE	0.440	0.526	2629.67	1382.05	0.231	1924.66	445.41
A_68_P27942257	chr11:78075584-78075628	NM_001002004:351	2610507B11Rik	INSIDE	0.369	0.544	2133.03	1160.71	0.201	1619.30	325.27
A_68_P27835439	chr11:59015720-59015764	NR_037964:191	2610507I01Rik	INSIDE	0.137	0.588	2042.86	1201.16	0.080	1539.89	123.82
A_68_P27835440	chr11:59015809-59015853	NR_037964:103	2610507I01Rik	INSIDE	0.256	0.488	1229.36	599.64	0.125	976.08	121.97
A_68_P30787361	chr16:57606603-57606647	NM_025599:356	2610528E23Rik	INSIDE	0.149	0.558	1787.63	997.60	0.083	1398.14	116.59
A_68_P21503641	chr2:106814392-106814436	NM_173750:-3866	2700007P21Rik	PROMOTER	0.341	0.712	3658.98	2605.11	0.243	2707.00	657.80
A_68_P32070030	chr19:32464044-32464088	NR_033198:361	2700046G09Rik	INSIDE	0.496	0.679	1243.17	844.35	0.337	1071.11	361.03
A_68_P25541690	chr7:140829066-140829110	NM_178115:-269	2700050L05Rik	PROMOTER	0.368	0.463	1801.90	834.44	0.170	1636.13	278.48
A_68_P29443675	chr14:20643061-20643105	NM_026528:-37	2700060E02Rik	PROMOTER	0.538	0.615	1171.55	720.77	0.331	1111.87	368.15
A_68_P32226224	chr19:60656314-60656358	NM_001172096:590	2700078E11Rik	INSIDE	0.263	0.528	2263.29	1194.88	0.139	1593.13	220.91
A_68_P21393923	chr2:84510529-84510573	NM_001033166:315	2700094K13Rik	INSIDE	0.343	0.538	1529.39	822.20	0.184	1088.64	200.69
A_68_P31456431	chr17:95149568-95149616	NR_024720:153	2700099C18Rik	INSIDE	0.260	0.642	1050.61	674.32	0.167	722.30	120.75
A_68_P21441000	chr2:94246926-94246970	NR_027831:85	2810002D19Rik	INSIDE	0.300	0.402	1299.08	521.91	0.121	963.26	116.22
A_68_P28853132	chr13:17786964-17787008	NM_025479:-260	2810021B07Rik	DIVERGENT_PROMOTER	0.156	0.737	2772.38	2041.88	0.115	1955.65	224.62
A_68_P27833210	chr11:58680690-58680734	NM_172403:-29	2810021J22Rik	PROMOTER	0.203	0.471	1495.18	704.17	0.095	1292.19	123.35
A_68_P22141947	chr3:53267624-53267668	NM_173382:-92	2810046L04Rik	DIVERGENT_PROMOTER	0.276	0.374	1367.28	511.93	0.103	1194.18	123.56
A_68_P27897804	chr11:69714837-69714881	NM_027419:-370	2810408A11Rik	DIVERGENT_PROMOTER	0.203	0.623	2830.13	1763.22	0.127	2153.95	272.76
A_68_P28182937	chr11:120046685-120046736	NR_030682:4460	2810410L24Rik	DOWNSTREAM	0.204	0.708	1312.95	929.89	0.144	990.91	143.01
A_68_P28182972	chr11:120050647-120050697	NR_030682:498	2810410L24Rik	INSIDE	0.255	0.531	1271.45	674.60	0.135	863.83	116.90
A_68_P28182973	chr11:120050796-120050840	NR_030682:352	2810410L24Rik	INSIDE	0.234	0.512	1211.69	620.02	0.120	1017.97	121.81
A_68_P25952415	chr8:73046804-73046848	NM_029366:253	2810422J05Rik	INSIDE	0.239	0.593	3218.37	1908.33	0.142	2295.64	325.10
A_68_P25952418	chr8:73047097-73047144	NM_029366:-41	2810422J05Rik	DIVERGENT_PROMOTER	0.220	0.459	1642.77	754.01	0.101	1173.97	118.38
A_68_P28788254	chr13:3477478-3477522	NR_015522:-48	2810429I04Rik	PROMOTER	0.275	0.649	3641.31	2362.40	0.178	2668.69	475.74
A_68_P28788255	chr13:3477597-3477641	NR_015522:70	2810429I04Rik	INSIDE	0.303	0.585	2232.57	1306.06	0.177	1586.41	281.05
A_68_P22863908	chr4:41660593-41660637	NM_027278:-65	2810432D09Rik	PROMOTER	0.650	0.535	1473.68	788.48	0.348	1281.32	445.32
A_68_P23545745	chr5:24606880-24606928	NR_027851:112	2900005J15Rik	INSIDE	0.260	0.644	954.35	614.72	0.167	713.29	119.41

ProbeName	Target position of probe on CpG island microarray	TargetID	GeneSymbol	CpG island Description	Ratio of relative methylation (TiO2-NP/Vehicle)	Sham group			TiO_2-H group		
						Relative methylation (Cy-5/Cy-3)	Cy-3 signal (Input)	Cy-5 signal (MeDIP)	Relative methylation (Cy-5/Cy-3)	Cy-3 signal (Input)	Cy-5 signal (MeDIP)
A_68_P23545746	chr5:24606967-24607011	NR_027851:196	2900005J15Rik	INSIDE	0.299	0.607	2727.40	1655.22	0.181	2023.02	366.75
A_68_P31114504	chr17:27270575-27270620	NM_026063:239	2900010M23Rik	INSIDE	0.625	0.666	2784.56	1854.97	0.416	2154.48	896.31
A_68_P23996379	chr5:113567220-113567264	NM_172884:25091	2900026A02Rik	INSIDE	0.229	0.720	1688.07	1214.90	0.165	1308.43	215.28
A_68_P23996380	chr5:113567336-113567380	NM_172884:24975	2900026A02Rik	INSIDE	0.597	0.491	1434.83	705.15	0.293	1109.33	325.36
A_68_P23996382	chr5:113567569-113567615	NM_172884:24741	2900026A02Rik	INSIDE	0.586	0.508	1928.01	979.69	0.298	1581.06	470.66
A_68_P20551799	chr1:120356444-120356488	NR_027901:-624	2900060B14Rik	PROMOTER	0.182	0.680	2315.07	1575.21	0.124	1653.86	204.26
A_68_P21528941	chr2:112295418-112295462	NM_133749:259	2900064A13Rik	INSIDE	0.208	0.657	1184.08	778.36	0.137	868.83	119.05
A_68_P27787096	chr11:49988189-49988233	NM_026543:-142	3010026O09Rik	PROMOTER	0.275	0.462	1569.17	725.65	0.127	1145.00	145.41
A_68_P27787098	chr11:49988398-49988442	NM_026543:68	3010026O09Rik	INSIDE	0.252	0.670	3617.47	2424.19	0.169	2600.39	438.38
A_68_P27787100	chr11:49988573-49988624	NM_026543:246	3010026O09Rik	INSIDE	0.484	0.595	1168.90	695.64	0.288	776.36	223.67
A_68_P23023123	chr4:74923898-74923942	NM_025849:270	3110001D03Rik	INSIDE	0.548	0.648	1123.40	728.35	0.355	848.05	301.35
A_68_P23023125	chr4:74924188-74924236	NM_025849:-22	3110001D03Rik	PROMOTER	0.191	0.638	1264.85	806.60	0.122	1023.28	124.89
A_68_P26973142	chr10:17742734-17742778	NM_028440:302	3110003A17Rik	INSIDE	0.289	0.605	3596.24	2174.83	0.175	2581.80	450.71
A_68_P20560376	chr1:122017920-122017975	NM_001172074:184	3110009E18Rik	INSIDE	0.072	1.646	1745.17	2873.17	0.118	1163.32	137.64
A_68_P22834880	chr4:35172594-35172638	NM_001081343:513	3110043O21Rik	INSIDE	0.283	0.592	889.38	526.11	0.167	743.22	124.48
A_68_P22834883	chr4:35172926-35172970	NM_001081343:181	3110043O21Rik	INSIDE	0.409	0.669	955.75	639.50	0.274	791.03	216.49
A_68_P23746644	chr5:64040795-64040839	NM_177006:475	3110047P20Rik	INSIDE	0.153	0.509	1926.17	981.37	0.078	1544.37	120.72
A_68_P27285911	chr10:80318512-80318556	NM_175195:515	3110056O03Rik	INSIDE	0.337	0.551	1278.89	704.99	0.186	997.38	185.43
A_68_P24358386	chr6:34827862-34827906	NM_001135611:181	3110062M04Rik	INSIDE	0.158	0.465	2119.36	984.89	0.073	1593.11	116.88
A_68_P24358388	chr6:34828039-34828083	NM_001135611:5	3110062M04Rik	INSIDE	0.354	0.388	2876.05	1117.23	0.138	2102.69	289.36
A_68_P31183534	chr17:42453113-42453157	NM_028474:239	3110082D06Rik	INSIDE	0.188	1.500	2124.97	3186.90	0.282	1389.09	391.23
A_68_P24135765	chr5:139936243-139936287	NM_028469:224	3110082I17Rik	INSIDE	0.157	1.365	4117.54	5621.09	0.214	2785.72	596.43
A_68_P25375960	chr7:109044724-109044768	NM_027532:-182	3200002M19Rik	PROMOTER	0.661	0.649	1096.91	712.10	0.429	1002.42	430.31
A_68_P21812326	chr2:163244652-163244697	NM_025699:532	3230401D17Rik	INSIDE	0.347	0.550	1403.57	771.87	0.191	1069.58	204.11
A_68_P28577068	chr12:81793936-81793980	NM_001177503:371	3830431G21Rik	INSIDE	0.390	0.530	1326.37	703.50	0.207	1003.11	207.54
A_68_P32050338	chr19:29038878-29038922	NM_198651:-256	4430402I18Rik	PROMOTER	0.309	0.540	1263.74	682.01	0.167	1227.41	204.57
A_68_P21977361	chr3:19795434-19795478	NR_027985:586	4632415L05Rik	INSIDE	0.068	0.670	3689.45	2470.52	0.046	2570.35	117.06
A_68_P25326410	chr7:99889553-99889597	NR_015510:395	4632427E13Rik	INSIDE	0.418	0.613	1591.85	975.17	0.256	1213.29	310.51
A_68_P25327110	chr7:100022539-100022587	NM_001080995:180	4632434I11Rik	INSIDE	0.262	0.488	1052.76	513.29	0.128	925.58	118.30
A_68_P25327115	chr7:100023177-100023221	NM_001080995:-456	4632434I11Rik	DIVERGENT_PROMOTER	0.153	1.432	4311.10	6174.69	0.219	2980.57	653.60
A_68_P24938724	chr6:148160617-148160668	NR_015615:-167	4732416N19Rik	PROMOTER	0.246	0.544	1132.18	616.32	0.134	902.71	120.87
A_68_P24938726	chr6:148160773-148160817	NR_015615:-15	4732416N19Rik	PROMOTER	0.322	0.722	4696.46	3388.84	0.233	3349.09	778.85
A_68_P25010196	chr7:26180415-26180459	NR_015569:4	4732471J01Rik	INSIDE	0.241	0.567	1132.31	641.65	0.136	846.53	115.51
A_68_P25010197	chr7:26180486-26180536	NR_015569:78	4732471J01Rik	INSIDE	0.297	0.409	1377.94	563.29	0.121	1038.30	126.07
A_68_P25010199	chr7:26180658-26180710	NR_015569:252	4732471J01Rik	INSIDE	0.276	0.586	917.67	537.74	0.162	731.17	118.37
A_68_P29050102	chr13:54666387-54666431	NM_133797:335	4833439L19Rik	INSIDE	0.215	0.664	3256.05	2160.82	0.143	2139.99	305.26
A_68_P29050105	chr13:54666706-54666750	NM_133797:15	4833439L19Rik	INSIDE	0.194	0.550	1363.30	749.81	0.107	1169.34	124.90
A_68_P28463393	chr12:58677002-58677046	NM_001037743:11923	4921506M07Rik	INSIDE	0.413	0.680	1762.12	1197.90	0.281	1675.53	470.49
A_68_P28463396	chr12:58677361-58677405	NM_001037743:12281	4921506M07Rik	INSIDE	0.337	0.690	2575.94	1778.36	0.233	1996.79	464.33
A_68_P28463397	chr12:58677434-58677478	NM_001037743:12355	4921506M07Rik	INSIDE	0.339	0.725	1649.62	1195.56	0.246	1211.69	298.10
A_68_P30560720	chr16:14159038-14159083	NM_001081154:307	4921513D23Rik	INSIDE	0.408	0.497	2073.99	1031.49	0.203	1636.44	332.16
A_68_P21770587	chr2:156137562-156137616	NM_027585:-620	4921517L17Rik	PROMOTER	0.189	0.483	1762.59	851.11	0.091	1271.12	116.03
A_68_P21900712	chr2:179711703-179711747	NR_033782:167	4921531C22Rik	INSIDE	0.525	0.563	1067.86	601.46	0.296	855.19	253.06
A_68_P27554557	chr11:3794614-3794660	NM_026150:493	4921536K21Rik	INSIDE	0.210	0.685	1094.46	750.24	0.144	853.87	122.70

ProbeName	Target position of probe on CpG island microarray	TargetID	GeneSymbol	CpG island Description	Ratio of relative methylation (TiO2-NP/Vehicle)	Sham group			TiO_2-H group		
						Relative methylation (Cy-5/Cy-3)	Cy-3 signal (Input)	Cy-5 signal (MeDIP)	Relative methylation (Cy-5/Cy-3)	Cy-3 signal (Input)	Cy-5 signal (MeDIP)
A_68_P27554558	chr11:3794697-3794745	NM_026150:409	4921536K21Rik	INSIDE	0.203	0.615	1690.84	1039.26	0.125	1225.11	153.16
A_68_P21631946	chr2:130665493-130665537	NM_029432:332	4930402H24Rik	INSIDE	0.097	0.579	2842.83	1647.26	0.056	2115.54	118.97
A_68_P20997485	chr2:9803030-9803074	NR_024257:180	4930412O13Rik	INSIDE	0.316	0.640	2306.32	1475.50	0.202	1708.55	345.24
A_68_P20997496	chr2:9804242-9804286	NR_024257:1392	4930412O13Rik	INSIDE	0.578	0.432	1238.32	535.15	0.250	906.54	226.47
A_68_P20997499	chr2:9804639-9804695	NR_024257:1794	4930412O13Rik	INSIDE	0.148	0.705	1524.16	1075.11	0.104	1142.43	119.23
A_68_P28755046	chr12:114394157-114394201	NM_134041:-453	4930427A07Rik	PROMOTER	0.271	0.339	2445.69	827.90	0.092	1889.02	173.14
A_68_P28755050	chr12:114394591-114394636	NM_134041:-18	4930427A07Rik	PROMOTER	0.308	0.424	2383.78	1010.51	0.131	1862.38	243.31
A_68_P28755052	chr12:114394756-114394800	NM_134041:147	4930427A07Rik	INSIDE	0.463	0.324	1702.21	551.06	0.150	1313.65	196.77
A_68_P22037673	chr3:32264387-32264431	NR_027966:-2	4930429B21Rik	PROMOTER	0.655	0.458	4313.59	1976.21	0.300	3350.07	1005.86
A_68_P30686473	chr16:38522456-38522500	NM_024273:269	4930455C21Rik	INSIDE	0.439	0.485	1655.14	803.08	0.213	1287.88	274.02
A_68_P30686477	chr16:38522888-38522932	NM_024273:-163	4930455C21Rik	PROMOTER	0.492	0.730	1769.00	1292.00	0.359	1288.50	462.83
A_68_P32388635	chrX:51019534-51019578	NM_029142:-8451	4930502E18Rik	PROMOTER	0.125	1.397	1691.96	2363.75	0.174	691.77	120.70
A_68_P31809744	chr18:70631379-70631435	NM_172967:728	4930503L19Rik	INSIDE	0.222	0.629	1113.79	700.24	0.139	864.49	120.41
A_68_P31809747	chr18:70631630-70631674	NM_172967:482	4930503L19Rik	INSIDE	0.350	0.589	1543.07	908.65	0.206	1154.66	237.68
A_68_P26085411	chr8:98330678-98330722	NR_015574:-29	4930513N10Rik	PROMOTER	0.103	0.695	7015.31	4878.62	0.071	4904.43	350.38
A_68_P26085412	chr8:98330809-98330853	NR_015574:101	4930513N10Rik	INSIDE	0.239	0.528	1077.22	569.02	0.126	955.36	120.83
A_68_P24006041	chr5:115224674-115224718	NR_027872:296	4930515G01Rik	INSIDE	0.483	0.522	1829.39	954.87	0.252	1493.79	376.31
A_68_P33012177	chr9_random:57060-57104	NR_027950:2390	4930526I15Rik	DOWNSTREAM	0.207	0.426	2559.74	1089.63	0.088	1905.00	167.60
A_68_P33012178	chr9_random:57139-57183	NR_027950:2468	4930526I15Rik	DOWNSTREAM	0.503	0.409	1912.33	781.62	0.206	1501.20	308.77
A_68_P33012180	chr9_random:57290-57334	NR_027950:2620	4930526I15Rik	DOWNSTREAM	0.295	0.671	1868.30	1253.84	0.198	1395.16	276.24
A_68_P33012181	chr9_random:57403-57447	NR_027950:2732	4930526I15Rik	DOWNSTREAM	0.414	0.557	1497.55	834.42	0.231	1276.19	294.67
A_68_P31099615	chr17:24941244-24941288	NM_029197:-60	4930528F23Rik	PROMOTER	0.578	0.577	956.95	552.02	0.333	928.45	309.45
A_68_P22552058	chr3:135101385-135101433	NR_030689:221	4930539J05Rik	INSIDE	0.231	0.462	1382.80	638.29	0.107	1089.09	116.36
A_68_P22552059	chr3:135101503-135101547	NR_030689:105	4930539J05Rik	INSIDE	0.196	0.472	1831.15	863.82	0.092	1272.31	117.43
A_68_P27910205	chr11:72028532-72028576	NM_001163728:-85	4930563E22Rik	PROMOTER	0.550	0.471	1501.69	707.05	0.259	1237.94	320.34
A_68_P27910207	chr11:72028699-72028750	NM_001163728:85	4930563E22Rik	INSIDE	0.597	0.520	1367.10	711.38	0.310	1077.14	334.38
A_68_P24091170	chr5:130384460-130384504	NM_026916:73	4930579G22Rik	INSIDE	0.530	0.567	2042.86	1157.47	0.300	1718.06	515.90
A_68_P25457394	chr7:125387430-125387474	NR_027879:32	4930583K01Rik	INSIDE	0.346	0.664	1675.75	1113.01	0.230	1298.59	298.55
A_68_P25457396	chr7:125387673-125387717	NR_027879:274	4930583K01Rik	INSIDE	0.541	0.431	2179.68	939.57	0.233	1564.34	364.96
A_68_P29613142	chr14:55203593-55203649	NM_028890:21125	4931414P19Rik	INSIDE	0.528	3.177	531.23	1687.54	1.678	473.37	794.39
A_68_P26132762	chr8:107813304-107813348	NM_001166394:102	4931428F04Rik	INSIDE	0.337	0.654	2913.44	1904.57	0.220	2154.06	474.43
A_68_P26132765	chr8:107813703-107813757	NM_001166394:-302	4931428F04Rik	PROMOTER	0.227	0.493	1401.74	691.31	0.112	1099.34	123.31
A_68_P21626541	chr2:129626864-129626913	NR_029452:307	4932416H05Rik	INSIDE	0.274	0.558	1137.42	634.55	0.153	793.43	121.37
A_68_P21626548	chr2:129627604-129627648	NR_029452:-431	4932416H05Rik	PROMOTER	0.609	0.560	2485.64	1392.84	0.342	1718.23	586.82
A_68_P21626549	chr2:129627707-129627751	NR_029452:-533	4932416H05Rik	PROMOTER	0.185	0.590	3038.78	1794.00	0.109	2191.57	239.36
A_68_P23720349	chr5:58108780-58108824	NR_015588:356	4932441J04Rik	INSIDE	0.208	0.355	2032.39	722.34	0.074	1583.11	116.77
A_68_P23720350	chr5:58108862-58108920	NR_015588:268	4932441J04Rik	INSIDE	0.310	0.437	1280.56	560.18	0.136	858.56	116.48
A_68_P23588492	chr5:33721019-33721068	NM_001081101:-301	4933407H18Rik	PROMOTER	0.182	0.576	1422.67	818.82	0.105	1132.97	118.89
A_68_P25834611	chr8:47248829-47248873	NM_025747:-6059	4933411K20Rik	PROMOTER	0.393	0.377	1891.58	713.22	0.148	1452.97	215.28
A_68_P22402613	chr3:106525217-106525262	NM_001039488:640	4933421E11Rik	INSIDE	0.465	0.674	3373.51	2272.66	0.313	2582.86	808.30
A_68_P30339073	chr15:74539555-74539599	NR_033197:-14	4933427E11Rik	PROMOTER	0.594	0.475	1781.36	846.98	0.282	1168.47	329.98
A_68_P25471459	chr7:128126104-128126148	NM_028955:104	4933427G17Rik	INSIDE	0.573	0.600	1261.10	756.18	0.344	1044.62	359.02
A_68_P25471460	chr7:128126217-128126263	NM_028955:218	4933427G17Rik	INSIDE	0.322	0.594	1391.28	826.12	0.191	1011.77	193.60
A_68_P22964960	chr4:62186324-62186371	NM_177607:-55	4933430I17Rik	DIVERGENT_PROMOTER	0.475	0.529	1219.13	644.59	0.251	848.41	212.90

ProbeName	Target position of probe on CpG island microarray	TargetID	GeneSymbol	CpG island Description	Ratio of relative methylation (TiO2-NP/Vehicle)	Sham group			TiO_2-H group		
						Relative methylation (Cy-5/Cy-3)	Cy-3 signal (Input)	Cy-5 signal (MeDIP)	Relative methylation (Cy-5/Cy-3)	Cy-3 signal (Input)	Cy-5 signal (MeDIP)
A_68_P22964961	chr4:62186403-62186447	NM_177607:22	4933430I17Rik	INSIDE	0.287	0.665	1107.30	735.94	0.191	996.80	190.04
A_68_P30367722	chr15:79489390-79489434	NM_029407:-26	4933432B09Rik	DIVERGENT_PROMOTER	0.426	0.502	1548.19	776.84	0.214	1181.94	252.65
A_68_P28710689	chr12:106923839-106923883	NM_178613:299	4933433P14Rik	INSIDE	0.196	0.704	1230.52	866.51	0.138	979.94	135.01
A_68_P26065123	chr8:94880083-94880127	NR_033641:-85	4933436C20Rik	DIVERGENT_PROMOTER	0.389	1.413	1248.29	1763.93	0.550	857.60	471.77
A_68_P26065129	chr8:94880803-94880847	NR_033641:-805	4933436C20Rik	DIVERGENT_PROMOTER	0.103	0.628	2822.65	1772.14	0.065	1886.33	122.16
A_68_P27842897	chr11:60231302-60231346	NM_025757:678	4933439F18Rik	INSIDE	0.654	0.655	1006.25	659.05	0.429	775.80	332.46
A_68_P22130347	chr3:51364112-51364156	NR_033624:456	5031434O11Rik	INSIDE	0.179	0.478	1863.67	890.38	0.085	1364.20	116.63
A_68_P21773772	chr2:156688598-156688643	NR_015591:61	5430405H02Rik	INSIDE	0.463	0.535	1153.63	617.68	0.248	995.21	246.50
A_68_P23929548	chr5:100858327-100858371	NR_034038:206	5430416N02Rik	INSIDE	0.250	0.310	2096.69	649.23	0.077	1558.90	120.54
A_68_P24908452	chr6:143048181-143048225	NM_001109688:425	5730419I09Rik	INSIDE	0.664	0.479	1960.53	938.78	0.318	1721.28	547.51
A_68_P24908455	chr6:143048545-143048589	NM_001109688:61	5730419I09Rik	INSIDE	0.261	1.471	3034.78	4463.86	0.384	2078.50	798.52
A_68_P24183686	chr5:149995738-149995782	NR_015478:-121	5730422E09Rik	DIVERGENT_PROMOTER	0.286	0.546	995.30	543.23	0.156	763.11	119.15
A_68_P32100295	chr19:38298465-38298509	NM_001081075:136	5730455O13Rik	INSIDE	0.361	0.520	1351.82	702.50	0.187	1104.35	206.90
A_68_P24449864	chr6:52264164-52264215	NR_038163:637	5730457N03Rik	INSIDE	0.327	0.527	1015.98	535.84	0.173	706.77	122.05
A_68_P24449865	chr6:52264311-52264355	NR_038163:494	5730457N03Rik	INSIDE	0.468	0.442	1882.45	831.31	0.207	1404.11	290.39
A_68_P33008134	chr12:18521060-18521104	NM_001033157:-233	5730507C01Rik	PROMOTER	0.422	0.675	2966.19	2001.15	0.285	2172.47	618.07
A_68_P27661783	chr11:26110740-26110784	NR_027973:-186	5730522E02Rik	PROMOTER	0.255	0.552	2000.73	1104.89	0.141	1605.63	226.18
A_68_P22900662	chr4:48552839-48552883	NM_001145924:-92	5730528L13Rik	PROMOTER	0.263	0.679	2390.51	1622.23	0.178	1855.54	330.55
A_68_P22900663	chr4:48552940-48552986	NM_028137:-408	5730528L13Rik	PROMOTER	0.239	0.632	924.22	584.41	0.151	784.92	118.41
A_68_P22900664	chr4:48553063-48553107	NM_028137:-286	5730528L13Rik	PROMOTER	0.357	0.645	1051.86	678.04	0.230	752.36	172.96
A_68_P24084069	chr5:129106010-129106054	NR_015517:530	5930412G12Rik	INSIDE	0.582	0.549	1411.44	774.39	0.319	1193.13	381.14
A_68_P24838569	chr6:128312716-128312760	NR_027360:102	5930416I19Rik	INSIDE	0.268	0.271	1924.42	521.64	0.073	1632.55	118.73
A_68_P21081717	chr2:26865712-26865756	NM_029862:249	5930434B04Rik	INSIDE	0.560	0.488	1458.96	711.61	0.273	1037.33	283.46
A_68_P29448465	chr14:21522444-21522488	NR_029446:-145	6230400D17Rik	PROMOTER	0.306	0.636	1615.46	1028.01	0.195	1214.50	236.56
A_68_P22775538	chr4:21858442-21858486	NM_175234:-8	6230409E13Rik	PROMOTER	0.517	0.568	1159.15	658.86	0.294	1039.90	305.84
A_68_P26812297	chr9:107888019-107888063	NM_026597:207	6230427J02Rik	INSIDE	0.370	0.566	1015.81	574.57	0.209	795.95	166.55
A_68_P20876215	chr1:182413252-182413296	NM_001081227:361	6330403A02Rik	INSIDE	0.657	0.646	793.94	512.70	0.425	663.82	281.80
A_68_P27904028	chr11:70846735-70846779	NM_134022:363	6330403K07Rik	INSIDE	0.649	0.578	1180.96	682.81	0.376	949.72	356.62
A_68_P27904030	chr11:70846911-70846955	NM_134022:187	6330403K07Rik	INSIDE	0.401	0.439	1364.46	599.38	0.176	1066.17	187.83
A_68_P24975023	chr7:13863906-13863950	NM_177312:142	6330408A02Rik	INSIDE	0.293	0.622	2851.13	1773.84	0.182	2030.50	369.65
A_68_P21707651	chr2:144353166-144353210	NM_172859:-54	6330439K17Rik	DIVERGENT_PROMOTER	0.304	0.336	2405.03	808.62	0.102	2017.05	206.21
A_68_P25459708	chr7:125856496-125856541	NM_182995:394	6330503K22Rik	INSIDE	0.170	0.584	2112.61	1234.70	0.099	1517.05	150.82
A_68_P25594223	chr7:149557894-149557939	NM_177265:248	6330512M04Rik	INSIDE	0.259	0.597	1039.87	621.10	0.155	751.83	116.47
A_68_P25594227	chr7:149558369-149558413	NM_177265:-226	6330512M04Rik	PROMOTER	0.362	1.697	3354.54	5691.01	0.614	2329.28	1430.01
A_68_P20153981	chr1:37486651-37486695	NM_198006:276	6330578E17Rik	INSIDE	0.505	0.626	2263.79	1417.08	0.316	1818.64	575.33
A_68_P28612719	chr12:88223642-88223689	NM_145836:2099	6430527G18Rik	INSIDE	0.468	0.594	1052.63	625.27	0.278	853.20	237.12
A_68_P28612732	chr12:88224969-88225013	NM_145836:774	6430527G18Rik	INSIDE	0.496	0.516	1454.63	750.69	0.256	1240.20	317.14
A_68_P28612738	chr12:88225828-88225872	NM_145836:-86	6430527G18Rik	PROMOTER	0.207	0.417	1685.17	702.72	0.086	1393.89	120.26
A_68_P28612794	chr12:88232860-88232910	NM_145836:-7120	6430527G18Rik	PROMOTER	0.221	0.481	4579.19	2202.59	0.106	3103.89	330.53
A_68_P28612795	chr12:88232987-88233039	NM_145836:-7248	6430527G18Rik	PROMOTER	0.234	0.631	1574.07	993.66	0.148	1198.97	177.21
A_68_P25581585	chr7:147164519-147164563	NM_001033465:114	6430531B16Rik	INSIDE	0.506	0.590	1228.92	724.51	0.298	839.09	250.14
A_68_P26217964	chr8:122637936-122637980	NM_001163762:-93	6430548M08Rik	PROMOTER	0.315	0.529	2610.81	1379.95	0.166	2055.91	341.82
A_68_P26217968	chr8:122638484-122638528	NM_001163762:455	6430548M08Rik	INSIDE	0.450	0.479	1376.07	658.56	0.216	1101.84	237.56
A_68_P24127313	chr5:138182525-138182569	NM_175521:-1320	6430598A04Rik	DIVERGENT_PROMOTER	0.105	1.887	6790.45	12814.33	0.198	4405.83	874.09

ProbeName	Target position of probe on CpG island microarray	TargetID	GeneSymbol	CpG island Description	Ratio of relative methylation (TiO2-NP/Vehicle)	Sham group			TiO_2-H group		
						Relative methylation (Cy-5/Cy-3)	Cy-3 signal (Input)	Cy-5 signal (MeDIP)	Relative methylation (Cy-5/Cy-3)	Cy-3 signal (Input)	Cy-5 signal (MeDIP)
A_68_P24127316	chr5:138182800-138182844	NM_175521:-1596	6430598A04Rik	DIVERGENT_PROMOTER	0.428	0.443	1275.80	565.77	0.190	1132.49	214.71
A_68_P22398702	chr3:105508064-105508108	NM_001163356:-289	6530418L21Rik	PROMOTER	0.183	0.382	1991.05	759.84	0.070	1677.78	117.45
A_68_P22398705	chr3:105508370-105508414	NM_001163356:17	6530418L21Rik	INSIDE	0.129	1.684	7314.69	12316.09	0.218	5002.22	1090.55
A_68_P22398706	chr3:105508483-105508532	NM_001163356:132	6530418L21Rik	INSIDE	0.070	1.990	1457.80	2901.24	0.139	890.76	123.87
A_68_P24760446	chr6:113554636-113554680	NM_133937:-107	6720456B07Rik	DIVERGENT_PROMOTER	0.245	0.601	951.85	572.19	0.147	792.84	116.55
A_68_P24760450	chr6:113555022-113555066	NM_133937:279	6720456B07Rik	INSIDE	0.657	0.482	3223.92	1555.53	0.317	2566.48	814.00
A_68_P29587990	chr14:49065856-49065910	NM_172600:-144	6720456H20Rik	PROMOTER	0.089	0.709	2798.46	1982.78	0.063	1907.49	120.25
A_68_P24618848	chr6:87863953-87863997	NM_173737:5	8430410A17Rik	INSIDE	0.560	0.378	2092.85	790.05	0.211	1739.85	367.76
A_68_P24865610	chr6:135148284-135148328	NM_028982:302	8430419L09Rik	INSIDE	0.312	0.588	7845.62	4616.42	0.183	5418.44	993.91
A_68_P24865611	chr6:135148370-135148414	NM_028982:388	8430419L09Rik	INSIDE	0.260	0.604	1708.37	1032.67	0.157	1212.46	190.53
A_68_P21754949	chr2:153269590-153269634	NM_001001986:603	8430427H17Rik	INSIDE	0.218	0.518	3142.39	1629.16	0.113	2335.58	264.12
A_68_P21755509	chr2:153355180-153355224	NM_001134300:505	8430427H17Rik	INSIDE	0.303	0.536	4321.53	2314.36	0.162	3184.50	517.36
A_68_P21755516	chr2:153356263-153356307	NM_001134300:-577	8430427H17Rik	PROMOTER	0.456	0.516	1139.99	587.97	0.235	889.87	209.24
A_68_P27085192	chr10:40403058-40403102	NM_177793:-7	9030224M15Rik	PROMOTER	0.547	0.682	1325.94	904.47	0.373	1157.92	431.89
A_68_P25459894	chr7:125884088-125884134	NM_027815:326	9030624J02Rik	INSIDE	0.176	0.618	3105.32	1918.00	0.109	2116.17	230.08
A_68_P31177904	chr17:41016953-41016997	NM_027834:533	9130008F23Rik	INSIDE	0.253	0.499	1212.22	604.69	0.126	944.15	119.26
A_68_P32141578	chr19:46073169-46073219	NM_198296:-216	9130011E15Rik	DIVERGENT_PROMOTER	0.364	0.540	1105.89	596.97	0.197	794.71	156.33
A_68_P25962138	chr8:75094798-75094842	NM_172396:127	9130011J15Rik	INSIDE	0.423	0.533	1353.42	721.20	0.225	960.95	216.45
A_68_P24463220	chr6:54379822-54379866	NR_033635:371	9130019P16Rik	INSIDE	0.171	0.493	1713.98	844.41	0.084	1433.43	120.68
A_68_P25509653	chr7:135381206-135381250	NM_177001:317	9130023H24Rik	INSIDE	0.153	0.636	2384.71	1516.26	0.097	1502.10	145.82
A_68_P25509654	chr7:135381359-135381407	NM_177001:163	9130023H24Rik	INSIDE	0.297	0.455	3341.32	1521.88	0.135	2245.49	303.74
A_68_P25509657	chr7:135381773-135381817	NM_177001:-249	9130023H24Rik	PROMOTER	0.634	0.692	2857.26	1976.81	0.439	2169.56	952.30
A_68_P20250392	chr1:57028581-57028634	NR_024325:295	9130024F11Rik	INSIDE	0.016	3.220	3574.32	11508.11	0.052	2359.54	121.70
A_68_P23693366	chr5:52581910-52581954	NR_015537:13	9230114K14Rik	INSIDE	0.246	0.669	895.12	599.22	0.164	748.74	123.07
A_68_P30475231	chr15:98397707-98397751	NR_028442:-26	9330020H09Rik	PROMOTER	0.603	0.522	2177.89	1136.61	0.315	1624.71	511.56
A_68_P21782538	chr2:158186969-158187016	NR_015463:253	9430008C03Rik	INSIDE	0.294	0.433	1475.14	638.46	0.127	1038.55	132.14
A_68_P21782540	chr2:158187149-158187193	NR_015463:75	9430008C03Rik	INSIDE	0.361	0.611	1703.82	1041.43	0.221	1310.07	289.15
A_68_P23442482	chr4:155484180-155484224	NM_145557:96	9430015G10Rik	INSIDE	0.409	0.431	1382.21	595.85	0.176	1104.16	194.66
A_68_P20252945	chr1:57463017-57463061	NM_001081181:-353	9430016H08Rik	PROMOTER	0.465	0.573	1971.74	1129.97	0.266	1549.91	413.03
A_68_P31464038	chr18:4635009-4635053	NM_001081963:104	9430020K01Rik	INSIDE	0.496	0.512	3171.82	1624.49	0.254	2380.57	605.03
A_68_P24455804	chr6:53237462-53237506	NR_015553:196	9430076C15Rik	INSIDE	0.362	0.441	1507.71	665.62	0.160	1198.21	191.54
A_68_P24455806	chr6:53237701-53237745	NR_015553:434	9430076C15Rik	INSIDE	0.239	0.670	2243.97	1503.01	0.160	1827.15	292.91
A_68_P24455810	chr6:53238161-53238205	NR_015553:894	9430076C15Rik	INSIDE	0.594	0.428	1853.46	793.26	0.254	1447.77	367.84
A_68_P24648066	chr6:92893011-92893055	NR_015530:2457	9530026P05Rik	INSIDE	0.384	0.659	1940.02	1277.78	0.253	1367.67	346.12
A_68_P24648067	chr6:92893117-92893161	NR_015530:2563	9530026P05Rik	INSIDE	0.146	0.552	2239.11	1235.64	0.081	1714.78	138.49
A_68_P23562834	chr5:28785352-28785396	NR_015562:-8149	9530036O11Rik	PROMOTER	0.445	0.664	5162.24	3429.44	0.296	3919.25	1159.80
A_68_P23562892	chr5:28791826-28791870	NR_015562:-1675	9530036O11Rik	PROMOTER	0.331	0.402	1583.43	637.04	0.133	1415.85	188.76
A_68_P24830747	chr6:127059111-127059169	NM_177003:430	9630033F20Rik	INSIDE	0.489	0.628	1497.81	940.76	0.307	1301.40	399.40
A_68_P25286562	chr7:91234889-91234933	NM_030728:105	9930013L23Rik	INSIDE	0.447	0.527	1669.55	879.18	0.235	1386.10	326.40
A_68_P25286566	chr7:91235318-91235362	NM_030728:-325	9930013L23Rik	PROMOTER	0.347	0.623	1614.11	1006.39	0.216	1140.03	246.58
A_68_P30261318	chr15:60654478-60654526	NR_030696:-17	9930014A18Rik	PROMOTER	0.272	0.528	1085.91	572.91	0.144	840.37	120.70
A_68_P30261322	chr15:60654906-60654950	NR_030696:409	9930014A18Rik	INSIDE	0.320	1.433	1224.73	1755.61	0.458	1001.38	458.70
A_68_P30261323	chr15:60655040-60655084	NR_030696:543	9930014A18Rik	INSIDE	0.616	0.454	1534.45	696.58	0.280	1241.21	347.00
A_68_P32054913	chr19:29879873-29879917	NM_172836:605	9930021J03Rik	INSIDE	0.538	0.475	2404.99	1143.19	0.256	1820.32	465.54

ProbeName	Target position of probe on CpG island microarray	TargetID	GeneSymbol	CpG island Description	Ratio of relative methylation (TiO2-NP/Vehicle)	Sham group			TiO_2-H group		
						Relative methylation (Cy-5/Cy-3)	Cy-3 signal (Input)	Cy-5 signal (MeDIP)	Relative methylation (Cy-5/Cy-3)	Cy-3 signal (Input)	Cy-5 signal (MeDIP)
A_68_P32054915	chr19:29880098-29880142	NM_172836:379	9930021J03Rik	INSIDE	0.555	0.614	866.77	532.58	0.341	733.85	250.22
A_68_P23271163	chr4:124614216-124614260	NM_177573:-53	9930104L06Rik	DIVERGENT_PROMOTER	0.640	0.656	1168.39	766.73	0.420	927.63	389.38
A_68_P27894430	chr11:69154065-69154109	NR_027827:-184	A030009H04Rik	PROMOTER	0.460	0.634	885.96	561.45	0.291	805.19	234.60
A_68_P20947700	chr1:194999554-194999598	NM_001160360:-86	A130010J15Rik	PROMOTER	0.253	0.501	1101.04	551.61	0.127	906.35	114.75
A_68_P20935795	chr1:192849358-192849402	NR_027298:151	A230020J21Rik	INSIDE	0.499	0.543	1577.88	856.18	0.271	1289.41	348.96
A_68_P20935796	chr1:192849520-192849564	NR_027298:313	A230020J21Rik	INSIDE	0.335	0.455	1278.55	582.32	0.153	988.28	150.85
A_68_P27835193	chr11:58977324-58977368	NM_173785:-102	A230051G13Rik	PROMOTER	0.641	0.710	1345.47	955.91	0.456	1036.07	472.19
A_68_P29964259	chr14:123312516-123312560	NM_145466:-151	A2ld1	PROMOTER	0.240	0.412	3027.30	1246.68	0.099	2385.40	236.01
A_68_P23456811	chr5:5664103-5664147	NM_172447:108	A330021E22Rik	INSIDE	0.362	0.682	1203.11	820.16	0.247	905.36	223.21
A_68_P23456813	chr5:5664349-5664400	NM_172447:-142	A330021E22Rik	PROMOTER	0.125	0.695	1930.87	1342.01	0.087	1419.02	123.07
A_68_P31961512	chr19:12670749-12670793	NR_015503:286	A330040F15Rik	INSIDE	0.651	0.371	1868.62	693.37	0.242	1442.61	348.69
A_68_P23293015	chr4:128444743-128444787	NM_001009819:8263	A3galt2	INSIDE	0.614	2.437	1056.13	2573.32	1.495	898.87	1343.67
A_68_P30387340	chr15:83081767-83081811	NM_001170954:373	A4galt	INSIDE	0.639	0.488	1467.01	715.23	0.311	1375.72	428.41
A_68_P31145426	chr17:32533638-32533682	NR_029458:-6298	A530088E08Rik	PROMOTER	0.250	0.616	890.06	548.63	0.154	770.84	118.86
A_68_P26920085	chr10:7382878-7382922	NR_030698:-268	A630066F11Rik	PROMOTER	0.623	0.672	1249.75	840.30	0.419	1076.44	450.76
A_68_P26920089	chr10:7383225-7383269	NR_030698:78	A630066F11Rik	INSIDE	0.292	0.506	2660.02	1345.58	0.148	2036.56	300.51
A_68_P28178207	chr11:119352476-119352520	NM_001164636:-162	A730011L01Rik	PROMOTER	0.386	0.699	1368.51	957.09	0.270	1069.81	288.84
A_68_P21632193	chr2:130732022-130732066	NR_015523:88	A730017L22Rik	INSIDE	0.487	0.448	1208.61	540.95	0.218	986.16	214.80
A_68_P28202319	chr12:4776061-4776106	NM_175431:-10	A830093I24Rik	DIVERGENT_PROMOTER	0.271	0.645	1217.67	785.47	0.175	1011.27	177.06
A_68_P31112025	chr17:26853064-26853108	NM_029870:492	A930001N09Rik	INSIDE	0.552	0.575	2484.45	1428.84	0.317	2103.29	667.25
A_68_P32053384	chr19:29596371-29596418	NR_015567:83	A930007I19Rik	INSIDE	0.473	0.570	972.26	554.23	0.269	784.74	211.41
A_68_P21487320	chr2:103963515-103963559	NM_026634:611	A930018P22Rik	INSIDE	0.213	0.547	1711.64	936.35	0.116	1350.53	157.27
A_68_P27081279	chr10:39618779-39618823	NR_033628:244	AA474331	INSIDE	0.444	0.601	1486.98	893.63	0.267	1342.12	357.96
A_68_P21071433	chr2:25187978-25188029	NR_027448:88	AA543186	INSIDE	0.205	0.617	1139.84	703.03	0.127	937.15	118.60
A_68_P24067080	chr5:125956003-125956050	NM_030210:-216	Aacs	PROMOTER	0.245	0.484	1225.23	593.39	0.119	1006.51	119.61
A_68_P20347693	chr1:74331033-74331077	NM_001190444:258	Aamp	INSIDE	0.280	0.431	1648.02	710.43	0.121	1313.97	158.37
A_68_P30214224	chr15:51871601-51871645	NM_175503:-30	Aard	PROMOTER	0.584	0.646	1498.65	967.89	0.377	1408.32	531.36
A_68_P30214228	chr15:51872101-51872145	NM_175503:470	Aard	INSIDE	0.356	0.516	1477.57	762.35	0.183	1221.35	224.04
A_68_P31201455	chr17:45643843-45643887	NM_198608:75	Aars2	INSIDE	0.372	0.561	1362.30	763.95	0.209	1028.70	214.79
A_68_P28073762	chr11:101278541-101278585	NM_144829:185	Aarsd1	INSIDE	0.588	0.663	2841.75	1882.70	0.389	2252.20	876.80
A_68_P28181983	chr11:119908193-119908237	NM_001198787:-5119	Aatk	PROMOTER	0.560	0.701	2711.60	1901.38	0.393	2101.40	825.63
A_68_P22921610	chr4:53173018-53173062	NM_013454:-273	Abca1	PROMOTER	0.149	0.597	1907.07	1138.50	0.089	1345.39	119.70
A_68_P31096791	chr17:24489119-24489163	NM_001039581:150	Abca3	INSIDE	0.503	0.575	1359.48	781.13	0.289	1045.09	302.20
A_68_P24059260	chr5:124540242-124540286	NM_019875:5543	Abcb9	INSIDE	0.648	2.562	3085.29	7904.10	1.661	2247.29	3731.71
A_68_P29940450	chr14:119105251-119105295	NM_001033336:169	Abcc4	INSIDE	0.302	0.501	1987.26	995.31	0.151	1713.82	259.17
A_68_P29940453	chr14:119105644-119105689	NM_001033336:-225	Abcc4	PROMOTER	0.388	0.553	937.89	518.53	0.215	799.90	171.59
A_68_P30587615	chr16:20426603-20426647	NM_013790:-157	Abcc5	PROMOTER	0.215	0.631	1192.94	752.32	0.136	911.87	123.67
A_68_P25097406	chr7:53435172-53435216	NM_011510:209	Abcc8	INSIDE	0.233	1.709	815.63	1393.61	0.397	472.43	187.76
A_68_P23542799	chr5:24082860-24082904	NM_001190443:403	Abcf2	INSIDE	0.350	0.633	2273.51	1438.78	0.221	1579.81	349.76
A_68_P23542801	chr5:24083043-24083087	NM_001190443:221	Abcf2	INSIDE	0.630	0.639	2154.11	1376.27	0.403	1492.52	601.00
A_68_P24482545	chr6:58590224-58590281	NM_011920:43587	Abcg2	INSIDE	0.154	0.712	1539.68	1095.49	0.109	1144.30	125.11
A_68_P31406130	chr17:85075448-85075492	NM_031884:6793	Abcg5	INSIDE	0.463	0.464	1145.15	530.85	0.215	984.79	211.54
A_68_P31406131	chr17:85075545-85075589	NM_031884:6697	Abcg5	INSIDE	0.619	0.430	1248.70	537.12	0.266	968.77	257.85
A_68_P24114259	chr5:135484848-135484896	NM_001190437:-149	Abhd11	DIVERGENT_PROMOTER	0.196	0.630	1325.81	834.96	0.123	990.99	122.26

ProbeName	Target position of probe on CpG island microarray	TargetID	GeneSymbol	CpG island Description	Ratio of relative methylation (TiO2-NP/Vehicle)	Sham group			TiO_2-H group		
						Relative methylation (Cy-5/Cy-3)	Cy-3 signal (Input)	Cy-5 signal (MeDIP)	Relative methylation (Cy-5/Cy-3)	Cy-3 signal (Input)	Cy-5 signal (MeDIP)
A_68_P24114260	chr5:135484941-135484991	NM_001190437:-55	Abhd11	DIVERGENT_PROMOTER	0.244	0.474	1464.32	694.49	0.116	1000.99	115.94
A_68_P24114264	chr5:135485504-135485548	NR_033794:201	Abhd11	INSIDE	0.508	0.669	1282.03	857.73	0.340	938.05	318.73
A_68_P24114265	chr5:135485654-135485698	NR_033794:351	Abhd11	INSIDE	0.574	0.617	1223.70	754.79	0.354	885.51	313.72
A_68_P21741660	chr2:150730004-150730048	NM_024465:441	Abhd12	INSIDE	0.522	0.663	3093.69	2050.35	0.346	2373.54	821.67
A_68_P21741662	chr2:150730240-150730284	NM_024465:205	Abhd12	INSIDE	0.560	0.552	1913.46	1055.45	0.309	1584.74	489.26
A_68_P25645837	chr8:9978074-9978118	NM_001081119:380	Abhd13	INSIDE	0.231	0.627	931.50	584.18	0.145	819.22	118.56
A_68_P27938080	chr11:77329296-77329340	NM_026185:700	Abhd15	INSIDE	0.369	0.522	1318.57	687.90	0.193	1028.42	198.06
A_68_P31491180	chr18:10707044-10707088	NM_134130:-372	Abhd3	PROMOTER	0.344	0.673	2149.10	1445.60	0.232	1657.30	383.91
A_68_P26888898	chr9:122261191-122261235	NM_026179:479	Abhd5	INSIDE	0.548	0.475	1220.49	580.11	0.260	1149.07	299.21
A_68_P29387299	chr14:8835518-8835562	NM_025341:125	Abhd6	INSIDE	0.496	0.496	1829.45	908.22	0.246	1383.90	341.01
A_68_P25956918	chr8:73987077-73987121	NM_022419:458	Abhd8	INSIDE	0.263	0.510	1074.65	548.49	0.134	913.13	122.63
A_68_P21061191	chr2:22895186-22895230	NM_001077190:552	Abi1	INSIDE	0.544	0.528	1339.40	707.62	0.287	1188.33	341.57
A_68_P21061195	chr2:22895595-22895639	NM_001077190:144	Abi1	INSIDE	0.207	0.689	1127.46	777.11	0.143	843.87	120.53
A_68_P21110672	chr2:31543979-31544023	NM_001112703:-75	Abl1	PROMOTER	0.426	0.339	2003.50	680.11	0.144	1675.62	242.06
A_68_P27932045	chr11:76250435-76250479	NM_198018:72465	Abr	INSIDE	0.271	0.626	3404.65	2130.45	0.169	2601.46	440.86
A_68_P27932458	chr11:76308565-76308609	NM_198018:14335	Abr	INSIDE	0.563	0.513	1670.04	856.81	0.289	1335.96	385.79
A_68_P27932871	chr11:76384753-76384797	NM_198895:102	Abr	INSIDE	0.368	0.676	4400.34	2973.23	0.248	3399.93	844.40
A_68_P28881306	chr13:23515429-23515473	NM_013924:285	Abt1	INSIDE	0.486	0.501	1304.49	654.11	0.244	1021.46	249.03
A_68_P24624507	chr6:88792017-88792061	NM_030251:-109	Abtb1	PROMOTER	0.220	1.629	9945.62	16204.31	0.358	6894.62	2468.28
A_68_P24624508	chr6:88792125-88792169	NM_030251:-217	Abtb1	PROMOTER	0.423	3.214	16049.16	51578.49	1.361	9621.76	13091.91
A_68_P24624509	chr6:88792201-88792245	NM_030251:-293	Abtb1	PROMOTER	0.174	1.570	5082.42	7978.09	0.273	3210.12	876.93
A_68_P21483985	chr2:103406320-103406364	NM_178890:-124	Abtb2	PROMOTER	0.179	1.477	3797.96	5609.94	0.264	2414.63	638.31
A_68_P26870853	chr9:119066197-119066250	NM_146230:-12	Acaa1b	PROMOTER	0.307	0.623	1274.20	793.86	0.191	1075.27	205.65
A_68_P31833164	chr18:74938725-74938769	NM_177470:-119	Acaa2	DIVERGENT_PROMOTER	0.241	0.502	2606.13	1308.81	0.121	1781.96	215.91
A_68_P31833165	chr18:74938885-74938929	NM_177470:41	Acaa2	INSIDE	0.291	0.576	3477.89	2001.80	0.168	2416.86	404.97
A_68_P26373431	chr9:26806964-26807008	NM_025862:148	Acad8	INSIDE	0.137	0.474	2559.24	1211.87	0.065	1811.77	117.56
A_68_P24007906	chr5:115568969-115569013	NM_007383:332	Acads	INSIDE	0.286	1.430	1816.65	2597.95	0.409	1297.06	531.05
A_68_P24007907	chr5:115569084-115569128	NM_007383:216	Acads	INSIDE	0.295	0.630	2283.44	1438.67	0.186	1678.89	311.69
A_68_P25258775	chr7:86198137-86198181	NM_007424:-210	Acan	PROMOTER	0.273	0.563	2377.74	1339.81	0.154	1854.72	285.27
A_68_P30646211	chr16:31200561-31200605	NM_030138:742	Acap2	INSIDE	0.456	0.483	2455.17	1186.49	0.220	1973.96	434.60
A_68_P31055160	chr17:13153437-13153481	NM_009338:133	Acat2	INSIDE	0.654	0.483	1171.71	566.14	0.316	1001.83	316.34
A_68_P31055162	chr17:13153233-13153278	NM_009338:336	Acat2	INSIDE	0.318	0.538	1930.12	1038.46	0.171	1383.85	236.51
A_68_P20877471	chr1:182656470-182656514	NM_133225:319	Acbd3	INSIDE	0.602	0.472	2000.22	943.87	0.284	1672.07	474.65
A_68_P21061281	chr2:22923971-22924015	NM_001102437:-599	Acbd5	PROMOTER	0.304	0.505	1195.66	604.01	0.154	906.21	139.11
A_68_P21061285	chr2:22924459-22924503	NM_001102437:-111	Acbd5	PROMOTER	0.570	0.701	2070.25	1452.17	0.400	1512.58	604.66
A_68_P21061287	chr2:22924684-22924728	NM_001102437:115	Acbd5	INSIDE	0.154	1.430	5212.07	7455.00	0.220	3718.61	816.61
A_68_P21061288	chr2:22924783-22924828	NM_001102437:214	Acbd5	INSIDE	0.024	1.918	4351.38	8347.05	0.045	2834.50	128.16
A_68_P27958467	chr11:80965329-80965373	NM_007384:1055	Accn1	INSIDE	0.478	0.656	1189.57	779.90	0.313	1067.78	334.62
A_68_P27958470	chr11:80965747-80965791	NM_007384:637	Accn1	INSIDE	0.389	0.643	2651.27	1705.25	0.250	2029.90	507.65
A_68_P27958481	chr11:80966909-80966953	NM_007384:-525	Accn1	PROMOTER	0.587	0.530	1433.96	760.52	0.311	1131.20	352.29
A_68_P25354923	chr7:105457417-105457461	NM_025408:599	Acer3	INSIDE	0.444	0.693	1705.31	1181.82	0.308	1299.88	399.96
A_68_P24124950	chr5:137730313-137730357	NM_009599:830	Ache	INSIDE	0.265	0.678	2136.29	1447.55	0.179	1641.91	294.45
A_68_P29613712	chr14:55305381-55305425	NM_001085473:366	Acin1	INSIDE	0.333	0.693	1185.67	821.88	0.231	961.80	222.15
A_68_P29613716	chr14:55305799-55305843	NM_001085473:-52	Acin1	PROMOTER	0.571	0.413	2027.70	837.31	0.236	1701.80	400.98

ProbeName	Target position of probe on CpG island microarray	TargetID	GeneSymbol	CpG island Description	Ratio of relative methylation (TiO2-NP/Vehicle)	Sham group			TiO_2-H group		
						Relative methylation (Cy-5/Cy-3)	Cy-3 signal (Input)	Cy-5 signal (MeDIP)	Relative methylation (Cy-5/Cy-3)	Cy-3 signal (Input)	Cy-5 signal (MeDIP)
A_68_P28068583	chr11:100389102-100389146	NM_001199296:190	Acly	INSIDE	0.195	0.399	1972.04	787.31	0.078	1513.68	118.02
A_68_P28068584	chr11:100389186-100389230	NM_001199296:106	Acly	INSIDE	0.359	0.592	1045.30	618.30	0.212	773.14	164.29
A_68_P24219278	chr6:6906039-6906086	NM_001077713:45	Acn9	INSIDE	0.373	0.491	1636.84	803.46	0.183	1192.51	218.16
A_68_P30380158	chr15:81702740-81702784	NM_080633:-130	Aco2	DIVERGENT_PROMOTER	0.346	0.562	1716.09	963.94	0.195	1293.49	251.63
A_68_P30380159	chr15:81702880-81702926	NM_080633:10	Aco2	INSIDE	0.285	0.550	1078.74	593.74	0.157	764.99	119.88
A_68_P23417005	chr4:151552435-151552479	NM_001146057:-7786	Acot7	PROMOTER	0.542	0.278	2371.44	659.43	0.151	1876.67	282.61
A_68_P23417006	chr4:151552589-151552633	NM_001146057:-7632	Acot7	PROMOTER	0.328	0.435	1700.38	738.82	0.143	1283.70	183.01
A_68_P32759042	chrX:151697320-151697364	NM_019736:300	Acot9	INSIDE	0.224	1.545	1448.06	2236.73	0.347	587.84	203.70
A_68_P21421302	chr2:91042978-91043022	NM_007387:-68	Acp2	DIVERGENT_PROMOTER	0.469	0.553	1544.97	853.77	0.259	1194.36	309.42
A_68_P22353007	chr3:96962730-96962774	NM_019800:53	Acp6	INSIDE	0.234	0.698	1586.71	1107.81	0.164	1317.97	215.73
A_68_P26749713	chr9:96789331-96789375	NM_153420:489	Acpl2	INSIDE	0.358	0.567	1211.53	687.33	0.203	931.50	189.06
A_68_P26749716	chr9:96789602-96789646	NM_153420:217	Acpl2	INSIDE	0.365	0.604	8480.39	5122.60	0.220	5920.23	1304.23
A_68_P26749722	chr9:96790253-96790298	NM_153420:-434	Acpl2	PROMOTER	0.519	0.510	1346.84	687.13	0.265	990.00	261.93
A_68_P26235836	chr8:125299500-125299544	NM_144932:118	Acsf3	INSIDE	0.269	1.433	3955.96	5667.01	0.386	2719.70	1048.79
A_68_P26235837	chr8:125299586-125299630	NM_144932:204	Acsf3	INSIDE	0.142	0.632	1690.76	1068.31	0.090	1295.99	116.19
A_68_P26235838	chr8:125299671-125299715	NM_144932:288	Acsf3	INSIDE	0.497	0.634	879.09	557.22	0.315	751.30	236.47
A_68_P27809324	chr11:54117377-54117421	NM_001033597:-307	Acsl6	PROMOTER	0.243	0.597	2608.15	1556.32	0.145	1796.55	260.30
A_68_P27809329	chr11:54117906-54117950	NM_001033597:223	Acsl6	INSIDE	0.380	0.691	3018.23	2086.45	0.262	2417.92	634.67
A_68_P21766024	chr2:155343697-155343741	NM_019811:-60	Acss2	DIVERGENT_PROMOTER	0.182	0.528	1762.34	931.09	0.096	1473.68	141.61
A_68_P26242184	chr8:126416045-126416089	NM_009606:2570	Acta1	INSIDE	0.279	0.610	4222.33	2574.56	0.170	3074.41	523.07
A_68_P28574814	chr12:81360288-81360334	NM_134156:1048	Actn1	INSIDE	0.315	0.606	2559.63	1550.15	0.191	1984.65	378.58
A_68_P28574815	chr12:81360407-81360451	NM_134156:930	Actn1	INSIDE	0.588	0.558	1566.46	874.72	0.328	1291.53	423.94
A_68_P25024594	chr7:29747163-29747207	NM_021895:115	Actn4	INSIDE	0.645	0.570	1895.95	1081.05	0.368	1565.62	575.77
A_68_P32143651	chr19:46469679-46469723	NM_016860:525	Actr1a	INSIDE	0.280	0.608	1106.56	672.40	0.170	919.00	156.10
A_68_P32143656	chr19:46470216-46470260	NM_016860:-13	Actr1a	DIVERGENT_PROMOTER	0.644	0.418	2256.12	942.36	0.269	1745.71	469.68
A_68_P20149940	chr1:36766481-36766525	NM_146107:268	Actr1b	INSIDE	0.358	0.442	2835.28	1253.82	0.158	2026.68	321.20
A_68_P23548816	chr5:25266039-25266083	NM_001004365:217	Actr3b	INSIDE	0.274	1.719	13031.31	22401.81	0.472	8506.81	4013.59
A_68_P21784026	chr2:158450747-158450791	NM_175419:120	Actr5	INSIDE	0.273	0.659	2060.57	1357.21	0.180	1368.50	246.22
A_68_P27332753	chr10:89194937-89194988	NM_025914:78	Actr6	INSIDE	0.168	0.521	1795.15	935.48	0.087	1382.16	120.83
A_68_P26872171	chr9:119312063-119312107	NM_007397:466	Acvr2b	INSIDE	0.079	0.717	2617.25	1877.42	0.057	2058.02	117.29
A_68_P26872302	chr9:119336590-119336634	NM_007397:24994	Acvr2b	INSIDE	0.612	2.514	1913.37	4810.10	1.537	1406.02	2161.67
A_68_P27680036	chr11:30549094-30549138	NM_029344:280	Acyp2	INSIDE	0.100	0.378	4018.16	1517.92	0.038	3253.51	122.83
A_68_P21814245	chr2:163575768-163575812	NM_007398:123	Ada	INSIDE	0.190	0.575	2172.96	1250.34	0.109	1562.42	170.93
A_68_P28081367	chr11:102635750-102635794	NM_001110778:13020	Adam11	INSIDE	0.258	0.657	858.17	564.16	0.169	742.98	125.83
A_68_P28081370	chr11:102636075-102636119	NM_001110778:13344	Adam11	INSIDE	0.233	0.526	1296.45	681.48	0.122	980.89	120.04
A_68_P27765617	chr11:45869706-45869750	NM_009616:240	Adam19	INSIDE	0.319	0.584	2450.04	1431.58	0.186	1938.75	361.00
A_68_P27765618	chr11:45869772-45869816	NM_009616:306	Adam19	INSIDE	0.641	0.640	1923.87	1232.24	0.411	1518.19	623.73
A_68_P20286847	chr1:63493039-63493083	NM_001177600:583	Adam23	INSIDE	0.564	0.590	2294.50	1354.14	0.333	1842.08	612.75
A_68_P30931274	chr16:85802529-85802573	NM_009621:810	Adamts1	INSIDE	0.578	0.515	1303.21	671.32	0.298	1028.77	306.50
A_68_P31149283	chr17:33661330-33661374	NM_172619:212	Adamts10	INSIDE	0.277	1.632	922.24	1505.21	0.452	739.29	334.16
A_68_P27179426	chr10:60708843-60708887	NM_001081127:27322	Adamts14	INSIDE	0.601	2.570	1597.10	4104.40	1.544	1165.79	1800.06
A_68_P27179599	chr10:60735806-60735850	NM_001081127:358	Adamts14	INSIDE	0.183	0.700	2886.75	2021.85	0.128	2179.58	278.88
A_68_P26397638	chr9:30729092-30729141	NM_001024139:921	Adamts15	INSIDE	0.325	0.630	1640.84	1034.07	0.205	1173.36	240.07
A_68_P26397642	chr9:30729620-30729675	NM_001024139:390	Adamts15	INSIDE	0.171	0.599	1573.92	943.39	0.102	1205.69	123.52

ProbeName	Target position of probe on CpG island microarray	TargetID	GeneSymbol	CpG island Description	Ratio of relative methylation (TiO2-NP/Vehicle)	Sham group			TiO_2-H group		
						Relative methylation (Cy-5/Cy-3)	Cy-3 signal (Input)	Cy-5 signal (MeDIP)	Relative methylation (Cy-5/Cy-3)	Cy-3 signal (Input)	Cy-5 signal (MeDIP)
A_68_P26397643	chr9:30729700-30729744	NM_001024139:315	Adamts15	INSIDE	0.504	0.569	1883.97	1072.40	0.287	1442.49	414.02
A_68_P26397644	chr9:30729778-30729822	NM_001024139:237	Adamts15	INSIDE	0.393	0.551	1105.34	609.40	0.217	921.87	199.88
A_68_P26397645	chr9:30729921-30729965	NM_001024139:95	Adamts15	INSIDE	0.292	0.587	2655.06	1559.55	0.172	1935.71	332.19
A_68_P25190563	chr7:73984782-73984826	NM_001033877:184	Adamts17	INSIDE	0.578	0.708	2513.85	1779.56	0.409	1968.77	805.09
A_68_P31744347	chr18:58995833-58995877	NM_175506:-563	Adamts19	PROMOTER	0.279	0.496	1144.12	567.40	0.138	863.25	119.34
A_68_P31744350	chr18:58996079-58996123	NM_175506:-317	Adamts19	PROMOTER	0.668	0.581	1351.03	784.35	0.388	1235.00	478.76
A_68_P27789380	chr11:50416439-50416486	NM_175643:876	Adamts2	INSIDE	0.176	0.474	1930.20	915.07	0.083	1400.30	116.75
A_68_P30931898	chr16:85900536-85900586	NM_011782:810	Adamts5	INSIDE	0.281	0.421	1248.03	525.97	0.118	984.46	116.54
A_68_P30931901	chr16:85900910-85900966	NM_011782:432	Adamts5	INSIDE	0.323	0.482	1808.46	872.30	0.156	1374.78	214.52
A_68_P26716243	chr9:90058080-90058124	NM_001003911:287	Adamts7	INSIDE	0.426	0.622	3260.24	2027.74	0.265	2504.27	662.96
A_68_P26716246	chr9:90058314-90058358	NM_001003911:521	Adamts7	INSIDE	0.245	0.578	1049.77	606.86	0.142	834.05	118.08
A_68_P25276874	chr7:89484048-89484092	NM_001190374:-133	Adamtsl3	PROMOTER	0.560	46.465	529.23	24590.75	26.006	626.01	16279.64
A_68_P25276880	chr7:89484688-89484732	NM_001190374:507	Adamtsl3	INSIDE	0.414	0.612	2844.93	1740.42	0.253	2171.37	549.74
A_68_P27282598	chr10:79804958-79805002	NM_001113548:6211	Adamtsl5	INSIDE	0.481	0.428	1589.39	680.46	0.206	1199.97	247.28
A_68_P27282601	chr10:79805419-79805466	NM_001113548:5749	Adamtsl5	INSIDE	0.054	2.077	5997.11	12454.74	0.113	3739.60	422.49
A_68_P27282647	chr10:79811365-79811409	NM_001113548:-195	Adamtsl5	DIVERGENT_PROMOTER	0.171	0.568	1666.32	946.24	0.097	1236.10	119.81
A_68_P22321220	chr3:89519673-89519729	NM_001038587:757	Adar	INSIDE	0.089	0.692	2887.04	1997.45	0.062	2104.34	130.19
A_68_P26171282	chr8:114516157-114516201	NM_013925:24	Adat1	INSIDE	0.237	0.667	1148.20	765.53	0.158	900.57	142.08
A_68_P23293919	chr4:128602230-128602274	NM_172875:37409	Adc	DOWNSTREAM	0.439	0.704	1371.14	965.02	0.309	1094.55	338.56
A_68_P23294134	chr4:128639387-128639431	NM_172875:253	Adc	INSIDE	0.305	0.550	936.37	514.76	0.168	720.57	120.90
A_68_P23294136	chr4:128639545-128639589	NM_172875:95	Adc	INSIDE	0.295	0.686	6057.50	4153.97	0.202	4127.09	835.52
A_68_P24383758	chr6:39523742-39523792	NM_178873:-108	Adck2	PROMOTER	0.306	0.501	1030.05	515.84	0.153	804.15	123.34
A_68_P24383760	chr6:39523904-39523948	NM_178873:52	Adck2	INSIDE	0.571	0.606	3931.43	2382.30	0.346	2869.75	992.80
A_68_P30349161	chr15:76406872-76406916	NM_172960:106	Adck5	INSIDE	0.390	0.474	2356.78	1117.91	0.185	1905.00	352.18
A_68_P27571287	chr11:6963887-6963931	NM_009622:417	Adcy1	INSIDE	0.528	0.561	1086.20	609.81	0.296	937.42	277.72
A_68_P29113346	chr13:69137647-69137691	NM_153534:751	Adcy2	INSIDE	0.535	0.440	1345.71	592.01	0.236	1189.35	280.10
A_68_P29113347	chr13:69137713-69137757	NM_153534:685	Adcy2	INSIDE	0.586	0.475	2924.73	1389.59	0.279	2510.56	699.32
A_68_P30668268	chr16:35154633-35154677	NM_001012765:-1067	Adcy5	PROMOTER	0.361	0.385	3579.63	1378.53	0.139	2871.51	399.14
A_68_P26039918	chr8:90796247-90796291	NM_007406:-33	Adcy7	PROMOTER	0.267	0.574	965.93	554.35	0.153	780.81	119.53
A_68_P30284259	chr15:64753330-64753385	NM_009623:501	Adcy8	INSIDE	0.164	0.614	1507.56	925.75	0.101	1182.61	119.05
A_68_P30284263	chr15:64753735-64753779	NM_009623:102	Adcy8	INSIDE	0.200	0.386	2013.85	777.96	0.077	1477.48	114.23
A_68_P30284265	chr15:64753968-64754012	NM_009623:-132	Adcy8	PROMOTER	0.664	0.712	2819.75	2007.06	0.472	2311.08	1091.54
A_68_P24469549	chr6:55401776-55401820	NM_001025372:-175	Adcyap1r1	PROMOTER	0.344	0.587	2809.37	1648.59	0.202	2108.33	425.67
A_68_P24469550	chr6:55401858-55401902	NM_001025372:-93	Adcyap1r1	PROMOTER	0.436	0.548	2826.73	1548.02	0.239	2086.40	498.17
A_68_P23595357	chr5:34959448-34959497	NM_001024458:43110	Add1	INSIDE	0.538	4.228	1353.64	5723.59	2.273	954.73	2170.37
A_68_P32180644	chr19:53216958-53217002	NM_001164100:-265	Add3	PROMOTER	0.201	0.580	4243.47	2462.55	0.117	3297.27	384.65
A_68_P32180645	chr19:53217074-53217118	NM_001164100:-149	Add3	PROMOTER	0.275	0.597	1953.21	1166.94	0.164	1459.23	239.73
A_68_P32180647	chr19:53217337-53217384	NM_013758:-1126	Add3	PROMOTER	0.286	0.567	1104.47	626.67	0.162	889.75	144.36
A_68_P22568796	chr3:138106098-138106142	NM_007410:-7	Adh5	PROMOTER	0.574	0.367	1963.40	720.78	0.211	1479.80	311.80
A_68_P28314428	chr12:29360059-29360103	NM_134052:9	Adi1	INSIDE	0.387	0.510	2484.60	1267.06	0.197	1921.23	379.24
A_68_P28314431	chr12:29360343-29360387	NM_134052:293	Adi1	INSIDE	0.244	0.469	1350.01	633.68	0.115	1064.12	121.95
A_68_P24792873	chr6:119339010-119339056	NM_197985:28469	Adipor2	INSIDE	0.445	0.561	2349.05	1317.51	0.249	1921.58	479.31
A_68_P21840065	chr2:168031845-168031889	NM_009628:696	Adnp	INSIDE	0.368	1.759	6730.07	11837.36	0.647	4875.54	3155.13
A_68_P31862682	chr18:80347987-80348031	NM_175028:213	Adnp2	INSIDE	0.497	0.641	6326.56	4058.41	0.319	4988.63	1590.41

ProbeName	Target position of probe on CpG island microarray	TargetID	GeneSymbol	CpG island Description	Ratio of relative methylation (TiO2-NP/Vehicle)	Sham group			TiO_2-H group		
						Relative methylation (Cy-5/Cy-3)	Cy-3 signal (Input)	Cy-5 signal (MeDIP)	Relative methylation (Cy-5/Cy-3)	Cy-3 signal (Input)	Cy-5 signal (MeDIP)
A_68_P27212532	chr10:67010792-67010836	NM_001005419:889	Ado	INSIDE	0.512	0.635	858.81	545.74	0.325	664.71	216.20
A_68_P27212539	chr10:67011459-67011503	NM_001005419:223	Ado	INSIDE	0.348	0.474	1953.05	926.63	0.165	1854.02	305.70
A_68_P20632244	chr1:136131312-136131356	NM_001008533:670	Adora1	INSIDE	0.364	0.529	1761.63	931.49	0.193	1462.88	281.90
A_68_P20632245	chr1:136131391-136131435	NM_001008533:592	Adora1	INSIDE	0.236	0.724	3114.22	2255.32	0.171	2208.47	377.27
A_68_P20632251	chr1:136132200-136132244	NM_001039510:-214	Adora1	PROMOTER	0.312	0.500	1936.92	968.66	0.156	1415.32	220.56
A_68_P20632252	chr1:136132290-136132334	NM_001039510:-304	Adora1	PROMOTER	0.126	0.486	2639.68	1282.81	0.061	1928.81	118.51
A_68_P27255924	chr10:74779536-74779580	NM_009630:-129	Adora2a	PROMOTER	0.343	0.525	1504.76	790.52	0.180	1360.65	245.11
A_68_P27255927	chr10:74779785-74779829	NM_009630:119	Adora2a	INSIDE	0.493	0.672	1237.39	831.22	0.331	943.47	312.53
A_68_P27255933	chr10:74780494-74780538	NM_009630:829	Adora2a	INSIDE	0.411	1.363	5453.48	7433.18	0.561	3819.41	2141.52
A_68_P27852924	chr11:62062701-62062745	NM_007413:237	Adora2b	INSIDE	0.299	0.603	1530.82	923.71	0.180	1165.56	210.17
A_68_P27852925	chr11:62062872-62062916	NM_007413:409	Adora2b	INSIDE	0.400	0.617	1152.32	711.48	0.247	829.07	204.77
A_68_P26552549	chr9:59138886-59138933	NM_028121:-469	Adpgk	PROMOTER	0.199	1.569	699.77	1097.66	0.312	432.76	135.16
A_68_P26552550	chr9:59139013-59139070	NM_028121:-337	Adpgk	PROMOTER	0.101	1.660	908.07	1507.08	0.168	688.46	115.73
A_68_P30686065	chr16:38452492-38452536	NM_007414:255	Adprh	INSIDE	0.196	0.558	2277.40	1270.88	0.109	1709.95	187.03
A_68_P27752256	chr11:43589831-43589875	NM_007416:59982	Adra1b	INSIDE	0.169	0.528	1676.76	885.01	0.089	1353.69	120.75
A_68_P21635701	chr2:131387590-131387634	NM_013460:409	Adra1d	INSIDE	0.233	0.736	10680.82	7860.54	0.172	7030.56	1207.73
A_68_P32186046	chr19:54119698-54119742	NM_007417:49	Adra2a	INSIDE	0.273	0.511	1098.89	561.95	0.140	866.41	120.92
A_68_P32186047	chr19:54119811-54119855	NM_007417:161	Adra2a	INSIDE	0.536	0.587	3096.72	1818.20	0.315	2514.63	791.42
A_68_P32186057	chr19:54120878-54120926	NM_007417:1231	Adra2a	INSIDE	0.168	0.536	1918.46	1027.47	0.090	1372.09	123.73
A_68_P32186059	chr19:54121110-54121154	NM_007417:1461	Adra2a	INSIDE	0.304	0.409	2147.85	877.83	0.124	1504.28	186.62
A_68_P32186060	chr19:54121227-54121271	NM_007417:1577	Adra2a	INSIDE	0.634	0.689	1199.32	826.91	0.437	916.79	400.61
A_68_P32186064	chr19:54121845-54121889	NM_007417:2195	Adra2a	INSIDE	0.582	0.467	4281.35	1999.14	0.272	3174.42	862.59
A_68_P21612948	chr2:127188974-127189018	NM_009633:-25	Adra2b	PROMOTER	0.303	0.563	1277.86	718.97	0.170	1108.29	188.80
A_68_P23599454	chr5:35621406-35621450	NM_007418:214	Adra2c	INSIDE	0.182	0.553	1598.51	883.50	0.100	1161.96	116.66
A_68_P32202579	chr19:56797245-56797289	NM_007419:405	Adrb1	INSIDE	0.323	0.619	1423.99	880.90	0.200	1004.83	200.82
A_68_P32202582	chr19:56797590-56797634	NM_007419:751	Adrb1	INSIDE	0.509	0.519	1228.43	637.95	0.265	789.68	208.88
A_68_P31762245	chr18:62340024-62340068	NM_007420:-433	Adrb2	PROMOTER	0.179	1.460	5578.55	8143.74	0.262	3947.58	1032.70
A_68_P25737279	chr8:28338376-28338420	NM_013462:1662	Adrb3	INSIDE	0.541	0.720	4041.98	2911.20	0.389	2792.44	1087.61
A_68_P25737280	chr8:28338452-28338496	NM_013462:1586	Adrb3	INSIDE	0.255	0.590	1032.95	609.49	0.150	815.87	122.62
A_68_P25737281	chr8:28338526-28338570	NM_013462:1512	Adrb3	INSIDE	0.304	0.722	2240.17	1618.18	0.220	1672.88	367.55
A_68_P25737282	chr8:28338659-28338703	NM_013462:1380	Adrb3	INSIDE	0.155	0.659	2667.12	1756.75	0.102	1824.16	186.68
A_68_P25737283	chr8:28338782-28338826	NM_013462:1256	Adrb3	INSIDE	0.565	0.478	1126.79	539.00	0.270	883.04	238.86
A_68_P31922428	chr19:4305887-4305931	NM_130863:47	Adrbk1	INSIDE	0.663	0.669	1755.30	1173.42	0.443	1417.95	628.83
A_68_P21901918	chr2:179906691-179906735	NM_019822:420	Adrm1	INSIDE	0.560	0.711	1492.54	1061.72	0.399	1260.83	502.47
A_68_P20861819	chr1:179726176-179726220	NM_007422:442	Adss	INSIDE	0.556	0.534	1026.39	548.56	0.297	842.72	250.47
A_68_P28751330	chr12:113858462-113858506	NM_007421:227	Adssl1	INSIDE	0.547	0.643	4984.36	3205.37	0.352	3561.95	1253.03
A_68_P27565299	chr11:5761759-5761803	NM_009636:-88	Aebp1	PROMOTER	0.244	0.742	2809.22	2083.64	0.181	1898.17	343.24
A_68_P20161228	chr1:38683615-38683660	NM_010678:38163	Aff3	INSIDE	0.157	0.686	3374.56	2315.49	0.108	2187.27	235.93
A_68_P20161231	chr1:38683942-38683986	NM_010678:37836	Aff3	INSIDE	0.191	0.433	1946.82	842.06	0.083	1422.82	117.60
A_68_P26240062	chr8:126001616-126001660	NM_054070:-123	Afg3l1	PROMOTER	0.404	0.494	2109.64	1041.20	0.199	1607.97	320.67
A_68_P20429507	chr1:91350965-91351009	NM_001037136:-399	Agap1	PROMOTER	0.420	0.601	947.41	568.97	0.252	814.03	205.32
A_68_P20429508	chr1:91351074-91351118	NM_001037136:-289	Agap1	PROMOTER	0.630	0.599	1031.65	617.65	0.377	954.07	359.59
A_68_P27534003	chr10:126528845-126528889	NM_001033263:12904	Agap2	INSIDE	0.607	0.585	2525.24	1476.80	0.355	1894.39	672.92
A_68_P21418961	chr2:90623244-90623288	NM_178755:366	Agbl2	INSIDE	0.266	0.655	832.57	545.04	0.174	673.62	117.42

ProbeName	Target position of probe on CpG island microarray	TargetID	GeneSymbol	CpG island Description	Ratio of relative methylation (TiO2-NP/Vehicle)	Sham group			TiO_2-H group		
						Relative methylation (Cy-5/Cy-3)	Cy-3 signal (Input)	Cy-5 signal (MeDIP)	Relative methylation (Cy-5/Cy-3)	Cy-3 signal (Input)	Cy-5 signal (MeDIP)
A_68_P21418962	chr2:90623430-90623474	NM_178755:552	Agbl2	INSIDE	0.136	1.458	737.57	1075.64	0.199	591.98	117.60
A_68_P20395056	chr1:82835794-82835840	NM_010472:-241	Agfg1	PROMOTER	0.228	0.582	1367.22	795.85	0.132	1049.33	139.00
A_68_P20395061	chr1:82836417-82836461	NM_010472:381	Agfg1	INSIDE	0.639	0.503	2044.72	1029.10	0.321	1736.66	558.30
A_68_P20395062	chr1:82836520-82836565	NM_010472:485	Agfg1	INSIDE	0.525	0.473	2355.63	1115.29	0.248	2053.78	510.21
A_68_P24127041	chr5:138125324-138125368	NM_178162:499	Agfg2	INSIDE	0.355	1.514	3297.19	4992.30	0.538	2537.47	1365.55
A_68_P24127047	chr5:138126002-138126046	NM_145566:-103	Agfg2	PROMOTER	0.102	0.612	2730.81	1672.50	0.063	1991.80	124.89
A_68_P22451304	chr3:116510918-116510962	NM_001081326:144	Agl	INSIDE	0.428	0.535	1294.85	692.82	0.229	1035.02	236.91
A_68_P25700847	chr8:18846041-18846085	NM_026792:-216	Agpat5	PROMOTER	0.292	0.411	1275.85	524.29	0.120	1017.50	121.91
A_68_P25700848	chr8:18846199-18846243	NM_026792:-58	Agpat5	PROMOTER	0.522	0.509	1102.65	561.01	0.265	892.11	236.84
A_68_P23931758	chr5:101275769-101275813	NM_172715:543	Agpat9	INSIDE	0.462	0.479	1329.45	636.65	0.221	1183.39	261.82
A_68_P26528457	chr9:54765090-54765134	NM_177351:16	Agphd1	INSIDE	0.378	0.546	1391.53	759.53	0.206	1175.08	242.26
A_68_P26528458	chr9:54765158-54765202	NM_177351:84	Agphd1	INSIDE	0.539	0.449	1164.67	523.52	0.242	912.64	221.10
A_68_P21763873	chr2:154899789-154899833	NM_016661:423	Ahcy	INSIDE	0.297	0.646	938.05	605.85	0.192	870.33	166.71
A_68_P22408052	chr3:107498784-107498828	NM_145542:660	Ahcyl1	INSIDE	0.304	0.538	963.70	518.10	0.164	739.87	121.09
A_68_P22408053	chr3:107498878-107498922	NM_145542:566	Ahcyl1	INSIDE	0.234	0.588	2456.61	1443.28	0.138	1764.56	243.08
A_68_P23315176	chr4:132567687-132567731	NM_146155:288	Ahdc1	INSIDE	0.172	1.466	1685.86	2471.02	0.251	1289.17	324.19
A_68_P23315178	chr4:132568014-132568058	NM_146155:616	Ahdc1	INSIDE	0.191	0.670	2242.42	1502.03	0.128	1618.48	207.13
A_68_P28615065	chr12:88607920-88607964	NM_146036:265	Ahsa1	INSIDE	0.122	0.638	2048.00	1306.59	0.078	1546.60	120.84
A_68_P27646188	chr11:23397444-23397495	NM_172391:477	Ahsa2	INSIDE	0.172	0.686	1131.32	775.95	0.118	1041.98	122.75
A_68_P21492617	chr2:104966415-104966459	NR_015462:231	AI314831	INSIDE	0.654	0.614	1321.80	812.21	0.402	1146.31	460.51
A_68_P31217295	chr17:48549255-48549304	NM_207219:-125	AI314976	DIVERGENT_PROMOTER	0.105	0.542	4840.56	2622.58	0.057	3366.25	191.33
A_68_P22862464	chr4:41445752-41445802	NM_001085515:4333	AI464131	INSIDE	0.231	0.610	1138.17	694.28	0.141	833.82	117.44
A_68_P30645469	chr16:31080852-31080896	NM_198626:644	AI480653	INSIDE	0.181	0.322	2651.92	854.51	0.058	2070.11	120.64
A_68_P27306489	chr10:84565593-84565637	NM_001013028:243	AI597468	INSIDE	0.209	0.401	1986.43	796.55	0.084	1449.41	121.45
A_68_P27306493	chr10:84565965-84566009	NM_001013028:615	AI597468	INSIDE	0.434	0.556	1941.87	1079.40	0.241	1589.97	383.45
A_68_P27257133	chr10:75023101-75023145	NM_198860:-47	AI646023	PROMOTER	0.236	0.516	1623.55	837.00	0.122	1313.90	160.10
A_68_P30473516	chr15:97998981-97999025	NM_177716:766	AI836003	INSIDE	0.440	0.387	1924.31	743.77	0.170	1490.62	253.52
A_68_P31928608	chr19:5425407-5425451	NM_134149:285	AI837181	INSIDE	0.245	0.497	1252.93	622.27	0.122	994.39	121.10
A_68_P31939553	chr19:7456891-7456935	NM_001033139:604	AI846148	INSIDE	0.243	0.574	1135.49	651.36	0.139	925.61	129.10
A_68_P31939559	chr19:7457501-7457545	NM_001033139:-6	AI846148	PROMOTER	0.614	0.510	1834.51	936.50	0.313	1487.51	466.18
A_68_P31939560	chr19:7457601-7457645	NM_001033139:-106	AI846148	PROMOTER	0.330	0.526	1069.98	563.05	0.173	900.28	156.12
A_68_P20774313	chr1:163806678-163806722	NM_172645:92	AI848100	INSIDE	0.367	0.580	4191.61	2429.48	0.213	2962.20	630.90
A_68_P21112312	chr2:31805862-31805906	NM_145144:62	Aif1l	INSIDE	0.184	0.505	1702.28	859.54	0.093	1254.23	116.52
A_68_P21112313	chr2:31806031-31806075	NM_145144:230	Aif1l	INSIDE	0.112	0.736	2051.78	1509.51	0.082	1448.79	119.03
A_68_P32365089	chrX:45866544-45866588	NM_012019:37	Aifm1	INSIDE	0.141	1.937	2558.66	4956.93	0.273	1047.74	285.92
A_68_P23320947	chr4:133637971-133638015	NM_001162970:13626	Aim1l	INSIDE	0.204	0.553	1238.36	685.15	0.113	1041.24	117.48
A_68_P31921206	chr19:4121378-4121422	NM_016666:4427	Aip	INSIDE	0.212	0.356	2112.87	751.28	0.075	1614.89	121.80
A_68_P27271774	chr10:77506231-77506275	NM_009646:73	Aire	INSIDE	0.602	0.694	2650.11	1838.64	0.418	2071.33	865.39
A_68_P23424431	chr4:152748069-152748119	NM_001099299:108845	Ajap1	INSIDE	0.214	2.353	842.68	1982.91	0.504	619.09	311.99
A_68_P23154898	chr4:101092046-101092090	NM_009647:-239	Ak4	PROMOTER	0.559	0.389	1620.81	630.52	0.218	1481.13	322.15
A_68_P23154900	chr4:101092304-101092348	NM_009647:19	Ak4	INSIDE	0.656	0.687	1672.17	1148.10	0.451	1431.01	645.01
A_68_P28002760	chr11:88725701-88725745	NM_001042541:178	Akap1	INSIDE	0.486	0.621	1989.26	1234.52	0.302	1676.77	505.57
A_68_P27851243	chr11:61743609-61743653	NM_019921:98	Akap10	INSIDE	0.352	0.516	2421.17	1248.76	0.182	1779.02	323.01
A_68_P29746359	chr14:78936378-78936422	NM_001164503:267	Akap11	INSIDE	0.518	0.603	1770.61	1066.91	0.312	1508.62	471.03

ProbeName	Target position of probe on CpG island microarray	TargetID	GeneSymbol	CpG island Description	Ratio of relative methylation (TiO2-NP/Vehicle)	Sham group			TiO_2-H group		
						Relative methylation (Cy-5/Cy-3)	Cy-3 signal (Input)	Cy-5 signal (MeDIP)	Relative methylation (Cy-5/Cy-3)	Cy-3 signal (Input)	Cy-5 signal (MeDIP)
A_68_P26913680	chr10:6079737-6079781	NM_031185:454	Akap12	INSIDE	0.292	0.593	2600.83	1542.13	0.173	2115.32	366.03
A_68_P26913681	chr10:6079865-6079909	NM_031185:326	Akap12	INSIDE	0.665	0.659	1348.73	889.00	0.438	1308.06	573.28
A_68_P28553309	chr12:77426063-77426107	NM_001101471:207	Akap5	INSIDE	0.172	0.552	2337.65	1289.53	0.095	1749.51	165.95
A_68_P31144953	chr17:32457424-32457468	NM_019774:652	Akap8	INSIDE	0.213	0.556	1507.99	838.82	0.118	1145.46	135.54
A_68_P31144955	chr17:32457753-32457797	NM_019774:324	Akap8	INSIDE	0.398	0.543	1365.85	741.62	0.216	1107.07	239.38
A_68_P31145137	chr17:32487319-32487363	NM_017476:182	Akap8l	INSIDE	0.087	1.577	3470.55	5473.20	0.137	2299.72	314.37
A_68_P31145138	chr17:32487412-32487456	NM_017476:88	Akap8l	INSIDE	0.099	0.749	4511.75	3377.65	0.074	2899.74	214.89
A_68_P31145139	chr17:32487576-32487627	NM_017476:-79	Akap8l	PROMOTER	0.322	0.500	1310.92	655.00	0.161	954.49	153.79
A_68_P22831249	chr4:34498566-34498610	NM_001007589:725	Akirin2	INSIDE	0.413	0.604	2742.82	1656.91	0.250	2173.52	542.87
A_68_P22970308	chr4:63071362-63071406	NM_001045514:-6905	Akna	PROMOTER	0.536	0.613	1618.74	991.68	0.328	1251.62	410.78
A_68_P24355040	chr6:34267503-34267547	NM_009658:-35	Akr1b3	PROMOTER	0.195	0.528	1484.47	784.17	0.103	1179.64	121.74
A_68_P25089660	chr7:52104564-52104618	NM_026270:-6	Akt1s1	DIVERGENT_PROMOTER	0.238	0.600	1141.66	685.17	0.143	811.48	115.94
A_68_P25089669	chr7:52105544-52105588	NM_026270:970	Akt1s1	INSIDE	0.303	0.509	1725.73	878.07	0.154	1072.00	165.37
A_68_P25089670	chr7:52105704-52105748	NM_026270:1130	Akt1s1	INSIDE	0.596	0.546	998.05	545.25	0.325	857.57	279.07
A_68_P25089687	chr7:52107857-52107901	NM_026270:3282	Akt1s1	INSIDE	0.639	2.612	1327.96	3469.01	1.668	938.04	1564.97
A_68_P25017993	chr7:28377186-28377230	NM_001110208:630	Akt2	INSIDE	0.274	0.697	2136.42	1489.41	0.191	1819.26	346.88
A_68_P20858578	chr1:179187386-179187430	NM_011785:-8510	Akt3	PROMOTER	0.336	0.610	1755.05	1071.10	0.205	1321.79	271.31
A_68_P20858579	chr1:179187522-179187574	NM_011785:-8650	Akt3	PROMOTER	0.070	1.543	4336.99	6692.37	0.108	2838.08	305.62
A_68_P22964916	chr4:62180749-62180793	NM_008525:327	Alad	INSIDE	0.192	0.537	1393.27	747.63	0.103	1143.27	117.60
A_68_P26802099	chr9:106150157-106150201	NM_020559:107	Alas1	INSIDE	0.653	0.720	1662.89	1196.94	0.470	1261.48	593.12
A_68_P30762456	chr16:52453372-52453416	NM_009655:-284	Alcam	PROMOTER	0.482	0.403	1841.83	741.46	0.194	1337.84	259.65
A_68_P22885108	chr4:45811962-45812007	NM_028270:91	Aldh1b1	INSIDE	0.030	1.585	5500.95	8720.82	0.047	3396.99	159.35
A_68_P24045427	chr5:122043186-122043230	NM_009656:625	Aldh2	INSIDE	0.431	0.585	1349.74	789.32	0.252	1113.59	280.38
A_68_P24045430	chr5:122043561-122043605	NM_009656:251	Aldh2	INSIDE	0.156	0.476	3110.23	1481.66	0.074	2240.34	165.99
A_68_P27847520	chr11:61081060-61081115	NM_007437:-458	Aldh3a2	PROMOTER	0.172	0.455	2115.04	961.48	0.078	1541.58	120.82
A_68_P28598366	chr12:85791678-85791722	NM_134042:200	Aldh6a1	INSIDE	0.121	1.332	7482.58	9964.34	0.161	4747.57	765.86
A_68_P20804634	chr1:169280619-169280663	NM_019993:519	Aldh9a1	INSIDE	0.052	1.869	4491.55	8394.50	0.097	2920.32	284.26
A_68_P25708993	chr8:23171282-23171328	NM_183142:113	Alg11	INSIDE	0.102	0.617	2628.99	1622.12	0.063	1888.49	118.64
A_68_P30421417	chr15:88649565-88649609	NM_001142357:162	Alg12	INSIDE	0.273	0.543	1595.05	866.27	0.148	1265.72	187.67
A_68_P30421419	chr15:88649879-88649923	NM_001142357:-152	Alg12	DIVERGENT_PROMOTER	0.235	0.665	2666.42	1773.58	0.156	1891.08	295.64
A_68_P32717148	chrX:140752921-140752965	NM_026247:434	Alg13	INSIDE	0.142	2.935	7059.89	20719.84	0.417	2859.28	1192.69
A_68_P22149462	chr3:54539562-54539606	NM_025442:124	Alg5	INSIDE	0.625	0.553	1629.65	901.92	0.346	1252.56	432.93
A_68_P26505579	chr9:50583333-50583384	NM_133981:-76	Alg9	PROMOTER	0.162	0.588	1731.65	1018.31	0.095	1203.41	114.79
A_68_P26505580	chr9:50583427-50583471	NM_133981:14	Alg9	INSIDE	0.337	0.698	2005.94	1400.85	0.235	1403.20	330.31
A_68_P31341458	chr17:72953415-72953461	NM_007439:209	Alk	INSIDE	0.166	0.460	2298.82	1057.21	0.076	1553.46	118.26
A_68_P21438173	chr2:93816579-93816623	NM_026944:34287	Alkbh3	DOWNSTREAM	0.391	0.698	1383.11	965.21	0.273	1073.71	293.35
A_68_P27843668	chr11:60351780-60351824	NM_172943:618	Alkbh5	INSIDE	0.486	0.593	2332.94	1383.25	0.288	1880.47	541.65
A_68_P27843675	chr11:60352579-60352623	NM_172943:1416	Alkbh5	INSIDE	0.440	0.495	1031.61	510.70	0.218	768.43	167.25
A_68_P27899782	chr11:70068529-70068574	NM_007440:292	Alox12	INSIDE	0.150	0.677	1773.54	1200.29	0.101	1165.93	118.12
A_68_P20262082	chr1:59176751-59176795	NM_001037812:-122	Als2cr4	PROMOTER	0.637	0.626	1856.35	1162.14	0.399	1222.12	487.50
A_68_P20262085	chr1:59176968-59177012	NM_001033449:-50	Als2cr4	PROMOTER	0.394	0.586	1764.61	1034.63	0.231	1379.76	318.35
A_68_P27404961	chr10:102491000-102491044	NM_172553:389	Alx1	INSIDE	0.261	0.510	1147.41	585.22	0.133	905.64	120.72
A_68_P22407408	chr3:107399047-107399091	NM_007441:1120	Alx3	INSIDE	0.394	0.699	1352.07	945.75	0.276	993.19	273.74
A_68_P22407409	chr3:107399162-107399206	NM_007441:1236	Alx3	INSIDE	0.383	0.530	2117.75	1122.27	0.203	1486.17	301.27

ProbeName	Target position of probe on CpG island microarray	TargetID	GeneSymbol	CpG island Description	Ratio of relative methylation (TiO2-NP/Vehicle)	Sham group			TiO_2-H group		
						Relative methylation (Cy-5/Cy-3)	Cy-3 signal (Input)	Cy-5 signal (MeDIP)	Relative methylation (Cy-5/Cy-3)	Cy-3 signal (Input)	Cy-5 signal (MeDIP)
A_68_P22407410	chr3:107399288-107399333	NM_007441:1362	Alx3	INSIDE	0.188	0.471	2328.02	1097.46	0.089	1540.32	136.72
A_68_P22407412	chr3:107399505-107399559	NM_007441:1584	Alx3	INSIDE	0.262	0.590	1020.05	601.42	0.155	781.24	120.71
A_68_P21424293	chr2:91570286-91570331	NM_001080754:14	Ambra1	INSIDE	0.186	0.427	1821.53	777.97	0.079	1444.12	114.72
A_68_P27083331	chr10:40021980-40022028	NM_009665:-10	Amd1	PROMOTER	0.209	0.490	1556.69	763.54	0.103	1316.87	135.25
A_68_P27083332	chr10:40022071-40022115	NM_009665:-98	Amd1	PROMOTER	0.498	0.615	1393.29	856.67	0.306	1096.09	335.78
A_68_P27083334	chr10:40022291-40022335	NM_009665:-318	Amd1	PROMOTER	0.453	0.540	1626.28	877.98	0.245	1254.26	306.87
A_68_P27083337	chr10:40022544-40022597	NM_009665:-576	Amd1	PROMOTER	0.253	0.449	1427.78	641.68	0.114	1062.36	120.88
A_68_P27083323	chr10:40020837-40020881	NM_007444:1134	Amd2	INSIDE	0.476	0.678	1594.46	1081.77	0.323	1282.78	414.38
A_68_P26074969	chr8:96535449-96535505	NM_011787:1064	Amfr	INSIDE	0.246	0.415	1454.74	603.24	0.102	1137.19	115.91
A_68_P26074973	chr8:96535729-96535781	NM_011787:786	Amfr	INSIDE	0.166	0.400	3487.11	1395.55	0.067	2481.13	165.01
A_68_P26074974	chr8:96535812-96535856	NM_011787:706	Amfr	INSIDE	0.477	0.589	2100.45	1236.68	0.281	1586.27	445.93
A_68_P27285631	chr10:80270128-80270172	NM_007445:2158	Amh	INSIDE	0.616	0.443	1418.57	628.69	0.273	984.36	268.53
A_68_P32710881	chrX:139400366-139400410	NM_019496:883	Ammecr1	INSIDE	0.107	2.310	1482.67	3425.50	0.246	578.71	142.39
A_68_P32710884	chrX:139400658-139400702	NM_019496:591	Ammecr1	INSIDE	0.187	2.202	1850.91	4076.51	0.412	808.53	333.31
A_68_P31599400	chr18:31919398-31919452	NM_153515:-109	Ammecr1l	PROMOTER	0.151	0.448	2364.51	1060.24	0.068	1841.00	124.67
A_68_P31599403	chr18:31919740-31919784	NM_153515:229	Ammecr1l	INSIDE	0.619	0.683	1004.46	686.51	0.423	796.59	337.11
A_68_P28743035	chr12:112509516-112509560	NM_033603:217	Amn	INSIDE	0.234	2.192	2467.95	5408.97	0.512	1719.17	880.66
A_68_P28743061	chr12:112512577-112512621	NM_033603:3277	Amn	INSIDE	0.523	0.681	3901.04	2654.67	0.356	2858.10	1018.09
A_68_P28743067	chr12:112513239-112513283	NM_033603:3939	Amn	INSIDE	0.659	0.444	2108.31	935.35	0.292	1697.05	496.39
A_68_P26318242	chr9:14419308-14419352	NM_001081395:114	Amotl1	INSIDE	0.132	0.390	2953.45	1152.58	0.052	2339.58	120.67
A_68_P26318243	chr9:14419424-14419468	NM_001081395:-2	Amotl1	PROMOTER	0.411	0.602	873.40	525.70	0.247	753.40	186.27
A_68_P22410426	chr3:107889144-107889188	NM_028779:379	Ampd2	INSIDE	0.272	0.392	1509.32	590.95	0.106	1141.85	121.45
A_68_P25415072	chr7:117911998-117912042	NM_009667:-4097	Ampd3	PROMOTER	0.360	0.535	1828.63	977.71	0.193	1638.59	315.51
A_68_P25415073	chr7:117912172-117912216	NM_009667:-3923	Ampd3	PROMOTER	0.590	0.708	1292.80	915.88	0.418	1120.65	468.80
A_68_P28118515	chr11:109287477-109287521	NM_025275:239	Amz2	INSIDE	0.154	0.616	1711.81	1054.24	0.095	1242.60	117.91
A_68_P28185536	chr11:120459969-120460013	NM_001038230:145	Anapc11	INSIDE	0.445	0.675	1691.06	1141.57	0.300	1408.93	423.21
A_68_P28185537	chr11:120460129-120460181	NM_001038230:309	Anapc11	INSIDE	0.204	0.523	1413.86	738.97	0.106	1145.06	121.88
A_68_P27171935	chr10:59465906-59465950	NM_025514:-68	Anapc16	DIVERGENT_PROMOTER	0.481	0.501	2530.57	1266.60	0.241	2123.48	510.99
A_68_P21071034	chr2:25127984-25128028	NM_175300:21	Anapc2	INSIDE	0.588	0.520	1814.36	943.09	0.305	1493.07	456.13
A_68_P24052416	chr5:123271272-123271316	NM_001042491:54	Anapc5	INSIDE	0.433	0.665	1214.00	806.95	0.288	1049.39	302.30
A_68_P30092464	chr15:27396409-27396457	NM_020332:1	Ank	INSIDE	0.174	1.764	1351.34	2383.72	0.308	918.98	282.77
A_68_P30092465	chr15:27396520-27396564	NM_020332:111	Ank	INSIDE	0.284	1.666	1809.05	3014.71	0.474	1329.51	630.30
A_68_P30092466	chr15:27396756-27396800	NM_020332:347	Ank	INSIDE	0.600	0.632	3710.37	2345.48	0.379	2786.22	1055.95
A_68_P30092468	chr15:27396965-27397009	NM_020332:555	Ank	INSIDE	0.250	0.418	1669.18	698.02	0.104	1301.10	135.88
A_68_P25714451	chr8:24168891-24168935	NM_031158:166	Ank1	INSIDE	0.152	0.525	2178.04	1142.55	0.080	1552.58	124.11
A_68_P27226083	chr10:69285075-69285119	NM_146005:288641	Ank3	INSIDE	0.606	2.579	1959.05	5052.22	1.563	1565.26	2446.25
A_68_P27226084	chr10:69285147-69285191	NM_146005:288713	Ank3	INSIDE	0.666	2.148	2256.07	4846.37	1.430	1692.90	2420.42
A_68_P27912780	chr11:72503452-72503496	NM_009671:-29	Ankfy1	PROMOTER	0.322	1.388	2307.87	3202.55	0.447	1454.93	649.83
A_68_P27912781	chr11:72503632-72503676	NM_009671:151	Ankfy1	INSIDE	0.123	0.621	2038.00	1264.73	0.077	1511.33	115.69
A_68_P31624558	chr18:36719345-36719389	NM_175375:-890	Ankhd1	PROMOTER	0.652	0.443	1562.32	691.96	0.289	1311.14	378.84
A_68_P31624566	chr18:36720188-36720232	NM_175375:-46	Ankhd1	PROMOTER	0.203	0.676	1116.52	754.30	0.137	874.25	119.67
A_68_P31624569	chr18:36720617-36720661	NM_175375:382	Ankhd1	INSIDE	0.448	0.678	2049.77	1388.83	0.303	1603.52	486.33
A_68_P26237598	chr8:125565114-125565166	NM_001081379:1044	Ankrd11	INSIDE	0.147	1.414	991.73	1401.99	0.208	769.56	160.26
A_68_P26237607	chr8:125566355-125566399	NM_001081379:-192	Ankrd11	PROMOTER	0.118	0.542	2633.19	1426.02	0.064	1914.55	122.42

ProbeName	Target position of probe on CpG island microarray	TargetID	GeneSymbol	CpG island Description	Ratio of relative methylation (TiO2-NP/Vehicle)	Sham group			TiO_2-H group		
						Relative methylation (Cy-5/Cy-3)	Cy-3 signal (Input)	Cy-5 signal (MeDIP)	Relative methylation (Cy-5/Cy-3)	Cy-3 signal (Input)	Cy-5 signal (MeDIP)
A_68_P26237608	chr8:125566474-125566518	NM_001081379:-312	Ankrd11	PROMOTER	0.262	0.647	2276.19	1472.92	0.170	1672.48	283.70
A_68_P22682773	chr3:157611083-157611131	NM_001013806:677	Ankrd13c	INSIDE	0.241	0.612	1000.60	612.51	0.148	782.41	115.64
A_68_P31922242	chr19:4282667-4282711	NM_026720:449	Ankrd13d	INSIDE	0.569	0.487	1226.66	597.09	0.277	847.48	234.89
A_68_P31922243	chr19:4282842-4282886	NM_026720:273	Ankrd13d	INSIDE	0.153	0.627	3628.31	2276.09	0.096	2599.10	249.14
A_68_P21007908	chr2:11699545-11699589	NM_177268:187	Ankrd16	INSIDE	0.102	0.663	2470.87	1638.24	0.067	1759.34	118.59
A_68_P21007913	chr2:11700124-11700174	NM_177268:769	Ankrd16	INSIDE	0.336	0.538	1281.37	689.48	0.181	928.52	167.72
A_68_P23882634	chr5:90794262-90794306	NM_030886:927	Ankrd17	INSIDE	0.305	0.569	5428.97	3088.89	0.173	3740.49	648.30
A_68_P23882635	chr5:90794338-90794382	NM_030886:851	Ankrd17	INSIDE	0.292	0.690	3054.67	2107.60	0.202	2236.75	451.10
A_68_P24787430	chr6:118512210-118512259	NM_001081112:40	Ankrd26	INSIDE	0.467	0.595	2127.73	1265.26	0.277	1523.83	422.85
A_68_P24787432	chr6:118512444-118512488	NM_001081112:-192	Ankrd26	PROMOTER	0.373	1.366	2015.02	2752.25	0.509	1533.02	780.91
A_68_P25050066	chr7:36371059-36371104	NM_178263:-184	Ankrd27	DIVERGENT_PROMOTER	0.311	0.585	2004.59	1171.95	0.182	1608.09	292.19
A_68_P25050069	chr7:36371356-36371409	NM_178263:117	Ankrd27	INSIDE	0.369	0.612	860.19	526.55	0.226	672.05	151.87
A_68_P29514241	chr14:32644334-32644378	NM_001024604:-755	Ankrd28	PROMOTER	0.645	0.404	1801.85	728.03	0.260	1310.82	341.36
A_68_P30113930	chr15:31296769-31296813	NM_001164441:724	Ankrd33b	INSIDE	0.295	0.568	2634.76	1497.79	0.168	2118.43	355.51
A_68_P22350123	chr3:96401551-96401595	NM_001024851:1014	Ankrd34a	INSIDE	0.659	0.357	1442.42	514.58	0.235	1071.90	251.88
A_68_P22350131	chr3:96402359-96402403	NM_001024851:1822	Ankrd34a	INSIDE	0.223	0.644	1039.90	669.76	0.144	816.36	117.23
A_68_P22350132	chr3:96402437-96402481	NM_001024851:1900	Ankrd34a	INSIDE	0.656	0.660	2265.19	1495.18	0.433	1805.69	781.54
A_68_P22350134	chr3:96402650-96402694	NM_001024851:2114	Ankrd34a	INSIDE	0.167	0.582	1875.84	1091.15	0.097	1435.18	139.49
A_68_P29223867	chr13:93195722-93195766	NM_175455:-179	Ankrd34b	PROMOTER	0.269	0.357	2515.33	897.35	0.096	2137.69	204.90
A_68_P25833743	chr8:47085442-47085486	NM_001039562:-260	Ankrd37	PROMOTER	0.237	0.688	2837.34	1953.45	0.163	2115.47	345.86
A_68_P25833744	chr8:47085514-47085558	NM_001039562:-332	Ankrd37	PROMOTER	0.162	0.659	6235.37	4107.47	0.107	4090.02	436.65
A_68_P20148960	chr1:36604078-36604122	NM_026241:-54	Ankrd39	PROMOTER	0.437	0.390	1415.38	551.80	0.170	1034.18	176.20
A_68_P28032140	chr11:94189670-94189714	NM_146024:378	Ankrd40	INSIDE	0.563	0.532	4088.05	2175.64	0.299	2843.43	851.43
A_68_P20239573	chr1:54982711-54982755	NM_001081433:499	Ankrd44	INSIDE	0.301	0.566	2291.09	1296.37	0.170	1823.89	310.36
A_68_P20239575	chr1:54982981-54983025	NM_001081433:229	Ankrd44	INSIDE	0.506	0.502	1728.21	868.19	0.254	1361.37	345.90
A_68_P20770261	chr1:163073112-163073156	NM_028664:292	Ankrd45	INSIDE	0.268	0.453	1294.84	586.81	0.121	972.84	118.12
A_68_P20770398	chr1:163101115-163101159	NM_028664:28294	Ankrd45	DOWNSTREAM	0.367	0.706	2741.32	1934.83	0.259	2206.06	571.18
A_68_P30138905	chr15:36426080-36426131	NM_175134:441	Ankrd46	INSIDE	0.249	0.446	1354.38	603.41	0.111	1065.59	118.07
A_68_P22069323	chr3:38354784-38354828	NM_001167883:28932	Ankrd50	INSIDE	0.607	2.767	733.56	2029.72	1.680	686.76	1153.66
A_68_P22069523	chr3:38383943-38383987	NM_001167883:-226	Ankrd50	PROMOTER	0.142	1.541	10968.42	16902.02	0.218	7499.82	1638.58
A_68_P23897548	chr5:93473182-93473226	NM_175270:844	Ankrd56	INSIDE	0.536	0.504	2230.22	1123.66	0.270	1844.26	498.19
A_68_P23897552	chr5:93473776-93473820	NM_175270:250	Ankrd56	INSIDE	0.323	0.413	1290.56	532.86	0.133	1022.51	136.49
A_68_P32321493	chrX:34588727-34588771	NM_173779:-91	Ankrd58	PROMOTER	0.114	1.501	4199.39	6302.27	0.171	1810.12	309.98
A_68_P32321494	chrX:34588841-34588885	NM_173779:23	Ankrd58	INSIDE	0.129	2.083	923.08	1923.07	0.268	435.96	116.80
A_68_P31119198	chr17:28046785-28046829	NM_181413:522	Anks1	INSIDE	0.288	0.366	1475.23	539.44	0.105	1139.79	119.99
A_68_P27333413	chr10:89335981-89336025	NM_001128086:-251	Anks1b	PROMOTER	0.373	0.631	849.39	535.69	0.235	736.94	173.20
A_68_P27334198	chr10:89512597-89512641	NM_001128086:176365	Anks1b	INSIDE	0.652	3.116	369.75	1152.02	2.032	365.58	743.02
A_68_P30512950	chr16:4964504-4964548	NM_028301:-196	Anks3	PROMOTER	0.263	0.484	3576.27	1730.55	0.127	2482.98	316.07
A_68_P30512951	chr16:4964673-4964717	NM_028301:-364	Anks3	PROMOTER	0.449	0.465	1447.41	672.81	0.209	1062.93	221.98
A_68_P26351649	chr9:22193381-22193425	NM_028390:248	Anln	INSIDE	0.581	0.534	2202.16	1176.53	0.311	1832.55	569.23
A_68_P26351650	chr9:22193454-22193498	NM_028390:174	Anln	INSIDE	0.228	0.582	1163.79	677.46	0.132	892.77	118.28
A_68_P26888668	chr9:122203107-122203151	NM_133979:364	Ano10	INSIDE	0.331	0.662	988.54	654.43	0.219	827.93	181.41
A_68_P26888672	chr9:122203588-122203632	NM_133979:-118	Ano10	PROMOTER	0.277	0.602	3002.32	1807.81	0.167	2427.02	404.99
A_68_P26888673	chr9:122203754-122203798	NM_133979:-284	Ano10	PROMOTER	0.326	1.412	6995.73	9875.00	0.460	4283.64	1970.44

ProbeName	Target position of probe on CpG island microarray	TargetID	GeneSymbol	CpG island Description	Ratio of relative methylation (TiO2-NP/Vehicle)	Sham group			TiO_2-H group		
						Relative methylation (Cy-5/Cy-3)	Cy-3 signal (Input)	Cy-5 signal (MeDIP)	Relative methylation (Cy-5/Cy-3)	Cy-3 signal (Input)	Cy-5 signal (MeDIP)
A_68_P25123372	chr7:58766676-58766720	NM_177694:300	Ano5	INSIDE	0.187	0.542	1536.46	832.27	0.101	1188.28	120.37
A_68_P30459963	chr15:95621546-95621590	NM_175344:295	Ano6	INSIDE	0.475	0.516	2300.58	1186.50	0.245	1680.35	411.74
A_68_P20455320	chr1:95297981-95298025	NM_207031:27530	Ano7	INSIDE	0.481	0.652	1115.64	727.50	0.313	903.76	283.23
A_68_P26569949	chr9:62188962-62189006	NM_009672:-165	Anp32a	PROMOTER	0.275	0.481	3395.66	1634.79	0.132	2513.84	332.86
A_68_P26569951	chr9:62189186-62189230	NM_009672:59	Anp32a	INSIDE	0.260	0.687	1240.19	851.51	0.178	844.25	150.60
A_68_P26569953	chr9:62189357-62189414	NM_009672:236	Anp32a	INSIDE	0.131	1.346	3294.51	4435.43	0.176	2266.30	399.76
A_68_P26569954	chr9:62189473-62189517	NM_009672:345	Anp32a	INSIDE	0.124	2.725	6282.22	17120.84	0.338	4433.06	1500.03
A_68_P26569955	chr9:62189553-62189597	NM_009672:425	Anp32a	INSIDE	0.288	2.218	7454.98	16534.36	0.638	4783.60	3051.89
A_68_P26569958	chr9:62189997-62190041	NM_009672:869	Anp32a	INSIDE	0.163	0.550	2442.60	1342.26	0.090	1711.46	153.46
A_68_P26569970	chr9:62191389-62191433	NM_009672:2261	Anp32a	INSIDE	0.256	0.376	1923.03	723.35	0.096	1579.92	151.98
A_68_P26569979	chr9:62192471-62192515	NM_009672:3343	Anp32a	INSIDE	0.183	0.455	1803.17	820.29	0.083	1410.16	117.37
A_68_P22346423	chr3:95733097-95733141	NM_023210:-61	Anp32e	PROMOTER	0.458	0.579	1126.65	652.73	0.265	910.30	241.52
A_68_P22346424	chr3:95733202-95733246	NM_023210:45	Anp32e	INSIDE	0.091	0.587	3302.07	1939.57	0.053	2484.57	132.32
A_68_P22346556	chr3:95755077-95755121	NM_023210:21919	Anp32e	DOWNSTREAM	0.345	0.701	3316.82	2325.71	0.242	2505.96	606.98
A_68_P29480018	chr14:26661840-26661884	NM_013469:222	Anxa11	INSIDE	0.316	0.637	1341.51	853.91	0.201	1069.02	215.21
A_68_P29480020	chr14:26662064-26662110	NM_013469:446	Anxa11	INSIDE	0.329	0.534	1751.87	934.91	0.176	1452.69	255.29
A_68_P22058283	chr3:36374398-36374443	NM_009673:389	Anxa5	INSIDE	0.460	0.527	1028.66	542.16	0.243	753.08	182.64
A_68_P22058284	chr3:36374544-36374588	NM_009673:243	Anxa5	INSIDE	0.124	0.607	2100.48	1274.01	0.075	1531.04	115.44
A_68_P22058285	chr3:36374705-36374749	NM_009673:83	Anxa5	INSIDE	0.356	0.531	2081.16	1105.66	0.189	1468.31	277.93
A_68_P22510975	chr3:127540105-127540149	NM_145964:284	Ap1ar	INSIDE	0.591	0.647	1969.64	1275.09	0.383	1545.85	591.83
A_68_P27560708	chr11:4887125-4887169	NM_007454:239	Ap1b1	INSIDE	0.489	0.697	4765.26	3323.48	0.341	3644.17	1241.86
A_68_P27560709	chr11:4887252-4887296	NM_007454:367	Ap1b1	INSIDE	0.431	0.512	2650.54	1356.04	0.221	1850.24	408.37
A_68_P27560710	chr11:4887330-4887374	NM_007454:445	Ap1b1	INSIDE	0.307	0.582	2095.62	1219.07	0.178	1429.47	255.06
A_68_P25960288	chr8:74763954-74763998	NM_007456:-54	Ap1m1	PROMOTER	0.224	0.419	1594.49	668.04	0.094	1274.85	119.73
A_68_P32795512	chrX:160346535-160346579	NM_026887:-535	Ap1s2	PROMOTER	0.098	1.570	2884.52	4528.49	0.155	1094.51	169.22
A_68_P32795517	chrX:160347213-160347257	NM_026887:143	Ap1s2	INSIDE	0.295	2.033	1837.35	3735.54	0.599	901.22	540.07
A_68_P32795520	chrX:160347620-160347664	NM_026887:551	Ap1s2	INSIDE	0.387	1.410	2493.78	3516.61	0.546	1204.55	657.69
A_68_P20377068	chr1:79668401-79668445	NM_183027:125	Ap1s3	INSIDE	0.523	0.705	2389.03	1683.13	0.369	1803.25	665.03
A_68_P25090207	chr7:52184463-52184510	NM_001077264:374	Ap2a1	INSIDE	0.243	0.639	1123.22	717.46	0.155	829.36	128.78
A_68_P25090210	chr7:52184875-52184919	NM_001077264:-36	Ap2a1	PROMOTER	0.487	0.497	1125.86	559.74	0.242	850.21	205.95
A_68_P25090211	chr7:52184951-52185005	NM_001077264:-118	Ap2a1	PROMOTER	0.254	0.636	1021.77	649.55	0.162	733.63	118.69
A_68_P27970639	chr11:83116144-83116188	NM_001035854:-32	Ap2b1	DIVERGENT_PROMOTER	0.266	0.337	1694.36	570.46	0.090	1348.12	120.78
A_68_P27970640	chr11:83116209-83116256	NM_001035854:34	Ap2b1	INSIDE	0.288	0.506	1137.54	576.09	0.146	795.64	115.97
A_68_P27970642	chr11:83116490-83116534	NM_001035854:314	Ap2b1	INSIDE	0.412	0.613	1542.09	945.94	0.253	1146.43	289.90
A_68_P29234318	chr13:95128761-95128805	NM_009680:-132	Ap3b1	PROMOTER	0.107	0.733	2427.48	1778.62	0.078	1583.23	124.13
A_68_P29234321	chr13:95129057-95129101	NM_009680:164	Ap3b1	INSIDE	0.300	0.707	1455.47	1028.55	0.212	1162.74	246.57
A_68_P29234322	chr13:95129157-95129201	NM_009680:264	Ap3b1	INSIDE	0.331	0.633	2204.00	1395.02	0.210	1681.11	352.45
A_68_P25712955	chr8:23915948-23915992	NM_001122820:156	Ap3m2	INSIDE	0.405	0.626	938.42	587.32	0.253	785.72	199.13
A_68_P21611139	chr2:126834542-126834586	NM_175550:118	Ap4e1	INSIDE	0.387	0.559	2168.56	1211.45	0.216	1837.20	397.00
A_68_P28431152	chr12:52792067-52792111	NM_021710:136	Ap4s1	INSIDE	0.350	0.599	1966.91	1177.34	0.209	1515.36	317.08
A_68_P32019520	chr19:23832652-23832703	NM_177034:-688	Apba1	PROMOTER	0.285	0.633	849.54	537.40	0.180	663.11	119.36
A_68_P23763025	chr5:67011439-67011483	NM_001201413:-1404	Apbb2	PROMOTER	0.180	0.645	5037.93	3247.43	0.116	4030.61	468.32
A_68_P31625260	chr18:36839170-36839214	NM_146085:-172	Apbb3	PROMOTER	0.231	1.575	1739.12	2739.35	0.364	1216.07	442.92
A_68_P29599143	chr14:51544686-51544730	NM_009687:85	Apex1	INSIDE	0.316	0.568	1054.30	599.15	0.180	808.85	145.22

ProbeName	Target position of probe on CpG island microarray	TargetID	GeneSymbol	CpG island Description	Ratio of relative methylation (TiO2-NP/Vehicle)	Sham group			TiO_2-H group		
						Relative methylation (Cy-5/Cy-3)	Cy-3 signal (Input)	Cy-5 signal (MeDIP)	Relative methylation (Cy-5/Cy-3)	Cy-3 signal (Input)	Cy-5 signal (MeDIP)
A_68_P22346233	chr3:95698306-95698355	NM_146134:353	Aph1a	INSIDE	0.236	0.541	1344.79	728.00	0.128	960.83	122.67
A_68_P26595209	chr9:66643358-66643402	NM_177583:-150	Aph1b	PROMOTER	0.226	0.460	1352.03	621.39	0.104	1192.41	123.65
A_68_P26595210	chr9:66643453-66643497	NM_177583:-244	Aph1b	PROMOTER	0.243	0.391	1839.09	719.38	0.095	1408.54	133.93
A_68_P26595212	chr9:66643626-66643670	NM_177583:-418	Aph1b	PROMOTER	0.273	0.575	2855.08	1642.69	0.157	2129.20	334.72
A_68_P26595214	chr9:66643808-66643852	NM_177583:-600	Aph1b	PROMOTER	0.348	0.523	1276.14	666.86	0.182	948.74	172.32
A_68_P26595468	chr9:66682422-66682466	NM_026674:69	Aph1c	INSIDE	0.154	0.471	3924.39	1849.39	0.073	2883.89	209.19
A_68_P26595469	chr9:66682493-66682537	NM_026674:-1	Aph1c	PROMOTER	0.616	0.722	1603.34	1157.12	0.445	1297.80	577.33
A_68_P26595470	chr9:66682593-66682637	NM_026674:-101	Aph1c	PROMOTER	0.524	0.575	3493.70	2008.35	0.301	2816.28	848.41
A_68_P26595471	chr9:66682687-66682731	NM_026674:-195	Aph1c	PROMOTER	0.172	0.609	1452.99	884.84	0.105	1197.01	125.27
A_68_P26595472	chr9:66682802-66682846	NM_026674:-311	Aph1c	PROMOTER	0.554	0.564	2116.14	1193.05	0.312	1592.91	497.13
A_68_P26595474	chr9:66683005-66683049	NM_026674:-513	Aph1c	PROMOTER	0.221	0.571	2018.53	1153.26	0.126	1774.12	223.63
A_68_P26595475	chr9:66683088-66683132	NM_026674:-597	Aph1c	PROMOTER	0.533	0.569	3058.67	1739.29	0.303	2286.37	693.61
A_68_P21441211	chr2:94277500-94277544	NM_007466:782	Api5	INSIDE	0.265	0.417	1244.17	518.83	0.111	1103.43	121.96
A_68_P21481086	chr2:102914077-102914121	NM_019735:267	Apip	INSIDE	0.642	0.564	964.81	544.20	0.362	866.22	313.63
A_68_P26399345	chr9:31019701-31019745	NM_001102455:-322	Aplp2	PROMOTER	0.612	0.534	1052.03	561.48	0.327	829.63	271.20
A_68_P22311543	chr3:87862306-87862350	NM_144897:89	Apoa1bp	INSIDE	0.667	0.475	2288.20	1085.86	0.317	1695.77	537.05
A_68_P30927982	chr16:85173354-85173398	NM_001198823:576	App	INSIDE	0.225	0.442	1681.20	742.41	0.100	1203.18	119.72
A_68_P27981860	chr11:85048235-85048279	NM_025825:366	Appbp2	INSIDE	0.297	0.566	1614.72	914.38	0.168	1238.42	208.60
A_68_P29484376	chr14:27783003-27783055	NM_145221:709	Appl1	INSIDE	0.092	0.665	2691.53	1789.55	0.061	1896.03	116.17
A_68_P29484385	chr14:27783967-27784011	NM_145221:-251	Appl1	PROMOTER	0.425	0.571	1575.58	900.31	0.243	1334.00	323.64
A_68_P26234301	chr8:125100388-125100432	NM_009698:397	Aprt	INSIDE	0.223	0.607	2351.89	1427.00	0.135	1568.82	211.96
A_68_P26234304	chr8:125100653-125100697	NM_009698:133	Aprt	INSIDE	0.115	0.744	4203.02	3125.02	0.086	3182.83	273.05
A_68_P26234306	chr8:125100834-125100878	NM_009698:-49	Aprt	PROMOTER	0.618	2.571	9686.04	24899.64	1.589	6517.89	10354.67
A_68_P26234307	chr8:125100918-125100973	NM_009698:-138	Aprt	PROMOTER	0.049	1.853	2008.98	3722.06	0.091	1303.90	119.25
A_68_P22858339	chr4:40650160-40650206	NM_025545:-233	Aptx	PROMOTER	0.291	0.556	3699.17	2057.41	0.162	2715.18	439.07
A_68_P25352107	chr7:104886338-104886382	NM_175105:397	Aqp11	INSIDE	0.180	0.737	2131.95	1571.00	0.133	1621.23	215.26
A_68_P25352110	chr7:104886718-104886762	NM_175105:17	Aqp11	INSIDE	0.328	0.687	3990.52	2741.02	0.226	3126.19	705.20
A_68_P25352112	chr7:104886923-104886967	NM_175105:-187	Aqp11	PROMOTER	0.268	0.580	1006.01	583.48	0.155	776.88	120.66
A_68_P22860480	chr4:41045173-41045217	NM_016689:22	Aqp3	INSIDE	0.234	0.480	1320.46	633.38	0.112	1077.59	120.80
A_68_P27835624	chr11:59040902-59040946	NM_001130408:740	Arf1	INSIDE	0.511	0.617	2545.03	1569.28	0.315	1884.92	593.33
A_68_P27835626	chr11:59041120-59041164	NM_001130408:522	Arf1	INSIDE	0.313	0.455	1172.79	533.81	0.143	912.01	130.09
A_68_P28088312	chr11:103827831-103827875	NM_007477:-332	Arf2	PROMOTER	0.180	0.522	1991.90	1038.99	0.094	1486.10	139.51
A_68_P30476324	chr15:98593039-98593083	NM_007478:489	Arf3	INSIDE	0.095	1.584	2749.81	4356.37	0.151	1835.59	277.20
A_68_P30476325	chr15:98593156-98593200	NM_007478:371	Arf3	INSIDE	0.434	0.661	4392.11	2903.33	0.287	3220.23	924.08
A_68_P30476329	chr15:98593570-98593614	NM_007478:-43	Arf3	PROMOTER	0.576	0.556	2638.09	1467.56	0.320	2215.02	709.90
A_68_P30476330	chr15:98593656-98593700	NM_007478:-129	Arf3	PROMOTER	0.225	0.595	1091.17	649.54	0.134	882.99	118.50
A_68_P30476331	chr15:98593814-98593858	NM_007478:-287	Arf3	PROMOTER	0.529	0.434	1932.01	839.28	0.230	1535.45	352.93
A_68_P29482769	chr14:27457424-27457468	NM_007479:-236	Arf4	PROMOTER	0.226	0.645	3679.84	2373.45	0.146	2565.68	374.55
A_68_P21907441	chr2:180701664-180701708	NM_001177707:-243	Arfgap1	PROMOTER	0.444	0.549	1007.37	552.64	0.244	750.71	182.98
A_68_P21421623	chr2:91105084-91105140	NM_001166024:-159	Arfgap2	PROMOTER	0.238	0.543	1243.22	674.75	0.129	926.35	119.58
A_68_P21421624	chr2:91105178-91105222	NM_001166024:-71	Arfgap2	PROMOTER	0.370	0.643	1051.41	676.43	0.238	865.43	206.21
A_68_P21421625	chr2:91105340-91105384	NM_001166024:91	Arfgap2	INSIDE	0.495	0.604	1291.45	779.89	0.299	948.79	283.61
A_68_P20026598	chr1:10222127-10222186	NM_001102430:595	Arfgef1	INSIDE	0.143	1.528	1118.22	1708.68	0.218	795.12	173.60
A_68_P20026601	chr1:10222483-10222527	NM_001102430:247	Arfgef1	INSIDE	0.181	0.586	1307.97	766.37	0.106	1126.76	119.19

ProbeName	Target position of probe on CpG island microarray	TargetID	GeneSymbol	CpG island Description	Ratio of relative methylation (TiO2-NP/Vehicle)	Sham group			TiO_2-H group		
						Relative methylation (Cy-5/Cy-3)	Cy-3 signal (Input)	Cy-5 signal (MeDIP)	Relative methylation (Cy-5/Cy-3)	Cy-3 signal (Input)	Cy-5 signal (MeDIP)
A_68_P22291856	chr3:84386605-84386649	NM_001081093:-79	Arfip1	PROMOTER	0.430	0.554	1121.05	621.23	0.238	1009.01	240.25
A_68_P22291860	chr3:84387008-84387052	NM_001081093:-483	Arfip1	PROMOTER	0.448	0.631	1815.87	1146.56	0.283	1516.57	428.80
A_68_P28567750	chr12:80231609-80231653	NM_009705:-144	Arg2	PROMOTER	0.499	0.548	1062.12	582.11	0.273	855.37	233.72
A_68_P21423815	chr2:91490335-91490379	NM_146124:-3008	Arhgap1	PROMOTER	0.433	0.569	991.08	563.92	0.246	809.93	199.52
A_68_P25484114	chr7:130514088-130514135	NM_001122640:-682	Arhgap17	DIVERGENT_PROMOTER	0.129	0.670	1932.37	1294.52	0.086	1376.75	118.92
A_68_P32117276	chr19:41876224-41876268	NM_001163495:328	Arhgap19	INSIDE	0.617	0.495	1654.12	818.59	0.305	1309.43	399.97
A_68_P26511318	chr9:51574321-51574365	NM_175535:886	Arhgap20	INSIDE	0.123	0.651	2394.91	1559.34	0.080	1801.54	143.76
A_68_P21052780	chr2:20889683-20889727	NM_001081364:-356	Arhgap21	DIVERGENT_PROMOTER	0.264	0.377	1771.21	667.53	0.099	1252.59	124.43
A_68_P29523611	chr14:34164707-34164753	NM_153800:134722	Arhgap22	INSIDE	0.115	1.439	1301.16	1872.93	0.166	902.64	149.45
A_68_P28050587	chr11:97302015-97302059	NM_021493:-9437	Arhgap23	PROMOTER	0.197	0.691	2072.10	1432.11	0.136	1536.39	208.76
A_68_P28050588	chr11:97302102-97302146	NM_021493:-9349	Arhgap23	PROMOTER	0.159	0.685	1509.05	1033.98	0.109	1098.69	119.55
A_68_P28050942	chr11:97361786-97361830	NM_021493:50335	Arhgap23	INSIDE	0.542	0.664	2050.85	1360.95	0.359	1674.22	601.87
A_68_P31637164	chr18:39152519-39152563	NM_175164:-258	Arhgap26	PROMOTER	0.410	0.725	1703.48	1234.31	0.297	1219.68	361.91
A_68_P28084858	chr11:103222146-103222190	NM_001205236:2838	Arhgap27	INSIDE	0.603	0.558	1261.80	703.64	0.337	945.53	318.21
A_68_P22479596	chr3:121656435-121656479	NM_172525:213	Arhgap29	INSIDE	0.650	0.600	1067.93	640.26	0.390	917.44	357.48
A_68_P25032018	chr7:31320141-31320185	NM_178252:-134	Arhgap33	PROMOTER	0.276	0.419	1337.10	559.89	0.116	1014.16	117.21
A_68_P32369309	chrX:46847140-46847184	NM_001081123:23536	Arhgap36	INSIDE	0.091	1.831	3852.82	7054.90	0.166	1444.40	240.23
A_68_P30350784	chr15:76648087-76648131	NM_001168288:492	Arhgap39	INSIDE	0.116	5.436	21824.95	118649.00	0.632	14501.69	9161.22
A_68_P30350785	chr15:76648219-76648263	NM_001168288:360	Arhgap39	INSIDE	0.248	1.361	6207.90	8446.78	0.337	4429.15	1492.51
A_68_P25007188	chr7:25689481-25689525	NM_001130152:138	Arhgef1	INSIDE	0.147	0.653	1911.53	1247.54	0.096	1358.90	130.04
A_68_P25677405	chr8:14911386-14911430	NM_001037736:-308	Arhgef10	PROMOTER	0.151	0.581	2206.29	1282.91	0.088	1550.03	136.06
A_68_P25677406	chr8:14911525-14911569	NM_001037736:-170	Arhgef10	PROMOTER	0.480	0.642	1548.94	993.75	0.308	1391.38	428.63
A_68_P25677407	chr8:14911603-14911647	NM_001037736:-92	Arhgef10	PROMOTER	0.514	0.531	2007.66	1065.07	0.273	1585.43	432.17
A_68_P26459683	chr9:42913398-42913453	NM_027144:376	Arhgef12	INSIDE	0.190	0.544	1537.57	835.73	0.103	1139.07	117.86
A_68_P23430517	chr4:153673893-153673937	NM_001112744:90	Arhgef16	INSIDE	0.427	0.712	1902.35	1355.25	0.304	1498.03	456.01
A_68_P23430519	chr4:153674176-153674220	NM_001112744:-194	Arhgef16	PROMOTER	0.275	0.426	3011.20	1283.74	0.117	2347.81	275.15
A_68_P25370485	chr7:108080406-108080454	NM_001081116:245	Arhgef17	INSIDE	0.307	0.324	1617.44	524.46	0.099	1238.33	123.19
A_68_P22314832	chr3:88425175-88425219	NM_001198912:-119	Arhgef2	PROMOTER	0.603	0.440	1225.51	538.80	0.265	984.79	261.27
A_68_P27534513	chr10:126626792-126626847	NM_001166413:60	Arhgef25	INSIDE	0.051	1.389	2672.40	3710.76	0.071	1699.16	120.72
A_68_P27534515	chr10:126627015-126627059	NM_001166413:-157	Arhgef25	PROMOTER	0.660	0.701	1586.51	1112.37	0.463	1074.10	497.23
A_68_P22186536	chr3:62143169-62143213	NM_001081295:492	Arhgef26	INSIDE	0.354	0.261	2043.42	533.31	0.092	1705.32	157.56
A_68_P29486554	chr14:28149566-28149610	NM_027871:98364	Arhgef3	INSIDE	0.344	0.625	1140.57	712.63	0.215	936.30	201.24
A_68_P31758245	chr18:61696023-61696067	NM_177828:146	Arhgef37	INSIDE	0.502	0.570	3424.01	1951.50	0.286	2888.38	826.87
A_68_P29604086	chr14:52604738-52604782	NM_001145921:253	Arhgef40	INSIDE	0.402	0.420	2403.91	1009.44	0.169	1831.28	309.16
A_68_P25657168	chr8:11727352-11727396	NM_001113517:-730	Arhgef7	PROMOTER	0.135	1.363	1967.00	2681.30	0.184	1363.99	250.51
A_68_P25657170	chr8:11727542-11727589	NM_001113517:-539	Arhgef7	PROMOTER	0.147	0.519	2257.04	1170.55	0.076	1612.50	123.06
A_68_P25657171	chr8:11727694-11727738	NM_001113517:-388	Arhgef7	PROMOTER	0.179	0.584	1503.22	877.26	0.104	1135.66	118.52
A_68_P25657379	chr8:11758150-11758194	NM_001113518:-157	Arhgef7	PROMOTER	0.300	0.631	5001.76	3157.28	0.189	3997.93	757.01
A_68_P23319375	chr4:133312530-133312574	NM_001080819:-3026	Arid1a	PROMOTER	0.375	0.432	1290.39	557.28	0.162	1118.56	181.29
A_68_P30462697	chr15:96118805-96118849	NM_175251:874	Arid2	INSIDE	0.613	2.922	759.14	2218.15	1.792	653.35	1170.67
A_68_P27279758	chr10:79389293-79389337	NM_007880:-502	Arid3a	PROMOTER	0.426	0.472	1199.10	565.47	0.201	934.58	187.72
A_68_P26544131	chr9:57684762-57684806	NM_019689:-2743	Arid3b	PROMOTER	0.307	0.536	1084.24	580.89	0.164	755.43	124.08
A_68_P22864000	chr4:41672774-41672818	NM_001017362:5378	Arid3c	INSIDE	0.315	0.641	798.47	511.82	0.202	684.75	138.37
A_68_P22864042	chr4:41678377-41678421	NM_001017362:-224	Arid3c	PROMOTER	0.421	1.491	3127.52	4663.60	0.627	2219.77	1392.00

ProbeName	Target position of probe on CpG island microarray	TargetID	GeneSymbol	CpG island Description	Ratio of relative methylation (TiO2-NP/Vehicle)	Sham group			TiO_2-H group		
						Relative methylation (Cy-5/Cy-3)	Cy-3 signal (Input)	Cy-5 signal (MeDIP)	Relative methylation (Cy-5/Cy-3)	Cy-3 signal (Input)	Cy-5 signal (MeDIP)
A_68_P22864043	chr4:41678560-41678610	NM_001017362:-410	Arid3c	PROMOTER	0.139	2.159	3516.81	7591.05	0.300	2035.89	611.32
A_68_P28523840	chr12:72116565-72116609	NM_001081195:-367	Arid4a	PROMOTER	0.138	0.721	2671.37	1926.18	0.100	2074.18	206.87
A_68_P28524149	chr12:72170902-72170957	NM_001081195:53976	Arid4a	INSIDE	0.637	3.138	1242.63	3899.38	1.997	930.82	1859.19
A_68_P28836955	chr13:14156050-14156094	NM_194262:14	Arid4b	INSIDE	0.228	0.499	1692.12	844.59	0.114	1468.23	166.81
A_68_P28836956	chr13:14156155-14156199	NM_194262:118	Arid4b	INSIDE	0.669	0.690	1919.69	1325.30	0.462	1563.90	722.57
A_68_P20147467	chr1:36364433-36364477	NM_001172205:-123	Arid5a	PROMOTER	0.364	0.592	997.95	590.96	0.215	902.68	194.44
A_68_P20147469	chr1:36364742-36364786	NM_001172205:187	Arid5a	INSIDE	0.179	0.533	1711.95	913.27	0.095	1292.59	123.34
A_68_P20147470	chr1:36364812-36364860	NM_001172205:259	Arid5a	INSIDE	0.178	0.711	1746.68	1241.52	0.127	1260.98	159.54
A_68_P29050148	chr13:54676323-54676367	NM_019968:-29	Arl10	DIVERGENT_PROMOTER	0.219	0.331	2013.17	665.54	0.072	1613.11	116.60
A_68_P29050149	chr13:54676413-54676457	NM_019968:61	Arl10	INSIDE	0.634	0.544	2082.33	1132.56	0.345	1714.75	591.28
A_68_P30811312	chr16:62846773-62846817	NM_026577:72	Arl13b	INSIDE	0.550	0.665	2678.06	1782.18	0.366	2119.09	775.27
A_68_P30811313	chr16:62846874-62846918	NM_026577:-30	Arl13b	DIVERGENT_PROMOTER	0.352	0.563	1751.08	985.69	0.198	1531.81	303.94
A_68_P29342438	chr13:114584476-114584520	NM_172595:-217	Arl15	PROMOTER	0.541	0.581	1224.91	711.98	0.315	961.95	302.75
A_68_P29342440	chr13:114584633-114584677	NM_172595:-61	Arl15	PROMOTER	0.240	0.709	2740.09	1942.55	0.170	1994.72	339.44
A_68_P28184735	chr11:120328666-120328710	NM_197995:226	Arl16	INSIDE	0.599	0.697	1341.45	935.31	0.418	1020.53	426.48
A_68_P26078867	chr8:97190919-97190966	NM_024269:-63	Arl2bp	PROMOTER	0.215	0.562	2225.49	1250.55	0.121	1533.88	185.36
A_68_P28075090	chr11:101526871-101526915	NM_025404:38	Arl4d	INSIDE	0.194	0.626	2054.59	1287.08	0.121	1449.01	175.80
A_68_P21219427	chr2:52279977-52280021	NM_182994:396	Arl5a	INSIDE	0.192	0.580	1441.71	836.71	0.111	1059.03	118.00
A_68_P24059367	chr5:124566387-124566431	NM_144509:292	Arl6ip4	INSIDE	0.344	0.407	1975.84	804.76	0.140	1547.73	217.04
A_68_P21224226	chr2:53051139-53051183	NM_022989:43	Arl6ip6	INSIDE	0.534	0.426	1242.49	529.75	0.228	1089.20	248.21
A_68_P20637766	chr1:137043169-137043217	NM_026823:-218	Arl8a	PROMOTER	0.643	0.668	1332.89	889.74	0.429	1043.44	447.63
A_68_P24735710	chr6:108733170-108733214	NM_026011:140	Arl8b	INSIDE	0.646	0.535	1523.38	815.54	0.346	1278.76	442.11
A_68_P25509652	chr7:135381090-135381137	NM_146205:243	Armc5	INSIDE	0.206	0.625	1325.94	828.76	0.129	959.78	123.47
A_68_P26764913	chr9:99469012-99469056	NM_028768:284	Armc8	INSIDE	0.235	0.503	1708.02	858.31	0.118	1403.53	165.42
A_68_P26764915	chr9:99469261-99469305	NM_028768:36	Armc8	INSIDE	0.591	0.507	1275.98	647.07	0.300	944.09	283.19
A_68_P32673102	chrX:131220969-131221013	NM_001202500:-6872	Armcx4	PROMOTER	0.157	1.696	2755.68	4674.73	0.267	1102.55	294.43
A_68_P25288544	chr7:91558102-91558146	NM_007488:345	Arnt2	INSIDE	0.228	0.557	1228.88	683.88	0.127	943.46	119.77
A_68_P25429183	chr7:120351014-120351062	NM_007489:60	Arntl	INSIDE	0.120	0.613	2021.53	1239.23	0.073	1635.69	120.17
A_68_P25429189	chr7:120351704-120351748	NM_007489:748	Arntl	INSIDE	0.292	0.529	1000.70	529.57	0.155	829.35	128.15
A_68_P25429192	chr7:120352049-120352095	NM_007489:1094	Arntl	INSIDE	0.316	0.677	1310.12	886.85	0.214	1060.90	226.75
A_68_P24050077	chr5:122841748-122841803	NM_019824:-161	Arpc3	PROMOTER	0.266	0.480	1637.78	786.52	0.128	1173.02	150.12
A_68_P24050080	chr5:122842069-122842113	NM_019824:154	Arpc3	INSIDE	0.400	0.718	4394.64	3154.81	0.287	3316.23	952.58
A_68_P24759116	chr6:113328203-113328247	NM_001170485:118	Arpc4	INSIDE	0.596	0.626	924.00	578.23	0.373	765.03	285.30
A_68_P21151655	chr2:38863832-38863876	NM_028809:196	Arpc5l	INSIDE	0.390	0.589	4486.65	2640.44	0.230	3453.30	793.23
A_68_P22029171	chr3:30494182-30494226	NM_029690:4588	Arpm1	DOWNSTREAM	0.438	0.628	1590.38	999.46	0.275	1188.76	327.46
A_68_P26834222	chr9:112137879-112137923	NM_001177619:-555	Arpp21	PROMOTER	0.487	0.497	1421.06	705.74	0.242	1267.75	306.45
A_68_P27900650	chr11:70245838-70245882	NM_145429:-294	Arrb2	PROMOTER	0.265	0.590	1112.28	656.73	0.156	1005.28	157.11
A_68_P27900654	chr11:70246223-70246267	NM_145429:90	Arrb2	INSIDE	0.260	0.482	1798.49	867.27	0.125	1423.92	178.46
A_68_P25954402	chr8:73362864-73362908	NM_027560:733	Arrdc2	INSIDE	0.353	0.510	1134.57	578.25	0.180	861.51	155.06
A_68_P25954403	chr8:73362971-73363015	NM_027560:627	Arrdc2	INSIDE	0.518	0.524	1499.61	785.72	0.272	1276.75	346.77
A_68_P25954407	chr8:73363491-73363535	NM_027560:107	Arrdc2	INSIDE	0.431	0.629	4802.10	3020.09	0.271	3083.84	836.07
A_68_P25954408	chr8:73363586-73363630	NM_027560:11	Arrdc2	INSIDE	0.456	0.684	1297.47	887.59	0.312	956.10	298.18
A_68_P23893979	chr5:92821310-92821354	NM_181728:60466	Art3	INSIDE	0.602	2.868	1474.08	4228.29	1.727	1177.63	2033.79
A_68_P23237356	chr4:117599653-117599697	NM_009711:2694	Artn	INSIDE	0.120	0.681	2787.19	1896.90	0.082	2092.35	171.28

ProbeName	Target position of probe on CpG island microarray	TargetID	GeneSymbol	CpG island Description	Ratio of relative methylation (TiO2-NP/Vehicle)	Sham group			TiO_2-H group		
						Relative methylation (Cy-5/Cy-3)	Cy-3 signal (Input)	Cy-5 signal (MeDIP)	Relative methylation (Cy-5/Cy-3)	Cy-3 signal (Input)	Cy-5 signal (MeDIP)
A_68_P23237358	chr4:117599846-117599890	NM_009711:2500	Artn	INSIDE	0.359	0.564	1056.45	596.23	0.202	882.24	178.51
A_68_P23237382	chr4:117602465-117602509	NM_009711:-118	Artn	PROMOTER	0.581	0.578	927.52	535.73	0.336	760.39	255.25
A_68_P30579427	chr16:18392999-18393043	NM_033474:44654	Arvcf	INSIDE	0.588	0.577	1495.70	862.72	0.339	1055.92	357.93
A_68_P25810705	chr8:42459691-42459735	NM_019734:339	Asah1	INSIDE	0.139	0.561	2521.91	1414.34	0.078	2001.07	155.57
A_68_P25810709	chr8:42460188-42460232	NM_019734:-159	Asah1	PROMOTER	0.221	0.564	2228.79	1257.40	0.124	1636.78	203.73
A_68_P30281417	chr15:64214477-64214523	NM_010026:-19	Asap1	PROMOTER	0.263	0.537	1075.83	577.34	0.141	854.13	120.74
A_68_P33008888	chr12:21117412-21117456	NM_001004364:-182	Asap2	PROMOTER	0.495	0.624	2182.03	1360.53	0.309	1852.20	572.08
A_68_P23332631	chr4:135762030-135762074	NM_001008232:-227	Asap3	PROMOTER	0.350	1.625	4946.21	8037.03	0.568	3366.45	1912.69
A_68_P23332632	chr4:135762182-135762226	NM_001008232:-75	Asap3	PROMOTER	0.543	2.821	18055.88	50943.65	1.533	12634.63	19363.60
A_68_P23332634	chr4:135762412-135762456	NM_001008232:155	Asap3	INSIDE	0.445	1.456	5422.86	7897.83	0.649	3741.92	2426.99
A_68_P23332636	chr4:135762606-135762650	NM_001008232:349	Asap3	INSIDE	0.378	0.542	1377.48	746.30	0.205	1042.38	213.43
A_68_P20443149	chr1:93437651-93437695	NM_023046:16	Asb1	INSIDE	0.341	0.604	982.35	593.63	0.206	826.95	170.48
A_68_P27129524	chr10:50312341-50312389	NM_001146089:-110	Ascc3	PROMOTER	0.245	0.369	1787.00	659.68	0.091	1316.22	119.18
A_68_P27129528	chr10:50312762-50312806	NM_001146089:310	Ascc3	INSIDE	0.402	0.639	3730.88	2382.81	0.257	3140.60	806.64
A_68_P27320001	chr10:86955807-86955851	NM_008553:577	Ascl1	INSIDE	0.604	0.595	2074.22	1235.00	0.359	1735.27	623.81
A_68_P25597927	chr7:150153952-150153996	NM_008554:1195	Ascl2	INSIDE	0.601	0.661	3359.94	2222.43	0.397	2505.57	995.60
A_68_P27143033	chr10:53316773-53316825	NM_025541:32	Asf1a	INSIDE	0.325	0.576	1402.87	808.05	0.187	1079.97	202.03
A_68_P26017291	chr8:86479163-86479209	NM_024184:-406	Asf1b	PROMOTER	0.463	0.562	1330.21	747.39	0.260	1026.62	267.35
A_68_P24222134	chr6:7643189-7643233	NM_012055:-28	Asns	PROMOTER	0.396	0.542	2387.82	1293.90	0.215	2052.41	440.34
A_68_P26797104	chr9:105297728-105297772	NM_001164828:-5367	Aste1	PROMOTER	0.326	0.267	2822.70	753.01	0.087	2039.80	177.66
A_68_P22987051	chr4:66064069-66064114	NM_019514:1426	Astn2	INSIDE	0.334	0.582	1421.91	827.13	0.194	1118.36	217.36
A_68_P21754296	chr2:153171121-153171165	NM_001039939:-732	Asxl1	PROMOTER	0.280	0.543	1780.67	966.24	0.152	1397.73	212.40
A_68_P21754298	chr2:153171422-153171466	NM_001039939:-430	Asxl1	PROMOTER	0.279	0.595	1114.15	663.06	0.166	835.84	138.76
A_68_P28195046	chr12:3427021-3427065	NM_172421:159	Asxl2	INSIDE	0.273	0.518	1125.85	583.05	0.141	956.58	135.04
A_68_P28195050	chr12:3427455-3427499	NM_172421:593	Asxl2	INSIDE	0.432	0.652	2722.75	1775.44	0.282	1964.95	554.15
A_68_P28195051	chr12:3427564-3427608	NM_172421:703	Asxl2	INSIDE	0.528	0.694	1675.94	1162.81	0.367	1311.65	480.87
A_68_P31549440	chr18:22503476-22503523	NM_001167777:-90	Asxl3	PROMOTER	0.227	1.545	1348.28	2083.47	0.350	980.71	343.59
A_68_P30246252	chr15:57966703-57966747	NM_027435:-87	Atad2	DIVERGENT_PROMOTER	0.273	0.396	1933.52	766.20	0.108	1613.12	174.67
A_68_P25522113	chr7:137663240-137663284	NM_013799:-209	Ate1	PROMOTER	0.373	0.399	1322.65	527.16	0.149	1146.31	170.55
A_68_P25522114	chr7:137663333-137663377	NM_013799:-301	Ate1	PROMOTER	0.357	0.465	1593.89	741.10	0.166	1271.71	211.32
A_68_P25522115	chr7:137663415-137663459	NM_013799:-383	Ate1	PROMOTER	0.373	0.500	2341.57	1170.99	0.186	1742.52	324.88
A_68_P21334155	chr2:73729976-73730020	NM_001025093:687	Atf2	INSIDE	0.149	1.442	3688.30	5319.20	0.216	2553.75	550.51
A_68_P21334161	chr2:73730794-73730838	NM_001025093:-131	Atf2	PROMOTER	0.621	0.592	1565.67	927.41	0.368	1036.41	381.19
A_68_P21334162	chr2:73730864-73730908	NM_001025093:-201	Atf2	PROMOTER	0.329	0.595	1886.52	1122.96	0.196	1416.68	277.07
A_68_P21334163	chr2:73730945-73730989	NM_001025093:-281	Atf2	PROMOTER	0.288	0.498	1166.27	581.04	0.143	875.44	125.49
A_68_P20936657	chr1:193006272-193006316	NM_007498:918	Atf3	INSIDE	0.290	0.486	1095.96	532.55	0.141	829.74	116.77
A_68_P20936658	chr1:193006394-193006440	NM_007498:796	Atf3	INSIDE	0.262	0.363	1570.51	570.60	0.095	1266.87	120.50
A_68_P20936667	chr1:193007287-193007331	NM_007498:-96	Atf3	PROMOTER	0.325	0.558	3284.36	1832.38	0.182	2456.72	446.10
A_68_P20936670	chr1:193007730-193007774	NM_007498:-540	Atf3	PROMOTER	0.121	0.524	2412.38	1263.17	0.064	1813.19	115.16
A_68_P30371083	chr15:80085473-80085517	NM_009716:-119	Atf4	PROMOTER	0.543	0.632	913.43	577.46	0.343	744.56	255.46
A_68_P30371086	chr15:80085860-80085904	NM_009716:269	Atf4	INSIDE	0.143	0.739	6418.96	4741.92	0.106	4567.50	482.91
A_68_P30371088	chr15:80086012-80086056	NM_009716:421	Atf4	INSIDE	0.232	0.672	1019.51	685.55	0.156	773.57	120.87
A_68_P30498878	chr15:102455997-102456041	NM_146065:-166	Atf7	PROMOTER	0.282	0.534	1176.12	627.93	0.151	1056.04	159.25
A_68_P30498879	chr15:102456094-102456138	NM_146065:-264	Atf7	PROMOTER	0.209	0.676	1260.49	851.60	0.141	942.59	133.37

ProbeName	Target position of probe on CpG island microarray	TargetID	GeneSymbol	CpG island Description	Ratio of relative methylation (TiO2-NP/Vehicle)	Sham group			TiO_2-H group		
						Relative methylation (Cy-5/Cy-3)	Cy-3 signal (Input)	Cy-5 signal (MeDIP)	Relative methylation (Cy-5/Cy-3)	Cy-3 signal (Input)	Cy-5 signal (MeDIP)
A_68_P30541084	chr16:10193396-10193440	NM_029253:420	Atf7ip2	INSIDE	0.398	0.608	995.72	605.68	0.242	813.64	197.17
A_68_P21424191	chr2:91550574-91550618	NM_145528:137	Atg13	INSIDE	0.178	0.473	1858.80	878.54	0.084	1387.62	116.93
A_68_P31933382	chr19:6241388-6241441	NM_194348:-253	Atg2a	DIVERGENT_PROMOTER	0.522	0.641	1685.53	1081.01	0.335	1362.54	456.31
A_68_P28710685	chr12:106923369-106923413	NM_029654:61	Atg2b	INSIDE	0.464	0.489	1650.17	807.24	0.227	1234.84	280.45
A_68_P30725228	chr16:45159441-45159485	NM_026402:521	Atg3	INSIDE	0.516	0.405	1313.07	532.40	0.209	1403.01	293.47
A_68_P27104595	chr10:43988364-43988408	NM_053069:223	Atg5	INSIDE	0.393	0.648	1331.05	862.37	0.255	1161.90	295.86
A_68_P24766251	chr6:114593227-114593277	NM_028835:110	Atg7	INSIDE	0.190	0.532	1515.19	805.42	0.101	1194.24	120.70
A_68_P24766252	chr6:114593333-114593377	NM_028835:212	Atg7	INSIDE	0.361	0.552	1095.39	605.07	0.200	843.59	168.36
A_68_P20331601	chr1:71603829-71603873	NM_026195:121	Atic	INSIDE	0.492	0.572	1087.42	622.15	0.282	942.67	265.52
A_68_P31380046	chr17:80294471-80294516	NM_019717:875	Atl2	INSIDE	0.256	0.423	1900.27	804.48	0.108	1656.10	179.57
A_68_P31380056	chr17:80295692-80295741	NM_178050:-253	Atl2	PROMOTER	0.249	0.670	1582.53	1060.85	0.167	1146.29	190.96
A_68_P26521201	chr9:53344702-53344746	NM_007499:52	Atm	INSIDE	0.371	0.563	1806.66	1017.29	0.209	1388.30	289.78
A_68_P26521203	chr9:53344856-53344900	NM_007499:-102	Atm	DIVERGENT_PROMOTER	0.523	0.480	1954.94	938.10	0.251	1532.71	384.40
A_68_P26199828	chr8:119467966-119468010	NM_177700:696	Atmin	INSIDE	0.286	0.586	1366.95	801.42	0.168	1053.01	176.42
A_68_P24816772	chr6:124696114-124696158	NM_007881:10369	Atn1	INSIDE	0.416	0.313	1682.71	526.81	0.130	1384.15	180.22
A_68_P24540589	chr6:72184688-72184732	NM_153778:861	Atoh8	INSIDE	0.344	0.409	3018.72	1233.51	0.140	2370.38	333.00
A_68_P24540591	chr6:72184914-72184958	NM_153778:635	Atoh8	INSIDE	0.657	0.687	2346.58	1611.95	0.451	1756.77	792.80
A_68_P27815711	chr11:55274535-55274579	NM_009720:84	Atox1	INSIDE	0.573	0.421	1520.34	640.64	0.241	1224.27	295.63
A_68_P22055030	chr3:35653187-35653231	NM_029570:149	Atp11b	INSIDE	0.335	0.485	2517.63	1220.74	0.163	1939.55	315.50
A_68_P30641650	chr16:30388204-30388248	NM_001128094:390	Atp13a3	INSIDE	0.453	0.669	2570.18	1719.18	0.303	1876.88	568.63
A_68_P30641651	chr16:30388272-30388316	NM_001128094:322	Atp13a3	INSIDE	0.161	0.634	1567.10	994.05	0.102	1204.28	122.65
A_68_P22376200	chr3:101408960-101409004	NM_144900:-402	Atp1a1	PROMOTER	0.131	0.564	2175.68	1226.08	0.074	1686.48	124.14
A_68_P25007799	chr7:25790496-25790540	NM_144921:396	Atp1a3	INSIDE	0.249	0.699	4545.61	3177.14	0.174	3043.34	530.46
A_68_P25007800	chr7:25790566-25790610	NM_144921:326	Atp1a3	INSIDE	0.209	0.658	5992.61	3945.69	0.138	4462.89	613.87
A_68_P25007802	chr7:25790777-25790821	NM_144921:116	Atp1a3	INSIDE	0.325	0.643	1282.34	824.20	0.209	1114.82	232.54
A_68_P27896074	chr11:69419192-69419236	NM_013415:248	Atp1b2	INSIDE	0.233	0.617	1655.94	1021.30	0.144	1303.26	187.19
A_68_P24050685	chr5:122952811-122952855	NM_009722:-598	Atp2a2	PROMOTER	0.354	0.571	2296.54	1311.45	0.202	1599.57	323.01
A_68_P27383830	chr10:98378401-98378445	NM_026482:637	Atp2b1	INSIDE	0.349	0.489	1129.78	552.80	0.171	981.17	167.42
A_68_P27383831	chr10:98378471-98378515	NM_026482:707	Atp2b1	INSIDE	0.198	0.586	1396.74	818.14	0.116	1044.50	121.02
A_68_P24763305	chr6:113991563-113991607	NM_009723:490	Atp2b2	INSIDE	0.178	2.059	2370.00	4879.63	0.366	1373.47	502.65
A_68_P26797752	chr9:105396915-105396959	NM_175025:511	Atp2c1	INSIDE	0.268	0.689	1785.32	1230.42	0.184	1314.36	242.36
A_68_P26797759	chr9:105397652-105397696	NM_175025:-227	Atp2c1	PROMOTER	0.302	0.667	2789.01	1860.65	0.202	2145.78	432.40
A_68_P31849681	chr18:78012952-78012997	NM_007505:468	Atp5a1	INSIDE	0.631	0.550	2082.15	1144.89	0.347	1835.81	636.47
A_68_P31849682	chr18:78013054-78013098	NM_007505:570	Atp5a1	INSIDE	0.530	0.666	6049.71	4029.27	0.353	4937.87	1742.18
A_68_P27281231	chr10:79604257-79604301	NM_025313:-781	Atp5d	DIVERGENT_PROMOTER	0.212	0.398	2124.86	845.47	0.084	1462.53	123.55
A_68_P30499085	chr15:102501421-102501465	NM_026468:36	Atp5g2	INSIDE	0.360	0.503	1158.87	583.29	0.181	975.92	176.77
A_68_P28153350	chr11:115274720-115274764	NM_027862:6491	Atp5h	DOWNSTREAM	0.626	0.324	1681.50	544.65	0.203	1313.19	266.33
A_68_P23971890	chr5:108863226-108863271	NM_007507:149	Atp5k	INSIDE	0.332	1.509	3721.60	5614.14	0.501	2764.16	1385.19
A_68_P23971891	chr5:108863299-108863343	NM_007507:77	Atp5k	INSIDE	0.257	0.700	3174.55	2223.18	0.180	2375.54	427.66
A_68_P23971892	chr5:108863393-108863437	NM_007507:-17	Atp5k	PROMOTER	0.219	0.542	1340.39	726.27	0.119	982.53	116.49
A_68_P32262593	chrX:12164837-12164881	NM_027439:-26	Atp6ap2	PROMOTER	0.212	1.548	1788.61	2768.02	0.328	654.60	214.96
A_68_P32262594	chrX:12164926-12164970	NM_027439:64	Atp6ap2	INSIDE	0.083	1.768	3157.66	5582.75	0.147	1146.07	168.05
A_68_P23237065	chr4:117559612-117559656	NM_033617:300	Atp6v0b	INSIDE	0.213	0.361	1985.78	716.44	0.077	1563.17	120.25
A_68_P23237067	chr4:117559785-117559829	NM_033617:128	Atp6v0b	INSIDE	0.073	0.749	4477.31	3352.79	0.055	2996.22	163.37

ProbeName	Target position of probe on CpG island microarray	TargetID	GeneSymbol	CpG island Description	Ratio of relative methylation (TiO2-NP/Vehicle)	Sham group			TiO_2-H group		
						Relative methylation (Cy-5/Cy-3)	Cy-3 signal (Input)	Cy-5 signal (MeDIP)	Relative methylation (Cy-5/Cy-3)	Cy-3 signal (Input)	Cy-5 signal (MeDIP)
A_68_P23237068	chr4:117559980-117560024	NM_033617:-68	Atp6v0b	PROMOTER	0.561	0.520	3287.93	1710.73	0.292	2579.57	752.50
A_68_P31095793	chr17:24306024-24306068	NR_037854:294	Atp6v0c-ps2	INSIDE	0.204	1.458	5335.23	7779.95	0.298	3518.06	1048.64
A_68_P31095796	chr17:24306345-24306389	NR_037854:-26	Atp6v0c-ps2	PROMOTER	0.426	0.579	2335.57	1352.31	0.247	1816.59	447.87
A_68_P26134606	chr8:108089561-108089605	NM_013477:358	Atp6v0d1	INSIDE	0.649	0.568	2506.60	1424.01	0.369	1834.28	676.18
A_68_P26134610	chr8:108089960-108090004	NM_013477:-42	Atp6v0d1	PROMOTER	0.190	0.562	1340.84	752.90	0.107	1105.59	117.94
A_68_P26134614	chr8:108090464-108090513	NM_013477:-548	Atp6v0d1	PROMOTER	0.056	1.824	1603.00	2924.67	0.103	1152.37	118.29
A_68_P31111771	chr17:26813342-26813386	NM_025272:24	Atp6v0e	INSIDE	0.638	0.477	1213.59	578.99	0.304	925.51	281.80
A_68_P31111772	chr17:26813424-26813468	NM_025272:106	Atp6v0e	INSIDE	0.351	0.352	2011.81	708.90	0.124	1616.33	200.07
A_68_P31111775	chr17:26813633-26813677	NM_025272:314	Atp6v0e	INSIDE	0.110	0.490	2647.43	1298.45	0.054	2132.37	114.84
A_68_P30150545	chr15:38591758-38591802	NM_025494:122	Atp6v1c1	INSIDE	0.620	0.584	1683.06	983.12	0.362	1387.33	502.20
A_68_P30150546	chr15:38591825-38591869	NM_025494:188	Atp6v1c1	INSIDE	0.451	0.473	1277.36	604.40	0.213	1026.16	218.75
A_68_P28566337	chr12:79962384-79962437	NM_023721:215	Atp6v1d	INSIDE	0.454	0.573	1049.72	601.31	0.260	785.99	204.55
A_68_P24800464	chr6:120772369-120772413	NM_007510:313	Atp6v1e1	INSIDE	0.505	0.573	2045.06	1172.60	0.289	1552.76	449.45
A_68_P31158465	chr17:35373569-35373613	NM_023179:50	Atp6v1g2	INSIDE	0.484	0.727	2241.25	1630.25	0.352	1591.45	560.35
A_68_P31776698	chr18:64819777-64819821	NM_001001488:856	Atp8b1	INSIDE	0.627	2.636	1212.06	3194.80	1.652	1067.03	1762.54
A_68_P31776699	chr18:64819875-64819919	NM_001001488:758	Atp8b1	INSIDE	0.416	0.645	1010.68	651.63	0.268	849.92	228.17
A_68_P31776706	chr18:64820510-64820554	NM_001001488:122	Atp8b1	INSIDE	0.506	0.644	1484.65	956.16	0.326	1323.99	431.07
A_68_P22322690	chr3:89767430-89767474	NM_001081182:-22	Atp8b2	PROMOTER	0.161	0.673	2283.87	1538.14	0.109	1457.85	158.39
A_68_P23312749	chr4:132089123-132089167	NM_007512:430	Atpif1	INSIDE	0.252	0.668	1867.63	1247.15	0.169	1405.01	236.85
A_68_P26818135	chr9:108976311-108976355	NM_172774:306	Atrip	INSIDE	0.237	0.444	1435.34	637.57	0.105	1131.70	119.01
A_68_P26818136	chr9:108976442-108976486	NM_172774:174	Atrip	INSIDE	0.288	0.607	3567.99	2164.71	0.175	2549.16	444.92
A_68_P26818137	chr9:108976539-108976583	NM_172774:78	Atrip	INSIDE	0.275	0.644	4735.75	3049.07	0.177	3476.43	615.89
A_68_P26818138	chr9:108976646-108976690	NM_172774:-30	Atrip	PROMOTER	0.201	0.688	2069.91	1423.61	0.139	1605.61	222.50
A_68_P26818139	chr9:108976760-108976804	NM_172774:-144	Atrip	PROMOTER	0.214	0.492	1577.61	775.75	0.105	1148.62	120.80
A_68_P21632199	chr2:130732765-130732810	NM_009730:556	Atrn	INSIDE	0.178	0.698	1194.82	834.42	0.125	938.74	116.92
A_68_P32208210	chr19:57684728-57684772	NM_181415:-773	Atrnl1	PROMOTER	0.225	0.598	1168.08	698.32	0.135	866.06	116.62
A_68_P32582138	chrX:103124420-103124464	NM_009530:269	Atrx	INSIDE	0.146	1.403	1563.93	2193.88	0.204	737.55	150.59
A_68_P29005200	chr13:46059987-46060031	NM_009124:337	Atxn1	INSIDE	0.321	0.681	2019.49	1376.26	0.219	1592.59	348.06
A_68_P29005206	chr13:46060896-46060940	NM_009124:-573	Atxn1	PROMOTER	0.289	0.533	1901.12	1013.24	0.154	1466.43	226.22
A_68_P26158730	chr8:112261101-112261147	NM_001080930:515	Atxn1l	INSIDE	0.172	1.711	788.99	1349.74	0.295	543.84	160.18
A_68_P24046036	chr5:122161287-122161331	NM_009125:-309	Atxn2	PROMOTER	0.409	0.670	2342.01	1570.29	0.274	1840.76	505.08
A_68_P25500900	chr7:133645875-133645919	NM_183020:920	Atxn2l	INSIDE	0.343	1.484	4817.28	7147.49	0.509	3484.37	1773.35
A_68_P25500902	chr7:133646038-133646082	NM_183020:756	Atxn2l	INSIDE	0.274	0.469	1481.07	695.32	0.128	1165.36	149.66
A_68_P22411287	chr3:108013581-108013625	NM_175183:250	Atxn7l2	INSIDE	0.207	0.553	1489.46	824.29	0.115	1006.84	115.32
A_68_P28078752	chr11:102158739-102158784	NM_001098836:-818	Atxn7l3	PROMOTER	0.309	0.699	1648.75	1152.08	0.216	1189.68	256.68
A_68_P32536866	chrX:91226350-91226409	NR_028320:12385	AU015836	DOWNSTREAM	0.587	3.387	761.39	2578.65	1.989	311.25	618.96
A_68_P23281852	chr4:126430875-126430919	NM_133886:-117	AU040320	PROMOTER	0.482	0.570	2215.34	1261.74	0.275	1570.02	431.24
A_68_P23281853	chr4:126430986-126431030	NM_133886:-5	AU040320	PROMOTER	0.342	0.470	1829.05	859.01	0.161	1333.78	214.08
A_68_P27291405	chr10:81590620-81590664	NM_001163064:-115	AU041133	PROMOTER	0.265	0.641	887.31	568.78	0.170	709.54	120.48
A_68_P23440848	chr4:155205726-155205770	NM_025338:-6	Aurkaip1	PROMOTER	0.248	0.382	1957.60	747.37	0.095	1497.45	141.64
A_68_P23440850	chr4:155205944-155205991	NM_025338:213	Aurkaip1	INSIDE	0.190	0.514	1693.57	870.12	0.097	1242.51	121.00
A_68_P27892698	chr11:68853700-68853744	NM_011496:-5422	Aurkb	PROMOTER	0.441	0.655	3670.83	2403.87	0.289	2532.99	730.83
A_68_P27892699	chr11:68853816-68853860	NM_011496:-5306	Aurkb	PROMOTER	0.238	0.632	1137.73	718.78	0.150	817.33	122.66
A_68_P27892735	chr11:68859244-68859288	NM_011496:122	Aurkb	INSIDE	0.171	0.425	3872.89	1647.01	0.073	2824.15	205.58

ProbeName	Target position of probe on CpG island microarray	TargetID	GeneSymbol	CpG island Description	Ratio of relative methylation (TiO2-NP/Vehicle)	Sham group			TiO_2-H group		
						Relative methylation (Cy-5/Cy-3)	Cy-3 signal (Input)	Cy-5 signal (MeDIP)	Relative methylation (Cy-5/Cy-3)	Cy-3 signal (Input)	Cy-5 signal (MeDIP)
A_68_P27892738	chr11:68859556-68859600	NM_011496:434	Aurkb	INSIDE	0.167	0.703	1338.53	941.36	0.117	999.22	117.24
A_68_P27892739	chr11:68859626-68859680	NM_011496:508	Aurkb	INSIDE	0.131	0.673	1749.18	1176.88	0.088	1324.96	116.93
A_68_P24098745	chr5:132014699-132014743	NM_177047:1003493	Auts2	INSIDE	0.640	0.421	1656.56	697.87	0.270	1591.66	429.26
A_68_P24104837	chr5:133017392-133017444	NM_177047:795	Auts2	INSIDE	0.098	1.484	1154.17	1712.27	0.145	835.43	121.01
A_68_P24104838	chr5:133017511-133017555	NM_177047:681	Auts2	INSIDE	0.466	0.677	1020.42	690.35	0.315	838.88	264.39
A_68_P24104846	chr5:133018497-133018541	NM_177047:-305	Auts2	PROMOTER	0.353	0.448	2293.45	1028.53	0.158	1553.00	245.62
A_68_P24104848	chr5:133018792-133018836	NM_177047:-601	Auts2	PROMOTER	0.347	0.585	1578.68	923.29	0.203	1180.31	239.25
A_68_P24104854	chr5:133019778-133019822	NM_177047:-1587	Auts2	PROMOTER	0.377	0.685	3249.49	2225.28	0.258	2618.90	676.35
A_68_P21630551	chr2:130406332-130406390	NM_009732:1917	Avp	DOWNSTREAM	0.178	1.730	1320.96	2285.70	0.308	911.32	280.29
A_68_P21630552	chr2:130406411-130406455	NM_009732:1845	Avp	INSIDE	0.170	0.685	1598.44	1095.20	0.116	1039.70	120.99
A_68_P27508736	chr10:121886005-121886049	NM_016847:472	Avpr1a	INSIDE	0.253	0.633	4461.36	2822.08	0.160	3436.78	549.54
A_68_P27508738	chr10:121886239-121886283	NM_016847:706	Avpr1a	INSIDE	0.386	0.565	1135.31	641.21	0.218	868.95	189.68
A_68_P29977061	chr15:3945633-3945677	NM_145930:98	AW549877	INSIDE	0.508	0.642	2560.84	1643.83	0.326	2170.23	708.28
A_68_P31108555	chr17:26275483-26275527	NM_001159598:-126	Axin1	PROMOTER	0.259	0.549	2182.74	1197.45	0.142	1680.47	238.80
A_68_P31108560	chr17:26276183-26276227	NM_001159598:574	Axin1	INSIDE	0.435	1.451	8023.27	11640.05	0.632	5667.44	3580.60
A_68_P28115679	chr11:108781544-108781588	NM_015732:-96	Axin2	PROMOTER	0.572	0.627	1909.56	1196.62	0.358	1757.95	630.21
A_68_P28115682	chr11:108781991-108782035	NM_015732:350	Axin2	INSIDE	0.518	0.453	2557.17	1159.25	0.235	2215.46	520.00
A_68_P28115684	chr11:108782304-108782348	NM_015732:664	Axin2	INSIDE	0.601	0.494	3204.51	1581.72	0.297	2628.61	779.86
A_68_P30149944	chr15:38448175-38448219	NM_001102458:825	Azin1	INSIDE	0.547	0.721	6662.80	4806.06	0.394	5041.57	1987.79
A_68_P25588372	chr7:148547330-148547384	NR_033532:3653	B230206H07Rik	INSIDE	0.367	4.016	965.36	3877.05	1.473	734.15	1081.55
A_68_P25588374	chr7:148547481-148547530	NR_033532:3504	B230206H07Rik	INSIDE	0.129	1.759	3300.83	5805.26	0.227	2256.96	512.73
A_68_P26948226	chr10:12642736-12642780	NR_038027:167	B230208H11Rik	INSIDE	0.541	0.614	959.35	589.19	0.332	836.80	277.82
A_68_P21073309	chr2:25463281-25463325	NM_001024616:664	B230208H17Rik	INSIDE	0.556	0.422	1283.25	541.75	0.235	1017.19	238.59
A_68_P21073310	chr2:25463379-25463423	NM_001024616:566	B230208H17Rik	INSIDE	0.420	0.461	2280.92	1051.03	0.193	1867.74	361.13
A_68_P23364698	chr4:141102196-141102247	NR_030695:145	B330016D10Rik	INSIDE	0.327	0.686	1102.77	757.05	0.224	841.11	188.80
A_68_P28836390	chr13:14046166-14046215	NM_178640:-750	B3galnt2	PROMOTER	0.208	0.471	1662.08	782.80	0.098	1194.51	117.01
A_68_P28836391	chr13:14046280-14046324	NM_178640:-638	B3galnt2	PROMOTER	0.190	0.545	1475.05	803.54	0.103	1131.74	116.94
A_68_P28836397	chr13:14046979-14047023	NM_178640:60	B3galnt2	INSIDE	0.243	0.301	2015.66	606.76	0.073	1578.47	115.55
A_68_P28836401	chr13:14047360-14047404	NM_178640:442	B3galnt2	INSIDE	0.473	0.498	1672.03	833.04	0.236	1311.99	308.99
A_68_P31151212	chr17:34088387-34088432	NM_019420:24	B3galt4	INSIDE	0.433	0.350	2374.68	831.92	0.152	1767.64	268.00
A_68_P27643056	chr11:22759939-22759983	NM_016888:-225	B3gnt2	PROMOTER	0.378	0.447	1284.53	574.01	0.169	1059.94	178.86
A_68_P25958415	chr8:74225497-74225541	NM_028189:181	B3gnt3	INSIDE	0.422	0.737	2143.45	1580.77	0.311	1563.69	486.64
A_68_P24056045	chr5:123958382-123958426	NM_198611:-2064	B3gnt4	PROMOTER	0.210	0.724	2214.58	1603.82	0.152	1378.04	209.88
A_68_P24056049	chr5:123958827-123958871	NM_198611:-1620	B3gnt4	PROMOTER	0.471	0.661	2449.16	1620.11	0.312	2061.15	642.77
A_68_P24056050	chr5:123958945-123958990	NM_198611:-1501	B3gnt4	PROMOTER	0.370	0.545	1243.02	677.99	0.202	988.45	199.35
A_68_P24056051	chr5:123959009-123959060	NM_198611:-1434	B3gnt4	PROMOTER	0.279	0.596	1020.64	608.48	0.166	712.01	118.30
A_68_P25354426	chr7:105342464-105342508	NM_001081167:5499	B3gnt6	INSIDE	0.309	0.544	1800.99	980.45	0.168	1510.86	254.19
A_68_P25354431	chr7:105343001-105343045	NM_001081167:4963	B3gnt6	INSIDE	0.384	0.645	1620.36	1045.61	0.248	1423.85	352.46
A_68_P25354432	chr7:105343075-105343119	NM_001081167:4889	B3gnt6	INSIDE	0.352	0.593	1579.08	937.11	0.209	1334.57	278.92
A_68_P20411442	chr1:88199826-88199871	NM_145222:53	B3gnt7	INSIDE	0.255	0.510	1093.64	557.52	0.130	927.82	120.66
A_68_P25010985	chr7:26412400-26412444	NM_001036740:-220	B3gnt8	PROMOTER	0.380	1.663	4751.97	7900.87	0.632	3194.05	2020.21
A_68_P26132516	chr8:107778018-107778071	NM_178879:1007	B3gnt9-ps	INSIDE	0.279	0.618	863.99	533.71	0.172	713.52	122.82
A_68_P26132522	chr8:107778665-107778709	NM_178879:365	B3gnt9-ps	INSIDE	0.405	0.596	1716.07	1023.62	0.242	1234.29	298.28
A_68_P28191918	chr11:121533998-121534047	NM_178664:443	B3gntl1	INSIDE	0.224	1.567	1903.51	2983.37	0.351	1321.69	464.28

ProbeName	Target position of probe on CpG island microarray	TargetID	GeneSymbol	CpG island Description	Ratio of relative methylation (TiO2-NP/Vehicle)	Sham group			TiO_2-H group		
						Relative methylation (Cy-5/Cy-3)	Cy-3 signal (Input)	Cy-5 signal (MeDIP)	Relative methylation (Cy-5/Cy-3)	Cy-3 signal (Input)	Cy-5 signal (MeDIP)
A_68_P28191919	chr11:121534104-121534151	NM_178664:338	B3gntl1	INSIDE	0.287	0.630	876.92	552.82	0.181	659.65	119.51
A_68_P31791363	chr18:67503527-67503573	NR_033214:207	B430212C06Rik	INSIDE	0.062	1.525	1796.39	2738.65	0.095	1274.39	120.93
A_68_P31791364	chr18:67503647-67503691	NR_033214:89	B430212C06Rik	INSIDE	0.359	0.645	4521.54	2917.14	0.231	3609.29	834.84
A_68_P27534317	chr10:126602065-126602109	NM_008080:-194	B4galnt1	PROMOTER	0.130	0.678	2554.98	1733.41	0.088	1791.88	158.48
A_68_P27534361	chr10:126607747-126607791	NM_027739:4882	B4galnt1	INSIDE	0.455	0.603	1381.62	833.06	0.274	1141.31	312.97
A_68_P27534362	chr10:126607844-126607888	NM_027739:4980	B4galnt1	INSIDE	0.206	1.623	5915.14	9597.99	0.335	4256.99	1425.98
A_68_P27534325	chr10:126602946-126602990	NM_027739:82	B4galnt1	INSIDE	0.667	0.488	1792.27	874.65	0.326	1549.71	504.52
A_68_P28041223	chr11:95720113-95720157	NM_008081:56051	B4galnt2	DOWNSTREAM	0.164	0.706	3433.64	2424.23	0.116	2318.82	268.57
A_68_P28041229	chr11:95720981-95721025	NM_008081:55183	B4galnt2	DOWNSTREAM	0.546	0.659	908.26	598.24	0.360	803.74	289.06
A_68_P28041231	chr11:95721147-95721199	NM_008081:55013	B4galnt2	DOWNSTREAM	0.129	0.632	1736.33	1097.68	0.082	1457.13	119.06
A_68_P24797813	chr6:120244449-120244493	NM_198884:107	B4galnt3	INSIDE	0.432	0.596	1611.33	960.49	0.258	1288.11	331.77
A_68_P25586435	chr7:148246749-148246793	NM_177897:-402	B4galnt4	PROMOTER	0.508	0.609	864.20	526.70	0.309	774.20	239.57
A_68_P22859202	chr4:40801217-40801261	NM_022305:-207	B4galt1	PROMOTER	0.256	0.655	5248.09	3439.66	0.168	3734.72	626.73
A_68_P20827006	chr1:173200478-173200522	NM_020579:42	B4galt3	INSIDE	0.214	0.567	1207.57	684.57	0.122	980.32	119.18
A_68_P20827008	chr1:173200751-173200795	NM_020579:314	B4galt3	INSIDE	0.161	0.461	2298.72	1059.57	0.074	1584.77	117.29
A_68_P21835087	chr2:167174410-167174454	NM_019835:246	B4galt5	INSIDE	0.334	0.599	1762.22	1055.89	0.200	1334.71	267.18
A_68_P31541309	chr18:20904102-20904146	NM_019737:781	B4galt6	INSIDE	0.630	0.559	3266.39	1827.17	0.352	2408.73	848.99
A_68_P31541315	chr18:20904732-20904776	NM_019737:151	B4galt6	INSIDE	0.496	0.483	3673.30	1774.40	0.239	2711.68	649.15
A_68_P24248435	chr6:13627675-13627719	NM_175312:270	B630005N14Rik	INSIDE	0.447	0.495	3160.95	1563.50	0.221	2444.29	540.72
A_68_P24248438	chr6:13628084-13628128	NM_175312:-140	B630005N14Rik	PROMOTER	0.604	0.604	3333.22	2014.92	0.365	2409.21	879.89
A_68_P23440041	chr4:155069180-155069224	NM_178699:752	B930041F14Rik	INSIDE	0.201	0.433	2290.82	992.66	0.087	1698.61	147.58
A_68_P28739136	chr12:111829407-111829460	NR_033340:-150	B930059L03Rik	PROMOTER	0.184	0.461	1918.56	884.50	0.085	1387.78	117.67
A_68_P28739140	chr12:111829834-111829881	NR_033340:274	B930059L03Rik	INSIDE	0.253	0.419	1487.94	622.74	0.106	1104.94	116.79
A_68_P25011291	chr7:26466045-26466089	NM_172148:-110	B9d2	DIVERGENT_PROMOTER	0.249	0.494	1170.17	577.54	0.123	1002.37	123.37
A_68_P24142832	chr5:141180716-141180760	NM_172724:-238	Baat1	DIVERGENT_PROMOTER	0.561	0.612	2843.49	1739.02	0.343	2029.52	696.21
A_68_P26476278	chr9:45646743-45646787	NM_001145947:153	Bace1	INSIDE	0.593	0.651	1643.77	1069.63	0.386	1365.52	527.25
A_68_P31936824	chr19:7016562-7016606	NM_007522:240	Bad	INSIDE	0.335	0.552	1852.09	1022.11	0.185	1346.95	248.86
A_68_P22859646	chr4:40894916-40894962	NM_001171739:389	Bag1	INSIDE	0.252	0.438	1370.07	600.23	0.110	1093.08	120.50
A_68_P22859648	chr4:40895242-40895286	NM_001171739:63	Bag1	INSIDE	0.208	0.672	1045.28	702.02	0.139	870.47	121.42
A_68_P25729004	chr8:26895328-26895372	NM_026121:331	Bag4	INSIDE	0.257	0.726	2088.94	1516.27	0.187	1593.79	297.73
A_68_P31157811	chr17:35271987-35272046	NM_057171:-170	Bag6	DIVERGENT_PROMOTER	0.237	0.590	913.18	538.76	0.140	835.90	116.77
A_68_P31157814	chr17:35272332-35272376	NM_057171:168	Bag6	INSIDE	0.527	0.550	1620.34	891.65	0.290	1254.70	363.55
A_68_P31157817	chr17:35272686-35272730	NM_057171:522	Bag6	INSIDE	0.161	0.451	2178.45	982.58	0.073	1588.13	115.64
A_68_P28183217	chr11:120094827-120094871	NM_198423:588	Bahcc1	INSIDE	0.254	0.580	1341.64	778.34	0.147	1091.17	160.66
A_68_P28183223	chr11:120095537-120095581	NM_198423:1298	Bahcc1	INSIDE	0.197	0.628	3099.37	1945.17	0.123	2157.04	266.27
A_68_P28183224	chr11:120095621-120095666	NM_198423:1383	Bahcc1	INSIDE	0.655	0.647	2464.21	1595.23	0.424	1712.63	726.58
A_68_P21566789	chr2:118726839-118726885	NM_001045523:-488	Bahd1	PROMOTER	0.043	4.027	1664.43	6703.31	0.172	953.62	164.43
A_68_P21566797	chr2:118728139-118728183	NM_001045523:810	Bahd1	INSIDE	0.301	0.538	3665.07	1970.45	0.162	2797.55	451.99
A_68_P21566798	chr2:118728254-118728298	NM_001045523:926	Bahd1	INSIDE	0.491	0.615	1793.63	1103.81	0.302	1414.19	427.61
A_68_P30337592	chr15:74346503-74346547	NM_174991:-101	Bai1	PROMOTER	0.137	0.677	1685.50	1141.21	0.093	1259.89	116.84
A_68_P30337596	chr15:74347238-74347282	NM_174991:635	Bai1	INSIDE	0.655	0.607	1736.25	1053.94	0.398	1380.84	549.00
A_68_P30337603	chr15:74348034-74348078	NM_174991:1431	Bai1	INSIDE	0.160	0.511	2107.94	1077.42	0.082	1478.80	120.66
A_68_P30337616	chr15:74349537-74349581	NM_174991:2933	Bai1	INSIDE	0.355	0.591	1613.67	954.27	0.210	1301.66	273.33
A_68_P30337992	chr15:74394645-74394689	NM_174991:48041	Bai1	INSIDE	0.483	0.665	1714.56	1140.18	0.321	1333.96	428.37

ProbeName	Target position of probe on CpG island microarray	TargetID	GeneSymbol	CpG island Description	Ratio of relative methylation (TiO2-NP/Vehicle)	Sham group			TiO_2-H group		
						Relative methylation (Cy-5/Cy-3)	Cy-3 signal (Input)	Cy-5 signal (MeDIP)	Relative methylation (Cy-5/Cy-3)	Cy-3 signal (Input)	Cy-5 signal (MeDIP)
A_68_P23299521	chr4:129662643-129662687	NM_001199696:343	Bai2	INSIDE	0.294	0.677	1184.37	801.57	0.199	840.45	167.23
A_68_P23299525	chr4:129662999-129663043	NM_001199696:699	Bai2	INSIDE	0.177	1.930	5092.59	9827.63	0.342	2909.27	994.51
A_68_P23299526	chr4:129663110-129663154	NM_001199696:811	Bai2	INSIDE	0.173	1.847	10078.00	18611.84	0.319	6203.11	1981.77
A_68_P20101397	chr1:25887463-25887509	NM_175642:-934	Bai3	PROMOTER	0.080	0.599	3280.46	1965.75	0.048	2529.25	121.29
A_68_P28181189	chr11:119803657-119803701	NM_001037754:-727	Baiap2	PROMOTER	0.341	0.550	2487.62	1367.46	0.187	1801.34	337.58
A_68_P28181190	chr11:119803788-119803832	NM_001037754:-595	Baiap2	PROMOTER	0.278	0.531	3460.85	1837.20	0.148	2541.24	374.90
A_68_P28181191	chr11:119803923-119803967	NM_001037754:-461	Baiap2	PROMOTER	0.657	0.387	1383.14	535.48	0.254	1139.96	290.04
A_68_P24160783	chr5:145118513-145118557	NM_025833:447	Baiap2l1	INSIDE	0.602	0.630	2141.54	1348.14	0.379	1824.01	691.42
A_68_P24160789	chr5:145119094-145119138	NM_025833:-135	Baiap2l1	PROMOTER	0.155	0.461	2338.47	1077.40	0.071	1862.46	132.88
A_68_P24160791	chr5:145119346-145119390	NM_025833:-387	Baiap2l1	PROMOTER	0.197	0.511	2737.46	1399.69	0.101	1963.30	197.57
A_68_P30365368	chr15:79091566-79091610	NM_177580:24351	Baiap2l2	INSIDE	0.348	0.686	2382.77	1635.65	0.239	2087.92	498.34
A_68_P31102614	chr17:25392741-25392785	NM_001163270:547	Baiap3	INSIDE	0.126	0.569	2446.22	1392.56	0.072	1652.46	118.29
A_68_P31102615	chr17:25392896-25392940	NM_001163270:391	Baiap3	INSIDE	0.187	0.710	1736.49	1232.94	0.133	1359.67	180.28
A_68_P31102616	chr17:25393043-25393087	NM_001163270:245	Baiap3	INSIDE	0.206	0.484	1772.69	858.10	0.100	1298.53	129.50
A_68_P31458877	chr18:3508057-3508101	NM_026505:124	Bambi	INSIDE	0.123	0.667	1882.72	1255.86	0.082	1447.38	119.13
A_68_P31458881	chr18:3508571-3508630	NM_026505:646	Bambi	INSIDE	0.143	0.715	1410.05	1008.83	0.102	1201.39	122.95
A_68_P31928284	chr19:5366667-5366713	NM_011793:-45	Banf1	DIVERGENT_PROMOTER	0.416	0.595	996.83	592.66	0.247	718.76	177.78
A_68_P21094090	chr2:28765327-28765371	NM_019446:6593	Barhl1	INSIDE	0.042	1.337	3319.03	4438.08	0.057	2144.44	121.85
A_68_P21094142	chr2:28771352-28771396	NM_019446:567	Barhl1	INSIDE	0.456	0.532	2671.45	1421.37	0.243	1808.28	438.59
A_68_P21094157	chr2:28773265-28773309	NM_001164186:-1326	Barhl1	PROMOTER	0.411	0.696	2445.75	1702.23	0.286	1840.68	527.06
A_68_P23961348	chr5:106886251-106886295	NM_001005477:913	Barhl2	INSIDE	0.135	0.591	2335.12	1379.35	0.080	1669.97	133.27
A_68_P23961352	chr5:106886727-106886771	NM_001005477:437	Barhl2	INSIDE	0.274	0.495	2262.70	1119.67	0.135	1646.56	222.88
A_68_P23961361	chr5:106887861-106887906	NM_001005477:-698	Barhl2	PROMOTER	0.108	0.687	2747.15	1888.55	0.074	2022.23	149.99
A_68_P29020643	chr13:48755179-48755223	NM_007526:-3204	Barx1	PROMOTER	0.394	0.473	1255.55	593.95	0.186	993.02	185.15
A_68_P29020701	chr13:48761510-48761554	NM_007526:3128	Barx1	INSIDE	0.131	0.583	2230.01	1299.99	0.077	1576.23	120.77
A_68_P29020720	chr13:48764426-48764470	NM_007526:6044	Barx1	DOWNSTREAM	0.415	0.533	3856.85	2055.47	0.221	2861.55	632.80
A_68_P26402553	chr9:31721424-31721468	NM_013800:-576	Barx2	PROMOTER	0.456	0.621	2599.53	1613.81	0.283	2212.96	626.13
A_68_P20936238	chr1:192922619-192922663	NM_030060:348	Batf3	INSIDE	0.245	0.611	1599.85	978.03	0.150	1215.48	182.03
A_68_P25093324	chr7:52722052-52722096	NM_007527:194	Bax	INSIDE	0.338	0.512	1886.42	965.17	0.173	1529.95	264.53
A_68_P25093327	chr7:52722315-52722359	NM_007527:-68	Bax	PROMOTER	0.210	0.440	1677.04	737.35	0.092	1334.59	123.19
A_68_P28450015	chr12:56085848-56085892	NM_013815:1453	Baz1a	INSIDE	0.365	0.680	2723.92	1851.08	0.248	2147.50	532.50
A_68_P24115147	chr5:135662582-135662626	NM_011714:-588	Baz1b	PROMOTER	0.390	0.583	2093.19	1220.19	0.227	1622.42	368.86
A_68_P24115149	chr5:135662813-135662862	NM_011714:-355	Baz1b	PROMOTER	0.290	0.480	1075.80	516.45	0.139	874.52	121.72
A_68_P24115153	chr5:135663286-135663330	NM_011714:116	Baz1b	INSIDE	0.476	0.659	1057.98	697.28	0.314	927.00	290.73
A_68_P27539540	chr10:127534403-127534447	NM_054078:4586	Baz2a	INSIDE	0.413	0.431	2288.05	985.78	0.178	2055.08	365.44
A_68_P24330581	chr6:30123488-30123532	NR_038124:-3112	BB283400	DIVERGENT_PROMOTER	0.175	0.640	3801.39	2431.18	0.112	2702.26	302.72
A_68_P24330585	chr6:30124034-30124078	NR_038124:-2566	BB283400	DIVERGENT_PROMOTER	0.223	0.446	1567.77	698.75	0.099	1168.43	116.05
A_68_P24330599	chr6:30125667-30125711	NR_038124:-934	BB283400	DIVERGENT_PROMOTER	0.529	0.471	1364.52	642.08	0.249	1005.33	250.05
A_68_P24330603	chr6:30126052-30126101	NR_038124:-546	BB283400	DIVERGENT_PROMOTER	0.238	0.596	1088.84	649.41	0.142	830.48	118.00
A_68_P24330604	chr6:30126170-30126214	NR_038124:-430	BB283400	DIVERGENT_PROMOTER	0.239	0.436	2105.06	916.86	0.104	1573.25	163.47
A_68_P24330605	chr6:30126242-30126286	NR_038124:-358	BB283400	DIVERGENT_PROMOTER	0.231	0.510	2454.40	1251.63	0.118	1994.97	234.89
A_68_P24980778	chr7:16897508-16897552	NM_133234:2599	Bbc3	INSIDE	0.650	0.515	1396.88	719.35	0.335	1236.23	413.85
A_68_P27448190	chr10:110735869-110735913	NM_027914:156	Bbs10	INSIDE	0.372	0.497	1259.28	626.04	0.185	941.39	173.95
A_68_P26075475	chr8:96622393-96622437	NM_026116:297	Bbs2	INSIDE	0.430	0.383	1914.53	733.23	0.165	1329.59	219.11

ProbeName	Target position of probe on CpG island microarray	TargetID	GeneSymbol	CpG island Description	Ratio of relative methylation (TiO2-NP/Vehicle)	Sham group			TiO_2-H group		
						Relative methylation (Cy-5/Cy-3)	Cy-3 signal (Input)	Cy-5 signal (MeDIP)	Relative methylation (Cy-5/Cy-3)	Cy-3 signal (Input)	Cy-5 signal (MeDIP)
A_68_P26352023	chr9:22280042-22280086	NM_178415:-94	Bbs9	DIVERGENT_PROMOTER	0.581	0.453	2385.20	1080.37	0.263	1848.16	486.01
A_68_P23284269	chr4:126921347-126921391	NM_030252:341	BC003266	INSIDE	0.564	0.460	1692.80	778.92	0.260	1393.23	361.55
A_68_P31102143	chr17:25321627-25321671	NM_183150:143	BC003965	INSIDE	0.356	0.693	1465.43	1015.66	0.247	1277.64	315.45
A_68_P21106324	chr2:30837544-30837588	NM_144885:-105	BC005624	DIVERGENT_PROMOTER	0.074	0.699	4223.37	2950.23	0.052	2853.65	147.36
A_68_P27279426	chr10:79337278-79337322	NM_001170935:79	BC005764	INSIDE	0.270	0.575	1006.84	578.86	0.155	765.90	118.75
A_68_P27279427	chr10:79337379-79337423	NM_001170935:-21	BC005764	DIVERGENT_PROMOTER	0.520	0.697	3505.25	2441.76	0.362	2603.48	942.18
A_68_P26920732	chr10:7500225-7500275	NM_145418:465	BC013529	INSIDE	0.051	0.720	4171.11	3004.32	0.037	3130.63	114.96
A_68_P23314122	chr4:132352279-132352323	NM_001033308:-21	BC013712	PROMOTER	0.257	0.524	1021.92	535.19	0.134	881.43	118.45
A_68_P28788962	chr13:3610060-3610104	NM_134063:272	BC016423	INSIDE	0.264	0.552	1324.53	730.87	0.146	1111.58	161.92
A_68_P26349632	chr9:21741348-21741392	NM_144935:-1347	BC018242	PROMOTER	0.405	0.437	1486.50	649.65	0.177	1174.97	207.88
A_68_P29122630	chr13:70776238-70776282	NM_144837:252	BC018507	INSIDE	0.152	0.658	4530.78	2981.85	0.100	3223.00	323.04
A_68_P26254625	chr8:128434525-128434569	NM_145608:197	BC021891	INSIDE	0.440	0.440	1331.18	586.11	0.194	1002.52	194.30
A_68_P32448984	chrX:67730148-67730192	NM_001033328:48	BC023829	INSIDE	0.130	1.472	1503.18	2212.90	0.191	603.15	115.41
A_68_P32448986	chrX:67730422-67730470	NM_001033328:-228	BC023829	PROMOTER	0.129	2.099	1192.19	2502.00	0.271	455.59	123.30
A_68_P28976946	chr13:41344925-41344972	NM_001135577:-264	BC024659	PROMOTER	0.252	0.491	1292.73	634.97	0.124	984.42	121.72
A_68_P28976947	chr13:41345064-41345108	NM_001135577:-126	BC024659	PROMOTER	0.479	0.526	2012.27	1059.02	0.252	1435.63	361.73
A_68_P28976951	chr13:41345598-41345642	NM_001135577:408	BC024659	INSIDE	0.151	0.571	2558.07	1461.88	0.086	1951.13	168.75
A_68_P22941606	chr4:56815104-56815148	NM_001081420:-74	BC026590	PROMOTER	0.240	0.624	2061.16	1286.76	0.150	1541.91	230.83
A_68_P21072311	chr2:25316393-25316437	NM_153557:200	BC029214	INSIDE	0.385	0.633	2621.56	1659.14	0.244	1888.00	460.39
A_68_P21072314	chr2:25316726-25316774	NM_153557:-136	BC029214	PROMOTER	0.260	0.557	1151.09	640.91	0.145	816.77	118.22
A_68_P27315615	chr10:86168388-86168432	NM_001003939:-145	BC030307	DIVERGENT_PROMOTER	0.294	0.445	1172.76	521.83	0.131	927.24	121.32
A_68_P25898923	chr8:61391591-61391635	NM_173411:1061	BC030500	INSIDE	0.354	0.612	1692.65	1035.71	0.217	1269.62	275.38
A_68_P28078450	chr11:102110241-102110285	NM_153544:67	BC030867	INSIDE	0.270	0.529	1044.74	552.68	0.143	859.44	122.64
A_68_P31834411	chr18:75165635-75165679	NM_001001181:103	BC031181	INSIDE	0.133	0.492	2926.85	1439.99	0.065	2239.99	146.57
A_68_P31834412	chr18:75165713-75165757	NM_001001181:181	BC031181	INSIDE	0.186	0.627	2602.69	1632.91	0.117	1798.45	210.31
A_68_P30684012	chr16:38089843-38089887	NR_033221:-518	BC031361	PROMOTER	0.433	0.387	1469.59	568.10	0.167	1398.26	234.14
A_68_P20878147	chr1:182781059-182781103	NM_145943:-201	BC031781	PROMOTER	0.230	0.459	2994.02	1372.79	0.105	2437.79	256.85
A_68_P20878149	chr1:182781262-182781306	NM_145943:3	BC031781	INSIDE	0.286	0.625	917.25	573.30	0.179	688.08	123.08
A_68_P24129541	chr5:138705342-138705401	NM_153161:-91	BC037034	PROMOTER	0.066	1.555	1655.49	2573.91	0.102	1150.94	117.60
A_68_P26229676	chr8:124431860-124431904	NM_001033485:150	BC048644	INSIDE	0.244	0.520	1210.66	628.95	0.127	932.86	118.29
A_68_P25032100	chr7:31337206-31337250	NM_183321:63	BC053749	INSIDE	0.211	0.678	1287.72	873.66	0.143	968.38	138.38
A_68_P25032103	chr7:31337454-31337505	NM_183321:-188	BC053749	DIVERGENT_PROMOTER	0.255	0.594	1222.36	725.97	0.152	829.22	125.79
A_68_P20786425	chr1:165924761-165924805	NM_201364:130	BC055324	INSIDE	0.634	0.637	5119.83	3263.62	0.404	3884.09	1570.60
A_68_P24004706	chr5:115021554-115021604	NM_001004180:3319	BC057022	INSIDE	0.100	1.572	2898.66	4556.83	0.157	1713.57	269.70
A_68_P24004707	chr5:115021632-115021676	NM_001004180:3395	BC057022	INSIDE	0.223	0.650	3315.15	2155.60	0.145	2225.50	322.35
A_68_P24004708	chr5:115021723-115021774	NM_001004180:3489	BC057022	INSIDE	0.300	0.522	1088.32	568.08	0.157	764.33	119.65
A_68_P21040894	chr2:18612064-18612108	NM_001001334:-8562	BC061194	PROMOTER	0.527	0.636	879.57	559.69	0.335	756.60	253.70
A_68_P21040966	chr2:18620478-18620522	NM_001001334:-148	BC061194	PROMOTER	0.295	0.461	1908.34	880.42	0.136	1521.56	207.18
A_68_P25616527	chr8:4206553-4206597	NM_207203:10738	BC068157	DOWNSTREAM	0.345	0.649	1674.15	1086.44	0.224	1142.10	256.05
A_68_P30425163	chr15:89259930-89259974	NR_004843:406	BC090627	INSIDE	0.195	0.544	3456.39	1881.49	0.106	2649.67	281.50
A_68_P22482404	chr3:122123031-122123075	NM_013867:355	Bcar3	INSIDE	0.651	0.688	1892.26	1301.59	0.448	1459.46	653.58
A_68_P24920736	chr6:144996969-144997013	NM_001024468:342	Bcat1	INSIDE	0.184	0.610	3431.78	2091.70	0.112	2598.47	292.15
A_68_P24920859	chr6:145024368-145024419	NM_007532:284	Bcat1	INSIDE	0.192	0.566	1492.74	844.31	0.108	1078.33	116.81
A_68_P25093837	chr7:52825440-52825491	NM_009737:-267	Bcat2	PROMOTER	0.092	1.931	1625.10	3138.28	0.177	1068.24	189.09

ProbeName	Target position of probe on CpG island microarray	TargetID	GeneSymbol	CpG island Description	Ratio of relative methylation (TiO2-NP/Vehicle)	Sham group			TiO_2-H group		
						Relative methylation (Cy-5/Cy-3)	Cy-3 signal (Input)	Cy-5 signal (MeDIP)	Relative methylation (Cy-5/Cy-3)	Cy-3 signal (Input)	Cy-5 signal (MeDIP)
A_68_P25093840	chr7:52825839-52825883	NM_009737:128	Bcat2	INSIDE	0.430	0.404	2126.05	858.33	0.174	1831.57	318.13
A_68_P25093841	chr7:52825908-52825952	NM_009737:198	Bcat2	INSIDE	0.143	0.524	2014.69	1054.89	0.075	1611.22	120.81
A_68_P25542189	chr7:140901181-140901225	NM_025392:187	Bccip	INSIDE	0.114	0.543	2814.16	1527.67	0.062	1986.22	123.34
A_68_P22611615	chr3:145587665-145587709	NM_009740:345	Bcl10	INSIDE	0.269	0.620	1349.91	837.35	0.167	1077.79	179.79
A_68_P27649060	chr11:23977147-23977191	NM_001159289:-887	Bcl11a	PROMOTER	0.223	0.636	1174.52	746.71	0.142	836.81	118.72
A_68_P28725020	chr12:109218453-109218497	NM_001079883:23150	Bcl11b	INSIDE	0.514	0.522	1741.54	909.57	0.269	1359.26	365.23
A_68_P28725222	chr12:109242213-109242257	NM_001079883:-610	Bcl11b	PROMOTER	0.241	0.588	1544.12	908.21	0.142	1218.37	172.55
A_68_P28725223	chr12:109242384-109242437	NM_001079883:-786	Bcl11b	PROMOTER	0.227	0.611	1322.10	807.96	0.139	977.03	135.75
A_68_P28725238	chr12:109244329-109244373	NM_001079883:-2726	Bcl11b	PROMOTER	0.515	0.644	1347.74	868.21	0.332	1134.29	376.64
A_68_P20509920	chr1:108609243-108609287	NM_009741:1603	Bcl2	INSIDE	0.490	0.662	1171.34	775.04	0.324	952.90	308.93
A_68_P20509928	chr1:108610270-108610327	NM_009741:569	Bcl2	INSIDE	0.150	0.622	1578.73	981.39	0.093	1230.71	115.06
A_68_P20509929	chr1:108610390-108610434	NM_009741:455	Bcl2	INSIDE	0.416	0.638	1687.46	1076.52	0.265	1125.49	298.72
A_68_P21617362	chr2:127951410-127951454	NM_009754:-341	Bcl2l11	PROMOTER	0.416	0.608	2537.30	1542.87	0.253	1871.35	473.37
A_68_P21617363	chr2:127951507-127951551	NM_009754:-245	Bcl2l11	PROMOTER	0.131	0.540	3192.45	1724.28	0.071	2353.84	166.18
A_68_P21617365	chr2:127951737-127951781	NM_009754:-15	Bcl2l11	PROMOTER	0.191	0.321	3559.69	1142.91	0.061	2582.36	157.98
A_68_P21617369	chr2:127952159-127952203	NM_009754:407	Bcl2l11	INSIDE	0.521	0.528	2129.39	1124.93	0.275	1695.44	466.84
A_68_P21617375	chr2:127952895-127952939	NM_009754:1143	Bcl2l11	INSIDE	0.150	0.717	2033.76	1458.34	0.107	1685.58	181.05
A_68_P21617370	chr2:127952278-127952322	NM_009754:527	Bcl2l11	INSIDE	0.668	0.557	1199.21	667.56	0.372	939.33	349.06
A_68_P24800510	chr6:120786535-120786579	NM_153516:309	Bcl2l13	INSIDE	0.326	0.375	2013.72	755.91	0.122	1678.70	205.41
A_68_P24995363	chr7:20395100-20395149	NM_033601:12980	Bcl3	INSIDE	0.246	0.482	1232.37	593.66	0.118	1010.24	119.59
A_68_P30605853	chr16:23983382-23983426	NM_009744:5294	Bcl6	INSIDE	0.237	0.545	1598.52	870.94	0.129	1300.39	168.24
A_68_P27899563	chr11:70040019-70040063	NM_007528:3260	Bcl6b	INSIDE	0.254	0.592	1588.96	941.28	0.150	1186.63	178.42
A_68_P27899564	chr11:70040111-70040155	NM_007528:3168	Bcl6b	INSIDE	0.242	0.548	1105.14	605.71	0.133	906.96	120.48
A_68_P27899586	chr11:70042779-70042828	NM_007528:497	Bcl6b	INSIDE	0.178	0.707	1860.62	1315.18	0.126	1418.07	178.73
A_68_P27899593	chr11:70043429-70043486	NM_007528:-157	Bcl6b	DIVERGENT_PROMOTER	0.048	1.531	2278.22	3486.99	0.073	1668.41	122.14
A_68_P24055179	chr5:123794010-123794061	NM_029850:-421	Bcl7a	PROMOTER	0.275	0.488	1083.41	529.08	0.134	900.64	121.10
A_68_P24055190	chr5:123795453-123795497	NM_029850:1018	Bcl7a	INSIDE	0.347	0.730	2433.72	1775.81	0.253	1715.66	434.55
A_68_P32260250	chrX:11654399-11654443	NM_029510:3259	Bcor	INSIDE	0.099	1.476	6046.26	8925.59	0.146	1946.65	284.92
A_68_P32260255	chrX:11655059-11655115	NM_029510:2593	Bcor	INSIDE	0.525	0.556	1059.70	589.72	0.292	446.82	130.49
A_68_P32260272	chrX:11657098-11657142	NM_029510:559	Bcor	INSIDE	0.047	1.527	7364.88	11244.67	0.072	2313.39	167.32
A_68_P32260332	chrX:11664013-11664057	NM_029510:-6355	Bcor	PROMOTER	0.576	0.588	1667.60	981.30	0.339	737.01	249.92
A_68_P32260348	chrX:11665608-11665652	NM_029510:-7951	Bcor	PROMOTER	0.319	0.565	1677.90	947.72	0.180	676.34	121.68
A_68_P32260568	chrX:11704944-11704992	NM_001168321:32513	Bcor	INSIDE	0.197	1.927	2757.03	5313.84	0.380	1025.70	389.31
A_68_P32260572	chrX:11705418-11705462	NM_001168321:32041	Bcor	INSIDE	0.467	0.572	1644.76	940.80	0.267	615.99	164.54
A_68_P32260574	chrX:11705655-11705699	NM_001168321:31805	Bcor	INSIDE	0.274	1.667	5079.86	8467.01	0.456	1843.53	841.12
A_68_P32260575	chrX:11705762-11705806	NM_001168321:31697	Bcor	INSIDE	0.120	2.661	5788.90	15404.08	0.320	2050.19	656.24
A_68_P32260576	chrX:11705862-11705906	NM_001168321:31597	Bcor	INSIDE	0.340	1.523	1805.39	2748.90	0.517	603.83	312.24
A_68_P32260619	chrX:11710953-11710997	NM_001168321:26507	Bcor	INSIDE	0.131	1.496	1663.66	2489.66	0.196	587.51	115.14
A_68_P32260636	chrX:11712923-11712967	NM_001168321:24537	Bcor	INSIDE	0.494	0.527	1614.31	851.15	0.261	660.37	172.06
A_68_P32364048	chrX:45696786-45696830	NM_178782:2274	Bcorl1	INSIDE	0.218	1.818	2008.87	3651.17	0.397	794.03	315.28
A_68_P32364049	chrX:45696991-45697035	NM_178782:2478	Bcorl1	INSIDE	0.573	3.269	2293.72	7497.48	1.872	912.24	1708.07
A_68_P32364050	chrX:45697107-45697151	NM_178782:2594	Bcorl1	INSIDE	0.383	9.469	12523.37	118577.60	3.626	5339.78	19361.94
A_68_P32364052	chrX:45697351-45697395	NM_178782:2838	Bcorl1	INSIDE	0.157	2.064	901.87	1861.49	0.324	365.05	118.27
A_68_P32364064	chrX:45698666-45698710	NM_178782:4154	Bcorl1	INSIDE	0.171	2.431	1583.53	3850.12	0.415	827.91	343.61

ProbeName	Target position of probe on CpG island microarray	TargetID	GeneSymbol	CpG island Description	Ratio of relative methylation (TiO2-NP/Vehicle)	Sham group			TiO_2-H group		
						Relative methylation (Cy-5/Cy-3)	Cy-3 signal (Input)	Cy-5 signal (MeDIP)	Relative methylation (Cy-5/Cy-3)	Cy-3 signal (Input)	Cy-5 signal (MeDIP)
A_68_P32364065	chrX:45698842-45698886	NM_178782:4330	Bcorl1	INSIDE	0.653	2.914	865.54	2521.90	1.904	483.93	921.37
A_68_P30647299	chr16:31430033-31430077	NM_001122683:1216	Bdh1	INSIDE	0.229	0.646	983.74	635.74	0.148	846.49	125.33
A_68_P21516613	chr2:109517788-109517832	NM_001048139:1757	Bdnf	INSIDE	0.441	0.689	1157.11	797.50	0.304	929.69	282.59
A_68_P21516740	chr2:109533518-109533562	NM_001048142:-179	Bdnf	PROMOTER	0.296	0.561	933.08	523.23	0.166	766.95	127.48
A_68_P26127081	chr8:106695023-106695067	NM_001141922:632	Bean1	INSIDE	0.556	0.638	1363.79	870.38	0.355	1289.77	458.06
A_68_P23767820	chr5:67817841-67817885	NM_001164806:1176	Bend4	INSIDE	0.074	2.214	4749.84	10514.43	0.165	2920.32	480.52
A_68_P23767821	chr5:67817925-67817969	NM_001164806:1092	Bend4	INSIDE	0.054	2.300	6069.92	13961.59	0.124	3932.74	487.66
A_68_P23210413	chr4:111087417-111087472	NM_026279:-166	Bend5	PROMOTER	0.435	0.554	1649.22	913.64	0.241	1311.55	315.76
A_68_P26023138	chr8:87531521-87531565	NM_001130193:6688	Best2	INSIDE	0.374	1.463	1331.75	1947.71	0.547	987.58	540.10
A_68_P25585263	chr7:148042305-148042349	NM_018742:-44	Bet1l	DIVERGENT_PROMOTER	0.292	0.591	2650.92	1565.37	0.172	2027.12	349.16
A_68_P21703962	chr2:143688269-143688313	NM_009751:619	Bfsp1	INSIDE	0.550	0.546	3013.50	1646.23	0.301	2402.58	722.05
A_68_P32678565	chrX:132420534-132420578	NM_001098222:167	Bhlhb9	INSIDE	0.670	2.230	1544.96	3445.00	1.494	797.82	1192.24
A_68_P24735111	chr6:108610435-108610479	NM_011498:-166	Bhlhe40	PROMOTER	0.328	0.495	1175.97	581.53	0.162	957.28	155.05
A_68_P24735177	chr6:108618758-108618808	NM_011498:8160	Bhlhe40	DOWNSTREAM	0.111	1.624	1047.87	1701.79	0.180	808.09	145.76
A_68_P24925475	chr6:145812338-145812382	NM_024469:1500	Bhlhe41	INSIDE	0.271	0.434	1331.73	577.76	0.118	1030.57	121.35
A_68_P24800932	chr6:120866913-120866957	NM_007544:-96	Bid	PROMOTER	0.329	0.520	1901.47	988.02	0.171	1443.08	246.66
A_68_P30388922	chr15:83357128-83357179	NM_007546:-138	Bik	PROMOTER	0.171	0.618	1537.68	950.90	0.106	1104.36	116.61
A_68_P30388925	chr15:83357441-83357485	NM_007546:171	Bik	INSIDE	0.507	0.664	1048.29	695.80	0.337	851.32	286.62
A_68_P30388929	chr15:83357855-83357899	NM_007546:585	Bik	INSIDE	0.277	0.586	880.06	515.70	0.163	715.98	116.36
A_68_P26289572	chr9:7837195-7837239	NM_007465:-1961	Birc2	PROMOTER	0.584	0.612	1049.29	642.09	0.357	770.67	275.28
A_68_P31351851	chr17:74927352-74927396	NM_007566:-260	Birc6	PROMOTER	0.278	0.526	2662.66	1400.83	0.146	2001.97	292.52
A_68_P31351852	chr17:74927433-74927479	NM_007566:-178	Birc6	PROMOTER	0.242	0.596	962.59	573.43	0.144	805.36	116.00
A_68_P31351858	chr17:74928310-74928354	NM_007566:698	Birc6	INSIDE	0.158	0.503	2795.33	1404.81	0.080	2107.81	167.89
A_68_P21777898	chr2:157391806-157391850	NM_016916:269	Blcap	INSIDE	0.525	0.533	1132.85	603.97	0.280	971.06	271.87
A_68_P25267240	chr7:87679859-87679903	NM_007550:4	Blm	INSIDE	0.443	0.388	2732.14	1061.30	0.172	2245.55	386.81
A_68_P32130738	chr19:44220823-44220867	NM_028607:92	Bloc1s2	INSIDE	0.584	0.550	1274.72	701.66	0.322	1090.56	350.70
A_68_P21611440	chr2:126896255-126896304	NM_026678:-113	Blvra	PROMOTER	0.490	0.663	2615.34	1733.34	0.325	2058.76	669.03
A_68_P21564754	chr2:118375138-118375182	NM_138313:254	Bmf	INSIDE	0.517	0.639	7210.45	4606.30	0.330	5323.14	1756.68
A_68_P21040880	chr2:18610461-18610505	NM_007552:11838	Bmi1	DOWNSTREAM	0.589	0.633	1224.93	775.07	0.373	1105.97	412.53
A_68_P21646218	chr2:133378320-133378364	NM_007553:-592	Bmp2	PROMOTER	0.511	0.700	1924.24	1346.76	0.357	1606.86	574.39
A_68_P21646220	chr2:133378597-133378641	NM_007553:-316	Bmp2	PROMOTER	0.549	0.608	1727.31	1050.49	0.334	1473.87	491.72
A_68_P29575951	chr14:47006999-47007043	NM_007554:3254	Bmp4	INSIDE	0.182	0.556	2634.16	1465.46	0.101	2039.64	206.07
A_68_P29575956	chr14:47007694-47007740	NM_007554:2558	Bmp4	INSIDE	0.087	2.096	1870.95	3921.78	0.183	1256.59	230.24
A_68_P29575957	chr14:47007792-47007836	NM_007554:2460	Bmp4	INSIDE	0.045	1.669	5683.48	9482.93	0.075	3733.09	279.16
A_68_P29575959	chr14:47007992-47008036	NM_007554:2260	Bmp4	INSIDE	0.102	0.743	2711.94	2014.71	0.076	1957.06	148.65
A_68_P29575961	chr14:47008247-47008291	NM_007554:2006	Bmp4	INSIDE	0.209	0.677	1030.31	697.63	0.142	852.97	120.73
A_68_P28959945	chr13:38437436-38437480	NM_007556:-126	Bmp6	PROMOTER	0.383	0.657	1024.36	673.30	0.252	703.83	177.12
A_68_P28959953	chr13:38438458-38438503	NM_007556:896	Bmp6	INSIDE	0.281	0.617	2412.64	1488.63	0.173	1731.52	299.91
A_68_P28959954	chr13:38438568-38438612	NM_007556:1006	Bmp6	INSIDE	0.455	0.672	2248.38	1510.00	0.306	1846.10	564.08
A_68_P21866700	chr2:172764763-172764807	NM_007557:1010	Bmp7	INSIDE	0.067	0.581	6091.26	3536.32	0.039	4480.39	175.43
A_68_P21866701	chr2:172764888-172764932	NM_007557:884	Bmp7	INSIDE	0.538	0.594	2080.97	1235.60	0.319	1501.23	479.30
A_68_P21866704	chr2:172765156-172765200	NM_007557:616	Bmp7	INSIDE	0.251	0.617	1113.69	687.29	0.155	799.62	123.96
A_68_P21866705	chr2:172765232-172765276	NM_007557:540	Bmp7	INSIDE	0.211	0.607	1805.03	1094.84	0.128	1376.69	175.80
A_68_P23262156	chr4:123019628-123019672	NM_007558:396	Bmp8a	INSIDE	0.148	0.694	2120.32	1471.63	0.103	1521.93	156.18

ProbeName	Target position of probe on CpG island microarray	TargetID	GeneSymbol	CpG island Description	Ratio of relative methylation (TiO2-NP/Vehicle)	Sham group			TiO_2-H group		
						Relative methylation (Cy-5/Cy-3)	Cy-3 signal (Input)	Cy-5 signal (MeDIP)	Relative methylation (Cy-5/Cy-3)	Cy-3 signal (Input)	Cy-5 signal (MeDIP)
A_68_P23262162	chr4:123020250-123020294	NM_007558:-226	Bmp8a	PROMOTER	0.607	0.528	1218.13	642.86	0.321	1020.81	327.24
A_68_P23260768	chr4:122782372-122782416	NM_007559:-13	Bmp8b	PROMOTER	0.594	0.539	1363.52	735.06	0.320	1135.02	363.35
A_68_P26356121	chr9:23026902-23026947	NM_028472:-595	Bmper	PROMOTER	0.369	1.643	1952.36	3208.29	0.606	1498.57	907.76
A_68_P26356133	chr9:23028290-23028334	NM_028472:793	Bmper	INSIDE	0.501	0.718	4984.49	3577.87	0.360	3648.44	1311.63
A_68_P29530302	chr14:35315095-35315147	NM_009758:612	Bmpr1a	INSIDE	0.482	0.599	1221.79	731.81	0.289	1077.15	310.83
A_68_P29530310	chr14:35316304-35316358	NM_009758:-598	Bmpr1a	PROMOTER	0.260	0.483	1204.06	581.45	0.126	927.24	116.61
A_68_P20265713	chr1:59820408-59820452	NM_007561:-1050	Bmpr2	PROMOTER	0.640	0.492	1359.20	669.18	0.315	1108.33	349.36
A_68_P20265717	chr1:59820840-59820884	NM_007561:-618	Bmpr2	PROMOTER	0.526	0.467	1127.96	526.30	0.245	983.90	241.53
A_68_P21073893	chr2:25562488-25562532	NM_023326:112	Bmyc	INSIDE	0.291	0.518	2191.14	1134.96	0.151	1771.29	267.16
A_68_P25275162	chr7:89137615-89137659	NM_007562:-451	Bnc1	PROMOTER	0.492	0.655	901.73	590.46	0.322	598.73	192.80
A_68_P23069990	chr4:84322037-84322088	NM_172870:-1072	Bnc2	PROMOTER	0.377	0.582	919.68	535.21	0.219	725.51	159.22
A_68_P31112417	chr17:26918235-26918282	NM_172149:235	Bnip1	INSIDE	0.226	0.574	1232.99	707.88	0.130	894.82	116.33
A_68_P29682515	chr14:67627653-67627697	NM_009761:40	Bnip3l	INSIDE	0.354	0.494	2395.59	1183.56	0.175	1697.55	297.00
A_68_P29682516	chr14:67627743-67627787	NM_009761:-50	Bnip3l	PROMOTER	0.506	0.648	1485.99	962.27	0.328	1149.10	376.56
A_68_P29682517	chr14:67627899-67627944	NM_009761:-207	Bnip3l	PROMOTER	0.131	0.540	2816.41	1521.44	0.071	2123.86	150.15
A_68_P27684135	chr11:31572000-31572044	NM_001024919:-160	Bod1	PROMOTER	0.521	0.500	1403.15	701.95	0.261	1110.76	289.66
A_68_P23637941	chr5:42235067-42235111	NM_001081422:466	Bod1l	INSIDE	0.608	0.500	1234.59	617.58	0.304	1086.26	330.62
A_68_P20456912	chr1:95582245-95582289	NM_016778:-4	Bok	PROMOTER	0.383	0.511	1064.21	543.39	0.196	812.05	158.91
A_68_P22348132	chr3:96001190-96001234	NM_026975:297	Bola1	INSIDE	0.209	0.545	1434.53	782.45	0.114	1022.17	116.65
A_68_P25501815	chr7:133839681-133839725	NM_175103:189	Bola2	INSIDE	0.601	0.393	1973.25	775.55	0.236	1391.41	328.75
A_68_P28936242	chr13:34129507-34129551	NM_026512:19	Bphl	INSIDE	0.649	0.542	1096.78	594.95	0.352	982.49	345.68
A_68_P28104944	chr11:106911768-106911812	NM_176850:81446	Bptf	INSIDE	0.554	2.532	46344.44	117337.80	1.402	36795.29	51603.19
A_68_P31152204	chr17:34255264-34255308	NR_037970:1559	Brd2	INSIDE	0.185	1.665	5401.10	8995.19	0.308	3536.85	1090.15
A_68_P31152205	chr17:34255338-34255382	NR_037970:1485	Brd2	INSIDE	0.653	0.581	1894.32	1101.27	0.380	1315.29	499.50
A_68_P31152216	chr17:34256835-34256882	NR_037970:-13	Brd2	PROMOTER	0.297	0.664	1305.39	866.95	0.197	878.61	173.17
A_68_P31152219	chr17:34257154-34257198	NR_037970:-331	Brd2	PROMOTER	0.242	0.558	2029.96	1132.29	0.135	1410.13	190.53
A_68_P31152223	chr17:34257573-34257617	NR_037970:-749	Brd2	PROMOTER	0.479	0.648	2483.84	1609.22	0.310	1780.17	552.63
A_68_P31152225	chr17:34257765-34257809	NR_037970:-941	Brd2	PROMOTER	0.283	0.657	5608.29	3685.58	0.186	3785.75	703.50
A_68_P27987612	chr11:86014370-86014414	NM_178309:303	Brip1	INSIDE	0.582	0.442	1329.23	586.89	0.257	1017.15	261.40
A_68_P27987613	chr11:86014523-86014567	NM_178309:151	Brip1	INSIDE	0.377	0.519	1442.15	748.02	0.195	1105.35	215.97
A_68_P27987617	chr11:86015036-86015080	NM_178309:-363	Brip1	PROMOTER	0.238	0.550	2619.06	1439.79	0.131	2060.48	270.07
A_68_P30010833	chr15:10415572-10415616	NM_026396:98	Brix1	INSIDE	0.166	0.685	1910.79	1308.77	0.113	1330.06	150.94
A_68_P28453879	chr12:56937597-56937641	NM_001037756:266	Brms1l	INSIDE	0.292	0.517	3907.17	2021.34	0.151	2900.31	438.81
A_68_P28454005	chr12:56962212-56962256	NM_001037756:24882	Brms1l	INSIDE	0.587	2.506	3126.73	7837.02	1.472	2390.91	3518.38
A_68_P20794610	chr1:167391699-167391743	NM_027430:382	Brp44	INSIDE	0.172	0.544	1884.09	1025.75	0.093	1323.72	123.61
A_68_P32957266	chr17:8477443-8477487	NM_018819:787	Brp44l	INSIDE	0.562	0.540	2045.56	1104.06	0.304	1646.50	499.84
A_68_P32957267	chr17:8477533-8477577	NM_018819:877	Brp44l	INSIDE	0.366	0.430	2294.45	985.74	0.157	1874.90	295.18
A_68_P32592614	chrX:106029534-106029578	NM_001081477:138	Brwd3	INSIDE	0.275	2.326	4733.30	11008.85	0.639	1847.91	1180.65
A_68_P32592615	chrX:106029602-106029650	NM_001081477:68	Brwd3	INSIDE	0.052	2.627	2774.50	7287.86	0.137	861.55	117.82
A_68_P23296722	chr4:129138895-129138941	NM_133889:-4	Bsdc1	PROMOTER	0.104	2.164	3236.95	7003.60	0.224	2243.55	503.07
A_68_P23296723	chr4:129138984-129139028	NM_133889:84	Bsdc1	INSIDE	0.217	1.938	4850.53	9399.00	0.420	3306.54	1388.52
A_68_P23296726	chr4:129139208-129139252	NM_133889:308	Bsdc1	INSIDE	0.281	0.461	1182.13	545.33	0.130	890.14	115.58
A_68_P27278359	chr10:79167367-79167411	NM_001077184:286	Bsg	INSIDE	0.212	0.438	1665.67	728.83	0.093	1262.70	117.39
A_68_P26445764	chr9:40684336-40684380	NM_178245:2149	Bsx	INSIDE	0.405	0.423	1901.12	803.37	0.171	1525.03	260.78

ProbeName	Target position of probe on CpG island microarray	TargetID	GeneSymbol	CpG island Description	Ratio of relative methylation (TiO2-NP/Vehicle)	Sham group			TiO_2-H group		
						Relative methylation (Cy-5/Cy-3)	Cy-3 signal (Input)	Cy-5 signal (MeDIP)	Relative methylation (Cy-5/Cy-3)	Cy-3 signal (Input)	Cy-5 signal (MeDIP)
A_68_P27308091	chr10:84849925-84849970	NM_028709:389	Btbd11	INSIDE	0.227	0.598	1118.31	668.60	0.136	904.39	122.55
A_68_P27308092	chr10:84850082-84850126	NM_028709:546	Btbd11	INSIDE	0.086	0.453	4449.39	2016.92	0.039	3007.86	117.16
A_68_P27308093	chr10:84850152-84850196	NM_028709:616	Btbd11	INSIDE	0.154	0.453	3317.82	1504.20	0.070	2310.99	160.95
A_68_P27308102	chr10:84851199-84851243	NM_028709:1662	Btbd11	INSIDE	0.124	0.604	2262.41	1366.82	0.075	1635.23	122.97
A_68_P27309451	chr10:85061404-85061448	NM_001017525:271	Btbd11	INSIDE	0.514	0.434	2082.85	903.63	0.223	1569.36	349.73
A_68_P28149796	chr11:114653639-114653683	NM_028055:3546	Btbd17	INSIDE	0.539	0.605	1012.98	612.71	0.326	895.07	292.07
A_68_P21673353	chr2:138082625-138082669	NM_001025431:327	Btbd3	INSIDE	0.347	0.439	2287.72	1004.93	0.152	1958.63	298.59
A_68_P21673355	chr2:138082825-138082869	NM_001025431:527	Btbd3	INSIDE	0.427	0.424	1382.18	586.22	0.181	991.91	179.61
A_68_P31134865	chr17:30712521-30712565	NM_027060:690	Btbd9	INSIDE	0.195	0.574	1343.54	770.52	0.112	1059.10	118.55
A_68_P29513184	chr14:32454218-32454262	NM_025295:-2	Btd	DIVERGENT_PROMOTER	0.374	0.584	1018.95	595.25	0.219	797.44	174.38
A_68_P27371449	chr10:96079420-96079468	NM_007569:-190	Btg1	PROMOTER	0.149	1.915	3073.84	5887.06	0.286	2093.55	598.18
A_68_P20631265	chr1:135975492-135975536	NM_007570:218	Btg2	INSIDE	0.223	0.494	1402.73	693.60	0.110	1049.13	115.80
A_68_P20631266	chr1:135975629-135975675	NM_007570:80	Btg2	INSIDE	0.572	0.519	1236.30	641.90	0.297	944.69	280.50
A_68_P32139026	chr19:45616482-45616535	NM_001037758:178285	Btrc	DOWNSTREAM	0.214	0.665	1005.56	668.88	0.142	866.88	123.16
A_68_P21615856	chr2:127657274-127657318	NM_001113179:299	Bub1	INSIDE	0.230	0.398	1552.78	617.92	0.091	1339.09	122.33
A_68_P21615857	chr2:127657373-127657417	NM_001113179:201	Bub1	INSIDE	0.536	0.610	1653.90	1008.38	0.327	1335.81	436.17
A_68_P21615858	chr2:127657441-127657493	NM_001113179:129	Bub1	INSIDE	0.202	0.694	1119.43	776.42	0.140	852.83	119.77
A_68_P21564939	chr2:118424101-118424145	NM_009773:176	Bub1b	INSIDE	0.187	2.521	1635.60	4123.18	0.471	1037.77	488.62
A_68_P31213160	chr17:47747908-47747952	NM_016859:511	Bysl	INSIDE	0.228	0.669	1659.15	1110.35	0.153	1158.95	177.21
A_68_P20258660	chr1:58449893-58449937	NM_025824:-65	Bzw1	PROMOTER	0.402	0.379	2338.44	886.59	0.152	1931.23	294.39
A_68_P20258664	chr1:58450590-58450634	NM_025824:633	Bzw1	INSIDE	0.392	0.644	1254.18	807.72	0.252	1054.82	266.06
A_68_P28356775	chr12:36883341-36883387	NM_025840:48	Bzw2	INSIDE	0.301	1.635	1872.76	3062.06	0.492	1394.80	686.87
A_68_P32040830	chr19:27504703-27504752	NR_027987:201	C030016D13Rik	INSIDE	0.135	0.698	1691.61	1180.67	0.094	1241.33	116.78
A_68_P31323854	chr17:69766606-69766650	NR_026848:842	C030034I22Rik	INSIDE	0.191	0.471	1746.19	823.00	0.090	1292.38	116.56
A_68_P31323858	chr17:69767042-69767093	NR_026848:1281	C030034I22Rik	INSIDE	0.191	0.684	1127.06	770.71	0.131	921.76	120.68
A_68_P31323859	chr17:69767217-69767270	NR_026848:1457	C030034I22Rik	INSIDE	0.271	0.588	1073.03	630.54	0.159	817.56	130.24
A_68_P24641803	chr6:91827853-91827908	NM_172730:-166	C130022K22Rik	PROMOTER	0.254	0.523	1199.69	627.78	0.133	911.33	120.90
A_68_P24641805	chr6:91828146-91828192	NM_172730:122	C130022K22Rik	INSIDE	0.216	0.569	1374.82	781.83	0.123	998.05	122.84
A_68_P20411732	chr1:88256159-88256203	NR_015507:32	C130036L24Rik	INSIDE	0.531	0.584	2833.67	1653.54	0.310	2282.19	706.71
A_68_P28596925	chr12:85533705-85533749	NM_001163502:1239	C130039O16Rik	INSIDE	0.192	0.530	1437.34	761.46	0.102	1168.46	118.93
A_68_P28596936	chr12:85534975-85535019	NM_001163502:-31	C130039O16Rik	PROMOTER	0.588	0.587	1200.40	704.15	0.345	1073.45	370.30
A_68_P24511775	chr6:65331286-65331330	NM_175524:21	C130060K24Rik	INSIDE	0.147	0.530	2081.02	1102.85	0.078	1554.64	120.73
A_68_P20898010	chr1:186706559-186706603	NM_178692:335	C130074G19Rik	INSIDE	0.215	0.629	1120.27	705.20	0.135	946.14	128.04
A_68_P27903680	chr11:70796542-70796586	NM_007573:-36	C1qbp	PROMOTER	0.393	0.533	1532.93	816.29	0.209	1280.18	267.89
A_68_P20561487	chr1:122239035-122239079	NM_207233:1898	C1ql2	INSIDE	0.332	0.481	1353.72	650.98	0.160	984.99	157.15
A_68_P20561488	chr1:122239104-122239151	NM_207233:1969	C1ql2	INSIDE	0.148	0.537	2023.78	1086.08	0.080	1559.66	124.08
A_68_P21013689	chr2:12931615-12931659	NM_153155:855	C1ql3	INSIDE	0.647	0.672	974.19	655.09	0.435	849.89	369.68
A_68_P21419446	chr2:90725823-90725867	NM_026161:-98	C1qtnf4	PROMOTER	0.304	0.498	1106.38	550.81	0.152	853.07	129.25
A_68_P23351276	chr4:138908601-138908645	NM_001039200:115	C230096C10Rik	INSIDE	0.608	0.625	1153.69	720.54	0.380	790.46	300.24
A_68_P31001857	chr16:98143725-98143769	NM_174847:497	C2cd2	INSIDE	0.383	0.689	1211.64	834.54	0.264	935.34	246.86
A_68_P26466977	chr9:44127873-44127917	NM_027909:471	C2cd2l	INSIDE	0.570	0.627	3836.44	2404.59	0.357	2957.77	1055.85
A_68_P26600871	chr9:67607518-67607562	NM_001081314:297	C2cd4b	INSIDE	0.345	0.513	2348.85	1204.41	0.177	1940.66	343.40
A_68_P27277825	chr10:79076430-79076474	NM_198614:311	C2cd4c	INSIDE	0.564	0.342	2022.44	691.42	0.193	1722.39	331.93
A_68_P22338542	chr3:94168357-94168405	NM_001136117:2015	C2cd4d	INSIDE	0.257	0.585	989.64	579.24	0.150	779.66	117.12

ProbeName	Target position of probe on CpG island microarray	TargetID	GeneSymbol	CpG island Description	Ratio of relative methylation (TiO2-NP/Vehicle)	Sham group			TiO_2-H group		
						Relative methylation (Cy-5/Cy-3)	Cy-3 signal (Input)	Cy-5 signal (MeDIP)	Relative methylation (Cy-5/Cy-3)	Cy-3 signal (Input)	Cy-5 signal (MeDIP)
A_68_P25022917	chr7:29415899-29415943	NM_175319:114	C330005M16Rik	INSIDE	0.437	0.520	1440.02	748.27	0.227	1222.21	277.84
A_68_P22733181	chr4:12016440-12016484	NM_198957:-54	C430048L16Rik	DIVERGENT_PROMOTER	0.133	0.608	3136.35	1906.29	0.081	2171.18	176.00
A_68_P32385503	chrX:50410575-50410619	NR_038184:-229	C430049B03Rik	PROMOTER	0.187	2.036	3303.44	6726.82	0.380	1329.00	505.02
A_68_P23230003	chr4:116269931-116269975	NR_029450:283	C530005A16Rik	INSIDE	0.439	0.628	1343.00	843.05	0.276	1099.52	303.25
A_68_P23818502	chr5:77286666-77286710	NM_001163793:17065	C530008M17Rik	INSIDE	0.281	1.592	1314.50	2092.35	0.447	973.85	434.84
A_68_P23818507	chr5:77287352-77287398	NM_001163793:17751	C530008M17Rik	INSIDE	0.586	2.378	1867.71	4441.86	1.394	1621.02	2258.91
A_68_P24818072	chr6:124946958-124947002	NM_001145927:-1474	C530028O21Rik	PROMOTER	0.215	0.679	5799.59	3935.88	0.146	4114.28	599.34
A_68_P24818074	chr6:124947113-124947157	NM_001145927:-1320	C530028O21Rik	PROMOTER	0.096	0.584	3975.60	2320.86	0.056	2612.11	145.96
A_68_P28153130	chr11:115235797-115235841	NM_175454:-6785	C630004H02Rik	DIVERGENT_PROMOTER	0.268	0.693	1465.38	1015.15	0.185	1237.82	229.54
A_68_P23295722	chr4:128916103-128916147	NM_001033189:9563	C77080	INSIDE	0.265	0.521	2413.50	1257.67	0.138	1941.47	268.53
A_68_P23295723	chr4:128916179-128916223	NM_001033189:9487	C77080	INSIDE	0.524	0.532	3414.15	1816.75	0.279	2652.26	739.66
A_68_P20408509	chr1:87690854-87690908	NM_133781:859	Cab39	INSIDE	0.249	0.498	1087.17	541.31	0.124	936.15	116.20
A_68_P20408511	chr1:87691108-87691154	NM_133781:1109	Cab39	INSIDE	0.172	0.546	1562.43	852.44	0.094	1248.16	117.17
A_68_P31497267	chr18:11997722-11997766	NM_001146287:-38	Cables1	PROMOTER	0.553	0.486	1665.49	808.73	0.268	1224.89	328.79
A_68_P24008375	chr5:115644484-115644528	NM_013879:-8376	Cabp1	PROMOTER	0.650	0.403	1287.34	519.41	0.262	1078.13	282.69
A_68_P27559363	chr11:4645990-4646034	NM_138948:769	Cabp7	INSIDE	0.270	0.621	1321.68	821.29	0.168	1083.20	182.06
A_68_P23151263	chr4:100450587-100450631	NM_198037:1325	Cachd1	INSIDE	0.237	0.653	1046.97	683.68	0.155	777.29	120.27
A_68_P21067988	chr2:24617711-24617766	NM_001042528:829	Cacna1b	INSIDE	0.245	0.592	1501.68	889.57	0.145	1151.69	166.89
A_68_P24791281	chr6:119057478-119057522	NM_001159533:707	Cacna1c	INSIDE	0.488	0.454	2298.28	1042.53	0.221	1910.78	423.18
A_68_P28033137	chr11:94334569-94334613	NM_001112813:922	Cacna1g	INSIDE	0.370	0.334	2173.74	726.93	0.124	1681.55	207.80
A_68_P30371303	chr15:80118250-80118294	NM_001044308:605	Cacna1i	INSIDE	0.520	0.557	1719.51	957.94	0.290	1356.02	392.85
A_68_P30371307	chr15:80118683-80118727	NM_001044308:1037	Cacna1i	INSIDE	0.586	0.546	1468.48	801.33	0.320	1132.34	362.15
A_68_P30371905	chr15:80202995-80203040	NM_001044308:85350	Cacna1i	INSIDE	0.619	6.483	113.92	738.50	4.011	142.16	570.23
A_68_P26808313	chr9:107301962-107302006	NM_001174047:-226	Cacna2d2	PROMOTER	0.189	0.654	1831.03	1197.03	0.124	1408.19	174.06
A_68_P26809310	chr9:107429107-107429155	NM_001174047:126920	Cacna2d2	INSIDE	0.226	0.430	1548.14	665.56	0.097	1204.64	117.14
A_68_P28053985	chr11:97883420-97883464	NM_001159320:-3756	Cacnb1	PROMOTER	0.403	0.437	1225.57	535.50	0.176	1003.72	176.92
A_68_P28053991	chr11:97884084-97884128	NM_145121:-165	Cacnb1	DIVERGENT_PROMOTER	0.582	0.583	1340.27	781.35	0.339	1030.71	349.81
A_68_P21221048	chr2:52531254-52531298	NM_001037099:825	Cacnb4	INSIDE	0.292	0.484	1120.73	542.76	0.141	907.44	128.19
A_68_P21221053	chr2:52531826-52531870	NM_001037099:253	Cacnb4	INSIDE	0.611	0.486	1392.83	676.68	0.297	1086.75	322.45
A_68_P28109099	chr11:107655337-107655381	NM_019431:420	Cacng4	INSIDE	0.318	0.648	1051.32	681.39	0.206	825.45	170.02
A_68_P28109102	chr11:107655644-107655688	NM_019431:112	Cacng4	INSIDE	0.484	0.556	1788.57	994.28	0.269	1320.97	355.36
A_68_P33016567	chr7:3424267-3424311	NM_133183:-1134	Cacng6	PROMOTER	0.460	0.707	1435.58	1015.24	0.325	1202.52	391.28
A_68_P33005171	chr7:3366221-3366265	NM_133189:29558	Cacng7	INSIDE	0.241	0.708	1610.43	1139.90	0.170	1209.08	205.95
A_68_P23576171	chr5:31357229-31357273	NM_023525:67	Cad	INSIDE	0.336	0.693	1630.74	1130.54	0.233	1334.36	310.43
A_68_P26486118	chr9:47338069-47338113	NM_001025600:-344	Cadm1	PROMOTER	0.530	0.478	1112.89	532.05	0.253	837.34	212.03
A_68_P26486122	chr9:47338579-47338623	NM_001025600:166	Cadm1	INSIDE	0.500	0.506	1109.75	561.40	0.253	835.76	211.30
A_68_P26486124	chr9:47338813-47338857	NM_001025600:400	Cadm1	INSIDE	0.593	0.689	1720.45	1185.19	0.409	1558.50	637.17
A_68_P26486125	chr9:47338950-47338994	NM_001025600:538	Cadm1	INSIDE	0.197	0.484	2492.18	1207.29	0.096	2010.82	192.05
A_68_P30836891	chr16:67620592-67620636	NM_001145977:539	Cadm2	INSIDE	0.655	0.618	1691.84	1046.08	0.405	1262.79	511.09
A_68_P30836892	chr16:67620668-67620712	NM_001145977:463	Cadm2	INSIDE	0.166	1.518	991.00	1504.13	0.251	720.76	181.25
A_68_P30836893	chr16:67620755-67620799	NM_001145977:377	Cadm2	INSIDE	0.281	0.666	1005.38	669.65	0.187	645.82	120.96
A_68_P20837649	chr1:175297409-175297453	NM_053199:396	Cadm3	INSIDE	0.346	0.488	2611.70	1273.63	0.169	1819.80	306.71
A_68_P20837653	chr1:175297800-175297844	NM_053199:4	Cadm3	INSIDE	0.235	0.486	3872.79	1882.95	0.114	2816.07	322.23
A_68_P29413818	chr14:13655644-13655688	NM_001042617:-73	Cadps	PROMOTER	0.171	0.411	2506.75	1031.03	0.070	2001.49	140.55

ProbeName	Target position of probe on CpG island microarray	TargetID	GeneSymbol	CpG island Description	Ratio of relative methylation (TiO2-NP/Vehicle)	Sham group			TiO_2-H group		
						Relative methylation (Cy-5/Cy-3)	Cy-3 signal (Input)	Cy-5 signal (MeDIP)	Relative methylation (Cy-5/Cy-3)	Cy-3 signal (Input)	Cy-5 signal (MeDIP)
A_68_P28680556	chr12:101437490-101437534	NM_009790:-238	Calm1	PROMOTER	0.361	0.354	2330.47	824.17	0.128	1909.05	243.64
A_68_P28680559	chr12:101437798-101437842	NM_009790:70	Calm1	INSIDE	0.545	0.438	1423.84	623.99	0.239	1086.39	259.37
A_68_P31423374	chr17:87846116-87846160	NM_007589:137	Calm2	INSIDE	0.259	0.559	3168.11	1772.10	0.145	2303.86	333.19
A_68_P24093836	chr5:130925066-130925110	NM_021371:427	Caln1	INSIDE	0.276	0.422	1512.80	638.21	0.117	1137.34	132.63
A_68_P22503486	chr3:126300384-126300428	NM_001025438:516	Camk2d	INSIDE	0.116	0.600	2437.09	1463.02	0.070	1683.84	117.34
A_68_P22503487	chr3:126300546-126300590	NM_001025438:678	Camk2d	INSIDE	0.391	0.431	2047.11	883.00	0.169	1532.12	258.42
A_68_P29449128	chr14:21612623-21612667	NM_001039138:666	Camk2g	INSIDE	0.641	0.738	3410.83	2517.97	0.473	2557.73	1209.41
A_68_P29449134	chr14:21613118-21613162	NM_001039138:170	Camk2g	INSIDE	0.529	0.294	1853.08	544.01	0.155	1427.08	221.52
A_68_P23345839	chr4:138009933-138009977	NM_025451:-1108	Camk2n1	PROMOTER	0.230	0.499	1350.39	673.19	0.114	1018.54	116.58
A_68_P23345849	chr4:138011542-138011586	NM_025451:502	Camk2n1	INSIDE	0.625	0.631	1900.64	1199.08	0.394	1338.71	528.10
A_68_P30588523	chr16:20619843-20619887	NM_028420:1487	Camk2n2	INSIDE	0.491	1.360	5028.18	6836.13	0.667	3604.50	2405.53
A_68_P30588528	chr16:20620501-20620545	NM_028420:829	Camk2n2	INSIDE	0.212	2.275	5785.35	13159.28	0.483	3595.98	1735.95
A_68_P30588530	chr16:20620757-20620801	NM_028420:573	Camk2n2	INSIDE	0.435	0.471	2118.12	998.07	0.205	1663.51	341.23
A_68_P30588535	chr16:20621603-20621647	NM_028420:-273	Camk2n2	DIVERGENT_PROMOTER	0.667	0.443	1624.09	719.47	0.295	1327.82	392.09
A_68_P30588537	chr16:20621774-20621818	NM_028420:-445	Camk2n2	DIVERGENT_PROMOTER	0.368	0.486	2447.06	1190.31	0.179	1934.82	346.13
A_68_P30588538	chr16:20621896-20621940	NM_028420:-567	Camk2n2	DIVERGENT_PROMOTER	0.229	0.430	2415.28	1037.65	0.099	1723.21	169.85
A_68_P30588539	chr16:20621997-20622043	NM_028420:-669	Camk2n2	DIVERGENT_PROMOTER	0.257	0.384	1628.91	626.14	0.099	1210.12	119.78
A_68_P30588540	chr16:20622060-20622119	NM_028420:-738	Camk2n2	DIVERGENT_PROMOTER	0.232	0.667	992.97	662.42	0.155	753.11	116.79
A_68_P30588542	chr16:20622241-20622285	NM_028420:-911	Camk2n2	DIVERGENT_PROMOTER	0.237	0.583	1427.54	831.71	0.138	1042.94	143.75
A_68_P30588544	chr16:20622405-20622449	NM_028420:-1075	Camk2n2	DIVERGENT_PROMOTER	0.170	0.624	1347.74	840.42	0.106	1107.61	117.49
A_68_P26811951	chr9:107838581-107838626	NM_145621:353	Camkv	INSIDE	0.297	0.469	1168.01	548.13	0.139	860.37	119.89
A_68_P21075525	chr2:25837701-25837745	NM_001115076:1080	Camsap1	INSIDE	0.210	0.619	5224.55	3234.22	0.130	3672.23	476.80
A_68_P21075526	chr2:25837853-25837902	NM_001115076:925	Camsap1	INSIDE	0.432	0.652	1123.64	733.04	0.282	904.28	254.82
A_68_P21075527	chr2:25838017-25838061	NM_001115076:764	Camsap1	INSIDE	0.162	0.734	3121.54	2289.68	0.119	2110.39	250.73
A_68_P21075528	chr2:25838113-25838157	NM_001115076:668	Camsap1	INSIDE	0.491	0.707	3109.82	2199.19	0.347	2264.66	786.34
A_68_P23410164	chr4:150482143-150482187	NM_001081557:753713	Camta1	INSIDE	0.347	0.648	3995.98	2590.53	0.225	2728.26	614.28
A_68_P23410166	chr4:150482290-150482334	NM_001081557:753565	Camta1	INSIDE	0.240	0.480	6637.68	3189.30	0.115	4849.80	559.19
A_68_P23410311	chr4:150503055-150503099	NM_001081557:732801	Camta1	INSIDE	0.316	0.605	1254.59	758.51	0.191	959.68	183.07
A_68_P23415140	chr4:151236795-151236839	NM_001081557:-939	Camta1	PROMOTER	0.500	0.631	1044.21	659.27	0.316	825.57	260.62
A_68_P27902011	chr11:70483347-70483401	NM_001190376:18233	Camta2	INSIDE	0.238	1.484	1534.86	2277.19	0.353	1109.53	392.16
A_68_P27902141	chr11:70501579-70501623	NM_001190376:7	Camta2	INSIDE	0.223	0.408	1899.04	774.26	0.091	1384.81	125.96
A_68_P27489860	chr10:118677501-118677545	NM_027994:-411	Cand1	PROMOTER	0.253	0.658	1529.32	1006.46	0.167	1147.21	191.22
A_68_P24772854	chr6:115724644-115724688	NM_025958:92	Cand2	INSIDE	0.104	1.742	3122.07	5438.23	0.182	2126.47	386.65
A_68_P27788009	chr11:50139294-50139338	NM_001110499:-141	Canx	PROMOTER	0.471	0.466	1378.00	642.66	0.220	1091.46	239.59
A_68_P23259659	chr4:122562951-122562995	NM_007598:152	Cap1	INSIDE	0.386	0.563	1933.48	1087.93	0.217	1356.49	294.41
A_68_P23259661	chr4:122563178-122563222	NM_007598:-76	Cap1	PROMOTER	0.165	0.622	1470.39	914.75	0.102	1174.15	120.25
A_68_P31932216	chr19:6015590-6015634	NM_007600:-365	Capn1	PROMOTER	0.297	0.502	2108.34	1058.29	0.149	1674.37	249.26
A_68_P31932217	chr19:6015773-6015817	NM_007600:-547	Capn1	PROMOTER	0.344	0.592	2441.53	1444.31	0.203	1880.58	382.65
A_68_P31202232	chr17:45767295-45767346	NM_001013767:28938	Capn11	INSIDE	0.201	0.545	1439.37	784.15	0.109	1084.88	118.51
A_68_P20887292	chr1:184447385-184447429	NM_009794:208	Capn2	INSIDE	0.214	0.529	1722.29	911.49	0.113	1424.86	161.52
A_68_P25354352	chr7:105326428-105326472	NM_007602:253	Capn5	INSIDE	0.392	0.666	2220.45	1478.16	0.261	1546.65	403.21
A_68_P24942635	chr6:148844880-148844924	NM_181541:-254	Caprin2	PROMOTER	0.288	0.636	1656.30	1053.88	0.183	1278.52	233.94
A_68_P22393605	chr3:104667362-104667406	NM_009797:39	Capza1	INSIDE	0.621	0.739	2537.34	1875.67	0.459	2024.34	928.75
A_68_P28025142	chr11:92961341-92961387	NM_028296:761	Car10	INSIDE	0.027	1.596	3914.31	6247.50	0.043	2760.61	119.50

ProbeName	Target position of probe on CpG island microarray	TargetID	GeneSymbol	CpG island Description	Ratio of relative methylation (TiO2-NP/Vehicle)	Sham group			TiO_2-H group		
						Relative methylation (Cy-5/Cy-3)	Cy-3 signal (Input)	Cy-5 signal (MeDIP)	Relative methylation (Cy-5/Cy-3)	Cy-3 signal (Input)	Cy-5 signal (MeDIP)
A_68_P28025143	chr11:92961409-92961453	NM_028296:827	Car10	INSIDE	0.168	1.628	4195.42	6831.32	0.273	2752.95	752.64
A_68_P27980570	chr11:84771638-84771682	NM_007607:405	Car4	INSIDE	0.173	0.465	2011.08	935.28	0.081	1491.58	120.23
A_68_P27980571	chr11:84771814-84771858	NM_007607:581	Car4	INSIDE	0.238	0.464	1397.64	648.16	0.110	1205.84	132.91
A_68_P26129050	chr8:107065100-107065144	NM_053070:416	Car7	INSIDE	0.623	0.616	1457.84	898.37	0.384	1326.11	509.07
A_68_P30362466	chr15:78632748-78632792	NM_130859:702	Card10	INSIDE	0.500	0.581	1544.53	897.58	0.290	1268.59	368.48
A_68_P25655856	chr8:11497567-11497611	NM_026995:21	Carkd	INSIDE	0.632	0.426	1901.22	809.80	0.269	1558.69	419.41
A_68_P31921523	chr19:4169744-4169788	NM_134148:5713	Carns1	INSIDE	0.213	0.718	2056.57	1476.48	0.153	1352.51	206.71
A_68_P29265994	chr13:100670562-100670606	NM_001081493:54	Cartpt	INSIDE	0.613	0.490	1785.71	874.59	0.300	1439.31	432.37
A_68_P24921625	chr6:145159463-145159508	NM_177222:5	Casc1	INSIDE	0.282	0.611	1361.87	831.85	0.172	1001.41	172.43
A_68_P21583233	chr2:121692537-121692581	NM_001205369:-147	Casc4	PROMOTER	0.168	0.650	1582.77	1029.49	0.109	1109.67	121.50
A_68_P24205537	chr6:4550857-4550901	NM_145398:-187	Casd1	PROMOTER	0.666	0.598	1044.51	624.91	0.398	851.82	339.45
A_68_P28155482	chr11:115674860-115674904	NM_080643:24	Caskin2	INSIDE	0.193	0.515	1341.42	691.00	0.099	1189.23	118.05
A_68_P22522754	chr3:129604639-129604686	NM_009811:320	Casp6	INSIDE	0.308	0.684	1224.78	837.20	0.210	872.90	183.60
A_68_P32200577	chr19:56471803-56471847	NM_007611:206	Casp7	INSIDE	0.200	0.544	2040.89	1110.40	0.109	1849.95	200.84
A_68_P22822455	chr4:32702059-32702108	NM_001122978:-364	Casp8ap2	PROMOTER	0.438	0.588	1196.61	703.15	0.257	1015.33	261.36
A_68_P22822463	chr4:32702764-32702814	NM_001122978:341	Casp8ap2	INSIDE	0.250	0.431	1458.07	627.89	0.108	1082.94	116.47
A_68_P23366007	chr4:141349370-141349414	NM_015733:-134	Casp9	DIVERGENT_PROMOTER	0.184	1.354	4764.33	6450.46	0.249	3352.77	836.26
A_68_P23366008	chr4:141349558-141349602	NM_015733:54	Casp9	INSIDE	0.525	3.292	6717.58	22113.85	1.728	4665.93	8061.83
A_68_P23396583	chr4:148179764-148179808	NM_027195:1286	Casz1	INSIDE	0.646	0.546	2151.18	1175.23	0.353	1743.54	615.11
A_68_P23396586	chr4:148180019-148180067	NM_027195:1542	Casz1	INSIDE	0.254	0.482	1461.78	704.77	0.122	1024.98	125.37
A_68_P23396591	chr4:148180645-148180689	NM_027195:2166	Casz1	INSIDE	0.247	0.605	1078.19	652.15	0.150	851.78	127.34
A_68_P23397203	chr4:148265241-148265285	NM_027195:86762	Casz1	INSIDE	0.323	0.745	3039.69	2264.27	0.241	2187.80	526.88
A_68_P23397714	chr4:148326567-148326611	NM_001159344:148088	Casz1	INSIDE	0.319	0.446	4925.34	2196.24	0.142	3600.04	511.90
A_68_P27170204	chr10:59165501-59165545	NM_144822:140	Cbara1	INSIDE	0.122	0.649	2515.02	1632.99	0.079	1963.89	155.06
A_68_P21760474	chr2:154262689-154262733	NM_009823:491	Cbfa2t2	INSIDE	0.462	0.638	1453.33	927.53	0.295	1239.88	365.91
A_68_P28331589	chr12:32183737-32183789	NM_134048:643	Cbll1	INSIDE	0.331	0.464	1148.20	533.15	0.154	969.88	149.27
A_68_P28331590	chr12:32183836-32183892	NM_134048:541	Cbll1	INSIDE	0.246	0.561	1151.20	645.59	0.138	906.59	125.16
A_68_P28331599	chr12:32184853-32184897	NM_134048:-469	Cbll1	PROMOTER	0.383	0.648	1114.88	722.24	0.248	898.13	223.06
A_68_P26035014	chr8:89994814-89994859	NM_019626:1655	Cbln1	INSIDE	0.169	0.540	1620.93	875.25	0.091	1331.72	121.85
A_68_P26035015	chr8:89994889-89994933	NM_019626:1581	Cbln1	INSIDE	0.254	0.579	1616.71	935.56	0.147	1169.52	171.59
A_68_P26035016	chr8:89994962-89995006	NM_019626:1507	Cbln1	INSIDE	0.163	0.547	1863.52	1019.39	0.089	1317.44	117.26
A_68_P26035027	chr8:89996039-89996083	NM_019626:431	Cbln1	INSIDE	0.313	0.497	2682.57	1334.19	0.156	1960.44	305.02
A_68_P31899538	chr18:86881037-86881081	NM_172633:-1381	Cbln2	PROMOTER	0.067	1.702	14900.30	25364.05	0.114	9814.59	1123.72
A_68_P31899539	chr18:86881114-86881158	NM_172633:-1303	Cbln2	PROMOTER	0.075	2.705	5819.18	15742.21	0.203	3236.54	658.59
A_68_P31899540	chr18:86881200-86881249	NM_172633:-1215	Cbln2	PROMOTER	0.033	3.469	1983.08	6878.59	0.114	1062.30	120.80
A_68_P31899554	chr18:86882710-86882754	NM_172633:293	Cbln2	INSIDE	0.337	0.554	1159.27	642.23	0.186	982.22	183.16
A_68_P21862266	chr2:171868455-171868499	NM_175631:490	Cbln4	INSIDE	0.220	0.476	1245.34	593.24	0.105	1123.41	117.97
A_68_P30976751	chr16:93608089-93608133	NM_007620:29	Cbr1	INSIDE	0.356	0.552	1337.60	738.25	0.196	1234.39	242.53
A_68_P30977032	chr16:93683403-93683447	NM_173047:-39	Cbr3	PROMOTER	0.558	0.567	1827.85	1036.24	0.316	1438.18	454.79
A_68_P30977033	chr16:93683476-93683520	NM_173047:35	Cbr3	INSIDE	0.575	0.313	1765.34	552.66	0.180	1377.70	248.14
A_68_P30977036	chr16:93683818-93683862	NM_173047:377	Cbr3	INSIDE	0.255	0.518	2863.07	1482.08	0.132	2135.60	281.40
A_68_P31140913	chr17:31773722-31773766	NM_178224:332	Cbs	INSIDE	0.265	0.493	1674.74	825.27	0.130	1345.15	175.42
A_68_P31140915	chr17:31773909-31773953	NM_178224:146	Cbs	INSIDE	0.312	0.708	2004.67	1420.17	0.221	1690.64	373.21
A_68_P31140916	chr17:31774060-31774104	NM_178224:-6	Cbs	PROMOTER	0.232	0.365	1673.06	610.58	0.085	1378.49	116.65

ProbeName	Target position of probe on CpG island microarray	TargetID	GeneSymbol	CpG island Description	Ratio of relative methylation (TiO2-NP/Vehicle)	Sham group			TiO_2-H group		
						Relative methylation (Cy-5/Cy-3)	Cy-3 signal (Input)	Cy-5 signal (MeDIP)	Relative methylation (Cy-5/Cy-3)	Cy-3 signal (Input)	Cy-5 signal (MeDIP)
A_68_P24444437	chr6:51420446-51420490	NM_007624:-146	Cbx3	DIVERGENT_PROMOTER	0.193	0.670	1005.84	674.35	0.129	914.19	118.11
A_68_P28175793	chr11:118951344-118951390	NM_007625:-3815	Cbx4	PROMOTER	0.353	1.551	4847.13	7516.25	0.547	3290.46	1798.99
A_68_P28175796	chr11:118951645-118951689	NM_007625:-4115	Cbx4	PROMOTER	0.313	0.570	1703.95	971.16	0.178	1352.97	241.16
A_68_P28175797	chr11:118951776-118951826	NM_007625:-4249	Cbx4	PROMOTER	0.465	0.712	1396.94	995.03	0.332	1080.57	358.28
A_68_P28175801	chr11:118952251-118952295	NM_007625:-4721	Cbx4	PROMOTER	0.184	0.488	2060.99	1006.73	0.090	1576.34	141.99
A_68_P28175804	chr11:118952556-118952600	NM_007625:-5027	Cbx4	PROMOTER	0.437	0.467	1441.59	672.56	0.204	1179.66	240.53
A_68_P30502657	chr15:103013103-103013147	NM_001110216:32659	Cbx5	DOWNSTREAM	0.663	0.512	1157.92	592.32	0.339	1091.68	370.28
A_68_P30369303	chr15:79763884-79763928	NM_144811:-830	Cbx7	PROMOTER	0.381	0.614	1036.84	637.03	0.234	902.92	211.45
A_68_P30369305	chr15:79764056-79764100	NM_144811:-1002	Cbx7	PROMOTER	0.243	0.517	1337.74	692.07	0.126	1058.17	133.11
A_68_P28175446	chr11:118902080-118902124	NM_013926:125	Cbx8	INSIDE	0.178	0.588	3311.35	1947.04	0.105	2201.83	230.95
A_68_P28175448	chr11:118902325-118902369	NM_013926:-119	Cbx8	PROMOTER	0.467	0.643	2083.81	1339.54	0.300	1467.55	440.81
A_68_P28175453	chr11:118902817-118902861	NM_013926:-611	Cbx8	PROMOTER	0.267	0.668	5932.39	3962.21	0.178	4121.47	734.38
A_68_P28175454	chr11:118902921-118902967	NM_013926:-717	Cbx8	PROMOTER	0.119	0.624	2397.33	1496.36	0.075	1616.81	120.46
A_68_P26018414	chr8:86671587-86671631	NM_145970:44	Cc2d1a	INSIDE	0.424	0.656	1816.16	1190.95	0.278	1331.89	370.06
A_68_P23196910	chr4:108292768-108292812	NM_177045:230	Cc2d1b	INSIDE	0.539	0.612	4166.43	2548.41	0.330	2976.81	981.07
A_68_P23196911	chr4:108292854-108292898	NM_177045:316	Cc2d1b	INSIDE	0.172	0.657	1710.43	1124.28	0.113	1164.67	131.62
A_68_P23196912	chr4:108293003-108293049	NM_177045:466	Cc2d1b	INSIDE	0.386	0.559	1352.55	755.60	0.215	918.88	197.95
A_68_P27187842	chr10:62254957-62255001	NM_026201:138	Ccar1	INSIDE	0.201	0.465	1658.33	771.11	0.093	1248.31	116.57
A_68_P31786168	chr18:66451122-66451166	NM_178793:348	Ccbe1	INSIDE	0.132	0.583	2758.49	1607.95	0.077	2137.94	164.38
A_68_P21101913	chr2:30061520-30061564	NM_172404:-323	Ccbl1	PROMOTER	0.223	0.406	3242.53	1316.38	0.091	2507.03	227.35
A_68_P21101914	chr2:30061663-30061707	NM_172404:-465	Ccbl1	PROMOTER	0.300	0.497	2037.06	1013.36	0.149	1516.18	226.33
A_68_P22592103	chr3:142364167-142364211	NM_173763:145	Ccbl2	INSIDE	0.233	0.573	2890.94	1655.59	0.133	2268.19	302.15
A_68_P26080248	chr8:97441798-97441842	NM_001033533:178	Ccdc102a	INSIDE	0.202	0.696	1400.12	974.39	0.141	1161.98	163.33
A_68_P27275483	chr10:78215546-78215590	NM_027630:244	Ccdc105	INSIDE	0.327	0.515	1222.98	630.21	0.168	988.39	166.34
A_68_P27275484	chr10:78215712-78215756	NM_027630:78	Ccdc105	INSIDE	0.181	0.558	2358.13	1314.68	0.101	1792.99	181.33
A_68_P24951853	chr7:5011977-5012021	NM_146178:3671	Ccdc106	INSIDE	0.463	0.432	1382.32	596.92	0.200	1177.84	235.28
A_68_P22871974	chr4:43506583-43506627	NM_001037913:368	Ccdc107	INSIDE	0.334	0.565	1528.06	862.84	0.188	1207.56	227.47
A_68_P22523142	chr3:129672517-129672561	NM_025779:586	Ccdc109b	INSIDE	0.392	0.505	2604.71	1315.65	0.198	1951.55	386.76
A_68_P27563901	chr11:5442216-5442260	NM_134033:-18	Ccdc117	PROMOTER	0.344	0.718	1718.50	1233.47	0.247	1349.64	333.42
A_68_P26825042	chr9:110559281-110559325	NM_028312:296	Ccdc12	INSIDE	0.482	0.617	2313.78	1428.18	0.297	1753.97	521.53
A_68_P29737459	chr14:77436746-77436790	NM_175369:190	Ccdc122	INSIDE	0.502	0.696	1125.01	783.27	0.350	1001.93	350.47
A_68_P29269562	chr13:101439843-101439887	NM_001168386:429	Ccdc125	INSIDE	0.258	0.498	1358.06	676.80	0.129	1221.28	157.21
A_68_P24432032	chr6:49269455-49269499	NM_175098:125	Ccdc126	INSIDE	0.313	0.599	1074.03	643.78	0.188	910.61	170.88
A_68_P24432033	chr6:49269637-49269686	NM_175098:310	Ccdc126	INSIDE	0.537	0.626	854.45	535.11	0.336	757.41	254.51
A_68_P26885914	chr9:121748048-121748092	NM_028384:509	Ccdc13	INSIDE	0.565	0.632	920.03	581.07	0.357	657.55	234.52
A_68_P26885918	chr9:121748458-121748502	NM_028384:99	Ccdc13	INSIDE	0.609	0.709	1349.44	956.93	0.432	1078.83	465.64
A_68_P26019094	chr8:86794146-86794190	NM_026350:91	Ccdc130	INSIDE	0.667	0.639	2432.78	1555.21	0.426	1849.34	788.59
A_68_P30381572	chr15:81958155-81958201	NM_172428:-173	Ccdc134	PROMOTER	0.161	0.544	2619.49	1425.71	0.088	1801.50	157.69
A_68_P30381575	chr15:81958547-81958591	NM_172428:217	Ccdc134	INSIDE	0.184	0.688	2056.79	1414.21	0.126	1528.30	193.02
A_68_P30381577	chr15:81958707-81958759	NM_172428:381	Ccdc134	INSIDE	0.040	1.497	3033.92	4542.02	0.060	2027.68	122.01
A_68_P28184671	chr11:120319473-120319517	NM_152807:52	Ccdc137	INSIDE	0.360	0.574	2047.05	1174.06	0.206	1632.56	336.62
A_68_P28184672	chr11:120319589-120319633	NM_152807:168	Ccdc137	INSIDE	0.155	0.666	1592.81	1060.55	0.103	1147.62	118.38
A_68_P26430108	chr9:37155823-37155867	NM_001081429:133	Ccdc15	INSIDE	0.176	0.675	1495.98	1009.74	0.119	1046.17	124.28
A_68_P23895411	chr5:93104067-93104111	NM_177230:65	Ccdc158	INSIDE	0.534	0.730	1930.12	1408.97	0.390	1535.13	598.00

ProbeName	Target position of probe on CpG island microarray	TargetID	GeneSymbol	CpG island Description	Ratio of relative methylation (TiO2-NP/Vehicle)	Sham group			TiO_2-H group		
						Relative methylation (Cy-5/Cy-3)	Cy-3 signal (Input)	Cy-5 signal (MeDIP)	Relative methylation (Cy-5/Cy-3)	Cy-3 signal (Input)	Cy-5 signal (MeDIP)
A_68_P23321454	chr4:133742757-133742801	NM_144527:222	Ccdc21	INSIDE	0.194	0.580	1512.87	877.58	0.112	1021.32	114.73
A_68_P23321458	chr4:133743204-133743248	NM_144527:-226	Ccdc21	PROMOTER	0.245	0.628	980.49	615.47	0.154	795.81	122.63
A_68_P23245373	chr4:119087968-119088012	NM_029286:136	Ccdc30	INSIDE	0.375	0.511	2076.30	1061.75	0.192	1777.26	340.50
A_68_P23245376	chr4:119088209-119088253	NM_029286:-104	Ccdc30	PROMOTER	0.341	0.653	1052.61	687.63	0.223	849.73	189.12
A_68_P21518697	chr2:109857970-109858014	NM_026613:19	Ccdc34	INSIDE	0.250	0.385	1651.79	636.17	0.096	1266.09	121.78
A_68_P27360780	chr10:94151776-94151822	NM_029852:264	Ccdc41	INSIDE	0.225	0.510	1519.35	774.16	0.114	1119.43	128.11
A_68_P28103527	chr11:106650485-106650529	NM_001166685:-59	Ccdc45	DIVERGENT_PROMOTER	0.190	0.524	1551.87	813.42	0.099	1214.68	120.75
A_68_P24617811	chr6:87680675-87680719	NM_001159697:-166	Ccdc48	PROMOTER	0.351	0.727	4984.09	3625.21	0.256	3364.11	859.84
A_68_P24617829	chr6:87682986-87683030	NM_001159697:2146	Ccdc48	INSIDE	0.363	0.632	1753.37	1108.15	0.229	1543.22	354.13
A_68_P28187623	chr11:120794145-120794189	NM_027745:20	Ccdc57	INSIDE	0.485	0.666	1253.90	834.77	0.323	1006.35	324.87
A_68_P28187624	chr11:120794237-120794281	NM_027745:-72	Ccdc57	PROMOTER	0.531	0.630	979.24	616.72	0.334	694.21	232.19
A_68_P28187625	chr11:120794313-120794357	NM_027745:-148	Ccdc57	PROMOTER	0.526	0.484	1751.38	848.39	0.255	1263.67	321.70
A_68_P30673619	chr16:36072197-36072241	NM_001159422:-9142	Ccdc58	PROMOTER	0.478	0.554	1163.45	644.47	0.265	915.06	242.18
A_68_P27418182	chr10:105278621-105278665	NM_025602:108	Ccdc59	INSIDE	0.348	0.601	1307.64	785.41	0.209	1008.25	210.65
A_68_P27228313	chr10:69652213-69652261	NM_001111121:92368	Ccdc6	INSIDE	0.590	3.830	1022.68	3916.45	2.259	799.74	1807.01
A_68_P24990312	chr7:19477759-19477803	NM_001033314:17973	Ccdc61	INSIDE	0.630	0.421	1511.52	636.40	0.265	1289.45	341.99
A_68_P24990418	chr7:19495547-19495597	NM_001033314:181	Ccdc61	INSIDE	0.202	0.598	1461.41	873.69	0.121	1130.10	136.73
A_68_P24011279	chr5:116181126-116181170	NM_001080808:420	Ccdc64	INSIDE	0.242	0.452	2184.71	988.45	0.109	1684.64	184.15
A_68_P26814948	chr9:108354920-108354964	NM_133744:-7906	Ccdc71	PROMOTER	0.258	0.682	2070.73	1412.54	0.176	1567.50	275.79
A_68_P26814949	chr9:108355021-108355065	NM_133744:-7806	Ccdc71	PROMOTER	0.275	0.418	1408.81	588.95	0.115	1134.04	130.56
A_68_P26814988	chr9:108363033-108363077	NM_133744:206	Ccdc71	INSIDE	0.172	0.549	1732.60	951.35	0.094	1255.02	118.20
A_68_P31373928	chr17:79234991-79235035	NM_181649:157	Ccdc75	INSIDE	0.490	0.608	1355.91	823.81	0.297	1059.03	314.99
A_68_P26128839	chr8:107033201-107033245	NM_180958:565	Ccdc79	INSIDE	0.544	0.619	1433.66	887.98	0.337	1088.20	366.73
A_68_P26467525	chr9:44225880-44225924	NM_201372:188	Ccdc84	INSIDE	0.433	0.632	3188.92	2014.72	0.274	2379.71	650.91
A_68_P26467527	chr9:44226161-44226205	NM_201372:-92	Ccdc84	PROMOTER	0.277	0.654	1166.34	762.83	0.181	1031.60	186.87
A_68_P28727042	chr12:109514039-109514083	NM_001159910:-433	Ccdc85c	PROMOTER	0.112	0.683	2271.74	1550.90	0.077	1539.47	118.17
A_68_P31953831	chr19:11023233-11023277	NM_023731:502	Ccdc86	INSIDE	0.408	0.643	889.16	571.31	0.262	716.46	187.98
A_68_P27674153	chr11:29274024-29274068	NM_176841:272	Ccdc88a	INSIDE	0.567	0.537	954.11	512.21	0.304	822.91	250.40
A_68_P31936460	chr19:6930124-6930168	NM_001081291:2555	Ccdc88b	INSIDE	0.459	0.654	2669.86	1745.79	0.300	2085.51	626.56
A_68_P28685558	chr12:102267375-102267419	NM_026681:-203	Ccdc88c	PROMOTER	0.270	0.407	1691.89	689.44	0.110	1231.55	135.74
A_68_P25325434	chr7:99709780-99709824	NM_001162918:144	Ccdc90b	INSIDE	0.647	0.703	1190.19	836.67	0.454	937.76	426.20
A_68_P24934669	chr6:147424894-147424938	NM_025911:524	Ccdc91	INSIDE	0.323	0.543	1210.07	657.52	0.175	1003.08	175.85
A_68_P31252978	chr17:56098506-56098550	NM_028381:-81	Ccdc94	PROMOTER	0.565	0.506	1125.72	569.95	0.286	834.62	238.95
A_68_P31252979	chr17:56098596-56098641	NM_028381:9	Ccdc94	INSIDE	0.300	0.628	842.30	529.27	0.189	638.62	120.51
A_68_P23607907	chr5:36827498-36827542	NM_025725:284	Ccdc96	INSIDE	0.385	0.615	1350.55	830.38	0.236	1084.30	256.36
A_68_P25011514	chr7:26503814-26503858	NM_028771:236	Ccdc97	INSIDE	0.185	0.624	1508.25	941.49	0.116	1041.96	120.40
A_68_P25011515	chr7:26503925-26503969	NM_028771:126	Ccdc97	INSIDE	0.437	0.397	2050.70	813.70	0.173	1600.72	277.58
A_68_P27702675	chr11:34646901-34646945	NM_027411:221	Ccdc99	INSIDE	0.216	0.342	1771.09	606.24	0.074	1558.20	114.97
A_68_P27702677	chr11:34647134-34647181	NM_027411:-14	Ccdc99	PROMOTER	0.227	0.567	1373.61	779.00	0.129	949.47	122.32
A_68_P26883803	chr9:121403379-121403423	NM_031161:1407	Cck	INSIDE	0.513	0.743	2674.70	1987.12	0.381	1869.53	711.97
A_68_P26883806	chr9:121403762-121403806	NM_031161:1023	Cck	INSIDE	0.203	0.591	2642.63	1560.77	0.120	1986.84	238.69
A_68_P25392269	chr7:112574333-112574377	NM_007627:21	Cckbr	INSIDE	0.204	0.744	3037.40	2261.06	0.152	2333.85	354.73
A_68_P25392273	chr7:112574745-112574790	NM_007627:434	Cckbr	INSIDE	0.133	1.597	1130.99	1806.52	0.213	807.15	172.02
A_68_P22058853	chr3:36470390-36470434	NM_009828:506	Ccna2	INSIDE	0.149	0.518	3345.40	1733.49	0.077	2152.20	166.69

ProbeName	Target position of probe on CpG island microarray	TargetID	GeneSymbol	CpG island Description	Ratio of relative methylation (TiO2-NP/Vehicle)	Sham group			TiO_2-H group		
						Relative methylation (Cy-5/Cy-3)	Cy-3 signal (Input)	Cy-5 signal (MeDIP)	Relative methylation (Cy-5/Cy-3)	Cy-3 signal (Input)	Cy-5 signal (MeDIP)
A_68_P22058854	chr3:36470470-36470514	NM_009828:426	Ccna2	INSIDE	0.142	0.588	5226.28	3071.76	0.083	3578.10	297.82
A_68_P25610226	chr7:152128254-152128298	NM_007631:-2446	Ccnd1	PROMOTER	0.662	0.407	1574.16	639.92	0.269	1284.45	345.41
A_68_P25610231	chr7:152128709-152128753	NM_007631:-2900	Ccnd1	PROMOTER	0.364	0.447	1161.38	519.42	0.163	858.28	139.89
A_68_P24831041	chr6:127100503-127100547	NM_009829:542	Ccnd2	INSIDE	0.155	0.605	1758.37	1063.19	0.094	1315.53	123.56
A_68_P24831080	chr6:127105235-127105279	NM_009829:-4190	Ccnd2	PROMOTER	0.640	0.712	1507.40	1073.68	0.456	1322.49	603.22
A_68_P21578342	chr2:120834147-120834191	NM_010761:25	Ccndbp1	INSIDE	0.207	0.497	1474.35	732.77	0.103	1131.62	116.70
A_68_P25065442	chr7:38892952-38892996	NM_007633:-465	Ccne1	PROMOTER	0.350	0.497	4907.98	2437.19	0.174	3349.10	581.82
A_68_P23898891	chr5:93696983-93697027	NM_007635:406	Ccng2	INSIDE	0.281	0.502	1240.12	622.20	0.141	890.36	125.31
A_68_P32111334	chr19:40906541-40906585	NM_172839:794	Ccnj	INSIDE	0.638	0.367	1696.24	621.81	0.234	1386.38	324.13
A_68_P27750956	chr11:43342693-43342737	NM_001045530:429	Ccnjl	INSIDE	0.486	0.645	2025.15	1305.63	0.313	1622.45	508.59
A_68_P22202884	chr3:65762093-65762137	NM_019937:33	Ccnl1	INSIDE	0.642	0.497	2814.84	1398.89	0.319	2196.65	700.89
A_68_P22202886	chr3:65762239-65762283	NM_019937:-113	Ccnl1	PROMOTER	0.516	0.713	1822.10	1298.53	0.368	1502.71	552.31
A_68_P22202887	chr3:65762326-65762370	NM_019937:-201	Ccnl1	PROMOTER	0.519	0.591	1278.90	756.02	0.307	1053.73	323.14
A_68_P23440735	chr4:155186739-155186783	NM_207678:163	Ccnl2	INSIDE	0.188	0.500	4519.25	2258.53	0.094	3204.71	300.89
A_68_P30475237	chr15:98398586-98398630	NM_009833:-541	Ccnt1	PROMOTER	0.270	0.497	1171.00	581.59	0.134	878.82	117.85
A_68_P20596873	chr1:129670505-129670549	NM_028399:-214	Ccnt2	PROMOTER	0.362	0.696	1897.84	1321.27	0.252	1332.43	335.56
A_68_P20596874	chr1:129670673-129670717	NM_028399:-46	Ccnt2	PROMOTER	0.636	0.622	2718.22	1689.58	0.395	2117.80	837.57
A_68_P31485624	chr18:9449444-9449488	NM_026484:682	Ccny	INSIDE	0.364	0.626	1079.22	675.59	0.228	843.35	192.32
A_68_P31485630	chr18:9450058-9450102	NM_026484:68	Ccny	INSIDE	0.263	0.645	1115.46	719.09	0.170	965.71	163.73
A_68_P28072387	chr11:101035328-101035372	NM_007721:1407	Ccr10	INSIDE	0.355	0.389	2889.98	1123.43	0.138	2081.47	287.14
A_68_P28072388	chr11:101035404-101035448	NM_007721:1331	Ccr10	INSIDE	0.637	0.568	1405.57	798.95	0.362	1007.15	364.74
A_68_P22128529	chr3:51028612-51028656	NM_009834:266	Ccrn4l	INSIDE	0.266	0.692	4112.17	2845.24	0.184	3149.48	578.75
A_68_P22128530	chr3:51028680-51028724	NM_009834:334	Ccrn4l	INSIDE	0.153	0.490	2018.20	989.37	0.075	1568.82	117.60
A_68_P27479232	chr10:116500293-116500343	NM_007636:552	Cct2	INSIDE	0.292	0.702	1693.56	1188.50	0.205	1169.03	239.89
A_68_P22312956	chr3:88101171-88101215	NM_009836:136	Cct3	INSIDE	0.306	0.528	1948.15	1028.32	0.162	1464.90	236.89
A_68_P27643749	chr11:22890683-22890727	NM_009837:112	Cct4	INSIDE	0.465	0.693	1394.15	966.78	0.322	1140.92	367.52
A_68_P24605098	chr6:85401665-85401709	NM_007638:188	Cct7	INSIDE	0.177	0.492	1677.19	825.62	0.087	1338.67	116.59
A_68_P30940063	chr16:87495606-87495650	NM_009840:486	Cct8	INSIDE	0.286	0.625	3419.51	2137.75	0.179	2726.76	487.36
A_68_P30940064	chr16:87495686-87495730	NM_009840:406	Cct8	INSIDE	0.131	0.663	1643.15	1089.66	0.087	1360.88	118.03
A_68_P24159039	chr5:144775595-144775639	NM_177682:106	Ccz1	INSIDE	0.351	0.622	2033.64	1265.35	0.219	1557.24	340.40
A_68_P26660362	chr9:78463468-78463512	NM_153098:138	Cd109	INSIDE	0.351	0.433	1680.35	727.12	0.152	1438.78	218.80
A_68_P26660364	chr9:78463666-78463710	NM_153098:336	Cd109	INSIDE	0.551	0.484	2473.96	1196.74	0.267	2093.29	558.35
A_68_P31625483	chr18:36885784-36885836	NM_009841:498	Cd14	INSIDE	0.253	0.719	1472.57	1059.13	0.182	1077.95	196.04
A_68_P27089717	chr10:41238672-41238716	NM_016898:-611	Cd164	PROMOTER	0.249	0.416	1409.04	586.62	0.104	1189.80	123.24
A_68_P27089727	chr10:41239601-41239645	NM_016898:317	Cd164	INSIDE	0.572	0.479	1117.35	535.70	0.274	1158.53	317.56
A_68_P27089728	chr10:41239688-41239732	NM_016898:405	Cd164	INSIDE	0.540	0.390	1355.81	529.30	0.211	1090.99	229.82
A_68_P27089721	chr10:41239063-41239107	NM_016898:-221	Cd164	PROMOTER	0.668	0.442	1478.47	654.22	0.295	1128.98	333.50
A_68_P27101048	chr10:43299118-43299162	NM_009846:166	Cd24a	INSIDE	0.516	0.599	2997.47	1794.56	0.309	2305.45	711.81
A_68_P31186622	chr17:43013421-43013465	NM_009847:-69	Cd2ap	PROMOTER	0.650	0.721	1637.03	1179.64	0.468	1520.31	712.00
A_68_P24992859	chr7:19945635-19945679	NM_145822:-824	Cd3eap	DIVERGENT_PROMOTER	0.311	0.402	3657.03	1470.39	0.125	2771.27	346.17
A_68_P20829088	chr1:173610061-173610105	NM_007649:-2103	Cd48	PROMOTER	0.562	2.889	2481.68	7170.39	1.625	2263.70	3677.64
A_68_P24820862	chr6:125444303-125444347	NM_007657:449	Cd9	INSIDE	0.433	0.582	1893.00	1102.63	0.252	1613.75	407.23
A_68_P29639450	chr14:60216642-60216686	NM_001168535:132	Cdadc1	INSIDE	0.581	0.529	1593.61	843.08	0.307	1313.86	403.51
A_68_P29639453	chr14:60216909-60216959	NM_001168535:-138	Cdadc1	PROMOTER	0.207	0.705	1290.49	909.87	0.146	858.38	125.57

ProbeName	Target position of probe on CpG island microarray	TargetID	GeneSymbol	CpG island Description	Ratio of relative methylation (TiO2-NP/Vehicle)	Sham group			TiO_2-H group		
						Relative methylation (Cy-5/Cy-3)	Cy-3 signal (Input)	Cy-5 signal (MeDIP)	Relative methylation (Cy-5/Cy-3)	Cy-3 signal (Input)	Cy-5 signal (MeDIP)
A_68_P21576978	chr2:120557105-120557149	NM_026891:127	Cdan1	INSIDE	0.660	0.514	2920.88	1501.20	0.339	2432.31	825.35
A_68_P22449310	chr3:116126022-116126067	NM_001080818:906	Cdc14a	INSIDE	0.556	0.599	1344.64	805.42	0.333	1127.01	375.04
A_68_P22449313	chr3:116126350-116126394	NM_001080818:578	Cdc14a	INSIDE	0.657	0.574	1449.04	832.43	0.377	1155.72	436.23
A_68_P23240385	chr4:118109464-118109508	NM_023223:462	Cdc20	INSIDE	0.187	0.679	1416.43	961.24	0.127	921.42	117.20
A_68_P23240388	chr4:118109735-118109779	NM_023223:192	Cdc20	INSIDE	0.269	0.510	5179.82	2642.88	0.137	3749.47	514.66
A_68_P31613997	chr18:34810920-34810964	NM_178347:448	Cdc23	INSIDE	0.587	0.539	1317.81	710.65	0.316	1280.28	405.10
A_68_P31614001	chr18:34811328-34811372	NM_178347:40	Cdc23	INSIDE	0.540	0.692	2518.82	1742.23	0.373	1822.46	680.62
A_68_P22964275	chr4:62069685-62069729	NM_139291:-49	Cdc26	DIVERGENT_PROMOTER	0.623	0.508	1093.77	555.39	0.316	846.94	268.04
A_68_P26345696	chr9:20954248-20954292	NM_016742:80	Cdc37	INSIDE	0.458	0.666	1284.61	855.41	0.305	942.60	287.57
A_68_P32050464	chr19:29064739-29064788	NM_025950:-220	Cdc37l1	PROMOTER	0.302	0.491	1312.98	644.98	0.148	1021.80	151.54
A_68_P32050469	chr19:29065228-29065272	NM_025950:267	Cdc37l1	INSIDE	0.164	0.678	2356.73	1598.79	0.111	1781.24	197.88
A_68_P20872964	chr1:181889645-181889689	NM_001033285:-1553	Cdc42bpa	PROMOTER	0.221	0.579	1329.05	769.35	0.128	1181.58	151.12
A_68_P28743715	chr12:112615120-112615164	NM_183016:787	Cdc42bpb	INSIDE	0.186	0.463	2455.96	1138.12	0.086	1861.06	160.55
A_68_P28743716	chr12:112615206-112615250	NM_183016:701	Cdc42bpb	INSIDE	0.258	0.476	3007.78	1430.22	0.123	2145.77	263.65
A_68_P31933965	chr19:6320530-6320574	NM_001033342:14096	Cdc42bpg	INSIDE	0.549	3.625	1485.09	5383.15	1.989	1314.89	2615.11
A_68_P30362683	chr15:78673475-78673519	NM_027219:420	Cdc42ep1	INSIDE	0.242	0.635	881.58	559.83	0.154	802.19	123.46
A_68_P31376859	chr17:79754745-79754789	NM_026514:-335	Cdc42ep3	PROMOTER	0.334	0.667	1932.72	1289.86	0.223	1495.43	333.82
A_68_P28143856	chr11:113613201-113613245	NM_001163346:-93	Cdc42ep4	PROMOTER	0.558	0.674	1217.12	820.91	0.376	970.52	364.96
A_68_P24948002	chr7:4103289-4103333	NM_021454:12991	Cdc42ep5	INSIDE	0.587	0.550	1204.32	662.79	0.323	994.85	321.59
A_68_P28059225	chr11:98769164-98769211	NM_001025779:-15	Cdc6	PROMOTER	0.092	1.435	3080.32	4421.42	0.132	1984.56	261.01
A_68_P28059226	chr11:98769291-98769335	NM_011799:-152	Cdc6	PROMOTER	0.196	1.887	10924.37	20613.09	0.371	7249.68	2687.05
A_68_P28059227	chr11:98769369-98769413	NM_011799:-74	Cdc6	PROMOTER	0.173	2.564	6792.48	17416.08	0.444	4223.49	1874.92
A_68_P28059228	chr11:98769471-98769515	NM_011799:28	Cdc6	INSIDE	0.344	1.407	1819.73	2560.78	0.484	1234.76	597.56
A_68_P28752827	chr12:114066991-114067046	NM_028023:582	Cdca4	INSIDE	0.353	0.487	1401.24	682.63	0.172	1284.88	221.07
A_68_P28752829	chr12:114067223-114067267	NM_028023:356	Cdca4	INSIDE	0.513	0.670	3014.77	2019.46	0.344	2247.10	772.13
A_68_P28752832	chr12:114067540-114067584	NM_028023:38	Cdca4	INSIDE	0.597	0.529	3303.95	1748.13	0.316	2474.00	781.15
A_68_P31932622	chr19:6085142-6085186	NM_026410:68	Cdca5	INSIDE	0.607	0.455	2496.54	1136.69	0.276	2019.11	557.75
A_68_P21325834	chr2:72314810-72314854	NM_025866:557	Cdca7	INSIDE	0.230	0.559	1315.79	734.89	0.128	934.23	119.79
A_68_P21325835	chr2:72314881-72314927	NM_025866:629	Cdca7	INSIDE	0.319	0.669	1191.25	796.55	0.214	945.63	201.98
A_68_P28776179	chr12:119081965-119082009	NM_146040:-347	Cdca7l	PROMOTER	0.530	0.596	922.78	549.92	0.316	763.66	241.31
A_68_P28776182	chr12:119082285-119082329	NM_146040:-27	Cdca7l	PROMOTER	0.663	0.391	1820.37	710.99	0.259	1573.66	407.71
A_68_P23271159	chr4:124613810-124613854	NM_026560:329	Cdca8	INSIDE	0.405	0.625	2290.39	1431.64	0.253	1723.95	436.25
A_68_P23271162	chr4:124614141-124614185	NM_026560:-1	Cdca8	DIVERGENT_PROMOTER	0.383	0.554	1874.20	1038.39	0.212	1341.22	284.79
A_68_P26140465	chr8:109127899-109127943	NM_009864:653	Cdh1	INSIDE	0.257	0.666	1669.23	1111.30	0.171	1176.17	201.07
A_68_P26207422	chr8:120807660-120807704	NM_019707:28	Cdh13	INSIDE	0.403	0.463	1396.49	647.17	0.187	1115.55	208.39
A_68_P26236513	chr8:125394326-125394370	NM_007662:22075	Cdh15	DOWNSTREAM	0.265	1.798	1979.57	3559.51	0.477	1523.30	726.93
A_68_P26236517	chr8:125394810-125394862	NM_007662:22563	Cdh15	DOWNSTREAM	0.404	1.403	1945.50	2728.65	0.566	1315.85	745.01
A_68_P31524846	chr18:16966832-16966876	NM_007664:704	Cdh2	INSIDE	0.661	0.602	908.10	546.70	0.398	793.88	315.98
A_68_P20501014	chr1:106890387-106890439	NM_011800:225017	Cdh20	INSIDE	0.276	1.800	766.92	1380.67	0.497	673.52	334.64
A_68_P21821717	chr2:164938019-164938063	NM_174988:122197	Cdh22	INSIDE	0.308	0.461	1976.94	910.90	0.142	1492.14	211.67
A_68_P21822531	chr2:165058849-165058897	NM_174988:1365	Cdh22	INSIDE	0.233	0.655	964.16	631.37	0.152	775.00	118.13
A_68_P21822534	chr2:165059212-165059256	NM_174988:1003	Cdh22	INSIDE	0.268	0.628	812.47	510.25	0.168	716.53	120.62
A_68_P21822538	chr2:165059682-165059726	NM_174988:533	Cdh22	INSIDE	0.556	0.576	2327.71	1341.58	0.320	1922.96	615.69
A_68_P27176087	chr10:60148828-60148872	NM_023370:10388	Cdh23	INSIDE	0.252	0.487	1481.03	721.42	0.123	1021.99	125.32

ProbeName	Target position of probe on CpG island microarray	TargetID	GeneSymbol	CpG island Description	Ratio of relative methylation (TiO2-NP/Vehicle)	Sham group			TiO_2-H group		
						Relative methylation (Cy-5/Cy-3)	Cy-3 signal (Input)	Cy-5 signal (MeDIP)	Relative methylation (Cy-5/Cy-3)	Cy-3 signal (Input)	Cy-5 signal (MeDIP)
A_68_P27176094	chr10:60149519-60149563	NM_023370:9698	Cdh23	INSIDE	0.458	0.684	2090.96	1430.59	0.313	1654.88	518.23
A_68_P27176098	chr10:60149935-60149979	NM_023370:9282	Cdh23	INSIDE	0.330	0.642	1312.10	842.19	0.212	1070.71	226.74
A_68_P27176102	chr10:60150451-60150495	NM_023370:8766	Cdh23	INSIDE	0.666	0.559	970.33	542.53	0.373	843.33	314.16
A_68_P29613437	chr14:55259966-55260010	NM_199470:213	Cdh24	INSIDE	0.569	0.679	3029.23	2055.85	0.386	2450.58	945.95
A_68_P26139991	chr8:109031801-109031850	NM_007665:-2965	Cdh3	PROMOTER	0.237	0.584	1016.71	593.83	0.138	846.48	117.03
A_68_P26140015	chr8:109035666-109035710	NM_001037809:876	Cdh3	INSIDE	0.305	0.590	1353.51	798.17	0.180	1129.42	203.15
A_68_P30026215	chr15:13103074-13103118	NM_007666:298	Cdh6	INSIDE	0.650	0.710	5232.29	3715.88	0.462	3839.97	1773.12
A_68_P30026217	chr15:13103232-13103276	NM_007666:140	Cdh6	INSIDE	0.391	0.672	4209.50	2829.81	0.263	2762.48	726.88
A_68_P20522333	chr1:111879695-111879739	NM_172853:-597	Cdh7	PROMOTER	0.549	0.625	2362.99	1475.73	0.343	1769.93	606.97
A_68_P25503359	chr7:134120190-134120234	NM_138754:311	Cdipt	INSIDE	0.279	0.616	881.62	542.65	0.172	694.98	119.22
A_68_P27223320	chr10:68815779-68815823	NM_007659:-140	Cdk1	PROMOTER	0.415	0.495	1876.26	927.88	0.205	1570.55	321.96
A_68_P26238593	chr8:125749038-125749082	NM_194444:320	Cdk10	INSIDE	0.556	0.520	1983.84	1032.21	0.289	1550.55	448.44
A_68_P23439688	chr4:154998849-154998893	NM_007661:-107	Cdk11b	PROMOTER	0.439	0.482	1142.17	550.31	0.212	905.93	191.67
A_68_P23439689	chr4:154998937-154998981	NM_007661:-19	Cdk11b	PROMOTER	0.272	0.528	1003.84	529.90	0.144	872.08	125.29
A_68_P23455496	chr5:5380045-5380094	NM_011074:182	Cdk14	INSIDE	0.170	0.572	1663.32	951.24	0.097	1270.44	123.40
A_68_P32289634	chrX:20265458-20265502	NM_011049:-138	Cdk16	PROMOTER	0.152	1.369	1922.20	2631.62	0.208	800.76	166.60
A_68_P20619646	chr1:134035792-134035836	NM_008795:448	Cdk18	INSIDE	0.302	0.491	1523.18	748.63	0.149	1239.82	184.16
A_68_P27083518	chr10:40068868-40068914	NM_001168304:-223	Cdk19	PROMOTER	0.234	0.416	1580.29	658.15	0.097	1201.49	116.90
A_68_P27542784	chr10:128141723-128141767	NM_016756:363	Cdk2	INSIDE	0.429	0.608	1592.16	968.65	0.261	1257.00	327.89
A_68_P27542787	chr10:128142015-128142063	NM_016756:69	Cdk2	INSIDE	0.230	0.446	1582.07	705.57	0.102	1206.47	123.58
A_68_P31921019	chr19:4097126-4097170	NM_026373:-202	Cdk2ap2	PROMOTER	0.579	0.598	2421.43	1448.75	0.347	1777.27	616.17
A_68_P31921020	chr19:4097226-4097270	NM_026373:-102	Cdk2ap2	PROMOTER	0.228	0.505	3118.73	1573.89	0.115	2145.91	246.50
A_68_P27533815	chr10:126500675-126500719	NM_009870:38	Cdk4	INSIDE	0.584	0.617	1119.52	690.68	0.360	881.74	317.42
A_68_P27533816	chr10:126500794-126500838	NM_009870:158	Cdk4	INSIDE	0.147	0.642	1855.86	1191.82	0.095	1239.12	117.31
A_68_P23541721	chr5:23929534-23929578	NM_007668:-208	Cdk5	DIVERGENT_PROMOTER	0.261	0.398	1823.91	725.47	0.104	1213.29	125.79
A_68_P27954544	chr11:80291421-80291465	NM_009871:895	Cdk5r1	INSIDE	0.566	0.593	1698.75	1008.11	0.336	1220.56	409.99
A_68_P20350882	chr1:74902485-74902529	NM_009872:904	Cdk5r2	INSIDE	0.449	0.526	1585.72	834.75	0.236	1345.40	317.89
A_68_P20350884	chr1:74902743-74902787	NM_009872:1162	Cdk5r2	INSIDE	0.103	0.618	2538.87	1568.75	0.064	1872.97	119.36
A_68_P23005993	chr4:70071160-70071204	NM_145990:219	Cdk5rap2	INSIDE	0.153	0.647	5103.18	3300.01	0.099	3635.49	359.90
A_68_P23005994	chr4:70071277-70071321	NM_145990:103	Cdk5rap2	INSIDE	0.628	0.572	1910.35	1093.22	0.360	1466.55	527.45
A_68_P23005998	chr4:70071674-70071724	NM_145990:-297	Cdk5rap2	PROMOTER	0.237	0.622	916.61	570.54	0.147	803.27	118.31
A_68_P28047526	chr11:96777751-96777795	NM_030248:23	Cdk5rap3	INSIDE	0.374	0.596	1501.27	895.07	0.223	1151.62	256.54
A_68_P28047528	chr11:96777981-96778025	NM_030248:-207	Cdk5rap3	PROMOTER	0.644	0.630	1635.48	1030.65	0.406	1163.80	471.95
A_68_P24167402	chr5:147042562-147042606	NM_153599:-666	Cdk8	DIVERGENT_PROMOTER	0.406	0.608	1352.14	821.47	0.246	1139.57	280.81
A_68_P24167577	chr5:147072524-147072578	NM_153599:29300	Cdk8	INSIDE	0.628	3.728	301.16	1122.79	2.340	284.14	664.76
A_68_P21117007	chr2:32567836-32567880	NM_130860:446	Cdk9	INSIDE	0.332	0.453	2551.54	1156.85	0.151	1980.24	298.43
A_68_P31125919	chr17:29230688-29230732	NM_007669:-6	Cdkn1a	PROMOTER	0.541	0.612	2807.15	1719.38	0.331	2049.09	678.77
A_68_P31125922	chr17:29231051-29231095	NM_007669:356	Cdkn1a	INSIDE	0.341	0.568	1745.25	992.08	0.194	1261.12	244.16
A_68_P31125923	chr17:29231167-29231211	NM_007669:472	Cdkn1a	INSIDE	0.256	0.468	1219.23	570.02	0.120	1002.53	119.97
A_68_P31125924	chr17:29231277-29231321	NM_007669:582	Cdkn1a	INSIDE	0.192	0.368	2451.01	903.09	0.071	1773.09	125.40
A_68_P31125930	chr17:29232003-29232047	NM_007669:1308	Cdkn1a	INSIDE	0.238	0.614	2904.24	1782.82	0.146	2167.87	316.25
A_68_P24864239	chr6:134871088-134871141	NM_009875:696	Cdkn1b	INSIDE	0.183	0.513	2205.53	1131.37	0.094	1269.46	119.28
A_68_P23094685	chr4:88922650-88922694	NM_001040654:5424	Cdkn2a	INSIDE	0.129	0.449	2482.98	1113.76	0.058	2022.74	117.38
A_68_P23094686	chr4:88922820-88922864	NM_001040654:5254	Cdkn2a	INSIDE	0.321	0.573	1402.32	803.61	0.184	1077.56	197.99

ProbeName	Target position of probe on CpG island microarray	TargetID	GeneSymbol	CpG island Description	Ratio of relative methylation (TiO2-NP/Vehicle)	Sham group			TiO_2-H group		
						Relative methylation (Cy-5/Cy-3)	Cy-3 signal (Input)	Cy-5 signal (MeDIP)	Relative methylation (Cy-5/Cy-3)	Cy-3 signal (Input)	Cy-5 signal (MeDIP)
A_68_P23094814	chr4:88941150-88941194	NM_009877:-649	Cdkn2a	PROMOTER	0.260	0.605	1710.14	1034.46	0.157	1371.20	215.88
A_68_P25843135	chr8:48798585-48798629	NM_172407:679	Cdkn2aip	INSIDE	0.230	0.618	1365.36	844.39	0.142	989.25	140.46
A_68_P25843138	chr8:48798881-48798925	NM_172407:383	Cdkn2aip	INSIDE	0.289	0.518	1190.71	616.78	0.150	980.32	146.98
A_68_P25843141	chr8:48799219-48799263	NM_172407:45	Cdkn2aip	INSIDE	0.573	0.616	3600.91	2218.30	0.353	2750.15	970.19
A_68_P27796702	chr11:51781404-51781448	NM_029976:294	Cdkn2aipnl	INSIDE	0.640	0.453	1411.50	638.77	0.290	1139.71	330.17
A_68_P23201931	chr4:109330348-109330392	NM_007671:7607	Cdkn2c	DOWNSTREAM	0.410	0.428	2457.97	1053.23	0.176	2044.63	359.05
A_68_P23202006	chr4:109338923-109338967	NM_007671:-967	Cdkn2c	PROMOTER	0.443	0.593	1474.25	874.55	0.263	1226.01	322.23
A_68_P23202007	chr4:109339070-109339115	NM_007671:-1115	Cdkn2c	PROMOTER	0.590	0.689	1117.88	770.49	0.407	936.38	380.95
A_68_P26346391	chr9:21095271-21095315	NM_009878:361	Cdkn2d	INSIDE	0.460	0.729	2772.31	2022.37	0.336	2033.06	682.14
A_68_P31678446	chr18:46887405-46887449	NM_033037:570	Cdo1	INSIDE	0.600	0.570	1542.13	879.69	0.342	1208.86	413.91
A_68_P31678447	chr18:46887518-46887562	NM_033037:456	Cdo1	INSIDE	0.261	0.746	5411.06	4034.95	0.194	3873.70	753.00
A_68_P31678448	chr18:46887631-46887675	NM_033037:344	Cdo1	INSIDE	0.422	0.460	2637.23	1212.62	0.194	1900.31	368.93
A_68_P25471450	chr7:128125237-128125281	NM_007672:568	Cdr2	INSIDE	0.597	0.664	1492.75	990.68	0.396	1161.96	460.64
A_68_P28153161	chr11:115243333-115243378	NM_001080929:126	Cdr2l	INSIDE	0.615	0.703	1343.15	943.86	0.432	1293.34	558.71
A_68_P21639430	chr2:132089195-132089239	NM_138651:224	Cds2	INSIDE	0.666	0.568	964.42	547.35	0.378	782.88	295.74
A_68_P21639431	chr2:132089279-132089323	NM_138651:308	Cds2	INSIDE	0.269	0.471	2740.33	1290.68	0.127	2096.93	265.86
A_68_P21639432	chr2:132089391-132089435	NM_138651:420	Cds2	INSIDE	0.194	0.679	2146.73	1458.04	0.132	1708.67	225.09
A_68_P26785468	chr9:103266904-103266948	NM_001134427:383	Cdv3	INSIDE	0.230	0.644	2629.97	1694.11	0.148	1895.91	280.58
A_68_P24172962	chr5:148115952-148116010	NM_007673:2845	Cdx2	INSIDE	0.156	0.580	1919.73	1113.43	0.091	1311.99	118.90
A_68_P24172965	chr5:148116273-148116317	NM_007673:2531	Cdx2	INSIDE	0.555	0.566	1928.69	1091.83	0.314	1504.76	472.42
A_68_P24172980	chr5:148117798-148117842	NM_007673:1005	Cdx2	INSIDE	0.198	0.439	1722.33	756.32	0.087	1366.22	118.65
A_68_P24995570	chr7:20437597-20437641	NM_001033419:9030	Ceacam16	INSIDE	0.138	0.716	1584.42	1134.90	0.099	1230.46	121.30
A_68_P25047606	chr7:35903706-35903750	NM_007678:-583	Cebpa	PROMOTER	0.110	0.681	2602.76	1772.23	0.075	1633.83	122.82
A_68_P21836878	chr2:167514004-167514048	NM_009883:-388	Cebpb	PROMOTER	0.416	0.532	1435.24	763.87	0.221	1226.46	271.50
A_68_P30567432	chr16:15887571-15887615	NM_007679:214	Cebpd	INSIDE	0.612	0.562	3420.96	1921.32	0.344	2518.76	865.88
A_68_P31374516	chr17:79336106-79336150	NM_001024806:282	Cebpz	INSIDE	0.520	0.672	1013.10	681.20	0.350	885.58	309.93
A_68_P31374517	chr17:79336211-79336255	NM_001024806:178	Cebpz	INSIDE	0.228	0.527	1260.97	664.02	0.120	1009.35	121.04
A_68_P24799552	chr6:120615622-120615666	NM_001128151:-794	Cecr2	PROMOTER	0.403	0.603	3552.63	2143.25	0.243	2839.95	689.74
A_68_P24799556	chr6:120616027-120616071	NM_001128151:-390	Cecr2	PROMOTER	0.100	0.687	3552.49	2441.58	0.069	2449.23	167.96
A_68_P24798989	chr6:120481488-120481532	NM_144815:-193	Cecr5	PROMOTER	0.598	0.628	1119.77	702.70	0.376	763.89	286.89
A_68_P24798990	chr6:120481585-120481629	NM_144815:-289	Cecr5	PROMOTER	0.560	0.325	1978.65	643.44	0.182	1646.51	299.76
A_68_P24798779	chr6:120442774-120442818	NM_033567:1029	Cecr6	INSIDE	0.285	0.473	2029.78	960.77	0.135	1580.40	213.41
A_68_P24798783	chr6:120443507-120443551	NM_033567:297	Cecr6	INSIDE	0.481	0.454	2166.85	982.79	0.218	1678.62	366.17
A_68_P31565720	chr18:25645973-25646017	NM_001146292:266490	Celf4	INSIDE	0.116	0.644	2386.56	1537.11	0.075	1600.31	119.46
A_68_P31567781	chr18:25913080-25913124	NM_001146292:-618	Celf4	PROMOTER	0.519	0.678	1334.73	904.99	0.352	1199.85	422.05
A_68_P30404227	chr15:85809018-85809063	NM_009886:55167	Celsr1	INSIDE	0.591	2.968	311.14	923.41	1.755	375.22	658.46
A_68_P26816838	chr9:108728469-108728513	NM_080437:-160	Celsr3	PROMOTER	0.517	0.409	3585.13	1465.05	0.211	2717.36	574.18
A_68_P26816846	chr9:108729372-108729416	NM_080437:744	Celsr3	INSIDE	0.199	0.521	3449.92	1796.89	0.104	2478.45	257.31
A_68_P22550732	chr3:134875583-134875627	NM_173762:78	Cenpe	INSIDE	0.391	0.608	1219.19	740.70	0.237	914.46	216.97
A_68_P22550733	chr3:134875756-134875800	NM_173762:252	Cenpe	INSIDE	0.649	0.648	991.59	642.34	0.420	773.26	324.99
A_68_P29270035	chr13:101545475-101545519	NM_021886:358	Cenph	INSIDE	0.198	0.517	3664.88	1893.96	0.103	2523.96	258.79
A_68_P29270037	chr13:101545636-101545680	NM_021886:196	Cenph	INSIDE	0.497	0.482	1991.03	959.10	0.239	1613.78	386.23
A_68_P26199702	chr8:119445672-119445716	NM_028131:55	Cenpn	INSIDE	0.546	0.614	949.24	582.54	0.335	819.18	274.50
A_68_P27854481	chr11:62352177-62352221	NM_028448:565	Cenpv	INSIDE	0.367	0.675	1792.10	1209.72	0.248	1317.70	326.44

ProbeName	Target position of probe on CpG island microarray	TargetID	GeneSymbol	CpG island Description	Ratio of relative methylation (TiO2-NP/Vehicle)	Sham group			TiO_2-H group		
						Relative methylation (Cy-5/Cy-3)	Cy-3 signal (Input)	Cy-5 signal (MeDIP)	Relative methylation (Cy-5/Cy-3)	Cy-3 signal (Input)	Cy-5 signal (MeDIP)
A_68_P21603779	chr2:125450959-125451003	NM_001081091:-131	Cep152	PROMOTER	0.584	0.440	1192.28	525.10	0.257	954.54	245.33
A_68_P26476239	chr9:45636484-45636528	NM_001081373:215	Cep164	INSIDE	0.419	0.600	4238.47	2542.52	0.251	2950.74	741.98
A_68_P27392329	chr10:99950462-99950506	NM_146009:-438	Cep290	DIVERGENT_PROMOTER	0.559	0.337	1814.92	610.94	0.188	1345.69	253.04
A_68_P20740951	chr1:157820596-157820640	NM_001039184:-233	Cep350	PROMOTER	0.608	0.675	1368.22	923.57	0.410	1014.32	416.26
A_68_P32099201	chr19:38129127-38129171	NM_028293:-366	Cep55	PROMOTER	0.275	1.940	12468.12	24188.29	0.533	7872.86	4194.58
A_68_P32099207	chr19:38129688-38129732	NM_001164362:179	Cep55	INSIDE	0.486	0.558	1634.87	912.57	0.271	1289.73	349.94
A_68_P26313776	chr9:13631112-13631156	NR_037622:363	Cep57	INSIDE	0.218	0.455	1704.09	774.90	0.099	1190.57	118.27
A_68_P26313777	chr9:13631232-13631276	NR_037622:243	Cep57	INSIDE	0.407	0.486	3391.82	1647.72	0.198	2734.82	540.70
A_68_P27630624	chr11:20149666-20149710	NM_172260:-261	Cep68	PROMOTER	0.583	0.701	1895.69	1328.52	0.409	1310.85	535.75
A_68_P31792968	chr18:67800428-67800477	NM_001081073:538	Cep76	INSIDE	0.172	0.670	1353.59	906.32	0.115	1046.08	120.56
A_68_P31977523	chr19:16058932-16058979	NM_198019:524	Cep78	INSIDE	0.128	0.673	1732.51	1166.79	0.086	1360.79	117.39
A_68_P30778447	chr16:55933919-55933975	NM_028815:1015	Cep97	INSIDE	0.222	0.440	1614.04	710.87	0.098	1224.26	119.86
A_68_P30778452	chr16:55934493-55934544	NM_028815:443	Cep97	INSIDE	0.196	0.551	1765.37	973.22	0.108	1137.71	123.13
A_68_P30778453	chr16:55934660-55934704	NM_028815:279	Cep97	INSIDE	0.323	0.616	1514.17	932.49	0.199	1096.48	218.02
A_68_P30778320	chr16:55905833-55905877	NM_028815:29107	Cep97	INSIDE	0.670	2.862	380.98	1090.21	1.918	356.28	683.46
A_68_P22402364	chr3:106350168-106350212	NM_133869:489	Cept1	INSIDE	0.350	0.711	2349.11	1670.19	0.249	1749.54	435.91
A_68_P22402367	chr3:106350620-106350664	NM_133869:37	Cept1	INSIDE	0.195	0.590	1253.08	739.59	0.115	1042.28	120.12
A_68_P30405649	chr15:86015668-86015712	NM_145475:881	Cerk	INSIDE	0.270	0.611	952.62	582.38	0.165	733.58	121.17
A_68_P32459814	chrX:70163539-70163583	NM_019405:123	Cetn2	INSIDE	0.274	1.811	1351.75	2447.48	0.496	519.22	257.64
A_68_P29177663	chr13:81922129-81922187	NM_007684:-132	Cetn3	PROMOTER	0.266	0.526	1070.28	563.33	0.140	883.45	123.60
A_68_P31929038	chr19:5491024-5491068	NM_007687:592	Cfl1	INSIDE	0.543	0.647	1064.32	688.27	0.351	871.14	306.02
A_68_P28449508	chr12:55963342-55963389	NM_007688:499	Cfl2	INSIDE	0.372	0.613	932.54	572.01	0.228	691.92	157.95
A_68_P28449511	chr12:55963765-55963809	NM_007688:78	Cfl2	INSIDE	0.654	0.440	1716.52	755.81	0.288	1482.68	426.90
A_68_P30822062	chr16:64851258-64851302	NM_178647:-630	Cggbp1	PROMOTER	0.404	0.519	3183.58	1650.75	0.210	2462.97	516.24
A_68_P22340672	chr3:94590209-94590253	NM_001037711:207	Cgn	INSIDE	0.556	0.730	1766.28	1288.82	0.406	1252.38	508.07
A_68_P22340673	chr3:94590302-94590346	NM_001037711:113	Cgn	INSIDE	0.566	0.543	4165.07	2260.24	0.307	3089.59	948.51
A_68_P22340676	chr3:94590702-94590746	NM_001037711:-287	Cgn	PROMOTER	0.283	0.421	1390.08	584.89	0.119	1001.79	119.45
A_68_P21569217	chr2:119176751-119176795	NM_026929:-205	Chac1	PROMOTER	0.183	0.519	1593.16	826.99	0.095	1215.22	115.31
A_68_P21569219	chr2:119176997-119177041	NM_026929:41	Chac1	INSIDE	0.353	0.604	2071.02	1251.04	0.213	1652.42	352.12
A_68_P31253494	chr17:56179975-56180019	NM_013733:158	Chaf1a	INSIDE	0.504	0.505	1012.57	511.72	0.255	732.23	186.66
A_68_P30978142	chr16:93884445-93884489	NM_028083:321	Chaf1b	INSIDE	0.237	0.529	2334.30	1234.14	0.126	1707.44	214.36
A_68_P24091083	chr5:130363235-130363294	NM_024166:76	Chchd2	INSIDE	0.218	0.527	1457.36	767.87	0.115	1093.40	125.35
A_68_P24347496	chr6:33009915-33009959	NM_025336:216	Chchd3	INSIDE	0.190	0.685	3361.95	2302.32	0.130	2480.97	322.69
A_68_P24347497	chr6:33010009-33010053	NM_025336:122	Chchd3	INSIDE	0.422	0.536	1123.39	602.45	0.226	879.35	198.89
A_68_P24639203	chr6:91422971-91423017	NM_133928:423	Chchd4	INSIDE	0.603	0.611	1014.68	619.91	0.368	765.80	282.03
A_68_P22695195	chr4:3866261-3866305	NM_001190322:222	Chchd7	INSIDE	0.236	0.518	3915.50	2027.86	0.122	2828.14	344.95
A_68_P31066946	chr17:15842783-15842827	NM_007690:874	Chd1	INSIDE	0.307	0.495	2613.42	1292.57	0.152	1867.71	283.26
A_68_P22355189	chr3:97413826-97413870	NM_026539:265	Chd1l	INSIDE	0.249	0.549	1079.94	592.37	0.137	976.17	133.40
A_68_P25229722	chr7:80682604-80682648	NM_001081345:4006	Chd2	INSIDE	0.197	0.581	2332.22	1353.87	0.114	1730.07	197.85
A_68_P27894657	chr11:69181886-69181930	NM_146019:1020	Chd3	INSIDE	0.255	0.602	1082.25	651.85	0.154	781.56	120.11
A_68_P27894658	chr11:69182004-69182048	NM_146019:902	Chd3	INSIDE	0.634	0.650	2150.42	1398.55	0.413	1538.82	634.86
A_68_P24818639	chr6:125046323-125046367	NM_145979:164	Chd4	INSIDE	0.272	0.298	2233.34	665.25	0.081	1657.90	134.33
A_68_P23418117	chr4:151712690-151712736	NM_001081376:-47	Chd5	PROMOTER	0.315	0.338	2320.97	783.66	0.106	1837.66	195.40
A_68_P21799027	chr2:160935428-160935473	NM_173368:-658	Chd6	PROMOTER	0.564	0.682	1001.83	682.95	0.385	776.01	298.51

ProbeName	Target position of probe on CpG island microarray	TargetID	GeneSymbol	CpG island Description	Ratio of relative methylation (TiO2-NP/Vehicle)	Sham group			TiO_2-H group		
						Relative methylation (Cy-5/Cy-3)	Cy-3 signal (Input)	Cy-5 signal (MeDIP)	Relative methylation (Cy-5/Cy-3)	Cy-3 signal (Input)	Cy-5 signal (MeDIP)
A_68_P22715816	chr4:8618846-8618890	NM_001081417:801	Chd7	INSIDE	0.582	0.588	1987.24	1168.28	0.342	1535.90	525.41
A_68_P22715818	chr4:8619237-8619281	NM_001081417:1191	Chd7	INSIDE	0.130	0.666	1707.28	1136.37	0.086	1404.23	121.23
A_68_P26055734	chr8:93431736-93431780	NM_177224:79025	Chd9	INSIDE	0.365	0.516	1586.60	818.66	0.188	1265.60	238.22
A_68_P26055745	chr8:93433140-93433184	NM_177224:80429	Chd9	INSIDE	0.358	0.393	2705.33	1062.18	0.140	2181.41	306.28
A_68_P26426474	chr9:36533941-36533985	NM_007691:281	Chek1	INSIDE	0.498	0.617	2866.35	1769.95	0.308	2181.68	671.28
A_68_P21642100	chr2:132607157-132607201	NM_007694:165	Chgb	INSIDE	0.192	0.591	2294.98	1357.31	0.113	1674.37	189.70
A_68_P32572441	chrX:100551713-100551757	NM_009767:-80	Chic1	PROMOTER	0.123	1.887	3718.74	7015.74	0.232	1422.98	329.87
A_68_P23807021	chr5:75440173-75440217	NM_028850:457	Chic2	INSIDE	0.242	0.641	857.81	549.48	0.155	792.60	122.78
A_68_P31919584	chr19:3851556-3851600	NM_001025566:-194	Chka	PROMOTER	0.312	0.613	1323.34	811.46	0.192	939.48	179.98
A_68_P31919590	chr19:3852379-3852423	NM_001025566:628	Chka	INSIDE	0.321	0.475	1951.50	927.80	0.152	1485.68	226.50
A_68_P20849636	chr1:177622240-177622284	NM_021350:-3778	Chml	PROMOTER	0.465	0.700	3019.19	2114.39	0.325	2048.46	666.65
A_68_P20849637	chr1:177622396-177622440	NM_021350:-3934	Chml	PROMOTER	0.409	0.736	3272.00	2406.68	0.301	2326.69	699.39
A_68_P21761657	chr2:154483014-154483064	NM_029362:277	Chmp4b	INSIDE	0.333	0.640	875.11	559.96	0.213	680.46	145.10
A_68_P21761658	chr2:154483094-154483138	NM_029362:355	Chmp4b	INSIDE	0.079	0.435	4970.80	2162.56	0.034	3535.80	121.94
A_68_P21761659	chr2:154483174-154483218	NM_029362:435	Chmp4b	INSIDE	0.317	0.423	1221.21	517.10	0.134	874.38	117.31
A_68_P21761660	chr2:154483260-154483304	NM_029362:521	Chmp4b	INSIDE	0.305	0.566	1615.67	914.49	0.173	1097.06	189.28
A_68_P22859653	chr4:40895771-40895815	NM_029814:207	Chmp5	INSIDE	0.159	0.656	2943.86	1930.79	0.104	2197.19	228.71
A_68_P26336205	chr9:18096816-18096860	NM_025844:128	Chordc1	INSIDE	0.306	0.710	1483.02	1053.65	0.217	1106.17	240.51
A_68_P30328265	chr15:72921187-72921231	NM_053068:367	Chrac1	INSIDE	0.642	0.448	1281.58	573.79	0.288	1000.24	287.65
A_68_P30589273	chr16:20733395-20733439	NM_009893:217	Chrd	INSIDE	0.535	0.496	1500.39	744.75	0.265	1252.52	332.43
A_68_P30589275	chr16:20733601-20733645	NM_009893:423	Chrd	INSIDE	0.439	0.591	1890.77	1118.12	0.260	1433.01	372.01
A_68_P30589277	chr16:20733808-20733856	NM_009893:633	Chrd	INSIDE	0.162	0.569	1908.64	1086.20	0.092	1314.14	121.00
A_68_P21425362	chr2:91761645-91761689	NM_007699:-679	Chrm4	PROMOTER	0.134	0.659	2073.81	1367.59	0.088	1417.75	124.99
A_68_P21425363	chr2:91761712-91761756	NM_007699:-611	Chrm4	PROMOTER	0.116	0.654	2283.50	1493.54	0.076	1559.52	118.08
A_68_P21425364	chr2:91761807-91761851	NM_007699:-517	Chrm4	PROMOTER	0.426	0.563	1003.42	564.90	0.240	792.85	190.09
A_68_P21425372	chr2:91762936-91762980	NM_007699:613	Chrm4	INSIDE	0.454	0.711	1921.16	1365.93	0.323	1516.66	489.31
A_68_P21425382	chr2:91763987-91764031	NM_007699:1663	Chrm4	INSIDE	0.206	0.561	1302.41	730.67	0.116	1165.03	134.91
A_68_P27897126	chr11:69609386-69609430	NM_009601:31	Chrnb1	INSIDE	0.512	0.661	1292.68	854.96	0.338	1008.82	341.48
A_68_P21429513	chr2:92440608-92440652	NM_023850:767	Chst1	INSIDE	0.252	0.689	1515.97	1044.00	0.173	1107.85	192.14
A_68_P24141722	chr5:140981721-140981765	NM_021528:180	Chst12	INSIDE	0.119	0.703	2447.89	1721.20	0.083	1826.94	152.43
A_68_P24141811	chr5:141000117-141000161	NM_021528:18576	Chst12	INSIDE	0.628	3.118	3628.19	11313.18	1.960	2600.06	5095.03
A_68_P25533789	chr7:139508721-139508765	NM_029935:96	Chst15	INSIDE	0.488	0.580	1655.12	959.62	0.283	1350.92	382.53
A_68_P25533790	chr7:139508854-139508902	NM_029935:-40	Chst15	PROMOTER	0.245	0.490	1193.59	585.00	0.120	1017.80	122.13
A_68_P27172917	chr10:59649185-59649231	NM_016803:32800	Chst3	INSIDE	0.605	2.849	1161.45	3308.62	1.725	971.49	1675.68
A_68_P27173114	chr10:59681401-59681445	NM_016803:586	Chst3	INSIDE	0.405	0.480	1633.42	783.31	0.194	1275.03	247.62
A_68_P26170766	chr8:114414003-114414047	NM_019950:20075	Chst5	INSIDE	0.536	0.485	2072.53	1004.22	0.260	1651.10	429.15
A_68_P26170767	chr8:114414110-114414154	NM_019950:19967	Chst5	INSIDE	0.181	0.495	1585.65	784.79	0.090	1403.18	125.96
A_68_P26170772	chr8:114414651-114414695	NM_019950:19427	Chst5	INSIDE	0.454	1.456	1248.02	1817.02	0.660	1085.04	716.47
A_68_P32287120	chrX:19637562-19637606	NM_021715:889	Chst7	INSIDE	0.238	1.576	1428.99	2251.80	0.375	622.90	233.31
A_68_P32287127	chrX:19638262-19638306	NM_021715:1589	Chst7	INSIDE	0.171	1.581	875.30	1383.85	0.271	432.07	117.11
A_68_P25044994	chr7:35460266-35460310	NM_175140:137442	Chst8	INSIDE	0.411	0.599	1228.10	735.14	0.246	994.25	244.50
A_68_P25045001	chr7:35460951-35460995	NM_175140:136758	Chst8	INSIDE	0.186	0.663	1687.40	1119.33	0.123	1184.68	145.82
A_68_P25045002	chr7:35461038-35461082	NM_175140:136670	Chst8	INSIDE	0.140	0.684	1690.55	1156.88	0.096	1216.16	116.90
A_68_P25045003	chr7:35461137-35461181	NM_175140:136572	Chst8	INSIDE	0.566	0.593	1404.10	832.32	0.336	1336.94	448.61

ProbeName	Target position of probe on CpG island microarray	TargetID	GeneSymbol	CpG island Description	Ratio of relative methylation (TiO2-NP/Vehicle)	Sham group			TiO_2-H group		
						Relative methylation (Cy-5/Cy-3)	Cy-3 signal (Input)	Cy-5 signal (MeDIP)	Relative methylation (Cy-5/Cy-3)	Cy-3 signal (Input)	Cy-5 signal (MeDIP)
A_68_P25045779	chr7:35597123-35597172	NM_175140:583	Chst8	INSIDE	0.062	0.700	3530.48	2471.14	0.043	2796.11	121.54
A_68_P25045781	chr7:35597428-35597472	NM_175140:280	Chst8	INSIDE	0.279	0.726	2068.81	1501.84	0.203	1430.67	290.07
A_68_P31746196	chr18:59334709-59334753	NM_001081328:-263	Chsy3	PROMOTER	0.639	0.574	2107.12	1208.92	0.366	1680.18	615.52
A_68_P31746205	chr18:59335935-59335979	NM_001081328:963	Chsy3	INSIDE	0.146	0.590	2367.87	1397.88	0.086	1681.82	144.95
A_68_P32130472	chr19:44181487-44181534	NM_001162410:457	Chuk	INSIDE	0.288	1.567	5209.02	8160.95	0.451	3712.27	1672.88
A_68_P28555790	chr12:77866473-77866523	NM_206534:-61	Churc1	PROMOTER	0.227	0.538	1321.97	711.28	0.122	1081.85	132.15
A_68_P28555792	chr12:77866643-77866687	NM_206534:105	Churc1	INSIDE	0.221	0.540	1305.10	705.30	0.119	1024.00	122.33
A_68_P21612302	chr2:127073420-127073472	NM_025296:106	Ciao1	INSIDE	0.130	0.530	2767.80	1465.57	0.069	1905.78	130.69
A_68_P25265479	chr7:87377398-87377442	NM_011870:82	Cib1	INSIDE	0.539	0.608	4253.81	2587.49	0.328	3241.80	1062.18
A_68_P25265480	chr7:87377466-87377510	NM_011870:14	Cib1	INSIDE	0.461	0.745	3446.33	2567.13	0.344	2908.06	999.31
A_68_P25009415	chr7:26066798-26066842	NM_001110131:-377	Cic	PROMOTER	0.247	0.707	2165.51	1531.25	0.174	1462.29	255.03
A_68_P31791362	chr18:67503417-67503461	NM_007702:221	Cidea	INSIDE	0.147	0.750	6607.30	4956.51	0.111	4295.64	475.23
A_68_P25948511	chr8:72409941-72409985	NM_026818:1329	Cilp2	INSIDE	0.480	0.526	1118.11	587.97	0.253	865.18	218.55
A_68_P25948512	chr8:72410079-72410123	NM_026818:1191	Cilp2	INSIDE	0.654	0.443	2700.09	1196.23	0.290	1845.91	535.14
A_68_P25948513	chr8:72410234-72410278	NM_026818:1035	Cilp2	INSIDE	0.270	0.530	1079.67	572.66	0.143	809.33	115.87
A_68_P25948520	chr8:72411249-72411293	NM_026818:21	Cilp2	INSIDE	0.150	0.690	2370.82	1636.94	0.104	1680.64	174.26
A_68_P25948527	chr8:72412099-72412143	NM_026818:-829	Cilp2	PROMOTER	0.494	0.726	1619.68	1176.51	0.359	1040.00	372.95
A_68_P25948535	chr8:72413120-72413164	NM_026818:-1851	Cilp2	PROMOTER	0.597	0.479	1235.17	591.11	0.286	957.46	273.46
A_68_P25948536	chr8:72413250-72413294	NM_026818:-1981	Cilp2	PROMOTER	0.375	0.550	2940.27	1616.09	0.206	2203.60	454.36
A_68_P27281378	chr10:79622976-79623020	NM_007705:-7587	Cirbp	PROMOTER	0.257	0.342	1867.22	638.63	0.088	1413.73	124.08
A_68_P27281431	chr10:79630462-79630506	NM_007705:-101	Cirbp	PROMOTER	0.151	0.509	2366.89	1205.70	0.077	1611.98	123.75
A_68_P26141981	chr8:109417694-109417738	NM_011574:177	Cirh1a	INSIDE	0.336	0.719	1877.59	1350.27	0.241	1458.53	352.21
A_68_P32567796	chrX:99445717-99445761	NM_007709:1370	Cited1	INSIDE	0.171	1.639	980.81	1607.14	0.281	426.80	119.91
A_68_P32567807	chrX:99447270-99447325	NM_007709:-189	Cited1	PROMOTER	0.113	1.565	1595.09	2496.11	0.177	687.09	121.34
A_68_P26971597	chr10:17443088-17443132	NM_010828:77	Cited2	INSIDE	0.092	0.727	4990.73	3629.41	0.067	3467.83	230.99
A_68_P26971602	chr10:17443634-17443678	NM_010828:623	Cited2	INSIDE	0.432	0.712	3830.13	2727.47	0.307	2974.75	914.53
A_68_P26971606	chr10:17444088-17444133	NM_010828:1077	Cited2	INSIDE	0.600	0.664	1726.83	1146.50	0.398	1241.37	494.69
A_68_P21114932	chr2:32218910-32218958	NM_028412:181	Ciz1	INSIDE	0.158	2.241	636.64	1426.73	0.354	398.04	141.03
A_68_P21580444	chr2:121181215-121181259	NM_009897:-3140	Ckmt1	DIVERGENT_PROMOTER	0.434	0.461	1306.01	601.82	0.200	1063.72	212.59
A_68_P22319259	chr3:89221857-89221906	NM_016904:332	Cks1b	INSIDE	0.165	1.891	3058.36	5784.20	0.312	2099.25	654.19
A_68_P22319260	chr3:89222015-89222059	NM_016904:177	Cks1b	INSIDE	0.183	1.871	3134.04	5864.79	0.343	2010.64	689.92
A_68_P29033454	chr13:51740459-51740503	NM_025415:-120	Cks2	PROMOTER	0.248	0.666	2797.00	1862.94	0.165	1960.06	323.12
A_68_P29033460	chr13:51741130-51741185	NM_025415:557	Cks2	INSIDE	0.162	0.568	1872.70	1063.29	0.092	1323.90	121.98
A_68_P20551449	chr1:120285275-120285326	NM_001081276:-334	Clasp1	PROMOTER	0.275	0.560	1224.74	686.25	0.154	880.76	135.94
A_68_P20551457	chr1:120286262-120286306	NM_001081276:650	Clasp1	INSIDE	0.363	0.575	2595.78	1492.91	0.209	2048.58	427.43
A_68_P20551458	chr1:120286341-120286385	NM_001081276:728	Clasp1	INSIDE	0.215	0.601	1367.14	821.79	0.129	1065.48	137.92
A_68_P24994312	chr7:20189858-20189902	NM_016680:-63	Clasrp	PROMOTER	0.274	0.537	1872.87	1006.07	0.147	1489.84	219.38
A_68_P22413865	chr3:108456942-108456986	NM_001177770:57	Clcc1	INSIDE	0.651	0.330	1817.88	600.02	0.215	1674.97	360.17
A_68_P31921845	chr19:4215539-4215583	NM_019952:1169	Clcf1	INSIDE	0.368	0.459	4158.15	1906.85	0.169	3338.13	563.15
A_68_P30589167	chr16:20716459-20716503	NM_009900:229	Clcn2	INSIDE	0.453	0.662	2648.65	1752.21	0.299	2376.23	711.54
A_68_P30589168	chr16:20716549-20716593	NM_009900:139	Clcn2	INSIDE	0.152	0.551	1863.85	1027.07	0.084	1407.81	118.24
A_68_P29941246	chr14:119254241-119254285	NM_001160099:91	Cldn10	INSIDE	0.561	0.580	1588.08	921.60	0.325	1242.28	404.29
A_68_P25782090	chr8:36889097-36889141	NM_027998:495	Cldn23	INSIDE	0.431	0.540	1860.05	1004.54	0.233	1281.82	298.48
A_68_P25782095	chr8:36889705-36889749	NM_027998:-113	Cldn23	PROMOTER	0.240	0.662	5066.24	3353.75	0.159	3789.35	601.43

ProbeName	Target position of probe on CpG island microarray	TargetID	GeneSymbol	CpG island Description	Ratio of relative methylation (TiO2-NP/Vehicle)	Sham group			TiO_2-H group		
						Relative methylation (Cy-5/Cy-3)	Cy-3 signal (Input)	Cy-5 signal (MeDIP)	Relative methylation (Cy-5/Cy-3)	Cy-3 signal (Input)	Cy-5 signal (MeDIP)
A_68_P30532560	chr16:8424805-8424849	NM_029070:403	Cldn26	INSIDE	0.626	0.568	1407.49	799.30	0.356	1205.20	428.55
A_68_P30532562	chr16:8424938-8424982	NM_029070:269	Cldn26	INSIDE	0.277	0.705	2714.55	1914.14	0.196	2026.78	396.46
A_68_P24114165	chr5:135462201-135462245	NM_009902:139	Cldn3	INSIDE	0.278	0.363	1646.86	597.77	0.101	1284.66	129.85
A_68_P30793143	chr16:58728472-58728525	NM_171826:342	Cldnd1	INSIDE	0.291	0.516	1065.71	549.69	0.150	790.94	118.52
A_68_P25086654	chr7:51559898-51559942	NM_009131:2409	Clec11a	INSIDE	0.282	0.664	848.05	563.18	0.187	640.56	119.90
A_68_P25086655	chr7:51560012-51560056	NM_009131:2295	Clec11a	INSIDE	0.564	0.584	1693.26	988.26	0.329	1372.86	451.94
A_68_P31193443	chr17:44325522-44325566	NM_172621:24	Clic5	INSIDE	0.615	0.487	1461.95	711.75	0.299	1172.02	350.96
A_68_P24057000	chr5:124133425-124133474	NM_019765:851	Clip1	INSIDE	0.154	0.570	1792.18	1020.80	0.088	1374.02	120.69
A_68_P22317612	chr3:88972806-88972850	NM_001163432:4102	Clk2	INSIDE	0.543	1.357	5688.68	7717.31	0.736	4168.93	3069.01
A_68_P26543677	chr9:57612772-57612816	NM_007713:873	Clk3	INSIDE	0.256	0.710	2611.59	1853.39	0.182	2075.87	377.17
A_68_P26543684	chr9:57613558-57613602	NM_007713:87	Clk3	INSIDE	0.225	0.523	1385.99	724.53	0.118	1029.36	121.22
A_68_P26543685	chr9:57613651-57613695	NM_007713:-5	Clk3	PROMOTER	0.366	0.424	2415.65	1025.21	0.155	1907.78	296.45
A_68_P27792932	chr11:51076708-51076752	NM_007714:58	Clk4	INSIDE	0.254	0.692	2234.37	1546.28	0.175	1465.41	257.09
A_68_P25351869	chr7:104845090-104845134	NM_023671:-88	Clns1a	PROMOTER	0.186	0.648	1149.10	745.08	0.120	978.81	117.90
A_68_P31259429	chr17:57129711-57129755	NM_017393:46	Clpp	INSIDE	0.065	1.638	5541.89	9077.77	0.106	3565.52	378.50
A_68_P31259430	chr17:57129793-57129837	NM_017393:128	Clpp	INSIDE	0.042	1.585	6366.32	10088.04	0.067	3872.02	259.37
A_68_P31259431	chr17:57129908-57129952	NM_017393:244	Clpp	INSIDE	0.215	0.600	1560.12	935.58	0.129	1124.24	144.91
A_68_P31259432	chr17:57130046-57130090	NM_017393:382	Clpp	INSIDE	0.268	0.612	979.87	599.61	0.164	764.50	125.31
A_68_P26586895	chr9:65142202-65142246	NM_001044389:123	Clpx	INSIDE	0.610	0.495	1320.21	653.66	0.302	993.50	300.26
A_68_P26586898	chr9:65142471-65142515	NM_001044389:391	Clpx	INSIDE	0.221	0.483	1308.51	632.29	0.107	1136.13	121.45
A_68_P23401325	chr4:148960914-148960958	NM_023051:190	Clstn1	INSIDE	0.215	0.624	4047.49	2524.53	0.134	3361.42	450.60
A_68_P23401327	chr4:148961132-148961176	NM_023051:408	Clstn1	INSIDE	0.065	0.743	3944.35	2928.84	0.049	2517.93	122.15
A_68_P23401328	chr4:148961215-148961259	NM_023051:490	Clstn1	INSIDE	0.114	1.709	9352.64	15982.37	0.195	6029.65	1178.52
A_68_P23401329	chr4:148961308-148961352	NM_023051:584	Clstn1	INSIDE	0.236	1.777	8284.43	14721.64	0.420	5276.83	2216.45
A_68_P22874797	chr4:44025966-44026010	NM_001080384:474	Clta	INSIDE	0.271	0.611	872.51	533.24	0.165	728.24	120.40
A_68_P22874798	chr4:44026078-44026122	NM_001080384:586	Clta	INSIDE	0.142	0.594	3563.10	2116.44	0.084	2508.59	211.54
A_68_P27990834	chr11:86571018-86571062	NM_001003908:-46	Cltc	PROMOTER	0.307	0.544	3606.28	1963.38	0.167	2688.21	449.14
A_68_P23222740	chr4:114659326-114659370	NM_025647:485	Cmpk1	INSIDE	0.472	0.487	1647.80	802.03	0.230	1205.52	276.76
A_68_P23222744	chr4:114659711-114659755	NM_025647:101	Cmpk1	INSIDE	0.554	0.469	2116.44	992.63	0.260	1720.59	446.81
A_68_P28302020	chr12:27154103-27154147	NM_020557:45	Cmpk2	INSIDE	0.359	0.521	1941.54	1011.62	0.187	1587.41	297.21
A_68_P26127989	chr8:106864528-106864572	NM_024217:57	Cmtm3	INSIDE	0.531	3.567	2705.88	9651.50	1.893	1636.22	3097.11
A_68_P26846411	chr9:114690663-114690707	NM_133978:263	Cmtm7	INSIDE	0.260	0.610	3026.72	1847.42	0.159	2275.93	361.86
A_68_P26846738	chr9:114752895-114752940	NM_027294:353	Cmtm8	INSIDE	0.224	0.402	2429.36	977.79	0.090	1935.55	174.75
A_68_P20880981	chr1:183282461-183282505	NM_001160211:-276	Cnih3	PROMOTER	0.191	0.578	1375.16	795.22	0.111	1102.05	121.91
A_68_P27280209	chr10:79451266-79451310	NM_007725:-56	Cnn2	DIVERGENT_PROMOTER	0.600	0.498	1202.49	598.45	0.299	900.74	269.17
A_68_P27280212	chr10:79451564-79451608	NM_007725:242	Cnn2	INSIDE	0.232	0.650	3682.96	2393.12	0.151	2593.38	391.22
A_68_P22476389	chr3:121130415-121130465	NM_028044:982	Cnn3	INSIDE	0.347	0.478	2206.32	1053.53	0.166	1572.16	260.25
A_68_P32145753	chr19:46835804-46835848	NM_001102471:-272	Cnnm2	PROMOTER	0.192	0.536	4318.38	2316.11	0.103	3203.51	330.69
A_68_P32145754	chr19:46835869-46835913	NM_001102471:-208	Cnnm2	PROMOTER	0.128	0.596	2133.60	1271.49	0.076	1611.46	123.22
A_68_P32145760	chr19:46836584-46836628	NM_001102471:508	Cnnm2	INSIDE	0.543	0.449	2306.30	1035.74	0.244	1821.43	444.46
A_68_P32145764	chr19:46837127-46837172	NM_001102471:1051	Cnnm2	INSIDE	0.353	0.643	939.54	603.88	0.227	684.27	155.44
A_68_P20148456	chr1:36528653-36528697	NM_033570:233	Cnnm4	INSIDE	0.184	0.572	1630.07	932.58	0.105	1172.89	123.35
A_68_P20148457	chr1:36528751-36528795	NM_033570:331	Cnnm4	INSIDE	0.422	0.482	2547.50	1228.37	0.203	1966.77	399.81
A_68_P23609440	chr5:37139337-37139381	NM_133724:560	Cno	INSIDE	0.389	0.426	1673.18	712.55	0.166	1430.27	236.80

ProbeName	Target position of probe on CpG island microarray	TargetID	GeneSymbol	CpG island Description	Ratio of relative methylation (TiO2-NP/Vehicle)	Sham group			TiO_2-H group		
						Relative methylation (Cy-5/Cy-3)	Cy-3 signal (Input)	Cy-5 signal (MeDIP)	Relative methylation (Cy-5/Cy-3)	Cy-3 signal (Input)	Cy-5 signal (MeDIP)
A_68_P26085416	chr8:98331248-98331292	NM_001205226:92	Cnot1	INSIDE	0.392	0.403	2325.01	938.12	0.158	1832.93	289.72
A_68_P26085422	chr8:98331940-98331984	NM_001205226:-600	Cnot1	PROMOTER	0.278	0.722	1947.02	1405.60	0.201	1396.11	280.41
A_68_P24359958	chr6:35083871-35083915	NM_001164412:-155	Cnot4	PROMOTER	0.286	0.570	1444.44	823.65	0.163	1111.87	181.03
A_68_P24359961	chr6:35084163-35084220	NM_001164412:-454	Cnot4	PROMOTER	0.152	0.699	1378.85	964.29	0.106	1083.88	115.12
A_68_P23904976	chr5:96591506-96591557	NM_144910:-522	Cnot6l	PROMOTER	0.222	1.477	2018.15	2981.57	0.329	1455.09	478.23
A_68_P27540747	chr10:127759432-127759482	NM_019953:-58	Cnpy2	PROMOTER	0.244	0.628	955.92	600.68	0.153	772.39	118.43
A_68_P22829261	chr4:34010768-34010823	NM_007726:-811	Cnr1	PROMOTER	0.255	0.574	1125.72	646.46	0.147	878.96	128.87
A_68_P27894312	chr11:69137170-69137214	NM_172560:183	Cntrob	INSIDE	0.613	0.477	1808.54	863.43	0.293	1303.35	381.55
A_68_P28071818	chr11:100943909-100943954	NM_027896:-7	Coasy	PROMOTER	0.525	0.618	1182.11	730.56	0.324	784.20	254.46
A_68_P21070567	chr2:25066910-25066954	NM_021393:77	Cobra1	INSIDE	0.587	0.613	2930.85	1797.52	0.360	2259.95	813.56
A_68_P28430601	chr12:52695077-52695121	NM_001198835:771	Coch	INSIDE	0.414	0.570	1734.46	988.83	0.236	1303.88	307.67
A_68_P28143204	chr11:113511459-113511505	NM_013581:640	Cog1	INSIDE	0.306	0.631	855.45	539.61	0.193	733.34	141.68
A_68_P28332516	chr12:32339734-32339779	NM_001163126:23	Cog5	INSIDE	0.324	0.516	1819.70	938.79	0.167	1454.03	243.41
A_68_P26665706	chr9:79566930-79566982	NM_007730:-471	Col12a1	PROMOTER	0.139	0.578	1800.42	1040.62	0.080	1449.70	116.37
A_68_P26665710	chr9:79567360-79567404	NM_007730:-897	Col12a1	PROMOTER	0.315	0.698	2718.00	1897.60	0.220	2040.62	448.42
A_68_P26665705	chr9:79566838-79566882	NM_007730:-375	Col12a1	PROMOTER	0.668	0.695	3230.21	2245.78	0.464	2319.51	1077.35
A_68_P27183505	chr10:61441491-61441535	NM_007731:344	Col13a1	INSIDE	0.289	0.464	1189.96	552.60	0.134	898.88	120.49
A_68_P27183507	chr10:61441706-61441750	NM_007731:128	Col13a1	INSIDE	0.253	0.649	1229.23	797.35	0.164	762.50	125.21
A_68_P22893293	chr4:47221194-47221238	NM_009928:333	Col15a1	INSIDE	0.451	0.710	7261.14	5151.80	0.320	5222.86	1672.20
A_68_P27266461	chr10:76628282-76628326	NM_009929:971	Col18a1	INSIDE	0.217	0.628	2195.41	1377.63	0.136	1733.46	235.82
A_68_P27266466	chr10:76628787-76628837	NM_009929:463	Col18a1	INSIDE	0.069	2.104	3474.49	7308.83	0.145	2390.09	346.92
A_68_P27793111	chr11:51103960-51104004	NM_153393:561	Col23a1	INSIDE	0.232	0.666	2226.32	1483.60	0.154	1682.83	259.68
A_68_P22968878	chr4:62875674-62875718	NM_025685:-749	Col27a1	PROMOTER	0.075	1.634	2729.17	4459.20	0.122	1704.67	207.86
A_68_P22968884	chr4:62876409-62876457	NM_025685:-13	Col27a1	PROMOTER	0.268	0.551	1119.91	616.53	0.148	808.31	119.35
A_68_P30472461	chr15:97832553-97832597	NM_001113515:2581	Col2a1	INSIDE	0.127	0.664	2322.67	1542.97	0.084	1548.26	130.13
A_68_P30472484	chr15:97835084-97835128	NM_001113515:49	Col2a1	INSIDE	0.489	0.643	1403.13	901.75	0.315	1122.06	352.90
A_68_P29246781	chr13:97313402-97313446	NM_001164222:735	Col4a3bp	INSIDE	0.533	0.540	2701.59	1460.20	0.288	2053.29	591.26
A_68_P21087493	chr2:27741935-27741979	NM_015734:12	Col5a1	INSIDE	0.639	0.633	1692.94	1071.61	0.405	1321.68	534.64
A_68_P21087495	chr2:27742216-27742260	NM_015734:294	Col5a1	INSIDE	0.167	0.528	2987.33	1576.89	0.088	2176.71	191.99
A_68_P27262972	chr10:76059240-76059284	NM_146007:26887	Col6a2	INSIDE	0.621	2.423	1159.92	2810.02	1.504	990.29	1489.82
A_68_P23254629	chr4:120712162-120712206	NM_007741:14	Col9a2	INSIDE	0.348	0.656	1641.29	1076.36	0.228	1340.93	305.89
A_68_P23254630	chr4:120712227-120712271	NM_007741:78	Col9a2	INSIDE	0.272	0.592	1082.22	640.95	0.161	890.79	143.52
A_68_P21904962	chr2:180333066-180333110	NM_009936:162	Col9a3	INSIDE	0.448	0.686	2689.73	1844.72	0.307	2069.64	636.14
A_68_P21904963	chr2:180333140-180333184	NM_009936:236	Col9a3	INSIDE	0.434	0.609	2667.80	1625.38	0.264	2106.33	556.86
A_68_P31679713	chr18:47118783-47118827	NM_178377:275	Commd10	INSIDE	0.271	0.626	1764.23	1104.33	0.170	1414.72	240.03
A_68_P29856056	chr14:102039512-102039556	NM_001033132:154	Commd6	INSIDE	0.444	0.524	2094.58	1098.22	0.233	1458.91	339.78
A_68_P29856057	chr14:102039624-102039668	NM_001033132:42	Commd6	INSIDE	0.287	0.454	4775.14	2167.54	0.130	3500.99	456.54
A_68_P25951436	chr8:72898442-72898486	NM_016685:1018	Comp	INSIDE	0.607	0.631	1820.77	1149.15	0.383	1374.59	526.43
A_68_P25951442	chr8:72899053-72899099	NM_016685:1630	Comp	INSIDE	0.628	0.579	2072.65	1200.28	0.364	1575.08	572.82
A_68_P25951443	chr8:72899164-72899208	NM_016685:1740	Comp	INSIDE	0.543	0.397	1681.33	668.26	0.216	1209.96	261.28
A_68_P25951452	chr8:72900302-72900346	NM_016685:2878	Comp	INSIDE	0.464	0.556	1257.80	699.23	0.258	989.21	255.21
A_68_P25951454	chr8:72900470-72900514	NM_016685:3046	Comp	INSIDE	0.238	0.641	2556.74	1640.01	0.153	1812.97	277.27
A_68_P29455223	chr14:22666866-22666910	NM_026965:1244	Comtd1	INSIDE	0.257	0.664	2081.90	1383.19	0.171	1466.44	250.26
A_68_P29455233	chr14:22668221-22668265	NM_026965:-110	Comtd1	PROMOTER	0.580	0.619	1182.20	732.35	0.359	892.59	320.72

ProbeName	Target position of probe on CpG island microarray	TargetID	GeneSymbol	CpG island Description	Ratio of relative methylation (TiO2-NP/Vehicle)	Sham group			TiO_2-H group		
						Relative methylation (Cy-5/Cy-3)	Cy-3 signal (Input)	Cy-5 signal (MeDIP)	Relative methylation (Cy-5/Cy-3)	Cy-3 signal (Input)	Cy-5 signal (MeDIP)
A_68_P25435064	chr7:121397732-121397776	NM_033370:440	Copb1	INSIDE	0.310	0.674	942.61	635.01	0.209	784.38	164.07
A_68_P25435069	chr7:121398243-121398287	NM_033370:-70	Copb1	PROMOTER	0.322	0.582	1570.16	913.29	0.187	1374.61	257.32
A_68_P21605247	chr2:125684679-125684728	NM_009939:51	Cops2	INSIDE	0.087	1.832	1477.37	2707.09	0.159	959.46	152.71
A_68_P23930021	chr5:100947315-100947359	NM_012001:9	Cops4	INSIDE	0.309	0.492	1167.19	573.85	0.152	881.98	133.94
A_68_P20412923	chr1:88483836-88483880	NM_172974:184	Cops7b	INSIDE	0.240	0.534	3097.12	1654.84	0.128	2250.29	288.46
A_68_P20437266	chr1:92500005-92500049	NM_133805:27	Cops8	INSIDE	0.648	0.582	1136.15	661.69	0.378	937.25	353.96
A_68_P20437269	chr1:92500284-92500337	NM_133805:311	Cops8	INSIDE	0.198	0.693	1048.40	726.11	0.137	890.24	121.83
A_68_P28047198	chr11:96723388-96723432	NM_019877:12221	Copz2	DOWNSTREAM	0.378	0.520	2137.91	1110.95	0.196	1668.77	327.66
A_68_P27540982	chr10:127805462-127805508	NM_001081040:1609	Coq10a	INSIDE	0.197	0.641	1315.01	843.25	0.126	963.91	121.86
A_68_P27540983	chr10:127805581-127805626	NM_001081040:1490	Coq10a	INSIDE	0.206	0.541	3351.38	1813.28	0.111	2483.56	276.82
A_68_P20240146	chr1:55109304-55109348	NM_001039710:-287	Coq10b	PROMOTER	0.572	0.604	1427.39	862.22	0.346	1131.81	391.22
A_68_P24008813	chr5:115729847-115729891	NM_026504:158	Coq5	INSIDE	0.306	0.650	876.70	569.85	0.199	685.97	136.62
A_68_P24008814	chr5:115729967-115730011	NM_026504:278	Coq5	INSIDE	0.126	0.506	2392.61	1209.75	0.064	1832.64	116.88
A_68_P25458764	chr7:125676519-125676570	NM_009940:283	Coq7	INSIDE	0.182	0.621	1310.99	813.62	0.113	1035.79	116.70
A_68_P25458767	chr7:125676796-125676840	NM_009940:9	Coq7	INSIDE	0.495	0.687	1716.01	1179.57	0.340	1340.19	455.98
A_68_P25458768	chr7:125676881-125676932	NM_009940:-79	Coq7	PROMOTER	0.235	0.623	973.65	606.34	0.147	802.68	117.65
A_68_P26079750	chr8:97362477-97362521	NM_026452:182	Coq9	INSIDE	0.543	0.377	3531.86	1332.20	0.205	2767.06	566.52
A_68_P23792212	chr5:72895290-72895334	NM_016869:135	Corin	INSIDE	0.520	0.496	1492.72	739.71	0.258	1165.66	300.32
A_68_P22889568	chr4:46614078-46614122	NM_001164804:701	Coro2a	INSIDE	0.088	0.724	2470.65	1788.66	0.064	1938.06	123.89
A_68_P22889569	chr4:46614182-46614226	NM_001164804:597	Coro2a	INSIDE	0.241	0.676	1630.99	1103.03	0.163	1072.11	174.96
A_68_P22889573	chr4:46614584-46614628	NM_001164804:195	Coro2a	INSIDE	0.525	0.541	1659.55	897.98	0.284	1346.16	382.59
A_68_P26571035	chr9:62384198-62384249	NM_175484:628	Coro2b	INSIDE	0.176	0.647	1360.55	880.36	0.114	1039.51	118.12
A_68_P27937691	chr11:77275964-77276008	NM_139128:-1428	Coro6	PROMOTER	0.202	0.539	4893.39	2636.77	0.109	3266.74	356.09
A_68_P30511519	chr16:4679291-4679335	NM_030205:408	Coro7	INSIDE	0.585	0.743	2750.91	2044.13	0.435	2371.87	1031.09
A_68_P30511523	chr16:4679735-4679779	NM_030205:-36	Coro7	DIVERGENT_PROMOTER	0.543	0.679	1880.36	1276.46	0.369	1458.73	537.79
A_68_P30511525	chr16:4679878-4679922	NM_030205:-180	Coro7	DIVERGENT_PROMOTER	0.356	0.547	1226.56	670.80	0.194	1036.47	201.52
A_68_P28581229	chr12:82585711-82585766	NM_025461:359	Cox16	INSIDE	0.221	0.487	1436.61	699.30	0.107	1130.79	121.39
A_68_P28581231	chr12:82586058-82586102	NM_025461:17	Cox16	INSIDE	0.206	0.430	1631.91	701.37	0.088	1341.97	118.54
A_68_P24135004	chr5:139821148-139821192	NM_197980:-50	Cox19	DIVERGENT_PROMOTER	0.088	0.636	4720.53	3002.72	0.056	3184.38	178.65
A_68_P26221733	chr8:123191825-123191869	NM_010926:166	Cox4nb	INSIDE	0.657	0.444	1290.41	573.13	0.292	1065.41	310.74
A_68_P26542211	chr9:57368765-57368824	NM_007747:-244	Cox5a	PROMOTER	0.250	0.550	940.51	517.00	0.137	876.65	120.48
A_68_P20149809	chr1:36748265-36748309	NM_009942:-45	Cox5b	DIVERGENT_PROMOTER	0.187	0.599	1609.22	963.51	0.112	1236.80	138.80
A_68_P20149810	chr1:36748331-36748375	NM_009942:21	Cox5b	INSIDE	0.356	0.628	2340.02	1468.56	0.223	1593.40	356.01
A_68_P24009173	chr5:115798570-115798614	NM_007748:372	Cox6a1	INSIDE	0.371	0.616	2290.27	1410.48	0.228	1743.29	397.93
A_68_P24009177	chr5:115799028-115799072	NM_007748:-86	Cox6a1	PROMOTER	0.349	0.603	1919.81	1157.42	0.210	1453.08	305.38
A_68_P24009178	chr5:115799183-115799231	NM_007748:-242	Cox6a1	PROMOTER	0.223	0.642	1190.71	764.41	0.143	831.42	118.85
A_68_P30135998	chr15:35867761-35867805	NM_053071:219	Cox6c	INSIDE	0.529	0.432	1432.50	618.45	0.229	1182.85	270.29
A_68_P26665914	chr9:79607348-79607392	NM_009945:290	Cox7a2	INSIDE	0.306	0.600	2208.00	1325.14	0.183	1604.11	294.20
A_68_P26665915	chr9:79607435-79607480	NM_009945:203	Cox7a2	INSIDE	0.202	0.652	1853.07	1208.60	0.132	1468.04	193.66
A_68_P29196857	chr13:86186094-86186138	NM_007749:284	Cox7c	INSIDE	0.650	0.505	1146.09	579.31	0.328	1081.14	355.01
A_68_P25272111	chr7:88599609-88599653	NM_007755:-68	Cpeb1	PROMOTER	0.276	0.444	2878.93	1277.00	0.123	2154.34	264.05
A_68_P25272118	chr7:88600431-88600478	NM_007755:-892	Cpeb1	PROMOTER	0.192	0.531	2124.81	1128.16	0.102	1537.68	156.71
A_68_P32093242	chr19:37093220-37093264	NM_198300:188541	Cpeb3	DOWNSTREAM	0.650	0.735	2543.40	1870.48	0.478	2135.53	1020.16
A_68_P32094198	chr19:37248988-37249032	NM_198300:32773	Cpeb3	INSIDE	0.388	0.743	3061.68	2274.48	0.288	2444.14	704.83

ProbeName	Target position of probe on CpG island microarray	TargetID	GeneSymbol	CpG island Description	Ratio of relative methylation (TiO2-NP/Vehicle)	Sham group			TiO_2-H group		
						Relative methylation (Cy-5/Cy-3)	Cy-3 signal (Input)	Cy-5 signal (MeDIP)	Relative methylation (Cy-5/Cy-3)	Cy-3 signal (Input)	Cy-5 signal (MeDIP)
A_68_P32094200	chr19:37249204-37249248	NM_198300:32557	Cpeb3	INSIDE	0.651	0.667	3503.09	2337.36	0.434	2782.03	1207.72
A_68_P32094201	chr19:37249291-37249335	NM_198300:32471	Cpeb3	INSIDE	0.146	0.533	4915.16	2618.02	0.078	4221.46	328.25
A_68_P27685427	chr11:31771207-31771257	NM_026252:-978	Cpeb4	PROMOTER	0.095	1.649	1143.34	1885.26	0.157	760.29	119.28
A_68_P21769581	chr2:155937720-155937764	NM_170588:-41	Cpne1	PROMOTER	0.375	0.652	1093.47	713.36	0.245	871.72	213.46
A_68_P22765602	chr4:19496630-19496674	NM_027769:599	Cpne3	INSIDE	0.293	0.649	793.67	514.74	0.190	700.35	132.94
A_68_P22765609	chr4:19497322-19497369	NM_027769:-94	Cpne3	DIVERGENT_PROMOTER	0.337	0.512	3128.24	1601.48	0.173	2299.02	396.63
A_68_P31126792	chr17:29375212-29375256	NM_153166:-499	Cpne5	PROMOTER	0.282	0.570	3122.91	1781.38	0.161	2326.13	373.64
A_68_P31126793	chr17:29375333-29375377	NM_153166:-619	Cpne5	PROMOTER	0.213	0.657	4373.03	2873.01	0.140	3311.27	463.04
A_68_P30431381	chr15:90509463-90509507	NM_025815:335	Cpne8	INSIDE	0.247	0.574	2229.14	1279.68	0.142	1778.65	251.86
A_68_P30431388	chr15:90510200-90510246	NM_025815:-403	Cpne8	PROMOTER	0.281	0.443	1325.88	587.98	0.124	1014.18	126.26
A_68_P30431389	chr15:90510273-90510317	NM_025815:-475	Cpne8	PROMOTER	0.297	0.635	897.45	569.88	0.189	639.64	120.75
A_68_P30792739	chr16:58670948-58670993	NM_007757:650	Cpox	INSIDE	0.189	0.680	1324.47	900.03	0.128	1009.72	129.66
A_68_P28688780	chr12:103213637-103213681	NM_016856:-525	Cpsf2	PROMOTER	0.226	0.477	1300.12	619.67	0.108	1093.07	117.81
A_68_P28688781	chr12:103213776-103213820	NM_016856:-385	Cpsf2	PROMOTER	0.352	0.697	2411.15	1679.69	0.245	1947.31	477.31
A_68_P28688787	chr12:103214342-103214386	NM_016856:181	Cpsf2	INSIDE	0.666	0.622	2682.38	1667.29	0.414	2038.61	843.77
A_68_P28279804	chr12:21292173-21292217	NM_018813:37	Cpsf3	INSIDE	0.511	0.635	852.53	541.29	0.325	694.88	225.55
A_68_P24164623	chr5:145928226-145928276	NM_178576:158	Cpsf4	INSIDE	0.210	0.495	1597.11	791.11	0.104	1135.02	118.17
A_68_P31951138	chr19:10599754-10599798	NM_001164272:43	Cpsf7	INSIDE	0.472	0.479	1594.89	764.44	0.226	1409.44	319.07
A_68_P23193625	chr4:107595986-107596030	NM_009949:186	Cpt2	INSIDE	0.541	0.570	1575.74	897.86	0.308	1261.84	388.93
A_68_P23601183	chr5:35868307-35868351	NM_153107:-53	Cpz	PROMOTER	0.267	0.577	2645.43	1527.42	0.154	1994.43	307.66
A_68_P26527663	chr9:54613548-54613592	NM_013496:956	Crabp1	INSIDE	0.393	0.518	1434.66	742.44	0.203	1097.56	223.21
A_68_P27364532	chr10:94786282-94786326	NM_009950:427	Cradd	INSIDE	0.435	0.404	1815.26	733.87	0.176	1427.80	251.19
A_68_P31100996	chr17:25150700-25150744	NM_020608:1453	Cramp1l	INSIDE	0.264	0.505	1505.33	760.87	0.133	1172.77	156.40
A_68_P31100997	chr17:25150808-25150852	NM_020608:1345	Cramp1l	INSIDE	0.422	0.664	3615.04	2399.44	0.280	2718.26	761.40
A_68_P31101000	chr17:25151186-25151230	NM_020608:967	Cramp1l	INSIDE	0.217	0.571	8431.38	4816.82	0.124	5935.64	736.09
A_68_P21103093	chr2:30270839-30270883	NM_007760:408	Crat	INSIDE	0.241	0.471	1612.70	759.28	0.114	1062.47	120.66
A_68_P31259843	chr17:57201503-57201547	NM_177638:-175	Crb3	DIVERGENT_PROMOTER	0.607	0.687	2382.63	1636.66	0.417	1768.01	737.28
A_68_P31259844	chr17:57201624-57201668	NM_177638:-53	Crb3	DIVERGENT_PROMOTER	0.401	0.600	1139.58	684.20	0.241	965.34	232.70
A_68_P31259845	chr17:57201740-57201784	NM_177638:63	Crb3	INSIDE	0.233	0.557	1288.48	718.25	0.130	950.80	123.39
A_68_P20293011	chr1:64579120-64579164	NM_001037726:-235	Creb1	PROMOTER	0.517	0.564	3850.61	2170.53	0.291	2688.19	783.60
A_68_P20293015	chr1:64579497-64579541	NM_001037726:141	Creb1	INSIDE	0.395	1.637	1498.43	2452.72	0.646	1044.74	674.70
A_68_P21425776	chr2:91815643-91815687	NM_011957:48663	Creb3l1	DOWNSTREAM	0.323	0.554	1548.19	858.35	0.179	1192.03	213.29
A_68_P21425958	chr2:91848432-91848476	NM_011957:15873	Creb3l1	INSIDE	0.544	0.461	1531.75	705.60	0.251	1151.80	288.78
A_68_P24372701	chr6:37391699-37391743	NM_178661:428	Creb3l2	INSIDE	0.504	0.562	1416.80	796.68	0.283	1123.79	318.38
A_68_P30508954	chr16:4213021-4213065	NM_001025432:362	Crebbp	INSIDE	0.526	0.674	2557.64	1725.06	0.355	2017.28	715.64
A_68_P25313622	chr7:97591232-97591276	NM_145151:-36	Crebzf	PROMOTER	0.214	0.476	1463.37	697.12	0.102	1187.97	121.08
A_68_P25313629	chr7:97592324-97592368	NM_145151:1056	Crebzf	INSIDE	0.335	0.576	3401.89	1959.86	0.193	2509.54	484.51
A_68_P25313631	chr7:97592513-97592557	NM_145151:1244	Crebzf	INSIDE	0.190	0.590	1691.83	998.28	0.112	1274.77	143.18
A_68_P30421424	chr15:88650529-88650573	NM_029720:475	Creld2	INSIDE	0.303	0.531	1051.01	558.34	0.161	858.22	138.19
A_68_P21976418	chr3:19594468-19594512	NM_205769:906	Crh	INSIDE	0.370	0.350	1613.65	564.62	0.130	1344.50	174.16
A_68_P28089251	chr11:103994664-103994708	NM_007762:491	Crhr1	INSIDE	0.257	0.456	2043.80	932.74	0.118	1551.33	182.30
A_68_P24467398	chr6:55067497-55067541	NM_009953:15448	Crhr2	INSIDE	0.290	0.491	1247.82	612.33	0.142	865.93	123.04
A_68_P24467402	chr6:55067829-55067873	NM_009953:15116	Crhr2	INSIDE	0.422	0.608	2457.72	1493.45	0.257	2070.59	531.50
A_68_P24467403	chr6:55067907-55067951	NM_009953:15038	Crhr2	INSIDE	0.259	0.413	1531.70	633.35	0.107	1247.41	133.69

ProbeName	Target position of probe on CpG island microarray	TargetID	GeneSymbol	CpG island Description	Ratio of relative methylation (TiO2-NP/Vehicle)	Sham group			TiO_2-H group		
						Relative methylation (Cy-5/Cy-3)	Cy-3 signal (Input)	Cy-5 signal (MeDIP)	Relative methylation (Cy-5/Cy-3)	Cy-3 signal (Input)	Cy-5 signal (MeDIP)
A_68_P31369877	chr17:78600790-78600834	NM_015800:1225	Crim1	INSIDE	0.468	0.707	1646.14	1163.88	0.331	1227.24	406.36
A_68_P28754915	chr12:114378792-114378837	NM_024223:368	Crip2	INSIDE	0.242	0.632	945.68	598.03	0.153	758.98	116.00
A_68_P31420723	chr17:87424816-87424860	NM_019936:-62	Cript	DIVERGENT_PROMOTER	0.225	0.716	1836.76	1314.24	0.161	1325.06	213.69
A_68_P31420724	chr17:87424991-87425037	NM_019936:114	Cript	INSIDE	0.094	1.411	2658.36	3751.73	0.133	1895.95	251.82
A_68_P27927584	chr11:75493253-75493297	NM_133656:463	Crk	INSIDE	0.231	0.688	2841.10	1953.86	0.159	2229.42	354.73
A_68_P30573854	chr16:17452586-17452630	NM_007764:529	Crkl	INSIDE	0.542	0.685	1684.53	1154.00	0.372	1284.92	477.48
A_68_P30573855	chr16:17452673-17452717	NM_007764:615	Crkl	INSIDE	0.176	0.673	1312.79	882.97	0.118	978.99	115.74
A_68_P25952213	chr8:73017547-73017591	NM_018827:514	Crlf1	INSIDE	0.414	0.663	1178.42	780.87	0.274	916.86	251.36
A_68_P25952216	chr8:73018033-73018077	NM_018827:1000	Crlf1	INSIDE	0.122	0.669	1916.75	1282.14	0.081	1472.21	119.92
A_68_P21715592	chr2:145760196-145760240	NM_025820:218	Crnkl1	INSIDE	0.327	0.435	1660.27	722.48	0.142	1238.30	176.21
A_68_P23361908	chr4:140616772-140616816	NM_001145958:-334	Crocc	PROMOTER	0.289	0.442	4975.18	2200.64	0.128	3735.03	476.92
A_68_P23470689	chr5:8997395-8997439	NM_023733:-270	Crot	PROMOTER	0.521	0.541	1008.41	545.45	0.282	794.17	223.71
A_68_P32121139	chr19:42505784-42505828	NM_145123:467	Crtac1	INSIDE	0.141	1.354	4979.47	6743.92	0.191	3461.59	662.13
A_68_P32121140	chr19:42505908-42505952	NM_145123:343	Crtac1	INSIDE	0.215	0.612	2052.70	1256.73	0.131	1546.30	203.26
A_68_P32121150	chr19:42506930-42506974	NM_145123:-679	Crtac1	PROMOTER	0.494	0.601	1071.34	643.49	0.297	792.14	235.11
A_68_P26844288	chr9:114277438-114277482	NM_019922:22370	Crtap	DOWNSTREAM	0.532	0.503	1497.39	753.86	0.268	1268.14	339.71
A_68_P21428538	chr2:92274073-92274117	NM_009963:132	Cry2	INSIDE	0.277	0.458	1293.82	591.94	0.127	926.38	117.21
A_68_P21428540	chr2:92274280-92274324	NM_009963:-76	Cry2	PROMOTER	0.447	0.485	2280.66	1106.33	0.217	1641.36	355.74
A_68_P20351100	chr1:74939365-74939409	NM_021541:323	Cryba2	INSIDE	0.505	0.611	960.87	587.30	0.309	797.41	246.27
A_68_P23543724	chr5:24256980-24257024	NM_153076:6659	Crygn	INSIDE	0.260	0.620	921.89	571.35	0.161	754.76	121.85
A_68_P29627745	chr14:58017455-58017499	NM_030004:-156	Cryl1	PROMOTER	0.467	0.497	1504.53	747.01	0.232	1267.21	293.90
A_68_P22663511	chr3:154259686-154259731	NM_009968:-267	Cryz	PROMOTER	0.155	0.472	2252.36	1062.45	0.073	1605.74	117.14
A_68_P30964660	chr16:91728640-91728684	NM_026994:385	Cryzl1	INSIDE	0.270	0.726	2596.26	1885.32	0.196	1729.11	338.75
A_68_P30964663	chr16:91729071-91729122	NM_026994:-49	Cryzl1	DIVERGENT_PROMOTER	0.604	0.597	1606.66	959.02	0.361	1232.55	444.72
A_68_P24850800	chr6:131338648-131338692	NM_011733:-202	Csda	PROMOTER	0.608	0.474	1407.49	667.06	0.288	1216.73	350.56
A_68_P22383903	chr3:102824344-102824388	NM_001161854:-102	Csde1	PROMOTER	0.376	0.647	808.74	523.50	0.243	705.15	171.62
A_68_P22383904	chr3:102824419-102824463	NM_001161854:-28	Csde1	PROMOTER	0.582	0.712	1739.02	1238.71	0.414	1408.55	583.84
A_68_P25944440	chr8:71258901-71258945	NM_172753:119	Csgalnact1	INSIDE	0.295	0.632	2416.83	1526.41	0.186	1907.58	355.01
A_68_P25693339	chr8:17534712-17534756	NM_053171:651	Csmd1	INSIDE	0.102	0.565	4990.32	2818.22	0.057	3377.19	194.11
A_68_P25693340	chr8:17534794-17534838	NM_053171:569	Csmd1	INSIDE	0.227	0.643	1476.56	950.06	0.146	1076.95	157.17
A_68_P25693341	chr8:17534942-17534988	NM_053171:421	Csmd1	INSIDE	0.109	0.534	3144.37	1680.06	0.058	2141.23	124.73
A_68_P31758331	chr18:61715432-61715480	NM_146087:221	Csnk1a1	INSIDE	0.267	0.536	1117.18	599.06	0.143	843.72	120.88
A_68_P30366491	chr15:79271911-79271956	NM_013767:554	Csnk1e	INSIDE	0.489	0.636	1677.27	1067.49	0.311	1333.40	414.71
A_68_P26590186	chr9:65757328-65757375	NM_173185:535	Csnk1g1	INSIDE	0.201	0.555	1332.98	739.79	0.112	1041.20	116.32
A_68_P27284377	chr10:80085062-80085106	NM_134002:-440	Csnk1g2	PROMOTER	0.404	0.629	1419.87	892.51	0.254	1060.64	269.20
A_68_P27284381	chr10:80085791-80085835	NM_001159591:-6588	Csnk1g2	PROMOTER	0.407	0.460	2111.74	970.58	0.187	1557.44	291.41
A_68_P27284382	chr10:80085896-80085940	NM_001159591:-6482	Csnk1g2	PROMOTER	0.240	0.400	2767.12	1106.66	0.096	1963.51	188.81
A_68_P27284472	chr10:80097247-80097291	NM_001159591:4868	Csnk1g2	INSIDE	0.625	2.677	2810.08	7522.39	1.673	1993.33	3334.83
A_68_P27284501	chr10:80101148-80101192	NM_001159591:8770	Csnk1g2	INSIDE	0.652	2.188	1736.53	3798.94	1.427	1321.42	1885.63
A_68_P31715729	chr18:54021378-54021423	NM_152809:-366	Csnk1g3	PROMOTER	0.133	0.698	1796.19	1253.59	0.093	1298.58	120.61
A_68_P31715738	chr18:54022583-54022627	NM_152809:838	Csnk1g3	INSIDE	0.081	1.393	8923.58	12431.41	0.113	5833.60	656.68
A_68_P31157712	chr17:35257797-35257841	NM_009975:574	Csnk2b	INSIDE	0.253	0.606	1049.24	635.32	0.153	839.51	128.42
A_68_P31157713	chr17:35257880-35257924	NM_009975:490	Csnk2b	INSIDE	0.150	0.559	2161.91	1208.66	0.084	1703.06	142.82
A_68_P31157714	chr17:35257967-35258011	NM_009975:404	Csnk2b	INSIDE	0.472	0.713	1646.62	1174.39	0.336	1354.79	455.74

ProbeName	Target position of probe on CpG island microarray	TargetID	GeneSymbol	CpG island Description	Ratio of relative methylation (TiO2-NP/Vehicle)	Sham group			TiO_2-H group		
						Relative methylation (Cy-5/Cy-3)	Cy-3 signal (Input)	Cy-5 signal (MeDIP)	Relative methylation (Cy-5/Cy-3)	Cy-3 signal (Input)	Cy-5 signal (MeDIP)
A_68_P31157715	chr17:35258104-35258148	NM_009975:266	Csnk2b	INSIDE	0.525	0.671	2018.35	1355.21	0.353	1461.97	515.49
A_68_P31157716	chr17:35258201-35258245	NM_009975:170	Csnk2b	INSIDE	0.248	0.679	3573.03	2425.02	0.168	2515.84	423.30
A_68_P26822740	chr9:110148381-110148425	NM_001166273:2116	Cspg5	INSIDE	0.243	0.733	5820.68	4268.36	0.178	4139.87	736.45
A_68_P26875482	chr9:119892235-119892279	NM_153287:1520	Csrnp1	INSIDE	0.288	0.343	3301.34	1132.11	0.099	2401.95	236.83
A_68_P30486141	chr15:100325298-100325342	NM_153407:350	Csrnp2	INSIDE	0.246	0.656	3590.26	2354.78	0.161	2892.42	466.83
A_68_P30486145	chr15:100325764-100325808	NM_153407:-116	Csrnp2	PROMOTER	0.623	0.631	1488.54	939.42	0.393	1158.38	455.09
A_68_P21706947	chr2:144219117-144219161	NM_181417:24368	Csrp2bp	INSIDE	0.536	2.854	879.23	2509.04	1.530	714.56	1093.14
A_68_P21732682	chr2:148700871-148700915	NM_009976:312	Cst3	INSIDE	0.450	0.434	1634.82	709.94	0.196	1360.69	266.14
A_68_P21732684	chr2:148701174-148701218	NM_009976:8	Cst3	INSIDE	0.102	0.467	3411.58	1592.02	0.047	2577.17	122.17
A_68_P31928179	chr19:5349318-5349363	NM_028623:234	Cst6	INSIDE	0.275	0.450	2286.28	1028.02	0.124	1811.51	223.91
A_68_P21863931	chr2:172196546-172196590	NM_024199:66	Cstf1	INSIDE	0.235	0.721	2329.05	1678.69	0.169	1783.98	302.07
A_68_P21489860	chr2:104430467-104430511	NM_001037326:-152	Cstf3	PROMOTER	0.146	0.705	2994.71	2109.85	0.103	2084.39	214.56
A_68_P25539028	chr7:140316412-140316456	NM_009980:-1268	Ctbp2	PROMOTER	0.474	0.420	1352.64	568.73	0.199	1173.11	233.91
A_68_P22614659	chr3:146113265-146113309	NM_028836:-170	Ctbs	PROMOTER	0.454	0.492	1523.72	748.91	0.223	1212.16	270.64
A_68_P26135025	chr8:108159869-108159913	NM_181322:-547	Ctcf	PROMOTER	0.275	0.481	1876.65	902.22	0.132	1343.38	177.67
A_68_P26135030	chr8:108160451-108160495	NM_181322:35	Ctcf	INSIDE	0.442	0.615	1113.39	684.51	0.271	911.29	247.39
A_68_P31864632	chr18:80665927-80665971	NM_026295:458	Ctdp1	INSIDE	0.457	0.543	2390.19	1297.01	0.248	1893.54	469.66
A_68_P31838819	chr18:75856123-75856171	NM_201354:1204	Ctif	INSIDE	0.187	0.587	1597.47	937.31	0.110	1322.73	145.18
A_68_P31838825	chr18:75856820-75856864	NM_201354:508	Ctif	INSIDE	0.351	0.478	1876.78	897.37	0.168	1354.08	227.25
A_68_P31616437	chr18:35278200-35278244	NM_009818:-343	Ctnna1	PROMOTER	0.425	0.613	1314.59	806.16	0.261	1136.59	296.11
A_68_P31616442	chr18:35278972-35279016	NM_009818:429	Ctnna1	INSIDE	0.211	0.530	2167.37	1148.16	0.112	1700.53	189.95
A_68_P27191665	chr10:63046368-63046418	NM_001164376:126134	Ctnna3	INSIDE	0.637	2.627	760.18	1996.91	1.674	593.57	993.89
A_68_P22941963	chr4:56877865-56877909	NM_018761:197	Ctnnal1	INSIDE	0.267	0.673	3539.80	2381.13	0.180	2605.56	468.37
A_68_P22941964	chr4:56877971-56878015	NM_018761:91	Ctnnal1	INSIDE	0.136	0.566	4259.49	2410.06	0.077	3282.49	251.89
A_68_P22941966	chr4:56878242-56878286	NM_018761:-181	Ctnnal1	PROMOTER	0.094	0.737	3378.25	2490.28	0.069	2376.21	164.66
A_68_P26880506	chr9:120843223-120843267	NM_001165902:549	Ctnnb1	INSIDE	0.422	0.512	1548.12	792.34	0.216	1269.45	274.37
A_68_P23400905	chr4:148892711-148892755	NM_023465:383	Ctnnbip1	INSIDE	0.188	0.664	2722.42	1808.01	0.125	2067.79	257.58
A_68_P21778673	chr2:157563323-157563367	NM_025680:208	Ctnnbl1	INSIDE	0.304	0.482	1643.11	792.00	0.147	1302.72	190.98
A_68_P21778674	chr2:157563392-157563436	NM_025680:278	Ctnnbl1	INSIDE	0.241	0.409	1607.35	657.19	0.098	1161.13	114.34
A_68_P30106579	chr15:30102117-30102161	NM_008729:-209	Ctnnd2	PROMOTER	0.282	0.616	1878.08	1157.70	0.174	1487.11	258.73
A_68_P30109622	chr15:30576916-30576960	NM_008729:474591	Ctnnd2	INSIDE	0.580	0.676	6571.73	4443.22	0.392	5192.23	2037.84
A_68_P30109623	chr15:30576998-30577042	NM_008729:474673	Ctnnd2	INSIDE	0.362	0.407	1353.05	550.99	0.148	980.81	144.78
A_68_P23252015	chr4:120242852-120242896	NM_016748:7	Ctps	INSIDE	0.606	0.510	1269.60	647.95	0.309	1139.09	352.38
A_68_P23252016	chr4:120243027-120243071	NM_016748:-167	Ctps	PROMOTER	0.170	0.552	2445.91	1351.29	0.094	1616.25	151.68
A_68_P25416680	chr7:118172636-118172680	NM_009431:194	Ctr9	INSIDE	0.460	0.650	2433.92	1581.60	0.299	1903.35	569.24
A_68_P25416834	chr7:118199064-118199108	NM_009431:26622	Ctr9	INSIDE	0.629	2.955	630.19	1862.12	1.860	559.19	1040.14
A_68_P21820017	chr2:164658603-164658647	NM_001038492:252	Ctsa	INSIDE	0.584	0.619	1168.57	723.19	0.361	836.37	302.12
A_68_P29659269	chr14:63741273-63741317	NM_007798:-7	Ctsb	PROMOTER	0.356	0.737	4175.57	3077.50	0.262	3408.61	893.77
A_68_P29659270	chr14:63741403-63741447	NM_007798:123	Ctsb	INSIDE	0.270	0.620	2316.59	1435.18	0.167	1751.69	293.15
A_68_P29659271	chr14:63741571-63741615	NM_007798:291	Ctsb	INSIDE	0.246	0.642	871.04	559.29	0.158	729.97	115.21
A_68_P29659272	chr14:63741639-63741683	NM_007798:359	Ctsb	INSIDE	0.302	0.570	985.32	561.73	0.172	849.82	146.38
A_68_P25301855	chr7:95426643-95426687	NM_009982:62	Ctsc	INSIDE	0.139	0.586	2010.20	1178.91	0.081	1438.24	117.22
A_68_P25594358	chr7:149573933-149573977	NM_009983:-179	Ctsd	PROMOTER	0.329	0.444	1208.99	536.50	0.146	972.25	141.86
A_68_P29098530	chr13:64471460-64471504	NM_009984:132	Ctsl	INSIDE	0.300	0.650	1179.67	766.37	0.195	936.16	182.74

ProbeName	Target position of probe on CpG island microarray	TargetID	GeneSymbol	CpG island Description	Ratio of relative methylation (TiO2-NP/Vehicle)	Sham group			TiO_2-H group		
						Relative methylation (Cy-5/Cy-3)	Cy-3 signal (Input)	Cy-5 signal (MeDIP)	Relative methylation (Cy-5/Cy-3)	Cy-3 signal (Input)	Cy-5 signal (MeDIP)
A_68_P29098531	chr13:64471536-64471580	NM_009984:56	Ctsl	INSIDE	0.261	0.546	1591.37	869.53	0.142	1296.56	184.68
A_68_P21875386	chr2:174264415-174264459	NM_022325:57	Ctsz	INSIDE	0.377	0.679	6326.25	4298.29	0.256	4237.74	1085.97
A_68_P22394781	chr3:104855923-104855967	NM_001163332:-73	Cttnbp2nl	PROMOTER	0.207	0.640	1291.37	826.03	0.133	925.04	122.58
A_68_P25083581	chr7:50931799-50931843	NM_145582:4420	Ctu1	INSIDE	0.669	2.401	2120.58	5091.69	1.606	1452.51	2332.23
A_68_P25616894	chr8:4258857-4258901	NM_183315:396	Ctxn1	INSIDE	0.666	0.634	1584.26	1005.21	0.422	1240.33	523.97
A_68_P27997845	chr11:87913051-87913095	NM_198013:425	Cuedc1	INSIDE	0.604	0.638	1295.02	826.82	0.386	1219.44	470.60
A_68_P27998412	chr11:88007125-88007169	NM_001172099:24080	Cuedc1	INSIDE	0.291	1.632	1600.20	2611.79	0.474	1254.77	595.27
A_68_P32143334	chr19:46413011-46413055	NM_001164290:118	Cuedc2	INSIDE	0.512	0.469	1230.91	577.32	0.240	1008.01	241.88
A_68_P31458279	chr18:3383133-3383177	NM_029402:-68	Cul2	PROMOTER	0.309	0.489	1043.64	510.51	0.151	794.34	119.90
A_68_P32328375	chrX:35918239-35918283	NM_001110142:11113	Cul4b	INSIDE	0.070	2.613	4453.97	11636.75	0.184	1912.55	351.27
A_68_P32328376	chrX:35918353-35918397	NM_001110142:10999	Cul4b	INSIDE	0.362	4.458	5368.72	23933.97	1.613	2521.92	4067.59
A_68_P32328377	chrX:35918417-35918470	NM_001110142:10930	Cul4b	INSIDE	0.052	3.025	1745.89	5281.59	0.156	739.79	115.73
A_68_P31207500	chr17:46683388-46683432	NM_001081335:-73	Cul9	PROMOTER	0.051	2.117	3755.77	7950.94	0.108	2256.59	243.96
A_68_P31113295	chr17:27076389-27076433	NM_026307:3	Cuta	INSIDE	0.635	0.361	1600.29	578.07	0.229	1269.62	291.24
A_68_P32128432	chr19:43827652-43827696	NM_001113562:162	Cutc	INSIDE	0.154	0.546	5862.75	3201.70	0.084	4363.22	367.43
A_68_P32128433	chr19:43827788-43827832	NM_001113562:298	Cutc	INSIDE	0.343	0.633	2293.03	1451.94	0.217	1730.26	375.31
A_68_P24122101	chr5:137043954-137043998	NM_009986:-675	Cux1	PROMOTER	0.339	0.571	1610.95	919.22	0.194	1380.86	267.45
A_68_P24047549	chr5:122398855-122398899	NM_007804:98958	Cux2	INSIDE	0.494	0.424	1593.28	674.77	0.209	1301.92	272.49
A_68_P26317494	chr9:14305171-14305215	NM_023153:130	Cwc15	INSIDE	0.174	0.589	2438.09	1436.86	0.102	2042.22	208.86
A_68_P21360374	chr2:77783936-77783980	NM_030560:452	Cwc22	INSIDE	0.267	0.466	2112.35	983.94	0.125	1760.28	219.18
A_68_P29292644	chr13:105607137-105607182	NM_026072:-126	Cwc27	DIVERGENT_PROMOTER	0.547	0.609	1368.52	833.90	0.333	1139.10	379.52
A_68_P30895371	chr16:78301854-78301898	NM_001025192:-59	Cxadr	PROMOTER	0.432	0.542	1773.20	961.47	0.234	1472.04	344.71
A_68_P30895372	chr16:78302001-78302045	NM_001025192:87	Cxadr	INSIDE	0.485	0.644	1640.50	1056.16	0.312	1285.44	401.09
A_68_P30895376	chr16:78302422-78302466	NM_001025192:509	Cxadr	INSIDE	0.559	0.599	1035.41	619.77	0.334	734.45	245.67
A_68_P24780081	chr6:117118598-117118642	NM_021704:68	Cxcl12	INSIDE	0.256	0.527	3385.70	1785.81	0.135	2469.10	333.23
A_68_P20601161	chr1:130487885-130487929	NM_009911:970	Cxcr4	INSIDE	0.500	0.419	2586.95	1084.96	0.210	1983.28	415.54
A_68_P22546441	chr3:133899193-133899237	NM_001004367:-3897	Cxxc4	PROMOTER	0.078	0.749	4240.34	3178.10	0.058	2918.18	169.53
A_68_P31620296	chr18:35989257-35989301	NM_133687:-193	Cxxc5	PROMOTER	0.502	0.421	1309.01	550.85	0.211	1045.22	221.00
A_68_P31620298	chr18:35989480-35989524	NM_133687:31	Cxxc5	INSIDE	0.058	0.711	6141.97	4366.36	0.041	4314.31	177.72
A_68_P31620305	chr18:35990289-35990333	NM_133687:839	Cxxc5	INSIDE	0.173	0.572	4581.05	2620.95	0.099	3103.76	307.44
A_68_P22411201	chr3:108003559-108003603	NM_001081320:172	Cyb561d1	INSIDE	0.147	0.723	2406.39	1739.12	0.106	1685.36	179.31
A_68_P27913314	chr11:72608834-72608878	NM_001024926:485	Cyb5d2	INSIDE	0.269	0.530	1076.15	570.73	0.142	841.16	119.81
A_68_P27913316	chr11:72608989-72609033	NM_001024926:331	Cyb5d2	INSIDE	0.553	0.692	1555.18	1076.84	0.383	1307.64	500.27
A_68_P26233347	chr8:124956923-124956967	NM_007806:-104	Cyba	PROMOTER	0.515	0.547	1100.64	602.03	0.282	904.73	255.00
A_68_P21317590	chr2:70956135-70956179	NM_028593:46	Cybrd1	INSIDE	0.472	0.418	3059.45	1277.99	0.197	2248.01	443.31
A_68_P21317591	chr2:70956240-70956284	NM_028593:152	Cybrd1	INSIDE	0.174	0.585	1607.24	939.63	0.102	1194.98	121.41
A_68_P21317592	chr2:70956339-70956383	NM_028593:250	Cybrd1	INSIDE	0.406	0.558	1737.90	970.02	0.226	1323.53	299.72
A_68_P24438772	chr6:50516320-50516364	NM_007808:131	Cycs	INSIDE	0.338	0.389	1792.99	697.19	0.131	1553.85	204.01
A_68_P30349757	chr15:76490222-76490266	NM_019396:247	Cyhr1	INSIDE	0.163	0.651	2882.75	1876.43	0.106	1975.22	208.99
A_68_P26042567	chr8:91220855-91220899	NM_001128170:-50	Cyld	PROMOTER	0.137	0.680	3877.03	2636.67	0.093	2731.99	254.67
A_68_P26042572	chr8:91221405-91221449	NM_001128169:484	Cyld	INSIDE	0.266	0.527	1386.38	730.10	0.140	990.49	138.50
A_68_P32097099	chr19:37772682-37772726	NM_007811:407	Cyp26a1	INSIDE	0.636	0.685	1552.26	1062.66	0.436	1380.23	601.39
A_68_P32097101	chr19:37772874-37772918	NM_007811:599	Cyp26a1	INSIDE	0.393	0.426	1213.97	517.08	0.167	1052.81	176.09
A_68_P32097109	chr19:37773781-37773827	NM_007811:1507	Cyp26a1	INSIDE	0.203	0.472	2573.06	1214.84	0.096	2008.95	192.74

ProbeName	Target position of probe on CpG island microarray	TargetID	GeneSymbol	CpG island Description	Ratio of relative methylation (TiO2-NP/Vehicle)	Sham group			TiO_2-H group		
						Relative methylation (Cy-5/Cy-3)	Cy-3 signal (Input)	Cy-5 signal (MeDIP)	Relative methylation (Cy-5/Cy-3)	Cy-3 signal (Input)	Cy-5 signal (MeDIP)
A_68_P24600267	chr6:84534196-84534247	NM_175475:9583	Cyp26b1	INSIDE	0.206	0.512	1498.50	766.72	0.105	1165.31	122.64
A_68_P24600268	chr6:84534331-84534375	NM_175475:9452	Cyp26b1	INSIDE	0.254	0.403	1425.21	574.31	0.102	1140.45	116.76
A_68_P24600270	chr6:84534486-84534530	NM_175475:9296	Cyp26b1	INSIDE	0.171	0.698	1418.90	989.91	0.119	1227.07	146.30
A_68_P24600279	chr6:84535601-84535645	NM_175475:8182	Cyp26b1	INSIDE	0.498	0.661	845.60	559.03	0.329	781.39	257.13
A_68_P24600305	chr6:84538923-84538970	NM_175475:4858	Cyp26b1	INSIDE	0.205	0.731	2470.27	1804.54	0.149	1827.62	273.18
A_68_P24600308	chr6:84539342-84539397	NM_175475:4435	Cyp26b1	INSIDE	0.182	0.694	1167.85	811.02	0.126	958.11	120.98
A_68_P24600309	chr6:84539419-84539463	NM_175475:4364	Cyp26b1	INSIDE	0.240	0.514	1259.93	647.70	0.124	976.92	120.77
A_68_P27533698	chr10:126486217-126486263	NM_010009:939	Cyp27b1	INSIDE	0.326	0.365	1690.55	617.35	0.119	1110.02	132.01
A_68_P27533699	chr10:126486293-126486337	NM_010009:1013	Cyp27b1	INSIDE	0.318	0.531	2346.75	1245.55	0.169	1762.54	297.73
A_68_P25436575	chr7:121706623-121706670	NM_177382:-160	Cyp2r1	PROMOTER	0.390	0.629	1074.68	675.45	0.245	783.63	191.86
A_68_P28727506	chr12:109591095-109591139	NM_010010:18526	Cyp46a1	INSIDE	0.357	0.489	1756.76	859.13	0.175	1359.43	237.62
A_68_P31145617	chr17:32589667-32589711	NM_177307:21	Cyp4f39	INSIDE	0.369	1.547	1792.63	2772.84	0.571	1301.42	743.55
A_68_P31145618	chr17:32589827-32589872	NM_177307:182	Cyp4f39	INSIDE	0.131	1.497	3862.24	5780.25	0.197	2548.25	501.03
A_68_P23448645	chr5:4104155-4104199	NM_020010:521	Cyp51	INSIDE	0.479	0.667	1310.96	874.53	0.319	1029.64	328.77
A_68_P23448648	chr5:4104578-4104622	NM_020010:97	Cyp51	INSIDE	0.233	0.382	1575.88	602.63	0.089	1302.24	115.83
A_68_P22609954	chr3:145312413-145312457	NM_010516:515	Cyr61	INSIDE	0.106	0.509	3045.06	1549.09	0.054	2202.75	119.22
A_68_P22609958	chr3:145312902-145312946	NM_010516:25	Cyr61	INSIDE	0.201	0.534	1382.71	737.81	0.107	1107.28	118.96
A_68_P22609959	chr3:145313007-145313055	NM_010516:-81	Cyr61	PROMOTER	0.444	0.515	1021.54	525.69	0.229	776.44	177.50
A_68_P22609986	chr3:145316237-145316281	NM_010516:-3309	Cyr61	PROMOTER	0.232	2.130	5851.95	12467.31	0.494	3845.62	1900.72
A_68_P24157330	chr5:144383580-144383624	NM_011182:287	Cyth3	INSIDE	0.518	0.607	2619.29	1588.92	0.314	2100.62	659.94
A_68_P27272488	chr10:77632611-77632655	NM_138601:-119	D10Jhu81e	PROMOTER	0.476	0.523	1479.67	774.53	0.249	1114.96	277.98
A_68_P27297057	chr10:82823167-82823211	NM_026579:223	D10Wsu102e	INSIDE	0.359	0.481	2993.75	1439.96	0.172	2206.63	380.57
A_68_P28143433	chr11:113546017-113546061	NM_177777:313	D11Wsu47e	INSIDE	0.319	0.698	1910.64	1333.85	0.222	1321.37	294.00
A_68_P28143423	chr11:113544774-113544818	NM_138598:669	D11Wsu99e	INSIDE	0.199	0.623	1170.45	729.50	0.124	947.60	117.73
A_68_P28143424	chr11:113544952-113544996	NM_138598:491	D11Wsu99e	INSIDE	0.389	0.525	2517.62	1321.72	0.204	2111.80	431.46
A_68_P29480508	chr14:26746521-26746565	NM_025311:194	D14Ertd449e	INSIDE	0.150	0.537	5034.47	2705.84	0.080	4056.60	326.20
A_68_P29480509	chr14:26746634-26746678	NM_025311:80	D14Ertd449e	INSIDE	0.219	0.513	3717.51	1907.51	0.112	2996.38	336.38
A_68_P29480513	chr14:26747056-26747103	NM_025311:-343	D14Ertd449e	PROMOTER	0.398	0.358	1735.25	621.67	0.143	1456.35	207.60
A_68_P30247880	chr15:58247520-58247564	NM_145959:520	D15Ertd621e	INSIDE	0.212	0.584	1212.14	707.47	0.124	937.07	116.08
A_68_P30896823	chr16:78577179-78577223	NM_025967:-287	D16Ertd472e	PROMOTER	0.645	0.681	1168.17	795.47	0.439	1059.91	465.78
A_68_P31254442	chr17:56323432-56323476	NM_080837:-111	D17Wsu104e	PROMOTER	0.344	0.454	2548.60	1158.08	0.156	2023.79	315.92
A_68_P31118681	chr17:27956946-27956990	NM_001033279:519	D17Wsu92e	INSIDE	0.551	0.485	2873.68	1393.55	0.267	2432.03	649.68
A_68_P32040826	chr19:27504142-27504186	NM_177474:146	D19Bwg1357e	INSIDE	0.288	0.614	4153.16	2551.77	0.177	3232.43	572.62
A_68_P32121689	chr19:42593058-42593107	NM_177464:-212	D19Ertd386e	PROMOTER	0.219	0.463	1829.26	847.56	0.101	1380.23	139.96
A_68_P32121691	chr19:42593347-42593391	NM_177464:74	D19Ertd386e	INSIDE	0.292	0.582	2611.01	1518.40	0.170	1878.54	318.97
A_68_P32224193	chr19:60302406-60302450	NM_029648:172	D19Ertd737e	INSIDE	0.155	0.696	1366.16	951.17	0.108	1088.68	117.33
A_68_P32145202	chr19:46728964-46729014	NM_001177813:31098	D19Wsu162e	INSIDE	0.605	3.450	2587.61	8926.64	2.086	1851.03	3860.42
A_68_P20166296	chr1:39592351-39592396	NM_028043:-273	D1Bwg0212e	PROMOTER	0.118	0.686	1771.19	1215.27	0.081	1484.78	120.16
A_68_P26132332	chr8:107748862-107748908	NM_145604:-203	D230025D16Rik	PROMOTER	0.135	0.694	1698.26	1178.06	0.094	1288.11	120.64
A_68_P21566329	chr2:118640037-118640081	NM_026412:320	D2Ertd750e	INSIDE	0.443	1.588	15642.42	24845.41	0.703	10913.54	7672.78
A_68_P21489013	chr2:104249944-104249988	NM_001033347:525	D430041D05Rik	INSIDE	0.186	0.631	2467.55	1556.27	0.117	1679.45	196.91
A_68_P29134764	chr13:72761714-72761758	NR_030701:4276	D430050G20	INSIDE	0.492	0.716	1701.25	1217.58	0.352	1327.54	467.01
A_68_P29134804	chr13:72766185-72766229	NR_030701:-194	D430050G20	DIVERGENT_PROMOTER	0.534	0.472	2227.09	1050.28	0.252	1665.29	419.72
A_68_P23362366	chr4:140695171-140695215	NM_001025608:519	D4Ertd22e	INSIDE	0.229	0.487	2705.91	1319.03	0.112	2048.62	229.04

ProbeName	Target position of probe on CpG island microarray	TargetID	GeneSymbol	CpG island Description	Ratio of relative methylation (TiO2-NP/Vehicle)	Sham group			TiO_2-H group		
						Relative methylation (Cy-5/Cy-3)	Cy-3 signal (Input)	Cy-5 signal (MeDIP)	Relative methylation (Cy-5/Cy-3)	Cy-3 signal (Input)	Cy-5 signal (MeDIP)
A_68_P23362368	chr4:140695411-140695455	NM_001025608:279	D4Ertd22e	INSIDE	0.143	0.680	1498.73	1019.43	0.097	1202.61	117.16
A_68_P23362370	chr4:140695629-140695673	NM_001025608:61	D4Ertd22e	INSIDE	0.359	0.621	2261.43	1403.38	0.223	1703.32	379.66
A_68_P23241428	chr4:118293645-118293692	NR_029469:-5340	D4Ertd617e	PROMOTER	0.575	0.694	1313.45	911.38	0.399	963.78	384.26
A_68_P27155060	chr10:55948706-55948750	NM_001033385:-233	D630037F22Rik	PROMOTER	0.601	0.666	895.17	596.40	0.400	671.00	268.64
A_68_P24775469	chr6:116158491-116158535	NM_026585:462	D6Wsu116e	INSIDE	0.634	0.541	1295.52	700.46	0.343	1087.33	372.83
A_68_P23262654	chr4:123088662-123088706	NM_001167918:470	D830031N03Rik	INSIDE	0.233	0.439	1564.50	686.89	0.102	1135.67	116.27
A_68_P23262655	chr4:123088845-123088889	NM_001167918:288	D830031N03Rik	INSIDE	0.201	0.680	2105.68	1432.35	0.137	1569.54	214.32
A_68_P23262660	chr4:123089459-123089503	NM_001167918:-326	D830031N03Rik	PROMOTER	0.248	0.563	1842.75	1037.08	0.140	1442.59	201.73
A_68_P26018981	chr8:86773349-86773396	NM_001007571:288	D8Ertd738e	INSIDE	0.302	0.479	1548.80	742.58	0.145	1177.15	170.63
A_68_P25783726	chr8:37158263-37158317	NM_172911:409	D8Ertd82e	INSIDE	0.248	0.643	896.11	575.93	0.159	750.27	119.60
A_68_P25409556	chr7:116847071-116847115	NM_020616:-158	D930014E17Rik	PROMOTER	0.384	0.502	2548.77	1279.57	0.193	1928.95	371.99
A_68_P22288630	chr3:83844564-83844615	NM_172681:-506	D930015E06Rik	PROMOTER	0.511	0.600	1416.59	850.62	0.307	1108.94	340.37
A_68_P27504659	chr10:121079155-121079199	NM_177335:420	D930020B18Rik	INSIDE	0.153	0.702	2104.99	1478.60	0.108	1535.01	165.34
A_68_P27795128	chr11:51463862-51463906	NR_027958:-571	D930048N14Rik	PROMOTER	0.182	0.468	1738.94	813.16	0.085	1365.94	116.28
A_68_P27795130	chr11:51464039-51464083	NR_027958:-395	D930048N14Rik	PROMOTER	0.063	0.712	3868.01	2755.52	0.045	2649.21	119.65
A_68_P27795131	chr11:51464219-51464263	NR_027958:-215	D930048N14Rik	PROMOTER	0.463	0.509	1038.22	528.74	0.236	791.80	186.73
A_68_P28528482	chr12:72932824-72932868	NM_026102:782	Daam1	INSIDE	0.286	0.443	1250.67	554.25	0.127	919.50	116.72
A_68_P29987885	chr15:6336369-6336413	NM_001037905:-357	Dab2	PROMOTER	0.191	0.683	1021.79	698.10	0.131	899.71	117.67
A_68_P21134666	chr2:35477528-35477572	NM_001114124:50	Dab2ip	INSIDE	0.289	0.413	1276.03	527.55	0.119	1055.63	126.06
A_68_P21134955	chr2:35517597-35517641	NM_001114124:40118	Dab2ip	INSIDE	0.126	0.466	3157.98	1473.09	0.059	2508.24	147.00
A_68_P28525478	chr12:72411253-72411297	NM_001190466:404	Dact1	INSIDE	0.274	0.603	1095.25	660.89	0.165	781.86	129.22
A_68_P31058439	chr17:14340006-14340050	NM_172826:810	Dact2	INSIDE	0.477	0.524	1451.59	760.53	0.250	1301.74	325.01
A_68_P29611635	chr14:54873344-54873388	NM_001113358:238	Dad1	INSIDE	0.366	0.544	2468.07	1343.80	0.199	1980.63	394.71
A_68_P26815510	chr9:108472099-108472144	NM_026378:-101	Dalrd3	PROMOTER	0.340	0.353	2690.95	951.19	0.120	2178.95	261.88
A_68_P30113000	chr15:31153773-31153829	NM_146057:-339	Dap	PROMOTER	0.430	0.513	1044.05	536.10	0.221	821.01	181.12
A_68_P30113001	chr15:31153906-31153950	NM_146057:-211	Dap	PROMOTER	0.255	0.454	1495.20	678.79	0.116	1103.09	127.77
A_68_P22316449	chr3:88754686-88754730	NM_001164533:-504	Dap3	PROMOTER	0.332	0.575	2457.68	1412.69	0.191	2067.19	394.06
A_68_P29086263	chr13:60704015-60704059	NM_134062:465	Dapk1	INSIDE	0.452	0.621	1345.19	834.79	0.281	1021.44	286.60
A_68_P26591484	chr9:66005801-66005855	NM_010019:-204	Dapk2	PROMOTER	0.118	1.566	4337.79	6795.09	0.185	2827.90	524.49
A_68_P26591487	chr9:66006201-66006245	NM_010019:190	Dapk2	INSIDE	0.104	0.727	2279.42	1657.08	0.076	1592.45	120.73
A_68_P27287961	chr10:80645653-80645705	NM_001190474:-73	Dapk3	PROMOTER	0.215	0.593	1201.87	712.63	0.127	965.21	122.92
A_68_P27288000	chr10:80652951-80652995	NM_007828:6310	Dapk3	INSIDE	0.327	1.782	1669.49	2975.08	0.583	1158.49	674.86
A_68_P20600402	chr1:130313507-130313551	NM_177445:465	Dars	INSIDE	0.325	0.686	1909.72	1310.50	0.223	1426.18	317.99
A_68_P20600403	chr1:130313627-130313671	NM_177445:345	Dars	INSIDE	0.520	0.421	1579.20	664.11	0.219	1326.39	289.93
A_68_P20600404	chr1:130313816-130313860	NM_177445:155	Dars	INSIDE	0.441	0.641	3398.23	2177.34	0.283	2341.08	662.18
A_68_P31150909	chr17:34045790-34045846	NM_007829:-571	Daxx	PROMOTER	0.158	0.468	2184.09	1023.15	0.074	1585.86	117.42
A_68_P31150915	chr17:34046487-34046531	NM_001199733:-37	Daxx	PROMOTER	0.036	1.513	3355.42	5076.31	0.054	2218.99	120.83
A_68_P30486705	chr15:100445276-100445320	NM_011873:-794	Dazap2	PROMOTER	0.507	0.408	1286.53	524.76	0.207	1011.88	209.35
A_68_P22998987	chr4:68614912-68614956	NM_019967:497	Dbc1	INSIDE	0.582	0.588	4040.92	2377.58	0.343	2851.93	976.91
A_68_P22998988	chr4:68615056-68615100	NM_019967:353	Dbc1	INSIDE	0.626	0.560	1044.33	584.64	0.351	699.53	245.32
A_68_P22998989	chr4:68615175-68615219	NM_019967:235	Dbc1	INSIDE	0.344	0.506	1069.98	541.88	0.174	790.43	137.54
A_68_P23467995	chr5:8422471-8422515	NM_001190717:224	Dbf4	INSIDE	0.301	0.385	1346.98	517.97	0.116	1040.47	120.50
A_68_P26240284	chr8:126038626-126038670	NM_001170976:707	Dbndd1	INSIDE	0.291	0.626	2142.22	1340.42	0.182	1685.82	306.92
A_68_P26240285	chr8:126038733-126038778	NM_001170976:600	Dbndd1	INSIDE	0.311	0.647	2796.26	1808.64	0.201	2111.46	424.65

ProbeName	Target position of probe on CpG island microarray	TargetID	GeneSymbol	CpG island Description	Ratio of relative methylation (TiO2-NP/Vehicle)	Sham group			TiO_2-H group		
						Relative methylation (Cy-5/Cy-3)	Cy-3 signal (Input)	Cy-5 signal (MeDIP)	Relative methylation (Cy-5/Cy-3)	Cy-3 signal (Input)	Cy-5 signal (MeDIP)
A_68_P26240286	chr8:126038854-126038898	NM_001170976:479	Dbndd1	INSIDE	0.468	0.633	1393.98	881.80	0.296	1074.10	317.89
A_68_P21818148	chr2:164311684-164311728	NM_026797:-248	Dbndd2	PROMOTER	0.353	0.450	1293.28	582.22	0.159	1044.82	166.10
A_68_P21818155	chr2:164312484-164312528	NM_001048229:-1104	Dbndd2	PROMOTER	0.365	0.680	1143.31	777.68	0.249	900.59	223.81
A_68_P27564954	chr11:5688709-5688754	NM_001146308:246	Dbnl	INSIDE	0.247	0.504	2049.45	1033.33	0.124	1644.25	204.69
A_68_P27564953	chr11:5688605-5688649	NM_001146308:141	Dbnl	INSIDE	0.670	0.695	2021.36	1403.86	0.465	1567.56	728.93
A_68_P25094588	chr7:52960776-52960821	NM_016974:182	Dbp	INSIDE	0.296	0.354	1557.36	551.37	0.105	1140.67	119.71
A_68_P25094601	chr7:52962152-52962196	NM_016974:1558	Dbp	INSIDE	0.508	0.503	1957.27	984.22	0.255	1589.42	405.91
A_68_P25113816	chr7:56890828-56890872	NM_001005232:1355	Dbx1	INSIDE	0.567	0.631	828.80	522.87	0.358	700.60	250.47
A_68_P25113824	chr7:56891950-56892001	NM_001005232:230	Dbx1	INSIDE	0.061	1.479	4110.71	6079.61	0.090	2549.02	229.67
A_68_P30459325	chr15:95485192-95485236	NM_207533:-12	Dbx2	PROMOTER	0.471	0.699	1706.24	1192.21	0.329	1140.96	375.23
A_68_P22882386	chr4:45355486-45355530	NM_153167:536	Dcaf10	INSIDE	0.141	0.673	1928.95	1298.60	0.095	1355.43	128.66
A_68_P22882387	chr4:45355569-45355613	NM_153167:618	Dcaf10	INSIDE	0.360	0.511	1542.50	788.61	0.184	1163.88	214.04
A_68_P29618485	chr14:56179012-56179056	NM_001199009:-725	Dcaf11	PROMOTER	0.166	0.603	1791.88	1081.12	0.100	1229.70	123.35
A_68_P29618486	chr14:56179164-56179210	NM_001199009:-573	Dcaf11	PROMOTER	0.175	0.715	2883.56	2062.36	0.125	2143.86	267.81
A_68_P22861616	chr4:41261724-41261768	NM_026893:188	Dcaf12	INSIDE	0.481	0.699	1489.47	1040.41	0.336	1226.08	411.88
A_68_P22861618	chr4:41261982-41262026	NM_026893:-70	Dcaf12	PROMOTER	0.169	0.636	1582.37	1006.29	0.108	1066.36	114.66
A_68_P32351253	chrX:42143286-42143330	NM_001190718:30	Dcaf12l1	INSIDE	0.112	1.381	1695.79	2341.11	0.155	744.88	115.23
A_68_P21317255	chr2:70893877-70893924	NM_001165982:100	Dcaf17	INSIDE	0.150	0.661	1606.01	1062.03	0.099	1230.69	121.85
A_68_P28593178	chr12:84861329-84861374	NM_030246:-64	Dcaf4	PROMOTER	0.509	0.659	1175.40	774.41	0.336	951.78	319.41
A_68_P28593179	chr12:84861421-84861470	NM_001165256:-5553	Dcaf4	PROMOTER	0.291	0.541	1017.28	550.51	0.157	825.27	129.77
A_68_P20794594	chr1:167389733-167389777	NM_028759:840	Dcaf6	INSIDE	0.245	0.493	1247.80	615.17	0.121	999.50	120.48
A_68_P20794596	chr1:167389914-167389958	NM_028759:658	Dcaf6	INSIDE	0.162	0.535	1923.31	1029.17	0.087	1345.78	116.58
A_68_P28099221	chr11:105898542-105898586	NM_027946:379	Dcaf7	INSIDE	0.184	0.534	1674.37	894.36	0.098	1210.96	119.27
A_68_P20831243	chr1:174078348-174078392	NM_153555:225	Dcaf8	INSIDE	0.180	0.543	1808.79	982.31	0.098	1229.52	120.15
A_68_P20831244	chr1:174078454-174078498	NM_153555:331	Dcaf8	INSIDE	0.457	0.637	1517.09	966.18	0.291	1265.46	368.01
A_68_P30791338	chr16:58408465-58408509	NM_028523:-161	Dcbld2	PROMOTER	0.206	0.524	3810.82	1998.41	0.108	2860.03	308.87
A_68_P28890407	chr13:25147770-25147817	NM_001195617:-79	Dcdc2a	PROMOTER	0.180	0.432	1824.85	789.13	0.078	1513.07	117.75
A_68_P28890408	chr13:25147877-25147921	NM_001195617:26	Dcdc2a	INSIDE	0.228	0.634	1612.26	1022.62	0.145	1252.37	181.08
A_68_P23873911	chr5:89193834-89193878	NM_007832:-181	Dck	PROMOTER	0.195	0.548	1875.09	1027.72	0.107	1519.10	162.08
A_68_P22305771	chr3:86723508-86723553	NM_001195496:1276	Dclk2	INSIDE	0.442	0.524	1049.98	550.62	0.232	924.09	214.13
A_68_P22305779	chr3:86724403-86724447	NM_001195496:382	Dclk2	INSIDE	0.539	0.310	2021.40	626.65	0.167	1718.49	286.96
A_68_P29506061	chr14:31292780-31292824	NM_133761:52	Dcp1a	INSIDE	0.255	0.561	1187.63	666.14	0.143	937.02	134.17
A_68_P29506063	chr14:31292967-31293011	NM_133761:238	Dcp1a	INSIDE	0.374	0.704	1577.12	1110.64	0.264	1229.62	324.21
A_68_P29506064	chr14:31293149-31293193	NM_133761:420	Dcp1a	INSIDE	0.411	0.548	1594.27	873.20	0.225	1172.68	263.71
A_68_P24791601	chr6:119125187-119125231	NM_001033379:-62	Dcp1b	PROMOTER	0.632	0.555	1294.89	718.25	0.351	1091.87	382.79
A_68_P31666410	chr18:44540001-44540048	NM_027490:-129	Dcp2	PROMOTER	0.230	0.612	1102.95	674.76	0.141	869.71	122.60
A_68_P31666413	chr18:44540296-44540340	NM_027490:165	Dcp2	INSIDE	0.419	0.438	2832.24	1241.26	0.184	2037.69	374.49
A_68_P31666414	chr18:44540418-44540462	NM_027490:287	Dcp2	INSIDE	0.403	0.471	1722.26	811.15	0.190	1330.43	252.26
A_68_P22318793	chr3:89154296-89154340	NM_029974:14843	Dcst1	INSIDE	0.350	0.467	1404.13	655.56	0.163	1222.06	199.72
A_68_P31751922	chr18:60686064-60686108	NM_026302:212	Dctn4	INSIDE	0.659	0.611	1171.49	715.57	0.403	921.77	371.06
A_68_P25477510	chr7:129276573-129276617	NM_021608:40	Dctn5	INSIDE	0.362	0.494	1506.41	744.10	0.179	1229.40	219.58
A_68_P25504836	chr7:134404187-134404231	NM_023203:-27	Dctpp1	PROMOTER	0.213	0.634	1960.28	1242.27	0.135	1478.54	199.20
A_68_P25667343	chr8:13288388-13288432	NM_001024504:-284	Dcun1d2	PROMOTER	0.244	0.326	1894.86	616.97	0.079	1556.70	123.69
A_68_P23797904	chr5:73881900-73881944	NM_001190733:-342	Dcun1d4	PROMOTER	0.583	0.535	1183.96	633.58	0.312	948.80	296.23

ProbeName	Target position of probe on CpG island microarray	TargetID	GeneSymbol	CpG island Description	Ratio of relative methylation (TiO2-NP/Vehicle)	Sham group			TiO_2-H group		
						Relative methylation (Cy-5/Cy-3)	Cy-3 signal (Input)	Cy-5 signal (MeDIP)	Relative methylation (Cy-5/Cy-3)	Cy-3 signal (Input)	Cy-5 signal (MeDIP)
A_68_P23797907	chr5:73882297-73882341	NM_001190733:54	Dcun1d4	INSIDE	0.269	0.510	1858.99	948.19	0.137	1504.47	206.78
A_68_P23797908	chr5:73882402-73882446	NM_001190733:160	Dcun1d4	INSIDE	0.107	0.495	3141.07	1554.79	0.053	2306.55	121.67
A_68_P28186436	chr11:120588411-120588455	NM_026428:163	Dcxr	INSIDE	0.167	0.732	3076.87	2253.17	0.122	2260.95	276.71
A_68_P31951638	chr19:10679902-10679946	NM_015735:-190	Ddb1	DIVERGENT_PROMOTER	0.261	0.460	2234.92	1027.13	0.120	1698.49	204.09
A_68_P21421465	chr2:91076866-91076910	NM_028119:335	Ddb2	INSIDE	0.260	0.573	1487.24	852.56	0.149	1141.67	170.00
A_68_P21421467	chr2:91077045-91077089	NM_028119:157	Ddb2	INSIDE	0.415	0.481	2729.92	1314.31	0.200	1977.93	395.17
A_68_P29571975	chr14:46276736-46276780	NM_001039106:1060	Ddhd1	INSIDE	0.395	0.513	2910.02	1493.49	0.203	2124.93	430.75
A_68_P27535081	chr10:126727643-126727687	NM_007837:-184	Ddit3	DIVERGENT_PROMOTER	0.217	0.702	2155.36	1513.65	0.152	1507.48	229.87
A_68_P27171637	chr10:59414321-59414365	NM_029083:176	Ddit4	INSIDE	0.438	0.426	1369.55	584.01	0.187	1046.06	195.23
A_68_P27171638	chr10:59414463-59414507	NM_029083:34	Ddit4	INSIDE	0.296	0.266	2024.13	537.73	0.079	1482.62	116.59
A_68_P27171640	chr10:59414686-59414730	NM_029083:-190	Ddit4	PROMOTER	0.643	0.621	1703.05	1058.42	0.400	1477.74	590.80
A_68_P27171642	chr10:59414981-59415025	NM_029083:-484	Ddit4	PROMOTER	0.152	0.481	2143.31	1031.59	0.073	1604.35	117.23
A_68_P30476581	chr15:98635925-98635969	NM_001013741:2410	Ddn	INSIDE	0.370	0.477	1136.62	541.64	0.176	853.54	150.62
A_68_P30476587	chr15:98636550-98636594	NM_001013741:1784	Ddn	INSIDE	0.156	0.636	1771.26	1126.18	0.099	1304.68	129.63
A_68_P30476595	chr15:98637338-98637382	NM_001013741:996	Ddn	INSIDE	0.587	0.527	1055.42	556.17	0.309	607.30	187.81
A_68_P31160531	chr17:35837972-35838016	NM_001198833:-459	Ddr1	PROMOTER	0.512	0.598	2415.69	1445.06	0.306	1771.13	542.49
A_68_P21631052	chr2:130490403-130490447	NM_029832:-43	Ddrgk1	DIVERGENT_PROMOTER	0.563	0.664	1731.04	1148.78	0.374	1368.53	511.45
A_68_P28248079	chr12:13255873-13255917	NM_134040:86	Ddx1	INSIDE	0.259	0.410	1363.21	558.60	0.106	1130.66	120.11
A_68_P26519508	chr9:53056259-53056305	NM_029936:-65	Ddx10	PROMOTER	0.193	0.701	2583.81	1810.45	0.135	2035.72	275.47
A_68_P26519509	chr9:53056349-53056393	NM_029936:-153	Ddx10	PROMOTER	0.653	0.537	1522.99	817.41	0.351	1233.91	432.66
A_68_P20567558	chr1:123464212-123464256	NM_025860:323	Ddx18	INSIDE	0.643	0.666	1561.56	1039.62	0.428	1340.21	573.95
A_68_P22398584	chr3:105490057-105490101	NM_017397:411	Ddx20	INSIDE	0.623	0.625	955.34	596.94	0.389	797.36	310.28
A_68_P27186920	chr10:62064870-62064914	NM_019553:154	Ddx21	INSIDE	0.262	0.630	1524.72	961.32	0.165	1259.10	207.79
A_68_P30475794	chr15:98493006-98493053	NM_001080981:291	Ddx23	INSIDE	0.209	0.639	2285.80	1460.93	0.134	1669.03	222.94
A_68_P30475796	chr15:98493283-98493327	NM_001080981:16	Ddx23	INSIDE	0.579	0.691	2075.61	1433.25	0.400	1564.93	625.98
A_68_P28697531	chr12:104664203-104664252	NM_020494:-150	Ddx24	DIVERGENT_PROMOTER	0.225	0.454	1592.75	723.07	0.102	1138.88	116.34
A_68_P32395899	chrX:53708448-53708501	NM_172779:459	Ddx26b	INSIDE	0.039	5.109	2071.50	10583.14	0.199	581.42	115.63
A_68_P32395900	chrX:53708549-53708594	NM_172779:556	Ddx26b	INSIDE	0.037	3.030	3147.19	9535.48	0.111	1084.40	120.86
A_68_P26016154	chr8:86238907-86238952	NM_197982:-146	Ddx39	PROMOTER	0.278	0.612	887.31	543.43	0.171	680.21	115.98
A_68_P26016155	chr8:86239004-86239050	NM_197982:-49	Ddx39	PROMOTER	0.264	0.436	1335.39	582.13	0.115	1047.94	120.51
A_68_P26016157	chr8:86239183-86239227	NM_197982:129	Ddx39	INSIDE	0.472	0.548	2210.33	1210.20	0.259	1900.00	491.19
A_68_P26016158	chr8:86239347-86239391	NM_197982:293	Ddx39	INSIDE	0.279	0.607	4677.60	2837.73	0.169	3307.24	559.31
A_68_P26016160	chr8:86239497-86239541	NM_197982:443	Ddx39	INSIDE	0.223	0.669	3311.86	2216.78	0.149	2072.39	309.11
A_68_P31158507	chr17:35378777-35378821	NM_019693:108	Ddx39b	INSIDE	0.337	0.374	2398.36	896.01	0.126	1755.58	220.71
A_68_P31158511	chr17:35379261-35379306	NM_019693:593	Ddx39b	INSIDE	0.190	0.665	1603.70	1067.12	0.126	1117.59	141.27
A_68_P32265724	chrX:12858330-12858374	NM_010028:205	Ddx3x	INSIDE	0.540	0.508	1080.21	548.94	0.274	445.91	122.29
A_68_P29335930	chr13:113442131-113442175	NM_001145885:366	Ddx4	INSIDE	0.645	2.608	2026.92	5286.71	1.681	1708.42	2872.61
A_68_P28100235	chr11:106078135-106078182	NM_028074:-81	Ddx42	DIVERGENT_PROMOTER	0.267	0.488	1267.69	618.10	0.130	900.17	117.27
A_68_P28100238	chr11:106078606-106078650	NM_028074:389	Ddx42	INSIDE	0.441	0.655	2883.28	1887.26	0.288	2163.76	624.04
A_68_P28100239	chr11:106078730-106078775	NM_028074:513	Ddx42	INSIDE	0.174	1.479	1082.85	1601.05	0.258	781.99	201.65
A_68_P26659373	chr9:78243366-78243412	NM_001191044:-195	Ddx43	PROMOTER	0.251	0.644	883.18	569.18	0.162	723.28	117.06
A_68_P29056202	chr13:55736711-55736755	NM_145975:345	Ddx46	INSIDE	0.196	0.649	4914.27	3191.70	0.127	3720.38	473.03
A_68_P29056203	chr13:55736834-55736882	NM_145975:471	Ddx46	INSIDE	0.188	0.572	1555.52	889.72	0.107	1118.25	120.08
A_68_P25950970	chr8:72826351-72826395	NM_001024922:-21	Ddx49	DIVERGENT_PROMOTER	0.155	0.543	2324.12	1261.95	0.084	1634.63	137.64

ProbeName	Target position of probe on CpG island microarray	TargetID	GeneSymbol	CpG island Description	Ratio of relative methylation (TiO2-NP/Vehicle)	Sham group			TiO_2-H group		
						Relative methylation (Cy-5/Cy-3)	Cy-3 signal (Input)	Cy-5 signal (MeDIP)	Relative methylation (Cy-5/Cy-3)	Cy-3 signal (Input)	Cy-5 signal (MeDIP)
A_68_P28103522	chr11:106650003-106650048	NM_007840:-217	Ddx5	DIVERGENT_PROMOTER	0.350	0.460	1898.40	874.02	0.161	1414.98	228.26
A_68_P27187161	chr10:62114275-62114319	NM_053183:-350	Ddx50	PROMOTER	0.193	0.560	1385.79	776.04	0.108	1152.33	124.81
A_68_P27187162	chr10:62114413-62114457	NM_053183:-488	Ddx50	PROMOTER	0.379	0.538	1677.62	902.00	0.204	1276.68	260.05
A_68_P23981089	chr5:111082753-111082797	NM_027156:305	Ddx51	INSIDE	0.395	1.415	2235.79	3162.99	0.559	1524.12	851.91
A_68_P24039809	chr5:121063031-121063075	NM_028041:-86	Ddx54	DIVERGENT_PROMOTER	0.428	0.505	1103.35	556.80	0.216	981.25	212.11
A_68_P24039813	chr5:121063452-121063497	NM_028041:336	Ddx54	INSIDE	0.614	0.453	1291.47	584.56	0.278	994.37	276.44
A_68_P26468507	chr9:44412404-44412448	NM_001110826:-548	Ddx6	PROMOTER	0.366	0.641	1400.24	897.94	0.235	1104.97	259.50
A_68_P25588174	chr7:148513704-148513752	NM_016874:-104	Deaf1	DIVERGENT_PROMOTER	0.272	0.531	1000.56	531.62	0.145	805.47	116.56
A_68_P25588255	chr7:148524764-148524808	NR_027770:-104	Deaf1	DIVERGENT_PROMOTER	0.656	0.645	1280.83	826.61	0.423	1036.09	438.71
A_68_P22752470	chr4:15872272-15872316	NM_026172:360	Decr1	INSIDE	0.361	0.715	5079.40	3632.75	0.258	4058.78	1046.83
A_68_P20827300	chr1:173259326-173259370	NM_001128609:73	Dedd	INSIDE	0.518	0.364	1930.26	703.37	0.189	1665.09	314.02
A_68_P25009058	chr7:26004203-26004247	NM_207677:654	Dedd2	INSIDE	0.520	0.672	3997.66	2685.31	0.349	2861.04	998.56
A_68_P25009061	chr7:26004576-26004627	NM_207677:277	Dedd2	INSIDE	0.241	0.694	1176.13	816.64	0.167	846.85	141.51
A_68_P25009064	chr7:26004847-26004897	NM_207677:6	Dedd2	INSIDE	0.188	0.446	2132.53	951.36	0.084	1541.43	129.58
A_68_P31121207	chr17:28360556-28360600	NM_027185:15856	Def6	INSIDE	0.116	0.624	2452.55	1530.13	0.072	1955.89	141.70
A_68_P26239891	chr8:125967011-125967055	NM_054046:147	Def8	INSIDE	0.205	0.738	3573.26	2635.91	0.151	2702.48	408.50
A_68_P28729567	chr12:109940312-109940358	NM_001171002:182	Degs2	INSIDE	0.209	1.574	1344.99	2117.06	0.329	1051.35	345.38
A_68_P28729569	chr12:109940441-109940485	NM_001171002:54	Degs2	INSIDE	0.432	0.678	1110.84	753.56	0.293	925.58	271.52
A_68_P29011725	chr13:47201899-47201943	NM_025900:-331	Dek	PROMOTER	0.279	0.384	1550.14	595.33	0.107	1081.93	115.95
A_68_P21147278	chr2:38142391-38142435	NM_146122:492	Dennd1a	INSIDE	0.557	0.697	4186.41	2917.72	0.388	3094.62	1200.72
A_68_P24383688	chr6:39508034-39508079	NM_172477:-223	Dennd2a	PROMOTER	0.407	0.588	1614.11	949.25	0.239	1128.93	270.38
A_68_P26584122	chr9:64659329-64659373	NM_001162917:533	Dennd4a	INSIDE	0.272	0.598	4331.66	2591.77	0.163	3237.48	527.72
A_68_P26584123	chr9:64659420-64659464	NM_001162917:625	Dennd4a	INSIDE	0.246	0.637	3599.08	2292.08	0.157	2799.30	438.41
A_68_P22324269	chr3:90068935-90068979	NM_201407:-1499	Dennd4b	PROMOTER	0.521	0.551	995.76	548.92	0.287	937.25	269.07
A_68_P24058178	chr5:124356959-124357007	NM_026603:-301	Denr	PROMOTER	0.242	0.604	1267.63	766.02	0.146	1001.66	146.57
A_68_P24058179	chr5:124357031-124357075	NM_026603:-231	Denr	PROMOTER	0.591	0.439	2534.45	1111.70	0.259	2046.88	530.57
A_68_P24058180	chr5:124357134-124357178	NM_026603:-127	Denr	PROMOTER	0.550	0.502	3004.11	1508.81	0.276	2477.22	684.61
A_68_P24058184	chr5:124357527-124357571	NM_026603:265	Denr	INSIDE	0.592	0.638	1275.27	813.70	0.377	1045.85	394.79
A_68_P29310804	chr13:109106277-109106321	NM_178683:-232	Depdc1b	PROMOTER	0.201	0.653	1030.65	673.28	0.131	890.20	116.69
A_68_P23585887	chr5:33206388-33206432	NM_177786:41	Depdc5	INSIDE	0.373	0.579	1412.99	817.53	0.216	1269.23	273.76
A_68_P30230393	chr15:54944017-54944061	NM_145470:1	Deptor	INSIDE	0.480	0.550	2257.27	1242.03	0.264	1758.95	464.63
A_68_P24880219	chr6:137703098-137703142	NM_172733:23	Dera	INSIDE	0.515	0.390	1596.78	622.99	0.201	1310.11	263.15
A_68_P24880220	chr6:137703212-137703256	NM_172733:137	Dera	INSIDE	0.304	0.674	1331.36	896.68	0.205	1083.34	222.02
A_68_P30244835	chr15:57723554-57723598	NM_024207:397	Derl1	INSIDE	0.425	0.522	2550.26	1331.81	0.222	2025.95	449.64
A_68_P30244838	chr15:57723989-57724033	NM_024207:-37	Derl1	PROMOTER	0.232	0.450	1669.72	750.80	0.104	1165.30	121.68
A_68_P27903933	chr11:70832700-70832744	NM_033562:43	Derl2	INSIDE	0.244	0.381	1754.26	668.50	0.093	1381.42	128.49
A_68_P27903934	chr11:70832798-70832842	NM_033562:-55	Derl2	DIVERGENT_PROMOTER	0.565	0.503	2204.32	1108.27	0.284	1714.27	486.74
A_68_P27259217	chr10:75355978-75356022	NM_024440:-142	Derl3	PROMOTER	0.233	1.510	6147.63	9284.02	0.353	4088.95	1441.76
A_68_P27259221	chr10:75356293-75356337	NM_024440:172	Derl3	INSIDE	0.465	0.597	1430.30	854.16	0.278	1172.62	325.43
A_68_P30348812	chr15:76342113-76342157	NM_010046:114	Dgat1	INSIDE	0.627	0.689	2411.73	1660.52	0.432	1886.27	814.94
A_68_P30576344	chr16:17891528-17891572	NM_001109750:271	Dgcr2	INSIDE	0.347	0.526	2446.86	1286.03	0.182	1913.85	349.27
A_68_P28003586	chr11:88921355-88921399	NM_019505:686	Dgke	INSIDE	0.205	0.488	1550.72	756.41	0.100	1202.65	120.29
A_68_P28003593	chr11:88922175-88922219	NM_019505:-134	Dgke	PROMOTER	0.091	0.724	4385.09	3174.27	0.066	3073.38	201.66
A_68_P30599073	chr16:22656907-22656951	NM_138650:376	Dgkg	INSIDE	0.208	0.398	1936.43	771.09	0.083	1520.32	125.83

ProbeName	Target position of probe on CpG island microarray	TargetID	GeneSymbol	CpG island Description	Ratio of relative methylation (TiO2-NP/Vehicle)	Sham group			TiO_2-H group		
						Relative methylation (Cy-5/Cy-3)	Cy-3 signal (Input)	Cy-5 signal (MeDIP)	Relative methylation (Cy-5/Cy-3)	Cy-3 signal (Input)	Cy-5 signal (MeDIP)
A_68_P29747596	chr14:79132794-79132838	NM_001081336:-7920	Dgkh	PROMOTER	0.444	0.562	3136.63	1762.77	0.250	2305.16	575.59
A_68_P21425684	chr2:91803378-91803422	NM_138306:320	Dgkz	INSIDE	0.592	0.472	1509.86	713.20	0.280	1290.92	361.18
A_68_P21425690	chr2:91804013-91804057	NM_138306:-314	Dgkz	PROMOTER	0.402	1.354	2042.05	2764.55	0.544	1585.34	862.03
A_68_P24593884	chr6:83447860-83447916	NM_001162521:9075	Dguok	INSIDE	0.149	1.778	555.57	987.75	0.264	445.81	117.71
A_68_P24593936	chr6:83456719-83456763	NM_001162521:223	Dguok	INSIDE	0.551	0.637	858.23	546.91	0.351	760.83	267.37
A_68_P25603239	chr7:151016135-151016179	NM_007856:7085	Dhcr7	INSIDE	0.399	0.597	1125.54	672.50	0.238	815.91	194.46
A_68_P25093408	chr7:52744206-52744250	NM_027903:-62	Dhdh	PROMOTER	0.592	0.591	1981.28	1171.77	0.350	1605.28	562.44
A_68_P30477181	chr15:98724879-98724923	NM_007857:4071	Dhh	INSIDE	0.580	0.550	1153.09	634.29	0.319	1038.42	331.31
A_68_P27941031	chr11:77845557-77845601	NM_183286:-236	Dhrs13	PROMOTER	0.579	0.745	3029.23	2257.21	0.432	2349.80	1014.47
A_68_P23379974	chr4:144482718-144482768	NM_011303:-297	Dhrs3	PROMOTER	0.150	1.494	2470.32	3689.51	0.224	1584.09	355.10
A_68_P33007791	chr4_random:128451-128495	NM_001033326:18671	Dhrsx	INSIDE	0.666	2.468	7810.77	19279.26	1.644	5480.38	9007.71
A_68_P33008825	chr4_random:109830-109874	NM_001033326:51	Dhrsx	INSIDE	0.397	0.470	1833.30	861.33	0.186	1457.07	271.74
A_68_P26821951	chr9:110003563-110003607	NM_133347:14502	Dhx30	INSIDE	0.501	0.497	1054.41	524.01	0.249	862.41	214.92
A_68_P26822030	chr9:110019511-110019555	NM_133347:-1446	Dhx30	PROMOTER	0.407	0.637	1377.60	877.71	0.260	1241.84	322.32
A_68_P27903825	chr11:70817620-70817664	NM_178367:292	Dhx33	INSIDE	0.253	0.384	2082.91	800.73	0.097	1763.97	171.74
A_68_P24066887	chr5:125914212-125914256	NM_203319:184	Dhx37	INSIDE	0.564	0.504	2121.66	1068.58	0.284	1769.97	503.12
A_68_P26157751	chr8:112089473-112089517	NM_178380:7	Dhx38	INSIDE	0.384	0.599	2610.74	1564.85	0.230	1737.08	399.73
A_68_P27991090	chr11:86620800-86620848	NM_026191:338	Dhx40	INSIDE	0.197	0.604	1360.71	821.31	0.119	990.68	118.08
A_68_P27991096	chr11:86621298-86621342	NM_026191:-158	Dhx40	PROMOTER	0.407	0.538	1239.12	666.15	0.219	916.71	200.56
A_68_P20726997	chr1:155334160-155334214	NM_007842:604	Dhx9	INSIDE	0.196	0.534	1455.22	776.93	0.105	1158.21	121.50
A_68_P24056167	chr5:123974295-123974339	NM_023232:-143	Diablo	PROMOTER	0.571	0.484	2310.52	1118.89	0.276	1973.22	545.19
A_68_P32651796	chrX:126284437-126284481	NM_172493:181	Diap2	INSIDE	0.046	2.019	3296.73	6655.66	0.094	1263.70	118.38
A_68_P32651797	chrX:126284617-126284662	NM_172493:362	Diap2	INSIDE	0.114	1.830	1684.44	3083.15	0.209	677.82	141.66
A_68_P32651800	chrX:126284923-126284967	NM_172493:667	Diap2	INSIDE	0.085	1.796	2452.36	4405.13	0.154	978.86	150.28
A_68_P29787827	chr14:87540778-87540822	NM_019670:121	Diap3	INSIDE	0.228	0.321	2073.67	665.05	0.073	1605.15	117.38
A_68_P29787828	chr14:87540919-87540963	NM_019670:-19	Diap3	PROMOTER	0.260	0.445	1272.45	566.38	0.116	1054.15	122.07
A_68_P28705225	chr12:105989571-105989615	NM_148948:570	Dicer1	INSIDE	0.549	0.404	1714.93	693.62	0.222	1382.82	307.15
A_68_P21905657	chr2:180436282-180436326	NM_175551:501	Dido1	INSIDE	0.623	0.561	2040.66	1144.36	0.349	1441.47	503.53
A_68_P21905698	chr2:180444588-180444638	NM_175551:-7807	Dido1	PROMOTER	0.136	0.472	2594.30	1225.42	0.064	1819.21	117.19
A_68_P21905703	chr2:180445288-180445335	NM_177852:-607	Dido1	DIVERGENT_PROMOTER	0.160	0.625	1566.59	979.57	0.100	1159.82	116.26
A_68_P28737547	chr12:111517314-111517358	NM_172119:-103	Dio3	DIVERGENT_PROMOTER	0.115	0.660	1901.27	1254.57	0.076	1540.42	116.86
A_68_P28737548	chr12:111517382-111517429	NM_172119:-34	Dio3	DIVERGENT_PROMOTER	0.292	0.621	840.39	522.19	0.182	664.86	120.84
A_68_P28737539	chr12:111516074-111516118	NR_002866:182	Dio3os	INSIDE	0.554	0.557	1383.04	769.67	0.308	1133.20	349.36
A_68_P28737540	chr12:111516152-111516199	NR_002866:103	Dio3os	INSIDE	0.324	0.378	1660.43	627.60	0.122	1385.18	169.57
A_68_P29038492	chr13:52625955-52625999	NM_001024474:229	Diras2	INSIDE	0.545	0.682	4342.23	2960.54	0.372	3238.56	1203.85
A_68_P30672033	chr16:35768842-35768886	NM_153550:578	Dirc2	INSIDE	0.457	0.605	1297.65	785.52	0.277	1184.21	327.64
A_68_P30672035	chr16:35769023-35769067	NM_153550:398	Dirc2	INSIDE	0.592	0.411	1741.59	714.97	0.243	1510.92	367.51
A_68_P20413587	chr1:88600174-88600218	NM_001172157:-182	Dis3l2	PROMOTER	0.157	0.542	1819.73	986.39	0.085	1376.97	117.52
A_68_P20413588	chr1:88600253-88600297	NM_001172157:-104	Dis3l2	PROMOTER	0.547	0.626	3588.05	2247.88	0.343	2757.39	945.60
A_68_P26249220	chr8:127578421-127578465	NM_174853:348	Disc1	INSIDE	0.626	0.636	1507.71	959.49	0.398	1281.30	510.18
A_68_P26505085	chr9:50507371-50507415	NM_178118:28697	Dixdc1	INSIDE	0.558	0.489	1050.32	513.12	0.273	769.66	209.88
A_68_P26505265	chr9:50536331-50536375	NM_178118:-263	Dixdc1	PROMOTER	0.462	0.709	1369.82	971.01	0.327	1114.03	364.82
A_68_P32470552	chrX:72341213-72341257	NM_001030307:42	Dkc1	INSIDE	0.084	1.365	2697.83	3683.43	0.114	1045.20	119.18
A_68_P25422972	chr7:119301935-119301979	NM_015814:615	Dkk3	INSIDE	0.204	2.165	9130.25	19767.68	0.443	5681.27	2514.80

ProbeName	Target position of probe on CpG island microarray	TargetID	GeneSymbol	CpG island Description	Ratio of relative methylation (TiO2-NP/Vehicle)	Sham group			TiO_2-H group		
						Relative methylation (Cy-5/Cy-3)	Cy-3 signal (Input)	Cy-5 signal (MeDIP)	Relative methylation (Cy-5/Cy-3)	Cy-3 signal (Input)	Cy-5 signal (MeDIP)
A_68_P29651206	chr14:62266896-62266940	NR_028264:34292	Dleu2	INSIDE	0.644	0.700	1475.22	1033.22	0.451	1259.92	568.46
A_68_P29651209	chr14:62267312-62267356	NR_028264:33876	Dleu2	INSIDE	0.463	0.361	2229.34	804.10	0.167	1935.60	322.97
A_68_P29651214	chr14:62267812-62267856	NR_028264:33376	Dleu2	INSIDE	0.128	1.640	4837.12	7933.72	0.210	3150.69	661.54
A_68_P29651216	chr14:62268043-62268087	NR_028264:33146	Dleu2	INSIDE	0.259	0.665	1283.83	854.01	0.172	1162.00	200.09
A_68_P29651217	chr14:62268108-62268159	NR_028264:33077	Dleu2	INSIDE	0.398	0.543	1443.25	783.46	0.216	1158.50	250.08
A_68_P29651431	chr14:62305946-62306001	NR_028264:-4763	Dleu2	PROMOTER	0.200	0.623	1314.06	819.32	0.125	978.36	122.26
A_68_P29651453	chr14:62308585-62308640	NR_028264:-7402	Dleu2	PROMOTER	0.626	0.658	1112.26	731.90	0.412	960.19	395.76
A_68_P29654694	chr14:62911407-62911451	NM_173419:388	Dleu7	INSIDE	0.314	0.609	1151.47	700.81	0.191	1040.00	198.47
A_68_P29654698	chr14:62911858-62911904	NM_173419:-64	Dleu7	PROMOTER	0.158	0.599	1463.37	877.18	0.095	1288.13	122.38
A_68_P30648664	chr16:31663664-31663708	NM_007862:-438	Dlg1	PROMOTER	0.392	0.458	3597.39	1647.10	0.180	2613.64	469.67
A_68_P30648665	chr16:31663764-31663808	NM_007862:-338	Dlg1	PROMOTER	0.291	0.436	1220.85	532.57	0.127	933.67	118.67
A_68_P27898577	chr11:69834947-69834991	NM_001109752:2862	Dlg4	INSIDE	0.648	0.513	1313.67	674.23	0.333	1020.02	339.38
A_68_P31330088	chr17:71012118-71012162	NM_001128181:101054	Dlgap1	INSIDE	0.453	1.496	2328.12	3482.04	0.678	1758.63	1192.07
A_68_P25672259	chr8:14096757-14096801	NM_001145965:904	Dlgap2	INSIDE	0.225	0.657	984.04	646.62	0.148	783.35	115.74
A_68_P29582178	chr14:48037787-48037833	NM_144553:272	Dlgap5	INSIDE	0.626	0.587	1015.43	596.15	0.368	726.20	266.99
A_68_P28732704	chr12:110691154-110691198	NM_001190703:-488	Dlk1	PROMOTER	0.196	0.561	1425.61	799.09	0.110	1181.70	129.80
A_68_P28732705	chr12:110691224-110691271	NM_001190703:-417	Dlk1	PROMOTER	0.174	0.564	1693.86	954.75	0.098	1255.25	123.15
A_68_P28732710	chr12:110691897-110691941	NM_001190703:254	Dlk1	INSIDE	0.478	0.502	2056.95	1033.52	0.240	1542.30	370.33
A_68_P31065256	chr17:15512298-15512342	NM_007865:467	Dll1	INSIDE	0.428	1.381	2803.51	3870.71	0.591	2321.68	1371.13
A_68_P31065259	chr17:15512604-15512648	NM_007865:161	Dll1	INSIDE	0.487	0.468	1288.39	603.05	0.228	926.33	211.00
A_68_P31065263	chr17:15513198-15513242	NM_007865:-433	Dll1	PROMOTER	0.221	0.505	1418.02	716.55	0.112	1091.38	121.76
A_68_P31065304	chr17:15517920-15517964	NM_007865:-5155	Dll1	PROMOTER	0.233	0.429	2393.26	1026.31	0.100	1839.54	183.79
A_68_P31065305	chr17:15517994-15518045	NM_007865:-5232	Dll1	PROMOTER	0.519	0.519	1623.07	842.03	0.269	1325.33	356.95
A_68_P21569027	chr2:119148109-119148156	NM_019454:-3387	Dll4	PROMOTER	0.237	0.551	1196.58	659.25	0.131	921.33	120.35
A_68_P21320095	chr2:71379350-71379394	NR_002854:-3424	Dlx1as	PROMOTER	0.390	0.546	2383.88	1302.44	0.213	1831.75	390.00
A_68_P21320122	chr2:71382362-71382406	NM_010054:2427	Dlx2	INSIDE	0.287	0.503	1577.81	794.03	0.145	1323.44	191.28
A_68_P21320135	chr2:71384020-71384064	NM_010054:769	Dlx2	INSIDE	0.325	0.585	2056.74	1203.82	0.190	1438.81	273.76
A_68_P21320136	chr2:71384105-71384149	NM_010054:685	Dlx2	INSIDE	0.249	0.479	2446.96	1171.31	0.119	1782.62	212.75
A_68_P28036768	chr11:94981457-94981501	NM_010055:48	Dlx3	INSIDE	0.330	0.663	6824.89	4524.71	0.219	4438.31	970.14
A_68_P28036771	chr11:94981735-94981779	NM_010055:326	Dlx3	INSIDE	0.160	0.694	1430.45	992.60	0.111	1064.15	118.07
A_68_P28036957	chr11:95006662-95006706	NM_007867:431	Dlx4	INSIDE	0.281	0.651	2321.49	1510.92	0.183	1744.75	318.99
A_68_P28036958	chr11:95006743-95006787	NM_007867:351	Dlx4	INSIDE	0.485	0.688	4489.99	3088.20	0.334	3238.82	1080.56
A_68_P24218863	chr6:6829517-6829561	NM_010056:2530	Dlx5	INSIDE	0.342	0.489	2406.66	1176.53	0.167	1698.33	284.26
A_68_P24218883	chr6:6831846-6831891	NM_010056:200	Dlx5	INSIDE	0.132	0.639	1804.65	1152.74	0.084	1392.22	117.51
A_68_P24218884	chr6:6831933-6831977	NM_010056:114	Dlx5	INSIDE	0.166	0.630	1786.61	1125.89	0.105	1214.20	127.35
A_68_P24218731	chr6:6813573-6813617	NM_010057:261	Dlx6	INSIDE	0.629	0.518	3828.75	1984.03	0.326	3140.88	1023.77
A_68_P23236051	chr4:117354667-117354711	NM_023178:142	Dmap1	INSIDE	0.348	0.512	2108.78	1079.77	0.178	1662.61	296.03
A_68_P23226777	chr4:115609072-115609120	NM_001025567:3435	Dmbx1	INSIDE	0.267	0.588	1195.49	702.94	0.157	928.86	145.93
A_68_P23226797	chr4:115611470-115611514	NM_001025567:1039	Dmbx1	INSIDE	0.203	0.604	2027.15	1225.38	0.123	1562.01	191.89
A_68_P23226799	chr4:115611753-115611802	NM_001025567:754	Dmbx1	INSIDE	0.279	0.640	982.15	628.68	0.179	665.18	118.81
A_68_P23226802	chr4:115612163-115612209	NM_001025567:345	Dmbx1	INSIDE	0.237	0.694	1164.16	808.42	0.164	882.65	145.18
A_68_P23226804	chr4:115612306-115612352	NM_001025567:203	Dmbx1	INSIDE	0.102	1.420	1220.94	1733.19	0.145	802.38	116.35
A_68_P32030084	chr19:25580234-25580278	NM_015826:61	Dmrt1	INSIDE	0.423	0.495	2646.19	1309.16	0.210	2197.89	460.46
A_68_P32030085	chr19:25580326-25580370	NM_015826:153	Dmrt1	INSIDE	0.494	0.668	1249.14	833.98	0.330	999.07	329.54

ProbeName	Target position of probe on CpG island microarray	TargetID	GeneSymbol	CpG island Description	Ratio of relative methylation (TiO2-NP/Vehicle)	Sham group			TiO_2-H group		
						Relative methylation (Cy-5/Cy-3)	Cy-3 signal (Input)	Cy-5 signal (MeDIP)	Relative methylation (Cy-5/Cy-3)	Cy-3 signal (Input)	Cy-5 signal (MeDIP)
A_68_P32030090	chr19:25580813-25580857	NM_015826:639	Dmrt1	INSIDE	0.393	0.736	4472.71	3292.67	0.289	3179.73	919.43
A_68_P32030091	chr19:25580881-25580925	NM_015826:707	Dmrt1	INSIDE	0.377	0.693	3461.68	2397.73	0.261	2630.53	687.09
A_68_P32031152	chr19:25747038-25747084	NM_145831:160	Dmrt2	INSIDE	0.310	0.686	1388.95	952.80	0.213	1035.27	220.16
A_68_P32030685	chr19:25680631-25680675	NM_177360:-4374	Dmrt3	PROMOTER	0.203	0.698	1697.10	1184.90	0.141	1261.40	178.39
A_68_P32030687	chr19:25680849-25680893	NM_177360:-4156	Dmrt3	PROMOTER	0.262	0.496	1556.60	772.47	0.130	1183.93	154.18
A_68_P32030716	chr19:25684536-25684580	NM_177360:-468	Dmrt3	PROMOTER	0.361	0.667	1409.09	939.16	0.241	1098.30	264.44
A_68_P23203726	chr4:109650077-109650121	NM_172296:-531	Dmrta2	PROMOTER	0.248	0.348	1771.24	616.01	0.086	1394.98	120.09
A_68_P23203729	chr4:109650347-109650391	NM_172296:-261	Dmrta2	PROMOTER	0.432	0.401	3107.48	1247.05	0.174	2233.97	387.65
A_68_P23203752	chr4:109652831-109652875	NM_172296:2223	Dmrta2	INSIDE	0.227	0.473	1258.70	594.89	0.107	1156.24	123.89
A_68_P23203773	chr4:109655219-109655263	NM_172296:4611	Dmrta2	INSIDE	0.526	0.569	1057.83	601.95	0.299	879.38	263.17
A_68_P23471509	chr5:9160963-9161011	NM_001110327:790	Dmtf1	INSIDE	0.311	0.645	1452.99	937.24	0.201	1079.13	216.46
A_68_P24991257	chr7:19661336-19661380	NM_010058:-190	Dmwd	PROMOTER	0.396	0.616	1112.54	684.81	0.244	889.48	217.03
A_68_P27188615	chr10:62411987-62412031	NM_177372:2232	Dna2	INSIDE	0.618	2.596	910.51	2363.28	1.605	760.06	1220.14
A_68_P28778248	chr12:119437069-119437113	NM_010060:426	Dnahc11	INSIDE	0.429	0.600	1767.16	1059.95	0.257	1351.87	347.71
A_68_P27895681	chr11:69359967-69360011	NM_001081330:2622	Dnahc2	INSIDE	0.190	0.551	1375.31	757.70	0.104	1123.98	117.42
A_68_P26025443	chr8:88079254-88079298	NM_019794:-106	Dnaja2	PROMOTER	0.618	0.555	1859.01	1031.32	0.343	1720.93	590.43
A_68_P26527191	chr9:54546796-54546840	NM_021422:-547	Dnaja4	PROMOTER	0.342	1.445	3453.02	4990.09	0.494	2623.13	1294.83
A_68_P26527199	chr9:54547693-54547737	NM_021422:349	Dnaja4	INSIDE	0.275	0.556	1005.14	558.46	0.153	768.88	117.48
A_68_P26015687	chr8:86132654-86132698	NM_018808:603	Dnajb1	INSIDE	0.605	0.595	1336.38	794.62	0.360	1176.46	423.12
A_68_P26015688	chr8:86132717-86132768	NM_018808:669	Dnajb1	INSIDE	0.198	0.503	1573.85	792.17	0.099	1170.32	116.43
A_68_P30600193	chr16:22857790-22857834	NM_001190804:-105	Dnajb11	DIVERGENT_PROMOTER	0.304	0.577	2072.81	1195.30	0.176	1427.93	250.66
A_68_P30600196	chr16:22858132-22858176	NM_001190804:237	Dnajb11	INSIDE	0.442	0.462	1650.30	763.25	0.204	1246.41	254.85
A_68_P20352840	chr1:75233023-75233067	NM_001159885:-232	Dnajb2	PROMOTER	0.260	0.555	1122.62	623.32	0.145	806.66	116.66
A_68_P20352842	chr1:75233266-75233310	NM_001159885:12	Dnajb2	INSIDE	0.113	0.677	2053.90	1391.18	0.077	1505.54	115.35
A_68_P22649279	chr3:151873278-151873322	NM_027287:-37	Dnajb4	DIVERGENT_PROMOTER	0.369	0.480	1350.69	648.44	0.177	1091.22	193.15
A_68_P22869096	chr4:42962948-42962992	NM_019874:-2995	Dnajb5	PROMOTER	0.195	0.729	3574.50	2607.49	0.142	2533.01	359.98
A_68_P22869099	chr4:42963311-42963355	NM_019874:-2633	Dnajb5	PROMOTER	0.540	0.712	1323.06	941.75	0.384	1081.19	415.56
A_68_P28396957	chr12:45310575-45310620	NM_013760:458	Dnajb9	INSIDE	0.545	0.405	1667.74	675.83	0.221	1374.69	303.33
A_68_P28396959	chr12:45310845-45310889	NM_013760:189	Dnajb9	INSIDE	0.351	0.633	1982.31	1255.39	0.222	1490.06	331.30
A_68_P28396961	chr12:45311078-45311122	NM_013760:-45	Dnajb9	PROMOTER	0.381	0.457	2172.07	993.00	0.174	1688.94	294.02
A_68_P28396962	chr12:45311155-45311199	NM_013760:-121	Dnajb9	PROMOTER	0.277	0.394	1422.76	560.34	0.109	1067.66	116.53
A_68_P23415476	chr4:151308731-151308775	NM_172704:924	Dnajc11	INSIDE	0.285	0.476	1138.93	542.64	0.136	972.31	132.01
A_68_P26790784	chr9:104164628-104164672	NM_001163026:610	Dnajc13	INSIDE	0.386	0.701	1651.99	1157.73	0.270	1188.76	321.22
A_68_P26790786	chr9:104164871-104164915	NM_001163026:368	Dnajc13	INSIDE	0.483	0.425	1834.27	778.78	0.205	1387.67	284.86
A_68_P26790789	chr9:104165231-104165275	NM_001163026:8	Dnajc13	INSIDE	0.294	0.439	2216.01	973.37	0.129	1782.78	230.03
A_68_P27543290	chr10:128242192-128242236	NM_028873:-517	Dnajc14	DIVERGENT_PROMOTER	0.234	0.612	1036.99	634.30	0.143	890.24	127.51
A_68_P27543295	chr10:128242767-128242811	NM_028873:57	Dnajc14	INSIDE	0.199	0.511	1583.52	809.79	0.102	1222.92	124.16
A_68_P23365986	chr4:141346174-141346218	NM_172338:363	Dnajc16	INSIDE	0.423	0.466	1787.12	833.14	0.197	1389.82	274.01
A_68_P21568394	chr2:119034600-119034644	NM_139139:-91	Dnajc17	PROMOTER	0.230	0.469	1812.04	850.67	0.108	1319.29	142.16
A_68_P21568396	chr2:119034800-119034844	NM_139139:-291	Dnajc17	PROMOTER	0.320	0.621	1302.31	808.98	0.199	1076.80	213.97
A_68_P31619631	chr18:35862603-35862647	NM_029669:174	Dnajc18	INSIDE	0.459	0.616	1100.69	677.79	0.283	794.98	224.68
A_68_P22046077	chr3:33979970-33980014	NM_001026211:256	Dnajc19	INSIDE	0.612	0.558	1122.87	626.97	0.342	729.58	249.15
A_68_P23527832	chr5:21290509-21290553	NM_009584:453	Dnajc2	INSIDE	0.437	0.671	1741.11	1167.70	0.293	1419.28	415.80
A_68_P23527834	chr5:21290784-21290828	NM_009584:177	Dnajc2	INSIDE	0.462	0.621	2982.31	1851.15	0.287	2410.18	690.52

ProbeName	Target position of probe on CpG island microarray	TargetID	GeneSymbol	CpG island Description	Ratio of relative methylation (TiO2-NP/Vehicle)	Sham group			TiO_2-H group		
						Relative methylation (Cy-5/Cy-3)	Cy-3 signal (Input)	Cy-5 signal (MeDIP)	Relative methylation (Cy-5/Cy-3)	Cy-3 signal (Input)	Cy-5 signal (MeDIP)
A_68_P30478214	chr15:98926989-98927033	NM_176835:-2904	Dnajc22	DIVERGENT_PROMOTER	0.378	0.414	1883.76	778.97	0.156	1542.55	241.04
A_68_P30478221	chr15:98927716-98927760	NM_176835:-2176	Dnajc22	DIVERGENT_PROMOTER	0.553	0.383	1523.30	583.61	0.212	1164.54	246.89
A_68_P30478222	chr15:98927785-98927829	NM_176835:-2108	Dnajc22	DIVERGENT_PROMOTER	0.337	0.662	1175.81	778.42	0.223	900.94	200.98
A_68_P30478224	chr15:98928096-98928143	NM_176835:-1795	Dnajc22	DIVERGENT_PROMOTER	0.217	2.356	920.59	2169.23	0.510	625.86	319.42
A_68_P28198624	chr12:4082843-4082887	NM_153082:291	Dnajc27	INSIDE	0.102	0.597	6184.51	3691.19	0.061	4158.08	253.91
A_68_P31937160	chr19:7062391-7062440	NM_020566:4347	Dnajc4	INSIDE	0.283	0.419	1358.98	569.90	0.119	987.10	117.28
A_68_P23155325	chr4:101169532-101169576	NM_198412:302	Dnajc6	INSIDE	0.367	0.589	1393.48	820.60	0.216	994.58	214.77
A_68_P23155326	chr4:101169630-101169674	NM_198412:400	Dnajc6	INSIDE	0.553	0.660	2483.52	1639.61	0.365	2019.88	737.18
A_68_P23155401	chr4:101181535-101181581	NM_001164584:978	Dnajc6	INSIDE	0.158	0.467	2248.25	1049.28	0.074	1648.03	121.68
A_68_P23155610	chr4:101223010-101223054	NM_001164583:-166	Dnajc6	PROMOTER	0.616	0.725	2314.37	1677.70	0.447	1849.94	826.56
A_68_P28069090	chr11:100480471-100480515	NM_019795:990	Dnajc7	INSIDE	0.572	0.689	4194.67	2891.82	0.394	3144.37	1239.33
A_68_P28069093	chr11:100480802-100480846	NM_019795:658	Dnajc7	INSIDE	0.411	0.700	1858.85	1301.35	0.288	1302.63	375.00
A_68_P23312760	chr4:132091448-132091492	NM_172400:-3	Dnajc8	DIVERGENT_PROMOTER	0.247	0.429	1410.70	604.57	0.106	1177.90	124.69
A_68_P29446595	chr14:21207859-21207903	NM_134081:252	Dnajc9	INSIDE	0.323	0.410	2167.14	887.53	0.132	1525.68	201.53
A_68_P21078047	chr2:26207480-26207524	NM_001139503:128	Dnlz	INSIDE	0.261	0.427	1395.73	596.10	0.111	1101.89	122.80
A_68_P21114877	chr2:32208336-32208380	NM_010065:466	Dnm1	INSIDE	0.182	0.439	2139.13	939.13	0.080	1545.52	123.35
A_68_P21114882	chr2:32208948-32208992	NM_010065:-146	Dnm1	DIVERGENT_PROMOTER	0.196	0.609	1283.73	781.66	0.119	1015.31	121.15
A_68_P26347068	chr9:21229822-21229866	NM_001039520:456	Dnm2	INSIDE	0.205	0.679	3523.93	2394.24	0.139	2488.52	346.23
A_68_P20778062	chr1:164407645-164407689	NM_001038619:495	Dnm3	INSIDE	0.098	0.553	4111.38	2271.78	0.054	3052.44	166.13
A_68_P32129171	chr19:43954139-43954183	NM_028029:32722	Dnmbp	INSIDE	0.471	0.428	3182.83	1361.88	0.201	2210.38	445.27
A_68_P20353308	chr1:75313628-75313672	NM_016878:-58	Dnpep	PROMOTER	0.193	0.586	2248.50	1318.67	0.113	1575.63	178.47
A_68_P20353314	chr1:75314278-75314322	NM_001110831:-88	Dnpep	PROMOTER	0.155	0.484	2326.43	1126.36	0.075	1794.33	134.37
A_68_P22481535	chr3:121977658-121977702	NM_153806:349	Dnttip2	INSIDE	0.627	0.644	2361.54	1521.84	0.404	1831.98	740.02
A_68_P25502555	chr7:133991327-133991371	NM_010069:282	Doc2a	INSIDE	0.542	0.526	1156.49	608.46	0.285	965.39	275.48
A_68_P27928205	chr11:75608766-75608811	NM_007873:771	Doc2b	INSIDE	0.633	0.689	1849.08	1273.82	0.436	1455.04	634.03
A_68_P27928209	chr11:75609329-75609373	NM_007873:209	Doc2b	INSIDE	0.324	0.409	2502.99	1024.49	0.132	1958.78	259.37
A_68_P20383167	chr1:80754578-80754622	NM_175291:528	Dock10	INSIDE	0.275	0.671	3548.93	2381.70	0.185	2794.36	515.67
A_68_P32315762	chrX:33428641-33428685	NM_001009947:-164	Dock11	PROMOTER	0.294	1.708	1636.37	2794.21	0.503	769.78	386.90
A_68_P26807068	chr9:107095792-107095844	NM_153413:38422	Dock3	INSIDE	0.467	5.465	905.88	4950.49	2.550	696.03	1774.54
A_68_P29688234	chr14:68551046-68551090	NM_177780:561	Dock5	INSIDE	0.585	0.514	1339.68	688.48	0.300	1156.66	347.46
A_68_P29688239	chr14:68551910-68551954	NM_177780:-303	Dock5	PROMOTER	0.609	0.568	924.51	525.56	0.346	817.83	283.14
A_68_P29957981	chr14:122196637-122196681	NM_134074:298	Dock9	INSIDE	0.216	0.504	2447.64	1233.96	0.109	1897.75	206.32
A_68_P24590977	chr6:82982750-82982794	NM_010070:693	Dok1	INSIDE	0.424	0.543	1563.64	848.90	0.230	1267.91	292.10
A_68_P23597968	chr5:35399719-35399763	NM_172708:8	Dok7	INSIDE	0.483	0.725	1681.12	1218.03	0.350	1334.37	466.66
A_68_P21102297	chr2:30141550-30141594	NM_177648:302	Dolk	INSIDE	0.240	0.584	1091.43	637.59	0.140	871.46	122.24
A_68_P21102299	chr2:30141747-30141791	NM_177648:106	Dolk	INSIDE	0.289	0.581	2654.39	1542.59	0.168	1932.29	324.04
A_68_P21102914	chr2:30247811-30247855	NM_020329:-103	Dolpp1	PROMOTER	0.613	0.489	4399.74	2149.48	0.299	3276.44	981.06
A_68_P31156008	chr17:34974068-34974113	NM_001163770:127	Dom3z	INSIDE	0.611	0.503	1152.02	579.63	0.307	772.23	237.39
A_68_P31156012	chr17:34974444-34974488	NM_001163770:503	Dom3z	INSIDE	0.487	0.622	1713.07	1065.59	0.303	1350.94	409.64
A_68_P27281160	chr10:79594459-79594503	NM_001195268:5652	Dos	INSIDE	0.639	0.649	1352.83	877.60	0.414	1025.43	424.80
A_68_P27281212	chr10:79601441-79601485	NM_001195268:-1330	Dos	PROMOTER	0.066	1.344	7195.75	9673.17	0.088	4601.83	405.26
A_68_P27281213	chr10:79601565-79601609	NM_001195268:-1454	Dos	PROMOTER	0.212	0.720	2801.43	2016.59	0.152	1810.65	275.80
A_68_P26467019	chr9:44135043-44135087	NM_007875:137	Dpagt1	INSIDE	0.480	0.539	1362.77	734.99	0.259	1126.21	291.65
A_68_P26467020	chr9:44135161-44135205	NM_007875:255	Dpagt1	INSIDE	0.106	0.571	2939.59	1679.83	0.061	1893.04	114.95

ProbeName	Target position of probe on CpG island microarray	TargetID	GeneSymbol	CpG island Description	Ratio of relative methylation (TiO2-NP/Vehicle)	Sham group			TiO_2-H group		
						Relative methylation (Cy-5/Cy-3)	Cy-3 signal (Input)	Cy-5 signal (MeDIP)	Relative methylation (Cy-5/Cy-3)	Cy-3 signal (Input)	Cy-5 signal (MeDIP)
A_68_P26137126	chr8:108502836-108502891	NM_027960:456	Dpep3	INSIDE	0.538	5.455	266.55	1454.01	2.936	209.99	616.48
A_68_P25026463	chr7:30089112-30089158	NM_013874:111	Dpf1	INSIDE	0.192	0.657	1116.74	733.80	0.126	926.69	116.75
A_68_P25026465	chr7:30089302-30089346	NM_013874:301	Dpf1	INSIDE	0.592	0.682	1670.30	1139.36	0.404	1269.35	512.71
A_68_P28593010	chr12:84827335-84827379	NM_058212:1302	Dpf3	INSIDE	0.222	2.186	3502.32	7657.51	0.486	2189.41	1063.70
A_68_P28593011	chr12:84827402-84827446	NM_058212:1234	Dpf3	INSIDE	0.053	1.542	8286.68	12778.79	0.081	5432.00	442.38
A_68_P29515525	chr14:32898520-32898564	NM_001047433:309	Dph3	INSIDE	0.494	0.402	2249.71	905.32	0.199	1744.50	347.04
A_68_P29515526	chr14:32898628-32898679	NM_001047433:198	Dph3	INSIDE	0.434	0.537	1318.22	708.39	0.233	1040.87	242.91
A_68_P22318234	chr3:89070459-89070503	NM_026767:7	Dpm3	INSIDE	0.441	0.535	2096.22	1121.85	0.236	1553.58	366.66
A_68_P22318235	chr3:89070608-89070657	NM_026767:159	Dpm3	INSIDE	0.164	0.715	1680.15	1200.62	0.117	1150.04	134.93
A_68_P20578314	chr1:125942313-125942357	NM_199021:-198	Dpp10	PROMOTER	0.220	0.698	2018.23	1408.33	0.153	1557.90	238.78
A_68_P20578315	chr1:125942392-125942436	NM_199021:-278	Dpp10	PROMOTER	0.356	0.463	2751.40	1273.57	0.165	2109.16	347.79
A_68_P31925905	chr19:4928339-4928383	NM_133803:-73	Dpp3	PROMOTER	0.118	0.740	2862.19	2119.12	0.087	2088.62	182.69
A_68_P26585431	chr9:64880028-64880087	NM_028906:-207	Dpp8	PROMOTER	0.293	0.440	1159.20	509.86	0.129	948.77	122.08
A_68_P26585436	chr9:64880684-64880728	NM_028906:442	Dpp8	INSIDE	0.467	0.699	1563.21	1093.18	0.326	1063.72	347.30
A_68_P26585437	chr9:64880753-64880797	NM_028906:510	Dpp8	INSIDE	0.202	0.730	2641.78	1929.15	0.148	1885.44	278.25
A_68_P25051005	chr7:36539482-36539526	NM_178704:-31	Dpy19l3	PROMOTER	0.556	0.541	2558.75	1384.64	0.301	2077.84	624.93
A_68_P22729160	chr4:11248943-11248991	NM_001081201:312	Dpy19l4	INSIDE	0.261	0.567	916.14	519.86	0.148	782.07	115.62
A_68_P30156152	chr15:39688848-39688892	NM_022722:146	Dpys	INSIDE	0.557	0.581	1804.16	1048.81	0.324	1202.43	389.35
A_68_P30156153	chr15:39688931-39688978	NM_022722:62	Dpys	INSIDE	0.146	0.705	1631.29	1149.86	0.103	1188.32	122.11
A_68_P29681786	chr14:67486350-67486394	NM_009955:1065	Dpysl2	INSIDE	0.541	0.609	4537.04	2763.69	0.330	3389.91	1117.68
A_68_P29681788	chr14:67486601-67486645	NM_009955:815	Dpysl2	INSIDE	0.088	1.990	6312.82	12565.59	0.175	4012.47	701.53
A_68_P29681795	chr14:67487454-67487502	NM_009955:-41	Dpysl2	DIVERGENT_PROMOTER	0.201	0.617	1264.24	780.41	0.124	974.26	120.62
A_68_P25575621	chr7:146271919-146271963	NM_011993:41	Dpysl4	INSIDE	0.390	0.561	2409.39	1351.91	0.219	1732.06	378.82
A_68_P23574103	chr5:31013991-31014035	NM_023047:-255	Dpysl5	PROMOTER	0.414	0.710	1574.89	1118.13	0.294	1144.85	336.89
A_68_P23970972	chr5:108697917-108697961	NM_026106:23	Dr1	INSIDE	0.151	0.408	3760.10	1533.47	0.062	2765.36	170.10
A_68_P23970975	chr5:108698202-108698246	NM_026106:309	Dr1	INSIDE	0.626	0.692	1479.65	1023.38	0.433	1373.15	594.14
A_68_P29047193	chr13:54150633-54150677	NM_010076:373	Drd1a	INSIDE	0.396	0.545	2490.00	1356.97	0.216	1903.02	411.08
A_68_P29047195	chr13:54150782-54150826	NM_010076:223	Drd1a	INSIDE	0.284	0.507	2712.96	1375.12	0.144	2034.05	293.08
A_68_P29047196	chr13:54150905-54150949	NM_010076:101	Drd1a	INSIDE	0.439	0.594	3805.06	2258.45	0.261	3019.15	786.63
A_68_P25587939	chr7:148477922-148477966	NM_007878:40	Drd4	INSIDE	0.364	0.465	1820.17	847.17	0.169	1494.82	252.94
A_68_P25587959	chr7:148480560-148480604	NM_007878:2678	Drd4	INSIDE	0.387	0.495	1097.57	543.76	0.192	917.90	175.85
A_68_P27551032	chr11:3166258-3166302	NM_007879:109	Drg1	INSIDE	0.172	0.690	3620.02	2497.50	0.118	2488.61	294.72
A_68_P26473573	chr9:45239483-45239530	NM_001081270:1131	Dscaml1	INSIDE	0.154	0.525	1944.60	1019.95	0.081	1516.86	122.16
A_68_P30981933	chr16:94747960-94748013	NM_007834:250	Dscr3	INSIDE	0.389	0.617	1236.96	762.84	0.240	982.57	235.93
A_68_P21774638	chr2:156832712-156832756	NM_025853:77	Dsn1	INSIDE	0.359	0.581	1832.53	1065.41	0.209	1547.20	322.82
A_68_P20137270	chr1:34234710-34234761	NM_133833:166066	Dst	INSIDE	0.651	3.787	212.75	805.64	2.467	206.01	508.22
A_68_P20938879	chr1:193399292-193399336	NM_029766:99	Dtl	INSIDE	0.564	0.410	2542.52	1042.82	0.231	1970.82	455.87
A_68_P24118893	chr5:136470543-136470598	NM_023742:-176	Dtx2	PROMOTER	0.274	0.615	983.42	604.71	0.169	910.04	153.37
A_68_P24118896	chr5:136470915-136470959	NM_023742:190	Dtx2	INSIDE	0.617	0.645	2522.89	1627.48	0.398	2062.38	821.34
A_68_P31961132	chr19:12575516-12575560	NM_172442:948	Dtx4	INSIDE	0.595	0.611	1741.39	1064.59	0.364	1392.13	506.25
A_68_P20457519	chr1:95698419-95698463	NM_001105667:71	Dtymk	INSIDE	0.304	0.502	1962.36	984.78	0.153	1438.06	219.73
A_68_P20457521	chr1:95698612-95698656	NM_001105667:-123	Dtymk	DIVERGENT_PROMOTER	0.371	0.592	1041.93	616.49	0.220	836.20	183.79
A_68_P21585577	chr2:122124337-122124381	NM_025777:-277	Duoxa2	DIVERGENT_PROMOTER	0.292	0.597	901.23	537.78	0.174	691.95	120.45
A_68_P29454308	chr14:22505922-22505966	NM_001013826:27854	Dupd1	INSIDE	0.588	0.575	1335.15	767.19	0.338	1088.89	367.69

ProbeName	Target position of probe on CpG island microarray	TargetID	GeneSymbol	CpG island Description	Ratio of relative methylation (TiO2-NP/Vehicle)	Sham group			TiO_2-H group		
						Relative methylation (Cy-5/Cy-3)	Cy-3 signal (Input)	Cy-5 signal (MeDIP)	Relative methylation (Cy-5/Cy-3)	Cy-3 signal (Input)	Cy-5 signal (MeDIP)
A_68_P28186829	chr11:120657297-120657341	NM_026824:391	Dus1l	INSIDE	0.235	0.603	1173.52	707.97	0.142	821.66	116.66
A_68_P28186830	chr11:120657372-120657416	NM_026824:315	Dus1l	INSIDE	0.480	0.659	2813.60	1854.72	0.316	2222.26	702.91
A_68_P28186831	chr11:120657508-120657552	NM_026824:179	Dus1l	INSIDE	0.640	0.632	861.08	544.18	0.404	746.98	301.96
A_68_P28186834	chr11:120657869-120657913	NM_026824:-181	Dus1l	PROMOTER	0.423	0.567	2245.52	1272.24	0.240	1847.20	442.92
A_68_P31258228	chr17:56904354-56904398	NM_144858:203	Dus3l	INSIDE	0.311	0.621	1871.83	1163.03	0.193	1359.56	262.92
A_68_P28332514	chr12:32339519-32339563	NM_028002:151	Dus4l	INSIDE	0.333	0.443	1334.63	591.46	0.148	1132.44	167.14
A_68_P28332515	chr12:32339615-32339664	NM_028002:52	Dus4l	INSIDE	0.272	0.567	1352.00	766.46	0.154	1092.96	168.75
A_68_P31110712	chr17:26645159-26645203	NM_013642:237	Dusp1	INSIDE	0.305	0.685	1711.07	1171.73	0.209	1326.08	277.13
A_68_P24607363	chr6:85911171-85911215	NM_028099:469	Dusp11	INSIDE	0.110	0.562	2551.77	1433.52	0.062	1891.29	116.92
A_68_P24607366	chr6:85911499-85911543	NM_028099:141	Dusp11	INSIDE	0.670	0.621	866.20	537.54	0.416	726.14	302.05
A_68_P27554562	chr11:3795169-3795213	NM_173745:-52	Dusp18	DIVERGENT_PROMOTER	0.292	0.588	1725.53	1014.44	0.172	1300.49	223.61
A_68_P21612763	chr2:127161943-127161987	NM_010090:70	Dusp2	INSIDE	0.527	0.415	1381.84	573.89	0.219	1181.19	258.47
A_68_P20834313	chr1:174563103-174563147	NM_026725:-19	Dusp23	PROMOTER	0.557	0.482	1550.12	747.21	0.268	1209.27	324.62
A_68_P28076812	chr11:101846260-101846304	NM_028207:-177	Dusp3	DIVERGENT_PROMOTER	0.660	0.493	1421.69	701.35	0.325	1094.23	356.06
A_68_P25776075	chr8:35869882-35869926	NM_176933:-759	Dusp4	PROMOTER	0.176	0.435	2041.21	888.65	0.077	1589.93	121.80
A_68_P25776076	chr8:35869953-35869997	NM_176933:-689	Dusp4	PROMOTER	0.654	0.571	2564.91	1464.14	0.373	1967.69	734.05
A_68_P25776082	chr8:35870564-35870608	NM_176933:-77	Dusp4	PROMOTER	0.222	0.683	2338.94	1597.35	0.151	1536.62	232.66
A_68_P25776088	chr8:35871210-35871254	NM_176933:569	Dusp4	INSIDE	0.637	0.602	2469.37	1486.92	0.384	1867.02	716.31
A_68_P25776089	chr8:35871359-35871403	NM_176933:717	Dusp4	INSIDE	0.125	0.701	2310.37	1619.92	0.088	1707.32	150.16
A_68_P25776095	chr8:35872064-35872108	NM_176933:1423	Dusp4	INSIDE	0.238	0.666	1972.23	1312.54	0.158	1699.59	268.82
A_68_P32183070	chr19:53603197-53603241	NM_001085390:-589	Dusp5	PROMOTER	0.299	0.465	1305.63	607.44	0.139	969.44	135.02
A_68_P32183081	chr19:53604393-53604437	NM_001085390:607	Dusp5	INSIDE	0.075	1.453	1606.62	2334.21	0.110	1087.04	119.17
A_68_P32183082	chr19:53604536-53604581	NM_001085390:751	Dusp5	INSIDE	0.198	0.691	1291.86	892.57	0.137	906.88	124.01
A_68_P27386030	chr10:98725559-98725613	NM_026268:-278	Dusp6	PROMOTER	0.152	0.577	1825.14	1052.84	0.087	1325.87	115.94
A_68_P27386037	chr10:98726254-98726298	NM_026268:412	Dusp6	INSIDE	0.523	0.555	1184.29	657.03	0.290	1010.52	292.97
A_68_P25592946	chr7:149281230-149281274	NM_008748:-63	Dusp8	PROMOTER	0.590	0.457	2086.08	952.95	0.269	1690.04	455.17
A_68_P21601160	chr2:125073182-125073226	NM_023595:-46	Dut	PROMOTER	0.329	0.733	4470.05	3276.61	0.241	3542.84	855.05
A_68_P21601161	chr2:125073250-125073294	NM_023595:22	Dut	INSIDE	0.346	0.695	2144.94	1490.24	0.240	1617.56	388.64
A_68_P21601162	chr2:125073336-125073380	NM_023595:108	Dut	INSIDE	0.116	0.746	3746.94	2796.44	0.087	2518.72	217.98
A_68_P27898423	chr11:69814128-69814172	NM_007888:23	Dvl2	INSIDE	0.265	0.528	2671.78	1409.79	0.140	1863.09	260.61
A_68_P24213705	chr6:5955978-5956022	NM_001191023:280362	Dync1i1	INSIDE	0.360	0.613	1716.96	1051.72	0.220	1371.21	302.34
A_68_P21318133	chr2:71050301-71050345	NM_001198877:320	Dync1i2	INSIDE	0.667	0.585	905.85	529.99	0.390	735.70	287.24
A_68_P24008924	chr5:115750457-115750502	NM_019682:520	Dynll1	INSIDE	0.376	0.625	2770.59	1732.93	0.235	2047.53	481.21
A_68_P25020569	chr7:28964516-28964561	NM_010092:26	Dyrk1b	INSIDE	0.487	0.644	954.38	614.76	0.314	868.98	272.77
A_68_P27487944	chr10:118305191-118305235	NM_001014390:747	Dyrk2	INSIDE	0.544	0.645	1276.23	823.73	0.351	1093.33	383.90
A_68_P20613762	chr1:133026314-133026358	NM_145508:8475	Dyrk3	INSIDE	0.646	2.760	1035.86	2858.97	1.782	860.35	1532.86
A_68_P20613814	chr1:133034401-133034445	NM_145508:389	Dyrk3	INSIDE	0.258	0.521	1882.84	980.99	0.135	1552.70	208.88
A_68_P26630496	chr9:72806623-72806667	NM_001163725:53	Dyx1c1	INSIDE	0.157	0.536	2529.38	1354.55	0.084	1858.00	156.40
A_68_P29941731	chr14:119324409-119324453	NM_025943:-2041	Dzip1	PROMOTER	0.550	0.475	1629.32	774.21	0.261	1344.65	351.27
A_68_P29941732	chr14:119324506-119324550	NM_025943:-2139	Dzip1	PROMOTER	0.588	0.519	1763.44	915.93	0.306	1422.85	434.86
A_68_P26765225	chr9:99530050-99530094	NM_028258:59	Dzip1l	INSIDE	0.667	0.573	1786.88	1024.12	0.382	1503.61	574.42
A_68_P26135514	chr8:108231844-108231888	NM_198299:202	E130303B06Rik	INSIDE	0.272	0.540	1060.09	572.21	0.147	789.68	115.78
A_68_P25051306	chr7:36587684-36587728	NR_033567:96	E130304I02Rik	INSIDE	0.587	0.600	1063.82	638.82	0.353	839.10	295.97
A_68_P27921507	chr11:74433104-74433148	NM_001013784:20	E130309D14Rik	INSIDE	0.385	0.588	2126.36	1249.32	0.226	1752.37	396.42

ProbeName	Target position of probe on CpG island microarray	TargetID	GeneSymbol	CpG island Description	Ratio of relative methylation (TiO2-NP/Vehicle)	Sham group			TiO_2-H group		
						Relative methylation (Cy-5/Cy-3)	Cy-3 signal (Input)	Cy-5 signal (MeDIP)	Relative methylation (Cy-5/Cy-3)	Cy-3 signal (Input)	Cy-5 signal (MeDIP)
A_68_P22903293	chr4:49072217-49072261	NM_178756:-95	E130309F12Rik	PROMOTER	0.038	1.987	5573.01	11074.82	0.075	3841.46	287.20
A_68_P22903297	chr4:49072600-49072644	NM_178756:289	E130309F12Rik	INSIDE	0.186	0.542	1581.49	857.66	0.101	1224.29	123.30
A_68_P22903299	chr4:49072794-49072838	NM_178756:483	E130309F12Rik	INSIDE	0.221	0.469	1377.16	646.06	0.104	1128.47	116.80
A_68_P21761231	chr2:154395665-154395709	NM_007891:-98	E2f1	PROMOTER	0.502	0.342	2061.10	704.35	0.172	1604.47	275.25
A_68_P28914774	chr13:30075826-30075881	NM_010093:2079	E2f3	INSIDE	0.191	0.573	1418.79	813.17	0.109	1115.78	121.98
A_68_P28914783	chr13:30077028-30077072	NM_010093:882	E2f3	INSIDE	0.295	0.653	1692.28	1105.35	0.193	1342.03	258.74
A_68_P28914786	chr13:30077338-30077382	NM_010093:572	E2f3	INSIDE	0.344	0.459	3660.62	1681.12	0.158	2711.94	428.15
A_68_P28914787	chr13:30077407-30077451	NM_010093:504	E2f3	INSIDE	0.406	0.611	2612.84	1596.12	0.248	2058.37	509.93
A_68_P28914791	chr13:30077924-30077972	NM_010093:-16	E2f3	PROMOTER	0.137	0.436	2865.18	1250.54	0.060	1910.86	114.42
A_68_P28914792	chr13:30078001-30078045	NM_010093:-90	E2f3	PROMOTER	0.302	0.519	1421.78	737.76	0.157	991.18	155.34
A_68_P21957442	chr3:14578468-14578519	NM_007892:-177	E2f5	PROMOTER	0.266	0.464	1625.94	754.46	0.123	1380.23	170.26
A_68_P28266976	chr12:16818150-16818194	NM_033270:402	E2f6	INSIDE	0.307	0.572	2104.39	1204.19	0.176	1746.15	306.56
A_68_P27445215	chr10:110182553-110182597	NM_178609:54	E2f7	INSIDE	0.512	0.436	1420.39	619.06	0.223	1097.74	244.80
A_68_P27445216	chr10:110182710-110182754	NM_178609:212	E2f7	INSIDE	0.421	0.462	1902.18	877.87	0.194	1517.45	294.85
A_68_P26659601	chr9:78290277-78290323	NM_173386:654	E330016A19Rik	INSIDE	0.249	0.541	1557.65	842.83	0.135	1237.15	166.67
A_68_P26659608	chr9:78291066-78291110	NM_173386:-134	E330016A19Rik	DIVERGENT_PROMOTER	0.256	0.648	1462.04	948.02	0.166	1212.77	201.24
A_68_P25505455	chr7:134536637-134536681	NM_198011:477	E430018J23Rik	INSIDE	0.504	0.521	993.18	517.43	0.263	860.95	226.19
A_68_P31097445	chr17:24592379-24592423	NM_007893:-144	E4f1	PROMOTER	0.068	0.746	3145.67	2346.73	0.051	2272.53	115.10
A_68_P29512329	chr14:32308482-32308526	NM_028932:240	Eaf1	INSIDE	0.646	0.459	1287.20	590.78	0.296	1039.79	308.20
A_68_P30677440	chr16:36828175-36828219	NM_001113401:149	Eaf2	INSIDE	0.448	0.380	1847.89	702.56	0.170	1422.12	242.02
A_68_P30677441	chr16:36828287-36828331	NM_001113401:37	Eaf2	INSIDE	0.661	0.640	806.69	516.26	0.423	706.70	299.12
A_68_P27756958	chr11:44433864-44433908	NM_007897:2251	Ebf1	INSIDE	0.097	0.602	2795.45	1682.18	0.058	2017.61	117.38
A_68_P29683688	chr14:67853251-67853295	NM_010095:1144	Ebf2	INSIDE	0.666	0.603	1322.62	797.41	0.401	1084.90	435.48
A_68_P29683700	chr14:67854699-67854743	NM_010095:2592	Ebf2	INSIDE	0.152	0.685	3435.06	2351.80	0.104	2541.11	264.34
A_68_P25565211	chr7:144506565-144506609	NM_001113414:-458	Ebf3	PROMOTER	0.413	0.566	1305.03	738.81	0.234	1040.78	243.47
A_68_P25565217	chr7:144507254-144507298	NM_001113414:-1148	Ebf3	PROMOTER	0.278	0.689	1139.32	785.53	0.192	912.45	174.99
A_68_P25565281	chr7:144514141-144514185	NM_001113414:-8034	Ebf3	PROMOTER	0.484	0.643	1717.13	1103.61	0.311	1446.85	449.80
A_68_P21628998	chr2:130120631-130120675	NM_001110513:-1022	Ebf4	PROMOTER	0.593	0.714	2592.44	1850.61	0.423	1876.95	794.32
A_68_P21629316	chr2:130187590-130187634	NM_001110513:65938	Ebf4	INSIDE	0.597	0.645	2161.58	1394.12	0.385	1725.52	664.14
A_68_P21629321	chr2:130188203-130188247	NM_001110513:66550	Ebf4	INSIDE	0.243	1.346	2219.49	2987.63	0.327	1802.35	589.92
A_68_P23241426	chr4:118293372-118293419	NM_026932:-8	Ebna1bp2	DIVERGENT_PROMOTER	0.195	0.638	1235.31	788.08	0.124	927.85	115.34
A_68_P32242173	chrX:7769712-7769759	NM_007898:903	Ebp	INSIDE	0.322	1.416	1649.64	2336.36	0.456	820.88	374.58
A_68_P32242175	chrX:7769957-7770003	NM_007898:658	Ebp	INSIDE	0.311	0.625	1578.04	986.37	0.195	626.18	121.86
A_68_P29649840	chr14:61979049-61979093	NM_026598:212	Ebpl	INSIDE	0.379	0.627	961.94	602.95	0.238	806.22	191.53
A_68_P23342688	chr4:137469440-137469484	NM_199307:51311	Ece1	INSIDE	0.463	0.548	1427.71	781.82	0.253	1358.60	344.25
A_68_P23342966	chr4:137514008-137514052	NM_199307:95879	Ece1	INSIDE	0.630	3.308	2803.57	9274.59	2.085	2035.51	4243.58
A_68_P30588475	chr16:20611479-20611531	NM_025462:-169	Ece2	DIVERGENT_PROMOTER	0.078	0.723	3023.77	2184.80	0.056	2057.09	116.13
A_68_P30588476	chr16:20611624-20611668	NM_025462:-27	Ece2	DIVERGENT_PROMOTER	0.159	0.612	1636.15	1001.51	0.098	1193.98	116.53
A_68_P27034329	chr10:29033110-29033154	NM_025855:-173	Echdc1	PROMOTER	0.243	0.529	4172.73	2208.30	0.128	3208.55	412.26
A_68_P25582340	chr7:147302368-147302412	NM_053119:-68	Echs1	DIVERGENT_PROMOTER	0.258	0.566	3901.65	2206.87	0.146	3156.08	459.78
A_68_P25582341	chr7:147302455-147302502	NM_053119:-156	Echs1	DIVERGENT_PROMOTER	0.265	0.562	1003.74	563.95	0.149	816.93	121.63
A_68_P31097235	chr17:24563756-24563800	NM_010023:151	Eci1	INSIDE	0.396	0.527	3129.78	1648.81	0.209	2436.59	508.04
A_68_P28941742	chr13:35086077-35086121	NM_001110331:-85	Eci2	PROMOTER	0.058	1.647	2213.86	3646.81	0.095	1375.37	131.01
A_68_P26543334	chr9:57556210-57556266	NM_153799:-137	Edc3	PROMOTER	0.219	0.668	1038.76	694.08	0.146	788.01	115.20

ProbeName	Target position of probe on CpG island microarray	TargetID	GeneSymbol	CpG island Description	Ratio of relative methylation (TiO2-NP/Vehicle)	Sham group			TiO_2-H group		
						Relative methylation (Cy-5/Cy-3)	Cy-3 signal (Input)	Cy-5 signal (MeDIP)	Relative methylation (Cy-5/Cy-3)	Cy-3 signal (Input)	Cy-5 signal (MeDIP)
A_68_P26543335	chr9:57556330-57556374	NM_153799:-23	Edc3	PROMOTER	0.639	0.622	1894.48	1178.32	0.398	1420.47	564.74
A_68_P26136484	chr8:108404850-108404894	NM_181594:22	Edc4	INSIDE	0.232	1.482	3712.53	5500.23	0.344	2542.61	874.56
A_68_P24735991	chr6:108779125-108779169	NM_138677:512	Edem1	INSIDE	0.534	0.594	884.04	524.77	0.317	706.55	224.11
A_68_P21767285	chr2:155555051-155555095	NM_145537:139	Edem2	INSIDE	0.221	0.422	1513.77	638.13	0.093	1302.04	121.37
A_68_P29204944	chr13:88961571-88961615	NM_001037987:516	Edil3	INSIDE	0.201	0.629	1172.78	738.12	0.126	911.57	115.06
A_68_P25310575	chr7:97128573-97128627	NM_021876:886	Eed	INSIDE	0.230	0.560	1210.82	678.16	0.129	895.85	115.59
A_68_P25310576	chr7:97128694-97128740	NM_021876:770	Eed	INSIDE	0.182	0.593	1592.32	944.22	0.108	1107.26	119.66
A_68_P26659810	chr9:78328608-78328655	NM_010106:900	Eef1a1	INSIDE	0.209	0.441	1641.13	723.39	0.092	1262.23	116.02
A_68_P26659811	chr9:78328698-78328742	NM_010106:811	Eef1a1	INSIDE	0.134	0.497	2332.21	1159.60	0.067	1815.37	121.26
A_68_P28962018	chr13:38750527-38750572	NM_025380:348	Eef1e1	INSIDE	0.473	0.529	1293.25	684.71	0.250	1003.15	251.03
A_68_P28962021	chr13:38750788-38750832	NM_025380:87	Eef1e1	INSIDE	0.291	0.640	1197.51	766.37	0.186	937.50	174.80
A_68_P25470622	chr7:127986473-127986517	NM_007908:98	Eef2k	INSIDE	0.342	0.620	2300.15	1425.40	0.212	1815.39	385.30
A_68_P25470627	chr7:127987068-127987112	NM_007908:694	Eef2k	INSIDE	0.252	0.659	1620.71	1068.33	0.166	1275.93	211.57
A_68_P20863709	chr1:180336540-180336584	NM_026626:551	Efcab2	INSIDE	0.170	0.458	3807.40	1743.50	0.078	2925.00	227.43
A_68_P25588981	chr7:148648103-148648147	NM_001025103:1132	Efcab4a	INSIDE	0.401	0.489	1279.57	625.46	0.196	951.70	186.53
A_68_P23146396	chr4:99496193-99496248	NM_145549:30	Efcab7	INSIDE	0.226	0.593	1339.13	793.48	0.134	923.42	123.66
A_68_P20416756	chr1:89160739-89160787	NM_028889:-176	Efhd1	PROMOTER	0.305	0.479	1156.38	553.94	0.146	810.92	118.64
A_68_P22318336	chr3:89084785-89084834	NM_010107:-1242	Efna1	PROMOTER	0.160	0.626	1735.52	1086.78	0.100	1236.35	123.95
A_68_P27281569	chr10:79649352-79649396	NM_007909:7148	Efna2	INSIDE	0.407	1.541	1256.56	1936.53	0.627	969.03	607.79
A_68_P27281570	chr10:79649434-79649479	NM_007909:7230	Efna2	INSIDE	0.588	2.645	5708.01	15096.17	1.556	3752.70	5837.50
A_68_P22318526	chr3:89120527-89120571	NM_010108:6253	Efna3	INSIDE	0.397	0.639	1485.97	948.79	0.254	1110.36	281.80
A_68_P22318527	chr3:89120595-89120646	NM_010108:6181	Efna3	INSIDE	0.197	0.642	1459.59	937.30	0.127	990.46	125.31
A_68_P22318558	chr3:89124366-89124410	NM_010108:2413	Efna3	INSIDE	0.333	1.492	4248.51	6336.96	0.496	3141.81	1559.10
A_68_P22318559	chr3:89124527-89124571	NM_010108:2253	Efna3	INSIDE	0.209	0.569	1246.80	709.24	0.119	1012.21	120.11
A_68_P22318571	chr3:89125906-89125950	NM_010108:873	Efna3	INSIDE	0.263	1.756	11254.09	19767.21	0.461	6990.29	3223.29
A_68_P31286837	chr17:63229431-63229475	NM_010109:1214	Efna5	INSIDE	0.444	0.545	1056.23	575.46	0.242	786.12	190.15
A_68_P31286845	chr17:63230423-63230467	NM_010109:222	Efna5	INSIDE	0.358	0.701	2262.00	1584.83	0.251	1591.00	399.54
A_68_P31286851	chr17:63231331-63231377	NM_010109:-688	Efna5	PROMOTER	0.173	0.682	1466.36	1000.36	0.118	1025.23	121.29
A_68_P32553698	chrX:96331493-96331537	NM_010110:46	Efnb1	INSIDE	0.143	1.438	1503.49	2161.96	0.206	785.68	161.92
A_68_P32553707	chrX:96332536-96332580	NM_010110:1090	Efnb1	INSIDE	0.055	2.793	1851.05	5170.11	0.155	777.30	120.18
A_68_P25637619	chr8:8660957-8661001	NM_010111:-205	Efnb2	PROMOTER	0.342	0.556	1755.35	976.85	0.190	1389.17	264.38
A_68_P25637620	chr8:8661042-8661086	NM_010111:-291	Efnb2	PROMOTER	0.384	0.429	1357.65	581.82	0.165	1001.31	164.86
A_68_P29615091	chr14:55545244-55545288	NM_010112:359	Efs	INSIDE	0.227	0.697	2996.73	2088.62	0.158	2246.55	354.71
A_68_P29615094	chr14:55545462-55545506	NM_010112:141	Efs	INSIDE	0.296	0.619	1572.86	974.21	0.183	1139.18	208.54
A_68_P27612234	chr11:16652279-16652323	NM_007912:95	Egfr	INSIDE	0.351	0.580	936.29	543.40	0.204	700.44	142.85
A_68_P26248660	chr8:127472125-127472169	NM_053207:1008	Egln1	INSIDE	0.405	0.419	1430.66	598.91	0.170	1160.96	197.05
A_68_P25015864	chr7:27951357-27951401	NM_053208:443	Egln2	INSIDE	0.537	0.503	3145.81	1582.63	0.270	2313.69	625.17
A_68_P25015867	chr7:27951865-27951909	NM_053208:-65	Egln2	PROMOTER	0.373	0.528	1116.01	589.21	0.197	856.52	168.89
A_68_P31615128	chr18:35020826-35020870	NM_007913:-12	Egr1	PROMOTER	0.157	0.471	2150.96	1012.61	0.074	1559.68	115.61
A_68_P27212431	chr10:66999287-66999333	NM_010118:-1306	Egr2	PROMOTER	0.167	0.567	1741.96	987.37	0.095	1249.12	118.39
A_68_P27212434	chr10:66999516-66999563	NM_010118:-1077	Egr2	PROMOTER	0.198	2.177	3937.19	8572.79	0.431	2827.98	1219.76
A_68_P29698995	chr14:70474363-70474407	NM_018781:-2867	Egr3	PROMOTER	0.139	0.548	1932.06	1058.90	0.076	1522.60	115.60
A_68_P29699000	chr14:70475029-70475073	NM_018781:-2201	Egr3	PROMOTER	0.375	0.542	1194.60	647.38	0.203	954.61	193.80
A_68_P29699002	chr14:70475220-70475264	NM_018781:-2009	Egr3	PROMOTER	0.233	0.626	1024.73	641.53	0.146	798.27	116.46

ProbeName	Target position of probe on CpG island microarray	TargetID	GeneSymbol	CpG island Description	Ratio of relative methylation (TiO2-NP/Vehicle)	Sham group			TiO_2-H group		
						Relative methylation (Cy-5/Cy-3)	Cy-3 signal (Input)	Cy-5 signal (MeDIP)	Relative methylation (Cy-5/Cy-3)	Cy-3 signal (Input)	Cy-5 signal (MeDIP)
A_68_P29699020	chr14:70477756-70477800	NM_018781:527	Egr3	INSIDE	0.368	0.632	2005.00	1266.39	0.232	1471.41	341.64
A_68_P29699021	chr14:70477881-70477925	NM_018781:651	Egr3	INSIDE	0.200	0.651	2839.06	1848.43	0.130	2319.54	301.92
A_68_P29699028	chr14:70478715-70478759	NM_018781:1485	Egr3	INSIDE	0.441	0.563	1355.65	763.34	0.248	993.42	246.71
A_68_P29699029	chr14:70478786-70478830	NM_018781:1557	Egr3	INSIDE	0.307	0.538	1017.69	547.72	0.165	699.15	115.53
A_68_P29699033	chr14:70479382-70479426	NM_018781:2153	Egr3	INSIDE	0.163	0.460	2042.28	938.77	0.075	1575.72	118.15
A_68_P31930375	chr19:5716486-5716534	NM_001114595:9807	Ehbp1l1	INSIDE	0.169	0.504	1845.37	929.82	0.085	1357.42	115.87
A_68_P31933631	chr19:6276479-6276524	NM_010119:-394	Ehd1	PROMOTER	0.221	0.476	1612.19	767.36	0.105	1143.85	120.40
A_68_P31933638	chr19:6277193-6277237	NM_010119:319	Ehd1	INSIDE	0.257	0.361	4704.13	1698.35	0.093	3546.43	328.91
A_68_P21573664	chr2:119979962-119980006	NM_133838:327	Ehd4	INSIDE	0.204	0.507	1361.52	690.49	0.104	1120.93	116.07
A_68_P21573667	chr2:119980437-119980486	NM_133838:-150	Ehd4	PROMOTER	0.212	0.650	1219.61	792.90	0.138	907.55	125.04
A_68_P21573668	chr2:119980528-119980572	NM_133838:-239	Ehd4	PROMOTER	0.160	0.456	2041.22	930.87	0.073	1609.40	117.49
A_68_P26426830	chr9:36604533-36604577	NM_007915:99	Ei24	INSIDE	0.245	0.560	1102.62	617.41	0.137	960.57	131.84
A_68_P28067416	chr11:100180895-100180948	NM_011508:-388	Eif1	PROMOTER	0.176	0.619	1706.07	1056.87	0.109	1144.17	125.07
A_68_P28067421	chr11:100181597-100181641	NM_011508:309	Eif1	INSIDE	0.505	0.388	2398.67	929.51	0.196	1907.61	373.50
A_68_P28067422	chr11:100181743-100181787	NM_011508:455	Eif1	INSIDE	0.261	0.526	1833.83	964.85	0.137	1286.42	176.57
A_68_P31928290	chr19:5367444-5367500	NM_027236:660	Eif1ad	INSIDE	0.579	0.459	1233.97	566.61	0.266	1159.37	308.41
A_68_P26878190	chr9:120401831-120401875	NM_026892:129	Eif1b	INSIDE	0.590	0.643	2200.97	1414.61	0.379	1671.60	634.25
A_68_P26878194	chr9:120402272-120402316	NM_026892:571	Eif1b	INSIDE	0.340	0.643	1232.01	792.49	0.219	908.79	198.62
A_68_P24158346	chr5:144578888-144578932	NM_013557:250	Eif2ak1	INSIDE	0.380	0.493	3030.88	1494.45	0.187	2286.94	428.01
A_68_P28602892	chr12:86560540-86560584	NM_145445:100	Eif2b2	INSIDE	0.203	0.534	1407.50	751.37	0.108	1091.84	118.30
A_68_P28602893	chr12:86560681-86560733	NM_145445:244	Eif2b2	INSIDE	0.163	0.438	2476.38	1084.81	0.071	1621.67	115.92
A_68_P28602894	chr12:86560864-86560908	NM_145445:424	Eif2b2	INSIDE	0.178	0.661	1526.31	1008.15	0.118	1129.99	132.96
A_68_P23232034	chr4:116691797-116691854	NM_175135:-187	Eif2b3	PROMOTER	0.226	0.465	1277.46	593.48	0.105	1150.60	120.98
A_68_P23576981	chr5:31495306-31495353	NM_001127355:183	Eif2b4	INSIDE	0.076	0.575	3657.33	2104.26	0.044	2694.09	118.19
A_68_P30328924	chr15:73014625-73014669	NM_153178:731	Eif2c2	INSIDE	0.307	0.542	1536.65	832.16	0.166	1283.33	213.28
A_68_P30328931	chr15:73015939-73015989	NM_153178:-587	Eif2c2	PROMOTER	0.152	0.665	1581.51	1051.35	0.101	1152.23	116.70
A_68_P28566344	chr12:79963184-79963228	NM_026114:148	Eif2s1	INSIDE	0.409	0.487	1532.72	745.90	0.199	1213.84	241.38
A_68_P21762927	chr2:154718076-154718120	NM_026030:544	Eif2s2	INSIDE	0.422	0.494	1295.85	640.47	0.209	1049.91	219.18
A_68_P21762928	chr2:154718164-154718208	NM_026030:456	Eif2s2	INSIDE	0.482	0.521	3504.72	1826.80	0.251	2641.39	663.62
A_68_P21762930	chr2:154718365-154718409	NM_026030:256	Eif2s2	INSIDE	0.514	0.404	1515.20	611.42	0.208	1128.13	234.09
A_68_P21762934	chr2:154718701-154718745	NM_026030:-80	Eif2s2	PROMOTER	0.634	0.632	983.75	622.14	0.401	747.45	299.69
A_68_P32227250	chr19:60866724-60866768	NM_010123:-150	Eif3a	PROMOTER	0.154	0.701	1395.48	977.90	0.108	1126.20	121.40
A_68_P24141307	chr5:140894611-140894655	NM_133916:-626	Eif3b	PROMOTER	0.611	0.418	1644.66	687.41	0.256	1499.27	383.16
A_68_P24141318	chr5:140895750-140895794	NM_133916:514	Eif3b	INSIDE	0.308	0.711	1372.71	975.79	0.219	1101.52	241.33
A_68_P25501141	chr7:133709739-133709783	NM_146200:120	Eif3c	INSIDE	0.449	0.584	3014.82	1759.84	0.262	2498.26	654.79
A_68_P25501142	chr7:133709848-133709892	NM_146200:10	Eif3c	INSIDE	0.231	0.669	1853.08	1239.80	0.155	1422.48	219.90
A_68_P25501143	chr7:133709945-133709993	NM_146200:-88	Eif3c	PROMOTER	0.408	0.609	935.98	570.37	0.248	786.54	195.38
A_68_P30356903	chr15:77800770-77800814	NM_018749:462	Eif3d	INSIDE	0.214	0.401	1814.73	726.86	0.086	1402.05	120.45
A_68_P30356904	chr15:77800920-77800964	NM_018749:312	Eif3d	INSIDE	0.169	0.429	2066.94	886.70	0.073	1592.48	115.63
A_68_P30356906	chr15:77801158-77801202	NM_018749:74	Eif3d	INSIDE	0.118	0.574	2446.97	1403.66	0.068	1774.70	120.56
A_68_P30356910	chr15:77801656-77801700	NM_018749:-424	Eif3d	PROMOTER	0.262	0.709	1621.73	1149.04	0.186	1294.92	240.82
A_68_P26344425	chr9:20702979-20703023	NM_016876:34	Eif3g	INSIDE	0.325	0.497	2891.30	1437.01	0.162	2273.68	367.67
A_68_P30213285	chr15:51696567-51696616	NM_080635:416	Eif3h	INSIDE	0.105	1.622	1322.73	2145.54	0.170	941.63	160.24
A_68_P21584024	chr2:121854878-121854922	NM_144545:542	Eif3j	INSIDE	0.228	0.531	1199.32	636.98	0.121	976.93	118.55

ProbeName	Target position of probe on CpG island microarray	TargetID	GeneSymbol	CpG island Description	Ratio of relative methylation (TiO2-NP/Vehicle)	Sham group			TiO_2-H group		
						Relative methylation (Cy-5/Cy-3)	Cy-3 signal (Input)	Cy-5 signal (MeDIP)	Relative methylation (Cy-5/Cy-3)	Cy-3 signal (Input)	Cy-5 signal (MeDIP)
A_68_P27896442	chr11:69485330-69485374	NM_001159375:573	Eif4a1	INSIDE	0.255	0.633	1529.93	969.07	0.161	1242.39	200.41
A_68_P20416436	chr1:89110508-89110552	NM_001039169:17	Eif4e2	INSIDE	0.306	0.609	1179.37	718.03	0.187	960.07	179.11
A_68_P24687559	chr6:99616974-99617018	NM_025829:-231	Eif4e3	PROMOTER	0.393	0.659	1635.27	1077.16	0.259	1160.44	300.29
A_68_P25737431	chr8:28371155-28371199	NM_007918:378	Eif4ebp1	INSIDE	0.103	0.601	2545.81	1529.03	0.062	1846.76	114.75
A_68_P27180612	chr10:60915666-60915710	NM_010124:-271	Eif4ebp2	PROMOTER	0.300	2.127	3037.71	6460.83	0.637	2245.62	1431.38
A_68_P25416985	chr7:118225708-118225752	NM_001040131:814	Eif4g2	INSIDE	0.351	0.601	1018.69	611.78	0.211	908.92	191.53
A_68_P28744646	chr12:112776044-112776088	NM_173363:-245	Eif5	PROMOTER	0.269	0.663	2828.18	1875.34	0.178	2241.52	399.96
A_68_P27897957	chr11:69734148-69734192	NM_001166590:-38	Eif5a	PROMOTER	0.556	0.655	936.87	613.40	0.364	849.72	309.06
A_68_P27897961	chr11:69734599-69734643	NM_001166596:-33	Eif5a	PROMOTER	0.406	0.463	1940.76	898.54	0.188	1546.60	290.79
A_68_P20157022	chr1:38055055-38055099	NM_198303:222	Eif5b	INSIDE	0.628	0.707	1327.07	938.31	0.444	1144.07	508.22
A_68_P21767847	chr2:155651782-155651827	NM_010579:857	Eif6	INSIDE	0.130	1.558	884.45	1377.62	0.202	573.61	115.78
A_68_P21767850	chr2:155652193-155652237	NM_010579:447	Eif6	INSIDE	0.347	0.554	4497.99	2492.59	0.192	3414.99	656.39
A_68_P21767852	chr2:155652392-155652439	NM_010579:246	Eif6	INSIDE	0.180	0.578	2736.92	1581.08	0.104	1938.08	201.22
A_68_P23103758	chr4:91041293-91041337	NM_010486:-2568	Elavl2	PROMOTER	0.177	0.728	2369.28	1724.79	0.129	1762.39	227.00
A_68_P23103759	chr4:91041388-91041432	NM_010486:-2664	Elavl2	PROMOTER	0.575	0.693	2443.92	1693.04	0.398	2132.77	848.93
A_68_P23103963	chr4:91067208-91067252	NM_207685:-555	Elavl2	PROMOTER	0.080	0.639	3163.57	2021.53	0.051	2263.49	115.40
A_68_P26350219	chr9:21855638-21855682	NM_010487:807	Elavl3	INSIDE	0.297	0.704	4462.12	3140.26	0.209	3223.27	674.40
A_68_P22128828	chr3:51079948-51080002	NM_023502:49935	Elf2	INSIDE	0.212	0.543	1349.36	732.60	0.115	1059.90	121.80
A_68_P22128829	chr3:51080053-51080101	NM_023502:49833	Elf2	INSIDE	0.198	0.430	1871.37	804.06	0.085	1416.45	120.23
A_68_P22128830	chr3:51080151-51080198	NM_023502:49735	Elf2	INSIDE	0.222	0.707	1359.74	961.45	0.157	893.64	140.22
A_68_P22128837	chr3:51081149-51081193	NM_023502:48739	Elf2	INSIDE	0.142	0.545	2634.44	1434.62	0.078	2014.56	156.16
A_68_P32364768	chrX:45807315-45807359	NM_019680:8973	Elf4	INSIDE	0.155	1.674	3291.28	5510.12	0.260	1215.78	315.94
A_68_P24138249	chr5:140383826-140383870	NM_175522:-48	Elfn1	PROMOTER	0.020	1.782	5297.62	9442.53	0.036	3315.72	118.02
A_68_P24138250	chr5:140383933-140383977	NM_175522:58	Elfn1	INSIDE	0.042	1.584	2700.46	4277.88	0.066	1760.59	116.87
A_68_P24138252	chr5:140384071-140384115	NM_175522:196	Elfn1	INSIDE	0.338	0.583	1399.90	815.47	0.197	1168.09	230.30
A_68_P24138259	chr5:140384979-140385023	NM_175522:1104	Elfn1	INSIDE	0.529	0.635	1341.42	851.77	0.336	1017.60	341.66
A_68_P24138260	chr5:140385057-140385101	NM_175522:1182	Elfn1	INSIDE	0.138	0.567	1989.09	1128.13	0.078	1511.23	117.91
A_68_P24138293	chr5:140389088-140389132	NM_175522:5214	Elfn1	INSIDE	0.145	0.524	2335.48	1223.21	0.076	1655.78	126.15
A_68_P30361561	chr15:78503090-78503140	NM_183141:45429	Elfn2	INSIDE	0.306	1.438	1384.09	1990.61	0.441	1048.42	461.94
A_68_P30361874	chr15:78543529-78543573	NM_183141:4993	Elfn2	INSIDE	0.659	0.436	2396.17	1045.86	0.287	1940.54	557.78
A_68_P27352991	chr10:92773177-92773221	NM_013508:706	Elk3	INSIDE	0.239	0.659	956.91	630.54	0.157	775.26	121.84
A_68_P20618861	chr1:133904468-133904512	NM_007923:309	Elk4	INSIDE	0.602	0.632	2302.05	1453.89	0.380	1819.24	691.20
A_68_P20618864	chr1:133904839-133904887	NM_007923:681	Elk4	INSIDE	0.257	0.515	1770.28	911.89	0.132	1351.53	179.07
A_68_P25952515	chr8:73062683-73062729	NM_007924:-867	Ell	PROMOTER	0.274	1.486	2985.15	4436.35	0.407	1808.70	736.48
A_68_P25952518	chr8:73062911-73062958	NM_007924:-639	Ell	PROMOTER	0.230	0.477	1401.11	667.69	0.109	1136.48	124.39
A_68_P25952523	chr8:73063414-73063459	NM_007924:-137	Ell	PROMOTER	0.339	0.622	1705.78	1060.79	0.211	1280.19	269.65
A_68_P25952525	chr8:73063833-73063877	NM_007924:281	Ell	INSIDE	0.559	0.535	1118.59	598.08	0.299	918.35	274.41
A_68_P29150850	chr13:75845171-75845215	NM_138953:261	Ell2	INSIDE	0.611	0.560	2148.41	1202.57	0.342	1824.14	623.46
A_68_P29150854	chr13:75845732-75845776	NM_138953:823	Ell2	INSIDE	0.206	0.632	2950.51	1864.78	0.130	2282.07	297.40
A_68_P29150855	chr13:75845818-75845862	NM_138953:909	Ell2	INSIDE	0.284	0.551	3812.96	2100.90	0.157	2775.00	434.36
A_68_P28865537	chr13:20182539-20182583	NM_080288:185	Elmo1	INSIDE	0.160	0.428	3567.37	1526.30	0.068	2815.55	192.78
A_68_P28865540	chr13:20183015-20183059	NM_080288:661	Elmo1	INSIDE	0.323	0.557	1580.09	880.66	0.180	1222.61	220.38
A_68_P28865541	chr13:20183122-20183166	NM_080288:769	Elmo1	INSIDE	0.401	0.362	2120.48	767.66	0.145	1712.97	248.82
A_68_P21823221	chr2:165151623-165151667	NM_207705:-9251	Elmo2	PROMOTER	0.583	0.534	1692.06	904.29	0.311	1298.13	404.31

ProbeName	Target position of probe on CpG island microarray	TargetID	GeneSymbol	CpG island Description	Ratio of relative methylation (TiO2-NP/Vehicle)	Sham group			TiO_2-H group		
						Relative methylation (Cy-5/Cy-3)	Cy-3 signal (Input)	Cy-5 signal (MeDIP)	Relative methylation (Cy-5/Cy-3)	Cy-3 signal (Input)	Cy-5 signal (MeDIP)
A_68_P21823222	chr2:165151741-165151785	NM_207705:-9369	Elmo2	PROMOTER	0.561	0.563	2297.23	1293.88	0.316	1698.80	536.64
A_68_P26685697	chr9:83699258-83699303	NM_001145974:569	Elovl4	INSIDE	0.200	1.549	758.58	1174.68	0.310	595.79	184.95
A_68_P26657025	chr9:77765481-77765525	NM_134255:331	Elovl5	INSIDE	0.575	0.572	998.33	571.27	0.329	842.64	277.24
A_68_P26657026	chr9:77765571-77765615	NM_134255:421	Elovl5	INSIDE	0.232	0.545	2279.31	1242.80	0.127	1766.07	223.49
A_68_P22520449	chr3:129236571-129236615	NM_130450:1289	Elovl6	INSIDE	0.516	0.523	1307.01	683.74	0.270	1073.46	289.89
A_68_P22520456	chr3:129237236-129237280	NM_130450:1955	Elovl6	INSIDE	0.493	0.615	1443.86	888.58	0.303	1109.90	336.77
A_68_P29310181	chr13:109005203-109005247	NM_029001:627	Elovl7	INSIDE	0.194	0.542	1712.22	928.73	0.105	1137.36	119.41
A_68_P29310182	chr13:109005275-109005324	NM_029001:702	Elovl7	INSIDE	0.198	0.580	1308.98	758.75	0.115	1013.94	116.38
A_68_P24123511	chr5:137358417-137358461	NM_024474:539	Emid2	INSIDE	0.249	1.441	6966.42	10040.89	0.358	4715.86	1690.59
A_68_P31333824	chr17:71660558-71660602	NM_145158:-275	Emilin2	PROMOTER	0.511	0.673	2729.26	1836.49	0.344	2302.06	791.75
A_68_P28672817	chr12:100138890-100138934	NM_001081191:782	Eml5	INSIDE	0.156	0.608	2431.43	1478.25	0.095	1894.60	179.73
A_68_P27677356	chr11:29924368-29924417	NM_146016:1641	Eml6	INSIDE	0.102	2.039	835.83	1704.43	0.208	598.55	124.56
A_68_P27677359	chr11:29924618-29924662	NM_146016:1393	Eml6	INSIDE	0.499	0.668	1041.80	695.63	0.333	875.65	291.61
A_68_P27677361	chr11:29924802-29924846	NM_146016:1209	Eml6	INSIDE	0.542	0.668	1041.82	695.84	0.362	987.53	357.17
A_68_P27677368	chr11:29925763-29925807	NM_146016:249	Eml6	INSIDE	0.244	0.608	987.77	600.81	0.148	830.95	123.13
A_68_P30541580	chr16:10313819-10313863	NM_007929:221	Emp2	INSIDE	0.083	1.402	7452.33	10446.84	0.117	5020.15	586.06
A_68_P24603555	chr6:85137374-85137418	NM_010131:-528	Emx1	PROMOTER	0.057	0.637	4927.83	3140.04	0.036	3613.56	131.38
A_68_P24603562	chr6:85138183-85138227	NM_010131:280	Emx1	INSIDE	0.627	0.674	1104.60	744.62	0.423	943.13	398.48
A_68_P32219573	chr19:59534086-59534130	NM_010132:929	Emx2	INSIDE	0.487	0.383	1447.80	554.09	0.186	1176.86	219.13
A_68_P32219644	chr19:59541958-59542009	NM_010132:8804	Emx2	DOWNSTREAM	0.401	0.501	1502.12	752.11	0.201	1161.64	233.43
A_68_P32219658	chr19:59543743-59543787	NM_010132:10585	Emx2	DOWNSTREAM	0.518	0.571	989.70	564.72	0.295	803.68	237.39
A_68_P32219504	chr19:59526023-59526072	NR_002863:7078	Emx2os	INSIDE	0.289	0.588	883.92	519.35	0.170	746.75	126.83
A_68_P32219507	chr19:59526323-59526367	NR_002863:6781	Emx2os	INSIDE	0.222	0.539	1342.15	723.59	0.120	1009.85	120.74
A_68_P20562764	chr1:122500570-122500618	NM_010133:1531	En1	INSIDE	0.221	0.507	1330.92	674.70	0.112	1069.33	120.02
A_68_P23561108	chr5:28492702-28492746	NM_010134:489	En2	INSIDE	0.590	0.582	1171.21	681.86	0.344	1050.16	360.88
A_68_P22863377	chr4:41586983-41587027	NM_027147:331	Enho	INSIDE	0.318	0.518	2096.37	1085.56	0.165	1714.52	282.75
A_68_P23404989	chr4:149610705-149610749	NM_023119:-579	Eno1	PROMOTER	0.253	0.699	4196.06	2931.02	0.177	3037.00	536.56
A_68_P23404992	chr4:149611119-149611164	NM_023119:-164	Eno1	PROMOTER	0.380	0.563	1922.55	1081.72	0.214	1383.68	295.56
A_68_P24816932	chr6:124719430-124719474	NM_013509:75	Eno2	INSIDE	0.621	0.617	3958.17	2442.02	0.383	2813.88	1078.75
A_68_P27901910	chr11:70469767-70469811	NM_001136062:-926	Eno3	DIVERGENT_PROMOTER	0.100	0.582	3006.37	1749.57	0.058	2087.66	121.08
A_68_P29737944	chr14:77557089-77557133	NM_172813:488	Enox1	INSIDE	0.096	0.619	2862.54	1772.12	0.060	2050.29	122.15
A_68_P27010235	chr10:24431971-24432016	NM_008813:-85	Enpp1	PROMOTER	0.211	1.551	4773.09	7404.26	0.328	3283.83	1077.11
A_68_P28175184	chr11:118860828-118860872	NM_001030291:11349	Enpp7	DOWNSTREAM	0.218	0.673	1798.49	1209.95	0.147	1236.34	181.69
A_68_P28175187	chr11:118861087-118861131	NM_001030291:11607	Enpp7	DOWNSTREAM	0.557	0.558	1312.41	732.47	0.311	959.99	298.16
A_68_P21071852	chr2:25251736-25251780	NM_009849:365	Entpd2	INSIDE	0.657	0.679	1003.25	681.27	0.446	736.79	328.75
A_68_P21740704	chr2:150574793-150574837	NM_172117:-2	Entpd6	PROMOTER	0.183	0.432	1946.95	841.04	0.079	1573.74	124.63
A_68_P32128057	chr19:43764144-43764188	NM_053103:-12	Entpd7	PROMOTER	0.220	0.437	1708.33	746.86	0.096	1331.71	128.00
A_68_P30180493	chr15:44259134-44259178	NM_175009:-500	Eny2	PROMOTER	0.453	0.422	1473.45	621.13	0.191	1181.77	225.44
A_68_P30378498	chr15:81415923-81415967	NM_177821:-699	Ep300	PROMOTER	0.446	0.582	2195.96	1278.50	0.260	1852.88	481.32
A_68_P30378500	chr15:81416070-81416114	NM_177821:-551	Ep300	PROMOTER	0.528	0.676	1310.47	885.42	0.357	1049.19	374.23
A_68_P30378506	chr15:81416769-81416813	NM_177821:147	Ep300	INSIDE	0.237	0.499	2553.78	1274.79	0.118	1810.22	214.05
A_68_P23981800	chr5:111199966-111200010	NM_029337:-252	Ep400	PROMOTER	0.272	0.306	1779.38	543.62	0.083	1446.64	120.14
A_68_P31418980	chr17:87152677-87152727	NM_010137:-501	Epas1	PROMOTER	0.233	0.437	1621.88	708.25	0.102	1324.91	134.92
A_68_P31418994	chr17:87154526-87154570	NM_010137:1345	Epas1	INSIDE	0.551	0.645	1176.05	758.34	0.356	976.78	347.35

ProbeName	Target position of probe on CpG island microarray	TargetID	GeneSymbol	CpG island Description	Ratio of relative methylation (TiO2-NP/Vehicle)	Sham group			TiO_2-H group		
						Relative methylation (Cy-5/Cy-3)	Cy-3 signal (Input)	Cy-5 signal (MeDIP)	Relative methylation (Cy-5/Cy-3)	Cy-3 signal (Input)	Cy-5 signal (MeDIP)
A_68_P31418995	chr17:87154659-87154703	NM_010137:1477	Epas1	INSIDE	0.462	0.551	1253.17	690.84	0.255	983.82	250.56
A_68_P21771129	chr2:156247015-156247059	NM_001003815:249	Epb4.1l1	INSIDE	0.239	0.591	3749.62	2215.37	0.141	2627.74	370.51
A_68_P21771130	chr2:156247152-156247196	NM_001003815:387	Epb4.1l1	INSIDE	0.143	1.853	2136.61	3959.67	0.264	1540.17	407.01
A_68_P31610604	chr18:34166101-34166145	NM_013512:738	Epb4.1l4a	INSIDE	0.428	0.570	2976.61	1696.24	0.244	2147.76	523.49
A_68_P31610611	chr18:34166774-34166818	NM_013512:64	Epb4.1l4a	INSIDE	0.152	0.690	1760.82	1215.32	0.105	1416.16	148.78
A_68_P31610612	chr18:34166849-34166893	NM_013512:-10	Epb4.1l4a	PROMOTER	0.568	0.602	1215.32	731.69	0.342	930.00	317.78
A_68_P22943680	chr4:57155729-57155773	NM_019427:278	Epb4.1l4b	INSIDE	0.276	0.484	1090.05	528.11	0.134	905.94	121.35
A_68_P20558185	chr1:121545078-121545122	NM_145506:477	Epb4.1l5	INSIDE	0.591	0.534	1283.66	686.03	0.316	1047.29	330.86
A_68_P20558188	chr1:121545438-121545482	NM_145506:117	Epb4.1l5	INSIDE	0.225	0.421	2282.47	960.48	0.095	1827.99	173.00
A_68_P29702477	chr14:71018142-71018186	NM_013514:11797	Epb4.9	INSIDE	0.353	0.619	1580.18	978.14	0.219	1214.78	265.80
A_68_P29702478	chr14:71018216-71018260	NM_013514:11723	Epb4.9	INSIDE	0.217	0.546	1416.88	774.30	0.118	1084.92	128.50
A_68_P31473763	chr18:6516415-6516459	NM_007935:-351	Epc1	PROMOTER	0.119	0.396	3517.30	1394.07	0.047	2585.96	122.12
A_68_P21202304	chr2:49307386-49307430	NM_172663:403	Epc2	INSIDE	0.396	0.453	2466.98	1117.64	0.179	1901.54	341.08
A_68_P28863232	chr13:19711147-19711191	NM_134065:531	Epdr1	INSIDE	0.324	0.475	1194.76	568.01	0.154	934.33	143.87
A_68_P23270838	chr4:124558750-124558797	NM_177671:-255	Epha10	PROMOTER	0.236	0.601	981.03	589.78	0.142	821.39	116.53
A_68_P23270847	chr4:124559714-124559758	NM_177671:708	Epha10	INSIDE	0.388	0.609	2152.03	1311.03	0.237	1656.04	391.91
A_68_P23270909	chr4:124571706-124571750	NM_177671:12700	Epha10	DOWNSTREAM	0.285	0.701	1547.42	1084.80	0.200	1080.50	215.98
A_68_P23270915	chr4:124572399-124572443	NM_177671:13392	Epha10	DOWNSTREAM	0.203	0.637	3074.52	1957.06	0.129	2382.37	307.28
A_68_P23363149	chr4:140847557-140847601	NM_010139:-9576	Epha2	PROMOTER	0.449	0.512	2369.62	1212.75	0.230	2188.99	503.54
A_68_P23363150	chr4:140847720-140847764	NM_010139:-9412	Epha2	PROMOTER	0.443	0.548	1558.73	853.96	0.243	1193.32	289.60
A_68_P23363213	chr4:140857120-140857165	NM_010139:-12	Epha2	PROMOTER	0.104	1.529	967.41	1478.91	0.159	724.37	115.36
A_68_P20366321	chr1:77511149-77511206	NM_007936:486	Epha4	INSIDE	0.298	0.640	861.99	552.09	0.191	639.72	122.06
A_68_P20366327	chr1:77511720-77511764	NM_007936:-79	Epha4	PROMOTER	0.425	0.615	5264.01	3234.77	0.261	3695.72	966.32
A_68_P30801798	chr16:60605990-60606034	NM_007938:-655	Epha6	PROMOTER	0.511	0.467	1476.39	689.57	0.239	1364.89	325.79
A_68_P22803175	chr4:28740439-28740495	NM_001122889:172	Epha7	INSIDE	0.151	0.657	2030.47	1333.44	0.099	1663.44	164.59
A_68_P22803176	chr4:28740507-28740557	NM_001122889:238	Epha7	INSIDE	0.245	0.609	1487.17	905.80	0.149	1091.94	162.87
A_68_P22803177	chr4:28740631-28740686	NM_001122889:364	Epha7	INSIDE	0.225	0.622	1204.78	749.81	0.140	860.74	120.32
A_68_P22803188	chr4:28741959-28742006	NM_001122889:1688	Epha7	INSIDE	0.610	0.475	1383.03	657.36	0.290	1197.30	347.26
A_68_P22803192	chr4:28742495-28742539	NM_001122889:2222	Epha7	INSIDE	0.280	0.395	2846.75	1124.65	0.110	2230.06	246.37
A_68_P26779719	chr9:102255984-102256028	NM_001168296:1017	Ephb1	INSIDE	0.231	0.492	1373.43	676.13	0.114	1048.05	119.08
A_68_P26779727	chr9:102256967-102257011	NM_001168296:35	Ephb1	INSIDE	0.177	0.607	4893.64	2968.88	0.107	3316.01	355.60
A_68_P23336240	chr4:136390937-136390981	NM_010142:892	Ephb2	INSIDE	0.572	0.459	1492.56	684.86	0.262	1120.00	293.71
A_68_P23336251	chr4:136392310-136392354	NM_010142:-482	Ephb2	PROMOTER	0.532	0.641	1804.03	1156.03	0.341	1498.37	510.35
A_68_P23335796	chr4:136327079-136327123	NM_010142:64750	Ephb2	INSIDE	0.669	2.233	1845.17	4120.32	1.493	1478.72	2207.49
A_68_P24125310	chr5:137791448-137791492	NM_001159571:134	Ephb4	INSIDE	0.343	0.541	3935.14	2126.96	0.186	3267.51	606.59
A_68_P24125311	chr5:137791525-137791569	NM_001159571:210	Ephb4	INSIDE	0.601	0.588	4521.60	2657.53	0.353	3480.29	1229.09
A_68_P24125355	chr5:137799146-137799190	NM_001159571:7832	Ephb4	INSIDE	0.655	2.823	615.22	1736.53	1.848	566.27	1046.42
A_68_P31144187	chr17:32326072-32326116	NM_001033163:314	Ephx3	INSIDE	0.455	0.659	856.19	564.20	0.300	710.80	212.91
A_68_P26940377	chr10:11063537-11063581	NM_010146:316	Epm2a	INSIDE	0.574	0.541	1361.70	736.85	0.311	1256.73	390.51
A_68_P24952006	chr7:5032174-5032218	NM_010147:290	Epn1	INSIDE	0.618	0.674	1203.37	811.37	0.417	1007.89	419.94
A_68_P28033281	chr11:94352708-94352752	NM_027984:8558	Epn3	INSIDE	0.418	0.713	1812.06	1292.17	0.298	1494.42	445.05
A_68_P20900748	chr1:187186729-187186773	NM_029735:-223	Eprs	PROMOTER	0.226	0.542	1199.24	649.65	0.122	981.95	120.28
A_68_P27996720	chr11:87684836-87684880	NM_007946:4180	Epx	INSIDE	0.602	2.771	723.69	2005.40	1.669	593.25	990.38
A_68_P28056287	chr11:98273671-98273715	NM_001003817:-105	Erbb2	PROMOTER	0.314	0.709	1513.44	1072.42	0.223	1206.13	268.51

ProbeName	Target position of probe on CpG island microarray	TargetID	GeneSymbol	CpG island Description	Ratio of relative methylation (TiO2-NP/Vehicle)	Sham group			TiO_2-H group		
						Relative methylation (Cy-5/Cy-3)	Cy-3 signal (Input)	Cy-5 signal (MeDIP)	Relative methylation (Cy-5/Cy-3)	Cy-3 signal (Input)	Cy-5 signal (MeDIP)
A_68_P28056288	chr11:98273791-98273835	NM_001003817:15	Erbb2	INSIDE	0.152	0.634	4465.48	2830.37	0.096	3259.86	313.39
A_68_P29287622	chr13:104710157-104710201	NM_001005868:416	Erbb2ip	INSIDE	0.447	0.588	906.33	532.94	0.263	751.27	197.52
A_68_P20317723	chr1:69154010-69154069	NM_010154:594	Erbb4	INSIDE	0.338	1.371	1345.01	1844.25	0.463	1261.90	584.09
A_68_P20317730	chr1:69154592-69154636	NM_010154:19	Erbb4	INSIDE	0.392	0.422	1426.54	601.74	0.165	1216.30	201.21
A_68_P29488027	chr14:28436033-28436077	NM_177814:427	Erc2	INSIDE	0.319	1.875	4506.26	8451.34	0.599	2846.84	1705.57
A_68_P24992779	chr7:19930361-19930405	NM_001127324:-37	Ercc1	PROMOTER	0.166	0.644	1912.46	1231.67	0.107	1333.04	142.09
A_68_P24992782	chr7:19930618-19930662	NM_001127324:221	Ercc1	INSIDE	0.132	0.641	2054.47	1316.54	0.085	1416.51	119.88
A_68_P29518106	chr14:33327086-33327130	NM_001081221:402	Ercc6	INSIDE	0.231	0.598	2380.00	1422.77	0.138	1768.56	244.24
A_68_P29309821	chr13:108948883-108948927	NM_028042:-27	Ercc8	DIVERGENT_PROMOTER	0.206	0.464	2242.55	1041.49	0.095	1640.73	156.61
A_68_P29309824	chr13:108949176-108949220	NM_028042:267	Ercc8	INSIDE	0.542	0.546	937.84	512.42	0.296	779.43	230.99
A_68_P31111041	chr17:26698638-26698682	NM_026170:204	Ergic1	INSIDE	0.239	0.443	1959.41	868.94	0.106	1422.68	150.48
A_68_P21768928	chr2:155834064-155834108	NM_025516:226	Ergic3	INSIDE	0.516	0.475	2318.92	1100.41	0.245	1700.86	416.77
A_68_P28576810	chr12:81744684-81744728	NM_007951:142	Erh	INSIDE	0.479	0.739	3291.02	2433.20	0.354	2654.68	939.45
A_68_P25672217	chr8:14090101-14090145	NM_001034862:205	Erich1	INSIDE	0.358	0.615	2309.18	1420.87	0.220	1697.74	374.12
A_68_P32130261	chr19:44144105-44144149	NM_145502:50	Erlin1	INSIDE	0.575	0.546	1589.93	868.54	0.314	1403.40	440.59
A_68_P32053973	chr19:29700840-29700884	NM_001081213:22048	Ermp1	INSIDE	0.638	3.951	5484.76	21669.41	2.521	4005.10	10097.35
A_68_P28101900	chr11:106349136-106349180	NM_023913:-48	Ern1	PROMOTER	0.421	0.508	2320.57	1179.85	0.214	1863.93	398.54
A_68_P30497082	chr15:102126996-102127044	NM_001014976:297	Espl1	INSIDE	0.172	0.619	1374.00	850.32	0.106	1125.71	119.88
A_68_P23416686	chr4:151500208-151500252	NM_019585:2804	Espn	INSIDE	0.246	0.673	2026.10	1363.71	0.166	1552.55	257.45
A_68_P23416730	chr4:151506015-151506059	NM_019585:-3002	Espn	PROMOTER	0.446	0.720	3184.73	2291.50	0.321	2404.06	771.46
A_68_P23416731	chr4:151506130-151506174	NM_019585:-3118	Espn	PROMOTER	0.261	0.742	3064.47	2273.83	0.193	2069.66	400.14
A_68_P22729550	chr4:11314133-11314177	NM_194055:-224	Esrp1	PROMOTER	0.414	0.637	3420.97	2180.31	0.264	2777.70	733.15
A_68_P22729554	chr4:11314649-11314704	NM_194055:-746	Esrp1	PROMOTER	0.265	0.627	1378.65	864.11	0.166	1081.67	179.97
A_68_P26137945	chr8:108660566-108660610	NM_176838:286	Esrp2	INSIDE	0.284	0.703	2587.98	1818.11	0.200	1926.67	384.79
A_68_P26137946	chr8:108660718-108660762	NM_176838:134	Esrp2	INSIDE	0.410	0.672	6302.10	4233.59	0.275	4092.88	1126.41
A_68_P31936680	chr19:6996479-6996523	NM_007953:-202	Esrra	PROMOTER	0.576	0.541	1686.14	911.97	0.312	1451.94	452.29
A_68_P28609997	chr12:87763078-87763133	NM_011934:512	Esrrb	INSIDE	0.232	0.539	1254.47	675.77	0.125	972.41	121.42
A_68_P28610001	chr12:87763573-87763617	NM_011934:1001	Esrrb	INSIDE	0.127	0.636	3568.00	2268.71	0.081	2543.10	205.08
A_68_P28610002	chr12:87763647-87763691	NM_011934:1075	Esrrb	INSIDE	0.363	0.588	2445.88	1437.16	0.213	1696.19	362.02
A_68_P32684275	chrX:133652900-133652944	NM_007957:1943	Esx1	INSIDE	0.182	1.491	2353.02	3509.45	0.272	820.08	223.06
A_68_P27541863	chr10:127962197-127962241	NM_011843:697	Esyt1	INSIDE	0.095	0.484	3415.01	1653.85	0.046	2501.62	115.69
A_68_P27541866	chr10:127962475-127962519	NM_011843:419	Esyt1	INSIDE	0.123	1.410	999.92	1409.50	0.174	685.70	119.16
A_68_P27541867	chr10:127962642-127962686	NM_011843:251	Esyt1	INSIDE	0.559	0.577	1870.60	1080.15	0.323	1422.16	459.21
A_68_P27618338	chr11:17853460-17853504	NM_026576:396	Etaa1	INSIDE	0.131	0.309	3631.53	1123.96	0.041	3010.45	122.49
A_68_P31615538	chr18:35091084-35091128	NM_144866:551	Etf1	INSIDE	0.510	0.632	2002.13	1264.58	0.322	1498.30	482.38
A_68_P31615540	chr18:35091234-35091284	NM_144866:399	Etf1	INSIDE	0.150	0.657	1663.12	1092.97	0.098	1185.57	116.58
A_68_P31615543	chr18:35091590-35091634	NM_144866:45	Etf1	INSIDE	0.246	0.471	1215.83	572.78	0.116	1023.61	118.81
A_68_P31615546	chr18:35092064-35092109	NM_144866:-429	Etf1	PROMOTER	0.150	0.482	2393.05	1153.68	0.072	1700.61	122.64
A_68_P26531721	chr9:55359505-55359549	NM_145615:524	Etfa	INSIDE	0.214	0.669	924.00	618.42	0.144	808.91	116.11
A_68_P26531723	chr9:55359728-55359772	NM_145615:300	Etfa	INSIDE	0.548	0.568	1555.05	882.77	0.311	1334.06	414.85
A_68_P25082380	chr7:50699539-50699583	NM_026695:48	Etfb	INSIDE	0.129	0.584	2169.19	1267.53	0.075	1600.96	120.22
A_68_P25082496	chr7:50720344-50720388	NM_026695:20854	Etfb	DOWNSTREAM	0.366	0.528	3554.86	1878.03	0.193	2621.67	506.75
A_68_P30988901	chr16:95923763-95923807	NM_011809:-229	Ets2	PROMOTER	0.455	0.704	1608.77	1131.97	0.320	1315.41	421.38
A_68_P30988904	chr16:95924063-95924107	NM_011809:71	Ets2	INSIDE	0.337	0.740	3358.88	2485.54	0.250	2387.72	596.09

ProbeName	Target position of probe on CpG island microarray	TargetID	GeneSymbol	CpG island Description	Ratio of relative methylation (TiO2-NP/Vehicle)	Sham group			TiO_2-H group		
						Relative methylation (Cy-5/Cy-3)	Cy-3 signal (Input)	Cy-5 signal (MeDIP)	Relative methylation (Cy-5/Cy-3)	Cy-3 signal (Input)	Cy-5 signal (MeDIP)
A_68_P22308548	chr3:87329844-87329888	NM_001083318:367	Etv3	INSIDE	0.504	0.497	1438.35	715.39	0.251	1087.17	272.68
A_68_P28075719	chr11:101646593-101646637	NM_008815:10	Etv4	INSIDE	0.264	0.387	1398.33	541.09	0.102	1162.02	118.63
A_68_P24859168	chr6:133985011-133985055	NM_007961:-692	Etv6	PROMOTER	0.339	0.684	2321.36	1588.07	0.232	1712.62	397.63
A_68_P23613114	chr5:37728265-37728310	NM_021292:-167	Evc	DIVERGENT_PROMOTER	0.177	0.579	1583.03	916.19	0.102	1174.62	120.25
A_68_P23969234	chr5:108298178-108298222	NM_007964:5926	Evi5	INSIDE	0.644	0.636	1399.66	890.76	0.410	1169.91	479.42
A_68_P25616318	chr8:4166571-4166615	NM_001039578:26	Evi5l	INSIDE	0.569	0.727	3763.31	2734.40	0.413	3072.69	1269.57
A_68_P25616319	chr8:4166672-4166716	NM_001039578:128	Evi5l	INSIDE	0.608	0.490	2932.47	1437.51	0.298	2388.07	711.77
A_68_P28158191	chr11:116099429-116099473	NM_025276:-45	Evpl	PROMOTER	0.390	0.689	1292.37	890.62	0.269	1148.19	308.81
A_68_P24449832	chr6:52260556-52260605	NM_007966:-2911	Evx1	PROMOTER	0.192	0.640	1276.58	816.42	0.123	967.19	118.81
A_68_P24449834	chr6:52260814-52260866	NM_007966:-2651	Evx1	PROMOTER	0.282	0.540	1031.45	557.14	0.153	762.12	116.28
A_68_P24449858	chr6:52263643-52263687	NM_007966:173	Evx1	INSIDE	0.651	0.440	1586.70	698.08	0.286	1311.23	375.36
A_68_P21338549	chr2:74494232-74494276	NM_007967:3222	Evx2	INSIDE	0.535	0.346	2116.62	731.62	0.185	1745.05	322.58
A_68_P21570230	chr2:119372732-119372776	NM_172857:609	Exd1	INSIDE	0.635	0.549	1903.16	1044.28	0.349	1615.08	563.12
A_68_P21570231	chr2:119372809-119372853	NM_172857:533	Exd1	INSIDE	0.525	0.591	1897.79	1122.21	0.310	1620.11	502.83
A_68_P28575796	chr12:81564448-81564492	NM_133798:389	Exd2	INSIDE	0.313	0.562	1070.64	601.76	0.176	932.09	164.02
A_68_P29144944	chr13:74346071-74346115	NM_177333:56	Exoc3	INSIDE	0.475	0.623	3066.97	1910.91	0.296	2497.47	739.39
A_68_P26132766	chr8:107813805-107813857	NM_177788:6168	Exoc3l	INSIDE	0.188	0.666	1471.32	979.44	0.125	948.32	118.81
A_68_P26132768	chr8:107814026-107814070	NM_177788:5950	Exoc3l	INSIDE	0.283	0.585	3362.80	1968.90	0.166	2244.27	371.46
A_68_P24602877	chr6:85019040-85019084	NM_177077:445	Exoc6b	INSIDE	0.468	0.490	1377.13	674.50	0.229	1065.92	244.36
A_68_P26248320	chr8:127420438-127420486	NM_198103:1143	Exoc8	INSIDE	0.290	1.552	928.99	1442.11	0.450	780.54	351.57
A_68_P26248321	chr8:127420511-127420555	NM_198103:1073	Exoc8	INSIDE	0.358	1.456	1115.06	1623.27	0.521	904.14	471.20
A_68_P32117991	chr19:42007492-42007537	NM_001164561:290	Exosc1	INSIDE	0.213	0.591	3443.25	2036.05	0.126	2579.87	324.38
A_68_P32117993	chr19:42007635-42007679	NM_001164561:148	Exosc1	INSIDE	0.455	0.493	1540.43	759.64	0.225	1328.72	298.39
A_68_P23395155	chr4:147932643-147932687	NM_016699:129	Exosc10	INSIDE	0.485	0.586	886.56	519.22	0.284	766.71	217.58
A_68_P25011168	chr7:26444266-26444310	NM_138586:117	Exosc5	INSIDE	0.290	0.569	3894.44	2214.29	0.165	2797.00	460.86
A_68_P26893205	chr9:123022399-123022443	NM_001081188:72	Exosc7	INSIDE	0.634	0.444	1766.43	783.61	0.281	1468.20	413.17
A_68_P26893206	chr9:123022552-123022596	NM_001081188:226	Exosc7	INSIDE	0.460	0.574	1057.47	606.69	0.264	795.13	209.92
A_68_P26893207	chr9:123022692-123022739	NM_001081188:367	Exosc7	INSIDE	0.114	2.117	1044.95	2211.92	0.242	763.78	184.76
A_68_P22149452	chr3:54538552-54538602	NM_001163570:710	Exosc8	INSIDE	0.447	0.611	1174.63	717.56	0.273	917.47	250.67
A_68_P22149457	chr3:54539042-54539086	NM_001163570:222	Exosc8	INSIDE	0.460	0.630	1649.62	1039.92	0.290	1283.20	372.50
A_68_P22149458	chr3:54539187-54539231	NM_001163570:78	Exosc8	INSIDE	0.494	0.560	2287.99	1281.08	0.276	1736.72	479.98
A_68_P22149459	chr3:54539268-54539312	NM_001163570:-4	Exosc8	DIVERGENT_PROMOTER	0.367	0.619	1633.62	1012.02	0.227	1454.55	330.87
A_68_P30221114	chr15:53176756-53176800	NM_010162:960	Ext1	INSIDE	0.243	0.723	3801.24	2749.66	0.176	2835.52	499.15
A_68_P30221120	chr15:53177342-53177386	NM_010162:374	Ext1	INSIDE	0.078	0.582	3731.61	2170.97	0.045	2615.44	118.71
A_68_P29671494	chr14:65696542-65696586	NM_018788:20379	Extl3	INSIDE	0.568	2.377	2904.76	6904.49	1.350	2132.45	2879.37
A_68_P29671617	chr14:65716487-65716531	NM_018788:435	Extl3	INSIDE	0.609	0.447	1700.67	759.39	0.272	1422.87	386.99
A_68_P28072730	chr11:101087587-101087631	NM_007970:156	Ezh1	INSIDE	0.439	0.537	2728.69	1465.05	0.235	2056.54	484.28
A_68_P24423931	chr6:47544220-47544264	NM_001146689:787	Ezh2	INSIDE	0.284	0.462	1665.91	770.07	0.131	1313.21	172.63
A_68_P24423937	chr6:47544969-47545013	NM_001146689:39	Ezh2	INSIDE	0.638	0.644	1090.33	701.77	0.410	976.73	400.80
A_68_P31022580	chr17:6986963-6987007	NM_009510:145	Ezr	INSIDE	0.490	0.450	1493.85	671.80	0.220	1262.16	277.97
A_68_P20827965	chr1:173367690-173367734	NM_172647:21	F11r	INSIDE	0.230	0.607	1412.46	858.03	0.139	1210.79	168.87
A_68_P20827968	chr1:173368006-173368050	NM_172647:337	F11r	INSIDE	0.464	0.415	2052.28	852.34	0.193	1636.65	315.13
A_68_P29241614	chr13:96388371-96388415	NM_010169:-4	F2r	PROMOTER	0.344	0.540	2019.99	1091.38	0.186	1478.25	275.00
A_68_P27289145	chr10:80826688-80826732	NM_028657:6396	F630110N24Rik	INSIDE	0.346	0.566	913.40	517.16	0.196	674.88	132.15

ProbeName	Target position of probe on CpG island microarray	TargetID	GeneSymbol	CpG island Description	Ratio of relative methylation (TiO2-NP/Vehicle)	Sham group			TiO_2-H group		
						Relative methylation (Cy-5/Cy-3)	Cy-3 signal (Input)	Cy-5 signal (MeDIP)	Relative methylation (Cy-5/Cy-3)	Cy-3 signal (Input)	Cy-5 signal (MeDIP)
A_68_P28340239	chr12:33832233-33832277	NR_015602:197	F730043M19Rik	INSIDE	0.239	0.626	1297.81	812.88	0.150	996.94	149.17
A_68_P32461568	chrX:70473551-70473595	NM_007978:-72	F8a	PROMOTER	0.319	1.492	1712.51	2555.71	0.476	617.47	293.75
A_68_P32461576	chrX:70474445-70474489	NM_007978:822	F8a	INSIDE	0.139	2.026	1120.02	2269.46	0.282	438.88	123.64
A_68_P27865450	chr11:64250200-64250244	NR_028445:-46	F930015N05Rik	PROMOTER	0.349	0.551	1324.97	729.95	0.192	1103.99	212.10
A_68_P26168090	chr8:113917279-113917323	NM_178086:421	Fa2h	INSIDE	0.420	0.691	1285.10	888.34	0.290	1056.43	306.48
A_68_P23227292	chr4:115689902-115689946	NM_010173:583	Faah	INSIDE	0.477	0.496	1430.53	709.23	0.236	1154.19	272.86
A_68_P23227295	chr4:115690207-115690251	NM_010173:279	Faah	INSIDE	0.525	0.549	2199.10	1207.29	0.288	2005.15	577.49
A_68_P23301437	chr4:129985225-129985269	NM_010174:-775	Fabp3	PROMOTER	0.312	0.537	3055.68	1641.66	0.168	2365.91	397.16
A_68_P23301440	chr4:129985621-129985680	NM_010174:-371	Fabp3	PROMOTER	0.022	2.767	2315.19	6404.98	0.062	1915.62	118.89
A_68_P23301441	chr4:129985717-129985761	NM_010174:-283	Fabp3	PROMOTER	0.143	2.006	3897.41	7818.90	0.286	2360.07	675.17
A_68_P21940380	chr3:10012548-10012592	NM_010634:-35	Fabp5	PROMOTER	0.197	0.731	3055.98	2235.36	0.144	2143.35	308.92
A_68_P31948839	chr19:10257741-10257785	NM_146094:385	Fads1	INSIDE	0.169	0.606	2386.06	1446.42	0.103	1881.63	192.95
A_68_P31948840	chr19:10257815-10257859	NM_146094:459	Fads1	INSIDE	0.197	0.627	2789.64	1750.15	0.124	2230.46	275.56
A_68_P31948448	chr19:10175771-10175815	NM_019699:201	Fads2	INSIDE	0.665	0.622	1068.35	664.43	0.414	824.89	341.19
A_68_P31948451	chr19:10176193-10176237	NM_019699:-221	Fads2	PROMOTER	0.346	0.488	1341.38	654.93	0.169	1059.29	178.79
A_68_P31948452	chr19:10176316-10176360	NM_019699:-345	Fads2	PROMOTER	0.409	0.572	1284.29	734.28	0.234	1045.44	244.33
A_68_P31948068	chr19:10116519-10116563	NM_021890:503	Fads3	INSIDE	0.178	0.600	1581.44	948.93	0.107	1133.36	120.95
A_68_P28152627	chr11:115158906-115158950	NM_178035:-68	Fads6	DIVERGENT_PROMOTER	0.529	0.592	1506.01	892.06	0.313	1283.10	402.22
A_68_P23202061	chr4:109349433-109349477	NM_007983:223	Faf1	INSIDE	0.426	0.363	1627.53	590.92	0.155	1325.49	204.86
A_68_P23202063	chr4:109349653-109349697	NM_007983:443	Faf1	INSIDE	0.294	0.447	2022.94	904.09	0.131	1611.81	211.86
A_68_P29050431	chr13:54723325-54723369	NM_178397:202	Faf2	INSIDE	0.200	0.592	1357.03	803.83	0.118	992.95	117.46
A_68_P25289617	chr7:91754287-91754332	NM_010176:143	Fah	INSIDE	0.038	1.364	3888.45	5304.05	0.052	2397.88	124.31
A_68_P31099851	chr17:24986997-24987041	NM_023480:229	Fahd1	INSIDE	0.273	0.608	945.82	575.36	0.166	731.33	121.44
A_68_P26761637	chr9:98886525-98886573	NM_001122851:-243	Faim	PROMOTER	0.300	0.342	1574.82	538.81	0.103	1150.49	118.07
A_68_P26761639	chr9:98886751-98886795	NM_001122851:-19	Faim	PROMOTER	0.351	0.484	3000.49	1451.66	0.170	2317.14	393.58
A_68_P26761641	chr9:98887034-98887078	NM_001122851:265	Faim	INSIDE	0.442	0.599	2072.05	1241.28	0.265	1863.64	493.75
A_68_P20612315	chr1:132774907-132774951	NM_026976:12575	Faim3	INSIDE	0.550	0.439	1576.37	691.78	0.241	1307.67	315.70
A_68_P20612316	chr1:132774985-132775029	NM_026976:12653	Faim3	INSIDE	0.509	0.366	1452.22	531.65	0.186	1143.31	213.05
A_68_P30512413	chr16:4879317-4879361	NM_145359:513	Fam100a	INSIDE	0.648	0.533	1151.90	614.17	0.346	819.29	283.16
A_68_P30512427	chr16:4880947-4880994	NM_145359:-1119	Fam100a	DIVERGENT_PROMOTER	0.341	0.480	1328.98	638.19	0.164	886.08	144.97
A_68_P21115862	chr2:32390731-32390776	NM_153560:-125	Fam102a	PROMOTER	0.493	0.363	1535.39	557.81	0.179	1201.38	215.36
A_68_P25273836	chr7:88907300-88907346	NM_025997:-516	Fam103a1	PROMOTER	0.447	0.581	1321.11	767.84	0.260	919.68	239.14
A_68_P25273840	chr7:88907783-88907827	NM_025997:-34	Fam103a1	PROMOTER	0.436	0.587	1312.42	770.10	0.256	1072.47	274.12
A_68_P25273842	chr7:88907926-88907970	NM_025997:110	Fam103a1	INSIDE	0.214	0.506	1396.06	705.75	0.108	1135.26	122.96
A_68_P25273844	chr7:88908139-88908183	NM_025997:322	Fam103a1	INSIDE	0.511	0.550	982.21	539.94	0.281	705.12	198.12
A_68_P30093915	chr15:27610984-27611028	NM_198301:247	Fam105a	INSIDE	0.651	0.684	2057.35	1406.39	0.445	1708.53	760.09
A_68_P21746892	chr2:151796494-151796538	NM_028666:3194	Fam110a	INSIDE	0.260	1.403	954.40	1339.09	0.364	679.62	247.63
A_68_P22702206	chr4:5571678-5571722	NM_173426:375	Fam110b	INSIDE	0.387	0.654	1996.63	1304.95	0.253	1615.37	408.64
A_68_P21629724	chr2:130250001-130250045	NM_178762:2	Fam113a	INSIDE	0.215	0.452	1530.22	692.37	0.097	1217.98	118.45
A_68_P21629726	chr2:130250214-130250258	NM_001114541:141	Fam113a	INSIDE	0.471	0.515	1386.82	714.78	0.243	1080.77	262.17
A_68_P27826023	chr11:57331958-57332002	NM_001168667:166	Fam114a2	INSIDE	0.267	0.523	3648.60	1906.86	0.140	2548.04	355.69
A_68_P30423843	chr15:89027122-89027166	NM_027081:-239	Fam116b	PROMOTER	0.195	0.559	1347.01	752.51	0.109	1113.67	121.29
A_68_P28038138	chr11:95198892-95198936	NM_172543:583	Fam117a	INSIDE	0.240	1.589	7638.24	12136.56	0.381	4870.98	1855.31
A_68_P20266463	chr1:59970586-59970630	NM_001037725:759	Fam117b	INSIDE	0.531	0.741	2514.61	1862.46	0.393	2202.29	865.86

ProbeName	Target position of probe on CpG island microarray	TargetID	GeneSymbol	CpG island Description	Ratio of relative methylation (TiO2-NP/Vehicle)	Sham group			TiO_2-H group		
						Relative methylation (Cy-5/Cy-3)	Cy-3 signal (Input)	Cy-5 signal (MeDIP)	Relative methylation (Cy-5/Cy-3)	Cy-3 signal (Input)	Cy-5 signal (MeDIP)
A_68_P26421597	chr9:35075221-35075265	NM_194257:110	Fam118b	INSIDE	0.493	0.560	1163.26	651.81	0.276	933.09	257.77
A_68_P32023816	chr19:24551478-24551522	NM_026520:464	Fam122a	INSIDE	0.264	0.556	1049.22	583.33	0.147	820.35	120.62
A_68_P32386814	chrX:50622922-50622968	NM_001166365:38	Fam122b	INSIDE	0.387	3.956	2049.73	8109.44	1.532	750.92	1150.54
A_68_P29644188	chr14:60997183-60997227	NM_001164705:82	Fam123a	INSIDE	0.656	0.550	1771.93	975.27	0.361	1433.61	517.39
A_68_P29644190	chr14:60997369-60997413	NM_001164705:268	Fam123a	INSIDE	0.343	0.602	1906.14	1146.59	0.206	1505.08	310.69
A_68_P25957388	chr8:74066580-74066624	NM_028617:-226	Fam125a	DIVERGENT_PROMOTER	0.654	0.603	1064.35	641.34	0.394	853.31	336.09
A_68_P25957393	chr8:74067094-74067138	NM_028617:288	Fam125a	INSIDE	0.383	0.717	2146.50	1538.78	0.274	1662.90	456.22
A_68_P25957394	chr8:74067191-74067235	NM_028617:384	Fam125a	INSIDE	0.248	1.361	4553.28	6199.19	0.337	2935.43	989.97
A_68_P23539565	chr5:23536003-23536047	NM_053090:477	Fam126a	INSIDE	0.542	0.540	2375.65	1283.56	0.293	1969.12	576.23
A_68_P23539567	chr5:23536236-23536280	NM_053090:243	Fam126a	INSIDE	0.580	0.532	1173.13	624.22	0.309	1009.61	311.81
A_68_P23539568	chr5:23536418-23536462	NM_053090:61	Fam126a	INSIDE	0.388	0.364	2141.71	779.58	0.141	1832.65	259.00
A_68_P20259678	chr1:58643163-58643207	NM_172513:-7	Fam126b	DIVERGENT_PROMOTER	0.152	0.600	1947.22	1167.79	0.091	1468.06	133.54
A_68_P25957748	chr8:74126235-74126279	NM_001166213:4712	Fam129c	INSIDE	0.473	0.476	1866.23	888.43	0.225	1409.07	317.59
A_68_P25957752	chr8:74126620-74126664	NM_001166213:5098	Fam129c	INSIDE	0.307	0.565	1846.74	1043.92	0.174	1386.55	240.96
A_68_P30589002	chr16:20693547-20693591	NM_133778:-1561	Fam131a	PROMOTER	0.442	1.654	4680.12	7740.39	0.730	3109.04	2270.27
A_68_P30589004	chr16:20693747-20693792	NM_133778:-1360	Fam131a	PROMOTER	0.290	0.623	818.12	509.47	0.180	636.34	114.76
A_68_P30589022	chr16:20695936-20695980	NM_133778:829	Fam131a	INSIDE	0.583	0.481	1123.15	539.78	0.280	885.61	248.13
A_68_P24397654	chr6:42274034-42274078	NM_001113327:583	Fam131b	INSIDE	0.236	0.478	2129.41	1016.84	0.112	1648.23	185.38
A_68_P24397655	chr6:42274146-42274190	NM_001113327:471	Fam131b	INSIDE	0.108	0.546	3761.64	2052.52	0.059	2354.77	138.42
A_68_P23363642	chr4:140924465-140924509	NM_001085513:352	Fam131c	INSIDE	0.519	0.558	2287.61	1275.69	0.289	1791.64	518.35
A_68_P23363728	chr4:140938466-140938511	NM_001085513:14354	Fam131c	INSIDE	0.207	0.668	2729.78	1822.27	0.138	1996.94	275.52
A_68_P23363729	chr4:140938544-140938588	NM_001085513:14432	Fam131c	INSIDE	0.210	0.349	2605.47	909.22	0.073	2026.32	148.37
A_68_P20442008	chr1:93262992-93263036	NM_173395:8	Fam132b	INSIDE	0.524	0.623	3507.65	2185.83	0.326	2714.21	885.50
A_68_P20352246	chr1:75139198-75139242	NM_170755:-139	Fam134a	DIVERGENT_PROMOTER	0.284	0.487	2793.58	1360.33	0.138	2206.11	305.33
A_68_P30083648	chr15:25772800-25772844	NM_001034851:-196	Fam134b	PROMOTER	0.614	0.680	2093.85	1424.58	0.418	1793.37	748.82
A_68_P28072082	chr11:100981134-100981178	NM_026501:-18	Fam134c	PROMOTER	0.347	0.560	1409.76	789.69	0.194	1110.88	215.98
A_68_P20091639	chr1:24106505-24106549	NM_026604:654	Fam135a	INSIDE	0.291	0.529	1272.08	672.54	0.154	1041.98	160.09
A_68_P24609727	chr6:86315530-86315574	NM_025591:-124	Fam136a	PROMOTER	0.339	0.705	6052.97	4269.05	0.239	4767.19	1140.80
A_68_P24609728	chr6:86315617-86315661	NM_025591:-38	Fam136a	PROMOTER	0.540	0.717	3758.40	2694.67	0.387	2969.11	1150.06
A_68_P20012308	chr1:6349244-6349288	NM_001195732:-145	Fam150a	PROMOTER	0.220	0.552	1876.32	1035.36	0.122	1483.36	180.38
A_68_P23195773	chr4:108056312-108056356	NM_001099303:-380	Fam159a	PROMOTER	0.248	0.608	882.16	535.92	0.151	768.56	116.00
A_68_P29292940	chr13:105653878-105653922	NM_029984:73	Fam159b	INSIDE	0.201	0.551	1329.81	732.42	0.111	1074.31	119.08
A_68_P29702362	chr14:70999309-70999353	NM_194345:312	Fam160b2	INSIDE	0.525	0.359	1598.94	574.24	0.188	1237.15	233.10
A_68_P30673613	chr16:36071461-36071505	NM_027342:119	Fam162a	INSIDE	0.122	0.572	3357.25	1921.90	0.070	2572.09	179.47
A_68_P25369176	chr7:107855309-107855353	NM_178764:115	Fam168a	INSIDE	0.485	0.525	1320.02	693.27	0.255	1090.90	278.11
A_68_P29249242	chr13:97842053-97842097	NM_001100458:477	Fam169a	INSIDE	0.262	1.449	7529.40	10913.83	0.380	4994.78	1897.35
A_68_P29249243	chr13:97842140-97842199	NM_001100458:572	Fam169a	INSIDE	0.044	1.885	2079.58	3920.02	0.084	1622.84	135.71
A_68_P28079561	chr11:102299305-102299349	NM_199200:9651	Fam171a2	INSIDE	0.388	0.678	1299.25	880.31	0.263	929.75	244.30
A_68_P28079628	chr11:102308536-102308580	NM_199200:419	Fam171a2	INSIDE	0.270	0.556	4410.99	2451.56	0.150	3202.38	479.82
A_68_P29160014	chr13:77847868-77847912	NM_001163419:-60	Fam172a	PROMOTER	0.135	0.727	2969.61	2157.54	0.098	2344.87	230.17
A_68_P29161996	chr13:78302959-78303003	NM_001163419:455030	Fam172a	INSIDE	0.523	0.708	1326.64	938.85	0.370	1070.86	396.19
A_68_P31106284	chr17:25929403-25929447	NM_145410:-195	Fam173a	PROMOTER	0.654	0.688	1331.38	916.06	0.450	1013.23	456.00
A_68_P24586428	chr6:81991724-81991768	NM_145570:125	Fam176a	INSIDE	0.637	0.633	1449.43	917.63	0.403	1219.75	491.76
A_68_P24586430	chr6:81991869-81991913	NM_145570:269	Fam176a	INSIDE	0.430	0.583	1250.34	729.02	0.251	1000.94	251.16

ProbeName	Target position of probe on CpG island microarray	TargetID	GeneSymbol	CpG island Description	Ratio of relative methylation (TiO2-NP/Vehicle)	Sham group			TiO_2-H group		
						Relative methylation (Cy-5/Cy-3)	Cy-3 signal (Input)	Cy-5 signal (MeDIP)	Relative methylation (Cy-5/Cy-3)	Cy-3 signal (Input)	Cy-5 signal (MeDIP)
A_68_P24586431	chr6:81991939-81991986	NM_145570:341	Fam176a	INSIDE	0.121	0.510	2562.49	1307.44	0.062	1949.39	120.16
A_68_P32135376	chr19:45005642-45005686	NM_001081225:56	Fam178a	INSIDE	0.151	0.690	1515.64	1046.26	0.104	1121.45	116.78
A_68_P32135377	chr19:45005799-45005843	NM_001081225:212	Fam178a	INSIDE	0.609	0.559	2677.66	1495.88	0.340	2182.19	741.87
A_68_P32135378	chr19:45005914-45005958	NM_001081225:328	Fam178a	INSIDE	0.162	1.620	2180.75	3532.95	0.262	1585.12	415.47
A_68_P32135379	chr19:45006013-45006057	NM_001081225:426	Fam178a	INSIDE	0.583	0.712	1613.59	1149.54	0.415	1325.25	550.36
A_68_P32135375	chr19:45005538-45005582	NM_001081225:-48	Fam178a	PROMOTER	0.667	0.425	1610.73	685.16	0.284	1193.66	338.90
A_68_P28493246	chr12:66067167-66067211	NM_177805:460	Fam179b	INSIDE	0.528	0.648	1451.03	940.29	0.342	1111.93	380.15
A_68_P28493248	chr12:66067408-66067452	NM_177805:702	Fam179b	INSIDE	0.119	0.562	2658.49	1493.43	0.067	1807.59	120.61
A_68_P28493249	chr12:66067546-66067594	NM_177805:842	Fam179b	INSIDE	0.172	0.632	1670.05	1055.36	0.109	1100.07	119.66
A_68_P25328172	chr7:100228249-100228299	NM_021427:-114	Fam181b	PROMOTER	0.228	0.622	1141.45	709.95	0.142	869.60	123.51
A_68_P25328181	chr7:100229343-100229387	NM_021427:976	Fam181b	INSIDE	0.061	0.716	4535.13	3247.78	0.044	3244.95	142.88
A_68_P21010978	chr2:12340776-12340820	NM_024185:289	Fam188a	INSIDE	0.553	0.697	1174.00	817.77	0.385	958.38	369.45
A_68_P25181446	chr7:72301608-72301652	NM_183087:-216	Fam189a1	PROMOTER	0.580	0.633	1533.31	971.24	0.368	1321.13	485.66
A_68_P25181447	chr7:72301734-72301778	NM_183087:-342	Fam189a1	PROMOTER	0.080	0.622	4566.13	2839.35	0.050	3019.94	150.74
A_68_P31106871	chr17:26005229-26005273	NM_026633:433	Fam195a	INSIDE	0.234	0.400	2342.18	936.32	0.094	1823.49	170.70
A_68_P25549532	chr7:142129376-142129420	NM_001143802:715	Fam196a	INSIDE	0.215	0.438	1618.87	708.29	0.094	1238.96	116.41
A_68_P32683850	chrX:133583924-133583968	NM_146261:-186	Fam199x	PROMOTER	0.108	1.697	1612.97	2736.65	0.183	817.88	149.56
A_68_P22392957	chr3:104561384-104561428	NM_183224:19059	Fam19a3	DOWNSTREAM	0.281	0.477	1131.90	540.11	0.134	907.71	121.49
A_68_P22392958	chr3:104561530-104561574	NM_183224:18913	Fam19a3	DOWNSTREAM	0.177	0.617	1349.72	833.05	0.109	1104.18	120.34
A_68_P30347858	chr15:76199296-76199340	NM_021555:-9	Fam203a	PROMOTER	0.290	0.590	980.36	578.65	0.171	721.73	123.65
A_68_P30347865	chr15:76200037-76200087	NM_021555:735	Fam203a	INSIDE	0.147	1.388	1576.99	2189.31	0.203	1228.77	250.05
A_68_P28120310	chr11:109583554-109583598	NM_153782:-6	Fam20a	PROMOTER	0.623	0.464	1323.81	613.60	0.289	1052.51	303.71
A_68_P28120311	chr11:109583661-109583705	NM_153782:-112	Fam20a	PROMOTER	0.453	0.419	1787.46	748.26	0.190	1397.89	265.27
A_68_P28120302	chr11:109582605-109582649	NM_153782:944	Fam20a	INSIDE	0.668	0.580	1847.27	1072.02	0.388	1591.69	616.80
A_68_P24131499	chr5:139230499-139230543	NM_030565:-514	Fam20c	PROMOTER	0.567	0.679	1326.13	900.41	0.385	1196.45	460.94
A_68_P24131511	chr5:139231842-139231886	NM_030565:830	Fam20c	INSIDE	0.250	0.417	1683.12	702.67	0.104	1268.17	132.25
A_68_P25960243	chr8:74743668-74743712	NM_026455:62	Fam32a	INSIDE	0.447	0.668	1673.26	1117.32	0.299	1291.04	385.48
A_68_P20863273	chr1:180248534-180248578	NM_025511:-727	Fam36a	PROMOTER	0.209	0.411	3704.05	1521.18	0.086	2683.04	230.25
A_68_P31769416	chr18:63546211-63546255	NM_001039485:605	Fam38b	INSIDE	0.653	0.626	1508.79	945.19	0.409	1172.54	479.91
A_68_P24290978	chr6:22305712-22305756	NM_138587:347	Fam3c	INSIDE	0.344	0.380	2552.66	970.80	0.131	2087.58	273.19
A_68_P22407674	chr3:107434065-107434109	NM_153563:542	Fam40a	INSIDE	0.111	1.436	1752.13	2516.68	0.159	1311.00	208.60
A_68_P22407676	chr3:107434272-107434316	NM_153563:334	Fam40a	INSIDE	0.457	0.655	836.35	548.10	0.299	731.28	218.93
A_68_P30642670	chr16:30599886-30599930	NM_177632:100	Fam43a	INSIDE	0.473	0.614	2985.20	1833.32	0.291	2009.95	584.06
A_68_P30642673	chr16:30600225-30600275	NM_177632:442	Fam43a	INSIDE	0.471	0.502	1239.68	621.77	0.236	1011.07	239.09
A_68_P32227328	chr19:60887531-60887575	NM_001167829:80	Fam45a	INSIDE	0.441	0.501	1986.74	995.16	0.221	1578.45	348.66
A_68_P32227330	chr19:60887752-60887797	NM_001167829:302	Fam45a	INSIDE	0.573	0.602	870.95	524.64	0.345	770.61	266.04
A_68_P26693192	chr9:85220596-85220642	NM_001160378:113	Fam46a	INSIDE	0.278	0.560	1460.86	818.73	0.156	1225.83	191.20
A_68_P26693197	chr9:85221191-85221249	NM_001160379:-463	Fam46a	PROMOTER	0.272	0.409	1273.95	521.15	0.111	1062.68	118.15
A_68_P23317810	chr4:133035888-133035933	NM_175307:-137	Fam46b	PROMOTER	0.349	0.567	1302.78	738.54	0.198	1004.61	198.83
A_68_P23317815	chr4:133036401-133036445	NM_175307:375	Fam46b	INSIDE	0.526	0.695	1689.50	1174.35	0.366	1246.47	455.87
A_68_P22369560	chr3:100293076-100293120	NM_001142952:17	Fam46c	INSIDE	0.428	0.481	4172.15	2006.74	0.206	3200.67	659.17
A_68_P22369562	chr3:100293420-100293464	NM_001142952:-327	Fam46c	PROMOTER	0.292	0.710	1428.78	1014.97	0.207	1178.39	244.24
A_68_P22149159	chr3:54496467-54496512	NM_019995:-537	Fam48a	PROMOTER	0.247	0.598	930.57	556.52	0.148	797.22	117.81
A_68_P22149161	chr3:54496698-54496742	NM_019995:-306	Fam48a	PROMOTER	0.274	0.725	1682.31	1220.29	0.199	1260.47	250.22

ProbeName	Target position of probe on CpG island microarray	TargetID	GeneSymbol	CpG island Description	Ratio of relative methylation (TiO2-NP/Vehicle)	Sham group			TiO_2-H group		
						Relative methylation (Cy-5/Cy-3)	Cy-3 signal (Input)	Cy-5 signal (MeDIP)	Relative methylation (Cy-5/Cy-3)	Cy-3 signal (Input)	Cy-5 signal (MeDIP)
A_68_P28242247	chr12:12269223-12269267	NM_001146119:300	Fam49a	INSIDE	0.183	0.696	1504.27	1046.98	0.128	1023.11	130.56
A_68_P30279146	chr15:63891463-63891507	NM_144846:526	Fam49b	INSIDE	0.200	0.702	1470.92	1032.91	0.141	1240.59	174.59
A_68_P32467455	chrX:71558602-71558649	NM_138607:254	Fam50a	INSIDE	0.140	1.791	1017.43	1822.19	0.251	474.27	118.97
A_68_P23590168	chr5:33972239-33972283	NM_178390:24	Fam53a	INSIDE	0.346	0.261	2591.49	675.40	0.090	1961.12	176.96
A_68_P25537048	chr7:140004752-140004796	NM_212473:105	Fam53b	INSIDE	0.422	0.395	1663.24	656.80	0.167	1453.98	242.25
A_68_P26986431	chr10:20067872-20067916	NM_027930:270	Fam54a	INSIDE	0.295	0.482	1155.15	557.13	0.142	860.19	122.49
A_68_P23323333	chr4:134090414-134090464	NM_029759:-1771	Fam54b	PROMOTER	0.218	0.419	1983.79	832.15	0.092	1316.62	120.50
A_68_P31544264	chr18:21458028-21458072	NM_001033445:590	Fam59a	INSIDE	0.194	0.374	2023.66	756.91	0.073	1645.96	119.50
A_68_P31544266	chr18:21458217-21458261	NM_001033445:402	Fam59a	INSIDE	0.206	0.338	2077.35	702.95	0.070	1666.76	116.30
A_68_P31544272	chr18:21459015-21459059	NM_001033445:-396	Fam59a	PROMOTER	0.303	0.644	2589.20	1668.63	0.195	2037.40	398.16
A_68_P23570722	chr5:30431999-30432043	NM_001167879:285	Fam59b	INSIDE	0.376	0.492	1931.89	950.09	0.185	1357.36	250.70
A_68_P24942889	chr6:148893354-148893400	NM_019643:1578	Fam60a	INSIDE	0.570	0.544	1192.04	648.66	0.310	907.54	281.66
A_68_P22343254	chr3:95087394-95087438	NM_133858:560	Fam63a	INSIDE	0.554	0.511	1412.47	721.45	0.283	1056.22	298.82
A_68_P26134825	chr8:108129199-108129243	NM_001081241:92	Fam65a	INSIDE	0.504	0.463	4343.91	2010.93	0.233	3434.23	801.16
A_68_P28888196	chr13:24730767-24730811	NM_001080381:272	Fam65b	INSIDE	0.150	0.540	1737.10	937.18	0.081	1443.13	117.13
A_68_P31890268	chr18:84889452-84889496	NM_173770:-159	Fam69c	PROMOTER	0.365	0.519	1894.09	983.56	0.190	1558.48	295.66
A_68_P25087743	chr7:51752229-51752273	NM_028169:293	Fam71e1	INSIDE	0.084	0.619	4174.24	2584.28	0.052	2598.44	135.66
A_68_P20616256	chr1:133424915-133424959	NM_175382:371	Fam72a	INSIDE	0.394	0.598	939.33	561.70	0.235	773.66	182.08
A_68_P21113205	chr2:31938737-31938781	NM_175511:467	Fam78a	INSIDE	0.327	0.594	2244.07	1333.68	0.194	1629.12	316.59
A_68_P21113207	chr2:31938899-31938943	NM_175511:305	Fam78a	INSIDE	0.527	0.442	3151.64	1393.05	0.233	2489.87	579.90
A_68_P21113214	chr2:31939681-31939725	NM_175511:-477	Fam78a	PROMOTER	0.622	0.593	1617.40	959.38	0.369	1278.06	471.64
A_68_P20802626	chr1:168930754-168930798	NM_001160261:-771	Fam78b	DIVERGENT_PROMOTER	0.409	0.544	4120.83	2241.58	0.223	3702.58	824.57
A_68_P20802627	chr1:168930921-168930965	NM_001160261:-605	Fam78b	DIVERGENT_PROMOTER	0.256	0.491	1108.28	544.22	0.126	962.92	121.06
A_68_P21784859	chr2:158593842-158593886	NM_027975:30	Fam83d	INSIDE	0.319	0.389	2629.73	1022.84	0.124	2015.92	250.46
A_68_P21784861	chr2:158594102-158594146	NM_027975:290	Fam83d	INSIDE	0.224	0.294	3051.25	896.00	0.066	2377.29	156.69
A_68_P30373739	chr15:80502384-80502428	NM_145986:130	Fam83f	INSIDE	0.434	0.643	1311.34	843.82	0.279	1111.32	310.20
A_68_P27849840	chr11:61498639-61498683	NM_178618:749	Fam83g	INSIDE	0.279	1.794	902.48	1619.19	0.500	636.82	318.52
A_68_P28252857	chr12:14158564-14158621	NM_029007:252	Fam84a	INSIDE	0.359	0.700	1266.96	887.11	0.252	789.74	198.64
A_68_P30261327	chr15:60655565-60655617	NM_001162926:1045	Fam84b	INSIDE	0.283	0.608	919.35	558.71	0.172	699.74	120.36
A_68_P30261328	chr15:60655675-60655719	NM_001162926:939	Fam84b	INSIDE	0.251	0.662	860.65	569.47	0.166	705.49	117.12
A_68_P30261333	chr15:60656265-60656309	NM_001162926:349	Fam84b	INSIDE	0.393	0.655	1042.70	683.29	0.258	911.55	234.94
A_68_P30261335	chr15:60656490-60656535	NM_001162926:123	Fam84b	INSIDE	0.334	0.514	1333.29	684.70	0.171	964.41	165.17
A_68_P26129630	chr8:107165289-107165337	NM_026753:316	Fam96b	INSIDE	0.084	1.360	1467.13	1995.89	0.114	1021.51	116.42
A_68_P26129635	chr8:107165709-107165753	NM_026753:-102	Fam96b	PROMOTER	0.127	0.686	1791.31	1228.27	0.087	1378.49	119.71
A_68_P26129637	chr8:107165970-107166027	NM_026753:-370	Fam96b	PROMOTER	0.259	0.606	935.90	567.32	0.157	739.15	115.93
A_68_P31357838	chr17:75951190-75951234	NM_133747:74	Fam98a	INSIDE	0.162	0.671	1504.50	1009.87	0.109	1106.13	120.44
A_68_P25025637	chr7:29940517-29940567	NM_001146023:687	Fam98c	INSIDE	0.181	0.556	1451.94	807.71	0.101	1163.45	117.14
A_68_P25025638	chr7:29940590-29940634	NM_001146023:617	Fam98c	INSIDE	0.517	0.684	3093.74	2114.94	0.353	2326.73	822.15
A_68_P31121749	chr17:28450313-28450357	NM_001163819:-140	Fance	PROMOTER	0.347	0.718	1420.58	1019.99	0.249	985.33	245.38
A_68_P31121750	chr17:28450482-28450526	NM_001163819:30	Fance	INSIDE	0.327	0.719	1777.05	1277.97	0.235	1349.73	317.35
A_68_P25125430	chr7:59116936-59116980	NM_001115087:679	Fancf	INSIDE	0.197	0.438	2024.92	887.03	0.086	1566.38	135.31
A_68_P25125435	chr7:59117580-59117624	NM_001115087:35	Fancf	INSIDE	0.387	0.389	1514.68	589.37	0.151	1241.29	186.88
A_68_P25260792	chr7:86537034-86537078	NM_145946:-167	Fanci	DIVERGENT_PROMOTER	0.119	0.577	3528.27	2035.24	0.069	2574.59	177.44
A_68_P25260795	chr7:86537392-86537436	NM_145946:191	Fanci	INSIDE	0.102	0.594	2596.27	1541.64	0.060	1943.83	117.34

ProbeName	Target position of probe on CpG island microarray	TargetID	GeneSymbol	CpG island Description	Ratio of relative methylation (TiO2-NP/Vehicle)	Sham group			TiO_2-H group		
						Relative methylation (Cy-5/Cy-3)	Cy-3 signal (Input)	Cy-5 signal (MeDIP)	Relative methylation (Cy-5/Cy-3)	Cy-3 signal (Input)	Cy-5 signal (MeDIP)
A_68_P27662609	chr11:26287534-26287589	NM_025923:478	Fancl	INSIDE	0.248	0.539	1119.39	603.35	0.134	880.25	117.62
A_68_P28493835	chr12:66176402-66176461	NM_178912:-161	Fancm	DIVERGENT_PROMOTER	0.489	0.558	1182.85	659.90	0.273	861.92	235.30
A_68_P28493836	chr12:66176491-66176538	NM_178912:-78	Fancm	DIVERGENT_PROMOTER	0.278	0.443	1198.26	530.99	0.123	978.67	120.62
A_68_P25430847	chr7:120657214-120657258	NM_026143:-139	Far1	PROMOTER	0.165	1.424	2227.29	3170.93	0.235	1609.66	377.51
A_68_P20456054	chr1:95408480-95408524	NM_145519:-178	Farp2	PROMOTER	0.480	0.465	1324.72	616.09	0.223	1060.35	236.71
A_68_P28947128	chr13:36209814-36209858	NM_001039189:557	Fars2	INSIDE	0.266	0.634	945.14	599.59	0.169	815.97	137.83
A_68_P28187037	chr11:120686073-120686117	NM_007988:-233	Fasn	PROMOTER	0.659	0.547	2938.89	1607.96	0.361	2277.31	820.98
A_68_P23541889	chr5:23950730-23950774	NM_023229:301	Fastk	INSIDE	0.530	0.581	1987.04	1153.56	0.308	1611.98	495.89
A_68_P29110676	chr13:68721046-68721090	NM_027123:-56	Fastkd3	DIVERGENT_PROMOTER	0.625	0.626	2254.47	1411.85	0.391	1937.62	758.54
A_68_P25827401	chr8:46036029-46036073	NM_001081286:489	Fat1	INSIDE	0.665	2.649	1533.08	4060.58	1.763	1253.18	2208.91
A_68_P25828056	chr8:46122297-46122344	NM_001081286:86759	Fat1	INSIDE	0.268	1.687	641.92	1082.79	0.453	567.83	257.12
A_68_P25828172	chr8:46136729-46136773	NM_001081286:101189	Fat1	INSIDE	0.638	2.472	3628.42	8967.89	1.576	2402.99	3787.61
A_68_P25828173	chr8:46136798-46136846	NM_001081286:101261	Fat1	INSIDE	0.567	2.570	2112.83	5429.90	1.457	1471.99	2144.67
A_68_P22071595	chr3:38784639-38784683	NM_183221:-1201	Fat4	PROMOTER	0.311	0.651	2694.91	1753.61	0.202	1968.38	398.25
A_68_P22071613	chr3:38786725-38786769	NM_183221:885	Fat4	INSIDE	0.458	0.447	1492.68	666.98	0.205	1218.04	249.52
A_68_P22071616	chr3:38786996-38787040	NM_183221:1157	Fat4	INSIDE	0.568	0.666	1415.98	943.31	0.378	1074.47	406.55
A_68_P22071617	chr3:38787100-38787144	NM_183221:1261	Fat4	INSIDE	0.529	0.639	1698.34	1084.82	0.338	1263.31	426.53
A_68_P22071618	chr3:38787168-38787212	NM_183221:1329	Fat4	INSIDE	0.205	0.479	1581.88	758.06	0.098	1187.12	116.78
A_68_P28157764	chr11:116029125-116029181	NM_172571:340	Fbf1	INSIDE	0.248	0.665	897.83	597.17	0.165	722.58	119.30
A_68_P28157765	chr11:116029229-116029273	NM_172571:242	Fbf1	INSIDE	0.363	0.488	2357.68	1150.60	0.177	1831.93	324.14
A_68_P28687868	chr12:103057400-103057444	NM_011812:-157	Fbln5	PROMOTER	0.250	0.451	1263.91	570.60	0.113	1050.30	118.38
A_68_P21603035	chr2:125331152-125331196	NM_007993:1000	Fbn1	INSIDE	0.385	0.407	1662.11	676.94	0.157	1295.90	203.23
A_68_P21603036	chr2:125331219-125331263	NM_007993:934	Fbn1	INSIDE	0.476	0.657	935.55	614.36	0.313	779.93	243.78
A_68_P31741004	chr18:58369796-58369845	NM_010181:-240	Fbn2	PROMOTER	0.401	0.672	1429.00	960.85	0.270	1189.50	320.74
A_68_P23979395	chr5:110815816-110815860	NM_028596:308	Fbrsl1	INSIDE	0.273	0.553	1392.97	770.15	0.151	1216.14	183.39
A_68_P23979843	chr5:110877476-110877520	NM_001142642:24	Fbrsl1	INSIDE	0.575	0.491	1786.34	877.14	0.283	1389.15	392.50
A_68_P23979845	chr5:110877631-110877675	NM_001142642:-130	Fbrsl1	PROMOTER	0.560	0.631	2505.81	1581.38	0.354	1994.69	705.30
A_68_P23979846	chr5:110877794-110877838	NM_001142642:-294	Fbrsl1	PROMOTER	0.331	0.624	3872.09	2414.63	0.206	2935.11	605.59
A_68_P24793341	chr6:119429106-119429150	NM_133940:-557	Fbxl14	PROMOTER	0.471	1.354	3221.50	4360.39	0.638	2446.36	1561.18
A_68_P24793349	chr6:119430121-119430165	NM_133940:457	Fbxl14	INSIDE	0.500	0.443	1249.63	553.10	0.221	952.77	211.01
A_68_P24793359	chr6:119431189-119431233	NM_133940:1525	Fbxl14	INSIDE	0.197	0.616	3279.68	2018.70	0.121	2399.85	291.28
A_68_P32143269	chr19:46402678-46402722	NM_133694:27	Fbxl15	INSIDE	0.592	0.569	950.84	541.30	0.337	834.76	281.20
A_68_P32143271	chr19:46402975-46403019	NM_133694:323	Fbxl15	INSIDE	0.433	0.654	1719.17	1123.56	0.283	1417.27	400.83
A_68_P32143272	chr19:46403086-46403130	NM_133694:435	Fbxl15	INSIDE	0.349	0.408	2457.72	1003.19	0.143	1919.29	273.60
A_68_P31106383	chr17:25945693-25945737	NM_001164225:-315	Fbxl16	PROMOTER	0.174	0.569	1926.85	1096.78	0.099	1409.88	139.80
A_68_P31106388	chr17:25946298-25946342	NM_001164225:291	Fbxl16	INSIDE	0.475	0.688	1554.55	1069.98	0.327	1248.05	407.99
A_68_P25507025	chr7:134889380-134889424	NM_172748:-886	Fbxl19	PROMOTER	0.418	0.589	3043.86	1792.94	0.246	1889.60	465.46
A_68_P25507036	chr7:134890617-134890661	NM_172748:350	Fbxl19	INSIDE	0.545	0.577	3130.41	1805.33	0.314	2168.27	681.06
A_68_P28054684	chr11:98010545-98010589	NM_028149:364	Fbxl20	INSIDE	0.120	0.546	3521.42	1921.09	0.066	2742.18	180.13
A_68_P28054685	chr11:98010627-98010671	NM_028149:282	Fbxl20	INSIDE	0.167	0.494	2679.41	1323.04	0.082	2056.77	169.66
A_68_P28054692	chr11:98011319-98011364	NM_028149:-411	Fbxl20	PROMOTER	0.120	0.423	3374.31	1427.53	0.051	2373.73	120.90
A_68_P29061785	chr13:56623742-56623786	NM_178674:-104	Fbxl21	PROMOTER	0.219	0.471	5436.07	2558.92	0.103	4378.98	451.91
A_68_P26593512	chr9:66359862-66359906	NM_175206:2516	Fbxl22	INSIDE	0.366	0.675	991.70	669.31	0.247	868.51	214.47
A_68_P30089764	chr15:26824671-26824715	NM_176959:627	Fbxl7	INSIDE	0.324	0.629	2508.25	1578.89	0.204	1946.34	397.18

ProbeName	Target position of probe on CpG island microarray	TargetID	GeneSymbol	CpG island Description	Ratio of relative methylation (TiO2-NP/Vehicle)	Sham group			TiO_2-H group		
						Relative methylation (Cy-5/Cy-3)	Cy-3 signal (Input)	Cy-5 signal (MeDIP)	Relative methylation (Cy-5/Cy-3)	Cy-3 signal (Input)	Cy-5 signal (MeDIP)
A_68_P26132610	chr8:107792581-107792625	NM_015821:4055	Fbxl8	INSIDE	0.340	0.605	1463.87	886.37	0.206	1127.49	232.42
A_68_P26132614	chr8:107793016-107793064	NM_015821:4493	Fbxl8	INSIDE	0.160	0.555	2094.84	1162.85	0.089	1370.76	121.96
A_68_P25023220	chr7:29501806-29501850	NM_015796:20	Fbxo17	INSIDE	0.557	0.656	1623.49	1064.23	0.365	1443.82	527.31
A_68_P25023288	chr7:29517538-29517582	NM_015796:15752	Fbxo17	INSIDE	0.510	0.588	912.14	536.30	0.300	817.40	245.08
A_68_P25023289	chr7:29517672-29517716	NM_015796:15886	Fbxo17	INSIDE	0.542	0.613	1326.90	813.96	0.332	1094.32	363.66
A_68_P21007903	chr2:11698976-11699020	NM_015792:156	Fbxo18	INSIDE	0.613	0.626	3801.92	2380.95	0.384	2789.82	1071.73
A_68_P21007905	chr2:11699155-11699199	NM_015792:-22	Fbxo18	DIVERGENT_PROMOTER	0.228	0.687	6451.61	4434.24	0.157	4539.72	711.85
A_68_P23392754	chr4:147534489-147534533	NM_176848:-266	Fbxo2	DIVERGENT_PROMOTER	0.244	0.459	1456.48	667.93	0.112	1069.47	119.76
A_68_P26529950	chr9:55057294-55057339	NM_028049:575	Fbxo22	INSIDE	0.240	0.582	1123.23	654.00	0.140	848.96	118.67
A_68_P21486778	chr2:103867915-103867959	NM_020593:-19	Fbxo3	PROMOTER	0.509	0.500	1250.65	625.27	0.254	1039.88	264.62
A_68_P21486780	chr2:103868083-103868131	NM_020593:151	Fbxo3	INSIDE	0.101	2.395	699.92	1676.08	0.243	495.55	120.36
A_68_P28470864	chr12:60320947-60320991	NM_001033156:-498	Fbxo33	PROMOTER	0.597	0.493	1475.94	727.36	0.294	1217.60	358.41
A_68_P20405972	chr1:84836507-84836554	NM_025386:115	Fbxo36	INSIDE	0.207	0.611	1513.41	924.52	0.126	1158.18	146.33
A_68_P31764313	chr18:62708319-62708363	NM_134136:57	Fbxo38	INSIDE	0.328	0.651	1387.03	902.87	0.214	988.00	211.00
A_68_P30651586	chr16:32246480-32246526	NM_173439:609	Fbxo45	INSIDE	0.221	0.664	938.83	623.10	0.147	791.97	116.26
A_68_P24991622	chr7:19717039-19717083	NM_175530:11853	Fbxo46	INSIDE	0.236	0.705	1814.17	1279.23	0.167	1475.60	245.86
A_68_P26905371	chr10:4541584-4541628	NM_025995:531	Fbxo5	INSIDE	0.195	0.422	1778.22	750.04	0.082	1423.04	117.12
A_68_P32920574	chr13:50513406-50513450	NM_175401:183	Fbxw17	INSIDE	0.291	0.407	1725.21	703.01	0.119	1387.43	164.75
A_68_P32139835	chr19:45734791-45734835	NM_013907:-129	Fbxw4	PROMOTER	0.594	0.573	1068.08	612.45	0.341	896.83	305.64
A_68_P26023481	chr8:87584180-87584224	NM_026791:185	Fbxw9	INSIDE	0.396	0.627	1561.81	978.68	0.248	1215.89	301.53
A_68_P22348573	chr3:96081761-96081812	NM_010186:16106	Fcgr1	DOWNSTREAM	0.258	0.428	1339.42	573.08	0.110	1067.05	117.67
A_68_P20825994	chr1:172994223-172994267	NM_010188:-4710	Fcgr3	PROMOTER	0.444	3.466	22821.82	79103.15	1.540	15614.41	24042.33
A_68_P20825998	chr1:172994760-172994804	NM_010188:-5248	Fcgr3	PROMOTER	0.196	0.557	6963.61	3879.52	0.109	5761.66	629.67
A_68_P20826007	chr1:172995883-172995927	NM_010188:-6370	Fcgr3	PROMOTER	0.475	0.324	7844.01	2538.94	0.154	6645.24	1021.34
A_68_P20826008	chr1:172995959-172996003	NM_010188:-6446	Fcgr3	PROMOTER	0.406	0.350	18198.05	6361.85	0.142	15322.08	2176.74
A_68_P29259682	chr13:99585022-99585066	NM_172591:360	Fcho2	INSIDE	0.100	0.530	3021.18	1600.17	0.053	2475.84	131.59
A_68_P31631189	chr18:38129379-38129423	NM_175684:-15	Fchsd1	PROMOTER	0.181	0.669	2092.14	1398.67	0.121	1673.93	202.19
A_68_P25371474	chr7:108256833-108256877	NM_001146010:-434	Fchsd2	PROMOTER	0.443	0.409	1624.10	664.34	0.181	1226.50	222.45
A_68_P25371480	chr7:108257652-108257696	NM_001146010:386	Fchsd2	INSIDE	0.018	1.469	6853.58	10064.94	0.026	4417.02	116.42
A_68_P20825266	chr1:172837419-172837463	NM_001029984:5632	Fcrlb	INSIDE	0.381	1.347	2671.36	3598.72	0.514	2081.09	1068.86
A_68_P20825279	chr1:172838779-172838823	NM_001029984:4272	Fcrlb	INSIDE	0.171	0.650	1961.31	1274.92	0.111	1504.44	167.14
A_68_P29659636	chr14:63796998-63797042	NM_010191:-390	Fdft1	PROMOTER	0.284	0.459	1214.26	557.87	0.130	912.10	118.93
A_68_P22317214	chr3:88905621-88905667	NM_134469:223	Fdps	INSIDE	0.113	0.705	2236.03	1576.61	0.080	1667.48	133.43
A_68_P31775663	chr18:64648900-64648944	NM_007998:-202	Fech	PROMOTER	0.348	0.574	2138.47	1228.40	0.200	1545.21	308.52
A_68_P31254845	chr17:56395400-56395444	NM_010192:-793	Fem1a	PROMOTER	0.527	0.490	1685.22	825.45	0.258	1313.88	338.91
A_68_P26572463	chr9:62658873-62658917	NM_010193:561	Fem1b	INSIDE	0.604	0.724	2162.60	1565.76	0.437	1624.45	710.21
A_68_P26572470	chr9:62659765-62659809	NM_010193:-331	Fem1b	PROMOTER	0.208	0.734	3215.67	2361.11	0.153	2271.39	347.13
A_68_P31677411	chr18:46685323-46685367	NM_173423:281	Fem1c	INSIDE	0.492	0.694	3789.43	2629.76	0.341	2702.09	922.10
A_68_P31677416	chr18:46685866-46685910	NM_173423:-263	Fem1c	PROMOTER	0.236	0.603	997.57	601.92	0.142	856.38	121.70
A_68_P20351018	chr1:74928813-74928857	NM_153111:3148	Fev	INSIDE	0.283	0.642	3070.75	1970.84	0.181	2408.62	436.84
A_68_P20351043	chr1:74931488-74931534	NM_153111:472	Fev	INSIDE	0.169	0.476	2040.17	971.48	0.080	1514.48	121.80
A_68_P20351044	chr1:74931604-74931648	NM_153111:356	Fev	INSIDE	0.365	0.652	3328.55	2168.66	0.238	2335.52	555.21
A_68_P20351048	chr1:74931966-74932010	NM_153111:-6	Fev	PROMOTER	0.427	0.656	1876.17	1230.17	0.280	1690.43	473.23
A_68_P20351050	chr1:74932235-74932279	NM_153111:-274	Fev	PROMOTER	0.173	2.211	6752.79	14933.72	0.383	4043.83	1549.35

ProbeName	Target position of probe on CpG island microarray	TargetID	GeneSymbol	CpG island Description	Ratio of relative methylation (TiO2-NP/Vehicle)	Sham group			TiO_2-H group		
						Relative methylation (Cy-5/Cy-3)	Cy-3 signal (Input)	Cy-5 signal (MeDIP)	Relative methylation (Cy-5/Cy-3)	Cy-3 signal (Input)	Cy-5 signal (MeDIP)
A_68_P24295385	chr6:23197965-23198012	NM_028462:276	Fezf1	INSIDE	0.097	0.674	2733.93	1843.83	0.066	1863.56	122.39
A_68_P24295386	chr6:23198116-23198160	NM_028462:126	Fezf1	INSIDE	0.093	0.733	3157.65	2316.08	0.069	2097.79	143.85
A_68_P24295435	chr6:23204086-23204130	NM_028462:-5844	Fezf1	PROMOTER	0.600	0.695	1354.26	940.63	0.417	1081.26	450.87
A_68_P29410629	chr14:13173646-13173690	NM_080433:4711	Fezf2	DOWNSTREAM	0.543	0.462	1147.86	529.98	0.250	1052.13	263.56
A_68_P29410630	chr14:13173724-13173771	NM_080433:4632	Fezf2	DOWNSTREAM	0.453	0.655	1059.17	693.65	0.296	846.28	250.92
A_68_P27357056	chr10:93498664-93498716	NM_053072:-55	Fgd6	DIVERGENT_PROMOTER	0.295	0.553	975.06	538.97	0.163	744.85	121.32
A_68_P27357059	chr10:93498957-93499001	NM_053072:233	Fgd6	INSIDE	0.267	0.491	1250.55	613.55	0.131	900.05	117.91
A_68_P27357060	chr10:93499035-93499079	NM_053072:311	Fgd6	INSIDE	0.525	0.653	1340.98	875.59	0.343	1185.84	406.51
A_68_P27897164	chr11:69614462-69614506	NM_010198:643	Fgf11	INSIDE	0.340	0.317	2021.17	640.82	0.108	1463.23	157.89
A_68_P27897167	chr11:69614867-69614911	NM_010198:239	Fgf11	INSIDE	0.129	0.646	2032.92	1312.96	0.083	1410.84	117.31
A_68_P27897170	chr11:69615173-69615217	NM_010198:-67	Fgf11	PROMOTER	0.387	0.494	1422.97	703.22	0.191	1141.34	218.27
A_68_P30630405	chr16:28445397-28445447	NM_183064:-109	Fgf12	PROMOTER	0.132	0.604	1991.04	1201.71	0.080	1536.08	122.51
A_68_P30630410	chr16:28445868-28445912	NM_183064:-577	Fgf12	PROMOTER	0.510	0.707	2576.61	1821.60	0.360	1966.69	708.89
A_68_P25609911	chr7:152081721-152081765	NM_008003:-694	Fgf15	PROMOTER	0.442	0.444	1253.54	556.52	0.196	989.14	193.93
A_68_P29702644	chr14:71041729-71041773	NM_008004:325	Fgf17	INSIDE	0.403	0.627	2188.74	1371.28	0.252	1859.32	469.04
A_68_P24830594	chr6:127030212-127030256	NM_022657:7315	Fgf23	INSIDE	0.562	0.643	796.56	512.55	0.362	643.98	232.88
A_68_P24830597	chr6:127030465-127030509	NM_022657:7567	Fgf23	INSIDE	0.545	0.659	3245.69	2139.62	0.359	2522.13	906.67
A_68_P25609547	chr7:152025243-152025287	NM_008007:748	Fgf3	INSIDE	0.539	0.604	1651.95	997.23	0.325	1374.01	446.97
A_68_P25609683	chr7:152047109-152047153	NM_010202:-160	Fgf4	PROMOTER	0.342	0.530	2799.23	1484.20	0.181	2301.08	416.77
A_68_P25609691	chr7:152047946-152047990	NM_010202:678	Fgf4	INSIDE	0.412	0.492	1642.75	808.69	0.203	1186.41	240.81
A_68_P23917030	chr5:98682994-98683038	NM_010203:-186	Fgf5	PROMOTER	0.395	0.553	1859.16	1027.23	0.218	1360.03	296.52
A_68_P23917031	chr5:98683201-98683245	NM_010203:20	Fgf5	INSIDE	0.175	0.699	1750.58	1224.45	0.122	1265.06	154.45
A_68_P23917033	chr5:98683430-98683474	NM_010203:250	Fgf5	INSIDE	0.327	0.615	1605.45	987.03	0.201	1412.65	283.62
A_68_P32140185	chr19:45811804-45811848	NM_001166361:5548	Fgf8	INSIDE	0.378	0.518	1130.07	584.84	0.196	886.64	173.41
A_68_P32140226	chr19:45817015-45817059	NM_001166361:338	Fgf8	INSIDE	0.480	0.537	1065.37	572.62	0.258	836.27	215.78
A_68_P29631501	chr14:58694549-58694593	NM_013518:3048	Fgf9	INSIDE	0.478	0.378	1966.29	743.61	0.181	1526.29	276.13
A_68_P29631506	chr14:58695032-58695076	NM_013518:3532	Fgf9	INSIDE	0.150	0.698	2848.64	1988.57	0.105	2150.26	225.35
A_68_P29631507	chr14:58695104-58695149	NM_013518:3604	Fgf9	INSIDE	0.221	0.553	1110.80	614.03	0.122	940.82	114.94
A_68_P25520717	chr7:137409140-137409185	NM_010207:1160	Fgfr2	INSIDE	0.083	1.362	2061.58	2807.24	0.113	1466.40	165.91
A_68_P23590777	chr5:34064147-34064191	NM_001163215:-204	Fgfr3	PROMOTER	0.354	0.542	989.97	536.39	0.192	732.60	140.47
A_68_P23590781	chr5:34064587-34064631	NM_001163216:-346	Fgfr3	PROMOTER	0.405	0.530	3266.43	1729.91	0.215	2438.35	523.55
A_68_P23590782	chr5:34064726-34064770	NM_001163216:-206	Fgfr3	PROMOTER	0.531	0.689	1161.14	800.35	0.366	854.02	312.68
A_68_P29053524	chr13:55254501-55254545	NM_008011:344	Fgfr4	INSIDE	0.406	1.439	1816.38	2614.22	0.584	1466.89	857.10
A_68_P23973608	chr5:109123109-109123155	NM_001164259:-115	Fgfrl1	PROMOTER	0.314	0.691	5348.29	3695.93	0.217	4106.77	890.10
A_68_P23973612	chr5:109123736-109123780	NM_001164259:511	Fgfrl1	INSIDE	0.255	0.415	1763.84	732.32	0.106	1305.41	138.36
A_68_P20849152	chr1:177555795-177555846	NM_010209:-54	Fh1	DIVERGENT_PROMOTER	0.066	1.365	2170.50	2961.80	0.090	1673.46	150.99
A_68_P22291215	chr3:84284164-84284208	NM_001205355:-773	Fhdc1	PROMOTER	0.387	0.475	3144.88	1494.82	0.184	2494.26	458.70
A_68_P22291219	chr3:84284726-84284770	NM_001033301:-387	Fhdc1	PROMOTER	0.666	0.447	2105.73	940.81	0.297	1601.10	476.27
A_68_P32397420	chrX:53985031-53985075	NM_001077361:-81	Fhl1	PROMOTER	0.595	2.929	2221.21	6506.14	1.743	1013.79	1767.47
A_68_P23269774	chr4:124377782-124377826	NM_010213:-138	Fhl3	DIVERGENT_PROMOTER	0.305	0.537	3301.51	1771.42	0.163	2410.10	393.97
A_68_P23269775	chr4:124377958-124378002	NM_010213:38	Fhl3	INSIDE	0.490	0.538	2623.76	1410.84	0.263	2064.18	543.47
A_68_P26133209	chr8:107871674-107871718	NM_177699:174	Fhod1	INSIDE	0.195	0.514	1692.58	869.66	0.100	1245.76	125.01
A_68_P31561531	chr18:24868543-24868587	NM_175276:619	Fhod3	INSIDE	0.149	0.620	1660.31	1029.07	0.092	1352.22	125.06
A_68_P21111659	chr2:31701359-31701403	NM_178887:145	Fibcd1	INSIDE	0.503	0.479	1794.74	859.85	0.241	1531.96	369.25

ProbeName	Target position of probe on CpG island microarray	TargetID	GeneSymbol	CpG island Description	Ratio of relative methylation (TiO2-NP/Vehicle)	Sham group			TiO_2-H group		
						Relative methylation (Cy-5/Cy-3)	Cy-3 signal (Input)	Cy-5 signal (MeDIP)	Relative methylation (Cy-5/Cy-3)	Cy-3 signal (Input)	Cy-5 signal (MeDIP)
A_68_P21111665	chr2:31702261-31702305	NM_178887:-757	Fibcd1	PROMOTER	0.515	0.479	2368.72	1135.18	0.247	1803.38	445.06
A_68_P23999715	chr5:114185534-114185578	NM_001010825:-234	Ficd	PROMOTER	0.327	0.464	2132.62	988.98	0.152	1495.81	226.98
A_68_P23999718	chr5:114185852-114185896	NM_001010825:84	Ficd	INSIDE	0.641	0.455	3096.15	1410.16	0.292	2435.03	711.35
A_68_P24607722	chr6:85978843-85978887	NM_012013:11680	Figla	DOWNSTREAM	0.374	0.504	1389.48	699.90	0.188	1073.81	202.30
A_68_P24607723	chr6:85978919-85978963	NM_012013:11756	Figla	DOWNSTREAM	0.164	0.503	1886.70	948.48	0.082	1424.37	117.35
A_68_P24607724	chr6:85978997-85979041	NM_012013:11834	Figla	DOWNSTREAM	0.294	0.395	1372.49	541.51	0.116	1018.25	118.25
A_68_P23803966	chr5:74931407-74931451	NM_001159573:-78	Fip1l1	DIVERGENT_PROMOTER	0.362	0.678	2028.43	1376.00	0.245	1754.68	430.30
A_68_P21477082	chr2:102290453-102290497	NM_010218:1475	Fjx1	INSIDE	0.135	1.859	930.80	1730.25	0.250	714.43	178.68
A_68_P21477083	chr2:102290541-102290585	NM_010218:1387	Fjx1	INSIDE	0.345	0.690	1870.10	1290.36	0.238	1501.93	357.29
A_68_P21477084	chr2:102290645-102290692	NM_010218:1281	Fjx1	INSIDE	0.422	0.535	1716.13	917.66	0.226	1340.04	302.63
A_68_P22963965	chr4:62021291-62021335	NM_001045528:270	Fkbp15	INSIDE	0.209	0.417	1761.10	734.52	0.087	1367.87	119.46
A_68_P25952440	chr8:73051499-73051543	NM_001111066:-121	Fkbp8	DIVERGENT_PROMOTER	0.335	0.630	1257.36	792.22	0.211	901.38	189.99
A_68_P22924772	chr4:53727484-53727528	NM_139309:453	Fktn	INSIDE	0.560	0.679	1666.83	1132.53	0.381	1370.51	521.66
A_68_P27839121	chr11:59623157-59623201	NM_146018:97	Flcn	INSIDE	0.665	0.491	2653.43	1302.98	0.327	1880.19	614.39
A_68_P26406144	chr9:32348552-32348596	NM_008026:379	Fli1	INSIDE	0.549	0.569	917.17	522.06	0.312	752.94	235.12
A_68_P26406146	chr9:32348818-32348862	NM_008026:113	Fli1	INSIDE	0.197	0.696	4697.42	3267.47	0.137	3409.21	468.18
A_68_P26406181	chr9:32353158-32353202	NM_008026:-4227	Fli1	PROMOTER	0.185	0.460	1778.13	818.45	0.085	1447.37	123.32
A_68_P32467037	chrX:71491458-71491502	NM_010227:393	Flna	INSIDE	0.126	1.843	3760.51	6930.66	0.232	1298.04	301.57
A_68_P32467038	chrX:71491563-71491607	NM_010227:289	Flna	INSIDE	0.152	1.538	2086.99	3209.16	0.234	721.94	168.92
A_68_P32467039	chrX:71491630-71491682	NM_010227:217	Flna	INSIDE	0.103	1.642	2153.63	3535.33	0.168	716.93	120.63
A_68_P29386050	chr14:8650925-8650970	NM_134080:477	Flnb	INSIDE	0.274	0.496	1103.67	547.52	0.136	868.91	117.93
A_68_P29386051	chr14:8651064-8651108	NM_134080:616	Flnb	INSIDE	0.136	0.522	2409.82	1257.60	0.071	1705.76	121.46
A_68_P29386052	chr14:8651174-8651222	NM_134080:728	Flnb	INSIDE	0.150	0.545	2078.34	1133.72	0.082	1452.92	118.54
A_68_P24326457	chr6:29383678-29383722	NM_001081185:548	Flnc	INSIDE	0.559	0.668	1801.03	1203.76	0.373	1262.17	471.32
A_68_P28656381	chr12:96933833-96933877	NM_201518:3419	Flrt2	INSIDE	0.470	0.524	1366.33	715.37	0.246	1140.47	280.64
A_68_P24175497	chr5:148537110-148537154	NM_010228:432	Flt1	INSIDE	0.420	0.617	2038.47	1257.73	0.259	1729.64	447.73
A_68_P24175503	chr5:148537797-148537841	NM_010228:-254	Flt1	PROMOTER	0.222	0.456	2386.15	1088.41	0.101	1826.77	185.09
A_68_P24173494	chr5:148211545-148211589	NM_010229:499	Flt3	INSIDE	0.580	0.536	1212.71	649.66	0.311	1069.32	332.11
A_68_P24173495	chr5:148211661-148211705	NM_010229:383	Flt3	INSIDE	0.291	0.651	1303.31	848.05	0.189	1103.09	208.52
A_68_P27783947	chr11:49447973-49448018	NM_008029:24815	Flt4	INSIDE	0.526	1.367	3184.53	4353.73	0.719	2326.58	1673.18
A_68_P31093565	chr17:23908024-23908068	NM_153791:512	Flywch1	INSIDE	0.200	0.580	1344.49	779.38	0.116	1002.94	116.44
A_68_P31093570	chr17:23908581-23908625	NM_153791:-44	Flywch1	PROMOTER	0.278	0.546	1754.60	957.21	0.152	1401.58	212.82
A_68_P20842586	chr1:176432169-176432213	NM_019445:235	Fmn2	INSIDE	0.229	0.618	1096.30	677.62	0.141	846.06	119.63
A_68_P20842587	chr1:176432270-176432314	NM_019445:337	Fmn2	INSIDE	0.204	0.704	4111.06	2893.75	0.144	2781.22	399.20
A_68_P20842589	chr1:176432473-176432517	NM_019445:539	Fmn2	INSIDE	0.123	0.446	3203.00	1427.30	0.055	2178.44	119.27
A_68_P20842590	chr1:176432550-176432594	NM_019445:617	Fmn2	INSIDE	0.181	0.638	1574.02	1004.34	0.115	1155.67	133.46
A_68_P28083893	chr11:103060694-103060738	NM_001077698:28265	Fmnl1	DOWNSTREAM	0.438	0.515	2513.03	1294.04	0.225	1828.85	412.35
A_68_P21221945	chr2:52717198-52717242	NM_172409:319	Fmnl2	INSIDE	0.340	0.583	2460.09	1434.58	0.198	1766.26	349.74
A_68_P21221946	chr2:52717355-52717399	NM_172409:475	Fmnl2	INSIDE	0.618	0.490	2710.32	1329.41	0.303	2104.41	638.12
A_68_P30479788	chr15:99200288-99200332	NM_011711:587	Fmnl3	INSIDE	0.503	0.512	1140.24	583.70	0.258	798.12	205.64
A_68_P30479790	chr15:99200609-99200653	NM_011711:267	Fmnl3	INSIDE	0.357	0.491	4277.39	2100.99	0.175	3175.08	557.23
A_68_P20332283	chr1:71699233-71699279	NM_010233:489	Fn1	INSIDE	0.297	0.563	930.89	524.18	0.167	694.08	116.20
A_68_P20332287	chr1:71699659-71699703	NM_010233:65	Fn1	INSIDE	0.322	0.637	2362.01	1505.54	0.205	1784.10	366.11
A_68_P20332288	chr1:71699744-71699788	NM_010233:-21	Fn1	PROMOTER	0.149	0.581	1889.71	1097.16	0.086	1357.67	117.40

ProbeName	Target position of probe on CpG island microarray	TargetID	GeneSymbol	CpG island Description	Ratio of relative methylation (TiO2-NP/Vehicle)	Sham group			TiO_2-H group		
						Relative methylation (Cy-5/Cy-3)	Cy-3 signal (Input)	Cy-5 signal (MeDIP)	Relative methylation (Cy-5/Cy-3)	Cy-3 signal (Input)	Cy-5 signal (MeDIP)
A_68_P20332289	chr1:71699909-71699953	NM_010233:-185	Fn1	PROMOTER	0.108	0.569	2794.25	1591.26	0.061	1927.05	118.48
A_68_P21107212	chr2:30996536-30996580	NM_001038700:970	Fnbp1	INSIDE	0.167	0.663	1847.81	1224.67	0.111	1328.38	146.85
A_68_P21107213	chr2:30996649-30996693	NM_001038700:858	Fnbp1	INSIDE	0.155	0.658	1885.44	1241.40	0.102	1219.02	124.70
A_68_P21107214	chr2:30996795-30996839	NM_001038700:712	Fnbp1	INSIDE	0.240	0.658	2276.84	1498.09	0.158	1765.69	278.45
A_68_P22483658	chr3:122321383-122321434	NM_001114665:1177	Fnbp1l	INSIDE	0.193	0.592	1512.14	894.47	0.114	1050.53	120.14
A_68_P29712961	chr14:72932323-72932367	NM_207636:177466	Fndc3a	DOWNSTREAM	0.246	0.458	1223.38	559.76	0.113	1044.89	117.72
A_68_P29713895	chr14:73110156-73110200	NM_207636:-368	Fndc3a	PROMOTER	0.158	0.678	1409.74	956.30	0.107	1165.62	125.23
A_68_P27810087	chr11:54251592-54251636	NM_173753:-66	Fnip1	PROMOTER	0.569	0.658	2478.66	1632.10	0.375	2100.32	787.14
A_68_P28604312	chr12:86814373-86814417	NM_010234:-456	Fos	PROMOTER	0.155	0.490	1847.43	904.56	0.076	1536.61	116.89
A_68_P28604396	chr12:86827240-86827284	NM_010234:12412	Fos	DOWNSTREAM	0.597	0.620	1083.29	671.61	0.370	897.62	332.01
A_68_P28604402	chr12:86827971-86828024	NM_010234:13147	Fos	DOWNSTREAM	0.158	0.618	3691.79	2280.92	0.097	2586.93	252.13
A_68_P24992574	chr7:19890287-19890331	NM_008036:5086	Fosb	INSIDE	0.185	0.518	2361.03	1223.60	0.096	1677.33	160.66
A_68_P24992575	chr7:19890451-19890495	NM_008036:4922	Fosb	INSIDE	0.194	0.507	2537.61	1286.21	0.099	1860.47	183.26
A_68_P24992615	chr7:19895551-19895595	NM_008036:-178	Fosb	PROMOTER	0.339	0.558	1114.98	622.51	0.189	933.85	176.96
A_68_P24992660	chr7:19905290-19905334	NM_008036:-9918	Fosb	PROMOTER	0.123	0.710	2042.26	1450.16	0.087	1557.15	135.59
A_68_P31928718	chr19:5447671-5447715	NM_010235:-5	Fosl1	PROMOTER	0.632	0.622	1066.72	663.68	0.393	950.71	373.71
A_68_P31928719	chr19:5447756-5447800	NM_010235:81	Fosl1	INSIDE	0.584	0.663	1175.44	778.85	0.387	992.25	383.70
A_68_P31928721	chr19:5447928-5447972	NM_010235:253	Fosl1	INSIDE	0.513	0.555	2362.33	1312.03	0.285	1703.15	485.09
A_68_P23582142	chr5:32435789-32435833	NM_008037:-3034	Fosl2	PROMOTER	0.156	0.658	1698.17	1117.73	0.103	1267.59	130.06
A_68_P23582162	chr5:32438904-32438948	NM_008037:82	Fosl2	INSIDE	0.652	0.693	1512.24	1047.93	0.451	1175.62	530.76
A_68_P28463161	chr12:58640100-58640144	NM_008259:6986	Foxa1	DOWNSTREAM	0.279	0.579	959.17	555.42	0.162	804.71	130.17
A_68_P28463221	chr12:58647081-58647125	NM_008259:6	Foxa1	INSIDE	0.606	0.499	1992.93	993.54	0.302	1599.98	483.68
A_68_P21727904	chr2:147870499-147870543	NM_010446:2185	Foxa2	INSIDE	0.558	0.632	2231.15	1409.17	0.353	1644.72	580.08
A_68_P21727905	chr2:147870580-147870624	NM_010446:2103	Foxa2	INSIDE	0.104	0.662	8649.96	5730.24	0.069	6086.19	419.37
A_68_P21727910	chr2:147871145-147871199	NM_010446:1533	Foxa2	INSIDE	0.212	0.488	1554.78	758.55	0.104	1116.50	115.72
A_68_P21727914	chr2:147871631-147871680	NM_010446:1050	Foxa2	INSIDE	0.296	0.644	871.17	561.09	0.191	628.51	119.74
A_68_P21727918	chr2:147872154-147872201	NM_010446:528	Foxa2	INSIDE	0.502	0.593	1016.49	602.73	0.298	746.00	222.03
A_68_P21727937	chr2:147874425-147874470	NM_010446:-1742	Foxa2	PROMOTER	0.385	0.672	1303.99	875.82	0.258	1089.74	281.64
A_68_P26612672	chr9:69607372-69607416	NM_022378:1353	Foxb1	INSIDE	0.360	0.654	1741.68	1139.07	0.236	1300.54	306.29
A_68_P26612681	chr9:69608333-69608380	NM_022378:391	Foxb1	INSIDE	0.262	0.575	1502.34	863.18	0.151	1310.12	197.56
A_68_P26612686	chr9:69609029-69609073	NM_022378:-303	Foxb1	PROMOTER	0.565	0.672	2272.22	1527.08	0.380	1816.04	689.95
A_68_P26612687	chr9:69609097-69609152	NM_022378:-377	Foxb1	PROMOTER	0.254	0.584	1028.12	600.77	0.149	776.19	115.31
A_68_P31982703	chr19:16947053-16947097	NM_008023:1246	Foxb2	INSIDE	0.235	0.394	1598.16	629.53	0.093	1266.31	117.41
A_68_P31982708	chr19:16947889-16947933	NM_008023:410	Foxb2	INSIDE	0.358	0.689	1490.93	1026.98	0.247	1092.31	269.29
A_68_P31982719	chr19:16949088-16949132	NM_008023:-790	Foxb2	PROMOTER	0.193	0.442	1831.95	810.28	0.085	1445.01	123.30
A_68_P28925094	chr13:31898196-31898240	NM_008592:-296	Foxc1	PROMOTER	0.586	0.561	1913.30	1073.09	0.329	1442.34	474.33
A_68_P28925105	chr13:31899742-31899786	NM_008592:1250	Foxc1	INSIDE	0.212	0.352	2137.96	753.60	0.075	1619.39	121.05
A_68_P26224615	chr8:123641830-123641874	NM_013519:1782	Foxc2	INSIDE	0.652	0.439	1379.86	606.37	0.286	1020.43	292.22
A_68_P29257118	chr13:99124161-99124205	NM_008242:-17	Foxd1	PROMOTER	0.402	0.510	1490.33	760.15	0.205	1157.08	237.16
A_68_P23222200	chr4:114574734-114574778	NM_008593:6747	Foxd2	DOWNSTREAM	0.594	0.415	1753.67	727.13	0.246	1345.87	331.71
A_68_P23222252	chr4:114581006-114581050	NM_008593:475	Foxd2	INSIDE	0.653	0.663	984.16	652.19	0.433	828.85	358.66
A_68_P23145500	chr4:99317555-99317599	NM_010425:-5413	Foxd3	PROMOTER	0.473	0.624	2532.12	1581.00	0.295	1875.23	553.52
A_68_P23145525	chr4:99320580-99320624	NM_010425:-2387	Foxd3	PROMOTER	0.567	0.458	1131.51	518.60	0.260	984.49	256.00
A_68_P23145544	chr4:99322690-99322737	NM_010425:-276	Foxd3	PROMOTER	0.299	0.633	841.55	532.51	0.189	638.07	120.81

ProbeName	Target position of probe on CpG island microarray	TargetID	GeneSymbol	CpG island Description	Ratio of relative methylation (TiO2-NP/Vehicle)	Sham group			TiO_2-H group		
						Relative methylation (Cy-5/Cy-3)	Cy-3 signal (Input)	Cy-5 signal (MeDIP)	Relative methylation (Cy-5/Cy-3)	Cy-3 signal (Input)	Cy-5 signal (MeDIP)
A_68_P23145554	chr4:99323682-99323726	NM_010425:715	Foxd3	INSIDE	0.189	0.694	4633.99	3218.02	0.131	3146.09	412.53
A_68_P32026410	chr19:24971751-24971795	NM_008022:4027	Foxd4	DOWNSTREAM	0.435	0.395	1349.91	532.92	0.172	1103.84	189.46
A_68_P32026412	chr19:24971921-24971965	NM_008022:3857	Foxd4	DOWNSTREAM	0.380	0.406	1833.59	744.71	0.154	1366.72	210.77
A_68_P32026439	chr19:24975135-24975179	NM_008022:643	Foxd4	INSIDE	0.666	0.698	2978.11	2077.88	0.465	2138.93	993.63
A_68_P22888257	chr4:46356845-46356889	NM_183298:-199	Foxe1	PROMOTER	0.375	0.620	1158.31	717.90	0.232	1056.51	245.61
A_68_P23222364	chr4:114598169-114598213	NM_015758:428	Foxe3	INSIDE	0.335	0.481	2199.74	1058.09	0.161	1748.13	281.77
A_68_P23222365	chr4:114598324-114598368	NM_015758:272	Foxe3	INSIDE	0.271	0.530	1483.79	787.09	0.143	1185.89	170.17
A_68_P23222366	chr4:114598399-114598443	NM_015758:198	Foxe3	INSIDE	0.326	0.370	2556.91	946.80	0.121	2042.06	246.38
A_68_P23222368	chr4:114598578-114598622	NM_015758:18	Foxe3	INSIDE	0.276	0.630	2720.76	1714.30	0.174	2103.50	366.27
A_68_P26224342	chr8:123606667-123606711	NM_010426:-1685	Foxf1a	PROMOTER	0.602	0.463	1434.04	663.83	0.279	1035.19	288.39
A_68_P26224353	chr8:123607977-123608021	NM_010426:-375	Foxf1a	PROMOTER	0.397	0.504	1890.38	952.54	0.200	1686.45	337.20
A_68_P28924020	chr13:31712001-31712045	NM_010225:-5662	Foxf2	PROMOTER	0.352	0.535	1077.89	576.66	0.188	951.86	179.28
A_68_P28924024	chr13:31712368-31712412	NM_010225:-5294	Foxf2	PROMOTER	0.522	0.634	1177.27	746.26	0.331	925.39	306.28
A_68_P28924076	chr13:31718416-31718460	NM_010225:754	Foxf2	INSIDE	0.268	0.525	1141.36	599.10	0.141	950.70	133.64
A_68_P28924078	chr13:31718670-31718714	NM_010225:1008	Foxf2	INSIDE	0.152	0.455	2481.07	1129.51	0.069	1703.53	118.07
A_68_P28924079	chr13:31718773-31718817	NM_010225:1110	Foxf2	INSIDE	0.607	0.700	4496.20	3149.18	0.425	3390.73	1440.90
A_68_P28924080	chr13:31718919-31718963	NM_010225:1256	Foxf2	INSIDE	0.407	0.689	3172.57	2185.00	0.281	2213.14	621.08
A_68_P28924098	chr13:31721161-31721205	NM_010225:3498	Foxf2	INSIDE	0.365	0.641	1875.27	1201.92	0.234	1376.93	321.71
A_68_P28924099	chr13:31721274-31721321	NM_010225:3613	Foxf2	INSIDE	0.315	0.404	1337.29	540.05	0.127	908.42	115.62
A_68_P28419951	chr12:50484189-50484235	NM_008241:219	Foxg1	INSIDE	0.143	0.663	1701.63	1128.29	0.095	1264.70	119.59
A_68_P28419964	chr12:50485980-50486024	NM_008241:2009	Foxg1	INSIDE	0.472	0.553	2152.69	1189.94	0.261	1712.87	446.76
A_68_P28419966	chr12:50486205-50486249	NM_008241:2233	Foxg1	INSIDE	0.311	0.427	1752.30	747.96	0.133	1278.08	169.42
A_68_P28419968	chr12:50486468-50486512	NM_008241:2497	Foxg1	INSIDE	0.085	0.715	3350.25	2393.93	0.061	2453.86	149.14
A_68_P28419969	chr12:50486658-50486702	NM_008241:2687	Foxg1	INSIDE	0.620	0.691	2377.01	1642.31	0.428	1716.69	735.02
A_68_P24533906	chr6:70906262-70906307	NM_001101464:-315	Foxi3	PROMOTER	0.159	0.633	3695.05	2340.27	0.101	2582.63	260.88
A_68_P24533907	chr6:70906387-70906431	NM_001101464:-191	Foxi3	PROMOTER	0.457	0.683	5098.82	3483.92	0.313	3316.49	1036.53
A_68_P24533908	chr6:70906504-70906548	NM_001101464:-73	Foxi3	PROMOTER	0.652	0.571	1723.25	984.33	0.373	1304.38	486.08
A_68_P24533909	chr6:70906592-70906636	NM_001101464:15	Foxi3	INSIDE	0.244	0.417	1622.51	676.55	0.102	1155.70	117.56
A_68_P24533914	chr6:70907055-70907100	NM_001101464:478	Foxi3	INSIDE	0.474	0.616	1034.28	637.53	0.292	821.83	240.22
A_68_P28158771	chr11:116195721-116195765	NM_008240:926	Foxj1	INSIDE	0.308	0.423	1299.01	549.84	0.130	965.13	125.85
A_68_P28158778	chr11:116196536-116196580	NM_008240:110	Foxj1	INSIDE	0.603	0.698	1889.70	1318.66	0.421	1406.04	591.32
A_68_P28158782	chr11:116197021-116197065	NM_008240:-374	Foxj1	PROMOTER	0.285	0.470	1201.62	564.88	0.134	868.88	116.34
A_68_P26224676	chr8:123649716-123649760	NM_008024:-1846	Foxl1	PROMOTER	0.178	0.486	1908.77	927.75	0.086	1476.05	127.61
A_68_P26224698	chr8:123652159-123652203	NM_008024:596	Foxl1	INSIDE	0.153	0.610	1808.61	1102.87	0.094	1324.91	123.89
A_68_P26224705	chr8:123653054-123653101	NM_008024:1493	Foxl1	INSIDE	0.498	0.652	1045.68	681.81	0.325	798.27	259.20
A_68_P26761439	chr9:98857310-98857354	NM_012020:1307	Foxl2	INSIDE	0.646	0.380	2619.83	996.83	0.246	2193.86	538.88
A_68_P26761442	chr9:98857658-98857702	NM_012020:1655	Foxl2	INSIDE	0.204	0.615	1224.55	752.82	0.125	962.15	120.56
A_68_P26761419	chr9:98855194-98855238	NR_003248:513	Foxl2os	INSIDE	0.258	0.554	995.23	551.29	0.143	826.08	117.92
A_68_P26761425	chr9:98855848-98855892	NR_003248:-141	Foxl2os	DIVERGENT_PROMOTER	0.379	0.439	1241.13	544.51	0.166	1052.26	175.12
A_68_P24838573	chr6:128313280-128313324	NM_008021:291	Foxm1	INSIDE	0.335	0.536	2103.41	1127.86	0.179	1542.37	276.72
A_68_P31427927	chr17:88841025-88841069	NM_180974:995	Foxn2	INSIDE	0.265	0.520	3096.73	1609.43	0.138	2419.52	332.78
A_68_P24002948	chr5:114724032-114724076	NM_148935:-284	Foxn4	PROMOTER	0.627	0.538	1086.21	584.61	0.338	861.39	290.73
A_68_P24002949	chr5:114724178-114724222	NM_148935:-430	Foxn4	PROMOTER	0.305	0.583	2697.56	1572.79	0.178	1877.98	334.28
A_68_P27094090	chr10:41997470-41997514	NM_019740:-944	Foxo3	PROMOTER	0.259	0.371	3378.20	1254.08	0.096	2531.14	243.64

ProbeName	Target position of probe on CpG island microarray	TargetID	GeneSymbol	CpG island Description	Ratio of relative methylation (TiO2-NP/Vehicle)	Sham group			TiO_2-H group		
						Relative methylation (Cy-5/Cy-3)	Cy-3 signal (Input)	Cy-5 signal (MeDIP)	Relative methylation (Cy-5/Cy-3)	Cy-3 signal (Input)	Cy-5 signal (MeDIP)
A_68_P23250455	chr4:119959495-119959539	NM_194060:350	Foxo6	INSIDE	0.175	0.463	1916.34	888.09	0.081	1501.80	121.52
A_68_P23250459	chr4:119960018-119960062	NM_194060:-174	Foxo6	PROMOTER	0.522	0.507	6242.18	3163.84	0.264	4501.15	1190.08
A_68_P31214659	chr17:48009732-48009776	NM_001110824:51827	Foxp4	INSIDE	0.284	1.607	1108.44	1780.99	0.456	915.59	417.32
A_68_P28923675	chr13:31650511-31650560	NM_008239:497	Foxq1	INSIDE	0.664	0.607	1112.82	675.23	0.403	946.63	381.14
A_68_P28923680	chr13:31651196-31651240	NM_008239:1180	Foxq1	INSIDE	0.190	0.435	1817.96	791.00	0.083	1430.00	118.14
A_68_P28923679	chr13:31651001-31651045	NM_008239:984	Foxq1	INSIDE	0.670	0.669	2172.00	1452.05	0.448	1561.09	698.77
A_68_P21116904	chr2:32549583-32549627	NM_010236:91	Fpgs	INSIDE	0.442	0.533	2061.20	1098.37	0.236	1755.02	413.65
A_68_P22666539	chr3:154756237-154756282	NM_029330:83	Fpgt	INSIDE	0.253	0.578	1023.23	591.59	0.146	824.71	120.69
A_68_P32117376	chr19:41904363-41904407	NM_008043:-75	Frat1	PROMOTER	0.628	0.617	1990.52	1228.56	0.387	1661.84	643.79
A_68_P32117486	chr19:41921651-41921695	NM_177603:950	Frat2	INSIDE	0.474	0.525	2050.18	1075.58	0.249	1582.09	393.70
A_68_P26000510	chr8:83135226-83135270	NM_001167898:311	Frem3	INSIDE	0.473	0.687	1682.60	1156.70	0.325	1417.04	460.90
A_68_P23018911	chr4:73659893-73659937	NM_172869:408	Frmd3	INSIDE	0.412	0.571	1375.71	786.02	0.235	1140.99	268.36
A_68_P20967779	chr2:4322142-4322186	NM_001177843:143	Frmd4a	INSIDE	0.207	0.513	1447.52	742.55	0.106	1134.20	120.21
A_68_P24674230	chr6:97567022-97567066	NM_145148:607	Frmd4b	INSIDE	0.469	0.572	1018.31	582.90	0.268	869.90	233.50
A_68_P24674231	chr6:97567120-97567164	NM_145148:509	Frmd4b	INSIDE	0.448	0.560	5348.50	2992.85	0.251	3784.83	949.10
A_68_P24674232	chr6:97567208-97567252	NM_145148:421	Frmd4b	INSIDE	0.499	0.668	1871.75	1250.40	0.333	1258.95	419.73
A_68_P32815027	chrX:164560836-164560880	NM_001033330:454307	Frmpd4	INSIDE	0.347	1.463	933.95	1366.66	0.508	557.14	283.14
A_68_P27479719	chr10:116584876-116584920	NM_177798:632	Frs2	INSIDE	0.626	0.734	2670.68	1961.34	0.460	1711.13	786.38
A_68_P31213681	chr17:47831661-47831705	NM_144939:-473	Frs3	DIVERGENT_PROMOTER	0.159	0.476	6187.53	2944.19	0.076	3978.62	301.12
A_68_P31213682	chr17:47831829-47831878	NM_144939:-302	Frs3	DIVERGENT_PROMOTER	0.156	0.518	2006.03	1039.56	0.081	1492.51	121.04
A_68_P23795929	chr5:73524595-73524639	NM_028194:123241	Fryl	INSIDE	0.621	2.907	717.25	2084.89	1.804	617.36	1113.74
A_68_P23796634	chr5:73647594-73647638	NM_028194:241	Fryl	INSIDE	0.612	0.645	854.45	551.52	0.395	702.24	277.44
A_68_P23796637	chr5:73647888-73647932	NM_028194:-53	Fryl	PROMOTER	0.399	0.474	1998.35	946.81	0.189	1621.76	306.32
A_68_P23796638	chr5:73648010-73648054	NM_028194:-175	Fryl	PROMOTER	0.492	0.432	1338.67	578.04	0.212	1027.83	218.19
A_68_P21374843	chr2:80287482-80287526	NM_011356:49	Frzb	INSIDE	0.247	0.517	1865.58	964.17	0.127	1511.34	192.69
A_68_P21374845	chr2:80287756-80287800	NM_011356:-225	Frzb	PROMOTER	0.587	0.598	1486.51	889.05	0.351	1228.64	431.51
A_68_P29346589	chr13:115249074-115249124	NM_008046:-160	Fst	PROMOTER	0.071	1.475	1858.64	2741.70	0.105	1247.68	130.53
A_68_P27278867	chr10:79240093-79240137	NM_031380:96	Fstl3	INSIDE	0.241	1.481	8370.81	12393.22	0.357	5246.39	1873.49
A_68_P27278868	chr10:79240243-79240287	NM_031380:246	Fstl3	INSIDE	0.596	2.279	5870.34	13380.16	1.358	3783.22	5138.65
A_68_P27800930	chr11:52578204-52578248	NM_177059:19	Fstl4	INSIDE	0.303	0.526	1767.90	929.20	0.159	1438.16	228.84
A_68_P27800933	chr11:52578613-52578657	NM_177059:427	Fstl4	INSIDE	0.336	0.529	2350.07	1242.50	0.178	1799.39	319.63
A_68_P27800935	chr11:52578820-52578864	NM_177059:635	Fstl4	INSIDE	0.264	0.604	2257.04	1363.23	0.159	1817.90	289.88
A_68_P31947674	chr19:10056973-10057017	NM_010239:-198	Fth1	PROMOTER	0.260	0.530	1611.95	854.66	0.138	1292.12	178.30
A_68_P26058236	chr8:93837616-93837660	NM_011936:215	Fto	INSIDE	0.630	0.579	1268.19	734.29	0.365	940.46	343.04
A_68_P26161392	chr8:112741955-112741999	NM_146215:117	Ftsjd1	INSIDE	0.323	0.714	3057.34	2182.95	0.231	2212.69	510.79
A_68_P22649280	chr3:151873415-151873459	NM_057172:15	Fubp1	INSIDE	0.232	0.596	2909.92	1734.84	0.138	1925.74	266.55
A_68_P21109954	chr2:31428423-31428467	NM_001033389:200	Fubp3	INSIDE	0.660	0.417	1273.80	531.61	0.275	1152.89	317.49
A_68_P23331228	chr4:135476820-135476864	NM_024243:202	Fuca1	INSIDE	0.324	0.616	1569.76	967.72	0.200	1295.05	258.75
A_68_P26165492	chr8:113426392-113426436	NM_181666:-55	Fuk	PROMOTER	0.670	0.566	1644.99	931.16	0.379	1458.43	553.00
A_68_P25508346	chr7:135110839-135110887	NM_139149:-130	Fus	PROMOTER	0.307	0.360	1514.75	544.96	0.111	1206.26	133.34
A_68_P29448410	chr14:21514889-21514933	NM_028428:721	Fut11	INSIDE	0.270	0.353	1663.01	586.40	0.095	1267.34	120.88
A_68_P29448411	chr14:21514995-21515039	NM_028428:827	Fut11	INSIDE	0.162	0.621	2215.40	1375.21	0.100	1696.01	170.16
A_68_P29448412	chr14:21515116-21515167	NM_028428:952	Fut11	INSIDE	0.306	0.604	948.23	572.44	0.185	646.75	119.45
A_68_P26319145	chr9:14556214-14556258	NM_010242:330	Fut4	INSIDE	0.499	0.513	1155.82	592.86	0.256	969.65	248.00

ProbeName	Target position of probe on CpG island microarray	TargetID	GeneSymbol	CpG island Description	Ratio of relative methylation (TiO2-NP/Vehicle)	Sham group			TiO_2-H group		
						Relative methylation (Cy-5/Cy-3)	Cy-3 signal (Input)	Cy-5 signal (MeDIP)	Relative methylation (Cy-5/Cy-3)	Cy-3 signal (Input)	Cy-5 signal (MeDIP)
A_68_P28558324	chr12:78338946-78338990	NM_016893:-1039	Fut8	PROMOTER	0.393	0.501	2557.23	1281.73	0.197	2032.50	400.01
A_68_P22791943	chr4:25726823-25726867	NM_010243:306	Fut9	INSIDE	0.251	0.613	3352.60	2053.88	0.154	2601.33	399.92
A_68_P22791944	chr4:25726907-25726951	NM_010243:222	Fut9	INSIDE	0.522	0.746	3942.61	2941.31	0.389	3012.64	1172.89
A_68_P25089991	chr7:52151549-52151593	NM_027376:122	Fuz	INSIDE	0.321	0.553	1968.66	1087.93	0.177	1562.48	277.33
A_68_P25089992	chr7:52151653-52151697	NM_027376:226	Fuz	INSIDE	0.291	0.609	1832.41	1115.85	0.178	1331.80	236.40
A_68_P32022550	chr19:24354685-24354736	NM_008044:366	Fxn	INSIDE	0.191	0.385	2407.72	926.92	0.074	1747.14	128.46
A_68_P25034759	chr7:31837288-31837332	NM_052991:2562	Fxyd1	INSIDE	0.205	0.516	1508.92	778.84	0.106	1191.40	126.36
A_68_P26897303	chr9:123761167-123761211	NM_001110253:-168	Fyco1	PROMOTER	0.418	0.378	2746.02	1037.60	0.158	2152.23	339.85
A_68_P27077934	chr10:39089328-39089372	NM_001122892:-254	Fyn	PROMOTER	0.485	1.459	9707.23	14159.60	0.708	6682.81	4732.36
A_68_P27077942	chr10:39090283-39090342	NM_001122892:708	Fyn	INSIDE	0.180	3.150	6416.29	20214.34	0.566	3781.55	2139.74
A_68_P30655031	chr16:32877641-32877685	NM_001159349:-207	Fyttd1	DIVERGENT_PROMOTER	0.247	0.679	999.43	678.38	0.168	908.31	152.40
A_68_P23451685	chr5:4757093-4757137	NM_021457:1102	Fzd1	INSIDE	0.634	0.616	1047.22	644.60	0.390	825.75	322.38
A_68_P24084080	chr5:129107459-129107503	NM_175284:500	Fzd10	INSIDE	0.460	0.627	951.34	596.55	0.289	832.58	240.41
A_68_P24084082	chr5:129107740-129107784	NM_175284:782	Fzd10	INSIDE	0.316	0.452	2566.07	1159.99	0.143	1964.88	281.06
A_68_P24084083	chr5:129107881-129107932	NM_175284:926	Fzd10	INSIDE	0.281	0.500	1019.37	509.94	0.141	844.22	118.86
A_68_P28080440	chr11:102467429-102467473	NM_020510:1706	Fzd2	INSIDE	0.360	0.368	2575.05	946.93	0.132	1850.97	244.83
A_68_P28080441	chr11:102467523-102467567	NM_020510:1800	Fzd2	INSIDE	0.214	0.443	1719.82	762.55	0.095	1281.40	121.74
A_68_P29672520	chr14:65881704-65881748	NM_021458:-426	Fzd3	DIVERGENT_PROMOTER	0.371	0.390	1823.79	710.57	0.145	1432.21	207.20
A_68_P25307421	chr7:96553016-96553060	NM_008055:163	Fzd4	INSIDE	0.383	0.416	2360.05	981.14	0.159	1688.85	269.13
A_68_P25307424	chr7:96553350-96553394	NM_008055:497	Fzd4	INSIDE	0.227	0.603	1060.52	639.19	0.137	849.41	116.30
A_68_P20294241	chr1:64781408-64781452	NM_001042659:2894	Fzd5	INSIDE	0.272	0.599	1039.29	622.94	0.163	784.86	127.76
A_68_P20294255	chr1:64783028-64783072	NM_001042659:1274	Fzd5	INSIDE	0.189	0.588	2056.42	1210.10	0.111	1543.33	172.02
A_68_P20294266	chr1:64784611-64784655	NM_001042659:-308	Fzd5	PROMOTER	0.295	0.531	1159.38	615.42	0.156	868.90	135.84
A_68_P20264182	chr1:59538765-59538809	NM_008057:-204	Fzd7	PROMOTER	0.330	0.652	1126.55	735.04	0.215	892.72	191.95
A_68_P20264196	chr1:59540370-59540414	NM_008057:1402	Fzd7	INSIDE	0.291	0.647	4951.91	3202.58	0.188	3716.89	699.77
A_68_P31484553	chr18:9213708-9213752	NM_008058:877	Fzd8	INSIDE	0.256	0.544	2788.80	1517.27	0.139	2149.47	299.46
A_68_P31484554	chr18:9213845-9213889	NM_008058:1013	Fzd8	INSIDE	0.205	0.504	1373.75	691.81	0.103	1130.15	116.60
A_68_P31484555	chr18:9214035-9214079	NM_008058:1203	Fzd8	INSIDE	0.302	0.639	3884.69	2482.56	0.193	2795.20	539.70
A_68_P31484557	chr18:9214262-9214306	NM_008058:1431	Fzd8	INSIDE	0.375	0.660	1436.17	947.72	0.248	1152.02	285.42
A_68_P31484558	chr18:9214394-9214440	NM_008058:1563	Fzd8	INSIDE	0.240	0.683	1065.65	728.21	0.164	751.82	123.30
A_68_P24115511	chr5:135726586-135726630	NM_010246:309	Fzd9	INSIDE	0.484	0.470	2130.42	1001.81	0.227	1662.56	378.18
A_68_P27289250	chr10:80840838-80840882	NM_019757:255	Fzr1	INSIDE	0.068	1.874	2237.73	4194.37	0.127	1493.69	189.10
A_68_P28429306	chr12:52449562-52449606	NM_001015099:368	G2e3	INSIDE	0.548	0.644	1112.50	716.32	0.353	1006.48	355.44
A_68_P23892218	chr5:92512396-92512440	NM_001080797:-475	G3bp2	PROMOTER	0.167	0.609	1476.13	898.72	0.102	1127.90	114.73
A_68_P23892220	chr5:92512615-92512659	NM_001080794:-53	G3bp2	PROMOTER	0.348	0.718	2229.52	1600.68	0.250	1610.20	402.39
A_68_P23892222	chr5:92512823-92512867	NM_011816:-83	G3bp2	PROMOTER	0.576	0.494	1504.75	742.84	0.285	1221.40	347.50
A_68_P27897193	chr11:69617732-69617776	NR_027913:584	G630025P09Rik	INSIDE	0.246	0.603	3433.80	2070.82	0.148	2527.28	374.87
A_68_P31157364	chr17:35202217-35202261	NR_001462:857	G6b	INSIDE	0.413	0.552	2616.09	1444.87	0.228	1893.95	432.00
A_68_P32468048	chrX:71674396-71674448	NM_008062:78	G6pdx	INSIDE	0.087	2.031	1862.12	3782.36	0.176	685.10	120.82
A_68_P28176851	chr11:119129170-119129216	NM_001159324:-88	Gaa	PROMOTER	0.187	0.682	1293.27	881.53	0.128	962.50	122.97
A_68_P26001978	chr8:83403405-83403452	NM_021356:950	Gab1	INSIDE	0.486	0.502	1639.93	822.60	0.244	1281.09	312.36
A_68_P26001979	chr8:83403513-83403557	NM_021356:844	Gab1	INSIDE	0.538	0.401	1993.30	800.17	0.216	1390.58	300.27
A_68_P27898357	chr11:69805074-69805118	NM_019749:225	Gabarap	INSIDE	0.121	0.715	6255.46	4472.78	0.087	4212.30	364.58
A_68_P24844081	chr6:129483283-129483327	NM_020590:95	Gabarapl1	INSIDE	0.414	0.481	1103.95	530.78	0.199	834.82	165.97

ProbeName	Target position of probe on CpG island microarray	TargetID	GeneSymbol	CpG island Description	Ratio of relative methylation (TiO2-NP/Vehicle)	Sham group			TiO_2-H group		
						Relative methylation (Cy-5/Cy-3)	Cy-3 signal (Input)	Cy-5 signal (MeDIP)	Relative methylation (Cy-5/Cy-3)	Cy-3 signal (Input)	Cy-5 signal (MeDIP)
A_68_P26171001	chr8:114464407-114464460	NM_026693:-184	Gabarapl2	DIVERGENT_PROMOTER	0.219	0.643	984.49	632.93	0.141	828.49	116.56
A_68_P31166955	chr17:37183328-37183372	NM_019439:440	Gabbr1	INSIDE	0.084	1.401	6152.17	8619.26	0.117	4556.94	535.18
A_68_P31166994	chr17:37188152-37188196	NM_019439:5264	Gabbr1	INSIDE	0.197	0.705	1408.17	993.25	0.139	1047.40	145.25
A_68_P30925866	chr16:84835027-84835071	NM_008065:-320	Gabpa	PROMOTER	0.327	0.631	3756.39	2371.05	0.206	2791.11	575.64
A_68_P30925869	chr16:84835358-84835402	NM_008065:12	Gabpa	INSIDE	0.413	0.551	1057.97	583.26	0.228	789.20	179.70
A_68_P21609430	chr2:126501185-126501229	NM_010249:16	Gabpb1	INSIDE	0.241	0.665	1458.02	969.02	0.160	1358.39	217.26
A_68_P21609431	chr2:126501251-126501295	NM_207669:-49	Gabpb1	PROMOTER	0.634	0.630	2080.23	1309.89	0.399	1921.36	767.08
A_68_P22342883	chr3:95021867-95021911	NM_029885:-24	Gabpb2	PROMOTER	0.210	0.533	2194.14	1168.55	0.112	1552.65	173.45
A_68_P22342884	chr3:95022045-95022089	NM_029885:-202	Gabpb2	PROMOTER	0.339	0.656	956.74	627.92	0.222	804.86	179.06
A_68_P23785052	chr5:71487399-71487443	NM_008066:-332	Gabra2	PROMOTER	0.594	0.547	1099.64	601.65	0.325	861.42	279.79
A_68_P25151146	chr7:64764649-64764693	NM_176942:709	Gabra5	INSIDE	0.415	0.485	1209.79	586.31	0.201	1112.35	223.50
A_68_P23787825	chr5:72092392-72092436	NM_008069:1160	Gabrb1	INSIDE	0.272	0.496	1200.92	595.31	0.135	904.97	121.84
A_68_P23787826	chr5:72092511-72092555	NM_008069:1278	Gabrb1	INSIDE	0.351	0.641	1713.97	1097.91	0.225	1363.63	307.01
A_68_P25151575	chr7:64847050-64847094	NM_008071:530	Gabrb3	INSIDE	0.447	0.689	3099.78	2136.00	0.308	2431.03	748.86
A_68_P23438376	chr4:154771887-154771931	NM_008072:270	Gabrd	INSIDE	0.162	0.539	1732.08	933.40	0.087	1431.60	124.88
A_68_P25150558	chr7:64642169-64642213	NM_008074:51	Gabrg3	INSIDE	0.355	0.651	1083.07	704.54	0.231	988.34	228.09
A_68_P21059044	chr2:22478107-22478151	NM_008078:282	Gad2	INSIDE	0.659	0.563	2920.10	1644.43	0.371	2196.90	814.91
A_68_P21059051	chr2:22478980-22479024	NM_008078:1156	Gad2	INSIDE	0.623	0.728	2507.45	1824.41	0.453	1952.96	885.11
A_68_P21059052	chr2:22479080-22479124	NM_008078:1256	Gad2	INSIDE	0.167	0.475	1994.87	948.28	0.079	1525.06	120.88
A_68_P24519505	chr6:66986497-66986541	NM_007836:883	Gadd45a	INSIDE	0.345	0.431	2343.74	1010.48	0.149	1850.66	274.91
A_68_P24519506	chr6:66986584-66986628	NM_007836:795	Gadd45a	INSIDE	0.492	0.572	3227.80	1846.34	0.282	2687.87	756.64
A_68_P24519510	chr6:66987027-66987071	NM_007836:353	Gadd45a	INSIDE	0.284	0.657	4073.48	2676.09	0.187	3289.26	613.92
A_68_P27286406	chr10:80392969-80393019	NM_008655:159	Gadd45b	INSIDE	0.244	0.460	1472.03	677.22	0.112	1063.21	119.17
A_68_P27286409	chr10:80393272-80393316	NM_008655:459	Gadd45b	INSIDE	0.272	0.492	3524.87	1733.92	0.134	2502.37	335.36
A_68_P29034749	chr13:51942380-51942424	NM_011817:359	Gadd45g	INSIDE	0.511	0.623	1565.13	975.81	0.318	1087.34	346.26
A_68_P29034751	chr13:51942528-51942572	NM_011817:507	Gadd45g	INSIDE	0.486	1.416	11702.50	16567.28	0.689	8042.87	5539.15
A_68_P29034755	chr13:51943120-51943164	NM_011817:1099	Gadd45g	INSIDE	0.478	0.402	1902.56	765.41	0.192	1503.63	288.92
A_68_P23973155	chr5:109058342-109058386	NM_153569:394	Gak	INSIDE	0.273	0.621	1598.27	991.75	0.169	1173.02	198.75
A_68_P23973156	chr5:109058413-109058457	NM_153569:324	Gak	INSIDE	0.519	0.606	4363.22	2646.10	0.315	3148.67	991.30
A_68_P28669119	chr12:99497006-99497050	NM_008079:519	Galc	INSIDE	0.341	0.571	927.29	529.74	0.195	704.18	137.34
A_68_P23331442	chr4:135519710-135519754	NM_178389:-1347	Gale	PROMOTER	0.164	0.634	4490.02	2844.55	0.104	2903.65	301.49
A_68_P28156837	chr11:115873664-115873708	NM_016905:347	Galk1	INSIDE	0.303	0.558	1834.42	1024.51	0.169	1392.43	235.32
A_68_P31559063	chr18:24365030-24365074	NM_001160404:558	Galnt1	INSIDE	0.122	0.448	2673.05	1196.27	0.055	2135.21	116.59
A_68_P27826626	chr11:57458476-57458520	NM_134189:-445	Galnt10	PROMOTER	0.324	0.668	1176.19	786.21	0.217	976.16	211.69
A_68_P27826627	chr11:57458543-57458587	NM_134189:-379	Galnt10	PROMOTER	0.238	0.571	1133.39	647.50	0.136	886.24	120.53
A_68_P27826629	chr11:57458793-57458837	NM_134189:-129	Galnt10	PROMOTER	0.400	0.608	3664.79	2229.96	0.244	2544.15	619.66
A_68_P22892660	chr4:47105120-47105164	NM_172693:318	Galnt12	INSIDE	0.470	0.618	991.77	612.74	0.290	756.44	219.62
A_68_P21229090	chr2:54288809-54288853	NM_173030:33	Galnt13	INSIDE	0.537	0.475	2104.72	998.70	0.255	1616.68	412.23
A_68_P21229089	chr2:54288691-54288735	NM_173030:-85	Galnt13	PROMOTER	0.668	0.640	1762.61	1128.40	0.428	1599.03	684.31
A_68_P31347822	chr17:74058856-74058900	NM_027864:913	Galnt14	INSIDE	0.406	0.542	1480.35	802.89	0.220	1057.71	232.90
A_68_P31347823	chr17:74058995-74059039	NM_027864:775	Galnt14	INSIDE	0.358	0.577	2749.71	1586.43	0.207	2015.92	416.95
A_68_P31347824	chr17:74059066-74059113	NM_027864:702	Galnt14	INSIDE	0.056	2.589	4127.46	10686.06	0.144	2551.03	367.54
A_68_P31347825	chr17:74059155-74059199	NM_027864:615	Galnt14	INSIDE	0.420	1.634	7514.33	12275.01	0.686	4948.64	3394.20
A_68_P26244129	chr8:126756057-126756101	NM_139272:785	Galnt2	INSIDE	0.362	0.568	2036.88	1156.20	0.205	1543.13	317.06

ProbeName	Target position of probe on CpG island microarray	TargetID	GeneSymbol	CpG island Description	Ratio of relative methylation (TiO2-NP/Vehicle)	Sham group			TiO_2-H group		
						Relative methylation (Cy-5/Cy-3)	Cy-3 signal (Input)	Cy-5 signal (MeDIP)	Relative methylation (Cy-5/Cy-3)	Cy-3 signal (Input)	Cy-5 signal (MeDIP)
A_68_P26244130	chr8:126756129-126756173	NM_139272:857	Galnt2	INSIDE	0.244	0.534	1334.06	711.97	0.130	917.18	119.50
A_68_P30487410	chr15:100559585-100559629	NM_001161768:-8698	Galnt6	PROMOTER	0.418	0.526	1537.56	808.37	0.220	1232.61	271.10
A_68_P30487413	chr15:100559897-100559941	NM_001161767:-111	Galnt6	PROMOTER	0.494	0.518	2203.93	1142.41	0.256	1760.26	450.33
A_68_P25892523	chr8:60131751-60131795	NM_001167981:80	Galnt7	INSIDE	0.472	0.557	2676.26	1490.97	0.263	2155.19	566.67
A_68_P31876066	chr18:82575283-82575327	NM_008082:865	Galr1	INSIDE	0.479	0.483	2065.44	997.01	0.231	1600.25	370.27
A_68_P31876071	chr18:82575755-82575799	NM_008082:393	Galr1	INSIDE	0.316	0.578	2794.97	1614.79	0.182	2046.50	373.42
A_68_P31876072	chr18:82575840-82575884	NM_008082:307	Galr1	INSIDE	0.274	0.550	2060.59	1133.84	0.151	1520.48	229.52
A_68_P28158431	chr11:116142573-116142617	NM_010254:342	Galr2	INSIDE	0.363	0.476	1565.55	745.74	0.173	1209.65	208.96
A_68_P28158433	chr11:116142782-116142826	NM_010254:552	Galr2	INSIDE	0.522	0.699	1461.04	1020.97	0.365	1225.86	447.11
A_68_P28158434	chr11:116142875-116142919	NM_010254:644	Galr2	INSIDE	0.628	0.435	1715.76	745.91	0.273	1323.62	361.38
A_68_P28158435	chr11:116142974-116143018	NM_010254:744	Galr2	INSIDE	0.329	0.538	3090.76	1662.50	0.177	2339.73	414.36
A_68_P28158436	chr11:116143049-116143093	NM_010254:818	Galr2	INSIDE	0.341	0.630	1348.14	848.69	0.215	926.83	199.12
A_68_P28158445	chr11:116144132-116144186	NM_010254:1906	Galr2	INSIDE	0.018	2.351	4288.22	10083.10	0.043	2941.95	125.80
A_68_P28158447	chr11:116144333-116144377	NM_010254:2102	Galr2	INSIDE	0.165	0.541	1652.92	894.54	0.089	1276.44	113.98
A_68_P28158449	chr11:116144557-116144601	NM_010254:2326	Galr2	INSIDE	0.287	0.402	1510.04	606.34	0.115	1072.64	123.66
A_68_P30364009	chr15:78873840-78873884	NM_015738:1548	Galr3	INSIDE	0.289	0.709	2482.84	1759.91	0.205	1813.34	372.01
A_68_P22864172	chr4:41702487-41702531	NM_016658:383	Galt	INSIDE	0.511	0.631	2104.90	1329.09	0.323	1595.48	515.14
A_68_P27282039	chr10:79723618-79723662	NM_010255:73	Gamt	INSIDE	0.624	0.614	1077.94	662.22	0.383	887.24	339.90
A_68_P27282040	chr10:79723724-79723768	NM_010255:-33	Gamt	DIVERGENT_PROMOTER	0.549	0.456	3792.04	1728.04	0.250	2988.07	747.36
A_68_P27282041	chr10:79723845-79723889	NM_010255:-153	Gamt	DIVERGENT_PROMOTER	0.608	0.660	1685.06	1111.51	0.401	1312.24	526.41
A_68_P26200994	chr8:119681587-119681631	NM_001081151:-426	Gan	PROMOTER	0.264	0.514	1034.07	531.36	0.136	861.90	117.07
A_68_P21575069	chr2:120229642-120229686	NM_172672:33	Ganc	INSIDE	0.166	0.594	2214.61	1315.25	0.098	1757.52	172.83
A_68_P24819099	chr6:125114766-125114820	NM_008084:809	Gapdh	INSIDE	0.144	0.713	1595.12	1137.65	0.103	1135.74	116.87
A_68_P24819105	chr6:125115461-125115505	NM_008084:119	Gapdh	INSIDE	0.268	0.720	6768.06	4873.16	0.193	4535.51	873.94
A_68_P25033089	chr7:31514898-31514944	NM_008085:9455	Gapdhs	INSIDE	0.238	0.571	1186.52	677.36	0.136	875.44	118.81
A_68_P21129593	chr2:34609363-34609407	NM_025709:1368	Gapvd1	INSIDE	0.548	0.546	3536.90	1930.95	0.299	2825.39	844.86
A_68_P22522345	chr3:129533741-129533785	NM_026578:552	Gar1	INSIDE	0.347	0.613	2300.94	1410.15	0.212	1679.40	356.72
A_68_P22522346	chr3:129533887-129533931	NM_026578:406	Gar1	INSIDE	0.405	0.598	2284.02	1365.84	0.242	1847.04	446.89
A_68_P24466829	chr6:54988330-54988374	NM_180678:358	Gars	INSIDE	0.435	0.550	2430.92	1338.08	0.239	1941.61	464.96
A_68_P29083946	chr13:60278080-60278124	NM_008086:794	Gas1	INSIDE	0.647	2.797	346.02	967.97	1.810	347.89	629.57
A_68_P27561149	chr11:4961389-4961433	NM_001190406:3920	Gas2l1	INSIDE	0.601	0.560	2634.12	1474.41	0.337	2254.87	758.77
A_68_P27561155	chr11:4962120-4962164	NM_001190406:3188	Gas2l1	INSIDE	0.319	0.639	1889.53	1208.16	0.204	1423.13	290.20
A_68_P27561172	chr11:4963946-4963990	NM_001190406:1362	Gas2l1	INSIDE	0.342	1.478	1500.20	2217.22	0.505	1179.52	596.05
A_68_P20769690	chr1:162964920-162964964	NR_002840:-354	Gas5	DIVERGENT_PROMOTER	0.288	0.565	4380.48	2475.69	0.163	3082.19	500.88
A_68_P25668519	chr8:13468177-13468227	NM_019521:26333	Gas6	INSIDE	0.545	2.755	986.63	2718.03	1.502	664.35	997.76
A_68_P25668520	chr8:13468316-13468360	NM_019521:26197	Gas6	INSIDE	0.609	2.535	1674.75	4245.58	1.545	1151.97	1779.25
A_68_P25668725	chr8:13494426-13494470	NM_019521:87	Gas6	INSIDE	0.382	0.445	3011.25	1340.42	0.170	2228.70	379.31
A_68_P24620284	chr6:88140062-88140106	NM_008090:-8573	Gata2	PROMOTER	0.623	0.709	1936.62	1372.21	0.441	1468.86	648.11
A_68_P24620285	chr6:88140131-88140179	NM_008090:-8503	Gata2	PROMOTER	0.468	0.648	2131.57	1381.31	0.303	1653.83	501.27
A_68_P24620291	chr6:88140777-88140827	NM_008090:-7855	Gata2	PROMOTER	0.140	2.317	2466.70	5716.21	0.325	1622.65	527.89
A_68_P24620313	chr6:88143671-88143715	NM_008090:-4965	Gata2	PROMOTER	0.643	0.694	1209.74	839.92	0.446	872.92	389.45
A_68_P24620314	chr6:88143770-88143814	NM_008090:-4865	Gata2	PROMOTER	0.376	0.539	2269.98	1224.54	0.203	1815.15	368.02
A_68_P24620341	chr6:88146759-88146803	NM_008090:-1877	Gata2	PROMOTER	0.337	0.552	923.90	510.19	0.186	654.05	121.79
A_68_P24620356	chr6:88148509-88148553	NM_008090:-127	Gata2	PROMOTER	0.419	0.469	1442.64	676.95	0.197	1340.33	263.69

ProbeName	Target position of probe on CpG island microarray	TargetID	GeneSymbol	CpG island Description	Ratio of relative methylation (TiO2-NP/Vehicle)	Sham group			TiO_2-H group		
						Relative methylation (Cy-5/Cy-3)	Cy-3 signal (Input)	Cy-5 signal (MeDIP)	Relative methylation (Cy-5/Cy-3)	Cy-3 signal (Input)	Cy-5 signal (MeDIP)
A_68_P24620360	chr6:88148968-88149012	NM_008090:333	Gata2	INSIDE	0.404	0.683	2322.07	1585.49	0.276	1713.63	472.62
A_68_P20997432	chr2:9796607-9796655	NM_008091:3597	Gata3	INSIDE	0.123	1.582	3687.92	5833.72	0.195	2669.74	519.95
A_68_P29660083	chr14:63864272-63864316	NM_008092:-197	Gata4	PROMOTER	0.275	0.528	3707.11	1958.58	0.145	2991.53	434.68
A_68_P29660085	chr14:63864436-63864480	NM_008092:-361	Gata4	PROMOTER	0.514	0.597	1016.09	606.78	0.307	842.41	258.44
A_68_P29660137	chr14:63870430-63870474	NM_008092:-6355	Gata4	PROMOTER	0.327	0.660	2536.61	1675.09	0.216	1862.65	402.08
A_68_P29660138	chr14:63870620-63870670	NM_008092:-6547	Gata4	PROMOTER	0.204	0.546	1439.97	785.91	0.111	1087.14	120.86
A_68_P21903066	chr2:180068973-180069017	NM_008093:390	Gata5	INSIDE	0.165	0.604	1416.62	855.66	0.099	1186.31	117.92
A_68_P21903068	chr2:180069255-180069300	NM_008093:107	Gata5	INSIDE	0.439	0.542	1319.22	715.58	0.238	976.06	232.35
A_68_P31493012	chr18:11048533-11048577	NM_010258:-3953	Gata6	PROMOTER	0.639	0.605	1283.71	777.13	0.387	1095.99	423.72
A_68_P31493048	chr18:11053045-11053099	NM_010258:565	Gata6	INSIDE	0.134	0.629	2098.04	1318.64	0.084	1449.86	122.10
A_68_P31493068	chr18:11055096-11055140	NM_010258:2611	Gata6	INSIDE	0.308	0.658	1794.84	1180.78	0.203	1390.45	281.95
A_68_P23446277	chr5:3647175-3647234	NM_026033:732	Gatad1	INSIDE	0.263	0.480	1156.23	555.11	0.126	942.21	118.86
A_68_P25949228	chr8:72520400-72520444	NM_001113345:-144	Gatad2a	PROMOTER	0.408	0.489	2940.06	1438.94	0.200	2316.97	463.12
A_68_P25949229	chr8:72520506-72520550	NM_001113345:-250	Gatad2a	PROMOTER	0.323	0.338	3200.59	1082.52	0.109	2626.20	286.70
A_68_P25949230	chr8:72520586-72520630	NM_001113345:-330	Gatad2a	PROMOTER	0.663	0.589	1082.30	637.93	0.391	865.19	338.15
A_68_P21587096	chr2:122436805-122436849	NM_025961:187	Gatm	INSIDE	0.578	0.637	1149.79	732.57	0.368	992.33	365.34
A_68_P22872581	chr4:43591992-43592036	NM_172692:-278	Gba2	PROMOTER	0.279	0.667	1750.32	1166.84	0.186	1386.88	258.22
A_68_P30849202	chr16:70314865-70314917	NM_028803:559	Gbe1	INSIDE	0.162	0.642	1707.73	1097.06	0.104	1161.22	120.54
A_68_P20432928	chr1:91819533-91819577	NM_010262:8197	Gbx2	DOWNSTREAM	0.448	0.565	3982.53	2251.11	0.253	3110.31	787.09
A_68_P20432979	chr1:91825300-91825344	NM_010262:2429	Gbx2	INSIDE	0.243	0.575	1071.25	616.12	0.140	893.18	124.67
A_68_P20433001	chr1:91827813-91827857	NM_010262:-83	Gbx2	PROMOTER	0.640	0.642	1031.45	662.60	0.411	724.71	297.74
A_68_P30363918	chr15:78861120-78861164	NM_013847:-161	Gcat	PROMOTER	0.541	0.470	2274.42	1068.97	0.254	1734.85	441.01
A_68_P28185092	chr11:120392138-120392182	NM_008101:120	Gcgr	INSIDE	0.329	0.476	2115.77	1007.90	0.157	1706.84	267.17
A_68_P27565570	chr11:5802331-5802375	NM_010292:47250	Gck	INSIDE	0.628	0.650	837.86	544.37	0.408	744.34	303.83
A_68_P31985484	chr19:17430865-17430909	NM_173442:271	Gcnt1	INSIDE	0.444	0.580	1236.59	717.45	0.258	1024.58	263.86
A_68_P29248565	chr13:97695108-97695152	NM_001166065:487	Gcnt4	INSIDE	0.264	0.497	2107.56	1047.39	0.131	1876.41	246.08
A_68_P21812431	chr2:163264311-163264355	NM_144891:130	Gdap1l1	INSIDE	0.525	0.540	1278.39	690.65	0.283	1056.04	299.37
A_68_P22367900	chr3:99966673-99966717	NM_010269:254	Gdap2	INSIDE	0.373	0.604	1259.37	760.79	0.225	960.25	216.33
A_68_P27543728	chr10:128328158-128328202	NM_010272:594	Gdf11	INSIDE	0.614	0.713	2342.09	1668.76	0.437	1895.11	828.69
A_68_P22722574	chr4:9787244-9787288	NM_013526:15748	Gdf6	INSIDE	0.417	0.641	1027.55	658.62	0.267	864.10	230.94
A_68_P28221291	chr12:8304878-8304922	NM_013527:3860	Gdf7	INSIDE	0.421	0.586	3303.52	1935.60	0.247	2268.27	559.55
A_68_P28221292	chr12:8305005-8305049	NM_013527:3734	Gdf7	INSIDE	0.240	0.471	3100.53	1461.60	0.113	2033.55	230.54
A_68_P28221299	chr12:8305679-8305723	NM_013527:3060	Gdf7	INSIDE	0.551	0.562	1176.39	661.38	0.310	942.05	291.95
A_68_P28221328	chr12:8308980-8309024	NM_013527:-242	Gdf7	PROMOTER	0.178	0.566	1528.93	864.66	0.101	1200.00	120.75
A_68_P28788493	chr13:3537834-3537878	NM_008112:536	Gdi2	INSIDE	0.384	0.541	1387.53	751.02	0.208	1088.91	226.22
A_68_P29996485	chr15:7761244-7761297	NM_010275:260	Gdnf	INSIDE	0.237	0.607	1081.00	655.95	0.144	811.07	116.54
A_68_P29996501	chr15:7762967-7763011	NM_010275:1978	Gdnf	INSIDE	0.489	0.364	2461.17	895.06	0.178	1707.36	303.57
A_68_P29996526	chr15:7765753-7765797	NM_010275:4764	Gdnf	INSIDE	0.395	0.703	1521.03	1069.87	0.278	1204.55	334.90
A_68_P25361477	chr7:106530129-106530173	NM_201352:92	Gdpd5	INSIDE	0.583	0.713	4847.48	3456.02	0.415	3705.98	1539.77
A_68_P25361482	chr7:106530807-106530853	NM_201352:772	Gdpd5	INSIDE	0.403	1.577	1363.77	2150.15	0.636	979.45	622.45
A_68_P24994093	chr7:20158292-20158342	NM_027189:376	Gemin7	INSIDE	0.217	0.433	1744.46	755.51	0.094	1311.11	123.25
A_68_P24994095	chr7:20158628-20158672	NM_027189:42	Gemin7	INSIDE	0.377	0.684	3085.38	2111.39	0.258	2288.35	590.73
A_68_P28082093	chr11:102754576-102754620	NM_010277:3916	Gfap	INSIDE	0.189	0.441	1791.23	789.73	0.084	1385.42	115.70
A_68_P23968407	chr5:108150057-108150101	NM_010278:3285	Gfi1	INSIDE	0.248	0.524	1114.27	583.38	0.130	902.75	117.05

ProbeName	Target position of probe on CpG island microarray	TargetID	GeneSymbol	CpG island Description	Ratio of relative methylation (TiO2-NP/Vehicle)	Sham group			TiO_2-H group		
						Relative methylation (Cy-5/Cy-3)	Cy-3 signal (Input)	Cy-5 signal (MeDIP)	Relative methylation (Cy-5/Cy-3)	Cy-3 signal (Input)	Cy-5 signal (MeDIP)
A_68_P23968409	chr5:108150354-108150398	NM_010278:2987	Gfi1	INSIDE	0.368	0.440	2694.53	1186.86	0.162	2359.37	381.96
A_68_P23968432	chr5:108153037-108153081	NM_010278:305	Gfi1	INSIDE	0.366	0.652	2715.19	1770.48	0.239	2039.13	486.52
A_68_P23968440	chr5:108154062-108154106	NM_010278:-721	Gfi1	PROMOTER	0.625	0.488	1271.79	620.83	0.305	1042.16	317.71
A_68_P23968443	chr5:108154493-108154537	NM_010278:-1151	Gfi1	PROMOTER	0.287	0.572	2172.92	1242.44	0.164	1848.78	303.22
A_68_P23968445	chr5:108154656-108154700	NM_010278:-1315	Gfi1	PROMOTER	0.219	0.620	1078.97	668.62	0.136	848.09	115.35
A_68_P23968446	chr5:108154743-108154789	NM_010278:-1403	Gfi1	PROMOTER	0.197	0.423	1821.50	770.49	0.083	1481.81	123.26
A_68_P23968454	chr5:108155866-108155910	NM_010278:-2525	Gfi1	PROMOTER	0.182	0.589	2544.34	1497.52	0.107	1957.54	210.20
A_68_P23968410	chr5:108150495-108150539	NM_010278:2847	Gfi1	INSIDE	0.669	0.649	1001.29	649.95	0.434	891.94	387.34
A_68_P29249639	chr13:97908050-97908094	NM_177266:181	Gfm2	INSIDE	0.416	0.475	1662.26	789.34	0.197	1181.89	233.24
A_68_P28989502	chr13:43398690-43398734	NM_001033399:829	Gfod1	INSIDE	0.296	0.541	2800.65	1514.17	0.160	1873.14	299.96
A_68_P28989503	chr13:43398780-43398824	NM_001033399:739	Gfod1	INSIDE	0.460	0.576	954.66	549.71	0.265	777.04	205.71
A_68_P26135758	chr8:108282379-108282423	NM_027469:107	Gfod2	INSIDE	0.454	0.610	1234.17	753.37	0.277	938.18	259.84
A_68_P27784885	chr11:49607988-49608032	NM_013529:354	Gfpt2	INSIDE	0.132	0.724	2312.34	1674.19	0.095	1774.82	169.11
A_68_P27784886	chr11:49608055-49608100	NM_013529:421	Gfpt2	INSIDE	0.180	0.638	2029.21	1294.67	0.115	1681.83	192.78
A_68_P27784888	chr11:49608283-49608327	NM_013529:648	Gfpt2	INSIDE	0.505	0.482	1537.48	740.55	0.243	1337.59	325.21
A_68_P32213509	chr19:58528941-58528985	NM_010279:-6	Gfra1	PROMOTER	0.318	0.585	1261.52	738.16	0.186	986.24	183.24
A_68_P32213510	chr19:58529067-58529111	NM_010279:-132	Gfra1	PROMOTER	0.263	0.492	1248.19	614.02	0.129	922.96	119.40
A_68_P32213514	chr19:58529502-58529546	NM_010279:-568	Gfra1	PROMOTER	0.156	0.544	2506.69	1362.64	0.085	1910.18	161.47
A_68_P32213516	chr19:58529694-58529738	NM_010279:-760	Gfra1	PROMOTER	0.552	0.699	3599.35	2514.57	0.386	2391.78	922.06
A_68_P29703998	chr14:71290922-71290966	NM_008115:1008	Gfra2	INSIDE	0.342	0.467	2053.29	958.58	0.160	1567.16	250.28
A_68_P31614303	chr18:34879926-34879970	NM_010280:93	Gfra3	INSIDE	0.422	0.459	2095.67	961.61	0.193	1705.92	329.96
A_68_P24466585	chr6:54942609-54942653	NM_026637:231	Ggct	INSIDE	0.524	0.482	1086.83	523.50	0.252	916.67	231.24
A_68_P24466586	chr6:54942741-54942785	NM_026637:99	Ggct	INSIDE	0.204	0.428	1823.74	781.34	0.088	1331.71	116.65
A_68_P22767952	chr4:19968906-19968950	NM_010281:-270	Ggh	PROMOTER	0.460	0.505	1565.98	790.84	0.232	1284.93	298.32
A_68_P22767953	chr4:19968999-19969043	NM_010281:-178	Ggh	PROMOTER	0.421	0.429	1570.69	673.59	0.181	1388.11	250.57
A_68_P25025742	chr7:29956544-29956588	NM_182696:1158	Ggn	INSIDE	0.379	0.497	1466.17	729.41	0.189	1024.49	193.18
A_68_P25025744	chr7:29956908-29956952	NM_182696:1522	Ggn	INSIDE	0.233	0.510	1654.80	844.00	0.119	1331.23	158.23
A_68_P27980049	chr11:84683380-84683424	NM_153144:838	Ggnbp2	INSIDE	0.177	0.632	1488.84	941.06	0.112	1155.09	129.12
A_68_P28836952	chr13:14155652-14155696	NM_010282:-6	Ggps1	DIVERGENT_PROMOTER	0.140	0.658	2971.62	1956.18	0.092	2215.76	203.63
A_68_P21133753	chr2:35317703-35317754	NM_001145821:-783	Ggta1	PROMOTER	0.217	0.610	1035.25	631.88	0.133	870.24	115.39
A_68_P29543501	chr14:37948355-37948400	NM_001199122:-52	Ghitm	PROMOTER	0.531	0.660	1103.99	728.97	0.351	924.81	324.34
A_68_P29543502	chr14:37948492-37948546	NM_078478:-10	Ghitm	PROMOTER	0.282	0.619	908.79	562.64	0.175	668.07	116.73
A_68_P22010765	chr3:27270773-27270817	NM_177330:522	Ghsr	INSIDE	0.255	0.630	5161.96	3250.36	0.161	3663.81	589.33
A_68_P22010766	chr3:27270872-27270916	NM_177330:622	Ghsr	INSIDE	0.565	0.694	2020.63	1402.78	0.392	1427.12	560.14
A_68_P22010767	chr3:27270950-27270994	NM_177330:700	Ghsr	INSIDE	0.304	0.673	2032.00	1367.20	0.205	1465.99	299.92
A_68_P22010769	chr3:27271192-27271236	NM_177330:942	Ghsr	INSIDE	0.278	0.629	1416.27	890.73	0.175	1126.14	197.20
A_68_P22010771	chr3:27271350-27271394	NM_177330:1100	Ghsr	INSIDE	0.102	0.673	3318.83	2235.15	0.069	2148.80	147.41
A_68_P22010772	chr3:27271476-27271522	NM_177330:1226	Ghsr	INSIDE	0.255	0.502	1159.36	581.81	0.128	899.87	114.94
A_68_P20417066	chr1:89223605-89223651	NM_001110212:25	Gigyf2	INSIDE	0.268	0.607	1552.15	942.01	0.163	1281.53	208.77
A_68_P24429122	chr6:48693166-48693210	NM_008376:4143	Gimap1	INSIDE	0.295	1.877	2991.48	5614.39	0.554	2024.80	1121.84
A_68_P26221252	chr8:123113160-123113204	NM_178856:-207	Gins2	PROMOTER	0.184	0.648	3138.23	2033.78	0.119	2273.75	271.27
A_68_P26084323	chr8:98157470-98157514	NM_030198:34	Gins3	INSIDE	0.490	0.644	1796.46	1157.20	0.316	1431.88	452.09
A_68_P25715599	chr8:24348136-24348180	NM_024240:-18	Gins4	PROMOTER	0.595	0.634	1208.93	765.98	0.377	871.37	328.63
A_68_P22649021	chr3:151828589-151828633	NM_016867:254	Gipc2	INSIDE	0.643	0.612	1020.89	625.26	0.394	880.87	347.06

ProbeName	Target position of probe on CpG island microarray	TargetID	GeneSymbol	CpG island Description	Ratio of relative methylation (TiO2-NP/Vehicle)	Sham group			TiO_2-H group		
						Relative methylation (Cy-5/Cy-3)	Cy-3 signal (Input)	Cy-5 signal (MeDIP)	Relative methylation (Cy-5/Cy-3)	Cy-3 signal (Input)	Cy-5 signal (MeDIP)
A_68_P27937914	chr11:77306484-77306528	NM_001004144:-407	Git1	DIVERGENT_PROMOTER	0.163	0.318	3524.28	1120.61	0.052	2433.98	126.21
A_68_P24005878	chr5:115199611-115199670	NM_001077360:23860	Git2	INSIDE	0.588	3.829	817.22	3129.23	2.252	735.92	1657.63
A_68_P29625860	chr14:57676319-57676363	NM_016975:442	Gja3	INSIDE	0.339	0.516	1227.11	632.98	0.175	1065.22	186.06
A_68_P23284717	chr4:127003796-127003840	NM_008126:2551	Gjb3	INSIDE	0.545	4.401	2507.42	11034.19	2.398	1882.09	4513.55
A_68_P27835269	chr11:58990423-58990467	NM_175452:5884	Gjc2	INSIDE	0.188	0.538	1437.85	773.29	0.101	1179.38	119.00
A_68_P28059766	chr11:98844320-98844364	NM_178596:-12	Gjd3	PROMOTER	0.240	0.634	2659.07	1686.37	0.152	1969.80	299.66
A_68_P29072412	chr13:58376159-58376204	NM_019832:-632	Gkap1	PROMOTER	0.276	1.706	2038.90	3477.83	0.471	1472.02	693.44
A_68_P26844443	chr9:114310274-114310318	NM_009752:60	Glb1	INSIDE	0.085	0.667	4000.44	2667.31	0.056	3204.12	180.90
A_68_P24226572	chr6:8459387-8459431	NM_133236:-187	Glcci1	PROMOTER	0.329	0.407	1976.94	804.00	0.134	1662.49	222.59
A_68_P32056973	chr19:30249514-30249558	NM_138595:372	Gldc	INSIDE	0.537	0.693	1365.76	946.23	0.372	1000.56	372.57
A_68_P26167379	chr8:113782539-113782583	NM_009149:542	Glg1	INSIDE	0.141	0.394	2867.48	1130.21	0.056	2154.21	119.57
A_68_P28842019	chr13:15554706-15554754	NM_008130:-825	Gli3	PROMOTER	0.308	0.622	874.17	544.10	0.191	729.07	139.60
A_68_P22874559	chr4:43970296-43970340	NM_027450:-255	Glipr2	PROMOTER	0.496	0.559	1771.03	989.51	0.277	1386.07	383.78
A_68_P22874562	chr4:43970796-43970840	NM_027450:245	Glipr2	INSIDE	0.203	0.687	2370.98	1629.69	0.139	1874.53	261.24
A_68_P23190566	chr4:107108029-107108073	NM_147221:727	Glis1	INSIDE	0.377	1.478	4788.96	7077.76	0.556	3651.27	2031.75
A_68_P32048646	chr19:28752584-28752628	NM_175459:1961	Glis3	INSIDE	0.345	0.616	1558.16	960.44	0.212	1266.66	269.05
A_68_P28707019	chr12:106271229-106271273	NM_028419:352	Glrx5	INSIDE	0.535	0.396	1445.97	572.75	0.212	1100.45	232.99
A_68_P24078732	chr5:128112557-128112601	NM_177005:-53	Glt1d1	PROMOTER	0.275	0.655	2080.08	1363.22	0.180	1648.89	296.76
A_68_P24078733	chr5:128112702-128112746	NM_177005:93	Glt1d1	INSIDE	0.299	0.428	1751.18	749.64	0.128	1530.19	195.95
A_68_P24078735	chr5:128112945-128112989	NM_177005:335	Glt1d1	INSIDE	0.154	0.586	1799.84	1054.40	0.090	1351.22	122.23
A_68_P24078736	chr5:128113050-128113094	NM_177005:441	Glt1d1	INSIDE	0.168	0.527	2456.25	1293.46	0.089	1862.68	164.85
A_68_P20720547	chr1:154247487-154247531	NM_177756:512	Glt25d2	INSIDE	0.532	0.630	1331.51	838.96	0.335	937.45	314.34
A_68_P22299771	chr3:85691284-85691328	NM_177130:134	Glt28d2	INSIDE	0.542	0.544	1148.12	624.12	0.295	976.14	287.65
A_68_P23441153	chr4:155243478-155243522	NM_024472:49	Gltpd1	INSIDE	0.496	0.652	897.42	585.11	0.323	694.65	224.60
A_68_P29529255	chr14:35124395-35124439	NM_008133:504	Glud1	INSIDE	0.225	0.419	1938.13	811.39	0.094	1549.72	146.20
A_68_P20729266	chr1:155746790-155746834	NM_008131:-262	Glul	PROMOTER	0.659	0.418	1419.85	593.41	0.276	1090.54	300.51
A_68_P30513459	chr16:5049380-5049424	NM_001079814:601	Glyr1	INSIDE	0.185	0.385	2033.48	782.98	0.071	1603.69	114.32
A_68_P25947454	chr8:71919340-71919384	NR_038043:81	Gm10033	INSIDE	0.142	0.631	1761.42	1111.03	0.090	1309.54	117.36
A_68_P24839024	chr6:128388816-128388862	NR_028592:64	Gm10069	INSIDE	0.128	0.586	1903.76	1115.11	0.075	1663.82	124.44
A_68_P24839025	chr6:128388883-128388942	NR_028592:138	Gm10069	INSIDE	0.224	0.491	1224.64	600.76	0.110	1049.15	115.49
A_68_P31469355	chr18:5592249-5592293	NR_033552:165	Gm10125	INSIDE	0.289	0.412	1288.43	530.38	0.119	982.72	116.95
A_68_P30347360	chr15:76124857-76124901	NM_001195537:15	Gm10345	INSIDE	0.530	0.563	1041.93	586.88	0.299	872.91	260.61
A_68_P30347361	chr15:76124944-76124988	NM_001195537:103	Gm10345	INSIDE	0.346	0.670	2218.48	1486.76	0.232	1701.76	394.89
A_68_P28771929	chr12:118392739-118392783	NR_033538:-3018	Gm10421	PROMOTER	0.518	0.513	1032.45	529.89	0.266	691.08	183.88
A_68_P20941835	chr1:193960556-193960600	NR_033536:-201	Gm10516	PROMOTER	0.599	0.631	1911.71	1206.98	0.378	1339.15	506.58
A_68_P20941839	chr1:193961046-193961090	NR_033536:289	Gm10516	INSIDE	0.166	0.538	4764.51	2565.43	0.090	3621.27	324.58
A_68_P25533778	chr7:139507346-139507390	NR_028578:22	Gm10584	INSIDE	0.275	0.483	3417.22	1651.74	0.133	2367.90	314.29
A_68_P27267010	chr10:76721714-76721758	NR_026944:232	Gm10941	INSIDE	0.338	0.602	1017.53	612.92	0.204	810.65	165.13
A_68_P31260063	chr17:57245094-57245138	NR_033508:249	Gm11110	INSIDE	0.159	0.691	1609.13	1112.43	0.110	1085.37	119.30
A_68_P22346156	chr3:95685923-95685967	NM_001033302:207	Gm129	INSIDE	0.189	0.555	2301.14	1276.79	0.105	1761.90	184.70
A_68_P22346159	chr3:95686322-95686366	NM_001033302:-193	Gm129	PROMOTER	0.428	0.460	1798.38	827.42	0.197	1429.60	281.50
A_68_P21565607	chr2:118528199-118528243	NM_001081971:1479	Gm1337	INSIDE	0.501	0.573	2083.12	1193.66	0.287	1618.38	464.29
A_68_P21565608	chr2:118528286-118528330	NM_001081971:1391	Gm1337	INSIDE	0.282	0.716	1588.94	1138.16	0.202	1307.35	264.36
A_68_P21565618	chr2:118529436-118529480	NM_001081971:241	Gm1337	INSIDE	0.442	0.518	1582.95	819.92	0.229	1202.22	275.24

ProbeName	Target position of probe on CpG island microarray	TargetID	GeneSymbol	CpG island Description	Ratio of relative methylation (TiO2-NP/Vehicle)	Sham group			TiO_2-H group		
						Relative methylation (Cy-5/Cy-3)	Cy-3 signal (Input)	Cy-5 signal (MeDIP)	Relative methylation (Cy-5/Cy-3)	Cy-3 signal (Input)	Cy-5 signal (MeDIP)
A_68_P21052789	chr2:20890869-20890913	NR_033225:390	Gm13375	INSIDE	0.352	0.551	1205.68	664.22	0.194	1039.22	201.53
A_68_P21438061	chr2:93796206-93796250	NM_001145034:1029	Gm13889	INSIDE	0.545	0.646	3243.11	2093.86	0.352	2326.97	818.79
A_68_P21438070	chr2:93797249-93797293	NM_001145034:-13	Gm13889	PROMOTER	0.237	0.404	1890.71	763.41	0.096	1359.35	130.06
A_68_P21569057	chr2:119151750-119151794	NR_030683:161	Gm14207	INSIDE	0.255	0.672	2166.85	1456.50	0.172	1612.44	276.87
A_68_P21569058	chr2:119151824-119151868	NR_030683:87	Gm14207	INSIDE	0.387	0.398	1735.41	690.56	0.154	1256.20	193.45
A_68_P21569059	chr2:119151913-119151957	NR_030683:-1	Gm14207	PROMOTER	0.313	0.610	2568.77	1565.77	0.191	1984.18	378.63
A_68_P25954808	chr8:73421306-73421350	NM_001111116:-17	Gm15427	PROMOTER	0.191	0.562	1680.38	945.20	0.107	1153.92	123.99
A_68_P28577473	chr12:81859415-81859459	NM_001008423:2266	Gm1568	INSIDE	0.603	3.825	273.66	1046.71	2.308	341.37	787.73
A_68_P30588159	chr16:20548466-20548510	NR_030670:141	Gm15760	INSIDE	0.343	0.695	1557.95	1083.45	0.238	1066.30	254.19
A_68_P24225086	chr6:8209405-8209449	NR_033518:139	Gm16039	INSIDE	0.113	0.622	5268.18	3276.99	0.070	3832.71	268.46
A_68_P24225087	chr6:8209485-8209529	NR_033518:219	Gm16039	INSIDE	0.506	0.565	2212.07	1250.54	0.286	1747.95	500.35
A_68_P27845445	chr11:60727478-60727528	NM_025294:-208	Gm16515	PROMOTER	0.196	0.655	1274.17	834.69	0.129	946.32	121.66
A_68_P27845563	chr11:60744900-60744958	NR_027800:441	Gm16516	INSIDE	0.218	0.557	1399.94	779.86	0.121	958.84	116.18
A_68_P27845566	chr11:60745169-60745219	NR_027800:175	Gm16516	INSIDE	0.280	0.512	1039.71	532.39	0.144	807.31	115.86
A_68_P27845568	chr11:60745336-60745380	NR_027800:11	Gm16516	INSIDE	0.202	0.424	1765.82	749.44	0.086	1337.97	114.76
A_68_P27278157	chr10:79132042-79132087	NM_148934:-90	Gm16517	PROMOTER	0.155	0.511	1817.73	928.53	0.079	1486.61	117.85
A_68_P23591962	chr5:34325727-34325771	NM_001033458:-374	Gm1673	PROMOTER	0.519	0.579	1267.64	734.52	0.301	1019.87	306.96
A_68_P23591965	chr5:34325967-34326011	NM_001033458:-134	Gm1673	PROMOTER	0.504	0.467	1675.98	783.04	0.235	1339.29	315.28
A_68_P23591967	chr5:34326256-34326300	NM_001033458:156	Gm1673	INSIDE	0.323	0.440	4056.81	1783.20	0.142	3021.30	429.15
A_68_P23591969	chr5:34326576-34326620	NM_001033458:476	Gm1673	INSIDE	0.154	0.456	2418.12	1102.67	0.070	1711.46	119.90
A_68_P23591970	chr5:34326722-34326766	NM_001033458:622	Gm1673	INSIDE	0.206	0.640	1317.32	843.64	0.132	919.47	121.41
A_68_P28744398	chr12:112723379-112723423	NM_001033248:634	Gm266	INSIDE	0.403	0.466	1750.51	815.19	0.188	1666.67	313.00
A_68_P26035031	chr8:89996648-89996692	NR_033430:-40	Gm2694	DIVERGENT_PROMOTER	0.602	0.465	1283.10	596.31	0.280	1145.03	320.35
A_68_P26035034	chr8:89997077-89997121	NR_033430:388	Gm2694	INSIDE	0.165	0.630	3175.04	2001.39	0.104	2212.28	229.59
A_68_P21046072	chr2:19579131-19579175	NR_033642:372	Gm3230	INSIDE	0.414	0.580	1816.74	1053.00	0.240	1498.43	359.31
A_68_P31063011	chr17:15115516-15115564	NM_001123367:236	Gm3417	INSIDE	0.066	0.493	5097.23	2514.76	0.033	3680.04	120.47
A_68_P31063013	chr17:15115758-15115802	NM_001123367:-4	Gm3417	DIVERGENT_PROMOTER	0.471	0.517	1851.68	957.30	0.243	1691.50	411.68
A_68_P25766297	chr8:33994011-33994055	NM_001177589:66466	Gm3985	DOWNSTREAM	0.268	0.596	969.08	577.10	0.160	761.22	121.61
A_68_P25766298	chr8:33994085-33994129	NM_001177589:66392	Gm3985	DOWNSTREAM	0.170	0.496	1919.57	952.71	0.084	1459.04	123.19
A_68_P25766299	chr8:33994276-33994320	NM_001177589:66200	Gm3985	DOWNSTREAM	0.438	0.680	2856.05	1940.82	0.298	2150.00	640.63
A_68_P25504680	chr7:134376542-134376586	NR_030674:80	Gm4532	INSIDE	0.624	0.614	1378.69	846.77	0.383	1128.24	432.07
A_68_P27327040	chr10:88193638-88193682	NM_001037166:186	Gm4925	INSIDE	0.210	0.682	1158.20	790.22	0.144	860.64	123.59
A_68_P27327041	chr10:88193707-88193757	NM_001037166:114	Gm4925	INSIDE	0.202	0.712	2460.09	1751.05	0.143	1849.12	265.29
A_68_P25362932	chr7:106774074-106774118	NM_001195529:1517	Gm4980	INSIDE	0.279	0.534	2137.54	1141.77	0.149	1580.08	235.53
A_68_P25362934	chr7:106774303-106774347	NM_001195529:1289	Gm4980	INSIDE	0.316	0.565	1208.77	682.81	0.178	990.43	176.61
A_68_P25362942	chr7:106775190-106775234	NM_001195529:401	Gm4980	INSIDE	0.533	0.469	1476.70	691.87	0.250	1077.85	269.29
A_68_P25362945	chr7:106775610-106775654	NM_001195529:-19	Gm4980	PROMOTER	0.221	0.596	1216.15	725.10	0.132	903.27	119.17
A_68_P20875108	chr1:182261016-182261060	NR_003623:-358	Gm5069	PROMOTER	0.504	0.427	2698.69	1151.73	0.215	2106.15	453.33
A_68_P20875111	chr1:182261385-182261429	NR_003623:-726	Gm5069	PROMOTER	0.504	0.628	1635.64	1027.29	0.317	1228.47	389.23
A_68_P20875116	chr1:182262065-182262109	NR_003623:-1406	Gm5069	PROMOTER	0.242	0.613	1056.39	648.05	0.149	815.81	121.22
A_68_P20875120	chr1:182262437-182262486	NR_003623:-1781	Gm5069	PROMOTER	0.227	0.471	1415.53	667.34	0.107	1133.67	121.35
A_68_P23440271	chr4:155109205-155109249	NM_001101506:314	Gm5151	INSIDE	0.249	0.664	1431.11	950.82	0.165	978.84	161.92
A_68_P24131920	chr5:139296987-139297031	NM_001195128:975	Gm5294	INSIDE	0.271	0.596	1815.72	1083.07	0.162	1460.19	235.83
A_68_P24131922	chr5:139297257-139297301	NM_001195128:1245	Gm5294	INSIDE	0.151	0.538	2285.55	1229.21	0.081	1741.04	141.62

ProbeName	Target position of probe on CpG island microarray	TargetID	GeneSymbol	CpG island Description	Ratio of relative methylation (TiO2-NP/Vehicle)	Sham group			TiO_2-H group		
						Relative methylation (Cy-5/Cy-3)	Cy-3 signal (Input)	Cy-5 signal (MeDIP)	Relative methylation (Cy-5/Cy-3)	Cy-3 signal (Input)	Cy-5 signal (MeDIP)
A_68_P28043171	chr11:96113156-96113200	NR_037977:205	Gm53	INSIDE	0.272	0.525	1062.94	557.89	0.143	813.18	116.18
A_68_P26912795	chr10:5914411-5914463	NR_002891:-2938	Gm5512	PROMOTER	0.350	0.445	1369.32	609.20	0.156	961.81	149.71
A_68_P25661163	chr8:12385271-12385315	NR_027975:-478	Gm5607	PROMOTER	0.618	0.694	1255.05	870.74	0.429	981.76	420.97
A_68_P25661487	chr8:12430676-12430720	NR_027975:44928	Gm5607	INSIDE	0.339	0.526	1959.63	1030.26	0.178	1552.09	276.81
A_68_P25661492	chr8:12431084-12431128	NR_027975:45336	Gm5607	INSIDE	0.570	0.713	3639.99	2594.08	0.406	2677.83	1087.00
A_68_P29338084	chr13:113784193-113784237	NM_001037914:139	Gm6320	INSIDE	0.321	0.575	1394.98	801.53	0.185	1268.59	234.19
A_68_P29338085	chr13:113784265-113784309	NM_001037914:211	Gm6320	INSIDE	0.281	0.575	1191.92	685.22	0.161	977.43	157.79
A_68_P21345229	chr2:75497988-75498032	NR_033513:664	Gm6793	INSIDE	0.510	0.486	2613.81	1269.29	0.248	2037.51	504.86
A_68_P21345230	chr2:75498077-75498131	NR_033513:758	Gm6793	INSIDE	0.234	0.519	1346.23	698.49	0.121	1003.81	121.69
A_68_P26508624	chr9:51087195-51087239	NM_001195681:-557	Gm684	PROMOTER	0.663	0.738	2252.09	1662.84	0.489	1680.64	822.31
A_68_P31162851	chr17:36304426-36304470	NM_001081032:1034	Gm8909	INSIDE	0.271	0.567	2648.62	1501.56	0.154	1891.47	291.08
A_68_P24326760	chr6:29424133-29424177	NM_001145360:2718	Gm9047	DOWNSTREAM	0.575	0.439	1592.23	699.21	0.252	1285.44	324.39
A_68_P31929497	chr19:5567328-5567372	NM_001033448:-723	Gm962	DIVERGENT_PROMOTER	0.210	0.396	1771.28	701.58	0.083	1400.99	116.79
A_68_P31929504	chr19:5568132-5568176	NM_001033448:81	Gm962	INSIDE	0.629	0.497	1629.72	810.65	0.313	1261.36	394.82
A_68_P26886025	chr9:121766341-121766385	NM_001112668:287	Gm9790	INSIDE	0.336	0.568	3536.66	2007.95	0.191	2439.06	465.53
A_68_P21073093	chr2:25433409-25433453	NM_001005424:2189	Gm996	INSIDE	0.284	0.705	1422.20	1002.37	0.201	1083.70	217.30
A_68_P21073096	chr2:25433798-25433842	NM_001005424:1799	Gm996	INSIDE	0.635	0.678	1034.05	701.41	0.431	814.96	351.21
A_68_P21073102	chr2:25434469-25434514	NM_001005424:1128	Gm996	INSIDE	0.336	1.548	1725.74	2672.20	0.520	1236.81	642.79
A_68_P21073106	chr2:25434968-25435012	NM_001005424:629	Gm996	INSIDE	0.377	0.563	1539.04	865.75	0.212	1109.75	235.27
A_68_P21073109	chr2:25435334-25435378	NM_001005424:263	Gm996	INSIDE	0.416	0.678	2103.14	1425.89	0.282	1549.36	437.37
A_68_P25150561	chr7:64642517-64642562	NR_033504:-102	Gm9962	DIVERGENT_PROMOTER	0.490	0.449	1807.40	810.77	0.220	1425.00	313.44
A_68_P25150562	chr7:64642665-64642709	NR_033504:45	Gm9962	INSIDE	0.100	0.526	3078.98	1620.38	0.053	2241.26	118.13
A_68_P24611769	chr6:86683533-86683577	NM_011818:-182	Gmcl1	PROMOTER	0.272	0.645	819.39	528.22	0.175	687.12	120.47
A_68_P21909705	chr2:181022801-181022845	NM_198169:-151	Gmeb2	PROMOTER	0.106	0.667	2462.12	1641.71	0.070	1723.44	121.47
A_68_P21909706	chr2:181022876-181022920	NM_198169:-227	Gmeb2	PROMOTER	0.432	0.624	1107.72	691.60	0.270	859.57	231.79
A_68_P29578549	chr14:47442107-47442163	NM_022023:-217	Gmfb	DIVERGENT_PROMOTER	0.201	0.504	1458.55	734.90	0.101	1152.78	116.58
A_68_P25021899	chr7:29221970-29222015	NM_022024:-473	Gmfg	DIVERGENT_PROMOTER	0.499	0.618	1029.93	636.38	0.308	901.62	277.76
A_68_P28888885	chr13:24853671-24853715	NM_020567:114	Gmnn	INSIDE	0.414	0.546	1466.72	800.57	0.226	1159.39	261.93
A_68_P28888886	chr13:24853737-24853781	NM_020567:48	Gmnn	INSIDE	0.623	0.652	3986.32	2598.08	0.406	3025.99	1229.46
A_68_P29002205	chr13:45602874-45602918	NM_025508:59	Gmpr	INSIDE	0.605	0.546	1397.33	763.28	0.330	1112.58	367.64
A_68_P22194396	chr3:63780003-63780047	NM_001033300:-40	Gmps	PROMOTER	0.408	0.683	1296.60	885.44	0.279	1021.69	284.75
A_68_P27290416	chr10:81008062-81008106	NM_010301:-293	Gna11	PROMOTER	0.315	0.629	1392.76	876.66	0.199	1151.07	228.51
A_68_P28118144	chr11:109223851-109223895	NM_010303:-235	Gna13	PROMOTER	0.316	0.700	2039.32	1427.59	0.221	1392.58	307.64
A_68_P28118149	chr11:109224381-109224425	NM_010303:295	Gna13	INSIDE	0.611	0.521	1186.78	618.62	0.318	840.02	267.48
A_68_P28118150	chr11:109224474-109224518	NM_010303:389	Gna13	INSIDE	0.490	0.588	2640.98	1552.30	0.288	2010.43	579.54
A_68_P31980262	chr19:16511319-16511363	NM_008137:1184	Gna14	INSIDE	0.075	0.685	3023.98	2070.76	0.051	2280.64	117.31
A_68_P23510640	chr5:17865451-17865500	NM_010305:756	Gnai1	INSIDE	0.258	0.524	1027.11	538.29	0.135	882.05	119.26
A_68_P26073621	chr8:96335410-96335454	NM_001113384:695	Gnao1	INSIDE	0.181	0.552	2769.36	1529.05	0.100	2015.45	200.99
A_68_P31978254	chr19:16207846-16207890	NM_008139:548	Gnaq	INSIDE	0.612	0.495	1558.38	771.01	0.303	1272.35	385.25
A_68_P31978257	chr19:16208260-16208304	NM_008139:962	Gnaq	INSIDE	0.537	0.350	1795.94	629.12	0.188	1381.92	260.20
A_68_P21874764	chr2:174156135-174156179	NM_001077510:567	Gnas	INSIDE	0.217	0.691	2930.84	2026.36	0.150	2257.92	339.15
A_68_P21874766	chr2:174156274-174156318	NM_001077510:707	Gnas	INSIDE	0.325	0.643	1812.77	1166.24	0.209	1502.48	313.93
A_68_P26810381	chr9:107578748-107578792	NM_008140:3153	Gnat1	INSIDE	0.373	0.650	3492.37	2268.41	0.242	2393.76	580.43
A_68_P26810382	chr9:107578840-107578884	NM_008140:3061	Gnat1	INSIDE	0.370	0.562	2572.85	1446.90	0.208	1862.26	387.15

ProbeName	Target position of probe on CpG island microarray	TargetID	GeneSymbol	CpG island Description	Ratio of relative methylation (TiO2-NP/Vehicle)	Sham group			TiO_2-H group		
						Relative methylation (Cy-5/Cy-3)	Cy-3 signal (Input)	Cy-5 signal (MeDIP)	Relative methylation (Cy-5/Cy-3)	Cy-3 signal (Input)	Cy-5 signal (MeDIP)
A_68_P23438946	chr4:154866208-154866252	NM_001160016:761	Gnb1	INSIDE	0.273	0.489	2009.40	983.07	0.134	1514.53	202.52
A_68_P30580078	chr16:18498994-18499038	NM_023120:53	Gnb1l	INSIDE	0.186	0.349	2572.54	898.62	0.065	2048.72	133.31
A_68_P24126241	chr5:137971926-137971970	NM_010312:2509	Gnb2	INSIDE	0.403	0.702	1875.13	1316.69	0.283	1528.56	432.65
A_68_P24126242	chr5:137972085-137972129	NM_010312:2351	Gnb2	INSIDE	0.518	0.587	1855.41	1088.88	0.304	1489.16	453.02
A_68_P24126250	chr5:137972865-137972909	NM_010312:1571	Gnb2	INSIDE	0.232	0.470	1789.81	841.55	0.109	1339.94	146.17
A_68_P24126253	chr5:137973169-137973213	NM_010312:1267	Gnb2	INSIDE	0.229	0.518	3088.52	1599.67	0.118	2325.88	275.27
A_68_P27779978	chr11:48613766-48613813	NM_008143:-72	Gnb2l1	PROMOTER	0.276	0.433	1548.56	670.53	0.120	1016.21	121.67
A_68_P22875166	chr4:44085073-44085117	NM_001190414:451	Gne	INSIDE	0.296	0.428	2122.03	909.19	0.127	1602.89	203.35
A_68_P31105766	chr17:25854648-25854692	NM_022422:554	Gng13	INSIDE	0.211	0.607	1896.77	1151.03	0.128	1384.17	177.16
A_68_P27286895	chr10:80477880-80477928	NM_010319:-234	Gng7	PROMOTER	0.191	0.491	1568.92	770.85	0.094	1250.67	117.50
A_68_P31162040	chr17:36117126-36117170	NM_008136:249	Gnl1	INSIDE	0.422	0.509	1276.95	650.32	0.215	977.43	210.30
A_68_P31162043	chr17:36117444-36117488	NM_008136:567	Gnl1	INSIDE	0.213	0.639	2631.89	1682.19	0.136	1877.78	255.71
A_68_P32743828	chrX:147451859-147451906	NM_198110:-17	Gnl3l	PROMOTER	0.210	1.520	1249.25	1899.02	0.320	439.86	140.58
A_68_P23778715	chr5:69983278-69983322	NM_001038015:224	Gnpda2	INSIDE	0.170	0.698	2200.94	1535.44	0.118	1745.74	206.77
A_68_P21114434	chr2:32143877-32143921	NM_001080968:126	Golga2	INSIDE	0.316	0.640	976.66	624.91	0.202	710.52	143.72
A_68_P21114435	chr2:32143977-32144021	NM_001080968:226	Golga2	INSIDE	0.105	0.725	2305.50	1670.42	0.076	1647.98	125.77
A_68_P23978152	chr5:110605959-110606003	NM_008146:261	Golga3	INSIDE	0.276	0.742	3679.06	2728.70	0.205	2822.51	577.60
A_68_P26867067	chr9:118468644-118468695	NM_018748:53234	Golga4	INSIDE	0.658	2.225	881.75	1962.18	1.465	798.09	1169.49
A_68_P25715748	chr8:24367539-24367583	NM_001042484:-8	Golga7	PROMOTER	0.413	0.613	2389.75	1464.08	0.253	1668.08	421.69
A_68_P32119983	chr19:42330356-42330400	NM_027694:150	Golga7b	INSIDE	0.288	0.578	1822.77	1053.90	0.167	1356.45	226.09
A_68_P22248622	chr3:75760179-75760223	NM_175193:553	Golim4	INSIDE	0.305	0.480	1318.91	632.79	0.146	1007.44	147.25
A_68_P30021004	chr15:12251953-12252003	NM_025673:728	Golph3	INSIDE	0.288	0.608	1180.07	717.61	0.175	896.26	157.00
A_68_P26875190	chr9:119846515-119846559	NM_028976:140	Gorasp1	INSIDE	0.336	0.582	3904.44	2271.27	0.196	2671.26	522.69
A_68_P28086531	chr11:103558931-103558975	NM_019650:72	Gosr2	INSIDE	0.602	0.456	1316.56	600.68	0.275	1112.71	305.66
A_68_P32127031	chr19:43599236-43599283	NM_010324:-164	Got1	PROMOTER	0.220	0.351	2021.86	709.33	0.077	1499.30	115.49
A_68_P25737190	chr8:28317205-28317249	NM_029674:-4207	Got1l1	PROMOTER	0.558	0.709	1356.61	961.52	0.396	1032.68	408.54
A_68_P26085825	chr8:98411955-98411999	NM_010325:289	Got2	INSIDE	0.334	0.623	1481.30	923.05	0.208	1115.16	232.23
A_68_P26085828	chr8:98412281-98412325	NM_010325:-37	Got2	PROMOTER	0.359	0.707	2729.09	1929.90	0.254	2056.67	521.77
A_68_P30641174	chr16:30309847-30309891	NM_008148:997	Gp5	INSIDE	0.287	0.678	1551.89	1052.21	0.194	1119.97	217.67
A_68_P30347594	chr15:76161371-76161427	NM_010331:-325	Gpaa1	PROMOTER	0.097	1.732	3798.95	6579.07	0.168	2617.21	439.45
A_68_P25048708	chr7:36103274-36103318	NM_026181:163	Gpatch1	INSIDE	0.551	0.600	2849.90	1709.53	0.331	2327.19	769.70
A_68_P28080240	chr11:102417615-102417659	NM_001159492:-164	Gpatch8	PROMOTER	0.188	0.733	2111.17	1546.99	0.138	1590.26	219.47
A_68_P29329254	chr13:112280052-112280096	NM_001122963:175	Gpbp1	INSIDE	0.294	1.470	5825.93	8562.02	0.432	3881.47	1675.84
A_68_P20451396	chr1:94727977-94728021	NM_016696:-264	Gpc1	PROMOTER	0.664	0.381	2725.04	1037.67	0.253	2281.88	577.30
A_68_P20451402	chr1:94728781-94728825	NM_016696:540	Gpc1	INSIDE	0.384	0.399	3973.72	1585.15	0.153	3108.58	475.88
A_68_P20451403	chr1:94728878-94728922	NM_016696:638	Gpc1	INSIDE	0.249	0.530	1071.57	568.12	0.132	880.34	116.38
A_68_P32383359	chrX:49965740-49965784	NM_016697:1389	Gpc3	INSIDE	0.167	1.414	1187.23	1678.79	0.237	505.04	119.52
A_68_P32380508	chrX:49517357-49517405	NM_008150:720	Gpc4	INSIDE	0.194	1.566	934.20	1463.14	0.304	381.37	116.13
A_68_P32380514	chrX:49518048-49518092	NM_008150:30	Gpc4	INSIDE	0.031	3.509	2434.58	8541.97	0.109	1060.70	115.78
A_68_P32380515	chrX:49518249-49518293	NM_008150:-170	Gpc4	PROMOTER	0.045	2.950	2257.23	6658.92	0.134	890.06	118.95
A_68_P29928901	chr14:117325143-117325187	NM_001079844:628	Gpc6	INSIDE	0.124	0.488	3170.00	1545.95	0.061	2569.44	155.61
A_68_P26847148	chr9:114842896-114842940	NM_175380:187	Gpd1l	INSIDE	0.442	0.646	1884.94	1217.44	0.285	1483.66	423.46
A_68_P21240630	chr2:57090894-57090938	NM_010274:124	Gpd2	INSIDE	0.226	0.409	1920.58	784.94	0.092	1393.48	128.66
A_68_P28563524	chr12:79327386-79327430	NM_145965:-233	Gphn	PROMOTER	0.295	0.680	1080.98	735.04	0.201	887.89	178.22

ProbeName	Target position of probe on CpG island microarray	TargetID	GeneSymbol	CpG island Description	Ratio of relative methylation (TiO2-NP/Vehicle)	Sham group			TiO_2-H group		
						Relative methylation (Cy-5/Cy-3)	Cy-3 signal (Input)	Cy-5 signal (MeDIP)	Relative methylation (Cy-5/Cy-3)	Cy-3 signal (Input)	Cy-5 signal (MeDIP)
A_68_P28563528	chr12:79327956-79328000	NM_145965:337	Gphn	INSIDE	0.250	0.623	931.00	579.71	0.156	749.76	116.75
A_68_P32239706	chrX:7274518-7274562	NM_173747:281	Gpkow	INSIDE	0.058	3.468	1824.03	6326.36	0.200	618.74	124.05
A_68_P32239707	chrX:7274604-7274648	NM_173747:367	Gpkow	INSIDE	0.189	2.356	1363.34	3212.02	0.446	587.75	262.05
A_68_P23318324	chr4:133140609-133140653	NM_133884:343	Gpn2	INSIDE	0.353	0.556	1487.26	826.96	0.196	1219.44	239.12
A_68_P23318326	chr4:133140776-133140820	NM_133884:511	Gpn2	INSIDE	0.404	0.640	1169.25	748.32	0.259	985.30	254.87
A_68_P32401469	chrX:54756662-54756706	NM_001033360:250	Gpr101	INSIDE	0.279	1.503	1133.46	1703.26	0.419	530.24	222.05
A_68_P21107277	chr2:31008194-31008238	NM_178760:381	Gpr107	INSIDE	0.491	0.521	1101.55	573.61	0.256	929.38	237.79
A_68_P31260713	chr17:57386929-57386973	NM_030084:114	Gpr108	INSIDE	0.431	0.560	1295.83	725.49	0.241	933.64	225.09
A_68_P31260715	chr17:57387144-57387195	NM_030084:-105	Gpr108	DIVERGENT_PROMOTER	0.215	0.558	1245.72	695.29	0.120	974.77	117.10
A_68_P24168905	chr5:147396571-147396615	NM_008151:-612	Gpr12	PROMOTER	0.177	0.445	1817.97	809.44	0.079	1519.45	119.57
A_68_P25580591	chr7:147020155-147020199	NM_177469:104	Gpr123	INSIDE	0.366	0.556	951.68	528.86	0.204	821.51	167.20
A_68_P25736445	chr8:28195931-28195975	NM_054044:-360	Gpr124	PROMOTER	0.630	0.559	1132.18	633.02	0.352	1019.07	359.21
A_68_P25736704	chr8:28232129-28232173	NM_054044:35838	Gpr124	INSIDE	0.368	1.449	1249.75	1810.38	0.533	1079.04	575.38
A_68_P23681255	chr5:50449451-50449501	NM_133911:759	Gpr125	INSIDE	0.358	0.666	1890.34	1259.88	0.238	1368.67	326.31
A_68_P26957818	chr10:14264701-14264745	NM_001002268:120	Gpr126	INSIDE	0.398	0.467	1511.94	705.41	0.186	1275.00	236.56
A_68_P28529999	chr12:73171616-73171660	NM_181752:340	Gpr135	INSIDE	0.198	0.679	5572.04	3785.44	0.134	3970.28	533.12
A_68_P28530000	chr12:73171697-73171741	NM_181752:260	Gpr135	INSIDE	0.441	0.671	2296.17	1540.35	0.296	1759.27	519.91
A_68_P28530004	chr12:73172194-73172239	NM_181752:-238	Gpr135	PROMOTER	0.534	0.608	1217.10	740.47	0.325	1036.36	336.95
A_68_P31936885	chr19:7026235-7026279	NM_001177361:-9316	Gpr137	PROMOTER	0.121	0.695	1914.49	1330.29	0.084	1427.45	119.85
A_68_P28833516	chr13:13485759-13485803	NM_031999:111	Gpr137b	INSIDE	0.555	0.623	1968.81	1226.19	0.346	1665.85	575.57
A_68_P28833511	chr13:12742044-12742088	NR_003568:596	Gpr137b-ps	INSIDE	0.079	0.555	4116.48	2283.77	0.044	3162.14	138.51
A_68_P28833514	chr13:12742271-12742315	NR_003568:370	Gpr137b-ps	INSIDE	0.274	0.479	1584.31	759.23	0.131	1308.74	171.55
A_68_P28833519	chr13:12742906-12742950	NR_003568:-266	Gpr137b-ps	PROMOTER	0.398	0.363	4391.82	1592.50	0.144	3385.93	488.41
A_68_P29569518	chr14:45840204-45840248	NM_027518:835	Gpr137c	INSIDE	0.329	0.680	1405.75	955.29	0.224	1019.18	227.96
A_68_P25462321	chr7:126328077-126328121	NM_001024138:-210	Gpr139	PROMOTER	0.634	0.569	1096.54	623.49	0.360	757.26	272.78
A_68_P32750743	chrX:149216712-149216756	NM_010951:271	Gpr143	INSIDE	0.139	1.605	1725.72	2770.55	0.223	657.78	146.37
A_68_P22187974	chr3:62407714-62407758	NM_177346:1326	Gpr149	INSIDE	0.159	0.555	1639.45	910.62	0.088	1377.95	121.42
A_68_P22187976	chr3:62407890-62407934	NM_177346:1150	Gpr149	INSIDE	0.298	0.714	1701.46	1214.37	0.213	1269.21	269.94
A_68_P21331222	chr2:73224458-73224502	NM_001190297:-25	Gpr155	PROMOTER	0.444	0.566	4010.01	2269.44	0.251	3134.06	787.82
A_68_P21331225	chr2:73224765-73224809	NM_001190297:-331	Gpr155	PROMOTER	0.325	0.588	2712.39	1594.02	0.191	2026.63	386.67
A_68_P21331226	chr2:73224914-73224958	NM_001190297:-481	Gpr155	PROMOTER	0.276	0.517	1051.40	543.58	0.143	819.19	116.82
A_68_P30683214	chr16:37916363-37916407	NM_153394:-197	Gpr156	PROMOTER	0.543	0.654	962.33	629.66	0.355	758.05	269.48
A_68_P23404232	chr4:149461503-149461547	NM_177366:-87	Gpr157	PROMOTER	0.244	1.620	10929.57	17710.04	0.396	7593.87	3007.82
A_68_P23404323	chr4:149475519-149475563	NM_177366:13929	Gpr157	INSIDE	0.349	1.558	2216.23	3452.56	0.543	1619.05	879.43
A_68_P23404324	chr4:149475624-149475668	NM_177366:14035	Gpr157	INSIDE	0.405	1.773	2904.68	5150.79	0.719	2051.82	1474.86
A_68_P21054862	chr2:21289571-21289618	NM_001004761:401	Gpr158	INSIDE	0.428	0.553	984.62	544.69	0.237	837.31	198.19
A_68_P22030513	chr3:30755322-30755366	NM_001134385:473	Gpr160	INSIDE	0.443	0.555	2154.68	1195.23	0.246	1568.11	385.21
A_68_P24817420	chr6:124811153-124811197	NM_013533:2761	Gpr162	INSIDE	0.650	2.824	1192.47	3368.01	1.834	939.70	1723.85
A_68_P24817442	chr6:124813681-124813725	NM_013533:233	Gpr162	INSIDE	0.369	0.432	2790.75	1206.46	0.159	2126.92	339.15
A_68_P30349000	chr15:76369261-76369305	NM_029643:-90	Gpr172b	DIVERGENT_PROMOTER	0.211	0.610	1310.06	798.80	0.128	921.05	118.29
A_68_P29936841	chr14:118536110-118536156	NM_021434:-216	Gpr180	DIVERGENT_PROMOTER	0.284	0.645	846.89	546.24	0.183	635.90	116.38
A_68_P24864114	chr6:134847635-134847686	NM_001167697:-9783	Gpr19	PROMOTER	0.303	0.666	1646.81	1096.63	0.202	1249.68	252.28
A_68_P24864116	chr6:134847884-134847928	NM_001167695:-73	Gpr19	PROMOTER	0.486	0.427	1300.51	555.03	0.207	1069.51	221.73
A_68_P20644872	chr1:138156299-138156343	NM_001101516:1130	Gpr25	INSIDE	0.478	0.513	1269.17	650.55	0.245	954.00	233.70

ProbeName	Target position of probe on CpG island microarray	TargetID	GeneSymbol	CpG island Description	Ratio of relative methylation (TiO2-NP/Vehicle)	Sham group			TiO_2-H group		
						Relative methylation (Cy-5/Cy-3)	Cy-3 signal (Input)	Cy-5 signal (MeDIP)	Relative methylation (Cy-5/Cy-3)	Cy-3 signal (Input)	Cy-5 signal (MeDIP)
A_68_P20644875	chr1:138156699-138156743	NM_001101516:730	Gpr25	INSIDE	0.236	0.409	2461.74	1005.72	0.096	1911.15	184.04
A_68_P25531562	chr7:139158754-139158798	NM_173410:634	Gpr26	INSIDE	0.222	0.404	1661.87	671.82	0.090	1302.92	117.10
A_68_P31953771	chr19:11015569-11015613	NM_009962:3941	Gpr44	INSIDE	0.346	0.585	1188.64	695.44	0.203	938.38	190.04
A_68_P20186163	chr1:43010333-43010377	NM_053107:638	Gpr45	INSIDE	0.088	1.770	10255.33	18152.41	0.156	6082.87	951.65
A_68_P32454737	chrX:68917956-68918000	NM_010340:1037	Gpr50	INSIDE	0.150	1.914	3135.75	6003.36	0.288	1138.91	328.04
A_68_P26803573	chr9:106367885-106367929	NM_001159652:365	Gpr62	INSIDE	0.284	0.609	1636.61	996.12	0.173	1176.95	203.18
A_68_P32779374	chrX:156828817-156828861	NM_001079847:217	Gpr64	INSIDE	0.114	1.648	1878.78	3095.79	0.189	749.51	141.29
A_68_P28684630	chr12:102137196-102137240	NM_001177674:516	Gpr68	INSIDE	0.236	0.446	1366.00	609.35	0.105	1112.33	117.31
A_68_P28684631	chr12:102137378-102137422	NM_001177674:334	Gpr68	INSIDE	0.598	0.509	1290.68	656.58	0.304	975.80	296.96
A_68_P28684632	chr12:102137444-102137488	NM_001177674:268	Gpr68	INSIDE	0.566	0.504	2787.51	1405.18	0.285	1916.13	546.92
A_68_P28684633	chr12:102137516-102137560	NM_001177674:196	Gpr68	INSIDE	0.152	0.514	2130.13	1094.36	0.078	1553.16	121.28
A_68_P22448211	chr3:115955166-115955210	NM_022427:1214	Gpr88	INSIDE	0.389	0.610	909.64	554.72	0.237	837.06	198.43
A_68_P22448216	chr3:115955553-115955597	NM_022427:828	Gpr88	INSIDE	0.265	0.697	1122.37	782.55	0.184	889.23	164.02
A_68_P22351744	chr3:96708735-96708782	NM_026229:463	Gpr89	INSIDE	0.235	0.518	1322.86	685.78	0.122	1099.69	133.83
A_68_P24864941	chr6:135015515-135015567	NM_181444:-139	Gprc5a	PROMOTER	0.167	0.528	1948.37	1028.07	0.088	1360.19	119.65
A_68_P28150124	chr11:114712661-114712705	NM_001110337:-163	Gprc5c	PROMOTER	0.281	0.342	1622.76	555.06	0.096	1266.11	121.86
A_68_P27897908	chr11:69727660-69727704	NM_019726:-11	Gps2	PROMOTER	0.655	0.503	2695.71	1356.48	0.330	1950.72	642.99
A_68_P27897910	chr11:69727956-69728000	NM_019726:285	Gps2	INSIDE	0.583	0.475	2358.46	1120.53	0.277	2000.33	553.62
A_68_P21077788	chr2:26171431-26171475	NM_001199147:-3837	Gpsm1	PROMOTER	0.186	0.695	2432.36	1691.13	0.130	1639.41	212.31
A_68_P30350021	chr15:76528223-76528267	NM_182805:1051	Gpt	INSIDE	0.536	0.499	2407.88	1201.20	0.267	1944.87	520.05
A_68_P26025053	chr8:88016777-88016821	NM_173866:283	Gpt2	INSIDE	0.434	0.679	1680.35	1140.21	0.294	1320.66	388.62
A_68_P26025056	chr8:88017117-88017161	NM_173866:623	Gpt2	INSIDE	0.088	0.679	3052.04	2071.98	0.060	2121.83	126.60
A_68_P26814363	chr9:108241800-108241844	NM_008160:412	Gpx1	INSIDE	0.263	0.526	1512.56	794.87	0.138	1164.10	160.62
A_68_P27812596	chr11:54716387-54716431	NM_008161:-19	Gpx3	PROMOTER	0.406	0.537	985.97	529.92	0.218	768.72	167.64
A_68_P27280641	chr10:79516441-79516485	NM_001037741:-319	Gpx4	PROMOTER	0.478	0.502	2844.34	1428.39	0.240	1949.29	468.24
A_68_P27280642	chr10:79516583-79516627	NM_001037741:-177	Gpx4	PROMOTER	0.165	0.306	3440.50	1053.80	0.051	2600.79	131.41
A_68_P23195896	chr4:108079185-108079229	NM_024198:112	Gpx7	INSIDE	0.161	0.629	1653.33	1039.44	0.101	1128.72	113.93
A_68_P25035344	chr7:31935750-31935794	NM_027898:297	Gramd1a	INSIDE	0.290	0.720	2585.09	1861.85	0.209	1791.82	374.37
A_68_P30404764	chr15:85888808-85888852	NM_001205353:-326	Gramd4	PROMOTER	0.184	0.509	1718.53	875.47	0.094	1288.34	121.02
A_68_P30490430	chr15:101055110-101055154	NM_019518:495	Grasp	INSIDE	0.255	0.420	1458.01	612.63	0.107	1122.94	120.32
A_68_P21283785	chr2:64860691-64860735	NM_016719:111	Grb14	INSIDE	0.267	0.583	1408.09	820.40	0.156	1085.28	168.77
A_68_P21283787	chr2:64861006-64861050	NM_016719:-205	Grb14	PROMOTER	0.339	0.576	1649.62	950.03	0.195	1437.21	280.90
A_68_P28154940	chr11:115569306-115569350	NM_008163:583	Grb2	INSIDE	0.250	0.475	1192.48	565.84	0.119	986.11	116.90
A_68_P28056595	chr11:98319477-98319521	NM_010346:11351	Grb7	DOWNSTREAM	0.284	0.545	2796.07	1523.32	0.155	2077.31	321.17
A_68_P24816729	chr6:124690602-124690649	NM_013535:472	Grcc10	INSIDE	0.150	0.716	1744.89	1249.61	0.107	1212.94	130.21
A_68_P24816733	chr6:124690988-124691032	NM_013535:87	Grcc10	INSIDE	0.297	0.555	943.86	524.02	0.165	731.22	120.76
A_68_P28266912	chr12:16807246-16807296	NM_015764:422	Greb1	INSIDE	0.213	0.662	1020.75	676.17	0.141	843.42	119.20
A_68_P21536545	chr2:113597825-113597869	NM_011824:959	Grem1	INSIDE	0.208	0.495	1505.72	745.64	0.103	1194.34	123.19
A_68_P21536546	chr2:113597904-113597948	NM_011824:879	Grem1	INSIDE	0.634	0.527	1857.10	978.87	0.334	1209.25	404.16
A_68_P23329301	chr4:135126386-135126430	NM_001013756:3127	Grhl3	INSIDE	0.454	0.536	1452.22	779.08	0.243	1232.79	300.01
A_68_P23329327	chr4:135129537-135129581	NM_001013756:-23	Grhl3	PROMOTER	0.639	0.499	1064.11	531.15	0.319	890.80	283.90
A_68_P32337350	chrX:38754572-38754616	NM_016886:114	Gria3	INSIDE	0.212	10.544	7878.95	83076.23	2.233	3793.06	8470.29
A_68_P32337351	chrX:38754697-38754743	NM_016886:240	Gria3	INSIDE	0.148	1.590	1415.23	2249.72	0.236	503.64	118.83
A_68_P29531949	chr14:35632705-35632749	NM_008166:-595	Grid1	PROMOTER	0.283	0.450	1149.77	517.11	0.127	930.75	118.36

ProbeName	Target position of probe on CpG island microarray	TargetID	GeneSymbol	CpG island Description	Ratio of relative methylation (TiO2-NP/Vehicle)	Sham group			TiO_2-H group		
						Relative methylation (Cy-5/Cy-3)	Cy-3 signal (Input)	Cy-5 signal (MeDIP)	Relative methylation (Cy-5/Cy-3)	Cy-3 signal (Input)	Cy-5 signal (MeDIP)
A_68_P29531954	chr14:35633533-35633577	NM_008166:233	Grid1	INSIDE	0.458	0.386	1652.93	637.33	0.177	1325.29	234.11
A_68_P29531957	chr14:35633864-35633908	NM_008166:565	Grid1	INSIDE	0.054	1.338	4410.16	5899.98	0.073	2791.40	203.22
A_68_P24501230	chr6:63206331-63206375	NM_008167:-498	Grid2	PROMOTER	0.620	0.455	1381.44	628.56	0.282	1105.87	311.82
A_68_P24501234	chr6:63206775-63206826	NM_008167:-50	Grid2	PROMOTER	0.229	0.614	1104.05	677.98	0.141	870.04	122.35
A_68_P24501236	chr6:63206960-63207004	NM_008167:132	Grid2	INSIDE	0.155	0.458	2045.42	937.19	0.071	1639.64	116.76
A_68_P24156212	chr5:144125779-144125823	NM_133355:214	Grid2ip	INSIDE	0.168	0.457	2012.18	919.98	0.077	1609.33	123.26
A_68_P24156213	chr5:144125893-144125937	NM_133355:328	Grid2ip	INSIDE	0.280	0.387	1715.24	664.49	0.108	1251.75	135.75
A_68_P24156215	chr5:144126161-144126205	NM_133355:596	Grid2ip	INSIDE	0.091	0.658	2795.15	1838.53	0.060	1986.64	118.49
A_68_P24156244	chr5:144131752-144131796	NM_133355:6188	Grid2ip	INSIDE	0.635	0.524	1618.60	848.49	0.333	1291.53	429.81
A_68_P27127190	chr10:49503177-49503221	NM_001111268:5362	Grik2	INSIDE	0.229	0.713	1333.07	950.48	0.163	1153.25	188.08
A_68_P23274589	chr4:125167651-125167695	NM_001081097:-402	Grik3	PROMOTER	0.660	0.456	1142.11	521.06	0.301	986.64	297.01
A_68_P23274592	chr4:125168108-125168152	NM_001081097:56	Grik3	INSIDE	0.320	0.504	1342.14	676.59	0.161	1178.43	190.25
A_68_P23274595	chr4:125168487-125168531	NM_001081097:434	Grik3	INSIDE	0.651	0.648	1355.65	879.12	0.422	1100.74	464.36
A_68_P26458363	chr9:42705778-42705822	NM_175481:46654	Grik4	INSIDE	0.302	0.618	5699.87	3523.12	0.186	3862.16	719.99
A_68_P26458693	chr9:42751946-42751990	NM_175481:486	Grik4	INSIDE	0.511	1.462	9798.38	14322.62	0.747	6431.69	4805.55
A_68_P26458695	chr9:42752086-42752130	NM_175481:346	Grik4	INSIDE	0.624	3.936	10800.17	42505.35	2.454	6227.14	15282.22
A_68_P21071264	chr2:25160798-25160842	NM_001177656:13863	Grin1	INSIDE	0.160	0.513	3294.87	1691.13	0.082	2379.11	195.48
A_68_P21071306	chr2:25166855-25166901	NM_001177656:7805	Grin1	INSIDE	0.328	0.573	1554.63	890.80	0.188	1255.65	236.24
A_68_P21071362	chr2:25174225-25174270	NM_001177656:436	Grin1	INSIDE	0.092	0.738	2525.73	1864.24	0.068	1722.66	116.66
A_68_P30540360	chr16:9994787-9994831	NM_008170:-2182	Grin2a	PROMOTER	0.136	0.567	1991.22	1129.72	0.077	1490.93	115.17
A_68_P24868563	chr6:135683062-135683116	NM_008171:440441	Grin2b	INSIDE	0.666	3.710	1775.75	6588.79	2.471	1180.46	2916.88
A_68_P24868564	chr6:135683145-135683189	NM_008171:440363	Grin2b	INSIDE	0.507	4.190	1333.07	5585.51	2.125	917.72	1950.39
A_68_P28152305	chr11:115111838-115111882	NM_010350:16697	Grin2c	INSIDE	0.283	0.446	2260.91	1009.37	0.126	1592.06	201.15
A_68_P28152427	chr11:115128177-115128221	NM_010350:359	Grin2c	INSIDE	0.382	0.620	2404.14	1491.55	0.237	1860.09	440.44
A_68_P25095384	chr7:53087940-53087984	NM_008172:34089	Grin2d	INSIDE	0.277	0.679	1134.09	769.55	0.188	911.54	171.57
A_68_P25095548	chr7:53121361-53121405	NM_008172:669	Grin2d	INSIDE	0.594	0.662	1402.91	929.37	0.394	1222.04	480.94
A_68_P22907353	chr4:49858612-49858656	NM_001033351:-681	Grin3a	PROMOTER	0.114	0.735	3348.02	2460.06	0.084	2248.59	188.77
A_68_P22907354	chr4:49858702-49858746	NM_001033351:-771	Grin3a	PROMOTER	0.603	0.582	1701.90	990.30	0.351	1147.95	402.82
A_68_P26622300	chr9:71333407-71333451	NM_001164793:-663	Grinl1a	PROMOTER	0.470	0.558	1220.55	681.52	0.262	1050.40	275.48
A_68_P26622303	chr9:71333818-71333862	NM_178602:-50	Grinl1a	PROMOTER	0.434	0.403	2113.33	851.67	0.175	1713.63	299.51
A_68_P23595699	chr5:35003466-35003510	NM_001080743:461	Grk4	INSIDE	0.645	0.613	1008.96	618.42	0.395	790.34	312.33
A_68_P29055020	chr13:55545946-55545990	NM_011938:-726	Grk6	PROMOTER	0.155	1.478	810.86	1198.23	0.229	513.73	117.72
A_68_P29055022	chr13:55546184-55546228	NM_011938:-488	Grk6	PROMOTER	0.103	0.639	2723.94	1741.07	0.066	1913.17	125.93
A_68_P29055023	chr13:55546318-55546369	NM_011938:-351	Grk6	PROMOTER	0.209	0.531	1579.54	838.79	0.111	1085.71	120.68
A_68_P29055029	chr13:55547182-55547226	NM_011938:510	Grk6	INSIDE	0.183	0.473	1651.20	780.85	0.087	1384.51	119.97
A_68_P24982506	chr7:17200789-17200833	NM_172739:-468	Grlf1	PROMOTER	0.320	0.468	3258.55	1524.24	0.150	2453.71	366.86
A_68_P26936713	chr10:10408926-10408970	NM_001114333:393181	Grm1	INSIDE	0.280	0.386	1984.50	765.94	0.108	1707.58	184.84
A_68_P26804413	chr9:106558146-106558190	NM_001160353:272	Grm2	INSIDE	0.564	0.574	2025.33	1162.75	0.324	1662.37	538.64
A_68_P31116575	chr17:27610002-27610046	NM_001013385:30225	Grm4	INSIDE	0.623	4.118	2069.05	8520.77	2.567	1431.83	3675.22
A_68_P31116576	chr17:27610068-27610113	NM_001013385:30159	Grm4	INSIDE	0.670	3.990	4281.32	17081.76	2.671	2957.50	7900.55
A_68_P24743884	chr6:110595643-110595687	NM_177328:73	Grm7	INSIDE	0.247	0.626	3444.15	2155.10	0.155	2719.05	420.72
A_68_P24318684	chr6:28083743-28083787	NM_008174:605	Grm8	INSIDE	0.609	0.544	1165.14	633.31	0.331	960.59	317.93
A_68_P24318697	chr6:28085365-28085421	NM_008174:-1023	Grm8	PROMOTER	0.370	0.524	1001.98	525.21	0.194	878.10	170.48
A_68_P23607762	chr5:36807361-36807405	NM_024478:-451	Grpel1	PROMOTER	0.172	0.684	1366.16	935.00	0.118	985.94	115.89

ProbeName	Target position of probe on CpG island microarray	TargetID	GeneSymbol	CpG island Description	Ratio of relative methylation (TiO2-NP/Vehicle)	Sham group			TiO_2-H group		
						Relative methylation (Cy-5/Cy-3)	Cy-3 signal (Input)	Cy-5 signal (MeDIP)	Relative methylation (Cy-5/Cy-3)	Cy-3 signal (Input)	Cy-5 signal (MeDIP)
A_68_P23607763	chr5:36807446-36807491	NM_024478:-365	Grpel1	PROMOTER	0.357	0.541	3092.77	1672.04	0.193	2365.57	457.16
A_68_P25095367	chr7:53085924-53085970	NM_153419:213	Grwd1	INSIDE	0.188	0.503	2503.33	1260.16	0.095	1743.64	165.37
A_68_P25095368	chr7:53086086-53086130	NM_153419:51	Grwd1	INSIDE	0.265	0.603	866.65	522.86	0.160	750.00	119.92
A_68_P28703487	chr12:105711415-105711459	NM_010351:10	Gsc	INSIDE	0.322	0.584	988.58	576.92	0.188	823.69	154.66
A_68_P30344599	chr15:75692883-75692927	NM_026960:136	Gsdmd	INSIDE	0.545	0.654	1225.36	800.98	0.356	892.64	317.78
A_68_P26220482	chr8:123011851-123011906	NM_198671:-887	Gse1	PROMOTER	0.233	0.659	1206.07	794.68	0.154	959.89	147.68
A_68_P26220486	chr8:123012400-123012445	NM_198671:-343	Gse1	PROMOTER	0.370	0.631	1511.00	953.06	0.233	993.55	231.90
A_68_P27915399	chr11:72951333-72951377	NM_010353:442	Gsg2	INSIDE	0.668	0.643	1027.53	660.99	0.430	834.13	358.28
A_68_P30684006	chr16:38089070-38089114	NM_019827:6	Gsk3b	INSIDE	0.422	0.648	2453.27	1589.87	0.274	1758.48	480.99
A_68_P21132485	chr2:35111777-35111821	NM_001206367:-80	Gsn	PROMOTER	0.391	0.613	2026.14	1242.11	0.240	1463.14	350.71
A_68_P21132488	chr2:35112210-35112254	NM_001206367:354	Gsn	INSIDE	0.540	0.606	1346.80	816.75	0.328	1093.01	358.22
A_68_P30547081	chr16:11253703-11253747	NM_001130008:694	Gspt1	INSIDE	0.304	0.491	2163.25	1062.04	0.149	1863.27	278.48
A_68_P30547086	chr16:11254244-11254288	NM_001130008:152	Gspt1	INSIDE	0.280	0.440	2981.65	1312.19	0.123	2199.17	271.37
A_68_P30547087	chr16:11254371-11254415	NM_001130008:26	Gspt1	INSIDE	0.577	0.614	3031.98	1860.73	0.354	2326.81	824.29
A_68_P21766452	chr2:155418422-155418466	NM_008180:-2	Gss	PROMOTER	0.447	0.552	1662.28	917.93	0.247	1326.07	327.25
A_68_P32152397	chr19:47939957-47940001	NM_030051:-56	Gsto2	PROMOTER	0.331	0.475	1378.65	655.14	0.157	998.53	156.94
A_68_P27258887	chr10:75299960-75300008	NM_010361:-2358	Gstt2	PROMOTER	0.491	0.664	1469.45	975.94	0.326	1161.91	379.01
A_68_P28614428	chr12:88487974-88488018	NM_010363:-671	Gstz1	PROMOTER	0.325	0.678	2336.86	1583.57	0.220	1908.15	420.14
A_68_P28614430	chr12:88488238-88488282	NM_010363:-407	Gstz1	PROMOTER	0.526	0.678	1114.53	755.34	0.356	977.63	348.19
A_68_P28614433	chr12:88488594-88488639	NM_010363:-51	Gstz1	PROMOTER	0.146	0.613	1975.54	1211.73	0.089	1383.70	123.75
A_68_P24172322	chr5:148001001-148001045	NM_008178:751	Gsx1	INSIDE	0.417	0.428	1713.77	732.81	0.178	1429.29	255.11
A_68_P23807224	chr5:75471627-75471671	NM_133256:23	Gsx2	INSIDE	0.292	0.511	2343.80	1198.27	0.149	1839.49	274.26
A_68_P23807225	chr5:75471780-75471824	NM_133256:177	Gsx2	INSIDE	0.081	0.449	4540.07	2039.43	0.037	3157.80	115.28
A_68_P23807226	chr5:75471874-75471918	NM_133256:271	Gsx2	INSIDE	0.263	0.572	2189.19	1251.64	0.150	1679.14	252.11
A_68_P23807227	chr5:75471945-75471989	NM_133256:341	Gsx2	INSIDE	0.649	0.448	1377.26	616.42	0.291	1001.14	290.99
A_68_P23807228	chr5:75472072-75472116	NM_133256:469	Gsx2	INSIDE	0.668	0.707	2024.06	1430.51	0.472	1638.57	773.77
A_68_P24757355	chr6:113027040-113027084	NR_027010:176	Gt(ROSA)26Sor	INSIDE	0.607	0.391	1817.88	711.35	0.238	1389.91	330.26
A_68_P28635712	chr12:92826632-92826677	NM_031391:1435	Gtf2a1	INSIDE	0.663	0.683	1034.83	706.39	0.453	948.48	429.29
A_68_P22592456	chr3:142428233-142428277	NM_145546:44	Gtf2b	INSIDE	0.450	0.642	847.40	544.06	0.289	819.62	236.77
A_68_P31259555	chr17:57150392-57150436	NM_133801:297	Gtf2f1	INSIDE	0.114	0.510	2824.94	1442.11	0.058	2016.32	117.47
A_68_P25101371	chr7:54051634-54051678	NM_008186:193	Gtf2h1	INSIDE	0.663	0.589	1266.18	745.54	0.391	959.92	374.94
A_68_P29268695	chr13:101262403-101262447	NM_022011:140	Gtf2h2	INSIDE	0.610	0.714	3258.92	2328.09	0.435	2502.66	1089.91
A_68_P24112565	chr5:134789851-134789895	NM_001080746:744	Gtf2i	INSIDE	0.402	0.714	2256.93	1612.52	0.287	1769.13	508.52
A_68_P24112568	chr5:134790208-134790252	NM_001080746:386	Gtf2i	INSIDE	0.617	0.617	2532.59	1563.75	0.381	2013.74	767.02
A_68_P24111875	chr5:134660308-134660352	NM_053266:423	Gtf2ird2	INSIDE	0.618	0.494	2700.07	1333.23	0.305	2069.94	631.95
A_68_P24111876	chr5:134660418-134660462	NM_053266:533	Gtf2ird2	INSIDE	0.299	0.639	1141.25	728.79	0.191	838.54	160.37
A_68_P25496221	chr7:132850823-132850867	NM_207239:358	Gtf3c1	INSIDE	0.239	0.572	4038.57	2311.41	0.137	2902.89	397.17
A_68_P23576909	chr5:31481980-31482025	NM_027901:515	Gtf3c2	INSIDE	0.327	0.613	948.93	581.83	0.201	714.94	143.48
A_68_P21093572	chr2:28695445-28695489	NM_001166033:414	Gtf3c4	INSIDE	0.490	0.542	1010.39	547.18	0.265	800.93	212.35
A_68_P21092001	chr2:28438373-28438417	NM_148928:405	Gtf3c5	INSIDE	0.121	0.596	2181.26	1299.59	0.072	1686.92	121.48
A_68_P27083090	chr10:39977304-39977348	NM_026113:145	Gtf3c6	INSIDE	0.660	0.706	1785.25	1261.18	0.466	1542.72	718.81
A_68_P30367930	chr15:79521605-79521649	NM_013818:301	Gtpbp1	INSIDE	0.639	0.354	2153.79	762.88	0.226	1851.95	418.93
A_68_P31205408	chr17:46297849-46297904	NM_001145979:-104	Gtpbp2	DIVERGENT_PROMOTER	0.243	0.545	1351.33	735.85	0.132	916.38	121.11
A_68_P25957090	chr8:74012768-74012812	NM_032544:789	Gtpbp3	INSIDE	0.227	0.615	1880.96	1156.69	0.140	1471.00	205.56

ProbeName	Target position of probe on CpG island microarray	TargetID	GeneSymbol	CpG island Description	Ratio of relative methylation (TiO2-NP/Vehicle)	Sham group			TiO_2-H group		
						Relative methylation (Cy-5/Cy-3)	Cy-3 signal (Input)	Cy-5 signal (MeDIP)	Relative methylation (Cy-5/Cy-3)	Cy-3 signal (Input)	Cy-5 signal (MeDIP)
A_68_P25957092	chr8:74012930-74012974	NM_032544:951	Gtpbp3	INSIDE	0.322	0.566	904.37	511.45	0.182	668.64	121.76
A_68_P21901246	chr2:179805450-179805494	NM_001083328:175	Gtpbp5	INSIDE	0.606	0.631	1197.29	756.00	0.382	882.63	337.49
A_68_P23977818	chr5:110536465-110536509	NM_145147:730	Gtpbp6	INSIDE	0.233	1.433	6337.49	9079.31	0.334	4296.42	1435.34
A_68_P23977819	chr5:110536642-110536688	NM_145147:552	Gtpbp6	INSIDE	0.191	1.714	11771.56	20172.69	0.327	7593.94	2486.50
A_68_P23977821	chr5:110536870-110536914	NM_145147:324	Gtpbp6	INSIDE	0.260	0.481	1316.34	632.71	0.125	935.37	116.70
A_68_P23778450	chr5:69947976-69948020	NM_172711:-182	Guf1	PROMOTER	0.189	0.647	2022.79	1308.46	0.122	1565.78	191.15
A_68_P20193920	chr1:44608775-44608819	NM_028450:281	Gulp1	INSIDE	0.596	0.647	1750.20	1133.25	0.386	1417.05	547.02
A_68_P20193921	chr1:44608865-44608909	NM_028450:371	Gulp1	INSIDE	0.443	0.456	2276.31	1037.24	0.202	1793.04	362.10
A_68_P30446023	chr15:93105424-93105468	NM_001033275:69	Gxylt1	INSIDE	0.512	0.475	1298.36	616.26	0.243	1064.07	258.39
A_68_P30446028	chr15:93106155-93106199	NM_001033275:-661	Gxylt1	PROMOTER	0.308	0.455	1295.48	589.24	0.140	915.10	128.11
A_68_P32507213	chrX:83022104-83022148	NM_008194:32	Gyk	INSIDE	0.136	3.630	4744.25	17219.68	0.495	1881.94	930.63
A_68_P21428049	chr2:92210891-92210935	NM_001166633:281	Gyltl1b	INSIDE	0.411	0.603	2316.06	1395.78	0.247	1656.29	409.93
A_68_P31603480	chr18:32719536-32719580	NM_001048207:130	Gypc	INSIDE	0.314	0.565	2544.38	1437.98	0.178	2085.18	370.43
A_68_P31603481	chr18:32719632-32719687	NM_001048207:29	Gypc	INSIDE	0.144	0.652	1678.83	1095.19	0.094	1275.87	119.70
A_68_P25093127	chr7:52690084-52690132	NM_030678:-100	Gys1	DIVERGENT_PROMOTER	0.139	0.698	1559.87	1088.21	0.097	1260.83	122.64
A_68_P24904452	chr6:142363210-142363254	NM_145572:58397	Gys2	DOWNSTREAM	0.503	0.413	1462.01	604.36	0.208	1226.68	255.18
A_68_P21731577	chr2:148506945-148506989	NM_028986:111	Gzf1	INSIDE	0.642	0.737	2375.53	1750.12	0.473	1693.37	800.84
A_68_P24619080	chr6:87930668-87930712	NM_198622:786	H1fx	INSIDE	0.488	0.604	2033.85	1228.42	0.295	1860.91	548.16
A_68_P24619081	chr6:87930736-87930780	NM_198622:718	H1fx	INSIDE	0.250	0.348	1708.96	594.22	0.087	1402.74	121.79
A_68_P31152954	chr17:34401924-34401968	NM_207105:1775	H2-Ab1	INSIDE	0.284	0.659	7394.39	4874.37	0.187	5405.93	1010.72
A_68_P24874512	chr6:136757288-136757338	NM_177688:544	H2afj	INSIDE	0.129	1.345	3864.97	5199.27	0.173	2528.08	438.36
A_68_P26467074	chr9:44143508-44143552	NM_010436:733	H2afx	INSIDE	0.568	0.539	1039.19	560.28	0.306	869.37	266.17
A_68_P22565463	chr3:137527117-137527161	NM_016750:-424	H2afz	PROMOTER	0.534	0.415	2737.64	1135.15	0.221	2273.51	503.52
A_68_P22565473	chr3:137528589-137528633	NM_016750:1048	H2afz	INSIDE	0.384	0.459	1411.87	647.69	0.176	1140.22	200.77
A_68_P31159075	chr17:35516634-35516678	NM_001143689:95	H2-Gs10	INSIDE	0.277	0.546	1820.45	993.97	0.151	1377.83	208.43
A_68_P31151371	chr17:34115239-34115283	NR_004446:476	H2-K2	INSIDE	0.407	0.456	1605.18	731.55	0.185	1385.24	256.72
A_68_P31151376	chr17:34115729-34115774	NR_004446:-15	H2-K2	DIVERGENT_PROMOTER	0.586	0.479	1184.32	567.85	0.281	896.88	252.05
A_68_P31151132	chr17:34077320-34077372	NM_010385:-58	H2-Ke2	DIVERGENT_PROMOTER	0.271	0.578	903.23	522.05	0.157	775.07	121.60
A_68_P31166661	chr17:37125639-37125683	NM_001115075:789	H2-M5	INSIDE	0.301	0.729	2275.44	1658.37	0.220	1770.73	388.90
A_68_P31158870	chr17:35457706-35457750	NM_010390:226	H2-Q1	INSIDE	0.603	0.547	1166.12	638.01	0.330	983.84	324.67
A_68_P31159261	chr17:35606907-35606951	NM_010391:-105	H2-Q10	PROMOTER	0.302	0.564	1180.89	666.38	0.170	1060.75	180.49
A_68_P20877853	chr1:182724319-182724363	NM_008210:19394	H3f3a	DOWNSTREAM	0.645	0.523	1074.31	561.60	0.337	838.95	282.98
A_68_P25186204	chr7:73224455-73224499	NM_024439:-58	H47	PROMOTER	0.308	0.449	1540.45	691.17	0.138	1180.55	163.11
A_68_P22531297	chr3:130974718-130974765	NM_008212:214	Hadh	INSIDE	0.151	0.468	2361.74	1105.35	0.071	1734.32	122.67
A_68_P23571063	chr5:30481709-30481753	NM_145558:-131	Hadhb	DIVERGENT_PROMOTER	0.206	0.550	1400.06	770.27	0.114	1102.29	125.21
A_68_P23571064	chr5:30481871-30481915	NM_145558:31	Hadhb	INSIDE	0.177	0.509	3436.32	1749.92	0.090	2549.02	229.29
A_68_P23571065	chr5:30481976-30482020	NM_145558:137	Hadhb	INSIDE	0.158	0.608	1512.98	919.33	0.096	1222.17	117.62
A_68_P23571066	chr5:30482087-30482136	NM_145558:250	Hadhb	INSIDE	0.271	0.517	1092.18	565.12	0.140	831.66	116.82
A_68_P23571067	chr5:30482177-30482221	NM_145558:337	Hadhb	INSIDE	0.395	0.651	971.59	632.21	0.257	751.39	193.05
A_68_P31099857	chr17:24987665-24987709	NM_001159626:75	Hagh	INSIDE	0.410	0.628	1229.78	771.74	0.257	1042.21	268.16
A_68_P27827732	chr11:57641072-57641118	NM_008213:4555	Hand1	DOWNSTREAM	0.050	1.535	4642.97	7127.85	0.077	2995.31	230.27
A_68_P27827763	chr11:57644849-57644893	NM_008213:779	Hand1	INSIDE	0.316	0.473	1204.13	569.65	0.150	795.06	118.89
A_68_P27827764	chr11:57644954-57644998	NM_008213:673	Hand1	INSIDE	0.239	0.588	1092.67	642.27	0.140	841.67	118.22
A_68_P25890515	chr8:59799750-59799801	NM_010402:-4	Hand2	PROMOTER	0.238	0.673	1746.30	1175.90	0.160	1357.53	217.41

ProbeName	Target position of probe on CpG island microarray	TargetID	GeneSymbol	CpG island Description	Ratio of relative methylation (TiO2-NP/Vehicle)	Sham group			TiO_2-H group		
						Relative methylation (Cy-5/Cy-3)	Cy-3 signal (Input)	Cy-5 signal (MeDIP)	Relative methylation (Cy-5/Cy-3)	Cy-3 signal (Input)	Cy-5 signal (MeDIP)
A_68_P28067607	chr11:100216626-100216670	NM_010404:807	Hap1	INSIDE	0.662	0.663	931.14	617.47	0.439	778.26	341.72
A_68_P28067610	chr11:100216965-100217009	NM_010404:469	Hap1	INSIDE	0.197	0.415	1901.36	788.50	0.082	1501.29	122.56
A_68_P28067614	chr11:100217488-100217532	NM_010404:-55	Hap1	PROMOTER	0.448	0.575	1133.08	651.98	0.258	869.63	224.19
A_68_P28067617	chr11:100217870-100217915	NM_010404:-437	Hap1	PROMOTER	0.266	0.581	1057.03	614.11	0.155	816.69	126.42
A_68_P22311349	chr3:87826614-87826658	NM_022031:4797	Hapln2	INSIDE	0.571	0.590	1205.66	710.99	0.337	1032.84	347.79
A_68_P22311351	chr3:87826838-87826882	NM_022031:4573	Hapln2	INSIDE	0.143	0.622	5199.28	3233.76	0.089	3528.41	314.89
A_68_P22311355	chr3:87827371-87827415	NM_022031:4041	Hapln2	INSIDE	0.459	0.567	2341.18	1326.68	0.260	1900.76	493.92
A_68_P22311356	chr3:87827542-87827586	NM_022031:3869	Hapln2	INSIDE	0.396	0.664	2761.76	1833.73	0.263	1981.97	520.78
A_68_P22311357	chr3:87827642-87827686	NM_022031:3769	Hapln2	INSIDE	0.507	0.621	1291.59	802.02	0.315	1091.23	343.72
A_68_P25949749	chr8:72610964-72611013	NM_177900:3561	Hapln4	INSIDE	0.131	0.627	1941.82	1218.02	0.082	1464.96	120.13
A_68_P21319207	chr2:71227345-71227389	NM_026115:50	Hat1	INSIDE	0.303	0.421	1296.44	545.81	0.128	1004.31	128.31
A_68_P31849647	chr18:78006501-78006545	NM_146089:-3	Haus1	DIVERGENT_PROMOTER	0.620	0.536	1708.20	915.30	0.332	1483.02	493.02
A_68_P21576348	chr2:120454904-120454948	NM_025475:19755	Haus2	DOWNSTREAM	0.244	0.697	3304.90	2303.35	0.170	2438.74	414.22
A_68_P23592734	chr5:34511815-34511865	NM_146159:233	Haus3	INSIDE	0.267	0.517	1113.92	576.21	0.138	872.87	120.52
A_68_P23592735	chr5:34511958-34512002	NM_146159:93	Haus3	INSIDE	0.360	0.510	2132.18	1087.18	0.184	1650.10	303.13
A_68_P31624334	chr18:36675661-36675705	NM_010415:-223	Hbegf	PROMOTER	0.590	0.491	2047.62	1006.16	0.290	1646.99	477.39
A_68_P22405342	chr3:107082078-107082122	NM_026774:325	Hbxip	INSIDE	0.625	0.467	1942.45	907.77	0.292	1409.57	411.88
A_68_P22405343	chr3:107082203-107082254	NM_026774:453	Hbxip	INSIDE	0.195	0.715	1392.62	996.07	0.139	971.40	135.42
A_68_P22405344	chr3:107082299-107082343	NM_026774:545	Hbxip	INSIDE	0.606	0.598	1435.14	858.53	0.363	1185.10	429.74
A_68_P32465786	chrX:71211813-71211857	NM_008224:-180	Hcfc1	PROMOTER	0.092	2.410	2382.95	5743.18	0.221	857.36	189.12
A_68_P32465787	chrX:71211906-71211951	NM_008224:-274	Hcfc1	PROMOTER	0.066	2.773	1632.69	4527.60	0.183	670.10	122.34
A_68_P27278459	chr10:79180444-79180488	NM_008226:1088	Hcn2	INSIDE	0.656	0.655	1170.31	766.08	0.429	940.59	403.81
A_68_P22317567	chr3:88963692-88963736	NM_008227:365	Hcn3	INSIDE	0.508	0.589	1460.91	861.08	0.299	1216.82	364.27
A_68_P26550024	chr9:58670000-58670044	NM_001081192:-1296	Hcn4	PROMOTER	0.105	0.624	2513.84	1568.23	0.066	1872.85	123.09
A_68_P23300617	chr4:129816677-129816721	NM_198959:-292	Hcrtr1	PROMOTER	0.606	0.656	1169.89	767.48	0.397	941.02	373.98
A_68_P23297198	chr4:129219482-129219532	NM_008228:384	Hdac1	INSIDE	0.080	1.379	1454.66	2006.33	0.110	1059.73	116.49
A_68_P24637118	chr6:91106557-91106606	NM_144919:-227	Hdac11	PROMOTER	0.586	0.551	945.08	520.85	0.323	768.25	248.10
A_68_P24637122	chr6:91107049-91107096	NM_144919:264	Hdac11	INSIDE	0.034	1.864	2902.03	5408.58	0.064	1920.46	122.60
A_68_P27067366	chr10:36694758-36694802	NM_008229:431	Hdac2	INSIDE	0.225	0.338	1989.68	672.65	0.076	1573.38	119.47
A_68_P31631084	chr18:38114429-38114473	NM_010411:192	Hdac3	INSIDE	0.045	2.035	2056.96	4186.80	0.091	1314.65	119.16
A_68_P31631086	chr18:38114645-38114689	NM_010411:-24	Hdac3	DIVERGENT_PROMOTER	0.640	0.468	2185.66	1021.91	0.299	1667.69	499.29
A_68_P32241075	chrX:7525044-7525088	NM_001130416:-51	Hdac6	PROMOTER	0.112	2.703	4241.32	11465.94	0.303	1528.29	462.44
A_68_P32241078	chrX:7525375-7525419	NM_001130416:-381	Hdac6	PROMOTER	0.113	1.621	1528.19	2476.52	0.183	636.95	116.45
A_68_P27043768	chr10:31033252-31033296	NM_027168:64	Hddc2	INSIDE	0.439	0.610	1176.36	717.39	0.267	887.91	237.48
A_68_P31253725	chr17:56218817-56218862	NM_008233:-240	Hdgfrp2	DIVERGENT_PROMOTER	0.230	2.071	10447.06	21635.33	0.476	6921.80	3296.94
A_68_P25274807	chr7:89078453-89078497	NM_013886:871	Hdgfrp3	INSIDE	0.241	0.657	1393.32	915.63	0.159	1078.97	171.14
A_68_P22964786	chr4:62163240-62163284	NM_024257:-28	Hdhd3	PROMOTER	0.209	0.526	4534.31	2384.03	0.110	3319.07	364.93
A_68_P20455838	chr1:95375093-95375137	NM_133808:271	Hdlbp	INSIDE	0.603	0.536	1707.94	915.49	0.323	1274.21	411.62
A_68_P28831963	chr13:12488087-12488132	NM_144835:468	Heatr1	INSIDE	0.448	0.645	1517.25	978.13	0.289	1224.65	353.51
A_68_P24133859	chr5:139625867-139625911	NM_001081265:-288	Heatr2	PROMOTER	0.493	3.006	10901.91	32770.22	1.481	6902.72	10221.18
A_68_P28432638	chr12:53072168-53072212	NM_177171:118	Heatr5a	INSIDE	0.648	0.462	1801.20	832.51	0.300	1625.66	487.10
A_68_P31373925	chr17:79234724-79234768	NM_001081179:-25	Heatr5b	DIVERGENT_PROMOTER	0.162	0.613	1688.00	1035.56	0.100	1290.76	128.53
A_68_P27973306	chr11:83567372-83567416	NM_145432:256	Heatr6	INSIDE	0.494	0.358	1511.56	541.48	0.177	1255.91	222.24
A_68_P27973307	chr11:83567440-83567484	NM_145432:324	Heatr6	INSIDE	0.529	0.647	1670.46	1080.41	0.342	1291.74	441.55

ProbeName	Target position of probe on CpG island microarray	TargetID	GeneSymbol	CpG island Description	Ratio of relative methylation (TiO2-NP/Vehicle)	Sham group			TiO_2-H group		
						Relative methylation (Cy-5/Cy-3)	Cy-3 signal (Input)	Cy-5 signal (MeDIP)	Relative methylation (Cy-5/Cy-3)	Cy-3 signal (Input)	Cy-5 signal (MeDIP)
A_68_P32090696	chr19:36628500-36628544	NM_001163471:-606	Hectd2	PROMOTER	0.187	0.620	3761.43	2332.90	0.116	2595.28	301.48
A_68_P32090697	chr19:36628656-36628700	NM_001163471:-450	Hectd2	PROMOTER	0.159	0.673	1365.57	918.67	0.107	1113.34	118.88
A_68_P32090698	chr19:36628741-36628785	NM_001163471:-366	Hectd2	PROMOTER	0.489	0.595	2248.85	1337.14	0.291	1664.63	483.79
A_68_P32090702	chr19:36629470-36629514	NM_001163471:364	Hectd2	INSIDE	0.404	0.272	2373.47	646.01	0.110	1953.97	214.88
A_68_P23231911	chr4:116668335-116668379	NM_175244:404	Hectd3	INSIDE	0.267	0.459	1858.83	853.81	0.123	1602.86	196.92
A_68_P23231912	chr4:116668443-116668487	NM_175244:512	Hectd3	INSIDE	0.174	0.669	2504.04	1675.53	0.116	1937.40	225.04
A_68_P27495202	chr10:119526727-119526771	NM_080446:23273	Helb	INSIDE	0.619	2.400	1052.84	2526.84	1.486	871.14	1294.48
A_68_P27495358	chr10:119549908-119549958	NM_080446:89	Helb	INSIDE	0.267	0.615	969.27	596.28	0.164	731.87	120.14
A_68_P23931498	chr5:101227159-101227203	NM_001081107:439	Helq	INSIDE	0.209	0.449	1418.54	637.57	0.094	1225.39	115.33
A_68_P25835333	chr8:47377718-47377762	NM_173789:2285	Helt	INSIDE	0.546	0.709	1376.17	975.11	0.387	1184.63	458.14
A_68_P25835354	chr8:47380103-47380147	NM_173789:-99	Helt	PROMOTER	0.145	0.659	1657.80	1092.20	0.095	1306.90	124.55
A_68_P28107584	chr11:107408339-107408383	NM_198298:-913	Helz	PROMOTER	0.448	0.609	1674.35	1019.42	0.273	1294.31	353.21
A_68_P26807872	chr9:107240552-107240596	NM_133984:107	Hemk1	INSIDE	0.242	0.372	1849.45	687.29	0.090	1339.56	120.37
A_68_P24483216	chr6:58781482-58781526	NM_028705:-2189	Herc3	PROMOTER	0.581	0.701	1869.26	1311.24	0.408	1596.32	650.82
A_68_P23416912	chr4:151533736-151533780	NM_008236:783	Hes2	INSIDE	0.328	0.644	2289.50	1473.88	0.211	1490.45	314.73
A_68_P23416916	chr4:151534184-151534228	NM_008236:1231	Hes2	INSIDE	0.177	0.454	2238.35	1016.34	0.080	1494.86	120.07
A_68_P23416918	chr4:151534429-151534473	NM_008236:1475	Hes2	INSIDE	0.418	0.526	1120.01	589.36	0.220	990.52	218.07
A_68_P23417743	chr4:151660802-151660846	NM_008237:4947	Hes3	INSIDE	0.234	0.655	1545.32	1011.76	0.153	1151.32	176.17
A_68_P23417747	chr4:151661148-151661192	NM_008237:4601	Hes3	INSIDE	0.608	0.535	2896.06	1550.47	0.325	2289.36	745.00
A_68_P23417749	chr4:151661314-151661358	NM_008237:4435	Hes3	INSIDE	0.221	0.659	1411.08	929.33	0.145	1140.56	165.80
A_68_P23417771	chr4:151664685-151664729	NM_008237:1065	Hes3	INSIDE	0.195	0.556	1313.42	729.88	0.108	1141.18	123.41
A_68_P20442334	chr1:93310020-93310067	NM_019479:-244	Hes6	PROMOTER	0.428	0.626	971.17	607.78	0.268	735.86	196.96
A_68_P28083427	chr11:102977675-102977719	NM_138753:58	Hexim1	INSIDE	0.175	0.511	1849.53	945.58	0.090	1352.84	121.25
A_68_P28083435	chr11:102978558-102978602	NM_138753:942	Hexim1	INSIDE	0.381	0.654	1619.45	1059.54	0.249	1327.14	331.08
A_68_P28083436	chr11:102978646-102978690	NM_138753:1030	Hexim1	INSIDE	0.171	0.598	1671.62	1000.41	0.102	1348.68	137.80
A_68_P28083441	chr11:102979217-102979261	NM_138753:1600	Hexim1	INSIDE	0.431	0.680	1269.78	863.31	0.293	1059.33	310.43
A_68_P28083512	chr11:102993647-102993691	NM_001130516:-984	Hexim2	PROMOTER	0.623	0.519	1413.27	733.25	0.323	1164.68	376.68
A_68_P28083514	chr11:102993838-102993882	NM_001130516:-792	Hexim2	PROMOTER	0.256	1.534	1317.26	2021.04	0.394	917.91	361.20
A_68_P28083515	chr11:102993907-102993951	NM_001130516:-724	Hexim2	PROMOTER	0.078	1.695	2730.81	4628.89	0.132	1621.76	214.70
A_68_P21933403	chr3:8664956-8665000	NM_010423:2060	Hey1	INSIDE	0.098	0.613	2701.20	1656.23	0.060	1964.09	118.45
A_68_P21933404	chr3:8665074-8665126	NM_010423:1938	Hey1	INSIDE	0.199	0.690	1111.30	766.70	0.138	884.71	121.66
A_68_P23964040	chr5:107354969-107355013	NM_177873:-81	Hfm1	PROMOTER	0.498	0.639	2141.64	1367.76	0.318	1834.41	583.29
A_68_P20945388	chr1:194597149-194597193	NM_144881:243	Hhat	INSIDE	0.219	0.555	1192.89	661.67	0.122	1010.54	122.83
A_68_P32095557	chr19:37509810-37509854	NM_008245:502	Hhex	INSIDE	0.498	0.365	2104.86	768.63	0.182	1796.54	326.42
A_68_P32095558	chr19:37509889-37509933	NM_008245:580	Hhex	INSIDE	0.471	0.509	1751.67	892.45	0.240	1582.63	379.54
A_68_P25997733	chr8:82581462-82581506	NM_020259:423	Hhip	INSIDE	0.333	0.541	1886.99	1021.05	0.180	1655.06	298.20
A_68_P28727214	chr12:109544952-109544996	NM_001044380:495	Hhipl1	INSIDE	0.550	0.429	1893.69	812.73	0.236	1451.64	342.78
A_68_P28727298	chr12:109557168-109557212	NM_001044380:12711	Hhipl1	INSIDE	0.664	4.705	1516.50	7134.36	3.125	1142.63	3570.46
A_68_P27924564	chr11:74979683-74979728	NM_001098203:1949	Hic1	INSIDE	0.410	0.384	1585.65	608.64	0.157	1227.08	193.10
A_68_P27924565	chr11:74979765-74979809	NM_001098203:1868	Hic1	INSIDE	0.448	0.469	1230.09	577.15	0.210	904.45	189.90
A_68_P27924588	chr11:74982421-74982465	NM_001098203:-788	Hic1	PROMOTER	0.294	0.429	2116.45	908.35	0.126	1711.04	216.24
A_68_P27924562	chr11:74979450-74979494	NM_001098203:2182	Hic1	INSIDE	0.670	0.487	1104.94	537.62	0.326	842.19	274.44
A_68_P30572652	chr16:17233443-17233487	NM_178922:-215	Hic2	PROMOTER	0.098	0.571	2791.23	1595.13	0.056	2187.85	122.38
A_68_P28540453	chr12:75008595-75008639	NM_010431:-237	Hif1a	PROMOTER	0.455	0.537	1693.26	909.27	0.244	1303.66	318.38

ProbeName	Target position of probe on CpG island microarray	TargetID	GeneSymbol	CpG island Description	Ratio of relative methylation (TiO2-NP/Vehicle)	Sham group			TiO_2-H group		
						Relative methylation (Cy-5/Cy-3)	Cy-3 signal (Input)	Cy-5 signal (MeDIP)	Relative methylation (Cy-5/Cy-3)	Cy-3 signal (Input)	Cy-5 signal (MeDIP)
A_68_P26466888	chr9:44113383-44113427	NM_172162:350	Hinfp	INSIDE	0.573	0.728	1790.13	1302.75	0.417	1557.83	649.66
A_68_P26466893	chr9:44113911-44113955	NM_172162:-178	Hinfp	PROMOTER	0.665	0.436	1731.62	755.54	0.290	1237.93	359.38
A_68_P24116584	chr5:135965213-135965257	NM_146001:55758	Hip1	INSIDE	0.586	0.687	1038.07	713.20	0.403	893.22	359.88
A_68_P24116811	chr5:136020789-136020833	NM_146001:182	Hip1	INSIDE	0.336	0.648	1656.74	1073.41	0.218	1180.12	256.84
A_68_P24116812	chr5:136020886-136020930	NM_146001:84	Hip1	INSIDE	0.193	0.516	1705.07	879.76	0.100	1283.89	127.92
A_68_P24116813	chr5:136021029-136021073	NM_146001:-58	Hip1	PROMOTER	0.204	0.560	1404.69	786.06	0.114	1097.20	125.40
A_68_P22388053	chr3:103594655-103594699	NM_010432:522	Hipk1	INSIDE	0.489	0.652	2358.97	1538.21	0.319	1716.38	546.89
A_68_P22388054	chr3:103594799-103594843	NM_010432:378	Hipk1	INSIDE	0.488	0.404	2251.66	909.39	0.197	1777.28	350.43
A_68_P22388055	chr3:103594904-103594948	NM_010432:272	Hipk1	INSIDE	0.606	0.677	1287.47	872.07	0.411	995.39	408.69
A_68_P24380022	chr6:38825884-38825928	NM_010433:283	Hipk2	INSIDE	0.251	0.613	909.00	556.86	0.154	797.67	122.74
A_68_P21489522	chr2:104333335-104333384	NM_001145824:1287	Hipk3	INSIDE	0.247	0.551	1078.04	593.61	0.136	855.10	116.07
A_68_P21489523	chr2:104333403-104333447	NM_001145824:1222	Hipk3	INSIDE	0.296	0.446	1339.26	596.69	0.132	1022.60	134.85
A_68_P25502648	chr7:134005213-134005270	NM_172746:-244	Hirip3	DIVERGENT_PROMOTER	0.181	0.659	1454.84	959.35	0.120	1046.73	125.14
A_68_P25502653	chr7:134005799-134005843	NM_172746:335	Hirip3	INSIDE	0.325	0.426	1549.80	659.52	0.138	1328.85	183.58
A_68_P28883386	chr13:23855470-23855521	NM_030609:-41	Hist1h1a	DIVERGENT_PROMOTER	0.155	0.727	4442.08	3230.36	0.113	3046.22	342.78
A_68_P28883199	chr13:23831258-23831302	NM_015786:605	Hist1h1c	INSIDE	0.194	0.450	1895.51	852.57	0.087	1428.11	124.46
A_68_P28882218	chr13:23654270-23654316	NM_178205:-58	Hist1h3e	PROMOTER	0.142	0.579	3050.22	1765.67	0.082	2142.61	175.81
A_68_P28882027	chr13:23627101-23627153	NM_145073:-160	Hist1h3g	PROMOTER	0.153	0.617	1652.11	1018.62	0.094	1282.28	120.61
A_68_P28883366	chr13:23853149-23853193	NM_178192:-144	Hist1h4a	PROMOTER	0.274	0.509	1158.81	589.30	0.140	857.37	119.61
A_68_P28882355	chr13:23673495-23673539	NM_175654:46	Hist1h4d	INSIDE	0.444	0.577	1228.26	708.59	0.256	898.56	230.00
A_68_P22348542	chr3:96073837-96073891	NM_175666:242	Hist2h2bb	INSIDE	0.239	0.591	1160.04	685.08	0.141	867.97	122.58
A_68_P22348231	chr3:96025351-96025395	NM_178214:329	Hist2h2be	INSIDE	0.491	0.348	2059.56	717.03	0.171	1615.94	276.38
A_68_P22348232	chr3:96025442-96025486	NM_178214:421	Hist2h2be	INSIDE	0.525	0.634	1337.54	848.64	0.333	1018.16	339.35
A_68_P27833729	chr11:58768398-58768446	NM_178218:236	Hist3h2a	INSIDE	0.389	0.527	1602.94	844.67	0.205	1168.73	239.53
A_68_P26954160	chr10:13686535-13686580	NM_010437:373	Hivep2	INSIDE	0.250	0.364	1693.23	616.19	0.091	1290.86	117.38
A_68_P26954163	chr10:13686795-13686839	NM_010437:632	Hivep2	INSIDE	0.364	0.539	1026.28	553.65	0.197	946.65	186.06
A_68_P23247367	chr4:119487484-119487528	NM_010657:224	Hivep3	INSIDE	0.497	0.581	1776.78	1032.20	0.289	1394.74	403.02
A_68_P27185478	chr10:61802859-61802903	NM_001146100:289	Hk1	INSIDE	0.316	0.517	1844.88	953.18	0.163	1390.06	227.01
A_68_P27185482	chr10:61803237-61803281	NM_001146100:-89	Hk1	DIVERGENT_PROMOTER	0.571	0.702	1982.39	1390.70	0.400	1654.77	662.31
A_68_P24589794	chr6:82723294-82723338	NM_013820:1132	Hk2	INSIDE	0.329	0.638	1871.92	1194.40	0.210	1528.37	321.29
A_68_P24589797	chr6:82723544-82723588	NM_013820:882	Hk2	INSIDE	0.634	0.700	1367.97	957.22	0.443	1086.57	481.83
A_68_P24589798	chr6:82723620-82723664	NM_013820:806	Hk2	INSIDE	0.259	0.608	1041.30	633.02	0.157	861.69	135.63
A_68_P24589801	chr6:82723921-82723966	NM_013820:505	Hk2	INSIDE	0.167	0.689	1467.27	1011.14	0.115	1037.19	119.63
A_68_P24589809	chr6:82724860-82724912	NM_013820:-438	Hk2	PROMOTER	0.147	0.594	1934.19	1149.04	0.087	1311.08	114.30
A_68_P20897165	chr1:186556818-186556862	NM_008250:-468	Hlx	PROMOTER	0.510	0.627	1028.58	644.62	0.320	926.83	296.27
A_68_P27289056	chr10:80813448-80813492	NM_001163166:-268	Hmg20b	DIVERGENT_PROMOTER	0.138	0.557	2214.07	1232.20	0.077	1578.63	120.94
A_68_P31117100	chr17:27692460-27692504	NM_001025427:-1036	Hmga1	PROMOTER	0.595	0.692	1553.21	1074.37	0.412	1403.69	577.94
A_68_P27497203	chr10:119851890-119851938	NM_010441:62077	Hmga2	INSIDE	0.619	3.041	1188.53	3614.42	1.883	922.28	1736.95
A_68_P27497666	chr10:119912069-119912113	NM_010441:1901	Hmga2	INSIDE	0.238	0.459	2402.95	1102.85	0.109	1805.91	197.24
A_68_P27497669	chr10:119912366-119912410	NM_010441:1603	Hmga2	INSIDE	0.391	0.640	1046.27	669.85	0.251	783.71	196.34
A_68_P32454250	chrX:68809345-68809389	NM_008253:199	Hmgb3	INSIDE	0.213	1.632	1137.69	1856.64	0.348	554.51	193.12
A_68_P23331344	chr4:135502377-135502421	NM_008254:31	Hmgcl	INSIDE	0.204	0.594	1240.26	736.22	0.121	952.96	115.63
A_68_P29247521	chr13:97440411-97440457	NM_008255:457	Hmgcr	INSIDE	0.219	0.510	1283.20	654.55	0.112	1052.96	117.56
A_68_P30991210	chr16:96349056-96349100	NM_008251:254	Hmgn1	INSIDE	0.151	0.564	2242.06	1264.82	0.085	1657.50	141.29

ProbeName	Target position of probe on CpG island microarray	TargetID	GeneSymbol	CpG island Description	Ratio of relative methylation (TiO2-NP/Vehicle)	Sham group			TiO_2-H group		
						Relative methylation (Cy-5/Cy-3)	Cy-3 signal (Input)	Cy-5 signal (MeDIP)	Relative methylation (Cy-5/Cy-3)	Cy-3 signal (Input)	Cy-5 signal (MeDIP)
A_68_P23320286	chr4:133523495-133523539	NM_016957:390	Hmgn2	INSIDE	0.300	0.453	3163.61	1431.56	0.136	2470.57	334.83
A_68_P26682080	chr9:83039935-83039979	NM_026122:258	Hmgn3	INSIDE	0.654	0.656	1783.67	1170.60	0.429	1385.19	594.39
A_68_P26682081	chr9:83040074-83040118	NM_026122:118	Hmgn3	INSIDE	0.497	0.640	1373.14	879.38	0.318	1083.76	344.67
A_68_P25971956	chr8:77617525-77617569	NM_010442:30	Hmox1	INSIDE	0.354	0.623	977.54	608.67	0.220	790.71	174.17
A_68_P30511777	chr16:4726653-4726697	NM_001136066:314	Hmox2	INSIDE	0.524	0.514	2757.78	1417.92	0.269	2341.07	630.63
A_68_P23600187	chr5:35731282-35731326	NM_010445:-461	Hmx1	PROMOTER	0.524	0.522	1697.52	886.83	0.274	1428.06	390.66
A_68_P23600193	chr5:35732106-35732150	NM_010445:363	Hmx1	INSIDE	0.281	0.444	3075.14	1366.56	0.125	2298.91	286.79
A_68_P25528903	chr7:138686351-138686395	NM_008257:-104	Hmx3	PROMOTER	0.426	0.430	1802.03	775.35	0.183	1528.16	280.03
A_68_P25528905	chr7:138686558-138686602	NM_008257:104	Hmx3	INSIDE	0.216	0.356	2359.46	839.52	0.077	1880.21	144.42
A_68_P25528910	chr7:138687411-138687455	NM_008257:956	Hmx3	INSIDE	0.579	0.611	2432.25	1486.29	0.354	1796.73	635.58
A_68_P28153961	chr11:115375325-115375369	NM_008258:338	Hn1	INSIDE	0.240	0.667	868.25	579.30	0.160	744.49	119.08
A_68_P28153966	chr11:115375918-115375967	NM_008258:-258	Hn1	PROMOTER	0.284	0.639	827.18	528.47	0.181	663.28	120.28
A_68_P31100623	chr17:25097367-25097411	NM_198937:180	Hn1l	INSIDE	0.523	0.461	1873.12	863.53	0.241	1688.36	406.87
A_68_P31100627	chr17:25097854-25097898	NM_198937:-308	Hn1l	PROMOTER	0.573	0.514	1415.31	728.17	0.295	1145.61	337.48
A_68_P27973894	chr11:83664886-83664930	NM_009330:538	Hnf1b	INSIDE	0.517	0.656	2460.11	1613.38	0.339	1948.79	660.51
A_68_P29071677	chr13:58230562-58230606	NM_029872:-667	Hnrnpa0	PROMOTER	0.302	0.622	3034.47	1887.17	0.188	2518.81	472.73
A_68_P29071679	chr13:58230820-58230864	NM_029872:-925	Hnrnpa0	PROMOTER	0.570	0.490	1513.66	741.79	0.279	1149.29	321.01
A_68_P24444427	chr6:51419364-51419408	NM_016806:507	Hnrnpa2b1	INSIDE	0.260	0.495	7818.60	3872.52	0.129	5293.75	682.31
A_68_P21345222	chr2:75497220-75497264	NM_053263:-73	Hnrnpa3	PROMOTER	0.619	0.489	1143.12	558.50	0.302	996.67	301.31
A_68_P29604764	chr14:52723955-52723999	NM_001170981:-273	Hnrnpc	DIVERGENT_PROMOTER	0.187	0.538	1609.29	865.54	0.100	1215.71	122.11
A_68_P23927128	chr5:100406693-100406737	NM_001077265:1243	Hnrnpd	INSIDE	0.342	0.657	1887.39	1240.47	0.225	1455.95	326.96
A_68_P23927130	chr5:100406931-100406976	NM_001077265:1004	Hnrnpd	INSIDE	0.492	0.521	1374.29	715.84	0.256	1040.83	266.89
A_68_P23927139	chr5:100407949-100407993	NM_001077265:-13	Hnrnpd	PROMOTER	0.429	0.391	2458.35	960.66	0.168	1988.35	333.70
A_68_P24783934	chr6:117857089-117857136	NM_001166428:-709	Hnrnpf	PROMOTER	0.210	0.593	1355.27	803.51	0.124	947.50	117.90
A_68_P24783942	chr6:117857933-117857977	NM_001166427:-9357	Hnrnpf	PROMOTER	0.243	0.508	1155.66	587.04	0.124	943.02	116.55
A_68_P24783944	chr6:117858262-117858321	NM_001166427:-9020	Hnrnpf	PROMOTER	0.196	0.619	1224.66	758.66	0.122	970.06	117.89
A_68_P24784005	chr6:117866930-117866974	NM_001166427:-359	Hnrnpf	PROMOTER	0.367	0.586	1905.99	1117.24	0.215	1450.07	312.04
A_68_P27788210	chr11:50191797-50191841	NM_021510:598	Hnrnph1	INSIDE	0.447	0.742	2420.16	1794.73	0.332	1721.94	571.11
A_68_P32672782	chrX:131135885-131135929	NM_019868:82	Hnrnph2	INSIDE	0.075	2.992	5115.46	15306.29	0.225	2082.20	468.59
A_68_P27189024	chr10:62485962-62486006	NM_001079824:613	Hnrnph3	INSIDE	0.541	0.692	2870.62	1986.57	0.375	2192.99	821.34
A_68_P29073062	chr13:58503740-58503784	NM_025279:115	Hnrnpk	INSIDE	0.626	0.613	898.64	550.43	0.384	755.18	289.67
A_68_P31150129	chr17:33822024-33822068	NM_001109913:357	Hnrnpm	INSIDE	0.162	0.528	1867.53	986.84	0.086	1526.06	130.80
A_68_P31150130	chr17:33822156-33822206	NM_001109913:223	Hnrnpm	INSIDE	0.111	0.476	3140.60	1495.74	0.053	2380.94	125.45
A_68_P23333247	chr4:135867486-135867530	NM_028871:618	Hnrnpr	INSIDE	0.171	0.698	3342.59	2331.65	0.119	2477.62	295.06
A_68_P25011771	chr7:26540127-26540178	NM_144922:-413	Hnrnpul1	PROMOTER	0.246	0.490	1203.20	590.03	0.121	1059.53	127.76
A_68_P31381041	chr17:80460590-80460634	NM_144802:1062	Hnrpll	INSIDE	0.367	0.428	2048.07	876.46	0.157	1597.30	250.70
A_68_P31381050	chr17:80461904-80461948	NM_144802:-252	Hnrpll	PROMOTER	0.583	0.604	1043.17	630.03	0.352	867.92	305.53
A_68_P25950855	chr8:72807002-72807046	NM_001146153:127	Homer3	INSIDE	0.436	0.610	3020.53	1843.68	0.266	2430.86	647.54
A_68_P29614704	chr14:55483280-55483329	NM_001177705:-309	Homez	PROMOTER	0.250	0.504	1164.74	586.74	0.126	937.73	118.15
A_68_P26023020	chr8:87509915-87509959	NM_001167991:-4557	Hook2	DIVERGENT_PROMOTER	0.288	0.510	1072.57	546.88	0.147	953.13	140.17
A_68_P26023052	chr8:87514587-87514631	NM_001167991:115	Hook2	INSIDE	0.424	0.642	1430.89	917.99	0.272	1070.13	290.93
A_68_P25730814	chr8:27229164-27229208	NM_207659:510	Hook3	INSIDE	0.561	0.465	1832.79	852.16	0.261	1488.23	388.33
A_68_P23819789	chr5:77524030-77524074	NM_001159900:240	Hopx	INSIDE	0.338	0.698	1624.76	1134.48	0.236	1430.51	337.68
A_68_P24449304	chr6:52184811-52184855	NM_008263:106	Hoxa10	INSIDE	0.247	0.543	1273.12	691.07	0.134	892.55	119.90

ProbeName	Target position of probe on CpG island microarray	TargetID	GeneSymbol	CpG island Description	Ratio of relative methylation (TiO2-NP/Vehicle)	Sham group			TiO_2-H group		
						Relative methylation (Cy-5/Cy-3)	Cy-3 signal (Input)	Cy-5 signal (MeDIP)	Relative methylation (Cy-5/Cy-3)	Cy-3 signal (Input)	Cy-5 signal (MeDIP)
A_68_P24449350	chr6:52190240-52190299	NM_008263:-5331	Hoxa10	PROMOTER	0.236	0.485	1311.09	636.18	0.114	1053.10	120.41
A_68_P24449398	chr6:52195787-52195837	NM_010450:-46	Hoxa11	PROMOTER	0.205	0.417	1997.63	832.36	0.086	1403.88	120.15
A_68_P24449402	chr6:52196249-52196297	NM_010450:-506	Hoxa11	PROMOTER	0.114	0.715	2020.02	1443.81	0.081	1473.78	119.83
A_68_P24449404	chr6:52196424-52196469	NM_010450:-680	Hoxa11	PROMOTER	0.362	0.568	1406.16	799.17	0.206	962.44	197.83
A_68_P24449395	chr6:52195431-52195475	NR_015348:211	Hoxa11as	INSIDE	0.472	0.477	1110.75	529.39	0.225	889.37	200.15
A_68_P24449462	chr6:52203025-52203069	NR_015348:7805	Hoxa11as	DOWNSTREAM	0.184	0.480	2973.37	1425.96	0.088	2472.11	218.04
A_68_P24448784	chr6:52122568-52122612	NM_010452:40476	Hoxa3	INSIDE	0.236	0.633	1727.18	1093.31	0.149	1299.10	193.89
A_68_P24448822	chr6:52127106-52127159	NM_010452:35934	Hoxa3	INSIDE	0.158	0.636	1596.56	1015.95	0.100	1238.86	124.26
A_68_P24448934	chr6:52142084-52142128	NM_008265:-404	Hoxa4	DIVERGENT_PROMOTER	0.588	0.393	1418.62	557.15	0.231	1199.19	277.16
A_68_P24449036	chr6:52153762-52153806	NM_010453:802	Hoxa5	INSIDE	0.329	0.520	1406.56	730.98	0.171	1068.76	182.49
A_68_P24449040	chr6:52154168-52154212	NM_010453:396	Hoxa5	INSIDE	0.620	0.622	3048.81	1897.39	0.386	2375.59	915.95
A_68_P24449041	chr6:52154245-52154298	NM_010453:315	Hoxa5	INSIDE	0.270	0.479	1220.24	584.92	0.129	921.20	119.11
A_68_P24449064	chr6:52156622-52156666	NM_010454:1979	Hoxa6	INSIDE	0.435	0.491	1095.46	538.22	0.214	920.02	196.71
A_68_P24449227	chr6:52175677-52175721	NM_010456:1671	Hoxa9	INSIDE	0.286	0.577	1083.34	625.13	0.165	906.20	149.66
A_68_P24449231	chr6:52176139-52176188	NM_010456:1206	Hoxa9	INSIDE	0.262	0.458	1245.49	570.57	0.120	974.02	116.97
A_68_P28044102	chr11:96227285-96227329	NM_008266:235	Hoxb1	INSIDE	0.150	0.716	3630.03	2599.93	0.108	2369.35	255.08
A_68_P28044105	chr11:96227597-96227641	NM_008266:547	Hoxb1	INSIDE	0.195	0.495	1785.36	884.48	0.097	1300.11	125.76
A_68_P28042988	chr11:96064572-96064616	NM_008267:8920	Hoxb13	DOWNSTREAM	0.148	0.673	2519.76	1696.56	0.100	1915.09	190.66
A_68_P28042990	chr11:96064768-96064813	NM_008267:9116	Hoxb13	DOWNSTREAM	0.234	0.726	4266.44	3097.35	0.170	3090.64	523.99
A_68_P28043946	chr11:96205491-96205535	NM_010458:430	Hoxb3	INSIDE	0.110	0.493	2866.05	1411.73	0.054	2193.26	118.74
A_68_P28043947	chr11:96205604-96205648	NM_010458:544	Hoxb3	INSIDE	0.422	0.531	2464.90	1309.04	0.224	1879.09	420.76
A_68_P28043948	chr11:96205685-96205729	NM_010458:624	Hoxb3	INSIDE	0.354	0.495	1421.11	703.43	0.175	1108.68	194.20
A_68_P28043737	chr11:96180501-96180545	NM_010459:942	Hoxb4	INSIDE	0.054	0.742	4134.06	3067.29	0.040	2834.86	114.11
A_68_P28043606	chr11:96165053-96165098	NM_008268:250	Hoxb5	INSIDE	0.135	0.595	2809.13	1671.01	0.080	1699.38	136.07
A_68_P28043569	chr11:96160876-96160920	NM_008269:414	Hoxb6	INSIDE	0.662	0.559	1057.54	590.81	0.370	903.37	334.06
A_68_P28043444	chr11:96145749-96145793	NM_010461:2552	Hoxb8	INSIDE	0.446	0.539	3451.12	1861.77	0.241	2677.95	644.60
A_68_P30501058	chr15:102797076-102797125	NM_010462:-126	Hoxc10	PROMOTER	0.388	0.430	1419.27	610.74	0.167	969.49	161.89
A_68_P30501066	chr15:102798113-102798164	NM_010462:912	Hoxc10	INSIDE	0.134	1.464	822.14	1203.90	0.196	593.66	116.37
A_68_P30500964	chr15:102785193-102785237	NM_001024842:258	Hoxc11	INSIDE	0.129	0.585	2165.22	1266.42	0.076	1605.07	121.34
A_68_P30500796	chr15:102765447-102765491	NM_010463:-1815	Hoxc12	PROMOTER	0.530	0.579	1629.59	944.23	0.307	1508.98	463.54
A_68_P30500861	chr15:102773323-102773367	NM_010463:6061	Hoxc12	DOWNSTREAM	0.653	0.703	1594.27	1121.45	0.460	1243.25	571.40
A_68_P30500862	chr15:102773465-102773509	NM_010463:6203	Hoxc12	DOWNSTREAM	0.102	0.686	2710.75	1859.78	0.070	1726.95	120.68
A_68_P30501631	chr15:102865426-102865470	NM_013553:623	Hoxc4	INSIDE	0.463	0.569	2610.99	1486.39	0.264	1955.37	515.30
A_68_P30501636	chr15:102866066-102866110	NM_013553:1263	Hoxc4	INSIDE	0.611	0.709	2052.98	1455.93	0.433	1530.93	663.51
A_68_P21339408	chr2:74600811-74600855	NM_010467:-204	Hoxd1	PROMOTER	0.528	0.558	2033.10	1134.00	0.295	1600.30	471.32
A_68_P21339410	chr2:74601035-74601079	NM_010467:20	Hoxd1	INSIDE	0.540	0.604	1186.35	716.54	0.326	957.69	312.34
A_68_P21339411	chr2:74601129-74601173	NM_010467:114	Hoxd1	INSIDE	0.234	0.471	3599.67	1695.18	0.110	2829.54	311.74
A_68_P21339415	chr2:74601471-74601515	NM_010467:456	Hoxd1	INSIDE	0.669	0.708	2539.99	1797.15	0.474	2083.43	986.57
A_68_P21338806	chr2:74526046-74526090	NM_013554:-3936	Hoxd10	PROMOTER	0.239	0.685	2057.06	1409.00	0.164	1567.47	257.10
A_68_P21338737	chr2:74517808-74517856	NM_008273:-2617	Hoxd11	PROMOTER	0.379	0.653	1564.43	1021.20	0.247	1084.94	268.44
A_68_P21338756	chr2:74520344-74520390	NM_008273:-83	Hoxd11	PROMOTER	0.242	0.554	1053.33	583.16	0.134	864.65	115.70
A_68_P21338645	chr2:74506594-74506638	NM_008275:250	Hoxd13	INSIDE	0.628	0.617	3313.08	2045.71	0.388	2518.88	976.32
A_68_P21339173	chr2:74570711-74570762	NM_010468:20687	Hoxd3	INSIDE	0.216	0.642	1038.19	666.11	0.139	849.83	117.73
A_68_P21339175	chr2:74570928-74570982	NM_010468:20905	Hoxd3	INSIDE	0.175	0.672	1839.81	1237.11	0.118	1054.44	124.16

ProbeName	Target position of probe on CpG island microarray	TargetID	GeneSymbol	CpG island Description	Ratio of relative methylation (TiO2-NP/Vehicle)	Sham group			TiO_2-H group		
						Relative methylation (Cy-5/Cy-3)	Cy-3 signal (Input)	Cy-5 signal (MeDIP)	Relative methylation (Cy-5/Cy-3)	Cy-3 signal (Input)	Cy-5 signal (MeDIP)
A_68_P21339241	chr2:74578701-74578745	NM_010468:28673	Hoxd3	INSIDE	0.437	0.593	1239.05	734.25	0.259	898.72	232.99
A_68_P21339293	chr2:74585053-74585097	NM_010468:35025	Hoxd3	INSIDE	0.245	0.569	4052.26	2305.23	0.139	2746.45	382.56
A_68_P21339294	chr2:74585119-74585163	NM_010468:35091	Hoxd3	INSIDE	0.344	0.683	1523.36	1039.69	0.235	1194.89	280.82
A_68_P21339318	chr2:74587859-74587910	NM_010468:37835	Hoxd3	DOWNSTREAM	0.271	0.535	990.54	530.01	0.145	835.08	121.03
A_68_P21339320	chr2:74588185-74588229	NM_010468:38157	Hoxd3	DOWNSTREAM	0.333	0.619	903.43	559.59	0.207	627.49	129.62
A_68_P21339129	chr2:74565400-74565445	NM_010469:5388	Hoxd4	INSIDE	0.460	0.542	1058.17	574.00	0.250	952.19	237.60
A_68_P21338894	chr2:74536266-74536310	NM_013555:469	Hoxd9	INSIDE	0.177	0.544	1477.23	803.63	0.096	1236.00	119.12
A_68_P28271876	chr12:17697378-17697423	NM_016677:-219	Hpcal1	PROMOTER	0.041	1.988	2184.05	4341.67	0.082	1451.54	118.99
A_68_P28271880	chr12:17697825-17697869	NM_016677:227	Hpcal1	INSIDE	0.470	0.636	900.56	572.63	0.299	796.02	238.04
A_68_P28271882	chr12:17698051-17698099	NM_016677:455	Hpcal1	INSIDE	0.031	1.447	3887.70	5626.17	0.045	2651.60	120.63
A_68_P23231145	chr4:116497325-116497369	NM_146256:-3233	Hpdl	PROMOTER	0.242	0.438	1410.27	617.73	0.106	1110.21	117.57
A_68_P32123366	chr19:42854178-42854227	NM_019424:264	Hps1	INSIDE	0.303	0.504	1169.93	590.13	0.153	865.39	132.45
A_68_P32141593	chr19:46078116-46078160	NM_176785:171	Hps6	INSIDE	0.220	0.376	1648.90	619.58	0.082	1429.07	117.87
A_68_P32141596	chr19:46078435-46078479	NM_176785:489	Hps6	INSIDE	0.054	0.718	5887.53	4228.23	0.039	4128.41	161.27
A_68_P30634660	chr16:29210031-29210075	NM_013751:272	Hrasls	INSIDE	0.327	2.119	4365.10	9249.79	0.692	3160.65	2188.69
A_68_P31940698	chr19:7686824-7686868	NM_025731:-212	Hrasls5	DIVERGENT_PROMOTER	0.335	0.574	1405.93	806.98	0.192	1006.23	193.20
A_68_P31940699	chr19:7686892-7686936	NM_025731:-144	Hrasls5	DIVERGENT_PROMOTER	0.403	0.677	2664.39	1803.92	0.273	1908.10	520.63
A_68_P29048047	chr13:54287555-54287599	NM_008286:79	Hrh2	INSIDE	0.519	0.549	1330.37	730.23	0.285	1098.78	313.18
A_68_P21901451	chr2:179838159-179838203	NM_133849:747	Hrh3	INSIDE	0.238	0.507	1884.94	955.40	0.121	1411.15	170.36
A_68_P24025031	chr5:118619337-118619381	NM_007545:-414	Hrk	PROMOTER	0.317	0.580	2371.16	1376.46	0.184	1975.67	363.65
A_68_P24025032	chr5:118619420-118619464	NM_007545:-330	Hrk	PROMOTER	0.317	0.498	2773.27	1380.12	0.158	2013.37	317.57
A_68_P24025033	chr5:118619514-118619558	NM_007545:-236	Hrk	PROMOTER	0.425	0.690	2723.58	1877.93	0.293	1906.88	558.84
A_68_P22603827	chr3:144232599-144232643	NM_011828:560	Hs2st1	INSIDE	0.574	0.592	1626.19	963.49	0.340	1213.11	412.60
A_68_P23627555	chr5:40036051-40036095	NM_010474:-202	Hs3st1	PROMOTER	0.375	0.628	1564.74	982.98	0.236	1171.99	276.35
A_68_P25473609	chr7:128536673-128536717	NM_001081327:885	Hs3st2	INSIDE	0.533	0.527	1665.04	877.49	0.281	1382.91	388.38
A_68_P25474217	chr7:128643986-128644031	NM_001081327:108199	Hs3st2	INSIDE	0.665	2.244	1612.51	3618.03	1.493	1081.08	1613.60
A_68_P27865436	chr11:64248735-64248779	NM_178870:-77	Hs3st3a1	PROMOTER	0.611	0.517	1450.49	750.17	0.316	1056.21	333.60
A_68_P27865440	chr11:64249134-64249178	NM_178870:323	Hs3st3a1	INSIDE	0.644	0.627	2304.24	1444.40	0.404	1736.33	700.61
A_68_P27862273	chr11:63732747-63732791	NM_018805:3018	Hs3st3b1	INSIDE	0.444	0.489	1102.67	539.65	0.217	913.95	198.59
A_68_P27862287	chr11:63734368-63734412	NM_018805:1396	Hs3st3b1	INSIDE	0.250	0.540	1175.52	634.46	0.135	884.21	119.39
A_68_P27862299	chr11:63735880-63735924	NM_018805:-116	Hs3st3b1	PROMOTER	0.393	0.443	2224.84	984.97	0.174	1748.73	304.15
A_68_P31099379	chr17:24889503-24889547	NM_001012402:-423	Hs3st6	PROMOTER	0.607	0.650	1028.18	668.07	0.394	828.92	326.80
A_68_P31099383	chr17:24890123-24890167	NM_001012402:197	Hs3st6	INSIDE	0.563	0.630	1409.88	887.85	0.354	1160.29	411.09
A_68_P20146390	chr1:36161035-36161084	NM_015818:35815	Hs6st1	INSIDE	0.569	2.857	777.68	2221.56	1.627	582.83	948.01
A_68_P32378022	chrX:49033742-49033786	NM_015819:-110	Hs6st2	PROMOTER	0.123	2.217	3471.88	7696.60	0.274	1469.37	401.91
A_68_P29942855	chr14:119537625-119537669	NM_015820:160	Hs6st3	INSIDE	0.226	0.665	2929.87	1949.12	0.151	2206.34	332.32
A_68_P26213861	chr8:121868279-121868323	NM_024219:-137	Hsbp1	PROMOTER	0.518	0.441	1227.41	541.85	0.229	953.94	218.26
A_68_P26213863	chr8:121868494-121868539	NM_024219:79	Hsbp1	INSIDE	0.556	0.491	1489.62	730.74	0.273	1214.04	330.94
A_68_P31863131	chr18:80433374-80433418	NM_001136181:10445	Hsbp1l1	INSIDE	0.453	0.493	1290.37	635.51	0.223	987.13	220.39
A_68_P26134292	chr8:108042458-108042502	NM_008289:-165	Hsd11b2	PROMOTER	0.326	0.425	1518.41	646.07	0.139	1128.21	156.61
A_68_P26134295	chr8:108042675-108042719	NM_008289:51	Hsd11b2	INSIDE	0.406	0.730	2677.10	1954.81	0.296	1979.05	586.14
A_68_P28071796	chr11:100941137-100941181	NM_010475:1434	Hsd17b1	INSIDE	0.532	0.657	1392.13	915.02	0.350	1086.16	379.97
A_68_P28071797	chr11:100941221-100941265	NM_010475:1518	Hsd17b1	INSIDE	0.376	0.473	1995.61	943.59	0.178	1426.12	253.51
A_68_P21439331	chr2:93998296-93998341	NM_019657:-252	Hsd17b12	PROMOTER	0.361	0.543	1429.19	775.83	0.196	1119.20	219.20

ProbeName	Target position of probe on CpG island microarray	TargetID	GeneSymbol	CpG island Description	Ratio of relative methylation (TiO2-NP/Vehicle)	Sham group			TiO_2-H group		
						Relative methylation (Cy-5/Cy-3)	Cy-3 signal (Input)	Cy-5 signal (MeDIP)	Relative methylation (Cy-5/Cy-3)	Cy-3 signal (Input)	Cy-5 signal (MeDIP)
A_68_P22957172	chr4:59594608-59594652	NM_024255:196	Hsdl2	INSIDE	0.225	0.569	1682.05	957.35	0.128	1278.50	164.03
A_68_P30348596	chr15:76307956-76308000	NM_008296:104	Hsf1	INSIDE	0.455	0.471	2542.92	1197.92	0.214	2038.31	437.16
A_68_P27315607	chr10:86167637-86167681	NM_011631:531	Hsp90b1	INSIDE	0.357	0.549	932.23	511.68	0.196	762.34	149.49
A_68_P27315611	chr10:86167975-86168019	NM_011631:193	Hsp90b1	INSIDE	0.363	0.537	1931.18	1037.46	0.195	1607.35	313.11
A_68_P32215807	chr19:58935699-58935743	NM_175199:-246	Hspa12a	PROMOTER	0.312	0.612	1871.08	1144.36	0.191	1301.11	248.26
A_68_P30880659	chr16:75766853-75766897	NM_030201:189	Hspa13	INSIDE	0.339	0.641	1975.32	1266.08	0.217	1430.94	311.12
A_68_P20962883	chr2:3429988-3430032	NM_015765:76	Hspa14	INSIDE	0.217	0.456	1304.03	594.09	0.099	1207.04	119.60
A_68_P28553824	chr12:77505751-77505795	NM_008301:416	Hspa2	INSIDE	0.286	0.465	1427.90	663.73	0.133	1109.00	147.26
A_68_P28553835	chr12:77507209-77507257	NM_008301:1876	Hspa2	INSIDE	0.291	0.577	2070.19	1195.39	0.168	1492.84	250.92
A_68_P27804084	chr11:53114187-53114231	NM_008300:-227	Hspa4	PROMOTER	0.489	0.540	1122.90	606.22	0.264	1038.25	274.20
A_68_P21129648	chr2:34627186-34627230	NM_001163434:-401	Hspa5	PROMOTER	0.177	0.707	1444.82	1022.11	0.125	1126.15	140.86
A_68_P21129649	chr2:34627324-34627368	NM_001163434:-263	Hspa5	PROMOTER	0.330	0.648	834.51	540.35	0.214	726.60	155.31
A_68_P21129653	chr2:34627681-34627725	NM_001163434:93	Hspa5	INSIDE	0.293	0.620	2180.82	1351.86	0.182	1776.45	322.79
A_68_P21129656	chr2:34628056-34628100	NM_001163434:469	Hspa5	INSIDE	0.299	0.482	1166.19	562.07	0.144	957.86	138.21
A_68_P21129657	chr2:34628148-34628192	NM_001163434:561	Hspa5	INSIDE	0.140	0.581	2196.73	1276.73	0.082	1521.06	124.03
A_68_P26445378	chr9:40608813-40608857	NM_031165:-521	Hspa8	PROMOTER	0.425	0.415	4533.39	1882.56	0.177	3146.44	555.59
A_68_P26445379	chr9:40608906-40608950	NM_031165:-427	Hspa8	PROMOTER	0.542	0.499	1949.01	972.53	0.271	1501.36	406.35
A_68_P26445381	chr9:40609139-40609183	NM_031165:-195	Hspa8	PROMOTER	0.400	0.691	1034.99	714.85	0.276	879.47	242.73
A_68_P31615691	chr18:35113624-35113674	NM_010481:357	Hspa9	INSIDE	0.153	0.436	2560.61	1117.66	0.067	1810.03	120.69
A_68_P24118365	chr5:136364296-136364340	NM_013560:530	Hspb1	INSIDE	0.224	0.692	2438.28	1688.50	0.155	1753.64	272.50
A_68_P23364007	chr4:140981800-140981844	NM_013868:5129	Hspb7	DOWNSTREAM	0.180	0.706	2165.62	1527.90	0.127	1598.95	202.64
A_68_P20240337	chr1:55144375-55144419	NM_010477:380	Hspd1	INSIDE	0.519	0.594	2443.80	1450.86	0.308	2004.29	617.99
A_68_P20240341	chr1:55144942-55144986	NM_008303:-27	Hspe1	DIVERGENT_PROMOTER	0.619	0.470	1872.90	879.42	0.291	1633.71	475.15
A_68_P20240344	chr1:55145241-55145285	NM_008303:271	Hspe1	INSIDE	0.650	0.475	1246.14	592.43	0.309	1020.33	315.22
A_68_P23339949	chr4:137024813-137024857	NM_008305:117	Hspg2	INSIDE	0.377	0.550	1992.29	1095.14	0.207	1580.28	327.18
A_68_P24186279	chr5:150438537-150438581	NM_013559:332	Hsph1	INSIDE	0.610	0.641	2614.15	1675.15	0.391	1852.45	724.07
A_68_P24186280	chr5:150438607-150438651	NM_013559:262	Hsph1	INSIDE	0.438	0.698	2606.55	1819.58	0.306	2104.52	643.74
A_68_P24186282	chr5:150438837-150438890	NM_013559:27	Hsph1	INSIDE	0.168	0.493	2255.05	1112.39	0.083	1597.19	132.49
A_68_P25114497	chr7:57014275-57014319	NM_001146049:-179	Htatip2	PROMOTER	0.147	0.632	1827.68	1155.55	0.093	1317.74	122.80
A_68_P32399258	chrX:54306421-54306465	NM_028242:-304	Htatsf1	PROMOTER	0.305	1.509	1586.32	2393.78	0.460	638.68	293.87
A_68_P29296002	chr13:106234796-106234840	NM_008308:1046	Htr1a	INSIDE	0.218	0.643	948.61	609.97	0.140	855.18	119.97
A_68_P26674970	chr9:81525437-81525481	NM_010482:701	Htr1b	INSIDE	0.131	1.657	1442.29	2389.37	0.216	1120.28	242.37
A_68_P26674977	chr9:81526156-81526200	NM_010482:-19	Htr1b	PROMOTER	0.333	0.609	1347.49	821.18	0.203	987.29	200.17
A_68_P23333862	chr4:135980297-135980341	NM_008309:880	Htr1d	INSIDE	0.215	0.525	2722.47	1430.04	0.113	2013.17	227.22
A_68_P32727713	chrX:143397691-143397735	NM_008312:657	Htr2c	INSIDE	0.139	2.080	4322.19	8992.22	0.289	1750.79	505.29
A_68_P32727714	chrX:143397800-143397847	NM_008312:768	Htr2c	INSIDE	0.091	2.948	1053.02	3104.47	0.268	438.49	117.56
A_68_P20567331	chr1:123424359-123424403	NM_010483:662	Htr5b	INSIDE	0.526	0.452	1275.69	576.62	0.238	1193.13	283.71
A_68_P23349538	chr4:138631686-138631730	NM_021358:-1004	Htr6	PROMOTER	0.459	0.532	1016.76	540.52	0.244	826.12	201.40
A_68_P32087893	chr19:36131475-36131520	NM_008315:353	Htr7	INSIDE	0.223	0.528	1413.08	746.73	0.118	997.15	117.36
A_68_P24591138	chr6:83004321-83004365	NM_019752:223	Htra2	INSIDE	0.205	0.536	1358.99	728.89	0.110	1125.58	123.95
A_68_P24591139	chr6:83004504-83004548	NM_019752:39	Htra2	INSIDE	0.305	0.427	1777.44	759.46	0.130	1307.76	170.57
A_68_P25724987	chr8:26149339-26149383	NM_001081187:74	Htra4	INSIDE	0.231	0.672	1954.50	1313.09	0.155	1484.86	230.09
A_68_P23596023	chr5:35104298-35104342	NM_010414:-68	Htt	PROMOTER	0.265	0.687	1410.34	968.64	0.182	1169.35	212.81
A_68_P27581115	chr11:8910943-8910987	NM_008316:176	Hus1	INSIDE	0.174	0.398	2247.94	893.58	0.069	1759.14	121.96

ProbeName	Target position of probe on CpG island microarray	TargetID	GeneSymbol	CpG island Description	Ratio of relative methylation (TiO2-NP/Vehicle)	Sham group			TiO_2-H group		
						Relative methylation (Cy-5/Cy-3)	Cy-3 signal (Input)	Cy-5 signal (MeDIP)	Relative methylation (Cy-5/Cy-3)	Cy-3 signal (Input)	Cy-5 signal (MeDIP)
A_68_P27581116	chr11:8911066-8911110	NM_008316:52	Hus1	INSIDE	0.273	0.739	2270.54	1677.16	0.201	1638.68	330.14
A_68_P32746462	chrX:148237878-148237922	NM_021523:76	Huwe1	INSIDE	0.205	1.411	1026.71	1449.13	0.289	400.33	115.65
A_68_P32746463	chrX:148237989-148238033	NM_021523:186	Huwe1	INSIDE	0.239	1.935	1360.84	2633.43	0.463	563.26	260.79
A_68_P32746464	chrX:148238102-148238146	NM_021523:300	Huwe1	INSIDE	0.036	2.197	4454.26	9785.52	0.079	1640.12	129.40
A_68_P32746467	chrX:148238346-148238395	NM_021523:546	Huwe1	INSIDE	0.095	2.035	1820.13	3704.42	0.194	592.67	115.02
A_68_P32746468	chrX:148238435-148238479	NM_021523:632	Huwe1	INSIDE	0.109	2.829	3242.16	9171.33	0.309	1114.26	343.91
A_68_P26809621	chr9:107471601-107471645	NM_010489:129	Hyal2	INSIDE	0.238	0.650	1680.29	1091.95	0.155	1121.80	173.51
A_68_P26809702	chr9:107483108-107483152	NM_178020:-496	Hyal3	PROMOTER	0.294	0.647	961.49	622.42	0.190	705.09	134.26
A_68_P26467268	chr9:44187370-44187414	NM_021395:-180	Hyou1	PROMOTER	0.378	0.624	891.20	555.89	0.236	721.21	170.23
A_68_P26467270	chr9:44187680-44187724	NM_021395:130	Hyou1	INSIDE	0.089	0.715	2770.75	1981.99	0.064	2056.23	131.22
A_68_P29026628	chr13:49777406-49777450	NM_172015:-70	Iars	PROMOTER	0.496	0.548	1193.14	653.29	0.272	896.64	243.65
A_68_P29026629	chr13:49777484-49777528	NM_172015:8	Iars	INSIDE	0.547	0.646	1137.31	735.03	0.354	945.69	334.59
A_68_P26695696	chr9:85642551-85642595	NM_001081282:369	Ibtk	INSIDE	0.239	0.442	1600.14	707.26	0.106	1319.07	139.30
A_68_P26345149	chr9:20836417-20836466	NM_008319:-40	Icam5	PROMOTER	0.348	0.531	1368.90	726.47	0.184	1163.21	214.58
A_68_P26658578	chr9:78023836-78023880	NM_019987:62814	Ick	DOWNSTREAM	0.607	0.721	1741.92	1256.45	0.438	1372.60	601.28
A_68_P27271936	chr10:77532518-77532562	NM_015790:428	Icosl	INSIDE	0.496	0.466	1122.48	522.87	0.231	925.33	213.67
A_68_P28153290	chr11:115265346-115265390	NM_026729:289	Ict1	INSIDE	0.281	0.584	2127.27	1242.61	0.164	1491.93	245.07
A_68_P28153291	chr11:115265419-115265463	NM_026729:361	Ict1	INSIDE	0.097	0.611	3100.80	1893.09	0.059	2044.59	121.42
A_68_P28293528	chr12:25783564-25783608	NM_010496:-2629	Id2	PROMOTER	0.177	0.597	1489.70	889.03	0.105	1139.54	120.04
A_68_P23332244	chr4:135691181-135691230	NM_008321:-8531	Id3	PROMOTER	0.171	1.592	548.78	873.47	0.272	421.95	114.84
A_68_P23332336	chr4:135703661-135703706	NM_008321:3947	Id3	DOWNSTREAM	0.441	0.505	1280.31	646.71	0.223	1010.68	225.39
A_68_P29018477	chr13:48357119-48357169	NM_031166:349	Id4	INSIDE	0.099	0.601	2733.85	1642.16	0.059	1996.37	118.19
A_68_P20296826	chr1:65225580-65225625	NM_010497:114	Idh1	INSIDE	0.147	0.530	2027.47	1073.80	0.078	1536.58	119.71
A_68_P26526551	chr9:54434255-54434299	NM_029573:-41	Idh3a	PROMOTER	0.366	0.643	3186.60	2048.03	0.235	2635.93	620.29
A_68_P26526554	chr9:54434551-54434595	NM_029573:255	Idh3a	INSIDE	0.478	0.328	1901.35	623.56	0.157	1481.35	232.28
A_68_P21628930	chr2:130109870-130109914	NM_130884:295	Idh3b	INSIDE	0.172	0.606	6715.17	4072.51	0.104	4499.49	469.42
A_68_P26020946	chr8:87180770-87180814	NM_010499:5959	Ier2	DOWNSTREAM	0.511	0.431	2241.58	965.52	0.220	1678.43	369.65
A_68_P26020977	chr8:87186135-87186179	NM_010499:595	Ier2	INSIDE	0.301	0.488	1879.99	917.11	0.147	1500.50	220.21
A_68_P26020978	chr8:87186231-87186275	NM_010499:499	Ier2	INSIDE	0.399	0.277	1961.30	542.96	0.110	1579.44	174.25
A_68_P26020979	chr8:87186333-87186377	NM_010499:397	Ier2	INSIDE	0.203	0.426	1628.62	694.60	0.086	1404.48	121.32
A_68_P20735832	chr1:156945633-156945677	NM_010500:1112	Ier5	INSIDE	0.497	0.656	1503.64	986.31	0.326	1275.79	415.95
A_68_P24818962	chr6:125095826-125095870	NM_001039669:580	Iffo1	INSIDE	0.439	0.591	2255.04	1333.38	0.260	1958.61	508.31
A_68_P23352769	chr4:139130258-139130302	NM_001205173:-354	Iffo2	PROMOTER	0.163	0.602	1547.58	931.48	0.098	1193.31	117.01
A_68_P23352770	chr4:139130352-139130396	NM_001205173:-260	Iffo2	PROMOTER	0.123	0.618	4871.11	3009.05	0.076	3422.52	259.95
A_68_P23352775	chr4:139130999-139131044	NM_001205173:387	Iffo2	INSIDE	0.320	0.390	1390.57	542.36	0.125	1300.49	162.46
A_68_P23352779	chr4:139131562-139131606	NM_001205173:950	Iffo2	INSIDE	0.426	0.661	998.98	660.53	0.282	773.34	217.78
A_68_P30962573	chr16:91372961-91373005	NM_001110498:-45	Ifnar2	PROMOTER	0.369	1.806	1740.37	3143.44	0.667	1253.02	835.76
A_68_P30963516	chr16:91547641-91547685	NM_008338:324	Ifngr2	INSIDE	0.520	0.638	1030.78	658.13	0.332	884.86	293.86
A_68_P28377236	chr12:40949170-40949214	NM_013562:584	Ifrd1	INSIDE	0.437	0.528	1354.55	715.20	0.231	1160.77	267.99
A_68_P28377242	chr12:40949783-40949827	NM_013562:-28	Ifrd1	PROMOTER	0.592	0.410	2672.45	1096.43	0.243	1962.72	476.90
A_68_P28377243	chr12:40949898-40949942	NM_013562:-144	Ifrd1	PROMOTER	0.393	0.573	1938.73	1110.44	0.225	1465.35	330.17
A_68_P26809753	chr9:107489861-107489907	NM_025903:-164	Ifrd2	PROMOTER	0.325	0.636	3494.17	2221.47	0.207	2465.15	509.70
A_68_P26809754	chr9:107489944-107489988	NM_025903:-82	Ifrd2	PROMOTER	0.301	0.503	1227.58	618.03	0.152	1004.00	152.27
A_68_P24773305	chr6:115803441-115803485	NM_001167763:-83	Ift122	DIVERGENT_PROMOTER	0.210	0.521	4045.60	2107.25	0.110	2545.14	279.03

ProbeName	Target position of probe on CpG island microarray	TargetID	GeneSymbol	CpG island Description	Ratio of relative methylation (TiO2-NP/Vehicle)	Sham group			TiO_2-H group		
						Relative methylation (Cy-5/Cy-3)	Cy-3 signal (Input)	Cy-5 signal (MeDIP)	Relative methylation (Cy-5/Cy-3)	Cy-3 signal (Input)	Cy-5 signal (MeDIP)
A_68_P27943941	chr11:78349868-78349912	NM_018854:-47	Ift20	DIVERGENT_PROMOTER	0.224	0.677	994.15	672.98	0.152	774.97	117.66
A_68_P30358260	chr15:78004836-78004880	NM_025931:-320	Ift27	PROMOTER	0.584	0.484	2458.65	1188.88	0.282	1884.91	532.31
A_68_P30746980	chr16:49699315-49699359	NM_028680:-70	Ift57	PROMOTER	0.481	0.699	3152.72	2204.33	0.336	2447.18	822.39
A_68_P23118613	chr4:94281110-94281154	NM_026319:-78	Ift74	PROMOTER	0.657	0.505	2146.78	1083.81	0.332	1823.82	605.34
A_68_P26586063	chr9:64989672-64989716	NM_008988:699	Igdcc3	INSIDE	0.204	0.657	1706.16	1121.37	0.134	1289.38	173.05
A_68_P26586064	chr9:64989806-64989850	NM_008988:833	Igdcc3	INSIDE	0.468	0.560	1358.97	761.54	0.262	1042.01	273.08
A_68_P26585801	chr9:64949612-64949656	NM_020043:333	Igdcc4	INSIDE	0.658	0.627	1439.01	901.83	0.412	1230.41	507.04
A_68_P25196754	chr7:75097245-75097289	NM_010513:124	Igf1r	INSIDE	0.396	0.473	3320.88	1569.80	0.187	2477.45	464.14
A_68_P25196760	chr7:75097984-75098028	NM_010513:864	Igf1r	INSIDE	0.269	0.593	1006.02	596.44	0.160	755.73	120.65
A_68_P25596355	chr7:149844923-149844967	NM_010514:-235	Igf2	DIVERGENT_PROMOTER	0.462	0.591	1490.03	880.28	0.273	1202.79	328.42
A_68_P25596366	chr7:149846243-149846296	NM_001122736:671	Igf2	INSIDE	0.265	0.565	1149.75	649.23	0.150	816.65	122.26
A_68_P25596368	chr7:149846445-149846489	NM_001122736:474	Igf2	INSIDE	0.577	0.370	1630.36	603.75	0.214	1179.04	252.07
A_68_P25596369	chr7:149846534-149846578	NM_001122736:384	Igf2	INSIDE	0.517	0.596	1727.31	1029.74	0.308	1472.01	453.38
A_68_P25596372	chr7:149846899-149846943	NM_001122736:20	Igf2	INSIDE	0.516	0.713	1993.94	1421.96	0.368	1828.72	673.07
A_68_P28041910	chr11:95866408-95866452	NM_009951:828	Igf2bp1	INSIDE	0.446	0.728	1676.59	1220.03	0.325	1247.96	405.26
A_68_P28041913	chr11:95866656-95866701	NM_009951:580	Igf2bp1	INSIDE	0.384	0.502	1598.54	802.29	0.193	1273.94	245.79
A_68_P28041914	chr11:95866847-95866891	NM_009951:390	Igf2bp1	INSIDE	0.619	0.704	1657.30	1166.79	0.436	1324.37	577.04
A_68_P28041918	chr11:95867212-95867256	NM_009951:24	Igf2bp1	INSIDE	0.492	0.525	1538.94	807.91	0.258	1191.60	307.57
A_68_P28041927	chr11:95868155-95868199	NM_009951:-918	Igf2bp1	PROMOTER	0.562	0.548	986.48	540.10	0.308	926.11	284.97
A_68_P28041932	chr11:95868837-95868881	NM_009951:-1600	Igf2bp1	PROMOTER	0.215	0.678	3053.63	2071.81	0.146	2236.41	326.97
A_68_P30596435	chr16:22163008-22163052	NM_183029:342	Igf2bp2	INSIDE	0.385	0.488	2271.92	1109.15	0.188	1725.01	324.49
A_68_P24431596	chr6:49163809-49163853	NM_023670:1123	Igf2bp3	INSIDE	0.552	0.528	1902.95	1004.54	0.291	1562.22	455.06
A_68_P24431635	chr6:49168267-49168311	NM_023670:-3335	Igf2bp3	PROMOTER	0.634	0.694	1529.37	1061.34	0.440	1187.27	522.47
A_68_P31053798	chr17:12962714-12962758	NM_010515:-164	Igf2r	PROMOTER	0.257	0.470	1654.62	777.03	0.121	1331.45	160.65
A_68_P20339086	chr1:72872062-72872107	NM_008342:1008	Igfbp2	INSIDE	0.390	0.451	1799.04	811.95	0.176	1379.23	242.69
A_68_P27572319	chr11:7114101-7114145	NM_008343:-196	Igfbp3	PROMOTER	0.595	0.390	1367.48	533.25	0.232	1066.04	247.33
A_68_P25082509	chr7:50721683-50721727	NM_001164518:23689	Iglon5	DOWNSTREAM	0.201	1.425	2700.72	3849.50	0.286	1906.16	544.92
A_68_P23356006	chr4:139665293-139665352	NM_198610:137404	Igsf21	INSIDE	0.173	0.509	1799.44	915.72	0.088	1372.39	120.92
A_68_P23356913	chr4:139800280-139800324	NM_198610:2424	Igsf21	INSIDE	0.134	0.551	2225.57	1226.01	0.074	1662.21	122.44
A_68_P22375208	chr3:101255145-101255189	NM_207205:74119	Igsf3	INSIDE	0.660	2.628	1387.42	3646.05	1.733	1171.12	2029.91
A_68_P20832289	chr1:174242640-174242684	NM_080419:125	Igsf8	INSIDE	0.373	0.710	3360.31	2386.34	0.265	2568.71	680.25
A_68_P20833276	chr1:174412036-174412080	NM_001145800:-285	Igsf9	PROMOTER	0.396	0.574	1317.37	756.65	0.228	1156.57	263.21
A_68_P26375207	chr9:27107032-27107084	NM_001033323:246	Igsf9b	INSIDE	0.114	0.563	2596.28	1460.71	0.064	1879.35	120.86
A_68_P26375211	chr9:27107465-27107509	NM_001033323:674	Igsf9b	INSIDE	0.597	0.520	2186.28	1137.52	0.310	1657.82	514.59
A_68_P26375217	chr9:27108097-27108141	NM_001033323:1306	Igsf9b	INSIDE	0.076	0.581	3690.51	2144.89	0.044	2698.86	118.66
A_68_P20351506	chr1:74997489-74997533	NM_010544:715	Ihh	INSIDE	0.354	0.591	2779.98	1643.39	0.210	2146.31	449.65
A_68_P20351509	chr1:74997858-74997902	NM_010544:345	Ihh	INSIDE	0.216	0.535	4688.95	2509.03	0.115	3681.84	425.12
A_68_P20351515	chr1:74998648-74998692	NM_010544:-445	Ihh	PROMOTER	0.190	0.582	1885.80	1097.69	0.111	1479.57	163.90
A_68_P31625592	chr18:36904491-36904538	NM_011879:205	Ik	INSIDE	0.136	0.678	1751.88	1188.45	0.092	1303.10	120.10
A_68_P27340037	chr10:90564728-90564779	NM_027078:18970	Ikbip	INSIDE	0.663	2.282	1345.04	3069.80	1.513	1012.31	1531.59
A_68_P25528244	chr7:138553556-138553600	NM_175115:414	Ikzf5	INSIDE	0.639	0.435	1298.19	564.92	0.278	1130.82	314.23
A_68_P27805842	chr11:53449954-53449998	NM_008355:-1772	Il13	PROMOTER	0.147	0.532	2695.44	1433.25	0.078	1983.97	155.09
A_68_P31759174	chr18:61852045-61852089	NM_019508:4478	Il17b	INSIDE	0.427	0.524	1291.49	676.38	0.224	892.38	199.63
A_68_P24798577	chr6:120413598-120413642	NM_008359:406	Il17ra	INSIDE	0.512	0.712	1682.85	1198.87	0.365	1554.18	566.60

ProbeName	Target position of probe on CpG island microarray	TargetID	GeneSymbol	CpG island Description	Ratio of relative methylation (TiO2-NP/Vehicle)	Sham group			TiO_2-H group		
						Relative methylation (Cy-5/Cy-3)	Cy-3 signal (Input)	Cy-5 signal (MeDIP)	Relative methylation (Cy-5/Cy-3)	Cy-3 signal (Input)	Cy-5 signal (MeDIP)
A_68_P24798580	chr6:120413892-120413937	NM_008359:700	Il17ra	INSIDE	0.207	0.566	1714.17	969.72	0.117	1354.93	158.53
A_68_P29503210	chr14:30821909-30821953	NM_019583:152	Il17rb	INSIDE	0.514	0.416	2050.39	853.93	0.214	1709.47	366.18
A_68_P24759769	chr6:113432843-113432887	NM_134159:11416	Il17rc	INSIDE	0.286	0.272	2040.65	555.79	0.078	1604.13	124.97
A_68_P29484662	chr14:27852087-27852131	NM_134437:-78	Il17rd	PROMOTER	0.296	0.552	2127.06	1175.11	0.164	1552.07	254.16
A_68_P29484663	chr14:27852219-27852263	NM_134437:54	Il17rd	INSIDE	0.557	0.516	1253.38	646.71	0.287	1027.00	295.15
A_68_P29484665	chr14:27852530-27852574	NM_134437:366	Il17rd	INSIDE	0.285	0.660	1194.43	787.81	0.188	981.10	184.21
A_68_P29484669	chr14:27852875-27852920	NM_134437:711	Il17rd	INSIDE	0.238	0.594	1284.13	763.21	0.142	940.23	133.05
A_68_P30621545	chr16:26581869-26581913	NM_134103:100	Il1rap	INSIDE	0.276	0.543	1535.69	834.13	0.150	1253.52	188.11
A_68_P20170820	chr1:40382866-40382910	NM_133193:1417	Il1rl2	INSIDE	0.613	0.599	1729.41	1035.11	0.367	1317.23	483.15
A_68_P26017803	chr8:86566236-86566280	NM_016671:216	Il27ra	INSIDE	0.543	0.727	3520.60	2560.29	0.395	2614.92	1033.06
A_68_P25089586	chr7:52095494-52095538	NM_010215:3859	Il4i1	INSIDE	0.341	0.638	2333.35	1487.96	0.218	1821.13	396.55
A_68_P21929617	chr3:7612894-7612938	NM_008371:511	Il7	INSIDE	0.485	0.523	1153.86	603.00	0.254	1067.39	270.70
A_68_P30676660	chr16:36694130-36694174	NM_134109:29	Ildr1	INSIDE	0.363	0.478	1591.35	760.33	0.173	1257.76	217.96
A_68_P22325452	chr3:90280402-90280446	NM_026374:302	Ilf2	INSIDE	0.145	0.691	1734.83	1198.46	0.100	1432.82	143.04
A_68_P26346746	chr9:21172514-21172558	NM_001042708:90	Ilf3	INSIDE	0.313	0.616	1991.46	1226.19	0.193	1459.73	281.48
A_68_P28382007	chr12:41750810-41750854	NM_053122:156	Immp2l	INSIDE	0.454	0.572	2021.80	1155.65	0.260	1681.86	436.50
A_68_P24538442	chr6:71781548-71781592	NM_029673:246	Immt	INSIDE	0.643	0.674	1186.21	799.41	0.433	1013.46	439.12
A_68_P24538443	chr6:71781629-71781673	NM_029673:326	Immt	INSIDE	0.217	0.595	1774.79	1056.80	0.129	1573.85	203.49
A_68_P24538445	chr6:71781870-71781919	NM_029673:570	Immt	INSIDE	0.180	0.503	1708.21	858.99	0.090	1305.46	118.05
A_68_P20138851	chr1:34496844-34496888	NM_178601:193	Imp4	INSIDE	0.123	0.647	1745.92	1129.72	0.079	1505.66	119.43
A_68_P31791018	chr18:67449050-67449094	NM_053261:196	Impa2	INSIDE	0.180	0.744	4171.49	3104.40	0.134	3098.51	414.33
A_68_P31791020	chr18:67449254-67449298	NM_053261:400	Impa2	INSIDE	0.027	1.454	5572.36	8102.62	0.040	3547.63	141.36
A_68_P22698894	chr4:4719763-4719807	NM_177730:669	Impad1	INSIDE	0.524	0.687	1867.04	1283.49	0.360	1425.36	513.73
A_68_P24325220	chr6:29161862-29161906	NM_011829:387	Impdh1	INSIDE	0.215	0.573	1491.16	854.85	0.123	969.70	119.71
A_68_P24325222	chr6:29162054-29162098	NM_011829:195	Impdh1	INSIDE	0.524	0.560	2574.26	1441.17	0.293	1716.21	503.33
A_68_P26815429	chr9:108462952-108462996	NM_011830:143	Impdh2	INSIDE	0.634	0.703	3132.35	2201.00	0.446	2559.36	1140.39
A_68_P23138265	chr4:98062281-98062325	NM_001005784:-214	Inadl	PROMOTER	0.173	0.541	1634.56	885.02	0.094	1289.39	120.79
A_68_P31947333	chr19:9973849-9973893	NM_016692:153	Incenp	INSIDE	0.456	0.477	1230.50	586.85	0.218	1017.30	221.28
A_68_P28751073	chr12:113827105-113827149	NM_198411:132	Inf2	INSIDE	0.554	0.528	1145.20	604.34	0.292	902.36	263.67
A_68_P25656209	chr8:11555685-11555729	NM_011919:-359	Ing1	DIVERGENT_PROMOTER	0.208	1.456	5723.71	8334.47	0.302	3509.69	1061.17
A_68_P25656212	chr8:11555954-11555998	NM_011919:-89	Ing1	DIVERGENT_PROMOTER	0.374	0.539	1788.90	964.77	0.202	1283.50	259.05
A_68_P25656213	chr8:11556085-11556129	NM_011919:41	Ing1	INSIDE	0.172	0.477	1844.84	879.72	0.082	1463.77	120.37
A_68_P25656225	chr8:11557852-11557899	NM_011919:1810	Ing1	INSIDE	0.563	0.540	1067.19	576.73	0.304	871.63	265.32
A_68_P20556963	chr1:121316999-121317043	NM_008381:1805	Inhbb	INSIDE	0.253	0.588	1608.71	945.72	0.148	1229.97	182.62
A_68_P20556978	chr1:121319090-121319134	NM_008381:-287	Inhbb	PROMOTER	0.609	0.628	862.01	541.47	0.383	748.04	286.33
A_68_P21569912	chr2:119303420-119303464	NM_026574:-77	Ino80	PROMOTER	0.294	0.436	3829.81	1671.27	0.128	2908.06	373.43
A_68_P20285211	chr1:63160025-63160069	NM_001081436:795	Ino80d	INSIDE	0.354	0.658	1881.88	1237.76	0.233	1522.45	354.36
A_68_P20285212	chr1:63160117-63160161	NM_001081436:703	Ino80d	INSIDE	0.501	0.624	979.00	610.57	0.312	833.36	260.19
A_68_P20229133	chr1:52874369-52874413	NM_008384:142	Inpp1	INSIDE	0.330	0.522	1016.33	530.28	0.172	755.61	130.05
A_68_P20229134	chr1:52874442-52874486	NM_008384:68	Inpp1	INSIDE	0.653	0.730	2012.80	1468.90	0.476	1592.47	758.51
A_68_P25577540	chr7:146574599-146574643	NM_001127363:-387	Inpp5a	PROMOTER	0.131	0.719	1925.47	1384.90	0.094	1298.73	122.41
A_68_P25577541	chr7:146574687-146574731	NM_001127363:-299	Inpp5a	PROMOTER	0.065	0.647	5097.15	3297.48	0.042	3693.72	155.70
A_68_P23270089	chr4:124428703-124428747	NM_008385:9603	Inpp5b	INSIDE	0.232	0.511	2225.93	1136.63	0.118	1843.02	218.23
A_68_P23270091	chr4:124428867-124428911	NM_008385:9767	Inpp5b	INSIDE	0.362	0.653	4855.24	3170.40	0.236	3669.14	867.58

ProbeName	Target position of probe on CpG island microarray	TargetID	GeneSymbol	CpG island Description	Ratio of relative methylation (TiO2-NP/Vehicle)	Sham group			TiO_2-H group		
						Relative methylation (Cy-5/Cy-3)	Cy-3 signal (Input)	Cy-5 signal (MeDIP)	Relative methylation (Cy-5/Cy-3)	Cy-3 signal (Input)	Cy-5 signal (MeDIP)
A_68_P25511565	chr7:135754795-135754839	NM_178641:-62	Inpp5f	PROMOTER	0.458	1.619	4253.80	6888.80	0.741	2914.50	2160.59
A_68_P27927255	chr11:75444500-75444544	NM_008916:1	Inpp5k	INSIDE	0.384	0.649	799.68	519.12	0.249	623.68	155.52
A_68_P27927256	chr11:75444673-75444717	NM_008916:173	Inpp5k	INSIDE	0.215	0.604	2194.17	1324.53	0.130	1601.96	207.50
A_68_P25375733	chr7:108986683-108986727	NM_001122739:-366	Inppl1	PROMOTER	0.314	0.672	1783.11	1198.28	0.211	1355.05	285.97
A_68_P23560507	chr5:28398167-28398211	NM_153526:237	Insig1	INSIDE	0.227	0.447	2884.31	1288.71	0.102	2063.02	209.57
A_68_P20566309	chr1:123224172-123224218	NM_133748:145	Insig2	INSIDE	0.557	0.487	1336.67	651.27	0.272	1040.86	282.63
A_68_P21717106	chr2:146048812-146048856	NM_016889:1102	Insm1	INSIDE	0.449	0.614	980.36	601.66	0.275	894.71	246.48
A_68_P28452661	chr12:56701135-56701179	NM_020287:1253	Insm2	INSIDE	0.484	0.448	1892.63	848.42	0.217	1554.72	336.97
A_68_P25613343	chr8:3279377-3279421	NM_010568:219	Insr	INSIDE	0.550	0.481	1718.27	825.72	0.264	1383.28	365.34
A_68_P22310076	chr3:87600886-87600930	NM_011832:36	Insrr	INSIDE	0.371	0.623	1828.87	1138.98	0.231	1451.56	335.52
A_68_P25944668	chr8:71317470-71317515	NM_027590:-360	Ints10	PROMOTER	0.539	0.605	1118.93	677.13	0.326	919.07	299.85
A_68_P27987929	chr11:86070620-86070664	NM_027421:428	Ints2	INSIDE	0.352	0.715	2228.60	1593.16	0.252	1750.76	440.53
A_68_P27987932	chr11:86070867-86070911	NM_027421:182	Ints2	INSIDE	0.603	0.513	1126.92	578.10	0.310	851.72	263.63
A_68_P22325243	chr3:90237709-90237753	NM_145540:-172	Ints3	PROMOTER	0.315	0.558	932.57	520.14	0.176	714.91	125.68
A_68_P29657354	chr14:63379882-63379926	NM_008715:45	Ints6	INSIDE	0.271	0.529	1051.19	555.92	0.143	831.51	119.01
A_68_P20938882	chr1:193399613-193399657	NM_178632:22	Ints7	INSIDE	0.481	0.496	1494.60	741.54	0.239	1079.60	257.53
A_68_P20938885	chr1:193399879-193399925	NM_178632:290	Ints7	INSIDE	0.593	0.450	1340.38	603.72	0.267	1058.05	282.71
A_68_P22728834	chr4:11181339-11181383	NM_178112:46	Ints8	INSIDE	0.250	0.522	1918.88	1001.82	0.130	1572.92	205.05
A_68_P26812406	chr9:107905251-107905295	NM_013785:294	Ip6k1	INSIDE	0.292	0.362	1542.06	558.32	0.106	1152.15	121.73
A_68_P27234954	chr10:70811019-70811063	NM_027184:500	Ipmk	INSIDE	0.301	0.628	4411.32	2769.91	0.189	3306.52	624.87
A_68_P27234955	chr10:70811089-70811133	NM_027184:570	Ipmk	INSIDE	0.417	0.432	2655.26	1145.79	0.180	1984.07	356.94
A_68_P27235134	chr10:70844276-70844325	NM_027184:33760	Ipmk	INSIDE	0.538	4.305	163.15	702.31	2.317	208.29	482.53
A_68_P23237284	chr4:117587755-117587799	NM_146152:-172	Ipo13	PROMOTER	0.262	0.488	2105.90	1028.05	0.128	1536.08	196.84
A_68_P29618998	chr14:56254267-56254311	NM_024267:227	Ipo4	INSIDE	0.203	0.579	1225.27	709.22	0.117	1024.29	120.17
A_68_P29619000	chr14:56254517-56254561	NM_024267:-23	Ipo4	PROMOTER	0.282	0.292	2453.01	716.88	0.083	1878.48	155.04
A_68_P29952573	chr14:121310169-121310213	NM_023579:-225	Ipo5	PROMOTER	0.567	0.540	973.95	525.93	0.306	813.28	249.18
A_68_P25411259	chr7:117161867-117161911	NM_181517:-50	Ipo7	PROMOTER	0.515	0.552	949.56	524.16	0.284	874.94	248.56
A_68_P24942331	chr6:148779822-148779866	NM_001081113:145	Ipo8	INSIDE	0.411	0.555	1755.36	974.80	0.228	1369.02	312.29
A_68_P23297601	chr4:129296491-129296543	NM_198026:-179	Iqcc	PROMOTER	0.273	0.658	823.66	542.29	0.180	657.74	118.15
A_68_P24142813	chr5:141177968-141178012	NM_028833:342	Iqce	INSIDE	0.292	0.567	1355.69	769.13	0.166	1178.25	195.26
A_68_P24142814	chr5:141178050-141178094	NM_028833:260	Iqce	INSIDE	0.272	0.508	2849.60	1446.95	0.138	2303.75	318.65
A_68_P22311640	chr3:87886128-87886172	NM_001033484:178	Iqgap3	INSIDE	0.327	0.640	1080.27	691.66	0.209	843.99	176.78
A_68_P24634427	chr6:90686065-90686109	NM_001134384:74031	Iqsec1	INSIDE	0.358	0.691	2105.16	1454.81	0.247	1692.06	418.36
A_68_P24634428	chr6:90686241-90686285	NM_001134384:73855	Iqsec1	INSIDE	0.579	0.678	2612.91	1772.44	0.393	2089.01	820.01
A_68_P24804130	chr6:121363049-121363093	NM_001033354:60626	Iqsec3	INSIDE	0.283	0.662	1510.78	1000.13	0.187	1176.93	220.64
A_68_P24804136	chr6:121363612-121363660	NM_001033354:60060	Iqsec3	INSIDE	0.394	1.459	861.78	1257.28	0.575	728.64	419.21
A_68_P26528146	chr9:54711666-54711710	NM_022655:127	Ireb2	INSIDE	0.236	0.537	2123.00	1139.14	0.127	1638.59	207.48
A_68_P27806467	chr11:53584134-53584178	NM_001159396:182	Irf1	INSIDE	0.594	0.577	2049.85	1182.05	0.343	1649.48	565.15
A_68_P25837684	chr8:47825681-47825725	NM_008391:604	Irf2	INSIDE	0.416	0.697	1597.32	1112.68	0.290	1181.44	342.31
A_68_P24990858	chr7:19589997-19590041	NM_178757:605	Irf2bp1	INSIDE	0.512	0.649	4180.13	2712.88	0.332	3243.74	1076.99
A_68_P26258430	chr8:129118076-129118120	NM_001164598:-762	Irf2bp2	PROMOTER	0.400	0.721	1718.04	1238.29	0.289	1460.35	421.48
A_68_P25090530	chr7:52253328-52253372	NM_016849:321	Irf3	INSIDE	0.334	0.476	1097.47	522.34	0.159	967.49	153.74
A_68_P28918889	chr13:30842632-30842676	NM_013674:1528	Irf4	INSIDE	0.241	0.560	2493.13	1395.72	0.135	1806.47	243.41
A_68_P20947552	chr1:194979319-194979363	NM_016851:35	Irf6	INSIDE	0.517	0.616	967.79	596.45	0.319	873.62	278.55

ProbeName	Target position of probe on CpG island microarray	TargetID	GeneSymbol	CpG island Description	Ratio of relative methylation (TiO2-NP/Vehicle)	Sham group			TiO_2-H group		
						Relative methylation (Cy-5/Cy-3)	Cy-3 signal (Input)	Cy-5 signal (MeDIP)	Relative methylation (Cy-5/Cy-3)	Cy-3 signal (Input)	Cy-5 signal (MeDIP)
A_68_P26222126	chr8:123259917-123259961	NM_008320:-337	Irf8	PROMOTER	0.653	0.582	1630.42	949.18	0.380	1302.79	495.12
A_68_P26222129	chr8:123260367-123260411	NM_008320:113	Irf8	INSIDE	0.342	0.452	1790.75	808.66	0.154	1437.85	222.07
A_68_P25005382	chr7:25316864-25316908	NM_153134:1220	Irgq	INSIDE	0.657	0.592	1466.50	868.70	0.389	1098.25	427.25
A_68_P25005383	chr7:25316968-25317015	NM_153134:1325	Irgq	INSIDE	0.103	0.685	2313.36	1585.67	0.070	1710.32	120.50
A_68_P20391651	chr1:82280179-82280223	NM_010570:7814	Irs1	INSIDE	0.658	0.653	5226.48	3411.42	0.430	3985.50	1712.03
A_68_P20391709	chr1:82286790-82286834	NM_010570:1202	Irs1	INSIDE	0.213	0.607	1052.84	638.66	0.129	882.24	114.25
A_68_P20391713	chr1:82287149-82287194	NM_010570:843	Irs1	INSIDE	0.260	0.563	1174.25	661.49	0.146	876.78	128.25
A_68_P20391714	chr1:82287221-82287265	NM_010570:772	Irs1	INSIDE	0.305	0.492	1356.43	668.02	0.150	930.38	139.95
A_68_P20391723	chr1:82288408-82288452	NM_010570:-416	Irs1	PROMOTER	0.563	0.412	1571.80	647.33	0.232	1306.74	303.13
A_68_P24126843	chr5:138086478-138086522	NM_010571:442	Irs3	INSIDE	0.541	0.522	1114.03	581.40	0.282	1075.10	303.33
A_68_P29130721	chr13:72100670-72100714	NM_010573:479	Irx1	INSIDE	0.611	0.650	1830.69	1189.90	0.397	1628.53	647.06
A_68_P29134809	chr13:72766875-72766919	NM_010574:471	Irx2	INSIDE	0.147	0.486	2334.06	1134.35	0.071	1873.96	133.64
A_68_P29134820	chr13:72768352-72768396	NM_010574:1949	Irx2	INSIDE	0.238	0.681	1921.84	1308.10	0.162	1670.26	270.98
A_68_P29134823	chr13:72768727-72768771	NM_010574:2323	Irx2	INSIDE	0.251	0.607	1084.09	658.22	0.152	791.69	120.70
A_68_P26061534	chr8:94325346-94325390	NM_008393:-95	Irx3	PROMOTER	0.142	0.667	3841.50	2562.64	0.095	2815.88	266.28
A_68_P29138699	chr13:73405898-73405942	NM_018885:7976	Irx4	INSIDE	0.268	0.514	1100.44	565.73	0.138	856.73	118.21
A_68_P26065130	chr8:94880881-94880925	NM_018826:-792	Irx5	DIVERGENT_PROMOTER	0.169	0.542	4847.98	2627.21	0.092	3183.37	292.31
A_68_P26065131	chr8:94880961-94881005	NM_018826:-712	Irx5	DIVERGENT_PROMOTER	0.180	0.569	2009.01	1142.78	0.103	1364.64	139.90
A_68_P26065134	chr8:94881423-94881467	NM_018826:-250	Irx5	DIVERGENT_PROMOTER	0.224	0.710	2622.75	1860.87	0.159	1783.03	282.91
A_68_P28600140	chr12:86114302-86114346	NM_028863:105	Isca2	INSIDE	0.102	1.409	3398.71	4789.39	0.144	2317.64	333.26
A_68_P29356812	chr13:117100315-117100359	NM_021459:-440	Isl1	PROMOTER	0.669	0.613	919.63	563.65	0.410	921.77	377.86
A_68_P26531858	chr9:55388757-55388801	NM_027397:-177	Isl2	PROMOTER	0.238	0.401	1516.64	608.33	0.096	1249.83	119.39
A_68_P26531866	chr9:55389590-55389634	NM_027397:657	Isl2	INSIDE	0.605	0.456	1252.05	571.38	0.276	1095.48	302.45
A_68_P26531904	chr9:55394018-55394062	NM_027397:5085	Isl2	DOWNSTREAM	0.427	0.331	1731.85	572.49	0.141	1133.06	159.91
A_68_P26531906	chr9:55394278-55394322	NM_027397:5345	Isl2	DOWNSTREAM	0.318	0.540	1898.94	1024.76	0.172	1440.71	247.57
A_68_P26546333	chr9:58046118-58046162	NM_001161535:3382	Islr2	INSIDE	0.176	0.407	3297.07	1343.18	0.072	2350.88	169.01
A_68_P26546335	chr9:58046426-58046470	NM_001161535:3074	Islr2	INSIDE	0.321	0.410	1624.17	665.74	0.132	1391.94	183.04
A_68_P26546339	chr9:58046840-58046884	NM_001161535:2660	Islr2	INSIDE	0.337	0.666	2409.98	1605.34	0.224	1693.87	379.84
A_68_P26546341	chr9:58046993-58047037	NM_001161535:2508	Islr2	INSIDE	0.257	0.594	930.73	552.98	0.153	796.38	121.54
A_68_P26546344	chr9:58047294-58047338	NM_001161535:2206	Islr2	INSIDE	0.244	0.661	1999.15	1322.25	0.162	1417.19	228.96
A_68_P26546363	chr9:58049670-58049721	NM_001161540:-89	Islr2	PROMOTER	0.289	1.612	3806.23	6134.04	0.466	2481.86	1156.46
A_68_P26546364	chr9:58049788-58049832	NM_001161540:-204	Islr2	PROMOTER	0.385	1.676	3041.84	5098.28	0.646	2084.52	1346.23
A_68_P21680283	chr2:139502974-139503018	NM_001126490:-917	Ism1	PROMOTER	0.286	0.499	2705.45	1349.99	0.142	2069.31	294.85
A_68_P21680286	chr2:139503286-139503330	NM_001126490:-605	Ism1	PROMOTER	0.390	0.557	2940.12	1637.95	0.217	2220.95	482.08
A_68_P28357924	chr12:37108183-37108227	NM_178629:99	Ispd	INSIDE	0.343	0.387	2597.33	1005.23	0.133	2013.78	267.18
A_68_P25952871	chr8:73119135-73119179	NM_023627:777	Isyna1	INSIDE	0.363	1.400	1995.11	2794.07	0.509	1512.14	769.49
A_68_P25952872	chr8:73119238-73119282	NM_023627:881	Isyna1	INSIDE	0.324	1.460	800.52	1168.77	0.473	778.01	367.89
A_68_P26026699	chr8:88364441-88364485	NM_028007:386	Itfg1	INSIDE	0.162	0.527	1729.81	911.22	0.085	1423.62	121.35
A_68_P28079666	chr11:102317339-102317383	NM_010575:13837	Itga2b	INSIDE	0.375	0.568	2920.77	1658.07	0.213	2054.87	437.17
A_68_P28079749	chr11:102328621-102328668	NM_010575:2553	Itga2b	INSIDE	0.136	0.669	2204.81	1475.73	0.091	1409.57	127.98
A_68_P28079750	chr11:102328735-102328779	NM_010575:2441	Itga2b	INSIDE	0.349	0.617	1324.91	817.38	0.215	995.03	214.03
A_68_P28036509	chr11:94937682-94937726	NM_013565:324	Itga3	INSIDE	0.245	0.446	1241.16	553.63	0.109	1125.20	122.77
A_68_P28036515	chr11:94938400-94938444	NM_013565:-394	Itga3	PROMOTER	0.206	0.532	1481.29	788.74	0.110	1079.50	118.57
A_68_P30503777	chr15:103196605-103196649	NM_010577:553	Itga5	INSIDE	0.457	0.686	1752.04	1201.09	0.314	1377.01	431.76

ProbeName	Target position of probe on CpG island microarray	TargetID	GeneSymbol	CpG island Description	Ratio of relative methylation (TiO2-NP/Vehicle)	Sham group			TiO_2-H group		
						Relative methylation (Cy-5/Cy-3)	Cy-3 signal (Input)	Cy-5 signal (MeDIP)	Relative methylation (Cy-5/Cy-3)	Cy-3 signal (Input)	Cy-5 signal (MeDIP)
A_68_P27543981	chr10:128370721-128370765	NM_008398:-126	Itga7	PROMOTER	0.597	0.515	1877.83	966.34	0.307	1326.14	407.68
A_68_P27543983	chr10:128370926-128370976	NM_008398:82	Itga7	INSIDE	0.191	0.597	1527.24	911.40	0.114	1089.23	123.86
A_68_P27543984	chr10:128371011-128371056	NM_008398:165	Itga7	INSIDE	0.268	0.318	1707.29	542.40	0.085	1374.66	116.90
A_68_P27543986	chr10:128371189-128371233	NM_008398:342	Itga7	INSIDE	0.307	0.442	2370.25	1048.13	0.136	1812.84	246.01
A_68_P21010259	chr2:12223211-12223256	NM_001001309:314	Itga8	INSIDE	0.528	0.686	1837.42	1260.25	0.362	1435.62	519.59
A_68_P21010261	chr2:12223501-12223545	NM_001001309:25	Itga8	INSIDE	0.228	0.609	1016.91	618.98	0.139	853.94	118.60
A_68_P28279800	chr12:21291772-21291816	NM_008403:304	Itgb1bp1	INSIDE	0.434	0.300	1716.13	514.83	0.130	1348.71	175.47
A_68_P28279803	chr12:21292038-21292083	NM_008403:38	Itgb1bp1	INSIDE	0.360	0.582	1324.20	770.93	0.210	996.58	208.90
A_68_P28156586	chr11:115836453-115836497	NM_001005608:436	Itgb4	INSIDE	0.587	0.690	1192.84	822.66	0.405	1019.21	412.34
A_68_P29717289	chr14:73784847-73784891	NM_008410:210	Itm2b	INSIDE	0.500	0.645	1090.62	703.48	0.323	962.78	310.73
A_68_P20409050	chr1:87791484-87791528	NM_022417:422	Itm2c	INSIDE	0.299	0.703	1596.53	1122.15	0.210	1179.45	247.88
A_68_P21571250	chr2:119568776-119568820	NM_146125:726	Itpka	INSIDE	0.500	0.466	1121.11	522.59	0.233	939.45	218.97
A_68_P25016220	chr7:28013751-28013795	NM_181593:-156	Itpkc	DIVERGENT_PROMOTER	0.317	0.561	2029.10	1138.64	0.178	1501.37	266.96
A_68_P32152775	chr19:47993384-47993428	NM_001001738:383	Itprip	INSIDE	0.421	0.671	1366.53	916.97	0.282	1158.75	327.20
A_68_P25458544	chr7:125634804-125634848	NM_001033380:663	Itpripl2	INSIDE	0.647	0.423	1217.89	514.67	0.273	962.69	263.22
A_68_P30964671	chr16:91730311-91730355	NM_001110276:717	Itsn1	INSIDE	0.321	0.667	3983.80	2655.88	0.214	2907.28	621.89
A_68_P30964672	chr16:91730394-91730438	NM_001110276:801	Itsn1	INSIDE	0.287	0.630	1288.50	812.30	0.181	1078.75	195.21
A_68_P20714215	chr1:153191431-153191475	NM_001039511:-175	Ivns1abp	PROMOTER	0.311	0.459	2192.95	1006.86	0.143	1657.53	236.40
A_68_P20714216	chr1:153191544-153191588	NM_001039511:-61	Ivns1abp	PROMOTER	0.658	0.439	1428.02	627.33	0.289	1103.53	318.97
A_68_P31601053	chr18:32227655-32227699	NM_173441:289	Iws1	INSIDE	0.459	0.618	1034.83	640.04	0.284	873.82	248.01
A_68_P21666831	chr2:136940212-136940256	NM_013822:2022	Jag1	INSIDE	0.500	0.531	1250.72	663.64	0.265	875.80	232.27
A_68_P24759516	chr6:113392769-113392813	NM_026365:228	Jagn1	INSIDE	0.262	0.447	1223.78	546.49	0.117	1001.61	117.06
A_68_P32051826	chr19:29325786-29325831	NM_001048177:-509	Jak2	PROMOTER	0.498	0.408	2178.12	887.94	0.203	1962.57	398.06
A_68_P23611439	chr5:37482314-37482358	NM_178394:40241	Jakmip1	INSIDE	0.433	0.619	2076.52	1285.31	0.268	1717.12	460.57
A_68_P25574931	chr7:146132875-146132921	NM_028708:466	Jakmip3	INSIDE	0.314	0.348	1561.07	543.97	0.109	1085.34	118.67
A_68_P30925477	chr16:84774396-84774440	NM_023844:51	Jam2	INSIDE	0.532	0.733	2998.51	2199.19	0.391	2229.88	870.81
A_68_P26374353	chr9:26962841-26962885	NM_023277:144	Jam3	INSIDE	0.480	0.597	940.40	561.62	0.287	786.31	225.60
A_68_P28997649	chr13:44825063-44825107	NM_001205044:-1058	Jarid2	PROMOTER	0.558	0.532	2829.26	1504.07	0.297	2181.41	647.35
A_68_P28997656	chr13:44825977-44826021	NM_001205044:-144	Jarid2	PROMOTER	0.268	0.506	1762.95	892.14	0.136	1405.33	190.89
A_68_P28997683	chr13:44829152-44829196	NM_001205043:2535	Jarid2	INSIDE	0.289	0.517	2630.47	1360.22	0.150	1836.55	274.76
A_68_P24381996	chr6:39156448-39156492	NM_001033430:302	Jhdm1d	INSIDE	0.172	0.497	4031.18	2004.31	0.085	2675.98	228.51
A_68_P28530099	chr12:73186645-73186689	NM_001205067:-159	Jkamp	DIVERGENT_PROMOTER	0.125	0.505	2580.37	1301.83	0.063	2039.39	128.45
A_68_P27836981	chr11:59263741-59263785	NM_001205068:216	Jmjd4	INSIDE	0.314	0.623	2470.69	1539.18	0.196	1856.16	362.91
A_68_P28161570	chr11:116704403-116704447	NM_033398:339	Jmjd6	INSIDE	0.498	0.734	2270.11	1665.81	0.366	1630.54	596.14
A_68_P21572764	chr2:119853205-119853250	NM_001114637:9	Jmjd7	INSIDE	0.631	0.580	1744.85	1011.54	0.366	1471.50	538.00
A_68_P21572765	chr2:119853360-119853404	NM_001114637:164	Jmjd7	INSIDE	0.559	0.471	1169.44	550.26	0.263	993.44	261.09
A_68_P20058158	chr1:17087056-17087100	NM_020604:892	Jph1	INSIDE	0.608	0.604	1157.64	699.31	0.367	976.90	358.60
A_68_P21811852	chr2:163165006-163165050	NM_021566:58681	Jph2	INSIDE	0.579	2.985	3279.26	9788.78	1.727	2219.38	3833.21
A_68_P21812066	chr2:163201241-163201292	NM_021566:22443	Jph2	INSIDE	0.485	1.413	1545.63	2183.51	0.685	1222.47	837.03
A_68_P26228499	chr8:124254393-124254437	NM_020605:-44	Jph3	PROMOTER	0.628	3.411	9864.62	33653.00	2.144	8324.80	17848.18
A_68_P26228503	chr8:124254738-124254782	NM_020605:302	Jph3	INSIDE	0.493	0.662	4592.01	3037.78	0.326	3475.80	1132.46
A_68_P26228504	chr8:124254834-124254878	NM_020605:398	Jph3	INSIDE	0.221	0.624	5034.56	3142.71	0.138	3573.54	492.61
A_68_P26228651	chr8:124276830-124276874	NM_020605:22394	Jph3	INSIDE	0.632	4.407	713.02	3142.37	2.786	541.11	1507.67
A_68_P26228658	chr8:124277668-124277712	NM_020605:23232	Jph3	INSIDE	0.607	3.557	2747.82	9774.66	2.159	1848.26	3989.51

ProbeName	Target position of probe on CpG island microarray	TargetID	GeneSymbol	CpG island Description	Ratio of relative methylation (TiO2-NP/Vehicle)	Sham group			TiO_2-H group		
						Relative methylation (Cy-5/Cy-3)	Cy-3 signal (Input)	Cy-5 signal (MeDIP)	Relative methylation (Cy-5/Cy-3)	Cy-3 signal (Input)	Cy-5 signal (MeDIP)
A_68_P29616405	chr14:55733423-55733480	NM_177049:2321	Jph4	INSIDE	0.427	7.920	2258.91	17890.37	3.382	1539.32	5205.58
A_68_P29616406	chr14:55733544-55733588	NM_177049:2206	Jph4	INSIDE	0.441	3.348	6057.86	20283.42	1.476	3752.85	5537.91
A_68_P29616408	chr14:55733743-55733787	NM_177049:2008	Jph4	INSIDE	0.300	0.666	2341.23	1558.41	0.200	1669.13	333.22
A_68_P22324048	chr3:90035364-90035413	NM_206924:-130	Jtb	PROMOTER	0.361	0.661	985.75	651.65	0.239	748.87	178.96
A_68_P22324049	chr3:90035487-90035531	NM_206924:-10	Jtb	PROMOTER	0.192	0.558	1541.99	860.32	0.107	1114.74	119.16
A_68_P29613101	chr14:55196302-55196346	NM_010590:174	Jub	INSIDE	0.596	0.558	3510.28	1958.95	0.333	2838.90	944.66
A_68_P29613102	chr14:55196396-55196440	NM_010590:80	Jub	INSIDE	0.387	0.605	1082.21	654.80	0.234	895.45	209.49
A_68_P29613103	chr14:55196489-55196533	NM_010590:-12	Jub	PROMOTER	0.267	0.694	1422.73	986.66	0.185	1056.41	195.74
A_68_P23120702	chr4:94717432-94717476	NM_010591:1459	Jun	INSIDE	0.585	0.436	1934.02	843.61	0.255	1533.51	391.60
A_68_P23120703	chr4:94717558-94717602	NM_010591:1333	Jun	INSIDE	0.286	0.666	949.37	632.09	0.191	754.96	143.85
A_68_P23120704	chr4:94717633-94717677	NM_010591:1259	Jun	INSIDE	0.643	0.606	1291.57	782.50	0.389	973.89	379.20
A_68_P23120706	chr4:94717919-94717966	NM_010591:971	Jun	INSIDE	0.151	0.574	2266.77	1300.54	0.087	1636.71	142.02
A_68_P23120716	chr4:94719068-94719112	NM_010591:-177	Jun	PROMOTER	0.402	0.423	1296.90	548.96	0.170	1043.85	177.68
A_68_P26022960	chr8:87502192-87502236	NM_008416:433	Junb	INSIDE	0.236	0.503	1916.97	964.93	0.119	1434.02	170.04
A_68_P26022961	chr8:87502281-87502328	NM_008416:343	Junb	INSIDE	0.209	0.546	1447.23	789.73	0.114	1027.33	117.18
A_68_P26022975	chr8:87504092-87504136	NM_008416:-1467	Junb	PROMOTER	0.498	0.681	1146.44	780.67	0.339	1015.43	344.21
A_68_P25953468	chr8:73223214-73223258	NM_010592:1599	Jund	INSIDE	0.627	0.512	1154.87	591.68	0.321	776.61	249.48
A_68_P28067851	chr11:100258744-100258788	NM_010593:338	Jup	INSIDE	0.570	0.483	1464.57	706.93	0.275	1199.55	330.09
A_68_P28067855	chr11:100259055-100259099	NM_010593:28	Jup	INSIDE	0.298	0.567	1550.90	879.40	0.169	1126.51	190.56
A_68_P32028425	chr19:25311521-25311565	NM_181404:-149	Kank1	PROMOTER	0.607	0.703	1355.53	953.17	0.426	1111.31	473.97
A_68_P32028428	chr19:25311817-25311861	NM_181404:147	Kank1	INSIDE	0.232	0.545	1629.53	887.99	0.126	1274.83	161.15
A_68_P32028430	chr19:25311947-25311991	NM_181404:277	Kank1	INSIDE	0.257	0.619	864.99	535.12	0.159	721.55	114.79
A_68_P32029540	chr19:25484871-25484921	NM_181404:173205	Kank1	INSIDE	0.454	3.394	1099.41	3731.11	1.542	863.42	1331.51
A_68_P26348961	chr9:21602785-21602833	NM_145611:182	Kank2	INSIDE	0.035	1.396	6997.57	9770.96	0.049	4304.21	209.97
A_68_P31150611	chr17:33955093-33955137	NM_030697:7647	Kank3	INSIDE	0.264	0.566	1159.37	656.27	0.149	953.33	142.35
A_68_P31929693	chr19:5610054-5610098	NR_037603:-369	Kat5	PROMOTER	0.416	0.475	1492.43	709.00	0.198	1082.98	214.21
A_68_P26920442	chr10:7446059-7446115	NM_011835:289	Katna1	INSIDE	0.174	0.630	1481.08	932.78	0.109	1104.77	120.80
A_68_P24182265	chr5:149740293-149740337	NM_153572:-91	Katnal1	PROMOTER	0.361	0.641	1163.83	745.85	0.231	980.94	226.82
A_68_P24182269	chr5:149740788-149740832	NM_153572:-587	Katnal1	PROMOTER	0.239	0.629	985.18	620.05	0.151	799.08	120.28
A_68_P26081252	chr8:97605501-97605545	NM_028805:422	Katnb1	INSIDE	0.615	2.298	1639.11	3766.25	1.412	1072.01	1513.75
A_68_P32136346	chr19:45154510-45154554	NM_178929:3904	Kazald1	DOWNSTREAM	0.305	0.521	983.17	512.38	0.159	772.46	122.97
A_68_P32136351	chr19:45155030-45155086	NM_178929:4430	Kazald1	DOWNSTREAM	0.049	1.432	2706.42	3875.13	0.070	1917.99	135.00
A_68_P25678155	chr8:15010976-15011020	NM_029116:-26	Kbtbd11	PROMOTER	0.275	0.533	1164.45	621.09	0.146	820.89	120.24
A_68_P25678160	chr8:15011568-15011612	NM_029116:566	Kbtbd11	INSIDE	0.066	0.715	3719.89	2659.04	0.047	2653.43	124.50
A_68_P25678163	chr8:15011860-15011904	NM_029116:858	Kbtbd11	INSIDE	0.391	0.478	1570.57	749.98	0.186	1240.73	231.39
A_68_P26885525	chr9:121687219-121687263	NM_028202:516	Kbtbd5	INSIDE	0.269	0.493	1103.61	543.89	0.133	940.62	124.74
A_68_P24660675	chr6:95067758-95067802	NM_001008785:-119	Kbtbd8	PROMOTER	0.631	0.493	1453.92	716.41	0.311	1223.34	380.35
A_68_P24660676	chr6:95067884-95067930	NM_001008785:7	Kbtbd8	INSIDE	0.423	0.501	1384.91	693.50	0.212	1067.31	226.27
A_68_P24828002	chr6:126593244-126593288	NM_010595:2553	Kcna1	INSIDE	0.060	0.726	3643.30	2644.31	0.043	2703.77	116.95
A_68_P24828015	chr6:126594566-126594610	NM_010595:1231	Kcna1	INSIDE	0.262	0.549	1578.22	866.29	0.144	1290.65	185.56
A_68_P24828016	chr6:126594679-126594723	NM_010595:1119	Kcna1	INSIDE	0.244	0.503	1603.48	807.19	0.123	1380.67	169.72
A_68_P24828020	chr6:126595162-126595206	NM_010595:635	Kcna1	INSIDE	0.437	0.607	1548.56	939.63	0.265	1216.48	322.52
A_68_P22404200	chr3:106904965-106905010	NM_008417:503	Kcna2	INSIDE	0.470	0.491	1476.98	725.75	0.231	1028.24	237.46
A_68_P22403737	chr3:106839605-106839649	NM_008418:547	Kcna3	INSIDE	0.184	0.548	3062.15	1679.45	0.101	2124.46	214.84

ProbeName	Target position of probe on CpG island microarray	TargetID	GeneSymbol	CpG island Description	Ratio of relative methylation (TiO2-NP/Vehicle)	Sham group			TiO_2-H group		
						Relative methylation (Cy-5/Cy-3)	Cy-3 signal (Input)	Cy-5 signal (MeDIP)	Relative methylation (Cy-5/Cy-3)	Cy-3 signal (Input)	Cy-5 signal (MeDIP)
A_68_P22403738	chr3:106839716-106839760	NM_008418:659	Kcna3	INSIDE	0.250	0.358	1902.64	680.64	0.089	1325.33	118.41
A_68_P27894382	chr11:69145803-69145849	NM_010599:6067	Kcnab3	INSIDE	0.211	0.615	1137.58	699.65	0.130	919.99	119.25
A_68_P25098817	chr7:53652411-53652455	NM_001112739:566	Kcnc1	INSIDE	0.550	0.504	2277.86	1147.36	0.277	1965.29	544.59
A_68_P25098818	chr7:53652536-53652580	NM_001112739:692	Kcnc1	INSIDE	0.407	0.400	1311.52	524.24	0.163	975.14	158.73
A_68_P25098820	chr7:53652793-53652837	NM_001112739:948	Kcnc1	INSIDE	0.493	0.725	2334.88	1692.51	0.357	1771.71	633.10
A_68_P27453320	chr10:111709075-111709123	NM_001025581:920	Kcnc2	INSIDE	0.592	0.552	990.86	547.40	0.327	709.97	232.23
A_68_P25088287	chr7:51847145-51847189	NM_008422:911	Kcnc3	INSIDE	0.423	0.462	2111.75	976.07	0.195	1448.34	282.91
A_68_P24284102	chr6:21164876-21164920	NM_019697:-1210	Kcnd2	PROMOTER	0.554	0.595	1729.34	1028.65	0.329	1273.09	419.27
A_68_P24284108	chr6:21165672-21165716	NM_019697:-414	Kcnd2	PROMOTER	0.246	0.424	1480.99	627.38	0.104	1327.55	138.59
A_68_P28269095	chr12:17182821-17182865	NM_201531:852	Kcnf1	INSIDE	0.450	0.449	1330.17	597.50	0.202	1061.87	214.59
A_68_P28269097	chr12:17183053-17183097	NM_201531:620	Kcnf1	INSIDE	0.536	0.706	1723.04	1217.03	0.378	1194.79	452.14
A_68_P28269098	chr12:17183229-17183273	NM_201531:444	Kcnf1	INSIDE	0.188	0.579	2611.73	1511.42	0.109	1961.17	213.40
A_68_P28269099	chr12:17183377-17183426	NM_201531:293	Kcnf1	INSIDE	0.263	0.449	1423.46	639.55	0.118	1023.25	121.10
A_68_P21840415	chr2:168094408-168094454	NM_001081134:401	Kcng1	INSIDE	0.590	4.346	2995.93	13019.33	2.564	1963.66	5034.52
A_68_P31863850	chr18:80559762-80559806	NM_001190373:1209	Kcng2	INSIDE	0.349	0.545	2077.46	1131.85	0.190	1568.32	297.86
A_68_P31400025	chr17:84030569-84030613	NM_153512:645	Kcng3	INSIDE	0.353	1.389	2456.92	3413.51	0.491	1758.85	863.10
A_68_P26215168	chr8:122157059-122157103	NM_025734:2500	Kcng4	INSIDE	0.651	2.357	818.18	1928.32	1.534	766.81	1176.59
A_68_P23541182	chr5:23857019-23857063	NM_013569:382	Kcnh2	INSIDE	0.408	0.563	1148.64	647.18	0.230	931.66	214.28
A_68_P30478813	chr15:99054103-99054147	NM_010601:-1282	Kcnh3	PROMOTER	0.286	0.493	1168.71	575.72	0.141	913.16	128.58
A_68_P28069993	chr11:100620866-100620910	NM_001081194:204	Kcnh4	INSIDE	0.263	0.627	1564.29	980.36	0.165	1237.72	203.74
A_68_P28547080	chr12:76277679-76277723	NM_172805:619	Kcnh5	INSIDE	0.280	0.403	1318.92	531.28	0.113	1070.99	120.62
A_68_P28099138	chr11:105881660-105881704	NM_001037712:12166	Kcnh6	INSIDE	0.269	0.698	3544.63	2474.23	0.188	2626.04	493.34
A_68_P27696175	chr11:33580643-33580689	NM_001190885:162919	Kcnip1	INSIDE	0.495	0.672	1483.55	996.39	0.333	1260.53	419.36
A_68_P21613745	chr2:127307805-127307849	NM_001111331:409	Kcnip3	INSIDE	0.650	0.441	2650.19	1168.26	0.287	2148.46	615.97
A_68_P20832488	chr1:174271323-174271367	NM_001039484:4	Kcnj10	INSIDE	0.335	0.681	2205.64	1501.20	0.228	1514.61	345.65
A_68_P21233127	chr2:55289729-55289773	NM_008426:184	Kcnj3	INSIDE	0.364	0.622	1796.23	1116.79	0.226	1390.13	314.43
A_68_P21233128	chr2:55289837-55289883	NM_008426:294	Kcnj3	INSIDE	0.358	0.376	1784.59	671.01	0.135	1342.96	180.69
A_68_P30366727	chr15:79316080-79316124	NM_008427:19569	Kcnj4	INSIDE	0.653	2.056	3781.89	7776.96	1.343	2686.58	3607.71
A_68_P30366869	chr15:79335593-79335637	NM_008427:57	Kcnj4	INSIDE	0.231	0.476	1403.09	668.18	0.110	1117.27	122.75
A_68_P30366871	chr15:79335939-79335983	NM_008427:-289	Kcnj4	PROMOTER	0.314	0.643	1312.02	843.23	0.202	1044.58	210.62
A_68_P24905346	chr6:142519942-142519986	NM_008428:-88	Kcnj8	PROMOTER	0.453	0.725	1854.73	1345.34	0.328	1478.94	485.44
A_68_P20832394	chr1:174256462-174256506	NM_008429:2910	Kcnj9	INSIDE	0.471	0.702	1531.96	1075.27	0.330	1106.62	365.65
A_68_P20832395	chr1:174256553-174256597	NM_008429:2820	Kcnj9	INSIDE	0.417	0.587	2183.54	1282.71	0.245	1782.70	436.35
A_68_P28670942	chr12:99812802-99812846	NM_029911:3326	Kcnk10	INSIDE	0.234	0.699	4832.58	3376.41	0.163	3451.33	563.71
A_68_P31425154	chr17:88197235-88197279	NM_199251:78	Kcnk12	INSIDE	0.551	0.642	1855.73	1190.91	0.354	1480.17	523.58
A_68_P31425157	chr17:88197480-88197524	NM_199251:-168	Kcnk12	PROMOTER	0.403	0.466	4375.97	2037.15	0.188	3471.60	651.98
A_68_P31425158	chr17:88197562-88197606	NM_199251:-250	Kcnk12	PROMOTER	0.374	0.571	1224.58	699.32	0.214	882.97	188.56
A_68_P28679266	chr12:101203176-101203220	NM_001164426:490	Kcnk13	INSIDE	0.224	0.548	1392.85	763.65	0.123	1007.56	123.49
A_68_P28679268	chr12:101203451-101203495	NM_001164426:764	Kcnk13	INSIDE	0.442	0.736	2083.21	1532.32	0.325	1587.95	516.39
A_68_P21814760	chr2:163679507-163679551	NM_001030292:43	Kcnk15	INSIDE	0.581	0.683	1483.07	1013.04	0.397	1168.80	463.49
A_68_P23573357	chr5:30891361-30891405	NM_010608:840	Kcnk3	INSIDE	0.502	0.581	1413.66	821.28	0.292	1082.69	315.65
A_68_P23573586	chr5:30924517-30924565	NM_010608:33998	Kcnk3	INSIDE	0.421	0.568	1124.37	638.72	0.239	915.88	219.27
A_68_P31936766	chr19:7008439-7008483	NM_008431:545	Kcnk4	INSIDE	0.349	0.559	2029.56	1134.32	0.195	1337.02	261.08
A_68_P31936768	chr19:7008741-7008785	NM_008431:243	Kcnk4	INSIDE	0.347	0.494	1948.28	962.37	0.172	1503.30	257.84

ProbeName	Target position of probe on CpG island microarray	TargetID	GeneSymbol	CpG island Description	Ratio of relative methylation (TiO2-NP/Vehicle)	Sham group			TiO_2-H group		
						Relative methylation (Cy-5/Cy-3)	Cy-3 signal (Input)	Cy-5 signal (MeDIP)	Relative methylation (Cy-5/Cy-3)	Cy-3 signal (Input)	Cy-5 signal (MeDIP)
A_68_P29445595	chr14:21000753-21000797	NM_021542:230	Kcnk5	INSIDE	0.388	0.502	1115.45	560.30	0.195	1023.65	199.39
A_68_P30325017	chr15:72376544-72376592	NM_001033876:141	Kcnk9	INSIDE	0.299	0.552	1254.57	692.97	0.165	989.60	163.53
A_68_P29469008	chr14:24823022-24823066	NM_010610:383	Kcnma1	INSIDE	0.301	0.601	2823.74	1698.02	0.181	1847.96	333.96
A_68_P31672396	chr18:45718914-45718958	NM_080465:-871	Kcnn2	PROMOTER	0.284	0.728	2275.19	1655.82	0.207	1551.17	320.94
A_68_P31672403	chr18:45719784-45719828	NM_080465:-1	Kcnn2	PROMOTER	0.122	0.682	3612.23	2463.14	0.083	2621.15	217.18
A_68_P25598743	chr7:150293354-150293398	NM_008434:218	Kcnq1	INSIDE	0.596	0.717	1531.70	1098.05	0.427	1262.71	539.38
A_68_P25599905	chr7:150482316-150482360	NR_001461:114	Kcnq1ot1	INSIDE	0.517	1.366	7091.04	9685.35	0.707	4961.85	3505.79
A_68_P30290896	chr15:66117380-66117424	NM_152923:384	Kcnq3	INSIDE	0.333	0.417	2616.11	1091.34	0.139	1995.45	277.20
A_68_P30290897	chr15:66117445-66117492	NM_152923:318	Kcnq3	INSIDE	0.162	0.628	1729.08	1085.51	0.102	1184.62	120.64
A_68_P23252660	chr4:120370742-120370786	NM_001081142:49017	Kcnq4	INSIDE	0.656	3.128	4023.88	12586.34	2.052	2870.52	5890.80
A_68_P23252895	chr4:120403760-120403804	NM_001081142:15999	Kcnq4	INSIDE	0.441	0.574	2018.15	1158.73	0.253	1578.47	399.81
A_68_P23252897	chr4:120404005-120404049	NM_001081142:15755	Kcnq4	INSIDE	0.197	0.550	1613.43	886.66	0.108	1095.09	118.31
A_68_P23253009	chr4:120419330-120419374	NM_001081142:429	Kcnq4	INSIDE	0.424	0.701	1634.38	1145.29	0.297	1213.62	360.38
A_68_P21816596	chr2:163993800-163993844	NM_008435:3027	Kcns1	INSIDE	0.496	0.552	1509.46	833.12	0.274	1233.01	337.56
A_68_P30130467	chr15:34767012-34767056	NM_181317:-101	Kcns2	PROMOTER	0.595	0.588	992.19	583.30	0.350	860.67	301.10
A_68_P30130468	chr15:34767120-34767164	NM_181317:7	Kcns2	INSIDE	0.183	0.442	1909.65	844.39	0.081	1400.70	113.65
A_68_P24326992	chr6:29457810-29457854	NM_001029985:120	Kcp	INSIDE	0.438	0.734	2819.38	2068.58	0.321	2183.45	700.91
A_68_P31515592	chr18:15223215-15223266	NM_001142731:-1149	Kctd1	PROMOTER	0.105	0.588	2969.77	1746.58	0.061	1962.40	120.61
A_68_P31515593	chr18:15223323-15223369	NM_001142731:-1255	Kctd1	PROMOTER	0.115	0.542	2658.26	1440.09	0.062	1974.93	123.08
A_68_P31516117	chr18:15308896-15308940	NM_134112:1037	Kctd1	INSIDE	0.453	0.679	1313.21	891.68	0.307	1025.35	315.20
A_68_P31516119	chr18:15309151-15309195	NM_134112:783	Kctd1	INSIDE	0.253	0.525	1732.70	909.46	0.133	1339.55	178.14
A_68_P31516120	chr18:15309236-15309280	NM_134112:697	Kctd1	INSIDE	0.204	0.585	2412.99	1412.22	0.120	1891.21	226.24
A_68_P31516121	chr18:15309314-15309358	NM_134112:619	Kctd1	INSIDE	0.444	0.498	1951.11	971.09	0.221	1433.41	316.92
A_68_P24003591	chr5:114830465-114830509	NM_001159941:28	Kctd10	INSIDE	0.594	0.449	1952.45	876.19	0.267	1600.68	426.94
A_68_P29863081	chr14:103380685-103380729	NM_177715:1148	Kctd12	INSIDE	0.082	0.624	3074.56	1918.04	0.051	2306.57	117.81
A_68_P25503046	chr7:134072788-134072832	NM_172747:418	Kctd13	INSIDE	0.608	0.679	1493.07	1014.50	0.413	1249.56	516.54
A_68_P25044855	chr7:35438249-35438293	NM_146188:-410	Kctd15	PROMOTER	0.526	0.476	1339.92	638.08	0.250	1162.42	291.04
A_68_P25044856	chr7:35438432-35438491	NM_146188:-601	Kctd15	PROMOTER	0.121	0.663	1966.91	1303.13	0.080	1459.30	116.61
A_68_P25044857	chr7:35438615-35438659	NM_146188:-776	Kctd15	PROMOTER	0.228	0.466	3075.38	1431.70	0.106	2089.06	221.55
A_68_P20256314	chr1:58026706-58026750	NR_027630:-360	Kctd18	PROMOTER	0.262	0.497	1256.59	623.96	0.130	934.48	121.78
A_68_P25349935	chr7:104480504-104480548	NM_001039039:-306	Kctd21	DIVERGENT_PROMOTER	0.289	0.299	1729.33	516.53	0.086	1377.68	118.82
A_68_P31095211	chr17:24210441-24210485	NM_027008:-10	Kctd5	PROMOTER	0.551	0.582	2467.49	1437.18	0.321	1824.41	585.38
A_68_P26520288	chr9:53192430-53192474	NM_212445:325	Kdelc2	INSIDE	0.638	0.632	1068.40	675.49	0.404	857.41	346.03
A_68_P24156414	chr5:144165340-144165384	NM_025841:-136	Kdelr2	PROMOTER	0.262	0.411	1449.80	595.41	0.108	1136.90	122.23
A_68_P30366911	chr15:79342352-79342396	NM_134090:-4463	Kdelr3	DIVERGENT_PROMOTER	0.264	0.657	3541.10	2327.67	0.173	2854.17	494.60
A_68_P30366913	chr15:79342515-79342567	NM_134090:-4297	Kdelr3	DIVERGENT_PROMOTER	0.259	0.605	1135.05	686.84	0.157	791.57	124.14
A_68_P23334804	chr4:136157993-136158037	NM_133872:624	Kdm1a	INSIDE	0.081	0.574	4248.67	2438.37	0.047	3328.66	155.49
A_68_P24053342	chr5:123439126-123439170	NM_001003953:-47	Kdm2b	PROMOTER	0.350	0.595	2810.70	1672.41	0.208	1999.93	416.43
A_68_P24537469	chr6:71583121-71583165	NM_001038695:-243	Kdm3a	PROMOTER	0.077	1.350	4220.43	5698.05	0.104	2899.62	300.82
A_68_P24537471	chr6:71583335-71583379	NM_001038695:-457	Kdm3a	PROMOTER	0.243	0.635	2180.31	1384.48	0.154	1665.30	257.21
A_68_P23019857	chr4:73898113-73898157	NM_144787:352	Kdm4c	INSIDE	0.187	0.580	1672.21	970.40	0.109	1586.42	172.49
A_68_P26317489	chr9:14304655-14304699	NM_173433:250	Kdm4d	INSIDE	0.177	0.575	2013.19	1156.58	0.101	1392.24	141.29
A_68_P24798110	chr6:120313882-120313931	NM_145997:-210	Kdm5a	DIVERGENT_PROMOTER	0.256	0.411	1599.14	656.93	0.105	1153.34	121.36
A_68_P20634005	chr1:136453383-136453427	NM_152895:-3350	Kdm5b	DIVERGENT_PROMOTER	0.483	0.595	1761.01	1048.22	0.287	1387.93	398.75

ProbeName	Target position of probe on CpG island microarray	TargetID	GeneSymbol	CpG island Description	Ratio of relative methylation (TiO2-NP/Vehicle)	Sham group			TiO_2-H group		
						Relative methylation (Cy-5/Cy-3)	Cy-3 signal (Input)	Cy-5 signal (MeDIP)	Relative methylation (Cy-5/Cy-3)	Cy-3 signal (Input)	Cy-5 signal (MeDIP)
A_68_P32281779	chrX:17739627-17739671	NM_009483:-144	Kdm6a	PROMOTER	0.659	0.414	2101.22	869.94	0.273	751.51	205.12
A_68_P27894946	chr11:69224331-69224375	NM_001017426:2825	Kdm6b	INSIDE	0.294	1.983	6938.02	13758.22	0.582	4494.01	2616.74
A_68_P27894955	chr11:69225357-69225401	NM_001017426:1799	Kdm6b	INSIDE	0.209	0.460	1724.87	792.89	0.096	1266.95	121.57
A_68_P20510205	chr1:108656151-108656195	NM_027534:147	Kdsr	INSIDE	0.532	0.685	1556.75	1066.07	0.364	1233.63	449.37
A_68_P20510206	chr1:108656242-108656286	NM_027534:55	Kdsr	INSIDE	0.537	0.574	2879.18	1652.62	0.308	2172.45	670.18
A_68_P23298314	chr4:129418594-129418638	NM_011317:910	Khdrbs1	INSIDE	0.519	0.525	1377.45	723.44	0.273	1242.32	338.57
A_68_P23298316	chr4:129418783-129418827	NM_011317:722	Khdrbs1	INSIDE	0.247	0.445	1345.62	599.40	0.110	1156.77	127.12
A_68_P23298318	chr4:129419063-129419107	NM_011317:442	Khdrbs1	INSIDE	0.195	0.411	1817.20	746.82	0.080	1428.52	114.25
A_68_P23298327	chr4:129420133-129420178	NM_011317:-629	Khdrbs1	PROMOTER	0.077	1.406	1879.44	2643.30	0.109	1298.73	141.46
A_68_P31259697	chr17:57171063-57171108	NM_010613:-155	Khsrp	PROMOTER	0.529	0.585	1395.41	816.58	0.310	1094.26	338.73
A_68_P31259698	chr17:57171184-57171228	NM_010613:-276	Khsrp	PROMOTER	0.093	0.563	3531.04	1987.89	0.052	2623.12	136.68
A_68_P28292712	chr12:25659767-25659811	NM_001081378:-8	Kidins220	PROMOTER	0.353	0.596	1392.40	829.48	0.211	1030.84	217.06
A_68_P32095250	chr19:37450569-37450613	NM_010615:-302	Kif11	PROMOTER	0.662	0.600	936.98	561.73	0.397	721.13	286.01
A_68_P32095251	chr19:37450692-37450736	NM_010615:-178	Kif11	PROMOTER	0.184	0.513	3322.40	1705.44	0.095	2397.48	226.61
A_68_P29010701	chr13:47026038-47026093	NM_010617:-978	Kif13a	PROMOTER	0.405	0.484	1767.47	854.99	0.196	1333.63	261.42
A_68_P29668829	chr14:65271192-65271238	NM_001081177:-153	Kif13b	PROMOTER	0.160	0.493	1794.03	885.17	0.079	1495.33	118.26
A_68_P29668831	chr14:65271409-65271453	NM_001081177:63	Kif13b	INSIDE	0.311	0.676	1231.23	832.23	0.210	936.30	196.54
A_68_P29668832	chr14:65271520-65271564	NM_001081177:175	Kif13b	INSIDE	0.363	0.514	2830.97	1454.50	0.186	2398.24	447.04
A_68_P20646087	chr1:138362972-138363016	NM_001081258:-1540	Kif14	PROMOTER	0.586	0.509	1568.30	797.99	0.298	1246.87	371.71
A_68_P28082319	chr11:102786133-102786177	NM_197959:284	Kif18b	INSIDE	0.100	0.605	4265.79	2579.55	0.061	2768.17	168.18
A_68_P28149601	chr11:114626740-114626784	NM_001102615:60	Kif19a	INSIDE	0.222	0.615	3182.32	1958.20	0.137	2326.78	318.00
A_68_P20453277	chr1:94998219-94998263	NM_001110315:202	Kif1a	INSIDE	0.170	0.622	1535.58	955.02	0.106	1147.99	121.25
A_68_P20453278	chr1:94998327-94998371	NM_001110315:94	Kif1a	INSIDE	0.293	0.550	2648.99	1458.18	0.161	1809.39	291.67
A_68_P20453279	chr1:94998401-94998445	NM_001110315:20	Kif1a	INSIDE	0.571	0.610	1585.84	967.91	0.348	1191.72	414.97
A_68_P23399813	chr4:148681750-148681794	NM_207682:35	Kif1b	INSIDE	0.396	0.601	2997.35	1800.31	0.238	2346.60	557.57
A_68_P23399815	chr4:148682002-148682046	NM_207682:-217	Kif1b	PROMOTER	0.272	0.665	4528.99	3012.98	0.181	3547.92	641.81
A_68_P31613845	chr18:34784223-34784267	NM_001166407:-33	Kif20a	PROMOTER	0.516	0.651	951.51	619.24	0.336	714.48	239.96
A_68_P32082884	chr19:34996996-34997040	NM_183046:171	Kif20b	INSIDE	0.575	0.534	1524.39	813.30	0.307	1149.54	352.94
A_68_P30433465	chr15:90879520-90879574	NM_001109040:833	Kif21a	INSIDE	0.301	0.443	1163.02	514.88	0.133	904.58	120.45
A_68_P30433474	chr15:90880547-90880591	NM_001109040:-189	Kif21a	PROMOTER	0.662	0.603	1332.70	803.69	0.399	1122.47	448.16
A_68_P20644037	chr1:138028308-138028352	NM_001039472:353	Kif21b	INSIDE	0.340	0.522	1919.86	1001.71	0.177	1596.07	282.84
A_68_P20644038	chr1:138028419-138028463	NM_001039472:463	Kif21b	INSIDE	0.543	0.566	1200.58	679.11	0.307	1107.51	340.33
A_68_P25503740	chr7:134185787-134185831	NM_145588:126	Kif22	INSIDE	0.305	0.638	1173.06	748.72	0.195	861.63	167.91
A_68_P26567939	chr9:61794038-61794082	NM_024245:546	Kif23	INSIDE	0.572	0.692	1333.40	922.34	0.395	1143.33	452.02
A_68_P26567942	chr9:61794410-61794454	NM_024245:174	Kif23	INSIDE	0.645	0.691	1252.07	865.46	0.446	1080.95	482.19
A_68_P28748127	chr12:113382080-113382124	NM_001097621:-2316	Kif26a	PROMOTER	0.450	0.280	1974.31	553.61	0.126	1580.17	199.56
A_68_P28748134	chr12:113382997-113383048	NM_001097621:-1396	Kif26a	PROMOTER	0.424	4.501	10309.02	46396.48	1.909	6069.03	11587.23
A_68_P28748138	chr12:113383612-113383656	NM_001097621:-784	Kif26a	PROMOTER	0.271	0.570	1017.07	579.61	0.154	743.14	114.67
A_68_P28748139	chr12:113383755-113383799	NM_001097621:-642	Kif26a	PROMOTER	0.274	0.645	1160.96	749.22	0.177	791.19	140.04
A_68_P20864285	chr1:180457620-180457665	NM_001161665:-1613	Kif26b	PROMOTER	0.211	0.685	1043.38	714.81	0.145	828.04	119.70
A_68_P20864287	chr1:180457911-180457955	NM_001161665:-1323	Kif26b	PROMOTER	0.164	0.495	2357.97	1166.98	0.081	1885.46	153.30
A_68_P20864288	chr1:180458040-180458084	NM_001161665:-1193	Kif26b	PROMOTER	0.197	0.677	1096.79	742.44	0.133	880.68	117.44
A_68_P20864291	chr1:180458344-180458388	NM_001161665:-889	Kif26b	PROMOTER	0.305	0.559	3546.14	1982.41	0.171	2609.64	445.47
A_68_P20864292	chr1:180458491-180458535	NM_001161665:-743	Kif26b	PROMOTER	0.182	0.614	1523.68	935.46	0.112	1084.76	121.40

ProbeName	Target position of probe on CpG island microarray	TargetID	GeneSymbol	CpG island Description	Ratio of relative methylation (TiO2-NP/Vehicle)	Sham group			TiO_2-H group		
						Relative methylation (Cy-5/Cy-3)	Cy-3 signal (Input)	Cy-5 signal (MeDIP)	Relative methylation (Cy-5/Cy-3)	Cy-3 signal (Input)	Cy-5 signal (MeDIP)
A_68_P20864294	chr1:180458639-180458683	NM_001161665:-595	Kif26b	PROMOTER	0.133	0.590	1901.92	1121.91	0.079	1535.15	120.81
A_68_P20864304	chr1:180459747-180459791	NM_001161665:513	Kif26b	INSIDE	0.308	0.641	2569.97	1646.84	0.197	1831.57	361.10
A_68_P29072860	chr13:58460543-58460587	NM_175214:-4341	Kif27	PROMOTER	0.351	0.430	4159.44	1787.57	0.151	3206.66	484.26
A_68_P27805436	chr11:53381255-53381299	NM_008443:396	Kif3a	INSIDE	0.576	0.531	1519.41	806.94	0.306	1138.73	348.14
A_68_P32560534	chrX:97821357-97821401	NM_008446:-25	Kif4	DIVERGENT_PROMOTER	0.259	1.624	1122.58	1823.48	0.422	487.74	205.59
A_68_P32560535	chrX:97821487-97821531	NM_008446:105	Kif4	INSIDE	0.118	2.407	3312.54	7972.91	0.283	1261.11	356.94
A_68_P27534777	chr10:126676555-126676599	NM_001039000:23843	Kif5a	INSIDE	0.381	1.451	909.64	1320.23	0.554	735.03	406.86
A_68_P27534886	chr10:126699975-126700019	NM_001039000:423	Kif5a	INSIDE	0.264	0.532	1181.40	628.45	0.140	850.79	119.49
A_68_P27534887	chr10:126700073-126700117	NM_001039000:325	Kif5a	INSIDE	0.322	0.635	3511.08	2229.25	0.205	2525.28	516.91
A_68_P21203200	chr2:49474749-49474793	NM_008449:-63	Kif5c	PROMOTER	0.626	0.582	1030.88	599.73	0.364	858.34	312.44
A_68_P31113145	chr17:27054067-27054111	NM_053173:53	Kifc5b	INSIDE	0.638	0.565	1547.45	873.86	0.360	1163.83	419.41
A_68_P27279732	chr10:79383697-79383741	NM_053244:4003	Kiss1r	INSIDE	0.504	0.690	2161.99	1492.60	0.348	1683.69	586.03
A_68_P27279736	chr10:79384102-79384146	NM_053244:4409	Kiss1r	INSIDE	0.106	0.654	2489.30	1627.83	0.069	1728.67	119.93
A_68_P27279737	chr10:79384185-79384238	NM_053244:4496	Kiss1r	INSIDE	0.177	1.533	1071.29	1642.47	0.272	799.13	217.16
A_68_P27279739	chr10:79384461-79384505	NM_053244:4767	Kiss1r	INSIDE	0.629	0.411	1414.74	581.63	0.259	1146.57	296.50
A_68_P27390318	chr10:99478125-99478169	NM_013598:-311	Kitl	PROMOTER	0.623	0.632	1060.59	670.45	0.394	856.22	336.99
A_68_P27390319	chr10:99478265-99478309	NM_013598:-171	Kitl	PROMOTER	0.419	0.515	1992.06	1025.00	0.215	1728.00	372.28
A_68_P24193751	chr5:151755711-151755755	NM_013823:551	Kl	INSIDE	0.544	0.537	1002.19	538.11	0.292	947.84	276.69
A_68_P31927054	chr19:5117908-5117952	NM_008451:478	Klc2	INSIDE	0.584	0.569	1030.60	585.96	0.332	772.93	256.46
A_68_P24993121	chr7:19989091-19989135	NM_146182:-3907	Klc3	DIVERGENT_PROMOTER	0.594	0.435	1409.77	612.81	0.258	1126.58	291.00
A_68_P28290821	chr12:25335787-25335837	NM_178357:-423	Klf11	PROMOTER	0.389	0.533	1034.45	551.52	0.207	785.97	162.93
A_68_P28290830	chr12:25336784-25336828	NM_178357:571	Klf11	INSIDE	0.235	1.611	1105.43	1781.36	0.379	863.67	326.94
A_68_P25174079	chr7:71036675-71036719	NM_021366:47105	Klf13	INSIDE	0.627	2.450	789.09	1933.59	1.537	659.13	1013.13
A_68_P25174410	chr7:71084241-71084285	NM_021366:-461	Klf13	PROMOTER	0.198	0.491	1760.19	863.53	0.097	1213.78	117.72
A_68_P24334773	chr6:30907971-30908017	NM_001135093:996	Klf14	INSIDE	0.291	0.529	1220.51	645.44	0.154	916.79	140.84
A_68_P24632567	chr6:90413191-90413235	NM_023184:593	Klf15	INSIDE	0.240	0.525	2516.21	1320.70	0.126	1879.15	237.05
A_68_P27284095	chr10:80039472-80039516	NM_078477:547	Klf16	INSIDE	0.247	0.599	1082.35	648.75	0.148	850.10	125.90
A_68_P27284101	chr10:80040251-80040295	NM_078477:-231	Klf16	PROMOTER	0.620	0.532	2891.93	1539.90	0.330	2218.23	732.89
A_68_P27284104	chr10:80040680-80040724	NM_078477:-661	Klf16	PROMOTER	0.553	0.607	2026.42	1230.08	0.336	1567.43	526.54
A_68_P25960687	chr8:74843237-74843281	NM_008452:298	Klf2	INSIDE	0.125	1.386	4459.19	6180.08	0.173	2925.50	507.23
A_68_P23752952	chr5:65195598-65195642	NM_008453:859	Klf3	INSIDE	0.273	0.420	1300.66	546.35	0.115	1043.43	119.55
A_68_P22934416	chr4:55543231-55543275	NM_010637:2095	Klf4	INSIDE	0.568	0.675	1338.12	903.88	0.383	1075.42	412.35
A_68_P22934418	chr4:55543459-55543503	NM_010637:1867	Klf4	INSIDE	0.489	0.538	1611.10	867.00	0.263	1261.18	332.22
A_68_P22934424	chr4:55544128-55544172	NM_010637:1197	Klf4	INSIDE	0.264	0.534	1468.63	784.65	0.141	1321.60	186.61
A_68_P22934427	chr4:55544464-55544508	NM_010637:861	Klf4	INSIDE	0.394	0.566	1638.41	926.60	0.223	1245.95	277.60
A_68_P29842694	chr14:99697586-99697630	NM_009769:-301	Klf5	PROMOTER	0.558	0.555	2008.47	1115.62	0.310	1623.46	502.74
A_68_P29842699	chr14:99698157-99698201	NM_009769:269	Klf5	INSIDE	0.599	0.627	1269.40	795.40	0.375	1196.09	448.72
A_68_P29842704	chr14:99698682-99698726	NM_009769:795	Klf5	INSIDE	0.273	0.515	1114.89	574.59	0.140	859.70	120.74
A_68_P20290945	chr1:64168690-64168734	NM_033563:-749	Klf7	PROMOTER	0.180	0.586	1484.49	870.28	0.105	1106.58	116.54
A_68_P32752564	chrX:149672338-149672390	NM_173780:-223	Klf8	PROMOTER	0.130	2.195	1003.62	2203.31	0.286	416.60	119.04
A_68_P32752567	chrX:149672709-149672753	NM_173780:143	Klf8	INSIDE	0.204	1.586	2576.06	4085.29	0.323	1015.29	328.05
A_68_P32016038	chr19:23209873-23209917	NM_010638:-5821	Klf9	PROMOTER	0.222	0.618	1116.76	690.02	0.137	856.02	117.19
A_68_P32016080	chr19:23215457-23215501	NM_010638:-237	Klf9	PROMOTER	0.637	0.486	1225.61	595.08	0.309	1048.53	324.25
A_68_P28513757	chr12:70398282-70398327	NM_027117:637	Klhdc2	INSIDE	0.073	3.260	3464.37	11292.49	0.239	2091.29	499.84

ProbeName	Target position of probe on CpG island microarray	TargetID	GeneSymbol	CpG island Description	Ratio of relative methylation (TiO2-NP/Vehicle)	Sham group			TiO_2-H group		
						Relative methylation (Cy-5/Cy-3)	Cy-3 signal (Input)	Cy-5 signal (MeDIP)	Relative methylation (Cy-5/Cy-3)	Cy-3 signal (Input)	Cy-5 signal (MeDIP)
A_68_P24932467	chr6:147040098-147040142	NM_001081237:524	Klhdc5	INSIDE	0.284	0.423	1233.08	521.35	0.120	989.84	118.70
A_68_P28068271	chr11:100333552-100333596	NM_172565:522	Klhl11	INSIDE	0.240	0.560	1198.84	671.53	0.134	917.33	123.31
A_68_P31545963	chr18:21811255-21811299	NM_001081403:-407	Klhl14	PROMOTER	0.294	0.461	1899.45	876.38	0.136	1439.61	195.37
A_68_P31545964	chr18:21811364-21811410	NM_001081403:-517	Klhl14	PROMOTER	0.179	0.623	1563.99	974.57	0.112	1184.81	132.50
A_68_P31545966	chr18:21811604-21811648	NM_001081403:-757	Klhl14	PROMOTER	0.051	1.495	4721.84	7059.15	0.076	3244.50	247.11
A_68_P31545967	chr18:21811707-21811752	NM_001081403:-860	Klhl14	PROMOTER	0.104	1.613	3155.93	5091.46	0.168	2050.86	344.17
A_68_P30575506	chr16:17759553-17759597	NM_145479:-139	Klhl22	PROMOTER	0.480	0.679	1589.70	1079.12	0.326	1274.65	415.05
A_68_P30575507	chr16:17759672-17759716	NM_145479:-19	Klhl22	PROMOTER	0.531	0.600	3488.89	2093.91	0.319	2907.37	926.01
A_68_P30575512	chr16:17760349-17760393	NM_145479:657	Klhl22	INSIDE	0.462	0.607	1018.38	618.37	0.280	861.84	241.62
A_68_P21311215	chr2:69660694-69660738	NM_177784:290	Klhl23	INSIDE	0.565	0.647	2109.52	1363.94	0.365	1700.98	621.06
A_68_P28206058	chr12:5380617-5380662	NM_001164493:1849	Klhl29	INSIDE	0.568	0.545	1529.67	834.43	0.310	1206.06	373.61
A_68_P28206067	chr12:5381571-5381617	NM_001164493:894	Klhl29	INSIDE	0.224	0.386	1891.09	730.58	0.086	1389.97	120.09
A_68_P28206069	chr12:5381867-5381911	NM_001164493:600	Klhl29	INSIDE	0.646	0.571	1469.22	839.34	0.369	1153.24	425.71
A_68_P28206071	chr12:5382006-5382050	NM_001164493:460	Klhl29	INSIDE	0.405	0.524	3088.99	1617.12	0.212	2432.86	515.70
A_68_P26655646	chr9:77502889-77502936	NM_172925:18374	Klhl31	INSIDE	0.159	0.706	1266.67	893.82	0.112	1058.36	118.95
A_68_P26216543	chr8:122394587-122394646	NM_146219:8412	Klhl36	INSIDE	0.092	2.460	687.92	1692.01	0.226	562.75	127.29
A_68_P23091882	chr4:88368088-88368132	NM_172871:302	Klhl9	INSIDE	0.549	0.575	1844.70	1060.45	0.316	1498.88	472.96
A_68_P25084097	chr7:51052779-51052823	NM_008940:-146	Klk8	PROMOTER	0.223	0.654	1079.39	705.65	0.146	821.74	119.64
A_68_P23226466	chr4:115563188-115563232	NM_001039124:6206	Kncn	DOWNSTREAM	0.409	0.556	1854.46	1030.70	0.227	1582.06	359.35
A_68_P23226467	chr4:115563355-115563399	NM_001039124:6372	Kncn	DOWNSTREAM	0.208	0.282	2582.53	729.15	0.059	2030.85	119.09
A_68_P24057384	chr5:124199713-124199761	NM_001042421:2	Kntc1	INSIDE	0.235	0.650	1032.62	671.61	0.153	791.13	120.72
A_68_P28104645	chr11:106861028-106861074	NM_010655:-211	Kpna2	PROMOTER	0.329	0.625	924.17	577.41	0.205	751.86	154.49
A_68_P29650241	chr14:62058563-62058607	NM_008466:200	Kpna3	INSIDE	0.403	0.405	1537.06	622.81	0.163	1438.25	235.02
A_68_P28049249	chr11:97049576-97049622	NM_008379:-392	Kpnb1	PROMOTER	0.350	0.517	3594.87	1860.24	0.181	2788.10	505.66
A_68_P24979758	chr7:16705347-16705391	NM_133727:144	Kptn	INSIDE	0.279	0.601	1547.69	929.93	0.168	1213.70	203.83
A_68_P24921910	chr6:145197794-145197838	NM_021284:935	Kras	INSIDE	0.356	0.734	1962.50	1439.52	0.261	1309.32	341.84
A_68_P24921912	chr6:145197978-145198027	NM_021284:749	Kras	INSIDE	0.145	0.721	2164.39	1561.55	0.105	1620.53	169.47
A_68_P31093374	chr17:23879287-23879335	NM_028416:3486	Kremen2	INSIDE	0.307	1.442	1137.02	1639.25	0.442	749.28	331.12
A_68_P26346365	chr9:21092135-21092179	NM_145416:257	Kri1	INSIDE	0.473	0.659	1141.12	752.12	0.312	934.66	291.63
A_68_P26346370	chr9:21092650-21092694	NM_145416:-259	Kri1	PROMOTER	0.269	0.549	2546.79	1398.83	0.148	1997.49	294.68
A_68_P27452133	chr10:111409980-111410024	NM_178610:252	Krr1	INSIDE	0.443	0.542	1265.23	685.94	0.240	884.31	212.47
A_68_P27452135	chr10:111410230-111410274	NM_178610:502	Krr1	INSIDE	0.165	1.345	2836.31	3815.35	0.222	1978.99	439.64
A_68_P28066376	chr11:100006643-100006687	NM_008471:569	Krt19	INSIDE	0.428	0.638	1553.77	991.90	0.273	1163.22	317.88
A_68_P28066377	chr11:100006745-100006792	NM_008471:465	Krt19	INSIDE	0.297	0.402	1333.60	536.05	0.120	1037.15	124.01
A_68_P30491859	chr15:101269185-101269229	NM_001003668:29	Krt83	INSIDE	0.239	0.535	2013.72	1078.12	0.128	1424.27	182.29
A_68_P27947413	chr11:78959127-78959171	NM_013571:708	Ksr1	INSIDE	0.292	0.652	3720.80	2427.69	0.190	2729.19	519.38
A_68_P27947415	chr11:78959320-78959364	NM_013571:514	Ksr1	INSIDE	0.099	0.690	2768.75	1909.62	0.068	1801.10	122.82
A_68_P27947420	chr11:78960035-78960079	NM_013571:-200	Ksr1	PROMOTER	0.177	0.373	2319.40	865.07	0.066	1805.20	119.02
A_68_P28516077	chr12:70825553-70825597	NM_145443:287	L2hgdh	INSIDE	0.570	0.620	1226.48	760.61	0.354	1048.37	370.75
A_68_P21809532	chr2:162769099-162769143	NM_001081338:-80	L3mbtl1	PROMOTER	0.359	0.395	2115.44	834.65	0.142	1587.72	225.04
A_68_P25310385	chr7:97089281-97089328	NM_026304:391	l7Rn6	INSIDE	0.171	0.728	3396.19	2474.01	0.124	2516.93	312.98
A_68_P20042289	chr1:13650498-13650542	NM_145381:70	Lactb2	INSIDE	0.364	0.613	1278.27	783.69	0.223	1015.13	226.65
A_68_P21902322	chr2:179959737-179959781	NM_001081171:806	Lama5	INSIDE	0.417	0.592	1520.26	899.44	0.246	1245.47	306.94
A_68_P21902323	chr2:179959846-179959890	NM_001081171:696	Lama5	INSIDE	0.336	0.279	2052.96	573.01	0.094	1737.11	162.97

ProbeName	Target position of probe on CpG island microarray	TargetID	GeneSymbol	CpG island Description	Ratio of relative methylation (TiO2-NP/Vehicle)	Sham group			TiO_2-H group		
						Relative methylation (Cy-5/Cy-3)	Cy-3 signal (Input)	Cy-5 signal (MeDIP)	Relative methylation (Cy-5/Cy-3)	Cy-3 signal (Input)	Cy-5 signal (MeDIP)
A_68_P21902326	chr2:179960119-179960164	NM_001081171:423	Lama5	INSIDE	0.448	0.542	1267.02	686.26	0.243	999.45	242.55
A_68_P20725330	chr1:155033404-155033448	NM_008485:151	Lamc2	INSIDE	0.545	0.571	1269.63	725.03	0.311	1053.56	327.89
A_68_P20306026	chr1:67085213-67085257	NM_001190984:174	Lancl1	INSIDE	0.570	0.592	3031.06	1795.81	0.338	2374.70	801.63
A_68_P28225275	chr12:8928468-8928512	NM_008640:378	Laptm4a	INSIDE	0.150	0.633	2476.22	1568.69	0.095	1910.40	182.00
A_68_P28225282	chr12:8929275-8929319	NM_008640:1184	Laptm4a	INSIDE	0.207	0.455	2872.67	1306.34	0.094	2023.07	190.15
A_68_P25965604	chr8:75876534-75876578	NM_010687:-101	Large	PROMOTER	0.617	0.495	1383.28	684.59	0.306	1184.68	361.93
A_68_P25965607	chr8:75876888-75876932	NM_010687:-455	Large	PROMOTER	0.580	0.640	3075.41	1967.19	0.371	2662.94	987.24
A_68_P25965612	chr8:75877517-75877561	NM_010687:-1083	Large	PROMOTER	0.326	0.325	2752.12	893.29	0.106	1869.82	197.79
A_68_P27828607	chr11:57822062-57822109	NM_028451:-480	Larp1	PROMOTER	0.172	0.630	1418.58	893.35	0.108	1154.91	124.84
A_68_P22082254	chr3:40753323-40753367	NM_001040399:-1208	Larp1b	PROMOTER	0.223	0.727	2704.73	1966.74	0.162	2073.35	336.45
A_68_P22082267	chr3:40754901-40754949	NM_001040399:372	Larp1b	INSIDE	0.297	0.561	1498.78	841.48	0.167	1141.79	190.57
A_68_P30483165	chr15:99803409-99803453	NM_001024526:220	Larp4	INSIDE	0.385	0.684	2794.77	1912.37	0.263	2103.85	553.97
A_68_P28814398	chr13:9092754-9092798	NM_172585:-374	Larp4b	PROMOTER	0.579	0.462	2798.39	1293.94	0.268	2228.87	596.23
A_68_P26560493	chr9:60561252-60561296	NM_026235:347	Larp6	INSIDE	0.229	0.463	1618.21	749.42	0.106	1254.34	133.27
A_68_P28052727	chr11:97661201-97661245	NM_010688:237	Lasp1	INSIDE	0.247	0.663	921.86	611.42	0.164	725.01	118.93
A_68_P30482198	chr15:99602344-99602388	NM_028015:580	Lass5	INSIDE	0.147	0.660	1573.99	1039.52	0.097	1216.34	118.16
A_68_P30482203	chr15:99603119-99603163	NM_028015:-194	Lass5	PROMOTER	0.396	0.596	2632.96	1568.15	0.236	2144.25	506.20
A_68_P21305449	chr2:68699678-68699722	NM_172856:87	Lass6	INSIDE	0.089	0.714	2771.14	1978.10	0.063	1875.64	118.97
A_68_P31343005	chr17:73267359-73267403	NM_029999:-264	Lbh	PROMOTER	0.244	0.389	1741.25	676.88	0.095	1292.48	122.46
A_68_P32137135	chr19:45298271-45298326	NM_010691:11428	Lbx1	DOWNSTREAM	0.265	0.492	1426.47	701.76	0.130	962.12	125.49
A_68_P32137138	chr19:45298661-45298705	NM_010691:11044	Lbx1	DOWNSTREAM	0.268	0.588	1886.38	1108.70	0.158	1325.49	209.14
A_68_P24591351	chr6:83036388-83036432	NM_010692:52	Lbx2	INSIDE	0.104	0.672	2691.68	1809.04	0.070	1704.80	119.69
A_68_P26683680	chr9:83341498-83341543	NM_029434:-6786	Lca5	PROMOTER	0.560	0.624	910.87	568.13	0.349	741.85	258.92
A_68_P30991545	chr16:96413628-96413672	NM_001001492:214	Lca5l	INSIDE	0.260	0.598	1019.46	609.31	0.155	960.75	149.18
A_68_P30991547	chr16:96413886-96413930	NM_001001492:-44	Lca5l	DIVERGENT_PROMOTER	0.196	0.543	2338.07	1269.88	0.106	1744.40	185.29
A_68_P31344183	chr17:73457385-73457429	NM_001081071:82	Lclat1	INSIDE	0.523	0.321	1799.39	577.36	0.168	1490.87	250.21
A_68_P31344185	chr17:73457680-73457725	NM_001081071:378	Lclat1	INSIDE	0.096	0.605	2613.75	1580.26	0.058	2063.89	119.58
A_68_P25484147	chr7:130521434-130521478	NM_025304:-39	Lcmt1	DIVERGENT_PROMOTER	0.271	0.411	1585.90	652.59	0.111	1099.91	122.53
A_68_P25484148	chr7:130521551-130521596	NM_025304:78	Lcmt1	INSIDE	0.239	0.470	1227.30	577.17	0.112	1032.20	115.86
A_68_P23658787	chr5:46247794-46247838	NM_172153:-25	Lcorl	PROMOTER	0.148	0.525	4074.36	2140.18	0.078	2739.42	213.26
A_68_P25101649	chr7:54101500-54101544	NM_001136069:-929	Ldha	PROMOTER	0.164	0.529	1557.57	823.47	0.087	1395.24	120.98
A_68_P21475553	chr2:102026602-102026646	NM_178886:-90	Ldlrad3	PROMOTER	0.212	0.633	1564.80	990.89	0.134	1390.68	186.51
A_68_P22530170	chr3:130814530-130814579	NM_010703:1166	Lef1	INSIDE	0.169	0.713	1306.94	931.43	0.121	1001.02	120.63
A_68_P22201239	chr3:65462965-65463009	NM_001166659:-7163	Lekr1	PROMOTER	0.246	0.638	1854.25	1182.19	0.157	1453.43	228.11
A_68_P27500772	chr10:120415468-120415512	NM_001081193:896	Lemd3	INSIDE	0.620	0.574	977.29	561.25	0.356	794.67	282.79
A_68_P26644014	chr9:75289095-75289140	NM_001039522:-213	Leo1	PROMOTER	0.425	0.669	1036.55	693.78	0.284	843.34	239.68
A_68_P23156636	chr4:101390083-101390127	NM_010704:93	Lepr	INSIDE	0.457	0.542	1020.14	552.64	0.247	822.63	203.49
A_68_P24817384	chr6:124806960-124807011	NM_013534:720	Leprel2	INSIDE	0.163	0.560	1692.23	947.48	0.091	1288.16	117.63
A_68_P24817385	chr6:124807053-124807099	NM_013534:629	Leprel2	INSIDE	0.305	0.543	992.44	539.18	0.165	751.11	124.31
A_68_P25727978	chr8:26707398-26707452	NM_173012:535	Letm2	INSIDE	0.156	0.694	1541.18	1069.57	0.108	1101.85	119.26
A_68_P25727980	chr8:26707531-26707578	NM_173012:405	Letm2	INSIDE	0.185	0.690	1228.19	846.90	0.128	992.94	127.01
A_68_P25727984	chr8:26707935-26707980	NM_173012:2	Letm2	INSIDE	0.384	0.742	5217.74	3873.84	0.285	3736.09	1064.50
A_68_P24142214	chr5:141082926-141082970	NM_008494:-346	Lfng	PROMOTER	0.240	0.299	2280.33	682.81	0.072	1610.79	115.87
A_68_P30363244	chr15:78758585-78758629	NM_008495:1452	Lgals1	INSIDE	0.250	0.499	1257.39	627.81	0.125	961.17	120.08

ProbeName	Target position of probe on CpG island microarray	TargetID	GeneSymbol	CpG island Description	Ratio of relative methylation (TiO2-NP/Vehicle)	Sham group			TiO_2-H group		
						Relative methylation (Cy-5/Cy-3)	Cy-3 signal (Input)	Cy-5 signal (MeDIP)	Relative methylation (Cy-5/Cy-3)	Cy-3 signal (Input)	Cy-5 signal (MeDIP)
A_68_P29581906	chr14:47993698-47993742	NM_001145953:186	Lgals3	INSIDE	0.625	0.638	1990.11	1270.59	0.399	1817.17	725.14
A_68_P28832392	chr13:12553513-12553557	NM_001199043:471	Lgals8	INSIDE	0.577	0.611	1021.01	623.80	0.352	870.26	306.72
A_68_P28832394	chr13:12553847-12553891	NM_001199043:137	Lgals8	INSIDE	0.285	0.707	1827.52	1291.24	0.201	1343.56	270.44
A_68_P21517947	chr2:109757830-109757874	NM_172671:49	Lgr4	INSIDE	0.667	0.625	1149.45	718.29	0.417	966.96	402.85
A_68_P27470613	chr10:115024518-115024562	NM_010195:296	Lgr5	INSIDE	0.389	0.548	1972.97	1081.22	0.213	1512.60	322.24
A_68_P20636647	chr1:136884516-136884560	NM_001033409:117315	Lgr6	INSIDE	0.249	0.558	1194.18	665.95	0.139	948.90	131.66
A_68_P22139474	chr3:52845318-52845362	NM_175386:-128	Lhfp	PROMOTER	0.485	0.698	2128.02	1485.84	0.339	1611.47	545.91
A_68_P29233347	chr13:94944386-94944430	NM_172589:116658	Lhfpl2	INSIDE	0.348	0.730	2550.73	1860.93	0.254	1880.91	477.32
A_68_P24758095	chr6:113143886-113143930	NM_177763:1470	Lhfpl4	INSIDE	0.424	0.550	930.66	511.41	0.233	748.36	174.18
A_68_P31123144	chr17:28712407-28712451	NM_026571:-235	Lhfpl5	PROMOTER	0.306	0.555	1443.17	800.38	0.170	1195.70	202.74
A_68_P25535615	chr7:139802512-139802556	NM_029609:209	Lhpp	INSIDE	0.250	0.677	1632.46	1104.72	0.169	1279.92	216.22
A_68_P27977992	chr11:84338560-84338604	NM_008498:454	Lhx1	INSIDE	0.575	0.719	1637.36	1176.99	0.413	1242.14	513.35
A_68_P21147620	chr2:38204034-38204078	NM_010710:-2771	Lhx2	PROMOTER	0.214	0.698	1219.42	850.76	0.149	904.37	134.90
A_68_P21147671	chr2:38210043-38210094	NM_010710:3241	Lhx2	INSIDE	0.548	0.563	946.02	532.52	0.308	580.27	178.99
A_68_P21147673	chr2:38210263-38210310	NM_010710:3459	Lhx2	INSIDE	0.325	0.537	2109.82	1133.30	0.174	1665.38	290.44
A_68_P21147687	chr2:38212141-38212185	NM_010710:5335	Lhx2	INSIDE	0.155	0.638	2348.15	1498.43	0.099	1624.60	161.05
A_68_P21147691	chr2:38212503-38212547	NM_010710:5697	Lhx2	INSIDE	0.592	0.527	1144.24	602.93	0.312	948.15	295.77
A_68_P21077003	chr2:26057606-26057650	NM_001039653:4448	Lhx3	INSIDE	0.466	0.441	2104.38	927.16	0.205	1689.06	346.69
A_68_P21077040	chr2:26061806-26061850	NM_001039653:248	Lhx3	INSIDE	0.587	0.674	1325.00	892.57	0.395	1011.50	400.02
A_68_P20739566	chr1:157584400-157584444	NM_010712:4735	Lhx4	INSIDE	0.459	0.420	2129.12	894.97	0.193	1586.13	306.27
A_68_P20739601	chr1:157588425-157588469	NM_010712:711	Lhx4	INSIDE	0.202	0.494	1652.57	816.53	0.100	1243.00	123.79
A_68_P20739602	chr1:157588540-157588584	NM_010712:595	Lhx4	INSIDE	0.211	0.559	1290.80	721.36	0.118	1000.12	118.05
A_68_P20739604	chr1:157588731-157588781	NM_010712:401	Lhx4	INSIDE	0.264	0.577	1144.34	660.76	0.152	772.62	117.78
A_68_P20739614	chr1:157590081-157590125	NM_010712:-945	Lhx4	PROMOTER	0.472	0.703	5881.13	4136.58	0.332	4653.92	1546.13
A_68_P24038671	chr5:120881905-120881949	NM_008499:32	Lhx5	INSIDE	0.184	0.728	1770.78	1289.00	0.134	1363.93	182.33
A_68_P24038672	chr5:120882014-120882058	NM_008499:142	Lhx5	INSIDE	0.486	0.436	1538.10	670.10	0.212	1325.14	280.69
A_68_P24038673	chr5:120882123-120882167	NM_008499:250	Lhx5	INSIDE	0.166	0.539	1776.36	957.52	0.089	1337.60	119.65
A_68_P24038700	chr5:120885515-120885559	NM_008499:3642	Lhx5	INSIDE	0.541	0.456	4240.57	1934.99	0.247	3343.32	825.08
A_68_P24038701	chr5:120885606-120885650	NM_008499:3734	Lhx5	INSIDE	0.471	0.399	2019.20	806.60	0.188	1501.33	282.27
A_68_P24038702	chr5:120885755-120885799	NM_008499:3882	Lhx5	INSIDE	0.236	0.574	2246.26	1288.75	0.135	1720.35	232.70
A_68_P24038703	chr5:120885821-120885873	NM_008499:3952	Lhx5	INSIDE	0.233	0.532	1193.21	635.22	0.124	972.07	120.79
A_68_P24038737	chr5:120890387-120890431	NM_008499:8514	Lhx5	INSIDE	0.181	0.572	1899.66	1086.15	0.104	1449.40	150.04
A_68_P21137726	chr2:35946774-35946818	NM_001083126:12784	Lhx6	INSIDE	0.202	0.448	2055.55	920.94	0.091	1429.55	129.42
A_68_P21137750	chr2:35949963-35950016	NM_001083126:9591	Lhx6	INSIDE	0.168	0.657	1695.06	1113.25	0.110	1196.99	132.17
A_68_P21137822	chr2:35958973-35959017	NM_001083126:586	Lhx6	INSIDE	0.202	0.429	2004.19	860.63	0.087	1632.53	141.74
A_68_P21137823	chr2:35959065-35959110	NM_001083126:493	Lhx6	INSIDE	0.272	0.451	1462.87	659.90	0.123	981.61	120.30
A_68_P21137825	chr2:35959267-35959311	NM_001083126:292	Lhx6	INSIDE	0.183	0.726	2268.44	1646.77	0.133	1756.97	233.27
A_68_P21137835	chr2:35960385-35960443	NM_001083126:-834	Lhx6	PROMOTER	0.168	0.634	1477.68	937.11	0.106	1118.38	119.09
A_68_P21137836	chr2:35960496-35960540	NM_001083126:-938	Lhx6	PROMOTER	0.401	1.336	8929.66	11925.85	0.536	6249.44	3346.87
A_68_P22661991	chr3:153992609-153992654	NM_010713:893	Lhx8	INSIDE	0.250	0.520	1089.44	566.10	0.130	925.76	120.41
A_68_P28099907	chr11:106022041-106022085	NM_172397:-606	Limd2	PROMOTER	0.260	0.509	1120.65	570.89	0.133	885.89	117.42
A_68_P24112795	chr5:135164074-135164118	NM_010717:364	Limk1	INSIDE	0.408	0.682	5503.00	3753.40	0.279	3918.82	1091.53
A_68_P24112796	chr5:135164221-135164265	NM_010717:218	Limk1	INSIDE	0.245	0.638	1035.06	660.74	0.157	745.45	116.75
A_68_P27163119	chr10:57785941-57785985	NM_026148:-251	Lims1	PROMOTER	0.396	0.680	1897.17	1290.62	0.269	1413.46	380.81

ProbeName	Target position of probe on CpG island microarray	TargetID	GeneSymbol	CpG island Description	Ratio of relative methylation (TiO2-NP/Vehicle)	Sham group			TiO_2-H group		
						Relative methylation (Cy-5/Cy-3)	Cy-3 signal (Input)	Cy-5 signal (MeDIP)	Relative methylation (Cy-5/Cy-3)	Cy-3 signal (Input)	Cy-5 signal (MeDIP)
A_68_P25092713	chr7:52625245-52625289	NM_011698:668	Lin7b	INSIDE	0.328	0.646	1494.01	964.68	0.212	1244.88	264.01
A_68_P25092716	chr7:52625642-52625686	NM_011698:270	Lin7b	INSIDE	0.140	0.512	2044.28	1047.66	0.072	1622.61	116.58
A_68_P22843571	chr4:36897285-36897329	NM_001165999:1471	Lingo2	INSIDE	0.282	0.649	1776.84	1152.69	0.183	1408.41	258.00
A_68_P25010078	chr7:26165068-26165112	NM_001039507:10035	Lipe	INSIDE	0.439	0.459	1245.19	571.35	0.201	898.86	181.09
A_68_P28155559	chr11:115685801-115685845	NM_145438:451	Llgl2	INSIDE	0.613	0.546	2563.04	1399.14	0.334	1948.82	651.73
A_68_P28155560	chr11:115685911-115685955	NM_145438:561	Llgl2	INSIDE	0.178	0.519	2565.41	1330.25	0.092	1869.48	172.17
A_68_P28155820	chr11:115719994-115720038	NM_145438:34645	Llgl2	DOWNSTREAM	0.386	0.695	1326.79	922.46	0.268	1040.21	279.24
A_68_P27495998	chr10:119664180-119664224	NM_025431:87	Llph	INSIDE	0.424	0.414	1261.65	522.47	0.176	993.23	174.57
A_68_P31784506	chr18:66162058-66162102	NM_027400:209	Lman1	INSIDE	0.354	0.575	1069.30	614.96	0.204	917.62	186.92
A_68_P31784508	chr18:66162318-66162362	NM_027400:-51	Lman1	PROMOTER	0.293	0.509	1077.33	548.48	0.149	878.66	130.92
A_68_P29054563	chr13:55463630-55463674	NM_025828:492	Lman2	INSIDE	0.534	0.582	2594.81	1511.19	0.311	1910.63	594.39
A_68_P20148353	chr1:36501896-36501940	NM_001013374:166	Lman2l	INSIDE	0.181	0.726	2408.16	1749.22	0.132	1650.16	217.32
A_68_P30477339	chr15:98748465-98748509	NM_029098:43	Lmbr1l	INSIDE	0.257	0.535	1280.29	684.76	0.138	1047.75	144.24
A_68_P30004453	chr15:9070451-9070495	NM_177178:147	Lmbrd2	INSIDE	0.245	0.418	1601.35	669.70	0.102	1191.30	121.89
A_68_P31104860	chr17:25716044-25716088	NM_029624:-52	Lmf1	DIVERGENT_PROMOTER	0.182	0.493	1763.91	870.04	0.090	1406.46	126.53
A_68_P31104861	chr17:25716207-25716251	NM_029624:110	Lmf1	INSIDE	0.177	0.492	2633.19	1294.44	0.087	1919.27	167.02
A_68_P31104862	chr17:25716282-25716326	NM_029624:186	Lmf1	INSIDE	0.577	0.708	1309.45	926.46	0.409	987.70	403.56
A_68_P30424715	chr15:89185807-89185851	NM_178919:262	Lmf2	INSIDE	0.555	0.665	1547.05	1028.76	0.369	1238.97	457.63
A_68_P30424716	chr15:89185877-89185921	NM_178919:192	Lmf2	INSIDE	0.273	0.364	1616.69	588.23	0.099	1185.44	117.79
A_68_P30656039	chr16:33062880-33062924	NM_172823:296	Lmln	INSIDE	0.605	0.441	2475.71	1090.93	0.266	1927.64	513.59
A_68_P31731707	chr18:56868451-56868495	NM_010721:1006	Lmnb1	INSIDE	0.522	0.653	1130.13	737.47	0.340	888.28	302.36
A_68_P27286341	chr10:80380635-80380679	NM_010722:334	Lmnb2	INSIDE	0.472	0.487	1673.95	814.70	0.230	1361.95	312.94
A_68_P27286342	chr10:80380790-80380834	NM_010722:178	Lmnb2	INSIDE	0.621	0.559	1287.32	720.17	0.347	1064.87	369.65
A_68_P21486502	chr2:103810841-103810885	NM_001142335:419	Lmo2	INSIDE	0.582	0.502	2107.53	1058.46	0.292	1592.02	465.02
A_68_P22601419	chr3:143865258-143865302	NM_001161770:16	Lmo4	INSIDE	0.156	0.603	2736.89	1650.83	0.094	1758.72	165.69
A_68_P29856566	chr14:102128766-102128810	NM_201529:-356	Lmo7	PROMOTER	0.506	0.395	1807.39	714.19	0.200	1485.16	296.82
A_68_P29856567	chr14:102128847-102128891	NM_201529:-276	Lmo7	PROMOTER	0.248	0.566	1138.18	644.40	0.140	966.86	135.63
A_68_P29856570	chr14:102129092-102129136	NM_201529:-30	Lmo7	PROMOTER	0.656	0.543	1325.98	719.70	0.356	1049.77	373.79
A_68_P20806518	chr1:169618425-169618469	NM_033652:-1242	Lmx1a	PROMOTER	0.353	0.668	873.48	583.44	0.236	656.47	154.73
A_68_P20806524	chr1:169619169-169619217	NM_033652:-496	Lmx1a	PROMOTER	0.481	0.492	1224.37	602.89	0.237	1036.42	245.71
A_68_P21122318	chr2:33436169-33436213	NM_010725:59841	Lmx1b	INSIDE	0.181	0.541	1521.56	822.65	0.098	1185.10	115.98
A_68_P21122320	chr2:33436378-33436430	NM_010725:59627	Lmx1b	INSIDE	0.157	0.511	2344.33	1197.65	0.080	1504.80	120.45
A_68_P21122321	chr2:33436455-33436499	NM_010725:59555	Lmx1b	INSIDE	0.240	0.472	1498.85	707.39	0.113	1062.56	120.34
A_68_P21122681	chr2:33487222-33487266	NM_010725:8787	Lmx1b	INSIDE	0.341	0.593	1550.97	919.68	0.202	1177.14	238.06
A_68_P21122747	chr2:33495004-33495053	NM_010725:1003	Lmx1b	INSIDE	0.202	0.543	1509.64	820.36	0.110	1078.16	118.27
A_68_P21122748	chr2:33495093-33495137	NM_010725:917	Lmx1b	INSIDE	0.386	0.530	1146.85	607.31	0.204	857.04	175.20
A_68_P21122757	chr2:33496008-33496052	NM_010725:1	Lmx1b	INSIDE	0.178	0.495	1718.67	851.39	0.088	1333.11	117.87
A_68_P21122772	chr2:33498002-33498046	NM_010725:-1993	Lmx1b	PROMOTER	0.316	0.547	2287.78	1251.52	0.173	1801.96	311.68
A_68_P21338177	chr2:74416496-74416545	NM_001110209:485	Lnp	INSIDE	0.182	0.694	1429.85	991.99	0.126	1201.84	151.70
A_68_P31074422	chr17:17761131-17761175	NM_172827:301	Lnpep	INSIDE	0.217	0.675	3437.73	2320.19	0.147	2441.17	357.67
A_68_P31074423	chr17:17761336-17761380	NM_172827:95	Lnpep	INSIDE	0.358	0.465	2106.22	978.49	0.166	1721.33	286.52
A_68_P31074431	chr17:17762209-17762253	NM_172827:-777	Lnpep	PROMOTER	0.318	0.525	2919.16	1531.48	0.167	2095.34	349.74
A_68_P31074433	chr17:17762462-17762506	NM_172827:-1031	Lnpep	PROMOTER	0.096	1.541	4977.27	7669.21	0.149	3293.10	489.09
A_68_P24171751	chr5:147888207-147888251	NM_080795:-80	Lnx2	DIVERGENT_PROMOTER	0.367	0.432	1415.22	610.72	0.159	1052.78	166.95

ProbeName	Target position of probe on CpG island microarray	TargetID	GeneSymbol	CpG island Description	Ratio of relative methylation (TiO2-NP/Vehicle)	Sham group			TiO_2-H group		
						Relative methylation (Cy-5/Cy-3)	Cy-3 signal (Input)	Cy-5 signal (MeDIP)	Relative methylation (Cy-5/Cy-3)	Cy-3 signal (Input)	Cy-5 signal (MeDIP)
A_68_P21037807	chr2:17949322-17949366	NR_028378:469	LOC100034739	INSIDE	0.199	0.653	1963.96	1282.65	0.130	1479.02	192.21
A_68_P20353555	chr1:75366170-75366222	NR_037976:5394	LOC100503248	INSIDE	0.361	1.945	2112.18	4107.44	0.702	1463.37	1026.86
A_68_P27533640	chr10:126478229-126478273	NM_001205036:200	LOC100504608	INSIDE	0.282	0.361	2077.96	751.15	0.102	1620.47	165.27
A_68_P31257378	chr17:56765895-56765939	NM_028782:410	Lonp1	INSIDE	0.226	0.533	1370.49	730.40	0.121	1002.40	120.89
A_68_P32318077	chrX:33870275-33870326	NM_028894:1897	Lonrf3	INSIDE	0.168	1.844	973.19	1794.96	0.309	378.95	117.19
A_68_P31708194	chr18:52689223-52689267	NM_010728:118	Lox	INSIDE	0.256	0.639	1116.91	714.20	0.164	922.54	151.29
A_68_P29696147	chr14:70008844-70008888	NM_033325:-416	Loxl2	PROMOTER	0.387	1.695	4811.56	8155.31	0.656	3027.77	1985.36
A_68_P29696148	chr14:70008926-70008970	NM_033325:-334	Loxl2	PROMOTER	0.053	1.760	4525.86	7966.21	0.092	2920.66	269.90
A_68_P29696150	chr14:70009101-70009145	NM_033325:-160	Loxl2	PROMOTER	0.197	0.598	4983.34	2978.11	0.118	3416.03	401.66
A_68_P29696151	chr14:70009216-70009260	NM_033325:-44	Loxl2	PROMOTER	0.185	0.694	2406.26	1671.06	0.128	1801.19	231.32
A_68_P32122223	chr19:42674559-42674603	NM_001164311:12716	Loxl4	INSIDE	0.390	1.433	1197.83	1716.78	0.559	945.09	528.24
A_68_P22951805	chr4:58566279-58566323	NM_172989:-135	Lpar1	PROMOTER	0.325	0.581	1485.20	862.66	0.189	1231.58	232.16
A_68_P26068286	chr8:95379269-95379313	NM_173014:42	Lpcat2	INSIDE	0.298	0.635	3188.41	2024.16	0.189	2268.79	429.12
A_68_P24816264	chr6:124612578-124612622	NM_145130:-521	Lpcat3	PROMOTER	0.283	0.591	1112.55	657.42	0.167	968.93	162.25
A_68_P24816270	chr6:124613220-124613264	NM_145130:121	Lpcat3	INSIDE	0.485	0.680	2281.06	1550.91	0.330	1601.01	528.04
A_68_P24816271	chr6:124613291-124613335	NM_145130:191	Lpcat3	INSIDE	0.067	0.552	4653.68	2567.20	0.037	3141.69	116.63
A_68_P26016963	chr8:86423548-86423592	NM_181039:-426	Lphn1	PROMOTER	0.211	1.819	1385.72	2520.14	0.384	862.35	330.90
A_68_P26016964	chr8:86423687-86423732	NM_181039:-287	Lphn1	PROMOTER	0.213	0.567	3680.21	2085.07	0.121	2727.19	329.79
A_68_P23839619	chr5:81758936-81758980	NM_198702:308341	Lphn3	INSIDE	0.396	1.822	2298.64	4187.16	0.722	1764.06	1273.14
A_68_P23842572	chr5:82223907-82223953	NM_198702:773313	Lphn3	INSIDE	0.596	2.820	711.71	2007.26	1.681	568.12	954.97
A_68_P27996401	chr11:87630681-87630732	NM_080420:8910	Lpo	INSIDE	0.297	0.421	1352.36	569.03	0.125	910.85	113.94
A_68_P22281944	chr3:82708169-82708225	NM_023624:-300	Lrat	PROMOTER	0.232	0.448	1438.07	644.30	0.104	1180.35	122.71
A_68_P22301529	chr3:86028379-86028423	NM_001077687:-211	Lrba	PROMOTER	0.386	0.501	2111.14	1058.28	0.193	1730.67	334.87
A_68_P29725078	chr14:75347229-75347273	NM_001033439:434	Lrch1	INSIDE	0.382	0.517	1295.66	669.60	0.198	1038.95	205.20
A_68_P29725079	chr14:75347435-75347479	NM_001033439:228	Lrch1	INSIDE	0.635	0.681	2126.09	1447.08	0.432	1683.23	727.52
A_68_P32730440	chrX:143988591-143988635	NM_001081173:12	Lrch2	INSIDE	0.062	2.353	2924.30	6880.77	0.146	1103.33	161.60
A_68_P30655247	chr16:32914173-32914217	NM_001081255:9	Lrch3	INSIDE	0.197	0.574	1413.09	811.09	0.113	1289.46	146.07
A_68_P24126716	chr5:138070623-138070667	NM_001168652:294	Lrch4	INSIDE	0.107	0.690	2647.20	1825.83	0.074	1832.18	135.32
A_68_P24126717	chr5:138070748-138070792	NM_001168652:420	Lrch4	INSIDE	0.239	0.619	3670.65	2271.46	0.148	2544.93	377.02
A_68_P31220005	chr17:49071091-49071135	NM_027452:-794	Lrfn2	PROMOTER	0.428	0.459	1700.17	780.47	0.196	1290.14	253.30
A_68_P31220014	chr17:49072235-49072279	NM_027452:350	Lrfn2	INSIDE	0.514	0.570	1560.05	889.24	0.293	1206.37	353.68
A_68_P28480019	chr12:62623999-62624043	NM_178714:-1598	Lrfn5	PROMOTER	0.107	0.697	2153.58	1500.76	0.075	1554.05	116.10
A_68_P31253992	chr17:56260257-56260301	NM_029796:1091	Lrg1	INSIDE	0.232	0.475	1515.77	720.31	0.110	1143.42	125.89
A_68_P24658386	chr6:94650061-94650105	NM_008377:57	Lrig1	INSIDE	0.467	0.722	2034.01	1468.57	0.337	1755.80	592.25
A_68_P22391857	chr3:104315282-104315333	NM_001025067:472	Lrig2	INSIDE	0.154	0.657	1835.53	1206.21	0.101	1503.48	152.02
A_68_P27528049	chr10:125403615-125403659	NM_177152:362	Lrig3	INSIDE	0.653	0.628	1397.99	877.79	0.410	1190.24	488.34
A_68_P27536901	chr10:127030734-127030787	NM_008512:27444	Lrp1	INSIDE	0.629	2.985	1284.50	3833.85	1.877	943.04	1770.01
A_68_P27536903	chr10:127031018-127031062	NM_008512:27164	Lrp1	INSIDE	0.652	2.582	1667.49	4305.84	1.683	1242.84	2091.36
A_68_P29612545	chr14:55082897-55082941	NM_022993:-65	Lrp10	PROMOTER	0.492	0.465	1962.67	912.81	0.229	1527.36	349.52
A_68_P21309972	chr2:69423381-69423427	NM_001081088:720	Lrp2	INSIDE	0.289	0.553	1038.24	574.28	0.160	761.29	121.55
A_68_P21309978	chr2:69424022-69424066	NM_001081088:80	Lrp2	INSIDE	0.361	0.507	1148.82	582.30	0.183	912.84	166.90
A_68_P21309980	chr2:69424250-69424294	NM_001081088:-148	Lrp2	PROMOTER	0.419	0.613	2816.66	1727.82	0.257	2232.51	574.45
A_68_P23192850	chr4:107475564-107475608	NM_001080926:723	Lrp8	INSIDE	0.354	0.541	1025.28	554.32	0.192	940.55	180.21
A_68_P23192855	chr4:107476214-107476258	NM_001080926:1373	Lrp8	INSIDE	0.185	0.344	2441.43	840.11	0.064	1828.68	116.58

ProbeName	Target position of probe on CpG island microarray	TargetID	GeneSymbol	CpG island Description	Ratio of relative methylation (TiO2-NP/Vehicle)	Sham group			TiO_2-H group		
						Relative methylation (Cy-5/Cy-3)	Cy-3 signal (Input)	Cy-5 signal (MeDIP)	Relative methylation (Cy-5/Cy-3)	Cy-3 signal (Input)	Cy-5 signal (MeDIP)
A_68_P23192856	chr4:107476288-107476332	NM_001080926:1447	Lrp8	INSIDE	0.493	0.692	1614.87	1117.23	0.341	1128.58	385.27
A_68_P23598367	chr5:35448339-35448383	NM_013587:-14	Lrpap1	PROMOTER	0.230	0.573	1196.14	685.54	0.132	920.87	121.64
A_68_P23598368	chr5:35448526-35448576	NM_013587:-204	Lrpap1	PROMOTER	0.175	0.675	1419.04	957.73	0.118	968.39	114.65
A_68_P31406961	chr17:85189534-85189578	NM_028233:570	Lrpprc	INSIDE	0.651	0.624	1016.38	634.34	0.407	766.49	311.60
A_68_P28513154	chr12:70269870-70269914	NM_001081406:92	Lrr1	INSIDE	0.360	0.440	1601.40	703.86	0.158	1276.28	202.13
A_68_P28513155	chr12:70269942-70269986	NM_001081406:164	Lrr1	INSIDE	0.563	0.646	1595.59	1030.61	0.364	1213.98	441.28
A_68_P30350131	chr15:76541158-76541202	NM_145471:11	Lrrc14	INSIDE	0.322	0.642	2574.57	1652.84	0.207	1800.54	372.39
A_68_P26826863	chr9:110856302-110856347	NM_028838:2276	Lrrc2	INSIDE	0.432	0.537	1024.14	549.76	0.232	762.17	176.69
A_68_P27180700	chr10:60938374-60938418	NM_153542:-184	Lrrc20	PROMOTER	0.269	0.603	1258.02	758.07	0.162	994.79	161.06
A_68_P30350168	chr15:76546311-76546355	NM_198119:6271	Lrrc24	INSIDE	0.419	0.423	1576.40	666.84	0.177	1146.32	203.17
A_68_P30350212	chr15:76553038-76553082	NM_198119:-457	Lrrc24	PROMOTER	0.366	0.490	1781.72	873.53	0.180	1217.47	218.75
A_68_P25576399	chr7:146398944-146398988	NM_001143755:-207	Lrrc27	PROMOTER	0.596	0.649	991.03	643.50	0.387	789.55	305.75
A_68_P27270904	chr10:77364916-77364963	NM_145152:342	Lrrc3	INSIDE	0.505	0.572	1069.92	612.22	0.289	833.59	240.67
A_68_P23374709	chr4:142939818-142939864	NM_001162983:188	Lrrc38	INSIDE	0.321	0.588	916.58	538.54	0.188	728.77	137.35
A_68_P24322954	chr6:28782175-28782219	NM_138682:-449	Lrrc4	PROMOTER	0.542	0.515	1533.76	790.29	0.280	1204.87	336.76
A_68_P22683292	chr3:157699623-157699667	NM_024194:-22	Lrrc40	DIVERGENT_PROMOTER	0.251	0.412	2396.71	987.11	0.103	1828.22	188.88
A_68_P22683294	chr3:157699823-157699867	NM_024194:178	Lrrc40	INSIDE	0.560	0.487	2499.03	1217.62	0.273	2043.10	557.29
A_68_P24055951	chr5:123939417-123939461	NM_001033461:105	Lrrc43	INSIDE	0.334	0.464	2379.21	1103.66	0.155	1826.63	283.02
A_68_P28048328	chr11:96902741-96902785	NM_027026:-79	Lrrc46	DIVERGENT_PROMOTER	0.363	0.485	1848.56	896.38	0.176	1751.10	308.14
A_68_P26214833	chr8:122099237-122099281	NM_026648:124	Lrrc50	INSIDE	0.354	0.650	2180.92	1416.83	0.230	1885.31	433.04
A_68_P25376177	chr7:109082594-109082648	NM_001162973:-249	Lrrc51	PROMOTER	0.250	0.657	1623.44	1066.72	0.164	1236.97	202.92
A_68_P25587299	chr7:148380231-148380275	NM_153777:-789	Lrrc56	PROMOTER	0.140	0.608	1949.57	1184.88	0.085	1391.28	118.56
A_68_P25587300	chr7:148380302-148380347	NM_153777:-717	Lrrc56	PROMOTER	0.342	0.583	3378.94	1968.59	0.199	2386.53	474.93
A_68_P21576250	chr2:120434989-120435033	NM_025657:41	Lrrc57	INSIDE	0.514	0.469	1152.32	540.37	0.241	995.00	239.74
A_68_P30682961	chr16:37869139-37869183	NM_177093:675	Lrrc58	INSIDE	0.347	0.540	2018.30	1088.96	0.187	1581.02	295.65
A_68_P28033987	chr11:94490892-94490936	NM_133807:-223	Lrrc59	PROMOTER	0.346	0.467	1648.54	769.91	0.162	1465.96	236.99
A_68_P28033990	chr11:94491386-94491430	NM_133807:271	Lrrc59	INSIDE	0.094	0.437	4001.82	1747.53	0.041	2873.50	118.49
A_68_P24427997	chr6:48504680-48504727	NM_001110160:-94	Lrrc61	PROMOTER	0.218	0.455	1528.41	695.21	0.099	1205.06	119.72
A_68_P29726036	chr14:75530896-75530940	NM_027581:-228	Lrrc63	DIVERGENT_PROMOTER	0.261	0.612	1030.64	630.82	0.160	946.64	151.42
A_68_P20025036	chr1:9957153-9957197	NM_145692:42027	Lrrc67	DOWNSTREAM	0.174	0.703	1286.63	905.00	0.123	993.52	121.82
A_68_P20025044	chr1:9958133-9958177	NM_145692:41047	Lrrc67	DOWNSTREAM	0.147	0.458	2357.32	1079.34	0.067	1700.36	114.20
A_68_P24994037	chr7:20147028-20147077	NM_199149:695	Lrrc68	INSIDE	0.364	0.492	1095.27	538.91	0.179	832.16	149.23
A_68_P24994044	chr7:20147793-20147837	NM_199149:-67	Lrrc68	PROMOTER	0.346	0.375	1459.04	547.60	0.130	1273.98	165.40
A_68_P23955160	chr5:105845255-105845299	NM_001033550:483	Lrrc8b	INSIDE	0.211	0.721	3690.54	2661.87	0.152	2789.03	423.94
A_68_P23955793	chr5:105948778-105948822	NM_133897:311	Lrrc8c	INSIDE	0.213	0.481	1484.06	714.32	0.103	1271.43	130.44
A_68_P23955794	chr5:105948943-105948987	NM_133897:475	Lrrc8c	INSIDE	0.280	0.579	1273.90	737.70	0.162	967.85	156.87
A_68_P23956788	chr5:106129546-106129590	NM_178701:-260	Lrrc8d	PROMOTER	0.123	0.655	1992.46	1305.40	0.080	1507.63	121.00
A_68_P23956961	chr5:106161428-106161472	NM_178701:31622	Lrrc8d	INSIDE	0.292	1.627	5444.91	8860.01	0.475	3636.37	1726.04
A_68_P21957225	chr3:14533992-14534036	NM_001163579:227	Lrrcc1	INSIDE	0.602	0.606	2893.89	1754.91	0.365	2195.12	801.46
A_68_P20440038	chr1:92949235-92949279	NM_001111311:-764	Lrrfip1	PROMOTER	0.246	0.534	1239.90	662.04	0.132	1002.65	131.96
A_68_P20440041	chr1:92949532-92949576	NM_001111311:-466	Lrrfip1	PROMOTER	0.185	0.693	1236.30	856.88	0.128	963.01	123.18
A_68_P26827665	chr9:111020395-111020439	NM_001164838:-198	Lrrfip2	PROMOTER	0.202	0.716	2023.28	1449.50	0.144	1379.14	199.20
A_68_P25187992	chr7:73532448-73532492	NM_146191:757	Lrrk1	INSIDE	0.308	0.500	1378.83	690.10	0.154	1171.14	180.40
A_68_P25187998	chr7:73533035-73533079	NM_146191:171	Lrrk1	INSIDE	0.619	0.453	2351.17	1064.01	0.280	2026.61	567.49

ProbeName	Target position of probe on CpG island microarray	TargetID	GeneSymbol	CpG island Description	Ratio of relative methylation (TiO2-NP/Vehicle)	Sham group			TiO_2-H group		
						Relative methylation (Cy-5/Cy-3)	Cy-3 signal (Input)	Cy-5 signal (MeDIP)	Relative methylation (Cy-5/Cy-3)	Cy-3 signal (Input)	Cy-5 signal (MeDIP)
A_68_P24728933	chr6:107479987-107480031	NM_008516:289	Lrrn1	INSIDE	0.509	0.571	2352.89	1344.39	0.291	1889.07	549.00
A_68_P20624273	chr1:134776795-134776839	NM_010732:-115	Lrrn2	PROMOTER	0.240	0.656	1544.42	1013.26	0.157	1141.21	179.35
A_68_P20624276	chr1:134777196-134777240	NM_010732:287	Lrrn2	INSIDE	0.544	0.629	1327.89	835.28	0.342	1101.54	376.70
A_68_P21642726	chr2:132704942-132704986	NM_177303:1634	Lrrn4	INSIDE	0.156	0.508	1990.75	1011.60	0.079	1456.84	115.15
A_68_P24563510	chr6:77193700-77193744	NM_028880:1012	Lrrtm1	INSIDE	0.305	0.625	2735.05	1709.24	0.191	2116.18	403.91
A_68_P24563511	chr6:77193808-77193857	NM_028880:1122	Lrrtm1	INSIDE	0.297	0.467	1187.23	554.94	0.139	858.30	119.33
A_68_P24563513	chr6:77193982-77194026	NM_028880:1294	Lrrtm1	INSIDE	0.177	0.457	1945.42	888.59	0.081	1482.37	119.94
A_68_P24563520	chr6:77194801-77194845	NM_028880:2112	Lrrtm1	INSIDE	0.229	0.567	1769.99	1003.29	0.130	1170.13	152.11
A_68_P24792499	chr6:119280504-119280548	NM_001172207:-1304	Lrtm2	PROMOTER	0.396	0.523	1726.40	903.05	0.207	1158.45	239.74
A_68_P24119630	chr5:136611720-136611770	NM_027891:200	Lrwd1	INSIDE	0.219	0.670	2503.87	1678.03	0.147	1750.50	256.69
A_68_P24119632	chr5:136611907-136611951	NM_027891:16	Lrwd1	INSIDE	0.180	0.435	2165.95	942.65	0.078	1614.95	126.51
A_68_P24119634	chr5:136612073-136612117	NM_027891:-150	Lrwd1	DIVERGENT_PROMOTER	0.246	0.500	1502.65	751.90	0.123	1087.69	133.96
A_68_P23278139	chr4:125773830-125773874	NM_001163266:-44	Lsm10	PROMOTER	0.428	0.502	1899.41	953.03	0.215	1425.24	306.22
A_68_P27765087	chr11:45757827-45757871	NM_028185:589	Lsm11	INSIDE	0.513	0.548	1017.78	557.34	0.281	781.41	219.55
A_68_P27765088	chr11:45757957-45758001	NM_028185:459	Lsm11	INSIDE	0.242	0.377	1609.20	606.79	0.091	1316.93	120.41
A_68_P27765091	chr11:45758223-45758267	NM_028185:193	Lsm11	INSIDE	0.573	0.376	1929.95	725.05	0.215	1567.62	337.36
A_68_P21900915	chr2:179759580-179759624	NM_177727:-89	Lsm14b	PROMOTER	0.607	0.659	901.20	593.66	0.400	874.32	349.57
A_68_P31156873	chr17:35118675-35118726	NM_001204274:-98	Lsm2	PROMOTER	0.343	0.633	1144.79	724.59	0.217	836.98	181.68
A_68_P31156874	chr17:35118802-35118846	NM_001204273:-250	Lsm2	PROMOTER	0.286	0.428	2081.23	890.56	0.122	1752.74	214.56
A_68_P31156878	chr17:35119267-35119311	NM_001204273:214	Lsm2	INSIDE	0.211	0.426	2163.90	921.01	0.090	1727.66	155.21
A_68_P24639527	chr6:91466074-91466119	NM_026309:68	Lsm3	INSIDE	0.214	0.481	1591.63	765.80	0.103	1182.52	121.51
A_68_P25953303	chr8:73197017-73197061	NM_015816:-91	Lsm4	PROMOTER	0.190	0.633	3049.94	1931.25	0.120	2058.76	247.77
A_68_P27285906	chr10:80317819-80317863	NM_025349:114	Lsm7	INSIDE	0.500	0.562	964.30	541.89	0.281	725.20	203.91
A_68_P27353694	chr10:92916374-92916418	NM_008517:256	Lta4h	INSIDE	0.281	0.461	1176.69	542.62	0.130	913.09	118.37
A_68_P31158189	chr17:35332625-35332669	NM_008518:1195	Ltb	INSIDE	0.267	0.396	1615.19	639.59	0.106	1192.92	126.15
A_68_P31158190	chr17:35332751-35332795	NM_008518:1321	Ltb	INSIDE	0.512	0.484	1166.87	564.21	0.248	895.35	221.65
A_68_P31930561	chr19:5742058-5742102	NM_008520:1177	Ltbp3	INSIDE	0.463	0.603	951.02	573.22	0.279	751.16	209.65
A_68_P25016713	chr7:28114386-28114430	NM_001113549:4259	Ltbp4	INSIDE	0.550	0.724	1877.58	1359.86	0.398	1365.19	543.97
A_68_P25016725	chr7:28115897-28115945	NM_001113549:2747	Ltbp4	INSIDE	0.558	0.616	1201.99	740.92	0.344	1039.46	357.39
A_68_P21571366	chr2:119584202-119584246	NM_008523:37	Ltk	INSIDE	0.570	0.636	1211.98	771.39	0.363	1037.16	376.53
A_68_P21571367	chr2:119584322-119584366	NM_008523:-83	Ltk	PROMOTER	0.397	0.476	1630.05	775.10	0.189	1333.23	251.49
A_68_P30939710	chr16:87432281-87432325	NM_001081068:549	Ltn1	INSIDE	0.366	0.682	1974.27	1347.44	0.250	1663.64	415.75
A_68_P31109175	chr17:26390511-26390556	NM_025881:679	Luc7l	INSIDE	0.153	0.671	1465.52	983.62	0.103	1192.28	122.58
A_68_P24377897	chr6:38501394-38501438	NM_001170849:-27	Luc7l2	PROMOTER	0.467	0.536	2359.55	1265.25	0.250	2165.71	541.85
A_68_P28032108	chr11:94182771-94182815	NM_026313:433	Luc7l3	INSIDE	0.328	1.743	7729.97	13474.87	0.571	4792.05	2736.04
A_68_P28032110	chr11:94183065-94183109	NM_026313:139	Luc7l3	INSIDE	0.532	0.714	1852.21	1323.37	0.380	1152.22	438.26
A_68_P28032111	chr11:94183206-94183250	NM_026313:-3	Luc7l3	DIVERGENT_PROMOTER	0.383	0.581	1257.23	730.56	0.223	890.55	198.18
A_68_P23334048	chr4:136018725-136018769	NM_024452:-6929	Luzp1	PROMOTER	0.277	0.553	1038.17	573.81	0.153	789.09	120.97
A_68_P22210126	chr3:67267629-67267673	NM_016753:179	Lxn	INSIDE	0.131	1.604	3191.32	5120.21	0.210	2275.08	476.80
A_68_P22210127	chr3:67267701-67267745	NM_016753:107	Lxn	INSIDE	0.157	1.760	6664.05	11727.54	0.276	4566.69	1259.04
A_68_P30340467	chr15:74786292-74786336	NM_001164040:779	Ly6e	INSIDE	0.282	0.517	1075.37	555.47	0.146	812.49	118.44
A_68_P21258085	chr2:60221190-60221234	NM_013825:76	Ly75	INSIDE	0.317	0.518	3628.14	1880.21	0.164	2695.63	442.78
A_68_P26021275	chr8:87229477-87229521	NM_008535:4143	Lyl1	DOWNSTREAM	0.268	1.834	5704.28	10461.67	0.492	3414.91	1680.44
A_68_P22693714	chr4:3605437-3605481	NM_001111096:191	Lyn	INSIDE	0.565	0.542	1002.80	543.31	0.306	827.53	253.41

ProbeName	Target position of probe on CpG island microarray	TargetID	GeneSymbol	CpG island Description	Ratio of relative methylation (TiO2-NP/Vehicle)	Sham group			TiO_2-H group		
						Relative methylation (Cy-5/Cy-3)	Cy-3 signal (Input)	Cy-5 signal (MeDIP)	Relative methylation (Cy-5/Cy-3)	Cy-3 signal (Input)	Cy-5 signal (MeDIP)
A_68_P20587181	chr1:127808737-127808781	NM_145100:33	Lypd1	INSIDE	0.099	1.824	2114.75	3858.07	0.181	1318.43	239.07
A_68_P23331511	chr4:135528428-135528472	NM_011942:59	Lypla2	INSIDE	0.392	0.473	1769.63	837.26	0.185	1552.06	287.58
A_68_P20905055	chr1:187941024-187941068	NM_146106:143	Lyplal1	INSIDE	0.519	0.471	2071.17	974.55	0.244	1586.16	387.40
A_68_P22823553	chr4:32887074-32887124	NM_175364:-135	Lyrm2	PROMOTER	0.439	0.454	1133.03	514.86	0.200	944.85	188.67
A_68_P27812424	chr11:54674270-54674317	NM_029327:-200	Lyrm7	DIVERGENT_PROMOTER	0.201	0.614	1145.31	703.43	0.123	973.58	120.06
A_68_P28834522	chr13:13682471-13682515	NM_010748:-183	Lyst	PROMOTER	0.161	0.482	2052.21	988.96	0.078	1560.66	121.27
A_68_P26884585	chr9:121550768-121550812	NM_026915:-106	Lyzl4	DIVERGENT_PROMOTER	0.392	0.586	1165.46	682.57	0.229	885.62	203.12
A_68_P26884586	chr9:121550854-121550898	NM_026915:-192	Lyzl4	DIVERGENT_PROMOTER	0.296	0.488	2617.71	1278.25	0.145	1836.94	265.46
A_68_P26884587	chr9:121550968-121551014	NM_026915:-306	Lyzl4	DIVERGENT_PROMOTER	0.134	0.413	3297.10	1363.09	0.055	2095.23	115.81
A_68_P26884588	chr9:121551071-121551115	NM_026915:-408	Lyzl4	DIVERGENT_PROMOTER	0.244	0.645	1522.36	981.64	0.158	1127.33	177.62
A_68_P23400758	chr4:148859457-148859501	NM_026963:37	Lzic	INSIDE	0.252	0.559	1110.84	621.30	0.141	857.81	120.70
A_68_P26896522	chr9:123626490-123626534	NM_033322:163	Lztfl1	INSIDE	0.209	0.509	1557.42	793.18	0.106	1194.78	127.24
A_68_P32135887	chr19:45090142-45090186	NM_001130526:-2460	Lzts2	PROMOTER	0.412	0.691	1347.91	930.96	0.285	1100.25	313.42
A_68_P22303234	chr3:86350689-86350733	NM_011839:1495	Mab21l2	INSIDE	0.371	0.692	3024.69	2091.84	0.257	2148.70	552.00
A_68_P22303238	chr3:86351126-86351170	NM_011839:1057	Mab21l2	INSIDE	0.658	0.321	1866.19	598.95	0.211	1307.52	276.08
A_68_P22303239	chr3:86351242-86351286	NM_011839:941	Mab21l2	INSIDE	0.492	0.598	1614.71	964.91	0.294	1195.95	351.85
A_68_P31938325	chr19:7240713-7240757	NM_134147:109477	Macrod1	INSIDE	0.494	0.601	1881.54	1131.71	0.297	1393.49	413.83
A_68_P21683843	chr2:140221161-140221205	NM_028387:12	Macrod2	INSIDE	0.270	0.509	1963.27	1000.00	0.138	1607.61	221.42
A_68_P24517586	chr6:66485635-66485679	NM_019499:195	Mad2l1	INSIDE	0.301	0.648	1159.02	751.05	0.195	1025.09	199.61
A_68_P23588230	chr5:33678025-33678069	NM_021500:-174	Maea	PROMOTER	0.286	0.374	1878.77	703.16	0.107	1386.19	148.30
A_68_P23588231	chr5:33678100-33678145	NM_021500:-98	Maea	PROMOTER	0.335	0.614	2204.00	1352.69	0.206	1881.81	387.36
A_68_P20799114	chr1:168169051-168169095	NM_175296:-197	Mael	PROMOTER	0.555	3.213	4234.87	13605.29	1.784	3466.50	6184.25
A_68_P20799115	chr1:168169115-168169174	NM_175296:-269	Mael	PROMOTER	0.507	5.741	186.23	1069.13	2.909	135.52	394.29
A_68_P26192671	chr8:118229614-118229663	NM_001025577:1156	Maf	INSIDE	0.279	0.518	2078.13	1076.01	0.144	1552.88	224.28
A_68_P26192682	chr8:118231333-118231382	NM_001025577:-563	Maf	PROMOTER	0.274	0.531	1446.93	768.66	0.146	1192.45	173.59
A_68_P26192689	chr8:118232168-118232225	NM_001025577:-1402	Maf	PROMOTER	0.192	0.462	1766.37	816.22	0.089	1352.51	119.73
A_68_P30347740	chr15:76181682-76181726	NM_001164607:-19	Mafl	DIVERGENT_PROMOTER	0.084	1.888	4164.69	7864.25	0.159	2780.52	442.59
A_68_P30347741	chr15:76181792-76181836	NM_001164607:91	Mafl	INSIDE	0.287	1.738	3682.37	6398.89	0.498	2466.15	1228.36
A_68_P30347742	chr15:76181910-76181954	NM_001164607:209	Mafl	INSIDE	0.314	0.671	1461.02	980.13	0.211	1140.91	240.20
A_68_P21794484	chr2:160191502-160191546	NM_010658:1277	Mafb	INSIDE	0.239	0.575	1209.16	695.07	0.137	908.40	124.75
A_68_P21794488	chr2:160191953-160191997	NM_010658:827	Mafb	INSIDE	0.306	0.551	2432.00	1340.48	0.168	1838.20	309.53
A_68_P21794498	chr2:160193025-160193069	NM_010658:-245	Mafb	PROMOTER	0.356	0.738	4337.06	3202.29	0.263	3253.79	855.16
A_68_P30365874	chr15:79178468-79178512	NM_010755:383	Maff	INSIDE	0.280	1.627	4199.84	6834.63	0.455	2838.74	1291.98
A_68_P30365876	chr15:79178645-79178689	NM_010755:559	Maff	INSIDE	0.056	0.744	4402.30	3275.28	0.041	2943.95	121.66
A_68_P24137624	chr5:140267260-140267304	NM_010757:-207	Mafk	PROMOTER	0.147	0.617	1768.63	1091.14	0.091	1360.91	123.35
A_68_P24137625	chr5:140267376-140267420	NM_010757:-91	Mafk	PROMOTER	0.234	0.548	8056.66	4414.12	0.128	5679.47	727.79
A_68_P21583616	chr2:121781508-121781552	NM_008545:298	Mageb3	INSIDE	0.179	0.561	2686.96	1508.04	0.100	1953.02	196.18
A_68_P32539359	chrX:91786297-91786345	NM_019791:1093	Maged1	INSIDE	0.110	2.611	1501.58	3920.54	0.287	566.67	162.83
A_68_P32539360	chrX:91786471-91786515	NM_019791:921	Maged1	INSIDE	0.191	1.882	1482.38	2789.20	0.359	567.37	203.64
A_68_P32539363	chrX:91786834-91786878	NM_019791:557	Maged1	INSIDE	0.214	1.969	2039.95	4016.39	0.422	861.74	363.89
A_68_P32539365	chrX:91787127-91787171	NM_019791:265	Maged1	INSIDE	0.290	1.612	4543.27	7325.31	0.468	1646.10	770.57
A_68_P32742984	chrX:147247606-147247650	NM_001199246:618	Maged2	INSIDE	0.219	2.249	3314.50	7454.73	0.492	1228.13	604.81
A_68_P32742985	chrX:147247678-147247726	NM_001199246:544	Maged2	INSIDE	0.037	3.682	2465.96	9080.68	0.135	876.03	117.91
A_68_P24655997	chr6:94232874-94232918	NM_001029850:1002	Magi1	INSIDE	0.264	0.504	1466.14	739.50	0.133	1225.95	163.36

ProbeName	Target position of probe on CpG island microarray	TargetID	GeneSymbol	CpG island Description	Ratio of relative methylation (TiO2-NP/Vehicle)	Sham group			TiO_2-H group		
						Relative methylation (Cy-5/Cy-3)	Cy-3 signal (Input)	Cy-5 signal (MeDIP)	Relative methylation (Cy-5/Cy-3)	Cy-3 signal (Input)	Cy-5 signal (MeDIP)
A_68_P24655998	chr6:94232997-94233041	NM_001029850:880	Magi1	INSIDE	0.245	0.606	946.93	573.81	0.148	867.92	128.81
A_68_P24656010	chr6:94234328-94234372	NM_001029850:-452	Magi1	PROMOTER	0.108	0.583	2365.89	1378.76	0.063	1820.99	114.56
A_68_P23513995	chr5:18733341-18733385	NM_001170746:499	Magi2	INSIDE	0.445	0.584	1843.20	1076.15	0.260	1232.77	320.52
A_68_P23193385	chr4:107552379-107552423	NM_010760:-17	Magoh	PROMOTER	0.344	0.587	1208.40	708.95	0.202	918.58	185.23
A_68_P25756476	chr8:32278992-32279036	NM_026453:182	Mak16	INSIDE	0.383	0.614	1140.32	699.86	0.235	959.02	225.20
A_68_P25756477	chr8:32279067-32279111	NM_026453:108	Mak16	INSIDE	0.356	0.664	1383.78	919.33	0.237	1017.34	240.89
A_68_P21614869	chr2:127481863-127481907	NM_001171187:547	Mal	INSIDE	0.456	0.626	1145.91	717.62	0.286	964.41	275.64
A_68_P31930911	chr19:5803095-5803139	NR_002847:-445	Malat1	PROMOTER	0.275	0.622	946.67	589.23	0.171	703.84	120.52
A_68_P31930913	chr19:5803254-5803298	NR_002847:-605	Malat1	PROMOTER	0.249	0.365	1861.18	679.93	0.091	1271.31	115.83
A_68_P27787816	chr11:50105944-50105992	NM_175334:-130	Maml1	PROMOTER	0.279	0.611	864.37	528.36	0.171	704.18	120.09
A_68_P27787817	chr11:50106087-50106131	NM_175334:-270	Maml1	PROMOTER	0.336	0.529	1025.84	543.18	0.178	817.07	145.50
A_68_P22133744	chr3:51906476-51906520	NM_001004176:2430	Maml3	INSIDE	0.351	0.675	1006.00	679.14	0.237	777.73	184.13
A_68_P22133753	chr3:51907587-51907631	NM_001004176:1320	Maml3	INSIDE	0.229	0.492	2548.76	1253.29	0.112	1797.82	202.21
A_68_P32451587	chrX:68303129-68303173	NM_001081354:-280	Mamld1	PROMOTER	0.159	2.534	1396.22	3538.14	0.403	613.24	247.39
A_68_P31296465	chr17:64951073-64951117	NM_008549:106	Man2a1	INSIDE	0.241	0.465	1684.05	783.28	0.112	1390.37	155.69
A_68_P25266321	chr7:87515732-87515779	NM_172903:506	Man2a2	INSIDE	0.180	0.695	2106.99	1463.86	0.125	1410.98	176.06
A_68_P25266322	chr7:87515822-87515866	NM_172903:417	Man2a2	INSIDE	0.191	0.681	4472.38	3046.27	0.130	3535.23	458.91
A_68_P22552361	chr3:135148732-135148776	NM_027288:180	Manba	INSIDE	0.318	0.553	1312.87	725.90	0.176	1150.75	202.41
A_68_P22793477	chr4:26273660-26273704	NM_172865:117	Manea	INSIDE	0.214	0.522	2042.56	1066.96	0.112	1569.08	175.51
A_68_P22793478	chr4:26273773-26273817	NM_172865:5	Manea	INSIDE	0.649	0.589	2829.43	1667.16	0.383	2197.29	840.60
A_68_P22793479	chr4:26273870-26273915	NM_172865:-93	Manea	PROMOTER	0.259	0.636	2350.69	1495.72	0.165	1838.38	302.63
A_68_P23270752	chr4:124539019-124539063	NM_001007573:375	Maneal	INSIDE	0.358	0.505	1459.73	736.51	0.181	1129.54	203.96
A_68_P26805729	chr9:106793805-106793862	NM_029103:436	Manf	INSIDE	0.150	0.695	1621.48	1126.14	0.104	1165.93	121.60
A_68_P26805730	chr9:106793972-106794020	NM_029103:273	Manf	INSIDE	0.162	0.726	1691.90	1229.03	0.117	1236.20	145.19
A_68_P26805732	chr9:106794296-106794340	NM_029103:-49	Manf	PROMOTER	0.314	0.556	969.69	538.82	0.174	714.03	124.51
A_68_P32276229	chrX:16195746-16195790	NM_173740:-1055	Maoa	PROMOTER	0.374	0.575	1194.71	687.20	0.215	536.94	115.36
A_68_P26227605	chr8:124109328-124109378	NM_026160:-5015	Map1lc3b	DIVERGENT_PROMOTER	0.272	0.591	995.82	588.63	0.161	733.41	118.12
A_68_P26227607	chr8:124109628-124109679	NM_026160:-4714	Map1lc3b	DIVERGENT_PROMOTER	0.265	0.620	943.16	584.78	0.164	724.21	118.92
A_68_P26227629	chr8:124114089-124114133	NM_026160:-257	Map1lc3b	PROMOTER	0.203	0.467	1645.46	768.07	0.095	1245.84	117.86
A_68_P26580968	chr9:64100873-64100917	NM_008927:518	Map2k1	INSIDE	0.152	0.433	2290.58	991.80	0.066	1770.03	116.82
A_68_P26580975	chr9:64101634-64101678	NM_008927:-244	Map2k1	PROMOTER	0.262	0.690	1916.99	1322.23	0.181	1631.38	295.05
A_68_P27287421	chr10:80568903-80568947	NM_023138:233	Map2k2	INSIDE	0.306	0.460	1148.19	528.02	0.141	830.12	116.64
A_68_P27845569	chr11:60745467-60745511	NM_008928:-70	Map2k3	DIVERGENT_PROMOTER	0.646	0.328	1578.99	517.62	0.212	1136.75	240.67
A_68_P25616729	chr8:4238967-4239011	NM_001042557:249	Map2k7	INSIDE	0.404	0.580	2908.83	1687.64	0.234	2062.11	483.10
A_68_P29330805	chr13:112598506-112598550	NM_011945:663	Map3k1	INSIDE	0.359	0.432	2163.06	934.12	0.155	1715.14	265.84
A_68_P31930244	chr19:5696279-5696323	NM_022012:7170	Map3k11	INSIDE	0.214	0.664	1221.58	811.28	0.142	884.26	125.39
A_68_P30498285	chr15:102340830-102340876	NM_001163643:6583	Map3k12	INSIDE	0.294	0.625	860.46	537.87	0.183	666.50	122.30
A_68_P30498330	chr15:102347068-102347112	NM_001163643:345	Map3k12	INSIDE	0.400	1.500	2499.08	3748.29	0.600	1607.42	964.83
A_68_P30498333	chr15:102347359-102347404	NM_001163643:54	Map3k12	INSIDE	0.347	0.557	1772.33	987.48	0.193	1335.19	258.25
A_68_P28084316	chr11:103128366-103128410	NM_016896:327	Map3k14	INSIDE	0.315	0.533	2663.72	1420.95	0.168	1903.82	320.35
A_68_P28084317	chr11:103128474-103128518	NM_016896:219	Map3k14	INSIDE	0.430	0.677	2901.94	1963.69	0.291	2093.48	609.49
A_68_P28084319	chr11:103128781-103128825	NM_016896:-87	Map3k14	PROMOTER	0.430	0.489	1810.48	884.93	0.210	1355.08	284.85
A_68_P28084320	chr11:103128905-103128949	NM_016896:-211	Map3k14	PROMOTER	0.257	0.471	1406.35	662.20	0.121	996.05	120.57
A_68_P32777675	chrX:156426686-156426730	NM_001163085:344	Map3k15	INSIDE	0.186	1.497	772.35	1155.89	0.278	431.33	119.92

ProbeName	Target position of probe on CpG island microarray	TargetID	GeneSymbol	CpG island Description	Ratio of relative methylation (TiO2-NP/Vehicle)	Sham group			TiO_2-H group		
						Relative methylation (Cy-5/Cy-3)	Cy-3 signal (Input)	Cy-5 signal (MeDIP)	Relative methylation (Cy-5/Cy-3)	Cy-3 signal (Input)	Cy-5 signal (MeDIP)
A_68_P31601524	chr18:32323271-32323316	NM_011946:551	Map3k2	INSIDE	0.401	0.591	1415.59	837.13	0.237	1152.26	273.12
A_68_P31051124	chr17:12511856-12511901	NM_011948:-352	Map3k4	PROMOTER	0.660	0.508	1315.65	668.69	0.335	1125.73	377.64
A_68_P22818617	chr4:32050724-32050768	NM_009316:-335	Map3k7	PROMOTER	0.408	0.377	1924.93	726.34	0.154	1466.92	225.63
A_68_P22818618	chr4:32050821-32050865	NM_009316:-239	Map3k7	PROMOTER	0.572	0.362	2234.04	809.83	0.207	1848.73	383.14
A_68_P31462759	chr18:4353279-4353323	NM_007746:-349	Map3k8	PROMOTER	0.316	0.508	2271.95	1155.14	0.161	1776.76	285.32
A_68_P31934085	chr19:6341468-6341512	NM_009006:241	Map4k2	INSIDE	0.486	0.671	1478.67	992.70	0.326	1237.50	403.43
A_68_P31383812	chr17:80976478-80976522	NM_001081357:150933	Map4k3	DOWNSTREAM	0.663	0.648	1188.54	770.31	0.430	947.73	407.50
A_68_P30423458	chr15:88977331-88977375	NM_011161:2684	Mapk11	INSIDE	0.206	0.627	1094.51	686.24	0.129	926.20	119.72
A_68_P30423403	chr15:88970637-88970681	NM_013871:475	Mapk12	INSIDE	0.459	0.565	1890.77	1067.54	0.259	1426.09	369.87
A_68_P30423406	chr15:88971030-88971074	NM_013871:81	Mapk12	INSIDE	0.281	0.716	2592.92	1857.68	0.201	1804.08	363.14
A_68_P30423408	chr15:88971259-88971303	NM_013871:-147	Mapk12	PROMOTER	0.151	0.661	1607.55	1062.25	0.100	1190.75	118.98
A_68_P30345342	chr15:75824398-75824446	NM_177922:224	Mapk15	INSIDE	0.242	0.550	1780.30	979.20	0.133	1385.91	184.43
A_68_P30345375	chr15:75828132-75828176	NM_177922:3956	Mapk15	INSIDE	0.318	0.516	1004.95	518.50	0.164	725.96	119.26
A_68_P30345377	chr15:75828316-75828360	NM_177922:4140	Mapk15	INSIDE	0.176	0.653	1469.85	960.03	0.115	1061.69	122.25
A_68_P29581489	chr14:47917977-47918021	NM_178684:10	Mapk1ip1l	INSIDE	0.311	0.520	1302.58	676.73	0.161	1067.60	172.28
A_68_P31829409	chr18:74224278-74224322	NM_172632:303	Mapk4	INSIDE	0.196	0.652	2563.25	1670.09	0.128	1920.54	245.38
A_68_P31829416	chr18:74225103-74225147	NM_172632:-521	Mapk4	PROMOTER	0.282	0.460	1360.36	626.05	0.130	1238.03	160.75
A_68_P27848679	chr11:61307617-61307661	NM_011841:67	Mapk7	INSIDE	0.178	0.647	1338.78	866.40	0.115	1014.69	116.64
A_68_P21428210	chr2:92229536-92229580	NM_001202445:3282	Mapk8ip1	INSIDE	0.216	0.541	1363.25	737.44	0.117	1008.86	117.90
A_68_P30425255	chr15:89284136-89284180	NM_021921:-183	Mapk8ip2	PROMOTER	0.550	0.697	4044.32	2817.95	0.383	2992.20	1146.30
A_68_P30425256	chr15:89284256-89284300	NM_021921:-63	Mapk8ip2	PROMOTER	0.124	1.457	2526.93	3681.12	0.180	1644.22	296.73
A_68_P31100481	chr17:25073380-25073424	NM_001163447:520	Mapk8ip3	INSIDE	0.430	0.494	1770.02	875.20	0.213	1269.58	270.08
A_68_P31556681	chr18:23910710-23910754	NM_001162942:-101	Mapre2	PROMOTER	0.619	0.570	1000.45	570.56	0.353	907.80	320.23
A_68_P31556969	chr18:23962757-23962801	NM_153058:308	Mapre2	INSIDE	0.073	1.884	6992.76	13172.49	0.138	4524.25	623.56
A_68_P28089839	chr11:104092302-104092349	NM_001038609:-424	Mapt	PROMOTER	0.234	0.649	848.16	550.81	0.152	767.68	116.83
A_68_P28089843	chr11:104092753-104092797	NM_001038609:25	Mapt	INSIDE	0.342	0.509	1566.11	797.20	0.174	1253.98	218.61
A_68_P28089844	chr11:104092991-104093035	NM_001038609:263	Mapt	INSIDE	0.391	1.364	2693.55	3672.88	0.533	1860.12	991.85
A_68_P28089845	chr11:104093103-104093147	NM_001038609:375	Mapt	INSIDE	0.368	0.451	1322.61	596.24	0.166	913.25	151.68
A_68_P28089849	chr11:104093607-104093651	NM_001038609:879	Mapt	INSIDE	0.234	0.384	1552.58	596.49	0.090	1337.82	120.28
A_68_P28089853	chr11:104094131-104094175	NM_001038609:1403	Mapt	INSIDE	0.566	0.580	2669.04	1548.50	0.328	1732.60	568.89
A_68_P31150248	chr17:33855451-33855495	NM_145486:143	Marchf2	INSIDE	0.461	0.518	1175.41	608.38	0.239	1107.68	264.24
A_68_P31733116	chr18:57085552-57085596	NM_177115:-372	Marchf3	PROMOTER	0.369	0.549	1476.92	810.67	0.203	1171.17	237.29
A_68_P20337462	chr1:72582708-72582752	NM_001045533:408	Marchf4	INSIDE	0.201	0.683	1949.89	1331.51	0.137	1454.01	199.79
A_68_P23297039	chr4:129191585-129191629	NM_010807:782	Marcksl1	INSIDE	0.215	0.451	2018.76	909.69	0.097	1618.42	156.86
A_68_P24993423	chr7:20043402-20043446	NM_172279:419	Mark4	INSIDE	0.245	0.476	3301.16	1572.63	0.117	2516.76	293.36
A_68_P24993425	chr7:20043646-20043690	NM_172279:175	Mark4	INSIDE	0.185	2.249	10218.67	22985.36	0.415	6641.72	2756.53
A_68_P24993428	chr7:20044025-20044069	NM_172279:-203	Mark4	PROMOTER	0.432	0.471	1471.90	693.97	0.204	1226.71	249.76
A_68_P27535211	chr10:126748493-126748537	NM_001003913:328	Mars	INSIDE	0.293	0.523	4633.52	2422.92	0.153	3347.90	513.77
A_68_P27535212	chr10:126748563-126748607	NM_001003913:258	Mars	INSIDE	0.291	0.641	1250.46	802.14	0.187	1011.00	188.76
A_68_P27535216	chr10:126748924-126748968	NM_001003913:-104	Mars	PROMOTER	0.214	0.513	1570.58	806.44	0.110	1103.63	121.01
A_68_P32119292	chr19:42222487-42222531	NM_183195:630	Marveld1	INSIDE	0.517	0.737	5617.74	4142.62	0.381	4129.04	1574.01
A_68_P32119295	chr19:42222796-42222840	NM_183195:940	Marveld1	INSIDE	0.209	0.625	1289.12	805.26	0.131	991.06	129.49
A_68_P26159871	chr8:112472495-112472551	NM_212447:13583	Marveld3	INSIDE	0.248	0.444	1317.34	585.43	0.110	1064.82	117.42
A_68_P26022615	chr8:87436867-87436911	NM_019945:24364	Mast1	INSIDE	0.539	0.557	1579.59	879.45	0.300	1196.93	359.25

ProbeName	Target position of probe on CpG island microarray	TargetID	GeneSymbol	CpG island Description	Ratio of relative methylation (TiO2-NP/Vehicle)	Sham group			TiO_2-H group		
						Relative methylation (Cy-5/Cy-3)	Cy-3 signal (Input)	Cy-5 signal (MeDIP)	Relative methylation (Cy-5/Cy-3)	Cy-3 signal (Input)	Cy-5 signal (MeDIP)
A_68_P26022619	chr8:87437298-87437343	NM_019945:23932	Mast1	INSIDE	0.252	0.612	1223.67	748.93	0.154	931.22	143.43
A_68_P26022686	chr8:87447681-87447725	NM_019945:13550	Mast1	INSIDE	0.518	2.821	2382.49	6719.96	1.461	1713.15	2503.61
A_68_P26022753	chr8:87461135-87461179	NM_019945:96	Mast1	INSIDE	0.044	1.797	5022.41	9027.06	0.078	3037.14	237.70
A_68_P25954140	chr8:73321012-73321061	NM_199308:-4704	Mast3	PROMOTER	0.379	3.708	4916.78	18230.35	1.407	3289.09	4627.57
A_68_P25954141	chr8:73321121-73321165	NM_199308:-4810	Mast3	PROMOTER	0.144	2.216	3856.19	8546.18	0.320	2589.98	828.89
A_68_P25954143	chr8:73321298-73321342	NM_199308:-4988	Mast3	PROMOTER	0.324	1.546	3449.19	5331.35	0.501	2349.00	1176.71
A_68_P25954146	chr8:73321652-73321696	NM_199308:-5342	Mast3	PROMOTER	0.305	0.487	1954.75	951.25	0.148	1418.66	210.64
A_68_P25954147	chr8:73321717-73321761	NM_199308:-5406	Mast3	PROMOTER	0.536	0.676	1483.89	1002.87	0.362	1063.48	384.95
A_68_P29284641	chr13:104125004-104125048	NM_175171:-454	Mast4	PROMOTER	0.577	0.664	1558.60	1034.88	0.383	1257.14	481.27
A_68_P30127810	chr15:34236528-34236572	NM_016762:115	Matn2	INSIDE	0.328	0.640	2207.22	1411.80	0.210	1655.60	346.92
A_68_P21817647	chr2:164229981-164230025	NM_013592:112	Matn4	INSIDE	0.391	0.476	1295.63	616.22	0.186	1035.52	192.49
A_68_P21817648	chr2:164230100-164230144	NM_013592:-8	Matn4	PROMOTER	0.626	0.426	1891.65	806.02	0.267	1543.94	412.07
A_68_P31618898	chr18:35721681-35721725	NM_010771:-109	Matr3	PROMOTER	0.583	0.687	1018.69	700.32	0.401	801.10	320.86
A_68_P28556953	chr12:78063293-78063337	NM_001146176:-79	Max	PROMOTER	0.194	1.514	2927.83	4433.05	0.293	2024.08	593.04
A_68_P28556954	chr12:78063431-78063475	NM_001146176:-217	Max	PROMOTER	0.214	0.552	1344.60	742.55	0.118	1011.22	119.36
A_68_P28556955	chr12:78063502-78063546	NM_001146176:-289	Max	PROMOTER	0.225	0.632	1880.31	1187.61	0.142	1410.83	200.35
A_68_P25503658	chr7:134170789-134170833	NM_010772:-817	Maz	PROMOTER	0.108	2.170	20497.19	44486.13	0.235	12606.55	2958.09
A_68_P27282971	chr10:79862699-79862743	NM_013595:-496	Mbd3	PROMOTER	0.356	0.579	916.64	530.98	0.206	663.01	136.66
A_68_P24129308	chr5:138635505-138635553	NM_177878:-13	Mblac1	PROMOTER	0.198	0.526	1362.28	716.73	0.104	1168.05	121.94
A_68_P24129310	chr5:138635652-138635696	NM_177878:133	Mblac1	INSIDE	0.464	0.517	1585.64	819.11	0.239	1251.92	299.75
A_68_P32375459	chrX:48558594-48558638	NM_134163:393	Mbnl3	INSIDE	0.135	1.594	2211.23	3523.80	0.215	885.86	190.30
A_68_P32375460	chrX:48558753-48558797	NM_134163:235	Mbnl3	INSIDE	0.073	1.444	3023.47	4366.83	0.106	1085.52	115.03
A_68_P28291795	chr12:25515888-25515932	NM_001083341:-553	Mboat2	PROMOTER	0.224	0.425	1799.10	765.28	0.095	1226.85	116.65
A_68_P24946853	chr7:3644138-3644182	NM_029934:967	Mboat7	INSIDE	0.274	0.511	1155.74	591.04	0.140	868.71	121.60
A_68_P24946854	chr7:3644206-3644250	NM_029934:899	Mboat7	INSIDE	0.222	0.523	2317.38	1211.72	0.116	1868.31	216.60
A_68_P24946855	chr7:3644343-3644387	NM_029934:763	Mboat7	INSIDE	0.235	0.516	2454.88	1266.28	0.121	2005.03	243.01
A_68_P24946857	chr7:3644688-3644732	NM_029934:417	Mboat7	INSIDE	0.513	0.400	1815.75	726.91	0.206	1469.67	302.04
A_68_P24946860	chr7:3645012-3645069	NM_029934:87	Mboat7	INSIDE	0.272	0.425	1259.44	535.12	0.116	1026.35	118.71
A_68_P32767188	chrX:154035988-154036043	NM_172307:632	Mbtps2	INSIDE	0.111	1.744	1575.08	2746.45	0.194	594.09	115.24
A_68_P26465868	chr9:43938018-43938064	NM_023061:-4700	Mcam	PROMOTER	0.285	0.654	1067.23	697.96	0.186	841.86	156.71
A_68_P26465869	chr9:43938100-43938144	NM_023061:-4618	Mcam	PROMOTER	0.579	0.651	1367.40	889.59	0.377	920.52	346.68
A_68_P26465908	chr9:43942942-43942986	NM_023061:224	Mcam	INSIDE	0.087	0.708	3512.73	2485.37	0.062	2331.60	143.60
A_68_P22882728	chr4:45421725-45421769	NM_001009949:-108	Mcart1	PROMOTER	0.544	0.460	1645.80	756.44	0.250	1276.62	319.43
A_68_P31669146	chr18:44972023-44972067	NM_001085373:-208	Mcc	PROMOTER	0.212	0.680	1072.62	729.22	0.144	950.32	136.99
A_68_P22056222	chr3:35899243-35899287	NM_023644:336	Mccc1	INSIDE	0.493	0.460	1174.19	539.96	0.227	919.31	208.37
A_68_P22056223	chr3:35899395-35899439	NM_023644:184	Mccc1	INSIDE	0.189	0.613	4751.54	2913.71	0.116	3569.33	414.04
A_68_P22056224	chr3:35899487-35899534	NM_023644:90	Mccc1	INSIDE	0.129	0.627	2218.23	1390.88	0.081	1506.04	121.77
A_68_P29266706	chr13:100785478-100785522	NM_030026:94	Mccc2	INSIDE	0.599	0.479	1263.17	604.47	0.286	969.08	277.61
A_68_P25664799	chr8:12915651-12915695	NM_178076:-220	Mcf2l	PROMOTER	0.099	0.713	2965.67	2115.78	0.071	1847.24	130.46
A_68_P25664801	chr8:12915981-12916035	NM_178076:116	Mcf2l	INSIDE	0.274	0.689	1297.83	894.77	0.189	959.41	181.19
A_68_P31422103	chr17:87665170-87665214	NM_139295:95	Mcfd2	INSIDE	0.476	0.560	4201.84	2354.54	0.267	2989.83	797.80
A_68_P24624871	chr6:88848565-88848609	NM_008564:188	Mcm2	INSIDE	0.208	0.552	1909.73	1053.30	0.115	1419.60	162.77
A_68_P27262211	chr10:75931408-75931452	NM_019434:-285	Mcm3ap	PROMOTER	0.395	0.628	2981.60	1872.43	0.248	2404.79	596.32
A_68_P27262214	chr10:75931729-75931773	NM_019434:35	Mcm3ap	INSIDE	0.450	0.287	2471.94	708.97	0.129	1970.94	254.13

ProbeName	Target position of probe on CpG island microarray	TargetID	GeneSymbol	CpG island Description	Ratio of relative methylation (TiO2-NP/Vehicle)	Sham group			TiO_2-H group		
						Relative methylation (Cy-5/Cy-3)	Cy-3 signal (Input)	Cy-5 signal (MeDIP)	Relative methylation (Cy-5/Cy-3)	Cy-3 signal (Input)	Cy-5 signal (MeDIP)
A_68_P30566358	chr16:15637618-15637664	NM_008565:-147	Mcm4	DIVERGENT_PROMOTER	0.266	0.442	1217.32	537.50	0.118	991.35	116.59
A_68_P25972025	chr8:77633597-77633641	NM_008566:192	Mcm5	INSIDE	0.575	0.451	1369.88	617.36	0.259	1024.82	265.60
A_68_P25972026	chr8:77633688-77633732	NM_008566:284	Mcm5	INSIDE	0.247	0.479	1134.94	543.88	0.118	977.78	115.69
A_68_P20600071	chr1:130256165-130256209	NM_008567:47	Mcm6	INSIDE	0.371	0.330	1786.80	589.58	0.122	1566.63	191.87
A_68_P27143272	chr10:53349951-53349995	NM_027830:273	Mcm9	INSIDE	0.362	1.819	5783.79	10523.23	0.659	3906.06	2574.75
A_68_P25512275	chr7:135883649-135883693	NM_145955:273	Mcmbp	INSIDE	0.501	0.600	1175.55	705.52	0.300	1019.38	306.24
A_68_P22612825	chr3:145812772-145812816	NM_026656:-2	Mcoln2	PROMOTER	0.227	0.553	1541.66	852.82	0.125	1200.17	150.48
A_68_P29154205	chr13:76522219-76522263	NM_030174:-168	Mctp1	PROMOTER	0.410	0.580	1388.94	805.24	0.237	1088.20	258.36
A_68_P31161217	chr17:35978332-35978376	NM_001010833:-88	Mdc1	DIVERGENT_PROMOTER	0.303	0.334	1856.65	620.90	0.101	1507.56	152.63
A_68_P31161220	chr17:35978699-35978752	NM_001010833:283	Mdc1	INSIDE	0.336	0.678	1164.75	789.22	0.227	855.22	194.47
A_68_P24258182	chr6:15671365-15671410	NM_175088:727	Mdfic	INSIDE	0.359	0.494	1383.43	683.28	0.177	1037.11	183.71
A_68_P31130151	chr17:29979095-29979139	NM_001081160:45711	Mdga1	INSIDE	0.310	0.737	3923.97	2892.50	0.228	2840.85	649.04
A_68_P31130153	chr17:29979350-29979394	NM_001081160:45455	Mdga1	INSIDE	0.183	0.619	1900.79	1177.29	0.113	1366.30	154.62
A_68_P31130218	chr17:29989237-29989281	NM_001081160:35569	Mdga1	INSIDE	0.219	1.358	2278.29	3093.88	0.297	1689.65	501.92
A_68_P31130465	chr17:30025201-30025252	NM_001081160:-399	Mdga1	PROMOTER	0.168	0.458	2153.69	986.16	0.077	1512.45	116.44
A_68_P31130472	chr17:30026258-30026302	NM_001081160:-1453	Mdga1	PROMOTER	0.152	0.662	2377.77	1573.85	0.101	1842.43	185.49
A_68_P31130473	chr17:30026432-30026476	NM_001081160:-1627	Mdga1	PROMOTER	0.197	0.660	1154.37	761.80	0.130	942.24	122.67
A_68_P31130474	chr17:30026534-30026578	NM_001081160:-1729	Mdga1	PROMOTER	0.470	0.578	1314.33	759.29	0.271	1054.23	286.10
A_68_P28504589	chr12:68321801-68321851	NM_001193266:1710	Mdga2	INSIDE	0.240	0.598	1030.45	616.64	0.144	804.32	115.52
A_68_P28504593	chr12:68322368-68322415	NM_001193266:1145	Mdga2	INSIDE	0.359	1.495	2753.81	4116.16	0.536	1988.65	1066.51
A_68_P24117829	chr5:136254539-136254583	NM_008617:42	Mdh2	INSIDE	0.194	0.537	1534.04	823.93	0.104	1230.67	128.20
A_68_P24117834	chr5:136255023-136255074	NM_008617:530	Mdh2	INSIDE	0.188	0.531	1482.95	787.91	0.100	1213.50	121.36
A_68_P27484948	chr10:117578776-117578823	NM_001162904:-43	Mdm1	PROMOTER	0.380	0.638	1123.74	716.89	0.242	880.61	213.36
A_68_P27482565	chr10:117147534-117147578	NM_010786:216	Mdm2	INSIDE	0.489	0.534	1301.76	695.21	0.261	1130.71	295.02
A_68_P20625164	chr1:134921568-134921624	NM_008575:329	Mdm4	INSIDE	0.211	0.476	1535.53	731.06	0.100	1151.70	115.67
A_68_P29619152	chr14:56279216-56279260	NM_023397:107	Mdp1	INSIDE	0.181	0.725	2116.29	1534.67	0.132	1468.61	193.27
A_68_P31827988	chr18:73975355-73975399	NM_145494:-330	Me2	PROMOTER	0.360	0.639	2932.55	1873.53	0.230	2291.93	527.23
A_68_P31208333	chr17:46818209-46818253	NM_010787:145	Mea1	INSIDE	0.156	0.716	1656.82	1185.83	0.111	1208.63	134.59
A_68_P22028766	chr3:30407046-30407090	NM_021442:1341	Mecom	INSIDE	0.207	0.554	1358.36	752.35	0.114	1029.61	117.86
A_68_P22028767	chr3:30407126-30407173	NM_021442:1260	Mecom	INSIDE	0.159	0.715	1488.17	1063.97	0.113	1059.84	120.29
A_68_P22028769	chr3:30407354-30407398	NM_021442:1033	Mecom	INSIDE	0.269	0.736	4058.31	2988.19	0.198	3247.42	643.40
A_68_P22028818	chr3:30413802-30413849	NM_021442:-5416	Mecom	PROMOTER	0.245	0.540	1131.26	610.42	0.132	897.32	118.46
A_68_P32466326	chrX:71330622-71330666	NM_010788:288	Mecp2	INSIDE	0.099	1.935	2987.87	5781.00	0.192	1034.32	198.18
A_68_P23308627	chr4:131399668-131399712	NM_025297:305	Mecr	INSIDE	0.153	0.516	1774.46	915.24	0.079	1464.90	115.24
A_68_P28054913	chr11:98054206-98054250	NM_013634:-8	Med1	PROMOTER	0.245	0.407	1488.16	606.27	0.100	1159.67	115.57
A_68_P32563202	chrX:98469396-98469440	NM_021521:-11	Med12	DIVERGENT_PROMOTER	0.129	1.795	3286.56	5899.12	0.231	1244.23	287.86
A_68_P27988567	chr11:86170969-86171013	NM_001080931:37	Med13	INSIDE	0.372	0.633	2475.01	1567.00	0.236	1888.51	445.29
A_68_P30575357	chr16:17722612-17722656	NM_001040683:389	Med15	INSIDE	0.285	0.581	1108.52	643.61	0.165	918.66	151.83
A_68_P30575358	chr16:17722742-17722786	NM_001040683:259	Med15	INSIDE	0.286	0.453	1994.93	904.08	0.130	1685.87	218.73
A_68_P23312412	chr4:132019695-132019742	NM_026039:118	Med18	INSIDE	0.583	0.639	1206.94	770.71	0.372	972.08	362.05
A_68_P23312413	chr4:132019816-132019860	NM_026039:-2	Med18	PROMOTER	0.220	0.510	1309.51	667.85	0.112	1033.51	116.04
A_68_P24929767	chr6:146591034-146591078	NM_025315:-44	Med21	DIVERGENT_PROMOTER	0.623	0.583	1030.87	600.65	0.363	804.04	292.07
A_68_P24929768	chr6:146591129-146591173	NM_025315:50	Med21	INSIDE	0.335	0.478	1517.62	725.11	0.160	1248.74	199.94
A_68_P25089970	chr7:52147618-52147662	NM_029365:96	Med25	INSIDE	0.159	0.434	2815.10	1223.08	0.069	2156.39	148.87

ProbeName	Target position of probe on CpG island microarray	TargetID	GeneSymbol	CpG island Description	Ratio of relative methylation (TiO2-NP/Vehicle)	Sham group			TiO_2-H group		
						Relative methylation (Cy-5/Cy-3)	Cy-3 signal (Input)	Cy-5 signal (MeDIP)	Relative methylation (Cy-5/Cy-3)	Cy-3 signal (Input)	Cy-5 signal (MeDIP)
A_68_P23657076	chr5:45911955-45911999	NM_025895:509	Med28	INSIDE	0.231	0.478	1401.70	669.62	0.110	1080.19	119.27
A_68_P29717921	chr14:73909953-73909997	NM_026119:119	Med4	INSIDE	0.540	0.453	1921.04	869.75	0.244	1616.83	395.26
A_68_P28581705	chr12:82696134-82696178	NM_027213:-211	Med6	PROMOTER	0.546	3.552	7665.01	27224.48	1.939	4821.74	9348.28
A_68_P27767867	chr11:46250322-46250367	NM_001104530:-104	Med7	PROMOTER	0.353	0.399	1342.28	535.92	0.141	1034.22	145.75
A_68_P27767868	chr11:46250398-46250442	NM_001104530:-28	Med7	PROMOTER	0.413	0.663	990.79	656.77	0.274	789.79	216.19
A_68_P23240178	chr4:118082173-118082217	NM_020000:253	Med8	INSIDE	0.245	1.336	7930.80	10595.11	0.327	4974.24	1627.96
A_68_P25193391	chr7:74516874-74516921	NM_001033713:847	Mef2a	INSIDE	0.270	0.382	1531.05	584.82	0.103	1173.78	120.84
A_68_P31734231	chr18:57292496-57292540	NM_001001979:-225	Megf10	PROMOTER	0.536	0.632	1758.89	1111.93	0.339	1408.68	477.32
A_68_P31734233	chr18:57292628-57292672	NM_001001979:-93	Megf10	PROMOTER	0.199	0.624	2747.69	1713.88	0.124	2036.81	252.76
A_68_P31734234	chr18:57292707-57292751	NM_001001979:-15	Megf10	PROMOTER	0.617	0.485	1416.03	687.00	0.299	1027.76	307.71
A_68_P26581610	chr9:64233768-64233812	NM_172522:358	Megf11	INSIDE	0.397	0.699	2374.21	1658.78	0.277	1758.64	487.33
A_68_P26581615	chr9:64234276-64234320	NM_172522:866	Megf11	INSIDE	0.343	0.588	1478.56	868.87	0.202	1282.68	258.60
A_68_P23006649	chr4:70195250-70195294	NM_172694:690	Megf9	INSIDE	0.445	0.493	1476.67	728.09	0.220	1292.16	283.64
A_68_P23006655	chr4:70196033-70196077	NM_172694:-92	Megf9	PROMOTER	0.526	0.513	1080.16	554.51	0.270	939.24	253.74
A_68_P23006657	chr4:70196174-70196218	NM_172694:-234	Megf9	PROMOTER	0.437	0.589	1971.72	1161.27	0.257	1607.21	413.20
A_68_P23006658	chr4:70196288-70196332	NM_172694:-348	Megf9	PROMOTER	0.627	0.437	1205.86	527.14	0.274	1004.73	275.61
A_68_P21548581	chr2:115686326-115686370	NM_001159569:204009	Meis2	DOWNSTREAM	0.344	0.430	1557.31	669.02	0.148	1174.48	173.63
A_68_P21550148	chr2:115887946-115888001	NM_001159569:2384	Meis2	INSIDE	0.167	0.631	1793.58	1132.21	0.105	1249.50	131.44
A_68_P21550149	chr2:115888052-115888096	NM_001159569:2283	Meis2	INSIDE	0.292	0.513	1690.71	867.77	0.150	1157.09	173.65
A_68_P21550150	chr2:115888204-115888259	NM_001159569:2126	Meis2	INSIDE	0.514	0.495	1260.07	624.13	0.255	1004.28	255.89
A_68_P24980081	chr7:16760697-16760741	NM_008627:-10	Meis3	PROMOTER	0.319	0.416	1308.51	543.77	0.133	1110.65	147.46
A_68_P31934033	chr19:6335488-6335532	NM_001168490:472	Men1	INSIDE	0.459	0.544	2134.24	1160.38	0.250	1686.74	421.39
A_68_P31934035	chr19:6335704-6335748	NM_001168490:688	Men1	INSIDE	0.472	0.386	3461.25	1336.00	0.182	2378.54	432.94
A_68_P24127563	chr5:138227997-138228041	NM_144913:-89	Mepce	DIVERGENT_PROMOTER	0.251	0.520	1295.01	673.71	0.131	955.63	125.01
A_68_P25285301	chr7:91032798-91032845	NM_030705:30	Mesdc1	INSIDE	0.189	0.632	1311.23	829.13	0.120	982.24	117.63
A_68_P25285304	chr7:91033159-91033207	NM_030705:-331	Mesdc1	DIVERGENT_PROMOTER	0.172	0.583	1623.03	945.70	0.100	1225.91	122.59
A_68_P25285350	chr7:91040523-91040567	NM_023403:35	Mesdc2	INSIDE	0.167	0.650	4459.46	2898.61	0.108	3202.79	347.19
A_68_P25285353	chr7:91040923-91040967	NM_023403:435	Mesdc2	INSIDE	0.343	0.742	3615.43	2682.89	0.254	2438.90	620.28
A_68_P28192098	chr11:121563706-121563750	NM_144797:-12	Metrnl	PROMOTER	0.412	0.530	1369.10	725.65	0.218	1193.02	260.61
A_68_P28192099	chr11:121563822-121563866	NM_144797:104	Metrnl	INSIDE	0.529	0.553	1960.77	1085.19	0.293	1643.80	481.06
A_68_P27533648	chr10:126479103-126479147	NM_010792:137	Mettl1	INSIDE	0.520	0.627	2175.66	1364.15	0.326	1711.38	558.10
A_68_P27533650	chr10:126479347-126479401	NM_010792:387	Mettl1	INSIDE	0.244	0.591	1156.34	683.10	0.144	809.82	116.79
A_68_P25537271	chr7:140044033-140044077	NM_028095:276	Mettl10	INSIDE	0.354	0.616	1386.53	854.61	0.218	1115.54	243.58
A_68_P20778371	chr1:164478469-164478518	NM_144877:-17	Mettl13	PROMOTER	0.158	0.603	1649.91	995.20	0.095	1268.58	120.94
A_68_P20778374	chr1:164478713-164478757	NM_144877:-258	Mettl13	PROMOTER	0.376	0.698	1673.31	1168.63	0.263	1374.55	360.91
A_68_P27922355	chr11:74584384-74584428	NM_026197:42	Mettl16	INSIDE	0.416	0.584	2413.79	1410.45	0.243	1906.66	463.72
A_68_P27922356	chr11:74584498-74584542	NM_026197:156	Mettl16	INSIDE	0.467	0.541	1514.82	819.63	0.253	1039.96	263.00
A_68_P27922357	chr11:74584686-74584730	NM_026197:344	Mettl16	INSIDE	0.094	1.562	1192.86	1863.45	0.147	942.42	138.16
A_68_P20293520	chr1:64664228-64664272	NM_025964:-508	Mettl21a	PROMOTER	0.428	0.546	1851.72	1011.72	0.234	1475.13	344.59
A_68_P30532723	chr16:8471005-8471050	NM_146247:122	Mettl22	INSIDE	0.539	0.587	1087.20	638.15	0.317	803.36	254.38
A_68_P30532724	chr16:8471150-8471194	NM_146247:267	Mettl22	INSIDE	0.167	0.473	2176.31	1028.99	0.079	1670.76	131.59
A_68_P21311538	chr2:69723596-69723640	NM_029280:43	Mettl5	INSIDE	0.237	0.361	1892.72	683.01	0.086	1449.82	124.08
A_68_P29512324	chr14:32307722-32307766	NM_025907:419	Mettl6	INSIDE	0.386	0.405	1352.21	548.05	0.157	1069.13	167.34
A_68_P29512326	chr14:32307928-32307972	NM_025907:213	Mettl6	INSIDE	0.257	0.637	3846.95	2451.42	0.164	2876.98	472.03

ProbeName	Target position of probe on CpG island microarray	TargetID	GeneSymbol	CpG island Description	Ratio of relative methylation (TiO2-NP/Vehicle)	Sham group			TiO_2-H group		
						Relative methylation (Cy-5/Cy-3)	Cy-3 signal (Input)	Cy-5 signal (MeDIP)	Relative methylation (Cy-5/Cy-3)	Cy-3 signal (Input)	Cy-5 signal (MeDIP)
A_68_P25471699	chr7:128177893-128177937	NM_021554:-44	Mettl9	PROMOTER	0.319	0.628	917.67	575.84	0.200	724.47	145.05
A_68_P22314345	chr3:88340114-88340159	NM_001029890:3820	Mex3a	INSIDE	0.359	0.669	1173.92	785.40	0.240	815.67	195.79
A_68_P25279939	chr7:90014474-90014518	NM_175366:-1346	Mex3b	PROMOTER	0.624	0.515	3217.05	1657.68	0.322	2574.17	827.85
A_68_P25279940	chr7:90014576-90014620	NM_175366:-1244	Mex3b	PROMOTER	0.321	0.602	1372.26	826.29	0.193	1055.62	203.99
A_68_P25279944	chr7:90015164-90015208	NM_175366:-656	Mex3b	PROMOTER	0.341	0.517	1609.49	832.10	0.176	1251.62	220.49
A_68_P25279951	chr7:90016308-90016352	NM_175366:488	Mex3b	INSIDE	0.204	0.519	1451.64	753.75	0.106	1151.48	121.92
A_68_P25279963	chr7:90017868-90017912	NM_175366:2048	Mex3b	INSIDE	0.405	0.636	1415.44	900.30	0.258	1133.24	291.99
A_68_P25279966	chr7:90018157-90018201	NM_175366:2336	Mex3b	INSIDE	0.466	0.598	945.76	565.74	0.279	699.85	195.23
A_68_P31826517	chr18:73733495-73733539	NM_001039214:1158	Mex3c	INSIDE	0.078	1.399	2843.39	3978.71	0.110	1972.83	216.63
A_68_P27282836	chr10:79844946-79844990	NM_198615:5428	Mex3d	INSIDE	0.638	2.635	587.52	1547.81	1.682	514.30	864.85
A_68_P27282871	chr10:79849463-79849507	NM_198615:912	Mex3d	INSIDE	0.268	0.520	1423.79	740.17	0.139	938.11	130.48
A_68_P23361560	chr4:140566716-140566760	NM_008546:187	Mfap2	INSIDE	0.485	0.496	1156.20	573.98	0.241	893.26	214.88
A_68_P25905383	chr8:63111766-63111810	NM_027756:132	Mfap3l	INSIDE	0.425	0.452	2114.35	955.46	0.192	1973.04	379.33
A_68_P20394350	chr1:82721424-82721468	NM_029409:-46	Mff	PROMOTER	0.216	0.666	1563.58	1041.16	0.144	1171.41	168.60
A_68_P25780627	chr8:36650640-36650684	NM_001081279:-189	Mfhas1	PROMOTER	0.170	0.626	1764.56	1105.26	0.106	1344.87	142.89
A_68_P25780634	chr8:36651604-36651648	NM_001081279:775	Mfhas1	INSIDE	0.406	0.482	1783.48	859.18	0.196	1341.49	262.70
A_68_P23391249	chr4:147279034-147279078	NM_133201:-128	Mfn2	PROMOTER	0.075	0.741	3922.36	2905.25	0.056	2626.60	146.34
A_68_P22210773	chr3:67386862-67386906	NM_025813:195	Mfsd1	INSIDE	0.431	0.436	1431.44	623.47	0.188	1098.19	206.25
A_68_P23260021	chr4:122638391-122638435	NM_029662:19	Mfsd2a	INSIDE	0.289	0.622	1411.09	877.56	0.180	1126.79	202.73
A_68_P30497015	chr15:102110224-102110272	NM_134100:362	Mfsd5	INSIDE	0.258	0.596	913.35	544.77	0.154	766.22	117.76
A_68_P27890038	chr11:68369674-68369718	NM_146004:9	Mfsd6l	INSIDE	0.263	0.598	1064.35	636.58	0.157	937.70	147.62
A_68_P23972003	chr5:108877737-108877781	NM_172883:152	Mfsd7a	INSIDE	0.389	1.422	4663.32	6629.61	0.553	3380.56	1869.85
A_68_P20935802	chr1:192850220-192850264	NM_001081259:-173	Mfsd7b	PROMOTER	0.639	0.549	952.00	523.08	0.351	736.89	258.77
A_68_P28605828	chr12:87087327-87087371	NM_145447:-140	Mfsd7c	PROMOTER	0.259	0.650	1866.08	1212.86	0.168	1477.13	248.37
A_68_P20593974	chr1:129101586-129101630	NM_145128:46	Mgat5	INSIDE	0.536	0.540	1337.71	722.43	0.290	1015.63	294.19
A_68_P20593981	chr1:129102201-129102245	NM_145128:660	Mgat5	INSIDE	0.631	0.377	2212.75	834.77	0.238	1756.59	417.87
A_68_P24623657	chr6:88674155-88674204	NM_001166249:-226	Mgll	PROMOTER	0.265	0.615	910.30	559.40	0.163	745.81	121.25
A_68_P20804906	chr1:169324243-169324289	NM_025569:-338	Mgst3	PROMOTER	0.173	0.684	1301.21	889.97	0.118	1005.60	119.00
A_68_P33003685	chr1_random:342475-342519	NM_177389:417	Mia3	INSIDE	0.665	3.332	950.43	3167.27	2.217	815.73	1808.72
A_68_P33003688	chr1_random:342766-342810	NM_177389:125	Mia3	INSIDE	0.421	0.625	3157.26	1973.34	0.263	2445.27	642.87
A_68_P33003690	chr1_random:342987-343031	NM_177389:-95	Mia3	PROMOTER	0.521	0.536	3386.71	1814.67	0.279	2521.96	703.67
A_68_P23990800	chr5:112657892-112657936	NR_003718:54	Miat	INSIDE	0.299	0.533	1405.27	749.37	0.160	1059.02	168.97
A_68_P25423373	chr7:119369629-119369673	NM_001193305:301	Mical2	INSIDE	0.559	0.707	2118.24	1496.69	0.395	1671.64	660.13
A_68_P25423375	chr7:119369796-119369840	NM_001193305:469	Mical2	INSIDE	0.356	0.633	1366.45	865.08	0.225	1036.64	233.62
A_68_P30364397	chr15:78940024-78940068	NM_177461:634	Micall1	INSIDE	0.639	0.394	1481.73	584.52	0.252	1163.41	293.34
A_68_P24137287	chr5:140212019-140212063	NM_174850:247	Micall2	INSIDE	0.250	0.440	2339.67	1029.16	0.110	1809.68	198.77
A_68_P24137290	chr5:140212320-140212364	NM_174850:-55	Micall2	PROMOTER	0.243	0.659	4096.08	2697.83	0.160	3099.99	496.06
A_68_P32252886	chrX:10294260-10294304	NM_001166635:-208	Mid1ip1	PROMOTER	0.228	1.749	3300.16	5771.56	0.400	1260.09	503.43
A_68_P32252888	chrX:10294568-10294612	NM_001166635:100	Mid1ip1	INSIDE	0.179	2.996	2297.32	6882.51	0.536	930.03	498.07
A_68_P32252894	chrX:10295292-10295336	NM_001166635:824	Mid1ip1	INSIDE	0.172	1.365	2864.83	3910.65	0.235	1086.57	255.37
A_68_P32252895	chrX:10295371-10295422	NM_001166635:906	Mid1ip1	INSIDE	0.134	1.498	1469.74	2202.29	0.201	584.01	117.45
A_68_P32252897	chrX:10295608-10295654	NM_001166635:1140	Mid1ip1	INSIDE	0.562	0.691	1207.34	834.01	0.388	552.43	214.53
A_68_P27281287	chr10:79611758-79611802	NM_021565:746	Midn	INSIDE	0.354	0.514	2491.43	1281.64	0.182	1725.91	314.69
A_68_P27281289	chr10:79611936-79611980	NM_021565:924	Midn	INSIDE	0.280	0.643	839.66	539.96	0.180	664.72	119.56

ProbeName	Target position of probe on CpG island microarray	TargetID	GeneSymbol	CpG island Description	Ratio of relative methylation (TiO2-NP/Vehicle)	Sham group			TiO_2-H group		
						Relative methylation (Cy-5/Cy-3)	Cy-3 signal (Input)	Cy-5 signal (MeDIP)	Relative methylation (Cy-5/Cy-3)	Cy-3 signal (Input)	Cy-5 signal (MeDIP)
A_68_P27281294	chr10:79612495-79612539	NM_021565:1482	Midn	INSIDE	0.144	0.575	3449.71	1982.84	0.083	2534.98	209.94
A_68_P27281295	chr10:79612639-79612683	NM_021565:1626	Midn	INSIDE	0.205	0.381	2238.43	853.50	0.078	1564.83	122.55
A_68_P27281301	chr10:79613466-79613510	NM_021565:2454	Midn	INSIDE	0.401	0.367	1465.23	537.97	0.147	1036.83	152.57
A_68_P27281304	chr10:79613780-79613824	NM_021565:2768	Midn	INSIDE	0.448	0.690	3230.35	2229.42	0.309	2156.27	667.21
A_68_P29330069	chr13:112476100-112476144	NM_172593:-263	Mier3	PROMOTER	0.332	0.706	1932.94	1364.94	0.235	1389.53	326.18
A_68_P29330079	chr13:112477883-112477927	NM_172593:1519	Mier3	INSIDE	0.310	0.295	2250.96	664.54	0.092	1566.96	143.38
A_68_P29330296	chr13:112515544-112515588	NM_172593:39181	Mier3	DOWNSTREAM	0.596	0.394	1640.41	646.06	0.235	1309.51	307.46
A_68_P28154418	chr11:115474202-115474246	NM_027162:-407	Mif4gd	PROMOTER	0.350	0.730	2201.37	1607.70	0.256	1623.92	415.03
A_68_P23391035	chr4:147242398-147242442	NM_001025365:408	Miip	INSIDE	0.381	0.537	1237.13	663.77	0.204	1010.63	206.44
A_68_P27901340	chr11:70376701-70376745	NM_001045959:340	Mink1	INSIDE	0.502	0.542	3265.26	1769.89	0.272	2467.73	671.72
A_68_P29646483	chr14:61403303-61403351	NM_027436:-76	Mipep	PROMOTER	0.363	0.587	1104.41	648.00	0.213	803.94	171.19
A_68_P28461539	chr12:58331901-58331948	NM_001164370:513	Mipol1	INSIDE	0.282	0.490	1182.98	579.77	0.138	918.97	126.93
A_68_P23154636	chr4:101028607-101028651	NR_029537:-8996	Mir101a	PROMOTER	0.186	0.564	3032.52	1710.82	0.105	2193.25	230.68
A_68_P32383983	chrX:50097448-50097492	NR_029657:-1726	Mir106a	PROMOTER	0.192	1.502	1034.70	1553.93	0.288	414.97	119.42
A_68_P24129157	chr5:138612451-138612495	NR_029658:-5426	Mir106b	PROMOTER	0.295	0.501	2923.33	1464.32	0.148	2009.96	297.20
A_68_P24129161	chr5:138612875-138612919	NR_029658:-5850	Mir106b	PROMOTER	0.427	0.734	4243.44	3116.55	0.314	2961.70	929.12
A_68_P24129162	chr5:138612940-138612988	NR_029658:-5918	Mir106b	PROMOTER	0.247	0.478	1434.04	685.99	0.118	1050.23	124.18
A_68_P28043703	chr11:96176215-96176259	NR_029784:-2242	Mir10a	PROMOTER	0.338	0.480	1343.98	645.55	0.162	1215.40	197.05
A_68_P32016082	chr19:23215759-23215803	NR_035423:-8140	Mir1192	PROMOTER	0.445	0.468	2544.85	1192.17	0.209	2104.26	438.85
A_68_P32016093	chr19:23217070-23217114	NR_035423:-6828	Mir1192	PROMOTER	0.255	0.536	1149.74	616.62	0.137	952.48	130.39
A_68_P21906900	chr2:180627890-180627934	NR_029538:-832	Mir124a-3	PROMOTER	0.370	0.452	2903.67	1311.40	0.167	2160.01	361.10
A_68_P21906910	chr2:180629073-180629117	NR_029538:350	Mir124a-3	DOWNSTREAM	0.139	0.570	1948.81	1110.72	0.079	1525.94	120.67
A_68_P21906911	chr2:180629180-180629224	NR_029538:458	Mir124a-3	DOWNSTREAM	0.420	0.570	2546.98	1450.56	0.239	2069.63	495.38
A_68_P27924627	chr11:74987037-74987081	NR_029546:-125	Mir132	PROMOTER	0.434	0.692	2353.48	1629.06	0.300	1962.89	589.02
A_68_P24443347	chr6:51220315-51220359	NR_029719:-427	Mir148a	PROMOTER	0.377	0.701	1229.88	862.39	0.264	928.52	245.22
A_68_P28047118	chr11:96711362-96711406	NR_029562:-322	Mir152	PROMOTER	0.156	0.708	1874.02	1327.33	0.110	1395.07	154.09
A_68_P22218630	chr3:68809433-68809477	NR_029529:-4239	Mir15b	PROMOTER	0.264	0.567	1025.55	580.98	0.150	802.95	120.16
A_68_P26018638	chr8:86707466-86707510	NR_029821:-4628	Mir181c	PROMOTER	0.609	0.570	1613.66	920.38	0.348	1165.56	405.13
A_68_P26345184	chr9:20841202-20841246	NR_035438:-4452	Mir1900	PROMOTER	0.152	0.572	1863.06	1065.66	0.087	1419.14	123.28
A_68_P26345185	chr9:20841278-20841322	NR_035438:-4528	Mir1900	PROMOTER	0.244	0.466	1128.85	526.57	0.114	1021.55	116.11
A_68_P26345197	chr9:20842972-20843016	NR_035438:-6222	Mir1900	PROMOTER	0.393	0.633	1129.22	715.19	0.249	905.51	225.62
A_68_P26345199	chr9:20843307-20843352	NR_035438:-6557	Mir1900	PROMOTER	0.349	0.485	1410.84	684.42	0.169	1051.61	178.19
A_68_P22314346	chr3:88340207-88340251	NR_035434:76	Mir1905	INSIDE	0.299	0.671	973.25	653.32	0.201	734.20	147.55
A_68_P27950592	chr11:79524502-79524546	NR_029579:-946	Mir193	PROMOTER	0.527	0.481	2598.95	1250.46	0.253	1890.95	479.02
A_68_P27950593	chr11:79524583-79524627	NR_029579:-866	Mir193	PROMOTER	0.139	0.587	2752.97	1614.84	0.081	1959.04	159.29
A_68_P27950599	chr11:79525184-79525228	NR_029579:-264	Mir193	PROMOTER	0.533	0.346	2199.70	761.37	0.184	1904.29	351.12
A_68_P27351981	chr10:92624097-92624141	NR_035452:-1411	Mir1931	PROMOTER	0.394	0.461	1435.67	661.71	0.182	1234.00	224.27
A_68_P28177556	chr11:119251329-119251373	NR_035453:-435	Mir1932	PROMOTER	0.212	0.618	1251.20	773.13	0.131	1163.40	152.46
A_68_P28177558	chr11:119251624-119251668	NR_035453:-139	Mir1932	PROMOTER	0.286	0.462	4271.84	1973.19	0.132	3223.29	425.76
A_68_P28377241	chr12:40949707-40949751	NR_035459:-330	Mir1938	PROMOTER	0.184	0.548	1762.34	966.09	0.101	1301.25	131.05
A_68_P30557333	chr16:13448464-13448508	NR_030549:-1129	Mir193b	PROMOTER	0.534	0.562	2408.52	1353.68	0.300	1803.85	541.31
A_68_P30557334	chr16:13448585-13448633	NR_030549:-1007	Mir193b	PROMOTER	0.185	0.700	1281.65	897.45	0.129	931.67	120.61
A_68_P30557335	chr16:13448659-13448703	NR_030549:-935	Mir193b	PROMOTER	0.517	0.585	3380.12	1976.05	0.302	2588.96	782.22
A_68_P29240037	chr13:96095372-96095416	NR_035461:-5140	Mir1940	PROMOTER	0.603	0.517	2115.72	1093.90	0.312	1826.87	569.57

ProbeName	Target position of probe on CpG island microarray	TargetID	GeneSymbol	CpG island Description	Ratio of relative methylation (TiO2-NP/Vehicle)	Sham group			TiO_2-H group		
						Relative methylation (Cy-5/Cy-3)	Cy-3 signal (Input)	Cy-5 signal (MeDIP)	Relative methylation (Cy-5/Cy-3)	Cy-3 signal (Input)	Cy-5 signal (MeDIP)
A_68_P29240038	chr13:96095444-96095488	NR_035461:-5068	Mir1940	PROMOTER	0.449	0.288	2883.13	830.94	0.129	1613.99	208.88
A_68_P30547089	chr16:11254525-11254569	NR_035466:-8	Mir1945	PROMOTER	0.334	0.565	961.64	543.77	0.189	806.61	152.19
A_68_P25025263	chr7:29870710-29870754	NR_035489:-2019	Mir1963	PROMOTER	0.651	2.608	4466.52	11647.53	1.698	2906.53	4936.17
A_68_P26134879	chr8:108137855-108137899	NR_035492:-1489	Mir1966	PROMOTER	0.369	0.547	1646.97	901.33	0.202	1453.38	293.57
A_68_P24449267	chr6:52180763-52180807	NR_029912:-620	Mir196b	PROMOTER	0.462	0.517	1553.06	802.41	0.239	1186.13	283.41
A_68_P27285700	chr10:80289932-80289982	NR_035503:-1585	Mir1982	PROMOTER	0.244	0.549	1267.57	695.71	0.134	870.10	116.62
A_68_P21114608	chr2:32167860-32167904	NR_029811:-6097	Mir199b	PROMOTER	0.061	0.545	5383.54	2932.25	0.033	3466.85	115.46
A_68_P22858433	chr4:40669503-40669547	NR_029594:-425	Mir207	PROMOTER	0.334	0.447	2107.54	941.45	0.149	1743.33	260.30
A_68_P27924617	chr11:74985774-74985818	NR_029794:-1093	Mir212	DIVERGENT_PROMOTER	0.215	0.495	2420.60	1198.01	0.106	1753.87	186.61
A_68_P27924619	chr11:74986101-74986145	NR_029794:-767	Mir212	DIVERGENT_PROMOTER	0.597	0.582	3777.86	2199.81	0.348	2972.84	1033.89
A_68_P27924623	chr11:74986635-74986679	NR_029794:-233	Mir212	DIVERGENT_PROMOTER	0.265	0.611	1122.06	685.33	0.162	738.36	119.43
A_68_P27924624	chr11:74986764-74986808	NR_029794:-103	Mir212	DIVERGENT_PROMOTER	0.370	0.589	886.91	522.01	0.218	675.07	146.83
A_68_P27924625	chr11:74986839-74986883	NR_029794:-29	Mir212	DIVERGENT_PROMOTER	0.658	0.565	2361.24	1333.63	0.372	2102.63	781.59
A_68_P27924626	chr11:74986914-74986958	NR_029794:47	Mir212	INSIDE	0.198	0.542	1497.93	811.22	0.107	1161.17	124.24
A_68_P31151557	chr17:34162040-34162084	NR_029800:-25	Mir219-1	PROMOTER	0.653	0.618	3317.33	2050.15	0.404	2651.31	1070.63
A_68_P31151558	chr17:34162154-34162198	NR_029800:-139	Mir219-1	PROMOTER	0.229	0.552	3826.60	2112.13	0.126	2635.08	333.09
A_68_P31151559	chr17:34162253-34162297	NR_029800:-237	Mir219-1	PROMOTER	0.168	0.640	2005.53	1284.41	0.108	1479.66	159.47
A_68_P31151560	chr17:34162324-34162368	NR_029800:-309	Mir219-1	PROMOTER	0.520	0.563	2440.44	1374.98	0.293	1678.09	491.25
A_68_P21099803	chr2:29701365-29701409	NR_029838:-139	Mir219-2	PROMOTER	0.305	0.588	2024.15	1190.18	0.179	1455.66	260.99
A_68_P21099809	chr2:29701916-29701960	NR_029838:-691	Mir219-2	PROMOTER	0.278	0.609	2809.15	1711.19	0.169	1983.89	336.01
A_68_P21099810	chr2:29702001-29702045	NR_029838:-775	Mir219-2	PROMOTER	0.522	0.532	1726.86	918.25	0.277	1250.61	346.80
A_68_P27926234	chr11:75275864-75275908	NR_029739:-1331	Mir22	PROMOTER	0.494	0.502	1393.46	699.44	0.248	1145.59	284.29
A_68_P26018712	chr8:86722479-86722523	NR_029740:-9916	Mir23a	PROMOTER	0.297	0.641	891.06	570.89	0.190	782.87	148.91
A_68_P20348304	chr1:74438995-74439039	NR_029743:-1867	Mir26b	PROMOTER	0.249	0.593	1025.19	607.94	0.148	823.03	121.73
A_68_P27992598	chr11:86922877-86922926	NR_029652:-3604	Mir301	PROMOTER	0.251	0.671	913.85	613.08	0.168	698.71	117.62
A_68_P27992600	chr11:86923122-86923170	NR_029652:-3359	Mir301	PROMOTER	0.045	2.344	1719.34	4029.52	0.107	1095.49	116.69
A_68_P27555852	chr11:4035495-4035539	NR_037220:-3850	Mir3060	PROMOTER	0.172	0.532	1746.82	929.11	0.092	1272.87	116.62
A_68_P28103510	chr11:106648827-106648871	NR_037224:-4775	Mir3064	PROMOTER	0.306	0.692	3660.83	2533.29	0.211	2637.22	557.72
A_68_P28103512	chr11:106648993-106649037	NR_037224:-4941	Mir3064	PROMOTER	0.318	0.560	914.62	512.44	0.178	720.70	128.57
A_68_P30722301	chr16:44558812-44558856	NR_037242:-592	Mir3081	PROMOTER	0.617	0.662	1582.35	1046.83	0.408	1317.74	538.01
A_68_P22312503	chr3:88018770-88018821	NR_037276:-297	Mir3093	PROMOTER	0.617	0.495	1146.59	567.86	0.306	811.73	248.22
A_68_P25015935	chr7:27963726-27963770	NR_037283:-2636	Mir3101	PROMOTER	0.480	0.709	1802.20	1277.94	0.341	1472.34	501.47
A_68_P26611007	chr9:69301684-69301728	NR_037296:-3044	Mir3109	PROMOTER	0.183	0.550	1407.39	774.53	0.101	1161.21	116.77
A_68_P29701297	chr14:70843346-70843390	NR_029802:52	Mir320	INSIDE	0.643	0.627	994.33	623.54	0.403	780.89	314.73
A_68_P29701298	chr14:70843425-70843469	NR_029802:130	Mir320	DOWNSTREAM	0.286	0.682	3204.31	2185.44	0.195	2370.08	462.40
A_68_P29701300	chr14:70843636-70843680	NR_029802:342	Mir320	DOWNSTREAM	0.291	0.415	1340.23	556.11	0.121	1026.45	123.74
A_68_P32385499	chrX:50410041-50410085	NR_029756:-2536	Mir322	PROMOTER	0.085	5.460	8292.22	45276.10	0.462	3869.76	1788.41
A_68_P24992976	chr7:19967388-19967432	NR_030759:-4581	Mir343	PROMOTER	0.469	0.538	1121.89	603.12	0.252	935.90	236.16
A_68_P20351170	chr1:74947336-74947380	NR_029876:-63	Mir375	PROMOTER	0.452	0.411	2496.18	1026.47	0.186	2081.25	386.55
A_68_P27935699	chr11:76891803-76891847	NR_030756:-150	Mir423	PROMOTER	0.234	0.649	2118.19	1374.49	0.152	1754.69	266.12
A_68_P30501455	chr15:102844938-102844988	NR_030526:-378	Mir615	PROMOTER	0.223	0.682	1955.63	1334.50	0.152	1482.50	225.64
A_68_P32240001	chrX:7327551-7327595	NR_030454:-2642	Mir684-1	DIVERGENT_PROMOTER	0.077	2.605	4060.53	10578.59	0.200	1527.46	304.95
A_68_P32240002	chrX:7327694-7327738	NR_030454:-2498	Mir684-1	DIVERGENT_PROMOTER	0.175	1.361	1699.90	2314.19	0.239	593.09	141.49
A_68_P29613301	chr14:55236421-55236465	NR_030457:-820	Mir686	PROMOTER	0.485	0.633	1569.75	994.38	0.307	1231.88	378.47

ProbeName	Target position of probe on CpG island microarray	TargetID	GeneSymbol	CpG island Description	Ratio of relative methylation (TiO2-NP/Vehicle)	Sham group			TiO_2-H group		
						Relative methylation (Cy-5/Cy-3)	Cy-3 signal (Input)	Cy-5 signal (MeDIP)	Relative methylation (Cy-5/Cy-3)	Cy-3 signal (Input)	Cy-5 signal (MeDIP)
A_68_P29613303	chr14:55236595-55236639	NR_030457:-994	Mir686	PROMOTER	0.281	0.556	1034.77	575.14	0.156	921.06	143.94
A_68_P29613305	chr14:55236808-55236852	NR_030457:-1208	Mir686	PROMOTER	0.556	0.656	1403.16	919.91	0.365	1204.35	439.34
A_68_P30499092	chr15:102502227-102502271	NR_030460:49	Mir688	INSIDE	0.347	0.636	921.97	586.04	0.220	662.62	145.96
A_68_P30499097	chr15:102502826-102502874	NR_030460:-553	Mir688	PROMOTER	0.205	0.601	1299.35	780.72	0.123	981.98	120.95
A_68_P30499098	chr15:102502893-102502944	NR_030460:-621	Mir688	PROMOTER	0.218	0.642	2372.14	1523.12	0.140	1712.84	239.71
A_68_P24424404	chr6:47763266-47763310	NR_030485:-9637	Mir704	PROMOTER	0.457	0.621	1463.61	908.59	0.284	1072.58	304.09
A_68_P24424405	chr6:47763384-47763429	NR_030485:-9755	Mir704	PROMOTER	0.147	0.427	2688.43	1148.66	0.063	1981.75	124.42
A_68_P24796451	chr6:119987387-119987431	NR_030487:-3079	Mir706	PROMOTER	0.135	0.530	2312.86	1226.42	0.071	1732.05	123.66
A_68_P24796456	chr6:119988018-119988063	NR_030487:-3711	Mir706	PROMOTER	0.271	0.640	2373.74	1519.40	0.173	1878.18	325.82
A_68_P24796460	chr6:119988597-119988641	NR_030487:-4289	Mir706	PROMOTER	0.205	0.575	1994.68	1147.28	0.118	1485.86	175.52
A_68_P25257803	chr7:86027612-86027656	NR_029826:-5528	Mir7-2	PROMOTER	0.592	0.595	1051.81	625.68	0.352	837.58	294.80
A_68_P27788128	chr11:50172669-50172713	NR_030529:-1309	Mir804	PROMOTER	0.257	0.575	1876.93	1079.45	0.148	1315.86	194.80
A_68_P27788132	chr11:50172976-50173028	NR_030529:-1621	Mir804	PROMOTER	0.180	0.654	1286.76	841.29	0.118	1003.26	118.06
A_68_P22312508	chr3:88019276-88019320	NR_029817:-221	Mir9-1	PROMOTER	0.588	0.675	3063.02	2066.37	0.397	2338.02	927.20
A_68_P22312509	chr3:88019343-88019387	NR_029817:-155	Mir9-1	PROMOTER	0.178	0.704	1355.53	953.77	0.125	941.17	118.08
A_68_P29187684	chr13:83884882-83884926	NR_029545:6486	Mir9-2	DOWNSTREAM	0.447	0.594	1137.36	675.25	0.265	980.16	260.10
A_68_P22317994	chr3:89031385-89031429	NR_030579:-286	Mir92b	DIVERGENT_PROMOTER	0.314	0.649	1222.94	793.11	0.204	839.30	170.98
A_68_P25261460	chr7:86649666-86649710	NR_029818:-461	Mir9-3	PROMOTER	0.526	0.659	866.11	571.20	0.347	708.77	245.94
A_68_P25261467	chr7:86650381-86650425	NR_029818:253	Mir9-3	DOWNSTREAM	0.173	0.587	1804.05	1058.83	0.101	1356.12	137.31
A_68_P29020009	chr13:48641246-48641290	NR_029725:-7627	Mirlet7a-1	PROMOTER	0.227	0.342	1970.65	673.30	0.078	1617.68	125.47
A_68_P27903938	chr11:70833216-70833260	NM_025993:126	Mis12	INSIDE	0.584	0.426	1984.46	845.79	0.249	1636.81	407.41
A_68_P27903939	chr11:70833307-70833351	NM_025993:216	Mis12	INSIDE	0.282	0.431	1217.58	524.45	0.121	981.65	119.22
A_68_P20156457	chr1:37947122-37947166	NM_026913:112	Mitd1	INSIDE	0.328	0.498	1063.49	529.09	0.163	719.51	117.41
A_68_P20877310	chr1:182626954-182626998	NM_013729:189	Mixl1	INSIDE	0.220	0.511	2298.80	1175.44	0.112	1768.12	198.67
A_68_P20877311	chr1:182627122-182627166	NM_013729:21	Mixl1	INSIDE	0.556	0.675	5597.45	3780.38	0.375	4215.78	1582.74
A_68_P20877313	chr1:182627309-182627353	NM_013729:-165	Mixl1	PROMOTER	0.554	0.623	3579.75	2230.22	0.345	2884.62	995.97
A_68_P20551218	chr1:120218342-120218386	NM_026472:-55	Mki67ip	PROMOTER	0.380	0.521	1265.95	659.71	0.198	1035.88	204.88
A_68_P20551221	chr1:120218602-120218646	NM_026472:205	Mki67ip	INSIDE	0.267	0.557	987.20	550.06	0.149	789.50	117.28
A_68_P21665425	chr2:136717109-136717162	NM_001141946:7	Mkks	INSIDE	0.221	0.647	1087.20	703.64	0.143	819.68	117.01
A_68_P23226096	chr4:115511583-115511632	NM_021461:-243	Mknk1	PROMOTER	0.269	0.576	1032.04	594.16	0.155	752.72	116.63
A_68_P23226097	chr4:115511687-115511731	NM_021461:-142	Mknk1	PROMOTER	0.464	0.598	2361.22	1413.00	0.278	1767.09	490.40
A_68_P24771973	chr6:115552217-115552261	NM_023290:283	Mkrn2	INSIDE	0.354	0.694	4289.30	2977.69	0.246	3154.72	776.37
A_68_P31475749	chr18:7002345-7002389	NM_177595:2411	Mkx	INSIDE	0.229	0.503	1655.81	832.28	0.115	1285.05	148.23
A_68_P24008164	chr5:115608110-115608154	NM_175403:53	Mlec	INSIDE	0.335	0.375	1561.27	585.26	0.125	1484.05	186.17
A_68_P24008165	chr5:115608256-115608300	NM_175403:-93	Mlec	PROMOTER	0.630	0.384	2003.34	770.11	0.242	1630.56	395.04
A_68_P30477055	chr15:98701709-98701753	NM_001033276:-116	Mll2	PROMOTER	0.337	0.432	1460.87	630.65	0.146	1215.27	176.94
A_68_P23547781	chr5:25003445-25003504	NM_001081383:1127	Mll3	INSIDE	0.230	0.613	1029.87	631.36	0.141	825.57	116.54
A_68_P23547782	chr5:25003584-25003633	NM_001081383:993	Mll3	INSIDE	0.272	0.448	1218.45	546.25	0.122	988.33	120.52
A_68_P23547790	chr5:25005052-25005096	NM_001081383:-473	Mll3	DIVERGENT_PROMOTER	0.211	0.431	1689.66	727.94	0.091	1313.71	119.23
A_68_P23536404	chr5:22938833-22938877	NM_026984:-1392	Mll5	PROMOTER	0.316	1.442	6877.37	9913.78	0.456	4615.31	2104.90
A_68_P23536409	chr5:22939362-22939412	NM_026984:-860	Mll5	PROMOTER	0.194	0.629	1221.10	768.58	0.122	1003.10	122.52
A_68_P23536410	chr5:22939501-22939545	NM_026984:-724	Mll5	PROMOTER	0.153	0.549	2304.38	1265.79	0.084	1735.97	145.75
A_68_P23536414	chr5:22940038-22940082	NM_026984:-186	Mll5	PROMOTER	0.529	0.407	1445.13	587.67	0.215	1076.97	231.55
A_68_P23536417	chr5:22940364-22940408	NM_026984:140	Mll5	INSIDE	0.221	0.425	3661.06	1556.77	0.094	2883.92	270.88

ProbeName	Target position of probe on CpG island microarray	TargetID	GeneSymbol	CpG island Description	Ratio of relative methylation (TiO2-NP/Vehicle)	Sham group			TiO_2-H group		
						Relative methylation (Cy-5/Cy-3)	Cy-3 signal (Input)	Cy-5 signal (MeDIP)	Relative methylation (Cy-5/Cy-3)	Cy-3 signal (Input)	Cy-5 signal (MeDIP)
A_68_P31259252	chr17:57075335-57075382	NM_022328:-547	Mllt1	PROMOTER	0.307	0.600	906.69	544.44	0.184	647.94	119.41
A_68_P21038062	chr2:17979342-17979387	NM_010804:2467	Mllt10	INSIDE	0.447	0.488	2359.60	1151.21	0.218	1771.60	386.70
A_68_P21038121	chr2:17986435-17986479	NM_010804:9559	Mllt10	INSIDE	0.231	0.529	2055.16	1088.18	0.122	1603.82	196.13
A_68_P23088441	chr4:87677667-87677717	NM_027326:1619	Mllt3	INSIDE	0.212	0.534	1325.92	708.62	0.113	1058.65	120.07
A_68_P23088443	chr4:87677902-87677949	NM_027326:1386	Mllt3	INSIDE	0.050	1.378	3132.38	4315.93	0.069	2132.62	147.74
A_68_P23088445	chr4:87678149-87678193	NM_027326:1141	Mllt3	INSIDE	0.097	1.463	1250.70	1829.75	0.142	851.33	121.23
A_68_P23088447	chr4:87678387-87678431	NM_027326:903	Mllt3	INSIDE	0.516	0.708	2138.29	1514.01	0.365	1765.23	645.04
A_68_P28071861	chr11:100948728-100948772	NM_001159384:147	Mlx	INSIDE	0.449	0.677	2486.91	1683.29	0.304	1903.81	578.81
A_68_P24055452	chr5:123845226-123845273	NM_177582:422	Mlxip	INSIDE	0.269	0.612	1275.11	780.85	0.165	976.05	161.01
A_68_P24114755	chr5:135583039-135583083	NM_021455:278	Mlxipl	INSIDE	0.595	0.605	1122.30	678.59	0.360	990.96	356.51
A_68_P24114757	chr5:135583228-135583272	NM_021455:468	Mlxipl	INSIDE	0.178	0.696	1265.56	880.64	0.124	935.18	115.78
A_68_P25993841	chr8:81819136-81819180	NM_133823:-303	Mmaa	PROMOTER	0.173	0.565	1655.96	936.07	0.098	1177.56	115.08
A_68_P25993842	chr8:81819240-81819295	NM_133823:-412	Mmaa	PROMOTER	0.167	0.521	1868.13	972.73	0.087	1329.92	115.54
A_68_P24004044	chr5:114894084-114894128	NM_029956:-70	Mmab	DIVERGENT_PROMOTER	0.554	0.504	1070.43	539.18	0.279	929.38	259.14
A_68_P28010558	chr11:90110449-90110493	NM_026178:-319	Mmd	PROMOTER	0.285	0.653	1230.30	802.83	0.186	966.13	179.74
A_68_P28010563	chr11:90111119-90111163	NM_026178:351	Mmd	INSIDE	0.182	0.537	1392.39	747.76	0.098	1216.44	119.17
A_68_P24152505	chr5:143084497-143084543	NM_175217:186	Mmd2	INSIDE	0.165	0.645	1646.80	1062.90	0.107	1105.43	117.73
A_68_P22190746	chr3:63099501-63099545	NM_008604:-271	Mme	PROMOTER	0.525	0.555	3999.99	2220.29	0.292	3196.57	931.94
A_68_P32396639	chrX:53851315-53851359	NM_146234:-240	Mmgt1	PROMOTER	0.090	1.981	2044.38	4050.73	0.179	969.54	173.19
A_68_P27855086	chr11:62462305-62462349	NM_175002:161	Mmgt2	INSIDE	0.273	0.670	1593.12	1067.87	0.183	1107.38	203.00
A_68_P27855153	chr11:62478528-62478583	NM_175002:16390	Mmgt2	INSIDE	0.582	2.520	1615.21	4069.55	1.466	1313.96	1926.60
A_68_P29612399	chr14:55051483-55051527	NM_008608:1064	Mmp14	INSIDE	0.293	0.657	2064.39	1357.11	0.193	1585.31	305.40
A_68_P26082839	chr8:97877231-97877275	NM_008609:1016	Mmp15	INSIDE	0.528	0.638	884.76	564.61	0.337	719.94	242.36
A_68_P26082840	chr8:97877404-97877448	NM_008609:1190	Mmp15	INSIDE	0.237	0.581	1182.59	687.06	0.137	876.63	120.49
A_68_P22758679	chr4:17780394-17780442	NM_019724:-210	Mmp16	PROMOTER	0.263	1.556	704.55	1096.48	0.410	614.72	251.83
A_68_P24089919	chr5:130089703-130089747	NM_011846:-364	Mmp17	PROMOTER	0.544	0.649	3126.10	2027.88	0.353	2301.27	812.81
A_68_P24089926	chr5:130090512-130090556	NM_011846:446	Mmp17	INSIDE	0.547	0.421	1943.77	818.03	0.230	1667.85	383.78
A_68_P26068080	chr8:95351388-95351432	NM_008610:184	Mmp2	INSIDE	0.624	0.530	1233.21	653.13	0.330	971.02	320.76
A_68_P23439841	chr4:155027081-155027125	NM_011985:391	Mmp23	INSIDE	0.621	0.721	3407.25	2456.30	0.448	2512.20	1125.54
A_68_P23439844	chr4:155027433-155027477	NM_011985:39	Mmp23	INSIDE	0.160	0.664	1677.61	1114.23	0.106	1142.94	121.64
A_68_P21767513	chr2:155601572-155601616	NM_010808:515	Mmp24	INSIDE	0.236	1.833	7977.50	14624.27	0.433	5165.28	2239.08
A_68_P21820676	chr2:164774969-164775013	NM_013599:1240	Mmp9	INSIDE	0.081	1.346	3257.25	4385.41	0.110	2138.71	234.40
A_68_P22785948	chr4:24423938-24423984	NM_199467:352	Mms22l	INSIDE	0.345	0.554	922.74	511.39	0.191	630.84	120.57
A_68_P23985659	chr5:111846818-111846862	NM_001081235:-345	Mn1	PROMOTER	0.537	0.453	1403.31	635.91	0.243	1104.49	268.77
A_68_P23985662	chr5:111847249-111847293	NM_001081235:85	Mn1	INSIDE	0.389	0.733	1915.89	1404.16	0.285	1480.94	422.52
A_68_P23985663	chr5:111847362-111847406	NM_001081235:199	Mn1	INSIDE	0.421	0.575	1005.17	578.01	0.242	781.17	189.07
A_68_P23985671	chr5:111848092-111848136	NM_001081235:929	Mn1	INSIDE	0.294	0.648	3672.91	2380.26	0.191	2489.23	474.22
A_68_P23985674	chr5:111848466-111848510	NM_001081235:1303	Mn1	INSIDE	0.377	0.643	1622.85	1042.79	0.242	1169.35	283.36
A_68_P22289190	chr3:83959655-83959699	NM_029797:32	Mnd1	INSIDE	0.625	0.562	2696.81	1514.78	0.351	2103.73	738.87
A_68_P22289192	chr3:83959834-83959879	NM_029797:-148	Mnd1	PROMOTER	0.241	0.577	1528.94	881.90	0.139	1204.95	167.29
A_68_P27922700	chr11:74645583-74645627	NM_010813:1179	Mnt	INSIDE	0.593	0.482	1154.05	556.61	0.286	1009.08	288.46
A_68_P27922753	chr11:74651662-74651706	NM_010813:7259	Mnt	INSIDE	0.265	0.537	1150.75	617.92	0.142	851.75	121.25
A_68_P27922757	chr11:74652026-74652070	NM_010813:7623	Mnt	INSIDE	0.276	0.595	1916.07	1140.92	0.164	1581.50	259.85
A_68_P23567756	chr5:29800752-29800798	NM_019944:4236	Mnx1	INSIDE	0.268	1.501	821.61	1232.99	0.402	556.60	223.56

ProbeName	Target position of probe on CpG island microarray	TargetID	GeneSymbol	CpG island Description	Ratio of relative methylation (TiO2-NP/Vehicle)	Sham group			TiO_2-H group		
						Relative methylation (Cy-5/Cy-3)	Cy-3 signal (Input)	Cy-5 signal (MeDIP)	Relative methylation (Cy-5/Cy-3)	Cy-3 signal (Input)	Cy-5 signal (MeDIP)
A_68_P23567792	chr5:29805215-29805259	NM_019944:-226	Mnx1	PROMOTER	0.432	0.649	2226.04	1445.38	0.281	1642.90	460.89
A_68_P28693441	chr12:103981601-103981648	NM_001142937:246	Moap1	INSIDE	0.195	0.666	1141.53	760.21	0.130	952.75	123.70
A_68_P24592786	chr6:83276392-83276436	NM_145571:382	Mobkl1b	INSIDE	0.550	0.506	1381.30	698.94	0.278	1160.57	322.90
A_68_P27284924	chr10:80164367-80164423	NM_172457:171	Mobkl2a	INSIDE	0.301	0.506	1100.89	556.99	0.153	794.12	121.12
A_68_P31222784	chr17:49567933-49567977	NM_020042:266	Mocs1	INSIDE	0.258	0.623	1012.45	631.16	0.161	833.11	134.07
A_68_P29348446	chr13:115608303-115608349	NM_001113374:-118	Mocs2	PROMOTER	0.221	0.625	1209.20	755.22	0.138	874.37	120.87
A_68_P29348448	chr13:115608501-115608545	NM_001113374:78	Mocs2	INSIDE	0.095	0.685	3500.56	2396.99	0.065	2555.47	166.97
A_68_P29348449	chr13:115608614-115608659	NM_001113374:192	Mocs2	INSIDE	0.325	0.651	973.07	633.63	0.211	793.11	167.72
A_68_P26811671	chr9:107804092-107804136	NM_028369:13655	Mon1a	INSIDE	0.612	0.601	994.45	597.82	0.368	768.57	282.58
A_68_P27553117	chr11:3549252-3549296	NM_001159288:-222	Morc2a	DIVERGENT_PROMOTER	0.428	0.415	1488.45	617.90	0.178	1220.63	217.11
A_68_P27553119	chr11:3549485-3549529	NM_198162:-9196	Morc2a	PROMOTER	0.440	0.401	2147.12	860.50	0.176	1708.81	301.18
A_68_P30977847	chr16:93832195-93832251	NM_001045529:-143	Morc3	PROMOTER	0.168	0.605	1539.24	931.50	0.101	1154.19	117.05
A_68_P32696393	chrX:136405600-136405644	NM_001193309:571	Morc4	INSIDE	0.290	1.434	3195.96	4582.39	0.415	1254.52	520.76
A_68_P32696394	chrX:136405677-136405721	NM_001193309:495	Morc4	INSIDE	0.101	1.642	2535.81	4164.73	0.166	1103.02	182.57
A_68_P32696398	chrX:136406178-136406222	NM_001193309:-7	Morc4	PROMOTER	0.168	1.980	2753.63	5451.06	0.332	1044.26	346.92
A_68_P26716008	chr9:90008880-90008924	NM_001039147:756	Morf4l1	INSIDE	0.530	0.675	5682.17	3835.14	0.358	4100.41	1466.62
A_68_P26716009	chr9:90009071-90009118	NM_001039147:564	Morf4l1	INSIDE	0.453	0.666	964.42	642.65	0.302	718.09	216.88
A_68_P26716011	chr9:90009342-90009386	NM_001039147:294	Morf4l1	INSIDE	0.264	1.700	6468.15	10998.33	0.448	4107.86	1841.19
A_68_P26716013	chr9:90009639-90009683	NM_001039147:-2	Morf4l1	PROMOTER	0.569	0.544	1835.28	998.48	0.310	1452.06	449.68
A_68_P32682200	chrX:133276451-133276498	NM_019768:-326	Morf4l2	PROMOTER	0.210	2.160	1277.76	2760.01	0.454	556.76	252.51
A_68_P24126542	chr5:138041887-138041931	NM_030037:360	Mospd3	INSIDE	0.420	0.710	3325.86	2360.03	0.298	2661.94	794.19
A_68_P24126543	chr5:138042018-138042062	NM_030037:228	Mospd3	INSIDE	0.647	0.583	1499.36	874.05	0.377	1140.88	430.06
A_68_P26204794	chr8:120325573-120325617	NM_026758:235	Mphosph6	INSIDE	0.652	0.561	1212.15	679.99	0.366	1011.10	369.93
A_68_P26204800	chr8:120326079-120326123	NM_026758:-271	Mphosph6	PROMOTER	0.375	0.560	1128.23	632.06	0.210	997.82	209.56
A_68_P26204801	chr8:120326174-120326218	NM_026758:-367	Mphosph6	PROMOTER	0.266	0.505	1151.05	581.37	0.135	911.11	122.60
A_68_P29624050	chr14:57287581-57287630	NM_023773:521	Mphosph8	INSIDE	0.103	1.468	1563.36	2294.48	0.151	1049.13	158.53
A_68_P26542420	chr9:57400417-57400461	NM_025837:121	Mpi	INSIDE	0.218	0.632	1306.03	824.99	0.138	889.75	122.52
A_68_P32470782	chrX:72376286-72376330	NM_008621:-20	Mpp1	PROMOTER	0.169	1.549	3268.88	5064.48	0.262	1340.62	350.77
A_68_P28077432	chr11:101948559-101948603	NM_016695:1249	Mpp2	INSIDE	0.231	0.591	1080.73	639.08	0.137	843.37	115.31
A_68_P28077443	chr11:101949878-101949922	NM_016695:-71	Mpp2	PROMOTER	0.233	0.589	3476.25	2048.62	0.138	2582.86	355.15
A_68_P28077065	chr11:101887780-101887824	NM_007863:467	Mpp3	INSIDE	0.138	0.486	2188.74	1063.17	0.067	1777.48	118.97
A_68_P28077066	chr11:101887900-101887944	NM_007863:347	Mpp3	INSIDE	0.499	0.653	1395.17	911.44	0.326	1050.26	342.28
A_68_P28565713	chr12:79849962-79850008	NM_019579:51	Mpp5	INSIDE	0.128	1.530	971.41	1486.55	0.196	804.23	157.64
A_68_P21501826	chr2:106534164-106534208	NM_029837:-1571	Mpped2	PROMOTER	0.255	0.694	2711.45	1882.62	0.177	2027.49	358.99
A_68_P21501839	chr2:106535501-106535545	NM_029837:-235	Mpped2	PROMOTER	0.467	0.609	1671.72	1017.64	0.284	1385.85	394.21
A_68_P27838142	chr11:59475681-59475726	NM_012027:-293	Mprip	PROMOTER	0.590	0.489	2725.32	1333.89	0.289	2117.86	611.96
A_68_P27838143	chr11:59475883-59475927	NM_012027:-92	Mprip	PROMOTER	0.291	0.608	848.32	515.51	0.177	678.35	119.82
A_68_P27838147	chr11:59476518-59476570	NM_012027:548	Mprip	INSIDE	0.078	1.462	1401.86	2049.00	0.114	1050.26	120.02
A_68_P30359737	chr15:78236916-78236960	NM_001162493:-203	Mpst	DIVERGENT_PROMOTER	0.253	0.601	1041.60	626.15	0.152	787.95	119.64
A_68_P30359739	chr15:78237110-78237154	NM_001162493:-9	Mpst	DIVERGENT_PROMOTER	0.395	0.517	1965.86	1015.55	0.204	1519.10	309.59
A_68_P30359767	chr15:78240574-78240618	NM_138670:3063	Mpst	INSIDE	0.401	0.682	1086.67	740.80	0.274	845.19	231.31
A_68_P30359769	chr15:78240781-78240825	NM_138670:3269	Mpst	INSIDE	0.381	0.617	1060.37	654.13	0.235	771.69	181.47
A_68_P30559668	chr16:13941247-13941291	NM_033564:481	Mpv17l	INSIDE	0.121	0.701	2164.81	1518.24	0.085	1616.77	137.42
A_68_P25953851	chr8:73284450-73284494	NM_183170:348	Mpv17l2	INSIDE	0.314	0.634	957.66	607.23	0.199	768.90	153.06

ProbeName	Target position of probe on CpG island microarray	TargetID	GeneSymbol	CpG island Description	Ratio of relative methylation (TiO2-NP/Vehicle)	Sham group			TiO_2-H group		
						Relative methylation (Cy-5/Cy-3)	Cy-3 signal (Input)	Cy-5 signal (MeDIP)	Relative methylation (Cy-5/Cy-3)	Cy-3 signal (Input)	Cy-5 signal (MeDIP)
A_68_P25953855	chr8:73284899-73284943	NM_183170:-100	Mpv17l2	PROMOTER	0.405	0.408	2453.08	1000.94	0.165	1787.56	295.51
A_68_P20795497	chr1:167564136-167564180	NM_001001880:514	Mpzl1	INSIDE	0.624	0.675	1095.66	739.84	0.421	918.17	386.86
A_68_P26319355	chr9:14588836-14588880	NM_018736:-292	Mre11a	DIVERGENT_PROMOTER	0.390	0.581	1999.35	1161.35	0.227	1453.26	329.26
A_68_P23609669	chr5:37187204-37187251	NM_026242:766	Mrfap1	INSIDE	0.199	0.677	1172.98	794.28	0.135	880.28	118.67
A_68_P23609675	chr5:37187966-37188010	NM_026242:5	Mrfap1	INSIDE	0.465	0.470	1789.03	840.97	0.219	1560.60	341.40
A_68_P26019014	chr8:86780727-86780772	NM_026423:474	Mri1	INSIDE	0.275	1.820	1382.34	2516.20	0.500	1033.50	516.86
A_68_P29630112	chr14:58445036-58445080	NM_026401:-17	Mrp63	DIVERGENT_PROMOTER	0.238	0.635	987.47	627.24	0.151	835.14	126.44
A_68_P23905255	chr5:96638963-96639007	NM_001039084:-149	Mrpl1	PROMOTER	0.502	0.715	1586.70	1133.80	0.359	1245.38	446.86
A_68_P23905257	chr5:96639210-96639261	NM_001039084:102	Mrpl1	INSIDE	0.283	0.603	1236.33	745.79	0.171	1003.15	171.15
A_68_P31926078	chr19:4962474-4962518	NM_025553:191	Mrpl11	INSIDE	0.329	0.617	1433.26	883.99	0.203	1195.39	242.70
A_68_P28184854	chr11:120345796-120345840	NM_027204:-164	Mrpl12	PROMOTER	0.308	0.415	1306.21	542.07	0.128	973.52	124.46
A_68_P30232953	chr15:55388796-55388840	NM_026759:49	Mrpl13	INSIDE	0.413	0.473	2660.21	1257.09	0.195	2051.67	400.62
A_68_P31202532	chr17:45823669-45823714	NM_026732:371	Mrpl14	INSIDE	0.253	0.738	2348.99	1732.95	0.187	1638.74	306.16
A_68_P25394533	chr7:112959197-112959241	NM_025301:383	Mrpl17	INSIDE	0.179	0.522	1705.03	890.18	0.093	1259.14	117.49
A_68_P25394534	chr7:112959304-112959348	NM_025301:275	Mrpl17	INSIDE	0.482	0.653	1215.91	794.16	0.315	937.64	295.07
A_68_P25394540	chr7:112959842-112959886	NM_025301:-263	Mrpl17	PROMOTER	0.062	0.675	4014.69	2708.01	0.042	2894.92	120.14
A_68_P31055436	chr17:13109119-13109167	NM_026310:-185	Mrpl18	DIVERGENT_PROMOTER	0.178	0.692	1534.51	1061.22	0.123	1131.07	139.01
A_68_P31055437	chr17:13109036-13109080	NM_026310:-101	Mrpl18	DIVERGENT_PROMOTER	0.161	0.518	1969.15	1019.98	0.083	1380.44	115.13
A_68_P31055438	chr17:13108951-13108995	NM_026310:-15	Mrpl18	DIVERGENT_PROMOTER	0.246	0.555	2004.58	1111.78	0.137	1378.10	188.36
A_68_P31055439	chr17:13108867-13108911	NM_026310:69	Mrpl18	INSIDE	0.306	0.391	1728.34	675.39	0.119	1306.05	155.93
A_68_P22310708	chr3:87723736-87723780	NM_026591:293	Mrpl24	INSIDE	0.185	0.613	1347.00	826.36	0.113	1023.99	116.13
A_68_P28839394	chr13:14705133-14705177	NM_029271:150	Mrpl32	INSIDE	0.661	0.567	912.05	517.10	0.375	814.12	305.27
A_68_P23579047	chr5:31916324-31916368	NM_025796:23	Mrpl33	INSIDE	0.295	0.611	1447.93	884.64	0.180	1142.65	205.92
A_68_P23188355	chr4:106739488-106739543	NM_025500:-44	Mrpl37	DIVERGENT_PROMOTER	0.281	0.506	1134.27	573.53	0.142	858.86	121.88
A_68_P23188357	chr4:106739700-106739748	NM_025500:-253	Mrpl37	DIVERGENT_PROMOTER	0.188	0.589	1769.24	1042.63	0.111	1397.62	154.83
A_68_P23188358	chr4:106739774-106739823	NM_025500:-327	Mrpl37	DIVERGENT_PROMOTER	0.351	0.625	3049.70	1907.51	0.219	2132.82	468.02
A_68_P28157575	chr11:115999733-115999777	NM_024177:428	Mrpl38	INSIDE	0.584	0.723	2131.30	1540.49	0.422	1658.02	700.46
A_68_P28157577	chr11:115999946-115999990	NM_024177:214	Mrpl38	INSIDE	0.398	0.493	3724.44	1836.53	0.196	2843.38	558.00
A_68_P26344986	chr9:20807641-20807685	NM_023167:482	Mrpl4	INSIDE	0.148	0.579	2912.47	1686.79	0.086	2153.49	184.72
A_68_P21069127	chr2:24830756-24830800	NM_001031808:-160	Mrpl41	DIVERGENT_PROMOTER	0.303	0.401	1398.91	561.51	0.122	991.06	120.44
A_68_P22039663	chr3:32635419-32635463	NM_029017:237	Mrpl47	INSIDE	0.220	0.602	1443.47	868.54	0.132	1078.72	142.90
A_68_P25368570	chr7:107731384-107731428	NM_198831:238	Mrpl48	INSIDE	0.087	0.577	3498.30	2017.03	0.050	2586.56	129.01
A_68_P25368688	chr7:107756761-107756805	NR_003559:-272	Mrpl48	PROMOTER	0.433	0.488	1437.47	702.13	0.212	1100.89	233.06
A_68_P24819300	chr6:125141938-125141982	NM_025595:-257	Mrpl51	DIVERGENT_PROMOTER	0.185	0.667	1176.60	784.24	0.124	953.32	117.85
A_68_P24591483	chr6:83058946-83058997	NM_026744:-130	Mrpl53	PROMOTER	0.247	0.554	1065.49	590.41	0.137	862.94	117.92
A_68_P24591488	chr6:83059493-83059537	NM_026744:413	Mrpl53	INSIDE	0.253	0.554	992.61	550.16	0.140	842.28	117.95
A_68_P27835445	chr11:59016315-59016359	NM_026035:318	Mrpl55	INSIDE	0.239	0.638	825.75	526.60	0.153	794.02	121.16
A_68_P22339047	chr3:94247097-94247141	NM_030116:-139	Mrpl9	DIVERGENT_PROMOTER	0.665	0.700	1718.75	1203.68	0.466	1528.25	711.60
A_68_P22339050	chr3:94247442-94247486	NM_030116:207	Mrpl9	INSIDE	0.467	0.706	2630.32	1858.27	0.330	2047.71	676.03
A_68_P22339052	chr3:94247698-94247742	NM_030116:463	Mrpl9	INSIDE	0.283	0.443	1707.10	755.57	0.125	1209.25	151.40
A_68_P20765301	chr1:162125216-162125275	NM_025474:-145	Mrps14	PROMOTER	0.175	0.647	2204.54	1426.05	0.113	1582.08	178.69
A_68_P23277861	chr4:125724192-125724236	NM_025544:43	Mrps15	INSIDE	0.339	0.569	1751.68	996.75	0.193	1354.29	261.39
A_68_P29446629	chr14:21212600-21212644	NM_025440:155	Mrps16	INSIDE	0.196	0.543	1403.41	761.67	0.106	1137.12	120.71
A_68_P29446632	chr14:21212949-21212993	NM_025440:-193	Mrps16	PROMOTER	0.354	0.325	2585.87	839.18	0.115	2020.51	232.14

ProbeName	Target position of probe on CpG island microarray	TargetID	GeneSymbol	CpG island Description	Ratio of relative methylation (TiO2-NP/Vehicle)	Sham group			TiO_2-H group		
						Relative methylation (Cy-5/Cy-3)	Cy-3 signal (Input)	Cy-5 signal (MeDIP)	Relative methylation (Cy-5/Cy-3)	Cy-3 signal (Input)	Cy-5 signal (MeDIP)
A_68_P31205113	chr17:46247925-46247969	NM_026768:-6	Mrps18a	PROMOTER	0.375	0.423	1745.63	739.22	0.159	1403.26	222.69
A_68_P31161697	chr17:36053340-36053384	NM_025878:-48	Mrps18b	DIVERGENT_PROMOTER	0.641	0.574	1546.78	887.48	0.368	1262.04	464.16
A_68_P23931503	chr5:101227718-101227762	NM_026826:-37	Mrps18c	DIVERGENT_PROMOTER	0.191	0.626	2457.91	1538.68	0.120	1769.91	211.62
A_68_P27998472	chr11:88017697-88017741	NM_024174:-201	Mrps23	PROMOTER	0.164	0.710	2072.68	1470.72	0.116	1545.59	179.32
A_68_P27998475	chr11:88018009-88018053	NM_024174:111	Mrps23	INSIDE	0.234	0.715	2166.43	1548.72	0.167	1692.33	282.84
A_68_P24643637	chr6:92133635-92133679	NM_025578:361	Mrps25	INSIDE	0.258	0.471	1218.06	574.11	0.121	998.31	121.24
A_68_P24643638	chr6:92133742-92133786	NM_025578:253	Mrps25	INSIDE	0.390	0.494	1982.78	978.66	0.192	1582.60	304.52
A_68_P29368030	chr13:119176079-119176123	NM_021556:-41	Mrps30	PROMOTER	0.241	0.488	1388.29	677.38	0.118	1022.53	120.20
A_68_P24385094	chr6:39760759-39760803	NM_001010930:155	Mrps33	INSIDE	0.253	0.499	1094.41	546.56	0.126	946.97	119.67
A_68_P24932172	chr6:146991149-146991196	NM_145573:-119	Mrps35	PROMOTER	0.205	0.650	968.71	629.59	0.133	916.27	122.09
A_68_P24932173	chr6:146991217-146991261	NM_145573:-53	Mrps35	PROMOTER	0.593	0.358	1804.95	645.93	0.212	1586.10	336.34
A_68_P29269917	chr13:101514369-101514413	NM_001190264:224	Mrps36	INSIDE	0.393	0.562	1483.16	833.55	0.221	1137.24	251.42
A_68_P21614449	chr2:127413281-127413325	NM_029963:141	Mrps5	INSIDE	0.376	0.672	1546.53	1039.02	0.253	1288.59	325.64
A_68_P21614450	chr2:127413402-127413446	NM_029963:263	Mrps5	INSIDE	0.481	0.702	1575.99	1106.42	0.337	1167.14	393.86
A_68_P30966735	chr16:92059004-92059048	NM_080456:446	Mrps6	INSIDE	0.288	0.491	1978.11	971.22	0.141	1636.30	231.32
A_68_P20185543	chr1:42908342-42908386	NM_023514:287	Mrps9	INSIDE	0.613	0.465	1569.81	729.99	0.285	1272.81	363.00
A_68_P20185544	chr1:42908420-42908464	NM_023514:365	Mrps9	INSIDE	0.328	0.678	1127.31	763.76	0.222	885.66	196.52
A_68_P21138065	chr2:35991976-35992020	NM_026422:81	Mrrf	INSIDE	0.290	0.380	1379.22	524.74	0.110	1059.61	116.93
A_68_P31424458	chr17:88071727-88071771	NM_008628:-148	Msh2	PROMOTER	0.349	0.557	1109.81	618.20	0.194	936.25	181.98
A_68_P29223483	chr13:93125415-93125459	NM_010829:-478	Msh3	PROMOTER	0.414	0.660	1694.35	1118.52	0.273	1384.23	377.98
A_68_P24009559	chr5:115886650-115886694	NM_008629:6979	Msi1	INSIDE	0.265	0.634	963.95	611.18	0.168	745.10	125.39
A_68_P24009561	chr5:115886828-115886872	NM_008629:7157	Msi1	INSIDE	0.389	0.420	1710.15	717.48	0.163	1361.24	222.06
A_68_P28058585	chr11:98656131-98656175	NM_028722:-930	Msl1	PROMOTER	0.112	0.556	2952.96	1640.98	0.062	2016.57	125.79
A_68_P28058586	chr11:98656221-98656265	NM_028722:-840	Msl1	PROMOTER	0.152	0.653	5236.24	3417.67	0.099	3424.99	339.57
A_68_P26772694	chr9:100979083-100979130	NM_001100451:656	Msl2	INSIDE	0.212	0.570	1325.78	756.27	0.121	1057.20	127.87
A_68_P27154483	chr10:55835222-55835266	NM_001163833:8522	Msl3l2	INSIDE	0.443	0.444	1207.43	535.66	0.197	935.63	184.04
A_68_P32543772	chrX:93291348-93291392	NM_010833:-13	Msn	PROMOTER	0.305	2.325	4448.28	10340.82	0.709	1856.50	1315.46
A_68_P29667754	chr14:65059716-65059760	NM_026322:15002	Msra	INSIDE	0.297	0.607	853.63	518.11	0.181	685.33	123.71
A_68_P21044522	chr2:19293408-19293452	NM_029619:168	Msrb2	INSIDE	0.269	0.576	2781.09	1603.02	0.155	1989.46	308.99
A_68_P23615663	chr5:38208346-38208390	NM_010835:7456	Msx1	DOWNSTREAM	0.303	1.765	3893.21	6873.03	0.535	2698.87	1444.12
A_68_P23615664	chr5:38208449-38208493	NM_010835:7354	Msx1	DOWNSTREAM	0.651	2.478	2092.20	5184.99	1.614	1476.33	2383.32
A_68_P23615749	chr5:38218796-38218844	NM_010835:-2996	Msx1	PROMOTER	0.097	0.700	2498.75	1748.87	0.068	1757.86	119.86
A_68_P29044037	chr13:53557736-53557785	NM_013601:10389	Msx2	DOWNSTREAM	0.367	0.659	1970.32	1299.01	0.242	1359.38	328.66
A_68_P25581948	chr7:147233969-147234013	NM_010836:997	Msx3	INSIDE	0.463	0.570	1680.05	957.30	0.264	1210.76	319.27
A_68_P25581952	chr7:147234431-147234477	NM_010836:533	Msx3	INSIDE	0.165	0.681	2794.24	1902.77	0.112	1971.31	221.10
A_68_P25581954	chr7:147234659-147234703	NM_010836:307	Msx3	INSIDE	0.578	0.504	2172.19	1094.67	0.291	1560.04	454.44
A_68_P25582013	chr7:147243953-147243997	NM_010836:-8987	Msx3	PROMOTER	0.126	0.660	2504.54	1653.55	0.083	1781.17	148.44
A_68_P26075899	chr8:96703109-96703153	NM_013602:142	Mt1	INSIDE	0.442	0.498	1026.81	511.41	0.220	911.68	200.51
A_68_P26075855	chr8:96696139-96696185	NM_008630:-355	Mt2	PROMOTER	0.273	0.652	1159.31	755.79	0.178	994.54	177.13
A_68_P26075858	chr8:96696472-96696516	NM_008630:-23	Mt2	PROMOTER	0.249	0.518	1334.06	691.64	0.129	1082.89	139.77
A_68_P26075859	chr8:96696623-96696667	NM_008630:127	Mt2	INSIDE	0.388	0.556	1281.29	712.61	0.216	1053.10	227.32
A_68_P26075861	chr8:96696797-96696851	NM_008630:307	Mt2	INSIDE	0.317	0.474	1129.70	535.59	0.150	913.33	137.27
A_68_P31400333	chr17:84105260-84105304	NM_001171053:-220	Mta3	PROMOTER	0.579	0.580	2863.75	1660.20	0.336	2363.73	793.91
A_68_P31400335	chr17:84105596-84105640	NM_001171053:116	Mta3	INSIDE	0.379	0.340	1941.82	660.09	0.129	1577.14	203.21

ProbeName	Target position of probe on CpG island microarray	TargetID	GeneSymbol	CpG island Description	Ratio of relative methylation (TiO2-NP/Vehicle)	Sham group			TiO_2-H group		
						Relative methylation (Cy-5/Cy-3)	Cy-3 signal (Input)	Cy-5 signal (MeDIP)	Relative methylation (Cy-5/Cy-3)	Cy-3 signal (Input)	Cy-5 signal (MeDIP)
A_68_P25092688	chr7:52622594-52622638	NR_027802:14	Mtag2	INSIDE	0.248	0.424	2052.66	870.38	0.105	1640.76	172.55
A_68_P25092691	chr7:52622936-52622980	NR_027802:356	Mtag2	INSIDE	0.253	0.565	1167.31	660.00	0.143	844.92	120.84
A_68_P25092692	chr7:52623070-52623114	NR_027802:490	Mtag2	INSIDE	0.234	0.589	1156.52	681.03	0.138	914.46	126.01
A_68_P23094197	chr4:88783582-88783626	NM_024433:331	Mtap	INSIDE	0.312	0.678	1560.00	1057.61	0.212	1202.35	254.49
A_68_P21579978	chr2:121114942-121114992	NM_032393:-371	Mtap1a	PROMOTER	0.361	0.485	1371.23	664.89	0.175	1114.24	195.15
A_68_P21579979	chr2:121115051-121115098	NM_032393:-263	Mtap1a	PROMOTER	0.258	0.454	1406.73	638.29	0.117	1024.38	119.88
A_68_P29263987	chr13:100285787-100285831	NM_008634:749	Mtap1b	INSIDE	0.413	0.593	1126.62	667.52	0.245	890.06	217.62
A_68_P25954854	chr8:73430001-73430045	NM_173013:150	Mtap1s	INSIDE	0.173	0.716	9666.59	6923.16	0.124	6411.88	792.74
A_68_P26821060	chr9:109834141-109834185	NM_001205331:-115	Mtap4	PROMOTER	0.641	0.661	1010.27	667.28	0.423	961.08	406.93
A_68_P26821062	chr9:109834457-109834501	NM_001205331:201	Mtap4	INSIDE	0.513	0.548	2387.42	1308.77	0.281	1888.78	530.86
A_68_P26821065	chr9:109834739-109834783	NM_001205331:483	Mtap4	INSIDE	0.601	0.735	2605.53	1914.22	0.441	1897.80	837.46
A_68_P26821068	chr9:109835124-109835175	NM_001205331:872	Mtap4	INSIDE	0.105	0.690	2234.59	1541.09	0.072	1615.81	116.74
A_68_P25360738	chr7:106415893-106415949	NM_001048167:-36	Mtap6	PROMOTER	0.212	0.709	1944.04	1378.28	0.151	1326.39	199.73
A_68_P25360744	chr7:106416416-106416460	NM_001043355:-417	Mtap6	PROMOTER	0.223	0.511	2418.34	1236.69	0.114	1740.19	198.36
A_68_P26985108	chr10:19868390-19868434	NM_001198635:-313	Mtap7	PROMOTER	0.630	0.627	960.34	601.97	0.395	830.65	328.19
A_68_P23278939	chr4:125911753-125911803	NM_001145970:21785	Mtap7d1	INSIDE	0.451	3.465	1273.29	4411.55	1.561	1031.98	1610.84
A_68_P23279071	chr4:125932970-125933014	NM_001145970:571	Mtap7d1	INSIDE	0.531	0.416	3133.26	1304.23	0.221	2249.58	497.33
A_68_P32774622	chrX:155853067-155853111	NM_001081124:579	Mtap7d2	INSIDE	0.255	1.575	2887.48	4549.12	0.401	1260.64	505.77
A_68_P32774623	chrX:155853132-155853176	NM_001081124:645	Mtap7d2	INSIDE	0.220	1.352	4018.89	5431.73	0.298	1763.61	525.16
A_68_P22278905	chr3:82162143-82162187	NM_001081230:171	Mtap9	INSIDE	0.392	0.487	1290.87	628.19	0.191	1051.49	200.76
A_68_P22278908	chr3:82162460-82162514	NM_001081230:493	Mtap9	INSIDE	0.266	0.442	1225.07	541.92	0.118	993.17	117.05
A_68_P30232954	chr15:55388910-55388954	NM_001168250:-30	Mtbp	DIVERGENT_PROMOTER	0.397	0.432	3116.90	1347.01	0.172	2372.98	407.34
A_68_P21419237	chr2:90687518-90687562	NM_019758:229	Mtch2	INSIDE	0.239	0.403	1534.02	617.76	0.096	1247.03	120.04
A_68_P21419238	chr2:90687606-90687650	NM_019758:317	Mtch2	INSIDE	0.578	0.484	1622.41	785.73	0.280	1170.82	327.70
A_68_P32471624	chrX:72661844-72661888	NM_010839:16	Mtcp1	INSIDE	0.246	1.875	2811.14	5270.45	0.461	1255.02	578.78
A_68_P30126794	chr15:34012255-34012299	NM_026002:-197	Mtdh	PROMOTER	0.281	0.703	2580.46	1815.24	0.197	1925.28	380.12
A_68_P30126800	chr15:34012857-34012901	NM_026002:405	Mtdh	INSIDE	0.233	0.566	1116.12	632.15	0.132	971.16	128.37
A_68_P20454694	chr1:95202421-95202465	NM_178051:5	Mterfd2	INSIDE	0.662	0.577	1626.22	938.73	0.382	1052.21	401.83
A_68_P23970077	chr5:108494745-108494789	NM_013827:6	Mtf2	INSIDE	0.503	0.472	1575.18	743.09	0.237	1182.80	280.55
A_68_P27555607	chr11:3996584-3996628	NM_026443:-1172	Mtfp1	PROMOTER	0.371	0.540	1683.71	909.01	0.200	1243.79	248.91
A_68_P27555611	chr11:3996953-3997010	NM_026443:-1547	Mtfp1	PROMOTER	0.233	0.678	934.18	633.01	0.158	748.48	118.13
A_68_P28552887	chr12:77356011-77356057	NM_138745:-184	Mthfd1	DIVERGENT_PROMOTER	0.309	0.550	1015.45	558.66	0.170	708.43	120.50
A_68_P23392000	chr4:147413032-147413076	NM_010840:-131	Mthfr	DIVERGENT_PROMOTER	0.233	0.683	2292.33	1565.34	0.159	1870.54	297.44
A_68_P26224549	chr8:123632350-123632394	NM_001166482:-93	Mthfsd	DIVERGENT_PROMOTER	0.452	0.640	1591.65	1018.46	0.289	1282.58	371.18
A_68_P27674921	chr11:29426434-29426478	NM_133767:0	Mtif2	INSIDE	0.592	0.496	1979.73	981.54	0.293	1667.20	489.31
A_68_P30020398	chr15:12135347-12135391	NM_172958:520	Mtmr12	INSIDE	0.254	0.433	1225.75	530.80	0.110	1062.33	116.77
A_68_P24758234	chr6:113187868-113187912	NM_026849:54	Mtmr14	INSIDE	0.082	0.677	2866.59	1939.61	0.055	2176.18	120.56
A_68_P27558533	chr11:4494164-4494208	NM_028860:632	Mtmr3	INSIDE	0.238	0.635	1033.93	656.51	0.151	815.70	123.39
A_68_P27994973	chr11:87405603-87405647	NM_133215:-94	Mtmr4	PROMOTER	0.496	1.489	5235.41	7798.09	0.739	3633.11	2683.93
A_68_P29643653	chr14:60884373-60884418	NM_144843:331	Mtmr6	INSIDE	0.154	0.610	1562.70	953.37	0.094	1241.19	116.93
A_68_P25806929	chr8:41719873-41719917	NM_001040699:252	Mtmr7	INSIDE	0.248	0.666	1759.44	1172.43	0.166	1171.81	193.98
A_68_P23394433	chr4:147822470-147822515	NM_020009:-198	Mtor	DIVERGENT_PROMOTER	0.214	0.631	1215.94	767.22	0.135	861.85	116.54
A_68_P24362050	chr6:35489064-35489123	NM_008098:795	Mtpn	INSIDE	0.346	0.658	817.74	538.03	0.228	744.07	169.54
A_68_P24362054	chr6:35489548-35489592	NM_008098:318	Mtpn	INSIDE	0.117	0.500	2604.67	1301.98	0.058	2118.06	123.82

ProbeName	Target position of probe on CpG island microarray	TargetID	GeneSymbol	CpG island Description	Ratio of relative methylation (TiO2-NP/Vehicle)	Sham group			TiO_2-H group		
						Relative methylation (Cy-5/Cy-3)	Cy-3 signal (Input)	Cy-5 signal (MeDIP)	Relative methylation (Cy-5/Cy-3)	Cy-3 signal (Input)	Cy-5 signal (MeDIP)
A_68_P24362056	chr6:35489772-35489816	NM_008098:94	Mtpn	INSIDE	0.180	0.428	1963.24	840.72	0.077	1668.73	128.37
A_68_P28831282	chr13:12350227-12350271	NM_001081128:19	Mtr	INSIDE	0.296	0.473	2220.29	1050.04	0.140	1540.42	215.54
A_68_P28831283	chr13:12350347-12350391	NM_001081128:-101	Mtr	PROMOTER	0.293	0.527	1009.78	532.47	0.155	763.32	117.96
A_68_P29110672	chr13:68720676-68720720	NM_172480:300	Mtrr	INSIDE	0.665	0.641	1667.98	1068.62	0.426	1104.70	470.95
A_68_P29110673	chr13:68720745-68720789	NM_172480:232	Mtrr	INSIDE	0.407	0.371	1931.95	716.22	0.151	1508.16	227.48
A_68_P29110674	chr13:68720849-68720893	NM_172480:128	Mtrr	INSIDE	0.346	0.543	6993.05	3796.75	0.188	5272.54	989.59
A_68_P29110675	chr13:68720936-68720980	NM_172480:40	Mtrr	INSIDE	0.340	0.574	1799.72	1033.33	0.195	1338.83	261.01
A_68_P22567224	chr3:137806385-137806429	NM_001163457:-54	Mttp	DIVERGENT_PROMOTER	0.167	0.709	1704.46	1209.28	0.118	1202.05	142.29
A_68_P24176700	chr5:148769468-148769512	NM_029920:595	Mtus2	INSIDE	0.407	0.526	1630.86	857.36	0.214	1291.13	276.17
A_68_P22317890	chr3:89017814-89017858	NM_001161824:421	Mtx1	INSIDE	0.203	0.737	4415.74	3253.73	0.150	2917.74	437.03
A_68_P22317892	chr3:89018127-89018171	NM_001161824:109	Mtx1	INSIDE	0.453	0.623	1854.34	1155.55	0.282	1487.06	419.54
A_68_P21339749	chr2:74664060-74664104	NM_016804:214	Mtx2	INSIDE	0.444	0.467	1450.60	677.58	0.208	1291.15	268.03
A_68_P29591677	chr14:49685584-49685628	NM_144535:-563	Mudeng	PROMOTER	0.601	0.570	2138.88	1220.06	0.343	1757.77	602.70
A_68_P28961838	chr13:38726587-38726631	NM_139063:370	Muted	INSIDE	0.146	0.650	1652.99	1074.70	0.095	1293.75	122.85
A_68_P28961839	chr13:38726652-38726696	NM_139063:304	Muted	INSIDE	0.402	0.532	1082.81	575.86	0.214	844.67	180.51
A_68_P26233415	chr8:124967303-124967347	NM_138656:-2	Mvd	PROMOTER	0.387	0.650	1192.88	775.28	0.251	953.11	239.48
A_68_P32181719	chr19:53384898-53384942	NM_001008542:-75	Mxi1	PROMOTER	0.317	0.645	2113.80	1363.71	0.205	1652.54	338.46
A_68_P32181743	chr19:53388066-53388111	NM_001008542:3093	Mxi1	INSIDE	0.308	0.429	1821.72	781.42	0.132	1669.23	220.66
A_68_P32181752	chr19:53389016-53389065	NM_001008542:4045	Mxi1	INSIDE	0.182	0.603	1309.82	789.28	0.109	1068.00	116.93
A_68_P23440918	chr4:155215643-155215688	NM_024263:1877	Mxra8	INSIDE	0.422	1.415	1342.30	1899.36	0.597	870.00	519.73
A_68_P26991374	chr10:20878723-20878777	NM_001198914:2040	Myb	INSIDE	0.216	0.623	1126.09	702.08	0.134	896.91	120.53
A_68_P20023668	chr1:9690963-9691016	NM_008651:-699	Mybl1	PROMOTER	0.316	0.610	918.92	560.66	0.193	638.57	123.00
A_68_P30267534	chr15:61819131-61819181	NM_001177352:2261	Myc	INSIDE	0.107	0.618	2362.38	1461.03	0.066	1787.71	117.95
A_68_P23265180	chr4:123581921-123581965	NM_019660:-313	Mycbp	PROMOTER	0.292	0.604	2027.64	1224.95	0.177	1616.30	285.42
A_68_P23265181	chr4:123582007-123582051	NM_019660:-227	Mycbp	PROMOTER	0.201	0.384	1828.94	702.29	0.077	1497.45	115.71
A_68_P28033523	chr11:94382882-94382926	NM_170671:-88	Mycbpap	PROMOTER	0.446	0.536	1774.96	951.92	0.239	1290.63	308.88
A_68_P28246294	chr12:12947123-12947167	NM_008709:1498	Mycn	INSIDE	0.203	0.485	3235.04	1570.37	0.099	2055.94	203.09
A_68_P28246315	chr12:12949549-12949593	NM_008709:-928	Mycn	PROMOTER	0.200	0.464	1639.65	760.27	0.093	1287.97	119.40
A_68_P26871809	chr9:119248880-119248924	NM_010851:256	Myd88	INSIDE	0.194	0.609	2528.26	1540.03	0.118	1924.18	227.84
A_68_P26871811	chr9:119249105-119249149	NM_010851:32	Myd88	INSIDE	0.575	0.397	1622.18	643.73	0.228	1414.08	322.78
A_68_P21600380	chr2:124949503-124949547	NM_001162417:-128	Myef2	PROMOTER	0.494	0.465	1835.58	853.12	0.229	1450.53	332.74
A_68_P20450137	chr1:94538493-94538537	NR_028108:11	Myeov2	INSIDE	0.428	0.339	3227.05	1092.73	0.145	2584.01	374.18
A_68_P30497294	chr15:102162316-102162360	NM_021713:199	Myg1	INSIDE	0.117	0.483	3419.15	1651.18	0.057	2543.67	144.28
A_68_P30497296	chr15:102162515-102162559	NM_021713:397	Myg1	INSIDE	0.110	0.703	2397.05	1684.13	0.078	1472.60	114.29
A_68_P27890684	chr11:68505119-68505163	NM_175260:-276	Myh10	PROMOTER	0.403	0.664	1027.75	682.38	0.268	863.61	231.22
A_68_P30356226	chr15:77672472-77672516	NM_022410:51	Myh9	INSIDE	0.213	0.556	1149.55	638.58	0.118	1012.00	119.81
A_68_P31331867	chr17:71340037-71340081	NM_023402:-202	Myl12b	PROMOTER	0.295	0.729	5895.03	4299.37	0.215	4404.46	949.13
A_68_P29001574	chr13:45485076-45485120	NM_153789:-12	Mylip	PROMOTER	0.530	0.531	1866.41	991.57	0.281	1653.44	465.34
A_68_P22029224	chr3:30501291-30501335	NM_030557:304	Mynn	INSIDE	0.394	0.491	1203.66	591.48	0.194	948.31	183.80
A_68_P22029225	chr3:30501383-30501427	NM_030557:396	Mynn	INSIDE	0.414	0.425	1483.23	631.03	0.176	1178.90	207.57
A_68_P30082171	chr15:25552353-25552397	NM_019472:70	Myo10	INSIDE	0.274	0.499	2761.04	1376.67	0.137	1900.34	259.65
A_68_P20224246	chr1:51972071-51972115	NM_001161817:726	Myo1b	INSIDE	0.259	0.508	1107.72	562.21	0.132	985.99	129.71
A_68_P20224249	chr1:51972342-51972397	NM_001161817:449	Myo1b	INSIDE	0.319	0.593	953.15	565.63	0.189	766.66	145.17
A_68_P20224252	chr1:51972666-51972710	NM_001161817:130	Myo1b	INSIDE	0.216	0.425	3979.41	1689.63	0.092	3162.87	289.48

ProbeName	Target position of probe on CpG island microarray	TargetID	GeneSymbol	CpG island Description	Ratio of relative methylation (TiO2-NP/Vehicle)	Sham group			TiO_2-H group		
						Relative methylation (Cy-5/Cy-3)	Cy-3 signal (Input)	Cy-5 signal (MeDIP)	Relative methylation (Cy-5/Cy-3)	Cy-3 signal (Input)	Cy-5 signal (MeDIP)
A_68_P27956333	chr11:80593206-80593250	NM_177390:299	Myo1d	INSIDE	0.460	0.582	1144.28	666.42	0.268	911.42	244.25
A_68_P26641837	chr9:74919720-74919769	NM_010864:732	Myo5a	INSIDE	0.250	0.452	1553.93	701.83	0.113	1136.12	128.18
A_68_P26642806	chr9:75079725-75079769	NM_001081322:-74	Myo5c	PROMOTER	0.103	0.649	2333.40	1513.45	0.067	1800.68	120.28
A_68_P26668158	chr9:80012869-80012913	NM_001039546:50	Myo6	INSIDE	0.237	0.485	1414.70	685.63	0.115	1096.58	125.96
A_68_P25353895	chr7:105259944-105259988	NM_008663:8037	Myo7a	INSIDE	0.530	0.700	1793.40	1255.01	0.371	1483.63	550.32
A_68_P26555224	chr9:59598868-59598912	NM_173018:-90	Myo9a	DIVERGENT_PROMOTER	0.074	0.531	4461.33	2370.67	0.039	3225.68	126.42
A_68_P26555228	chr9:59599330-59599374	NM_173018:372	Myo9a	INSIDE	0.401	0.623	1581.72	985.65	0.250	1278.00	319.75
A_68_P25955963	chr8:73796755-73796799	NM_001142322:164	Myo9b	INSIDE	0.315	0.581	1757.69	1021.08	0.183	1279.05	233.95
A_68_P25955964	chr8:73796836-73796880	NM_001142322:246	Myo9b	INSIDE	0.427	0.699	2481.41	1734.07	0.299	1887.62	563.56
A_68_P25955965	chr8:73796966-73797010	NM_001142322:376	Myo9b	INSIDE	0.151	0.700	2562.07	1792.89	0.106	1903.58	201.38
A_68_P25098703	chr7:53632108-53632154	NM_010866:287	Myod1	INSIDE	0.309	0.465	1338.94	623.18	0.144	922.48	132.79
A_68_P25098706	chr7:53632477-53632521	NM_010866:655	Myod1	INSIDE	0.436	0.656	3452.43	2263.98	0.286	2667.74	762.17
A_68_P29448044	chr14:21459988-21460032	NM_021508:15752	Myoz1	DOWNSTREAM	0.307	0.627	2642.88	1657.82	0.193	2136.78	411.42
A_68_P29448045	chr14:21460170-21460214	NM_021508:15570	Myoz1	DOWNSTREAM	0.210	0.570	1308.25	746.19	0.120	1075.45	129.03
A_68_P29448046	chr14:21460257-21460301	NM_021508:15484	Myoz1	DOWNSTREAM	0.190	0.607	1437.59	872.07	0.115	1176.43	135.38
A_68_P29448048	chr14:21460427-21460474	NM_021508:15312	Myoz1	DOWNSTREAM	0.369	0.591	1254.51	740.98	0.218	1043.25	227.15
A_68_P24990787	chr7:19577120-19577164	NM_145579:549	Mypop	INSIDE	0.133	0.688	2030.37	1397.06	0.092	1293.77	118.59
A_68_P24990788	chr7:19577261-19577305	NM_145579:689	Mypop	INSIDE	0.313	0.560	1902.02	1065.02	0.175	1477.16	258.81
A_68_P23120295	chr4:94645754-94645798	NM_177239:15	Mysm1	INSIDE	0.552	0.623	1198.81	746.32	0.344	1053.70	362.31
A_68_P23120296	chr4:94645934-94645983	NM_177239:-167	Mysm1	PROMOTER	0.296	0.501	1093.13	547.24	0.148	809.97	119.88
A_68_P28037993	chr11:95170830-95170877	NM_001195003:707	Myst2	INSIDE	0.245	0.679	1249.80	848.64	0.166	987.41	163.98
A_68_P28037994	chr11:95170942-95170986	NM_001195003:596	Myst2	INSIDE	0.091	0.712	2425.63	1726.09	0.065	1875.50	121.79
A_68_P28037998	chr11:95171463-95171507	NM_001195003:76	Myst2	INSIDE	0.518	0.604	1913.35	1155.83	0.313	1555.98	487.26
A_68_P29453022	chr14:22318726-22318770	NM_001205241:-243	Myst4	PROMOTER	0.476	0.652	2715.22	1769.97	0.310	2259.17	700.97
A_68_P29453025	chr14:22319120-22319164	NM_017479:136	Myst4	INSIDE	0.354	0.658	2124.63	1398.55	0.233	1702.60	396.53
A_68_P29453027	chr14:22319300-22319344	NM_017479:316	Myst4	INSIDE	0.613	0.706	2117.96	1496.31	0.433	1647.68	713.30
A_68_P29453032	chr14:22319904-22319948	NM_017479:920	Myst4	INSIDE	0.260	0.602	1831.59	1101.96	0.156	1439.74	225.28
A_68_P24974337	chr7:13629455-13629499	NM_145819:10637	Mzf1	INSIDE	0.556	0.460	1369.40	629.44	0.255	1254.65	320.46
A_68_P30567336	chr16:15863446-15863492	NM_029354:-53	Mzt2	PROMOTER	0.222	0.549	1337.26	734.18	0.122	990.04	120.68
A_68_P30567338	chr16:15863650-15863696	NM_029354:-257	Mzt2	PROMOTER	0.248	0.550	1210.20	665.90	0.137	876.81	119.89
A_68_P24191781	chr5:151396250-151396294	NM_133898:828	N4bp2l1	INSIDE	0.242	0.498	1290.90	642.22	0.120	982.44	118.33
A_68_P24191782	chr5:151396383-151396427	NM_133898:696	N4bp2l1	INSIDE	0.597	0.551	1231.69	678.95	0.329	1028.45	338.20
A_68_P32465509	chrX:71167300-71167346	NM_001177965:-39	Naa10	PROMOTER	0.156	1.382	1542.19	2131.35	0.215	558.92	120.40
A_68_P22129524	chr3:51220706-51220750	NM_053089:791	Naa15	INSIDE	0.179	0.681	1206.14	821.71	0.122	1011.94	123.47
A_68_P24044318	chr5:121847841-121847885	NM_172722:-128	Naa25	PROMOTER	0.348	0.665	1669.70	1109.92	0.232	1353.75	313.59
A_68_P30719817	chr16:44140456-44140500	NM_028108:557	Naa50	INSIDE	0.299	0.464	1161.17	538.28	0.139	895.49	124.12
A_68_P20227604	chr1:52556860-52556904	NM_008667:410	Nab1	INSIDE	0.186	0.483	2120.26	1025.04	0.090	1499.80	135.05
A_68_P27539152	chr10:127472516-127472560	NM_001113199:137	Naca	INSIDE	0.661	0.541	1584.53	857.07	0.357	1252.56	447.78
A_68_P27539157	chr10:127473205-127473249	NM_001113199:825	Naca	INSIDE	0.254	0.470	1170.38	549.74	0.119	1011.14	120.70
A_68_P27539158	chr10:127473288-127473332	NM_001113199:909	Naca	INSIDE	0.260	0.610	881.38	537.32	0.159	729.80	115.75
A_68_P21076058	chr2:25915846-25915890	NM_001037098:32048	Nacc2	INSIDE	0.585	3.005	760.70	2285.96	1.759	618.06	1087.18
A_68_P21076059	chr2:25915973-25916017	NM_001037098:31922	Nacc2	INSIDE	0.546	3.135	557.06	1746.57	1.712	472.85	809.35
A_68_P30382864	chr15:82169262-82169306	NM_008669:-28	Naga	DIVERGENT_PROMOTER	0.154	0.637	1530.88	975.38	0.098	1260.29	123.30
A_68_P24595550	chr6:83745123-83745167	NM_001164187:-7	Nagk	PROMOTER	0.576	0.700	1824.13	1277.32	0.404	1476.84	595.96

ProbeName	Target position of probe on CpG island microarray	TargetID	GeneSymbol	CpG island Description	Ratio of relative methylation (TiO2-NP/Vehicle)	Sham group			TiO_2-H group		
						Relative methylation (Cy-5/Cy-3)	Cy-3 signal (Input)	Cy-5 signal (MeDIP)	Relative methylation (Cy-5/Cy-3)	Cy-3 signal (Input)	Cy-5 signal (MeDIP)
A_68_P24595553	chr6:83745392-83745436	NM_001164187:263	Nagk	INSIDE	0.186	0.681	1256.74	855.76	0.127	922.81	116.90
A_68_P28071732	chr11:100931187-100931231	NM_013792:-199	Naglu	PROMOTER	0.541	0.389	1752.33	682.15	0.211	1435.44	302.28
A_68_P28071735	chr11:100931508-100931552	NM_013792:123	Naglu	INSIDE	0.589	0.655	3157.25	2067.87	0.386	2575.22	993.59
A_68_P28077768	chr11:102007243-102007287	NM_178053:-415	Nags	PROMOTER	0.315	0.517	1829.39	945.43	0.163	1325.77	215.72
A_68_P28338223	chr12:33504900-33504944	NM_021524:-277	Nampt	PROMOTER	0.235	0.713	1627.55	1160.45	0.168	1281.67	214.98
A_68_P32226999	chr19:60832637-60832681	NM_178421:769	Nanos1	INSIDE	0.620	0.686	1576.41	1081.76	0.425	1332.58	566.66
A_68_P32227001	chr19:60832863-60832907	NM_178421:995	Nanos1	INSIDE	0.142	0.467	2123.47	992.04	0.066	1757.05	116.56
A_68_P24979588	chr7:16683856-16683900	NM_025898:-113	Napa	PROMOTER	0.483	0.549	1638.19	898.82	0.265	1405.09	372.68
A_68_P24979593	chr7:16684397-16684441	NM_025898:427	Napa	INSIDE	0.616	0.536	1795.96	962.56	0.330	1447.41	477.97
A_68_P21731904	chr2:148557983-148558027	NM_019632:152	Napb	INSIDE	0.421	0.604	3761.23	2270.02	0.254	2478.43	630.22
A_68_P23527432	chr5:21207260-21207304	NM_178728:-119	Napepld	PROMOTER	0.628	0.666	3288.87	2189.42	0.418	2663.73	1112.75
A_68_P30344675	chr15:75714543-75714587	NM_172607:10347	Naprt1	DOWNSTREAM	0.206	0.704	1351.77	951.47	0.145	1014.95	146.84
A_68_P30344676	chr15:75714693-75714737	NM_172607:10197	Naprt1	DOWNSTREAM	0.613	0.702	1537.91	1080.22	0.431	1142.02	491.74
A_68_P30344754	chr15:75724482-75724526	NM_172607:407	Naprt1	INSIDE	0.278	0.655	1813.88	1187.79	0.182	1479.30	269.17
A_68_P30344755	chr15:75724584-75724628	NM_172607:305	Naprt1	INSIDE	0.379	0.701	5491.86	3848.68	0.265	3965.40	1052.51
A_68_P30344756	chr15:75724658-75724702	NM_172607:231	Naprt1	INSIDE	0.240	0.606	3756.08	2274.42	0.145	2887.78	419.72
A_68_P26610633	chr9:69245586-69245639	NM_145618:-192	Narg2	PROMOTER	0.181	0.673	1114.09	750.22	0.122	952.46	115.92
A_68_P26610636	chr9:69245924-69245968	NM_145618:142	Narg2	INSIDE	0.407	0.384	5793.84	2227.06	0.156	4240.45	663.08
A_68_P31775847	chr18:64676070-64676114	NM_001142950:119	Nars	INSIDE	0.409	0.572	2345.12	1342.55	0.234	1852.25	434.23
A_68_P31775848	chr18:64676151-64676195	NM_001142950:39	Nars	INSIDE	0.255	0.503	1203.13	605.41	0.129	921.01	118.39
A_68_P21485377	chr2:103601079-103601123	NM_153126:307	Nat10	INSIDE	0.477	0.590	1023.16	603.68	0.281	767.63	216.03
A_68_P21485378	chr2:103601161-103601205	NM_153126:225	Nat10	INSIDE	0.398	0.618	3637.53	2248.47	0.246	2544.68	625.73
A_68_P21485380	chr2:103601403-103601448	NM_153126:-18	Nat10	PROMOTER	0.292	0.351	1473.67	517.41	0.102	1179.32	120.82
A_68_P30506990	chr16:3884336-3884380	NM_029090:-260	Nat15	PROMOTER	0.511	0.549	2044.21	1122.05	0.281	1551.00	435.45
A_68_P23592016	chr5:34338666-34338710	NM_001001985:56	Nat8l	INSIDE	0.317	0.731	2467.43	1804.16	0.232	2011.04	465.66
A_68_P23592028	chr5:34340360-34340404	NM_001001985:1750	Nat8l	INSIDE	0.193	0.513	2354.20	1207.24	0.099	1755.93	173.82
A_68_P20640025	chr1:137429239-137429283	NM_173437:52672	Nav1	INSIDE	0.533	2.979	2700.63	8045.90	1.589	1960.85	3116.36
A_68_P25111230	chr7:56501781-56501825	NM_175272:244	Nav2	INSIDE	0.350	0.442	2139.56	945.58	0.155	1491.83	231.03
A_68_P25111235	chr7:56502306-56502350	NM_175272:770	Nav2	INSIDE	0.377	0.475	2103.45	998.59	0.179	1602.07	287.07
A_68_P22157975	chr3:55986717-55986761	NM_030595:885	Nbea	INSIDE	0.234	0.481	1636.72	786.77	0.112	1228.86	138.21
A_68_P26824826	chr9:110532797-110532841	NM_183276:23847	Nbeal2	INSIDE	0.648	2.812	2334.25	6563.42	1.822	1637.31	2983.00
A_68_P23349653	chr4:138648926-138648970	NM_008675:-63	Nbl1	PROMOTER	0.289	0.597	1053.71	628.70	0.173	887.58	153.14
A_68_P22752527	chr4:15885475-15885519	NM_013752:383	Nbn	INSIDE	0.595	0.493	1053.71	519.57	0.293	793.14	232.50
A_68_P26500006	chr9:49605230-49605274	NM_001081445:1922	Ncam1	INSIDE	0.549	0.306	2266.14	694.34	0.168	1697.68	285.48
A_68_P25949908	chr8:72636524-72636568	NM_007789:8197	Ncan	INSIDE	0.367	0.582	1171.23	681.59	0.213	918.64	195.95
A_68_P24819297	chr6:125141590-125141635	NM_146171:-8	Ncapd2	DIVERGENT_PROMOTER	0.123	0.585	2401.71	1405.14	0.072	1778.15	127.85
A_68_P24819298	chr6:125141713-125141757	NM_146171:-130	Ncapd2	DIVERGENT_PROMOTER	0.630	0.547	1865.58	1021.21	0.345	1373.52	473.75
A_68_P24819299	chr6:125141841-125141885	NM_146171:-258	Ncapd2	DIVERGENT_PROMOTER	0.176	0.434	2165.18	938.70	0.076	1579.73	120.74
A_68_P26373646	chr9:26838450-26838494	NM_178113:713	Ncapd3	INSIDE	0.292	0.575	1380.17	793.06	0.168	1032.90	173.47
A_68_P23657846	chr5:46060767-46060811	NM_019438:-375	Ncapg	PROMOTER	0.231	0.607	2072.32	1257.71	0.140	1388.98	194.68
A_68_P28767700	chr12:117643877-117643921	NM_133762:24	Ncapg2	INSIDE	0.142	2.324	8194.30	19044.50	0.330	5306.27	1749.19
A_68_P30650229	chr16:31948693-31948737	NM_026554:83	Ncbp2	INSIDE	0.361	0.627	861.15	539.74	0.226	686.61	155.30
A_68_P23281848	chr4:126430490-126430534	NM_011986:161	Ncdn	INSIDE	0.467	0.557	1957.44	1090.81	0.260	1608.46	418.21
A_68_P22009787	chr3:27081688-27081732	NM_178772:-215	Nceh1	PROMOTER	0.576	0.702	2056.24	1443.40	0.404	1651.64	667.85

ProbeName	Target position of probe on CpG island microarray	TargetID	GeneSymbol	CpG island Description	Ratio of relative methylation (TiO2-NP/Vehicle)	Sham group			TiO_2-H group		
						Relative methylation (Cy-5/Cy-3)	Cy-3 signal (Input)	Cy-5 signal (MeDIP)	Relative methylation (Cy-5/Cy-3)	Cy-3 signal (Input)	Cy-5 signal (MeDIP)
A_68_P24112020	chr5:134697723-134697767	NM_010876:7751	Ncf1	INSIDE	0.205	1.742	3557.52	6197.13	0.357	2328.54	831.60
A_68_P20188963	chr1:43503219-43503263	NM_010879:645	Nck2	INSIDE	0.529	0.657	2062.99	1356.04	0.347	1601.98	556.67
A_68_P20188964	chr1:43503293-43503346	NM_010879:724	Nck2	INSIDE	0.161	0.474	2440.58	1157.83	0.077	1623.29	124.31
A_68_P20590467	chr1:128389351-128389395	NM_172484:132	Nckap5	INSIDE	0.496	0.592	1414.93	837.30	0.293	1179.18	346.01
A_68_P30480286	chr15:99288275-99288319	NM_001001884:-117	Nckap5l	PROMOTER	0.357	0.659	2150.11	1417.18	0.235	1582.58	371.87
A_68_P30480289	chr15:99288589-99288642	NM_001001884:-436	Nckap5l	PROMOTER	0.232	0.587	1160.68	681.02	0.136	876.01	119.15
A_68_P27290092	chr10:80958931-80958975	NM_134009:156	Ncln	INSIDE	0.597	0.544	3936.53	2141.26	0.325	2844.15	923.92
A_68_P27290096	chr10:80959275-80959319	NM_134009:-188	Ncln	PROMOTER	0.247	0.505	1653.89	834.60	0.125	1263.69	157.70
A_68_P21826986	chr2:165818945-165818989	NM_008679:830	Ncoa3	INSIDE	0.165	1.974	6308.14	12453.14	0.327	4331.62	1414.75
A_68_P29515925	chr14:32978942-32978986	NM_019744:-13	Ncoa4	PROMOTER	0.488	0.384	1559.27	598.63	0.188	1418.60	266.00
A_68_P21821252	chr2:164859772-164859816	NM_144892:485	Ncoa5	INSIDE	0.612	0.593	930.67	552.14	0.363	804.09	291.83
A_68_P21821258	chr2:164860465-164860509	NM_144892:-207	Ncoa5	PROMOTER	0.303	0.546	1543.78	842.66	0.165	1208.50	199.80
A_68_P27041530	chr10:30522669-30522713	NM_172495:223	Ncoa7	INSIDE	0.554	0.548	1606.18	880.11	0.304	1306.02	396.82
A_68_P21107803	chr2:31101353-31101397	NM_019681:-68	Ncs1	PROMOTER	0.664	0.440	1396.79	614.35	0.292	1184.54	345.76
A_68_P21107807	chr2:31101894-31101938	NM_019681:474	Ncs1	INSIDE	0.151	0.584	1890.14	1103.28	0.088	1415.86	125.00
A_68_P21070911	chr2:25110830-25110874	NM_001082476:82	Ndor1	INSIDE	0.268	0.703	1591.65	1119.44	0.188	1185.44	223.34
A_68_P30295267	chr15:66800261-66800306	NM_008681:920	Ndrg1	INSIDE	0.131	0.714	5724.36	4086.71	0.094	3879.17	363.39
A_68_P30295270	chr15:66800532-66800576	NM_008681:649	Ndrg1	INSIDE	0.459	0.395	1964.70	775.97	0.181	1550.23	280.93
A_68_P30295276	chr15:66801140-66801184	NM_008681:41	Ndrg1	INSIDE	0.177	0.434	3852.04	1672.29	0.077	3098.07	238.36
A_68_P30295277	chr15:66801273-66801317	NM_008681:-91	Ndrg1	PROMOTER	0.652	0.392	1920.63	753.31	0.256	1429.87	365.71
A_68_P29603748	chr14:52533567-52533611	NM_001145959:-425	Ndrg2	DIVERGENT_PROMOTER	0.407	0.480	1560.30	749.06	0.196	1153.20	225.46
A_68_P20449354	chr1:94370335-94370379	NM_024197:-21	Ndufa10	PROMOTER	0.570	0.602	980.29	589.81	0.343	669.35	229.49
A_68_P20449355	chr1:94370405-94370449	NM_024197:-91	Ndufa10	PROMOTER	0.534	0.626	889.06	556.74	0.334	644.07	215.30
A_68_P31257925	chr17:56857454-56857498	NM_027244:292	Ndufa11	INSIDE	0.285	0.710	2636.33	1871.06	0.202	1931.98	390.66
A_68_P25948617	chr8:72426740-72426784	NM_023312:-305	Ndufa13	PROMOTER	0.466	0.541	1871.68	1012.20	0.252	1402.86	353.32
A_68_P25948618	chr8:72426813-72426857	NM_023312:-377	Ndufa13	PROMOTER	0.267	0.444	2442.87	1083.58	0.118	1777.55	210.55
A_68_P31625586	chr18:36903899-36903943	NM_010885:282	Ndufa2	INSIDE	0.568	0.515	1076.74	554.72	0.293	943.88	276.09
A_68_P31625587	chr18:36904013-36904057	NM_010885:168	Ndufa2	INSIDE	0.488	0.706	2629.35	1855.44	0.344	1958.35	674.10
A_68_P31625589	chr18:36904175-36904219	NM_010885:6	Ndufa2	INSIDE	0.246	0.535	1190.46	637.10	0.132	897.44	118.28
A_68_P24302042	chr6:24477224-24477272	NM_026614:439	Ndufa5	INSIDE	0.258	0.498	1296.76	646.39	0.129	1005.79	129.36
A_68_P24302044	chr6:24477440-24477484	NM_026614:225	Ndufa5	INSIDE	0.277	0.567	3081.87	1747.08	0.157	2370.37	372.60
A_68_P29309818	chr13:108948386-108948430	NM_001127346:411	Ndufaf2	INSIDE	0.259	0.540	2197.54	1186.04	0.140	1671.17	233.20
A_68_P26815490	chr9:108469907-108469951	NM_023247:-255	Ndufaf3	DIVERGENT_PROMOTER	0.115	0.441	3490.45	1538.00	0.051	2500.22	126.94
A_68_P26815491	chr9:108470017-108470061	NM_023247:-365	Ndufaf3	DIVERGENT_PROMOTER	0.173	0.533	1780.83	948.68	0.092	1342.15	123.59
A_68_P26815492	chr9:108470126-108470170	NM_023247:-475	Ndufaf3	DIVERGENT_PROMOTER	0.155	1.401	2272.14	3182.58	0.217	1525.04	331.05
A_68_P22788124	chr4:24825414-24825458	NM_026742:207	Ndufaf4	INSIDE	0.355	0.524	2453.41	1284.42	0.186	1765.45	328.39
A_68_P31099229	chr17:24861250-24861296	NM_026684:61	Ndufb10	INSIDE	0.177	0.711	2065.37	1468.64	0.126	1496.96	188.66
A_68_P22856642	chr4:40226159-40226203	NM_001033305:221	Ndufb6	INSIDE	0.596	0.436	2086.76	910.02	0.260	1462.95	380.38
A_68_P32132944	chr19:44629890-44629934	NM_026061:-7	Ndufb8	DIVERGENT_PROMOTER	0.522	0.648	876.50	568.25	0.338	721.40	244.16
A_68_P22129477	chr3:51212635-51212679	NM_025523:221	Ndufc1	INSIDE	0.621	0.612	1563.98	957.33	0.380	1247.67	473.94
A_68_P22129478	chr3:51212746-51212790	NM_025523:109	Ndufc1	INSIDE	0.282	0.487	2313.80	1126.57	0.137	1725.09	237.16
A_68_P22129479	chr3:51212850-51212894	NM_025523:5	Ndufc1	INSIDE	0.460	0.562	1329.95	747.02	0.258	1022.55	264.13
A_68_P25350314	chr7:104548897-104548941	NM_024220:406	Ndufc2	INSIDE	0.474	0.708	1873.10	1326.63	0.336	1456.45	488.82
A_68_P20285508	chr1:63223074-63223120	NM_001160038:300	Ndufs1	INSIDE	0.248	0.586	1008.39	591.00	0.145	822.94	119.39

ProbeName	Target position of probe on CpG island microarray	TargetID	GeneSymbol	CpG island Description	Ratio of relative methylation (TiO2-NP/Vehicle)	Sham group			TiO_2-H group		
						Relative methylation (Cy-5/Cy-3)	Cy-3 signal (Input)	Cy-5 signal (MeDIP)	Relative methylation (Cy-5/Cy-3)	Cy-3 signal (Input)	Cy-5 signal (MeDIP)
A_68_P20826868	chr1:173177287-173177331	NM_153064:-65	Ndufs2	DIVERGENT_PROMOTER	0.279	0.570	910.55	519.24	0.159	736.87	117.21
A_68_P20826869	chr1:173177373-173177425	NM_153064:-155	Ndufs2	DIVERGENT_PROMOTER	0.143	0.695	1648.83	1145.93	0.099	1175.65	116.93
A_68_P29139136	chr13:73466105-73466159	NM_010888:-202	Ndufs6	DIVERGENT_PROMOTER	0.264	0.636	1318.25	838.33	0.168	1001.16	168.12
A_68_P22748787	chr4:15076098-15076142	NM_178617:158	Necab1	INSIDE	0.331	0.639	4194.84	2680.58	0.212	3139.91	664.84
A_68_P21761151	chr2:154384393-154384437	NM_021546:175	Necab3	INSIDE	0.324	1.466	1381.50	2025.28	0.475	1131.57	537.67
A_68_P24810880	chr6:122824802-122824859	NM_026267:256	Necap1	INSIDE	0.365	0.520	1498.27	778.43	0.190	1145.87	217.19
A_68_P23362016	chr4:140633999-140634043	NM_025383:240	Necap2	INSIDE	0.313	0.457	1134.93	518.54	0.143	939.91	134.60
A_68_P23362017	chr4:140634100-140634144	NM_025383:138	Necap2	INSIDE	0.267	0.685	1299.10	890.49	0.183	962.95	176.55
A_68_P27560503	chr11:4847990-4848034	NM_010904:55	Nefh	INSIDE	0.542	0.436	2764.76	1204.94	0.236	2271.19	536.02
A_68_P29689072	chr14:68701679-68701726	NM_010910:-238	Nefl	PROMOTER	0.172	1.428	1057.46	1510.57	0.245	821.26	201.25
A_68_P29689075	chr14:68701929-68701973	NM_010910:10	Nefl	INSIDE	0.312	0.676	3074.89	2078.21	0.211	2344.48	495.11
A_68_P29689076	chr14:68702020-68702064	NM_010910:102	Nefl	INSIDE	0.263	0.609	1345.69	819.39	0.160	980.66	156.81
A_68_P29689081	chr14:68702832-68702876	NM_010910:914	Nefl	INSIDE	0.504	0.601	1916.37	1151.87	0.303	1448.18	438.39
A_68_P29689083	chr14:68703035-68703085	NM_010910:1120	Nefl	INSIDE	0.286	0.585	1767.07	1033.39	0.167	1351.09	226.08
A_68_P29689305	chr14:68743026-68743070	NM_008691:13	Nefm	INSIDE	0.288	0.449	5041.87	2261.94	0.129	4106.09	529.88
A_68_P29689306	chr14:68743165-68743210	NM_008691:-126	Nefm	PROMOTER	0.158	0.541	1964.20	1062.64	0.086	1348.78	115.38
A_68_P22674486	chr3:156225270-156225314	NM_177274:370	Negr1	INSIDE	0.223	0.573	2338.36	1338.93	0.128	1657.76	212.05
A_68_P26797102	chr9:105297505-105297549	NM_172461:91	Nek11	INSIDE	0.607	0.404	1654.00	667.97	0.245	1435.81	352.11
A_68_P29508895	chr14:31764856-31764900	NM_011849:205	Nek4	INSIDE	0.327	0.545	1412.27	769.22	0.178	1179.76	209.82
A_68_P29508896	chr14:31764964-31765008	NM_011849:313	Nek4	INSIDE	0.435	0.666	1633.10	1088.41	0.290	1378.71	399.28
A_68_P25115904	chr7:57230774-57230819	NM_001037906:77	Nell1	INSIDE	0.281	1.345	2100.10	2825.20	0.378	1473.11	556.36
A_68_P20937428	chr1:193141556-193141600	NM_025424:419	Nenf	INSIDE	0.172	0.636	1542.92	981.71	0.109	1296.83	141.61
A_68_P22310997	chr3:87774900-87774944	NM_016701:-92	Nes	PROMOTER	0.220	0.611	1215.89	743.21	0.134	933.88	125.51
A_68_P22310998	chr3:87774969-87775013	NM_016701:-24	Nes	PROMOTER	0.293	0.474	2870.67	1359.97	0.139	2059.17	285.72
A_68_P32148153	chr19:47254029-47254073	NM_021360:741	Neurl1a	INSIDE	0.274	0.719	2005.46	1442.43	0.197	1556.76	307.34
A_68_P32148154	chr19:47254136-47254180	NM_021360:849	Neurl1a	INSIDE	0.543	6.887	11150.18	76794.00	3.742	7444.01	27859.02
A_68_P21820019	chr2:164658808-164658852	NM_001082974:266	Neurl2	INSIDE	0.445	0.576	2329.81	1342.62	0.256	1695.38	434.57
A_68_P21820020	chr2:164658912-164658956	NM_001082974:162	Neurl2	INSIDE	0.581	0.624	2133.92	1331.60	0.363	1653.66	600.01
A_68_P21820025	chr2:164659401-164659445	NM_001082974:-326	Neurl2	PROMOTER	0.324	0.551	2199.28	1212.46	0.178	1636.93	292.07
A_68_P20147268	chr1:36326573-36326617	NM_153408:3676	Neurl3	INSIDE	0.570	3.028	2584.59	7827.19	1.726	1746.11	3013.23
A_68_P27897807	chr11:69715139-69715183	NM_001013414:-219	Neurl4	DIVERGENT_PROMOTER	0.602	0.370	2029.20	751.82	0.223	1519.97	338.98
A_68_P27897808	chr11:69715226-69715270	NM_001013414:-131	Neurl4	DIVERGENT_PROMOTER	0.220	0.490	1412.54	692.69	0.108	1118.73	120.59
A_68_P27897812	chr11:69715753-69715797	NM_001013414:395	Neurl4	INSIDE	0.655	0.546	1649.75	900.96	0.358	1233.32	441.15
A_68_P28055687	chr11:98188542-98188588	NM_010895:2395	Neurod2	INSIDE	0.204	0.561	1453.35	815.91	0.115	1156.57	132.64
A_68_P28055689	chr11:98188746-98188790	NM_010895:2191	Neurod2	INSIDE	0.316	0.447	1637.45	732.22	0.141	1423.29	201.24
A_68_P28055765	chr11:98199818-98199862	NM_010895:-8881	Neurod2	PROMOTER	0.338	0.529	1873.82	992.10	0.179	1372.85	245.61
A_68_P29060115	chr13:56353410-56353454	NM_010896:92	Neurog1	INSIDE	0.601	0.682	2951.80	2013.31	0.410	2345.20	961.68
A_68_P22509724	chr3:127336069-127336113	NM_009718:28	Neurog2	INSIDE	0.283	0.382	1512.09	576.95	0.108	1244.62	134.27
A_68_P22509733	chr3:127337069-127337113	NM_009718:1028	Neurog2	INSIDE	0.394	0.536	1400.00	750.34	0.211	1146.46	242.10
A_68_P27184368	chr10:61595655-61595699	NM_009719:-161	Neurog3	PROMOTER	0.391	0.630	2160.43	1360.12	0.246	1755.04	431.48
A_68_P27184376	chr10:61596437-61596481	NM_009719:621	Neurog3	INSIDE	0.214	0.611	4051.69	2475.51	0.131	2544.56	332.73
A_68_P27184377	chr10:61596546-61596590	NM_009719:731	Neurog3	INSIDE	0.138	0.702	4953.55	3479.40	0.097	3214.96	312.33
A_68_P26143994	chr8:109817474-109817518	NM_018823:127	Nfat5	INSIDE	0.268	0.602	904.00	544.53	0.161	757.62	122.26
A_68_P31866297	chr18:80904269-80904314	NM_001164111:621	Nfatc1	INSIDE	0.597	0.618	1196.16	738.86	0.369	1035.19	381.56

ProbeName	Target position of probe on CpG island microarray	TargetID	GeneSymbol	CpG island Description	Ratio of relative methylation (TiO2-NP/Vehicle)	Sham group			TiO_2-H group		
						Relative methylation (Cy-5/Cy-3)	Cy-3 signal (Input)	Cy-5 signal (MeDIP)	Relative methylation (Cy-5/Cy-3)	Cy-3 signal (Input)	Cy-5 signal (MeDIP)
A_68_P31866349	chr18:80910082-80910126	NM_001164110:-294	Nfatc1	PROMOTER	0.434	0.568	2641.35	1501.07	0.247	2097.65	517.57
A_68_P31866356	chr18:80910683-80910727	NM_001164110:-894	Nfatc1	PROMOTER	0.098	0.682	2648.16	1806.05	0.067	1876.69	125.67
A_68_P21842414	chr2:168415753-168415797	NM_001136073:-83	Nfatc2	PROMOTER	0.593	0.657	2148.69	1411.51	0.390	1208.10	470.89
A_68_P21842484	chr2:168426628-168426674	NM_001037178:505	Nfatc2	INSIDE	0.320	0.543	1560.13	847.85	0.174	1209.19	210.28
A_68_P21842485	chr2:168426812-168426856	NM_001037178:321	Nfatc2	INSIDE	0.562	0.424	1492.40	632.27	0.238	1299.45	309.63
A_68_P21842487	chr2:168427037-168427082	NM_001037178:96	Nfatc2	INSIDE	0.583	0.632	2127.20	1343.38	0.368	1665.96	612.90
A_68_P29620047	chr14:56442581-56442635	NM_001168346:-1023	Nfatc4	PROMOTER	0.242	0.553	1077.63	595.69	0.134	908.41	121.71
A_68_P28047030	chr11:96690616-96690669	NM_001130451:-5201	Nfe2l1	PROMOTER	0.231	0.568	1541.58	875.05	0.131	1166.96	152.97
A_68_P21345533	chr2:75542035-75542079	NM_010902:642	Nfe2l2	INSIDE	0.519	0.523	1396.72	730.30	0.271	1119.48	303.92
A_68_P21345534	chr2:75542163-75542207	NM_010902:514	Nfe2l2	INSIDE	0.557	0.483	2548.48	1229.88	0.269	2074.48	557.51
A_68_P21345538	chr2:75542631-75542675	NM_010902:46	Nfe2l2	INSIDE	0.235	1.890	1017.95	1923.79	0.445	717.11	318.98
A_68_P21345540	chr2:75542919-75542963	NM_010902:-242	Nfe2l2	PROMOTER	0.219	1.443	9326.85	13462.96	0.317	5986.99	1895.76
A_68_P24444195	chr6:51382942-51382986	NM_010903:296	Nfe2l3	INSIDE	0.260	0.527	1729.63	911.74	0.137	1332.69	182.34
A_68_P23134122	chr4:97412574-97412618	NM_001122952:163963	Nfia	INSIDE	0.188	0.361	2286.25	825.06	0.068	1694.82	114.91
A_68_P23134315	chr4:97438906-97438950	NM_001122953:-5388	Nfia	PROMOTER	0.323	0.625	892.54	557.84	0.202	796.05	160.61
A_68_P23134317	chr4:97439073-97439121	NM_001122953:-5220	Nfia	PROMOTER	0.247	1.496	979.20	1464.48	0.370	748.59	276.95
A_68_P23057173	chr4:82150827-82150874	NM_001113209:362	Nfib	INSIDE	0.158	0.512	1950.75	997.92	0.081	1438.31	115.99
A_68_P23057176	chr4:82151139-82151183	NM_001113209:52	Nfib	INSIDE	0.273	0.700	1996.42	1398.11	0.191	1418.21	271.28
A_68_P23057181	chr4:82151702-82151757	NM_001113209:-517	Nfib	PROMOTER	0.562	0.645	1116.94	719.93	0.363	898.38	325.68
A_68_P23057187	chr4:82152716-82152762	NM_001113209:-1526	Nfib	PROMOTER	0.118	1.803	1003.91	1810.13	0.213	665.53	141.99
A_68_P26021786	chr8:87295957-87296001	NM_001081981:2290	Nfix	INSIDE	0.618	0.439	1489.51	653.41	0.271	1071.33	290.47
A_68_P26021880	chr8:87308048-87308097	NM_001081981:-9804	Nfix	PROMOTER	0.293	0.697	1850.78	1289.55	0.204	1327.75	271.33
A_68_P26021884	chr8:87308434-87308479	NM_001081982:15783	Nfix	INSIDE	0.251	0.402	1616.46	649.56	0.101	1163.98	117.58
A_68_P26021885	chr8:87308549-87308593	NM_001081982:15669	Nfix	INSIDE	0.234	0.667	3124.48	2083.98	0.156	2177.25	340.14
A_68_P32143069	chr19:46379412-46379456	NM_019408:-750	Nfkb2	PROMOTER	0.449	0.447	2686.41	1201.59	0.201	2112.99	424.67
A_68_P32143106	chr19:46383535-46383579	NM_001177370:3137	Nfkb2	INSIDE	0.631	0.567	1753.43	994.37	0.358	1356.57	485.49
A_68_P25023455	chr7:29551632-29551677	NM_010908:-111	Nfkbib	DIVERGENT_PROMOTER	0.358	0.514	1476.67	759.70	0.184	1146.83	211.30
A_68_P25031432	chr7:31206971-31207015	NM_172142:-1330	Nfkbid	DIVERGENT_PROMOTER	0.227	0.654	1051.60	688.17	0.148	792.05	117.47
A_68_P31201772	chr17:45692808-45692853	NM_008690:166	Nfkbie	INSIDE	0.406	0.497	1713.85	852.26	0.202	1237.60	249.77
A_68_P31201773	chr17:45692924-45692968	NM_008690:282	Nfkbie	INSIDE	0.188	0.472	1942.55	917.53	0.089	1348.68	119.77
A_68_P26400217	chr9:31194116-31194160	NM_172766:362	Nfrkb	INSIDE	0.285	0.582	1195.13	695.01	0.166	970.54	160.97
A_68_P31217285	chr17:48548234-48548282	NM_001110832:887	Nfya	INSIDE	0.604	0.543	1238.29	672.28	0.328	995.94	326.52
A_68_P31217293	chr17:48549071-48549115	NM_001110832:53	Nfya	INSIDE	0.508	0.742	5210.63	3868.13	0.377	3695.38	1394.35
A_68_P27293629	chr10:82226162-82226206	NM_010914:702	Nfyb	INSIDE	0.408	0.567	1982.74	1125.03	0.232	1672.39	387.54
A_68_P27293631	chr10:82226680-82226728	NM_010914:182	Nfyb	INSIDE	0.153	0.717	1409.74	1010.22	0.110	1117.02	122.44
A_68_P28614145	chr12:88443030-88443074	NM_022414:437	Ngb	INSIDE	0.272	0.554	5083.73	2817.64	0.151	3776.37	570.08
A_68_P28614148	chr12:88443473-88443517	NM_022414:-5	Ngb	PROMOTER	0.407	0.415	1799.48	746.75	0.169	1681.23	284.09
A_68_P29615709	chr14:55633702-55633746	NM_026890:-566	Ngdn	PROMOTER	0.347	0.683	1576.78	1076.28	0.237	1163.61	275.85
A_68_P20418214	chr1:89406592-89406636	NM_019867:323	Ngef	INSIDE	0.338	0.484	3915.00	1895.24	0.164	3017.43	493.49
A_68_P28039686	chr11:95446719-95446763	NM_033217:2272	Ngfr	INSIDE	0.380	0.690	1630.84	1125.96	0.262	1171.77	307.46
A_68_P28039696	chr11:95448025-95448069	NM_033217:966	Ngfr	INSIDE	0.344	0.554	1706.15	945.41	0.191	1239.07	236.27
A_68_P29428096	chr14:17075774-17075818	NM_021504:-6031	Ngly1	PROMOTER	0.347	0.351	1599.30	561.67	0.122	1270.14	154.81
A_68_P20352130	chr1:75121478-75121522	NM_029342:300	Nhej1	INSIDE	0.547	0.505	1453.54	733.90	0.276	1120.07	309.27
A_68_P20352131	chr1:75121569-75121613	NM_029342:210	Nhej1	INSIDE	0.599	0.565	1094.85	618.55	0.338	909.93	307.82

ProbeName	Target position of probe on CpG island microarray	TargetID	GeneSymbol	CpG island Description	Ratio of relative methylation (TiO2-NP/Vehicle)	Sham group			TiO_2-H group		
						Relative methylation (Cy-5/Cy-3)	Cy-3 signal (Input)	Cy-5 signal (MeDIP)	Relative methylation (Cy-5/Cy-3)	Cy-3 signal (Input)	Cy-5 signal (MeDIP)
A_68_P20352132	chr1:75121636-75121680	NM_029342:142	Nhej1	INSIDE	0.139	0.526	2274.82	1195.65	0.073	1641.83	120.23
A_68_P22378546	chr3:101816277-101816321	NM_178777:2231	Nhlh2	INSIDE	0.206	0.441	1622.57	715.38	0.091	1330.59	121.14
A_68_P22378553	chr3:101816844-101816888	NM_178777:2799	Nhlh2	INSIDE	0.395	0.497	2536.22	1261.46	0.197	2171.96	426.83
A_68_P29011093	chr13:47106234-47106282	NM_175340:3961	Nhlrc1	DOWNSTREAM	0.604	3.761	2716.80	10217.71	2.271	1867.85	4242.03
A_68_P29011114	chr13:47109579-47109623	NM_175340:619	Nhlrc1	INSIDE	0.397	0.517	1150.56	595.40	0.206	823.92	169.45
A_68_P29011119	chr13:47110041-47110085	NM_175340:157	Nhlrc1	INSIDE	0.383	0.608	5479.66	3332.96	0.233	4146.81	966.73
A_68_P32201474	chr19:56623518-56623562	NM_025811:790	Nhlrc2	INSIDE	0.177	0.666	1445.69	963.43	0.118	1061.21	125.12
A_68_P31107419	chr17:26088437-26088481	NM_001039038:-6582	Nhlrc4	PROMOTER	0.649	0.705	1543.19	1087.78	0.458	1224.04	560.00
A_68_P31107425	chr17:26089202-26089246	NM_001039038:-7348	Nhlrc4	PROMOTER	0.252	0.645	1005.11	648.02	0.163	717.95	116.80
A_68_P27794923	chr11:51433377-51433421	NM_026631:124	Nhp2	INSIDE	0.129	0.688	5485.20	3774.02	0.089	3748.07	333.08
A_68_P30381130	chr15:81877398-81877442	NM_011482:608	Nhp2l1	INSIDE	0.315	0.708	1465.01	1036.69	0.223	1070.02	238.28
A_68_P30381132	chr15:81877600-81877644	NM_011482:406	Nhp2l1	INSIDE	0.376	0.482	2791.11	1346.58	0.181	2334.99	423.32
A_68_P32786682	chrX:158346088-158346143	NM_001081052:251607	Nhs	INSIDE	0.064	2.986	2136.10	6377.93	0.192	816.00	156.77
A_68_P28833747	chr13:13530554-13530598	NM_010917:708	Nid1	INSIDE	0.243	0.598	969.85	579.59	0.145	838.52	121.56
A_68_P29443159	chr14:20570751-20570795	NM_008695:294	Nid2	INSIDE	0.512	0.519	1191.32	618.11	0.266	971.94	258.12
A_68_P29023783	chr13:49283461-49283505	NM_013610:567	Ninj1	INSIDE	0.286	0.600	926.87	555.99	0.171	682.64	116.94
A_68_P25144263	chr7:63217306-63217365	NM_023647:485	Nipa2	INSIDE	0.274	0.462	1255.49	580.62	0.127	952.08	120.51
A_68_P25144264	chr7:63217409-63217453	NM_023647:390	Nipa2	INSIDE	0.185	0.625	1332.57	832.52	0.115	1047.59	120.90
A_68_P25144265	chr7:63217530-63217574	NM_023647:268	Nipa2	INSIDE	0.250	0.567	979.05	554.68	0.142	867.32	122.89
A_68_P25144267	chr7:63217811-63217855	NM_023647:-12	Nipa2	DIVERGENT_PROMOTER	0.583	0.572	1925.68	1102.39	0.334	1670.82	557.86
A_68_P23328803	chr4:135050627-135050671	NM_028995:-229	Nipal3	DIVERGENT_PROMOTER	0.401	0.553	1557.06	861.22	0.222	1180.18	261.60
A_68_P23328805	chr4:135050806-135050850	NM_028995:-409	Nipal3	DIVERGENT_PROMOTER	0.484	0.603	1098.79	663.09	0.292	856.25	249.85
A_68_P30000041	chr15:8393675-8393723	NM_027707:765	Nipbl	INSIDE	0.597	0.572	1360.81	777.79	0.341	1211.29	413.04
A_68_P30000046	chr15:8394229-8394273	NM_027707:213	Nipbl	INSIDE	0.138	0.424	2560.38	1085.56	0.059	2056.75	120.77
A_68_P22920589	chr4:53024957-53025001	NM_025623:183	Nipsnap3b	INSIDE	0.113	1.672	4718.76	7888.37	0.190	3202.14	607.07
A_68_P22920590	chr4:53025131-53025175	NM_025623:357	Nipsnap3b	INSIDE	0.113	1.461	10345.19	15116.54	0.166	6698.52	1109.63
A_68_P29510443	chr14:32019604-32019648	NM_022656:386	Nisch	INSIDE	0.417	0.679	1573.09	1067.41	0.283	1272.08	360.07
A_68_P30784592	chr16:57167576-57167629	NM_023175:-157	Nit2	PROMOTER	0.236	0.637	1034.90	659.23	0.150	801.68	120.37
A_68_P27050630	chr10:32610114-32610158	NM_001013411:-415	Nkain2	PROMOTER	0.418	0.558	3230.91	1803.75	0.233	2528.35	589.83
A_68_P22771642	chr4:20705490-20705534	NM_172987:303	Nkain3	INSIDE	0.612	0.652	1130.86	737.18	0.399	956.36	381.83
A_68_P22771645	chr4:20705806-20705850	NM_172987:-13	Nkain3	PROMOTER	0.505	3.486	10184.38	35497.85	1.759	7168.92	12613.22
A_68_P29142608	chr13:73984841-73984885	NM_028186:217	Nkd2	INSIDE	0.500	0.591	1343.61	794.71	0.296	1034.83	306.07
A_68_P24993777	chr7:20108952-20108996	NM_027116:4895	Nkpd1	INSIDE	0.134	0.706	1525.27	1076.21	0.094	1239.64	117.04
A_68_P24993784	chr7:20109810-20109854	NM_027116:5753	Nkpd1	INSIDE	0.171	0.455	1981.77	901.20	0.078	1583.94	123.46
A_68_P26885085	chr9:121628083-121628127	NM_010918:-194	Nktr	PROMOTER	0.566	0.471	1500.13	706.12	0.267	1164.19	310.30
A_68_P25535541	chr7:139791496-139791540	NM_009123:-198	Nkx1-2	PROMOTER	0.237	0.396	1501.30	595.07	0.094	1264.16	118.71
A_68_P28457439	chr12:57634754-57634798	NM_001146198:1317	Nkx2-1	INSIDE	0.605	0.467	1609.36	751.63	0.283	1341.25	379.02
A_68_P28457446	chr12:57635707-57635751	NM_001146198:365	Nkx2-1	INSIDE	0.624	0.522	1497.94	781.79	0.325	1359.08	442.38
A_68_P28457454	chr12:57636900-57636944	NM_001146198:-829	Nkx2-1	PROMOTER	0.395	0.568	2415.54	1372.50	0.225	1730.29	388.75
A_68_P28457455	chr12:57637056-57637100	NM_001146198:-985	Nkx2-1	PROMOTER	0.261	0.679	2448.65	1661.89	0.177	1953.15	346.47
A_68_P28457458	chr12:57637325-57637369	NM_001146198:-1253	Nkx2-1	PROMOTER	0.270	0.455	1415.43	644.37	0.123	970.61	119.24
A_68_P21722732	chr2:147010130-147010174	NR_030769:334	Nkx2-2as	INSIDE	0.287	0.629	1165.97	732.92	0.181	992.36	179.16
A_68_P31112704	chr17:26975881-26975926	NM_008700:2607	Nkx2-5	INSIDE	0.305	0.598	3038.79	1816.48	0.182	2319.96	422.79
A_68_P31112724	chr17:26978218-26978262	NM_008700:270	Nkx2-5	INSIDE	0.651	0.639	2373.49	1517.28	0.416	2003.61	833.68

ProbeName	Target position of probe on CpG island microarray	TargetID	GeneSymbol	CpG island Description	Ratio of relative methylation (TiO2-NP/Vehicle)	Sham group			TiO_2-H group		
						Relative methylation (Cy-5/Cy-3)	Cy-3 signal (Input)	Cy-5 signal (MeDIP)	Relative methylation (Cy-5/Cy-3)	Cy-3 signal (Input)	Cy-5 signal (MeDIP)
A_68_P31112749	chr17:26981132-26981187	NM_008700:-2649	Nkx2-5	PROMOTER	0.229	0.613	1091.76	668.73	0.140	865.35	121.26
A_68_P31112751	chr17:26981297-26981344	NM_008700:-2810	Nkx2-5	PROMOTER	0.193	0.683	2648.97	1808.06	0.132	1957.72	257.63
A_68_P31112795	chr17:26986945-26986989	NM_008700:-8456	Nkx2-5	PROMOTER	0.487	0.596	1721.05	1025.00	0.290	1291.32	374.77
A_68_P31112797	chr17:26987123-26987182	NM_008700:-8642	Nkx2-5	PROMOTER	0.178	1.689	594.11	1003.18	0.301	401.73	120.88
A_68_P31112801	chr17:26987616-26987660	NM_008700:-9128	Nkx2-5	PROMOTER	0.217	0.583	1235.25	719.91	0.126	966.81	122.03
A_68_P28457818	chr12:57714241-57714285	NM_008701:9	Nkx2-9	INSIDE	0.521	0.542	1256.19	680.95	0.283	992.38	280.45
A_68_P29694907	chr14:69808925-69808969	NM_010921:198	Nkx3-1	INSIDE	0.590	0.638	1546.71	986.36	0.376	1204.94	453.06
A_68_P29694908	chr14:69809045-69809089	NM_010921:318	Nkx3-1	INSIDE	0.333	0.617	1285.66	793.18	0.206	1005.25	206.76
A_68_P23637406	chr5:42153189-42153233	NM_007524:2249	Nkx3-2	INSIDE	0.195	0.442	1980.72	874.64	0.086	1472.33	127.09
A_68_P23637423	chr5:42155238-42155282	NM_007524:199	Nkx3-2	INSIDE	0.132	0.503	2263.37	1138.25	0.066	1817.30	120.68
A_68_P23637424	chr5:42155415-42155459	NM_007524:23	Nkx3-2	INSIDE	0.236	0.668	5005.43	3344.39	0.158	3885.17	612.69
A_68_P23637425	chr5:42155503-42155547	NM_007524:-65	Nkx3-2	PROMOTER	0.491	0.530	2223.40	1178.35	0.260	1664.94	432.94
A_68_P23936294	chr5:102088064-102088108	NM_144955:5644	Nkx6-1	DOWNSTREAM	0.636	0.667	1836.63	1225.65	0.424	1509.94	640.58
A_68_P23936297	chr5:102088485-102088535	NM_144955:5220	Nkx6-1	INSIDE	0.191	0.702	1248.69	876.50	0.134	929.87	124.68
A_68_P23936330	chr5:102092926-102092970	NM_144955:782	Nkx6-1	INSIDE	0.209	0.313	2159.98	675.14	0.065	1813.16	118.67
A_68_P25578887	chr7:146767017-146767061	NR_027857:1658	Nkx6-2	INSIDE	0.325	0.674	1031.65	695.50	0.219	841.23	184.36
A_68_P25578888	chr7:146767137-146767194	NR_027857:1531	Nkx6-2	INSIDE	0.215	1.487	770.38	1145.54	0.320	626.56	200.61
A_68_P25578898	chr7:146768350-146768394	NR_027857:324	Nkx6-2	INSIDE	0.368	0.584	1587.11	927.23	0.215	1164.07	250.52
A_68_P25578925	chr7:146771094-146771138	NR_027857:-2420	Nkx6-2	PROMOTER	0.240	0.634	1013.49	642.24	0.152	773.00	117.77
A_68_P27969034	chr11:82721872-82721916	NM_145431:3	Nle1	INSIDE	0.168	0.593	1655.59	981.13	0.100	1210.24	120.73
A_68_P22006131	chr3:26230237-26230281	NM_138666:573	Nlgn1	INSIDE	0.613	0.426	1343.22	571.62	0.261	998.03	260.19
A_68_P29288685	chr13:104899639-104899683	NM_029447:34	Nln	INSIDE	0.267	0.502	3750.88	1882.96	0.134	2717.95	364.88
A_68_P20723959	chr1:154802284-154802330	NM_175460:76	Nmnat2	INSIDE	0.166	0.705	1302.24	918.59	0.117	1007.86	117.88
A_68_P20961506	chr2:3200909-3200953	NM_008708:-629	Nmt2	PROMOTER	0.474	0.537	1726.64	927.27	0.255	1287.86	328.03
A_68_P20961508	chr2:3201225-3201269	NM_008708:-313	Nmt2	PROMOTER	0.473	0.732	2027.47	1483.22	0.346	1469.18	508.10
A_68_P29372955	chr13:120197789-120197833	NM_008710:8	Nnt	INSIDE	0.330	0.607	2162.56	1313.26	0.201	1778.37	356.82
A_68_P26144743	chr8:109948649-109948693	NM_026277:268	Nob1	INSIDE	0.472	0.543	964.11	523.06	0.256	858.72	220.12
A_68_P32103772	chr19:38893658-38893702	NM_021315:47	Noc3l	INSIDE	0.253	0.551	3680.11	2025.94	0.139	2647.97	368.75
A_68_P27180398	chr10:60881315-60881364	NM_013611:620	Nodal	INSIDE	0.210	0.553	1653.01	914.21	0.116	1045.46	121.46
A_68_P28004879	chr11:89160026-89160082	NM_008711:3819	Nog	DOWNSTREAM	0.153	0.688	1607.67	1106.36	0.105	1171.61	123.17
A_68_P28004885	chr11:89160628-89160672	NM_008711:3223	Nog	DOWNSTREAM	0.640	0.524	1607.08	841.78	0.335	1359.69	455.88
A_68_P28004913	chr11:89163892-89163940	NM_008711:-43	Nog	PROMOTER	0.212	0.658	1124.21	740.24	0.139	882.21	122.93
A_68_P28105683	chr11:107050715-107050759	NM_001161329:-41	Nol11	PROMOTER	0.254	0.453	1295.12	586.91	0.115	1151.89	132.70
A_68_P31552999	chr18:23198421-23198469	NM_001161483:-1280	Nol4	PROMOTER	0.310	0.584	900.37	526.13	0.181	656.88	119.04
A_68_P23567511	chr5:29761679-29761723	NM_001033457:494	Nom1	INSIDE	0.271	0.725	1879.56	1363.20	0.197	1471.14	289.57
A_68_P25096453	chr7:53289096-53289140	NM_153057:53	Nomo1	INSIDE	0.378	0.555	1276.68	708.95	0.210	1041.00	218.28
A_68_P25096457	chr7:53289487-53289535	NM_153057:445	Nomo1	INSIDE	0.171	0.658	1670.73	1098.71	0.112	1259.93	141.59
A_68_P32564062	chrX:98625083-98625127	NM_023144:115	Nono	INSIDE	0.133	2.727	2528.62	6895.70	0.363	1121.06	406.96
A_68_P32564063	chrX:98625189-98625233	NM_023144:221	Nono	INSIDE	0.117	1.638	1939.09	3175.27	0.191	774.86	148.30
A_68_P24818880	chr6:125082083-125082127	NM_138747:204	Nop2	INSIDE	0.541	0.519	4512.78	2343.53	0.281	3630.37	1020.18
A_68_P24818881	chr6:125082179-125082232	NM_138747:305	Nop2	INSIDE	0.207	0.648	1343.94	871.34	0.134	892.32	120.00
A_68_P21628852	chr2:130099893-130099937	NM_024193:-233	Nop56	PROMOTER	0.661	0.545	1054.58	575.08	0.360	795.64	286.66
A_68_P20823476	chr1:172519433-172519477	NM_001109985:526	Nos1ap	INSIDE	0.239	0.668	1001.56	668.97	0.160	765.96	122.22
A_68_P27946028	chr11:78738513-78738557	NM_010927:4174	Nos2	INSIDE	0.253	0.622	1056.79	656.90	0.157	908.50	143.06

ProbeName	Target position of probe on CpG island microarray	TargetID	GeneSymbol	CpG island Description	Ratio of relative methylation (TiO2-NP/Vehicle)	Sham group			TiO_2-H group		
						Relative methylation (Cy-5/Cy-3)	Cy-3 signal (Input)	Cy-5 signal (MeDIP)	Relative methylation (Cy-5/Cy-3)	Cy-3 signal (Input)	Cy-5 signal (MeDIP)
A_68_P21079208	chr2:26358390-26358438	NM_008714:928	Notch1	INSIDE	0.281	0.633	1567.20	991.68	0.178	1075.31	191.03
A_68_P22357701	chr3:97817894-97817938	NM_010928:456	Notch2	INSIDE	0.077	0.623	3547.43	2209.09	0.048	2398.52	115.09
A_68_P31143793	chr17:32250971-32251015	NM_008716:52805	Notch3	DOWNSTREAM	0.141	0.589	1923.80	1133.46	0.083	1442.95	120.04
A_68_P31143941	chr17:32280371-32280415	NM_008716:23405	Notch3	INSIDE	0.629	0.633	1371.65	868.42	0.398	1249.43	497.62
A_68_P24990613	chr7:19542978-19543022	NM_001029877:31764	Nova2	INSIDE	0.498	0.658	1894.30	1246.22	0.328	1379.28	452.06
A_68_P24990653	chr7:19548237-19548281	NM_001029877:37022	Nova2	INSIDE	0.584	0.596	864.84	515.06	0.348	716.69	249.28
A_68_P24990654	chr7:19548318-19548362	NM_001029877:37104	Nova2	INSIDE	0.368	0.671	2332.29	1564.36	0.247	1919.15	473.29
A_68_P31099062	chr17:24833399-24833444	NM_027988:243	Noxo1	INSIDE	0.255	0.530	1205.75	639.44	0.135	910.91	123.16
A_68_P24981699	chr7:17060079-17060123	NM_008718:2029	Npas1	INSIDE	0.198	0.694	1308.38	908.12	0.137	1075.54	147.48
A_68_P24981705	chr7:17060954-17060998	NM_008718:1153	Npas1	INSIDE	0.460	0.485	3392.34	1645.38	0.223	2717.13	606.84
A_68_P24981706	chr7:17061083-17061127	NM_008718:1025	Npas1	INSIDE	0.548	0.582	1004.12	584.56	0.319	874.17	279.13
A_68_P24981708	chr7:17061429-17061473	NM_008718:679	Npas1	INSIDE	0.264	0.541	1054.92	570.50	0.143	813.15	116.19
A_68_P24981712	chr7:17061914-17061958	NM_008718:193	Npas1	INSIDE	0.397	0.712	2890.67	2056.71	0.283	2241.41	633.52
A_68_P20164265	chr1:39250200-39250244	NM_008719:-894	Npas2	PROMOTER	0.608	0.623	2167.63	1350.98	0.379	1748.67	662.81
A_68_P20164271	chr1:39251178-39251222	NM_008719:84	Npas2	INSIDE	0.481	0.378	2295.88	868.81	0.182	1697.49	309.03
A_68_P31926194	chr19:4989799-4989843	NM_153553:151	Npas4	INSIDE	0.204	0.494	1799.08	889.29	0.101	1230.56	124.04
A_68_P26521206	chr9:53345098-53345142	NM_001081152:-31	Npat	DIVERGENT_PROMOTER	0.665	0.515	1476.63	761.04	0.343	1089.94	373.51
A_68_P20010809	chr1:5906460-5906507	NM_010342:996	Npbwr1	INSIDE	0.653	2.274	1096.84	2494.24	1.484	766.36	1137.20
A_68_P20010810	chr1:5906552-5906596	NM_010342:905	Npbwr1	INSIDE	0.369	1.711	2495.38	4269.75	0.631	1896.36	1195.84
A_68_P20010813	chr1:5906992-5907041	NM_010342:463	Npbwr1	INSIDE	0.268	0.595	1239.20	737.49	0.159	945.14	150.64
A_68_P20010816	chr1:5907329-5907377	NM_010342:127	Npbwr1	INSIDE	0.220	0.567	1229.17	697.00	0.124	966.44	120.31
A_68_P20010817	chr1:5907429-5907473	NM_010342:29	Npbwr1	INSIDE	0.152	0.543	1572.48	854.32	0.082	1421.97	117.09
A_68_P31499628	chr18:12394947-12394991	NM_008720:-73	Npc1	PROMOTER	0.246	0.446	1984.24	885.73	0.110	1492.97	163.80
A_68_P28600137	chr12:86113873-86113917	NM_023409:168	Npc2	INSIDE	0.566	0.524	3879.45	2034.68	0.297	3086.46	916.64
A_68_P28600138	chr12:86114028-86114072	NM_023409:12	Npc2	INSIDE	0.264	0.361	2097.68	758.08	0.096	1556.27	148.71
A_68_P30368842	chr15:79664125-79664169	NM_001013360:617	Npcd	INSIDE	0.198	0.707	1360.74	961.81	0.140	834.02	116.60
A_68_P30368844	chr15:79664362-79664415	NM_001013360:375	Npcd	INSIDE	0.104	1.771	891.14	1578.34	0.184	635.42	116.64
A_68_P30368846	chr15:79664558-79664602	NM_001013360:183	Npcd	INSIDE	0.339	0.432	1901.98	822.09	0.147	1392.49	204.25
A_68_P30368850	chr15:79664958-79665002	NM_001013360:-217	Npcd	PROMOTER	0.193	0.619	1309.22	811.01	0.119	975.64	116.47
A_68_P28049717	chr11:97141114-97141159	NM_008942:754	Npepps	INSIDE	0.293	1.593	6250.36	9956.81	0.467	4024.92	1879.83
A_68_P23419143	chr4:151852331-151852375	NM_153424:102	Nphp4	INSIDE	0.186	0.687	2407.37	1653.22	0.128	1760.58	225.08
A_68_P25031675	chr7:31250465-31250509	NR_004443:124	Nphs1as	INSIDE	0.222	0.708	2299.80	1628.55	0.157	1832.80	287.65
A_68_P27693121	chr11:33063184-33063228	NM_008722:-150	Npm1	PROMOTER	0.117	0.440	3131.95	1379.56	0.052	2250.00	116.41
A_68_P32140280	chr19:45823401-45823445	NM_008723:631	Npm3	INSIDE	0.292	0.529	1686.66	892.00	0.154	1223.51	188.69
A_68_P20413377	chr1:88566126-88566173	NM_010933:999	Nppc	INSIDE	0.171	0.656	1373.05	901.25	0.112	1121.32	125.56
A_68_P20413379	chr1:88566390-88566434	NM_010933:736	Nppc	INSIDE	0.450	0.533	1446.19	770.17	0.240	1313.60	314.67
A_68_P20413386	chr1:88567063-88567107	NM_010933:64	Nppc	INSIDE	0.596	0.569	1056.02	600.65	0.339	904.77	306.88
A_68_P22325407	chr3:90269682-90269726	NM_008727:84	Npr1	INSIDE	0.415	0.463	2335.39	1081.29	0.192	1864.58	358.54
A_68_P22872798	chr4:43644462-43644506	NM_173788:-322	Npr2	PROMOTER	0.145	0.685	3693.85	2530.88	0.099	2601.01	258.44
A_68_P22872799	chr4:43644616-43644660	NM_173788:-168	Npr2	PROMOTER	0.476	1.511	3992.20	6032.33	0.719	2609.02	1875.02
A_68_P22872800	chr4:43644731-43644775	NM_173788:-54	Npr2	PROMOTER	0.288	1.511	5436.31	8213.47	0.435	3337.09	1451.11
A_68_P30018889	chr15:11834455-11834499	NM_001039181:953	Npr3	INSIDE	0.301	0.597	4118.83	2458.74	0.180	3024.13	543.61
A_68_P30018898	chr15:11835366-11835410	NM_001039181:41	Npr3	INSIDE	0.515	0.443	2915.23	1291.08	0.228	2417.64	551.50
A_68_P30018903	chr15:11836061-11836105	NM_001039181:-653	Npr3	PROMOTER	0.495	0.722	1765.01	1275.08	0.357	1463.43	523.00

ProbeName	Target position of probe on CpG island microarray	TargetID	GeneSymbol	CpG island Description	Ratio of relative methylation (TiO2-NP/Vehicle)	Sham group			TiO_2-H group		
						Relative methylation (Cy-5/Cy-3)	Cy-3 signal (Input)	Cy-5 signal (MeDIP)	Relative methylation (Cy-5/Cy-3)	Cy-3 signal (Input)	Cy-5 signal (MeDIP)
A_68_P28178576	chr11:119408102-119408146	NM_008730:1010	Nptx1	INSIDE	0.276	0.650	1013.18	658.50	0.179	666.32	119.32
A_68_P28178579	chr11:119408457-119408501	NM_008730:656	Nptx1	INSIDE	0.136	0.696	1871.48	1302.93	0.095	1301.95	123.21
A_68_P24161584	chr5:145307316-145307360	NM_016789:583	Nptx2	INSIDE	0.291	0.593	925.50	548.83	0.173	691.26	119.32
A_68_P30368650	chr15:79634238-79634282	NM_030689:879	Nptxr	INSIDE	0.552	0.707	1287.74	910.09	0.390	1115.47	435.02
A_68_P25934101	chr8:69212003-69212047	NM_016708:-31	Npy5r	DIVERGENT_PROMOTER	0.261	0.691	2268.25	1566.88	0.180	1775.21	319.68
A_68_P28058441	chr11:98635083-98635127	NM_145434:1452	Nr1d1	INSIDE	0.562	0.495	1093.90	541.80	0.278	1028.08	286.08
A_68_P29437679	chr14:19071747-19071791	NM_011584:-148	Nr1d2	PROMOTER	0.292	1.714	1886.57	3233.78	0.501	1321.71	662.59
A_68_P27357765	chr10:93610485-93610538	NM_011629:-164	Nr2c1	PROMOTER	0.090	1.434	2855.08	4093.69	0.130	1944.92	252.30
A_68_P24643145	chr6:92042439-92042483	NM_011630:1049	Nr2c2	INSIDE	0.572	0.443	1697.54	752.66	0.254	1205.20	305.84
A_68_P27095710	chr10:42303461-42303505	NM_152229:-88	Nr2e1	PROMOTER	0.564	0.594	1771.51	1053.13	0.335	1429.46	479.08
A_68_P27095770	chr10:42311388-42311432	NM_152229:-8016	Nr2e1	PROMOTER	0.648	0.515	1520.31	782.29	0.333	1203.04	401.11
A_68_P29162267	chr13:78334860-78334904	NM_010151:3361	Nr2f1	INSIDE	0.062	1.413	7189.05	10158.16	0.088	4645.59	408.31
A_68_P29162321	chr13:78342782-78342832	NM_010151:-4563	Nr2f1	PROMOTER	0.224	0.582	1214.72	707.18	0.130	912.49	119.04
A_68_P29162323	chr13:78342968-78343012	NM_010151:-4747	Nr2f1	PROMOTER	0.523	0.627	1214.20	761.07	0.328	1068.19	350.34
A_68_P25210773	chr7:77502893-77502937	NM_009697:2565	Nr2f2	INSIDE	0.419	0.456	1637.13	746.81	0.191	1360.60	260.11
A_68_P25956587	chr8:73900036-73900080	NM_010150:5793	Nr2f6	INSIDE	0.557	0.568	1405.79	798.22	0.316	1202.23	380.34
A_68_P25956629	chr8:73904896-73904940	NM_010150:933	Nr2f6	INSIDE	0.256	0.680	2607.97	1773.22	0.174	1807.81	314.99
A_68_P31640473	chr18:39648559-39648603	NM_008173:-1681	Nr3c1	PROMOTER	0.472	1.405	4421.18	6213.11	0.664	3063.80	2033.46
A_68_P25979818	chr8:79426066-79426110	NM_001083906:-318	Nr3c2	DIVERGENT_PROMOTER	0.226	0.646	2683.35	1734.08	0.146	1859.18	271.02
A_68_P25979819	chr8:79426229-79426273	NM_001083906:-156	Nr3c2	DIVERGENT_PROMOTER	0.224	0.633	1069.13	676.55	0.142	853.53	121.06
A_68_P21240002	chr2:56966945-56966989	NM_013613:483	Nr4a2	INSIDE	0.234	0.598	3165.16	1891.41	0.140	2717.55	380.54
A_68_P21240006	chr2:56967343-56967388	NM_013613:84	Nr4a2	INSIDE	0.353	0.638	2028.46	1294.99	0.226	1598.35	360.64
A_68_P22898162	chr4:48057954-48057998	NM_015743:-6143	Nr4a3	PROMOTER	0.212	0.645	2007.57	1293.93	0.137	1556.05	212.88
A_68_P22898164	chr4:48058319-48058363	NM_015743:-5779	Nr4a3	PROMOTER	0.617	0.558	2232.91	1246.65	0.345	1848.83	637.15
A_68_P22898179	chr4:48059988-48060034	NM_015743:-4109	Nr4a3	PROMOTER	0.250	0.586	1083.45	634.62	0.147	834.97	122.48
A_68_P21150007	chr2:38565998-38566044	NM_139051:4042	Nr5a1	INSIDE	0.254	0.572	1063.30	608.59	0.145	811.01	117.88
A_68_P20648914	chr1:138845211-138845255	NM_001159769:4975	Nr5a2	INSIDE	0.266	0.467	1245.99	581.75	0.124	956.08	118.87
A_68_P20648919	chr1:138845800-138845844	NM_001159769:4385	Nr5a2	INSIDE	0.535	0.399	1742.35	696.01	0.214	1534.85	327.94
A_68_P21151178	chr2:38780768-38780812	NM_010264:1191	Nr6a1	INSIDE	0.430	0.727	3471.77	2523.33	0.312	2582.24	806.87
A_68_P21151183	chr2:38781338-38781382	NM_010264:621	Nr6a1	INSIDE	0.595	0.691	2248.49	1553.46	0.411	1773.06	729.37
A_68_P21070361	chr2:25036829-25036873	NM_025980:573	Nrarp	INSIDE	0.285	0.691	2563.98	1771.64	0.197	1831.98	361.15
A_68_P21070362	chr2:25036916-25036960	NM_025980:661	Nrarp	INSIDE	0.282	0.637	3173.71	2022.47	0.180	2317.54	416.33
A_68_P21070363	chr2:25037051-25037095	NM_025980:795	Nrarp	INSIDE	0.182	0.638	1694.75	1080.83	0.116	1259.37	145.94
A_68_P27211072	chr10:66748247-66748295	NM_001036293:-241	Nrbf2	PROMOTER	0.226	0.496	1248.05	618.48	0.112	1043.39	116.87
A_68_P30346012	chr15:75920669-75920713	NM_144847:-247	Nrbp2	PROMOTER	0.552	0.670	1346.58	901.54	0.369	932.08	344.37
A_68_P23198683	chr4:108673036-108673080	NM_146150:-351	Nrd1	PROMOTER	0.355	0.582	4518.05	2629.68	0.206	3154.75	650.95
A_68_P23198686	chr4:108673421-108673465	NM_146150:33	Nrd1	INSIDE	0.080	0.626	3248.33	2032.43	0.050	2490.66	124.05
A_68_P31622626	chr18:36356438-36356482	NM_001167891:354	Nrg2	INSIDE	0.478	0.582	1174.01	683.70	0.279	994.54	277.10
A_68_P31622630	chr18:36357100-36357144	NM_001167891:-308	Nrg2	PROMOTER	0.511	0.452	1860.39	840.59	0.231	1443.43	333.15
A_68_P29554793	chr14:40285528-40285572	NM_001190187:826	Nrg3	INSIDE	0.375	0.638	1011.76	645.17	0.239	788.74	188.77
A_68_P30883587	chr16:76293685-76293730	NM_173440:79587	Nrip1	INSIDE	0.465	1.378	2289.28	3155.19	0.640	1674.90	1072.31
A_68_P30883588	chr16:76293765-76293824	NM_173440:79500	Nrip1	INSIDE	0.192	1.687	1078.55	1819.69	0.324	767.18	248.48
A_68_P25410004	chr7:116925392-116925436	NM_020610:-355	Nrip3	PROMOTER	0.547	0.676	1933.21	1307.24	0.370	1507.09	557.46
A_68_P32691811	chrX:135448869-135448913	NM_013724:-78	Nrk	PROMOTER	0.157	2.243	12204.76	27377.88	0.353	4834.37	1705.29

ProbeName	Target position of probe on CpG island microarray	TargetID	GeneSymbol	CpG island Description	Ratio of relative methylation (TiO2-NP/Vehicle)	Sham group			TiO_2-H group		
						Relative methylation (Cy-5/Cy-3)	Cy-3 signal (Input)	Cy-5 signal (MeDIP)	Relative methylation (Cy-5/Cy-3)	Cy-3 signal (Input)	Cy-5 signal (MeDIP)
A_68_P32691812	chrX:135449017-135449063	NM_013724:72	Nrk	INSIDE	0.094	3.143	1481.01	4654.61	0.294	570.56	167.80
A_68_P31161346	chr17:35998590-35998640	NM_134122:352	Nrm	INSIDE	0.415	1.623	3958.59	6423.63	0.673	2470.63	1663.50
A_68_P28950724	chr13:36818604-36818663	NM_153529:7690	Nrn1	INSIDE	0.182	0.540	1648.09	890.21	0.098	1233.86	121.18
A_68_P26268769	chr8:130881368-130881412	NM_008737:-1582	Nrp1	PROMOTER	0.381	0.592	1392.25	824.88	0.225	1178.98	265.85
A_68_P26268777	chr8:130882335-130882384	NM_008737:-613	Nrp1	PROMOTER	0.253	0.610	995.81	607.04	0.154	779.14	119.94
A_68_P26268778	chr8:130882433-130882477	NM_008737:-518	Nrp1	PROMOTER	0.481	0.712	1356.25	965.21	0.342	1017.05	347.82
A_68_P26268784	chr8:130883235-130883284	NM_008737:287	Nrp1	INSIDE	0.153	0.498	2154.51	1073.27	0.076	1558.54	118.92
A_68_P26268785	chr8:130883347-130883403	NM_008737:402	Nrp1	INSIDE	0.250	0.516	1285.24	663.39	0.129	895.01	115.67
A_68_P31258135	chr17:56890739-56890783	NM_008738:6193	Nrtn	INSIDE	0.301	1.430	1022.35	1462.25	0.430	808.62	347.81
A_68_P31934664	chr19:6428736-6428780	NM_001205234:10021	Nrxn2	INSIDE	0.328	0.603	1095.93	660.62	0.198	815.98	161.49
A_68_P31934666	chr19:6428995-6429039	NM_001205234:10279	Nrxn2	INSIDE	0.594	2.293	5391.29	12362.88	1.363	3511.12	4786.12
A_68_P31935004	chr19:6481505-6481549	NM_001205234:62789	Nrxn2	INSIDE	0.655	2.154	3802.31	8188.49	1.411	2531.56	3573.21
A_68_P31935408	chr19:6531356-6531400	NM_001205234:112641	Nrxn2	INSIDE	0.668	0.583	1603.83	935.12	0.389	1334.63	519.47
A_68_P21744134	chr2:151319923-151319967	NM_198326:-99	Nsfl1c	PROMOTER	0.660	0.649	897.33	582.70	0.428	697.84	298.98
A_68_P23617866	chr5:38550212-38550259	NM_010942:471	Nsg1	INSIDE	0.297	0.618	826.48	510.65	0.183	640.65	117.53
A_68_P23617870	chr5:38550639-38550683	NM_010942:46	Nsg1	INSIDE	0.195	0.577	1256.40	725.24	0.112	1185.89	133.22
A_68_P22705746	chr4:6380618-6380662	NM_010945:778	Nsmaf	INSIDE	0.291	0.598	1501.04	897.31	0.174	1239.58	215.96
A_68_P22705747	chr4:6380739-6380783	NM_010945:658	Nsmaf	INSIDE	0.405	0.415	1617.03	671.38	0.168	1348.70	226.72
A_68_P25494915	chr7:132634916-132634960	NM_026330:118	Nsmce1	INSIDE	0.538	0.678	2798.73	1897.41	0.365	2099.17	765.53
A_68_P25494916	chr7:132635012-132635056	NM_026330:22	Nsmce1	INSIDE	0.201	0.540	2314.21	1249.49	0.109	1645.48	178.56
A_68_P25522280	chr7:137690501-137690545	NM_001162855:373	Nsmce4a	INSIDE	0.503	0.564	2401.25	1353.98	0.284	1791.02	508.46
A_68_P23760679	chr5:66651534-66651578	NM_027602:193	Nsun7	INSIDE	0.211	2.423	3500.23	8481.48	0.510	2272.03	1159.24
A_68_P23261316	chr4:122878552-122878598	NM_001085502:-221	Nt5c1a	PROMOTER	0.212	0.667	1290.84	860.65	0.141	883.84	125.03
A_68_P32147202	chr19:47090004-47090048	NM_001164363:-347	Nt5c2	PROMOTER	0.387	0.484	1367.29	661.09	0.187	1103.59	206.66
A_68_P32147204	chr19:47090203-47090247	NM_001164363:-545	Nt5c2	PROMOTER	0.187	0.710	1808.50	1284.59	0.133	1471.62	195.03
A_68_P28068130	chr11:100302472-100302516	NM_026561:-91	Nt5c3l	DIVERGENT_PROMOTER	0.300	0.677	1646.00	1113.66	0.203	1214.32	246.45
A_68_P27056941	chr10:34138297-34138341	NM_176968:16	Nt5dc1	INSIDE	0.139	1.588	5026.10	7980.69	0.220	3697.00	813.78
A_68_P30559115	chr16:13819208-13819252	NM_010946:-139	Ntan1	PROMOTER	0.448	0.613	2042.89	1252.94	0.275	1488.62	409.32
A_68_P25092989	chr7:52671296-52671340	NM_198190:2254	Ntf5	INSIDE	0.259	1.361	2359.88	3212.89	0.352	1741.58	612.93
A_68_P31098617	chr17:24769589-24769637	NM_008743:-14	Nthl1	DIVERGENT_PROMOTER	0.259	0.438	1343.19	587.77	0.113	1033.40	116.93
A_68_P26388504	chr9:29219516-29219560	NM_172290:551176	Ntm	INSIDE	0.534	0.666	1955.15	1303.07	0.356	1485.82	528.84
A_68_P27889080	chr11:68198460-68198504	NM_008744:1846	Ntn1	INSIDE	0.389	0.596	1947.38	1160.66	0.232	1552.36	359.66
A_68_P27889095	chr11:68200015-68200060	NM_008744:291	Ntn1	INSIDE	0.139	0.675	1757.64	1186.31	0.094	1243.46	116.80
A_68_P22422573	chr3:109946012-109946056	NM_001163348:356	Ntng1	INSIDE	0.251	0.544	1642.08	893.53	0.137	1231.93	168.45
A_68_P22422579	chr3:109946712-109946764	NM_001163348:-348	Ntng1	PROMOTER	0.292	0.564	929.84	524.30	0.164	728.06	119.75
A_68_P22422580	chr3:109946888-109946938	NM_001163348:-522	Ntng1	PROMOTER	0.338	0.560	2088.91	1170.50	0.189	1590.07	301.02
A_68_P21095672	chr2:29050439-29050483	NM_133500:53100	Ntng2	INSIDE	0.645	0.730	2161.13	1577.79	0.471	1866.60	878.51
A_68_P21096074	chr2:29107607-29107664	NM_133500:-4075	Ntng2	PROMOTER	0.158	1.553	2861.42	4444.43	0.245	1933.41	473.66
A_68_P21096076	chr2:29107825-29107869	NM_133500:-4286	Ntng2	PROMOTER	0.297	0.598	1790.03	1071.17	0.178	1394.41	247.93
A_68_P22310058	chr3:87598739-87598783	NM_001033124:324	Ntrk1	INSIDE	0.636	0.691	2699.07	1863.82	0.439	2215.10	972.96
A_68_P29075345	chr13:58907821-58907865	NM_008745:-114	Ntrk2	PROMOTER	0.391	0.692	1892.71	1310.54	0.271	1464.42	396.74
A_68_P29075352	chr13:58908396-58908440	NM_001025074:-775	Ntrk2	PROMOTER	0.489	0.659	2536.05	1670.02	0.322	1999.16	644.05
A_68_P25256092	chr7:85723225-85723269	NM_008746:-522	Ntrk3	PROMOTER	0.438	0.647	1011.10	654.18	0.283	766.67	217.27
A_68_P25256093	chr7:85723547-85723591	NM_008746:-844	Ntrk3	PROMOTER	0.261	0.650	1106.75	719.75	0.169	839.19	142.23

ProbeName	Target position of probe on CpG island microarray	TargetID	GeneSymbol	CpG island Description	Ratio of relative methylation (TiO2-NP/Vehicle)	Sham group			TiO_2-H group		
						Relative methylation (Cy-5/Cy-3)	Cy-3 signal (Input)	Cy-5 signal (MeDIP)	Relative methylation (Cy-5/Cy-3)	Cy-3 signal (Input)	Cy-5 signal (MeDIP)
A_68_P25256094	chr7:85723616-85723660	NM_008746:-914	Ntrk3	PROMOTER	0.208	0.471	1632.25	768.13	0.098	1205.76	117.77
A_68_P28265956	chr12:16660397-16660441	NM_008747:143	Ntsr2	INSIDE	0.621	0.465	1150.11	535.29	0.289	961.02	277.93
A_68_P20620659	chr1:134213013-134213057	NM_001195025:333	Nuak2	INSIDE	0.501	0.617	1276.45	786.99	0.309	1170.21	361.54
A_68_P23543337	chr5:24191628-24191672	NM_016736:18	Nub1	INSIDE	0.203	1.600	4781.80	7649.06	0.324	3187.57	1034.03
A_68_P31100079	chr17:25023157-25023201	NM_011956:117	Nubp2	INSIDE	0.426	0.469	5167.57	2423.85	0.200	3511.20	701.20
A_68_P25093530	chr7:52765419-52765466	NM_001163662:336	Nucb1	INSIDE	0.242	0.652	1067.29	695.72	0.158	768.33	121.05
A_68_P25448400	chr7:123648290-123648334	NM_016773:425	Nucb2	INSIDE	0.124	1.395	4436.86	6188.51	0.173	2668.75	460.58
A_68_P20618261	chr1:133806870-133806914	NM_001145804:-142	Nucks1	PROMOTER	0.120	0.645	2518.02	1623.65	0.077	1776.11	136.92
A_68_P20618262	chr1:133806972-133807021	NM_001145804:-38	Nucks1	PROMOTER	0.059	0.573	4903.36	2808.04	0.034	3475.83	116.53
A_68_P24140929	chr5:140808002-140808046	NM_008637:149	Nudt1	INSIDE	0.265	1.402	1716.18	2405.38	0.371	1358.22	504.11
A_68_P29718025	chr14:73924796-73924840	NM_172527:23231	Nudt15	INSIDE	0.174	0.663	5473.03	3631.03	0.115	3869.57	445.76
A_68_P30512786	chr16:4939254-4939298	NM_025839:166	Nudt16l1	INSIDE	0.194	0.710	2106.79	1495.70	0.137	1362.72	187.33
A_68_P22350765	chr3:96512213-96512257	NM_001162925:249	Nudt17	INSIDE	0.344	0.488	1912.80	934.13	0.168	1486.44	249.62
A_68_P29702205	chr14:70977218-70977273	NM_153136:-408	Nudt18	PROMOTER	0.219	0.494	1447.28	714.31	0.108	1079.99	116.63
A_68_P26075123	chr8:96560510-96560557	NM_026623:406	Nudt21	INSIDE	0.289	0.604	1360.52	822.02	0.175	1087.56	189.83
A_68_P31117522	chr17:27759599-27759649	NM_019837:773	Nudt3	INSIDE	0.212	0.571	1376.47	786.56	0.121	952.80	115.26
A_68_P22063505	chr3:37318090-37318134	NM_153561:400	Nudt6	INSIDE	0.446	0.595	1766.22	1050.41	0.265	1459.60	387.04
A_68_P31920554	chr19:4000379-4000423	NM_025529:-179	Nudt8	PROMOTER	0.307	0.519	1609.25	834.54	0.159	1305.69	207.58
A_68_P26242531	chr8:126472768-126472821	NM_172288:371	Nup133	INSIDE	0.180	0.589	2114.34	1245.39	0.106	1447.79	153.73
A_68_P26242536	chr8:126473255-126473299	NM_172288:-111	Nup133	PROMOTER	0.201	0.322	2254.66	726.10	0.065	1823.86	117.95
A_68_P26242539	chr8:126473498-126473548	NM_172288:-357	Nup133	PROMOTER	0.154	0.579	1680.69	973.87	0.089	1301.58	116.11
A_68_P29009464	chr13:46822934-46822978	NM_175749:262	Nup153	INSIDE	0.648	0.433	1908.64	826.99	0.281	1480.98	416.03
A_68_P21418379	chr2:90519075-90519128	NM_021512:1730	Nup160	INSIDE	0.198	0.639	1306.12	834.87	0.127	920.60	116.71
A_68_P21102303	chr2:30142233-30142277	NM_198304:302	Nup188	INSIDE	0.325	0.431	2753.10	1187.89	0.140	2026.23	284.10
A_68_P24360135	chr6:35127937-35127981	NM_027513:343	Nup205	INSIDE	0.634	0.579	1249.82	724.04	0.368	1063.37	390.85
A_68_P21112482	chr2:31830089-31830133	NM_172268:141	Nup214	INSIDE	0.217	0.462	1430.40	660.43	0.100	1205.86	120.66
A_68_P30397616	chr15:84753564-84753608	NM_016714:-271	Nup50	PROMOTER	0.177	0.446	2041.68	911.47	0.079	1585.46	125.19
A_68_P23894274	chr5:92863985-92864029	NM_183392:219	Nup54	INSIDE	0.160	0.580	1668.34	968.02	0.093	1240.45	115.41
A_68_P23894275	chr5:92864051-92864096	NM_183392:152	Nup54	INSIDE	0.227	0.635	5751.09	3654.74	0.144	4115.74	592.88
A_68_P23894277	chr5:92864276-92864320	NM_183392:-73	Nup54	PROMOTER	0.364	0.633	1620.81	1026.47	0.231	1304.06	301.01
A_68_P32697227	chrX:136596984-136597028	NM_001081668:101	Nup62cl	INSIDE	0.223	1.655	1194.86	1977.34	0.370	551.75	203.93
A_68_P25089408	chr7:52071427-52071471	NM_001171024:-291	Nup62-il4i1	DIVERGENT_PROMOTER	0.261	0.708	1442.12	1021.06	0.185	1029.63	190.19
A_68_P27903590	chr11:70783209-70783253	NM_001083331:234	Nup88	INSIDE	0.194	0.483	1721.41	831.37	0.093	1290.37	120.59
A_68_P27903591	chr11:70783309-70783353	NM_001083331:134	Nup88	INSIDE	0.252	0.561	1888.83	1060.44	0.142	1364.85	193.26
A_68_P26076074	chr8:96738369-96738413	NM_172410:-110	Nup93	PROMOTER	0.365	0.640	1151.63	737.03	0.234	880.41	205.75
A_68_P25377673	chr7:109358261-109358306	NM_022979:351	Nup98	INSIDE	0.267	0.374	1583.05	591.34	0.100	1224.31	121.96
A_68_P27137647	chr10:52137521-52137565	NM_030250:190	Nus1	INSIDE	0.464	0.651	5850.61	3808.45	0.302	4439.83	1339.71
A_68_P27137650	chr10:52137866-52137910	NM_030250:536	Nus1	INSIDE	0.600	0.572	1525.12	872.76	0.343	1337.33	459.15
A_68_P31943410	chr19:8831910-8831954	NM_016813:340	Nxf1	INSIDE	0.216	0.419	1761.24	738.39	0.091	1322.43	119.97
A_68_P29031122	chr13:51266973-51267017	NM_029173:601	Nxnl2	INSIDE	0.232	0.644	2163.55	1392.28	0.149	1691.08	252.45
A_68_P27536452	chr10:126971104-126971148	NM_183297:489	Nxph4	INSIDE	0.045	1.402	12909.02	18098.38	0.064	7582.89	483.68
A_68_P27536453	chr10:126971246-126971290	NM_183297:347	Nxph4	INSIDE	0.444	0.595	1610.34	957.72	0.264	1084.93	286.51
A_68_P27536461	chr10:126972343-126972387	NM_183297:-749	Nxph4	PROMOTER	0.183	0.538	2326.13	1251.91	0.098	1517.35	149.09
A_68_P32099497	chr19:38171760-38171804	NM_181748:214	O3far1	INSIDE	0.307	0.500	1215.27	607.68	0.153	973.89	149.46

ProbeName	Target position of probe on CpG island microarray	TargetID	GeneSymbol	CpG island Description	Ratio of relative methylation (TiO2-NP/Vehicle)	Sham group			TiO_2-H group		
						Relative methylation (Cy-5/Cy-3)	Cy-3 signal (Input)	Cy-5 signal (MeDIP)	Relative methylation (Cy-5/Cy-3)	Cy-3 signal (Input)	Cy-5 signal (MeDIP)
A_68_P32099500	chr19:38172076-38172120	NM_181748:530	O3far1	INSIDE	0.568	0.513	2719.67	1395.86	0.291	2141.75	624.03
A_68_P26460519	chr9:43047271-43047315	NM_178644:607	Oaf	INSIDE	0.219	0.536	1851.00	991.97	0.118	1439.47	169.19
A_68_P26460523	chr9:43047879-43047923	NM_178644:-1	Oaf	PROMOTER	0.188	0.422	2116.38	893.93	0.079	1577.08	125.36
A_68_P26460524	chr9:43048046-43048090	NM_178644:-169	Oaf	PROMOTER	0.148	0.597	2265.97	1352.33	0.088	1894.99	167.05
A_68_P27285693	chr10:80289215-80289268	NM_008753:-159	Oaz1	PROMOTER	0.341	0.565	1467.24	829.33	0.193	1028.09	198.41
A_68_P26588950	chr9:65524268-65524312	NM_010952:-64	Oaz2-ps	PROMOTER	0.650	0.614	1028.55	631.76	0.399	831.67	331.97
A_68_P20221655	chr1:51535170-51535214	NM_028696:51	Obfc2a	INSIDE	0.191	0.468	1704.65	797.87	0.090	1345.45	120.43
A_68_P20221656	chr1:51535340-51535384	NM_028696:-119	Obfc2a	PROMOTER	0.410	0.578	4764.54	2752.21	0.237	3808.61	902.69
A_68_P27541262	chr10:127846864-127846908	NM_027257:-34	Obfc2b	DIVERGENT_PROMOTER	0.138	0.649	3690.52	2394.30	0.090	2625.23	235.69
A_68_P27835021	chr11:58948977-58949031	NM_001171512:873	Obscn	INSIDE	0.257	0.532	1261.38	670.51	0.137	870.24	118.93
A_68_P27835027	chr11:58949650-58949694	NM_001171512:205	Obscn	INSIDE	0.511	0.537	1404.96	753.92	0.274	1265.11	346.76
A_68_P20354397	chr1:75483176-75483220	NM_178884:19829	Obsl1	INSIDE	0.189	0.684	1499.12	1026.11	0.129	938.26	121.49
A_68_P20354542	chr1:75501748-75501792	NM_178884:1257	Obsl1	INSIDE	0.524	0.390	2008.01	782.44	0.204	1542.57	314.94
A_68_P20354543	chr1:75501853-75501897	NM_178884:1153	Obsl1	INSIDE	0.379	0.484	1584.19	766.92	0.183	1259.54	231.06
A_68_P20354544	chr1:75501965-75502010	NM_178884:1040	Obsl1	INSIDE	0.082	0.567	3667.31	2080.00	0.046	2724.69	126.61
A_68_P20354547	chr1:75502266-75502310	NM_178884:739	Obsl1	INSIDE	0.581	0.691	1324.77	915.62	0.402	1148.24	461.21
A_68_P29269006	chr13:101321942-101321987	NM_008756:489	Ocln	INSIDE	0.121	1.621	1050.20	1701.96	0.196	752.48	147.50
A_68_P29269011	chr13:101322401-101322445	NM_008756:31	Ocln	INSIDE	0.194	0.430	1613.59	694.20	0.084	1469.64	122.93
A_68_P29269014	chr13:101322735-101322779	NM_008756:-303	Ocln	PROMOTER	0.224	1.359	5204.64	7074.70	0.305	3479.69	1059.69
A_68_P29269015	chr13:101322806-101322850	NM_008756:-375	Ocln	PROMOTER	0.068	1.923	3702.50	7119.37	0.132	2243.73	295.18
A_68_P21100055	chr2:29745964-29746008	NM_001177662:404	Odf2	INSIDE	0.201	0.609	1174.99	715.00	0.122	987.70	120.84
A_68_P21100057	chr2:29746300-29746344	NM_001177662:740	Odf2	INSIDE	0.268	0.593	962.80	571.25	0.159	767.46	122.15
A_68_P22606646	chr3:144781460-144781506	NM_001162538:-70	Odf2l	PROMOTER	0.544	0.598	2282.71	1365.36	0.325	1816.47	590.70
A_68_P22606647	chr3:144781543-144781587	NM_025714:1	Odf2l	INSIDE	0.431	0.515	2321.62	1194.95	0.222	1838.55	408.21
A_68_P30424871	chr15:89208941-89208985	NM_001013022:723	Odf3b	INSIDE	0.187	1.450	2442.21	3540.10	0.271	1608.19	435.68
A_68_P30424873	chr15:89209271-89209315	NM_001013022:393	Odf3b	INSIDE	0.532	0.701	5150.98	3609.14	0.373	3802.05	1416.55
A_68_P30424875	chr15:89209486-89209530	NM_001013022:177	Odf3b	INSIDE	0.332	0.576	1115.25	642.53	0.191	902.01	172.46
A_68_P25848475	chr8:49640835-49640879	NM_001145937:119188	Odz3	INSIDE	0.393	0.623	1717.71	1069.53	0.245	1357.53	332.13
A_68_P25848476	chr8:49641034-49641078	NM_001145937:118988	Odz3	INSIDE	0.326	1.385	4524.73	6265.24	0.452	3103.08	1401.44
A_68_P25343523	chr7:103360337-103360381	NM_011858:1212	Odz4	INSIDE	0.589	0.648	1218.86	789.75	0.382	970.32	370.61
A_68_P27567705	chr11:6191666-6191710	NM_010956:89	Ogdh	INSIDE	0.312	0.472	2945.64	1389.37	0.147	2126.37	313.31
A_68_P27567706	chr11:6191780-6191824	NM_010956:203	Ogdh	INSIDE	0.321	0.474	2299.39	1088.84	0.152	1778.73	270.62
A_68_P27567707	chr11:6191869-6191913	NM_010956:291	Ogdh	INSIDE	0.124	0.481	6923.48	3326.88	0.060	5190.09	309.95
A_68_P27567708	chr11:6192040-6192084	NM_010956:463	Ogdh	INSIDE	0.464	0.572	2473.11	1415.47	0.265	2093.52	555.48
A_68_P26075129	chr8:96561318-96561362	NM_001093757:243	Ogfod1	INSIDE	0.518	0.721	2131.22	1536.21	0.373	1534.30	572.41
A_68_P21904887	chr2:180324446-180324490	NM_031373:357	Ogfr	INSIDE	0.420	0.370	1864.70	690.24	0.155	1417.92	220.43
A_68_P20088284	chr1:23390288-23390332	NM_001081079:-296	Ogfrl1	PROMOTER	0.335	0.497	1281.57	637.48	0.167	1031.91	171.96
A_68_P24758833	chr6:113277046-113277100	NM_010957:103	Ogg1	INSIDE	0.060	1.477	1972.22	2912.53	0.088	1364.63	120.44
A_68_P21089693	chr2:28061497-28061541	NM_001038612:310	Olfm1	INSIDE	0.274	0.276	2136.67	589.05	0.076	1666.45	125.96
A_68_P26343617	chr9:20530951-20530995	NM_173777:1686	Olfm2	INSIDE	0.441	0.539	1137.97	613.48	0.238	820.60	195.07
A_68_P26343619	chr9:20531260-20531304	NM_173777:1376	Olfm2	INSIDE	0.179	0.522	1783.14	930.71	0.094	1271.45	118.88
A_68_P26343622	chr9:20531703-20531748	NM_173777:933	Olfm2	INSIDE	0.472	0.370	2747.40	1015.49	0.174	2246.34	391.89
A_68_P30474567	chr15:98268138-98268187	NM_146457:262	Olfr282	INSIDE	0.593	3.048	743.38	2265.76	1.809	609.24	1101.89
A_68_P31167786	chr17:37334030-37334074	NM_001011518:859	Olfr94	INSIDE	0.504	0.627	1326.07	831.18	0.316	1130.14	356.97

ProbeName	Target position of probe on CpG island microarray	TargetID	GeneSymbol	CpG island Description	Ratio of relative methylation (TiO2-NP/Vehicle)	Sham group			TiO_2-H group		
						Relative methylation (Cy-5/Cy-3)	Cy-3 signal (Input)	Cy-5 signal (MeDIP)	Relative methylation (Cy-5/Cy-3)	Cy-3 signal (Input)	Cy-5 signal (MeDIP)
A_68_P30961940	chr16:91269862-91269906	NM_016968:-129	Olig1	PROMOTER	0.453	0.521	1364.64	711.16	0.236	1158.15	273.66
A_68_P30961944	chr16:91270214-91270258	NM_016968:223	Olig1	INSIDE	0.472	0.457	1283.92	587.13	0.216	1004.69	216.65
A_68_P30961955	chr16:91271343-91271387	NM_016968:1351	Olig1	INSIDE	0.381	0.573	2374.04	1361.14	0.218	1834.52	400.26
A_68_P30961642	chr16:91220360-91220404	NM_016967:-5412	Olig2	PROMOTER	0.236	0.632	1864.85	1177.71	0.149	1449.49	215.84
A_68_P30961643	chr16:91220441-91220485	NM_016967:-5332	Olig2	PROMOTER	0.447	0.632	1232.36	778.97	0.283	856.94	242.19
A_68_P30961690	chr16:91226016-91226061	NM_016967:244	Olig2	INSIDE	0.532	0.696	1090.68	758.73	0.370	925.55	342.54
A_68_P30961691	chr16:91226111-91226155	NM_016967:338	Olig2	INSIDE	0.598	0.611	1189.41	726.43	0.365	904.05	330.23
A_68_P30961694	chr16:91226413-91226457	NM_016967:640	Olig2	INSIDE	0.639	0.545	2814.15	1535.09	0.349	2091.87	729.27
A_68_P30961704	chr16:91227772-91227816	NM_016967:2000	Olig2	INSIDE	0.130	0.577	2070.35	1193.89	0.075	1565.33	117.67
A_68_P26980660	chr10:19077044-19077088	NM_053008:722	Olig3	INSIDE	0.335	0.729	7665.22	5584.24	0.244	5365.06	1310.44
A_68_P26980662	chr10:19077212-19077256	NM_053008:890	Olig3	INSIDE	0.204	0.493	1482.76	731.55	0.101	1131.98	114.07
A_68_P23165462	chr4:102986330-102986374	NM_025909:-98	Oma1	PROMOTER	0.252	0.375	1524.11	571.70	0.094	1250.13	117.95
A_68_P23165464	chr4:102986645-102986689	NM_025909:216	Oma1	INSIDE	0.479	0.613	996.25	610.96	0.294	859.86	252.48
A_68_P26640621	chr9:74709991-74710035	NM_008262:285	Onecut1	INSIDE	0.117	0.739	6982.63	5156.91	0.086	4531.49	390.53
A_68_P26640622	chr9:74710098-74710142	NM_008262:393	Onecut1	INSIDE	0.568	0.691	2058.51	1423.29	0.393	1578.58	620.07
A_68_P26640627	chr9:74710721-74710765	NM_008262:1015	Onecut1	INSIDE	0.421	0.493	2826.27	1394.05	0.208	2042.42	424.55
A_68_P26640630	chr9:74711042-74711086	NM_008262:1337	Onecut1	INSIDE	0.460	0.364	1579.97	575.84	0.167	1299.53	217.67
A_68_P26640631	chr9:74711117-74711161	NM_008262:1411	Onecut1	INSIDE	0.201	0.644	1047.88	674.85	0.129	900.21	116.27
A_68_P26640636	chr9:74711696-74711740	NM_008262:1991	Onecut1	INSIDE	0.131	0.727	1690.21	1229.19	0.095	1204.16	114.72
A_68_P31774685	chr18:64499703-64499747	NM_194268:-293	Onecut2	PROMOTER	0.260	0.642	2246.19	1442.58	0.167	1660.70	277.03
A_68_P31774689	chr18:64500435-64500479	NM_194268:439	Onecut2	INSIDE	0.594	0.667	3296.23	2200.05	0.396	2371.46	940.06
A_68_P31774691	chr18:64500742-64500786	NM_194268:747	Onecut2	INSIDE	0.304	0.612	2694.80	1648.87	0.186	1986.83	369.33
A_68_P27283558	chr10:79957724-79957768	NM_139226:96	Onecut3	INSIDE	0.241	0.669	1296.30	867.20	0.161	1054.45	169.95
A_68_P27283560	chr10:79958022-79958066	NM_139226:394	Onecut3	INSIDE	0.395	0.559	2970.49	1661.40	0.221	2195.37	484.90
A_68_P26381828	chr9:28211224-28211268	NM_177906:612393	Opcml	INSIDE	0.312	0.621	1967.48	1221.50	0.194	1602.77	310.66
A_68_P32552413	chrX:96086058-96086102	NM_052976:244	Ophn1	INSIDE	0.226	1.785	1041.69	1858.89	0.403	475.40	191.68
A_68_P32552414	chrX:96086168-96086212	NM_052976:134	Ophn1	INSIDE	0.195	1.373	1448.29	1988.71	0.268	619.69	166.07
A_68_P20849640	chr1:177622720-177622764	NM_010098:-21	Opn3	DIVERGENT_PROMOTER	0.360	0.638	877.37	559.53	0.230	784.04	180.03
A_68_P23310402	chr4:131700231-131700275	NM_013622:149	Oprd1	INSIDE	0.463	0.397	2616.83	1037.78	0.183	2323.33	426.26
A_68_P25507194	chr7:134913194-134913238	NM_198424:-112	Orai3	PROMOTER	0.301	0.468	1119.03	523.95	0.141	870.90	122.76
A_68_P25507195	chr7:134913265-134913309	NM_198424:-42	Orai3	PROMOTER	0.296	0.416	2043.31	849.01	0.123	1541.67	189.67
A_68_P25507196	chr7:134913351-134913395	NM_198424:44	Orai3	INSIDE	0.632	0.601	964.23	579.78	0.380	772.09	293.24
A_68_P25507198	chr7:134913587-134913631	NM_198424:280	Orai3	INSIDE	0.390	0.574	1146.27	658.43	0.224	942.27	210.99
A_68_P20259229	chr1:58561716-58561760	NM_001025378:-3478	Orc2	PROMOTER	0.488	0.613	1220.41	748.30	0.299	1036.45	310.21
A_68_P21199928	chr2:48804507-48804551	NR_033442:259	Orc4	INSIDE	0.319	0.641	1113.59	713.64	0.204	871.92	178.04
A_68_P21199929	chr2:48804619-48804663	NR_033442:147	Orc4	INSIDE	0.509	0.697	1869.92	1302.45	0.354	1442.42	511.04
A_68_P26024230	chr8:87823989-87824033	NM_001163791:480	Orc6	INSIDE	0.577	0.591	1130.55	668.43	0.341	913.64	311.66
A_68_P20231215	chr1:53354231-53354281	NM_145517:318	Ormdl1	INSIDE	0.406	0.534	1129.37	603.42	0.217	980.16	212.39
A_68_P28057359	chr11:98448749-98448807	NM_025661:-219	Ormdl3	PROMOTER	0.272	0.380	1387.33	527.27	0.104	1146.88	118.76
A_68_P27534166	chr10:126558114-126558159	NM_001171026:80	Os9	INSIDE	0.101	1.364	3748.51	5114.02	0.138	2553.80	353.06
A_68_P31958766	chr19:12040692-12040739	NM_001033174:382	Osbp	INSIDE	0.210	0.549	2021.86	1109.81	0.115	1485.17	171.21
A_68_P27553885	chr11:3661656-3661712	NM_152818:102222	Osbp2	INSIDE	0.275	0.462	1229.09	567.60	0.127	985.74	125.03
A_68_P30656618	chr16:33185004-33185048	NM_176840:-130	Osbpl11	PROMOTER	0.240	0.654	1195.62	782.21	0.157	877.80	137.67
A_68_P24438209	chr6:50405431-50405477	NM_001163645:715	Osbpl3	INSIDE	0.275	0.562	1096.61	616.10	0.155	803.08	124.16

ProbeName	Target position of probe on CpG island microarray	TargetID	GeneSymbol	CpG island Description	Ratio of relative methylation (TiO2-NP/Vehicle)	Sham group			TiO_2-H group		
						Relative methylation (Cy-5/Cy-3)	Cy-3 signal (Input)	Cy-5 signal (MeDIP)	Relative methylation (Cy-5/Cy-3)	Cy-3 signal (Input)	Cy-5 signal (MeDIP)
A_68_P24438215	chr6:50406155-50406199	NM_001163645:-7	Osbpl3	PROMOTER	0.198	0.635	2993.58	1901.56	0.126	2327.92	293.40
A_68_P21350014	chr2:76244191-76244236	NM_145525:-381	Osbpl6	PROMOTER	0.386	0.500	1498.18	748.41	0.193	1241.20	239.14
A_68_P21350016	chr2:76244453-76244497	NM_145525:-120	Osbpl6	PROMOTER	0.328	0.405	1936.43	783.94	0.133	1603.37	213.03
A_68_P21350022	chr2:76245198-76245242	NM_145525:626	Osbpl6	INSIDE	0.303	0.669	3267.75	2185.75	0.202	2481.34	502.37
A_68_P21350023	chr2:76245283-76245327	NM_145525:710	Osbpl6	INSIDE	0.167	0.389	2506.16	976.01	0.065	2061.98	133.94
A_68_P23199842	chr4:108874395-108874439	NM_133885:461	Osbpl9	INSIDE	0.295	0.672	3598.78	2417.08	0.198	2579.37	511.68
A_68_P23277932	chr4:125735548-125735592	NM_172701:-238	Oscp1	PROMOTER	0.117	0.700	2061.16	1443.65	0.082	1485.22	122.08
A_68_P23277936	chr4:125736057-125736104	NM_172701:272	Oscp1	INSIDE	0.157	2.357	6203.43	14621.64	0.370	4070.21	1506.50
A_68_P22752865	chr4:15941230-15941274	NM_145950:-228	Osgin2	PROMOTER	0.220	0.591	1076.26	635.82	0.130	910.26	118.49
A_68_P30132589	chr15:35223344-35223388	NM_054049:-2500	Osr2	PROMOTER	0.196	0.499	1353.64	675.98	0.098	1179.00	115.20
A_68_P30132611	chr15:35225684-35225737	NM_054049:-156	Osr2	PROMOTER	0.318	0.636	811.22	516.13	0.202	588.59	118.99
A_68_P30132613	chr15:35225986-35226030	NM_054049:142	Osr2	INSIDE	0.625	0.547	1390.27	760.71	0.342	1015.68	347.19
A_68_P23575370	chr5:31209922-31209966	NM_001134692:151	Ost4	INSIDE	0.179	0.473	4382.49	2073.94	0.085	2858.10	241.87
A_68_P23618576	chr5:38668524-38668568	NM_172709:-96	Otop1	PROMOTER	0.240	0.573	2753.29	1577.43	0.137	2031.58	279.02
A_68_P23618580	chr5:38668967-38669011	NM_172709:346	Otop1	INSIDE	0.285	0.445	2069.17	921.27	0.127	1547.19	196.57
A_68_P28152867	chr11:115195826-115195870	NM_027132:-199	Otop3	PROMOTER	0.124	0.539	2165.40	1167.89	0.067	1730.15	115.63
A_68_P28152870	chr11:115196117-115196161	NM_027132:91	Otop3	INSIDE	0.334	0.530	1083.19	574.39	0.177	850.05	150.77
A_68_P29237525	chr13:95645453-95645497	NM_011021:-107	Otp	PROMOTER	0.499	0.567	1406.86	797.81	0.283	1267.55	358.50
A_68_P29237543	chr13:95647408-95647452	NM_011021:1849	Otp	INSIDE	0.152	0.566	1800.61	1019.69	0.086	1393.42	120.19
A_68_P29237593	chr13:95652973-95653017	NM_011021:7413	Otp	INSIDE	0.492	0.526	1211.10	636.50	0.259	1074.15	277.83
A_68_P29237603	chr13:95654247-95654300	NM_011021:8692	Otp	DOWNSTREAM	0.182	0.589	1535.76	904.01	0.107	1129.08	121.13
A_68_P21046080	chr2:19580505-19580552	NM_027715:840	Otud1	INSIDE	0.093	1.398	1215.23	1698.91	0.130	1014.23	131.51
A_68_P25995582	chr8:82163121-82163165	NM_001081164:-432	Otud4	PROMOTER	0.176	0.474	2162.96	1025.66	0.084	1395.84	116.61
A_68_P25171969	chr7:70590173-70590217	NM_130880:537	Otud7a	INSIDE	0.283	0.433	1666.98	721.11	0.123	1482.49	181.72
A_68_P25173477	chr7:70902398-70902442	NM_130880:312763	Otud7a	INSIDE	0.326	0.460	1275.32	586.99	0.150	1055.58	158.56
A_68_P22347475	chr3:95908828-95908872	NM_001025613:401	Otud7b	INSIDE	0.237	0.639	4257.97	2719.83	0.151	2976.07	449.84
A_68_P27638836	chr11:21893026-21893070	NM_011023:8606	Otx1	DOWNSTREAM	0.512	0.605	1749.74	1058.92	0.310	1436.46	444.96
A_68_P27638868	chr11:21896875-21896919	NM_011023:4758	Otx1	INSIDE	0.578	0.602	908.17	547.01	0.348	724.67	252.19
A_68_P27638869	chr11:21896943-21896987	NM_011023:4690	Otx1	INSIDE	0.268	0.539	1447.03	779.37	0.144	1141.53	164.71
A_68_P27638907	chr11:21901433-21901477	NM_011023:200	Otx1	INSIDE	0.318	0.392	1595.62	626.26	0.125	1330.84	166.15
A_68_P27638910	chr11:21901664-21901708	NM_011023:-32	Otx1	PROMOTER	0.376	0.456	1923.97	876.51	0.171	1427.76	244.86
A_68_P29589360	chr14:49282109-49282153	NM_144841:417	Otx2	INSIDE	0.520	0.524	1009.66	528.59	0.272	850.24	231.39
A_68_P29589383	chr14:49284720-49284764	NM_144841:-2195	Otx2	DIVERGENT_PROMOTER	0.311	0.452	2371.68	1071.39	0.141	1728.89	243.22
A_68_P29589384	chr14:49284797-49284844	NM_144841:-2273	Otx2	DIVERGENT_PROMOTER	0.212	0.442	1758.34	776.68	0.094	1301.11	121.78
A_68_P27924663	chr11:74992086-74992130	NM_027136:202	Ovca2	INSIDE	0.374	0.551	1536.71	847.08	0.206	1192.92	246.00
A_68_P31929450	chr19:5560018-5560062	NM_019935:535	Ovol1	INSIDE	0.365	0.507	2049.44	1040.05	0.185	1613.64	299.18
A_68_P21706642	chr2:144156624-144156668	NM_152947:452	Ovol2	INSIDE	0.659	0.463	1783.17	826.18	0.305	1626.83	496.36
A_68_P21706649	chr2:144157501-144157545	NM_152947:-424	Ovol2	PROMOTER	0.481	0.484	1533.54	742.41	0.233	1176.65	273.94
A_68_P30165307	chr15:41278629-41278673	NM_001130166:-377	Oxr1	PROMOTER	0.181	0.506	1656.22	838.61	0.092	1344.99	123.31
A_68_P24754179	chr6:112439818-112439862	NM_001081147:-38	Oxtr	PROMOTER	0.243	0.504	1737.96	875.89	0.122	1551.07	189.66
A_68_P24051803	chr5:123158016-123158060	NM_011026:473	P2rx4	INSIDE	0.469	0.538	1178.68	634.53	0.252	925.97	233.59
A_68_P22180929	chr3:60807059-60807103	NM_008772:364	P2ry1	INSIDE	0.384	0.717	2585.02	1852.22	0.275	2083.51	573.97
A_68_P25370974	chr7:108160323-108160367	NM_008773:161	P2ry2	INSIDE	0.381	0.573	1520.90	870.99	0.218	1226.47	267.58
A_68_P25370975	chr7:108160402-108160446	NM_008773:81	P2ry2	INSIDE	0.342	0.538	2527.30	1359.55	0.184	2030.92	373.19

ProbeName	Target position of probe on CpG island microarray	TargetID	GeneSymbol	CpG island Description	Ratio of relative methylation (TiO2-NP/Vehicle)	Sham group			TiO_2-H group		
						Relative methylation (Cy-5/Cy-3)	Cy-3 signal (Input)	Cy-5 signal (MeDIP)	Relative methylation (Cy-5/Cy-3)	Cy-3 signal (Input)	Cy-5 signal (MeDIP)
A_68_P28185372	chr11:120433823-120433867	NM_011032:406	P4hb	INSIDE	0.644	0.651	1657.79	1079.68	0.420	1330.17	558.10
A_68_P28185376	chr11:120434174-120434218	NM_011032:54	P4hb	INSIDE	0.401	0.405	1978.78	800.88	0.162	1637.81	265.81
A_68_P27542070	chr10:128002461-128002505	NM_011119:508	Pa2g4	INSIDE	0.213	0.622	1273.81	792.64	0.133	907.46	120.41
A_68_P27542071	chr10:128002533-128002577	NM_011119:436	Pa2g4	INSIDE	0.137	0.727	5194.48	3777.12	0.100	3678.94	366.69
A_68_P30139524	chr15:36538383-36538427	NM_008774:324	Pabpc1	INSIDE	0.153	0.710	8352.49	5929.71	0.109	5975.04	650.28
A_68_P31117691	chr17:27792800-27792847	NM_011861:197	Pacsin1	INSIDE	0.097	1.781	2537.19	4517.62	0.173	1782.84	309.16
A_68_P31117836	chr17:27822046-27822090	NM_178365:-130	Pacsin1	PROMOTER	0.366	0.466	1736.60	809.51	0.171	1241.91	212.04
A_68_P21421554	chr2:91097131-91097175	NM_028733:-333	Pacsin3	PROMOTER	0.115	0.629	2507.47	1577.11	0.072	1691.59	122.47
A_68_P23360936	chr4:140462092-140462151	NM_008812:-153	Padi2	PROMOTER	0.148	1.833	508.54	932.20	0.270	430.89	116.52
A_68_P25021608	chr7:29178020-29178064	NM_019458:28	Paf1	INSIDE	0.485	0.602	1810.55	1090.50	0.292	1319.52	385.55
A_68_P25009541	chr7:26082496-26082540	NM_008776:456	Pafah1b3	INSIDE	0.400	0.438	1706.65	746.98	0.175	1461.72	256.05
A_68_P25009542	chr7:26082633-26082677	NM_008776:320	Pafah1b3	INSIDE	0.191	0.620	1313.21	813.87	0.118	1054.27	124.82
A_68_P23819018	chr5:77380092-77380136	NM_025939:-321	Paics	PROMOTER	0.610	0.694	2442.61	1695.65	0.423	1747.29	739.64
A_68_P23819019	chr5:77380180-77380224	NM_025939:-233	Paics	PROMOTER	0.253	0.599	2057.85	1231.88	0.152	1576.69	238.91
A_68_P31619153	chr18:35758686-35758732	NM_026420:388	Paip2	INSIDE	0.272	0.448	1311.64	587.93	0.122	989.62	120.73
A_68_P24595788	chr6:83781684-83781728	NM_146169:29	Paip2b	INSIDE	0.319	0.442	1996.92	883.24	0.141	1585.98	223.94
A_68_P24595789	chr6:83781804-83781857	NM_146169:-95	Paip2b	PROMOTER	0.177	0.494	1921.04	948.76	0.087	1366.72	119.32
A_68_P21565424	chr2:118503074-118503118	NM_001145854:115	Pak6	INSIDE	0.549	0.425	1545.02	657.06	0.233	1355.55	316.31
A_68_P26017700	chr8:86550149-86550193	NM_028877:4798	Palm3	INSIDE	0.392	1.362	4053.05	5519.11	0.533	2798.18	1491.77
A_68_P20474865	chr1:99991588-99991632	NM_013626:599	Pam	INSIDE	0.237	0.516	2373.73	1225.88	0.123	1819.31	222.91
A_68_P32082682	chr19:34953665-34953709	NM_023792:-1279	Pank1	PROMOTER	0.123	0.687	1815.11	1246.87	0.084	1436.69	120.99
A_68_P21634174	chr2:131087984-131088028	NM_153501:-229	Pank2	PROMOTER	0.255	0.605	979.32	592.66	0.154	756.60	116.58
A_68_P27708453	chr11:35582983-35583027	NM_145962:8	Pank3	INSIDE	0.196	0.442	1834.62	811.64	0.087	1387.60	120.16
A_68_P30422878	chr15:88898359-88898403	NM_001002005:8225	Panx2	INSIDE	0.474	2.966	1936.80	5744.91	1.406	1405.19	1975.56
A_68_P29228004	chr13:93962351-93962395	NM_133905:-134	Papd4	PROMOTER	0.394	0.345	2639.04	910.03	0.136	2030.41	275.52
A_68_P26039502	chr8:90722771-90722830	NM_001164497:-311	Papd5	PROMOTER	0.235	0.596	1067.36	635.66	0.140	879.73	123.07
A_68_P24152138	chr5:143005802-143005846	NM_019943:206	Papolb	INSIDE	0.347	1.795	1155.09	2073.25	0.622	916.43	569.98
A_68_P32071012	chr19:32670221-32670265	NM_001201470:38	Papss2	INSIDE	0.140	0.694	1559.72	1082.10	0.097	1288.82	125.51
A_68_P32071016	chr19:32670611-32670655	NM_001201470:428	Papss2	INSIDE	0.262	0.565	905.97	511.93	0.148	817.77	121.14
A_68_P20074998	chr1:20880874-20880920	NM_028829:194	Paqr8	INSIDE	0.574	0.697	1920.11	1338.10	0.400	1499.85	599.83
A_68_P26742491	chr9:95459602-95459646	NM_198414:-611	Paqr9	PROMOTER	0.221	0.494	3459.56	1710.48	0.109	2436.14	266.52
A_68_P26742492	chr9:95459743-95459787	NM_198414:-471	Paqr9	PROMOTER	0.261	0.585	2114.75	1237.23	0.152	1786.35	272.26
A_68_P26742504	chr9:95461303-95461354	NM_198414:1093	Paqr9	INSIDE	0.613	2.623	1333.75	3498.63	1.608	977.47	1572.11
A_68_P26261063	chr8:129588776-129588820	NM_001122850:843	Pard3	INSIDE	0.513	0.555	1085.47	602.93	0.285	750.86	213.86
A_68_P26261064	chr8:129588848-129588893	NM_001122850:915	Pard3	INSIDE	0.268	0.728	3734.54	2719.01	0.195	2724.24	531.59
A_68_P21839348	chr2:167905989-167906034	NM_021409:-492	Pard6b	PROMOTER	0.266	0.582	2128.01	1238.18	0.155	1636.37	253.01
A_68_P21839349	chr2:167906063-167906107	NM_021409:-419	Pard6b	PROMOTER	0.639	0.437	1735.05	758.43	0.280	1323.04	369.83
A_68_P21839459	chr2:167924216-167924266	NM_021409:17737	Pard6b	INSIDE	0.486	3.416	584.47	1996.35	1.659	535.97	889.17
A_68_P31861993	chr18:80244305-80244349	NM_053117:693	Pard6g	INSIDE	0.284	0.562	1948.38	1094.44	0.160	1442.57	230.37
A_68_P31861995	chr18:80244514-80244558	NM_053117:903	Pard6g	INSIDE	0.315	0.662	1985.56	1314.53	0.208	1482.63	308.80
A_68_P31041951	chr17:11033242-11033286	NM_016694:15	Park2	INSIDE	0.388	0.416	1724.79	716.92	0.161	1429.97	230.49
A_68_P30587017	chr16:20302514-20302558	NM_001005767:-101	Parl	PROMOTER	0.547	0.730	2179.68	1592.07	0.400	1745.38	697.47
A_68_P30558433	chr16:13668026-13668070	NM_028761:215	Parn	INSIDE	0.160	0.541	1759.66	952.82	0.087	1443.54	124.88
A_68_P24832846	chr6:127403694-127403738	NM_181402:-24	Parp11	PROMOTER	0.138	0.672	1613.64	1084.01	0.093	1365.72	126.94

ProbeName	Target position of probe on CpG island microarray	TargetID	GeneSymbol	CpG island Description	Ratio of relative methylation (TiO2-NP/Vehicle)	Sham group			TiO_2-H group		
						Relative methylation (Cy-5/Cy-3)	Cy-3 signal (Input)	Cy-5 signal (MeDIP)	Relative methylation (Cy-5/Cy-3)	Cy-3 signal (Input)	Cy-5 signal (MeDIP)
A_68_P26586478	chr9:65063416-65063460	NM_177460:942	Parp16	INSIDE	0.538	0.609	1142.28	695.48	0.328	1012.99	332.08
A_68_P26554372	chr9:59464905-59464949	NM_001205239:-164	Parp6	PROMOTER	0.634	0.673	1353.16	910.63	0.427	1197.47	510.94
A_68_P23185679	chr4:106323706-106323750	NM_001083887:55	Pars2	INSIDE	0.163	0.566	2228.52	1261.77	0.092	1438.05	132.66
A_68_P23185680	chr4:106323776-106323820	NM_001083887:125	Pars2	INSIDE	0.308	0.455	3076.60	1399.50	0.140	2264.92	317.68
A_68_P23185681	chr4:106323879-106323923	NM_001083887:227	Pars2	INSIDE	0.137	0.584	2148.44	1254.58	0.080	1505.26	120.70
A_68_P20454938	chr1:95239228-95239272	NM_080850:115	Pask	INSIDE	0.244	0.722	3157.92	2279.07	0.176	2475.54	436.55
A_68_P20454941	chr1:95239500-95239544	NM_080850:-157	Pask	DIVERGENT_PROMOTER	0.107	0.473	3446.19	1631.35	0.050	2631.85	132.78
A_68_P27551111	chr11:3188684-3188734	NM_019574:-1751	Patz1	PROMOTER	0.308	0.466	1570.97	731.51	0.143	1116.85	160.23
A_68_P27551119	chr11:3189697-3189741	NM_019574:-741	Patz1	PROMOTER	0.552	0.697	1944.77	1355.02	0.385	1623.76	625.05
A_68_P21723787	chr2:147191374-147191418	NM_008780:667	Pax1	INSIDE	0.129	0.702	1777.16	1247.03	0.091	1304.96	118.15
A_68_P32134165	chr19:44825072-44825116	NM_011037:-6789	Pax2	PROMOTER	0.172	1.527	2932.08	4477.73	0.263	1876.06	492.54
A_68_P32134210	chr19:44830843-44830902	NM_011037:-1011	Pax2	PROMOTER	0.165	0.489	1882.38	920.56	0.081	1460.59	118.05
A_68_P32134211	chr19:44831069-44831120	NM_011037:-789	Pax2	PROMOTER	0.239	0.579	1189.72	689.32	0.138	867.34	120.06
A_68_P32134218	chr19:44831888-44831933	NM_011037:27	Pax2	INSIDE	0.429	0.555	3005.41	1667.93	0.238	2302.50	547.97
A_68_P32134234	chr19:44833782-44833828	NM_011037:1921	Pax2	INSIDE	0.139	1.435	851.73	1221.82	0.200	596.65	119.30
A_68_P32134814	chr19:44910682-44910726	NM_011037:78821	Pax2	INSIDE	0.254	0.557	1819.58	1012.69	0.141	1503.55	212.19
A_68_P32134817	chr19:44911075-44911119	NM_011037:79213	Pax2	INSIDE	0.207	0.665	3155.10	2098.20	0.137	2086.37	286.87
A_68_P32134818	chr19:44911149-44911193	NM_011037:79287	Pax2	INSIDE	0.154	0.626	6495.60	4068.15	0.096	4693.29	452.72
A_68_P20370345	chr1:78186294-78186338	NM_001159520:7395	Pax3	INSIDE	0.205	0.389	2393.57	931.76	0.080	2085.41	166.70
A_68_P20370400	chr1:78193226-78193270	NM_001159520:463	Pax3	INSIDE	0.285	0.398	2203.97	877.86	0.114	1787.04	203.17
A_68_P20370402	chr1:78193529-78193573	NM_001159520:161	Pax3	INSIDE	0.652	0.595	1505.07	895.51	0.388	1258.59	488.35
A_68_P20370404	chr1:78193792-78193836	NM_001159520:-103	Pax3	PROMOTER	0.252	0.338	2355.52	795.38	0.085	2047.23	173.97
A_68_P20370459	chr1:78200539-78200583	NM_001159520:-6849	Pax3	PROMOTER	0.536	0.683	1263.90	862.82	0.366	1074.63	393.14
A_68_P22878366	chr4:44676950-44676994	NM_008782:46340	Pax5	INSIDE	0.453	0.427	2104.81	898.28	0.193	1633.81	316.01
A_68_P22878640	chr4:44716235-44716287	NM_008782:7052	Pax5	INSIDE	0.107	0.735	2237.57	1644.59	0.079	1541.99	121.19
A_68_P22878698	chr4:44723640-44723686	NM_008782:-350	Pax5	PROMOTER	0.320	0.538	1095.77	589.27	0.172	901.83	155.05
A_68_P21495956	chr2:105515369-105515413	NM_013627:-1211	Pax6	DIVERGENT_PROMOTER	0.493	0.451	1145.93	516.28	0.222	925.68	205.79
A_68_P21495957	chr2:105515455-105515507	NM_013627:-1121	Pax6	DIVERGENT_PROMOTER	0.291	0.604	1261.47	761.99	0.176	908.46	159.49
A_68_P21496012	chr2:105522220-105522268	NM_013627:5643	Pax6	INSIDE	0.208	0.658	989.65	651.41	0.137	886.93	121.32
A_68_P21495905	chr2:105509058-105509107	NR_002867:1405	Pax6os1	INSIDE	0.183	0.725	1950.25	1414.48	0.133	1395.68	185.05
A_68_P21495906	chr2:105509202-105509246	NR_002867:1263	Pax6os1	INSIDE	0.254	0.687	1818.45	1249.49	0.175	1512.12	264.40
A_68_P21495913	chr2:105509959-105510003	NR_002867:507	Pax6os1	INSIDE	0.502	0.575	1299.46	747.59	0.289	1042.77	301.29
A_68_P21495934	chr2:105512616-105512660	NR_002867:-2151	Pax6os1	DIVERGENT_PROMOTER	0.458	0.519	1891.55	980.89	0.238	1399.98	332.81
A_68_P23354400	chr4:139377667-139377712	NM_011039:11194	Pax7	INSIDE	0.653	0.693	2436.09	1687.37	0.452	1808.82	817.53
A_68_P23354401	chr4:139377795-139377839	NM_011039:11067	Pax7	INSIDE	0.090	0.624	3088.81	1927.55	0.056	2075.44	117.12
A_68_P23354456	chr4:139384273-139384317	NM_011039:4589	Pax7	INSIDE	0.369	0.593	1252.20	742.35	0.219	1007.13	220.38
A_68_P23354457	chr4:139384411-139384455	NM_011039:4451	Pax7	INSIDE	0.450	0.584	995.36	581.26	0.263	732.20	192.58
A_68_P23354482	chr4:139387475-139387522	NM_011039:1385	Pax7	INSIDE	0.278	0.520	1020.59	530.29	0.145	863.91	124.90
A_68_P23354494	chr4:139389036-139389081	NM_011039:-175	Pax7	PROMOTER	0.502	0.643	1596.62	1027.05	0.323	1247.57	402.67
A_68_P28458266	chr12:57793635-57793679	NM_011041:-2969	Pax9	PROMOTER	0.481	0.582	1359.23	791.15	0.280	1150.77	322.38
A_68_P28458297	chr12:57797636-57797681	NM_011041:1033	Pax9	INSIDE	0.201	0.481	1608.53	774.33	0.097	1229.54	118.93
A_68_P28458300	chr12:57797914-57797958	NM_011041:1311	Pax9	INSIDE	0.153	0.445	2475.66	1102.51	0.068	1790.79	122.04
A_68_P29675623	chr14:66424674-66424718	NM_023209:-51	Pbk	PROMOTER	0.280	0.597	1015.90	606.75	0.167	859.53	143.56
A_68_P31154665	chr17:34729645-34729690	NM_017463:252	Pbx2	INSIDE	0.182	0.709	1466.36	1039.81	0.129	925.73	119.51

ProbeName	Target position of probe on CpG island microarray	TargetID	GeneSymbol	CpG island Description	Ratio of relative methylation (TiO2-NP/Vehicle)	Sham group			TiO_2-H group		
						Relative methylation (Cy-5/Cy-3)	Cy-3 signal (Input)	Cy-5 signal (MeDIP)	Relative methylation (Cy-5/Cy-3)	Cy-3 signal (Input)	Cy-5 signal (MeDIP)
A_68_P25948236	chr8:72356467-72356516	NM_001024954:-111	Pbx4	PROMOTER	0.327	0.419	1294.35	542.72	0.137	1029.47	141.14
A_68_P25948238	chr8:72356673-72356717	NM_001024954:92	Pbx4	INSIDE	0.204	0.486	1389.65	675.11	0.099	1155.72	114.80
A_68_P24610518	chr6:86475728-86475772	NM_011865:409	Pcbp1	INSIDE	0.197	0.480	2496.08	1198.88	0.094	1985.48	187.55
A_68_P24610519	chr6:86475835-86475879	NM_011865:303	Pcbp1	INSIDE	0.627	0.549	1089.32	598.13	0.344	781.72	268.95
A_68_P30498007	chr15:102301316-102301361	NM_001103165:276	Pcbp2	INSIDE	0.542	0.688	1724.37	1187.17	0.373	1255.17	468.11
A_68_P27265099	chr10:76424280-76424324	NM_021568:390	Pcbp3	INSIDE	0.557	0.670	1142.64	765.24	0.373	972.69	362.62
A_68_P26803472	chr9:106355981-106356025	NM_021567:-185	Pcbp4	PROMOTER	0.371	0.633	3803.95	2409.24	0.235	2799.94	658.50
A_68_P29962216	chr14:122933648-122933692	NM_144844:121	Pcca	INSIDE	0.349	0.590	1001.64	591.10	0.206	778.44	160.54
A_68_P29962218	chr14:122933824-122933868	NM_144844:297	Pcca	INSIDE	0.242	0.347	1782.42	618.16	0.084	1398.41	117.43
A_68_P31632700	chr18:38371037-38371082	NM_029357:-1643	Pcdh1	PROMOTER	0.342	0.613	1613.63	988.37	0.209	1201.91	251.75
A_68_P31632701	chr18:38371127-38371171	NM_029357:-1732	Pcdh1	PROMOTER	0.451	0.621	1033.23	641.43	0.280	816.26	228.68
A_68_P31633050	chr18:38428628-38428672	NM_017378:15405	Pcdh12	INSIDE	0.649	2.673	409.14	1093.71	1.736	411.17	713.73
A_68_P29773644	chr14:84845775-84845819	NM_001013753:2427	Pcdh17	INSIDE	0.363	0.422	2016.17	850.23	0.153	1558.98	238.72
A_68_P29773648	chr14:84846227-84846278	NM_001013753:2883	Pcdh17	INSIDE	0.156	0.714	1627.35	1162.52	0.111	1097.28	122.18
A_68_P29773654	chr14:84846918-84846962	NM_001013753:3571	Pcdh17	INSIDE	0.147	0.570	3206.13	1827.43	0.084	2164.94	180.99
A_68_P29773655	chr14:84847043-84847087	NM_001013753:3695	Pcdh17	INSIDE	0.340	0.515	1272.98	656.18	0.175	1056.78	185.20
A_68_P23720369	chr5:58111053-58111097	NM_018764:1815	Pcdh7	INSIDE	0.342	0.626	2357.92	1475.45	0.214	1774.01	379.78
A_68_P23720370	chr5:58111147-58111191	NM_018764:1909	Pcdh7	INSIDE	0.321	0.426	3975.06	1691.86	0.137	3268.28	446.41
A_68_P23720372	chr5:58111337-58111385	NM_018764:2101	Pcdh7	INSIDE	0.255	0.561	1135.39	637.20	0.143	856.38	122.59
A_68_P23720373	chr5:58111459-58111503	NM_018764:2221	Pcdh7	INSIDE	0.279	0.417	1401.92	583.99	0.116	1053.95	122.50
A_68_P23720374	chr5:58111556-58111604	NM_018764:2321	Pcdh7	INSIDE	0.197	0.565	1511.59	854.41	0.112	1098.63	122.57
A_68_P29753328	chr14:80168173-80168220	NM_001042726:2923	Pcdh8	INSIDE	0.138	0.588	2210.42	1300.81	0.081	1591.26	129.65
A_68_P29753340	chr14:80169561-80169605	NM_001042726:1537	Pcdh8	INSIDE	0.305	0.490	1698.45	832.13	0.149	1298.54	193.99
A_68_P29753373	chr14:80173634-80173678	NM_001042726:-2537	Pcdh8	PROMOTER	0.320	0.556	1738.60	966.09	0.178	1417.39	252.02
A_68_P31626274	chr18:37107885-37107929	NM_007766:-4436	Pcdha4	PROMOTER	0.409	1.496	948.35	1419.04	0.612	808.66	495.14
A_68_P31626368	chr18:37121882-37121926	NM_007767:-5505	Pcdha6	PROMOTER	0.387	1.503	820.70	1233.71	0.582	675.98	393.51
A_68_P31626861	chr18:37249454-37249501	NM_001003671:-312	Pcdhac1	PROMOTER	0.281	0.621	990.47	615.09	0.175	820.09	143.12
A_68_P31627037	chr18:37303374-37303418	NM_001003672:-226	Pcdhac2	PROMOTER	0.505	0.615	1109.08	682.28	0.311	974.39	302.66
A_68_P31627038	chr18:37303465-37303509	NM_001003672:-136	Pcdhac2	PROMOTER	0.472	0.722	1640.17	1184.88	0.341	1425.13	486.14
A_68_P31629954	chr18:37927868-37927912	NM_033595:2657	Pcdhga12	INSIDE	0.626	2.467	799.41	1972.00	1.544	724.94	1119.06
A_68_P25326015	chr7:99817991-99818041	NM_029078:406	Pcf11	INSIDE	0.134	0.614	2628.65	1613.87	0.082	1783.14	147.02
A_68_P25326019	chr7:99818550-99818594	NM_029078:-150	Pcf11	PROMOTER	0.201	0.433	1873.39	811.66	0.087	1358.15	118.48
A_68_P24591296	chr6:83028488-83028532	NM_197992:127	Pcgf1	INSIDE	0.347	0.334	2005.96	669.57	0.116	1555.03	180.15
A_68_P28052172	chr11:97560234-97560280	NM_001163307:442	Pcgf2	INSIDE	0.269	0.365	1830.49	668.55	0.098	1345.75	132.28
A_68_P32089879	chr19:36483936-36483983	NM_029508:30403	Pcgf5	INSIDE	0.319	0.629	1456.26	915.91	0.201	1108.73	222.72
A_68_P25666151	chr8:13104540-13104584	NM_178708:781	Pcid2	INSIDE	0.172	0.596	1830.38	1090.26	0.103	1440.76	147.81
A_68_P25666157	chr8:13105151-13105195	NM_178708:171	Pcid2	INSIDE	0.479	0.530	1273.97	675.04	0.254	1099.59	279.25
A_68_P21820250	chr2:164704772-164704816	NM_146129:-73	Pcif1	PROMOTER	0.171	0.620	1393.16	864.05	0.106	1130.88	120.09
A_68_P21820255	chr2:164705460-164705507	NM_146129:616	Pcif1	INSIDE	0.641	0.500	1254.20	627.53	0.321	880.58	282.51
A_68_P29618377	chr14:56159128-56159172	NM_028994:48	Pck2	INSIDE	0.196	0.562	1662.31	934.91	0.110	1178.46	129.72
A_68_P25809871	chr8:42325399-42325443	NM_023662:308	Pcm1	INSIDE	0.584	0.450	1836.14	826.68	0.263	1441.26	379.25
A_68_P21913061	chr2:181572625-181572669	NM_153594:39	Pcmtd2	INSIDE	0.189	0.464	1747.52	810.57	0.088	1337.42	117.03
A_68_P21913062	chr2:181572751-181572795	NM_153594:165	Pcmtd2	INSIDE	0.338	0.523	2130.87	1115.14	0.177	1539.97	272.28
A_68_P21639372	chr2:132078905-132078949	NM_011045:-10	Pcna	PROMOTER	0.220	0.479	1561.67	748.74	0.105	1221.16	128.56

ProbeName	Target position of probe on CpG island microarray	TargetID	GeneSymbol	CpG island Description	Ratio of relative methylation (TiO2-NP/Vehicle)	Sham group			TiO_2-H group		
						Relative methylation (Cy-5/Cy-3)	Cy-3 signal (Input)	Cy-5 signal (MeDIP)	Relative methylation (Cy-5/Cy-3)	Cy-3 signal (Input)	Cy-5 signal (MeDIP)
A_68_P21639373	chr2:132078980-132079024	NM_011045:-86	Pcna	DIVERGENT_PROMOTER	0.537	0.674	1327.11	894.74	0.362	1078.25	390.50
A_68_P30778928	chr16:56029876-56029920	NM_001024622:-68	Pcnp	PROMOTER	0.427	0.540	1618.80	874.03	0.230	1205.16	277.78
A_68_P20826590	chr1:173126271-173126315	NM_025557:107	Pcp4l1	INSIDE	0.353	0.406	1496.51	607.24	0.143	1375.90	196.87
A_68_P32240897	chrX:7499587-7499631	NM_013892:2661	Pcsk1n	INSIDE	0.390	1.478	1634.44	2416.47	0.577	700.79	404.31
A_68_P32240898	chrX:7499680-7499725	NM_013892:2755	Pcsk1n	INSIDE	0.059	2.926	2124.50	6217.11	0.173	806.98	139.48
A_68_P32240899	chrX:7499781-7499825	NM_013892:2855	Pcsk1n	INSIDE	0.198	1.476	3780.26	5580.09	0.292	1500.72	438.65
A_68_P27282471	chr10:79786234-79786278	NM_008793:5962	Pcsk4	INSIDE	0.442	0.580	1614.05	936.13	0.257	1117.69	286.72
A_68_P31988616	chr19:17911313-17911358	NM_001190483:787	Pcsk5	INSIDE	0.466	0.599	1225.76	734.57	0.279	1047.43	292.23
A_68_P25184944	chr7:73007570-73007614	NM_011048:571	Pcsk6	INSIDE	0.335	0.459	2435.49	1117.45	0.154	1976.39	303.45
A_68_P23184552	chr4:106136609-106136653	NM_153565:300	Pcsk9	INSIDE	0.297	0.606	1091.54	661.02	0.180	828.68	148.97
A_68_P23184553	chr4:106136681-106136725	NM_153565:228	Pcsk9	INSIDE	0.236	0.585	2619.25	1531.96	0.138	1790.12	246.69
A_68_P28009091	chr11:89864227-89864271	NM_008796:-40	Pctp	PROMOTER	0.107	1.961	1527.77	2996.19	0.210	1137.92	239.47
A_68_P31923508	chr19:4510485-4510529	NM_001162946:35	Pcx	INSIDE	0.328	0.570	1057.80	603.33	0.187	953.77	178.46
A_68_P31923511	chr19:4510848-4510892	NM_001162946:399	Pcx	INSIDE	0.494	0.652	1093.31	712.58	0.322	931.84	299.86
A_68_P24609843	chr6:86338939-86338994	NM_025823:8178	Pcyox1	INSIDE	0.510	3.848	666.54	2564.55	1.963	552.45	1084.53
A_68_P30652429	chr16:32431312-32431356	NM_001163160:19	Pcyt1a	INSIDE	0.338	0.624	1228.22	766.59	0.211	906.99	191.58
A_68_P30652430	chr16:32431382-32431427	NM_001163160:89	Pcyt1a	INSIDE	0.328	0.516	1340.09	691.10	0.169	1008.66	170.45
A_68_P28185658	chr11:120478932-120478976	NM_024229:250	Pcyt2	INSIDE	0.304	0.441	1269.98	560.42	0.134	936.43	125.46
A_68_P24164493	chr5:145900637-145900681	NM_001033313:300	Pdap1	INSIDE	0.271	0.483	1518.33	733.67	0.131	1248.49	163.74
A_68_P25050419	chr7:36432218-36432262	NM_019746:261	Pdcd5	INSIDE	0.495	0.524	1773.00	928.48	0.259	1469.40	381.18
A_68_P29145567	chr13:74454737-74454781	NM_011051:16	Pdcd6	INSIDE	0.622	0.375	1578.31	592.15	0.233	1210.49	282.42
A_68_P26587155	chr9:65194859-65194910	NM_016688:1010	Pdcd7	INSIDE	0.090	0.674	2960.66	1995.37	0.060	2000.98	120.88
A_68_P21141628	chr2:37214555-37214599	NM_026176:276	Pdcl	INSIDE	0.120	0.666	6876.50	4579.52	0.080	4719.56	378.34
A_68_P21141629	chr2:37214726-37214772	NM_026176:104	Pdcl	INSIDE	0.228	0.656	929.43	609.90	0.150	810.06	121.39
A_68_P20163206	chr1:39044427-39044471	NM_026850:-210	Pdcl3	PROMOTER	0.494	0.423	3066.50	1298.39	0.209	2276.08	476.23
A_68_P31029178	chr17:8995181-8995225	NM_011866:593	Pde10a	INSIDE	0.463	0.718	1523.98	1094.69	0.333	1280.21	425.82
A_68_P31029179	chr17:8995332-8995389	NM_011866:751	Pde10a	INSIDE	0.256	0.381	1595.08	607.53	0.098	1245.60	121.50
A_68_P29482952	chr14:27488079-27488123	NM_178668:1232	Pde12	INSIDE	0.363	0.480	1875.53	900.54	0.174	1450.80	252.82
A_68_P30504522	chr15:103333324-103333368	NM_008800:-382	Pde1b	PROMOTER	0.587	0.563	5703.82	3213.64	0.331	4299.93	1422.32
A_68_P25373531	chr7:108599782-108599826	NR_026574:-17	Pde2a	PROMOTER	0.365	0.492	4645.45	2283.91	0.179	3315.28	594.43
A_68_P25373532	chr7:108599866-108599910	NR_026574:67	Pde2a	INSIDE	0.408	0.510	2866.26	1461.57	0.208	2125.20	441.78
A_68_P25373934	chr7:108653145-108653191	NR_026574:53347	Pde2a	INSIDE	0.424	0.629	892.80	561.63	0.267	682.21	181.86
A_68_P25435754	chr7:121559761-121559805	NM_011055:1015	Pde3b	INSIDE	0.473	0.659	838.96	553.00	0.312	774.19	241.19
A_68_P26345766	chr9:20969493-20969537	NM_183408:-643	Pde4a	PROMOTER	0.508	0.715	1602.54	1145.24	0.363	1262.67	458.37
A_68_P26345767	chr9:20969656-20969700	NM_183408:-479	Pde4a	PROMOTER	0.453	0.615	2161.01	1329.88	0.279	1648.17	459.14
A_68_P26345850	chr9:20988460-20988504	NM_183408:18325	Pde4a	INSIDE	0.592	0.536	1152.31	617.18	0.317	1038.81	329.58
A_68_P22356948	chr3:97692392-97692436	NM_001039376:216	Pde4dip	INSIDE	0.649	0.684	2626.34	1796.39	0.444	2051.58	910.16
A_68_P22356950	chr3:97692610-97692655	NM_001039376:-2	Pde4dip	PROMOTER	0.180	0.581	1515.51	880.91	0.105	1143.04	119.58
A_68_P22356952	chr3:97692829-97692873	NM_001039376:-220	Pde4dip	PROMOTER	0.565	0.581	1173.35	681.71	0.328	892.32	292.93
A_68_P22484165	chr3:122432345-122432393	NM_153422:293	Pde5a	INSIDE	0.241	0.718	1869.49	1342.96	0.173	1351.58	233.54
A_68_P22484166	chr3:122432487-122432536	NM_153422:436	Pde5a	INSIDE	0.267	0.522	1141.46	595.92	0.140	846.25	118.12
A_68_P20412903	chr1:88479253-88479300	NM_008801:-200	Pde6d	DIVERGENT_PROMOTER	0.218	0.585	1102.76	644.58	0.127	917.18	116.84
A_68_P25270819	chr7:88358500-88358544	NM_008803:-167	Pde8a	PROMOTER	0.666	0.579	1813.83	1049.79	0.386	1581.86	609.83
A_68_P31139420	chr17:31523449-31523493	NM_001163748:292	Pde9a	INSIDE	0.649	0.538	1767.14	951.56	0.349	1459.95	509.92

ProbeName	Target position of probe on CpG island microarray	TargetID	GeneSymbol	CpG island Description	Ratio of relative methylation (TiO2-NP/Vehicle)	Sham group			TiO_2-H group		
						Relative methylation (Cy-5/Cy-3)	Cy-3 signal (Input)	Cy-5 signal (MeDIP)	Relative methylation (Cy-5/Cy-3)	Cy-3 signal (Input)	Cy-5 signal (MeDIP)
A_68_P31139421	chr17:31523560-31523605	NM_001163748:404	Pde9a	INSIDE	0.507	0.698	1798.13	1254.35	0.354	1297.10	459.05
A_68_P26142833	chr8:109572050-109572094	NM_026513:442	Pdf	INSIDE	0.617	0.486	1244.49	605.28	0.300	975.92	293.09
A_68_P26142834	chr8:109572130-109572174	NM_026513:362	Pdf	INSIDE	0.203	0.667	2418.04	1612.21	0.135	1921.62	260.15
A_68_P24132961	chr5:139469670-139469719	NM_008808:1213	Pdgfa	INSIDE	0.419	0.450	1236.09	555.78	0.188	951.74	179.09
A_68_P24132967	chr5:139470355-139470399	NM_008808:531	Pdgfa	INSIDE	0.399	0.437	1789.36	781.96	0.174	1451.55	253.04
A_68_P30369725	chr15:79844779-79844823	NM_011057:438	Pdgfb	INSIDE	0.457	0.450	1476.58	664.16	0.206	1097.39	225.69
A_68_P22271765	chr3:80840549-80840593	NM_019971:233	Pdgfc	INSIDE	0.307	0.655	4758.85	3117.57	0.201	4044.76	812.21
A_68_P26282994	chr9:6168621-6168669	NM_027924:33	Pdgfd	INSIDE	0.572	0.493	1583.15	781.21	0.282	1232.96	348.13
A_68_P26282995	chr9:6168715-6168768	NM_027924:130	Pdgfd	INSIDE	0.438	0.570	1318.34	751.53	0.250	1144.98	285.94
A_68_P26282996	chr9:6168807-6168852	NM_027924:218	Pdgfd	INSIDE	0.263	0.506	1127.69	570.19	0.133	862.98	114.97
A_68_P22582958	chr3:140874353-140874397	NM_008811:945	Pdha2	INSIDE	0.623	2.348	2156.64	5063.04	1.462	1808.41	2643.97
A_68_P29388217	chr14:9005728-9005772	NM_024221:-244	Pdhb	PROMOTER	0.495	0.695	1410.05	979.64	0.344	1176.69	404.65
A_68_P21580767	chr2:121239698-121239742	NM_007952:83	Pdia3	INSIDE	0.488	0.464	1856.14	861.06	0.226	1526.10	345.63
A_68_P21580771	chr2:121240143-121240187	NM_007952:527	Pdia3	INSIDE	0.327	0.376	2675.08	1004.86	0.123	2061.48	253.29
A_68_P30670426	chr16:35490448-35490492	NM_028295:489	Pdia5	INSIDE	0.256	0.596	2155.10	1283.41	0.153	1781.12	272.00
A_68_P30670427	chr16:35490517-35490561	NM_028295:421	Pdia5	INSIDE	0.198	0.487	1977.23	963.58	0.097	1496.50	144.72
A_68_P28269480	chr12:17273418-17273462	NM_027959:40	Pdia6	INSIDE	0.510	0.651	2058.29	1340.09	0.332	1785.25	592.77
A_68_P28269482	chr12:17273597-17273641	NM_027959:218	Pdia6	INSIDE	0.465	0.497	1277.94	634.88	0.231	976.51	225.42
A_68_P21322065	chr2:71710952-71710996	NM_172665:-354	Pdk1	PROMOTER	0.639	0.668	901.20	601.73	0.427	682.81	291.47
A_68_P21322066	chr2:71711031-71711075	NM_172665:-276	Pdk1	PROMOTER	0.438	0.474	2335.40	1106.96	0.207	1733.94	359.64
A_68_P21322074	chr2:71711897-71711941	NM_172665:590	Pdk1	INSIDE	0.645	0.552	1129.30	623.13	0.356	993.73	353.69
A_68_P32107646	chr19:40345679-40345723	NM_016861:406	Pdlim1	INSIDE	0.651	0.346	2015.46	697.52	0.225	1704.07	384.19
A_68_P32107650	chr19:40346140-40346184	NM_016861:-56	Pdlim1	PROMOTER	0.378	0.425	1347.60	572.21	0.161	1066.57	171.25
A_68_P29699779	chr14:70576599-70576643	NM_145978:859	Pdlim2	INSIDE	0.593	0.557	3028.68	1686.77	0.330	2376.19	784.84
A_68_P22590679	chr3:142058808-142058852	NM_001190852:-170	Pdlim5	PROMOTER	0.315	0.562	1240.68	697.58	0.177	985.94	174.41
A_68_P29055509	chr13:55614601-55614645	NM_001114088:178	Pdlim7	INSIDE	0.383	0.398	3015.47	1199.10	0.152	2314.74	352.41
A_68_P26129290	chr8:107115528-107115572	NM_001024606:183	Pdp2	INSIDE	0.612	0.632	3062.32	1936.90	0.387	2437.09	943.68
A_68_P21752417	chr2:152841063-152841107	NM_178939:35	Pdrg1	INSIDE	0.268	0.557	1741.01	969.95	0.149	1317.68	196.70
A_68_P21752418	chr2:152841222-152841266	NM_178939:-125	Pdrg1	PROMOTER	0.513	0.656	1014.82	665.35	0.336	837.41	281.53
A_68_P24192245	chr5:151476526-151476570	NM_175310:147	Pds5b	INSIDE	0.264	0.473	2267.39	1073.05	0.125	1818.86	227.40
A_68_P24172758	chr5:148081815-148081859	NM_008814:130	Pdx1	INSIDE	0.217	0.637	2676.54	1705.06	0.138	1822.81	251.55
A_68_P24172759	chr5:148081959-148082003	NM_008814:274	Pdx1	INSIDE	0.138	0.733	3678.81	2696.06	0.101	2571.85	259.42
A_68_P30559565	chr16:13903341-13903391	NM_001039533:-138	Pdxdc1	PROMOTER	0.343	0.446	1252.10	558.00	0.153	897.76	137.13
A_68_P30363155	chr15:78745009-78745053	NM_020271:682	Pdxp	INSIDE	0.337	0.682	2950.08	2011.01	0.229	1987.34	456.04
A_68_P32464979	chrX:71069627-71069676	NM_001029868:657	Pdzd4	INSIDE	0.194	1.750	1041.83	1823.36	0.339	435.07	147.53
A_68_P32464981	chrX:71069831-71069875	NM_001029868:456	Pdzd4	INSIDE	0.171	1.688	1122.43	1894.74	0.288	528.09	152.05
A_68_P30441118	chr15:92227846-92227890	NM_001164593:628	Pdzrn4	INSIDE	0.319	0.533	1499.69	799.03	0.170	1237.59	210.44
A_68_P20335742	chr1:72301442-72301486	NM_023523:29424	Pecr	DOWNSTREAM	0.618	0.387	2902.68	1123.65	0.239	2134.24	510.80
A_68_P31926004	chr19:4942668-4942712	NM_172835:402	Peli3	INSIDE	0.322	0.727	3180.37	2313.19	0.234	2465.11	577.80
A_68_P31926007	chr19:4942924-4942968	NM_172835:146	Peli3	INSIDE	0.192	1.330	6473.47	8606.76	0.255	4962.52	1267.16
A_68_P29350210	chr13:115880236-115880280	NM_134058:108	Pelo	INSIDE	0.571	0.428	1365.78	584.99	0.244	1293.53	316.15
A_68_P27900561	chr11:70223294-70223338	NM_029231:217	Pelp1	INSIDE	0.273	0.661	1311.25	866.90	0.180	1027.67	185.49
A_68_P27900565	chr11:70223680-70223724	NM_029231:-169	Pelp1	PROMOTER	0.604	0.542	3105.07	1684.38	0.327	2393.70	783.69
A_68_P27900566	chr11:70223756-70223800	NM_029231:-245	Pelp1	PROMOTER	0.264	0.691	2541.05	1756.22	0.183	1812.48	330.89

ProbeName	Target position of probe on CpG island microarray	TargetID	GeneSymbol	CpG island Description	Ratio of relative methylation (TiO2-NP/Vehicle)	Sham group			TiO_2-H group		
						Relative methylation (Cy-5/Cy-3)	Cy-3 signal (Input)	Cy-5 signal (MeDIP)	Relative methylation (Cy-5/Cy-3)	Cy-3 signal (Input)	Cy-5 signal (MeDIP)
A_68_P27840489	chr11:59859589-59859648	NM_008819:-9277	Pemt	PROMOTER	0.033	2.790	2341.77	6534.34	0.093	1602.80	148.31
A_68_P32135820	chr19:45081022-45081066	NM_153796:-3	Peo1	DIVERGENT_PROMOTER	0.418	0.651	2097.28	1365.42	0.272	1519.11	413.74
A_68_P21706810	chr2:144194546-144194590	NM_001164813:-150	Pet117	PROMOTER	0.327	0.472	1145.94	541.12	0.155	963.15	148.85
A_68_P23445965	chr5:3596035-3596079	NM_027777:-9	Pex1	PROMOTER	0.553	0.527	1240.26	653.58	0.291	1005.49	292.76
A_68_P23445966	chr5:3596177-3596221	NM_027777:133	Pex1	INSIDE	0.207	0.601	1327.02	798.14	0.125	969.34	120.85
A_68_P27970616	chr11:83112191-83112235	NM_134025:267	Pex12	INSIDE	0.105	0.507	2952.27	1498.13	0.053	2221.79	118.43
A_68_P27970618	chr11:83112471-83112515	NM_134025:-13	Pex12	DIVERGENT_PROMOTER	0.281	0.631	2669.29	1685.21	0.177	1858.05	329.27
A_68_P27970619	chr11:83112556-83112601	NM_134025:-99	Pex12	DIVERGENT_PROMOTER	0.251	0.482	1413.18	681.67	0.121	991.90	120.15
A_68_P23398660	chr4:148473749-148473793	NM_019781:151	Pex14	INSIDE	0.132	0.512	2249.34	1152.53	0.068	1731.93	116.97
A_68_P26951868	chr10:13272953-13272998	NM_001164195:-27	Pex3	PROMOTER	0.217	0.613	1178.92	723.07	0.133	901.64	119.76
A_68_P24815138	chr6:124364754-124364798	NM_008995:309	Pex5	INSIDE	0.094	0.726	2860.12	2077.77	0.068	1976.67	135.04
A_68_P24815139	chr6:124364839-124364883	NM_008995:225	Pex5	INSIDE	0.306	0.508	4295.46	2180.83	0.156	3282.57	510.53
A_68_P27892526	chr11:68821691-68821735	NM_001159519:250	Pfas	INSIDE	0.260	0.679	1985.15	1347.58	0.177	1438.44	253.91
A_68_P21853418	chr2:170322093-170322137	NM_001013369:187	Pfdn4	INSIDE	0.187	0.465	1684.48	783.27	0.087	1350.47	117.58
A_68_P21853525	chr2:170336931-170336975	NM_001110152:223	Pfdn4	INSIDE	0.084	0.631	3612.00	2280.58	0.053	2589.43	137.14
A_68_P21853526	chr2:170337089-170337133	NM_001110152:381	Pfdn4	INSIDE	0.422	0.689	5002.07	3447.40	0.291	3516.04	1022.07
A_68_P20611336	chr1:132612197-132612241	NM_001162416:-1065	Pfkfb2	PROMOTER	0.632	0.719	1983.00	1426.16	0.454	1578.71	717.52
A_68_P20611337	chr1:132612275-132612319	NM_001162416:-1143	Pfkfb2	PROMOTER	0.531	0.423	1200.60	507.44	0.225	1033.62	232.06
A_68_P21006480	chr2:11423558-11423602	NM_001177752:137	Pfkfb3	INSIDE	0.103	0.622	2601.96	1619.63	0.064	1816.90	116.65
A_68_P21006492	chr2:11424861-11424905	NM_001177752:-1165	Pfkfb3	PROMOTER	0.651	0.537	1161.10	623.36	0.349	958.88	335.11
A_68_P27271611	chr10:77471981-77472025	NM_008826:539	Pfkl	INSIDE	0.329	0.529	1010.60	534.75	0.174	660.99	114.95
A_68_P27271615	chr10:77472561-77472605	NM_008826:-41	Pfkl	PROMOTER	0.545	0.580	1289.63	748.22	0.316	1077.71	340.70
A_68_P30473040	chr15:97923295-97923339	NM_001163488:297	Pfkm	INSIDE	0.373	0.398	1591.36	632.58	0.148	1158.36	171.82
A_68_P27901889	chr11:70467270-70467315	NM_011072:860	Pfn1	INSIDE	0.140	0.714	2299.71	1641.87	0.100	1771.30	177.23
A_68_P27901894	chr11:70467828-70467872	NM_011072:302	Pfn1	INSIDE	0.367	0.660	1700.64	1122.36	0.242	1120.07	271.19
A_68_P32117863	chr19:41986389-41986433	NM_023418:50	Pgam1	INSIDE	0.268	0.485	2687.15	1302.79	0.130	2042.45	265.75
A_68_P25377819	chr7:109384967-109385014	NM_145583:13403	Pgap2	INSIDE	0.562	2.859	888.88	2541.17	1.608	785.22	1262.72
A_68_P26245546	chr8:126957920-126957964	NM_171824:-106	Pgbd5	PROMOTER	0.501	0.410	1437.98	590.23	0.206	1129.08	232.08
A_68_P23399007	chr4:148540337-148540381	NM_001081274:458	Pgd	INSIDE	0.369	0.494	3376.20	1667.68	0.182	2673.45	487.18
A_68_P23399010	chr4:148540735-148540779	NM_001081274:60	Pgd	INSIDE	0.126	0.529	2356.26	1247.01	0.067	1780.51	118.91
A_68_P31676055	chr18:46440555-46440599	NM_172627:-72	Pggt1b	PROMOTER	0.535	0.619	1297.61	802.79	0.331	1152.58	381.29
A_68_P25957670	chr8:74115606-74115650	NM_025396:-454	Pgls	PROMOTER	0.410	0.602	4980.29	3000.61	0.247	3676.46	907.74
A_68_P25957673	chr8:74115916-74115960	NM_025396:-144	Pgls	PROMOTER	0.506	0.377	3086.45	1162.11	0.191	2465.23	470.05
A_68_P25957674	chr8:74115987-74116031	NM_025396:-74	Pgls	PROMOTER	0.650	0.586	1398.98	819.98	0.381	1063.00	404.72
A_68_P25957680	chr8:74116556-74116600	NM_025396:496	Pgls	INSIDE	0.289	0.479	1111.20	531.95	0.138	852.96	118.07
A_68_P25957709	chr8:74120139-74120192	NM_025396:4083	Pgls	INSIDE	0.266	0.620	1070.78	664.12	0.165	861.19	142.27
A_68_P23749118	chr5:64484258-64484302	NM_025700:92	Pgm1	INSIDE	0.582	0.435	1405.88	610.91	0.253	1098.24	277.77
A_68_P25366405	chr7:107375733-107375777	NM_027629:-362	Pgm2l1	PROMOTER	0.179	0.686	1188.00	814.92	0.123	969.53	119.37
A_68_P25366411	chr7:107376423-107376469	NM_027629:330	Pgm2l1	INSIDE	0.360	0.548	1061.66	581.90	0.197	888.06	175.38
A_68_P26700012	chr9:86465331-86465375	NM_001163746:97	Pgm3	INSIDE	0.242	0.436	1471.64	641.64	0.105	1155.40	121.71
A_68_P32026208	chr19:24936124-24936168	NM_175013:186	Pgm5	INSIDE	0.540	0.581	1036.10	602.43	0.314	837.92	263.05
A_68_P31097499	chr17:24607735-24607779	NM_025954:339	Pgp	INSIDE	0.653	0.483	1351.18	652.43	0.315	1073.26	338.44
A_68_P25953243	chr8:73183392-73183436	NM_023217:166	Pgpep1	INSIDE	0.271	0.620	1913.18	1185.96	0.168	1408.27	236.84
A_68_P26293544	chr9:8900444-8900488	NM_008829:634	Pgr	INSIDE	0.309	0.392	1565.31	613.31	0.121	1155.13	139.75

ProbeName	Target position of probe on CpG island microarray	TargetID	GeneSymbol	CpG island Description	Ratio of relative methylation (TiO2-NP/Vehicle)	Sham group			TiO_2-H group		
						Relative methylation (Cy-5/Cy-3)	Cy-3 signal (Input)	Cy-5 signal (MeDIP)	Relative methylation (Cy-5/Cy-3)	Cy-3 signal (Input)	Cy-5 signal (MeDIP)
A_68_P32546910	chrX:94267800-94267844	NM_001033361:-112	Pgr15l	PROMOTER	0.251	1.589	842.15	1338.09	0.399	490.63	195.86
A_68_P32319271	chrX:34138375-34138419	NM_016783:177	Pgrmc1	INSIDE	0.229	2.513	5728.76	14398.84	0.576	2272.59	1309.61
A_68_P32319273	chrX:34138558-34138602	NM_016783:361	Pgrmc1	INSIDE	0.358	1.404	1678.06	2355.96	0.502	670.44	336.80
A_68_P32319275	chrX:34138849-34138893	NM_016783:651	Pgrmc1	INSIDE	0.048	4.730	1509.48	7140.44	0.225	545.32	122.94
A_68_P22082999	chr3:40886268-40886325	NM_027558:672	Pgrmc2	INSIDE	0.184	0.576	1627.36	937.55	0.106	1189.47	126.33
A_68_P22083009	chr3:40887619-40887663	NM_027558:-672	Pgrmc2	PROMOTER	0.638	0.606	1226.19	743.43	0.387	1036.50	400.86
A_68_P28987052	chr13:43011760-43011804	NM_001005740:206833	Phactr1	INSIDE	0.438	0.656	2490.44	1634.25	0.287	2091.09	601.14
A_68_P28987053	chr13:43011826-43011870	NM_001005740:206899	Phactr1	INSIDE	0.462	0.574	1020.04	585.88	0.265	896.87	237.93
A_68_P26951409	chr10:13193710-13193754	NM_001195065:470	Phactr2	INSIDE	0.142	0.516	3272.04	1686.83	0.073	2460.01	179.63
A_68_P21888794	chr2:177877318-177877362	NM_028806:703	Phactr3	INSIDE	0.304	0.532	1007.53	536.11	0.162	746.47	120.70
A_68_P31731073	chr18:56722210-56722254	NM_001162989:10	Phax	INSIDE	0.339	0.611	1265.55	772.81	0.207	1014.37	209.81
A_68_P24816532	chr6:124662438-124662482	NM_007531:154	Phb2	INSIDE	0.270	0.649	854.19	554.53	0.175	692.18	121.25
A_68_P24808210	chr6:122289199-122289250	NM_001042623:-2191	Phc1	PROMOTER	0.261	0.615	853.00	524.41	0.161	758.78	121.84
A_68_P24808212	chr6:122289513-122289557	NM_001042623:-2501	Phc1	PROMOTER	0.240	0.588	3941.43	2316.93	0.141	2493.67	351.46
A_68_P23292280	chr4:128331714-128331758	NM_001195130:-209	Phc2	PROMOTER	0.193	0.539	1414.75	762.41	0.104	1160.28	120.80
A_68_P23292761	chr4:128405549-128405608	NM_001195083:764	Phc2	INSIDE	0.343	0.648	904.44	586.46	0.222	564.26	125.50
A_68_P31113238	chr17:27070074-27070118	NM_009343:25	Phf1	INSIDE	0.580	0.473	1647.89	778.89	0.274	1296.92	355.43
A_68_P31062916	chr17:15097920-15097964	NM_024250:295	Phf10	INSIDE	0.464	0.653	6295.84	4112.61	0.303	4914.93	1490.71
A_68_P31062919	chr17:15098706-15098750	NM_024250:-491	Phf10	PROMOTER	0.292	1.357	6416.10	8704.67	0.397	4682.72	1856.94
A_68_P27940743	chr11:77796384-77796428	NM_174852:89	Phf12	INSIDE	0.396	0.423	4734.44	2004.16	0.168	3592.41	602.73
A_68_P27940753	chr11:77797545-77797589	NM_174852:1249	Phf12	INSIDE	0.449	0.475	1363.64	647.40	0.213	995.82	212.38
A_68_P23415879	chr4:151368860-151368904	NM_172705:1406	Phf13	INSIDE	0.118	0.591	2449.97	1449.12	0.070	1678.49	116.66
A_68_P23415890	chr4:151370343-151370387	NM_172705:-76	Phf13	PROMOTER	0.151	0.431	2263.75	974.70	0.065	1857.21	121.13
A_68_P23415895	chr4:151370916-151370960	NM_172705:-650	Phf13	PROMOTER	0.256	0.546	1141.95	623.62	0.140	837.73	117.06
A_68_P27796178	chr11:51669754-51669800	NM_199299:1207	Phf15	INSIDE	0.215	1.880	904.15	1700.07	0.404	548.44	221.76
A_68_P27796185	chr11:51670574-51670621	NM_199299:386	Phf15	INSIDE	0.122	0.697	1875.77	1307.39	0.085	1377.92	116.85
A_68_P27796196	chr11:51671860-51671916	NM_199299:-905	Phf15	PROMOTER	0.281	0.635	916.81	581.81	0.179	685.66	122.42
A_68_P21769996	chr2:156022460-156022504	NM_172674:100	Phf20	INSIDE	0.274	0.405	1428.89	578.54	0.111	1123.25	124.64
A_68_P30397289	chr15:84684008-84684052	NM_001081166:2529	Phf21b	INSIDE	0.574	0.711	2450.67	1742.93	0.408	1998.97	815.55
A_68_P30397292	chr15:84684469-84684513	NM_001081166:2069	Phf21b	INSIDE	0.239	0.669	978.25	654.78	0.160	764.34	122.42
A_68_P30397294	chr15:84684736-84684780	NM_001081166:1801	Phf21b	INSIDE	0.391	0.463	1726.92	800.27	0.181	1439.78	261.16
A_68_P30397300	chr15:84685696-84685740	NM_001081166:841	Phf21b	INSIDE	0.161	1.372	1438.57	1974.32	0.221	931.51	205.95
A_68_P30397302	chr15:84685989-84686033	NM_001081166:549	Phf21b	INSIDE	0.505	0.526	1246.85	655.29	0.265	978.96	259.70
A_68_P30397310	chr15:84686778-84686823	NM_001081166:-241	Phf21b	PROMOTER	0.483	0.691	3869.34	2672.26	0.334	2983.60	995.79
A_68_P27898393	chr11:69810295-69810339	NM_030064:1044	Phf23	INSIDE	0.255	1.753	3199.51	5608.95	0.446	2134.93	952.71
A_68_P29510753	chr14:32064135-32064185	NM_027949:244	Phf7	INSIDE	0.404	0.534	960.26	513.16	0.216	777.89	167.83
A_68_P32569611	chrX:99839237-99839281	NM_008832:327	Phka1	INSIDE	0.056	2.794	4375.23	12225.17	0.156	1577.23	246.39
A_68_P27449324	chr10:110943649-110943693	NM_009344:329	Phlda1	INSIDE	0.654	0.539	1081.33	582.49	0.352	903.72	318.26
A_68_P25601371	chr7:150687585-150687629	NM_009434:823	Phlda2	INSIDE	0.305	0.640	2217.40	1418.70	0.195	1596.89	311.32
A_68_P20641447	chr1:137662511-137662555	NM_013750:-129	Phlda3	PROMOTER	0.194	1.773	1946.44	3451.63	0.345	1457.90	502.66
A_68_P26469278	chr9:44541688-44541732	NM_153537:1567	Phldb1	INSIDE	0.309	1.578	1314.26	2074.23	0.488	966.00	471.28
A_68_P26469281	chr9:44542040-44542084	NM_153537:1215	Phldb1	INSIDE	0.427	0.714	4272.68	3050.03	0.305	3273.45	998.91
A_68_P26469282	chr9:44542109-44542153	NM_153537:1147	Phldb1	INSIDE	0.122	0.617	2150.08	1326.41	0.075	1559.08	117.66
A_68_P30728923	chr16:45844322-45844366	NM_153412:147	Phldb2	INSIDE	0.168	0.559	4648.33	2598.67	0.094	3381.44	318.14

ProbeName	Target position of probe on CpG island microarray	TargetID	GeneSymbol	CpG island Description	Ratio of relative methylation (TiO2-NP/Vehicle)	Sham group			TiO_2-H group		
						Relative methylation (Cy-5/Cy-3)	Cy-3 signal (Input)	Cy-5 signal (MeDIP)	Relative methylation (Cy-5/Cy-3)	Cy-3 signal (Input)	Cy-5 signal (MeDIP)
A_68_P20506801	chr1:108068058-108068102	NM_133821:-365	Phlpp1	PROMOTER	0.335	0.566	2301.60	1302.82	0.190	1736.91	329.71
A_68_P20506802	chr1:108068165-108068215	NM_133821:-255	Phlpp1	PROMOTER	0.283	0.571	3301.60	1885.10	0.162	2325.73	376.11
A_68_P20506813	chr1:108069803-108069847	NM_133821:1379	Phlpp1	INSIDE	0.257	0.405	1464.44	592.73	0.104	1114.65	115.93
A_68_P28041006	chr11:95685266-95685311	NM_153104:-525	Phospho1	PROMOTER	0.275	0.550	939.14	516.98	0.152	796.29	120.65
A_68_P28041049	chr11:95691702-95691746	NM_153104:5911	Phospho1	INSIDE	0.206	0.379	1814.12	687.98	0.078	1469.47	114.85
A_68_P28041052	chr11:95692092-95692136	NM_153104:6301	Phospho1	INSIDE	0.458	0.647	1197.54	774.64	0.296	953.21	282.31
A_68_P25375581	chr7:108966881-108966925	NM_008887:76	Phox2a	INSIDE	0.095	0.713	2481.75	1769.57	0.068	1898.03	128.44
A_68_P25375584	chr7:108967279-108967328	NM_008887:477	Phox2a	INSIDE	0.222	3.232	2387.91	7718.57	0.718	1716.21	1232.01
A_68_P25375613	chr7:108970639-108970683	NM_008887:3834	Phox2a	INSIDE	0.241	0.595	947.93	564.05	0.143	821.31	117.77
A_68_P21073068	chr2:25430283-25430334	NM_029293:83	Phpt1	INSIDE	0.220	0.705	2050.79	1446.58	0.155	1397.62	216.77
A_68_P25587561	chr7:148414503-148414549	NM_001081118:-160	Phrf1	DIVERGENT_PROMOTER	0.234	0.675	1003.84	677.39	0.158	746.82	117.88
A_68_P27230700	chr10:70061466-70061510	NM_001162846:-5928	Phyhipl	PROMOTER	0.275	0.519	1106.01	573.61	0.143	910.16	129.83
A_68_P31127302	chr17:29459217-29459261	NM_023734:3412	Pi16	INSIDE	0.555	2.465	2975.74	7336.30	1.369	1879.24	2572.35
A_68_P31127304	chr17:29459396-29459440	NM_023734:3592	Pi16	INSIDE	0.664	3.213	1425.74	4581.14	2.133	1020.44	2176.48
A_68_P31127333	chr17:29463770-29463814	NM_023734:7966	Pi16	INSIDE	0.298	0.554	1109.54	614.43	0.165	771.49	127.28
A_68_P32118990	chr19:42165379-42165423	NM_145501:476	Pi4k2a	INSIDE	0.091	0.705	3132.84	2209.61	0.064	2332.25	149.57
A_68_P32118992	chr19:42165554-42165602	NM_145501:654	Pi4k2a	INSIDE	0.359	0.670	1833.56	1228.50	0.241	1273.81	306.48
A_68_P26573398	chr9:62828411-62828455	NM_019663:254	Pias1	INSIDE	0.652	0.686	3303.73	2264.88	0.447	2612.06	1167.71
A_68_P26573403	chr9:62828953-62828997	NM_019663:-288	Pias1	PROMOTER	0.237	0.442	2496.30	1103.02	0.105	2046.95	214.24
A_68_P22350678	chr3:96500622-96500667	NM_001165949:-354	Pias3	PROMOTER	0.260	0.490	1885.22	923.57	0.127	1452.62	185.08
A_68_P27287756	chr10:80616365-80616414	NM_021501:14076	Pias4	DOWNSTREAM	0.645	3.573	1617.64	5780.39	2.304	1166.31	2686.76
A_68_P29841642	chr14:99498547-99498591	NM_029454:-83	Pibf1	PROMOTER	0.222	0.601	1780.94	1070.43	0.133	1437.93	191.69
A_68_P32798240	chrX:160857773-160857818	NM_011081:77	Piga	INSIDE	0.052	1.666	3449.95	5746.86	0.086	1373.40	118.01
A_68_P32798241	chrX:160857957-160858011	NM_011081:266	Piga	INSIDE	0.102	1.457	2146.44	3126.36	0.149	807.01	120.17
A_68_P23971131	chr5:108741769-108741813	NM_001081234:-153	Pigg	PROMOTER	0.565	0.577	1542.46	889.51	0.326	1240.48	403.92
A_68_P22652203	chr3:152377356-152377400	NM_025662:315	Pigk	INSIDE	0.326	0.501	1096.58	549.24	0.163	905.32	147.86
A_68_P20832748	chr1:174307013-174307057	NM_026234:373	Pigm	INSIDE	0.297	0.667	1648.01	1099.60	0.198	1331.03	263.79
A_68_P27942688	chr11:78142334-78142378	NM_201406:433	Pigs	INSIDE	0.239	0.704	1187.31	835.77	0.168	891.88	149.88
A_68_P21818226	chr2:164323071-164323115	NM_133779:68	Pigt	INSIDE	0.388	0.636	1906.00	1212.07	0.247	1528.24	377.07
A_68_P21818227	chr2:164323199-164323243	NM_133779:196	Pigt	INSIDE	0.503	0.627	1939.02	1216.47	0.315	1421.70	448.25
A_68_P21818228	chr2:164323274-164323322	NM_133779:274	Pigt	INSIDE	0.232	0.474	1533.57	726.45	0.110	1144.52	125.71
A_68_P24479722	chr6:57642005-57642049	NM_025574:46	Pigy	INSIDE	0.355	0.466	1127.04	524.97	0.166	982.62	162.68
A_68_P30650142	chr16:31933808-31933852	NM_172822:-106	Pigz	PROMOTER	0.463	0.582	1865.83	1086.52	0.270	1336.72	360.78
A_68_P30650143	chr16:31933937-31933981	NM_172822:22	Pigz	INSIDE	0.282	0.517	1181.84	610.58	0.146	846.76	123.55
A_68_P29274934	chr13:102464406-102464450	NM_001024955:-1826	Pik3r1	PROMOTER	0.656	0.608	966.97	588.13	0.399	900.07	359.29
A_68_P29275477	chr13:102537731-102537775	NM_001077495:420	Pik3r1	INSIDE	0.477	0.489	1109.14	542.56	0.233	910.65	212.37
A_68_P29275481	chr13:102538276-102538320	NM_001077495:-126	Pik3r1	PROMOTER	0.203	0.522	2880.19	1503.99	0.106	2349.49	248.86
A_68_P25953920	chr8:73293490-73293534	NM_008841:7099	Pik3r2	INSIDE	0.349	0.679	1182.80	803.20	0.237	970.57	230.28
A_68_P25953979	chr8:73300434-73300478	NM_008841:155	Pik3r2	INSIDE	0.355	0.389	1578.56	614.04	0.138	1324.32	183.13
A_68_P23228358	chr4:115894280-115894324	NM_181585:-216	Pik3r3	PROMOTER	0.154	0.481	1913.43	920.52	0.074	1611.99	119.44
A_68_P23228359	chr4:115894431-115894475	NM_181585:-66	Pik3r3	PROMOTER	0.365	0.529	1566.05	829.17	0.193	1213.12	234.41
A_68_P31128228	chr17:29627044-29627088	NM_008842:-923	Pim1	PROMOTER	0.318	0.649	4174.97	2710.75	0.207	2967.55	613.15
A_68_P31128243	chr17:29629131-29629176	NM_008842:1164	Pim1	INSIDE	0.131	0.642	1937.35	1244.54	0.084	1438.02	120.82
A_68_P30421714	chr15:88692421-88692465	NM_145478:-181	Pim3	PROMOTER	0.110	0.545	3841.18	2094.99	0.060	2759.09	165.65

ProbeName	Target position of probe on CpG island microarray	TargetID	GeneSymbol	CpG island Description	Ratio of relative methylation (TiO2-NP/Vehicle)	Sham group			TiO_2-H group		
						Relative methylation (Cy-5/Cy-3)	Cy-3 signal (Input)	Cy-5 signal (MeDIP)	Relative methylation (Cy-5/Cy-3)	Cy-3 signal (Input)	Cy-5 signal (MeDIP)
A_68_P30421723	chr15:88693456-88693500	NM_145478:855	Pim3	INSIDE	0.640	0.693	1434.73	994.80	0.444	1103.85	489.91
A_68_P28052462	chr11:97605811-97605862	NM_054051:182	Pip4k2b	INSIDE	0.238	0.493	1477.68	728.81	0.117	1019.80	119.71
A_68_P28052466	chr11:97606492-97606536	NM_054051:-496	Pip4k2b	PROMOTER	0.276	0.584	2592.54	1514.05	0.161	1842.31	297.34
A_68_P22342239	chr3:94910572-94910617	NM_008847:186	Pip5k1a	INSIDE	0.243	0.663	904.30	599.63	0.161	732.92	118.25
A_68_P22342241	chr3:94910847-94910891	NM_008847:-88	Pip5k1a	DIVERGENT_PROMOTER	0.241	0.617	967.92	596.73	0.148	820.56	121.76
A_68_P27288652	chr10:80755895-80755939	NM_001146687:200	Pip5k1c	INSIDE	0.485	0.427	2716.23	1159.04	0.207	2212.44	458.22
A_68_P23585503	chr5:33127970-33128014	NM_177298:283	Pisd	INSIDE	0.192	0.467	1687.07	787.39	0.089	1325.96	118.62
A_68_P23585505	chr5:33128122-33128166	NM_177298:131	Pisd	INSIDE	0.416	0.458	1904.84	871.63	0.191	1459.14	278.02
A_68_P27926998	chr11:75401820-75401864	NM_008850:233	Pitpna	INSIDE	0.200	0.663	1335.14	885.01	0.133	1121.74	148.86
A_68_P28107246	chr11:107330411-107330455	NM_145823:1602	Pitpnc1	INSIDE	0.272	0.588	3975.75	2339.59	0.160	3186.82	509.66
A_68_P28107248	chr11:107330701-107330745	NM_145823:1312	Pitpnc1	INSIDE	0.141	0.532	2114.44	1124.23	0.075	1579.74	118.72
A_68_P28107252	chr11:107331040-107331084	NM_145823:972	Pitpnc1	INSIDE	0.520	0.607	2393.36	1453.50	0.316	1967.44	621.57
A_68_P28107255	chr11:107331346-107331390	NM_145823:666	Pitpnc1	INSIDE	0.408	0.658	1864.59	1226.24	0.268	1561.88	419.27
A_68_P28107256	chr11:107331453-107331497	NM_145823:560	Pitpnc1	INSIDE	0.390	0.646	918.10	592.76	0.252	663.38	166.88
A_68_P24059409	chr5:124571234-124571278	NM_011256:95171	Pitpnm2	INSIDE	0.628	3.626	631.12	2288.74	2.276	551.76	1255.76
A_68_P24059427	chr5:124573452-124573496	NM_011256:92953	Pitpnm2	INSIDE	0.653	2.145	1806.45	3873.94	1.401	1175.89	1647.64
A_68_P28802625	chr13:6547192-6547238	NM_145131:-188	Pitrm1	PROMOTER	0.595	2.377	6069.01	14426.27	1.415	3902.82	5521.78
A_68_P29057249	chr13:55926961-55927005	NM_011097:5804	Pitx1	INSIDE	0.278	0.626	2167.75	1356.45	0.174	1561.70	271.78
A_68_P29057250	chr13:55927065-55927109	NM_011097:5700	Pitx1	INSIDE	0.271	0.589	1041.08	612.90	0.160	760.85	121.46
A_68_P29057257	chr13:55927883-55927927	NM_011097:4882	Pitx1	INSIDE	0.279	0.664	1735.85	1151.83	0.185	1409.77	261.10
A_68_P29057274	chr13:55929838-55929882	NM_011097:2926	Pitx1	INSIDE	0.246	0.515	1751.44	902.75	0.127	1017.60	129.18
A_68_P29057276	chr13:55930039-55930083	NM_011097:2726	Pitx1	INSIDE	0.492	0.714	2090.57	1493.64	0.352	1604.25	564.22
A_68_P29057299	chr13:55932917-55932961	NM_011097:-152	Pitx1	PROMOTER	0.543	0.491	1163.44	570.69	0.266	928.53	247.14
A_68_P29057342	chr13:55937765-55937809	NM_011097:-5000	Pitx1	PROMOTER	0.186	0.583	1825.86	1064.61	0.109	1410.67	153.31
A_68_P22518619	chr3:128902745-128902789	NM_011098:-69	Pitx2	PROMOTER	0.265	0.629	896.46	563.85	0.167	737.64	122.95
A_68_P22518620	chr3:128902934-128902978	NM_001042504:115	Pitx2	INSIDE	0.520	0.691	2387.47	1650.08	0.359	1963.44	705.65
A_68_P22518658	chr3:128907203-128907247	NM_001042502:-9630	Pitx2	PROMOTER	0.153	0.574	1612.92	925.36	0.088	1370.17	120.64
A_68_P22518740	chr3:128916656-128916700	NM_001042502:-176	Pitx2	PROMOTER	0.168	0.372	2754.32	1024.02	0.062	2008.37	125.52
A_68_P22518744	chr3:128917174-128917220	NM_001042502:342	Pitx2	INSIDE	0.272	0.664	1138.17	755.21	0.180	885.98	159.89
A_68_P22518745	chr3:128917350-128917394	NM_001042502:518	Pitx2	INSIDE	0.098	0.713	2440.81	1739.12	0.070	1670.43	116.53
A_68_P22518747	chr3:128917569-128917613	NM_001042502:736	Pitx2	INSIDE	0.376	0.418	1871.32	782.01	0.157	1453.58	228.38
A_68_P22518759	chr3:128919062-128919106	NM_001042502:2230	Pitx2	INSIDE	0.177	0.544	2054.18	1116.66	0.096	1399.82	134.47
A_68_P22518783	chr3:128921672-128921716	NM_001042502:4840	Pitx2	INSIDE	0.600	0.588	887.59	522.06	0.353	736.64	259.96
A_68_P32142233	chr19:46210854-46210898	NM_008852:11939	Pitx3	INSIDE	0.468	0.469	2790.16	1308.54	0.219	2201.20	483.12
A_68_P32142236	chr19:46211273-46211317	NM_008852:11521	Pitx3	INSIDE	0.224	1.389	2293.90	3186.51	0.311	1545.80	480.40
A_68_P32142240	chr19:46211708-46211752	NM_008852:11085	Pitx3	INSIDE	0.345	0.537	1189.29	638.22	0.185	973.75	180.21
A_68_P32142306	chr19:46221925-46221969	NM_008852:869	Pitx3	INSIDE	0.241	0.402	4293.47	1727.85	0.097	3312.00	320.83
A_68_P32142315	chr19:46223136-46223180	NM_008852:-343	Pitx3	DIVERGENT_PROMOTER	0.239	0.409	1947.09	796.59	0.098	1441.84	140.82
A_68_P32555654	chrX:96665945-96665989	NM_001083110:108	Pja1	INSIDE	0.270	1.528	1401.52	2140.98	0.413	586.99	242.14
A_68_P31295068	chr17:64681131-64681175	NM_001025309:71	Pja2	INSIDE	0.510	0.442	2533.20	1119.03	0.225	2101.75	473.18
A_68_P31295073	chr17:64681710-64681754	NM_001025309:-509	Pja2	PROMOTER	0.310	0.447	1995.51	892.86	0.139	1482.72	205.45
A_68_P31612701	chr18:34569089-34569133	NM_001163004:34	Pkd2l2	INSIDE	0.586	0.478	1227.35	587.13	0.280	1201.01	336.66
A_68_P21928444	chr3:7366471-7366516	NM_008862:-110	Pkia	PROMOTER	0.604	0.582	1249.51	726.84	0.352	978.78	344.14
A_68_P21813660	chr2:163484302-163484346	NM_001164053:131	Pkig	INSIDE	0.477	0.560	986.37	552.78	0.267	905.78	242.04

ProbeName	Target position of probe on CpG island microarray	TargetID	GeneSymbol	CpG island Description	Ratio of relative methylation (TiO2-NP/Vehicle)	Sham group			TiO_2-H group		
						Relative methylation (Cy-5/Cy-3)	Cy-3 signal (Input)	Cy-5 signal (MeDIP)	Relative methylation (Cy-5/Cy-3)	Cy-3 signal (Input)	Cy-5 signal (MeDIP)
A_68_P26554636	chr9:59505260-59505304	NM_011099:868	Pkm2	INSIDE	0.257	0.623	1228.93	765.27	0.160	997.34	159.31
A_68_P26554815	chr9:59528234-59528278	NM_011099:23842	Pkm2	DOWNSTREAM	0.103	0.582	2631.43	1531.54	0.060	1948.29	117.21
A_68_P26554816	chr9:59528311-59528358	NM_011099:23920	Pkm2	DOWNSTREAM	0.167	0.386	2290.30	885.07	0.065	1808.73	116.99
A_68_P31093271	chr17:23863763-23863807	NM_023058:482	Pkmyt1	INSIDE	0.261	0.619	3258.03	2016.07	0.161	2439.27	393.82
A_68_P26016104	chr8:86223190-86223234	NM_177262:-134	Pkn1	PROMOTER	0.499	0.574	1098.93	630.83	0.286	899.64	257.49
A_68_P22593191	chr3:142545216-142545260	NM_178654:-270	Pkn2	PROMOTER	0.590	0.365	1598.84	582.80	0.215	1338.37	287.70
A_68_P31140479	chr17:31701231-31701275	NM_016670:-465	Pknox1	PROMOTER	0.597	0.542	1130.48	613.27	0.324	828.28	268.31
A_68_P26429011	chr9:36953245-36953299	NM_001029838:1579	Pknox2	INSIDE	0.338	0.468	1242.53	581.44	0.158	990.49	156.59
A_68_P26429014	chr9:36953463-36953512	NM_001029838:1364	Pknox2	INSIDE	0.130	0.716	1838.18	1316.95	0.093	1397.56	130.05
A_68_P20642548	chr1:137815415-137815459	NM_019645:165	Pkp1	INSIDE	0.231	0.496	1469.62	729.61	0.115	1129.56	129.38
A_68_P20642549	chr1:137815494-137815538	NM_019645:85	Pkp1	INSIDE	0.207	0.556	1379.07	766.37	0.115	1012.58	116.56
A_68_P20642550	chr1:137815675-137815719	NM_019645:-95	Pkp1	PROMOTER	0.160	0.550	3846.71	2116.20	0.088	2820.11	247.88
A_68_P20642552	chr1:137815854-137815898	NM_019645:-275	Pkp1	PROMOTER	0.510	0.611	2166.92	1323.26	0.311	1704.79	530.71
A_68_P22522614	chr3:129581824-129581868	NM_183423:-2255	Pla2g12a	PROMOTER	0.500	1.369	3800.75	5201.39	0.684	2739.15	1873.33
A_68_P30365773	chr15:79158644-79158688	NM_001199023:135	Pla2g6	INSIDE	0.258	0.677	4985.11	3376.22	0.175	3564.43	622.24
A_68_P30365774	chr15:79158797-79158841	NM_001199023:-17	Pla2g6	PROMOTER	0.372	0.609	1039.23	633.31	0.227	829.12	187.89
A_68_P29480406	chr14:26722448-26722492	NM_207229:-91	Plac9	PROMOTER	0.460	0.565	3136.69	1773.15	0.260	2624.34	681.90
A_68_P29480407	chr14:26722558-26722602	NM_207229:-201	Plac9	PROMOTER	0.281	0.690	7465.52	5150.73	0.194	6608.79	1282.54
A_68_P24873768	chr6:136610320-136610368	NM_025806:70	Plbd1	INSIDE	0.183	0.594	1276.49	757.71	0.109	1089.98	118.30
A_68_P28083253	chr11:102941713-102941757	NM_152813:21238	Plcd3	INSIDE	0.291	0.571	954.33	545.03	0.166	734.64	122.25
A_68_P28083254	chr11:102941888-102941932	NM_152813:21062	Plcd3	INSIDE	0.281	0.719	1777.53	1277.53	0.202	1219.75	246.12
A_68_P26203093	chr8:120022311-120022355	NM_172285:142	Plcg2	INSIDE	0.436	0.441	1297.85	572.58	0.192	1146.51	220.34
A_68_P23435437	chr4:154357997-154358041	NM_001113360:27075	Plch2	INSIDE	0.192	1.597	480.09	766.75	0.306	444.58	136.23
A_68_P20241613	chr1:55463553-55463597	NM_001114663:785	Plcl1	INSIDE	0.390	0.622	819.18	509.57	0.243	692.34	168.12
A_68_P23977768	chr5:110530421-110530465	NR_027665:865	Plcxd1	INSIDE	0.284	0.388	1783.69	692.33	0.110	1272.40	140.06
A_68_P22014196	chr3:27837925-27837969	NM_001164056:345	Pld1	INSIDE	0.640	0.604	1984.92	1198.54	0.386	1581.36	610.92
A_68_P27901224	chr11:70353932-70353976	NM_008876:289	Pld2	INSIDE	0.347	0.608	2218.30	1348.45	0.211	1849.13	390.19
A_68_P27901225	chr11:70354037-70354081	NM_008876:393	Pld2	INSIDE	0.239	0.674	1192.74	803.98	0.161	959.31	154.27
A_68_P20852905	chr1:178205134-178205178	NM_176916:287	Pld5	INSIDE	0.054	0.732	4045.40	2961.82	0.039	3061.41	120.17
A_68_P20852906	chr1:178205311-178205355	NM_176916:111	Pld5	INSIDE	0.175	0.620	3447.14	2137.91	0.109	2578.68	280.07
A_68_P30346620	chr15:76012400-76012444	NM_201394:13718	Plec	INSIDE	0.594	2.759	550.95	1520.08	1.640	466.16	764.39
A_68_P30346763	chr15:76028781-76028825	NM_201393:-163	Plec	PROMOTER	0.208	0.582	1177.22	685.59	0.121	1012.57	122.73
A_68_P30346947	chr15:76059642-76059686	NM_201385:263	Plec	INSIDE	0.294	0.451	1296.40	584.06	0.132	922.68	122.12
A_68_P30346951	chr15:76060156-76060200	NM_201385:-251	Plec	PROMOTER	0.508	0.478	1602.64	765.41	0.242	1309.91	317.54
A_68_P30346974	chr15:76062654-76062698	NM_001163542:-868	Plec	PROMOTER	0.180	0.463	1944.49	901.27	0.083	1415.25	117.86
A_68_P30346979	chr15:76063160-76063204	NM_001163542:-1374	Plec	PROMOTER	0.237	0.575	1117.57	642.64	0.136	991.08	134.88
A_68_P30346980	chr15:76063274-76063318	NM_001163542:-1488	Plec	PROMOTER	0.356	0.316	3643.12	1151.50	0.112	2691.64	302.46
A_68_P28566639	chr12:80013104-80013148	NM_013738:-5201	Plek2	PROMOTER	0.249	0.622	1004.53	624.80	0.155	860.67	133.32
A_68_P25725384	chr8:26212812-26212856	NM_031257:-551	Plekha2	PROMOTER	0.412	0.703	3332.86	2343.63	0.290	2682.89	776.75
A_68_P21351698	chr2:76513834-76513878	NM_031256:485	Plekha3	INSIDE	0.260	0.603	1034.89	623.53	0.157	927.69	145.59
A_68_P25093689	chr7:52793682-52793726	NM_148927:12005	Plekha4	INSIDE	0.187	0.630	1765.63	1112.17	0.118	1337.90	157.64
A_68_P25093690	chr7:52793837-52793881	NM_148927:12159	Plekha4	INSIDE	0.294	0.540	1520.91	820.86	0.159	1147.84	182.08
A_68_P20140986	chr1:34906861-34906905	NM_145516:79	Plekhb2	INSIDE	0.348	0.565	3188.57	1800.24	0.196	2510.90	493.37
A_68_P22727582	chr4:10935187-10935231	NM_175175:-442	Plekhf2	PROMOTER	0.380	0.447	1915.38	856.58	0.170	1445.89	245.94

ProbeName	Target position of probe on CpG island microarray	TargetID	GeneSymbol	CpG island Description	Ratio of relative methylation (TiO2-NP/Vehicle)	Sham group			TiO_2-H group		
						Relative methylation (Cy-5/Cy-3)	Cy-3 signal (Input)	Cy-5 signal (MeDIP)	Relative methylation (Cy-5/Cy-3)	Cy-3 signal (Input)	Cy-5 signal (MeDIP)
A_68_P25021470	chr7:29157527-29157571	NM_001083912:133	Plekhg2	INSIDE	0.546	0.452	1942.28	876.99	0.246	1514.20	373.18
A_68_P28072366	chr11:101032330-101032374	NM_146030:264	Plekhh3	INSIDE	0.115	0.623	4359.82	2716.47	0.072	3095.40	221.70
A_68_P27285553	chr10:80260510-80260554	NM_023900:839	Plekhj1	INSIDE	0.454	0.603	2912.80	1757.28	0.274	2105.75	576.33
A_68_P27285558	chr10:80261134-80261187	NM_023900:211	Plekhj1	INSIDE	0.173	0.625	1549.63	967.88	0.108	1119.19	120.71
A_68_P23365306	chr4:141219776-141219820	NM_001033150:232	Plekhm2	INSIDE	0.525	0.343	1595.14	546.53	0.180	1260.13	226.86
A_68_P29322931	chr13:111185370-111185414	NM_152804:141	Plk2	INSIDE	0.336	0.427	1874.97	800.72	0.143	1439.94	206.36
A_68_P29322934	chr13:111185727-111185771	NM_152804:497	Plk2	INSIDE	0.231	0.658	2584.00	1700.99	0.152	1944.48	295.33
A_68_P23232644	chr4:116805889-116805940	NM_013807:643	Plk3	INSIDE	0.098	1.433	2102.86	3012.87	0.140	1484.68	208.44
A_68_P23232647	chr4:116806257-116806301	NM_013807:279	Plk3	INSIDE	0.320	0.519	1158.08	600.85	0.166	940.16	155.99
A_68_P22081523	chr3:40604233-40604277	NM_011495:382	Plk4	INSIDE	0.245	0.572	2379.25	1360.46	0.140	2032.66	284.53
A_68_P27282698	chr10:79819083-79819127	NM_183152:-99	Plk5	DIVERGENT_PROMOTER	0.173	0.580	1689.64	980.34	0.101	1210.77	121.87
A_68_P26079047	chr8:97219704-97219748	NM_026385:416	Pllp	INSIDE	0.330	0.562	2488.97	1399.13	0.186	1934.41	359.31
A_68_P26079048	chr8:97219780-97219824	NM_026385:340	Pllp	INSIDE	0.177	0.477	2353.33	1123.46	0.085	1746.55	147.88
A_68_P26727063	chr9:92437135-92437179	NM_001142916:96	Plod2	INSIDE	0.259	0.406	1465.54	595.12	0.105	1253.35	131.64
A_68_P24123988	chr5:137462758-137462802	NM_011962:-108	Plod3	PROMOTER	0.217	0.741	3574.70	2648.17	0.161	2601.49	418.05
A_68_P24123992	chr5:137463167-137463211	NM_011962:300	Plod3	INSIDE	0.218	0.530	1554.59	824.02	0.115	1028.86	118.78
A_68_P24123993	chr5:137463233-137463277	NM_011962:366	Plod3	INSIDE	0.474	0.553	1095.74	606.16	0.262	781.43	204.85
A_68_P22282899	chr3:82859431-82859475	NM_016784:-7	Plrg1	DIVERGENT_PROMOTER	0.580	0.691	1792.03	1238.57	0.401	1438.42	576.72
A_68_P22282900	chr3:82859514-82859558	NM_016784:77	Plrg1	INSIDE	0.210	0.651	1292.96	841.12	0.136	912.32	124.39
A_68_P21820163	chr2:164683274-164683318	NM_011125:-88	Pltp	PROMOTER	0.215	0.543	2459.64	1334.68	0.117	1895.58	221.18
A_68_P25957205	chr8:74031894-74031938	NM_032398:3752	Plvap	INSIDE	0.629	0.623	2304.19	1436.26	0.392	1844.09	722.71
A_68_P25957206	chr8:74031971-74032015	NM_032398:3676	Plvap	INSIDE	0.462	0.617	1776.78	1096.06	0.285	1370.90	390.45
A_68_P20956471	chr1:196446176-196446220	NM_008882:176	Plxna2	INSIDE	0.647	0.518	2147.75	1112.04	0.335	1680.28	562.98
A_68_P20956472	chr1:196446255-196446299	NM_008882:254	Plxna2	INSIDE	0.298	0.634	1595.91	1012.02	0.189	1163.51	219.86
A_68_P20956473	chr1:196446359-196446403	NM_008882:358	Plxna2	INSIDE	0.360	0.514	1066.96	548.68	0.185	944.67	175.10
A_68_P26818269	chr9:108998498-108998542	NM_172775:571	Plxnb1	INSIDE	0.123	0.660	2060.00	1360.27	0.081	1481.71	120.42
A_68_P30423566	chr15:88991488-88991534	NM_001159521:12770	Plxnb2	INSIDE	0.523	3.344	930.56	3111.62	1.748	714.34	1248.70
A_68_P26546675	chr9:58094820-58094867	NM_008884:2750	Pml	INSIDE	0.406	0.442	1835.96	811.92	0.180	1371.08	246.26
A_68_P27857781	chr11:62946689-62946733	NM_008885:1699	Pmp22	INSIDE	0.228	0.715	2391.88	1709.08	0.163	1719.60	280.20
A_68_P21078344	chr2:26245012-26245056	NM_173180:167	Pmpca	INSIDE	0.258	0.683	3723.02	2544.40	0.176	2491.77	439.28
A_68_P23527586	chr5:21243231-21243275	NM_028431:275	Pmpcb	INSIDE	0.420	0.735	5394.70	3965.09	0.308	3955.20	1219.56
A_68_P24158565	chr5:144671053-144671097	NM_008886:205	Pms2	INSIDE	0.270	0.418	1317.53	550.65	0.113	1036.82	117.10
A_68_P32464004	chrX:70904998-70905042	NM_001199351:173	Pnck	INSIDE	0.151	2.664	9345.08	24897.50	0.401	3853.71	1545.52
A_68_P20347698	chr1:74331582-74331626	NM_025580:-3	Pnkd	DIVERGENT_PROMOTER	0.575	0.566	1536.65	870.03	0.325	1145.16	372.56
A_68_P25089727	chr7:52112663-52112707	NM_021549:134	Pnkp	INSIDE	0.249	0.428	1476.56	631.94	0.106	1097.26	116.85
A_68_P29681978	chr14:67528942-67528986	NM_175498:-1080	Pnma2	PROMOTER	0.579	0.659	2916.67	1920.97	0.381	2155.40	821.28
A_68_P29681981	chr14:67529271-67529315	NM_175498:-752	Pnma2	PROMOTER	0.635	0.474	1667.09	790.64	0.301	1256.84	378.67
A_68_P24984095	chr7:17530028-17530072	NM_001099636:20	Pnmal2	INSIDE	0.521	0.651	1539.60	1001.72	0.339	1231.81	417.29
A_68_P24984097	chr7:17530264-17530308	NM_001099636:256	Pnmal2	INSIDE	0.263	0.483	1145.78	552.86	0.127	970.38	123.37
A_68_P24984112	chr7:17532039-17532083	NM_001099636:2030	Pnmal2	INSIDE	0.249	0.542	1291.60	700.57	0.135	1078.49	145.47
A_68_P24984115	chr7:17532440-17532484	NM_001099636:2432	Pnmal2	INSIDE	0.200	0.503	1456.66	732.44	0.101	1171.19	117.94
A_68_P28056108	chr11:98248019-98248063	NM_008890:95	Pnmt	INSIDE	0.605	0.728	1962.01	1429.14	0.440	1588.81	699.84
A_68_P25614335	chr8:3515288-3515332	NM_001122818:-73	Pnpla6	PROMOTER	0.479	0.619	2298.49	1423.24	0.297	1726.09	512.10
A_68_P28397152	chr12:45370530-45370574	NM_026164:412	Pnpla8	INSIDE	0.166	0.655	1701.23	1113.60	0.109	1094.96	119.04

ProbeName	Target position of probe on CpG island microarray	TargetID	GeneSymbol	CpG island Description	Ratio of relative methylation (TiO2-NP/Vehicle)	Sham group			TiO_2-H group		
						Relative methylation (Cy-5/Cy-3)	Cy-3 signal (Input)	Cy-5 signal (MeDIP)	Relative methylation (Cy-5/Cy-3)	Cy-3 signal (Input)	Cy-5 signal (MeDIP)
A_68_P28397153	chr12:45370604-45370657	NM_026164:490	Pnpla8	INSIDE	0.157	0.609	1835.36	1116.95	0.095	1290.54	123.05
A_68_P22825899	chr4:33334854-33334898	NM_001033225:886	Pnrc1	INSIDE	0.234	0.334	2113.96	707.02	0.078	1737.46	136.20
A_68_P27385035	chr10:98569435-98569486	NM_027740:-344	Poc1b	PROMOTER	0.320	0.651	1178.64	767.44	0.208	885.98	184.52
A_68_P24624715	chr6:88824256-88824300	NM_176973:-240	Podxl2	PROMOTER	0.253	1.524	3710.00	5653.87	0.385	2664.39	1026.48
A_68_P22340913	chr3:94641242-94641288	NM_001165948:-224	Pogz	PROMOTER	0.140	0.694	2141.72	1485.92	0.097	1462.10	141.58
A_68_P32535129	chrX:90877426-90877470	NM_008892:46	Pola1	INSIDE	0.197	1.708	1785.58	3049.75	0.337	646.78	218.00
A_68_P25088092	chr7:51803991-51804035	NM_011131:173	Pold1	INSIDE	0.159	0.697	2838.95	1977.55	0.111	1874.27	207.66
A_68_P30386866	chr15:82979570-82979614	NM_178627:174	Poldip3	INSIDE	0.524	0.447	1219.72	545.34	0.234	986.78	231.29
A_68_P22964956	chr4:62185874-62185918	NM_021498:152	Pole3	INSIDE	0.071	1.756	4791.00	8414.54	0.125	3104.51	389.18
A_68_P25261269	chr7:86610660-86610704	NM_017462:477	Polg	INSIDE	0.526	0.352	2361.12	829.94	0.185	1736.33	321.23
A_68_P31205623	chr17:46339517-46339561	NM_030715:36	Polh	INSIDE	0.629	0.670	2337.58	1565.75	0.421	1891.15	797.00
A_68_P23592737	chr5:34512192-34512236	NM_181857:-117	Poln	PROMOTER	0.103	0.682	7047.68	4804.10	0.070	4710.00	330.17
A_68_P24538901	chr6:71859383-71859431	NM_009088:360	Polr1a	INSIDE	0.276	0.687	1229.99	844.63	0.189	871.66	165.02
A_68_P21623005	chr2:128926868-128926912	NM_009086:159	Polr1b	INSIDE	0.540	0.619	1011.25	626.33	0.334	806.44	269.47
A_68_P22880641	chr4:45031688-45031732	NM_022811:214	Polr1e	INSIDE	0.185	0.492	1716.64	844.91	0.091	1324.33	120.69
A_68_P22880642	chr4:45031769-45031813	NM_022811:294	Polr1e	INSIDE	0.217	0.562	1262.24	709.54	0.122	958.42	116.91
A_68_P23820897	chr5:77739417-77739462	NM_153798:-69	Polr2b	DIVERGENT_PROMOTER	0.243	0.520	1813.02	943.61	0.127	1398.18	177.01
A_68_P23820898	chr5:77739527-77739571	NM_153798:40	Polr2b	INSIDE	0.314	0.429	1266.53	542.83	0.135	898.13	120.83
A_68_P23820901	chr5:77739843-77739887	NM_153798:356	Polr2b	INSIDE	0.397	0.647	1612.02	1043.01	0.257	1284.16	330.01
A_68_P26079860	chr8:97381962-97382011	NM_009090:637	Polr2c	INSIDE	0.266	0.467	1201.30	560.87	0.124	927.62	115.20
A_68_P25030468	chr7:31017900-31017944	NM_027259:830	Polr2i	INSIDE	0.340	0.513	1782.34	914.06	0.174	1180.51	205.75
A_68_P25589079	chr7:148660855-148660899	NM_025593:176	Polr2l	INSIDE	0.326	1.338	11219.53	15007.40	0.436	8052.58	3511.85
A_68_P22350868	chr3:96531418-96531462	NM_028925:-78	Polr3c	DIVERGENT_PROMOTER	0.525	0.567	1474.70	835.95	0.297	1356.25	403.34
A_68_P29701296	chr14:70843268-70843313	NM_025945:-12	Polr3d	DIVERGENT_PROMOTER	0.407	0.631	2190.63	1383.25	0.257	1603.41	411.96
A_68_P21707654	chr2:144353449-144353493	NM_029763:-10	Polr3f	DIVERGENT_PROMOTER	0.515	0.383	3800.11	1457.21	0.197	3178.40	627.48
A_68_P22350096	chr3:96397949-96397993	NM_027241:111	Polr3gl	INSIDE	0.526	0.538	2987.97	1608.27	0.283	1981.79	561.16
A_68_P30380501	chr15:81756859-81756907	NM_030229:-239	Polr3h	PROMOTER	0.300	0.498	1063.02	529.56	0.150	797.95	119.37
A_68_P21913217	chr2:181598855-181598899	NM_025901:-188	Polr3k	PROMOTER	0.366	0.499	1025.36	511.73	0.183	723.84	132.32
A_68_P21913219	chr2:181599054-181599098	NM_025901:12	Polr3k	INSIDE	0.179	0.607	1461.74	887.60	0.109	1166.18	126.95
A_68_P21913222	chr2:181599335-181599379	NM_025901:292	Polr3k	INSIDE	0.183	0.588	1352.22	794.67	0.108	1083.96	116.61
A_68_P28197875	chr12:3960097-3960141	NM_008895:5168	Pomc	INSIDE	0.439	0.486	1905.28	926.48	0.213	1632.12	348.09
A_68_P28197876	chr12:3960172-3960216	NM_008895:5244	Pomc	INSIDE	0.215	0.617	1693.45	1044.97	0.132	1304.22	172.63
A_68_P28197877	chr12:3960273-3960317	NM_008895:5344	Pomc	INSIDE	0.453	0.503	2050.65	1031.25	0.228	1527.45	347.64
A_68_P24176163	chr5:148672587-148672634	NM_025624:407	Pomp	INSIDE	0.146	0.529	2026.06	1071.92	0.077	1571.59	121.26
A_68_P24176178	chr5:148674084-148674130	NM_025624:1903	Pomp	INSIDE	0.650	2.514	645.38	1622.69	1.635	512.61	838.38
A_68_P21114132	chr2:32091883-32091928	NM_145145:-297	Pomt1	PROMOTER	0.165	0.584	1824.95	1065.34	0.096	1301.02	125.19
A_68_P28614436	chr12:88488867-88488911	NM_153415:-36	Pomt2	PROMOTER	0.278	0.508	1893.67	961.70	0.141	1559.62	220.35
A_68_P24008582	chr5:115688344-115688391	NM_026398:2508	Pop5	INSIDE	0.217	0.659	1079.88	711.64	0.143	833.10	118.98
A_68_P24126055	chr5:137943429-137943477	NM_028753:205	Pop7	INSIDE	0.424	0.637	970.48	618.59	0.270	965.90	261.13
A_68_P24126056	chr5:137943558-137943602	NM_028753:77	Pop7	INSIDE	0.454	0.587	983.88	578.02	0.267	845.07	225.53
A_68_P24117359	chr5:136164594-136164638	NM_008898:-467	Por	PROMOTER	0.455	0.638	1615.63	1031.25	0.291	1284.71	373.31
A_68_P24117365	chr5:136165147-136165191	NM_008898:85	Por	INSIDE	0.332	0.379	3793.96	1439.74	0.126	2873.72	362.20
A_68_P32242263	chrX:7783783-7783827	NM_016913:-153	Porcn	PROMOTER	0.111	1.546	2695.29	4165.80	0.172	1037.07	178.63
A_68_P31252241	chr17:55851663-55851707	NM_028370:242	Pot1b	INSIDE	0.078	0.678	4114.54	2791.39	0.053	2979.72	157.39

ProbeName	Target position of probe on CpG island microarray	TargetID	GeneSymbol	CpG island Description	Ratio of relative methylation (TiO2-NP/Vehicle)	Sham group			TiO_2-H group		
						Relative methylation (Cy-5/Cy-3)	Cy-3 signal (Input)	Cy-5 signal (MeDIP)	Relative methylation (Cy-5/Cy-3)	Cy-3 signal (Input)	Cy-5 signal (MeDIP)
A_68_P31252242	chr17:55851800-55851847	NM_028370:103	Pot1b	INSIDE	0.280	0.616	2320.37	1429.39	0.173	1799.85	310.91
A_68_P31252243	chr17:55851934-55851978	NM_028370:-30	Pot1b	PROMOTER	0.218	0.671	919.36	616.51	0.146	846.60	123.75
A_68_P20797862	chr1:167933482-167933526	NM_011137:-739	Pou2f1	PROMOTER	0.287	0.503	2291.28	1152.49	0.144	1791.86	258.47
A_68_P23269520	chr4:124335988-124336032	NM_011141:1122	Pou3f1	INSIDE	0.263	0.410	1436.85	589.49	0.108	1120.14	120.65
A_68_P23269524	chr4:124336535-124336579	NM_011141:1668	Pou3f1	INSIDE	0.515	0.447	1919.96	858.72	0.230	1485.26	341.83
A_68_P22778417	chr4:22404692-22404736	NM_008899:10564	Pou3f2	DOWNSTREAM	0.264	0.510	1777.13	906.10	0.135	1488.14	200.52
A_68_P22778505	chr4:22414595-22414639	NM_008899:662	Pou3f2	INSIDE	0.157	1.647	4813.48	7929.94	0.259	3343.69	864.41
A_68_P22778510	chr4:22415241-22415285	NM_008899:16	Pou3f2	INSIDE	0.582	0.557	1936.78	1078.50	0.324	1608.27	521.51
A_68_P22778520	chr4:22416842-22416887	NM_008899:-1586	Pou3f2	PROMOTER	0.444	0.539	1130.02	609.17	0.240	929.50	222.69
A_68_P22778523	chr4:22417085-22417134	NM_008899:-1831	Pou3f2	PROMOTER	0.167	0.506	1795.29	908.22	0.084	1394.05	117.56
A_68_P22778525	chr4:22417373-22417424	NM_008899:-2120	Pou3f2	PROMOTER	0.210	0.693	1839.83	1275.14	0.145	1322.69	192.24
A_68_P20184671	chr1:42754188-42754232	NM_008900:220	Pou3f3	INSIDE	0.480	0.646	959.00	619.22	0.310	929.95	288.27
A_68_P20184674	chr1:42754743-42754787	NM_008900:774	Pou3f3	INSIDE	0.613	0.631	1701.20	1073.53	0.387	1494.22	578.41
A_68_P20184676	chr1:42754950-42754994	NM_008900:982	Pou3f3	INSIDE	0.384	0.530	1020.23	540.91	0.204	815.91	166.23
A_68_P20184679	chr1:42755285-42755329	NM_008900:1316	Pou3f3	INSIDE	0.259	0.342	1670.37	572.10	0.089	1297.84	115.19
A_68_P32598389	chrX:108010047-108010091	NM_008901:265	Pou3f4	INSIDE	0.044	5.620	6802.72	38228.22	0.246	2678.51	659.05
A_68_P32598390	chrX:108010197-108010241	NM_008901:415	Pou3f4	INSIDE	0.207	1.766	1988.11	3511.50	0.366	770.87	282.44
A_68_P25988740	chr8:80959381-80959425	NM_138944:1149	Pou4f2	INSIDE	0.099	2.163	15337.40	33180.84	0.215	9247.74	1985.87
A_68_P25988742	chr8:80959567-80959624	NM_138944:956	Pou4f2	INSIDE	0.198	0.557	1502.78	837.75	0.111	1094.76	121.00
A_68_P31655262	chr18:42553965-42554009	NM_138945:-264	Pou4f3	PROMOTER	0.536	0.470	2878.95	1352.84	0.252	2437.99	614.19
A_68_P31655264	chr18:42554220-42554264	NM_138945:-8	Pou4f3	PROMOTER	0.248	0.516	2157.75	1113.05	0.128	1627.84	208.28
A_68_P31655265	chr18:42554339-42554383	NM_138945:110	Pou4f3	INSIDE	0.306	0.476	4057.89	1932.58	0.146	2789.92	406.78
A_68_P31655275	chr18:42555427-42555471	NM_138945:1198	Pou4f3	INSIDE	0.296	0.603	906.72	546.45	0.179	676.84	120.93
A_68_P29336879	chr13:113591191-113591235	NM_008903:82	Ppap2a	INSIDE	0.346	0.400	2048.00	818.63	0.138	1487.73	205.49
A_68_P23176982	chr4:104830286-104830330	NM_080555:357	Ppap2b	INSIDE	0.204	0.588	2653.85	1560.48	0.120	1893.15	226.67
A_68_P27277400	chr10:78996361-78996405	NM_015817:150	Ppap2c	INSIDE	0.383	1.507	11061.78	16667.18	0.577	7272.18	4196.60
A_68_P25514699	chr7:136400655-136400699	NM_001080963:69	Ppapdc1a	INSIDE	0.143	0.548	1757.12	963.73	0.079	1485.47	116.63
A_68_P25728675	chr8:26830785-26830829	NM_028000:287	Ppapdc1b	INSIDE	0.161	0.576	1848.70	1064.49	0.093	1341.83	124.14
A_68_P30402716	chr15:85566360-85566404	NM_011144:177	Ppara	INSIDE	0.448	0.641	1474.90	945.52	0.287	1247.55	358.42
A_68_P24770497	chr6:115311740-115311784	NM_001127330:524	Pparg	INSIDE	0.505	0.586	2380.61	1394.49	0.296	1937.70	573.60
A_68_P24770498	chr6:115311955-115311999	NM_001127330:738	Pparg	INSIDE	0.275	0.496	1107.89	549.64	0.137	905.46	123.74
A_68_P31757530	chr18:61558577-61558621	NM_133249:1487	Ppargc1b	INSIDE	0.106	0.699	3221.43	2251.85	0.074	2165.58	160.16
A_68_P31757537	chr18:61559490-61559534	NM_133249:573	Ppargc1b	INSIDE	0.341	0.406	1984.91	805.32	0.138	1502.22	207.54
A_68_P21909033	chr2:180922121-180922165	NM_025598:95	Ppdpf	INSIDE	0.215	0.683	2411.20	1648.05	0.147	1744.28	256.12
A_68_P21909038	chr2:180922669-180922713	NM_025598:643	Ppdpf	INSIDE	0.357	0.441	1594.20	703.42	0.158	1328.23	209.25
A_68_P25607698	chr7:151739256-151739302	NM_001033319:356	Ppfia1	INSIDE	0.129	0.554	2015.67	1117.52	0.071	1612.47	115.05
A_68_P25092648	chr7:52616935-52616979	NM_029741:5433	Ppfia3	INSIDE	0.647	0.546	1744.66	952.09	0.353	1343.74	474.37
A_68_P20632576	chr1:136195378-136195427	NM_001144855:34103	Ppfia4	INSIDE	0.332	1.667	1077.95	1797.29	0.554	866.36	479.93
A_68_P20632578	chr1:136195654-136195698	NM_001144855:33829	Ppfia4	INSIDE	0.396	0.708	1305.46	924.84	0.281	1092.82	306.60
A_68_P20632580	chr1:136195843-136195887	NM_001144855:33641	Ppfia4	INSIDE	0.612	0.668	1158.23	773.15	0.409	951.89	388.86
A_68_P20632581	chr1:136195963-136196007	NM_001144855:33521	Ppfia4	INSIDE	0.530	0.643	2492.75	1602.60	0.341	1852.97	631.25
A_68_P24931167	chr6:146837167-146837211	NM_001170433:173	Ppfibp1	INSIDE	0.655	0.692	2211.77	1530.32	0.453	1846.75	836.77
A_68_P27568393	chr11:6315493-6315540	NM_008907:-356	Ppia	PROMOTER	0.168	0.679	1431.78	972.87	0.114	1044.41	119.53
A_68_P27568396	chr11:6315839-6315883	NM_008907:-12	Ppia	PROMOTER	0.416	0.551	1712.42	943.39	0.229	1413.81	323.65

ProbeName	Target position of probe on CpG island microarray	TargetID	GeneSymbol	CpG island Description	Ratio of relative methylation (TiO2-NP/Vehicle)	Sham group			TiO_2-H group		
						Relative methylation (Cy-5/Cy-3)	Cy-3 signal (Input)	Cy-5 signal (MeDIP)	Relative methylation (Cy-5/Cy-3)	Cy-3 signal (Input)	Cy-5 signal (MeDIP)
A_68_P29479230	chr14:26513644-26513688	NM_134084:11	Ppif	INSIDE	0.195	0.482	1816.19	875.31	0.094	1342.43	126.31
A_68_P31126966	chr17:29400812-29400856	NM_026845:82	Ppil1	INSIDE	0.456	0.638	856.35	546.57	0.291	691.51	201.38
A_68_P20258949	chr1:58502448-58502495	NM_027351:-141	Ppil3	DIVERGENT_PROMOTER	0.290	0.508	1199.71	609.82	0.147	786.45	115.84
A_68_P26920793	chr10:7512764-7512808	NM_026141:95	Ppil4	INSIDE	0.537	0.520	1395.72	725.17	0.279	1023.24	285.45
A_68_P20472798	chr1:99666127-99666173	NM_173760:519	Ppip5k2	INSIDE	0.269	0.477	1076.17	513.79	0.128	923.54	118.59
A_68_P20472799	chr1:99666240-99666284	NM_173760:407	Ppip5k2	INSIDE	0.123	0.628	2042.56	1281.87	0.077	1514.72	117.17
A_68_P30513991	chr16:5132312-5132356	NM_008909:240	Ppl	INSIDE	0.622	0.653	5131.29	3349.38	0.406	3921.83	1592.24
A_68_P30513992	chr16:5132390-5132436	NM_008909:162	Ppl	INSIDE	0.282	0.468	1156.38	540.83	0.132	902.55	119.09
A_68_P27982131	chr11:85124251-85124295	NM_016910:-483	Ppm1d	PROMOTER	0.394	0.440	1279.88	563.48	0.173	1059.51	183.63
A_68_P27982135	chr11:85124644-85124688	NM_016910:-89	Ppm1d	PROMOTER	0.232	0.470	1328.03	624.62	0.109	1120.50	122.48
A_68_P27982140	chr11:85125306-85125350	NM_016910:573	Ppm1d	INSIDE	0.089	0.734	2455.57	1802.06	0.066	1804.02	118.41
A_68_P27993894	chr11:87171685-87171729	NM_177167:790	Ppm1e	INSIDE	0.329	0.528	3696.59	1953.44	0.174	2917.15	507.29
A_68_P23577162	chr5:31523190-31523234	NM_008014:-294	Ppm1g	PROMOTER	0.273	0.517	1770.88	914.90	0.141	1231.46	173.56
A_68_P27509905	chr10:122115624-122115668	NM_176919:-171	Ppm1h	PROMOTER	0.335	0.507	1496.26	758.69	0.170	1244.74	211.29
A_68_P27509909	chr10:122116007-122116051	NM_176919:211	Ppm1h	INSIDE	0.615	0.681	1353.95	922.59	0.419	1184.77	496.89
A_68_P22393105	chr3:104583807-104583851	NM_027982:-145	Ppm1j	DIVERGENT_PROMOTER	0.293	0.463	1284.55	594.17	0.135	951.62	128.90
A_68_P22393108	chr3:104584185-104584229	NM_027982:233	Ppm1j	INSIDE	0.489	0.626	2432.93	1522.23	0.306	1881.45	575.45
A_68_P22393113	chr3:104584603-104584647	NM_027982:651	Ppm1j	INSIDE	0.630	0.655	1650.21	1080.34	0.413	1328.57	548.04
A_68_P22220260	chr3:69120137-69120181	NM_178726:-681	Ppm1l	PROMOTER	0.658	0.580	1565.18	907.78	0.382	1400.32	534.54
A_68_P22220267	chr3:69120847-69120891	NM_178726:29	Ppm1l	INSIDE	0.434	0.545	2515.36	1370.78	0.236	1904.22	450.07
A_68_P22220270	chr3:69121308-69121352	NM_178726:491	Ppm1l	INSIDE	0.443	0.526	1482.27	779.49	0.233	1208.68	281.64
A_68_P24992435	chr7:19864459-19864503	NM_177691:918	Ppm1n	INSIDE	0.240	0.634	2121.13	1345.56	0.152	1615.66	246.37
A_68_P25367283	chr7:107520379-107520423	NM_028292:6	Ppme1	INSIDE	0.192	0.673	3551.19	2389.95	0.129	2503.89	323.89
A_68_P31921665	chr19:4191850-4191903	NM_031868:-297	Ppp1ca	DIVERGENT_PROMOTER	0.384	0.618	906.79	560.73	0.238	670.71	159.33
A_68_P31921667	chr19:4192216-4192260	NM_031868:65	Ppp1ca	INSIDE	0.409	0.625	2319.46	1450.06	0.256	1886.44	482.61
A_68_P31921669	chr19:4192588-4192632	NM_031868:437	Ppp1ca	INSIDE	0.248	0.333	1914.53	638.15	0.083	1396.65	115.60
A_68_P24048830	chr5:122607910-122607954	NM_013636:-355	Ppp1cc	PROMOTER	0.242	0.542	2194.06	1189.79	0.131	1630.19	214.19
A_68_P31161701	chr17:36053710-36053754	NM_175934:-123	Ppp1r10	DIVERGENT_PROMOTER	0.353	0.690	1629.09	1124.65	0.244	1295.11	315.80
A_68_P31161702	chr17:36053831-36053875	NM_175934:-3	Ppp1r10	DIVERGENT_PROMOTER	0.569	0.672	3400.04	2283.76	0.382	2415.44	923.69
A_68_P27430001	chr10:107598764-107598808	NM_027892:-669	Ppp1r12a	PROMOTER	0.318	0.448	3455.67	1549.30	0.143	2476.59	353.05
A_68_P27430005	chr10:107599244-107599288	NM_027892:-189	Ppp1r12a	PROMOTER	0.345	0.445	1358.46	604.42	0.153	1248.57	191.50
A_68_P31937055	chr19:7048934-7048978	NM_008889:-581	Ppp1r14b	DIVERGENT_PROMOTER	0.141	0.712	1970.00	1401.84	0.100	1417.99	142.16
A_68_P31937064	chr19:7050018-7050063	NM_008889:503	Ppp1r14b	INSIDE	0.244	0.593	1205.82	714.66	0.144	853.58	123.19
A_68_P31937065	chr19:7050107-7050153	NM_008889:593	Ppp1r14b	INSIDE	0.206	0.620	1412.19	875.28	0.128	955.26	121.88
A_68_P26918731	chr10:6980219-6980263	NM_133485:151	Ppp1r14c	INSIDE	0.194	0.696	1819.25	1266.44	0.135	1409.70	190.15
A_68_P25093636	chr7:52781291-52781335	NM_008654:326	Ppp1r15a	INSIDE	0.494	0.592	1683.46	996.36	0.292	1416.98	414.25
A_68_P25093637	chr7:52781402-52781446	NM_008654:214	Ppp1r15a	INSIDE	0.301	0.543	1675.58	909.14	0.163	1291.61	210.70
A_68_P30349852	chr15:76501941-76501985	NM_033371:-147	Ppp1r16a	DIVERGENT_PROMOTER	0.252	0.572	917.75	525.14	0.144	815.00	117.53
A_68_P21784240	chr2:158492976-158493020	NM_001159662:128	Ppp1r16b	INSIDE	0.521	0.498	1902.05	946.70	0.259	1560.31	404.36
A_68_P21784241	chr2:158493133-158493177	NM_001159662:284	Ppp1r16b	INSIDE	0.062	1.522	5115.00	7784.87	0.095	3433.40	324.83
A_68_P28055832	chr11:98209796-98209843	NM_144828:-232	Ppp1r1b	PROMOTER	0.220	1.502	2044.06	3070.04	0.330	1364.32	449.94
A_68_P28055835	chr11:98210174-98210218	NM_144828:145	Ppp1r1b	INSIDE	0.415	0.626	3132.75	1960.48	0.260	2366.43	614.59
A_68_P28055837	chr11:98210403-98210447	NM_144828:373	Ppp1r1b	INSIDE	0.291	0.565	4091.51	2311.88	0.164	2917.39	479.12
A_68_P28055838	chr11:98210489-98210533	NM_144828:459	Ppp1r1b	INSIDE	0.614	0.536	3461.83	1855.57	0.329	2814.69	926.37

ProbeName	Target position of probe on CpG island microarray	TargetID	GeneSymbol	CpG island Description	Ratio of relative methylation (TiO2-NP/Vehicle)	Sham group			TiO_2-H group		
						Relative methylation (Cy-5/Cy-3)	Cy-3 signal (Input)	Cy-5 signal (MeDIP)	Relative methylation (Cy-5/Cy-3)	Cy-3 signal (Input)	Cy-5 signal (MeDIP)
A_68_P32238975	chrX:7151016-7151060	NM_138605:208	Ppp1r3f	INSIDE	0.222	2.207	5680.18	12533.80	0.491	2241.81	1100.11
A_68_P32238979	chrX:7151677-7151727	NM_138605:-456	Ppp1r3f	DIVERGENT_PROMOTER	0.098	1.949	2287.73	4458.78	0.191	834.31	159.24
A_68_P28946217	chr13:36061405-36061450	NM_029628:1653	Ppp1r3g	INSIDE	0.417	0.388	1422.88	552.68	0.162	1181.97	191.50
A_68_P28035915	chr11:94852120-94852164	NM_172261:-383	Ppp1r9b	PROMOTER	0.259	0.656	2162.62	1419.34	0.170	1618.29	275.48
A_68_P31084397	chr17:21082131-21082175	NM_016891:-265	Ppp2r1a	PROMOTER	0.244	0.565	1103.92	623.25	0.138	871.77	120.28
A_68_P31084400	chr17:21082518-21082562	NM_016891:123	Ppp2r1a	INSIDE	0.251	0.523	1275.90	667.73	0.131	953.66	125.31
A_68_P29682921	chr14:67691110-67691154	NM_001205188:176	Ppp2r2a	INSIDE	0.305	0.434	5722.88	2483.43	0.132	4602.45	608.34
A_68_P23610087	chr5:37260107-37260152	NM_172994:321	Ppp2r2c	INSIDE	0.273	0.501	1025.47	514.19	0.137	886.49	121.48
A_68_P23610088	chr5:37260189-37260233	NM_172994:402	Ppp2r2c	INSIDE	0.495	0.675	1768.04	1193.35	0.334	1317.65	440.50
A_68_P23610099	chr5:37261384-37261428	NM_172994:1598	Ppp2r2c	INSIDE	0.339	0.521	2486.87	1296.63	0.177	1877.70	331.55
A_68_P25574424	chr7:146038574-146038618	NM_026391:528	Ppp2r2d	INSIDE	0.287	0.623	1761.29	1097.13	0.179	1484.25	265.10
A_68_P33012162	chr9_random:55248-55292	NM_011154:-238	Ppp2r3d	PROMOTER	0.409	0.688	1410.70	970.21	0.282	1027.25	289.29
A_68_P33012166	chr9_random:55669-55721	NM_011154:-662	Ppp2r3d	PROMOTER	0.120	1.474	3323.84	4900.44	0.177	2200.93	388.78
A_68_P21103105	chr2:30272089-30272133	NM_138748:541	Ppp2r4	INSIDE	0.406	0.636	3968.58	2525.95	0.258	2830.49	731.40
A_68_P20937890	chr1:193221506-193221550	NM_144880:-608	Ppp2r5a	PROMOTER	0.088	0.739	2514.81	1859.29	0.065	1834.82	119.50
A_68_P28738223	chr12:111685895-111685939	NM_001135001:528	Ppp2r5c	INSIDE	0.453	0.484	1922.49	930.95	0.219	1473.75	323.19
A_68_P28549414	chr12:76696397-76696441	NM_012024:769	Ppp2r5e	INSIDE	0.586	0.546	1307.02	713.63	0.320	1038.60	332.58
A_68_P28549419	chr12:76697049-76697093	NM_012024:117	Ppp2r5e	INSIDE	0.481	0.664	5537.38	3677.83	0.319	3978.85	1271.15
A_68_P28549421	chr12:76697259-76697303	NM_012024:-93	Ppp2r5e	PROMOTER	0.536	0.576	3414.71	1966.81	0.309	2422.58	748.13
A_68_P28549422	chr12:76697372-76697416	NM_012024:-207	Ppp2r5e	PROMOTER	0.669	0.614	4111.37	2525.91	0.411	3356.09	1379.87
A_68_P22558734	chr3:136332458-136332502	NM_008913:-1253	Ppp3ca	PROMOTER	0.270	0.434	2000.90	867.72	0.117	1569.79	184.00
A_68_P22558738	chr3:136332932-136332976	NM_008913:-779	Ppp3ca	PROMOTER	0.638	0.400	1495.87	597.87	0.255	1261.42	321.47
A_68_P22558741	chr3:136333499-136333554	NM_008913:-207	Ppp3ca	PROMOTER	0.459	3.774	706.40	2666.24	1.734	568.58	985.80
A_68_P22558744	chr3:136333820-136333864	NM_008913:109	Ppp3ca	INSIDE	0.323	0.507	1318.47	668.10	0.164	975.43	159.85
A_68_P22558749	chr3:136334580-136334624	NM_008913:869	Ppp3ca	INSIDE	0.287	0.530	2584.21	1369.02	0.152	2083.54	316.64
A_68_P25502251	chr7:133935372-133935418	NM_019674:591	Ppp4c	INSIDE	0.237	0.573	1612.56	924.09	0.136	1215.37	164.85
A_68_P21870533	chr2:173411101-173411145	NR_027957:73918	Ppp4r1l-ps	INSIDE	0.271	0.517	1178.09	608.66	0.140	889.54	124.38
A_68_P28698040	chr12:104770943-104770987	NM_028980:190	Ppp4r4	INSIDE	0.515	0.546	2406.01	1314.29	0.281	1969.38	553.65
A_68_P31918147	chr19:3575682-3575726	NM_001164159:45	Ppp6r3	INSIDE	0.294	0.652	4055.20	2645.44	0.192	2867.08	549.30
A_68_P30380961	chr15:81846636-81846680	NM_134095:-88	Pppde2	DIVERGENT_PROMOTER	0.052	0.697	7707.50	5372.11	0.036	5343.73	194.99
A_68_P32141864	chr19:46131303-46131347	NM_001081214:296	Pprc1	INSIDE	0.379	0.651	3419.68	2226.53	0.247	2531.00	624.54
A_68_P32141867	chr19:46131714-46131758	NM_001081214:708	Pprc1	INSIDE	0.292	0.549	3380.16	1854.29	0.160	2755.75	441.96
A_68_P24049493	chr5:122735201-122735245	NM_177242:816	Pptc7	INSIDE	0.269	0.579	3182.05	1842.25	0.156	2339.06	364.23
A_68_P31863202	chr18:80453578-80453622	NM_001164423:556	Pqlc1	INSIDE	0.246	0.576	1681.61	968.09	0.142	1349.25	191.28
A_68_P23351037	chr4:138866350-138866394	NM_145384:243	Pqlc2	INSIDE	0.438	0.452	1914.53	864.68	0.198	1516.22	299.80
A_68_P23351038	chr4:138866454-138866498	NM_145384:139	Pqlc2	INSIDE	0.387	0.613	1241.01	761.20	0.237	955.93	226.87
A_68_P25265835	chr7:87439504-87439548	NM_145150:176	Prc1	INSIDE	0.297	0.555	1443.64	800.97	0.165	1176.43	193.63
A_68_P21432195	chr2:92885557-92885608	NM_001177536:719	Prdm11	INSIDE	0.442	0.339	1532.01	519.27	0.150	1208.33	180.83
A_68_P21110347	chr2:31491119-31491163	NM_001123362:-4416	Prdm12	PROMOTER	0.289	0.605	2072.75	1254.06	0.175	1628.91	285.24
A_68_P21110351	chr2:31491732-31491776	NM_001123362:-3802	Prdm12	PROMOTER	0.464	0.703	1657.37	1165.20	0.326	1360.18	443.40
A_68_P21110355	chr2:31492078-31492122	NM_001123362:-3456	Prdm12	PROMOTER	0.192	0.488	1523.58	743.79	0.094	1243.51	116.81
A_68_P21110357	chr2:31492430-31492474	NM_001123362:-3104	Prdm12	PROMOTER	0.167	0.396	2719.63	1075.85	0.066	1892.23	125.35
A_68_P21110358	chr2:31492494-31492545	NM_001123362:-3037	Prdm12	PROMOTER	0.219	0.655	1499.15	981.63	0.143	1196.84	171.26
A_68_P21110359	chr2:31492659-31492703	NM_001123362:-2876	Prdm12	PROMOTER	0.210	0.527	1536.65	809.63	0.110	1149.29	126.98

ProbeName	Target position of probe on CpG island microarray	TargetID	GeneSymbol	CpG island Description	Ratio of relative methylation (TiO2-NP/Vehicle)	Sham group			TiO_2-H group		
						Relative methylation (Cy-5/Cy-3)	Cy-3 signal (Input)	Cy-5 signal (MeDIP)	Relative methylation (Cy-5/Cy-3)	Cy-3 signal (Input)	Cy-5 signal (MeDIP)
A_68_P21110387	chr2:31495883-31495927	NM_001123362:348	Prdm12	INSIDE	0.623	0.525	1190.86	625.01	0.327	996.20	325.67
A_68_P21110488	chr2:31509792-31509836	NM_001123362:14258	Prdm12	INSIDE	0.310	0.686	2682.89	1840.93	0.213	2519.68	535.54
A_68_P22774390	chr4:21616242-21616286	NM_001080771:-3154	Prdm13	PROMOTER	0.555	0.433	1791.33	775.75	0.240	1502.19	361.15
A_68_P20039230	chr1:13116543-13116587	NM_001081209:680	Prdm14	INSIDE	0.402	0.572	3613.44	2067.32	0.230	2620.44	603.08
A_68_P23433031	chr4:154008979-154009030	NM_001177995:1978	Prdm16	INSIDE	0.222	0.463	1534.85	710.42	0.103	1160.07	119.40
A_68_P23433034	chr4:154009287-154009331	NM_001177995:1674	Prdm16	INSIDE	0.504	0.539	2032.43	1095.75	0.271	1647.95	447.39
A_68_P23433035	chr4:154009415-154009459	NM_001177995:1546	Prdm16	INSIDE	0.218	0.516	1439.28	742.62	0.112	1070.32	120.25
A_68_P23433043	chr4:154010163-154010214	NM_001177995:794	Prdm16	INSIDE	0.066	0.736	3679.81	2707.03	0.048	2470.24	119.77
A_68_P23373719	chr4:142751586-142751630	NM_001081355:51004	Prdm2	INSIDE	0.414	0.600	2265.89	1359.50	0.248	1795.27	445.77
A_68_P23374046	chr4:142802052-142802101	NM_001081355:536	Prdm2	INSIDE	0.356	0.623	1062.06	661.45	0.222	812.48	180.01
A_68_P23374047	chr4:142802126-142802170	NM_001081355:464	Prdm2	INSIDE	0.463	0.452	2303.49	1040.16	0.209	1772.34	370.54
A_68_P23374051	chr4:142802693-142802737	NM_001081355:-102	Prdm2	PROMOTER	0.596	0.531	1841.54	977.22	0.316	1580.36	499.43
A_68_P27311090	chr10:85379118-85379174	NM_181650:544	Prdm4	INSIDE	0.234	0.624	1012.52	631.82	0.146	816.68	119.27
A_68_P27311093	chr10:85379465-85379509	NM_181650:204	Prdm4	INSIDE	0.364	0.420	1790.11	751.12	0.153	1539.97	235.30
A_68_P31713373	chr18:53622483-53622527	NM_001033281:-1695	Prdm6	PROMOTER	0.381	0.623	1481.10	922.66	0.237	1180.35	279.88
A_68_P31713378	chr18:53623569-53623613	NM_001033281:-609	Prdm6	PROMOTER	0.141	0.720	1801.75	1296.47	0.101	1308.97	132.57
A_68_P31713386	chr18:53624258-53624302	NM_001033281:81	Prdm6	INSIDE	0.564	0.373	2439.14	909.74	0.210	1964.38	412.96
A_68_P23916638	chr5:98614079-98614123	NM_029947:4213	Prdm8	INSIDE	0.423	0.666	938.20	624.44	0.282	770.31	217.06
A_68_P23916648	chr5:98615578-98615622	NM_029947:5713	Prdm8	INSIDE	0.115	1.436	3010.13	4323.03	0.165	2076.03	343.14
A_68_P23916649	chr5:98615733-98615777	NM_029947:5867	Prdm8	INSIDE	0.139	1.483	5168.37	7663.67	0.206	3478.63	718.01
A_68_P31066337	chr17:15701209-15701253	NM_144809:-943	Prdm9	PROMOTER	0.386	0.448	1781.88	799.01	0.173	1543.38	266.84
A_68_P31066339	chr17:15701385-15701429	NM_144809:-1119	Prdm9	PROMOTER	0.178	0.554	1517.87	840.84	0.099	1188.33	117.49
A_68_P23230433	chr4:116358143-116358188	NM_011034:-38	Prdx1	PROMOTER	0.215	0.514	1789.23	919.01	0.110	1275.88	140.81
A_68_P26022893	chr8:87493563-87493607	NM_011563:38	Prdx2	INSIDE	0.132	0.692	1815.95	1256.13	0.091	1297.37	118.31
A_68_P31936609	chr19:6983898-6983942	NM_012021:215	Prdx5	INSIDE	0.610	0.577	1095.19	631.95	0.352	840.94	295.98
A_68_P31936610	chr19:6984035-6984079	NM_012021:79	Prdx5	INSIDE	0.258	0.530	1234.11	654.53	0.137	872.43	119.46
A_68_P23575728	chr5:31262393-31262437	NM_016703:320	Preb	INSIDE	0.252	0.403	2398.80	967.07	0.102	1849.15	187.96
A_68_P23575729	chr5:31262539-31262583	NM_016703:174	Preb	INSIDE	0.556	0.435	1957.97	852.60	0.242	1574.30	381.42
A_68_P27109366	chr10:44786725-44786769	NM_011156:-273	Prep	PROMOTER	0.469	0.630	1146.19	722.10	0.295	977.61	288.59
A_68_P27110003	chr10:44877999-44878045	NM_011156:91003	Prep	INSIDE	0.666	2.654	526.78	1397.88	1.768	385.35	681.48
A_68_P21831536	chr2:166539405-166539449	NM_177782:-94	Prex1	PROMOTER	0.294	2.047	14771.77	30234.45	0.602	9815.80	5904.42
A_68_P21831539	chr2:166539726-166539770	NM_177782:-416	Prex1	PROMOTER	0.568	0.452	1321.79	597.79	0.257	1027.42	263.70
A_68_P20029520	chr1:10983953-10983997	NM_029525:429	Prex2	INSIDE	0.562	0.439	1493.77	655.96	0.247	1230.28	303.83
A_68_P30447264	chr15:93331165-93331209	NM_001033217:95136	Prickle1	INSIDE	0.466	1.561	2699.48	4212.75	0.727	2023.69	1470.26
A_68_P30447393	chr15:93350085-93350134	NM_001033217:76213	Prickle1	INSIDE	0.304	0.582	1032.40	600.49	0.177	940.59	166.16
A_68_P20134288	chr1:33726517-33726564	NM_008922:63	Prim2	INSIDE	0.608	0.507	1021.62	518.28	0.308	697.23	215.01
A_68_P28696316	chr12:104480086-104480133	NM_133364:247	Prima1	INSIDE	0.119	1.830	4927.57	9019.29	0.217	3356.33	729.43
A_68_P29982188	chr15:5093945-5093989	NM_001013367:106	Prkaa1	INSIDE	0.516	0.276	2168.50	598.03	0.142	1691.22	240.81
A_68_P23176715	chr4:104782702-104782746	NM_178143:-221	Prkaa2	PROMOTER	0.554	0.429	2125.66	912.00	0.238	1625.92	386.73
A_68_P30476734	chr15:98661277-98661332	NM_016781:635	Prkag1	INSIDE	0.170	0.617	1471.75	907.61	0.105	1094.16	114.87
A_68_P30476735	chr15:98661367-98661411	NM_016781:551	Prkag1	INSIDE	0.492	0.550	1437.00	789.77	0.270	1160.60	313.62
A_68_P30476737	chr15:98661589-98661633	NM_016781:329	Prkag1	INSIDE	0.270	0.494	1404.42	693.69	0.134	975.81	130.30
A_68_P23545730	chr5:24605328-24605372	NM_145401:1110	Prkag2	INSIDE	0.169	1.411	1249.85	1763.50	0.238	925.14	220.40
A_68_P23545731	chr5:24605464-24605510	NM_145401:974	Prkag2	INSIDE	0.231	1.809	2890.53	5229.58	0.418	1905.40	795.52

ProbeName	Target position of probe on CpG island microarray	TargetID	GeneSymbol	CpG island Description	Ratio of relative methylation (TiO2-NP/Vehicle)	Sham group			TiO_2-H group		
						Relative methylation (Cy-5/Cy-3)	Cy-3 signal (Input)	Cy-5 signal (MeDIP)	Relative methylation (Cy-5/Cy-3)	Cy-3 signal (Input)	Cy-5 signal (MeDIP)
A_68_P23545735	chr5:24605832-24605876	NM_145401:606	Prkag2	INSIDE	0.193	0.495	1571.63	777.85	0.095	1228.51	117.24
A_68_P20350233	chr1:74795245-74795297	NM_153744:259	Prkag3	INSIDE	0.312	1.415	3788.79	5360.00	0.442	2741.39	1211.94
A_68_P28119819	chr11:109511596-109511640	NM_021880:-644	Prkar1a	PROMOTER	0.573	0.538	1105.98	594.70	0.308	836.18	257.86
A_68_P28119821	chr11:109511777-109511821	NM_021880:-464	Prkar1a	PROMOTER	0.189	0.633	1457.86	922.16	0.120	974.94	116.81
A_68_P28119824	chr11:109512145-109512189	NM_021880:-96	Prkar1a	PROMOTER	0.280	0.460	1170.83	538.40	0.129	901.30	116.04
A_68_P24133760	chr5:139605384-139605428	NM_008923:300	Prkar1b	INSIDE	0.136	0.574	2248.19	1289.39	0.078	1659.87	129.63
A_68_P26816178	chr9:108595255-108595302	NM_008924:805	Prkar2a	INSIDE	0.491	0.645	804.21	518.88	0.317	700.23	222.05
A_68_P28334786	chr12:32745694-32745738	NM_011158:428	Prkar2b	INSIDE	0.271	0.650	1100.48	715.17	0.176	804.41	141.51
A_68_P28112590	chr11:108204958-108205002	NM_011101:222	Prkca	INSIDE	0.547	0.716	1635.90	1170.74	0.391	1247.44	488.36
A_68_P29507007	chr14:31438823-31438867	NM_011103:550	Prkcd	INSIDE	0.629	2.446	6147.13	15036.60	1.538	3438.11	5286.19
A_68_P31415134	chr17:86566646-86566690	NM_011104:-456	Prkce	PROMOTER	0.254	0.623	1912.25	1190.80	0.158	1313.18	207.50
A_68_P31415139	chr17:86567438-86567482	NM_011104:336	Prkce	INSIDE	0.239	0.696	1353.34	941.55	0.167	1035.38	172.52
A_68_P31415145	chr17:86568034-86568078	NM_011104:932	Prkce	INSIDE	0.245	0.519	1143.65	593.22	0.127	926.35	117.61
A_68_P31415146	chr17:86568164-86568208	NM_011104:1062	Prkce	INSIDE	0.529	0.635	1508.75	957.50	0.336	1104.97	370.74
A_68_P22031194	chr3:30894990-30895034	NM_008857:320	Prkci	INSIDE	0.311	0.483	2784.80	1344.04	0.150	2328.63	348.98
A_68_P22031195	chr3:30895073-30895117	NM_008857:402	Prkci	INSIDE	0.246	0.701	4152.45	2909.57	0.172	3217.84	554.14
A_68_P21004674	chr2:11093675-11093725	NM_008859:-308	Prkcq	PROMOTER	0.192	0.533	1475.35	786.25	0.102	1162.51	118.87
A_68_P23438160	chr4:154735910-154735954	NM_008860:-432	Prkcz	PROMOTER	0.285	0.346	1560.54	540.03	0.099	1188.92	117.20
A_68_P28426163	chr12:51750413-51750457	NM_008858:-224	Prkd1	PROMOTER	0.435	0.452	1637.89	740.09	0.197	1377.70	270.95
A_68_P24983572	chr7:17428015-17428059	NM_178900:-377	Prkd2	PROMOTER	0.456	0.494	2352.31	1161.71	0.225	1719.73	387.17
A_68_P32066438	chr19:31838454-31838498	NM_001013833:1047	Prkg1	INSIDE	0.143	0.715	1475.93	1054.89	0.102	1131.58	115.42
A_68_P21351535	chr2:76485641-76485685	NM_011871:389	Prkra	INSIDE	0.247	0.492	1386.02	682.37	0.122	1100.87	134.11
A_68_P24119913	chr5:136674484-136674528	NM_025774:318	Prkrip1	INSIDE	0.205	0.674	2938.19	1979.41	0.138	2100.59	290.08
A_68_P24119914	chr5:136674559-136674603	NM_025774:244	Prkrip1	INSIDE	0.559	0.609	2183.08	1330.26	0.341	1509.25	514.25
A_68_P32479503	chrX:75040859-75040905	NM_016979:417	Prkx	INSIDE	0.088	1.916	1957.16	3750.13	0.169	710.61	120.44
A_68_P32479506	chrX:75041133-75041177	NM_016979:145	Prkx	INSIDE	0.232	1.361	2009.54	2735.05	0.316	788.05	249.02
A_68_P32225520	chr19:60543550-60543594	NM_201615:635	Prlhr	INSIDE	0.304	0.643	1273.50	819.03	0.196	1015.53	198.76
A_68_P25090466	chr7:52241494-52241538	NM_019830:186	Prmt1	INSIDE	0.550	0.582	2394.80	1392.61	0.320	1757.31	561.92
A_68_P25090472	chr7:52242228-52242277	NM_019830:-550	Prmt1	PROMOTER	0.258	0.440	1220.67	536.62	0.114	1032.71	117.24
A_68_P27260807	chr10:75700398-75700442	NM_001077638:190	Prmt2	INSIDE	0.083	0.601	3581.04	2152.35	0.050	2395.19	119.62
A_68_P27260808	chr10:75700481-75700530	NM_001077638:105	Prmt2	INSIDE	0.272	0.594	2200.79	1307.97	0.162	1495.66	242.19
A_68_P29612846	chr14:55136095-55136139	NM_013768:191	Prmt5	INSIDE	0.185	0.554	1520.75	842.86	0.102	1203.87	123.19
A_68_P26138376	chr8:108735052-108735096	NM_145404:121	Prmt7	INSIDE	0.423	0.530	1753.45	929.97	0.224	1499.64	336.49
A_68_P24834758	chr6:127717838-127717882	NM_201371:887	Prmt8	INSIDE	0.258	0.454	1249.15	567.32	0.117	1041.14	121.95
A_68_P24834763	chr6:127718286-127718330	NM_201371:439	Prmt8	INSIDE	0.293	0.528	1007.47	531.79	0.155	788.32	122.03
A_68_P24834768	chr6:127718939-127718983	NM_201371:-213	Prmt8	PROMOTER	0.628	0.541	1630.42	881.85	0.340	1227.04	416.85
A_68_P21637464	chr2:131735522-131735570	NM_011170:-117	Prnp	PROMOTER	0.388	0.723	1662.95	1202.84	0.280	1338.97	375.41
A_68_P21637466	chr2:131735690-131735734	NM_011170:49	Prnp	INSIDE	0.454	0.529	1511.50	800.01	0.240	1246.66	299.47
A_68_P30577452	chr16:18089136-18089180	NM_011172:125	Prodh	INSIDE	0.339	0.406	1428.07	579.57	0.138	1030.07	141.71
A_68_P24617030	chr6:87540705-87540749	NM_021381:-31	Prokr1	PROMOTER	0.456	0.625	3507.50	2192.63	0.285	2620.70	747.45
A_68_P21640034	chr2:132210975-132211019	NM_144944:187	Prokr2	INSIDE	0.347	0.605	1847.94	1118.73	0.210	1484.92	311.82
A_68_P23649331	chr5:44491778-44491823	NM_001163584:-5793	Prom1	PROMOTER	0.639	0.449	2239.47	1004.57	0.286	1784.79	511.32
A_68_P21630920	chr2:130468079-130468124	NM_197945:438	Prosapip1	INSIDE	0.332	1.646	1718.94	2829.56	0.546	1116.46	609.97
A_68_P25736232	chr8:28153444-28153488	NM_001039077:440	Prosc	INSIDE	0.580	0.637	1710.49	1089.82	0.370	1291.68	477.72

ProbeName	Target position of probe on CpG island microarray	TargetID	GeneSymbol	CpG island Description	Ratio of relative methylation (TiO2-NP/Vehicle)	Sham group			TiO_2-H group		
						Relative methylation (Cy-5/Cy-3)	Cy-3 signal (Input)	Cy-5 signal (MeDIP)	Relative methylation (Cy-5/Cy-3)	Cy-3 signal (Input)	Cy-5 signal (MeDIP)
A_68_P22346028	chr3:95659528-95659572	NM_027541:126	Prpf3	INSIDE	0.387	0.590	2572.21	1516.67	0.228	1976.81	451.55
A_68_P24946434	chr7:3581622-3581669	NM_001159714:59	Prpf31	INSIDE	0.284	0.510	1092.30	557.24	0.145	808.75	117.27
A_68_P23196701	chr4:108251917-108251961	NM_172697:3	Prpf38a	INSIDE	0.111	0.589	2768.15	1630.15	0.065	2036.75	133.26
A_68_P28940997	chr13:34966103-34966147	NM_013830:-1238	Prpf4b	PROMOTER	0.334	0.645	834.93	538.32	0.216	691.14	148.99
A_68_P28940998	chr13:34966207-34966251	NM_013830:-1134	Prpf4b	PROMOTER	0.295	0.510	3080.41	1569.79	0.150	2126.73	319.29
A_68_P28941006	chr13:34967366-34967410	NM_013830:26	Prpf4b	INSIDE	0.267	0.446	1303.47	581.96	0.119	1031.54	123.01
A_68_P21911590	chr2:181335967-181336011	NM_133701:-35	Prpf6	DIVERGENT_PROMOTER	0.487	0.665	1374.26	913.55	0.324	983.56	318.63
A_68_P30477985	chr15:98885628-98885672	NM_001163588:46	Prph	INSIDE	0.432	0.736	3231.96	2379.82	0.318	2337.44	743.16
A_68_P32699217	chrX:136991521-136991565	NM_021463:401	Prps1	INSIDE	0.091	1.930	2446.42	4721.17	0.176	954.07	168.25
A_68_P32699218	chrX:136991656-136991701	NM_021463:537	Prps1	INSIDE	0.132	1.782	2722.01	4849.49	0.235	1221.70	286.79
A_68_P32699219	chrX:136991758-136991802	NM_021463:639	Prps1	INSIDE	0.123	1.590	2272.75	3614.55	0.195	864.05	168.70
A_68_P28159660	chr11:116351347-116351391	NM_026364:292	Prpsap1	INSIDE	0.419	0.654	1739.53	1136.92	0.274	1497.75	409.90
A_68_P24462649	chr6:54277015-54277059	NM_030024:31	Prr15	INSIDE	0.474	0.460	1777.85	817.43	0.218	1426.55	311.18
A_68_P31701194	chr18:51277202-51277246	NM_001081224:-327	Prr16	PROMOTER	0.262	0.422	2592.04	1093.66	0.111	1981.48	219.32
A_68_P32957124	chr17:8533875-8533919	NM_178774:293	Prr18	INSIDE	0.178	0.444	1898.57	842.57	0.079	1453.11	114.99
A_68_P32957129	chr17:8534343-8534387	NM_178774:761	Prr18	INSIDE	0.518	0.626	1114.38	697.44	0.324	1032.46	334.91
A_68_P24980575	chr7:16858057-16858101	NM_001136270:963	Prr24	INSIDE	0.523	0.678	1582.48	1072.73	0.354	1159.31	410.80
A_68_P24980579	chr7:16858527-16858571	NM_001136270:493	Prr24	INSIDE	0.267	0.639	11345.83	7252.67	0.171	7850.37	1341.64
A_68_P24980582	chr7:16858850-16858896	NM_001136270:169	Prr24	INSIDE	0.526	3.083	7046.42	21723.01	1.620	4740.82	7681.36
A_68_P31162035	chr17:36116527-36116571	NM_145487:-136	Prr3	PROMOTER	0.274	0.516	2389.16	1231.91	0.141	1976.97	279.50
A_68_P30396225	chr15:84511839-84511883	NM_146061:433	Prr5	INSIDE	0.510	0.501	2069.43	1036.24	0.255	1674.23	427.60
A_68_P21473125	chr2:101636609-101636660	NM_001083810:1230	Prr5l	INSIDE	0.205	0.639	1193.94	763.32	0.131	910.97	119.39
A_68_P21473128	chr2:101637014-101637058	NM_001083810:828	Prr5l	INSIDE	0.541	0.708	2475.47	1753.79	0.383	1980.21	759.31
A_68_P29055170	chr13:55565739-55565783	NM_001030296:133	Prr7	INSIDE	0.652	0.555	1054.47	585.21	0.362	875.87	317.12
A_68_P29055175	chr13:55566226-55566270	NM_001030296:621	Prr7	INSIDE	0.413	0.641	997.70	639.06	0.265	859.80	227.64
A_68_P31735610	chr18:57514422-57514466	NM_028447:58	Prrc1	INSIDE	0.428	0.533	3461.36	1846.17	0.228	2669.48	609.54
A_68_P31158044	chr17:35302111-35302155	NM_001199044:-310	Prrc2a	PROMOTER	0.220	0.658	2606.73	1714.01	0.145	1756.43	253.85
A_68_P32481889	chrX:75828853-75828902	NM_001164275:244	Prrg1	INSIDE	0.136	1.555	1385.57	2154.44	0.212	576.68	122.29
A_68_P32481890	chrX:75828993-75829037	NM_001164275:107	Prrg1	INSIDE	0.059	1.411	4045.61	5706.80	0.083	1508.92	125.58
A_68_P25090923	chr7:52309399-52309443	NM_022999:7602	Prrg2	INSIDE	0.211	0.414	2016.49	834.08	0.087	1331.76	116.05
A_68_P25090924	chr7:52309475-52309519	NM_022999:7526	Prrg2	INSIDE	0.334	0.547	1987.85	1086.59	0.182	1528.63	278.69
A_68_P32456129	chrX:69208413-69208457	NM_001081135:75	Prrg3	INSIDE	0.101	2.052	2479.58	5089.18	0.208	973.52	202.13
A_68_P31154934	chr17:34767881-34767925	NM_030890:1272	Prrt1	INSIDE	0.148	0.432	5022.36	2168.37	0.064	3732.73	239.24
A_68_P31154945	chr17:34769052-34769096	NM_030890:2444	Prrt1	INSIDE	0.419	0.664	859.65	571.17	0.279	659.58	183.75
A_68_P20782631	chr1:165242518-165242562	NM_011127:1241	Prrx1	INSIDE	0.503	0.648	2463.38	1597.12	0.326	1983.37	647.09
A_68_P21105567	chr2:30701245-30701289	NM_009116:380	Prrx2	INSIDE	0.487	0.536	1063.78	569.84	0.261	841.23	219.68
A_68_P22488128	chr3:123149644-123149696	NM_008939:-160	Prss12	PROMOTER	0.184	0.674	1356.81	913.87	0.124	975.11	120.81
A_68_P22488130	chr3:123149844-123149888	NM_008939:36	Prss12	INSIDE	0.614	0.572	1247.95	713.78	0.351	1075.79	377.71
A_68_P22488132	chr3:123150107-123150151	NM_008939:298	Prss12	INSIDE	0.636	0.483	1826.65	883.10	0.307	1507.45	463.51
A_68_P28875775	chr13:22098833-22098877	NM_019429:2756	Prss16	INSIDE	0.164	0.624	1531.19	955.62	0.102	1187.08	121.26
A_68_P28875777	chr13:22099045-22099089	NM_019429:2544	Prss16	INSIDE	0.467	0.536	1015.41	544.28	0.250	861.09	215.62
A_68_P25308131	chr7:96665783-96665827	NM_029614:292	Prss23	INSIDE	0.322	0.621	1100.59	683.16	0.200	944.67	188.58
A_68_P25508223	chr7:135079560-135079608	NM_001081374:10655	Prss36	INSIDE	0.232	0.516	1289.27	665.11	0.120	976.47	116.85
A_68_P26629702	chr9:72654701-72654745	NM_175485:-358	Prtg	PROMOTER	0.147	0.718	4221.62	3030.89	0.106	3133.78	330.92

ProbeName	Target position of probe on CpG island microarray	TargetID	GeneSymbol	CpG island Description	Ratio of relative methylation (TiO2-NP/Vehicle)	Sham group			TiO_2-H group		
						Relative methylation (Cy-5/Cy-3)	Cy-3 signal (Input)	Cy-5 signal (MeDIP)	Relative methylation (Cy-5/Cy-3)	Cy-3 signal (Input)	Cy-5 signal (MeDIP)
A_68_P31983175	chr19:17030736-17030792	NM_181348:157	Prune2	INSIDE	0.087	1.416	1322.36	1872.16	0.124	965.87	119.36
A_68_P25017607	chr7:28293097-28293141	NM_019412:8776	Prx	INSIDE	0.239	0.704	2034.55	1431.90	0.168	1479.08	249.09
A_68_P31621133	chr18:36124466-36124510	NM_028707:0	Psd2	INSIDE	0.226	0.673	1959.00	1317.70	0.152	1477.38	224.43
A_68_P31621135	chr18:36124744-36124788	NM_028707:278	Psd2	INSIDE	0.431	0.516	1453.25	750.11	0.222	1191.44	264.85
A_68_P20874690	chr1:182186271-182186316	NM_001128605:138	Psen2	INSIDE	0.539	0.630	2751.06	1733.59	0.340	2283.29	775.66
A_68_P23062643	chr4:83131639-83131686	NM_133948:632	Psip1	INSIDE	0.262	0.465	1213.13	564.02	0.122	1010.70	123.17
A_68_P23062644	chr4:83131741-83131785	NM_133948:532	Psip1	INSIDE	0.359	0.513	1767.20	906.00	0.184	1327.78	244.70
A_68_P26136633	chr8:108424693-108424737	NM_173432:341	Pskh1	INSIDE	0.320	0.553	2032.17	1122.90	0.177	1481.30	262.11
A_68_P25435182	chr7:121419370-121419414	NM_011965:238	Psma1	INSIDE	0.166	0.501	1843.87	923.12	0.083	1452.26	120.89
A_68_P25435184	chr7:121419570-121419614	NM_011965:38	Psma1	INSIDE	0.146	0.703	1512.13	1063.11	0.103	1137.00	116.81
A_68_P25435185	chr7:121419707-121419751	NM_011965:-98	Psma1	PROMOTER	0.391	0.724	2831.07	2050.70	0.283	2139.61	605.66
A_68_P26528578	chr9:54798480-54798524	NM_011966:-163	Psma4	PROMOTER	0.648	0.718	1755.06	1259.28	0.465	1265.83	588.51
A_68_P22411525	chr3:108059789-108059833	NM_011967:-33	Psma5	PROMOTER	0.623	0.488	1245.34	607.81	0.304	1061.12	322.83
A_68_P22411526	chr3:108059982-108060035	NM_011967:165	Psma5	INSIDE	0.281	0.631	1111.74	702.05	0.177	808.70	143.42
A_68_P22411529	chr3:108060296-108060340	NM_011967:475	Psma5	INSIDE	0.364	0.576	1211.32	697.48	0.210	1021.59	214.14
A_68_P28451628	chr12:56499606-56499653	NM_011968:-182	Psma6	PROMOTER	0.115	0.677	2272.71	1538.34	0.078	1774.29	138.57
A_68_P28451629	chr12:56499790-56499834	NM_011968:1	Psma6	INSIDE	0.450	0.659	975.07	642.35	0.297	837.49	248.53
A_68_P21901044	chr2:179776594-179776638	NM_011969:491	Psma7	INSIDE	0.540	0.609	3878.26	2362.91	0.329	2914.69	958.39
A_68_P31065957	chr17:15635193-15635237	NM_011185:26	Psmb1	INSIDE	0.419	0.610	1107.75	675.79	0.255	837.69	213.89
A_68_P23281392	chr4:126354680-126354732	NM_011970:-180	Psmb2	PROMOTER	0.149	0.548	1907.60	1045.23	0.081	1430.29	116.46
A_68_P28071923	chr11:100956472-100956516	NM_008949:221	Psmc3ip	INSIDE	0.185	0.690	1160.43	800.63	0.127	903.95	115.20
A_68_P27954273	chr11:80241942-80241986	NM_178616:-152	Psmd11	PROMOTER	0.641	0.624	1819.27	1135.26	0.400	1508.78	603.12
A_68_P27954278	chr11:80242652-80242696	NM_178616:558	Psmd11	INSIDE	0.289	0.570	2297.37	1309.55	0.165	1732.48	285.19
A_68_P28107295	chr11:107340618-107340662	NM_025894:-201	Psmd12	DIVERGENT_PROMOTER	0.447	0.554	2323.51	1288.02	0.248	1839.94	456.38
A_68_P28107297	chr11:107340918-107340962	NM_025894:99	Psmd12	INSIDE	0.388	0.458	2343.59	1072.77	0.178	1712.82	304.09
A_68_P28107298	chr11:107341035-107341079	NM_025894:215	Psmd12	INSIDE	0.598	0.445	1765.52	785.03	0.266	1435.59	381.52
A_68_P28057822	chr11:98544211-98544255	NM_009439:365	Psmd3	INSIDE	0.198	0.537	1431.08	768.90	0.106	1182.55	125.60
A_68_P28057823	chr11:98544328-98544372	NM_009439:483	Psmd3	INSIDE	0.363	0.643	1843.14	1185.27	0.233	1357.88	316.61
A_68_P22341989	chr3:94846418-94846462	NM_008951:27	Psmd4	INSIDE	0.434	0.561	1911.42	1072.31	0.243	1493.72	363.34
A_68_P29618605	chr14:56197304-56197348	NM_011189:-4	Psme1	PROMOTER	0.228	0.455	2276.43	1036.04	0.104	1636.18	170.03
A_68_P27680413	chr11:30672528-30672572	NM_134013:776	Psme4	INSIDE	0.253	0.643	816.93	525.46	0.163	750.05	122.16
A_68_P24137873	chr5:140302698-140302742	NM_025604:77	Psmg3	INSIDE	0.469	0.585	1517.65	888.49	0.275	1208.26	331.73
A_68_P25528062	chr7:138514868-138514912	NM_001039534:231	Pstk	INSIDE	0.570	0.612	1795.30	1099.46	0.349	1420.24	496.16
A_68_P25528063	chr7:138515003-138515047	NM_001039534:365	Pstk	INSIDE	0.413	0.557	1266.05	704.56	0.230	941.94	216.26
A_68_P31849787	chr18:78033303-78033347	NM_013831:36	Pstpip2	INSIDE	0.227	0.557	3770.04	2098.95	0.126	2881.75	364.01
A_68_P31849792	chr18:78033867-78033918	NM_013831:604	Pstpip2	INSIDE	0.069	1.383	3351.66	4636.44	0.096	2396.56	230.19
A_68_P27279289	chr10:79317369-79317415	NM_001077363:43	Ptbp1	INSIDE	0.145	0.643	3440.87	2213.88	0.093	2369.15	221.00
A_68_P22467630	chr3:119485493-119485539	NM_019550:790	Ptbp2	INSIDE	0.532	0.711	1604.82	1140.42	0.378	1134.49	428.65
A_68_P29094157	chr13:63664712-63664756	NM_008957:2094	Ptch1	INSIDE	0.531	0.641	1748.04	1119.74	0.340	1306.15	444.22
A_68_P29094161	chr13:63665111-63665155	NM_008957:1696	Ptch1	INSIDE	0.584	0.636	1075.76	683.74	0.371	814.33	302.29
A_68_P29094165	chr13:63665588-63665632	NM_008957:1218	Ptch1	INSIDE	0.288	0.668	891.25	595.20	0.193	622.39	119.82
A_68_P29094168	chr13:63665973-63666017	NM_008957:834	Ptch1	INSIDE	0.480	0.677	1363.51	922.75	0.325	1064.54	345.90
A_68_P29094234	chr13:63674256-63674300	NM_008957:-7450	Ptch1	PROMOTER	0.345	0.536	2331.34	1249.73	0.185	1792.86	331.49
A_68_P32760541	chrX:152057603-152057648	NM_001093750:245	Ptchd1	INSIDE	0.307	1.655	2211.33	3659.14	0.508	896.80	455.50

ProbeName	Target position of probe on CpG island microarray	TargetID	GeneSymbol	CpG island Description	Ratio of relative methylation (TiO2-NP/Vehicle)	Sham group			TiO_2-H group		
						Relative methylation (Cy-5/Cy-3)	Cy-3 signal (Input)	Cy-5 signal (MeDIP)	Relative methylation (Cy-5/Cy-3)	Cy-3 signal (Input)	Cy-5 signal (MeDIP)
A_68_P32760557	chrX:152059508-152059553	NM_001093750:-1660	Ptchd1	PROMOTER	0.496	0.613	1047.90	641.91	0.304	410.56	124.87
A_68_P23393507	chr4:147661524-147661570	NM_001083342:528	Ptchd2	INSIDE	0.172	0.693	1340.86	929.17	0.119	1016.98	121.46
A_68_P23393508	chr4:147661631-147661675	NM_001083342:422	Ptchd2	INSIDE	0.220	0.525	1671.57	877.18	0.116	1087.12	125.78
A_68_P23393509	chr4:147661752-147661796	NM_001083342:300	Ptchd2	INSIDE	0.179	0.704	3244.74	2285.80	0.126	2490.23	314.76
A_68_P32071912	chr19:32830438-32830489	NM_008960:-1603	Pten	PROMOTER	0.110	0.509	2568.07	1306.01	0.056	2138.97	120.13
A_68_P32071916	chr19:32830947-32830991	NM_008960:-1098	Pten	PROMOTER	0.115	0.687	5738.49	3942.91	0.079	3993.79	314.99
A_68_P32071917	chr19:32831118-32831162	NM_008960:-926	Pten	PROMOTER	0.285	0.460	1600.62	735.67	0.131	1213.86	159.15
A_68_P32071923	chr19:32831726-32831770	NM_008960:-318	Pten	PROMOTER	0.465	0.486	2005.36	974.46	0.226	1531.13	345.72
A_68_P21044976	chr2:19367626-19367673	NM_018809:360	Ptf1a	INSIDE	0.490	0.656	1479.15	970.04	0.321	1286.41	413.57
A_68_P21044977	chr2:19367764-19367808	NM_018809:497	Ptf1a	INSIDE	0.232	0.402	3542.83	1422.71	0.093	2799.56	261.23
A_68_P21044982	chr2:19368271-19368321	NM_018809:1007	Ptf1a	INSIDE	0.314	0.649	1022.44	663.67	0.204	734.30	149.56
A_68_P29568494	chr14:45608721-45608765	NM_008964:957	Ptger2	INSIDE	0.501	0.520	2224.91	1156.07	0.260	1586.01	412.60
A_68_P29568495	chr14:45608828-45608874	NM_008964:1065	Ptger2	INSIDE	0.119	0.650	2329.74	1514.40	0.077	1565.49	120.82
A_68_P29568499	chr14:45609164-45609208	NM_008964:1401	Ptger2	INSIDE	0.266	0.518	1160.21	601.11	0.138	859.22	118.22
A_68_P29568500	chr14:45609358-45609402	NM_008964:1595	Ptger2	INSIDE	0.610	0.611	1117.69	683.42	0.373	827.85	308.64
A_68_P29982792	chr15:5192574-5192618	NM_001136079:1086	Ptger4	INSIDE	0.574	0.609	1175.28	715.29	0.349	1048.64	366.06
A_68_P21115151	chr2:32251028-32251072	NM_133783:-359	Ptges2	DIVERGENT_PROMOTER	0.365	0.588	1314.57	772.59	0.214	1013.72	217.17
A_68_P21115161	chr2:32252153-32252197	NM_133783:765	Ptges2	INSIDE	0.136	0.594	2144.51	1273.43	0.081	1420.51	114.72
A_68_P21115162	chr2:32252222-32252274	NM_133783:839	Ptges2	INSIDE	0.199	0.677	1509.10	1021.34	0.135	1036.54	139.73
A_68_P21834485	chr2:167066151-167066195	NM_008968:-135	Ptgis	PROMOTER	0.112	1.544	7613.76	11758.96	0.174	5179.26	899.08
A_68_P21138680	chr2:36086050-36086094	NM_008969:127	Ptgs1	INSIDE	0.275	0.424	2870.32	1216.90	0.117	2218.77	258.87
A_68_P26825470	chr9:110629961-110630005	NM_001083936:15132	Pth1r	INSIDE	0.187	1.773	1441.41	2556.16	0.332	951.18	316.00
A_68_P26825587	chr9:110645381-110645425	NM_001083936:-288	Pth1r	PROMOTER	0.199	0.714	2429.62	1735.16	0.142	1721.31	244.66
A_68_P26825589	chr9:110645592-110645636	NM_001083936:-500	Pth1r	PROMOTER	0.580	0.411	1991.10	817.54	0.238	1559.66	371.21
A_68_P26825596	chr9:110646504-110646548	NM_001083935:-336	Pth1r	PROMOTER	0.428	0.556	1547.35	860.35	0.238	1146.31	272.69
A_68_P26825597	chr9:110646599-110646643	NM_001083935:-430	Pth1r	PROMOTER	0.545	0.686	2045.01	1402.42	0.374	1634.41	611.20
A_68_P25091647	chr7:52436916-52436960	NM_053256:574	Pth2	INSIDE	0.051	1.638	5199.99	8515.05	0.083	3282.43	273.82
A_68_P24933419	chr6:147212900-147212944	NM_008970:-315	Pthlh	PROMOTER	0.240	0.597	1054.80	629.68	0.143	825.32	118.20
A_68_P30330337	chr15:73253106-73253150	NM_007982:493	Ptk2	INSIDE	0.111	0.566	5635.53	3191.39	0.063	4147.93	259.69
A_68_P31207969	chr17:46765813-46765857	NM_175168:619	Ptk7	INSIDE	0.591	0.685	1632.20	1117.64	0.405	1424.66	576.74
A_68_P31207970	chr17:46765913-46765957	NM_175168:519	Ptk7	INSIDE	0.487	0.587	1359.07	797.16	0.286	1009.10	288.38
A_68_P31207971	chr17:46765981-46766025	NM_175168:451	Ptk7	INSIDE	0.203	0.523	2923.74	1528.13	0.106	2021.64	214.17
A_68_P31207972	chr17:46766049-46766093	NM_175168:383	Ptk7	INSIDE	0.522	0.489	3103.20	1518.78	0.255	2376.47	606.87
A_68_P31207973	chr17:46766171-46766215	NM_175168:261	Ptk7	INSIDE	0.392	0.571	1376.75	786.16	0.224	1101.03	246.77
A_68_P20412581	chr1:88421464-88421508	NM_008972:-1824	Ptma	PROMOTER	0.265	0.557	1663.73	927.10	0.148	1350.33	199.57
A_68_P20412615	chr1:88425095-88425139	NM_008972:1806	Ptma	INSIDE	0.393	0.704	1399.14	985.11	0.277	1200.12	332.10
A_68_P20412616	chr1:88425208-88425252	NM_008972:1920	Ptma	INSIDE	0.119	0.612	2756.72	1688.08	0.073	1995.33	145.61
A_68_P21019038	chr2:13976735-13976779	NM_001012396:906	Ptpla	INSIDE	0.261	0.441	1408.43	620.68	0.115	1048.47	120.55
A_68_P21019039	chr2:13976814-13976858	NM_001012396:826	Ptpla	INSIDE	0.417	0.459	2459.88	1129.22	0.192	1945.14	372.64
A_68_P26585376	chr9:64868976-64869020	NM_021345:526	Ptplad1	INSIDE	0.411	0.593	1041.52	618.04	0.244	901.82	220.16
A_68_P26585377	chr9:64869052-64869096	NM_021345:450	Ptplad1	INSIDE	0.372	0.622	923.15	574.18	0.231	836.12	193.53
A_68_P21838402	chr2:167757994-167758038	NM_011201:190	Ptpn1	INSIDE	0.132	0.707	3742.12	2644.22	0.093	2602.61	242.23
A_68_P21838403	chr2:167758113-167758157	NM_011201:308	Ptpn1	INSIDE	0.240	0.639	2010.74	1284.23	0.153	1447.22	221.38
A_68_P23524106	chr5:20561482-20561526	NM_011203:111	Ptpn12	INSIDE	0.202	0.431	2473.67	1066.31	0.087	1985.19	172.77

ProbeName	Target position of probe on CpG island microarray	TargetID	GeneSymbol	CpG island Description	Ratio of relative methylation (TiO2-NP/Vehicle)	Sham group			TiO_2-H group		
						Relative methylation (Cy-5/Cy-3)	Cy-3 signal (Input)	Cy-5 signal (MeDIP)	Relative methylation (Cy-5/Cy-3)	Cy-3 signal (Input)	Cy-5 signal (MeDIP)
A_68_P23946409	chr5:103854106-103854150	NM_011204:-82	Ptpn13	PROMOTER	0.560	0.619	1473.05	912.23	0.347	1290.53	447.50
A_68_P20927660	chr1:191552263-191552307	NM_008976:138	Ptpn14	INSIDE	0.188	0.720	1453.97	1046.44	0.135	1248.33	168.54
A_68_P20927662	chr1:191552465-191552509	NM_008976:340	Ptpn14	INSIDE	0.623	0.503	1091.33	548.52	0.313	858.56	268.81
A_68_P20927663	chr1:191552534-191552578	NM_008976:410	Ptpn14	INSIDE	0.585	0.627	1222.18	765.79	0.367	994.35	364.73
A_68_P20138941	chr1:34516537-34516581	NM_011206:-32	Ptpn18	PROMOTER	0.649	0.553	1213.68	671.17	0.359	976.21	350.26
A_68_P20138943	chr1:34516740-34516784	NM_011206:172	Ptpn18	INSIDE	0.217	0.471	3441.33	1619.67	0.102	2469.09	252.00
A_68_P20138944	chr1:34516811-34516856	NM_011206:243	Ptpn18	INSIDE	0.311	0.497	2255.29	1121.63	0.155	1747.96	270.79
A_68_P31793418	chr18:67883645-67883689	NM_001127177:609	Ptpn2	INSIDE	0.568	0.573	2730.29	1565.13	0.326	2353.85	766.35
A_68_P26823553	chr9:110310358-110310402	NM_001081043:334	Ptpn23	INSIDE	0.395	0.521	1283.21	669.17	0.206	1086.75	223.83
A_68_P26823555	chr9:110310525-110310569	NM_001081043:168	Ptpn23	INSIDE	0.324	0.428	1786.44	765.15	0.139	1351.38	187.53
A_68_P20559034	chr1:121734234-121734278	NM_019933:-608	Ptpn4	PROMOTER	0.354	0.690	1451.93	1001.36	0.244	1060.00	258.69
A_68_P21629881	chr2:130276040-130276084	NM_001163688:49	Ptpra	INSIDE	0.518	0.626	5416.56	3392.06	0.324	3935.00	1275.44
A_68_P21629885	chr2:130276440-130276484	NM_001163688:449	Ptpra	INSIDE	0.254	0.453	1365.05	618.74	0.115	1192.92	137.52
A_68_P25553144	chr7:142729766-142729810	NM_011212:282	Ptpre	INSIDE	0.530	0.582	1821.22	1059.71	0.308	1581.80	487.82
A_68_P23239264	chr4:117933534-117933578	NM_011213:30446	Ptprf	INSIDE	0.586	0.622	2082.68	1295.04	0.364	1607.52	585.38
A_68_P23239474	chr4:117963100-117963144	NM_011213:880	Ptprf	INSIDE	0.458	0.464	2532.80	1174.08	0.212	1971.67	418.30
A_68_P29405444	chr14:12386788-12386841	NM_008981:748	Ptprg	INSIDE	0.232	0.674	1055.50	711.71	0.157	757.52	118.69
A_68_P29405446	chr14:12386981-12387025	NM_008981:936	Ptprg	INSIDE	0.087	0.565	3968.43	2242.14	0.049	2981.08	147.26
A_68_P21417952	chr2:90419722-90419766	NM_008982:1060	Ptprj	INSIDE	0.222	0.464	1393.67	646.07	0.103	1167.97	120.06
A_68_P27027630	chr10:27794848-27794893	NM_008983:245	Ptprk	INSIDE	0.218	0.474	1775.42	841.13	0.103	1262.62	130.46
A_68_P31311916	chr17:67703103-67703147	NM_008984:675	Ptprm	INSIDE	0.518	0.531	1401.92	744.02	0.275	1148.81	315.56
A_68_P31311919	chr17:67703480-67703524	NM_008984:297	Ptprm	INSIDE	0.386	0.444	1802.80	799.88	0.171	1378.61	235.92
A_68_P31311921	chr17:67703645-67703689	NM_008984:133	Ptprm	INSIDE	0.484	0.637	1627.31	1036.43	0.308	1495.69	461.41
A_68_P28768145	chr12:117724630-117724674	NM_011215:460	Ptprn2	INSIDE	0.278	0.424	1564.92	664.12	0.118	1091.87	129.03
A_68_P24877028	chr6:137201005-137201049	NM_001164401:207	Ptpro	INSIDE	0.489	0.607	2148.51	1304.75	0.297	1693.93	502.81
A_68_P31256335	chr17:56601867-56601911	NM_011218:14015	Ptprs	INSIDE	0.319	0.480	1171.26	561.67	0.153	790.94	121.16
A_68_P31256423	chr17:56613777-56613821	NM_011218:2105	Ptprs	INSIDE	0.566	0.725	2457.40	1782.69	0.411	1862.73	764.70
A_68_P31256434	chr17:56615114-56615158	NM_011218:767	Ptprs	INSIDE	0.046	1.406	10793.29	15174.52	0.064	6437.47	412.14
A_68_P31256438	chr17:56615517-56615561	NM_011218:365	Ptprs	INSIDE	0.295	0.397	1320.98	524.04	0.117	1033.69	120.88
A_68_P23308584	chr4:131392713-131392757	NM_001083119:1459	Ptpru	INSIDE	0.122	4.185	11964.07	50074.15	0.512	7209.21	3692.26
A_68_P23308589	chr4:131393487-131393532	NM_001083119:684	Ptpru	INSIDE	0.389	1.782	9005.35	16046.43	0.693	5751.07	3988.01
A_68_P23308590	chr4:131393615-131393659	NM_001083119:557	Ptpru	INSIDE	0.224	1.430	2572.74	3680.22	0.321	1833.59	587.85
A_68_P23308591	chr4:131393677-131393729	NM_001083119:491	Ptpru	INSIDE	0.141	1.655	2144.36	3549.76	0.233	1535.45	357.65
A_68_P23308592	chr4:131393872-131393916	NM_001083119:299	Ptpru	INSIDE	0.405	1.432	2326.39	3331.75	0.580	1769.52	1025.80
A_68_P24293210	chr6:22825825-22825869	NM_001081306:345	Ptprz1	INSIDE	0.328	0.540	1458.38	787.56	0.177	1115.51	197.31
A_68_P28071134	chr11:100820206-100820250	NM_008986:11703	Ptrf	INSIDE	0.410	0.593	2523.39	1495.35	0.243	1817.82	441.98
A_68_P28071211	chr11:100830670-100830714	NM_008986:1239	Ptrf	INSIDE	0.195	1.562	7198.03	11241.73	0.305	4664.73	1420.47
A_68_P28071212	chr11:100830764-100830808	NM_008986:1145	Ptrf	INSIDE	0.265	1.721	8144.08	14014.31	0.457	5451.22	2490.51
A_68_P21117413	chr2:32631242-32631286	NM_178595:-76	Ptrh1	DIVERGENT_PROMOTER	0.274	0.405	1300.18	526.14	0.111	1049.75	116.57
A_68_P23301758	chr4:130219950-130220000	NM_001159603:701	Pum1	INSIDE	0.397	0.586	1032.44	605.24	0.232	893.17	207.63
A_68_P28223702	chr12:8680649-8680696	NM_001160221:-267	Pum2	PROMOTER	0.165	0.697	1143.84	796.86	0.115	1045.99	120.19
A_68_P28223705	chr12:8681057-8681101	NM_030723:-408	Pum2	PROMOTER	0.601	0.620	1390.83	862.72	0.373	982.46	366.01
A_68_P27568682	chr11:6374896-6374940	NM_011221:1002	Purb	INSIDE	0.353	0.344	4121.48	1416.41	0.121	2880.64	349.66
A_68_P27568683	chr11:6374982-6375033	NM_011221:913	Purb	INSIDE	0.288	0.450	1239.90	558.31	0.130	910.10	117.95

ProbeName	Target position of probe on CpG island microarray	TargetID	GeneSymbol	CpG island Description	Ratio of relative methylation (TiO2-NP/Vehicle)	Sham group			TiO_2-H group		
						Relative methylation (Cy-5/Cy-3)	Cy-3 signal (Input)	Cy-5 signal (MeDIP)	Relative methylation (Cy-5/Cy-3)	Cy-3 signal (Input)	Cy-5 signal (MeDIP)
A_68_P23981857	chr5:111209299-111209343	NM_001025561:295	Pus1	INSIDE	0.604	0.690	2104.92	1453.25	0.417	1734.51	723.61
A_68_P23538312	chr5:23289160-23289204	NM_178403:297	Pus7	INSIDE	0.304	0.336	2362.92	793.83	0.102	1704.18	174.13
A_68_P30452932	chr15:94373863-94373910	NM_172437:52	Pus7l	INSIDE	0.418	0.680	997.57	678.65	0.284	698.60	198.48
A_68_P26463567	chr9:43552486-43552530	NM_021424:-150	Pvrl1	PROMOTER	0.167	0.447	2032.39	908.61	0.075	1611.49	120.60
A_68_P26463574	chr9:43553274-43553318	NM_021424:638	Pvrl1	INSIDE	0.242	0.476	1881.60	894.91	0.115	1558.53	179.03
A_68_P26463575	chr9:43553383-43553427	NM_021424:746	Pvrl1	INSIDE	0.289	0.468	1102.54	516.48	0.136	899.37	121.89
A_68_P26463577	chr9:43553595-43553639	NM_021424:958	Pvrl1	INSIDE	0.344	0.566	943.26	534.19	0.195	727.92	141.86
A_68_P26463578	chr9:43553721-43553771	NM_021424:1088	Pvrl1	INSIDE	0.162	0.686	1618.63	1111.18	0.111	1081.26	120.43
A_68_P26464018	chr9:43611946-43611990	NM_021424:59310	Pvrl1	INSIDE	0.369	1.592	2626.63	4180.82	0.588	2022.89	1189.23
A_68_P24995078	chr7:20334418-20334462	NM_008990:482	Pvrl2	INSIDE	0.441	1.457	4703.29	6852.41	0.643	2952.10	1897.15
A_68_P24995079	chr7:20334543-20334587	NM_008990:358	Pvrl2	INSIDE	0.462	0.691	1180.67	815.86	0.319	933.98	298.01
A_68_P24995081	chr7:20334787-20334831	NM_008990:114	Pvrl2	INSIDE	0.507	0.647	2243.68	1452.11	0.328	1670.83	547.76
A_68_P24995083	chr7:20334983-20335030	NM_008990:-84	Pvrl2	PROMOTER	0.181	1.931	6073.00	11724.05	0.350	3805.69	1332.55
A_68_P30732552	chr16:46496367-46496411	NM_021496:692	Pvrl3	INSIDE	0.128	0.394	3493.19	1377.76	0.050	2607.68	131.51
A_68_P25576662	chr7:146434859-146434912	NM_001033206:505	Pwwp2b	INSIDE	0.250	0.548	1233.31	676.32	0.137	886.07	121.68
A_68_P20878432	chr1:182834386-182834430	NM_133705:4	Pycr2	INSIDE	0.316	0.464	1487.39	690.74	0.147	1191.12	174.93
A_68_P20878433	chr1:182834482-182834526	NM_133705:100	Pycr2	INSIDE	0.167	0.622	1580.89	983.05	0.104	1154.60	120.26
A_68_P30344972	chr15:75751799-75751843	NM_025412:170	Pycrl	INSIDE	0.593	0.610	1528.07	932.19	0.362	1219.50	441.14
A_68_P21740960	chr2:150612370-150612414	NM_153781:-139	Pygb	PROMOTER	0.214	0.551	1328.47	731.88	0.118	995.95	117.64
A_68_P21740962	chr2:150612685-150612729	NM_153781:175	Pygb	INSIDE	0.456	0.406	1854.77	752.86	0.185	1403.78	259.68
A_68_P28519206	chr12:71328237-71328281	NM_133198:412	Pygl	INSIDE	0.199	0.564	1439.20	811.22	0.112	1124.78	126.31
A_68_P28519207	chr12:71328317-71328361	NM_133198:332	Pygl	INSIDE	0.098	0.733	2449.40	1795.97	0.072	1678.78	120.54
A_68_P28519208	chr12:71328463-71328507	NM_133198:186	Pygl	INSIDE	0.052	1.798	6304.49	11338.04	0.093	3743.06	346.85
A_68_P26630337	chr9:72772909-72772953	NM_028116:-526	Pygo1	PROMOTER	0.217	0.746	4817.15	3595.57	0.162	3665.08	593.01
A_68_P26630338	chr9:72772983-72773027	NM_028116:-452	Pygo1	PROMOTER	0.550	0.586	2539.61	1488.02	0.322	1916.71	617.62
A_68_P26630344	chr9:72773728-72773772	NM_028116:294	Pygo1	INSIDE	0.610	0.607	1909.56	1159.73	0.371	1458.27	540.37
A_68_P22319356	chr3:89234182-89234226	NM_026869:-554	Pygo2	PROMOTER	0.495	0.461	1171.65	540.19	0.228	971.62	221.59
A_68_P23656662	chr5:45841524-45841568	NM_024236:-78	Qdpr	DIVERGENT_PROMOTER	0.543	0.446	1395.00	622.19	0.242	1152.90	279.07
A_68_P23656663	chr5:45841705-45841749	NM_024236:-258	Qdpr	DIVERGENT_PROMOTER	0.157	0.492	1967.61	968.18	0.077	1556.45	120.01
A_68_P31375198	chr17:79451766-79451811	NM_027455:543	Qpct	INSIDE	0.227	0.551	1256.17	692.22	0.125	989.53	123.97
A_68_P24991720	chr7:19734447-19734491	NM_026111:77	Qpctl	INSIDE	0.460	0.398	1844.63	734.75	0.183	1385.49	253.69
A_68_P26815242	chr9:108420124-108420168	NM_001114119:729	Qrich1	INSIDE	0.418	0.543	3343.99	1816.39	0.227	2647.85	600.78
A_68_P26815243	chr9:108420248-108420296	NM_001114119:855	Qrich1	INSIDE	0.255	0.546	1230.81	672.17	0.139	896.46	124.67
A_68_P21490975	chr2:104656460-104656504	NM_001123327:371	Qser1	INSIDE	0.160	0.714	1495.29	1068.21	0.114	1076.84	122.90
A_68_P21490982	chr2:104657364-104657415	NM_001123327:-536	Qser1	PROMOTER	0.216	0.577	1357.06	782.79	0.124	942.23	117.13
A_68_P20740064	chr1:157659559-157659603	NM_001024945:449	Qsox1	INSIDE	0.374	0.659	3760.37	2479.17	0.246	2848.85	701.65
A_68_P20740065	chr1:157659643-157659687	NM_001024945:365	Qsox1	INSIDE	0.464	0.440	2015.71	886.96	0.204	1703.67	348.10
A_68_P20740066	chr1:157659774-157659818	NM_001024945:233	Qsox1	INSIDE	0.616	0.630	2087.28	1315.94	0.389	1556.18	604.64
A_68_P21077271	chr2:26092393-26092439	NM_153559:524	Qsox2	INSIDE	0.183	0.644	1176.75	758.17	0.118	989.76	116.45
A_68_P26347006	chr9:21216708-21216752	NM_021888:450	Qtrt1	INSIDE	0.615	0.495	1285.34	636.11	0.304	1167.96	355.36
A_68_P29696767	chr14:70107122-70107166	NM_001146012:243	R3hcc1	INSIDE	0.179	0.480	2629.67	1261.18	0.086	2007.08	172.62
A_68_P20598626	chr1:129999945-129999989	NM_181750:84	R3hdm1	INSIDE	0.179	0.680	1163.91	791.07	0.121	967.60	117.56
A_68_P27630331	chr11:20101120-20101164	NM_008996:-462	Rab1	PROMOTER	0.503	0.633	1313.60	830.93	0.318	915.64	291.46
A_68_P27630333	chr11:20101414-20101458	NM_008996:-168	Rab1	PROMOTER	0.241	0.648	1231.38	797.88	0.156	941.83	146.80

ProbeName	Target position of probe on CpG island microarray	TargetID	GeneSymbol	CpG island Description	Ratio of relative methylation (TiO2-NP/Vehicle)	Sham group			TiO_2-H group		
						Relative methylation (Cy-5/Cy-3)	Cy-3 signal (Input)	Cy-5 signal (MeDIP)	Relative methylation (Cy-5/Cy-3)	Cy-3 signal (Input)	Cy-5 signal (MeDIP)
A_68_P27630334	chr11:20101506-20101552	NM_008996:-76	Rab1	PROMOTER	0.238	0.669	2440.74	1632.71	0.159	1960.15	312.42
A_68_P27630335	chr11:20101626-20101670	NM_008996:44	Rab1	INSIDE	0.488	0.626	976.58	611.62	0.306	867.42	265.32
A_68_P28194541	chr12:3309848-3309892	NM_016676:99	Rab10	INSIDE	0.488	0.569	2174.95	1238.12	0.278	1733.52	481.18
A_68_P31150390	chr17:33896983-33897027	NM_008997:427	Rab11b	INSIDE	0.352	0.542	3620.11	1963.30	0.191	2748.19	524.49
A_68_P25737017	chr8:28284572-28284616	NM_001080813:524	Rab11fip1	INSIDE	0.497	0.587	4001.98	2348.08	0.291	3137.30	914.17
A_68_P31107657	chr17:26122228-26122272	NM_153140:36555	Rab11fip3	DOWNSTREAM	0.602	0.534	1205.51	643.56	0.321	1061.30	341.07
A_68_P31107662	chr17:26122929-26122973	NM_153140:35855	Rab11fip3	DOWNSTREAM	0.247	0.499	1311.97	654.16	0.123	948.80	116.92
A_68_P31108117	chr17:26204538-26204582	NM_001162868:1562	Rab11fip3	INSIDE	0.327	0.619	1921.83	1189.49	0.203	1359.32	275.42
A_68_P31108119	chr17:26204737-26204781	NM_001162868:1364	Rab11fip3	INSIDE	0.492	0.685	1720.41	1179.28	0.337	1446.61	487.65
A_68_P31108129	chr17:26205895-26205939	NM_001162868:206	Rab11fip3	INSIDE	0.210	0.412	1898.53	782.04	0.087	1363.38	118.20
A_68_P31108130	chr17:26205973-26206017	NM_001162868:128	Rab11fip3	INSIDE	0.481	0.682	2004.10	1366.87	0.328	1539.18	505.40
A_68_P31108134	chr17:26206603-26206648	NM_001162868:-503	Rab11fip3	PROMOTER	0.346	0.643	1198.34	770.82	0.223	907.53	202.24
A_68_P27949792	chr11:79404418-79404462	NM_175543:-273	Rab11fip4	PROMOTER	0.571	0.648	1053.99	683.13	0.370	778.13	288.13
A_68_P27950225	chr11:79474212-79474256	NM_175543:69521	Rab11fip4	INSIDE	0.431	0.657	1815.22	1192.45	0.283	1417.94	401.57
A_68_P24604717	chr6:85324325-85324369	NM_001003955:282	Rab11fip5	INSIDE	0.242	0.368	1593.78	585.86	0.089	1316.00	117.27
A_68_P24604720	chr6:85324839-85324883	NM_001003955:-232	Rab11fip5	PROMOTER	0.439	0.719	1662.08	1194.98	0.316	1279.46	404.09
A_68_P31306702	chr17:66868481-66868525	NM_024448:508	Rab12	INSIDE	0.311	0.641	2832.09	1816.20	0.199	2142.21	426.63
A_68_P31306703	chr17:66868585-66868629	NM_024448:404	Rab12	INSIDE	0.272	0.538	1759.09	945.83	0.146	1430.87	209.26
A_68_P31306704	chr17:66868707-66868751	NM_024448:282	Rab12	INSIDE	0.548	0.368	2046.32	752.61	0.201	1460.20	294.13
A_68_P31306706	chr17:66869156-66869200	NM_024448:-168	Rab12	PROMOTER	0.394	0.563	2387.55	1344.99	0.222	1888.64	419.35
A_68_P21132195	chr2:35055732-35055776	NM_026697:886	Rab14	INSIDE	0.580	0.630	1690.96	1065.90	0.366	1391.18	508.67
A_68_P28556142	chr12:77923792-77923838	NM_134050:-303	Rab15	PROMOTER	0.193	0.495	1621.93	803.03	0.096	1254.91	120.04
A_68_P21871015	chr2:173485606-173485650	NM_024436:283	Rab22a	INSIDE	0.326	0.522	1322.49	689.96	0.170	1079.65	183.49
A_68_P31097945	chr17:24670178-24670222	NM_177375:492	Rab26	INSIDE	0.271	0.509	1134.52	577.72	0.138	872.29	120.48
A_68_P26630975	chr9:72892792-72892836	NM_023635:143	Rab27a	INSIDE	0.522	0.587	2110.75	1239.23	0.307	1765.63	541.52
A_68_P31302581	chr17:66121665-66121709	NM_133685:406	Rab31	INSIDE	0.524	0.714	2274.34	1623.87	0.374	1834.79	687.09
A_68_P31302583	chr17:66121848-66121892	NM_133685:222	Rab31	INSIDE	0.366	0.430	1523.83	655.05	0.157	1090.70	171.37
A_68_P26936200	chr10:10277608-10277652	NM_026405:375	Rab32	INSIDE	0.372	0.664	4550.56	3021.23	0.247	3475.21	859.00
A_68_P22129911	chr3:51288253-51288297	NM_016858:387	Rab33b	INSIDE	0.370	0.582	1602.37	932.44	0.215	1357.65	291.97
A_68_P22129913	chr3:51288449-51288493	NM_016858:583	Rab33b	INSIDE	0.405	0.485	1658.46	804.34	0.196	1337.61	262.75
A_68_P22129914	chr3:51288525-51288569	NM_016858:659	Rab33b	INSIDE	0.435	0.480	2286.28	1096.36	0.208	1743.88	363.60
A_68_P27941848	chr11:78002243-78002287	NM_033475:15	Rab34	INSIDE	0.108	0.606	2856.48	1729.90	0.065	2086.50	136.39
A_68_P28151618	chr11:115015440-115015484	NM_021411:-29	Rab37	PROMOTER	0.367	13.375	4190.60	56049.64	4.911	3112.74	15287.51
A_68_P26522114	chr9:53513817-53513861	NM_175562:499	Rab39	INSIDE	0.442	0.703	1514.20	1064.49	0.311	1219.90	379.01
A_68_P26522115	chr9:53513955-53513999	NM_175562:361	Rab39	INSIDE	0.517	0.736	2541.23	1870.45	0.380	2054.21	781.16
A_68_P25953808	chr8:73279185-73279229	NM_001166399:629	Rab3a	INSIDE	0.495	0.628	1646.81	1034.74	0.311	1179.88	367.20
A_68_P26349534	chr9:21722752-21722796	NM_031874:-209	Rab3d	PROMOTER	0.398	0.403	1561.47	629.94	0.160	1150.25	184.58
A_68_P20597392	chr1:129765401-129765445	NM_178690:73	Rab3gap1	INSIDE	0.256	0.626	1752.15	1097.06	0.160	1416.28	226.66
A_68_P27478501	chr10:116386814-116386861	NM_001003950:599	Rab3ip	INSIDE	0.204	0.670	1135.79	761.11	0.137	870.62	119.07
A_68_P27478502	chr10:116386947-116386991	NM_001003950:468	Rab3ip	INSIDE	0.669	0.509	1179.86	600.15	0.340	942.38	320.46
A_68_P28190285	chr11:121249100-121249144	NM_139147:443	Rab40b	INSIDE	0.640	0.592	2051.82	1214.50	0.379	1551.46	588.11
A_68_P28190286	chr11:121249176-121249220	NM_139147:367	Rab40b	INSIDE	0.454	0.532	1436.73	764.37	0.242	1136.18	274.43
A_68_P28190288	chr11:121249326-121249370	NM_139147:217	Rab40b	INSIDE	0.600	0.634	866.71	549.64	0.380	758.05	288.27
A_68_P24618280	chr6:87761089-87761133	NM_133717:-1362	Rab43	PROMOTER	0.313	0.552	1168.34	645.17	0.173	907.92	156.73

ProbeName	Target position of probe on CpG island microarray	TargetID	GeneSymbol	CpG island Description	Ratio of relative methylation (TiO2-NP/Vehicle)	Sham group			TiO_2-H group		
						Relative methylation (Cy-5/Cy-3)	Cy-3 signal (Input)	Cy-5 signal (MeDIP)	Relative methylation (Cy-5/Cy-3)	Cy-3 signal (Input)	Cy-5 signal (MeDIP)
A_68_P24618289	chr6:87762138-87762182	NM_001039394:-387	Rab43	PROMOTER	0.259	1.426	4902.32	6989.80	0.369	3081.41	1138.39
A_68_P25015938	chr7:27964028-27964072	NM_029391:-148	Rab4b	PROMOTER	0.197	1.609	6446.80	10373.43	0.318	4121.72	1309.59
A_68_P27542730	chr10:128133098-128133142	NM_177411:204	Rab5b	INSIDE	0.421	0.635	1145.72	728.04	0.267	1055.13	282.18
A_68_P25368679	chr7:107755747-107755795	NM_001163663:-329	Rab6	PROMOTER	0.130	0.489	2520.81	1231.91	0.064	1815.48	115.33
A_68_P25368680	chr7:107755893-107755937	NM_001163663:-185	Rab6	PROMOTER	0.430	0.707	3728.15	2634.93	0.304	2431.82	739.73
A_68_P25368681	chr7:107756005-107756054	NM_001163663:-70	Rab6	PROMOTER	0.511	0.514	1615.80	830.53	0.263	1380.20	362.52
A_68_P26783903	chr9:103014881-103014925	NM_173781:499	Rab6b	INSIDE	0.579	0.656	1862.64	1222.49	0.380	1492.34	567.03
A_68_P20618103	chr1:133763971-133764015	NM_144875:139	Rab7l1	INSIDE	0.660	0.596	1245.37	742.01	0.393	1040.68	409.25
A_68_P25959941	chr8:74685000-74685047	NM_023126:-75	Rab8a	PROMOTER	0.038	1.500	3214.79	4822.37	0.056	2138.57	120.77
A_68_P26595983	chr9:66767400-66767448	NM_173413:88	Rab8b	INSIDE	0.240	0.492	1447.64	712.61	0.118	997.94	117.73
A_68_P32806533	chrX:162917564-162917608	NM_019773:213	Rab9	INSIDE	0.365	1.482	2254.55	3340.86	0.541	975.33	527.39
A_68_P25500411	chr7:133572604-133572648	NM_030566:346	Rabep2	INSIDE	0.631	0.375	1911.48	716.57	0.236	1552.20	367.06
A_68_P21129807	chr2:34655295-34655339	NM_145522:-5	Rabepk	PROMOTER	0.127	0.607	2249.73	1365.76	0.077	1530.40	117.81
A_68_P21142143	chr2:37298714-37298758	NM_146121:-68	Rabgap1	DIVERGENT_PROMOTER	0.188	0.570	1347.67	767.67	0.107	1132.51	121.25
A_68_P21142145	chr2:37298992-37299036	NM_001033960:-8759	Rabgap1	PROMOTER	0.509	0.687	1727.49	1186.17	0.349	1324.23	462.48
A_68_P21142186	chr2:37308087-37308131	NM_001033960:335	Rabgap1	INSIDE	0.306	0.685	1593.12	1091.59	0.209	1255.07	262.75
A_68_P20768470	chr1:162722306-162722353	NM_013862:740	Rabgap1l	INSIDE	0.174	1.430	1370.04	1959.76	0.249	1051.90	261.79
A_68_P20768471	chr1:162722421-162722468	NM_013862:625	Rabgap1l	INSIDE	0.047	1.347	2476.00	3334.55	0.064	1913.93	122.20
A_68_P29619512	chr14:56340802-56340846	NM_019519:189	Rabggta	INSIDE	0.153	1.548	4599.91	7122.05	0.237	2634.55	624.59
A_68_P29619513	chr14:56340877-56340921	NM_019519:115	Rabggta	INSIDE	0.419	1.425	12228.98	17424.16	0.597	7695.71	4593.86
A_68_P22659666	chr3:153576060-153576104	NM_001163478:-152	Rabggtb	PROMOTER	0.598	0.434	1389.17	602.76	0.260	1050.59	272.66
A_68_P20633689	chr1:136391258-136391302	NM_145510:44	Rabif	INSIDE	0.552	0.449	1167.36	523.97	0.248	924.39	229.20
A_68_P30426166	chr15:89421992-89422040	NM_026817:338	Rabl2	INSIDE	0.276	0.526	1238.52	651.34	0.145	816.93	118.47
A_68_P24156971	chr5:144288853-144288897	NM_009007:-13	Rac1	PROMOTER	0.560	0.666	2639.95	1758.58	0.373	1981.84	738.77
A_68_P28186389	chr11:120582623-120582667	NM_133223:-137	Rac3	PROMOTER	0.453	0.512	2079.93	1065.18	0.232	1557.23	361.05
A_68_P30010836	chr15:10415930-10415974	NM_011232:164	Rad1	INSIDE	0.656	0.690	1309.92	903.80	0.452	1088.47	492.49
A_68_P24755176	chr6:112646414-112646458	NM_001167730:228	Rad18	INSIDE	0.421	0.605	1133.64	685.89	0.255	962.43	245.03
A_68_P30213987	chr15:51823056-51823100	NM_009009:228	Rad21	INSIDE	0.581	0.625	1189.79	743.37	0.363	1281.63	465.47
A_68_P22933513	chr4:55363588-55363632	NM_009011:697	Rad23b	INSIDE	0.206	0.722	3535.60	2554.47	0.149	2988.87	444.07
A_68_P27806201	chr11:53520531-53520577	NM_009012:267	Rad50	INSIDE	0.297	0.617	1174.44	724.17	0.183	1066.68	195.06
A_68_P24829778	chr6:126889168-126889219	NM_009013:380	Rad51ap1	INSIDE	0.166	0.500	2037.07	1018.09	0.083	1424.51	118.04
A_68_P24829780	chr6:126889329-126889373	NM_009013:223	Rad51ap1	INSIDE	0.429	0.705	3601.47	2537.98	0.302	2733.45	825.52
A_68_P27994099	chr11:87217797-87217841	NM_053269:179	Rad51c	INSIDE	0.257	0.544	949.30	516.49	0.140	860.86	120.27
A_68_P22730294	chr4:11486345-11486389	NM_001039556:248	Rad54b	INSIDE	0.222	0.604	2029.09	1225.36	0.134	1473.31	197.23
A_68_P24152254	chr5:143026387-143026435	NM_178702:621	Radil	INSIDE	0.114	1.559	2185.39	3406.71	0.178	1407.95	250.40
A_68_P21867064	chr2:172826009-172826053	NM_175112:413	Rae1	INSIDE	0.616	0.612	1108.55	678.63	0.377	967.90	365.17
A_68_P26997165	chr10:21879836-21879886	NM_009016:1446	Raet1a	INSIDE	0.332	0.691	1210.19	835.66	0.230	953.47	218.83
A_68_P27840802	chr11:59919053-59919097	NM_009021:560	Rai1	INSIDE	0.325	0.609	4194.73	2555.13	0.198	3321.06	658.23
A_68_P27840803	chr11:59919168-59919212	NM_009021:676	Rai1	INSIDE	0.168	0.674	5339.83	3600.58	0.113	4163.27	470.39
A_68_P27840876	chr11:59928639-59928683	NM_009021:10146	Rai1	INSIDE	0.265	0.523	2954.70	1544.03	0.138	2300.34	318.32
A_68_P27841324	chr11:59989338-59989382	NM_001037764:35777	Rai1	INSIDE	0.477	0.623	1199.73	746.89	0.297	978.58	290.76
A_68_P27841330	chr11:59989954-59989998	NM_001037764:36393	Rai1	INSIDE	0.610	0.405	3478.04	1409.63	0.247	2566.04	634.55
A_68_P27841333	chr11:59990255-59990299	NM_001037764:36693	Rai1	INSIDE	0.390	0.377	1620.98	611.52	0.147	1174.81	172.69
A_68_P20557410	chr1:121400955-121400999	NM_022327:383	Ralb	INSIDE	0.228	0.470	1289.95	606.52	0.107	1098.83	117.85

ProbeName	Target position of probe on CpG island microarray	TargetID	GeneSymbol	CpG island Description	Ratio of relative methylation (TiO2-NP/Vehicle)	Sham group			TiO_2-H group		
						Relative methylation (Cy-5/Cy-3)	Cy-3 signal (Input)	Cy-5 signal (MeDIP)	Relative methylation (Cy-5/Cy-3)	Cy-3 signal (Input)	Cy-5 signal (MeDIP)
A_68_P20557411	chr1:121401081-121401125	NM_022327:257	Ralb	INSIDE	0.543	0.592	3035.96	1797.70	0.322	2369.74	762.06
A_68_P20557414	chr1:121401510-121401554	NM_022327:-173	Ralb	PROMOTER	0.140	0.616	1876.08	1155.68	0.086	1397.04	120.39
A_68_P20557415	chr1:121401606-121401656	NM_022327:-271	Ralb	PROMOTER	0.364	0.498	1170.49	582.38	0.181	884.83	160.09
A_68_P31303253	chr17:66234412-66234456	NM_001198949:-171	Ralbp1	PROMOTER	0.478	0.647	1063.99	688.12	0.309	889.87	274.93
A_68_P31303254	chr17:66234502-66234546	NM_001198949:-261	Ralbp1	PROMOTER	0.581	0.565	1492.42	842.92	0.328	1169.84	384.02
A_68_P21091470	chr2:28369078-28369122	NM_001145834:414	Ralgds	INSIDE	0.300	0.445	1702.64	757.67	0.133	1231.55	164.31
A_68_P20745868	chr1:158870188-158870239	NM_001159965:-456	Ralgps2	PROMOTER	0.121	0.713	1773.03	1264.17	0.086	1351.45	116.36
A_68_P21762356	chr2:154616308-154616364	NM_001139511:-509	Raly	PROMOTER	0.149	0.574	2066.36	1187.01	0.086	1455.79	124.90
A_68_P21762357	chr2:154616385-154616429	NM_001139511:-439	Raly	PROMOTER	0.574	0.530	5463.18	2897.66	0.304	4104.39	1249.13
A_68_P21762361	chr2:154616920-154616964	NM_001139511:97	Raly	INSIDE	0.390	0.495	1287.23	637.07	0.193	1030.27	199.03
A_68_P21762363	chr2:154617145-154617189	NM_001139511:321	Raly	INSIDE	0.381	0.604	2141.14	1293.22	0.230	1803.78	415.27
A_68_P21952653	chr3:13472808-13472852	NM_001163328:1176	Ralyl	INSIDE	0.662	0.669	2071.17	1384.94	0.442	1586.41	701.86
A_68_P21952656	chr3:13473217-13473261	NM_001163328:1584	Ralyl	INSIDE	0.177	0.658	1229.02	809.30	0.117	1061.28	123.71
A_68_P28072822	chr11:101107992-101108036	NM_019444:367	Ramp2	INSIDE	0.427	0.617	1396.03	861.11	0.263	1052.20	276.94
A_68_P30578587	chr16:18248121-18248165	NM_011239:645	Ranbp1	INSIDE	0.201	0.579	1146.06	664.12	0.117	1005.90	117.32
A_68_P27695167	chr11:33413838-33413882	NM_023146:-114	Ranbp17	PROMOTER	0.619	0.547	1762.37	964.25	0.339	1472.20	498.76
A_68_P31257591	chr17:56812887-56812931	NM_027933:229	Ranbp3	INSIDE	0.203	0.329	2100.47	690.05	0.067	1813.72	120.85
A_68_P32054982	chr19:29887617-29887661	NM_177721:-174	Ranbp6	PROMOTER	0.215	0.635	1252.81	794.95	0.136	978.17	133.14
A_68_P30379305	chr15:81560318-81560371	NM_001146174:5	Rangap1	INSIDE	0.173	0.717	1472.99	1056.85	0.124	946.00	117.49
A_68_P22399296	chr3:105603567-105603617	NM_145541:662	Rap1a	INSIDE	0.281	0.550	973.04	534.79	0.154	755.33	116.69
A_68_P27921006	chr11:74335943-74335987	NM_001015046:67696	Rap1gap2	INSIDE	0.594	0.532	2306.35	1226.08	0.316	1703.70	538.42
A_68_P27921011	chr11:74336607-74336651	NM_001015046:67032	Rap1gap2	INSIDE	0.482	0.673	3342.51	2250.51	0.324	2475.59	802.64
A_68_P27921014	chr11:74336964-74337008	NM_001015046:66674	Rap1gap2	INSIDE	0.229	0.567	1792.23	1016.13	0.130	1253.56	162.62
A_68_P27921382	chr11:74403457-74403501	NM_001015046:182	Rap1gap2	INSIDE	0.401	0.564	1202.64	678.74	0.227	932.96	211.38
A_68_P22572455	chr3:138738714-138738758	NM_001040690:-573	Rap1gds1	PROMOTER	0.656	0.377	1539.73	581.21	0.248	1233.78	305.44
A_68_P29950631	chr14:120878349-120878398	NM_029519:691	Rap2a	INSIDE	0.290	0.569	1022.09	582.08	0.165	727.34	120.08
A_68_P22182580	chr3:61168280-61168324	NM_028712:-126	Rap2b	PROMOTER	0.244	0.606	1397.27	846.36	0.148	1139.91	168.73
A_68_P32374343	chrX:48371227-48371271	NM_172413:-53	Rap2c	PROMOTER	0.303	5.261	4264.93	22436.89	1.595	2010.66	3206.85
A_68_P32374347	chrX:48371964-48372008	NM_172413:-791	Rap2c	PROMOTER	0.190	1.458	1877.31	2737.70	0.277	894.54	247.55
A_68_P32374349	chrX:48372156-48372200	NM_172413:-983	Rap2c	PROMOTER	0.161	1.550	916.33	1420.77	0.249	484.48	120.83
A_68_P20269871	chr1:60624074-60624118	NM_001045513:-487	Raph1	PROMOTER	0.346	0.454	1323.52	601.18	0.157	1077.61	169.44
A_68_P28059432	chr11:98798988-98799032	NM_001177302:-21	Rara	PROMOTER	0.479	0.604	1672.00	1009.06	0.289	1168.93	337.96
A_68_P28059598	chr11:98821127-98821171	NM_001176528:-636	Rara	PROMOTER	0.311	0.634	1923.06	1218.81	0.197	1477.29	291.35
A_68_P29430092	chr14:17408235-17408279	NM_011243:-532	Rarb	PROMOTER	0.319	0.438	2884.54	1262.51	0.140	2205.74	308.24
A_68_P30496838	chr15:102077585-102077629	NM_001042727:-823	Rarg	PROMOTER	0.593	0.414	1630.74	674.50	0.245	1351.07	331.20
A_68_P30496839	chr15:102077744-102077788	NM_001042727:-983	Rarg	PROMOTER	0.627	0.484	1077.53	522.05	0.304	882.94	268.37
A_68_P30496840	chr15:102077921-102077968	NM_001042727:-1161	Rarg	PROMOTER	0.238	0.454	1342.61	609.51	0.108	1082.86	116.92
A_68_P22210464	chr3:67319384-67319428	NM_001164763:39	Rarres1	INSIDE	0.129	0.715	5444.87	3895.74	0.092	4132.73	380.10
A_68_P22210465	chr3:67319575-67319619	NM_001164763:-151	Rarres1	PROMOTER	0.563	0.605	1333.46	806.95	0.341	1058.70	360.81
A_68_P26748149	chr9:96531046-96531090	NM_053268:854	Rasa2	INSIDE	0.197	0.591	1442.12	852.04	0.117	1172.93	136.65
A_68_P26748151	chr9:96531280-96531324	NM_053268:620	Rasa2	INSIDE	0.616	0.558	2267.55	1265.27	0.344	2134.17	733.36
A_68_P25669972	chr8:13677124-13677168	NM_009025:441	Rasa3	INSIDE	0.254	0.689	2615.30	1803.09	0.175	1884.79	329.83
A_68_P24119438	chr5:136578137-136578181	NM_133914:18373	Rasa4	INSIDE	0.553	0.659	1416.44	932.98	0.364	1122.29	409.03
A_68_P24119440	chr5:136578358-136578403	NM_133914:18595	Rasa4	INSIDE	0.294	0.548	1458.41	798.68	0.161	1075.99	172.99

ProbeName	Target position of probe on CpG island microarray	TargetID	GeneSymbol	CpG island Description	Ratio of relative methylation (TiO2-NP/Vehicle)	Sham group			TiO_2-H group		
						Relative methylation (Cy-5/Cy-3)	Cy-3 signal (Input)	Cy-5 signal (MeDIP)	Relative methylation (Cy-5/Cy-3)	Cy-3 signal (Input)	Cy-5 signal (MeDIP)
A_68_P24040026	chr5:121098911-121098955	NM_013832:102	Rasal1	INSIDE	0.206	1.637	3219.68	5269.97	0.338	2523.31	851.68
A_68_P24040027	chr5:121099008-121099052	NM_013832:200	Rasal1	INSIDE	0.081	2.528	1026.21	2594.46	0.204	595.00	121.17
A_68_P24040029	chr5:121099253-121099297	NM_013832:444	Rasal1	INSIDE	0.253	0.361	1675.88	604.30	0.091	1277.89	116.66
A_68_P24040031	chr5:121099444-121099488	NM_013832:636	Rasal1	INSIDE	0.275	0.569	1124.74	640.49	0.157	921.54	144.51
A_68_P23922675	chr5:99681822-99681866	NM_145839:102	Rasgef1b	INSIDE	0.128	0.513	2574.10	1321.64	0.066	1827.96	119.74
A_68_P26714904	chr9:89805072-89805116	NM_001039655:482	Rasgrf1	INSIDE	0.376	0.641	1055.56	676.09	0.241	881.96	212.28
A_68_P26714905	chr9:89805194-89805241	NM_001039655:605	Rasgrf1	INSIDE	0.237	0.593	1021.30	605.32	0.141	834.71	117.30
A_68_P26714893	chr9:89803729-89803773	NM_001039655:-862	Rasgrf1	PROMOTER	0.669	0.659	856.26	564.24	0.441	715.42	315.48
A_68_P21557415	chr2:117168712-117168756	NM_011246:-121	Rasgrp1	PROMOTER	0.434	0.597	1661.08	991.78	0.259	1394.04	361.06
A_68_P31934460	chr19:6398732-6398778	NM_011242:-1828	Rasgrp2	PROMOTER	0.578	0.465	1130.32	525.27	0.268	897.33	240.83
A_68_P31934468	chr19:6399999-6400043	NM_011242:-562	Rasgrp2	PROMOTER	0.317	0.548	4238.66	2323.82	0.174	3283.47	570.34
A_68_P25094132	chr7:52884421-52884465	NM_028544:1536	Rasip1	INSIDE	0.536	1.350	2985.70	4029.90	0.724	2220.03	1606.46
A_68_P27971294	chr11:83223607-83223651	NM_001013386:55	Rasl10b	INSIDE	0.390	0.597	1329.78	794.26	0.233	967.02	225.31
A_68_P24170389	chr5:147656737-147656781	NM_026864:112	Rasl11a	INSIDE	0.560	0.649	1181.89	767.18	0.364	1037.70	377.39
A_68_P24170392	chr5:147657058-147657102	NM_026864:434	Rasl11a	INSIDE	0.293	0.666	1475.99	983.08	0.195	1211.41	236.39
A_68_P24170393	chr5:147657202-147657261	NM_026864:585	Rasl11a	INSIDE	0.262	0.628	862.86	542.00	0.165	744.37	122.61
A_68_P23801766	chr5:74590964-74591008	NM_026878:-364	Rasl11b	PROMOTER	0.269	0.565	2245.94	1269.74	0.152	1672.29	254.39
A_68_P23801769	chr5:74591333-74591377	NM_026878:4	Rasl11b	INSIDE	0.309	0.462	1675.56	773.71	0.143	1358.00	194.04
A_68_P26809513	chr9:107456377-107456421	NM_019713:-588	Rassf1	PROMOTER	0.203	0.686	1131.58	775.79	0.139	839.72	116.60
A_68_P26809514	chr9:107456460-107456504	NM_019713:-504	Rassf1	PROMOTER	0.291	0.569	1935.92	1101.47	0.166	1313.80	217.82
A_68_P26809521	chr9:107457493-107457537	NM_019713:528	Rassf1	INSIDE	0.598	0.495	1189.45	588.53	0.296	931.76	275.71
A_68_P25427824	chr7:120098233-120098277	NM_175279:779	Rassf10	INSIDE	0.277	0.501	2614.57	1308.78	0.139	2103.27	292.12
A_68_P27503751	chr10:120912576-120912620	NM_138956:708	Rassf3	INSIDE	0.529	0.636	2735.34	1740.44	0.337	2085.38	702.08
A_68_P24777649	chr6:116623707-116623752	NM_178045:125	Rassf4	INSIDE	0.359	0.617	1306.76	805.96	0.221	923.35	204.47
A_68_P24777650	chr6:116623776-116623820	NM_178045:56	Rassf4	INSIDE	0.168	0.732	1966.25	1439.87	0.123	1437.39	176.71
A_68_P25587465	chr7:148401434-148401478	NM_025886:-302	Rassf7	DIVERGENT_PROMOTER	0.166	0.605	1453.65	879.77	0.100	1148.80	115.26
A_68_P25587466	chr7:148401511-148401555	NM_025886:-226	Rassf7	DIVERGENT_PROMOTER	0.249	0.482	3154.33	1521.92	0.120	1867.66	224.16
A_68_P26345409	chr9:20896597-20896641	NR_038081:-166	Raver1-fdx1l	PROMOTER	0.165	0.695	1766.46	1226.87	0.114	1290.26	147.57
A_68_P23152967	chr4:100741468-100741512	NM_183024:-152	Raver2	PROMOTER	0.338	0.552	1400.97	773.23	0.187	1240.37	231.50
A_68_P23152972	chr4:100742061-100742105	NM_183024:440	Raver2	INSIDE	0.122	2.092	3660.61	7659.09	0.255	2427.75	618.92
A_68_P23152973	chr4:100742175-100742219	NM_183024:554	Raver2	INSIDE	0.123	2.177	3486.44	7590.62	0.268	2492.45	667.52
A_68_P20011680	chr1:6204723-6204767	NM_009826:2	Rb1cc1	INSIDE	0.433	0.385	1750.61	674.21	0.167	1388.61	231.75
A_68_P25481826	chr7:130114107-130114151	NM_175023:51	Rbbp6	INSIDE	0.317	0.383	1654.05	634.13	0.121	1313.93	159.46
A_68_P32790792	chrX:159198218-159198262	NM_009031:-63	Rbbp7	PROMOTER	0.229	2.049	3263.63	6685.96	0.469	1471.76	690.26
A_68_P32790799	chrX:159199053-159199097	NM_009031:771	Rbbp7	INSIDE	0.094	2.219	4538.78	10072.80	0.208	2097.62	435.96
A_68_P31496430	chr18:11816616-11816661	NM_001081223:288	Rbbp8	INSIDE	0.140	0.659	1681.30	1108.46	0.092	1244.74	114.89
A_68_P21748795	chr2:152158169-152158213	NM_001083921:-29	Rbck1	PROMOTER	0.424	0.622	1354.24	841.74	0.264	1240.43	326.89
A_68_P30518123	chr16:5885155-5885211	NM_021477:297	Rbfox1	INSIDE	0.274	0.595	1352.88	805.35	0.163	1019.62	166.57
A_68_P30520835	chr16:6349110-6349154	NM_021477:464247	Rbfox1	INSIDE	0.283	0.648	1817.20	1176.68	0.183	1424.51	260.77
A_68_P28172264	chr11:118430533-118430577	NM_001024931:340332	Rbfox3	INSIDE	0.522	0.513	1315.00	674.32	0.268	984.46	263.47
A_68_P28172265	chr11:118430608-118430652	NM_001024931:340256	Rbfox3	INSIDE	0.293	0.490	1592.34	780.22	0.143	1158.14	166.16
A_68_P28174642	chr11:118771162-118771206	NM_001024931:-298	Rbfox3	PROMOTER	0.254	0.604	1069.81	646.22	0.154	769.17	118.08
A_68_P26056692	chr8:93594415-93594471	NM_011250:450	Rbl2	INSIDE	0.182	0.594	1386.35	823.65	0.108	1123.90	121.48
A_68_P32289235	chrX:20194111-20194155	NM_001167775:-496	Rbm10	PROMOTER	0.311	1.498	1293.19	1937.09	0.466	657.26	305.99

ProbeName	Target position of probe on CpG island microarray	TargetID	GeneSymbol	CpG island Description	Ratio of relative methylation (TiO2-NP/Vehicle)	Sham group			TiO_2-H group		
						Relative methylation (Cy-5/Cy-3)	Cy-3 signal (Input)	Cy-5 signal (MeDIP)	Relative methylation (Cy-5/Cy-3)	Cy-3 signal (Input)	Cy-5 signal (MeDIP)
A_68_P32289247	chrX:20195452-20195505	NM_001167776:594	Rbm10	INSIDE	0.093	1.501	2255.84	3386.70	0.139	849.63	118.40
A_68_P32289248	chrX:20195553-20195607	NM_001167776:696	Rbm10	INSIDE	0.143	2.026	1692.56	3428.57	0.291	715.96	208.02
A_68_P30879835	chr16:75593202-75593246	NM_198302:89	Rbm11	INSIDE	0.322	0.573	1181.66	676.84	0.185	981.41	181.14
A_68_P31925255	chr19:4811527-4811571	NM_019869:86	Rbm14	INSIDE	0.543	0.553	1317.37	728.30	0.300	1027.16	308.37
A_68_P31925256	chr19:4811668-4811714	NM_019869:-56	Rbm14	PROMOTER	0.183	0.522	1427.01	744.50	0.096	1211.67	115.81
A_68_P31925257	chr19:4811827-4811871	NM_019869:-214	Rbm14	PROMOTER	0.384	0.487	1962.26	954.80	0.187	1835.14	342.81
A_68_P22405706	chr3:107135381-107135431	NM_001045807:801	Rbm15	INSIDE	0.217	0.465	1542.54	716.97	0.101	1153.45	116.49
A_68_P22405708	chr3:107135601-107135645	NM_001045807:585	Rbm15	INSIDE	0.540	0.544	1421.81	774.13	0.294	1190.38	349.86
A_68_P22405711	chr3:107135861-107135907	NM_001045807:323	Rbm15	INSIDE	0.236	0.524	1429.53	748.48	0.123	1113.74	137.33
A_68_P22405717	chr3:107136584-107136628	NM_001045807:-399	Rbm15	PROMOTER	0.556	0.481	1819.97	875.19	0.267	1356.02	362.69
A_68_P26805684	chr9:106788066-106788110	NM_175402:1243	Rbm15b	INSIDE	0.266	0.639	849.13	542.77	0.170	678.03	115.28
A_68_P26805685	chr9:106788200-106788244	NM_175402:1109	Rbm15b	INSIDE	0.492	0.598	1088.56	651.41	0.294	830.57	244.54
A_68_P21138067	chr2:35992191-35992235	NM_001159635:12	Rbm18	INSIDE	0.629	0.469	1495.94	701.46	0.295	1171.16	345.47
A_68_P31752099	chr18:60720832-60720876	NM_025776:415	Rbm22	INSIDE	0.313	0.499	1170.38	583.96	0.156	907.54	141.52
A_68_P29007689	chr13:46513571-46513615	NM_001081425:-76	Rbm24	PROMOTER	0.219	0.535	1288.04	689.07	0.117	1001.11	117.15
A_68_P26260675	chr8:129494647-129494691	NM_172762:311	Rbm34	INSIDE	0.263	0.642	889.38	570.92	0.169	708.92	119.68
A_68_P26260677	chr8:129494964-129495008	NM_172762:-7	Rbm34	PROMOTER	0.469	0.470	1410.04	663.32	0.221	1205.66	266.11
A_68_P21867219	chr2:172847604-172847648	NM_019547:224	Rbm38	INSIDE	0.513	0.711	1925.78	1370.09	0.365	1461.18	532.79
A_68_P21867220	chr2:172847673-172847717	NM_019547:292	Rbm38	INSIDE	0.235	0.365	2237.16	817.21	0.086	1560.60	134.19
A_68_P21769937	chr2:156005990-156006034	NM_133242:-36	Rbm39	PROMOTER	0.357	0.439	1650.17	724.25	0.157	1330.90	208.68
A_68_P25032745	chr7:31435198-31435242	NM_133693:27	Rbm42	INSIDE	0.411	0.643	1186.15	762.97	0.264	930.76	246.05
A_68_P22281783	chr3:82680573-82680617	NM_001146328:-189	Rbm46	PROMOTER	0.484	1.399	1858.55	2600.42	0.678	1455.68	986.26
A_68_P31924875	chr19:4756268-4756312	NM_025717:-234	Rbm4b	PROMOTER	0.492	0.689	1893.55	1304.09	0.339	1588.78	538.28
A_68_P26811064	chr9:107673153-107673200	NM_148930:157	Rbm5	INSIDE	0.252	0.666	1035.06	688.89	0.168	748.98	125.57
A_68_P26811065	chr9:107673263-107673307	NM_148930:49	Rbm5	INSIDE	0.261	0.581	3298.25	1915.74	0.151	2540.08	384.63
A_68_P32401097	chrX:54646265-54646313	NR_029425:-75	Rbmx	PROMOTER	0.128	1.422	1637.78	2328.45	0.182	653.37	118.78
A_68_P32365931	chrX:46048088-46048132	NM_173376:-70	Rbmx2	PROMOTER	0.376	0.657	1113.81	731.76	0.247	507.14	125.23
A_68_P26758565	chr9:98325194-98325238	NM_011254:1837	Rbp1	INSIDE	0.667	0.517	2718.74	1405.67	0.345	2031.16	700.18
A_68_P21817622	chr2:164226486-164226530	NM_009036:-2185	Rbpjl	PROMOTER	0.149	0.562	2891.71	1624.92	0.084	2178.00	182.49
A_68_P25771679	chr8:35040712-35040756	NM_001042674:-421	Rbpms	PROMOTER	0.104	0.673	2574.00	1732.31	0.070	1861.78	130.03
A_68_P26588674	chr9:65477102-65477146	NM_028030:-1264	Rbpms2	PROMOTER	0.562	0.542	1527.94	828.87	0.305	1209.09	368.88
A_68_P26588678	chr9:65477711-65477764	NM_028030:-651	Rbpms2	PROMOTER	0.364	0.429	1876.50	804.27	0.156	1567.11	244.47
A_68_P23328509	chr4:134989508-134989552	NM_022980:190	Rcan3	INSIDE	0.364	0.663	1405.51	931.59	0.241	1138.84	275.03
A_68_P23328512	chr4:134989887-134989933	NM_022980:-190	Rcan3	PROMOTER	0.393	0.620	1491.84	925.02	0.244	1175.09	286.59
A_68_P23328514	chr4:134990088-134990132	NM_022980:-390	Rcan3	PROMOTER	0.579	0.637	1470.26	936.68	0.369	1113.42	410.39
A_68_P23328515	chr4:134990204-134990248	NM_022980:-506	Rcan3	PROMOTER	0.489	0.703	1738.96	1222.08	0.344	1410.08	484.53
A_68_P23311883	chr4:131901216-131901260	NM_001197082:427	Rcc1	INSIDE	0.424	0.600	1049.23	629.95	0.255	818.84	208.65
A_68_P23359686	chr4:140258987-140259035	NM_173867:1623	Rcc2	INSIDE	0.203	0.601	1281.87	770.88	0.122	962.24	117.72
A_68_P26533942	chr9:55889666-55889710	NM_011992:37	Rcn2	INSIDE	0.180	0.433	1976.80	855.38	0.078	1529.11	118.78
A_68_P28741728	chr12:112276886-112276930	NM_198023:-1100	Rcor1	PROMOTER	0.457	0.494	1409.67	696.75	0.226	1096.84	247.53
A_68_P28741734	chr12:112278458-112278502	NM_198023:472	Rcor1	INSIDE	0.290	0.583	1825.48	1065.11	0.169	1419.55	240.15
A_68_P20053922	chr1:16095556-16095600	NM_133832:-384	Rdh10	DIVERGENT_PROMOTER	0.506	0.546	1711.47	933.66	0.276	1379.27	380.66
A_68_P30350127	chr15:76540848-76540892	NM_058214:119	Recql4	INSIDE	0.511	0.668	1775.08	1186.16	0.342	1352.78	462.13
A_68_P24537772	chr6:71657975-71658019	NM_178608:322	Reep1	INSIDE	0.110	0.506	2540.77	1285.70	0.056	2130.18	119.01

ProbeName	Target position of probe on CpG island microarray	TargetID	GeneSymbol	CpG island Description	Ratio of relative methylation (TiO2-NP/Vehicle)	Sham group			TiO_2-H group		
						Relative methylation (Cy-5/Cy-3)	Cy-3 signal (Input)	Cy-5 signal (MeDIP)	Relative methylation (Cy-5/Cy-3)	Cy-3 signal (Input)	Cy-5 signal (MeDIP)
A_68_P24537774	chr6:71658275-71658319	NM_178608:622	Reep1	INSIDE	0.172	0.446	1735.98	773.43	0.077	1525.65	116.74
A_68_P27209845	chr10:66560085-66560129	NM_001204915:-370	Reep3	PROMOTER	0.316	0.725	1973.20	1431.51	0.229	1480.80	338.96
A_68_P27647502	chr11:23670649-23670693	NM_009044:300	Rel	INSIDE	0.486	0.652	2102.83	1371.24	0.317	1788.84	566.53
A_68_P24994423	chr7:20214968-20215012	NM_009046:-203	Relb	PROMOTER	0.542	0.679	1367.16	927.81	0.368	1126.93	414.69
A_68_P31631094	chr18:38115681-38115725	NM_153793:490	Rell2	INSIDE	0.064	0.658	3935.67	2591.41	0.042	2784.17	117.36
A_68_P23531277	chr5:21851025-21851070	NM_011261:-524	Reln	PROMOTER	0.651	0.661	1497.21	990.22	0.430	1294.20	556.98
A_68_P21750258	chr2:152452780-152452824	NM_009047:59	Rem1	INSIDE	0.351	0.587	986.89	579.11	0.206	873.31	179.83
A_68_P21750259	chr2:152452978-152453022	NM_009047:257	Rem1	INSIDE	0.192	0.588	1810.97	1065.00	0.113	1342.19	151.80
A_68_P21750261	chr2:152453185-152453229	NM_009047:463	Rem1	INSIDE	0.645	0.444	1860.67	826.16	0.287	1469.94	421.20
A_68_P32465510	chrX:71167458-71167506	NM_001164704:8707	Renbp	INSIDE	0.101	2.040	1651.28	3369.26	0.205	574.49	118.03
A_68_P32465511	chrX:71167531-71167575	NM_001164704:8637	Renbp	INSIDE	0.077	1.563	2571.84	4020.36	0.120	1067.20	127.67
A_68_P24428256	chr6:48551764-48551808	NM_001079901:7905	Repin1	DOWNSTREAM	0.097	0.571	3897.33	2224.12	0.056	2850.56	158.25
A_68_P24428257	chr6:48551937-48551981	NM_001079901:8077	Repin1	DOWNSTREAM	0.535	0.531	4169.93	2214.12	0.284	3189.15	905.31
A_68_P23407014	chr4:149944046-149944090	NM_001085492:288044	Rere	INSIDE	0.597	0.590	2454.17	1448.36	0.352	1861.77	655.57
A_68_P23820628	chr5:77694649-77694693	NM_011263:152	Rest	INSIDE	0.598	0.528	1795.51	947.91	0.316	1573.14	496.55
A_68_P24785568	chr6:118147500-118147544	NM_001080780:240	Ret	INSIDE	0.094	2.388	10979.76	26219.25	0.225	6636.61	1490.45
A_68_P24785569	chr6:118147581-118147630	NM_001080780:157	Ret	INSIDE	0.031	2.450	3790.76	9288.09	0.077	2407.56	184.55
A_68_P27080344	chr10:39452278-39452323	NM_011264:335	Rev3l	INSIDE	0.443	0.567	1488.42	844.16	0.251	1117.16	280.88
A_68_P27283982	chr10:80023920-80023964	NM_025852:363	Rexo1	INSIDE	0.524	0.385	2003.75	771.99	0.202	1626.95	328.65
A_68_P27968801	chr11:82684323-82684367	NM_001164570:-98	Rffl	PROMOTER	0.454	0.455	1610.47	732.36	0.207	1231.36	254.43
A_68_P31985662	chr19:17468432-17468476	NM_019437:-78	Rfk	PROMOTER	0.472	0.417	3102.35	1293.67	0.197	2617.78	514.97
A_68_P31985663	chr19:17468572-17468616	NM_019437:62	Rfk	INSIDE	0.446	0.493	2174.07	1070.79	0.219	1675.67	367.73
A_68_P26017900	chr8:86590423-86590471	NM_009055:-288	Rfx1	PROMOTER	0.219	0.467	1542.89	720.55	0.102	1141.34	116.58
A_68_P32044142	chr19:28086118-28086162	NM_011265:-484	Rfx3	PROMOTER	0.244	0.695	3564.25	2477.90	0.170	2644.48	449.18
A_68_P26628305	chr9:72379707-72379757	NM_001033536:-314	Rfx7	PROMOTER	0.324	0.657	823.83	540.96	0.213	566.59	120.64
A_68_P30778981	chr16:56037605-56037649	NM_029092:261	Rg9mtd1	INSIDE	0.467	0.420	1245.39	522.66	0.196	997.37	195.53
A_68_P20722064	chr1:154472045-154472089	NM_016846:175	Rgl1	INSIDE	0.228	0.518	2141.40	1108.91	0.118	1617.76	190.72
A_68_P20722065	chr1:154472108-154472163	NM_016846:106	Rgl1	INSIDE	0.268	0.398	1496.32	596.14	0.107	1195.18	127.54
A_68_P31151052	chr17:34067414-34067462	NM_009059:600	Rgl2	INSIDE	0.039	4.551	5324.40	24230.36	0.179	3132.35	559.83
A_68_P31151053	chr17:34067535-34067579	NM_009059:718	Rgl2	INSIDE	0.130	2.716	4198.16	11404.20	0.352	2488.69	877.13
A_68_P31067730	chr17:15962490-15962534	NM_178615:1038	Rgmb	INSIDE	0.645	0.417	2151.37	897.58	0.269	1632.10	439.16
A_68_P31067774	chr17:15967950-15967994	NM_178615:-4422	Rgmb	PROMOTER	0.592	0.579	2910.60	1684.44	0.342	2239.36	766.97
A_68_P25510686	chr7:135561251-135561295	NM_026418:414	Rgs10	INSIDE	0.648	0.707	1356.19	958.84	0.458	1082.84	496.08
A_68_P31108918	chr17:26340491-26340535	NM_001081069:606	Rgs11	INSIDE	0.504	0.612	1512.53	925.60	0.309	1224.05	377.86
A_68_P31108916	chr17:26340305-26340349	NM_001081069:420	Rgs11	INSIDE	0.670	0.588	1233.03	724.99	0.394	1057.55	416.67
A_68_P23597153	chr5:35292578-35292624	NM_173402:504	Rgs12	INSIDE	0.239	1.933	751.95	1453.25	0.463	581.31	268.95
A_68_P21912147	chr2:181428245-181428289	NM_026446:363	Rgs19	INSIDE	0.597	0.587	1635.67	959.55	0.350	1612.89	565.09
A_68_P20681326	chr1:145851560-145851609	NM_009061:-305	Rgs2	PROMOTER	0.314	0.576	1052.78	606.92	0.181	772.29	139.63
A_68_P20008180	chr1:5008925-5008969	NM_021374:561	Rgs20	INSIDE	0.256	0.312	1988.23	619.88	0.080	1559.71	124.60
A_68_P28587835	chr12:83718196-83718240	NM_015812:194	Rgs6	INSIDE	0.637	0.584	1606.29	937.43	0.372	1389.97	517.07
A_68_P29294057	chr13:105844812-105844856	NM_029879:176	Rgs7bp	INSIDE	0.252	0.459	1256.61	576.18	0.116	1031.29	119.37
A_68_P20391888	chr1:82313214-82313260	NM_029777:83	Rhbdd1	INSIDE	0.239	0.479	1322.67	633.46	0.115	1026.43	117.54
A_68_P28160473	chr11:116485490-116485534	NM_001167680:54	Rhbdf2	INSIDE	0.634	0.579	1372.87	794.50	0.367	1102.26	404.22
A_68_P22312700	chr3:88044849-88044893	NM_021375:13736	Rhbg	DOWNSTREAM	0.300	0.675	1054.10	712.01	0.203	878.09	177.91

ProbeName	Target position of probe on CpG island microarray	TargetID	GeneSymbol	CpG island Description	Ratio of relative methylation (TiO2-NP/Vehicle)	Sham group			TiO_2-H group		
						Relative methylation (Cy-5/Cy-3)	Cy-3 signal (Input)	Cy-5 signal (MeDIP)	Relative methylation (Cy-5/Cy-3)	Cy-3 signal (Input)	Cy-5 signal (MeDIP)
A_68_P22312701	chr3:88044948-88044992	NM_021375:13636	Rhbg	DOWNSTREAM	0.482	0.400	1768.14	707.26	0.193	1340.77	258.59
A_68_P22312702	chr3:88045112-88045156	NM_021375:13472	Rhbg	DOWNSTREAM	0.352	0.647	817.10	528.52	0.228	713.72	162.42
A_68_P22312797	chr3:88058696-88058740	NM_021375:-112	Rhbg	PROMOTER	0.427	0.518	1100.66	569.84	0.221	865.57	191.15
A_68_P25262216	chr7:86762430-86762474	NM_019799:91	Rhcg	INSIDE	0.627	0.550	1137.87	626.33	0.345	872.04	301.05
A_68_P23544150	chr5:24348013-24348057	NM_053075:145	Rheb	INSIDE	0.302	0.447	2693.22	1203.31	0.135	1930.51	260.66
A_68_P30477113	chr15:98711795-98711839	NM_026967:29	Rhebl1	INSIDE	0.590	0.638	2410.21	1538.84	0.376	1874.16	705.52
A_68_P29152215	chr13:76080947-76080991	NM_028493:304	Rhobtb3	INSIDE	0.261	0.649	1542.39	1000.33	0.169	1357.99	229.92
A_68_P24054149	chr5:123591328-123591372	NM_175092:-8712	Rhof	PROMOTER	0.467	0.477	1636.68	781.23	0.223	1258.59	280.63
A_68_P24054157	chr5:123592214-123592258	NM_175092:-9598	Rhof	PROMOTER	0.275	0.734	2218.10	1628.65	0.202	1766.95	357.29
A_68_P25377911	chr7:109398265-109398309	NM_019566:346	Rhog	INSIDE	0.157	0.505	1887.92	952.76	0.079	1453.13	115.33
A_68_P25377920	chr7:109399308-109399352	NM_019566:-698	Rhog	PROMOTER	0.379	0.677	2603.19	1762.26	0.256	1904.52	488.06
A_68_P30343544	chr15:75543867-75543911	NM_001163465:9171	Rhpn1	INSIDE	0.600	2.680	2917.76	7820.67	1.607	2077.65	3339.03
A_68_P25048759	chr7:36119164-36119208	NM_027897:-69	Rhpn2	PROMOTER	0.296	0.480	1957.54	939.90	0.142	1650.02	234.47
A_68_P25048761	chr7:36119403-36119447	NM_027897:169	Rhpn2	INSIDE	0.278	0.678	1219.41	826.55	0.188	828.80	156.00
A_68_P25048763	chr7:36119695-36119739	NM_027897:461	Rhpn2	INSIDE	0.342	0.501	1703.30	852.57	0.171	1380.89	236.65
A_68_P25406004	chr7:116226565-116226609	NM_001038624:252	Ric3	INSIDE	0.668	0.517	1077.58	556.82	0.345	787.70	272.01
A_68_P25585273	chr7:148043403-148043447	NM_053194:129	Ric8	INSIDE	0.379	0.593	1126.28	667.47	0.224	898.41	201.57
A_68_P21217119	chr2:51928961-51929008	NM_175238:628	Rif1	INSIDE	0.103	3.352	1710.96	5735.83	0.345	897.51	309.75
A_68_P24061766	chr5:124981229-124981273	NM_021430:150	Rilpl1	INSIDE	0.516	0.406	1536.95	624.37	0.210	1115.47	233.86
A_68_P24061481	chr5:124927981-124928025	NM_030259:242	Rilpl2	INSIDE	0.143	0.696	3170.36	2206.78	0.100	2288.85	227.81
A_68_P30572549	chr16:17209535-17209579	NM_001033338:1329	Rimbp3	INSIDE	0.297	0.528	4076.18	2153.37	0.157	2939.18	460.39
A_68_P30572550	chr16:17209655-17209699	NM_001033338:1449	Rimbp3	INSIDE	0.141	0.747	4156.80	3106.86	0.106	2778.69	293.50
A_68_P30572559	chr16:17210640-17210686	NM_001033338:2435	Rimbp3	INSIDE	0.268	0.627	1739.47	1091.01	0.168	1341.45	225.66
A_68_P23245808	chr4:119165389-119165433	NM_177572:-207	Rimkla	PROMOTER	0.225	0.712	1299.82	924.89	0.160	1104.98	177.24
A_68_P20083594	chr1:22540800-22540844	NM_001012623:271741	Rims1	INSIDE	0.335	0.662	1033.37	683.92	0.222	850.72	188.60
A_68_P20085263	chr1:22812453-22812497	NM_001012623:89	Rims1	INSIDE	0.131	2.441	2858.24	6977.07	0.320	1868.49	597.44
A_68_P20085267	chr1:22813048-22813092	NM_001012623:-507	Rims1	PROMOTER	0.234	0.714	2083.88	1488.27	0.167	1498.07	250.84
A_68_P30152564	chr15:39028639-39028683	NM_053271:-1217	Rims2	PROMOTER	0.469	0.685	1621.62	1110.15	0.321	1135.29	364.41
A_68_P28958208	chr13:38129074-38129118	NM_024242:239	Riok1	INSIDE	0.233	0.718	3482.16	2499.46	0.168	2431.95	407.54
A_68_P22753437	chr4:16090490-16090534	NM_138952:133	Ripk2	INSIDE	0.584	0.534	3360.68	1795.39	0.312	2760.93	861.70
A_68_P31000873	chr16:97984845-97984902	NM_023663:489	Ripk4	INSIDE	0.318	0.584	961.88	561.91	0.186	623.95	115.79
A_68_P31000876	chr16:97985140-97985184	NM_023663:200	Ripk4	INSIDE	0.181	0.434	2082.83	904.70	0.079	1521.97	119.59
A_68_P31000877	chr16:97985236-97985280	NM_023663:104	Ripk4	INSIDE	0.258	0.585	1650.93	965.70	0.151	1308.98	197.71
A_68_P31000878	chr16:97985324-97985368	NM_023663:16	Ripk4	INSIDE	0.609	0.500	2313.74	1156.23	0.304	1915.25	582.80
A_68_P26702535	chr9:86910781-86910825	NM_001037907:431	Ripply2	INSIDE	0.355	0.696	2712.26	1887.62	0.247	2398.42	591.77
A_68_P22315262	chr3:88520847-88520891	NM_001163310:93	Rit1	INSIDE	0.420	0.670	1279.17	857.61	0.281	1095.06	308.12
A_68_P22315263	chr3:88520957-88521001	NM_001163310:203	Rit1	INSIDE	0.162	0.399	4190.98	1670.30	0.065	3125.81	202.04
A_68_P32575423	chrX:101176377-101176421	NM_011276:225	Rlim	INSIDE	0.137	1.409	1738.02	2448.18	0.193	705.74	136.32
A_68_P32575425	chrX:101176563-101176615	NM_011276:35	Rlim	INSIDE	0.188	1.645	907.54	1493.05	0.310	399.65	123.90
A_68_P29073051	chr13:58502488-58502536	NR_031761:-1096	Rmi1	DIVERGENT_PROMOTER	0.314	0.444	1268.57	563.20	0.139	1019.57	142.17
A_68_P29073052	chr13:58502691-58502735	NR_031761:-896	Rmi1	DIVERGENT_PROMOTER	0.374	0.699	1409.37	984.66	0.261	1116.26	291.50
A_68_P29073064	chr13:58503963-58504007	NM_001168248:27	Rmi1	INSIDE	0.513	0.455	2328.87	1060.36	0.233	1774.24	414.23
A_68_P24536421	chr6:71389996-71390040	NM_024288:613	Rmnd5a	INSIDE	0.497	0.495	2162.93	1071.61	0.246	1714.27	422.27
A_68_P24536429	chr6:71391138-71391182	NM_024288:-529	Rmnd5a	PROMOTER	0.151	0.542	1918.14	1039.65	0.082	1435.06	117.56

ProbeName	Target position of probe on CpG island microarray	TargetID	GeneSymbol	CpG island Description	Ratio of relative methylation (TiO2-NP/Vehicle)	Sham group			TiO_2-H group		
						Relative methylation (Cy-5/Cy-3)	Cy-3 signal (Input)	Cy-5 signal (MeDIP)	Relative methylation (Cy-5/Cy-3)	Cy-3 signal (Input)	Cy-5 signal (MeDIP)
A_68_P24536430	chr6:71391224-71391268	NM_024288:-615	Rmnd5a	PROMOTER	0.657	0.712	1828.65	1301.86	0.468	1256.83	587.82
A_68_P26022868	chr8:87489665-87489709	NM_027187:224	Rnaseh2a	INSIDE	0.659	0.554	2412.21	1337.52	0.366	1650.17	603.15
A_68_P27899668	chr11:70051980-70052024	NM_173742:1352	Rnasek	INSIDE	0.271	0.662	2107.64	1396.09	0.179	1454.29	260.82
A_68_P30475880	chr15:98507518-98507562	NM_172612:352	Rnd1	INSIDE	0.500	0.509	2553.07	1299.78	0.255	2075.69	528.77
A_68_P30475881	chr15:98507653-98507697	NM_172612:218	Rnd1	INSIDE	0.139	0.618	2848.29	1759.93	0.086	2208.97	189.56
A_68_P28074045	chr11:101329337-101329381	NM_009708:-293	Rnd2	DIVERGENT_PROMOTER	0.329	0.513	1295.32	664.66	0.169	1087.51	183.31
A_68_P21212081	chr2:51003539-51003583	NM_028810:1071	Rnd3	INSIDE	0.225	0.673	4864.49	3271.81	0.151	3367.42	509.13
A_68_P21212090	chr2:51004592-51004636	NM_028810:17	Rnd3	INSIDE	0.313	0.287	2117.20	608.34	0.090	1693.31	152.40
A_68_P21212092	chr2:51004822-51004866	NM_028810:-213	Rnd3	PROMOTER	0.396	0.586	1113.91	653.02	0.232	521.16	120.98
A_68_P23201330	chr4:109148797-109148841	NM_013876:292	Rnf11	INSIDE	0.162	0.553	1864.00	1030.94	0.090	1367.47	122.82
A_68_P32322124	chrX:34731543-34731587	NM_153503:227	Rnf113a1	INSIDE	0.343	1.725	2033.00	3506.19	0.592	837.31	495.53
A_68_P32322125	chrX:34731608-34731653	NM_153503:293	Rnf113a1	INSIDE	0.264	1.436	1448.26	2079.46	0.379	565.57	214.43
A_68_P21835806	chr2:167317742-167317786	NM_030743:-380	Rnf114	PROMOTER	0.255	0.540	1085.20	585.49	0.137	883.32	121.38
A_68_P21835857	chr2:167329794-167329838	NM_030743:11672	Rnf114	INSIDE	0.110	0.551	2461.40	1355.01	0.061	1908.26	115.58
A_68_P27278803	chr10:79229502-79229546	NM_144528:142	Rnf126	INSIDE	0.531	0.634	2469.59	1565.00	0.337	1791.62	602.94
A_68_P27278806	chr10:79229855-79229899	NR_027505:-190	Rnf126	PROMOTER	0.217	0.677	3395.37	2299.16	0.147	2693.21	396.10
A_68_P22165084	chr3:57539624-57539668	NM_011883:-341	Rnf13	PROMOTER	0.543	0.444	1615.48	717.98	0.241	1336.18	322.20
A_68_P22165085	chr3:57539702-57539746	NM_011883:-263	Rnf13	PROMOTER	0.477	0.526	2000.56	1052.59	0.251	1715.08	430.87
A_68_P22165088	chr3:57540040-57540084	NM_011883:75	Rnf13	INSIDE	0.194	0.571	1254.86	716.61	0.111	1041.16	115.48
A_68_P31542489	chr18:21160241-21160286	NM_019706:422	Rnf138	INSIDE	0.134	0.490	2635.47	1291.67	0.066	1794.92	117.62
A_68_P31542490	chr18:21160400-21160444	NM_019706:581	Rnf138	INSIDE	0.420	0.671	2509.27	1684.57	0.282	2009.07	566.87
A_68_P28301679	chr12:27099772-27099816	NM_001081977:327	Rnf144a	INSIDE	0.470	0.623	3090.85	1926.55	0.293	2339.86	685.71
A_68_P29011796	chr13:47217864-47217923	NM_146042:-195	Rnf144b	PROMOTER	0.183	0.549	1568.74	861.46	0.101	1200.27	120.67
A_68_P29011800	chr13:47218349-47218393	NM_146042:282	Rnf144b	INSIDE	0.481	0.690	1064.79	735.20	0.332	727.15	241.26
A_68_P27756338	chr11:44332144-44332188	NM_028862:-299	Rnf145	PROMOTER	0.186	0.637	1078.96	687.45	0.119	1001.17	118.78
A_68_P20166592	chr1:39633542-39633586	NM_001033135:628	Rnf149	INSIDE	0.306	0.477	1657.94	791.46	0.146	1444.19	210.71
A_68_P26011762	chr8:85387432-85387476	NM_177378:200	Rnf150	INSIDE	0.144	0.373	2953.75	1100.78	0.054	2359.11	126.34
A_68_P26011763	chr8:85387565-85387609	NM_177378:332	Rnf150	INSIDE	0.449	0.656	2195.76	1439.35	0.294	1638.72	482.49
A_68_P28159196	chr11:116273617-116273661	NM_027258:708	Rnf157	INSIDE	0.593	0.710	1436.28	1019.53	0.421	1107.91	466.62
A_68_P24541329	chr6:72312280-72312324	NM_025607:73	Rnf181	INSIDE	0.308	0.662	938.17	621.52	0.204	767.13	156.66
A_68_P28991172	chr13:43711132-43711176	NM_183204:-11	Rnf182	PROMOTER	0.277	0.583	891.90	519.95	0.161	742.80	119.85
A_68_P27833616	chr11:58751821-58751865	NM_022423:566	Rnf187	INSIDE	0.529	0.642	846.79	543.99	0.340	674.29	229.19
A_68_P30137684	chr15:36212197-36212241	NM_013923:684	Rnf19a	INSIDE	0.527	0.559	1358.96	759.71	0.295	1087.80	320.36
A_68_P23294616	chr4:128725929-128725973	NM_029219:-9564	Rnf19b	PROMOTER	0.170	0.668	1735.83	1159.41	0.114	1393.20	158.41
A_68_P23294682	chr4:128735948-128735992	NM_029219:456	Rnf19b	INSIDE	0.628	0.564	3204.02	1808.51	0.355	2596.62	920.73
A_68_P23294683	chr4:128736055-128736099	NM_029219:562	Rnf19b	INSIDE	0.333	0.527	1567.38	825.96	0.176	1141.09	200.34
A_68_P20715112	chr1:153348264-153348308	NM_011277:-333	Rnf2	PROMOTER	0.600	0.662	1196.30	791.90	0.397	978.14	388.20
A_68_P22906128	chr4:49645277-49645321	NM_001163263:367	Rnf20	INSIDE	0.242	0.440	1702.79	748.41	0.107	1139.29	121.41
A_68_P23417944	chr4:151687118-151687162	NM_001033489:5594	Rnf207	INSIDE	0.170	0.716	3158.26	2259.78	0.122	2062.98	251.32
A_68_P23417945	chr4:151687256-151687300	NM_001033489:5456	Rnf207	INSIDE	0.520	0.527	1833.26	965.97	0.274	1332.43	365.40
A_68_P23417946	chr4:151687386-151687430	NM_001033489:5326	Rnf207	INSIDE	0.583	0.395	1871.00	738.27	0.230	1446.89	332.85
A_68_P23417961	chr4:151689896-151689940	NM_001033489:2816	Rnf207	INSIDE	0.096	1.485	1945.18	2888.13	0.142	1178.34	167.47
A_68_P26476664	chr9:45714791-45714835	NM_178709:148	Rnf214	INSIDE	0.158	0.448	2329.57	1044.43	0.071	1776.66	125.70
A_68_P26476668	chr9:45715123-45715167	NM_178709:-184	Rnf214	PROMOTER	0.315	0.693	1634.38	1132.30	0.219	1365.83	298.51

ProbeName	Target position of probe on CpG island microarray	TargetID	GeneSymbol	CpG island Description	Ratio of relative methylation (TiO2-NP/Vehicle)	Sham group			TiO_2-H group		
						Relative methylation (Cy-5/Cy-3)	Cy-3 signal (Input)	Cy-5 signal (MeDIP)	Relative methylation (Cy-5/Cy-3)	Cy-3 signal (Input)	Cy-5 signal (MeDIP)
A_68_P24155046	chr5:143874559-143874603	NM_080561:119	Rnf216	INSIDE	0.287	0.548	1427.99	782.23	0.157	1146.26	180.05
A_68_P27045330	chr10:31328554-31328598	NM_001146349:955	Rnf217	INSIDE	0.467	0.463	2002.46	926.91	0.216	1624.02	351.12
A_68_P23233430	chr4:116961488-116961532	NM_025739:208010	Rnf220	INSIDE	0.190	0.674	1272.30	857.73	0.128	1015.19	129.73
A_68_P23233548	chr4:116977181-116977225	NM_025739:192318	Rnf220	INSIDE	0.595	2.418	2800.74	6770.84	1.437	1868.70	2685.92
A_68_P23234667	chr4:117156923-117156969	NM_025739:12574	Rnf220	INSIDE	0.235	0.434	1231.67	533.99	0.102	1145.56	116.51
A_68_P23234771	chr4:117169165-117169209	NM_025739:334	Rnf220	INSIDE	0.357	0.406	1995.83	810.02	0.145	1517.42	219.67
A_68_P21634641	chr2:131178781-131178836	NM_178607:-180	Rnf24	PROMOTER	0.269	0.471	1229.72	579.43	0.127	936.00	118.59
A_68_P20349521	chr1:74647661-74647705	NM_021313:289	Rnf25	INSIDE	0.175	0.607	5399.84	3280.06	0.106	4086.00	433.52
A_68_P29618701	chr14:56210506-56210552	NM_194346:-98	Rnf31	DIVERGENT_PROMOTER	0.257	0.490	1201.07	588.59	0.126	969.33	122.31
A_68_P24052509	chr5:123299880-123299924	NM_030564:-294	Rnf34	PROMOTER	0.445	0.663	969.86	643.23	0.295	778.82	230.07
A_68_P24052512	chr5:123300221-123300265	NM_030564:46	Rnf34	INSIDE	0.366	0.692	1037.33	717.42	0.253	891.48	225.95
A_68_P31166377	chr17:37079998-37080042	NM_001099632:25	Rnf39	INSIDE	0.419	0.577	2136.79	1233.36	0.242	1870.24	452.19
A_68_P23593807	chr5:34678844-34678888	NM_011278:-172	Rnf4	PROMOTER	0.621	0.616	1394.05	859.07	0.383	1193.81	457.20
A_68_P23593812	chr5:34679442-34679486	NM_011278:426	Rnf4	INSIDE	0.642	0.533	1152.43	614.50	0.342	904.59	309.47
A_68_P23593813	chr5:34679537-34679581	NM_011278:520	Rnf4	INSIDE	0.194	0.453	1658.92	750.71	0.088	1396.42	122.33
A_68_P23593815	chr5:34679727-34679773	NM_011278:712	Rnf4	INSIDE	0.321	0.566	1040.25	588.55	0.181	829.14	150.44
A_68_P25506338	chr7:134732367-134732411	NM_172281:177	Rnf40	INSIDE	0.332	0.539	3684.14	1985.86	0.179	2754.98	492.71
A_68_P25506340	chr7:134732564-134732608	NM_172281:375	Rnf40	INSIDE	0.340	1.706	2715.64	4631.89	0.579	1880.58	1089.29
A_68_P27541276	chr10:127848724-127848768	NM_001164237:75	Rnf41	INSIDE	0.285	0.346	1482.41	512.45	0.098	1202.87	118.41
A_68_P29050859	chr13:54795572-54795616	NR_027395:-273	Rnf44	DIVERGENT_PROMOTER	0.500	0.552	2845.53	1571.61	0.276	2362.35	652.97
A_68_P31154741	chr17:34740094-34740143	NM_019403:388	Rnf5	INSIDE	0.194	1.967	1283.64	2525.31	0.381	901.01	343.39
A_68_P31154750	chr17:34741259-34741304	NM_019403:-775	Rnf5	DIVERGENT_PROMOTER	0.255	0.651	2081.62	1354.46	0.166	1539.66	255.00
A_68_P31154751	chr17:34741404-34741448	NM_019403:-920	Rnf5	DIVERGENT_PROMOTER	0.379	0.539	1148.62	619.50	0.204	897.67	183.55
A_68_P26747275	chr9:96378576-96378624	NM_011279:414	Rnf7	INSIDE	0.272	0.621	908.83	564.60	0.169	723.69	122.09
A_68_P25587156	chr7:148354570-148354614	NM_001172101:-7	Rnh1	PROMOTER	0.380	0.629	1970.53	1239.78	0.239	1596.49	381.57
A_68_P32075617	chr19:33466407-33466451	NM_001146342:357	Rnls	INSIDE	0.278	0.605	1223.58	740.14	0.168	977.74	164.39
A_68_P27930798	chr11:76057462-76057506	NM_183263:247	Rnmtl1	INSIDE	0.361	0.646	1022.85	660.68	0.233	792.69	184.63
A_68_P31097159	chr17:24551794-24551838	NM_001080127:197	Rnps1	INSIDE	0.594	0.663	1629.48	1080.65	0.394	1406.48	553.71
A_68_P22301214	chr3:85946197-85946241	NR_004418:-1609	Rnu73b	PROMOTER	0.644	0.562	960.79	540.23	0.362	815.47	295.20
A_68_P31488616	chr18:10181915-10181959	NM_009071:-146	Rock1	PROMOTER	0.354	0.396	1867.11	738.84	0.140	1558.91	218.12
A_68_P22957025	chr4:59561479-59561533	NM_144904:730	Rod1	INSIDE	0.107	0.559	2486.69	1389.22	0.060	1947.88	116.26
A_68_P22957030	chr4:59562123-59562167	NM_144904:92	Rod1	INSIDE	0.634	0.596	2044.82	1218.47	0.378	1536.07	579.98
A_68_P30513214	chr16:5013320-5013364	NM_133185:304	Rogdi	INSIDE	0.385	0.715	2269.83	1622.83	0.275	1581.98	435.04
A_68_P30114432	chr15:31383420-31383464	NM_145852:2	Ropn1l	INSIDE	0.572	0.367	1671.99	614.10	0.210	1297.00	272.53
A_68_P26605676	chr9:68502227-68502277	NM_013646:644	Rora	INSIDE	0.138	0.712	1565.16	1113.89	0.098	1176.29	115.52
A_68_P26605678	chr9:68502422-68502466	NM_013646:836	Rora	INSIDE	0.363	0.530	2074.71	1099.56	0.193	1711.75	329.55
A_68_P26609890	chr9:69138794-69138840	NM_013646:637208	Rora	INSIDE	0.374	0.598	1305.17	780.20	0.223	1018.95	227.73
A_68_P26609891	chr9:69138894-69138938	NM_013646:637308	Rora	INSIDE	0.409	0.527	2100.37	1107.77	0.215	1636.20	352.54
A_68_P23313958	chr4:132324242-132324286	NM_011284:-10	Rpa2	PROMOTER	0.235	0.623	1899.16	1182.91	0.146	1397.23	204.59
A_68_P23313959	chr4:132324309-132324353	NM_011284:56	Rpa2	INSIDE	0.227	1.401	2229.35	3122.60	0.318	1580.26	503.03
A_68_P23313962	chr4:132324543-132324587	NM_011284:290	Rpa2	INSIDE	0.196	0.603	3888.61	2346.23	0.119	2862.84	339.39
A_68_P24225084	chr6:8209188-8209232	NM_026632:-69	Rpa3	DIVERGENT_PROMOTER	0.102	1.348	4137.62	5578.65	0.138	2556.86	352.94
A_68_P20304094	chr1:66747456-66747500	NM_025683:12	Rpe	INSIDE	0.608	0.534	1472.35	785.78	0.325	1219.74	395.84
A_68_P20304095	chr1:66747574-66747618	NM_025683:130	Rpe	INSIDE	0.489	0.364	2463.11	897.32	0.178	2142.67	381.80

ProbeName	Target position of probe on CpG island microarray	TargetID	GeneSymbol	CpG island Description	Ratio of relative methylation (TiO2-NP/Vehicle)	Sham group			TiO_2-H group		
						Relative methylation (Cy-5/Cy-3)	Cy-3 signal (Input)	Cy-5 signal (MeDIP)	Relative methylation (Cy-5/Cy-3)	Cy-3 signal (Input)	Cy-5 signal (MeDIP)
A_68_P22615129	chr3:146183762-146183806	NM_027371:603	Rpf1	INSIDE	0.305	0.649	994.91	646.00	0.198	927.29	183.38
A_68_P22615131	chr3:146184129-146184173	NM_027371:237	Rpf1	INSIDE	0.232	0.683	1735.20	1185.85	0.159	1443.96	229.10
A_68_P29604814	chr14:52738859-52738903	NM_001168515:8303	Rpgrip1	INSIDE	0.372	0.434	1490.82	647.28	0.162	1149.00	185.61
A_68_P24533018	chr6:70741721-70741765	NM_009075:427	Rpia	INSIDE	0.173	0.510	2330.13	1188.80	0.088	1759.33	155.01
A_68_P26237918	chr8:125626137-125626182	NM_016738:-90	Rpl13	PROMOTER	0.372	0.569	1555.77	885.91	0.212	1140.64	241.46
A_68_P25091294	chr7:52384091-52384135	NM_009438:-7	Rpl13a	PROMOTER	0.215	0.508	1412.29	716.96	0.109	1088.05	118.93
A_68_P25091295	chr7:52384233-52384280	NM_009438:-151	Rpl13a	PROMOTER	0.323	0.453	1250.08	566.51	0.146	818.09	119.58
A_68_P25091298	chr7:52384568-52384612	NM_009438:-485	Rpl13a	PROMOTER	0.321	0.469	1320.06	619.18	0.151	1028.48	154.80
A_68_P24170315	chr5:147644587-147644631	NM_019647:143	Rpl21	INSIDE	0.273	0.428	1357.42	580.44	0.117	1039.87	121.44
A_68_P28052672	chr11:97643844-97643889	NM_022891:-113	Rpl23	PROMOTER	0.398	0.606	1390.97	842.99	0.241	1054.91	254.30
A_68_P27891995	chr11:68714810-68714854	NM_009080:-235	Rpl26	PROMOTER	0.398	0.694	3449.52	2393.87	0.276	2457.62	679.51
A_68_P28073901	chr11:101303846-101303890	NM_011289:310	Rpl27	INSIDE	0.273	0.501	1070.54	536.15	0.137	879.78	120.31
A_68_P25408483	chr7:116662453-116662497	NM_011975:-234	Rpl27a	PROMOTER	0.324	0.709	2713.74	1924.57	0.230	1975.60	454.47
A_68_P25408484	chr7:116662619-116662663	NM_011975:-68	Rpl27a	PROMOTER	0.513	0.618	1778.81	1099.42	0.317	1300.08	411.92
A_68_P24950541	chr7:4744529-4744573	NM_009081:-16	Rpl28	DIVERGENT_PROMOTER	0.476	0.725	1844.12	1336.34	0.345	1373.30	474.08
A_68_P30128575	chr15:34372745-34372789	NM_009083:214	Rpl30	INSIDE	0.422	0.412	1394.28	574.75	0.174	1238.70	215.54
A_68_P30128578	chr15:34373065-34373109	NM_001163485:-55	Rpl30	DIVERGENT_PROMOTER	0.148	0.543	1890.07	1027.02	0.081	1500.12	121.03
A_68_P20165368	chr1:39425278-39425322	NM_053257:605	Rpl31	INSIDE	0.191	1.493	6710.42	10020.69	0.286	4482.70	1281.82
A_68_P21151628	chr2:38860218-38860262	NM_025592:411	Rpl35	INSIDE	0.100	0.656	3001.82	1968.35	0.066	2335.39	153.75
A_68_P30655991	chr16:33056419-33056463	NM_001130485:-98	Rpl35a	DIVERGENT_PROMOTER	0.318	0.467	1287.12	601.27	0.149	957.31	142.25
A_68_P30655996	chr16:33056959-33057003	NM_021338:381	Rpl35a	INSIDE	0.331	0.584	1016.65	593.81	0.193	742.77	143.60
A_68_P31257289	chr17:56753149-56753198	NM_018730:356	Rpl36	INSIDE	0.100	0.657	2368.40	1555.93	0.066	1774.54	116.93
A_68_P31257290	chr17:56753266-56753310	NM_018730:471	Rpl36	INSIDE	0.318	0.500	1535.88	768.11	0.159	1069.97	170.13
A_68_P32672687	chrX:131120488-131120543	NM_019865:323	Rpl36a	INSIDE	0.124	2.125	1292.59	2746.20	0.264	453.43	119.72
A_68_P32672688	chrX:131120583-131120633	NM_019865:416	Rpl36a	INSIDE	0.214	1.483	1086.35	1610.71	0.317	406.96	128.91
A_68_P32672689	chrX:131120700-131120748	NM_019865:532	Rpl36a	INSIDE	0.113	1.830	1594.58	2918.44	0.206	565.96	116.71
A_68_P28513220	chr12:70284706-70284750	NM_025589:326	Rpl36al	INSIDE	0.177	0.702	1282.42	900.25	0.124	998.76	123.94
A_68_P28513223	chr12:70285038-70285082	NM_025589:-6	Rpl36al	DIVERGENT_PROMOTER	0.084	0.690	2835.31	1956.33	0.058	2031.47	118.31
A_68_P29982086	chr15:5066083-5066136	NM_026069:-503	Rpl37	DIVERGENT_PROMOTER	0.251	0.503	1450.34	729.04	0.126	1017.12	128.52
A_68_P29982088	chr15:5066265-5066309	NM_026069:-326	Rpl37	DIVERGENT_PROMOTER	0.266	0.347	1734.07	602.29	0.092	1268.43	117.01
A_68_P29982091	chr15:5066504-5066548	NM_026069:-86	Rpl37	DIVERGENT_PROMOTER	0.660	0.527	1313.48	692.63	0.348	1118.21	389.18
A_68_P28149048	chr11:114530351-114530395	NM_001048058:278	Rpl38	INSIDE	0.238	0.607	1349.48	819.36	0.145	1087.82	157.45
A_68_P32321655	chrX:34625187-34625231	NM_026055:-29	Rpl39	PROMOTER	0.077	1.761	2145.06	3777.63	0.135	881.35	118.80
A_68_P27541975	chr10:127986066-127986114	NM_018860:134	Rpl41	INSIDE	0.053	1.609	2185.75	3517.88	0.085	1372.94	116.61
A_68_P24043181	chr5:121654660-121654704	NM_011290:173	Rpl6	INSIDE	0.095	0.538	2971.47	1598.61	0.051	2277.71	116.39
A_68_P24043182	chr5:121654805-121654849	NM_011290:317	Rpl6	INSIDE	0.416	0.525	1530.53	802.85	0.218	1243.30	271.58
A_68_P24043184	chr5:121654997-121655041	NM_011290:509	Rpl6	INSIDE	0.535	0.663	1199.86	795.04	0.354	982.68	348.03
A_68_P30351089	chr15:76734484-76734528	NM_012053:6	Rpl8	INSIDE	0.216	2.013	4053.37	8160.62	0.434	2534.05	1100.09
A_68_P30351090	chr15:76734597-76734641	NM_012053:118	Rpl8	INSIDE	0.235	1.752	3291.12	5766.71	0.412	2381.31	980.56
A_68_P30351093	chr15:76734840-76734884	NM_012053:362	Rpl8	INSIDE	0.266	0.623	1058.14	658.88	0.166	738.40	122.43
A_68_P26567740	chr9:61761969-61762013	NM_018853:327	Rplp1	INSIDE	0.431	0.585	2880.40	1683.88	0.252	2159.08	544.13
A_68_P26567741	chr9:61762053-61762098	NM_018853:242	Rplp1	INSIDE	0.368	0.673	2655.14	1786.03	0.247	2042.06	505.38
A_68_P26567746	chr9:61762612-61762656	NM_018853:-317	Rplp1	PROMOTER	0.209	0.563	2224.76	1252.02	0.118	1738.35	204.80
A_68_P25588866	chr7:148633186-148633239	NM_026020:-336	Rplp2	DIVERGENT_PROMOTER	0.400	0.587	1498.29	879.31	0.235	1091.39	255.97

ProbeName	Target position of probe on CpG island microarray	TargetID	GeneSymbol	CpG island Description	Ratio of relative methylation (TiO2-NP/Vehicle)	Sham group			TiO_2-H group		
						Relative methylation (Cy-5/Cy-3)	Cy-3 signal (Input)	Cy-5 signal (MeDIP)	Relative methylation (Cy-5/Cy-3)	Cy-3 signal (Input)	Cy-5 signal (MeDIP)
A_68_P31560661	chr18:24687904-24687948	NM_144861:779	Rprd1a	INSIDE	0.283	0.622	1662.70	1033.75	0.176	1292.73	227.47
A_68_P31560663	chr18:24688162-24688206	NM_144861:521	Rprd1a	INSIDE	0.402	0.580	1300.04	753.96	0.233	1070.85	249.46
A_68_P31560664	chr18:24688268-24688312	NM_144861:415	Rprd1a	INSIDE	0.352	0.523	1270.97	664.86	0.184	1047.90	192.85
A_68_P21780562	chr2:157854036-157854080	NM_027434:-471	Rprd1b	PROMOTER	0.270	0.435	1452.64	632.44	0.118	1121.18	131.91
A_68_P21780568	chr2:157854743-157854787	NM_027434:235	Rprd1b	INSIDE	0.509	0.595	2441.06	1452.79	0.303	1892.12	573.15
A_68_P22345831	chr3:95622735-95622779	NM_001081293:120	Rprd2	INSIDE	0.493	0.687	2580.20	1771.66	0.339	1837.65	622.59
A_68_P22345833	chr3:95622982-95623029	NM_001081293:-129	Rprd2	PROMOTER	0.229	0.515	2375.11	1222.59	0.118	1766.61	208.20
A_68_P22345836	chr3:95623356-95623400	NM_001081293:-502	Rprd2	PROMOTER	0.658	0.625	918.79	574.31	0.411	845.16	347.61
A_68_P22345837	chr3:95623518-95623562	NM_001081293:-664	Rprd2	PROMOTER	0.610	0.691	1853.12	1280.45	0.422	1463.67	617.43
A_68_P21227533	chr2:53937683-53937727	NM_023396:259	Rprm	INSIDE	0.271	0.421	1379.77	581.23	0.114	1078.45	123.26
A_68_P31117588	chr17:27771794-27771838	NM_025963:371	Rps10	INSIDE	0.292	0.554	999.66	554.01	0.162	735.73	119.24
A_68_P27005216	chr10:23507028-23507072	NM_011295:-35	Rps12	DIVERGENT_PROMOTER	0.227	0.325	1968.49	640.42	0.074	1604.57	118.75
A_68_P31753488	chr18:60934010-60934054	NM_020600:-217	Rps14	PROMOTER	0.362	0.383	1605.60	615.28	0.139	1190.38	165.27
A_68_P31753490	chr18:60934321-60934365	NM_020600:93	Rps14	INSIDE	0.441	0.608	1030.88	626.42	0.268	766.85	205.72
A_68_P27282239	chr10:79755107-79755151	NM_009091:-47	Rps15	PROMOTER	0.617	0.562	1324.96	744.01	0.346	943.82	326.75
A_68_P27282240	chr10:79755289-79755333	NM_009091:135	Rps15	INSIDE	0.535	0.667	1450.87	967.98	0.357	1121.97	400.75
A_68_P25021324	chr7:29135677-29135721	NM_013647:-9	Rps16	PROMOTER	0.192	0.697	2240.25	1562.32	0.134	1498.69	200.78
A_68_P25007067	chr7:25669258-25669302	NM_023133:-452	Rps19	PROMOTER	0.318	0.571	1920.01	1096.01	0.182	1364.94	247.75
A_68_P25007068	chr7:25669351-25669396	NM_023133:-359	Rps19	PROMOTER	0.647	0.695	2395.65	1665.50	0.450	1762.43	793.30
A_68_P26467468	chr9:44215837-44215881	NM_024266:62	Rps25	INSIDE	0.545	0.472	3137.36	1480.95	0.257	2248.34	578.92
A_68_P32567638	chrX:99384443-99384487	NM_009094:-754	Rps4x	PROMOTER	0.142	1.502	1346.64	2022.92	0.214	561.33	120.06
A_68_P31024850	chr17:7374080-7374124	NM_011299:-361	Rps6ka2	PROMOTER	0.160	0.483	2850.15	1375.90	0.077	2241.33	173.47
A_68_P31024851	chr17:7374165-7374209	NM_011299:-277	Rps6ka2	PROMOTER	0.446	0.713	3137.62	2236.78	0.318	2286.50	726.99
A_68_P31024853	chr17:7374468-7374512	NM_011299:27	Rps6ka2	INSIDE	0.474	0.528	1322.33	697.66	0.250	1141.11	285.31
A_68_P32773804	chrX:155693758-155693802	NM_148945:-271	Rps6ka3	PROMOTER	0.530	3.452	4698.70	16218.21	1.829	2372.12	4339.42
A_68_P32773807	chrX:155694248-155694292	NM_148945:219	Rps6ka3	INSIDE	0.065	1.597	3251.45	5192.77	0.104	1141.61	118.64
A_68_P31936299	chr19:6904994-6905038	NM_019924:10075	Rps6ka4	INSIDE	0.651	0.447	1447.70	646.70	0.291	1061.00	308.55
A_68_P31936308	chr19:6906036-6906086	NM_019924:9031	Rps6ka4	INSIDE	0.539	3.598	2399.74	8634.04	1.940	1578.13	3060.96
A_68_P31936365	chr19:6914837-6914881	NM_019924:233	Rps6ka4	INSIDE	0.134	0.578	2018.64	1167.04	0.078	1500.59	116.39
A_68_P28683722	chr12:101962780-101962824	NM_153587:436	Rps6ka5	INSIDE	0.391	0.589	1867.99	1100.53	0.230	1460.70	336.55
A_68_P28683724	chr12:101963187-101963231	NM_153587:30	Rps6ka5	INSIDE	0.490	0.480	2493.49	1195.92	0.235	2088.58	491.28
A_68_P32599603	chrX:108577244-108577288	NM_025949:74288	Rps6ka6	INSIDE	0.479	0.559	1003.25	560.77	0.268	437.18	117.08
A_68_P27989555	chr11:86358098-86358142	NM_001114334:189	Rps6kb1	INSIDE	0.513	0.518	1411.96	731.79	0.266	1152.60	306.25
A_68_P24946916	chr7:3655703-3655755	NM_029767:86	Rps9	INSIDE	0.149	0.670	1740.31	1165.76	0.100	1222.34	121.70
A_68_P24946917	chr7:3655779-3655833	NM_029767:164	Rps9	INSIDE	0.180	0.571	1422.66	812.06	0.103	1166.18	120.11
A_68_P21567499	chr2:118860959-118861006	NM_173450:457	Rpusd2	INSIDE	0.285	0.431	1203.70	518.34	0.123	946.80	116.40
A_68_P20348978	chr1:74551985-74552034	NM_021383:-624	Rqcd1	PROMOTER	0.154	0.670	1504.09	1007.87	0.103	1193.56	123.26
A_68_P20348981	chr1:74552352-74552396	NM_021383:-259	Rqcd1	PROMOTER	0.252	0.664	2065.16	1371.79	0.168	1351.71	226.66
A_68_P20348983	chr1:74552547-74552591	NM_021383:-65	Rqcd1	PROMOTER	0.382	0.440	3658.22	1610.76	0.168	2627.28	441.91
A_68_P20348986	chr1:74552917-74552961	NM_021383:305	Rqcd1	INSIDE	0.119	0.604	3884.63	2347.81	0.072	2909.90	209.55
A_68_P20348987	chr1:74553038-74553082	NM_021383:427	Rqcd1	INSIDE	0.410	0.622	1929.98	1200.79	0.255	1468.88	374.64
A_68_P23080556	chr4:86221599-86221643	NM_178376:44	Rraga	INSIDE	0.235	0.491	1057.19	518.78	0.115	995.50	114.97
A_68_P25434394	chr7:121261406-121261450	NM_025846:-133	Rras2	PROMOTER	0.409	0.699	1787.85	1250.07	0.286	1426.98	408.44
A_68_P21704900	chr2:143837439-143837493	NM_024281:-467	Rrbp1	PROMOTER	0.249	0.608	879.19	534.45	0.151	813.55	123.01

ProbeName	Target position of probe on CpG island microarray	TargetID	GeneSymbol	CpG island Description	Ratio of relative methylation (TiO2-NP/Vehicle)	Sham group			TiO_2-H group		
						Relative methylation (Cy-5/Cy-3)	Cy-3 signal (Input)	Cy-5 signal (MeDIP)	Relative methylation (Cy-5/Cy-3)	Cy-3 signal (Input)	Cy-5 signal (MeDIP)
A_68_P28956734	chr13:37918572-37918616	NM_001177868:-186	Rreb1	PROMOTER	0.294	0.423	4304.25	1818.77	0.124	3030.87	376.08
A_68_P25378791	chr7:109590355-109590403	NM_009103:170	Rrm1	INSIDE	0.239	0.573	1106.47	633.78	0.137	878.19	120.27
A_68_P25378792	chr7:109590509-109590553	NM_009103:322	Rrm1	INSIDE	0.273	0.658	1384.13	911.23	0.180	903.12	162.28
A_68_P25378793	chr7:109590580-109590624	NM_009103:394	Rrm1	INSIDE	0.456	0.540	6626.39	3578.50	0.246	4678.80	1151.19
A_68_P28291145	chr12:25393139-25393183	NM_009104:42	Rrm2	INSIDE	0.237	0.429	2583.12	1107.31	0.101	1970.28	199.87
A_68_P28291147	chr12:25393381-25393425	NM_009104:284	Rrm2	INSIDE	0.386	0.626	2599.15	1627.70	0.242	1944.45	469.87
A_68_P28291148	chr12:25393461-25393505	NM_009104:364	Rrm2	INSIDE	0.625	0.701	1467.99	1029.07	0.438	1087.94	476.86
A_68_P28291149	chr12:25393568-25393620	NM_009104:476	Rrm2	INSIDE	0.148	0.719	1770.14	1272.33	0.106	1182.39	125.76
A_68_P30146825	chr15:37890467-37890516	NM_199476:319	Rrm2b	INSIDE	0.118	0.694	2097.80	1456.49	0.082	1460.65	119.90
A_68_P30146826	chr15:37890566-37890610	NM_199476:222	Rrm2b	INSIDE	0.561	0.592	2331.50	1379.43	0.332	1642.59	545.08
A_68_P30146831	chr15:37891066-37891112	NM_199476:-278	Rrm2b	PROMOTER	0.533	0.634	998.11	632.36	0.338	902.89	304.89
A_68_P30558909	chr16:13780665-13780710	NM_001039521:-104	Rrn3	PROMOTER	0.150	0.610	1611.25	982.63	0.092	1332.71	122.28
A_68_P27274091	chr10:77875465-77875509	NM_010925:302	Rrp1	INSIDE	0.100	0.543	3127.89	1699.28	0.055	2218.82	121.00
A_68_P32117790	chr19:41970534-41970578	NM_199447:87	Rrp12	INSIDE	0.096	1.475	5616.48	8284.03	0.141	3624.66	511.70
A_68_P25394039	chr7:112885744-112885788	NM_133951:133	Rrp8	INSIDE	0.451	0.447	1606.94	718.98	0.202	1312.88	264.64
A_68_P26803653	chr9:106379642-106379686	NM_145620:25	Rrp9	INSIDE	0.503	0.511	1815.86	927.42	0.257	1356.06	348.63
A_68_P20022705	chr1:9535306-9535350	NM_021511:-160	Rrs1	PROMOTER	0.634	0.525	1044.51	548.22	0.333	846.96	281.96
A_68_P28033654	chr11:94410127-94410174	NM_001013381:371	Rsad1	INSIDE	0.322	0.576	1069.18	615.52	0.186	777.58	144.35
A_68_P22388655	chr3:103718078-103718124	NM_172684:58	Rsbn1	INSIDE	0.592	0.308	1704.85	525.40	0.183	1451.51	264.92
A_68_P22388660	chr3:103718569-103718613	NM_172684:548	Rsbn1	INSIDE	0.305	0.619	849.22	525.31	0.189	611.07	115.40
A_68_P23523572	chr5:20457823-20457867	NM_001080977:-204	Rsbn1l	PROMOTER	0.221	0.564	1162.73	655.66	0.124	995.22	123.80
A_68_P23523573	chr5:20457890-20457941	NM_001080977:-275	Rsbn1l	PROMOTER	0.261	0.601	951.08	571.17	0.157	736.57	115.32
A_68_P25351155	chr7:104728838-104728882	NM_001081267:455	Rsf1	INSIDE	0.501	0.578	2138.36	1236.69	0.290	1872.34	542.59
A_68_P30546792	chr16:11203231-11203275	NM_025546:133	Rsl1d1	INSIDE	0.253	0.698	2707.74	1889.84	0.177	2152.81	380.38
A_68_P30546793	chr16:11203333-11203377	NM_025546:31	Rsl1d1	INSIDE	0.144	0.673	1570.09	1056.73	0.097	1216.18	118.06
A_68_P31138742	chr17:31413976-31414020	NM_025290:303	Rsph1	INSIDE	0.278	0.483	1230.30	594.82	0.135	968.81	130.39
A_68_P23271479	chr4:124664198-124664242	NM_138683:547	Rspo1	INSIDE	0.286	1.542	9361.65	14434.57	0.442	6803.05	3005.24
A_68_P23271482	chr4:124664450-124664494	NM_138683:799	Rspo1	INSIDE	0.589	0.534	1364.90	729.21	0.314	1093.02	343.75
A_68_P23271503	chr4:124667328-124667372	NM_138683:3677	Rspo1	INSIDE	0.068	1.366	4346.41	5935.84	0.092	2711.92	250.02
A_68_P23271505	chr4:124667492-124667536	NM_138683:3841	Rspo1	INSIDE	0.156	0.622	1647.29	1024.08	0.097	1286.56	124.97
A_68_P22207931	chr3:66789980-66790024	NM_025822:409	Rsrc1	INSIDE	0.223	0.484	1667.13	806.64	0.108	1515.52	163.79
A_68_P24057380	chr5:124199269-124199317	NM_001005525:129	Rsrc2	INSIDE	0.302	0.593	1203.73	713.96	0.179	876.61	156.88
A_68_P22449707	chr3:116211186-116211230	NM_025517:-115	Rtcd1	DIVERGENT_PROMOTER	0.326	0.545	1821.44	993.37	0.178	1535.89	273.01
A_68_P24591630	chr6:83085934-83085978	NM_009106:-1121	Rtkn	PROMOTER	0.127	0.606	2364.55	1432.02	0.077	1647.87	127.14
A_68_P24591631	chr6:83086000-83086044	NM_009106:-1055	Rtkn	PROMOTER	0.400	0.655	1225.70	802.41	0.262	828.16	216.99
A_68_P24591678	chr6:83092414-83092458	NM_009106:5359	Rtkn	INSIDE	0.510	0.544	1362.14	740.92	0.277	1054.37	292.49
A_68_P28531916	chr12:73509185-73509229	NM_153457:720	Rtn1	INSIDE	0.517	0.679	1209.17	820.57	0.351	909.19	319.29
A_68_P24992460	chr7:19868360-19868404	NM_001025364:-8065	Rtn2	PROMOTER	0.548	0.493	1960.99	966.53	0.270	1462.25	394.66
A_68_P31940077	chr19:7557297-7557341	NM_053076:453	Rtn3	INSIDE	0.408	1.623	3523.70	5720.20	0.662	2531.91	1675.36
A_68_P27675734	chr11:29592809-29592856	NM_194052:-65	Rtn4	PROMOTER	0.103	1.871	969.72	1814.44	0.194	622.03	120.41
A_68_P27675741	chr11:29593764-29593808	NM_194051:13	Rtn4	INSIDE	0.170	0.606	3599.56	2180.48	0.103	2723.51	280.55
A_68_P30577646	chr16:18127970-18128014	NM_022982:194	Rtn4r	INSIDE	0.190	0.524	1336.50	700.59	0.100	1200.85	119.71
A_68_P27924763	chr11:75006899-75006943	NM_177708:-574	Rtn4rl1	DIVERGENT_PROMOTER	0.458	0.689	3535.73	2436.76	0.316	2509.06	792.08
A_68_P27924766	chr11:75007358-75007402	NM_177708:-114	Rtn4rl1	DIVERGENT_PROMOTER	0.557	0.556	1115.18	619.93	0.310	883.17	273.44

ProbeName	Target position of probe on CpG island microarray	TargetID	GeneSymbol	CpG island Description	Ratio of relative methylation (TiO2-NP/Vehicle)	Sham group			TiO_2-H group		
						Relative methylation (Cy-5/Cy-3)	Cy-3 signal (Input)	Cy-5 signal (MeDIP)	Relative methylation (Cy-5/Cy-3)	Cy-3 signal (Input)	Cy-5 signal (MeDIP)
A_68_P27924775	chr11:75008355-75008401	NM_177708:884	Rtn4rl1	INSIDE	0.274	0.413	1547.24	638.93	0.113	1025.14	116.19
A_68_P27924778	chr11:75008653-75008697	NM_177708:1180	Rtn4rl1	INSIDE	0.449	0.669	1853.26	1239.36	0.300	1264.66	379.64
A_68_P27924902	chr11:75027241-75027285	NM_177708:19768	Rtn4rl1	INSIDE	0.368	0.546	1933.57	1055.77	0.201	1441.23	289.59
A_68_P21395229	chr2:84728472-84728517	NM_199223:-1645	Rtn4rl2	PROMOTER	0.218	0.511	1371.91	700.70	0.111	1068.19	118.67
A_68_P28079293	chr11:102255152-102255196	NM_016759:426	Rundc3a	INSIDE	0.246	0.585	1027.28	600.56	0.144	855.32	123.21
A_68_P23468799	chr5:8623093-8623137	NM_198620:-162	Rundc3b	PROMOTER	0.524	0.551	1394.30	768.90	0.289	1118.35	323.01
A_68_P30970267	chr16:92606200-92606244	NM_001111023:91351	Runx1	INSIDE	0.516	1.408	3130.00	4407.72	0.726	2354.96	1709.68
A_68_P30970947	chr16:92697153-92697197	NM_001111023:399	Runx1	INSIDE	0.406	0.515	2685.30	1382.40	0.209	2016.68	421.81
A_68_P31196983	chr17:44871711-44871755	NM_001145920:1865	Runx2	INSIDE	0.639	0.512	1047.42	536.45	0.327	806.88	264.25
A_68_P23327084	chr4:134731141-134731185	NM_019732:54603	Runx3	INSIDE	0.180	0.672	1193.06	801.36	0.121	988.42	119.79
A_68_P22871359	chr4:43395245-43395289	NM_001037709:413	Rusc2	INSIDE	0.172	0.676	1726.81	1166.66	0.116	1393.29	162.11
A_68_P26700014	chr9:86465671-86465715	NM_001145968:33	Rwdd2a	INSIDE	0.516	0.585	4142.67	2424.58	0.302	3140.75	949.10
A_68_P25842224	chr8:48619355-48619399	NM_203507:378	Rwdd4a	INSIDE	0.184	0.430	2782.07	1197.41	0.079	2011.19	159.67
A_68_P25842225	chr8:48619443-48619487	NM_203507:466	Rwdd4a	INSIDE	0.169	0.677	1489.95	1009.30	0.114	1051.65	120.14
A_68_P30013954	chr15:10965913-10965957	NM_178717:1789	Rxfp3	INSIDE	0.188	0.711	1397.06	992.87	0.133	1076.55	143.56
A_68_P30013955	chr15:10966024-10966068	NM_178717:1677	Rxfp3	INSIDE	0.300	0.692	1201.59	831.41	0.208	816.46	169.56
A_68_P30013961	chr15:10966774-10966818	NM_178717:927	Rxfp3	INSIDE	0.157	0.697	4372.73	3046.88	0.109	3396.17	371.03
A_68_P21085997	chr2:27532005-27532049	NM_011305:-694	Rxra	PROMOTER	0.097	0.539	3354.66	1807.88	0.052	2324.06	121.18
A_68_P21085998	chr2:27532103-27532149	NM_011305:-594	Rxra	PROMOTER	0.307	0.619	1738.58	1075.62	0.190	1309.57	248.37
A_68_P31151620	chr17:34169430-34169477	NM_001205215:159	Rxrb	INSIDE	0.211	0.635	1134.78	720.41	0.134	909.88	121.64
A_68_P25025137	chr7:29849427-29849471	NM_009109:60722	Ryr1	INSIDE	0.585	3.562	488.34	1739.48	2.085	447.61	933.23
A_68_P28830400	chr13:12198333-12198377	NM_023868:858	Ryr2	INSIDE	0.601	0.548	1628.34	891.62	0.329	1428.77	470.24
A_68_P22336825	chr3:93324681-93324725	NM_016740:285	S100a11	INSIDE	0.492	0.475	1116.92	529.99	0.234	890.27	208.04
A_68_P22336828	chr3:93324960-93325004	NM_016740:565	S100a11	INSIDE	0.603	0.580	1343.79	779.27	0.350	1075.51	375.97
A_68_P26344889	chr9:20780299-20780343	NM_010333:917	S1pr2	INSIDE	0.258	0.333	1678.98	558.95	0.086	1407.01	121.05
A_68_P26344892	chr9:20780634-20780678	NM_010333:581	S1pr2	INSIDE	0.150	0.721	2810.39	2025.90	0.108	2201.03	237.29
A_68_P26344895	chr9:20780976-20781020	NM_010333:239	S1pr2	INSIDE	0.627	0.379	1860.91	705.48	0.238	1518.23	360.79
A_68_P26344897	chr9:20781232-20781276	NM_010333:-17	S1pr2	PROMOTER	0.434	1.480	2047.13	3029.62	0.643	1398.76	899.34
A_68_P29032313	chr13:51504178-51504222	NM_010101:214	S1pr3	INSIDE	0.260	0.719	3156.51	2268.83	0.187	2171.75	406.64
A_68_P26346140	chr9:21048545-21048589	NM_053190:4321	S1pr5	INSIDE	0.403	0.535	1135.96	608.15	0.216	1006.93	217.15
A_68_P26346146	chr9:21049162-21049206	NM_053190:3703	S1pr5	INSIDE	0.414	0.484	1981.32	959.66	0.200	1636.69	327.93
A_68_P25100828	chr7:53965985-53966029	NM_030233:15	Saal1	INSIDE	0.627	0.632	1392.36	880.50	0.396	1171.80	464.41
A_68_P26895475	chr9:123438639-123438695	NM_030692:-333	Sacm1l	PROMOTER	0.251	0.413	1389.88	574.54	0.104	1135.50	117.92
A_68_P26044784	chr8:91566566-91566616	NM_021390:1471	Sall1	INSIDE	0.469	0.628	1205.93	756.78	0.294	986.55	290.39
A_68_P26044789	chr8:91567168-91567212	NM_021390:871	Sall1	INSIDE	0.205	0.554	1453.85	805.70	0.114	1052.79	119.57
A_68_P26044795	chr8:91567813-91567857	NM_021390:227	Sall1	INSIDE	0.526	0.330	1868.36	616.44	0.173	1602.95	277.95
A_68_P31868104	chr18:81182542-81182586	NM_178280:753	Sall3	INSIDE	0.650	0.534	1422.10	759.78	0.348	1067.28	370.91
A_68_P31868115	chr18:81184119-81184163	NM_178280:-823	Sall3	PROMOTER	0.627	0.600	2579.41	1548.67	0.377	2060.63	776.29
A_68_P30224201	chr15:53733924-53733974	NM_177225:43	Samd12	INSIDE	0.244	0.505	2498.86	1262.95	0.123	1701.81	209.48
A_68_P28036069	chr11:94871187-94871231	NM_146025:16	Samd14	INSIDE	0.429	0.511	1098.60	560.89	0.219	888.67	194.42
A_68_P29578867	chr14:47501948-47501992	NM_001037221:-669	Samd4	PROMOTER	0.281	0.414	1606.07	665.62	0.117	1240.06	144.55
A_68_P29578888	chr14:47504466-47504510	NM_001037221:1849	Samd4	INSIDE	0.537	0.459	2559.16	1174.53	0.246	2128.73	524.34
A_68_P25021896	chr7:29221702-29221746	NM_175021:-514	Samd4b	DIVERGENT_PROMOTER	0.583	0.559	1178.30	658.97	0.326	988.86	322.20
A_68_P29454672	chr14:22570199-22570243	NM_026283:468	Samd8	INSIDE	0.175	0.658	1611.64	1059.89	0.115	1149.32	132.07

ProbeName	Target position of probe on CpG island microarray	TargetID	GeneSymbol	CpG island Description	Ratio of relative methylation (TiO2-NP/Vehicle)	Sham group			TiO_2-H group		
						Relative methylation (Cy-5/Cy-3)	Cy-3 signal (Input)	Cy-5 signal (MeDIP)	Relative methylation (Cy-5/Cy-3)	Cy-3 signal (Input)	Cy-5 signal (MeDIP)
A_68_P21775423	chr2:156960457-156960501	NM_001139520:480	Samhd1	INSIDE	0.408	0.568	1117.04	634.74	0.232	1007.64	233.45
A_68_P30393146	chr15:84022703-84022747	NM_178614:62	Samm50	INSIDE	0.421	0.447	1409.97	629.72	0.188	1155.30	217.45
A_68_P30393147	chr15:84022777-84022821	NM_178614:136	Samm50	INSIDE	0.406	0.691	3994.97	2761.52	0.280	3060.42	858.38
A_68_P31598893	chr18:31794423-31794469	NM_172965:410	Sap130	INSIDE	0.108	0.710	2044.22	1450.72	0.076	1568.02	119.73
A_68_P27827566	chr11:57614919-57614965	NM_001081168:-196	Sap30l	PROMOTER	0.172	1.782	1897.59	3381.73	0.306	1183.28	361.79
A_68_P27181743	chr10:61143343-61143387	NM_009120:296	Sar1a	INSIDE	0.566	0.631	2216.17	1399.40	0.358	1667.68	596.47
A_68_P27943642	chr11:78304280-78304324	NM_001168521:6954	Sarm1	INSIDE	0.602	0.680	1333.95	906.64	0.409	1015.98	415.37
A_68_P27943646	chr11:78304705-78304749	NM_001168521:6530	Sarm1	INSIDE	0.210	0.508	1438.88	731.17	0.107	1130.88	120.88
A_68_P27543378	chr10:128259182-128259239	NM_025364:384	Sarnp	INSIDE	0.231	0.497	1334.36	662.70	0.115	1044.10	120.03
A_68_P22412728	chr3:108247875-108247920	NM_001204979:280	Sars	INSIDE	0.191	0.559	1433.83	800.91	0.107	1107.48	118.39
A_68_P22412729	chr3:108247976-108248020	NM_001204979:179	Sars	INSIDE	0.144	0.620	1784.22	1106.24	0.089	1331.89	119.05
A_68_P22412732	chr3:108248246-108248294	NM_001204979:-93	Sars	PROMOTER	0.093	0.667	2721.42	1816.43	0.062	1934.76	120.72
A_68_P31928437	chr19:5388969-5389013	NM_016882:-287	Sart1	PROMOTER	0.282	0.529	1120.54	592.66	0.149	886.23	131.98
A_68_P26926728	chr10:8606159-8606207	NM_175155:-314	Sash1	PROMOTER	0.219	0.538	1448.43	778.96	0.118	1026.36	121.00
A_68_P22450159	chr3:116297548-116297592	NM_028349:-355	Sass6	PROMOTER	0.430	0.525	1549.58	813.48	0.226	1281.98	289.46
A_68_P22450166	chr3:116298284-116298328	NM_028349:381	Sass6	INSIDE	0.145	0.590	1968.04	1161.25	0.085	1393.24	119.11
A_68_P32758876	chrX:151651017-151651061	NM_009121:-86	Sat1	PROMOTER	0.284	1.456	2986.18	4346.66	0.413	1226.63	506.19
A_68_P20250374	chr1:57026684-57026728	NM_139146:1472	Satb2	INSIDE	0.590	0.551	962.45	530.59	0.325	833.78	271.05
A_68_P20250375	chr1:57026759-57026807	NM_139146:1396	Satb2	INSIDE	0.431	0.673	1459.32	981.68	0.290	1126.26	326.72
A_68_P20250386	chr1:57027876-57027920	NM_139146:280	Satb2	INSIDE	0.367	0.721	4515.25	3255.16	0.264	2873.17	759.41
A_68_P24092946	chr5:130731035-130731079	NM_023248:276	Sbds	INSIDE	0.172	0.644	5110.85	3292.95	0.111	3901.95	432.30
A_68_P24092947	chr5:130731194-130731238	NM_023248:116	Sbds	INSIDE	0.175	0.649	1450.87	941.34	0.113	1059.46	119.97
A_68_P30424508	chr15:89145163-89145207	NM_001081030:558	Sbf1	INSIDE	0.558	0.538	1198.03	644.62	0.300	1067.07	320.46
A_68_P24061230	chr5:124874812-124874856	NM_001081203:1089	Sbno1	INSIDE	0.169	0.571	3945.66	2252.86	0.096	2651.15	255.52
A_68_P24061239	chr5:124875960-124876004	NM_001081203:-59	Sbno1	PROMOTER	0.423	0.464	2926.54	1357.27	0.196	2319.38	454.59
A_68_P27280974	chr10:79564978-79565022	NM_183426:447	Sbno2	INSIDE	0.382	1.813	2958.89	5365.60	0.692	2160.66	1494.97
A_68_P27280977	chr10:79565295-79565339	NM_183426:131	Sbno2	INSIDE	0.452	0.463	3369.65	1558.55	0.209	2384.72	498.98
A_68_P27280979	chr10:79565571-79565615	NM_183426:-145	Sbno2	PROMOTER	0.189	0.530	1545.81	818.67	0.100	1157.92	115.89
A_68_P26454290	chr9:42072563-42072607	NM_172769:-201	Sc5d	PROMOTER	0.557	0.466	2173.31	1012.46	0.259	1775.26	460.30
A_68_P25090667	chr7:52271089-52271133	NM_001008422:509	Scaf1	INSIDE	0.525	0.597	935.41	558.13	0.313	703.40	220.26
A_68_P25090672	chr7:52271527-52271571	NM_001008422:71	Scaf1	INSIDE	0.297	0.495	1154.40	571.19	0.147	814.15	119.77
A_68_P25090673	chr7:52271610-52271654	NM_001008422:-13	Scaf1	DIVERGENT_PROMOTER	0.234	0.503	1312.83	660.29	0.118	1005.47	118.24
A_68_P30463768	chr15:96290631-96290675	NM_028148:622	Scaf11	INSIDE	0.212	0.646	1225.33	792.11	0.137	899.46	123.32
A_68_P30463774	chr15:96291694-96291742	NM_028148:-444	Scaf11	PROMOTER	0.294	0.501	1219.43	610.39	0.147	812.36	119.44
A_68_P30463776	chr15:96291913-96291959	NM_028148:-662	Scaf11	PROMOTER	0.230	0.690	1041.08	718.10	0.158	769.02	121.80
A_68_P31003193	chr17:3114560-3114614	NM_134123:-385	Scaf8	PROMOTER	0.269	0.397	1860.62	738.51	0.107	1441.08	154.09
A_68_P31003198	chr17:3115122-3115166	NM_134123:173	Scaf8	INSIDE	0.263	0.617	1848.61	1141.12	0.162	1633.59	264.93
A_68_P27284239	chr10:80065786-80065830	NM_019575:182	Scamp4	INSIDE	0.263	0.374	1973.96	738.89	0.098	1545.77	152.22
A_68_P29676509	chr14:66572807-66572857	NM_172604:-251	Scara3	PROMOTER	0.110	1.425	1005.72	1433.29	0.156	762.96	119.34
A_68_P24066362	chr5:125821382-125821426	NM_001205082:60	Scarb1	INSIDE	0.117	0.693	3135.41	2172.49	0.081	2016.71	164.03
A_68_P24066363	chr5:125821560-125821604	NM_001205082:-118	Scarb1	PROMOTER	0.248	2.001	3253.79	6510.19	0.497	1989.18	988.87
A_68_P30575777	chr16:17802822-17802869	NM_153790:5471	Scarf2	INSIDE	0.319	0.660	996.82	658.39	0.211	732.58	154.45
A_68_P30575818	chr16:17807580-17807624	NM_153790:10228	Scarf2	INSIDE	0.437	0.702	1450.27	1018.03	0.306	1203.90	368.91
A_68_P30575819	chr16:17807668-17807712	NM_153790:10316	Scarf2	INSIDE	0.328	0.515	3124.31	1609.55	0.169	2519.47	425.42

ProbeName	Target position of probe on CpG island microarray	TargetID	GeneSymbol	CpG island Description	Ratio of relative methylation (TiO2-NP/Vehicle)	Sham group			TiO_2-H group		
						Relative methylation (Cy-5/Cy-3)	Cy-3 signal (Input)	Cy-5 signal (MeDIP)	Relative methylation (Cy-5/Cy-3)	Cy-3 signal (Input)	Cy-5 signal (MeDIP)
A_68_P24819294	chr6:125141350-125141400	NR_028517:-4925	Scarna10	PROMOTER	0.408	0.615	1712.69	1053.88	0.251	1327.18	332.98
A_68_P24819295	chr6:125141439-125141483	NR_028517:-5011	Scarna10	PROMOTER	0.392	0.538	2141.51	1152.69	0.211	1553.71	328.17
A_68_P22413314	chr3:108357099-108357143	NR_028538:200	Scarna2	DOWNSTREAM	0.226	0.642	1098.59	705.00	0.145	833.93	120.84
A_68_P32131187	chr19:44322861-44322905	NM_024450:45105	Scd3	DOWNSTREAM	0.579	0.483	1336.55	645.79	0.280	1158.84	324.23
A_68_P28429502	chr12:52478398-52478449	NM_029825:-143	Scfd1	PROMOTER	0.232	0.411	1649.56	678.52	0.095	1260.64	120.27
A_68_P28429504	chr12:52478611-52478655	NM_029825:66	Scfd1	INSIDE	0.254	0.602	1313.85	791.57	0.153	910.79	139.23
A_68_P26645323	chr9:75531947-75531992	NM_001164790:-106	Scg3	PROMOTER	0.258	0.342	1926.36	658.56	0.088	1402.14	123.81
A_68_P22215786	chr3:68297563-68297607	NR_036665:-530	Schip1	PROMOTER	0.450	0.428	3152.94	1348.73	0.193	2472.58	476.28
A_68_P22215791	chr3:68298276-68298322	NR_036665:184	Schip1	INSIDE	0.236	0.512	1328.75	680.03	0.121	1000.96	121.08
A_68_P22215796	chr3:68298884-68298928	NR_036665:792	Schip1	INSIDE	0.160	0.506	5750.71	2909.70	0.081	4159.49	336.40
A_68_P22215801	chr3:68299336-68299380	NR_036665:1244	Schip1	INSIDE	0.131	0.728	1665.00	1211.78	0.095	1256.49	119.50
A_68_P23251106	chr4:120078426-120078470	NM_001159630:563	Scmh1	INSIDE	0.379	0.600	1674.00	1004.47	0.227	1603.57	364.74
A_68_P26442128	chr9:40076808-40076852	NM_001083917:30	Scn3b	INSIDE	0.162	0.495	2012.11	996.61	0.080	1520.69	121.77
A_68_P26873361	chr9:119488531-119488575	NM_021544:-418	Scn5a	PROMOTER	0.227	0.637	1505.63	959.34	0.145	1164.02	168.23
A_68_P30489277	chr15:100877902-100877949	NM_011323:111224	Scn8a	DOWNSTREAM	0.492	0.572	1566.30	896.17	0.281	1222.01	343.79
A_68_P30489324	chr15:100883675-100883719	NM_011323:116995	Scn8a	DOWNSTREAM	0.561	0.627	1675.76	1050.43	0.352	1580.13	556.04
A_68_P25475987	chr7:129008610-129008655	NM_011325:8	Scnn1b	INSIDE	0.296	0.444	2237.84	992.61	0.131	1674.89	219.88
A_68_P25475988	chr7:129008709-129008753	NM_011325:106	Scnn1b	INSIDE	0.646	0.428	1252.92	536.83	0.277	933.48	258.50
A_68_P30424832	chr15:89204085-89204129	NM_001111288:143	Sco2	INSIDE	0.191	0.587	1327.50	779.70	0.112	1073.59	120.28
A_68_P24464120	chr6:54516036-54516080	NM_027268:318	Scrn1	INSIDE	0.261	0.611	2879.70	1758.99	0.159	2081.78	331.40
A_68_P24464121	chr6:54516133-54516177	NM_027268:222	Scrn1	INSIDE	0.187	0.562	1461.21	820.71	0.105	1132.37	119.20
A_68_P30348858	chr15:76348557-76348601	NM_130893:3981	Scrt1	INSIDE	0.346	0.480	1591.67	763.81	0.166	1138.79	189.10
A_68_P30348859	chr15:76348629-76348673	NM_130893:3909	Scrt1	INSIDE	0.339	0.476	1845.93	877.89	0.161	1351.96	218.04
A_68_P30348874	chr15:76350196-76350240	NM_130893:2341	Scrt1	INSIDE	0.088	1.855	10808.14	20049.85	0.163	6370.92	1038.67
A_68_P30348901	chr15:76353383-76353427	NM_130893:-845	Scrt1	PROMOTER	0.246	0.440	1857.06	816.83	0.108	1307.90	141.70
A_68_P21747623	chr2:151921158-151921202	NM_001160410:13916	Scrt2	INSIDE	0.264	1.355	2032.50	2753.88	0.358	1383.66	495.61
A_68_P31120663	chr17:28278567-28278611	NM_001004366:-882	Scube3	PROMOTER	0.412	0.452	1146.06	518.47	0.187	957.24	178.55
A_68_P31120667	chr17:28279215-28279259	NM_001004366:-234	Scube3	PROMOTER	0.623	0.574	2709.46	1553.88	0.357	2254.63	804.92
A_68_P30348454	chr15:76288272-76288316	NM_198885:427	Scx	INSIDE	0.606	0.519	1891.00	981.97	0.315	1523.61	479.34
A_68_P31930772	chr19:5770953-5770997	NM_023912:427	Scyl1	INSIDE	0.520	0.655	1920.96	1257.37	0.340	1369.97	466.12
A_68_P27332529	chr10:89148969-89149013	NM_198021:40	Scyl2	INSIDE	0.531	0.697	1423.12	991.24	0.370	1195.74	441.94
A_68_P23893504	chr5:92738668-92738712	NM_172713:360	Sdad1	INSIDE	0.293	0.723	2304.22	1666.04	0.212	1683.50	357.23
A_68_P28224313	chr12:8777876-8777920	NM_011519:-303	Sdc1	PROMOTER	0.286	0.478	1159.22	553.62	0.137	887.67	121.34
A_68_P28224314	chr12:8777965-8778009	NM_011519:-215	Sdc1	PROMOTER	0.229	0.578	2685.91	1552.20	0.132	2166.00	286.47
A_68_P28224317	chr12:8778225-8778269	NM_011519:45	Sdc1	INSIDE	0.561	0.563	2360.37	1329.96	0.316	1718.40	543.22
A_68_P28224330	chr12:8779493-8779537	NM_011519:1313	Sdc1	INSIDE	0.189	0.480	1837.77	882.41	0.091	1368.81	123.90
A_68_P28224331	chr12:8779590-8779634	NM_011519:1411	Sdc1	INSIDE	0.377	0.650	3084.75	2005.47	0.245	2341.09	573.27
A_68_P21817911	chr2:164268487-164268531	NM_011521:180	Sdc4	INSIDE	0.304	0.522	1281.44	668.61	0.159	1064.40	169.09
A_68_P27942191	chr11:78059485-78059537	NM_009143:263	Sdf2	INSIDE	0.249	0.519	1254.55	650.62	0.129	936.65	120.74
A_68_P27942192	chr11:78059616-78059660	NM_009143:391	Sdf2	INSIDE	0.532	0.569	1964.93	1117.66	0.303	1486.96	450.02
A_68_P30572149	chr16:17132425-17132469	NM_022324:30	Sdf2l1	INSIDE	0.638	0.561	2784.40	1561.61	0.358	2256.50	807.17
A_68_P30572152	chr16:17132713-17132758	NM_022324:-259	Sdf2l1	PROMOTER	0.343	0.652	972.55	634.34	0.223	716.56	160.11
A_68_P23441760	chr4:155367175-155367219	NM_011341:174	Sdf4	INSIDE	0.300	0.443	3597.37	1592.29	0.133	2849.65	378.39
A_68_P23441761	chr4:155367293-155367337	NM_011341:292	Sdf4	INSIDE	0.307	0.477	1271.30	606.18	0.146	983.95	143.92

ProbeName	Target position of probe on CpG island microarray	TargetID	GeneSymbol	CpG island Description	Ratio of relative methylation (TiO2-NP/Vehicle)	Sham group			TiO_2-H group		
						Relative methylation (Cy-5/Cy-3)	Cy-3 signal (Input)	Cy-5 signal (MeDIP)	Relative methylation (Cy-5/Cy-3)	Cy-3 signal (Input)	Cy-5 signal (MeDIP)
A_68_P29145778	chr13:74487536-74487580	NM_023281:130	Sdha	INSIDE	0.416	0.479	1256.33	601.51	0.199	969.17	193.08
A_68_P28145824	chr11:113926254-113926298	NM_172800:989	Sdk2	INSIDE	0.415	0.423	2654.77	1123.94	0.176	1979.25	347.60
A_68_P28145837	chr11:113927724-113927772	NM_172800:-483	Sdk2	PROMOTER	0.213	0.457	1717.83	785.36	0.097	1261.79	122.97
A_68_P29620551	chr14:56518869-56518914	NM_001082975:178	Sdr39u1	INSIDE	0.176	0.632	1687.88	1066.23	0.111	1049.63	116.66
A_68_P29620552	chr14:56519022-56519066	NM_001082975:25	Sdr39u1	INSIDE	0.315	0.709	3748.22	2658.11	0.223	2769.29	618.42
A_68_P29620554	chr14:56519301-56519352	NM_001082975:-257	Sdr39u1	PROMOTER	0.238	0.523	1255.69	657.31	0.124	952.39	118.51
A_68_P25269485	chr7:88092809-88092853	NM_019951:-394	Sec11a	PROMOTER	0.307	0.634	826.34	523.49	0.194	636.34	123.60
A_68_P28163122	chr11:116976258-116976302	NR_029459:-205	Sec14l1	PROMOTER	0.220	0.621	2834.40	1759.68	0.136	2013.85	274.88
A_68_P21078762	chr2:26300450-26300494	NM_153125:264	Sec16a	INSIDE	0.312	0.477	1159.54	553.10	0.149	797.89	118.81
A_68_P21078765	chr2:26300767-26300811	NM_153125:-52	Sec16a	PROMOTER	0.352	0.704	3435.76	2419.82	0.248	2631.82	652.80
A_68_P21707834	chr2:144381520-144381564	NM_019787:-470	Sec23b	DIVERGENT_PROMOTER	0.491	0.524	1561.14	817.77	0.257	1207.69	310.56
A_68_P29448256	chr14:21493458-21493502	NM_001168273:-62	Sec24c	DIVERGENT_PROMOTER	0.631	0.497	1200.16	596.51	0.313	1039.54	325.78
A_68_P29448257	chr14:21493552-21493596	NM_001168273:32	Sec24c	INSIDE	0.592	0.524	1383.50	725.40	0.310	1162.57	360.68
A_68_P29448259	chr14:21493732-21493776	NM_001168273:212	Sec24c	INSIDE	0.313	0.592	2499.08	1480.28	0.186	1952.88	362.43
A_68_P20976256	chr2:5816062-5816106	NM_021305:315	Sec61a2	INSIDE	0.226	0.459	1341.45	615.26	0.104	1155.91	120.02
A_68_P22030177	chr3:30692267-30692311	NM_027016:491	Sec62	INSIDE	0.472	0.442	1312.52	580.65	0.209	1143.66	239.01
A_68_P22030179	chr3:30692442-30692486	NM_027016:667	Sec62	INSIDE	0.493	0.596	2371.11	1413.16	0.294	1916.45	563.10
A_68_P27096527	chr10:42481255-42481299	NM_153055:-25	Sec63	PROMOTER	0.571	0.585	1212.18	709.24	0.334	1047.00	349.96
A_68_P27096531	chr10:42481959-42482003	NM_153055:679	Sec63	INSIDE	0.168	2.489	2830.36	7045.36	0.418	1743.99	728.62
A_68_P27096532	chr10:42482055-42482099	NM_153055:775	Sec63	INSIDE	0.114	1.824	10066.38	18363.01	0.207	6271.40	1298.66
A_68_P29033515	chr13:51747268-51747312	NM_029279:208	Secisbp2	INSIDE	0.208	0.435	1823.23	793.52	0.091	1354.61	122.85
A_68_P23699207	chr5:53604122-53604166	NM_172710:547	Sel1l3	INSIDE	0.399	0.533	1774.42	945.20	0.212	1363.90	289.58
A_68_P29502937	chr14:30781713-30781757	NM_019979:169	Selk	INSIDE	0.563	0.654	2837.27	1854.71	0.368	2160.24	794.56
A_68_P29502939	chr14:30781960-30782004	NM_019979:417	Selk	INSIDE	0.387	0.457	1368.15	625.57	0.177	965.93	170.93
A_68_P26809843	chr9:107501498-107501542	NM_009153:6213	Sema3b	INSIDE	0.549	3.968	607.40	2409.97	2.178	440.27	958.74
A_68_P26809957	chr9:107514932-107514976	NM_001042779:-3382	Sema3b	PROMOTER	0.317	0.678	2737.19	1855.43	0.215	2093.27	449.55
A_68_P26809960	chr9:107515313-107515362	NM_001042779:-3765	Sema3b	PROMOTER	0.228	0.430	1862.68	801.38	0.098	1275.80	125.33
A_68_P23482832	chr5:12383399-12383443	NM_028882:255	Sema3d	INSIDE	0.451	0.596	900.43	536.53	0.269	755.21	202.92
A_68_P25265199	chr7:87331654-87331698	NM_013659:-50	Sema4b	PROMOTER	0.343	0.535	1137.27	608.78	0.184	971.67	178.54
A_68_P25265202	chr7:87331967-87332011	NM_013659:262	Sema4b	INSIDE	0.262	0.668	1106.61	739.41	0.175	963.33	168.38
A_68_P25265270	chr7:87343737-87343781	NM_013659:12032	Sema4b	INSIDE	0.253	0.604	1204.20	726.96	0.153	871.69	133.15
A_68_P20149045	chr1:36613770-36613814	NM_001126047:1434	Sema4c	INSIDE	0.252	0.701	1920.32	1346.18	0.176	1297.88	228.83
A_68_P20149046	chr1:36613936-36613984	NM_001126047:1266	Sema4c	INSIDE	0.307	0.465	1165.59	542.06	0.143	881.56	125.81
A_68_P20149048	chr1:36614178-36614222	NM_001126047:1026	Sema4c	INSIDE	0.266	0.466	1230.25	573.10	0.124	939.14	116.27
A_68_P20149057	chr1:36615326-36615370	NM_001126047:-122	Sema4c	PROMOTER	0.222	0.600	1039.55	624.11	0.133	895.19	119.36
A_68_P20149051	chr1:36614518-36614562	NM_001126047:686	Sema4c	INSIDE	0.669	0.645	1130.29	728.96	0.431	949.38	409.44
A_68_P29034450	chr13:51888603-51888647	NM_013660:389	Sema4d	INSIDE	0.175	0.539	1663.93	896.28	0.094	1284.85	121.24
A_68_P29034451	chr13:51888679-51888723	NM_013660:313	Sema4d	INSIDE	0.284	0.409	1636.17	668.85	0.116	1261.66	146.71
A_68_P32135692	chr19:45063608-45063652	NM_011976:-203	Sema4g	PROMOTER	0.384	0.700	3727.06	2609.07	0.269	2764.75	742.89
A_68_P30118688	chr15:32175071-32175115	NM_009154:525	Sema5a	INSIDE	0.438	0.645	1312.52	846.02	0.283	1106.30	312.62
A_68_P30118694	chr15:32175744-32175788	NM_009154:1199	Sema5a	INSIDE	0.265	0.377	1639.40	618.15	0.100	1315.41	131.64
A_68_P30670707	chr16:35542109-35542153	NM_013661:683	Sema5b	INSIDE	0.206	0.690	5977.77	4122.35	0.142	4231.33	601.94
A_68_P30670708	chr16:35542234-35542279	NM_013661:809	Sema5b	INSIDE	0.303	0.691	2015.11	1392.01	0.209	1485.20	310.35
A_68_P30670710	chr16:35542481-35542525	NM_013661:1055	Sema5b	INSIDE	0.333	0.693	1104.16	765.26	0.231	916.15	211.69

ProbeName	Target position of probe on CpG island microarray	TargetID	GeneSymbol	CpG island Description	Ratio of relative methylation (TiO2-NP/Vehicle)	Sham group			TiO_2-H group		
						Relative methylation (Cy-5/Cy-3)	Cy-3 signal (Input)	Cy-5 signal (MeDIP)	Relative methylation (Cy-5/Cy-3)	Cy-3 signal (Input)	Cy-5 signal (MeDIP)
A_68_P30670711	chr16:35542576-35542620	NM_013661:1151	Sema5b	INSIDE	0.091	0.449	3881.02	1744.02	0.041	3032.25	123.37
A_68_P31682227	chr18:47526193-47526237	NM_018744:2308	Sema6a	INSIDE	0.283	0.385	1381.04	532.08	0.109	1066.25	116.36
A_68_P31682228	chr18:47526263-47526314	NM_018744:2234	Sema6a	INSIDE	0.221	0.401	1677.78	672.30	0.089	1322.60	117.19
A_68_P31682231	chr18:47526609-47526653	NM_018744:1892	Sema6a	INSIDE	0.345	0.668	2256.66	1506.77	0.230	1764.30	406.24
A_68_P31254139	chr17:56278984-56279028	NM_013662:-5731	Sema6b	PROMOTER	0.378	0.495	2403.85	1190.58	0.187	1795.25	336.05
A_68_P31254157	chr17:56281353-56281397	NM_001130456:-1608	Sema6b	PROMOTER	0.414	0.648	3569.53	2312.29	0.268	2533.73	678.80
A_68_P31254158	chr17:56281426-56281470	NM_001130456:-1682	Sema6b	PROMOTER	0.404	0.581	2799.57	1627.69	0.235	2130.76	500.62
A_68_P31254208	chr17:56288089-56288133	NM_001130456:-8344	Sema6b	PROMOTER	0.524	2.602	6117.85	15916.37	1.362	4071.00	5545.04
A_68_P22342532	chr3:94964402-94964446	NM_011351:-3871	Sema6c	PROMOTER	0.195	0.548	1503.41	823.23	0.107	1130.11	120.72
A_68_P22342533	chr3:94964490-94964535	NM_011351:-3783	Sema6c	PROMOTER	0.227	0.645	1517.74	979.46	0.146	1204.60	176.39
A_68_P22342542	chr3:94965500-94965544	NM_011351:-2773	Sema6c	PROMOTER	0.535	0.571	1346.41	768.17	0.305	1051.43	320.68
A_68_P21597425	chr2:124435761-124435806	NM_172537:-248	Sema6d	PROMOTER	0.406	0.615	871.15	535.88	0.249	650.35	162.24
A_68_P30473047	chr15:97923923-97923967	NM_144851:-108	Senp1	PROMOTER	0.665	0.647	1402.42	907.98	0.430	1117.88	481.06
A_68_P30595530	chr16:22009516-22009560	NM_029457:-18	Senp2	PROMOTER	0.156	0.564	1753.46	989.64	0.088	1411.18	124.37
A_68_P27896512	chr11:69494853-69494903	NM_030702:594	Senp3	INSIDE	0.517	0.570	1478.72	843.10	0.295	1087.25	320.45
A_68_P27896515	chr11:69495196-69495240	NM_030702:254	Senp3	INSIDE	0.289	0.519	2388.39	1238.44	0.150	1846.44	276.67
A_68_P26667556	chr9:79915402-79915446	NM_146003:715	Senp6	INSIDE	0.524	0.580	1184.12	686.23	0.303	1003.97	304.68
A_68_P23696202	chr5:53060801-53060845	NM_172490:118	Sepsecs	INSIDE	0.577	0.299	1850.27	553.64	0.173	1378.19	237.74
A_68_P27167591	chr10:58684147-58684191	NM_001024910:427	Sept10	INSIDE	0.213	0.481	1967.67	946.89	0.102	1442.77	147.74
A_68_P26365988	chr9:25059616-25059661	NM_001205367:-401	Sept7	PROMOTER	0.348	0.689	1101.77	759.06	0.240	955.50	229.17
A_68_P26365996	chr9:25060486-25060530	NM_001205367:469	Sept7	INSIDE	0.661	0.556	1312.34	729.21	0.367	1069.71	392.71
A_68_P28164067	chr11:117127295-117127344	NM_001113487:-240	Sept9	PROMOTER	0.281	0.545	1091.92	594.59	0.153	785.44	120.04
A_68_P28164072	chr11:117127975-117128019	NM_001113487:437	Sept9	INSIDE	0.281	0.414	1960.82	810.94	0.116	1462.50	170.22
A_68_P28164253	chr11:117151685-117151737	NM_001113487:24151	Sept9	INSIDE	0.447	4.153	316.04	1312.54	1.858	334.15	620.91
A_68_P28164528	chr11:117193656-117193700	NM_001113488:52	Sept9	INSIDE	0.547	0.472	1732.47	818.41	0.258	1274.35	329.23
A_68_P21580974	chr2:121275109-121275153	NM_011354:167	Serf2	INSIDE	0.504	0.508	1930.91	981.26	0.256	1715.70	439.52
A_68_P27160893	chr10:57252088-57252132	NM_019760:225	Serinc1	INSIDE	0.584	0.598	2679.26	1601.93	0.349	2042.17	713.07
A_68_P27160897	chr10:57252403-57252448	NM_019760:-90	Serinc1	PROMOTER	0.349	0.466	1189.08	554.31	0.163	942.99	153.44
A_68_P32926581	chr13:93381448-93381492	NM_172588:378	Serinc5	INSIDE	0.273	0.505	1092.29	552.12	0.138	893.39	123.25
A_68_P20378194	chr1:79855540-79855584	NM_009255:-322	Serpine2	PROMOTER	0.250	0.599	1703.55	1020.23	0.150	1455.57	218.36
A_68_P25017512	chr7:28271931-28271975	NM_018820:-19	Sertad1	PROMOTER	0.279	0.622	2163.16	1344.97	0.173	1769.61	306.95
A_68_P27631990	chr11:20443463-20443507	NM_021372:229	Sertad2	INSIDE	0.613	0.689	1889.90	1302.35	0.422	1447.47	611.00
A_68_P27632486	chr11:20531998-20532042	NM_001038625:41	Sertad2	INSIDE	0.284	0.465	1105.30	514.01	0.132	894.76	118.22
A_68_P25017446	chr7:28258643-28258687	NM_133210:-194	Sertad3	PROMOTER	0.665	0.425	1727.41	734.53	0.283	1521.32	430.17
A_68_P20945779	chr1:194661281-194661325	NM_001177794:16639	Sertad4	DOWNSTREAM	0.470	0.428	1399.41	598.48	0.201	1143.28	229.72
A_68_P27091227	chr10:41530014-41530058	NM_001162908:-343	Sesn1	DIVERGENT_PROMOTER	0.517	0.586	1123.65	658.05	0.303	858.74	260.18
A_68_P27091228	chr10:41530095-41530139	NM_001162908:-263	Sesn1	DIVERGENT_PROMOTER	0.232	0.568	2939.31	1670.16	0.132	2179.70	287.93
A_68_P23312646	chr4:132065845-132065889	NM_144907:505	Sesn2	INSIDE	0.259	0.589	985.42	580.54	0.153	759.13	116.03
A_68_P23312649	chr4:132066191-132066235	NM_144907:159	Sesn2	INSIDE	0.278	0.617	870.56	537.41	0.171	695.75	119.24
A_68_P23312650	chr4:132066273-132066317	NM_144907:77	Sesn2	INSIDE	0.657	0.640	1179.23	754.89	0.420	958.86	403.05
A_68_P26316370	chr9:14080581-14080626	NM_030261:-141	Sesn3	PROMOTER	0.114	0.604	2863.27	1729.16	0.069	1960.62	135.26
A_68_P26316371	chr9:14080770-14080814	NM_030261:48	Sesn3	INSIDE	0.478	0.559	1900.92	1061.75	0.267	1585.58	423.54
A_68_P26316376	chr9:14081279-14081323	NM_030261:556	Sesn3	INSIDE	0.398	0.552	1345.98	743.47	0.220	1037.83	228.33
A_68_P21101101	chr2:29918413-29918457	NM_001204875:-3559	Set	PROMOTER	0.546	0.653	878.99	574.01	0.357	637.51	227.30

ProbeName	Target position of probe on CpG island microarray	TargetID	GeneSymbol	CpG island Description	Ratio of relative methylation (TiO2-NP/Vehicle)	Sham group			TiO_2-H group		
						Relative methylation (Cy-5/Cy-3)	Cy-3 signal (Input)	Cy-5 signal (MeDIP)	Relative methylation (Cy-5/Cy-3)	Cy-3 signal (Input)	Cy-5 signal (MeDIP)
A_68_P21101103	chr2:29918555-29918605	NM_001204875:-3413	Set	PROMOTER	0.307	0.409	1398.57	572.36	0.125	1078.77	135.35
A_68_P21101128	chr2:29921642-29921686	NM_001204875:-329	Set	PROMOTER	0.455	1.532	2617.19	4010.37	0.697	1966.23	1369.69
A_68_P31857881	chr18:79305640-79305684	NM_053099:468	Setbp1	INSIDE	0.474	0.628	5152.73	3235.64	0.298	3688.43	1097.86
A_68_P25507247	chr7:134920536-134920580	NM_178029:-344	Setd1a	PROMOTER	0.481	0.341	3262.41	1113.63	0.164	2654.59	435.65
A_68_P28726320	chr12:109416772-109416817	NM_028262:700	Setd3	INSIDE	0.178	0.516	1860.07	959.94	0.092	1310.87	120.28
A_68_P24757362	chr6:113027834-113027882	NM_028385:226	Setd5	INSIDE	0.171	0.685	1325.64	907.99	0.117	1037.87	121.43
A_68_P26084862	chr8:98240139-98240183	NM_001035123:348	Setd6	INSIDE	0.452	0.669	1856.95	1242.26	0.303	1431.39	433.21
A_68_P26084863	chr8:98240210-98240254	NM_001035123:420	Setd6	INSIDE	0.374	0.665	1819.01	1209.95	0.249	1391.57	346.00
A_68_P24061289	chr5:124890348-124890392	NM_030241:432	Setd8	INSIDE	0.499	0.534	4435.36	2367.73	0.266	3424.01	912.46
A_68_P21095246	chr2:28980261-28980312	NM_198033:-225	Setx	PROMOTER	0.317	0.507	1048.02	531.44	0.161	734.92	118.04
A_68_P21095247	chr2:28980444-28980489	NM_198033:-45	Setx	PROMOTER	0.225	0.478	3232.84	1546.02	0.108	2561.81	276.11
A_68_P27940380	chr11:77744288-77744332	NM_021286:-134	Sez6	DIVERGENT_PROMOTER	0.236	0.644	2433.21	1565.98	0.152	1867.18	283.94
A_68_P27940386	chr11:77745058-77745102	NM_021286:636	Sez6	INSIDE	0.306	0.543	1094.97	595.09	0.166	867.73	144.19
A_68_P23993052	chr5:113005975-113006019	NM_019982:209	Sez6l	INSIDE	0.286	0.594	2040.56	1211.37	0.170	1645.50	279.06
A_68_P23993053	chr5:113006095-113006139	NM_019982:89	Sez6l	INSIDE	0.536	0.541	3984.37	2155.95	0.290	3078.01	892.33
A_68_P23993055	chr5:113006360-113006404	NM_019982:-177	Sez6l	PROMOTER	0.431	0.471	1700.24	800.51	0.203	1326.27	268.88
A_68_P31934219	chr19:6363498-6363542	NM_001110791:-169	Sf1	PROMOTER	0.617	0.631	1450.81	916.02	0.389	1059.31	412.36
A_68_P31934220	chr19:6363615-6363659	NM_001110791:-53	Sf1	PROMOTER	0.204	0.566	2062.29	1168.25	0.115	1363.93	157.53
A_68_P27285561	chr10:80261463-80261507	NM_013651:5	Sf3a2	INSIDE	0.225	0.461	1503.32	692.75	0.104	1146.08	118.93
A_68_P23269837	chr4:124386139-124386183	NM_029157:-5944	Sf3a3	PROMOTER	0.464	0.728	3183.11	2317.38	0.338	2323.93	785.44
A_68_P20240053	chr1:55084178-55084222	NM_031179:122	Sf3b1	INSIDE	0.226	0.444	1384.17	614.65	0.100	1143.74	114.79
A_68_P20240054	chr1:55084325-55084372	NM_031179:-26	Sf3b1	PROMOTER	0.334	0.467	1224.59	572.11	0.156	1049.46	163.67
A_68_P22347940	chr3:95976314-95976359	NM_153053:-136	Sf3b4	PROMOTER	0.419	0.579	1023.64	592.25	0.242	971.97	235.52
A_68_P26948616	chr10:12728440-12728484	NM_175102:207	Sf3b5	INSIDE	0.507	0.432	2366.77	1022.64	0.219	1933.08	423.55
A_68_P26948617	chr10:12728567-12728611	NM_175102:333	Sf3b5	INSIDE	0.277	0.474	1273.32	603.31	0.131	1126.82	147.89
A_68_P26948618	chr10:12728670-12728714	NM_175102:437	Sf3b5	INSIDE	0.442	0.533	2051.96	1092.68	0.235	1666.12	391.82
A_68_P29507574	chr14:31528289-31528333	NM_001166531:-42	Sfmbt1	PROMOTER	0.643	0.676	961.67	650.19	0.435	775.40	337.34
A_68_P21000515	chr2:10296432-10296476	NM_001198809:2392	Sfmbt2	INSIDE	0.278	0.559	1165.84	652.28	0.156	966.25	150.45
A_68_P21420761	chr2:90955447-90955493	NM_011355:18517	Sfpi1	INSIDE	0.211	0.616	2220.61	1367.15	0.130	1646.00	213.32
A_68_P22286829	chr3:83568459-83568506	NM_009144:-1760	Sfrp2	PROMOTER	0.291	0.395	1409.56	556.86	0.115	1077.22	124.04
A_68_P22286848	chr3:83570629-83570673	NM_009144:408	Sfrp2	INSIDE	0.441	0.580	3003.67	1742.06	0.256	2242.65	574.07
A_68_P22286849	chr3:83570700-83570744	NM_009144:480	Sfrp2	INSIDE	0.506	0.704	2903.66	2044.40	0.357	2270.81	809.60
A_68_P22286850	chr3:83570771-83570815	NM_009144:550	Sfrp2	INSIDE	0.479	0.595	1282.53	762.50	0.285	914.27	260.51
A_68_P28863266	chr13:19714869-19714913	NM_016687:-153	Sfrp4	DIVERGENT_PROMOTER	0.534	0.699	1695.05	1184.97	0.373	1424.99	531.78
A_68_P28863267	chr13:19714988-19715032	NM_016687:-33	Sfrp4	DIVERGENT_PROMOTER	0.576	0.589	1211.63	713.85	0.340	957.44	325.14
A_68_P24089364	chr5:130007431-130007475	NM_172276:347	Sfswap	INSIDE	0.217	0.422	1659.11	700.53	0.091	1319.24	120.65
A_68_P24089368	chr5:130007907-130007951	NM_172276:823	Sfswap	INSIDE	0.359	0.505	1512.54	763.12	0.181	1144.55	207.54
A_68_P20793340	chr1:167124392-167124436	NM_145512:150	Sft2d2	INSIDE	0.656	0.691	1278.22	883.79	0.453	1099.45	498.32
A_68_P29047306	chr13:54167631-54167675	NM_027324:439	Sfxn1	INSIDE	0.163	0.584	1716.87	1002.57	0.095	1252.42	119.43
A_68_P24604447	chr6:85282597-85282641	NM_178639:798	Sfxn5	INSIDE	0.303	0.371	2852.15	1057.76	0.112	2147.87	241.55
A_68_P24604448	chr6:85282737-85282781	NM_178639:658	Sfxn5	INSIDE	0.257	0.485	1204.68	584.81	0.125	958.93	119.47
A_68_P24604449	chr6:85282873-85282917	NM_178639:522	Sfxn5	INSIDE	0.316	0.519	1896.78	984.59	0.164	1488.04	243.94
A_68_P28035860	chr11:94840370-94840414	NM_009161:-2751	Sgca	PROMOTER	0.287	0.710	1261.96	896.40	0.204	952.35	194.21
A_68_P28035861	chr11:94840510-94840554	NM_009161:-2891	Sgca	PROMOTER	0.521	0.655	892.65	584.79	0.341	755.20	257.88

ProbeName	Target position of probe on CpG island microarray	TargetID	GeneSymbol	CpG island Description	Ratio of relative methylation (TiO2-NP/Vehicle)	Sham group			TiO_2-H group		
						Relative methylation (Cy-5/Cy-3)	Cy-3 signal (Input)	Cy-5 signal (MeDIP)	Relative methylation (Cy-5/Cy-3)	Cy-3 signal (Input)	Cy-5 signal (MeDIP)
A_68_P23162218	chr4:102432046-102432090	NM_144906:-899	Sgip1	PROMOTER	0.538	0.433	1480.49	640.74	0.233	1296.96	301.71
A_68_P26995733	chr10:21602269-21602313	NM_001161845:301	Sgk1	INSIDE	0.266	0.534	1247.06	666.50	0.142	1019.87	144.79
A_68_P26996378	chr10:21714127-21714171	NM_011361:-323	Sgk1	PROMOTER	0.431	0.625	1978.07	1236.73	0.270	1444.33	389.40
A_68_P32070012	chr19:32462008-32462052	NM_144792:914	Sgms1	INSIDE	0.465	0.636	2021.00	1284.89	0.295	1504.70	444.42
A_68_P20370950	chr1:78306691-78306735	NM_001004173:-208	Sgpp2	PROMOTER	0.274	0.506	2193.77	1109.05	0.139	1814.46	251.35
A_68_P20370954	chr1:78307126-78307170	NM_001004173:228	Sgpp2	INSIDE	0.372	0.570	1032.22	588.69	0.212	541.15	114.83
A_68_P23997297	chr5:113716248-113716292	NR_027934:-6649	Sgsm1	PROMOTER	0.347	0.691	2844.68	1966.65	0.240	2188.28	525.11
A_68_P23997299	chr5:113716482-113716526	NR_027934:-6883	Sgsm1	PROMOTER	0.206	0.632	1542.14	974.42	0.130	1093.21	142.15
A_68_P23997436	chr5:113739937-113739981	NM_172718:-152	Sgsm1	PROMOTER	0.235	0.668	1154.23	770.79	0.157	1014.52	158.92
A_68_P27923087	chr11:74709868-74709912	NM_197943:692	Sgsm2	INSIDE	0.507	0.520	1107.95	576.24	0.264	894.69	236.03
A_68_P29288686	chr13:104899839-104899883	NM_144838:-9	Sgtb	DIVERGENT_PROMOTER	0.140	0.641	2931.60	1878.98	0.090	2053.70	183.87
A_68_P29288687	chr13:104899925-104899970	NM_144838:78	Sgtb	INSIDE	0.527	0.658	3487.76	2296.13	0.347	2668.97	926.00
A_68_P29288689	chr13:104900121-104900165	NM_144838:273	Sgtb	INSIDE	0.398	0.583	3354.69	1956.35	0.232	2336.34	542.22
A_68_P25500723	chr7:133618294-133618338	NM_011363:328	Sh2b1	INSIDE	0.575	0.689	1287.82	887.81	0.396	1062.67	420.92
A_68_P24120066	chr5:136707860-136707905	NM_018825:12891	Sh2b2	INSIDE	0.601	2.852	1914.56	5460.69	1.713	1297.49	2222.73
A_68_P24120067	chr5:136707928-136707972	NM_018825:12823	Sh2b2	INSIDE	0.531	2.580	2576.89	6648.62	1.370	1811.15	2481.20
A_68_P24120068	chr5:136708025-136708069	NM_018825:12727	Sh2b2	INSIDE	0.529	2.946	1957.15	5765.04	1.560	1345.02	2097.57
A_68_P24046791	chr5:122286329-122286373	NM_008507:460	Sh2b3	INSIDE	0.557	0.520	1493.26	776.52	0.290	1281.16	371.15
A_68_P23344730	chr4:137806358-137806402	NM_001099631:55	Sh2d5	INSIDE	0.509	0.413	2445.43	1010.44	0.210	1774.98	373.19
A_68_P23594807	chr5:34868707-34868751	NM_011893:296	Sh3bp2	INSIDE	0.150	2.222	4823.82	10720.52	0.333	3204.04	1067.04
A_68_P20427307	chr1:90967569-90967615	NM_133816:556	Sh3bp4	INSIDE	0.218	0.556	2192.39	1219.94	0.121	1649.27	199.62
A_68_P20427310	chr1:90967840-90967891	NM_133816:829	Sh3bp4	INSIDE	0.229	0.686	1010.05	693.00	0.157	747.24	117.40
A_68_P29512055	chr14:32248911-32248955	NM_011894:287	Sh3bp5	INSIDE	0.171	0.372	2699.92	1005.53	0.064	1824.95	116.34
A_68_P27830409	chr11:58144073-58144117	NM_001161338:-125	Sh3bp5l	DIVERGENT_PROMOTER	0.324	0.681	1229.07	836.78	0.220	858.93	189.18
A_68_P31253475	chr17:56176053-56176097	NM_013664:-75	Sh3gl1	DIVERGENT_PROMOTER	0.550	0.553	4830.53	2671.43	0.304	3637.50	1106.08
A_68_P23072588	chr4:84851180-84851224	NM_019535:-157	Sh3gl2	PROMOTER	0.527	0.624	863.04	538.62	0.329	831.01	273.33
A_68_P32775563	chrX:156064795-156064839	NM_001135727:-523	Sh3kbp1	PROMOTER	0.152	1.691	1378.43	2330.66	0.257	510.94	131.07
A_68_P32775570	chrX:156065828-156065872	NM_001135727:511	Sh3kbp1	INSIDE	0.292	1.517	2708.20	4109.38	0.443	1044.62	462.77
A_68_P32775571	chrX:156065929-156065973	NM_001135727:611	Sh3kbp1	INSIDE	0.262	2.135	1953.68	4170.95	0.559	864.18	482.74
A_68_P32150072	chr19:47538682-47538726	NM_001164717:197	Sh3pxd2a	INSIDE	0.655	0.608	1522.14	924.88	0.398	1241.86	493.98
A_68_P27688273	chr11:32248073-32248117	NM_177364:284	Sh3pxd2b	INSIDE	0.643	0.738	4627.14	3415.56	0.475	3784.82	1797.77
A_68_P25908463	chr8:63702506-63702553	NM_021506:-438	Sh3rf1	PROMOTER	0.292	0.459	1306.91	599.66	0.134	1163.87	156.17
A_68_P27165500	chr10:58275328-58275372	NM_172788:-756	Sh3rf3	PROMOTER	0.329	0.424	1488.26	630.44	0.140	1073.55	149.81
A_68_P27165510	chr10:58276430-58276477	NM_172788:347	Sh3rf3	INSIDE	0.161	0.566	2763.45	1563.59	0.091	1999.48	182.00
A_68_P27165511	chr10:58276497-58276541	NM_172788:412	Sh3rf3	INSIDE	0.470	0.573	1509.66	865.21	0.270	1204.93	324.88
A_68_P28328450	chr12:31596678-31596722	NM_013709:167	Sh3yl1	INSIDE	0.433	0.687	1307.89	898.56	0.297	1149.46	341.59
A_68_P25605218	chr7:151361386-151361430	NM_001113373:-16	Shank2	PROMOTER	0.424	0.512	2129.65	1090.85	0.217	1528.90	332.09
A_68_P30425525	chr15:89329138-89329182	NM_021423:-1127	Shank3	PROMOTER	0.394	0.589	1347.03	794.03	0.232	1002.00	232.85
A_68_P27277986	chr10:79100770-79100814	NM_001024539:-129	Shc2	PROMOTER	0.431	0.504	1121.40	564.70	0.217	942.73	204.84
A_68_P29033147	chr13:51663043-51663087	NM_009167:-611	Shc3	PROMOTER	0.218	0.502	1396.09	701.39	0.109	1078.14	117.96
A_68_P29033148	chr13:51663167-51663218	NM_009167:-739	Shc3	PROMOTER	0.213	0.559	1402.04	784.25	0.119	985.98	117.27
A_68_P21604382	chr2:125548878-125548922	NM_199022:984	Shc4	INSIDE	0.565	0.409	1504.09	614.77	0.231	1178.89	272.42
A_68_P25618723	chr8:4779387-4779431	NM_011369:126	Shcbp1	INSIDE	0.506	0.369	1739.58	641.96	0.187	1489.84	278.05
A_68_P31253054	chr17:56109780-56109824	NM_001159523:-102	Shd	PROMOTER	0.611	0.544	2327.83	1266.72	0.333	1596.29	530.99

ProbeName	Target position of probe on CpG island microarray	TargetID	GeneSymbol	CpG island Description	Ratio of relative methylation (TiO2-NP/Vehicle)	Sham group			TiO_2-H group		
						Relative methylation (Cy-5/Cy-3)	Cy-3 signal (Input)	Cy-5 signal (MeDIP)	Relative methylation (Cy-5/Cy-3)	Cy-3 signal (Input)	Cy-5 signal (MeDIP)
A_68_P31253055	chr17:56109896-56109940	NM_001159523:14	Shd	INSIDE	0.564	0.518	4610.58	2387.13	0.292	3559.17	1038.80
A_68_P21585949	chr2:122174591-122174635	NM_001013829:20042	Shf	DOWNSTREAM	0.266	0.532	1138.26	605.30	0.142	932.96	132.20
A_68_P23768841	chr5:67998640-67998694	NM_001033415:-455	Shisa3	PROMOTER	0.215	0.548	1358.69	745.24	0.118	1042.23	123.18
A_68_P27877798	chr11:66340379-66340423	NM_001034874:-772	Shisa6	PROMOTER	0.477	0.563	2271.08	1279.17	0.269	1743.23	468.57
A_68_P24950722	chr7:4781562-4781606	NM_172737:14714	Shisa7	INSIDE	0.605	2.517	2137.86	5380.02	1.523	1644.26	2504.79
A_68_P24950723	chr7:4781645-4781689	NM_172737:14632	Shisa7	INSIDE	0.491	2.830	3360.14	9507.66	1.390	2245.47	3120.90
A_68_P24950803	chr7:4795789-4795833	NM_172737:488	Shisa7	INSIDE	0.271	0.408	1569.89	641.13	0.110	1181.68	130.56
A_68_P24950806	chr7:4796112-4796158	NM_172737:164	Shisa7	INSIDE	0.121	1.807	1285.74	2323.97	0.219	728.10	159.74
A_68_P24950807	chr7:4796189-4796233	NM_172737:88	Shisa7	INSIDE	0.126	1.434	3560.55	5105.55	0.180	2393.68	432.05
A_68_P30550103	chr16:11983989-11984033	NM_001174086:-195	Shisa9	PROMOTER	0.236	0.466	1546.59	721.02	0.110	1081.69	118.95
A_68_P30550105	chr16:11984315-11984359	NM_001174086:131	Shisa9	INSIDE	0.440	0.578	2001.15	1155.68	0.254	1514.02	385.06
A_68_P25016883	chr7:28140851-28140895	NM_138676:155	Shkbp1	INSIDE	0.430	0.495	3331.24	1648.27	0.213	2575.41	547.99
A_68_P27844938	chr11:60624796-60624840	NM_009171:-51	Shmt1	PROMOTER	0.379	0.654	3482.45	2278.99	0.248	2969.94	736.22
A_68_P32185502	chr19:54019941-54019985	NM_019658:597	Shoc2	INSIDE	0.534	0.645	1907.01	1229.57	0.344	1454.47	500.38
A_68_P32185503	chr19:54020036-54020080	NM_019658:693	Shoc2	INSIDE	0.304	0.576	1786.87	1029.32	0.175	1393.04	243.57
A_68_P22207866	chr3:66782104-66782148	NM_013665:3567	Shox2	INSIDE	0.325	0.738	3000.19	2215.07	0.240	2068.51	496.71
A_68_P22207872	chr3:66782957-66783001	NM_013665:2715	Shox2	INSIDE	0.216	0.639	1015.17	648.97	0.138	890.26	122.84
A_68_P27915802	chr11:73012820-73012864	NM_029031:-141	Shpk	DIVERGENT_PROMOTER	0.328	0.550	1385.83	762.72	0.180	1062.94	191.67
A_68_P27915806	chr11:73013237-73013281	NM_029031:275	Shpk	INSIDE	0.537	0.571	1243.69	710.72	0.307	961.60	295.21
A_68_P27915807	chr11:73013310-73013355	NM_029031:349	Shpk	INSIDE	0.282	0.649	903.19	586.29	0.183	628.50	114.93
A_68_P24693485	chr6:100620652-100620698	NM_181590:477	Shq1	INSIDE	0.335	0.657	899.49	590.54	0.220	840.75	185.09
A_68_P27804858	chr11:53270789-53270833	NM_027917:104	Shroom1	INSIDE	0.576	0.401	1370.05	549.13	0.231	1092.37	252.36
A_68_P27804859	chr11:53270871-53270915	NM_027917:186	Shroom1	INSIDE	0.625	0.380	1868.92	710.80	0.238	1462.56	347.79
A_68_P27804893	chr11:53276844-53276888	NM_027917:6160	Shroom1	INSIDE	0.519	0.595	906.20	539.18	0.309	811.85	250.84
A_68_P27804916	chr11:53280008-53280053	NM_027917:9324	Shroom1	INSIDE	0.195	0.707	2162.09	1527.52	0.138	1797.46	247.22
A_68_P32234209	chrX:5977910-5977954	NM_001040459:670	Shroom4	INSIDE	0.467	1.426	3548.57	5061.29	0.666	1349.60	899.01
A_68_P32796352	chrX:160514293-160514337	NM_009173:-333	Siah1b	PROMOTER	0.221	1.672	1531.25	2560.23	0.369	718.98	265.23
A_68_P22169677	chr3:58496154-58496198	NM_009174:134	Siah2	INSIDE	0.368	0.501	1256.52	629.58	0.184	1022.76	188.36
A_68_P22169678	chr3:58496243-58496287	NM_009174:46	Siah2	INSIDE	0.590	0.678	1708.09	1158.64	0.400	1488.06	595.39
A_68_P22169679	chr3:58496414-58496458	NM_009174:-126	Siah2	PROMOTER	0.504	0.590	905.23	534.20	0.297	801.03	238.01
A_68_P22169681	chr3:58496575-58496620	NM_009174:-287	Siah2	PROMOTER	0.468	0.542	1220.30	661.62	0.254	847.22	215.09
A_68_P30720961	chr16:44332811-44332855	NM_001159419:119	Sidt1	INSIDE	0.211	0.514	1403.51	721.28	0.108	1057.45	114.47
A_68_P22864088	chr4:41687513-41687565	NM_011014:648	Sigmar1	INSIDE	0.605	0.638	872.40	556.37	0.386	665.26	256.65
A_68_P22864089	chr4:41687680-41687724	NM_011014:484	Sigmar1	INSIDE	0.275	0.517	2643.94	1365.98	0.142	1826.63	259.12
A_68_P31142312	chr17:31992898-31992942	NM_010831:-183	Sik1	PROMOTER	0.362	0.545	1357.06	739.91	0.197	1298.38	256.01
A_68_P31142314	chr17:31993086-31993130	NM_010831:-371	Sik1	PROMOTER	0.172	0.504	1973.59	994.89	0.087	1737.94	150.89
A_68_P31142315	chr17:31993161-31993205	NM_010831:-445	Sik1	PROMOTER	0.124	0.520	2504.48	1302.91	0.065	1876.04	121.20
A_68_P26506990	chr9:50816646-50816690	NM_178710:510	Sik2	INSIDE	0.421	0.666	1460.48	972.49	0.280	1189.96	333.71
A_68_P26477291	chr9:45821396-45821440	NM_027498:516	Sik3	INSIDE	0.464	0.484	1634.85	790.55	0.225	1437.46	322.78
A_68_P27131116	chr10:50614810-50614854	NM_011376:-624	Sim1	PROMOTER	0.319	0.516	2206.95	1138.45	0.164	1878.92	308.99
A_68_P27131118	chr10:50615055-50615099	NM_011376:-380	Sim1	PROMOTER	0.620	0.677	1119.43	757.96	0.419	930.01	390.13
A_68_P27131168	chr10:50620782-50620826	NM_011376:5348	Sim1	INSIDE	0.175	0.712	1361.61	969.35	0.124	978.40	121.59
A_68_P30979305	chr16:94083673-94083718	NM_011377:-1809	Sim2	PROMOTER	0.333	0.645	851.23	549.43	0.215	704.95	151.40
A_68_P30979311	chr16:94084490-94084534	NM_011377:-992	Sim2	PROMOTER	0.520	0.552	1013.62	559.29	0.287	862.01	247.44

ProbeName	Target position of probe on CpG island microarray	TargetID	GeneSymbol	CpG island Description	Ratio of relative methylation (TiO2-NP/Vehicle)	Sham group			TiO_2-H group		
						Relative methylation (Cy-5/Cy-3)	Cy-3 signal (Input)	Cy-5 signal (MeDIP)	Relative methylation (Cy-5/Cy-3)	Cy-3 signal (Input)	Cy-5 signal (MeDIP)
A_68_P30979318	chr16:94085768-94085812	NM_011377:286	Sim2	INSIDE	0.502	0.551	1614.65	889.06	0.276	1302.63	360.09
A_68_P30979319	chr16:94085864-94085908	NM_011377:382	Sim2	INSIDE	0.381	0.402	2426.59	975.03	0.153	1954.83	299.27
A_68_P30979328	chr16:94087095-94087143	NM_011377:1614	Sim2	INSIDE	0.227	0.487	1307.82	636.43	0.110	1033.86	114.14
A_68_P30979595	chr16:94346850-94346894	NM_011377:261368	Sim2	INSIDE	0.164	0.696	2233.47	1553.92	0.114	1799.55	205.68
A_68_P30979597	chr16:94347042-94347086	NM_011377:261560	Sim2	INSIDE	0.473	0.532	2893.16	1538.32	0.251	2255.31	566.90
A_68_P26540068	chr9:56920704-56920750	NM_001110350:-3456	Sin3a	PROMOTER	0.086	0.589	3349.84	1973.10	0.051	2397.22	122.00
A_68_P26540093	chr9:56924054-56924108	NM_001110350:-102	Sin3a	PROMOTER	0.117	0.635	2431.69	1544.02	0.075	1730.32	129.05
A_68_P26540098	chr9:56924586-56924630	NM_001110350:426	Sin3a	INSIDE	0.559	0.712	3459.83	2462.91	0.398	2827.68	1125.53
A_68_P25962573	chr8:75247186-75247230	NM_001113248:22	Sin3b	INSIDE	0.459	0.500	3570.22	1785.07	0.230	2824.00	648.27
A_68_P25962580	chr8:75247857-75247901	NM_001113248:692	Sin3b	INSIDE	0.667	0.564	1820.03	1027.17	0.376	1543.99	580.83
A_68_P31929909	chr19:5654397-5654451	NM_001164480:9283	Sipa1	INSIDE	0.159	0.540	1876.73	1013.72	0.086	1353.16	116.39
A_68_P31929913	chr19:5654718-5654762	NM_001164480:8967	Sipa1	INSIDE	0.260	0.609	1556.45	947.97	0.159	948.49	150.47
A_68_P28585118	chr12:83270099-83270143	NM_001167983:-882	Sipa1l1	PROMOTER	0.447	0.569	2305.16	1312.09	0.255	1793.11	456.73
A_68_P28585128	chr12:83271163-83271207	NM_001167983:182	Sipa1l1	INSIDE	0.271	0.268	2096.62	562.41	0.073	1638.21	119.11
A_68_P25023456	chr7:29551693-29551750	NM_001122765:-49	Sirt2	DIVERGENT_PROMOTER	0.215	0.529	1418.61	750.93	0.114	1041.82	118.62
A_68_P25023458	chr7:29551865-29551909	NM_001122765:116	Sirt2	INSIDE	0.627	0.640	1524.09	974.95	0.401	1180.08	473.61
A_68_P25585438	chr7:148067601-148067645	NM_001127351:146	Sirt3	INSIDE	0.251	0.485	2710.95	1314.97	0.122	2100.29	255.63
A_68_P25585439	chr7:148067707-148067751	NM_001127351:40	Sirt3	INSIDE	0.212	0.648	4502.68	2916.34	0.137	3126.92	429.10
A_68_P25585441	chr7:148067881-148067925	NM_001127351:-134	Sirt3	PROMOTER	0.425	0.664	2052.05	1362.14	0.282	1698.79	479.75
A_68_P28185710	chr11:120485805-120485849	NM_153056:490	Sirt7	INSIDE	0.563	0.544	1310.04	712.60	0.306	1028.17	314.88
A_68_P28751515	chr12:113883521-113883565	NM_001161737:504	Siva1	INSIDE	0.100	0.686	2619.46	1797.55	0.068	1845.59	126.25
A_68_P28535261	chr12:74146417-74146461	NM_009189:1261	Six1	INSIDE	0.197	0.492	1985.22	975.89	0.097	1337.98	129.75
A_68_P28535262	chr12:74146492-74146537	NM_009189:1185	Six1	INSIDE	0.170	0.666	2732.43	1820.79	0.113	1938.38	219.78
A_68_P28535264	chr12:74146664-74146708	NM_009189:1013	Six1	INSIDE	0.294	0.589	1124.15	662.40	0.173	841.12	145.60
A_68_P28535265	chr12:74146791-74146835	NM_009189:887	Six1	INSIDE	0.144	0.419	2479.21	1037.84	0.060	2086.28	125.71
A_68_P28535273	chr12:74147578-74147622	NM_009189:99	Six1	INSIDE	0.249	0.691	1666.59	1152.26	0.172	1355.58	233.54
A_68_P28535335	chr12:74154555-74154606	NM_009189:-6881	Six1	PROMOTER	0.312	0.700	1656.51	1158.88	0.218	1212.57	264.27
A_68_P31412092	chr17:86084426-86084470	NM_011380:3146	Six2	INSIDE	0.439	0.648	2333.49	1511.04	0.284	1636.79	465.01
A_68_P31412093	chr17:86084523-86084567	NM_011380:3050	Six2	INSIDE	0.302	0.590	1185.81	700.21	0.178	970.62	172.83
A_68_P31412111	chr17:86086474-86086527	NM_011380:1094	Six2	INSIDE	0.247	0.516	1216.26	627.03	0.127	937.53	119.16
A_68_P31412112	chr17:86086622-86086666	NM_011380:950	Six2	INSIDE	0.509	0.627	1292.35	809.94	0.319	1152.84	367.85
A_68_P31412148	chr17:86091318-86091363	NM_011380:-3746	Six2	PROMOTER	0.180	0.475	1908.55	906.61	0.085	1365.48	116.60
A_68_P31412149	chr17:86091406-86091450	NM_011380:-3834	Six2	PROMOTER	0.249	0.609	942.97	574.15	0.152	787.60	119.40
A_68_P31412155	chr17:86092046-86092090	NM_011380:-4474	Six2	PROMOTER	0.282	0.451	1703.10	768.03	0.127	1391.83	176.85
A_68_P31411759	chr17:86020803-86020847	NM_011381:651	Six3	INSIDE	0.654	0.595	2238.44	1332.71	0.389	1756.08	683.90
A_68_P31411765	chr17:86021432-86021477	NM_011381:1281	Six3	INSIDE	0.092	0.658	2687.41	1769.21	0.060	2029.65	122.78
A_68_P31411656	chr17:86007613-86007657	NR_038085:10102	Six3os1	INSIDE	0.270	0.552	1036.40	571.73	0.149	835.90	124.37
A_68_P31411711	chr17:86014433-86014477	NR_038085:3282	Six3os1	INSIDE	0.461	0.645	1685.01	1087.39	0.297	1367.96	406.57
A_68_P31411737	chr17:86017485-86017529	NR_038085:230	Six3os1	INSIDE	0.186	0.508	1666.57	847.22	0.094	1286.03	121.31
A_68_P31411739	chr17:86017697-86017743	NR_038085:16	Six3os1	INSIDE	0.208	0.478	1661.34	794.86	0.099	1227.89	122.08
A_68_P28535622	chr12:74213314-74213358	NM_011382:896	Six4	INSIDE	0.098	0.458	5157.11	2363.02	0.045	4099.58	183.87
A_68_P28535623	chr12:74213467-74213511	NM_011382:744	Six4	INSIDE	0.179	0.632	1480.46	935.19	0.113	1084.65	122.46
A_68_P28535628	chr12:74213900-74213944	NM_011382:310	Six4	INSIDE	0.362	0.564	2662.05	1501.38	0.204	2072.53	422.87
A_68_P28534661	chr12:74040784-74040828	NM_011384:-125	Six6	PROMOTER	0.147	0.603	2438.40	1471.16	0.089	1838.01	163.19

ProbeName	Target position of probe on CpG island microarray	TargetID	GeneSymbol	CpG island Description	Ratio of relative methylation (TiO2-NP/Vehicle)	Sham group			TiO_2-H group		
						Relative methylation (Cy-5/Cy-3)	Cy-3 signal (Input)	Cy-5 signal (MeDIP)	Relative methylation (Cy-5/Cy-3)	Cy-3 signal (Input)	Cy-5 signal (MeDIP)
A_68_P28534662	chr12:74040869-74040913	NM_011384:-41	Six6	PROMOTER	0.286	0.575	2021.53	1162.50	0.164	1466.62	240.87
A_68_P28534667	chr12:74041473-74041517	NM_011384:563	Six6	INSIDE	0.201	0.707	1249.09	883.01	0.142	1026.02	145.52
A_68_P31830207	chr18:74367434-74367478	NM_001164355:16	Ska1	INSIDE	0.330	0.675	1542.09	1040.85	0.223	1152.40	256.59
A_68_P24447786	chr6:51962237-51962281	NM_018773:290	Skap2	INSIDE	0.383	0.510	1892.49	965.77	0.195	1579.61	308.51
A_68_P24447788	chr6:51962397-51962441	NM_018773:130	Skap2	INSIDE	0.272	0.583	1040.59	606.93	0.159	746.70	118.55
A_68_P24447792	chr6:51962699-51962743	NM_018773:-172	Skap2	PROMOTER	0.176	0.552	1572.06	867.38	0.097	1213.14	118.04
A_68_P23436702	chr4:154524641-154524685	NM_011385:71982	Ski	DOWNSTREAM	0.228	0.707	2730.88	1930.75	0.161	1921.47	309.69
A_68_P22031732	chr3:30992663-30992708	NM_001039090:-1297	Skil	PROMOTER	0.175	0.538	1678.20	903.20	0.094	1223.13	114.95
A_68_P22031733	chr3:30992733-30992791	NM_001039090:-1220	Skil	PROMOTER	0.312	0.395	1342.75	530.78	0.123	1018.17	125.63
A_68_P22031742	chr3:30994277-30994321	NM_001039090:316	Skil	INSIDE	0.433	0.684	5333.16	3650.43	0.296	4142.42	1227.29
A_68_P29337690	chr13:113717502-113717546	NM_028151:64	Skiv2l2	INSIDE	0.300	0.574	986.30	565.81	0.172	703.81	121.22
A_68_P26574086	chr9:62990509-62990553	NM_172446:4257	Skor1	INSIDE	0.140	1.471	2598.37	3822.72	0.205	1699.93	349.32
A_68_P26574110	chr9:62993652-62993696	NM_172446:1113	Skor1	INSIDE	0.157	0.575	1920.92	1104.72	0.090	1307.67	117.97
A_68_P31844546	chr18:77098668-77098712	NM_001109743:3547	Skor2	INSIDE	0.441	0.418	1625.46	679.48	0.185	1476.19	272.42
A_68_P27798062	chr11:52045287-52045331	NM_011543:-188	Skp1a	PROMOTER	0.552	0.536	1812.67	971.02	0.296	1514.98	448.31
A_68_P25989169	chr8:81033142-81033186	NM_029736:-69	Slc10a7	DIVERGENT_PROMOTER	0.156	0.647	2714.74	1756.46	0.101	2096.02	211.97
A_68_P25989172	chr8:81033404-81033456	NM_029736:197	Slc10a7	INSIDE	0.067	1.382	3021.95	4175.13	0.093	2170.20	202.18
A_68_P30485728	chr15:100253426-100253470	NM_008732:38	Slc11a2	INSIDE	0.625	0.409	2542.12	1039.85	0.256	2195.62	561.25
A_68_P30485731	chr15:100253716-100253768	NM_008732:-256	Slc11a2	PROMOTER	0.496	0.653	1447.85	944.76	0.324	1067.65	345.79
A_68_P30485733	chr15:100253963-100254009	NM_008732:-500	Slc11a2	PROMOTER	0.178	0.606	2749.82	1665.72	0.108	2146.70	231.20
A_68_P21820737	chr2:164786214-164786258	NM_020333:-7251	Slc12a5	PROMOTER	0.454	0.449	1368.56	615.03	0.204	1130.89	230.65
A_68_P29141940	chr13:73901479-73901523	NM_011390:356	Slc12a7	INSIDE	0.064	0.574	4587.60	2633.57	0.037	3231.45	118.90
A_68_P27910560	chr11:72080885-72080933	NM_001004148:-802	Slc13a5	PROMOTER	0.136	0.700	1720.46	1203.84	0.095	1331.47	126.50
A_68_P24078667	chr5:128097745-128097789	NM_133895:-4	Slc15a4	PROMOTER	0.234	0.396	1600.03	634.00	0.093	1259.71	117.05
A_68_P22392363	chr3:104442146-104442191	NM_009196:-422	Slc16a1	PROMOTER	0.275	0.374	1638.66	613.26	0.103	1156.77	119.10
A_68_P22392368	chr3:104442587-104442631	NM_009196:18	Slc16a1	INSIDE	0.522	0.566	1703.49	964.24	0.296	1295.31	382.87
A_68_P22392373	chr3:104443206-104443250	NM_009196:638	Slc16a1	INSIDE	0.249	0.452	1320.86	596.50	0.112	1091.23	122.48
A_68_P27082611	chr10:39861509-39861553	NM_001114332:530	Slc16a10	INSIDE	0.294	0.612	888.49	543.96	0.180	686.30	123.72
A_68_P27899473	chr11:70028070-70028114	NM_153081:681	Slc16a11	INSIDE	0.653	0.449	1495.14	671.05	0.293	1262.84	370.40
A_68_P27899519	chr11:70034182-70034226	NM_172371:292	Slc16a13	INSIDE	0.287	0.677	2138.90	1448.28	0.194	1471.59	286.19
A_68_P28187724	chr11:120812178-120812222	NM_001038653:1820	Slc16a3	INSIDE	0.282	0.610	2676.51	1632.44	0.172	1910.64	329.15
A_68_P28187725	chr11:120812339-120812383	NM_001038653:1980	Slc16a3	INSIDE	0.586	0.481	1844.74	887.19	0.282	1489.49	419.73
A_68_P28118784	chr11:109333745-109333789	NM_134038:777	Slc16a6	INSIDE	0.270	0.454	1520.70	691.02	0.123	1224.16	150.33
A_68_P28118795	chr11:109335066-109335110	NM_001029842:-178	Slc16a6	PROMOTER	0.494	0.603	1130.37	681.16	0.298	940.74	280.31
A_68_P26660234	chr9:78435451-78435495	NM_172773:362	Slc17a5	INSIDE	0.173	0.605	1398.56	846.38	0.105	1128.57	118.50
A_68_P25091604	chr7:52431430-52431474	NM_182993:12162	Slc17a7	INSIDE	0.094	2.939	8630.71	25361.52	0.277	4801.50	1331.85
A_68_P32218217	chr19:59335443-59335487	NM_172523:97	Slc18a2	INSIDE	0.437	0.375	1951.34	730.94	0.164	1600.79	261.74
A_68_P29517796	chr14:33276471-33276515	NM_021712:1544	Slc18a3	INSIDE	0.312	0.704	4136.47	2914.05	0.220	2772.07	609.41
A_68_P29517797	chr14:33276567-33276611	NM_021712:1448	Slc18a3	INSIDE	0.067	0.684	4420.12	3025.44	0.046	3068.08	140.23
A_68_P29517799	chr14:33276809-33276853	NM_021712:1206	Slc18a3	INSIDE	0.330	0.644	1387.88	893.81	0.212	1161.94	246.74
A_68_P20788006	chr1:166178784-166178828	NM_054087:-379	Slc19a2	PROMOTER	0.267	0.630	1163.80	733.02	0.168	891.55	150.13
A_68_P20788007	chr1:166178862-166178906	NM_054087:-301	Slc19a2	PROMOTER	0.229	0.623	2213.34	1379.14	0.143	1587.80	227.00
A_68_P20788009	chr1:166179184-166179228	NM_054087:21	Slc19a2	INSIDE	0.282	0.531	3660.11	1942.01	0.150	2936.10	439.37
A_68_P32049530	chr19:28909696-28909740	NM_009199:63	Slc1a1	INSIDE	0.223	0.399	2660.52	1061.46	0.089	1677.45	149.03

ProbeName	Target position of probe on CpG island microarray	TargetID	GeneSymbol	CpG island Description	Ratio of relative methylation (TiO2-NP/Vehicle)	Sham group			TiO_2-H group		
						Relative methylation (Cy-5/Cy-3)	Cy-3 signal (Input)	Cy-5 signal (MeDIP)	Relative methylation (Cy-5/Cy-3)	Cy-3 signal (Input)	Cy-5 signal (MeDIP)
A_68_P21478263	chr2:102499439-102499483	NM_001077514:621	Slc1a2	INSIDE	0.349	0.426	1244.38	530.24	0.149	1051.03	156.14
A_68_P30001154	chr15:8586390-8586434	NM_148938:74395	Slc1a3	INSIDE	0.520	4.194	624.39	2618.46	2.182	622.94	1359.41
A_68_P27630977	chr11:20232784-20232830	NM_018861:-90	Slc1a4	PROMOTER	0.374	0.620	1423.10	882.05	0.232	999.64	231.85
A_68_P24983214	chr7:17367558-17367602	NM_009201:886	Slc1a5	INSIDE	0.580	0.568	2163.86	1229.99	0.330	1549.68	511.26
A_68_P21623514	chr2:129024077-129024121	NM_001159593:-410	Slc20a1	PROMOTER	0.504	0.576	2595.13	1495.79	0.291	1903.21	553.12
A_68_P21623519	chr2:129024601-129024645	NM_001159593:114	Slc20a1	INSIDE	0.525	0.599	2687.80	1610.63	0.314	1991.40	625.96
A_68_P27807567	chr11:53793320-53793364	NM_019723:187	Slc22a21	INSIDE	0.217	0.558	1200.61	669.52	0.121	1023.84	123.87
A_68_P28938190	chr13:34436028-34436072	NM_001033167:1001	Slc22a23	INSIDE	0.256	0.619	1229.32	761.07	0.158	921.92	145.95
A_68_P28938203	chr13:34437339-34437383	NM_001033167:-309	Slc22a23	PROMOTER	0.235	0.671	944.05	633.18	0.158	787.18	124.33
A_68_P27807843	chr11:53841465-53841509	NM_019687:106	Slc22a4	INSIDE	0.311	0.656	1669.02	1095.08	0.204	1261.63	257.68
A_68_P28689579	chr12:103367433-103367477	NM_172152:-174	Slc24a4	PROMOTER	0.227	0.522	2343.86	1222.49	0.118	1721.88	203.96
A_68_P24039146	chr5:120961225-120961269	NM_001177594:46	Slc24a6	INSIDE	0.394	0.528	3211.48	1697.21	0.208	2575.83	536.78
A_68_P24039147	chr5:120961336-120961380	NM_001177594:158	Slc24a6	INSIDE	0.319	0.473	1197.17	566.24	0.151	803.21	121.16
A_68_P30576622	chr16:17927537-17927581	NM_153150:754	Slc25a1	INSIDE	0.448	0.688	2021.54	1390.20	0.308	1549.55	477.85
A_68_P21319124	chr2:71205525-71205575	NM_172436:61	Slc25a12	INSIDE	0.334	0.604	1138.58	688.07	0.202	846.80	170.93
A_68_P30377509	chr15:81191134-81191178	NM_011399:39	Slc25a17	INSIDE	0.116	0.650	2295.39	1492.02	0.076	1681.37	127.13
A_68_P28154494	chr11:115488974-115489018	NM_026071:457	Slc25a19	INSIDE	0.433	0.633	862.56	546.39	0.274	711.16	195.15
A_68_P28154497	chr11:115489343-115489387	NM_026071:89	Slc25a19	INSIDE	0.234	0.519	2614.44	1356.14	0.122	1961.26	238.46
A_68_P25588799	chr7:148623331-148623375	NM_001177576:-2859	Slc25a22	PROMOTER	0.639	0.564	2863.01	1613.45	0.360	2015.33	725.84
A_68_P31259825	chr17:57198936-57198980	NM_025877:328	Slc25a23	INSIDE	0.554	0.488	2654.48	1296.22	0.271	2082.08	563.67
A_68_P31259829	chr17:57199355-57199400	NM_025877:-91	Slc25a23	DIVERGENT_PROMOTER	0.400	0.589	2001.97	1180.00	0.236	1472.23	347.15
A_68_P24657050	chr6:94450275-94450319	NM_026255:-11	Slc25a26	PROMOTER	0.148	0.659	1733.51	1141.55	0.098	1259.85	123.03
A_68_P32127976	chr19:43748957-43749001	NM_145156:393	Slc25a28	INSIDE	0.169	0.510	1858.26	947.25	0.086	1352.81	116.60
A_68_P32127987	chr19:43750198-43750243	NM_145156:-849	Slc25a28	PROMOTER	0.155	0.696	1895.01	1319.27	0.108	1366.48	147.51
A_68_P29729987	chr14:76186477-76186521	NM_026232:346	Slc25a30	INSIDE	0.221	0.461	2324.93	1071.35	0.102	1708.26	174.13
A_68_P29729988	chr14:76186558-76186602	NM_026232:264	Slc25a30	INSIDE	0.601	0.525	1223.05	642.53	0.316	965.31	304.96
A_68_P23402407	chr4:149147586-149147630	NM_027460:768	Slc25a33	INSIDE	0.614	0.586	1141.88	668.64	0.360	946.72	340.62
A_68_P26750963	chr9:97011708-97011752	NM_138756:-270	Slc25a36	PROMOTER	0.275	0.510	1489.71	759.94	0.140	1255.09	175.96
A_68_P25834905	chr8:47296158-47296202	NM_007450:183	Slc25a4	INSIDE	0.427	0.363	3243.34	1176.72	0.155	2452.80	380.33
A_68_P25950463	chr8:72735887-72735931	NM_001007570:272	Slc25a42	INSIDE	0.348	0.612	1770.31	1082.71	0.213	1267.46	269.44
A_68_P25950467	chr8:72736364-72736408	NM_001007570:-206	Slc25a42	PROMOTER	0.254	0.717	2209.13	1583.69	0.182	1728.52	314.58
A_68_P25950469	chr8:72736531-72736577	NM_001007570:-374	Slc25a42	PROMOTER	0.174	0.598	1531.97	915.90	0.104	1155.40	119.93
A_68_P25950471	chr8:72736718-72736765	NM_001007570:-561	Slc25a42	PROMOTER	0.359	0.590	1101.61	650.02	0.212	813.20	172.39
A_68_P23973505	chr5:109106557-109106601	NM_174870:-1910	Slc26a1	PROMOTER	0.052	1.822	1939.53	3534.19	0.094	1277.76	120.45
A_68_P23973506	chr5:109106670-109106714	NM_174870:-2024	Slc26a1	PROMOTER	0.270	1.498	4716.65	7067.46	0.405	2790.84	1130.22
A_68_P28177384	chr11:119217045-119217089	NM_178743:196	Slc26a11	INSIDE	0.609	0.605	1646.79	996.71	0.369	1314.26	484.36
A_68_P28332015	chr12:32244658-32244702	NM_011867:154	Slc26a4	INSIDE	0.275	0.575	1918.56	1103.79	0.158	1386.50	219.63
A_68_P25957539	chr8:74093493-74093537	NM_011977:689	Slc27a1	INSIDE	0.263	0.656	965.00	633.10	0.173	689.33	118.92
A_68_P25957540	chr8:74093690-74093734	NM_011977:887	Slc27a1	INSIDE	0.450	0.570	1188.11	677.45	0.257	973.27	249.67
A_68_P25957549	chr8:74094641-74094686	NM_011977:1838	Slc27a1	INSIDE	0.449	0.487	1901.62	925.32	0.218	1411.90	308.25
A_68_P21608668	chr2:126379204-126379249	NM_011978:467	Slc27a2	INSIDE	0.399	0.514	2438.81	1252.84	0.205	1823.73	373.99
A_68_P21608669	chr2:126379290-126379337	NM_011978:554	Slc27a2	INSIDE	0.174	0.554	1744.95	967.04	0.096	1200.39	115.56
A_68_P21099540	chr2:29658503-29658547	NM_011989:325	Slc27a4	INSIDE	0.589	0.635	2505.89	1590.11	0.374	2038.73	761.84
A_68_P31926382	chr19:5023935-5023979	NM_007854:-49	Slc29a2	PROMOTER	0.312	0.617	2154.83	1330.51	0.193	1509.83	290.69

ProbeName	Target position of probe on CpG island microarray	TargetID	GeneSymbol	CpG island Description	Ratio of relative methylation (TiO2-NP/Vehicle)	Sham group			TiO_2-H group		
						Relative methylation (Cy-5/Cy-3)	Cy-3 signal (Input)	Cy-5 signal (MeDIP)	Relative methylation (Cy-5/Cy-3)	Cy-3 signal (Input)	Cy-5 signal (MeDIP)
A_68_P31926383	chr19:5024065-5024109	NM_007854:81	Slc29a2	INSIDE	0.208	0.736	4823.55	3549.49	0.153	3181.33	487.98
A_68_P31926384	chr19:5024260-5024304	NM_007854:277	Slc29a2	INSIDE	0.594	0.524	1656.91	868.73	0.312	1423.90	443.74
A_68_P31926386	chr19:5024497-5024541	NM_007854:513	Slc29a2	INSIDE	0.251	0.647	910.24	589.06	0.162	715.85	116.11
A_68_P27176491	chr10:60215496-60215540	NM_023596:12	Slc29a3	INSIDE	0.319	0.461	4027.72	1857.59	0.147	2857.79	420.10
A_68_P27176493	chr10:60215697-60215744	NM_023596:-190	Slc29a3	PROMOTER	0.232	0.573	1256.27	719.52	0.133	891.49	118.71
A_68_P24152941	chr5:143177960-143178004	NM_146257:-72	Slc29a4	PROMOTER	0.658	2.270	11641.00	26427.90	1.495	7524.11	11247.90
A_68_P23243656	chr4:118781563-118781607	NM_011400:235	Slc2a1	INSIDE	0.613	0.601	1382.72	830.48	0.368	1175.39	432.70
A_68_P21824257	chr2:165329532-165329576	NM_130451:77	Slc2a10	INSIDE	0.365	0.542	1306.20	707.64	0.198	1231.11	243.71
A_68_P26998899	chr10:22365156-22365200	NM_178934:362	Slc2a12	INSIDE	0.323	0.737	2954.87	2176.30	0.238	2393.14	568.74
A_68_P26998900	chr10:22365258-22365302	NM_178934:464	Slc2a12	INSIDE	0.216	0.713	3308.36	2358.11	0.154	2497.69	384.56
A_68_P30436281	chr15:91402869-91402913	NM_001033633:802	Slc2a13	INSIDE	0.185	0.441	1931.15	851.47	0.081	1457.54	118.77
A_68_P20901342	chr1:187278951-187278995	NM_001033286:246	Slc30a10	INSIDE	0.435	0.414	1573.57	650.90	0.180	1299.23	233.87
A_68_P23322291	chr4:133899284-133899328	NM_001039677:322	Slc30a2	INSIDE	0.319	0.547	1470.19	804.25	0.174	1230.34	214.66
A_68_P23576445	chr5:31395426-31395470	NM_011773:452	Slc30a3	INSIDE	0.266	0.622	5256.90	3269.71	0.165	3941.63	652.25
A_68_P23576446	chr5:31395554-31395598	NM_011773:324	Slc30a3	INSIDE	0.239	0.588	2122.58	1248.39	0.140	1595.07	224.02
A_68_P23576450	chr5:31396001-31396045	NM_011773:-122	Slc30a3	PROMOTER	0.288	1.336	4328.71	5783.69	0.384	2888.85	1109.73
A_68_P23576461	chr5:31397375-31397419	NM_011773:-1496	Slc30a3	PROMOTER	0.151	0.674	1658.04	1117.04	0.102	1144.17	116.34
A_68_P31351306	chr17:74795028-74795072	NM_144798:79	Slc30a6	INSIDE	0.289	0.614	1014.56	622.62	0.177	846.16	150.14
A_68_P22446821	chr3:115710285-115710329	NM_023214:18	Slc30a7	INSIDE	0.552	0.621	1370.18	850.22	0.342	1049.51	359.32
A_68_P23767118	chr5:67698039-67698083	NM_178651:-135	Slc30a9	PROMOTER	0.306	0.696	2507.38	1744.43	0.213	1754.00	372.85
A_68_P22963968	chr4:62021675-62021719	NM_175090:-38	Slc31a1	DIVERGENT_PROMOTER	0.275	0.486	2719.08	1322.52	0.134	1958.93	262.22
A_68_P21783924	chr2:158436419-158436463	NM_009508:-53	Slc32a1	PROMOTER	0.224	0.451	2642.45	1192.06	0.101	2041.59	205.90
A_68_P21783925	chr2:158436516-158436560	NM_009508:45	Slc32a1	INSIDE	0.253	0.622	2370.41	1474.89	0.158	1913.02	301.52
A_68_P21783932	chr2:158437350-158437394	NM_009508:879	Slc32a1	INSIDE	0.094	0.748	6245.23	4670.35	0.071	4035.16	285.01
A_68_P21783953	chr2:158439712-158439756	NM_009508:3241	Slc32a1	INSIDE	0.153	0.524	4106.70	2153.66	0.080	2916.93	233.58
A_68_P21783954	chr2:158439815-158439861	NM_009508:3345	Slc32a1	INSIDE	0.106	0.599	2771.22	1658.63	0.063	1913.30	121.06
A_68_P21783955	chr2:158439931-158439975	NM_009508:3459	Slc32a1	INSIDE	0.144	0.708	1510.80	1068.98	0.102	1160.61	118.27
A_68_P21783956	chr2:158440051-158440100	NM_009508:3582	Slc32a1	INSIDE	0.313	0.568	940.57	534.46	0.178	693.97	123.49
A_68_P23698174	chr5:53440423-53440467	NM_011402:-147	Slc34a2	PROMOTER	0.238	0.704	1995.35	1404.58	0.167	1649.27	276.23
A_68_P32240689	chrX:7461201-7461245	NM_001083937:-147	Slc35a2	PROMOTER	0.131	2.395	2067.91	4953.34	0.314	730.87	229.37
A_68_P32240693	chrX:7461619-7461664	NM_001083937:272	Slc35a2	INSIDE	0.267	1.363	1457.12	1985.99	0.365	620.73	226.31
A_68_P32240694	chrX:7461707-7461754	NM_001083937:361	Slc35a2	INSIDE	0.047	2.503	2768.65	6928.78	0.116	1019.91	118.76
A_68_P22450818	chr3:116414578-116414622	NM_144902:598	Slc35a3	INSIDE	0.337	0.498	2384.90	1187.78	0.168	1934.40	324.78
A_68_P30725222	chr16:45158683-45158727	NM_028756:82	Slc35a5	INSIDE	0.648	0.611	843.81	515.92	0.396	757.76	300.05
A_68_P28038461	chr11:95245810-95245864	NM_016752:-399	Slc35b1	PROMOTER	0.241	0.677	948.26	642.06	0.163	735.47	120.24
A_68_P28963695	chr13:39052121-39052165	NM_001170430:264	Slc35b3	INSIDE	0.206	0.352	2444.68	861.62	0.073	1875.39	136.47
A_68_P28963696	chr13:39052204-39052248	NM_001170430:180	Slc35b3	INSIDE	0.211	0.424	2156.19	914.27	0.089	1634.57	146.09
A_68_P28963699	chr13:39052582-39052626	NM_001170430:-198	Slc35b3	PROMOTER	0.363	0.673	1247.85	839.95	0.244	981.07	239.84
A_68_P23165023	chr4:102888044-102888088	NM_177732:-577	Slc35d1	PROMOTER	0.651	0.694	1415.28	982.17	0.452	1125.28	508.43
A_68_P26983365	chr10:19569569-19569613	NM_029529:1675	Slc35d3	INSIDE	0.588	0.572	1545.29	883.45	0.336	1185.10	398.63
A_68_P26983369	chr10:19569958-19570002	NM_029529:1285	Slc35d3	INSIDE	0.492	0.503	1367.23	688.20	0.248	1009.08	249.76
A_68_P26983375	chr10:19570710-19570754	NM_029529:533	Slc35d3	INSIDE	0.245	0.632	2887.57	1824.08	0.155	2183.68	338.17
A_68_P26983378	chr10:19571100-19571144	NM_029529:143	Slc35d3	INSIDE	0.096	0.615	2845.12	1750.59	0.059	2155.76	127.27
A_68_P25961696	chr8:75015878-75015926	NM_177766:611	Slc35e1	INSIDE	0.261	0.406	1478.90	600.55	0.106	1216.53	128.73

ProbeName	Target position of probe on CpG island microarray	TargetID	GeneSymbol	CpG island Description	Ratio of relative methylation (TiO2-NP/Vehicle)	Sham group			TiO_2-H group		
						Relative methylation (Cy-5/Cy-3)	Cy-3 signal (Input)	Cy-5 signal (MeDIP)	Relative methylation (Cy-5/Cy-3)	Cy-3 signal (Input)	Cy-5 signal (MeDIP)
A_68_P25961699	chr8:75016300-75016344	NM_177766:191	Slc35e1	INSIDE	0.258	0.351	1716.66	603.10	0.091	1381.07	125.22
A_68_P23439556	chr4:154976380-154976427	NM_177186:879	Slc35e2	INSIDE	0.217	0.497	1428.41	710.37	0.108	1090.57	117.81
A_68_P27554681	chr11:3813626-3813670	NM_153142:1019	Slc35e4	INSIDE	0.179	0.665	1359.73	904.61	0.119	1012.87	120.44
A_68_P27138497	chr10:52410862-52410906	NM_178675:578	Slc35f1	INSIDE	0.317	0.561	1223.00	686.52	0.178	1010.72	179.68
A_68_P27138498	chr10:52410952-52410996	NM_178675:668	Slc35f1	INSIDE	0.510	0.542	982.61	532.30	0.277	855.59	236.59
A_68_P26522400	chr9:53619652-53619696	NM_028060:333	Slc35f2	INSIDE	0.578	0.536	1415.28	758.62	0.310	1195.29	370.62
A_68_P26256736	chr8:128822425-128822469	NM_175434:-32	Slc35f3	PROMOTER	0.357	0.573	3684.64	2113.05	0.205	2919.91	597.22
A_68_P20585378	chr1:127457195-127457239	NM_028787:-376	Slc35f5	PROMOTER	0.398	0.549	1232.48	677.04	0.218	1037.55	226.65
A_68_P20585379	chr1:127457276-127457320	NM_028787:-294	Slc35f5	PROMOTER	0.273	0.655	826.55	541.37	0.179	704.50	125.89
A_68_P20585380	chr1:127457355-127457399	NM_028787:-216	Slc35f5	PROMOTER	0.606	0.427	1335.57	570.20	0.259	1127.49	291.80
A_68_P31138852	chr17:31433001-31433045	NM_153062:595	Slc37a1	INSIDE	0.259	0.647	1554.79	1006.32	0.167	1278.81	214.11
A_68_P31138855	chr17:31433416-31433460	NM_153062:1011	Slc37a1	INSIDE	0.260	0.535	1291.17	690.63	0.139	864.92	120.08
A_68_P26467387	chr9:44205049-44205093	NM_008063:-1188	Slc37a4	PROMOTER	0.391	0.721	1626.23	1172.93	0.282	1467.19	414.12
A_68_P30464705	chr15:96471903-96471947	NM_134086:469	Slc38a1	INSIDE	0.379	0.501	1147.95	575.10	0.190	897.07	170.44
A_68_P30464706	chr15:96472084-96472128	NM_134086:287	Slc38a1	INSIDE	0.643	0.621	1270.19	788.24	0.399	996.93	397.86
A_68_P30465070	chr15:96529072-96529122	NM_175121:1033	Slc38a2	INSIDE	0.346	0.434	1176.92	510.40	0.150	921.32	138.35
A_68_P30465139	chr15:96539817-96539861	NM_175121:-9709	Slc38a2	PROMOTER	0.233	0.578	1594.16	920.82	0.135	1284.27	173.18
A_68_P22324142	chr3:90052208-90052252	NM_013901:117	Slc39a1	INSIDE	0.438	0.508	1286.91	654.27	0.223	1118.40	248.91
A_68_P21420492	chr2:90910618-90910662	NM_026721:-262	Slc39a13	PROMOTER	0.123	0.611	2065.79	1262.26	0.075	1559.32	117.02
A_68_P29700849	chr14:70750941-70750985	NM_001135152:-2722	Slc39a14	PROMOTER	0.457	0.737	2377.00	1752.80	0.337	1666.45	561.36
A_68_P31151612	chr17:34168426-34168470	NM_001077709:187	Slc39a7	INSIDE	0.282	0.441	1242.25	547.48	0.124	937.67	116.51
A_68_P31151613	chr17:34168502-34168546	NM_001077709:111	Slc39a7	INSIDE	0.548	0.588	1203.11	706.85	0.322	898.48	289.29
A_68_P31943153	chr19:8787770-8787814	NM_008577:595	Slc3a2	INSIDE	0.534	0.676	1585.38	1072.11	0.361	1172.38	423.18
A_68_P31943156	chr19:8788183-8788228	NM_008577:182	Slc3a2	INSIDE	0.300	0.649	1373.09	891.78	0.195	937.37	182.55
A_68_P31943157	chr19:8788272-8788316	NM_008577:93	Slc3a2	INSIDE	0.176	0.613	1539.03	943.38	0.108	1082.47	116.78
A_68_P20617850	chr1:133724396-133724440	NM_173865:-170	Slc41a1	PROMOTER	0.143	0.648	3597.66	2331.67	0.093	2661.22	247.15
A_68_P21394884	chr2:84679887-84679931	NM_024497:-571	Slc43a1	PROMOTER	0.267	0.395	1482.17	585.71	0.105	1143.40	120.42
A_68_P21395065	chr2:84706336-84706380	NM_001083809:25576	Slc43a1	DOWNSTREAM	0.180	0.526	3371.98	1774.51	0.095	2424.73	229.96
A_68_P21395068	chr2:84706724-84706775	NM_001083809:25967	Slc43a1	DOWNSTREAM	0.114	0.673	2322.84	1563.25	0.077	1539.46	117.95
A_68_P27926646	chr11:75344654-75344698	NM_001199284:-519	Slc43a2	PROMOTER	0.240	0.383	1726.94	661.76	0.092	1306.77	120.23
A_68_P27926647	chr11:75344750-75344794	NM_001199284:-423	Slc43a2	PROMOTER	0.212	0.614	3844.62	2361.91	0.130	2777.15	361.76
A_68_P27926653	chr11:75345380-75345424	NM_001199283:-211	Slc43a2	PROMOTER	0.417	0.433	3188.26	1381.53	0.181	2533.47	457.61
A_68_P22659972	chr3:153636247-153636291	NM_001081263:-131	Slc44a5	PROMOTER	0.609	0.592	962.49	569.93	0.361	806.88	291.12
A_68_P22659976	chr3:153636770-153636814	NM_001081263:393	Slc44a5	INSIDE	0.523	0.537	1862.95	1001.08	0.281	1449.80	407.80
A_68_P20618601	chr1:133860103-133860147	NM_145977:-7061	Slc45a3	PROMOTER	0.252	0.605	1023.46	619.57	0.152	844.77	128.65
A_68_P27943491	chr11:78279298-78279342	NM_026740:118	Slc46a1	INSIDE	0.388	0.632	956.21	604.78	0.245	777.95	190.79
A_68_P27943493	chr11:78279470-78279514	NM_026740:290	Slc46a1	INSIDE	0.369	0.544	1583.58	861.37	0.201	1242.60	249.24
A_68_P24176374	chr5:148706152-148706196	NM_027872:204	Slc46a3	INSIDE	0.621	0.561	1055.09	592.00	0.349	888.81	309.76
A_68_P27848102	chr11:61191658-61191702	NM_026183:-103	Slc47a1	PROMOTER	0.507	1.406	3890.51	5468.60	0.713	3248.35	2315.81
A_68_P21631237	chr2:130522606-130522650	NM_001081162:627	Slc4a11	INSIDE	0.158	0.616	2096.96	1291.76	0.098	1671.59	163.02
A_68_P23541735	chr5:23931232-23931276	NM_009207:205	Slc4a2	INSIDE	0.502	0.591	1220.94	721.48	0.297	1048.39	311.24
A_68_P20354817	chr1:75543227-75543271	NM_009208:408	Slc4a3	INSIDE	0.430	0.555	4935.41	2738.41	0.239	3596.93	858.77
A_68_P29421302	chr14:15535329-15535377	NM_001033270:-186	Slc4a7	PROMOTER	0.349	0.445	1301.27	579.43	0.156	976.97	151.93
A_68_P23576135	chr5:31350810-31350854	NM_177870:-380	Slc5a6	PROMOTER	0.473	0.577	1275.52	735.41	0.273	903.48	246.57

ProbeName	Target position of probe on CpG island microarray	TargetID	GeneSymbol	CpG island Description	Ratio of relative methylation (TiO2-NP/Vehicle)	Sham group			TiO_2-H group		
						Relative methylation (Cy-5/Cy-3)	Cy-3 signal (Input)	Cy-5 signal (MeDIP)	Relative methylation (Cy-5/Cy-3)	Cy-3 signal (Input)	Cy-5 signal (MeDIP)
A_68_P31247136	chr17:54437767-54437811	NM_022025:571	Slc5a7	INSIDE	0.522	0.671	1318.86	885.17	0.351	1023.01	358.71
A_68_P31247137	chr17:54437878-54437922	NM_022025:459	Slc5a7	INSIDE	0.206	0.676	1031.82	697.71	0.139	837.54	116.66
A_68_P31247139	chr17:54438039-54438089	NM_022025:295	Slc5a7	INSIDE	0.064	0.690	3657.85	2522.83	0.044	2637.98	116.63
A_68_P27327919	chr10:88349039-88349083	NM_145423:324	Slc5a8	INSIDE	0.109	0.716	4081.81	2921.07	0.078	3003.97	234.05
A_68_P27327920	chr10:88349166-88349210	NM_145423:452	Slc5a8	INSIDE	0.643	0.410	1537.83	630.03	0.263	1071.91	282.16
A_68_P27327921	chr10:88349290-88349347	NM_145423:582	Slc5a8	INSIDE	0.223	0.641	1035.31	663.66	0.143	815.56	116.52
A_68_P24764866	chr6:114232993-114233037	NM_178703:386	Slc6a1	INSIDE	0.471	0.533	1506.24	803.05	0.251	1206.57	302.85
A_68_P24763849	chr6:114081414-114081459	NM_172890:202	Slc6a11	INSIDE	0.268	0.444	2197.67	974.80	0.119	1641.03	195.42
A_68_P24763851	chr6:114081597-114081641	NM_172890:384	Slc6a11	INSIDE	0.607	0.648	1224.23	793.89	0.394	1041.94	410.18
A_68_P26896298	chr9:123587851-123587896	NM_139142:77	Slc6a20a	INSIDE	0.668	0.415	3054.71	1267.00	0.277	2432.20	673.39
A_68_P25115500	chr7:57167143-57167187	NM_148931:95	Slc6a5	INSIDE	0.618	0.599	1270.30	760.81	0.370	959.40	355.32
A_68_P31755042	chr18:61173520-61173564	NM_201353:311	Slc6a7	INSIDE	0.196	0.528	1881.91	994.11	0.103	1465.17	151.44
A_68_P32464060	chrX:70918500-70918544	NM_001142809:51	Slc6a8	INSIDE	0.242	1.628	1115.81	1816.54	0.394	465.66	183.51
A_68_P23236660	chr4:117508118-117508162	NM_008135:278	Slc6a9	INSIDE	0.123	1.455	3112.36	4528.70	0.179	2055.57	367.46
A_68_P23236661	chr4:117508209-117508255	NM_008135:370	Slc6a9	INSIDE	0.022	3.433	4780.88	16413.51	0.077	3061.80	235.89
A_68_P23236666	chr4:117508743-117508787	NM_008135:902	Slc6a9	INSIDE	0.208	1.603	1739.84	2789.62	0.333	1369.60	455.94
A_68_P24179198	chr5:149211250-149211294	NM_007513:208	Slc7a1	INSIDE	0.098	0.678	2631.10	1784.16	0.066	1868.57	124.19
A_68_P22032968	chr3:31208905-31208949	NM_172861:315	Slc7a14	INSIDE	0.238	0.438	1510.22	661.38	0.104	1201.77	125.33
A_68_P30574557	chr16:17576828-17576872	NM_144852:-86	Slc7a4	PROMOTER	0.455	0.647	1637.39	1060.01	0.295	1311.48	386.28
A_68_P26229668	chr8:124430812-124430856	NM_011404:752	Slc7a5	INSIDE	0.229	0.475	2550.79	1211.51	0.109	1869.54	203.48
A_68_P26229670	chr8:124431063-124431107	NM_011404:502	Slc7a5	INSIDE	0.584	0.556	1446.85	804.54	0.325	967.08	314.19
A_68_P26229672	chr8:124431324-124431368	NM_011404:240	Slc7a5	INSIDE	0.243	0.312	2401.66	749.30	0.076	1603.84	121.68
A_68_P28580849	chr12:82433588-82433632	NM_001167920:557	Slc8a3	INSIDE	0.615	0.666	997.56	663.93	0.410	772.99	316.54
A_68_P23317226	chr4:132925776-132925820	NM_016981:112	Slc9a1	INSIDE	0.444	0.553	2591.34	1434.14	0.246	2238.00	549.81
A_68_P29144761	chr13:74304464-74304508	NM_001081060:45524	Slc9a3	DOWNSTREAM	0.641	0.647	1075.95	695.72	0.415	865.52	358.83
A_68_P28151694	chr11:115024434-115024480	NM_012030:-198	Slc9a3r1	PROMOTER	0.171	0.626	1464.21	916.70	0.107	1140.40	121.85
A_68_P28151695	chr11:115024532-115024576	NM_012030:-100	Slc9a3r1	PROMOTER	0.285	0.480	1527.52	733.96	0.137	1217.10	166.40
A_68_P28151701	chr11:115025012-115025056	NM_012030:380	Slc9a3r1	INSIDE	0.252	0.379	1605.23	608.54	0.095	1196.98	114.20
A_68_P21835385	chr2:167247037-167247081	NM_148929:-162	Slc9a8	PROMOTER	0.484	0.664	1773.84	1178.65	0.321	1353.62	435.08
A_68_P21835387	chr2:167247383-167247427	NM_148929:184	Slc9a8	INSIDE	0.127	0.351	3404.37	1196.55	0.045	2679.10	119.63
A_68_P25235396	chr7:81699348-81699401	NM_001038643:292	Slco3a1	INSIDE	0.243	0.664	1024.28	679.64	0.161	769.19	124.14
A_68_P20038592	chr1:12981645-12981695	NM_172841:-454	Slco5a1	PROMOTER	0.462	0.568	965.89	548.33	0.262	832.08	218.07
A_68_P20038594	chr1:12981919-12981963	NM_172841:-724	Slco5a1	PROMOTER	0.391	0.619	903.30	558.79	0.242	770.40	186.25
A_68_P20038597	chr1:12982588-12982632	NM_172841:-1394	Slco5a1	PROMOTER	0.574	0.592	1150.49	680.83	0.340	942.04	320.02
A_68_P20038598	chr1:12982722-12982766	NM_172841:-1528	Slco5a1	PROMOTER	0.230	0.707	2334.38	1649.89	0.162	1690.97	274.48
A_68_P32116948	chr19:41817676-41817720	NM_015748:648	Slit1	INSIDE	0.386	0.671	4698.14	3152.91	0.259	3613.87	936.75
A_68_P32116953	chr19:41818287-41818331	NM_015748:38	Slit1	INSIDE	0.299	1.381	5569.52	7689.30	0.412	4138.15	1705.80
A_68_P23668849	chr5:48375003-48375047	NM_178804:631	Slit2	INSIDE	0.328	0.737	2370.43	1748.07	0.242	1695.31	409.93
A_68_P23668851	chr5:48375153-48375197	NM_178804:781	Slit2	INSIDE	0.125	0.662	2755.17	1823.56	0.083	1884.78	155.78
A_68_P23668871	chr5:48377683-48377731	NM_178804:3313	Slit2	INSIDE	0.236	0.450	1498.39	673.99	0.106	1081.97	114.74
A_68_P27704162	chr11:34934585-34934629	NM_011412:-351	Slit3	PROMOTER	0.344	0.467	1586.99	740.41	0.161	1269.13	203.90
A_68_P29892688	chr14:109310708-109310754	NM_199065:2726	Slitrk1	INSIDE	0.242	0.479	1492.99	715.24	0.116	1061.43	122.83
A_68_P32427540	chrX:61528858-61528902	NM_178740:1291	Slitrk4	INSIDE	0.152	1.779	2360.34	4199.69	0.270	913.40	247.07
A_68_P32427552	chrX:61530169-61530213	NM_178740:-19	Slitrk4	PROMOTER	0.281	1.914	10588.74	20265.47	0.537	4113.15	2209.98

ProbeName	Target position of probe on CpG island microarray	TargetID	GeneSymbol	CpG island Description	Ratio of relative methylation (TiO2-NP/Vehicle)	Sham group			TiO_2-H group		
						Relative methylation (Cy-5/Cy-3)	Cy-3 signal (Input)	Cy-5 signal (MeDIP)	Relative methylation (Cy-5/Cy-3)	Cy-3 signal (Input)	Cy-5 signal (MeDIP)
A_68_P32427553	chrX:61530261-61530305	NM_178740:-111	Slitrk4	PROMOTER	0.222	2.360	6864.58	16201.24	0.523	2553.62	1336.04
A_68_P29904270	chr14:112074911-112074955	NM_198865:596	Slitrk5	INSIDE	0.188	0.529	1531.50	810.51	0.100	1155.28	115.06
A_68_P32150751	chr19:47654747-47654794	NM_001164639:262	Slk	INSIDE	0.224	0.664	1068.65	709.14	0.149	886.09	131.91
A_68_P29482226	chr14:27354098-27354142	NM_032008:-894	Slmap	PROMOTER	0.062	0.662	5259.13	3482.21	0.041	3537.96	144.74
A_68_P29482227	chr14:27354230-27354274	NM_032008:-1026	Slmap	PROMOTER	0.199	0.534	3110.16	1661.18	0.106	2184.68	231.92
A_68_P31791992	chr18:67624679-67624723	NM_144867:198	Slmo1	INSIDE	0.437	0.430	1964.17	844.63	0.188	1581.87	297.14
A_68_P27750513	chr11:43247484-43247528	NM_148673:274	Slu7	INSIDE	0.474	0.604	1473.17	889.54	0.286	1210.37	346.10
A_68_P25501810	chr7:133839074-133839118	NM_029420:201	Slx1b	INSIDE	0.528	0.651	1914.41	1247.04	0.344	1490.69	512.50
A_68_P30507721	chr16:4003615-4003659	NM_177472:-1956	Slx4	PROMOTER	0.454	0.362	1657.73	600.28	0.164	1289.18	211.83
A_68_P25994483	chr8:81923506-81923561	NM_008539:-166	Smad1	PROMOTER	0.250	0.535	1100.58	588.80	0.134	899.64	120.24
A_68_P31841966	chr18:76400802-76400846	NM_010754:-754	Smad2	PROMOTER	0.385	0.482	2841.35	1370.16	0.186	2146.79	399.04
A_68_P31841969	chr18:76401128-76401172	NM_010754:-428	Smad2	PROMOTER	0.657	0.581	3962.36	2300.73	0.382	3061.97	1168.17
A_68_P26578199	chr9:63605284-63605328	NM_016769:495	Smad3	INSIDE	0.217	0.487	1865.39	909.37	0.106	1437.25	151.98
A_68_P26578203	chr9:63605811-63605855	NM_016769:-31	Smad3	PROMOTER	0.457	0.727	3276.63	2383.28	0.332	2138.08	710.59
A_68_P31827294	chr18:73863805-73863852	NM_008540:-433	Smad4	PROMOTER	0.080	0.742	2742.81	2035.67	0.059	2018.84	119.36
A_68_P26579680	chr9:63870057-63870101	NM_008542:-212	Smad6	PROMOTER	0.185	0.547	2258.91	1236.31	0.101	1610.69	162.89
A_68_P26579681	chr9:63870209-63870254	NM_008542:-365	Smad6	PROMOTER	0.138	0.631	2199.25	1387.07	0.087	1305.69	113.99
A_68_P31836526	chr18:75526561-75526605	NM_001042660:-436	Smad7	PROMOTER	0.521	0.462	2780.33	1284.39	0.241	2151.86	517.83
A_68_P31836532	chr18:75527265-75527309	NM_001042660:268	Smad7	INSIDE	0.257	0.355	2708.69	960.49	0.091	2229.45	203.56
A_68_P31836541	chr18:75528547-75528591	NM_001042660:1550	Smad7	INSIDE	0.227	0.433	1579.90	684.75	0.098	1186.66	116.88
A_68_P22149789	chr3:54589967-54590012	NM_019483:30486	Smad9	INSIDE	0.545	2.793	751.23	2098.47	1.523	693.79	1056.72
A_68_P22149839	chr3:54598091-54598144	NM_019483:38614	Smad9	INSIDE	0.588	3.121	567.72	1771.93	1.836	545.08	1000.84
A_68_P32036067	chr19:26678985-26679030	NM_011416:-642	Smarca2	PROMOTER	0.088	1.741	5326.76	9275.55	0.153	3434.24	525.22
A_68_P32036080	chr19:26680365-26680409	NM_011416:737	Smarca2	INSIDE	0.507	0.571	1200.32	685.53	0.290	959.91	278.16
A_68_P32036081	chr19:26680484-26680528	NM_011416:857	Smarca2	INSIDE	0.495	0.463	1318.06	610.05	0.229	1232.79	282.36
A_68_P32036084	chr19:26680907-26680951	NM_011416:1279	Smarca2	INSIDE	0.096	0.602	2879.04	1733.99	0.058	2012.12	116.74
A_68_P32036086	chr19:26681225-26681269	NM_011416:1597	Smarca2	INSIDE	0.147	0.496	2365.78	1174.36	0.073	1580.24	115.13
A_68_P32036090	chr19:26681592-26681637	NM_011416:1965	Smarca2	INSIDE	0.321	1.760	1990.52	3503.15	0.564	1480.23	835.27
A_68_P32036092	chr19:26681843-26681887	NM_011416:2215	Smarca2	INSIDE	0.382	0.738	2246.36	1657.71	0.282	1648.69	464.78
A_68_P32036095	chr19:26682223-26682267	NM_011416:2595	Smarca2	INSIDE	0.293	0.657	1770.36	1162.95	0.192	1363.01	261.99
A_68_P27259390	chr10:75384205-75384249	NM_001161853:133	Smarcb1	INSIDE	0.500	0.330	1647.38	542.89	0.165	1498.10	246.72
A_68_P26822087	chr9:110034313-110034357	NM_009211:-193	Smarcc1	PROMOTER	0.411	0.545	978.56	533.48	0.224	789.66	176.88
A_68_P30481816	chr15:99532450-99532494	NM_031842:-245	Smarcd1	PROMOTER	0.298	0.641	1203.61	771.11	0.191	906.16	172.95
A_68_P28100612	chr11:106134531-106134575	NM_001130187:-266	Smarcd2	DIVERGENT_PROMOTER	0.148	0.717	2817.48	2019.75	0.106	1950.45	206.37
A_68_P23542947	chr5:24104268-24104313	NM_025891:3530	Smarcd3	INSIDE	0.294	0.462	1776.32	820.22	0.136	1418.76	192.66
A_68_P23542950	chr5:24104639-24104683	NM_025891:3160	Smarcd3	INSIDE	0.371	0.697	3373.47	2352.53	0.259	2692.22	697.16
A_68_P23542951	chr5:24104726-24104770	NM_025891:3072	Smarcd3	INSIDE	0.200	0.603	2177.16	1312.59	0.121	1575.82	190.31
A_68_P32747584	chrX:148450937-148450989	NM_019710:-8	Smc1a	DIVERGENT_PROMOTER	0.116	1.495	1800.44	2691.20	0.174	692.72	120.34
A_68_P32747588	chrX:148451460-148451504	NM_019710:512	Smc1a	INSIDE	0.083	2.064	2069.27	4271.13	0.171	779.51	133.28
A_68_P30398810	chr15:84962157-84962201	NM_080470:209	Smc1b	INSIDE	0.313	1.612	890.62	1435.72	0.504	837.68	422.05
A_68_P22917597	chr4:52452103-52452147	NM_008017:4	Smc2	INSIDE	0.409	0.450	1247.91	561.74	0.184	1050.69	193.22
A_68_P22917598	chr4:52452191-52452235	NM_008017:92	Smc2	INSIDE	0.563	0.530	1960.76	1039.04	0.298	1727.26	514.92
A_68_P32016818	chr19:23347992-23348036	NM_153808:353	Smc5	INSIDE	0.177	0.502	1825.29	915.41	0.089	1399.17	124.51
A_68_P32016820	chr19:23348207-23348251	NM_153808:139	Smc5	INSIDE	0.191	0.612	1393.84	852.92	0.117	1030.85	120.76

ProbeName	Target position of probe on CpG island microarray	TargetID	GeneSymbol	CpG island Description	Ratio of relative methylation (TiO2-NP/Vehicle)	Sham group			TiO_2-H group		
						Relative methylation (Cy-5/Cy-3)	Cy-3 signal (Input)	Cy-5 signal (MeDIP)	Relative methylation (Cy-5/Cy-3)	Cy-3 signal (Input)	Cy-5 signal (MeDIP)
A_68_P30370942	chr15:80064643-80064687	NM_178719:155	Smcr7l	INSIDE	0.659	0.560	1635.09	915.58	0.369	1271.94	469.21
A_68_P30370943	chr15:80064789-80064834	NM_178719:302	Smcr7l	INSIDE	0.446	0.655	1700.27	1113.63	0.292	1248.21	364.91
A_68_P27844725	chr11:60591042-60591086	NM_175491:38	Smcr8	INSIDE	0.268	0.531	1262.52	670.68	0.143	1045.08	148.93
A_68_P27844726	chr11:60591168-60591212	NM_175491:164	Smcr8	INSIDE	0.589	0.522	1251.94	653.71	0.308	882.45	271.43
A_68_P27844727	chr11:60591282-60591326	NM_175491:278	Smcr8	INSIDE	0.243	0.570	1093.53	623.35	0.138	862.45	119.33
A_68_P27673275	chr11:29072187-29072231	NM_134034:-698	Smek2	PROMOTER	0.358	0.319	1615.34	515.58	0.114	1184.98	135.40
A_68_P27673276	chr11:29072349-29072395	NM_134034:-534	Smek2	PROMOTER	0.504	0.498	1991.74	991.42	0.251	1411.18	353.74
A_68_P27673282	chr11:29072968-29073015	NM_134034:85	Smek2	INSIDE	0.406	0.639	949.47	606.88	0.260	796.75	206.96
A_68_P27673287	chr11:29073384-29073428	NM_134034:500	Smek2	INSIDE	0.332	0.541	2795.39	1511.34	0.180	1975.10	354.87
A_68_P27673288	chr11:29073469-29073513	NM_134034:584	Smek2	INSIDE	0.224	0.563	2869.12	1616.49	0.126	2088.09	263.61
A_68_P20723677	chr1:154749026-154749070	NM_001005507:728	Smg7	INSIDE	0.193	1.370	1351.08	1850.67	0.265	992.60	263.05
A_68_P20723689	chr1:154750745-154750789	NM_001005507:-990	Smg7	PROMOTER	0.213	0.560	1196.19	669.59	0.119	1019.43	121.35
A_68_P32182252	chr19:53465097-53465141	NM_172429:-55	Smndc1	PROMOTER	0.139	0.640	1952.58	1249.18	0.089	1351.06	119.87
A_68_P32182254	chr19:53465262-53465306	NM_172429:-221	Smndc1	PROMOTER	0.132	1.442	4708.15	6791.25	0.190	2951.27	561.30
A_68_P32182255	chr19:53465351-53465395	NM_172429:-309	Smndc1	PROMOTER	0.316	1.720	2194.67	3775.73	0.543	1466.27	796.49
A_68_P24328151	chr6:29686313-29686357	NM_176996:838	Smo	INSIDE	0.521	0.490	1382.36	677.01	0.255	1119.40	285.82
A_68_P24328152	chr6:29686394-29686438	NM_176996:920	Smo	INSIDE	0.354	0.516	2644.16	1363.26	0.182	1900.79	346.72
A_68_P24328153	chr6:29686461-29686505	NM_176996:986	Smo	INSIDE	0.265	0.565	1568.63	886.99	0.150	1095.05	163.87
A_68_P28578875	chr12:82127694-82127738	NM_001146217:-78	Smoc1	PROMOTER	0.442	0.664	1497.68	994.72	0.294	1107.58	325.50
A_68_P28578880	chr12:82128213-82128257	NM_001146217:440	Smoc1	INSIDE	0.240	0.443	1418.74	628.52	0.106	1109.31	117.80
A_68_P31058944	chr17:14416346-14416390	NM_022315:-144	Smoc2	PROMOTER	0.496	0.549	1622.83	891.43	0.272	1123.03	305.84
A_68_P21635216	chr2:131318279-131318323	NM_001177833:703	Smox	INSIDE	0.610	0.636	1055.27	671.38	0.388	808.80	313.76
A_68_P27162285	chr10:57514583-57514627	NM_020561:255	Smpdl3a	INSIDE	0.520	0.534	1247.20	665.50	0.277	986.73	273.53
A_68_P27162286	chr10:57514696-57514740	NM_020561:369	Smpdl3a	INSIDE	0.282	0.475	1179.15	559.67	0.134	929.07	124.20
A_68_P32766763	chrX:153929218-153929262	NM_009214:738	Sms	INSIDE	0.302	1.425	1456.92	2076.00	0.431	606.78	261.41
A_68_P27911311	chr11:72224533-72224583	NM_177776:657	Smtnl2	INSIDE	0.067	1.615	2028.51	3276.09	0.108	1455.58	157.84
A_68_P27911313	chr11:72224759-72224803	NM_177776:435	Smtnl2	INSIDE	0.393	0.562	1527.09	858.89	0.221	1231.22	271.86
A_68_P27911317	chr11:72225169-72225213	NM_177776:25	Smtnl2	INSIDE	0.441	0.403	1735.43	698.68	0.177	1513.33	268.61
A_68_P22858652	chr4:40704660-40704704	NM_021535:236	Smu1	INSIDE	0.570	0.652	2674.12	1742.33	0.372	2167.75	805.33
A_68_P22858653	chr4:40704800-40704844	NM_021535:96	Smu1	INSIDE	0.257	0.522	2101.67	1097.71	0.134	1600.01	215.05
A_68_P30502555	chr15:102999581-102999625	NM_027885:-5887	Smug1	PROMOTER	0.385	0.569	3410.54	1941.02	0.219	2647.80	580.82
A_68_P20870655	chr1:181447900-181447944	NM_027188:212	Smyd3	INSIDE	0.307	0.486	1813.14	881.71	0.149	1454.39	216.91
A_68_P20870657	chr1:181448104-181448148	NM_027188:8	Smyd3	INSIDE	0.254	0.573	1056.77	605.15	0.146	806.52	117.39
A_68_P24604963	chr6:85382060-85382104	NM_144918:113	Smyd5	INSIDE	0.632	0.612	1158.73	708.80	0.387	828.46	320.23
A_68_P21836027	chr2:167363971-167364015	NM_011427:266	Snai1	INSIDE	0.150	0.528	2122.27	1120.19	0.079	1607.15	127.17
A_68_P21576016	chr2:120393619-120393664	NM_001177792:235	Snap23	INSIDE	0.358	0.463	1332.55	617.44	0.166	1159.23	192.38
A_68_P28540842	chr12:75065446-75065490	NM_178392:-48	Snapc1	PROMOTER	0.146	0.520	2184.93	1136.69	0.076	1599.66	121.17
A_68_P28540843	chr12:75065592-75065636	NM_178392:98	Snapc1	INSIDE	0.402	0.389	1905.69	741.84	0.157	1618.55	253.52
A_68_P28540844	chr12:75065711-75065755	NM_178392:216	Snapc1	INSIDE	0.650	0.627	1423.89	893.06	0.408	1175.17	479.01
A_68_P23062262	chr4:83063627-83063671	NM_029949:1	Snapc3	INSIDE	0.632	0.477	1983.02	945.11	0.301	1568.90	472.77
A_68_P21078286	chr2:26236121-26236165	NM_172339:31	Snapc4	INSIDE	0.194	0.520	1546.42	803.53	0.101	1186.67	119.36
A_68_P26580558	chr9:64027298-64027342	NM_183316:217	Snapc5	INSIDE	0.534	0.572	2819.00	1612.87	0.305	2279.26	696.29
A_68_P31709379	chr18:52927884-52927928	NM_001199154:109	Sncaip	INSIDE	0.587	0.598	3074.14	1837.96	0.351	2558.60	897.60
A_68_P28042071	chr11:95896081-95896125	NM_033568:-128	Snf8	PROMOTER	0.238	0.480	1180.69	567.05	0.114	1023.13	117.03

ProbeName	Target position of probe on CpG island microarray	TargetID	GeneSymbol	CpG island Description	Ratio of relative methylation (TiO2-NP/Vehicle)	Sham group			TiO_2-H group		
						Relative methylation (Cy-5/Cy-3)	Cy-3 signal (Input)	Cy-5 signal (MeDIP)	Relative methylation (Cy-5/Cy-3)	Cy-3 signal (Input)	Cy-5 signal (MeDIP)
A_68_P22488497	chr3:123211440-123211484	NR_028574:-208	Snhg8	PROMOTER	0.121	0.544	2563.94	1393.82	0.066	1843.59	121.55
A_68_P23271975	chr4:124744170-124744214	NM_175246:256	Snip1	INSIDE	0.318	0.459	1387.67	636.97	0.146	1112.09	162.20
A_68_P23271976	chr4:124744252-124744296	NM_175246:338	Snip1	INSIDE	0.095	0.656	2594.54	1702.23	0.062	1892.47	117.77
A_68_P30546003	chr16:11066050-11066094	NM_009223:-318	Snn	PROMOTER	0.367	0.487	2775.35	1351.27	0.179	2112.81	377.92
A_68_P24090829	chr5:130293530-130293574	NR_003681:-6884	Snora15	PROMOTER	0.502	0.646	1032.41	666.54	0.324	844.36	273.80
A_68_P28744648	chr12:112776290-112776334	NR_033168:-2844	Snora28	PROMOTER	0.159	0.535	4270.42	2286.59	0.085	3019.34	257.39
A_68_P28744656	chr12:112777247-112777291	NR_033168:-1888	Snora28	PROMOTER	0.285	0.489	1390.09	679.70	0.139	1202.16	167.57
A_68_P30475041	chr15:98364232-98364276	NR_034052:-7364	Snora2b	PROMOTER	0.465	0.582	4763.06	2773.17	0.271	3439.02	931.88
A_68_P20285517	chr1:63224065-63224115	NR_028558:-1506	Snora41	PROMOTER	0.211	0.661	1079.27	713.94	0.140	870.30	121.74
A_68_P27569489	chr11:6525837-6525881	NR_034042:-5436	Snora5c	PROMOTER	0.262	0.498	3956.12	1972.10	0.131	2837.34	371.25
A_68_P27569490	chr11:6525943-6525987	NR_034042:-5542	Snora5c	PROMOTER	0.254	0.385	2827.29	1088.72	0.098	2041.96	200.00
A_68_P24773074	chr6:115758230-115758281	NR_028546:-134	Snora7a	PROMOTER	0.167	0.586	1450.14	849.60	0.098	1209.77	118.50
A_68_P24773075	chr6:115758321-115758365	NR_028546:-221	Snora7a	PROMOTER	0.261	0.422	1285.48	542.41	0.110	1070.96	117.83
A_68_P28101955	chr11:106362323-106362370	NR_030703:42	Snord104	INSIDE	0.174	0.626	3342.72	2092.04	0.109	2440.65	265.07
A_68_P21628855	chr2:130100231-130100275	NR_028547:-998	Snord110	PROMOTER	0.066	0.675	3651.49	2464.71	0.044	2749.58	121.58
A_68_P25362154	chr7:106631883-106631927	NR_002172:-462	Snord15a	PROMOTER	0.190	0.480	2397.94	1152.03	0.091	1735.84	158.48
A_68_P25362155	chr7:106631960-106632004	NR_002172:-540	Snord15a	PROMOTER	0.636	0.445	1544.64	686.94	0.283	1260.32	356.51
A_68_P25091293	chr7:52383927-52383971	NR_000002:-1115	Snord32a	PROMOTER	0.216	0.496	2853.82	1416.27	0.107	2008.95	215.02
A_68_P31834370	chr18:75160579-75160623	NR_028552:-121	Snord58b	PROMOTER	0.283	0.716	2081.82	1491.03	0.203	1612.49	326.96
A_68_P32401092	chrX:54645722-54645768	NR_002903:-1058	Snord61	PROMOTER	0.236	1.548	1608.93	2491.29	0.365	688.02	251.46
A_68_P32401095	chrX:54646102-54646148	NR_002903:-1438	Snord61	PROMOTER	0.136	1.529	1540.62	2355.91	0.209	624.49	130.23
A_68_P26237919	chr8:125626230-125626274	NR_028128:-705	Snord68	PROMOTER	0.250	0.303	2213.47	670.78	0.076	1631.05	123.52
A_68_P29982096	chr15:5067068-5067116	NR_028091:-1328	Snord72	PROMOTER	0.246	0.580	980.55	568.62	0.143	815.50	116.45
A_68_P20411726	chr1:88255561-88255605	NR_002851:-2680	Snord82	PROMOTER	0.552	0.724	2936.10	2125.00	0.400	2155.77	861.95
A_68_P20024912	chr1:9934064-9934108	NR_004410:-1462	Snord87	PROMOTER	0.267	0.519	3610.77	1875.67	0.139	2681.81	371.53
A_68_P27779980	chr11:48613984-48614028	NR_028563:-1528	Snord96a	PROMOTER	0.286	0.484	1131.53	548.14	0.139	850.77	118.00
A_68_P27779981	chr11:48614053-48614098	NR_028563:-1459	Snord96a	PROMOTER	0.251	0.560	1688.88	946.06	0.141	1321.04	185.84
A_68_P27779983	chr11:48614301-48614357	NR_028563:-1206	Snord96a	PROMOTER	0.179	0.631	1358.53	857.39	0.113	1073.11	121.33
A_68_P21745018	chr2:151457749-151457793	NM_198214:499	Snph	INSIDE	0.379	0.513	1905.37	977.81	0.195	1525.99	296.87
A_68_P27687370	chr11:32105281-32105325	NM_030093:-112	Snrnp25	DIVERGENT_PROMOTER	0.128	0.712	5116.91	3643.70	0.091	3504.23	318.35
A_68_P27687371	chr11:32105349-32105400	NM_030093:-40	Snrnp25	DIVERGENT_PROMOTER	0.227	0.697	1684.48	1174.71	0.158	1181.82	186.84
A_68_P25092883	chr7:52650953-52650997	NM_009224:43	Snrnp70	INSIDE	0.661	0.674	1737.70	1170.49	0.445	1471.32	655.14
A_68_P25092884	chr7:52651057-52651101	NM_009224:-61	Snrnp70	PROMOTER	0.246	0.682	1805.87	1230.80	0.168	1483.35	248.75
A_68_P25016043	chr7:27980751-27980801	NM_001046637:3	Snrpa	INSIDE	0.227	0.682	976.34	666.12	0.155	786.18	121.56
A_68_P25016046	chr7:27981095-27981139	NM_001046637:-337	Snrpa	PROMOTER	0.228	0.556	2592.11	1441.01	0.127	1811.49	230.09
A_68_P25186101	chr7:73205809-73205860	NM_021336:613	Snrpa1	INSIDE	0.321	0.520	1014.95	527.50	0.167	724.25	120.79
A_68_P21628394	chr2:130004810-130004854	NM_009225:268	Snrpb	INSIDE	0.242	0.439	1231.64	540.40	0.106	1112.82	118.16
A_68_P31118766	chr17:27976996-27977040	NM_011432:-13	Snrpc	PROMOTER	0.444	0.525	2298.67	1206.29	0.233	1889.17	439.82
A_68_P24991727	chr7:19735291-19735345	NM_026943:132	Snrpd2	INSIDE	0.112	0.687	1994.22	1370.38	0.077	1564.65	120.20
A_68_P27256923	chr10:74980993-74981047	NM_026095:234	Snrpd3	INSIDE	0.507	0.481	1409.53	678.13	0.244	1050.05	256.07
A_68_P20628459	chr1:135506499-135506543	NM_009227:337	Snrpe	INSIDE	0.273	0.660	4416.18	2913.10	0.180	3339.08	600.44
A_68_P24609765	chr6:86321486-86321530	NM_026506:-25	Snrpg	PROMOTER	0.541	0.561	980.67	550.01	0.304	827.07	251.16
A_68_P21760344	chr2:154233694-154233738	NM_009228:104	Snta1	INSIDE	0.532	0.555	1883.34	1044.62	0.295	1697.06	500.75
A_68_P30234984	chr15:55738403-55738447	NM_016667:80	Sntb1	INSIDE	0.251	0.584	1983.17	1158.14	0.147	1406.89	206.35

ProbeName	Target position of probe on CpG island microarray	TargetID	GeneSymbol	CpG island Description	Ratio of relative methylation (TiO2-NP/Vehicle)	Sham group			TiO_2-H group		
						Relative methylation (Cy-5/Cy-3)	Cy-3 signal (Input)	Cy-5 signal (MeDIP)	Relative methylation (Cy-5/Cy-3)	Cy-3 signal (Input)	Cy-5 signal (MeDIP)
A_68_P26142250	chr8:109460044-109460088	NM_009229:417	Sntb2	INSIDE	0.452	0.514	1474.45	758.31	0.233	1244.93	289.51
A_68_P20022048	chr1:9289699-9289746	NM_027671:89	Sntg1	INSIDE	0.269	0.571	937.05	534.97	0.154	776.36	119.29
A_68_P20022049	chr1:9289822-9289866	NM_027671:-33	Sntg1	PROMOTER	0.370	0.527	1337.96	704.86	0.195	1107.80	216.19
A_68_P24445012	chr6:51542432-51542476	NM_001127348:47933	Snx10	DOWNSTREAM	0.317	0.610	1280.18	781.43	0.193	957.39	185.08
A_68_P32562915	chrX:98417474-98417518	NM_001110311:406	Snx12	INSIDE	0.252	2.320	2372.61	5505.41	0.585	1069.45	626.07
A_68_P28351013	chr12:35731615-35731659	NM_001014973:-224	Snx13	PROMOTER	0.546	0.624	967.64	604.04	0.341	954.36	325.42
A_68_P26710070	chr9:88334164-88334211	NM_172926:-398	Snx14	PROMOTER	0.141	0.522	2405.68	1255.27	0.074	1962.30	144.84
A_68_P29341537	chr13:114407731-114407779	NM_130796:1018	Snx18	INSIDE	0.289	0.539	1023.50	551.54	0.156	768.99	119.62
A_68_P26394906	chr9:30234770-30234814	NM_028874:-121	Snx19	PROMOTER	0.529	0.634	2493.71	1580.00	0.335	2136.38	716.08
A_68_P26591018	chr9:65917336-65917381	NM_001025612:180	Snx22	INSIDE	0.194	0.615	1246.66	766.68	0.119	971.79	115.90
A_68_P31712046	chr18:53405025-53405069	NM_029394:-269	Snx24	PROMOTER	0.169	0.490	1942.13	951.15	0.083	1472.37	122.03
A_68_P22339767	chr3:94386067-94386111	NM_029721:536	Snx27	INSIDE	0.439	0.611	6243.91	3812.84	0.268	4648.40	1244.74
A_68_P22339768	chr3:94386146-94386190	NM_029721:456	Snx27	INSIDE	0.396	0.513	10731.28	5502.96	0.203	7576.74	1538.58
A_68_P22339769	chr3:94386307-94386351	NM_029721:296	Snx27	INSIDE	0.321	0.349	4540.45	1585.08	0.112	3089.74	346.00
A_68_P22958420	chr4:59819277-59819321	NM_172468:777	Snx30	INSIDE	0.453	0.594	1284.35	762.54	0.269	988.56	265.67
A_68_P22958421	chr4:59819390-59819434	NM_172468:891	Snx30	INSIDE	0.293	0.701	1625.10	1138.38	0.205	1357.73	278.35
A_68_P31929158	chr19:5510644-5510688	NM_001024560:-177	Snx32	PROMOTER	0.125	0.551	2326.60	1281.65	0.069	1788.48	123.46
A_68_P31929160	chr19:5510818-5510862	NM_001024560:-351	Snx32	PROMOTER	0.329	1.631	6529.73	10647.34	0.537	4546.26	2442.33
A_68_P30656968	chr16:33251336-33251380	NM_080557:-183	Snx4	PROMOTER	0.523	0.611	1409.78	860.70	0.320	1120.48	358.00
A_68_P30656972	chr16:33251779-33251823	NM_080557:259	Snx4	INSIDE	0.599	0.405	1770.76	717.61	0.243	1379.56	334.67
A_68_P31019372	chr17:5840861-5840905	NM_025664:-497	Snx9	PROMOTER	0.549	0.626	1071.86	671.50	0.344	818.74	281.71
A_68_P27098028	chr10:42740979-42741023	NM_175407:153336	Sobp	INSIDE	0.307	0.630	2639.78	1664.18	0.194	2234.42	432.43
A_68_P27098034	chr10:42741868-42741912	NM_175407:152446	Sobp	INSIDE	0.280	0.645	1619.54	1043.79	0.181	1356.07	244.96
A_68_P30544452	chr16:10784908-10784952	NM_009896:699	Socs1	INSIDE	0.246	0.522	4884.69	2550.00	0.129	3844.45	494.19
A_68_P30544457	chr16:10785415-10785459	NM_009896:193	Socs1	INSIDE	0.484	0.417	2700.03	1126.63	0.202	2124.78	428.99
A_68_P30544461	chr16:10785857-10785901	NM_009896:-249	Socs1	PROMOTER	0.368	0.595	1490.91	887.75	0.219	1122.29	245.84
A_68_P27365025	chr10:94878000-94878046	NM_001168657:-105	Socs2	PROMOTER	0.071	1.614	1570.75	2535.47	0.115	1049.86	120.93
A_68_P27365026	chr10:94878069-94878116	NM_001168657:-175	Socs2	PROMOTER	0.155	1.339	2319.06	3105.68	0.207	1617.09	335.02
A_68_P27365032	chr10:94878721-94878765	NM_001168656:-624	Socs2	PROMOTER	0.238	0.542	1075.34	582.41	0.129	951.34	122.87
A_68_P28168277	chr11:117829266-117829310	NM_007707:1392	Socs3	INSIDE	0.306	0.439	1397.72	613.99	0.134	1080.52	145.26
A_68_P28168283	chr11:117829826-117829870	NM_007707:832	Socs3	INSIDE	0.324	0.656	1019.69	668.44	0.212	822.72	174.58
A_68_P28168284	chr11:117829892-117829936	NM_007707:766	Socs3	INSIDE	0.115	0.673	1956.73	1317.12	0.077	1544.55	119.17
A_68_P28168285	chr11:117830022-117830066	NM_007707:636	Socs3	INSIDE	0.274	0.580	1701.31	987.33	0.159	1192.17	189.29
A_68_P28168286	chr11:117830189-117830233	NM_007707:470	Socs3	INSIDE	0.473	0.485	1355.41	657.76	0.230	1091.57	250.74
A_68_P28168289	chr11:117830449-117830493	NM_007707:210	Socs3	INSIDE	0.410	0.498	1705.64	849.37	0.204	1381.55	281.93
A_68_P29581356	chr14:47896866-47896910	NM_080843:71	Socs4	INSIDE	0.334	0.538	2225.72	1196.94	0.180	1783.39	320.60
A_68_P29581357	chr14:47896981-47897025	NM_080843:185	Socs4	INSIDE	0.451	0.405	1761.55	714.21	0.183	1369.28	250.22
A_68_P31421207	chr17:87507571-87507615	NM_019654:574	Socs5	INSIDE	0.220	0.603	1102.86	664.52	0.133	865.96	114.97
A_68_P31909280	chr18:89063162-89063206	NM_018821:415	Socs6	INSIDE	0.382	0.706	2132.44	1504.63	0.269	1772.95	477.74
A_68_P28050111	chr11:97223439-97223483	NM_138657:-404	Socs7	PROMOTER	0.251	0.486	1061.15	515.54	0.122	1010.41	123.40
A_68_P28050118	chr11:97224447-97224491	NM_138657:604	Socs7	INSIDE	0.291	0.494	1273.59	629.35	0.144	1088.65	156.52
A_68_P30955583	chr16:90220671-90220715	NM_011434:-294	Sod1	PROMOTER	0.170	0.566	1703.51	963.76	0.096	1208.35	116.14
A_68_P31054933	chr17:13200672-13200716	NM_013671:-10	Sod2	PROMOTER	0.427	0.497	1484.39	737.22	0.212	1248.75	264.61
A_68_P30964217	chr16:91660217-91660261	NM_019973:12170	Son	INSIDE	0.631	2.702	598.42	1617.13	1.705	534.39	910.91

ProbeName	Target position of probe on CpG island microarray	TargetID	GeneSymbol	CpG island Description	Ratio of relative methylation (TiO2-NP/Vehicle)	Sham group			TiO_2-H group		
						Relative methylation (Cy-5/Cy-3)	Cy-3 signal (Input)	Cy-5 signal (MeDIP)	Relative methylation (Cy-5/Cy-3)	Cy-3 signal (Input)	Cy-5 signal (MeDIP)
A_68_P32109309	chr19:40588021-40588065	NM_178362:184	Sorbs1	INSIDE	0.573	0.468	1716.44	804.03	0.269	1455.53	390.82
A_68_P29699882	chr14:70591597-70591641	NM_011366:15826	Sorbs3	INSIDE	0.142	0.740	4672.18	3458.45	0.105	3122.61	329.25
A_68_P32168786	chr19:50752601-50752645	NM_021377:480	Sorcs1	INSIDE	0.554	0.620	1509.19	935.50	0.343	1213.94	416.53
A_68_P23607365	chr5:36740222-36740266	NM_030889:544	Sorcs2	INSIDE	0.433	0.596	1044.48	622.61	0.258	879.21	227.18
A_68_P21585133	chr2:122060313-122060357	NM_146126:-240	Sord	PROMOTER	0.541	0.546	1617.85	884.11	0.296	1450.86	429.30
A_68_P26453528	chr9:41931891-41931935	NM_011436:460	Sorl1	INSIDE	0.545	0.463	1655.88	766.03	0.252	1376.90	347.07
A_68_P31383391	chr17:80878801-80878845	NM_009231:971	Sos1	INSIDE	0.182	0.509	1623.90	826.37	0.093	1268.82	117.54
A_68_P31383392	chr17:80878882-80878927	NM_009231:889	Sos1	INSIDE	0.130	0.617	1928.79	1189.92	0.080	1500.61	120.37
A_68_P31383394	chr17:80879061-80879105	NM_009231:711	Sos1	INSIDE	0.334	0.444	2345.35	1041.49	0.149	1857.25	275.85
A_68_P25661253	chr8:12396004-12396049	NM_009233:508	Sox1	INSIDE	0.554	0.488	1936.47	945.71	0.271	1446.16	391.54
A_68_P25661254	chr8:12396094-12396138	NM_009233:598	Sox1	INSIDE	0.374	0.523	1735.04	908.20	0.196	1348.89	263.86
A_68_P25661257	chr8:12396498-12396542	NM_009233:1002	Sox1	INSIDE	0.467	0.699	2548.57	1781.43	0.326	1820.57	594.08
A_68_P28307234	chr12:28023843-28023887	NM_009234:3719	Sox11	INSIDE	0.650	0.673	1241.04	834.66	0.437	1084.51	474.32
A_68_P28307238	chr12:28024309-28024353	NM_009234:3253	Sox11	INSIDE	0.643	0.665	1290.02	857.63	0.427	1168.43	499.42
A_68_P28307239	chr12:28024428-28024472	NM_009234:3133	Sox11	INSIDE	0.283	0.476	3106.48	1479.64	0.135	2467.91	332.14
A_68_P28307260	chr12:28026977-28027021	NM_009234:585	Sox11	INSIDE	0.236	0.492	2068.26	1018.46	0.116	1588.87	184.55
A_68_P28307269	chr12:28028213-28028257	NM_009234:-651	Sox11	PROMOTER	0.201	0.597	2510.47	1499.70	0.120	1675.13	200.76
A_68_P21749116	chr2:152222489-152222533	NM_011438:1272	Sox12	INSIDE	0.248	0.715	1820.46	1302.14	0.178	1253.43	222.55
A_68_P21749117	chr2:152222565-152222609	NM_011438:1196	Sox12	INSIDE	0.324	0.582	2264.60	1317.36	0.188	1759.69	331.63
A_68_P26766610	chr9:99771206-99771250	NM_011440:5361	Sox14	DOWNSTREAM	0.276	1.584	2458.98	3895.31	0.438	1555.38	681.07
A_68_P26766650	chr9:99775559-99775603	NM_011440:1009	Sox14	INSIDE	0.498	0.698	1933.57	1350.29	0.348	1542.75	536.85
A_68_P26766651	chr9:99775638-99775682	NM_011440:929	Sox14	INSIDE	0.242	0.592	1159.00	686.08	0.143	996.97	142.58
A_68_P26766654	chr9:99775916-99775960	NM_011440:651	Sox14	INSIDE	0.355	0.531	1665.50	883.77	0.188	1293.35	243.31
A_68_P26766655	chr9:99776052-99776096	NM_011440:515	Sox14	INSIDE	0.245	0.660	996.38	657.82	0.162	762.66	123.39
A_68_P20005798	chr1:4482441-4482485	NM_011441:4032	Sox17	INSIDE	0.615	0.428	1368.50	585.40	0.263	1140.95	300.06
A_68_P20005837	chr1:4486985-4487029	NM_011441:-512	Sox17	PROMOTER	0.342	0.587	1511.13	887.02	0.201	1226.86	246.02
A_68_P20005839	chr1:4487234-4487278	NM_011441:-762	Sox17	PROMOTER	0.292	0.467	1142.06	533.59	0.137	911.72	124.50
A_68_P20005842	chr1:4487520-4487564	NM_011441:-1048	Sox17	PROMOTER	0.264	0.628	2618.18	1643.63	0.166	1927.02	319.87
A_68_P21911961	chr2:181405887-181405931	NM_009236:437	Sox18	INSIDE	0.206	0.419	1844.05	773.02	0.086	1340.17	115.64
A_68_P22049384	chr3:34548485-34548529	NM_011443:-420	Sox2	PROMOTER	0.180	0.689	2288.19	1577.17	0.124	1629.69	202.39
A_68_P22049389	chr3:34549058-34549109	NM_011443:157	Sox2	INSIDE	0.250	0.374	1498.19	560.90	0.094	1236.66	115.88
A_68_P22049390	chr3:34549137-34549181	NM_011443:232	Sox2	INSIDE	0.532	0.527	2314.83	1219.76	0.280	1864.55	522.61
A_68_P22049393	chr3:34549575-34549619	NM_011443:670	Sox2	INSIDE	0.359	0.676	1895.61	1282.33	0.243	1431.82	348.20
A_68_P29937400	chr14:118625343-118625387	NM_177753:10888	Sox21	DOWNSTREAM	0.572	0.509	1005.32	511.31	0.291	804.61	233.91
A_68_P29937467	chr14:118633748-118633793	NM_177753:2482	Sox21	INSIDE	0.118	0.747	3815.18	2850.82	0.089	2628.25	232.63
A_68_P29937474	chr14:118634627-118634671	NM_177753:1604	Sox21	INSIDE	0.268	0.593	905.42	536.49	0.159	755.00	119.69
A_68_P29937481	chr14:118635351-118635395	NM_177753:880	Sox21	INSIDE	0.107	1.819	962.28	1750.05	0.194	699.66	135.63
A_68_P29937483	chr14:118635646-118635690	NM_177753:584	Sox21	INSIDE	0.471	0.649	2099.79	1362.56	0.305	1634.50	499.23
A_68_P22049509	chr3:34562671-34562720	NR_015580:103393	Sox2ot	INSIDE	0.137	0.636	1901.02	1208.17	0.087	1400.57	122.27
A_68_P22049514	chr3:34563117-34563161	NR_015580:103836	Sox2ot	INSIDE	0.403	0.484	2066.20	999.89	0.195	1574.89	307.29
A_68_P22049518	chr3:34563441-34563498	NR_015580:104167	Sox2ot	INSIDE	0.270	0.425	1444.16	613.38	0.115	1024.52	117.35
A_68_P32415806	chrX:58146341-58146385	NM_009237:243	Sox3	INSIDE	0.254	2.374	1679.56	3986.93	0.602	802.85	483.50
A_68_P32415812	chrX:58146994-58147038	NM_009237:-411	Sox3	PROMOTER	0.060	1.992	2303.86	4589.02	0.119	996.00	118.31
A_68_P32415814	chrX:58147306-58147350	NM_009237:-723	Sox3	PROMOTER	0.309	1.551	3090.46	4792.71	0.479	1344.24	644.52

ProbeName	Target position of probe on CpG island microarray	TargetID	GeneSymbol	CpG island Description	Ratio of relative methylation (TiO2-NP/Vehicle)	Sham group			TiO_2-H group		
						Relative methylation (Cy-5/Cy-3)	Cy-3 signal (Input)	Cy-5 signal (MeDIP)	Relative methylation (Cy-5/Cy-3)	Cy-3 signal (Input)	Cy-5 signal (MeDIP)
A_68_P28909015	chr13:29050121-29050165	NM_009238:-4591	Sox4	PROMOTER	0.379	0.568	1790.08	1017.56	0.216	1321.89	285.13
A_68_P28909029	chr13:29051502-29051546	NM_009238:-5973	Sox4	PROMOTER	0.370	0.592	1691.19	1001.67	0.219	1214.69	266.31
A_68_P29664621	chr14:64562219-64562263	NM_011446:-302	Sox7	PROMOTER	0.287	0.675	1774.40	1198.17	0.194	1488.83	288.88
A_68_P31104790	chr17:25707537-25707581	NM_011447:73	Sox8	INSIDE	0.474	0.513	1839.25	943.00	0.243	1423.75	346.34
A_68_P31104791	chr17:25707616-25707662	NM_011447:-7	Sox8	DIVERGENT_PROMOTER	0.293	0.529	1282.74	678.92	0.155	1010.72	156.57
A_68_P28137823	chr11:112643047-112643091	NM_011448:-455	Sox9	PROMOTER	0.503	0.674	943.84	635.97	0.339	792.57	268.85
A_68_P28137824	chr11:112643225-112643274	NM_011448:-274	Sox9	PROMOTER	0.238	0.598	1143.53	683.66	0.142	851.53	121.00
A_68_P28137827	chr11:112643586-112643636	NM_011448:87	Sox9	INSIDE	0.284	0.588	894.94	526.10	0.167	709.87	118.32
A_68_P28137834	chr11:112644282-112644326	NM_011448:781	Sox9	INSIDE	0.578	0.511	1460.42	745.79	0.295	1178.99	347.99
A_68_P28137844	chr11:112645388-112645432	NM_011448:1887	Sox9	INSIDE	0.338	0.507	1581.52	801.59	0.171	1241.06	212.67
A_68_P28137851	chr11:112646227-112646271	NM_011448:2725	Sox9	INSIDE	0.652	0.476	3114.77	1483.97	0.310	2500.51	776.27
A_68_P28137853	chr11:112646459-112646503	NM_011448:2957	Sox9	INSIDE	0.475	0.635	5799.83	3681.65	0.302	4110.70	1239.86
A_68_P28137854	chr11:112646538-112646582	NM_011448:3037	Sox9	INSIDE	0.310	1.454	16942.70	24640.93	0.451	11286.38	5085.82
A_68_P28137855	chr11:112646610-112646655	NM_011448:3109	Sox9	INSIDE	0.313	1.546	7635.10	11803.18	0.484	5030.24	2436.94
A_68_P30497693	chr15:102235932-102235976	NM_013672:-792	Sp1	PROMOTER	0.483	0.563	1706.22	960.11	0.272	1277.79	347.43
A_68_P28047901	chr11:96839052-96839100	NM_001080964:-74	Sp2	PROMOTER	0.096	2.050	940.01	1927.24	0.198	612.72	121.04
A_68_P21328878	chr2:72817077-72817121	NM_001098425:240	Sp3	INSIDE	0.294	0.405	3871.91	1567.02	0.119	2908.63	345.72
A_68_P21328890	chr2:72819073-72819117	NM_001018042:-591	Sp3	PROMOTER	0.380	0.477	1207.64	576.04	0.181	1084.35	196.48
A_68_P28778756	chr12:119539812-119539856	NM_001166385:79	Sp4	INSIDE	0.522	0.452	1511.26	683.41	0.236	1125.00	265.51
A_68_P28778765	chr12:119540713-119540757	NM_001166385:-821	Sp4	PROMOTER	0.315	0.479	3913.42	1875.75	0.151	2958.73	446.12
A_68_P28048202	chr11:96883898-96883943	NM_031183:9038	Sp6	INSIDE	0.067	2.296	2943.93	6759.36	0.154	1974.68	304.04
A_68_P30497455	chr15:102189021-102189065	NM_130458:7660	Sp7	INSIDE	0.633	0.631	899.42	567.58	0.399	732.98	292.80
A_68_P28781843	chr12:120081478-120081528	NM_177082:-3299	Sp8	PROMOTER	0.207	0.633	1141.75	722.26	0.131	945.26	123.88
A_68_P28781844	chr12:120081565-120081609	NM_177082:-3215	Sp8	PROMOTER	0.372	0.462	2662.37	1228.89	0.172	2012.03	345.67
A_68_P21330516	chr2:73110924-73110977	NM_001005343:968	Sp9	INSIDE	0.236	0.599	1160.10	694.45	0.142	946.97	134.01
A_68_P21330519	chr2:73111290-73111334	NM_001005343:1330	Sp9	INSIDE	0.525	0.451	1831.50	826.07	0.237	1456.82	345.17
A_68_P21330529	chr2:73112421-73112465	NM_001005343:2460	Sp9	INSIDE	0.491	0.660	3662.17	2418.17	0.324	2776.38	899.26
A_68_P27942517	chr11:78114890-78114934	NM_017407:-180	Spag5	PROMOTER	0.240	0.552	2688.70	1483.43	0.132	2027.92	268.46
A_68_P30570619	chr16:16829103-16829147	NM_015773:332	Spag6	INSIDE	0.155	0.527	4089.76	2155.27	0.082	3015.83	245.92
A_68_P31351018	chr17:74737917-74737961	NM_001162870:-388	Spast	PROMOTER	0.343	0.531	1832.83	973.25	0.182	1583.59	288.44
A_68_P29645527	chr14:61253344-61253393	NM_001033272:-197	Spata13	PROMOTER	0.219	1.637	789.50	1292.71	0.358	597.57	214.18
A_68_P21835810	chr2:167318513-167318557	NM_170756:-160	Spata2	PROMOTER	0.206	0.443	2442.29	1082.11	0.091	1742.88	158.77
A_68_P28033247	chr11:94347425-94347469	NM_144827:-822	Spata20	PROMOTER	0.467	0.635	896.09	569.16	0.297	743.17	220.44
A_68_P20409777	chr1:87914871-87914915	NM_027029:-3624	Spata3	PROMOTER	0.314	0.683	2260.61	1543.56	0.215	1734.78	372.44
A_68_P23212277	chr4:111392547-111392591	NM_026470:-46	Spata6	PROMOTER	0.579	0.722	2450.04	1768.02	0.418	1935.81	808.79
A_68_P23212280	chr4:111392824-111392869	NM_026470:232	Spata6	INSIDE	0.490	0.438	1747.77	766.17	0.215	1483.13	318.46
A_68_P28671225	chr12:99866511-99866555	NM_178914:139	Spata7	INSIDE	0.477	0.714	2053.80	1466.08	0.341	1525.59	519.84
A_68_P30478329	chr15:98956569-98956613	NM_139140:-685	Spats2	PROMOTER	0.259	0.401	2401.18	963.44	0.104	1683.67	175.17
A_68_P20254931	chr1:57831531-57831575	NM_144882:-152	Spats2l	PROMOTER	0.232	0.580	2911.19	1689.19	0.135	2333.82	314.72
A_68_P21307667	chr2:69043827-69043871	NM_001199123:399	Spc25	INSIDE	0.336	0.484	1184.98	573.10	0.163	914.60	148.81
A_68_P27851931	chr11:61890435-61890479	NM_001029936:-142	Specc1	PROMOTER	0.500	3.261	4090.40	13339.98	1.631	2596.31	4235.81
A_68_P27255276	chr10:74674681-74674725	NM_153406:-115	Specc1l	PROMOTER	0.280	0.566	2185.57	1237.33	0.158	1688.67	267.56
A_68_P20353582	chr1:75371730-75371774	NM_007463:-119	Speg	DIVERGENT_PROMOTER	0.296	0.693	2142.52	1484.62	0.205	1623.95	333.44
A_68_P20353638	chr1:75379009-75379053	NM_001085370:-3154	Speg	PROMOTER	0.173	0.692	7252.21	5019.83	0.120	4874.62	583.64

ProbeName	Target position of probe on CpG island microarray	TargetID	GeneSymbol	CpG island Description	Ratio of relative methylation (TiO2-NP/Vehicle)	Sham group			TiO_2-H group		
						Relative methylation (Cy-5/Cy-3)	Cy-3 signal (Input)	Cy-5 signal (MeDIP)	Relative methylation (Cy-5/Cy-3)	Cy-3 signal (Input)	Cy-5 signal (MeDIP)
A_68_P20353660	chr1:75381669-75381713	NM_001085370:-494	Speg	PROMOTER	0.466	1.573	5132.50	8074.70	0.733	3395.55	2488.68
A_68_P20353690	chr1:75385333-75385377	NM_001085370:3170	Speg	INSIDE	0.277	0.669	2015.09	1348.33	0.185	1596.50	296.11
A_68_P20353933	chr1:75419052-75419096	NM_001085370:36890	Speg	INSIDE	0.514	0.669	3596.88	2407.46	0.344	2505.44	862.47
A_68_P20353934	chr1:75419154-75419198	NM_001085370:36992	Speg	INSIDE	0.403	1.343	4896.31	6574.99	0.541	3345.01	1810.88
A_68_P23364637	chr4:141092753-141092798	NM_019763:1737	Spen	INSIDE	0.207	0.516	2508.16	1294.92	0.107	1890.18	201.74
A_68_P26587801	chr9:65309057-65309101	NM_138584:335	Spg21	INSIDE	0.145	0.610	3316.04	2023.07	0.089	2586.25	229.16
A_68_P28159860	chr11:116394577-116394621	NM_011451:-1455	Sphk1	PROMOTER	0.249	0.421	1380.55	581.67	0.105	1138.93	119.52
A_68_P28159866	chr11:116395212-116395256	NM_011451:-819	Sphk1	PROMOTER	0.333	0.717	1422.35	1020.19	0.239	1032.44	246.66
A_68_P25094661	chr7:52968968-52969012	NM_020011:1587	Sphk2	INSIDE	0.662	2.561	1056.76	2706.28	1.695	892.29	1512.06
A_68_P25094672	chr7:52970422-52970466	NM_020011:133	Sphk2	INSIDE	0.581	0.611	1761.61	1077.13	0.355	1348.74	479.06
A_68_P25094675	chr7:52970796-52970843	NM_020011:-242	Sphk2	PROMOTER	0.170	0.709	1390.39	986.07	0.120	974.67	117.26
A_68_P25094689	chr7:52973005-52973055	NM_020011:-2453	Sphk2	PROMOTER	0.221	0.394	1649.80	650.58	0.087	1387.81	120.90
A_68_P30721030	chr16:44347912-44347958	NM_144550:421	Spice1	INSIDE	0.261	1.462	1597.91	2336.91	0.381	1233.66	470.42
A_68_P25026345	chr7:30066535-30066579	NM_001082548:440	Spint2	INSIDE	0.550	0.718	1768.27	1268.84	0.395	1463.88	577.69
A_68_P26239286	chr8:125857009-125857053	NM_172287:418	Spire2	INSIDE	0.437	0.417	2644.30	1103.38	0.182	2061.87	376.19
A_68_P28555624	chr12:77810657-77810701	NM_013675:856	Spnb1	INSIDE	0.645	0.303	2647.47	801.05	0.195	2209.99	431.34
A_68_P28555630	chr12:77811318-77811362	NM_013675:194	Spnb1	INSIDE	0.384	0.669	1808.55	1209.90	0.257	1421.57	365.08
A_68_P28555634	chr12:77811742-77811790	NM_013675:-232	Spnb1	PROMOTER	0.267	0.632	901.05	569.83	0.169	716.22	121.15
A_68_P31924553	chr19:4712434-4712478	NM_021287:1234	Spnb3	INSIDE	0.450	0.671	1349.97	905.28	0.302	1028.50	310.31
A_68_P31924554	chr19:4712508-4712552	NM_021287:1308	Spnb3	INSIDE	0.147	0.633	2518.67	1593.51	0.093	1762.68	164.01
A_68_P31924555	chr19:4712589-4712633	NM_021287:1388	Spnb3	INSIDE	0.468	0.565	1249.14	706.20	0.265	1019.90	270.06
A_68_P25016925	chr7:28146852-28146896	NM_001199235:32651	Spnb4	INSIDE	0.537	0.575	1653.97	950.83	0.309	1471.23	453.96
A_68_P25017007	chr7:28160302-28160347	NM_001199235:19201	Spnb4	INSIDE	0.425	0.702	1568.81	1101.78	0.298	1289.57	384.55
A_68_P27911821	chr11:72302535-72302580	NM_153060:849	Spns2	INSIDE	0.487	0.643	1352.13	869.12	0.313	1065.32	333.18
A_68_P29070326	chr13:58009122-58009166	NM_001166464:549	Spock1	INSIDE	0.506	0.524	1246.19	653.13	0.265	1059.14	280.90
A_68_P29070327	chr13:58009211-58009255	NM_001166464:461	Spock1	INSIDE	0.262	0.611	949.70	580.03	0.160	775.08	124.21
A_68_P28038653	chr11:95275248-95275292	NM_025287:-126	Spop	PROMOTER	0.638	2.705	2310.60	6250.27	1.726	1571.40	2712.43
A_68_P24007309	chr5:115462025-115462071	NM_029012:516	Sppl3	INSIDE	0.340	2.073	1933.68	4008.61	0.705	1553.83	1094.99
A_68_P24603257	chr6:85087734-85087778	NM_011467:2	Spr	INSIDE	0.118	0.636	1909.44	1213.74	0.075	1589.35	119.51
A_68_P21556051	chr2:116946577-116946621	NM_033524:-587	Spred1	PROMOTER	0.334	0.440	3528.51	1554.07	0.147	2790.48	410.47
A_68_P27628894	chr11:19820464-19820508	NM_033523:-3958	Spred2	PROMOTER	0.665	2.904	294.59	855.42	1.932	379.16	732.65
A_68_P27628921	chr11:19825017-19825061	NM_033523:594	Spred2	INSIDE	0.468	0.469	4522.81	2122.40	0.220	3561.26	781.88
A_68_P29879699	chr14:106294307-106294351	NM_011897:1708	Spry2	INSIDE	0.273	0.616	3758.28	2313.71	0.168	2666.38	448.47
A_68_P29879712	chr14:106295768-106295812	NM_011897:246	Spry2	INSIDE	0.577	0.442	1487.24	657.86	0.255	1204.45	307.63
A_68_P29879718	chr14:106296554-106296598	NM_011897:-540	Spry2	PROMOTER	0.214	0.522	1234.23	643.73	0.111	1053.87	117.43
A_68_P30496206	chr15:101966579-101966623	NM_001033277:46	Spryd3	INSIDE	0.151	0.345	2743.26	945.31	0.052	2330.38	121.35
A_68_P30496207	chr15:101966685-101966729	NM_001033277:-60	Spryd3	DIVERGENT_PROMOTER	0.235	0.490	1236.78	605.57	0.115	1013.36	116.77
A_68_P27540138	chr10:127650212-127650256	NM_025716:-1384	Spryd4	PROMOTER	0.460	0.631	1205.34	761.02	0.290	963.34	279.75
A_68_P23403479	chr4:149328561-149328605	NM_029035:533	Spsb1	INSIDE	0.255	0.493	1250.73	616.65	0.126	927.62	116.73
A_68_P24817040	chr6:124755640-124755684	NM_013539:-3296	Spsb2	PROMOTER	0.314	0.475	1229.87	584.16	0.149	957.83	142.90
A_68_P26750480	chr9:96918879-96918923	NM_145134:-126	Spsb4	PROMOTER	0.348	0.445	1561.18	694.96	0.155	1344.12	208.26
A_68_P28615264	chr12:88640362-88640406	NM_011479:88796	Sptlc2	DOWNSTREAM	0.260	0.742	4012.53	2977.58	0.193	2737.51	527.21
A_68_P28615265	chr12:88640437-88640481	NM_011479:88722	Sptlc2	DOWNSTREAM	0.264	0.635	3336.21	2118.25	0.167	2605.01	436.06
A_68_P28615271	chr12:88641179-88641223	NM_011479:87980	Sptlc2	DOWNSTREAM	0.299	0.614	3622.78	2225.65	0.184	2774.53	509.31

ProbeName	Target position of probe on CpG island microarray	TargetID	GeneSymbol	CpG island Description	Ratio of relative methylation (TiO2-NP/Vehicle)	Sham group			TiO_2-H group		
						Relative methylation (Cy-5/Cy-3)	Cy-3 signal (Input)	Cy-5 signal (MeDIP)	Relative methylation (Cy-5/Cy-3)	Cy-3 signal (Input)	Cy-5 signal (MeDIP)
A_68_P25102496	chr7:54263191-54263240	NM_175318:569	Spty2d1	INSIDE	0.259	0.641	1605.62	1028.44	0.166	1192.96	197.82
A_68_P25102502	chr7:54263847-54263895	NM_175318:-86	Spty2d1	PROMOTER	0.152	0.552	1815.47	1001.40	0.084	1421.04	119.51
A_68_P25102503	chr7:54263926-54263970	NM_175318:-164	Spty2d1	PROMOTER	0.372	0.709	1940.59	1375.01	0.264	1561.52	412.11
A_68_P21587980	chr2:122590894-122590938	NM_001162503:-178	Sqrdl	PROMOTER	0.348	0.511	2170.79	1109.61	0.178	1708.45	303.97
A_68_P31415000	chr17:86544304-86544360	NM_030133:183	Srbd1	INSIDE	0.264	0.612	954.25	583.60	0.162	748.11	120.92
A_68_P28051144	chr11:97393113-97393157	NM_018873:43306	Srcin1	INSIDE	0.210	0.620	1230.03	762.31	0.130	902.66	117.54
A_68_P28051297	chr11:97413036-97413080	NM_018873:23382	Srcin1	INSIDE	0.623	0.633	817.06	517.33	0.394	635.78	250.71
A_68_P28051299	chr11:97413226-97413270	NM_018873:23192	Srcin1	INSIDE	0.593	0.608	889.92	541.50	0.361	809.41	291.86
A_68_P24118744	chr5:136438405-136438449	NM_001160366:11920	Srcrb4d	INSIDE	0.114	0.543	3947.13	2142.47	0.062	2645.93	163.34
A_68_P24118745	chr5:136438525-136438569	NM_001160366:11800	Srcrb4d	INSIDE	0.663	0.660	4244.66	2802.70	0.438	3021.76	1323.39
A_68_P29116718	chr13:69749922-69749966	NM_175283:397	Srd5a1	INSIDE	0.611	0.491	1683.52	826.10	0.300	1354.43	406.03
A_68_P29116719	chr13:69750004-69750048	NM_175283:315	Srd5a1	INSIDE	0.499	0.542	2348.08	1273.45	0.270	1991.53	538.44
A_68_P29116720	chr13:69750085-69750129	NM_175283:235	Srd5a1	INSIDE	0.294	0.327	1824.07	596.40	0.096	1215.67	116.70
A_68_P29116723	chr13:69750440-69750484	NM_175283:-121	Srd5a1	DIVERGENT_PROMOTER	0.244	0.533	2400.14	1279.76	0.130	1674.04	218.05
A_68_P31349752	chr17:74397147-74397191	NM_053188:88	Srd5a2	INSIDE	0.271	0.405	1263.86	512.28	0.110	1087.52	119.43
A_68_P27841612	chr11:60024194-60024238	NM_011480:9890	Srebf1	INSIDE	0.272	0.365	1444.68	527.32	0.099	1170.03	116.38
A_68_P27841685	chr11:60033832-60033876	NM_011480:252	Srebf1	INSIDE	0.210	0.430	1969.99	846.79	0.090	1551.22	139.74
A_68_P27841686	chr11:60033953-60033997	NM_011480:132	Srebf1	INSIDE	0.537	0.480	1636.00	785.54	0.258	1292.43	333.03
A_68_P30381700	chr15:81977560-81977604	NM_033218:-116	Srebf2	PROMOTER	0.268	0.552	1464.31	808.90	0.148	1024.78	151.75
A_68_P30381703	chr15:81977883-81977927	NM_033218:206	Srebf2	INSIDE	0.122	0.735	7136.20	5242.94	0.090	4976.80	445.44
A_68_P29286846	chr13:104563824-104563877	NM_172592:-9188	Srek1	PROMOTER	0.193	0.570	1311.67	747.61	0.110	1110.43	122.08
A_68_P29286848	chr13:104564132-104564176	NM_172592:-9492	Srek1	PROMOTER	0.631	0.560	1868.39	1046.08	0.354	1663.54	588.15
A_68_P29292646	chr13:105607347-105607391	NM_026075:50	Srek1ip1	INSIDE	0.471	0.441	1745.54	769.69	0.208	1192.85	247.69
A_68_P31207576	chr17:46692058-46692102	NM_020493:1031	Srf	INSIDE	0.623	0.725	3690.55	2677.11	0.452	2324.42	1050.17
A_68_P31207578	chr17:46692314-46692358	NM_020493:775	Srf	INSIDE	0.158	0.667	1514.02	1009.94	0.106	1123.57	118.57
A_68_P27506870	chr10:121484975-121485019	NM_001081037:-747	Srgap1	PROMOTER	0.635	0.710	1308.03	929.24	0.451	1135.09	512.18
A_68_P20616231	chr1:133422247-133422291	NM_001081011:1670	Srgap2	INSIDE	0.299	0.412	2073.72	854.41	0.123	1549.42	191.09
A_68_P20616241	chr1:133423301-133423345	NM_001081011:616	Srgap2	INSIDE	0.269	0.467	1393.83	650.69	0.125	1000.15	125.44
A_68_P20616242	chr1:133423393-133423437	NM_001081011:524	Srgap2	INSIDE	0.375	0.521	2544.03	1324.36	0.195	2056.63	401.09
A_68_P20616246	chr1:133423879-133423923	NM_001081011:38	Srgap2	INSIDE	0.165	0.750	5910.20	4432.85	0.123	4115.64	507.99
A_68_P23395328	chr4:147965305-147965349	NM_009272:-295	Srm	PROMOTER	0.165	0.681	2347.57	1598.71	0.112	1660.23	186.30
A_68_P31612300	chr18:34490731-34490775	NM_025527:-46	Srp19	PROMOTER	0.246	0.570	1033.65	589.20	0.140	865.75	121.36
A_68_P28158411	chr11:116135230-116135274	NM_146032:279	Srp68	INSIDE	0.158	1.446	2201.26	3183.70	0.229	1480.33	338.74
A_68_P23819151	chr5:77403818-77403862	NM_025691:115	Srp72	INSIDE	0.315	0.455	1208.35	549.44	0.143	903.88	129.65
A_68_P20885446	chr1:184055324-184055368	NM_012058:479	Srp9	INSIDE	0.272	0.440	2506.19	1103.56	0.120	2066.48	247.19
A_68_P31123423	chr17:28758242-28758293	NM_016795:1132	Srpk1	INSIDE	0.170	0.582	1647.29	959.35	0.099	1251.97	123.87
A_68_P31123424	chr17:28758327-28758378	NM_016795:1047	Srpk1	INSIDE	0.282	0.529	1613.63	853.61	0.149	1237.90	184.37
A_68_P31123427	chr17:28758620-28758664	NM_016795:757	Srpk1	INSIDE	0.332	0.652	2257.58	1471.67	0.217	1739.13	376.59
A_68_P26421279	chr9:35018970-35019014	NM_026130:205	Srpr	INSIDE	0.248	0.458	1282.93	587.07	0.113	1084.90	123.11
A_68_P23328077	chr4:134908288-134908332	NM_001130477:819	Srrm1	INSIDE	0.329	0.459	2385.61	1096.13	0.151	1846.87	279.42
A_68_P23328078	chr4:134908468-134908512	NM_001130477:639	Srrm1	INSIDE	0.298	0.722	1561.86	1128.40	0.215	1225.60	263.61
A_68_P31093747	chr17:23944056-23944100	NM_175229:3925	Srrm2	INSIDE	0.467	1.416	1224.30	1733.34	0.661	1023.49	676.73
A_68_P31093841	chr17:23956616-23956662	NM_175229:16485	Srrm2	INSIDE	0.659	2.506	862.34	2161.44	1.652	613.74	1013.66
A_68_P24016052	chr5:117041312-117041356	NM_026886:492	Srrm4	INSIDE	0.243	0.597	1087.61	649.40	0.145	809.76	117.66

ProbeName	Target position of probe on CpG island microarray	TargetID	GeneSymbol	CpG island Description	Ratio of relative methylation (TiO2-NP/Vehicle)	Sham group			TiO_2-H group		
						Relative methylation (Cy-5/Cy-3)	Cy-3 signal (Input)	Cy-5 signal (MeDIP)	Relative methylation (Cy-5/Cy-3)	Cy-3 signal (Input)	Cy-5 signal (MeDIP)
A_68_P24125091	chr5:137748534-137748578	NM_001109909:346	Srrt	INSIDE	0.262	0.429	3285.78	1408.74	0.112	2266.19	254.16
A_68_P27997502	chr11:87860747-87860791	NM_001078167:-404	Srsf1	PROMOTER	0.315	0.580	1194.48	693.03	0.183	953.83	174.32
A_68_P23330841	chr4:135411994-135412038	NM_001080387:10	Srsf10	INSIDE	0.405	0.543	2145.64	1166.05	0.220	1597.74	351.61
A_68_P23330845	chr4:135412711-135412755	NM_001080387:726	Srsf10	INSIDE	0.130	0.704	2020.04	1421.74	0.091	1484.32	135.30
A_68_P22683258	chr3:157694818-157694868	NM_001093753:-124	Srsf11	PROMOTER	0.339	0.643	1098.09	706.04	0.218	903.15	196.63
A_68_P22825735	chr4:33296468-33296513	NM_177774:525	Srsf12	INSIDE	0.312	0.744	2829.04	2105.95	0.232	2326.52	540.72
A_68_P28161646	chr11:116714298-116714343	NM_011358:88	Srsf2	INSIDE	0.225	0.417	1703.04	710.00	0.094	1296.28	121.79
A_68_P28161648	chr11:116714471-116714515	NM_011358:-84	Srsf2	DIVERGENT_PROMOTER	0.186	0.551	1366.73	753.46	0.102	1158.23	118.49
A_68_P28578485	chr12:82046655-82046699	NM_001079694:186	Srsf5	INSIDE	0.183	0.392	2396.97	939.51	0.072	1897.39	136.07
A_68_P31381748	chr17:80606129-80606175	NM_001195485:493	Srsf7	INSIDE	0.171	1.517	4212.40	6389.69	0.260	2651.26	689.30
A_68_P31381751	chr17:80606502-80606546	NM_001195485:121	Srsf7	INSIDE	0.544	0.617	1660.75	1024.34	0.335	1280.02	429.18
A_68_P31381753	chr17:80606810-80606854	NM_001195485:-187	Srsf7	DIVERGENT_PROMOTER	0.191	0.533	1673.20	891.30	0.102	1304.47	132.97
A_68_P21747680	chr2:151931345-151931389	NM_029688:-99	Srxn1	PROMOTER	0.595	0.646	2846.43	1838.72	0.385	2308.07	887.70
A_68_P21747681	chr2:151931454-151931498	NM_029688:11	Srxn1	INSIDE	0.551	0.690	2167.54	1495.96	0.380	1752.81	666.04
A_68_P21901050	chr2:179777293-179777337	NM_178750:127	Ss18l1	INSIDE	0.336	0.482	2057.89	992.10	0.162	1514.67	245.47
A_68_P21311407	chr2:69699641-69699685	NM_001110145:44	Ssb	INSIDE	0.655	0.470	1500.34	705.40	0.308	1358.31	418.06
A_68_P29218722	chr13:91600765-91600809	NM_024186:85	Ssbp2	INSIDE	0.112	0.614	3203.40	1967.63	0.069	2229.34	153.62
A_68_P29218728	chr13:91601752-91601796	NM_024186:1073	Ssbp2	INSIDE	0.400	0.631	1627.78	1026.95	0.252	1284.93	323.86
A_68_P23187158	chr4:106584284-106584328	NM_023672:232	Ssbp3	INSIDE	0.536	0.538	1632.94	877.89	0.288	1352.29	389.68
A_68_P25952901	chr8:73122626-73122673	NM_133772:9564	Ssbp4	INSIDE	0.601	2.646	2179.14	5766.93	1.591	1340.71	2133.65
A_68_P25952984	chr8:73133754-73133798	NM_133772:-1563	Ssbp4	DIVERGENT_PROMOTER	0.165	0.728	1875.54	1364.47	0.120	1483.08	178.43
A_68_P25952997	chr8:73135574-73135625	NM_133772:-3386	Ssbp4	DIVERGENT_PROMOTER	0.236	0.521	1205.98	627.73	0.123	1006.37	123.76
A_68_P24001332	chr5:114443223-114443267	NM_198109:522	Ssh1	INSIDE	0.587	0.614	974.48	598.35	0.360	898.80	323.69
A_68_P27936387	chr11:77029678-77029722	NM_177710:-226	Ssh2	PROMOTER	0.445	0.529	1386.73	733.42	0.235	1221.69	287.33
A_68_P28957974	chr13:38085985-38086029	NM_025965:53	Ssr1	INSIDE	0.620	0.464	1318.20	611.17	0.287	1236.17	355.27
A_68_P22199716	chr3:65196136-65196183	NM_026155:316	Ssr3	INSIDE	0.366	1.567	1433.03	2244.91	0.573	1122.68	643.02
A_68_P22199717	chr3:65196220-65196264	NM_026155:233	Ssr3	INSIDE	0.169	0.720	6823.25	4911.23	0.122	5046.74	613.64
A_68_P32464768	chrX:71032322-71032366	NM_001166480:-22	Ssr4	DIVERGENT_PROMOTER	0.414	1.428	2322.49	3316.10	0.591	898.89	531.58
A_68_P21395950	chr2:84877269-84877313	NM_182990:-67	Ssrp1	DIVERGENT_PROMOTER	0.129	0.655	4429.49	2899.56	0.084	3164.63	267.09
A_68_P21395951	chr2:84877360-84877404	NM_001136081:-225	Ssrp1	PROMOTER	0.514	0.739	3665.82	2709.21	0.380	2642.53	1004.21
A_68_P21395954	chr2:84877657-84877701	NM_001136081:71	Ssrp1	INSIDE	0.579	0.680	1147.42	780.43	0.394	841.63	331.42
A_68_P21395956	chr2:84877845-84877892	NM_001136081:261	Ssrp1	INSIDE	0.163	1.640	519.38	851.92	0.268	445.15	119.23
A_68_P31930503	chr19:5731362-5731406	NM_020491:337	Sssca1	INSIDE	0.440	0.723	2204.80	1595.08	0.318	1671.91	531.85
A_68_P31930504	chr19:5731477-5731521	NM_020491:223	Sssca1	INSIDE	0.543	0.578	1028.03	593.89	0.314	770.02	241.55
A_68_P31930506	chr19:5731655-5731699	NM_020491:45	Sssca1	INSIDE	0.474	0.485	2931.98	1423.06	0.230	2146.46	493.78
A_68_P28466194	chr12:59314126-59314170	NM_009216:1358	Sstr1	INSIDE	0.266	0.523	1498.91	784.15	0.139	1114.45	155.35
A_68_P28143023	chr11:113480461-113480507	NM_001042606:-5086	Sstr2	PROMOTER	0.252	0.400	1490.33	595.89	0.101	1226.66	123.47
A_68_P21729941	chr2:148220875-148220919	NM_009219:-216	Sstr4	PROMOTER	0.375	0.598	880.40	526.78	0.224	729.88	163.82
A_68_P21729944	chr2:148221334-148221378	NM_009219:244	Sstr4	INSIDE	0.211	0.419	1936.96	811.30	0.088	1419.29	125.50
A_68_P30377732	chr15:81229723-81229767	NM_133726:380	St13	INSIDE	0.482	0.642	1394.93	895.13	0.309	1246.24	385.22
A_68_P30377735	chr15:81230102-81230146	NM_133726:0	St13	INSIDE	0.283	0.741	7066.61	5237.90	0.210	4964.21	1042.96
A_68_P30377736	chr15:81230239-81230283	NM_133726:-136	St13	DIVERGENT_PROMOTER	0.511	0.636	1652.89	1051.20	0.325	1243.42	404.08
A_68_P30296502	chr15:67007203-67007248	NM_009177:1219	St3gal1	INSIDE	0.096	0.590	2928.70	1727.49	0.057	2120.27	120.20
A_68_P30296503	chr15:67007295-67007349	NM_009177:1122	St3gal1	INSIDE	0.192	0.628	1380.77	867.23	0.121	989.96	119.33

ProbeName	Target position of probe on CpG island microarray	TargetID	GeneSymbol	CpG island Description	Ratio of relative methylation (TiO2-NP/Vehicle)	Sham group			TiO_2-H group		
						Relative methylation (Cy-5/Cy-3)	Cy-3 signal (Input)	Cy-5 signal (MeDIP)	Relative methylation (Cy-5/Cy-3)	Cy-3 signal (Input)	Cy-5 signal (MeDIP)
A_68_P30296504	chr15:67007424-67007469	NM_009177:998	St3gal1	INSIDE	0.129	0.738	3133.21	2312.57	0.095	2072.70	197.79
A_68_P30296506	chr15:67007655-67007699	NM_009177:768	St3gal1	INSIDE	0.267	0.461	1360.77	627.89	0.123	996.11	122.60
A_68_P30296519	chr15:67008963-67009007	NM_009177:-540	St3gal1	PROMOTER	0.234	1.397	6262.09	8746.16	0.327	3972.83	1297.23
A_68_P26165834	chr8:113494088-113494132	NM_009179:50346	St3gal2	INSIDE	0.398	0.677	1222.12	827.61	0.269	901.87	242.81
A_68_P23238475	chr4:117807044-117807088	NM_009176:429	St3gal3	INSIDE	0.116	0.714	2675.68	1910.93	0.083	2011.02	166.26
A_68_P30792107	chr16:58523074-58523118	NM_018784:329	St3gal6	INSIDE	0.543	0.578	1177.04	680.78	0.314	897.75	281.89
A_68_P28160910	chr11:116555525-116555569	NM_009180:428	St6galnac2	INSIDE	0.224	0.553	1129.88	624.68	0.124	965.65	119.80
A_68_P21116356	chr2:32462749-32462793	NM_001025310:290	St6galnac6	INSIDE	0.653	0.443	2138.72	946.90	0.289	1637.68	473.42
A_68_P22393610	chr3:104667906-104667950	NM_153091:-70	St7l	DIVERGENT_PROMOTER	0.465	0.373	1659.75	618.42	0.173	1471.45	254.75
A_68_P24907674	chr6:142912449-142912500	NM_011374:498	St8sia1	INSIDE	0.206	0.641	1077.96	690.69	0.132	910.95	120.33
A_68_P24907678	chr6:142912941-142912985	NM_011374:10	St8sia1	INSIDE	0.512	4.197	2260.30	9487.15	2.149	1804.81	3878.40
A_68_P25232260	chr7:81157728-81157772	NM_009181:818	St8sia2	INSIDE	0.155	0.486	2003.93	974.16	0.075	1564.10	117.54
A_68_P25232261	chr7:81157848-81157892	NM_009181:698	St8sia2	INSIDE	0.253	0.679	2235.50	1518.32	0.172	1503.65	257.94
A_68_P25232272	chr7:81159033-81159078	NM_009181:-487	St8sia2	PROMOTER	0.593	0.522	3343.28	1744.06	0.310	2614.63	809.38
A_68_P31774240	chr18:64425610-64425654	NM_009182:11620	St8sia3	INSIDE	0.464	0.426	2867.14	1221.38	0.198	2108.09	416.43
A_68_P20464815	chr1:97564271-97564315	NM_009183:-121	St8sia4	PROMOTER	0.263	0.602	3080.86	1854.12	0.158	2195.52	347.78
A_68_P31846732	chr18:77493370-77493414	NM_013666:68807	St8sia5	INSIDE	0.649	2.348	2710.63	6364.55	1.524	1865.98	2843.82
A_68_P21018168	chr2:13714756-13714800	NM_145838:369	St8sia6	INSIDE	0.350	0.542	2894.52	1568.46	0.190	2206.39	418.34
A_68_P28054167	chr11:97913713-97913757	NM_146028:1042	Stac2	INSIDE	0.380	0.687	2016.37	1385.58	0.261	1470.46	384.09
A_68_P28054175	chr11:97914595-97914639	NM_146028:160	Stac2	INSIDE	0.433	0.528	3682.99	1946.43	0.229	2753.77	630.78
A_68_P26770646	chr9:100543212-100543261	NM_009282:-805	Stag1	PROMOTER	0.207	0.547	1360.75	744.75	0.113	1029.76	116.60
A_68_P26770648	chr9:100543417-100543473	NM_009282:-597	Stag1	PROMOTER	0.290	0.551	990.61	545.74	0.160	756.42	120.75
A_68_P32341131	chrX:39503134-39503178	NM_021465:-721	Stag2	PROMOTER	0.117	1.760	3510.26	6179.39	0.206	1499.73	308.32
A_68_P21221438	chr2:52601606-52601650	NM_019667:-445	Stam2	PROMOTER	0.373	0.581	1188.08	689.77	0.217	877.37	190.01
A_68_P21221439	chr2:52601733-52601777	NM_019667:-571	Stam2	PROMOTER	0.157	0.671	1572.17	1054.98	0.105	1138.67	119.99
A_68_P32078821	chr19:34266845-34266890	NM_029682:108	Stambpl1	INSIDE	0.104	2.001	2573.12	5149.74	0.208	1651.50	343.86
A_68_P25372612	chr7:108470032-108470076	NM_019990:222	Stard10	INSIDE	0.119	0.634	1962.15	1244.37	0.075	1567.90	118.15
A_68_P25372613	chr7:108470115-108470159	NM_019990:304	Stard10	INSIDE	0.602	0.601	1988.33	1195.02	0.362	1657.57	599.40
A_68_P28055911	chr11:98219606-98219650	NM_021547:-69	Stard3	PROMOTER	0.589	0.669	1966.85	1314.93	0.394	1854.60	730.57
A_68_P31606709	chr18:33373631-33373675	NM_133774:-182	Stard4	PROMOTER	0.278	0.506	1136.21	575.08	0.141	878.34	123.59
A_68_P31809752	chr18:70632290-70632335	NM_029019:114	Stard6	INSIDE	0.101	1.505	1184.65	1783.13	0.151	786.22	119.10
A_68_P21612443	chr2:127096615-127096659	NM_139308:672	Stard7	INSIDE	0.234	0.575	2492.29	1433.71	0.135	2089.87	281.79
A_68_P28071037	chr11:100800754-100800798	NM_011486:49	Stat3	INSIDE	0.372	0.701	1543.79	1082.81	0.261	1181.06	307.97
A_68_P28070587	chr11:100721732-100721776	NM_001164062:-43	Stat5a	PROMOTER	0.204	0.531	1426.91	758.02	0.108	1076.18	116.36
A_68_P28070590	chr11:100721997-100722041	NM_001164062:221	Stat5a	INSIDE	0.293	0.592	1196.98	709.14	0.173	945.49	164.00
A_68_P21833056	chr2:166821240-166821285	NM_001109905:516	Stau1	INSIDE	0.570	0.595	1196.97	711.91	0.339	986.92	334.35
A_68_P21833062	chr2:166821989-166822037	NM_001109905:-234	Stau1	PROMOTER	0.166	0.659	1422.26	937.41	0.110	1113.63	122.03
A_68_P27682712	chr11:31269004-31269048	NM_011491:1035	Stc2	INSIDE	0.410	0.590	897.61	529.27	0.242	837.64	202.36
A_68_P27682716	chr11:31269467-31269511	NM_011491:573	Stc2	INSIDE	0.560	0.561	1058.09	594.10	0.314	903.97	284.24
A_68_P27682732	chr11:31271512-31271556	NM_011491:-1473	Stc2	PROMOTER	0.664	0.474	2672.53	1266.60	0.315	1977.73	622.37
A_68_P27682733	chr11:31271650-31271694	NM_011491:-1611	Stc2	PROMOTER	0.253	0.472	1361.46	642.53	0.120	1033.56	123.55
A_68_P27689370	chr11:32433541-32433585	NM_009288:297	Stk10	INSIDE	0.563	0.618	1245.10	768.86	0.348	942.19	327.51
A_68_P27281036	chr10:79578010-79578054	NM_011492:-1248	Stk11	PROMOTER	0.199	0.605	1344.72	813.44	0.120	959.33	115.55
A_68_P27281037	chr10:79578104-79578148	NM_011492:-1154	Stk11	PROMOTER	0.649	0.530	1828.89	969.27	0.344	1251.59	430.62

ProbeName	Target position of probe on CpG island microarray	TargetID	GeneSymbol	CpG island Description	Ratio of relative methylation (TiO2-NP/Vehicle)	Sham group			TiO_2-H group		
						Relative methylation (Cy-5/Cy-3)	Cy-3 signal (Input)	Cy-5 signal (MeDIP)	Relative methylation (Cy-5/Cy-3)	Cy-3 signal (Input)	Cy-5 signal (MeDIP)
A_68_P27281038	chr10:79578186-79578230	NM_011492:-1072	Stk11	PROMOTER	0.341	0.659	910.75	600.38	0.225	707.91	159.15
A_68_P20354640	chr1:75518210-75518265	NM_027886:134	Stk11ip	INSIDE	0.164	0.460	2161.70	993.42	0.076	1600.44	120.99
A_68_P20352666	chr1:75207636-75207680	NM_011494:255	Stk16	INSIDE	0.350	0.663	1477.59	979.32	0.232	1157.58	268.18
A_68_P20233542	chr1:53841734-53841778	NM_133810:303	Stk17b	INSIDE	0.637	0.424	1870.82	793.03	0.270	1607.60	433.96
A_68_P31156002	chr17:34973346-34973390	NM_019442:480	Stk19	INSIDE	0.544	0.616	1454.28	895.82	0.335	1323.89	444.02
A_68_P20456743	chr1:95532366-95532410	NM_021537:-84	Stk25	PROMOTER	0.258	0.638	1464.50	934.08	0.165	1204.52	198.39
A_68_P20456744	chr1:95532440-95532484	NM_021537:-158	Stk25	PROMOTER	0.240	0.551	2664.75	1468.30	0.132	1994.89	264.16
A_68_P30132016	chr15:35085389-35085433	NM_019635:151	Stk3	INSIDE	0.212	0.427	2475.64	1056.78	0.091	1865.54	168.98
A_68_P30132017	chr15:35085586-35085630	NM_019635:-47	Stk3	PROMOTER	0.295	0.488	4603.04	2246.98	0.144	3782.78	544.91
A_68_P28740640	chr12:112082039-112082083	NM_011973:-2911	Stk30	DIVERGENT_PROMOTER	0.498	0.699	1298.54	908.06	0.348	1025.62	356.89
A_68_P25576304	chr7:146373808-146373852	NM_021302:563	Stk32c	INSIDE	0.370	0.526	1714.22	901.08	0.195	1413.47	274.93
A_68_P25576402	chr7:146399285-146399329	NM_001162540:-100	Stk32c	PROMOTER	0.323	0.472	2110.43	995.44	0.152	1549.76	236.26
A_68_P21626533	chr2:129625984-129626034	NM_001038635:-244	Stk35	PROMOTER	0.131	0.624	2138.25	1333.29	0.082	1454.08	118.73
A_68_P21626534	chr2:129626071-129626115	NM_001038635:-160	Stk35	PROMOTER	0.217	0.676	1252.10	846.38	0.146	905.72	132.61
A_68_P21626535	chr2:129626154-129626199	NM_001038635:-76	Stk35	PROMOTER	0.229	0.638	1788.07	1140.54	0.146	1277.63	186.81
A_68_P31125470	chr17:29144780-29144824	NM_134115:80	Stk38	INSIDE	0.339	0.543	1290.56	700.74	0.184	959.77	176.47
A_68_P21303499	chr2:68309684-68309728	NM_016866:332	Stk39	INSIDE	0.577	0.458	1598.00	731.37	0.264	1365.24	360.73
A_68_P21815998	chr2:163900092-163900136	NM_021420:201	Stk4	INSIDE	0.616	0.425	1468.72	623.74	0.262	1215.31	317.93
A_68_P23322939	chr4:134024154-134024198	NM_019641:-58	Stmn1	PROMOTER	0.248	0.677	1098.48	743.38	0.168	909.59	152.39
A_68_P23322943	chr4:134024577-134024621	NM_019641:364	Stmn1	INSIDE	0.156	0.645	2161.19	1392.93	0.100	1533.24	153.95
A_68_P21133064	chr2:35192370-35192414	NM_013515:137	Stom	INSIDE	0.160	0.652	1567.37	1021.47	0.104	1175.73	122.53
A_68_P22869602	chr4:43044144-43044188	NM_023231:90	Stoml2	INSIDE	0.375	0.633	1196.06	757.30	0.237	941.35	223.35
A_68_P28635988	chr12:92885896-92885940	NM_175367:138958	Ston2	INSIDE	0.548	2.984	964.38	2877.66	1.635	863.57	1412.37
A_68_P25840857	chr8:48373280-48373324	NM_001114311:1454	Stox2	INSIDE	0.174	0.625	1857.49	1160.78	0.108	1276.11	138.37
A_68_P25840860	chr8:48373600-48373648	NM_001114311:1132	Stox2	INSIDE	0.175	0.512	1732.66	887.09	0.090	1330.12	119.39
A_68_P25840861	chr8:48373757-48373801	NM_001114311:978	Stox2	INSIDE	0.265	0.530	3849.13	2041.53	0.141	3167.91	445.20
A_68_P28186320	chr11:120574795-120574839	NM_016665:265	Stra13	INSIDE	0.304	0.653	1701.86	1111.63	0.199	1269.57	252.10
A_68_P28186321	chr11:120574903-120574948	NM_016665:156	Stra13	INSIDE	0.177	0.546	3576.16	1952.29	0.096	2694.71	259.95
A_68_P28186322	chr11:120575013-120575057	NM_016665:47	Stra13	INSIDE	0.247	0.477	2062.63	984.56	0.118	1300.03	153.53
A_68_P28100097	chr11:106055038-106055082	NM_028126:-198	Strada	PROMOTER	0.276	0.564	2868.73	1617.07	0.156	2206.11	343.13
A_68_P24880085	chr6:137683395-137683454	NM_011499:-178	Strap	PROMOTER	0.191	0.550	1376.24	757.23	0.105	1134.99	119.24
A_68_P24880090	chr6:137684038-137684082	NM_011499:458	Strap	INSIDE	0.402	0.565	2008.73	1135.59	0.227	1503.42	341.29
A_68_P31373355	chr17:79135650-79135694	NM_011500:228	Strn	INSIDE	0.256	0.648	2416.13	1566.20	0.166	1760.83	292.30
A_68_P26426682	chr9:36575102-36575146	NM_008408:39	Stt3a	INSIDE	0.277	0.596	1931.40	1150.44	0.165	1437.89	237.04
A_68_P26426686	chr9:36575536-36575582	NM_008408:-395	Stt3a	PROMOTER	0.132	0.475	2701.04	1283.39	0.063	1951.49	122.58
A_68_P31106579	chr17:25969550-25969594	NM_019719:734	Stub1	INSIDE	0.309	0.732	2880.62	2110.02	0.227	2057.03	466.08
A_68_P31106580	chr17:25969693-25969737	NM_019719:592	Stub1	INSIDE	0.149	0.531	2140.17	1135.90	0.079	1526.64	120.86
A_68_P31106586	chr17:25970234-25970278	NM_019719:50	Stub1	INSIDE	0.574	0.606	1580.31	957.97	0.348	1193.25	415.48
A_68_P31106589	chr17:25970569-25970614	NM_019719:-285	Stub1	PROMOTER	0.600	0.508	1234.01	627.21	0.305	958.01	291.94
A_68_P23616976	chr5:38430129-38430184	NM_026959:-317	Stx18	PROMOTER	0.262	0.520	1055.52	548.62	0.136	867.02	117.95
A_68_P23616979	chr5:38430477-38430521	NM_026959:25	Stx18	INSIDE	0.236	0.529	2805.20	1483.16	0.125	2117.72	264.34
A_68_P23616980	chr5:38430589-38430633	NM_026959:137	Stx18	INSIDE	0.176	0.537	1651.76	887.73	0.095	1235.71	117.17
A_68_P24114328	chr5:135499558-135499602	NM_016801:139	Stx1a	INSIDE	0.512	0.650	1259.02	818.09	0.333	1013.75	337.49
A_68_P24114432	chr5:135517814-135517858	NM_016801:18395	Stx1a	INSIDE	0.446	1.396	2378.55	3320.29	0.623	1640.68	1022.52

ProbeName	Target position of probe on CpG island microarray	TargetID	GeneSymbol	CpG island Description	Ratio of relative methylation (TiO2-NP/Vehicle)	Sham group			TiO_2-H group		
						Relative methylation (Cy-5/Cy-3)	Cy-3 signal (Input)	Cy-5 signal (MeDIP)	Relative methylation (Cy-5/Cy-3)	Cy-3 signal (Input)	Cy-5 signal (MeDIP)
A_68_P24114433	chr5:135517901-135517945	NM_016801:18481	Stx1a	INSIDE	0.196	1.446	2560.20	3703.22	0.283	1720.37	487.41
A_68_P24114434	chr5:135517995-135518039	NM_016801:18575	Stx1a	INSIDE	0.233	2.067	2582.59	5339.45	0.482	1589.18	766.62
A_68_P31958008	chr19:11892927-11892972	NM_011502:434	Stx3	INSIDE	0.213	0.548	1367.45	749.55	0.117	1038.29	121.12
A_68_P31958015	chr19:11893696-11893748	NM_011502:-339	Stx3	PROMOTER	0.274	0.644	895.93	577.08	0.176	690.83	121.89
A_68_P25507645	chr7:134985062-134985106	NM_009294:-237	Stx4a	PROMOTER	0.287	0.454	1433.00	651.08	0.130	1154.76	150.48
A_68_P25507650	chr7:134985680-134985724	NM_009294:381	Stx4a	INSIDE	0.284	0.495	1378.96	682.77	0.140	986.11	138.42
A_68_P25507651	chr7:134985781-134985825	NM_009294:481	Stx4a	INSIDE	0.513	0.509	1031.52	524.89	0.261	745.01	194.56
A_68_P20736172	chr1:157005611-157005658	NM_021433:-198	Stx6	PROMOTER	0.321	0.538	1076.13	578.77	0.173	864.75	149.38
A_68_P27006965	chr10:23869254-23869298	NM_016797:154	Stx7	INSIDE	0.338	0.311	1938.48	603.50	0.105	1686.79	177.73
A_68_P21117852	chr2:32701638-32701686	NM_009295:1090	Stxbp1	INSIDE	0.231	0.465	1469.55	683.45	0.108	1104.27	118.87
A_68_P28012997	chr11:90499182-90499226	NM_011505:218	Stxbp4	INSIDE	0.657	0.648	1058.56	686.34	0.426	718.26	306.10
A_68_P30680167	chr16:37384851-37384895	NM_001114611:172	Stxbp5l	INSIDE	0.566	0.629	1870.26	1176.79	0.356	1586.46	565.03
A_68_P28402110	chr12:46174903-46174947	NM_144552:546	Stxbp6	INSIDE	0.223	0.691	2217.95	1531.70	0.154	1492.17	229.35
A_68_P29570219	chr14:45970863-45970907	NM_019637:24	Styx	INSIDE	0.221	0.507	3184.68	1614.61	0.112	2349.79	263.33
A_68_P24117827	chr5:136254305-136254349	NM_029659:-71	Styxl1	DIVERGENT_PROMOTER	0.433	0.535	1742.21	931.42	0.231	1395.04	322.94
A_68_P32143669	chr19:46471505-46471549	NM_001025391:119	Sufu	INSIDE	0.227	0.568	3769.01	2140.86	0.129	2680.27	344.84
A_68_P25949498	chr8:72567097-72567141	NM_027481:407	Sugp1	INSIDE	0.210	0.674	1080.25	727.73	0.141	883.59	124.97
A_68_P25949499	chr8:72567227-72567271	NM_027481:537	Sugp1	INSIDE	0.616	0.530	1543.33	817.97	0.327	1335.24	436.21
A_68_P25950579	chr8:72758186-72758230	NM_001168290:84	Sugp2	INSIDE	0.200	0.491	1547.57	759.29	0.098	1268.99	124.75
A_68_P28154079	chr11:115397941-115397990	NM_133354:-421	Sumo2	PROMOTER	0.142	0.550	1981.25	1090.26	0.078	1531.33	119.91
A_68_P27269198	chr10:77068646-77068690	NM_019929:-310	Sumo3	PROMOTER	0.315	0.606	1287.95	780.64	0.191	1038.66	198.12
A_68_P24134127	chr5:139676250-139676294	NM_024451:-351	Sun1	PROMOTER	0.215	0.545	3313.35	1806.29	0.117	2218.98	259.55
A_68_P24134128	chr5:139676346-139676390	NM_024451:-255	Sun1	PROMOTER	0.312	0.728	2664.34	1940.32	0.227	1851.12	420.40
A_68_P24134129	chr5:139676494-139676538	NM_024451:-107	Sun1	PROMOTER	0.225	0.479	2168.67	1039.52	0.108	1474.69	159.26
A_68_P24134132	chr5:139676747-139676791	NM_024451:145	Sun1	INSIDE	0.226	0.529	4932.70	2610.57	0.120	3774.58	451.15
A_68_P27542562	chr10:128106343-128106387	NM_173733:4610	Suox	DOWNSTREAM	0.452	0.510	1549.51	790.34	0.231	1277.90	294.62
A_68_P29605233	chr14:52816767-52816813	NM_033618:124	Supt16h	INSIDE	0.257	0.546	1965.22	1073.44	0.140	1373.23	192.89
A_68_P29605238	chr14:52817188-52817232	NM_033618:-296	Supt16h	PROMOTER	0.629	0.713	1878.41	1339.91	0.449	1684.43	755.82
A_68_P31197213	chr17:44914413-44914457	NM_178652:315	Supt3h	INSIDE	0.276	0.523	1541.87	807.13	0.145	1330.11	192.22
A_68_P27995859	chr11:87550663-87550719	NM_009296:-376	Supt4h1	PROMOTER	0.220	0.549	1312.14	719.86	0.120	981.68	118.25
A_68_P27995863	chr11:87551226-87551270	NM_009296:182	Supt4h1	INSIDE	0.532	0.552	1604.92	885.25	0.293	1244.19	364.88
A_68_P25021279	chr7:29123215-29123259	NM_013676:502	Supt5h	INSIDE	0.377	0.553	3336.64	1843.66	0.208	2427.40	505.73
A_68_P21081135	chr2:26772103-26772147	NM_013677:-74	Surf1	PROMOTER	0.181	0.625	4877.48	3050.33	0.113	3438.21	389.04
A_68_P21081136	chr2:26772191-26772235	NM_013677:-162	Surf1	PROMOTER	0.184	0.553	2078.85	1149.54	0.102	1443.85	147.07
A_68_P21081137	chr2:26772296-26772346	NM_013677:-270	Surf1	PROMOTER	0.222	0.469	1525.77	716.25	0.104	1092.17	113.86
A_68_P21081132	chr2:26771837-26771881	NM_013678:-82	Surf2	PROMOTER	0.218	0.581	1757.38	1021.63	0.127	1228.82	155.81
A_68_P21081016	chr2:26758519-26758565	NM_009298:-209	Surf6	PROMOTER	0.216	0.444	1587.69	705.71	0.096	1208.72	115.86
A_68_P22956443	chr4:59450910-59450954	NM_001163288:573	Susd1	INSIDE	0.509	0.730	6056.34	4421.95	0.372	4740.52	1762.26
A_68_P32241629	chrX:7651043-7651087	NM_011514:822	Suv39h1	INSIDE	0.072	1.414	2929.62	4142.89	0.102	1185.63	120.42
A_68_P32241630	chrX:7651107-7651151	NM_011514:758	Suv39h1	INSIDE	0.258	1.452	3468.64	5035.74	0.375	1484.14	556.46
A_68_P20962665	chr2:3391654-3391704	NM_022724:580	Suv39h2	INSIDE	0.242	0.594	1232.45	732.22	0.144	848.70	122.23
A_68_P20962671	chr2:3392446-3392492	NM_022724:-210	Suv39h2	PROMOTER	0.519	0.521	984.43	513.13	0.270	782.82	211.70
A_68_P31919099	chr19:3767561-3767605	NM_001167884:162	Suv420h1	INSIDE	0.534	0.520	1448.19	752.98	0.278	1192.43	331.36
A_68_P31919102	chr19:3767890-3767935	NM_001167884:492	Suv420h1	INSIDE	0.582	0.368	1882.44	692.33	0.214	1476.78	316.13

ProbeName	Target position of probe on CpG island microarray	TargetID	GeneSymbol	CpG island Description	Ratio of relative methylation (TiO2-NP/Vehicle)	Sham group			TiO_2-H group		
						Relative methylation (Cy-5/Cy-3)	Cy-3 signal (Input)	Cy-5 signal (MeDIP)	Relative methylation (Cy-5/Cy-3)	Cy-3 signal (Input)	Cy-5 signal (MeDIP)
A_68_P31919105	chr19:3768272-3768316	NM_001167884:874	Suv420h1	INSIDE	0.312	0.488	1204.30	588.05	0.152	1004.06	153.05
A_68_P27952008	chr11:79806189-79806233	NM_001163018:-397	Suz12	PROMOTER	0.441	0.572	915.55	524.12	0.252	779.33	196.59
A_68_P29243708	chr13:96731321-96731365	NM_029210:171190	Sv2c	INSIDE	0.323	0.541	1120.33	605.63	0.175	969.44	169.49
A_68_P29243709	chr13:96731427-96731477	NM_029210:171080	Sv2c	INSIDE	0.207	0.697	1088.88	758.67	0.144	842.99	121.36
A_68_P24001850	chr5:114541225-114541269	NM_026805:143	Svop	INSIDE	0.577	4.049	564.96	2287.46	2.338	415.58	971.69
A_68_P32791361	chrX:159326363-159326407	NM_025932:10	Syap1	INSIDE	0.645	5.291	1302.62	6892.50	3.411	603.29	2058.07
A_68_P26022397	chr8:87396203-87396247	NM_001168244:68	Syce2	INSIDE	0.174	1.579	8319.12	13136.59	0.275	5430.36	1492.67
A_68_P23325609	chr4:134487161-134487205	NM_026780:288	Syf2	INSIDE	0.156	0.486	2314.27	1123.92	0.076	1624.03	122.75
A_68_P32290626	chrX:20497844-20497888	NM_001110780:178	Syn1	INSIDE	0.149	1.424	1314.25	1871.98	0.213	567.33	120.62
A_68_P28550371	chr12:76920085-76920143	NM_001005510:810	Syne2	INSIDE	0.178	0.419	2072.35	868.40	0.075	1623.89	121.31
A_68_P31020008	chr17:5941641-5941685	NM_001113352:383	Synj2	INSIDE	0.428	0.643	1695.65	1090.26	0.275	1397.27	384.47
A_68_P25195706	chr7:74904045-74904089	NM_183312:562	Synm	INSIDE	0.538	0.435	1667.21	724.89	0.234	1472.48	344.67
A_68_P25195709	chr7:74904507-74904551	NM_183312:100	Synm	INSIDE	0.633	0.428	3143.10	1344.47	0.271	2529.27	684.46
A_68_P31752323	chr18:60755666-60755710	NM_177340:28271	Synpo	INSIDE	0.220	0.694	2519.68	1747.97	0.153	2012.45	306.92
A_68_P31752537	chr18:60784125-60784169	NM_177340:-187	Synpo	PROMOTER	0.368	0.713	2813.16	2005.75	0.262	2269.24	595.25
A_68_P32239400	chrX:7215668-7215712	NM_009305:-15	Syp	PROMOTER	0.085	1.890	8689.40	16423.24	0.161	3233.06	521.89
A_68_P32239401	chrX:7215812-7215857	NM_009305:129	Syp	INSIDE	0.330	1.564	3404.39	5325.71	0.517	1357.55	701.23
A_68_P32239402	chrX:7215889-7215933	NM_009305:205	Syp	INSIDE	0.146	2.741	2039.56	5590.66	0.400	797.29	319.04
A_68_P28339006	chr12:33638754-33638798	NM_013635:-33	Sypl	PROMOTER	0.601	0.482	1530.53	737.06	0.290	1198.52	347.09
A_68_P22411405	chr3:108029325-108029369	NM_008596:171	Sypl2	INSIDE	0.577	0.591	3969.92	2348.18	0.341	3288.41	1121.94
A_68_P22411407	chr3:108029536-108029580	NM_008596:-41	Sypl2	PROMOTER	0.329	0.624	909.47	567.79	0.206	664.03	136.49
A_68_P21818004	chr2:164287014-164287058	NM_025575:566	Sys1	INSIDE	0.402	0.517	1359.86	703.50	0.208	1188.15	247.05
A_68_P31923329	chr19:4476649-4476693	NM_134164:473	Syt12	INSIDE	0.209	0.584	1908.85	1114.19	0.122	1492.52	181.69
A_68_P20946924	chr1:194862258-194862302	NM_181546:-388	Syt14	PROMOTER	0.303	1.558	5136.92	8003.02	0.472	3535.96	1667.82
A_68_P20634930	chr1:136605441-136605485	NM_009307:62205	Syt2	INSIDE	0.209	0.623	1887.85	1177.05	0.130	1439.02	187.36
A_68_P20634931	chr1:136605584-136605628	NM_009307:62349	Syt2	INSIDE	0.149	0.569	1850.55	1053.27	0.085	1400.73	118.94
A_68_P22386738	chr3:103379153-103379197	NM_018800:-29	Syt6	PROMOTER	0.138	0.547	3752.83	2051.75	0.075	2824.36	212.93
A_68_P22386744	chr3:103379702-103379746	NM_018800:521	Syt6	INSIDE	0.626	0.631	840.40	530.69	0.395	752.89	297.67
A_68_P31932349	chr19:6047162-6047206	NM_001164709:40	Syvn1	INSIDE	0.114	0.633	1976.36	1251.08	0.072	1616.46	116.79
A_68_P31932352	chr19:6047496-6047540	NM_001164709:374	Syvn1	INSIDE	0.491	0.502	1959.48	983.17	0.246	1510.55	372.35
A_68_P23240175	chr4:118081682-118081726	NM_198170:164	Szt2	INSIDE	0.422	0.619	2031.40	1257.39	0.261	1720.99	449.84
A_68_P32958525	chr17:8627413-8627457	NM_009309:147	T	INSIDE	0.436	0.669	1899.94	1271.80	0.292	1570.06	458.17
A_68_P26921705	chr10:7675095-7675141	NM_138667:803	Tab2	INSIDE	0.248	0.490	1294.80	634.17	0.121	1001.60	121.49
A_68_P26921714	chr10:7676666-7676718	NM_138667:-771	Tab2	PROMOTER	0.175	0.629	1484.26	933.95	0.110	1229.81	135.45
A_68_P25725927	chr8:26311587-26311631	NM_177089:313	Tacc1	INSIDE	0.185	0.387	2292.71	887.34	0.072	1693.11	121.27
A_68_P23590346	chr5:34000962-34001006	NM_001040435:189	Tacc3	INSIDE	0.150	0.562	2048.93	1151.90	0.085	1386.66	117.30
A_68_P23590347	chr5:34001167-34001211	NM_001040435:393	Tacc3	INSIDE	0.138	0.532	2087.73	1110.09	0.074	1585.54	116.58
A_68_P23590348	chr5:34001258-34001302	NM_001040435:485	Tacc3	INSIDE	0.280	0.502	1566.40	786.82	0.141	1212.27	170.46
A_68_P28099381	chr11:105927583-105927627	NM_027346:184	Taco1	INSIDE	0.519	0.617	2035.81	1255.81	0.320	1585.00	507.13
A_68_P24521962	chr6:67485386-67485430	NM_020047:408	Tacstd2	INSIDE	0.265	0.563	1048.17	590.62	0.150	817.80	122.34
A_68_P24521963	chr6:67485504-67485548	NM_020047:290	Tacstd2	INSIDE	0.182	0.625	1355.82	847.36	0.114	1084.56	123.42
A_68_P24521964	chr6:67485578-67485622	NM_020047:216	Tacstd2	INSIDE	0.317	0.706	1491.28	1053.38	0.224	1233.32	276.08
A_68_P20799887	chr1:168309926-168309970	NM_030245:651	Tada1	INSIDE	0.357	0.628	1726.09	1083.70	0.224	1385.60	310.29
A_68_P23607899	chr5:36826309-36826353	NM_001170454:604	Tada2b	INSIDE	0.232	0.687	2737.89	1879.93	0.159	1874.62	298.03

ProbeName	Target position of probe on CpG island microarray	TargetID	GeneSymbol	CpG island Description	Ratio of relative methylation (TiO2-NP/Vehicle)	Sham group			TiO_2-H group		
						Relative methylation (Cy-5/Cy-3)	Cy-3 signal (Input)	Cy-5 signal (MeDIP)	Relative methylation (Cy-5/Cy-3)	Cy-3 signal (Input)	Cy-5 signal (MeDIP)
A_68_P32564413	chrX:98728110-98728154	NM_001081008:59	Taf1	INSIDE	0.197	1.551	837.42	1298.49	0.306	376.49	115.13
A_68_P27971715	chr11:83286236-83286295	NM_027427:-344	Taf15	DIVERGENT_PROMOTER	0.185	0.604	1459.98	881.99	0.112	1097.66	122.81
A_68_P26215000	chr8:122128943-122128988	NM_021441:175	Taf1c	INSIDE	0.141	0.699	1865.55	1304.40	0.099	1397.70	137.69
A_68_P32147457	chr19:47142031-47142081	NM_177342:-181	Taf5	PROMOTER	0.153	0.465	2668.17	1239.73	0.071	1920.01	136.51
A_68_P32147505	chr19:47149484-47149530	NM_177342:7269	Taf5	INSIDE	0.601	3.208	269.63	865.01	1.927	289.71	558.29
A_68_P31023476	chr17:7165875-7165919	NM_147155:-391	Tagap1	PROMOTER	0.075	0.662	10591.97	7015.01	0.050	7779.16	388.33
A_68_P20833435	chr1:174430368-174430412	NM_178598:14	Tagln2	INSIDE	0.259	0.561	1076.93	604.25	0.145	856.45	124.37
A_68_P20833437	chr1:174430507-174430560	NM_178598:157	Tagln2	INSIDE	0.211	0.632	1185.26	748.97	0.133	872.30	116.31
A_68_P20833440	chr1:174430851-174430895	NM_178598:496	Tagln2	INSIDE	0.208	1.526	3322.33	5068.85	0.318	2115.33	672.02
A_68_P23223128	chr4:114729001-114729045	NM_011527:-3109	Tal1	PROMOTER	0.248	0.628	2838.27	1782.73	0.156	2026.60	315.49
A_68_P23223130	chr4:114729210-114729264	NM_011527:-2895	Tal1	PROMOTER	0.247	0.599	1091.58	653.85	0.148	812.11	120.04
A_68_P23223132	chr4:114729449-114729493	NM_011527:-2661	Tal1	PROMOTER	0.255	0.725	4478.27	3247.27	0.185	3204.40	593.51
A_68_P23223137	chr4:114730092-114730136	NM_011527:-2017	Tal1	PROMOTER	0.402	1.506	1521.71	2292.42	0.606	1280.29	776.17
A_68_P28096909	chr11:105451661-105451705	NM_181071:383	Tanc2	INSIDE	0.424	0.670	1636.27	1096.28	0.284	1449.26	411.89
A_68_P21265453	chr2:61494424-61494471	NM_001164071:63294	Tank	DOWNSTREAM	0.465	0.591	1437.71	849.42	0.275	955.14	262.41
A_68_P21265457	chr2:61494997-61495041	NM_001164071:63865	Tank	DOWNSTREAM	0.176	0.610	1551.97	946.09	0.107	1221.48	130.74
A_68_P25502831	chr7:134028530-134028574	NM_001163774:-71	Taok2	PROMOTER	0.215	0.532	1210.85	643.63	0.114	1093.88	124.80
A_68_P24018824	chr5:117569883-117569927	NM_001199685:-233	Taok3	DIVERGENT_PROMOTER	0.146	0.565	1899.34	1073.81	0.083	1433.26	118.63
A_68_P24018832	chr5:117570657-117570701	NM_001199685:541	Taok3	INSIDE	0.313	1.384	2333.12	3228.26	0.433	1762.46	763.32
A_68_P31152609	chr17:34324546-34324590	NM_001161730:68	Tap1	INSIDE	0.507	0.532	2606.50	1387.87	0.270	2083.49	562.90
A_68_P31152610	chr17:34324622-34324668	NM_001161730:144	Tap1	INSIDE	0.150	0.577	2120.30	1223.20	0.086	1547.66	133.51
A_68_P31150992	chr17:34056973-34057020	NM_001025313:574	Tapbp	INSIDE	0.263	0.458	1400.82	641.17	0.120	1005.80	120.94
A_68_P31151041	chr17:34066176-34066220	NM_001025313:9776	Tapbp	INSIDE	0.256	1.682	1534.38	2580.59	0.431	1104.55	476.40
A_68_P30498347	chr15:102349032-102349076	NM_009319:378	Tarbp2	INSIDE	0.255	0.498	1317.88	656.61	0.127	1019.98	129.51
A_68_P23395556	chr4:148000439-148000483	NM_001003898:645	Tardbp	INSIDE	0.457	0.400	1581.02	632.18	0.183	1092.36	199.45
A_68_P30016102	chr15:11329112-11329156	NM_033074:279	Tars	INSIDE	0.435	1.477	2294.80	3389.60	0.643	1792.16	1152.62
A_68_P23416141	chr4:151406800-151406844	NM_031867:5777	Tas1r1	INSIDE	0.579	2.881	2323.06	6692.06	1.668	1697.79	2831.10
A_68_P21682255	chr2:139892211-139892255	NM_175225:264	Tasp1	INSIDE	0.272	0.556	1569.48	873.04	0.152	1259.13	190.81
A_68_P30251030	chr15:58765208-58765252	NM_175151:55	Tatdn1	INSIDE	0.548	0.598	1946.93	1163.77	0.327	1508.81	493.79
A_68_P30251031	chr15:58765278-58765322	NM_175151:-15	Tatdn1	DIVERGENT_PROMOTER	0.343	0.550	962.61	529.46	0.189	846.52	159.61
A_68_P24760939	chr6:113646833-113646877	NM_001033463:-638	Tatdn2	PROMOTER	0.268	0.607	1884.49	1144.62	0.163	1714.85	278.69
A_68_P24760940	chr6:113646921-113646965	NM_001033463:-550	Tatdn2	PROMOTER	0.643	0.310	1943.88	603.41	0.200	1473.06	294.21
A_68_P24760947	chr6:113647724-113647768	NM_001033463:254	Tatdn2	INSIDE	0.649	0.524	1236.48	648.06	0.340	1076.77	366.41
A_68_P20936014	chr1:192886534-192886578	NM_001163421:255	Tatdn3	INSIDE	0.125	0.724	2061.60	1492.45	0.090	1561.98	140.93
A_68_P20936015	chr1:192886699-192886743	NM_001163421:91	Tatdn3	INSIDE	0.260	0.715	1415.22	1011.79	0.186	1138.24	211.49
A_68_P24452213	chr6:52663843-52663887	NM_025816:142	Tax1bp1	INSIDE	0.402	0.633	1114.69	706.07	0.255	971.26	247.36
A_68_P27915644	chr11:72991108-72991152	NM_029564:546	Tax1bp3	INSIDE	0.561	0.614	1118.93	687.16	0.344	937.63	322.86
A_68_P23749448	chr5:64550705-64550749	NM_019636:-723	Tbc1d1	PROMOTER	0.098	0.588	2519.70	1480.59	0.058	2083.97	119.96
A_68_P23749449	chr5:64550826-64550870	NM_019636:-601	Tbc1d1	PROMOTER	0.584	0.723	1945.85	1406.56	0.422	1543.93	651.55
A_68_P23749880	chr5:64621862-64621906	NM_019636:70435	Tbc1d1	INSIDE	0.548	0.474	1482.14	702.26	0.260	1194.78	310.27
A_68_P25504557	chr7:134351496-134351540	NM_144522:464	Tbc1d10b	INSIDE	0.322	0.584	1091.21	637.29	0.188	691.62	130.07
A_68_P32103865	chr19:38911994-38912038	NM_145952:948	Tbc1d12	INSIDE	0.201	0.409	1826.24	746.26	0.082	1472.60	120.68
A_68_P21101533	chr2:29989110-29989161	NM_146252:-255	Tbc1d13	DIVERGENT_PROMOTER	0.470	0.705	1526.27	1075.63	0.331	1120.23	370.66
A_68_P21101536	chr2:29989398-29989442	NM_146252:30	Tbc1d13	INSIDE	0.454	0.639	1289.64	824.39	0.290	948.16	275.09

ProbeName	Target position of probe on CpG island microarray	TargetID	GeneSymbol	CpG island Description	Ratio of relative methylation (TiO2-NP/Vehicle)	Sham group			TiO_2-H group		
						Relative methylation (Cy-5/Cy-3)	Cy-3 signal (Input)	Cy-5 signal (MeDIP)	Relative methylation (Cy-5/Cy-3)	Cy-3 signal (Input)	Cy-5 signal (MeDIP)
A_68_P21101537	chr2:29989486-29989530	NM_146252:118	Tbc1d13	INSIDE	0.261	0.307	1950.56	598.97	0.080	1516.43	121.56
A_68_P23608243	chr5:36872903-36872947	NM_001113364:348	Tbc1d14	INSIDE	0.226	0.564	1122.37	633.34	0.128	919.62	117.39
A_68_P23608588	chr5:36925165-36925210	NM_133910:-141	Tbc1d14	PROMOTER	0.135	0.507	2254.95	1143.40	0.068	1693.06	115.56
A_68_P27468585	chr10:114688513-114688557	NM_025706:15	Tbc1d15	INSIDE	0.081	1.643	1762.21	2894.45	0.133	1156.78	153.80
A_68_P27468586	chr10:114688584-114688628	NM_025706:-57	Tbc1d15	PROMOTER	0.110	1.338	3399.19	4547.68	0.147	2431.45	357.55
A_68_P25089653	chr7:52103806-52103850	NM_001042655:621	Tbc1d17	INSIDE	0.102	0.696	2513.65	1748.40	0.071	1809.48	128.72
A_68_P23702720	chr5:54200876-54200920	NM_144517:33	Tbc1d19	INSIDE	0.301	0.709	1440.75	1020.80	0.213	1099.03	234.00
A_68_P22889891	chr4:46663047-46663091	NM_198664:3	Tbc1d2	INSIDE	0.609	0.618	2097.59	1295.27	0.376	1552.68	584.26
A_68_P22889897	chr4:46663588-46663636	NM_198664:-541	Tbc1d2	PROMOTER	0.407	0.662	982.47	650.35	0.269	767.27	206.69
A_68_P30405820	chr15:86044641-86044685	NM_145476:-226	Tbc1d22a	PROMOTER	0.347	0.565	2300.99	1300.40	0.196	1734.31	339.90
A_68_P30405821	chr15:86044775-86044819	NM_145476:-92	Tbc1d22a	PROMOTER	0.584	0.707	1291.53	913.22	0.413	1017.61	420.45
A_68_P30405823	chr15:86045014-86045058	NM_145476:148	Tbc1d22a	INSIDE	0.431	1.568	5816.42	9120.58	0.677	3995.28	2702.95
A_68_P30405824	chr15:86045163-86045207	NM_145476:296	Tbc1d22a	INSIDE	0.227	1.886	5680.44	10712.98	0.428	3911.71	1672.61
A_68_P31096038	chr17:24342160-24342204	NM_001163847:325	Tbc1d24	INSIDE	0.145	1.358	5357.45	7277.47	0.197	3351.03	659.28
A_68_P31096050	chr17:24343435-24343480	NM_001163847:-950	Tbc1d24	PROMOTER	0.082	0.687	3700.30	2541.32	0.056	2675.83	150.10
A_68_P31096072	chr17:24345615-24345659	NM_001163847:-3129	Tbc1d24	PROMOTER	0.180	0.609	1317.18	802.77	0.110	1082.52	118.90
A_68_P31096074	chr17:24345798-24345842	NM_001163847:-3313	Tbc1d24	PROMOTER	0.185	0.625	1172.76	733.37	0.115	1013.58	116.95
A_68_P32242102	chrX:7752757-7752806	NM_001166437:526	Tbc1d25	INSIDE	0.173	1.792	950.57	1703.61	0.311	377.75	117.32
A_68_P26717017	chr9:90165120-90165164	NM_194334:465	Tbc1d2b	INSIDE	0.575	0.698	2545.27	1777.14	0.402	1817.95	730.18
A_68_P26717018	chr9:90165190-90165234	NM_194334:395	Tbc1d2b	INSIDE	0.364	0.392	1692.75	663.34	0.143	1481.25	211.49
A_68_P26717019	chr9:90165367-90165411	NM_194334:219	Tbc1d2b	INSIDE	0.453	0.582	1498.80	872.68	0.264	1262.42	332.92
A_68_P29855885	chr14:102007169-102007213	NM_001081278:1218	Tbc1d4	INSIDE	0.234	0.626	2309.75	1445.57	0.146	1716.71	251.11
A_68_P29855886	chr14:102007246-102007295	NM_001081278:1138	Tbc1d4	INSIDE	0.420	0.592	1030.36	609.95	0.249	715.80	178.15
A_68_P29855890	chr14:102007762-102007806	NM_001081278:624	Tbc1d4	INSIDE	0.368	0.515	2418.56	1245.80	0.190	1945.13	368.82
A_68_P29855894	chr14:102008105-102008149	NM_001081278:282	Tbc1d4	INSIDE	0.109	0.544	2654.64	1444.88	0.059	2059.87	121.67
A_68_P31232584	chr17:51318828-51318872	NM_028162:-176	Tbc1d5	PROMOTER	0.666	0.618	1991.74	1231.86	0.412	1666.78	686.17
A_68_P28988729	chr13:43266743-43266787	NM_025935:-36	Tbc1d7	PROMOTER	0.486	0.678	1334.18	905.10	0.330	1075.69	354.84
A_68_P28988730	chr13:43266919-43266963	NM_025935:-212	Tbc1d7	PROMOTER	0.078	0.624	3490.74	2177.97	0.049	2524.62	122.59
A_68_P28988731	chr13:43267019-43267063	NM_025935:-312	Tbc1d7	PROMOTER	0.185	0.602	1345.41	809.27	0.112	1080.68	120.50
A_68_P26013519	chr8:85689979-85690023	NM_001111304:750	Tbc1d9	INSIDE	0.635	0.650	1327.87	862.60	0.413	1162.47	479.86
A_68_P25030456	chr7:31016600-31016644	NM_025548:426	Tbcb	INSIDE	0.253	0.602	1977.07	1191.00	0.152	1410.42	214.77
A_68_P25030460	chr7:31017038-31017082	NM_025548:-12	Tbcb	DIVERGENT_PROMOTER	0.356	0.683	1744.35	1192.22	0.243	1387.00	337.08
A_68_P31209391	chr17:47027623-47027667	NM_178385:75	Tbcc	INSIDE	0.264	0.514	1767.64	909.38	0.136	1393.69	189.26
A_68_P30600190	chr16:22857403-22857447	NM_001081368:218	Tbccd1	INSIDE	0.300	0.504	1833.25	923.31	0.151	1422.85	215.19
A_68_P22538542	chr3:132347071-132347115	NM_173032:-15	Tbck	DIVERGENT_PROMOTER	0.483	0.530	2348.87	1245.55	0.256	1857.29	475.36
A_68_P22538543	chr3:132347141-132347185	NM_173032:55	Tbck	INSIDE	0.538	0.648	1433.46	928.75	0.348	1176.50	410.00
A_68_P22538544	chr3:132347238-132347282	NM_173032:153	Tbck	INSIDE	0.147	0.539	1856.66	1000.80	0.079	1473.03	116.98
A_68_P22538545	chr3:132347325-132347369	NM_173032:239	Tbck	INSIDE	0.242	0.564	1141.22	644.05	0.137	872.71	119.33
A_68_P21986756	chr3:21974577-21974636	NM_030732:-967	Tbl1xr1	PROMOTER	0.187	0.445	1879.65	836.66	0.083	1462.30	122.04
A_68_P21986757	chr3:21974664-21974711	NM_030732:-886	Tbl1xr1	PROMOTER	0.279	0.374	1515.46	566.59	0.104	1158.01	120.71
A_68_P21986761	chr3:21975073-21975117	NM_030732:-479	Tbl1xr1	PROMOTER	0.353	0.519	2249.55	1167.90	0.183	1855.31	339.58
A_68_P21266386	chr2:61643664-61643708	NM_009322:1177	Tbr1	INSIDE	0.235	0.370	1751.30	648.82	0.087	1354.19	118.14
A_68_P21266387	chr2:61643809-61643853	NM_009322:1321	Tbr1	INSIDE	0.378	0.411	3510.27	1442.32	0.155	2675.82	415.74
A_68_P21266443	chr2:61650440-61650484	NM_009322:7953	Tbr1	INSIDE	0.457	0.568	1269.99	721.43	0.260	934.88	242.89

ProbeName	Target position of probe on CpG island microarray	TargetID	GeneSymbol	CpG island Description	Ratio of relative methylation (TiO2-NP/Vehicle)	Sham group			TiO_2-H group		
						Relative methylation (Cy-5/Cy-3)	Cy-3 signal (Input)	Cy-5 signal (MeDIP)	Relative methylation (Cy-5/Cy-3)	Cy-3 signal (Input)	Cy-5 signal (MeDIP)
A_68_P26431942	chr9:37464709-37464753	NM_025289:167	Tbrg1	INSIDE	0.474	0.558	919.30	513.02	0.265	693.35	183.39
A_68_P26431943	chr9:37464781-37464825	NM_025289:95	Tbrg1	INSIDE	0.244	0.463	1252.33	580.45	0.113	1042.16	117.71
A_68_P30385311	chr15:82730377-82730421	NR_027799:-1507	Tbrg3	PROMOTER	0.548	0.707	1452.20	1026.17	0.388	1282.99	497.26
A_68_P27569494	chr11:6526399-6526444	NM_001130457:-351	Tbrg4	PROMOTER	0.285	0.562	990.46	556.67	0.160	735.74	117.74
A_68_P30580553	chr16:18581703-18581747	NM_011532:5338	Tbx1	DOWNSTREAM	0.408	0.656	1584.96	1040.01	0.267	1296.88	346.80
A_68_P30580616	chr16:18588788-18588832	NM_011532:-1748	Tbx1	PROMOTER	0.595	0.415	1778.89	738.00	0.247	1371.09	338.49
A_68_P30580619	chr16:18589104-18589148	NM_011532:-2064	Tbx1	PROMOTER	0.561	0.697	2396.69	1671.42	0.391	1860.59	727.67
A_68_P30580630	chr16:18590465-18590509	NM_011532:-3424	Tbx1	PROMOTER	0.262	0.636	1959.90	1246.80	0.167	1509.33	251.50
A_68_P26706396	chr9:87624611-87624655	NM_023814:1463	Tbx18	INSIDE	0.202	0.428	1771.06	757.16	0.086	1387.00	119.78
A_68_P26706400	chr9:87625023-87625067	NM_023814:1051	Tbx18	INSIDE	0.635	0.436	1733.04	755.80	0.277	1521.64	421.40
A_68_P26706408	chr9:87625899-87625944	NM_023814:174	Tbx18	INSIDE	0.159	0.689	1381.98	952.27	0.110	1081.58	118.54
A_68_P26706479	chr9:87634192-87634246	NM_023814:-8123	Tbx18	PROMOTER	0.279	0.466	1263.18	588.70	0.130	937.83	121.87
A_68_P26706481	chr9:87634446-87634490	NM_023814:-8373	Tbx18	PROMOTER	0.426	0.636	1528.24	972.50	0.271	1240.49	336.63
A_68_P26706484	chr9:87634686-87634730	NM_023814:-8613	Tbx18	PROMOTER	0.517	0.338	1611.29	544.04	0.175	1292.66	225.65
A_68_P27985513	chr11:85643144-85643194	NM_009324:-2948	Tbx2	PROMOTER	0.194	0.576	1622.49	933.82	0.112	1070.78	119.62
A_68_P27985539	chr11:85646087-85646145	NM_009324:0	Tbx2	INSIDE	0.072	1.485	1523.17	2261.27	0.107	1092.65	116.97
A_68_P27985540	chr11:85646214-85646259	NM_009324:120	Tbx2	INSIDE	0.091	1.680	960.96	1614.69	0.154	766.00	117.73
A_68_P27985547	chr11:85647100-85647150	NM_009324:1008	Tbx2	INSIDE	0.598	2.876	710.64	2044.07	1.719	539.30	926.94
A_68_P27985579	chr11:85650619-85650663	NM_009324:4524	Tbx2	INSIDE	0.245	0.454	1767.83	802.03	0.111	1126.42	125.20
A_68_P27985638	chr11:85657554-85657604	NM_009324:11462	Tbx2	DOWNSTREAM	0.221	0.635	2955.37	1878.11	0.141	2099.88	295.11
A_68_P26363429	chr9:24578655-24578699	NM_194263:71	Tbx20	INSIDE	0.337	0.556	1024.81	569.50	0.187	912.00	170.62
A_68_P28048726	chr11:96976651-96976695	NM_019507:-27	Tbx21	PROMOTER	0.513	0.478	1637.70	783.49	0.246	1353.46	332.43
A_68_P24033990	chr5:120120273-120120317	NM_011535:-383	Tbx3	PROMOTER	0.261	0.394	1541.12	606.70	0.103	1242.43	127.87
A_68_P24034078	chr5:120130938-120130982	NM_011535:10283	Tbx3	INSIDE	0.448	0.550	1811.26	996.52	0.247	1513.80	373.50
A_68_P24034079	chr5:120131042-120131086	NM_011535:10387	Tbx3	INSIDE	0.360	0.706	2245.57	1586.31	0.255	1559.85	397.14
A_68_P24035159	chr5:120317204-120317248	NM_011537:32555	Tbx5	INSIDE	0.307	1.519	3033.48	4606.35	0.467	1973.23	920.98
A_68_P21912031	chr2:181414879-181414923	NM_009326:-114	Tcea2	DIVERGENT_PROMOTER	0.253	0.574	1062.47	609.89	0.145	804.11	116.95
A_68_P21912033	chr2:181415118-181415162	NM_009326:126	Tcea2	INSIDE	0.410	0.504	1224.04	616.75	0.206	930.26	192.01
A_68_P21912034	chr2:181415230-181415274	NM_009326:238	Tcea2	INSIDE	0.189	0.419	2077.63	871.07	0.079	1646.34	130.75
A_68_P23332903	chr4:135804530-135804574	NM_011542:681	Tcea3	INSIDE	0.071	1.422	4105.23	5839.24	0.101	2502.04	252.30
A_68_P23332904	chr4:135804629-135804673	NM_011542:779	Tcea3	INSIDE	0.252	1.454	8931.15	12984.54	0.367	6266.69	2298.48
A_68_P31655787	chr18:42671213-42671257	NM_001039474:94	Tcerg1	INSIDE	0.514	0.558	2278.01	1270.94	0.287	1850.43	530.33
A_68_P25571660	chr7:145588945-145588989	NM_183289:447	Tcerg1l	INSIDE	0.243	0.622	2394.00	1488.74	0.151	2010.74	303.62
A_68_P30385400	chr15:82742401-82742445	NM_001114140:142	Tcf20	INSIDE	0.189	1.406	5304.86	7456.46	0.266	3667.11	976.41
A_68_P26239528	chr8:125898007-125898051	NM_001037877:294	Tcf25	INSIDE	0.229	0.415	1480.75	613.93	0.095	1293.94	122.71
A_68_P26239532	chr8:125898381-125898425	NM_001037877:668	Tcf25	INSIDE	0.268	2.679	2303.14	6169.57	0.719	1727.72	1241.70
A_68_P24543641	chr6:72739120-72739164	NM_001079822:-192	Tcf7l1	PROMOTER	0.638	0.497	3199.68	1591.50	0.317	2630.69	834.83
A_68_P32196175	chr19:55817146-55817190	NM_001142918:869	Tcf7l2	INSIDE	0.246	0.608	2512.88	1527.96	0.150	1820.57	272.60
A_68_P28974118	chr13:40818211-40818255	NM_001122948:7580	Tcfap2a	INSIDE	0.225	0.595	1079.99	642.85	0.134	889.72	119.32
A_68_P28974163	chr13:40823956-40824000	NM_001122948:1834	Tcfap2a	INSIDE	0.443	1.386	4339.19	6013.21	0.614	3160.68	1941.24
A_68_P28974165	chr13:40824171-40824219	NM_001122948:1618	Tcfap2a	INSIDE	0.623	0.702	1295.96	909.52	0.437	1130.91	494.40
A_68_P28974205	chr13:40828820-40828871	NM_001122948:-3033	Tcfap2a	PROMOTER	0.153	0.716	1524.23	1091.89	0.110	1085.20	119.10
A_68_P28974206	chr13:40828928-40828972	NM_001122948:-3138	Tcfap2a	PROMOTER	0.320	0.595	1180.07	702.09	0.191	875.83	166.86
A_68_P28974207	chr13:40829034-40829090	NM_001122948:-3250	Tcfap2a	PROMOTER	0.215	0.599	1215.48	727.99	0.129	918.48	118.31

ProbeName	Target position of probe on CpG island microarray	TargetID	GeneSymbol	CpG island Description	Ratio of relative methylation (TiO2-NP/Vehicle)	Sham group			TiO_2-H group		
						Relative methylation (Cy-5/Cy-3)	Cy-3 signal (Input)	Cy-5 signal (MeDIP)	Relative methylation (Cy-5/Cy-3)	Cy-3 signal (Input)	Cy-5 signal (MeDIP)
A_68_P28974244	chr13:40833219-40833263	NM_011547:-4048	Tcfap2a	PROMOTER	0.177	0.669	1395.70	934.25	0.119	1036.11	122.84
A_68_P28974246	chr13:40833476-40833520	NM_011547:-4306	Tcfap2a	PROMOTER	0.636	0.687	1336.42	917.93	0.437	1185.03	517.86
A_68_P20066611	chr1:19199816-19199872	NM_001025305:-2290	Tcfap2b	PROMOTER	0.543	0.645	1427.03	920.39	0.350	1101.28	385.88
A_68_P21864922	chr2:172376858-172376902	NM_001159696:390	Tcfap2c	INSIDE	0.243	0.596	1295.26	771.39	0.145	996.76	144.38
A_68_P23281702	chr4:126409701-126409745	NM_198960:3791	Tcfap2e	INSIDE	0.396	0.646	816.79	527.67	0.256	636.94	163.10
A_68_P23281723	chr4:126412326-126412376	NM_198960:1163	Tcfap2e	INSIDE	0.230	0.514	1321.82	679.86	0.119	1017.52	120.60
A_68_P30510672	chr16:4541712-4541756	NM_031182:17986	Tcfap4	DOWNSTREAM	0.465	0.579	1939.95	1122.57	0.269	1486.61	399.77
A_68_P30510783	chr16:4558483-4558531	NM_031182:1214	Tcfap4	INSIDE	0.224	0.644	1002.74	645.57	0.144	820.88	118.54
A_68_P30510784	chr16:4558577-4558621	NM_031182:1122	Tcfap4	INSIDE	0.244	0.518	1220.51	631.83	0.126	1044.20	131.77
A_68_P30510785	chr16:4558716-4558760	NM_031182:982	Tcfap4	INSIDE	0.509	0.429	2293.06	983.82	0.218	1732.25	378.00
A_68_P30510786	chr16:4558818-4558862	NM_031182:880	Tcfap4	INSIDE	0.094	0.496	3581.30	1774.60	0.046	2543.23	118.19
A_68_P30510787	chr16:4558890-4558934	NM_031182:808	Tcfap4	INSIDE	0.424	0.614	7233.98	4440.62	0.260	4912.91	1279.00
A_68_P30510788	chr16:4558960-4559004	NM_031182:738	Tcfap4	INSIDE	0.224	0.646	4333.57	2798.33	0.145	3231.19	467.51
A_68_P30486394	chr15:100382394-100382438	NM_033476:-38	Tcfcp2	PROMOTER	0.199	0.611	2300.39	1405.40	0.122	1870.95	227.84
A_68_P20552784	chr1:120524752-120524796	NM_023755:253	Tcfcp2l1	INSIDE	0.435	0.553	1832.94	1014.46	0.241	1488.49	358.16
A_68_P31213960	chr17:47874243-47874287	NM_001161723:-615	Tcfeb	PROMOTER	0.659	0.682	2676.72	1826.49	0.450	2273.36	1022.84
A_68_P31213967	chr17:47875064-47875108	NM_001161723:207	Tcfeb	INSIDE	0.177	0.697	1858.49	1295.83	0.123	1342.55	165.59
A_68_P22336392	chr3:93247658-93247702	NM_001163098:1429	Tchh	INSIDE	0.272	0.510	1225.25	625.43	0.139	869.52	120.72
A_68_P22336394	chr3:93247847-93247891	NM_001163098:1617	Tchh	INSIDE	0.114	0.530	2607.52	1382.63	0.060	1967.41	118.88
A_68_P22336402	chr3:93248746-93248790	NM_001163098:2517	Tchh	INSIDE	0.618	0.715	1596.61	1141.44	0.442	1243.86	549.45
A_68_P22336405	chr3:93249236-93249280	NM_001163098:3007	Tchh	INSIDE	0.461	0.638	1908.11	1216.85	0.294	1482.56	435.74
A_68_P22336416	chr3:93250348-93250392	NM_001163098:4119	Tchh	INSIDE	0.320	0.503	1545.93	777.32	0.161	1305.03	210.06
A_68_P22336429	chr3:93251869-93251913	NM_001163098:5639	Tchh	INSIDE	0.285	0.544	3864.49	2102.53	0.155	2807.29	435.78
A_68_P24005588	chr5:115158042-115158088	NM_029992:277	Tchp	INSIDE	0.082	0.733	2868.20	2103.62	0.060	2061.91	124.42
A_68_P31120331	chr17:28216591-28216635	NM_013687:917	Tcp11	INSIDE	0.669	0.714	1324.09	945.25	0.477	1091.45	521.14
A_68_P26814193	chr9:108208141-108208185	NM_133986:120	Tcta	INSIDE	0.154	0.478	2565.09	1226.13	0.073	1787.83	131.22
A_68_P30652355	chr16:32420130-32420180	NM_025329:367	Tctex1d2	INSIDE	0.278	0.415	1761.15	730.98	0.115	1293.59	149.40
A_68_P32203265	chr19:56896867-56896911	NM_001002238:-3810	Tdrd1	DIVERGENT_PROMOTER	0.327	0.652	2194.29	1431.00	0.214	1674.06	357.47
A_68_P22338873	chr3:94216925-94216977	NM_028307:-289	Tdrkh	PROMOTER	0.345	0.614	1227.17	753.72	0.212	819.41	173.68
A_68_P22338874	chr3:94217114-94217158	NM_028307:-103	Tdrkh	PROMOTER	0.314	0.599	1901.27	1139.41	0.188	1400.04	263.38
A_68_P22338876	chr3:94217322-94217366	NM_028307:105	Tdrkh	INSIDE	0.156	1.368	9561.43	13081.72	0.214	6500.13	1391.10
A_68_P25425954	chr7:119822563-119822607	NM_001166584:-249	Tead1	PROMOTER	0.591	0.696	1116.05	776.37	0.411	827.84	340.06
A_68_P25425956	chr7:119822840-119822884	NM_001166584:29	Tead1	INSIDE	0.344	0.469	1119.16	524.58	0.161	856.78	138.13
A_68_P25091864	chr7:52471284-52471328	NM_011565:184	Tead2	INSIDE	0.236	1.385	3920.49	5428.73	0.327	2823.33	923.91
A_68_P31121931	chr17:28478403-28478447	NM_001204156:8867	Tead3	INSIDE	0.543	0.478	1439.41	687.77	0.260	1169.74	303.73
A_68_P24838282	chr6:128250695-128250739	NM_001080979:115	Tead4	INSIDE	0.318	0.286	7210.52	2061.40	0.091	5771.82	524.63
A_68_P24160097	chr5:144984259-144984304	NM_027410:166	Tecpr1	INSIDE	0.240	0.586	2682.15	1571.99	0.141	1836.21	258.47
A_68_P26015637	chr8:86118596-86118640	NM_027179:-228	Tecr	PROMOTER	0.580	0.569	1785.36	1015.03	0.330	1281.21	422.42
A_68_P30379763	chr15:81642166-81642210	NM_017376:345	Tef	INSIDE	0.577	4.365	785.27	3427.49	2.518	798.52	2010.95
A_68_P31104079	chr17:25611509-25611553	NM_027951:2996	Tekt4	INSIDE	0.290	0.538	2290.27	1232.73	0.156	1651.83	258.06
A_68_P30542067	chr16:10395304-10395348	NM_001099275:215	Tekt5	INSIDE	0.664	0.689	1815.48	1251.03	0.458	1387.89	635.07
A_68_P31101668	chr17:25252658-25252702	NM_027880:170	Telo2	INSIDE	0.278	0.569	902.12	513.41	0.158	731.53	115.79
A_68_P26082617	chr8:97844517-97844561	NM_028532:9026	Tepp	INSIDE	0.247	0.715	3769.19	2693.51	0.176	2829.49	499.21
A_68_P26082618	chr8:97844602-97844646	NM_028532:9112	Tepp	INSIDE	0.218	0.488	1408.76	687.38	0.106	1097.57	116.57

ProbeName	Target position of probe on CpG island microarray	TargetID	GeneSymbol	CpG island Description	Ratio of relative methylation (TiO2-NP/Vehicle)	Sham group			TiO_2-H group		
						Relative methylation (Cy-5/Cy-3)	Cy-3 signal (Input)	Cy-5 signal (MeDIP)	Relative methylation (Cy-5/Cy-3)	Cy-3 signal (Input)	Cy-5 signal (MeDIP)
A_68_P22349233	chr3:96218809-96218853	NR_001579:-74	Terc	PROMOTER	0.276	0.669	2629.17	1759.65	0.185	2121.83	392.50
A_68_P20052698	chr1:15795876-15795920	NM_009352:160	Terf1	INSIDE	0.453	0.461	1961.90	903.56	0.209	1491.05	311.09
A_68_P26143093	chr8:109619905-109619949	NM_009353:519	Terf2	INSIDE	0.203	0.656	2194.17	1440.32	0.133	1586.25	211.56
A_68_P29141046	chr13:73764254-73764302	NM_009354:-170	Tert	PROMOTER	0.265	1.714	4654.65	7976.14	0.454	2975.72	1350.69
A_68_P29141047	chr13:73764398-73764442	NM_009354:-28	Tert	PROMOTER	0.164	1.730	2630.18	4549.43	0.284	1706.64	484.87
A_68_P24265240	chr6:17015458-17015502	NM_207176:332	Tes	INSIDE	0.585	0.500	1068.22	534.25	0.293	888.76	260.11
A_68_P22871718	chr4:43455695-43455739	NM_011571:568	Tesk1	INSIDE	0.358	0.440	1213.47	533.81	0.157	959.00	150.94
A_68_P22871719	chr4:43455836-43455880	NM_011571:710	Tesk1	INSIDE	0.208	0.547	1291.16	706.00	0.114	1078.75	122.79
A_68_P27188397	chr10:62349624-62349668	NM_027384:-6884	Tet1	PROMOTER	0.580	0.470	1401.47	658.65	0.273	1197.01	326.31
A_68_P27188402	chr10:62350034-62350078	NM_027384:-7294	Tet1	PROMOTER	0.240	0.498	1184.98	590.54	0.119	979.41	116.97
A_68_P24593530	chr6:83391357-83391401	NM_183138:294	Tet3	INSIDE	0.268	0.553	2332.34	1290.74	0.148	1475.98	218.98
A_68_P28102660	chr11:106473862-106473908	NM_198292:360	Tex2	INSIDE	0.093	0.613	3115.84	1908.84	0.057	2226.23	127.47
A_68_P26804660	chr9:106587656-106587700	NM_011573:320	Tex264	INSIDE	0.608	0.587	1043.54	612.71	0.357	872.80	311.83
A_68_P26804665	chr9:106588204-106588248	NM_011573:-228	Tex264	PROMOTER	0.384	0.535	3120.21	1668.26	0.205	2397.02	492.12
A_68_P26628115	chr9:72339784-72339828	NM_009359:-43	Tex9	PROMOTER	0.518	0.579	922.11	534.25	0.300	796.02	239.04
A_68_P27234342	chr10:70700324-70700368	NM_009360:446	Tfam	INSIDE	0.158	0.561	3070.69	1723.97	0.089	2439.78	216.40
A_68_P31006134	chr17:3557457-3557501	NM_146074:235	Tfb1m	INSIDE	0.598	0.658	2450.16	1611.56	0.393	1941.65	763.90
A_68_P31006135	chr17:3557579-3557623	NM_146074:113	Tfb1m	INSIDE	0.232	0.563	1307.09	735.85	0.131	902.22	117.79
A_68_P20870808	chr1:181476368-181476413	NM_008249:8	Tfb2m	INSIDE	0.121	0.557	3069.15	1710.86	0.068	2181.28	147.28
A_68_P30782603	chr16:56717699-56717743	NM_019678:-253	Tfg	PROMOTER	0.377	0.639	1044.09	666.87	0.241	911.00	219.11
A_68_P23991446	chr5:112755370-112755414	NM_018783:4	Tfip11	INSIDE	0.360	0.584	1880.97	1097.98	0.210	1351.56	284.16
A_68_P24946431	chr7:3581260-3581308	NM_023524:202	Tfpt	INSIDE	0.280	0.468	1175.23	549.55	0.131	910.45	119.17
A_68_P30653345	chr16:32608966-32609010	NM_011638:7	Tfrc	INSIDE	0.280	0.544	1547.28	841.30	0.152	1262.45	192.09
A_68_P30653349	chr16:32609415-32609459	NM_011638:455	Tfrc	INSIDE	0.361	0.620	1844.75	1144.05	0.224	1438.90	321.89
A_68_P30653350	chr16:32609484-32609540	NM_011638:531	Tfrc	INSIDE	0.239	0.555	1029.36	571.46	0.132	871.59	115.44
A_68_P24608768	chr6:86145664-86145708	NM_031199:442	Tgfa	INSIDE	0.201	0.461	1548.42	713.33	0.092	1238.73	114.54
A_68_P20908721	chr1:188528261-188528311	NM_009367:1585	Tgfb2	INSIDE	0.181	0.709	1323.93	938.52	0.128	953.64	122.07
A_68_P22894258	chr4:47365465-47365509	NM_009370:-690	Tgfbr1	PROMOTER	0.334	0.574	1331.23	764.62	0.192	892.00	171.18
A_68_P22894259	chr4:47365606-47365650	NM_009370:-548	Tgfbr1	PROMOTER	0.149	1.682	4487.99	7547.65	0.251	2946.64	740.06
A_68_P22894260	chr4:47365694-47365738	NM_009370:-460	Tgfbr1	PROMOTER	0.055	1.745	2338.09	4078.79	0.095	1466.34	139.55
A_68_P22894262	chr4:47366025-47366069	NM_009370:-130	Tgfbr1	PROMOTER	0.573	0.726	2531.27	1837.14	0.416	2232.68	929.32
A_68_P31331157	chr17:71203092-71203136	NM_001164074:-242	Tgif1	PROMOTER	0.297	0.622	1196.16	744.42	0.185	981.70	181.46
A_68_P24542638	chr6:72566666-72566710	NM_009444:54	Tgoln2	INSIDE	0.618	0.711	2406.13	1709.67	0.439	1943.97	854.01
A_68_P24542639	chr6:72566738-72566782	NM_009444:-18	Tgoln2	PROMOTER	0.565	0.570	1142.68	651.42	0.322	947.30	305.29
A_68_P21875215	chr2:174241468-174241512	NM_020580:186	Th1l	INSIDE	0.277	0.694	1417.70	983.20	0.192	1072.06	206.01
A_68_P26136326	chr8:108379372-108379416	NM_021513:392	Thap11	INSIDE	0.612	0.724	1593.83	1153.63	0.443	1325.04	586.55
A_68_P23891540	chr5:92391970-92392018	NR_028429:87	Thap6	INSIDE	0.217	0.598	1218.87	728.40	0.130	950.96	123.28
A_68_P30574321	chr16:17530648-17530693	NM_026909:475	Thap7	INSIDE	0.321	0.704	1900.52	1338.88	0.226	1359.50	307.82
A_68_P30574323	chr16:17530866-17530910	NM_026909:257	Thap7	INSIDE	0.569	0.514	1312.67	675.16	0.293	1058.94	310.03
A_68_P27765165	chr11:45768563-45768620	NM_001080969:405	Thg1l	INSIDE	0.271	0.507	1104.46	559.49	0.138	839.87	115.48
A_68_P24534825	chr6:71094013-71094057	NM_001033929:336	Thnsl2	INSIDE	0.550	0.529	2103.11	1112.05	0.291	1601.07	465.56
A_68_P31487558	chr18:9957999-9958043	NM_153552:-157	Thoc1	PROMOTER	0.649	0.503	1773.60	891.31	0.326	1342.73	437.60
A_68_P32340124	chrX:39264668-39264712	NM_001033422:388	Thoc2	INSIDE	0.085	2.072	1610.88	3338.30	0.176	677.99	119.38
A_68_P32340125	chrX:39264784-39264828	NM_001033422:272	Thoc2	INSIDE	0.469	1.577	6527.99	10294.78	0.740	2716.10	2010.36

ProbeName	Target position of probe on CpG island microarray	TargetID	GeneSymbol	CpG island Description	Ratio of relative methylation (TiO2-NP/Vehicle)	Sham group			TiO_2-H group		
						Relative methylation (Cy-5/Cy-3)	Cy-3 signal (Input)	Cy-5 signal (MeDIP)	Relative methylation (Cy-5/Cy-3)	Cy-3 signal (Input)	Cy-5 signal (MeDIP)
A_68_P32340126	chrX:39264963-39265012	NM_001033422:91	Thoc2	INSIDE	0.109	3.117	2622.57	8173.43	0.341	866.85	295.27
A_68_P32340127	chrX:39265129-39265173	NM_001033422:-72	Thoc2	PROMOTER	0.294	2.007	3746.06	7518.66	0.590	1500.11	884.89
A_68_P27560196	chr11:4795414-4795458	NM_172438:91	Thoc5	INSIDE	0.499	0.703	1912.31	1344.39	0.351	1524.05	534.85
A_68_P31092955	chr17:23810698-23810747	NM_001008425:15	Thoc6	INSIDE	0.213	0.505	1823.42	921.40	0.108	1354.68	145.90
A_68_P31092956	chr17:23810793-23810838	NM_001008425:-78	Thoc6	PROMOTER	0.443	0.613	2258.70	1383.83	0.271	1685.84	457.61
A_68_P29418914	chr14:14793253-14793298	NM_025435:464	Thoc7	INSIDE	0.257	1.810	1746.58	3161.01	0.465	1344.66	625.92
A_68_P29418923	chr14:14794239-14794283	NM_025435:-521	Thoc7	PROMOTER	0.407	0.537	1962.94	1053.66	0.218	1739.95	379.84
A_68_P28058193	chr11:98603344-98603393	NM_178060:182	Thra	INSIDE	0.281	0.339	1682.81	571.28	0.095	1271.26	121.06
A_68_P23278807	chr4:125879742-125879788	NM_146153:190	Thrap3	INSIDE	0.169	0.589	2022.15	1191.30	0.100	1432.91	142.76
A_68_P25465170	chr7:126863806-126863854	NM_145585:449	Thumpd1	INSIDE	0.220	0.546	1174.82	641.66	0.120	972.79	116.95
A_68_P25465171	chr7:126863923-126863967	NM_145585:335	Thumpd1	INSIDE	0.153	0.457	2540.49	1162.22	0.070	1716.53	120.47
A_68_P26465329	chr9:43851804-43851848	NM_009382:360	Thy1	INSIDE	0.294	0.683	1981.00	1352.86	0.201	1441.04	289.23
A_68_P25510944	chr7:135603768-135603821	NM_009383:1233	Tial1	INSIDE	0.219	0.551	1134.54	625.30	0.120	977.11	117.70
A_68_P25510946	chr7:135603923-135603972	NM_009383:1080	Tial1	INSIDE	0.176	0.540	1590.33	858.61	0.095	1259.21	119.69
A_68_P25510961	chr7:135605591-135605635	NM_009383:-585	Tial1	PROMOTER	0.368	0.536	1014.76	543.51	0.197	867.11	170.87
A_68_P31004585	chr17:3327130-3327174	NM_001122998:580	Tiam2	INSIDE	0.334	0.526	1005.00	528.23	0.176	833.81	146.50
A_68_P31931515	chr19:5893867-5893911	NM_198634:219	Tigd3	INSIDE	0.291	0.584	1366.26	798.07	0.170	1106.96	187.96
A_68_P30344891	chr15:75741091-75741135	NM_178646:948	Tigd5	INSIDE	0.402	0.694	2910.45	2020.64	0.279	2088.38	583.52
A_68_P30344892	chr15:75741160-75741204	NM_178646:1018	Tigd5	INSIDE	0.340	0.640	3566.60	2282.79	0.218	2745.60	597.69
A_68_P27286214	chr10:80363023-80363067	NM_013895:670	Timm13	INSIDE	0.305	0.591	2288.03	1351.38	0.180	1692.35	305.26
A_68_P27286219	chr10:80363610-80363656	NM_013895:82	Timm13	INSIDE	0.636	0.464	1142.84	529.86	0.295	897.89	264.69
A_68_P27286220	chr10:80363685-80363729	NM_013895:8	Timm13	INSIDE	0.192	0.394	2090.39	823.04	0.075	1737.21	130.99
A_68_P20638618	chr1:137210111-137210155	NM_011590:182	Timm17a	INSIDE	0.549	0.528	2434.31	1285.29	0.290	1764.74	511.79
A_68_P20638619	chr1:137210199-137210243	NM_011590:94	Timm17a	INSIDE	0.370	0.694	1718.46	1192.95	0.257	1320.26	339.28
A_68_P20638621	chr1:137210401-137210445	NM_011590:-108	Timm17a	PROMOTER	0.138	0.737	4696.09	3461.85	0.102	3208.97	325.98
A_68_P25617012	chr8:4275589-4275633	NM_011592:295	Timm44	INSIDE	0.389	0.352	1784.11	628.53	0.137	1462.47	200.17
A_68_P25617013	chr8:4275737-4275784	NM_011592:145	Timm44	INSIDE	0.207	1.605	1445.75	2321.02	0.333	1021.57	339.75
A_68_P25617015	chr8:4275885-4275929	NM_011592:-1	Timm44	PROMOTER	0.418	0.466	1571.46	731.62	0.194	1206.45	234.55
A_68_P25021123	chr7:29097166-29097212	NM_025616:-123	Timm50	PROMOTER	0.138	0.611	3272.01	1999.33	0.084	2232.88	188.39
A_68_P26504538	chr9:50412116-50412160	NM_013897:133	Timm8b	INSIDE	0.324	0.599	3069.78	1838.08	0.194	2210.71	428.87
A_68_P26504539	chr9:50412320-50412364	NM_013897:337	Timm8b	INSIDE	0.121	0.619	2527.45	1565.30	0.075	1687.83	126.48
A_68_P26504540	chr9:50412429-50412473	NM_013897:445	Timm8b	INSIDE	0.528	0.635	957.49	607.85	0.335	808.13	270.70
A_68_P28524523	chr12:72237247-72237292	NM_001024853:393	Timm9	INSIDE	0.274	0.417	1285.30	536.13	0.114	1046.67	119.56
A_68_P27313251	chr10:85763083-85763128	NM_011595:-51	Timp3	PROMOTER	0.313	0.553	2251.16	1244.53	0.173	1867.17	322.71
A_68_P29619287	chr14:56300293-56300337	NM_145705:340	Tinf2	INSIDE	0.290	0.359	1619.23	581.11	0.104	1191.14	123.90
A_68_P22200380	chr3:65331656-65331707	NM_178892:-687	Tiparp	PROMOTER	0.387	0.468	1309.30	612.30	0.181	1067.33	193.00
A_68_P22200389	chr3:65332655-65332699	NM_178892:308	Tiparp	INSIDE	0.273	0.582	1856.08	1080.83	0.159	1515.63	241.15
A_68_P26421200	chr9:35007442-35007486	NM_001177847:26	Tirap	INSIDE	0.194	0.332	2449.66	813.48	0.064	1934.19	124.68
A_68_P26421202	chr9:35007602-35007646	NM_001177846:-74	Tirap	PROMOTER	0.657	0.573	1140.89	653.58	0.376	972.80	366.21
A_68_P29506485	chr14:31362134-31362189	NM_009388:-173	Tkt	PROMOTER	0.232	0.524	1355.15	709.45	0.122	979.44	119.11
A_68_P23012911	chr4:71860767-71860811	NM_011599:1165	Tle1	INSIDE	0.370	0.453	1503.64	681.47	0.167	1141.27	191.14
A_68_P23012918	chr4:71861537-71861581	NM_011599:395	Tle1	INSIDE	0.251	0.423	1877.73	793.71	0.106	1584.44	168.14
A_68_P27290579	chr10:81033785-81033829	NM_019725:-4470	Tle2	PROMOTER	0.514	0.602	3634.26	2189.17	0.310	2801.37	867.32
A_68_P27290580	chr10:81033862-81033910	NM_019725:-4390	Tle2	PROMOTER	0.259	0.485	1276.90	619.11	0.125	922.00	115.67

ProbeName	Target position of probe on CpG island microarray	TargetID	GeneSymbol	CpG island Description	Ratio of relative methylation (TiO2-NP/Vehicle)	Sham group			TiO_2-H group		
						Relative methylation (Cy-5/Cy-3)	Cy-3 signal (Input)	Cy-5 signal (MeDIP)	Relative methylation (Cy-5/Cy-3)	Cy-3 signal (Input)	Cy-5 signal (MeDIP)
A_68_P27290695	chr10:81050342-81050386	NM_019725:12088	Tle2	INSIDE	0.374	1.682	2059.96	3464.99	0.629	1360.62	856.45
A_68_P26564381	chr9:61220364-61220408	NM_001083927:214	Tle3	INSIDE	0.338	0.459	1810.81	831.47	0.155	1404.45	218.13
A_68_P26564383	chr9:61220517-61220561	NM_001083927:366	Tle3	INSIDE	0.343	0.567	1269.16	720.00	0.195	923.94	179.79
A_68_P26564385	chr9:61220827-61220871	NM_001083927:676	Tle3	INSIDE	0.185	0.471	1635.56	770.05	0.087	1315.33	114.78
A_68_P26564391	chr9:61221577-61221621	NM_001083927:1426	Tle3	INSIDE	0.224	0.561	1189.27	667.05	0.126	919.45	115.66
A_68_P31969894	chr19:14672643-14672687	NM_011600:-191	Tle4	PROMOTER	0.547	0.448	1528.45	684.76	0.245	1323.42	324.57
A_68_P27290791	chr10:81063724-81063768	NM_053254:-101	Tle6	DIVERGENT_PROMOTER	0.528	0.588	1477.87	869.72	0.311	1247.37	387.83
A_68_P32113650	chr19:41280684-41280728	NM_011904:558	Tll2	INSIDE	0.556	0.416	2341.26	973.11	0.231	1880.05	434.34
A_68_P32113654	chr19:41281095-41281139	NM_011904:148	Tll2	INSIDE	0.583	0.324	1788.52	579.01	0.189	1515.62	285.94
A_68_P32113659	chr19:41281622-41281669	NM_011904:-381	Tll2	PROMOTER	0.572	0.506	1114.07	563.53	0.289	704.58	203.69
A_68_P32136713	chr19:45218072-45218116	NM_021901:-7110	Tlx1	PROMOTER	0.388	0.621	874.63	543.49	0.241	668.88	161.26
A_68_P32136793	chr19:45227850-45227894	NM_021901:2668	Tlx1	INSIDE	0.156	0.650	5489.08	3566.55	0.101	3798.26	384.68
A_68_P32136797	chr19:45228297-45228341	NM_021901:3114	Tlx1	INSIDE	0.286	0.574	1014.95	582.93	0.164	765.50	125.67
A_68_P24591244	chr6:83019585-83019631	NM_009392:611	Tlx2	INSIDE	0.211	1.357	2903.42	3939.31	0.287	2041.11	585.21
A_68_P24591245	chr6:83019654-83019698	NM_009392:543	Tlx2	INSIDE	0.157	0.470	2183.16	1026.90	0.074	1742.88	128.83
A_68_P24591254	chr6:83020596-83020640	NM_009392:-399	Tlx2	DIVERGENT_PROMOTER	0.505	0.608	3648.78	2219.56	0.307	2681.41	823.38
A_68_P24591261	chr6:83021489-83021533	NM_009392:-1291	Tlx2	DIVERGENT_PROMOTER	0.628	0.418	1760.59	735.92	0.262	1491.91	391.43
A_68_P27693350	chr11:33102180-33102224	NM_019916:1386	Tlx3	INSIDE	0.620	2.714	6440.35	17478.25	1.684	4142.87	6974.99
A_68_P27693351	chr11:33102254-33102303	NM_019916:1310	Tlx3	INSIDE	0.642	4.137	2358.31	9756.09	2.658	1525.60	4054.39
A_68_P27693353	chr11:33102552-33102596	NM_019916:1014	Tlx3	INSIDE	0.175	0.564	1488.77	839.17	0.098	1262.33	124.20
A_68_P27693363	chr11:33103831-33103878	NM_019916:-266	Tlx3	PROMOTER	0.221	0.453	1492.55	676.80	0.100	1176.29	117.83
A_68_P27693368	chr11:33104357-33104401	NM_019916:-790	Tlx3	PROMOTER	0.248	0.378	1811.26	684.73	0.094	1335.83	125.43
A_68_P25724847	chr8:26127928-26127972	NM_027194:268	Tm2d2	INSIDE	0.143	0.479	2579.85	1236.96	0.069	1716.41	117.76
A_68_P25724849	chr8:26128143-26128187	NM_027194:482	Tm2d2	INSIDE	0.506	0.472	1720.20	811.15	0.239	1345.00	321.23
A_68_P25184109	chr7:72838329-72838373	NM_026795:48	Tm2d3	INSIDE	0.528	0.507	2105.01	1066.45	0.268	1576.93	421.87
A_68_P25184111	chr7:72838614-72838658	NM_026795:334	Tm2d3	INSIDE	0.400	0.349	1726.28	602.90	0.140	1348.93	188.30
A_68_P32114042	chr19:41337768-41337812	NM_133352:704	Tm9sf3	INSIDE	0.138	0.645	1884.33	1215.40	0.089	1308.07	116.34
A_68_P27495880	chr10:119645922-119645966	NM_026617:63	Tmbim4	INSIDE	0.300	0.511	3248.99	1658.80	0.153	2547.88	389.68
A_68_P30479872	chr15:99223536-99223580	NM_001171035:-91	Tmbim6	PROMOTER	0.174	0.487	2046.14	997.19	0.085	1450.77	123.22
A_68_P25459062	chr7:125727948-125727992	NM_172476:230	Tmc7	INSIDE	0.426	0.646	1991.21	1286.03	0.275	1416.69	389.44
A_68_P28167195	chr11:117644840-117644884	NM_001195088:891	Tmc8	INSIDE	0.107	1.657	2818.32	4670.59	0.178	1961.96	348.37
A_68_P20620971	chr1:134257276-134257320	NM_178874:30560	Tmcc2	INSIDE	0.611	3.302	7294.02	24086.94	2.019	4591.99	9270.90
A_68_P20620972	chr1:134257371-134257415	NM_178874:30466	Tmcc2	INSIDE	0.623	3.221	3614.04	11639.46	2.005	2309.75	4632.10
A_68_P20804420	chr1:169238624-169238668	NM_001039483:-154	Tmco1	PROMOTER	0.623	0.542	1321.98	716.47	0.338	1038.25	350.73
A_68_P26140912	chr8:109207098-109207142	NM_173037:153	Tmco7	INSIDE	0.483	0.570	1022.10	582.28	0.275	966.40	266.05
A_68_P26347560	chr9:21314771-21314829	NM_010744:-170	Tmed1	PROMOTER	0.187	0.661	1363.31	901.58	0.124	965.41	119.26
A_68_P26713770	chr9:89599836-89599880	NM_025360:23	Tmed3	INSIDE	0.579	0.526	1640.26	863.54	0.305	1456.47	443.97
A_68_P27567652	chr11:6174705-6174749	NM_134020:114	Tmed4	INSIDE	0.053	1.414	2618.98	3702.34	0.075	1607.48	120.52
A_68_P23970433	chr5:108561687-108561731	NM_028876:-98	Tmed5	DIVERGENT_PROMOTER	0.591	0.563	1286.53	724.64	0.333	1064.53	354.31
A_68_P31677780	chr18:46757146-46757190	NM_025698:21	Tmed7	INSIDE	0.621	0.524	1358.72	712.29	0.325	1254.78	408.22
A_68_P28614747	chr12:88540890-88540934	NM_001033475:267	Tmed8	INSIDE	0.388	0.434	1256.05	544.69	0.168	968.60	163.07
A_68_P22900943	chr4:48598769-48598813	NM_021436:726	Tmeff1	INSIDE	0.177	2.012	3128.67	6294.60	0.357	2112.15	753.81
A_68_P20218132	chr1:50984895-50984939	NM_019790:550	Tmeff2	INSIDE	0.188	0.533	1535.11	818.16	0.100	1207.67	121.27
A_68_P28077838	chr11:102017610-102017654	NM_029649:89	Tmem101	INSIDE	0.211	0.537	1320.23	708.54	0.113	1019.49	115.32

ProbeName	Target position of probe on CpG island microarray	TargetID	GeneSymbol	CpG island Description	Ratio of relative methylation (TiO2-NP/Vehicle)	Sham group			TiO_2-H group		
						Relative methylation (Cy-5/Cy-3)	Cy-3 signal (Input)	Cy-5 signal (MeDIP)	Relative methylation (Cy-5/Cy-3)	Cy-3 signal (Input)	Cy-5 signal (MeDIP)
A_68_P24245776	chr6:13020122-13020168	NM_027992:386	Tmem106b	INSIDE	0.084	1.468	2774.21	4072.07	0.124	1719.67	213.26
A_68_P27892867	chr11:68884346-68884390	NM_025838:58	Tmem107	INSIDE	0.202	0.364	2524.41	919.11	0.074	1745.29	128.44
A_68_P27892868	chr11:68884481-68884525	NM_025838:192	Tmem107	INSIDE	0.313	0.504	1047.51	528.00	0.158	754.41	119.00
A_68_P27892869	chr11:68884583-68884627	NM_025838:294	Tmem107	INSIDE	0.301	0.451	1590.84	718.13	0.136	1179.13	160.48
A_68_P26787848	chr9:103663928-103663974	NM_178638:217	Tmem108	INSIDE	0.140	0.520	2130.84	1107.00	0.073	1676.23	121.91
A_68_P26787851	chr9:103664317-103664362	NM_178638:-172	Tmem108	PROMOTER	0.425	0.641	1147.40	735.64	0.272	971.25	264.61
A_68_P27845263	chr11:60691958-60692002	NM_001168507:560	Tmem11	INSIDE	0.224	0.273	5352.00	1462.81	0.061	4513.81	276.73
A_68_P27845268	chr11:60692459-60692503	NM_001168507:60	Tmem11	INSIDE	0.209	0.462	1528.73	705.52	0.096	1235.84	118.93
A_68_P29508103	chr14:31638633-31638679	NM_028839:-123	Tmem110	PROMOTER	0.474	0.641	1383.44	886.42	0.304	1115.78	338.92
A_68_P24760052	chr6:113481369-113481416	NM_175101:240	Tmem111	INSIDE	0.334	0.605	898.06	543.45	0.202	731.96	147.75
A_68_P26809359	chr9:107436884-107436928	NM_019704:631	Tmem115	INSIDE	0.388	0.468	1517.33	710.48	0.182	1120.49	203.73
A_68_P26809364	chr9:107437458-107437502	NM_019704:1205	Tmem115	INSIDE	0.456	0.565	1452.86	820.34	0.258	1061.04	273.42
A_68_P24117665	chr5:136219665-136219716	NM_172541:352	Tmem120a	INSIDE	0.229	0.419	1817.89	762.17	0.096	1454.78	139.72
A_68_P24053751	chr5:123526467-123526511	NM_001039723:205	Tmem120b	INSIDE	0.263	0.472	1791.91	846.46	0.124	1346.43	167.23
A_68_P24053752	chr5:123526651-123526695	NM_001039723:389	Tmem120b	INSIDE	0.633	0.294	1826.77	537.00	0.186	1374.88	255.77
A_68_P21612305	chr2:127073731-127073775	NM_175145:42	Tmem127	INSIDE	0.619	0.476	1722.70	819.82	0.295	1265.67	372.99
A_68_P21612309	chr2:127074111-127074155	NM_175145:422	Tmem127	INSIDE	0.228	0.615	2264.80	1391.86	0.140	2102.52	294.53
A_68_P23590340	chr5:34000396-34000440	NM_026698:63	Tmem129	INSIDE	0.529	0.552	1864.36	1029.78	0.292	1445.13	422.26
A_68_P23590341	chr5:34000502-34000546	NM_026698:-43	Tmem129	DIVERGENT_PROMOTER	0.190	0.444	1953.75	866.77	0.084	1407.78	118.48
A_68_P20151298	chr1:36995764-36995812	NM_018872:584	Tmem131	INSIDE	0.249	0.669	936.42	626.07	0.166	709.28	117.87
A_68_P20151307	chr1:36996847-36996891	NM_018872:-496	Tmem131	PROMOTER	0.334	0.430	1668.97	718.43	0.144	1315.97	188.97
A_68_P24076583	chr5:127721966-127722010	NM_175432:-207	Tmem132c	PROMOTER	0.253	0.405	1679.29	680.32	0.102	1140.43	116.78
A_68_P24076587	chr5:127722499-127722543	NM_175432:325	Tmem132c	INSIDE	0.426	0.613	2255.62	1381.85	0.261	1684.29	439.13
A_68_P25307027	chr7:96487209-96487253	NM_028343:67	Tmem135	INSIDE	0.292	0.414	3962.50	1640.35	0.121	3189.47	386.18
A_68_P26459754	chr9:42924375-42924419	NM_001034863:257	Tmem136	INSIDE	0.507	0.585	906.18	530.44	0.297	705.80	209.28
A_68_P25033088	chr7:31514777-31514828	NM_027215:-249	Tmem147	PROMOTER	0.335	0.447	1556.57	695.32	0.150	1142.35	171.09
A_68_P28975560	chr13:41111770-41111814	NM_025387:174	Tmem14c	INSIDE	0.280	0.644	1020.72	657.77	0.181	830.66	150.02
A_68_P24541278	chr6:72305613-72305657	NM_144916:158	Tmem150a	INSIDE	0.150	0.711	4004.53	2846.26	0.107	2735.04	292.03
A_68_P23928043	chr5:100587765-100587824	NM_182841:1033	Tmem150c	INSIDE	0.249	0.482	1184.56	570.70	0.120	965.70	115.97
A_68_P31926780	chr19:5082631-5082675	NM_001001885:2825	Tmem151a	INSIDE	0.618	2.379	950.57	2261.20	1.469	759.18	1115.50
A_68_P31926801	chr19:5085238-5085283	NM_001001885:217	Tmem151a	INSIDE	0.656	0.555	1342.06	744.73	0.364	1171.07	426.35
A_68_P26894025	chr9:123168633-123168677	NM_001002267:1253	Tmem158	INSIDE	0.647	0.704	1365.83	961.32	0.455	1099.05	500.15
A_68_P24981572	chr7:17038213-17038257	NM_026938:107	Tmem160	INSIDE	0.246	0.522	1183.90	617.87	0.128	901.40	115.50
A_68_P29189772	chr13:84361804-84361848	NM_175187:-74	Tmem161b	PROMOTER	0.330	0.647	2401.18	1552.43	0.213	1935.16	412.29
A_68_P20596467	chr1:129573905-129573949	NM_028135:672	Tmem163	INSIDE	0.241	0.393	1527.15	600.07	0.095	1231.66	116.79
A_68_P20596468	chr1:129574040-129574084	NM_028135:536	Tmem163	INSIDE	0.542	0.553	1420.07	785.55	0.300	1096.41	328.69
A_68_P20596471	chr1:129574483-129574527	NM_028135:94	Tmem163	INSIDE	0.501	0.699	1868.43	1306.48	0.350	1589.87	557.18
A_68_P20596472	chr1:129574599-129574643	NM_028135:-22	Tmem163	PROMOTER	0.522	0.693	1917.07	1328.71	0.362	1445.26	522.72
A_68_P20596473	chr1:129574700-129574744	NM_028135:-124	Tmem163	PROMOTER	0.243	0.480	1539.93	739.68	0.117	1033.16	120.45
A_68_P32709178	chrX:139116482-139116526	NM_001199360:236	Tmem164	INSIDE	0.457	1.485	2611.94	3879.23	0.678	1209.09	820.00
A_68_P32709179	chrX:139116571-139116615	NM_001199360:324	Tmem164	INSIDE	0.111	3.814	3928.28	14980.50	0.421	1741.08	733.83
A_68_P29211816	chr13:90229293-90229337	NM_025335:43	Tmem167	INSIDE	0.580	0.594	2440.41	1450.01	0.345	1874.10	646.27
A_68_P29211817	chr13:90229380-90229424	NM_025335:131	Tmem167	INSIDE	0.657	0.513	1928.84	989.87	0.337	1446.10	487.23
A_68_P29211818	chr13:90229540-90229584	NM_025335:291	Tmem167	INSIDE	0.627	0.577	1913.81	1104.10	0.362	1438.59	520.70

ProbeName	Target position of probe on CpG island microarray	TargetID	GeneSymbol	CpG island Description	Ratio of relative methylation (TiO2-NP/Vehicle)	Sham group			TiO_2-H group		
						Relative methylation (Cy-5/Cy-3)	Cy-3 signal (Input)	Cy-5 signal (MeDIP)	Relative methylation (Cy-5/Cy-3)	Cy-3 signal (Input)	Cy-5 signal (MeDIP)
A_68_P22413376	chr3:108365347-108365394	NM_026198:14	Tmem167b	INSIDE	0.125	0.560	2408.04	1347.79	0.070	1649.43	114.99
A_68_P20559395	chr1:121809675-121809721	NM_175106:47	Tmem177	INSIDE	0.168	0.682	1364.17	931.04	0.115	1013.87	116.17
A_68_P28750631	chr12:113749099-113749143	NM_178915:251	Tmem179	INSIDE	0.306	0.671	1006.20	675.06	0.205	723.82	148.36
A_68_P28326654	chr12:31269275-31269319	NM_172049:-11	Tmem18	PROMOTER	0.240	0.441	2546.94	1123.07	0.106	1977.26	209.37
A_68_P28326655	chr12:31269403-31269447	NM_172049:117	Tmem18	INSIDE	0.305	0.464	1240.91	575.78	0.141	847.55	119.87
A_68_P32143397	chr19:46423267-46423311	NM_029186:-8081	Tmem180	PROMOTER	0.267	0.449	1558.92	699.81	0.120	1280.80	153.60
A_68_P30572941	chr16:17276967-17277011	NM_177473:596	Tmem191c	INSIDE	0.438	0.513	1018.12	522.72	0.225	875.56	197.04
A_68_P30572946	chr16:17277489-17277533	NM_177473:1118	Tmem191c	INSIDE	0.200	0.586	1278.86	749.53	0.117	1042.69	122.32
A_68_P30572948	chr16:17277681-17277725	NM_177473:1310	Tmem191c	INSIDE	0.411	0.419	1794.88	752.09	0.172	1424.21	245.03
A_68_P30572953	chr16:17278195-17278239	NM_177473:1824	Tmem191c	INSIDE	0.576	0.630	1052.54	663.41	0.363	834.60	302.99
A_68_P20354344	chr1:75476279-75476323	NM_177056:194	Tmem198	INSIDE	0.530	0.438	2068.17	905.45	0.232	1589.86	369.02
A_68_P20354345	chr1:75476353-75476397	NM_177056:268	Tmem198	INSIDE	0.360	0.629	2696.14	1696.53	0.227	1869.52	423.62
A_68_P32008155	chr19:21853166-21853210	NM_001033759:357	Tmem2	INSIDE	0.334	0.550	1254.36	689.67	0.184	1016.56	186.79
A_68_P23309106	chr4:131476303-131476347	NM_001201367:-1361	Tmem200b	PROMOTER	0.551	0.672	1491.79	1002.37	0.370	1377.04	509.77
A_68_P23309110	chr4:131476820-131476864	NM_001201367:-843	Tmem200b	PROMOTER	0.311	0.637	1115.05	710.08	0.198	968.65	191.54
A_68_P31320371	chr17:69186413-69186457	NM_001206661:-41	Tmem200c	PROMOTER	0.468	0.599	1833.77	1098.41	0.281	1413.42	396.60
A_68_P21070912	chr2:25110973-25111017	NM_177344:36	Tmem203	INSIDE	0.464	0.493	1972.73	973.27	0.229	1573.11	360.21
A_68_P21070914	chr2:25111268-25111312	NM_177344:332	Tmem203	INSIDE	0.272	0.607	3220.74	1954.68	0.165	2146.38	354.06
A_68_P20937460	chr1:193149822-193149866	NM_025864:1	Tmem206	INSIDE	0.500	0.606	936.75	567.97	0.303	746.32	226.24
A_68_P20937462	chr1:193150005-193150049	NM_025864:183	Tmem206	INSIDE	0.388	0.710	2924.08	2076.58	0.276	2035.80	561.01
A_68_P23575088	chr5:31172158-31172203	NM_144525:161	Tmem214	INSIDE	0.201	0.602	2368.37	1425.35	0.121	1444.20	174.87
A_68_P22857437	chr4:40420294-40420338	NM_001166009:154	Tmem215	INSIDE	0.550	0.522	1267.86	662.26	0.287	1098.66	315.69
A_68_P31951351	chr19:10630157-10630201	NM_026798:550	Tmem216	INSIDE	0.402	1.573	3244.90	5105.54	0.633	2382.85	1508.24
A_68_P31128446	chr17:29686489-29686534	NM_001162901:27	Tmem217	INSIDE	0.060	1.482	1893.32	2805.17	0.089	1368.66	121.21
A_68_P25957479	chr8:74082789-74082840	NM_001100462:-44	Tmem221	PROMOTER	0.222	0.678	1055.74	715.89	0.150	820.15	123.37
A_68_P23316764	chr4:132833345-132833389	NM_025667:339	Tmem222	INSIDE	0.253	0.501	1394.89	698.19	0.127	980.37	124.30
A_68_P23316765	chr4:132833435-132833480	NM_025667:248	Tmem222	INSIDE	0.482	0.581	2523.92	1466.58	0.280	2020.68	566.19
A_68_P24304147	chr6:24906093-24906137	NM_177013:11	Tmem229a	INSIDE	0.300	0.461	2128.04	981.74	0.139	1780.30	246.61
A_68_P28567141	chr12:80108301-80108345	NM_178745:-58	Tmem229b	PROMOTER	0.151	0.376	2864.45	1077.51	0.057	2224.36	126.66
A_68_P26170969	chr8:114457634-114457678	NM_001033321:35	Tmem231	INSIDE	0.376	0.613	1831.69	1122.60	0.230	1433.18	330.11
A_68_P26469618	chr9:44607091-44607135	NM_027865:187	Tmem25	INSIDE	0.232	0.566	6036.59	3415.64	0.131	4438.21	582.03
A_68_P32741829	chrX:146893580-146893628	NM_001164683:89	Tmem29	INSIDE	0.397	0.687	1246.33	855.90	0.273	436.00	118.86
A_68_P30595257	chr16:21947236-21947280	NM_025693:367	Tmem41a	INSIDE	0.413	0.705	1335.69	942.24	0.291	1141.59	332.69
A_68_P30595258	chr16:21947351-21947395	NM_025693:253	Tmem41a	INSIDE	0.270	0.710	4519.94	3208.62	0.192	3570.10	684.14
A_68_P30642144	chr16:30510918-30510962	NM_172614:39724	Tmem44	DOWNSTREAM	0.116	0.534	2371.78	1267.35	0.062	1918.45	119.23
A_68_P30642392	chr16:30550884-30550928	NM_172614:-242	Tmem44	PROMOTER	0.429	0.604	2111.36	1275.91	0.259	1651.61	428.19
A_68_P32488665	chrX:78316348-78316392	NM_138751:388	Tmem47	INSIDE	0.083	2.290	4701.99	10769.55	0.190	1725.36	328.11
A_68_P32488666	chrX:78316434-78316478	NM_138751:474	Tmem47	INSIDE	0.323	1.684	2769.27	4664.43	0.545	987.39	537.79
A_68_P23367600	chr4:141640361-141640405	NM_145402:-163	Tmem51	PROMOTER	0.374	0.680	2337.67	1589.41	0.254	1770.14	449.70
A_68_P23367601	chr4:141640480-141640524	NM_145402:-283	Tmem51	PROMOTER	0.203	0.504	1862.95	939.53	0.102	1420.14	145.43
A_68_P23233186	chr4:116924691-116924735	NM_026837:119	Tmem53	INSIDE	0.555	0.523	1081.98	565.55	0.290	914.03	265.04
A_68_P23189068	chr4:106851270-106851320	NM_029565:60	Tmem59	INSIDE	0.163	0.533	2035.46	1084.33	0.087	1406.85	122.20
A_68_P23189069	chr4:106851457-106851501	NM_029565:244	Tmem59	INSIDE	0.194	0.601	2464.22	1481.85	0.117	1760.27	205.58
A_68_P21578169	chr2:120802714-120802767	NM_175285:-57	Tmem62	DIVERGENT_PROMOTER	0.220	0.468	1291.18	604.77	0.103	1148.64	118.44

ProbeName	Target position of probe on CpG island microarray	TargetID	GeneSymbol	CpG island Description	Ratio of relative methylation (TiO2-NP/Vehicle)	Sham group			TiO_2-H group		
						Relative methylation (Cy-5/Cy-3)	Cy-3 signal (Input)	Cy-5 signal (MeDIP)	Relative methylation (Cy-5/Cy-3)	Cy-3 signal (Input)	Cy-5 signal (MeDIP)
A_68_P20878652	chr1:182872565-182872613	NM_144794:-60	Tmem63a	PROMOTER	0.196	0.478	1818.60	870.04	0.094	1294.30	121.41
A_68_P20878657	chr1:182873054-182873098	NM_144794:428	Tmem63a	INSIDE	0.481	0.689	1123.22	773.44	0.331	846.78	280.24
A_68_P31202520	chr17:45822206-45822252	NM_198167:939	Tmem63b	INSIDE	0.196	0.619	1416.25	876.64	0.121	994.70	120.65
A_68_P31202521	chr17:45822303-45822347	NM_198167:843	Tmem63b	INSIDE	0.107	0.597	3648.58	2178.39	0.064	2543.60	161.86
A_68_P22749335	chr4:15193708-15193752	NM_181401:764	Tmem64	INSIDE	0.448	0.676	1452.89	982.25	0.303	1103.47	334.06
A_68_P22733170	chr4:12015068-12015112	NM_177861:14	Tmem67	INSIDE	0.372	0.333	1848.60	616.16	0.124	1452.29	180.16
A_68_P22693141	chr4:3501940-3501984	NM_028097:-47	Tmem68	DIVERGENT_PROMOTER	0.611	0.637	1267.27	807.37	0.390	883.29	344.10
A_68_P31108361	chr17:26250094-26250138	NM_021793:-144	Tmem8	PROMOTER	0.172	0.673	1159.93	780.64	0.115	1061.02	122.53
A_68_P25588179	chr7:148514181-148514225	NR_024509:174	Tmem80	INSIDE	0.202	0.369	1949.27	719.31	0.075	1554.11	115.81
A_68_P21621499	chr2:128643821-128643865	NM_028248:-196	Tmem87b	PROMOTER	0.516	0.683	1950.87	1332.09	0.352	1528.32	538.48
A_68_P23440601	chr4:155158362-155158406	NM_001033394:1599	Tmem88b	INSIDE	0.279	0.443	1265.33	560.57	0.124	941.84	116.58
A_68_P21736743	chr2:149656066-149656110	NM_001085521:-430	Tmem90b	PROMOTER	0.612	0.634	1383.81	877.21	0.388	1006.57	390.19
A_68_P21736744	chr2:149656175-149656219	NM_001085521:-322	Tmem90b	PROMOTER	0.549	0.414	1518.06	628.69	0.227	1125.00	255.93
A_68_P21736750	chr2:149656913-149656958	NM_001085521:417	Tmem90b	INSIDE	0.344	0.547	1978.60	1081.48	0.188	1688.29	317.19
A_68_P27915635	chr11:72990099-72990143	NM_001168470:390	Tmem93	INSIDE	0.255	0.511	1762.61	901.27	0.131	1335.15	174.41
A_68_P27944034	chr11:78364333-78364383	NM_133706:-121	Tmem97	PROMOTER	0.184	0.628	1454.68	913.75	0.115	1053.69	121.58
A_68_P24671557	chr6:97129251-97129295	NM_001081111:-154	Tmf1	PROMOTER	0.341	0.658	1851.91	1218.68	0.224	1396.22	313.41
A_68_P26644959	chr9:75458604-75458648	NM_016711:506	Tmod2	INSIDE	0.398	0.426	2977.71	1268.02	0.170	2449.97	415.69
A_68_P26644654	chr9:75406902-75406946	NM_016963:540	Tmod3	INSIDE	0.618	0.550	1637.02	900.32	0.340	1383.47	469.90
A_68_P27340413	chr10:90634459-90634503	NM_001080129:-153	Tmpo	PROMOTER	0.637	0.532	1631.07	868.20	0.339	1314.71	445.66
A_68_P30999921	chr16:97832578-97832622	NM_015775:202	Tmprss2	INSIDE	0.325	0.387	1770.06	685.77	0.126	1448.29	182.21
A_68_P27392328	chr10:99950341-99950385	NM_001110013:-381	Tmtc3	DIVERGENT_PROMOTER	0.366	0.500	3863.71	1931.11	0.183	2898.74	530.85
A_68_P28078648	chr11:102145969-102146013	NM_028076:-284	Tmub2	PROMOTER	0.105	0.621	5025.74	3119.72	0.065	3714.55	241.90
A_68_P28078649	chr11:102146096-102146140	NM_028076:-156	Tmub2	PROMOTER	0.506	0.657	1328.67	872.90	0.332	1108.92	368.39
A_68_P28078652	chr11:102146344-102146398	NM_028076:96	Tmub2	INSIDE	0.142	2.534	2094.28	5307.20	0.360	1324.01	476.80
A_68_P28520552	chr12:71554055-71554099	NM_028339:-64	Tmx1	PROMOTER	0.232	0.645	936.06	603.94	0.150	787.49	117.81
A_68_P28520554	chr12:71554239-71554283	NM_028339:120	Tmx1	INSIDE	0.241	0.426	1369.19	583.75	0.103	1131.56	116.17
A_68_P21652098	chr2:134469794-134469838	NM_029148:41	Tmx4	INSIDE	0.653	0.491	1576.93	775.03	0.321	1270.80	407.65
A_68_P21652099	chr2:134469945-134469989	NM_029148:-109	Tmx4	PROMOTER	0.184	0.575	1436.16	825.16	0.106	1144.01	121.00
A_68_P27943940	chr11:78349786-78349836	NM_001159392:-48	Tnfaip1	DIVERGENT_PROMOTER	0.269	0.604	1091.68	659.27	0.162	739.47	120.15
A_68_P28744113	chr12:112680513-112680557	NM_009396:-337	Tnfaip2	PROMOTER	0.154	0.680	2124.48	1444.75	0.105	1578.48	165.41
A_68_P28744144	chr12:112683687-112683731	NM_009396:2837	Tnfaip2	INSIDE	0.315	0.641	1159.38	742.68	0.202	841.52	169.59
A_68_P28744145	chr12:112683846-112683890	NM_009396:2997	Tnfaip2	INSIDE	0.371	0.520	2026.59	1053.27	0.193	1632.63	314.96
A_68_P26978748	chr10:18734800-18734844	NM_001166402:-3053	Tnfaip3	PROMOTER	0.538	0.417	3438.18	1432.36	0.224	2664.50	596.84
A_68_P22342446	chr3:94944251-94944296	NM_027206:2009	Tnfaip8l2	INSIDE	0.462	3.566	1855.17	6614.92	1.649	1356.31	2235.99
A_68_P22342447	chr3:94944410-94944458	NM_027206:1848	Tnfaip8l2	INSIDE	0.626	5.217	2252.92	11753.35	3.264	1502.71	4904.67
A_68_P31092979	chr17:23813505-23813549	NM_001161746:890	Tnfrsf12a	INSIDE	0.245	0.733	2153.76	1578.53	0.180	1562.27	281.07
A_68_P31092987	chr17:23814347-23814391	NM_001161746:48	Tnfrsf12a	INSIDE	0.439	0.568	2157.24	1224.67	0.249	1778.86	443.64
A_68_P30382206	chr15:82054633-82054677	NM_028075:112	Tnfrsf13c	INSIDE	0.308	0.409	1535.29	628.63	0.126	1098.26	138.39
A_68_P29647965	chr14:61656646-61656690	NM_001164155:156	Tnfrsf19	INSIDE	0.296	0.424	1805.28	765.52	0.125	1451.09	182.03
A_68_P23382179	chr4:144836289-144836333	NM_011610:463	Tnfrsf1b	INSIDE	0.379	0.710	2172.27	1541.23	0.269	1510.46	406.10
A_68_P31187140	chr17:43154341-43154385	NM_178589:859	Tnfrsf21	INSIDE	0.223	1.504	3996.57	6010.79	0.335	2823.24	946.31
A_68_P22015871	chr3:28161528-28161576	NM_001163009:-583	Tnik	PROMOTER	0.208	0.685	1661.47	1138.35	0.142	1103.60	157.16
A_68_P22015879	chr3:28162505-28162549	NM_001163009:391	Tnik	INSIDE	0.360	0.558	1455.69	812.90	0.201	1111.03	223.27

ProbeName	Target position of probe on CpG island microarray	TargetID	GeneSymbol	CpG island Description	Ratio of relative methylation (TiO2-NP/Vehicle)	Sham group			TiO_2-H group		
						Relative methylation (Cy-5/Cy-3)	Cy-3 signal (Input)	Cy-5 signal (MeDIP)	Relative methylation (Cy-5/Cy-3)	Cy-3 signal (Input)	Cy-5 signal (MeDIP)
A_68_P23594730	chr5:34856051-34856097	NM_139064:554	Tnip2	INSIDE	0.325	1.737	1122.27	1949.46	0.565	867.02	490.08
A_68_P23594734	chr5:34856584-34856628	NM_139064:22	Tnip2	INSIDE	0.530	0.519	2578.87	1339.05	0.275	2021.01	555.90
A_68_P25777210	chr8:36028138-36028182	NM_175091:584	Tnks	INSIDE	0.236	0.523	3263.36	1707.65	0.123	2635.24	324.98
A_68_P21396039	chr2:84888658-84888702	NM_001081260:-1936	Tnks1bp1	PROMOTER	0.136	0.598	3705.39	2214.45	0.082	2725.49	222.19
A_68_P21396040	chr2:84888732-84888776	NM_001081260:-1862	Tnks1bp1	PROMOTER	0.320	0.685	1542.69	1056.32	0.219	1141.47	250.15
A_68_P29260160	chr13:99660969-99661013	NM_001048267:7	Tnpo1	INSIDE	0.379	0.400	1452.88	581.14	0.152	1244.49	188.74
A_68_P26023333	chr8:87561242-87561290	NM_001122843:200	Tnpo2	INSIDE	0.334	5.335	1922.75	10257.39	1.781	1145.10	2039.63
A_68_P26023334	chr8:87561401-87561445	NM_001122843:356	Tnpo2	INSIDE	0.118	2.630	15621.84	41083.68	0.311	10145.26	3155.63
A_68_P26023335	chr8:87561478-87561522	NM_001122843:434	Tnpo2	INSIDE	0.218	2.482	6585.83	16344.37	0.540	4234.41	2286.58
A_68_P26023338	chr8:87561856-87561900	NM_001122843:812	Tnpo2	INSIDE	0.308	0.563	1020.00	574.25	0.173	736.69	127.64
A_68_P26023339	chr8:87561977-87562021	NM_001122843:932	Tnpo2	INSIDE	0.127	0.513	2537.94	1303.00	0.065	1860.45	121.30
A_68_P24327616	chr6:29559131-29559176	NM_177296:454	Tnpo3	INSIDE	0.087	0.652	2501.68	1630.35	0.056	2038.32	115.08
A_68_P24327617	chr6:29559260-29559304	NM_177296:325	Tnpo3	INSIDE	0.137	0.735	6423.12	4720.37	0.101	4616.03	464.23
A_68_P24327619	chr6:29559522-29559566	NM_177296:63	Tnpo3	INSIDE	0.474	0.545	1375.40	749.19	0.258	1119.01	289.03
A_68_P24153541	chr5:143578202-143578246	NM_001122730:842	Tnrc18	INSIDE	0.276	0.445	1329.77	591.54	0.123	985.09	121.07
A_68_P24153542	chr5:143578302-143578350	NM_001122730:740	Tnrc18	INSIDE	0.135	0.577	2329.17	1344.42	0.078	1499.81	116.77
A_68_P24153543	chr5:143578378-143578435	NM_001122730:660	Tnrc18	INSIDE	0.189	0.700	1269.33	888.91	0.132	874.65	115.63
A_68_P24153575	chr5:143582152-143582196	NM_001122730:-3108	Tnrc18	PROMOTER	0.603	0.599	1074.49	643.17	0.361	846.95	305.86
A_68_P30374035	chr15:80541596-80541640	NM_144812:-124	Tnrc6b	PROMOTER	0.114	0.595	2247.59	1336.71	0.068	1723.90	117.03
A_68_P30374040	chr15:80542264-80542308	NM_144812:544	Tnrc6b	INSIDE	0.495	0.647	2328.40	1505.81	0.320	1688.43	540.25
A_68_P30374041	chr15:80542355-80542399	NM_144812:634	Tnrc6b	INSIDE	0.216	0.675	2383.47	1608.99	0.146	1698.77	247.47
A_68_P30374042	chr15:80542447-80542491	NM_144812:726	Tnrc6b	INSIDE	0.291	0.565	2485.99	1404.12	0.164	1757.12	288.84
A_68_P28166392	chr11:117517198-117517249	NM_198022:1621	Tnrc6c	INSIDE	0.135	0.677	1899.77	1286.31	0.091	1297.93	118.68
A_68_P20346581	chr1:74115086-74115133	NM_027884:55912	Tns1	INSIDE	0.594	0.542	1191.98	645.89	0.322	865.66	278.39
A_68_P20346582	chr1:74115236-74115286	NM_027884:55761	Tns1	INSIDE	0.585	0.486	1637.65	796.65	0.284	1178.01	335.06
A_68_P20346773	chr1:74144944-74144988	NM_027884:26055	Tns1	INSIDE	0.445	0.456	1362.03	621.17	0.203	1054.48	213.78
A_68_P28031414	chr11:94071733-94071777	NM_009427:-1013	Tob1	PROMOTER	0.304	0.584	957.13	559.06	0.178	830.53	147.57
A_68_P28031420	chr11:94072519-94072563	NM_009427:-227	Tob1	PROMOTER	0.112	0.690	1945.63	1342.85	0.077	1587.01	122.52
A_68_P30380064	chr15:81688492-81688536	NM_020507:242	Tob2	INSIDE	0.141	0.691	1994.19	1377.13	0.097	1532.96	149.05
A_68_P30380073	chr15:81689400-81689446	NM_020507:-666	Tob2	PROMOTER	0.289	0.544	1004.28	546.65	0.157	757.58	119.12
A_68_P30380080	chr15:81690283-81690327	NM_020507:-1548	Tob2	PROMOTER	0.473	0.481	2092.30	1006.96	0.228	1728.62	393.52
A_68_P30380081	chr15:81690360-81690404	NM_020507:-1626	Tob2	PROMOTER	0.303	0.448	1756.42	786.79	0.136	1382.89	187.56
A_68_P23231032	chr4:116480068-116480112	NM_026654:74	Toe1	INSIDE	0.396	0.637	1622.39	1033.81	0.252	1277.20	322.39
A_68_P25591626	chr7:149088429-149088473	NM_023764:-139	Tollip	PROMOTER	0.655	0.646	1001.20	646.50	0.423	830.74	351.32
A_68_P27842502	chr11:60166254-60166298	NM_001039092:131	Tom1l2	INSIDE	0.286	0.524	2049.56	1073.20	0.150	1540.13	230.37
A_68_P27842504	chr11:60166452-60166496	NM_001039092:-67	Tom1l2	DIVERGENT_PROMOTER	0.465	0.454	1311.65	595.13	0.211	1114.16	234.83
A_68_P27842505	chr11:60166539-60166583	NM_001039092:-153	Tom1l2	DIVERGENT_PROMOTER	0.404	0.361	1587.91	572.60	0.146	1187.68	172.87
A_68_P24994882	chr7:20300214-20300266	NM_001109748:538	Tomm40	INSIDE	0.257	0.597	1233.87	737.16	0.154	810.04	124.51
A_68_P24994886	chr7:20300585-20300629	NM_001109748:172	Tomm40	INSIDE	0.125	0.549	2347.38	1289.31	0.069	1698.43	116.58
A_68_P24994888	chr7:20300800-20300844	NM_001109748:-44	Tomm40	PROMOTER	0.309	0.703	2686.35	1888.44	0.217	1825.38	396.24
A_68_P24994892	chr7:20301207-20301251	NM_001109748:-450	Tomm40	PROMOTER	0.452	0.561	1535.10	861.57	0.254	1205.67	305.93
A_68_P22881129	chr4:45120647-45120691	NM_001099675:317	Tomm5	INSIDE	0.580	0.725	3108.26	2254.27	0.421	2339.66	984.34
A_68_P31213633	chr17:47825295-47825344	NM_001164729:16	Tomm6	INSIDE	0.312	0.539	1512.89	815.78	0.168	1072.92	180.78
A_68_P31213635	chr17:47825554-47825598	NM_001164729:-241	Tomm6	DIVERGENT_PROMOTER	0.251	0.696	1225.87	852.85	0.175	968.48	169.39

ProbeName	Target position of probe on CpG island microarray	TargetID	GeneSymbol	CpG island Description	Ratio of relative methylation (TiO2-NP/Vehicle)	Sham group			TiO_2-H group		
						Relative methylation (Cy-5/Cy-3)	Cy-3 signal (Input)	Cy-5 signal (MeDIP)	Relative methylation (Cy-5/Cy-3)	Cy-3 signal (Input)	Cy-5 signal (MeDIP)
A_68_P31213636	chr17:47825672-47825716	NM_001164729:-359	Tomm6	DIVERGENT_PROMOTER	0.437	0.614	1449.34	889.49	0.268	1090.21	292.12
A_68_P30349611	chr15:76470372-76470416	NM_183091:-35	Tonsl	PROMOTER	0.412	1.668	9193.56	15333.36	0.687	5983.93	4113.26
A_68_P21796145	chr2:160471648-160471692	NM_009408:38	Top1	INSIDE	0.180	0.712	1596.72	1136.89	0.128	1198.05	153.55
A_68_P21796146	chr2:160471717-160471761	NM_009408:106	Top1	INSIDE	0.120	0.735	2672.00	1965.25	0.088	1902.34	167.62
A_68_P29428727	chr14:17197508-17197553	NM_009409:-189	Top2b	PROMOTER	0.221	0.554	1431.70	792.82	0.123	1177.86	144.42
A_68_P29428735	chr14:17198392-17198436	NM_009409:695	Top2b	INSIDE	0.370	0.674	1504.02	1014.20	0.249	1100.21	274.36
A_68_P30570744	chr16:16870950-16870994	NM_011624:-11	Top3b	DIVERGENT_PROMOTER	0.288	0.325	1641.88	533.84	0.094	1394.13	130.41
A_68_P26785090	chr9:103207295-103207339	NM_176979:-340	Topbp1	PROMOTER	0.147	0.388	2743.91	1064.70	0.057	2150.14	122.56
A_68_P22856581	chr4:40216815-40216859	NM_134097:38	Topors	INSIDE	0.340	0.655	831.30	544.49	0.223	700.62	156.13
A_68_P21106251	chr2:30822965-30823009	NM_144884:452	Tor1a	INSIDE	0.489	0.476	3023.40	1439.48	0.233	2264.47	527.50
A_68_P21106145	chr2:30808853-30808897	NM_133673:354	Tor1b	INSIDE	0.316	0.420	1700.27	714.36	0.133	1334.02	177.11
A_68_P21117288	chr2:32612911-32612955	NM_152800:142	Tor2a	INSIDE	0.599	0.668	1061.59	709.04	0.400	876.03	350.28
A_68_P20744278	chr1:158604428-158604472	NM_023141:20	Tor3a	INSIDE	0.567	0.435	1221.33	531.24	0.247	1085.85	267.91
A_68_P22708706	chr4:6918269-6918313	NM_145711:-420	Tox	PROMOTER	0.094	0.752	5768.87	4337.10	0.071	3833.26	270.81
A_68_P26052838	chr8:92871596-92871645	NM_172913:531	Tox3	INSIDE	0.505	0.698	2166.30	1512.29	0.353	1706.60	601.80
A_68_P26696144	chr9:85736977-85737021	NM_001164792:540	Tpbg	INSIDE	0.373	0.555	3333.29	1850.00	0.207	2445.55	505.91
A_68_P26696146	chr9:85737161-85737205	NM_001164792:724	Tpbg	INSIDE	0.234	0.718	8510.89	6108.73	0.168	5688.51	954.17
A_68_P21935172	chr3:9004150-9004194	NM_001025263:343	Tpd52	INSIDE	0.503	0.587	3003.09	1762.66	0.295	2286.45	674.61
A_68_P27044450	chr10:31165279-31165323	NM_009413:427	Tpd52l1	INSIDE	0.444	1.523	2312.79	3521.38	0.675	1632.74	1102.72
A_68_P27044452	chr10:31165585-31165629	NM_009413:121	Tpd52l1	INSIDE	0.264	0.445	1375.58	611.90	0.118	1068.33	125.54
A_68_P26596713	chr9:66891367-66891411	NM_001164252:290	Tpm1	INSIDE	0.411	0.633	1916.73	1214.25	0.260	1718.65	447.69
A_68_P26596750	chr9:66896100-66896144	NM_001164252:-4444	Tpm1	PROMOTER	0.597	0.443	1696.14	752.00	0.264	1218.54	322.29
A_68_P26596751	chr9:66896180-66896228	NM_001164252:-4526	Tpm1	PROMOTER	0.573	0.467	1734.19	810.51	0.268	1328.51	355.62
A_68_P22872184	chr4:43536297-43536341	NM_009416:-58	Tpm2	PROMOTER	0.591	0.647	1978.44	1279.19	0.382	1525.80	582.66
A_68_P22872187	chr4:43536672-43536716	NM_009416:-434	Tpm2	PROMOTER	0.617	0.497	1160.65	576.55	0.306	917.73	281.16
A_68_P22323355	chr3:89883388-89883433	NM_022314:6795	Tpm3	INSIDE	0.491	0.404	1330.90	537.16	0.198	1163.31	230.75
A_68_P22323363	chr3:89884285-89884329	NM_022314:7691	Tpm3	INSIDE	0.398	0.365	1427.82	521.52	0.145	1038.00	150.77
A_68_P25959791	chr8:74658901-74658954	NM_001001491:-263	Tpm4	PROMOTER	0.167	1.576	620.44	977.71	0.263	457.97	120.45
A_68_P25959798	chr8:74659599-74659643	NM_001001491:430	Tpm4	INSIDE	0.204	0.518	3464.10	1794.52	0.105	2558.82	269.81
A_68_P20191545	chr1:43990635-43990679	NM_009418:-195	Tpp2	PROMOTER	0.386	0.587	1071.02	628.56	0.226	849.56	192.28
A_68_P24624900	chr6:88852416-88852460	NM_011906:194	Tpra1	INSIDE	0.619	0.412	1630.70	672.01	0.255	1328.12	338.55
A_68_P23429475	chr4:153535140-153535184	NM_026388:-369	Tprgl	DIVERGENT_PROMOTER	0.222	0.693	3013.74	2088.44	0.154	2198.31	338.44
A_68_P21070952	chr2:25118487-25118531	NM_175286:391	Tprn	INSIDE	0.561	0.472	2594.30	1223.22	0.264	1909.58	505.07
A_68_P24092036	chr5:130555652-130555696	NM_001130476:319	Tpst1	INSIDE	0.290	0.699	1632.32	1140.96	0.203	1334.75	270.58
A_68_P23991126	chr5:112706061-112706105	NM_009419:356	Tpst2	INSIDE	0.359	0.547	2099.86	1148.95	0.197	1642.33	322.96
A_68_P29730260	chr14:76244587-76244631	NM_009429:-454	Tpt1	PROMOTER	0.556	0.620	1882.04	1166.11	0.345	1509.21	519.98
A_68_P29730261	chr14:76244654-76244698	NM_009429:-386	Tpt1	PROMOTER	0.339	0.504	2082.32	1050.12	0.171	1574.00	269.35
A_68_P24431872	chr6:49213460-49213504	NM_198102:569	Tra2a	INSIDE	0.611	0.663	1617.45	1072.19	0.405	1375.13	556.98
A_68_P24431873	chr6:49213588-49213632	NM_198102:441	Tra2a	INSIDE	0.215	0.626	2953.52	1847.72	0.134	2091.01	280.83
A_68_P24431874	chr6:49213690-49213734	NM_198102:339	Tra2a	INSIDE	0.481	0.437	2489.43	1086.95	0.210	1956.38	410.72
A_68_P24431878	chr6:49214131-49214175	NM_198102:-101	Tra2a	PROMOTER	0.660	0.547	1378.24	753.33	0.361	1225.96	442.04
A_68_P30596854	chr16:22265994-22266038	NM_009186:-14	Tra2b	PROMOTER	0.551	0.416	2931.55	1220.49	0.230	2189.19	502.54
A_68_P30596855	chr16:22266102-22266148	NM_009186:-122	Tra2b	PROMOTER	0.497	0.469	1763.69	826.70	0.233	1298.29	302.60
A_68_P26132557	chr8:107783610-107783659	NM_001033161:4860	Tradd	INSIDE	0.309	0.384	1335.67	513.41	0.119	1041.01	123.64

ProbeName	Target position of probe on CpG island microarray	TargetID	GeneSymbol	CpG island Description	Ratio of relative methylation (TiO2-NP/Vehicle)	Sham group			TiO_2-H group		
						Relative methylation (Cy-5/Cy-3)	Cy-3 signal (Input)	Cy-5 signal (MeDIP)	Relative methylation (Cy-5/Cy-3)	Cy-3 signal (Input)	Cy-5 signal (MeDIP)
A_68_P21072876	chr2:25402210-25402254	NM_009422:182	Traf2	INSIDE	0.357	0.682	2963.88	2022.28	0.244	2354.67	574.25
A_68_P27941695	chr11:77979180-77979227	NM_009423:-151	Traf4	PROMOTER	0.282	0.538	1100.62	591.94	0.152	926.94	140.73
A_68_P31097896	chr17:24664666-24664710	NM_001172113:195	Traf7	INSIDE	0.557	0.341	5166.04	1761.38	0.190	3885.73	738.42
A_68_P24044263	chr5:121835304-121835348	NM_001163470:298	Trafd1	INSIDE	0.558	0.489	1698.87	830.23	0.273	1368.54	373.35
A_68_P26812057	chr9:107853467-107853511	NM_011634:195	Traip	INSIDE	0.284	0.576	971.65	559.31	0.163	761.10	124.36
A_68_P20261290	chr1:59030112-59030156	NM_172406:192	Trak2	INSIDE	0.173	0.576	1347.13	775.38	0.099	1223.76	121.66
A_68_P20261291	chr1:59030219-59030263	NM_172406:86	Trak2	INSIDE	0.545	0.467	2678.36	1252.08	0.255	2055.05	523.99
A_68_P22492342	chr3:124024204-124024248	NM_146140:272	Tram1l1	INSIDE	0.183	0.634	1498.47	949.44	0.116	1189.22	138.15
A_68_P20076127	chr1:21068999-21069043	NM_133252:270	Tram2	INSIDE	0.301	0.699	3103.23	2169.79	0.210	2237.15	470.26
A_68_P20076128	chr1:21069108-21069152	NM_133252:160	Tram2	INSIDE	0.358	0.673	900.90	606.18	0.241	699.67	168.66
A_68_P30508110	chr16:4077547-4077591	NM_026508:242	Trap1	INSIDE	0.226	0.516	4083.99	2108.96	0.116	3328.19	387.58
A_68_P27273026	chr10:77707095-77707139	NM_001081055:271	Trappc10	INSIDE	0.203	0.411	2015.47	827.41	0.083	1647.53	137.20
A_68_P27273027	chr10:77707175-77707219	NM_001081055:191	Trappc10	INSIDE	0.486	0.604	988.87	596.80	0.293	877.04	257.23
A_68_P26467466	chr9:44215609-44215655	NM_021789:-1	Trappc4	DIVERGENT_PROMOTER	0.269	0.448	1284.89	576.13	0.121	983.79	118.56
A_68_P25615117	chr8:3676454-3676498	NM_025701:0	Trappc5	INSIDE	0.556	0.418	1651.13	689.43	0.232	1207.39	280.25
A_68_P24993673	chr7:20094233-20094277	NM_025960:177	Trappc6a	INSIDE	0.110	0.688	2221.60	1528.73	0.076	1595.84	121.13
A_68_P24993674	chr7:20094360-20094415	NM_025960:310	Trappc6a	INSIDE	0.331	0.575	908.18	522.04	0.190	633.11	120.39
A_68_P30255505	chr15:59479171-59479215	NM_144549:-1016	Trib1	PROMOTER	0.444	0.602	942.82	567.58	0.267	726.19	193.97
A_68_P30255508	chr15:59479606-59479650	NM_144549:-580	Trib1	PROMOTER	0.206	0.566	1128.83	638.86	0.117	1002.69	117.03
A_68_P30255521	chr15:59480987-59481031	NM_144549:800	Trib1	INSIDE	0.644	0.711	2941.36	2089.94	0.458	2130.32	975.09
A_68_P30255522	chr15:59481111-59481155	NM_144549:924	Trib1	INSIDE	0.307	0.739	3058.17	2261.46	0.227	2131.78	483.22
A_68_P28261127	chr12:15821624-15821668	NM_144551:1945	Trib2	INSIDE	0.472	0.419	2338.01	980.55	0.198	1791.67	354.29
A_68_P28261128	chr12:15821694-15821738	NM_144551:1875	Trib2	INSIDE	0.553	0.685	3081.72	2110.61	0.379	2537.93	960.76
A_68_P28261129	chr12:15821863-15821907	NM_144551:1707	Trib2	INSIDE	0.261	0.514	2868.64	1474.94	0.134	2134.33	286.42
A_68_P24459339	chr6:53768981-53769025	NM_025817:1817	Tril	INSIDE	0.306	0.465	1127.31	524.46	0.142	852.50	121.18
A_68_P24459340	chr6:53769060-53769104	NM_025817:1737	Tril	INSIDE	0.185	0.613	4396.63	2697.18	0.114	2944.14	334.33
A_68_P24459344	chr6:53769514-53769558	NM_025817:1283	Tril	INSIDE	0.475	1.411	4056.02	5722.18	0.670	2907.24	1947.23
A_68_P24459352	chr6:53770524-53770568	NM_025817:273	Tril	INSIDE	0.225	0.675	1095.07	739.25	0.152	870.73	132.26
A_68_P24459354	chr6:53770780-53770824	NM_025817:17	Tril	INSIDE	0.465	0.507	1818.07	922.10	0.236	1314.72	310.32
A_68_P27833860	chr11:58791364-58791408	NM_053168:-231	Trim11	PROMOTER	0.208	0.587	1611.99	946.59	0.122	1170.48	142.80
A_68_P24374361	chr6:37820805-37820849	NM_145076:16	Trim24	INSIDE	0.308	0.697	3366.12	2345.10	0.215	2760.08	592.80
A_68_P24374366	chr6:37821755-37821801	NM_145076:968	Trim24	INSIDE	0.246	0.494	1248.19	616.73	0.122	990.00	120.32
A_68_P28003255	chr11:88860965-88861009	NM_009546:270	Trim25	INSIDE	0.464	0.654	1701.70	1112.18	0.304	1229.69	373.28
A_68_P28871637	chr13:21292413-21292457	NM_009054:20607	Trim27	DOWNSTREAM	0.433	0.518	1248.32	647.22	0.225	964.05	216.53
A_68_P28871638	chr13:21292545-21292595	NM_009054:20743	Trim27	DOWNSTREAM	0.346	0.415	1486.03	616.88	0.144	1132.32	162.54
A_68_P25393298	chr7:112766252-112766301	NM_018880:15737	Trim3	INSIDE	0.583	3.635	900.14	3272.04	2.120	687.53	1457.72
A_68_P25393401	chr7:112781612-112781656	NM_018880:379	Trim3	INSIDE	0.229	2.580	12493.00	32226.47	0.591	7813.22	4616.60
A_68_P25388365	chr7:111650415-111650462	NM_001167828:5925	Trim30d	INSIDE	0.170	0.630	1362.31	857.84	0.107	1107.63	118.37
A_68_P22982497	chr4:65265989-65266033	NM_001161782:-9	Trim32	PROMOTER	0.114	0.610	2629.47	1605.00	0.070	1876.15	130.92
A_68_P22982499	chr4:65266160-65266204	NM_001161782:163	Trim32	INSIDE	0.205	2.166	12466.27	26998.36	0.443	8254.48	3659.90
A_68_P22982500	chr4:65266278-65266324	NM_001161782:281	Trim32	INSIDE	0.372	4.813	8083.80	38907.65	1.792	5043.12	9035.68
A_68_P22385237	chr3:103084452-103084496	NM_001079830:1259	Trim33	INSIDE	0.490	0.698	2203.17	1538.33	0.342	1451.29	496.79
A_68_P31675570	chr18:46358067-46358111	NM_001170855:384	Trim36	INSIDE	0.501	0.620	1180.61	732.54	0.311	987.23	306.77
A_68_P31675571	chr18:46358139-46358185	NM_001170855:310	Trim36	INSIDE	0.082	0.694	2673.28	1855.88	0.057	2181.95	123.51

ProbeName	Target position of probe on CpG island microarray	TargetID	GeneSymbol	CpG island Description	Ratio of relative methylation (TiO2-NP/Vehicle)	Sham group			TiO_2-H group		
						Relative methylation (Cy-5/Cy-3)	Cy-3 signal (Input)	Cy-5 signal (MeDIP)	Relative methylation (Cy-5/Cy-3)	Cy-3 signal (Input)	Cy-5 signal (MeDIP)
A_68_P31675646	chr18:46371668-46371712	NM_178872:571	Trim36	INSIDE	0.361	0.384	2178.47	836.93	0.139	1795.39	248.86
A_68_P27780095	chr11:48630257-48630301	NM_145377:615	Trim41	INSIDE	0.366	0.693	3398.98	2354.41	0.253	2424.95	614.69
A_68_P27780097	chr11:48630503-48630547	NM_145377:369	Trim41	INSIDE	0.238	0.608	3970.23	2413.06	0.144	3080.04	444.73
A_68_P27780098	chr11:48630599-48630643	NM_145377:273	Trim41	INSIDE	0.329	0.491	1747.72	857.96	0.161	1283.11	207.09
A_68_P21476827	chr2:102241389-102241433	NM_020267:-353	Trim44	PROMOTER	0.524	0.700	1758.05	1230.21	0.366	1369.47	501.80
A_68_P22372021	chr3:100726069-100726113	NM_001165953:-325	Trim45	PROMOTER	0.228	0.724	3143.26	2274.43	0.165	2238.33	369.09
A_68_P22372025	chr3:100726540-100726584	NM_001165953:147	Trim45	INSIDE	0.198	1.549	5632.82	8726.45	0.307	3943.95	1209.26
A_68_P22218907	chr3:68848966-68849010	NM_025863:-324	Trim59	PROMOTER	0.276	0.478	1197.29	571.94	0.132	927.40	122.43
A_68_P23293635	chr4:128561085-128561129	NM_178110:-277	Trim62	PROMOTER	0.322	0.694	1071.89	743.45	0.224	794.08	177.50
A_68_P23293641	chr4:128561860-128561904	NM_178110:499	Trim62	INSIDE	0.545	0.667	2352.63	1569.34	0.364	1936.20	703.94
A_68_P28157536	chr11:115992427-115992471	NM_178802:-6	Trim65	PROMOTER	0.243	0.472	1327.99	626.75	0.115	1088.63	124.89
A_68_P26247722	chr8:127316654-127316700	NM_198632:-242	Trim67	PROMOTER	0.256	0.448	1189.03	533.27	0.115	1006.93	115.71
A_68_P26247723	chr8:127316765-127316809	NM_198632:-132	Trim67	PROMOTER	0.130	0.582	2110.45	1229.32	0.076	1611.02	122.21
A_68_P26247724	chr8:127316847-127316891	NM_198632:-50	Trim67	PROMOTER	0.222	0.629	2976.08	1871.69	0.140	2437.95	340.98
A_68_P26247733	chr8:127317798-127317842	NM_198632:902	Trim67	INSIDE	0.636	0.644	2690.72	1732.42	0.409	2128.66	871.68
A_68_P26247735	chr8:127318077-127318121	NM_198632:1180	Trim67	INSIDE	0.178	0.382	2268.52	865.46	0.068	1732.63	117.89
A_68_P27780212	chr11:48650835-48650879	NM_053166:11217	Trim7	INSIDE	0.325	0.690	1523.39	1050.85	0.224	1105.36	247.47
A_68_P27780214	chr11:48651115-48651159	NM_053166:11497	Trim7	INSIDE	0.153	0.541	2002.24	1083.94	0.083	1477.48	122.07
A_68_P27780290	chr11:48663076-48663120	NM_053166:23459	Trim7	INSIDE	0.577	2.957	4750.74	14048.07	1.706	3224.86	5501.84
A_68_P25508614	chr7:135153815-135153862	NM_001079932:5947	Trim72	INSIDE	0.618	2.597	1699.77	4413.51	1.604	1251.21	2007.20
A_68_P32144293	chr19:46574614-46574658	NM_053100:-1501	Trim8	PROMOTER	0.368	0.699	2968.85	2076.71	0.257	2146.07	552.02
A_68_P28520017	chr12:71447587-71447631	NM_001110202:993	Trim9	INSIDE	0.504	0.429	1306.83	560.48	0.216	1053.01	227.62
A_68_P28520022	chr12:71448035-71448079	NM_001110202:545	Trim9	INSIDE	0.351	0.558	2268.98	1265.65	0.196	1524.69	298.25
A_68_P28520026	chr12:71448401-71448445	NM_001110202:179	Trim9	INSIDE	0.381	0.608	867.71	527.28	0.232	729.99	169.06
A_68_P31260730	chr17:57388800-57388844	NM_134125:-51	Trip10	DIVERGENT_PROMOTER	0.392	0.608	3559.47	2163.18	0.238	2651.86	631.60
A_68_P20405961	chr1:84835228-84835272	NM_133975:629	Trip12	INSIDE	0.203	1.596	6729.40	10740.45	0.324	4499.56	1458.85
A_68_P20405966	chr1:84835679-84835723	NM_133975:179	Trip12	INSIDE	0.448	0.673	1931.43	1299.19	0.301	1759.24	530.16
A_68_P20405967	chr1:84835781-84835825	NM_133975:77	Trip12	INSIDE	0.111	0.583	2472.51	1442.66	0.065	1833.90	118.44
A_68_P29143148	chr13:74074758-74074802	NM_027182:435	Trip13	INSIDE	0.205	2.800	7233.64	20250.81	0.575	4920.66	2830.36
A_68_P29143150	chr13:74074924-74074968	NM_027182:269	Trip13	INSIDE	0.491	0.462	3704.86	1709.81	0.227	2739.37	620.89
A_68_P31936613	chr19:6984303-6984347	NM_001166370:137	Trmt112	INSIDE	0.244	0.473	2017.31	954.86	0.116	1482.48	171.54
A_68_P31936614	chr19:6984396-6984443	NM_001166370:232	Trmt112	INSIDE	0.272	0.397	1504.42	597.01	0.108	1094.70	118.25
A_68_P31936615	chr19:6984479-6984523	NM_001166370:313	Trmt112	INSIDE	0.351	0.384	1480.45	568.66	0.135	1145.18	154.21
A_68_P30250626	chr15:58704469-58704513	NM_026642:287	Trmt12	INSIDE	0.334	0.599	984.69	590.16	0.200	773.72	154.87
A_68_P30578596	chr16:18249157-18249201	NM_001080999:203	Trmt2a	INSIDE	0.480	0.671	1791.48	1202.58	0.322	1420.71	457.65
A_68_P28536527	chr12:74387503-74387547	NM_029580:174	Trmt5	INSIDE	0.239	0.548	1058.22	579.94	0.131	899.35	117.74
A_68_P21642319	chr2:132641532-132641578	NM_175113:236	Trmt6	INSIDE	0.180	0.537	1786.05	958.61	0.097	1240.30	119.83
A_68_P23311786	chr4:131885241-131885285	NM_027925:191	Trnau1ap	INSIDE	0.234	0.647	1547.09	1000.95	0.151	1252.65	189.76
A_68_P23311790	chr4:131885707-131885751	NM_027925:-275	Trnau1ap	PROMOTER	0.646	0.615	1618.76	995.18	0.397	1438.11	570.86
A_68_P23317939	chr4:133054412-133054456	NM_001081156:31	Trnp1	INSIDE	0.257	0.640	2171.98	1389.98	0.164	1634.57	268.81
A_68_P23317940	chr4:133054507-133054551	NM_001081156:-63	Trnp1	PROMOTER	0.141	0.584	2921.82	1705.71	0.083	2092.08	172.62
A_68_P32742644	chrX:147091720-147091764	NM_001002272:237	Tro	INSIDE	0.324	1.623	1668.43	2707.75	0.526	803.83	423.10
A_68_P30478054	chr15:98904927-98904971	NM_001162506:-455	Troap	PROMOTER	0.281	0.675	1353.53	913.56	0.189	969.68	183.59
A_68_P21579903	chr2:121096750-121096794	NM_013735:350	Trp53bp1	INSIDE	0.386	1.499	10760.68	16131.09	0.578	7597.34	4390.72

ProbeName	Target position of probe on CpG island microarray	TargetID	GeneSymbol	CpG island Description	Ratio of relative methylation (TiO2-NP/Vehicle)	Sham group			TiO_2-H group		
						Relative methylation (Cy-5/Cy-3)	Cy-3 signal (Input)	Cy-5 signal (MeDIP)	Relative methylation (Cy-5/Cy-3)	Cy-3 signal (Input)	Cy-5 signal (MeDIP)
A_68_P21579905	chr2:121097022-121097066	NM_013735:78	Trp53bp1	INSIDE	0.452	0.566	2441.59	1381.63	0.256	2012.26	514.89
A_68_P20886548	chr1:184339548-184339592	NM_173378:273	Trp53bp2	INSIDE	0.535	0.618	889.01	549.56	0.331	808.57	267.35
A_68_P21433077	chr2:93028116-93028160	NM_001025246:398	Trp53i11	INSIDE	0.158	0.454	2258.93	1026.01	0.072	1723.33	123.44
A_68_P21433078	chr2:93028237-93028293	NM_001025246:524	Trp53i11	INSIDE	0.070	1.431	1844.01	2638.20	0.100	1150.48	114.97
A_68_P21433080	chr2:93028458-93028502	NM_001025246:740	Trp53i11	INSIDE	0.216	0.666	1377.28	917.76	0.144	1043.00	149.90
A_68_P22728289	chr4:11083171-11083217	NM_001199105:-393	Trp53inp1	PROMOTER	0.292	0.528	1082.70	572.05	0.155	783.13	121.02
A_68_P22728294	chr4:11083669-11083713	NM_001199105:103	Trp53inp1	INSIDE	0.663	0.473	1751.11	828.77	0.314	1542.51	484.14
A_68_P22728295	chr4:11083786-11083830	NM_001199105:221	Trp53inp1	INSIDE	0.560	0.563	1881.95	1060.08	0.316	1447.62	457.02
A_68_P22728296	chr4:11083878-11083922	NM_001199105:313	Trp53inp1	INSIDE	0.408	0.443	2227.67	986.41	0.181	1725.74	311.95
A_68_P21765246	chr2:155207391-155207435	NM_178111:-179	Trp53inp2	PROMOTER	0.252	0.659	3385.42	2231.52	0.166	2563.74	425.32
A_68_P21765252	chr2:155208027-155208072	NM_178111:458	Trp53inp2	INSIDE	0.389	0.631	1550.89	978.36	0.246	1258.39	309.06
A_68_P21765289	chr2:155212485-155212529	NM_178111:4915	Trp53inp2	INSIDE	0.494	1.369	3149.57	4313.11	0.677	2137.30	1446.26
A_68_P21831888	chr2:166612033-166612077	NM_023815:-7212	Trp53rk	PROMOTER	0.129	3.650	3562.89	13005.70	0.471	2209.23	1041.49
A_68_P26743634	chr9:95650227-95650271	NM_011643:529	Trpc1	INSIDE	0.269	0.644	1039.10	669.39	0.173	881.73	152.71
A_68_P22145981	chr3:53959830-53959874	NM_016984:-173	Trpc4	PROMOTER	0.482	0.592	1367.19	808.74	0.285	1112.73	317.09
A_68_P21767075	chr2:155517577-155517621	NM_001163452:522	Trpc4ap	INSIDE	0.185	0.745	5254.94	3915.07	0.137	3538.16	486.40
A_68_P29064233	chr13:56989277-56989325	NM_012035:7649	Trpc7	INSIDE	0.598	3.530	1275.13	4500.65	2.110	856.97	1807.91
A_68_P32010023	chr19:22213713-22213757	NM_001035239:128	Trpm3	INSIDE	0.207	0.633	1092.36	691.78	0.131	890.32	116.73
A_68_P32010024	chr19:22213827-22213871	NM_001035239:242	Trpm3	INSIDE	0.296	0.576	1073.25	617.71	0.170	900.06	153.11
A_68_P25092468	chr7:52588927-52588971	NM_175130:202	Trpm4	INSIDE	0.536	0.543	2576.90	1399.29	0.291	1828.71	532.36
A_68_P31992848	chr19:18824278-18824322	NM_153417:-172	Trpm6	PROMOTER	0.224	0.525	1331.08	698.30	0.118	1033.08	121.53
A_68_P30207180	chr15:50721578-50721632	NM_032000:-17	Trps1	PROMOTER	0.035	1.391	3787.97	5268.18	0.049	2339.62	114.92
A_68_P30207182	chr15:50721909-50721953	NM_032000:-343	Trps1	PROMOTER	0.191	0.533	2195.85	1171.01	0.102	1747.98	177.69
A_68_P30207183	chr15:50721969-50722024	NM_032000:-409	Trps1	PROMOTER	0.315	0.446	1413.75	629.93	0.140	1070.76	150.24
A_68_P24162461	chr5:145528855-145528900	NM_001081362:-783	Trrap	DIVERGENT_PROMOTER	0.345	0.692	1276.24	883.62	0.239	930.82	222.09
A_68_P24162465	chr5:145529208-145529252	NM_001081362:-430	Trrap	DIVERGENT_PROMOTER	0.299	0.611	1448.64	885.12	0.183	1176.65	214.85
A_68_P21092313	chr2:28496774-28496818	NM_022887:34	Tsc1	INSIDE	0.452	0.459	1582.59	726.76	0.208	1399.56	290.56
A_68_P29733545	chr14:76814748-76814792	NM_207652:-857	Tsc22d1	PROMOTER	0.552	0.670	1552.87	1040.69	0.370	1208.06	446.93
A_68_P29733550	chr14:76815819-76815868	NM_207652:216	Tsc22d1	INSIDE	0.337	0.659	1206.39	794.62	0.222	889.10	197.56
A_68_P22168170	chr3:58220169-58220213	NM_001081229:580	Tsc22d2	INSIDE	0.240	0.426	1533.22	653.35	0.102	1154.07	117.94
A_68_P22168171	chr3:58220304-58220348	NM_001081229:716	Tsc22d2	INSIDE	0.230	0.512	2654.64	1358.59	0.118	1937.31	228.40
A_68_P22168174	chr3:58220683-58220727	NM_001081229:1094	Tsc22d2	INSIDE	0.306	0.620	1368.55	849.10	0.190	1397.48	265.58
A_68_P22168178	chr3:58221125-58221170	NM_001081229:1537	Tsc22d2	INSIDE	0.232	0.391	1634.68	639.56	0.091	1344.83	121.81
A_68_P32700055	chrX:137132673-137132722	NM_001077364:2364	Tsc22d3	INSIDE	0.135	1.560	1367.22	2133.18	0.211	569.62	119.94
A_68_P32700071	chrX:137134752-137134796	NM_001077364:287	Tsc22d3	INSIDE	0.398	3.957	5767.82	22822.44	1.575	2608.73	4107.70
A_68_P32700072	chrX:137134843-137134887	NM_001077364:197	Tsc22d3	INSIDE	0.339	7.001	3913.06	27395.46	2.371	1903.95	4514.37
A_68_P24946865	chr7:3645526-3645571	NM_024168:-561	Tsen34	PROMOTER	0.235	0.434	1474.09	640.26	0.102	1181.30	120.83
A_68_P24946869	chr7:3645931-3645975	NM_024168:-157	Tsen34	PROMOTER	0.181	0.722	2688.92	1942.53	0.131	1972.03	258.20
A_68_P28155496	chr11:115676513-115676557	NM_029557:482	Tsen54	INSIDE	0.222	0.409	1575.38	644.19	0.091	1320.59	120.08
A_68_P28155497	chr11:115676636-115676680	NM_029557:606	Tsen54	INSIDE	0.433	0.595	975.44	580.67	0.258	697.46	179.61
A_68_P20156312	chr1:37921635-37921679	NM_207228:268	Tsga10	INSIDE	0.589	0.734	2196.50	1611.88	0.432	1827.78	789.63
A_68_P24333613	chr6:30643690-30643734	NM_031998:-30	Tsga14	PROMOTER	0.233	0.486	1368.04	664.28	0.113	1098.58	124.28
A_68_P21848231	chr2:169459132-169459176	NM_080455:9	Tshz2	INSIDE	0.233	0.442	1443.67	638.70	0.103	1160.36	119.75
A_68_P32572889	chrX:100643328-100643372	NR_002844:16495	Tsix	INSIDE	0.425	3.366	7222.62	24314.54	1.431	2843.03	4068.66

ProbeName	Target position of probe on CpG island microarray	TargetID	GeneSymbol	CpG island Description	Ratio of relative methylation (TiO2-NP/Vehicle)	Sham group			TiO_2-H group		
						Relative methylation (Cy-5/Cy-3)	Cy-3 signal (Input)	Cy-5 signal (MeDIP)	Relative methylation (Cy-5/Cy-3)	Cy-3 signal (Input)	Cy-5 signal (MeDIP)
A_68_P25355274	chr7:105509904-105509948	NM_001168539:-88	Tsku	PROMOTER	0.561	0.488	1169.56	570.41	0.273	1020.35	279.06
A_68_P20551183	chr1:120207585-120207629	NM_011650:103	Tsn	INSIDE	0.648	0.539	1932.44	1042.17	0.349	1603.20	560.27
A_68_P24287930	chr6:21801755-21801799	NM_173007:739	Tspan12	INSIDE	0.325	0.579	2836.66	1641.35	0.188	2156.87	406.19
A_68_P24287932	chr6:21801985-21802029	NM_173007:509	Tspan12	INSIDE	0.538	0.517	2203.92	1138.52	0.278	1836.71	510.11
A_68_P24287933	chr6:21802094-21802138	NM_173007:399	Tspan12	INSIDE	0.575	0.711	1841.57	1309.71	0.409	1520.64	622.07
A_68_P28356086	chr12:36768656-36768700	NM_025359:387	Tspan13	INSIDE	0.240	0.624	4176.37	2605.91	0.150	3185.85	477.85
A_68_P28356088	chr12:36768830-36768874	NM_025359:213	Tspan13	INSIDE	0.199	0.604	3258.81	1968.73	0.120	2354.42	282.63
A_68_P29560572	chr14:41780482-41780526	NM_145928:-408	Tspan14	PROMOTER	0.189	0.561	1341.96	753.37	0.106	1101.77	117.12
A_68_P29560588	chr14:41779590-41779634	NM_145928:484	Tspan14	INSIDE	0.581	0.520	1838.70	955.94	0.302	1504.30	454.60
A_68_P29560589	chr14:41779724-41779768	NM_145928:350	Tspan14	INSIDE	0.652	0.589	1931.70	1138.56	0.384	1656.26	636.34
A_68_P29560592	chr14:41780099-41780143	NM_145928:-24	Tspan14	PROMOTER	0.598	0.567	1621.41	918.61	0.339	1295.42	438.68
A_68_P27184874	chr10:61693685-61693729	NM_197996:260	Tspan15	INSIDE	0.372	0.456	2279.58	1039.91	0.170	1826.28	310.29
A_68_P21434081	chr2:93174589-93174633	NM_183180:34	Tspan18	INSIDE	0.644	0.636	822.85	523.40	0.409	729.18	298.47
A_68_P22382654	chr3:102538511-102538562	NM_027533:-156	Tspan2	PROMOTER	0.289	0.588	1057.44	621.30	0.170	758.81	128.76
A_68_P22382659	chr3:102539232-102539276	NM_027533:562	Tspan2	INSIDE	0.468	0.485	3086.63	1495.77	0.227	2452.08	556.29
A_68_P25589127	chr7:148667760-148667804	NM_053082:6644	Tspan4	INSIDE	0.242	1.392	4036.73	5617.31	0.337	2621.74	883.40
A_68_P22570423	chr3:138406072-138406116	NM_019571:936	Tspan5	INSIDE	0.298	0.557	5007.03	2790.18	0.166	3779.51	627.44
A_68_P32749010	chrX:148776690-148776734	NM_029836:315	Tspyl2	INSIDE	0.232	1.354	4174.90	5653.56	0.315	1736.77	546.61
A_68_P27056226	chr10:34017317-34017361	NM_030203:112	Tspyl4	INSIDE	0.281	0.464	1299.68	602.88	0.130	1003.98	130.86
A_68_P27056228	chr10:34017531-34017575	NM_030203:326	Tspyl4	INSIDE	0.617	0.630	1544.81	972.47	0.388	1238.09	480.83
A_68_P27056229	chr10:34017638-34017682	NM_030203:434	Tspyl4	INSIDE	0.239	0.614	1028.06	631.73	0.147	805.14	118.10
A_68_P30125732	chr15:33617536-33617580	NM_001085421:80	Tspyl5	INSIDE	0.516	0.643	818.41	525.87	0.331	724.43	240.09
A_68_P30125734	chr15:33617702-33617758	NM_001085421:-92	Tspyl5	PROMOTER	0.345	0.439	1748.06	767.52	0.151	1378.11	208.73
A_68_P28314942	chr12:29436931-29436975	NM_201357:260	Tssc1	INSIDE	0.220	0.577	1189.84	686.99	0.127	895.52	113.69
A_68_P25598461	chr7:150254989-150255033	NM_001115085:-262	Tssc4	PROMOTER	0.376	0.561	1030.85	577.91	0.211	694.88	146.50
A_68_P25598465	chr7:150255389-150255433	NM_001115085:138	Tssc4	INSIDE	0.117	0.590	3081.52	1817.23	0.069	2287.58	158.45
A_68_P25948612	chr8:72426018-72426066	NM_032004:-71	Tssk6	PROMOTER	0.505	0.627	991.05	621.52	0.317	751.56	238.08
A_68_P25948613	chr8:72426109-72426153	NM_032004:17	Tssk6	INSIDE	0.431	0.712	3179.15	2263.23	0.307	2270.51	697.43
A_68_P22887152	chr4:46151235-46151279	NM_173033:91	Tstd2	INSIDE	0.576	0.516	2509.76	1294.07	0.297	1766.84	525.10
A_68_P31206979	chr17:46592282-46592326	NM_001162864:32320	Ttbk1	INSIDE	0.321	0.442	2903.34	1284.53	0.142	2013.32	286.35
A_68_P31206981	chr17:46592555-46592609	NM_001162864:32042	Ttbk1	INSIDE	0.392	1.717	3863.90	6635.69	0.673	2504.05	1685.58
A_68_P31206982	chr17:46592667-46592712	NM_001162864:31935	Ttbk1	INSIDE	0.392	1.403	1399.62	1963.16	0.549	1059.83	582.08
A_68_P21577444	chr2:120675934-120675978	NM_001024856:198	Ttbk2	INSIDE	0.272	0.591	955.67	564.61	0.161	771.32	124.01
A_68_P27752129	chr11:43561327-43561371	NM_133795:127	Ttc1	INSIDE	0.268	0.579	1558.11	901.90	0.155	1154.64	179.09
A_68_P22044439	chr3:33698918-33698962	NM_025978:-176	Ttc14	PROMOTER	0.562	0.275	2184.87	601.13	0.155	1810.25	279.75
A_68_P21117412	chr2:32631071-32631115	NM_177384:-189	Ttc16	DIVERGENT_PROMOTER	0.234	0.368	1711.38	628.97	0.086	1341.61	115.48
A_68_P26875193	chr9:119846837-119846881	NM_028735:135	Ttc21a	INSIDE	0.427	0.588	1305.35	767.49	0.251	976.75	245.21
A_68_P23984910	chr5:111714358-111714402	NM_024477:405559	Ttc28	INSIDE	0.289	1.503	3161.06	4751.77	0.435	2197.69	954.94
A_68_P29982442	chr15:5135931-5135975	NM_026213:393	Ttc33	INSIDE	0.215	0.512	2213.53	1132.96	0.110	1641.21	180.60
A_68_P26469621	chr9:44607463-44607507	NM_138951:3550	Ttc36	INSIDE	0.220	0.554	2445.69	1355.52	0.122	1759.58	214.34
A_68_P23200902	chr4:109079361-109079406	NM_153392:-321	Ttc39a	PROMOTER	0.284	0.620	851.96	528.47	0.176	666.06	117.44
A_68_P23200903	chr4:109079476-109079520	NM_153392:-206	Ttc39a	PROMOTER	0.437	0.469	1459.99	684.90	0.205	1153.72	236.25
A_68_P23061865	chr4:82969752-82969796	NM_027238:319	Ttc39b	INSIDE	0.354	0.615	1060.46	651.68	0.217	895.67	194.79
A_68_P31501878	chr18:12802774-12802818	NM_028341:755	Ttc39c	INSIDE	0.223	0.558	1396.43	778.72	0.124	992.46	123.39

ProbeName	Target position of probe on CpG island microarray	TargetID	GeneSymbol	CpG island Description	Ratio of relative methylation (TiO2-NP/Vehicle)	Sham group			TiO_2-H group		
						Relative methylation (Cy-5/Cy-3)	Cy-3 signal (Input)	Cy-5 signal (MeDIP)	Relative methylation (Cy-5/Cy-3)	Cy-3 signal (Input)	Cy-5 signal (MeDIP)
A_68_P23185865	chr4:106351237-106351283	NM_001172073:31	Ttc4	INSIDE	0.292	0.596	911.39	542.97	0.174	694.48	121.01
A_68_P28581895	chr12:82732734-82732780	NM_001033149:401	Ttc9	INSIDE	0.144	0.544	2004.45	1091.41	0.078	1490.13	116.75
A_68_P25018371	chr7:28438749-28438793	NM_028417:-172	Ttc9b	PROMOTER	0.154	0.678	3610.47	2446.11	0.104	2537.41	264.46
A_68_P22372357	chr3:100773219-100773269	NM_001013026:342	Ttf2	INSIDE	0.226	0.574	1209.86	695.00	0.130	914.88	118.99
A_68_P22372358	chr3:100773375-100773419	NM_001013026:190	Ttf2	INSIDE	0.222	0.456	1603.65	731.80	0.101	1212.38	122.64
A_68_P22372359	chr3:100773528-100773572	NM_001013026:36	Ttf2	INSIDE	0.242	0.379	1562.60	591.71	0.092	1281.75	117.52
A_68_P26685860	chr9:83728064-83728108	NM_001110265:-209	Ttk	PROMOTER	0.251	0.642	1466.07	941.56	0.161	1124.79	180.98
A_68_P30388853	chr15:83341230-83341274	NM_178869:85	Ttll1	INSIDE	0.316	0.708	2039.81	1444.84	0.224	1502.97	336.53
A_68_P30388854	chr15:83341412-83341456	NM_178869:-97	Ttll1	PROMOTER	0.503	0.526	2594.20	1364.28	0.264	1902.91	502.95
A_68_P23441977	chr4:155409010-155409054	NM_029264:15894	Ttll10	INSIDE	0.600	0.598	988.82	590.88	0.358	850.24	304.68
A_68_P20349750	chr1:74708445-74708489	NM_001014974:139	Ttll4	INSIDE	0.522	0.412	2042.66	842.41	0.215	1604.77	345.34
A_68_P28042483	chr11:95985716-95985760	NM_172799:-9361	Ttll6	PROMOTER	0.512	0.665	958.90	637.64	0.341	944.08	321.73
A_68_P30422257	chr15:88785384-88785428	NM_172818:-558	Ttll8	PROMOTER	0.144	0.634	1698.15	1077.19	0.091	1346.29	122.97
A_68_P28149096	chr11:114537102-114537146	NM_053273:343	Ttyh2	INSIDE	0.338	0.584	1186.00	692.48	0.197	917.64	181.19
A_68_P25405561	chr7:116154611-116154655	NM_021885:239	Tub	INSIDE	0.528	0.428	1254.12	536.30	0.226	1062.98	240.08
A_68_P20352742	chr1:75216148-75216195	NM_009447:-343	Tuba4a	PROMOTER	0.252	0.615	929.77	572.00	0.155	785.47	121.96
A_68_P20352744	chr1:75216331-75216375	NM_009447:-524	Tuba4a	PROMOTER	0.502	0.657	1219.81	801.55	0.330	938.84	309.65
A_68_P20352745	chr1:75216487-75216531	NM_009447:-680	Tuba4a	PROMOTER	0.099	0.597	2801.56	1673.20	0.059	2084.90	123.34
A_68_P28936499	chr13:34169593-34169637	NM_009450:263	Tubb2a	INSIDE	0.434	0.707	3359.06	2376.05	0.307	2486.24	763.25
A_68_P28936501	chr13:34169775-34169819	NM_009450:81	Tubb2a	INSIDE	0.507	0.539	1638.25	882.70	0.273	1315.64	359.39
A_68_P28936822	chr13:34222312-34222356	NM_023716:-111	Tubb2b	PROMOTER	0.070	1.945	5328.16	10364.36	0.137	3246.00	445.09
A_68_P28936823	chr13:34222460-34222504	NM_023716:-259	Tubb2b	PROMOTER	0.095	1.885	9046.41	17054.26	0.179	5512.25	986.47
A_68_P21070645	chr2:25079554-25079599	NM_146116:646	Tubb2c	INSIDE	0.409	0.491	1200.14	589.19	0.201	968.76	194.37
A_68_P21070648	chr2:25079927-25079971	NM_146116:274	Tubb2c	INSIDE	0.286	0.550	2642.39	1453.91	0.157	2034.13	319.71
A_68_P21070649	chr2:25080027-25080071	NM_146116:174	Tubb2c	INSIDE	0.409	0.478	1412.01	675.35	0.196	1110.29	217.23
A_68_P21070651	chr2:25080168-25080218	NM_146116:30	Tubb2c	INSIDE	0.255	0.536	1514.11	810.85	0.137	1178.49	161.17
A_68_P21070652	chr2:25080248-25080297	NM_146116:-50	Tubb2c	PROMOTER	0.409	0.423	1560.81	659.82	0.173	1088.84	188.17
A_68_P21070655	chr2:25080672-25080722	NM_146116:-474	Tubb2c	PROMOTER	0.277	0.375	1654.74	620.62	0.104	1188.67	123.65
A_68_P26239786	chr8:125935222-125935266	NM_023279:-219	Tubb3	PROMOTER	0.654	0.589	1583.80	932.19	0.385	1419.24	546.12
A_68_P31259987	chr17:57226887-57226931	NM_009451:297	Tubb4	INSIDE	0.279	0.424	2760.55	1170.13	0.118	2071.49	245.36
A_68_P31259988	chr17:57226985-57227029	NM_009451:199	Tubb4	INSIDE	0.555	0.642	1049.31	673.97	0.357	849.33	302.91
A_68_P31161200	chr17:35975270-35975315	NM_011655:-46	Tubb5	DIVERGENT_PROMOTER	0.164	0.643	1931.59	1241.20	0.105	1407.87	147.99
A_68_P31791610	chr18:67551054-67551098	NM_026473:692	Tubb6	INSIDE	0.433	0.494	1698.06	838.42	0.214	1422.44	304.23
A_68_P28072085	chr11:100981492-100981536	NM_134024:70	Tubg1	INSIDE	0.181	0.493	2031.08	1001.35	0.089	1424.27	126.75
A_68_P28072252	chr11:101017412-101017456	NM_134028:237	Tubg2	INSIDE	0.480	0.569	1803.32	1026.03	0.273	1412.35	385.83
A_68_P25581865	chr7:147222114-147222158	NM_133755:113	Tubgcp2	INSIDE	0.670	0.693	2465.37	1707.82	0.464	2077.08	963.56
A_68_P25143365	chr7:63049393-63049437	NM_146190:-103	Tubgcp5	PROMOTER	0.536	1.392	4737.08	6594.67	0.747	3718.33	2776.19
A_68_P30423271	chr15:88953473-88953517	NM_001163319:86	Tubgcp6	INSIDE	0.185	2.593	4119.29	10679.64	0.481	2792.71	1342.48
A_68_P30423272	chr15:88953588-88953632	NM_001163319:-30	Tubgcp6	PROMOTER	0.283	1.407	1974.38	2777.34	0.398	1327.48	528.47
A_68_P25500776	chr7:133630670-133630714	NM_001163713:-176	Tufm	PROMOTER	0.425	0.554	1769.13	980.78	0.236	1292.11	304.30
A_68_P25500777	chr7:133630795-133630839	NM_001163713:-52	Tufm	PROMOTER	0.371	0.604	2306.18	1393.18	0.224	1675.38	375.58
A_68_P25500779	chr7:133631050-133631094	NM_001163713:204	Tufm	INSIDE	0.184	0.520	1427.60	741.82	0.095	1224.95	116.81
A_68_P27553116	chr11:3549120-3549164	NR_002321:-331	Tug1	DIVERGENT_PROMOTER	0.583	0.551	1689.80	930.29	0.321	1397.58	448.47
A_68_P24838521	chr6:128305667-128305711	NM_011657:181	Tulp3	INSIDE	0.145	0.746	6524.39	4868.60	0.108	4634.17	501.95

ProbeName	Target position of probe on CpG island microarray	TargetID	GeneSymbol	CpG island Description	Ratio of relative methylation (TiO2-NP/Vehicle)	Sham group			TiO_2-H group		
						Relative methylation (Cy-5/Cy-3)	Cy-3 signal (Input)	Cy-5 signal (MeDIP)	Relative methylation (Cy-5/Cy-3)	Cy-3 signal (Input)	Cy-5 signal (MeDIP)
A_68_P23111977	chr4:93002257-93002301	NM_026954:-76	Tusc1	PROMOTER	0.623	0.432	1974.38	853.07	0.269	1522.63	410.16
A_68_P26809576	chr9:107465904-107465952	NM_019742:343	Tusc2	INSIDE	0.239	0.642	1442.79	925.64	0.154	1125.52	172.87
A_68_P25798745	chr8:40068907-40068951	NM_030254:8	Tusc3	INSIDE	0.257	0.501	2686.88	1347.04	0.129	2025.98	261.31
A_68_P26801828	chr9:106105584-106105628	NM_011876:168	Twf2	INSIDE	0.598	0.689	1170.24	806.50	0.412	1168.39	481.73
A_68_P28344354	chr12:34642575-34642619	NM_011658:61	Twist1	INSIDE	0.479	0.582	2241.59	1305.33	0.279	1874.20	522.72
A_68_P28344357	chr12:34643155-34643199	NM_011658:641	Twist1	INSIDE	0.208	0.519	2328.00	1207.52	0.108	1817.89	196.54
A_68_P20444769	chr1:93698784-93698828	NM_007855:753	Twist2	INSIDE	0.465	0.527	1498.21	789.11	0.245	1092.66	267.35
A_68_P20444771	chr1:93699116-93699160	NM_007855:1085	Twist2	INSIDE	0.534	0.530	1369.72	726.43	0.283	1052.66	298.32
A_68_P23297755	chr4:129318416-129318460	NM_001199695:-129	Txlna	PROMOTER	0.333	0.698	1677.30	1170.21	0.232	1301.70	302.60
A_68_P32791133	chrX:159266782-159266826	NM_178935:527	Txlng	INSIDE	0.229	2.150	4533.73	9746.57	0.493	1915.65	945.02
A_68_P32791135	chrX:159266933-159266977	NM_178935:377	Txlng	INSIDE	0.234	2.256	4704.49	10614.11	0.527	2013.52	1061.48
A_68_P22948240	chr4:57968866-57968910	NM_011660:395	Txn1	INSIDE	0.618	0.562	1046.69	588.20	0.347	910.36	316.27
A_68_P30356630	chr15:77759323-77759367	NM_019913:80	Txn2	INSIDE	0.491	0.516	1660.39	857.00	0.253	1292.87	327.33
A_68_P30356632	chr15:77759535-77759579	NM_019913:-132	Txn2	PROMOTER	0.387	0.738	4818.93	3558.05	0.286	3258.39	931.89
A_68_P30546420	chr16:11134105-11134152	NM_029582:497	Txndc11	INSIDE	0.124	0.494	2990.76	1478.87	0.062	2120.31	130.46
A_68_P30546421	chr16:11134214-11134258	NM_029582:389	Txndc11	INSIDE	0.214	0.540	1251.05	675.46	0.116	1053.64	121.97
A_68_P29056625	chr13:55816369-55816420	NM_175150:384	Txndc15	INSIDE	0.215	0.578	1333.07	770.55	0.124	943.04	117.21
A_68_P29569510	chr14:45839181-45839225	NM_172597:-133	Txndc16	DIVERGENT_PROMOTER	0.453	0.628	1068.30	671.23	0.284	530.92	150.99
A_68_P31771006	chr18:63851716-63851760	NM_016792:275	Txnl1	INSIDE	0.180	0.338	2655.78	897.07	0.061	2002.14	121.86
A_68_P31771008	chr18:63851869-63851913	NM_016792:123	Txnl1	INSIDE	0.500	0.624	967.36	604.02	0.312	795.19	248.03
A_68_P26157753	chr8:112089724-112089768	NM_175646:-139	Txnl4b	DIVERGENT_PROMOTER	0.442	0.496	1241.52	615.33	0.219	986.23	215.99
A_68_P27294064	chr10:82321765-82321809	NM_001042513:-164	Txnrd1	PROMOTER	0.407	0.407	1520.91	618.76	0.166	1187.54	196.62
A_68_P27294068	chr10:82322231-82322275	NM_001042514:-833	Txnrd1	PROMOTER	0.455	0.527	1103.64	581.49	0.240	897.92	215.07
A_68_P30579666	chr16:18426377-18426421	NM_013711:-111	Txnrd2	PROMOTER	0.223	0.561	1220.57	684.81	0.125	1009.17	126.49
A_68_P24629441	chr6:89594015-89594059	NM_001178058:55	Txnrd3	INSIDE	0.248	0.656	1009.04	662.17	0.163	805.31	131.26
A_68_P24629444	chr6:89594337-89594382	NM_001178058:378	Txnrd3	INSIDE	0.323	0.633	850.49	538.49	0.205	606.75	124.15
A_68_P26345603	chr9:20935690-20935734	NM_001205312:7	Tyk2	INSIDE	0.522	0.447	1299.32	581.22	0.234	950.51	222.05
A_68_P26345605	chr9:20935853-20935897	NM_001205312:-155	Tyk2	PROMOTER	0.215	0.430	1723.99	740.76	0.092	1306.52	120.43
A_68_P26345606	chr9:20935934-20935978	NM_001205312:-237	Tyk2	PROMOTER	0.183	0.523	2169.75	1135.59	0.096	1687.23	161.85
A_68_P27181839	chr10:61158255-61158299	NM_027912:15	Tysnd1	INSIDE	0.421	0.715	1529.10	1092.85	0.301	1262.60	380.16
A_68_P27181840	chr10:61158435-61158479	NM_027912:195	Tysnd1	INSIDE	0.539	0.559	1478.70	826.44	0.301	1225.02	369.29
A_68_P31962486	chr19:12869204-12869248	NR_024093:1387	U05342	INSIDE	0.505	0.456	2112.45	964.22	0.230	1734.61	399.72
A_68_P31962498	chr19:12870780-12870828	NR_024093:-191	U05342	DIVERGENT_PROMOTER	0.145	0.672	1627.79	1093.84	0.098	1213.82	118.51
A_68_P31962499	chr19:12870892-12870947	NR_024093:-306	U05342	DIVERGENT_PROMOTER	0.221	0.665	1256.85	835.66	0.147	878.84	129.33
A_68_P24951860	chr7:5012759-5012803	NM_001205231:-964	U2af2	PROMOTER	0.035	1.731	3897.00	6746.08	0.061	2578.09	157.88
A_68_P24951861	chr7:5012862-5012906	NM_001205231:-860	U2af2	PROMOTER	0.050	1.386	5584.12	7737.96	0.069	3565.50	246.70
A_68_P24951866	chr7:5013371-5013415	NM_001205231:-352	U2af2	PROMOTER	0.511	0.525	1150.93	604.71	0.268	918.34	246.56
A_68_P24951867	chr7:5013463-5013510	NM_001205231:-258	U2af2	PROMOTER	0.204	0.628	1088.31	683.19	0.128	913.11	117.03
A_68_P20820926	chr1:172105452-172105507	NM_133806:-402	Uap1	PROMOTER	0.205	0.535	1269.42	679.15	0.110	1086.78	119.01
A_68_P25952345	chr8:73033987-73034031	NM_019883:258	Uba52	INSIDE	0.531	0.523	1817.14	951.17	0.278	1421.48	395.29
A_68_P23862543	chr5:86601868-86601912	NM_172712:-122	Uba6	PROMOTER	0.398	0.692	2387.01	1652.51	0.275	1949.46	536.57
A_68_P21075774	chr2:25877393-25877437	NM_133835:-134	Ubac1	PROMOTER	0.473	0.587	929.27	545.17	0.277	735.12	203.83
A_68_P26447519	chr9:40964965-40965009	NM_176860:591	Ubash3b	INSIDE	0.344	0.430	1488.87	640.59	0.148	1269.45	188.16
A_68_P26447525	chr9:40965613-40965657	NM_176860:-57	Ubash3b	PROMOTER	0.615	0.411	1287.73	529.80	0.253	1161.68	293.92

ProbeName	Target position of probe on CpG island microarray	TargetID	GeneSymbol	CpG island Description	Ratio of relative methylation (TiO2-NP/Vehicle)	Sham group			TiO_2-H group		
						Relative methylation (Cy-5/Cy-3)	Cy-3 signal (Input)	Cy-5 signal (MeDIP)	Relative methylation (Cy-5/Cy-3)	Cy-3 signal (Input)	Cy-5 signal (MeDIP)
A_68_P27854524	chr11:62364466-62364510	NM_011664:-517	Ubb	PROMOTER	0.556	0.626	3728.27	2333.43	0.348	2821.54	981.17
A_68_P27854525	chr11:62364572-62364616	NM_011664:-411	Ubb	PROMOTER	0.247	0.331	1984.84	657.46	0.082	1445.63	118.38
A_68_P27854526	chr11:62364648-62364692	NM_011664:-335	Ubb	PROMOTER	0.206	0.344	2463.59	847.60	0.071	1806.92	128.23
A_68_P24066623	chr5:125869716-125869760	NM_019639:649	Ubc	INSIDE	0.134	0.634	6152.72	3902.70	0.085	4499.47	382.72
A_68_P24066628	chr5:125870372-125870416	NM_019639:-7	Ubc	PROMOTER	0.427	0.549	1991.27	1093.22	0.234	1505.86	352.80
A_68_P32320542	chrX:34414678-34414722	NM_019668:340	Ube2a	INSIDE	0.172	1.843	976.13	1798.99	0.317	387.01	122.84
A_68_P27796900	chr11:51813767-51813811	NM_009458:180	Ube2b	INSIDE	0.289	0.391	1321.77	517.13	0.113	1046.13	118.28
A_68_P21819632	chr2:164595210-164595259	NM_026785:-194	Ube2c	PROMOTER	0.192	0.507	1812.01	919.57	0.098	1389.77	135.69
A_68_P27234634	chr10:70747646-70747690	NM_145420:342	Ube2d1	INSIDE	0.376	0.444	1934.49	859.07	0.167	1590.62	265.77
A_68_P22552061	chr3:135101698-135101742	NM_025356:-2	Ube2d3	DIVERGENT_PROMOTER	0.446	0.590	1701.83	1003.69	0.263	1466.57	385.37
A_68_P22552063	chr3:135102173-135102217	NM_025356:472	Ube2d3	INSIDE	0.368	0.625	1947.42	1216.38	0.230	1437.53	330.82
A_68_P29438120	chr14:19159804-19159848	NM_009455:4532	Ube2e1	INSIDE	0.499	1.438	13529.53	19455.29	0.718	9696.69	6961.56
A_68_P29440562	chr14:19726350-19726394	NM_144839:-231	Ube2e2	PROMOTER	0.345	0.574	1097.38	629.74	0.198	820.14	162.60
A_68_P29440563	chr14:19726488-19726532	NM_144839:-369	Ube2e2	PROMOTER	0.092	2.790	14911.27	41604.55	0.257	9652.93	2478.26
A_68_P27269296	chr10:77085151-77085195	NM_019803:107	Ube2g2	INSIDE	0.307	0.484	1202.52	581.61	0.149	974.75	144.97
A_68_P24331348	chr6:30254521-30254565	NM_001169576:-3	Ube2h	PROMOTER	0.250	0.632	978.60	618.42	0.158	768.99	121.58
A_68_P24974288	chr7:13622741-13622791	NM_145578:561	Ube2m	INSIDE	0.587	3.217	3408.62	10964.70	1.887	2139.26	4036.39
A_68_P24974296	chr7:13623790-13623834	NM_001168469:-193	Ube2m	PROMOTER	0.236	0.505	1239.19	625.40	0.119	1003.09	119.35
A_68_P24974297	chr7:13623923-13623967	NM_001168469:-325	Ube2m	PROMOTER	0.296	0.616	967.88	596.50	0.182	764.16	139.33
A_68_P27365510	chr10:94977758-94977802	NM_080560:-15	Ube2n	PROMOTER	0.544	0.448	1157.40	518.61	0.244	935.78	228.11
A_68_P28160204	chr11:116442979-116443023	NM_173755:-239	Ube2o	PROMOTER	0.346	0.696	1128.49	785.63	0.241	927.90	223.53
A_68_P29117520	chr13:69878183-69878227	NM_001145162:571	Ube2ql1	INSIDE	0.388	0.642	1526.90	979.63	0.249	1303.06	324.09
A_68_P24950633	chr7:4763653-4763698	NM_133777:267	Ube2s	INSIDE	0.320	0.696	2552.57	1776.55	0.223	1803.54	401.60
A_68_P24950639	chr7:4764364-4764408	NM_133777:-444	Ube2s	PROMOTER	0.366	1.628	13007.66	21180.35	0.597	8763.23	5227.49
A_68_P20636517	chr1:136859020-136859067	NM_026024:-110	Ube2t	DIVERGENT_PROMOTER	0.423	0.447	1648.84	736.46	0.189	1452.07	274.19
A_68_P20636519	chr1:136859233-136859277	NM_026024:101	Ube2t	INSIDE	0.412	0.519	1782.14	924.96	0.214	1524.67	325.64
A_68_P21836562	chr2:167456829-167456873	NM_023230:655	Ube2v1	INSIDE	0.369	0.640	7254.47	4646.42	0.236	5275.91	1247.68
A_68_P25158144	chr7:66483973-66484017	NM_173010:-125	Ube3a	PROMOTER	0.348	0.462	1876.60	866.42	0.161	1602.23	257.60
A_68_P25158152	chr7:66484943-66484987	NM_001033962:843	Ube3a	INSIDE	0.256	0.485	1627.14	789.31	0.124	1358.78	168.87
A_68_P24003597	chr5:114830959-114831004	NM_054093:366	Ube3b	INSIDE	0.411	0.529	4098.81	2167.91	0.218	3145.46	684.31
A_68_P26470628	chr9:44773616-44773660	NM_145400:45	Ube4a	INSIDE	0.279	0.615	1197.04	736.45	0.172	834.72	143.46
A_68_P23400515	chr4:148800617-148800661	NM_022022:102	Ube4b	INSIDE	0.122	0.452	3107.56	1405.64	0.055	2084.13	114.58
A_68_P23400518	chr4:148800981-148801025	NM_022022:-262	Ube4b	PROMOTER	0.453	0.412	1376.42	567.31	0.187	1115.89	208.26
A_68_P23394410	chr4:147818444-147818488	NM_027873:394	Ubiad1	INSIDE	0.594	0.710	1967.05	1395.74	0.421	1672.34	704.34
A_68_P22314484	chr3:88358116-88358160	NM_033526:501	Ubqln4	INSIDE	0.137	0.544	2132.16	1160.56	0.074	1560.54	115.96
A_68_P31210122	chr17:47147092-47147136	NM_001177374:367	Ubr2	INSIDE	0.198	0.375	2160.65	809.81	0.074	1648.66	122.20
A_68_P31210125	chr17:47147460-47147504	NM_001177374:-1	Ubr2	PROMOTER	0.340	0.467	1693.61	790.74	0.159	1214.67	192.81
A_68_P21311590	chr2:69735990-69736034	NM_001081548:710	Ubr3	INSIDE	0.359	1.511	6847.83	10349.14	0.543	5104.77	2769.63
A_68_P30147669	chr15:38007832-38007883	NM_001081359:751	Ubr5	INSIDE	0.239	0.477	1329.57	633.72	0.114	1067.81	121.71
A_68_P30147677	chr15:38008870-38008917	NM_001081359:-285	Ubr5	PROMOTER	0.194	0.582	1400.60	815.45	0.113	1131.24	127.50
A_68_P32118291	chr19:42056116-42056160	NM_145500:-114	Ubtd1	DIVERGENT_PROMOTER	0.438	0.640	2745.78	1757.67	0.281	2175.59	610.50
A_68_P32118296	chr19:42056888-42056932	NM_145500:658	Ubtd1	INSIDE	0.209	0.569	1433.39	815.35	0.119	1000.59	118.92
A_68_P27688940	chr11:32354802-32354846	NM_173784:-547	Ubtd2	PROMOTER	0.126	0.492	2761.76	1358.95	0.062	1967.27	122.45
A_68_P27688949	chr11:32355960-32356004	NM_173784:611	Ubtd2	INSIDE	0.422	0.650	2519.59	1637.60	0.274	1965.94	539.05

ProbeName	Target position of probe on CpG island microarray	TargetID	GeneSymbol	CpG island Description	Ratio of relative methylation (TiO2-NP/Vehicle)	Sham group			TiO_2-H group		
						Relative methylation (Cy-5/Cy-3)	Cy-3 signal (Input)	Cy-5 signal (MeDIP)	Relative methylation (Cy-5/Cy-3)	Cy-3 signal (Input)	Cy-5 signal (MeDIP)
A_68_P28078911	chr11:102180801-102180845	NM_011551:-412	Ubtf	PROMOTER	0.446	0.652	1379.17	898.69	0.291	1087.62	316.34
A_68_P28078915	chr11:102181318-102181368	NM_011551:-932	Ubtf	PROMOTER	0.099	1.396	1276.09	1781.33	0.139	869.46	120.73
A_68_P22704455	chr4:6118180-6118224	NM_026534:-49	Ubxn2b	PROMOTER	0.407	0.671	1912.76	1284.31	0.274	1666.37	455.81
A_68_P20599491	chr1:130140934-130140978	NM_026390:199	Ubxn4	INSIDE	0.522	0.677	2087.79	1413.90	0.353	1703.00	601.59
A_68_P20599492	chr1:130141015-130141059	NM_026390:279	Ubxn4	INSIDE	0.459	0.650	4640.72	3016.78	0.299	3399.53	1014.81
A_68_P20599496	chr1:130141494-130141539	NM_026390:759	Ubxn4	INSIDE	0.351	0.521	1427.11	743.99	0.183	1232.59	225.43
A_68_P30651942	chr16:32332504-32332548	NM_177633:189	Ubxn7	INSIDE	0.220	0.435	1879.98	816.89	0.096	1483.41	141.93
A_68_P25769987	chr8:34752626-34752684	NM_178648:-206	Ubxn8	PROMOTER	0.250	0.593	977.36	580.01	0.148	796.30	117.94
A_68_P29856116	chr14:102053116-102053160	NM_016723:-45	Uchl3	PROMOTER	0.477	0.678	1252.29	848.85	0.323	1035.20	334.63
A_68_P20680089	chr1:145624837-145624881	NM_001159866:451	Uchl5	INSIDE	0.199	0.635	1275.72	810.46	0.126	960.30	121.29
A_68_P20827191	chr1:173224816-173224860	NM_025388:275	Ufc1	INSIDE	0.642	0.613	2240.32	1372.98	0.394	1628.28	641.14
A_68_P24124995	chr5:137735672-137735716	NM_027356:-202	Ufsp1	PROMOTER	0.340	1.516	984.83	1492.78	0.515	738.67	380.39
A_68_P22955051	chr4:59202692-59202736	NM_011673:293	Ugcg	INSIDE	0.545	0.563	1620.86	912.76	0.307	1262.37	387.28
A_68_P22955052	chr4:59202854-59202909	NM_011673:460	Ugcg	INSIDE	0.191	0.493	2628.45	1296.85	0.094	1261.71	119.09
A_68_P23756354	chr5:65826840-65826884	NM_009466:219	Ugdh	INSIDE	0.329	0.477	1619.17	772.85	0.157	1266.07	199.08
A_68_P23756359	chr5:65827367-65827411	NM_009466:-307	Ugdh	PROMOTER	0.415	0.455	1287.76	586.48	0.189	1071.15	202.44
A_68_P27636579	chr11:21270946-21270990	NM_139297:-86	Ugp2	PROMOTER	0.413	0.627	3347.53	2100.29	0.259	2781.75	720.34
A_68_P27636581	chr11:21271108-21271152	NM_139297:-248	Ugp2	PROMOTER	0.658	0.547	1452.35	794.69	0.360	1201.24	432.29
A_68_P22500445	chr3:125641287-125641331	NM_011674:160	Ugt8a	INSIDE	0.336	0.490	3569.56	1747.63	0.164	2752.41	452.21
A_68_P31255135	chr17:56442788-56442838	NM_001111078:-923	Uhrf1	PROMOTER	0.223	0.671	1245.67	835.51	0.150	764.21	114.41
A_68_P31255137	chr17:56443079-56443123	NM_001111078:-635	Uhrf1	PROMOTER	0.531	0.361	2256.96	815.72	0.192	1682.86	323.24
A_68_P31255140	chr17:56443504-56443548	NM_001111078:-209	Uhrf1	PROMOTER	0.179	0.492	2188.59	1076.76	0.088	1546.69	136.25
A_68_P31118858	chr17:27993704-27993749	NM_001080769:275	Uhrf1bp1	INSIDE	0.230	0.517	1782.60	921.56	0.119	1305.18	155.06
A_68_P31118859	chr17:27993828-27993872	NM_001080769:399	Uhrf1bp1	INSIDE	0.203	0.657	3498.82	2298.77	0.133	2559.01	340.95
A_68_P27332827	chr10:89208343-89208387	NM_029166:629	Uhrf1bp1l	INSIDE	0.517	0.602	2053.76	1236.54	0.311	1498.78	466.31
A_68_P27332828	chr10:89208416-89208468	NM_029166:707	Uhrf1bp1l	INSIDE	0.232	0.575	1160.00	667.26	0.133	881.77	117.45
A_68_P27850821	chr11:61668019-61668063	NM_013881:554	Ulk2	INSIDE	0.469	0.615	921.91	566.85	0.289	894.06	258.08
A_68_P26882495	chr9:121186443-121186487	NM_177589:-174	Ulk4	PROMOTER	0.302	0.724	1738.14	1258.88	0.219	1360.39	297.54
A_68_P30660864	chr16:33966979-33967023	NM_009471:89	Umps	INSIDE	0.400	0.552	1618.29	893.67	0.221	1283.96	283.85
A_68_P30660865	chr16:33967049-33967093	NM_009471:19	Umps	INSIDE	0.206	0.507	2569.49	1301.49	0.104	2231.05	232.45
A_68_P27942765	chr11:78157408-78157455	NM_011676:408	Unc119	INSIDE	0.135	0.599	1847.99	1106.68	0.081	1445.23	116.78
A_68_P24007965	chr5:115577169-115577225	NM_175352:7788	Unc119b	INSIDE	0.627	3.482	399.10	1389.78	2.182	342.83	748.07
A_68_P24007966	chr5:115577255-115577310	NM_175352:7702	Unc119b	INSIDE	0.649	5.250	1870.32	9818.65	3.406	1256.12	4278.53
A_68_P25958199	chr8:74194916-74194960	NM_001029873:718	Unc13a	INSIDE	0.198	0.511	3020.20	1544.04	0.101	2116.64	214.29
A_68_P25958201	chr8:74195222-74195266	NM_001029873:412	Unc13a	INSIDE	0.385	0.429	1736.62	744.39	0.165	1310.45	216.39
A_68_P22869764	chr4:43071791-43071835	NM_001081413:-43	Unc13b	PROMOTER	0.404	0.501	1272.86	637.85	0.202	1133.30	229.43
A_68_P29052391	chr13:55057276-55057320	NM_153131:6506	Unc5a	INSIDE	0.248	0.494	1243.66	614.83	0.123	944.51	115.98
A_68_P22584284	chr3:141128445-141128489	NM_009472:-61	Unc5c	PROMOTER	0.370	0.575	2698.73	1551.80	0.213	2117.13	450.32
A_68_P25747454	chr8:30329630-30329674	NM_153135:456	Unc5d	INSIDE	0.114	0.658	2688.28	1768.87	0.075	1939.09	145.04
A_68_P28694453	chr12:104187317-104187361	NM_001081017:270	Unc79	INSIDE	0.135	0.658	1775.46	1169.04	0.089	1317.48	116.97
A_68_P28694455	chr12:104187509-104187553	NM_001081017:462	Unc79	INSIDE	0.477	0.648	1703.01	1103.15	0.309	1328.01	409.98
A_68_P28694456	chr12:104187580-104187626	NM_001081017:534	Unc79	INSIDE	0.292	0.413	1388.25	573.76	0.121	1017.38	122.81
A_68_P20302824	chr1:66515076-66515120	NM_175510:78	Unc80	INSIDE	0.537	0.442	2680.25	1183.87	0.237	2131.57	505.42
A_68_P24136132	chr5:140016559-140016603	NM_013702:-3271	Uncx	PROMOTER	0.543	0.700	2479.04	1736.39	0.381	1900.50	723.36

ProbeName	Target position of probe on CpG island microarray	TargetID	GeneSymbol	CpG island Description	Ratio of relative methylation (TiO2-NP/Vehicle)	Sham group			TiO_2-H group		
						Relative methylation (Cy-5/Cy-3)	Cy-3 signal (Input)	Cy-5 signal (MeDIP)	Relative methylation (Cy-5/Cy-3)	Cy-3 signal (Input)	Cy-5 signal (MeDIP)
A_68_P24136133	chr5:140016708-140016752	NM_013702:-3121	Uncx	PROMOTER	0.230	0.701	1942.94	1362.01	0.161	1361.68	219.39
A_68_P24136136	chr5:140017148-140017192	NM_013702:-2681	Uncx	PROMOTER	0.205	0.410	1855.71	761.57	0.084	1437.44	121.11
A_68_P24136159	chr5:140020492-140020536	NM_013702:663	Uncx	INSIDE	0.554	0.493	1501.07	740.30	0.273	1128.42	308.57
A_68_P24136178	chr5:140022739-140022790	NM_013702:2913	Uncx	INSIDE	0.143	0.547	1889.50	1033.10	0.078	1509.11	118.01
A_68_P24136182	chr5:140023230-140023274	NM_013702:3401	Uncx	INSIDE	0.664	0.389	1861.09	723.76	0.258	1418.20	366.30
A_68_P24136194	chr5:140024878-140024927	NM_013702:5051	Uncx	DOWNSTREAM	0.263	0.366	1679.94	615.64	0.096	1316.56	126.70
A_68_P24002090	chr5:114580992-114581036	NM_011677:-150	Ung	PROMOTER	0.203	0.552	1463.58	808.59	0.112	1104.62	123.89
A_68_P24002094	chr5:114581485-114581529	NM_011677:342	Ung	INSIDE	0.562	0.697	2466.04	1718.55	0.391	1766.12	691.36
A_68_P24002095	chr5:114581570-114581614	NM_011677:428	Ung	INSIDE	0.452	0.669	1632.59	1092.65	0.303	1191.18	360.56
A_68_P31102171	chr17:25325763-25325807	NM_001197024:440	Unkl	INSIDE	0.626	0.446	1153.78	514.61	0.279	1027.76	287.03
A_68_P31102173	chr17:25325952-25325996	NM_001197024:630	Unkl	INSIDE	0.448	1.411	4361.39	6154.12	0.632	2988.51	1887.80
A_68_P31102174	chr17:25326044-25326091	NM_001197024:723	Unkl	INSIDE	0.287	2.199	2892.25	6361.48	0.630	1885.87	1188.62
A_68_P20976549	chr2:5872087-5872131	NM_001081132:-406	Upf2	DIVERGENT_PROMOTER	0.412	0.537	2244.35	1204.12	0.221	1687.54	373.12
A_68_P25670550	chr8:13785336-13785380	NM_025924:-256	Upf3a	PROMOTER	0.164	0.418	2270.42	949.67	0.069	1798.70	123.76
A_68_P25670556	chr8:13785998-13786042	NM_025924:406	Upf3a	INSIDE	0.431	0.710	2540.02	1802.82	0.306	1946.37	595.74
A_68_P32321786	chrX:34650071-34650115	NM_026573:225	Upf3b	INSIDE	0.338	1.475	1569.57	2315.84	0.499	628.25	313.59
A_68_P21768465	chr2:155755929-155755973	NM_018888:96	Uqcc	INSIDE	0.300	0.548	2307.00	1265.32	0.164	1686.87	277.31
A_68_P26817223	chr9:108838835-108838879	NM_025407:196	Uqcrc1	INSIDE	0.378	0.569	3102.80	1764.39	0.215	2284.53	491.08
A_68_P26817225	chr9:108838988-108839032	NM_025407:350	Uqcrc1	INSIDE	0.251	0.665	1543.53	1026.21	0.167	1080.27	180.40
A_68_P25469455	chr7:127778730-127778774	NM_025899:50	Uqcrc2	INSIDE	0.600	0.556	1663.72	924.96	0.333	1301.62	433.87
A_68_P25469458	chr7:127779197-127779246	NM_025899:519	Uqcrc2	INSIDE	0.274	0.480	1509.41	723.99	0.131	1071.55	140.69
A_68_P27804721	chr11:53244209-53244253	NM_025352:103	Uqcrq	INSIDE	0.656	0.376	1433.16	539.11	0.247	1209.03	298.46
A_68_P27804727	chr11:53244769-53244814	NM_025352:-458	Uqcrq	DIVERGENT_PROMOTER	0.136	0.472	2385.36	1124.72	0.064	1820.09	117.07
A_68_P30959118	chr16:90810399-90810443	NM_029497:238	Urb1	INSIDE	0.567	0.716	1794.66	1285.74	0.406	1464.42	594.60
A_68_P30959121	chr16:90810687-90810731	NM_029497:-50	Urb1	PROMOTER	0.085	0.749	5604.64	4196.73	0.064	3815.21	243.76
A_68_P27564839	chr11:5662438-5662482	NM_001077661:-81	Urgcp	PROMOTER	0.291	0.643	1337.79	860.34	0.187	1214.75	227.24
A_68_P21099685	chr2:29682982-29683026	NM_026615:96	Urm1	INSIDE	0.241	0.515	1175.62	605.07	0.124	967.59	119.94
A_68_P25542187	chr7:140900886-140900932	NM_009479:70	Uros	INSIDE	0.374	0.654	1344.15	878.77	0.244	1112.54	271.69
A_68_P25956520	chr8:73891092-73891136	NM_025917:-1	Use1	PROMOTER	0.360	0.672	1224.42	823.05	0.242	876.07	212.00
A_68_P20827836	chr1:173341700-173341744	NM_009480:-89	Usf1	PROMOTER	0.387	0.638	846.99	540.07	0.247	766.48	189.14
A_68_P25034254	chr7:31741889-31741933	NM_011680:-88	Usf2	PROMOTER	0.521	0.456	1801.66	821.91	0.238	1564.91	372.27
A_68_P25034259	chr7:31742709-31742753	NM_011680:-908	Usf2	PROMOTER	0.150	0.626	1646.18	1029.94	0.094	1299.90	121.88
A_68_P23141221	chr4:98590064-98590123	NM_146144:-407	Usp1	PROMOTER	0.257	0.460	1246.18	573.60	0.118	1016.12	120.01
A_68_P23141222	chr4:98590182-98590226	NM_146144:-296	Usp1	PROMOTER	0.249	0.389	2799.55	1088.64	0.097	1963.66	190.30
A_68_P32289745	chrX:20281291-20281340	NM_145628:281	Usp11	INSIDE	0.061	3.297	1735.70	5722.13	0.202	648.10	131.15
A_68_P32289746	chrX:20281381-20281426	NM_145628:369	Usp11	INSIDE	0.120	4.915	2187.48	10750.72	0.587	841.62	494.36
A_68_P32289747	chrX:20281555-20281599	NM_145628:542	Usp11	INSIDE	0.083	1.484	3365.04	4992.15	0.123	1300.83	159.74
A_68_P32289749	chrX:20281744-20281788	NM_145628:732	Usp11	INSIDE	0.294	1.410	2905.22	4097.29	0.415	1322.34	549.07
A_68_P22039930	chr3:32716759-32716803	NM_001013024:233	Usp13	INSIDE	0.369	0.588	1373.60	807.09	0.217	1076.73	233.35
A_68_P31487964	chr18:10029653-10029701	NM_001038589:471	Usp14	INSIDE	0.079	1.842	6195.31	11413.37	0.145	4093.49	592.67
A_68_P31487968	chr18:10030044-10030088	NM_001038589:81	Usp14	INSIDE	0.478	0.718	2964.47	2127.98	0.343	2212.20	758.69
A_68_P27513158	chr10:122634206-122634250	NM_027604:-249	Usp15	PROMOTER	0.306	0.705	4044.48	2850.09	0.216	3043.05	657.08
A_68_P27513159	chr10:122634312-122634356	NM_027604:-355	Usp15	PROMOTER	0.547	0.569	1198.25	681.30	0.311	905.95	281.84
A_68_P30939796	chr16:87455183-87455227	NM_024258:-25	Usp16	PROMOTER	0.365	0.514	1171.02	601.72	0.188	1168.48	219.09

ProbeName	Target position of probe on CpG island microarray	TargetID	GeneSymbol	CpG island Description	Ratio of relative methylation (TiO2-NP/Vehicle)	Sham group			TiO_2-H group		
						Relative methylation (Cy-5/Cy-3)	Cy-3 signal (Input)	Cy-5 signal (MeDIP)	Relative methylation (Cy-5/Cy-3)	Cy-3 signal (Input)	Cy-5 signal (MeDIP)
A_68_P32975183	chr9:108393525-108393569	NM_027804:540	Usp19	INSIDE	0.281	0.533	1036.90	552.26	0.149	786.82	117.61
A_68_P32975184	chr9:108393603-108393647	NM_027804:618	Usp19	INSIDE	0.145	0.448	2553.08	1142.68	0.065	1982.21	128.47
A_68_P21106327	chr2:30837787-30837831	NM_028846:10	Usp20	INSIDE	0.151	0.628	1694.49	1063.48	0.095	1276.90	120.83
A_68_P27846980	chr11:60988451-60988495	NM_001004143:89	Usp22	INSIDE	0.096	0.605	2922.75	1769.03	0.058	2122.12	122.84
A_68_P27846981	chr11:60988586-60988630	NM_001004143:-47	Usp22	PROMOTER	0.395	0.591	3307.59	1953.18	0.233	2575.10	600.24
A_68_P27846982	chr11:60988669-60988713	NM_001004143:-129	Usp22	PROMOTER	0.174	0.704	1374.08	967.17	0.123	978.49	119.89
A_68_P23183545	chr4:105988267-105988311	NM_183225:-529	Usp24	PROMOTER	0.232	1.519	1236.60	1878.81	0.353	961.88	339.71
A_68_P30887980	chr16:77014501-77014545	NM_013918:209	Usp25	INSIDE	0.258	0.617	1777.23	1096.07	0.159	1478.42	235.57
A_68_P26494988	chr9:48793866-48793910	NM_175482:399	Usp28	INSIDE	0.229	0.611	2573.08	1571.55	0.140	1976.90	276.95
A_68_P26494989	chr9:48793954-48794001	NM_175482:488	Usp28	INSIDE	0.478	0.544	958.06	521.24	0.260	769.11	199.94
A_68_P26593987	chr9:66439772-66439816	NM_144937:993	Usp3	INSIDE	0.666	0.520	1052.45	547.37	0.347	874.53	303.08
A_68_P27981394	chr11:84953213-84953257	NM_001029934:223	Usp32	INSIDE	0.204	0.729	5481.35	3997.05	0.149	4000.64	594.10
A_68_P27981396	chr11:84953706-84953750	NM_001029934:-271	Usp32	PROMOTER	0.213	0.477	1455.31	694.32	0.102	1174.14	119.28
A_68_P28170438	chr11:118151273-118151317	NM_001033528:264	Usp36	INSIDE	0.265	0.690	2983.32	2058.58	0.183	2067.49	377.70
A_68_P28170440	chr11:118151534-118151578	NM_001033528:2	Usp36	INSIDE	0.235	0.345	1889.19	652.00	0.081	1482.46	120.12
A_68_P26002691	chr8:83538819-83538865	NM_027554:-37	Usp38	PROMOTER	0.115	0.544	2694.02	1466.30	0.063	2055.40	129.13
A_68_P26814406	chr9:108250276-108250320	NM_011678:137	Usp4	INSIDE	0.566	0.419	1705.18	714.12	0.237	1419.62	336.31
A_68_P26814408	chr9:108250538-108250582	NM_011678:399	Usp4	INSIDE	0.373	0.377	1442.99	543.97	0.141	1142.13	160.51
A_68_P26814409	chr9:108250644-108250688	NM_011678:505	Usp4	INSIDE	0.287	0.698	3306.90	2307.48	0.200	2490.18	499.03
A_68_P20421183	chr1:89905073-89905117	NM_001033291:32	Usp40	INSIDE	0.189	0.702	3735.32	2620.52	0.133	2823.27	374.41
A_68_P27355935	chr10:93296839-93296883	NM_183199:2561	Usp44	INSIDE	0.317	0.467	2048.29	957.04	0.148	1560.01	231.12
A_68_P27356012	chr10:93309036-93309080	NM_183199:14759	Usp44	INSIDE	0.655	3.048	1192.22	3633.63	1.996	1052.88	2101.65
A_68_P22774764	chr4:21703668-21703712	NM_152825:274	Usp45	INSIDE	0.220	0.416	1567.03	651.39	0.092	1257.44	115.25
A_68_P23340748	chr4:137150166-137150210	NM_130879:85	Usp48	INSIDE	0.230	0.638	1200.76	765.53	0.147	1080.33	158.73
A_68_P23340749	chr4:137150280-137150324	NM_130879:199	Usp48	INSIDE	0.589	0.550	1095.82	602.36	0.324	964.16	312.11
A_68_P24817207	chr6:124779297-124779341	NM_013700:147	Usp5	INSIDE	0.158	0.485	2622.24	1273.09	0.077	1796.26	137.57
A_68_P24817209	chr6:124779503-124779547	NM_013700:-59	Usp5	DIVERGENT_PROMOTER	0.302	0.503	1165.94	586.78	0.152	782.00	118.66
A_68_P24817210	chr6:124779618-124779662	NM_013700:-175	Usp5	DIVERGENT_PROMOTER	0.171	0.594	2734.83	1623.20	0.102	1951.57	198.61
A_68_P22485523	chr3:122686980-122687024	NM_133857:363	Usp53	INSIDE	0.261	0.520	1295.91	673.84	0.135	1054.22	142.82
A_68_P22485526	chr3:122687380-122687424	NM_133857:-37	Usp53	PROMOTER	0.645	0.448	3046.44	1364.61	0.289	2356.61	680.60
A_68_P22485530	chr3:122687874-122687932	NM_133857:-537	Usp53	PROMOTER	0.192	0.674	1165.29	785.53	0.129	901.34	116.42
A_68_P26924746	chr10:8238018-8238062	NM_177387:583	Ust	INSIDE	0.505	0.612	1224.44	749.46	0.309	964.32	297.83
A_68_P26924749	chr10:8238335-8238379	NM_177387:267	Ust	INSIDE	0.419	0.480	2967.43	1423.15	0.201	2436.12	489.69
A_68_P26924750	chr10:8238430-8238474	NM_177387:171	Ust	INSIDE	0.613	0.633	1249.79	791.26	0.388	1122.33	435.37
A_68_P25581334	chr7:147129626-147129670	NM_009482:-106	Utf1	PROMOTER	0.363	0.470	3752.43	1762.33	0.170	2832.98	482.46
A_68_P25581335	chr7:147129738-147129782	NM_009482:6	Utf1	INSIDE	0.340	0.504	2430.42	1224.45	0.171	1909.32	326.69
A_68_P25581338	chr7:147130178-147130222	NM_009482:446	Utf1	INSIDE	0.508	0.603	1427.31	861.20	0.306	1137.09	348.46
A_68_P25581339	chr7:147130322-147130366	NM_009482:590	Utf1	INSIDE	0.212	0.737	3285.27	2420.71	0.156	2239.97	350.38
A_68_P25581341	chr7:147130605-147130649	NM_009482:872	Utf1	INSIDE	0.085	1.655	11064.98	18312.08	0.141	6853.57	967.55
A_68_P23269743	chr4:124370663-124370707	NM_026031:114	Utp11l	INSIDE	0.312	0.637	1271.91	810.07	0.198	1062.86	210.87
A_68_P32363625	chrX:45609838-45609889	NM_028276:-247	Utp14a	PROMOTER	0.079	3.156	1161.87	3666.93	0.248	518.54	128.66
A_68_P32363627	chrX:45610039-45610084	NM_028276:-49	Utp14a	PROMOTER	0.536	0.539	1200.53	647.27	0.289	404.71	116.99
A_68_P29256601	chr13:99032893-99032937	NM_178918:33	Utp15	INSIDE	0.482	0.569	2426.49	1380.02	0.274	1982.66	543.99
A_68_P23872778	chr5:88983098-88983142	NM_023054:-387	Utp3	PROMOTER	0.232	0.583	1209.59	705.76	0.135	960.89	130.11

ProbeName	Target position of probe on CpG island microarray	TargetID	GeneSymbol	CpG island Description	Ratio of relative methylation (TiO2-NP/Vehicle)	Sham group			TiO_2-H group		
						Relative methylation (Cy-5/Cy-3)	Cy-3 signal (Input)	Cy-5 signal (MeDIP)	Relative methylation (Cy-5/Cy-3)	Cy-3 signal (Input)	Cy-5 signal (MeDIP)
A_68_P23872839	chr5:88993614-88993669	NM_023054:10134	Utp3	DOWNSTREAM	0.299	0.459	1123.80	515.85	0.137	847.87	116.46
A_68_P26947989	chr10:12588953-12588997	NM_011682:-7441	Utrn	PROMOTER	0.558	0.531	974.37	517.69	0.296	848.90	251.55
A_68_P20191089	chr1:43884192-43884236	NM_026430:339	Uxs1	INSIDE	0.476	0.662	867.69	574.69	0.315	768.64	242.48
A_68_P32290858	chrX:20539135-20539180	NM_013840:-53	Uxt	DIVERGENT_PROMOTER	0.171	2.121	1155.39	2450.36	0.362	473.19	171.34
A_68_P23409803	chr4:150432014-150432058	NM_009498:26	Vamp3	INSIDE	0.636	0.349	1524.16	532.27	0.222	1315.57	292.32
A_68_P20778488	chr1:164500535-164500579	NM_016796:-402	Vamp4	PROMOTER	0.228	0.611	1052.93	643.04	0.139	869.56	120.86
A_68_P20778493	chr1:164501104-164501148	NM_016796:168	Vamp4	INSIDE	0.469	0.563	2489.20	1400.49	0.264	2284.57	602.27
A_68_P24541408	chr6:72330305-72330349	NM_001080742:136	Vamp5	INSIDE	0.199	0.553	3609.20	1994.65	0.110	2799.63	308.22
A_68_P24541411	chr6:72330618-72330663	NM_001080742:-178	Vamp5	PROMOTER	0.336	0.706	1265.76	894.05	0.237	952.72	226.24
A_68_P31156940	chr17:35137702-35137746	NM_011690:-127	Vars	PROMOTER	0.611	0.658	1243.88	819.07	0.402	1014.05	407.80
A_68_P31160312	chr17:35804249-35804293	NM_175137:267	Vars2	INSIDE	0.639	0.659	2132.19	1404.93	0.421	1507.83	635.08
A_68_P28611531	chr12:88019465-88019515	NM_177354:-159	Vash1	PROMOTER	0.515	0.684	1095.39	748.84	0.352	803.68	282.97
A_68_P28611533	chr12:88019760-88019804	NM_177354:133	Vash1	INSIDE	0.374	0.304	2327.26	706.63	0.114	2022.48	229.66
A_68_P20935543	chr1:192802468-192802514	NM_144879:387	Vash2	INSIDE	0.239	0.557	1140.56	635.48	0.133	899.07	119.93
A_68_P24992393	chr7:19856667-19856716	NM_009499:512	Vasp	INSIDE	0.162	1.495	1320.58	1974.31	0.242	944.98	228.85
A_68_P24992403	chr7:19857732-19857776	NM_009499:-551	Vasp	PROMOTER	0.452	0.450	2584.13	1162.00	0.203	1938.30	393.84
A_68_P28074035	chr11:101327600-101327644	NM_012037:-109	Vat1	DIVERGENT_PROMOTER	0.377	0.555	2583.48	1432.77	0.209	1999.11	417.91
A_68_P31260843	chr17:57415026-57415070	NM_001163815:-3474	Vav1	PROMOTER	0.616	0.575	2460.19	1414.40	0.354	2166.22	766.78
A_68_P21084619	chr2:27281633-27281677	NM_009500:691	Vav2	INSIDE	0.513	0.596	2520.63	1502.35	0.306	1744.29	533.62
A_68_P21084623	chr2:27282256-27282300	NM_009500:67	Vav2	INSIDE	0.473	0.558	3145.39	1754.33	0.264	2375.05	626.26
A_68_P32217657	chr19:59240762-59240806	NM_009501:3735	Vax1	INSIDE	0.331	0.488	1580.67	770.74	0.162	1310.75	211.81
A_68_P32217661	chr19:59241208-59241252	NM_009501:3289	Vax1	INSIDE	0.621	0.630	866.15	545.71	0.391	762.10	298.23
A_68_P32217702	chr19:59246036-59246083	NM_009501:-1540	Vax1	PROMOTER	0.368	1.763	2005.02	3535.34	0.650	1394.70	906.05
A_68_P24595035	chr6:83661384-83661428	NM_011912:149	Vax2	INSIDE	0.341	0.577	1338.25	772.15	0.197	927.73	182.45
A_68_P24595030	chr6:83660707-83660751	NR_002873:198	Vax2os1	INSIDE	0.351	0.734	2299.57	1688.85	0.258	1778.87	458.13
A_68_P29449769	chr14:21748618-21748662	NM_009502:-14	Vcl	PROMOTER	0.413	0.610	3674.94	2241.40	0.252	2828.59	712.86
A_68_P22869408	chr4:43013304-43013349	NM_009503:53	Vcp	INSIDE	0.123	0.560	2539.12	1421.82	0.069	1820.77	125.42
A_68_P20023918	chr1:9737605-9737655	NM_173443:833	Vcpip1	INSIDE	0.289	0.585	1069.26	625.55	0.169	683.09	115.38
A_68_P20023926	chr1:9738516-9738560	NM_173443:-75	Vcpip1	PROMOTER	0.463	0.472	1517.67	716.53	0.219	1190.45	260.22
A_68_P27798797	chr11:52174094-52174138	NM_011694:-500	Vdac1	PROMOTER	0.406	0.650	1764.27	1146.33	0.264	1315.69	346.88
A_68_P30471848	chr15:97738946-97738998	NM_009504:-245	Vdr	PROMOTER	0.164	0.620	1669.97	1035.46	0.102	1209.23	123.13
A_68_P30471849	chr15:97739034-97739078	NM_009504:-329	Vdr	PROMOTER	0.479	0.585	1964.43	1148.28	0.280	1583.43	443.33
A_68_P31204657	chr17:46167697-46167741	NM_001110266:-5606	Vegfa	PROMOTER	0.204	0.642	2436.41	1563.93	0.131	1890.76	247.36
A_68_P31204662	chr17:46168262-46168306	NM_001110266:-6172	Vegfa	PROMOTER	0.655	0.559	1009.01	563.57	0.366	853.50	312.15
A_68_P31204666	chr17:46168644-46168688	NM_001110266:-6554	Vegfa	PROMOTER	0.255	0.501	1374.64	688.78	0.128	997.78	127.64
A_68_P31204667	chr17:46168826-46168870	NM_001110266:-6736	Vegfa	PROMOTER	0.278	0.407	1714.38	697.41	0.113	1245.65	140.94
A_68_P25877023	chr8:55163035-55163079	NM_009506:171	Vegfc	INSIDE	0.648	0.603	2737.76	1650.89	0.391	2305.21	901.17
A_68_P27357053	chr10:93498402-93498447	NM_172538:69	Vezt	INSIDE	0.181	0.651	1418.07	923.77	0.118	1056.89	124.90
A_68_P27357055	chr10:93498582-93498626	NM_172538:-111	Vezt	DIVERGENT_PROMOTER	0.187	0.591	3740.14	2209.91	0.111	2827.06	313.16
A_68_P30826536	chr16:65815148-65815192	NM_028572:-707	Vgll3	PROMOTER	0.624	0.624	1090.77	680.92	0.390	952.90	371.16
A_68_P24760554	chr6:113574432-113574476	NM_009507:440	Vhl	INSIDE	0.476	0.671	2660.19	1784.19	0.319	2034.11	649.02
A_68_P21017265	chr2:13496059-13496103	NM_011701:143	Vim	INSIDE	0.463	0.494	1039.25	512.94	0.228	894.88	204.34
A_68_P21017267	chr2:13496235-13496279	NM_011701:319	Vim	INSIDE	0.165	0.620	1830.73	1134.17	0.102	1388.10	142.16
A_68_P21017268	chr2:13496366-13496411	NM_011701:451	Vim	INSIDE	0.451	0.547	1184.67	647.92	0.247	982.22	242.37

ProbeName	Target position of probe on CpG island microarray	TargetID	GeneSymbol	CpG island Description	Ratio of relative methylation (TiO2-NP/Vehicle)	Sham group			TiO_2-H group		
						Relative methylation (Cy-5/Cy-3)	Cy-3 signal (Input)	Cy-5 signal (MeDIP)	Relative methylation (Cy-5/Cy-3)	Cy-3 signal (Input)	Cy-5 signal (MeDIP)
A_68_P26884795	chr9:121578529-121578573	NM_011703:26717	Vipr1	INSIDE	0.531	0.618	2321.23	1433.59	0.328	1719.39	563.48
A_68_P28765897	chr12:117316101-117316145	NM_009511:-73	Vipr2	PROMOTER	0.089	0.738	2223.11	1641.65	0.066	1824.72	120.40
A_68_P24091295	chr5:130418466-130418510	NM_001001327:506	Vkorc1l1	INSIDE	0.594	0.448	1611.24	721.77	0.266	1259.09	334.89
A_68_P32039604	chr19:27293281-27293330	NM_001161420:1796	Vldlr	INSIDE	0.305	0.474	1209.02	572.49	0.144	833.43	120.26
A_68_P31257916	chr17:56856619-56856667	NM_178926:-298	Vmac	PROMOTER	0.172	3.875	7531.15	29185.39	0.666	4225.32	2812.82
A_68_P31257917	chr17:56856713-56856757	NM_178926:-390	Vmac	PROMOTER	0.168	2.180	6992.44	15243.02	0.367	4178.40	1532.51
A_68_P31257918	chr17:56856812-56856859	NM_178926:-491	Vmac	PROMOTER	0.178	0.711	1313.12	933.32	0.126	919.68	116.17
A_68_P26805303	chr9:106724052-106724097	NM_001015507:-232	Vprbp	PROMOTER	0.151	0.630	1992.32	1254.31	0.095	1611.18	153.39
A_68_P26805304	chr9:106724237-106724281	NM_001015507:-48	Vprbp	PROMOTER	0.507	0.710	1469.79	1043.29	0.360	1103.93	397.48
A_68_P26805312	chr9:106725024-106725068	NM_001015507:740	Vprbp	INSIDE	0.291	0.639	1814.16	1159.24	0.186	1451.16	269.83
A_68_P26467229	chr9:44169555-44169599	NM_027889:177	Vps11	INSIDE	0.261	0.687	2489.70	1710.48	0.180	1802.70	323.81
A_68_P26467230	chr9:44169640-44169684	NM_027889:91	Vps11	INSIDE	0.345	0.616	3114.82	1920.10	0.213	2334.67	497.19
A_68_P30133048	chr15:35300905-35300956	NM_177151:-370	Vps13b	PROMOTER	0.403	0.492	1070.04	526.44	0.198	848.32	168.32
A_68_P21568805	chr2:119114248-119114292	NM_172269:-207	Vps18	PROMOTER	0.187	0.463	1877.48	869.97	0.086	1371.56	118.54
A_68_P21568807	chr2:119114429-119114473	NM_172269:-27	Vps18	PROMOTER	0.612	0.443	1270.63	562.46	0.271	882.92	239.10
A_68_P27186311	chr10:61949916-61949968	NM_133672:-389	Vps26a	PROMOTER	0.061	1.371	1933.92	2651.77	0.084	1414.91	118.56
A_68_P24049902	chr5:122804456-122804500	NM_019780:57	Vps29	INSIDE	0.514	0.688	2582.45	1776.29	0.353	1990.30	703.43
A_68_P24056367	chr5:124022745-124022789	NM_029929:258	Vps33a	INSIDE	0.400	0.514	1120.84	576.62	0.206	884.82	181.92
A_68_P24056370	chr5:124023089-124023133	NM_029929:-86	Vps33a	PROMOTER	0.546	0.646	1251.04	808.41	0.353	1059.59	373.89
A_68_P26024221	chr8:87823033-87823077	NM_022997:342	Vps35	INSIDE	0.580	0.564	1183.96	668.26	0.327	1087.68	356.17
A_68_P26024222	chr8:87823113-87823157	NM_022997:262	Vps35	INSIDE	0.112	0.527	3767.11	1984.30	0.059	2728.73	161.17
A_68_P26024223	chr8:87823249-87823293	NM_022997:126	Vps35	INSIDE	0.385	0.705	4010.05	2828.26	0.271	3021.53	820.03
A_68_P31952189	chr19:10763284-10763328	NM_181403:2	Vps37c	INSIDE	0.332	0.460	1485.26	682.69	0.153	1210.67	184.90
A_68_P31952190	chr19:10763362-10763406	NM_181403:80	Vps37c	INSIDE	0.360	0.419	2333.19	977.40	0.151	1928.16	290.51
A_68_P21574793	chr2:120178464-120178508	NM_147153:383	Vps39	INSIDE	0.273	0.459	1625.73	745.88	0.125	1311.91	164.56
A_68_P27635753	chr11:21139469-21139513	NM_139061:599	Vps54	INSIDE	0.551	0.630	1069.12	673.57	0.347	883.52	306.69
A_68_P30592418	chr16:21423272-21423316	NM_001081366:104	Vps8	INSIDE	0.381	0.490	1413.78	693.21	0.187	1241.36	232.03
A_68_P28712490	chr12:107248322-107248369	NM_001029843:-127	Vrk1	PROMOTER	0.268	0.492	1236.22	608.73	0.132	913.09	120.62
A_68_P28712491	chr12:107248439-107248483	NM_001029843:-12	Vrk1	PROMOTER	0.417	0.553	1385.42	765.60	0.230	1094.21	251.90
A_68_P24019925	chr5:117769743-117769788	NM_001033311:491	Vsig10	INSIDE	0.196	0.476	1496.85	712.00	0.093	1262.77	117.99
A_68_P25075267	chr7:48155196-48155240	NM_021387:571	Vstm2b	INSIDE	0.587	0.600	1537.76	922.13	0.352	1083.43	381.25
A_68_P25075288	chr7:48157851-48157898	NM_021387:3227	Vstm2b	INSIDE	0.473	0.630	1247.51	786.47	0.298	905.97	270.08
A_68_P21780068	chr2:157767229-157767273	NM_198627:26862	Vstm2l	INSIDE	0.422	0.597	1879.46	1122.76	0.252	1501.75	379.02
A_68_P21740453	chr2:150514165-150514209	NM_054068:687	Vsx1	INSIDE	0.182	0.599	1922.73	1151.76	0.109	1459.65	158.99
A_68_P28599059	chr12:85910912-85910956	NM_007701:133	Vsx2	INSIDE	0.107	0.642	4496.75	2888.33	0.069	3263.32	223.78
A_68_P28599061	chr12:85911065-85911109	NM_007701:285	Vsx2	INSIDE	0.486	0.611	1566.71	957.78	0.297	1357.31	403.32
A_68_P26958515	chr10:14425185-14425230	NM_025418:88	Vta1	INSIDE	0.313	0.701	1707.62	1197.33	0.220	1278.69	280.73
A_68_P26958516	chr10:14425277-14425321	NM_025418:-3	Vta1	PROMOTER	0.231	0.633	1890.19	1195.71	0.146	1397.50	204.00
A_68_P28567992	chr12:80273136-80273180	NM_016800:287	Vti1b	INSIDE	0.499	0.647	2254.18	1459.04	0.323	1866.96	602.41
A_68_P28567993	chr12:80273243-80273287	NM_016800:181	Vti1b	INSIDE	0.283	0.629	1090.67	686.21	0.178	868.86	154.77
A_68_P23440510	chr4:155144347-155144391	NM_147776:4302	Vwa1	INSIDE	0.096	1.780	4304.34	7660.18	0.171	2725.87	465.45
A_68_P23440513	chr4:155144647-155144691	NM_147776:4002	Vwa1	INSIDE	0.630	0.560	1129.99	633.04	0.353	937.60	331.16
A_68_P23440545	chr4:155148332-155148376	NM_147776:316	Vwa1	INSIDE	0.210	0.618	1236.84	764.72	0.130	912.07	118.50
A_68_P23440546	chr4:155148502-155148549	NM_147776:145	Vwa1	INSIDE	0.134	0.746	3334.18	2486.17	0.100	2021.41	201.88

ProbeName	Target position of probe on CpG island microarray	TargetID	GeneSymbol	CpG island Description	Ratio of relative methylation (TiO2-NP/Vehicle)	Sham group			TiO_2-H group		
						Relative methylation (Cy-5/Cy-3)	Cy-3 signal (Input)	Cy-5 signal (MeDIP)	Relative methylation (Cy-5/Cy-3)	Cy-3 signal (Input)	Cy-5 signal (MeDIP)
A_68_P23440547	chr4:155148630-155148674	NM_147776:18	Vwa1	INSIDE	0.521	0.662	2450.19	1622.79	0.345	1828.98	630.97
A_68_P32203589	chr19:56948890-56948934	NM_172840:7	Vwa2	INSIDE	0.661	0.555	1557.43	863.66	0.367	1261.95	462.79
A_68_P30588339	chr16:20591420-20591465	NM_182636:1788	Vwa5b2	INSIDE	0.299	0.507	1289.65	653.54	0.151	985.18	149.07
A_68_P27589657	chr11:11016303-11016347	NM_177033:2306	Vwc2	INSIDE	0.469	0.643	1224.08	786.97	0.302	1031.46	311.26
A_68_P27589658	chr11:11016375-11016419	NM_177033:2378	Vwc2	INSIDE	0.308	0.325	1628.81	529.54	0.100	1221.17	122.11
A_68_P29531321	chr14:35487082-35487126	NM_001004436:-9	Wapal	PROMOTER	0.524	0.495	3713.31	1839.03	0.259	3076.43	798.07
A_68_P29531325	chr14:35487686-35487738	NM_001004436:599	Wapal	INSIDE	0.234	0.416	1647.16	684.76	0.097	1240.23	120.52
A_68_P29531327	chr14:35487897-35487941	NM_001004436:805	Wapal	INSIDE	0.529	0.629	1321.86	831.18	0.333	1027.48	341.99
A_68_P22362400	chr3:98945013-98945057	NM_027462:22	Wars2	INSIDE	0.647	0.586	1480.70	867.95	0.379	1232.09	467.18
A_68_P22362401	chr3:98945093-98945137	NM_027462:102	Wars2	INSIDE	0.615	0.591	1894.53	1120.60	0.363	1479.21	537.67
A_68_P22362402	chr3:98945208-98945252	NM_027462:218	Wars2	INSIDE	0.429	0.445	1547.83	688.13	0.191	1188.20	226.60
A_68_P27086283	chr10:40603268-40603315	NM_031877:-48	Wasf1	DIVERGENT_PROMOTER	0.331	0.549	1332.25	731.06	0.182	1022.21	185.78
A_68_P27086284	chr10:40603346-40603390	NM_031877:29	Wasf1	INSIDE	0.586	0.617	1123.31	692.57	0.361	904.19	326.61
A_68_P23315907	chr4:132686541-132686585	NM_153423:15	Wasf2	INSIDE	0.302	0.494	1284.81	635.26	0.149	1019.39	152.15
A_68_P24097663	chr5:131782424-131782475	NM_145218:943	Wbscr17	INSIDE	0.525	0.541	947.64	512.44	0.284	790.04	224.30
A_68_P24097670	chr5:131783250-131783294	NM_145218:120	Wbscr17	INSIDE	0.369	0.747	4369.97	3265.77	0.275	3230.56	889.72
A_68_P24097673	chr5:131783669-131783713	NM_145218:-298	Wbscr17	PROMOTER	0.584	0.547	1383.22	756.17	0.319	1156.88	369.14
A_68_P24114550	chr5:135539661-135539705	NM_025375:298	Wbscr22	INSIDE	0.192	0.495	2603.69	1290.00	0.095	2108.83	200.85
A_68_P24113930	chr5:135408345-135408389	NM_024479:124	Wbscr27	INSIDE	0.201	0.706	2367.41	1671.21	0.142	1727.80	244.89
A_68_P20377582	chr1:79758038-79758082	NM_001111279:284	Wdfy1	INSIDE	0.575	0.683	4103.31	2801.99	0.393	2836.70	1114.77
A_68_P23621601	chr5:38952237-38952281	NM_011715:576	Wdr1	INSIDE	0.280	0.536	1344.69	721.07	0.150	1059.34	159.06
A_68_P23621602	chr5:38952317-38952368	NM_011715:492	Wdr1	INSIDE	0.253	0.362	1821.20	659.43	0.092	1336.12	122.32
A_68_P23621603	chr5:38952428-38952472	NM_011715:384	Wdr1	INSIDE	0.568	0.543	1462.37	794.23	0.308	1059.71	326.78
A_68_P28730612	chr12:110132421-110132465	NM_177602:-39	Wdr25	DIVERGENT_PROMOTER	0.346	0.584	2508.95	1464.63	0.202	1879.81	379.22
A_68_P20880187	chr1:183140858-183140911	NM_145514:1225	Wdr26	INSIDE	0.068	1.852	1729.94	3204.00	0.126	1230.71	155.21
A_68_P20880189	chr1:183141032-183141076	NM_145514:1055	Wdr26	INSIDE	0.250	0.475	1247.65	593.23	0.119	978.33	116.08
A_68_P22964621	chr4:62131699-62131744	NM_023597:185	Wdr31	INSIDE	0.207	1.446	1591.49	2301.38	0.299	1144.37	341.99
A_68_P28225579	chr12:8980872-8980920	NM_001159527:90	Wdr35	INSIDE	0.156	0.649	1687.23	1094.60	0.101	1151.98	116.56
A_68_P28813401	chr13:8870209-8870253	NM_172445:58	Wdr37	INSIDE	0.308	0.697	4478.10	3121.35	0.215	3238.33	695.05
A_68_P31140216	chr17:31649029-31649073	NM_021322:382	Wdr4	INSIDE	0.384	0.655	1367.68	895.97	0.252	1170.53	294.66
A_68_P31335318	chr17:71966149-71966193	NM_175639:616	Wdr43	INSIDE	0.542	0.536	1731.96	927.84	0.290	1479.80	429.51
A_68_P32300610	chrX:23270059-23270108	NM_175180:-159	Wdr44	PROMOTER	0.068	1.739	2450.06	4260.32	0.118	1028.19	121.48
A_68_P32300615	chrX:23270584-23270630	NM_175180:364	Wdr44	INSIDE	0.422	0.627	1040.85	652.64	0.264	459.39	121.46
A_68_P32239781	chrX:7291710-7291754	NM_172372:-7642	Wdr45	PROMOTER	0.126	1.812	1260.22	2284.02	0.228	529.90	120.67
A_68_P31151137	chr17:34077801-34077845	NM_020603:155	Wdr46	INSIDE	0.483	0.707	1352.91	956.04	0.341	1024.41	349.80
A_68_P22413520	chr3:108394071-108394115	NM_181400:-103	Wdr47	PROMOTER	0.381	0.485	1558.91	755.65	0.185	1300.47	240.11
A_68_P22413525	chr3:108394707-108394751	NM_181400:533	Wdr47	INSIDE	0.435	0.486	2053.26	997.28	0.211	1599.90	338.04
A_68_P22413527	chr3:108395008-108395052	NM_181400:835	Wdr47	INSIDE	0.212	0.674	1234.98	832.66	0.143	958.45	137.22
A_68_P26874898	chr9:119803985-119804029	NM_026236:-6	Wdr48	PROMOTER	0.401	0.449	1258.18	565.02	0.180	961.70	173.28
A_68_P26874902	chr9:119804502-119804546	NM_026236:512	Wdr48	INSIDE	0.223	0.440	1385.24	609.24	0.098	1180.24	115.63
A_68_P21085075	chr2:27370596-27370640	NM_080848:-48	Wdr5	PROMOTER	0.268	0.704	5083.37	3577.92	0.189	3778.18	712.26
A_68_P21085240	chr2:27395797-27395841	NM_080848:25152	Wdr5	DOWNSTREAM	0.274	0.468	1597.32	747.42	0.128	1382.51	177.34
A_68_P30651594	chr16:32247382-32247433	NM_026898:69	Wdr53	INSIDE	0.116	0.636	2138.23	1359.01	0.074	1626.32	119.77
A_68_P30673430	chr16:36041581-36041625	NM_027113:327	Wdr5b	INSIDE	0.302	0.628	1457.42	915.53	0.190	1119.50	212.43

ProbeName	Target position of probe on CpG island microarray	TargetID	GeneSymbol	CpG island Description	Ratio of relative methylation (TiO2-NP/Vehicle)	Sham group			TiO_2-H group		
						Relative methylation (Cy-5/Cy-3)	Cy-3 signal (Input)	Cy-5 signal (MeDIP)	Relative methylation (Cy-5/Cy-3)	Cy-3 signal (Input)	Cy-5 signal (MeDIP)
A_68_P26815581	chr9:108480573-108480617	NM_031392:407	Wdr6	INSIDE	0.229	1.755	3847.46	6751.21	0.402	2617.85	1051.32
A_68_P26815583	chr9:108480765-108480809	NM_031392:215	Wdr6	INSIDE	0.343	0.751	4941.07	3708.80	0.258	3387.87	873.24
A_68_P25030741	chr7:31065286-31065332	NM_146186:132	Wdr62	INSIDE	0.179	0.561	1506.21	844.99	0.101	1176.12	118.34
A_68_P30244924	chr15:57743688-57743732	NM_001167679:-43	Wdr67	PROMOTER	0.665	0.704	1461.45	1028.98	0.468	1213.96	568.55
A_68_P20198785	chr1:45852285-45852329	NM_028599:-39	Wdr75	PROMOTER	0.574	0.557	1204.36	670.33	0.319	1004.44	320.88
A_68_P22400150	chr3:105761819-105761870	NM_027432:-571	Wdr77	PROMOTER	0.178	0.456	1956.63	891.26	0.081	1557.28	126.30
A_68_P22400156	chr3:105762584-105762628	NM_027432:191	Wdr77	INSIDE	0.390	0.258	3120.47	803.89	0.100	2417.29	242.77
A_68_P23164319	chr4:102786672-102786716	NM_146254:210	Wdr78	INSIDE	0.436	0.438	2729.97	1196.26	0.191	2013.77	384.95
A_68_P23164320	chr4:102786738-102786782	NM_146254:144	Wdr78	INSIDE	0.317	0.528	2194.19	1157.74	0.167	1559.94	260.85
A_68_P23429333	chr4:153515623-153515667	NM_021499:-836	Wdr8	DIVERGENT_PROMOTER	0.119	0.723	2820.28	2039.80	0.086	1905.93	164.32
A_68_P23429335	chr4:153515771-153515815	NM_021499:-688	Wdr8	DIVERGENT_PROMOTER	0.308	0.637	2009.41	1280.38	0.196	1424.96	279.41
A_68_P27926185	chr11:75268208-75268252	NM_138950:-11	Wdr81	DIVERGENT_PROMOTER	0.591	0.590	998.65	589.12	0.349	725.60	252.95
A_68_P27926186	chr11:75268311-75268355	NM_138950:-113	Wdr81	DIVERGENT_PROMOTER	0.499	0.685	1489.05	1019.78	0.341	1045.26	356.93
A_68_P21069043	chr2:24817956-24818000	NM_026044:37	Wdr85	INSIDE	0.364	0.709	2243.20	1589.40	0.258	1843.84	475.47
A_68_P23543621	chr5:24236647-24236691	NM_001081441:-170	Wdr86	PROMOTER	0.161	0.650	1490.59	968.61	0.105	1131.20	118.30
A_68_P28549762	chr12:76770236-76770280	NM_028203:266	Wdr89	INSIDE	0.447	0.735	2446.61	1797.08	0.328	1847.07	606.24
A_68_P30246279	chr15:57972958-57973002	NM_029734:-10	Wdyhv1	DIVERGENT_PROMOTER	0.289	0.601	950.62	571.54	0.174	671.27	116.80
A_68_P25411817	chr7:117265589-117265633	NM_009516:38	Wee1	INSIDE	0.511	0.375	1760.24	660.82	0.192	1550.17	297.36
A_68_P22970353	chr4:63077625-63077669	NM_001008797:16710	Whrn	INSIDE	0.626	2.656	3032.62	8053.90	1.661	2170.31	3605.79
A_68_P22970869	chr4:63155665-63155709	NM_001008791:1299	Whrn	INSIDE	0.113	0.742	3050.37	2264.25	0.084	2223.68	186.60
A_68_P22970878	chr4:63156551-63156595	NM_001008791:413	Whrn	INSIDE	0.397	0.613	3917.18	2399.98	0.243	2938.54	714.69
A_68_P25728415	chr8:26787130-26787174	NM_001081269:36397	Whsc1l1	INSIDE	0.293	1.637	516.31	845.41	0.479	525.16	251.72
A_68_P23591876	chr5:34278835-34278879	NM_011914:51	Whsc2	INSIDE	0.333	0.638	1238.52	789.59	0.213	1098.24	233.43
A_68_P23591878	chr5:34279071-34279115	NM_011914:-185	Whsc2	PROMOTER	0.647	0.718	1656.32	1189.28	0.465	1213.21	563.55
A_68_P23591879	chr5:34279139-34279183	NM_011914:-253	Whsc2	PROMOTER	0.148	0.534	2089.38	1115.94	0.079	1486.76	117.47
A_68_P23591880	chr5:34279319-34279363	NM_011914:-433	Whsc2	PROMOTER	0.274	0.496	1170.62	580.93	0.136	1024.26	139.05
A_68_P27543029	chr10:128185074-128185118	NM_001170869:-481	Wibg	PROMOTER	0.316	0.494	1122.91	554.87	0.156	771.19	120.26
A_68_P21332187	chr2:73367126-73367170	NM_153138:319	Wipf1	INSIDE	0.575	0.654	865.10	565.43	0.376	827.83	311.21
A_68_P21332193	chr2:73367805-73367850	NM_153138:-360	Wipf1	PROMOTER	0.575	0.529	1822.11	964.58	0.304	1528.39	464.90
A_68_P28119630	chr11:109472954-109472998	NM_145940:-273	Wipi1	PROMOTER	0.273	0.702	9981.09	7006.85	0.192	7927.89	1522.00
A_68_P24152596	chr5:143105941-143105985	NM_178398:425	Wipi2	INSIDE	0.181	0.475	1697.21	806.27	0.086	1383.16	119.16
A_68_P29023537	chr13:49242028-49242072	NM_029361:1333	Wnk2	INSIDE	0.493	0.553	1026.73	567.95	0.273	760.28	207.34
A_68_P28072948	chr11:101126562-101126606	NM_175638:4704	Wnk4	INSIDE	0.463	0.619	977.66	605.58	0.287	880.52	252.40
A_68_P28072950	chr11:101126785-101126842	NM_175638:4933	Wnk4	INSIDE	0.279	0.449	1167.52	524.22	0.125	943.22	118.08
A_68_P30476456	chr15:98619939-98619989	NM_021279:-323	Wnt1	PROMOTER	0.171	1.510	678.62	1024.83	0.259	452.29	117.00
A_68_P30476457	chr15:98620052-98620096	NM_021279:-213	Wnt1	PROMOTER	0.235	0.743	3250.75	2414.27	0.175	2345.77	409.61
A_68_P30476474	chr15:98622103-98622147	NM_021279:1837	Wnt1	INSIDE	0.230	0.554	1776.70	984.99	0.127	1383.80	176.20
A_68_P30476477	chr15:98622390-98622434	NM_021279:2125	Wnt1	INSIDE	0.168	0.584	3621.06	2114.93	0.098	2609.51	256.64
A_68_P30476480	chr15:98622772-98622816	NM_021279:2507	Wnt1	INSIDE	0.244	2.247	1342.39	3015.79	0.547	940.63	514.86
A_68_P30476370	chr15:98602905-98602961	NM_011718:5649	Wnt10b	INSIDE	0.658	0.648	983.34	637.23	0.426	799.76	340.80
A_68_P30476373	chr15:98603197-98603241	NM_011718:5363	Wnt10b	INSIDE	0.274	0.531	973.68	517.09	0.146	820.13	119.40
A_68_P30476409	chr15:98608492-98608536	NM_011718:67	Wnt10b	INSIDE	0.642	0.733	1990.71	1460.02	0.471	1581.59	744.93
A_68_P30476411	chr15:98608721-98608765	NM_011718:-161	Wnt10b	PROMOTER	0.440	0.662	1704.65	1127.74	0.291	1299.68	378.15
A_68_P25358180	chr7:105983274-105983321	NM_009519:-4057	Wnt11	PROMOTER	0.238	0.690	1387.69	958.00	0.164	1012.62	166.19

ProbeName	Target position of probe on CpG island microarray	TargetID	GeneSymbol	CpG island Description	Ratio of relative methylation (TiO2-NP/Vehicle)	Sham group			TiO_2-H group		
						Relative methylation (Cy-5/Cy-3)	Cy-3 signal (Input)	Cy-5 signal (MeDIP)	Relative methylation (Cy-5/Cy-3)	Cy-3 signal (Input)	Cy-5 signal (MeDIP)
A_68_P22394202	chr3:104764921-104764965	NM_009520:-315	Wnt2b	PROMOTER	0.143	0.703	1711.20	1202.13	0.101	1180.85	118.91
A_68_P22394204	chr3:104765246-104765290	NM_009520:-641	Wnt2b	PROMOTER	0.172	0.649	1303.97	846.77	0.112	1058.28	118.00
A_68_P28087055	chr11:103635334-103635379	NM_009521:-132	Wnt3	PROMOTER	0.629	0.568	1040.45	590.64	0.357	884.95	316.13
A_68_P28087056	chr11:103635473-103635522	NM_009521:9	Wnt3	INSIDE	0.155	0.606	1689.48	1023.53	0.094	1284.16	120.59
A_68_P28087330	chr11:103671857-103671901	NM_009521:36390	Wnt3	INSIDE	0.579	0.650	1416.30	921.02	0.377	1132.06	426.34
A_68_P28087340	chr11:103672887-103672931	NM_009521:37420	Wnt3	INSIDE	0.377	1.537	5118.67	7865.00	0.580	3885.97	2253.23
A_68_P28087343	chr11:103673531-103673575	NM_009521:38064	Wnt3	INSIDE	0.670	4.641	2433.84	11295.21	3.107	1734.84	5390.88
A_68_P27836025	chr11:59103487-59103531	NM_009522:745	Wnt3a	INSIDE	0.456	0.276	2543.26	702.85	0.126	2027.03	255.69
A_68_P29493855	chr14:29319194-29319238	NM_009524:558	Wnt5a	INSIDE	0.335	0.439	1319.01	578.59	0.147	1027.20	150.84
A_68_P29493860	chr14:29319799-29319843	NM_009524:1162	Wnt5a	INSIDE	0.131	0.700	5111.08	3578.10	0.092	3653.85	336.16
A_68_P29493861	chr14:29319923-29319970	NM_009524:1288	Wnt5a	INSIDE	0.212	0.649	1142.28	740.90	0.138	884.44	121.88
A_68_P30401337	chr15:85374375-85374419	NM_001163634:34104	Wnt7b	INSIDE	0.661	2.597	787.40	2045.23	1.717	678.17	1164.31
A_68_P30401588	chr15:85407657-85407702	NM_001163634:821	Wnt7b	INSIDE	0.256	0.407	1563.41	636.09	0.104	1152.95	120.14
A_68_P30401593	chr15:85408317-85408361	NM_001163634:162	Wnt7b	INSIDE	0.248	0.484	1858.71	898.85	0.120	1460.05	174.80
A_68_P27836130	chr11:59119814-59119858	NM_139298:-595	Wnt9a	PROMOTER	0.246	0.582	1039.44	604.57	0.143	836.70	119.79
A_68_P30991292	chr16:96367120-96367164	NM_207301:117	Wrb	INSIDE	0.252	0.677	2934.92	1987.61	0.171	2052.27	350.11
A_68_P28931376	chr13:32893858-32893902	NM_030215:-18	Wrnip1	PROMOTER	0.592	0.442	1884.77	833.81	0.262	1466.74	384.43
A_68_P28931377	chr13:32893930-32893974	NM_030215:54	Wrnip1	INSIDE	0.521	0.400	1803.71	721.31	0.208	1417.83	295.59
A_68_P28931385	chr13:32894778-32894822	NM_030215:902	Wrnip1	INSIDE	0.534	0.472	1235.22	583.52	0.252	899.07	226.98
A_68_P28931386	chr13:32894845-32894889	NM_030215:968	Wrnip1	INSIDE	0.203	0.404	2034.57	821.24	0.082	1543.77	126.66
A_68_P28931387	chr13:32894954-32894998	NM_030215:1078	Wrnip1	INSIDE	0.384	0.617	1980.72	1221.55	0.237	1549.04	367.09
A_68_P28931388	chr13:32895025-32895071	NM_030215:1150	Wrnip1	INSIDE	0.175	0.571	1916.49	1094.34	0.100	1294.07	129.40
A_68_P27947945	chr11:79068066-79068110	NM_019653:109	Wsb1	INSIDE	0.259	0.695	1869.44	1298.95	0.180	1284.56	231.41
A_68_P21492627	chr2:104967572-104967616	NM_144783:909	Wt1	INSIDE	0.283	0.572	1330.60	761.18	0.162	1090.85	176.72
A_68_P31054974	chr17:13185825-13185876	NM_175394:-445	Wtap	PROMOTER	0.204	0.600	1131.54	679.26	0.122	997.11	122.05
A_68_P31054976	chr17:13185515-13185559	NM_175394:-131	Wtap	PROMOTER	0.658	0.434	1511.65	656.75	0.286	1108.73	317.01
A_68_P25042335	chr7:34917422-34917466	NM_207212:843	Wtip	INSIDE	0.285	0.712	3656.96	2605.57	0.203	2868.96	581.90
A_68_P25042342	chr7:34918494-34918538	NM_207212:-229	Wtip	PROMOTER	0.364	0.525	2836.45	1489.36	0.191	2244.05	429.00
A_68_P26184809	chr8:117045830-117045874	NM_019573:82301	Wwox	INSIDE	0.644	2.694	1201.00	3235.35	1.734	870.81	1509.62
A_68_P26184811	chr8:117045980-117046026	NM_019573:82451	Wwox	INSIDE	0.644	2.941	853.03	2508.99	1.895	616.44	1168.19
A_68_P22164429	chr3:57379088-57379132	NM_133784:688	Wwtr1	INSIDE	0.253	0.664	947.44	629.49	0.168	774.59	130.00
A_68_P22164435	chr3:57379593-57379637	NM_133784:184	Wwtr1	INSIDE	0.505	0.490	1214.79	595.55	0.248	1018.93	252.32
A_68_P27563772	chr11:5421438-5421482	NM_013842:491	Xbp1	INSIDE	0.426	0.461	1736.71	800.62	0.196	1362.88	267.49
A_68_P20002773	chr1:3662046-3662090	NM_001011874:-489	Xkr4	PROMOTER	0.068	0.648	3911.41	2534.36	0.044	2772.36	121.88
A_68_P20002775	chr1:3662278-3662322	NM_001011874:-721	Xkr4	PROMOTER	0.233	0.536	1206.69	647.03	0.125	940.71	117.56
A_68_P25701519	chr8:18950799-18950843	NM_001113350:153	Xkr5	INSIDE	0.295	0.550	1900.50	1045.48	0.162	1597.83	258.98
A_68_P29662310	chr14:64225813-64225857	NM_173393:468	Xkr6	INSIDE	0.525	0.541	2910.96	1575.77	0.284	2322.28	659.56
A_68_P22887518	chr4:46209177-46209222	NM_011728:-16	Xpa	PROMOTER	0.268	0.601	882.51	530.59	0.161	745.71	120.32
A_68_P22887519	chr4:46209270-46209318	NM_011728:-111	Xpa	PROMOTER	0.391	0.602	964.46	580.45	0.236	743.18	175.04
A_68_P30377738	chr15:81230455-81230499	NM_177310:-141	Xpnpep3	DIVERGENT_PROMOTER	0.425	0.585	3206.19	1874.83	0.248	2430.25	603.33
A_68_P27644761	chr11:23155625-23155669	NM_001035226:-394	Xpo1	PROMOTER	0.150	0.428	2430.62	1040.22	0.064	1822.87	116.67
A_68_P25499299	chr7:133343490-133343534	NM_028816:410	Xpo6	INSIDE	0.535	0.735	3441.86	2530.75	0.394	2583.45	1016.96
A_68_P29703308	chr14:71165727-71165771	NM_023045:687	Xpo7	INSIDE	0.398	0.702	1752.43	1229.46	0.279	1340.90	374.38
A_68_P29703310	chr14:71165976-71166021	NM_023045:437	Xpo7	INSIDE	0.596	0.516	1046.55	540.47	0.308	816.33	251.35

ProbeName	Target position of probe on CpG island microarray	TargetID	GeneSymbol	CpG island Description	Ratio of relative methylation (TiO2-NP/Vehicle)	Sham group			TiO_2-H group		
						Relative methylation (Cy-5/Cy-3)	Cy-3 signal (Input)	Cy-5 signal (MeDIP)	Relative methylation (Cy-5/Cy-3)	Cy-3 signal (Input)	Cy-5 signal (MeDIP)
A_68_P27504460	chr10:121043018-121043071	NM_001081056:20328	Xpot	INSIDE	0.605	2.799	725.45	2030.52	1.694	566.97	960.58
A_68_P23548555	chr5:25211490-25211534	NM_020570:103	Xrcc2	INSIDE	0.556	0.450	1279.43	575.45	0.250	1054.18	263.46
A_68_P28746254	chr12:113052004-113052048	NM_028875:26	Xrcc3	INSIDE	0.402	0.647	4008.98	2594.96	0.260	3067.97	797.87
A_68_P29211814	chr13:90229104-90229154	NM_028012:85	Xrcc4	INSIDE	0.334	0.629	1117.32	703.03	0.210	843.60	177.21
A_68_P30380963	chr15:81846798-81846842	NM_010247:22	Xrcc6	INSIDE	0.246	0.470	2002.64	941.12	0.116	1437.61	166.13
A_68_P26744825	chr9:95854980-95855036	NM_011916:-170	Xrn1	PROMOTER	0.256	0.688	1726.58	1187.18	0.176	1312.62	231.39
A_68_P26744827	chr9:95855160-95855204	NM_011916:4	Xrn1	INSIDE	0.204	0.648	1124.55	728.52	0.132	905.68	119.45
A_68_P21721814	chr2:146838725-146838769	NM_011917:-49	Xrn2	PROMOTER	0.622	0.549	1238.50	679.61	0.342	930.84	317.94
A_68_P21721820	chr2:146839259-146839303	NM_011917:485	Xrn2	INSIDE	0.248	0.466	1205.21	562.07	0.116	1026.95	118.86
A_68_P21721821	chr2:146839369-146839413	NM_011917:595	Xrn2	INSIDE	0.353	0.440	1608.97	707.14	0.155	1283.99	199.43
A_68_P25364623	chr7:107065859-107065903	NM_001164258:58153	Xrra1	INSIDE	0.382	0.552	1279.25	705.79	0.211	1061.22	223.43
A_68_P28034315	chr11:94538289-94538333	NM_145828:497	Xylt2	INSIDE	0.446	0.588	3266.40	1920.22	0.262	2479.69	650.03
A_68_P26290457	chr9:8004110-8004154	NM_001171147:464	Yap1	INSIDE	0.327	0.634	3063.78	1942.36	0.207	2456.14	508.50
A_68_P30568843	chr16:16302936-16302980	NM_198246:-99	Yars2	PROMOTER	0.345	0.536	1519.30	813.81	0.185	1283.94	237.00
A_68_P27898020	chr11:69749025-69749069	NM_016875:-354	Ybx2	PROMOTER	0.550	0.566	2117.23	1197.87	0.311	1787.40	556.56
A_68_P30572230	chr16:17147040-17147084	NM_026940:3	Ydjc	INSIDE	0.611	0.601	883.12	530.49	0.367	695.06	255.05
A_68_P30572235	chr16:17147510-17147562	NM_026940:477	Ydjc	INSIDE	0.213	0.470	1873.92	880.30	0.100	1217.96	121.97
A_68_P30572236	chr16:17147632-17147677	NM_026940:595	Ydjc	INSIDE	0.276	0.504	1137.45	573.08	0.139	826.87	115.16
A_68_P27480106	chr10:116661162-116661206	NM_026570:379	Yeats4	INSIDE	0.362	0.398	1735.68	690.14	0.144	1541.08	221.81
A_68_P23584758	chr5:32913772-32913816	NM_001205132:251	Yes1	INSIDE	0.423	0.343	1952.00	669.02	0.145	1562.07	226.56
A_68_P31926817	chr19:5088108-5088153	NM_026553:-407	Yif1a	DIVERGENT_PROMOTER	0.381	0.622	1033.89	643.35	0.237	751.85	178.03
A_68_P25026074	chr7:30023055-30023109	NM_001110201:-259	Yif1b	DIVERGENT_PROMOTER	0.126	0.731	1790.00	1308.71	0.092	1284.30	118.20
A_68_P25026079	chr7:30023546-30023590	NM_001110201:227	Yif1b	INSIDE	0.228	0.623	1092.98	680.64	0.142	863.65	122.69
A_68_P31644531	chr18:40378658-40378702	NM_023311:373	Yipf5	INSIDE	0.197	0.714	2126.77	1517.65	0.141	1486.92	209.42
A_68_P31644532	chr18:40378757-40378801	NM_023311:275	Yipf5	INSIDE	0.163	1.778	6506.46	11569.67	0.289	4385.35	1267.87
A_68_P31644533	chr18:40378844-40378888	NM_023311:187	Yipf5	INSIDE	0.054	1.878	14054.89	26395.33	0.102	8501.30	866.43
A_68_P27565815	chr11:5856146-5856195	NM_019661:410	Ykt6	INSIDE	0.288	0.510	1006.23	512.70	0.147	829.23	121.49
A_68_P27992046	chr11:86806680-86806725	NM_001005341:562	Ypel2	INSIDE	0.286	0.717	2621.91	1881.13	0.205	2013.03	413.64
A_68_P27992047	chr11:86806779-86806823	NM_001005341:464	Ypel2	INSIDE	0.413	0.672	4891.83	3288.84	0.278	3710.47	1030.84
A_68_P27992048	chr11:86806873-86806917	NM_001005341:370	Ypel2	INSIDE	0.276	0.558	1442.28	804.89	0.154	1067.92	164.24
A_68_P23865421	chr5:87233222-87233266	NM_177680:-270	Ythdc1	PROMOTER	0.303	0.584	2462.73	1439.18	0.177	1878.03	332.25
A_68_P23865423	chr5:87233404-87233448	NM_177680:-88	Ythdc1	PROMOTER	0.503	0.719	1949.41	1400.99	0.362	1550.02	560.57
A_68_P31669248	chr18:44988658-44988702	NM_001163013:362	Ythdc2	INSIDE	0.567	0.428	1645.12	704.73	0.243	1200.46	291.34
A_68_P31669249	chr18:44988742-44988786	NM_001163013:446	Ythdc2	INSIDE	0.141	0.709	1587.57	1126.27	0.100	1245.48	124.69
A_68_P21907109	chr2:180655229-180655273	NM_173761:391	Ythdf1	INSIDE	0.579	0.732	4037.06	2954.50	0.423	3038.38	1286.42
A_68_P21962519	chr3:16083394-16083438	NM_001145919:234	Ythdf3	INSIDE	0.498	0.479	1491.03	713.64	0.238	1208.42	287.88
A_68_P27927819	chr11:75547027-75547071	NM_009536:660	Ywhae	INSIDE	0.199	0.574	4961.05	2848.34	0.114	3732.30	425.80
A_68_P24118436	chr5:136379600-136379644	NM_018871:30889	Ywhag	DOWNSTREAM	0.403	0.634	1135.29	719.87	0.256	915.76	234.26
A_68_P24118491	chr5:136387249-136387293	NM_018871:23241	Ywhag	INSIDE	0.563	2.731	1500.76	4098.78	1.538	1042.21	1602.46
A_68_P24118624	chr5:136409884-136409928	NM_018871:605	Ywhag	INSIDE	0.568	0.668	3287.17	2196.31	0.380	2580.55	979.53
A_68_P23586719	chr5:33362030-33362074	NM_011738:588	Ywhah	INSIDE	0.592	0.440	1903.90	837.08	0.260	1523.40	396.55
A_68_P28280594	chr12:21423400-21423444	NM_011739:-125	Ywhaq	PROMOTER	0.599	0.456	1720.19	783.97	0.273	1289.76	351.84
A_68_P28280595	chr12:21423493-21423537	NM_011739:-217	Ywhaq	PROMOTER	0.488	0.524	1861.72	976.25	0.256	1439.01	368.48
A_68_P28280597	chr12:21423661-21423705	NM_011739:-385	Ywhaq	PROMOTER	0.142	0.515	2174.82	1120.31	0.073	1666.47	121.62

ProbeName	Target position of probe on CpG island microarray	TargetID	GeneSymbol	CpG island Description	Ratio of relative methylation (TiO2-NP/Vehicle)	Sham group			TiO_2-H group		
						Relative methylation (Cy-5/Cy-3)	Cy-3 signal (Input)	Cy-5 signal (MeDIP)	Relative methylation (Cy-5/Cy-3)	Cy-3 signal (Input)	Cy-5 signal (MeDIP)
A_68_P30140504	chr15:36723408-36723452	NM_011740:863	Ywhaz	INSIDE	0.225	0.498	2368.50	1179.93	0.112	1691.16	189.74
A_68_P30140507	chr15:36723784-36723828	NM_011740:487	Ywhaz	INSIDE	0.274	0.637	810.77	516.51	0.175	688.21	120.32
A_68_P28729922	chr12:110031214-110031258	NM_009537:-284	Yy1	PROMOTER	0.500	0.706	3619.11	2555.48	0.353	2766.67	976.39
A_68_P28729928	chr12:110032252-110032296	NM_009537:754	Yy1	INSIDE	0.078	0.702	3053.77	2143.40	0.054	2223.67	121.00
A_68_P31886106	chr18:84257584-84257628	NM_146090:57	Zadh2	INSIDE	0.394	0.377	3025.06	1141.33	0.149	2306.11	342.56
A_68_P31886140	chr18:84261455-84261501	NM_146090:3929	Zadh2	INSIDE	0.190	0.706	1682.30	1187.94	0.134	1350.96	181.01
A_68_P29240034	chr13:96094965-96095009	NM_028106:-205	Zbed3	PROMOTER	0.277	0.450	1360.48	611.58	0.124	1094.16	136.16
A_68_P30421093	chr15:88582624-88582670	NM_181412:506	Zbed4	INSIDE	0.223	0.526	1384.33	728.62	0.117	1044.77	122.49
A_68_P28553596	chr12:77471058-77471102	NM_178744:-172	Zbtb1	DIVERGENT_PROMOTER	0.409	0.552	2284.85	1261.68	0.226	1750.16	395.07
A_68_P30778633	chr16:55973743-55973787	NM_173026:-152	Zbtb11	PROMOTER	0.338	0.639	1822.62	1164.11	0.216	1614.97	348.49
A_68_P31156341	chr17:35031034-35031078	NM_198886:-447	Zbtb12	PROMOTER	0.474	0.628	960.50	602.87	0.297	786.37	233.74
A_68_P26494148	chr9:48643704-48643758	NM_001033324:320	Zbtb16	INSIDE	0.185	0.589	1411.78	832.08	0.109	1080.11	117.65
A_68_P26494154	chr9:48644306-48644350	NM_001033324:-278	Zbtb16	PROMOTER	0.545	0.533	1469.34	782.75	0.291	1137.61	330.51
A_68_P23364201	chr4:141020235-141020279	NM_009541:19669	Zbtb17	INSIDE	0.575	2.984	2071.46	6182.02	1.717	1603.27	2752.63
A_68_P21121521	chr2:33286475-33286519	NM_001085507:348	Zbtb34	INSIDE	0.503	0.511	1156.35	591.23	0.257	874.47	224.73
A_68_P27896952	chr11:69579759-69579803	NM_029348:367	Zbtb4	INSIDE	0.334	0.685	1333.10	913.29	0.229	1059.80	242.41
A_68_P20662734	chr1:141319149-141319193	NM_172643:211	Zbtb41	INSIDE	0.349	0.476	1372.99	652.86	0.166	1054.20	175.15
A_68_P28751782	chr12:113917645-113917689	NM_001100460:616	Zbtb42	INSIDE	0.506	0.733	2166.76	1587.69	0.371	1635.97	606.63
A_68_P28751784	chr12:113917844-113917888	NM_001100460:816	Zbtb42	INSIDE	0.284	0.714	2093.79	1494.32	0.202	1495.49	302.64
A_68_P21121777	chr2:33324228-33324287	NM_001025594:-205	Zbtb43	PROMOTER	0.286	0.607	860.01	521.62	0.174	684.62	118.83
A_68_P24974109	chr7:13593102-13593146	NM_001024699:2025	Zbtb45	INSIDE	0.455	4.787	1387.92	6643.87	2.179	1483.31	3232.05
A_68_P24974124	chr7:13594887-13594931	NM_001024699:241	Zbtb45	INSIDE	0.392	1.550	8940.84	13859.75	0.607	5657.47	3435.24
A_68_P24974125	chr7:13594994-13595038	NM_001024699:133	Zbtb45	INSIDE	0.473	1.355	3694.32	5006.44	0.640	2654.12	1699.85
A_68_P24974126	chr7:13595184-13595228	NM_001024699:-57	Zbtb45	PROMOTER	0.634	0.606	1005.90	609.91	0.384	753.09	289.51
A_68_P24974127	chr7:13595301-13595345	NM_001024699:-173	Zbtb45	PROMOTER	0.383	0.567	2246.83	1274.90	0.217	1400.51	304.31
A_68_P21910759	chr2:181195175-181195219	NM_028125:-1065	Zbtb46	PROMOTER	0.251	0.383	1798.01	688.86	0.096	1403.35	135.02
A_68_P23618267	chr5:38611613-38611657	NM_029162:33	Zbtb49	INSIDE	0.564	0.467	1488.70	695.63	0.264	1235.93	325.87
A_68_P27287608	chr10:80598003-80598047	NM_010731:-991	Zbtb7a	PROMOTER	0.200	0.470	1658.99	779.85	0.094	1258.52	118.28
A_68_P27287609	chr10:80598116-80598160	NM_010731:-877	Zbtb7a	PROMOTER	0.283	0.701	1844.23	1292.99	0.198	1148.37	227.51
A_68_P27287622	chr10:80599571-80599615	NM_010731:577	Zbtb7a	INSIDE	0.303	0.454	1161.77	527.20	0.137	874.92	120.18
A_68_P22319094	chr3:89195751-89195795	NM_009565:1353	Zbtb7b	INSIDE	0.412	0.477	1482.92	707.26	0.196	1153.77	226.49
A_68_P22319099	chr3:89196394-89196438	NM_009565:709	Zbtb7b	INSIDE	0.217	0.461	1592.61	734.56	0.100	1281.94	128.06
A_68_P22319130	chr3:89199983-89200027	NM_009565:-2879	Zbtb7b	PROMOTER	0.097	1.370	3604.55	4939.84	0.132	2314.19	306.42
A_68_P31840894	chr18:76219449-76219494	NM_145356:239640	Zbtb7c	INSIDE	0.244	0.543	1301.60	707.11	0.133	914.90	121.46
A_68_P23296400	chr4:129054411-129054455	NM_028603:840	Zbtb8a	INSIDE	0.136	0.622	4062.61	2525.45	0.085	3035.03	257.52
A_68_P23296404	chr4:129055173-129055217	NM_028603:78	Zbtb8a	INSIDE	0.368	0.554	1797.67	995.39	0.204	1518.39	309.05
A_68_P31113562	chr17:27109927-27109971	NM_001005916:-175	Zbtb9	PROMOTER	0.643	0.410	1334.08	547.14	0.264	1046.96	276.11
A_68_P27541962	chr10:127984459-127984503	NM_134003:320	Zc3h10	INSIDE	0.402	0.565	1308.93	738.91	0.227	1006.35	228.22
A_68_P27541963	chr10:127984584-127984628	NM_134003:194	Zc3h10	INSIDE	0.172	1.435	2103.89	3018.19	0.247	1405.73	346.62
A_68_P32542578	chrX:92907030-92907074	NM_001034907:36	Zc3h12b	INSIDE	0.566	2.857	1730.47	4944.09	1.617	835.50	1351.21
A_68_P21388692	chr2:83484476-83484520	NM_026934:-236	Zc3h15	PROMOTER	0.469	0.602	1674.83	1008.09	0.282	1220.88	344.55
A_68_P26232972	chr8:124900602-124900646	NM_001029993:109	Zc3h18	INSIDE	0.442	0.388	1718.34	666.76	0.171	1401.42	240.18
A_68_P30344504	chr15:75672213-75672257	NM_172121:104	Zc3h3	INSIDE	0.644	0.488	1641.17	800.63	0.314	1138.16	357.45
A_68_P30344506	chr15:75672397-75672441	NM_172121:-80	Zc3h3	PROMOTER	0.624	0.469	1120.24	524.92	0.292	858.42	250.86

ProbeName	Target position of probe on CpG island microarray	TargetID	GeneSymbol	CpG island Description	Ratio of relative methylation (TiO2-NP/Vehicle)	Sham group			TiO_2-H group		
						Relative methylation (Cy-5/Cy-3)	Cy-3 signal (Input)	Cy-5 signal (MeDIP)	Relative methylation (Cy-5/Cy-3)	Cy-3 signal (Input)	Cy-5 signal (MeDIP)
A_68_P30344507	chr15:75672464-75672508	NM_172121:-148	Zc3h3	PROMOTER	0.273	0.426	1350.14	574.93	0.116	1049.25	121.93
A_68_P24981256	chr7:16985178-16985232	NM_198631:-1340	Zc3h4	PROMOTER	0.318	0.373	1449.53	540.10	0.118	1015.44	120.28
A_68_P21622285	chr2:128793081-128793125	NM_178404:-35	Zc3h6	PROMOTER	0.494	0.506	1903.96	964.00	0.250	1312.20	328.14
A_68_P21622286	chr2:128793161-128793205	NM_178404:45	Zc3h6	INSIDE	0.384	0.683	1457.58	995.55	0.262	1061.84	278.46
A_68_P30546657	chr16:11175989-11176033	NM_145931:476	Zc3h7a	INSIDE	0.205	0.644	2402.16	1546.32	0.132	1818.70	240.31
A_68_P30546660	chr16:11176366-11176410	NM_145931:98	Zc3h7a	INSIDE	0.412	0.489	1495.01	730.64	0.201	1359.83	273.69
A_68_P21622217	chr2:128769546-128769590	NM_020594:188	Zc3h8	INSIDE	0.404	0.554	1092.21	605.07	0.224	984.96	220.62
A_68_P24376836	chr6:38304039-38304083	NM_028421:543	Zc3hav1	INSIDE	0.198	0.594	1904.89	1132.26	0.118	1492.70	175.90
A_68_P24376841	chr6:38304658-38304702	NM_028421:-77	Zc3hav1	PROMOTER	0.268	0.419	1499.53	628.88	0.112	1192.29	134.02
A_68_P27804179	chr11:53138214-53138260	NM_026479:46	Zcchc10	INSIDE	0.297	0.393	1423.03	559.29	0.117	1016.64	118.57
A_68_P27804180	chr11:53138329-53138380	NM_026479:164	Zcchc10	INSIDE	0.181	0.432	2399.18	1036.96	0.078	1702.79	133.06
A_68_P23196088	chr4:108131810-108131854	NM_175472:-198	Zcchc11	PROMOTER	0.314	0.516	1517.11	783.36	0.162	1236.15	200.63
A_68_P23196100	chr4:108133295-108133339	NM_175472:1286	Zcchc11	INSIDE	0.414	0.646	1484.72	958.90	0.267	1125.04	300.94
A_68_P26228075	chr8:124175832-124175876	NM_080855:-21	Zcchc14	PROMOTER	0.343	0.463	2103.87	975.09	0.159	1537.21	244.55
A_68_P26228083	chr8:124177031-124177079	NM_080855:-1221	Zcchc14	PROMOTER	0.290	0.456	1346.16	613.25	0.132	940.16	124.30
A_68_P20505868	chr1:107886770-107886814	NM_001122675:-190	Zcchc2	PROMOTER	0.327	0.533	1625.06	865.63	0.174	1208.30	210.16
A_68_P21749217	chr2:152239906-152239950	NM_175126:852	Zcchc3	INSIDE	0.343	0.609	1741.48	1060.96	0.209	1346.73	281.21
A_68_P21749218	chr2:152239987-152240031	NM_175126:772	Zcchc3	INSIDE	0.295	0.353	1958.83	690.72	0.104	1453.63	151.39
A_68_P21749219	chr2:152240057-152240101	NM_175126:702	Zcchc3	INSIDE	0.606	0.611	1622.07	991.19	0.370	1373.36	508.34
A_68_P29081868	chr13:59923934-59923978	NM_153538:552	Zcchc6	INSIDE	0.371	0.380	2426.51	922.15	0.141	2050.26	289.17
A_68_P22878935	chr4:44769408-44769452	NM_138590:0	Zcchc7	INSIDE	0.270	0.432	1307.13	564.76	0.117	1023.21	119.21
A_68_P24057205	chr5:124170298-124170343	NM_027494:733	Zcchc8	INSIDE	0.441	0.346	2295.83	793.54	0.153	1866.38	284.69
A_68_P24057206	chr5:124170389-124170437	NM_027494:641	Zcchc8	INSIDE	0.238	0.470	1373.75	645.48	0.112	1055.47	117.79
A_68_P24057208	chr5:124170620-124170665	NM_027494:411	Zcchc8	INSIDE	0.239	0.479	1258.50	602.59	0.115	1007.44	115.37
A_68_P29220644	chr13:91947270-91947314	NM_145453:9	Zcchc9	INSIDE	0.636	0.501	1717.52	860.32	0.319	1415.02	450.99
A_68_P21101316	chr2:29949011-29949055	NM_001037762:123	Zdhhc12	INSIDE	0.290	0.613	1506.51	923.59	0.178	1068.77	190.18
A_68_P25108145	chr7:56044314-56044358	NM_028031:-36	Zdhhc13	PROMOTER	0.222	0.662	2869.26	1900.27	0.147	2287.55	336.69
A_68_P32578102	chrX:101865933-101865986	NM_175358:444	Zdhhc15	INSIDE	0.113	1.649	1438.59	2372.71	0.187	669.18	125.04
A_68_P32578103	chrX:101866009-101866059	NM_175358:369	Zdhhc15	INSIDE	0.055	2.727	2259.62	6162.57	0.149	806.45	120.13
A_68_P23318625	chr4:133189081-133189125	NM_001017968:242	Zdhhc18	INSIDE	0.591	0.727	1867.74	1358.58	0.430	1424.77	612.52
A_68_P29630483	chr14:58509439-58509485	NM_029492:-363	Zdhhc20	PROMOTER	0.179	1.573	650.04	1022.74	0.282	553.45	156.16
A_68_P23059228	chr4:82505265-82505309	NM_026647:279	Zdhhc21	INSIDE	0.594	0.362	1506.93	545.23	0.215	1274.73	273.96
A_68_P31925602	chr19:4878554-4878607	NM_001168516:-87	Zdhhc24	DIVERGENT_PROMOTER	0.280	0.366	1495.19	546.60	0.102	1195.49	122.29
A_68_P24156072	chr5:144090683-144090727	NM_028379:201	Zdhhc4	INSIDE	0.300	0.663	2941.05	1949.64	0.199	1987.61	394.70
A_68_P24156073	chr5:144090815-144090859	NM_028379:69	Zdhhc4	INSIDE	0.577	0.577	3562.81	2054.34	0.333	3003.39	999.47
A_68_P21394205	chr2:84554801-84554845	NM_144887:499	Zdhhc5	INSIDE	0.401	0.723	2712.89	1962.11	0.290	2037.85	590.93
A_68_P21394211	chr2:84555629-84555673	NM_144887:-329	Zdhhc5	PROMOTER	0.341	0.439	4734.44	2076.53	0.150	3532.02	528.54
A_68_P26217896	chr8:122625507-122625560	NM_133967:-161	Zdhhc7	PROMOTER	0.305	0.442	1251.84	553.33	0.135	997.37	134.37
A_68_P30578493	chr16:18234733-18234777	NM_172151:475	Zdhhc8	INSIDE	0.195	0.653	1295.80	845.62	0.127	898.37	114.44
A_68_P31469352	chr18:5591749-5591796	NM_011546:-116	Zeb1	PROMOTER	0.364	0.627	1055.27	661.74	0.228	870.77	198.86
A_68_P24135897	chr5:139960495-139960539	NM_001159908:-71	Zfand2a	PROMOTER	0.329	0.677	1149.23	777.68	0.223	972.75	216.67
A_68_P20352371	chr1:75165078-75165131	NM_001159905:-115	Zfand2b	PROMOTER	0.302	0.425	1698.12	721.94	0.128	1230.07	157.78
A_68_P31131059	chr17:30141179-30141223	NM_148926:-831	Zfand3	PROMOTER	0.152	0.563	1954.31	1101.06	0.086	1466.94	125.93
A_68_P31131060	chr17:30141259-30141303	NM_148926:-751	Zfand3	PROMOTER	0.321	0.624	1409.90	879.83	0.200	1049.12	209.91

ProbeName	Target position of probe on CpG island microarray	TargetID	GeneSymbol	CpG island Description	Ratio of relative methylation (TiO2-NP/Vehicle)	Sham group			TiO_2-H group		
						Relative methylation (Cy-5/Cy-3)	Cy-3 signal (Input)	Cy-5 signal (MeDIP)	Relative methylation (Cy-5/Cy-3)	Cy-3 signal (Input)	Cy-5 signal (MeDIP)
A_68_P27469300	chr10:114821835-114821879	NM_001033261:-158	Zfc3h1	DIVERGENT_PROMOTER	0.174	0.588	1675.89	985.64	0.102	1184.83	121.21
A_68_P27469304	chr10:114822217-114822261	NM_001033261:224	Zfc3h1	INSIDE	0.569	0.723	1971.17	1424.91	0.411	1642.50	675.30
A_68_P29616217	chr14:55710378-55710422	NM_001039198:485	Zfhx2	INSIDE	0.257	0.671	3657.23	2453.69	0.173	2586.56	446.60
A_68_P26152538	chr8:111238302-111238346	NM_007496:-219	Zfhx3	PROMOTER	0.595	0.679	1081.35	734.45	0.404	944.21	381.59
A_68_P26152542	chr8:111238708-111238758	NM_007496:189	Zfhx3	INSIDE	0.042	1.352	3471.69	4694.99	0.057	2100.28	120.40
A_68_P24129051	chr5:138596643-138596687	NM_019747:308	Zfp113	INSIDE	0.252	0.542	1896.16	1027.19	0.137	1377.73	188.16
A_68_P24129053	chr5:138596838-138596882	NM_019747:112	Zfp113	INSIDE	0.581	0.696	1327.08	923.33	0.404	1009.66	407.96
A_68_P24155561	chr5:143997211-143997255	NM_177681:346	Zfp12	INSIDE	0.552	0.717	2810.31	2014.94	0.396	2164.43	857.11
A_68_P31092534	chr17:23729724-23729768	NM_011747:6708	Zfp13	INSIDE	0.388	0.519	1338.86	695.11	0.201	1137.36	229.06
A_68_P25029683	chr7:30836003-30836047	NM_011748:391	Zfp14	INSIDE	0.565	0.643	1081.36	695.68	0.364	755.83	274.92
A_68_P20349425	chr1:74634360-74634404	NM_029888:220	Zfp142	INSIDE	0.617	0.449	2720.49	1222.32	0.277	2035.70	564.19
A_68_P25411540	chr7:117204605-117204649	NM_009281:-589	Zfp143	PROMOTER	0.256	0.524	1155.24	605.15	0.134	899.50	120.40
A_68_P25411544	chr7:117205106-117205158	NM_009281:-83	Zfp143	PROMOTER	0.048	1.934	1882.54	3640.72	0.092	1349.52	124.02
A_68_P25411546	chr7:117205275-117205334	NM_009281:89	Zfp143	INSIDE	0.110	2.701	481.41	1300.06	0.298	390.63	116.42
A_68_P30657579	chr16:33381453-33381497	NM_011749:614	Zfp148	INSIDE	0.542	0.588	1881.85	1107.05	0.319	1357.88	432.86
A_68_P24130229	chr5:138882742-138882786	NM_028130:61	Zfp157	INSIDE	0.191	0.575	1298.43	746.98	0.110	1082.33	119.03
A_68_P24130230	chr5:138882841-138882885	NM_028130:159	Zfp157	INSIDE	0.302	0.705	1946.79	1372.41	0.213	1620.02	345.40
A_68_P24130232	chr5:138883088-138883132	NM_028130:407	Zfp157	INSIDE	0.638	0.559	1125.68	628.75	0.356	895.87	319.00
A_68_P31323658	chr17:69731780-69731824	NM_009547:-1515	Zfp161	PROMOTER	0.238	0.587	1532.71	899.39	0.140	1122.30	156.80
A_68_P31323669	chr17:69733337-69733381	NM_009547:41	Zfp161	INSIDE	0.163	0.592	1683.35	996.60	0.096	1209.57	116.53
A_68_P31323661	chr17:69732210-69732254	NM_009547:-1085	Zfp161	PROMOTER	0.669	0.661	1152.64	761.38	0.442	970.05	428.87
A_68_P26891988	chr9:122812318-122812362	NM_001177505:14752	Zfp167	DOWNSTREAM	0.346	0.693	1775.77	1230.14	0.240	1231.48	295.22
A_68_P26891990	chr9:122812568-122812612	NM_001177505:15002	Zfp167	DOWNSTREAM	0.342	0.659	1992.99	1312.63	0.226	1603.40	361.64
A_68_P29019863	chr13:48608843-48608887	NM_001164576:-59	Zfp169	PROMOTER	0.621	0.678	1016.38	688.98	0.421	884.39	372.36
A_68_P32460116	chrX:70232552-70232596	NM_001109043:-103	Zfp185	PROMOTER	0.084	1.652	2553.62	4219.84	0.138	1147.58	158.86
A_68_P22905511	chr4:49534082-49534127	NM_145547:16	Zfp189	INSIDE	0.375	0.545	1540.35	838.91	0.204	1299.90	265.54
A_68_P31092273	chr17:23684002-23684046	NM_001033496:17169	Zfp213	DOWNSTREAM	0.254	0.524	1933.43	1012.25	0.133	1395.18	185.18
A_68_P31092274	chr17:23684082-23684126	NM_001033496:17089	Zfp213	DOWNSTREAM	0.605	0.578	1127.59	651.49	0.349	909.99	317.95
A_68_P31092275	chr17:23684173-23684217	NM_001033496:16999	Zfp213	DOWNSTREAM	0.438	0.694	1466.01	1017.79	0.304	1135.55	345.69
A_68_P31092406	chr17:23701243-23701288	NM_001033496:-72	Zfp213	PROMOTER	0.215	0.467	1510.69	705.92	0.100	1222.53	122.54
A_68_P21851437	chr2:169959061-169959105	NM_001033299:-2362	Zfp217	PROMOTER	0.199	0.543	1373.10	745.41	0.108	1100.18	118.92
A_68_P21851439	chr2:169959243-169959287	NM_001033299:-2544	Zfp217	PROMOTER	0.426	0.618	1178.79	728.96	0.264	949.40	250.32
A_68_P24786878	chr6:118405236-118405280	NM_028335:266	Zfp248	INSIDE	0.380	0.605	1310.10	791.99	0.230	973.42	223.41
A_68_P30350970	chr15:76701410-76701454	NM_001007568:433	Zfp251	INSIDE	0.581	0.542	1337.07	724.17	0.315	1079.66	339.70
A_68_P30350971	chr15:76701517-76701566	NM_001007568:324	Zfp251	INSIDE	0.460	0.482	1470.61	708.58	0.222	1068.10	236.65
A_68_P26478900	chr9:46081259-46081303	NM_011752:134	Zfp259	INSIDE	0.370	0.476	1359.42	646.66	0.176	1243.16	218.91
A_68_P26342513	chr9:20264224-20264268	NM_011753:358	Zfp26	INSIDE	0.456	0.465	1580.60	735.40	0.212	1286.51	272.85
A_68_P30506408	chr16:3744081-3744125	NM_148924:4	Zfp263	INSIDE	0.171	0.548	2998.60	1643.21	0.094	2308.15	215.88
A_68_P26342786	chr9:20326146-20326190	NM_001082485:-305	Zfp266	PROMOTER	0.297	0.434	5985.65	2600.73	0.129	4200.66	542.84
A_68_P26342787	chr9:20326238-20326285	NM_001082485:-398	Zfp266	PROMOTER	0.233	0.635	1069.12	678.69	0.148	807.82	119.60
A_68_P32462127	chrX:70588115-70588159	NM_001160229:177	Zfp275	INSIDE	0.007	8.116	5707.12	46321.67	0.056	2110.42	117.44
A_68_P26238820	chr8:125778693-125778737	NM_020497:620	Zfp276	INSIDE	0.359	0.630	1063.44	669.45	0.226	921.19	207.92
A_68_P24955196	chr7:6335606-6335650	NM_175247:-399	Zfp28	PROMOTER	0.242	0.734	4728.37	3468.38	0.177	3321.74	588.85
A_68_P24955199	chr7:6335923-6335967	NM_175247:-83	Zfp28	PROMOTER	0.189	0.472	1659.57	783.58	0.089	1335.68	119.43

ProbeName	Target position of probe on CpG island microarray	TargetID	GeneSymbol	CpG island Description	Ratio of relative methylation (TiO2-NP/Vehicle)	Sham group			TiO_2-H group		
						Relative methylation (Cy-5/Cy-3)	Cy-3 signal (Input)	Cy-5 signal (MeDIP)	Relative methylation (Cy-5/Cy-3)	Cy-3 signal (Input)	Cy-5 signal (MeDIP)
A_68_P27855686	chr11:62603073-62603117	NM_138949:-175	Zfp286	PROMOTER	0.288	0.383	1745.56	669.02	0.110	1299.64	143.42
A_68_P27855417	chr11:62545135-62545179	NM_133208:-2561	Zfp287	PROMOTER	0.466	0.701	1194.57	837.43	0.327	992.98	324.31
A_68_P31002111	chr16:98183558-98183609	NM_001081684:203	Zfp295	INSIDE	0.280	0.548	1006.51	551.09	0.153	810.57	124.40
A_68_P24994119	chr7:20162872-20162916	NM_022409:259	Zfp296	INSIDE	0.181	0.392	2668.91	1045.35	0.071	1836.11	129.85
A_68_P27902560	chr11:70577738-70577782	NM_177565:-188	Zfp3	PROMOTER	0.222	0.618	2629.42	1625.00	0.137	1961.89	268.95
A_68_P27902563	chr11:70578032-70578076	NM_177565:106	Zfp3	INSIDE	0.548	0.405	1453.52	587.98	0.222	1276.75	282.92
A_68_P24155790	chr5:144031255-144031299	NM_017467:425	Zfp316	INSIDE	0.172	0.612	1471.81	900.28	0.105	1108.29	116.69
A_68_P24155791	chr5:144031328-144031372	NM_017467:351	Zfp316	INSIDE	0.076	0.449	4921.70	2209.77	0.034	3505.98	119.72
A_68_P24155794	chr5:144031725-144031769	NM_017467:-45	Zfp316	PROMOTER	0.466	0.561	1936.11	1086.82	0.262	1548.00	404.81
A_68_P28881066	chr13:23461022-23461066	NM_001111107:27	Zfp322a	INSIDE	0.572	0.657	2833.68	1861.60	0.376	2303.79	866.40
A_68_P28881068	chr13:23461198-23461242	NM_001111107:-149	Zfp322a	PROMOTER	0.344	0.589	1652.29	972.74	0.202	1327.03	268.56
A_68_P24973875	chr7:13551224-13551268	NM_178732:34	Zfp324	INSIDE	0.409	0.515	1331.45	685.54	0.210	1045.44	219.91
A_68_P24973048	chr7:13388705-13388749	NM_026046:15483	Zfp329	DOWNSTREAM	0.466	0.697	1307.60	911.89	0.325	1083.63	351.86
A_68_P24973049	chr7:13388821-13388865	NM_026046:15367	Zfp329	DOWNSTREAM	0.144	0.674	1664.69	1121.87	0.097	1183.86	114.95
A_68_P24973050	chr7:13388889-13388933	NM_026046:15299	Zfp329	DOWNSTREAM	0.247	0.691	1425.62	984.65	0.171	1036.10	176.71
A_68_P24973141	chr7:13403835-13403879	NM_026046:353	Zfp329	INSIDE	0.340	0.530	1288.31	682.69	0.180	1080.60	194.90
A_68_P26011369	chr8:85298048-85298092	NM_145600:-45	Zfp330	PROMOTER	0.360	0.351	1589.13	558.40	0.127	1315.68	166.53
A_68_P21823563	chr2:165213394-165213441	NM_178411:342	Zfp334	INSIDE	0.200	0.484	2097.41	1015.15	0.097	1669.03	161.23
A_68_P21820483	chr2:164736035-164736086	NM_199027:1190	Zfp335	INSIDE	0.297	0.551	1094.05	603.28	0.164	734.59	120.43
A_68_P29053295	chr13:55206803-55206847	NM_012017:155	Zfp346	INSIDE	0.276	0.603	4070.91	2454.59	0.166	2887.16	480.19
A_68_P27792049	chr11:50872760-50872804	NM_009329:24	Zfp354a	INSIDE	0.393	0.466	1271.52	592.67	0.183	1116.60	204.33
A_68_P27790811	chr11:50641126-50641170	NM_013922:85	Zfp354c	INSIDE	0.646	0.575	2899.51	1666.58	0.372	2099.59	780.08
A_68_P25614206	chr8:3493424-3493468	NM_080461:309	Zfp358	INSIDE	0.554	0.352	2119.63	746.48	0.195	1681.69	327.95
A_68_P25021520	chr7:29164259-29164303	NM_011756:-33	Zfp36	PROMOTER	0.466	0.666	1620.87	1079.14	0.310	1331.10	413.27
A_68_P29102609	chr13:65380152-65380198	NM_178364:13	Zfp369	INSIDE	0.174	0.544	1622.39	882.81	0.095	1278.17	121.07
A_68_P28573854	chr12:81216498-81216557	NM_007564:-2527	Zfp36l1	PROMOTER	0.278	0.613	903.02	553.40	0.171	697.81	119.01
A_68_P31403191	chr17:84585735-84585779	NM_001001806:1531	Zfp36l2	INSIDE	0.561	0.595	1047.30	623.28	0.334	877.89	293.28
A_68_P31403203	chr17:84587080-84587124	NM_001001806:185	Zfp36l2	INSIDE	0.516	0.305	2182.82	664.69	0.157	1708.21	268.44
A_68_P31403204	chr17:84587274-84587318	NM_001001806:-9	Zfp36l2	PROMOTER	0.321	0.316	3508.71	1107.40	0.101	2651.07	268.22
A_68_P22963156	chr4:61869252-61869305	NM_009554:302	Zfp37	INSIDE	0.235	1.663	3280.03	5454.28	0.390	2359.03	920.14
A_68_P22963157	chr4:61869358-61869402	NM_009554:200	Zfp37	INSIDE	0.265	0.617	3214.07	1982.33	0.164	2353.55	384.85
A_68_P24818152	chr6:124958758-124958812	NM_175557:-1038	Zfp384	PROMOTER	0.104	0.621	2468.36	1533.90	0.065	1888.64	122.54
A_68_P24818153	chr6:124958879-124958923	NM_175557:-922	Zfp384	PROMOTER	0.517	0.505	1347.18	679.78	0.261	1055.62	275.37
A_68_P30503432	chr15:103144711-103144755	NM_013866:25785	Zfp385a	INSIDE	0.657	2.208	1289.63	2847.64	1.450	944.34	1368.97
A_68_P30503505	chr15:103153548-103153598	NM_013866:16945	Zfp385a	INSIDE	0.079	2.034	4335.45	8817.79	0.160	2853.66	457.12
A_68_P28069222	chr11:100501295-100501339	NM_177790:10691	Zfp385c	INSIDE	0.604	0.612	1173.82	718.80	0.370	897.51	331.73
A_68_P28765737	chr12:117285901-117285952	NM_001004066:-8	Zfp386	PROMOTER	0.229	0.483	1357.82	655.37	0.110	1100.32	121.55
A_68_P29672988	chr14:65977163-65977207	NM_199029:-328	Zfp395	PROMOTER	0.164	0.706	1716.30	1210.95	0.116	1424.76	164.68
A_68_P29672990	chr14:65977418-65977462	NM_199029:-72	Zfp395	PROMOTER	0.650	0.548	1234.36	676.50	0.356	1021.31	363.56
A_68_P29672993	chr14:65977836-65977880	NM_199029:346	Zfp395	INSIDE	0.445	0.659	1259.26	829.55	0.293	1109.18	325.14
A_68_P31889607	chr18:84758993-84759052	NM_001033341:-126	Zfp407	PROMOTER	0.301	0.290	1917.24	556.95	0.087	1370.17	119.82
A_68_P30342959	chr15:75447008-75447052	NM_001044718:-84	Zfp41	PROMOTER	0.332	0.545	2116.98	1152.76	0.181	1691.87	305.67
A_68_P30342960	chr15:75447074-75447118	NM_001044718:-18	Zfp41	PROMOTER	0.662	0.658	5546.32	3649.34	0.436	3908.26	1702.44
A_68_P30342961	chr15:75447233-75447277	NM_001044718:140	Zfp41	INSIDE	0.625	0.412	1561.99	644.23	0.258	1111.18	286.26

ProbeName	Target position of probe on CpG island microarray	TargetID	GeneSymbol	CpG island Description	Ratio of relative methylation (TiO2-NP/Vehicle)	Sham group			TiO_2-H group		
						Relative methylation (Cy-5/Cy-3)	Cy-3 signal (Input)	Cy-5 signal (MeDIP)	Relative methylation (Cy-5/Cy-3)	Cy-3 signal (Input)	Cy-5 signal (MeDIP)
A_68_P31149741	chr17:33766122-33766166	NM_026712:108	Zfp414	INSIDE	0.479	0.612	906.13	554.98	0.294	758.17	222.56
A_68_P31149742	chr17:33766187-33766231	NM_026712:172	Zfp414	INSIDE	0.291	0.445	6747.39	3002.33	0.129	5296.35	685.64
A_68_P25028818	chr7:30645166-30645210	NM_172740:191	Zfp420	INSIDE	0.253	0.492	1317.60	648.40	0.124	990.70	123.34
A_68_P25028819	chr7:30645245-30645299	NM_172740:275	Zfp420	INSIDE	0.302	0.593	957.43	567.41	0.179	679.63	121.45
A_68_P24777346	chr6:116578724-116578768	NM_026057:249	Zfp422	INSIDE	0.282	0.553	3244.64	1793.90	0.156	2317.78	361.06
A_68_P26036154	chr8:90184192-90184236	NM_033327:299280	Zfp423	DOWNSTREAM	0.291	0.573	7295.16	4182.97	0.167	4961.94	828.90
A_68_P26036157	chr8:90184552-90184597	NM_033327:298920	Zfp423	DOWNSTREAM	0.166	0.616	1439.35	886.32	0.102	1138.78	116.10
A_68_P26038366	chr8:90484574-90484618	NM_033327:-1102	Zfp423	PROMOTER	0.391	0.702	2889.02	2027.03	0.275	2132.18	585.63
A_68_P26038371	chr8:90485107-90485151	NM_033327:-1634	Zfp423	PROMOTER	0.517	0.517	1355.59	700.29	0.267	1180.36	315.23
A_68_P26038378	chr8:90485888-90485932	NM_033327:-2416	Zfp423	PROMOTER	0.292	0.628	2432.62	1528.64	0.183	1809.04	331.51
A_68_P26038381	chr8:90486316-90486360	NM_033327:-2844	Zfp423	PROMOTER	0.508	0.687	1521.67	1045.01	0.349	1154.13	402.49
A_68_P25005239	chr7:25292778-25292822	NM_146183:695	Zfp428	INSIDE	0.344	0.540	2038.45	1099.97	0.185	1600.26	296.62
A_68_P31468070	chr18:5334710-5334760	NM_178722:-297	Zfp438	PROMOTER	0.131	2.465	1927.74	4752.17	0.324	1133.78	366.87
A_68_P31468071	chr18:5334781-5334825	NM_178722:-365	Zfp438	PROMOTER	0.262	1.714	6621.53	11347.19	0.449	4557.49	2044.96
A_68_P24954373	chr7:6124443-6124487	NM_001146024:350	Zfp444	INSIDE	0.315	1.391	1459.96	2030.37	0.438	1044.12	456.88
A_68_P20135165	chr1:33871553-33871598	NM_133817:-303	Zfp451	PROMOTER	0.173	0.688	1734.76	1194.10	0.119	1370.44	163.41
A_68_P24427172	chr6:48389196-48389240	NM_001085415:5871	Zfp467	INSIDE	0.549	1.351	4264.31	5759.59	0.741	2680.08	1985.71
A_68_P25089142	chr7:52003877-52003921	NM_178734:89	Zfp473	INSIDE	0.295	0.530	1007.57	533.75	0.156	772.53	120.81
A_68_P24165132	chr5:146041465-146041509	NM_001081431:-2725	Zfp498	PROMOTER	0.581	0.514	1170.54	602.02	0.299	1047.64	313.04
A_68_P24165134	chr5:146041748-146041800	NM_001081431:-2437	Zfp498	PROMOTER	0.171	0.552	1481.40	817.50	0.094	1286.94	121.57
A_68_P29455869	chr14:22804369-22804413	NM_145459:4433	Zfp503	INSIDE	0.313	0.658	1731.42	1140.05	0.206	1176.21	242.46
A_68_P29455892	chr14:22807139-22807185	NM_145459:1661	Zfp503	INSIDE	0.144	0.636	1611.16	1024.55	0.092	1288.71	118.05
A_68_P29455893	chr14:22807230-22807274	NM_145459:1571	Zfp503	INSIDE	0.160	0.467	2433.51	1135.41	0.075	1945.26	145.44
A_68_P29455907	chr14:22808867-22808911	NM_145459:-65	Zfp503	PROMOTER	0.399	0.569	1604.07	911.92	0.227	1214.52	275.57
A_68_P25581872	chr7:147222785-147222830	NM_027201:518	Zfp511	INSIDE	0.198	0.307	2758.23	848.04	0.061	1939.46	118.02
A_68_P23577059	chr5:31504739-31504783	NM_175311:-84	Zfp513	PROMOTER	0.238	0.402	1702.97	685.42	0.096	1268.39	121.69
A_68_P31879113	chr18:83078968-83079012	NM_183033:-1280	Zfp516	PROMOTER	0.447	1.389	7263.72	10088.54	0.621	4919.39	3053.47
A_68_P31879118	chr18:83079545-83079600	NM_183033:-698	Zfp516	PROMOTER	0.263	0.436	1472.32	642.13	0.115	1117.63	128.16
A_68_P31879119	chr18:83079657-83079701	NM_183033:-592	Zfp516	PROMOTER	0.577	0.687	1555.85	1069.20	0.397	1190.30	472.38
A_68_P31879121	chr18:83079861-83079905	NM_183033:-388	Zfp516	PROMOTER	0.234	0.632	975.37	616.53	0.148	821.38	121.34
A_68_P31879129	chr18:83080856-83080900	NM_001177464:-3145	Zfp516	PROMOTER	0.201	1.487	1836.25	2730.14	0.298	1301.81	388.17
A_68_P31086419	chr17:21669765-21669813	NM_144515:-2714	Zfp52	PROMOTER	0.244	0.595	1120.49	666.87	0.145	853.51	124.17
A_68_P31509663	chr18:14128717-14128761	NM_145492:2504	Zfp521	INSIDE	0.647	0.709	1369.05	970.87	0.459	1248.48	573.06
A_68_P31509666	chr18:14129064-14129108	NM_145492:2156	Zfp521	INSIDE	0.224	1.826	1431.31	2614.02	0.409	1103.50	451.41
A_68_P24951572	chr7:4966899-4966945	NM_025324:-187	Zfp524	DIVERGENT_PROMOTER	0.233	0.450	1318.63	593.58	0.105	1108.13	116.21
A_68_P31782009	chr18:65741080-65741124	NM_207255:1219	Zfp532	INSIDE	0.345	0.494	1236.74	611.02	0.170	956.13	162.83
A_68_P25063497	chr7:38554739-38554783	NM_172385:11	Zfp536	INSIDE	0.254	0.366	1505.87	551.57	0.093	1250.12	116.48
A_68_P25063501	chr7:38555245-38555289	NM_172385:-495	Zfp536	PROMOTER	0.363	0.675	1965.18	1325.54	0.245	1624.38	397.88
A_68_P25063515	chr7:38556810-38556854	NM_172385:-2061	Zfp536	PROMOTER	0.370	0.645	1075.96	693.54	0.239	811.94	193.89
A_68_P25063517	chr7:38557044-38557088	NM_172385:-2295	Zfp536	PROMOTER	0.587	0.596	1286.86	767.42	0.350	1140.18	399.32
A_68_P25063531	chr7:38558593-38558648	NM_172385:-3849	Zfp536	PROMOTER	0.280	0.398	1341.66	534.25	0.111	1078.38	120.20
A_68_P25504685	chr7:134377078-134377122	NM_146201:144	Zfp553	INSIDE	0.351	0.595	3038.36	1807.63	0.209	2190.21	457.91
A_68_P26337051	chr9:18277941-18277985	NM_028935:41	Zfp558	INSIDE	0.134	1.754	5047.42	8850.97	0.235	3329.71	780.91
A_68_P25029850	chr7:30875471-30875515	NM_152814:37	Zfp566	INSIDE	0.332	0.561	1419.16	795.82	0.186	1058.68	196.97

ProbeName	Target position of probe on CpG island microarray	TargetID	GeneSymbol	CpG island Description	Ratio of relative methylation (TiO2-NP/Vehicle)	Sham group			TiO_2-H group		
						Relative methylation (Cy-5/Cy-3)	Cy-3 signal (Input)	Cy-5 signal (MeDIP)	Relative methylation (Cy-5/Cy-3)	Cy-3 signal (Input)	Cy-5 signal (MeDIP)
A_68_P31166723	chr17:37139521-37139565	NR_033137:-1068	Zfp57	PROMOTER	0.402	0.568	1340.99	761.64	0.228	962.23	219.44
A_68_P25008231	chr7:25862140-25862187	NM_001168506:-60	Zfp574	DIVERGENT_PROMOTER	0.222	0.680	1118.80	760.76	0.151	820.12	123.60
A_68_P24951776	chr7:5003006-5003050	NM_026900:-105	Zfp580	PROMOTER	0.190	0.514	3152.43	1619.80	0.097	2211.55	215.50
A_68_P24951777	chr7:5003100-5003144	NM_026900:-11	Zfp580	PROMOTER	0.423	0.626	1500.18	938.54	0.264	1245.68	329.43
A_68_P24951780	chr7:5003473-5003517	NM_026900:361	Zfp580	INSIDE	0.300	1.634	1369.84	2238.52	0.491	1043.17	511.69
A_68_P25269627	chr7:88137961-88138005	NM_178707:-587	Zfp592	PROMOTER	0.529	0.475	1775.37	842.83	0.251	1519.33	381.72
A_68_P30506931	chr16:3872466-3872510	NM_001033159:-114	Zfp597	PROMOTER	0.268	0.594	2790.27	1656.08	0.159	2087.26	331.93
A_68_P26351028	chr9:22064295-22064339	NM_181419:23	Zfp599	INSIDE	0.604	0.523	1647.92	861.06	0.316	1267.56	400.15
A_68_P25018704	chr7:28516297-28516346	NM_009560:-106	Zfp60	PROMOTER	0.410	0.489	1102.47	539.29	0.201	827.88	166.06
A_68_P25018706	chr7:28516596-28516644	NM_009560:193	Zfp60	INSIDE	0.261	0.635	947.15	601.13	0.166	751.37	124.48
A_68_P24972086	chr7:13063602-13063648	NM_001039951:-29	Zfp606	PROMOTER	0.097	0.731	2191.81	1602.35	0.071	1688.84	120.13
A_68_P31722243	chr18:55147517-55147561	NM_175751:2296	Zfp608	INSIDE	0.444	0.413	1266.85	523.68	0.184	982.38	180.48
A_68_P31722245	chr18:55147700-55147744	NM_175751:2112	Zfp608	INSIDE	0.349	0.653	1413.12	922.57	0.228	1056.52	240.77
A_68_P25068287	chr7:46799838-46799882	NM_001004139:26725	Zfp619	DOWNSTREAM	0.193	0.585	1305.08	763.29	0.113	1030.77	116.47
A_68_P27781837	chr11:49016791-49016837	NM_001024846:-187	Zfp62	PROMOTER	0.155	1.535	747.40	1147.24	0.238	520.00	123.51
A_68_P27781838	chr11:49016880-49016924	NM_001024846:-99	Zfp62	PROMOTER	0.268	0.682	4759.34	3244.03	0.182	3846.82	701.43
A_68_P30084535	chr15:25913713-25913757	NM_144523:-386	Zfp622	PROMOTER	0.320	0.589	1339.31	788.35	0.188	1004.97	189.42
A_68_P30084537	chr15:25913916-25913960	NM_144523:-182	Zfp622	PROMOTER	0.307	0.740	3944.02	2917.47	0.227	2881.16	654.34
A_68_P30084538	chr15:25914029-25914073	NM_144523:-70	Zfp622	PROMOTER	0.324	0.545	2913.87	1587.63	0.177	2220.02	392.50
A_68_P22038452	chr3:32409336-32409380	NM_001161818:-113	Zfp639	PROMOTER	0.209	0.497	1877.11	932.54	0.104	1464.39	151.70
A_68_P23962876	chr5:107125079-107125123	NM_026856:749	Zfp644	INSIDE	0.593	0.299	2493.51	746.29	0.178	2052.64	364.35
A_68_P20730831	chr1:156052030-156052074	NM_001204908:3736	Zfp648	INSIDE	0.580	0.635	1837.98	1167.92	0.368	1387.16	511.01
A_68_P20730836	chr1:156052430-156052474	NM_001204908:4136	Zfp648	INSIDE	0.402	0.615	1327.37	815.96	0.247	1041.18	257.49
A_68_P24164945	chr5:145992341-145992385	NM_001083958:-221	Zfp655	PROMOTER	0.443	0.599	1108.91	664.54	0.265	960.77	255.03
A_68_P24164946	chr5:145992407-145992453	NM_001083958:-153	Zfp655	PROMOTER	0.168	0.497	1853.86	920.47	0.083	1411.20	117.60
A_68_P24164951	chr5:145993056-145993100	NM_028298:451	Zfp655	INSIDE	0.471	0.556	1210.41	672.50	0.262	1057.89	277.07
A_68_P25082976	chr7:50817634-50817683	NM_001008549:-81	Zfp658	PROMOTER	0.111	0.621	2416.77	1500.29	0.069	1777.42	122.54
A_68_P25082977	chr7:50817749-50817793	NM_001008549:31	Zfp658	INSIDE	0.176	0.563	1600.81	900.83	0.099	1206.00	119.75
A_68_P25082978	chr7:50817824-50817869	NM_001008549:107	Zfp658	INSIDE	0.161	0.595	1693.69	1008.10	0.096	1244.50	119.40
A_68_P21823447	chr2:165194226-165194272	NM_001005425:-6629	Zfp663	PROMOTER	0.410	0.586	1286.83	753.90	0.240	937.90	225.02
A_68_P24954796	chr7:6237873-6237917	NM_001024928:-287	Zfp667	PROMOTER	0.233	0.626	982.30	614.46	0.146	794.62	115.95
A_68_P25507823	chr7:135019837-135019881	NM_146259:479	Zfp668	INSIDE	0.619	0.639	1201.88	768.49	0.396	966.17	382.61
A_68_P24130914	chr5:139060935-139060979	NM_001044747:15	Zfp68	INSIDE	0.494	0.723	1924.95	1391.16	0.357	1499.59	535.79
A_68_P22341850	chr3:94818782-94818826	NM_030074:356	Zfp687	INSIDE	0.546	0.581	1044.57	606.94	0.317	792.04	251.19
A_68_P22341859	chr3:94819900-94819945	NM_030074:-762	Zfp687	PROMOTER	0.530	0.544	1255.30	682.68	0.288	1007.23	290.23
A_68_P25505644	chr7:134592579-134592625	NM_175163:70	Zfp689	INSIDE	0.656	0.528	1421.16	749.72	0.346	1004.56	347.43
A_68_P23254146	chr4:120624021-120624065	NM_001005788:264	Zfp69	INSIDE	0.519	0.454	1515.26	688.54	0.236	1260.87	297.48
A_68_P27830258	chr11:58120752-58120796	NM_001040686:204	Zfp692	INSIDE	0.624	0.616	1151.09	708.82	0.384	948.30	364.10
A_68_P22360090	chr3:98231396-98231440	NM_172863:45015	Zfp697	INSIDE	0.233	0.571	1128.96	644.47	0.133	894.34	118.88
A_68_P22360097	chr3:98232384-98232428	NM_172863:46003	Zfp697	INSIDE	0.160	0.498	1747.31	870.94	0.080	1458.40	116.57
A_68_P30351004	chr15:76709899-76709943	NM_145916:215	Zfp7	INSIDE	0.217	0.596	1210.58	721.99	0.130	900.39	116.73
A_68_P25735800	chr8:28087824-28087868	NM_001101502:39	Zfp703	INSIDE	0.261	0.608	2335.99	1420.43	0.159	1711.28	271.44
A_68_P30141816	chr15:36937216-36937260	NM_026521:-81	Zfp706	PROMOTER	0.477	0.588	2500.93	1471.67	0.281	1874.31	526.37
A_68_P25264702	chr7:87226863-87226912	NM_175433:55774	Zfp710	INSIDE	0.341	1.610	1303.70	2098.42	0.549	951.92	522.55

ProbeName	Target position of probe on CpG island microarray	TargetID	GeneSymbol	CpG island Description	Ratio of relative methylation (TiO2-NP/Vehicle)	Sham group			TiO_2-H group		
						Relative methylation (Cy-5/Cy-3)	Cy-3 signal (Input)	Cy-5 signal (MeDIP)	Relative methylation (Cy-5/Cy-3)	Cy-3 signal (Input)	Cy-5 signal (MeDIP)
A_68_P32603683	chrX:109714707-109714751	NM_177747:594	Zfp711	INSIDE	0.314	1.682	1399.18	2352.86	0.527	652.40	343.98
A_68_P29107216	chr13:67161978-67162022	NM_001166218:106	Zfp712	INSIDE	0.496	0.614	1579.81	969.42	0.304	1241.60	378.03
A_68_P25081793	chr7:50568397-50568441	NM_027264:213	Zfp715	INSIDE	0.644	0.507	5156.42	2612.85	0.326	4186.34	1366.36
A_68_P30496553	chr15:102034646-102034690	NM_153194:-4029	Zfp740	PROMOTER	0.443	0.497	1135.27	564.41	0.220	894.13	196.99
A_68_P25505329	chr7:134507375-134507419	NM_175560:12168	Zfp747	DOWNSTREAM	0.226	0.581	2164.11	1257.35	0.131	1676.62	220.09
A_68_P25505268	chr7:134488780-134488824	NM_146202:26	Zfp768	INSIDE	0.294	0.541	5020.78	2714.09	0.159	3484.95	553.49
A_68_P21538596	chr2:114027103-114027147	NM_175466:44	Zfp770	INSIDE	0.374	0.555	1476.51	818.98	0.207	1188.32	246.40
A_68_P25504739	chr7:134387875-134387919	NM_177362:-143	Zfp771	PROMOTER	0.445	0.593	1789.04	1060.87	0.264	1419.08	374.74
A_68_P25504797	chr7:134398221-134398265	NM_177362:10203	Zfp771	INSIDE	0.469	0.439	1289.62	566.25	0.206	989.47	203.64
A_68_P27291280	chr10:81233564-81233608	NM_199062:301	Zfp781	INSIDE	0.279	0.672	3324.63	2233.59	0.187	2733.51	511.68
A_68_P24951727	chr7:4990151-4990195	NM_001039532:-124	Zfp784	PROMOTER	0.590	0.495	1695.96	839.82	0.292	1174.83	343.22
A_68_P24954290	chr7:6106969-6107013	NM_001013012:583	Zfp787	INSIDE	0.263	0.636	2127.44	1353.98	0.167	1612.30	269.57
A_68_P21382117	chr2:81893227-81893271	NM_175513:-566	Zfp804a	PROMOTER	0.256	0.626	4735.49	2962.82	0.160	3531.01	566.57
A_68_P31148787	chr17:33495685-33495729	NM_207541:117	Zfp81	INSIDE	0.166	0.496	1792.46	889.21	0.082	1430.98	117.67
A_68_P26158503	chr8:112229246-112229291	NM_001167946:-180	Zfp821	PROMOTER	0.209	0.587	1375.93	808.02	0.123	967.55	118.73
A_68_P25670892	chr8:13869760-13869804	NM_181854:142	Zfp828	INSIDE	0.588	0.622	1248.35	776.91	0.366	942.02	344.73
A_68_P27968102	chr11:82578176-82578220	NM_025884:352	Zfp830	INSIDE	0.441	0.519	2850.36	1480.36	0.229	2169.05	496.36
A_68_P28740679	chr12:112088312-112088356	NM_001199785:-154	Zfp839	DIVERGENT_PROMOTER	0.211	0.522	1567.46	818.57	0.110	1114.40	122.80
A_68_P28740680	chr12:112088415-112088460	NM_001199785:-51	Zfp839	DIVERGENT_PROMOTER	0.549	0.750	4590.79	3442.32	0.412	3358.40	1383.67
A_68_P26343012	chr9:20385783-20385830	NM_172919:35	Zfp846	INSIDE	0.421	0.679	981.98	666.79	0.286	714.64	204.52
A_68_P24951608	chr7:4971880-4971924	NM_001033383:-75	Zfp865	PROMOTER	0.211	0.610	1354.54	825.75	0.128	1101.01	141.39
A_68_P25947576	chr8:72149308-72149352	NM_001045553:-256	Zfp868	PROMOTER	0.639	0.748	3835.41	2867.80	0.478	3162.71	1511.47
A_68_P25947577	chr8:72149392-72149438	NM_001045553:-340	Zfp868	PROMOTER	0.138	0.704	1744.78	1227.91	0.097	1254.48	121.53
A_68_P31147012	chr17:32925128-32925172	NM_172458:82	Zfp871	INSIDE	0.281	0.683	1074.16	733.40	0.192	783.56	150.47
A_68_P25959007	chr8:74432656-74432700	NM_001166645:174	Zfp882	INSIDE	0.539	0.635	820.64	521.05	0.342	739.49	252.94
A_68_P26139674	chr8:108939165-108939209	NM_011764:-52	Zfp90	PROMOTER	0.248	0.631	2362.80	1491.34	0.157	1857.77	291.06
A_68_P26139675	chr8:108939273-108939317	NM_011764:56	Zfp90	INSIDE	0.299	0.494	1046.46	516.55	0.148	842.17	124.23
A_68_P26139677	chr8:108939449-108939493	NM_011764:232	Zfp90	INSIDE	0.569	0.531	1320.09	700.60	0.302	1016.72	306.91
A_68_P25004262	chr7:25101544-25101588	NM_001199321:119	Zfp94	INSIDE	0.470	0.719	2015.64	1448.31	0.338	1391.49	470.18
A_68_P24958144	chr7:7073964-7074008	NM_172738:201	Zfp954	INSIDE	0.547	0.594	2113.52	1255.26	0.325	1698.24	551.27
A_68_P24958145	chr7:7074049-7074093	NM_172738:117	Zfp954	INSIDE	0.368	0.624	1752.32	1094.30	0.230	1266.05	290.61
A_68_P25947839	chr8:72273658-72273702	NM_001200023:181	Zfp963	INSIDE	0.652	0.357	2152.55	767.40	0.232	1797.64	417.73
A_68_P25947618	chr8:72178919-72178963	NM_001177527:486	Zfp964	INSIDE	0.424	0.654	853.37	558.16	0.277	599.49	166.07
A_68_P26232278	chr8:124805221-124805265	NM_009569:-798	Zfpm1	PROMOTER	0.287	0.706	2890.92	2040.33	0.203	2183.91	442.96
A_68_P26232281	chr8:124805601-124805645	NM_009569:-418	Zfpm1	PROMOTER	0.654	3.113	7093.42	22079.62	2.037	4233.43	8622.50
A_68_P26232299	chr8:124808193-124808241	NM_009569:2176	Zfpm1	INSIDE	0.240	0.490	1186.44	581.57	0.118	988.73	116.35
A_68_P30160396	chr15:40486463-40486507	NM_011766:-103	Zfpm2	PROMOTER	0.085	0.606	3340.84	2023.81	0.052	2247.96	115.78
A_68_P30160401	chr15:40487098-40487142	NM_011766:533	Zfpm2	INSIDE	0.169	0.680	1427.21	970.91	0.115	1061.15	121.88
A_68_P30160402	chr15:40487224-40487274	NM_011766:661	Zfpm2	INSIDE	0.103	0.733	2682.53	1967.08	0.075	1773.98	133.72
A_68_P30019878	chr15:12047227-12047275	NM_011767:-355	Zfr	PROMOTER	0.165	1.476	2159.40	3187.97	0.243	1475.39	359.07
A_68_P30019880	chr15:12047515-12047559	NM_011767:-69	Zfr	PROMOTER	0.251	0.641	961.01	615.57	0.161	749.52	120.32
A_68_P21568392	chr2:119034387-119034433	NM_001164827:-45	Zfyve19	PROMOTER	0.302	0.484	1173.15	567.34	0.146	828.70	121.06
A_68_P28746257	chr12:113052309-113052356	NM_026752:-48	Zfyve21	DIVERGENT_PROMOTER	0.234	0.514	3051.94	1568.17	0.120	2361.82	284.15
A_68_P21910191	chr2:181100855-181100899	NM_001048148:749	Zgpat	INSIDE	0.207	0.519	2327.42	1208.76	0.108	1971.85	212.37

ProbeName	Target position of probe on CpG island microarray	TargetID	GeneSymbol	CpG island Description	Ratio of relative methylation (TiO2-NP/Vehicle)	Sham group			TiO_2-H group		
						Relative methylation (Cy-5/Cy-3)	Cy-3 signal (Input)	Cy-5 signal (MeDIP)	Relative methylation (Cy-5/Cy-3)	Cy-3 signal (Input)	Cy-5 signal (MeDIP)
A_68_P30245925	chr15:57908333-57908377	NM_001042438:-310	Zhx1	PROMOTER	0.448	0.556	4504.29	2502.28	0.249	3468.84	863.28
A_68_P30245927	chr15:57908540-57908584	NM_001042438:-518	Zhx1	PROMOTER	0.604	0.659	906.18	597.41	0.398	684.63	272.56
A_68_P21797520	chr2:160698110-160698154	NM_177263:594	Zhx3	INSIDE	0.121	0.556	4546.46	2527.45	0.067	3220.19	217.36
A_68_P26721507	chr9:91259790-91259834	NM_009573:825	Zic1	INSIDE	0.635	0.461	1301.45	600.40	0.293	1053.38	308.39
A_68_P29961926	chr14:122873265-122873309	NM_009574:-1319	Zic2	DIVERGENT_PROMOTER	0.496	0.613	2803.90	1718.23	0.304	2119.16	644.41
A_68_P29961928	chr14:122873448-122873492	NM_009574:-1135	Zic2	DIVERGENT_PROMOTER	0.256	0.512	2477.24	1268.52	0.131	1979.82	259.46
A_68_P29961931	chr14:122873762-122873806	NM_009574:-821	Zic2	DIVERGENT_PROMOTER	0.215	0.639	1897.42	1212.25	0.137	1310.39	179.67
A_68_P29961944	chr14:122875451-122875495	NM_009574:867	Zic2	INSIDE	0.478	0.595	2523.21	1501.09	0.284	1988.30	565.48
A_68_P32403901	chrX:55283389-55283441	NM_009575:-390	Zic3	PROMOTER	0.123	2.087	1230.12	2567.23	0.256	471.82	120.76
A_68_P32403906	chrX:55284045-55284104	NM_009575:270	Zic3	INSIDE	0.074	4.497	1428.96	6425.88	0.331	542.29	179.57
A_68_P26721618	chr9:91273756-91273800	NM_009576:9969	Zic4	INSIDE	0.387	0.678	1231.49	835.37	0.263	838.63	220.15
A_68_P29961839	chr14:122863102-122863146	NM_022987:1756	Zic5	INSIDE	0.352	0.604	916.24	552.97	0.212	774.75	164.57
A_68_P29961850	chr14:122864514-122864560	NM_022987:344	Zic5	INSIDE	0.085	1.788	1092.78	1954.43	0.152	792.19	120.29
A_68_P22953710	chr4:58956479-58956523	NM_001099323:1	Zkscan16	INSIDE	0.566	0.608	1084.88	660.06	0.344	862.70	296.98
A_68_P27837321	chr11:59319755-59319799	NM_001130529:366	Zkscan17	INSIDE	0.426	0.460	1368.04	629.36	0.196	1124.79	220.27
A_68_P27837323	chr11:59320124-59320168	NM_001130529:-4	Zkscan17	PROMOTER	0.130	0.660	1810.35	1194.00	0.085	1387.29	118.52
A_68_P28872662	chr13:21494612-21494656	NM_001145778:-10	Zkscan3	PROMOTER	0.365	0.728	2642.27	1922.65	0.266	2039.20	541.83
A_68_P27873629	chr11:65620598-65620642	NM_026107:-125	Zkscan6	PROMOTER	0.212	0.473	1604.04	758.35	0.100	1386.18	138.84
A_68_P31625899	chr18:36953656-36953708	NM_025594:106	Zmat2	INSIDE	0.199	0.651	1267.50	825.40	0.129	927.30	120.06
A_68_P25717772	chr8:24780109-24780156	NM_177086:0	Zmat4	INSIDE	0.183	0.708	1720.10	1217.42	0.130	1233.41	160.02
A_68_P29477470	chr14:26276881-26276925	NM_183208:-1768	Zmiz1	PROMOTER	0.520	0.358	1595.70	571.13	0.186	1111.08	206.81
A_68_P29478592	chr14:26426602-26426646	NM_183208:147954	Zmiz1	INSIDE	0.421	0.445	1472.58	655.05	0.187	1061.12	198.80
A_68_P29478593	chr14:26426711-26426755	NM_183208:148062	Zmiz1	INSIDE	0.232	0.562	1087.34	611.02	0.131	894.35	116.79
A_68_P29478594	chr14:26426870-26426915	NM_183208:148222	Zmiz1	INSIDE	0.353	0.525	2428.51	1275.42	0.185	1639.02	303.99
A_68_P23283385	chr4:126738210-126738254	NM_026670:144	Zmym1	INSIDE	0.387	0.361	2545.42	917.71	0.139	2042.09	284.75
A_68_P32564022	chrX:98614921-98614970	NM_001177985:308	Zmym3	INSIDE	0.140	1.403	1636.79	2296.51	0.197	767.63	150.92
A_68_P23282963	chr4:126645533-126645577	NM_001114399:-387	Zmym4	PROMOTER	0.261	0.562	972.88	547.04	0.147	814.24	119.47
A_68_P29624841	chr14:57429933-57429977	NM_144842:599	Zmym5	INSIDE	0.372	0.460	1489.81	685.86	0.171	1148.41	196.90
A_68_P28818473	chr13:9762982-9763026	NM_001199141:1556	Zmynd11	INSIDE	0.077	0.664	5357.19	3558.42	0.051	3894.44	200.43
A_68_P28818474	chr13:9763095-9763139	NM_001199141:1444	Zmynd11	INSIDE	0.171	0.476	2344.92	1117.22	0.081	1766.11	143.52
A_68_P28818475	chr13:9763163-9763207	NM_001199141:1376	Zmynd11	INSIDE	0.524	0.567	1210.13	686.07	0.297	1053.98	313.34
A_68_P21826274	chr2:165671183-165671231	NM_027230:38982	Zmynd8	INSIDE	0.559	4.337	1961.36	8505.97	2.425	1400.38	3396.47
A_68_P24465593	chr6:54766531-54766575	NM_199143:-357	Znrf2	PROMOTER	0.401	0.670	2222.15	1489.45	0.269	1707.36	459.35
A_68_P24465594	chr6:54766604-54766648	NM_199143:-283	Znrf2	PROMOTER	0.410	0.537	1840.31	987.56	0.220	1516.27	333.72
A_68_P27563481	chr11:5345058-5345102	NM_001080924:-230	Znrf3	PROMOTER	0.190	0.554	3769.09	2087.22	0.105	2964.57	311.38
A_68_P31256669	chr17:56651021-56651065	NM_011483:864	Znrf4	INSIDE	0.340	0.683	2859.56	1951.67	0.232	2164.65	503.03
A_68_P31256670	chr17:56651120-56651164	NM_011483:764	Znrf4	INSIDE	0.298	0.618	1002.25	619.27	0.184	778.98	143.56
A_68_P28057143	chr11:98412469-98412513	NM_001166494:80	Zpbp2	INSIDE	0.246	0.505	1163.05	587.02	0.124	991.07	123.27
A_68_P28057144	chr11:98412585-98412629	NM_001166494:196	Zpbp2	INSIDE	0.269	0.457	2698.52	1233.87	0.123	2073.75	255.43
A_68_P32795837	chrX:160396347-160396391	NM_009453:230	Zrsr2	INSIDE	0.229	1.434	1335.87	1915.14	0.328	616.79	202.08
A_68_P24972904	chr7:13365807-13365851	NM_001017955:-4821	Zscan18	PROMOTER	0.389	0.658	2850.42	1874.64	0.256	2132.69	545.96
A_68_P23291908	chr4:128262900-128262946	NM_177758:24420	Zscan20	INSIDE	0.520	3.081	1828.81	5635.01	1.601	1362.16	2181.09
A_68_P23291909	chr4:128263071-128263115	NM_177758:24250	Zscan20	INSIDE	0.582	2.509	1907.70	4786.37	1.460	1270.91	1855.73
A_68_P24128899	chr5:138558291-138558335	NM_001044703:180	Zscan21	INSIDE	0.455	0.563	2698.96	1520.41	0.256	2005.35	514.03

ProbeName	Target position of probe on CpG island microarray	TargetID	GeneSymbol	CpG island Description	Ratio of relative methylation (TiO2-NP/Vehicle)	Sham group			TiO_2-H group		
						Relative methylation (Cy-5/Cy-3)	Cy-3 signal (Input)	Cy-5 signal (MeDIP)	Relative methylation (Cy-5/Cy-3)	Cy-3 signal (Input)	Cy-5 signal (MeDIP)
A_68_P24973507	chr7:13483057-13483103	NM_001001447:-83	Zscan22	PROMOTER	0.284	0.539	1225.76	660.50	0.153	1031.70	157.85
A_68_P21579347	chr2:120996224-120996271	NM_178889:638	Zscan29	INSIDE	0.292	0.364	1531.16	556.88	0.106	1135.92	120.64
A_68_P21579349	chr2:120996373-120996418	NM_178889:490	Zscan29	INSIDE	0.480	0.666	1797.60	1197.59	0.320	1294.63	413.66
A_68_P21819854	chr2:164630477-164630534	NM_178375:-108	Zswim3	DIVERGENT_PROMOTER	0.192	0.437	2240.88	978.95	0.084	1423.24	119.47
A_68_P26018919	chr8:86760604-86760650	NM_172503:315	Zswim4	INSIDE	0.175	2.010	653.32	1313.04	0.351	491.37	172.66
A_68_P26018926	chr8:86761267-86761311	NM_172503:-347	Zswim4	PROMOTER	0.602	0.548	1005.10	550.67	0.330	789.16	260.26
A_68_P27054330	chr10:33670738-33670782	NM_028287:258	Zufsp	INSIDE	0.176	0.608	1478.15	899.10	0.107	1126.53	120.72
A_68_P26495379	chr9:48863756-48863800	NM_012039:93	Zw10	INSIDE	0.537	0.706	1299.77	917.03	0.379	1151.55	436.01
A_68_P27241481	chr10:72117558-72117607	NM_025635:-60	Zwint	PROMOTER	0.343	0.635	823.34	522.85	0.218	684.27	149.03
A_68_P27241482	chr10:72117682-72117727	NM_025635:62	Zwint	INSIDE	0.208	0.550	2538.04	1397.16	0.114	1784.68	203.97
A_68_P27241483	chr10:72117792-72117836	NM_025635:172	Zwint	INSIDE	0.145	0.593	1715.60	1017.51	0.086	1370.75	117.82
A_68_P24631969	chr6:90320325-90320370	NM_030260:860	Zxdc	INSIDE	0.655	0.533	1114.63	594.15	0.349	916.81	320.28
A_68_P24631971	chr6:90320525-90320569	NM_030260:1059	Zxdc	INSIDE	0.619	0.692	1515.49	1048.08	0.428	1144.78	489.68
A_68_P23195050	chr4:107890175-107890219	NM_001167936:331	Zyg11a	INSIDE	0.160	0.678	2288.46	1552.64	0.109	2011.46	218.44
A_68_P24397781	chr6:42299527-42299571	NM_011777:-278	Zyx	PROMOTER	0.557	0.595	988.78	588.11	0.331	793.73	262.99
A_68_P24397786	chr6:42300193-42300237	NM_011777:388	Zyx	INSIDE	0.324	0.509	3123.25	1591.09	0.165	2268.68	374.48
A_68_P22650340	chr3:152059616-152059660	NM_198416:-19	Zzz3	PROMOTER	0.386	0.628	997.79	626.69	0.242	807.48	195.68
