# Supplementary material for: Effects of Prenatal Exposure to Titanium Dioxide Nanoparticles on DNA Methylation and Gene Expression Profile in the Mouse Brain
Source: Front Toxicol. 2021 Oct 8;3:705910. doi: 10.3389/ftox.2021.705910 (PMC8915839; doi:10.3389/ftox.2021.705910)
Supplement: Supplementary file 11 [file Table13.PDF]

Supplementary Table 13.

MeSH terms enriched in the genes that showed differential expression accompanied by altered DNA methylation commonly in between male (M) and female (F) offspring in the TiO_2-H group.

ID	MeSH term	Sex	Enrichment factor	p-value
D019070	Cell Lineage	M	6.21	< 0.001
		F	5.19	0.001
D013234	Stem Cells	M	4.83	0.002
		F	4.71	0.001
D018398	Homeodomain Proteins	M	4.19	< 0.001
		F	3.88	< 0.001
D009024	Morphogenesis	M	3.32	0.018
		F	4.44	< 0.001
D018507	Gene Expression Regulation, Developmental	M	3.13	< 0.001
		F	3.92	< 0.001
D009419	Nerve Tissue Proteins	M	3.09	0.013
		F	2.58	0.030
D017403	In Situ Hybridization	M	3.07	0.005
		F	4.17	< 0.001
D004622	Embryo, Mammalian	M	3.01	0.003
		F	3.91	< 0.001
D014157	Transcription Factors	M	2.76	0.009
		F	4.33	< 0.001

The enrichment factor for each category was defined as described in Materials and Methods. Statistical analysis was performed using Fisher's exact test with hypergeometric distribution and the level of statistical significance was set at $p < 0.05$.
