# Supplementary material for: Effects of Prenatal Exposure to Titanium Dioxide Nanoparticles on DNA Methylation and Gene Expression Profile in the Mouse Brain
Source: Front Toxicol. 2021 Oct 8;3:705910. doi: 10.3389/ftox.2021.705910 (PMC8915839; doi:10.3389/ftox.2021.705910)
Supplement: Supplementary file 12 [file Table8.pdf]

Supplementary Table 8

The mRNAs that expression levels were increased (A: ≥ 1.5-fold) or decreased (B: ≤ 0.67-fold) by prenatal TiO_2-NP treatment in the brain of male offspring determined by SurePrint G3 Mouse GE 8 × 60 K Microarray.

(A)

Genbank Accession	GeneSymbol	Fold change (TiO_2-H vs Sham)
NM_026489	Hormad1	67.275
NM_146401	Olfr1305	66.857
NM_008089	Gata1	66.579
NM_007947	Lcn5	65.345
NM_029144	4930503B20Rik	57.083
NM_001114678	Gm5072	49.317
BC119564	Obox3	48.068
XM_987873	6530409C15Rik	36.631
NR_028311	Gm4371	36.328
NM_010554	Il1a	35.728
NM_199366	Gal3st2	30.654
NM_001104644	Vmn2r53	29.878
NM_001163721	1190007F08Rik	28.071
NM_207574	Olfr1383	27.877
NM_019473	Olfr155	26.965
NM_026328	Reg4	26.853
NM_138746	Fam50b	26.853
NR_037972	BB014433	25.759
NM_008117	Gh	25.440
NM_015767	Ttpa	25.124
NM_181564	Dsg4	24.319
NM_183278	Fam25c	23.769
AB049605	Cav2	23.736
NM_007972	F10	23.442
AK054182	Esr1	23.023
AK043214	Mtus1	22.054
CA947298	Gdap5	21.766
NM_025467	Gkn2	20.521
NM_013468	Ankrd1	20.140
NM_001168318	Scara5	18.687
NM_010935	Npy6r	18.610
NR_001463	Xist	18.240
NM_133996	Apon	18.202
NM_145157	Defb19	17.655
NM_146411	Olfr462	17.472
NM_183113	4932414N04Rik	17.280
NM_007775	Crygc	17.208
NM_025731	Hrasls5	17.184
NM_025721	Spesp1	17.125
NR_030675	D730005E14Rik	17.006
AK079222	Gm13219	17.006
NM_183131	4930451I11Rik	16.668
NM_001033283	Gm97	16.518
NM_007777	Cryge	16.359
NM_029755	Calcoco2	15.802
NM_147102	Olfr552	15.617
AK018600	9130007G19Rik	15.595
NM_026108	Hdhd1a	15.359
NM_001011815	Olfr533	15.327
NM_031250	Ucn3	15.242
NM_146328	Olfr110	15.242
NM_007543	Ceacam2	15.012
NM_001029893	Psg26	14.980
NM_007617	Cav3	14.231
AK020115	Adamtsl1	13.852
XM_001475610	Mcg1038069	13.775
NM_009220	Ssty1	13.371
NM_182928	Adm2	13.068
NM_026599	Cgnl1	13.059
NM_134192	Vmn1r228	13.032
NM_001164725	Fcrl6	12.879
NR_045159	4933427I22Rik	12.799
NM_028826	Sept14	12.641
NM_001037247	Defb36	12.363
NM_134183	Vmn1r21	12.126
NM_175369	Ccdc122	12.101
NM_146924	Olfr476	12.084
NM_001134458	Il9r	11.852
NM_001033356	Ntn5	11.819
NM_146582	Olfr1046	11.705
NM_001100465	Rhox2h	11.640
NM_053152	Klra22	11.624
NM_175408	Tmem139	11.559
NM_198628	Gm711	11.511
AK052446	Pigv	11.464
NM_198623	Ubqln3	11.337
AK132924	1700012B07Rik	11.189
NM_008336	Ifnab	11.181
NM_009047	Rem1	11.096
AK014899	4921515G04Rik	11.066
NM_175531	Mrgprb2	10.883
AK142339	Stab2	10.733
NM_001080769	Uhrf1bp1	10.703
NM_001102584	Vmn2r114	10.652
NM_027304	H1fnt	10.498
XR_104915	Gm3831	10.411
NM_001163621	Apol6	10.396
NM_011120	Prl7d1	10.360
CB202205	AI449212	10.204
NM_207276	Defb21	10.063
NM_010359	Gstm3	9.945
NR_040538	1700007F19Rik	9.911
XR_141004	Gm10744	9.877
NM_178047	Prom2	9.822
BC160365	Olfr105-ps	9.788
NM_146700	Olfr1453	9.768
NM_001081027	Kcnt2	9.761
NM_146946	Olfr50	9.667
NM_001190310	Clec4b1	9.441
NM_021782	Il21	9.337

Genbank Accession	GeneSymbol	Fold change (TiO$_2$-H vs Sham)
NM_146821	Olfr629	9.221
AU024546	AU024546	9.177
NM_001039118	Klra33	9.057
NM_027128	Odam	9.038
AK005775	Ankrd36	8.938
NM_001127686	Hbb-bh2	8.914
XR_141939	Gm10555	8.907
NM_001185040	Gm5885	8.858
AK030785	C78197	8.834
NM_013728	Olfr154	8.809
NM_027960	Dpep3	8.797
XM_001480781	Gm4553	8.791
NM_025621	2310050C09Rik	8.730
AK042136	Gm5103	8.652
NM_027914	Bbs10	8.628
AK086943	Gm6980	8.616
NM_008189	Guca1a	8.604
NM_029351	Krtap9-3	8.580
NM_029379	Tmem225	8.509
NM_172842	Lax1	8.462
BC096608	Gm9975	8.421
NM_147037	Olfr1413	8.346
NM_146456	Olfr92	8.311
AK017086	4933436I20Rik	8.265
NM_001177524	Gml	8.242
NM_001102662	Skint1	8.219
NM_146763	Olfr1406	8.039
NR_038045	Gm10440	7.950
NM_001100444	Gm5934	7.754
CD766363	1810013D15Rik	7.738
NM_001005247	Hps5	7.595
NM_029587	1700012A03Rik	7.574
AK049799	E130307A14Rik	7.568
NM_145424	BC089597	7.553
NR_045404	G630055G22Rik	7.506
NM_001085516	Gm12794	7.501
XR_141235	D830044D21Rik	7.423
NM_007646	Cd38	7.397
AK144055	Gm2814	7.280
AK016679	4933406B15Rik	7.265
NM_013542	Gzmb	7.185
NM_178924	Upk1b	7.155
NM_001013810	Gm5591	7.106
NM_146847	Olfr1090	7.062
AK043165	A730062M13Rik	6.936
XR_140618	Ptprd	6.931
NM_001142631	Spdya	6.926
NM_007675	Ceacam10	6.840
NR_033355	5430416O09Rik	6.807
NM_022879	Myl7	6.797
XM_003086474	A630081J09Rik	6.639
BC075721	Xpr1	6.616
NM_001252661	5830473C10Rik	6.575
NM_144834	Serpina10	6.575

Genbank Accession	GeneSymbol	Fold change (TiO$_2$-H vs Sham)
NM_199152	Obscn	6.566
NM_028550	1700074P13Rik	6.539
AK005108	1500002C15Rik	6.494
NM_177669	Skint11	6.489
NR_040663	4930509K18Rik	6.386
NM_010780	Cma1	6.378
XR_105027	A230083G16Rik	6.351
NM_001102607	Col6a6	6.338
NM_025867	Serpinb11	6.333
NM_001101588	Cyp4f40	6.298
NM_053077	Slc45a2	6.294
NM_001033131	Krtdap	6.272
NM_001195579	Gm15070	6.203
NM_146020	Gltpd2	6.160
NM_178715	Tmem30b	6.080
NR_015568	4933406F09Rik	6.080
NM_001037928	Gm11992	6.071
NM_013868	Hspb7	6.025
NM_145146	Afm	5.988
NR_040523	1700025N23Rik	5.971
CA481107	AA617406	5.963
AK033690	9130221J18Rik	5.942
NR_045831	4933408N05Rik	5.909
NM_011207	Ptpn3	5.909
NM_147039	Olfr1414	5.881
NR_040610	1700040N02Rik	5.873
NM_177589	Ulk4	5.816
NM_001039223	Gm14137	5.804
XR_106616	Gm11735	5.788
AK016370	Cdk14	5.780
XR_104956	Katnal1	5.780
NM_207674	Olfr1082	5.772
AK077051	4932439E07Rik	5.752
AK076789	4930458A03Rik	5.724
NM_021440	BC100451	5.669
NM_175439	Mars2	5.629
NM_001163192	Ly6g6f	5.618
NM_199468	Zcchc5	5.610
NM_138953	Ell2	5.560
NM_008361	Il1b	5.552
NM_027010	Crygf	5.529
AK049156	Gm6846	5.514
NM_026136	4930449I24Rik	5.457
NM_001033235	Trim40	5.453
AK087175	Slc23a2	5.441
NM_001048219	Nlrp9a	5.367
AK080477	6030451C04Rik	5.363
NM_016869	Corin	5.329
NM_001159934	Oas1h	5.293
NM_138674	Pkhd1l1	5.282
NM_008803	Pde8a	5.274
AK082335	Adam22	5.263
AK016636	4933403J19Rik	5.216
AK016158	4930557B15Rik	5.195

Genbank Accession	GeneSymbol	Fold change (TiO_2-H vs Sham)
NM_153143	Kctd11	5.187
NM_177316	Fpr-rs6	5.180
NM_008694	Ngp	5.166
AK077314	Gm5447	5.141
NM_007538	Opn1sw	5.116
NM_177361	Ifna12	5.098
NM_029692	Upp2	5.084
NM_030098	Rnase6	5.081
NR_045930	1700092K14Rik	5.067
NM_001037502	Defb28	5.025
AK018581	9130001E16Rik	5.021
AK162803	Dleu2	5.014
NM_178687	Cd226	5.014
NM_001001807	Olfr279	5.014
XM_486266	4921528I07Rik	5.011
BC152873	Vmn2r66	4.986
NM_009802	Car6	4.959
NM_183224	Fam19a3	4.955
NM_033593	Pcdhga10	4.945
AA985897	AI195470	4.931
NM_001146107	Gm10696	4.925
AK021364	E130101E03Rik	4.914
NM_146762	Olfr1013	4.911
NM_008006	Fgf2	4.884
BG068955	AU024245	4.877
NM_146360	Olfr574	4.877
AK134699	4931406P16Rik	4.874
XM_003086833	Gpr112	4.874
AK138241	Gm10489	4.874
NM_001162928	Pom121l2	4.850
NM_001099314	Msmp	4.837
NM_175649	Tnfrsf26	4.803
BG066474	C79562	4.790
NM_001039241	LOC100045026	4.787
NM_027340	Lipn	4.783
NM_172944	Itgae	4.780
AK007554	1810019N24Rik	4.767
NM_183039	Defb40	4.760
AK016337	4930580E04Rik	4.760
NM_001010834	Slc10a5	4.754
AK051876	D230014I24Rik	4.754
NM_027222	2010001M09Rik	4.717
NM_021358	Htr6	4.695
NR_040633	4930555B11Rik	4.682
NM_001011836	Olfr1129	4.669
NM_146366	Olfr1093	4.665
NM_010388	H2-DMb2	4.652
NR_040596	4930455H04Rik	4.643
NM_001130444	Hras1	4.640
NR_045825	1700025K24Rik	4.640
NM_029550	Keg1	4.595
AK007098	1700097M23Rik	4.592
XM_906502	Gm1096	4.569
NM_001081954	Dux	4.550

Genbank Accession	GeneSymbol	Fold change (TiO_2-H vs Sham)
NM_173739	Galntl4	4.541
NM_172881	Ugt2b35	4.497
NM_028584	Marveld3	4.482
NR_045810	4933408J17Rik	4.432
NM_001170632	Fcamr	4.423
NM_174846	Glyctk	4.386
NM_030610	Fgf20	4.302
NM_030728	9930013L23Rik	4.296
NM_009645	Aicda	4.228
NM_001007463	Spag8	4.213
NM_001111143	Cym	4.208
NM_153077	Il1f10	4.202
NM_020282	Nqo2	4.179
NM_031189	Myog	4.167
NM_013797	Slco1a1	4.132
AK082849	Mcf2l	4.124
XR_104924	Gm9903	4.098
NM_173379	Leprel1	4.067
AK045665	1700008O03Rik	4.059
NM_146755	Olfr551	4.056
NM_013913	Angptl3	4.014
AK135677	Gm12633	3.986
AK172246	Ipmk	3.953
NM_153510	Pilra	3.883
NR_045119	Gm18756	3.872
NM_134163	Mbnl3	3.866
AK090265	G630030J09Rik	3.848
AU018794	AU018794	3.842
NM_001081391	Csmd3	3.826
NM_172463	Sned1	3.818
NM_029245	Ankrd53	3.771
NM_019680	Elf4	3.742
NM_026331	Slc25a37	3.732
NM_138600	Aldh7a1	3.714
NM_029112	Morn3	3.663
AK054344	E330017L17Rik	3.660
NM_001033243	Ccdc114	3.658
NM_001081388	Rimbp2	3.633
NM_001005508	Arhgap30	3.615
NM_001081127	Adamts14	3.565
NM_133226	Pdzd3	3.553
NM_028370	Pot1b	3.548
NM_007989	Foxh1	3.511
XM_621555	Olfr1175-ps	3.502
AK087815	E330024J20Rik	3.502
NM_022411	Slc13a2	3.494
NM_010928	Notch2	3.482
NM_001081211	Ptafr	3.430
AK005727	1700007J24Rik	3.366
NM_001033365	Gm628	3.366
NM_001034907	Zc3h12b	3.350
AK034105	9330156P08Rik	3.331
NM_021400	Prg4	3.322
NM_020574	Kcne3	3.322

Genbank Accession	GeneSymbol	Fold change (TiO_2-H vs Sham)
NM_198961	Vmn2r43	3.313
BC116230	Ankrd16	3.308
NM_027137	Lce1d	3.299
NM_010230	Fmn1	3.285
NM_172420	Ppp1r1c	3.276
NM_010191	Fdft1	3.247
NM_001081200	Crnn	3.240
NM_001177408	Gm15319	3.233
NM_147031	Olfr1122	3.224
NM_020028	Lpar2	3.224
NM_013684	Tbp	3.222
AK143978	Frmd4b	3.213
NM_013501	Cryaa	3.200
XM_003084652	LOC100502592	3.180
NM_001081239	Lilra5	3.165
NM_001034859	Gm4841	3.158
NM_177353	Slc9a7	3.149
NM_213728	Krt72-ps	3.102
NM_175449	Fam26f	3.089
NM_013878	Cabp2	3.089
NM_177397	Atp6v1g3	3.055
NM_020259	Hhip	3.053
NM_001160403	Il1rapl1	3.031
NM_019410	Pfn2	3.023
NM_010548	Il10	3.019
AK042649	Gm9869	3.015
NM_172603	Phf11	2.986
NM_015785	Zpbp	2.975
NM_009948	Cpt1b	2.975
NM_027600	4921504E06Rik	2.971
CB196702	AA536887	2.969
NM_021545	Naip7	2.963
NM_175362	Card11	2.944
NM_028638	Gadl1	2.936
NM_008237	Hes3	2.932
NM_172799	Ttll6	2.926
AK155789	Gm10802	2.922
NM_145536	BC020535	2.916
NM_172807	Ppwd1	2.908
AK052659	Gm12273	2.906
NM_001013784	E130309D14Rik	2.890
XR_104959	2310047D07Rik	2.880
NM_001163141	Krtap24-1	2.862
AK045799	Fhad1	2.856
NM_205795	Mrgprb4	2.848
NM_178882	D2hgdh	2.834
NM_001122899	Lepr	2.815
NM_145223	Alms1	2.815
NM_001113351	Synj2	2.805
AI853652	D030036P13Rik	2.803
AK032506	6430573P05Rik	2.791
NM_008011	Fgfr4	2.789
NM_146878	Olfr30	2.784
XM_916431	Skint5	2.784

Genbank Accession	GeneSymbol	Fold change (TiO_2-H vs Sham)
AK047256	B930042K01Rik	2.782
AF396877	Dst	2.755
NM_011776	Zp3	2.743
NM_020272	Pik3cg	2.742
NM_146549	Olfr786	2.706
NM_001080820	Cass4	2.704
AK048022	C130030K03Rik	2.702
NR_015613	1700029M20Rik	2.694
BC002139	1700052N19Rik	2.680
NM_001025353	Gm6040	2.670
NM_146559	Olfr868	2.667
NM_180958	Ccdc79	2.661
NM_001099297	Duox1	2.659
NM_001034893	Zfp936	2.657
AK017177	5033417F24Rik	2.652
NM_010827	Msc	2.646
NM_172507	Sh3bgrl2	2.637
NM_010213	Fhl3	2.632
AK052165	LOC100036540	2.623
NM_026054	2810474O19Rik	2.623
AK015366	4930442P07Rik	2.610
NM_153600	Ttc26	2.606
NM_001004193	Rhox8	2.603
CD772809	1700092C02Rik	2.599
AK041682	Pds5b	2.599
NM_028918	Ttc25	2.581
NM_011891	Sgcd	2.578
NR_045865	9230009I02Rik	2.574
NM_133213	Xpnpep2	2.570
NM_020036	Calm4	2.567
NM_011290	Rpl6	2.567
XR_140816	Gm9653	2.562
NM_024263	Mxra8	2.558
NM_023114	Apoc3	2.544
AK020962	B230110G15Rik	2.532
AK145442	Mllt3	2.519
NM_001039119	Defb33	2.519
NM_001098269	Gm10375	2.518
AK013575	Lias	2.511
XR_140805	Gm10648	2.504
NM_010730	Anxa1	2.500
AK029371	Ablim1	2.474
NR_038172	4931403G20Rik	2.467
NM_001162901	Tmem217	2.467
NM_030709	Tmprss5	2.464
NM_001163539	5430427O19Rik	2.461
NM_001081396	Wdr67	2.461
NM_183282	Actl9	2.452
NM_011382	Six4	2.444
NM_028057	Cyb5r1	2.437
NM_032004	Tssk6	2.425
NM_007831	Dcc	2.415
NM_010620	Kif15	2.415
NM_029416	Klf17	2.410

Genbank Accession	GeneSymbol	Fold change (TiO_2-H vs Sham)
XR_141359	2310026I22Rik	2.407
CF746308	D5Buc30e	2.402
NM_001013759	Gas2l2	2.388
AK144774	Gm16686	2.385
NM_201617	Gnas	2.382
NM_133207	Kcnh7	2.375
AK044656	Esyt1	2.351
XR_141351	AA667203	2.346
NM_028399	Ccnt2	2.329
XM_138722	Fam81b	2.328
NM_001177630	Etl4	2.323
NM_175418	Mybpc1	2.321
NM_080729	Il25	2.318
NM_146235	Ercc6l	2.317
NM_001101431	2510049J12Rik	2.317
AK144510	1810059H22Rik	2.312
NM_173416	BC068281	2.309
NR_001584	Speer8-ps1	2.305
NM_001145978	Parp4	2.297
NM_007870	Dnase1l3	2.297
AK080282	D10Ertd755e	2.289
NM_028598	2410076I21Rik	2.286
AK044654	C230012O17Rik	2.277
NM_009256	Serpinb9	2.275
NM_153055	Sec63	2.275
NM_001081499	Tbc1d8b	2.267
NM_028022	Gatsl3	2.263
NM_001081377	Pcdh9	2.261
NM_177866	Catsper4	2.261
AK085971	Tbck	2.258
NR_033209	Gm4792	2.249
NM_007914	Ehf	2.249
AK047818	A730009E18Rik	2.247
NM_007776	Crygd	2.244
NM_001105076	Vmn2r46	2.230
NM_008455	Klkb1	2.228
NM_001205102	Lrit3	2.228
NM_199446	Phkb	2.228
NM_024438	Dusp19	2.222
NM_133239	Crb1	2.219
NM_177358	Zfp945	2.219
NM_001204233	Spp1	2.219
NM_133730	Krt25	2.213
AK042233	A630073K07Rik	2.213
NM_019684	Srpk3	2.213
NM_023377	Stard5	2.205
AK018390	8430406P12Rik	2.202
NM_054066	Plcz1	2.201
XR_140553	LOC100862344	2.186
NM_007659	Cdk1	2.180
NM_001034097	BC096441	2.180
NM_153582	Cmtm4	2.173
AK051763	9430038I01Rik	2.167
AK051017	Asb7	2.166

Genbank Accession	GeneSymbol	Fold change (TiO_2-H vs Sham)
NM_007868	Dmd	2.163
AK045831	E230008O15Rik	2.157
AK038793	Sfxn5	2.152
NM_029324	1700018C11Rik	2.152
AI851140	C130093G08Rik	2.148
AK141853	4930590A17Rik	2.144
NM_153137	Traf3ip3	2.142
NR_033549	Gm11190	2.141
NM_177674	2010015L04Rik	2.136
NM_001013832	Gpr31b	2.133
NM_001243760	Ciita	2.132
AK036325	Syn3	2.118
NM_177582	Mlxip	2.118
NM_178786	Skint4	2.117
NM_001081201	Dpy19l4	2.111
NM_010707	Lgals6	2.108
NM_029362	Chmp4b	2.107
NM_020519	Slurp1	2.105
NM_001195693	Plscr5	2.104
NM_009646	Aire	2.102
NM_001007222	Tdpoz2	2.102
NM_001001130	Zfp85-rs1	2.101
NM_206822	Olfr10	2.099
AK052374	Pde3a	2.095
NM_177307	Cyp4f39	2.094
NM_207683	Pik3c2g	2.092
NM_027462	Wars2	2.092
NR_002874	Has2as	2.086
AK149431	Bhmt	2.086
NM_028890	4931414P19Rik	2.083
NM_172286	6430548M08Rik	2.081
C78535	D7Ertd183e	2.078
NM_146618	Olfr297	2.072
NM_146974	Olfr1262	2.071
NM_178899	Hepacam2	2.071
NM_009965	Cryba1	2.069
NM_145229	AY074887	2.068
NM_001081012	4930473A06Rik	2.066
AK036921	9930024M15Rik	2.062
NM_008747	Ntsr2	2.053
NM_001025585	Kcnj6	2.048
NM_146042	Rnf144b	2.048
NM_015820	Hs6st3	2.046
NM_007774	Cryga	2.043
NM_170671	Mycbpap	2.042
NM_008103	Gcm1	2.042
NM_030740	Vmn1r56	2.032
NM_029315	Pou5f2	2.031
NM_174876	Impg2	2.028
NM_199364	Lzts1	2.027
AK144547	Exoc6b	2.024
AK138828	Tdrd5	2.022
NM_001038590	Cldn19	2.021
AK164818	Slc44a1	2.018

Genbank Accession	GeneSymbol	Fold change (TiO_2-H vs Sham)
NM_029347	Fggy	2.015
NM_027630	Ccdc105	2.014
AK046275	4930588A03Rik	2.010
NM_001033769	B020031M17Rik	2.008
AK139339	Spata2l	2.004
NM_016700	Mapk8	2.004
NM_008434	Kcnq1	2.001
NM_010104	Edn1	2.001
AK036480	Disp2	1.948
NM_010461	Hoxb8	1.945
NM_146877	Olfr1395	1.939
NM_001039658	Mtl5	1.937
NM_008879	Lcp1	1.936
NM_001033126	Cd27	1.929
NM_001025385	Tas2r137	1.927
AK041660	D5Ertd798e	1.927
BC027633	Cd79a	1.925
AK047423	Odz1	1.923
NM_001113209	Nfib	1.919
AK049967	Myt1l	1.915
NM_009010	Rad23a	1.912
NM_001077514	Slc1a2	1.911
NM_001013365	Osm	1.907
NM_183151	Mid1	1.897
NM_011347	Selp	1.897
XM_916080	4930438A08Rik	1.896
NM_008726	Nppb	1.895
NM_146513	Olfr95	1.891
AK036290	AU015680	1.889
NM_001166636	Gm4312	1.887
NM_019775	Cpb2	1.887
NM_009502	Vcl	1.869
NM_023821	Cmya5	1.865
NM_001163145	1810041L15Rik	1.861
AK081411	B230216N24Rik	1.848
BE650233	D9Ertd115e	1.847
NR_040614	4930533B01Rik	1.842
AK049181	Pik3c2b	1.834
NM_016694	Park2	1.831
NM_028882	Sema3d	1.820
NM_009695	Apoc2	1.814
NM_029685	1700113H08Rik	1.794
AK044519	Stard7	1.788
AK016698	4933406K04Rik	1.783
NM_009767	Chic1	1.781
NR_045080	3300005D01Rik	1.780
NM_001081175	Itpkb	1.774
NM_001017427	Rasef	1.769
AK082713	C230094B09Rik	1.765
NM_009035	Rbpj	1.762
NM_153079	Nmur2	1.761
NM_001169153	Cd300lf	1.759
NM_194336	Gbp6	1.758
NM_053273	Ttyh2	1.747

Genbank Accession	GeneSymbol	Fold change (TiO_2-H vs Sham)
AK145592	Gm10204	1.747
NM_029210	Sv2c	1.733
NM_001033411	Gm826	1.728
NM_016667	Sntb1	1.720
NM_146370	Olfr47	1.697
NM_023755	Tfcp2l1	1.697
NM_013877	Cabp5	1.693
BC036324	Akna	1.692
AK142040	Gm2590	1.684
AK090034	Gm3161	1.677
AK034586	9430011C21Rik	1.676
NM_019536	Dnahc10	1.671
NM_133365	Dnahc5	1.670
NM_010029	Ddx4	1.668
NM_021403	Srrm3	1.659
NM_027551	Klhl30	1.657
NM_207262	Abpe	1.656
NM_013473	Anxa8	1.656
NR_003649	Gm5434	1.646
AK165152	Tmem29	1.639
AK008925	Mettl7a1	1.631
NM_153588	Mkl2	1.630
NR_038008	B230209K01Rik	1.630
NM_178662	Atcay	1.628
NM_146577	Olfr1043	1.627
NM_010628	Kif9	1.610
NM_010299	Gm2a	1.609
NM_013848	Ermap	1.605
NM_001167891	Nrg2	1.604
AK002639	Memo1	1.604
NM_001012402	Hs3st6	1.603
AK083760	Kif6	1.602
NM_008664	Myom2	1.601
NM_001146007	Trim12c	1.599
XM_914998	Gm9804	1.591
NM_172418	Mamstr	1.591
NM_177340	Synpo	1.588
NM_177145	Pde4dip	1.586
NM_176965	Efcab5	1.576
NM_008895	Pomc	1.576
NR_045446	4933438K21Rik	1.572
AK088035	C230085N15Rik	1.569
NM_001167581	Gsdmcl1	1.568
XM_003085800	Gm3908	1.568
NM_183183	Gprin3	1.564
AK141640	BC055308	1.560
AK035028	Fat3	1.560
NM_001174107	Map3k9	1.559
NM_026754	Ucma	1.558
NR_026561	Gm8884	1.556
NM_144940	Uroc1	1.555
AK048766	Vps13c	1.555
NM_177607	4933430I17Rik	1.553
NM_146981	Olfr1260	1.545

Genbank Accession	GeneSymbol	Fold change (TiO_2-H vs Sham)
NM_145210	Oas1e	1.541
AK048874	Ncrna00085	1.540
NM_001136056	Cntfr	1.538
NM_021365	Xlr4b	1.537
AK146348	Gm2022	1.535
NM_013589	Ltbp2	1.533
NM_001134697	Ctxn3	1.527
NM_021609	Ccbp2	1.526
NM_022434	Cyp4f14	1.525
NR_040534	Gm10548	1.523
NM_144909	Gckr	1.522
NM_023476	Tinagl1	1.519
NM_001177951	Rpgr	1.519
AK084110	D130095D21Rik	1.506
NR_037978	E030025P04Rik	1.505
NM_172932	Nlgn3	1.503
AK045009	B130019D13Rik	1.501
NM_019835	B4galt5	1.500

(B)

Genbank Accession	GeneSymbol	Fold change (TiO_2-H vs Sham)
NM_012011	Eif2s3y	0.002
NM_012008	Ddx3y	0.005
NM_147099	Olfr616	0.007
NM_146693	Olfr1462	0.008
NM_027046	Ccdc54	0.009
NM_144936	Tmem45b	0.009
NM_001011530	Olfr723	0.010
NR_040732	4833428L15Rik	0.010
NM_175343	Chdh	0.011
NM_001004061	Fam170a	0.011
NR_028426	Gm5129	0.012
NM_146505	Olfr148	0.012
NM_007704	Inadl	0.012
NM_008532	Epcam	0.013
NM_146254	Wdr78	0.013
NM_001097979	Hist1h2bq	0.014
NM_011964	Psg19	0.015
NM_134202	Vmn1r233	0.015
NM_025738	Cypt1	0.016
NM_033616	Csprs	0.016
NM_001160386	Dnahc7b	0.017
NM_001040089	Rhox3f	0.018
NM_011684	Vmn1r45	0.019
NM_029042	4930435E12Rik	0.020
NM_177841	4932418E24Rik	0.022
NM_181990	Cmtm1	0.022
NM_008693	Klk1b3	0.023
NM_001256042	Hsf4	0.024
NM_009694	Apobec2	0.024
AK015323	4930435F18Rik	0.024
NM_177265	6330512M04Rik	0.025
NM_001011532	Olfr1037	0.026
NM_031382	Tex16	0.026
NM_133698	Hrnr	0.027
BC042707	Prok1	0.027
NM_029415	Slc10a6	0.027
AK015488	4930459I23Rik	0.027
NM_009043	Reg2	0.028
NM_145389	BC016579	0.028
NM_001011802	Olfr1318	0.028
NM_019404	Avpr2	0.029
NM_147108	Olfr979	0.029
NM_031164	F13b	0.029
NM_146899	Olfr1219	0.030
XR_140635	Mup-ps12	0.030
NM_001205037	Gm11544	0.031
NM_001033339	Mmp25	0.031
XM_136255	Gm4845	0.031
NM_207176	Tes	0.032
NM_008205	H2-M9	0.032
NR_037997	2310015D24Rik	0.033
NM_027321	Lrrc39	0.033
AK006305	1700024J04Rik	0.034

Genbank Accession	GeneSymbol	Fold change (TiO_2-H vs Sham)
NM_001013575	Olfr112	0.034
NR_003639	1700013N18Rik	0.034
NM_181407	Me3	0.035
NM_027058	1700017D01Rik	0.037
NR_003643	1700123L14Rik	0.037
BG069230	D3Ertd711e	0.038
NM_009641	Angpt4	0.039
NM_001085543	Gm14347	0.039
NR_045474	2310081J21Rik	0.039
NM_053088	Ifitm5	0.040
AK015516	Gm10620	0.041
AK140479	Dnahc6	0.041
NM_008024	Foxl1	0.042
NM_001164613	Atp13a4	0.044
NM_001113387	Myl1	0.044
NM_027397	Isl2	0.045
NM_031368	Bglap-rs1	0.045
BC048590	Cep120	0.045
NR_033532	B230206H07Rik	0.045
NM_177083	B430306N03Rik	0.046
AK014721	4833419A21Rik	0.046
NM_207248	4930433I11Rik	0.046
NM_146198	Slc5a11	0.046
NM_029959	Lcn9	0.047
NR_040639	Gm13490	0.047
AK133037	Gm10473	0.047
NR_033556	A630010A05Rik	0.048
BC028561	Gm568	0.048
NM_001013765	Zscan4c	0.049
NM_009693	Apob	0.049
AK139732	Gm4316	0.049
NM_134179	Vmn1r23	0.050
NM_001146022	Wdfy4	0.052
AK028479	Pvrl4	0.052
NM_146932	Olfr802	0.052
NM_033325	Loxl2	0.055
NM_001033459	Gm1679	0.055
AK035916	9630017O17	0.056
NM_205821	Mrgpra6	0.056
NM_029367	Spaca3	0.057
AK040963	1700123M08Rik	0.057
NM_145713	Hist1h1d	0.058
NM_144813	Slc24a1	0.059
NM_029054	4930443G12Rik	0.059
NM_001039114	Acsbg2	0.060
NM_206973	Gpr152	0.063
AK043688	LOC432500	0.063
NM_024264	Cyp27a1	0.063
NM_146284	Olfr780	0.063
NM_201360	Cyp2d12	0.063
BY714998	4930452L12Rik	0.064
NM_177081	Ptpn7	0.064
NM_001136181	Hsbp1l1	0.064

Genbank Accession	GeneSymbol	Fold change (TiO$_2$-H vs Sham)
NR_045468	1700095A21Rik	0.064
AK164319	Foxp2	0.065
NM_026906	Cts3	0.065
NR_045740	A630020A06	0.065
NM_007815	Cyp2c29	0.067
NM_001083618	Ttll9	0.067
AK016012	4930540M05Rik	0.068
NM_177703	Fbxw19	0.068
AK034506	Gm9876	0.070
NM_001079929	Ccdc154	0.070
NM_001243024	Gm3763	0.070
NM_146934	Olfr46	0.071
NM_027886	Stk11ip	0.072
NM_133192	Npffr2	0.073
AK041212	Trib1	0.076
NM_011406	Slc8a1	0.077
NM_008814	Pdx1	0.078
BC021607	Akr1c20	0.078
AK017087	Cpvl	0.079
NM_001081351	A430107O13Rik	0.079
XR_104835	A830029E22Rik	0.080
AK140217	Fgfr3	0.080
NM_010216	Figf	0.080
NM_207261	Kcnk18	0.080
BG083254	AU015621	0.082
NM_146948	Olfr342	0.082
AK030033	Gm7167	0.082
NM_198711	Col25a1	0.082
BU554875	2010001M06Rik	0.083
NM_001085549	Gm12824	0.083
NM_147034	Olfr713	0.083
NM_008037	Fosl2	0.083
NM_175020	Prss58	0.084
AK015532	4930470F04Rik	0.084
AK087703	Agxt2l2	0.085
NM_177744	Apol10a	0.086
NR_023846	Peg3as	0.091
CA463412	4933408A14Rik	0.091
NM_181753	Opn5	0.091
NM_008493	Lep	0.092
AK156176	Gm10926	0.093
NM_146136	Slc16a4	0.093
AK019889	6430710C18Rik	0.093
AK030805	A730021G18Rik	0.093
BG075595	AW556556	0.093
NM_028784	F13a1	0.094
NM_008604	Mme	0.094
NR_034074	Etd	0.094
NM_009995	Cyp21a1	0.098
AK081233	C030017G13Rik	0.098
NM_008266	Hoxb1	0.100
NM_183096	Ttc29	0.100
AK040044	Skint10	0.100
XM_001472632	LOC100044656	0.104

Genbank Accession	GeneSymbol	Fold change (TiO$_2$-H vs Sham)
NM_175501	Adamts12	0.105
NM_207538	Mrgprb5	0.105
NM_008192	Gucy2e	0.105
BC023070	5430437J10Rik	0.106
XR_106339	4632411P08Rik	0.106
NM_008938	Prph2	0.108
BC132243	1810009J06Rik	0.108
AK046115	A930016O22Rik	0.109
NR_045920	4933404G15Rik	0.111
XM_003086636	Gm5737	0.111
C77618	D15Ertd55e	0.111
NM_008270	Hoxb9	0.114
AK077007	1700014D04Rik	0.114
NM_009285	Stc1	0.114
NM_001033455	Ccdc27	0.114
NM_026097	Rffl	0.114
AK039060	Gm5441	0.116
XM_003086830	Gm2483	0.116
NM_146305	Olfr420	0.121
NR_027970	0610008F07Rik	0.121
AK076465	Sdr16c6	0.122
NM_177471	Ccdc69	0.122
NR_045360	2210409D07Rik	0.123
NM_007751	Cox8b	0.123
NM_025271	Actl7b	0.124
AK142244	Atr	0.125
U04807	Flt3l	0.125
NM_001104580	Vmn2r116	0.129
NM_001104575	Vmn2r112	0.130
NM_001013013	Dhrs7c	0.131
NR_024513	Gm5	0.131
NM_027631	4931407G18Rik	0.132
BF531481	2010320M18Rik	0.132
NM_010115	Egfbp2	0.133
NM_026105	1700093K21Rik	0.136
NM_146457	Olfr282	0.138
NM_178666	Themis	0.138
NM_001037748	LOC380994	0.139
AK016151	4930556N08Rik	0.139
NM_133208	Zfp287	0.139
NM_001004194	Nlrp4e	0.140
NM_183249	1100001G20Rik	0.140
NM_007640	Cd1d2	0.140
NM_053085	Tcf23	0.142
AK005715	1700007H22Rik	0.142
NM_007511	Atp7b	0.143
AK140467	BC048594	0.144
NM_028994	Pck2	0.146
AK052613	Dab2	0.146
NM_021408	Ush2a	0.148
NM_001013751	Syna	0.149
NM_020496	Tbx20	0.150
NM_001018031	Otol1	0.151
NR_045953	4930432J09Rik	0.151

Genbank Accession	GeneSymbol	Fold change (TiO_2-H vs Sham)
NM_134210	Vmn1r81	0.151
NM_146708	Olfr402	0.151
NM_146461	Olfr1209	0.152
NM_009724	Atp4b	0.152
NM_001013371	Dtx3l	0.152
NM_146050	Oit1	0.152
NM_021467	Tnni1	0.154
XM_001475533	Olfr687	0.155
NM_009484	Uty	0.156
NM_028533	1700065D16Rik	0.157
AK014883	4921513I08Rik	0.158
CB575032	2310007O11Rik	0.158
NM_145548	Cyp2j13	0.159
NM_001166494	Zpbp2	0.159
NM_001111318	Cldn24	0.160
NM_153106	Padi6	0.160
AK030052	4930455J16Rik	0.161
NM_172931	Hsf3	0.163
NM_010743	Il1rl1	0.164
NM_025487	1700011A15Rik	0.165
NM_001161855	4933416C03Rik	0.165
NM_020563	Abph	0.166
NR_027707	Gm5166	0.167
AK013707	Hivep3	0.169
NM_001199332	LOC100041550	0.169
NM_146623	Olfr357	0.169
NM_001012766	Ear12	0.170
NM_011667	Ube1y1	0.170
NM_212487	Krt78	0.170
NM_011088	Pira11	0.170
NM_177921	E230019M04Rik	0.172
NR_040327	E130018N17Rik	0.172
NM_011283	Rp1	0.173
NM_028300	Pih1d2	0.174
NM_001130693	Gucy2d	0.175
NM_144906	Sgip1	0.175
NR_033584	Gm10280	0.176
AK087331	Vps4b	0.176
AI851680	A830021K08Rik	0.176
NR_045878	9030404E10Rik	0.176
XR_141514	LOC100048891	0.177
NM_001166064	Syde2	0.178
NR_040462	1700034P13Rik	0.179
AK016278	4930572G02Rik	0.179
NM_008639	Mtnr1a	0.180
NM_001160265	Cyp2w1	0.183
NM_001081378	Kidins220	0.183
XM_001472451	Gm2102	0.184
AK146791	Ewsr1	0.185
NM_011800	Cdh20	0.186
AK164374	Aldh16a1	0.188
AK144269	Lemd1	0.188
NM_183104	4931429L15Rik	0.188
NM_001033461	Lrrc43	0.190

Genbank Accession	GeneSymbol	Fold change (TiO_2-H vs Sham)
NM_030720	Gpr84	0.190
XM_001478213	Gm7166	0.196
NM_001252485	Inca1	0.196
NM_001101501	Gm15104	0.197
NM_021423	Shank3	0.198
NM_019985	Clec1b	0.199
NM_023132	Renbp	0.199
AK002748	0610033M10Rik	0.200
AK037533	5830408C22Rik	0.201
NM_001195074	Kcnmb3	0.203
AK041796	Cog5	0.206
NM_026450	Zfp169	0.208
NM_001195091	Gm6537	0.209
NM_207131	Cebpe	0.211
NM_015743	Nr4a3	0.214
NM_008596	Sypl2	0.215
NM_053130	Pcdhb5	0.217
NM_018865	Wisp1	0.217
NM_008976	Ptpn14	0.218
NR_045357	4930545H06Rik	0.223
NM_015830	Solh	0.227
NM_019809	Pdlim5	0.231
AF178753	Olfr1077-ps1	0.233
NM_018738	Igtp	0.233
NM_028130	Zfp157	0.233
AK133213	Gm13547	0.241
AK050354	Gm14168	0.243
NM_001163625	Sult6b1	0.244
NM_010089	Usp17l5	0.246
NM_147010	Olfr1052	0.249
NM_028004	Ttn	0.250
NR_033596	5730416F02Rik	0.253
NM_008557	Fxyd3	0.255
XM_894578	Gm6993	0.257
NR_040686	8430423G03Rik	0.259
NM_144491	Dph1	0.261
XM_003086808	LOC100040235	0.261
AI604347	Gm11570	0.262
NM_009027	Rasgrf2	0.263
NM_001163810	1700008P20Rik	0.264
NM_145153	Oas1f	0.264
NM_008333	Ifna11	0.269
NM_011077	Phex	0.270
NM_021607	Ncstn	0.275
NM_009463	Ucp1	0.276
AK015936	4930529I22Rik	0.280
NR_046029	LOC629206	0.282
NM_027411	Ccdc99	0.283
NM_177335	D930020B18Rik	0.284
NM_001162381	Fam166b	0.284
NM_012010	Eif2s3x	0.284
NM_011310	S100a3	0.287
NM_177233	Fam19a4	0.288
AK147615	LOC100047044	0.289

Genbank Accession	GeneSymbol	Fold change (TiO_2-H vs Sham)
NM_001012322	Sctr	0.290
NM_001033380	Itpripl2	0.292
NM_016767	Batf	0.293
NM_008981	Ptprg	0.293
AK161464	A930030B08Rik	0.295
NR_015604	E130112N10Rik	0.296
NM_183182	BC055111	0.301
NM_001033785	Akap14	0.302
NM_027126	Hfe2	0.304
NM_001033415	Shisa3	0.306
NM_001111079	Uhrf1	0.310
NR_045743	D930007P13Rik	0.312
NM_019448	Dnmt3l	0.313
NM_144532	Cabp4	0.315
NM_007628	Ccna1	0.316
NM_001007581	2810408M09Rik	0.316
NM_001009550	Mup21	0.318
NM_001042653	Oip5	0.320
NM_029714	Catsperg2	0.322
NM_172451	Galnt6	0.322
NM_182783	Fam167b	0.323
NR_003519	Pisd-ps2	0.324
NM_008100	Gcg	0.325
NM_009385	Nkx2-1	0.325
NM_013488	Cd4	0.325
NM_008703	Nmbr	0.326
NM_029821	1190003J15Rik	0.328
NR_028385	Gm10190	0.328
NM_145933	St6gal1	0.328
BC016220	4930432F04Rik	0.329
NM_212473	Fam53b	0.329
NM_144789	Prdm15	0.331
NM_011720	Wnt8b	0.336
AK019760	Mccc2	0.337
NM_022311	Tcte2	0.338
NM_012012	Exo1	0.340
NM_001031621	Abca17	0.341
NM_013731	Sgk2	0.343
NM_207268	Ccdc87	0.354
NM_174851	Il28ra	0.358
NM_008262	Onecut1	0.359
NM_009422	Traf2	0.361
NM_030560	Cwc22	0.366
NM_053075	Rheb	0.367
NM_008545	Mageb3	0.369
NM_153502	Ankrd23	0.378
NM_001081174	6330545A04Rik	0.379
NM_007860	Dio1	0.382
AK014289	Rbm33	0.384
NM_010207	Fgfr2	0.384
NM_027444	Bbx	0.388
AK031795	BB070754	0.389
NM_009567	Zfp93	0.389
NM_028360	Ttc19	0.391

Genbank Accession	GeneSymbol	Fold change (TiO_2-H vs Sham)
NM_021274	Cxcl10	0.396
NM_026622	3110057O12Rik	0.396
NM_177282	Mical2	0.402
NM_027815	9030624J02Rik	0.402
NM_023680	Tnfrsf22	0.402
AK163097	4933407K13Rik	0.404
NM_007473	Aqp7	0.404
NM_008588	Mesp1	0.407
NM_031391	Gtf2a1	0.410
NM_001012309	Ccdc55	0.411
NM_001081332	Slc9a5	0.412
NM_009701	Aqp5	0.417
NM_010275	Gdnf	0.422
NR_045892	Gm10538	0.423
XR_106299	Gm7467	0.426
NM_001114174	Fam189a2	0.426
BC028660	Gm15498	0.427
NM_028164	1600014C23Rik	0.427
NM_029847	Arsk	0.433
NM_011176	St14	0.436
NM_030682	Tlr1	0.438
NM_028873	Dnajc14	0.439
NM_178909	Wdr92	0.440
NM_011724	Xirp1	0.451
NM_144795	Pycr1	0.451
NM_001033337	Ttc38	0.453
AK017326	5430420F09Rik	0.454
NM_053115	Acox2	0.456
NM_008184	Gstm6	0.457
NM_172610	Mpped1	0.462
NR_040758	0610040F04Rik	0.463
NM_030739	Vmn1r58	0.466
AK016980	Gm9856	0.468
NM_028608	Glipr1	0.470
NM_009672	Anp32a	0.472
NM_029317	Izumo2	0.472
XR_035095	4930594M22Rik	0.475
NM_007649	Cd48	0.477
NM_001033247	Wdr52	0.479
AK140373	Fam196a	0.479
NM_001033300	Gmps	0.480
AF034610	Nasp	0.480
NM_019732	Runx3	0.484
NM_172831	E230025N22Rik	0.487
NM_029381	Tex22	0.487
NM_009863	Cdc7	0.488
NM_009936	Col9a3	0.491
AK179187	Sort1	0.492
NM_010163	Ext2	0.496
NM_146106	Lyplal1	0.498
NM_008362	Il1r1	0.498
NR_015529	R74862	0.500
NM_001081160	Mdga1	0.500
NM_134095	Pppde2	0.500

Genbank Accession	GeneSymbol	Fold change (TiO_2-H vs Sham)
NM_177759	Ccdc60	0.501
NM_001081399	Prss33	0.501
NM_172578	Mis18bp1	0.501
NM_009416	Tpm2	0.502
NM_175164	Arhgap26	0.506
NM_010558	Il5	0.525
NM_009608	Actc1	0.526
NM_009202	Slc22a1	0.529
NM_144854	ORF63	0.530
NM_138654	5033411D12Rik	0.534
AK163481	Kdm5b	0.537
AK142889	Pde3b	0.539
NM_001205236	Arhgap27	0.542
NM_007739	Col8a1	0.551
NM_133762	Ncapg2	0.556
XM_621386	Gm5958	0.562
NM_007423	Afp	0.563
NM_009943	Cox6a2	0.568
XM_003689333	1810030J14Rik	0.569
NM_007424	Acan	0.571
NM_027057	Wdfy1	0.579
NM_022318	Popdc2	0.584
NM_009398	Tnfaip6	0.586
NM_015783	Isg15	0.586
AK078505	Tmem220	0.586
AF031663	Strm	0.590
NM_007691	Chek1	0.595
NM_029095	Hhatl	0.600
NM_026222	Ccdc39	0.603
AK084992	D430022A14Rik	0.604
NM_001113548	Adamtsl5	0.626
NM_001190332	Frmd7	0.629
AK015935	Orly	0.634
NM_011139	Pou2f3	0.635
NM_053247	Lyve1	0.636
NM_177190	Tcp11l1	0.636
NR_045841	4933407G14Rik	0.639
NM_001123394	Ang3	0.639
NM_009185	Stil	0.639
NM_170727	Scgb3a1	0.646
NM_001162980	1700024P16Rik	0.646
NM_009160	Sftpd	0.650
AK049968	Anapc7	0.651
NM_177200	Svopl	0.652
NM_001135093	Klf14	0.654
NM_028352	Pgm3	0.658
NM_001098267	Ttll2	0.659
NM_001159693	Zar1l	0.659
NM_018746	Itih4	0.661
AK137878	A430071A18Rik	0.661
NM_173429	Zfp775	0.664
NM_020033	Ankrd2	0.665
