# Supplementary material for: Effects of Prenatal Exposure to Titanium Dioxide Nanoparticles on DNA Methylation and Gene Expression Profile in the Mouse Brain
Source: Front Toxicol. 2021 Oct 8;3:705910. doi: 10.3389/ftox.2021.705910 (PMC8915839; doi:10.3389/ftox.2021.705910)
Supplement: Supplementary file 13 [file Table10.PDF]

Supplementary Table 10.
The genes of which expression levels were increased with decreased methylation of CpG island in the brain of male (A) and female (B) offspring.

(A)

GenBank Accession	GeneSymbol	Target position of probe on CpG island microarray	mRNA	DNA methylation
			Fold change	Fold change of relative methylation
NM_029685	1700113H08Rik	chr10:86521172-86521216	1.795	0.254
NM_001163145	1810041L15Rik	chr15:84276781-84276825	1.861	0.188
NM_001163145	1810041L15Rik	chr15:84236917-84236961	1.861	0.626
NM_027222	2010001M09Rik	chr18:35812025-35812069	4.719	0.644
NM_001081012	4930473A06Rik	chr4:83171213-83171257	2.066	0.624
NM_030728	9930013L23Rik	chr7:91234999-91235044	4.297	0.625
NM_001098225	Adam22	chr5:8367164-8367208	5.264	0.348
NM_001098225	Adam22	chr5:8368117-8368161	5.264	0.439
NM_001098225	Adam22	chr5:8368197-8368241	5.264	0.466
NM_001098225	Adam22	chr5:8368019-8368063	5.264	0.600
NM_138600	Aldh7a1	chr18:56721947-56721991	3.714	0.128
NM_001127338	Aldh7a1	chr18:56732842-56732886	3.714	0.129
NM_138600	Aldh7a1	chr18:56722024-56722068	3.714	0.338
NM_001127338	Aldh7a1	chr18:56732742-56732786	3.714	0.529
NM_029245	Ankrd53	chr6:83713011-83713056	3.772	0.379
NM_019835	B4galt5	chr2:167174904-167174948	1.501	0.376
NM_019835	B4galt5	chr2:167174992-167175036	1.501	0.383
NM_027914	Bbs10	chr10:110735503-110735547	8.628	0.224
NM_027630	Ccdc105	chr10:78215712-78215756	2.015	0.497
NM_180958	Ccdc79	chr8:107033201-107033245	2.662	0.599
NM_026599	Cgnl1	chr9:71619304-71619348	13.060	0.450
NM_009767	Chic1	chrX:100551806-100551850	1.781	0.184
NM_153582	Cmtm4	chr8:106919324-106919368	2.174	0.356
NM_016869	Corin	chr5:72895109-72895153	5.331	0.462
NM_016869	Corin	chr5:72895290-72895334	5.331	0.466
NM_177307	Cyp4f39	chr17:32589667-32589711	2.094	0.120
NM_177307	Cyp4f39	chr17:32589827-32589872	2.094	0.193
NM_177307	Cyp4f39	chr17:32629460-32629505	2.094	0.623
NM_001145885	Ddx4	chr13:113442131-113442175	1.668	0.416
NM_001145885	Ddx4	chr13:113442236-113442280	1.668	0.579
NR_028264	Dleu2	chr14:62301793-62301837	5.017	0.133
NR_028264	Dleu2	chr14:62300610-62300654	5.017	0.418
NR_028264	Dleu2	chr14:62305865-62305909	5.017	0.483
NR_028264	Dleu2	chr14:62301504-62301548	5.017	0.490
NR_028264	Dleu2	chr14:62300358-62300402	5.017	0.568
NM_027960	Dpep3	chr8:108503069-108503114	8.802	0.237
NM_027960	Dpep3	chr8:108502919-108502963	8.802	0.255
NM_027960	Dpep3	chr8:108503182-108503226	8.802	0.278
NM_001081201	Dpy19l4	chr4:11248834-11248879	2.112	0.642
NR_038037	E130307A14Rik	chr10:39451377-39451421	7.574	0.405
NM_138953	Ell2	chr13:75844848-75844893	5.561	0.282
NM_138953	Ell2	chr13:75845818-75845862	5.561	0.395
NM_011843	Esyt1	chr10:127962373-127962417	2.351	0.431
NM_177077	Exoc6b	chr6:85019299-85019343	2.024	0.645
NM_010213	Fhl3	chr4:124378137-124378181	2.632	0.363
NM_010213	Fhl3	chr4:124377782-124377826	2.632	0.457

GenBank Accession	GeneSymbol	Target position of probe on CpG island microarray	mRNA	DNA methylation
			Fold change	Fold change of relative methylation
NM_010213	Fhl3	chr4:124377958-124378002	2.632	0.649
NM_145148	Frmd4b	chr6:97567120-97567164	3.213	0.530
NM_173739	Galntl4	chr7:118923736-118923780	4.541	0.332
NM_173739	Galntl4	chr7:118923422-118923466	4.541	0.631
NM_028022	Gatsl3	chr11:4118442-4118486	2.264	0.398
NM_028022	Gatsl3	chr11:4118164-4118208	2.264	0.482
NM_028022	Gatsl3	chr11:4118542-4118586	2.264	0.658
NM_010299	Gm2a	chr11:54911498-54911542	1.609	0.483
NM_010299	Gm2a	chr11:54911411-54911457	1.609	0.653
NM_008237	Hes3	chr4:151664569-151664614	2.933	0.629
NM_008237	Hes3	chr4:151660365-151660409	2.933	0.645
NM_020259	Hhip	chr8:82581462-82581506	3.053	0.586
NM_010461	Hoxb8	chr11:96144310-96144354	1.946	0.326
NM_010461	Hoxb8	chr11:96145448-96145492	1.946	0.639
NM_001005247	Hps5	chr7:54051013-54051057	7.599	0.637
NM_001012402	Hs3st6	chr17:24889759-24889803	1.604	0.433
NM_015820	Hs6st3	chr14:119537211-119537255	2.048	0.592
NM_015820	Hs6st3	chr14:119537426-119537470	2.048	0.635
NM_013868	Hspb7	chr4:140981800-140981844	6.025	0.323
NM_021358	Htr6	chr4:138630943-138630988	4.696	0.418
NM_021358	Htr6	chr4:138631065-138631109	4.696	0.443
NM_021358	Htr6	chr4:138630332-138630376	4.696	0.618
NM_027184	Ipmk	chr10:70810954-70810998	3.953	0.342
NM_153572	Katnal1	chr5:149740161-149740215	5.780	0.471
NM_153572	Katnal1	chr5:149739992-149740036	5.780	0.504
NM_177052	Kif6	chr17:49754556-49754600	1.602	0.393
NM_173379	Leprel1	chr16:26105726-26105770	4.070	0.232
NM_013589	Ltbp2	chr12:86217110-86217154	1.533	0.520
NM_013589	Ltbp2	chr12:86217266-86217310	1.533	0.546
NM_001174107	Map3k9	chr12:82881553-82881597	1.560	0.325
NM_175439	Mars2	chr1:55294197-55294241	5.632	0.427
NM_212447	Marveld3	chr8:112485994-112486038	4.482	0.346
NM_178076	Mcf2l	chr8:12915822-12915866	4.126	0.428
NM_133771	Memo1	chr17:74695143-74695187	1.604	0.011
NM_001122667	Mkl2	chr16:13256418-13256462	1.631	0.350
NM_181860	Mkl2	chr16:13257041-13257085	1.631	0.419
NM_027326	Mllt3	chr4:87677993-87678037	2.520	0.170
NM_001005863	Mtus1	chr8:42218752-42218796	22.060	0.250
NM_001005863	Mtus1	chr8:42219374-42219418	22.060	0.650
NM_001162909	Ncrna00085	chr17:17975189-17975234	1.541	0.444
NM_001113209	Nfib	chr4:82151139-82151183	1.919	0.391
NM_001113209	Nfib	chr4:82150339-82150383	1.919	0.517
NM_010928	Notch2	chr3:97817894-97817938	3.483	0.512
NM_001167891	Nrg2	chr18:36178281-36178325	1.605	0.422
NM_001167891	Nrg2	chr18:36177796-36177840	1.605	0.441
NM_001167891	Nrg2	chr18:36356271-36356315	1.605	0.490
NM_001167891	Nrg2	chr18:36178193-36178239	1.605	0.556
NM_001167891	Nrg2	chr18:36227658-36227702	1.605	0.599
NM_001033356	Ntn5	chr7:52949587-52949631	11.819	0.291
NM_001171512	Obscn	chr11:58949650-58949694	6.566	0.272
NM_001171512	Obscn	chr11:58947926-58947970	6.566	0.380
NM_018779	Pde3a	chr6:141198621-141198665	2.096	0.348

GenBank Accession	GeneSymbol	Target position of probe on CpG island microarray	mRNA	DNA methylation
			Fold change	Fold change of relative methylation
NM_018779	Pde3a	chr6:141198105-141198149	2.096	0.409
NM_019410	Pfn2	chr3:57651977-57652021	3.023	0.579
NM_001145955	Pigv	chr4:133228762-133228806	11.464	0.409
NM_001099276	Pik3c2b	chr1:134942691-134942735	1.835	0.499
NM_001099276	Pik3c2b	chr1:134942880-134942924	1.835	0.505
NM_008895	Pomc	chr12:3960273-3960317	1.576	0.498
NM_011207	Ptpn3	chr4:57313204-57313248	5.912	0.459
NM_001017427	Rasef	chr4:73436995-73437039	1.770	0.189
NM_009035	Rbpj	chr5:53980937-53980981	1.762	0.451
NM_001080927	Rbpj	chr5:53982361-53982405	1.762	0.525
NM_001081388	Rimbp2	chr5:129287634-129287678	3.633	0.487
NM_153055	Sec63	chr10:42482055-42482099	2.276	0.054
NM_153055	Sec63	chr10:42481959-42482003	2.276	0.275
NM_178639	Sfxn5	chr6:85283005-85283049	2.154	0.363
NM_172507	Sh3bgrl2	chr9:83442213-83442257	2.638	0.187
NM_011382	Six4	chr12:74213314-74213358	2.445	0.104
NM_011382	Six4	chr12:74213900-74213944	2.445	0.292
NM_011382	Six4	chr12:74213467-74213511	2.445	0.620
NM_001077514	Slc1a2	chr2:102498900-102498944	1.912	0.580
NM_026331	Slc25a37	chr14:69902363-69902407	3.734	0.327
NM_026331	Slc25a37	chr14:69902277-69902321	3.734	0.421
NM_001159633	Slc44a1	chr4:53453754-53453798	2.019	0.197
NM_177353	Slc9a7	chrX:19869101-19869145	3.150	0.554
NM_139308	Stard7	chr2:127096105-127096149	1.788	0.582
NM_029210	Sv2c	chr13:96902535-96902579	1.733	0.343
NM_029210	Sv2c	chr13:96901056-96901100	1.733	0.583
NM_029210	Sv2c	chr13:96902709-96902753	1.733	0.620
NM_001113352	Synj2	chr17:5941768-5941812	2.071	0.232
NM_001113352	Synj2	chr17:5941641-5941685	2.071	0.521
NM_177340	Synpo	chr18:60755666-60755710	1.588	0.377
NM_177340	Synpo	chr18:60784125-60784169	1.588	0.508
NM_173032	Tbck	chr3:132347071-132347115	2.259	0.592
NM_001134741	Tdrd5	chr1:158233625-158233669	2.023	0.289
NM_032004	Tssk6	chr8:72426109-72426153	2.425	0.524
NM_001080769	Uhrf1bp1	chr17:27993453-27993497	10.705	0.429
NM_001080769	Uhrf1bp1	chr17:27993828-27993872	10.705	0.432
NM_009502	Vcl	chr14:21748700-21748744	1.869	0.429
NM_009502	Vcl	chr14:21748618-21748662	1.869	0.636
NM_177184	Vps13c	chr9:67688155-67688199	1.556	0.334
NM_027462	Wars2	chr3:98945208-98945252	2.093	0.433
NR_001463	Xist	chrX:100677336-100677380	15.895	0.394
NR_001463	Xist	chrX:100677473-100677517	15.895	0.483
NM_011273	Xpr1	chr1:157264084-157264128	6.617	0.327
NM_011273	Xpr1	chr1:157264675-157264719	6.617	0.360
NM_011273	Xpr1	chr1:157264468-157264512	6.617	0.456
NM_011273	Xpr1	chr1:157264567-157264611	6.617	0.620
NM_001110254	Zfp945	chr17:23013560-23013604	2.220	0.067
NM_001110254	Zfp945	chr17:23013653-23013697	2.220	0.478

(B)

GenBank Accession	GeneSymbol	Target position of probe on CpG island microarray	mRNA	DNA methylation
			Fold change	Fold change of relative methylation
NM_001163145	1810041L15Rik	chr15:84237151-84237195	1.885	0.495
NM_001163145	1810041L15Rik	chr15:84237076-84237131	1.885	0.649
NM_173750	2700007P21Rik	chr2:106814392-106814436	2.617	0.341
NM_001172074	3110009E18Rik	chr1:122017920-122017975	2.128	0.072
NM_177607	4933430I17Rik	chr4:62186324-62186371	4.136	0.475
NM_177607	4933430I17Rik	chr4:62186403-62186447	4.136	0.287
NR_015553	9430076C15Rik	chr6:53237462-53237506	1.764	0.362
NR_015553	9430076C15Rik	chr6:53237701-53237745	1.764	0.239
NR_015553	9430076C15Rik	chr6:53238161-53238205	1.764	0.594
NR_015567	A930007I19Rik	chr19:29596371-29596418	4.062	0.473
NM_001190374	Adamtsl3	chr7:89484688-89484732	2.556	0.414
NM_001190374	Adamtsl3	chr7:89484048-89484092	2.556	0.560
NM_001205236	Arhgap27	chr11:103222146-103222190	1.825	0.603
NM_033474	Arvcf	chr16:18392999-18393043	6.117	0.588
NM_001167777	Asxl3	chr18:22503476-22503523	4.025	0.227
NM_001001488	Atp8b1	chr18:64819875-64819919	7.239	0.416
NM_001001488	Atp8b1	chr18:64819777-64819821	7.239	0.627
NM_001001488	Atp8b1	chr18:64820510-64820554	7.239	0.506
NM_177047	Auts2	chr5:133017511-133017555	1.937	0.466
NM_177047	Auts2	chr5:133019778-133019822	1.937	0.377
NM_177047	Auts2	chr5:133018497-133018541	1.937	0.353
NM_177047	Auts2	chr5:133018792-133018836	1.937	0.347
NM_177047	Auts2	chr5:133017392-133017444	1.937	0.098
NM_177047	Auts2	chr5:132014699-132014743	1.937	0.640
NM_001170935	BC005764	chr10:79337278-79337322	3.347	0.270
NM_001170935	BC005764	chr10:79337379-79337423	3.347	0.520
NM_001163502	C130039O16Rik	chr12:85533705-85533749	2.057	0.192
NM_001163502	C130039O16Rik	chr12:85534975-85535019	2.057	0.588
NM_027411	Ccdc99	chr11:34647134-34647181	1.686	0.227
NM_027411	Ccdc99	chr11:34646901-34646945	1.686	0.216
NM_153098	Cd109	chr9:78463468-78463512	3.833	0.351
NM_153098	Cd109	chr9:78463666-78463710	3.833	0.551
NM_001080818	Cdc14a	chr3:116126350-116126394	2.749	0.657
NM_001080818	Cdc14a	chr3:116126022-116126067	2.749	0.556
NM_023370	Cdh23	chr10:60149519-60149563	5.268	0.458
NM_023370	Cdh23	chr10:60149935-60149979	5.268	0.330
NM_023370	Cdh23	chr10:60148828-60148872	5.268	0.252
NM_023370	Cdh23	chr10:60150451-60150495	5.268	0.666
NM_009767	Chic1	chrX:100551713-100551757	2.065	0.123
NM_001163126	Cog5	chr12:32339734-32339779	3.725	0.324
NM_152809	Csnk1g3	chr18:54021378-54021423	2.684	0.133
NM_152809	Csnk1g3	chr18:54022583-54022627	2.684	0.081
NM_030560	Cwc22	chr2:77783936-77783980	1.568	0.267
NM_001081320	Cyb561d1	chr3:108003559-108003603	2.752	0.147
NM_010009	Cyp27b1	chr10:126486217-126486263	2.281	0.326
NM_010009	Cyp27b1	chr10:126486293-126486337	2.281	0.318
NM_177382	Cyp2r1	chr7:121706623-121706670	2.235	0.390
NM_177307	Cyp4f39	chr17:32589667-32589711	2.595	0.369

GenBank Accession	GeneSymbol	Target position of probe on CpG island microarray	mRNA	DNA methylation
			Fold change	Fold change of relative methylation
NM_177307	Cyp4f39	chr17:32589827-32589872	2.595	0.131
NM_177335	D930020B18Rik	chr10:121079155-121079199	5.549	0.153
NM_022722	Dpys	chr15:39688931-39688978	2.097	0.146
NM_022722	Dpys	chr15:39688848-39688892	2.097	0.557
NM_133833	Dst	chr1:34234710-34234761	1.755	0.651
NM_001039169	Eif4e2	chr1:89110508-89110552	1.697	0.306
NM_053103	Entpd7	chr19:43764144-43764188	3.598	0.220
NM_019585	Espn	chr4:151506015-151506059	4.381	0.446
NM_019585	Espn	chr4:151506130-151506174	4.381	0.261
NM_019585	Espn	chr4:151500208-151500252	4.381	0.246
NM_011934	Esrrb	chr12:87763647-87763691	7.950	0.363
NM_011934	Esrrb	chr12:87763078-87763133	7.950	0.232
NM_011934	Esrrb	chr12:87763573-87763617	7.950	0.127
NM_173395	Fam132b	chr1:93262992-93263036	7.995	0.524
NM_015796	Fbxo17	chr7:29501806-29501850	2.333	0.557
NM_015796	Fbxo17	chr7:29517672-29517716	2.333	0.542
NM_015796	Fbxo17	chr7:29517538-29517582	2.333	0.510
NM_008007	Fgf3	chr7:152025243-152025287	1.988	0.539
NM_008029	Flt4	chr11:49447973-49448018	49.368	0.526
NM_001042659	Fzd5	chr1:64784611-64784655	2.214	0.295
NM_001042659	Fzd5	chr1:64781408-64781452	2.214	0.272
NM_001042659	Fzd5	chr1:64783028-64783072	2.214	0.189
NM_008092	Gata4	chr14:63870430-63870474	4.013	0.327
NM_008092	Gata4	chr14:63864272-63864316	4.013	0.275
NM_008092	Gata4	chr14:63870620-63870670	4.013	0.204
NM_008092	Gata4	chr14:63864436-63864480	4.013	0.514
NM_010275	Gdnf	chr15:7762967-7763011	1.850	0.489
NM_010275	Gdnf	chr15:7765753-7765797	1.850	0.395
NM_010275	Gdnf	chr15:7761244-7761297	1.850	0.237
NR_033538	Gm10421	chr12:118392739-118392783	2.215	0.518
NM_133984	Hemk1	chr9:107240552-107240596	2.094	0.242
NM_145073	Hist1h3g	chr13:23627101-23627153	2.588	0.153
NM_008263	Hoxa10	chr6:52184811-52184855	12.744	0.247
NM_008263	Hoxa10	chr6:52190240-52190299	12.744	0.236
NM_010450	Hoxa11	chr6:52196424-52196469	3.888	0.362
NM_010450	Hoxa11	chr6:52195787-52195837	3.888	0.205
NM_010450	Hoxa11	chr6:52196249-52196297	3.888	0.114
NM_010469	Hoxd4	chr2:74565400-74565445	2.396	0.460
NM_025731	Hrasls5	chr19:7686892-7686936	19.535	0.403
NM_025731	Hrasls5	chr19:7686824-7686868	19.535	0.335
NM_015820	Hs6st3	chr14:119537625-119537669	1.672	0.226
NM_008308	Htr1a	chr13:106234796-106234840	1.978	0.218
NM_001033380	Itpripl2	chr7:125634804-125634848	1.802	0.647
NM_029116	Kbtbd11	chr8:15011860-15011904	3.795	0.391
NM_029116	Kbtbd11	chr8:15010976-15011020	3.795	0.275
NM_029116	Kbtbd11	chr8:15011568-15011612	3.795	0.066
NM_033325	Loxl2	chr14:70008844-70008888	2.706	0.387
NM_033325	Loxl2	chr14:70009101-70009145	2.706	0.197
NM_033325	Loxl2	chr14:70009216-70009260	2.706	0.185
NM_033325	Loxl2	chr14:70008926-70008970	2.706	0.053

GenBank Accession	GeneSymbol	Target position of probe on CpG island microarray	mRNA	DNA methylation
			Fold change	Fold change of relative methylation
NM_026915	Lyzl4	chr9:121550768-121550812	4.136	0.392
NM_026915	Lyzl4	chr9:121550854-121550898	4.136	0.296
NM_026915	Lyzl4	chr9:121551071-121551115	4.136	0.244
NM_026915	Lyzl4	chr9:121550968-121551014	4.136	0.134
NM_001081160	Mdga1	chr17:30026534-30026578	1.750	0.470
NM_001081160	Mdga1	chr17:29979095-29979139	1.750	0.310
NM_001081160	Mdga1	chr17:29989237-29989281	1.750	0.219
NM_001081160	Mdga1	chr17:30026432-30026476	1.750	0.197
NM_001081160	Mdga1	chr17:29979350-29979394	1.750	0.183
NM_001081160	Mdga1	chr17:30025201-30025252	1.750	0.168
NM_001081160	Mdga1	chr17:30026258-30026302	1.750	0.152
NM_133201	Mfn2	chr4:147279034-147279078	1.549	0.075
NM_001193305	Mical2	chr7:119369796-119369840	2.543	0.356
NM_001193305	Mical2	chr7:119369629-119369673	2.543	0.559
NM_010866	Myod1	chr7:53632477-53632521	3.446	0.436
NM_010866	Myod1	chr7:53632108-53632154	3.446	0.309
NM_009123	Nkx1-2	chr7:139791496-139791540	8.157	0.237
NM_008710	Nnt	chr13:120197789-120197833	2.662	0.330
NM_015743	Nr4a3	chr4:48059988-48060034	3.304	0.250
NM_015743	Nr4a3	chr4:48057954-48057998	3.304	0.212
NM_015743	Nr4a3	chr4:48058319-48058363	3.304	0.617
NM_010264	Nr6a1	chr2:38780768-38780812	2.374	0.430
NM_010264	Nr6a1	chr2:38781338-38781382	2.374	0.595
NM_013724	Nrk	chrX:135448869-135448913	2.036	0.157
NM_013724	Nrk	chrX:135449017-135449063	2.036	0.094
NM_013622	Oprd1	chr4:131700231-131700275	2.871	0.463
NM_011026	P2rx4	chr5:123158016-123158060	2.234	0.469
NM_021409	Pard6b	chr2:167924216-167924266	2.603	0.486
NM_021409	Pard6b	chr2:167905989-167906034	2.603	0.266
NM_021409	Pard6b	chr2:167906063-167906107	2.603	0.639
NM_016694	Park2	chr17:11033242-11033286	2.194	0.388
NM_001163746	Pgm3	chr9:86465331-86465375	2.133	0.242
NM_028370	Pot1b	chr17:55851800-55851847	4.193	0.280
NM_028370	Pot1b	chr17:55851934-55851978	4.193	0.218
NM_028370	Pot1b	chr17:55851663-55851707	4.193	0.078
NM_001127330	Pparg	chr6:115311955-115311999	3.654	0.275
NM_001127330	Pparg	chr6:115311740-115311784	3.654	0.505
NM_181348	Prune2	chr19:17030736-17030792	44.190	0.087
NM_008960	Pten	chr19:32831726-32831770	1.877	0.465
NM_008960	Pten	chr19:32831118-32831162	1.877	0.285
NM_008960	Pten	chr19:32830947-32830991	1.877	0.115
NM_008960	Pten	chr19:32830438-32830489	1.877	0.110
NM_001038624	Ric3	chr7:116226565-116226609	5.438	0.668
NM_001177868	Rreb1	chr13:37918572-37918616	2.609	0.294
NM_172788	Sh3rf3	chr10:58276497-58276541	1.623	0.470
NM_172788	Sh3rf3	chr10:58275328-58275372	1.623	0.329
NM_172788	Sh3rf3	chr10:58276430-58276477	1.623	0.161
NM_001033415	Shisa3	chr5:67998640-67998694	2.976	0.215
NM_001077514	Slc1a2	chr2:102499439-102499483	1.670	0.349
NM_138584	Spg21	chr9:65309057-65309101	1.876	0.145

GenBank Accession	GeneSymbol	Target position of probe on CpG island microarray	mRNA	DNA methylation
			Fold change	Fold change of relative methylation
NM_139308	Stard7	chr2:127096615-127096659	3.769	0.234
NM_001113352	Synj2	chr17:5941641-5941685	3.461	0.428
NM_009307	Syt2	chr1:136605441-136605485	4.560	0.209
NM_009307	Syt2	chr1:136605584-136605628	4.560	0.149
NM_181071	Tanc2	chr11:105451661-105451705	1.543	0.424
NM_028876	Tmed5	chr5:108561687-108561731	3.416	0.591
NM_207228	Tsga10	chr1:37921635-37921679	1.638	0.589
NM_029264	Ttll10	chr4:155409010-155409054	4.449	0.600
NM_183225	Usp24	chr4:105988267-105988311	21.299	0.232
NM_011718	Wnt10b	chr15:98608721-98608765	8.259	0.440
NM_011718	Wnt10b	chr15:98603197-98603241	8.259	0.274
NM_011718	Wnt10b	chr15:98602905-98602961	8.259	0.658
NM_011718	Wnt10b	chr15:98608492-98608536	8.259	0.642
NM_144515	Zfp52	chr17:21669765-21669813	2.077	0.244
NM_181419	Zfp599	chr9:22064295-22064339	2.002	0.604
NM_177263	Zhx3	chr2:160698110-160698154	2.137	0.121
